# *Plurigon*: three dimensional visualization and classification of high-dimensionality data

**DOI:** 10.3389/fphys.2013.00190

**Published:** 2013-07-22

**Authors:** Bronwen Martin, Hongyu Chen, Caitlin M. Daimon, Wayne Chadwick, Sana Siddiqui, Stuart Maudsley

**Affiliations:** ^1^Metabolism Unit, Laboratory of Clinical Investigation, National Institute on Aging, National Institutes of HealthBaltimore, MD, USA; ^2^Receptor Pharmacology Unit, Laboratory of Neuroscience, National Institute on Aging, National Institutes of HealthBaltimore, MD, USA

**Keywords:** *Plurigon*, three dimensional, data visualization, data classification, multivariate data vectors, algorithms, systems biology, bioinformatics

## Abstract

High-dimensionality data is rapidly becoming the norm for biomedical sciences and many other analytical disciplines. Not only is the collection and processing time for such data becoming problematic, but it has become increasingly difficult to form a comprehensive appreciation of high-dimensionality data. Though data analysis methods for coping with multivariate data are well-documented in technical fields such as computer science, little effort is currently being expended to condense data vectors that exist beyond the realm of physical space into an easily interpretable and aesthetic form. To address this important need, we have developed *Plurigon*, a data visualization and classification tool for the integration of high-dimensionality visualization algorithms with a user-friendly, interactive graphical interface. Unlike existing data visualization methods, which are focused on an ensemble of data points, *Plurigon* places a strong emphasis upon the visualization of a single data point and its determining characteristics. Multivariate data vectors are represented in the form of a deformed sphere with a distinct topology of hills, valleys, plateaus, peaks, and crevices. The gestalt structure of the resultant *Plurigon* object generates an easily-appreciable model. User interaction with the *Plurigon* is extensive; zoom, rotation, axial and vector display, feature extraction, and anaglyph stereoscopy are currently supported. With *Plurigon* and its ability to analyze high-complexity data, we hope to see a unification of biomedical and computational sciences as well as practical applications in a wide array of scientific disciplines. Increased accessibility to the analysis of high-dimensionality data may increase the number of new discoveries and breakthroughs, ranging from drug screening to disease diagnosis to medical literature mining.

## Introduction

With the advent of large-scale repositories of scientific knowledge and the increasing prevalence of information science, researchers from multiple disciplines are often faced with the task of collecting, manipulating, and disseminating high-dimensionality data. Currently, the model of choice for dealing with such data is the vector space model, a system of representing any entity with a list of identifiers in vector form. For example, high-throughput screening assay data from PubChem presents each compound in their database as a 881-dimensional binary vector representing the absence or presence of various features (elements, ring systems, atom pairing, nearest neighbors) in the compound. Similarly, a list of experimental results from any test subject can be equated to a vector with a dimensionality equal to the cardinality of the number of tests performed.

Such a model is undoubtedly invaluable for its condensation of data into a computable space. However, with increasing dimensionality, various numerical analysis challenges such as sparseness, statistical insignificance, and computational difficulty arise (Bellman, 1957). For classification, Vapnik-Chervonenkis theory and the Hughes effect state that theoretical classification rate decreases as dimensionality increases (Hughes, 1968). To address these issues, data is often preprocessed with feature extraction and dimensionality reduction techniques. Reducing dimensionality allows data to be processed more quickly and leads to reductions in noise, sparseness, and redundancy.

Additionally, as vectors often extend the scope of physical space, data visualization becomes an issue. Unfortunately, since presenting information in graphical ways is not necessary for our ability to extract answers, the field of data visualization lags far behind its sister disciplines of data analysis and data mining (Fayyad et al., 2002). This occurrence has led to a system in which only highly-specialized experts in esoteric technicalities can interpret the data at hand. If high-dimensionality data hopes to gain a larger audience however, advanced data visualization is instrumental in allowing end users with a rudimentary understanding of complex mathematics to interpret the graphical metaphors of the data at hand.

There are currently several excellent examples of high-dimensionality data visualization software including Ggobi (http://www.ggobi.org/), Visumap (http://www.visumap.net/) and Iris from Ayasdi (http://www.visumap.net/). It is clear from the applications of these programs (Wurtele et al., 2003; Landauer et al., 2004; Nicolau et al., 2011; Lum et al., 2013) that the inclusion of the exquisite capacity for human-centric visual appreciation and recognition into complex data analysis is a fertile ground for future research into high-dimensionality data interpretation. *Plurigon* serves to further this current interest and provide a synergistic alternative to these already useful applications.

*Plurigon* provides unification of high-dimensionality algorithms with visual human interfaces by converting a vector that exceeds physical space into an easily interpretable and highly interactive three-dimensional object. Feature extraction can then be performed on the *Plurigon*, either visually or computationally, for classification or machine learning. The usage of these features for data analysis is unexplored but promising due to the ease of *Plurigon* visual interpretation. The most unique trait of the *Plurigon* is that it is currently the only data visualization technique that places an emphasis on individual data vectors as opposed to an ensemble of different data vectors. This aspect of *Plurigon* provides an alternative to other forms of high-dimensional data visualizers. For example, genes or proteins are likely to act in two different modes, at times there may be strong individual actions (e.g., amyloid precursor protein mutations in Alzheimer's disease; Maudsley and Mattson, 2006) while at other times a specific gene/protein may act in a collective manner with other genes/proteins (Mootha et al., 2003). In most physiological systems a combination of these two functional modes is likely to be apparent, and especially in the presence of relatively few data points, *Plurigon* may provide a valuable alternative to ensemble visualization. In addition, the actual physiological actions of gene transcripts or proteins are highly contextual, i.e., a gene or a protein may possess a wide range of potential functionalities, but depending on the activity of other functionally-related or physically proximal factors, this spectrum of activity may be both qualitatively and quantitatively affected. By creating a data-derived physical object we intend to allow the influence of each individual piece of data with each other to create a form that encodes all potential interactions via the revelation of a recognizable series of topologies. These structures therefore may be characteristic of the actual “gestalt” output of the altered series of genes/proteins in the physiological paradigm.

With the *Plurigon*, data mining and knowledge discovery are more easily accessible to everyone—providing for integrated solutions between the biological and computational sciences. Increased accessibility to the analysis of high-dimensionality data may increase the number of new discoveries and breakthroughs in science, ranging from drug screening to literature mining.

## Materials and methods

### Determination of vertices on the *Plurigon*

For an input of *n*-values corresponding to a vector in *n*-dimensional space, a *Plurigon* can be generated without loss as a set of spherical coordinates (*r*, θ, φ). While the radius captures the magnitude of each value θ, φ captures its location in the original vector. Transformation of a data set into a *Plurigon* structure requires three steps: generation of a prototype structure with equal radii, remapping every point in the prototype to reflect actual data values, and iterative smoothing of the resulting *Plurigon* to remove sharp edges and unaesthetic qualities.

The vertices of the prototype *Plurigon* are generated by spacing *n* points on the prototype's circumsphere as far as possible. Unfortunately, this is a non-trivial task. Due to Euclid's proof that there are only five platonic solids, perfectly spaced points on a cube can only be achieved for dimensions 4, 6, 8, 12, and 20. In all other dimensions, perfect spacing cannot be achieved; however, there are a number of methods for approximating a distribution that minimizes the variance in distance between points. It is important to note that the naïve method of choosing points at equally spaced intervals of θ and φ is insufficient because data points are much more concentrated near the sphere's poles (Cook, 1957). As such, current methods for spacing vertices on a sphere include hypercube rejection, creation of a simulation involving electron repulsion, and spiral tracing (Smith, 1984; Rakhmanov et al., 1995; Saff and Kujilaars, 1997; Thomsen, 2007).

**Figure 1:**
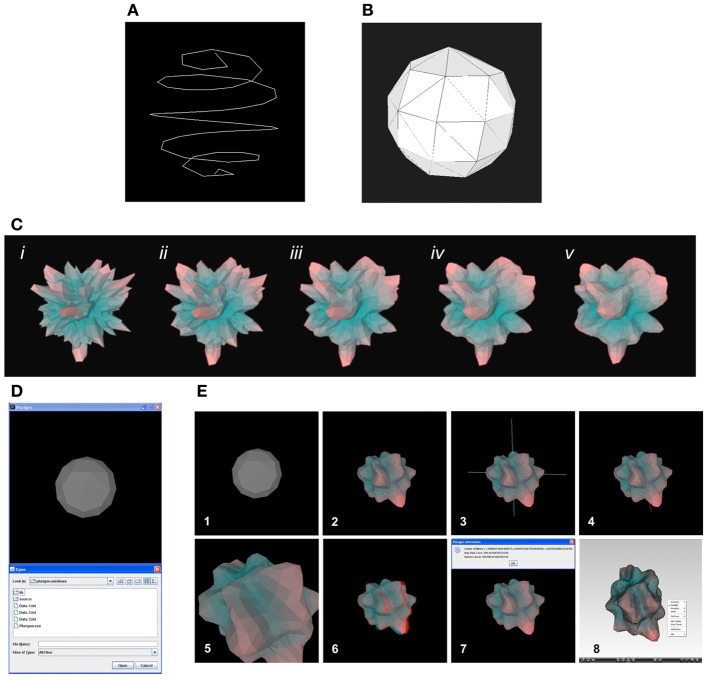
**Generation of *Plurigon* structure and its general manipulation. (A)** Initial *Plurigon* backbone creation. An illustration of vertex placement through spiral tracing. A set of 50 points was placed on the sphere at approximately equal distances from each other. **(B)** *Plurigon* polygonal basic structure. Convex hull generated from the vertices shown in **(A)**. This is the completed version of the *Plurigon* prototype; radial distance to the core remains constant for all vertices. **(C)** Laplacian smoothing in progress. Data was taken from a subset of gene expression values from murine genomic expression data. Iterations shown are *i, ii, iii, iv*, and *v*, afterwards, the movement of points becomes negligible, so iteration is stopped. **(D)** Initial *Plurigon* interface. The basic start-up *Plurigon* is depicted in an image window. **(E)** Simple and advanced *Plurigon* operations. Pressing “o” initiates the ability to choose a specific file to be depicted (1). Loading and pre-processing of data text file results in the generation of the basic color-coded *Plurigon* (2). Rotation of the *Plurigon* in all three dimensions is achieved using the up/down and left/right cursor keys. Addition of any other visualization features onto the *Plurigon* does not affect the rotational capacity. Pressing “x” generates the superimposition of *x, y*, and *z* axes onto the *Plurigon* (3). Pressing “x” while the three axes are present toggles the axes off. This action format is conserved for all other forms of *Plurigon* visualization. Pressing “c” superimposes the vector position for the *Plurigon* center of mass (COM) (4). This COM is represented by a red line. Pressing “+” or “−” generates an ability to zoom in and out of the *Plurigon* (5). A 3-dimensional (3-D) viewing version of the *Plurigon* is generated by pressing the number “3” (6). Pressing “3” again while in the 3-D mode removes this visualization format. Simple output of basic *Plurigon* structural information is achieved by pressing “i” (7). The ability to save a TIFF picture file of the window view of the *Plurigon* is achieved by pressing “s.” For each of the functions, sequential superimposition upon the *Plurigon* can be achieved using the respective key functions. For export to further 3-D viewing applications a.vrml/.wrl file of the *Plurigon* can be generated by pressing “v” (8). The image depicted is viewed using a Cortona-3D viewing application (www.cortona3d.com/Products/Viewer/Cortona-3D-Viewer.aspx).

For its ability to run in linear time, we use a slight improvement, created by Thomsen (2007), upon the methodology developed by Saff et al. for spacing points (Saff and Kujilaars, 1997), in which a larger spacing between the highest and lowest point better promotes point sparseness. This method falls into the category of spiral tracing, where a spiral is constructed with the endpoints as the sphere's poles and vertices placed at equal distances along the line segment (Figure 1A).

### Generation of a polygonal mesh from the vertices

Optimal generation of faces from the prototype's vertices requires performing the Delaunay triangulation on the set of points (Delaunay, 1934; Lee and Schachter, 1980). Briefly, the Delaunay triangulation of a set of points, P, in two dimensions is defined as the triangulation, T, in which no point in P rests in any circumcircle of any triangle in T. Although Delaunay triangulation is always possible in two dimensions, when considering extensions to higher dimensions, triangulations are often impossible or not unique. Fortunately, in the case of points of equal radius from a sphere's center of mass (COM), Delaunay triangulations are not only always possible, but also computable via taking the convex hull of the collection of points. Since there are no points within the sphere, a convex hull should encompass all vertices on the *Plurigon* prototype.

Generation of a convex hull in n-dimensions is well documented in the field of computer science. Since the inception of the Jarvis march (O(n^2^)) method for computing convex hulls in 1973, a variety of algorithms have been discovered and employed with much lower time-complexity (Graham, 1972; Jarvis, 1973). Here we use the QuickHull (O(n log n)) divide-and-conquer algorithm outlined in Barber et al. for the construction of the prototype's triangular faces (Barber et al., 1996). The resulting three dimensional polyhedron contains *n* vertices, *n* triangular faces, and can be contained in a circumsphere such that all vertices rest on its surface (Figure 1B).

### Creation of the *Plurigon* from a prototype

The purpose of creating a prototype is to ensure that the number of vertices on the resulting *Plurigon* is the same as *n*, the dimensionality of the data vector, during calculation of the convex hull. This was calculated with a unit vector to ensure that no points are located in the interior of the sphere. After calculation of the convex hull, the radii, r, of the prototype must be replaced by the individual input data values. The resulting surface should still be continuous, though during this phase it will likely be excessively turbulent and undulating for aesthetic and interpretable viewing.

To address this issue, the surface of the *Plurigon* must be smoothed and normalized until distinct topographic features, i.e., troughs, hills, peaks, and crevices, can be viewed. To this end, iterative Laplacian smoothing is applied to the polygonal mesh. Laplacian smoothing is a widely used technique in a variety of scientific fields (Briere and George, 1995; Amenta et al., 1997; Canann et al., 1997). Since its inception, many optimizations and improvements have improved the aesthetic outcomes of the smoothing process. Most notably, it has become possible to smooth a surface while maintaining Delaunay triangulation (Herrmann, 1976; Field, 1988; George and Borouchaki, 1998). Since maintenance of the *Plurigon's* Delaunay triangulation is crucial, a version of the smoothing algorithm outlined in (Field, 1988) was used. After a certain amount of iterations, where the movement of vertices becomes negligible, iteration is terminated and a highly smooth surface with distinct contours can be observed (Figure 1C).

### Extraction of rudimentary features

As an example of *Plurigon's* feature extraction capabilities, the latest release of *Plurigon* facilitates the automated calculation of a small number of built-in global features. The COM is calculated by converting spherical coordinates to cartesian coordinates and then computing the mean of the *x*-,*y*-, and *z*-values. Average radius to the *Plurigon's* core, i.e., the origin, is calculated by the mean of all radial values. Finally, surface area is computed by applying Heron's formula to each of the *Plurigon's n* faces. Other advanced features that can be extracted from *Plurigons* that may facilitate integration with other interaction-based technologies such as tangible user interfaces (TUIs) (Ratti et al., 2004), but are not included in the program for their length of compute time include: angular momentum for spinning *Plurigons*, linear momentum for moving *Plurigons*, and recognition of specific local analogous features (valleys, hills, peaks, crevices) with automated pattern recognition. With ease of human visualization and interpretation, however, specific feature extraction algorithms can be generated to suit the specific experiment at hand. It is interesting to note, however, that feature extraction may be infeasible for certain applications. Despite the superficial simplicity of *Plurigon* structures for the visual appreciation of complex datasets, simple machine-based feature extractions can rapidly become computationally impractical. For example, the triangulation of the *Plurigon* into tetrahedrons cannot be computed in polynomial time and is NP-hard (Freund and Orlin, 1985).

### Functional features of *Plurigon*

The *Plurigon* interface exists as a Java application in either Windows, Mac OSX, or Linux formats. *Plurigon* is freely available for download from the National Institute on Aging/National Institutes of Health website (http://www.irp.nia.nih.gov/bioinformatics/plurigon.html) (Figure S1). For review purposes, we have uploaded a Windows and Mac version of *Plurigon* for the reviewers and editor to test. The *Plurigon* application can be controlled entirely by keyboard (Figures 1D,E). Pressing “o” initiates the ability to choose a specific file to be depicted (Figure 1D). Loading and pre-processing of data text file results in the generation of the basic *Plurigon* (Figure 1E). Rotation of the *Plurigon* in all three dimensions is achieved using the up/down and left/right cursor keys. Addition of any other visualization features onto the *Plurigon* does not affect the rotational capacity. Pressing “x” generates the superimposition of color-coded (pink, yellow and blue) x, y, and z axes onto the *Plurigon*. Pressing “x” while the three axes are present toggles the axes off. This action format is conserved for all other forms of *Plurigon* visualization. Pressing “c” superimposes the vector position for the *Plurigon* COM. This COM is represented by a red line. Pressing “+” or “−”generates the ability to zoom in and out of the *Plurigon*. A 3-dimensional (3-D) viewing version of the *Plurigon* can be generated by pressing the number “3.” Pressing “3” again while in the 3-D mode removes this visualization format. Output of basic *Plurigon* structural information is achieved by pressing “i.” The output information box details the three dimensional coordinates of the calculated COM (x, y, z—format), the average radius of the plurigon structure from the central core of the platform (Avg.Rad.Core) and the total plurigon surface area (Surface Area). For future versions of *Plurigon* additional feature extraction tools will be developed (see Conclusions). The ability to save a TIFF picture file of the window view of the *Plurigon* is achieved by pressing “s.” For each of the functions, sequential superimposition upon the *Plurigon* can be achieved using the respective key functions. For export to further 3-D viewing applications a.vrml/.wrl file of the *Plurigon* can be generated by pressing “v.” The image depicted can be viewed using a Cortona-3D viewing application (www.cortona3d.com/Products/Viewer/Cortona-3D-Viewer.aspx).

### Murine hypothalamic transcriptomic investigation

Wildtype C57BL6 mice were housed and employed in accordance with the Animal Care and Use Committee (ACUC) regulations at the NIH National Institute on Aging. Briefly, mice, three per age (3, 6, 12, and 18 months of age) and gender group (male, female), were humanely sacrificed and their hypothalamic tissue was excised rapidly and snap frozen for Illumina Fluorescent Gene Array analysis as described previously (Martin et al., 2012b).

### Heterozygous genotype transcriptomic investigation

Four month old male wildtype C57BL6 (WT) and G protein-coupled receptor kinase-interacting transcript 2 (GIT2)-heterozygous (GIT2^−/+^, aka HET) mice were housed and employed in accordance with the ACUC regulations at the National Institute on Aging. Briefly, multiple mice from both genotype groups (WT or HET) were humanely sacrificed and tissue extracts from the following organs were prepared for gene array analysis: hypothalamus, hippocampus, skeletal muscle, liver, pituitary gland and testes. For *Plurigon* analysis a similar tissue-based range of transcriptomic data was simultaneously analyzed for both genotypes under study, i.e., WT and HET.

### Anti-neurodegenerative transcriptomic investigation

Clonal human neuronal cells, SH-SY5Y, were employed to study the pro-neurotrophic actions of the tri-cyclic antidepressant amitriptyline (AMI). SH-SY5Y cells (American Type Culture Collection) were maintained in a humidified 5% CO_2_ atmosphere at 37°C as described previously (Chadwick et al., 2010). We have previously demonstrated that in an aging-neurodegenerative murine model AMI exerts strongly neurotrophic pharmacological activity. We therefore employed *Plurigon* to investigate and analogize the activity of AMI compared to endogenous classical neurotrophic peptides such as brain-derived neurotrophic factor (BDNF) and nerve growth factor (NGF). Transcriptomic responses to AMI (10 nM), BDNF (10 ng/mL) or NGF (10 ng/mL) stimulation (8 h) of SH-SY5Y cells were assessed as previously described (Martin et al., 2009; Chadwick et al., 2012). AMI-hydrochloride, BDNF and NGF were all obtained from Sigma Aldrich (St. Louis, MO). In addition to assessing the transcriptomic responses of these human neuronal cells to AMI, BDNF, and NGF we also assessed the same activity responses in SH-SY5Y cells pre-treated with a chronic minimal peroxide exposure protocol designed to mimic age-related neurodegeneration (Chadwick et al., 2010; Martin et al., 2012a). This oxidative insult consists of a chronic (7 days) exposure to a survivable and minimal concentration (10 nM) of the oxidizing agent hydrogen peroxide. Transcriptomic responses to these three ligands, AMI, BDNF, and NGF, were measured as described previously. In addition to transcriptomic effects, protein expression profiles for various proteins were assessed using selective antibody-based western blotting and immunoprecipitation procedures described previously (Maudsley et al., 2000). Western blotting procedures were as performed previously (Martin et al., 2009): the sources of the primary antibodies employed in this study are detailed in Table S1. Subcellular fractionation of SH-SY5Y cell proteins was performed to separate intracellular proteins between Golgi and endoplasmic reticular compartments as described in Ko and Puglielli (2009).

## Results and discussion

### Description of plurigon generation and the user interface

The *Plurigon* software application aims to facilitate the transformation of high-volume data into a simpler, more appreciable structure. We term this resultant three-dimensional structure a “plurigon.” To create this data structure we use spiral tracing, where a spiral is first generated with the endpoints as the sphere's poles and vertices placed at equal distances along the line segment (Figure 1A). We then use a divide-and-conquer algorithm to generate a solid figure with triangular faces on the three-dimensional solid contained in a circumsphere with all the vertices resting on its surface (Figure 1B). After data input and data-magnitude color-coding, surface smoothening and normalization is applied to generate a more aesthetic *Plurigon* with more easily-appreciable contoured topography (Figure 1C). A detailed description of the *Plurigon*-generating computational steps is outlined in the Materials and Methods section and a flowchart of the functional data transition through *Plurigon* is depicted in Figure S2. *Plurigon* is available as a lightweight, standalone Java application, and as with other visualizers such as Ggobi, is available in versions for Windows, Mac OSX, and Linux. Untagged data can be uploaded into the *Plurigon* program with a.txt file containing precisely three floating-point numbers per line delimited by newlines. For direct comparison of comparable data, e.g., similar denominating factors with variable numerators, appropriate pre-processing should be performed by the user. Memory and computational requirements for *Plurigon* are markedly low. The algorithm for *Plurigon* generation outlined in the Materials and Methods section is highly scalable because of its logarithmic time-complexity. As a result, graphics rendering is typically smooth even on very large data sets (~20—40,000 features) and with low-end computers (<256 MB Random Access Memory). Rendering is typically instantaneous with frame rates of over 30 in practice. *Plurigon* is accessible completely from the keyboard. User functionality for visualization, portability, and interaction is extensive, as described in section Functional Features of *Plurigon*. Plurigons are automatically normalized to emphasize differences within data sets. Additionally, peaks, plateaus, and troughs within the *Plurigon* are color-coded with varying shades of red (highest value scores), gray, and cyan (lowest value scores), respectively.

### Visualization of high dimensionality data sets

Visualization of high-dimensionality data sets provides for the clear communication of information, unites various scientific disciplines, and makes otherwise arcane or highly-cryptic information more easily accessible. The current state of data visualization revolves around the representation of data matrices—an ensemble of data points. For example, when data is sparse and slight information loss is not an issue, a common practice is to perform Principal Components Analysis (PCA) to extract three or fewer components and then graph the resulting data points in a three-dimensional scatterplot (Wold et al., 1987; Kim et al., 2007). Other methods have been developed for even larger data sets. Two online visualization suites, Mondrian (http://mondrian.pentaho.com/) and Ggobi, both employ polylines with parallel coordinates, a common method for visualizing multivariate data by drawing n equally-spaced, parallel, vertical lines and plotting data along each line (Swayne et al., 1998; Theus, 2002; Buja et al., 2003; Cook and Swayne, 2007). Despite the considerable attention given to the relationships between data vectors, each individual vector is often neglected. For example, Ggobi represents a single data vector as a linear chain, which can quickly become chaotic and unintelligible once dimensionality exceeds a certain threshold. *Plurigon* provides for an alternative visualization mechanism by placing the emphasis not on the ensemble of data points, but rather on the individual data points themselves. The chaos of a single Ggobi linear chain is not present in the compact, spherical representation of the *Plurigon* even with dimensionalities in the tens of thousands. The effective employment of multiple distinct strategies for data investigation is likely to provide adequate solutions for a wide range of scientists. For *Plurigon* emphasis is dedicated to the prominence of a single, high-dimensionality data vector, whether it be a test subject, a compound, a document, or a gene. Strong deformities in topography could potentially be identified as important features. Despite the emphasis on individual points, however, there still exists a capacity for visualizing multiple subsets of vectors in a data matrix by comparing multiple *Plurigons* simultaneously for structural analogies or by subtracting or averaging data vectors with a data preprocessor before visualization.

### Practical employment of *Plurigon*

As *Plurigon* represents a versatile tool that can be applied to multiple forms of biomedical investigation we chose to use it to interrogate three data from three distinct but functionally-related sets of transcriptomic data. The three experimental paradigms we used to investigate the implementation of Plurigon are all associated with the study of aging and age-related neurodegeneration. The first paradigm (Plurigon Investigation of Murine Hypothalamic Aging Profiles) involves scrutiny of gender-based aging in the hypothalamus (a core central nervous system tissue for the control of aging). The second paradigm (Plurigon Investigation of Complex Murine Genotypic Profiles) challenges the ability of *Plurigon* to identify and classify large transcriptomic datasets from animal tissues from wildtype and heterozygous transgenic mice with reduced expression of a protein crucial for aging, GIT2 (G protein-coupled receptor kinase-interactor 2: Chadwick et al., 2012). The final paradigm (Plurigon Investigation of Novel Drug-Response Profiles) uses *Plurigon* to investigate novel anti-neurodegenerative drug mechanisms.

#### Plurigon investigation of murine hypothalamic aging profiles

**Figure 2:**
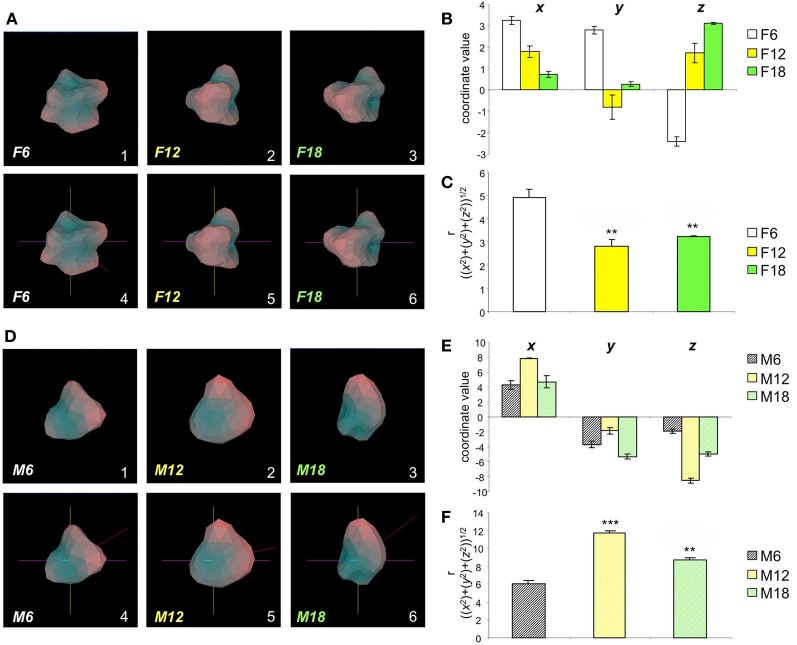
**Aging-related structural alteration in hypothalamic *Plurigons*. (A)** Panels 1, 2, and 3 represent plurigons created from hypothalamic transcriptome data from female mice of 6 (F6), 12 (F12), and 18 (F18) months of age, respectively. Panels 4, 5, and 6 indicate the identical coordinate nature of the three plurigons in 1–3. **(B)** Coordinate values, *x, y*, and *z* for the center or mass (COM) for the F6, F12, and F18 plurigons. **(C)** Calculation of *r*, (((*x*^2^) + (*y*^2^) + (*z*^2^))^1/2^), for F6, F12, and F18 plurigons. Statistical significance (^*^p < 0.05, ^**^p < 0.01, ^***^p < 0.001) was measured with GraphPad Prism (Student's *t*-test) for F12 and F18 relative to F6. **(D)** Panels 1, 2, and 3 represent plurigons created from hypothalamic transcriptome data from male mice of 6 (M6), 12 (M12), and 18 (M18) months of age, respectively. Panels 4, 5, and 6 indicate the identical coordinate nature of the three plurigons in 1–3. **(E)** Coordinate values, *x, y*, and *z* for the center or mass (COM) for the M6, M12, and M18 plurigons. **(F)** Calculation of *r*, (((*x*^2^) + (*y*^2^) + (*z*^2^))^1/2^), for M6, M12, and M18 plurigons. Statistical significance (^**^p < 0.01, ^***^p < 0.001) was measured with GraphPad Prism (Student's *t*-test) for M12 and M18 relative to M6.

We have previously identified the hypothalamus as one of the most important physiological loci for both aging and neurodegenerative activities (Chadwick et al., 2012; Martin et al., 2012b). In the current study, we employed hypothalamic gene transcriptomic data from wildtype mice, of both genders, across a broad age range (6, 12, 18 months) (Figure 2; Tables S2–S4 Female, Tables S5–S7 Male). After uploading of the transcripts consistently regulated across all three mouse ages (compared to 3 month old controls) for both genders there is a clear hypothalamic *Plurigon* structural “*evolution*” with the increasing age (Figure 2A: Female; Figure 2D: Male). As a simple indicator of the utility of our *Plurigon* program, we have just taken one output function, i.e., the COM calculation, to demonstrate an analytical function of our program. Using the Cartesian r, transform for the three spherical coordinates (*x*,*y*,*z*) we found that there are statistically significant changes (*p* < 0.05 see Figures 2; B,C Female, E,F Male) in the COM output with age in both females and males. Therefore, with relatively simple feature extraction *Plurigon* is able to generate easily-appreciable models of simple transcriptomic data associated with highly complex biological functions such as aging. The addition of further feature extraction modes will undoubtedly aid further structural differentiation pipelines.

#### Plurigon investigation of complex murine genotypic profiles

**Figure 3:**
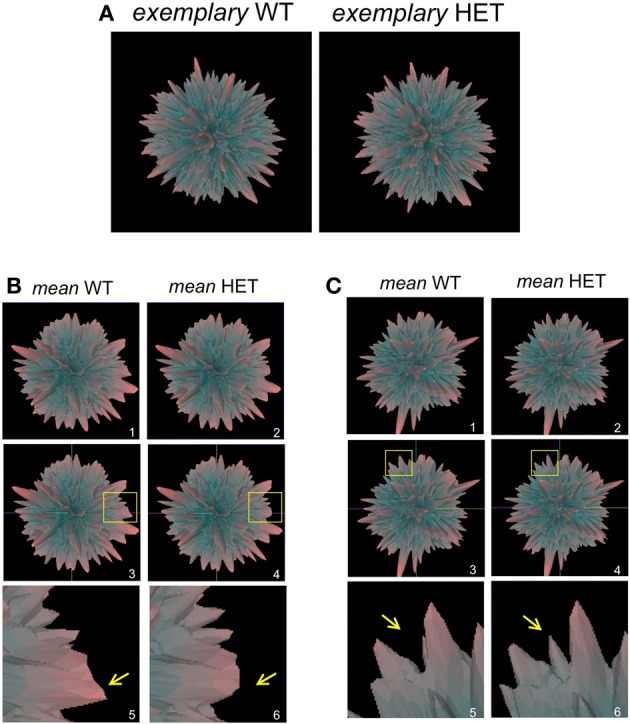
***Plurigon*-mediated investigation into genotypic data patterns. (A)** Exemplary plurigons generated from either a wildtype C576Bl6 mouse (WT) or a GIT2^−/+^ heterozygous mouse (HET). **(B)** Mean WT (panel 1) and HET plurigons (panel 2). Panels 3 and 4 indicate the identical coordinate nature of the two mean plurigons in panels 1 and 2. The yellow inset box indicates a region of structural idiosyncrasy between the mean WT and HET plurigons. This yellow inset box is expanded in panels 5 and 6 with associated arrows highlighting the structural variation. **(C)** Representation of a secondary structural variation in mean WT or HET plurigons. The panel numbering and content is identical to **(B)**.

**Figure 4:**
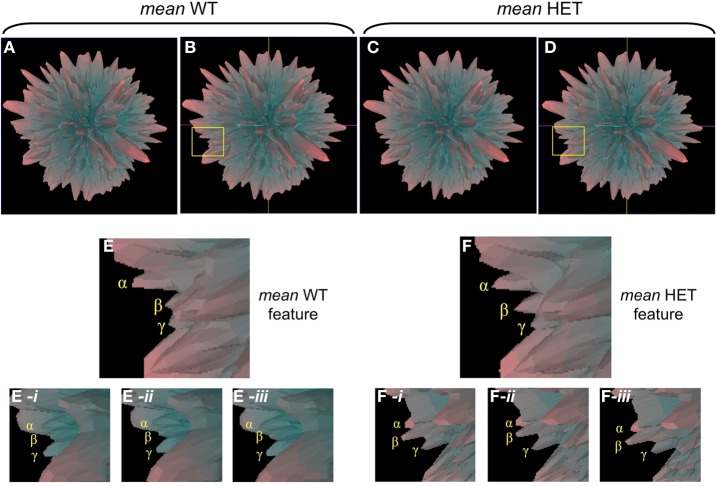
**Individual plurigon basis for structural variation in mean plurigon structures. (A)** Mean WT (**B**-coordinate location version) and mean HET (**C,D**-coordinate location version) are depicted with a region of structural diversity between them highlighted in the yellow inset box (**B**-WT, **D**-HET). Panels **(E)** and **(F)** indicate these expanded regions from the yellow inset boxes in **(B)** and **(D)**. The three projecting features creating the structural diversity are denoted as α, β, and γ. For the feature highlighted in **(E)**, three individual WT plurigon regions **(E-*i*–E-*iii*)** containing and potentially underlying the three projecting features (α, β, γ) are indicated. For the same generic feature indicated in the HET mean plurigon in panel **(F)**, three additional HET plurigon regions **(F-*i*–F-*iii*)** containing and potentially underlying the three projecting features (α, β, γ) are indicated.

In addition to employing *Plurigon* for relatively small datasets we also introduced a considerably larger data burden to *Plurigon* to assess its ability to separate highly complex transcriptomic data. To this end we also endeavored to assess whether *Plurigon* could effect a data group separation that was not achievable using PCA to three dimensions. We have therefore compared two heterogeneous, but functionally-related, groups of data, i.e., transcriptomic data from multiple murine tissues from either wildtype (WT) mice or mice with a heterozygous condition for the aging-regulator, GIT2 (Chadwick et al., 2012). GIT2 has been demonstrated to exert multiple cellular effects in mice (Schmalzigaug et al., 2009; Chadwick et al., 2010, 2012; Wang et al., 2012) therefore reduction in the expression of this important gene is likely to cause systemic effects in the animal, therefore there should be multiple differences in the transcriptomes between WT (*n* = 17) and the HET (*n* = 19) mice. Therefore, we employed *Plurigon* to attempt to separate these two sets of data (Table S8 WT, Table S9 HET). For these two genotypic examples our data corpus for each set has a dimensionality of over 20,000. Generating and scrutinizing individual plurigons for each mouse dataset (36 in total), therefore we initially chose to investigate whether systemic differences could be visually distinguished between “mean” plurigons for each genotype (Figure 3). We found that visual user scrutiny of both exemplary individual and the mean plurigons for each genotype facilitated the identification of complex and genotype-specific structural features (Figures 3A–C, S3–S7). We were also able to re-trace the origins of structural features observed in the mean plurigons back to individual animal plurigon data (Figure 4). Using visual user-based scrutiny it is clear that additional levels of data description can be generated with three dimensional plurigons, i.e., structural encryption and topological analogy. The presence or absence of view-obscuring factors can present or reveal distinctive features to the viewer that are discriminatory between complex datasets (Figure 3). Visual scrutiny is particularly good at quickly disseminating critical differences from less significant differences, even in large data sets. *Plurigon* outperforms PCA in this regard by virtue of being lossless. Distinctive features on the *Plurigon* can theoretically be mapped back to the individual dimensions by a second pass through the generation process. In this respect a counterpart for PCA does not exist.

**Figure 5:**
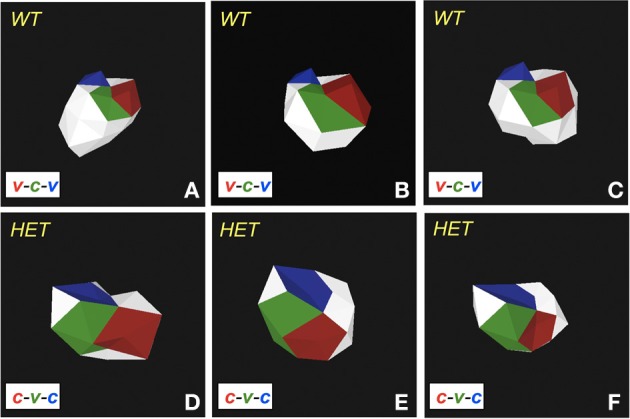
**Topographical data analysis with *Plurigon*. (A–C)** Exemplary topologically color-coded, and dimensionally-reduced, plurigons from the WT data corpus. The three demonstrated plurigons demonstrate a structural tripartite motif of convex [(v) red]—concave [(c) green]—convex [(v) blue]. **(D–F)** Exemplary topologically color-coded, and dimensionally-reduced, plurigons from the HET data corpus. The three demonstrated plurigons demonstrate a structural tripartite motif of concave [(c) red]—convex [(v) green]—concave [(c) blue].

We also found that structural topologies were also useful as characteristic agents, especially when compared to PCA-based analyses. Upon applying standard PCA algorithms, down to three dimensions, to the 36 mouse datasets we found the two distinct groups, WT or HET were non-linearly separable (Table S10: Figure S8). Unfortunately one of the main drawbacks of PCA is its inability to handle non-linearity, however human visual objects categorization however can help in this issue. Hence while PCA was unable to distinguish between the two groups we found that running a linear SVM (support vector machine) classifier on the genotypic data perfectly separates the two groups in 36 dimensions, suggesting that there are discriminatory structural plurigon features even in this reduced state. As the plurigons themselves are highly complex structures we computationally discovered structural motifs that support this genotype discrimination. Firstly we reduced the complexity of the WT/HET plurigons (with PCA) down to 36 dimensions for simpler visualization. Secondly we algorithmically selected three structural regions that could result in near-perfect separation of the genotypic data. Analyzing the three dimensional nature, convex (denoted by *v*) or concave (denoted by *c*), of the three specific regions we found that their relationship to each other engendered the near-perfect data separation (Figure 5). The order of structure of these three faces (highlighted in red, green or blue) was therefore able to strongly discriminate the WT group (*v-v-c* or *v-c-v*) from the HET group (*c-v-c* or *v-v-v*) (Figure 5: Table S11). Therefore, in this circumstance it seems that using the structural nature and topographical data extraction of *Plurigon* can offer some assistance where classical three dimensional PCA is limited. Linked to this, one important feature of *Plurigon* is that it can retain an arbitrary number of dimensions (in this case 36) while visualizable PCA is limited to two or three dimensions. Both PCA- and Plurigon-mediated analysis possess benefits and flaws and the implementation of them will depend on the eventual user preference, nature of the dataset and also the specific questions asked of the data.

#### Plurigon investigation of novel drug-response profiles

**Figure 6:**
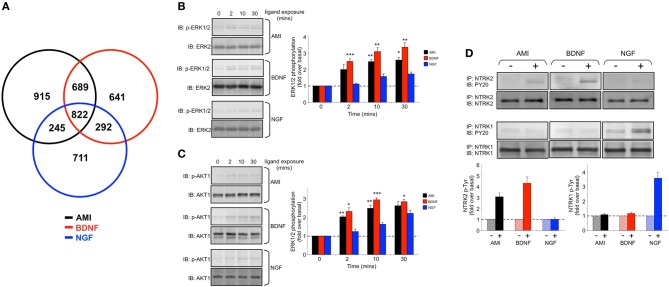
**Amitriptyline demonstrated functional signaling similarities with brain-derived neurotrophic factor in human neuronal cells. (A)** Venn diagram analysis of the gene transcripts significantly altered in their expression (compared to vehicle-treated control) in human SH-SY5Y cells 8 h after stimulation with amitriptyline (AMI-black), brain-derived neurotrophic factor (BDNF-red) and nerve growth factor (NGF-blue). **(B)** Time course data for AMI (10 nM)-, BDNF (10 ng/mL)- or NGF (10 ng/mL)-mediated extracellular signal-regulated kinase 1/2 (ERK1/2) phosphorylation which denotes kinase activation (measured with p-ERK1/2 phosphospecific antibody). Statistical significance (Student's *t*-test) (^*^p < 0.05, ^**^p < 0.01, ^***^p < 0.001) for AMI and BDNF compared to NGF was measured at each time point from three independent experiments. **(C)** Time course data for AMI-, BDNF- or NGF-mediated v-akt murine thymoma viral oncogene homolog 1 (AKT1) phosphorylation which denotes kinase activation (measured with p-AKT1 phosphospecific antibody). Statistical significance (Student's *t*-test) (^*^p < 0.05, ^**^p < 0.01, ^***^p < 0.001) for AMI and BDNF compared to NGF was measured at each time point from three independent experiments. **(D)** Treatment of SH-SY5Y cells (20 min) with AMI (10 nM) or BDNF (10 ng/ml) results in the increase of phosphotyrosine content [measured using anti-phosphotyrosine (p-Tyr) antibody—PY20] of immunoprecipitated (IP) NTRK2, while only NTRK1 is significantly phosphorylated by NGF.

**Figure 7:**
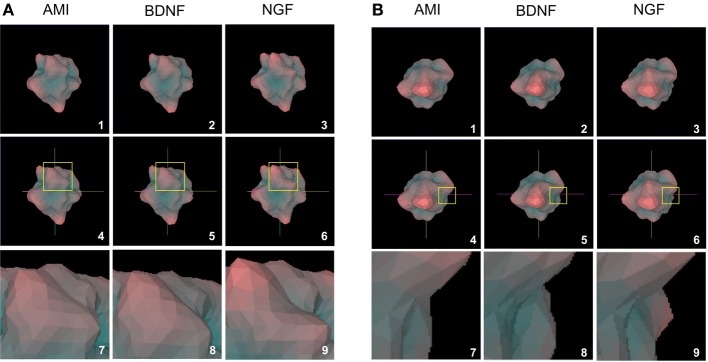
**Structural analogy between AMI and BDNF plurigons. (A)** Panels 1, 2, and 3 depict representative plurigons for AMI-, BDNF- and NGF-stimulated transcriptomic datasets. Panels 4–6 depict the coordinate locations for plurigons in panels 1–3. In each plurigon window (4–6) a region of structural divergence between the plurigon structures in highlighted in a yellow box. This region is expanded (panels 7–9) to demonstrate the similarity in structural region between AMI and BDNF and their diversity from NGF. **(B)** Distinct secondary example of structural analogy between AMI and BDNF plurigons compared to NGF. The panel enumeration pattern is identical to **(A)**.

Over the past decade our appreciation of the potential complexity of drug responses has necessitated the development of higher-density drug-response data (Maudsley et al., 2005, 2011b; Luttrell and Kenakin, 2011). Potentially one of the most efficient ways of generating this high-density drug response data is the use of transcriptomics (Ruf et al., 2003; Gesty-Palmer et al., 2013). It is now known that many “small molecule” drugs, for G protein-coupled receptors or other targets, can indeed exert strong transcriptomic and genomic responses (Golan et al., 2009; Chadwick et al., 2011) that were previously considered to be solely regulated by growth factor receptor systems. We have previously shown that the small molecule tricyclic AMI is able to exert beneficial therapeutic actions in aged mice that possess a pathophysiological signature of Alzheimer's disease (Oddo et al., 2003; Chadwick et al., 2011). The actions of AMI seemed to be strongly associated with the activation of neurotrophin-like signaling pathways. Neurotrophins are large endogenous growth factors that, via activation of their cognate receptors (of the receptor tyrosine kinase family), entrain strongly neuroprotective signaling pathways (e.g., v-akt murine thymoma viral oncogene homolog 1 (AKT-1) phosphorylation) as well as regulating signaling pathways (e.g., extracellular signal-regulated kinase 1/2 (ERK1/2) phosphorylation) and the expression of markers (post-synaptic density protein 95 (PSD95)) linked with neuronal development. The three primary neurotrophin ligands are NGF, BDNF, and neurotrophin-3 (NT3). Respectively these ligands activate their cognate neurotrophic tyrosine kinase receptors (NTRK), i.e., NTRK1, NTRK2 and NTRK3. The majority of research into these neurotrophin systems has focused on the therapeutic benefits of NGF and BDNF signaling. In Alzheimer's disease animals we previously found that the beneficial actions of AMI appeared to stem from its ability to mimic the actions of BDNF, rather than that of NGF. This finding was unexpected as AMI is considered a “small” drug molecule (0.277 kDa) while BDNF is approximately 13 kDa in mass, therefore despite their completely different physico-chemical properties they seemed to share strong functional analogy in the central nervous system of aged and diseased mice. We therefore chose to investigate, at the transcriptomic response level, whether *Plurigon* would be able to assist in the characterization of structures indicative of specific forms of pharmacological activity, such as neuroprotective and neurodevelopmental mechanisms. Stimulation of human clonal neuronal cells, SH-SY5Y, with AMI, BDNF or NGF followed by RNA extraction (8 h later) and microarray analysis revealed significant transcriptomic activity of all three ligands (AMI-Table S12, BDNF-Table S13, NGF-Table S14) compared to vehicle-treated cells. When we compared these three transcriptomic effects we again found that indeed the genomic response patterns to AMI and BDNF were more similar to each other than AMI to NGF or even BDNF to NGF (Figure 6A). Mirroring this transcriptomic similarity between AMI and BDNF we also found that at the cellular signaling level the response patterns for ERK1/2 (Figure 6B) and AKT-1 phosphorylation (Figure 6C) (kinases linked to neurodevelopmental and neuroprotective activities, respectively) to AMI and BDNF were similar in magnitude and temporal nature to each other and differential to the signaling patterns of NGF in these cells. Connected to these findings we were also able to demonstrate that only cellular treatment (20 min) with AMI and BDNF resulted in the increase in phosphotyrosine content of immunoprecipitated NTRK2 (cognate receptor for BDNF) (Figure 6D). In contrast, NGF cellular stimulation (20 min) resulted in the increase of phosphotyrosine content of the NTRK1 receptor (Figure 6D). These data therefore demonstrate that despite considerable physico-chemical differences AMI is able to mimic many of the functional effects of BDNF. We then assessed whether this distinction could be translated into distinct *Plurigon* structures. Using transcripts commonly activated between AMI, BDNF and NGF at the 8 h time point (Table S15-AMI, Table S16-BDNF, Table S17-NGF) we found after creating the plurigons for these three ligands that strong visual distinctions were apparent (Figures 7A,B, S9, S10). In each of these example plurigons the structural idiosyncrasies demonstrated a strong similarity between the AMI and BDNF structures, both of which were distinguishable from the NGF structures. With respect to the potential molecular mechanism of BDNF mimicry and the therapeutic effects observed in Alzheimer's disease animals (Chadwick et al., 2011) we found that chronic cellular treatment (24 h) with AMI resulted in a disruption of the cellular disposition (measured using subcellular biochemical separation) of components (anterior pharynx defective 1 homolog A (APH1A) and nicastrin (NCSTN)) of the amyloid precursor protein (APP) processing complex, γ-secretase (Figure S11A). Aberrant processing of APP is a hallmark of the Alzheimer's disease pathological process. In addition to changes in cellular distribution of components of the γ-secretase complex we found alterations in NTRK2 and the multifunctional adapter protein β-arrestin 2 (ARRB2). We subsequently found that acute AMI treatment (20 min) affected the physical interaction between NTRK2 and APP, APH1A and ARRB2 (Figure S11B), suggesting that changes in this multiprotein complex may be associated with the beneficial therapeutic actions of AMI.

**Figure 8:**
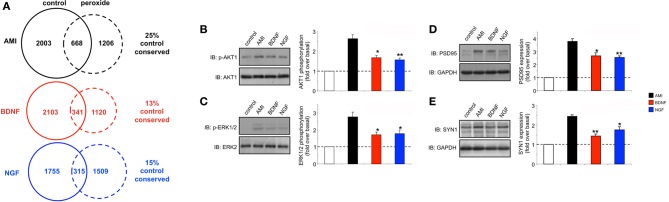
**Chronic low-dose peroxide treatment of human neuronal cells differentially affects stimulatory ligand behavior. (A)** Venn analysis of significant gene transcriptome regulatory activity for AMI, BDNF and NGF both in control SH-SY5Y neurons (treated with vehicle) and SH-SY5Y neurons previously exposed to 10 nM hydrogen peroxide for 7 days (peroxide). The percentage of transcription conservation between control and peroxide states is indicated for AMI, BDNF, and NGF. **(B)** AMI (20 min treatment) possesses a significantly better ability to activate AKT1 and ERK1/2 **(C)** in peroxide pre-treated cells compared to BDNF (red bars) or NGF (blue bars). AMI (48 h treatment) also possessed a significantly greater ability to elevate the expression of PSD95 **(D)** and synapsin I **(E)** in peroxide pre-treated cells compared to BDNF or NGF. Statistical significance was measured over three independent experiments with a Student's *t*-test (^*^p < 0.05, ^**^p < 0.01).

**Figure 9:**
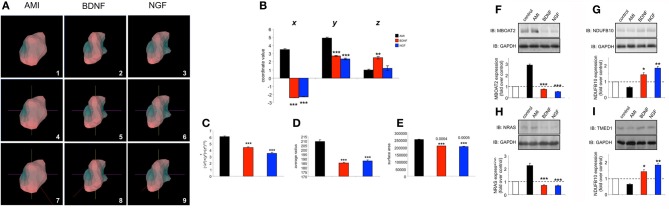
**Chronic peroxide treatment of human neuronal cells reveals idiosyncratic AMI-derived plurigon structure and selective protein expression regulation. (A)** Plurigon structures for AMI (panel 1), BDNF (panel 2) and NGF (panel 3)-mediated transcriptional activity in peroxide-treated SH-SY5Y cells. Panels 4–6 indicate the identical coordinate locations for the three plurigon structures in panels 1–3. The red COM vector line for each of the plurigon structures is depicted in panels 7–9. The COM vector line for the AMI plurigon is clearly distinct from the two more similar BDNF and NGF COM vector lines. **(B)** COM coordinate analysis for AMI, BDNF and NGF plurigon structures. AMI-derived plurigons demonstrate a significantly different *r* calculation **(C)**, average radius from core **(D)** and surface area **(E)** compared to BDNF- and NGF-derived plurigon structures. AMI treatment (48 h) of peroxide-exposed SH-SY5Y cells engenders an idiosyncratic, and statistically distinct, cellular expression pattern, compared to BDNF and NGF, of MBOAT2 **(F)**, NDUFB10 **(G)**, NRAS **(H),** and TMED1 **(I)**. Experiments in panels **(F**–**I)** were performed in triplicate and statistical significance was assessed using a Student's *t*-test (^*^p < 0.05, ^**^p < 0.01, ^***^p < 0.001).

BDNF possesses pharmacological activities that are highly sought after for novel pharmacotherapeutics for aging-related neurodegenerative disorders such as Alzheimer's disease (Mattson et al., 2004; Nagahara and Tuszynski, 2011). However, many therapeutic strategies are not evaluated in appropriate pharmacogenomic settings, i.e., most neurotherapeutic activity is assessed in non-diseased, young healthy tissues or cells. The genomic and transcriptomic effects of aging and disease are considerable and can exert potent effects on drug efficacy (Lesko and Schmidt, 2012; Liou et al., 2012). We recently developed an *in vitro* mechanism to artificially “age” neuronal cells lines (Chadwick et al., 2010) as the extraction and analysis of central nervous tissues from aged experimental animals is highly problematical due to cellular stress and rapid degradation. We therefore applied this minimal peroxide exposure (7 days, 10 nM hydrogen peroxide treatment) to SH-SY5Y cells and repeated the AMI, BDNF and NGF transcriptomic stimulation protocols (Table S18-AMI, Table S19-BDNF, Table S20-NGF). Upon inspection of the transcriptomic responses in these “aged,” peroxide treated cells we found that in the “aged” cells AMI was able to activate a transcriptomic response more similar (25% conserved) to that in non-peroxide-treated control cells than either BDNF (13% conserved) or NGF (15% conserved) (Figure 8A). Perhaps linked to this we found that AMI possessed a superior ability, compared to BDNF or NGF (all 10 min stimulation), to activate neuroprotective (AKT1, Figure 8B) and neurodevelopmental (ERK, Figure 8C) signaling functions. In addition to these rapid signaling effects of AMI we found that with longer-term exposure (48 h), AMI demonstrated an enhanced ability, compared to BDNF or NGF, to increase the expression of the neurosynaptic developmental markers (indicative of neurotrophin-like activity), post-synaptic density 95 (PSD95) and synapsin I (SYN1) (Figures 8D,E, respectively). To investigate whether this cellular context, and ligand-specific, alteration of pharmacological activity could be associated with plurigon structure we generated comparable plurigons from transcripts commonly activated by AMI, BDNF and NGF in these peroxide-treated “aged” cells (Table S21-AMI, Table S22-BDNF, Table S23-NGF) (Figure 9A). In the “aged” neuronal cells the AMI plurigon structures now demonstrated a more singular structure compared to those for BDNF or NGF (Figures 9B–E: COM coordinates, r, average radius, surface area). As we had previously shown that the actions of AMI appear to mimic the beneficial capacities of BDNF we applied bioinformatic annotation to the AMI-controlled transcripts more similarly regulated (ranked by expression score similarity) to BDNF as opposed to NGF (Table S24). Using the NIH DAVID Analytical Suite (http://david.abcc.ncifcrf.gov/) to generate KEGG (Kyoto Encyclopedia of Genes and Genomes (http://www.genome.jp/kegg/) pathway output from these AMI-regulated, BDNF-like, transcripts, we found that the only signaling pathway significantly populated (*p* < 0.05) by this gene list was the hsa047223: Neurotrophin signaling pathway (http://www.genome.jp/)(kegg-bin/show_pathway?hsa04722) (Table S25).

We assessed, at the protein level, these cell context-specific actions of AMI, by validating several transcript protein products of genes differentially regulated by all three ligands that showed a preference for BDNF-like activity (Table S24). We found that 48 h after exposure, AMI was able to cause highly selective and unique alterations in the expression of MBOAT2 (membrane bound O-acyltransferase domain containing 2: Figure 9F), NDUFB10 (NADH dehydrogenase (ubiquinone) 1 beta subcomplex, 10: Figure 9G), NRAS (neuroblastoma RAS viral (v-ras) oncogene homolog: Figure 9H) and TMED1 (transmembrane emp24 protein transport domain containing 1: Figure 9I). Therefore, with respect to the actions of AMI in the “aged” neuronal tissue, we have seen that a strong alteration in its plurigon structure (Figure 9) is associated with selective signaling (Figure 8) and protein expression changes (Figure 9) while still retaining a strongly pro-neurotrophic signaling activity (Table S25). Therefore, *Plurigon*, in association with classical protein biochemistry and informatic techniques is able to assist in the identification of activity patterns of “small molecule” compounds that possess a strong neuroprotective activity even in aged/damaged neuronal tissue.

## Conclusions

Given the increasing use of high-dimensionality data in many disciplines, including specifically biomedical research, the development of our application *Plurigon* serves as a mechanism to assist human-aided feature extraction. *Plurigon* may also be capable of bringing a larger audience to high-dimensionality data and represents a mechanism to open doors for integrated solutions to biological and biomedical problems. *Plurigon* also serves as a potential alternative to other standard forms of complex data analysis such as PCA and also as a complementary system to other excellent high-dimensionality suites such as Ggobi and Ayasdi Iris. One specific feature of *Plurigon* that may assist in comparative studies is its strong standardized structural platform. This is in contrast to the more “freeform” nature of visualizers such as Iris. A strong expected structural platform may facilitate simpler comparisons between minimally-distinct datasets, but in other circumstances it is also possible that a more freeform topological data interpretation may be superior. The nature of the data corpus, the personal preferences of the user and the required form of answers from the data should all combine to influence the choice of application used. Due to its ability to condense multivariate data into a visually interpretable form, *Plurigon* can lay the groundwork for novel methods, experimental design, and new discoveries in a variety of scientific fields ranging from molecular biology to computational linguistics, genomics to proteomics, bioinformatics to pharmacology.

**Figure 10:**
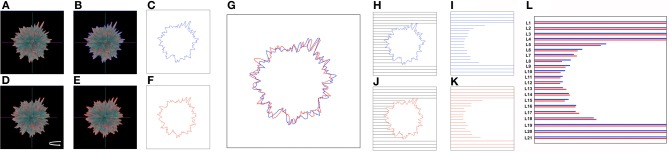
**Future developments for *Plurigon* feature output and analysis.** Outline recognition of cryptic feature generation. As Plurigon generates a reliable-oriented three-dimensional figure it is likely that surface features may indeed have an impact upon the appreciable visual nature of the plurigon through obscuring other data points. Such an effect therefore generates an entirely new level of data that other applications may not due to their free-form structure or lack of three-dimensional rendering. To attempt to quantify this effect, and also create additional extractable data features for detailed data classification, we propose the following methodology. **(A)** A single exemplary plurigon is chosen and for a given set of coordinates for the axes an outline of the edge of plurigon can be generated, indicated by the presence of the blue line in panel **(B)**. This trace can then be extracted in **(C)**. With the same exemplary plurigon as in **(A)**, slight rotation around the vertical y axis **(D)** helps generate a novel plurigon outline (**E**—red line) that also can be extracted **(F)**. Even with small rotations (in axis required) the plurigon outline can clearly change [**G**—superimposition of **(C)** and **(F)** traces], due to both loss of features from the visual field or also via obscuration of newly-oriented features with respect to the plane of the viewer. To extract quantifiable features from such effects line-length scanning can be used from any direction required in the extracted trace box. In this case scanning line-length is determined by the termination of the scan line (black) at the blue **(H)** or red **(I)** plurigon trace outline. Extraction and matching of these scan lines [**J**,**K**; compared with minor vertical shift to improve comparison in **(L)**] can be used to discriminate divergent features of the plurigon based on this perspective-regulated analysis. The number of numerical features such as this can be easily scaled up or down based on the plurigon rotation angles and the orientation and density of the trace outline scan lines.

**Figure 11:**
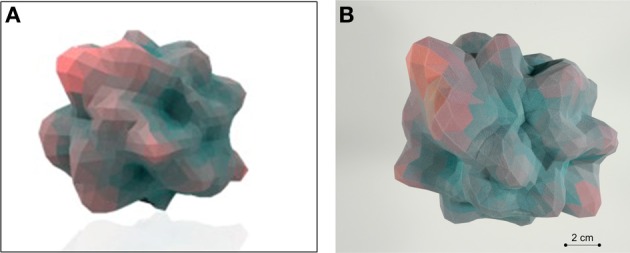
**Tangible-user interface outputs for *Plurigon*. (A)** Rendering of an exemplary *Plurigon* structure from a.wrl format file. **(B)** Actual physical 3-dimensional print-out of a *Plurigon* for potential TUI (tangible user interface) applications and topological analysis.

Here we have described the generation and implementation, in three varied but synergistic biomedical work paradigms, i.e., hypothalamic age patterns, genotypic analysis of age-modifying proteins such as GIT2 (Chadwick et al., 2012) and the pharmacogenomic investigation of small molecule neurotrophic ligands. Our current appreciation of the full gamut of *Plurigons'* utility will no doubt be expanded once the application is transferred to a diverse range of investigators in different disciplines. Therefore, it is highly likely, and desirable, that further significant advancements in analysis, classification and feature extraction will be made in the future. The relative simplicity of the Plurigon concept underlies the enormous potential for comparative data analysis. For example we propose that outline-transition analysis, i.e., the effect of feature “*crypticism*” (obscuration or revelation of outline features based on differential visual aspects and scale: Figure 10) in the plurigon may be an interesting avenue for further feature extraction. Another example for potential future development for *Plurigon* data outputs is the synergism of physical *plurigon* structures with TUIs. Hence, in addition to the standard *Plurigon* output file formats, the derived.vrml or.wrl files can be employed for physical 3-dimensional rendering/printing of a plurigon of choice (Figure 11). The generation of an actual physical output of *Plurigon*, with 3-dimensional printing using a Z Corp ZPrinter 650 (http://www.sculpteo.com/en/) may allow future users to easily connect their high-dimensionality data to machine-learning applications using TUIs (Ishii et al., 2012) that can extract more physically-relevant object data outputs.

When data dimensionality reaches a certain threshold, it becomes challenging even for advanced computers to process such data. Aside from the sheer infeasibility of computation, problems of noise, sparseness and statistical insignificance increase dramatically as the number of dimensions increases. To this end, a common practice is to preprocess data with feature extraction and dimensionality reduction techniques such as PCA, Latent Semantic Analysis (LSA), or Semidefinite Embedding (SDE). A collective group of different feature extraction methods can be effective for the classification of a broad spectrum of entities (Orlov et al., 2008). Extraction is often performed after various mathematical transforms have been applied to the original data set to increase the number of total usable features. As such, feature extraction from *Plurigon* may therefore potentially be useful as additional input features for applied machine learning algorithms. One potential advantage that *Plurigon* possesses is the ability for humans to easily participate in the genesis of diverse feature extraction processes from familiar and potentially tangible sources. With efficient data visualization, a coordination of efforts can be made for the efficient extraction of features either directly from the *Plurigon* structure, or from the original data set itself.

One potential important venue of future research for validating the use of “data texturizers” such as *Plurigon*, or the excellent Ayasdi Iris (Lum et al., 2013), as tools for visualization and classification is the field of drug screening and discovery. In our current work we have attempted to demonstrate that transitional changes in plurigon structures are tightly associated with complex changes in gene expression patterns, cellular signaling activity and protein-protein interaction. In addition to this we found that pharmacogenomic alterations in ligand (AMI) activity were mirrored in the subtle changes in plurigon structure (Figure 9) and that these changes were strongly associated with the maintenance of desirable drug activity patterns (Table S25). In addition to aiding pharmacological research by interpreting functional signaling patterns, the implementation of *Plurigon* to disease diagnoses and classification may be an important future use. Many disease processes, e.g., hypertension, Alzheimer's disease or diabetes (Maudsley et al., 2011a), are often considered in a monolithic sense. While the eventual diagnosis, e.g., elevated blood pressure, compromised memory function, or excess glucose in the urine, may seem to unify patients presenting those data, it has been steadily proven over recent decades, assisted by assay multiplexing and high-content analytical tools, that these disorders can be fractionated into multiple distinct subtypes (Maudsley et al., 2011a; Israel et al., 2012; Kota et al., 2012; Zeller et al., 2012). Therefore, rather than considering them singular entities they are actually the output function of hyper-complex molecular signatures. As the ability to gather genome- or proteome-wide levels of patient data becomes a reality, our need to analyze and classify disease sub-groups becomes ever more important. The ability to separate these complex disease phenotypes will significantly enhance our ability to differentially and specifically diagnose and treat these various “sub-conditions” more effectively compared to monolithic treatment strategies. *Plurigon* therefore may be able to assist in generating a readily comparable platform for patient data classification and disease spectrum analysis.

With the increasing prevalence of the Semantic Web (http://www.w3.org/standards/semanticweb/) and drug-target databases such as PubChem, Linked Open Drug Data (LODD), and Chem2Bio2RDF, data mining for novel drug discovery is particularly promising (Shadbolt et al., 2006; Chen et al., 2010; Samwald et al., 2011; Wang et al., 2011). Using mass analytical techniques such as genomics and protein mass spectrometry, the generation of high-dimensionality data is now routine in biomedical science. Currently, a significant degree of informatics development has taken place for the compilation of multivariate drug-target response data in high-throughput assays, standardized binary representation of compounds (PubChem fingerprints), and rudimentary feature extraction on compounds (atom pair similarities) (Cao et al., 2008). Therefore, at the present time, with the creation of high-dimensionality data analyzers, such as *Plurigon*, Ggobi and Iris following the widespread implementation of mass data collection pipelines (genome/proteome-wide), we may soon see the generation of highly predictive and nuanced structure-activity-relationships (SAR) specifically modeled against pathophysiological scenarios. We have already pioneered this approach, in a considerably low-scale manner, for the discovery and whole-animal SAR analysis of palliative agents for Huntington's disease (Martin et al., 2012b). In this study we were able to predict whole-animal therapeutic efficacy (using multiple biomedical indices) from complementary pathophysiological-pharmacogenomic-pattern investigation. In the future therefore, small molecular nuances may be able to be linked to subtle plurigon deformations induced by subtle “systems-level” transcriptional or proteomic responses in the experimental animal or even in clinical patients.

It is important to note that data texturizers such as *Plurigon* should rationally be employed, in a user-defined manner, alongside, and not instead of, conventional data analysis methods such as PCA, LSA, and SDE. Some of these techniques though do possess inherent deficiencies, such as PCA's inability to handle complex non-linearity, while in contrast many groups are beginning to appreciate the tremendous categorizing power of the human eye linked to our excellent shape-recognition capacities. We have shown with our present data that there may indeed be circumstances where novel applications such as *Plurigon* can assist where the implementation of PCA is not ideal. However, the specific use of one tool or application over another is entirely dependent on a combination of user preference and suitability of the datasets in use. With potential future user-created *Plurigon* developments additional and novel extracted features may serve as useful discriminatory identifiers in cases where traditional features may not.

### Conflict of interest statement

The authors declare that the research was conducted in the absence of any commercial or financial relationships that could be construed as a potential conflict of interest.

## Supplementary material

Figure S1 **Website platform for *Plurigon* application home**. The *Plurigon* application is available in Windows-PC, Mac OSX and Linux formats.

Figure S2 **Flowchart diagram for the generation and operation of the *Plurigon* application**.

Figure S3 **Structural diversity between mean WT and mean HET plurigon structures**. Panels 1 and 2 depict mean WT and HET plurigons, respectively. Panels 3 and 4 demonstrate the identical coordinate location of the plurigons in panels 1 and 2. The yellow inset box highlights the region of structural diversity between WT and HET. Panels 5 and 6 depict expanded views of the highlighted box in panels 3 and 4. Yellow arrows indicate the regions of structural diversity.

Figure S4 **Structural diversity between mean WT and mean HET plurigon structures**. Panels 1 and 2 depict mean WT and HET plurigons, respectively. Panels 3 and 4 demonstrate the identical coordinate location of the plurigons in panels 1 and 2. The yellow inset box highlights the region of structural diversity between WT and HET. Panels 5 and 6 depict expanded views of the highlighted box in panels 3 and 4. Yellow arrows indicate the regions of structural diversity.

Figure S5 **Structural diversity between mean WT and mean HET plurigon structures**. Panels 1 and 2 depict mean WT and HET plurigons, respectively. Panels 3 and 4 demonstrate the identical coordinate location of the plurigons in panels 1 and 2. The yellow inset box highlights the region of structural diversity between WT and HET. Panels 5 and 6 depict expanded views of the highlighted box in panels 3 and 4. Yellow arrows indicate the regions of structural diversity.

Figure S6 **Structural diversity between mean WT and mean HET plurigon structures**. Panels 1 and 2 depict mean WT and HET plurigons, respectively. Panels 3 and 4 demonstrate the identical coordinate location of the plurigons in panels 1 and 2. The yellow inset box highlights the region of structural diversity between WT and HET. Panels 5 and 6 depict expanded views of the highlighted box in panels 3 and 4. Yellow arrows indicate the regions of structural diversity.

Figure S7 **Structural diversity between mean WT and mean HET plurigon structures**. Panels 1 and 2 depict mean WT and HET plurigons, respectively. Panels 3 and 4 demonstrate the identical coordinate location of the plurigons in panels 1 and 2. The yellow inset box highlights the region of structural diversity between WT and HET. Panels 5 and 6 depict expanded views of the highlighted box in panels 3 and 4. Yellow arrows indicate the regions of structural diversity.

Figure S8 **PCA-mediated alignment of WT and HET murine multi-tissue transcriptomic data**. The three-dimensional PCA plot depicts the data points provided in Table S10. Red plus signs indicate WT data and the blue crosses indicate HET data.

Figure S9 **Structural diversity between AMI, BDNF and NGF plurigon structures**. Panels 1, 2 and 3 depict AMI-, BDNF- and NGF-derived plurigons, respectively. Panels 4, 5 and 6 demonstrate the identical coordinate location of the plurigons in panels 1–3. The yellow inset box highlights the region of structural diversity between the three plurigons. Panels 7–9 depict expanded views of the highlighted box containing the feature demonstrating similarity between AMI and BDNF compared to NGF in panels 4–6.

Figure S10 **Structural diversity between AMI, BDNF and NGF plurigon structures**. Panels 1, 2 and 3 depict AMI-, BDNF- and NGF-derived plurigons, respectively. Panels 4, 5 and 6 demonstrate the identical coordinate location of the plurigons in panels 1–3. The yellow inset box highlights the region of structural diversity between the three plurigons. Panels 7–9 depict expanded views of the highlighted box containing the feature demonstrating similarity between AMI and BDNF compared to NGF in panels 4–6.

Figure S11 **AMI-mediated subcellular expression and protein-protein interaction changes in human neuronal cells**. **(A)** Subcellular fractionation (fraction lanes 1–9: Golgi region = VAMP4-enriched lanes 4–8; endoplasmic reticulum region = calreticulin-enriched lanes 1–5) of human SH-SY5Y cells treated with AMI for 48 h reveals alterations in expression patterns of the amyloid precursor protein (APP) processing factors nicastrin (NCSTN) and anterior pharynx defective 1 homolog A (APH1A), the NTRK2 receptor and the multifunctional adaptor protein β-arrestin 2 (ARRB2). **(B)** AMI treatment (10 nM) of SH-SY5Y cells results in the altered co-immunoprecipitation of APP (reduced), APH1A (increased) and ARRB2 (increased) with the NTRK2 receptor (representative western blots and associated histograms below). Experiments in panel **(B)** were performed in triplicate and statistical significance was assessed using a Student's *t*-test (^*^*p* < 0.05, ^**^*p* < 0.01, ^***^*p* < 0.001).

Table S1 **Primary antibody sources and employed dilution factors for selective western blotting**.

Table S2 **Conserved hypothalamic transcriptomic expression data from 6 month old female mice**. The transcriptomic data from separate (F6-1, F6-2, F6-3) 6 month old mice was compared to 3 month old gender-matched control hypothalamic expression values.

Table S3 **Conserved hypothalamic transcriptomic expression data from 12 month old female mice**. The transcriptomic data from separate (F12-1, F12-2, F12-3) 12 month old mice was compared to 3 month old gender-matched control hypothalamic expression values.

Table S4 **Conserved hypothalamic transcriptomic expression data from 18 month old female mice**. The transcriptomic data from separate (F18-1, F18-2, F18-3) 18 month old mice was compared to 3 month old gender-matched control hypothalamic expression values.

Table S5 **Conserved hypothalamic transcriptomic expression data from 6 month old male mice**. The transcriptomic data from separate (M6-1, M6-2, M6-3) 6 month old mice was compared to 3 month old gender-matched control hypothalamic expression values.

Table S6 **Conserved hypothalamic transcriptomic expression data from 12 month old male mice**. The transcriptomic data from separate (M12-1, M12-2, M12-3) 12 month old mice was compared to 3 month old gender-matched control hypothalamic expression values.

Table S7 **Conserved hypothalamic transcriptomic expression data from 18 month old male mice**. The transcriptomic data from separate (M18-1, M18-2, M18-3) 18 month old mice was compared to 3 month old gender-matched control hypothalamic expression values.

Table S8 **Wildtype (WT) murine transcriptomic data from multiple isolated tissues**. Datasets are arranged alphanumerically with the following nomenclature: WT1 to WT16.

Table S9 **GIT2 heterozygous (HET) murine transcriptomic data from multiple isolated tissues**. Datasets are arranged alphanumerically with the following nomenclature: HET1 to HET19.

Table S10 **Standard PCA analysis of WT and HET genotypic datasets**. PCA was applied to reduce data dimensionality to three primary dimensions, D1, D2 and D3. Using these three dimensions the two genotype groups appeared non-separable.

Table S11 **Plurigon structural concavity/convexity analysis**. The structural nature of three coincident facets of each dataset plurigon are color coded (red, green or blue) and their physical nature (convex (VEX) or concave (CAVE)) is denoted.

Table S12 **Human neuronal cell (SH-SY5Y) transcriptomic effects induced by amitriptyline exposure**. For each amitriptyline (AMI) regulated transcript the official Gene Symbol, z-ratio and transcript description are indicated.

Table S13 **Human neuronal cell (SH-SY5Y) transcriptomic effects induced by brain-derived neurotrophic factor exposure**. For each brain-derived neurotrophic factor (BDNF) regulated transcript the official Gene Symbol, z-ratio and transcript description are indicated.

Table S14 **Human neuronal cell (SH-SY5Y) transcriptomic effects induced by nerve growth factor exposure**. For each nerve growth factor (NGF) regulated transcript the official Gene Symbol, z-ratio and transcript description are indicated.

Table S15 **Conserved neuronal transcriptomic expression data from amitriptyline-treated clonal human cells**. Transcriptomic gene regulation was measured from amitriptyline (AMI)-treated SH-SY5Y neuronal cell batches (AMI-1, AMI-2, AMI-3). The transcriptomic data from the three separate experiments was compared to gene expression levels in vehicle (phosphate-buffered saline)-treated control SH-SY5Y cells.

Table S16 **Conserved neuronal transcriptomic expression data from brain-derived neurotrophic factor-treated clonal human cells**. Transcriptomic gene regulation was measured from brain-derived neurotrophic factor (BDNF)-treated SH-SY5Y neuronal cell batches (BDNF-1, BDNF-2, BDNF-3). The transcriptomic data from the three separate experiments was compared to gene expression levels in vehicle (phosphate-buffered saline)-treated control SH-SY5Y cells.

Table S17 **Conserved neuronal transcriptomic expression data from nerve growth factor-treated clonal human cells**. Transcriptomic gene regulation was measured from nerve growth factor (NGF)-treated SH-SY5Y neuronal cell batches (NGF-1, NGF-2, NGF-3). The transcriptomic data from the three separate experiments was compared to gene expression levels in vehicle (phosphate-buffered saline)-treated control SH-SY5Y cells.

Table S18 **Transcriptomic effects of amitriptyline in human neuronal cells (SH-SY5Y) cells pre-treated with chronic minimal levels of hydrogen peroxide**. For each amitriptyline (AMI) regulated transcript in the peroxide-treated SH-SY5Y cells the official Gene Symbol, z-ratio and transcript description are indicated.

Table S19 **Transcriptomic effects of brain-derived neurotrophic factor in human neuronal cells (SH-SY5Y) cells pre-treated with chronic minimal levels of hydrogen peroxide**. For each brain-derived neurotrophic factor (BDNF) regulated transcript in the peroxide-treated SH-SY5Y cells the official Gene Symbol, z-ratio and transcript description are indicated.

Table S20 **Transcriptomic effects of nerve growth factor in human neuronal cells (SH-SY5Y) cells pre-treated with chronic minimal levels of hydrogen peroxide**. For each nerve growth factor (NGF) regulated transcript in the peroxide-treated SH-SY5Y cells the official Gene Symbol, z-ratio and transcript description are indicated.

Table S21 **Conserved neuronal transcriptomic expression data from pre-peroxidated amitriptyline-treated clonal human cells**. Transcriptomic gene regulation was measured from amitriptyline (AMI)-treated SH-SY5Y neuronal cell batches (AMI-1, AMI-2, AMI-3) that were pre-conditioned to mimic oxidative neurodegeneration with a continuous 7 day treatment with 10 nM hydrogen peroxide. The transcriptomic data from the three separate experiments was compared to gene expression levels in vehicle (phosphate-buffered saline)-treated control SH-SY5Y cells that were also treated with 10 nM hydrogen peroxide as well.

Table S22 **Conserved neuronal transcriptomic expression data from pre-peroxidated brain-derived neurotrophic factor-treated clonal human cells**. Transcriptomic gene regulation was measured from brain-derived neurotrophic factor (BDNF)-treated SH-SY5Y neuronal cell batches (BDNF-1, BDNF-2, BDNF-3) that were pre-conditioned to mimic oxidative neurodegeneration with a continuous 7 day treatment with 10 nM hydrogen peroxide. The transcriptomic data from the three separate experiments was compared to gene expression levels in vehicle (phosphate-buffered saline)-treated control SH-SY5Y cells that were also treated with 10 nM hydrogen peroxide as well.

Table S23 **Conserved neuronal transcriptomic expression data from pre-peroxidated nerve growth factor-treated clonal human cells**. Transcriptomic gene regulation was measured from nerve growth factor (NGF)-treated SH-SY5Y neuronal cell batches (NGF-1, NGF-2, NGF-3) that were pre-conditioned to mimic oxidative neurodegeneration with a continuous 7 day treatment with 10 nM hydrogen peroxide. The transcriptomic data from the three separate experiments was compared to gene expression levels in vehicle (phosphate-buffered saline)-treated control SH-SY5Y cells that were also treated with 10 nM hydrogen peroxide as well.

Table S24 **Similarity score calculations for BDNF-like AMI-regulated transcripts**. A relative similarity score for AMI-regulated transcripts that possessed a regulatory mode more akin to BDNF than compared to NGF was calculated as the follows. The modulus of the ((AMI score-NGF score)/(AMI score-BDNF score)) was obtained and transcripts possessing a modulus of over 1 were considered to be more AMI-BDNF correlated than AMI-NGF correlated.

Table S25 **KEGG pathway analysis for AMI-regulated, BDNF-like, transcripts in peroxide-treated human neuronal cells**. Using the NIH-DAVID Bioinformatic Suite (http://david.abcc.ncifcrf.gov/) we annotated the AMI-regulated, BDNF-like, transcripts stimulated by AMI treatment of artificially “aged” neuronal tissue. For each human signaling pathway the probability of geneset enrichment (*P*-Value), the enrichment relative to a scaled input from the human genome (Fold Enrichment) and the specific genes in the input list that populate (*n* ≥ 2) the assessed pathways. The only significantly populated signaling pathway was the hsa047223: neurotrophin signaling pathway.
