# Supplementary figures and images for: Plurigon: three dimensional visualization and classification of high-dimensionality data
Source: Front Physiol. 2013 Jul 22;4:190. doi: 10.3389/fphys.2013.00190 (PMC3717481; doi:10.3389/fphys.2013.00190)

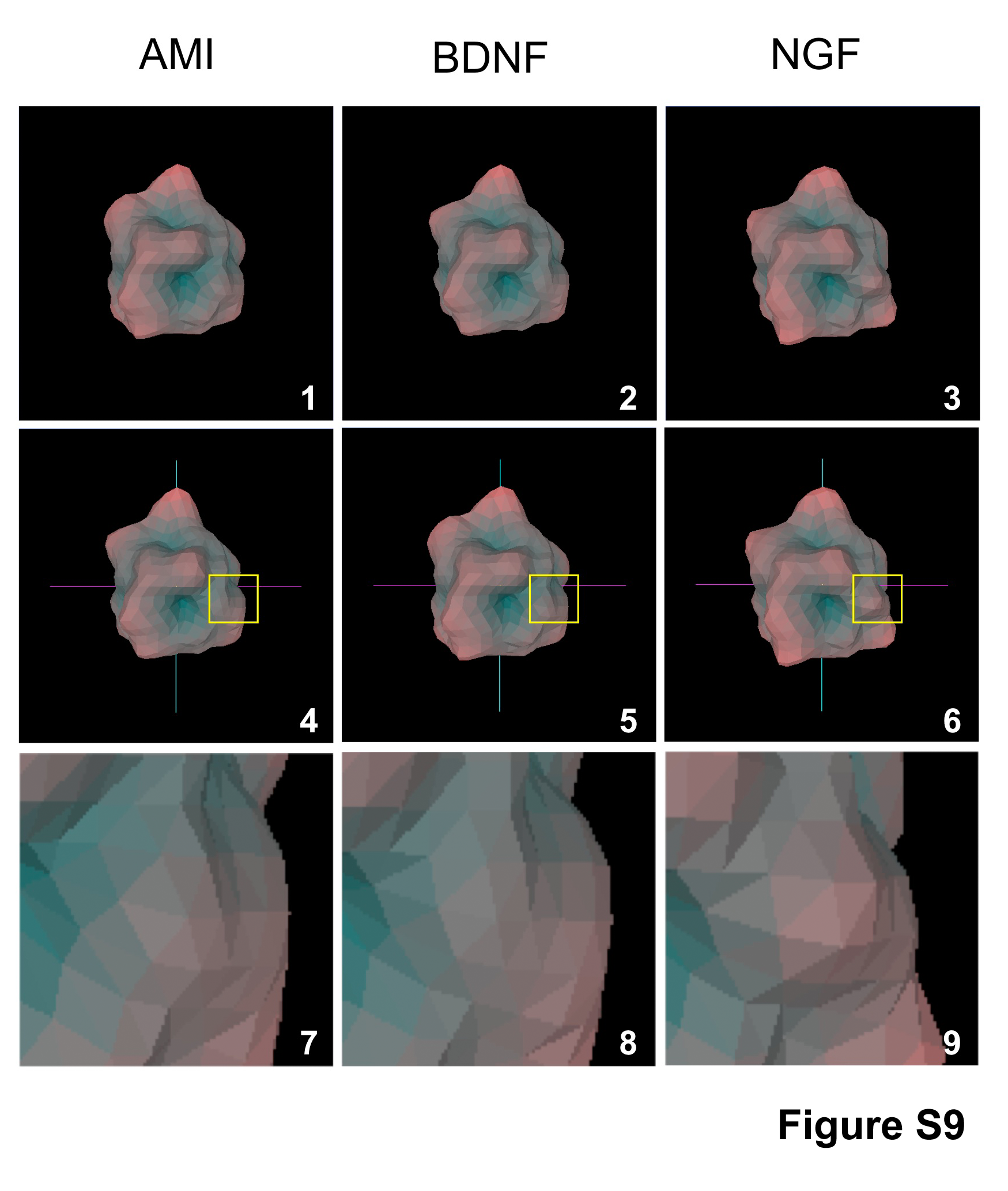

Supplement: Figure S1 — Website platform for Plurigon application home. The Plurigon application is available in Windows-PC, Mac OSX and Linux formats. [file 46440_Maudsley_Data_Sheet_1.ZIP › Figure-S9.tif]

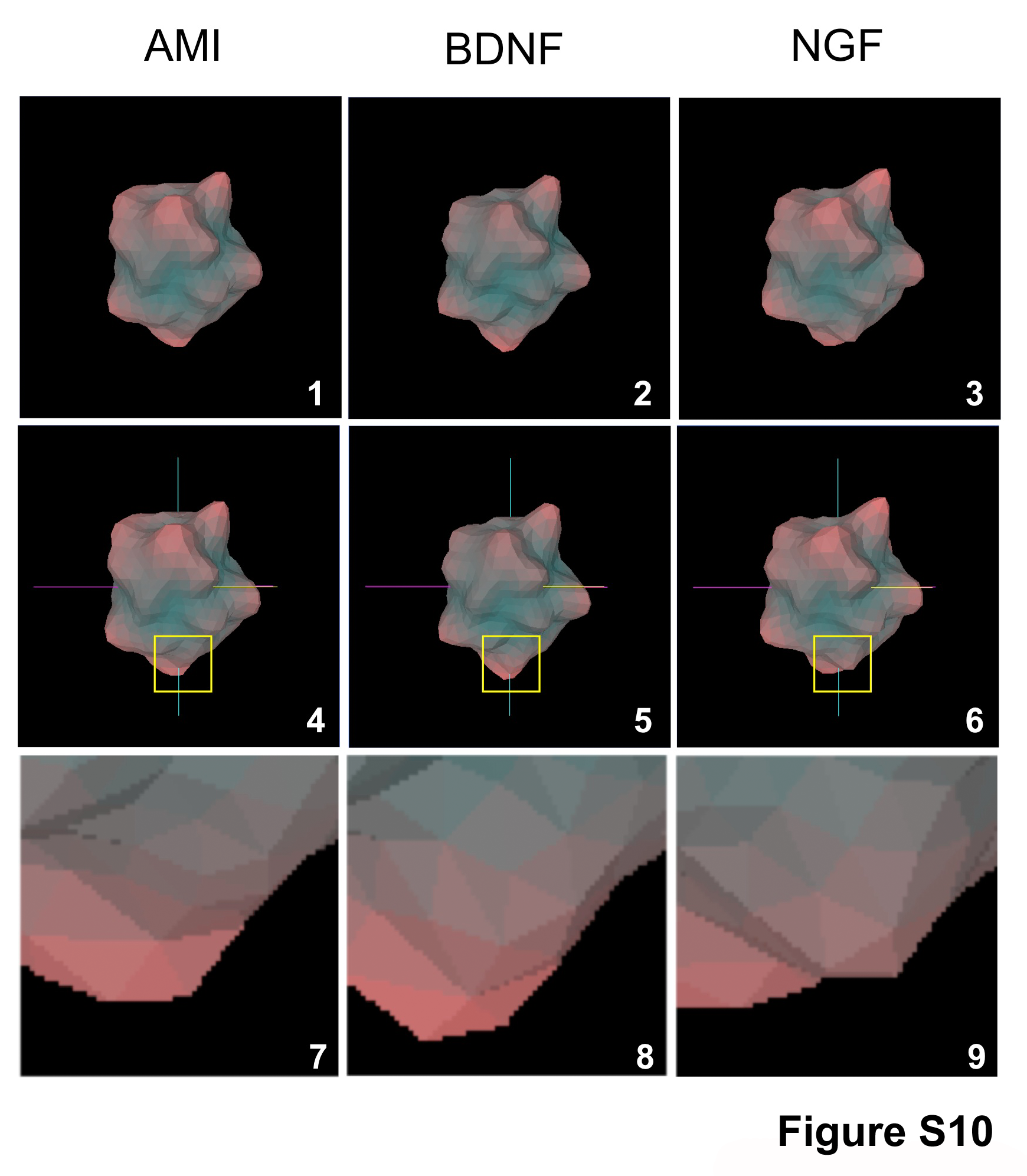

Supplement: Figure S1 — Website platform for Plurigon application home. The Plurigon application is available in Windows-PC, Mac OSX and Linux formats. [file 46440_Maudsley_Data_Sheet_1.ZIP › Figure-S10.tif]

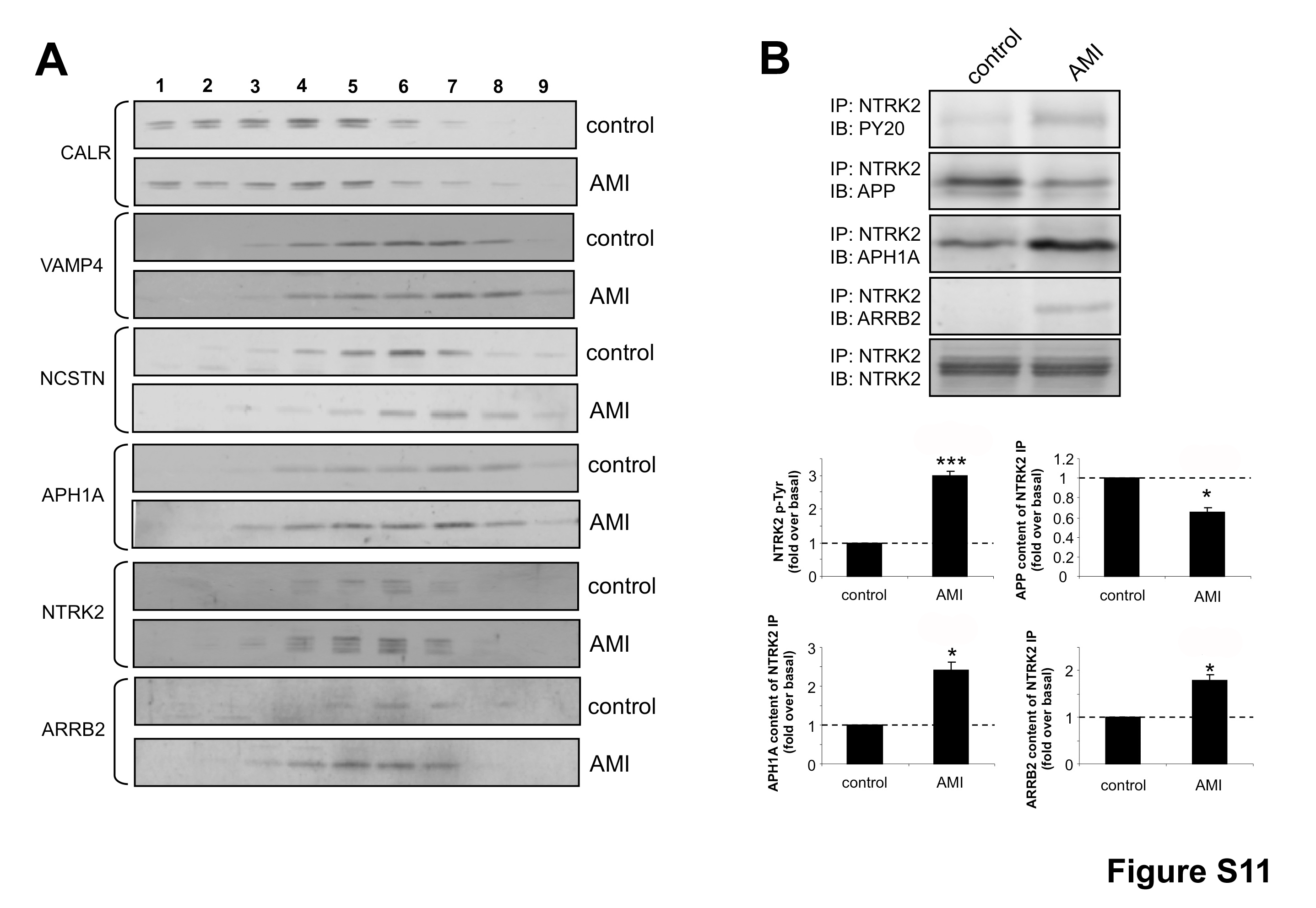

Supplement: Figure S1 — Website platform for Plurigon application home. The Plurigon application is available in Windows-PC, Mac OSX and Linux formats. [file 46440_Maudsley_Data_Sheet_1.ZIP › Figure-S11.tif]

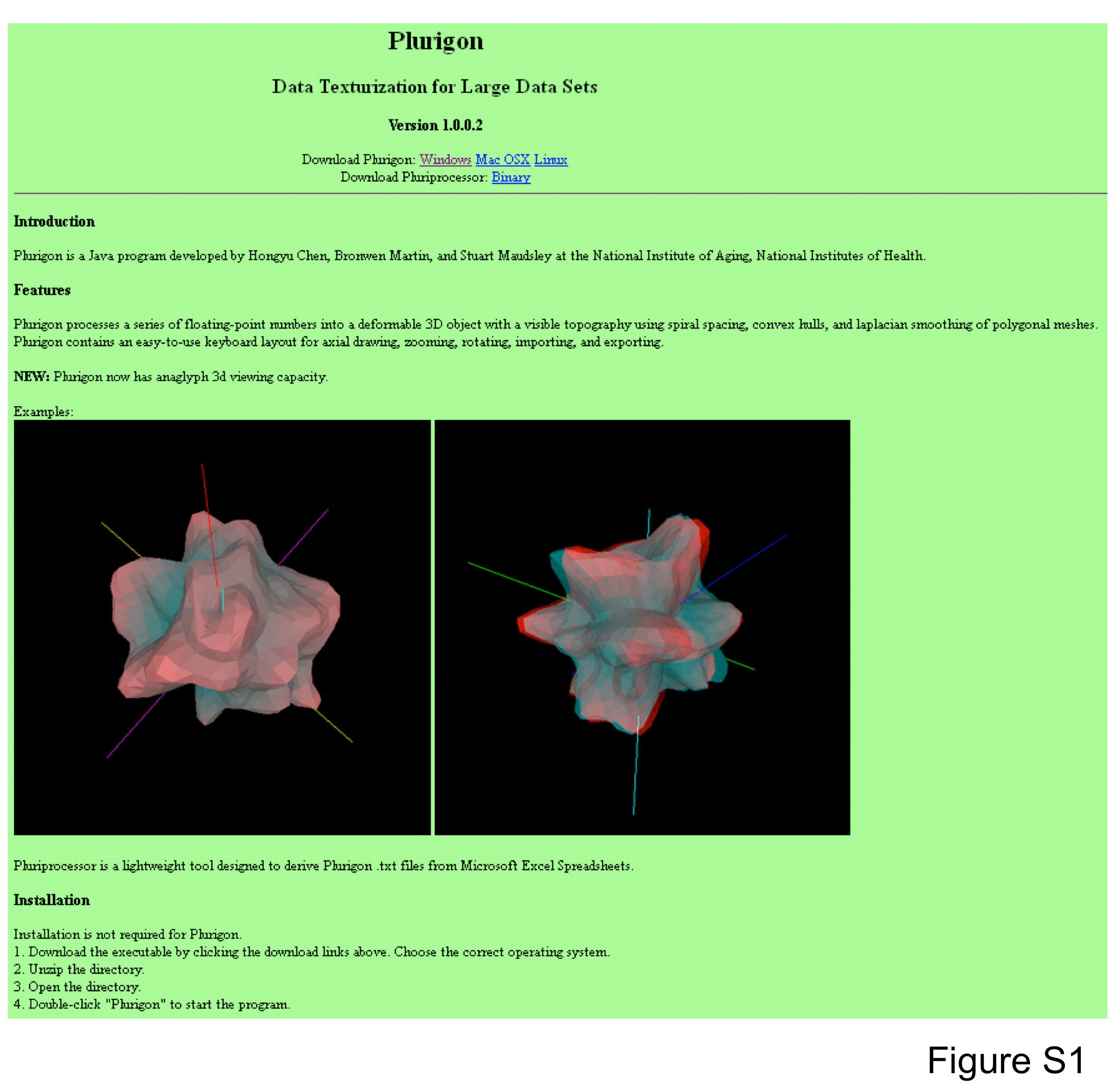

Supplement: Figure S1 — Website platform for Plurigon application home. The Plurigon application is available in Windows-PC, Mac OSX and Linux formats. [file 46440_Maudsley_Data_Sheet_1.ZIP › Figure-S1.tif]

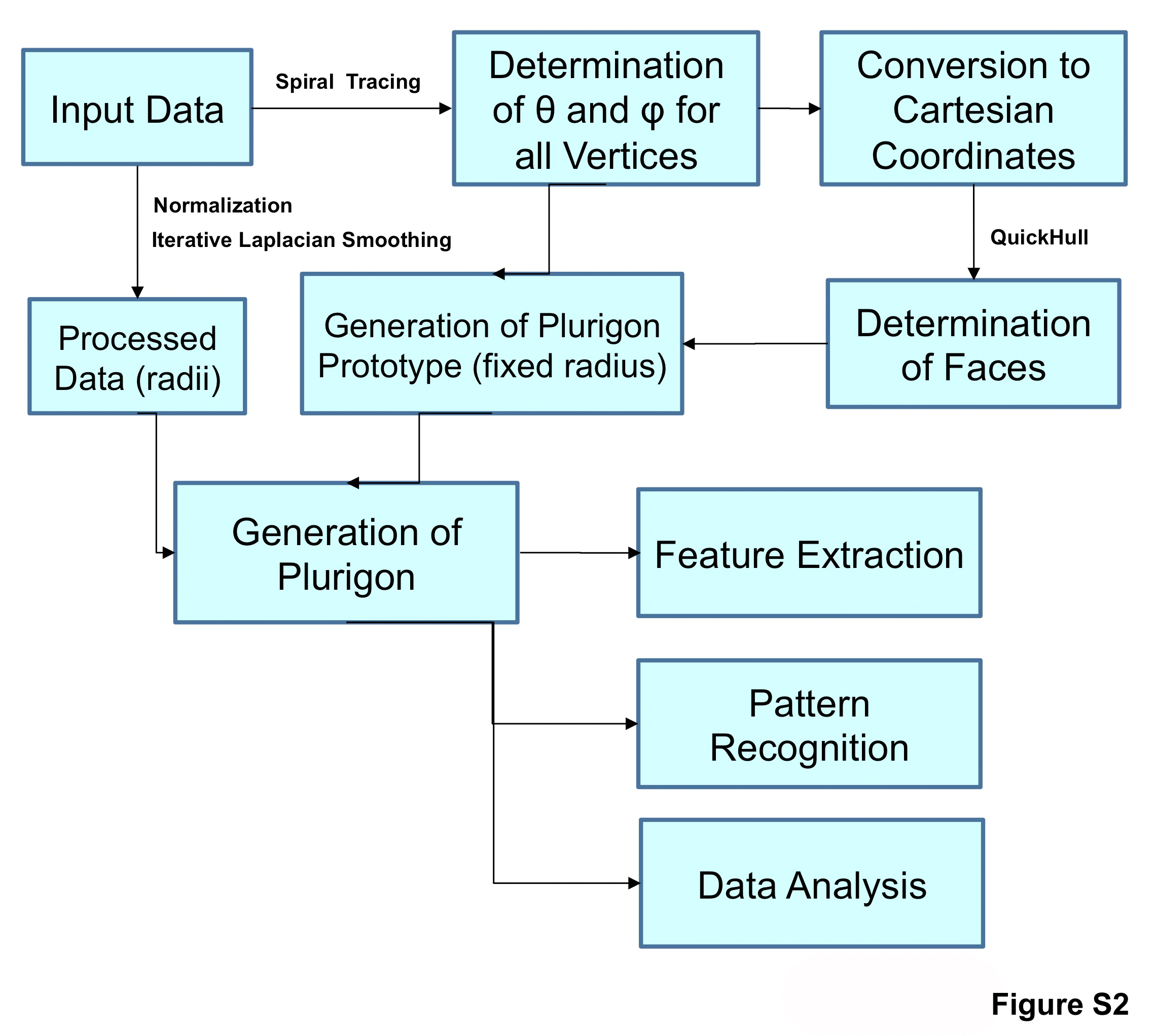

Supplement: Figure S1 — Website platform for Plurigon application home. The Plurigon application is available in Windows-PC, Mac OSX and Linux formats. [file 46440_Maudsley_Data_Sheet_1.ZIP › Figure-S2.tif]

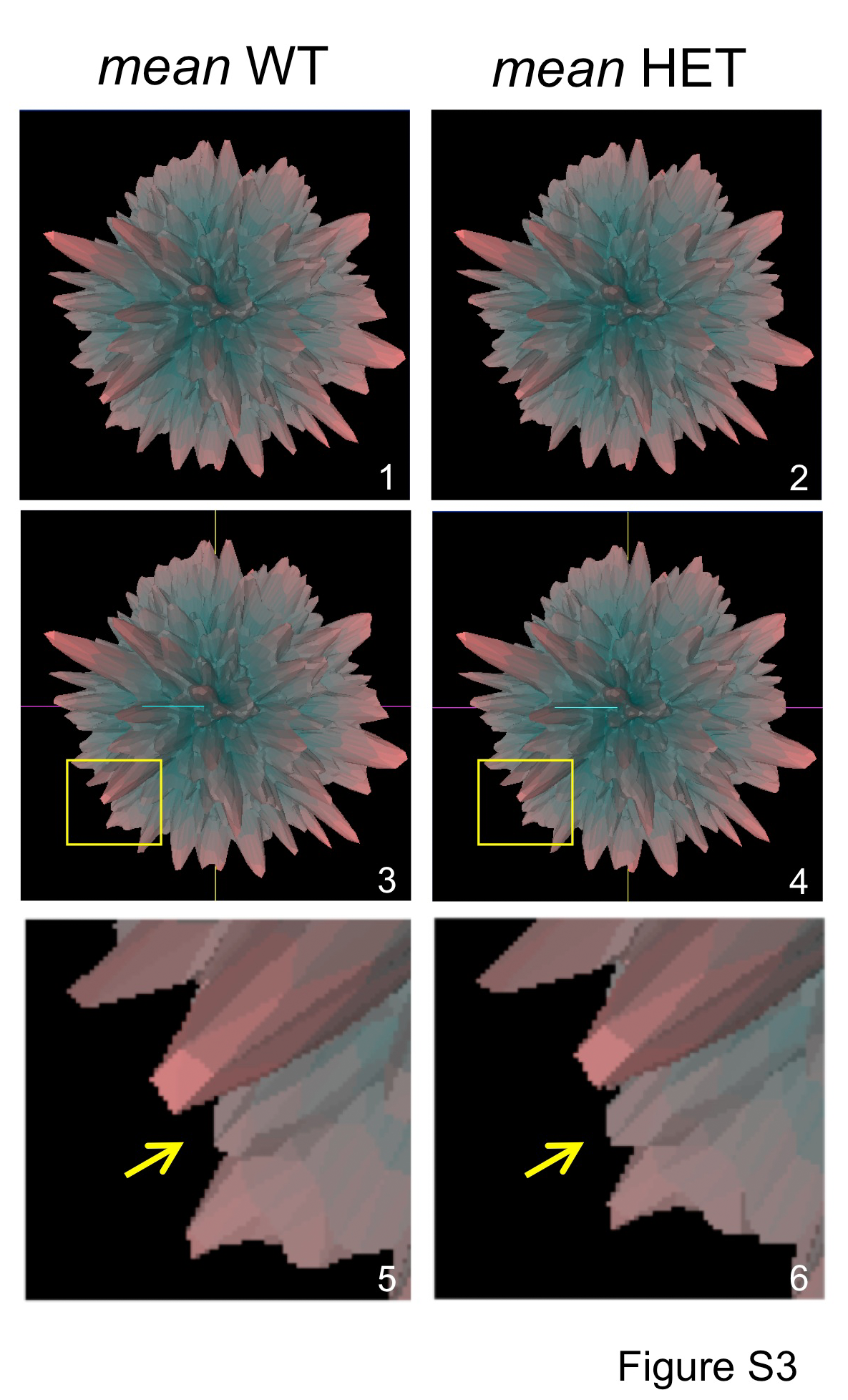

Supplement: Figure S1 — Website platform for Plurigon application home. The Plurigon application is available in Windows-PC, Mac OSX and Linux formats. [file 46440_Maudsley_Data_Sheet_1.ZIP › Figure-S3.tif]

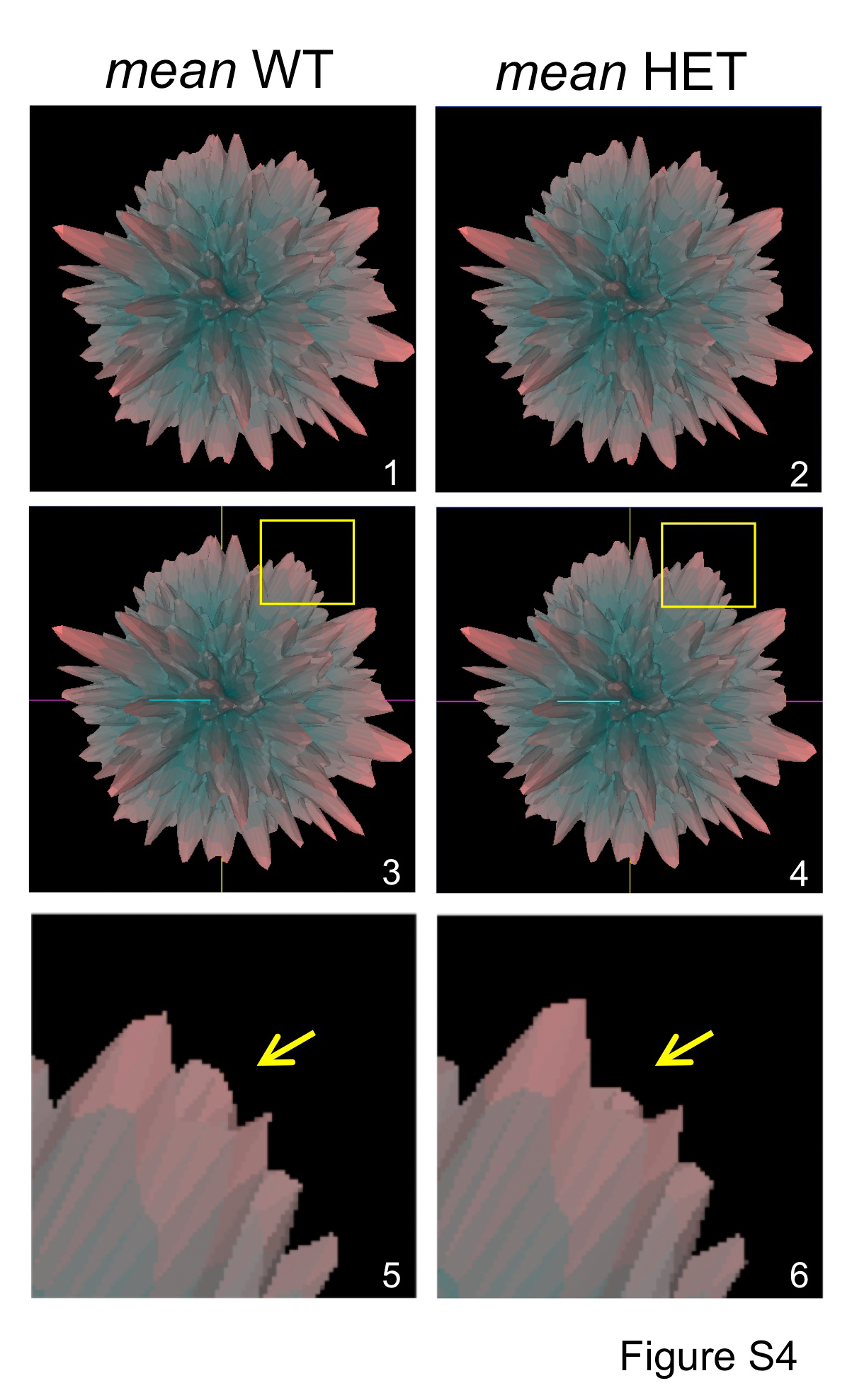

Supplement: Figure S1 — Website platform for Plurigon application home. The Plurigon application is available in Windows-PC, Mac OSX and Linux formats. [file 46440_Maudsley_Data_Sheet_1.ZIP › Figure-S4.tif]

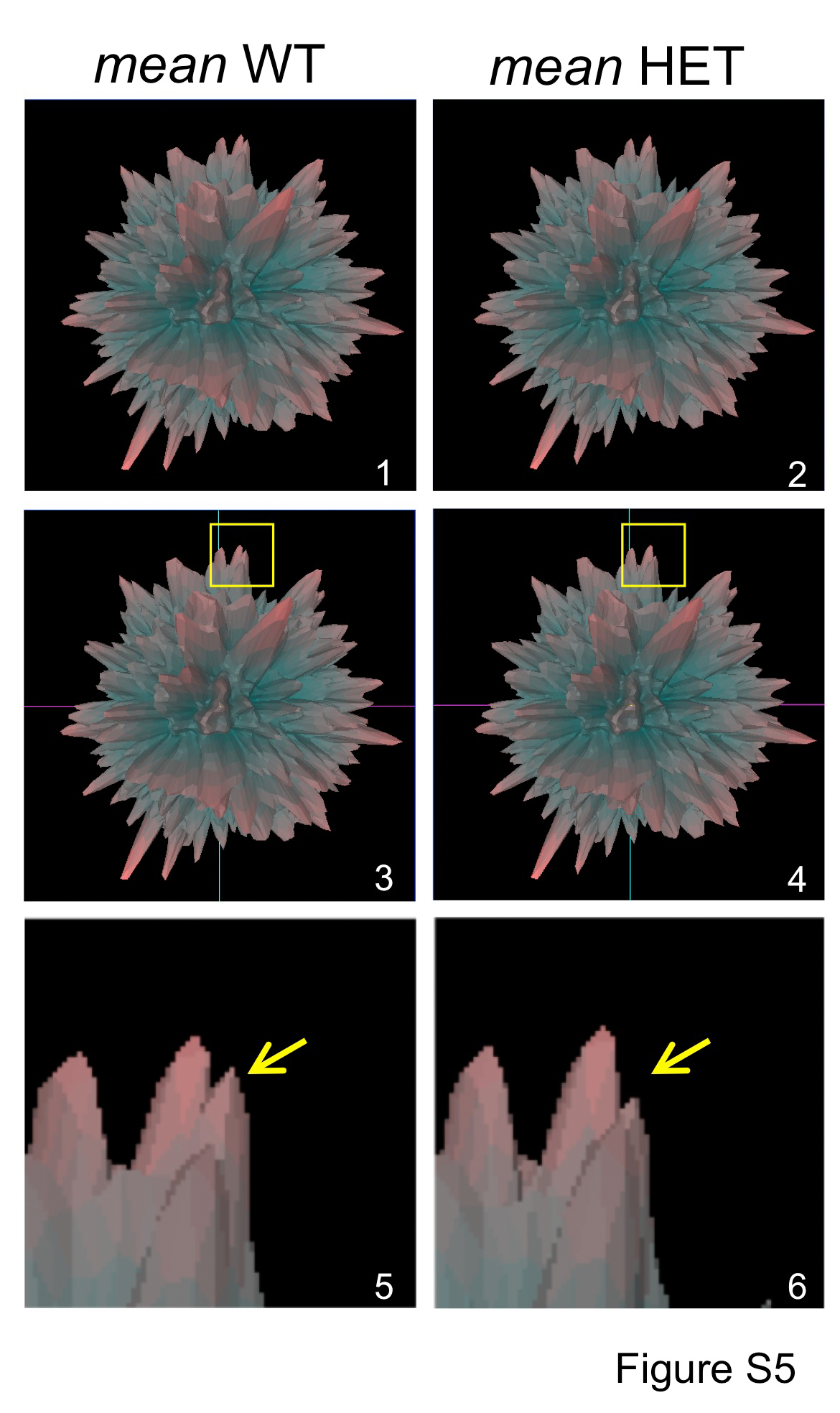

Supplement: Figure S1 — Website platform for Plurigon application home. The Plurigon application is available in Windows-PC, Mac OSX and Linux formats. [file 46440_Maudsley_Data_Sheet_1.ZIP › Figure-S5.tif]

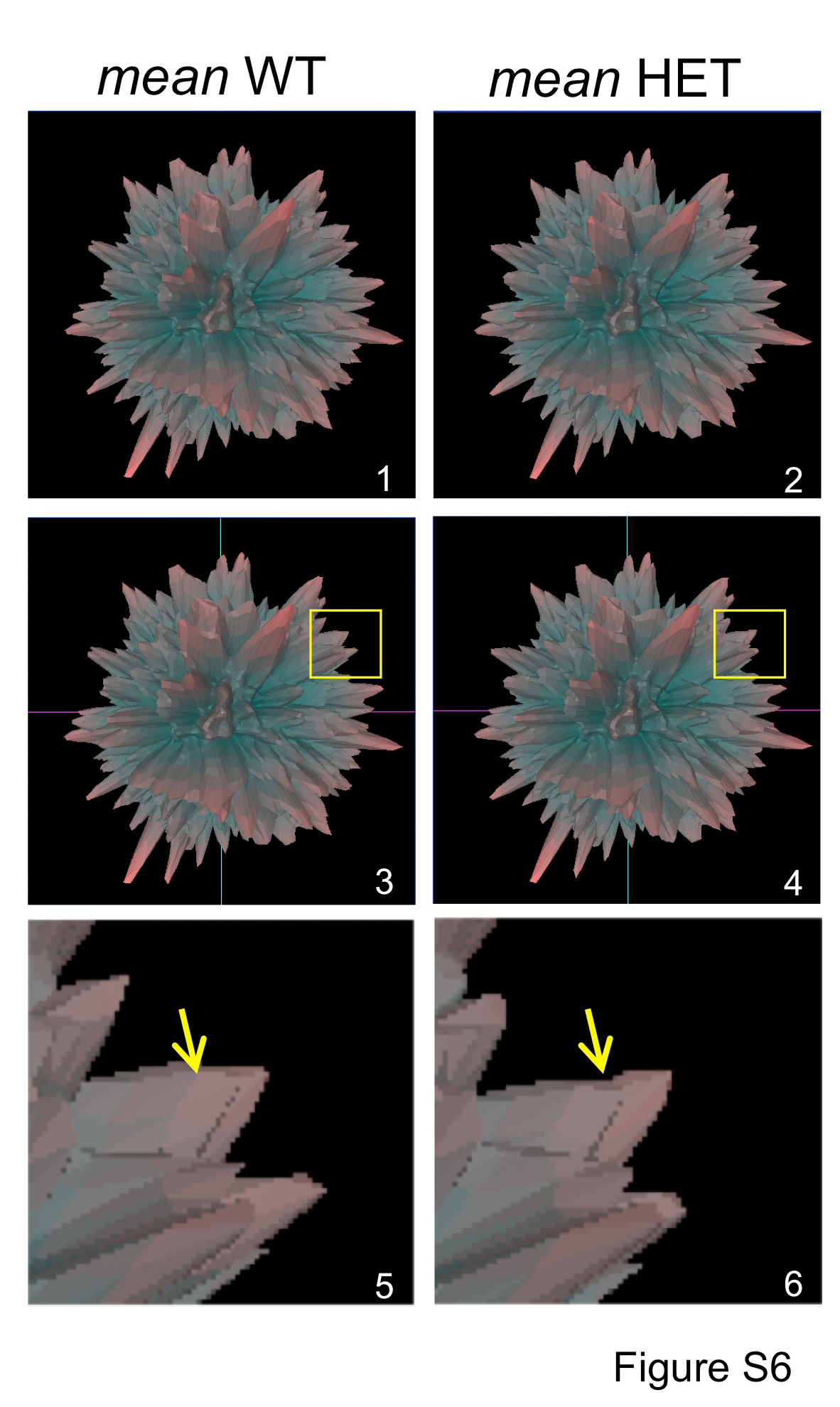

Supplement: Figure S1 — Website platform for Plurigon application home. The Plurigon application is available in Windows-PC, Mac OSX and Linux formats. [file 46440_Maudsley_Data_Sheet_1.ZIP › Figure-S6.tif]

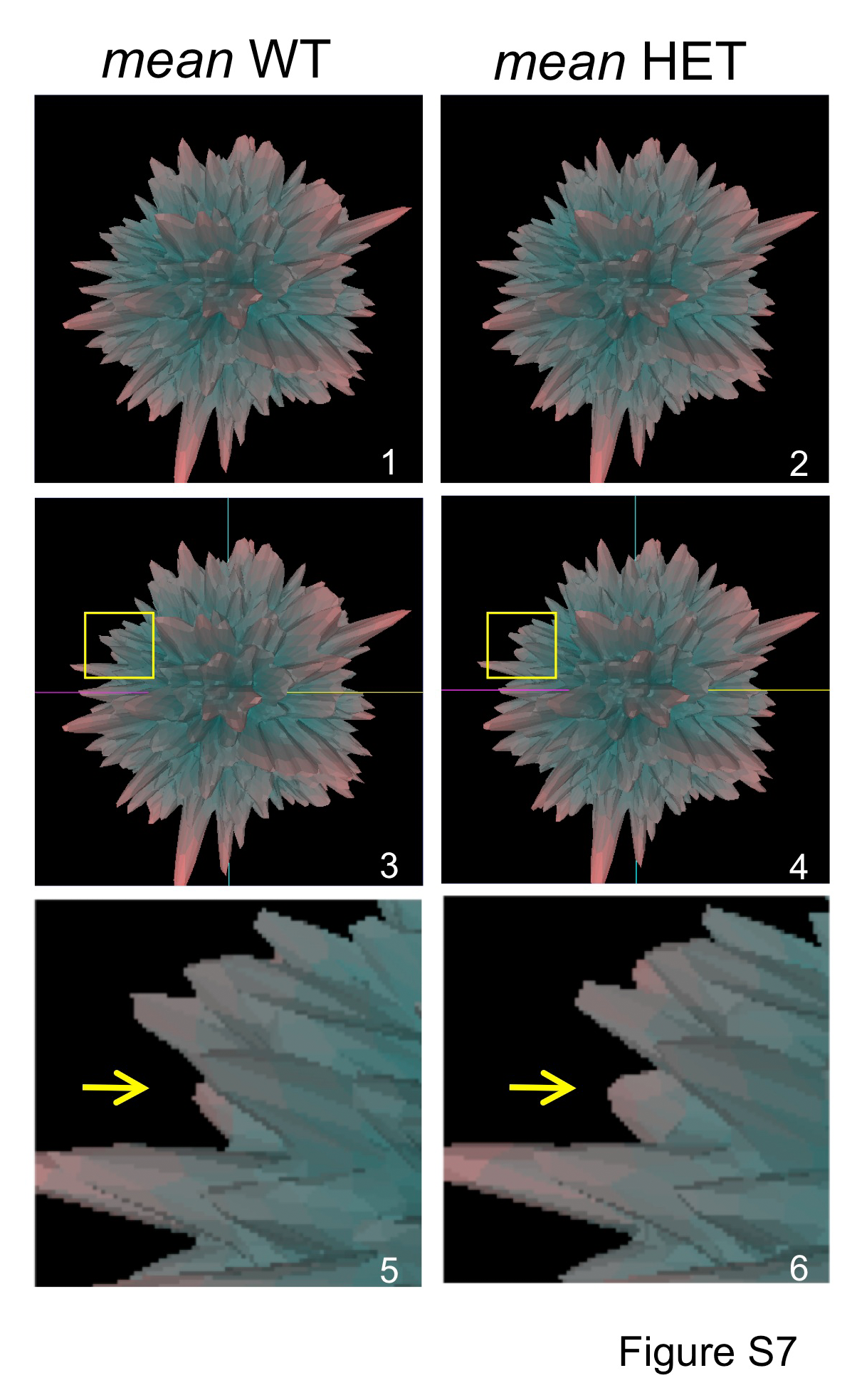

Supplement: Figure S1 — Website platform for Plurigon application home. The Plurigon application is available in Windows-PC, Mac OSX and Linux formats. [file 46440_Maudsley_Data_Sheet_1.ZIP › Figure-S7.tif]

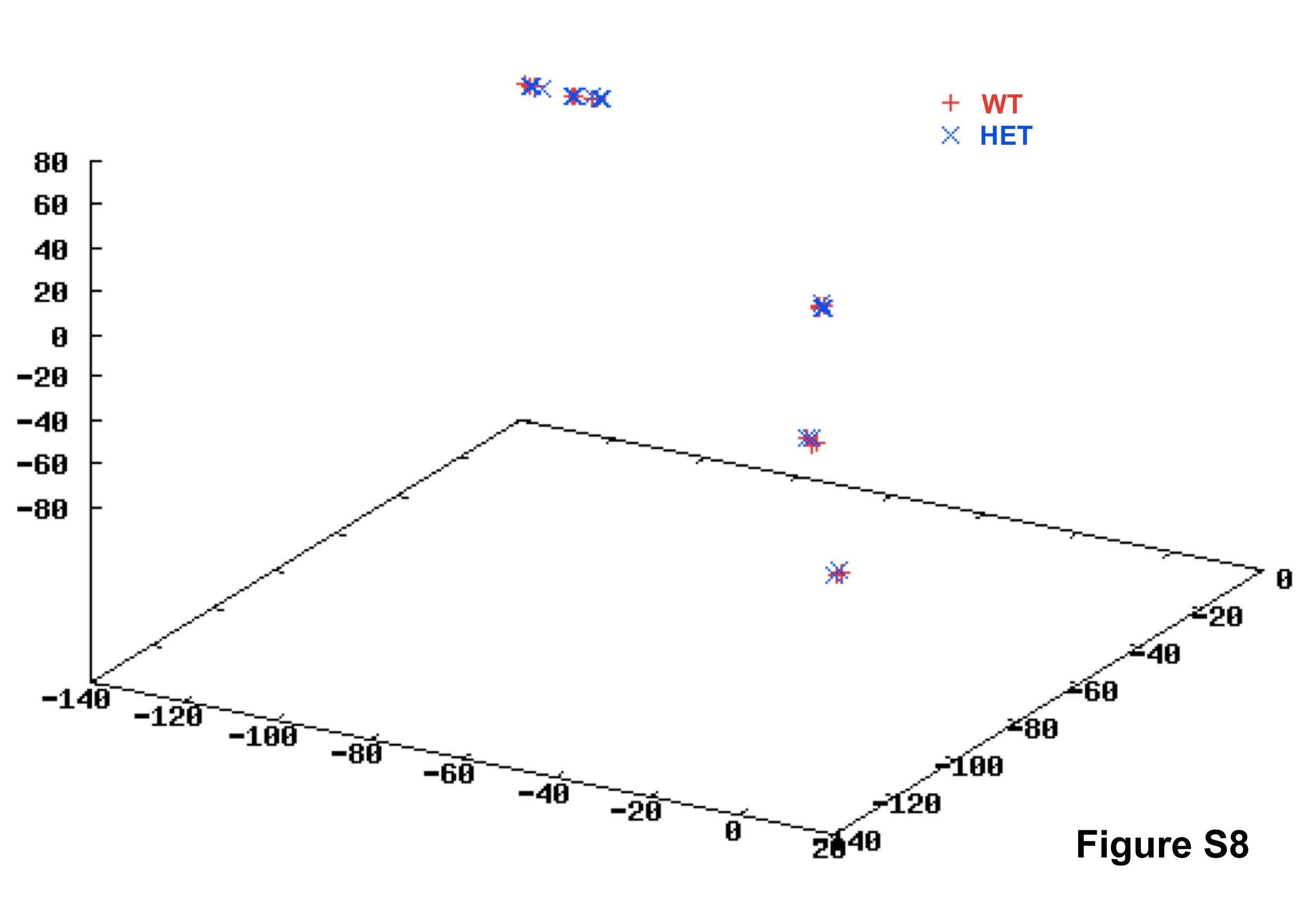

Supplement: Figure S1 — Website platform for Plurigon application home. The Plurigon application is available in Windows-PC, Mac OSX and Linux formats. [file 46440_Maudsley_Data_Sheet_1.ZIP › Figure-S8.tif]
