# Supplementary material for: Plurigon: three dimensional visualization and classification of high-dimensionality data
Source: Front Physiol. 2013 Jul 22;4:190. doi: 10.3389/fphys.2013.00190 (PMC3717481; doi:10.3389/fphys.2013.00190)
Supplement: Table S1 — Primary antibody sources and employed dilution factors for selective western blotting. [file 46440_Maudsley_Data_Sheet_2.ZIP › 46440_Maudsley_Table_6.DOC]

**Table S6. Conserved hypothalamic transcriptomic expression data from 12 month old male mice**. The transcriptomic data from separate (M12-1, M12-2, M12-3) 6 month old mice was compared to 3 month old gender-matched control hypothalamic expression values.

| **Gene Symbol** | **M12-1** | **M12-2** | **M12-3** |
| --- | --- | --- | --- |
| 2210016L21Rik | -2.04 | -1.87 | -2.22 |
| 2310047C04Rik | -1.69 | -1.52 | -1.88 |
| 9030612M13Rik | -1.83 | -1.71 | -1.96 |
| Acbd4 | 1.73 | 1.59 | 1.89 |
| Acta1 | -2.43 | -2.25 | -2.62 |
| Add1 | -2.44 | -2.28 | -2.61 |
| Adss | -2.67 | -2.47 | -2.88 |
| Akr1a4 | 1.98 | 1.58 | 2.48 |
| Arf3 | 1.90 | 1.76 | 2.05 |
| Arfip2 | 2.00 | 1.98 | 2.02 |
| Arl3 | 1.84 | 1.34 | 2.52 |
| Arl6ip2 | -1.86 | -1.42 | -2.44 |
| Armc6 | -2.38 | -2.33 | -2.43 |
| Atp1b2 | 2.81 | 2.40 | 3.29 |
| Atxn1 | -1.74 | -1.63 | -1.86 |
| B2m | 8.02 | 6.80 | 9.46 |
| BC021395 | -1.65 | -1.63 | -1.67 |
| Bdh1 | -3.18 | -2.65 | -3.82 |
| Bmpr1a | 1.86 | 1.46 | 2.36 |
| C1qc | 5.03 | 4.41 | 5.73 |
| Cacnb4 | -3.06 | -2.76 | -3.40 |
| Cdh8 | -1.67 | -1.52 | -1.84 |
| Chordc1 | -3.64 | -3.37 | -3.93 |
| Copg | 2.46 | 2.44 | 2.48 |
| Csde1 | 1.56 | 1.39 | 1.75 |
| Csnrp3 | -2.43 | -2.06 | -2.87 |
| Cxadr | -2.27 | -2.25 | -2.29 |
| Ddx6 | -2.43 | -1.91 | -3.09 |
| Dennd3 | 2.19 | 1.90 | 2.52 |
| Dmn | -1.67 | -1.59 | -1.75 |
| Dusp1 | 2.00 | 1.89 | 2.12 |
| Dync1li1 | -2.10 | -1.89 | -2.33 |
| E430025E21Rik | -2.60 | -2.43 | -2.78 |
| Ebna1bp2 | -2.24 | -1.91 | -2.62 |
| Eef1d | 1.89 | 1.51 | 2.36 |
| Efha1 | -1.71 | -1.29 | -2.27 |
| Egr1 | 5.69 | 5.47 | 5.92 |
| Eid2 | -1.83 | -1.61 | -2.09 |
| Eif4g2 | -2.72 | -2.25 | -3.29 |
| Emg1 | -3.77 | -3.40 | -4.18 |
| Erdr1 | -3.73 | -2.50 | -5.56 |
| Fbxo8 | -1.52 | -1.42 | -1.63 |
| Fibcd1 | -1.55 | -1.37 | -1.75 |
| Fos | 6.06 | 5.77 | 6.36 |
| Gabarap | 2.72 | 2.62 | 2.83 |
| Gabrb2 | -2.51 | -2.35 | -2.69 |
| Galnt1 | -1.57 | -1.10 | -2.25 |
| Glrb | -3.73 | -3.55 | -3.92 |
| Gpm6a | -2.21 | -1.74 | -2.81 |
| Gprc5b | -2.49 | -2.15 | -2.89 |
| Hap1 | -1.84 | -1.66 | -2.04 |
| Hspa2 | 2.55 | 2.30 | 2.83 |
| Hspd1 | -3.01 | -2.41 | -3.76 |
| Id2 | 2.01 | 1.88 | 2.15 |
| Ihpk1 | 2.31 | 1.72 | 3.10 |
| Insig1 | -2.58 | -2.48 | -2.68 |
| Junb | 4.32 | 4.28 | 4.36 |
| Khdrbs1 | -1.75 | -1.65 | -1.86 |
| Klhl9 | -2.15 | -2.05 | -2.26 |
| Lmna | 2.20 | 2.00 | 2.42 |
| LOC381629 | 1.67 | 1.46 | 1.90 |
| Lrp1 | -2.40 | -2.35 | -2.45 |
| Mboat2 | -4.29 | -3.18 | -5.79 |
| Mdh1 | -2.70 | -2.43 | -3.00 |
| Mgll | -2.18 | -1.76 | -2.70 |
| Mllt3 | -1.80 | -1.65 | -1.96 |
| Mlycd | -3.28 | -2.76 | -3.90 |
| Morc3 | -2.62 | -2.15 | -3.20 |
| Mrrf | -2.62 | -2.59 | -2.65 |
| Msrb2 | -2.43 | -2.43 | -2.43 |
| Mt3 | 3.35 | 3.22 | 3.48 |
| Mtap1b | -2.68 | -2.41 | -2.97 |
| Mtap2 | -2.26 | -2.19 | -2.33 |
| Mtmr1 | -1.82 | -1.63 | -2.04 |
| Ndufa6 | 1.77 | 1.34 | 2.34 |
| Nhlrc2 | -2.51 | -2.18 | -2.89 |
| Nsg2 | 1.78 | 1.60 | 1.98 |
| Nt5c2 | -1.85 | -1.83 | -1.87 |
| Nus1 | -2.24 | -2.09 | -2.40 |
| Ociad1 | 2.51 | 2.44 | 2.59 |
| Odz3 | -2.69 | -2.40 | -3.01 |
| Opa1 | -1.66 | -1.55 | -1.78 |
| OTTMUSG00000017677 | -1.86 | -1.63 | -2.12 |
| Otud6b | -2.00 | -1.85 | -2.16 |
| Papd1 | -2.52 | -2.02 | -3.15 |
| Pcdh9 | -3.72 | -3.35 | -4.13 |
| Pfn2 | -1.96 | -1.81 | -2.12 |
| Pnpla8 | -1.62 | -1.57 | -1.67 |
| Ppp1cb | -2.12 | -1.91 | -2.35 |
| Ppp1r14a | -2.38 | -2.29 | -2.48 |
| Psmb7 | 2.41 | 2.32 | 2.51 |
| Ptpn11 | 1.80 | 1.58 | 2.05 |
| Pum2 | -2.22 | -2.09 | -2.35 |
| Rab5a | -1.89 | -1.80 | -1.98 |
| Rasgrf1 | 1.72 | 1.61 | 1.84 |
| Rasl11b | 2.06 | 2.02 | 2.10 |
| Rbm18 | -1.72 | -1.64 | -1.81 |
| Rbm45 | -2.15 | -1.84 | -2.52 |
| Rbm9 | -3.34 | -3.01 | -3.71 |
| Rnf14 | -1.58 | -1.52 | -1.64 |
| Rps16 | 3.19 | 2.70 | 3.76 |
| Rps9 | 2.89 | 2.68 | 3.12 |
| Sgip1 | -1.68 | -1.63 | -1.73 |
| Sgtb | 2.11 | 1.83 | 2.43 |
| Slc25a36 | -1.83 | -1.61 | -2.09 |
| Slc7a14 | -9.40 | -8.95 | -9.87 |
| Snx14 | 2.54 | 1.92 | 3.35 |
| Spcs1 | 2.12 | 2.10 | 2.14 |
| Spsb1 | 3.42 | 2.78 | 4.21 |
| Stk19 | -1.64 | -1.58 | -1.71 |
| Stt3a | -1.63 | -1.60 | -1.66 |
| Tceal6 | -1.53 | -1.37 | -1.71 |
| Tmem9 | 1.77 | 1.64 | 1.91 |
| Tpd52l2 | -1.59 | -1.31 | -1.92 |
| Trappc3 | 1.62 | 1.60 | 1.64 |
| Trmt6 | -1.75 | -1.26 | -2.43 |
| Ube3a | -3.17 | -3.05 | -3.30 |
| Uqcrh | 2.01 | 1.50 | 2.69 |
| Usp8 | -1.91 | -1.87 | -1.95 |
| Vasn | 2.63 | 2.39 | 2.89 |
| Vhlh | -1.69 | -1.52 | -1.88 |
| Vps25 | -1.67 | -1.61 | -1.74 |
| Vps37b | -2.25 | -1.91 | -2.66 |
| Vstm2a | -1.53 | -1.49 | -1.58 |
| Vti1a | -1.52 | -1.25 | -1.85 |
| Wdr23 | -2.05 | -2.01 | -2.09 |
| Wdr37 | -1.86 | -1.79 | -1.93 |
| Yme1l1 | -2.99 | -2.77 | -3.23 |
| Zc3h18 | -3.68 | -3.41 | -3.97 |
| Zeb2 | -3.20 | -3.02 | -3.39 |
| Zfp91 | -1.75 | -1.75 | -1.75 |
