# Supplementary material for: Plurigon: three dimensional visualization and classification of high-dimensionality data
Source: Front Physiol. 2013 Jul 22;4:190. doi: 10.3389/fphys.2013.00190 (PMC3717481; doi:10.3389/fphys.2013.00190)
Supplement: Table S1 — Primary antibody sources and employed dilution factors for selective western blotting. [file 46440_Maudsley_Data_Sheet_2.ZIP › 46440_Maudsley_Table_7.DOC]

**Table S7. Conserved hypothalamic transcriptomic expression data from 18 month old male mice**. The transcriptomic data from separate (M18-1, M18-2, M18-3) 6 month old mice was compared to 3 month old gender-matched control hypothalamic expression values.

| **Gene Symbol** | **M18-1** | **M18-2** | **M18-3** |
| --- | --- | --- | --- |
| 2210016L21Rik | -1.96 | -1.78 | -1.87 |
| 2310047C04Rik | -4.45 | -3.31 | -3.84 |
| 9030612M13Rik | -18.42 | -2.06 | -6.16 |
| Acbd4 | 5.37 | 0.67 | 1.89 |
| Acta1 | -4.12 | -1.76 | -2.69 |
| Add1 | -6.37 | -0.96 | -2.47 |
| Adss | -4.32 | -2.81 | -3.48 |
| Akr1a4 | 2.43 | 1.04 | 1.59 |
| Arf3 | 6.50 | 0.81 | 2.29 |
| Arfip2 | -5.68 | -1.08 | -2.48 |
| Arl3 | 7.83 | 1.78 | 3.73 |
| Arl6ip2 | -6.41 | -0.91 | -2.41 |
| Armc6 | -2.11 | -1.49 | -1.77 |
| Atp1b2 | 2.41 | 1.10 | 1.63 |
| Atxn1 | -4.00 | -3.63 | -3.81 |
| B2m | 9.92 | 2.23 | 4.70 |
| BC021395 | -6.17 | -2.16 | -3.65 |
| Bdh1 | -5.46 | -1.24 | -2.60 |
| Bmpr1a | -1.91 | -1.91 | -1.91 |
| C1qc | 4.80 | 2.98 | 3.78 |
| Cacnb4 | -14.27 | -3.75 | -7.32 |
| Cdh8 | -3.78 | -0.81 | -1.75 |
| Chordc1 | 2.06 | 1.90 | 1.98 |
| Copg | 4.90 | 1.53 | 2.74 |
| Csde1 | -18.08 | -2.54 | -6.77 |
| Csnrp3 | -5.53 | -1.78 | -3.14 |
| Cxadr | -2.46 | -1.86 | -2.14 |
| Ddx6 | -7.54 | -6.71 | -7.11 |
| Dennd3 | -3.02 | -1.48 | -2.11 |
| Dmn | -10.49 | -1.98 | -4.56 |
| Dusp1 | -5.97 | -4.76 | -5.33 |
| Dync1li1 | -2.89 | -2.04 | -2.43 |
| E430025E21Rik | -5.94 | -1.11 | -2.57 |
| Ebna1bp2 | 2.27 | 2.02 | 2.14 |
| Eef1d | 5.33 | 0.97 | 2.27 |
| Efha1 | 4.77 | 1.06 | 2.25 |
| Egr1 | -19.37 | -2.03 | -6.27 |
| Eid2 | -3.71 | -0.75 | -1.67 |
| Eif4g2 | -5.11 | -1.02 | -2.28 |
| Emg1 | 2.19 | 1.32 | 1.70 |
| Erdr1 | 7.43 | 4.20 | 5.59 |
| Fbxo8 | -3.53 | -0.80 | -1.68 |
| Fibcd1 | -2.11 | -1.62 | -1.85 |
| Fos | -26.06 | -2.84 | -8.60 |
| Gabarap | 6.55 | 1.49 | 3.12 |
| Gabrb2 | -5.36 | -1.30 | -2.64 |
| Galnt1 | -3.50 | -1.47 | -2.27 |
| Glrb | -3.01 | -2.32 | -2.64 |
| Gpm6a | -2.23 | -1.69 | -1.94 |
| Gprc5b | 2.65 | 1.19 | 1.78 |
| Hap1 | 3.19 | 0.72 | 1.51 |
| Hspa2 | 2.64 | 1.93 | 2.26 |
| Hspd1 | 5.24 | 0.55 | 1.70 |
| Id2 | -2.70 | -2.45 | -2.57 |
| Ihpk1 | 1.80 | 1.57 | 1.68 |
| Insig1 | -2.69 | -1.18 | -1.78 |
| Junb | -5.19 | -3.32 | -4.15 |
| Khdrbs1 | -3.47 | -3.08 | -3.27 |
| Klhl9 | -2.70 | -1.88 | -2.25 |
| Lmna | -2.01 | -1.69 | -1.84 |
| LOC381629 | 1.82 | 1.31 | 1.54 |
| Lrp1 | -5.78 | -0.74 | -2.07 |
| Mboat2 | -3.57 | -1.39 | -2.23 |
| Mdh1 | -8.98 | -1.29 | -3.40 |
| Mgll | 3.31 | 1.02 | 1.84 |
| Mllt3 | -8.99 | -2.27 | -4.52 |
| Mlycd | -2.48 | -1.84 | -2.14 |
| Morc3 | -4.99 | -0.53 | -1.63 |
| Mrrf | -2.03 | -1.71 | -1.86 |
| Msrb2 | 2.31 | 1.34 | 1.76 |
| Mt3 | 10.81 | 2.76 | 5.46 |
| Mtap1b | -16.17 | -2.29 | -6.08 |
| Mtap2 | -4.13 | -2.52 | -3.23 |
| Mtmr1 | -2.37 | -1.19 | -1.68 |
| Ndufa6 | 3.80 | 1.54 | 2.42 |
| Nhlrc2 | -4.18 | -1.08 | -2.12 |
| Nsg2 | 3.19 | 2.11 | 2.59 |
| Nt5c2 | -3.61 | -2.59 | -3.06 |
| Nus1 | -7.54 | -1.40 | -3.25 |
| Ociad1 | 2.11 | 1.42 | 1.73 |
| Odz3 | -6.55 | -3.76 | -4.96 |
| Opa1 | -3.18 | -2.49 | -2.81 |
| OTTMUSG00000017677 | -4.62 | -0.82 | -1.95 |
| Otud6b | -3.84 | -1.85 | -2.67 |
| Papd1 | -2.54 | -1.02 | -1.61 |
| Pcdh9 | -8.63 | -5.71 | -7.02 |
| Pfn2 | -3.53 | -3.20 | -3.36 |
| Pnpla8 | -5.66 | -0.99 | -2.37 |
| Ppp1cb | -7.83 | -1.57 | -3.51 |
| Ppp1r14a | 2.79 | 1.55 | 2.08 |
| Psmb7 | 3.84 | 2.31 | 2.98 |
| Ptpn11 | 3.07 | 1.79 | 2.34 |
| Pum2 | -7.18 | -1.80 | -3.59 |
| Rab5a | -5.81 | -1.12 | -2.55 |
| Rasgrf1 | -8.10 | -2.05 | -4.07 |
| Rasl11b | -3.97 | -0.72 | -1.69 |
| Rbm18 | -4.82 | -1.22 | -2.42 |
| Rbm45 | -3.03 | -2.46 | -2.73 |
| Rbm9 | -10.41 | -6.66 | -8.33 |
| Rnf14 | -4.69 | -2.32 | -3.30 |
| Rps16 | 2.05 | 1.86 | 1.95 |
| Rps9 | 2.48 | 1.84 | 2.14 |
| Sgip1 | -2.40 | -1.28 | -1.75 |
| Sgtb | -3.21 | -1.28 | -2.03 |
| Slc25a36 | -3.71 | -2.04 | -2.75 |
| Slc7a14 | -7.13 | -3.54 | -5.02 |
| Snx14 | -5.76 | -2.02 | -3.41 |
| Spcs1 | 2.19 | 2.07 | 2.13 |
| Spsb1 | -3.67 | -0.66 | -1.56 |
| Stk19 | 3.13 | 2.14 | 2.59 |
| Stt3a | -13.84 | -0.82 | -3.36 |
| Tceal6 | 2.63 | 1.71 | 2.12 |
| Tmem9 | 2.65 | 2.00 | 2.30 |
| Tpd52l2 | -2.62 | -1.91 | -2.24 |
| Trappc3 | 1.62 | 1.44 | 1.53 |
| Trmt6 | -5.16 | -1.74 | -3.00 |
| Ube3a | -7.25 | -2.37 | -4.14 |
| Uqcrh | 7.32 | 0.49 | 1.90 |
| Usp8 | -3.82 | -0.87 | -1.82 |
| Vasn | -2.10 | -1.90 | -2.00 |
| Vhlh | -4.63 | -1.10 | -2.26 |
| Vps25 | 5.23 | 0.91 | 2.18 |
| Vps37b | -2.27 | -1.75 | -1.99 |
| Vstm2a | -6.26 | -1.77 | -3.33 |
| Vti1a | -4.74 | -0.77 | -1.91 |
| Wdr23 | -3.08 | -1.79 | -2.35 |
| Wdr37 | -5.78 | -1.60 | -3.04 |
| Yme1l1 | -10.13 | -2.17 | -4.69 |
| Zc3h18 | -3.65 | -2.58 | -3.07 |
| Zeb2 | -5.31 | -2.52 | -3.66 |
| Zfp91 | -7.53 | -2.22 | -4.09 |
