# Supplementary material for: Plurigon: three dimensional visualization and classification of high-dimensionality data
Source: Front Physiol. 2013 Jul 22;4:190. doi: 10.3389/fphys.2013.00190 (PMC3717481; doi:10.3389/fphys.2013.00190)
Supplement: Table S1 — Primary antibody sources and employed dilution factors for selective western blotting. [file 46440_Maudsley_Data_Sheet_2.ZIP › 46440_Maudsley_Table_1.DOC]

**Table S1. Primary antibody sources and employed dilution factors for selective western blotting.**

| **Primary Antibody** | **Source** | **Dilution Used** |
| --- | --- | --- |
|  |  |  |
| Phosphorylated ERK1/2 (active) | Cell Signaling Technology (Danvers, MA) | 1:2000 |
| ERK2 (non-phosphorylated) | Santa Cruz Biotechnology (Santa Cruz, CA) | 1:4000 |
| Phosphorylated Akt-1 (active) | Cell Signaling Technology (Danvers, MA) | 1:2000 |
| Akt-1 (non-phosphorylated) | Cell Signaling Technology (Danvers, MA) | 1:2000 |
| Anti-phosphotyrosine (PY20) | Santa Cruz Biotechnology (Santa Cruz, CA) | 1:1000 |
| Synapsin I | Sigma Aldrich (St. Louis, MO) | 1:1000 |
| Posy-synaptic density 95kDA protein (PSD-95) | EMD-Millipore (Billerica, MA) | 1:1000 |
| Neurotrophic tyrosine kinase, receptor, type 2 (NTRK2) | Abcam (Cambridge, MA) | 1:2000 |
| Amyloid precursor protein (APP) | Cell Signaling Technology (Danvers, MA) | 1:500 |
| Anterior pharynx defective 1 homolog A (APH1A) | Abcam (Cambridge, MA) | 1:500 |
| Beta-arrestin 2 | Santa Cruz Biotechnology (Santa Cruz, CA) | 1:2000 |
| Calreticulin | Cell Signaling Technology (Danvers, MA) | 1:3000 |
| Vesicle associated membrane protein 4 (VAMP4) | Sigma Aldrich (St. Louis, MO) | 1:1000 |
| Nicastrin | Santa Cruz Biotechnology (Santa Cruz, CA) | 1:500 |
| Glyceraldehyde 3-phosphate dehydrogenase (GAPDH) | Santa Cruz Biotechnology (Santa Cruz, CA) | 1:1000 |
| Membrane bound O-acyltransferase domain containing 2 (MBOAT2) | Abcam (Cambridge, MA) | 1:500 |
| NADH dehydrogenase [ubiquinone] 1 beta subcomplex subunit 10 (NDUFB10) | Sigma Aldrich (St. Louis, MO) | 1:750 |
| Neuroblastoma RAS viral (v-ras) oncogene homolog (NRAS) | Santa Cruz Biotechnology (Santa Cruz, CA) | 1:2000 |
| Transmembrane emp24 domain-containing protein 1 (TMED1) | Santa Cruz Biotechnology (Santa Cruz, CA) | 1:1000 |
