# Supplementary material for: Plurigon: three dimensional visualization and classification of high-dimensionality data
Source: Front Physiol. 2013 Jul 22;4:190. doi: 10.3389/fphys.2013.00190 (PMC3717481; doi:10.3389/fphys.2013.00190)
Supplement: Table S1 — Primary antibody sources and employed dilution factors for selective western blotting. [file 46440_Maudsley_Data_Sheet_2.ZIP › 46440_Maudsley_Table_4.DOC]

**Table S4. Conserved hypothalamic transcriptomic expression data from 18 month old female mice**. The transcriptomic data from separate (F18-1, F18-2, F18-3) 18 month old mice was compared to 3 month old gender-matched control hypothalamic expression values.

| **Gene Symbol** | **F18-1** | **F18-2** | **F18-3** |
| --- | --- | --- | --- |
| 0610037L13Rik | -4.41 | -4.95 | -4.67 |
| 1110049F12Rik | -3.04 | -4.09 | -3.53 |
| 1700021F05Rik | -2.92 | -3.04 | -2.98 |
| 1700021K19Rik | -1.61 | -2.02 | -1.80 |
| 1700025K23Rik | -1.87 | -2.14 | -2.00 |
| 1810027O10Rik | -2.48 | -3.56 | -2.97 |
| 1810035L17Rik | -1.42 | -1.94 | -1.66 |
| 2010200O16Rik | -1.72 | -1.86 | -1.79 |
| 2310003L22Rik | -2.11 | -2.60 | -2.34 |
| 2510012J08Rik | 3.79 | 5.64 | 4.62 |
| 2610204L23Rik | -1.58 | -2.76 | -2.09 |
| 2700055A20Rik | -2.28 | -2.66 | -2.46 |
| 4432405B04Rik | -2.82 | -2.88 | -2.85 |
| 4833426J09Rik | -1.87 | -2.22 | -2.04 |
| 4930570C03Rik | -1.96 | -2.33 | -2.14 |
| 4933426M11Rik | -2.69 | -2.91 | -2.80 |
| 5730589K01Rik | -1.69 | -2.16 | -1.91 |
| 6720456B07Rik | 2.10 | 2.72 | 2.39 |
| A230106M15Rik | -1.64 | -1.91 | -1.77 |
| A730098D12Rik | -2.90 | -3.44 | -3.16 |
| Aamp | -1.86 | -2.29 | -2.06 |
| AB182283 | -1.99 | -2.19 | -2.09 |
| Abtb1 | -1.58 | -1.81 | -1.69 |
| Acot1 | -2.94 | -3.62 | -3.26 |
| Acsl6 | -2.30 | -3.36 | -2.78 |
| Acta2 | -2.25 | -3.24 | -2.70 |
| Agxt2l1 | -4.71 | -5.00 | -4.85 |
| Ak3 | -2.14 | -2.18 | -2.16 |
| Als2cr13 | 1.97 | 2.34 | 2.15 |
| Ankrd12 | -4.82 | -5.02 | -4.92 |
| Ap2m1 | -1.52 | -2.18 | -1.82 |
| Arl3 | -2.60 | -2.87 | -2.73 |
| Arl4a | -1.52 | -2.42 | -1.92 |
| Arl4c | -2.14 | -2.64 | -2.38 |
| Armc10 | -2.05 | -2.53 | -2.28 |
| Armcx2 | -2.01 | -2.66 | -2.31 |
| Atf2 | -1.78 | -2.00 | -1.89 |
| Atp5sl | -1.93 | -2.51 | -2.20 |
| Atp6v0d1 | -1.62 | -2.18 | -1.88 |
| Atpaf2 | -2.52 | -3.00 | -2.75 |
| Atxn10 | 1.99 | 2.28 | 2.13 |
| AW551984 | -3.88 | -4.20 | -4.04 |
| B230209C24Rik | -2.09 | -2.81 | -2.42 |
| BC008155 | -2.81 | -3.40 | -3.09 |
| BC030476 | -3.07 | -4.80 | -3.84 |
| BC055107 | 1.64 | 1.81 | 1.72 |
| BC087945 | -4.49 | -4.86 | -4.67 |
| Bcap31 | 2.35 | 2.45 | 2.40 |
| Bgn | -1.73 | -1.83 | -1.78 |
| Bid | -2.13 | -2.35 | -2.24 |
| Bin1 | -1.59 | -1.96 | -1.77 |
| Brf1 | -2.38 | -2.88 | -2.62 |
| Bud13 | -1.61 | -2.32 | -1.93 |
| C2 | -2.49 | -2.85 | -2.66 |
| Calb2 | -1.13 | -2.07 | -1.53 |
| Car14 | 3.18 | 3.38 | 3.28 |
| Ccdc127 | -2.44 | -3.17 | -2.78 |
| Cd200 | -1.48 | -1.79 | -1.63 |
| Cd63 | -1.33 | -1.95 | -1.61 |
| Cd83 | -1.85 | -2.32 | -2.07 |
| Cdc37l1 | -1.37 | -1.78 | -1.56 |
| Cdkn1a | -1.95 | -2.41 | -2.17 |
| Cenpb | 4.08 | 4.59 | 4.33 |
| Chpf | -2.53 | -2.63 | -2.58 |
| Chpt1 | -1.97 | -2.43 | -2.19 |
| Clptm1l | -1.03 | -4.39 | -2.13 |
| Cnpy3 | -2.03 | -2.77 | -2.37 |
| Cotl1 | -2.11 | -2.99 | -2.51 |
| Cox4nb | -1.90 | -2.47 | -2.17 |
| Cox6a1 | -1.66 | -2.48 | -2.03 |
| Cpne6 | -2.41 | -3.25 | -2.80 |
| Crbn | -2.82 | -3.05 | -2.93 |
| Csde1 | -1.77 | -2.38 | -2.05 |
| Ctgf | 3.42 | 5.34 | 4.27 |
| D10Wsu102e | -2.47 | -2.88 | -2.67 |
| D14Ertd449e | -2.98 | -3.29 | -3.13 |
| Dctn1 | -2.52 | -3.57 | -3.00 |
| Ddx1 | -1.58 | -1.94 | -1.75 |
| Ddx17 | -3.25 | -4.23 | -3.71 |
| Ddx24 | -1.33 | -2.00 | -1.63 |
| Ddx6 | -2.87 | -4.00 | -3.39 |
| Dedd2 | -3.86 | -5.46 | -4.59 |
| Denr | 2.45 | 2.70 | 2.57 |
| Dnajb6 | 1.77 | 2.59 | 2.14 |
| Dtna | -1.67 | -2.53 | -2.06 |
| Dusp1 | -1.50 | -1.78 | -1.63 |
| EG232599 | -2.75 | -2.91 | -2.83 |
| EG277333 | -2.72 | -3.42 | -3.05 |
| EG434858 | 4.05 | 4.21 | 4.13 |
| Eif1a | -3.68 | -3.68 | -3.68 |
| Eif2b1 | -2.67 | -2.83 | -2.75 |
| Eif3i | -2.22 | -3.58 | -2.82 |
| Eif3s8 | -3.35 | -4.20 | -3.75 |
| Emd | -2.07 | -2.74 | -2.38 |
| Ercc5 | 2.14 | 4.51 | 3.11 |
| Extl3 | -2.71 | -3.52 | -3.09 |
| Fads3 | -2.06 | -2.14 | -2.10 |
| Fbxo38 | -1.50 | -1.88 | -1.68 |
| Fig4 | 2.85 | 3.39 | 3.11 |
| Fmn2 | -1.69 | -1.86 | -1.77 |
| Fnip1 | -1.50 | -1.66 | -1.58 |
| Frs3 | -1.36 | -2.13 | -1.70 |
| Gadd45a | -1.47 | -1.97 | -1.70 |
| Gcs1 | -1.91 | -1.99 | -1.95 |
| Ghitm | 2.47 | 2.78 | 2.62 |
| Ghsr | -1.29 | -2.11 | -1.65 |
| Gja4 | -3.39 | -4.26 | -3.80 |
| Glud1 | -5.03 | -6.31 | -5.63 |
| Gmcl1 | 1.62 | 2.37 | 1.96 |
| Gnai2 | -2.71 | -2.99 | -2.85 |
| Gnaz | -2.04 | -2.29 | -2.16 |
| Gnl3 | -3.60 | -3.68 | -3.64 |
| Golga2 | 2.10 | 2.72 | 2.39 |
| Golim4 | -1.65 | -1.86 | -1.75 |
| Gpc1 | -2.13 | -3.01 | -2.53 |
| Gpld1 | -3.46 | -5.59 | -4.40 |
| Gpr85 | -1.94 | -2.14 | -2.04 |
| Gtdc1 | -1.64 | -1.64 | -1.64 |
| Gtf2h3 | -3.04 | -3.41 | -3.22 |
| H13 | -1.51 | -1.70 | -1.60 |
| Hdac5 | -3.00 | -3.37 | -3.18 |
| Hddc2 | -1.86 | -2.33 | -2.08 |
| Hdhd2 | -1.43 | -2.12 | -1.74 |
| Heph | -3.11 | -3.76 | -3.42 |
| Hgsnat | -2.09 | -2.85 | -2.44 |
| Hist1h1c | -2.54 | -2.91 | -2.72 |
| Hn1 | -1.53 | -1.92 | -1.71 |
| Hnrpa1 | -2.24 | -2.57 | -2.40 |
| Hnrph1 | -1.63 | -1.97 | -1.79 |
| Hps6 | -2.03 | -2.11 | -2.07 |
| Hr | -3.42 | -3.56 | -3.49 |
| Hrsp12 | -1.49 | -2.04 | -1.74 |
| Hsd17b12 | -1.82 | -2.13 | -1.97 |
| Hspb6 | -2.96 | -3.72 | -3.32 |
| Hspd1 | -1.63 | -1.94 | -1.78 |
| Htf9c | -1.64 | -2.32 | -1.95 |
| Insig1 | -4.21 | -5.86 | -4.97 |
| Irs2 | -2.15 | -2.46 | -2.30 |
| Itpk1 | -1.61 | -1.77 | -1.69 |
| Kctd6 | -1.63 | -1.83 | -1.73 |
| Keap1 | 4.02 | 4.02 | 4.02 |
| Klhdc4 | 2.47 | 2.93 | 2.69 |
| Larp6 | -1.51 | -2.79 | -2.05 |
| Lasp1 | -1.88 | -2.71 | -2.26 |
| Lcmt1 | -1.38 | -1.83 | -1.59 |
| Ldb2 | 4.60 | 5.77 | 5.15 |
| Lhfp | -2.27 | -3.50 | -2.82 |
| Lig1 | -2.26 | -2.94 | -2.58 |
| Lingo2 | -2.02 | -2.27 | -2.14 |
| Lman1 | -1.74 | -2.03 | -1.88 |
| LOC100043391 | -1.50 | -1.54 | -1.52 |
| LOC100044177 | -1.70 | -1.87 | -1.78 |
| LOC100044468 | 2.66 | 3.34 | 2.98 |
| LOC100044566 | -1.94 | -2.89 | -2.37 |
| LOC100045882 | -4.51 | -5.76 | -5.10 |
| LOC100045981 | -2.66 | -3.34 | -2.98 |
| LOC100046056 | 2.08 | 2.47 | 2.27 |
| LOC100046953 | 2.09 | 2.26 | 2.17 |
| LOC100047856 | -2.36 | -3.07 | -2.69 |
| LOC100048221 | -1.65 | -2.53 | -2.04 |
| LOC333331 | -2.94 | -3.24 | -3.09 |
| LOC669168 | -4.18 | -4.18 | -4.18 |
| Lrrc8a | -2.28 | -2.42 | -2.35 |
| Lsm12 | 1.96 | 2.38 | 2.16 |
| Map1lc3b | -2.24 | -2.61 | -2.42 |
| Mapk1 | -8.06 | -8.38 | -8.22 |
| Mapk6 | -1.53 | -1.85 | -1.68 |
| Mat2a | -2.81 | -3.27 | -3.03 |
| Mat2b | -2.23 | -2.74 | -2.47 |
| Matk | -1.30 | -1.87 | -1.56 |
| Mboat2 | -2.36 | -2.46 | -2.41 |
| Mboat5 | 3.25 | 3.66 | 3.45 |
| Mfsd2 | -3.40 | -3.89 | -3.64 |
| Mier1 | 1.70 | 1.84 | 1.77 |
| Mkrn2 | -2.26 | -4.88 | -3.32 |
| Mospd1 | -3.46 | -3.46 | -3.46 |
| Mrm1 | -2.46 | -2.87 | -2.66 |
| Mrpl28 | -1.89 | -2.45 | -2.15 |
| Mrpl36 | -1.82 | -2.13 | -1.97 |
| Mrpl47 | -2.08 | -2.20 | -2.14 |
| Mt2 | 2.03 | 2.37 | 2.19 |
| Mtap1b | -1.59 | -1.63 | -1.61 |
| Mycbp2 | 1.48 | 2.65 | 1.98 |
| Nbl1 | -2.02 | -2.35 | -2.18 |
| Ndufb3 | -2.04 | -2.08 | -2.06 |
| Ndufb9 | -1.87 | -2.10 | -1.98 |
| Nelf | 1.66 | 1.93 | 1.79 |
| Nhlrc1 | -3.07 | -3.13 | -3.10 |
| Nol11 | -2.10 | -2.78 | -2.42 |
| Nsbp1 | -1.77 | -2.47 | -2.09 |
| Nt5c2 | -2.67 | -2.77 | -2.72 |
| Nudcd3 | 2.11 | 2.19 | 2.15 |
| Nudc-ps1 | 2.28 | 2.46 | 2.37 |
| Odf2 | -2.48 | -3.75 | -3.05 |
| Ogn | -1.63 | -2.04 | -1.82 |
| Ola1 | -1.52 | -2.15 | -1.81 |
| Paf1 | -1.98 | -1.98 | -1.98 |
| Pafah1b1 | -2.04 | -2.16 | -2.10 |
| Paip2b | -1.70 | -1.74 | -1.72 |
| Pak3 | -4.37 | -5.38 | -4.85 |
| Panx1 | -2.48 | -3.82 | -3.08 |
| Papd1 | -2.51 | -3.56 | -2.99 |
| Pcbd1 | -2.67 | -2.83 | -2.75 |
| Pcyox1 | -1.95 | -2.71 | -2.30 |
| Pcyt1a | -1.82 | -2.16 | -1.98 |
| Pdgfra | 2.02 | 2.44 | 2.22 |
| Pdlim4 | 2.89 | 3.07 | 2.98 |
| Pebp1 | -2.03 | -2.41 | -2.21 |
| Pex19 | -1.74 | -2.68 | -2.16 |
| Pglyrp1 | -2.32 | -2.81 | -2.55 |
| Phlda3 | -3.16 | -3.83 | -3.48 |
| Pik3cb | -2.84 | -3.76 | -3.27 |
| Pldn | -1.34 | -2.20 | -1.72 |
| Pnpla2 | -2.08 | -2.94 | -2.47 |
| Ppap2b | -2.83 | -3.18 | -3.00 |
| Ppm1a | -1.63 | -1.76 | -1.69 |
| Ppp1r10 | -1.75 | -2.27 | -1.99 |
| Ppp1r3f | -2.41 | -2.41 | -2.41 |
| Ppp2r4 | -2.53 | -3.01 | -2.76 |
| Prf1 | -1.97 | -2.61 | -2.27 |
| Prkcz | -4.41 | -9.28 | -6.40 |
| Prnp | 1.85 | 2.24 | 2.04 |
| Prr3 | -1.86 | -2.21 | -2.03 |
| Prrc1 | -2.27 | -2.41 | -2.34 |
| Psip1 | 3.24 | 3.85 | 3.53 |
| Psmd11 | -2.06 | -2.82 | -2.41 |
| Psmd8 | 3.58 | 5.51 | 4.44 |
| Ptcd3 | -1.81 | -2.35 | -2.06 |
| Rab18 | -2.77 | -3.42 | -3.08 |
| Rab3b | -2.03 | -2.11 | -2.07 |
| Rab5a | -1.44 | -1.65 | -1.54 |
| Ramp2 | 3.84 | 4.08 | 3.96 |
| Rars | -2.80 | -3.03 | -2.91 |
| Rbak | -1.74 | -1.74 | -1.74 |
| Rbbp7 | -1.62 | -2.73 | -2.10 |
| Rbm14 | -1.44 | -1.62 | -1.53 |
| Rcl1 | 1.97 | 2.09 | 2.03 |
| Rdbp | -3.36 | -4.52 | -3.90 |
| Rdh14 | -2.67 | -3.84 | -3.20 |
| Rela | -3.64 | -4.09 | -3.86 |
| Rnf113a1 | 3.36 | 3.77 | 3.56 |
| Rnf14 | 2.05 | 2.30 | 2.17 |
| Rnmt | -3.10 | -3.16 | -3.13 |
| Rnuxa | -1.56 | -1.68 | -1.62 |
| Rpia | -2.83 | -2.89 | -2.86 |
| Rps11 | 2.08 | 2.70 | 2.37 |
| Rps25 | -4.16 | -5.40 | -4.74 |
| Rrn3 | -2.40 | -2.50 | -2.45 |
| Ruvbl1 | -2.18 | -2.26 | -2.22 |
| Sc4mol | -2.35 | -2.49 | -2.42 |
| Scarb2 | 1.92 | 1.92 | 1.92 |
| Scotin | 2.03 | 2.24 | 2.13 |
| Scp2 | -1.76 | -2.50 | -2.10 |
| Sdc3 | -4.89 | -7.40 | -6.02 |
| Sdhb | -2.18 | -2.40 | -2.29 |
| Sdpr | -1.68 | -2.34 | -1.98 |
| Sec61a2 | -3.43 | -3.43 | -3.43 |
| Sec63 | -2.54 | -2.70 | -2.62 |
| Seh1l | -2.07 | -4.23 | -2.96 |
| Serinc3 | -1.55 | -1.71 | -1.63 |
| Serpini1 | -4.10 | -4.26 | -4.18 |
| Sesn1 | -1.85 | -2.54 | -2.17 |
| Sfrs16 | -2.60 | -4.39 | -3.38 |
| Siah1a | -5.26 | -5.48 | -5.37 |
| Slc12a9 | -1.56 | -1.62 | -1.59 |
| Slc22a5 | -4.80 | -5.00 | -4.90 |
| Slc25a16 | -2.36 | -3.29 | -2.79 |
| Slc35b1 | -2.97 | -4.00 | -3.45 |
| Slco4a1 | -1.32 | -1.87 | -1.57 |
| Smox | -2.65 | -2.98 | -2.81 |
| Snrpd3 | -2.53 | -3.23 | -2.86 |
| Sorcs3 | 2.72 | 3.05 | 2.88 |
| Sox8 | 1.46 | 1.86 | 1.65 |
| Spp1 | -1.74 | -2.18 | -1.95 |
| Spsb1 | -1.90 | -2.09 | -1.99 |
| Srebf2 | -1.88 | -2.03 | -1.95 |
| Srpr | 1.30 | 2.04 | 1.63 |
| Srxn1 | -2.31 | -2.75 | -2.52 |
| Strap | -1.44 | -1.80 | -1.61 |
| Sult1a1 | -2.68 | -2.84 | -2.76 |
| Suv420h1 | -1.58 | -2.48 | -1.98 |
| Syt11 | -2.31 | -2.75 | -2.52 |
| Tbl1x | -1.58 | -1.71 | -1.64 |
| Tceal5 | 1.69 | 1.79 | 1.74 |
| Tfam | 1.52 | 1.56 | 1.54 |
| Tgfbr3 | -2.65 | -2.98 | -2.81 |
| Tgm2 | -2.34 | -3.14 | -2.71 |
| Tgoln1 | 2.04 | 2.51 | 2.26 |
| Tgs1 | 1.60 | 1.83 | 1.71 |
| Timp4 | 1.60 | 2.05 | 1.81 |
| Tmem183a | 2.33 | 3.03 | 2.66 |
| Tmem204 | -2.80 | -3.70 | -3.22 |
| Tpi1 | -1.76 | -1.84 | -1.80 |
| Tpm3 | -1.42 | -1.60 | -1.51 |
| Trp53bp1 | 1.32 | 1.84 | 1.56 |
| Trp53inp1 | -4.54 | -5.29 | -4.90 |
| Tsc22d3 | -1.99 | -2.45 | -2.21 |
| Tsg101 | 1.93 | 1.97 | 1.95 |
| Twsg1 | -2.79 | -4.42 | -3.51 |
| Uap1 | 2.33 | 3.25 | 2.75 |
| Ube2g1 | -2.40 | -2.40 | -2.40 |
| Ubtd2 | -1.76 | -1.86 | -1.81 |
| Uck2 | -2.17 | -2.25 | -2.21 |
| Use1 | -1.62 | -1.75 | -1.68 |
| Usp1 | -1.44 | -1.87 | -1.64 |
| Uvrag | -1.51 | -1.80 | -1.65 |
| Vamp1 | -1.62 | -2.99 | -2.20 |
| Vamp3 | 1.36 | 1.68 | 1.51 |
| Vangl2 | -1.46 | -1.89 | -1.66 |
| Vps41 | -1.66 | -1.66 | -1.66 |
| Wdr61 | -3.28 | -3.48 | -3.38 |
| Wfdc1 | -1.47 | -1.62 | -1.54 |
| Yme1l1 | -1.60 | -2.07 | -1.82 |
| Zbtb22 | -2.30 | -2.89 | -2.58 |
| Zdhhc12 | -1.30 | -2.00 | -1.61 |
| Zdhhc3 | -2.02 | -3.00 | -2.46 |
| Zfp292 | 1.97 | 2.22 | 2.09 |
| Zfyve27 | 4.19 | 5.16 | 4.65 |
| Zkscan6 | -1.73 | -1.77 | -1.75 |
