# Supplementary material for: Plurigon: three dimensional visualization and classification of high-dimensionality data
Source: Front Physiol. 2013 Jul 22;4:190. doi: 10.3389/fphys.2013.00190 (PMC3717481; doi:10.3389/fphys.2013.00190)
Supplement: Table S1 — Primary antibody sources and employed dilution factors for selective western blotting. [file 46440_Maudsley_Data_Sheet_2.ZIP › 46440_Maudsley_Table_5.DOC]

**Table S5. Conserved hypothalamic transcriptomic expression data from 6 month old male mice**. The transcriptomic data from separate (M6-1, M6-2, M6-3) 6 month old mice was compared to 3 month old gender-matched control hypothalamic expression values.

| **Gene Symbol** | **M6-1** | **M6-2** | **M6-3** |
| --- | --- | --- | --- |
| 2210016L21Rik | 1.76 | 1.76 | 1.76 |
| 2310047C04Rik | -2.15 | -2.09 | -2.21 |
| 9030612M13Rik | -2.20 | -0.73 | -6.60 |
| Acbd4 | -1.81 | -0.65 | -5.07 |
| Acta1 | -1.75 | -1.14 | -2.68 |
| Add1 | -1.90 | -0.82 | -4.43 |
| Adss | -1.52 | -1.30 | -1.78 |
| Akr1a4 | 3.48 | 2.56 | 4.73 |
| Arf3 | 1.54 | 0.61 | 3.88 |
| Arfip2 | -2.08 | -0.87 | -4.95 |
| Arl3 | 1.80 | 1.05 | 3.10 |
| Arl6ip2 | -2.44 | -1.10 | -5.42 |
| Armc6 | -2.35 | -1.96 | -2.82 |
| Atp1b2 | 2.57 | 1.90 | 3.47 |
| Atxn1 | -1.69 | -1.59 | -1.79 |
| B2m | 2.43 | 1.12 | 5.27 |
| BC021395 | -2.02 | -1.40 | -2.91 |
| Bdh1 | -1.52 | -0.87 | -2.64 |
| Bmpr1a | -2.73 | -1.77 | -4.20 |
| C1qc | 1.60 | 1.31 | 1.95 |
| Cacnb4 | -2.51 | -1.43 | -4.42 |
| Cdh8 | -2.02 | -0.91 | -4.48 |
| Chordc1 | -1.62 | -1.54 | -1.70 |
| Copg | 1.57 | 0.73 | 3.39 |
| Csde1 | -1.97 | -0.81 | -4.81 |
| Csnrp3 | -2.43 | -1.35 | -4.37 |
| Cxadr | -2.66 | -2.42 | -2.93 |
| Ddx6 | -2.71 | -2.66 | -2.76 |
| Dennd3 | -2.35 | -1.62 | -3.41 |
| Dmn | -1.60 | -0.71 | -3.60 |
| Dusp1 | -5.11 | -4.82 | -5.42 |
| Dync1li1 | -1.90 | -1.58 | -2.28 |
| E430025E21Rik | -2.56 | -1.06 | -6.20 |
| Ebna1bp2 | -1.54 | -1.29 | -1.83 |
| Eef1d | 1.93 | 1.00 | 3.72 |
| Efha1 | -1.72 | -0.86 | -3.44 |
| Egr1 | -6.29 | -1.97 | -20.07 |
| Eid2 | -2.09 | -0.91 | -4.79 |
| Eif4g2 | -2.25 | -1.10 | -4.59 |
| Emg1 | -3.27 | -2.55 | -4.19 |
| Erdr1 | -2.99 | -2.37 | -3.77 |
| Fbxo8 | -1.82 | -0.89 | -3.71 |
| Fibcd1 | -1.61 | -1.38 | -1.88 |
| Fos | -8.98 | -2.81 | -28.74 |
| Gabarap | 2.15 | 1.00 | 4.62 |
| Gabrb2 | -1.91 | -1.01 | -3.63 |
| Galnt1 | -1.50 | -1.09 | -2.06 |
| Glrb | -2.87 | -2.31 | -3.56 |
| Gpm6a | -2.36 | -1.74 | -3.21 |
| Gprc5b | -2.57 | -1.81 | -3.65 |
| Hap1 | 3.44 | 2.04 | 5.81 |
| Hspa2 | 1.81 | 1.46 | 2.24 |
| Hspd1 | -1.53 | -0.51 | -4.62 |
| Id2 | -4.56 | -3.71 | -5.61 |
| Ihpk1 | 1.82 | 1.80 | 1.84 |
| Insig1 | -1.94 | -1.43 | -2.64 |
| Junb | -4.73 | -3.35 | -6.67 |
| Khdrbs1 | -1.50 | -1.43 | -1.58 |
| Klhl9 | -2.28 | -1.81 | -2.87 |
| Lmna | -1.84 | -1.79 | -1.90 |
| LOC381629 | 2.03 | 1.71 | 2.42 |
| Lrp1 | -2.39 | -0.82 | -6.98 |
| Mboat2 | -1.79 | -1.21 | -2.65 |
| Mdh1 | -2.77 | -1.07 | -7.20 |
| Mgll | 1.73 | 0.80 | 3.74 |
| Mllt3 | -1.74 | -0.76 | -4.00 |
| Mlycd | -2.38 | -1.97 | -2.88 |
| Morc3 | -3.15 | -1.01 | -9.86 |
| Mrrf | -1.93 | -1.91 | -1.95 |
| Msrb2 | -1.71 | -1.32 | -2.22 |
| Mt3 | 2.58 | 1.25 | 5.31 |
| Mtap1b | 1.77 | 0.56 | 5.61 |
| Mtap2 | -2.17 | -1.82 | -2.58 |
| Mtmr1 | -1.95 | -1.51 | -2.52 |
| Ndufa6 | 1.71 | 1.02 | 2.86 |
| Nhlrc2 | -1.61 | -0.90 | -2.87 |
| Nsg2 | 1.76 | 1.53 | 2.02 |
| Nt5c2 | -3.18 | -2.56 | -3.94 |
| Nus1 | -2.93 | -1.40 | -6.15 |
| Ociad1 | 2.61 | 2.33 | 2.92 |
| Odz3 | -2.45 | -2.15 | -2.79 |
| Opa1 | -2.18 | -2.16 | -2.20 |
| OTTMUSG00000017677 | -1.67 | -0.56 | -4.96 |
| Otud6b | -2.78 | -1.79 | -4.31 |
| Papd1 | -2.58 | -1.71 | -3.90 |
| Pcdh9 | -1.73 | -1.41 | -2.13 |
| Pfn2 | 1.83 | 1.81 | 1.85 |
| Pnpla8 | -1.68 | -0.65 | -4.35 |
| Ppp1cb | -3.21 | -1.54 | -6.68 |
| Ppp1r14a | -2.39 | -1.85 | -3.08 |
| Psmb7 | 1.75 | 1.39 | 2.21 |
| Ptpn11 | -2.29 | -2.03 | -2.59 |
| Pum2 | -2.56 | -1.55 | -4.22 |
| Rab5a | -3.20 | -1.43 | -7.17 |
| Rasgrf1 | 3.43 | 1.84 | 6.38 |
| Rasl11b | -1.78 | -0.71 | -4.47 |
| Rbm18 | -2.01 | -1.04 | -3.88 |
| Rbm45 | -1.94 | -1.90 | -1.98 |
| Rbm9 | -2.91 | -1.88 | -4.51 |
| Rnf14 | -1.92 | -1.43 | -2.57 |
| Rps16 | 1.79 | 1.54 | 2.08 |
| Rps9 | 4.32 | 3.72 | 5.01 |
| Sgip1 | -1.57 | -1.14 | -2.17 |
| Sgtb | -2.19 | -1.44 | -3.33 |
| Slc25a36 | -1.63 | -1.26 | -2.10 |
| Slc7a14 | -2.75 | -1.86 | -4.07 |
| Snx14 | -2.17 | -1.61 | -2.93 |
| Spcs1 | 1.87 | 1.80 | 1.94 |
| Spsb1 | -2.33 | -1.12 | -4.85 |
| Stk19 | 1.89 | 1.49 | 2.40 |
| Stt3a | -1.81 | -0.42 | -7.87 |
| Tceal6 | 2.08 | 1.58 | 2.75 |
| Tmem9 | 2.02 | 1.59 | 2.57 |
| Tpd52l2 | -2.21 | -1.99 | -2.45 |
| Trappc3 | 1.91 | 1.77 | 2.06 |
| Trmt6 | -3.99 | -2.85 | -5.59 |
| Ube3a | -4.42 | -2.21 | -8.84 |
| Uqcrh | 3.86 | 1.17 | 12.70 |
| Usp8 | -1.70 | -0.78 | -3.69 |
| Vasn | -2.06 | -1.72 | -2.47 |
| Vhlh | -4.24 | -1.93 | -9.33 |
| Vps25 | 2.19 | 0.98 | 4.88 |
| Vps37b | -2.65 | -2.43 | -2.89 |
| Vstm2a | -1.86 | -1.05 | -3.29 |
| Vti1a | -2.38 | -1.01 | -5.62 |
| Wdr23 | -3.04 | -2.08 | -4.44 |
| Wdr37 | -1.60 | -0.79 | -3.23 |
| Yme1l1 | -2.94 | -1.34 | -6.47 |
| Zc3h18 | -2.23 | -1.64 | -3.03 |
| Zeb2 | -1.84 | -1.31 | -2.58 |
| Zfp91 | -2.33 | -1.34 | -4.05 |
