# Supplementary material for: Plurigon: three dimensional visualization and classification of high-dimensionality data
Source: Front Physiol. 2013 Jul 22;4:190. doi: 10.3389/fphys.2013.00190 (PMC3717481; doi:10.3389/fphys.2013.00190)
Supplement: Table S8 — Wildtype (WT) murine transcriptomic data from multiple isolated tissues. Datasets are arranged alphanumerically with the following nomenclature: WT1 to WT16. [file 46440_Maudsley_Data_Sheet_3.ZIP › Table-S8.doc]

**Table S8. Wildtype (WT) murine transcriptomic data from multiple isolated tissues.** Datasets are arranged alphanumerically with the following nomenclature: WT1 to WT17.

| **Gene Symbol** | **WT1** |
| --- | --- |
| 0610006I08Rik | -0.39951 |
| 0610006I08Rik | -0.30863 |
| 0610007C21Rik | -0.33853 |
| 0610007C21Rik | 0.08059 |
| 0610007P08Rik | 3.43652 |
| 0610007P14Rik | -0.59856 |
| 0610007P22Rik | -0.55004 |
| 0610009B22Rik | 0.23048 |
| 0610009D07Rik | -0.54256 |
| 0610009O20Rik | -0.152 |
| 0610010E21Rik | 1.58182 |
| 0610010K06Rik | -0.55079 |
| 0610010K14Rik | 0.67252 |
| 0610012A05Rik | -0.31094 |
| 0610012D14Rik | -0.64645 |
| 0610012G03Rik | -0.45463 |
| 0610012H03Rik | 0.38055 |
| 0610025P10Rik | -0.68647 |
| 0610031J06Rik | -0.50323 |
| 0610031J06Rik | 0.7597 |
| 0610031J06Rik | 1.69068 |
| 0610037D15Rik | -0.42666 |
| 0610037L13Rik | 0.04163 |
| 0610037L13Rik | 0.27189 |
| 0610038F07Rik | 0.13451 |
| 0610040J01Rik | 1.36441 |
| 0710008K08Rik | 0.37151 |
| 0910001A06Rik | 1.74146 |
| 0910001A06Rik | -0.68821 |
| 0910001L09Rik | 0.2973 |
| 1110001A07Rik | 2.18255 |
| 1110001J03Rik | 1.28288 |
| 1110002B05Rik | -0.65062 |
| 1110002N22Rik | 1.11399 |
| 1110003E01Rik | 1.57315 |
| 1110006G06Rik | -0.20972 |
| 1110007A13Rik | -0.64146 |
| 1110007A13Rik | 0.23016 |
| 1110007C09Rik | 2.09941 |
| 1110007L15Rik | 1.54025 |
| 1110007L15Rik | 2.31915 |
| 1110007L15Rik | -0.56975 |
| 1110007L15Rik | -0.07325 |
| 1110007M04Rik | 0.04673 |
| 1110008F13Rik | 0.2865 |
| 1110008J03Rik | 1.58682 |
| 1110008J03Rik | 0.00914 |
| 1110008J03Rik | -0.30747 |
| 1110008P14Rik | -0.61855 |
| 1110012D08Rik | -0.67008 |
| 1110012J17Rik | -0.67867 |
| 1110012L19Rik | 0.1147 |
| 1110012L19Rik | -0.23174 |
| 1110012M11Rik | 3.1946 |
| 1110013L07Rik | -0.62667 |
| 1110017D15Rik | -0.80919 |
| 1110017D15Rik | 0.75337 |
| 1110017D15Rik | -0.68821 |
| 1110018G07Rik | -0.57129 |
| 1110018J18Rik | -0.28246 |
| 1110018J18Rik | 0.20741 |
| 1110019N10Rik | 0.5031 |
| 1110019N10Rik | 0.40236 |
| 1110020C03Rik | 2.27036 |
| 1110020P15Rik | -0.33674 |
| 1110020P15Rik | -0.75044 |
| 1110031B06Rik | -0.40526 |
| 1110031I02Rik | 0.91397 |
| 1110032A13Rik | -0.43589 |
| 1110032A13Rik | -0.74671 |
| 1110032E23Rik | 0.18442 |
| 1110034A24Rik | 0.91336 |
| 1110036O03Rik | 0.70315 |
| 1110038D17Rik | -0.76644 |
| 1110038F14Rik | 0.41689 |
| 1110038F14Rik | -0.05512 |
| 1110039B18Rik | -0.67522 |
| 1110039B18Rik | -0.60252 |
| 1110039B18Rik | -0.07727 |
| 1110048D14Rik | 3.72282 |
| 1110049B09Rik | 0.16188 |
| 1110049B09Rik | -0.08177 |
| 1110049F12Rik | 0.05454 |
| 1110049F12Rik | -0.67522 |
| 1110057K04Rik | -0.51905 |
| 1110057K04Rik | -0.17915 |
| 1110057K04Rik | 2.0798 |
| 1110058L19Rik | 0.58235 |
| 1110059E24Rik | -0.39507 |
| 1110059G10Rik | -0.25267 |
| 1110067D22Rik | -0.72558 |
| 1110067I12Rik | -0.10274 |
| 1190002A17Rik | 0.80717 |
| 1190002H23Rik | 1.47643 |
| 1190002N15Rik | -0.81418 |
| 1190003J15Rik | -0.61613 |
| 1190003M12Rik | -0.09311 |
| 1190005F20Rik | -0.47722 |
| 1190005I06Rik | 1.70265 |
| 1190005P17Rik | 0.11942 |
| 1190005P17Rik | -0.13751 |
| 1190007F08Rik | 0.74954 |
| 1190017O12Rik | 3.40092 |
| 1190020J12Rik | -0.18923 |
| 1200002N14Rik | -0.64812 |
| 1200003C05Rik | -0.39888 |
| 1200003C05Rik | 0.09446 |
| 1200003C05Rik | -0.61855 |
| 1200003I07Rik | -0.73746 |
| 1200009F10Rik | -0.34211 |
| 1200009I06Rik | 3.57463 |
| 1200009I06Rik | -0.5322 |
| 1200009O22Rik | -0.04035 |
| 1200011O22Rik | 2.85507 |
| 1200013B08Rik | 0.14829 |
| 1200013P24Rik | -0.53885 |
| 1200013P24Rik | 1.13174 |
| 1200014J11Rik | 2.56822 |
| 1200015F23Rik | -0.55681 |
| 1200015F23Rik | 0.58431 |
| 1200015N20Rik | 0.61629 |
| 1200016B10Rik | -0.48768 |
| 1200016B10Rik | -0.64229 |
| 1200016D23Rik | 3.22172 |
| 1300001I01Rik | 1.8618 |
| 1300007L22Rik | 2.81001 |
| 1300010M03Rik | 0.16567 |
| 1300013J15Rik | 0.1252 |
| 1300013J15Rik | -0.7274 |
| 1300014I06Rik | -0.24295 |
| 1300017J02Rik | -0.11528 |
| 1300018I05Rik | -0.33734 |
| 1300018J18Rik | -0.47584 |
| 1500001L15Rik | -0.01779 |
| 1500001M20Rik | -0.18367 |
| 1500001M20Rik | 1.85492 |
| 1500001M20Rik | -0.56516 |
| 1500002O20Rik | -0.30574 |
| 1500003O03Rik | 1.47428 |
| 1500005A01Rik | 0.31357 |
| 1500010J02Rik | 1.32245 |
| 1500010J02Rik | 0.3849 |
| 1500011H22Rik | -0.35234 |
| 1500012F01Rik | -0.36759 |
| 1500015O10Rik | -0.12043 |
| 1500019C06Rik | 0.83584 |
| 1500019G21Rik | 1.43056 |
| 1500031L02Rik | 2.17863 |
| 1500032D16Rik | -0.6365 |
| 1500032D16Rik | 0.02372 |
| 1500032D16Rik | 0.58737 |
| 1500032L24Rik | -0.45802 |
| 1500041J02Rik | 2.3209 |
| 1500041N16Rik | -0.74393 |
| 1520401A03Rik | -0.35416 |
| 1600002K03Rik | -0.16671 |
| 1600012H06Rik | -0.74578 |
| 1600012H06Rik | 0.92924 |
| 1600014C10Rik | -0.67265 |
| 1600016N20Rik | -0.72558 |
| 1600021P15Rik | 0.58453 |
| 1600027N09Rik | 0.05609 |
| 1600029D21Rik | 2.21748 |
| 1700001C02Rik | 0.03807 |
| 1700001C19Rik | -0.25976 |
| 1700001E04Rik | -0.62098 |
| 1700001J03Rik | 1.64439 |
| 1700001O22Rik | 1.89281 |
| 1700001O22Rik | -0.49119 |
| 1700001P01Rik | 0.32667 |
| 1700003F12Rik | 1.07668 |
| 1700003M02Rik | 0.61227 |
| 1700007G11Rik | 2.40702 |
| 1700007I06Rik | 0.15319 |
| 1700007K09Rik | 0.34441 |
| 1700008F21Rik | -0.30402 |
| 1700008I05Rik | 0.14302 |
| 1700008P20Rik | 0.85929 |
| 1700009P17Rik | 0.39354 |
| 1700010A17Rik | -0.64146 |
| 1700010A17Rik | 0.3895 |
| 1700010D01Rik | 0.13842 |
| 1700010M22Rik | -0.35963 |
| 1700010M22Rik | -0.11809 |
| 1700011A15Rik | -0.70138 |
| 1700011F03Rik | 0.55435 |
| 1700011F14Rik | 2.82013 |
| 1700011H14Rik | 0.36737 |
| 1700011K15Rik | -0.09356 |
| 1700012A03Rik | 0.38191 |
| 1700012B09Rik | 0.88766 |
| 1700012G19Rik | -0.31036 |
| 1700012H05Rik | -0.46963 |
| 1700012H17Rik | 0.45712 |
| 1700012L04Rik | -0.46757 |
| 1700013B16Rik | 0.62217 |
| 1700013G24Rik | -0.47307 |
| 1700013N18Rik | -0.47792 |
| 1700013N18Rik | 0.21364 |
| 1700015E13Rik | -0.6121 |
| 1700016C15Rik | -0.41882 |
| 1700016D06Rik | -0.29091 |
| 1700016G05Rik | 1.30955 |
| 1700016K19Rik | 0.27005 |
| 1700016K19Rik | -0.57436 |
| 1700017N19Rik | 2.69796 |
| 1700018B24Rik | -0.72377 |
| 1700018C11Rik | 1.41203 |
| 1700018F24Rik | -0.33972 |
| 1700019D03Rik | -0.40719 |
| 1700019E19Rik | -0.56516 |
| 1700019G17Rik | -0.19942 |
| 1700019H03Rik | -0.61451 |
| 1700019L03Rik | 0.06305 |
| 1700019M22Rik | 2.65738 |
| 1700019N19Rik | -0.55079 |
| 1700020C11Rik | -0.41428 |
| 1700020D05Rik | -0.59068 |
| 1700020L24Rik | -0.11668 |
| 1700020N01Rik | 0.2682 |
| 1700020O03Rik | 2.57443 |
| 1700021C14Rik | -0.16968 |
| 1700021F05Rik | 2.04639 |
| 1700021K02Rik | -0.17765 |
| 1700021K02Rik | 2.46085 |
| 1700021K19Rik | 0.43093 |
| 1700022C21Rik | -0.2061 |
| 1700023B02Rik | 1.09753 |
| 1700023B02Rik | 3.1802 |
| 1700023E05Rik | -0.47307 |
| 1700023E05Rik | -0.36085 |
| 1700023I07Rik | 0.20147 |
| 1700023M03Rik | -0.18166 |
| 1700024G13Rik | -0.66242 |
| 1700025D03Rik | -0.73471 |
| 1700025E21Rik | 0.95862 |
| 1700025F22Rik | 0.31562 |
| 1700025G04Rik | -0.46142 |
| 1700025K23Rik | -0.57129 |
| 1700026D08Rik | -0.30805 |
| 1700026L06Rik | -0.2603 |
| 1700027A23Rik | 0.96297 |
| 1700027D21Rik | -0.743 |
| 1700027J05Rik | -0.70404 |
| 1700027N10Rik | -0.78465 |
| 1700028K03Rik | -0.59147 |
| 1700029F12Rik | 0.47205 |
| 1700029F12Rik | -0.64645 |
| 1700029G01Rik | 2.50816 |
| 1700029H14Rik | 0.22823 |
| 1700029H14Rik | -0.69346 |
| 1700029I01Rik | 0.85913 |
| 1700029I08Rik | 2.6208 |
| 1700029I15Rik | -0.51616 |
| 1700029J07Rik | 0.30562 |
| 1700029P11Rik | 0.45134 |
| 1700029P11Rik | 1.55259 |
| 1700029P11Rik | -0.62667 |
| 1700030J22Rik | 1.76382 |
| 1700030K09Rik | -0.80127 |
| 1700034E13Rik | 0.25299 |
| 1700034H14Rik | -0.46006 |
| 1700034H14Rik | -0.18468 |
| 1700034H14Rik | 1.48745 |
| 1700034H14Rik | 1.89926 |
| 1700034I23Rik | 1.97061 |
| 1700034I23Rik | 0.99955 |
| 1700034O15Rik | 0.69331 |
| 1700036D21Rik | 2.52539 |
| 1700037C18Rik | 0.78725 |
| 1700037C18Rik | 1.66557 |
| 1700037H04Rik | 3.91556 |
| 1700039E15Rik | -0.61532 |
| 1700040I03Rik | -0.25485 |
| 1700042B14Rik | 1.10201 |
| 1700045I19Rik | -0.58599 |
| 1700047I17Rik1 | -0.08267 |
| 1700047I17Rik1 | -0.09814 |
| 1700049G17Rik | -0.34751 |
| 1700049K14Rik | -0.55681 |
| 1700049L16Rik | 1.82688 |
| 1700052N19Rik | 0.49567 |
| 1700052N19Rik | 0.77898 |
| 1700054O13Rik | 0.00093 |
| 1700055M20Rik | 2.165 |
| 1700057G04Rik | 1.61321 |
| 1700057K13Rik | 0.58976 |
| 1700058C13Rik | -0.78369 |
| 1700060H10Rik | 2.90822 |
| 1700061G19Rik | -0.67953 |
| 1700063I17Rik | 2.74528 |
| 1700065I17Rik | -0.17167 |
| 1700065O13Rik | -0.75887 |
| 1700065O13Rik | 0.07301 |
| 1700067C01Rik | -0.30805 |
| 1700067K01Rik | -0.48768 |
| 1700067P10Rik | 0.20741 |
| 1700071K01Rik | -0.64146 |
| 1700072E05Rik | -0.11855 |
| 1700072E05Rik | 1.47386 |
| 1700072E05Rik | 2.77515 |
| 1700073E17Rik | 0.21886 |
| 1700080E11Rik | -0.80127 |
| 1700081D17Rik | -0.24726 |
| 1700081D17Rik | 0.83551 |
| 1700084C01Rik | 0.72101 |
| 1700084C01Rik | 2.21051 |
| 1700088E04Rik | -0.2092 |
| 1700093K21Rik | 2.13435 |
| 1700094C09Rik | 1.04045 |
| 1700095K08Rik | 1.67128 |
| 1700102P08Rik | -0.02201 |
| 1700108M19Rik | 0.16223 |
| 1700109H08Rik | -0.7523 |
| 1700112C13Rik | -0.32024 |
| 1700113I22Rik | -0.217 |
| 1700113I22Rik | 0.86943 |
| 1700113O17Rik | 0.35035 |
| 1700120B06Rik | -0.73931 |
| 1700123D08Rik | -0.23174 |
| 1700123K08Rik | -0.10644 |
| 1700123L14Rik | 1.74554 |
| 1700123O20Rik | -0.43258 |
| 1700123O20Rik | -0.7779 |
| 1700127D06Rik | -0.35295 |
| 1700129C05Rik | -0.36146 |
| 1700129I15Rik | -0.51978 |
| 1810007M14Rik | 0.56266 |
| 1810007P19Rik | -0.70938 |
| 1810007P19Rik | 3.72491 |
| 1810008A18Rik | 0.38977 |
| 1810008A18Rik | -0.30402 |
| 1810009A15Rik | 0.10776 |
| 1810009A15Rik | -0.17316 |
| 1810009N02Rik | 1.91566 |
| 1810009O10Rik | -0.30517 |
| 1810009O10Rik | 1.37306 |
| 1810010M01Rik | 2.01898 |
| 1810011O10Rik | 0.47697 |
| 1810014F10Rik | -0.50895 |
| 1810015A11Rik | 1.23897 |
| 1810015C04Rik | -0.70849 |
| 1810019J16Rik | -0.48348 |
| 1810020D17Rik | -0.63157 |
| 1810020G14Rik | -0.68908 |
| 1810020G14Rik | 3.64318 |
| 1810021J13Rik | -0.65819 |
| 1810021J13Rik | -0.10274 |
| 1810022C23Rik | -0.18216 |
| 1810026J23Rik | 2.20569 |
| 1810027O10Rik | 1.82468 |
| 1810030J14Rik | -0.33318 |
| 1810030N24Rik | -0.6565 |
| 1810030N24Rik | -0.73838 |
| 1810030N24Rik | -0.5899 |
| 1810031K17Rik | 2.82999 |
| 1810035L17Rik | 2.96324 |
| 1810035L17Rik | 0.09297 |
| 1810037C20Rik | -0.50609 |
| 1810043G02Rik | 0.44957 |
| 1810044A24Rik | 0.30946 |
| 1810046J19Rik | 1.47577 |
| 1810048J11Rik | 0.03013 |
| 1810049H13Rik | 1.68562 |
| 1810049H19Rik | 0.82515 |
| 1810055E12Rik | -0.02666 |
| 1810055E12Rik | 1.41105 |
| 1810055E12Rik | -0.5448 |
| 1810055G02Rik | 0.648 |
| 1810063B05Rik | -0.4933 |
| 1810064F22Rik | 0.35848 |
| 1810065E05Rik | -0.16869 |
| 1810073G14Rik | -0.71565 |
| 1810074P20Rik | 1.7684 |
| 1810074P20Rik | -0.70938 |
| 2010001J22Rik | -0.39064 |
| 2010001J22Rik | -0.16129 |
| 2010001M09Rik | 0.39434 |
| 2010003J03Rik | -0.00154 |
| 2010003O18Rik | 1.24196 |
| 2010004A03Rik | -0.69433 |
| 2010005H15Rik | -0.24888 |
| 2010005J08Rik | -0.66496 |
| 2010005J08Rik | -0.68821 |
| 2010005J08Rik | -0.15834 |
| 2010007H12Rik | 0.12231 |
| 2010007H12Rik | -0.15443 |
| 2010011I20Rik | 0.65994 |
| 2010012O05Rik | 0.4826 |
| 2010100O12Rik | -0.49542 |
| 2010100O12Rik | 0.4286 |
| 2010106G01Rik | 2.89064 |
| 2010106G01Rik | 0.76617 |
| 2010107E04Rik | 0.77029 |
| 2010107G12Rik | -0.63239 |
| 2010109I03Rik | -0.6283 |
| 2010110K16Rik | 0.19482 |
| 2010111I01Rik | -0.54108 |
| 2010200O16Rik | -0.25159 |
| 2010301N04Rik | 0.92443 |
| 2010305A19Rik | 1.65112 |
| 2010305C02Rik | -0.31383 |
| 2010305C02Rik | 1.71352 |
| 2010308M01Rik | 0.94913 |
| 2010311D03Rik | -0.56593 |
| 2010316F05Rik | 1.03445 |
| 2010321M09Rik | -0.05555 |
| 2010321M09Rik | -0.35841 |
| 2200001I15Rik | 1.42366 |
| 2210009G21Rik | -0.38123 |
| 2210009G21Rik | -0.69433 |
| 2210009G21Rik | 0.53151 |
| 2210010B09Rik | -0.00195 |
| 2210010L05Rik | 3.8114 |
| 2210012G02Rik | -0.45666 |
| 2210012G02Rik | -0.01151 |
| 2210012G02Rik | -0.75044 |
| 2210012G02Rik | 0.29491 |
| 2210016L21Rik | 0.01199 |
| 2210016L21Rik | 4.48422 |
| 2210016L21Rik | -0.48838 |
| 2210021J22Rik | 2.78717 |
| 2210023G05Rik | -0.47307 |
| 2210023G05Rik | 0.81637 |
| 2210407C18Rik | -0.62098 |
| 2210412D01Rik | 0.2221 |
| 2210417D09Rik | 0.90292 |
| 2210418O10Rik | 1.72337 |
| 2300002D11Rik | 0.68865 |
| 2300002D11Rik | 0.03371 |
| 2300002M23Rik | -0.6073 |
| 2300005B03Rik | -0.76265 |
| 2310001A20Rik | -0.59461 |
| 2310002B06Rik | 0.02492 |
| 2310003C23Rik | 0.27618 |
| 2310003F16Rik | -0.2543 |
| 2310003H01Rik | 0.30088 |
| 2310003L22Rik | 1.19149 |
| 2310003L22Rik | -0.66837 |
| 2310004I24Rik | -0.57359 |
| 2310005E10Rik | -0.10228 |
| 2310005E10Rik | -0.57359 |
| 2310005G13Rik | 0.71856 |
| 2310005N01Rik | 0.30946 |
| 2310005N03Rik | 2.06117 |
| 2310005P05Rik | -0.02964 |
| 2310006J04Rik | -0.23227 |
| 2310007A19Rik | -0.39824 |
| 2310007B03Rik | -0.46894 |
| 2310007F21Rik | -0.30345 |
| 2310007F21Rik | -0.07235 |
| 2310008H04Rik | -0.55908 |
| 2310008H09Rik | -0.74671 |
| 2310008H09Rik | -0.49826 |
| 2310008M10Rik | 2.04161 |
| 2310009E04Rik | 0.27495 |
| 2310010B21Rik | -0.28078 |
| 2310010M24Rik | 2.22995 |
| 2310011J03Rik | 0.17117 |
| 2310014L17Rik | -0.49826 |
| 2310016C08Rik | -0.61855 |
| 2310016C08Rik | -0.81218 |
| 2310016C16Rik | 1.68364 |
| 2310016C16Rik | 2.33423 |
| 2310016C16Rik | 1.44153 |
| 2310016E02Rik | 1.63845 |
| 2310016M24Rik | 1.60835 |
| 2310021P13Rik | 0.4531 |
| 2310022A10Rik | -0.6081 |
| 2310022B05Rik | 1.72438 |
| 2310031L18Rik | 0.80854 |
| 2310031L18Rik | 0.61439 |
| 2310033E01Rik | -0.5448 |
| 2310033K02Rik | -0.51039 |
| 2310033P09Rik | -0.44388 |
| 2310035C23Rik | -0.26304 |
| 2310035C23Rik | -0.33081 |
| 2310035K24Rik | -0.5954 |
| 2310036D22Rik | 1.56093 |
| 2310036O22Rik | -0.42143 |
| 2310037I24Rik | 1.253 |
| 2310037I24Rik | 1.23252 |
| 2310038H17Rik | -0.2603 |
| 2310039E09Rik | 0.36875 |
| 2310039H08Rik | 2.76044 |
| 2310040C09Rik | 0.27342 |
| 2310042D19Rik | 0.12735 |
| 2310044G17Rik | -0.51833 |
| 2310044H10Rik | 1.59761 |
| 2310045A20Rik | -0.61774 |
| 2310046K01Rik | 0.26604 |
| 2310046O06Rik | -0.43523 |
| 2310047C04Rik | -0.37873 |
| 2310047D13Rik | -0.42077 |
| 2310047D13Rik | 0.66455 |
| 2310047M10Rik | 1.71696 |
| 2310047M10Rik | 0.00668 |
| 2310047O13Rik | -0.27743 |
| 2310056P07Rik | 0.95746 |
| 2310057J16Rik | 1.16704 |
| 2310058J06Rik | -0.75981 |
| 2310061C15Rik | -0.59619 |
| 2310061F22Rik | -0.69521 |
| 2310061F22Rik | -0.61936 |
| 2310065K24Rik | 3.35609 |
| 2310065K24Rik | -0.03949 |
| 2310066E14Rik | -0.62993 |
| 2310067B10Rik | -0.49049 |
| 2310076L09Rik | 0.84048 |
| 2310076L09Rik | -0.7005 |
| 2310079F23Rik | -0.48 |
| 2310079N02Rik | 1.34027 |
| 2400001E08Rik | 1.80389 |
| 2400003C14Rik | 1.84179 |
| 2400003C14Rik | -0.39127 |
| 2400003C14Rik | -0.63075 |
| 2400006N03Rik | 0.37068 |
| 2400010D15Rik | 1.82649 |
| 2410001C21Rik | -0.2791 |
| 2410001C21Rik | 1.10846 |
| 2410001C21Rik | 1.83978 |
| 2410002F23Rik | -0.52196 |
| 2410002F23Rik | 3.60839 |
| 2410002F23Rik | -0.44857 |
| 2410002I01Rik | 2.57633 |
| 2410002O22Rik | -0.02921 |
| 2410003P15Rik | 0.10151 |
| 2410004A20Rik | 1.54395 |
| 2410004B18Rik | 1.21246 |
| 2410004L22Rik | -0.44187 |
| 2410005O16Rik | 1.15679 |
| 2410008K03Rik | 0.33415 |
| 2410014A08Rik | 1.05365 |
| 2410014A08Rik | -0.54405 |
| 2410014A08Rik | 0.96312 |
| 2410014A08Rik | 0.53774 |
| 2410015M20Rik | 0.71724 |
| 2410015N17Rik | 2.14804 |
| 2410016F19Rik | 1.08849 |
| 2410016F19Rik | 1.37878 |
| 2410016O06Rik | -0.2478 |
| 2410017P07Rik | 1.28929 |
| 2410018C17Rik | -0.83542 |
| 2410018G20Rik | 0.54802 |
| 2410018M08Rik | 0.14689 |
| 2410019A14Rik | -0.49542 |
| 2410019G02Rik | 2.00237 |
| 2410022L05Rik | 0.64759 |
| 2410025L10Rik | 0.7413 |
| 2410042D21Rik | -0.62179 |
| 2410042D21Rik | 0.36737 |
| 2410066E13Rik | -0.52561 |
| 2410081M15Rik | 1.01128 |
| 2410091C18Rik | -0.7076 |
| 2410116G06Rik | 2.35618 |
| 2410127E18Rik | -0.50466 |
| 2410127L17Rik | 1.52516 |
| 2410127L17Rik | -0.66327 |
| 2410129H14Rik | 0.95804 |
| 2410137M14Rik | 2.19995 |
| 2410146L05Rik | -0.45734 |
| 2410166I05Rik | -0.30978 |
| 2500002L14Rik | 0.5611 |
| 2500003M10Rik | -0.19278 |
| 2500003M10Rik | -0.76929 |
| 2500003M10Rik | -0.8202 |
| 2510002A14Rik | 0.31912 |
| 2510003E04Rik | -0.81218 |
| 2510006D16Rik | -0.56669 |
| 2510009E07Rik | -0.84261 |
| 2510010F15Rik | -0.30574 |
| 2510012J08Rik | 0.95848 |
| 2510039O18Rik | 0.13948 |
| 2510040D07Rik | -0.59461 |
| 2510048L02Rik | -0.19993 |
| 2510049I19Rik | -0.9089 |
| 2600001B17Rik | -0.62585 |
| 2600009E05Rik | -0.02624 |
| 2600009E05Rik | -0.60332 |
| 2600010E01Rik | -0.6565 |
| 2600011E07Rik | -0.01234 |
| 2600011E07Rik | 1.9415 |
| 2610002J02Rik | -0.57977 |
| 2610002M06Rik | -0.61613 |
| 2610003J06Rik | -0.36759 |
| 2610016F04Rik | -0.78562 |
| 2610019A05Rik | 1.40311 |
| 2610019P18Rik | -0.61855 |
| 2610020C11Rik | 3.24059 |
| 2610020H08Rik | -0.64146 |
| 2610020H08Rik | 4.11246 |
| 2610020H08Rik | -0.43523 |
| 2610020O08Rik | 2.2745 |
| 2610021K21Rik | 2.25751 |
| 2610021K21Rik | 1.03632 |
| 2610024G14Rik | -0.18116 |
| 2610024G14Rik | 1.19037 |
| 2610024G14Rik | -0.99605 |
| 2610027C15Rik | 1.99938 |
| 2610027C15Rik | -0.90668 |
| 2610027L16Rik | 0.96341 |
| 2610028A01Rik | 1.29415 |
| 2610028H24Rik | -0.31267 |
| 2610029G23Rik | -0.81518 |
| 2610029G23Rik | 1.47569 |
| 2610034E13Rik | -0.44187 |
| 2610034E13Rik | 1.57322 |
| 2610034M16Rik | 2.18737 |
| 2610036D13Rik | 0.79718 |
| 2610039C10Rik | 0.42679 |
| 2610042L04Rik | -0.54929 |
| 2610044O15Rik | -0.39253 |
| 2610101N10Rik | 0.62698 |
| 2610101N10Rik | -0.82524 |
| 2610103J23Rik | -0.54182 |
| 2610110G12Rik | 1.96853 |
| 2610200G18Rik | -0.45666 |
| 2610204K14Rik | 2.56815 |
| 2610204L23Rik | -0.72105 |
| 2610204M08Rik | -0.86559 |
| 2610205E22Rik | 3.33802 |
| 2610207I05Rik | -0.17415 |
| 2610208M17Rik | -0.20507 |
| 2610209A20Rik | -0.44254 |
| 2610209A20Rik | 2.03059 |
| 2610209M04Rik | -0.59777 |
| 2610301B20Rik | 0.36043 |
| 2610301F02Rik | 3.51057 |
| 2610304G08Rik | -0.48978 |
| 2610305D13Rik | -0.78175 |
| 2610318N02Rik | -0.73655 |
| 2610507B11Rik | -0.55757 |
| 2610507B11Rik | -0.6523 |
| 2610510B01Rik | -0.02285 |
| 2610510J17Rik | -0.29944 |
| 2610524A10Rik | -0.45938 |
| 2610524H06Rik | 1.60555 |
| 2610528E23Rik | -0.54331 |
| 2610528J11Rik | 1.09678 |
| 2610528O22Rik | 2.64836 |
| 2700007P21Rik | -0.01234 |
| 2700007P21Rik | -0.34691 |
| 2700038C09Rik | 2.52923 |
| 2700038C09Rik | 1.30975 |
| 2700050L05Rik | -0.89133 |
| 2700055A20Rik | 0.84345 |
| 2700060E02Rik | 1.36898 |
| 2700062C07Rik | -0.42929 |
| 2700062C07Rik | 1.71435 |
| 2700078K21Rik | -0.47446 |
| 2700081O15Rik | 2.0235 |
| 2700087H15Rik | 0.7272 |
| 2700094K13Rik | -0.45328 |
| 2700097O09Rik | 1.28452 |
| 2810002N01Rik | 0.67767 |
| 2810002O09Rik | -0.59147 |
| 2810003C17Rik | -0.17316 |
| 2810004N20Rik | -0.84364 |
| 2810004N23Rik | 0.52781 |
| 2810008M24Rik | 0.76096 |
| 2810021B07Rik | 1.31341 |
| 2810021J22Rik | 0.76868 |
| 2810021J22Rik | -0.71925 |
| 2810022L02Rik | -0.63568 |
| 2810022L02Rik | -0.62504 |
| 2810022L02Rik | -0.76549 |
| 2810030E01Rik | 2.07714 |
| 2810046L04Rik | -0.42535 |
| 2810046M22Rik | -0.60093 |
| 2810403A07Rik | 0.67351 |
| 2810405J04Rik | -0.68908 |
| 2810405K02Rik | -0.2061 |
| 2810408A11Rik | -0.25104 |
| 2810408M09Rik | 1.84772 |
| 2810410C14Rik | -0.88807 |
| 2810410P22Rik | 1.48426 |
| 2810410P22Rik | -0.93704 |
| 2810417H13Rik | -0.81618 |
| 2810422O20Rik | 2.31524 |
| 2810428I15Rik | -0.38561 |
| 2810432D09Rik | 0.02532 |
| 2810432L12Rik | 0.84756 |
| 2810439F02Rik | 0.19181 |
| 2810439F02Rik | -0.37438 |
| 2810441K11Rik | -0.21492 |
| 2810452K22Rik | -0.80423 |
| 2810453I06Rik | -0.71206 |
| 2810453I06Rik | 0.81841 |
| 2810470D21Rik | -0.73105 |
| 2810485I05Rik | -0.54704 |
| 2900006K08Rik | 1.44213 |
| 2900010M23Rik | 0.7468 |
| 2900011O08Rik | 2.75108 |
| 2900011O08Rik | 1.99919 |
| 2900024C23Rik | -0.66327 |
| 2900024O10Rik | -0.75699 |
| 2900026A02Rik | 0.04673 |
| 2900046G09Rik | -0.58833 |
| 2900062L11Rik | -0.30345 |
| 2900064A13Rik | 0.37041 |
| 2900073G15Rik | -0.15785 |
| 2900092C05Rik | -0.94623 |
| 3000004C01Rik | -0.83747 |
| 3000004C01Rik | -0.76265 |
| 3000004C01Rik | -0.58599 |
| 3010003L21Rik | -0.78854 |
| 3010026O09Rik | 1.48957 |
| 3110001A13Rik | -0.64728 |
| 3110001D03Rik | -0.84882 |
| 3110004L20Rik | -0.81318 |
| 3110007F17Rik | -0.87837 |
| 3110009E18Rik | 2.16961 |
| 3110023B02Rik | -0.33913 |
| 3110035E14Rik | 0.65348 |
| 3110035E14Rik | 1.53429 |
| 3110037I16Rik | 0.85783 |
| 3110040N11Rik | 0.2084 |
| 3110040N11Rik | 0.38055 |
| 3110043J09Rik | -0.80919 |
| 3110043J09Rik | 1.77416 |
| 3110043J09Rik | -0.70227 |
| 3110048E14Rik | -0.33913 |
| 3110049J23Rik | -0.66667 |
| 3110050N22Rik | -0.38185 |
| 3110050N22Rik | 1.01279 |
| 3110056O03Rik | -0.42077 |
| 3110057O12Rik | 1.4742 |
| 3110057O12Rik | -0.16276 |
| 3110070M22Rik | -0.11481 |
| 3110082I17Rik | 0.46933 |
| 3200001I04Rik | 0.72007 |
| 3200002M19Rik | -0.20972 |
| 3200002M19Rik | 0.45888 |
| 3300001G02Rik | -0.53441 |
| 3300001P08Rik | 0.55571 |
| 3300001P08Rik | -0.00113 |
| 3300001P08Rik | 0.1605 |
| 3300002I08Rik | -0.02412 |
| 3321401G04Rik | -0.01863 |
| 3322402L07Rik | -0.65819 |
| 3632451O06Rik | 1.36852 |
| 3830402I07Rik | 0.60653 |
| 3830406C13Rik | 0.83118 |
| 3830406C13Rik | -0.93134 |
| 3830406C13Rik | 2.84961 |
| 3830408P04Rik | 2.50622 |
| 3930401K13Rik | -0.45734 |
| 4121402D02Rik | 2.09482 |
| 4121402D02Rik | -0.2376 |
| 4430402I18Rik | 1.00964 |
| 4432405B04Rik | 0.36405 |
| 4432416J03Rik | 0.13451 |
| 4631426J05Rik | 0.74093 |
| 4631427C17Rik | -0.33793 |
| 4632404H12Rik | 1.36273 |
| 4632404H12Rik | 3.45181 |
| 4632404H12Rik | -0.67608 |
| 4632404H22Rik | -0.61693 |
| 4632411B12Rik | -0.75417 |
| 4632411B12Rik | 0.21299 |
| 4632412N22Rik | 2.77178 |
| 4632415K11Rik | 1.50253 |
| 4632417K18Rik | 1.53118 |
| 4632417N05Rik | 0.66635 |
| 4632419K20Rik | 1.42296 |
| 4632434I11Rik | 1.49559 |
| 4732415M23Rik | 0.76384 |
| 4732418C07Rik | -0.20404 |
| 4732418C07Rik | 0.27709 |
| 4732418C07Rik | -0.69171 |
| 4732429D16Rik | 2.05352 |
| 4732456N10Rik | -0.42339 |
| 4732471D19Rik | -0.32024 |
| 4732473B16Rik | -0.58288 |
| 4732479N06Rik | -0.95318 |
| 4732479N06Rik | -0.06259 |
| 4732496O08Rik | 1.29556 |
| 4733401H18Rik | 0.30503 |
| 4831426I19Rik | 0.40076 |
| 4833418A01Rik | -0.08946 |
| 4833420G17Rik | 0.37316 |
| 4833421E05Rik | -0.62341 |
| 4833424O15Rik | 1.46159 |
| 4833424O15Rik | -0.68213 |
| 4833426J09Rik | -0.44857 |
| 4833426J09Rik | -0.43258 |
| 4833427G06Rik | 1.05155 |
| 4833436C18Rik | -0.26359 |
| 4833439L19Rik | -0.43523 |
| 4833439L19Rik | 1.08571 |
| 4833439L19Rik | 1.75518 |
| 4833442J19Rik | -0.06524 |
| 4833446K15Rik | -0.77502 |
| 4921501E09Rik | -0.10366 |
| 4921504E06Rik | -0.79341 |
| 4921504I05Rik | 2.50181 |
| 4921505C17Rik | -0.44722 |
| 4921505C17Rik | -0.5011 |
| 4921507P07Rik | 1.71083 |
| 4921511H03Rik | 0.82431 |
| 4921511H03Rik | 0.03847 |
| 4921511K06Rik | 0.36238 |
| 4921511K06Rik | 0.14865 |
| 4921515J06Rik | 0.01767 |
| 4921517D21Rik | -0.53073 |
| 4921517D22Rik | 1.98912 |
| 4921520G13Rik | 2.05744 |
| 4921521F21Rik | 2.1438 |
| 4921521F21Rik | 0.27862 |
| 4921523A10Rik | 0.46735 |
| 4921523A10Rik | -0.77119 |
| 4921523A10Rik | -0.39951 |
| 4921525H12Rik | -0.33793 |
| 4921528G01Rik | -0.68908 |
| 4921528I01Rik | -0.6523 |
| 4921530G04Rik | -0.4339 |
| 4921530L21Rik | -0.67265 |
| 4921530L21Rik | 1.24622 |
| 4921536K21Rik | -0.44121 |
| 4921537P18Rik | 0.61206 |
| 4922502D21Rik | 0.00381 |
| 4922503N01Rik | -0.68647 |
| 4922503N01Rik | 0.68689 |
| 4922505E12Rik | -0.2478 |
| 4922505G16Rik | 3.9655 |
| 4922505G16Rik | -0.64478 |
| 4930401F20Rik | -0.71206 |
| 4930402H24Rik | 2.31829 |
| 4930403C10Rik | 0.28378 |
| 4930403L05Rik | 0.15737 |
| 4930403O06Rik | 0.14513 |
| 4930404H21Rik | 1.10499 |
| 4930404N11Rik | -0.55908 |
| 4930408O21Rik | -0.81618 |
| 4930408O21Rik | -0.56975 |
| 4930412F15Rik | 0.13522 |
| 4930415L06Rik | -0.71385 |
| 4930418G15Rik | 0.17458 |
| 4930427A07Rik | -0.00195 |
| 4930428D18Rik | 0.05221 |
| 4930428E23Rik | 1.96494 |
| 4930430D24Rik | -0.43788 |
| 4930430E16Rik | -0.64812 |
| 4930430E16Rik | -0.41169 |
| 4930431L04Rik | -0.47101 |
| 4930432H15Rik | -0.31209 |
| 4930432K21Rik | -0.60252 |
| 4930432O21Rik | 0.42938 |
| 4930432O21Rik | 0.0483 |
| 4930438O05Rik | -0.30402 |
| 4930438O05Rik | -0.61693 |
| 4930449I24Rik | 0.38625 |
| 4930451C15Rik | -0.40079 |
| 4930451I11Rik | 0.04673 |
| 4930452B06Rik | 0.18745 |
| 4930453N24Rik | 0.61248 |
| 4930455C21Rik | -0.73105 |
| 4930455F23Rik | 0.00422 |
| 4930467M19Rik | 3.96431 |
| 4930470P17Rik | -0.68299 |
| 4930473A06Rik | 1.97855 |
| 4930473A06Rik | -0.66157 |
| 4930473A06Rik | -0.66667 |
| 4930474N05Rik | 1.06083 |
| 4930478A21Rik | 2.29357 |
| 4930481F22Rik | 0.05648 |
| 4930481F22Rik | 0.33901 |
| 4930481M05 | -0.76076 |
| 4930486L24Rik | 1.29677 |
| 4930502E18Rik | 1.09352 |
| 4930503B20Rik | 2.4558 |
| 4930503L19Rik | -0.64395 |
| 4930504E06Rik | -0.31325 |
| 4930504E06Rik | 3.32064 |
| 4930504O13Rik | -0.42143 |
| 4930506M07Rik | 0.41898 |
| 4930507C10Rik | -0.58833 |
| 4930511I11Rik | 1.6201 |
| 4930511J11Rik | -0.46757 |
| 4930515G01Rik | 1.47643 |
| 4930518C23Rik | -0.03691 |
| 4930519F16Rik | 3.84933 |
| 4930519G04Rik | -0.35355 |
| 4930519N13Rik | 0.97775 |
| 4930519N16Rik | -0.13751 |
| 4930522H14Rik | -0.45126 |
| 4930524B15Rik | 2.40711 |
| 4930524B15Rik | 0.77189 |
| 4930524E20Rik | -0.26249 |
| 4930526D03Rik | -0.72922 |
| 4930535B03Rik | 1.81552 |
| 4930535E21Rik | -0.55229 |
| 4930538K18Rik | 0.1504 |
| 4930539E08Rik | -0.62422 |
| 4930540L03Rik | -0.5759 |
| 4930544G11Rik | 0.18273 |
| 4930544G21Rik | 2.06029 |
| 4930547C10Rik | 2.07274 |
| 4930547N16Rik | -0.46483 |
| 4930549C01Rik | -0.13704 |
| 4930550C14Rik | -0.44722 |
| 4930550L24Rik | 1.83839 |
| 4930555G01Rik | 0.75645 |
| 4930555I21Rik | 0.55953 |
| 4930562D19Rik | -0.25757 |
| 4930562D19Rik | -0.11201 |
| 4930563D23Rik | -0.76929 |
| 4930563P21Rik | 0.91014 |
| 4930570C03Rik | -0.47653 |
| 4930572J05Rik | 2.53157 |
| 4930572J05Rik | 0.39542 |
| 4930572J05Rik | 0.42912 |
| 4930573I19Rik | -0.26469 |
| 4930578I06Rik | 2.63324 |
| 4930579C15Rik | 2.58189 |
| 4930579E17Rik | 0.48673 |
| 4930579E17Rik | -0.58677 |
| 4930579J09Rik | -0.17366 |
| 4930583H14Rik | 0.1525 |
| 4930590J08Rik | 0.25923 |
| 4930595M18Rik | 0.73446 |
| 4930596D02Rik | -0.64812 |
| 4930599N23Rik | 0.48479 |
| 4931406B18Rik | -0.6523 |
| 4931406C07Rik | -0.54554 |
| 4931406H21Rik | -0.4027 |
| 4931406I20Rik | 1.18288 |
| 4931406I20Rik | 0.35764 |
| 4931406P16Rik | 2.26203 |
| 4931407G18Rik | -0.42797 |
| 4931408A02Rik | -0.84364 |
| 4931408C20Rik | 2.29774 |
| 4931408C20Rik | 0.04986 |
| 4931409K22Rik | 1.69303 |
| 4931417E11Rik | -0.78659 |
| 4931417G12Rik | -0.77694 |
| 4931428L18Rik | 1.6505 |
| 4931432M23Rik | -0.38812 |
| 4931433A01Rik | -0.54554 |
| 4931440B09Rik | 2.15264 |
| 4931440F15Rik | -0.66667 |
| 4931440F15Rik | -0.53073 |
| 4931440L10Rik | 0.40661 |
| 4932409I22Rik | 2.62415 |
| 4932411E22Rik | -0.67867 |
| 4932411G14Rik | 0.04163 |
| 4932412H11Rik | 2.37669 |
| 4932413O14Rik | 2.29804 |
| 4932414J04Rik | 0.61269 |
| 4932414N04Rik | 0.39863 |
| 4932418E24Rik | 0.32522 |
| 4932431H17Rik | 0.74772 |
| 4932431H17Rik | -0.6081 |
| 4932441K18Rik | 3.1424 |
| 4932441K18Rik | 0.38436 |
| 4932442K08Rik | 0.40608 |
| 4932702K14Rik | 0.46685 |
| 4933400A11Rik | -0.43788 |
| 4933400C05Rik | -0.21387 |
| 4933400E14Rik | 1.35776 |
| 4933402J07Rik | 0.19848 |
| 4933402N03Rik | -0.63321 |
| 4933402P03Rik | 0.75735 |
| 4933403G14Rik | -0.78175 |
| 4933404M02Rik | -0.56593 |
| 4933405O20Rik | -0.24564 |
| 4933406M09Rik | 0.7597 |
| 4933407N01Rik | 0.77491 |
| 4933407N01Rik | -0.58055 |
| 4933407N01Rik | 0.55998 |
| 4933407P14Rik | -0.22224 |
| 4933407P14Rik | 2.74188 |
| 4933407P14Rik | 1.08546 |
| 4933409N07Rik | 1.46536 |
| 4933411G06Rik | -0.21076 |
| 4933411K20Rik | -0.20352 |
| 4933412E14Rik | -0.24295 |
| 4933413G11Rik | 0.02933 |
| 4933415F23Rik | 3.52798 |
| 4933416C03Rik | -0.57822 |
| 4933417A18Rik | -0.64812 |
| 4933417E01Rik | -0.50466 |
| 4933421E11Rik | -0.57668 |
| 4933421E11Rik | 1.00619 |
| 4933421E11Rik | 1.34515 |
| 4933422H20Rik | -0.71206 |
| 4933424B01Rik | -0.34571 |
| 4933424B01Rik | -0.32492 |
| 4933424B01Rik | -0.58288 |
| 4933425O20Rik | 1.54694 |
| 4933426M11Rik | -0.39001 |
| 4933427D14Rik | 0.70641 |
| 4933428G09Rik | 3.70764 |
| 4933428G20Rik | -0.71117 |
| 4933429E10Rik | 2.35739 |
| 4933429E10Rik | -0.3092 |
| 4933430I17Rik | 0.6251 |
| 4933434E20Rik | -0.55379 |
| 4933434E20Rik | -0.72105 |
| 4933434E20Rik | -0.30805 |
| 4933434I06Rik | 0.16808 |
| 4933436I01Rik | 0.22694 |
| 4933437F05Rik | 1.34094 |
| 4933439C20Rik | 0.48673 |
| 4933440H19Rik | 0.93835 |
| 5031414D18Rik | -0.6097 |
| 5031414D18Rik | -0.31907 |
| 5033411D12Rik | -0.5322 |
| 5033414D02Rik | -0.5606 |
| 5033414K04Rik | -0.16227 |
| 5133400G04Rik | 1.57143 |
| 5133400G04Rik | -0.68126 |
| 5133400G04Rik | 1.88696 |
| 5133401N09Rik | -0.50895 |
| 5230400G24Rik | -0.63321 |
| 5230400G24Rik | 1.56725 |
| 5330401P04Rik | 1.18243 |
| 5330417C22Rik | 0.80785 |
| 5330431N19Rik | -0.6856 |
| 5330431N19Rik | 0.16223 |
| 5330431N19Rik | -0.63321 |
| 5330437I02Rik | 2.43537 |
| 5330439C02Rik | 0.37123 |
| 5330439J01Rik | -0.62912 |
| 5330439J01Rik | -0.07325 |
| 5430407P10Rik | -0.38686 |
| 5430432M24Rik | -0.50895 |
| 5430432M24Rik | -0.66496 |
| 5430432M24Rik | 1.07528 |
| 5430435G22Rik | 3.36212 |
| 5430437P03Rik | 1.25416 |
| 5530601I19Rik | -0.64812 |
| 5630401D24Rik | -0.19737 |
| 5630401D24Rik | -0.65146 |
| 5630401D24Rik | 1.62419 |
| 5730403M16Rik | -0.26634 |
| 5730409E04Rik | 1.4032 |
| 5730410E15Rik | 0.65773 |
| 5730410E15Rik | 0.73483 |
| 5730410I19Rik | -0.79439 |
| 5730410I19Rik | 0.6394 |
| 5730410I19Rik | -0.37253 |
| 5730419I09Rik | -0.44857 |
| 5730437N04Rik | -0.69521 |
| 5730446C15Rik | -0.40912 |
| 5730449L18Rik | 1.60942 |
| 5730449L18Rik | -0.44588 |
| 5730449L18Rik | -0.41622 |
| 5730453I16Rik | 0.64881 |
| 5730453I16Rik | -0.11809 |
| 5730469M10Rik | 1.68509 |
| 5730472N09Rik | 0.38109 |
| 5730494M16Rik | -0.67094 |
| 5730494N06Rik | -0.03992 |
| 5730502D15Rik | 2.57411 |
| 5730507A09Rik | 2.24045 |
| 5730507A09Rik | -0.61451 |
| 5730528L13Rik | 1.26066 |
| 5730589K01Rik | 0.77527 |
| 5730593F17Rik | 0.20378 |
| 5730596K20Rik | -0.62017 |
| 5730596K20Rik | 0.05493 |
| 5730596K20Rik | -0.20198 |
| 5730596K20Rik | -0.06702 |
| 5830403L16Rik | 1.19991 |
| 5830404H04Rik | 0.8505 |
| 5830405N20Rik | -0.56212 |
| 5830415F09Rik | -0.70493 |
| 5830415L20Rik | -0.46757 |
| 5830417C01Rik | -0.39064 |
| 5830426I05Rik | -0.43324 |
| 5830427D03Rik | -0.26524 |
| 5830434P21Rik | -0.27187 |
| 5830435K17Rik | -0.69258 |
| 5830457O10Rik | -0.58288 |
| 5830457O10Rik | -0.75887 |
| 5830457O10Rik | -0.39443 |
| 5830467P10Rik | -0.41363 |
| 5830468K18Rik | 3.59819 |
| 5830472M02Rik | -0.26579 |
| 5830472M02Rik | 0.57245 |
| 5830472M02Rik | 0.43658 |
| 5930416I19Rik | -0.62993 |
| 5930431H10 | -0.71745 |
| 5930434B04Rik | -0.76644 |
| 5930434B04Rik | 0.02813 |
| 5930434B04Rik | -0.1209 |
| 6030405A18Rik | -0.3976 |
| 6030408B16Rik | -0.45396 |
| 6030408B16Rik | 0.12268 |
| 6030419C18Rik | -0.6523 |
| 6030429G01Rik | 0.39809 |
| 6030443O07Rik | 0.65773 |
| 6030446N20Rik | 0.11507 |
| 6030458C11Rik | 0.75699 |
| 6230409E13Rik | 2.28056 |
| 6230409E13Rik | 0.52198 |
| 6230416J20Rik | -0.66157 |
| 6230427J02Rik | 0.69001 |
| 6330403K07Rik | 0.75118 |
| 6330406I15Rik | -0.09128 |
| 6330406I15Rik | 0.58322 |
| 6330407J23Rik | -0.06039 |
| 6330408A02Rik | -0.61855 |
| 6330409N04Rik | -0.51543 |
| 6330416G13Rik | -0.31151 |
| 6330439K17Rik | 1.55601 |
| 6330442E10Rik | 0.12664 |
| 6330442E10Rik | -0.77406 |
| 6330500D04Rik | 2.95858 |
| 6330503C03Rik | -0.77215 |
| 6330503K22Rik | -0.48628 |
| 6330503K22Rik | -0.66242 |
| 6330503K22Rik | -0.65062 |
| 6330505N24Rik | 3.32986 |
| 6330512M04Rik | -0.4621 |
| 6330534C20Rik | -0.33496 |
| 6330548G22Rik | -0.56975 |
| 6330569M22Rik | -0.6097 |
| 6330577E15Rik | -0.69785 |
| 6330578E17Rik | 0.7106 |
| 6332401O19Rik | -0.67265 |
| 6430411K18Rik | 2.38995 |
| 6430502M16Rik | 0.76384 |
| 6430510M02Rik | 1.58115 |
| 6430517E21Rik | -0.76454 |
| 6430526N21Rik | -0.53811 |
| 6430527G18Rik | 1.17946 |
| 6430527G18Rik | 0.36543 |
| 6430531B16Rik | -0.6565 |
| 6430537H07Rik | -0.5278 |
| 6430537H07Rik | 0.97061 |
| 6430548M08Rik | 4.55228 |
| 6430550H21Rik | 1.94608 |
| 6430571L13Rik | -0.15785 |
| 6430573F11Rik | 0.71686 |
| 6430598A04Rik | 0.36792 |
| 6430604K15Rik | 2.44135 |
| 6430706D22Rik | -0.51183 |
| 6530404N21Rik | -0.10228 |
| 6530406M24Rik | -0.66752 |
| 6530418L21Rik | 2.58 |
| 6530418L21Rik | 1.88149 |
| 6530418L21Rik | 3.3064 |
| 6530421E24Rik | -0.48348 |
| 6620401K05Rik | -0.38623 |
| 6720456B07Rik | -0.66582 |
| 6720456B07Rik | -0.4027 |
| 6720456H20Rik | -0.77886 |
| 6720456H20Rik | 0.22307 |
| 6720458F09Rik | 0.45662 |
| 6720463M24Rik | -0.09311 |
| 6720467C03Rik | 2.24218 |
| 6720467C03Rik | 0.22791 |
| 6820408C15Rik | 0.08097 |
| 7420416P09Rik | -0.65146 |
| 7530404M11Rik | -0.45058 |
| 8030411F24Rik | 0.01767 |
| 8030451F13Rik | -0.31849 |
| 8030451F13Rik | 1.56387 |
| 8030451F13Rik | 1.38209 |
| 8430408G22Rik | 0.34696 |
| 8430410A17Rik | -0.1872 |
| 8430410K20Rik | 0.09185 |
| 8430415E04Rik | -0.5821 |
| 8430415E04Rik | -0.41105 |
| 8430419L09Rik | -0.65146 |
| 8430427H17Rik | 1.05927 |
| 8430432M10Rik | -0.14039 |
| 9030025P20Rik | 0.29222 |
| 9030025P20Rik | -0.43523 |
| 9030025P20Rik | 0.04791 |
| 9030205A07Rik | -0.58599 |
| 9030221M09Rik | 1.04905 |
| 9030224M15Rik | 0.96946 |
| 9030227G01Rik | -0.51327 |
| 9030409G11Rik | 0.10629 |
| 9030418K01Rik | 0.58541 |
| 9030425E11Rik | -0.03519 |
| 9030607L17Rik | 3.15767 |
| 9030607L17Rik | -0.42863 |
| 9030611O19Rik | 0.26481 |
| 9030612M13Rik | -0.64229 |
| 9030617O03Rik | 0.63611 |
| 9030624J02Rik | 0.11325 |
| 9030625A04Rik | 0.84739 |
| 9130005N14Rik | 0.20642 |
| 9130011E15Rik | 0.53911 |
| 9130011J15Rik | -0.18518 |
| 9130014G24Rik | 0.3353 |
| 9130017N09Rik | -0.07369 |
| 9130206N08Rik | 1.66058 |
| 9130211I03Rik | 3.68936 |
| 9130213B05Rik | -0.65146 |
| 9130213B05Rik | 1.03992 |
| 9130218O11Rik | 0.41426 |
| 9130221H12Rik | 3.25193 |
| 9130227C08Rik | -0.77406 |
| 9130401M01Rik | -0.268 |
| 9130404D08Rik | -0.58833 |
| 9130404D08Rik | 1.52091 |
| 9130404D14Rik | -0.52342 |
| 9130422G05Rik | -0.4587 |
| 9230104L09Rik | -0.57205 |
| 9330129D05Rik | 1.04098 |
| 9330134C04Rik | -0.23121 |
| 9330176C04Rik | -0.64478 |
| 9330186A19Rik | 0.60951 |
| 9430015G10Rik | 0.64942 |
| 9430015G10Rik | 3.72091 |
| 9430023L20Rik | 0.71686 |
| 9430025C20Rik | -0.22803 |
| 9430028L06Rik | 0.05726 |
| 9430029K10Rik | 0.11543 |
| 9430031J16Rik | 0.45461 |
| 9430034D17Rik | 0.12986 |
| 9430057O19Rik | 0.8897 |
| 9430069J07Rik | 2.39123 |
| 9430077A04Rik | 0.20971 |
| 9430079M16Rik | -0.18066 |
| 9530015I07Rik | 0.75118 |
| 9530027K23Rik | 3.95112 |
| 9530058B02Rik | 0.71969 |
| 9530058B02Rik | 0.24828 |
| 9530068E07Rik | -0.54929 |
| 9530077C05Rik | 0.78128 |
| 9530096D07Rik | 1.29364 |
| 9530096D07Rik | -0.75044 |
| 9630015D15Rik | 0.58889 |
| 9630020C08Rik | 1.03258 |
| 9630028B13Rik | -0.36821 |
| 9630031F12Rik | -0.46552 |
| 9630050M13Rik | -0.49684 |
| 9630058J23Rik | 0.07072 |
| 9830163H01Rik | -0.53 |
| 9830169C18Rik | -0.51255 |
| 9930013L23Rik | -0.51543 |
| 9930021D14Rik | -0.66582 |
| 9930023K05Rik | -0.57668 |
| 9930023K05Rik | 0.79527 |
| 9930104L06Rik | -0.0922 |
| a | 1.64776 |
| A030003K21Rik | -0.55984 |
| A030007L17Rik | 0.29432 |
| A030007L17Rik | -0.62912 |
| A030007L17Rik | 0.27892 |
| A030007L17Rik | 1.36161 |
| A030009H04Rik | 1.91387 |
| A030010B05Rik | 0.68141 |
| A030012M09Rik | 3.09332 |
| A130010J15Rik | 0.25079 |
| A130038H09Rik | 1.69297 |
| A130042E20Rik | -0.81518 |
| A130090K04Rik | 0.52502 |
| A130092J06Rik | 0.31533 |
| A230050P20Rik | -0.45058 |
| A230051G13Rik | -0.50752 |
| A230054D04Rik | 1.96202 |
| A230067G21Rik | -0.55757 |
| A230069A22Rik | 1.1162 |
| A230074B11Rik | -0.17765 |
| A230074B11Rik | 0.12951 |
| A230074B11Rik | -0.25376 |
| A230106M15Rik | -0.20096 |
| A230106N23Rik | 2.05858 |
| A230107C01Rik | 1.28808 |
| A2bp1 | -0.7495 |
| A2bp1 | 1.0833 |
| A330021E22Rik | -0.71835 |
| A330021E22Rik | 2.03506 |
| A330049M08Rik | 1.23521 |
| A330080J22Rik | 0.69564 |
| A3galt2 | 2.17163 |
| A430005L14Rik | -0.7779 |
| A430005L14Rik | 1.74567 |
| A430005L14Rik | 2.01815 |
| A430005L14Rik | -0.57668 |
| A430033K04Rik | -0.48279 |
| A430079E08 | -0.2505 |
| A430083B19Rik | -0.35355 |
| A430083B19Rik | 1.31971 |
| A430107D22Rik | -0.37562 |
| A430107P09Rik | 1.41504 |
| A430110N23Rik | 0.03926 |
| A4galt | 0.09148 |
| A4gnt | 0.66074 |
| A530050D06Rik | -0.67436 |
| A530082C11Rik | 0.93924 |
| A530082C11Rik | 0.70161 |
| A530088H08Rik | -0.63321 |
| A530088H08Rik | 1.50084 |
| A530098C11Rik | 1.34162 |
| A630042L21Rik | -0.5954 |
| A630054L15Rik | -0.40912 |
| A630077B13Rik | -0.43722 |
| A630095E13Rik | 1.19408 |
| A730008H23Rik | 0.08774 |
| A730008L03Rik | 1.95772 |
| A730008L03Rik | -0.37006 |
| A730017C20Rik | 0.16361 |
| A730024F05Rik | -0.68386 |
| A730037C10Rik | -0.12751 |
| A730042J05Rik | 0.43812 |
| A730098D12Rik | 1.05103 |
| A830006F12Rik | 0.03172 |
| A830007P12Rik | -0.48908 |
| A830053O21Rik | -0.55606 |
| A830059I20Rik | -0.72467 |
| A830073O21Rik | -0.34032 |
| A830080D01Rik | 2.09512 |
| A830093I24Rik | -0.47515 |
| A930001N09Rik | -0.29545 |
| A930005I04Rik | -0.29716 |
| A930008G19Rik | 0.03926 |
| A930010I20Rik | 0.56779 |
| A930016P21Rik | 1.23671 |
| A930018P22Rik | 1.60317 |
| A930024E05Rik | 1.27807 |
| A930025D01Rik | -0.24081 |
| A930025D01Rik | 2.32534 |
| A930025D01Rik | -0.64812 |
| A930034L06Rik | -0.53 |
| A930038C07Rik | 0.47402 |
| A930041I02Rik | -0.59619 |
| AA388235 | -0.38123 |
| AA407270 | 0.81229 |
| AA407659 | 0.68219 |
| AA407659 | 0.65994 |
| AA408296 | 0.36071 |
| AA408296 | -0.76738 |
| AA408296 | -0.57977 |
| AA409316 | -0.61371 |
| AA415398 | -0.64478 |
| AA467197 | 1.2336 |
| AA536717 | -0.70849 |
| AA536717 | -0.6065 |
| AA536749 | -0.41882 |
| AA536749 | -0.268 |
| AA536749 | -0.48908 |
| AA536749 | 0.80614 |
| AA673488 | -0.26359 |
| AA691260 | 0.72139 |
| AA792892 | -0.56669 |
| AA881470 | -0.58521 |
| AA881470 | 1.29606 |
| AA960436 | 2.4029 |
| AA960436 | -0.40976 |
| Aaas | 1.56912 |
| Aaas | -0.30116 |
| Aacs | 0.88876 |
| Aadacl1 | -0.60093 |
| Aak1 | -0.06436 |
| Aak1 | 0.74387 |
| Aamp | -0.48488 |
| Aard | -0.11341 |
| Aars | -0.60252 |
| Aars2 | -0.51978 |
| Aarsd1 | 0.2308 |
| Aasdh | -0.40398 |
| Aatf | -0.67179 |
| Aatf | 1.24983 |
| Aatk | -0.5448 |
| AB112350 | 2.11734 |
| AB182283 | -0.1603 |
| AB182283 | 1.39709 |
| Abat | 3.0955 |
| Abat | -0.45126 |
| Abat | -0.51616 |
| Abca14 | -0.31616 |
| Abca14 | 1.47627 |
| Abca15 | -0.72831 |
| Abca15 | -0.42404 |
| Abca15 | -0.332 |
| Abca15 | 1.63727 |
| Abca17 | -0.16079 |
| Abca2 | -0.13895 |
| Abca3 | -0.77886 |
| Abca4 | -0.73196 |
| Abca4 | -0.12846 |
| Abca5 | 0.82481 |
| Abca6 | -0.50967 |
| Abca6 | -0.12751 |
| Abca7 | -0.56822 |
| Abca7 | 0.82616 |
| Abca8a | 0.57245 |
| Abca8b | -0.59225 |
| Abca9 | -0.45463 |
| Abca9 | -0.65482 |
| Abca9 | 1.44932 |
| Abcb11 | -0.76076 |
| Abcb1b | 1.24207 |
| Abcb4 | 1.16738 |
| Abcb6 | -0.71655 |
| Abcb8 | 0.90153 |
| Abcb8 | -0.71565 |
| Abcb8 | -0.55606 |
| Abcb9 | -0.79831 |
| Abcb9 | 0.21755 |
| Abcc1 | -0.6097 |
| Abcc10 | -0.67179 |
| Abcc10 | 1.40954 |
| Abcc12 | -0.22066 |
| Abcc3 | 0.61902 |
| Abcc5 | -0.71565 |
| Abcc6 | 2.45314 |
| Abcc8 | -0.47515 |
| Abcc9 | -0.19227 |
| Abcc9 | 0.08097 |
| Abcd1 | -0.44187 |
| Abcd2 | -0.58055 |
| Abcd2 | -0.43589 |
| Abcd3 | 0.85132 |
| Abcd4 | -0.38749 |
| Abcd4 | 0.13058 |
| Abce1 | -0.42535 |
| Abcf1 | -0.77886 |
| Abcf1 | 0.26512 |
| Abcf1 | 2.94959 |
| Abcf2 | 2.02751 |
| Abcf2 | -0.63981 |
| Abcf2 | 0.09297 |
| Abcf2 | -0.35659 |
| Abcf3 | -0.74393 |
| Abcg1 | 1.44684 |
| Abcg4 | 2.18653 |
| Abcg5 | -0.62912 |
| Abhd1 | 2.10911 |
| Abhd1 | 4.31723 |
| Abhd10 | -0.35659 |
| Abhd11 | 0.89142 |
| Abhd12 | -0.44321 |
| Abhd14a | 0.87438 |
| Abhd14b | 0.25455 |
| Abhd2 | 3.38106 |
| Abhd2 | 1.80557 |
| Abhd2 | 1.30268 |
| Abhd3 | 0.24954 |
| Abhd4 | 0.62217 |
| Abhd5 | -0.37624 |
| Abhd6 | -0.6226 |
| Abhd7 | 0.25767 |
| Abhd7 | 0.09446 |
| Abhd8 | -0.25976 |
| Abi1 | -0.39317 |
| Abi1 | -0.73655 |
| Abi2 | 0.14231 |
| Abi2 | -0.11574 |
| Abi2 | -0.63981 |
| Abi3 | 1.05391 |
| Abi3bp | 3.71367 |
| Abi3bp | 0.21266 |
| Abl1 | 0.98441 |
| Ablim1 | 0.96268 |
| Ablim2 | 0.3693 |
| Ablim2 | -0.52927 |
| Ablim3 | -0.73013 |
| Ablim3 | 0.88136 |
| Abp1 | 0.3693 |
| Abr | -0.58443 |
| Abr | -0.78272 |
| Abtb1 | 0.19582 |
| Acaa1a | 1.69636 |
| Acaa1b | -0.59304 |
| Acaa2 | 0.77898 |
| Acaa2 | -0.51471 |
| Acaa2 | 1.24877 |
| Acaa2 | 0.78795 |
| Acad10 | 0.6553 |
| Acad10 | -0.05118 |
| Acad8 | -0.56975 |
| Acad8 | 0.4371 |
| Acad9 | -0.38623 |
| Acad9 | 0.58671 |
| Acadl | 1.33295 |
| Acadl | 1.70414 |
| Acadm | -0.5553 |
| Acads | -0.6804 |
| Acads | 0.56266 |
| Acadsb | 2.94795 |
| Acadvl | -0.55833 |
| Acan | 4.07061 |
| Acat2 | -0.5176 |
| Acbd3 | -0.56136 |
| Acbd4 | 1.62496 |
| Acbd4 | 0.50238 |
| Acbd5 | -0.63733 |
| Acbd6 | 1.99727 |
| Accn1 | 1.7062 |
| Accn1 | -0.54854 |
| Accn2 | 0.98116 |
| Accn3 | 1.05783 |
| Accn3 | 0.88136 |
| Accs | -0.73013 |
| Acd | 0.20014 |
| Ace | 1.11301 |
| Ace | 2.13593 |
| Ache | -0.29602 |
| Acin1 | -0.6081 |
| Acly | 4.55924 |
| Acn9 | -0.63321 |
| Aco1 | -0.46757 |
| Aco2 | -0.61291 |
| Acot1 | -0.65314 |
| Acot1 | -0.64478 |
| Acot10 | -0.77311 |
| Acot11 | -0.27132 |
| Acot11 | -0.33793 |
| Acot2 | -0.62504 |
| Acot2 | -0.36513 |
| Acot3 | 0.79196 |
| Acot5 | -0.51111 |
| Acot7 | -0.59777 |
| Acot7 | -0.15394 |
| Acot7 | -0.65314 |
| Acot7 | -0.77406 |
| Acot8 | 0.0124 |
| Acot8 | -0.50824 |
| Acot8 | -0.71027 |
| Acot9 | 2.73421 |
| Acox2 | -0.13179 |
| Acox3 | 0.96672 |
| Acox3 | -0.39064 |
| Acoxl | -0.73838 |
| Acp2 | -0.07727 |
| Acp6 | -0.38373 |
| Acpl2 | -0.5899 |
| Acpp | 2.28914 |
| Acr | 1.06524 |
| Acr | -0.69083 |
| Acrbp | -0.08946 |
| Acrbp | -0.50824 |
| Acrv1 | -0.41299 |
| Acsbg1 | -0.74578 |
| Acsbg1 | -0.12799 |
| Acsbg2 | -0.53885 |
| Acsf3 | -0.71655 |
| Acsl1 | -0.50537 |
| Acsl3 | 2.07675 |
| Acsl3 | 0.14443 |
| Acsl4 | -0.4926 |
| Acsl4 | 0.60611 |
| Acsl5 | -0.17765 |
| Acsl6 | 2.3485 |
| Acsl6 | 0.78181 |
| Acsl6 | -0.45261 |
| Acsm3 | 1.21861 |
| Acss1 | 4.19432 |
| Acss2 | -0.23546 |
| Acss2 | -0.47931 |
| Acta1 | -0.51183 |
| Acta2 | -0.69697 |
| Acta2 | 0.71856 |
| Acta2 | 0.33816 |
| Acta2 | -0.54704 |
| Actb | 0.65732 |
| Actb | -0.31036 |
| Actb | 0.56801 |
| Actb | 0.93998 |
| Actb | 0.50477 |
| Actg2 | -0.61451 |
| Actl6a | 0.30562 |
| Actl6b | -0.56899 |
| Actl6b | 1.70491 |
| Actl6b | -0.36268 |
| Actl6b | 1.15327 |
| Actl7a | -0.72649 |
| Actl7b | -0.02243 |
| Actn1 | -0.53663 |
| Actn2 | 2.19622 |
| Actn3 | -0.46347 |
| Actn3 | -0.48279 |
| Actn4 | 0.77011 |
| Actn4 | 1.12679 |
| Actn4 | 1.16193 |
| Actr10 | -0.32727 |
| Actr10 | -0.6089 |
| Actr1a | -0.39951 |
| Actr1b | -0.3831 |
| Actr3 | 0.36737 |
| Actr3b | -0.6097 |
| Actr5 | 0.60205 |
| Actr5 | -0.49472 |
| Actr6 | -0.10968 |
| Actrt1 | 3.03594 |
| Actrt2 | 0.52665 |
| Acvr1 | 0.00463 |
| Acvr1c | -0.09128 |
| Acvr2a | 1.4465 |
| Acvr2b | -0.32904 |
| Acvrl1 | 0.76814 |
| Acy1 | -0.2543 |
| Acy1l2 | -0.67522 |
| Acy3 | 2.32393 |
| Ada | -0.46074 |
| Adal | 2.36295 |
| Adal | 1.65167 |
| Adam11 | -0.34271 |
| Adam11 | 0.1643 |
| Adam12 | 0.88168 |
| Adam15 | -0.54704 |
| Adam17 | 0.8179 |
| Adam1b | -0.62667 |
| Adam1b | -0.52123 |
| Adam21 | -0.7617 |
| Adam22 | 1.96168 |
| Adam22 | -0.37749 |
| Adam22 | 0.4877 |
| Adam23 | 0.49879 |
| Adam24 | 1.47254 |
| Adam26a | 0.34696 |
| Adam28 | 1.69883 |
| Adam29 | 0.96254 |
| Adam29 | 2.00312 |
| Adam3 | 0.00504 |
| Adam30 | -0.18418 |
| Adam32 | -0.63898 |
| Adam33 | -0.60252 |
| Adam39 | 0.24418 |
| Adam4 | 0.2865 |
| Adam4 | 0.84295 |
| Adam4 | -0.61613 |
| Adam5 | -0.51399 |
| Adam6 | -0.61532 |
| Adam8 | -0.41428 |
| Adam8 | -0.68647 |
| Adam9 | 2.50081 |
| Adamdec1 | 0.98172 |
| Adamts1 | 0.35624 |
| Adamts13 | -0.2819 |
| Adamts15 | -0.60252 |
| Adamts16 | -0.35841 |
| Adamts17 | 0.34129 |
| Adamts18 | -0.35719 |
| Adamts19 | 0.92126 |
| Adamts2 | 0.38463 |
| Adamts2 | -0.21909 |
| Adamts2 | 1.22938 |
| Adamts20 | -0.2614 |
| Adamts20 | 0.29969 |
| Adamts4 | 0.90384 |
| Adamts7 | 0.29132 |
| Adamtsl2 | -0.61532 |
| Adamtsl4 | 0.77029 |
| Adamtsl5 | -0.38938 |
| Adar | -0.21596 |
| Adar | -0.69433 |
| Adar | 1.35427 |
| Adarb1 | -0.5644 |
| Adarb1 | 0.64002 |
| Adarb1 | -0.67351 |
| Adat1 | 0.30503 |
| Adc | 0.32203 |
| Adc | -0.67781 |
| Adck1 | -0.59698 |
| Adck2 | 2.4988 |
| Adck4 | -0.60411 |
| Adck5 | 0.38625 |
| Adcy1 | 0.43144 |
| Adcy10 | 0.93746 |
| Adcy2 | 0.72757 |
| Adcy4 | 1.30368 |
| Adcy4 | 0.16567 |
| Adcy6 | 1.09828 |
| Adcy7 | 1.24292 |
| Adcy8 | -0.11248 |
| Adcy9 | 1.99473 |
| Adcyap1 | 2.78125 |
| Adcyap1r1 | 1.85596 |
| Add1 | -0.64895 |
| Add1 | 0.04438 |
| Add1 | -0.03605 |
| Add1 | 2.11259 |
| Add3 | 2.01023 |
| Add3 | 3.91548 |
| Add3 | 0.45537 |
| Adfp | -0.67608 |
| Adh1 | -0.41557 |
| Adh1 | 0.73872 |
| Adh4 | 0.70679 |
| Adh5 | 0.23847 |
| Adh7 | 0.10003 |
| Adhfe1 | 0.48552 |
| Adi1 | 0.65024 |
| Adipoq | -0.72649 |
| Adipor1 | -0.67522 |
| Adipor2 | -0.26634 |
| Adk | 0.67232 |
| Adk | -0.58521 |
| Adm | -0.51978 |
| Adm2 | -0.37438 |
| Adnp | 0.33644 |
| Ado | -0.10459 |
| Adora1 | -0.75417 |
| Adora1 | 0.96283 |
| Adora1 | 1.86468 |
| Adora2b | 2.03172 |
| Adora3 | -0.34511 |
| Adpgk | 1.81985 |
| Adprh | -0.17465 |
| Adprhl1 | -0.44521 |
| Adprhl1 | -0.36882 |
| Adprhl1 | 2.03785 |
| Adprhl2 | 1.28594 |
| Adra1a | 0.43041 |
| Adra1a | 0.25861 |
| Adra1d | -0.48 |
| Adra2a | -0.63981 |
| Adra2c | 0.42211 |
| Adra2c | -0.38749 |
| Adrb1 | 0.95761 |
| Adrb2 | -0.55004 |
| Adrbk1 | -0.21335 |
| Adrbk2 | -0.31325 |
| Adrm1 | 0.78672 |
| Adsl | -0.7274 |
| Adss | 1.28084 |
| Adssl1 | 0.25518 |
| Adssl1 | 1.98987 |
| Adssl1 | -0.332 |
| Aebp1 | -0.75137 |
| Aebp2 | -0.55379 |
| Aes | -0.34331 |
| Afap1 | 0.66855 |
| Afap1 | 0.33844 |
| Afap1 | -0.15883 |
| Afap1l1 | 0.69254 |
| Aff1 | 0.54983 |
| Aff4 | -0.61774 |
| Afg3l1 | -0.2478 |
| Afg3l1 | -0.7274 |
| Afg3l1 | 2.61418 |
| Afg3l1 | -0.63898 |
| Afg3l2 | 2.52205 |
| Afmid | -0.6226 |
| Aftph | 1.13714 |
| Agbl1 | 1.21784 |
| Ager | 1.03632 |
| Ager | -0.18822 |
| Aggf1 | -0.54331 |
| Aggf1 | -0.41752 |
| Aggf1 | -0.77886 |
| Agpat1 | -0.66157 |
| Agpat2 | 1.89649 |
| Agpat3 | -0.77598 |
| Agpat4 | -0.51833 |
| Agpat5 | -0.3179 |
| Agpat6 | -0.53073 |
| Agpat6 | 0.88625 |
| Agpat7 | 0.68904 |
| Agpat9 | -0.72105 |
| Agpat9 | 0.62028 |
| Agps | -0.67608 |
| Agps | 0.15494 |
| Agrn | -0.42732 |
| Agrp | -0.51327 |
| Agt | 1.19465 |
| Agtpbp1 | -0.33853 |
| Agtr1a | -0.35598 |
| Agtrap | -0.45193 |
| Agtrl1 | 2.02053 |
| Agxt2l1 | -0.04294 |
| Agxt2l1 | 0.44577 |
| Agxt2l2 | 1.01975 |
| Agxt2l2 | -0.49472 |
| Agxt2l2 | 2.45051 |
| Agxt2l2 | 0.00627 |
| Ahctf1 | -0.32199 |
| Ahcy | -0.25593 |
| Ahcyl1 | -0.39633 |
| Ahdc1 | 1.01894 |
| Ahi1 | -0.41557 |
| Ahnak | -0.71655 |
| Ahnak | -0.06657 |
| Ahnak2 | -0.50395 |
| Ahrr | -0.55681 |
| Ahsa1 | -0.54929 |
| Ahsa2 | 1.18833 |
| AI118078 | -0.30632 |
| AI132487 | 0.1851 |
| AI314180 | -0.67608 |
| AI314976 | -0.51905 |
| AI316807 | -0.17915 |
| AI317395 | -0.33022 |
| AI413582 | 1.35926 |
| AI413782 | 0.03092 |
| AI427515 | -0.43788 |
| AI428936 | 1.21872 |
| AI449175 | 0.74552 |
| AI449175 | -0.46074 |
| AI450540 | 1.39808 |
| AI451557 | -0.37253 |
| AI451617 | 0.49543 |
| AI467606 | 1.03204 |
| AI480653 | -0.45463 |
| AI504432 | -0.53959 |
| AI553587 | -0.14328 |
| AI593442 | -0.73196 |
| AI593442 | -0.51183 |
| AI593442 | -0.75887 |
| AI595366 | 0.02532 |
| AI595406 | 0.99788 |
| AI597468 | -0.68126 |
| AI597479 | -0.46483 |
| AI606181 | 0.4526 |
| AI646023 | 1.58616 |
| AI661453 | 0.95221 |
| AI662250 | 2.32468 |
| AI747699 | 0.5107 |
| AI836003 | -0.55984 |
| AI837181 | -0.62667 |
| AI842396 | -0.51978 |
| AI846148 | -0.22856 |
| AI847670 | -0.57282 |
| AI851790 | -0.25976 |
| AI894139 | 0.38436 |
| AI987692 | 2.81527 |
| AI987692 | -0.2752 |
| AI987944 | -0.19075 |
| Aicda | 1.85465 |
| Aif1 | -0.15054 |
| Aif1 | -0.55908 |
| Aif1 | 1.15632 |
| Aifm1 | -0.46963 |
| Aifm1 | 4.3352 |
| Aifm2 | -0.58599 |
| Aifm2 | -0.60491 |
| Aifm2 | 0.8487 |
| Aifm3 | 2.9321 |
| Aig1 | -0.2328 |
| Aig1-pending | 0.60675 |
| Aim1 | 0.137 |
| Aim2 | -0.46142 |
| Aip | -0.56288 |
| Ak1 | -0.33972 |
| AK122209 | -0.6121 |
| AK122525 | 1.04905 |
| AK129128 | -0.33615 |
| AK129302 | -0.5821 |
| AK129302 | -0.19278 |
| AK129302 | 0.09855 |
| AK129341 | -0.47515 |
| AK162044 | -0.59461 |
| Ak2 | -0.65903 |
| Ak2 | 0.72477 |
| Ak2 | 0.51212 |
| Ak3 | 1.45788 |
| Ak3 | 4.16225 |
| Ak3l1 | 0.2774 |
| Ak5 | 0.21853 |
| Akap1 | 0.80682 |
| Akap1 | -0.49119 |
| Akap1 | -0.00568 |
| Akap10 | -0.67008 |
| Akap10 | -0.01109 |
| Akap12 | -0.42339 |
| Akap14 | -0.74857 |
| Akap2 | 0.85718 |
| Akap2 | -0.44588 |
| Akap3 | 1.81945 |
| Akap4 | 0.3646 |
| Akap4 | -0.53441 |
| Akap4 | -0.57129 |
| Akap4 | 0.15772 |
| Akap6 | -0.52634 |
| Akap7 | 3.15045 |
| Akap8 | 2.07456 |
| Akap8l | 2.98509 |
| Akap9 | -0.68908 |
| Akap9 | -0.48558 |
| Akap9 | 2.22366 |
| Akna | -0.60014 |
| Akp2 | -0.77886 |
| Akp5 | 1.17039 |
| Akr1a4 | 1.20058 |
| Akr1a4 | -0.47239 |
| Akr1b3 | -0.67008 |
| Akr1b8 | 0.77046 |
| Akr1c12 | 0.05687 |
| Akr1c14 | 0.13022 |
| Akr1c14 | -0.48418 |
| Akr1c18 | 1.02733 |
| Akr1c19 | 1.30138 |
| Akr1e1 | -0.29204 |
| Akr7a5 | -0.39253 |
| Akr7a5 | 0.69622 |
| Akr7a5 | 0.86236 |
| Akt1s1 | -0.6804 |
| Akt3 | 1.22613 |
| Akt3 | 3.64923 |
| Aktip | -0.64645 |
| Alad | -0.41299 |
| Alas1 | -0.58912 |
| Alas2 | -0.51111 |
| Alcam | -0.34511 |
| Aldh16a1 | 0.93597 |
| Aldh18a1 | -0.60173 |
| Aldh1a1 | -0.09174 |
| Aldh1a2 | 0.28529 |
| Aldh1a2 | -0.50039 |
| Aldh1a7 | -0.17018 |
| Aldh1b1 | 1.61071 |
| Aldh1l1 | -0.53737 |
| Aldh1l2 | -0.85717 |
| Aldh1l2 | -0.34151 |
| Aldh2 | 0.99982 |
| Aldh3a1 | -0.55757 |
| Aldh3a2 | -0.36024 |
| Aldh3b1 | 0.05376 |
| Aldh3b1 | -0.54108 |
| Aldh3b1 | 0.414 |
| Aldh4a1 | 0.75409 |
| Aldh5a1 | 0.36377 |
| Aldh5a1 | 2.22116 |
| Aldh6a1 | 0.47917 |
| Aldh8a1 | 0.47303 |
| Aldh8a1 | 2.42999 |
| Aldoa | -0.39001 |
| Aldoc | 3.29816 |
| Alg1 | 1.28787 |
| Alg10b | 2.05144 |
| Alg11 | 2.33391 |
| Alg11 | 1.87342 |
| Alg12 | 0.47131 |
| Alg12 | 0.16533 |
| Alg14 | -0.24403 |
| Alg2 | -0.07862 |
| Alg3 | 0.04517 |
| Alg3 | -0.42535 |
| Alg3 | -0.09906 |
| Alg5 | 1.44855 |
| Alg5 | -0.48978 |
| Alg5 | 0.79475 |
| Alg8 | -0.6365 |
| Alg8 | -0.55833 |
| Alg8 | -0.6565 |
| Alg8 | -0.07191 |
| Alg9 | 0.56623 |
| Alg9 | 1.4198 |
| Alg9 | 0.90369 |
| Alk | -0.51183 |
| Alkbh2 | -0.49896 |
| Alkbh3 | -0.56975 |
| Alkbh4 | -0.59619 |
| Alkbh5 | -0.4587 |
| Alkbh7 | -0.72467 |
| Alkbh8 | 1.72337 |
| Allc | -0.19839 |
| Allc | 1.22243 |
| Alox12 | -0.55681 |
| Alox12b | 0.83965 |
| Alox12e | 1.50341 |
| Alox15 | 0.73872 |
| Alox5ap | 1.2593 |
| Alox8 | -0.69083 |
| Aloxe3 | -0.49119 |
| Alpk3 | 0.18611 |
| Als2 | 2.67308 |
| Als2 | -0.63486 |
| Als2 | -0.65146 |
| Als2cl | -0.65566 |
| Als2cr13 | 1.20648 |
| Als2cr2 | -0.32492 |
| Als2cr2 | -0.40334 |
| Als2cr4 | 0.57377 |
| Als2cr4 | 0.40767 |
| Als2cr4 | -0.28022 |
| Alx3 | 1.00066 |
| Amac1 | -0.53737 |
| Ambp | 1.44213 |
| Ambra1 | -0.18872 |
| Ambra1 | -0.75417 |
| Amd2 | 0.74717 |
| Amdhd2 | 0.39273 |
| Amdhd2 | -0.89897 |
| Amfr | -0.3023 |
| Amh | -0.15151 |
| Amhr2 | 2.86774 |
| Amhr2 | 2.45946 |
| Amhr2 | 0.43838 |
| Amigo1 | 1.35219 |
| Amigo1 | 0.18172 |
| Amigo2 | -0.5011 |
| Amigo3 | 0.96283 |
| Ammecr1l | -0.41752 |
| Amn | -0.68734 |
| Amotl2 | 2.52607 |
| Ampd1 | 2.24441 |
| Ampd1 | 1.52225 |
| Ampd2 | 1.74109 |
| Amph | -0.78659 |
| Amph | 0.76617 |
| Amy1 | -0.74578 |
| Amz2 | -0.51039 |
| Anapc10 | 2.09689 |
| Anapc11 | -0.51688 |
| Anapc13 | -0.60093 |
| Anapc2 | 0.48406 |
| Anapc4 | -0.1867 |
| Anapc5 | 0.8947 |
| Anapc5 | -0.63157 |
| Anapc5 | -0.17167 |
| Anapc7 | 0.40316 |
| Ang | 0.45083 |
| Angel1 | -0.42404 |
| Angel2 | -0.31209 |
| Angpt1 | 1.9434 |
| Angpt2 | -0.15932 |
| Angpt2 | -0.00568 |
| Angptl1 | 0.24133 |
| Angptl2 | 1.33989 |
| Angptl3 | 0.66095 |
| Angptl4 | -0.66157 |
| Angptl7 | -0.67694 |
| Ank | 1.32782 |
| Ank1 | -0.39127 |
| Ank2 | 0.7624 |
| Ank2 | -0.01737 |
| Ank2 | -0.1872 |
| Ank3 | -0.7076 |
| Ank3 | -0.57822 |
| Ank3 | 1.54717 |
| Ankar | -0.56136 |
| Ankar | 0.07415 |
| Ankfy1 | 0.54756 |
| Ankle2 | -0.74393 |
| Ankle2 | 2.80731 |
| Ankle2 | 0.30739 |
| Ankmy1 | 0.2871 |
| Ankmy1 | -0.62017 |
| Ankmy1 | -0.48348 |
| Ankmy2 | 1.47262 |
| Ankmy2 | 0.93508 |
| Ankrd10 | -0.63239 |
| Ankrd12 | -0.46074 |
| Ankrd12 | -0.56975 |
| Ankrd13a | 0.64228 |
| Ankrd13b | -0.11015 |
| Ankrd13d | -0.63898 |
| Ankrd17 | 0.63114 |
| Ankrd17 | -0.46689 |
| Ankrd17 | -0.49684 |
| Ankrd22 | -0.58677 |
| Ankrd23 | 1.10561 |
| Ankrd24 | 0.50166 |
| Ankrd25 | 0.99982 |
| Ankrd27 | 3.01403 |
| Ankrd34a | 0.10923 |
| Ankrd35 | -0.68473 |
| Ankrd36 | -0.64478 |
| Ankrd37 | 0.34158 |
| Ankrd39 | 0.82481 |
| Ankrd40 | -0.39443 |
| Ankrd40 | -0.72467 |
| Ankrd40 | -0.05293 |
| Ankrd46 | 2.06248 |
| Ankrd47 | 0.35736 |
| Ankrd49 | -0.20559 |
| Ankrd5 | 3.00278 |
| Ankrd50 | -0.48418 |
| Ankrd53 | 0.57598 |
| Ankrd54 | 0.27709 |
| Ankrd54 | 0.04044 |
| Ankrd54 | 0.18442 |
| Ankrd56 | -0.22382 |
| Ankrd58 | -0.6089 |
| Ankrd6 | -0.54405 |
| Ankrd6 | 1.56665 |
| Ankrd6 | -0.28134 |
| Ankrd7 | 2.01765 |
| Ankrd9 | 0.71478 |
| Anks1 | 2.20876 |
| Anks3 | 4.4043 |
| Anks3 | 1.18867 |
| Anks6 | 2.07774 |
| Anks6 | 1.46461 |
| Ankzf1 | -0.61613 |
| Ankzf1 | 0.41584 |
| Anln | -0.60173 |
| Anln | -0.41947 |
| Anp32a | -0.58599 |
| Anp32a | -0.17965 |
| Anp32a | 0.26759 |
| Anp32e | -0.32492 |
| Anpep | 1.78988 |
| Antxr1 | 1.93746 |
| Antxr1 | 0.78637 |
| Antxrl | 2.79416 |
| Anxa1 | -0.64812 |
| Anxa11 | 3.13792 |
| Anxa11 | -0.50824 |
| Anxa2 | -0.41493 |
| Anxa3 | 0.16774 |
| Anxa3 | 3.34287 |
| Anxa4 | -0.02879 |
| Anxa5 | -0.58055 |
| Anxa6 | -0.68996 |
| Anxa8 | -0.70493 |
| Anxa8 | 0.50095 |
| Anxa9 | -0.66327 |
| Aoah | 0.40529 |
| Aoc2 | 0.38679 |
| Aoc2 | 1.72255 |
| Aoc3 | -0.45531 |
| Aof1 | -0.72831 |
| Aof1 | -0.68299 |
| Aof2 | -0.76929 |
| Aox3 | -0.29488 |
| Aox3l1 | -0.04857 |
| Ap1b1 | -0.0922 |
| Ap1g1 | -0.33555 |
| Ap1g1 | -0.72196 |
| Ap1g2 | 1.23736 |
| Ap1gbp1 | -0.4807 |
| Ap1gbp1 | 1.29455 |
| Ap1m1 | -0.09768 |
| Ap1m2 | 0.17356 |
| Ap1m2 | -0.63815 |
| Ap1m2 | 1.13029 |
| Ap1m2 | 0.41558 |
| Ap1s1 | -0.45734 |
| Ap1s1 | 0.98342 |
| Ap1s1 | 1.68265 |
| Ap1s3 | 0.63672 |
| Ap2a1 | -0.71835 |
| Ap2a1 | -0.67265 |
| Ap2a2 | -0.67008 |
| Ap2b1 | 1.21609 |
| Ap2b1 | -0.16375 |
| Ap2b1 | -0.36024 |
| Ap2b1 | -0.34992 |
| Ap2m1 | 1.95674 |
| Ap2s1 | -0.64229 |
| Ap2s1 | 1.06885 |
| Ap2s1 | -0.67522 |
| Ap3b1 | -0.65566 |
| Ap3b1 | 2.08953 |
| Ap3b2 | 2.48678 |
| Ap3d1 | 1.42226 |
| Ap3m1 | 0.7588 |
| Ap3m2 | 3.20911 |
| Ap3s2 | -0.49472 |
| Ap3s2 | -0.05819 |
| Ap4b1 | 0.82784 |
| Ap4m1 | 0.31912 |
| Ap4m1 | -0.74857 |
| Ap4m1 | -0.61451 |
| Ap4s1 | 1.15221 |
| Apaf1 | -0.19075 |
| Apaf1 | 0.2976 |
| Apba1 | -0.61613 |
| Apba2 | -0.33022 |
| Apba2 | 0.00422 |
| Apba3 | -0.64645 |
| Apbb1 | -0.70227 |
| Apbb1ip | 0.62573 |
| Apbb1ip | 0.08774 |
| Apbb1ip | -0.62422 |
| Apbb2 | -0.05249 |
| Apbb3 | -0.217 |
| Apc | -0.66582 |
| Apc2 | -0.80028 |
| Apcdd1 | 0.39595 |
| Apeh | -0.6089 |
| Apex1 | -0.48348 |
| Aph1a | 0.16464 |
| Aph1b | 2.878 |
| Apip | 1.62243 |
| Apip | 0.18138 |
| Apitd1 | -0.5606 |
| Aplp1 | -0.4621 |
| Aplp2 | 1.31597 |
| Apoa1 | 1.53258 |
| Apoa1bp | -0.32727 |
| Apoa2 | -0.2603 |
| Apoa4 | 1.56942 |
| Apob48r | -0.59304 |
| Apobec1 | 0.18408 |
| Apobec3 | -0.17865 |
| Apoc1 | -0.66582 |
| Apoc1 | -0.17117 |
| Apoc2 | 1.42664 |
| Apoc3 | -0.34872 |
| Apoe | 2.46187 |
| Apof | -0.6565 |
| Apoh | -0.48418 |
| Apol7c | 0.90091 |
| Apol9b | 0.16945 |
| App | -0.43788 |
| Appbp1 | -0.34691 |
| Appbp1 | 0.51471 |
| Appbp2 | 0.78163 |
| Appl1 | -0.33734 |
| Appl2 | 1.1173 |
| Aprin | 0.31357 |
| Aprt | -0.65903 |
| Aptx | -0.68213 |
| Aqp1 | -0.74485 |
| Aqp1 | -0.69258 |
| Aqp1 | -0.72015 |
| Aqp11 | 0.88057 |
| Aqp11 | -0.6565 |
| Aqp3 | -0.37998 |
| Aqp4 | -0.75511 |
| Aqp5 | 1.23865 |
| Aqp7 | 2.27346 |
| Aqp7 | 0.9637 |
| Aqp8 | -0.65062 |
| Aqp8 | 1.49898 |
| Aqp9 | 0.45235 |
| Aqr | -0.70315 |
| Aqr | -0.17665 |
| Ar | -0.26304 |
| Araf | 0.20708 |
| Araf | 1.56327 |
| Arbp | -0.29545 |
| Arc | 1.05181 |
| Arcn1 | 0.13877 |
| Ard1 | 0.02171 |
| Ard1 | -0.5644 |
| Areg | -0.65482 |
| Arf2 | -0.26469 |
| Arf3 | 0.06497 |
| Arf5 | -0.2392 |
| Arf6 | -0.77694 |
| Arfgap1 | 1.07361 |
| Arfgap1 | 0.0209 |
| Arfgap1 | -0.71655 |
| Arfgap2 | -0.51399 |
| Arfgap2 | 1.82897 |
| Arfgap2 | -0.35598 |
| Arfgap2 | -0.51039 |
| Arfgap3 | -0.54704 |
| Arfip2 | -0.49967 |
| Arfip2 | -0.07235 |
| Arfip2 | -0.70582 |
| Arfrp1 | 0.17493 |
| Arfrp1 | -0.12279 |
| Arg1 | 1.5516 |
| Arg2 | 0.29701 |
| Arglu1 | 2.84809 |
| Arhgap1 | -0.72196 |
| Arhgap1 | 3.50969 |
| Arhgap1 | 2.43274 |
| Arhgap10 | -0.02539 |
| Arhgap12 | -0.17665 |
| Arhgap15 | -0.66072 |
| Arhgap15 | -0.48558 |
| Arhgap17 | -0.15102 |
| Arhgap18 | -0.04035 |
| Arhgap19 | 0.47966 |
| Arhgap20 | 0.20774 |
| Arhgap22 | -0.17765 |
| Arhgap24 | 0.72026 |
| Arhgap24 | -0.25757 |
| Arhgap25 | -0.36146 |
| Arhgap26 | 0.86042 |
| Arhgap27 | -0.4339 |
| Arhgap28 | 0.45988 |
| Arhgap29 | 0.20444 |
| Arhgap30 | 0.95045 |
| Arhgap4 | 0.70908 |
| Arhgap6 | -0.07503 |
| Arhgap8 | 2.68628 |
| Arhgap9 | -0.54256 |
| Arhgdib | 1.48097 |
| Arhgdig | 1.21762 |
| Arhgef1 | 0.94102 |
| Arhgef1 | 0.94146 |
| Arhgef10 | 1.89229 |
| Arhgef10l | -0.59619 |
| Arhgef10l | 1.69584 |
| Arhgef15 | -0.43854 |
| Arhgef18 | 1.41795 |
| Arhgef19 | 0.0588 |
| Arhgef2 | 2.75537 |
| Arhgef3 | -0.75044 |
| Arhgef4 | -0.37998 |
| Arhgef6 | 0.99173 |
| Arid2 | 3.70297 |
| Arid3a | 0.13771 |
| Arid3b | 2.05568 |
| Arid4b | -0.77119 |
| Arid5a | -0.54033 |
| Arid5b | -0.50537 |
| Arih1 | -0.02964 |
| Arih2 | -0.47931 |
| Arl1 | 3.39623 |
| Arl11 | -0.46826 |
| Arl16 | 2.65012 |
| Arl2 | -0.48768 |
| Arl2bp | -0.54779 |
| Arl2bp | 1.36852 |
| Arl2bp | 0.11397 |
| Arl3 | -0.1009 |
| Arl3 | -0.40462 |
| Arl4a | -0.58132 |
| Arl4a | 0.73242 |
| Arl4c | -0.71745 |
| Arl5a | -0.72196 |
| Arl5b | 0.73742 |
| Arl6 | 0.12484 |
| Arl6ip1 | 0.57598 |
| Arl6ip2 | 0.48576 |
| Arl6ip2 | 1.9722 |
| Arl6ip2 | -0.64229 |
| Arl6ip2 | 0.50357 |
| Arl6ip4 | -0.29375 |
| Arl6ip4 | -0.48558 |
| Arl6ip5 | 1.12011 |
| Arl6ip5 | -0.08357 |
| Arl8a | 0.80631 |
| Arl8b | -0.64478 |
| Armc10 | 2.2906 |
| Armc3 | 2.04572 |
| Armc6 | 2.53978 |
| Armc6 | -0.7779 |
| Armc8 | 0.70027 |
| Armc8 | -0.70227 |
| Armc8 | 1.04456 |
| Armc9 | -0.22119 |
| Armc9 | -0.59383 |
| Armcx1 | -0.55079 |
| Armcx2 | -0.30345 |
| Armcx3 | 0.04908 |
| Armcx5 | 3.31895 |
| Armcx6 | 1.05705 |
| Armcx6 | 0.06995 |
| Armetl1 | 0.97061 |
| Arnt | 2.52033 |
| Arnt | -0.51039 |
| Arnt2 | -0.11061 |
| Arntl | 0.32551 |
| Arpc1a | 0.2051 |
| Arpc1a | -0.70404 |
| Arpc1a | -0.53147 |
| Arpc1b | -0.3919 |
| Arpc1b | 2.9573 |
| Arpc1b | -0.55079 |
| Arpc3 | 0.19815 |
| Arpc4 | -0.68908 |
| Arpc5 | -0.38185 |
| Arpc5 | -0.579 |
| Arpc5l | -0.26085 |
| Arpp19 | 0.43966 |
| Arpp21 | -0.15054 |
| Arpp21 | -0.68908 |
| Arrb1 | -0.02032 |
| Arrb1 | -0.60252 |
| Arrb1 | -0.20817 |
| Arrb1 | -0.36146 |
| Arrb2 | -0.66837 |
| Arrdc1 | -0.18872 |
| Arrdc2 | 0.07946 |
| Arrdc3 | 0.62989 |
| Arrdc4 | 1.21959 |
| Ars2 | 1.9042 |
| Ars2 | 1.94856 |
| Ars2 | -0.5278 |
| Arsa | -0.426 |
| Arsb | -0.65482 |
| Arsb | -0.39951 |
| Arsg | -0.07683 |
| Arsi | 1.62799 |
| Arsj | -0.54182 |
| Arsj | 2.32334 |
| Art1 | -0.78369 |
| Art2b | 1.83015 |
| Art3 | -0.52561 |
| Art3 | -0.62017 |
| Art4 | 0.06151 |
| Art5 | -0.01444 |
| Artn | 0.55908 |
| Artn | 0.6549 |
| Arv1 | -0.67179 |
| As3mt | -0.50824 |
| As3mt | 1.2791 |
| Asah1 | 0.0353 |
| Asah1 | 1.81614 |
| Asah2 | -0.15005 |
| Asah3 | -0.00113 |
| Asah3l | 0.87087 |
| Asb1 | -0.43258 |
| Asb1 | -0.6073 |
| Asb1 | -0.30058 |
| Asb12 | -0.72831 |
| Asb13 | -0.17715 |
| Asb15 | -0.73379 |
| Asb17 | 0.48867 |
| Asb2 | 0.10776 |
| Asb3 | -0.59304 |
| Asb3 | -0.62667 |
| Asb4 | 1.49096 |
| Asb4 | 0.63466 |
| Asb6 | -0.73655 |
| Asb6 | -0.77502 |
| Asb7 | -0.71117 |
| Asb7 | -0.59147 |
| Asb8 | -0.35902 |
| Asb9 | -0.55606 |
| Ascc1 | 0.65934 |
| Ascc1 | -0.47792 |
| Ascc2 | 0.38788 |
| Ascc2 | -0.4621 |
| Ascc2 | 2.02218 |
| Ascc2 | -0.35537 |
| Ascc3l1 | 2.56777 |
| Ascl1 | 2.0559 |
| Ascl2 | -0.66327 |
| Asf1a | -0.71925 |
| Asf1b | -0.7274 |
| Asgr1 | -0.47239 |
| Ash1l | -0.29148 |
| Ash1l | 1.78623 |
| Ash1l | 0.80305 |
| Ash2l | -0.61936 |
| Ash2l | -0.5759 |
| Asl | -0.65903 |
| Asl | 1.06031 |
| Asna1 | -0.72286 |
| Asns | 1.60267 |
| Asns | 0.18172 |
| Asns | 1.51926 |
| Asnsd1 | -0.84158 |
| Asnsd1 | -0.46963 |
| Asph | -0.57129 |
| Asph | -0.62422 |
| Asph | -0.52927 |
| Asph | 2.84528 |
| Asph | -0.40847 |
| Asphd1 | 1.17568 |
| Asphd2 | -0.62912 |
| Aspm | -0.54033 |
| Asprv1 | 2.73747 |
| Aspscr1 | 2.24451 |
| Aspscr1 | 0.35484 |
| Aspscr1 | 0.50572 |
| Asrgl1 | -0.78465 |
| Ass1 | -0.34211 |
| Aste1 | -0.2118 |
| Astn1 | 0.08361 |
| Astn2 | 3.93751 |
| Asz1 | 0.12628 |
| Atad1 | 1.78829 |
| Atad2 | -0.68908 |
| Atad3a | -0.29318 |
| Atad4 | 2.56911 |
| Atad4 | 0.73335 |
| Atcay | 1.84467 |
| Ate1 | 1.20382 |
| Ate1 | 0.25361 |
| Atf2 | -0.42273 |
| Atf2 | 0.11434 |
| Atf2 | 0.22113 |
| Atf3 | -0.72922 |
| Atf4 | -0.53885 |
| Atf4 | 2.47893 |
| Atf5 | -0.57436 |
| Atf7 | -0.64478 |
| Atf7ip | 0.27679 |
| Atg10 | -0.43456 |
| Atg10 | -0.28022 |
| Atg12 | -0.13417 |
| Atg16l1 | 0.64779 |
| Atg2a | 0.25767 |
| Atg2a | 1.61185 |
| Atg2b | -0.19126 |
| Atg2b | -0.64478 |
| Atg3 | -0.61291 |
| Atg3 | -0.71475 |
| Atg4b | -0.37811 |
| Atg4c | -0.18518 |
| Atg4c | 2.14744 |
| Atg4d | -0.80324 |
| Atg5 | -0.12279 |
| Atg5 | 1.10821 |
| Atg7 | 0.08135 |
| Atg9b | -0.09952 |
| Athl1 | -0.52269 |
| Atic | 3.05089 |
| Atic | 0.03847 |
| Atic | -0.63157 |
| Atm | -0.76929 |
| Atm | 1.42261 |
| Atmin | 0.32 |
| Atmin | -0.40015 |
| Atmin | -0.49967 |
| Atn1 | 0.10629 |
| Atoh8 | 0.62719 |
| Atox1 | -0.65146 |
| Atp10a | 0.78356 |
| Atp10d | -0.78659 |
| Atp11c | -0.49896 |
| Atp11c | -0.59304 |
| Atp12a | -0.45396 |
| Atp13a1 | -0.6105 |
| Atp13a1 | 0.06766 |
| Atp13a2 | -0.4933 |
| Atp13a4 | -0.55379 |
| Atp13a5 | -0.08901 |
| Atp1a1 | -0.63239 |
| Atp1a2 | 1.84948 |
| Atp1b1 | -0.5606 |
| Atp1b2 | -0.39253 |
| Atp1b3 | -0.53589 |
| Atp2a1 | 1.48302 |
| Atp2a2 | 0.2973 |
| Atp2a2 | 2.23297 |
| Atp2a3 | 1.23219 |
| Atp2a3 | 1.342 |
| Atp2a3 | 3.79458 |
| Atp2b1 | -0.70671 |
| Atp2b2 | -0.70671 |
| Atp2b2 | -0.10876 |
| Atp2b2 | -0.19482 |
| Atp2b3 | -0.72558 |
| Atp2b3 | 1.92001 |
| Atp2b4 | 1.58535 |
| Atp2b4 | 0.23432 |
| Atp2c1 | 0.42029 |
| Atp2c1 | 3.0312 |
| Atp5a1 | 0.39165 |
| Atp5b | 2.32488 |
| Atp5c1 | 0.28891 |
| Atp5d | 1.14251 |
| Atp5e | 0.56423 |
| Atp5f1 | -0.79635 |
| Atp5g2 | 1.19588 |
| Atp5g3 | 0.42419 |
| Atp5h | -0.65566 |
| Atp5j | -0.63321 |
| Atp5j | -0.08087 |
| Atp5j2 | 0.07034 |
| Atp5l | 2.13018 |
| Atp5o | -0.66496 |
| Atp5s | 0.87629 |
| Atp5sl | 0.30621 |
| Atp6ap1 | 0.22113 |
| Atp6ap2 | -0.76929 |
| Atp6ap2 | 2.55456 |
| Atp6ap2 | 0.11032 |
| Atp6ap2 | -0.55304 |
| Atp6v0a1 | -0.78562 |
| Atp6v0a1 | 0.76528 |
| Atp6v0a2 | -0.75605 |
| Atp6v0b | 1.9583 |
| Atp6v0d1 | -0.49049 |
| Atp6v0d1 | 0.16119 |
| Atp6v0d1 | -0.68473 |
| Atp6v0d1 | -0.4587 |
| Atp6v0e | -0.25376 |
| Atp6v0e2 | -0.58288 |
| Atp6v1a | 0.68121 |
| Atp6v1b1 | -0.29318 |
| Atp6v1b2 | 1.00495 |
| Atp6v1c1 | -0.78272 |
| Atp6v1c2 | -0.77982 |
| Atp6v1d | -0.18872 |
| Atp6v1e1 | -0.58443 |
| Atp6v1e2 | 1.87637 |
| Atp6v1g1 | 0.62301 |
| Atp6v1g2 | 0.7588 |
| Atp6v1g2 | 0.3071 |
| Atp6v1h | 2.22116 |
| Atp7a | -0.25593 |
| Atp8a1 | 0.55277 |
| Atp8a2 | -0.27855 |
| Atp8b1 | 2.11672 |
| Atp8b3 | -0.59856 |
| Atp8b3 | -0.59777 |
| Atp8b3 | 0.19482 |
| Atp8b3 | 0.27587 |
| Atp9a | 0.41689 |
| Atp9a | 3.47931 |
| Atp9b | 2.78896 |
| Atp9b | 1.03285 |
| Atpaf1 | -0.44254 |
| Atpaf2 | -0.71565 |
| Atpaf2 | 0.05065 |
| Atpbd1b | 0.46163 |
| Atpbd1c | 0.43196 |
| Atpbd3 | -0.50824 |
| Atpif1 | 1.28696 |
| Atrx | 0.05803 |
| Atxn1 | 2.42673 |
| Atxn10 | 0.65893 |
| Atxn2 | 1.29344 |
| Atxn2l | -0.56136 |
| Atxn3 | -0.71117 |
| Atxn3 | 0.31474 |
| Atxn3 | 0.3495 |
| Atxn7l1 | 2.76481 |
| Atxn7l2 | -0.5278 |
| AU014645 | -0.80621 |
| AU017455 | 2.12635 |
| AU017455 | -0.46415 |
| AU018778 | 1.41238 |
| AU018829 | -0.38875 |
| AU019823 | 2.59415 |
| AU021034 | -0.05031 |
| AU021092 | 1.75378 |
| AU021838 | -0.58132 |
| AU022252 | 1.26265 |
| AU022751 | -0.52927 |
| AU022870 | 1.57738 |
| AU023871 | -0.02751 |
| AU040320 | 2.6463 |
| AU040320 | -0.53663 |
| AU040320 | -0.5606 |
| AU040829 | 0.7144 |
| AU042651 | -0.73105 |
| Aup1 | 0.753 |
| Aup1 | 0.20047 |
| Aurka | -0.07727 |
| Aurkaip1 | -0.42929 |
| Aurkc | -0.43589 |
| Auts2 | -0.21648 |
| Auts2 | 0.1147 |
| AV028368 | 0.34072 |
| AV249152 | 0.11434 |
| AV249152 | 2.73619 |
| Aven | 0.95935 |
| Avil | 1.49518 |
| Avp | -0.37129 |
| Avpi1 | 0.4161 |
| Avpi1 | 0.46212 |
| Avpr1a | -0.58365 |
| AW049604 | -0.53515 |
| AW061290 | 0.23528 |
| AW111922 | -0.58521 |
| AW121567 | 0.66355 |
| AW125753 | -0.24996 |
| AW146242 | 0.7087 |
| AW146299 | 0.54551 |
| AW146299 | 1.60634 |
| AW540478 | -0.46826 |
| AW544981 | -0.48558 |
| AW548124 | -0.5448 |
| AW549877 | -0.57359 |
| AW551984 | 0.23464 |
| AW551984 | -0.17765 |
| AW551984 | -0.49049 |
| AW555464 | 0.78725 |
| Axin2 | -0.2791 |
| Axl | 0.95833 |
| Axud1 | 0.22855 |
| Azgp1 | -0.1677 |
| Azgp1 | 2.08868 |
| Azi1 | 3.33302 |
| Azi2 | 1.41874 |
| Azin1 | -0.24564 |
| B020018G12Rik | -0.24027 |
| B230118H07Rik | 0.75355 |
| B230120H23Rik | 0.72589 |
| B230206H07Rik | 2.62004 |
| B230209C24Rik | 0.43479 |
| B230217C12Rik | -0.35416 |
| B230219D22Rik | -0.73288 |
| B230312A22Rik | -0.54331 |
| B230312I18Rik | -0.58132 |
| B230317C12Rik | -0.69962 |
| B230317C12Rik | -0.2451 |
| B230339H12Rik | 1.29556 |
| B230339M05Rik | 1.47037 |
| B230339M05Rik | 2.46278 |
| B230340J04Rik | -0.54331 |
| B230342M21Rik | 1.96984 |
| B230373P09Rik | -0.64562 |
| B230378H13Rik | 0.40899 |
| B230380D07Rik | 0.43376 |
| B230396O12Rik | -0.0382 |
| B230399E16Rik | -0.24349 |
| B2m | 1.63692 |
| B2m | 0.82851 |
| B3galnt1 | -0.52488 |
| B3galnt2 | -0.44454 |
| B3galt1 | 1.28574 |
| B3galt4 | -0.35902 |
| B3galt4 | -0.73931 |
| B3galt5 | -0.75137 |
| B3galt6 | -0.29148 |
| B3gat1 | 1.23122 |
| B3gat1 | 1.59935 |
| B3gat1 | 0.41426 |
| B3gat2 | -0.45328 |
| B3gat3 | 0.75264 |
| B3gat3 | 1.76103 |
| B3gnt1 | 0.26604 |
| B3gnt3 | -0.12609 |
| B3gnt5 | -0.14811 |
| B3gnt7 | -0.65062 |
| B3gnt8 | 0.23304 |
| B3gnt8 | 0.50904 |
| B430119L13Rik | 0.48454 |
| B4galnt3 | -0.10783 |
| B4galnt4 | -0.21596 |
| B4galt1 | -0.70227 |
| B4galt3 | -0.34631 |
| B4galt3 | -0.65398 |
| B4galt4 | 0.09818 |
| B4galt7 | 2.55747 |
| B630005N14Rik | 1.3554 |
| B830045N13Rik | 2.27793 |
| B930006L02Rik | -0.24188 |
| B930011P16Rik | -0.34932 |
| B930037P14Rik | -0.52196 |
| B930041F14Rik | -0.25976 |
| B930041G04 | 0.8875 |
| B930076A02 | -0.64063 |
| B9d1 | 1.70665 |
| B9d2 | 1.74752 |
| Baalc | 0.58541 |
| Baalc | -0.51399 |
| Bace1 | 1.45653 |
| Bace2 | -0.74393 |
| Bace2 | -0.71565 |
| Bace2 | 2.21659 |
| Bach1 | 0.16292 |
| Bach2 | -0.51543 |
| Bad | 1.70852 |
| Bag1 | -0.43258 |
| Bag2 | 0.36543 |
| Bag3 | 0.72963 |
| Bag4 | -0.63075 |
| Bag5 | 1.84084 |
| Bahcc1 | 1.54287 |
| Bahd1 | 0.2558 |
| Bai1 | 0.67351 |
| Bai2 | 0.59904 |
| Bai2 | 3.97842 |
| Bai3 | -0.23973 |
| Baiap2 | -0.36636 |
| Baiap2 | 1.34267 |
| Banf1 | -0.2478 |
| Banf1 | -0.45802 |
| Banf1 | -0.5821 |
| Banf1 | -0.43921 |
| Banf2 | 0.06919 |
| Bap1 | 1.34382 |
| Bard1 | 2.28376 |
| Barx2 | 0.90554 |
| Basp1 | 1.19824 |
| Bat1a | -0.10783 |
| Bat1a | -0.57052 |
| Bat2 | -0.33972 |
| Bat3 | -0.66327 |
| Bat4 | 3.70382 |
| Bat4 | -0.50323 |
| Bat5 | -0.55079 |
| Batf | 0.90815 |
| Bax | -0.64645 |
| Baz1a | -0.4587 |
| Baz1b | 2.77859 |
| Baz2a | -0.32434 |
| BB014433 | -0.49826 |
| BB128963 | 0.29402 |
| BB146404 | -0.54704 |
| Bbc3 | 0.55773 |
| Bbc3 | 0.86397 |
| Bbox1 | -0.56288 |
| Bbs1 | 0.18846 |
| Bbs12 | 0.84081 |
| Bbs2 | 2.74359 |
| Bbs4 | -0.43258 |
| Bbs5 | -0.52927 |
| Bbs7 | -0.27799 |
| Bbs9 | -0.37253 |
| Bbs9 | -0.00195 |
| Bbx | -0.25159 |
| BC002163 | 0.31854 |
| BC002199 | -0.10644 |
| BC002216 | 0.17458 |
| BC003236 | 0.13806 |
| BC003266 | -0.36636 |
| BC003885 | 0.61986 |
| BC003885 | 0.50214 |
| BC003993 | 1.24643 |
| BC003993 | -0.43324 |
| BC004004 | -0.6105 |
| BC004044 | -0.59935 |
| BC004044 | -0.0136 |
| BC004728 | 0.30414 |
| BC004728 | -0.53663 |
| BC005537 | 1.62567 |
| BC005682 | -0.49119 |
| BC005764 | -0.69785 |
| BC006662 | 1.80649 |
| BC006779 | -0.71475 |
| BC006779 | 1.8886 |
| BC008155 | 1.84799 |
| BC008163 | -0.37998 |
| BC008163 | 0.75191 |
| BC010462 | 0.25455 |
| BC011248 | -0.43589 |
| BC011248 | -0.72286 |
| BC011487 | -0.62748 |
| BC013491 | -0.63321 |
| BC013491 | 0.07946 |
| BC013529 | 0.40023 |
| BC013712 | -0.24081 |
| BC016201 | -0.34391 |
| BC016423 | -0.79733 |
| BC016495 | 0.6547 |
| BC017158 | 1.8306 |
| BC017612 | 1.65542 |
| BC017643 | 0.25892 |
| BC017647 | 1.22014 |
| BC018242 | 2.9048 |
| BC018242 | -0.72196 |
| BC018465 | 0.91397 |
| BC020002 | -0.32024 |
| BC020535 | 0.29491 |
| BC021381 | -0.45261 |
| BC021395 | -0.72196 |
| BC021614 | -0.59698 |
| BC021790 | 2.95683 |
| BC021891 | -0.29318 |
| BC022224 | -0.24834 |
| BC022687 | 2.64498 |
| BC023055 | -0.58599 |
| BC023151 | 1.23575 |
| BC023744 | 2.54899 |
| BC023814 | 1.07872 |
| BC023829 | 2.37208 |
| BC024561 | 0.22113 |
| BC024814 | -0.71206 |
| BC024868 | -0.51543 |
| BC024997 | -0.01695 |
| BC025076 | 2.08715 |
| BC025546 | -0.37129 |
| BC025575 | 0.74625 |
| BC025920 | -0.35416 |
| BC026439 | -0.38749 |
| BC026585 | 3.5413 |
| BC026590 | -0.6113 |
| BC026590 | -0.51833 |
| BC027072 | 0.14935 |
| BC027231 | -0.16079 |
| BC027231 | -0.4926 |
| BC027231 | 0.63611 |
| BC027344 | -0.5448 |
| BC028528 | 1.0188 |
| BC028528 | 1.35926 |
| BC029169 | 1.0526 |
| BC029169 | 4.40039 |
| BC029214 | 1.9417 |
| BC030183 | 1.10363 |
| BC030307 | -0.09128 |
| BC030476 | -0.77406 |
| BC030499 | -0.25485 |
| BC030500 | 0.96471 |
| BC030867 | -0.49826 |
| BC031181 | -0.86453 |
| BC031353 | 0.57532 |
| BC031575 | -0.62017 |
| BC031748 | -0.64728 |
| BC031781 | 0.06074 |
| BC031853 | -0.03049 |
| BC032265 | 1.71032 |
| BC034076 | -0.17067 |
| BC037034 | 0.49351 |
| BC037594 | -0.67179 |
| BC038156 | -0.46142 |
| BC038286 | -0.5899 |
| BC038613 | -0.55154 |
| BC038822 | 0.4111 |
| BC038822 | -0.22066 |
| BC038822 | -0.48838 |
| BC038925 | -0.71296 |
| BC039093 | -0.12373 |
| BC040758 | 0.14935 |
| BC043098 | -0.35841 |
| BC043118 | 0.62468 |
| BC043301 | -0.35476 |
| BC046331 | 0.69486 |
| BC046404 | 1.10288 |
| BC048355 | -0.59304 |
| BC048502 | -0.19431 |
| BC048546 | 0.4013 |
| BC048546 | 1.68443 |
| BC048562 | 2.09271 |
| BC048644 | -0.55606 |
| BC048651 | -0.55379 |
| BC048671 | -0.28527 |
| BC049635 | 0.16602 |
| BC049635 | 0.8747 |
| BC049730 | 1.66146 |
| BC049762 | 3.78274 |
| BC049806 | 0.04123 |
| BC049806 | -0.30402 |
| BC049806 | -0.46006 |
| BC050196 | 3.24582 |
| BC050210 | -0.81818 |
| BC050777 | 0.39943 |
| BC050811 | -0.17815 |
| BC050811 | 0.02009 |
| BC051019 | -0.42273 |
| BC051070 | 1.76587 |
| BC051142 | 1.43688 |
| BC051227 | 1.44299 |
| BC051628 | 2.6869 |
| BC051665 | 1.54969 |
| BC052484 | -0.51471 |
| BC053749 | 1.27107 |
| BC053749 | 2.34 |
| BC054059 | -0.69346 |
| BC055107 | 0.72214 |
| BC055111 | -0.21283 |
| BC055324 | 0.62301 |
| BC055368 | 0.84608 |
| BC056474 | -0.53811 |
| BC056923 | 0.08549 |
| BC057022 | 0.96095 |
| BC057371 | 2.20251 |
| BC057371 | -0.62585 |
| BC057371 | 1.5279 |
| BC057552 | -0.63075 |
| BC057627 | 0.23496 |
| BC057893 | -0.62179 |
| BC060267 | 0.71041 |
| BC060615 | 3.18554 |
| BC060632 | 1.0944 |
| BC061039 | 1.21201 |
| BC061237 | -0.04424 |
| BC062115 | 1.39537 |
| BC062650 | -0.52488 |
| BC063749 | 0.5751 |
| BC064033 | -0.25485 |
| BC065085 | 0.56868 |
| BC065397 | -0.1209 |
| BC066028 | -0.70671 |
| BC066107 | 1.62792 |
| BC067047 | 0.79942 |
| BC067068 | 0.06035 |
| BC068157 | 1.4756 |
| BC068281 | -0.20096 |
| BC072620 | 2.92456 |
| BC085271 | 2.4617 |
| BC087945 | -0.33793 |
| BC088983 | 2.06858 |
| BC089491 | -0.59304 |
| BC099439 | 1.34458 |
| BC107230 | -0.34812 |
| Bcam | 1.73505 |
| Bcan | 1.25025 |
| Bcan | -0.26634 |
| Bcap29 | -0.6523 |
| Bcap29 | -0.72015 |
| Bcap31 | -0.2864 |
| Bcap31 | -0.42208 |
| Bcar1 | -0.43258 |
| Bcar3 | -0.78369 |
| Bcas1 | 0.7212 |
| Bcas2 | -0.78369 |
| Bcas3 | 3.13629 |
| Bcat1 | 0.77082 |
| Bcat2 | -0.59777 |
| Bccip | -0.34992 |
| Bcdin3d | -0.1672 |
| Bcdo2 | -0.66072 |
| Bckdha | -0.7274 |
| Bckdhb | -0.16276 |
| Bckdk | 0.25079 |
| Bckdk | -0.71296 |
| Bcl10 | 2.5716 |
| Bcl11a | 0.48649 |
| Bcl11b | 2.63037 |
| Bcl2 | 0.2263 |
| Bcl2 | -0.43655 |
| Bcl2l1 | 0.98342 |
| Bcl2l1 | 0.753 |
| Bcl2l1 | -0.74393 |
| Bcl2l10 | -0.79341 |
| Bcl2l11 | -0.59856 |
| Bcl2l11 | -0.6226 |
| Bcl2l11 | 0.25892 |
| Bcl2l12 | 3.31573 |
| Bcl2l13 | -0.37998 |
| Bcl2l14 | 0.48746 |
| Bcl2l14 | 0.46362 |
| Bcl2l2 | -0.69433 |
| Bcl2l2 | 0.51659 |
| Bcl3 | -0.44121 |
| Bcl6 | -0.27021 |
| Bcl6b | 0.82111 |
| Bcl7a | 0.62301 |
| Bcl7b | -0.11902 |
| Bcl9l | -0.50895 |
| Bclaf1 | -0.34211 |
| Bclaf1 | -0.57436 |
| Bclp2 | -0.32141 |
| Bcmo1 | -0.70315 |
| Bcor | -0.30173 |
| Bcor | 0.62594 |
| Bcorl1 | -0.05249 |
| Bcs1l | 1.11779 |
| Bdh1 | 1.84584 |
| Bdh2 | -0.25267 |
| Bdkrb1 | -0.62504 |
| Bdnf | 0.45411 |
| Bdnf | -0.50895 |
| Becn1 | -0.54331 |
| Becn1 | -0.54108 |
| Bet1 | -0.10413 |
| Bet1l | 1.56979 |
| Bex2 | 3.43728 |
| Bex2 | -0.44321 |
| Bex2 | 1.34324 |
| Bex4 | -0.13847 |
| Bex4 | 2.33257 |
| Bex6 | -0.74671 |
| Bfar | -0.72286 |
| Bfar | 1.06446 |
| Bglap2 | 1.58454 |
| Bgn | 1.57419 |
| Bhlhb2 | 3.4881 |
| Bhlhb5 | -0.35476 |
| Bhlhb8 | 0.04752 |
| Bhlhb8 | 1.20804 |
| Bhlhb9 | 1.39012 |
| Bhmt | -0.05075 |
| Bhmt2 | -0.56975 |
| Bicc1 | -0.12279 |
| Bicd1 | -0.45058 |
| Bicd2 | 2.40214 |
| Bicd2 | 0.9719 |
| Bid | -0.59935 |
| Bid | -0.15394 |
| Bik | 1.36665 |
| Bin1 | 1.55851 |
| Bin1 | -0.11809 |
| Bin1 | 0.29222 |
| Bin3 | 0.59279 |
| Birc2 | -0.72649 |
| Birc2 | -0.24834 |
| Birc2 | -0.152 |
| Birc4 | 1.41725 |
| Birc5 | 0.11543 |
| Birc5 | 2.57011 |
| Birc5 | 0.57134 |
| Blcap | 1.45128 |
| Blm | -0.49542 |
| Blm | 3.64088 |
| Blmh | 0.0205 |
| Blnk | -0.70138 |
| Bloc1s1 | -0.45802 |
| Bloc1s3 | -0.71835 |
| Blvra | 0.07339 |
| Blvrb | 0.83998 |
| Bmf | 1.97186 |
| Bmi1 | 1.3372 |
| Bmp1 | -0.44454 |
| Bmp1 | -0.0728 |
| Bmp1 | 0.20279 |
| Bmp1 | 1.4032 |
| Bmp15 | 1.54625 |
| Bmp2k | 1.30118 |
| Bmp2k | -0.0881 |
| Bmp3 | 0.85653 |
| Bmp4 | 1.37565 |
| Bmp6 | -0.62504 |
| Bmp7 | 2.41289 |
| Bmp8a | 0.93149 |
| Bmper | -0.69521 |
| Bmpr1a | 2.04532 |
| Bmpr1a | 1.02111 |
| Bmpr1b | 1.45721 |
| Bmpr1b | 1.86126 |
| Bmpr2 | -0.7076 |
| Bms1 | -0.71835 |
| Bnc1 | 1.9421 |
| Bnc2 | -0.01109 |
| Bnc2 | 0.42107 |
| Bnip1 | -0.59777 |
| Bnip2 | 0.95498 |
| Bnip3l | 1.84733 |
| Bnipl | 2.10669 |
| Bnipl | -0.6057 |
| Bok | 0.1698 |
| Bok | -0.66072 |
| Bola1 | 1.47478 |
| Bola2 | 1.93891 |
| Bola3 | -0.75044 |
| Boll | 1.48253 |
| Bop1 | -0.10136 |
| Bpi | -0.05468 |
| Bpil1 | 1.85333 |
| Bpil3 | -0.59777 |
| Bpnt1 | -0.63239 |
| Bpnt1 | -0.55229 |
| Bpnt1 | -0.39697 |
| Bpnt1 | -0.66411 |
| Brap | -0.7617 |
| Brca2 | 0.30385 |
| Brca2 | -0.68126 |
| Brcc3 | -0.4933 |
| Brcc3 | -0.74764 |
| Brcc3 | 1.76804 |
| Brd2 | 2.48997 |
| Brd2 | -0.63321 |
| Brd2 | 0.80184 |
| Brd4 | 1.86071 |
| Brd4 | 0.72176 |
| Brd8 | -0.66667 |
| Brd8 | -0.71475 |
| Brd9 | 2.13931 |
| Brd9 | -0.35053 |
| Brdt | -0.58132 |
| Brdt | -0.69785 |
| Bre | 0.30798 |
| Bre | -0.06835 |
| Bre | -0.43126 |
| Brf1 | 2.13766 |
| Brf2 | 1.77524 |
| Bri3 | 2.63235 |
| Brip1 | -0.6073 |
| Brms1 | 1.64879 |
| Brp16 | 0.64861 |
| Brp16 | 0.00299 |
| Brp17 | 2.24469 |
| Brp44 | -0.60411 |
| Brpf1 | 0.02773 |
| Brpf1 | -0.44254 |
| Brs3 | -0.45938 |
| Brsk1 | -0.5606 |
| Brsk2 | -0.72831 |
| Brsk2 | -0.73471 |
| Brunol4 | -0.69609 |
| Brunol4 | -0.68996 |
| Brwd1 | -0.67522 |
| Brwd1 | -0.51543 |
| Brwd2 | 0.99103 |
| Bscl2 | 0.04044 |
| Bsdc1 | -0.63075 |
| Bsg | 0.57972 |
| Bsg | 1.14772 |
| Bspry | 0.22015 |
| Bst2 | -0.7523 |
| Btbd1 | 2.0933 |
| Btbd10 | -0.64812 |
| Btbd11 | 0.74808 |
| Btbd12 | -0.04078 |
| Btbd14a | -0.70493 |
| Btbd14b | -0.6065 |
| Btbd16 | -0.2478 |
| Btbd3 | 0.22984 |
| Btbd3 | -0.22329 |
| Btbd6 | 2.79497 |
| Btbd9 | 1.03819 |
| Btc | -0.55455 |
| Btd | 2.5311 |
| Btf3 | -0.67008 |
| Btg1 | 1.09151 |
| Btg1 | 0.06651 |
| Btg3 | -0.67522 |
| Btg3 | -0.46415 |
| Btg4 | 0.77438 |
| Btk | -0.46415 |
| Btla | -0.80423 |
| Btrc | -0.17465 |
| Btrc | -0.49755 |
| Btrc | 2.02941 |
| Bub1b | -0.14957 |
| Bub3 | 1.33999 |
| Bud13 | 1.02016 |
| Bud31 | -0.10366 |
| Bves | 1.00454 |
| Bxdc1 | 1.05063 |
| Bxdc1 | -0.43788 |
| Bxdc2 | 0.16774 |
| Bxdc2 | 2.25287 |
| Bxdc5 | 1.51839 |
| Bxdc5 | 2.10431 |
| Bxdc5 | -0.49472 |
| Bzrap1 | 0.45134 |
| Bzrpl1 | -0.51399 |
| Bzw1 | -0.62341 |
| Bzw2 | 2.8493 |
| Bzw2 | 0.97003 |
| C030006K11Rik | 0.84477 |
| C030011O14Rik | -0.62098 |
| C030014K22Rik | 0.05919 |
| C030018G13Rik | 2.08953 |
| C030030A07Rik | -0.67781 |
| C030039L03Rik | 0.57355 |
| C030045D06Rik | 0.02532 |
| C030045D06Rik | -0.56516 |
| C030046I01Rik | -0.60014 |
| C030048B08Rik | -0.5954 |
| C030048B08Rik | -0.55379 |
| C130022K22Rik | 1.71747 |
| C130032J12Rik | 1.89171 |
| C130034I18Rik | -0.56745 |
| C130038G02Rik | -0.57668 |
| C130060K24Rik | 3.94321 |
| C130073D16Rik | 1.82264 |
| C130074G19Rik | -0.84571 |
| C130090K23Rik | -0.69171 |
| C130090K23Rik | 2.84604 |
| C1galt1 | 0.22178 |
| C1galt1c1 | -0.13036 |
| C1qa | -0.16621 |
| C1qb | 0.54688 |
| C1qbp | 1.47212 |
| C1qc | -0.74857 |
| C1qdc2 | 0.55751 |
| C1ql2 | -0.48 |
| C1ql3 | 0.54141 |
| C1qtnf1 | 0.37618 |
| C1qtnf2 | 0.97632 |
| C1qtnf3 | 2.19433 |
| C1qtnf3 | -0.47101 |
| C1qtnf3 | 1.46226 |
| C1qtnf4 | 0.91944 |
| C1qtnf6 | -0.43854 |
| C1qtnf7 | -0.52488 |
| C1qtnf9 | 0.56065 |
| C1rl | 0.70046 |
| C1s | 4.12421 |
| C1s | 0.30503 |
| C2 | -0.29488 |
| C230021P08Rik | 0.97175 |
| C230029F24Rik | -0.63733 |
| C230052I12Rik | -0.44588 |
| C230052I12Rik | 0.90784 |
| C230055K05Rik | 3.21875 |
| C230071H18Rik | -0.3069 |
| C230078M08Rik | -0.68386 |
| C230078M14Rik | -0.66923 |
| C230090D14 | 0.03926 |
| C230093N12Rik | 0.63858 |
| C230096C10Rik | -0.30517 |
| C2cd3 | 0.86252 |
| C3 | -0.59225 |
| C330001K17Rik | 0.75027 |
| C330005M16Rik | -0.72558 |
| C330016O10Rik | -0.74023 |
| C330019G07Rik | -0.33734 |
| C330023M02Rik | -0.66496 |
| C330027C09Rik | -0.57513 |
| C430003P19Rik | -0.71745 |
| C430004E15Rik | -0.03348 |
| C4b | 0.1807 |
| C530028I08Rik | 2.55857 |
| C530028I08Rik | -0.33377 |
| C530028O21Rik | 0.03052 |
| C530043G21Rik | -0.47515 |
| C530044N13Rik | 3.68008 |
| C6 | 0.34356 |
| C630004H02Rik | -0.49542 |
| C630004H02Rik | -0.02666 |
| C630007B19Rik | -0.30001 |
| C630028N24Rik | 0.98906 |
| C630035N08Rik | -0.46963 |
| C630035N08Rik | -0.64812 |
| C730025P13Rik | -0.64146 |
| C730027P07Rik | -0.17915 |
| C78339 | -0.11015 |
| C79127 | 3.39838 |
| C79267 | 0.30028 |
| C79407 | -0.79635 |
| C79407 | 3.25876 |
| C80008 | 1.62356 |
| C80913 | 0.28559 |
| C85492 | 1.07592 |
| C85492 | -0.66242 |
| C85492 | -0.45396 |
| C85627 | -0.06657 |
| C87436 | 3.26532 |
| C87436 | 1.85372 |
| C87499 | -0.7523 |
| C8b | 1.93555 |
| C8g | 2.20517 |
| C9 | 2.5423 |
| C9 | 0.97404 |
| C920005C14Rik | 3.83969 |
| Cab39l | 0.26171 |
| Cabc1 | 0.76546 |
| Cables2 | -0.56516 |
| Cabp2 | -0.31325 |
| Cabp7 | 0.8793 |
| Cabyr | 2.19641 |
| Cabyr | 1.80267 |
| Cachd1 | 1.35841 |
| Cacna1a | -0.6097 |
| Cacna1b | -0.45599 |
| Cacna1b | 0.20971 |
| Cacna1b | 0.31269 |
| Cacna1d | -0.55154 |
| Cacna1g | -0.56975 |
| Cacna1h | 2.35491 |
| Cacna2d1 | 0.67371 |
| Cacna2d3 | -0.50252 |
| Cacnb1 | -0.64979 |
| Cacnb1 | -0.52051 |
| Cacnb2 | 2.66417 |
| Cacnb2 | 0.18172 |
| Cacnb3 | -0.39633 |
| Cacnb3 | -0.41299 |
| Cacnb3 | 2.28286 |
| Cacnb3 | -0.65734 |
| Cacnb4 | 1.36591 |
| Cacnb4 | 2.30078 |
| Cacng2 | 1.05181 |
| Cacng3 | -0.5068 |
| Cacng3 | -0.20507 |
| Cacng4 | 0.95455 |
| Cacng5 | -0.67094 |
| Cacng5 | -0.15394 |
| Cacng5 | -0.54704 |
| Cacng7 | 1.96901 |
| Cacng8 | -0.42143 |
| Cacybp | -0.16424 |
| Cad | -0.30978 |
| Cadm2 | -0.62017 |
| Cadm3 | -0.33377 |
| Cadps | -0.32492 |
| Cadps2 | 1.98656 |
| Cadps2 | 0.43787 |
| Cage1 | -0.27132 |
| Calb2 | 1.23768 |
| Calb2 | -0.5954 |
| Calca | 0.92186 |
| Calca | 1.16472 |
| Calcoco1 | -0.68996 |
| Cald1 | 1.98099 |
| Cald1 | -0.66242 |
| Calm2 | 2.19973 |
| Calm3 | -0.5068 |
| Calml4 | -0.51688 |
| Caln1 | 0.11069 |
| Calr | -0.32845 |
| Calr3 | -0.6283 |
| Calr3 | -0.36085 |
| Calr3 | -0.49684 |
| Calr4 | 1.66071 |
| Calu | -0.4059 |
| Calu | -0.05775 |
| Calu | 0.93746 |
| Caly | -0.67265 |
| Camk1 | 2.30542 |
| Camk1d | 1.16877 |
| Camk1g | 0.45788 |
| Camk1g | 1.29273 |
| Camk2a | -0.70404 |
| Camk2a | 0.5398 |
| Camk2a | 0.26295 |
| Camk2b | -0.42273 |
| Camk2d | -0.16325 |
| Camk2d | 1.05574 |
| Camk2d | -0.48488 |
| Camk2g | 0.9595 |
| Camk2g | 1.09202 |
| Camk2n1 | 0.82431 |
| Camk4 | 0.52874 |
| Camkk1 | 1.1979 |
| Camkk2 | 2.1509 |
| Camkk2 | 1.56582 |
| Camkv | 0.26574 |
| Caml | -0.17167 |
| Camp | 2.04754 |
| Camsap1l1 | 1.94265 |
| Camta2 | -0.15638 |
| Cant1 | -0.36944 |
| Cant1 | -0.53811 |
| Cap1 | -0.34092 |
| Capg | -0.48279 |
| Capg | -0.59225 |
| Capn1 | -0.04597 |
| Capn10 | -0.52561 |
| Capn11 | 0.05259 |
| Capn2 | 2.27696 |
| Capn2 | -0.62667 |
| Capn2 | 0.35428 |
| Capn3 | 0.27587 |
| Capn5 | 0.26481 |
| Capn6 | -0.49472 |
| Capn7 | -0.6283 |
| Capn8 | 1.19801 |
| Capns1 | -0.52342 |
| Caprin1 | -0.78175 |
| Caprin1 | -0.45734 |
| Capsl | -0.47722 |
| Capza1 | 1.51284 |
| Capza3 | -0.11201 |
| Capzb | -0.47792 |
| Capzb | 1.52186 |
| Capzb | 1.89376 |
| Car11 | -0.39697 |
| Car12 | 0.2777 |
| Car13 | 1.23467 |
| Car14 | -0.09083 |
| Car15 | -0.24241 |
| Car2 | -0.34151 |
| Car3 | 0.33272 |
| Car4 | 0.12843 |
| Car5b | -0.44187 |
| Car5b | -0.38812 |
| Car5b | -0.15932 |
| Car7 | -0.67953 |
| Car9 | 2.09132 |
| Card10 | 0.16464 |
| Card11 | -0.2791 |
| Carhsp1 | 2.86081 |
| Carm1 | 0.19215 |
| Cart | 2.78263 |
| Cartpt | 1.00163 |
| Casc1 | 1.72495 |
| Casc1 | 2.40681 |
| Casc1 | 4.37675 |
| Casc3 | -0.26579 |
| Casc4 | 0.02211 |
| Casc4 | -0.53811 |
| Casc4 | -0.32669 |
| Cask | -0.6113 |
| Caskin1 | -0.33734 |
| Caskin1 | 1.35408 |
| Caskin1 | -0.5644 |
| Caskin2 | -0.76738 |
| Casp1 | -0.37935 |
| Casp2 | 1.86038 |
| Casp4 | -0.6057 |
| Casp4 | -0.50395 |
| Casp4 | 0.56801 |
| Casp6 | -0.62341 |
| Casp6 | -0.39507 |
| Casp7 | 0.32261 |
| Casp8 | 1.31883 |
| Casp8 | -0.59383 |
| Casp9 | 0.60376 |
| Casq2 | -0.47239 |
| Cass4 | 0.2777 |
| Cast | -0.64645 |
| Cat | 0.16602 |
| Catsper1 | 3.65486 |
| Catsper2 | -0.69521 |
| Catsper3 | -0.58365 |
| Catsper4 | 1.07361 |
| Cav1 | -0.68821 |
| Cav2 | 0.8659 |
| Cbara1 | 2.0765 |
| Cbfa2t3h | -0.44991 |
| Cbfa2t3h | -0.17216 |
| Cbfb | -0.70404 |
| Cbfb | -0.13369 |
| Cbfb | 0.35736 |
| Cbl | 0.25674 |
| Cblb | -0.74485 |
| Cblc | -0.28527 |
| Cbln1 | 0.89158 |
| Cbln2 | 0.44424 |
| Cbln4 | 2.40205 |
| Cbr1 | 0.16395 |
| Cbr2 | -0.67351 |
| Cbr3 | 0.83949 |
| Cbr3 | -0.49119 |
| Cbs | -0.76359 |
| Cbs | 1.28043 |
| Cbwd1 | 1.18334 |
| Cbx2 | 1.24739 |
| Cbx5 | 0.48892 |
| Cbx7 | 0.36737 |
| Cbx8 | 0.51659 |
| Cby1 | -0.41428 |
| Cc2d1a | -0.43126 |
| Cc2d1b | -0.63898 |
| Cc2d2a | 1.67395 |
| Ccar1 | -0.40847 |
| Ccbe1 | -0.54033 |
| Ccbl1 | 1.25257 |
| Ccbl1 | 1.20359 |
| Ccbl2 | -0.25812 |
| Ccbp2 | 0.29701 |
| Ccdc100 | 0.04869 |
| Ccdc101 | -0.30805 |
| Ccdc108 | 0.40449 |
| Ccdc109a | -0.02539 |
| Ccdc109a | 1.38547 |
| Ccdc109b | 1.70343 |
| Ccdc11 | -0.72558 |
| Ccdc110 | 0.01767 |
| Ccdc111 | -0.61613 |
| Ccdc113 | -0.6073 |
| Ccdc114 | 0.51565 |
| Ccdc115 | 0.14759 |
| Ccdc116 | -0.31732 |
| Ccdc117 | -0.15297 |
| Ccdc12 | -0.75137 |
| Ccdc120 | 2.25952 |
| Ccdc123 | -0.50039 |
| Ccdc124 | 0.67411 |
| Ccdc125 | 0.14865 |
| Ccdc126 | -0.55079 |
| Ccdc127 | 0.49495 |
| Ccdc127 | 2.72597 |
| Ccdc13 | -0.48838 |
| Ccdc130 | 0.70065 |
| Ccdc131 | 0.29581 |
| Ccdc132 | 1.28115 |
| Ccdc134 | -0.32082 |
| Ccdc137 | 2.85723 |
| Ccdc137 | -0.5068 |
| Ccdc18 | -0.5644 |
| Ccdc21 | 2.61787 |
| Ccdc23 | -0.62667 |
| Ccdc25 | 0.15459 |
| Ccdc27 | -0.43921 |
| Ccdc27 | 0.19682 |
| Ccdc27 | -0.67781 |
| Ccdc28a | -0.2791 |
| Ccdc28b | -0.32904 |
| Ccdc28b | -0.71206 |
| Ccdc3 | 1.13618 |
| Ccdc32 | 0.24512 |
| Ccdc38 | -0.67179 |
| Ccdc39 | 0.13129 |
| Ccdc40 | -0.29431 |
| Ccdc41 | 2.67357 |
| Ccdc42 | -0.57205 |
| Ccdc43 | 0.3229 |
| Ccdc45 | -0.45396 |
| Ccdc46 | 0.50429 |
| Ccdc46 | -0.51616 |
| Ccdc47 | 1.96338 |
| Ccdc49 | -0.48139 |
| Ccdc5 | -0.65482 |
| Ccdc50 | -0.5068 |
| Ccdc51 | 2.73436 |
| Ccdc53 | 2.23522 |
| Ccdc53 | -0.32963 |
| Ccdc53 | 0.36266 |
| Ccdc54 | 0.47476 |
| Ccdc56 | 0.28166 |
| Ccdc57 | 0.10886 |
| Ccdc58 | -0.44121 |
| Ccdc59 | -0.80324 |
| Ccdc59 | -0.3314 |
| Ccdc64 | -0.38498 |
| Ccdc65 | 1.97167 |
| Ccdc65 | 0.80819 |
| Ccdc66 | 0.76707 |
| Ccdc67 | -0.8344 |
| Ccdc68 | 0.07681 |
| Ccdc70 | -0.69083 |
| Ccdc71 | -0.29773 |
| Ccdc73 | -0.63981 |
| Ccdc77 | 1.60238 |
| Ccdc79 | 2.44083 |
| Ccdc80 | -0.71655 |
| Ccdc82 | 0.73945 |
| Ccdc84 | 2.22831 |
| Ccdc85a | -0.73838 |
| Ccdc85b | 0.62133 |
| Ccdc86 | 0.9484 |
| Ccdc87 | -0.579 |
| Ccdc88a | -0.85403 |
| Ccdc88b | 0.88515 |
| Ccdc88c | -0.44321 |
| Ccdc90b | 0.7597 |
| Ccdc91 | 0.56935 |
| Ccdc92 | -0.91001 |
| Ccdc93 | -0.48348 |
| Ccdc94 | 3.0833 |
| Ccdc95 | 1.0607 |
| Ccdc96 | 3.06791 |
| Ccdc97 | 1.4241 |
| Ccdc98 | 0.75699 |
| Ccdc99 | 0.25455 |
| Cchcr1 | -0.77406 |
| Ccin | -0.15346 |
| Cck | 1.02057 |
| Cckbr | -0.5821 |
| Ccl11 | -0.48 |
| Ccl17 | -0.75417 |
| Ccl19 | -0.82423 |
| Ccl21b | -0.60332 |
| Ccl21c | 1.97624 |
| Ccl25 | -0.55908 |
| Ccl25 | -0.24564 |
| Ccl27 | 0.05065 |
| Ccl27 | 0.70277 |
| Ccl28 | 1.24845 |
| Ccl3 | -0.55079 |
| Ccl4 | 1.18708 |
| Ccl4 | 0.05919 |
| Ccl5 | 2.70361 |
| Ccl6 | -0.71296 |
| Ccl7 | -0.49472 |
| Ccl9 | -0.6283 |
| Ccm2 | -0.41363 |
| Ccna1 | 1.86938 |
| Ccnb1 | -0.8202 |
| Ccnb1 | -0.56745 |
| Ccnb3 | 0.3646 |
| Ccnc | -0.28583 |
| Ccnd1 | -0.62098 |
| Ccnd1 | -0.02497 |
| Ccnd1 | 0.97917 |
| Ccnd2 | -0.49613 |
| Ccnd3 | -0.52196 |
| Ccnd3 | -0.85612 |
| Ccndbp1 | 1.62876 |
| Ccne1 | -0.28471 |
| Ccne2 | 1.45399 |
| Ccnf | -0.39888 |
| Ccng1 | -0.8202 |
| Ccng1 | -0.48488 |
| Ccng1 | -0.5759 |
| Ccng2 | 1.06795 |
| Ccnk | 0.09743 |
| Ccnl1 | -0.61532 |
| Ccno | 0.0642 |
| Ccr4 | 1.32733 |
| Ccr5 | -0.63733 |
| Ccr5 | -0.71745 |
| Ccr5 | 0.83368 |
| Ccr6 | 1.30687 |
| Ccr6 | 2.1528 |
| Ccrk | 1.36768 |
| Ccrk | 2.3156 |
| Ccrk | -0.14618 |
| Ccrk | 2.18293 |
| Ccrl2 | -0.83952 |
| Ccrn4l | 0.1511 |
| Ccrn4l | 0.65994 |
| Ccs | 1.90322 |
| Cct2 | -0.56516 |
| Cct3 | 1.45238 |
| Cct3 | -0.68734 |
| Cct4 | -0.29488 |
| Cct5 | -0.6113 |
| Cct6a | -0.56136 |
| Cct6b | -0.82828 |
| Cct7 | -0.05863 |
| Cct8 | -0.3314 |
| Cd109 | 0.40952 |
| Cd109 | 3.04705 |
| Cd14 | 0.22404 |
| Cd151 | -0.02116 |
| Cd151 | -0.47722 |
| Cd151 | -0.75417 |
| Cd160 | -0.11388 |
| Cd163 | -0.78079 |
| Cd164 | -0.64895 |
| Cd177 | 0.7996 |
| Cd180 | 1.84101 |
| Cd1d1 | -0.48978 |
| Cd2 | 0.9503 |
| Cd200 | -0.74393 |
| Cd200 | -0.62341 |
| Cd226 | -0.78369 |
| Cd247 | -0.05951 |
| Cd248 | 0.95935 |
| Cd248 | -0.6073 |
| Cd27 | -0.6283 |
| Cd274 | -0.73655 |
| Cd276 | -0.80423 |
| Cd2bp2 | 1.05247 |
| Cd300lg | -0.35841 |
| Cd320 | -0.51039 |
| Cd34 | -0.67008 |
| Cd3d | -0.4621 |
| Cd3eap | 0.67054 |
| Cd3g | -0.62017 |
| Cd4 | 0.86783 |
| Cd40 | -0.00693 |
| Cd40 | -0.09906 |
| Cd44 | 2.33926 |
| Cd44 | -0.68734 |
| Cd44 | -0.45599 |
| Cd44 | -0.9089 |
| Cd47 | -0.03092 |
| Cd52 | 0.08886 |
| Cd53 | -0.64812 |
| Cd55 | 2.59207 |
| Cd59a | -0.48768 |
| Cd59b | 1.26723 |
| Cd59b | -0.43324 |
| Cd6 | 0.18644 |
| Cd6 | -0.65734 |
| Cd6 | -0.03648 |
| Cd63 | -0.48628 |
| Cd63 | 0.01646 |
| Cd63 | -0.87516 |
| Cd68 | -0.22962 |
| Cd74 | -0.27799 |
| Cd74 | -0.62422 |
| Cd79b | -0.75324 |
| Cd79b | 0.30828 |
| Cd81 | -0.63157 |
| Cd82 | -0.81418 |
| Cd83 | -0.83032 |
| Cd84 | -0.69697 |
| Cd86 | -0.2451 |
| Cd86 | -0.74578 |
| Cd86 | -0.54033 |
| Cd8b1 | 2.37898 |
| Cd9 | 0.36294 |
| Cd93 | -0.26195 |
| Cd96 | 0.46784 |
| Cd97 | 1.78859 |
| Cd97 | -0.53515 |
| Cd97 | -0.83235 |
| Cd99l2 | -0.46415 |
| Cdan1 | -0.71027 |
| Cdc123 | 1.72596 |
| Cdc14b | -0.6065 |
| Cdc14b | -0.70938 |
| Cdc14b | 0.81654 |
| Cdc16 | -0.2819 |
| Cdc20 | -0.84468 |
| Cdc20 | 1.72589 |
| Cdc20 | 0.97974 |
| Cdc20 | 0.05376 |
| Cdc23 | -0.57745 |
| Cdc25a | -0.41234 |
| Cdc25b | -0.69785 |
| Cdc25c | -0.22856 |
| Cdc25c | -0.7523 |
| Cdc27 | 1.14701 |
| Cdc2a | 0.77064 |
| Cdc2l1 | -0.82221 |
| Cdc2l1 | -0.60411 |
| Cdc2l5 | 3.68446 |
| Cdc2l6 | 2.70075 |
| Cdc34 | -0.32786 |
| Cdc37 | 0.74185 |
| Cdc37l1 | 1.58218 |
| Cdc37l1 | 1.91633 |
| Cdc37l1 | -0.48628 |
| Cdc37l1 | -0.09585 |
| Cdc42 | 1.83783 |
| Cdc42bpb | 1.35794 |
| Cdc42ep1 | 0.34696 |
| Cdc42ep2 | -0.50252 |
| Cdc42ep3 | -0.54554 |
| Cdc42ep4 | 2.89507 |
| Cdc42ep5 | -0.80919 |
| Cdc42se1 | 0.24954 |
| Cdc42se1 | -0.49049 |
| Cdc45l | 0.21983 |
| Cdc5l | 0.30444 |
| Cdc5l | 1.75908 |
| Cdc5l | -0.28358 |
| Cdc5l | 0.85197 |
| Cdc6 | 0.65044 |
| Cdc7 | 0.54802 |
| Cdc73 | 0.99005 |
| Cdca2 | -0.55984 |
| Cdca3 | 0.18206 |
| Cdca3 | -0.12279 |
| Cdca4 | -0.60093 |
| Cdca4 | 1.18015 |
| Cdca4 | 0.84197 |
| Cdca5 | 3.87214 |
| Cdca7 | -0.04857 |
| Cdca8 | 0.12051 |
| Cdcp1 | -0.35476 |
| Cdgap | -0.72649 |
| Cdh1 | -0.6097 |
| Cdh11 | -0.71565 |
| Cdh11 | -0.21962 |
| Cdh12 | 1.84212 |
| Cdh13 | 1.16912 |
| Cdh13 | -0.09037 |
| Cdh16 | -0.35234 |
| Cdh22 | -0.70404 |
| Cdh23 | -0.58288 |
| Cdh23 | 0.36321 |
| Cdh23 | -0.65314 |
| Cdh24 | 0.8793 |
| Cdh26 | 0.26016 |
| Cdh3 | 0.89951 |
| Cdh5 | -0.25648 |
| Cdh6 | -0.52342 |
| Cdh7 | -0.76076 |
| Cdh8 | 0.28921 |
| Cdh8 | -0.61613 |
| Cdh8 | -0.45058 |
| Cdipt | -0.08087 |
| Cdipt | -0.46552 |
| Cdk10 | -0.66242 |
| Cdk10 | 0.27005 |
| Cdk10 | -0.60411 |
| Cdk2 | 0.86735 |
| Cdk2 | 0.96427 |
| Cdk2 | 0.54983 |
| Cdk2 | -0.2328 |
| Cdk2ap2 | -0.49542 |
| Cdk3 | 0.33673 |
| Cdk5 | -0.70849 |
| Cdk5r1 | -0.32904 |
| Cdk5rap1 | -0.69521 |
| Cdk5rap2 | 2.19122 |
| Cdk5rap3 | 1.76798 |
| Cdk6 | -0.6073 |
| Cdk8 | -0.73105 |
| Cdk9 | -0.66923 |
| Cdkal1 | -0.32727 |
| Cdkl1 | -0.50039 |
| Cdkl2 | -0.41105 |
| Cdkl2 | -0.20507 |
| Cdkl2 | -0.70227 |
| Cdkl2 | 1.03859 |
| Cdkl3 | 1.76072 |
| Cdkl4 | 1.28706 |
| Cdkn1a | 0.29999 |
| Cdkn1a | 1.59427 |
| Cdkn1a | -0.43987 |
| Cdkn1b | -0.23866 |
| Cdkn1b | -0.44187 |
| Cdkn1c | -0.17665 |
| Cdkn2a | 1.35766 |
| Cdkn2a | 0.16153 |
| Cdkn2aipnl | 0.16877 |
| Cdkn2b | -0.53368 |
| Cdkn2c | -0.15297 |
| Cdkn2c | 0.05609 |
| Cdkn2d | 1.2838 |
| Cdo1 | 0.65328 |
| Cdr2 | 0.73465 |
| Cdrt4 | -0.50323 |
| Cds1 | 0.96946 |
| Cds2 | -0.33377 |
| Cdsn | 0.49807 |
| Cdt1 | 1.63581 |
| Cdv3 | -0.34211 |
| Cdv3 | 2.92144 |
| Cdyl | 0.01565 |
| Cdyl | 0.71818 |
| Cdyl | -0.37935 |
| Cdyl2 | -0.59147 |
| Ceacam1 | 0.61312 |
| Ceacam13 | -0.12893 |
| Ceacam2 | -0.71206 |
| Cebpa | 0.55028 |
| Cebpb | -0.62179 |
| Cecr5 | 0.56601 |
| Cecr6 | -0.02921 |
| Celsr1 | -0.36513 |
| Celsr2 | 0.0588 |
| Celsr3 | 1.47684 |
| Cend1 | 0.07681 |
| Cenpa | 1.64453 |
| Cenpb | -0.39633 |
| Cenph | 0.71648 |
| Cenpi | -0.63568 |
| Cenpj | -0.58677 |
| Cenpk | 0.21886 |
| Cenpl | 1.63706 |
| Cenpl | -0.68213 |
| Cenpm | -0.01151 |
| Cenpm | -0.56593 |
| Cenpm | -0.65566 |
| Cenpn | 1.60892 |
| Cenpp | -0.69083 |
| Cenpq | 0.92729 |
| Cenpt | -0.65146 |
| Centa1 | 0.07605 |
| Centa1 | 1.28268 |
| Centa2 | -0.64478 |
| Centb5 | 2.36021 |
| Centb5 | -0.07013 |
| Centd2 | 1.83044 |
| Centd2 | 0.13629 |
| Centd3 | 0.1598 |
| Centg1 | 1.9903 |
| Centg1 | 0.09185 |
| Centg2 | -0.45463 |
| Centg2 | 0.34555 |
| Centg3 | 0.35091 |
| Centg3 | -0.50824 |
| Cep135 | 1.99492 |
| Cep164 | -0.48209 |
| Cep170 | 2.33735 |
| Cep55 | -0.49826 |
| Cep57 | -0.6073 |
| Cep57 | -0.01821 |
| Cep63 | 0.45235 |
| Cep68 | -0.44454 |
| Cep70 | 2.47603 |
| Cep72 | -0.19839 |
| Cep78 | 1.78941 |
| Cep97 | 0.26543 |
| Cept1 | -0.15736 |
| Cercam | -0.49542 |
| Ces3 | -0.10736 |
| Cetn2 | -0.24081 |
| Cetn3 | 2.70389 |
| Cetn4 | -0.31151 |
| Cetn4 | 0.64922 |
| Cfd | 1.20237 |
| Cfdp1 | 1.22841 |
| Cfh | 1.16715 |
| Cfhr1 | -0.64229 |
| Cfi | -0.2791 |
| Cfl1 | 0.25267 |
| Cfl2 | -0.65819 |
| Cfl2 | 1.37989 |
| Cfp | 1.12824 |
| Cftr | 0.28135 |
| Cga | 0.76366 |
| Cggbp1 | 0.16257 |
| Cgnl1 | 2.26093 |
| Cgrrf1 | 2.45011 |
| Ch25h | 0.51424 |
| Chac1 | 1.94573 |
| Chad | 1.29928 |
| Chaf1a | 0.41216 |
| Chaf1b | 0.828 |
| Chat | 1.54102 |
| Chchd1 | 0.95235 |
| Chchd2 | -0.22276 |
| Chchd4 | -0.4104 |
| Chchd5 | 0.86654 |
| Chchd6 | 2.35729 |
| Chchd7 | -0.37438 |
| Chchd8 | 0.08586 |
| Chd1 | 0.7726 |
| Chd1 | -0.25757 |
| Chd1l | -0.54331 |
| Chd1l | -0.07862 |
| Chd4 | -0.48488 |
| Chd4 | 0.51612 |
| Chd4 | 2.46634 |
| Chd5 | -0.60252 |
| Chd8 | 0.46933 |
| Chdh | -0.09448 |
| Chdh | 2.79197 |
| Chek1 | -0.64562 |
| Chek1 | -0.41363 |
| Chek2 | -0.25267 |
| Chek2 | -0.0061 |
| Cherp | -0.38875 |
| Chfr | -0.65398 |
| Chfr | -0.78079 |
| Chfr | -0.59147 |
| Chga | 1.43775 |
| Chga | 0.75173 |
| Chga | -0.06171 |
| Chgb | -0.77119 |
| Chi3l1 | 3.46419 |
| Chi3l3 | 2.06754 |
| Chi3l3 | 1.66564 |
| Chia | 2.35204 |
| Chia | 0.53567 |
| Chic1 | 4.2929 |
| Chic2 | -0.24027 |
| Chic2 | 0.11942 |
| Chit1 | 1.08051 |
| Chka | 1.3344 |
| Chka | -0.66327 |
| Chka | 2.66807 |
| Chkb | -0.38185 |
| Chl1 | -0.69962 |
| Chl1 | -0.49542 |
| Chl1 | 0.61227 |
| Chm | -0.77311 |
| Chmp1b | -0.51111 |
| Chmp2a | 2.29973 |
| Chmp2a | 1.17935 |
| Chmp2b | -0.73931 |
| Chmp4b | -0.07057 |
| Chmp5 | 0.00995 |
| Chmp7 | 2.37224 |
| Chmp7 | -0.77406 |
| Chmp7 | -0.42077 |
| Chn2 | 0.26543 |
| Chodl | 1.45196 |
| Chordc1 | 1.86365 |
| Chpf | 1.88918 |
| Chpt1 | -0.70138 |
| Chpt1 | -0.57513 |
| Chpt1 | -0.46552 |
| Chrac1 | 1.52665 |
| Chrd | 1.64893 |
| Chrm1 | -0.21962 |
| Chrm2 | -0.01737 |
| Chrm3 | 0.88735 |
| Chrna1 | -0.24564 |
| Chrna4 | -0.38185 |
| Chrna4 | 0.66675 |
| Chrna4 | -0.52488 |
| Chrna5 | -0.48558 |
| Chrna6 | -0.67608 |
| Chrna6 | 0.55751 |
| Chrna7 | -0.73655 |
| Chrna9 | -0.20301 |
| Chrnb1 | 0.33873 |
| Chrnb1 | 1.59949 |
| Chrnb2 | -0.81218 |
| Chrnb2 | -0.6065 |
| Chrnb3 | -0.50395 |
| Chrnb3 | -0.70315 |
| Chrnb3 | 0.61481 |
| Chrne | -0.1938 |
| Chst1 | 0.3895 |
| Chst10 | 1.56597 |
| Chst12 | 0.89765 |
| Chst14 | -0.57513 |
| Chst2 | 1.5727 |
| Chst3 | 0.34781 |
| Chst3 | -0.36575 |
| Chst7 | 1.68818 |
| Chst8 | 1.63246 |
| Chtf18 | -0.66327 |
| Chuk | -0.41428 |
| Churc1 | 1.39447 |
| Ciao1 | 3.01866 |
| Ciapin1 | 1.91191 |
| Cib1 | 0.15075 |
| Cib2 | 2.61413 |
| Cib2 | -0.4059 |
| Cic | 1.03766 |
| Cidea | -0.09265 |
| Cideb | -0.28022 |
| Cidec | 2.33586 |
| Ciita | 0.96946 |
| Cirbp | 3.32682 |
| Cirh1a | -0.53663 |
| Cisd1 | -0.62098 |
| Cish | -0.56516 |
| Cit | 2.53918 |
| Cited1 | 3.60315 |
| Cited2 | -0.36085 |
| Cited4 | -0.2691 |
| Ciz1 | 1.16006 |
| Ciz1 | -0.68821 |
| Ckap2l | -0.29091 |
| Ckap5 | -0.11621 |
| Ckb | 1.32859 |
| Cklf | -0.64895 |
| Cklf | -0.24403 |
| Ckmt1 | 0.32029 |
| Ckmt2 | -0.01444 |
| Ckmt2 | -0.57436 |
| Ckt2 | -0.50323 |
| Ckt2 | 0.67193 |
| Clca5 | 2.00013 |
| Clcc1 | 0.08774 |
| Clcf1 | 0.80956 |
| Clcf1 | -0.17965 |
| Clcf1 | 0.27892 |
| Clcn1 | -0.40015 |
| Clcn2 | -0.51183 |
| Clcn3 | 1.88654 |
| Clcn3 | -0.3633 |
| Clcn3 | 2.62018 |
| Clcn4-2 | -0.57205 |
| Clcn7 | -0.58055 |
| Clcnka | -0.41557 |
| Cldn1 | -0.55004 |
| Cldn10 | 1.26899 |
| Cldn10 | 0.92232 |
| Cldn10 | 0.6359 |
| Cldn11 | 2.34191 |
| Cldn12 | -0.66072 |
| Cldn15 | 0.12915 |
| Cldn15 | -0.52853 |
| Cldn17 | -0.57822 |
| Cldn2 | -0.42863 |
| Cldn23 | 0.63672 |
| Cldn3 | -0.35659 |
| Cldn4 | 1.70175 |
| Cldn5 | -0.51183 |
| Cldn6 | -0.26469 |
| Cldn9 | 0.68357 |
| Cldnd1 | -0.35963 |
| Cldnd2 | -0.09037 |
| Clec11a | 0.21135 |
| Clec16a | -0.81218 |
| Clec1a | -0.76265 |
| Clec2d | 1.66139 |
| Clec2e | -0.48698 |
| Clec3b | 0.17765 |
| Clec4a1 | 2.08519 |
| Clec4a3 | 2.22171 |
| Clec4b1 | 0.3495 |
| Clec4b1 | 1.91735 |
| Clec4d | -0.72105 |
| Clec4g | 0.22855 |
| Clec4n | 0.68062 |
| Clec7a | 1.46962 |
| Clecsf12 | -0.43788 |
| Clgn | 0.26759 |
| Clic1 | 1.6196 |
| Clic3 | -0.63321 |
| Clic4 | 0.35147 |
| Clic6 | 2.31334 |
| Clip1 | 0.45838 |
| Clip1 | 3.08838 |
| Clip4 | -0.64395 |
| Clk1 | 0.99899 |
| Clk2 | 1.7353 |
| Clk2 | -0.66837 |
| Clk2 | -0.65734 |
| Clk3 | 0.9103 |
| Clk4 | 1.20571 |
| Clk4 | -0.70404 |
| Clk4 | -0.82727 |
| Clm3 | 0.71003 |
| Clm3 | 1.26484 |
| Clmn | -0.59225 |
| Clmn | -0.64979 |
| Cln3 | -0.51543 |
| Cln5 | 0.33873 |
| Cln6 | 1.05796 |
| Cln6 | -0.35902 |
| Cln8 | -0.83032 |
| Clns1a | -0.44454 |
| Clns1a | -0.07503 |
| Clns1a | 1.67302 |
| Clock | -0.19891 |
| Clock | -0.07057 |
| Clp1 | -0.43258 |
| Clpb | 1.87138 |
| Clpp | 0.0588 |
| Clptm1 | 1.3997 |
| Clptm1l | -0.69258 |
| Clpx | 1.72608 |
| Clpx | -0.0464 |
| Clrn1 | 0.37261 |
| Clspn | -0.68386 |
| Clspn | 0.92909 |
| Clspn | 1.23079 |
| Clstn1 | 2.97578 |
| Clstn3 | -0.8509 |
| Clstn3 | 0.48162 |
| Clta | -0.11996 |
| Cltb | 0.42159 |
| Cluap1 | 0.83118 |
| Cma1 | 2.03565 |
| Cma1 | 0.50119 |
| Cmas | 0.49278 |
| Cmbl | -0.58599 |
| Cml1 | -0.57977 |
| Cml3 | -0.58443 |
| Cml4 | 1.8979 |
| Cmpk | -0.69258 |
| Cmtm2a | 3.13448 |
| Cmtm2b | 0.77721 |
| Cmtm4 | -0.73931 |
| Cmtm4 | -0.7993 |
| Cmtm5 | -0.55154 |
| Cmtm6 | -0.58599 |
| Cmtm7 | 4.36269 |
| Cmtm7 | 0.32696 |
| Cmtm7 | 3.34873 |
| Cmtm8 | -0.76454 |
| Cmtm8 | 1.68575 |
| Cmya1 | -0.63157 |
| CN716893 | 1.29797 |
| Cnbp2 | 1.76097 |
| Cnbp2 | -0.45058 |
| Cndp1 | 1.32089 |
| Cnga4 | -0.47515 |
| Cnih | -0.20455 |
| Cnih3 | 0.16361 |
| Cnih4 | 1.34162 |
| Cnksr2 | 1.59245 |
| Cnksr2 | -0.13226 |
| Cnksr3 | -0.03092 |
| Cnn1 | 1.48524 |
| Cnn2 | -0.8212 |
| Cnn2 | 0.52641 |
| Cnnm1 | 2.06493 |
| Cnnm1 | -0.34511 |
| Cnnm2 | -0.27076 |
| Cnnm3 | -0.60332 |
| Cno | -0.60252 |
| Cnot10 | -0.01151 |
| Cnot2 | -0.02751 |
| Cnot2 | 0.66054 |
| Cnot2 | 0.91899 |
| Cnot2 | -0.40398 |
| Cnot3 | 1.09653 |
| Cnot3 | 0.93254 |
| Cnot4 | -0.73471 |
| Cnot4 | 1.63177 |
| Cnot4 | -0.33437 |
| Cnot6l | -0.51255 |
| Cnot6l | -0.46757 |
| Cnot7 | -0.76076 |
| Cnp | 0.02009 |
| Cnp | -0.05337 |
| Cnpy2 | 2.40645 |
| Cnpy3 | -0.72558 |
| Cnr1 | -0.13274 |
| Cnr1 | -0.74023 |
| Cntd1 | 0.14266 |
| Cntd1 | 3.78748 |
| Cntfr | -0.24726 |
| Cntln | -0.53885 |
| Cntn1 | 1.48319 |
| Cntn4 | 0.53313 |
| Cntn6 | 1.00509 |
| Cntnap1 | -0.6523 |
| Cntnap2 | 0.82077 |
| Cntnap4 | 2.94854 |
| Cntnap4 | 0.75064 |
| Coasy | 0.52711 |
| Cobl | 1.42156 |
| Cobll1 | -0.79733 |
| Cobll1 | -0.21335 |
| Coch | -0.15981 |
| Cog1 | 1.97259 |
| Cog2 | -0.46552 |
| Cog3 | -0.62341 |
| Cog4 | 0.00381 |
| Cog6 | 1.88149 |
| Cog6 | -0.51543 |
| Cog8 | -0.26855 |
| Coil | 3.11473 |
| Coil | -0.3578 |
| Col11a1 | 2.00452 |
| Col12a1 | 1.29091 |
| Col14a1 | 3.05626 |
| Col15a1 | -0.66327 |
| Col16a1 | 2.09651 |
| Col17a1 | -0.29318 |
| Col17a1 | -0.58443 |
| Col17a1 | 3.18207 |
| Col18a1 | 0.69911 |
| Col18a1 | -0.46757 |
| Col1a1 | -0.64728 |
| Col1a2 | 0.59366 |
| Col24a1 | 0.55368 |
| Col24a1 | -0.70849 |
| Col25a1 | 1.54771 |
| Col25a1 | 1.92627 |
| Col25a1 | 1.63636 |
| Col27a1 | -0.64395 |
| Col28a1 | -0.22645 |
| Col2a1 | -0.78854 |
| Col2a1 | -0.56822 |
| Col3a1 | 1.03952 |
| Col3a1 | -0.55229 |
| Col4a1 | 0.0205 |
| Col4a2 | 0.13165 |
| Col4a3 | -0.38561 |
| Col4a3bp | 1.86495 |
| Col4a3bp | -0.73746 |
| Col4a3bp | 0.11325 |
| Col4a4 | 1.72331 |
| Col4a4 | 0.76725 |
| Col4a5 | -0.12326 |
| Col4a6 | 1.17809 |
| Col4a6 | -0.60252 |
| Col5a1 | -0.36944 |
| Col5a3 | 0.52827 |
| Col6a1 | 2.50503 |
| Col6a1 | 0.83185 |
| Col6a1 | 0.87978 |
| Col6a2 | -0.51616 |
| Col7a1 | -0.4717 |
| Col8a2 | -0.426 |
| Col9a2 | -0.02201 |
| Colec12 | 0.40236 |
| Commd1 | 0.73279 |
| Commd1 | -0.71835 |
| Commd10 | -0.46415 |
| Commd2 | -0.56212 |
| Commd2 | -0.43788 |
| Commd4 | -0.08629 |
| Commd5 | -0.71385 |
| Commd6 | 1.31538 |
| Commd8 | -0.45193 |
| Commd9 | -0.56288 |
| Comp | -0.55154 |
| Comtd1 | -0.10274 |
| Copa | -0.53294 |
| Copb2 | -0.34511 |
| Cope | -0.69258 |
| Cope | -0.57436 |
| Cope | -0.78465 |
| Cope | 0.78532 |
| Copg | 0.0124 |
| Copg | -0.75793 |
| Copg | -0.47032 |
| Copg | 0.35596 |
| Cops2 | -0.57359 |
| Cops3 | -0.78659 |
| Cops4 | -0.61451 |
| Cops4 | 1.6548 |
| Cops5 | 1.88484 |
| Cops5 | 0.71724 |
| Cops6 | 1.11938 |
| Cops7a | 0.00176 |
| Cops7a | -0.60014 |
| Cops7a | -0.21439 |
| Cops7b | -0.72558 |
| Cops7b | 3.71634 |
| Cops8 | 1.46862 |
| Copz1 | 0.01565 |
| Copz2 | -0.55908 |
| Copz2 | -0.39064 |
| Copz2 | 3.84702 |
| Copz2 | 0.20312 |
| Coq10b | 0.5539 |
| Coq10b | 0.66934 |
| Coq2 | -0.1009 |
| Coq3 | 2.31321 |
| Coq3 | -0.6804 |
| Coq4 | -0.26689 |
| Coq4 | 2.00177 |
| Coq5 | 1.04721 |
| Coq5 | -0.60252 |
| Coq5 | 2.06126 |
| Coq6 | 0.46387 |
| Coq6 | 0.18408 |
| Coq7 | -0.51039 |
| Coq9 | -0.67008 |
| Corin | 0.08399 |
| Coro1a | 2.13152 |
| Coro1a | -0.61774 |
| Coro1a | -0.2614 |
| Coro1a | 2.91252 |
| Coro1b | -0.40783 |
| Coro1b | -0.72196 |
| Coro1c | 0.8099 |
| Coro2a | 1.40132 |
| Coro2a | -0.36513 |
| Coro2b | -0.19993 |
| Coro2b | 1.85968 |
| Coro6 | 2.34252 |
| Coro7 | 2.0434 |
| Cort | -0.67781 |
| Cort | -0.48558 |
| Cotl1 | -0.69873 |
| Cova1 | -0.00776 |
| Cox10 | -0.38435 |
| Cox11 | 1.79825 |
| Cox15 | -0.65062 |
| Cox17 | -0.55004 |
| Cox18 | -0.73655 |
| Cox18 | -0.4059 |
| Cox19 | 2.20168 |
| Cox4i1 | 0.92564 |
| Cox4nb | -0.8202 |
| Cox4nb | 0.21037 |
| Cox5a | -0.42404 |
| Cox5b | 2.0714 |
| Cox6a1 | -0.26966 |
| Cox6a1 | -0.75044 |
| Cox6a2 | 3.39303 |
| Cox6b1 | -0.37811 |
| Cox6b1 | 2.20868 |
| Cox6b2 | 0.15598 |
| Cox6b2 | -0.74023 |
| Cox6c | 0.6228 |
| Cox7a1 | -0.02709 |
| Cox7a2 | -0.46552 |
| Cox7a2l | 0.37453 |
| Cox7b | -0.72922 |
| Cox7b2 | -0.64312 |
| Cox8b | -0.49049 |
| Cox8c | 0.87135 |
| Cp | -0.61693 |
| Cpa1 | -0.20455 |
| Cpa2 | 0.17083 |
| Cpa2 | 2.2497 |
| Cpa3 | 0.38027 |
| Cpa3 | 0.10261 |
| Cpa3 | -0.67522 |
| Cpa4 | 1.1998 |
| Cpa5 | -0.59068 |
| Cpa6 | -0.69962 |
| Cpb2 | 0.01077 |
| Cpeb1 | 2.60231 |
| Cpeb2 | 0.14724 |
| Cpeb3 | -0.28358 |
| Cpeb4 | 1.61435 |
| Cphx | 2.88632 |
| Cplx1 | -0.28358 |
| Cplx2 | 0.98413 |
| Cpn1 | 0.59063 |
| Cpne2 | -0.62422 |
| Cpne4 | 1.80632 |
| Cpne5 | -0.74764 |
| Cpne6 | 0.5446 |
| Cpne7 | 2.32724 |
| Cpne8 | 2.3067 |
| Cpne8 | -0.03691 |
| Cpne9 | -0.26634 |
| Cpsf1 | 1.12279 |
| Cpsf1 | -0.53589 |
| Cpsf2 | 0.05376 |
| Cpsf3l | 1.16808 |
| Cpsf4 | 1.81236 |
| Cpsf4l | 0.15737 |
| Cpsf4l | -0.74578 |
| Cpsf6 | 0.02492 |
| Cpsf6 | -0.80127 |
| Cpt1b | 1.80932 |
| Cpt1b | -0.63239 |
| Cpt1c | -0.29602 |
| Cpt2 | -0.22803 |
| Cpxcr1 | -0.73196 |
| Cpxm1 | 0.15215 |
| Cpxm2 | -0.53515 |
| Cpz | -0.6365 |
| Crabp2 | -0.51616 |
| Crat | 1.76756 |
| Crat | -0.03007 |
| Crat | -0.69697 |
| Crb1 | 0.78479 |
| Crbn | -0.19176 |
| Crbn | 0.10519 |
| Crbn | 4.20619 |
| Crcp | -0.41428 |
| Creb1 | -0.63486 |
| Creb1 | -0.66837 |
| Creb3 | 1.98684 |
| Creb3l1 | -0.6565 |
| Creb3l2 | -0.12562 |
| Creb3l2 | 3.37022 |
| Creb3l3 | -0.67351 |
| Creb3l4 | 1.67282 |
| Creb5 | 1.44599 |
| Crebl1 | -0.13656 |
| Crebl2 | 0.74203 |
| Creg2 | 2.46241 |
| Creld1 | -0.76738 |
| Creld2 | 0.16464 |
| Creld2 | 0.4286 |
| Crem | 0.96514 |
| Crem | 2.52715 |
| Crem | 1.01812 |
| Crem | 0.87167 |
| Crhbp | -0.36821 |
| Crhr1 | -0.68821 |
| Crim1 | 0.78216 |
| Crim2 | -0.41687 |
| Crip2 | -0.8344 |
| Crip3 | 1.02774 |
| Crip3 | 1.50108 |
| Cript | -0.68386 |
| Cript | -0.37067 |
| Crisp2 | -0.47515 |
| Crispld2 | -0.52269 |
| Crk | -0.29887 |
| Crk | 3.20341 |
| Crk | -0.65988 |
| Crkl | 1.33034 |
| Crkrs | 0.23496 |
| Crkrs | 4.22539 |
| Crlf3 | 1.28053 |
| Crlf3 | -0.61451 |
| Crls1 | -0.67608 |
| Crls1 | 0.77082 |
| Crls1 | -0.79831 |
| Crls1 | -0.71296 |
| Crmp1 | 1.29596 |
| Crmp1 | 0.25267 |
| Crnkl1 | -0.56212 |
| Crocc | 0.34725 |
| Crry | 3.03581 |
| Crry | 0.65631 |
| Crtac1 | 1.69002 |
| Crtam | 1.12691 |
| Crtap | -0.1032 |
| Crtap | 0.33987 |
| Crtc1 | -0.71027 |
| Crtc3 | -0.12609 |
| Crxos1 | 0.54163 |
| Cry2 | 1.58608 |
| Cryab | -0.29035 |
| Cryba2 | -0.16819 |
| Cryba4 | -0.44857 |
| Crybb1 | -0.25376 |
| Crybb2 | 0.47007 |
| Crybb3 | -0.76265 |
| Crygn | -0.66752 |
| Crygn | -0.79635 |
| Cryl1 | -0.69083 |
| Crym | 1.03499 |
| Cryz | 0.66755 |
| Cryz | 1.34487 |
| Cryz | 0.20642 |
| Cryzl1 | -0.51905 |
| Cryzl1 | -0.12184 |
| Cs | 2.47035 |
| Csad | -0.6057 |
| Csda | -0.66327 |
| Csda | 0.97746 |
| Csda | -0.20455 |
| Csdc2 | 1.13785 |
| Csde1 | 1.97965 |
| Csde1 | 0.13948 |
| Csde1 | 0.82212 |
| Cse1l | -0.48628 |
| Csf1 | 1.65494 |
| Csf1r | -0.3261 |
| Csf1r | 0.09297 |
| Csf2ra | -0.41817 |
| Csgalnact1 | -0.70315 |
| Csgalnact2 | -0.01948 |
| Csk | 2.16085 |
| Csl | -0.21492 |
| Csl | 0.49519 |
| Csmd1 | 0.45988 |
| Csnk1d | -0.18015 |
| Csnk1d | 1.39248 |
| Csnk1d | 1.22895 |
| Csnk1d | 1.66645 |
| Csnk1d | -0.13036 |
| Csnk1e | 0.74644 |
| Csnk1e | -0.1938 |
| Csnk1g1 | 0.54346 |
| Csnk1g1 | -0.53073 |
| Csnk1g1 | -0.51183 |
| Csnk1g2 | -0.79635 |
| Csnk1g2 | 1.31272 |
| Csnk1g2 | 1.3617 |
| Csnk1g3 | -0.4919 |
| Csnk2a1 | -0.20662 |
| Csnk2a2 | 2.42949 |
| Csnk2a2 | 0.26419 |
| Csnrp2 | -0.51543 |
| Csnrp3 | 0.7624 |
| Cspg4 | -0.11481 |
| Cspg5 | 1.49226 |
| Cspp1 | 0.80425 |
| Csprs | 0.66014 |
| Csrp1 | 0.76599 |
| Csrp2 | -0.67265 |
| Csrp2bp | 0.93284 |
| Cst11 | 0.02813 |
| Cst3 | 1.18957 |
| Cst8 | 2.36798 |
| Cst9 | 0.75663 |
| Cstad | 3.12436 |
| Cstb | -0.58365 |
| Cstf1 | 3.9372 |
| Cstf2 | 0.34611 |
| Cstf3 | -0.34391 |
| Cstf3 | 0.2645 |
| Cstl1 | 0.19415 |
| Ctage5 | -0.01402 |
| Ctcf | 0.65994 |
| Ctdp1 | -0.60411 |
| Ctdp1 | -0.49896 |
| Ctdp1 | 0.4161 |
| Ctdsp1 | 1.46737 |
| Ctdsp1 | 1.95894 |
| Ctdsp1 | -0.61451 |
| Ctdsp2 | -0.73196 |
| Ctdspl | -0.47515 |
| Ctdspl2 | -0.32727 |
| Ctdspl2 | 0.13129 |
| Ctf1 | -0.32434 |
| Ctgf | -0.49401 |
| Ctla4 | -0.4919 |
| Ctnna1 | -0.27021 |
| Ctnna3 | -0.07862 |
| Ctnnal1 | -0.41493 |
| Ctnnb1 | -0.67608 |
| Ctnnb1 | -0.19075 |
| Ctnnb1 | 0.93344 |
| Ctnnbip1 | -0.46552 |
| Ctnnbl1 | 0.59322 |
| Ctnnbl1 | -0.4306 |
| Ctnnd2 | -0.59147 |
| Ctnnd2 | 0.24449 |
| Ctnnd2 | -0.47515 |
| Ctns | -0.49472 |
| Ctns | 0.04241 |
| Ctps | -0.55304 |
| Ctps | -0.17465 |
| Ctps | 0.51377 |
| Ctps2 | -0.41622 |
| Ctps2 | 0.86991 |
| Ctps2 | 0.35792 |
| Ctr9 | -0.53589 |
| Ctrb1 | -0.57205 |
| Ctrl | 1.74665 |
| Ctsa | -0.08629 |
| Ctsa | -0.27687 |
| Ctsa | -0.41557 |
| Ctsa | -0.23121 |
| Ctsb | 0.59947 |
| Ctsc | 1.81489 |
| Ctsc | 2.70256 |
| Ctsc | -0.62017 |
| Ctse | -0.67522 |
| Ctsf | 1.18595 |
| Ctsh | -0.42535 |
| Ctsk | -0.07862 |
| Ctsl | -0.81518 |
| Ctso | 0.2156 |
| Ctsr | 0.18611 |
| Ctss | -0.66496 |
| Ctsw | -0.29488 |
| Ctsz | -0.14618 |
| Ctsz | -0.15005 |
| Cttn | -0.82221 |
| Cttn | 1.51308 |
| Cttnbp2nl | -0.42404 |
| Ctxn1 | -0.12562 |
| Cuedc1 | 0.31152 |
| Cuedc2 | 0.37891 |
| Cugbp1 | 1.21762 |
| Cugbp1 | -0.47861 |
| Cugbp1 | 1.91304 |
| Cugbp1 | -0.39317 |
| Cugbp2 | -0.28415 |
| Cugbp2 | -0.24295 |
| Cul1 | 1.13318 |
| Cul2 | 1.23972 |
| Cul2 | -0.54182 |
| Cul3 | -0.4027 |
| Cul4a | 0.56668 |
| Cul4a | 0.02332 |
| Cul4a | -0.58132 |
| Cul4b | 1.18379 |
| Cul5 | 0.06151 |
| Cul7 | 0.31093 |
| Cuta | 0.64002 |
| Cuta | -0.54779 |
| Cuta | -0.63157 |
| Cutc | -0.30632 |
| Cutc | -0.1428 |
| Cux1 | 0.71478 |
| Cux1 | -0.36821 |
| Cux1 | -0.6226 |
| Cux1 | 2.09655 |
| Cux1 | 1.6798 |
| Cux2 | 3.49098 |
| Cux2 | 0.19282 |
| Cuzd1 | -0.2392 |
| Cwf19l2 | 0.18307 |
| Cx3cl1 | -0.11294 |
| Cx3cr1 | 2.59195 |
| Cxadr | 0.66655 |
| Cxadr | -0.66411 |
| Cxadr | -0.22909 |
| Cxcl1 | 1.12133 |
| Cxcl12 | -0.70138 |
| Cxcl12 | -0.61371 |
| Cxcl12 | 1.65487 |
| Cxcl13 | -0.12846 |
| Cxcl14 | 0.10629 |
| Cxcl14 | -0.76076 |
| Cxcl15 | 1.08203 |
| Cxcl16 | -0.19329 |
| Cxcl17 | 1.01539 |
| Cxcl4 | -0.53147 |
| Cxcl9 | -0.62341 |
| Cxcr3 | -0.74208 |
| Cxcr4 | 0.4394 |
| Cxcr5 | -0.66327 |
| Cxcr7 | -0.35659 |
| Cxx1a | -0.52488 |
| Cxx1b | -0.52123 |
| Cxx1c | -0.4059 |
| Cxxc1 | -0.6283 |
| Cxxc1 | -0.62098 |
| Cxxc4 | -0.16375 |
| Cxxc4 | 2.81565 |
| Cyb5 | 0.45436 |
| Cyb561 | -0.22014 |
| Cyb561d1 | 1.10102 |
| Cyb561d1 | 1.90363 |
| Cyb561d2 | 1.14607 |
| Cyb5b | 0.48746 |
| Cyb5d1 | 2.16473 |
| Cyb5d2 | 1.4583 |
| Cyb5d2 | -0.68734 |
| Cyb5r1 | 0.7144 |
| Cyb5r2 | -0.58755 |
| Cyb5r3 | -0.67867 |
| Cyb5r3 | 0.74589 |
| Cyb5r3 | 2.95029 |
| Cyb5r4 | 0.30028 |
| Cyba | 0.09706 |
| Cybasc3 | -0.38686 |
| Cyc1 | -0.75511 |
| Cycs | 2.48209 |
| Cycs | -0.6283 |
| Cyfip1 | 0.82195 |
| Cyfip2 | 1.29697 |
| Cyfip2 | 0.81365 |
| Cygb | -0.62993 |
| Cyhr1 | 0.42523 |
| Cyhr1 | 3.17008 |
| Cyhr1 | -0.60252 |
| Cyhr1 | -0.7005 |
| Cyld | -0.67008 |
| Cyln2 | 3.652 |
| Cyp11a1 | -0.30747 |
| Cyp11b2 | 0.17595 |
| Cyp17a1 | -0.65398 |
| Cyp19a1 | -0.37562 |
| Cyp1b1 | -0.54704 |
| Cyp20a1 | -0.59068 |
| Cyp24a1 | -0.66667 |
| Cyp26a1 | 1.36665 |
| Cyp26b1 | -0.64645 |
| Cyp27a1 | -0.32845 |
| Cyp27a1 | -0.16325 |
| Cyp27a1 | -0.44924 |
| Cyp2a12 | -0.69171 |
| Cyp2a5 | 2.1675 |
| Cyp2a5 | -0.59147 |
| Cyp2b10 | -0.20713 |
| Cyp2c55 | 3.44067 |
| Cyp2d10 | 1.10375 |
| Cyp2d22 | -0.6121 |
| Cyp2d26 | 0.13344 |
| Cyp2d26 | -0.31499 |
| Cyp2d26 | 1.42252 |
| Cyp2e1 | 2.21814 |
| Cyp2f2 | -0.55379 |
| Cyp2j11 | 1.34439 |
| Cyp2j9 | -0.3831 |
| Cyp2s1 | -0.43921 |
| Cyp2u1 | -0.62912 |
| Cyp39a1 | -0.38123 |
| Cyp3a13 | 0.22015 |
| Cyp3a13 | -0.74485 |
| Cyp46a1 | -0.39127 |
| Cyp4b1 | 0.4923 |
| Cyp4f13 | 0.86799 |
| Cyp4f14 | 1.22232 |
| Cyp4f15 | 1.17373 |
| Cyp4f16 | -0.46074 |
| Cyp4f16 | -0.67094 |
| Cyp4f16 | 1.37602 |
| Cyp4f18 | 2.29142 |
| Cyp4f39 | -0.47584 |
| Cyp4v3 | 1.11485 |
| Cyp51 | 0.01767 |
| Cyp7b1 | -0.68126 |
| Cypt10 | -0.67008 |
| Cypt2 | -0.21128 |
| Cypt3 | 0.92834 |
| Cypt6 | 2.1916 |
| Cypt8 | 0.30503 |
| Cypt9 | 0.29342 |
| Cyr61 | -0.33793 |
| Cyr61 | -0.46963 |
| Cysltr1 | -0.51111 |
| Cytl1 | 0.58541 |
| D030011O10Rik | 1.42244 |
| D030013I16Rik | 1.9785 |
| D030018L15Rik | 2.68535 |
| D030028O16Rik | -0.47032 |
| D030051N19Rik | 0.30177 |
| D030056L22Rik | -0.6856 |
| D030070L09Rik | -0.08901 |
| D030070L09Rik | -0.35659 |
| D030070L09Rik | -0.1677 |
| D030070L09Rik | 0.36903 |
| D030074E01Rik | 2.0315 |
| D0H4S114 | 0.06228 |
| D0H8S2298E | -0.07907 |
| D0HXS9928E | -0.45126 |
| D0HXS9928E | -0.71835 |
| D10627 | -0.57745 |
| D10Bwg1364e | -0.45666 |
| D10Bwg1379e | 0.25455 |
| D10Ertd322e | 0.72888 |
| D10Ertd610e | 0.70468 |
| D10Ertd610e | -0.62912 |
| D10Ertd610e | 1.0974 |
| D10Ertd641e | -0.4587 |
| D10Jhu81e | 3.45501 |
| D10Wsu102e | 1.21795 |
| D10Wsu52e | -0.22856 |
| D11Bwg0517e | 2.05144 |
| D11Wsu47e | 0.13201 |
| D11Wsu47e | 0.53705 |
| D12Ertd551e | 3.42672 |
| D12Ertd551e | -0.50039 |
| D12Ertd553e | -0.64395 |
| D12Ertd647e | -0.17615 |
| D12Ertd647e | -0.67867 |
| D130040H23Rik | 0.27953 |
| D130043K22Rik | -0.64312 |
| D130059P03Rik | 1.90851 |
| D13Ertd608e | -0.64645 |
| D14Ertd171e | 0.8418 |
| D14Ertd231e | -0.46142 |
| D14Ertd449e | -0.70404 |
| D14Ertd500e | -0.72558 |
| D14Ertd668e | -0.71385 |
| D14Ertd668e | 1.96572 |
| D15Ertd621e | -0.54182 |
| D15Ertd682e | -0.6073 |
| D15Mgi27 | -0.78369 |
| D15Wsu169e | 0.36377 |
| D15Wsu75e | 1.03311 |
| D16Ertd472e | -0.60332 |
| D16H22S680E | 0.49399 |
| D17H6S56E-5 | 0.75699 |
| D17Wsu104e | 0.4676 |
| D17Wsu92e | -0.67608 |
| D19Bwg1357e | -0.73379 |
| D19Ertd721e | -0.5278 |
| D19Ertd737e | -0.57668 |
| D19Wsu162e | 0.11397 |
| D1Bwg0212e | 1.58211 |
| D1Bwg0212e | 1.90265 |
| D1Pas1 | -0.44321 |
| D230007K08Rik | -0.01527 |
| D230014K01Rik | -0.55984 |
| D2Ertd750e | 1.07335 |
| D2Ertd750e | 0.12087 |
| D330014H01Rik | -0.4717 |
| D330017J20Rik | -0.31325 |
| D330028D13Rik | 0.97433 |
| D330045A20Rik | 0.23496 |
| D330045A20Rik | 0.9202 |
| D330045A20Rik | 0.51046 |
| D3Bwg0562e | -0.61693 |
| D3Ertd300e | -0.62585 |
| D3Ertd751e | 1.59376 |
| D3Ucla1 | 1.83493 |
| D430015B01Rik | -0.68386 |
| D430028G21Rik | -0.47515 |
| D430039N05Rik | -0.63981 |
| D430041B17Rik | -0.50609 |
| D430042O09Rik | -0.71835 |
| D4Bwg0951e | 0.51753 |
| D4Bwg1540e | -0.05162 |
| D4Ertd22e | -0.46278 |
| D4Ertd22e | -0.60411 |
| D4Wsu114e | -0.36268 |
| D4Wsu132e | 3.52349 |
| D4Wsu132e | -0.16869 |
| D530004J12Rik | -0.69609 |
| D530033C11Rik | 1.60928 |
| D5Ertd135e | 3.60719 |
| D5Ertd579e | 3.63491 |
| D5Wsu178e | -0.18317 |
| D630003M21Rik | -0.74857 |
| D630003M21Rik | 0.55975 |
| D630014A15Rik | -0.62993 |
| D630023F18Rik | 0.05415 |
| D630042P16Rik | -0.63157 |
| D630048P19Rik | -0.29887 |
| D6Ertd474e | -0.5644 |
| D6Mm5e | 1.95781 |
| D6Wsu116e | 0.1807 |
| D6Wsu163e | -0.48279 |
| D6Wsu163e | -0.64645 |
| D6Wsu163e | -0.1867 |
| D6Wsu176e | 2.29567 |
| D6Wsu176e | -0.51183 |
| D730039F16Rik | 0.01158 |
| D730039F16Rik | 0.87007 |
| D7Bwg0611e | -0.62422 |
| D830014E11Rik | -0.5448 |
| D830030K20Rik | -0.01151 |
| D830030K20Rik | -0.33496 |
| D830050J10Rik | 0.80339 |
| D8Ertd738e | 1.76066 |
| D8Ertd82e | 0.21788 |
| D930001I22Rik | -0.71565 |
| D930005D10Rik | -0.29204 |
| D930014E17Rik | 1.66342 |
| D930015E06Rik | -0.49049 |
| D930020B18Rik | 2.83592 |
| D930028F11Rik | -0.42404 |
| D930049A15Rik | 1.06498 |
| D9Ertd280e | 0.20378 |
| D9Ertd392e | 2.03713 |
| D9Ertd402e | -0.7495 |
| D9Wsu20e | 1.37019 |
| Daam1 | -0.66327 |
| Daam2 | 0.9388 |
| Dab1 | -0.63403 |
| Dab1 | 1.14926 |
| Dab1 | 1.39483 |
| Dab2 | -0.59068 |
| Dab2 | -0.09768 |
| Dab2ip | 0.62468 |
| Dact1 | 1.70594 |
| Dact2 | 0.70908 |
| Dad1 | 1.56874 |
| Daf2 | 0.38027 |
| Dag1 | 1.21135 |
| Dag1 | -0.69785 |
| Dagla | -0.37067 |
| Daglb | 1.24877 |
| Dak | -0.49826 |
| Dalrd3 | 1.77333 |
| Dap | -0.64063 |
| Dap3 | 1.00605 |
| Dap3 | -0.00154 |
| Dap3 | 0.36654 |
| Dap3 | 0.43067 |
| Dapk1 | -0.67781 |
| Dapk1 | 0.99565 |
| Dapk2 | -0.32551 |
| Dapk2 | -0.31965 |
| Dapl1 | 3.2815 |
| Dapp1 | -0.67953 |
| Dapp1 | -0.62993 |
| Darc | -0.61613 |
| Dars | -0.52051 |
| Dars | -0.48139 |
| Daxx | -0.80028 |
| Dazap1 | 1.98418 |
| Dazap1 | -0.40719 |
| Dazap2 | 0.46312 |
| Dazl | -0.66496 |
| Dbc1 | 1.81466 |
| Dbf4 | -0.35659 |
| Dbi | 2.67168 |
| Dbi | -0.61693 |
| Dbil5 | -0.51039 |
| Dbn1 | 0.02532 |
| Dbndd1 | -0.44857 |
| Dbndd2 | -0.63486 |
| Dbndd2 | 0.1674 |
| Dbndd2 | -0.66242 |
| Dbnl | 0.63135 |
| Dbp | -0.59461 |
| Dbpht2 | 1.4114 |
| Dbr1 | 0.90508 |
| Dbt | -0.07907 |
| Dcakd | -0.41428 |
| Dcbld1 | 1.27838 |
| Dcbld2 | 0.44881 |
| Dci | -0.05031 |
| Dck | 0.78269 |
| Dclk1 | -0.19329 |
| Dclk2 | 0.98695 |
| Dclk3 | -0.09722 |
| Dclre1b | 0.93657 |
| Dclre1b | -0.55229 |
| Dclre1c | -0.6105 |
| Dclre1c | -0.43258 |
| Dclre1c | -0.49684 |
| Dcn | -0.68734 |
| Dcn | 0.62343 |
| Dcp1a | 1.13582 |
| Dcp1a | 1.14914 |
| Dcp1a | 0.93403 |
| Dcp1a | -0.37253 |
| Dcp1b | 0.35147 |
| Dcp1b | 1.30028 |
| Dcpp1 | -0.62341 |
| Dcpp2 | -0.72831 |
| Dcpp2 | -0.10228 |
| Dcpp3 | -0.71117 |
| Dcps | -0.63321 |
| Dcps | -0.69785 |
| Dct | 0.88357 |
| Dctd | 2.5477 |
| Dctn1 | 1.03405 |
| Dctn2 | -0.24349 |
| Dctn2 | 1.21543 |
| Dctn2 | 0.37261 |
| Dctn3 | -0.6283 |
| Dctn3 | -0.74115 |
| Dctn4 | 0.23624 |
| Dctn4 | -0.21857 |
| Dctn5 | 0.68042 |
| Dctn6 | -0.19431 |
| Dcun1d2 | 0.72402 |
| Dcun1d3 | -0.66411 |
| Dcun1d4 | -0.14135 |
| Dcun1d4 | 1.69681 |
| Dcun1d5 | 1.85311 |
| Dcx | 0.66815 |
| Dcx | 0.52665 |
| Dcxr | 1.02962 |
| Dcxr | 0.25267 |
| Ddah1 | -0.66752 |
| Ddah2 | -0.00319 |
| Ddb1 | 0.13344 |
| Ddb2 | -0.65988 |
| Ddb2 | 0.26512 |
| Ddb2 | -0.53663 |
| Ddc | -0.579 |
| Ddc8 | 1.25426 |
| Ddef2 | -0.64979 |
| Ddef2 | -0.02201 |
| Ddefl1 | 0.03092 |
| Ddhd1 | 0.77934 |
| Ddhd1 | -0.58288 |
| Ddi2 | -0.11809 |
| Ddit3 | -0.20559 |
| Ddit3 | 0.92322 |
| Ddit4 | 2.00777 |
| Ddit4l | -0.29261 |
| Ddo | 1.80891 |
| Ddo | 0.47279 |
| Ddo | 2.76375 |
| Ddost | 3.26978 |
| Ddr1 | -0.24726 |
| Ddr2 | 1.20704 |
| Ddt | 0.84295 |
| Ddx1 | -0.48279 |
| Ddx1 | 1.77661 |
| Ddx17 | 0.8961 |
| Ddx17 | -0.35537 |
| Ddx17 | 0.41636 |
| Ddx19a | -0.30001 |
| Ddx19b | -0.40334 |
| Ddx19b | -0.14957 |
| Ddx20 | 0.07262 |
| Ddx21 | -0.43921 |
| Ddx24 | -0.52707 |
| Ddx24 | 2.88986 |
| Ddx24 | 0.57884 |
| Ddx25 | 0.47476 |
| Ddx26b | -0.61855 |
| Ddx27 | -0.42077 |
| Ddx28 | 0.19148 |
| Ddx3x | 0.05221 |
| Ddx3y | -0.65482 |
| Ddx4 | -0.62017 |
| Ddx4 | 0.83717 |
| Ddx41 | 0.63135 |
| Ddx46 | 0.62028 |
| Ddx47 | -0.19635 |
| Ddx47 | -0.12941 |
| Ddx47 | 1.54947 |
| Ddx49 | 3.21206 |
| Ddx5 | -0.67265 |
| Ddx50 | -0.58833 |
| Ddx50 | 3.36114 |
| Ddx51 | 0.96037 |
| Ddx52 | -0.45938 |
| Ddx54 | 2.12222 |
| Ddx58 | -0.56822 |
| Ddx6 | 0.92684 |
| Ddx6 | -0.44388 |
| Ddx6 | -0.32024 |
| Ddx6 | -0.64645 |
| Deadc1 | -0.75793 |
| Deb1 | 0.19515 |
| Decr1 | 0.96687 |
| Decr2 | 1.50325 |
| Decr2 | -0.15492 |
| Dedd | 0.61797 |
| Dedd | -0.6065 |
| Dedd2 | -0.57513 |
| Def6 | 0.77313 |
| Def8 | 0.67074 |
| Defb11 | 0.66974 |
| Defb14 | -0.32845 |
| Defb15 | -0.03777 |
| Defb19 | 0.71535 |
| Defb20 | 1.05953 |
| Defb21 | -0.60332 |
| Defb25 | -0.46826 |
| Defb26 | 0.81297 |
| Defb30 | -0.39253 |
| Defb36 | 0.35428 |
| Defb36 | -0.41947 |
| Defb4 | -0.56975 |
| Defcr20 | 2.7368 |
| Defcr6 | -0.71745 |
| Defcr-rs7 | 0.88782 |
| Degs1 | 1.05992 |
| Degs2 | -0.71296 |
| Dek | 0.34922 |
| Dek | 0.149 |
| Dennd1a | -0.61613 |
| Dennd1c | -0.12279 |
| Dennd2a | 0.99369 |
| Dennd3 | -0.25485 |
| Denr | -0.67522 |
| Denr | 1.84373 |
| Depdc1b | -0.2791 |
| Depdc5 | 1.34611 |
| Depdc5 | -0.57977 |
| Depdc6 | -0.55379 |
| Depdc6 | 0.83518 |
| Dera | -0.48698 |
| Derl2 | 1.14902 |
| Derl3 | 1.63796 |
| Des | -0.3023 |
| Det1 | 1.83783 |
| Dexi | 0.03052 |
| Dexi | 2.39395 |
| Dffa | 1.70136 |
| Dfna5h | 0.27679 |
| Dgat1 | 0.70984 |
| Dgat2 | -0.51905 |
| Dgcr2 | -0.15248 |
| Dgcr2 | 2.06226 |
| Dgcr6 | 0.3124 |
| Dgcr6 | 2.50038 |
| Dgcr8 | 2.78578 |
| Dgka | -0.64728 |
| Dgka | 0.32406 |
| Dgkb | -0.75887 |
| Dgke | -0.82524 |
| Dgkg | -0.33734 |
| Dgkh | -0.71745 |
| Dgki | 0.30444 |
| Dgkk | 1.60612 |
| Dgkq | 0.37809 |
| Dgkz | 2.19743 |
| Dgkz | 0.56824 |
| Dgkz | 2.79078 |
| Dguok | 3.30728 |
| Dhcr24 | -0.23174 |
| Dhcr24 | 0.26728 |
| Dhcr24 | 2.60626 |
| Dhcr7 | -0.2344 |
| Dhdds | -0.50967 |
| Dhdds | 1.03177 |
| Dhdh | 3.12744 |
| Dhdh | -0.01569 |
| Dhdh | 2.69231 |
| Dhh | 0.67054 |
| Dhodh | -0.35537 |
| Dhps | 0.61481 |
| Dhrs1 | 0.53198 |
| Dhrs4 | 0.35876 |
| Dhrs7 | -0.55004 |
| Dhrs7 | 1.68252 |
| Dhrs7 | -0.42797 |
| Dhrs7b | -0.37811 |
| Dhrs7b | -0.55229 |
| Dhrs7b | 0.35288 |
| Dhrsx | -0.426 |
| Dhrsx | 0.14407 |
| Dhtkd1 | 2.85587 |
| Dhx15 | 0.24512 |
| Dhx15 | 0.8015 |
| Dhx16 | -0.58912 |
| Dhx29 | -0.47101 |
| Dhx30 | 0.62573 |
| Dhx32 | 0.35904 |
| Dhx33 | 0.01888 |
| Dhx36 | -0.26855 |
| Dhx37 | -0.06436 |
| Dhx38 | -0.54256 |
| Dhx57 | -0.49684 |
| Dhx58 | -0.50181 |
| Dhx8 | -0.45396 |
| Dhx9 | 1.23068 |
| Diablo | -0.63157 |
| Diap1 | -0.73563 |
| Diap3 | 2.7143 |
| Dicer1 | -0.47653 |
| Dido1 | -0.29659 |
| Dido1 | 1.35964 |
| Dido1 | 0.89329 |
| Dio1 | -0.64562 |
| Dio1 | 1.86814 |
| Dio3 | -0.41299 |
| Dip2b | 0.19882 |
| Dip2b | 0.29312 |
| Diras1 | 1.13342 |
| Diras2 | -0.24996 |
| Dirc2 | -0.54405 |
| Dis3 | 0.29072 |
| Dis3l | 0.36903 |
| Dis3l2 | 0.52641 |
| Disp1 | 1.42191 |
| Dixdc1 | 1.73717 |
| Dkk3 | 0.29551 |
| Dkk3 | -0.47239 |
| Dkk3 | -0.17366 |
| Dkkl1 | -0.60332 |
| Dkkl1 | 0.87311 |
| Dlat | 1.77727 |
| Dlc1 | -0.33972 |
| Dld | -0.62748 |
| Dleu7 | -0.58288 |
| Dlg1 | 0.85767 |
| Dlg2 | 1.36469 |
| Dlg3 | 2.41044 |
| Dlg4 | -0.65734 |
| Dlg7 | -0.52561 |
| Dlgap1 | -0.52051 |
| Dlgap2 | -0.52415 |
| Dlgap2 | -0.68734 |
| Dlgap2 | 0.15494 |
| Dlgap3 | 0.13735 |
| Dlgap4 | -0.72105 |
| Dlgap4 | -0.21024 |
| Dlgap4 | 0.61923 |
| Dlgap4 | 0.52035 |
| Dlk1 | -0.5899 |
| Dlk2 | 0.11725 |
| Dll1 | 0.18779 |
| Dlst | 0.53682 |
| Dlx1 | -0.64645 |
| Dlx1as | 0.43196 |
| Dlx2 | -0.01779 |
| Dlx3 | 3.12439 |
| Dmap1 | 1.56424 |
| Dmbt1 | 0.47917 |
| Dmkn | -0.47931 |
| Dmkn | 1.32723 |
| Dmkn | -0.69258 |
| Dmn | 1.45204 |
| Dmn | -0.58677 |
| Dmpk | 0.99857 |
| Dmrt2 | 0.37096 |
| Dmrt3 | -0.25593 |
| Dmrta1 | 0.34158 |
| Dmrta2 | 0.32348 |
| Dmrtc1a | 1.33228 |
| Dmrtc1a | 0.85702 |
| Dmrtc1a | 0.69873 |
| Dmrtc1b | -0.08765 |
| Dmrtc2 | -0.76265 |
| Dmtf1 | -0.66837 |
| Dmtf1 | -0.73746 |
| Dmtf1 | 2.07503 |
| Dmwd | 0.12664 |
| Dmwd | 2.15217 |
| Dmwd | 0.0588 |
| Dnahc11 | 0.22404 |
| Dnahc2 | -0.50752 |
| Dnahc5 | 1.26567 |
| Dnahc8 | -0.05249 |
| Dnaic1 | 0.20873 |
| Dnaic1 | 1.25647 |
| Dnaja1 | -0.66667 |
| Dnaja2 | 0.3258 |
| Dnaja2 | -0.71117 |
| Dnaja3 | -0.63898 |
| Dnajb1 | -0.42666 |
| Dnajb10 | -0.59461 |
| Dnajb12 | 0.51518 |
| Dnajb13 | 0.71269 |
| Dnajb3 | 1.15079 |
| Dnajb4 | -0.33913 |
| Dnajb4 | 3.11957 |
| Dnajb5 | 1.68812 |
| Dnajb6 | -0.20507 |
| Dnajb6 | 0.18577 |
| Dnajb7 | 1.23003 |
| Dnajb8 | 4.23533 |
| Dnajb9 | 0.66974 |
| Dnajc10 | 1.0949 |
| Dnajc10 | 0.76564 |
| Dnajc12 | 0.37946 |
| Dnajc14 | -0.43722 |
| Dnajc15 | -0.60173 |
| Dnajc16 | 0.15005 |
| Dnajc17 | -0.6105 |
| Dnajc18 | 1.32118 |
| Dnajc19 | -0.58912 |
| Dnajc19 | -0.55379 |
| Dnajc2 | 1.48729 |
| Dnajc2 | -0.21335 |
| Dnajc3a | -0.65398 |
| Dnajc4 | 0.41453 |
| Dnajc5 | -0.44722 |
| Dnajc5b | -0.11434 |
| Dnajc5g | -0.4662 |
| Dnajc6 | 0.70736 |
| Dnajc6 | -0.42012 |
| Dnajc7 | -0.32786 |
| Dnajc8 | 0.02853 |
| Dnalc1 | 0.95615 |
| Dnali1 | 1.64101 |
| Dnase1l1 | -0.44857 |
| Dnase1l2 | -0.44722 |
| Dnase1l2 | -0.21387 |
| Dnase2a | 3.54358 |
| Dnd1 | 0.1252 |
| Dner | -0.57668 |
| Dnm | 0.17288 |
| Dnm1 | 0.43735 |
| Dnm1l | -0.65314 |
| Dnm1l | 0.85001 |
| Dnmbp | -0.3069 |
| Dnmt1 | -0.1603 |
| Dnmt3b | -0.14521 |
| Dnmt3l | -0.6283 |
| Dnmt3l | -0.15785 |
| Dnttip1 | -0.2254 |
| Doc2a | -0.16918 |
| Doc2b | 1.12388 |
| Doc2b | -0.67608 |
| Dock1 | -0.66242 |
| Dock3 | -0.26524 |
| Dock4 | -0.71206 |
| Dock6 | 0.12628 |
| Dock8 | 0.14935 |
| Dock9 | -0.23653 |
| Dok1 | -0.22382 |
| Dok1 | -0.42535 |
| Dok2 | -0.66667 |
| Dok3 | 1.17039 |
| Dok4 | -0.65819 |
| Dok5 | 1.62215 |
| Dok5 | -0.24564 |
| Dok7 | 0.36432 |
| Dolk | 0.10298 |
| Dolpp1 | 0.53244 |
| Dolpp1 | 1.19138 |
| Dolpp1 | 1.91119 |
| Dom3z | -0.54108 |
| Dom3z | -0.70138 |
| Donson | -0.71475 |
| Donson | 2.65069 |
| Dos | -0.13512 |
| Dos | -0.52123 |
| Dot1l | -0.19635 |
| Dpagt1 | 0.76114 |
| Dpep1 | 0.77597 |
| Dpep2 | 0.31093 |
| Dpep2 | 2.32439 |
| Dpep3 | 1.86868 |
| Dpf1 | 0.19081 |
| Dpf1 | 1.31696 |
| Dpf3 | 0.71969 |
| Dph2 | -0.7076 |
| Dph2 | -0.14714 |
| Dph2 | -0.54629 |
| Dph3 | 2.10898 |
| Dph3 | -0.32199 |
| Dph3 | -0.34872 |
| Dph4 | -0.20404 |
| Dpm1 | -0.67694 |
| Dpm1 | 1.21367 |
| Dpm2 | 0.06689 |
| Dpm3 | 0.11069 |
| Dpp10 | -0.70938 |
| Dpp3 | -0.51616 |
| Dpp3 | -0.68821 |
| Dpp4 | -0.49472 |
| Dpp6 | 3.45177 |
| Dpp7 | 0.54049 |
| Dpp8 | -0.66496 |
| Dpp9 | -0.6065 |
| Dpp9 | -0.35537 |
| Dppa3 | 0.48527 |
| Dppa4 | 0.80459 |
| Dppa5 | 1.12157 |
| Dppa5 | 2.23413 |
| Dpt | 1.47585 |
| Dpy30 | -0.53 |
| Dpyd | -0.68647 |
| Dpysl2 | -0.37006 |
| Dpysl2 | 1.10673 |
| Dpysl2 | 1.24569 |
| Dpysl3 | -0.24942 |
| Dpysl4 | 1.18652 |
| Dpysl5 | 1.01921 |
| Drbp1 | -0.4621 |
| Drctnnb1a | -0.55154 |
| Drd1a | -0.62422 |
| Drd2 | -0.13704 |
| Drd4 | -0.67351 |
| Drd5 | 0.84887 |
| Drg1 | 1.76961 |
| Drg1 | -0.56822 |
| Drg2 | -0.31616 |
| Drp2 | 3.70905 |
| Dscam | -0.6065 |
| Dscam | 1.50957 |
| Dscc1 | 0.58519 |
| Dscr1 | 0.36654 |
| Dscr1l2 | -0.5011 |
| Dscr2 | 3.53583 |
| Dscr3 | -0.58288 |
| Dscr3 | 1.23413 |
| Dscr6 | -0.08087 |
| Dsg2 | 0.24512 |
| Dsg2 | -0.20868 |
| Dsn1 | -0.59777 |
| Dsp | -0.55154 |
| Dst | -0.63898 |
| Dst | 0.54711 |
| Dst | 1.07502 |
| Dstn | 0.25767 |
| Dtd1 | -0.02327 |
| Dtl | 0.25393 |
| Dtna | -0.51543 |
| Dtna | 1.05404 |
| Dtna | -0.4926 |
| Dtna | 0.89423 |
| Dtnb | 2.16961 |
| Dtnbp1 | -0.26524 |
| Dtwd1 | 1.58674 |
| Dtx1 | -0.56593 |
| Dtx2 | 0.71136 |
| Dtx2 | -0.45193 |
| Dtx2 | -0.42273 |
| Dtx4 | 0.3071 |
| Dtx4 | -0.19942 |
| Dtymk | -0.04078 |
| Dus1l | 1.64343 |
| Dus1l | -0.53959 |
| Dus2l | -0.50323 |
| Dus3l | -0.44521 |
| Dus3l | 1.92459 |
| Dus3l | 0.7212 |
| Dus4l | 0.19749 |
| Dusp1 | 2.52494 |
| Dusp11 | 1.43783 |
| Dusp11 | 0.35456 |
| Dusp13 | 1.20526 |
| Dusp13 | 0.33873 |
| Dusp13 | 0.12664 |
| Dusp14 | -0.41882 |
| Dusp15 | -0.39507 |
| Dusp15 | 3.15782 |
| Dusp16 | 1.8541 |
| Dusp16 | 0.51777 |
| Dusp16 | 0.59474 |
| Dusp18 | 0.16257 |
| Dusp19 | -0.14473 |
| Dusp2 | 2.36402 |
| Dusp2 | 0.05648 |
| Dusp21 | -0.35598 |
| Dusp22 | 0.87183 |
| Dusp23 | -0.31907 |
| Dusp26 | 0.42679 |
| Dusp28 | 1.35823 |
| Dusp3 | 1.5232 |
| Dusp3 | -0.2603 |
| Dusp3 | -0.46552 |
| Dusp4 | -0.62179 |
| Dusp4 | -0.43192 |
| Dusp4 | -0.43589 |
| Dusp6 | -0.10366 |
| Dusp6 | 0.3464 |
| Dusp6 | -0.53959 |
| Dusp7 | -0.23387 |
| Dusp8 | 0.83401 |
| Dut | 0.32203 |
| Duxbl | 3.1842 |
| Duxbl | -0.46278 |
| Dvl1 | -0.26195 |
| Dvl1 | -0.54929 |
| Dvl2 | -0.36759 |
| DXBwg1396e | -0.67608 |
| Dydc2 | -0.4919 |
| Dym | -0.68647 |
| Dync1h1 | 0.58279 |
| Dync1i1 | -0.63898 |
| Dync1i1 | -0.29488 |
| Dync1i2 | 0.35792 |
| Dync1li1 | -0.45126 |
| Dync1li2 | 2.49945 |
| Dync2li1 | 0.0209 |
| Dynll1 | 0.19181 |
| Dynll2 | 0.10813 |
| Dynlrb1 | -0.50181 |
| Dynlrb2 | -0.11061 |
| Dynlt3 | 1.5753 |
| Dyrk1a | -0.51688 |
| Dyrk1b | -0.30173 |
| Dyrk1b | -0.07057 |
| Dyrk2 | 4.24328 |
| Dyrk2 | -0.10228 |
| Dyrk3 | -0.67265 |
| Dyrk4 | 0.11252 |
| Dysf | 0.77951 |
| Dysfip1 | -0.65314 |
| Dzip1 | 2.0966 |
| E030010A14Rik | -0.61291 |
| E030034P13Rik | -0.236 |
| E130009J12Rik | -0.38812 |
| E130012A19Rik | -0.41947 |
| E130014J05Rik | 2.60012 |
| E130016E03Rik | 0.29252 |
| E130203B14Rik | -0.64229 |
| E130203B14Rik | 0.40023 |
| E130303B06Rik | -0.77406 |
| E130306D19Rik | 0.63197 |
| E130308A19Rik | -0.67008 |
| E130309D02Rik | -0.47032 |
| E130309D02Rik | -0.332 |
| E130309D14Rik | 1.27086 |
| E130309F12Rik | -0.58677 |
| E130309F12Rik | 1.88303 |
| E130309F12Rik | -0.34151 |
| E230008N13Rik | 2.89076 |
| E2f1 | 1.3631 |
| E2f2 | 0.92849 |
| E2f2 | -0.57977 |
| E2f3 | -0.57668 |
| E2f4 | -0.31732 |
| E2f5 | 3.02838 |
| E2f6 | -0.49755 |
| E2f6 | 0.66215 |
| E2f6 | -0.77215 |
| E330016A19Rik | -0.03519 |
| E330017A01Rik | 0.59861 |
| E330018D03Rik | -0.56975 |
| E330021D16Rik | 0.4877 |
| E330034G19Rik | -0.64812 |
| E330034G19Rik | -0.58521 |
| E330036I19Rik | 2.04728 |
| E430002D04Rik | -0.54629 |
| E430002G05Rik | -0.59147 |
| E430018J23Rik | -0.61693 |
| E430025E21Rik | 1.09553 |
| E430028B21Rik | -0.72286 |
| E4f1 | 1.1535 |
| Eaf1 | -0.33972 |
| Ear11 | 0.22855 |
| Ear2 | -0.22434 |
| Ear4 | -0.1486 |
| Ears2 | 2.53764 |
| Ebag9 | 0.54688 |
| Ebf2 | 0.45184 |
| Ebf3 | -0.30402 |
| Ebf4 | 1.59318 |
| Ebi2 | 0.79873 |
| Ebi3 | -0.64895 |
| Ebna1bp2 | 2.47915 |
| Ebna1bp2 | 1.05757 |
| Ebp | -0.44121 |
| Ebpl | 0.03886 |
| Ebpl | 0.90692 |
| Ecd | 0.09297 |
| Ecd | 0.14443 |
| Ece1 | 1.01238 |
| Ece1 | 1.98808 |
| Ece2 | -0.63075 |
| Ecel1 | 1.6686 |
| Ech1 | -0.72831 |
| Echdc1 | 0.97361 |
| Echdc2 | 1.49356 |
| Echdc2 | -0.63486 |
| Echdc2 | 0.01727 |
| Echdc3 | 0.18239 |
| Echs1 | 1.66557 |
| Ecm1 | 0.3568 |
| Ecm1 | -0.46826 |
| Ecsit | 0.30887 |
| Eda2r | 1.6601 |
| Edaradd | 0.56043 |
| Edc4 | 0.37151 |
| Edem1 | 2.09761 |
| Edem2 | -0.54629 |
| Edem2 | 0.6251 |
| Edem2 | 1.26557 |
| Edem2 | -0.65146 |
| Edf1 | -0.79341 |
| Edg2 | -0.39001 |
| Edg5 | 0.91229 |
| Edg7 | 1.58469 |
| Edg8 | 0.37864 |
| Edg8 | -0.06524 |
| Edil3 | 1.37075 |
| Edn1 | 0.38869 |
| Edn2 | -0.24134 |
| Ednra | -0.42797 |
| Ednrb | 2.3069 |
| Ednrb | 0.89236 |
| Ednrb | -0.73563 |
| Eed | 0.72158 |
| Eef1a1 | -0.78272 |
| Eef1a1 | 1.24696 |
| Eef1a2 | 0.70142 |
| Eef1a2 | -0.67265 |
| Eef1a2 | 1.0686 |
| Eef1b2 | -0.62912 |
| Eef1b2 | 0.91732 |
| Eef1b2 | 1.05744 |
| Eef1d | 2.76712 |
| Eef1d | 0.79214 |
| Eef1d | 0.47819 |
| Eef1d | 1.14666 |
| Eef1e1 | 1.35162 |
| Eef1g | -0.7076 |
| Eef2 | 0.14548 |
| Eef2 | 0.50047 |
| Eef2 | 0.4195 |
| Eef2k | 0.17048 |
| Eefsec | -0.01737 |
| Efcab1 | -0.09952 |
| Efcab2 | -0.27298 |
| Efcab2 | 2.75786 |
| Efcab3 | -0.76265 |
| Efcab3 | 1.19341 |
| Efcab3 | 0.01969 |
| Efcab4a | 0.90276 |
| Efcab4a | -0.66496 |
| Efcab5 | -0.53 |
| Efemp2 | 0.87724 |
| Efha1 | 1.45314 |
| Efha1 | -0.72196 |
| Efhc2 | 0.36543 |
| Efhd1 | 0.78602 |
| Efna1 | 1.45 |
| Efna3 | -0.8293 |
| Efna4 | -0.71745 |
| Efna4 | 0.00217 |
| Efna5 | 1.31232 |
| Efnb1 | 0.80973 |
| Efnb2 | -0.09174 |
| Efnb3 | -0.32199 |
| Efr3a | -0.83235 |
| Efs | -0.32199 |
| Eftud1 | 1.02544 |
| Eftud2 | 0.44805 |
| Eftud2 | -0.5322 |
| Eftud2 | -0.29318 |
| EG194588 | 0.08737 |
| EG208426 | -0.65903 |
| EG210155 | 0.53313 |
| EG212225 | 1.56033 |
| EG214321 | 0.37782 |
| EG232599 | 0.88199 |
| EG237009 | -0.34331 |
| EG237009 | -0.55229 |
| EG237300 | 0.34866 |
| EG238829 | 1.30458 |
| EG240916 | 1.96703 |
| EG245297 | -0.43722 |
| EG245297 | 2.83105 |
| EG245376 | -0.64229 |
| EG245376 | -0.58755 |
| EG272350 | -0.53368 |
| EG277333 | 1.06744 |
| EG317677 | -0.12232 |
| EG328264 | -0.61774 |
| EG328280 | 0.1147 |
| EG328644 | -0.75699 |
| EG328839 | 0.7326 |
| EG330305 | -0.72196 |
| EG330503 | -0.6057 |
| EG330513 | -0.58599 |
| EG333669 | -0.01737 |
| EG368203 | -0.3046 |
| EG380907 | -0.52561 |
| EG381438 | 1.75592 |
| EG382109 | 1.7841 |
| EG382109 | -0.6081 |
| EG382109 | -0.64562 |
| EG384639 | 0.11942 |
| EG384813 | 3.45346 |
| EG384813 | 1.79598 |
| EG384814 | -0.84468 |
| EG385328 | 0.65793 |
| EG408196 | 0.11688 |
| EG432825 | 0.7043 |
| EG432879 | 1.27879 |
| EG432939 | 0.63549 |
| EG433016 | 1.99049 |
| EG433182 | -0.4027 |
| EG433224 | 2.15802 |
| EG433923 | 2.42694 |
| EG434171 | 0.99215 |
| EG434197 | 0.72739 |
| EG434280 | -0.58599 |
| EG434459 | 2.44973 |
| EG434459 | -0.43722 |
| EG434858 | 0.74991 |
| EG435391 | -0.6073 |
| EG436332 | -0.42339 |
| EG545047 | 2.1237 |
| EG545056 | 0.2051 |
| EG545253 | 1.65317 |
| EG545370 | 1.42296 |
| EG545758 | 1.83683 |
| EG545861 | -0.67781 |
| EG545963 | -0.23493 |
| EG546166 | 2.77285 |
| EG546282 | 1.07923 |
| EG546325 | 1.45501 |
| EG546347 | -0.44454 |
| EG547109 | -0.71565 |
| EG574403 | 1.7162 |
| EG622320 | -0.22645 |
| EG622339 | -0.70404 |
| EG624866 | 1.59978 |
| EG625054 | -0.56364 |
| EG625424 | 0.19582 |
| EG630499 | 0.42185 |
| EG633640 | 0.69215 |
| EG638695 | 2.21296 |
| EG654453 | -0.66411 |
| EG665186 | 0.50071 |
| EG665378 | -0.38812 |
| EG667977 | 2.98917 |
| EG668668 | -0.26359 |
| Egf | 1.19093 |
| Egfbp2 | -0.75511 |
| Egfl7 | 1.72741 |
| Egfl7 | 0.22404 |
| Egflam | 1.38447 |
| Egflam | 1.18026 |
| Egfr | -0.6105 |
| Egfr | -0.34751 |
| Egfr | 0.56423 |
| Egfr | -0.56288 |
| Egln1 | 0.02291 |
| Egln1 | -0.42143 |
| Egln1 | -0.8072 |
| Egln2 | 0.32377 |
| Egln3 | 1.21773 |
| Egr1 | 0.13983 |
| Egr2 | -0.2344 |
| Egr3 | 2.14592 |
| Egr4 | -0.75605 |
| Ehbp1 | 1.59281 |
| Ehbp1l1 | -0.61855 |
| Ehd1 | -0.63321 |
| Ehd2 | 0.2976 |
| Ehd3 | 0.35147 |
| Ehd4 | 1.17396 |
| Ehmt1 | -0.6523 |
| Ehmt2 | -0.36146 |
| Ehmt2 | -0.58521 |
| Ei24 | -0.36513 |
| Eid1 | 1.03926 |
| Eid2 | 2.67728 |
| Eid3 | -0.69873 |
| Eif1a | -0.16523 |
| Eif1b | 0.53036 |
| Eif2a | 1.71044 |
| Eif2ak1 | -0.64478 |
| Eif2ak2 | -0.63239 |
| Eif2b1 | 1.57121 |
| Eif2b1 | 2.75958 |
| Eif2b2 | 0.47991 |
| Eif2b4 | 1.76303 |
| Eif2b5 | -0.42994 |
| Eif2b5 | 0.97161 |
| Eif2b5 | -0.58443 |
| Eif2c2 | 0.35091 |
| Eif2c3 | -0.57668 |
| Eif2c3 | -0.4339 |
| Eif2c3 | -0.64895 |
| Eif2c3 | 0.90168 |
| Eif2c4 | 1.38547 |
| Eif2s2 | 0.22888 |
| Eif2s2 | -0.71925 |
| Eif2s3x | 0.41426 |
| Eif2s3y | -0.69346 |
| Eif3b | -0.31557 |
| Eif3b | -0.35295 |
| Eif3d | 0.51259 |
| Eif3eip | -0.05599 |
| Eif3eip | 0.26295 |
| Eif3f | -0.47307 |
| Eif3g | 1.13498 |
| Eif3i | -0.01527 |
| Eif3i | 1.18106 |
| Eif3i | 1.8404 |
| Eif3k | 0.04595 |
| Eif3s1 | -0.54779 |
| Eif3s10 | -0.37562 |
| Eif3s10 | -0.49119 |
| Eif3s4 | 0.95045 |
| Eif3s8 | 2.78345 |
| Eif4a1 | 0.11507 |
| Eif4a1 | 0.81416 |
| Eif4a2 | -0.66072 |
| Eif4a3 | -0.46826 |
| Eif4b | 1.88479 |
| Eif4e | -0.27298 |
| Eif4e1b | -0.56593 |
| Eif4e1b | 0.50857 |
| Eif4e2 | -0.72196 |
| Eif4e2 | -0.6057 |
| Eif4e3 | -0.2118 |
| Eif4e3 | 0.45863 |
| Eif4e3 | 1.07834 |
| Eif4ebp1 | -0.49755 |
| Eif4ebp2 | 2.38967 |
| Eif4el3 | 1.57775 |
| Eif4enif1 | -0.1242 |
| Eif4enif1 | -0.05819 |
| Eif4g1 | 0.36071 |
| Eif4g2 | -0.33674 |
| Eif4g2 | 0.2356 |
| Eif4h | -0.64063 |
| Eif5 | -0.13465 |
| Eif5 | -0.53073 |
| Eif5 | 0.71705 |
| Eif5a | -0.52707 |
| Eif5a | -0.6856 |
| Eif6 | 0.31474 |
| Ela1 | 3.0783 |
| Ela2 | -0.21857 |
| Ela2a | 1.44923 |
| Ela3b | -0.59856 |
| Elac2 | 3.09618 |
| Elac2 | 2.83688 |
| Elac2 | 0.73093 |
| Elavl1 | -0.39127 |
| Elavl2 | -0.38623 |
| Elavl2 | -0.77311 |
| Elavl3 | 2.226 |
| Elavl4 | 3.94531 |
| Elavl4 | -0.02964 |
| Elf1 | 1.97022 |
| Elf2 | 0.07491 |
| Elf2 | 0.70641 |
| Elf2 | -0.78854 |
| Elf2 | -0.07102 |
| Elf2 | 2.59202 |
| Elf3 | -0.43854 |
| Elf3 | 1.36264 |
| Elf3 | 1.51212 |
| Elf5 | -0.82828 |
| Elfn1 | -0.25159 |
| Elfn2 | -0.3023 |
| Elk3 | 1.9351 |
| Elk3 | 1.91981 |
| Elk3 | 0.45612 |
| Ell | 0.35484 |
| Ell3 | -0.07235 |
| Elmo1 | 0.99047 |
| Elmo1 | -0.3831 |
| Elmo1 | 0.7212 |
| Elmo1 | -0.36085 |
| Elmo1 | -0.20972 |
| Elmo1 | 1.30915 |
| Elmo1 | 2.39172 |
| Elmo1 | 0.27953 |
| Elmo1 | -0.53737 |
| Elmo1 | 0.71041 |
| Elmo1 | -0.77694 |
| Elmo1 | -0.46483 |
| Elmo1 | -0.45531 |
| Elmo2 | -0.32434 |
| Elmo2 | -0.29659 |
| Elmo3 | 1.20792 |
| Elmo3 | -0.6365 |
| Elmod1 | -0.7779 |
| Eln | -0.63733 |
| Elof1 | -0.49755 |
| Elovl1 | -0.78659 |
| Elovl1 | 0.28499 |
| Elovl1 | -0.58288 |
| Elovl1 | -0.12656 |
| Elovl2 | -0.79439 |
| Elovl4 | 0.06266 |
| Elovl5 | 1.06537 |
| Elovl6 | 0.58453 |
| Elp2 | 0.74717 |
| Elp3 | -0.63898 |
| Elp3 | 1.41592 |
| Eltd1 | 1.10152 |
| Emb | -0.6097 |
| Emcn | 1.44522 |
| Emd | -0.13704 |
| Emd | -0.44321 |
| Emg1 | 2.14576 |
| Emg1 | -0.05162 |
| Emid1 | -0.48 |
| Emid2 | -0.05819 |
| Emilin1 | 1.14985 |
| Emilin2 | -0.05206 |
| Eml1 | -0.4933 |
| Eml3 | -0.53737 |
| Eml3 | 0.02332 |
| Eml4 | -0.43921 |
| Eml5 | 1.30278 |
| Emp1 | -0.50824 |
| Emp2 | -0.56212 |
| Emp3 | -0.75699 |
| Emr1 | -0.42077 |
| Emr1 | 2.13673 |
| Emr1 | 1.22439 |
| En2 | 3.63954 |
| Enah | 0.65308 |
| Enc1 | 0.68121 |
| Endod1 | 1.18788 |
| Endogl1 | -0.61613 |
| Endogl1 | 0.48964 |
| Eng | 4.2225 |
| Eno1 | -0.27353 |
| Eno2 | -0.47307 |
| Eno3 | 1.79047 |
| Enoph1 | 0.43581 |
| Enox1 | 0.04123 |
| Enpp1 | 0.05415 |
| Enpp1 | 2.00163 |
| Enpp2 | 2.53621 |
| Enpp4 | -0.38123 |
| Enpp5 | -0.1603 |
| Enpp6 | -0.12373 |
| Ensa | 2.46679 |
| Ensa | -0.26249 |
| ENSMUSG00000043795 | -0.54779 |
| ENSMUSG00000053178 | -0.81718 |
| ENSMUSG00000068790 | -0.48139 |
| Entpd2 | 1.0073 |
| Entpd3 | 1.97235 |
| Entpd4 | 0.71231 |
| Entpd5 | -0.22645 |
| Entpd5 | 1.29354 |
| Entpd5 | 0.28166 |
| Entpd6 | 0.59214 |
| Entpd7 | 1.96011 |
| Ep300 | -0.23546 |
| Ep400 | -0.29318 |
| Epas1 | -0.85403 |
| Epb4.1 | 0.93508 |
| Epb4.1l1 | 0.83816 |
| Epb4.1l1 | 0.77527 |
| Epb4.1l1 | 1.80105 |
| Epb4.1l2 | 0.79925 |
| Epb4.1l3 | 0.41479 |
| Epb4.1l3 | 0.23432 |
| Epb4.1l3 | 0.97218 |
| Epb4.1l4a | -0.53885 |
| Epb4.1l5 | -0.15005 |
| Epb4.1l5 | 0.01929 |
| Epb4.9 | -0.59147 |
| Epb4.9 | 0.49254 |
| Epc1 | 1.13534 |
| Epc1 | 0.55593 |
| Epc1 | 1.54717 |
| Epdr1 | -0.78756 |
| Epgn | 1.12194 |
| Epha1 | 1.41069 |
| Epha1 | -0.67008 |
| Epha3 | 0.37618 |
| Epha3 | -0.42404 |
| Epha3 | 1.54502 |
| Epha4 | -0.69962 |
| Epha5 | 0.42367 |
| Epha6 | 0.09111 |
| Epha7 | -0.04337 |
| Epha7 | -0.23068 |
| Epha7 | 2.71044 |
| Ephb1 | -0.39697 |
| Ephb2 | 2.1381 |
| Ephb4 | 0.99187 |
| Ephb6 | 0.96514 |
| Ephx1 | -0.54033 |
| Ephx2 | -0.41169 |
| Epm2a | 0.12412 |
| Epm2aip1 | 1.5075 |
| Epm2aip1 | 0.75735 |
| Epm2aip1 | -0.31732 |
| Epn1 | -0.54854 |
| Epn2 | 0.37754 |
| Epn2 | -0.7005 |
| Epn2 | 1.02855 |
| Epn2 | -0.61774 |
| Epn2 | 0.69254 |
| Epor | 0.64453 |
| Eprs | -0.58521 |
| Eprs | -0.68473 |
| Eps15 | 0.35512 |
| Eps15l1 | -0.4919 |
| Eps8 | 1.34334 |
| Eps8l1 | -0.57977 |
| Eps8l2 | -0.1242 |
| Eps8l2 | -0.63898 |
| Eps8l3 | 1.69244 |
| Epsti1 | -0.52342 |
| Epsti1 | 1.90545 |
| Eraf | 0.77775 |
| Eral1 | 0.17731 |
| Ercc1 | 1.67148 |
| Ercc2 | 0.12376 |
| Ercc3 | -0.12941 |
| Ercc3 | 1.7432 |
| Ercc4 | -0.69521 |
| Ercc4 | 2.89445 |
| Ercc5 | -0.11201 |
| Ercc5 | -0.54854 |
| Ercc8 | -0.56593 |
| Ercc8 | 0.53359 |
| Erdr1 | -0.53589 |
| Erf | -0.5899 |
| Erf | -0.04727 |
| Ergic1 | 1.06368 |
| Ergic2 | 0.92428 |
| Ergic2 | 0.17765 |
| Ergic3 | 0.2533 |
| Ergic3 | 2.07149 |
| Erh | -0.30632 |
| Erich1 | -0.67867 |
| Erlin1 | 0.69331 |
| Erlin2 | 0.09706 |
| Erlin2 | 2.28348 |
| Ermap | -0.45666 |
| Ermp1 | -0.68908 |
| Ern1 | 1.32636 |
| Ern2 | 1.86376 |
| Ero1lb | -0.51039 |
| Erp29 | -0.36636 |
| Errfi1 | 1.88383 |
| Es22 | -0.49049 |
| Esam1 | 1.80978 |
| Esco2 | 0.20971 |
| Esd | -0.16473 |
| Esd | 1.83195 |
| Esd | 1.78084 |
| Esd | -0.70315 |
| Esm1 | -0.29944 |
| Espl1 | -0.22224 |
| Espn | 1.05391 |
| Espn | 1.69746 |
| Espn | 0.16119 |
| Espn | -0.57359 |
| Esr1 | -0.04684 |
| Esr2 | 0.81535 |
| Esr2 | -0.33318 |
| Esr2 | -0.38373 |
| Esrra | -0.49119 |
| Esrrb | 0.64902 |
| Etaa1 | 1.27981 |
| Etf1 | 2.41828 |
| Etfa | -0.47653 |
| Etfa | 1.52154 |
| Etfa | -0.34992 |
| Etfb | 0.6549 |
| Etfb | 1.44265 |
| Etfdh | 0.42159 |
| Ethe1 | -0.20044 |
| Etnk2 | -0.13512 |
| Ets1 | 1.06161 |
| Ets2 | -0.33022 |
| Etsrp71 | -0.36513 |
| Etv1 | -0.59383 |
| Etv1 | 2.87696 |
| Etv4 | -0.10968 |
| Etv5 | -0.61936 |
| Etv6 | -0.73563 |
| Evc | -0.44789 |
| Evc2 | -0.25376 |
| Evi1 | 0.96181 |
| Evi1 | 0.54278 |
| Evi2a | -0.63403 |
| Evi2a | -0.42143 |
| Evi5 | 0.34243 |
| Evi5l | -0.77598 |
| Evl | -0.50181 |
| Evx1 | -0.28246 |
| Exdl1 | -0.62422 |
| Exdl2 | -0.77694 |
| Exdl2 | 1.2899 |
| Exdl2 | 1.55859 |
| Exoc2 | -0.12656 |
| Exoc3 | -0.40143 |
| Exoc3l | -0.14957 |
| Exoc4 | 1.60892 |
| Exoc7 | -0.47861 |
| Exoc8 | -0.5606 |
| Exod1 | -0.45802 |
| Exosc1 | 0.15807 |
| Exosc10 | 1.12764 |
| Exosc2 | -0.20765 |
| Exosc2 | -0.73379 |
| Exosc4 | -0.19533 |
| Exosc5 | -0.37998 |
| Exosc6 | 1.81368 |
| Exosc7 | -0.54405 |
| Exosc8 | -0.42797 |
| Ext1 | -0.86137 |
| Ext2 | -0.0136 |
| Extl1 | 1.99327 |
| Extl2 | 0.53567 |
| Extl3 | 1.36628 |
| Extl3 | 2.95568 |
| Eya1 | -0.48978 |
| Eya1 | 0.15737 |
| Eya2 | -0.4306 |
| Eya3 | -0.47307 |
| Eya3 | 1.64694 |
| Eya3 | -0.77406 |
| Eya4 | -0.5448 |
| Ezh1 | 2.90402 |
| Ezh2 | 1.27632 |
| Ezh2 | -0.78465 |
| F10 | -0.4926 |
| F11 | -0.61613 |
| F11 | -0.58365 |
| F11r | -0.75417 |
| F13a1 | -0.61936 |
| F2r | 0.87565 |
| F2rl1 | 0.11688 |
| F2rl3 | -0.30978 |
| F430201B04Rik | 0.37481 |
| F5 | 1.7368 |
| F630043A04Rik | -0.79341 |
| F730014I05Rik | 0.10482 |
| F730047E07Rik | 0.70813 |
| F730047E07Rik | 0.03411 |
| F730047E07Rik | -0.36882 |
| F8 | 0.11397 |
| F8 | 1.85267 |
| F8 | -0.67436 |
| F8a | 1.60137 |
| Fa2h | -0.13465 |
| Faah | -0.31557 |
| Faah | 0.34328 |
| Fabp3 | -0.7076 |
| Fabp5 | 1.29616 |
| Fabp9 | 1.95821 |
| Fadd | 2.02414 |
| Fadd | 1.74425 |
| Fadd | 1.6647 |
| Fads1 | 0.69467 |
| Fads2 | 0.98286 |
| Fads3 | -0.67094 |
| Fads6 | 0.24196 |
| Faf1 | 1.00163 |
| Faf1 | -0.54033 |
| Fah | -0.71027 |
| Fahd1 | 0.69776 |
| Fahd2a | 0.35764 |
| Fahd2a | -0.39824 |
| Fahd2a | 0.84985 |
| Fahd2a | 0.85083 |
| Faim | -0.33318 |
| Faim | -0.5899 |
| Faim2 | -0.10366 |
| Faim2 | 1.59841 |
| Faim3 | 2.60065 |
| Fancc | 1.14179 |
| Fancc | -0.6523 |
| Fancd2 | 0.38109 |
| Fancg | -0.70138 |
| Fanci | 0.20774 |
| Fancl | 0.21168 |
| Fancl | -0.54108 |
| Fank1 | 0.20411 |
| Fank1 | -0.3938 |
| Fap | -0.26689 |
| Fars2 | -0.17266 |
| Farsa | -0.50466 |
| Farsb | -0.33615 |
| Farsb | -0.23546 |
| Fas | -0.0199 |
| Fas | 0.01767 |
| Fasl | -0.38123 |
| Fastk | 0.71516 |
| Fastk | -0.64312 |
| Fastkd1 | 0.29551 |
| Fastkd5 | 0.63404 |
| Fat1 | 1.0505 |
| Fat4 | 0.11215 |
| Fbf1 | 0.06651 |
| Fblim1 | -0.80919 |
| Fbln1 | 0.22501 |
| Fbln1 | 0.20971 |
| Fbln1 | -0.62667 |
| Fbln2 | 0.23048 |
| Fbln2 | -0.54331 |
| Fbn1 | -0.66752 |
| Fbp1 | 2.09297 |
| Fbp2 | 0.06574 |
| Fbxl10 | 0.17117 |
| Fbxl12 | 0.70755 |
| Fbxl12 | 0.73074 |
| Fbxl12 | 0.67786 |
| Fbxl12 | 1.67054 |
| Fbxl13 | 0.02452 |
| Fbxl13 | -0.17266 |
| Fbxl14 | 0.00093 |
| Fbxl15 | 0.97461 |
| Fbxl18 | -0.19993 |
| Fbxl18 | 1.27951 |
| Fbxl21 | 0.87549 |
| Fbxl22 | 0.50833 |
| Fbxl4 | -0.77598 |
| Fbxl5 | -0.51039 |
| Fbxl6 | -0.51543 |
| Fbxl6 | -0.57436 |
| Fbxl8 | -0.05687 |
| Fbxo15 | 0.43221 |
| Fbxo16 | -0.66837 |
| Fbxo18 | -0.22382 |
| Fbxo18 | 0.10776 |
| Fbxo2 | 0.89858 |
| Fbxo21 | 0.95862 |
| Fbxo25 | -0.24618 |
| Fbxo27 | -0.09814 |
| Fbxo28 | 0.35428 |
| Fbxo3 | -0.54629 |
| Fbxo3 | 0.96138 |
| Fbxo30 | 2.13042 |
| Fbxo31 | 0.9813 |
| Fbxo31 | 0.44297 |
| Fbxo32 | 1.41442 |
| Fbxo32 | -0.48209 |
| Fbxo32 | -0.55004 |
| Fbxo33 | 0.20147 |
| Fbxo34 | -0.73655 |
| Fbxo34 | -0.63898 |
| Fbxo34 | 0.45913 |
| Fbxo36 | -0.02497 |
| Fbxo38 | 0.26109 |
| Fbxo38 | -0.51833 |
| Fbxo4 | -0.5068 |
| Fbxo42 | -0.07414 |
| Fbxo44 | 2.42984 |
| Fbxo6 | -0.30517 |
| Fbxo7 | -0.71385 |
| Fbxo8 | -0.44454 |
| Fbxo9 | 0.30592 |
| Fbxo9 | 0.00422 |
| Fbxw12 | 1.73612 |
| Fbxw13 | -0.78369 |
| Fbxw15 | 0.31122 |
| Fbxw16 | 0.26419 |
| Fbxw17 | 0.6093 |
| Fbxw19 | -0.5068 |
| Fbxw2 | 2.49864 |
| Fbxw4 | 0.96051 |
| Fbxw4 | -0.3046 |
| Fbxw5 | -0.51327 |
| Fbxw7 | 0.20312 |
| Fbxw7 | -0.03007 |
| Fbxw7 | 0.78233 |
| Fbxw8 | -0.59147 |
| Fcer1g | 0.78707 |
| Fcer2a | 0.22404 |
| Fcf1 | -0.69083 |
| Fcgr2b | 2.47801 |
| Fcgr2b | -0.02412 |
| Fcgr2b | -0.51327 |
| Fcgr3 | -0.67351 |
| Fcgr4 | 2.39556 |
| Fcgrt | -0.5176 |
| Fcho1 | -0.69785 |
| Fcho2 | -0.05162 |
| Fchsd1 | -0.05687 |
| Fchsd2 | 0.25455 |
| Fcna | 0.59214 |
| Fcrla | 2.26771 |
| Fcrla | 0.37727 |
| Fcrla | 2.78868 |
| Fdps | 1.21334 |
| Fdxr | -0.44789 |
| Fdxr | 1.93419 |
| Fem1b | 2.94481 |
| Fem1c | -0.37376 |
| Fem1c | 1.76515 |
| Fen1 | -0.57282 |
| Fermt2 | 1.60397 |
| Fermt3 | 0.37508 |
| Fert2 | 1.40024 |
| Fert2 | -0.57205 |
| Fes | 2.08554 |
| Fetub | -0.47792 |
| Fev | 0.33157 |
| Fez1 | 0.37206 |
| Fez1 | 0.70008 |
| Fez2 | -0.59856 |
| Fezf2 | 2.73971 |
| Ffar2 | -0.45463 |
| Fgd2 | -0.7495 |
| Fgd4 | 2.75428 |
| Fgd4 | -0.76549 |
| Fgd5 | -0.6226 |
| Fgd6 | 0.66994 |
| Fgf1 | -0.56136 |
| Fgf1 | 3.04753 |
| Fgf10 | -0.11015 |
| Fgf10 | -0.77598 |
| Fgf12 | 1.08229 |
| Fgf12 | 0.80356 |
| Fgf13 | 0.32464 |
| Fgf13 | 1.69668 |
| Fgf17 | 1.43325 |
| Fgf21 | 2.38217 |
| Fgf22 | 1.07014 |
| Fgf22 | 0.80373 |
| Fgf5 | 1.06381 |
| Fgf7 | -0.67351 |
| Fgf8 | 0.57994 |
| Fgf9 | 0.70258 |
| Fgfbp3 | -0.13943 |
| Fgfr1 | -0.40719 |
| Fgfr1op | -0.34992 |
| Fgfr1op2 | -0.73196 |
| Fgfr1op2 | 0.56445 |
| Fgfr2 | 0.21102 |
| Fgfr2 | -0.50537 |
| Fgfr3 | -0.32375 |
| Fgfrl1 | -0.66242 |
| Fgl2 | 1.11092 |
| Fh1 | 0.34837 |
| Fhad1 | 2.27748 |
| Fhit | 3.09975 |
| Fhl1 | 2.17573 |
| Fhl1 | 0.84952 |
| Fhl2 | 1.04681 |
| Fhl4 | -0.81018 |
| Fhl5 | 1.53351 |
| Fhod1 | 2.71195 |
| Fhod3 | -0.35719 |
| Fibcd1 | 1.65167 |
| Fibp | 0.37918 |
| Figf | -0.8212 |
| Figla | -0.57205 |
| Fign | -0.61693 |
| Fignl1 | -0.70938 |
| Fip1l1 | -0.24672 |
| Fis1 | 1.56956 |
| Fis1 | -0.65482 |
| Fiz1 | -0.27576 |
| Fjx1 | 0.84559 |
| Fkbp10 | 2.00926 |
| Fkbp11 | 0.28378 |
| Fkbp14 | -0.53 |
| Fkbp1a | 1.09977 |
| Fkbp1b | 0.46237 |
| Fkbp2 | -0.24349 |
| Fkbp2 | -0.19024 |
| Fkbp4 | -0.35537 |
| Fkbp5 | 0.04438 |
| Fkbp6 | 1.30278 |
| Fkbp7 | 1.9344 |
| Fkbp8 | 0.69738 |
| Fkbp9 | -0.3069 |
| Fkbpl | 0.57201 |
| Fkbpl | -0.5448 |
| Fkhl18 | -0.17415 |
| Fkrp | 0.96615 |
| Fktn | -0.22803 |
| Flad1 | 0.00832 |
| Flcn | 1.87797 |
| Flcn | -0.39317 |
| Fli1 | -0.35053 |
| Flii | 1.59935 |
| Flna | 2.87427 |
| Flot1 | 0.30562 |
| Flot1 | 0.15842 |
| Flot2 | -0.45802 |
| Flot2 | 1.70472 |
| Flot2 | 0.82431 |
| Flrt1 | 1.70652 |
| Flrt2 | -0.41299 |
| Flrt3 | -0.73196 |
| Flt1 | 0.21527 |
| Flt4 | 1.19498 |
| Flt4 | -0.54182 |
| Flvcr2 | 1.75854 |
| Flywch2 | 0.50023 |
| Fmn2 | -0.20147 |
| Fmn2 | -0.44121 |
| Fmnl1 | 0.0588 |
| Fmnl3 | 1.63865 |
| Fmnl3 | 0.73186 |
| Fmo1 | -0.61855 |
| Fmo2 | 1.19408 |
| Fmo5 | 0.07946 |
| Fmod | -0.44254 |
| Fmr1nb | 0.85491 |
| Fmr1nb | -0.73471 |
| Fn3k | 1.37195 |
| Fn3k | -0.09083 |
| Fnbp1 | 0.79457 |
| Fnbp1 | 1.81437 |
| Fnbp1 | 1.23736 |
| Fnbp1l | 2.62584 |
| Fnbp4 | -0.62179 |
| Fndc3 | -0.11668 |
| Fndc3a | 1.64218 |
| Fndc3b | 0.25923 |
| Fndc4 | 3.38369 |
| Fndc5 | 1.91119 |
| Fndc5 | -0.44857 |
| Fndc7 | -0.46006 |
| Fndc7 | -0.45734 |
| Fndc8 | -0.32845 |
| Fndc8 | -0.3046 |
| Fnip1 | -0.64229 |
| Fnip1 | -0.83542 |
| Fnta | -0.58132 |
| Fnta | -0.33615 |
| Fntb | -0.27021 |
| Folr1 | 0.92488 |
| Folr1 | 0.54779 |
| Folr2 | 2.04567 |
| Fos | 1.30148 |
| Fosb | -0.55304 |
| Foxa1 | 2.27245 |
| Foxa2 | -0.56516 |
| Foxa3 | -0.21024 |
| Foxc1 | -0.55681 |
| Foxd2 | 2.28489 |
| Foxg1 | 0.36294 |
| Foxh1 | 0.82784 |
| Foxh1 | 0.65853 |
| Foxj1 | -0.10968 |
| Foxj2 | -0.77502 |
| Foxj3 | 1.54617 |
| Foxj3 | -0.60332 |
| Foxj3 | 2.34635 |
| Foxj3 | 0.43633 |
| Foxj3 | 0.5669 |
| Foxk1 | -0.37811 |
| Foxk1 | 0.08586 |
| Foxk1 | 1.25867 |
| Foxl2 | -0.67694 |
| Foxm1 | -0.18771 |
| Foxm1 | -0.40398 |
| Foxn2 | 3.12608 |
| Foxn3 | -0.55455 |
| Foxo1 | 1.82025 |
| Foxo6 | -0.67694 |
| Foxo6 | -0.57822 |
| Foxp1 | -0.48418 |
| Foxp2 | -0.09265 |
| Foxp2 | -0.73471 |
| Foxp2 | 0.27097 |
| Foxp3 | 0.3997 |
| Foxp4 | 1.70239 |
| Foxq1 | 1.63671 |
| Foxred1 | -0.71117 |
| Foxred1 | -0.13417 |
| Foxred1 | 0.00668 |
| Foxred1 | 0.55908 |
| Foxred1 | -0.19584 |
| Foxred2 | 0.85945 |
| Fpgs | 0.58235 |
| Fpgs | 0.39783 |
| Fpgs | -0.36513 |
| Fpr2 | -0.75137 |
| Frag1 | 0.00176 |
| Frag1 | 1.68812 |
| Frag1 | -0.77502 |
| Frap1 | 3.31383 |
| Fras1 | 2.64816 |
| Fras1 | -0.42666 |
| Frat1 | 0.89485 |
| Frat1 | -0.42863 |
| Frat2 | 0.755 |
| Freq | 1.75683 |
| Freq | 0.29222 |
| Freq | -0.56975 |
| Frg1 | -0.9223 |
| Frk | -0.29887 |
| Frmd3 | -0.77215 |
| Frmd6 | -0.54779 |
| Frmd8 | 2.19095 |
| Frmpd4 | -0.57359 |
| Frrs1 | -0.23493 |
| Frs2 | 1.15749 |
| Frs3 | 1.39094 |
| Fryl | -0.15492 |
| Fryl | 0.16188 |
| Frzb | 0.43376 |
| Fscn1 | 3.93801 |
| Fscn2 | -0.34932 |
| Fscn3 | -0.61371 |
| Fsd1 | 0.39139 |
| Fsd2 | 1.06692 |
| Fshb | 0.16015 |
| Fshr | 3.3341 |
| Fshr | -0.51183 |
| Fshr | -0.33793 |
| Fsip1 | 0.63672 |
| Fst | -0.31965 |
| Fstl1 | -0.88807 |
| Fstl4 | -0.01905 |
| Fstl5 | -0.51471 |
| Fth1 | -0.76834 |
| Fth1 | 1.12655 |
| Ftmt | 0.72739 |
| Fto | -0.22066 |
| Ftsj1 | -0.36759 |
| Ftsj3 | -0.64478 |
| Fubp1 | -0.70493 |
| Fubp3 | 0.62385 |
| Fuk | 3.21278 |
| Fuk | 1.6031 |
| Fundc1 | -0.41363 |
| Fundc1 | 2.20033 |
| Fundc2 | 1.14417 |
| Furin | -0.76929 |
| Furin | -0.79635 |
| Fus | 1.90918 |
| Fusip1 | 2.50444 |
| Fusip1 | -0.57668 |
| Fusip1 | -0.60332 |
| Fut10 | 1.37842 |
| Fut10 | 0.61418 |
| Fut2 | -0.43854 |
| Fut4 | 0.3997 |
| Fut8 | 0.2682 |
| Fut8 | -0.04597 |
| Fut8 | 0.38381 |
| Fvt1 | -0.53959 |
| Fxc1 | -0.21909 |
| Fxn | -0.01276 |
| Fxn | -0.42929 |
| Fxyd1 | 2.45037 |
| Fxyd2 | 3.52814 |
| Fxyd2 | 0.01036 |
| Fxyd2 | -0.67781 |
| Fxyd3 | -0.70227 |
| Fxyd4 | -0.41493 |
| Fxyd4 | -0.62179 |
| Fxyd5 | 2.03852 |
| Fxyd5 | -0.04553 |
| Fxyd5 | -0.11061 |
| Fxyd6 | 0.4483 |
| Fxyd7 | -0.53885 |
| Fyb | -0.6565 |
| Fyco1 | -0.5011 |
| Fyn | 2.75743 |
| Fyttd1 | -0.08403 |
| Fzd1 | 0.05337 |
| Fzd10 | -0.68126 |
| Fzd10 | -0.5011 |
| Fzd2 | -0.66837 |
| Fzd3 | 1.15971 |
| Fzd4 | -0.71206 |
| Fzd5 | -0.67436 |
| Fzd6 | -0.31094 |
| Fzd7 | -0.54629 |
| Fzd8 | 1.02016 |
| Fzr1 | 1.5071 |
| Fzr1 | 0.90754 |
| Fzr1 | 0.83368 |
| G0s2 | -0.73931 |
| G3bp1 | -0.34391 |
| G3bp1 | -0.59619 |
| G3bp2 | 0.06957 |
| G3bp2 | -0.05599 |
| G3bp2 | 1.71249 |
| G430022H21Rik | 0.87883 |
| G430055L02Rik | -0.81818 |
| G6pc | -0.13608 |
| G6pc2 | 0.09743 |
| G6pc2 | -0.6065 |
| G6pc3 | 0.94324 |
| G6pd2 | -0.36821 |
| G6pd2 | -0.55004 |
| G6pdx | -0.47653 |
| Gaa | -0.0438 |
| Gab1 | 2.13261 |
| Gab2 | 1.62813 |
| Gab2 | 1.48606 |
| Gab2 | 1.65848 |
| Gab3 | -0.71745 |
| Gab3 | -0.39317 |
| Gab3 | -0.61532 |
| Gabarap | 0.00381 |
| Gabarapl1 | 1.05679 |
| Gabarapl2 | 0.72683 |
| Gabbr1 | -0.55004 |
| Gabpa | -0.47792 |
| Gabpa | -0.66242 |
| Gabpb1 | -0.21492 |
| Gabpb1 | -0.09037 |
| Gabpb2 | -0.44321 |
| Gabra3 | -0.25104 |
| Gabra4 | 1.9661 |
| Gabrb1 | -0.16424 |
| Gabrb2 | -0.71925 |
| Gabrb3 | -0.58912 |
| Gabrb3 | -0.31441 |
| Gabrd | -0.6081 |
| Gabrd | -0.18923 |
| Gabrg1 | 2.01037 |
| Gabrg2 | -0.19686 |
| Gabrg2 | -0.70227 |
| Gabrg2 | -0.52927 |
| Gabrg2 | -0.32727 |
| Gabrp | -0.38435 |
| Gabrp | -0.56899 |
| Gad1 | -0.70138 |
| Gad1 | -0.6121 |
| Gadd45a | 1.78564 |
| Gadd45a | 0.80373 |
| Gadd45b | -0.14424 |
| Gadd45g | 1.61442 |
| Gadd45g | 1.10623 |
| Gak | -0.08538 |
| Gal | 0.46735 |
| Gal3st1 | -0.24726 |
| Gal3st2 | 0.15633 |
| Gal3st3 | 0.68591 |
| Galc | -0.47101 |
| Galc | 0.36682 |
| Gale | -0.56822 |
| Gale | -0.73013 |
| Galk1 | -0.43854 |
| Galm | 2.6762 |
| Galns | 0.69157 |
| Galnt1 | 0.32725 |
| Galnt1 | -0.18015 |
| Galnt1 | 0.91914 |
| Galnt1 | -0.46142 |
| Galnt10 | 0.60632 |
| Galnt10 | 0.22952 |
| Galnt10 | 0.68337 |
| Galnt11 | -0.35234 |
| Galnt12 | -0.48628 |
| Galnt13 | -0.7523 |
| Galnt2 | 1.35181 |
| Galnt2 | 1.62384 |
| Galnt2 | -0.52269 |
| Galnt3 | 1.18288 |
| Galnt9 | -0.32024 |
| Galntl2 | 0.4826 |
| Galntl4 | -0.47446 |
| Galntl5 | 2.67267 |
| Galt | 1.40186 |
| Galt | -0.23387 |
| Gamt | 2.92623 |
| Gan | -0.68734 |
| Ganab | -0.51543 |
| Ganab | 1.05011 |
| Ganab | 1.14108 |
| Ganc | 1.66078 |
| Gap43 | -0.25702 |
| Gapdh | 0.99662 |
| Gapdhs | -0.23333 |
| Gapvd1 | 2.61516 |
| Gapvd1 | 0.62217 |
| Garnl3 | 0.11325 |
| Garnl4 | -0.51833 |
| Gars | 1.97196 |
| Gart | -0.53441 |
| Gart | -0.79243 |
| Gas2l1 | 0.23272 |
| Gas2l1 | 3.25403 |
| Gas2l1 | 1.88558 |
| Gas2l1 | 2.47843 |
| Gas6 | -0.59856 |
| Gas7 | 1.01948 |
| Gas7 | -0.4926 |
| Gas8 | -0.36821 |
| Gata2 | -0.22276 |
| Gata4 | -0.57745 |
| Gata6 | -0.48418 |
| Gatad1 | -0.62585 |
| Gatad1 | -0.02582 |
| Gatad2b | 1.31578 |
| Gats | -0.44722 |
| Gats | 3.95709 |
| Gba | -0.00568 |
| Gba2 | 1.15913 |
| Gbas | 1.86981 |
| Gbf1 | -0.69433 |
| Gbl | -0.35598 |
| Gbl | -0.4662 |
| Gbl | -0.28246 |
| Gbp2 | -0.36698 |
| Gbp3 | -0.68734 |
| Gbp3 | -0.48418 |
| Gbp6 | 1.65895 |
| Gbp6 | 2.68663 |
| Gbx2 | 2.25273 |
| Gca | -0.34751 |
| Gca | -0.46483 |
| Gcap14 | 0.69602 |
| Gcap14 | 0.956 |
| Gcc1 | -0.62017 |
| Gcc2 | -0.35963 |
| Gcdh | 0.51871 |
| Gch1 | -0.80028 |
| Gch1 | 2.91805 |
| Gchfr | 1.09428 |
| Gck | -0.58443 |
| Gclm | 0.28529 |
| Gcn5l2 | 1.27437 |
| Gcnt1 | 3.80092 |
| Gcnt1 | -0.59461 |
| Gcnt2 | 1.96969 |
| Gcnt2 | -0.28922 |
| Gcs1 | -0.57822 |
| Gda | 0.383 |
| Gdap1 | -0.71925 |
| Gdap1 | 0.74972 |
| Gdap1l1 | 1.74239 |
| Gdap1l1 | -0.7495 |
| Gde1 | 1.85234 |
| Gde1 | 1.25773 |
| Gdf10 | -0.73563 |
| Gdf15 | 0.76312 |
| Gdf3 | 0.38652 |
| Gdf9 | 2.30556 |
| Gdi1 | 0.6301 |
| Gdi1 | 1.09126 |
| Gdi1 | 1.11018 |
| Gdi1 | 0.4111 |
| Gdi1 | 3.87021 |
| Gdi2 | -0.37998 |
| Gdnf | -0.42535 |
| Gdpd1 | 1.08038 |
| Gdpd2 | 1.26181 |
| Gdpd2 | 0.16395 |
| Gdpd3 | -0.09814 |
| Gdpd4 | -0.4717 |
| Gdpd5 | -0.44588 |
| Gemin4 | -0.41557 |
| Gemin4 | 0.51988 |
| Gemin4 | 0.2859 |
| Gemin5 | 1.11632 |
| Gemin6 | -0.63075 |
| Gemin6 | -0.60491 |
| Gemin7 | 0.13983 |
| Gemin7 | -0.79831 |
| Gfap | 0.11106 |
| Gfer | -0.58521 |
| Gfi1 | -0.59619 |
| Gfm1 | -0.07191 |
| Gfod1 | -0.58443 |
| Gfod2 | 0.79492 |
| Gfpt2 | -0.04207 |
| Gfra1 | -0.34092 |
| Gfra4 | -0.36821 |
| Gga1 | 1.45102 |
| Gga1 | 0.09372 |
| Gga1 | -0.5278 |
| Gga3 | -0.14908 |
| Ggcx | -0.1209 |
| Ggcx | 0.23208 |
| Ggcx | -0.84468 |
| Ggn | -0.61613 |
| Ggn | 2.2134 |
| Ggnbp1 | -0.50252 |
| Ggnbp2 | 1.29344 |
| Ggt5 | 1.16935 |
| Ggt7 | -0.49119 |
| Ggta1 | 0.23815 |
| Ggtla1 | 0.00011 |
| Gh | -0.59777 |
| Ghitm | -0.6065 |
| Ghr | 0.06074 |
| Ghr | 0.17219 |
| Ghrh | -0.46757 |
| Ghrhr | 1.36404 |
| Ghrl | -0.72922 |
| Ghrl | -0.67351 |
| Ghsr | 1.03231 |
| Ghsr | -0.375 |
| Gif | -0.46552 |
| Gigyf1 | 3.79492 |
| Gimap1 | 0.41793 |
| Gimap4 | -0.51833 |
| Gimap5 | -0.77311 |
| Gimap6 | 2.81623 |
| Gimap8 | -0.49401 |
| Gimap9 | -0.69785 |
| Gins3 | 1.84595 |
| Gins4 | -0.54256 |
| Gipc1 | 0.24607 |
| Gipc1 | -0.06171 |
| Gipc1 | 1.01224 |
| Gipc2 | -0.45802 |
| Git1 | -0.41493 |
| Git2 | -0.68996 |
| Git2 | 0.32116 |
| Git2 | 0.42679 |
| Gja1 | 0.30533 |
| Gja4 | 0.84394 |
| Gja5 | -0.56288 |
| Gja5 | 3.17298 |
| Gja6 | 0.33473 |
| Gja7 | -0.64395 |
| Gjb1 | 0.14266 |
| Gjb2 | -0.67781 |
| Gjb2 | -0.6113 |
| Gjb3 | 1.58894 |
| Gjb6 | 1.85377 |
| Gjc2 | 1.09076 |
| Gjc2 | 0.16705 |
| Gjd2 | 0.92744 |
| Gjd2 | 0.39729 |
| Gjd3 | -0.73379 |
| Gk2 | -0.66496 |
| Gk5 | -0.70671 |
| Gkap1 | 1.49039 |
| Gkap1 | 1.78994 |
| Glb1 | 1.42882 |
| Glb1l2 | -0.64229 |
| Glcci1 | -0.37253 |
| Gldc | 0.92984 |
| Gldc | -0.70315 |
| Gldn | -0.23068 |
| Gle1 | 0.05726 |
| Glg1 | 0.65308 |
| Glipr2 | 1.78859 |
| Glis1 | 1.71613 |
| Glo1 | -0.66411 |
| Gloxd1 | 2.16972 |
| Glp2r | -0.04727 |
| Glra2 | 2.00661 |
| Glrb | 0.91351 |
| Glrp1 | -0.72922 |
| Glrx | -0.57436 |
| Glrx2 | 1.04523 |
| Glrx2 | -0.64146 |
| Glrx2 | -0.77982 |
| Glrx3 | 0.0483 |
| Glrx5 | -0.63321 |
| Gls2 | 1.73643 |
| Gls2 | 1.78564 |
| Glt25d1 | 0.22113 |
| Glt25d2 | -0.57205 |
| Glt25d2 | 0.28891 |
| Glt6d1 | 1.17889 |
| Glt8d1 | -0.06436 |
| Glt8d1 | -0.53811 |
| Glt8d1 | 0.30147 |
| Glt8d2 | 1.76792 |
| Glt8d3 | -0.43722 |
| Gltp | 1.00107 |
| Gltscr1 | -0.66923 |
| Glud1 | 2.51216 |
| Glud1 | -0.1486 |
| Glul | -0.60332 |
| Glyctk | 0.2979 |
| Glyctk | -0.5899 |
| Glyctk | -0.71475 |
| Gm1006 | -0.47792 |
| Gm1006 | -0.53663 |
| Gm1019 | -0.42666 |
| Gm1040 | 0.67431 |
| Gm1070 | 3.93851 |
| Gm1082 | -0.15981 |
| Gm114 | -0.44991 |
| Gm114 | 0.70123 |
| Gm1141 | -0.65062 |
| Gm128 | 2.0319 |
| Gm129 | -0.31499 |
| Gm129 | -0.13895 |
| Gm1322 | -0.82423 |
| Gm136 | 1.06524 |
| Gm136 | 0.79769 |
| Gm1381 | -0.73288 |
| Gm1527 | -0.56212 |
| Gm1568 | 0.87518 |
| Gm1574 | 0.8793 |
| Gm1574 | -0.53294 |
| Gm1604 | -0.63486 |
| Gm1604 | -0.71925 |
| Gm166 | -0.6856 |
| Gm166 | -0.53368 |
| Gm1661 | -0.01653 |
| Gm1673 | -0.05031 |
| Gm1673 | -0.0922 |
| Gm1698 | 3.01449 |
| Gm1821 | -0.6365 |
| Gm1943 | -0.77215 |
| Gm1965 | -0.54182 |
| Gm1967 | -0.09585 |
| Gm1967 | 0.94619 |
| Gm216 | -0.82423 |
| Gm239 | 2.3354 |
| Gm249 | 1.23725 |
| Gm266 | 2.23941 |
| Gm2a | 0.08286 |
| Gm347 | -0.13895 |
| Gm382 | -0.29716 |
| Gm410 | 0.2372 |
| Gm428 | -0.29375 |
| Gm443 | -0.06746 |
| Gm484 | -0.54629 |
| Gm50 | -0.57745 |
| Gm525 | -0.48768 |
| Gm525 | 0.05764 |
| Gm527 | -0.22434 |
| Gm527 | 0.65064 |
| Gm561 | -0.61371 |
| Gm561 | -0.81318 |
| Gm597 | -0.52853 |
| Gm603 | 2.07873 |
| Gm609 | -0.73838 |
| Gm614 | -0.2092 |
| Gm614 | -0.77119 |
| Gm648 | 1.07502 |
| Gm648 | 1.33681 |
| Gm71 | -0.5278 |
| Gm711 | 0.78567 |
| Gm732 | 1.1345 |
| Gm732 | -0.56669 |
| Gm767 | 1.4594 |
| Gm773 | 1.64087 |
| Gm773 | -0.75511 |
| Gm784 | 1.10276 |
| Gm813 | -0.6365 |
| Gm813 | -0.47931 |
| Gm817 | -0.60014 |
| Gm867 | -0.18317 |
| Gm884 | -0.4919 |
| Gm889 | 0.59582 |
| Gm906 | -0.83235 |
| Gm93 | -0.2864 |
| Gm93 | -0.46415 |
| Gm944 | -0.62422 |
| Gm949 | 0.02332 |
| Gm962 | 1.49615 |
| Gm962 | 2.69743 |
| Gm97 | -0.32082 |
| Gm97 | 1.15115 |
| Gmcl1 | 0.69312 |
| Gmcl1 | -0.74578 |
| Gmcl1 | 2.05379 |
| Gmds | -0.38623 |
| Gmeb2 | 1.61157 |
| Gmfg | -0.69083 |
| Gmfg | -0.80819 |
| Gmfg | 1.39003 |
| Gmip | 1.10301 |
| Gmip | -0.73563 |
| Gmppa | 1.41874 |
| Gmppa | -0.47239 |
| Gmppb | 1.56364 |
| Gmpr | 2.71115 |
| Gmpr2 | -0.61693 |
| Gna11 | -0.48908 |
| Gna13 | 0.11761 |
| Gna14 | -0.51616 |
| Gnai2 | 1.51411 |
| Gnao1 | -0.61613 |
| Gnao1 | -0.52561 |
| Gnaq | 0.486 |
| Gnaq | -0.71027 |
| Gna-rs1 | -0.65903 |
| Gnas | -0.47239 |
| Gnas | -0.15687 |
| Gnas | -0.28752 |
| Gnas | -0.24942 |
| Gnas | -0.06127 |
| Gnat1 | -0.69521 |
| Gnat2 | 2.89004 |
| Gnaz | 2.21796 |
| Gnb1 | -0.4662 |
| Gnb1l | 2.01157 |
| Gnb2 | -0.43722 |
| Gnb2l1 | -0.49472 |
| Gnb3 | 0.09297 |
| Gnb4 | 0.35147 |
| Gnb4 | 1.57775 |
| Gnb5 | 0.89517 |
| Gnb5 | 1.422 |
| Gne | -0.55606 |
| Gng10 | 0.83998 |
| Gng11 | 1.70884 |
| Gng13 | 2.42594 |
| Gng2 | -0.27132 |
| Gng2 | 0.88025 |
| Gng3 | 0.51093 |
| Gng4 | -0.67436 |
| Gng7 | -0.81318 |
| Gng8 | -0.40206 |
| Gngt2 | 0.35372 |
| Gnl2 | -0.58365 |
| Gnl2 | 0.03132 |
| Gnl3 | -0.06613 |
| Gnl3 | -0.32024 |
| Gnl3 | -0.6121 |
| Gnl3l | 1.39826 |
| Gnl3l | -0.70227 |
| Gnmt | -0.50395 |
| Gnmt | 0.1898 |
| Gnpat | -0.58833 |
| Gnpda1 | -0.22066 |
| Gnpda1 | -0.71565 |
| Gnpda1 | -0.17216 |
| Gnpda2 | 1.09765 |
| Gnptab | -0.64979 |
| Gnptg | 1.23316 |
| Gnptg | 0.23048 |
| Gnrhr | -0.44254 |
| Gns | 1.24961 |
| Golga1 | 0.67093 |
| Golga2 | -0.58521 |
| Golga2 | -0.71385 |
| Golga2 | -0.45666 |
| Golga2 | 1.14678 |
| Golga2 | 1.54908 |
| Golga3 | 0.7876 |
| Golga4 | -0.54108 |
| Golga5 | 0.22178 |
| Golga7 | 0.73945 |
| Golim4 | -0.4919 |
| Golm1 | -0.2344 |
| Golm1 | 0.19315 |
| Golph3l | -0.31094 |
| Golt1a | -0.32024 |
| Golt1b | 0.36238 |
| Gopc | -0.46963 |
| Gorasp1 | -0.54256 |
| Gorasp2 | 1.00743 |
| Gorasp2 | -0.08629 |
| Gorasp2 | -0.50466 |
| Gorasp2 | 0.32667 |
| Gosr1 | -0.53441 |
| Gosr2 | 1.77655 |
| Gosr2 | 2.17971 |
| Gosr2 | 1.11901 |
| Gosr2 | 3.02149 |
| Got1l1 | -0.57052 |
| Got2 | 0.27495 |
| Gp1bb | -0.71835 |
| Gp38 | 1.09139 |
| Gp5 | 0.00832 |
| Gp9 | -0.45463 |
| Gpaa1 | 1.45323 |
| Gpam | -0.20662 |
| Gpatch1 | 1.71473 |
| Gpatch2 | -0.18267 |
| Gpatch3 | -0.48 |
| Gpatch4 | -0.7495 |
| Gpbp1 | 0.50833 |
| Gpbp1 | -0.76644 |
| Gpbp1l1 | 3.12778 |
| Gpc1 | -0.80522 |
| Gpc3 | 0.41453 |
| Gpc3 | 1.39736 |
| Gpc4 | 1.51783 |
| Gpc5 | 0.58279 |
| Gpc5 | 2.96153 |
| Gpc5 | 0.85896 |
| Gpd1 | -0.73563 |
| Gpd2 | 2.24641 |
| Gpd2 | 1.26171 |
| Gper | -0.80127 |
| Gpha2 | 0.20708 |
| Gphb5 | 0.03172 |
| Gphn | 0.68826 |
| Gphn | 0.8774 |
| Gphn | -0.48418 |
| Gpihbp1 | -0.20713 |
| Gpkow | -0.70404 |
| Gpld1 | -0.38875 |
| Gpld1 | 0.24323 |
| Gpm6a | 0.61333 |
| Gpm6a | -0.73196 |
| Gpm6b | 0.51683 |
| Gpnmb | -0.0322 |
| Gpnmb | -0.7617 |
| Gpr1 | -0.21857 |
| Gpr1 | 2.7683 |
| Gpr103 | 1.86906 |
| Gpr107 | -0.60014 |
| Gpr108 | 0.56244 |
| Gpr108 | -0.70671 |
| Gpr108 | -0.49401 |
| Gpr109a | -0.43987 |
| Gpr113 | 0.09036 |
| Gpr113 | 1.73299 |
| Gpr114 | -0.72377 |
| Gpr12 | 2.39529 |
| Gpr12 | -0.72467 |
| Gpr120 | -0.17316 |
| Gpr123 | -0.6565 |
| Gpr124 | 0.24449 |
| Gpr128 | 2.7632 |
| Gpr135 | 0.79144 |
| Gpr137 | 1.11129 |
| Gpr137b | 0.96672 |
| Gpr141 | -0.18619 |
| Gpr146 | -0.37315 |
| Gpr146 | 0.18239 |
| Gpr149 | 0.702 |
| Gpr151 | -0.54331 |
| Gpr153 | -0.56745 |
| Gpr156 | 1.76647 |
| Gpr156 | -0.65398 |
| Gpr158 | -0.2691 |
| Gpr162 | -0.02497 |
| Gpr162 | -0.49401 |
| Gpr17 | 2.28893 |
| Gpr172b | 0.68787 |
| Gpr172b | 0.09818 |
| Gpr176 | 0.16945 |
| Gpr177 | -0.21962 |
| Gpr18 | 3.02861 |
| Gpr180 | 0.99579 |
| Gpr182 | 2.25695 |
| Gpr182 | -0.32669 |
| Gpr19 | 1.36301 |
| Gpr20 | -0.45193 |
| Gpr21 | -0.48418 |
| Gpr22 | 0.40422 |
| Gpr23 | 0.92443 |
| Gpr26 | -0.63157 |
| Gpr27 | 1.34142 |
| Gpr27 | 2.38214 |
| Gpr27 | -0.57977 |
| Gpr34 | 0.38381 |
| Gpr34 | 2.72541 |
| Gpr37l1 | -0.63403 |
| Gpr45 | -0.46415 |
| Gpr6 | -0.02836 |
| Gpr64 | 0.32058 |
| Gpr65 | 1.17419 |
| Gpr68 | -0.60411 |
| Gpr75 | 1.82417 |
| Gpr81 | 2.43955 |
| Gpr83 | 0.24954 |
| Gpr85 | -0.39127 |
| Gpr85 | -0.80028 |
| Gpr89 | 0.8774 |
| Gpr89 | -0.47722 |
| Gpr97 | -0.74393 |
| Gprasp1 | -0.73471 |
| Gprasp1 | 1.25573 |
| Gprc2a-rs5 | 1.0439 |
| Gprc2a-rs5 | 0.12664 |
| Gprc5a | -0.67265 |
| Gprc5b | 2.72607 |
| Gprc5c | -0.39317 |
| Gprk6 | -0.44521 |
| Gprk6 | 0.6747 |
| Gps1 | -0.27576 |
| Gps1 | -0.48418 |
| Gps1 | -0.5322 |
| Gps1 | -0.44857 |
| Gps2 | 0.84213 |
| Gpsm1 | 1.03392 |
| Gpsm2 | -0.47376 |
| Gpsm3 | -0.22909 |
| Gpsn2 | -0.49472 |
| Gpsn2 | 0.01686 |
| Gpsn2 | 0.52455 |
| Gpt1 | -0.5899 |
| Gpt2 | 0.7457 |
| Gpx1 | 1.90108 |
| Gpx2 | 1.15091 |
| Gpx3 | -0.28358 |
| Gpx4 | 0.59344 |
| Gpx4 | 1.84595 |
| Gpx6 | -0.47376 |
| Gpx7 | -0.58443 |
| Gramd1a | -0.27576 |
| Gramd1b | -0.43589 |
| Gramd2 | -0.40143 |
| Gramd2 | -0.18317 |
| Gramd3 | -0.47653 |
| Gramd4 | -0.29091 |
| Grap2 | -0.40462 |
| Grasp | -0.62912 |
| Grasp | 0.03172 |
| Grb10 | 0.1525 |
| Grb14 | -0.70671 |
| Grb14 | 0.7904 |
| Grb2 | -0.55606 |
| Grb7 | 0.49711 |
| Grcc10 | 1.38227 |
| Greb1 | 0.03092 |
| Greb1 | 1.22287 |
| Grem1 | -0.2819 |
| Grem2 | -0.3938 |
| Grhl1 | 1.03512 |
| Grhl2 | -0.33259 |
| Grhpr | -0.31616 |
| Gria1 | -0.57359 |
| Gria1 | -0.63321 |
| Gria1 | -0.5954 |
| Gria2 | 1.95634 |
| Gria2 | -0.40143 |
| Gria3 | -0.58521 |
| Gria3 | -0.03519 |
| Gria3 | -0.17765 |
| Grid2 | 2.12046 |
| Grik1 | -0.22434 |
| Grik2 | 1.41848 |
| Grik4 | 2.28444 |
| Grik5 | -0.50609 |
| Grin1 | 1.90799 |
| Grin1 | 1.46083 |
| Grin2a | 2.15233 |
| Grin2b | 1.74041 |
| Grin2c | 0.58475 |
| Grin3b | -0.65903 |
| Grina | -0.6365 |
| Grip1 | 3.02386 |
| Grip1 | -0.01151 |
| Grip1 | -0.68473 |
| Gripap1 | -0.4933 |
| Gripap1 | -0.62912 |
| Grit | -0.57977 |
| Grk4 | 1.34114 |
| Grk4 | -0.66411 |
| Grk4 | 1.70252 |
| Grk5 | -0.58599 |
| Grlf1 | -0.47307 |
| Grm1 | 2.51034 |
| Grm7 | -0.48209 |
| Grm8 | 0.15598 |
| Grn | 0.88215 |
| Grp | -0.57205 |
| Grpel1 | 0.49013 |
| Grpr | -0.75887 |
| Grsf1 | 1.08406 |
| Grtp1 | -0.41947 |
| Grwd1 | -0.28471 |
| Grwd1 | -0.45802 |
| Gsbs | 0.23656 |
| Gsc | -0.79537 |
| Gsdma1 | -0.41752 |
| Gsdmdc1 | 0.87151 |
| Gsdmdc1 | -0.21283 |
| Gsdmdc1 | -0.09722 |
| Gse1 | -0.83644 |
| Gsg1 | -0.75137 |
| Gsg1 | 0.58541 |
| Gsg1 | -0.29431 |
| Gsg2 | -0.34331 |
| Gsk3a | 0.55232 |
| Gsk3b | -0.19942 |
| Gsn | 1.17706 |
| Gspt1 | -0.57745 |
| Gspt2 | -0.46757 |
| Gss | -0.73746 |
| Gss | -0.65819 |
| Gss | 1.3873 |
| Gsta2 | -0.43456 |
| Gsta3 | 1.71696 |
| Gsta3 | -0.0003 |
| Gsta4 | -0.22698 |
| Gstcd | 0.53544 |
| Gstk1 | -0.63898 |
| Gstm1 | 0.16292 |
| Gstm1 | -0.13417 |
| Gstm2 | -0.37873 |
| Gstm2 | 0.32203 |
| Gstm2 | 3.31255 |
| Gstm4 | 1.59906 |
| Gstm5 | 0.21201 |
| Gstm6 | -0.46415 |
| Gstm6 | -0.66327 |
| Gsto1 | 3.79985 |
| Gsto2 | -0.48418 |
| Gsto2 | 0.05182 |
| Gstp1 | -0.48698 |
| Gstp1 | -0.77406 |
| Gstp2 | -0.2752 |
| Gstt1 | 0.81433 |
| Gstt2 | 0.7855 |
| Gstt3 | -0.31209 |
| Gstt4 | 0.05221 |
| Gstz1 | 0.27066 |
| Gtdc1 | -0.81318 |
| Gtf2a1 | 1.07579 |
| Gtf2e1 | 0.34781 |
| Gtf2e2 | -0.63403 |
| Gtf2e2 | 1.86386 |
| Gtf2f1 | 0.29312 |
| Gtf2f2 | -0.18367 |
| Gtf2h1 | 0.72757 |
| Gtf2h1 | 1.11889 |
| Gtf2h2 | 0.94206 |
| Gtf2h3 | -0.6105 |
| Gtf2h4 | 0.22694 |
| Gtf2h5 | 1.72678 |
| Gtf2ird1 | -0.61774 |
| Gtf2ird1 | 1.35653 |
| Gtf2ird2 | 1.24324 |
| Gtf2ird2 | 0.38896 |
| Gtf2ird2 | 0.08549 |
| Gtf3a | -0.09448 |
| Gtf3a | 0.68317 |
| Gtf3c1 | 0.5173 |
| Gtf3c2 | 0.67034 |
| Gtf3c3 | 0.34583 |
| Gtf3c4 | 1.41786 |
| Gtf3c4 | -0.38373 |
| Gtf3c5 | -0.53811 |
| Gtf3c5 | -0.33081 |
| Gtf3c5 | -0.03092 |
| Gtl3 | -0.29887 |
| Gtpbp1 | 1.52954 |
| Gtpbp2 | -0.11294 |
| Gtpbp3 | -0.55833 |
| Gtpbp4 | -0.6856 |
| Gtpbp5 | 0.19081 |
| Gtpbp6 | -0.65146 |
| Gtpbp8 | 1.94737 |
| Gtpbp8 | 0.24228 |
| Gtrgeo22 | 1.7197 |
| Gtse1 | -0.51688 |
| Gtse1 | -0.17465 |
| Gtse1 | -0.62912 |
| Gtsf1l | 0.13415 |
| Guca1a | -0.45599 |
| Guca1b | -0.52051 |
| Guca2a | 2.82483 |
| Guca2b | 0.38408 |
| Gucy1a2 | 1.82649 |
| Gucy1a3 | -0.59304 |
| Gucy1a3 | 0.22275 |
| Gucy1a3 | -0.22645 |
| Gucy1b3 | 1.46495 |
| Guk1 | 1.86549 |
| Gulo | 2.36134 |
| Gulp1 | -0.54182 |
| Gusb | -0.46415 |
| Gusb | 0.19349 |
| Gusb | -0.69346 |
| Gvin1 | 1.41096 |
| Gyg | 1.27024 |
| Gyg | 0.75391 |
| Gykl1 | 0.86413 |
| Gyltl1b | -0.59777 |
| Gyltl1b | 0.88766 |
| Gypa | 0.29342 |
| Gypc | -0.59304 |
| Gys1 | -0.12515 |
| Gys3 | 1.57024 |
| Gzf1 | -0.77886 |
| Gzf1 | -0.42339 |
| Gzmd | 2.79364 |
| Gzmd | 0.90754 |
| Gzmk | -0.2025 |
| Gzmm | -0.72922 |
| Gzmn | 1.12461 |
| H13 | -0.46074 |
| H13 | 2.51454 |
| H13 | -0.42929 |
| H13 | -0.59619 |
| H19 | 1.57775 |
| H1f0 | -0.56593 |
| H1fnt | -0.1356 |
| H1foo | 0.16602 |
| H1fx | -0.6283 |
| H1fx | -0.58755 |
| H2-Aa | 0.46511 |
| H2-Ab1 | 1.05168 |
| H2-Ab1 | 1.35095 |
| H2-Ab1 | 0.35316 |
| H2afj | -0.23227 |
| H2afx | 2.68599 |
| H2afy | -0.59935 |
| H2afz | 0.58693 |
| H2-Bl | 0.09781 |
| H2-DMa | 0.9813 |
| H2-DMb1 | -0.78079 |
| H2-Eb1 | -0.14087 |
| H2-K1 | -0.66923 |
| H2-Ke6 | -0.21962 |
| H2-M10.3 | 0.10959 |
| H2-M2 | 0.76168 |
| H2-M3 | 0.15633 |
| H2-Oa | 2.01064 |
| H2-Ob | 0.97988 |
| H2-Q1 | 1.04655 |
| H2-Q2 | -0.19278 |
| H2-Q7 | 0.11688 |
| H2-Q8 | -0.50395 |
| H2-T10 | -0.73746 |
| H2-T23 | 0.42782 |
| H2-T23 | -0.63403 |
| H2-T24 | 3.49814 |
| H2-Tw3 | -0.25593 |
| H3f3a | 0.35568 |
| H3f3a | -0.37624 |
| H3f3b | 0.58954 |
| H47 | 2.5684 |
| H47 | -0.70493 |
| H6pd | -0.14618 |
| Habp4 | -0.69171 |
| Habp4 | 2.47204 |
| Hace1 | -0.7076 |
| Hacl1 | -0.78659 |
| Hadhb | -0.59225 |
| Hagh | -0.3806 |
| Haghl | 2.90545 |
| Haghl | 4.08291 |
| Hal | 2.56446 |
| Hamp2 | -0.62179 |
| Hao1 | -0.80522 |
| Hao3 | -0.34812 |
| Hap1 | 0.2533 |
| Hap1 | 0.52757 |
| Hap1 | 1.01825 |
| Hapln1 | -0.07817 |
| Hapln2 | -0.47792 |
| Hapln4 | 1.19217 |
| Harbi1 | -0.3023 |
| Hars | 0.95659 |
| Has1 | -0.66752 |
| Hat1 | -0.50752 |
| Havcr1 | -0.58132 |
| Havcr2 | 1.61833 |
| Hax1 | -0.61451 |
| Hba-a1 | -0.76076 |
| Hbb-b1 | -0.53811 |
| Hbegf | -0.49967 |
| Hbp1 | -0.68299 |
| Hbp1 | 1.17246 |
| Hbs1l | -0.67608 |
| Hbs1l | -0.75324 |
| Hbs1l | 0.06382 |
| Hbs1l | 0.52734 |
| Hc | -0.53959 |
| Hccs | -0.41817 |
| Hcfc1 | -0.47653 |
| Hcfc1r1 | 1.58843 |
| Hcfc2 | 3.15364 |
| Hcls1 | -0.47515 |
| Hcn2 | 2.32924 |
| Hcn3 | 1.43861 |
| Hcn3 | 1.08292 |
| Hcr | -0.76549 |
| Hcst | -0.10136 |
| Hdac10 | -0.5759 |
| Hdac11 | -0.11715 |
| Hdac2 | -0.56516 |
| Hdac3 | -0.68647 |
| Hdac4 | -0.40847 |
| Hdac5 | -0.08403 |
| Hdac6 | -0.09722 |
| Hdac7 | 2.76564 |
| Hdac8 | -0.0322 |
| Hdac9 | -0.74764 |
| Hdc | -0.71565 |
| Hddc2 | -0.37376 |
| Hddc2 | -0.45463 |
| Hdgf | -0.37315 |
| Hdgf | -0.49472 |
| Hdgf | -0.63568 |
| Hdgf | 0.12484 |
| Hdgfl1 | -0.21387 |
| Hdgfrp2 | -0.54929 |
| Hdhd1a | 2.6187 |
| Hdhd2 | -0.34812 |
| Hdhd2 | 1.17843 |
| Hdhd2 | 0.09929 |
| Hdlbp | -0.17216 |
| Hdlbp | 1.87899 |
| Heatr1 | 1.85069 |
| Heatr1 | -0.64645 |
| Heatr3 | -0.14957 |
| Heatr5a | -0.59068 |
| Hebp1 | 3.22402 |
| Hebp2 | -0.67179 |
| Heca | 1.40329 |
| Hectd2 | -0.22434 |
| Hectd2 | 2.36651 |
| Hectd3 | 0.2051 |
| Hecw2 | 0.26357 |
| Hecw2 | 0.1147 |
| Hecw2 | -0.67867 |
| Heg1 | 2.61265 |
| Hel308 | 0.93074 |
| Helb | -0.47515 |
| Helb | -0.13751 |
| Helz | -0.44722 |
| Hemgn | 1.06937 |
| Hemk1 | -0.67867 |
| Hemt1 | 0.46834 |
| Hepacam | 1.39934 |
| Heph | -0.09494 |
| Heph | 0.33099 |
| Heph | -0.3046 |
| Heph | -0.31849 |
| Heph | 1.79528 |
| Herc2 | -0.44924 |
| Herc3 | -0.34571 |
| Herc3 | 0.77242 |
| Herc4 | -0.4919 |
| Herc4 | 0.74534 |
| Herpud1 | 0.37178 |
| Herpud1 | 1.00038 |
| Herpud2 | -0.17266 |
| Herpud2 | -0.5176 |
| Herpud2 | 0.92005 |
| Herpud2 | -0.27576 |
| Hes1 | -0.35113 |
| Hes5 | -0.64645 |
| Hes5 | -0.44789 |
| Hes6 | -0.74023 |
| Hes6 | 0.82043 |
| Hes6 | 1.18197 |
| Hexa | -0.58288 |
| Hexb | 0.57223 |
| Hexdc | 1.11314 |
| Hexdc | 2.31829 |
| Hexim1 | 3.59164 |
| Hexim2 | 1.87406 |
| Hey1 | 3.59072 |
| Hey2 | 1.35945 |
| Hey2 | 1.84932 |
| Heyl | 0.80905 |
| Hfe | -0.20301 |
| Hfe | -0.04164 |
| Hgfac | -0.48139 |
| Hgs | 0.53128 |
| Hgs | 0.39863 |
| Hgsnat | 1.23122 |
| Hhat | 2.28273 |
| Hhat | -0.70404 |
| Hhat | -0.35476 |
| Hhatl | -0.59068 |
| Hhex | -0.20868 |
| Hhip | -0.28583 |
| Hibadh | 0.79873 |
| Hic1 | -0.41557 |
| Hic2 | -0.6073 |
| Hif1a | 2.50932 |
| Hif3a | -0.73013 |
| Hif3a | 2.78049 |
| Higd1a | 0.11579 |
| Higd1b | -0.25593 |
| Higd2a | 1.23241 |
| Hils1 | -0.50181 |
| Hint1 | 2.3256 |
| Hint2 | -0.57205 |
| Hint3 | 0.68709 |
| Hint3 | -0.27021 |
| Hip1 | 0.47476 |
| Hip1r | -0.35234 |
| Hip2 | 0.46088 |
| Hipk1 | 1.93806 |
| Hipk2 | -0.7005 |
| Hipk3 | 2.16038 |
| Hipk4 | 0.30118 |
| Hira | -0.48768 |
| Hira | 1.94285 |
| Hirip3 | 0.58453 |
| Hisppd1 | -0.42732 |
| Hist1h1b | -0.43722 |
| Hist1h1c | -0.47101 |
| Hist1h2aa | -0.59461 |
| Hist1h2ac | -0.2118 |
| Hist1h2ad | 1.87524 |
| Hist1h2ae | -0.52342 |
| Hist1h2af | 2.24738 |
| Hist1h2ag | -0.69521 |
| Hist1h2ah | 1.16611 |
| Hist1h2ak | -0.52561 |
| Hist1h2an | -0.17865 |
| Hist1h2ao | -0.39888 |
| Hist1h2bc | -0.68996 |
| Hist1h2be | -0.53959 |
| Hist1h2bf | 1.96945 |
| Hist1h2bg | 1.3713 |
| Hist1h2bh | 1.76804 |
| Hist1h2bj | -0.61693 |
| Hist1h2bk | -0.42143 |
| Hist1h2bm | 1.02989 |
| Hist1h2bn | 0.93657 |
| Hist1h3a | 2.20416 |
| Hist1h3d | -0.63568 |
| Hist1h3e | -0.37191 |
| Hist1h3f | -0.48488 |
| Hist1h4c | 1.4082 |
| Hist1h4d | 0.29701 |
| Hist1h4f | 0.53221 |
| Hist1h4h | 2.05683 |
| Hist1h4i | 2.74163 |
| Hist1h4j | -0.25921 |
| Hist1h4k | -0.50537 |
| Hist1h4m | 1.12703 |
| Hist2h2aa1 | 0.39246 |
| Hist2h2ab | -0.0003 |
| Hist2h2ac | -0.4662 |
| Hist2h2bb | -0.11201 |
| Hist2h2be | 0.26419 |
| Hist2h3b | -0.49826 |
| Hist2h3c1 | -0.61855 |
| Hist2h3c1 | -0.53589 |
| Hist2h4 | 0.48333 |
| Hist3h2a | -0.72286 |
| Hist3h2bb | -0.39317 |
| Hist4h4 | 1.65664 |
| Hk1 | -0.47101 |
| Hk3 | -0.80127 |
| Hkdc1 | -0.70315 |
| Hlcs | 0.86123 |
| Hlf | 0.50214 |
| Hltf | 2.75743 |
| Hlx | 0.54232 |
| Hmg20a | 2.34802 |
| Hmg20a | -0.70582 |
| Hmg20b | -0.22066 |
| Hmga1 | -0.65566 |
| Hmgb2 | 0.83634 |
| Hmgb2l1 | 1.8134 |
| Hmgb2l1 | 0.91899 |
| Hmgb4 | -0.2025 |
| Hmgcl | -0.50609 |
| Hmgcl | 0.64514 |
| Hmgcl | 2.63669 |
| Hmgcll1 | -0.46894 |
| Hmgcs2 | -0.75605 |
| Hmgn1 | 1.78971 |
| Hmgn2 | -0.57745 |
| Hmgn3 | -0.07638 |
| Hmgn3 | 1.08735 |
| Hmha1 | -0.48348 |
| Hmox1 | 0.64473 |
| Hmox2 | -0.48279 |
| Hmx1 | -0.51039 |
| Hmx1 | 0.08436 |
| Hn1 | 1.18935 |
| Hn1 | 1.02679 |
| Hn1l | -0.03992 |
| Hnf1b | 0.91045 |
| Hnf1b | -0.65146 |
| Hnf4a | 1.33073 |
| Hnrnpc | 1.73599 |
| Hnrpa1 | 0.88907 |
| Hnrpa1 | -0.38812 |
| Hnrpab | 1.99139 |
| Hnrpc | 3.83499 |
| Hnrpdl | -0.51833 |
| Hnrpdl | -0.50967 |
| Hnrpf | -0.5278 |
| Hnrph1 | -0.59698 |
| Hnrph1 | -0.62017 |
| Hnrph1 | 1.08925 |
| Hnrph1 | 0.32464 |
| Hnrph1 | -0.43788 |
| Hnrph2 | -0.60093 |
| Hnrph3 | 0.37563 |
| Hnrpk | 1.56304 |
| Hnrpl | 0.13022 |
| Hnrpll | 0.48162 |
| Hnrpll | -0.28809 |
| Hnrpm | -0.74208 |
| Hnrpm | -0.64063 |
| Hnrpm | 3.0968 |
| Hnrpul1 | 0.75045 |
| Hnrpul1 | 0.6044 |
| Hnrpul1 | -0.45126 |
| Hnrpul1 | 1.23295 |
| Hnrpul1 | -0.12609 |
| Hnt | 0.03728 |
| Homer1 | 1.0642 |
| Homer1 | -0.70938 |
| Homer1 | -0.4306 |
| Homer1 | -0.68213 |
| Homez | -0.36759 |
| Homez | 1.34792 |
| Hook1 | -0.02412 |
| Hook1 | -0.6105 |
| Hook1 | 3.36559 |
| Hook1 | 0.46685 |
| Hook2 | -0.19176 |
| Hormad2 | -0.51833 |
| Hoxa13 | -0.52415 |
| Hoxa2 | -0.73655 |
| Hoxa2 | -0.09083 |
| Hoxa4 | -0.11809 |
| Hoxa5 | 0.02733 |
| Hoxa7 | 1.59106 |
| Hoxa9 | 1.61413 |
| Hoxa9 | 1.85394 |
| Hoxb2 | -0.38875 |
| Hoxb3 | 2.90023 |
| Hoxb3 | -0.55079 |
| Hoxb4 | -0.6565 |
| Hoxb5 | -0.62098 |
| Hoxb6 | -0.54108 |
| Hoxb7 | -0.45328 |
| Hoxc10 | 1.15021 |
| Hoxc10 | 1.71881 |
| Hoxc6 | 2.49684 |
| Hoxc9 | -0.51039 |
| Hoxd10 | -0.43788 |
| Hoxd3 | 2.59672 |
| Hoxd4 | 0.09111 |
| Hoxd8 | 0.64596 |
| Hoxd9 | 0.5462 |
| Hp | 2.0153 |
| Hp | 0.28981 |
| Hp | 0.67549 |
| Hp1bp3 | 1.49761 |
| Hpca | -0.5553 |
| Hpcal1 | -0.47653 |
| Hpcal1 | 1.82891 |
| Hpcal4 | -0.57205 |
| Hpgd | -0.64562 |
| Hpn | -0.16227 |
| Hpn | -0.03734 |
| Hprt1 | 2.23973 |
| Hprt1 | 2.21958 |
| Hprt1 | -0.59856 |
| Hps1 | 1.52312 |
| Hps3 | -0.26634 |
| Hps3 | -0.40015 |
| Hps4 | -0.57282 |
| Hps5 | -0.22066 |
| Hps6 | -0.75699 |
| Hpse | -0.58521 |
| Hpx | 2.19527 |
| Hr | -0.36821 |
| Hr | -0.42012 |
| Hras1 | 1.97759 |
| Hrasls | -0.57668 |
| Hrasls5 | 3.35138 |
| Hrasls5 | -0.2328 |
| Hrb | -0.55455 |
| Hrb | 0.24418 |
| Hrbl | -0.65566 |
| Hrbl | 1.24399 |
| Hrc | 1.28665 |
| Hrg | -0.51255 |
| Hrh3 | 0.39461 |
| Hrk | 1.81139 |
| Hrmt1l2 | 2.48744 |
| Hrsp12 | 0.13522 |
| Hrsp12 | -0.78369 |
| Hrsp12 | 0.35176 |
| Hs1bp3 | 2.18109 |
| Hs2st1 | 2.14692 |
| Hs3st1 | -0.55079 |
| Hs3st3a1 | 0.61839 |
| Hs3st3b1 | 2.82978 |
| Hs3st6 | 1.12509 |
| Hs6st1 | -0.36513 |
| Hs6st2 | -0.6081 |
| Hs6st2 | 0.81042 |
| Hs6st3 | 2.24663 |
| Hsbp1 | 0.53336 |
| Hsd11b1 | -0.6523 |
| Hsd11b1 | -0.40462 |
| Hsd11b1 | -0.50895 |
| Hsd11b1 | 0.18678 |
| Hsd11b2 | -0.05424 |
| Hsd17b1 | -0.65062 |
| Hsd17b10 | -0.64895 |
| Hsd17b11 | -0.5278 |
| Hsd17b11 | -0.64645 |
| Hsd17b11 | 2.3279 |
| Hsd17b12 | 0.75155 |
| Hsd17b2 | -0.55229 |
| Hsd17b3 | -0.09585 |
| Hsd17b4 | -0.77215 |
| Hsd17b7 | 2.07989 |
| Hsd3b1 | -0.51688 |
| Hsd3b2 | -0.5759 |
| Hsd3b2 | -0.5322 |
| Hsd3b4 | 0.20047 |
| Hsd3b7 | -0.54256 |
| Hsd3b7 | -0.40462 |
| Hsdl1 | -0.59935 |
| Hsf1 | -0.09539 |
| Hsf4 | -0.69962 |
| Hsf5 | -0.5322 |
| Hsn2 | 1.41123 |
| Hsp105 | 0.08961 |
| Hsp90aa1 | -0.55004 |
| Hsp90ab1 | 0.57664 |
| Hsp90ab1 | 1.45111 |
| Hsp90ab1 | 1.47046 |
| Hsp90b1 | -0.67608 |
| Hspa12b | 2.01953 |
| Hspa1a | 0.82649 |
| Hspa1l | 1.62939 |
| Hspa2 | -0.68821 |
| Hspa2 | 1.17522 |
| Hspa4 | -0.64063 |
| Hspa4l | -0.54779 |
| Hspa5 | 0.71932 |
| Hspa9 | -0.44588 |
| Hspa9 | 1.2233 |
| Hspb1 | -0.5899 |
| Hspb2 | 1.2296 |
| Hspb3 | -0.39127 |
| Hspb6 | -0.54331 |
| Hspb7 | -0.13512 |
| Hspb8 | -0.27799 |
| Hspbap1 | -0.58599 |
| Hspd1 | 0.34498 |
| Hspd1 | -0.3046 |
| Hspe1 | 2.63751 |
| Hspg2 | -0.12846 |
| Htatip | 0.65571 |
| Htatip | 1.21091 |
| Htatip2 | -0.07013 |
| Htatip2 | -0.42732 |
| Htatsf1 | 0.04123 |
| Htf9c | -0.18015 |
| Htr1a | -0.75793 |
| Htr1b | -0.53368 |
| Htr1d | -0.57282 |
| Htr1f | -0.63075 |
| Htr1f | -0.35355 |
| Htr2b | 1.71677 |
| Htr3a | 0.15842 |
| Htr5b | 0.19649 |
| Htr7 | -0.63981 |
| Htra1 | 2.94628 |
| Htra1 | 1.66044 |
| Htra2 | -0.00113 |
| Htra3 | 1.97442 |
| Htra3 | 0.68454 |
| Htt | 1.98618 |
| Hus1 | 1.18618 |
| Hus1 | 1.36824 |
| Hus1 | 0.35091 |
| Huwe1 | 2.10827 |
| Hvcn1 | 0.08662 |
| Hvcn1 | 0.58235 |
| Hyal1 | 0.4923 |
| Hyal2 | -0.72196 |
| Hyal3 | -0.39888 |
| Hyal5 | 0.22759 |
| Hyal6 | -0.7076 |
| Hydin | -0.33022 |
| Hyi | -0.34932 |
| Hyou1 | -0.32845 |
| Iah1 | 2.06016 |
| Iap | -0.10876 |
| Iars | -0.7005 |
| Iars | 0.53475 |
| Iars2 | 0.5878 |
| Ibtk | 2.46878 |
| Ica1 | -0.6365 |
| Ica1l | -0.45126 |
| Icam1 | 0.70622 |
| Icam2 | -0.66582 |
| Icam4 | 1.65167 |
| Icam5 | -0.4104 |
| Ick | 0.06727 |
| Icmt | -0.67522 |
| Ict1 | 2.18748 |
| Id1 | -0.31907 |
| Id2 | -0.72196 |
| Id2 | 1.21367 |
| Id3 | 1.65139 |
| Id4 | -0.34511 |
| Ide | -0.21024 |
| Idh1 | 1.61428 |
| Idh2 | 1.95821 |
| Idh3a | -0.38686 |
| Idh3b | -0.2025 |
| Idh3g | 1.49607 |
| Idh3g | 2.3644 |
| Idi2 | -0.5068 |
| Ids | 0.03926 |
| Ids | 1.67054 |
| Ids | 0.92549 |
| Idua | 1.10772 |
| Ier3 | -0.57513 |
| Ier3ip1 | -0.63733 |
| Ier5l | 1.77333 |
| Ifi202b | -0.72558 |
| Ifi202b | 0.64228 |
| Ifi27 | 0.56445 |
| Ifi30 | -0.40015 |
| Ifi30 | -0.32316 |
| Ifi35 | 0.47991 |
| Ifi47 | 3.77548 |
| Ifit2 | 1.67094 |
| Ifit2 | -0.35659 |
| Ifit3 | -0.46483 |
| Ifit3 | 1.67114 |
| Ifitm1 | -0.62098 |
| Ifitm1 | 0.33387 |
| Ifitm1 | -0.41882 |
| Ifitm2 | 1.12364 |
| Ifitm3 | 1.32343 |
| Ifitm5 | 0.02372 |
| Ifitm6 | -0.65146 |
| Ifna1 | -0.1032 |
| Ifna6 | -0.53663 |
| Ifnar2 | 0.966 |
| Ifnar2 | 2.70302 |
| Ifnar2 | 3.07378 |
| Ifngr1 | 0.20147 |
| Ifngr2 | -0.14039 |
| Ifngr2 | -0.39253 |
| Ifngr2 | -0.18569 |
| Ifnz | -0.37749 |
| Ifrd1 | 0.09111 |
| Ifrg15 | -0.01109 |
| Ift122 | -0.18872 |
| Ift140 | -0.28358 |
| Ift140 | 0.17014 |
| Ift140 | -0.48558 |
| Ift172 | -0.17117 |
| Ift172 | 1.58388 |
| Ift172 | -0.7495 |
| Ift20 | -0.44924 |
| Ift20 | 1.5286 |
| Ift20 | -0.63075 |
| Ift52 | 1.54149 |
| Ift80 | 2.41632 |
| Ift81 | 0.20938 |
| Ift81 | 1.34343 |
| Ift81 | -0.38435 |
| Ift88 | 1.30846 |
| Igbp1 | 0.57598 |
| Igbp1b | -0.45126 |
| Igf1 | -0.28809 |
| Igf1 | -0.04424 |
| Igf1r | 0.78462 |
| Igf2 | -0.4919 |
| Igf2bp1 | -0.54629 |
| Igf2bp2 | -0.63486 |
| Igf2bp3 | 2.57074 |
| Igf2bp3 | 3.41853 |
| Igf2r | -0.32669 |
| Igf2r | -0.35174 |
| Igfals | 0.02412 |
| Igfbp2 | -0.5448 |
| Igfbp2 | 0.48867 |
| Igfbp3 | -0.66411 |
| Igfbp4 | 0.79144 |
| Igfbp4 | -0.6105 |
| Igfbp5 | 1.51125 |
| Igfbp6 | -0.76738 |
| Igfbp7 | -0.76265 |
| Igfbpl1 | 0.79162 |
| Igsf1 | -0.55304 |
| Igsf1 | -0.6089 |
| Igsf1 | 2.1371 |
| Igsf1 | -0.03562 |
| Igsf11 | 0.24828 |
| Igsf11 | -0.29261 |
| Igsf11 | -0.5553 |
| Igsf21 | -0.73379 |
| Igsf3 | -0.56593 |
| Igsf4a | 0.47868 |
| Igsf5 | -0.53147 |
| Igsf8 | -0.3069 |
| Igsf9 | -0.42666 |
| Igtp | 1.57908 |
| Ihh | 0.98187 |
| Ihpk1 | 1.36814 |
| Ihpk2 | 0.21755 |
| Ihpk2 | 0.94825 |
| Iigp2 | 0.57201 |
| Ik | 0.82616 |
| Ikbkap | -0.44388 |
| Ikbkb | -0.84882 |
| Ikbke | 0.69195 |
| Ikbkg | 1.61406 |
| Ikbkg | -0.69697 |
| Il10ra | 0.00832 |
| Il10rb | -0.49613 |
| Il11 | 1.04615 |
| Il11ra1 | 0.65389 |
| Il12a | 0.68689 |
| Il13 | -0.53737 |
| Il13ra1 | -0.51905 |
| Il13ra2 | -0.64728 |
| Il15 | -0.14087 |
| Il15 | 1.06963 |
| Il15 | -0.37438 |
| Il15ra | -0.5553 |
| Il15ra | 0.33473 |
| Il15ra | 0.98172 |
| Il16 | 0.59625 |
| Il17d | 1.09063 |
| Il17d | -0.62585 |
| Il17ra | -0.45938 |
| Il17rb | -0.73471 |
| Il17rc | -0.71296 |
| Il17rc | -0.53663 |
| Il17rc | -0.5954 |
| Il17rd | 2.135 |
| Il17rd | 1.97191 |
| Il17re | 0.59538 |
| Il17re | 0.02653 |
| Il17re | 1.96324 |
| Il18 | 2.50235 |
| Il18bp | -0.67008 |
| Il18r1 | 2.33657 |
| Il1b | -0.66752 |
| Il1r1 | 1.43887 |
| Il1rap | -0.67351 |
| Il1rap | 1.09903 |
| Il1rap | -0.67781 |
| Il1rap | 1.28022 |
| Il1rap | 0.12376 |
| Il1rl1l | 1.00301 |
| Il1rl2 | 0.61269 |
| Il20ra | -0.41557 |
| Il20rb | -0.58055 |
| Il20rb | -0.51255 |
| Il23a | 0.62447 |
| Il27ra | -0.73563 |
| Il28ra | -0.6121 |
| Il28ra | 0.22855 |
| Il28ra | 1.45017 |
| Il31ra | 0.648 |
| Il33 | -0.16523 |
| Il3ra | -0.38435 |
| Il4i1 | 2.03493 |
| Il6ra | -0.22962 |
| Il6st | 0.68298 |
| Il7 | 2.28855 |
| Ildr1 | 0.91336 |
| Ildr1 | 0.13735 |
| Ilf2 | 0.35932 |
| Ilf3 | -0.42404 |
| Ilf3 | 0.29641 |
| Ilf3 | 0.55255 |
| Ilf3 | 0.98611 |
| Ilk | 1.14891 |
| Ilkap | -0.63981 |
| Ilvbl | 0.71818 |
| Ilvbl | -0.39697 |
| Ilvbl | -0.18518 |
| Ilvbl | -0.70315 |
| Immp2l | -0.05907 |
| Immt | 3.10752 |
| Imp3 | 0.5462 |
| Imp4 | -0.15883 |
| Imp4 | -0.13608 |
| Impa2 | 3.16039 |
| Impact | 0.92954 |
| Impad1 | 0.81569 |
| Impdh1 | 0.18036 |
| Impdh2 | 0.45033 |
| Ina | -0.06303 |
| Inadl | -0.48 |
| Inadl | 0.24512 |
| Incenp | -0.2254 |
| Indo | 2.05529 |
| Ing2 | -0.31674 |
| Ing3 | 0.54938 |
| Ing3 | 0.95717 |
| Ing3 | 0.12304 |
| Ing4 | 2.12042 |
| Ing4 | -0.85927 |
| Ing4 | -0.5176 |
| Ing5 | -0.07369 |
| Inha | -0.47101 |
| Inhba | 0.08097 |
| Inmt | 1.63316 |
| Inoc1 | -0.39507 |
| Inpp4a | -0.4587 |
| Inpp4a | -0.22382 |
| Inpp4a | 0.475 |
| Inpp4b | 1.02855 |
| Inpp5a | -0.55154 |
| Inpp5d | -0.56593 |
| Inpp5e | 0.34328 |
| Inpp5f | -0.55757 |
| Inppl1 | 3.78692 |
| Inppl1 | 0.27922 |
| Inppl1 | -0.75044 |
| Ins2 | -0.55455 |
| Insc | 0.06651 |
| Insig1 | -0.80324 |
| Insig1 | 2.45895 |
| Insig1 | 0.27342 |
| Insig2 | 0.45762 |
| Insig2 | -0.37129 |
| Insl3 | 1.65358 |
| Insl5 | 0.00586 |
| Insl6 | -0.30402 |
| Insm1 | 0.42029 |
| Ints1 | -0.24888 |
| Ints12 | -0.68213 |
| Ints3 | 0.12087 |
| Ints3 | 1.35209 |
| Ints4 | 0.95148 |
| Ints5 | -0.31674 |
| Ints7 | 1.20036 |
| Ints9 | -0.45126 |
| Ints9 | -0.47446 |
| Ipmk | -0.72286 |
| Ipmk | 2.18664 |
| Ipo13 | -0.09265 |
| Ipo4 | -0.7274 |
| Ipo5 | 2.3001 |
| Ipp | -0.46278 |
| Ippk | -0.55004 |
| Iqcb1 | 0.04359 |
| Iqcb1 | 0.74277 |
| Iqcd | 0.02573 |
| Iqce | 0.01077 |
| Iqcf3 | 0.41347 |
| Iqcf4 | -0.55833 |
| Iqgap1 | 0.6753 |
| Iqgap2 | 0.76546 |
| Iqgap3 | 2.25123 |
| Iqsec2 | 0.67885 |
| Iqsec3 | -0.43324 |
| Irak1 | -0.67608 |
| Irak1bp1 | -0.22856 |
| Irak2 | 1.17361 |
| Irak2 | 0.28861 |
| Irak3 | 0.84641 |
| Irak4 | 0.48015 |
| Irak4 | -0.0881 |
| Irak4 | -0.54629 |
| Ireb2 | -0.53368 |
| Irf1 | -0.65903 |
| Irf1 | -0.7076 |
| Irf1 | -0.59147 |
| Irf1 | 0.38842 |
| Irf2 | -0.60093 |
| Irf2bp1 | -0.67694 |
| Irf3 | 0.86461 |
| Irf3 | 1.05496 |
| Irf3 | 2.3204 |
| Irf4 | -0.54256 |
| Irf5 | 1.55889 |
| Irf6 | -0.63075 |
| Irf6 | 2.18534 |
| Irf7 | 0.99047 |
| Irf8 | 0.07377 |
| Irf9 | 0.02211 |
| Irf9 | 2.5833 |
| Irgm | 2.90948 |
| Irgm | -0.62912 |
| Irgq | 1.15221 |
| Irgq | -0.5954 |
| Irgq | -0.17316 |
| Irs2 | 0.00873 |
| Irs3 | 0.06228 |
| Irx2 | -0.69433 |
| Irx3 | -0.71565 |
| Irx5 | 0.6966 |
| Isca1 | -0.48698 |
| Iscu | -0.45126 |
| Isg20 | -0.08946 |
| Isg20l1 | -0.16129 |
| Isg20l2 | -0.7274 |
| Isl1 | -0.38498 |
| Islr2 | 2.08698 |
| Isy1 | -0.17915 |
| Isyna1 | 1.96187 |
| Itfg2 | 0.21495 |
| Itfg3 | 2.20711 |
| Itga1 | -0.54033 |
| Itga11 | 0.34243 |
| Itga11 | 0.81773 |
| Itga2b | 1.25437 |
| Itga3 | -0.55908 |
| Itga5 | -0.54854 |
| Itga5 | -0.3806 |
| Itga6 | 1.93851 |
| Itga7 | -0.30402 |
| Itgad | 1.27138 |
| Itgad | -0.73655 |
| Itgad | -0.42143 |
| Itgae | 1.4161 |
| Itgae | -0.62748 |
| Itgae | 0.98709 |
| Itgav | 1.81848 |
| Itgb1 | 1.91812 |
| Itgb1 | 0.34809 |
| Itgb1bp1 | 0.64351 |
| Itgb3bp | -0.39127 |
| Itgb4 | 0.99746 |
| Itgb4 | -0.43921 |
| Itgb5 | -0.26359 |
| Itgb6 | 4.29079 |
| Itgb6 | 0.89329 |
| Itgb6 | 1.79563 |
| Itgb7 | -0.49049 |
| Itgbl1 | -0.24134 |
| Itih2 | -0.32786 |
| Itih3 | 1.61506 |
| Itih5 | 0.08886 |
| Itih5 | -0.70671 |
| Itk | -0.62748 |
| Itlnb | 0.47303 |
| Itm2a | 0.99117 |
| Itm2b | -0.00568 |
| Itm2b | 0.32955 |
| Itpk1 | -0.73379 |
| Itpka | -0.6121 |
| Itpkc | -0.48348 |
| Itpr1 | -0.66327 |
| Itpr2 | -0.45396 |
| Itpr3 | -0.29716 |
| Itsn1 | 2.09385 |
| Itsn2 | -0.69873 |
| Ivd | -0.27242 |
| Ivns1abp | -0.21752 |
| Ivns1abp | 0.50238 |
| Iws1 | -0.32551 |
| Iyd | 1.19082 |
| Izumo1 | 1.53437 |
| Jag1 | -0.53368 |
| Jag2 | 0.42185 |
| Jagn1 | -0.20713 |
| Jak1 | -0.4339 |
| Jak2 | 0.87518 |
| Jak2 | -0.46552 |
| Jak3 | 2.08545 |
| Jak3 | 0.0128 |
| Jakmip1 | 0.02452 |
| Jam2 | -0.41363 |
| Jam2 | 0.81467 |
| Jam2 | 2.86232 |
| Jam2 | 2.99703 |
| Jam3 | 0.03052 |
| Jam4 | 0.52011 |
| Jarid1b | 0.96903 |
| Jarid1c | -0.46963 |
| Jarid1d | 3.21586 |
| Jazf1 | -0.6283 |
| Jdp2 | -0.41687 |
| Jmjd1a | -0.27465 |
| Jmjd1a | -0.31209 |
| Jmjd1a | -0.74857 |
| Jmjd2a | -0.28246 |
| Jmjd2b | 0.21788 |
| Jmjd2b | -0.51978 |
| Jmjd2c | -0.5448 |
| Jmjd2d | -0.85927 |
| Jmjd3 | 0.15075 |
| Jmjd3 | 0.01483 |
| Jmjd5 | -0.65482 |
| Jmjd6 | -0.60491 |
| Jmy | 2.81481 |
| Josd1 | -0.14232 |
| Josd2 | -0.6226 |
| Josd3 | -0.42666 |
| Josd3 | 3.0886 |
| Jph1 | -0.7495 |
| Jph2 | -0.47101 |
| Jph3 | 2.58563 |
| Jph4 | 0.50143 |
| Jph4 | -0.45938 |
| Jtb | 0.79942 |
| Jtv1 | -0.68734 |
| Jtv1 | -0.6365 |
| Jun | 2.20127 |
| Junb | 0.15668 |
| Jund1 | 1.96489 |
| Jup | -0.26689 |
| Kal1 | 2.94638 |
| Kalrn | -0.35659 |
| Kank1 | -0.19993 |
| Kank3 | -0.15687 |
| Kank4 | 0.0432 |
| Kank4 | 1.68726 |
| Kank4 | -0.17465 |
| Kars | 0.67589 |
| Katna1 | -0.68734 |
| Katnal1 | 1.91258 |
| Katnb1 | -0.72105 |
| Kazald1 | 1.06769 |
| Kbtbd2 | -0.09356 |
| Kbtbd2 | -0.56136 |
| Kbtbd4 | -0.42929 |
| Kbtbd4 | -0.68126 |
| Kbtbd7 | 0.20642 |
| Kbtbd7 | -0.55681 |
| Kbtbd8 | 0.42938 |
| Kcmf1 | -0.37624 |
| Kcna1 | -0.44388 |
| Kcna2 | 3.6772 |
| Kcna3 | 0.4526 |
| Kcna5 | -0.24295 |
| Kcna6 | 1.87872 |
| Kcnab1 | -0.63486 |
| Kcnab1 | -0.38938 |
| Kcnab1 | 1.2174 |
| Kcnab2 | -0.6565 |
| Kcnab3 | 1.12121 |
| Kcnb1 | -0.71835 |
| Kcnc1 | -0.29944 |
| Kcnc1 | 0.07529 |
| Kcnc4 | -0.45193 |
| Kcnc4 | 0.25173 |
| Kcnd2 | 0.11179 |
| Kcne1 | -0.55229 |
| Kcne1l | -0.49684 |
| Kcne2 | -0.33437 |
| Kcne4 | -0.77406 |
| Kcnf1 | -0.36575 |
| Kcng4 | -0.58833 |
| Kcnh1 | 2.63844 |
| Kcnh2 | 1.99445 |
| Kcnh2 | -0.62422 |
| Kcnh3 | 0.17356 |
| Kcnh5 | -0.15248 |
| Kcnip1 | -0.44454 |
| Kcnip2 | -0.51327 |
| Kcnip2 | 1.13893 |
| Kcnip2 | -0.0322 |
| Kcnip3 | 2.97824 |
| Kcnip4 | -0.38812 |
| Kcnj12 | -0.40526 |
| Kcnj16 | 2.33169 |
| Kcnj2 | 1.51395 |
| Kcnj3 | -0.86242 |
| Kcnj4 | 0.12556 |
| Kcnj6 | 0.14654 |
| Kcnj6 | 1.95516 |
| Kcnj8 | 1.43938 |
| Kcnj9 | 1.98494 |
| Kcnk1 | 0.53105 |
| Kcnk10 | 0.75318 |
| Kcnk12 | -0.61451 |
| Kcnk13 | -0.31849 |
| Kcnk2 | -0.69697 |
| Kcnk2 | 0.88057 |
| Kcnk3 | -0.3179 |
| Kcnk4 | 1.24217 |
| Kcnk5 | 0.65732 |
| Kcnk5 | 0.39165 |
| Kcnk6 | 0.1497 |
| Kcnk6 | -0.04337 |
| Kcnk9 | 1.69668 |
| Kcnma1 | -0.29035 |
| Kcnmb1 | -0.33259 |
| Kcnmb4 | 1.66866 |
| Kcnmb4 | 0.43221 |
| Kcnn1 | 2.01245 |
| Kcnn2 | -0.55154 |
| Kcnn4 | 0.43633 |
| Kcnq1 | 0.28347 |
| Kcnq2 | -0.19942 |
| Kcnq2 | 1.66335 |
| Kcnq3 | 0.66615 |
| Kcnq5 | -0.45261 |
| Kcnrg | -0.46826 |
| Kcnrg | -0.12704 |
| Kcns1 | 0.0128 |
| Kcns2 | 2.57541 |
| Kcnt1 | -0.35174 |
| Kcnu1 | 0.25861 |
| Kcnv1 | 0.63363 |
| Kcnv2 | -0.01821 |
| Kctd10 | -0.52853 |
| Kctd11 | 0.65105 |
| Kctd12 | -0.60014 |
| Kctd12b | 1.48712 |
| Kctd12b | 0.43633 |
| Kctd14 | -0.65819 |
| Kctd14 | -0.82625 |
| Kctd15 | -0.40334 |
| Kctd18 | 1.02949 |
| Kctd19 | 0.60162 |
| Kctd2 | 0.1504 |
| Kctd20 | -0.41299 |
| Kctd20 | -0.80819 |
| Kctd3 | 0.10261 |
| Kctd4 | -0.63568 |
| Kctd5 | -0.01611 |
| Kctd5 | 1.2283 |
| Kctd6 | 1.27375 |
| Kctd6 | 0.97204 |
| Kctd9 | -0.26085 |
| Kdelc1 | -0.61532 |
| Kdelc1 | 1.88442 |
| Kdelr2 | -0.72649 |
| Kdelr3 | 0.65024 |
| Kdr | -0.73931 |
| Keap1 | -0.17366 |
| Keap1 | -0.34751 |
| Keap1 | 0.24512 |
| Keap1 | -0.59777 |
| Kera | -0.2344 |
| Khdc1c | 3.35714 |
| Khdrbs1 | -0.34391 |
| Khdrbs2 | -0.30345 |
| Khdrbs3 | 0.47476 |
| Khk | -0.39507 |
| Khk | 0.59257 |
| Khsrp | 0.75988 |
| Kif11 | -0.60411 |
| Kif13a | 2.24369 |
| Kif14 | -0.36452 |
| Kif15 | -0.0538 |
| Kif17 | 0.57355 |
| Kif17 | -0.38561 |
| Kif18a | -0.57745 |
| Kif1a | 1.00977 |
| Kif1b | -0.65566 |
| Kif1b | 2.79384 |
| Kif1b | 0.70296 |
| Kif1b | 0.19148 |
| Kif1b | -0.55984 |
| Kif1b | 1.26484 |
| Kif1c | 2.15884 |
| Kif21a | -0.49472 |
| Kif21b | -0.70849 |
| Kif22 | 0.04163 |
| Kif23 | 0.43684 |
| Kif24 | 3.51661 |
| Kif27 | -0.15054 |
| Kif2a | -0.51399 |
| Kif2b | 1.81597 |
| Kif2c | -0.48209 |
| Kif3a | -0.48 |
| Kif3a | 3.78385 |
| Kif3b | 0.77757 |
| Kif3c | -0.29318 |
| Kif3c | -0.75511 |
| Kif4 | 0.18611 |
| Kif4 | 0.22404 |
| Kif4 | 0.74479 |
| Kif5a | -0.84158 |
| Kif5a | -0.29488 |
| Kif5a | 2.65543 |
| Kif5b | 1.69238 |
| Kif5c | 0.32811 |
| Kif9 | -0.80127 |
| Kifap3 | 1.59732 |
| Kifap3 | 0.61544 |
| Kifap3 | 0.32435 |
| Kifc2 | -0.68126 |
| Kifc2 | 0.41453 |
| Kifc3 | 0.2874 |
| Kin | -0.6365 |
| Kirrel3 | -0.6073 |
| Kiss1 | -0.55606 |
| Kiss1r | 0.33444 |
| Kitl | -0.70315 |
| Kl | -0.35476 |
| Klb | -0.69083 |
| Klc1 | 1.90928 |
| Klc1 | 0.91198 |
| Klc1 | -0.7617 |
| Klc2 | 0.02733 |
| Klc2 | 0.31357 |
| Klc4 | 2.60507 |
| Klf1 | 0.51377 |
| Klf10 | -0.11061 |
| Klf12 | -0.44454 |
| Klf12 | -0.00113 |
| Klf12 | 1.07809 |
| Klf13 | -0.68647 |
| Klf16 | -0.44857 |
| Klf17 | -0.01905 |
| Klf17 | 1.30756 |
| Klf2 | 0.58235 |
| Klf4 | -0.09037 |
| Klf4 | 0.66875 |
| Klf4 | -0.27076 |
| Klf5 | -0.62422 |
| Klf6 | 0.34979 |
| Klf6 | -0.32082 |
| Klf7 | -0.70404 |
| Klf9 | 1.12412 |
| Klhdc1 | 1.3762 |
| Klhdc1 | 0.35596 |
| Klhdc1 | -0.72558 |
| Klhdc2 | -0.6365 |
| Klhdc3 | -0.64812 |
| Klhdc4 | 2.18599 |
| Klhdc4 | 1.46796 |
| Klhdc4 | 0.17083 |
| Klhdc8b | -0.76076 |
| Klhdc9 | 0.15668 |
| Klhl10 | -0.78175 |
| Klhl11 | -0.55455 |
| Klhl12 | -0.72377 |
| Klhl14 | -0.39064 |
| Klhl15 | 0.06881 |
| Klhl15 | 0.67113 |
| Klhl17 | 0.50928 |
| Klhl2 | -0.8212 |
| Klhl21 | 3.04981 |
| Klhl22 | -0.23333 |
| Klhl22 | 0.69776 |
| Klhl23 | 1.24505 |
| Klhl24 | 0.90492 |
| Klhl24 | 0.91183 |
| Klhl25 | -0.56899 |
| Klhl26 | -0.49896 |
| Klhl26 | 0.48089 |
| Klhl30 | 2.06344 |
| Klhl31 | -0.54929 |
| Klhl32 | 1.1315 |
| Klhl5 | 0.8875 |
| Klhl5 | -0.50252 |
| Klhl6 | 3.646 |
| Klhl6 | -0.70138 |
| Klhl7 | -0.67694 |
| Klhl8 | 0.13842 |
| Klhl9 | 1.03952 |
| Klhl9 | -0.53959 |
| Klk1 | -0.72467 |
| Klk11 | 1.20203 |
| Klk11 | -0.73838 |
| Klk15 | -0.24188 |
| Klk1b1 | -0.70671 |
| Klk1b11 | 0.23464 |
| Klk1b16 | 0.1807 |
| Klk1b21 | 1.35879 |
| Klk1b21 | -0.40334 |
| Klk1b22 | -0.28865 |
| Klk1b24 | -0.41622 |
| Klk1b26 | 1.48598 |
| Klk1b27 | -0.7274 |
| Klk1b27 | -0.64812 |
| Klk1b4 | -0.74115 |
| Klk1b4 | 1.8315 |
| Klk1b5 | -0.76076 |
| Klk1b8 | -0.11574 |
| Klk1b9 | 0.47917 |
| Klk6 | -0.19126 |
| Klk8 | -0.52634 |
| Klkb1 | -0.27743 |
| Klra17 | -0.33674 |
| Klra4 | -0.63733 |
| Klrd1 | -0.49826 |
| Kndc1 | 1.16854 |
| Kng1 | -0.04337 |
| Kng1 | -0.36513 |
| Kng2 | 1.1257 |
| Kns2 | 0.75934 |
| Kns2 | -0.53147 |
| Kntc1 | 1.501 |
| Kpna1 | 0.68689 |
| Kpna2 | -0.74857 |
| Kpna3 | -0.7993 |
| Kpna6 | 0.44399 |
| Kpnb1 | 0.0821 |
| Kptn | -0.29773 |
| Kras | 1.85207 |
| Kras | -0.59935 |
| Krba1 | -0.65314 |
| Krba1 | 1.91474 |
| Krba1 | -0.56899 |
| Krcc1 | -0.51399 |
| Kremen | 3.46677 |
| Kremen1 | -0.74115 |
| Kremen2 | -0.68821 |
| Krit1 | -0.57052 |
| Krit1 | -0.24403 |
| Krr1 | 0.4013 |
| Krr1 | -0.01193 |
| Krr1 | -0.43788 |
| Krt10 | 3.21041 |
| Krt12 | -0.42143 |
| Krt13 | 0.83982 |
| Krt14 | -0.20765 |
| Krt17 | -0.72286 |
| Krt18 | -0.4919 |
| Krt19 | 0.36294 |
| Krt2 | -0.66752 |
| Krt20 | 0.8601 |
| Krt222 | -0.87409 |
| Krt23 | -0.03519 |
| Krt27 | -0.18015 |
| Krt32 | 2.04246 |
| Krt4 | 2.18752 |
| Krt7 | -0.28583 |
| Krt73 | 2.31497 |
| Krt75 | 0.34356 |
| Krt78 | -0.79439 |
| Krt79 | -0.5821 |
| Krt8 | -0.69697 |
| Krt84 | -0.48209 |
| Krt86 | -0.6804 |
| Krt86 | 1.30766 |
| Krt9 | -0.57822 |
| Krtap16-4 | 0.15842 |
| Krtap8-2 | -0.66582 |
| Krtcap2 | -0.01402 |
| Krtcap3 | 1.30627 |
| Ksr2 | -0.51111 |
| Ktelc1 | 0.49158 |
| Kti12 | 1.26463 |
| Ktn1 | 0.94825 |
| Ktn1 | -0.78175 |
| Ky | -0.37749 |
| L1cam | 2.36751 |
| L2hgdh | 0.79648 |
| L3mbtl2 | 1.1495 |
| L3mbtl3 | -0.79146 |
| l7Rn6 | 1.36516 |
| Lace1 | 1.34534 |
| Lad1 | -0.7523 |
| Lag3 | 2.89805 |
| Lage3 | 0.45285 |
| Lama1 | -0.50752 |
| Lama2 | 0.63135 |
| Lamb1-1 | -0.68386 |
| Lamb2 | 2.21396 |
| Lamb3 | -0.67608 |
| Lamc1 | 2.06095 |
| Lamc2 | -0.44054 |
| Lamc2 | 1.86809 |
| Lamc2 | 0.62864 |
| Lamc3 | 0.19181 |
| Lamp1 | 0.55773 |
| Lamp2 | -0.14328 |
| Lamp2 | -0.71206 |
| Lamp2 | -0.28527 |
| Lamp2 | -0.34631 |
| Lancl1 | -0.66752 |
| Lancl1 | -0.12373 |
| Lancl1 | 0.26728 |
| Laptm4a | -0.77694 |
| Laptm4b | -0.68908 |
| Laptm4b | -0.47307 |
| Laptm5 | 0.68865 |
| Large | -0.60173 |
| Larp2 | 1.91515 |
| Larp5 | 0.97604 |
| Larp6 | 0.07529 |
| Larp6 | 0.1504 |
| Lars | 0.52315 |
| Lars2 | -0.60332 |
| Las1l | -0.6105 |
| Lasp1 | 0.70889 |
| Lasp1 | -0.31907 |
| Lasp1 | -0.73105 |
| Lass1 | 0.87629 |
| Lass2 | -0.06879 |
| Lass2 | -0.58755 |
| Lass2 | -0.58677 |
| Lass4 | 0.08361 |
| Lass4 | -0.6121 |
| Lass5 | -0.05731 |
| Lass6 | -0.14135 |
| Lat2 | -0.66411 |
| Lat2 | -0.42012 |
| Lats2 | -0.62422 |
| Lbh | 0.01118 |
| Lbh | -0.39824 |
| Lbp | -0.67781 |
| Lbr | -0.63075 |
| Lbx2 | -0.50537 |
| Lca5 | 1.2309 |
| Lca5 | -0.42666 |
| Lca5 | 0.49879 |
| Lcat | -0.10505 |
| Lcat | 0.22855 |
| Lce1c | 0.24922 |
| Lck | -0.51616 |
| Lcmt1 | -0.15346 |
| Lcmt1 | 1.5484 |
| Lcmt1 | -0.6057 |
| Lcn10 | -0.53737 |
| Lcn12 | 1.46478 |
| Lcn13 | 1.13917 |
| Lcn2 | -0.31209 |
| Lcn3 | -0.61774 |
| Lcn8 | -0.07102 |
| Lcor | 0.70085 |
| Lcp1 | -0.53294 |
| Ldb1 | -0.50252 |
| Ldb2 | 0.95848 |
| Ldb2 | 0.55953 |
| Ldha | -0.39443 |
| Ldhc | -0.71117 |
| Ldhc | -0.31674 |
| Ldhc | -0.6365 |
| Ldhd | 1.13726 |
| Ldhd | -0.58288 |
| Ldoc1 | 2.50246 |
| Ldoc1l | 3.11381 |
| Lef1 | -0.60491 |
| Lef1 | 0.02171 |
| Lefty1 | 0.44221 |
| Lefty2 | -0.56516 |
| Lemd2 | 2.90489 |
| Leng1 | 2.93071 |
| Leng8 | 2.10619 |
| Leng9 | -0.69521 |
| Leo1 | -0.19839 |
| Lep | 1.52985 |
| Lepre1 | -0.20352 |
| Lepre1 | -0.69258 |
| Lepre1 | 2.1147 |
| Leprel1 | -0.27298 |
| Leprel2 | -0.02412 |
| Leprotl1 | 1.30518 |
| Letm1 | 1.13833 |
| Letm1 | 3.08973 |
| Letm1 | 1.71581 |
| Letmd1 | 0.95045 |
| Lgals1 | -0.11201 |
| Lgals2 | 3.03646 |
| Lgals3 | -0.63075 |
| Lgals3bp | 0.62823 |
| Lgals4 | -0.45463 |
| Lgals4 | 2.42284 |
| Lgals6 | -0.81018 |
| Lgals7 | -0.46826 |
| Lgals8 | -0.48348 |
| Lgals8 | 0.50572 |
| Lgals9 | 3.68828 |
| Lgals9 | 0.35512 |
| Lgi1 | 1.45306 |
| Lgi1 | 1.19891 |
| Lgi2 | -0.66837 |
| Lgi3 | -0.68386 |
| Lgi4 | -0.5322 |
| Lgmn | 1.88749 |
| Lgr5 | 2.21921 |
| Lgtn | 0.06689 |
| Lgtn | -0.36085 |
| Lhb | -0.5821 |
| Lhcgr | 1.28135 |
| Lhcgr | -0.47239 |
| Lhfp | 2.68916 |
| Lhfp | -0.31094 |
| Lhfp | 0.14654 |
| Lhfpl1 | 1.35105 |
| Lhfpl2 | -0.77982 |
| Lhx1 | 2.37202 |
| Lhx2 | -0.81818 |
| Lhx3 | -0.48 |
| Lhx4 | -0.80819 |
| Lhx9 | -0.7993 |
| Lhx9 | 0.13557 |
| Lias | 2.02896 |
| Lias | 2.10906 |
| Lias | 3.06549 |
| Lifr | -0.38686 |
| Lig1 | -0.78659 |
| Lig1 | -0.00568 |
| Lig1 | 0.99885 |
| Lig3 | -0.65398 |
| Lig4 | 0.11761 |
| Lima1 | -0.35234 |
| Lima1 | 0.89796 |
| Limd1 | -0.51255 |
| Limd2 | -0.61936 |
| Lime1 | 0.06842 |
| Limk1 | -0.7005 |
| Limk2 | -0.72649 |
| Limk2 | 1.81809 |
| Lims2 | 1.8547 |
| Lims2 | -0.15102 |
| Lims2 | -0.14521 |
| Lin54 | -0.66411 |
| Lin7b | 1.38163 |
| Lin7b | 1.0577 |
| Lin7b | -0.1867 |
| Lin7b | -0.36391 |
| Lin7c | -0.57745 |
| Lincr | 2.04108 |
| Lincr | -0.84882 |
| Lingo1 | -0.52196 |
| Lingo2 | -0.88483 |
| Lingo2 | 1.13678 |
| Lingo4 | -0.4926 |
| Lip1 | -0.01863 |
| Lipe | -0.35234 |
| Lipf | 0.42393 |
| Liph | 1.49226 |
| Liph | -0.45599 |
| Litaf | 1.88733 |
| Lix1 | -0.3806 |
| Lix1 | -0.67351 |
| Lix1 | -0.28922 |
| Lix1 | 1.59623 |
| Llgl1 | 0.37645 |
| Llgl2 | 0.09483 |
| Lman1 | 0.61439 |
| Lman2 | 0.2356 |
| Lman2l | -0.65398 |
| Lman2l | 1.32752 |
| Lmbr1 | -0.67436 |
| Lmbr1l | -0.66582 |
| Lmcd1 | -0.67781 |
| Lmcd1 | -0.68821 |
| Lmcd1 | -0.57745 |
| Lmf1 | -0.02964 |
| Lmf2 | -0.61532 |
| Lmna | 3.24734 |
| Lmna | 0.50381 |
| Lmna | -0.19839 |
| Lmna | -0.62017 |
| Lmna | -0.71385 |
| Lmnb1 | -0.78659 |
| Lmnb1 | -0.67265 |
| Lmnb2 | -0.7005 |
| Lmo2 | 1.06834 |
| Lmo3 | -0.06924 |
| Lmo3 | -0.44857 |
| Lmo4 | -0.63981 |
| Lmod1 | 1.75243 |
| Lmtk2 | 0.40952 |
| Lmtk3 | 0.67648 |
| Lmx1a | -0.45193 |
| Lmx1b | -0.25976 |
| Lnx1 | -0.25159 |
| Lnx2 | 0.50595 |
| Lnx2 | -0.42469 |
| LOC100038882 | -0.55229 |
| LOC100038984 | -0.26085 |
| LOC100039087 | -0.17665 |
| LOC100039227 | 2.68107 |
| LOC100039479 | -0.46689 |
| LOC100039532 | -0.37191 |
| LOC100039571 | -0.59304 |
| LOC100039728 | -0.76834 |
| LOC100040573 | -0.38123 |
| LOC100040592 | 1.91838 |
| LOC100040601 | -0.72286 |
| LOC100040799 | -0.18418 |
| LOC100040899 | 0.5865 |
| LOC100040919 | -0.23174 |
| LOC100041103 | -0.67781 |
| LOC100041290 | 0.25455 |
| LOC100041463 | 0.0711 |
| LOC100041483 | 1.62707 |
| LOC100041500 | -0.63075 |
| LOC100041504 | 0.58038 |
| LOC100041703 | -0.13179 |
| LOC100041722 | 1.83386 |
| LOC100041725 | -0.67953 |
| LOC100041835 | 2.65106 |
| LOC100042179 | 0.23176 |
| LOC100042427 | -0.41493 |
| LOC100042777 | -0.64146 |
| LOC100043257 | -0.64728 |
| LOC100043391 | -0.50039 |
| LOC100043671 | 3.29385 |
| LOC100043822 | 3.21731 |
| LOC100043986 | 0.06689 |
| LOC100044087 | 0.3284 |
| LOC100044089 | 3.36997 |
| LOC100044101 | 0.02813 |
| LOC100044103 | -0.50537 |
| LOC100044103 | -0.57052 |
| LOC100044122 | -0.48488 |
| LOC100044133 | 0.99857 |
| LOC100044159 | -0.0881 |
| LOC100044170 | 2.80751 |
| LOC100044172 | -0.23387 |
| LOC100044177 | -0.68299 |
| LOC100044190 | -0.37624 |
| LOC100044204 | -0.22909 |
| LOC100044210 | 0.02653 |
| LOC100044221 | -0.58055 |
| LOC100044257 | 1.97912 |
| LOC100044275 | -0.61532 |
| LOC100044289 | 2.97976 |
| LOC100044292 | -0.152 |
| LOC100044294 | 0.16119 |
| LOC100044298 | 0.94442 |
| LOC100044322 | -0.59856 |
| LOC100044324 | 1.05809 |
| LOC100044324 | -0.77311 |
| LOC100044363 | -0.32492 |
| LOC100044363 | -0.3806 |
| LOC100044439 | 1.47742 |
| LOC100044468 | 0.36599 |
| LOC100044475 | 1.49704 |
| LOC100044538 | 1.17373 |
| LOC100044566 | -0.01067 |
| LOC100044576 | -0.54033 |
| LOC100044636 | 0.80939 |
| LOC100044692 | -0.04467 |
| LOC100044708 | 2.19179 |
| LOC100044736 | 0.58584 |
| LOC100044756 | -0.33615 |
| LOC100044776 | 0.56579 |
| LOC100044779 | 1.61278 |
| LOC100044812 | -0.71385 |
| LOC100044829 | 0.19482 |
| LOC100044862 | 1.06122 |
| LOC100044873 | 0.51141 |
| LOC100044934 | 1.61271 |
| LOC100045005 | -0.15394 |
| LOC100045019 | -0.93475 |
| LOC100045040 | -0.24081 |
| LOC100045098 | 2.81494 |
| LOC100045228 | 1.78232 |
| LOC100045241 | 0.27036 |
| LOC100045250 | -0.73563 |
| LOC100045280 | 0.99103 |
| LOC100045300 | -0.30747 |
| LOC100045304 | -0.45938 |
| LOC100045343 | -0.05249 |
| LOC100045359 | 0.47402 |
| LOC100045359 | -0.54929 |
| LOC100045403 | 0.09111 |
| LOC100045413 | 2.54357 |
| LOC100045419 | 1.56319 |
| LOC100045430 | -0.49896 |
| LOC100045439 | 1.69159 |
| LOC100045491 | 0.19282 |
| LOC100045505 | -0.14908 |
| LOC100045522 | 0.24607 |
| LOC100045542 | 0.77845 |
| LOC100045551 | -0.61291 |
| LOC100045567 | -0.48418 |
| LOC100045607 | 1.64094 |
| LOC100045617 | -0.4926 |
| LOC100045644 | 0.2988 |
| LOC100045697 | 0.31796 |
| LOC100045780 | -0.06083 |
| LOC100045782 | 1.74973 |
| LOC100045877 | 0.38 |
| LOC100045882 | -0.61855 |
| LOC100045887 | -0.66496 |
| LOC100045950 | -0.05731 |
| LOC100045963 | 1.12121 |
| LOC100045981 | 0.25236 |
| LOC100046003 | 0.77527 |
| LOC100046035 | -0.15736 |
| LOC100046035 | 2.2737 |
| LOC100046039 | 0.12376 |
| LOC100046044 | -0.67179 |
| LOC100046049 | 0.36737 |
| LOC100046056 | 1.31558 |
| LOC100046068 | 1.13462 |
| LOC100046080 | -0.61371 |
| LOC100046081 | 0.16292 |
| LOC100046120 | -0.43126 |
| LOC100046128 | 0.80682 |
| LOC100046129 | 0.77916 |
| LOC100046163 | -0.26469 |
| LOC100046163 | -0.59619 |
| LOC100046207 | -0.80819 |
| LOC100046207 | -0.65819 |
| LOC100046207 | 0.05687 |
| LOC100046232 | -0.49755 |
| LOC100046254 | 1.18912 |
| LOC100046320 | 1.17591 |
| LOC100046393 | -0.30632 |
| LOC100046406 | -0.32199 |
| LOC100046457 | 0.23624 |
| LOC100046518 | -0.65482 |
| LOC100046568 | 1.33141 |
| LOC100046586 | 3.21182 |
| LOC100046608 | 1.60166 |
| LOC100046608 | 0.21102 |
| LOC100046608 | 0.1234 |
| LOC100046608 | 1.79533 |
| LOC100046616 | -0.34271 |
| LOC100046641 | 0.71288 |
| LOC100046650 | 0.96442 |
| LOC100046690 | 3.46132 |
| LOC100046690 | 0.16843 |
| LOC100046741 | 0.58628 |
| LOC100046744 | -0.64395 |
| LOC100046744 | -0.65903 |
| LOC100046746 | 1.40695 |
| LOC100046775 | 1.60749 |
| LOC100046781 | -0.62667 |
| LOC100046781 | -0.09448 |
| LOC100046792 | -0.51183 |
| LOC100046796 | 0.24512 |
| LOC100046802 | 1.40006 |
| LOC100046841 | -0.62098 |
| LOC100046855 | -0.80225 |
| LOC100046883 | 0.36516 |
| LOC100046891 | 1.0934 |
| LOC100046898 | -0.55833 |
| LOC100046918 | -0.75887 |
| LOC100046953 | -0.48978 |
| LOC100046959 | -0.77024 |
| LOC100046996 | 0.92804 |
| LOC100047009 | -0.76359 |
| LOC100047009 | 0.27587 |
| LOC100047012 | -0.70493 |
| LOC100047028 | -0.6073 |
| LOC100047046 | 1.7693 |
| LOC100047052 | 1.16239 |
| LOC100047052 | 1.37389 |
| LOC100047155 | -0.47584 |
| LOC100047155 | -0.47032 |
| LOC100047167 | -0.38373 |
| LOC100047173 | -0.2254 |
| LOC100047184 | 1.64267 |
| LOC100047200 | 0.12986 |
| LOC100047214 | 1.09076 |
| LOC100047214 | 0.20411 |
| LOC100047238 | 2.36349 |
| LOC100047261 | 2.10632 |
| LOC100047323 | -0.90558 |
| LOC100047323 | 1.91658 |
| LOC100047339 | 0.07908 |
| LOC100047353 | 3.48316 |
| LOC100047368 | 1.19296 |
| LOC100047369 | 1.13462 |
| LOC100047369 | -0.37624 |
| LOC100047391 | 0.28981 |
| LOC100047393 | -0.17865 |
| LOC100047427 | -0.59304 |
| LOC100047480 | 1.84644 |
| LOC100047490 | 1.769 |
| LOC100047579 | 0.79283 |
| LOC100047583 | -0.71027 |
| LOC100047606 | -0.49967 |
| LOC100047615 | -0.20096 |
| LOC100047619 | -0.36759 |
| LOC100047634 | 1.84738 |
| LOC100047651 | -0.76644 |
| LOC100047653 | 1.8792 |
| LOC100047659 | -0.55984 |
| LOC100047674 | 0.29162 |
| LOC100047707 | -0.09998 |
| LOC100047717 | 1.71785 |
| LOC100047749 | 0.84936 |
| LOC100047762 | -0.70938 |
| LOC100047776 | -0.82727 |
| LOC100047794 | 0.03768 |
| LOC100047800 | 0.76222 |
| LOC100047810 | -0.67781 |
| LOC100047815 | -0.85717 |
| LOC100047816 | -0.67094 |
| LOC100047834 | -0.26689 |
| LOC100047837 | 0.93701 |
| LOC100047837 | -0.02032 |
| LOC100047841 | -0.5278 |
| LOC100047856 | 0.82178 |
| LOC100047870 | -0.53663 |
| LOC100047911 | -0.24027 |
| LOC100047934 | 1.98803 |
| LOC100047935 | -0.78465 |
| LOC100047937 | 2.26382 |
| LOC100047937 | 0.08586 |
| LOC100047958 | 0.41872 |
| LOC100047963 | -0.5821 |
| LOC100047963 | 0.19549 |
| LOC100047998 | -0.63321 |
| LOC100048046 | -0.07369 |
| LOC100048046 | -0.16079 |
| LOC100048049 | -0.6523 |
| LOC100048049 | -0.7495 |
| LOC100048049 | 1.01962 |
| LOC100048056 | 2.5454 |
| LOC100048071 | -0.56136 |
| LOC100048076 | -0.49613 |
| LOC100048083 | -0.24888 |
| LOC100048105 | 2.39108 |
| LOC100048105 | 0.44475 |
| LOC100048187 | 1.83341 |
| LOC100048251 | -0.42143 |
| LOC100048295 | 0.1698 |
| LOC100048299 | -0.37006 |
| LOC100048301 | 1.07181 |
| LOC100048313 | -0.67953 |
| LOC100048331 | 0.46586 |
| LOC100048332 | 2.02076 |
| LOC100048346 | 0.93924 |
| LOC100048372 | 2.40437 |
| LOC100048384 | 0.13948 |
| LOC100048413 | -0.0382 |
| LOC100048436 | -0.4621 |
| LOC100048445 | 1.20459 |
| LOC100048461 | -0.46894 |
| LOC100048480 | 2.3893 |
| LOC100048483 | -0.82524 |
| LOC100048504 | 0.0711 |
| LOC100048530 | -0.18822 |
| LOC100048554 | 2.95482 |
| LOC100048556 | -0.60491 |
| LOC100048589 | 1.56537 |
| LOC100048613 | 0.59625 |
| LOC100048622 | 2.49991 |
| LOC100048645 | 0.46908 |
| LOC100048684 | 0.47155 |
| LOC100048703 | 2.281 |
| LOC100048710 | 2.66707 |
| LOC100048721 | -0.65988 |
| LOC100048721 | -0.52342 |
| LOC100048726 | 0.73428 |
| LOC100048733 | -0.7617 |
| LOC100048816 | -0.55004 |
| LOC100048832 | 0.7195 |
| LOC100048832 | -0.39507 |
| LOC100048858 | 0.1004 |
| LOC192758 | 0.7413 |
| LOC194227 | -0.42012 |
| LOC212390 | -0.86559 |
| LOC216443 | -0.22382 |
| LOC216963 | -0.53737 |
| LOC217341 | 2.88002 |
| LOC218963 | -0.55379 |
| LOC225922 | -0.68647 |
| LOC233529 | -0.73655 |
| LOC234374 | -0.48838 |
| LOC235497 | -0.67436 |
| LOC236749 | -0.5759 |
| LOC237408 | 0.76939 |
| LOC237877 | 0.79994 |
| LOC238829 | -0.46415 |
| LOC241593 | -0.82221 |
| LOC244595 | 1.3841 |
| LOC245263 | -0.53959 |
| LOC329575 | 0.23208 |
| LOC329575 | 0.86927 |
| LOC333331 | 2.92609 |
| LOC381066 | -0.65988 |
| LOC381302 | 0.87215 |
| LOC381375 | -0.18518 |
| LOC381483 | 0.30562 |
| LOC381629 | 2.24189 |
| LOC381801 | 0.66535 |
| LOC381936 | -0.41947 |
| LOC382010 | 1.07271 |
| LOC382044 | 1.47453 |
| LOC382106 | -0.23546 |
| LOC382106 | -0.72286 |
| LOC382133 | -0.10413 |
| LOC384806 | -0.57129 |
| LOC384806 | -0.73105 |
| LOC386486 | 1.67368 |
| LOC433479 | -0.69433 |
| LOC433801 | 3.16677 |
| LOC433886 | -0.74857 |
| LOC434172 | 0.73372 |
| LOC434172 | -0.64395 |
| LOC434864 | -0.71745 |
| LOC434960 | 3.08261 |
| LOC435023 | -0.64979 |
| LOC435337 | -0.81618 |
| LOC435565 | -0.53811 |
| LOC436177 | -0.82423 |
| LOC544988 | -0.41687 |
| LOC544988 | -0.11715 |
| LOC545013 | 0.93269 |
| LOC545013 | -0.77119 |
| LOC545056 | -0.40462 |
| LOC545238 | -0.23174 |
| LOC545703 | 1.07974 |
| LOC545732 | 2.83489 |
| LOC545952 | 0.0557 |
| LOC546015 | -0.09906 |
| LOC546015 | -0.28527 |
| LOC546723 | 1.58572 |
| LOC546840 | 1.68351 |
| LOC547323 | 0.52128 |
| LOC547343 | -0.63568 |
| LOC620807 | -0.67781 |
| LOC621823 | 0.12484 |
| LOC621824 | 0.15424 |
| LOC622404 | -0.78951 |
| LOC622665 | 2.20352 |
| LOC624855 | -0.73931 |
| LOC625480 | 0.54049 |
| LOC626152 | -0.52927 |
| LOC628053 | 1.54709 |
| LOC631002 | 0.0349 |
| LOC631784 | -0.81919 |
| LOC632431 | 1.89868 |
| LOC639396 | -0.41493 |
| LOC640963 | -0.55757 |
| LOC641240 | -0.54182 |
| LOC654426 | 1.1998 |
| LOC654467 | 1.0828 |
| LOC654494 | -0.42143 |
| LOC654842 | -0.31616 |
| LOC665250 | -0.34511 |
| LOC666676 | -0.579 |
| LOC666904 | 2.56991 |
| LOC667250 | 0.80132 |
| LOC667370 | 1.05142 |
| LOC667609 | 0.39622 |
| LOC668492 | 0.2988 |
| LOC668837 | 2.61651 |
| LOC669168 | -0.19891 |
| LOC669660 | 0.34214 |
| LOC670044 | -0.64478 |
| LOC671564 | 1.2836 |
| LOC672705 | 3.21557 |
| LOC674706 | 0.64351 |
| LOC675228 | 0.771 |
| LOC675567 | -0.41169 |
| LOC675567 | 2.63519 |
| LOC675813 | 3.13929 |
| LOC676420 | 0.58715 |
| LOC676420 | -0.51905 |
| LOC677144 | -0.24672 |
| LOC677205 | 1.53056 |
| LOC677317 | -0.40079 |
| LOC677319 | 1.25951 |
| LOC677528 | 0.99927 |
| LOC732482 | 0.47525 |
| Loh11cr2a | 0.22598 |
| Loh12cr1 | 1.87127 |
| Loh12cr1 | 0.07681 |
| Lonp2 | -0.36391 |
| Lonrf1 | 1.88415 |
| Lonrf2 | -0.54108 |
| Lor | -0.37998 |
| Lox | 0.58693 |
| Loxl1 | -0.64645 |
| Lpar2 | 0.51282 |
| Lpar2 | 1.0158 |
| Lpar3 | -0.2061 |
| Lpcat2 | -0.53515 |
| Lpcat2 | 2.94824 |
| Lpcat2 | -0.74764 |
| Lpgat1 | 1.88367 |
| Lphn1 | -0.66667 |
| Lphn3 | -0.17216 |
| Lpin1 | -0.39001 |
| Lpin1 | -0.7779 |
| Lpin2 | 2.17855 |
| Lpin3 | 1.62447 |
| Lpl | -0.68386 |
| Lpxn | -0.77502 |
| Lrat | -0.76834 |
| Lrba | 2.31071 |
| Lrba | 1.03819 |
| Lrch1 | 0.48235 |
| Lrch4 | 2.34603 |
| Lrch4 | 1.67522 |
| Lrdd | -0.64562 |
| Lrfn1 | -0.74671 |
| Lrfn2 | -0.12751 |
| Lrfn3 | -0.32904 |
| Lrfn4 | 2.05608 |
| Lrg1 | -0.74208 |
| Lrig1 | 0.09521 |
| Lrig3 | 1.97119 |
| Lrmp | -0.77886 |
| Lrp1 | -0.49401 |
| Lrp10 | -0.58677 |
| Lrp11 | -0.63568 |
| Lrp12 | 1.30168 |
| Lrp12 | -0.9223 |
| Lrp4 | -0.66667 |
| Lrp5 | 0.8505 |
| Lrp8 | -0.26414 |
| Lrp8 | -0.46963 |
| Lrpap1 | -0.77886 |
| Lrpprc | -0.41687 |
| Lrpprc | -0.64645 |
| Lrpprc | -0.27632 |
| Lrrc1 | -0.31674 |
| Lrrc1 | -0.82524 |
| Lrrc1 | 1.30667 |
| Lrrc10 | -0.72377 |
| Lrrc15 | 1.07156 |
| Lrrc16b | 1.45374 |
| Lrrc16b | -0.4621 |
| Lrrc18 | 2.47411 |
| Lrrc2 | 1.80845 |
| Lrrc20 | 1.79522 |
| Lrrc20 | -0.14328 |
| Lrrc23 | -0.73746 |
| Lrrc23 | 0.36626 |
| Lrrc24 | 0.84789 |
| Lrrc26 | 0.95848 |
| Lrrc28 | -0.26855 |
| Lrrc29 | 0.74662 |
| Lrrc3 | 0.96802 |
| Lrrc30 | 1.6049 |
| Lrrc33 | -0.54629 |
| Lrrc34 | 1.53654 |
| Lrrc34 | 1.14012 |
| Lrrc36 | 0.5795 |
| Lrrc39 | -0.5899 |
| Lrrc39 | 0.51589 |
| Lrrc4 | -0.34331 |
| Lrrc40 | -0.53737 |
| Lrrc41 | -0.6121 |
| Lrrc43 | 0.95309 |
| Lrrc44 | -0.80919 |
| Lrrc45 | -0.55079 |
| Lrrc46 | -0.08856 |
| Lrrc48 | -0.73931 |
| Lrrc48 | 2.31228 |
| Lrrc48 | 0.67173 |
| Lrrc49 | 1.52044 |
| Lrrc49 | 3.23703 |
| Lrrc49 | 2.94132 |
| Lrrc4c | 1.07783 |
| Lrrc50 | 3.47894 |
| Lrrc50 | -0.41947 |
| Lrrc52 | -0.62504 |
| Lrrc52 | 0.96773 |
| Lrrc56 | 0.39031 |
| Lrrc57 | -0.08042 |
| Lrrc59 | -0.40334 |
| Lrrc6 | -0.52051 |
| Lrrc7 | 1.05888 |
| Lrrc8 | -0.61291 |
| Lrrc8a | 2.00517 |
| Lrrc8b | -0.09722 |
| Lrrc8c | 0.30355 |
| Lrrc8d | -0.2092 |
| Lrrc9 | 0.23592 |
| Lrrfip1 | 0.50047 |
| Lrrfip1 | 1.5487 |
| Lrriq2 | -0.33555 |
| Lrriq2 | 2.02478 |
| Lrrk1 | 0.70065 |
| Lrrk2 | -0.40143 |
| Lrrn1 | 1.41777 |
| Lrrn2 | -0.49826 |
| Lrrn3 | 1.00093 |
| Lrrtm1 | -0.56822 |
| Lrrtm2 | -0.56593 |
| Lrrtm3 | 0.91091 |
| Lrsam1 | -0.29148 |
| Lrsam1 | -0.4059 |
| Lrtm1 | 0.42912 |
| Lrtm1 | 0.48479 |
| Lsamp | -0.39127 |
| Lsamp | -0.27242 |
| Lsg1 | -0.64645 |
| Lsg1 | 0.02251 |
| Lsm10 | 1.0964 |
| Lsm11 | 0.65348 |
| Lsm12 | 3.52467 |
| Lsm12 | -0.71117 |
| Lsm12 | 0.66675 |
| Lsm14a | 1.44983 |
| Lsm14b | -0.78562 |
| Lsm14b | -0.13991 |
| Lsm14b | 0.34243 |
| Lsm2 | 1.82592 |
| Lsm2 | 1.15103 |
| Lsm3 | -0.56745 |
| Lsm4 | 2.46281 |
| Lsm5 | -0.31732 |
| Lsm6 | 1.42594 |
| Lsm6 | 1.15597 |
| Lsm8 | -0.4027 |
| Lsr | -0.8946 |
| Lst1 | -0.62098 |
| Ltb4dh | -0.36513 |
| Ltb4r1 | -0.66496 |
| Ltb4r2 | 1.51704 |
| Ltbp1 | 0.18104 |
| Ltbp1 | 0.79301 |
| Ltbp2 | -0.36821 |
| Ltbp3 | 2.26062 |
| Ltbp3 | -0.51327 |
| Ltbp4 | -0.45463 |
| Ltbp4 | -0.5068 |
| Ltbp4 | 2.22201 |
| Ltbr | 2.07404 |
| Ltc4s | -0.48978 |
| Ltf | 0.25361 |
| Ltf | -0.53737 |
| Ltk | 0.25236 |
| Ltk | -0.27632 |
| Ltv1 | -0.22014 |
| Luc7l | -0.22382 |
| Luc7l | -0.09128 |
| Luc7l2 | 0.26264 |
| Luc7l2 | -0.22856 |
| Lum | 1.75884 |
| Luzp1 | 2.19319 |
| Luzp1 | -0.76834 |
| Luzp1 | -0.68821 |
| Luzp2 | 0.05298 |
| Lxn | -0.41105 |
| Ly6a | 1.9253 |
| Ly6c1 | -0.74764 |
| Ly6d | 1.06407 |
| Ly6e | -0.46006 |
| Ly6f | 0.15494 |
| Ly6g6c | 0.33186 |
| Ly6g6d | 0.10556 |
| Ly6g6e | 0.10703 |
| Ly6k | -0.72105 |
| Ly86 | 0.28499 |
| Ly9 | -0.58443 |
| Ly9 | 2.22322 |
| Ly96 | 0.88546 |
| Lyar | -0.69609 |
| Lyl1 | 2.33913 |
| Lynx1 | 0.43838 |
| Lynx1 | 1.56597 |
| Lypd1 | -0.07459 |
| Lypd3 | -0.7617 |
| Lypd4 | 0.25642 |
| Lypd5 | -0.4339 |
| Lypd6 | 0.95862 |
| Lypla1 | -0.66752 |
| Lypla1 | 0.29432 |
| Lypla2 | 0.35624 |
| Lypla3 | -0.72558 |
| Lyplal1 | -0.46963 |
| Lyrm1 | -0.61693 |
| Lyrm2 | -0.31267 |
| Lyrm2 | 0.80356 |
| Lyrm5 | -0.30402 |
| Lysmd1 | -0.579 |
| Lysmd1 | -0.16129 |
| Lysmd2 | 0.20807 |
| Lysmd4 | 0.58845 |
| Lyst | -0.64728 |
| Lyve1 | -0.1672 |
| Lyz | 0.16499 |
| Lyz1 | 1.21751 |
| Lyz1 | -0.49401 |
| Lyz2 | 3.53041 |
| Lyzl1 | 3.02308 |
| Lyzl4 | 1.96285 |
| Lyzl6 | 3.96943 |
| Lyzs | -0.11949 |
| Lzic | -0.45126 |
| Lztr1 | -0.63898 |
| Lzts2 | -0.55304 |
| M6pr | 0.69293 |
| M6pr | -0.44388 |
| M6prbp1 | 0.76006 |
| Mab21l2 | 0.83034 |
| Macrod1 | -0.46415 |
| Mad1l1 | -0.49755 |
| Mad2l1 | 1.15959 |
| Mad2l1bp | -0.71475 |
| Mad2l1bp | -0.57745 |
| Mad2l1bp | 2.80601 |
| Mad2l2 | -0.84261 |
| Madcam1 | 0.39327 |
| Madd | 0.10445 |
| Maea | 0.85197 |
| Maf | 0.22015 |
| Maf1 | -0.4717 |
| Mafg | 1.33941 |
| Mag | -0.40783 |
| Magea2 | 1.32956 |
| Magea5 | -0.57977 |
| Mageb3 | -0.59935 |
| Mageb3 | -0.3976 |
| Maged1 | 0.81908 |
| Maged2 | 0.17561 |
| Magee1 | 0.09781 |
| Mageh1 | -0.41234 |
| Magi1 | -0.64812 |
| Magi1 | 0.89951 |
| Magi1 | -0.58833 |
| Magi2 | -0.57668 |
| Magi3 | -0.19533 |
| Magi3 | -0.72196 |
| Magix | -0.48348 |
| Magmas | 3.15549 |
| Magoh | 0.87215 |
| Magoh | -0.37624 |
| Magohb | 1.60684 |
| Mak | -0.45734 |
| Mak10 | 0.56579 |
| Mak10 | -0.11762 |
| Mal | 1.95472 |
| Mal | 0.63507 |
| Mall | -0.75417 |
| Malt1 | 0.54711 |
| Mamdc2 | 0.35372 |
| Maml1 | -0.7005 |
| Maml3 | 0.98977 |
| Man1a | 1.0895 |
| Man1a2 | -0.6283 |
| Man1a2 | 1.60152 |
| Man1b1 | 2.84446 |
| Man1c1 | 0.29222 |
| Man1c1 | -0.39951 |
| Man2a1 | 2.90916 |
| Man2a2 | 1.52414 |
| Man2a2 | 1.87803 |
| Man2a2 | 0.56065 |
| Man2b1 | -0.15492 |
| Man2b2 | 0.88184 |
| Man2c1 | -0.68647 |
| Manba | -0.5606 |
| Manba | 2.19516 |
| Manbal | -0.06613 |
| Manea | -0.46757 |
| Mansc1 | 0.75445 |
| Maob | 1.89297 |
| Map1lc3a | -0.33259 |
| Map1lc3a | -0.16968 |
| Map1lc3b | 1.75041 |
| Map2k1 | 1.46117 |
| Map2k2 | 0.35372 |
| Map2k2 | -0.58755 |
| Map2k3 | 1.03365 |
| Map2k4 | -0.72467 |
| Map2k6 | 0.69331 |
| Map2k6 | -0.06259 |
| Map2k6 | -0.0136 |
| Map2k7 | 0.68317 |
| Map2k7 | 0.05143 |
| Map2k7 | -0.55004 |
| Map3k1 | 0.64207 |
| Map3k11 | 0.35372 |
| Map3k12 | 3.41457 |
| Map3k12 | 0.80597 |
| Map3k12 | 0.86895 |
| Map3k14 | 2.79224 |
| Map3k3 | 0.2682 |
| Map3k6 | 2.7382 |
| Map3k7 | 0.59106 |
| Map3k7ip1 | 0.44577 |
| Map3k7ip1 | -0.47446 |
| Map3k7ip1 | -0.73013 |
| Map3k7ip2 | -0.62341 |
| Map3k7ip3 | -0.24888 |
| Map3k8 | 2.12537 |
| Map3k9 | 0.22436 |
| Map4k2 | 3.13321 |
| Map4k4 | -0.70138 |
| Map4k5 | 0.95279 |
| Map4k5 | 0.66934 |
| Mapbpip | -0.31616 |
| Mapk1 | 1.64301 |
| Mapk1 | -0.2864 |
| Mapk1 | -0.56822 |
| Mapk10 | 0.84789 |
| Mapk10 | 0.14019 |
| Mapk11 | 0.78392 |
| Mapk12 | 0.03926 |
| Mapk12 | -0.62017 |
| Mapk12 | 0.26543 |
| Mapk13 | -0.71745 |
| Mapk13 | -0.743 |
| Mapk14 | 0.06305 |
| Mapk15 | 0.42263 |
| Mapk15 | -0.26249 |
| Mapk15 | -0.5553 |
| Mapk1ip1 | 1.75035 |
| Mapk1ip1 | -0.55606 |
| Mapk1ip1 | 2.26977 |
| Mapk3 | -0.57668 |
| Mapk3 | 1.35294 |
| Mapk4 | 1.01156 |
| Mapk6 | 1.20615 |
| Mapk6 | 0.30503 |
| Mapk6 | -0.58912 |
| Mapk8 | -0.59461 |
| Mapk8ip1 | -0.36698 |
| Mapk8ip1 | 2.88258 |
| Mapk8ip2 | -0.14908 |
| Mapk8ip3 | 2.50522 |
| Mapk8ip3 | 0.33157 |
| Mapk9 | 0.80063 |
| Mapk9 | -0.05337 |
| Mapk9 | 1.0084 |
| Mapkap1 | 1.00509 |
| Mapkap1 | -0.53147 |
| Mapkapk2 | 0.07605 |
| Mapkapk3 | 0.20312 |
| Mapkbp1 | 0.13557 |
| Mapre1 | -0.69609 |
| Mapre2 | -0.62179 |
| Mapre2 | -0.55908 |
| Mapre3 | -0.57745 |
| Mapt | -0.50895 |
| Mapt | 0.41374 |
| Mapt | -0.65146 |
| Mapt | -0.64895 |
| March1 | -0.38373 |
| March2 | -0.69171 |
| March2 | 0.76042 |
| March4 | -0.43655 |
| March4 | -0.05555 |
| March6 | -0.47584 |
| March7 | -0.64478 |
| March8 | 2.96872 |
| March10 | -0.4306 |
| Marcks | 0.41636 |
| Marcksl1 | -0.00942 |
| Marcksl1 | -0.20662 |
| Marcksl1 | -0.16227 |
| Mare | 0.25549 |
| Mark1 | 2.33068 |
| Mark2 | -0.02794 |
| Mark2 | 1.01525 |
| Mark3 | 0.10776 |
| Mark3 | -0.5176 |
| Mark4 | 0.16084 |
| Mars | 0.52198 |
| Mars2 | -0.45734 |
| Marveld3 | -0.53 |
| Marveld3 | 2.31331 |
| Mas1 | -0.49967 |
| Masp1 | -0.66411 |
| Masp2 | -0.65819 |
| Mast1 | -0.20044 |
| Mast1 | -0.07548 |
| Mast2 | -0.01737 |
| Mast2 | 0.42393 |
| Mat1a | -0.04944 |
| Mat2a | 0.26078 |
| Mat2a | 1.62412 |
| Mat2a | 0.63549 |
| Mat2a | -0.53368 |
| Mat2b | -0.6073 |
| Mat2b | -0.77502 |
| Matk | 0.22404 |
| Matk | 0.44221 |
| Matn4 | -0.51039 |
| Matn4 | -0.63568 |
| Matn4 | 2.19592 |
| Matr3 | 0.37864 |
| Max | -0.40398 |
| Maz | -0.67522 |
| Mb | 0.74093 |
| Mbc2 | -0.50181 |
| Mbd1 | -0.6089 |
| Mbd1 | -0.49896 |
| Mbd3 | 1.06355 |
| Mbd3l2 | -0.0986 |
| Mbd4 | 0.68591 |
| Mbd5 | -0.41299 |
| Mbd6 | 2.48378 |
| Mbip | -0.27743 |
| Mbl2 | 0.06459 |
| Mbnl1 | -0.81718 |
| Mbnl2 | 0.17663 |
| Mbnl2 | -0.58521 |
| Mboat1 | 0.25393 |
| Mboat2 | -0.48 |
| Mboat2 | 1.75848 |
| Mboat2 | 1.94061 |
| Mboat2 | -0.66157 |
| Mboat5 | -0.71925 |
| Mboat5 | 1.44761 |
| Mbp | -0.60491 |
| Mbp | -0.75981 |
| Mbp | -0.14957 |
| Mbtd1 | 0.93164 |
| Mbtps1 | -0.67265 |
| Mbtps1 | -0.72105 |
| Mbtps1 | 1.80139 |
| Mbtps2 | 0.71648 |
| Mc3r | -0.12704 |
| Mc4r | 0.33644 |
| Mc5r | -0.1009 |
| Mcam | -0.6856 |
| Mcam | 1.80139 |
| Mcam | 0.40449 |
| Mcat | 0.54824 |
| Mcc | -0.60093 |
| Mccc1 | -0.46894 |
| Mcee | -0.37376 |
| Mcf2l | 0.00914 |
| Mcf2l | 3.7128 |
| Mcf2l | -0.19686 |
| Mcfd2 | -0.60491 |
| Mchr1 | -0.36391 |
| Mcl1 | -0.67179 |
| Mcm10 | -0.34812 |
| Mcm10 | -0.61693 |
| Mcm2 | 2.54445 |
| Mcm3 | 3.20764 |
| Mcm4 | 1.57782 |
| Mcm5 | -0.68908 |
| Mcm6 | -0.81818 |
| Mcm6 | 0.04123 |
| Mcm6 | 0.6207 |
| Mcm7 | 0.59754 |
| Mcoln1 | 0.27097 |
| Mcoln2 | 1.75567 |
| Mcoln3 | 0.31034 |
| Mcph1 | -0.41493 |
| Mcph1 | 0.17151 |
| Mcph1 | 1.32791 |
| Mcph1 | 0.67213 |
| Mcpt4 | 0.69873 |
| Mcrs1 | -0.68213 |
| Mcrs1 | -0.52634 |
| Mcrs1 | 3.39014 |
| Mcts1 | -0.66752 |
| Mdfic | -0.6065 |
| Mdga1 | 1.57218 |
| Mdga2 | -0.51978 |
| Mdga2 | 0.84048 |
| Mdh1 | -0.50824 |
| Mdh1 | 0.45033 |
| Mdh1b | -0.75981 |
| Mdh2 | -0.69697 |
| Mdk | 1.15994 |
| Mdk | 3.87602 |
| Mdm1 | 0.2356 |
| Mdm1 | -0.70493 |
| Mdm1 | 2.36553 |
| Mdm1 | -0.63733 |
| Mdm1 | -0.58833 |
| Mdm1 | -0.59147 |
| Mdm1 | -0.77694 |
| Mdm1 | 1.35323 |
| Mdm1 | -0.21335 |
| Mdm2 | -0.24726 |
| Mdm2 | -0.31965 |
| Mdm4 | 0.05764 |
| Mdn1 | 0.31737 |
| Me2 | -0.31616 |
| Me3 | 2.14084 |
| Mea1 | -0.6226 |
| Mecp2 | -0.04164 |
| Mecr | 0.57576 |
| Med1 | 0.20378 |
| Med1 | -0.59619 |
| Med10 | 1.5261 |
| Med11 | 0.90292 |
| Med11 | 0.42782 |
| Med12 | 0.75391 |
| Med15 | 0.87023 |
| Med15 | 1.92093 |
| Med15 | 0.55255 |
| Med16 | 0.97218 |
| Med18 | -0.38749 |
| Med18 | 1.49088 |
| Med19 | -0.41947 |
| Med21 | -0.76549 |
| Med22 | -0.27743 |
| Med22 | 0.20114 |
| Med23 | -0.53073 |
| Med23 | 2.2076 |
| Med24 | 1.97754 |
| Med25 | -0.41947 |
| Med25 | -0.33318 |
| Med26 | 0.02211 |
| Med27 | -0.18518 |
| Med28 | -0.5606 |
| Med30 | 1.32762 |
| Med31 | 2.15407 |
| Med4 | -0.03476 |
| Med6 | 0.30857 |
| Med7 | 1.39628 |
| Med8 | -0.37811 |
| Med8 | 1.8223 |
| Med9 | -0.73563 |
| Mef2b | 1.41211 |
| Mef2b | -0.70671 |
| Mef2c | -0.50609 |
| Mef2c | 1.84329 |
| Mef2c | -0.37067 |
| Megf10 | -0.51183 |
| Megf11 | -0.65566 |
| Megf11 | -0.35598 |
| Megf11 | -0.7076 |
| Megf11 | 2.4961 |
| Megf6 | -0.43788 |
| Megf9 | -0.57668 |
| Mei1 | 2.53189 |
| Meis1 | 1.4183 |
| Meis2 | -0.73746 |
| Meis3 | 1.34401 |
| Melk | 2.1003 |
| Memo1 | -0.20198 |
| Men1 | 2.54017 |
| Men1 | -0.71027 |
| Meox1 | -0.56212 |
| Meox2 | 1.39898 |
| Mep1b | -0.4926 |
| Mepce | -0.22171 |
| Mertk | -0.77982 |
| Mesdc1 | -0.2691 |
| Mesp1 | -0.87409 |
| Mest | -0.41493 |
| Mest | -0.78951 |
| Mest | -0.30747 |
| Metap1 | 2.25461 |
| Metap2 | -0.67179 |
| Metap2 | 0.68003 |
| Metapl1 | -0.40462 |
| Metrn | -0.64979 |
| Metrnl | -0.19431 |
| Metrnl | -0.58132 |
| Mett11d1 | -0.28809 |
| Mettl1 | -0.67694 |
| Mettl1 | 0.63672 |
| Mettl2 | -0.6523 |
| Mettl3 | -0.59461 |
| Mettl4 | -0.4807 |
| Mettl6 | -0.69873 |
| Mettl8 | 0.84378 |
| Mettl9 | -0.47792 |
| Mettl9 | -0.12467 |
| Mex3a | 3.27527 |
| Mex3a | 0.20477 |
| Mex3c | 0.20675 |
| Mfap1b | -0.40655 |
| Mfap2 | 1.91494 |
| Mfap2 | 0.43093 |
| Mfap2 | -0.12799 |
| Mfap3 | 3.37003 |
| Mfap3 | 2.00745 |
| Mfap3l | -0.42732 |
| Mfap3l | -0.12609 |
| Mfap4 | 0.83949 |
| Mfap5 | 3.27271 |
| Mfge8 | 1.60116 |
| Mfge8 | 0.14584 |
| Mfi2 | -0.04078 |
| Mfn1 | -0.55757 |
| Mfn1 | 0.80871 |
| Mfn2 | -0.53441 |
| Mfng | -0.55154 |
| Mfrp | -0.44321 |
| Mfsd1 | -0.61451 |
| Mfsd10 | -0.5553 |
| Mfsd10 | 2.75348 |
| Mfsd11 | 1.52375 |
| Mfsd2 | 4.73868 |
| Mfsd3 | -0.46826 |
| Mfsd4 | 0.6547 |
| Mfsd7 | -0.64063 |
| Mfsd8 | 0.94353 |
| Mfsd9 | 0.56846 |
| Mga | -0.15638 |
| Mgat1 | 0.39809 |
| Mgat3 | 1.40015 |
| Mgat4b | 0.60632 |
| Mgat4b | -0.44254 |
| Mgat5 | -0.63403 |
| MGC107098 | 1.15185 |
| MGC117608 | -0.66837 |
| MGC118210 | 1.07399 |
| MGC18837 | 0.77473 |
| MGC41689 | -0.60332 |
| MGC67181 | -0.63981 |
| Mgea6 | 0.63796 |
| Mgl1 | -0.76834 |
| Mgl2 | 0.59171 |
| Mgll | 2.23239 |
| Mgll | -0.64478 |
| Mgll | -0.52196 |
| Mgmt | 0.31357 |
| Mgp | 0.64902 |
| Mgrn1 | 3.27862 |
| Mgst1 | -0.58055 |
| Mgst1 | -0.42732 |
| Mgst2 | 0.97704 |
| Mgst3 | 0.94913 |
| Mgst3 | -0.80919 |
| Mia1 | -0.31907 |
| Mia3 | -0.11809 |
| Mib1 | 2.4687 |
| Mib2 | 0.67252 |
| Mic2l1 | -0.06569 |
| Mical1 | 1.19756 |
| Mical1 | 0.09669 |
| Mical1 | -0.63321 |
| Mical2 | 0.6749 |
| Mical3 | 1.47932 |
| Micall2 | -0.58912 |
| Micall2 | 0.00791 |
| Mid1 | 1.67628 |
| Mid1 | 0.20741 |
| Mid1ip1 | -0.12467 |
| Midn | -0.23653 |
| Midn | 1.26202 |
| Mier1 | 0.59904 |
| Mier1 | -0.21544 |
| Mif | -0.10274 |
| Mif4gd | -0.6089 |
| Mif4gd | -0.54405 |
| Mill2 | 1.86392 |
| Mill2 | -0.24456 |
| Mina | 0.04359 |
| Mink1 | -0.42797 |
| Mink1 | -0.6523 |
| Minpp1 | 1.17866 |
| Minpp1 | -0.743 |
| Miox | -0.42339 |
| Mipep | 3.70139 |
| Mir16 | -0.8509 |
| Mist1 | -0.45193 |
| Mitf | -0.56975 |
| Mizf | -0.32845 |
| Mki67ip | -0.55606 |
| Mkks | 0.10335 |
| Mkks | 1.6912 |
| Mkl1 | 2.02533 |
| Mkl2 | 0.63734 |
| Mkl2 | -0.54256 |
| Mkl2 | -0.67351 |
| Mkln1 | 1.1321 |
| Mknk1 | -0.67608 |
| Mknk2 | 0.65105 |
| Mkrn1 | 1.4652 |
| Mkrn2 | -0.4306 |
| Mkrn3 | 0.61755 |
| Mkx | 0.73927 |
| Mlana | 0.13983 |
| Mlf1 | -0.71296 |
| Mlf1 | -0.41752 |
| Mlf2 | 2.25503 |
| Mlf2 | -0.57822 |
| Mlh1 | -0.63157 |
| Mlh3 | 1.3143 |
| Mlkl | -0.47931 |
| Mllt1 | 0.20609 |
| Mllt1 | 0.32406 |
| Mllt1 | -0.64728 |
| Mllt10 | -0.13847 |
| Mllt11 | -0.05731 |
| Mllt3 | -0.25539 |
| Mllt3 | -0.64895 |
| Mllt3 | -0.51978 |
| Mlp | -0.66837 |
| Mlstd2 | 1.1774 |
| Mlx | 0.43761 |
| Mlx | -0.7495 |
| Mlxipl | -0.72649 |
| Mlycd | -0.16968 |
| Mmab | -0.44521 |
| Mmab | 0.23528 |
| Mmd | 0.33071 |
| Mmd2 | 1.31971 |
| Mme | -0.23653 |
| Mme | 1.64039 |
| Mmel1 | 0.82649 |
| Mmp10 | -0.44991 |
| Mmp11 | -0.17565 |
| Mmp12 | 3.05636 |
| Mmp13 | 1.1045 |
| Mmp13 | 1.35794 |
| Mmp14 | 1.76804 |
| Mmp15 | -0.6804 |
| Mmp16 | -0.69083 |
| Mmp17 | -0.27076 |
| Mmp1a | 0.34753 |
| Mmp1a | 0.26512 |
| Mmp2 | 0.57774 |
| Mmp23 | -0.78854 |
| Mmp24 | 1.41654 |
| Mmp3 | 2.20935 |
| Mmp3 | -0.55455 |
| Mmp3 | 0.93612 |
| Mmp9 | -0.56364 |
| Mmrn2 | -0.05731 |
| Mnat1 | 0.73483 |
| Mnat1 | -0.62585 |
| Mnd1 | 0.08361 |
| Mns1 | -0.43655 |
| Mnt | -0.12137 |
| Mobk1b | 1.3942 |
| Mobkl2a | -0.44454 |
| Mobkl2b | -0.77024 |
| Mobkl2c | -0.67265 |
| Mobkl3 | -0.67265 |
| Mobp | 0.20246 |
| Mobp | -0.60093 |
| Mobp | 2.80001 |
| Mocos | -0.58521 |
| Mocs1 | 0.64371 |
| Mocs2 | -0.36575 |
| Mod1 | -0.67781 |
| Mog | 2.10886 |
| Mog | -0.29773 |
| Mon1a | 0.58344 |
| Mon1a | 1.61584 |
| Mon1b | 0.3646 |
| Mon1b | -0.4587 |
| Mon1b | 0.20312 |
| Morc2a | -0.03135 |
| Morc2b | 0.31533 |
| Morc3 | -0.8946 |
| Morf4l1 | -0.79635 |
| Morf4l1 | 0.49591 |
| Morf4l2 | -0.39697 |
| Morn2 | 1.95049 |
| Morn3 | 0.62906 |
| Morn3 | -0.04207 |
| Mos | -0.33437 |
| Mosc2 | -0.75605 |
| Mospd1 | -0.36636 |
| Mospd1 | 1.52375 |
| Mospd1 | -0.66496 |
| Mospd3 | -0.579 |
| Mospd3 | -0.64979 |
| Mov10 | 1.51093 |
| Mov10l1 | 1.71556 |
| Moxd1 | 0.83667 |
| Moxd1 | -0.64979 |
| Mpdu1 | 0.47721 |
| Mpdz | -0.55229 |
| Mpdz | -0.71475 |
| Mpg | -0.34992 |
| Mpg | 2.02396 |
| Mphosph6 | -0.61613 |
| Mpnd | 0.16945 |
| Mpo | 0.73056 |
| Mpo | 1.23618 |
| Mpp1 | -0.74671 |
| Mpp2 | 0.38625 |
| Mpp3 | 0.01199 |
| Mpp3 | 2.5234 |
| Mpp5 | 2.1693 |
| Mpp6 | 2.46139 |
| Mppe1 | -0.00071 |
| Mpped1 | -0.1603 |
| Mpst | 1.12921 |
| Mpv17 | 0.15946 |
| Mpv17 | -0.63239 |
| Mpv17 | 1.07796 |
| Mpv17 | -0.68126 |
| Mpv17l | 0.25923 |
| Mpv17l | 0.26604 |
| Mpv17l | 2.88176 |
| Mpz | 1.04191 |
| Mr1 | 0.65064 |
| Mrap | 2.27499 |
| Mras | -0.35416 |
| Mrc1 | -0.74393 |
| Mrc2 | -0.16869 |
| Mrc2 | 1.09803 |
| Mre11a | 0.19014 |
| Mre11a | -0.59147 |
| Mre11a | -0.55984 |
| Mreg | 0.57598 |
| Mrgpra2 | -0.54256 |
| Mrgpra2 | 2.33355 |
| Mrgprb2 | -0.13656 |
| Mrgpre | -0.52269 |
| Mrgprf | -0.76549 |
| Mrgprg | 0.21951 |
| Mrm1 | -0.70227 |
| Mro | -0.66072 |
| Mrp63 | 0.0128 |
| Mrpl1 | 0.94353 |
| Mrpl1 | -0.83952 |
| Mrpl10 | -0.4717 |
| Mrpl10 | 2.79939 |
| Mrpl11 | -0.55079 |
| Mrpl12 | -0.65062 |
| Mrpl13 | 0.28226 |
| Mrpl13 | 0.84887 |
| Mrpl14 | -0.51183 |
| Mrpl15 | 1.00743 |
| Mrpl16 | -0.63403 |
| Mrpl17 | 1.19037 |
| Mrpl18 | 0.32754 |
| Mrpl2 | -0.22962 |
| Mrpl20 | -0.67867 |
| Mrpl22 | -0.41234 |
| Mrpl23 | 0.42912 |
| Mrpl24 | -0.49896 |
| Mrpl27 | -0.54108 |
| Mrpl28 | -0.71475 |
| Mrpl3 | 0.37316 |
| Mrpl3 | -0.56364 |
| Mrpl30 | 0.30385 |
| Mrpl33 | -0.46963 |
| Mrpl33 | -0.6105 |
| Mrpl34 | 1.63037 |
| Mrpl36 | -0.70938 |
| Mrpl37 | 0.51636 |
| Mrpl37 | -0.08312 |
| Mrpl38 | 0.78339 |
| Mrpl39 | -0.27799 |
| Mrpl40 | -0.30978 |
| Mrpl43 | 1.47676 |
| Mrpl43 | 0.73835 |
| Mrpl45 | -0.56669 |
| Mrpl46 | 0.10372 |
| Mrpl47 | -0.61291 |
| Mrpl47 | 0.40502 |
| Mrpl48 | -0.03863 |
| Mrpl48 | 0.37041 |
| Mrpl48 | -0.38185 |
| Mrpl49 | 2.09246 |
| Mrpl49 | -0.62748 |
| Mrpl49 | 2.15798 |
| Mrpl49 | 1.10722 |
| Mrpl50 | -0.64812 |
| Mrpl51 | 0.44628 |
| Mrpl51 | -0.34992 |
| Mrpl51 | -0.4807 |
| Mrpl52 | -0.2392 |
| Mrpl53 | 0.27801 |
| Mrpl54 | 0.13913 |
| Mrpl55 | -0.13226 |
| Mrpl9 | -0.48908 |
| Mrps10 | 0.77402 |
| Mrps10 | 2.11018 |
| Mrps11 | -0.40719 |
| Mrps11 | 0.82077 |
| Mrps12 | -0.56899 |
| Mrps14 | 0.70104 |
| Mrps15 | -0.66411 |
| Mrps16 | -0.27409 |
| Mrps17 | -0.47722 |
| Mrps18a | -0.34691 |
| Mrps18b | 0.52292 |
| Mrps18b | 0.97803 |
| Mrps18c | -0.54331 |
| Mrps2 | -0.59383 |
| Mrps21 | -0.34331 |
| Mrps22 | 1.13869 |
| Mrps23 | 2.20405 |
| Mrps24 | 1.18584 |
| Mrps25 | -0.46689 |
| Mrps25 | 0.343 |
| Mrps25 | 1.561 |
| Mrps26 | 0.30414 |
| Mrps26 | -0.42732 |
| Mrps27 | 2.70968 |
| Mrps28 | -0.64478 |
| Mrps30 | 0.62133 |
| Mrps31 | 1.85547 |
| Mrps31 | -0.34511 |
| Mrps31 | -0.72196 |
| Mrps33 | 0.27953 |
| Mrps33 | -0.78951 |
| Mrps34 | 0.82212 |
| Mrps35 | 1.6049 |
| Mrps5 | 3.02604 |
| Mrps6 | -0.64562 |
| Mrps7 | -0.66157 |
| Mrps7 | 0.87788 |
| Mrps9 | -0.2254 |
| Mrrf | 0.49061 |
| Mrrf | 0.10629 |
| Mrto4 | -0.50967 |
| Mrvi1 | -0.30173 |
| Ms4a1 | 3.77819 |
| Ms4a10 | -0.49119 |
| Ms4a13 | 1.85525 |
| Ms4a5 | 0.94324 |
| Ms4a6d | -0.41622 |
| Ms4a6d | 0.58889 |
| Ms4a7 | -0.62667 |
| Ms4a8a | 1.79866 |
| Msc | -0.44187 |
| Msh2 | 0.07072 |
| Msh3 | 1.45509 |
| Msh3 | -0.40783 |
| Msh4 | 1.19273 |
| Msh5 | 0.83584 |
| Msh5 | -0.5954 |
| Msh6 | 0.48357 |
| Msh6 | 0.88735 |
| Msi2 | -0.1872 |
| Msi2h | 0.54301 |
| Msln | -0.17765 |
| Msn | -0.49472 |
| Msr2 | -0.74485 |
| Msrb2 | -0.59698 |
| Msrb2 | 3.75475 |
| Msrb3 | 1.56739 |
| Mst1r | -0.68126 |
| Msto1 | -0.78562 |
| Msx1 | 2.6383 |
| Mt1 | -0.15443 |
| Mt3 | 0.88986 |
| Mta1 | 0.15737 |
| Mta2 | 0.47451 |
| Mta3 | 1.20759 |
| Mta3 | 0.6549 |
| Mtag2 | 2.22688 |
| Mtap | -0.70671 |
| Mtap | -0.11762 |
| Mtap1b | 0.00995 |
| Mtap1b | -0.2691 |
| Mtap1b | 0.94751 |
| Mtap1b | 1.31912 |
| Mtap1s | -0.67953 |
| Mtap2 | 1.06381 |
| Mtap2 | -0.02624 |
| Mtap2 | -0.34872 |
| Mtap4 | 0.9106 |
| Mtap6 | 1.5297 |
| Mtap6 | -0.66242 |
| Mtap7 | 3.04182 |
| Mtap7d1 | -0.63568 |
| Mtap7d1 | -0.67867 |
| Mtap7d2 | -0.6089 |
| Mtap7d3 | 1.94781 |
| Mtbp | 0.60312 |
| Mtbp | 2.21592 |
| Mtbp | 0.05648 |
| Mtch1 | 2.42855 |
| Mtch1 | 0.1004 |
| Mtch2 | -0.7495 |
| Mtch2 | -0.2092 |
| Mtch2 | -0.19075 |
| Mtcp1 | 0.18644 |
| Mtdh | 1.27643 |
| Mterf | 4.10003 |
| Mterf | -0.66837 |
| Mterfd1 | -0.4306 |
| Mterfd2 | -0.25757 |
| Mterfd2 | 2.29669 |
| Mterfd2 | 3.59527 |
| Mterfd3 | -0.30058 |
| Mterfd3 | -0.31907 |
| Mtf1 | 0.25861 |
| Mtf1 | 1.90809 |
| Mtf2 | -0.67781 |
| Mtfmt | 0.33473 |
| Mtfmt | -0.68734 |
| Mtg1 | 0.07681 |
| Mthfd1 | 0.67213 |
| Mthfd2 | -0.19891 |
| Mthfs | -0.39443 |
| Mthfsd | -0.60173 |
| Mthfsd | -0.19839 |
| Mtif3 | -0.66667 |
| Mtl5 | 0.54049 |
| Mtl5 | 1.76611 |
| Mtmr1 | -0.41557 |
| Mtmr11 | -0.64645 |
| Mtmr11 | -0.80621 |
| Mtmr12 | -0.05249 |
| Mtmr14 | 0.48454 |
| Mtmr14 | 1.595 |
| Mtmr14 | -0.48628 |
| Mtmr14 | -0.75511 |
| Mtmr2 | -0.52488 |
| Mtmr2 | -0.61451 |
| Mtmr2 | -0.04814 |
| Mtmr3 | 0.01443 |
| Mtmr4 | 0.17833 |
| Mtmr9 | 0.82481 |
| Mto1 | 0.3229 |
| Mtpn | -0.40206 |
| Mtrf1 | -0.73013 |
| Mtrf1 | 0.27983 |
| Mtrf1l | -0.62585 |
| Mtrr | -0.49684 |
| Mtss1 | -0.67008 |
| Mttp | 2.39294 |
| Mtus1 | -0.152 |
| Mtus1 | -0.4919 |
| Mtvr2 | 0.5865 |
| Mtvr2 | -0.56288 |
| Mtx1 | 0.41005 |
| Mtx1 | -0.22434 |
| Mtx1 | 3.27969 |
| Mtx2 | -0.02794 |
| Muc1 | 3.375 |
| Muc1 | -0.23866 |
| Muc1 | -0.39001 |
| Muc1 | 0.10519 |
| Muc13 | 0.00873 |
| Muc5ac | 3.63608 |
| Mug2 | 0.26047 |
| Mug2 | 0.87438 |
| Mug4 | -0.8344 |
| Mum1 | -0.17515 |
| Mup1 | 2.23854 |
| Mup2 | -0.44254 |
| Mup2 | 1.67867 |
| Mup3 | 0.43196 |
| Mupcdh | 0.40741 |
| Mus81 | -0.57822 |
| Mus81 | 1.922 |
| Mustn1 | -0.69171 |
| Mustn1 | 3.2349 |
| Muted | 2.16676 |
| Muted | 0.65934 |
| Mutyh | 0.66415 |
| Mvd | -0.76549 |
| Mvp | -0.64895 |
| Mx2 | -0.6856 |
| Mxd1 | 2.72324 |
| Mxd4 | -0.66752 |
| Mxi1 | -0.44388 |
| Mxra7 | 1.44454 |
| Mxra8 | 0.95775 |
| Mxra8 | -0.47376 |
| Myadm | 1.59048 |
| Mybbp1a | -0.27132 |
| Mybbp1a | 0.60055 |
| Mybbp1a | 1.85119 |
| Mybl2 | 0.05337 |
| Mybpc2 | -0.37129 |
| Mybpc3 | -0.66837 |
| Myc | -0.59383 |
| Mycbp | 0.62656 |
| Mycbp2 | -0.7495 |
| Mycbp2 | -0.24456 |
| Mycbpap | -0.53885 |
| Mycl1 | -0.37562 |
| Myd116 | 0.72701 |
| Myd88 | -0.75324 |
| Myef2 | -0.35659 |
| Myg1 | 2.30448 |
| Myg1 | 0.04713 |
| Myg1 | 2.51889 |
| Myh10 | -0.32375 |
| Myh11 | 2.03037 |
| Myh8 | 1.76611 |
| Myh9 | 1.63602 |
| Myl1 | 1.00412 |
| Myl2 | -0.56745 |
| Myl4 | -0.35659 |
| Myl6 | -0.05468 |
| Myl6b | -0.53 |
| Mylc2b | -0.61855 |
| Mylc2b | -0.6856 |
| Mylc2b | -0.63815 |
| Mylc2pl | -0.13799 |
| Mylk | 1.77189 |
| Mylk | 0.97661 |
| Mynn | -0.50824 |
| Myo10 | -0.81018 |
| Myo10 | 1.39021 |
| Myo15 | 0.08097 |
| Myo18a | 1.55061 |
| Myo1b | -0.70493 |
| Myo1c | -0.51039 |
| Myo1c | 0.61502 |
| Myo1f | 0.22145 |
| Myo1g | -0.34211 |
| Myo3b | -0.45058 |
| Myo5a | 0.2725 |
| Myo5b | -0.6804 |
| Myo6 | -0.48418 |
| Myo7a | 1.87979 |
| Myo9a | -0.17266 |
| Myo9b | -0.53515 |
| Myo9b | 0.53405 |
| Myoc | -0.45599 |
| Myocd | -0.21962 |
| Myocd | 0.91259 |
| Myom1 | -0.34631 |
| Myom1 | 4.76048 |
| Myrip | -0.41622 |
| Mysm1 | 1.97355 |
| Myst1 | 2.3327 |
| Myst2 | -0.31325 |
| Myst4 | -0.60252 |
| Myst4 | -0.4717 |
| Myst4 | 0.09409 |
| Myt1 | 0.69796 |
| Myt1l | -0.30287 |
| Myt1l | 0.05842 |
| Mzf1 | -0.16473 |
| Mzf1 | -0.58912 |
| N28178 | -0.19482 |
| N4bp2 | 0.67034 |
| N6amt1 | -0.75511 |
| N6amt1 | -0.24672 |
| N6amt2 | -0.18317 |
| Naalad2 | 0.1851 |
| Nab1 | -0.59856 |
| Nab1 | -0.60093 |
| Nab1 | -0.5322 |
| Nab1 | -0.48908 |
| Nab2 | 2.31848 |
| Naca | -0.10829 |
| Naca | -0.77215 |
| Naca | -0.55681 |
| Nadk | 0.98934 |
| Nadsyn1 | 0.13272 |
| Nadsyn1 | -0.15687 |
| Naga | -0.65988 |
| Nagk | 1.39943 |
| Nagk | 0.41689 |
| Nagk | -0.54331 |
| Nagk | -0.67008 |
| Naglu | -0.07325 |
| Nagpa | 2.52931 |
| Nags | -0.62585 |
| Nags | 0.68357 |
| Naif1 | 1.94429 |
| Naif1 | -0.69697 |
| Naip5 | -0.53959 |
| Nalp10 | -0.63733 |
| Nampt | -0.53737 |
| Nanos1 | -0.67008 |
| Nanos2 | -0.63321 |
| Nans | 2.1879 |
| Nap1l1 | -0.56136 |
| Nap1l1 | -0.63568 |
| Nap1l3 | -0.0382 |
| Nap1l4 | -0.18468 |
| Nap1l5 | -0.44121 |
| Napa | 3.46479 |
| Napa | -0.03263 |
| Napa | 1.84528 |
| Napb | -0.42012 |
| Napepld | -0.02666 |
| Napg | 0.97075 |
| Napg | 0.13058 |
| Naprt1 | 0.0124 |
| Naprt1 | -0.4104 |
| Napsa | -0.0451 |
| Napsa | -0.19993 |
| Napsa | 0.94161 |
| Narf | -0.48978 |
| Narfl | 1.96314 |
| Narg1 | -0.27353 |
| Narg1 | -0.56975 |
| Narg1l | 1.41777 |
| Narg1l | -0.37873 |
| Narg1l | 2.18232 |
| Narg2 | -0.40655 |
| Nars | 0.23624 |
| Nars2 | -0.17715 |
| Nasp | -0.58365 |
| Nasp | -0.20147 |
| Nasp | -0.6523 |
| Nasp | -0.58599 |
| Nat1 | -0.76359 |
| Nat10 | -0.26195 |
| Nat14 | 0.7811 |
| Nat2 | -0.59461 |
| Nat5 | -0.37253 |
| Nat5 | -0.6105 |
| Nat6 | 1.69837 |
| Nat6 | 3.06765 |
| Nav1 | 1.25983 |
| Nav1 | -0.71296 |
| Nbl1 | 0.29132 |
| Nbr1 | 1.05313 |
| Nbr1 | 0.58541 |
| Ncald | -0.14714 |
| Ncam1 | -0.44521 |
| Ncam1 | 0.44017 |
| Ncam2 | 0.60781 |
| Ncan | 1.32421 |
| Ncapd2 | 1.98504 |
| Ncapd2 | 0.09929 |
| Ncapd2 | 0.49975 |
| Ncapd3 | 1.74505 |
| Ncapg2 | -0.426 |
| Ncapg2 | 2.82218 |
| Ncaph | 0.55796 |
| Ncaph2 | 1.46092 |
| Ncaph2 | -0.48838 |
| Ncaph2 | -0.63239 |
| Ncbp2 | 0.72701 |
| Ncbp2 | 0.2221 |
| Ncdn | 1.11987 |
| Ncdn | -0.42143 |
| Ncdn | -0.77502 |
| Ncdn | -0.70582 |
| Ncf4 | -0.2344 |
| Nck1 | 0.11725 |
| Nckap1 | 0.63218 |
| Nckap1l | 0.55255 |
| Nckipsd | 0.79353 |
| Ncl | -0.46963 |
| Ncl | -0.42732 |
| Ncoa2 | 1.1889 |
| Ncoa2 | 3.18814 |
| Ncoa4 | 0.11906 |
| Ncoa5 | 0.53452 |
| Ncoa6 | -0.71385 |
| Ncoa6 | -0.6097 |
| Ncoa6 | -0.5448 |
| Ncor1 | -0.60093 |
| Ncstn | 2.53097 |
| Ncstn | 0.75064 |
| Ncstn | 2.16218 |
| Ndc80 | -0.69785 |
| Nde1 | 1.74918 |
| Nde1 | 0.6076 |
| Ndel1 | 3.42812 |
| Ndfip1 | 1.78859 |
| Ndfip2 | -0.6523 |
| Ndg2 | -0.6226 |
| Ndn | -0.35476 |
| Ndor1 | -0.71565 |
| Ndor1 | 2.2326 |
| Ndor1 | -0.37624 |
| Ndrg1 | -0.51183 |
| Ndrg2 | 0.07756 |
| Ndrg3 | -0.12326 |
| Ndrg4 | -0.56899 |
| Ndrl | 2.21917 |
| Ndst1 | -0.29659 |
| Ndst1 | -0.4807 |
| Ndst4 | -0.3179 |
| Ndufa1 | -0.44054 |
| Ndufa10 | 1.48957 |
| Ndufa12 | 1.28135 |
| Ndufa13 | -0.6057 |
| Ndufa2 | 2.00223 |
| Ndufa3 | 0.04517 |
| Ndufa4 | -0.37438 |
| Ndufa5 | 2.4554 |
| Ndufa5 | 0.45537 |
| Ndufa6 | -0.32082 |
| Ndufa7 | 0.45008 |
| Ndufa8 | -0.71835 |
| Ndufa8 | -0.53441 |
| Ndufa9 | 0.92458 |
| Ndufaf1 | 1.82987 |
| Ndufb10 | -0.24726 |
| Ndufb11 | -0.65734 |
| Ndufb2 | 0.06228 |
| Ndufb3 | -0.6089 |
| Ndufb4 | 0.41242 |
| Ndufb5 | -0.68647 |
| Ndufb6 | 0.80322 |
| Ndufb6 | 1.7586 |
| Ndufb7 | 0.7869 |
| Ndufb8 | 1.07412 |
| Ndufb9 | -0.3831 |
| Ndufc1 | -0.32904 |
| Ndufc1 | -0.33081 |
| Ndufc2 | 0.88168 |
| Ndufs2 | 0.38761 |
| Ndufs2 | 1.124 |
| Ndufs3 | 2.61478 |
| Ndufs4 | 1.36329 |
| Ndufs4 | -0.63075 |
| Ndufs4 | -0.66923 |
| Ndufs4 | 0.97832 |
| Ndufs6 | 1.90819 |
| Ndufs7 | 0.92247 |
| Ndufs8 | -0.73563 |
| Ndufv1 | -0.4717 |
| Ndufv2 | 0.91716 |
| Necab1 | 3.03339 |
| Necab2 | 0.9164 |
| Necab3 | 2.43487 |
| Necap1 | 0.41426 |
| Necap1 | 0.09446 |
| Necap1 | 1.84777 |
| Necap2 | -0.50752 |
| Nedd1 | 2.41543 |
| Nedd4 | 0.61881 |
| Nedd4l | 0.14478 |
| Nedd8 | -0.29318 |
| Nedd9 | -0.46826 |
| Nedd9 | -0.6856 |
| Nefm | -0.77694 |
| Negr1 | -0.44655 |
| Negr1 | 1.2638 |
| Neil1 | -0.6081 |
| Neil2 | 1.5311 |
| Nek11 | 0.86429 |
| Nek3 | -0.5899 |
| Nek3 | -0.53589 |
| Nek3 | 2.60257 |
| Nek4 | 0.83998 |
| Nek5 | 1.55137 |
| Nek6 | -0.66923 |
| Nek8 | -0.4919 |
| Nek9 | 1.01156 |
| Nek9 | 0.87007 |
| Nelf | -0.55606 |
| Nelf | -0.22382 |
| Nell2 | -0.45058 |
| Nell2 | -0.63486 |
| Nell2 | -0.12751 |
| Nell2 | 1.07143 |
| Nenf | -0.66327 |
| Neo1 | -0.19993 |
| Nes | 1.59019 |
| Net1 | -0.7076 |
| Net1 | -0.59461 |
| Neto1 | 0.43144 |
| Neto1 | -0.72558 |
| Neto1 | 1.82965 |
| Neu1 | -0.60252 |
| Neu2 | -0.49542 |
| Neu3 | -0.4662 |
| Neu4 | 1.36945 |
| Neu4 | -0.6523 |
| Neu4 | 1.22352 |
| Neurl | -0.76549 |
| Neurod1 | 0.76671 |
| Neurod2 | 1.63302 |
| Neurod4 | -0.68386 |
| Neurog1 | 0.93299 |
| Neurog2 | 2.0995 |
| Nf1 | -0.62422 |
| Nfasc | -0.67436 |
| Nfasc | 2.68628 |
| Nfasc | -0.70227 |
| Nfat5 | 0.73298 |
| Nfat5 | -0.09311 |
| Nfatc1 | -0.44454 |
| Nfatc1 | 0.6146 |
| Nfatc2 | 0.23144 |
| Nfatc2 | 1.44616 |
| Nfatc2ip | -0.6073 |
| Nfatc3 | -0.73013 |
| Nfatc4 | -0.6065 |
| Nfe2 | 1.48261 |
| Nfe2l1 | -0.33615 |
| Nfe2l2 | -0.3092 |
| Nfe2l3 | -0.32963 |
| Nfib | 1.20014 |
| Nfic | 0.12951 |
| Nfic | 0.91014 |
| Nfic | -0.6105 |
| Nfix | -0.33555 |
| Nfix | 0.2979 |
| Nfkb1 | -0.20713 |
| Nfkb2 | -0.3919 |
| Nfkbia | -0.50323 |
| Nfkbib | 0.64555 |
| Nfkbid | 1.01853 |
| Nfkbie | 1.95118 |
| Nfkbil1 | -0.44722 |
| Nfkbil2 | 1.97408 |
| Nfkbiz | 0.82296 |
| Nfrkb | -0.47515 |
| Nfrkb | 1.0577 |
| Nfs1 | 0.80614 |
| Nfs1 | 2.02501 |
| Nfs1 | 0.09111 |
| Nfu1 | -0.1009 |
| Nfx1 | -0.7523 |
| Nfya | 1.33131 |
| Nfyb | -0.0464 |
| Nfyc | 1.31351 |
| Ngb | 0.85637 |
| Ngb | 0.33272 |
| Ngef | 1.54056 |
| Ngfb | -0.35659 |
| Ngfb | -0.22276 |
| Ngfr | -0.72649 |
| Ngfrap1 | -0.56516 |
| Ngp | -0.56593 |
| Nhedc1 | -0.23866 |
| Nhej1 | -0.11621 |
| Nhlrc1 | 0.86365 |
| Nhlrc2 | -0.4926 |
| Nhlrc2 | -0.71296 |
| Nhp2l1 | 1.61207 |
| Nhsl1 | -0.73379 |
| Niban | -0.73655 |
| Nicn1 | -0.6113 |
| Nicn1 | 3.06296 |
| Nid1 | 0.05648 |
| Nid2 | 0.60269 |
| Nif3l1 | -0.68908 |
| Nin | 0.13022 |
| Ninj1 | -0.31965 |
| Ninj2 | -0.67781 |
| Nip7 | -0.31616 |
| Nipa1 | 1.26442 |
| Nipbl | -0.35234 |
| Nipbl | 3.35101 |
| Nipsnap1 | -0.11715 |
| Nipsnap3a | -0.77694 |
| Nipsnap3a | 0.26326 |
| Nisch | 0.25861 |
| Nit1 | 0.14759 |
| Nit2 | -0.22276 |
| Nit2 | -0.65314 |
| Nkain3 | -0.22751 |
| Nkain4 | -0.84468 |
| Nkd2 | -0.68386 |
| Nkg7 | -0.13895 |
| Nkiras1 | -0.6804 |
| Nkiras2 | 1.97196 |
| Nkrf | 0.55187 |
| Nktr | -0.11809 |
| Nkx1-2 | 0.92035 |
| Nkx2-1 | -0.3938 |
| Nkx2-2 | -0.85298 |
| Nkx2-2 | -0.20147 |
| Nkx2-3 | -0.86453 |
| Nkx2-4 | 0.9786 |
| Nkx2-6 | 2.79157 |
| Nkx3-1 | -0.22856 |
| Nkx6-1 | 2.14128 |
| Nle1 | 0.60845 |
| Nlgn1 | 0.8812 |
| Nlgn2 | 0.87295 |
| Nlk | -0.60252 |
| Nln | -0.28865 |
| Nln | -0.59777 |
| Nln | -0.82524 |
| Nlrp14 | 0.43863 |
| Nlrp4a | -0.30632 |
| Nlrp4c | 2.42349 |
| Nlrp5 | -0.1356 |
| Nlrp5 | 1.81746 |
| Nlrp5 | -0.38623 |
| Nlrp9a | -0.44722 |
| Nlrp9c | 0.65389 |
| Nlrx1 | -0.16424 |
| Nlrx1 | 0.08624 |
| Nmb | 0.42185 |
| Nmbr | -0.49542 |
| Nmd3 | -0.78175 |
| Nme1 | 0.63155 |
| Nme2 | 1.9722 |
| Nme2 | -0.70227 |
| Nme2 | -0.35963 |
| Nme3 | 1.06044 |
| Nme4 | 2.47848 |
| Nme4 | 0.29551 |
| Nme4 | -0.77406 |
| Nme5 | 0.62948 |
| Nme7 | -0.23493 |
| Nme7 | 0.72832 |
| Nme7 | -0.63239 |
| Nme7 | 2.30907 |
| Nme7 | 0.76617 |
| Nmnat1 | 0.88798 |
| Nmnat2 | -0.42273 |
| Nmnat3 | 1.09365 |
| Nmral1 | 0.27953 |
| Nmt1 | -0.32786 |
| Nmu | -0.6226 |
| Nmur2 | 0.00463 |
| Nnat | -0.34872 |
| Nnat | -0.80225 |
| Nnmt | 0.09223 |
| Nnmt | -0.72286 |
| Nob1 | -0.48978 |
| Nob1 | 1.16541 |
| Nobox | 0.72701 |
| Noc3l | -0.3578 |
| Noc4l | -0.14232 |
| Noc4l | -0.68821 |
| Nod1 | 0.43299 |
| Nog | -0.69171 |
| Nol1 | 1.76109 |
| Nol1 | -0.57668 |
| Nol1 | 2.53294 |
| Nol10 | 0.18307 |
| Nol11 | -0.31499 |
| Nol12 | 2.08186 |
| Nol14 | 3.27854 |
| Nol3 | -0.51978 |
| Nol4 | 1.65915 |
| Nol4 | 0.45386 |
| Nol4 | 0.78128 |
| Nol5 | 1.8683 |
| Nol5a | 3.35558 |
| Nol6 | 0.00463 |
| Nol7 | -0.63321 |
| Nol9 | -0.62098 |
| Nola1 | 3.20769 |
| Nola2 | -0.25976 |
| Nola3 | -0.61451 |
| Nola3 | 0.66934 |
| Nolc1 | 1.06769 |
| Nomo1 | -0.55455 |
| Nono | 4.0439 |
| Nope | -0.6121 |
| Nos1 | 1.12679 |
| Nos1ap | -0.50752 |
| Nos2 | 1.79942 |
| Nos3 | 0.01077 |
| Nos3 | -0.62504 |
| Nos3 | -0.68299 |
| Nosip | 0.41531 |
| Nosip | -0.3957 |
| Nosip | -0.23866 |
| Nostrin | 0.91213 |
| Notch1 | -0.68908 |
| Notch3 | 0.62112 |
| Notch4 | -0.50967 |
| Notch4 | 1.14298 |
| Notum | -0.67781 |
| Nova2 | 1.01703 |
| Nox4 | 0.73761 |
| Noxo1 | -0.40847 |
| Noxo1 | 2.37579 |
| Npal2 | 0.52315 |
| Npas1 | 0.83783 |
| Npas2 | -0.28022 |
| Npas4 | -0.06171 |
| Npb | -0.41493 |
| Npc2 | 1.35757 |
| Npcd | 1.42384 |
| Npcd | -0.72831 |
| Npdc1 | 0.2985 |
| Npepl1 | 2.68339 |
| Npff | -0.59461 |
| Nphp1 | -0.6097 |
| Nphp1 | -0.79831 |
| Nphp3 | -0.28696 |
| Nphp4 | -0.71925 |
| Nphs2 | -0.55229 |
| Nploc4 | 0.70965 |
| Npm1 | -0.5954 |
| Npm2 | -0.39888 |
| Npm3 | -0.37562 |
| Npm3 | 0.63611 |
| Npm3-ps1 | 1.05129 |
| Npr1 | 2.26984 |
| Npr2 | -0.31616 |
| Npr3 | -0.36391 |
| Npsr1 | -0.4306 |
| Nptn | -0.56669 |
| Nptx1 | -0.59856 |
| Nptx2 | 0.53105 |
| Nptxr | 2.27419 |
| Npy | 0.07756 |
| Npy1r | -0.40655 |
| Npy2r | -0.00984 |
| Npy5r | -0.63981 |
| Nqo1 | -0.64229 |
| Nqo2 | 1.31123 |
| Nqo2 | -0.65734 |
| Nqo2 | -0.66923 |
| Nr1d1 | 0.26943 |
| Nr1d2 | -0.55757 |
| Nr1h2 | -0.68996 |
| Nr1h2 | -0.15054 |
| Nr1h3 | -0.56364 |
| Nr1h4 | -0.77406 |
| Nr1h5 | 0.48284 |
| Nr1i2 | -0.39507 |
| Nr2c1 | 2.3238 |
| Nr2c2ap | 1.34534 |
| Nr2e1 | 3.67136 |
| Nr2f2 | -0.72467 |
| Nr2f2 | -0.39443 |
| Nr2f6 | 0.02573 |
| Nr2f6 | 0.8842 |
| Nr4a2 | 0.5784 |
| Nr4a3 | 0.81365 |
| Nr4a3 | -0.17117 |
| Nr5a1 | -0.69697 |
| Nr5a2 | 1.17373 |
| Nr5a2 | 2.44112 |
| Nr5a2 | 0.14478 |
| Nr6a1 | -0.22119 |
| Nradd | 0.32927 |
| Nrap | -0.45666 |
| Nrarp | 0.27618 |
| Nras | -0.37935 |
| Nrbf2 | -0.66242 |
| Nrbp1 | -0.1603 |
| Nrbp2 | 3.46088 |
| Nrcam | 1.09227 |
| Nrd1 | -0.51978 |
| Nrf1 | -0.71745 |
| Nrg1 | -0.30345 |
| Nrg3 | -0.73105 |
| Nrg4 | 0.15876 |
| Nrgn | 1.99304 |
| Nrip2 | -0.53589 |
| Nrip3 | 0.93627 |
| Nrl | -0.41234 |
| Nrm | -0.01905 |
| Nrn1 | -0.62585 |
| Nrn1l | -0.72196 |
| Nrp1 | -0.46347 |
| Nrp1 | 1.30637 |
| Nrp2 | 0.6966 |
| Nrp2 | -0.39507 |
| Nrsn2 | -0.36882 |
| Nrsn2 | -0.13751 |
| Nrtn | -0.11481 |
| Nrxn1 | 0.64555 |
| Nrxn1 | 3.13267 |
| Nrxn1 | 1.24526 |
| Nrxn2 | 0.92096 |
| Nrxn3 | -0.41234 |
| Nrxn3 | -0.09998 |
| Nsbp1 | 0.5359 |
| Nsd1 | -0.6065 |
| Nsd1 | -0.46757 |
| Nsdhl | -0.64562 |
| Nsdhl | 4.65737 |
| Nsdhl | -0.74578 |
| Nsf | -0.64312 |
| Nsfl1c | -0.54256 |
| Nsg1 | 0.63093 |
| Nsg2 | 0.12015 |
| Nsl1 | -0.14714 |
| Nsmaf | -0.70315 |
| Nsmaf | -0.46278 |
| Nsmce1 | -0.52123 |
| Nsmce2 | -0.44722 |
| Nsmce2 | 2.47617 |
| Nsmce2 | -0.18015 |
| Nsun2 | -0.53737 |
| Nsun4 | -0.44187 |
| Nsun5 | 2.45494 |
| Nsun5 | 0.61755 |
| Nsun5 | -0.60411 |
| Nsun5 | -0.45666 |
| Nt5c | -0.66327 |
| Nt5c1b | -0.57282 |
| Nt5c2 | 1.04085 |
| Nt5c2 | -0.46074 |
| Nt5c2 | -0.6073 |
| Nt5c3 | 0.7106 |
| Nt5c3l | -0.61291 |
| Nt5c3l | -0.7617 |
| Nt5dc3 | -0.20301 |
| Nt5dc3 | -0.76454 |
| Nt5dc3 | 1.75567 |
| Nt5e | -0.58055 |
| Nt5e | -0.65819 |
| Nt5m | -0.53515 |
| Nt5m | 0.63259 |
| Ntan1 | -0.33555 |
| Ntf3 | -0.15687 |
| Nthl1 | 0.18644 |
| Ntn2l | -0.51471 |
| Ntn2l | 0.14724 |
| Ntn4 | -0.2118 |
| Ntng1 | -0.38686 |
| Ntng1 | -0.5068 |
| Ntng1 | 0.68767 |
| Ntrk2 | 0.31883 |
| Ntrk2 | 0.02573 |
| Ntrk2 | -0.14232 |
| Ntrk3 | 1.48138 |
| Ntrk3 | 1.17212 |
| Ntrk3 | 0.53866 |
| Nts | 0.56534 |
| Ntsr1 | -0.37438 |
| Ntsr2 | 2.40639 |
| Nuak1 | -0.62912 |
| Nuak1 | -0.46074 |
| Nuak2 | 1.64659 |
| Nub1 | -0.08719 |
| Nubp1 | -0.12609 |
| Nubp2 | -0.12609 |
| Nubp2 | 1.20293 |
| Nubp2 | 1.58923 |
| Nucb1 | 0.2208 |
| Nucb1 | 0.34385 |
| Nucks1 | -0.09631 |
| Nudc | -0.66496 |
| Nudc | -0.28752 |
| Nudcd2 | -0.30116 |
| Nudcd3 | 1.26025 |
| Nudc-ps1 | 1.13893 |
| Nudt1 | 0.38598 |
| Nudt10 | 0.68435 |
| Nudt11 | 0.90075 |
| Nudt12 | 0.48576 |
| Nudt13 | 1.11534 |
| Nudt14 | 0.76006 |
| Nudt16l1 | -0.46826 |
| Nudt18 | 1.42725 |
| Nudt19 | -0.43589 |
| Nudt19 | 0.67589 |
| Nudt2 | -0.27187 |
| Nudt21 | -0.50323 |
| Nudt22 | 0.55142 |
| Nudt4 | 1.52241 |
| Nudt5 | 1.24249 |
| Nudt5 | 0.02412 |
| Nudt6 | 0.2874 |
| Nudt7 | -0.53441 |
| Nudt8 | -0.22962 |
| Nudt9 | -0.63321 |
| Nufip1 | -0.66157 |
| Numa1 | -0.22014 |
| Numb | 0.51894 |
| Numb | 0.05026 |
| Numb | -0.72831 |
| Numb | 0.8992 |
| Numbl | -0.1559 |
| Nup107 | -0.12279 |
| Nup133 | -0.48768 |
| Nup133 | 1.72929 |
| Nup155 | 0.81416 |
| Nup155 | -0.00278 |
| Nup160 | -0.51978 |
| Nup210 | -0.24564 |
| Nup37 | 2.26704 |
| Nup43 | 2.04103 |
| Nup50 | -0.58132 |
| Nup50 | -0.26524 |
| Nup50 | 1.26816 |
| Nup62 | 1.34296 |
| Nup85 | -0.55681 |
| Nup88 | 2.21563 |
| Nup88 | -0.55681 |
| Nup88 | -0.37873 |
| Nup93 | -0.42994 |
| Nup98 | 0.92443 |
| Nup98 | 0.09446 |
| Nupl1 | -0.02921 |
| Nupl1 | -0.53073 |
| Nupl1 | -0.59856 |
| Nupl2 | -0.60173 |
| Nupr1 | 0.08774 |
| Nus1 | 1.95418 |
| Nusap1 | 0.04359 |
| Nut | 0.31122 |
| Nutf2 | -0.31732 |
| Nvl | -0.56822 |
| Nwd1 | -0.19635 |
| Nxf1 | -0.64812 |
| Nxf2 | -0.72105 |
| Nxf3 | -0.49401 |
| Nxph1 | 1.78688 |
| Nxph2 | -0.30402 |
| Nxph3 | 1.40463 |
| Nxt1 | 0.44246 |
| Nxt1 | 1.07117 |
| Nxt2 | -0.08267 |
| Nyx | -0.52561 |
| Oaf | -0.36759 |
| Oas1b | 0.71307 |
| Oas1b | -0.55229 |
| Oas1c | -0.43655 |
| Oas1d | 0.51683 |
| Oas1d | -0.22276 |
| Oas1e | 0.48186 |
| Oas1g | -0.62667 |
| Oas1g | 1.21102 |
| Oas2 | -0.01067 |
| Oasl1 | 0.86927 |
| Oasl2 | 0.09818 |
| Oasl2 | -0.48348 |
| Oat | 0.49591 |
| Oaz2 | -0.75324 |
| Oaz3 | -0.63981 |
| Obfc1 | 0.23176 |
| Obfc2a | -0.56593 |
| Obfc2b | -0.29944 |
| Obfc2b | 1.09803 |
| Obox1 | -0.43921 |
| Obox5 | -0.6065 |
| Obrgrp | -0.08584 |
| Obsl1 | -0.34812 |
| Oc90 | -0.64229 |
| Oca2 | -0.05819 |
| Ociad1 | -0.24241 |
| Ociad1 | 0.05065 |
| Ociad2 | -0.7076 |
| Ocln | 1.0969 |
| Ocrl | 0.10996 |
| Odc1 | 2.65304 |
| Odf1 | -0.43854 |
| Odf2 | -0.67351 |
| Odf2 | -0.22276 |
| Odf2 | -0.6113 |
| Odf2 | -0.17915 |
| Odf3 | -0.65398 |
| Odf3l1 | -0.4717 |
| Odf4 | 2.89902 |
| Odf4 | -0.36024 |
| Odz1 | -0.75511 |
| Odz3 | -0.33913 |
| Odz3 | -0.09174 |
| Odz4 | -0.15932 |
| Og9x | -0.19126 |
| Ogdh | -0.63157 |
| Ogfod1 | -0.69697 |
| Ogfod2 | 1.3285 |
| Ogfr | 1.10981 |
| Ogfrl1 | -0.6105 |
| Ogg1 | 0.11106 |
| Ogn | 2.54196 |
| Ogn | 0.57134 |
| Ogn | -0.71655 |
| Ogt | -0.40143 |
| Ogt | 0.17083 |
| Ogt | 1.06537 |
| Oit1 | 1.14191 |
| Oit3 | -0.55757 |
| Ola1 | 0.74369 |
| Olfm1 | 0.95877 |
| Olfm1 | -0.64895 |
| Olfm2 | -0.67608 |
| Olfm2 | 0.82565 |
| Olfm3 | 0.39004 |
| Olfm3 | -0.10413 |
| Olfm4 | 2.26592 |
| Olfml1 | -0.52488 |
| Olfml2b | -0.55681 |
| Olfml3 | 1.57538 |
| Olfr100 | -0.38938 |
| Olfr1006 | 1.97509 |
| Olfr1012 | -0.45666 |
| Olfr1020 | -0.47239 |
| Olfr1065 | 1.30438 |
| Olfr1094 | 0.9637 |
| Olfr1099 | -0.46278 |
| Olfr1105 | 3.5808 |
| Olfr1112 | 1.83878 |
| Olfr1113 | -0.25376 |
| Olfr1189 | 0.0642 |
| Olfr1197 | -0.25812 |
| Olfr1198 | 1.63407 |
| Olfr1249 | -0.34271 |
| Olfr1280 | -0.42863 |
| Olfr133 | 0.05143 |
| Olfr1330 | -0.56899 |
| Olfr134 | 0.2679 |
| Olfr1340 | -0.44789 |
| Olfr1384 | 3.22833 |
| Olfr1386 | -0.69873 |
| Olfr1390 | -0.18923 |
| Olfr1394 | -0.7779 |
| Olfr1412 | 2.17511 |
| Olfr1437 | 0.14829 |
| Olfr144 | 1.78042 |
| Olfr1443 | 1.37491 |
| Olfr1443 | -0.63486 |
| Olfr1459 | -0.41557 |
| Olfr1500 | 3.79694 |
| Olfr1508 | -0.62667 |
| Olfr166 | -0.55154 |
| Olfr168 | 1.43981 |
| Olfr206 | 0.88892 |
| Olfr213 | -0.28471 |
| Olfr215 | 0.20246 |
| Olfr232 | 0.34413 |
| Olfr257 | -0.2092 |
| Olfr267 | -0.426 |
| Olfr272 | -0.35537 |
| Olfr303 | 2.4294 |
| Olfr310 | 3.93701 |
| Olfr352 | -0.76738 |
| Olfr371 | 0.7904 |
| Olfr378 | 0.96974 |
| Olfr419 | 1.24792 |
| Olfr437 | -0.63815 |
| Olfr464 | -0.79635 |
| Olfr47 | 0.80305 |
| Olfr49 | -0.86877 |
| Olfr503 | -0.73931 |
| Olfr51 | 2.46009 |
| Olfr536 | 1.95664 |
| Olfr538 | -0.70938 |
| Olfr54 | -0.74578 |
| Olfr550 | 0.42627 |
| Olfr574 | -0.67781 |
| Olfr591 | 3.38449 |
| Olfr593 | -0.14424 |
| Olfr608 | 2.04032 |
| Olfr638 | -0.64063 |
| Olfr65 | 2.07989 |
| Olfr655 | 0.76528 |
| Olfr658 | -0.34812 |
| Olfr665 | -0.13608 |
| Olfr675 | 1.14322 |
| Olfr691 | 0.67133 |
| Olfr692 | 0.81297 |
| Olfr702 | 0.25954 |
| Olfr724 | -0.03177 |
| Olfr725 | -0.35355 |
| Olfr748 | -0.02921 |
| Olfr786 | 0.49326 |
| Olfr802 | 1.41874 |
| Olfr824 | 0.10666 |
| Olfr91 | 1.51077 |
| Olfr910 | 2.6156 |
| Olfr920 | -0.75887 |
| Olfr924 | 1.87819 |
| Olfr952 | 1.54518 |
| Olfr965 | -0.25213 |
| Olfr982 | -0.15834 |
| Olfr983 | 0.25016 |
| Olfr984 | -0.54854 |
| Olig1 | -0.46689 |
| Olig3 | -0.78951 |
| Oma1 | -0.17465 |
| Omd | 0.31474 |
| Omg | 0.01929 |
| Omt2b | -0.64645 |
| Onecut2 | -0.60252 |
| Onecut3 | -0.76265 |
| Onecut3 | 1.76436 |
| Oog4 | -0.21962 |
| Oosp1 | -0.48488 |
| Opa1 | -0.05555 |
| Opa1 | 1.00343 |
| Ophn1 | 1.43775 |
| Oplah | 1.4822 |
| Opn1mw | -0.00361 |
| Opn3 | -0.48348 |
| Oprl1 | 2.84727 |
| Oprl1 | -0.74023 |
| Oprl1 | 1.01894 |
| Oprs1 | -0.3092 |
| Optc | -0.08674 |
| Optn | -0.54629 |
| Optn | 0.13272 |
| Orai1 | -0.50466 |
| Orai3 | -0.50395 |
| Oraov1 | 0.20971 |
| Orc2l | 1.26181 |
| Orc3l | -0.63981 |
| Orc5l | -0.59777 |
| Orc5l | -0.72922 |
| Orc6l | 0.98667 |
| ORF19 | 1.94861 |
| ORF28 | -0.69258 |
| ORF34 | -0.06746 |
| ORF61 | 0.32927 |
| ORF63 | 0.30533 |
| ORF9 | -0.27298 |
| ORF9 | 0.00258 |
| ORF9 | -0.66157 |
| Orm1 | 1.31183 |
| Ormdl2 | 0.1518 |
| Ormdl3 | 0.76564 |
| Os9 | -0.67436 |
| Osbp2 | 0.00052 |
| Osbpl10 | 2.63014 |
| Osbpl11 | 0.94751 |
| Osbpl2 | -0.28696 |
| Osbpl3 | 0.19081 |
| Osbpl3 | 0.19215 |
| Osbpl6 | 2.49959 |
| Osbpl8 | -0.18216 |
| Osbpl9 | -0.58833 |
| Oscar | -0.41687 |
| Osgep | 0.77757 |
| Osgepl1 | 1.33729 |
| Osgin1 | 1.0276 |
| Osm | -0.77215 |
| Osmr | 1.35973 |
| Osr1 | 2.01097 |
| Osr2 | -0.43523 |
| Ostf1 | 2.86967 |
| Ostm1 | -0.65734 |
| Otof | 0.63176 |
| Otop1 | 0.01848 |
| Otop2 | -0.37687 |
| Ott | 0.93909 |
| Ott | 1.98992 |
| OTTMUSG00000000421 | 1.67568 |
| OTTMUSG00000000971 | -0.06746 |
| OTTMUSG00000000990 | 0.66555 |
| OTTMUSG00000001305 | -0.12515 |
| OTTMUSG00000002038 | 1.19206 |
| OTTMUSG00000004461 | -0.50395 |
| OTTMUSG00000004551 | -0.6565 |
| OTTMUSG00000005148 | 2.36059 |
| OTTMUSG00000005491 | -0.42994 |
| OTTMUSG00000008540 | 1.66672 |
| OTTMUSG00000010009 | 2.62882 |
| OTTMUSG00000010328 | 1.72577 |
| OTTMUSG00000015762 | -0.65566 |
| OTTMUSG00000015859 | 1.01032 |
| OTTMUSG00000016293 | 0.01848 |
| OTTMUSG00000016790 | -0.66242 |
| OTTMUSG00000016790 | 1.19958 |
| OTTMUSG00000017677 | -0.42732 |
| OTTMUSG00000020946 | 0.33071 |
| Otub1 | -0.31616 |
| Otub2 | -0.15541 |
| Otub2 | -0.64229 |
| Otub2 | -0.65314 |
| Otud5 | -0.33674 |
| Otud6b | 0.84131 |
| Otud7b | 0.27005 |
| Otud7b | 1.70478 |
| Otx1 | -0.53147 |
| Otx2 | -0.54854 |
| Ovca2 | -0.75417 |
| Ovgp1 | 1.66954 |
| Ovgp1 | 0.28951 |
| Ovol1 | -0.47446 |
| Ovol2 | 1.00315 |
| Ovol2 | 0.15703 |
| Oxct1 | -0.46142 |
| Oxct2a | -0.65903 |
| Oxct2b | 2.06047 |
| Oxgr1 | 1.94643 |
| Oxnad1 | -0.76454 |
| Oxr1 | 0.05803 |
| Oxr1 | -0.76265 |
| Oxt | -0.63898 |
| P140 | -0.10597 |
| P2rx2 | -0.44454 |
| P2rx3 | 2.66363 |
| P2rx4 | 0.17458 |
| P2rx4 | -0.62993 |
| P2rx5 | 0.30887 |
| P2rx7 | -0.76549 |
| P2rx7 | 2.88973 |
| P2ry1 | 1.64225 |
| P2ry12 | -0.58288 |
| P2ry13 | 2.88414 |
| P2ry14 | -0.61936 |
| P2ry14 | -0.63733 |
| P2ry5 | -0.61371 |
| P2ry6 | 0.65085 |
| P42pop | -0.22224 |
| P4ha1 | 0.26974 |
| P4ha2 | -0.30001 |
| P4hb | 1.15079 |
| Pa2g4 | -0.50395 |
| Pabpc1 | 1.57009 |
| Pabpc1 | -0.60411 |
| Pabpc1 | 0.13415 |
| Pabpc2 | 0.08511 |
| Pabpc4 | -0.31849 |
| Pabpn1 | -0.17316 |
| Pabpnl1 | 0.25393 |
| Pacrg | 2.07538 |
| Pacs1 | -0.35719 |
| Pacs2 | 1.65766 |
| Pacsin1 | -0.31907 |
| Pacsin1 | 2.37324 |
| Pacsin1 | -0.11668 |
| Pacsin2 | 1.9608 |
| Pacsin2 | -0.74208 |
| Pacsin3 | 0.37645 |
| Pacsin3 | 0.93537 |
| Padi1 | 1.92047 |
| Padi2 | -0.32845 |
| Padi6 | 0.46685 |
| Paf1 | -0.39507 |
| Paf1 | 0.70679 |
| Pafah1b1 | -0.03049 |
| Pafah1b1 | 0.12807 |
| Pafah1b3 | -0.29318 |
| Pafah2 | -0.03391 |
| Pafah2 | -0.61371 |
| Paip1 | -0.51471 |
| Paip2 | 1.79936 |
| Paip2b | -0.64979 |
| Paip2b | 0.81942 |
| Pak1 | -0.59856 |
| Pak3 | 1.79323 |
| Pak4 | 0.10556 |
| Pak6 | 2.7985 |
| Pak7 | -0.75605 |
| Palld | 0.47549 |
| Palm | 1.57352 |
| Palm | -0.6057 |
| Palm2 | -0.52488 |
| Palmd | -0.51616 |
| Palmd | 1.65807 |
| Palmd | 1.72463 |
| Pam | 3.14672 |
| Pank1 | -0.02158 |
| Pank1 | -0.58288 |
| Pank2 | 2.07611 |
| Pank3 | 1.74214 |
| Pank4 | 0.69931 |
| Panx1 | 1.53608 |
| Panx3 | -0.66923 |
| Paox | -0.71117 |
| Paox | 1.16762 |
| Paox | 1.46979 |
| Paox | 3.44497 |
| Papd1 | -0.67265 |
| Papd1 | -0.29204 |
| Papd4 | -0.31907 |
| Papln | 1.06394 |
| Papola | 1.0999 |
| Papolb | -0.67867 |
| Papolg | -0.36146 |
| Papss1 | -0.47722 |
| Papss1 | -0.77215 |
| Papss2 | 0.3393 |
| Paqr3 | 0.59517 |
| Paqr6 | -0.06039 |
| Paqr7 | -0.60411 |
| Paqr9 | -0.7523 |
| Paqr9 | -0.51833 |
| Paqr9 | 1.86115 |
| Pard3 | -0.10274 |
| Pard3 | -0.55984 |
| Pard6a | -0.61936 |
| Pard6a | 0.83518 |
| Pard6b | -0.25159 |
| Pard6g | -0.22856 |
| Park7 | -0.5448 |
| Parl | 1.18493 |
| Parp1 | 0.68415 |
| Parp1 | -0.21857 |
| Parp1 | 2.37914 |
| Parp12 | 1.09865 |
| Parp14 | -0.0922 |
| Parp16 | -0.52415 |
| Parp2 | -0.4717 |
| Parp2 | -0.74485 |
| Parp3 | 0.64535 |
| Parp6 | -0.64478 |
| Parp8 | 0.15075 |
| Pars2 | -0.17366 |
| Pars2 | -0.56212 |
| Parva | -0.52415 |
| Parvg | -0.6856 |
| Pask | 0.30857 |
| Patl1 | -0.67436 |
| Patz1 | -0.5448 |
| Pawr | -0.60252 |
| Pax8 | -0.60252 |
| Paxip1 | -0.73471 |
| Pbk | 2.17395 |
| Pbld | -0.03092 |
| Pbp2 | 0.20675 |
| Pbrm1 | 0.14935 |
| Pbrm1 | -0.57745 |
| Pbx1 | -0.70938 |
| Pbx1 | -0.1069 |
| Pbx2 | 0.72925 |
| Pbx2 | 0.39676 |
| Pbx2 | -0.55908 |
| Pbx3 | -0.50752 |
| Pbx3 | 0.90353 |
| Pbx4 | 1.77033 |
| Pcaf | -0.14473 |
| Pcaf | 0.50357 |
| Pcbd1 | 2.61706 |
| Pcbd2 | -0.57977 |
| Pcbp1 | -0.74023 |
| Pcbp2 | -0.72467 |
| Pcbp3 | -0.6523 |
| Pcca | 0.94531 |
| Pcca | -0.40847 |
| Pccb | -0.24456 |
| Pccb | -0.03648 |
| Pcdh1 | 0.51235 |
| Pcdh10 | -0.75044 |
| Pcdh10 | 2.26206 |
| Pcdh10 | 3.58115 |
| Pcdh15 | 1.29787 |
| Pcdh17 | -0.44588 |
| Pcdh20 | 1.75114 |
| Pcdh21 | -0.8212 |
| Pcdh7 | 4.06147 |
| Pcdh7 | 1.81672 |
| Pcdh8 | -0.6057 |
| Pcdh9 | 0.38652 |
| Pcdha10 | -0.05731 |
| Pcdha11 | 0.73446 |
| Pcdha2 | 0.29432 |
| Pcdha6 | 4.75173 |
| Pcdhac1 | 2.11721 |
| Pcdhac2 | 1.17361 |
| Pcdhb1 | -0.56669 |
| Pcdhb10 | 2.14028 |
| Pcdhb12 | 1.48409 |
| Pcdhb14 | 0.85848 |
| Pcdhb19 | 0.37918 |
| Pcdhb20 | -0.6105 |
| Pcdhb22 | -0.87837 |
| Pcdhb3 | -0.51616 |
| Pcdhb7 | -0.51905 |
| Pcdhb8 | -0.18872 |
| Pcdhga10 | -0.20455 |
| Pcdhga12 | -0.71027 |
| Pcdhga4 | -0.06613 |
| Pcdhga7 | -0.06569 |
| Pcdhga8 | 2.10523 |
| Pcdhgb2 | 0.00381 |
| Pcdhgb6 | 1.30796 |
| Pcdhgc3 | 1.97653 |
| Pcdhgc4 | -0.332 |
| Pcdhgc5 | 2.70154 |
| Pcf11 | 1.59848 |
| Pcgf1 | -0.80225 |
| Pcgf2 | 0.08323 |
| Pcgf3 | 1.69127 |
| Pcgf5 | -0.65062 |
| Pcgf6 | -0.54331 |
| Pck1 | 0.27801 |
| Pclo | 0.17765 |
| Pclo | 3.29871 |
| Pcmt1 | 0.16188 |
| Pcmt1 | -0.2691 |
| Pcmt1 | -0.44388 |
| Pcmtd1 | -0.34631 |
| Pcmtd2 | 0.01807 |
| Pcna | -0.38123 |
| Pcnt | 0.70046 |
| Pcnx | -0.67008 |
| Pcnxl2 | -0.5176 |
| Pcnxl2 | 0.12951 |
| Pcolce | 1.99859 |
| Pcolce2 | 0.15703 |
| Pcolce2 | 0.4082 |
| Pcsk1n | 2.98199 |
| Pcsk2 | 1.22461 |
| Pcsk2 | -0.70227 |
| Pcsk2 | 1.42672 |
| Pcsk2 | 1.72734 |
| Pcsk4 | -0.13179 |
| Pcsk4 | -0.59383 |
| Pcsk4 | 0.3068 |
| Pcsk4 | -0.18872 |
| Pcsk4 | 2.26276 |
| Pcsk9 | 1.2087 |
| Pctk1 | -0.32024 |
| Pcx | -0.18267 |
| Pcyox1 | -0.34391 |
| Pcyox1l | -0.56516 |
| Pcyt1a | -0.69962 |
| Pcyt1b | -0.55908 |
| Pcyt1b | 0.71818 |
| Pcyt2 | 0.21788 |
| Pcyt2 | 0.08436 |
| Pcyt2 | 0.07643 |
| Pdcd10 | -0.18317 |
| Pdcd2 | -0.4059 |
| Pdcd2l | 1.47965 |
| Pdcd4 | 1.66927 |
| Pdcd5 | 0.3373 |
| Pdcd5 | -0.42208 |
| Pdcd6ip | 1.52233 |
| Pdcl | -0.53 |
| Pdcl3 | 0.77296 |
| Pddc1 | 0.2862 |
| Pde10a | -0.44321 |
| Pde1a | -0.58365 |
| Pde1a | 0.01929 |
| Pde1a | 1.30776 |
| Pde1b | 1.9289 |
| Pde1c | 0.07034 |
| Pde2a | -0.11621 |
| Pde3a | -0.79243 |
| Pde4a | 2.52818 |
| Pde4a | -0.34211 |
| Pde4a | -0.73838 |
| Pde4a | -0.39253 |
| Pde4b | 0.54824 |
| Pde4d | -0.45126 |
| Pde4dip | 0.96283 |
| Pde4dip | -0.5821 |
| Pde4dip | -0.44187 |
| Pde4dip | 0.38164 |
| Pde4dip | -0.20559 |
| Pde5a | 0.90415 |
| Pde6a | -0.58288 |
| Pde6d | -0.40912 |
| Pde6d | -0.58365 |
| Pde6d | -0.70938 |
| Pde6d | 2.59131 |
| Pde6h | -0.27353 |
| Pde7a | -0.40015 |
| Pdgfa | -0.6121 |
| Pdgfb | 2.81357 |
| Pdgfc | -0.26745 |
| Pdgfra | 3.05504 |
| Pdgfrb | -0.55304 |
| Pdgfrl | 0.96946 |
| Pdha1 | 1.42986 |
| Pdhb | 0.03728 |
| Pdhb | -0.45734 |
| Pdia3 | -0.64645 |
| Pdia4 | 0.29342 |
| Pdia5 | -0.49896 |
| Pdia5 | -0.21962 |
| Pdia6 | -0.22066 |
| Pdik1l | 0.25173 |
| Pdk1 | 1.68641 |
| Pdk2 | -0.57129 |
| Pdk3 | -0.39824 |
| Pdk4 | 0.15459 |
| Pdlim1 | 0.50071 |
| Pdlim2 | 0.58606 |
| Pdlim2 | -0.77406 |
| Pdlim2 | 2.64952 |
| Pdlim2 | -0.65734 |
| Pdlim3 | 1.05757 |
| Pdlim3 | -0.25702 |
| Pdlim4 | -0.58833 |
| Pdlim5 | 2.6283 |
| Pdlim5 | -0.4306 |
| Pdlim7 | -0.69433 |
| Pdpk1 | -0.41557 |
| Pdpk1 | 2.80137 |
| Pdrg1 | -0.76738 |
| Pdss1 | -0.29602 |
| Pdxdc1 | 1.22494 |
| Pdxdc1 | 2.16657 |
| Pdxk | -0.05162 |
| Pdxp | -0.69962 |
| Pdyn | -0.5448 |
| Pdyn | 1.04708 |
| Pdzd11 | -0.52707 |
| Pdzd11 | 0.00217 |
| Pdzd11 | -0.00361 |
| Pdzd8 | 1.41424 |
| Pdzk1 | 0.05648 |
| Pdzk1ip1 | -0.15981 |
| Pdzrn3 | -0.37873 |
| Pdzrn3 | 1.22646 |
| Pea15 | -0.77694 |
| Pear1 | 0.69834 |
| Pear1 | 0.51683 |
| Pebp1 | -0.58912 |
| Pecam1 | -0.29887 |
| Pecam1 | -0.72377 |
| Peci | 0.21037 |
| Pef1 | 0.354 |
| Peg10 | -0.51833 |
| Peg3 | 0.00381 |
| Peli1 | -0.73013 |
| Peli1 | 0.2862 |
| Peli2 | -0.25702 |
| Peli3 | 0.0349 |
| Pelo | -0.10644 |
| Pelp1 | -0.65146 |
| Pemt | 1.62553 |
| Peo1 | 2.78165 |
| Pepd | 1.45662 |
| Per1 | -0.66072 |
| Per1 | -0.62667 |
| Per2 | 0.39085 |
| Per2 | -0.78079 |
| Per2 | 0.94545 |
| Per3 | 0.16223 |
| Perld1 | 0.84098 |
| Pes1 | -0.45666 |
| Pet112l | -0.26359 |
| Pet112l | 0.39327 |
| Pet112l | 1.18742 |
| Pet112l | 0.4082 |
| Pex11a | 0.48479 |
| Pex11a | -0.80621 |
| Pex11a | -0.6113 |
| Pex11b | -0.75793 |
| Pex11c | -0.32024 |
| Pex14 | -0.67953 |
| Pex16 | 1.67215 |
| Pex19 | -0.10228 |
| Pex19 | -0.62667 |
| Pex2 | 0.78128 |
| Pex26 | 0.91595 |
| Pex3 | 0.95206 |
| Pex3 | 2.5195 |
| Pex5 | -0.08087 |
| Pex5 | -0.10505 |
| Pex5 | -0.71925 |
| Pex5l | -0.26524 |
| Pex6 | 0.17048 |
| Pex6 | 1.12945 |
| Pex6 | -0.12704 |
| Pex7 | -0.55757 |
| Pfdn1 | -0.60014 |
| Pfdn2 | 0.55661 |
| Pfdn4 | 0.26264 |
| Pfdn5 | -0.4933 |
| Pfdn5 | 1.51117 |
| Pfkfb2 | 2.55117 |
| Pfkfb2 | -0.44789 |
| Pfkfb4 | 0.24038 |
| Pfkl | 1.3344 |
| Pfkl | -0.35174 |
| Pfkm | -0.14135 |
| Pfkm | 3.37896 |
| Pfkm | -0.11294 |
| Pfkp | -0.77982 |
| Pfkp | 0.10593 |
| Pfkp | 0.91853 |
| Pfn1 | 3.26994 |
| Pfn1 | -0.65988 |
| Pfn2 | 0.70181 |
| Pfn2 | 2.01921 |
| Pfn2 | -0.10551 |
| Pfn3 | 1.22309 |
| Pfn4 | -0.32258 |
| Pftk1 | -0.46074 |
| Pga5 | 1.39997 |
| Pgam1 | -0.17465 |
| Pgam2 | 0.76653 |
| Pgam5 | 1.42515 |
| Pgbd5 | -0.27021 |
| Pgcp | -0.56516 |
| Pgd | 0.702 |
| Pgk1 | -0.15005 |
| Pgk2 | -0.59935 |
| Pgls | 1.8792 |
| Pglyrp1 | -0.3092 |
| Pglyrp4 | -0.35659 |
| Pgm2 | -0.53885 |
| Pgm2l1 | -0.07772 |
| Pgm3 | -0.67436 |
| Pgm5 | 0.52198 |
| Pgm5 | -0.16227 |
| Pgpep1 | -0.7523 |
| Pgpep1 | -0.01779 |
| Pgr | 0.19715 |
| Pgrmc1 | -0.67522 |
| Pgs1 | -0.19839 |
| Phactr1 | -0.54182 |
| Phactr1 | -0.47792 |
| Phactr1 | -0.38185 |
| Phactr4 | -0.68908 |
| Phb2 | 0.18846 |
| Phc1 | -0.76359 |
| Phc1 | -0.67179 |
| Phc2 | 1.67761 |
| Phca | -0.1009 |
| Phca | 2.76779 |
| Phex | 1.19104 |
| Phf1 | 1.47684 |
| Phf11 | 2.08451 |
| Phf11 | -0.55079 |
| Phf12 | 1.03752 |
| Phf12 | 0.73056 |
| Phf13 | -0.1867 |
| Phf13 | -0.56593 |
| Phf14 | 0.33759 |
| Phf16 | -0.49049 |
| Phf17 | -0.42339 |
| Phf19 | 1.61598 |
| Phf2 | 0.75572 |
| Phf20l1 | 2.18355 |
| Phf21a | 0.19782 |
| Phf5a | 1.77506 |
| Phf6 | 1.26442 |
| Phf7 | 2.62185 |
| Phgdh | 2.19004 |
| Phka1 | 2.03727 |
| Phka1 | -0.15785 |
| Phka2 | -0.21283 |
| Phka2 | -0.46826 |
| Phka2 | -0.43258 |
| Phkb | -0.23706 |
| Phkg1 | 0.8375 |
| Phkg2 | 0.26109 |
| Phlda1 | 1.31262 |
| Phlda2 | 1.19678 |
| Phlda3 | 0.79752 |
| Phlda3 | 0.2988 |
| Phldb1 | -0.48348 |
| Phldb1 | -0.42404 |
| Phlpp | -0.59619 |
| Phospho1 | 1.45459 |
| Phospho2 | -0.21752 |
| Phox2a | -0.20301 |
| Phox2b | 2.1168 |
| Phpt1 | 0.96974 |
| Phtf1 | -0.75511 |
| Phtf2 | -0.48558 |
| Phyhip | -0.56516 |
| Pi16 | -0.66923 |
| Pi4k2b | 0.16774 |
| Pi4k2b | 2.91326 |
| Pi4ka | 1.85064 |
| Pi4kb | -0.33259 |
| Pi4kb | 1.82841 |
| Pias1 | 0.6301 |
| Pias2 | 0.08172 |
| Pias3 | 0.58431 |
| Pias3 | 1.03926 |
| Pias4 | 0.42601 |
| Pibf1 | -0.57282 |
| Pibf1 | -0.60491 |
| Picalm | -0.22962 |
| Pick1 | 1.43012 |
| Pick1 | 0.76904 |
| Pif1 | -0.07102 |
| Piga | 0.94531 |
| Piga | 2.77802 |
| Pigb | 1.59267 |
| Pigc | -0.47376 |
| Pigf | -0.73655 |
| Pigh | -0.11201 |
| Pigk | -0.5068 |
| Pigk | 0.38164 |
| Pigl | 0.127 |
| Pign | -0.4717 |
| Pigo | 2.39294 |
| Pigo | -0.64063 |
| Pigo | 0.37151 |
| Pigp | -0.6856 |
| Pigp | 2.95332 |
| Pigq | -0.68996 |
| Pigt | 1.28155 |
| Pigx | -0.5176 |
| Pigy | -0.77694 |
| Pigyl | 1.54579 |
| Pigz | -0.25213 |
| Pih1d1 | 0.56534 |
| Pik3ap1 | 0.9637 |
| Pik3c2a | -0.46347 |
| Pik3c3 | -0.55079 |
| Pik3ca | 0.57906 |
| Pik3cb | -0.30058 |
| Pik3cd | 1.74233 |
| Pik3cg | -0.52342 |
| Pik3ip1 | -0.47653 |
| Pik3r1 | -0.69697 |
| Pik3r1 | 0.25361 |
| Pik3r1 | -0.06702 |
| Pik3r2 | 0.48843 |
| Pik3r2 | 0.82984 |
| Pik3r3 | -0.41752 |
| Pik3r4 | 1.76091 |
| Pik3r6 | 1.96085 |
| Pik3r6 | -0.7005 |
| Pim1 | -0.02794 |
| Pim2 | 0.127 |
| Pim2 | 1.57411 |
| Pim3 | 0.0209 |
| Pip4k2a | 1.95453 |
| Pip4k2b | 2.35986 |
| Pip4k2b | 0.93254 |
| Pip4k2c | -0.1938 |
| Pip5k1a | -0.73196 |
| Pip5k1a | -0.46006 |
| Pip5k1b | -0.56822 |
| Pip5kl1 | -0.37998 |
| Pipox | -0.3976 |
| Pir | 2.13508 |
| Pira11 | 1.74097 |
| Pira4 | 2.0914 |
| Pisd | -0.57205 |
| Pit1 | -0.11762 |
| Pitpna | 1.24356 |
| Pitpnb | 0.9129 |
| Pitpnc1 | 3.67466 |
| Pitpnm1 | -0.33496 |
| Pitpnm2 | 2.54006 |
| Pitpnm3 | 0.96037 |
| Pitx1 | 0.41453 |
| Pitx2 | -0.78369 |
| Pitx2 | -0.35053 |
| Pitx2 | -0.58677 |
| Pitx2 | 3.9328 |
| Piwil1 | -0.67094 |
| Piwil2 | -0.51255 |
| Piwil4 | 1.36029 |
| Pja1 | -0.50609 |
| Pja2 | 0.84559 |
| Pja2 | 2.05885 |
| Pkd1 | -0.17865 |
| Pkd1l3 | 2.59748 |
| Pkd2 | -0.52051 |
| Pkd2l1 | -0.56593 |
| Pkd2l2 | -1.03973 |
| Pkdrej | 0.01727 |
| Pkhd1l1 | 1.81363 |
| Pkhd1l1 | 1.33489 |
| Pkia | 0.44754 |
| Pkib | 2.30599 |
| Pkib | -0.72558 |
| Pkig | -0.31094 |
| Pkig | -0.43324 |
| Pkig | -0.34271 |
| Pkm2 | 0.60012 |
| Pkmyt1 | -0.2691 |
| Pkn1 | -0.55681 |
| Pkn1 | 1.44205 |
| Pkn2 | 1.33518 |
| Pkn2 | 0.03172 |
| Pkn2 | 1.71288 |
| Pkn3 | 0.08248 |
| Pknox1 | 3.00479 |
| Pknox1 | 2.69822 |
| Pknox2 | -0.21231 |
| Pknox2 | 1.46503 |
| Pknox2 | 0.9481 |
| Pknox2 | -0.40847 |
| Pknox2 | 1.68753 |
| Pkp1 | 1.04668 |
| Pkp2 | -0.64478 |
| Pkp2 | 0.09148 |
| Pkp2 | 1.10499 |
| Pkp3 | -0.83542 |
| Pla1a | -0.49049 |
| Pla1a | -0.62504 |
| Pla1a | -0.74671 |
| Pla2g10 | -0.72196 |
| Pla2g12a | -0.72831 |
| Pla2g1b | -0.24027 |
| Pla2g2c | -0.57745 |
| Pla2g2d | 0.54323 |
| Pla2g2f | -0.5068 |
| Pla2g4c | -0.37315 |
| Pla2g4d | -0.47792 |
| Pla2g4e | -0.65314 |
| Pla2g4f | 2.11251 |
| Pla2g5 | 2.12227 |
| Pla2g6 | 0.58366 |
| Pla2g7 | 0.15354 |
| Plaa | 0.67767 |
| Plac1 | -0.61774 |
| Plac1l | -0.34032 |
| Plac8 | 0.127 |
| Plag1 | 0.74056 |
| Plag1 | 3.23916 |
| Plagl2 | -0.55984 |
| Plau | 0.00954 |
| Plb1 | 0.33214 |
| Plcb2 | 1.67967 |
| Plcb3 | 1.01073 |
| Plcb4 | 0.13344 |
| Plcd1 | -0.62422 |
| Plcd3 | -0.61936 |
| Plcd4 | -0.69697 |
| Plcd4 | -0.36085 |
| Plcd4 | -0.67608 |
| Plce1 | 2.43216 |
| Plcg2 | 0.51259 |
| Plch2 | 0.26974 |
| Plcl2 | 0.38842 |
| Plcxd1 | 0.29671 |
| Plcxd1 | -0.37129 |
| Plcz1 | 3.05483 |
| Pld1 | 1.20637 |
| Pld2 | -0.65062 |
| Pld3 | 0.29162 |
| Pld3 | 0.55796 |
| Pld4 | 0.74534 |
| Pld5 | -0.47722 |
| Pldn | -0.39317 |
| Plec1 | 0.55975 |
| Plec1 | 0.07794 |
| Plec1 | 0.21886 |
| Plec1 | 0.04084 |
| Plek | 2.52773 |
| Plek2 | -0.65819 |
| Plekha1 | 1.5474 |
| Plekha1 | 0.29611 |
| Plekha2 | 0.81942 |
| Plekha3 | -0.53368 |
| Plekha4 | 2.77983 |
| Plekha4 | -0.68126 |
| Plekha5 | -0.54629 |
| Plekha6 | -0.59698 |
| Plekha7 | -0.45261 |
| Plekha8 | -0.92455 |
| Plekhb1 | 0.41058 |
| Plekhb2 | -0.07727 |
| Plekhf1 | -0.08946 |
| Plekhf1 | -0.14473 |
| Plekhf2 | -0.60252 |
| Plekhf2 | 2.36365 |
| Plekhg2 | 1.14832 |
| Plekhg2 | -0.16572 |
| Plekhg2 | 1.38812 |
| Plekhg3 | 1.87229 |
| Plekhg3 | 0.65186 |
| Plekhg4 | -0.18518 |
| Plekhg5 | -0.58443 |
| Plekhg6 | -0.53294 |
| Plekhj1 | 2.4165 |
| Plekhm1 | 0.24323 |
| Plekhm3 | -0.47101 |
| Plekho2 | 1.42506 |
| Plin | -0.63486 |
| Plk1 | 0.57554 |
| Plk2 | -0.29091 |
| Plk3 | 1.04853 |
| Plk4 | 0.43787 |
| Pllp | -0.55379 |
| Plod1 | 2.51551 |
| Plod2 | -0.09356 |
| Plod3 | 0.08399 |
| Plp1 | -0.6226 |
| Plp2 | 0.9686 |
| Plrg1 | -0.38938 |
| Pls3 | -0.75699 |
| Plscr1 | 1.90928 |
| Plscr1 | 0.69331 |
| Plscr3 | -0.8385 |
| Plscr4 | 0.33387 |
| Pltp | -0.52123 |
| Plunc | 0.75627 |
| Plvap | -0.46278 |
| Plxdc1 | 0.88247 |
| Plxdc1 | 1.85728 |
| Plxna2 | -0.64146 |
| Plxnb1 | -0.54779 |
| Plxnb3 | -0.50466 |
| Plxnc1 | 0.55683 |
| Plxnd1 | -0.02666 |
| Pmaip1 | -0.47722 |
| Pmch | -0.37749 |
| Pmepa1 | 0.03886 |
| Pmf1 | 2.01999 |
| Pmfbp1 | -0.46074 |
| Pml | 1.3463 |
| Pml | -0.4926 |
| Pmm1 | -0.61936 |
| Pmm1 | -0.04467 |
| Pmm2 | -0.28471 |
| Pmm2 | -0.21283 |
| Pmm2 | 0.61692 |
| Pmm2 | 0.89485 |
| Pmp2 | 0.08286 |
| Pmp22 | -0.47446 |
| Pmp22 | -0.67867 |
| Pmpca | 1.4567 |
| Pmpcb | 0.33616 |
| Pms2 | -0.7076 |
| Pmvk | -0.59068 |
| Pnck | 2.10519 |
| Pnkd | -0.69521 |
| Pnkd | 0.49447 |
| Pnkp | 0.13379 |
| Pnkp | 3.28326 |
| Pnliprp1 | -0.54182 |
| Pnma1 | -0.42863 |
| Pnma2 | 0.44932 |
| Pno1 | 1.59492 |
| Pnpla2 | 0.97533 |
| Pnpla6 | 0.94043 |
| Pnpla7 | -0.61451 |
| Pnpla8 | -0.25921 |
| Pnpla8 | -0.25702 |
| Pnpo | 1.73997 |
| Pnpo | -0.14232 |
| Pnrc2 | 0.67252 |
| Podn | -0.6105 |
| Podxl | -0.48488 |
| Podxl2 | 0.01402 |
| Pof1b | 0.82481 |
| Pofut1 | 0.41584 |
| Pofut1 | -0.65482 |
| Pofut2 | -0.62993 |
| Pogz | -0.76549 |
| Pogz | 0.55638 |
| Pola2 | 0.01483 |
| Polb | 1.10326 |
| Pold1 | -0.64895 |
| Pold2 | -0.71296 |
| Pold3 | 1.31981 |
| Pold4 | -0.69258 |
| Poldip2 | -0.6121 |
| Poldip3 | -0.53294 |
| Poldip3 | 0.39112 |
| Pole | -0.07593 |
| Pole3 | -0.33555 |
| Pole3 | -0.87516 |
| Pole4 | -0.31732 |
| Polg | 1.55031 |
| Polg2 | 2.37211 |
| Polh | -0.78562 |
| Poli | -0.52707 |
| Polk | -0.76454 |
| Poll | 2.41914 |
| Poll | 1.62869 |
| Polm | 2.94693 |
| Polm | -0.32669 |
| Poln | 2.27474 |
| Polq | -0.64812 |
| Polr1e | 0.05958 |
| Polr2d | 1.50397 |
| Polr2d | -0.50967 |
| Polr2d | -0.73931 |
| Polr2d | -0.6097 |
| Polr2e | -0.79048 |
| Polr2e | 0.0642 |
| Polr2f | 1.17752 |
| Polr2g | -0.73013 |
| Polr2h | 0.54983 |
| Polr2j | -0.66667 |
| Polr3a | -0.42929 |
| Polr3a | -0.75699 |
| Polr3b | -0.37873 |
| Polr3d | 2.26431 |
| Polr3e | 3.28151 |
| Polr3g | 0.19682 |
| Polr3h | -0.51543 |
| Polr3h | 0.14196 |
| Polr3h | -0.65062 |
| Polr3k | -0.7005 |
| Polr3k | 1.42085 |
| Polrmt | 0.78269 |
| Pols | -0.35659 |
| Pom121 | -0.77598 |
| Pomc | -0.77502 |
| Pomgnt1 | -0.39317 |
| Pomgnt1 | 2.73082 |
| Pomp | 2.08165 |
| Pomt1 | 2.539 |
| Pomt1 | -0.43258 |
| Pomt2 | -0.76359 |
| Pon1 | 1.19093 |
| Pon2 | 1.10351 |
| Pon3 | 0.27709 |
| Pon3 | -0.25812 |
| Pop4 | 1.13941 |
| Pop5 | 0.2308 |
| Pop5 | -0.55984 |
| Pop7 | 0.80408 |
| Popdc2 | -0.64812 |
| Popdc2 | 1.33624 |
| Popdc3 | -0.66923 |
| Por | 0.36432 |
| Por | -0.39507 |
| Porcn | 0.22791 |
| Porcn | -0.2328 |
| Pou2af1 | 0.76582 |
| Pou2f1 | -0.24942 |
| Pou2f1 | -0.45938 |
| Pou2f2 | -0.4587 |
| Pou3f1 | -0.48488 |
| Pou3f3 | 0.31063 |
| Pou5f1 | 1.55069 |
| Pou5f1 | -0.16325 |
| Pou6f1 | 0.74185 |
| Ppa1 | 1.4985 |
| Ppa2 | 0.97533 |
| Ppa2 | 2.07391 |
| Ppa2 | -0.79048 |
| Ppap2a | -0.79048 |
| Ppap2a | 2.27935 |
| Ppap2a | -0.76549 |
| Ppap2b | -0.63239 |
| Ppap2b | 1.33681 |
| Ppap2c | -0.6073 |
| Ppapdc1 | 1.595 |
| Ppapdc1a | 1.65242 |
| Ppapdc2 | -0.78854 |
| Ppapdc3 | 0.61986 |
| Ppapdc3 | 2.73961 |
| Pparbp | -0.65062 |
| Pparg | -0.75137 |
| Ppargc1a | 3.86587 |
| Ppargc1b | 1.41105 |
| Ppat | 1.15174 |
| Ppcs | -0.24027 |
| Ppef2 | 0.05803 |
| Ppfia3 | 2.5787 |
| Ppfia3 | -0.2603 |
| Ppfibp1 | -0.85717 |
| Ppfibp2 | 1.63281 |
| Ppfibp2 | 1.73337 |
| Ppia | 0.20147 |
| Ppic | -0.23333 |
| Ppic | -0.6523 |
| Ppid | 0.14584 |
| Ppie | -0.15248 |
| Ppie | -0.45193 |
| Ppif | -0.84882 |
| Ppil2 | -0.36575 |
| Ppil3 | -0.41363 |
| Ppl | 1.03217 |
| Ppm1a | -0.63075 |
| Ppm1a | -0.05075 |
| Ppm1a | -0.54331 |
| Ppm1b | 0.5649 |
| Ppm1b | -0.63981 |
| Ppm1b | 0.31533 |
| Ppm1e | -0.81718 |
| Ppm1e | -0.71655 |
| Ppm1f | 1.96741 |
| Ppm1f | -0.71565 |
| Ppm1f | 4.34998 |
| Ppm1g | 0.00832 |
| Ppm1h | 0.66695 |
| Ppm1j | -0.67351 |
| Ppm1k | 0.98948 |
| Ppm1l | -0.39888 |
| Ppm1l | -0.82423 |
| Ppm1m | -0.52196 |
| Ppm1m | 0.56957 |
| Ppm2c | 0.31063 |
| Ppme1 | -0.73838 |
| Ppnr | 1.45712 |
| Ppox | 0.63383 |
| Ppox | -0.33318 |
| Ppp1ca | -0.63403 |
| Ppp1ca | 0.36903 |
| Ppp1ca | 0.25424 |
| Ppp1cb | 0.08059 |
| Ppp1cb | 0.02773 |
| Ppp1cc | -0.55984 |
| Ppp1r10 | 1.37065 |
| Ppp1r10 | 0.49133 |
| Ppp1r11 | 0.84015 |
| Ppp1r12c | -0.36698 |
| Ppp1r13b | 1.91679 |
| Ppp1r13b | -0.31674 |
| Ppp1r13b | 2.48256 |
| Ppp1r14a | 1.36991 |
| Ppp1r14a | -0.66242 |
| Ppp1r14c | 2.08079 |
| Ppp1r14d | 2.59276 |
| Ppp1r15b | -0.07503 |
| Ppp1r16a | -0.70227 |
| Ppp1r1a | -0.7495 |
| Ppp1r1b | 1.27622 |
| Ppp1r1b | 0.82145 |
| Ppp1r2 | -0.91112 |
| Ppp1r3c | -0.48139 |
| Ppp1r3f | -0.42208 |
| Ppp1r8 | -0.57513 |
| Ppp1r9a | -0.46757 |
| Ppp1r9b | 0.05143 |
| Ppp2ca | -0.39001 |
| Ppp2cb | -0.5644 |
| Ppp2cz | -0.38435 |
| Ppp2r1a | -0.46006 |
| Ppp2r1b | -0.28022 |
| Ppp2r2b | 0.74607 |
| Ppp2r2b | 3.38106 |
| Ppp2r2b | 0.93478 |
| Ppp2r2c | -0.24456 |
| Ppp2r2c | 0.85507 |
| Ppp2r2d | 0.37973 |
| Ppp2r3c | 0.24576 |
| Ppp2r4 | -0.75417 |
| Ppp2r5b | 1.53429 |
| Ppp2r5c | -0.08448 |
| Ppp2r5c | 0.62719 |
| Ppp2r5c | -0.48 |
| Ppp2r5d | 0.18678 |
| Ppp2r5d | -0.82221 |
| Ppp2r5d | -0.2983 |
| Ppp2r5d | 1.01073 |
| Ppp2r5e | -0.48628 |
| Ppp2r5e | 0.16602 |
| Ppp3ca | -0.29659 |
| Ppp3ca | -0.79831 |
| Ppp3ca | -0.34451 |
| Ppp3cb | -1.03336 |
| Ppp3cc | -0.56822 |
| Ppp3r1 | -0.62179 |
| Ppp4c | -0.77119 |
| Ppp4r1 | -0.85822 |
| Ppp4r1 | 1.13402 |
| Ppp4r1 | -0.69785 |
| Ppp4r1l | 0.09781 |
| Ppp5c | -0.46006 |
| Ppp5c | -0.62017 |
| Ppp6c | 0.41689 |
| Pps | 0.49013 |
| Ppt1 | 0.07832 |
| Ppt2 | -0.41299 |
| Pptc7 | -0.41428 |
| Pqbp1 | 0.22501 |
| Pqlc1 | -0.1677 |
| Pqlc2 | -0.82625 |
| Pqlc3 | 0.42912 |
| Pqlc3 | 0.43067 |
| Pqlc3 | 1.51173 |
| Pqlc3 | -0.57205 |
| Pramef12 | 0.84295 |
| Prap1 | 1.10499 |
| Prc1 | -0.78175 |
| Prcp | -0.10505 |
| Prdm10 | 1.33989 |
| Prdm4 | -0.42929 |
| Prdm8 | -0.3261 |
| Prdm9 | -0.54405 |
| Prdx1 | 1.2174 |
| Prdx2 | -0.34271 |
| Prdx2 | 2.36453 |
| Prdx2 | -0.29944 |
| Prdx3 | 0.33816 |
| Prdx4 | -0.71745 |
| Prdx5 | 0.38869 |
| Prdx5 | -1.00585 |
| Prdx6 | -0.579 |
| Prdx6-rs1 | -0.25921 |
| Prdx6-rs1 | 1.25447 |
| Preb | 0.12807 |
| Preb | 1.99478 |
| Prei3 | 0.38191 |
| Prei4 | 1.1646 |
| Prei4 | -0.15054 |
| Prelp | 1.60785 |
| Prep | -0.53 |
| Prep | -0.42797 |
| Prf1 | -0.16375 |
| Prickle1 | 0.05337 |
| Prickle2 | -0.2819 |
| Prickle3 | -0.34992 |
| Prickle4 | -0.41105 |
| Prim2 | -0.27409 |
| Prim2 | -0.28134 |
| Prima1 | 2.27256 |
| Prkaa2 | 1.06653 |
| Prkaa2 | -0.21335 |
| Prkab1 | 0.48381 |
| Prkab2 | -0.03476 |
| Prkaca | 1.91186 |
| Prkaca | -0.81818 |
| Prkacb | 0.94501 |
| Prkag1 | -0.55681 |
| Prkag2 | -0.83134 |
| Prkar1a | 0.44272 |
| Prkar1b | -0.51905 |
| Prkar2b | 0.30296 |
| Prkca | 0.61439 |
| Prkcb1 | -0.90447 |
| Prkcbp1 | 1.07936 |
| Prkcbp1 | -0.91669 |
| Prkcc | -0.07325 |
| Prkcd | 0.99857 |
| Prkcdbp | 3.4485 |
| Prkcdbp | -0.76359 |
| Prkce | -0.38749 |
| Prkch | -0.45396 |
| Prkcq | -0.77024 |
| Prkcsh | -0.1867 |
| Prkcz | -0.13226 |
| Prkcz | -0.00527 |
| Prkcz | -0.4104 |
| Prkd1 | -0.61774 |
| Prkd1 | -0.73196 |
| Prkd2 | 0.3759 |
| Prkd3 | -0.28078 |
| Prkd3 | 3.68121 |
| Prkd3 | 0.90013 |
| Prkg1 | -0.71565 |
| Prkg2 | -0.25104 |
| Prkra | 0.14935 |
| Prkrip1 | -0.73288 |
| Prkrip1 | -0.48838 |
| Prkrir | 1.62897 |
| Prkx | 0.97333 |
| Prkx | 0.85815 |
| Prl | -0.23973 |
| Prlr | -0.58132 |
| Prlr | -0.16671 |
| Prm1 | 2.31051 |
| Prm3 | 2.71662 |
| Prmt2 | -0.64895 |
| Prmt2 | -0.19482 |
| Prmt3 | -0.41687 |
| Prmt5 | -0.3976 |
| Prmt5 | -0.16178 |
| Prmt5 | -0.53147 |
| Prmt7 | 0.43863 |
| Prmt7 | 0.4335 |
| Prmt7 | -0.56212 |
| Prmt7 | 0.00586 |
| Prmt8 | -0.18619 |
| Prnp | -0.08538 |
| Prnpip1 | -0.58288 |
| Prnpip1 | -0.33734 |
| Prnpip1 | -0.80819 |
| Proc | 1.09815 |
| Proc | 2.4812 |
| Procr | 1.99422 |
| Prodh | 0.84015 |
| Prok2 | -0.83032 |
| Prok2 | -0.76834 |
| Prokr1 | 0.83418 |
| Prokr2 | 0.14759 |
| Prom1 | -0.77982 |
| Prom2 | 0.44221 |
| Prom2 | 2.20726 |
| Pros1 | -0.29318 |
| Prosapip1 | 0.34668 |
| Prosapip1 | -0.81818 |
| Prosapip1 | -0.65819 |
| Prosc | 0.58081 |
| Prosc | -0.31383 |
| Prosc | -0.14521 |
| Prox1 | -0.44722 |
| Prox1 | 2.44144 |
| Prpf18 | -0.39443 |
| Prpf19 | -0.07503 |
| Prpf19 | -0.76929 |
| Prpf19 | 3.31192 |
| Prpf3 | -0.64645 |
| Prpf3 | 0.2973 |
| Prpf3 | 2.45574 |
| Prpf3 | 0.16945 |
| Prpf3 | -0.57205 |
| Prpf31 | -0.17815 |
| Prpf38a | -0.7495 |
| Prpf38a | -0.82221 |
| Prpf38a | 0.02893 |
| Prpf38b | 3.53834 |
| Prpf39 | 1.0311 |
| Prpf40a | -0.55304 |
| Prpf40a | 1.48048 |
| Prpf40b | -0.3578 |
| Prpf40b | -0.74485 |
| Prpf40b | -0.25159 |
| Prpf6 | 0.67391 |
| Prpf8 | -0.57205 |
| Prpf8 | -0.37067 |
| Prph2 | 0.06343 |
| Prps1 | -0.5278 |
| Prps1l1 | -0.73013 |
| Prps2 | -0.24403 |
| Prpsap2 | -0.55455 |
| Prpsap2 | 1.29928 |
| Prr11 | -0.84261 |
| Prr13 | -0.6065 |
| Prr14 | -0.17865 |
| Prr15 | 2.13987 |
| Prr16 | -0.38373 |
| Prr18 | 0.56311 |
| Prr19 | 1.56492 |
| Prr3 | 1.22938 |
| Prr7 | 0.41479 |
| Prrc1 | 2.7078 |
| Prrg2 | 0.12664 |
| Prrt1 | -0.84571 |
| Prrt1 | -0.3919 |
| Prrt1 | -0.40783 |
| Prrt3 | -0.75605 |
| Prrx1 | 0.18745 |
| Prrx1 | -0.12232 |
| Prrx1 | -0.17715 |
| Prss21 | -0.84055 |
| Prss23 | 0.68376 |
| Prss27 | -0.06039 |
| Prss28 | -0.10136 |
| Prss29 | 1.87781 |
| Prss29 | 0.51589 |
| Prss34 | -0.45599 |
| Prss35 | 1.66618 |
| Prss36 | -0.426 |
| Prss7 | -0.58521 |
| Prss8 | 0.15633 |
| Prss8 | 1.42208 |
| Prtn3 | 1.23832 |
| Prune | 0.8228 |
| Prune | -0.02582 |
| Prune | 1.67148 |
| Prune | 2.44726 |
| Psap | -0.63403 |
| Psat1 | 0.42679 |
| Psca | 0.48406 |
| Pscd1 | -0.75605 |
| Pscd2 | 2.49801 |
| Pscd3 | 0.49085 |
| Pscdbp | -0.50039 |
| Psd2 | 0.00217 |
| Psd3 | 1.22657 |
| Psd4 | 0.32782 |
| Psen1 | -0.63898 |
| Psen2 | 0.57245 |
| Psenen | -0.37811 |
| Psg19 | 1.93047 |
| Psg23 | -0.59383 |
| Psg27 | 0.39112 |
| Psip1 | -0.69609 |
| Psip1 | 2.84427 |
| Pskh1 | 2.74808 |
| Psma2 | 0.64535 |
| Psma4 | 0.34015 |
| Psma5 | 0.58954 |
| Psma7 | 2.8951 |
| Psma7 | -0.29773 |
| Psmb1 | -0.70315 |
| Psmb10 | 1.38593 |
| Psmb10 | -0.39633 |
| Psmb2 | -0.68386 |
| Psmb2 | -0.20559 |
| Psmb3 | -0.21387 |
| Psmb4 | 0.46387 |
| Psmb5 | -0.18822 |
| Psmb6 | -0.36821 |
| Psmb7 | -0.40334 |
| Psmb7 | -0.84261 |
| Psmb7 | -0.52634 |
| Psmb8 | -0.08992 |
| Psmb9 | 1.71153 |
| Psmc1 | 0.97447 |
| Psmc2 | -0.8509 |
| Psmc3 | -0.86348 |
| Psmc3ip | -0.7779 |
| Psmc4 | 1.48508 |
| Psmc5 | -0.87089 |
| Psmc5 | 0.95571 |
| Psmd1 | 0.19081 |
| Psmd10 | -0.86032 |
| Psmd10 | -0.32258 |
| Psmd10 | -0.46074 |
| Psmd11 | -0.50252 |
| Psmd11 | 1.74702 |
| Psmd12 | 2.12284 |
| Psmd12 | 1.64906 |
| Psmd13 | 0.84657 |
| Psmd14 | 2.02428 |
| Psmd2 | 1.28431 |
| Psmd2 | 0.48722 |
| Psmd4 | -0.1938 |
| Psmd5 | -0.53147 |
| Psmd6 | 0.60611 |
| Psmd6 | 0.75136 |
| Psmd7 | -0.60411 |
| Psmd7 | 1.79808 |
| Psmd8 | 0.33873 |
| Psmd9 | 1.42594 |
| Psmd9 | 0.29162 |
| Psme1 | -0.29944 |
| Psme1 | 0.35764 |
| Psme2 | 1.02585 |
| Psme3 | 0.45562 |
| Psme4 | -0.81718 |
| Psme4 | -0.66923 |
| Psmf1 | 0.62885 |
| Psmf1 | -0.5644 |
| Psmg2 | 2.33163 |
| Psmg2 | 2.08724 |
| Psmg3 | 2.29794 |
| Psors1c2 | 2.20243 |
| Psp | 0.77242 |
| Pspc1 | -0.31674 |
| Pstpip1 | 0.43735 |
| Pstpip2 | 0.84739 |
| Ptbp1 | -0.65566 |
| Ptbp1 | -0.06215 |
| Ptcd3 | 2.7235 |
| Ptch1 | 1.53351 |
| Ptch2 | -0.81618 |
| Ptdss2 | 0.80081 |
| Pten | 3.47969 |
| Pter | -0.62912 |
| Ptgds | 1.10351 |
| Ptgds2 | 2.29899 |
| Ptgds2 | 0.68337 |
| Ptger2 | -0.65734 |
| Ptger2 | -0.7495 |
| Ptger3 | -0.21283 |
| Ptges | -0.55908 |
| Ptges | -0.25213 |
| Ptges2 | 0.3353 |
| Ptges3 | 1.0695 |
| Ptgfr | 0.00914 |
| Ptgir | -0.73838 |
| Ptgis | -0.5011 |
| Ptgs1 | -0.71475 |
| Ptgs1 | -0.90337 |
| Pth2 | -0.46483 |
| Pth2r | -0.47584 |
| Pthlh | 0.20147 |
| Pthr1 | -0.87409 |
| Pthr1 | -0.88483 |
| Pthr1 | 0.20114 |
| Ptk2 | 0.96095 |
| Ptk2b | 2.61284 |
| Ptk7 | -0.67781 |
| Ptms | 0.30533 |
| Ptn | -0.7005 |
| Ptp4a2 | -0.10829 |
| Ptp4a2 | 1.78629 |
| Ptp4a2 | -0.8293 |
| Ptp4a2 | -0.72196 |
| Ptp4a3 | -0.53663 |
| Ptp4a3 | -0.14908 |
| Ptpdc1 | 0.95877 |
| Ptpdc1 | 0.50548 |
| Ptpdc1 | 0.02291 |
| Ptpla | -0.53294 |
| Ptpla | 0.1605 |
| Ptplad1 | -0.37624 |
| Ptplad2 | 0.27922 |
| Ptplb | -0.44388 |
| Ptpn1 | -0.64812 |
| Ptpn11 | -0.3976 |
| Ptpn11 | -0.09174 |
| Ptpn11 | -0.20972 |
| Ptpn12 | -0.61693 |
| Ptpn12 | -0.54554 |
| Ptpn12 | -0.2819 |
| Ptpn12 | -0.82625 |
| Ptpn12 | 0.24607 |
| Ptpn14 | 1.0847 |
| Ptpn18 | 2.64047 |
| Ptpn20 | 1.84876 |
| Ptpn21 | -0.34631 |
| Ptpn22 | -0.86771 |
| Ptpn5 | 2.07989 |
| Ptpn6 | -0.83644 |
| Ptpn6 | -0.10551 |
| Ptpra | -0.37624 |
| Ptprb | -0.09356 |
| Ptprd | 1.10301 |
| Ptprd | -0.40912 |
| Ptpre | -0.17266 |
| Ptpre | -0.00278 |
| Ptprf | 0.72963 |
| Ptprf | -0.79439 |
| Ptprg | 0.34979 |
| Ptprj | -0.66667 |
| Ptprk | 0.96687 |
| Ptprk | -0.05951 |
| Ptprn | 1.44889 |
| Ptprn2 | -0.26745 |
| Ptpro | 0.9083 |
| Ptpro | -0.76834 |
| Ptprq | 1.20215 |
| Ptprr | 1.8051 |
| Ptprr | 1.62292 |
| Ptprs | 0.45008 |
| Ptprs | -0.76929 |
| Ptprt | -0.10829 |
| Ptpru | 0.83916 |
| Ptprv | -0.54033 |
| Ptrf | -0.61532 |
| Ptrh1 | 3.23552 |
| Ptrh1 | -0.5278 |
| Ptrh2 | 1.76484 |
| Pts | -0.65146 |
| Pts | -0.64312 |
| Pttg1 | 0.64677 |
| Pttg1ip | 1.93233 |
| Ptx3 | 1.02369 |
| Ptx3 | -0.41169 |
| Puf60 | 1.65922 |
| Puf60 | -0.85717 |
| Pum1 | -0.0438 |
| Pum2 | -0.47653 |
| Pum2 | 0.20543 |
| Punc | 1.93941 |
| Purb | -0.743 |
| Purg | 1.23413 |
| Pus1 | 0.24985 |
| Pus1 | -0.78562 |
| Pus10 | -0.06259 |
| Pus10 | 0.73223 |
| Pus10 | 0.56043 |
| Pus3 | -0.80819 |
| Pus7l | -0.18418 |
| Pvalb | -0.45802 |
| Pvr | 0.5762 |
| Pvr | -0.74578 |
| Pvrl1 | 1.95698 |
| Pvrl2 | -0.11294 |
| Pvrl2 | 2.58514 |
| Pvrl2 | 0.99551 |
| Pvrl3 | -0.66327 |
| Pvrl3 | 1.26879 |
| Pvrl3 | -0.60332 |
| Pwp1 | 2.45471 |
| Pwp2 | -0.01948 |
| Pwwp2b | -0.61613 |
| Pxdn | 1.56116 |
| Pxmp2 | 0.93746 |
| Pxmp4 | -0.27855 |
| Pxn | -0.66072 |
| Pxt1 | 0.17458 |
| Pycr2 | 1.79106 |
| Pycrl | 2.3875 |
| Pygb | -0.40847 |
| Pygl | -0.18872 |
| Pygl | 0.05803 |
| Pygm | -0.44121 |
| Pygo1 | -0.60014 |
| Pygo2 | 0.32955 |
| Pyroxd1 | -0.71565 |
| Pyy | -0.8072 |
| Qars | 0.0213 |
| Qdpr | 0.28317 |
| Qpct | 0.78812 |
| Qprt | -0.30287 |
| Qrich2 | 1.44787 |
| Qrich2 | 1.66611 |
| Qrsl1 | 1.25605 |
| Qsox1 | -0.6856 |
| Qtrt1 | 0.20905 |
| R3hdm1 | 3.33354 |
| R3hdm1 | -0.6121 |
| R3hdm2 | 0.07339 |
| R74862 | -0.27076 |
| Rab1 | 0.21755 |
| Rab10 | 1.69616 |
| Rab10 | -0.90779 |
| Rab11a | 0.02653 |
| Rab11b | 0.47229 |
| Rab11fip2 | 0.95659 |
| Rab11fip3 | -0.53663 |
| Rab11fip5 | 0.97275 |
| Rab11fip5 | 1.37694 |
| Rab11fip5 | 1.11938 |
| Rab12 | 0.44982 |
| Rab13 | 1.06148 |
| Rab13 | -0.06215 |
| Rab14 | -0.43258 |
| Rab15 | 0.24765 |
| Rab17 | 3.17181 |
| Rab17 | -0.66242 |
| Rab18 | 0.54049 |
| Rab19 | -0.57436 |
| Rab19 | 0.92879 |
| Rab1b | 0.96687 |
| Rab1b | -0.51978 |
| Rab1b | 0.03092 |
| Rab21 | 0.80682 |
| Rab22a | 1.51013 |
| Rab22a | -0.48628 |
| Rab23 | 1.02504 |
| Rab24 | -0.53147 |
| Rab24 | 0.03965 |
| Rab24 | -0.00817 |
| Rab25 | 1.44076 |
| Rab27a | 2.3644 |
| Rab27b | 1.43818 |
| Rab28 | -0.52269 |
| Rab28 | 0.89376 |
| Rab2a | 1.95821 |
| Rab31 | 2.58472 |
| Rab32 | -0.29204 |
| Rab33a | -0.63815 |
| Rab33b | -0.20765 |
| Rab34 | -0.16129 |
| Rab34 | 2.91972 |
| Rab34 | 0.0345 |
| Rab35 | 0.16602 |
| Rab36 | -0.61613 |
| Rab38 | -0.13369 |
| Rab38 | -0.63403 |
| Rab39 | -0.66752 |
| Rab39b | -0.50181 |
| Rab3a | -0.35355 |
| Rab3b | -0.08765 |
| Rab3d | -0.57513 |
| Rab3gap1 | -0.73746 |
| Rab3gap2 | -0.49119 |
| Rab3il1 | -0.43192 |
| Rab3il1 | -0.21283 |
| Rab3ip | -0.44991 |
| Rab40b | -0.55455 |
| Rab40c | -0.14473 |
| Rab43 | -0.66242 |
| Rab4a | 0.18002 |
| Rab4b | 1.10648 |
| Rab5a | 2.10573 |
| Rab5a | -0.57282 |
| Rab5b | -0.70315 |
| Rab5c | 0.03331 |
| Rab6 | -0.51543 |
| Rab6 | 0.07908 |
| Rab6 | -0.43126 |
| Rab6b | -0.5322 |
| Rab6ip1 | 0.6749 |
| Rab7 | 0.39756 |
| Rab7 | 2.185 |
| Rab7 | -0.69962 |
| Rab7l1 | -0.50609 |
| Rab8a | -0.21024 |
| Rab8a | -0.59225 |
| Rab8b | -0.28302 |
| Rab8b | 0.0034 |
| Rab9 | -0.5068 |
| Rab9 | 0.51117 |
| Rab9b | 1.80932 |
| Rabac1 | 0.66295 |
| Rabac1 | 2.67575 |
| Rabep1 | -0.5553 |
| Rabep1 | -0.3938 |
| Rabep2 | -0.39824 |
| Rabgap1 | 0.25142 |
| Rabgap1 | -0.25976 |
| Rabgap1l | -0.03135 |
| Rabgap1l | -0.12467 |
| Rabgef1 | -0.22276 |
| Rabggta | 0.48576 |
| Rabggtb | -0.60411 |
| Rabif | 0.82094 |
| Rabif | -0.40719 |
| Rabl2a | 2.44262 |
| Rabl2a | -0.5954 |
| Rabl3 | -0.42732 |
| Rabl4 | -0.51833 |
| Rabl4 | 0.34611 |
| Rabl5 | 0.72477 |
| Rac1 | 0.99871 |
| Rac2 | -0.67694 |
| Rac3 | 1.08748 |
| Rac3 | -0.00651 |
| Rad1 | -0.74578 |
| Rad17 | 1.08546 |
| Rad18 | -0.32551 |
| Rad23a | -0.43655 |
| Rad23a | 0.04163 |
| Rad23a | -0.51039 |
| Rad23b | -0.06039 |
| Rad50 | 1.9628 |
| Rad51 | -0.3831 |
| Rad51 | -0.64312 |
| Rad51ap1 | 0.47377 |
| Rad51c | 0.66095 |
| Rad51l3 | 0.58475 |
| Rad51l3 | 2.08554 |
| Rad51l3 | -0.68908 |
| Rad51l3 | 2.03848 |
| Rad52 | -0.43324 |
| Rad54l | -0.00776 |
| Rad9 | -0.06303 |
| Rad9 | -0.51471 |
| Rad9 | -0.48838 |
| Rad9b | -0.51543 |
| Rae1 | -0.50395 |
| Raet1b | -0.73105 |
| Raet1b | 0.19649 |
| Raf1 | -0.34391 |
| Rag1 | 0.37316 |
| Rag1ap1 | -0.77502 |
| Rage | -0.66752 |
| Rage | -0.60093 |
| Rai1 | -0.32727 |
| Rai12 | 0.10593 |
| Rai12 | 0.34498 |
| Rai12 | -0.12184 |
| Rai12 | 2.73293 |
| Rai14 | -0.40912 |
| Rala | 2.04394 |
| Ralb | -0.03734 |
| Ralbp1 | -0.53441 |
| Ralgds | 1.49939 |
| Ralgps1 | 2.02127 |
| Ralgps1 | 0.44323 |
| Ralgps1 | -0.13417 |
| Ralgps2 | 0.3373 |
| Ralgps2 | 0.01443 |
| Raly | 1.69941 |
| Raly | -0.51543 |
| Ralyl | 2.16594 |
| Ramp1 | -0.20662 |
| Ramp2 | -0.61291 |
| Ramp3 | 1.98041 |
| Ramp3 | -0.01569 |
| Ran | -0.69171 |
| Ranbp1 | -0.03391 |
| Ranbp10 | 0.62196 |
| Ranbp17 | -0.52196 |
| Ranbp2 | -0.72015 |
| Ranbp3l | 0.60589 |
| Ranbp6 | 1.79953 |
| Ranbp9 | 2.06671 |
| Rangap1 | -0.55833 |
| Rangrf | 1.12461 |
| Rap1b | -0.54331 |
| Rap1gap | 3.22272 |
| Rap2a | -0.69609 |
| Rap2b | -0.63486 |
| Rap2c | 0.16499 |
| Rap2c | -0.7005 |
| Rap2c | 0.20938 |
| Rap2ip | 1.46134 |
| Rapgef1 | 0.26202 |
| Rapgef1 | -0.35234 |
| Rapgef1 | -0.20096 |
| Rapgef1 | -0.39507 |
| Rapgef3 | -0.56669 |
| Rapgef4 | 1.0564 |
| Rapgef5 | 2.48706 |
| Rapgef6 | -0.63486 |
| Rapgefl1 | 0.92774 |
| Rapgefl1 | -0.27353 |
| Rapsn | -0.70138 |
| Rapsn | -0.15541 |
| Rara | 0.67252 |
| Rarb | -0.68386 |
| Rarb | 0.28287 |
| Rarg | -0.62504 |
| Rarg | -0.29318 |
| Rarg | -0.45802 |
| Rarres2 | 2.19599 |
| Rars | -0.73379 |
| Rars | -0.78079 |
| Rars2 | -0.5899 |
| Rars2 | 1.68707 |
| Rars2 | -0.31209 |
| Rasa1 | -0.50466 |
| Rasa2 | -0.45396 |
| Rasal1 | -0.32141 |
| Rasd1 | 1.78617 |
| Rasef | -0.49684 |
| Rasgef1b | -0.2603 |
| Rasgef1b | -0.58365 |
| Rasgef1c | -0.12799 |
| Rasgef1c | -0.62179 |
| Rasgrf1 | -0.66242 |
| Rasgrf1 | 0.24576 |
| Rasgrf1 | -0.69258 |
| Rasgrf1 | -0.10783 |
| Rasgrp1 | -0.46963 |
| Rasgrp1 | -0.6065 |
| Rasgrp1 | -0.43921 |
| Rasgrp3 | 0.22469 |
| Rasip1 | 0.51565 |
| Rasl10a | -0.3831 |
| Rasl10b | 1.14985 |
| Rasl10b | -0.72105 |
| Rasl11a | 0.44982 |
| Rasl11b | 0.83584 |
| Rasl11b | 1.56514 |
| Rasl12 | 1.05809 |
| Rasl2-9 | -0.47515 |
| Rassf1 | 1.93926 |
| Rassf1 | -0.50967 |
| Rassf10 | -0.36513 |
| Rassf2 | -0.44054 |
| Rassf2 | -0.34992 |
| Rassf3 | 2.4087 |
| Rassf4 | -0.03434 |
| Rassf4 | 0.55074 |
| Rassf4 | -0.25593 |
| Rassf4 | -0.45261 |
| Rassf5 | 0.44374 |
| Rassf7 | -0.53 |
| Rassf7 | 2.04469 |
| Raver1 | -0.09311 |
| Raver2 | 0.37206 |
| Rb1 | 0.34243 |
| Rb1 | 0.75663 |
| Rbak | -0.26469 |
| Rbak | -0.44857 |
| Rbbp5 | -0.2344 |
| Rbbp5 | -0.15492 |
| Rbbp6 | -0.52415 |
| Rbbp7 | -0.42469 |
| Rbbp7 | 1.20759 |
| Rbbp9 | -0.68908 |
| Rbck1 | -0.27187 |
| Rbed1 | -0.72286 |
| Rbed1 | -0.26414 |
| Rbj | -0.37624 |
| Rbks | -0.27855 |
| Rbl1 | 0.59236 |
| Rbl2 | 0.33587 |
| Rbm10 | 1.66348 |
| Rbm10 | -0.63239 |
| Rbm10 | 2.23133 |
| Rbm11 | -0.72831 |
| Rbm12 | 3.14797 |
| Rbm12 | 2.07387 |
| Rbm12 | 2.06462 |
| Rbm12 | 0.13983 |
| Rbm13 | -0.50824 |
| Rbm13 | 1.34877 |
| Rbm14 | -0.45734 |
| Rbm14 | 0.62844 |
| Rbm15 | 1.37445 |
| Rbm16 | 1.03979 |
| Rbm18 | -0.64812 |
| Rbm18 | -0.29431 |
| Rbm19 | 0.26326 |
| Rbm24 | 1.54786 |
| Rbm26 | -0.42012 |
| Rbm28 | 1.86343 |
| Rbm28 | 0.75735 |
| Rbm3 | -0.2752 |
| Rbm35a | -0.50824 |
| Rbm38 | 0.07034 |
| Rbm38 | -0.53294 |
| Rbm38 | -0.31441 |
| Rbm4 | 1.10895 |
| Rbm41 | 0.32754 |
| Rbm42 | 0.17458 |
| Rbm42 | 0.07832 |
| Rbm43 | -0.47722 |
| Rbm45 | -0.56745 |
| Rbm47 | 0.39246 |
| Rbm47 | -0.49755 |
| Rbm47 | 0.9637 |
| Rbm47 | 2.60531 |
| Rbm4b | -0.77119 |
| Rbm5 | -0.16869 |
| Rbm5 | 1.89654 |
| Rbm6 | 0.00995 |
| Rbm6 | 2.41376 |
| Rbm6 | 2.40126 |
| Rbm8a | -0.40143 |
| Rbm9 | -0.35841 |
| Rbm9 | -0.37873 |
| Rbms1 | 0.5611 |
| Rbms1 | -0.53959 |
| Rbms2 | 1.97787 |
| Rbms2 | -0.6105 |
| Rbms3 | 0.99201 |
| Rbmx | 1.07643 |
| Rbmy1a1 | 1.0642 |
| Rbp1 | -0.34932 |
| Rbp7 | 2.96002 |
| Rbpms | 2.5087 |
| Rbpms | 1.22526 |
| Rbpms | -0.55379 |
| Rbpms2 | 2.21444 |
| Rbx1 | 0.08474 |
| Rbx1 | -0.52561 |
| Rc3h1 | 1.14843 |
| Rcan1 | 0.09185 |
| Rcan2 | -0.52634 |
| Rcan2 | 1.46436 |
| Rcan2 | -0.70582 |
| Rcan3 | -0.6365 |
| Rcbtb2 | -0.73288 |
| Rcbtb2 | -0.68821 |
| Rcc1 | 0.13664 |
| Rccd1 | 1.12011 |
| Rce1 | -0.03906 |
| Rchy1 | 0.13129 |
| Rcl1 | 1.8724 |
| Rcl1 | -0.59935 |
| Rcl1 | 0.61839 |
| Rcn3 | -0.12326 |
| Rcor1 | 0.21495 |
| Rcor2 | 0.08059 |
| Rcsd1 | 2.08707 |
| Rdbp | -0.3919 |
| Rdbp | 0.02573 |
| Rdh11 | -0.4621 |
| Rdh12 | 1.15503 |
| Rdh14 | -0.54182 |
| Rdh14 | -0.23015 |
| Rdh5 | -0.01151 |
| Rdh7 | 0.45587 |
| Rdh9 | 2.05946 |
| Rdm1 | -0.6804 |
| Rec8 | 1.70755 |
| Reck | 0.35288 |
| Recql | 0.99802 |
| Recql4 | -0.80127 |
| Recql5 | 1.25679 |
| Reep1 | 2.2241 |
| Reep2 | -0.26359 |
| Reep3 | 3.31207 |
| Reep4 | -0.42404 |
| Reep5 | -0.34812 |
| Reep6 | 1.54525 |
| Reep6 | 1.92383 |
| Refbp2 | -0.45599 |
| Reg1 | -0.29148 |
| Rela | -0.72015 |
| Relb | 0.63093 |
| Rell1 | 1.24367 |
| Rell2 | 2.43679 |
| Rell2 | 1.42384 |
| Reln | 2.21492 |
| Relt | 0.2084 |
| Relt | 0.74424 |
| Rem1 | -0.05512 |
| Rem2 | 2.49167 |
| Renbp | 0.95265 |
| Repin1 | -0.72467 |
| Repin1 | 0.13022 |
| Reps1 | -0.34271 |
| Reps1 | 0.04044 |
| Rer1 | 0.3071 |
| Rer1 | 0.14654 |
| Rerg | 0.38598 |
| Resp18 | 1.40015 |
| Rest | -0.37811 |
| Ret | 1.89271 |
| Retnla | -0.81118 |
| Retnlg | 0.2725 |
| Retsat | -0.53737 |
| Retsat | -0.41234 |
| Rev1 | 1.56814 |
| Rev3l | 0.73279 |
| Rex3 | 2.42708 |
| Rexo1 | -0.20301 |
| Rexo1 | -0.74857 |
| Rexo2 | 3.30308 |
| Rfc1 | 2.63847 |
| Rfc2 | 2.121 |
| Rfc3 | -0.71565 |
| Rfc4 | -0.1032 |
| Rfc4 | -0.59304 |
| Rfc5 | 0.61881 |
| Rfesd | 1.11852 |
| Rffl | 1.31597 |
| Rffl | -0.65314 |
| Rffl | 3.00587 |
| Rffl | -0.79439 |
| Rffl | -0.50323 |
| Rfng | 3.39182 |
| Rfpl4 | 2.39717 |
| Rfpl4 | -0.36759 |
| Rft1 | 0.17048 |
| Rftn1 | 0.00668 |
| Rftn2 | 1.07834 |
| Rftn2 | 2.27377 |
| Rfwd2 | -0.64979 |
| Rfwd3 | 0.3582 |
| Rfwd3 | 1.01471 |
| Rfx1 | 1.32499 |
| Rfx1 | 1.21905 |
| Rfx2 | 1.33373 |
| Rfx3 | -0.7523 |
| Rfx4 | -0.47376 |
| Rfx4 | 0.54529 |
| Rfx4 | -0.42273 |
| Rfxank | 2.65985 |
| Rfxap | 2.87202 |
| Rfxdc1 | 1.16808 |
| Rg9mtd1 | -0.77886 |
| Rg9mtd2 | 0.983 |
| Rgag4 | -0.17415 |
| Rgl1 | 1.70717 |
| Rgl2 | -0.45396 |
| Rgl2 | -0.6065 |
| Rgl3 | 1.42305 |
| Rgma | 0.93523 |
| Rgmb | 2.73845 |
| Rgmb | 2.57773 |
| Rgnef | 2.1189 |
| Rgs1 | -0.70849 |
| Rgs1 | -0.47653 |
| Rgs1 | -0.73196 |
| Rgs10 | -0.59147 |
| Rgs12 | 3.00833 |
| Rgs14 | 2.79591 |
| Rgs16 | 0.36875 |
| Rgs17 | 1.44162 |
| Rgs2 | 1.60476 |
| Rgs3 | 0.29102 |
| Rgs3 | 1.15174 |
| Rgs3 | -0.3179 |
| Rgs3 | 1.13905 |
| Rgs3 | -0.81318 |
| Rgs3 | -0.82221 |
| Rgs3 | -0.53147 |
| Rgs4 | 3.76748 |
| Rgs5 | -0.04251 |
| Rgs6 | -0.12467 |
| Rgs7 | 0.73705 |
| Rgs7bp | 1.49193 |
| Rgs8 | -0.01444 |
| Rgs9 | 2.15612 |
| Rgs9bp | -0.76454 |
| Rhag | 3.27707 |
| Rhbdd1 | 1.22906 |
| Rhbdd3 | -0.64895 |
| Rhbdf1 | 2.7632 |
| Rhbdf2 | -0.4104 |
| Rhbdl1 | -0.4621 |
| Rhbdl2 | 2.35172 |
| Rhbdl3 | 0.26728 |
| Rhbdl7 | 0.71535 |
| Rhcg | 1.87957 |
| Rheb | 0.67034 |
| Rhebl1 | -0.57359 |
| Rhob | -0.25267 |
| Rhobtb2 | 1.20437 |
| Rhobtb2 | 1.24643 |
| Rhobtb2 | -0.74671 |
| Rhobtb2 | 1.4471 |
| Rhobtb3 | 0.80597 |
| Rhoc | -0.73196 |
| Rhoc | 0.41898 |
| Rhod | 3.68649 |
| Rhof | 0.3353 |
| Rhog | -0.16819 |
| Rhoj | -0.80324 |
| Rhot1 | 1.30488 |
| Rhot2 | 2.00279 |
| Rhot2 | 0.08021 |
| Rhot2 | 0.12735 |
| Rhou | -0.5176 |
| Rhov | -0.23387 |
| Rhox11 | -0.7076 |
| Rhox12 | 0.8469 |
| Rhox3a | -0.55681 |
| Rhox3f | 1.84512 |
| Rhox4d | -0.58755 |
| Rhox5 | -0.84571 |
| Rhox8 | -0.81418 |
| Rhpn1 | 0.64473 |
| Rhpn1 | -0.17117 |
| Rhpn1 | 0.2722 |
| Ribc1 | 0.15389 |
| Ric3 | 1.34487 |
| Ric3 | -0.54554 |
| Ric3 | 1.99643 |
| Ric8b | -0.74115 |
| Ric8b | -0.01276 |
| Rif1 | 0.31854 |
| Rilp | -0.75605 |
| Rilpl1 | 0.33272 |
| Rilpl1 | -0.55079 |
| Rilpl2 | 0.4417 |
| Rims1 | 0.18239 |
| Rims2 | -0.52196 |
| Rims3 | 2.30552 |
| Rin1 | -0.37253 |
| Rin2 | -0.59619 |
| Rin2 | -0.73105 |
| Rin2 | 0.91244 |
| Rin3 | -0.67436 |
| Riok1 | 0.21918 |
| Riok1 | 1.8058 |
| Riok3 | -0.58599 |
| Riok3 | 0.00954 |
| Ripk1 | -0.1677 |
| Ripk2 | 0.01199 |
| Ripk4 | 2.45174 |
| Ripk5 | 1.00121 |
| Rit1 | -0.28583 |
| Rit2 | -0.42273 |
| Rlbp1 | -0.35902 |
| Rlbp1l1 | 1.65651 |
| Rlbp1l1 | -0.70671 |
| Rln1 | 1.33903 |
| Rltpr | 1.03016 |
| Rmnd1 | 1.47784 |
| Rmnd5b | -0.64979 |
| Rmnd5b | 0.5088 |
| Rnase1 | 1.46804 |
| Rnase4 | -0.64728 |
| Rnase4 | 1.35851 |
| Rnase6 | -0.62667 |
| Rnaseh1 | 0.3353 |
| Rnaseh1 | -0.50039 |
| Rnaseh2c | 0.26913 |
| Rnasek | -0.44924 |
| Rnasen | 1.10077 |
| Rnaset2 | -0.02624 |
| Rnaset2b | -0.58288 |
| Rnd2 | 0.34158 |
| Rnd3 | 1.66954 |
| Rnf10 | -0.08901 |
| Rnf103 | -0.68473 |
| Rnf11 | -0.71745 |
| Rnf11 | -0.3314 |
| Rnf11 | -0.5954 |
| Rnf111 | -0.63157 |
| Rnf113a1 | 1.56055 |
| Rnf113a2 | 3.67735 |
| Rnf12 | -0.52707 |
| Rnf121 | -0.52707 |
| Rnf123 | -0.65398 |
| Rnf125 | -0.08946 |
| Rnf125 | -0.31036 |
| Rnf128 | -0.40976 |
| Rnf133 | 2.95334 |
| Rnf135 | 2.2209 |
| Rnf135 | 0.44119 |
| Rnf135 | -0.56822 |
| Rnf138 | 1.92632 |
| Rnf138 | -0.20972 |
| Rnf138 | -0.42535 |
| Rnf138 | 1.42515 |
| Rnf139 | 0.13237 |
| Rnf139 | -0.48838 |
| Rnf14 | -0.57513 |
| Rnf14 | -0.69258 |
| Rnf14 | -0.24726 |
| Rnf141 | 4.0548 |
| Rnf141 | 2.03803 |
| Rnf144a | -0.33734 |
| Rnf145 | -0.83235 |
| Rnf150 | -0.62504 |
| Rnf166 | 0.1504 |
| Rnf167 | -0.49119 |
| Rnf167 | -0.41557 |
| Rnf167 | -0.19431 |
| Rnf167 | -0.67008 |
| Rnf167 | -0.03092 |
| Rnf168 | 0.01646 |
| Rnf17 | 0.9379 |
| Rnf170 | -0.78659 |
| Rnf181 | 2.94889 |
| Rnf182 | 0.70315 |
| Rnf182 | 1.35606 |
| Rnf183 | -0.55455 |
| Rnf185 | 0.33358 |
| Rnf185 | 0.04752 |
| Rnf185 | 1.92078 |
| Rnf185 | 0.12915 |
| Rnf186 | 2.46898 |
| Rnf19a | -0.81518 |
| Rnf2 | 1.00426 |
| Rnf20 | 1.01539 |
| Rnf207 | 1.32421 |
| Rnf208 | 3.34783 |
| Rnf214 | 0.38027 |
| Rnf215 | -0.45802 |
| Rnf216 | 2.3082 |
| Rnf216 | -0.46074 |
| Rnf219 | 0.2221 |
| Rnf24 | -0.6565 |
| Rnf24 | 0.51871 |
| Rnf25 | -0.32024 |
| Rnf25 | -0.73838 |
| Rnf25 | -0.08132 |
| Rnf26 | -0.83134 |
| Rnf31 | 1.65664 |
| Rnf32 | 1.18117 |
| Rnf34 | -0.15492 |
| Rnf34 | -0.12043 |
| Rnf34 | 3.82605 |
| Rnf38 | 1.58498 |
| Rnf38 | 2.39322 |
| Rnf38 | 3.53245 |
| Rnf4 | -0.69171 |
| Rnf4 | -0.15492 |
| Rnf40 | -0.78272 |
| Rnf41 | -0.37067 |
| Rnf41 | -0.50395 |
| Rnf41 | -0.41299 |
| Rnf44 | -0.77215 |
| Rnf5 | -0.45599 |
| Rnf6 | -0.25702 |
| Rnf6 | -0.77024 |
| Rnf8 | -0.31965 |
| Rnf8 | -0.76265 |
| Rngtt | -0.0986 |
| Rnmt | 1.63344 |
| Rnmtl1 | 0.58824 |
| Rnpc2 | -0.02201 |
| Rnpep | -0.76265 |
| Rnpepl1 | 0.44246 |
| Rnps1 | -0.48209 |
| Rnps1 | 0.20147 |
| Rnu6 | -0.59068 |
| Rnuxa | 0.20279 |
| Rnuxa | -0.7076 |
| Robo1 | -0.79831 |
| Robo2 | -0.53811 |
| Robo4 | -0.06127 |
| Rock1 | -0.24888 |
| Rock2 | 0.77686 |
| Rock2 | 0.00627 |
| Rod1 | 0.64963 |
| Rogdi | -0.2864 |
| Rom1 | 0.92443 |
| Ropn1 | 0.34781 |
| Ror1 | -0.48978 |
| Ror2 | 1.42094 |
| Rora | 1.41123 |
| Rorb | -0.77024 |
| Rorb | -0.47653 |
| Rorc | -0.62341 |
| Rorc | 2.95068 |
| Ros1 | -0.01026 |
| Rp23-297j14.5 | 2.07222 |
| Rp23-438h3.2 | 0.67668 |
| rp9 | -0.34631 |
| rp9 | 0.91076 |
| Rpa1 | -0.22856 |
| Rpa2 | -0.4587 |
| Rpa2 | -0.64229 |
| Rpa3 | 1.0249 |
| Rpain | 1.14678 |
| Rpap1 | -0.7076 |
| Rpap1 | 0.86091 |
| Rpap2 | -0.65062 |
| Rpap2 | 0.27709 |
| Rpap3 | 1.1822 |
| Rpe | -0.30805 |
| Rpgr | -0.44655 |
| Rpgrip1 | 1.536 |
| Rpgrip1l | 3.44906 |
| Rph3a | -0.17366 |
| Rph3al | 1.05679 |
| Rpia | 0.09632 |
| Rpl12 | -0.32963 |
| Rpl13a | -0.48768 |
| Rpl18 | 0.1898 |
| Rpl19 | 0.94781 |
| Rpl21 | -0.84675 |
| Rpl21 | 1.11105 |
| Rpl22 | 0.47155 |
| Rpl22l1 | -0.64063 |
| Rpl23 | 0.98906 |
| Rpl23 | 0.10298 |
| Rpl23 | 0.08924 |
| Rpl24 | 0.72701 |
| Rpl27 | -0.64979 |
| Rpl27a | -0.63733 |
| Rpl28 | -0.4587 |
| Rpl29 | -0.60093 |
| Rpl29 | 0.13735 |
| Rpl3 | -0.63486 |
| Rpl30 | 0.64718 |
| Rpl31 | -0.73105 |
| Rpl31 | -0.48418 |
| Rpl34 | 2.59599 |
| Rpl35 | -0.03777 |
| Rpl36a | -0.22487 |
| Rpl36al | -0.28696 |
| Rpl37 | -0.58755 |
| Rpl38 | 0.16395 |
| Rpl38 | -0.07817 |
| Rpl39 | 0.53728 |
| Rpl39l | 1.7 |
| Rpl3l | 1.61107 |
| Rpl4 | -0.64979 |
| Rpl41 | 0.51941 |
| Rpl7a | -0.53294 |
| Rpl7l1 | 0.79908 |
| Rpl7l1 | 1.14938 |
| Rpl9 | -0.57052 |
| Rplp1 | -0.81118 |
| Rpn2 | 0.47795 |
| Rpo1-1 | -0.08403 |
| Rpo1-3 | 0.39112 |
| Rpo1-3 | 1.90119 |
| Rpo1-4 | 2.3329 |
| Rpo1-4 | 0.32174 |
| Rpo1-4 | -0.52051 |
| Rpo2tc1 | 2.13476 |
| Rpp21 | -0.17018 |
| Rpp25 | -0.17815 |
| Rpp30 | -0.50609 |
| Rpp38 | 2.00879 |
| Rpp40 | -0.07907 |
| Rprm | 3.18807 |
| Rprml | -0.69083 |
| Rps10 | -0.88375 |
| Rps11 | 1.2027 |
| Rps12 | 0.22533 |
| Rps13 | -0.27855 |
| Rps14 | 1.60368 |
| Rps15 | -0.60173 |
| Rps15a | -0.79831 |
| Rps16 | -0.58365 |
| Rps19 | -0.48348 |
| Rps19 | 0.09818 |
| Rps19bp1 | -0.76644 |
| Rps2 | 1.20626 |
| Rps21 | 0.32261 |
| Rps24 | 3.76255 |
| Rps24 | 0.52152 |
| Rps25 | 0.21983 |
| Rps26 | -0.68386 |
| Rps27a | 0.13593 |
| Rps27a | 0.80201 |
| Rps27l | 0.35007 |
| Rps3 | 0.33644 |
| Rps3 | 2.02099 |
| Rps3a | -0.48768 |
| Rps4x | -0.54929 |
| Rps4y2 | 0.07415 |
| Rps5 | -0.06259 |
| Rps6 | 0.70545 |
| Rps6ka1 | 1.5796 |
| Rps6ka3 | 3.43869 |
| Rps6ka4 | -0.55455 |
| Rps6ka5 | -0.19737 |
| Rps6kb1 | 2.13979 |
| Rps6kl1 | -0.31209 |
| Rps7 | 1.39907 |
| Rps8 | 0.94766 |
| Rps9 | 0.07567 |
| Rps9 | -0.41299 |
| Rpsa | -0.92794 |
| Rpusd1 | 0.87946 |
| Rpusd4 | 0.42705 |
| Rqcd1 | -0.82322 |
| Rrad | 2.06919 |
| Rragb | 3.25014 |
| Rragc | -0.86877 |
| Rragd | -0.6804 |
| Rras | -0.49755 |
| Rras2 | -0.5322 |
| Rrbp1 | -0.14811 |
| Rrbp1 | 0.44552 |
| Rreb1 | -0.35113 |
| Rreb1 | -0.49119 |
| Rrh | 0.61227 |
| Rrm1 | -0.41882 |
| Rrm2 | 2.01535 |
| Rrm2 | -0.71206 |
| Rrm2b | 0.49061 |
| Rrn3 | 1.59667 |
| Rrp1 | -0.53959 |
| Rrp12 | -0.08357 |
| Rrp15 | 1.41945 |
| Rrp1b | -0.34691 |
| Rrp1b | -0.06569 |
| Rrp1b | 0.82532 |
| Rrp9 | -0.54033 |
| Rs1 | -0.58599 |
| Rsad1 | -0.58365 |
| Rsad2 | 1.04813 |
| Rsf1 | 2.4054 |
| Rshl1 | -0.48279 |
| Rshl2a | -0.18418 |
| Rsl1d1 | 0.10666 |
| Rsl1d1 | -0.17018 |
| Rsn | 0.08286 |
| Rsph1 | 0.09706 |
| Rspo1 | -0.22698 |
| Rspo2 | -0.59777 |
| Rspo3 | 1.73331 |
| Rspo4 | -0.37624 |
| Rspry1 | -0.41622 |
| Rsrc1 | 1.7291 |
| Rsrc1 | -0.28471 |
| Rsrc2 | 3.53501 |
| Rsu1 | -0.74485 |
| Rtbdn | -0.18518 |
| Rtel1 | -0.46074 |
| Rtel1 | -0.61774 |
| Rtkn | 0.76743 |
| Rtn1 | 0.25236 |
| Rtn1 | -0.48908 |
| Rtn1 | 0.13664 |
| Rtn1 | 2.18802 |
| Rtn2 | 0.93269 |
| Rtn3 | -0.03177 |
| Rtn4 | -0.80522 |
| Rtn4 | -0.89897 |
| Rtn4 | -0.32669 |
| Rtn4ip1 | 0.0557 |
| Rtn4r | 0.21102 |
| Rtn4rl1 | -0.49049 |
| Rtn4rl2 | 2.98278 |
| Rtp1 | -0.51183 |
| Rtp4 | -0.66667 |
| Rttn | -0.54033 |
| Rufy1 | 0.69176 |
| Rufy2 | 1.5888 |
| Rufy3 | 2.0792 |
| Rufy3 | 0.8375 |
| Rufy3 | 0.46462 |
| Rufy4 | -0.55833 |
| Rundc1 | -0.61693 |
| Rundc3b | -0.59304 |
| Runx1 | -0.45734 |
| Runx1t1 | 0.29192 |
| Runx1t1 | -0.06879 |
| Runx2 | 2.72226 |
| Rusc1 | 0.78742 |
| Rusc2 | -0.57745 |
| Rusc2 | -0.52927 |
| Ruvbl1 | 0.35652 |
| Ruvbl1 | -0.65398 |
| Ruvbl1 | 1.68864 |
| Ruvbl2 | 1.3201 |
| Rwdd1 | -0.66242 |
| Rwdd1 | 2.25921 |
| Rwdd3 | -0.22592 |
| Rwdd3 | 1.46151 |
| Rwdd4a | -0.55455 |
| Rxfp1 | 0.95775 |
| Rxfp3 | 0.11725 |
| Rxra | -0.08087 |
| Rxrb | -0.17715 |
| Rxrb | -0.03562 |
| Rxrg | -0.83542 |
| Rya3 | 0.41058 |
| Ryk | -0.14957 |
| Ryk | -0.56593 |
| Ryr1 | -0.10182 |
| Ryr1 | 1.15244 |
| Ryr1 | 0.77916 |
| Ryr3 | -0.73471 |
| S100a1 | 0.07681 |
| S100a10 | -0.78951 |
| S100a11 | -0.09814 |
| S100a13 | 0.30769 |
| S100a16 | -0.48698 |
| S100a4 | -0.65398 |
| S100a6 | 0.67648 |
| S100a8 | -0.3919 |
| S100a9 | -0.01193 |
| S100b | 1.79831 |
| S100pbp | 0.60012 |
| S100pbp | -0.56669 |
| S3-12 | 1.99313 |
| Saa3 | -0.35841 |
| Sac3d1 | -0.23653 |
| Sacm1l | -0.15638 |
| Sacs | -0.55757 |
| Sae1 | 0.02532 |
| Sae1 | 0.19415 |
| Sae1 | -0.63733 |
| Sag | -0.07459 |
| Sall1 | -0.78951 |
| Sall1 | -0.39443 |
| Sall2 | -0.7076 |
| Sall4 | 0.59171 |
| Sall4 | 1.99919 |
| Samd10 | -0.68386 |
| Samd11 | 0.31533 |
| Samd12 | -0.61936 |
| Samd14 | 0.86703 |
| Samd4 | -0.4919 |
| Samd4 | 3.38131 |
| Samd4b | 0.43889 |
| Samd5 | -0.52269 |
| Samd7 | -0.69697 |
| Samd8 | 0.14759 |
| Samd9l | -0.60093 |
| Samhd1 | 0.04202 |
| Samm50 | 1.11166 |
| Samsn1 | -0.52853 |
| Sap130 | -0.64063 |
| Sap30 | 1.56025 |
| Sap30bp | 2.84887 |
| Sap30l | -0.7274 |
| Saps1 | -0.2061 |
| Saps2 | 1.8475 |
| Saps3 | -0.47515 |
| Saps3 | 1.37084 |
| Sar1a | -0.74115 |
| Sar1b | 0.66875 |
| Sar1b | -0.50967 |
| Sarm1 | -0.59068 |
| Sars | 0.20047 |
| Sars | 2.57788 |
| Sars2 | -0.68126 |
| Sart1 | 1.53793 |
| Sart3 | -0.54331 |
| Sas | 0.24006 |
| Sass6 | -0.48698 |
| Sat2 | -0.68996 |
| Satb1 | 1.40132 |
| Sav1 | 3.32216 |
| Sbds | 1.00191 |
| Sbf1 | -0.62667 |
| Sbk | 2.7855 |
| Sbno1 | -0.27242 |
| Sbno2 | -0.27576 |
| Sc4mol | -0.37315 |
| Sc5d | -0.72015 |
| Scaf1 | -0.74671 |
| Scamp1 | 2.05321 |
| Scamp2 | -0.55984 |
| Scamp3 | 1.55373 |
| Scamp4 | -0.4587 |
| Scamp5 | 2.36207 |
| Scamp5 | 4.19491 |
| Scand1 | 0.64084 |
| Scap | 0.42237 |
| Scaper | 0.35091 |
| Scara3 | -0.36207 |
| Scara5 | 1.95561 |
| Scarb1 | 1.47536 |
| Scarb2 | -0.55984 |
| Scarf1 | -0.26249 |
| Scarf2 | 0.09966 |
| Sccpdh | -0.60173 |
| Scd1 | -0.26085 |
| Scd2 | 0.14829 |
| Scd4 | -0.56288 |
| Scel | -0.69785 |
| Scg2 | 0.08361 |
| Scg3 | -0.43192 |
| Scg5 | -0.61855 |
| Scgn | 0.44602 |
| Scgn | -0.70582 |
| Scgn | 1.70562 |
| Schip1 | 1.28645 |
| Schip1 | 0.83435 |
| Scin | -0.45531 |
| scl0003251.1_21 | 1.83688 |
| Sclt1 | -0.53073 |
| Scly | -0.64312 |
| Scmh1 | -0.66496 |
| Scmh1 | -0.2025 |
| Scml2 | 1.46176 |
| Scml4 | -0.40976 |
| Scn10a | 1.48385 |
| Scn11a | -0.79831 |
| Scn1a | 0.16945 |
| Scn2b | 1.3462 |
| Scn3b | -0.34211 |
| Scn3b | 1.34229 |
| Scn3b | -0.51688 |
| Scn3b | -0.62748 |
| Scn4b | -0.77119 |
| Scn5a | 0.55841 |
| Scn7a | -0.70227 |
| Scn8a | 0.08399 |
| Scnm1 | -0.0003 |
| Scnm1 | 0.88562 |
| Scnn1a | -0.69171 |
| Scnn1b | 0.32435 |
| Scnn1g | -0.43324 |
| Scoc | 2.6837 |
| Scoc | 1.28716 |
| Scotin | -0.61371 |
| Scotin | -0.70671 |
| Scotin | -0.14183 |
| Scp2 | 0.44399 |
| Scp2 | 1.53592 |
| Scp2 | -0.65062 |
| Scrg1 | -0.6365 |
| Scrib | 2.0798 |
| Scrib | 1.2283 |
| Scrn1 | -0.11528 |
| Scrn1 | -0.1867 |
| Scrn1 | -0.41234 |
| Scrn2 | -0.67608 |
| Sct | -0.54256 |
| Sctr | 0.77863 |
| Scube1 | 1.52821 |
| Scube2 | 1.33575 |
| Scube3 | 0.24765 |
| Scube3 | -0.73379 |
| Scx | 0.3759 |
| Scye1 | -0.50537 |
| Scyl1 | 1.05875 |
| Scyl1bp1 | 0.74662 |
| Scyl3 | 3.53825 |
| Scyl3 | -0.6226 |
| Sdad1 | -0.37687 |
| Sdc1 | 1.64584 |
| Sdc1 | 0.25486 |
| Sdc2 | -0.38686 |
| Sdc3 | -0.47101 |
| Sdc4 | 4.51118 |
| Sdcbp | 2.13249 |
| Sdcbp | 3.32076 |
| Sdcbp2 | -0.03519 |
| Sdcbp2 | 0.28226 |
| Sdccag1 | 0.21168 |
| Sdccag1 | -0.743 |
| Sdccag3 | -0.52342 |
| Sdccag3 | 0.53082 |
| Sdf2 | -0.72649 |
| Sdf2 | -0.81018 |
| Sdf4 | -0.4306 |
| Sdhb | -0.42273 |
| Sdhb | 2.80825 |
| Sdhb | -0.5644 |
| Sdhc | -0.67179 |
| Sdhd | 0.83551 |
| Sdk1 | 0.75645 |
| Sdpr | -0.49401 |
| Sds | -0.35659 |
| Sdsl | -0.76738 |
| Sec1 | -0.7076 |
| Sec11a | -0.6365 |
| Sec11c | 0.96528 |
| Sec13 | 2.45634 |
| Sec14l1 | 0.89889 |
| Sec14l1 | 1.23057 |
| Sec14l1 | 0.18577 |
| Sec14l2 | 2.91111 |
| Sec14l3 | 0.05415 |
| Sec14l3 | -0.76359 |
| Sec14l4 | 0.18812 |
| Sec15l1 | 0.02813 |
| Sec16a | 0.17697 |
| Sec16b | -0.16869 |
| Sec22a | -0.38812 |
| Sec22b | -0.57205 |
| Sec22c | 1.40284 |
| Sec22c | -0.78369 |
| Sec23a | 2.59707 |
| Sec23ip | 0.40103 |
| Sec24b | 0.24164 |
| Sec24c | 0.24101 |
| Sec24d | 3.02833 |
| Sec61a1 | -0.6113 |
| Sec61a2 | -0.30173 |
| Sec61a2 | 1.07451 |
| Sec61b | 0.2614 |
| Sec61b | 2.91059 |
| Sec61g | -0.23653 |
| Sec63 | 0.39085 |
| Sec63 | -0.59698 |
| Secisbp2 | -0.6365 |
| Sectm1b | -0.51183 |
| Seh1l | -0.14473 |
| Seh1l | 1.40517 |
| Sel1l | -0.6073 |
| Sel1l | -0.23015 |
| Sel1l | 0.27618 |
| Sel1l2 | 1.11559 |
| Sel1l2 | 1.59514 |
| Selenbp2 | 0.58671 |
| Selk | 2.24545 |
| Sell | 3.39438 |
| Selp | -0.49049 |
| Selplg | -0.72831 |
| Sema3a | -0.52488 |
| Sema3b | -0.06259 |
| Sema3b | 0.25642 |
| Sema3b | 2.32419 |
| Sema3e | -0.64395 |
| Sema3f | 1.15562 |
| Sema3f | 0.2263 |
| Sema3g | -0.43523 |
| Sema4a | -0.0986 |
| Sema4b | -0.65314 |
| Sema4d | 1.29677 |
| Sema4f | -0.79243 |
| Sema4g | 1.97215 |
| Sema5a | -0.70138 |
| Sema5a | -0.64645 |
| Sema5b | 2.95622 |
| Sema6b | -0.87837 |
| Sema6b | 0.8922 |
| Sema6b | -0.07191 |
| Sema6d | 0.45134 |
| Sema6d | -0.57822 |
| Sema7a | -0.20765 |
| Sema7a | -0.63815 |
| Senp1 | -0.59225 |
| Senp2 | -0.37253 |
| Senp3 | 0.30769 |
| Senp5 | -0.39443 |
| Senp5 | -0.38623 |
| Senp5 | 0.43787 |
| Senp6 | -0.45261 |
| Senp6 | 0.96888 |
| Senp7 | 1.08432 |
| Senp7 | -0.6121 |
| Senp7 | 2.16504 |
| Senp8 | 2.30051 |
| Sephs1 | -0.64478 |
| Sephs2 | 0.07072 |
| Sepm | -0.62504 |
| Sepn1 | 0.3682 |
| Sepp1 | 0.07567 |
| Sepp1 | 0.17254 |
| Sepsecs | -0.62912 |
| Sept2 | -0.33318 |
| Sept3 | 3.15077 |
| Sept3 | -0.39697 |
| Sept5 | -0.06127 |
| Sept6 | 1.09402 |
| Sept6 | -0.56822 |
| Sept7 | 0.06727 |
| Sept8 | -0.66411 |
| Sept9 | 0.28287 |
| Sept10 | 0.55571 |
| Sept10 | -0.79733 |
| Sept12 | -0.08493 |
| Sept15 | 1.33585 |
| Sepw1 | -0.48908 |
| Sepx1 | -0.80621 |
| Sepx1 | 0.4353 |
| Serac1 | 0.09111 |
| Serbp1 | 0.07339 |
| Serbp1 | 1.57031 |
| Serbp1 | -0.02243 |
| Serf1 | -0.7005 |
| Serf2 | 1.45873 |
| Sergef | -0.11061 |
| Serhl | -0.30173 |
| Serhl | 0.74662 |
| Serinc1 | 1.40794 |
| Serinc2 | 1.67748 |
| Serinc3 | -0.3578 |
| Serpina1a | 1.99962 |
| Serpina1a | 0.20774 |
| Serpina1b | -0.17665 |
| Serpina1b | -0.23174 |
| Serpina1d | 0.64739 |
| Serpina1e | -0.05599 |
| Serpina3b | -0.5278 |
| Serpina3c | 0.72925 |
| Serpina3g | 0.28378 |
| Serpina3h | 1.77983 |
| Serpina3k | 1.39628 |
| Serpina3n | -0.88267 |
| Serpina5 | -0.18267 |
| Serpina5 | 2.86384 |
| Serpina7 | -0.71565 |
| Serpina9 | 0.0711 |
| Serpinb11 | 1.11522 |
| Serpinb12 | 1.14024 |
| Serpinb1a | 0.95411 |
| Serpinb3a | -0.26469 |
| Serpinb6a | -0.42535 |
| Serpinb6b | 0.08549 |
| Serpinb6c | -0.48418 |
| Serpind1 | -0.57282 |
| Serpine2 | 0.18307 |
| Serpinf1 | -0.44121 |
| Serping1 | -0.66667 |
| Serpinh1 | -0.78562 |
| Serpini1 | 1.0704 |
| Sertad1 | -0.13656 |
| Sertad2 | 0.59171 |
| Sertad2 | -0.38123 |
| Sertad3 | -0.72377 |
| Sertad4 | 3.79302 |
| Sesn1 | -0.21024 |
| Sesn1 | -0.07191 |
| Sesn2 | -0.67351 |
| Sesn3 | -0.12846 |
| Setd1b | -0.45126 |
| Setd2 | -0.60411 |
| Setd3 | 1.27684 |
| Setd4 | 1.00964 |
| Setd4 | 0.86139 |
| Setd5 | 2.56896 |
| Setd6 | -0.76738 |
| Setd6 | 0.18307 |
| Setd7 | 1.83392 |
| Setd7 | -0.64146 |
| Setd8 | 1.19835 |
| Setd8 | -0.58755 |
| Setd8 | -0.57282 |
| Setdb1 | 0.62698 |
| Setdb2 | -0.25702 |
| Setmar | -0.58521 |
| Setx | -0.3938 |
| Sez6 | -0.44121 |
| Sez6l | 0.96239 |
| Sez6l2 | -0.58365 |
| Sez6l2 | -0.52269 |
| Sf1 | 3.09294 |
| Sf1 | -0.37624 |
| Sf3a1 | -0.10505 |
| Sf3a2 | 1.47436 |
| Sf3a3 | 0.87199 |
| Sf3b1 | -0.73379 |
| Sf3b2 | -0.55681 |
| Sf3b3 | 1.04284 |
| Sf3b4 | 0.22855 |
| Sf3b4 | -0.54854 |
| Sf3b5 | -0.2864 |
| Sf4 | 0.5359 |
| Sfi1 | 0.89033 |
| Sfi1 | -0.61291 |
| Sfmbt1 | 0.59474 |
| Sfmbt1 | 0.42159 |
| Sfmbt1 | 1.1528 |
| Sfmbt2 | -0.57052 |
| Sfrp1 | -0.75324 |
| Sfrp2 | 0.18745 |
| Sfrp4 | 1.95948 |
| Sfrp5 | -0.61291 |
| Sfrs1 | 2.9812 |
| Sfrs1 | -0.77502 |
| Sfrs11 | 1.23682 |
| Sfrs12 | 0.01524 |
| Sfrs14 | 0.91883 |
| Sfrs16 | 0.02009 |
| Sfrs2 | 1.09063 |
| Sfrs2 | 2.12017 |
| Sfrs2 | -0.76076 |
| Sfrs3 | 0.27862 |
| Sfrs3 | -0.64312 |
| Sfrs4 | 2.06702 |
| Sfrs5 | -0.57129 |
| Sfrs5 | -0.49119 |
| Sfrs6 | 3.87403 |
| Sfrs7 | -0.57052 |
| Sfrs8 | 2.03344 |
| Sfrs9 | -0.7005 |
| Sft2d2 | 0.46685 |
| Sft2d2 | -0.43589 |
| Sft2d2 | -0.69697 |
| Sftpc | -0.00776 |
| Sftpd | -0.63239 |
| Sfxn1 | 1.87304 |
| Sfxn1 | -0.0003 |
| Sfxn3 | -0.70493 |
| Sfxn3 | -0.64229 |
| Sfxn4 | 1.38273 |
| Sfxn4 | -0.53515 |
| Sfxn5 | 0.67707 |
| Sgca | -0.44187 |
| Sgca | 0.84789 |
| Sgcb | 1.78292 |
| Sgcb | -0.61693 |
| Sgcz | -0.65988 |
| Sgip1 | 1.6578 |
| Sgip1 | -0.19533 |
| Sgip1 | 0.64146 |
| Sgip1 | -0.68299 |
| Sgip1 | -0.33734 |
| Sgk1 | -0.78465 |
| Sgk2 | 1.78185 |
| Sgk2 | -0.77598 |
| Sgk3 | 1.65023 |
| Sgk3 | 0.51212 |
| Sgk3 | -0.69962 |
| Sgk3 | -0.85612 |
| Sgk3 | 1.10994 |
| Sgms2 | 0.04241 |
| Sgol1 | -0.65566 |
| Sgpl1 | 1.68106 |
| Sgpl1 | -0.6565 |
| Sgpp1 | 1.58012 |
| Sgpp2 | -0.53959 |
| Sgsm1 | -0.74023 |
| Sgsm2 | 0.24922 |
| Sgsm3 | 1.03351 |
| Sgta | -0.04553 |
| Sgtb | -0.29035 |
| Sgtb | 0.95819 |
| Sh2b1 | 2.00796 |
| Sh2b1 | 0.58038 |
| Sh2b3 | -0.65988 |
| Sh2d2a | -0.48978 |
| Sh3bgrl | -0.70671 |
| Sh3bgrl3 | 1.74468 |
| Sh3bp2 | -0.0003 |
| Sh3bp5 | 1.55008 |
| Sh3bp5l | -0.76549 |
| Sh3gl1 | 2.00429 |
| Sh3gl2 | -0.69258 |
| Sh3gl2 | -0.21335 |
| Sh3gl2 | -0.65903 |
| Sh3gl3 | 0.48892 |
| Sh3glb1 | -0.52196 |
| Sh3glb2 | -0.43324 |
| Sh3kbp1 | 0.02573 |
| Sh3kbp1 | 0.30857 |
| Sh3kbp1 | -0.72196 |
| Sh3kbp1 | 1.78717 |
| Sh3md4 | 0.84295 |
| Sh3md4 | -0.69785 |
| Sh3pxd2a | 0.40608 |
| Sh3pxd2b | -0.38938 |
| Sh3rf1 | -0.61451 |
| Sh3rf2 | -0.26085 |
| Sh3tc1 | -0.80621 |
| Sh3tc2 | -0.57205 |
| Sh3yl1 | -0.65566 |
| Shank2 | -0.6565 |
| Sharpin | -0.61855 |
| Shbg | -0.45396 |
| Shc1 | 1.14262 |
| Shc1 | 1.30727 |
| Shc3 | -0.22856 |
| Shcbp1 | -0.21439 |
| Shd | -0.51978 |
| She | -0.3023 |
| Shf | -0.43722 |
| Shf | 0.56757 |
| Shf | -0.43324 |
| Shfm1 | -0.3633 |
| Shh | 0.77313 |
| Shisa2 | -0.1677 |
| Shisa3 | 0.39809 |
| Shisa4 | -0.04684 |
| Shkbp1 | 2.31254 |
| Shkbp1 | -0.77215 |
| Shmt1 | -0.27409 |
| Shmt2 | -0.35234 |
| Shoc2 | 1.73879 |
| Shox2 | -0.26469 |
| Shprh | 0.89625 |
| Shq1 | -0.40143 |
| Shroom3 | -0.47584 |
| Shroom3 | 0.04359 |
| Si | -0.42077 |
| Si | 0.53452 |
| Siah1a | 2.99327 |
| Siah1b | 0.64022 |
| Siah1b | -0.46689 |
| Siah1b | -0.86983 |
| Siah1b | 1.20192 |
| Siah2 | 0.03768 |
| Siat7f | -0.07057 |
| Siat9 | 1.70188 |
| Sidt1 | 0.77969 |
| Sidt2 | -0.48139 |
| Sidt2 | -0.14424 |
| Sidt2 | 0.00873 |
| Sigirr | -0.37006 |
| Siglec1 | -0.0464 |
| Siglech | 0.03926 |
| Siglech | -0.17067 |
| Sil1 | -0.25593 |
| Sin3a | -0.78272 |
| Sin3b | 0.10593 |
| Sip1 | 1.02774 |
| Sip1 | -0.04901 |
| Sipa1 | -0.2478 |
| Sipa1l1 | 0.15946 |
| Sirpa | -0.5176 |
| Sirpa | -0.2328 |
| Sirpb1 | -0.33081 |
| Sirt1 | -0.43324 |
| Sirt2 | -0.48698 |
| Sirt3 | 0.41976 |
| Sirt5 | -0.53073 |
| Sirt5 | 1.39275 |
| Sirt6 | -0.52196 |
| Sirt7 | -0.26304 |
| Siva1 | 0.09185 |
| Six1 | 0.31737 |
| Six2 | 0.24544 |
| Six3 | 2.30128 |
| Six5 | -0.55304 |
| Six6os1 | 2.23195 |
| Skap2 | -0.71745 |
| Skil | -0.49049 |
| Skiv2l | 0.34015 |
| Skiv2l | 0.08886 |
| Skiv2l2 | -0.43788 |
| Skp1a | 2.00121 |
| Skp2 | 1.10759 |
| Skp2 | 0.06343 |
| Skp2 | 4.23253 |
| Sla | 0.40369 |
| Slain1 | -0.3976 |
| Slain2 | -0.20507 |
| Slamf7 | 1.40481 |
| Slamf8 | 1.97128 |
| Slamf9 | 0.11252 |
| Slc10a3 | 4.02234 |
| Slc10a4 | 0.79769 |
| Slc10a6 | -0.63239 |
| Slc10a6 | -0.34812 |
| Slc10a7 | 1.18606 |
| Slc10a7 | -0.42469 |
| Slc10a7 | -0.71475 |
| Slc11a1 | -0.51399 |
| Slc11a1 | 1.89365 |
| Slc11a1 | 1.62278 |
| Slc11a1 | 1.25972 |
| Slc11a2 | 1.52508 |
| Slc12a2 | 0.36903 |
| Slc12a2 | 2.17248 |
| Slc12a3 | -0.56212 |
| Slc12a5 | -0.64063 |
| Slc12a6 | -0.60173 |
| Slc12a7 | -0.20455 |
| Slc12a8 | -0.74578 |
| Slc12a9 | 0.03965 |
| Slc12a9 | -0.55681 |
| Slc13a2 | -0.64478 |
| Slc13a3 | -0.65482 |
| Slc13a3 | -0.01737 |
| Slc13a4 | -0.50537 |
| Slc14a1 | 2.72745 |
| Slc14a1 | 0.38164 |
| Slc14a1 | -0.64063 |
| Slc14a2 | -0.78756 |
| Slc14a2 | -0.7779 |
| Slc15a2 | -0.8072 |
| Slc15a3 | -0.34451 |
| Slc15a4 | -0.33318 |
| Slc15a4 | -0.81518 |
| Slc16a10 | -0.67522 |
| Slc16a11 | 1.90892 |
| Slc16a4 | 1.99224 |
| Slc16a6 | -0.30517 |
| Slc16a6 | -0.71117 |
| Slc16a6 | 1.71403 |
| Slc16a8 | 0.66575 |
| Slc16a9 | -0.68647 |
| Slc17a2 | -0.07593 |
| Slc17a5 | -0.70849 |
| Slc17a6 | -0.3919 |
| Slc17a7 | 0.26078 |
| Slc17a8 | -0.62748 |
| Slc18a2 | 1.08419 |
| Slc19a1 | -0.20868 |
| Slc19a2 | -0.74578 |
| Slc1a1 | 1.6798 |
| Slc1a1 | -0.05951 |
| Slc1a2 | 1.53577 |
| Slc1a2 | 0.02813 |
| Slc1a2 | 1.33883 |
| Slc1a2 | 0.64514 |
| Slc1a3 | 1.00909 |
| Slc1a4 | -0.54405 |
| Slc1a6 | -0.32024 |
| Slc1a7 | 0.54779 |
| Slc20a1 | -0.61291 |
| Slc22a1 | -0.01151 |
| Slc22a12 | -0.44187 |
| Slc22a13 | -0.0648 |
| Slc22a14 | 0.76814 |
| Slc22a17 | -0.58443 |
| Slc22a17 | 1.04059 |
| Slc22a18 | -0.38185 |
| Slc22a21 | -0.1428 |
| Slc22a3 | 2.53952 |
| Slc22a4 | -0.58677 |
| Slc22a5 | 0.44552 |
| Slc22a5 | -0.54554 |
| Slc22a5 | -0.7523 |
| Slc22a6 | 0.11397 |
| Slc22a7 | 0.37261 |
| Slc22a8 | -0.01193 |
| Slc23a2 | 0.64432 |
| Slc23a3 | 2.01544 |
| Slc24a1 | 1.36441 |
| Slc24a3 | -0.41105 |
| Slc24a4 | -0.57129 |
| Slc24a5 | 0.28196 |
| Slc24a6 | 1.61221 |
| Slc25a1 | -0.19686 |
| Slc25a1 | -0.62912 |
| Slc25a10 | 2.82371 |
| Slc25a11 | -0.51833 |
| Slc25a11 | -0.14183 |
| Slc25a11 | 0.83849 |
| Slc25a12 | 1.52884 |
| Slc25a14 | -0.45193 |
| Slc25a15 | 0.8206 |
| Slc25a15 | 0.67668 |
| Slc25a16 | 0.04634 |
| Slc25a17 | -0.34391 |
| Slc25a18 | 0.89485 |
| Slc25a19 | -0.38749 |
| Slc25a19 | 3.25917 |
| Slc25a20 | 1.20293 |
| Slc25a20 | -0.67436 |
| Slc25a23 | -0.76549 |
| Slc25a24 | -0.47307 |
| Slc25a24 | -0.05468 |
| Slc25a25 | 0.1598 |
| Slc25a26 | -0.4621 |
| Slc25a27 | 1.12242 |
| Slc25a28 | -0.72105 |
| Slc25a29 | 1.66267 |
| Slc25a3 | -0.23653 |
| Slc25a3 | -0.15492 |
| Slc25a30 | -0.6073 |
| Slc25a32 | -0.68299 |
| Slc25a33 | -0.84055 |
| Slc25a34 | -0.07013 |
| Slc25a35 | -0.37315 |
| Slc25a36 | 0.84279 |
| Slc25a37 | -0.55757 |
| Slc25a38 | -0.67867 |
| Slc25a38 | 3.22768 |
| Slc25a38 | 1.60317 |
| Slc25a39 | -0.25866 |
| Slc25a41 | -0.28809 |
| Slc25a42 | 0.33071 |
| Slc25a44 | 1.02111 |
| Slc25a45 | 1.90597 |
| Slc25a46 | 0.34072 |
| Slc25a5 | -0.30402 |
| Slc26a4 | -0.40976 |
| Slc26a6 | -0.4933 |
| Slc26a7 | -0.37935 |
| Slc26a8 | -0.63403 |
| Slc27a1 | -0.66157 |
| Slc27a2 | 0.73724 |
| Slc27a3 | -0.60411 |
| Slc27a4 | 0.52432 |
| Slc27a6 | -0.46483 |
| Slc29a1 | -0.83952 |
| Slc29a2 | -0.55681 |
| Slc29a3 | -0.74023 |
| Slc29a4 | 1.20804 |
| Slc2a1 | 0.92594 |
| Slc2a10 | -0.27465 |
| Slc2a10 | -0.56975 |
| Slc2a13 | -0.26634 |
| Slc2a3 | 1.253 |
| Slc2a4 | -0.579 |
| Slc2a5 | -0.71655 |
| Slc2a6 | -0.6565 |
| Slc2a8 | 0.10923 |
| Slc2a8 | -0.29035 |
| Slc2a8 | 0.92443 |
| Slc2a9 | -0.3633 |
| Slc30a1 | 0.42808 |
| Slc30a10 | 0.18375 |
| Slc30a3 | -0.28752 |
| Slc30a5 | 0.95118 |
| Slc30a5 | -0.48628 |
| Slc30a6 | -0.74857 |
| Slc30a7 | 3.05904 |
| Slc30a7 | 1.49526 |
| Slc30a9 | -0.45328 |
| Slc31a1 | 0.04044 |
| Slc31a2 | 1.16099 |
| Slc32a1 | 0.13522 |
| Slc34a2 | -0.50537 |
| Slc35a1 | 0.01686 |
| Slc35a2 | 0.7883 |
| Slc35a3 | -0.40143 |
| Slc35a4 | -0.35963 |
| Slc35a5 | 2.08949 |
| Slc35a5 | 2.15609 |
| Slc35b1 | 0.27465 |
| Slc35b2 | 0.62885 |
| Slc35b3 | 2.26093 |
| Slc35b3 | 1.7951 |
| Slc35b3 | -0.45666 |
| Slc35b4 | -0.5176 |
| Slc35c1 | -0.46552 |
| Slc35c1 | 1.65902 |
| Slc35c2 | -0.64728 |
| Slc35c2 | -0.54256 |
| Slc35c2 | -0.2819 |
| Slc35e1 | -0.62017 |
| Slc35e3 | -0.52342 |
| Slc35e4 | -0.32551 |
| Slc35f1 | -0.16621 |
| Slc35f2 | 1.4827 |
| Slc35f3 | -0.36944 |
| Slc35f4 | -0.13943 |
| Slc35f4 | -0.67008 |
| Slc36a1 | 0.37508 |
| Slc36a3 | 1.14167 |
| Slc37a1 | -0.56364 |
| Slc37a3 | -0.31849 |
| Slc38a1 | 0.34044 |
| Slc38a10 | 2.26945 |
| Slc38a11 | -0.217 |
| Slc38a2 | 0.12051 |
| Slc38a2 | -0.5011 |
| Slc38a2 | -0.60093 |
| Slc38a3 | -0.59856 |
| Slc38a4 | 1.17258 |
| Slc38a5 | -0.07997 |
| Slc38a8 | 0.97661 |
| Slc38a9 | 1.23036 |
| Slc38a9 | -0.80423 |
| Slc39a10 | -0.69785 |
| Slc39a11 | -0.07862 |
| Slc39a12 | 0.45863 |
| Slc39a13 | -0.3179 |
| Slc39a13 | -0.16227 |
| Slc39a14 | 0.52152 |
| Slc39a3 | -0.11108 |
| Slc39a3 | -0.59935 |
| Slc39a4 | -0.68299 |
| Slc39a5 | -0.10366 |
| Slc39a5 | -0.36024 |
| Slc39a5 | 1.95019 |
| Slc39a6 | -0.73013 |
| Slc39a7 | -0.63815 |
| Slc39a7 | -0.34451 |
| Slc39a7 | -0.52634 |
| Slc39a8 | -0.25267 |
| Slc3a2 | 0.64657 |
| Slc40a1 | -0.1486 |
| Slc41a2 | 0.11252 |
| Slc41a3 | 3.3281 |
| Slc41a3 | -0.6365 |
| Slc41a3 | -0.65146 |
| Slc43a2 | 1.59157 |
| Slc43a2 | -0.46006 |
| Slc44a1 | -0.41947 |
| Slc44a1 | 3.26896 |
| Slc44a2 | -0.66837 |
| Slc44a2 | 1.10164 |
| Slc44a2 | -0.14376 |
| Slc44a3 | -0.71117 |
| Slc44a4 | 2.80607 |
| Slc45a1 | -0.87196 |
| Slc45a2 | -0.22224 |
| Slc45a3 | 0.05104 |
| Slc45a4 | -0.2791 |
| Slc46a1 | 0.85686 |
| Slc46a3 | -0.45802 |
| Slc47a1 | -0.47584 |
| Slc4a1 | -0.76929 |
| Slc4a1ap | -0.6081 |
| Slc4a2 | 1.82717 |
| Slc4a3 | -0.28358 |
| Slc4a3 | 0.0642 |
| Slc4a3 | -0.26085 |
| Slc4a3 | 0.53682 |
| Slc4a8 | -0.45802 |
| Slc4a8 | 0.23496 |
| Slc5a4a | -0.52707 |
| Slc5a4b | -0.57745 |
| Slc5a5 | 0.00545 |
| Slc5a6 | -0.49684 |
| Slc5a7 | 1.16204 |
| Slc5a8 | 1.6381 |
| Slc5a9 | 0.54437 |
| Slc6a1 | -0.45193 |
| Slc6a11 | -0.04467 |
| Slc6a12 | -0.49613 |
| Slc6a13 | -0.91446 |
| Slc6a15 | 1.43731 |
| Slc6a15 | 0.56712 |
| Slc6a17 | -0.53073 |
| Slc6a2 | 0.67806 |
| Slc6a4 | -0.17515 |
| Slc6a6 | -0.16819 |
| Slc6a7 | -0.73471 |
| Slc6a8 | -0.08629 |
| Slc6a9 | -0.03007 |
| Slc7a1 | 0.52198 |
| Slc7a10 | -0.2752 |
| Slc7a11 | -0.74857 |
| Slc7a14 | 2.52115 |
| Slc7a14 | 0.49133 |
| Slc7a14 | 0.94309 |
| Slc7a2 | 1.95477 |
| Slc7a3 | -0.58912 |
| Slc7a4 | -0.64063 |
| Slc7a6 | 0.39996 |
| Slc7a6os | -0.77598 |
| Slc7a7 | 1.07297 |
| Slc7a7 | 2.05946 |
| Slc7a8 | 0.26728 |
| Slc7a8 | 2.77654 |
| Slc8a1 | 0.10849 |
| Slc8a2 | -0.82423 |
| Slc8a2 | 0.67074 |
| Slc9a1 | 1.81133 |
| Slc9a10 | -0.29148 |
| Slc9a3r1 | -0.13417 |
| Slc9a3r2 | 0.96658 |
| Slc9a4 | -0.89788 |
| Slc9a6 | -0.95435 |
| Slc9a6 | 2.17453 |
| Slc9a8 | -0.73655 |
| Slc9a8 | 1.42585 |
| Slc9a8 | -0.52707 |
| Slc9a8 | 3.33919 |
| Slc9a9 | -0.08267 |
| Slco1a4 | -0.32669 |
| Slco1c1 | 0.70774 |
| Slco2a1 | -0.78175 |
| Slco2b1 | -0.78951 |
| Slco3a1 | -0.39951 |
| Slco3a1 | 0.09297 |
| Slco3a1 | -0.77406 |
| Slco4a1 | 1.14595 |
| Slfnl1 | -0.81818 |
| Slit1 | 0.48819 |
| Slit2 | -0.55379 |
| Slit3 | 0.60162 |
| Slitrk1 | 0.81637 |
| Slitrk3 | -0.42208 |
| Slitrk4 | 1.87175 |
| Slitrk5 | 0.31122 |
| Slitrk5 | -0.75137 |
| Slitrk6 | 0.08399 |
| Slk | -0.41169 |
| Slk | 1.45991 |
| Slmap | 1.27468 |
| Slmap | -0.2614 |
| Slmap | -0.60014 |
| Slmo1 | -0.51688 |
| Slmo2 | -0.4919 |
| Sln | -0.82221 |
| Slpi | 0.09297 |
| Sltm | 0.20609 |
| Sltm | 2.76966 |
| Slu7 | 1.17499 |
| Slu7 | 1.29717 |
| Sly | 3.28509 |
| Smad1 | -0.72286 |
| Smad1 | -0.67351 |
| Smad2 | 1.30468 |
| Smad3 | 1.68093 |
| Smad5 | 0.44932 |
| Smad5 | 1.72753 |
| Smad6 | -0.84675 |
| Smap2 | -0.47653 |
| Smap2 | -0.47931 |
| Smap2 | 1.60036 |
| Smarca1 | -0.76454 |
| Smarca2 | -0.73746 |
| Smarca2 | -0.67351 |
| Smarca4 | 2.20946 |
| Smarcad1 | 0.41584 |
| Smarcal1 | 0.24828 |
| Smarcb1 | -0.05819 |
| Smarcc1 | 0.33901 |
| Smarcc1 | -0.48558 |
| Smarcc2 | 0.9428 |
| Smarcd1 | 2.06296 |
| Smarcd1 | 0.61291 |
| Smarcd1 | 3.63176 |
| Smarcd2 | 1.43325 |
| Smarcd3 | -0.63486 |
| Smarce1 | 0.44577 |
| Smarce1 | 0.98625 |
| Smc1a | 0.97689 |
| Smc1a | -0.50967 |
| Smc1a | -0.07638 |
| Smc1a | -0.85194 |
| Smc1b | -0.65734 |
| Smc3 | 2.01824 |
| Smc5l1 | 0.67944 |
| Smc6 | 0.73279 |
| Smchd1 | 0.27709 |
| Smcp | -0.42797 |
| Smcr7 | 2.08724 |
| Smcr7l | 1.47137 |
| Smek1 | 0.46038 |
| Smek2 | -0.43722 |
| Smek2 | 2.47016 |
| Smg5 | -0.10136 |
| Smg6 | -0.2543 |
| Smn1 | 0.4483 |
| Smo | 0.61015 |
| Smo | -0.8212 |
| Smo | -0.59935 |
| Smoc1 | 1.71358 |
| Smox | -0.35537 |
| Smox | 0.40076 |
| Smox | 2.04362 |
| Smox | 1.95172 |
| Smox | 2.36481 |
| Smpd1 | 3.52454 |
| Smpd1 | 2.42526 |
| Smpd1 | 0.47131 |
| Smpd2 | 1.70736 |
| Smpd3 | 0.13308 |
| Smpd4 | 0.61944 |
| Smpdl3a | 0.9621 |
| Smpdl3b | -0.3179 |
| Smpx | 1.82008 |
| Smtn | 2.21366 |
| Smtn | -0.5606 |
| Smtn | 0.99411 |
| Smtnl1 | 1.95236 |
| Smtnl2 | 0.41426 |
| Smu1 | -0.42535 |
| Smu1 | 0.82043 |
| Smug1 | 1.31212 |
| Smurf1 | -0.02794 |
| Smyd1 | 0.47229 |
| Smyd2 | -0.68126 |
| Smyd2 | 1.85372 |
| Smyd3 | 1.98127 |
| Smyd4 | 0.34781 |
| Smyd5 | -0.41752 |
| Smyd5 | 0.58388 |
| Snai1 | 0.09297 |
| Snai3 | 1.51799 |
| Snap23 | 0.10886 |
| Snap29 | -0.25485 |
| Snap91 | -0.46483 |
| Snap91 | -0.55606 |
| Snapc1 | -0.58288 |
| Snapc3 | -0.5448 |
| Snapc3 | 1.02585 |
| Snapc3 | 0.02653 |
| Snapc3 | -0.09906 |
| Snapc4 | 1.5014 |
| Snapin | 0.4417 |
| Snca | 0.64677 |
| Snca | 0.57796 |
| Snca | -0.16424 |
| Sncaip | -0.66582 |
| Sncg | 0.53567 |
| Sncg | 4.44607 |
| Snd1 | -0.27576 |
| Snf1lk | 0.94957 |
| Snf1lk2 | -0.19024 |
| Snf8 | 0.10482 |
| Snip1 | -0.65903 |
| Snip1 | -0.6226 |
| Snn | 1.9358 |
| Snora65 | -0.36513 |
| Snph | -0.80621 |
| Snrk | -0.47653 |
| Snrk | -0.26085 |
| Snrk | 0.11834 |
| Snrp70 | -0.09128 |
| Snrpa | -0.30574 |
| Snrpb | -0.5759 |
| Snrpb | -0.75324 |
| Snrpd1 | -0.5954 |
| Snrpd1 | -0.64812 |
| Snrpd2 | -0.72922 |
| Snrpd3 | 0.06266 |
| Snrpg | -0.08992 |
| Snta1 | -0.56516 |
| Sntb2 | -0.69873 |
| Sntb2 | 1.06614 |
| Sntg2 | -0.18771 |
| Snupn | 2.58931 |
| Snurf | 2.71803 |
| Snurf | 1.63581 |
| Snx1 | -0.57205 |
| Snx10 | -0.69962 |
| Snx11 | -0.24403 |
| Snx12 | -0.2983 |
| Snx13 | 1.0547 |
| Snx14 | 0.63549 |
| Snx14 | -0.72558 |
| Snx14 | -0.4717 |
| Snx15 | -0.52634 |
| Snx16 | -0.13656 |
| Snx17 | -0.42666 |
| Snx17 | -0.02116 |
| Snx18 | -0.20096 |
| Snx19 | 1.46879 |
| Snx19 | -0.62504 |
| Snx2 | 1.61911 |
| Snx2 | -0.71475 |
| Snx20 | -0.51471 |
| Snx21 | -0.45328 |
| Snx22 | 2.70291 |
| Snx24 | -0.6365 |
| Snx25 | 1.09765 |
| Snx25 | -0.52196 |
| Snx26 | 1.89076 |
| Snx29 | -0.74023 |
| Snx3 | -0.39633 |
| Snx30 | -0.69083 |
| Snx33 | 1.52123 |
| Snx4 | 1.05888 |
| Snx6 | -0.25648 |
| Snx7 | -0.19482 |
| Snx8 | -0.58055 |
| Snx9 | -0.63239 |
| Soat1 | 1.08267 |
| Socs2 | 1.84744 |
| Socs3 | -0.73288 |
| Socs4 | -0.13036 |
| Socs5 | -0.44521 |
| Socs6 | -0.13847 |
| Socs7 | -0.32727 |
| Socs7 | -0.16227 |
| Sod1 | -0.11294 |
| Sod2 | 0.99131 |
| Sod3 | 0.87883 |
| Sohlh1 | 0.96917 |
| Sohlh1 | -0.70849 |
| Son | 0.02813 |
| Sorbs1 | -0.1672 |
| Sorbs2 | 3.23462 |
| Sorbs3 | 0.57045 |
| Sorcs1 | 1.595 |
| Sorcs1 | -0.67608 |
| Sorcs2 | 1.73124 |
| Sorcs3 | 2.49858 |
| Sorcs3 | -0.63321 |
| Sord | 1.18482 |
| Sorl1 | 0.3554 |
| Sort1 | -0.41882 |
| Sort1 | -0.69258 |
| Sort1 | -0.51255 |
| Sort1 | 0.98695 |
| Sostdc1 | 0.54323 |
| Sox12 | 0.74699 |
| Sox13 | -0.78175 |
| Sox17 | 1.41733 |
| Sox17 | -0.77215 |
| Sox18 | 0.5593 |
| Sox2 | 1.19913 |
| Sox2 | 0.51259 |
| Sox21 | 0.47721 |
| Sox21 | 0.24133 |
| Sox30 | 1.54172 |
| Sox5 | -0.43126 |
| Sox5 | -0.71385 |
| Sox5 | -0.41882 |
| Sox5 | 0.25705 |
| Sox7 | -0.78756 |
| Sox7 | 0.25079 |
| Sox8 | -0.4662 |
| Sox9 | 0.7043 |
| Sp100 | 2.11964 |
| Sp140 | -0.00901 |
| Sp2 | 2.86615 |
| Sp4 | -0.28696 |
| Sp5 | -0.69258 |
| Sp6 | 0.83916 |
| Sp7 | 2.48248 |
| Sp8 | -0.70493 |
| Sp9 | 0.09892 |
| Spaca3 | 1.43437 |
| Spaca4 | 1.12993 |
| Spag1 | 3.54341 |
| Spag1 | 0.8523 |
| Spag16 | 0.05919 |
| Spag16 | 0.60183 |
| Spag4 | 1.51467 |
| Spag4 | -0.72015 |
| Spag4l | 0.29581 |
| Spag4l | -0.54854 |
| Spag5 | -0.08312 |
| Spag5 | -0.13751 |
| Spag6 | -0.72196 |
| Spag6 | -0.70138 |
| Spag7 | 0.30236 |
| Spag8 | 1.77488 |
| Spag9 | 0.8588 |
| Spag9 | -0.22909 |
| Spag9 | -0.05512 |
| Spag9 | -0.18518 |
| Spam1 | -0.27632 |
| Spam1 | -0.0538 |
| Sparc | -0.25485 |
| Sparc | -0.68908 |
| Sparcl1 | -0.10459 |
| Spast | -0.47307 |
| Spast | -0.69873 |
| Spast | -0.47032 |
| Spata16 | -0.42929 |
| Spata16 | 2.48854 |
| Spata17 | -0.54033 |
| Spata17 | -0.30116 |
| Spata19 | 0.24228 |
| Spata2 | 1.09715 |
| Spata20 | -0.44655 |
| Spata20 | -0.7523 |
| Spata21 | -0.55833 |
| Spata22 | 0.9202 |
| Spata2L | 1.65753 |
| Spata3 | 2.26683 |
| Spata3 | -0.5011 |
| Spata3 | -0.01527 |
| Spata3 | 3.49636 |
| Spata4 | -0.70849 |
| Spata6 | 0.48211 |
| Spata7 | -0.02285 |
| Spata7 | 0.9847 |
| Spatc1 | -0.6105 |
| Spatc1 | -0.23706 |
| Spats1 | -0.64312 |
| Spc24 | -0.52707 |
| Spc24 | 0.60824 |
| Spc25 | -0.68996 |
| Spc25 | -0.4807 |
| Spcs1 | -0.62667 |
| Spcs1 | 0.12735 |
| Spcs3 | -0.07772 |
| Spdef | 0.03886 |
| Specc1l | 0.2725 |
| Speer1-ps1 | 0.50904 |
| Speer1-ps1 | -0.2983 |
| Speer2 | 0.27066 |
| Speer2 | 3.30374 |
| Speer3 | 0.36516 |
| Speer4b | -0.04207 |
| Speer4d | -0.68647 |
| Speer4f | 1.16193 |
| Speer6-ps1 | 0.0642 |
| Spef1 | -0.23387 |
| Spen | 0.93239 |
| Spert | -0.45802 |
| Spesp1 | -0.7005 |
| Spg20 | -0.26249 |
| Spg21 | 0.36349 |
| Spg7 | 2.89963 |
| Spg7 | 0.29461 |
| Sphk1 | -0.36268 |
| Sphk2 | 0.40263 |
| Sphk2 | -0.50537 |
| Spic | 1.39085 |
| Spin | -0.83747 |
| Spin2 | 0.2645 |
| Spink10 | -0.86032 |
| Spink2 | 2.38331 |
| Spink4 | 0.09929 |
| Spink8 | 0.7726 |
| Spinlw1 | -0.16819 |
| Spinlw1 | -0.76738 |
| Spinlw1 | 1.4989 |
| Spint1 | -0.80819 |
| Spint2 | 0.44602 |
| Spire1 | -0.18317 |
| Spire1 | -0.79831 |
| Spire1 | -0.27855 |
| Spire2 | 1.49275 |
| Spna1 | -0.50466 |
| Spnb1 | -0.67522 |
| Spnb2 | 1.39664 |
| Spnb2 | 0.54915 |
| Spnb2 | -0.67436 |
| Spnb2 | 1.31272 |
| Spnb3 | -0.34092 |
| Spnb4 | -0.75137 |
| Spns1 | -0.68473 |
| Spo11 | 0.93403 |
| Spo11 | 1.24004 |
| Spock1 | 1.14215 |
| Spock2 | 0.60824 |
| Spock3 | 1.42637 |
| Spon1 | 0.30444 |
| Spon2 | -0.63815 |
| Spop | 2.16285 |
| Spp1 | -0.55229 |
| Spp2 | -0.38123 |
| Sppl3 | -0.72831 |
| Spr | 1.44445 |
| Spred1 | 1.95256 |
| Spred2 | 1.09665 |
| Spred2 | 2.49929 |
| Sprn | 0.18239 |
| Sprn | 0.58606 |
| Sprr2a | 0.34583 |
| Sprr2a | 0.51988 |
| Sprr2f | -0.57513 |
| Spry3 | 0.66215 |
| Spry4 | 1.76816 |
| Spryd4 | 1.33053 |
| Spsb1 | -0.75793 |
| Spsb2 | 0.01565 |
| Spsb3 | -0.16079 |
| Spsb3 | -0.15834 |
| Spsb4 | 1.02328 |
| Sptlc1 | -0.75887 |
| Sptlc2 | -0.73471 |
| Spz1 | 0.29969 |
| Sqle | -0.11528 |
| Sqle | 1.37325 |
| Sqrdl | -0.55833 |
| Sqstm1 | -0.70671 |
| Sra1 | -0.45328 |
| Sra1 | 0.86107 |
| Src | 1.83358 |
| Srd5a1 | -0.73563 |
| Srd5a2 | 0.06651 |
| Srd5a3 | 0.66135 |
| Srd5a3 | 0.87994 |
| Srd5a3 | -0.73105 |
| Srebf1 | -0.48698 |
| Srebf2 | 1.56281 |
| Srf | 0.75082 |
| Srf | 2.492 |
| Srfbp1 | -0.39697 |
| Srgn | 0.08886 |
| Srgn | 0.46933 |
| Sri | -0.5068 |
| Sri | -0.63239 |
| Sri | -0.17765 |
| Srm | -0.55681 |
| Srm | 0.75155 |
| Srm | 1.4183 |
| Srp14 | 3.64837 |
| Srp19 | -0.19635 |
| Srp19 | -0.73105 |
| Srp54 | 0.32493 |
| Srp68 | 2.13204 |
| Srp72 | 0.82565 |
| Srp9 | -0.76644 |
| Srp9 | -0.20972 |
| Srpk1 | 0.89345 |
| Srpk2 | 2.22553 |
| Srpk3 | 0.45461 |
| Srpr | 1.43912 |
| Srprb | 0.65044 |
| Srprb | -0.73013 |
| Srpx | -0.46347 |
| Srpx | 0.79144 |
| Srpx2 | 0.85637 |
| Srpx2 | 1.84727 |
| Srpx2 | -0.43126 |
| Srr | 0.69351 |
| Srrd | -0.68473 |
| Srrm1 | 0.01524 |
| Srrm2 | -0.68213 |
| Srrm2 | 0.79249 |
| Srrp | 1.68192 |
| Srxn1 | -0.54256 |
| Srxn1 | 3.40505 |
| Srxn1 | 0.24922 |
| Ss18 | 2.35951 |
| Ss18 | -0.11996 |
| Ss18l1 | -0.55004 |
| Ss18l1 | 2.03915 |
| Ss18l1 | 1.21751 |
| Ssbp1 | 2.0068 |
| Ssbp2 | -0.61613 |
| Ssbp2 | 0.354 |
| Ssbp3 | -0.49401 |
| Ssbp3 | 0.72869 |
| Ssbp4 | 1.14808 |
| Ssfa2 | -0.64979 |
| Ssh2 | 2.65272 |
| Ssh3 | 0.18002 |
| Ssh3 | 0.60248 |
| Ssna1 | -0.29488 |
| Sspn | 2.44262 |
| Ssr2 | -0.66667 |
| Ssr3 | 0.11361 |
| Ssr4 | 0.20971 |
| Ssrp1 | 0.86863 |
| Sssca1 | 0.91716 |
| Sssca1 | 0.02291 |
| Sst | 0.44424 |
| Sstr1 | 0.18239 |
| Sstr5 | 2.15707 |
| Ssty2 | -0.77886 |
| Ssu72 | -0.6097 |
| Ssx2ip | 1.47428 |
| Ssxb2 | -0.69697 |
| Ssxb5 | -0.13847 |
| St13 | 1.5149 |
| St14 | -0.16819 |
| St14 | 2.885 |
| St18 | -0.65734 |
| St3gal1 | -0.35295 |
| St3gal2 | 0.34866 |
| St3gal2 | -0.22592 |
| St3gal3 | 0.60951 |
| St3gal3 | -0.69433 |
| St3gal4 | 1.09453 |
| St3gal5 | -0.17765 |
| St3gal6 | -0.69083 |
| St5 | 0.53957 |
| St5 | -0.67351 |
| St6gal1 | 0.81518 |
| St6galnac2 | -0.2864 |
| St6galnac3 | 0.69059 |
| St6galnac4 | -0.2328 |
| St6galnac4 | 2.05841 |
| St6galnac4 | -0.11762 |
| St6galnac5 | -0.37873 |
| St6galnac6 | 0.01848 |
| St6galnac6 | -0.69785 |
| St7 | 0.78777 |
| St7l | 1.09214 |
| St7l | 0.63445 |
| St8sia1 | -0.82727 |
| St8sia1 | 0.95877 |
| St8sia2 | -0.30116 |
| St8sia3 | -0.71925 |
| St8sia4 | 1.20304 |
| St8sia4 | -0.55229 |
| St8sia5 | -0.16178 |
| Stab1 | -0.11528 |
| Stab2 | -0.1209 |
| Stac | -0.57668 |
| Stac2 | -0.46757 |
| Stac3 | -0.21648 |
| Stag1 | -0.23866 |
| Stag2 | 1.11412 |
| Stag2 | -0.5011 |
| Stag3 | 3.60893 |
| Stam | -0.53147 |
| Stam | 2.38728 |
| Stam | 0.18273 |
| Stam2 | 0.03212 |
| Stam2 | -0.40398 |
| Stambp | -0.29261 |
| Stambpl1 | 0.67826 |
| Stap1 | 3.28374 |
| Stap2 | -0.45261 |
| Star | 1.57195 |
| Stard10 | -0.64478 |
| Stard10 | 3.45338 |
| Stard13 | -0.38623 |
| Stard3 | -0.65988 |
| Stard3nl | -0.63239 |
| Stard4 | 0.29521 |
| Stard5 | 0.2645 |
| Stard5 | -0.35476 |
| Stard6 | 2.59646 |
| Stard7 | -0.60014 |
| Stard7 | 0.18307 |
| Stard8 | 2.75757 |
| Stard8 | -0.6565 |
| Stat1 | -0.00817 |
| Stat1 | 0.52827 |
| Stat2 | 0.70526 |
| Stat3 | 0.05687 |
| Stat3 | -0.6073 |
| Stat3 | -0.11996 |
| Stat4 | -0.47722 |
| Stat4 | 2.13237 |
| Stat5a | 0.65571 |
| Stat5a | 0.2614 |
| Stat6 | -0.48418 |
| Statip1 | -0.43655 |
| Stau1 | 2.82163 |
| Stau2 | 0.63425 |
| Stbd1 | -0.20404 |
| Stc2 | 1.66927 |
| Steap1 | 2.02869 |
| Steap2 | -0.45531 |
| Steap3 | -0.50181 |
| Stfa1 | -0.61532 |
| Stfa2 | -0.73288 |
| Stim1 | -0.76265 |
| Stip1 | -0.48768 |
| Stk11 | 0.22436 |
| Stk11ip | -0.46483 |
| Stk17b | 1.09402 |
| Stk19 | 0.79022 |
| Stk19 | 1.91996 |
| Stk19 | -0.31849 |
| Stk22b | 1.50886 |
| Stk22s1 | -0.72922 |
| Stk24 | -0.41234 |
| Stk25 | 0.82381 |
| Stk25 | 2.99587 |
| Stk3 | -0.0199 |
| Stk31 | -0.56975 |
| Stk32a | 2.26498 |
| Stk32b | 0.45083 |
| Stk32c | 0.2973 |
| Stk35 | 1.79334 |
| Stk35 | 0.65166 |
| Stk36 | -0.36513 |
| Stk38l | 0.85116 |
| Stk39 | -0.62017 |
| Stk4 | 0.06266 |
| Stk40 | 0.29102 |
| Stmn1 | -0.73471 |
| Stmn2 | -0.6226 |
| Stmn3 | 0.23879 |
| Stmn4 | -0.41882 |
| Stmn4 | -0.37191 |
| Stoml1 | 0.90122 |
| Stoml1 | -0.32551 |
| Stoml1 | 0.2107 |
| Stoml2 | 0.38761 |
| Stoml3 | -0.50466 |
| Stra13 | 0.24639 |
| Stra13 | 0.42445 |
| Stra13 | 0.67371 |
| Stra6 | -0.68386 |
| Strap | 0.86058 |
| Strbp | -0.54256 |
| Strn4 | 0.33186 |
| Strn4 | -0.68473 |
| Stt3a | 1.16958 |
| Stt3b | -0.66411 |
| Stx12 | -0.64812 |
| Stx17 | 2.78466 |
| Stx18 | 0.53313 |
| Stx1a | 0.04123 |
| Stx2 | 0.34413 |
| Stx3 | -0.21492 |
| Stx3 | -0.64229 |
| Stx4a | -0.08856 |
| Stx5a | -0.22592 |
| Stx5a | 0.09743 |
| Stx6 | -0.61774 |
| Stx7 | -0.48838 |
| Stx8 | -0.62422 |
| Stx8 | -0.11762 |
| Stx8 | -0.45734 |
| Stx8 | 0.89189 |
| Stxbp1 | 2.6646 |
| Stxbp1 | -0.23493 |
| Stxbp1 | 0.66115 |
| Stxbp2 | 1.32956 |
| Stxbp4 | -0.59777 |
| Stxbp4 | 1.904 |
| Stxbp5l | -0.61371 |
| Stxbp5l | -0.54331 |
| Styx | -0.64895 |
| Styxl1 | -0.72377 |
| Styxl1 | 0.19981 |
| Styxl1 | 1.91093 |
| Suclg1 | 0.4013 |
| Suclg1 | 0.27892 |
| Suclg2 | 2.60374 |
| Suclg2 | -0.73746 |
| Suds3 | -0.45058 |
| Sufu | -0.67781 |
| Suhw2 | 0.27557 |
| Suhw4 | 1.72873 |
| Sulf1 | 0.96759 |
| Sult1a1 | -0.0986 |
| Sult1c2 | 2.35038 |
| Sult1d1 | 0.30118 |
| Sult1d1 | -0.68473 |
| Sult2b1 | -0.55079 |
| Sult2b1 | 0.16015 |
| Sult4a1 | -0.03177 |
| Sult5a1 | 0.77491 |
| Sult5a1 | 2.12696 |
| Sumf2 | 1.14286 |
| Sumo1 | 0.13451 |
| Sumo1 | -0.13991 |
| Sumo1 | 0.7606 |
| Sumo2 | 0.10224 |
| Sumo3 | -0.31209 |
| Sumo3 | 0.80201 |
| Sunc1 | 1.67342 |
| Suox | -0.38623 |
| Supt3h | -0.43589 |
| Supt4h1 | 1.23886 |
| Supt4h2 | 0.75735 |
| Supt5h | -0.06039 |
| Supt5h | 0.0588 |
| Supt6h | -0.66411 |
| Supv3l1 | 2.09004 |
| Surf1 | -0.43788 |
| Surf1 | 0.23783 |
| Surf2 | 2.27752 |
| Surf4 | -0.61371 |
| Susd2 | -0.4919 |
| Susd3 | 1.7509 |
| Susd4 | 1.79563 |
| Suv39h1 | -0.58833 |
| Suv420h1 | 0.25236 |
| Suv420h1 | 0.64166 |
| Suv420h1 | 2.99809 |
| Suv420h2 | 2.42826 |
| Suz12 | 1.45586 |
| Sv2a | -0.83134 |
| Sv2a | 4.59469 |
| Sv2b | 0.21102 |
| Sval2 | 0.73613 |
| Svep1 | 0.15703 |
| Svil | 0.17969 |
| Svil | 1.84611 |
| Svop | -0.75887 |
| Svopl | 0.04556 |
| Svs4 | 0.45637 |
| Svs5 | -0.81818 |
| Svs6 | 2.29411 |
| Swap70 | -0.44121 |
| Swap70 | -0.47515 |
| Swap70 | 0.96917 |
| Syap1 | 2.28924 |
| Syap1 | -0.70493 |
| Sybl1 | -0.4339 |
| Syce2 | -0.45666 |
| Sycp1 | -0.71565 |
| Syf2 | -0.73471 |
| Syf2 | 0.96499 |
| Syn1 | -0.35053 |
| Syn1 | -0.23068 |
| Syn2 | -0.42143 |
| Sync | -0.64562 |
| Syncrip | -0.61371 |
| Syne1 | 1.67322 |
| Syne1 | -0.6523 |
| Syngr1 | -0.51543 |
| Syngr1 | 2.58749 |
| Syngr1 | 1.43792 |
| Syngr1 | 2.78259 |
| Syngr2 | -0.58912 |
| Syngr2 | 0.24006 |
| Syngr2 | -0.12988 |
| Syngr3 | 1.05286 |
| Syngr4 | 3.16502 |
| Syngr4 | -0.58443 |
| Syngr4 | -0.59068 |
| Synj2 | -0.40783 |
| Synj2bp | 2.12586 |
| Synj2bp | 1.01443 |
| Synpo2l | -0.80127 |
| Syp | -0.43523 |
| Sypl | -0.69697 |
| Sypl | 1.14867 |
| Sypl2 | 2.03718 |
| Sys1 | -0.60332 |
| Syt1 | 1.00329 |
| Syt11 | -0.61371 |
| Syt11 | -0.44454 |
| Syt12 | -0.36759 |
| Syt13 | 0.27983 |
| Syt15 | 0.12304 |
| Syt16 | -0.76644 |
| Syt3 | 0.48527 |
| Syt4 | 1.32684 |
| Syt5 | 0.65328 |
| Syt9 | 0.43453 |
| Sytl2 | -0.5954 |
| Sytl3 | 0.16395 |
| Sytl3 | 0.27983 |
| Sytl3 | 0.94413 |
| Sytl4 | -0.11855 |
| Sytl5 | 0.55683 |
| Syvn1 | -0.51183 |
| Taar2 | -0.17216 |
| Taar3 | 1.78185 |
| Taar4 | 0.69757 |
| Tac1 | -0.07414 |
| Tacc1 | -0.1938 |
| Tacc1 | 0.40661 |
| Tacr3 | 0.7862 |
| Tacr3 | -0.37191 |
| Tacstd2 | -0.69609 |
| Tada1l | 1.24228 |
| Tada2l | 0.06689 |
| Tada2l | -0.32258 |
| Tada3l | 0.29162 |
| Tada3l | 2.41867 |
| Taf10 | -0.21128 |
| Taf11 | 1.26536 |
| Taf12 | -0.64229 |
| Taf12 | -0.58365 |
| Taf12 | 2.38596 |
| Taf13 | -0.37562 |
| Taf1c | 0.57112 |
| Taf3 | -0.29375 |
| Taf5 | -0.63321 |
| Taf5 | -0.03476 |
| Taf5 | -0.55908 |
| Taf5l | -0.375 |
| Taf6 | 0.37096 |
| Taf6 | 0.13735 |
| Taf6l | -0.47515 |
| Taf7l | 0.74918 |
| Taf9 | -0.11621 |
| Taf9 | -0.58833 |
| Taf9 | -0.28922 |
| Taf9b | -0.55455 |
| Tagap | 2.82767 |
| Tagap | 2.01097 |
| Tagln | 2.68094 |
| Tagln3 | 0.13022 |
| Tal1 | -0.49967 |
| Tal2 | 0.90369 |
| Taldo1 | 3.38518 |
| Tank | -0.09631 |
| Tank | -0.48488 |
| Tank | 0.69815 |
| Tap1 | 0.94206 |
| Tap2 | -0.48279 |
| Tapbp | 1.74264 |
| Tapbp | -0.31965 |
| Tapbpl | 1.98898 |
| Tapbpl | -0.60491 |
| Tapt1 | -0.34451 |
| Tarbp2 | -0.49896 |
| Tarbp2 | -0.52927 |
| Tardbp | -0.63321 |
| Tardbp | 2.69664 |
| Tardbp | 1.09227 |
| Tars | -0.64312 |
| Tarsl2 | 0.55187 |
| Tarsl2 | 1.29998 |
| Tas1r1 | 0.87087 |
| Tasp1 | -0.51905 |
| Tatdn2 | 1.10351 |
| Tax1bp1 | 0.22339 |
| Tax1bp3 | 1.2336 |
| Tax1bp3 | 0.99662 |
| Taz | -0.66496 |
| Taz | -0.35174 |
| Tbc1d1 | 0.3554 |
| Tbc1d10a | -0.59225 |
| Tbc1d10a | 0.28166 |
| Tbc1d10c | 2.00144 |
| Tbc1d12 | 3.7469 |
| Tbc1d13 | 0.04752 |
| Tbc1d14 | 0.32667 |
| Tbc1d15 | -0.51978 |
| Tbc1d16 | -0.7005 |
| Tbc1d17 | 0.56912 |
| Tbc1d19 | -0.57668 |
| Tbc1d2 | 1.68582 |
| Tbc1d20 | -0.65482 |
| Tbc1d22a | -0.57513 |
| Tbc1d22b | -0.47307 |
| Tbc1d23 | 2.25333 |
| Tbc1d24 | -0.66327 |
| Tbc1d25 | 0.45988 |
| Tbc1d2b | -0.59068 |
| Tbc1d5 | -0.46894 |
| Tbc1d5 | -0.47861 |
| Tbc1d7 | -0.70493 |
| Tbc1d7 | -0.52488 |
| Tbc1d8 | -0.47861 |
| Tbc1d9 | -0.38561 |
| Tbc1d9 | -0.50395 |
| Tbc1d9b | -0.04467 |
| Tbca | 4.53229 |
| Tbcb | 0.68513 |
| Tbcc | -0.24456 |
| Tbcd | 0.43812 |
| Tbcel | -0.37315 |
| Tbk1 | 1.93726 |
| Tbkbp1 | -0.26579 |
| Tbl1x | 2.06117 |
| Tbl1xr1 | -0.61936 |
| Tbl2 | 2.64273 |
| Tbl3 | -0.83032 |
| Tbp | -0.08629 |
| Tbpl1 | -0.6121 |
| Tbr1 | -0.53589 |
| Tbrg1 | -0.44857 |
| Tbrg1 | -0.45058 |
| Tbrg1 | -0.57129 |
| Tbrg4 | 1.53569 |
| Tbrg4 | -0.41947 |
| Tbx1 | 0.81671 |
| Tbx19 | -0.6856 |
| Tbx2 | 0.99802 |
| Tbx22 | -0.61291 |
| Tbx3 | 2.53657 |
| Tbx3 | 0.93373 |
| Tbx5 | 0.10556 |
| Tbx6 | -0.42666 |
| Tbxa2r | 1.73356 |
| Tbxa2r | -0.67436 |
| Tcam1 | 1.13126 |
| Tcam1 | -0.73288 |
| Tcam1 | -0.66242 |
| Tcap | 1.0695 |
| Tcea2 | 0.82901 |
| Tcea3 | 0.2976 |
| Tceal1 | 0.08886 |
| Tceal1 | 0.19782 |
| Tceal3 | -0.68821 |
| Tceal5 | -0.61532 |
| Tceal6 | 0.60461 |
| Tceal6 | -0.41817 |
| Tceal8 | -0.40015 |
| Tceb1 | 0.35316 |
| Tceb2 | -0.05687 |
| Tceb2 | 1.56183 |
| Tceb3 | -0.26689 |
| Tcerg1 | -0.28583 |
| Tcf12 | -0.33793 |
| Tcf12 | 1.9583 |
| Tcf15 | 1.58814 |
| Tcf19 | -0.53294 |
| Tcf20 | -0.31325 |
| Tcf20 | 0.06035 |
| Tcf20 | 0.67786 |
| Tcf20 | -0.40206 |
| Tcf20 | -0.90007 |
| Tcf21 | -0.59619 |
| Tcf23 | -0.78854 |
| Tcf25 | -0.8293 |
| Tcf25 | 0.30207 |
| Tcf25 | -0.13083 |
| Tcf3 | 0.55706 |
| Tcf3 | 0.75209 |
| Tcf4 | -0.67436 |
| Tcf7 | -0.5821 |
| Tcf7 | -0.0438 |
| Tcf7l2 | 3.16748 |
| Tcf7l2 | 1.60382 |
| Tcfap2b | -0.5954 |
| Tcfap2b | 0.91183 |
| Tcfap2c | -0.579 |
| Tcfap2c | 1.07104 |
| Tcfcp2 | -0.67436 |
| Tcfe3 | 0.95001 |
| Tcfl5 | 1.1822 |
| Tchp | 1.91909 |
| Tcirg1 | -0.31499 |
| Tcirg1 | 2.55979 |
| Tcirg1 | -0.71206 |
| Tcirg1 | -0.58443 |
| Tcl1 | 2.93498 |
| Tcl1 | 0.26604 |
| Tcl1b2 | 0.40476 |
| Tcl1b2 | -0.34631 |
| Tcl1b3 | 0.06804 |
| Tcl1b4 | 0.52943 |
| Tcn2 | -0.11528 |
| Tcof1 | -0.65314 |
| Tcp1 | 0.73465 |
| Tcp1 | -0.42994 |
| Tcp10a | 0.69234 |
| Tcp10a | 1.37906 |
| Tcp11 | 0.09297 |
| Tcstv1 | -0.7274 |
| Tcte3 | -0.69346 |
| Tctex1d1 | 0.93164 |
| Tctex1d2 | -0.20147 |
| Tctex1d4 | -0.45058 |
| Tctn3 | -0.2344 |
| Tctn3 | -0.46894 |
| Tctn3 | 1.43645 |
| Tdg | -0.40912 |
| Tdp1 | -0.12232 |
| Tdpoz1 | 1.48753 |
| Tdpoz2 | 1.15854 |
| Tdrd1 | -0.74485 |
| Tdrd12 | 0.64616 |
| Tdrd3 | 0.49879 |
| Tdrd3 | -0.04164 |
| Tdrd3 | 0.4826 |
| Tdrd6 | -0.60252 |
| Tdrd7 | 1.82178 |
| Tdrkh | 0.4483 |
| Tead2 | 0.05415 |
| Tec | 0.98187 |
| Tecta | 2.18378 |
| Tef | 2.17221 |
| Tef | -0.77982 |
| Tegt | 0.6093 |
| Tek | 1.44795 |
| Tekt1 | -0.88159 |
| Tekt1 | -0.19227 |
| Tekt3 | -0.87196 |
| Tenc1 | 3.33643 |
| Tenc1 | -0.35537 |
| Terf2 | -0.6065 |
| Terf2 | -0.35053 |
| Terf2ip | 1.41105 |
| Terf2ip | 0.61713 |
| Terf2ip | 1.01142 |
| Terf2ip | -0.07817 |
| Tes | -0.6804 |
| Tes | -0.7495 |
| Tes | -0.75605 |
| Tesc | 0.10003 |
| Tesk1 | -0.73563 |
| Tesp1 | 1.10164 |
| Tesp2 | 0.44323 |
| Tessp2 | 1.86836 |
| Tessp3 | -0.66667 |
| Tex10 | -0.55004 |
| Tex10 | 0.58322 |
| Tex101 | -0.25104 |
| Tex11 | 2.30854 |
| Tex13 | -0.67522 |
| Tex14 | -0.426 |
| Tex16 | -0.42404 |
| Tex19 | 0.82532 |
| Tex2 | -0.63898 |
| Tex2 | -0.64146 |
| Tex22 | 1.56784 |
| Tex24 | -0.73196 |
| Tex261 | -0.5553 |
| Tex261 | 0.66954 |
| Tex264 | 1.3143 |
| Tex264 | -0.80028 |
| Tex9 | 0.07415 |
| Tfam | -0.69346 |
| Tfam | 0.53705 |
| Tfb1m | -0.67953 |
| Tfb2m | 2.58109 |
| Tfdp1 | 2.14736 |
| Tfdp2 | 0.73446 |
| Tfdp2 | 2.66669 |
| Tfg | 3.44201 |
| Tfip11 | 0.60909 |
| Tfpi | 0.15842 |
| Tfpt | 1.38016 |
| Tfpt | -0.30001 |
| Tfpt | 1.91325 |
| Tfrc | 0.55571 |
| Tfrc | -0.66752 |
| Tg | -0.01695 |
| Tgfb1 | -0.66923 |
| Tgfb1i1 | 1.45009 |
| Tgfb1i1 | -0.67351 |
| Tgfb2 | -0.10413 |
| Tgfb3 | -0.33674 |
| Tgfbi | -0.58055 |
| Tgfbi | 0.63114 |
| Tgfbr1 | -0.17765 |
| Tgfbr1 | -0.55154 |
| Tgfbr1 | -0.6073 |
| Tgfbr2 | 2.25461 |
| Tgfbr2 | -0.80919 |
| Tgfbr3 | 1.07079 |
| Tgfbr3 | 1.53855 |
| Tgfbrap1 | -0.6065 |
| Tgif1 | -0.7495 |
| Tgif1 | -0.64229 |
| Tgif1 | 0.41898 |
| Tgif2 | 0.59041 |
| Tgif2lx | 2.16438 |
| Tgm2 | -0.67608 |
| Tgm4 | 0.30177 |
| Tgoln1 | -0.14424 |
| Tgs1 | -0.64229 |
| Tgs1 | -0.00984 |
| Th1l | 1.00135 |
| Tha1 | -0.5606 |
| Thada | 2.47795 |
| Thada | 0.2051 |
| Thap1 | -0.16178 |
| Thap1 | -0.31907 |
| Thap1 | -0.71117 |
| Thap11 | 0.84295 |
| Thap3 | -0.54629 |
| Thap4 | -0.59461 |
| Thap7 | -0.78756 |
| Thbd | 0.54688 |
| Thbs1 | -0.77886 |
| Thbs2 | 0.49711 |
| Thbs3 | -0.05162 |
| Thbs3 | -0.35598 |
| Theg | -0.14328 |
| Theg | -0.03135 |
| Theg | 0.6165 |
| Them2 | 0.79092 |
| Them4 | -0.67522 |
| Them5 | -0.51978 |
| Thg1l | 0.39809 |
| Thnsl1 | 0.53728 |
| Thnsl2 | -0.6113 |
| Thnsl2 | 0.77313 |
| Thoc1 | 1.47029 |
| Thoc3 | -0.57436 |
| Thoc4 | -0.26966 |
| Thoc4 | 0.29491 |
| Thoc5 | -0.23813 |
| Thoc6 | 1.93148 |
| Thop1 | 0.73446 |
| Thra | 0.00873 |
| Thra | 1.03378 |
| Thrap2 | 1.60217 |
| Thrap3 | 0.78935 |
| Thrsp | -0.22962 |
| Thsd1 | 0.35512 |
| Thsd4 | 2.84893 |
| Thsd4 | -0.62179 |
| Thsd7b | 0.93358 |
| Thsd7b | -0.03391 |
| Thsd7b | -0.4717 |
| Thtpa | -0.57205 |
| Thumpd1 | 2.78743 |
| Thumpd3 | -0.64895 |
| Thy1 | 0.1525 |
| Thyn1 | -0.62993 |
| Tia1 | -0.743 |
| Tial1 | -0.69346 |
| Tial1 | 0.21462 |
| Tiam1 | -0.11762 |
| Tiam2 | -0.23387 |
| Ticam1 | 0.62322 |
| Tie1 | 1.94672 |
| Tigd2 | 1.28635 |
| Tigd3 | 1.92037 |
| Timeless | -0.72467 |
| Timm10 | 0.12015 |
| Timm13 | 2.75007 |
| Timm17a | -0.42208 |
| Timm17b | 0.42653 |
| Timm22 | -0.13417 |
| Timm44 | 1.72829 |
| Timm44 | 3.54507 |
| Timm44 | -0.53147 |
| Timm50 | 3.50571 |
| Timm8a1 | -0.59304 |
| Timm8a2 | 1.63997 |
| Timm8a2 | -0.56364 |
| Timm8b | -0.59698 |
| Timm8b | -0.35963 |
| Timm9 | 1.47444 |
| Timm9 | 1.06044 |
| Timm9 | -0.46552 |
| Timp1 | -0.07013 |
| Timp1 | 0.06497 |
| Timp2 | -0.39507 |
| Timp3 | 0.95906 |
| Timp3 | -0.27132 |
| Timp4 | -0.53515 |
| Tinagl | -0.69609 |
| Tipin | -0.13656 |
| Tipin | -0.44454 |
| Tirap | -0.50967 |
| Tirap | 2.75796 |
| Tirap | -0.5821 |
| Tjap1 | -0.66667 |
| Tjap1 | 1.30448 |
| Tjap1 | -0.4027 |
| Tjp1 | 4.10737 |
| Tjp2 | -0.37749 |
| Tjp3 | 0.68141 |
| Tjp3 | 0.50166 |
| Tk1 | -0.63733 |
| Tk2 | 3.86669 |
| Tkt | 2.19194 |
| Tkt | 1.9359 |
| Tkt | 3.01291 |
| Tktl1 | -0.51688 |
| Tktl1 | -0.3092 |
| Tktl2 | 0.96744 |
| Tlcd1 | 0.80425 |
| Tle1 | -0.72105 |
| Tle1 | -0.70671 |
| Tle2 | 1.25279 |
| Tle3 | -0.3633 |
| Tle3 | -0.68386 |
| Tle3 | 0.30355 |
| Tle4 | 0.3229 |
| Tle4 | 1.64191 |
| Tle4 | 1.68476 |
| Tle6 | 2.76289 |
| Tlk1 | -0.44187 |
| Tlk2 | 0.9205 |
| Tln1 | -0.51978 |
| Tln2 | -0.1242 |
| Tlr13 | 3.20605 |
| Tlr2 | 0.56912 |
| Tlr3 | -0.72558 |
| Tlr4 | 0.62343 |
| Tlr6 | 0.23942 |
| Tlr7 | 2.74536 |
| Tm2d2 | -0.17865 |
| Tm2d3 | 1.47701 |
| Tm2d3 | -0.46278 |
| Tm4sf4 | -0.7523 |
| Tm4sf5 | 1.75671 |
| Tm6sf1 | 0.33358 |
| Tm6sf2 | 0.34837 |
| Tm9sf2 | 1.79176 |
| Tm9sf2 | -0.62585 |
| Tm9sf2 | 2.42396 |
| Tm9sf3 | -0.51833 |
| Tm9sf4 | 0.96744 |
| Tm9sf4 | 2.41724 |
| Tmbim1 | -0.54629 |
| Tmbim4 | -0.73746 |
| Tmc1 | -0.56593 |
| Tmc4 | 0.42107 |
| Tmc5 | -0.44521 |
| Tmc6 | 1.24356 |
| Tmc6 | -0.11248 |
| Tmc6 | 2.89796 |
| Tmc7 | 1.1798 |
| Tmcc2 | 3.05341 |
| Tmcc3 | 1.62602 |
| Tmco3 | -0.47861 |
| Tmco3 | -0.29035 |
| Tmco4 | -0.48698 |
| Tmco4 | -0.69785 |
| Tmco5 | -0.57052 |
| Tmco6 | 1.01307 |
| Tmco6 | -0.14328 |
| Tmco7 | -0.78369 |
| Tmed1 | 0.73074 |
| Tmed1 | -0.57513 |
| Tmed10 | -0.63981 |
| Tmed2 | -0.05731 |
| Tmed3 | -0.6097 |
| Tmed3 | 2.01769 |
| Tmed4 | 1.09 |
| Tmed4 | -0.69346 |
| Tmed4 | 2.88936 |
| Tmed4 | -0.63157 |
| Tmed8 | 1.2129 |
| Tmed8 | 1.90509 |
| Tmed9 | -0.32669 |
| Tmed9 | -0.48628 |
| Tmeff2 | -0.27743 |
| Tmem10 | -0.07102 |
| Tmem100 | 1.38401 |
| Tmem101 | -0.67265 |
| Tmem102 | -0.53073 |
| Tmem102 | -0.53589 |
| Tmem106a | -0.68473 |
| Tmem106c | 1.36161 |
| Tmem107 | 2.50208 |
| Tmem107 | -0.45734 |
| Tmem107 | -0.15054 |
| Tmem108 | 1.00024 |
| Tmem109 | 3.78949 |
| Tmem11 | 1.17281 |
| Tmem110 | -0.68126 |
| Tmem111 | 0.96167 |
| Tmem111 | -0.15883 |
| Tmem111 | 1.99572 |
| Tmem115 | 0.23592 |
| Tmem116 | -0.21962 |
| Tmem116 | -0.73471 |
| Tmem117 | -0.26579 |
| Tmem118 | -0.76359 |
| Tmem119 | 1.22678 |
| Tmem120a | 1.24654 |
| Tmem120b | 1.5022 |
| Tmem121 | 0.53613 |
| Tmem125 | -0.47239 |
| Tmem126a | 1.49623 |
| Tmem126b | 0.1888 |
| Tmem127 | -0.6065 |
| Tmem128 | 0.39756 |
| Tmem130 | 0.00135 |
| Tmem131 | 0.75355 |
| Tmem132a | 0.60525 |
| Tmem132a | 0.16084 |
| Tmem132c | 0.47525 |
| Tmem132d | 1.29536 |
| Tmem132e | -0.76359 |
| Tmem134 | -0.32316 |
| Tmem134 | 1.08899 |
| Tmem135 | -0.58055 |
| Tmem136 | 1.61762 |
| Tmem138 | 1.52052 |
| Tmem139 | -0.11902 |
| Tmem140 | -0.37129 |
| Tmem141 | -0.10366 |
| Tmem143 | -0.08403 |
| Tmem143 | 0.95323 |
| Tmem143 | -0.60252 |
| Tmem143 | -0.14424 |
| Tmem144 | 0.64187 |
| Tmem146 | 2.58573 |
| Tmem147 | -0.21283 |
| Tmem14c | 0.4445 |
| Tmem14c | 0.06035 |
| Tmem14c | 1.63761 |
| Tmem150 | 1.92098 |
| Tmem150 | 0.4013 |
| Tmem151a | -0.13036 |
| Tmem154 | -0.28415 |
| Tmem158 | -0.62993 |
| Tmem159 | 1.64701 |
| Tmem159 | -0.44521 |
| Tmem160 | -0.6121 |
| Tmem160 | -0.37253 |
| Tmem161a | -0.76929 |
| Tmem162 | -0.19635 |
| Tmem164 | 1.38803 |
| Tmem166 | -0.10551 |
| Tmem167 | -0.12515 |
| Tmem168 | 0.02532 |
| Tmem168 | -0.77024 |
| Tmem168 | -0.42666 |
| Tmem169 | 2.58179 |
| Tmem16a | 1.40087 |
| Tmem16b | 1.5339 |
| Tmem16d | -0.03863 |
| Tmem16e | 0.24828 |
| Tmem16g | -0.10876 |
| Tmem16j | 1.92429 |
| Tmem16k | -0.14957 |
| Tmem17 | 1.32411 |
| Tmem17 | -0.426 |
| Tmem170 | 2.07938 |
| Tmem176a | -0.62912 |
| Tmem176a | 2.3256 |
| Tmem176b | -0.58599 |
| Tmem177 | 0.42963 |
| Tmem178 | 1.69577 |
| Tmem179b | -0.67867 |
| Tmem180 | 0.44374 |
| Tmem183a | 0.94501 |
| Tmem183a | 0.10003 |
| Tmem184a | -0.70671 |
| Tmem184a | 0.43221 |
| Tmem184b | 0.50477 |
| Tmem185b | 0.15075 |
| Tmem186 | -0.69785 |
| Tmem19 | 0.37836 |
| Tmem192 | -0.53147 |
| Tmem198 | 1.20092 |
| Tmem199 | 1.75133 |
| Tmem2 | 1.97764 |
| Tmem2 | 0.46237 |
| Tmem2 | 0.98681 |
| Tmem201 | -0.47376 |
| Tmem204 | -0.62993 |
| Tmem205 | -0.38875 |
| Tmem208 | -0.53737 |
| Tmem209 | -0.58521 |
| Tmem209 | -0.42797 |
| Tmem23 | 0.27312 |
| Tmem25 | 0.00545 |
| Tmem25 | -0.3633 |
| Tmem25 | -0.49049 |
| Tmem27 | 0.26604 |
| Tmem32 | 0.39192 |
| Tmem33 | -0.6283 |
| Tmem33 | 0.47279 |
| Tmem34 | 0.89594 |
| Tmem35 | 0.75355 |
| Tmem38a | -0.34391 |
| Tmem38b | 0.84295 |
| Tmem38b | 0.03212 |
| Tmem39a | 1.09878 |
| Tmem40 | 0.55006 |
| Tmem41a | 0.21821 |
| Tmem41b | 1.74202 |
| Tmem42 | -0.50752 |
| Tmem43 | -0.76454 |
| Tmem43 | 2.68232 |
| Tmem44 | -0.47584 |
| Tmem45a | 1.14973 |
| Tmem45b | -0.65398 |
| Tmem47 | 1.70472 |
| Tmem47 | 3.09555 |
| Tmem48 | -0.20096 |
| Tmem49 | -0.21752 |
| Tmem49 | -0.68821 |
| Tmem49 | -0.3261 |
| Tmem49 | 0.69428 |
| Tmem5 | -0.36146 |
| Tmem50a | -0.23333 |
| Tmem50b | -0.57668 |
| Tmem50b | -0.52634 |
| Tmem51 | 0.53475 |
| Tmem53 | 0.26913 |
| Tmem53 | -0.00444 |
| Tmem53 | 0.33816 |
| Tmem54 | 0.60675 |
| Tmem55a | -0.25159 |
| Tmem55b | -0.77406 |
| Tmem56 | 2.04976 |
| Tmem57 | -0.29261 |
| Tmem59 | 1.04218 |
| Tmem59l | -0.44857 |
| Tmem60 | 1.18322 |
| Tmem62 | -0.5954 |
| Tmem63a | 0.03886 |
| Tmem63b | -0.1938 |
| Tmem63b | 0.15529 |
| Tmem63c | -0.36698 |
| Tmem65 | 3.33502 |
| Tmem66 | -0.03863 |
| Tmem66 | -0.62912 |
| Tmem66 | -0.52707 |
| Tmem68 | 1.397 |
| Tmem69 | -0.06702 |
| Tmem70 | 0.58453 |
| Tmem71 | 2.53391 |
| Tmem74 | -0.08946 |
| Tmem74 | -0.67781 |
| Tmem74 | 2.05744 |
| Tmem77 | 0.02332 |
| Tmem77 | 2.30071 |
| Tmem77 | -0.62098 |
| Tmem79 | -0.50395 |
| Tmem8 | -0.03777 |
| Tmem8 | -0.65398 |
| Tmem8 | 1.02571 |
| Tmem81 | -0.13131 |
| Tmem82 | 0.03649 |
| Tmem85 | 0.20345 |
| Tmem85 | 1.65283 |
| Tmem86a | 0.78409 |
| Tmem86b | -0.74208 |
| Tmem87a | -0.45193 |
| Tmem88 | -0.64395 |
| Tmem9 | -0.71385 |
| Tmem90a | 0.20576 |
| Tmem91 | -0.57205 |
| Tmem93 | 0.80631 |
| Tmem97 | 1.43524 |
| Tmem98 | 3.84194 |
| Tmem98 | 0.40076 |
| Tmem9b | 2.56015 |
| Tmem9b | 1.92784 |
| Tmem9b | -0.37935 |
| Tmie | 2.81779 |
| Tmod1 | -0.6283 |
| Tmod2 | -0.20662 |
| Tmod2 | 0.06074 |
| Tmod3 | -0.63815 |
| Tmod4 | 2.9624 |
| Tmod4 | 0.80956 |
| Tmod4 | 2.47363 |
| Tmpo | -0.54108 |
| Tmpo | 2.21054 |
| Tmpo | 2.90536 |
| Tmprss11a | -0.70582 |
| Tmprss11c | -0.53073 |
| Tmprss13 | -0.51688 |
| Tmprss13 | -0.3976 |
| Tmprss2 | -0.05863 |
| Tmprss4 | -0.52561 |
| Tmprss4 | -0.48908 |
| Tmprss4 | 1.73155 |
| Tmprss7 | -0.5553 |
| Tmprss9 | 0.70142 |
| Tmsb10 | -0.35053 |
| Tmsb10 | -0.12751 |
| Tmsb10 | 1.04615 |
| Tmsb4x | 0.34356 |
| Tmtc2 | -0.58677 |
| Tmtc4 | -0.5176 |
| Tmub1 | 0.17867 |
| Tmub1 | 0.63342 |
| Tnc | 0.24006 |
| Tnc | 1.45331 |
| Tnc | 1.98175 |
| Tnc | -0.36759 |
| Tnfaip1 | 1.24121 |
| Tnfaip2 | -0.25376 |
| Tnfaip2 | -0.33437 |
| Tnfaip2 | 1.21905 |
| Tnfaip3 | -0.47653 |
| Tnfaip8 | 0.8601 |
| Tnfaip8l2 | 2.57945 |
| Tnfaip8l2 | 1.46108 |
| Tnfaip8l3 | -0.71925 |
| Tnfrsf10b | 1.04694 |
| Tnfrsf10b | -0.53959 |
| Tnfrsf10b | 2.08583 |
| Tnfrsf11a | 1.73255 |
| Tnfrsf11b | 0.43376 |
| Tnfrsf12a | -0.84055 |
| Tnfrsf13c | -0.67608 |
| Tnfrsf19 | 0.71117 |
| Tnfrsf19 | 1.83055 |
| Tnfrsf1a | -0.37006 |
| Tnfrsf1a | -0.3957 |
| Tnfrsf1a | -0.6113 |
| Tnfrsf1b | 1.45034 |
| Tnfrsf21 | 0.64494 |
| Tnfrsf21 | -0.6097 |
| Tnfrsf22 | -0.2392 |
| Tnfrsf22 | -0.52269 |
| Tnfrsf25 | 1.71339 |
| Tnfrsf4 | -0.6226 |
| Tnfsf13b | -0.02921 |
| Tnfsf13b | 0.97775 |
| Tnip1 | 0.05726 |
| Tnip1 | -0.68299 |
| Tnip1 | -0.32316 |
| Tnip1 | 2.82925 |
| Tnip2 | 3.09816 |
| Tnks | -0.03476 |
| Tnks1bp1 | -0.47931 |
| Tnnc1 | -0.13704 |
| Tnnc2 | 0.06228 |
| Tnni2 | 1.46184 |
| Tnni3 | 1.27961 |
| Tnnt1 | 1.47594 |
| Tnp1 | 0.84328 |
| Tnp2 | 3.67099 |
| Tnpo1 | 0.50071 |
| Tnpo2 | -0.57436 |
| Tnr | 0.61142 |
| Tnrc15 | -0.71117 |
| Tnrc18 | -0.61613 |
| Tnrc18 | -0.57668 |
| Tnrc18 | -0.73288 |
| Tnrc6a | 2.12603 |
| Tnrc6a | 0.36765 |
| Tnrc6b | -0.83338 |
| Tnrc6b | -0.66327 |
| Tnrc6c | -0.10136 |
| Tns4 | 2.62688 |
| Tnxb | 0.66535 |
| Tnxb | -0.69609 |
| Tnxb | 3.84847 |
| Tob1 | -0.47861 |
| Tob2 | 0.34441 |
| Toe1 | -0.42273 |
| Toe1 | -0.4339 |
| Tollip | 3.15144 |
| Tollip | 1.1004 |
| Tom1 | 1.79662 |
| Tom1l2 | 1.62031 |
| Tom1l2 | -0.41493 |
| Tom1l2 | -0.52051 |
| Tomm22 | 2.67398 |
| Tomm34 | -0.45463 |
| Tomm34 | 1.30786 |
| Tomm7 | 0.66115 |
| Tomm70a | -0.43722 |
| Top1 | -0.49684 |
| Top1mt | -0.54108 |
| Top2a | -0.75324 |
| Top3a | 1.10673 |
| Top3b | -0.27855 |
| Topors | 2.32014 |
| Tor1a | -0.48348 |
| Tor1a | -0.41687 |
| Tor1aip1 | -0.12704 |
| Tor1aip2 | -0.28978 |
| Tor1aip2 | -0.31965 |
| Tor1b | 1.91057 |
| Tor3a | 0.17969 |
| Tox3 | 0.83201 |
| Tpbg | 1.96294 |
| Tpcn1 | 2.05661 |
| Tpcn1 | -0.44924 |
| Tpcn2 | 1.0954 |
| Tpd52 | -0.81818 |
| Tpd52l1 | -0.49755 |
| Tpd52l1 | -0.75605 |
| Tpd52l2 | -0.55004 |
| Tph2 | -0.24349 |
| Tpi1 | 1.50782 |
| Tpi1 | 1.87288 |
| Tpi1 | 0.10409 |
| Tpk1 | -0.51111 |
| Tpk1 | 0.01767 |
| Tpk1 | -0.60252 |
| Tpm1 | 1.61691 |
| Tpm2 | -0.4717 |
| Tpm2 | 1.42401 |
| Tpm3 | -0.70404 |
| Tpm3 | 0.87645 |
| Tpm4 | 0.12484 |
| Tpo | 1.54233 |
| Tpp1 | 0.343 |
| Tpp2 | 1.0695 |
| Tppp | 0.32377 |
| Tppp | -0.66327 |
| Tppp3 | -0.63898 |
| Tpr | -0.6226 |
| Tpra40 | -0.63403 |
| Tprgl | -0.63733 |
| Tprgl | 1.77189 |
| Tprkb | -0.46552 |
| Tprkb | 2.80803 |
| Tpsab1 | 2.15399 |
| Tpst1 | 1.8194 |
| Tpst2 | -0.70938 |
| Tpte | -0.54108 |
| Tpte | -0.16424 |
| Tpx2 | -0.62179 |
| Tra2a | -0.35295 |
| Trabd | 0.19749 |
| Trabd | -0.72558 |
| Traf1 | -0.63733 |
| Traf2 | -0.71027 |
| Traf3 | 1.38712 |
| Traf3 | -0.14424 |
| Traf3ip1 | -0.50323 |
| Traf3ip2 | 1.50397 |
| Traf4 | 0.01483 |
| Traf4 | 2.53309 |
| Traf4 | 0.17799 |
| Traf4 | 3.18264 |
| Traf5 | 0.81416 |
| Traf6 | -0.64146 |
| Traf6 | -0.70227 |
| Traf6 | -0.5899 |
| Traf7 | 1.94379 |
| Trafd1 | -0.71655 |
| Trafd1 | -0.56822 |
| Trafd1 | -0.88807 |
| Traip | 1.20515 |
| Trak1 | -0.33496 |
| Trak2 | -0.75137 |
| Trak2 | 0.23496 |
| Trak2 | 0.84031 |
| Tram1 | -0.72649 |
| Tram1 | -0.22487 |
| Tram1 | -0.66667 |
| Tram2 | -0.40783 |
| Tram2 | -0.41882 |
| Trap1 | -0.59225 |
| Trappc1 | 0.53128 |
| Trappc1 | 0.84772 |
| Trappc2 | 1.30348 |
| Trappc2l | 0.65389 |
| Trappc3 | 2.37942 |
| Trappc3 | -0.76738 |
| Trappc3 | -0.39253 |
| Trappc3 | 1.10388 |
| Trappc4 | -0.55606 |
| Trappc4 | -0.37749 |
| Trappc5 | -0.51039 |
| Trappc6a | 0.13522 |
| Trat1 | 2.42166 |
| Trcg1 | -0.16671 |
| Trdmt1 | -0.58599 |
| Trdmt1 | 1.65875 |
| Trem2 | -0.42535 |
| Treml1 | 0.66735 |
| Treml2 | 1.50686 |
| Treml4 | -0.62504 |
| Trerf1 | 3.93649 |
| Trerf1 | 0.88876 |
| Trex1 | -0.70849 |
| Trex1 | 0.28257 |
| Trex2 | -0.27187 |
| Trf | 0.39461 |
| Trfp | -0.35234 |
| Trfr2 | -0.6057 |
| Trfr2 | 0.34809 |
| Trh | 1.08634 |
| Trhde | -0.27966 |
| Trhr | -0.49613 |
| Triap1 | -0.38812 |
| Trib2 | 0.85197 |
| Trib3 | -0.24403 |
| Trib3 | -0.67694 |
| Trim11 | 3.39549 |
| Trim16 | 1.61605 |
| Trim17 | -0.45396 |
| Trim2 | -0.68821 |
| Trim2 | -0.62667 |
| Trim21 | 0.48722 |
| Trim21 | 1.66732 |
| Trim23 | 0.2874 |
| Trim23 | -0.68734 |
| Trim23 | -0.3261 |
| Trim25 | -0.74578 |
| Trim26 | -0.74485 |
| Trim28 | -0.80621 |
| Trim3 | 2.22443 |
| Trim3 | -0.32786 |
| Trim30 | -0.68473 |
| Trim32 | -0.62098 |
| Trim32 | 0.82077 |
| Trim33 | 1.52555 |
| Trim33 | 0.52035 |
| Trim33 | 0.00504 |
| Trim35 | -0.00113 |
| Trim36 | -0.42273 |
| Trim36 | -0.59698 |
| Trim36 | 0.21201 |
| Trim37 | 0.44272 |
| Trim38 | 1.30298 |
| Trim38 | -0.66837 |
| Trim39 | 2.06854 |
| Trim40 | 1.10994 |
| Trim41 | 1.31301 |
| Trim42 | -0.43987 |
| Trim44 | 0.49567 |
| Trim45 | -0.66496 |
| Trim45 | 1.29 |
| Trim46 | 1.61392 |
| Trim47 | 0.31621 |
| Trim54 | -0.53368 |
| Trim56 | -0.74485 |
| Trim59 | 0.20675 |
| Trim61 | 0.30503 |
| Trim62 | 0.65024 |
| Trim67 | -0.64146 |
| Trim68 | 2.10385 |
| Trim69 | -0.09539 |
| Trim72 | -0.52269 |
| Trim8 | 0.07377 |
| Trim9 | -0.40462 |
| Trim9 | -0.70138 |
| Trim9 | 0.55638 |
| Trim9 | -0.58288 |
| Triml1 | -0.06569 |
| Triobp | -0.09083 |
| Triobp | -0.69433 |
| Trip10 | -0.00568 |
| Trip12 | -0.4104 |
| Trip12 | 3.20737 |
| Trip12 | 1.24696 |
| Trip6 | -0.65146 |
| Trip6 | 0.10996 |
| Trit1 | -0.38686 |
| Trmt1 | 1.73262 |
| Trmt12 | 1.68 |
| Trmu | -0.73288 |
| Tro | -0.7076 |
| Tro | 0.29611 |
| Tro | -0.02454 |
| Trove2 | 0.08511 |
| Trp53 | 0.20774 |
| Trp53bp1 | -0.70671 |
| Trp53bp1 | -0.49684 |
| Trp53bp2 | -0.44857 |
| Trp53i11 | -0.16424 |
| Trp53i11 | -0.3938 |
| Trp53inp1 | -0.5606 |
| Trp53inp1 | 1.40033 |
| Trp53inp2 | 0.55142 |
| Trp53rk | -0.18317 |
| Trp63 | -0.70227 |
| Trpc1 | -0.21439 |
| Trpc1 | -0.63815 |
| Trpc2 | 0.73353 |
| Trpc3 | -0.46483 |
| Trpc4 | 0.12664 |
| Trpc4ap | -0.54929 |
| Trpc4ap | -0.77406 |
| Trpc5 | -0.53737 |
| Trpc6 | 0.11397 |
| Trpc7 | 1.09277 |
| Trpd52l3 | -0.76929 |
| Trpm1 | -0.29488 |
| Trpm2 | -0.54108 |
| Trpm3 | 0.21918 |
| Trpm3 | -0.17018 |
| Trpm3 | -0.54108 |
| Trpm3 | -0.77311 |
| Trpm3 | 1.97504 |
| Trpm4 | -0.72467 |
| Trpm6 | -0.54256 |
| Trpm6 | -0.19993 |
| Trpm6 | 0.83135 |
| Trpm8 | 0.24512 |
| Trps1 | -0.86453 |
| Trpt1 | 2.16097 |
| Trpt1 | 0.37645 |
| Trpt1 | -0.42077 |
| Trpv2 | 1.04734 |
| Trpv4 | -0.33853 |
| Trpv6 | 0.35708 |
| Trrap | 1.57434 |
| Trub1 | 3.47314 |
| Trub1 | 4.41436 |
| Trub1 | -0.31732 |
| Trub2 | 1.81093 |
| Tsc1 | -0.12232 |
| Tsc2 | -0.3806 |
| Tsc2 | 0.41374 |
| Tsc22d1 | 0.83135 |
| Tsc22d1 | 0.4195 |
| Tsc22d2 | -0.78272 |
| Tsc22d3 | 0.62698 |
| Tsc22d3 | -0.70849 |
| Tsc22d4 | -0.44521 |
| Tsen2 | 1.36207 |
| Tsen34 | -0.29887 |
| Tsen54 | -0.33259 |
| Tsfm | -0.58755 |
| Tsg101 | -0.71655 |
| Tsga10 | 2.78622 |
| Tsga10 | -0.75605 |
| Tsga14 | -0.75511 |
| Tsga8 | -0.70938 |
| Tshb | 1.3862 |
| Tshr | 0.42002 |
| Tshz3 | -0.5606 |
| Tsku | -0.44054 |
| Tsn | -0.63321 |
| Tsnaxip1 | 1.45712 |
| Tsnaxip1 | -0.64146 |
| Tspan1 | 0.06074 |
| Tspan11 | -0.70582 |
| Tspan12 | -0.52051 |
| Tspan13 | -0.72196 |
| Tspan14 | 0.0483 |
| Tspan17 | 1.39275 |
| Tspan17 | 0.16223 |
| Tspan18 | 0.79839 |
| Tspan18 | 1.10276 |
| Tspan2 | 0.149 |
| Tspan3 | -0.20559 |
| Tspan31 | 0.73779 |
| Tspan33 | -0.41493 |
| Tspan6 | 0.4005 |
| Tspan6 | 1.72954 |
| Tspan7 | -0.03007 |
| Tspan8 | -0.62422 |
| Tspo | -0.27855 |
| Tspo | 2.71316 |
| Tspo | -0.78272 |
| Tspyl1 | 3.1087 |
| Tspyl3 | 0.06343 |
| Tsr1 | 1.38447 |
| Tsr2 | -0.4059 |
| Tsr2 | 1.58263 |
| Tsr2 | 0.31005 |
| Tssc1 | -0.28134 |
| Tssc1 | -0.68299 |
| Tssc1 | -0.1672 |
| Tssc1 | 1.3501 |
| Tssc1 | 0.24922 |
| Tssc4 | 1.3961 |
| Tssc4 | -0.54405 |
| Tssk4 | -0.3831 |
| Tssk5 | 1.18584 |
| Tssk6 | -0.49472 |
| Tst | 0.04634 |
| Tsta3 | -0.2328 |
| Tsx | -0.22909 |
| Ttbk2 | -0.52051 |
| Ttc1 | -0.58677 |
| Ttc12 | -0.76454 |
| Ttc13 | 0.92217 |
| Ttc14 | -0.65398 |
| Ttc16 | -0.33437 |
| Ttc17 | 0.67786 |
| Ttc19 | 3.91281 |
| Ttc19 | 0.82263 |
| Ttc21a | 0.92171 |
| Ttc22 | 0.77863 |
| Ttc23 | -0.12799 |
| Ttc24 | 0.73909 |
| Ttc24 | 1.33652 |
| Ttc26 | 0.97504 |
| Ttc27 | 2.52937 |
| Ttc29 | 1.63865 |
| Ttc29 | -0.65482 |
| Ttc3 | 1.86164 |
| Ttc3 | -0.60173 |
| Ttc3 | -0.07593 |
| Ttc3 | -0.75324 |
| Ttc3 | 0.12123 |
| Ttc3 | 1.72936 |
| Ttc30a1 | 0.12807 |
| Ttc30a2 | -0.09402 |
| Ttc33 | 0.33214 |
| Ttc33 | 0.01443 |
| Ttc33 | 1.71479 |
| Ttc35 | 1.93304 |
| Ttc4 | 3.22336 |
| Ttc5 | -0.5644 |
| Ttc5 | 1.07528 |
| Ttc5 | 0.24796 |
| Ttc7 | -0.42666 |
| Ttc8 | -0.51905 |
| Ttc8 | 1.28074 |
| Ttf2 | -0.02158 |
| Ttk | -0.80423 |
| Ttl | -0.28246 |
| Ttll1 | 0.77242 |
| Ttll1 | 0.39139 |
| Ttll1 | 0.67232 |
| Ttll10 | 0.19749 |
| Ttll11 | -0.236 |
| Ttll13 | -0.45531 |
| Ttll4 | 3.49208 |
| Ttll5 | -0.743 |
| Ttll5 | 1.08937 |
| Ttll6 | 2.78951 |
| Ttll8 | 2.8472 |
| Ttll9 | 1.03083 |
| Ttn | 1.47693 |
| Ttn | -0.43655 |
| Ttr | -0.217 |
| Ttyh1 | 0.26943 |
| Ttyh1 | 2.79648 |
| Ttyh2 | -0.32551 |
| Ttyh2 | -0.18872 |
| Ttyh3 | -0.70227 |
| Tub | -0.4059 |
| Tub | -0.74857 |
| Tuba1a | 0.4353 |
| Tuba1b | -0.65062 |
| Tuba3a | -0.72649 |
| Tuba3a | 1.37795 |
| Tuba3a | -0.83952 |
| Tuba3a | -0.60332 |
| Tuba3b | 3.60527 |
| Tuba4a | 0.81042 |
| Tuba6 | -0.53811 |
| Tuba8 | 3.18841 |
| Tubb2b | -0.36391 |
| Tubb2b | 0.14231 |
| Tubb2b | -0.24996 |
| Tubb2b | 1.45459 |
| Tubb2b | 2.66172 |
| Tubb2c | -0.72105 |
| Tubb4 | -0.71117 |
| Tubb5 | -0.63815 |
| Tubb6 | 1.90498 |
| Tubd1 | 2.17974 |
| Tubd1 | -0.49049 |
| Tube1 | -0.81818 |
| Tubg1 | -0.6565 |
| Tubgcp2 | 1.29968 |
| Tubgcp4 | 1.27427 |
| Tufm | -0.61613 |
| Tuft1 | -0.54033 |
| Tug1 | 0.94913 |
| Tulp1 | -0.45328 |
| Tulp2 | 2.02755 |
| Tulp3 | 2.9069 |
| Tulp4 | 1.36591 |
| Tusc2 | -0.64395 |
| Tusc2 | 0.81688 |
| Tusc4 | 0.38625 |
| Tut1 | -0.75699 |
| Twf2 | 0.42107 |
| Twist1 | -0.72377 |
| Twist2 | -0.1867 |
| Twistnb | 0.73372 |
| Twistnb | 1.36972 |
| Twsg1 | -0.67522 |
| Txk | -0.63733 |
| Txlna | -0.62422 |
| Txn1 | 1.58535 |
| Txn2 | 0.58322 |
| Txndc1 | -0.42273 |
| Txndc11 | 1.05391 |
| Txndc11 | -0.41752 |
| Txndc11 | 3.67791 |
| Txndc12 | 1.82745 |
| Txndc13 | -0.24188 |
| Txndc13 | 2.95566 |
| Txndc15 | 1.70201 |
| Txndc2 | -0.78465 |
| Txndc5 | -0.79831 |
| Txndc8 | -0.57822 |
| Txndc9 | 0.07148 |
| Txnip | 1.67155 |
| Txnl2 | 0.44602 |
| Txnl4 | -0.67867 |
| Txnl4a | 2.38177 |
| Txnl4a | -0.6804 |
| Txnl4a | 1.10375 |
| Txnl4b | -0.07593 |
| Txnrd2 | -0.67179 |
| Txnrd3 | -0.73655 |
| Tyk2 | 1.24686 |
| Tyk2 | 1.62756 |
| Tyk2 | 1.69479 |
| Tyki | 0.20873 |
| Tyms | -0.00734 |
| Tyms | 0.09148 |
| Tyms | -0.78659 |
| Tyms-ps | 0.3693 |
| Tyro3 | -0.65566 |
| Tyrobp | 1.61684 |
| Tyw1 | -0.19635 |
| Tyw1 | 0.47917 |
| U2af1l4 | 2.86921 |
| U2af2 | 0.12807 |
| U2af2 | -0.49472 |
| Uaca | -0.24942 |
| Uap1 | 0.20905 |
| Uap1l1 | -0.41493 |
| Uba1 | 0.20345 |
| Uba1 | -0.60173 |
| Uba2 | -0.63239 |
| Uba5 | 0.21364 |
| Ubac1 | -0.7005 |
| Ubac1 | 1.55114 |
| Ubac1 | -0.69433 |
| Ubac2 | 0.51683 |
| Ubap1 | 1.89181 |
| Ubap2 | -0.72922 |
| Ubap2l | -0.24349 |
| Ubap2l | 0.59625 |
| Ubap2l | -0.80028 |
| Ubb | 3.69018 |
| Ubd | -0.04337 |
| Ube1c | -0.68734 |
| Ube1l | -0.1603 |
| Ube1x | -0.10551 |
| Ube2a | 2.06244 |
| Ube2b | 1.47312 |
| Ube2d1 | -0.64979 |
| Ube2d2 | -0.54033 |
| Ube2d3 | -0.71835 |
| Ube2e1 | -0.16473 |
| Ube2e2 | -0.36268 |
| Ube2e3 | 3.3403 |
| Ube2f | -0.72558 |
| Ube2f | -0.45463 |
| Ube2f | 0.28861 |
| Ube2g1 | 1.3687 |
| Ube2g1 | -0.6523 |
| Ube2g1 | -0.47101 |
| Ube2g2 | -0.43456 |
| Ube2i | 0.36543 |
| Ube2j1 | -0.27743 |
| Ube2j2 | 2.482 |
| Ube2j2 | 0.37563 |
| Ube2j2 | 0.62259 |
| Ube2k | 2.04411 |
| Ube2k | 0.55368 |
| Ube2l3 | 1.96397 |
| Ube2l3 | 0.46312 |
| Ube2l6 | 0.92156 |
| Ube2m | -0.06171 |
| Ube2n | -0.64979 |
| Ube2n | -0.73563 |
| Ube2o | -0.37376 |
| Ube2q1 | -0.57745 |
| Ube2q1 | -0.68734 |
| Ube2q2 | -0.46552 |
| Ube2q2 | 1.83582 |
| Ube2q2 | -0.5759 |
| Ube2r2 | 1.88037 |
| Ube2s | 1.925 |
| Ube2t | 0.28771 |
| Ube2u | 1.44949 |
| Ube2u | -0.69083 |
| Ube3a | 0.14619 |
| Ube3a | -0.37935 |
| Ube3b | -0.6565 |
| Ube3b | -0.21752 |
| Ube3c | -0.54554 |
| Ube4a | -0.32786 |
| Ube4b | 0.34498 |
| Ubfd1 | 1.02625 |
| Ubiad1 | 1.2232 |
| Ubie | -0.70227 |
| Ubl3 | 1.54709 |
| Ubl4 | -0.52269 |
| Ubl4b | -0.68386 |
| Ubl5 | -0.44857 |
| Ubl7 | -0.63239 |
| Ublcp1 | -0.60491 |
| Ubox5 | -0.40719 |
| Ubox5 | -0.79048 |
| Ubp1 | -0.54405 |
| Ubp1 | -0.6283 |
| Ubqln1 | -0.50323 |
| Ubqln1 | -0.27966 |
| Ubqln1 | 1.04958 |
| Ubqln3 | -0.64895 |
| Ubqln4 | -0.57436 |
| Ubqlnl | -0.68734 |
| Ubr1 | 1.8342 |
| Ubr5 | -0.21805 |
| Ubtd1 | -0.79831 |
| Ubtd2 | 1.19037 |
| Ubtd2 | -0.53959 |
| Ubtd2 | -0.12279 |
| Ubtf | -0.31325 |
| Ubxd1 | -0.16079 |
| Ubxd1 | -0.70315 |
| Ubxd1 | -0.23333 |
| Ubxd3 | 0.83783 |
| Ubxd4 | -0.35659 |
| Ubxd5 | 0.82582 |
| Ubxd8 | -0.68647 |
| Uchl1 | -0.3919 |
| Uchl3 | -0.43921 |
| Uchl5 | -0.44991 |
| Uchl5 | 0.28347 |
| Uchl5 | -0.11481 |
| Uck1 | 0.09781 |
| Uck1 | 1.42733 |
| Uck1 | 0.90338 |
| Uck1 | 0.05337 |
| Uck2 | -0.24564 |
| Uck2 | -0.44789 |
| Uck2 | 0.22533 |
| Uevld | 0.34498 |
| Ufc1 | -0.42535 |
| Ufsp1 | 1.09139 |
| Ufsp2 | 0.83617 |
| Ugcg | 0.03013 |
| Ugp2 | 1.02895 |
| Ugt1a10 | -0.6081 |
| Ugt1a6a | 2.07877 |
| Ugt1a6a | 0.68474 |
| Ugt1a6a | 0.11507 |
| Ugt3a2 | 0.09521 |
| Ugt8a | 0.89392 |
| Uhmk1 | -0.37811 |
| Uhrf1 | 1.07451 |
| Uhrf1bp1 | -0.51327 |
| Uhrf1bp1l | -0.68996 |
| Uhrf2 | -0.54629 |
| Uhrf2 | 0.27189 |
| Uhrf2 | -0.38686 |
| Uhrf2 | 1.16251 |
| Uimc1 | 1.73668 |
| Ulk1 | -0.39824 |
| Ulk1 | 0.62322 |
| Ulk2 | 1.37703 |
| Ulk2 | -0.67265 |
| Umodl1 | -0.54033 |
| Umps | -0.46415 |
| Umps | -0.72377 |
| Umps | -0.58443 |
| Unc119 | 0.648 |
| Unc119 | -0.41428 |
| Unc119b | -0.65566 |
| Unc119b | 0.10261 |
| Unc119b | -0.1209 |
| Unc13b | 1.44787 |
| Unc13c | -0.58055 |
| Unc13d | -0.45328 |
| Unc45a | -0.25648 |
| Unc45a | 2.42393 |
| Unc45b | -0.37067 |
| Unc5a | -0.39951 |
| Unc5a | 1.58189 |
| Unc5b | 1.90472 |
| Unc5c | 1.41601 |
| Unc84a | -0.72196 |
| Unc84a | 1.08241 |
| Unc84b | -0.50537 |
| Unc93a | -0.22014 |
| Unc93b1 | -0.58132 |
| Unc93b1 | -0.48209 |
| Ung | -0.43523 |
| Ung | 1.73811 |
| Unk | 0.10629 |
| Unkl | -0.3069 |
| Uox | 0.7685 |
| Upb1 | -0.21544 |
| Upb1 | -0.47584 |
| Upb1 | -0.11902 |
| Upf1 | -0.73379 |
| Upf1 | -0.56669 |
| Upf1 | -0.11154 |
| Upf2 | 0.8831 |
| Upf3a | 1.09315 |
| Upk1a | -0.7076 |
| Upk1a | -0.71565 |
| Upk1b | -0.58677 |
| Upk2 | -0.50609 |
| Upk3a | 0.81348 |
| Upk3b | -0.3938 |
| Upk3b | 1.6125 |
| Upk3b | 0.95323 |
| Upp1 | 2.69233 |
| Upp1 | -0.36391 |
| Upp1 | -0.05075 |
| Upp2 | 1.18037 |
| Upp2 | 2.80953 |
| Upp2 | 1.33883 |
| Upp2 | 0.48235 |
| Uqcc | 2.33754 |
| Uqcr | 0.95717 |
| Uqcrc1 | -0.77215 |
| Uqcrc2 | 0.39916 |
| Uqcrh | 0.59646 |
| Urm1 | -0.69962 |
| Urod | 1.30298 |
| Uros | -0.46963 |
| Uros | -0.26195 |
| Use1 | 0.48746 |
| Use1 | -0.78079 |
| Use1 | -0.60332 |
| Usf1 | 1.2442 |
| Usf1 | -0.55757 |
| Usf2 | 0.83849 |
| Usf2 | -0.45396 |
| Ush1c | -0.55606 |
| Ush1g | 0.63652 |
| Ush2a | -0.81618 |
| Ush3a | -0.41622 |
| Ushbp1 | 1.47519 |
| Usmg5 | -0.65062 |
| Uso1 | 0.96326 |
| Usp1 | 1.79604 |
| Usp1 | -0.63486 |
| Usp1 | -0.53737 |
| Usp10 | 0.06727 |
| Usp10 | 0.50238 |
| Usp10 | -0.17117 |
| Usp11 | 0.6228 |
| Usp11 | 1.18912 |
| Usp12 | -0.80919 |
| Usp12 | -0.34092 |
| Usp13 | 0.22533 |
| Usp14 | -0.04727 |
| Usp15 | -0.74857 |
| Usp16 | 0.78304 |
| Usp18 | -0.56822 |
| Usp18 | 3.09768 |
| Usp19 | 1.2119 |
| Usp2 | -0.80324 |
| Usp2 | 0.95688 |
| Usp2 | 1.29324 |
| Usp20 | -0.27409 |
| Usp21 | 1.14167 |
| Usp22 | -0.51183 |
| Usp25 | -0.24241 |
| Usp28 | 1.52233 |
| Usp29 | 0.04752 |
| Usp3 | -0.51183 |
| Usp3 | -0.69521 |
| Usp33 | 1.35323 |
| Usp37 | -0.6804 |
| Usp38 | -0.5068 |
| Usp38 | 1.03204 |
| Usp39 | -0.31441 |
| Usp39 | -0.18569 |
| Usp39 | -0.62341 |
| Usp39 | -0.6081 |
| Usp4 | 0.76796 |
| Usp4 | -0.6065 |
| Usp43 | 2.11172 |
| Usp45 | 0.51659 |
| Usp46 | 1.23972 |
| Usp47 | 1.53445 |
| Usp48 | -0.1938 |
| Usp49 | 0.15946 |
| Usp5 | 0.82465 |
| Usp50 | 1.23435 |
| Usp52 | 0.50857 |
| Usp52 | -0.0648 |
| Usp52 | -0.03177 |
| Usp53 | 2.22131 |
| Usp6nl | 2.6794 |
| Usp6nl | -0.46415 |
| Usp7 | 0.1674 |
| Usp8 | 0.51353 |
| Usp8 | 0.37453 |
| Usp9x | 2.9512 |
| Usp9y | 0.47328 |
| Uspl1 | -0.70938 |
| Utp11l | 1.0686 |
| Utp15 | -0.55304 |
| Utp6 | 0.07681 |
| Utx | -0.07727 |
| Utx | -0.13369 |
| Uty | 1.23714 |
| Uvrag | -0.41493 |
| Uxt | 1.19521 |
| Uxt | 2.47296 |
| V1ra2 | 0.85588 |
| V1rd2 | 0.09966 |
| V1rd20 | 1.91243 |
| V1rd21 | -0.62179 |
| V2r8 | 1.43757 |
| Vac14 | 3.62488 |
| Vamp1 | -0.71296 |
| Vamp1 | -0.6365 |
| Vamp2 | -0.37811 |
| Vamp3 | 0.43735 |
| Vamp3 | -0.1938 |
| Vamp4 | -0.83747 |
| Vamp5 | -0.51688 |
| Vamp8 | 0.45737 |
| Vamp8 | -0.47584 |
| Vangl2 | -0.37998 |
| Vapa | -0.50537 |
| Vapb | 3.09259 |
| Vars | -0.6073 |
| Vars | 1.7784 |
| Vars | 0.91534 |
| Vars2 | 1.09013 |
| Vash1 | -0.35416 |
| Vash1 | -0.59935 |
| Vasn | -0.34391 |
| Vasp | 0.10335 |
| Vat1 | 0.14443 |
| Vav1 | 0.64002 |
| Vav2 | 2.45551 |
| Vav3 | 0.71724 |
| Vax1 | -0.09722 |
| Vbp1 | -0.09631 |
| Vcam1 | 0.62447 |
| Vcl | -0.50395 |
| Vcp | -0.72558 |
| Vcpip1 | 2.08898 |
| Vdac1 | -0.65734 |
| Vdac2 | -0.45058 |
| Vdac3 | 0.76725 |
| Vdr | -0.24134 |
| Vdr | 0.38544 |
| Vdr | 0.75464 |
| Vegfa | 0.68728 |
| Vegfa | -0.56822 |
| Vegfb | 2.58425 |
| Vegfc | -0.03734 |
| Vezf1 | -0.72377 |
| Vezt | 0.19582 |
| Vgf | 2.02528 |
| Vgll2 | -0.27576 |
| Vgll4 | 1.9785 |
| Vhlh | 1.03164 |
| Vil1 | 1.06355 |
| Vill | -0.51833 |
| Vim | -0.6804 |
| Vip | 1.99441 |
| Vit | -0.54854 |
| Vkorc1 | -0.58288 |
| Vkorc1 | 0.31942 |
| Vkorc1 | -0.06259 |
| Vkorc1l1 | 1.91776 |
| Vkorc1l1 | 0.7615 |
| Vkorc1l1 | 0.43914 |
| Vldlr | -0.58443 |
| Vldlr | -0.31383 |
| Vldlr | -0.06569 |
| Vldlr | -0.47446 |
| Vldlr | 1.06988 |
| Vmn2r81 | 0.48015 |
| Vmo1 | -0.44187 |
| Vnn3 | -0.07057 |
| Vnn3 | 0.21201 |
| Vpreb1 | -0.41428 |
| Vpreb3 | 0.40899 |
| Vps11 | -0.04035 |
| Vps13a | -0.73379 |
| Vps16 | 1.00936 |
| Vps16 | -0.55757 |
| Vps16 | -0.5954 |
| Vps16 | -0.71027 |
| Vps18 | 0.96903 |
| Vps24 | 0.84641 |
| Vps25 | -0.0451 |
| Vps25 | -0.62912 |
| Vps26 | -0.46894 |
| Vps26a | -0.60173 |
| Vps26b | -0.49896 |
| Vps28 | 1.14583 |
| Vps29 | 0.3596 |
| Vps29 | -0.79635 |
| Vps29 | -0.6097 |
| Vps33a | 3.02435 |
| Vps33a | -0.02116 |
| Vps33b | 0.71516 |
| Vps33b | 2.55558 |
| Vps35 | 0.58235 |
| Vps36 | 0.4676 |
| Vps37b | -0.82221 |
| Vps37c | -0.51183 |
| Vps37d | 1.38766 |
| Vps39 | -0.36636 |
| Vps39 | -0.72105 |
| Vps41 | 1.23929 |
| Vps41 | -0.14328 |
| Vps45 | -0.57745 |
| Vps4a | -0.56288 |
| Vps4b | 0.79856 |
| Vps53 | -0.76549 |
| Vps53 | 0.89485 |
| Vps53 | 0.78356 |
| Vps54 | -0.19686 |
| Vps72 | 0.4666 |
| Vrk1 | 1.39619 |
| Vrk1 | -0.53368 |
| Vrk2 | 2.7571 |
| Vrk3 | -0.6523 |
| Vsig2 | -0.3046 |
| Vsig4 | -0.78854 |
| Vsnl1 | 3.61057 |
| Vstm2a | 1.40311 |
| Vstm2a | -0.35963 |
| Vstm2a | 0.27158 |
| Vstm2b | 0.23368 |
| Vsx2 | -0.61855 |
| Vti1a | -0.42012 |
| Vti1a | 2.27269 |
| Vti1a | 0.02372 |
| Vti1b | 1.38639 |
| Vti1b | -0.74115 |
| Vtn | -0.1242 |
| Vwa2 | 2.46091 |
| Vwa3a | -0.70493 |
| Vwf | -0.58132 |
| Wars | -0.80127 |
| Wars | -0.72558 |
| Wars | -0.49119 |
| Wars | -0.02624 |
| Wars2 | 2.5834 |
| Wars2 | -0.62748 |
| Was | -0.66667 |
| Wasf1 | -0.41817 |
| Wasf2 | 0.26388 |
| Wasf3 | -0.0451 |
| Wasl | 1.97157 |
| Wbp1 | -0.40526 |
| Wbp1 | -0.09722 |
| Wbp11 | 0.41268 |
| Wbp2 | 2.59143 |
| Wbp2nl | 0.38598 |
| Wbp4 | 0.44577 |
| Wbp5 | -0.04727 |
| Wbp7 | -0.64645 |
| Wbscr16 | 1.15831 |
| Wbscr18 | -0.55379 |
| Wbscr22 | -0.01653 |
| Wbscr22 | 0.16877 |
| Wbscr22 | -0.6073 |
| Wbscr27 | 1.18856 |
| Wbscr28 | -0.29375 |
| Wbscr28 | 0.13629 |
| Wdfy1 | 0.2084 |
| Wdfy3 | -0.14763 |
| Wdhd1 | 0.23368 |
| Wdr1 | -0.54704 |
| Wdr1 | 1.71906 |
| Wdr13 | -0.55681 |
| Wdr13 | -0.67008 |
| Wdr16 | -0.71117 |
| Wdr17 | 1.54017 |
| Wdr18 | -0.66752 |
| Wdr20a | 0.8908 |
| Wdr20b | 0.2865 |
| Wdr21 | -0.29148 |
| Wdr22 | -0.38938 |
| Wdr23 | 1.74979 |
| Wdr24 | -0.16819 |
| Wdr27 | 1.51348 |
| Wdr3 | 0.71497 |
| Wdr31 | -0.65314 |
| Wdr31 | -0.28078 |
| Wdr31 | 0.15354 |
| Wdr32 | 0.58103 |
| Wdr33 | 1.57938 |
| Wdr34 | -0.42208 |
| Wdr37 | 0.71667 |
| Wdr37 | -0.49613 |
| Wdr37 | -0.75981 |
| Wdr37 | 0.16153 |
| Wdr38 | 0.06574 |
| Wdr41 | 0.18239 |
| Wdr45 | 0.14513 |
| Wdr45l | -0.51039 |
| Wdr45l | 1.70091 |
| Wdr46 | -0.2392 |
| Wdr46 | -0.7005 |
| Wdr47 | -0.32904 |
| Wdr5 | -0.78369 |
| Wdr51b | -0.5068 |
| Wdr53 | -0.67867 |
| Wdr54 | -0.08403 |
| Wdr55 | -0.33022 |
| Wdr59 | -0.68821 |
| Wdr5b | 0.68728 |
| Wdr6 | -0.3179 |
| Wdr60 | -0.33318 |
| Wdr61 | -0.6565 |
| Wdr62 | 0.31357 |
| Wdr63 | 0.14689 |
| Wdr66 | -0.47515 |
| Wdr67 | 0.99982 |
| Wdr68 | 0.51282 |
| Wdr69 | 1.6841 |
| Wdr70 | -0.54033 |
| Wdr70 | -0.5821 |
| Wdr74 | 0.45134 |
| Wdr75 | -0.79341 |
| Wdr75 | 1.71658 |
| Wdr76 | -0.40847 |
| Wdr77 | -0.42469 |
| Wdr78 | 0.47155 |
| Wdr8 | 1.49947 |
| Wdr8 | -0.62504 |
| Wdr9 | 0.36377 |
| Wdr93 | -0.3314 |
| Wdsof1 | -0.69258 |
| Wfdc1 | -0.71745 |
| Wfdc10 | 1.44119 |
| Wfdc10 | 0.51424 |
| Wfdc12 | 0.93029 |
| Wfdc15b | -0.62504 |
| Wfdc15b | -0.63733 |
| Wfdc2 | -0.2451 |
| Wfdc3 | -0.39317 |
| Wfdc6a | -0.6523 |
| Wfdc6b | 0.40608 |
| Wfdc6b | -0.80225 |
| Wfikkn2 | -0.39317 |
| Wfs1 | -0.32316 |
| Whdc1 | 0.60845 |
| Whrn | -0.73931 |
| Whrn | -0.36759 |
| Whsc2 | -0.24564 |
| Wif1 | -0.32492 |
| Wipf1 | 1.54916 |
| Wipf2 | -0.23706 |
| Wipi1 | 1.52743 |
| Wipi1 | 0.17765 |
| Wipi1 | -0.59935 |
| Wipi2 | 0.00832 |
| Wipi2 | -0.64895 |
| Wipi2 | 0.57664 |
| Wisp1 | 0.74148 |
| Wisp1 | 1.48786 |
| Wisp2 | 1.57128 |
| Wiz | 4.70505 |
| Wiz | 0.04359 |
| Wnk1 | -0.72015 |
| Wnk1 | 0.36543 |
| Wnk2 | -0.21544 |
| Wnk4 | 0.4923 |
| Wnt10a | -0.29773 |
| Wnt10b | 1.26442 |
| Wnt16 | -0.2603 |
| Wnt16 | -0.41169 |
| Wnt16 | 1.5718 |
| Wnt2 | -0.11715 |
| Wnt3a | -0.02285 |
| Wnt4 | -0.58833 |
| Wnt4 | 0.36682 |
| Wnt5a | -0.17216 |
| Wnt5a | 0.87581 |
| Wnt5b | 2.15193 |
| Wnt6 | 0.83783 |
| Wnt7a | 1.0633 |
| Wnt7b | 1.31262 |
| Wnt7b | 0.21135 |
| Wrb | 0.06804 |
| Wrn | -0.5606 |
| Wsb1 | 0.74222 |
| Wsb2 | -0.16572 |
| Wscd1 | 1.60569 |
| Wscd2 | 2.17774 |
| Wtap | 2.91934 |
| Wtip | -0.03949 |
| Wwc1 | -0.56516 |
| Wwc1 | -0.54854 |
| Wwc2 | 3.75777 |
| Wwc2 | -0.74393 |
| Wwox | -0.44054 |
| Wwox | -0.55984 |
| Wwp1 | 2.35089 |
| Wwp2 | 1.06692 |
| Wwp2 | -0.09998 |
| Wwp2 | -0.03434 |
| Wwtr1 | 1.34066 |
| X99384 | 0.7144 |
| Xab1 | -0.61613 |
| Xab1 | 0.44856 |
| Xbp1 | -0.62017 |
| Xdh | 0.75391 |
| Xist | 0.31386 |
| Xist | 0.05958 |
| Xk | -0.53147 |
| Xkr4 | 1.76212 |
| Xkr5 | -0.61774 |
| Xkr6 | 0.8275 |
| Xkr8 | 0.40476 |
| Xkrx | 0.19014 |
| Xlr4a | 2.29946 |
| Xpc | 3.08495 |
| Xpnpep1 | 2.94223 |
| Xpnpep1 | 0.60845 |
| Xpnpep1 | 0.29671 |
| Xpnpep2 | 0.90846 |
| Xpnpep2 | 1.89234 |
| Xpnpep3 | -0.69171 |
| Xpo4 | -0.73379 |
| Xpo4 | -0.66923 |
| Xpo5 | -0.55379 |
| Xpo6 | -0.58755 |
| Xpo7 | 1.93062 |
| Xpo7 | -0.73471 |
| Xpr1 | -0.46826 |
| Xrcc1 | 1.47303 |
| Xrcc2 | 1.33508 |
| Xrcc2 | -0.26085 |
| Xrcc3 | 0.78145 |
| Xrcc6 | 2.69524 |
| Xrcc6 | -0.41947 |
| Xrcc6 | 2.20876 |
| Xrn1 | 2.03145 |
| Xrn2 | 1.49704 |
| Xylb | -0.50824 |
| Xylt2 | -0.71206 |
| Yaf2 | 0.42963 |
| Yap1 | 0.60781 |
| Ybx2 | 1.12267 |
| Ybx2 | -0.67008 |
| Yeats2 | 2.95038 |
| Yeats4 | 0.74662 |
| Yeats4 | 0.01118 |
| Yif1a | -0.35476 |
| Yif1a | -0.66411 |
| Yif1a | 1.40365 |
| Yif1b | 1.20281 |
| Yipf1 | 2.43836 |
| Yipf2 | 1.922 |
| Yipf3 | 0.22855 |
| Yipf3 | -0.05775 |
| Yipf3 | 3.36876 |
| Yipf4 | 1.74746 |
| Yipf6 | -0.72831 |
| Yipf6 | -0.12846 |
| Yipf6 | -0.24618 |
| Yipf6 | -0.54629 |
| Yipf7 | -0.37562 |
| Ykt6 | 0.12087 |
| Ykt6 | 2.59084 |
| Ylpm1 | 1.83145 |
| Yme1l1 | -0.50609 |
| Yme1l1 | 0.07453 |
| Yme1l1 | -0.75699 |
| Yod1 | 1.25584 |
| Ypel1 | -0.8212 |
| Ypel3 | -0.73746 |
| Ypel3 | -0.48838 |
| Ypel4 | 2.80471 |
| Ypel4 | -0.48698 |
| Ypel5 | 0.26604 |
| Yrdc | -0.32199 |
| Ythdf1 | 1.54456 |
| Ythdf2 | -0.84468 |
| Ythdf2 | -0.2376 |
| Ythdf3 | 0.32319 |
| Ythdf3 | -0.43192 |
| Ywhab | -0.25648 |
| Ywhab | -0.83235 |
| Ywhae | -0.52488 |
| Ywhag | 1.12254 |
| Ywhah | 1.39979 |
| Ywhaq | -0.53441 |
| Ywhaz | -0.48628 |
| Yy2 | 0.21495 |
| Zadh1 | -0.72196 |
| Zadh2 | 1.3701 |
| Zan | -0.6121 |
| Zap70 | 0.55006 |
| Zap70 | 2.5087 |
| Zbbx | -0.24241 |
| Zbed4 | 0.13451 |
| Zbp1 | 1.05234 |
| Zbtb17 | 0.54301 |
| Zbtb2 | 1.49453 |
| Zbtb22 | -0.73838 |
| Zbtb24 | -0.50395 |
| Zbtb3 | -0.79243 |
| Zbtb32 | 0.99019 |
| Zbtb33 | -0.96136 |
| Zbtb38 | 0.52408 |
| Zbtb39 | -0.80423 |
| Zbtb40 | 0.15772 |
| Zbtb41 | 1.1535 |
| Zbtb43 | 0.00504 |
| Zbtb45 | -0.42797 |
| Zbtb46 | 0.28044 |
| Zbtb5 | -0.56516 |
| Zbtb7a | 0.62802 |
| Zbtb7c | -0.85298 |
| Zbtb8 | 1.17923 |
| Zbtb9 | 0.20014 |
| Zbtb9 | -0.51688 |
| Zc3h10 | -0.55908 |
| Zc3h11a | -0.61855 |
| Zc3h12a | 1.81311 |
| Zc3h12b | -0.7274 |
| Zc3h13 | -0.44722 |
| Zc3h14 | -0.30805 |
| Zc3h14 | 2.82565 |
| Zc3h15 | -0.05599 |
| Zc3h18 | -0.74023 |
| Zc3h3 | 2.34384 |
| Zc3h3 | -0.61451 |
| Zc3h3 | 0.17458 |
| Zc3h6 | 2.05167 |
| Zc3h7a | -0.62017 |
| Zc3h7a | 0.81195 |
| Zc3h8 | 3.22095 |
| Zc3hc1 | -0.32845 |
| Zc3hc1 | -0.41622 |
| Zc3hc1 | -0.4059 |
| Zc3hc1 | -0.05337 |
| Zcchc12 | -0.68386 |
| Zcchc14 | 2.93964 |
| Zcchc16 | -0.33377 |
| Zcchc17 | -0.71835 |
| Zcchc17 | 2.64229 |
| Zcchc18 | -0.77119 |
| Zcchc18 | 1.04615 |
| Zcchc3 | 2.42302 |
| Zcchc3 | 2.84066 |
| Zcchc3 | -0.65398 |
| Zcchc5 | -0.42666 |
| Zcchc6 | -0.50466 |
| Zcchc8 | -0.55757 |
| Zcchc8 | 0.6274 |
| Zcchc8 | 0.753 |
| Zcchc9 | -0.81718 |
| Zcrb1 | -0.17865 |
| Zcwpw1 | 0.99982 |
| Zdhhc1 | 0.09818 |
| Zdhhc12 | 0.13735 |
| Zdhhc12 | -0.01863 |
| Zdhhc13 | 1.21554 |
| Zdhhc13 | -0.8344 |
| Zdhhc13 | -0.62017 |
| Zdhhc14 | -0.58521 |
| Zdhhc15 | -0.69171 |
| Zdhhc16 | -0.79635 |
| Zdhhc16 | -0.73196 |
| Zdhhc17 | -0.71475 |
| Zdhhc19 | 0.93939 |
| Zdhhc19 | -0.75793 |
| Zdhhc21 | 2.53291 |
| Zdhhc24 | 1.3141 |
| Zdhhc3 | 0.3849 |
| Zdhhc3 | 0.05026 |
| Zdhhc4 | 0.14548 |
| Zdhhc6 | 1.42208 |
| Zdhhc7 | 1.0307 |
| Zdhhc9 | -0.67179 |
| Zeb1 | -0.27855 |
| Zeb2 | -0.53147 |
| Zer1 | 0.73779 |
| Zf | 0.6359 |
| Zfa | -0.67608 |
| Zfand2a | -0.67094 |
| Zfand2b | 0.15772 |
| Zfand2b | 2.03798 |
| Zfand2b | 2.38746 |
| Zfand2b | 0.13735 |
| Zfand3 | -0.78659 |
| Zfand6 | 1.3903 |
| Zfand6 | -0.61693 |
| Zfat | 3.16487 |
| Zfh4 | 2.16395 |
| Zfhx2 | 1.36935 |
| Zfhx2 | -0.37067 |
| Zfhx3 | 0.19615 |
| Zfhx3 | 2.15201 |
| Zfml | 1.53623 |
| Zfp1 | -0.37624 |
| Zfp101 | 2.39328 |
| Zfp105 | -0.48978 |
| Zfp106 | 0.54072 |
| Zfp106 | 0.72944 |
| Zfp109 | 0.43607 |
| Zfp11 | 0.32927 |
| Zfp119 | -0.70849 |
| Zfp12 | -0.77598 |
| Zfp128 | -0.69873 |
| Zfp13 | -0.38812 |
| Zfp131 | -0.01905 |
| Zfp142 | 0.24418 |
| Zfp143 | 1.4556 |
| Zfp148 | -0.74764 |
| Zfp160 | 0.13237 |
| Zfp161 | -0.45058 |
| Zfp161 | -0.22276 |
| Zfp169 | -0.08584 |
| Zfp179 | 0.68709 |
| Zfp182 | -0.59147 |
| Zfp184 | -0.25757 |
| Zfp185 | 0.13522 |
| Zfp187 | -0.10182 |
| Zfp187 | -0.24834 |
| Zfp189 | -0.09128 |
| Zfp191 | 1.38383 |
| Zfp2 | -0.44254 |
| Zfp2 | -0.29431 |
| Zfp202 | -0.14039 |
| Zfp207 | 2.46783 |
| Zfp212 | -0.52415 |
| Zfp212 | -0.43523 |
| Zfp212 | 1.48933 |
| Zfp212 | -0.69873 |
| Zfp213 | -0.27409 |
| Zfp217 | 0.3373 |
| Zfp219 | -0.53 |
| Zfp235 | -0.21128 |
| Zfp236 | -0.55304 |
| Zfp236 | 0.75009 |
| Zfp238 | -0.14569 |
| Zfp238 | 0.62948 |
| Zfp238 | -0.13512 |
| Zfp238 | -0.18317 |
| Zfp238 | -0.55229 |
| Zfp239 | -0.6365 |
| Zfp251 | -0.88699 |
| Zfp259 | -0.4339 |
| Zfp26 | -0.28134 |
| Zfp263 | 0.69969 |
| Zfp27 | -0.62748 |
| Zfp27 | -0.58055 |
| Zfp275 | 1.15959 |
| Zfp276 | -0.65734 |
| Zfp277 | -0.51616 |
| Zfp277 | 0.61923 |
| Zfp280c | 1.30508 |
| Zfp281 | 2.12782 |
| Zfp282 | -0.3314 |
| Zfp286 | -0.76076 |
| Zfp288 | -0.24564 |
| Zfp292 | 1.1412 |
| Zfp292 | -0.59856 |
| Zfp295 | 0.23464 |
| Zfp296 | -0.74393 |
| Zfp296 | 2.42299 |
| Zfp3 | 3.12151 |
| Zfp30 | 3.3351 |
| Zfp313 | 0.01077 |
| Zfp313 | -0.41882 |
| Zfp316 | 0.11434 |
| Zfp316 | -0.63568 |
| Zfp318 | -0.51978 |
| Zfp322a | -0.0728 |
| Zfp324 | 3.02509 |
| Zfp326 | 2.42163 |
| Zfp326 | 0.54209 |
| Zfp326 | 0.63466 |
| Zfp329 | -0.46826 |
| Zfp329 | 2.98034 |
| Zfp330 | -0.42469 |
| Zfp330 | 2.14464 |
| Zfp334 | 2.2076 |
| Zfp335 | 0.95877 |
| Zfp339 | -0.36452 |
| Zfp346 | 1.1766 |
| Zfp346 | -0.24618 |
| Zfp35 | 0.29909 |
| Zfp354a | -0.59777 |
| Zfp36 | 0.54346 |
| Zfp365 | -0.85194 |
| Zfp367 | 1.08887 |
| Zfp367 | -0.11388 |
| Zfp36l1 | -0.76454 |
| Zfp36l1 | -0.17715 |
| Zfp36l1 | -0.7005 |
| Zfp36l2 | 0.58431 |
| Zfp37 | 1.2245 |
| Zfp371 | -0.05775 |
| Zfp382 | 0.8667 |
| Zfp385a | -0.87409 |
| Zfp385a | -0.03648 |
| Zfp385c | -0.64312 |
| Zfp386 | 0.38734 |
| Zfp386 | -0.69609 |
| Zfp39 | 0.15529 |
| Zfp393 | 1.51791 |
| Zfp395 | -0.54779 |
| Zfp397 | 1.21828 |
| Zfp398 | 0.36626 |
| Zfp398 | -0.48 |
| Zfp398 | -0.72196 |
| Zfp41 | 1.53056 |
| Zfp41 | -0.10644 |
| Zfp410 | -0.91893 |
| Zfp410 | -0.82524 |
| Zfp410 | -0.41687 |
| Zfp414 | -0.6565 |
| Zfp418 | -0.00071 |
| Zfp42 | 0.29611 |
| Zfp423 | 0.09929 |
| Zfp423 | 0.55863 |
| Zfp428 | 0.46611 |
| Zfp428 | -0.50537 |
| Zfp446 | 0.93552 |
| Zfp446 | -0.33437 |
| Zfp449 | -0.68908 |
| Zfp451 | 1.42524 |
| Zfp457 | 1.0939 |
| Zfp46 | 1.2077 |
| Zfp467 | 2.72452 |
| Zfp472 | -0.54331 |
| Zfp473 | -0.4933 |
| Zfp473 | 2.31214 |
| Zfp474 | -0.62341 |
| Zfp474 | -0.08267 |
| Zfp512 | 1.74746 |
| Zfp513 | 0.42938 |
| Zfp516 | -0.03863 |
| Zfp52 | 1.3961 |
| Zfp521 | -0.53368 |
| Zfp523 | -0.04597 |
| Zfp523 | 0.61439 |
| Zfp524 | -0.03092 |
| Zfp526 | 1.25162 |
| Zfp532 | -0.17415 |
| Zfp532 | 2.10252 |
| Zfp536 | 0.7904 |
| Zfp551 | 1.03056 |
| Zfp553 | 0.11325 |
| Zfp553 | -0.09677 |
| Zfp560 | 0.93179 |
| Zfp566 | 1.62644 |
| Zfp57 | -0.6283 |
| Zfp574 | 0.1888 |
| Zfp579 | 1.31716 |
| Zfp592 | -0.73746 |
| Zfp593 | 1.59492 |
| Zfp595 | 0.00135 |
| Zfp598 | 2.00405 |
| Zfp60 | 0.29252 |
| Zfp606 | 0.83285 |
| Zfp607 | 1.55586 |
| Zfp608 | 1.12981 |
| Zfp609 | -0.22803 |
| Zfp61 | 0.53636 |
| Zfp612 | -0.65146 |
| Zfp617 | 1.57664 |
| Zfp619 | 1.41733 |
| Zfp62 | -0.13895 |
| Zfp622 | 0.17697 |
| Zfp623 | -0.28527 |
| Zfp629 | 1.95201 |
| Zfp637 | -0.02794 |
| Zfp639 | -0.53589 |
| Zfp64 | 0.23272 |
| Zfp64 | -0.28583 |
| Zfp64 | -0.30116 |
| Zfp641 | 0.59883 |
| Zfp644 | 0.13415 |
| Zfp646 | 0.9161 |
| Zfp653 | 3.84092 |
| Zfp654 | 2.4638 |
| Zfp654 | 0.53475 |
| Zfp655 | -0.68386 |
| Zfp661 | -0.59225 |
| Zfp667 | -0.39824 |
| Zfp668 | 2.61694 |
| Zfp67 | -0.61774 |
| Zfp672 | 1.9042 |
| Zfp68 | 2.51745 |
| Zfp68 | -0.43788 |
| Zfp68 | 1.74227 |
| Zfp687 | -0.56899 |
| Zfp688 | -0.51833 |
| Zfp689 | -0.84055 |
| Zfp69 | -0.85403 |
| Zfp691 | 0.65631 |
| Zfp692 | -0.77502 |
| Zfp692 | 2.21218 |
| Zfp704 | 2.67633 |
| Zfp704 | 0.99089 |
| Zfp706 | 1.41442 |
| Zfp709 | 1.01375 |
| Zfp709 | -0.19635 |
| Zfp710 | -0.31732 |
| Zfp711 | -0.07593 |
| Zfp715 | -0.6804 |
| Zfp71-rs1 | -0.49542 |
| Zfp74 | 1.04324 |
| Zfp75 | 1.93329 |
| Zfp75 | 1.9115 |
| Zfp75 | 2.91503 |
| Zfp750 | -0.67953 |
| Zfp758 | 1.33614 |
| Zfp760 | -0.15736 |
| Zfp764 | -0.76929 |
| Zfp768 | -0.41363 |
| Zfp770 | 2.82948 |
| Zfp771 | -0.82423 |
| Zfp771 | 0.68943 |
| Zfp78 | -0.6226 |
| Zfp78 | 0.95309 |
| Zfp780b | -0.50466 |
| Zfp786 | 1.64625 |
| Zfp787 | -0.63239 |
| Zfp787 | -0.77215 |
| Zfp790 | -0.27021 |
| Zfp804a | -0.08312 |
| Zfp809 | -0.8946 |
| Zfp810 | 0.34866 |
| Zfp811 | 1.47195 |
| Zfp817 | 0.9608 |
| Zfp819 | 0.09892 |
| Zfp82 | 1.10487 |
| Zfp825 | 1.50301 |
| Zfp828 | 1.29998 |
| Zfp85-rs1 | -0.02243 |
| Zfp87 | 2.28407 |
| Zfp87 | -0.51111 |
| Zfp90 | -0.54629 |
| Zfp91 | -0.48768 |
| Zfp91-cntf | 0.97275 |
| Zfp92 | -0.19635 |
| Zfp93 | 0.82162 |
| Zfp93 | 1.63671 |
| Zfp94 | 2.48931 |
| Zfp97 | 2.2591 |
| Zfpl1 | 2.79001 |
| Zfpm2 | -0.30747 |
| Zfr | -0.55379 |
| Zfx | -0.58055 |
| Zfx | 0.57112 |
| Zfy1 | 1.58447 |
| Zfyve1 | -0.45261 |
| Zfyve16 | -0.79243 |
| Zfyve19 | 0.89719 |
| Zfyve20 | -0.46552 |
| Zfyve21 | 0.68337 |
| Zfyve26 | 0.84015 |
| Zfyve27 | -0.09128 |
| Zgpat | 0.26697 |
| Zgpat | 1.32333 |
| Zhx1 | -0.77024 |
| Zhx1 | -0.65819 |
| Zhx1 | -0.50252 |
| Zhx2 | -0.64979 |
| Zhx3 | -0.71925 |
| Zic1 | -0.46689 |
| Zic2 | 3.73678 |
| Zic3 | -0.39633 |
| Zic4 | -0.75044 |
| Zkscan1 | -0.69609 |
| Zkscan14 | 0.12268 |
| Zkscan17 | -0.7495 |
| Zkscan3 | 0.6867 |
| Zkscan6 | -0.88375 |
| Zkscan6 | 1.35209 |
| Zmat1 | -0.67608 |
| Zmat2 | -0.66327 |
| Zmat3 | 0.96181 |
| Zmat4 | 1.11461 |
| Zmat5 | 1.5202 |
| Zmiz1 | -0.6283 |
| Zmiz1 | -0.71385 |
| Zmiz2 | 0.60547 |
| Zmiz2 | -0.58132 |
| Zmpste24 | 0.14407 |
| Zmpste24 | 0.85197 |
| Zmpste24 | 0.07794 |
| Zmym3 | -0.09539 |
| Zmym3 | -0.06524 |
| Zmym5 | 0.2614 |
| Zmym6 | 3.66417 |
| Zmynd10 | -0.56288 |
| Zmynd11 | 0.71837 |
| Zmynd11 | 3.01305 |
| Zmynd11 | -0.47792 |
| Zmynd12 | -0.22329 |
| Zmynd15 | -0.77119 |
| Znhit2 | -0.18216 |
| Znhit3 | -0.67522 |
| Znhit4 | -0.14473 |
| Znrd1 | -0.07369 |
| Znrd1 | -0.48628 |
| Znrd1 | 0.2208 |
| Znrf1 | 1.1535 |
| Znrf2 | -0.47931 |
| Znrf3 | -0.48838 |
| Znrf4 | -0.17117 |
| Zp1 | 4.09953 |
| Zp3 | -0.57359 |
| Zp3r | -0.56364 |
| Zpbp | -0.2392 |
| Zpbp2 | -0.59383 |
| Zpbp2 | 3.44743 |
| Zranb1 | 1.9828 |
| Zranb2 | -0.69083 |
| Zranb3 | 1.11632 |
| Zrsr2 | -0.05075 |
| Zscan10 | -0.34932 |
| Zscan12 | 0.13557 |
| Zscan2 | -0.3957 |
| Zscan2 | -0.52927 |
| Zscan2 | 0.3447 |
| Zscan20 | 1.65746 |
| Zscan20 | 1.00647 |
| Zscan21 | 0.22469 |
| Zscan21 | -0.34691 |
| Zswim1 | 0.17901 |
| Zswim2 | 0.09111 |
| Zswim3 | -0.7005 |
| Zswim4 | -0.4306 |
| Zswim5 | 2.32077 |
| Zw10 | -0.04597 |
| Zw10 | 0.59387 |
| Zw10 | -0.67436 |
| Zwilch | 0.5019 |
| Zxda | 0.02853 |
| Zxdc | 1.85738 |
| Zxdc | 0.51495 |
| Zxdc | -0.26579 |
| Zyg11b | 0.80459 |
| Zyx | 0.01443 |
| Zzz3 | 1.49096 |
| Zzz3 | -0.68734 |
| Zzz3 | 1.62087 |
| Zzz3 | 0.04359 |

| **Gene Symbol** | **WT2** |
| --- | --- |
| 0610006I08Rik | -0.6078826 |
| 0610006I08Rik | -0.4247081 |
| 0610007C21Rik | -0.4349258 |
| 0610007C21Rik | 0.0384734 |
| 0610007P08Rik | 3.3676018 |
| 0610007P14Rik | -0.5404234 |
| 0610007P22Rik | -0.5126918 |
| 0610009B22Rik | 0.2697735 |
| 0610009D07Rik | -0.4891939 |
| 0610009O20Rik | -0.2613993 |
| 0610010E21Rik | 1.494704 |
| 0610010K06Rik | -0.6411824 |
| 0610010K14Rik | 0.613022 |
| 0610012A05Rik | -0.2768901 |
| 0610012D14Rik | -0.4623126 |
| 0610012G03Rik | -0.4844313 |
| 0610012H03Rik | 0.4157412 |
| 0610025P10Rik | -0.7374211 |
| 0610031J06Rik | -0.4796938 |
| 0610031J06Rik | 0.8619495 |
| 0610031J06Rik | 1.5702708 |
| 0610037D15Rik | -0.3330233 |
| 0610037L13Rik | 0.1231618 |
| 0610037L13Rik | 0.1141361 |
| 0610038F07Rik | 0.065022 |
| 0610040J01Rik | 1.1935 |
| 0710008K08Rik | 0.3102297 |
| 0910001A06Rik | 1.2272362 |
| 0910001A06Rik | -0.6196344 |
| 0910001L09Rik | 0.7932907 |
| 1110001A07Rik | 2.2035157 |
| 1110001J03Rik | 1.3886495 |
| 1110002B05Rik | -0.621213 |
| 1110002N22Rik | 1.2443869 |
| 1110003E01Rik | 1.4033392 |
| 1110006G06Rik | 0.087222 |
| 1110007A13Rik | -0.6918527 |
| 1110007A13Rik | 0.0111175 |
| 1110007C09Rik | 2.1455773 |
| 1110007L15Rik | 1.6241606 |
| 1110007L15Rik | 2.3881537 |
| 1110007L15Rik | -0.6047745 |
| 1110007L15Rik | -0.1669684 |
| 1110007M04Rik | 0.1610388 |
| 1110008F13Rik | 0.2056172 |
| 1110008J03Rik | 1.7982188 |
| 1110008J03Rik | -0.0046685 |
| 1110008J03Rik | -0.2109405 |
| 1110008P14Rik | -0.6251716 |
| 1110012D08Rik | -0.7205024 |
| 1110012J17Rik | -0.772244 |
| 1110012L19Rik | 0.2030924 |
| 1110012L19Rik | -0.0342121 |
| 1110012M11Rik | 3.0840331 |
| 1110013L07Rik | -0.675762 |
| 1110017D15Rik | -0.6180586 |
| 1110017D15Rik | 0.7631033 |
| 1110017D15Rik | -0.661605 |
| 1110018G07Rik | -0.7920825 |
| 1110018J18Rik | -0.4196424 |
| 1110018J18Rik | 0.2187575 |
| 1110019N10Rik | 0.6562544 |
| 1110019N10Rik | 0.4254204 |
| 1110020C03Rik | 2.300286 |
| 1110020P15Rik | -0.7178598 |
| 1110020P15Rik | -0.6468541 |
| 1110031B06Rik | -0.4310805 |
| 1110031I02Rik | 1.0336663 |
| 1110032A13Rik | -0.2768901 |
| 1110032A13Rik | -0.7546641 |
| 1110032E23Rik | -0.0106605 |
| 1110034A24Rik | 0.9270119 |
| 1110036O03Rik | 0.6803757 |
| 1110038D17Rik | -0.6724109 |
| 1110038F14Rik | 0.2735806 |
| 1110038F14Rik | 0.1845724 |
| 1110039B18Rik | -0.5520525 |
| 1110039B18Rik | -0.685891 |
| 1110039B18Rik | -0.1574819 |
| 1110048D14Rik | 3.6157898 |
| 1110049B09Rik | -0.0042705 |
| 1110049B09Rik | 0.0262468 |
| 1110049F12Rik | 0.228642 |
| 1110049F12Rik | -0.7056296 |
| 1110057K04Rik | -0.4400784 |
| 1110057K04Rik | 0.035813 |
| 1110057K04Rik | 2.1262874 |
| 1110058L19Rik | 0.4059568 |
| 1110059E24Rik | -0.4177502 |
| 1110059G10Rik | -0.1877094 |
| 1110067D22Rik | -0.7038959 |
| 1110067I12Rik | -0.0797805 |
| 1190002A17Rik | 0.7926344 |
| 1190002H23Rik | 1.4300804 |
| 1190002N15Rik | -0.8084689 |
| 1190003J15Rik | -0.5447667 |
| 1190003M12Rik | 0.1653337 |
| 1190005F20Rik | -0.4530907 |
| 1190005I06Rik | 1.7752745 |
| 1190005P17Rik | 0.0886588 |
| 1190005P17Rik | -0.1847865 |
| 1190007F08Rik | 0.7728873 |
| 1190017O12Rik | 3.3750958 |
| 1190020J12Rik | -0.0223592 |
| 1200002N14Rik | -0.675762 |
| 1200003C05Rik | -0.5050222 |
| 1200003C05Rik | 0.1842497 |
| 1200003C05Rik | -0.5668034 |
| 1200003I07Rik | -0.8523209 |
| 1200009F10Rik | -0.4362112 |
| 1200009I06Rik | 3.5972848 |
| 1200009I06Rik | -0.6525619 |
| 1200009O22Rik | -0.1612645 |
| 1200011O22Rik | 2.6071198 |
| 1200013B08Rik | 0.1450006 |
| 1200013P24Rik | -0.6363491 |
| 1200013P24Rik | 0.9277184 |
| 1200014J11Rik | 2.4418631 |
| 1200015F23Rik | -0.516903 |
| 1200015F23Rik | 0.3585684 |
| 1200015N20Rik | 0.6098099 |
| 1200016B10Rik | -0.6055506 |
| 1200016B10Rik | -0.6583061 |
| 1200016D23Rik | 3.3497192 |
| 1300001I01Rik | 1.8466557 |
| 1300007L22Rik | 2.6737401 |
| 1300010M03Rik | 0.4327957 |
| 1300013J15Rik | 0.02355 |
| 1300013J15Rik | -0.7187398 |
| 1300014I06Rik | 0.0411259 |
| 1300017J02Rik | -0.0841189 |
| 1300018I05Rik | -0.3261678 |
| 1300018J18Rik | -0.4817211 |
| 1500001L15Rik | -0.1872216 |
| 1500001M20Rik | -0.3070227 |
| 1500001M20Rik | 1.7086569 |
| 1500001M20Rik | -0.560874 |
| 1500002O20Rik | -0.4349258 |
| 1500003O03Rik | 1.5079204 |
| 1500005A01Rik | 0.1266092 |
| 1500010J02Rik | 1.4791502 |
| 1500010J02Rik | 0.2732883 |
| 1500011H22Rik | -0.0239849 |
| 1500012F01Rik | -0.4987953 |
| 1500015O10Rik | -0.1523067 |
| 1500019C06Rik | 0.7790737 |
| 1500019G21Rik | 1.2207322 |
| 1500031L02Rik | 2.1745888 |
| 1500032D16Rik | -0.4830752 |
| 1500032D16Rik | 0.0036537 |
| 1500032D16Rik | 0.8215449 |
| 1500032L24Rik | -0.5015575 |
| 1500041J02Rik | 2.3577727 |
| 1500041N16Rik | -0.675762 |
| 1520401A03Rik | -0.4756528 |
| 1600002K03Rik | 0.3484073 |
| 1600012H06Rik | -0.7275873 |
| 1600012H06Rik | 1.1797286 |
| 1600014C10Rik | -0.659954 |
| 1600016N20Rik | -0.6094407 |
| 1600021P15Rik | 0.4162401 |
| 1600027N09Rik | -0.1065761 |
| 1600029D21Rik | 2.2965912 |
| 1700001C02Rik | -0.0504024 |
| 1700001C19Rik | -0.0724529 |
| 1700001E04Rik | -0.5389803 |
| 1700001J03Rik | 1.8741092 |
| 1700001O22Rik | 1.8723806 |
| 1700001O22Rik | -0.5682918 |
| 1700001P01Rik | 0.367037 |
| 1700003F12Rik | 1.0011943 |
| 1700003M02Rik | 0.4860332 |
| 1700007G11Rik | 2.2368653 |
| 1700007I06Rik | -0.0806465 |
| 1700007K09Rik | 0.5633818 |
| 1700008F21Rik | -0.3776984 |
| 1700008I05Rik | -0.0754629 |
| 1700008P20Rik | 0.7762366 |
| 1700009P17Rik | 0.3845141 |
| 1700010A17Rik | -0.6315417 |
| 1700010A17Rik | 0.6138232 |
| 1700010D01Rik | 0.020845 |
| 1700010M22Rik | -0.3492256 |
| 1700010M22Rik | -0.1717494 |
| 1700011A15Rik | -0.7873192 |
| 1700011F03Rik | 0.647811 |
| 1700011F14Rik | 2.5651246 |
| 1700011H14Rik | 0.365719 |
| 1700011K15Rik | -0.2561188 |
| 1700012A03Rik | 0.5716029 |
| 1700012B09Rik | 0.7045915 |
| 1700012G19Rik | -0.3273067 |
| 1700012H05Rik | -0.5411459 |
| 1700012H17Rik | -0.4304412 |
| 1700012L04Rik | -0.423439 |
| 1700013B16Rik | 0.6949466 |
| 1700013G24Rik | -0.5433166 |
| 1700013N18Rik | -0.4919267 |
| 1700013N18Rik | 0.6176194 |
| 1700015E13Rik | -0.6607792 |
| 1700016C15Rik | -0.4576898 |
| 1700016D06Rik | -0.289915 |
| 1700016G05Rik | 1.3066576 |
| 1700016K19Rik | 0.2567672 |
| 1700016K19Rik | -0.6732475 |
| 1700017N19Rik | 2.5984483 |
| 1700018B24Rik | -0.7099784 |
| 1700018C11Rik | 1.6080579 |
| 1700018F24Rik | -0.2545407 |
| 1700019D03Rik | -0.4636377 |
| 1700019E19Rik | -0.4736392 |
| 1700019G17Rik | -0.0828152 |
| 1700019H03Rik | -0.5795346 |
| 1700019L03Rik | 0.11239 |
| 1700019M22Rik | 2.6317305 |
| 1700019N19Rik | -0.5462192 |
| 1700020C11Rik | -0.399043 |
| 1700020D05Rik | -0.4530907 |
| 1700020L24Rik | -0.1921118 |
| 1700020N01Rik | 0.1158789 |
| 1700020O03Rik | 2.5240969 |
| 1700021C14Rik | -0.0142749 |
| 1700021F05Rik | 2.1455773 |
| 1700021K02Rik | -0.3273067 |
| 1700021K02Rik | 2.4948758 |
| 1700021K19Rik | 0.4219578 |
| 1700022C21Rik | -0.2624591 |
| 1700023B02Rik | 1.1589959 |
| 1700023B02Rik | 3.3254762 |
| 1700023E05Rik | -0.5296561 |
| 1700023E05Rik | -0.6039992 |
| 1700023I07Rik | 0.1300435 |
| 1700023M03Rik | -0.3278767 |
| 1700024G13Rik | -0.671575 |
| 1700025D03Rik | -0.6749231 |
| 1700025E21Rik | 0.9110381 |
| 1700025F22Rik | 0.2817261 |
| 1700025G04Rik | -0.4221717 |
| 1700025K23Rik | -0.4629749 |
| 1700026D08Rik | -0.3568485 |
| 1700026L06Rik | -0.483753 |
| 1700027A23Rik | 1.1936051 |
| 1700027D21Rik | -0.6204233 |
| 1700027J05Rik | -0.675762 |
| 1700027N10Rik | -0.6918527 |
| 1700028K03Rik | -0.5924485 |
| 1700029F12Rik | 0.4046946 |
| 1700029F12Rik | -0.5727721 |
| 1700029G01Rik | 2.3624015 |
| 1700029H14Rik | 0.1460114 |
| 1700029H14Rik | -0.7546641 |
| 1700029I01Rik | 0.5380402 |
| 1700029I08Rik | 2.5594985 |
| 1700029I15Rik | -0.5985904 |
| 1700029J07Rik | 0.3593657 |
| 1700029P11Rik | -0.4027534 |
| 1700029P11Rik | 1.6674002 |
| 1700029P11Rik | -0.588631 |
| 1700030J22Rik | 1.7442936 |
| 1700030K09Rik | -0.7939949 |
| 1700034E13Rik | 0.2715326 |
| 1700034H14Rik | -0.3544961 |
| 1700034H14Rik | -0.4253433 |
| 1700034H14Rik | 1.7454329 |
| 1700034H14Rik | 1.9321922 |
| 1700034I23Rik | 2.1230755 |
| 1700034I23Rik | 0.9016359 |
| 1700034O15Rik | 0.505664 |
| 1700036D21Rik | 2.3777551 |
| 1700037C18Rik | 0.8240844 |
| 1700037C18Rik | 1.7048535 |
| 1700037H04Rik | 3.6940456 |
| 1700039E15Rik | -0.6665762 |
| 1700040I03Rik | -0.5382596 |
| 1700042B14Rik | 1.2668544 |
| 1700045I19Rik | -0.5653173 |
| 1700047I17Rik1 | 0.1137872 |
| 1700047I17Rik1 | -0.0058637 |
| 1700049G17Rik | -0.3645364 |
| 1700049K14Rik | -0.621213 |
| 1700049L16Rik | 1.9028072 |
| 1700052N19Rik | 0.3814087 |
| 1700052N19Rik | 0.7653055 |
| 1700054O13Rik | -0.0470575 |
| 1700055M20Rik | 2.2901509 |
| 1700057G04Rik | 1.381683 |
| 1700057K13Rik | 0.5732801 |
| 1700058C13Rik | -0.7666552 |
| 1700060H10Rik | 2.8443968 |
| 1700061G19Rik | -0.7666552 |
| 1700063I17Rik | 2.7303146 |
| 1700065I17Rik | 0.0879407 |
| 1700065O13Rik | -0.6782836 |
| 1700065O13Rik | 0.1842497 |
| 1700067C01Rik | -0.4077246 |
| 1700067K01Rik | -0.3347454 |
| 1700067P10Rik | 0.1527212 |
| 1700071K01Rik | -0.6039992 |
| 1700072E05Rik | -0.0466402 |
| 1700072E05Rik | 1.4603581 |
| 1700072E05Rik | 2.8511353 |
| 1700073E17Rik | 0.012681 |
| 1700080E11Rik | -0.7117239 |
| 1700081D17Rik | -0.3965778 |
| 1700081D17Rik | 0.7578299 |
| 1700084C01Rik | 0.6600662 |
| 1700084C01Rik | 2.1383965 |
| 1700088E04Rik | -0.1141882 |
| 1700093K21Rik | 2.1282983 |
| 1700094C09Rik | 0.6031486 |
| 1700095K08Rik | 1.8850943 |
| 1700102P08Rik | -0.1305236 |
| 1700108M19Rik | 0.425914 |
| 1700109H08Rik | -0.6867403 |
| 1700112C13Rik | -0.381927 |
| 1700113I22Rik | -0.2640512 |
| 1700113I22Rik | 0.8930157 |
| 1700113O17Rik | 0.3937665 |
| 1700120B06Rik | -0.6387625 |
| 1700123D08Rik | -0.1809039 |
| 1700123K08Rik | -0.240461 |
| 1700123L14Rik | 1.913166 |
| 1700123O20Rik | -0.5050222 |
| 1700123O20Rik | -0.7959114 |
| 1700127D06Rik | -0.3353201 |
| 1700129C05Rik | -0.3651305 |
| 1700129I15Rik | -0.519016 |
| 1810007M14Rik | 0.59378 |
| 1810007P19Rik | -0.6476673 |
| 1810007P19Rik | 3.5325224 |
| 1810008A18Rik | 0.2926641 |
| 1810008A18Rik | -0.2184828 |
| 1810009A15Rik | 0.0756444 |
| 1810009A15Rik | 0.0300855 |
| 1810009N02Rik | 1.7736208 |
| 1810009O10Rik | -0.4926112 |
| 1810009O10Rik | 1.4145918 |
| 1810010M01Rik | 1.8898584 |
| 1810011O10Rik | 0.4271467 |
| 1810014F10Rik | -0.6315417 |
| 1810015A11Rik | 1.135198 |
| 1810015C04Rik | -0.6909986 |
| 1810019J16Rik | -0.3193641 |
| 1810020D17Rik | -0.5623526 |
| 1810020G14Rik | -0.6086613 |
| 1810020G14Rik | 3.4948333 |
| 1810021J13Rik | -0.67074 |
| 1810021J13Rik | 0.0052302 |
| 1810022C23Rik | -0.1305236 |
| 1810026J23Rik | 2.294398 |
| 1810027O10Rik | 1.8434962 |
| 1810030J14Rik | -0.3154189 |
| 1810030N24Rik | -0.6799686 |
| 1810030N24Rik | -0.6867403 |
| 1810030N24Rik | -0.6574832 |
| 1810031K17Rik | 2.6299217 |
| 1810035L17Rik | 2.8452523 |
| 1810035L17Rik | 0.1567233 |
| 1810037C20Rik | -0.5022494 |
| 1810043G02Rik | 0.2187575 |
| 1810044A24Rik | 0.3285593 |
| 1810046J19Rik | 1.5256091 |
| 1810048J11Rik | 0.1656632 |
| 1810049H13Rik | 1.6043365 |
| 1810049H19Rik | 0.8022666 |
| 1810055E12Rik | 0.1293577 |
| 1810055E12Rik | 1.309342 |
| 1810055E12Rik | -0.6094407 |
| 1810055G02Rik | 0.392231 |
| 1810063B05Rik | -0.4583488 |
| 1810064F22Rik | 0.4014048 |
| 1810065E05Rik | -0.1717494 |
| 1810073G14Rik | -0.7030303 |
| 1810074P20Rik | 1.8374008 |
| 1810074P20Rik | -0.6640872 |
| 2010001J22Rik | -0.3849596 |
| 2010001J22Rik | -0.2629895 |
| 2010001M09Rik | 0.4488117 |
| 2010003J03Rik | 0.1358519 |
| 2010003O18Rik | 1.3352159 |
| 2010004A03Rik | -0.6102207 |
| 2010005H15Rik | -0.3335969 |
| 2010005J08Rik | -0.5268064 |
| 2010005J08Rik | -0.6935633 |
| 2010005J08Rik | -0.2828365 |
| 2010007H12Rik | -0.1132893 |
| 2010007H12Rik | 0.0350514 |
| 2010011I20Rik | 0.5279646 |
| 2010012O05Rik | 0.3494823 |
| 2010100O12Rik | -0.4946678 |
| 2010100O12Rik | 0.2732883 |
| 2010106G01Rik | 2.916037 |
| 2010106G01Rik | 1.0352971 |
| 2010107E04Rik | 0.807448 |
| 2010107G12Rik | -0.6251716 |
| 2010109I03Rik | -0.6275551 |
| 2010110K16Rik | 0.0940264 |
| 2010111I01Rik | -0.588631 |
| 2010200O16Rik | -0.2629895 |
| 2010301N04Rik | 0.9567664 |
| 2010305A19Rik | 1.6784452 |
| 2010305C02Rik | -0.4550589 |
| 2010305C02Rik | 1.6597313 |
| 2010308M01Rik | 0.9456196 |
| 2010311D03Rik | -0.8143244 |
| 2010316F05Rik | 1.2098554 |
| 2010321M09Rik | 0.0165779 |
| 2010321M09Rik | -0.5239657 |
| 2200001I15Rik | 1.3721659 |
| 2210009G21Rik | -0.4221717 |
| 2210009G21Rik | -0.7073666 |
| 2210009G21Rik | 0.8201133 |
| 2210010B09Rik | 0.1046665 |
| 2210010L05Rik | 3.7214976 |
| 2210012G02Rik | -0.5071074 |
| 2210012G02Rik | -0.2134476 |
| 2210012G02Rik | -0.7374211 |
| 2210012G02Rik | 0.1134381 |
| 2210016L21Rik | 0.3082636 |
| 2210016L21Rik | 4.2611602 |
| 2210016L21Rik | -0.4394327 |
| 2210021J22Rik | 2.7470011 |
| 2210023G05Rik | -0.514795 |
| 2210023G05Rik | 0.8953615 |
| 2210407C18Rik | -0.4623126 |
| 2210412D01Rik | 0.5314819 |
| 2210417D09Rik | 1.0858635 |
| 2210418O10Rik | 1.6184099 |
| 2300002D11Rik | 0.4398682 |
| 2300002D11Rik | 0.2519907 |
| 2300002M23Rik | -0.6078826 |
| 2300005B03Rik | -0.7258108 |
| 2310001A20Rik | -0.5022494 |
| 2310002B06Rik | 0.2735806 |
| 2310003C23Rik | 0.9555347 |
| 2310003F16Rik | -0.1990029 |
| 2310003H01Rik | 0.3427423 |
| 2310003L22Rik | 1.2362033 |
| 2310003L22Rik | -0.7240379 |
| 2310004I24Rik | -0.6063272 |
| 2310005E10Rik | -0.1155382 |
| 2310005E10Rik | -0.53682 |
| 2310005G13Rik | 0.7452886 |
| 2310005N01Rik | 0.5063411 |
| 2310005N03Rik | 2.3190169 |
| 2310005P05Rik | 0.0911677 |
| 2310006J04Rik | -0.1296082 |
| 2310007A19Rik | -0.4844313 |
| 2310007B03Rik | -0.398426 |
| 2310007F21Rik | -0.2363311 |
| 2310007F21Rik | -0.1980152 |
| 2310008H04Rik | -0.5218411 |
| 2310008H09Rik | -0.8401478 |
| 2310008H09Rik | -0.7187398 |
| 2310008M10Rik | 1.9732424 |
| 2310009E04Rik | -0.0420633 |
| 2310010B21Rik | -0.1684 |
| 2310010M24Rik | 2.0816148 |
| 2310011J03Rik | 0.1745152 |
| 2310014L17Rik | -0.5579238 |
| 2310016C08Rik | -0.6427992 |
| 2310016C08Rik | -0.8162847 |
| 2310016C16Rik | 1.4469741 |
| 2310016C16Rik | 2.5003783 |
| 2310016C16Rik | 1.5899042 |
| 2310016E02Rik | 1.448559 |
| 2310016M24Rik | 1.6118301 |
| 2310021P13Rik | 0.5667637 |
| 2310022A10Rik | -0.527518 |
| 2310022B05Rik | 1.8257619 |
| 2310031L18Rik | 1.0065158 |
| 2310031L18Rik | 0.8940427 |
| 2310033E01Rik | -0.6347438 |
| 2310033K02Rik | -0.4083479 |
| 2310033P09Rik | -0.4596681 |
| 2310035C23Rik | -0.3746901 |
| 2310035C23Rik | -0.0038726 |
| 2310035K24Rik | -0.5871086 |
| 2310036D22Rik | 1.7108483 |
| 2310036O22Rik | -0.5225487 |
| 2310037I24Rik | 1.2032241 |
| 2310037I24Rik | 0.9452041 |
| 2310038H17Rik | -0.3451476 |
| 2310039E09Rik | 0.1932412 |
| 2310039H08Rik | 2.6789796 |
| 2310040C09Rik | 0.5956207 |
| 2310042D19Rik | 0.3155448 |
| 2310044G17Rik | -0.0034749 |
| 2310044H10Rik | 1.7181541 |
| 2310045A20Rik | -0.6690721 |
| 2310046K01Rik | 0.2024601 |
| 2310046O06Rik | -0.4452608 |
| 2310047C04Rik | -0.671575 |
| 2310047D13Rik | -0.4215387 |
| 2310047D13Rik | 0.9019267 |
| 2310047M10Rik | 1.7331096 |
| 2310047M10Rik | -0.1555966 |
| 2310047O13Rik | -0.2661783 |
| 2310056P07Rik | 1.0327869 |
| 2310057J16Rik | 1.0422887 |
| 2310058J06Rik | -0.7047623 |
| 2310061C15Rik | -0.4803691 |
| 2310061F22Rik | -0.7196206 |
| 2310061F22Rik | -0.53682 |
| 2310065K24Rik | 3.2672291 |
| 2310065K24Rik | -0.1259561 |
| 2310066E14Rik | -0.7320439 |
| 2310067B10Rik | -0.4981061 |
| 2310076L09Rik | 1.0589233 |
| 2310076L09Rik | -0.6952772 |
| 2310079F23Rik | -0.4844313 |
| 2310079N02Rik | 1.3756071 |
| 2400001E08Rik | 1.8581296 |
| 2400003C14Rik | 1.5202878 |
| 2400003C14Rik | -0.5916838 |
| 2400003C14Rik | -0.6699057 |
| 2400006N03Rik | 0.4516933 |
| 2400010D15Rik | 1.8000958 |
| 2410001C21Rik | -0.3849596 |
| 2410001C21Rik | 1.0005432 |
| 2410001C21Rik | 1.8652335 |
| 2410002F23Rik | -0.6063272 |
| 2410002F23Rik | 3.4808776 |
| 2410002F23Rik | -0.524675 |
| 2410002I01Rik | 2.624302 |
| 2410002O22Rik | 0.2381151 |
| 2410003P15Rik | 0.0482866 |
| 2410004A20Rik | 1.4984572 |
| 2410004B18Rik | 1.3955625 |
| 2410004L22Rik | -0.5239657 |
| 2410005O16Rik | 1.0869292 |
| 2410008K03Rik | 0.2468876 |
| 2410014A08Rik | 1.2320835 |
| 2410014A08Rik | -0.5705292 |
| 2410014A08Rik | 0.9866274 |
| 2410014A08Rik | 0.5711831 |
| 2410015M20Rik | 0.6154236 |
| 2410015N17Rik | 2.1797678 |
| 2410016F19Rik | 1.2470653 |
| 2410016F19Rik | 1.4261114 |
| 2410016O06Rik | -0.0086587 |
| 2410017P07Rik | 1.3221897 |
| 2410018C17Rik | -0.772244 |
| 2410018G20Rik | 0.6023378 |
| 2410018M08Rik | 0.2727035 |
| 2410019A14Rik | -0.5593977 |
| 2410019G02Rik | 2.0177321 |
| 2410022L05Rik | 0.7374696 |
| 2410025L10Rik | 0.7896751 |
| 2410042D21Rik | -0.6315417 |
| 2410042D21Rik | 0.3524319 |
| 2410066E13Rik | -0.629945 |
| 2410081M15Rik | 1.1377741 |
| 2410091C18Rik | -0.5916838 |
| 2410116G06Rik | 2.3080872 |
| 2410127E18Rik | -0.4139775 |
| 2410127L17Rik | 1.4977827 |
| 2410127L17Rik | -0.7546641 |
| 2410129H14Rik | 1.1169558 |
| 2410137M14Rik | 2.1799083 |
| 2410146L05Rik | -0.5939801 |
| 2410166I05Rik | -0.3187994 |
| 2500002L14Rik | 0.486264 |
| 2500003M10Rik | -0.1828431 |
| 2500003M10Rik | -0.8133458 |
| 2500003M10Rik | -0.8553901 |
| 2510002A14Rik | 0.1819878 |
| 2510003E04Rik | -0.8045864 |
| 2510006D16Rik | -0.6315417 |
| 2510009E07Rik | -0.8212043 |
| 2510010F15Rik | -0.1780029 |
| 2510012J08Rik | 0.8299298 |
| 2510039O18Rik | 0.2582547 |
| 2510040D07Rik | -0.6492959 |
| 2510048L02Rik | -0.4504732 |
| 2510049I19Rik | -0.8813822 |
| 2600001B17Rik | -0.5571878 |
| 2600009E05Rik | -0.2342733 |
| 2600009E05Rik | -0.7099784 |
| 2600010E01Rik | -0.8045864 |
| 2600011E07Rik | 0.1007797 |
| 2600011E07Rik | 1.9459177 |
| 2610002J02Rik | -0.4465611 |
| 2610002M06Rik | -0.5985904 |
| 2610003J06Rik | -0.4381427 |
| 2610016F04Rik | -0.7564985 |
| 2610019A05Rik | 1.7459451 |
| 2610019P18Rik | -0.6918527 |
| 2610020C11Rik | 2.9715428 |
| 2610020H08Rik | -0.6624316 |
| 2610020H08Rik | 3.8553583 |
| 2610020H08Rik | -0.4550589 |
| 2610020O08Rik | 2.1249443 |
| 2610021K21Rik | 2.0550147 |
| 2610021K21Rik | 1.0006735 |
| 2610024G14Rik | -0.2981523 |
| 2610024G14Rik | 1.5100674 |
| 2610024G14Rik | -0.9504195 |
| 2610027C15Rik | 2.0485347 |
| 2610027C15Rik | -0.8271434 |
| 2610027L16Rik | 1.1436824 |
| 2610028A01Rik | 1.0667031 |
| 2610028H24Rik | -0.3238941 |
| 2610029G23Rik | -0.879275 |
| 2610029G23Rik | 1.4946288 |
| 2610034E13Rik | -0.588631 |
| 2610034E13Rik | 1.7456036 |
| 2610034M16Rik | 2.1997098 |
| 2610036D13Rik | 0.997542 |
| 2610039C10Rik | 0.3986116 |
| 2610042L04Rik | -0.5675473 |
| 2610044O15Rik | -0.1911316 |
| 2610101N10Rik | 0.8684604 |
| 2610101N10Rik | -0.9618819 |
| 2610103J23Rik | -0.5112925 |
| 2610110G12Rik | 2.1430925 |
| 2610200G18Rik | -0.4926112 |
| 2610204K14Rik | 2.3658647 |
| 2610204L23Rik | -0.7741146 |
| 2610204M08Rik | -0.8331221 |
| 2610205E22Rik | 3.5403469 |
| 2610207I05Rik | -0.086732 |
| 2610208M17Rik | -0.2164653 |
| 2610209A20Rik | -0.4304412 |
| 2610209A20Rik | 2.0237269 |
| 2610209M04Rik | -0.4215387 |
| 2610301B20Rik | 0.3213831 |
| 2610301F02Rik | 3.3387118 |
| 2610304G08Rik | -0.5161998 |
| 2610305D13Rik | -0.6387625 |
| 2610318N02Rik | -0.7464551 |
| 2610507B11Rik | -0.3131722 |
| 2610507B11Rik | -0.7694452 |
| 2610510B01Rik | -0.4114711 |
| 2610510J17Rik | 0.02355 |
| 2610524A10Rik | -0.4662939 |
| 2610524H06Rik | 1.5952342 |
| 2610528E23Rik | -0.4939818 |
| 2610528J11Rik | 1.0941195 |
| 2610528O22Rik | 2.551516 |
| 2700007P21Rik | -0.1337346 |
| 2700007P21Rik | -0.4330011 |
| 2700038C09Rik | 2.5419601 |
| 2700038C09Rik | 1.3145031 |
| 2700050L05Rik | -0.7759892 |
| 2700055A20Rik | 0.6674532 |
| 2700060E02Rik | 1.4411652 |
| 2700062C07Rik | -0.4368546 |
| 2700062C07Rik | 1.6957557 |
| 2700078K21Rik | -0.4878306 |
| 2700081O15Rik | 2.0937563 |
| 2700087H15Rik | 0.6965917 |
| 2700094K13Rik | -0.4844313 |
| 2700097O09Rik | 1.4730067 |
| 2810002N01Rik | 0.0095513 |
| 2810002O09Rik | -0.644419 |
| 2810003C17Rik | -0.2550665 |
| 2810004N20Rik | -0.8574421 |
| 2810004N23Rik | 0.4352408 |
| 2810008M24Rik | 0.5841059 |
| 2810021B07Rik | 1.3958984 |
| 2810021J22Rik | 1.087639 |
| 2810021J22Rik | -0.7949526 |
| 2810022L02Rik | -0.524675 |
| 2810022L02Rik | -0.5750206 |
| 2810022L02Rik | -0.6117828 |
| 2810030E01Rik | 2.2360737 |
| 2810046L04Rik | -0.5418689 |
| 2810046M22Rik | -0.5978204 |
| 2810403A07Rik | 0.8043751 |
| 2810405J04Rik | -0.7537483 |
| 2810405K02Rik | -0.3131722 |
| 2810408A11Rik | -0.3301605 |
| 2810408M09Rik | 1.7543826 |
| 2810410C14Rik | -0.7769279 |
| 2810410P22Rik | 1.4182843 |
| 2810410P22Rik | -0.8761234 |
| 2810417H13Rik | -0.891993 |
| 2810422O20Rik | 2.3995625 |
| 2810428I15Rik | -0.2672438 |
| 2810432D09Rik | -0.0018859 |
| 2810432L12Rik | 0.7799064 |
| 2810439F02Rik | 0.7688516 |
| 2810439F02Rik | -0.521134 |
| 2810441K11Rik | -0.7835267 |
| 2810452K22Rik | -0.8182493 |
| 2810453I06Rik | -0.9301482 |
| 2810453I06Rik | 0.6241748 |
| 2810470D21Rik | -0.560874 |
| 2810485I05Rik | -0.3250302 |
| 2900006K08Rik | 1.4869986 |
| 2900010M23Rik | 0.7267609 |
| 2900011O08Rik | 2.5776052 |
| 2900011O08Rik | 1.9438197 |
| 2900024C23Rik | -0.7196206 |
| 2900024O10Rik | -0.5623526 |
| 2900026A02Rik | 0.3102297 |
| 2900046G09Rik | -0.6395684 |
| 2900062L11Rik | -0.2493003 |
| 2900064A13Rik | 0.9925614 |
| 2900073G15Rik | -0.2044547 |
| 2900092C05Rik | -0.8212043 |
| 3000004C01Rik | -0.880328 |
| 3000004C01Rik | -0.7806927 |
| 3000004C01Rik | -0.6141311 |
| 3010003L21Rik | -0.7882699 |
| 3010026O09Rik | 1.5147876 |
| 3110001A13Rik | -0.6892929 |
| 3110001D03Rik | -0.880328 |
| 3110004L20Rik | -0.8026514 |
| 3110007F17Rik | -0.8271434 |
| 3110009E18Rik | 2.1154103 |
| 3110023B02Rik | -0.303686 |
| 3110035E14Rik | 0.5964375 |
| 3110035E14Rik | 1.5020455 |
| 3110037I16Rik | -0.0985859 |
| 3110040N11Rik | 0.2510922 |
| 3110040N11Rik | 0.3601623 |
| 3110043J09Rik | -0.815304 |
| 3110043J09Rik | 1.6248117 |
| 3110043J09Rik | -0.6607792 |
| 3110048E14Rik | -0.5260954 |
| 3110049J23Rik | -0.569037 |
| 3110050N22Rik | -0.4563734 |
| 3110050N22Rik | 1.2479563 |
| 3110056O03Rik | -0.4596681 |
| 3110057O12Rik | 1.7364507 |
| 3110057O12Rik | -0.0999127 |
| 3110070M22Rik | -0.1570102 |
| 3110082I17Rik | 0.5939847 |
| 3200001I04Rik | 0.9404815 |
| 3200002M19Rik | -0.0941773 |
| 3200002M19Rik | 0.4581434 |
| 3300001G02Rik | -0.6024505 |
| 3300001P08Rik | 0.5931656 |
| 3300001P08Rik | 0.2362893 |
| 3300001P08Rik | 0.3675636 |
| 3300002I08Rik | -0.0639079 |
| 3321401G04Rik | -0.0338008 |
| 3322402L07Rik | -0.6550192 |
| 3632451O06Rik | 1.2025999 |
| 3830402I07Rik | 0.5231057 |
| 3830406C13Rik | 1.0278718 |
| 3830406C13Rik | -0.8351238 |
| 3830406C13Rik | 2.6726845 |
| 3830408P04Rik | 2.4346145 |
| 3930401K13Rik | -0.4885119 |
| 4121402D02Rik | 2.29514 |
| 4121402D02Rik | -0.2059473 |
| 4430402I18Rik | 0.900035 |
| 4432405B04Rik | 0.3174951 |
| 4432416J03Rik | 0.0897349 |
| 4631426J05Rik | -0.7004385 |
| 4631427C17Rik | -0.1070221 |
| 4632404H12Rik | 1.5226972 |
| 4632404H12Rik | 3.2552322 |
| 4632404H12Rik | -0.7266986 |
| 4632404H22Rik | -0.6649161 |
| 4632411B12Rik | -0.8401478 |
| 4632411B12Rik | 0.1300435 |
| 4632412N22Rik | 2.766116 |
| 4632415K11Rik | 1.5631189 |
| 4632417K18Rik | 1.7416107 |
| 4632417N05Rik | 0.4676149 |
| 4632419K20Rik | 1.4144273 |
| 4632434I11Rik | 1.6835819 |
| 4732415M23Rik | 0.7083846 |
| 4732418C07Rik | -0.1218652 |
| 4732418C07Rik | 0.2943791 |
| 4732418C07Rik | -0.56606 |
| 4732429D16Rik | 2.0283944 |
| 4732456N10Rik | -0.4015149 |
| 4732471D19Rik | -0.3645364 |
| 4732473B16Rik | -0.6607792 |
| 4732479N06Rik | -0.6909986 |
| 4732479N06Rik | -0.0038726 |
| 4732496O08Rik | 1.4239983 |
| 4733401H18Rik | 0.3467923 |
| 4831426I19Rik | 0.4473675 |
| 4833418A01Rik | 0.0068039 |
| 4833420G17Rik | 0.1948374 |
| 4833421E05Rik | -0.5705292 |
| 4833424O15Rik | 1.5564717 |
| 4833424O15Rik | -0.5878695 |
| 4833426J09Rik | -0.3837453 |
| 4833426J09Rik | -0.56606 |
| 4833427G06Rik | 1.2958392 |
| 4833436C18Rik | -0.2731265 |
| 4833439L19Rik | -0.3776984 |
| 4833439L19Rik | 1.1326145 |
| 4833439L19Rik | 1.7429817 |
| 4833442J19Rik | -0.1775204 |
| 4833446K15Rik | -0.8594987 |
| 4921501E09Rik | -0.261929 |
| 4921504E06Rik | -0.815304 |
| 4921504I05Rik | 2.5285901 |
| 4921505C17Rik | -0.5112925 |
| 4921505C17Rik | -0.4623126 |
| 4921507P07Rik | 1.5477208 |
| 4921511H03Rik | -0.0681702 |
| 4921511H03Rik | 0.0426382 |
| 4921511K06Rik | 0.347331 |
| 4921511K06Rik | 0.1630236 |
| 4921515J06Rik | -0.0715947 |
| 4921517D21Rik | -0.5008661 |
| 4921517D22Rik | 2.0347729 |
| 4921520G13Rik | 2.0284775 |
| 4921521F21Rik | 1.9511415 |
| 4921521F21Rik | -0.0690251 |
| 4921523A10Rik | 0.613022 |
| 4921523A10Rik | -0.6892929 |
| 4921523A10Rik | -0.4298024 |
| 4921525H12Rik | -0.2704477 |
| 4921528G01Rik | -0.621213 |
| 4921528I01Rik | -0.6724109 |
| 4921530G04Rik | -0.1355747 |
| 4921530L21Rik | -0.6220033 |
| 4921530L21Rik | 1.3371895 |
| 4921536K21Rik | -0.4885119 |
| 4921537P18Rik | 0.5426026 |
| 4922502D21Rik | 0.021232 |
| 4922503N01Rik | -0.709107 |
| 4922503N01Rik | 0.5688709 |
| 4922505E12Rik | -0.2624591 |
| 4922505G16Rik | 3.7701071 |
| 4922505G16Rik | -0.5653173 |
| 4930401F20Rik | -0.6682394 |
| 4930402H24Rik | 2.3173904 |
| 4930403C10Rik | 0.3180516 |
| 4930403L05Rik | 0.1961124 |
| 4930403O06Rik | 0.1600447 |
| 4930404H21Rik | 1.2364038 |
| 4930404N11Rik | -0.4164908 |
| 4930408O21Rik | -0.7320439 |
| 4930408O21Rik | -0.5140934 |
| 4930412F15Rik | -0.0889154 |
| 4930415L06Rik | -0.7338327 |
| 4930418G15Rik | -0.0554432 |
| 4930427A07Rik | -0.345729 |
| 4930428D18Rik | 0.1446634 |
| 4930428E23Rik | 1.948647 |
| 4930430D24Rik | -0.4987953 |
| 4930430E16Rik | -0.7694452 |
| 4930430E16Rik | -0.5462192 |
| 4930431L04Rik | -0.4736392 |
| 4930432H15Rik | -0.1114942 |
| 4930432K21Rik | -0.5282301 |
| 4930432O21Rik | 0.2368983 |
| 4930432O21Rik | 0.0664946 |
| 4930438O05Rik | -0.2451298 |
| 4930438O05Rik | -0.7047623 |
| 4930449I24Rik | 0.6306829 |
| 4930451C15Rik | -0.3849596 |
| 4930451I11Rik | 0.0044423 |
| 4930452B06Rik | 0.354304 |
| 4930453N24Rik | 0.9450655 |
| 4930455C21Rik | -0.685891 |
| 4930455F23Rik | -0.2976008 |
| 4930467M19Rik | 3.9230861 |
| 4930470P17Rik | -0.8281371 |
| 4930473A06Rik | 1.8599861 |
| 4930473A06Rik | -0.7537483 |
| 4930473A06Rik | -0.7428306 |
| 4930474N05Rik | 0.9757128 |
| 4930478A21Rik | 2.2006027 |
| 4930481F22Rik | 0.2629982 |
| 4930481F22Rik | 0.4497733 |
| 4930481M05 | -0.6032245 |
| 4930486L24Rik | 1.2768757 |
| 4930502E18Rik | 1.1701775 |
| 4930503B20Rik | 2.473157 |
| 4930503L19Rik | -0.6419904 |
| 4930504E06Rik | -0.2683105 |
| 4930504E06Rik | 3.3134292 |
| 4930504O13Rik | -0.3716918 |
| 4930506M07Rik | 0.0997168 |
| 4930507C10Rik | -0.4898763 |
| 4930511I11Rik | 1.7346665 |
| 4930511J11Rik | -0.4769978 |
| 4930515G01Rik | 1.4457835 |
| 4930518C23Rik | -0.1660152 |
| 4930519F16Rik | 3.7677163 |
| 4930519G04Rik | -0.287731 |
| 4930519N13Rik | 1.0146319 |
| 4930519N16Rik | -0.01508 |
| 4930522H14Rik | -0.4830752 |
| 4930524B15Rik | 2.3859216 |
| 4930524B15Rik | 0.7759022 |
| 4930524E20Rik | -0.0841189 |
| 4930526D03Rik | -0.7428306 |
| 4930535B03Rik | 1.5467984 |
| 4930535E21Rik | -0.7064977 |
| 4930538K18Rik | 0.0576224 |
| 4930539E08Rik | -0.4729689 |
| 4930540L03Rik | -0.5105936 |
| 4930544G11Rik | 0.0741867 |
| 4930544G21Rik | 2.037287 |
| 4930547C10Rik | 2.0272714 |
| 4930547N16Rik | -0.5375395 |
| 4930549C01Rik | -0.3764939 |
| 4930550C14Rik | -0.5993611 |
| 4930550L24Rik | 1.752916 |
| 4930555G01Rik | 0.498637 |
| 4930555I21Rik | 0.4929299 |
| 4930562D19Rik | -0.3463109 |
| 4930562D19Rik | -0.0110613 |
| 4930563D23Rik | -0.6624316 |
| 4930563P21Rik | 0.9901925 |
| 4930570C03Rik | -0.5513212 |
| 4930572J05Rik | 2.5376277 |
| 4930572J05Rik | 0.3914623 |
| 4930572J05Rik | 0.350556 |
| 4930573I19Rik | -0.267777 |
| 4930578I06Rik | 2.5677076 |
| 4930579C15Rik | 2.4952222 |
| 4930579E17Rik | 0.6636726 |
| 4930579E17Rik | -0.7117239 |
| 4930579J09Rik | -0.2174735 |
| 4930583H14Rik | 0.3484073 |
| 4930590J08Rik | 0.0602192 |
| 4930595M18Rik | 0.7069415 |
| 4930596D02Rik | -0.7117239 |
| 4930599N23Rik | 0.5092694 |
| 4931406B18Rik | -0.7275873 |
| 4931406C07Rik | -0.4756528 |
| 4931406H21Rik | -0.423439 |
| 4931406I20Rik | 1.3780938 |
| 4931406I20Rik | 0.3832215 |
| 4931406P16Rik | 2.2622854 |
| 4931407G18Rik | -0.4919267 |
| 4931408A02Rik | -0.7347285 |
| 4931408C20Rik | 2.3261507 |
| 4931408C20Rik | 0.206877 |
| 4931409K22Rik | 1.5159638 |
| 4931417E11Rik | -0.7275873 |
| 4931417G12Rik | -0.7731788 |
| 4931428L18Rik | 1.8073521 |
| 4931432M23Rik | -0.4871497 |
| 4931433A01Rik | -0.5513212 |
| 4931440B09Rik | 2.3383973 |
| 4931440F15Rik | -0.6251716 |
| 4931440F15Rik | -0.4939818 |
| 4931440L10Rik | 0.4760549 |
| 4932409I22Rik | 2.5264658 |
| 4932411E22Rik | -0.5909196 |
| 4932411G14Rik | 0.2199991 |
| 4932412H11Rik | 2.3487342 |
| 4932413O14Rik | 2.2794917 |
| 4932414J04Rik | 0.531043 |
| 4932414N04Rik | 0.5690813 |
| 4932418E24Rik | 0.176145 |
| 4932431H17Rik | 0.8300872 |
| 4932431H17Rik | -0.6339422 |
| 4932441K18Rik | 3.0786676 |
| 4932441K18Rik | 0.2456827 |
| 4932442K08Rik | 0.8105104 |
| 4932702K14Rik | 0.6608266 |
| 4933400A11Rik | -0.365725 |
| 4933400C05Rik | -0.0884783 |
| 4933400E14Rik | 1.2759169 |
| 4933402J07Rik | 0.2477903 |
| 4933402N03Rik | -0.5985904 |
| 4933402P03Rik | 0.5499408 |
| 4933403G14Rik | -0.8055555 |
| 4933404M02Rik | -0.7564985 |
| 4933405O20Rik | -0.3439858 |
| 4933406M09Rik | 0.9234708 |
| 4933407N01Rik | 0.8759724 |
| 4933407N01Rik | -0.6259654 |
| 4933407N01Rik | 0.5838989 |
| 4933407P14Rik | -0.0296978 |
| 4933407P14Rik | 2.5733054 |
| 4933407P14Rik | 0.9742385 |
| 4933409N07Rik | 1.5188243 |
| 4933411G06Rik | -0.3255988 |
| 4933411K20Rik | 0.157056 |
| 4933412E14Rik | -0.2461706 |
| 4933413G11Rik | -0.0268368 |
| 4933415F23Rik | 3.4668196 |
| 4933416C03Rik | -0.6078826 |
| 4933417A18Rik | -0.6699057 |
| 4933417E01Rik | -0.3445665 |
| 4933421E11Rik | -0.6259654 |
| 4933421E11Rik | 0.968317 |
| 4933421E11Rik | 1.2111965 |
| 4933422H20Rik | -0.6766018 |
| 4933424B01Rik | -0.2683105 |
| 4933424B01Rik | -0.3216262 |
| 4933424B01Rik | -0.6387625 |
| 4933425O20Rik | 1.5561205 |
| 4933426M11Rik | -0.4381427 |
| 4933427D14Rik | 0.4264073 |
| 4933428G09Rik | 3.6148707 |
| 4933428G20Rik | -0.6259654 |
| 4933429E10Rik | 2.3319241 |
| 4933429E10Rik | -0.4709612 |
| 4933430I17Rik | 0.5482196 |
| 4933434E20Rik | -0.644419 |
| 4933434E20Rik | -0.6460417 |
| 4933434E20Rik | -0.36098 |
| 4933434I06Rik | 0.3688789 |
| 4933436I01Rik | 0.0990074 |
| 4933437F05Rik | 1.4897257 |
| 4933439C20Rik | 0.5582851 |
| 4933440H19Rik | 0.9258802 |
| 5031414D18Rik | -0.4885119 |
| 5031414D18Rik | -0.4177502 |
| 5033411D12Rik | -0.628351 |
| 5033414D02Rik | -0.5289428 |
| 5033414K04Rik | -0.0763248 |
| 5133400G04Rik | 1.5871934 |
| 5133400G04Rik | -0.5513212 |
| 5133400G04Rik | 1.8235725 |
| 5133401N09Rik | -0.2545407 |
| 5230400G24Rik | -0.6032245 |
| 5230400G24Rik | 1.6607951 |
| 5330401P04Rik | 1.0920038 |
| 5330417C22Rik | 0.6202075 |
| 5330431N19Rik | -0.7161024 |
| 5330431N19Rik | 0.3288342 |
| 5330431N19Rik | -0.6204233 |
| 5330437I02Rik | 2.2882534 |
| 5330439C02Rik | 0.4548047 |
| 5330439J01Rik | -0.6484812 |
| 5330439J01Rik | -0.0698808 |
| 5430407P10Rik | -0.365725 |
| 5430432M24Rik | -0.5411459 |
| 5430432M24Rik | -0.7750514 |
| 5430432M24Rik | 0.8990147 |
| 5430435G22Rik | 3.4116842 |
| 5430437P03Rik | 1.4016724 |
| 5530601I19Rik | -0.5765228 |
| 5630401D24Rik | -0.3627564 |
| 5630401D24Rik | -0.6624316 |
| 5630401D24Rik | 1.8444146 |
| 5730403M16Rik | -0.2169692 |
| 5730409E04Rik | 1.2694668 |
| 5730410E15Rik | 0.8253515 |
| 5730410E15Rik | 0.6971394 |
| 5730410I19Rik | -0.8231796 |
| 5730410I19Rik | 0.9229029 |
| 5730410I19Rik | -0.4537463 |
| 5730419I09Rik | -0.3801123 |
| 5730437N04Rik | -0.5765228 |
| 5730446C15Rik | -0.4171203 |
| 5730449L18Rik | 1.7132115 |
| 5730449L18Rik | -0.5675473 |
| 5730449L18Rik | -0.4629749 |
| 5730453I16Rik | 0.7069415 |
| 5730453I16Rik | -0.1650631 |
| 5730469M10Rik | 1.7446356 |
| 5730472N09Rik | 0.293522 |
| 5730494M16Rik | -0.7685143 |
| 5730494N06Rik | -0.1857597 |
| 5730502D15Rik | 2.5057496 |
| 5730507A09Rik | 2.326688 |
| 5730507A09Rik | -0.7222684 |
| 5730528L13Rik | 1.0943543 |
| 5730589K01Rik | 0.6063845 |
| 5730593F17Rik | 0.4984094 |
| 5730596K20Rik | -0.6071046 |
| 5730596K20Rik | 0.1099397 |
| 5730596K20Rik | -0.3405095 |
| 5730596K20Rik | -0.2953981 |
| 5830403L16Rik | 1.4838827 |
| 5830404H04Rik | 1.0219125 |
| 5830405N20Rik | -0.5513212 |
| 5830415F09Rik | -0.6969943 |
| 5830415L20Rik | -0.521134 |
| 5830417C01Rik | -0.4008963 |
| 5830426I05Rik | -0.2683105 |
| 5830427D03Rik | -0.283379 |
| 5830434P21Rik | -0.1337346 |
| 5830435K17Rik | -0.8045864 |
| 5830457O10Rik | -0.6063272 |
| 5830457O10Rik | -0.7987938 |
| 5830457O10Rik | -0.3978095 |
| 5830467P10Rik | -0.4433139 |
| 5830468K18Rik | 3.4931537 |
| 5830472M02Rik | -0.4278887 |
| 5830472M02Rik | 0.5286251 |
| 5830472M02Rik | 0.5312625 |
| 5930416I19Rik | -0.6009045 |
| 5930431H10 | -0.7583367 |
| 5930434B04Rik | -0.7302586 |
| 5930434B04Rik | 0.2709467 |
| 5930434B04Rik | -0.232733 |
| 6030405A18Rik | -0.5140934 |
| 6030408B16Rik | -0.3831388 |
| 6030408B16Rik | 0.1385724 |
| 6030419C18Rik | -0.6509274 |
| 6030429G01Rik | 0.7425163 |
| 6030443O07Rik | 0.8630127 |
| 6030446N20Rik | 0.1600447 |
| 6030458C11Rik | 0.7631033 |
| 6230409E13Rik | 2.3501016 |
| 6230409E13Rik | 0.4244325 |
| 6230416J20Rik | -0.7073666 |
| 6230427J02Rik | 0.7610656 |
| 6330403K07Rik | 0.6755281 |
| 6330406I15Rik | -0.0272449 |
| 6330406I15Rik | 0.7323945 |
| 6330407J23Rik | -0.0445569 |
| 6330408A02Rik | -0.6566611 |
| 6330409N04Rik | -0.5404234 |
| 6330416G13Rik | -0.3758922 |
| 6330439K17Rik | 1.284129 |
| 6330442E10Rik | 0.0770998 |
| 6330442E10Rik | -0.8182493 |
| 6330500D04Rik | 2.9519355 |
| 6330503C03Rik | -0.7920825 |
| 6330503K22Rik | -0.5303699 |
| 6330503K22Rik | -0.7082364 |
| 6330503K22Rik | -0.67074 |
| 6330505N24Rik | 3.1394629 |
| 6330512M04Rik | -0.5085003 |
| 6330534C20Rik | -0.2904618 |
| 6330548G22Rik | -0.5418689 |
| 6330569M22Rik | -0.5425925 |
| 6330577E15Rik | -0.7013016 |
| 6330578E17Rik | 0.7547537 |
| 6332401O19Rik | -0.6884412 |
| 6430411K18Rik | 2.2525534 |
| 6430502M16Rik | 0.872825 |
| 6430510M02Rik | 1.5255365 |
| 6430517E21Rik | -0.6995762 |
| 6430526N21Rik | -0.5720238 |
| 6430527G18Rik | 1.1391152 |
| 6430527G18Rik | 0.6098099 |
| 6430531B16Rik | -0.7816364 |
| 6430537H07Rik | -0.6259654 |
| 6430537H07Rik | 0.8991606 |
| 6430548M08Rik | 4.5010064 |
| 6430550H21Rik | 2.0382743 |
| 6430571L13Rik | -0.0158859 |
| 6430573F11Rik | 0.7942742 |
| 6430598A04Rik | 0.6764623 |
| 6430604K15Rik | 2.3584354 |
| 6430706D22Rik | -0.5001753 |
| 6530404N21Rik | -0.0321573 |
| 6530406M24Rik | -0.569037 |
| 6530418L21Rik | 2.411779 |
| 6530418L21Rik | 1.8640368 |
| 6530418L21Rik | 3.0985493 |
| 6530421E24Rik | -0.6331414 |
| 6620401K05Rik | -0.4368546 |
| 6720456B07Rik | -0.6833481 |
| 6720456B07Rik | -0.4465611 |
| 6720456H20Rik | -0.8441872 |
| 6720456H20Rik | 0.3208287 |
| 6720458F09Rik | 0.3213831 |
| 6720463M24Rik | -0.1079148 |
| 6720467C03Rik | 2.1338978 |
| 6720467C03Rik | 0.2062473 |
| 6820408C15Rik | 0.0189079 |
| 7420416P09Rik | -0.6411824 |
| 7530404M11Rik | -0.5282301 |
| 8030411F24Rik | -0.0668894 |
| 8030451F13Rik | -0.4209062 |
| 8030451F13Rik | 1.2295621 |
| 8030451F13Rik | 1.4091485 |
| 8430408G22Rik | 0.3158237 |
| 8430410A17Rik | -0.1833286 |
| 8430410K20Rik | 0.2332382 |
| 8430415E04Rik | -0.6180586 |
| 8430415E04Rik | -0.5015575 |
| 8430419L09Rik | -0.7266986 |
| 8430427H17Rik | 1.0896471 |
| 8430432M10Rik | -0.0529193 |
| 9030025P20Rik | 0.2564694 |
| 9030025P20Rik | -0.429164 |
| 9030025P20Rik | 0.0277843 |
| 9030205A07Rik | -0.5901561 |
| 9030221M09Rik | 0.8383932 |
| 9030224M15Rik | 0.724108 |
| 9030227G01Rik | -0.5282301 |
| 9030409G11Rik | -0.0014891 |
| 9030418K01Rik | 0.7782402 |
| 9030425E11Rik | 0.1092384 |
| 9030607L17Rik | 3.0868449 |
| 9030607L17Rik | -0.6055506 |
| 9030611O19Rik | 0.2537849 |
| 9030612M13Rik | -0.6782836 |
| 9030617O03Rik | 0.6680189 |
| 9030624J02Rik | 0.3940221 |
| 9030625A04Rik | 0.5697124 |
| 9130005N14Rik | 0.1433134 |
| 9130011E15Rik | 0.539781 |
| 9130011J15Rik | -0.0789153 |
| 9130014G24Rik | 0.4393822 |
| 9130017N09Rik | -0.086732 |
| 9130206N08Rik | 1.6649757 |
| 9130211I03Rik | 3.7908387 |
| 9130213B05Rik | -0.6039992 |
| 9130213B05Rik | 1.2797458 |
| 9130218O11Rik | 0.6497369 |
| 9130221H12Rik | 2.9921572 |
| 9130227C08Rik | -0.685891 |
| 9130401M01Rik | -0.2976008 |
| 9130404D08Rik | -0.4796938 |
| 9130404D08Rik | 1.6245513 |
| 9130404D14Rik | -0.5675473 |
| 9130422G05Rik | -0.3627564 |
| 9230104L09Rik | -0.6032245 |
| 9330129D05Rik | 0.9689919 |
| 9330134C04Rik | -0.2587552 |
| 9330176C04Rik | -0.6347438 |
| 9330186A19Rik | 0.5004557 |
| 9430015G10Rik | 0.9284244 |
| 9430015G10Rik | 3.7665547 |
| 9430023L20Rik | 0.9336316 |
| 9430025C20Rik | -0.269913 |
| 9430028L06Rik | 0.035813 |
| 9430029K10Rik | 0.3601623 |
| 9430031J16Rik | 0.5233271 |
| 9430034D17Rik | 0.1810166 |
| 9430057O19Rik | 0.771376 |
| 9430069J07Rik | 2.4196707 |
| 9430077A04Rik | 0.1064276 |
| 9430079M16Rik | -0.303686 |
| 9530015I07Rik | 0.7281726 |
| 9530027K23Rik | 3.9961363 |
| 9530058B02Rik | 0.942707 |
| 9530058B02Rik | 0.2184469 |
| 9530068E07Rik | -0.5630929 |
| 9530077C05Rik | 0.5486502 |
| 9530096D07Rik | 1.0801581 |
| 9530096D07Rik | -0.6987148 |
| 9630015D15Rik | 0.5713931 |
| 9630020C08Rik | 0.9263048 |
| 9630028B13Rik | -0.4616507 |
| 9630031F12Rik | -0.483753 |
| 9630050M13Rik | -0.477671 |
| 9630058J23Rik | 0.01852 |
| 9830163H01Rik | -0.4349258 |
| 9830169C18Rik | -0.6484812 |
| 9930013L23Rik | -0.5947468 |
| 9930021D14Rik | -0.6674074 |
| 9930023K05Rik | -0.6484812 |
| 9930023K05Rik | 0.7004187 |
| 9930104L06Rik | -0.2109405 |
| a | 1.6891744 |
| A030003K21Rik | -0.56606 |
| A030007L17Rik | 0.3345871 |
| A030007L17Rik | -0.6961353 |
| A030007L17Rik | 0.1957938 |
| A030007L17Rik | 1.1278776 |
| A030009H04Rik | 1.8583305 |
| A030010B05Rik | 0.2399371 |
| A030012M09Rik | 3.0818185 |
| A130010J15Rik | 0.3829628 |
| A130038H09Rik | 1.6647266 |
| A130042E20Rik | -0.7213849 |
| A130090K04Rik | 1.0581905 |
| A130092J06Rik | 0.5262007 |
| A230050P20Rik | -0.4285261 |
| A230051G13Rik | -0.4823979 |
| A230054D04Rik | 1.9985594 |
| A230067G21Rik | -0.5454927 |
| A230069A22Rik | 1.0800388 |
| A230074B11Rik | -0.2373618 |
| A230074B11Rik | 0.3598969 |
| A230074B11Rik | -0.4669592 |
| A230106M15Rik | -0.2184828 |
| A230106N23Rik | 1.9524993 |
| A230107C01Rik | 1.1670433 |
| A2bp1 | -0.659954 |
| A2bp1 | 0.9173474 |
| A330021E22Rik | -0.6094407 |
| A330021E22Rik | 2.0180264 |
| A330049M08Rik | 1.4894988 |
| A330080J22Rik | 0.892722 |
| A3galt2 | 2.3524243 |
| A430005L14Rik | -0.7816364 |
| A430005L14Rik | 1.7554529 |
| A430005L14Rik | 1.9374041 |
| A430005L14Rik | -0.53682 |
| A430033K04Rik | -0.5924485 |
| A430079E08 | -0.0968199 |
| A430083B19Rik | -0.4636377 |
| A430083B19Rik | 1.3129395 |
| A430107D22Rik | -0.1364961 |
| A430107P09Rik | 1.3925341 |
| A430110N23Rik | -0.0219533 |
| A4galt | -0.0871683 |
| A4gnt | 0.725347 |
| A530050D06Rik | -0.6509274 |
| A530082C11Rik | 0.850941 |
| A530082C11Rik | 0.810993 |
| A530088H08Rik | -0.6918527 |
| A530088H08Rik | 1.3450409 |
| A530098C11Rik | 1.011806 |
| A630042L21Rik | -0.6355461 |
| A630054L15Rik | -0.4511268 |
| A630077B13Rik | -0.5780274 |
| A630095E13Rik | 1.1629198 |
| A730008H23Rik | 0.0068039 |
| A730008L03Rik | 1.9709857 |
| A730008L03Rik | -0.4504732 |
| A730017C20Rik | -0.0681702 |
| A730024F05Rik | -0.64523 |
| A730037C10Rik | -0.1259561 |
| A730042J05Rik | 0.5204442 |
| A730098D12Rik | 1.1637895 |
| A830006F12Rik | 0.1127395 |
| A830007P12Rik | -0.6110014 |
| A830053O21Rik | -0.5232569 |
| A830059I20Rik | -0.8172665 |
| A830073O21Rik | -0.5810443 |
| A830080D01Rik | 2.4485067 |
| A830093I24Rik | -0.5742705 |
| A930001N09Rik | -0.0715947 |
| A930005I04Rik | -0.4240733 |
| A930008G19Rik | -0.0179038 |
| A930010I20Rik | 0.7399095 |
| A930016P21Rik | 1.7144497 |
| A930018P22Rik | 1.6205707 |
| A930024E05Rik | 1.2791725 |
| A930025D01Rik | -0.5520525 |
| A930025D01Rik | 2.2099252 |
| A930025D01Rik | -0.6032245 |
| A930034L06Rik | -0.4478632 |
| A930038C07Rik | 0.4497733 |
| A930041I02Rik | -0.5112925 |
| AA388235 | -0.3837453 |
| AA407270 | 0.9379713 |
| AA407659 | 0.7947656 |
| AA407659 | 0.8847567 |
| AA408296 | 0.6485819 |
| AA408296 | -0.675762 |
| AA408296 | -0.6016772 |
| AA409316 | -0.6995762 |
| AA415398 | -0.6196344 |
| AA467197 | 1.026354 |
| AA536717 | -0.64523 |
| AA536717 | -0.5848297 |
| AA536749 | -0.3227595 |
| AA536749 | -0.2373618 |
| AA536749 | -0.5239657 |
| AA536749 | 0.8601241 |
| AA673488 | -0.2064453 |
| AA691260 | 0.6367539 |
| AA792892 | -0.6063272 |
| AA881470 | -0.5183111 |
| AA881470 | 1.3625471 |
| AA960436 | 2.0999965 |
| AA960436 | -0.3387764 |
| Aaas | 1.5748927 |
| Aaas | -0.3574375 |
| Aacs | 0.5828634 |
| Aadacl1 | -0.6583061 |
| Aak1 | 0.1453377 |
| Aak1 | 0.6927485 |
| Aamp | -0.3722907 |
| Aard | -0.1030162 |
| Aars | -0.6251716 |
| Aars2 | -0.5978204 |
| Aarsd1 | 0.2131495 |
| Aasdh | -0.3159814 |
| Aatf | -0.7939949 |
| Aatf | 1.0505828 |
| Aatk | -0.5411459 |
| AB112350 | 2.2640785 |
| AB182283 | -0.2629895 |
| AB182283 | 1.2002031 |
| Abat | 3.0029304 |
| Abat | -0.467625 |
| Abat | -0.5418689 |
| Abca14 | -0.4851101 |
| Abca14 | 1.4597327 |
| Abca15 | -0.7788083 |
| Abca15 | -0.6782836 |
| Abca15 | -0.3533222 |
| Abca15 | 1.6858961 |
| Abca17 | -0.1565388 |
| Abca2 | -0.115088 |
| Abca3 | -0.8411559 |
| Abca4 | -0.7510064 |
| Abca4 | -0.3335969 |
| Abca5 | 0.6610166 |
| Abca6 | -0.4563734 |
| Abca6 | -0.2953981 |
| Abca7 | -0.67074 |
| Abca7 | 0.672907 |
| Abca8a | 0.6351911 |
| Abca8b | -0.7038959 |
| Abca9 | -0.2571724 |
| Abca9 | -0.6935633 |
| Abca9 | 1.5403866 |
| Abcb11 | -0.7901742 |
| Abcb1b | 1.206235 |
| Abcb4 | 1.190868 |
| Abcb6 | -0.7510064 |
| Abcb8 | 0.9463117 |
| Abcb8 | -0.7491831 |
| Abcb8 | -0.53682 |
| Abcb9 | -0.7161024 |
| Abcb9 | 0.2706535 |
| Abcc1 | -0.5520525 |
| Abcc10 | -0.7143485 |
| Abcc10 | 1.5862427 |
| Abcc12 | -0.3849596 |
| Abcc3 | 0.756464 |
| Abcc5 | -0.6001325 |
| Abcc6 | 2.2908653 |
| Abcc8 | -0.5126918 |
| Abcc9 | -0.1406543 |
| Abcc9 | 0.3345871 |
| Abcd1 | -0.4743099 |
| Abcd2 | -0.7030303 |
| Abcd2 | -0.3831388 |
| Abcd3 | 0.6898092 |
| Abcd4 | -0.3831388 |
| Abcd4 | 0.1679666 |
| Abce1 | -0.4202741 |
| Abcf1 | -0.7863696 |
| Abcf1 | 0.4720792 |
| Abcf1 | 2.9720527 |
| Abcf2 | 1.9362536 |
| Abcf2 | -0.6427992 |
| Abcf2 | 0.0467838 |
| Abcf2 | -0.2210109 |
| Abcf3 | -0.6566611 |
| Abcg1 | 1.043283 |
| Abcg4 | 2.3842718 |
| Abcg5 | -0.4864693 |
| Abhd1 | 2.2114187 |
| Abhd1 | 4.2954281 |
| Abhd10 | -0.3687035 |
| Abhd11 | 0.9807872 |
| Abhd12 | -0.5126918 |
| Abhd14a | 0.8854972 |
| Abhd14b | 0.1310713 |
| Abhd2 | 3.2389474 |
| Abhd2 | 1.7748338 |
| Abhd2 | 1.2313782 |
| Abhd3 | 0.3863207 |
| Abhd4 | 0.6003076 |
| Abhd5 | -0.2976008 |
| Abhd6 | -0.6607792 |
| Abhd7 | -0.0664629 |
| Abhd7 | 0.1238523 |
| Abhd8 | -0.0301072 |
| Abi1 | -0.5653173 |
| Abi1 | -0.6892929 |
| Abi2 | 0.3304816 |
| Abi2 | 0.0792785 |
| Abi2 | -0.7675843 |
| Abi3 | 0.8924283 |
| Abi3bp | 3.7871967 |
| Abi3bp | -0.0588195 |
| Abl1 | 0.9627641 |
| Ablim1 | 0.4454381 |
| Ablim2 | 0.3037534 |
| Ablim2 | -0.5579238 |
| Ablim3 | -0.8401478 |
| Ablim3 | 0.9260218 |
| Abp1 | 0.226489 |
| Abr | -0.6395684 |
| Abr | -0.7555808 |
| Abtb1 | 0.4132425 |
| Acaa1a | 1.7371404 |
| Acaa1b | -0.6024505 |
| Acaa2 | 0.9505908 |
| Acaa2 | -0.5810443 |
| Acaa2 | 1.2296631 |
| Acaa2 | 0.8985771 |
| Acad10 | 0.7042294 |
| Acad10 | -0.0789153 |
| Acad8 | -0.6944198 |
| Acad8 | 0.3725515 |
| Acad9 | -0.1872216 |
| Acad9 | 0.59378 |
| Acadl | 1.3291785 |
| Acadl | 1.9110848 |
| Acadm | -0.6275551 |
| Acads | -0.6419904 |
| Acads | 0.8302447 |
| Acadsb | 2.9433612 |
| Acadvl | -0.4164908 |
| Acan | 3.8701169 |
| Acat2 | -0.6558398 |
| Acbd3 | -0.5282301 |
| Acbd4 | 1.5790117 |
| Acbd4 | 0.4758215 |
| Acbd5 | -0.6690721 |
| Acbd6 | 1.8577278 |
| Accn1 | 1.8201736 |
| Accn1 | -0.6411824 |
| Accn2 | 1.2158231 |
| Accn3 | 1.0618486 |
| Accn3 | 0.8327598 |
| Accs | -0.6987148 |
| Acd | -0.0750323 |
| Ace | 0.9367135 |
| Ace | 2.1437143 |
| Ache | -0.3492256 |
| Acin1 | -0.6782836 |
| Acly | 4.489395 |
| Acn9 | -0.8084689 |
| Aco1 | -0.5218411 |
| Aco2 | -0.6315417 |
| Acot1 | -0.661605 |
| Acot1 | -0.593214 |
| Acot10 | -0.7675843 |
| Acot11 | -0.5071074 |
| Acot11 | -0.3284471 |
| Acot2 | -0.5564524 |
| Acot2 | -0.3687035 |
| Acot3 | 0.8649837 |
| Acot5 | -0.5183111 |
| Acot7 | -0.5161998 |
| Acot7 | -0.1970286 |
| Acot7 | -0.7410238 |
| Acot7 | -0.7816364 |
| Acot8 | -0.0605125 |
| Acot8 | -0.5697828 |
| Acot8 | -0.6591297 |
| Acot9 | 2.7006425 |
| Acox2 | -0.1641119 |
| Acox3 | 0.9522418 |
| Acox3 | -0.6024505 |
| Acoxl | -0.7930382 |
| Acp2 | -0.3627564 |
| Acp6 | -0.588631 |
| Acpl2 | -0.5924485 |
| Acpp | 2.4434369 |
| Acr | 1.26404 |
| Acr | -0.6867403 |
| Acrbp | -0.1679226 |
| Acrbp | -0.6395684 |
| Acrv1 | -0.4830752 |
| Acsbg1 | -0.7968711 |
| Acsbg1 | 0.0056239 |
| Acsbg2 | -0.4682912 |
| Acsf3 | -0.6909986 |
| Acsl1 | -0.4851101 |
| Acsl3 | 2.0263137 |
| Acsl3 | 0.415242 |
| Acsl4 | -0.5527843 |
| Acsl4 | 0.5847265 |
| Acsl5 | -0.4133502 |
| Acsl6 | 2.2484616 |
| Acsl6 | 0.8124392 |
| Acsl6 | -0.4381427 |
| Acsm3 | 1.3635049 |
| Acss1 | 4.0842153 |
| Acss2 | -0.303686 |
| Acss2 | -0.6371529 |
| Acta1 | -0.5802891 |
| Acta2 | -0.8212043 |
| Acta2 | 0.8853491 |
| Acta2 | -0.4323604 |
| Acta2 | -0.5112925 |
| Actb | 0.6613965 |
| Actb | -0.2645825 |
| Actb | 0.5707632 |
| Actb | 1.182074 |
| Actb | 0.4917841 |
| Actg2 | -0.5947468 |
| Actl6a | 0.1074827 |
| Actl6b | -0.5727721 |
| Actl6b | 1.8856303 |
| Actl6b | -0.5404234 |
| Actl6b | 1.1029983 |
| Actl7a | -0.7901742 |
| Actl7b | 0.1200478 |
| Actn1 | -0.547674 |
| Actn2 | 2.2349841 |
| Actn3 | -0.5462192 |
| Actn3 | -0.6918527 |
| Actn4 | 0.8755235 |
| Actn4 | 1.4197571 |
| Actn4 | 1.2358022 |
| Actr10 | -0.3698976 |
| Actr10 | -0.4374984 |
| Actr1a | -0.2932009 |
| Actr1b | -0.4317203 |
| Actr3 | 0.3443645 |
| Actr3b | -0.6133477 |
| Actr5 | 0.6320574 |
| Actr5 | -0.4796938 |
| Actr6 | 0.1120403 |
| Actrt1 | 3.0102464 |
| Actrt2 | 0.5281848 |
| Acvr1 | 0.1852174 |
| Acvr1c | 0.1399295 |
| Acvr2a | 1.7187994 |
| Acvr2b | -0.4563734 |
| Acvrl1 | 0.6587339 |
| Acy1 | -0.4864693 |
| Acy1l2 | -0.685891 |
| Acy3 | 2.3238788 |
| Ada | -0.560874 |
| Adal | 2.2813764 |
| Adal | 1.6632309 |
| Adam11 | -0.479019 |
| Adam11 | 0.0388528 |
| Adam12 | 1.0203846 |
| Adam15 | -0.4898763 |
| Adam17 | 0.6437532 |
| Adam1b | -0.7675843 |
| Adam1b | -0.4609894 |
| Adam21 | -0.8084689 |
| Adam22 | 1.9677456 |
| Adam22 | -0.1799359 |
| Adam22 | 0.526642 |
| Adam23 | 0.4327957 |
| Adam24 | 1.3425501 |
| Adam26a | 0.283751 |
| Adam28 | 1.717802 |
| Adam29 | 0.913481 |
| Adam29 | 1.9591228 |
| Adam3 | -0.1518377 |
| Adam30 | -0.0854245 |
| Adam32 | -0.682502 |
| Adam33 | -0.6149153 |
| Adam39 | 0.2030924 |
| Adam4 | 0.192602 |
| Adam4 | 0.9255971 |
| Adam4 | -0.6331414 |
| Adam5 | -0.5346648 |
| Adam6 | -0.6509274 |
| Adam8 | -0.4215387 |
| Adam8 | -0.661605 |
| Adam9 | 2.6652807 |
| Adamdec1 | 0.7244622 |
| Adamts1 | 0.2709467 |
| Adamts13 | -0.4682912 |
| Adamts15 | -0.468958 |
| Adamts16 | -0.2225313 |
| Adamts17 | 0.25766 |
| Adamts18 | -0.2169692 |
| Adamts19 | 1.1148931 |
| Adamts2 | 0.3572379 |
| Adamts2 | -0.3193641 |
| Adamts2 | 1.0225483 |
| Adamts20 | -0.3716918 |
| Adamts20 | 0.1983392 |
| Adamts4 | 1.0155292 |
| Adamts7 | 0.2227862 |
| Adamtsl2 | -0.5893933 |
| Adamtsl4 | 0.7445963 |
| Adamtsl5 | -0.6039992 |
| Adar | -0.2893685 |
| Adar | -0.7293673 |
| Adar | 1.449034 |
| Adarb1 | -0.6411824 |
| Adarb1 | 0.7226899 |
| Adarb1 | -0.7437354 |
| Adat1 | 0.260926 |
| Adc | -0.6071046 |
| Adc | -0.6884412 |
| Adck1 | -0.519016 |
| Adck2 | 2.2404519 |
| Adck4 | -0.6009045 |
| Adck5 | 0.4381661 |
| Adcy1 | 0.5807887 |
| Adcy10 | 0.9210549 |
| Adcy2 | 1.1047403 |
| Adcy4 | 1.2775462 |
| Adcy4 | 0.1439887 |
| Adcy6 | 1.090944 |
| Adcy7 | 1.5708239 |
| Adcy8 | -0.5057167 |
| Adcy9 | 2.0102986 |
| Adcyap1 | 2.5904048 |
| Adcyap1r1 | 1.6654113 |
| Add1 | -0.8007205 |
| Add1 | 0.0460315 |
| Add1 | -0.0227654 |
| Add1 | 1.805224 |
| Add3 | 2.1352645 |
| Add3 | 3.7934378 |
| Add3 | 0.4046946 |
| Adfp | -0.5772748 |
| Adh1 | -0.3734896 |
| Adh1 | 0.9622205 |
| Adh4 | 0.9127632 |
| Adh5 | 0.1826346 |
| Adh7 | 0.2059323 |
| Adhfe1 | 0.4492926 |
| Adi1 | 0.5915251 |
| Adipoq | -0.6220033 |
| Adipor1 | -0.7013016 |
| Adipor2 | -0.514795 |
| Adk | 0.5874109 |
| Adk | -0.5802891 |
| Adm | -0.5176068 |
| Adm2 | -0.4342837 |
| Adnp | 0.3909495 |
| Ado | -0.1052395 |
| Adora1 | -0.8472287 |
| Adora1 | 1.19989 |
| Adora1 | 1.9503712 |
| Adora2b | 2.2100611 |
| Adora3 | -0.5571878 |
| Adpgk | 1.7408672 |
| Adprh | -0.1476276 |
| Adprhl1 | -0.3278767 |
| Adprhl1 | -0.0972611 |
| Adprhl1 | 2.1042856 |
| Adprhl2 | 1.2737079 |
| Adra1a | 0.3617534 |
| Adra1a | 0.2474895 |
| Adra1d | -0.5765228 |
| Adra2a | -0.7410238 |
| Adra2c | 0.4673794 |
| Adra2c | -0.5218411 |
| Adrb1 | 1.1146637 |
| Adrb2 | -0.5720238 |
| Adrbk1 | -0.2440902 |
| Adrbk2 | -0.4071017 |
| Adrm1 | 0.9348936 |
| Adsl | -0.7258108 |
| Adss | 1.4444323 |
| Adssl1 | 0.1852174 |
| Adssl1 | 2.0214678 |
| Adssl1 | -0.4387875 |
| Aebp1 | -0.6791257 |
| Aebp2 | -0.640375 |
| Aes | -0.3250302 |
| Afap1 | 0.7554382 |
| Afap1 | 0.3269083 |
| Afap1 | -0.0793478 |
| Afap1l1 | 0.574745 |
| Aff1 | 0.8637713 |
| Aff4 | -0.5549834 |
| Afg3l1 | -0.2758132 |
| Afg3l1 | -0.7064977 |
| Afg3l1 | 2.4007174 |
| Afg3l1 | -0.6259654 |
| Afg3l2 | 2.5815699 |
| Afmid | -0.7134728 |
| Aftph | 1.1321644 |
| Agbl1 | 1.6099129 |
| Ager | 0.7210919 |
| Ager | -0.3910558 |
| Aggf1 | -0.5433166 |
| Aggf1 | -0.342246 |
| Aggf1 | -0.7731788 |
| Agpat1 | -0.477671 |
| Agpat2 | 1.931637 |
| Agpat3 | -0.6808123 |
| Agpat4 | -0.6468541 |
| Agpat5 | -0.2871859 |
| Agpat6 | -0.5425925 |
| Agpat6 | 0.8308741 |
| Agpat7 | 0.6909125 |
| Agpat9 | -0.6492959 |
| Agpat9 | 1.0268602 |
| Agps | -0.6039992 |
| Agps | 0.0752802 |
| Agrn | -0.342246 |
| Agrp | -0.6766018 |
| Agt | 1.6525647 |
| Agtpbp1 | -0.0537598 |
| Agtr1a | -0.4102205 |
| Agtrap | -0.4603285 |
| Agtrl1 | 2.046663 |
| Agxt2l1 | -0.0972611 |
| Agxt2l1 | 0.45337 |
| Agxt2l2 | 1.0335407 |
| Agxt2l2 | -0.519016 |
| Agxt2l2 | 2.5103838 |
| Agxt2l2 | -0.0884783 |
| Ahctf1 | -0.3014685 |
| Ahcy | 0.1000712 |
| Ahcyl1 | -0.3330233 |
| Ahdc1 | 1.1328394 |
| Ahi1 | -0.2910089 |
| Ahnak | -0.6484812 |
| Ahnak | -0.1784858 |
| Ahnak2 | -0.5462192 |
| Ahrr | -0.4994851 |
| Ahsa1 | -0.5043282 |
| Ahsa2 | 1.1870643 |
| AI118078 | -0.3734896 |
| AI132487 | 0.0441479 |
| AI314180 | -0.6892929 |
| AI314976 | -0.5653173 |
| AI316807 | -0.4623126 |
| AI317395 | -0.3347454 |
| AI413582 | 1.4798387 |
| AI413782 | 0.025092 |
| AI427515 | -0.4932962 |
| AI428936 | 1.2911356 |
| AI449175 | 0.5732801 |
| AI449175 | -0.4446114 |
| AI450540 | 1.3453073 |
| AI451557 | -0.5008661 |
| AI451617 | 0.4384095 |
| AI467606 | 0.8982853 |
| AI480653 | -0.3227595 |
| AI504432 | -0.5491311 |
| AI553587 | -0.2054495 |
| AI593442 | -0.7464551 |
| AI593442 | -0.5787807 |
| AI593442 | -0.7648 |
| AI595366 | 0.121087 |
| AI595406 | 0.8921344 |
| AI597468 | -0.6492959 |
| AI597479 | -0.5955143 |
| AI606181 | 0.4993195 |
| AI646023 | 1.8781952 |
| AI661453 | 1.0182157 |
| AI662250 | 2.2207311 |
| AI747699 | 0.4249266 |
| AI836003 | -0.2409785 |
| AI837181 | -0.6315417 |
| AI842396 | -0.5119919 |
| AI846148 | -0.6032245 |
| AI847670 | -0.6291476 |
| AI851790 | -0.2079412 |
| AI894139 | 0.5909092 |
| AI987692 | 3.0717199 |
| AI987692 | -0.4146052 |
| AI987944 | -0.2440902 |
| Aicda | 1.918348 |
| Aif1 | -0.2291492 |
| Aif1 | -0.6117828 |
| Aif1 | 1.1155812 |
| Aifm1 | -0.4830752 |
| Aifm1 | 4.2652212 |
| Aifm2 | -0.6251716 |
| Aifm2 | -0.2731265 |
| Aifm2 | 0.9747748 |
| Aifm3 | 2.6378744 |
| Aig1 | -0.0034749 |
| Aig1-pending | 0.4853406 |
| Aim1 | 0.1186603 |
| Aim2 | -0.5638338 |
| Aip | -0.659954 |
| Ak1 | -0.4709612 |
| AK122209 | -0.6624316 |
| AK122525 | 1.1477841 |
| AK129128 | -0.203461 |
| AK129302 | -0.7064977 |
| AK129302 | -0.1132893 |
| AK129302 | 0.4330405 |
| AK129341 | -0.3527358 |
| AK162044 | -0.6379573 |
| Ak2 | -0.6774423 |
| Ak2 | 0.7271141 |
| Ak2 | 0.5508002 |
| Ak3 | 1.4637122 |
| Ak3 | 4.1755063 |
| Ak3l1 | 0.2423608 |
| Ak5 | 0.2024601 |
| Akap1 | 0.8228156 |
| Akap1 | -0.3154189 |
| Akap1 | 0.1217791 |
| Akap10 | -0.5586605 |
| Akap10 | -0.0533395 |
| Akap12 | -0.4362112 |
| Akap14 | -0.7713101 |
| Akap2 | 0.8387052 |
| Akap2 | -0.470293 |
| Akap3 | 1.8301247 |
| Akap4 | 0.2932361 |
| Akap4 | -0.3922799 |
| Akap4 | -0.5520525 |
| Akap4 | 0.0961645 |
| Akap6 | -0.425979 |
| Akap7 | 3.1118631 |
| Akap8 | 1.9897077 |
| Akap8l | 2.7994718 |
| Akap9 | -0.6901453 |
| Akap9 | -0.4355682 |
| Akap9 | 2.0657652 |
| Akna | -0.6379573 |
| Akp2 | -0.7374211 |
| Akp5 | 1.1636808 |
| Akr1a4 | 1.396402 |
| Akr1a4 | -0.3873931 |
| Akr1b3 | -0.6918527 |
| Akr1b8 | 0.7031423 |
| Akr1c12 | 0.0430158 |
| Akr1c14 | 0.368353 |
| Akr1c14 | -0.3405095 |
| Akr1c18 | 1.3075841 |
| Akr1c19 | 1.3802319 |
| Akr1e1 | -0.5133923 |
| Akr7a5 | -0.3142948 |
| Akr7a5 | 1.2489453 |
| Akr7a5 | 0.8759724 |
| Akt1s1 | -0.7134728 |
| Akt3 | 1.3099887 |
| Akt3 | 3.5302619 |
| Aktip | -0.5579238 |
| Alad | -0.2998089 |
| Alas1 | -0.6558398 |
| Alas2 | -0.5232569 |
| Alcam | -0.1862468 |
| Aldh16a1 | 0.9450655 |
| Aldh18a1 | -0.4439624 |
| Aldh1a1 | -0.1970286 |
| Aldh1a2 | 0.4692622 |
| Aldh1a2 | -0.6078826 |
| Aldh1a7 | 0.0008882 |
| Aldh1b1 | 1.8552622 |
| Aldh1l1 | -0.4570314 |
| Aldh1l2 | -0.7949526 |
| Aldh1l2 | -0.3233266 |
| Aldh2 | 1.2655939 |
| Aldh3a1 | -0.5260954 |
| Aldh3a2 | -0.6055506 |
| Aldh3b1 | -0.1061304 |
| Aldh3b1 | -0.4446114 |
| Aldh3b1 | -0.1751113 |
| Aldh4a1 | 0.7812372 |
| Aldh5a1 | 0.5921407 |
| Aldh5a1 | 1.9487378 |
| Aldh6a1 | 0.6144237 |
| Aldh8a1 | 0.1938801 |
| Aldh8a1 | 2.8271981 |
| Aldoa | -0.3813217 |
| Aldoc | 2.79896 |
| Alg1 | 1.1965435 |
| Alg10b | 2.0122897 |
| Alg11 | 2.2427477 |
| Alg11 | 1.6408757 |
| Alg12 | 0.3099491 |
| Alg12 | 0.1823112 |
| Alg14 | -0.3886123 |
| Alg2 | -0.1485615 |
| Alg3 | -0.0264288 |
| Alg3 | -0.4583488 |
| Alg3 | 0.1057236 |
| Alg5 | 1.473469 |
| Alg5 | -0.5909196 |
| Alg5 | 0.6526181 |
| Alg8 | -0.6909986 |
| Alg8 | -0.5795346 |
| Alg8 | -0.6395684 |
| Alg8 | 0.1025485 |
| Alg9 | 0.7069415 |
| Alg9 | 1.4804503 |
| Alg9 | 1.1020678 |
| Alk | -0.630743 |
| Alkbh2 | -0.3267371 |
| Alkbh3 | -0.4994851 |
| Alkbh4 | -0.5772748 |
| Alkbh5 | -0.5871086 |
| Alkbh7 | -0.6574832 |
| Alkbh8 | 1.476928 |
| Allc | -0.2114414 |
| Allc | 1.0662189 |
| Alox12 | -0.3728899 |
| Alox12b | 0.9532034 |
| Alox12e | 1.7072919 |
| Alox15 | 0.9802544 |
| Alox5ap | 1.5692328 |
| Alox8 | -0.6484812 |
| Aloxe3 | -0.4209062 |
| Alpk3 | 0.2638849 |
| Als2 | 2.8103967 |
| Als2 | -0.5901561 |
| Als2 | -0.6969943 |
| Als2cl | -0.6657458 |
| Als2cr13 | 1.2666606 |
| Als2cr2 | -0.4164908 |
| Als2cr2 | -0.4583488 |
| Als2cr4 | 0.7464988 |
| Als2cr4 | 0.4444719 |
| Als2cr4 | -0.3746901 |
| Alx3 | 1.2091325 |
| Amac1 | -0.5098953 |
| Ambp | 1.5257545 |
| Ambra1 | -0.2255797 |
| Ambra1 | -0.671575 |
| Amd2 | 0.8304021 |
| Amdhd2 | 0.4531307 |
| Amdhd2 | -0.8281371 |
| Amfr | -0.3381994 |
| Amh | -0.0094589 |
| Amhr2 | 2.8463583 |
| Amhr2 | 2.3218412 |
| Amhr2 | 0.3238736 |
| Amigo1 | 1.5332805 |
| Amigo1 | 0.1197011 |
| Amigo2 | -0.6591297 |
| Amigo3 | 1.1278776 |
| Ammecr1l | -0.4570314 |
| Amn | -0.6583061 |
| Amotl2 | 2.649496 |
| Ampd1 | 2.5266091 |
| Ampd1 | 1.5949651 |
| Ampd2 | 1.7151565 |
| Amph | -0.7231527 |
| Amph | 0.8677057 |
| Amy1 | -0.6791257 |
| Amz2 | -0.5893933 |
| Anapc10 | 2.0891058 |
| Anapc11 | -0.5955143 |
| Anapc13 | -0.5105936 |
| Anapc2 | 0.4413246 |
| Anapc4 | 0.1036081 |
| Anapc5 | 0.6973219 |
| Anapc5 | -0.6995762 |
| Anapc5 | 0.1643444 |
| Anapc7 | 0.163354 |
| Ang | 0.4666723 |
| Angel1 | -0.5447667 |
| Angel2 | -0.3861756 |
| Angpt1 | 1.9499178 |
| Angpt2 | -0.3746901 |
| Angpt2 | 0.1728825 |
| Angptl1 | 0.141623 |
| Angptl2 | 1.4634007 |
| Angptl3 | 0.9116135 |
| Angptl4 | -0.5078036 |
| Angptl7 | -0.6732475 |
| Ank | 1.2368047 |
| Ank1 | -0.3199291 |
| Ank2 | 0.6833459 |
| Ank2 | 0.0243213 |
| Ank2 | -0.2472126 |
| Ank3 | -0.6133477 |
| Ank3 | -0.5375395 |
| Ank3 | 1.4148384 |
| Ankar | -0.5564524 |
| Ankar | -0.2199988 |
| Ankfy1 | 0.2289491 |
| Ankle2 | -0.8074967 |
| Ankle2 | 2.8535143 |
| Ankle2 | 0.4192278 |
| Ankmy1 | 0.3896661 |
| Ankmy1 | -0.7222684 |
| Ankmy1 | -0.5397016 |
| Ankmy2 | 1.5496335 |
| Ankmy2 | 1.4084037 |
| Ankrd10 | -0.6180586 |
| Ankrd12 | -0.5549834 |
| Ankrd12 | -0.7099784 |
| Ankrd13a | 0.5038558 |
| Ankrd13b | 0.0369541 |
| Ankrd13d | -0.7329379 |
| Ankrd17 | 0.7308136 |
| Ankrd17 | -0.3445665 |
| Ankrd17 | -0.4491672 |
| Ankrd22 | -0.5447667 |
| Ankrd23 | 1.020512 |
| Ankrd24 | 0.7691886 |
| Ankrd25 | 1.1003205 |
| Ankrd27 | 2.983485 |
| Ankrd34a | 0.1646743 |
| Ankrd35 | -0.7161024 |
| Ankrd36 | -0.7320439 |
| Ankrd37 | 0.3280093 |
| Ankrd39 | 1.0955273 |
| Ankrd40 | -0.3515642 |
| Ankrd40 | -0.6141311 |
| Ankrd40 | -0.1397286 |
| Ankrd46 | 2.0373693 |
| Ankrd47 | 0.2426633 |
| Ankrd49 | -0.0433093 |
| Ankrd5 | 2.9293906 |
| Ankrd50 | -0.5498606 |
| Ankrd53 | 0.7878619 |
| Ankrd54 | 0.1916422 |
| Ankrd54 | 0.137893 |
| Ankrd54 | 0.1938801 |
| Ankrd56 | -0.2592834 |
| Ankrd58 | -0.6411824 |
| Ankrd6 | -0.2998089 |
| Ankrd6 | 1.6538261 |
| Ankrd6 | -0.3364707 |
| Ankrd7 | 1.8053305 |
| Ankrd9 | 0.7235765 |
| Anks1 | 2.2069307 |
| Anks3 | 4.2388667 |
| Anks3 | 1.1754485 |
| Anks6 | 2.026147 |
| Anks6 | 1.4763141 |
| Ankzf1 | -0.6509274 |
| Ankzf1 | 0.5640169 |
| Anln | -0.5091975 |
| Anln | -0.5513212 |
| Anp32a | -0.4932962 |
| Anp32a | -0.412097 |
| Anp32a | 0.141623 |
| Anp32e | -0.3916676 |
| Anpep | 2.077885 |
| Antxr1 | 1.9851835 |
| Antxr1 | 0.0377141 |
| Antxrl | 2.7207761 |
| Anxa1 | -0.6665762 |
| Anxa11 | 3.1690186 |
| Anxa11 | -0.2839218 |
| Anxa2 | -0.284465 |
| Anxa3 | 0.1751675 |
| Anxa3 | 3.4132018 |
| Anxa4 | -0.1980152 |
| Anxa5 | -0.5447667 |
| Anxa6 | -0.6732475 |
| Anxa8 | -0.6468541 |
| Anxa8 | 0.8188388 |
| Anxa9 | -0.5418689 |
| Aoah | 0.5038558 |
| Aoc2 | 0.3438241 |
| Aoc2 | 1.8430367 |
| Aoc3 | -0.3221927 |
| Aof1 | -0.6867403 |
| Aof1 | -0.6969943 |
| Aof2 | -0.7629486 |
| Aox3 | -0.5765228 |
| Aox3l1 | -0.1727087 |
| Ap1b1 | -0.0915425 |
| Ap1g1 | -0.3114909 |
| Ap1g1 | -0.6355461 |
| Ap1g2 | 1.1748047 |
| Ap1gbp1 | -0.4563734 |
| Ap1gbp1 | 1.3826207 |
| Ap1m1 | -0.1061304 |
| Ap1m2 | 0.2567672 |
| Ap1m2 | -0.5268064 |
| Ap1m2 | 1.0058686 |
| Ap1m2 | 0.4692622 |
| Ap1s1 | -0.5668034 |
| Ap1s1 | 1.0531668 |
| Ap1s1 | 1.7159216 |
| Ap1s3 | 0.8188388 |
| Ap2a1 | -0.7694452 |
| Ap2a1 | -0.5140934 |
| Ap2a2 | -0.685891 |
| Ap2b1 | 1.10833 |
| Ap2b1 | -0.2311954 |
| Ap2b1 | -0.2981523 |
| Ap2b1 | -0.3539089 |
| Ap2m1 | 1.9611855 |
| Ap2s1 | -0.7620243 |
| Ap2s1 | 1.2178718 |
| Ap2s1 | -0.6624316 |
| Ap3b1 | -0.5947468 |
| Ap3b1 | 2.0976904 |
| Ap3b2 | 2.2219052 |
| Ap3d1 | 1.2553473 |
| Ap3m1 | 0.8191576 |
| Ap3m2 | 3.0423839 |
| Ap3s2 | -0.6291476 |
| Ap3s2 | 0.116575 |
| Ap4b1 | 0.9187752 |
| Ap4m1 | 0.3227676 |
| Ap4m1 | -0.7143485 |
| Ap4m1 | -0.640375 |
| Ap4s1 | 1.3073989 |
| Apaf1 | -0.2276177 |
| Apaf1 | 0.3860628 |
| Apba1 | -0.4636377 |
| Apba2 | -0.2779684 |
| Apba2 | 0.1207407 |
| Apba3 | -0.5802891 |
| Apbb1 | -0.7583367 |
| Apbb1ip | 0.2175143 |
| Apbb1ip | 0.0150212 |
| Apbb1ip | -0.5268064 |
| Apbb2 | 0.2030924 |
| Apbb3 | -0.2358162 |
| Apc | -0.77975 |
| Apc2 | -0.7258108 |
| Apcdd1 | 0.2125242 |
| Apeh | -0.5947468 |
| Apex1 | -0.5901561 |
| Aph1a | -0.0187123 |
| Aph1b | 2.7888412 |
| Apip | 1.6281902 |
| Apip | 0.4483306 |
| Apitd1 | -0.3971935 |
| Aplp1 | -0.4946678 |
| Aplp2 | 1.3094344 |
| Apoa1 | 1.4603581 |
| Apoa1bp | -0.1965357 |
| Apoa2 | -0.1255007 |
| Apoa4 | 1.6146653 |
| Apob48r | -0.6047745 |
| Apobec1 | 0.1705918 |
| Apobec3 | -0.2959483 |
| Apoc1 | -0.6909986 |
| Apoc1 | -0.2159616 |
| Apoc2 | 1.4489549 |
| Apoc3 | -0.3165443 |
| Apoe | 2.4252502 |
| Apof | -0.597051 |
| Apoh | -0.516903 |
| Apol7c | 0.807125 |
| Apol9b | 0.0169666 |
| App | -0.4926112 |
| Appbp1 | -0.358027 |
| Appbp1 | 0.4298529 |
| Appbp2 | 0.7824001 |
| Appl1 | -0.4563734 |
| Appl2 | 1.6480088 |
| Aprin | 0.5365142 |
| Aprt | -0.6674074 |
| Aptx | -0.6766018 |
| Aqp1 | -0.7713101 |
| Aqp1 | -0.7778676 |
| Aqp1 | -0.6690721 |
| Aqp11 | 0.8953615 |
| Aqp11 | -0.6476673 |
| Aqp3 | -0.5630929 |
| Aqp4 | -0.7169807 |
| Aqp5 | 1.2165407 |
| Aqp7 | 2.1646051 |
| Aqp7 | 1.2170529 |
| Aqp8 | -0.7056296 |
| Aqp8 | 1.5275694 |
| Aqp9 | 0.3071382 |
| Aqr | -0.6517443 |
| Aqr | -0.346893 |
| Ar | -0.3047969 |
| Araf | 0.1897196 |
| Araf | 1.5932139 |
| Arbp | -0.2064453 |
| Arc | 1.1268593 |
| Arcn1 | 0.0760085 |
| Ard1 | -0.0243917 |
| Ard1 | -0.5339476 |
| Areg | -0.640375 |
| Arf2 | -0.1881975 |
| Arf3 | 0.1764706 |
| Arf5 | -0.4616507 |
| Arf6 | -0.7759892 |
| Arfgap1 | 1.2843191 |
| Arfgap1 | 0.0900933 |
| Arfgap1 | -0.7629486 |
| Arfgap2 | -0.5985904 |
| Arfgap2 | 1.7960689 |
| Arfgap2 | -0.4459107 |
| Arfgap2 | -0.1429727 |
| Arfgap3 | -0.588631 |
| Arfip2 | -0.5579238 |
| Arfip2 | 0.0471597 |
| Arfip2 | -0.7082364 |
| Arfrp1 | 0.1989744 |
| Arfrp1 | -0.0284705 |
| Arg1 | 1.7245275 |
| Arg2 | 0.2969454 |
| Arglu1 | 2.7392626 |
| Arhgap1 | -0.7178598 |
| Arhgap1 | 3.5807414 |
| Arhgap1 | 2.4042829 |
| Arhgap10 | -0.0118637 |
| Arhgap12 | 0.0107262 |
| Arhgap15 | -0.7143485 |
| Arhgap15 | -0.2184828 |
| Arhgap17 | -0.3165443 |
| Arhgap18 | -0.1660152 |
| Arhgap19 | 0.4809418 |
| Arhgap20 | 0.4039365 |
| Arhgap22 | -0.2296603 |
| Arhgap24 | 0.937133 |
| Arhgap24 | -0.341088 |
| Arhgap25 | -0.4027534 |
| Arhgap26 | 0.8803008 |
| Arhgap27 | -0.4177502 |
| Arhgap28 | 0.1406073 |
| Arhgap29 | -0.0130685 |
| Arhgap30 | 1.4901794 |
| Arhgap4 | 0.8832738 |
| Arhgap6 | 0.0583651 |
| Arhgap8 | 2.6232081 |
| Arhgap9 | -0.479019 |
| Arhgdib | 1.3303531 |
| Arhgdig | 1.019237 |
| Arhgef1 | 0.8266168 |
| Arhgef1 | 0.7703671 |
| Arhgef10 | 1.9520469 |
| Arhgef10l | -0.5795346 |
| Arhgef10l | 1.6147311 |
| Arhgef15 | -0.4763251 |
| Arhgef18 | 1.3939655 |
| Arhgef19 | 0.0587363 |
| Arhgef2 | 2.9190851 |
| Arhgef3 | -0.7574171 |
| Arhgef4 | -0.5682918 |
| Arhgef6 | 1.1876993 |
| Arid2 | 3.5233596 |
| Arid3a | 0.08362 |
| Arid3b | 1.9398382 |
| Arid4b | -0.6468541 |
| Arid5a | -0.6227943 |
| Arid5b | -0.5772748 |
| Arih1 | -0.0546011 |
| Arih2 | -0.3922799 |
| Arl1 | 3.346065 |
| Arl11 | -0.5161998 |
| Arl16 | 2.6295595 |
| Arl2 | -0.561613 |
| Arl2bp | -0.6275551 |
| Arl2bp | 1.1680171 |
| Arl2bp | 0.2341546 |
| Arl3 | -0.1012414 |
| Arl3 | -0.1901525 |
| Arl4a | -0.4146052 |
| Arl4a | 0.5933704 |
| Arl4c | -0.6291476 |
| Arl5a | -0.7108508 |
| Arl5b | 0.9747748 |
| Arl6 | -0.4114711 |
| Arl6ip1 | 0.8654379 |
| Arl6ip2 | 0.4031777 |
| Arl6ip2 | 2.1943682 |
| Arl6ip2 | -0.5825565 |
| Arl6ip2 | 0.5874109 |
| Arl6ip4 | -0.3916676 |
| Arl6ip4 | -0.4433139 |
| Arl6ip5 | 1.1124807 |
| Arl6ip5 | -0.0836841 |
| Arl8a | 0.774396 |
| Arl8b | -0.6460417 |
| Armc10 | 2.3049748 |
| Armc3 | 2.0677966 |
| Armc6 | 2.5032764 |
| Armc6 | -0.67074 |
| Armc8 | 0.6964091 |
| Armc8 | -0.5418689 |
| Armc8 | 0.9006175 |
| Armc9 | -0.30758 |
| Armc9 | -0.5043282 |
| Armcx1 | -0.6251716 |
| Armcx2 | -0.2342733 |
| Armcx3 | 0.2140866 |
| Armcx5 | 3.111701 |
| Armcx6 | 1.1667184 |
| Armcx6 | 0.0701657 |
| Armetl1 | 0.9038144 |
| Arnt | 2.5682553 |
| Arnt | -0.4974174 |
| Arnt2 | -0.2164653 |
| Arntl | 0.266245 |
| Arpc1a | 0.2193785 |
| Arpc1a | -0.77975 |
| Arpc1a | -0.527518 |
| Arpc1b | -0.2976008 |
| Arpc1b | 2.8494512 |
| Arpc1b | -0.5484022 |
| Arpc3 | 0.0616997 |
| Arpc4 | -0.5947468 |
| Arpc5 | -0.3486419 |
| Arpc5 | -0.5433166 |
| Arpc5l | -0.3439858 |
| Arpp19 | 0.6035537 |
| Arpp21 | -0.2477341 |
| Arpp21 | -0.7703772 |
| Arrb1 | -0.1043495 |
| Arrb1 | -0.5071074 |
| Arrb1 | -0.3284471 |
| Arrb1 | -0.524675 |
| Arrb2 | -0.6591297 |
| Arrdc1 | -0.2094396 |
| Arrdc2 | -0.086296 |
| Arrdc3 | 0.470437 |
| Arrdc4 | 1.2609242 |
| Ars2 | 1.8430877 |
| Ars2 | 2.0554585 |
| Ars2 | -0.5795346 |
| Arsa | -0.5071074 |
| Arsb | -0.5491311 |
| Arsb | -0.1277803 |
| Arsg | 0.1348296 |
| Arsi | 1.7964993 |
| Arsj | -0.5015575 |
| Arsj | 2.3853068 |
| Art1 | -0.7930382 |
| Art2b | 1.7031248 |
| Art3 | -0.477671 |
| Art3 | -0.6799686 |
| Art4 | -0.0698808 |
| Art5 | 0.0886588 |
| Artn | 0.3738595 |
| Artn | 0.765982 |
| Arv1 | -0.6699057 |
| As3mt | -0.4177502 |
| As3mt | 1.3348565 |
| Asah1 | -0.0118637 |
| Asah1 | 1.7787372 |
| Asah2 | -0.2210109 |
| Asah3 | 0.080728 |
| Asah3l | 0.7947656 |
| Asb1 | -0.4039936 |
| Asb1 | -0.547674 |
| Asb1 | -0.4629749 |
| Asb12 | -0.7675843 |
| Asb13 | -0.2645825 |
| Asb15 | -0.7134728 |
| Asb17 | 0.4652564 |
| Asb2 | -0.0609362 |
| Asb3 | -0.5411459 |
| Asb3 | -0.53682 |
| Asb4 | 1.5789432 |
| Asb4 | 0.6176194 |
| Asb6 | -0.7401218 |
| Asb6 | -0.5833136 |
| Asb7 | -0.7455476 |
| Asb7 | -0.685891 |
| Asb8 | -0.4330011 |
| Asb9 | -0.5675473 |
| Ascc1 | 0.7970551 |
| Ascc1 | -0.4439624 |
| Ascc2 | 0.0134618 |
| Ascc2 | -0.5057167 |
| Ascc2 | 1.7868198 |
| Ascc2 | -0.4783448 |
| Ascc3l1 | 2.9181888 |
| Ascl1 | 2.0160902 |
| Ascl2 | -0.709107 |
| Asf1a | -0.7275873 |
| Asf1b | -0.7788083 |
| Asgr1 | -0.5855887 |
| Ash1l | -0.2964988 |
| Ash1l | 1.7930504 |
| Ash1l | 0.8699678 |
| Ash2l | -0.630743 |
| Ash2l | -0.7030303 |
| Asl | -0.8421652 |
| Asl | 1.0827775 |
| Asna1 | -0.7601786 |
| Asns | 1.5871934 |
| Asns | 0.2989364 |
| Asns | 1.6085883 |
| Asnsd1 | -0.8065256 |
| Asnsd1 | -0.3503942 |
| Asph | -0.5447667 |
| Asph | -0.7437354 |
| Asph | -0.5133923 |
| Asph | 2.7622997 |
| Asph | -0.3250302 |
| Asphd1 | 1.3121106 |
| Asphd2 | -0.5008661 |
| Aspm | -0.6501113 |
| Asprv1 | 2.7303146 |
| Aspscr1 | 2.2770357 |
| Aspscr1 | 0.4413246 |
| Aspscr1 | 0.6215981 |
| Asrgl1 | -0.7997566 |
| Ass1 | -0.429164 |
| Aste1 | -0.1537152 |
| Astn1 | -0.0946172 |
| Astn2 | 4.0662102 |
| Asz1 | 0.228642 |
| Atad1 | 1.8601364 |
| Atad2 | -0.7213849 |
| Atad3a | -0.1886859 |
| Atad4 | 2.3571672 |
| Atad4 | 0.4403539 |
| Atcay | 1.8150775 |
| Ate1 | 1.1613959 |
| Ate1 | 0.2049867 |
| Atf2 | -0.527518 |
| Atf2 | 0.020845 |
| Atf2 | 0.1816642 |
| Atf3 | -0.7401218 |
| Atf4 | -0.5939801 |
| Atf4 | 2.4401287 |
| Atf5 | -0.5064118 |
| Atf7 | -0.6607792 |
| Atf7ip | 0.1473574 |
| Atg10 | -0.4368546 |
| Atg10 | -0.5133923 |
| Atg12 | 0.0701657 |
| Atg16l1 | 0.7629337 |
| Atg2a | 0.3133107 |
| Atg2a | 1.2877338 |
| Atg2b | -0.3603886 |
| Atg2b | -0.5454927 |
| Atg3 | -0.5375395 |
| Atg3 | -0.6102207 |
| Atg4b | -0.4851101 |
| Atg4c | -0.4272516 |
| Atg4c | 1.9191932 |
| Atg4d | -0.7620243 |
| Atg5 | -0.0280618 |
| Atg5 | 0.5697124 |
| Atg7 | 0.0672301 |
| Atg9b | -0.2220242 |
| Athl1 | -0.4058572 |
| Atic | 3.1066365 |
| Atic | -0.0487284 |
| Atic | -0.6427992 |
| Atm | -0.6149153 |
| Atm | 1.3719073 |
| Atmin | 0.3751656 |
| Atmin | -0.4871497 |
| Atmin | -0.6987148 |
| Atn1 | 0.2375069 |
| Atoh8 | 0.8686113 |
| Atox1 | -0.5668034 |
| Atp10a | 0.9926928 |
| Atp10d | -0.7939949 |
| Atp11c | -0.6133477 |
| Atp11c | -0.6063272 |
| Atp12a | -0.3752909 |
| Atp13a1 | -0.6436087 |
| Atp13a1 | -0.0114624 |
| Atp13a2 | -0.6867403 |
| Atp13a4 | -0.4420183 |
| Atp13a5 | -0.2220242 |
| Atp1a1 | -0.6978541 |
| Atp1a2 | 1.785514 |
| Atp1b1 | -0.6525619 |
| Atp1b2 | -0.4304412 |
| Atp1b3 | -0.5505906 |
| Atp2a1 | 1.5636068 |
| Atp2a2 | 0.1071311 |
| Atp2a2 | 2.2356116 |
| Atp2a3 | 1.1611779 |
| Atp2a3 | 1.2908526 |
| Atp2a3 | 3.7901936 |
| Atp2b1 | -0.6624316 |
| Atp2b2 | -0.8007205 |
| Atp2b2 | -0.1030162 |
| Atp2b2 | -0.3849596 |
| Atp2b3 | -0.6909986 |
| Atp2b3 | 1.9646724 |
| Atp2b4 | 1.5005521 |
| Atp2b4 | 0.3285593 |
| Atp2c1 | 0.6067881 |
| Atp2c1 | 3.0590112 |
| Atp5a1 | 0.2323208 |
| Atp5b | 2.31046 |
| Atp5c1 | 0.5306039 |
| Atp5d | 1.0591674 |
| Atp5e | 0.5538016 |
| Atp5f1 | -0.7392207 |
| Atp5g2 | 1.325827 |
| Atp5g3 | 0.5345484 |
| Atp5h | -0.8321229 |
| Atp5j | -0.5985904 |
| Atp5j | -0.1523067 |
| Atp5j2 | 0.1938801 |
| Atp5l | 1.9228463 |
| Atp5o | -0.623586 |
| Atp5s | 1.2064422 |
| Atp5sl | 0.226489 |
| Atp6ap1 | 0.20404 |
| Atp6ap2 | -0.7284769 |
| Atp6ap2 | 2.6919762 |
| Atp6ap2 | 0.0300855 |
| Atp6ap2 | -0.4021339 |
| Atp6v0a1 | -0.7694452 |
| Atp6v0a1 | 0.880003 |
| Atp6v0a2 | -0.7320439 |
| Atp6v0b | 1.8428834 |
| Atp6v0d1 | -0.5787807 |
| Atp6v0d1 | 0.1412846 |
| Atp6v0d1 | -0.7873192 |
| Atp6v0d1 | -0.3935058 |
| Atp6v0e | -0.3996603 |
| Atp6v0e2 | -0.6525619 |
| Atp6v1a | 0.5462792 |
| Atp6v1b1 | -0.283379 |
| Atp6v1b2 | 0.9348936 |
| Atp6v1c1 | -0.7675843 |
| Atp6v1c2 | -0.8667342 |
| Atp6v1d | -0.2291492 |
| Atp6v1e1 | -0.4570314 |
| Atp6v1e2 | 1.6860785 |
| Atp6v1g1 | 0.610011 |
| Atp6v1g2 | 0.7418219 |
| Atp6v1g2 | 0.1656632 |
| Atp6v1h | 2.3248361 |
| Atp7a | -0.4323604 |
| Atp8a1 | 0.6800037 |
| Atp8a2 | -0.2921042 |
| Atp8b1 | 2.0387675 |
| Atp8b3 | -0.7788083 |
| Atp8b3 | -0.6550192 |
| Atp8b3 | 0.1758193 |
| Atp8b3 | 0.2969454 |
| Atp9a | 0.6235809 |
| Atp9a | 3.3414014 |
| Atp9b | 2.5886448 |
| Atp9b | 1.1909734 |
| Atpaf1 | -0.5498606 |
| Atpaf2 | -0.7620243 |
| Atpaf2 | -0.1205056 |
| Atpbd1b | 0.6229865 |
| Atpbd1c | 0.5228842 |
| Atpbd3 | -0.3758922 |
| Atpif1 | 1.2218534 |
| Atrx | 0.1741889 |
| Atxn1 | 2.3904916 |
| Atxn10 | 0.95745 |
| Atxn2 | 1.6655357 |
| Atxn2l | -0.5638338 |
| Atxn3 | -0.7583367 |
| Atxn3 | 0.3762092 |
| Atxn3 | 0.534111 |
| Atxn7l1 | 2.6793426 |
| Atxn7l2 | -0.5498606 |
| AU014645 | -0.7117239 |
| AU017455 | 2.2718498 |
| AU017455 | -0.5183111 |
| AU018778 | 1.5290911 |
| AU018829 | -0.3098127 |
| AU019823 | 2.3577727 |
| AU021034 | -0.0321573 |
| AU021092 | 1.8302801 |
| AU021838 | -0.6094407 |
| AU022252 | 1.3964859 |
| AU022751 | -0.8605288 |
| AU022870 | 1.4970328 |
| AU023871 | 0.0950961 |
| AU040320 | 2.7465896 |
| AU040320 | -0.4317203 |
| AU040320 | -0.7108508 |
| AU040829 | 0.6406491 |
| AU042651 | -0.7073666 |
| Aup1 | 0.9842428 |
| Aup1 | 0.2255647 |
| Aurka | -0.0114624 |
| Aurkaip1 | -0.3009149 |
| Aurkc | -0.4563734 |
| Auts2 | -0.3347454 |
| Auts2 | 0.2857714 |
| AV028368 | 0.489718 |
| AV249152 | -0.1173414 |
| AV249152 | 1.9533581 |
| Aven | 0.9530661 |
| Avil | 1.3971568 |
| Avp | -0.345729 |
| Avpi1 | 0.6980515 |
| Avpi1 | 0.707122 |
| Avpr1a | -0.4783448 |
| AW049604 | -0.3928927 |
| AW061290 | 0.3486761 |
| AW111922 | -0.6102207 |
| AW121567 | 0.7096454 |
| AW125753 | -0.3142948 |
| AW146242 | 0.7187786 |
| AW146299 | 0.6831605 |
| AW146299 | 1.6188031 |
| AW540478 | -0.5601355 |
| AW544981 | -0.5638338 |
| AW548124 | -0.4803691 |
| AW549877 | -0.4736392 |
| AW551984 | 0.157721 |
| AW551984 | -0.309254 |
| AW551984 | -0.6460417 |
| AW555464 | 0.9569031 |
| Axin2 | -0.1722289 |
| Axl | 1.2248027 |
| Axud1 | 0.3023393 |
| Azgp1 | -0.132816 |
| Azgp1 | 2.0793788 |
| Azi1 | 3.3575717 |
| Azi2 | 1.512873 |
| Azin1 | -0.3959626 |
| B020018G12Rik | -0.4102205 |
| B230118H07Rik | 0.7799064 |
| B230120H23Rik | 0.7414745 |
| B230206H07Rik | 2.7367881 |
| B230209C24Rik | 0.5764163 |
| B230217C12Rik | -0.3675109 |
| B230219D22Rik | -0.7082364 |
| B230312A22Rik | -0.5871086 |
| B230312I18Rik | -0.6395684 |
| B230317C12Rik | -0.5218411 |
| B230317C12Rik | -0.2661783 |
| B230339H12Rik | 1.384238 |
| B230339M05Rik | 1.5065852 |
| B230339M05Rik | 2.3620286 |
| B230340J04Rik | -0.67074 |
| B230342M21Rik | 2.0102986 |
| B230373P09Rik | -0.6541994 |
| B230378H13Rik | 0.3876088 |
| B230380D07Rik | 0.7212696 |
| B230396O12Rik | 0.0380938 |
| B230399E16Rik | -0.2074423 |
| B2m | 1.5644425 |
| B2m | 0.9883457 |
| B3galnt1 | -0.6533803 |
| B3galnt2 | -0.6624316 |
| B3galt1 | 1.2263244 |
| B3galt4 | -0.4158618 |
| B3galt4 | -0.7365227 |
| B3galt5 | -0.7528334 |
| B3galt6 | -0.4095959 |
| B3gat1 | 1.0412932 |
| B3gat1 | 1.5421723 |
| B3gat1 | 0.3604277 |
| B3gat2 | -0.4696252 |
| B3gat3 | 0.8868285 |
| B3gat3 | 1.7991846 |
| B3gnt1 | 0.1151822 |
| B3gnt3 | -0.1123913 |
| B3gnt5 | 0.0044423 |
| B3gnt7 | -0.675762 |
| B3gnt8 | 0.1092384 |
| B3gnt8 | 0.5555122 |
| B430119L13Rik | 0.4943029 |
| B4galnt3 | -0.1236811 |
| B4galnt4 | 0.0886588 |
| B4galt1 | -0.6094407 |
| B4galt3 | -0.3381994 |
| B4galt3 | -0.682502 |
| B4galt4 | 0.2462854 |
| B4galt7 | 2.5997488 |
| B630005N14Rik | 1.1929742 |
| B830045N13Rik | 2.439892 |
| B930006L02Rik | -0.3025766 |
| B930011P16Rik | -0.2094396 |
| B930037P14Rik | -0.5098953 |
| B930041F14Rik | 0.2227862 |
| B930041G04 | 1.0628216 |
| B930076A02 | -0.6952772 |
| B9d1 | 1.8098993 |
| B9d2 | 1.7155097 |
| Baalc | 0.3909495 |
| Baalc | -0.4796938 |
| Bace1 | 1.3644617 |
| Bace2 | -0.6944198 |
| Bace2 | -0.7685143 |
| Bace2 | 2.338574 |
| Bach1 | -0.1717494 |
| Bach2 | -0.3639427 |
| Bad | 1.9834374 |
| Bag1 | -0.3233266 |
| Bag2 | 0.3947887 |
| Bag3 | 1.0272397 |
| Bag4 | -0.7134728 |
| Bag5 | 2.1517594 |
| Bahcc1 | 1.5351522 |
| Bahd1 | 0.2059323 |
| Bai1 | 0.1810166 |
| Bai2 | 0.466908 |
| Bai2 | 2.8121902 |
| Bai3 | -0.470293 |
| Baiap2 | -0.2763515 |
| Baiap2 | 1.3876334 |
| Banf1 | -0.2205047 |
| Banf1 | -0.4756528 |
| Banf1 | -0.6509274 |
| Banf1 | -0.4967292 |
| Banf2 | 0.1018414 |
| Bap1 | 1.3649832 |
| Bard1 | 2.3894626 |
| Barx2 | 0.9285655 |
| Basp1 | 1.3888188 |
| Bat1a | -0.1397286 |
| Bat1a | -0.569037 |
| Bat2 | -0.4102205 |
| Bat3 | -0.6724109 |
| Bat4 | 3.5524009 |
| Bat4 | -0.6086613 |
| Bat5 | -0.6227943 |
| Batf | 0.8560033 |
| Bax | -0.8192332 |
| Baz1a | -0.477671 |
| Baz1b | 2.8842675 |
| Baz2a | -0.3928927 |
| BB014433 | -0.4946678 |
| BB128963 | 0.4194763 |
| BB146404 | -0.5955143 |
| Bbc3 | 0.6497369 |
| Bbc3 | 1.1123657 |
| Bbox1 | -0.5675473 |
| Bbs1 | 0.0369541 |
| Bbs12 | 1.1288947 |
| Bbs2 | 2.6282587 |
| Bbs4 | -0.289915 |
| Bbs5 | -0.4349258 |
| Bbs7 | -0.3171076 |
| Bbs9 | -0.5454927 |
| Bbs9 | -0.2795881 |
| Bbx | -0.359207 |
| BC002163 | 0.4760549 |
| BC002199 | -0.3233266 |
| BC002216 | 0.0258621 |
| BC003236 | 0.1334648 |
| BC003266 | -0.3165443 |
| BC003885 | 0.6476182 |
| BC003885 | 0.619213 |
| BC003993 | 1.3321572 |
| BC003993 | -0.4960416 |
| BC004004 | -0.4974174 |
| BC004044 | -0.6039992 |
| BC004044 | 0.0546455 |
| BC004728 | 0.1941993 |
| BC004728 | -0.6086613 |
| BC005537 | 1.8566216 |
| BC005682 | -0.5218411 |
| BC005764 | -0.7455476 |
| BC006662 | 1.8714903 |
| BC006779 | -0.6387625 |
| BC006779 | 1.8666277 |
| BC008155 | 1.8784899 |
| BC008163 | -0.448515 |
| BC008163 | 1.0321583 |
| BC010462 | 0.2402404 |
| BC011248 | -0.5571878 |
| BC011248 | -0.6275551 |
| BC011487 | -0.6850426 |
| BC013491 | -0.5218411 |
| BC013491 | 0.0908097 |
| BC013529 | 0.4848786 |
| BC013712 | -0.2709829 |
| BC016201 | -0.2998089 |
| BC016423 | -0.8231796 |
| BC016495 | 0.6298965 |
| BC017158 | 1.7335135 |
| BC017612 | 1.8085735 |
| BC017643 | 0.5428193 |
| BC017647 | 1.3818535 |
| BC018242 | 2.7997011 |
| BC018242 | -0.8431756 |
| BC018465 | 0.8341715 |
| BC020002 | -0.2149552 |
| BC020535 | 0.2828838 |
| BC021381 | 0.1000712 |
| BC021395 | -0.5043282 |
| BC021614 | -0.7392207 |
| BC021790 | 2.9709889 |
| BC021891 | -0.4317203 |
| BC022224 | -0.23891 |
| BC022687 | 2.3935438 |
| BC023055 | -0.6157001 |
| BC023151 | 1.3439742 |
| BC023744 | 2.6277036 |
| BC023814 | 1.1588866 |
| BC023829 | 2.486346 |
| BC024561 | 0.3993742 |
| BC024814 | -0.7134728 |
| BC024868 | -0.5425925 |
| BC024997 | -0.2250709 |
| BC025076 | 2.1220273 |
| BC025546 | -0.1891745 |
| BC025575 | 0.6483892 |
| BC025920 | -0.1182443 |
| BC026439 | -0.2337596 |
| BC026585 | 3.5650266 |
| BC026590 | -0.483753 |
| BC026590 | -0.5253849 |
| BC027072 | 0.2474895 |
| BC027231 | -0.1401913 |
| BC027231 | -0.6071046 |
| BC027231 | 0.874475 |
| BC027344 | -0.5727721 |
| BC028528 | 1.3087872 |
| BC028528 | 1.3220986 |
| BC029169 | 0.9368534 |
| BC029169 | 4.2944539 |
| BC029214 | 1.8594847 |
| BC030183 | 0.9964957 |
| BC030307 | -0.0058637 |
| BC030476 | -0.824169 |
| BC030499 | -0.000696 |
| BC030500 | 0.9547126 |
| BC030867 | -0.536101 |
| BC031181 | -0.8761234 |
| BC031353 | 0.7878619 |
| BC031575 | -0.6047745 |
| BC031748 | -0.7648 |
| BC031781 | 0.1881142 |
| BC031853 | -0.0170961 |
| BC032265 | 1.7451482 |
| BC034076 | -0.3603886 |
| BC037034 | 0.4559985 |
| BC037594 | -0.623586 |
| BC038156 | -0.5140934 |
| BC038286 | -0.6640872 |
| BC038613 | -0.4310805 |
| BC038822 | 0.382704 |
| BC038822 | -0.3020223 |
| BC038822 | -0.5593977 |
| BC038925 | -0.7930382 |
| BC039093 | -0.0643332 |
| BC040758 | 0.2629982 |
| BC043098 | -0.5268064 |
| BC043118 | 0.8308741 |
| BC043301 | -0.3904443 |
| BC046331 | 0.7367713 |
| BC046404 | 1.0509524 |
| BC048355 | -0.6395684 |
| BC048502 | -0.0354472 |
| BC048546 | 0.462181 |
| BC048546 | 1.5822251 |
| BC048562 | 2.0847827 |
| BC048644 | -0.4682912 |
| BC048651 | -0.6331414 |
| BC048671 | -0.1168902 |
| BC049635 | 0.1402685 |
| BC049635 | 0.8911052 |
| BC049730 | -0.6665762 |
| BC049762 | 3.6667799 |
| BC049806 | 0.016189 |
| BC049806 | -0.3527358 |
| BC049806 | -0.5098953 |
| BC050196 | 3.1692993 |
| BC050210 | -0.7056296 |
| BC050777 | 0.5108423 |
| BC050811 | 0.0297024 |
| BC050811 | 0.2252564 |
| BC051019 | -0.4071017 |
| BC051070 | 1.9226125 |
| BC051142 | 1.270529 |
| BC051227 | 1.4648015 |
| BC051628 | 2.6804307 |
| BC051665 | 1.5457329 |
| BC052484 | -0.5043282 |
| BC053749 | 1.1089076 |
| BC053749 | 2.3617417 |
| BC054059 | -0.7410238 |
| BC055107 | 0.6437532 |
| BC055111 | -0.289915 |
| BC055324 | 0.4490522 |
| BC055368 | 0.8455406 |
| BC056474 | -0.516903 |
| BC056923 | 0.0157999 |
| BC057022 | 0.9211972 |
| BC057371 | 2.0080905 |
| BC057371 | -0.6909986 |
| BC057371 | 1.5953687 |
| BC057552 | -0.7863696 |
| BC057627 | 0.268011 |
| BC057893 | -0.5840713 |
| BC060267 | 0.8726749 |
| BC060615 | 2.7706817 |
| BC060632 | 1.0783677 |
| BC061039 | 1.2414015 |
| BC061237 | -0.025206 |
| BC062115 | 1.4030894 |
| BC062650 | -0.5015575 |
| BC063749 | 0.5907038 |
| BC064033 | -0.269913 |
| BC065085 | 0.5671855 |
| BC065397 | -0.0789153 |
| BC066028 | -0.7601786 |
| BC066107 | 1.9106112 |
| BC067047 | 0.6685843 |
| BC067068 | 0.3174951 |
| BC068157 | 1.6314269 |
| BC068281 | -0.2629895 |
| BC072620 | -0.4342837 |
| BC085271 | 2.4324683 |
| BC087945 | -0.1494963 |
| BC088983 | 2.0482501 |
| BC089491 | -0.5893933 |
| BC099439 | 1.3753495 |
| BC107230 | -0.2394267 |
| Bcam | 1.6054014 |
| Bcan | 1.5095497 |
| Bcan | -0.412097 |
| Bcap29 | -0.6808123 |
| Bcap29 | -0.7108508 |
| Bcap31 | -0.3313045 |
| Bcap31 | -0.4616507 |
| Bcar1 | -0.5623526 |
| Bcar3 | -0.7004385 |
| Bcas1 | 0.3241499 |
| Bcas2 | -0.6978541 |
| Bcas3 | 3.0492268 |
| Bcat1 | 0.9200582 |
| Bcat2 | -0.7125979 |
| Bccip | -0.2736632 |
| Bcdin3d | -0.0504024 |
| Bcdo2 | -0.5833136 |
| Bckdha | -0.7082364 |
| Bckdhb | -0.0211419 |
| Bckdk | 0.1951563 |
| Bckdk | -0.6086613 |
| Bcl10 | 2.2076121 |
| Bcl11a | 0.4364609 |
| Bcl11b | 2.6075349 |
| Bcl2 | 0.4979541 |
| Bcl2 | -0.5955143 |
| Bcl2l1 | 0.9213395 |
| Bcl2l1 | 0.7603854 |
| Bcl2l1 | -0.7510064 |
| Bcl2l10 | -0.7275873 |
| Bcl2l11 | -0.3849596 |
| Bcl2l11 | -0.7284769 |
| Bcl2l11 | 0.3868361 |
| Bcl2l12 | 3.2816321 |
| Bcl2l13 | -0.329589 |
| Bcl2l14 | 0.534767 |
| Bcl2l14 | 0.4624179 |
| Bcl2l2 | -0.7347285 |
| Bcl2l2 | 0.3313042 |
| Bcl3 | -0.5036347 |
| Bcl6 | -0.2561188 |
| Bcl6b | 0.4841851 |
| Bcl7a | 0.734498 |
| Bcl7b | -0.090228 |
| Bcl9l | -0.4871497 |
| Bclaf1 | -0.3376228 |
| Bclaf1 | -0.6541994 |
| Bclp2 | -0.2399437 |
| Bcmo1 | -0.5855887 |
| Bcor | -0.217978 |
| Bcor | 0.5306039 |
| Bcorl1 | 0.0293191 |
| Bcs1l | 1.1512064 |
| Bdh1 | 1.8080427 |
| Bdh2 | -0.0694529 |
| Bdkrb1 | -0.5542498 |
| Bdnf | 0.4680859 |
| Bdnf | -0.5513212 |
| Becn1 | -0.4439624 |
| Becn1 | -0.6740849 |
| Bet1 | -0.0395766 |
| Bet1l | 1.6820562 |
| Bex2 | 3.3995591 |
| Bex2 | -0.4336422 |
| Bex2 | 1.2983689 |
| Bex4 | -0.2910089 |
| Bex4 | 2.3667498 |
| Bex6 | -0.7620243 |
| Bfar | -0.7583367 |
| Bfar | 1.0142471 |
| Bglap2 | 1.4169731 |
| Bgn | 1.6161124 |
| Bhlhb2 | 3.5343017 |
| Bhlhb5 | -0.4374984 |
| Bhlhb8 | 0.1620317 |
| Bhlhb8 | 1.2236851 |
| Bhlhb9 | 1.5729641 |
| Bhmt | -0.0002997 |
| Bhmt2 | -0.5232569 |
| Bicc1 | 0.0886588 |
| Bicd1 | -0.346893 |
| Bicd2 | 2.2388414 |
| Bicd2 | 1.0089713 |
| Bid | -0.7187398 |
| Bid | -0.3521498 |
| Bik | 1.5122093 |
| Bin1 | 1.5596265 |
| Bin1 | -0.2435708 |
| Bin1 | 0.3548381 |
| Bin3 | 0.5445509 |
| Birc2 | -0.6204233 |
| Birc2 | -0.1684 |
| Birc2 | -0.1579539 |
| Birc4 | 1.4141806 |
| Birc5 | 0.3759484 |
| Birc5 | 2.4549463 |
| Birc5 | 0.8272488 |
| Blcap | 1.3668926 |
| Blm | -0.4342837 |
| Blm | 3.5446655 |
| Blmh | 0.0342892 |
| Blnk | -0.7750514 |
| Bloc1s1 | -0.468958 |
| Bloc1s3 | -0.8492621 |
| Blvra | 0.1074827 |
| Blvrb | 0.6182173 |
| Bmf | 1.9755816 |
| Bmi1 | 1.4885147 |
| Bmp1 | -0.3639427 |
| Bmp1 | 0.7013274 |
| Bmp1 | 0.0839809 |
| Bmp1 | 1.5378807 |
| Bmp15 | 1.6307805 |
| Bmp2k | 1.3516788 |
| Bmp2k | -0.0358593 |
| Bmp3 | 0.8837189 |
| Bmp4 | 1.4511681 |
| Bmp6 | -0.5542498 |
| Bmp7 | 2.488916 |
| Bmp8a | 1.1645498 |
| Bmper | -0.6833481 |
| Bmpr1a | 2.1456137 |
| Bmpr1a | 0.9723586 |
| Bmpr1b | 0.8792579 |
| Bmpr1b | 1.6665306 |
| Bmpr2 | -0.6740849 |
| Bms1 | -0.7013016 |
| Bnc1 | 1.7668091 |
| Bnc2 | 0.0803658 |
| Bnc2 | 0.1709194 |
| Bnip1 | -0.4253433 |
| Bnip2 | 1.2108872 |
| Bnip3l | 1.8749476 |
| Bnipl | 2.2191857 |
| Bnipl | -0.5802891 |
| Bok | 0.2075062 |
| Bok | -0.6987148 |
| Bola1 | 1.4832735 |
| Bola2 | 2.0409426 |
| Bola3 | -0.8251594 |
| Boll | 1.3185376 |
| Bop1 | 0.0048363 |
| Bpi | -0.1196004 |
| Bpil1 | 1.7775295 |
| Bpil3 | -0.6419904 |
| Bpnt1 | -0.5772748 |
| Bpnt1 | -0.659954 |
| Bpnt1 | -0.5218411 |
| Bpnt1 | -0.6363491 |
| Brap | -0.8094422 |
| Brca2 | 0.1813404 |
| Brca2 | -0.7844733 |
| Brcc3 | -0.5008661 |
| Brcc3 | -0.7930382 |
| Brcc3 | 1.9259724 |
| Brd2 | 2.4386282 |
| Brd2 | -0.6347438 |
| Brd2 | 0.8692144 |
| Brd4 | 1.9814251 |
| Brd4 | 0.9458965 |
| Brd8 | -0.7021655 |
| Brd8 | -0.5810443 |
| Brd9 | 2.2843525 |
| Brd9 | -0.2953981 |
| Brdt | -0.5197214 |
| Brdt | -0.6918527 |
| Bre | 0.2756239 |
| Bre | -0.1114942 |
| Bre | -0.4939818 |
| Brf1 | 1.9070031 |
| Brf2 | 1.7204982 |
| Bri3 | 2.7524085 |
| Brip1 | -0.64523 |
| Brms1 | 1.5884821 |
| Brp16 | 0.5467108 |
| Brp16 | -0.1003554 |
| Brp17 | 2.2166941 |
| Brp44 | -0.5705292 |
| Brpf1 | -0.086296 |
| Brpf1 | -0.4355682 |
| Brs3 | -0.3807168 |
| Brsk1 | -0.4682912 |
| Brsk2 | -0.6347438 |
| Brsk2 | -0.7196206 |
| Brunol4 | -0.7930382 |
| Brunol4 | -0.7574171 |
| Brwd1 | -0.6525619 |
| Brwd1 | -0.2921042 |
| Brwd2 | 0.9747748 |
| Bscl2 | -0.1546554 |
| Bsdc1 | -0.6850426 |
| Bsg | 0.5441183 |
| Bsg | 1.3239193 |
| Bspry | 0.3389457 |
| Bst2 | -0.7258108 |
| Btbd1 | 2.0487379 |
| Btbd10 | -0.5742705 |
| Btbd11 | 0.2727035 |
| Btbd12 | -0.0243917 |
| Btbd14a | -0.7648 |
| Btbd14b | -0.6550192 |
| Btbd16 | -0.1457628 |
| Btbd3 | 0.2946646 |
| Btbd3 | -0.1857597 |
| Btbd6 | 2.7060217 |
| Btbd9 | 1.0497198 |
| Btc | -0.6766018 |
| Btd | 2.4584068 |
| Btf3 | -0.6387625 |
| Btg1 | 0.9541641 |
| Btg1 | 0.080728 |
| Btg3 | -0.6291476 |
| Btg3 | -0.5795346 |
| Btg4 | 0.845231 |
| Btk | -0.4960416 |
| Btla | -0.7064977 |
| Btrc | -0.3025766 |
| Btrc | -0.5810443 |
| Btrc | 1.8415543 |
| Bub1b | -0.429164 |
| Bub3 | 1.370354 |
| Bud13 | 0.8294572 |
| Bud31 | 0.0418824 |
| Bves | 1.2525951 |
| Bxdc1 | 0.9206279 |
| Bxdc1 | -0.4008963 |
| Bxdc2 | 0.0546455 |
| Bxdc2 | 2.4086518 |
| Bxdc5 | 1.4634786 |
| Bxdc5 | 2.2339924 |
| Bxdc5 | -0.6157001 |
| Bzrap1 | 0.4526518 |
| Bzrpl1 | -0.5978204 |
| Bzw1 | -0.5601355 |
| Bzw2 | 2.9310688 |
| Bzw2 | 1.0303957 |
| C030006K11Rik | 0.1806926 |
| C030011O14Rik | -0.5697828 |
| C030014K22Rik | 0.025092 |
| C030018G13Rik | 1.9885788 |
| C030030A07Rik | -0.5425925 |
| C030039L03Rik | 0.575581 |
| C030045D06Rik | 0.1764706 |
| C030045D06Rik | -0.6961353 |
| C030046I01Rik | -0.5535167 |
| C030048B08Rik | -0.5557176 |
| C030048B08Rik | -0.4616507 |
| C130022K22Rik | 2.0305947 |
| C130032J12Rik | 2.039055 |
| C130034I18Rik | -0.527518 |
| C130038G02Rik | -0.5050222 |
| C130060K24Rik | 3.8223701 |
| C130073D16Rik | 1.7537624 |
| C130074G19Rik | -0.7703772 |
| C130090K23Rik | -0.7125979 |
| C130090K23Rik | 2.7774329 |
| C1galt1 | 0.1228163 |
| C1galt1c1 | -0.7293673 |
| C1qa | -0.1731887 |
| C1qb | 0.6483892 |
| C1qbp | 1.4325018 |
| C1qc | -0.7455476 |
| C1qdc2 | 0.8035647 |
| C1ql2 | -0.2860966 |
| C1ql3 | 0.3288342 |
| C1qtnf1 | 0.2582547 |
| C1qtnf2 | 1.2686936 |
| C1qtnf3 | 2.2943671 |
| C1qtnf3 | -0.5535167 |
| C1qtnf3 | 1.4748542 |
| C1qtnf4 | 1.2033281 |
| C1qtnf6 | -0.3574375 |
| C1qtnf7 | -0.4368546 |
| C1qtnf9 | 0.5540156 |
| C1rl | 0.5964375 |
| C1s | 3.9373159 |
| C1s | 0.3720278 |
| C2 | -0.1926022 |
| C230021P08Rik | 1.1556031 |
| C230029F24Rik | -0.5909196 |
| C230052I12Rik | -0.3651305 |
| C230052I12Rik | 0.9703401 |
| C230055K05Rik | 3.3402995 |
| C230071H18Rik | -0.177038 |
| C230078M08Rik | -0.6533803 |
| C230078M14Rik | -0.6724109 |
| C230090D14 | 0.0154106 |
| C230093N12Rik | 0.7554382 |
| C230096C10Rik | -0.3204944 |
| C2cd3 | 0.8337011 |
| C3 | -0.6149153 |
| C330001K17Rik | 0.6233828 |
| C330005M16Rik | -0.6732475 |
| C330016O10Rik | -0.6315417 |
| C330019G07Rik | -0.4524356 |
| C330023M02Rik | -0.5863483 |
| C330027C09Rik | -0.5325148 |
| C430003P19Rik | -0.6468541 |
| C430004E15Rik | -0.0078593 |
| C4b | 0.1060757 |
| C530028I08Rik | 2.6271269 |
| C530028I08Rik | -0.4407246 |
| C530028O21Rik | 0.5795416 |
| C530043G21Rik | -0.569037 |
| C530044N13Rik | 3.5825093 |
| C6 | 0.4624179 |
| C630004H02Rik | -0.5712762 |
| C630004H02Rik | 0.2274123 |
| C630007B19Rik | -0.2144524 |
| C630028N24Rik | 1.0591674 |
| C630035N08Rik | -0.5015575 |
| C630035N08Rik | -0.6484812 |
| C730025P13Rik | -0.7428306 |
| C730027P07Rik | 0.0723612 |
| C78339 | -0.1378801 |
| C79127 | 3.3407443 |
| C79267 | 0.5443347 |
| C79407 | -0.782581 |
| C79407 | 3.1209189 |
| C80008 | 1.6165067 |
| C80913 | 0.3947887 |
| C85492 | 1.0846778 |
| C85492 | -0.573521 |
| C85492 | -0.527518 |
| C85627 | 0.0012837 |
| C87436 | 3.1821669 |
| C87436 | 1.8773598 |
| C87499 | -0.7500943 |
| C8b | 1.8551615 |
| C8g | 2.0195389 |
| C9 | 2.5791391 |
| C9 | 1.1216366 |
| C920005C14Rik | 3.9805613 |
| Cab39l | 0.2903724 |
| Cabc1 | 0.663862 |
| Cables2 | -0.5183111 |
| Cabp2 | -0.3807168 |
| Cabp7 | 0.9762483 |
| Cabyr | 2.2287845 |
| Cabyr | 1.8201212 |
| Cachd1 | 1.2969644 |
| Cacna1a | -0.6411824 |
| Cacna1b | -0.4146052 |
| Cacna1b | 0.0875815 |
| Cacna1b | 0.3614885 |
| Cacna1d | -0.5389803 |
| Cacna1g | -0.5750206 |
| Cacna1h | 2.3020663 |
| Cacna2d1 | 0.690361 |
| Cacna2d3 | -0.629945 |
| Cacnb1 | -0.6141311 |
| Cacnb1 | -0.4656292 |
| Cacnb2 | 2.7132156 |
| Cacnb2 | 0.1855398 |
| Cacnb3 | -0.4803691 |
| Cacnb3 | -0.4058572 |
| Cacnb3 | 2.2319062 |
| Cacnb3 | -0.6901453 |
| Cacnb4 | 1.2613143 |
| Cacnb4 | 2.2983789 |
| Cacng2 | 0.907578 |
| Cacng3 | -0.5825565 |
| Cacng3 | -0.2608698 |
| Cacng4 | 1.2142835 |
| Cacng5 | -0.5985904 |
| Cacng5 | -0.2265981 |
| Cacng5 | -0.6039992 |
| Cacng7 | 1.915101 |
| Cacng8 | -0.3318771 |
| Cacybp | -0.2119425 |
| Cad | -0.2779684 |
| Cadm2 | -0.7047623 |
| Cadm3 | -0.0106605 |
| Cadps | -0.468958 |
| Cadps2 | 2.1939187 |
| Cadps2 | 0.2118985 |
| Cage1 | -0.4590082 |
| Calb2 | 1.2807005 |
| Calb2 | -0.6791257 |
| Calca | 1.0995039 |
| Calca | 1.295933 |
| Calcoco1 | -0.7392207 |
| Cald1 | 1.888694 |
| Cald1 | -0.6371529 |
| Calm2 | 2.2715961 |
| Calm3 | -0.5303699 |
| Calml4 | -0.6808123 |
| Caln1 | 0.0369541 |
| Calr | -0.346893 |
| Calr3 | -0.623586 |
| Calr3 | -0.3586168 |
| Calr3 | -0.5325148 |
| Calr4 | 1.5205071 |
| Calu | -0.4987953 |
| Calu | -0.0919811 |
| Calu | 1.2789813 |
| Caly | -0.6952772 |
| Camk1 | 2.1032152 |
| Camk1d | 0.9882136 |
| Camk1g | 0.3572379 |
| Camk1g | 1.3792062 |
| Camk2a | -0.7537483 |
| Camk2a | 0.7033235 |
| Camk2a | 0.2435702 |
| Camk2b | -0.4046144 |
| Camk2d | -0.203461 |
| Camk2d | 1.0087131 |
| Camk2d | -0.47163 |
| Camk2g | 0.887715 |
| Camk2g | 1.3563402 |
| Camk2n1 | 0.5591365 |
| Camk4 | 0.29495 |
| Camkk1 | 1.164224 |
| Camkk2 | 2.1287446 |
| Camkk2 | 1.683094 |
| Camkv | -0.0235782 |
| Caml | -0.2089399 |
| Camp | 2.1184241 |
| Camsap1l1 | 2.1587268 |
| Camta2 | -0.0066614 |
| Cant1 | -0.4669592 |
| Cant1 | -0.5232569 |
| Cap1 | -0.4089717 |
| Capg | -0.5232569 |
| Capg | -0.6574832 |
| Capn1 | 0.1290146 |
| Capn10 | -0.6259654 |
| Capn11 | 0.2420582 |
| Capn2 | 2.2094495 |
| Capn2 | -0.6047745 |
| Capn2 | 0.2444761 |
| Capn3 | 0.420966 |
| Capn5 | 0.1500433 |
| Capn6 | -0.4763251 |
| Capn7 | -0.5253849 |
| Capn8 | 1.1326145 |
| Capns1 | -0.5586605 |
| Caprin1 | -0.685891 |
| Caprin1 | -0.5098953 |
| Capsl | -0.4576898 |
| Capza1 | 1.3776655 |
| Capza3 | -0.0720237 |
| Capzb | -0.5382596 |
| Capzb | 1.4178748 |
| Capzb | 1.8490941 |
| Car11 | -0.3284471 |
| Car12 | 0.2920917 |
| Car13 | 1.2649144 |
| Car14 | -0.0854245 |
| Car15 | -0.0520796 |
| Car2 | -0.4656292 |
| Car3 | 0.6231847 |
| Car4 | 0.122125 |
| Car5b | -0.4298024 |
| Car5b | -0.2114414 |
| Car5b | -0.4052356 |
| Car7 | -0.4919267 |
| Car9 | 1.9603789 |
| Card10 | 0.3116315 |
| Card11 | -0.4285261 |
| Carhsp1 | 2.5034235 |
| Carm1 | 0.3540368 |
| Cart | 2.6131515 |
| Cartpt | 0.8619495 |
| Casc1 | 1.8732206 |
| Casc1 | 2.3014528 |
| Casc1 | 4.2631115 |
| Casc3 | -0.1485615 |
| Casc4 | 0.0316164 |
| Casc4 | -0.5720238 |
| Casc4 | -0.4478632 |
| Cask | -0.7320439 |
| Caskin1 | -0.5253849 |
| Caskin1 | 1.5974513 |
| Caskin1 | -0.4729689 |
| Caskin2 | -0.7117239 |
| Casp1 | -0.4878306 |
| Casp2 | 1.9691674 |
| Casp4 | -0.6624316 |
| Casp4 | -0.5268064 |
| Casp4 | 0.6075949 |
| Casp6 | -0.7464551 |
| Casp6 | -0.2850085 |
| Casp7 | 0.1938801 |
| Casp8 | 1.5626308 |
| Casp8 | -0.6102207 |
| Casp9 | 0.5482196 |
| Casq2 | -0.4570314 |
| Cass4 | 0.2150227 |
| Cast | -0.6436087 |
| Cat | 0.1248872 |
| Catsper1 | 3.6098453 |
| Catsper2 | -0.6833481 |
| Catsper3 | -0.5993611 |
| Catsper4 | 0.9700706 |
| Cav1 | -0.5863483 |
| Cav2 | 0.6932985 |
| Cbara1 | 1.7415535 |
| Cbfa2t3h | -0.479019 |
| Cbfa2t3h | -0.0815134 |
| Cbfb | -0.6690721 |
| Cbfb | -0.1736689 |
| Cbfb | 0.4079725 |
| Cbl | 0.4783853 |
| Cblb | -0.663259 |
| Cblc | -0.2044547 |
| Cbln1 | 0.8564621 |
| Cbln2 | 0.502044 |
| Cbln4 | 2.4411014 |
| Cbr1 | 0.0940264 |
| Cbr2 | -0.6323412 |
| Cbr3 | 0.8380812 |
| Cbr3 | -0.569037 |
| Cbs | -0.7222684 |
| Cbs | 1.3929553 |
| Cbwd1 | 1.3974083 |
| Cbx2 | 1.4272472 |
| Cbx5 | 0.7968918 |
| Cbx7 | 0.2347651 |
| Cbx8 | 0.4640748 |
| Cby1 | -0.3669152 |
| Cc2d1a | -0.3059091 |
| Cc2d1b | -0.5296561 |
| Cc2d2a | 1.6163096 |
| Ccar1 | -0.5962823 |
| Ccbe1 | -0.6227943 |
| Ccbl1 | 1.2964957 |
| Ccbl1 | 1.1550547 |
| Ccbl2 | -0.1598442 |
| Ccbp2 | 0.3116315 |
| Ccdc100 | -0.0030774 |
| Ccdc101 | -0.4919267 |
| Ccdc108 | 0.3337675 |
| Ccdc109a | 0.1839269 |
| Ccdc109a | 1.3035931 |
| Ccdc109b | 1.8249806 |
| Ccdc11 | -0.7500943 |
| Ccdc110 | -0.0054652 |
| Ccdc111 | -0.56606 |
| Ccdc113 | -0.7365227 |
| Ccdc114 | 0.5865858 |
| Ccdc115 | 0.292378 |
| Ccdc116 | -0.4439624 |
| Ccdc117 | -0.1309816 |
| Ccdc12 | -0.7648 |
| Ccdc120 | 2.2334961 |
| Ccdc123 | -0.4152333 |
| Ccdc124 | 0.3497508 |
| Ccdc125 | -0.0058637 |
| Ccdc126 | -0.5527843 |
| Ccdc127 | 0.3819271 |
| Ccdc127 | 2.7472629 |
| Ccdc13 | -0.4749811 |
| Ccdc130 | 0.6940314 |
| Ccdc131 | 0.6907287 |
| Ccdc132 | 1.4024229 |
| Ccdc134 | -0.3801123 |
| Ccdc137 | 2.730105 |
| Ccdc137 | -0.5727721 |
| Ccdc18 | -0.4981061 |
| Ccdc21 | 2.5628095 |
| Ccdc23 | -0.4722992 |
| Ccdc25 | 0.1314136 |
| Ccdc27 | -0.547674 |
| Ccdc27 | 0.3526996 |
| Ccdc27 | -0.675762 |
| Ccdc28a | -0.2571724 |
| Ccdc28b | -0.2446098 |
| Ccdc28b | -0.7482729 |
| Ccdc3 | 1.2215477 |
| Ccdc32 | 0.4420519 |
| Ccdc38 | -0.6791257 |
| Ccdc39 | 0.2030924 |
| Ccdc40 | -0.4629749 |
| Ccdc41 | 2.6631482 |
| Ccdc42 | -0.5978204 |
| Ccdc43 | 0.449533 |
| Ccdc45 | -0.345729 |
| Ccdc46 | 0.340846 |
| Ccdc46 | -0.6047745 |
| Ccdc47 | 2.1016073 |
| Ccdc49 | -0.4736392 |
| Ccdc5 | -0.7731788 |
| Ccdc50 | -0.5119919 |
| Ccdc51 | 2.5889792 |
| Ccdc53 | 2.1701259 |
| Ccdc53 | -0.4317203 |
| Ccdc53 | 0.5327974 |
| Ccdc54 | 0.9090213 |
| Ccdc56 | 0.3202739 |
| Ccdc57 | 0.2417555 |
| Ccdc58 | -0.5057167 |
| Ccdc59 | -0.6995762 |
| Ccdc59 | -0.3886123 |
| Ccdc64 | -0.2688443 |
| Ccdc65 | 1.9746555 |
| Ccdc65 | 0.7432102 |
| Ccdc66 | 0.7069415 |
| Ccdc67 | -0.8231796 |
| Ccdc68 | 0.028552 |
| Ccdc70 | -0.7161024 |
| Ccdc71 | -0.6227943 |
| Ccdc73 | -0.5317993 |
| Ccdc77 | 1.7168623 |
| Ccdc79 | 2.3630321 |
| Ccdc80 | -0.6525619 |
| Ccdc82 | 0.7842244 |
| Ccdc84 | 2.2155807 |
| Ccdc85a | -0.7311508 |
| Ccdc85b | 0.4697323 |
| Ccdc86 | 0.8103495 |
| Ccdc87 | -0.5579238 |
| Ccdc88a | -0.7125979 |
| Ccdc88b | 0.8850529 |
| Ccdc88c | -0.4330011 |
| Ccdc90b | 0.613623 |
| Ccdc91 | 0.7888514 |
| Ccdc92 | -0.7769279 |
| Ccdc93 | -0.6436087 |
| Ccdc94 | 2.8246112 |
| Ccdc95 | 1.1837759 |
| Ccdc96 | 3.0706373 |
| Ccdc97 | 1.4429994 |
| Ccdc98 | 0.7367713 |
| Ccdc99 | 0.4746537 |
| Cchcr1 | -0.6978541 |
| Ccin | 0.0538997 |
| Cck | 0.9511414 |
| Cckbr | -0.6055506 |
| Ccl11 | -0.5433166 |
| Ccl17 | -0.7638738 |
| Ccl19 | -0.7108508 |
| Ccl21b | -0.6347438 |
| Ccl21c | 1.9627071 |
| Ccl25 | -0.6259654 |
| Ccl25 | -0.1603174 |
| Ccl27 | 0.0509104 |
| Ccl27 | 0.7830639 |
| Ccl28 | 1.2632621 |
| Ccl3 | -0.5579238 |
| Ccl4 | 1.2289559 |
| Ccl4 | -0.2019725 |
| Ccl5 | 2.5928942 |
| Ccl6 | -0.8271434 |
| Ccl7 | -0.5389803 |
| Ccl9 | -0.5645752 |
| Ccm2 | -0.6180586 |
| Ccna1 | 1.7961227 |
| Ccnb1 | -0.7329379 |
| Ccnb1 | -0.5855887 |
| Ccnb3 | 0.5288452 |
| Ccnc | -0.3722907 |
| Ccnd1 | -0.5962823 |
| Ccnd1 | -0.3109312 |
| Ccnd1 | 1.1842009 |
| Ccnd2 | -0.4323604 |
| Ccnd3 | -0.5433166 |
| Ccnd3 | -0.8133458 |
| Ccndbp1 | 1.6503529 |
| Ccne1 | 0.022778 |
| Ccne2 | 1.5014485 |
| Ccnf | -0.467625 |
| Ccng1 | -0.6501113 |
| Ccng1 | -0.561613 |
| Ccng1 | -0.4967292 |
| Ccng2 | 1.190868 |
| Ccnk | 0.3213831 |
| Ccnl1 | -0.5498606 |
| Ccno | -0.0946172 |
| Ccr4 | 1.4519573 |
| Ccr5 | -0.4413712 |
| Ccr5 | -0.6411824 |
| Ccr5 | 0.6116181 |
| Ccr6 | 1.3750058 |
| Ccr6 | 2.00741 |
| Ccrk | 1.3667192 |
| Ccrk | 2.2631824 |
| Ccrk | -0.3227595 |
| Ccrk | 2.0912015 |
| Ccrl2 | -0.8750753 |
| Ccrn4l | 0.2137743 |
| Ccrn4l | 0.5828634 |
| Ccs | 1.7946148 |
| Cct2 | -0.597051 |
| Cct3 | 1.2913242 |
| Cct3 | -0.8172665 |
| Cct4 | -0.3405095 |
| Cct5 | -0.623586 |
| Cct6a | -0.467625 |
| Cct6b | -0.7703772 |
| Cct7 | -0.0146774 |
| Cct8 | -0.3758922 |
| Cd109 | 0.338402 |
| Cd109 | 3.1076642 |
| Cd14 | 0.0879407 |
| Cd151 | -0.5015575 |
| Cd151 | -0.5901561 |
| Cd151 | -0.7769279 |
| Cd160 | -0.1490288 |
| Cd163 | -0.9301482 |
| Cd164 | -0.7030303 |
| Cd177 | 0.8376129 |
| Cd180 | 1.8854354 |
| Cd1d1 | -0.6315417 |
| Cd2 | 1.0420399 |
| Cd200 | -0.8143244 |
| Cd200 | -0.5924485 |
| Cd226 | -0.8143244 |
| Cd247 | -0.3064657 |
| Cd248 | 1.2853638 |
| Cd248 | -0.4400784 |
| Cd27 | -0.4336422 |
| Cd274 | -0.7266986 |
| Cd276 | -0.6867403 |
| Cd2bp2 | 0.9385297 |
| Cd300lg | -0.1565388 |
| Cd320 | -0.2672438 |
| Cd34 | -0.5962823 |
| Cd3d | -0.7038959 |
| Cd3eap | 0.7765708 |
| Cd3g | -0.7482729 |
| Cd4 | 1.0214035 |
| Cd40 | 0.1375532 |
| Cd40 | -0.0685976 |
| Cd44 | 2.1115532 |
| Cd44 | -0.7741146 |
| Cd44 | -0.5638338 |
| Cd44 | -0.8533428 |
| Cd47 | 0.1060757 |
| Cd52 | 0.3801117 |
| Cd53 | -0.5520525 |
| Cd55 | 2.4566782 |
| Cd59a | -0.6574832 |
| Cd59b | 1.000022 |
| Cd59b | -0.3704953 |
| Cd6 | 0.1507135 |
| Cd6 | -0.3586168 |
| Cd6 | -0.399043 |
| Cd63 | -0.5339476 |
| Cd63 | 0.0886588 |
| Cd63 | -0.9607291 |
| Cd68 | -0.3953477 |
| Cd74 | -0.1443667 |
| Cd74 | -0.663259 |
| Cd79b | -0.6901453 |
| Cd79b | 0.1780968 |
| Cd81 | -0.7222684 |
| Cd82 | -0.7601786 |
| Cd83 | -0.8007205 |
| Cd84 | -0.8202182 |
| Cd86 | -0.278508 |
| Cd86 | -0.6978541 |
| Cd86 | -0.5705292 |
| Cd8b1 | 2.3882652 |
| Cd9 | 0.5843128 |
| Cd93 | -0.5411459 |
| Cd96 | 0.6785143 |
| Cd97 | 1.8878197 |
| Cd97 | -0.4926112 |
| Cd97 | -0.7703772 |
| Cd99l2 | -0.3910558 |
| Cdan1 | -0.6525619 |
| Cdc123 | 1.8749969 |
| Cdc14b | -0.6460417 |
| Cdc14b | -0.6690721 |
| Cdc14b | 0.7473622 |
| Cdc16 | -0.5498606 |
| Cdc20 | -0.8761234 |
| Cdc20 | 1.7028861 |
| Cdc20 | 1.0002826 |
| Cdc20 | -0.0170961 |
| Cdc23 | -0.7064977 |
| Cdc25a | -0.2603407 |
| Cdc25b | -0.5675473 |
| Cdc25c | -0.2210109 |
| Cdc25c | -0.7882699 |
| Cdc27 | 1.3649832 |
| Cdc2a | 0.8127602 |
| Cdc2l1 | -0.7446411 |
| Cdc2l1 | -0.7038959 |
| Cdc2l5 | 3.5678252 |
| Cdc2l6 | 2.5826257 |
| Cdc34 | -0.2059473 |
| Cdc37 | 0.6435595 |
| Cdc37l1 | 1.5068821 |
| Cdc37l1 | 1.9151953 |
| Cdc37l1 | -0.3886123 |
| Cdc37l1 | -0.1584261 |
| Cdc42 | 1.8938736 |
| Cdc42bpb | 1.2787901 |
| Cdc42ep1 | 0.3000721 |
| Cdc42ep2 | -0.4939818 |
| Cdc42ep3 | -0.5289428 |
| Cdc42ep4 | 2.7986775 |
| Cdc42ep5 | -0.7806927 |
| Cdc42se1 | 0.1973855 |
| Cdc42se1 | -0.451781 |
| Cdc45l | 0.1361924 |
| Cdc5l | 0.5239911 |
| Cdc5l | 1.7726272 |
| Cdc5l | -0.5091975 |
| Cdc5l | 0.7605555 |
| Cdc6 | 0.7092854 |
| Cdc7 | 0.644334 |
| Cdc73 | 0.9937435 |
| Cdca2 | -0.4381427 |
| Cdca3 | -0.3434055 |
| Cdca3 | -0.2477341 |
| Cdca4 | -0.4851101 |
| Cdca4 | 1.1180997 |
| Cdca4 | 0.8783631 |
| Cdca5 | 3.7192152 |
| Cdca7 | 0.1446634 |
| Cdca8 | 0.1699362 |
| Cdcp1 | -0.3916676 |
| Cdgap | -0.7491831 |
| Cdh1 | -0.675762 |
| Cdh11 | -0.7205024 |
| Cdh11 | -0.2154583 |
| Cdh12 | 1.7371404 |
| Cdh13 | 1.3696628 |
| Cdh13 | -0.0954976 |
| Cdh16 | -0.284465 |
| Cdh22 | -0.7266986 |
| Cdh23 | -0.5638338 |
| Cdh23 | 0.3975937 |
| Cdh23 | -0.6188462 |
| Cdh24 | 0.9802544 |
| Cdh26 | 0.4281317 |
| Cdh3 | 0.7710399 |
| Cdh5 | -0.2265981 |
| Cdh6 | -0.5682918 |
| Cdh7 | -0.7258108 |
| Cdh8 | 0.3564387 |
| Cdh8 | -0.6460417 |
| Cdh8 | -0.3544961 |
| Cdipt | 0.0361935 |
| Cdipt | -0.3527358 |
| Cdk10 | -0.7437354 |
| Cdk10 | 0.3147077 |
| Cdk10 | -0.5697828 |
| Cdk2 | 0.9385297 |
| Cdk2 | 1.2981817 |
| Cdk2 | 0.3896661 |
| Cdk2 | -0.1712702 |
| Cdk2ap2 | -0.6583061 |
| Cdk3 | 0.183281 |
| Cdk5 | -0.6558398 |
| Cdk5r1 | -0.4368546 |
| Cdk5rap1 | -0.6969943 |
| Cdk5rap2 | 2.0951082 |
| Cdk5rap3 | 1.9560199 |
| Cdk6 | -0.6047745 |
| Cdk8 | -0.6509274 |
| Cdk9 | -0.6918527 |
| Cdkal1 | -0.2353016 |
| Cdkl1 | -0.5126918 |
| Cdkl2 | -0.342246 |
| Cdkl2 | -0.3216262 |
| Cdkl2 | -0.6419904 |
| Cdkl2 | 0.9785206 |
| Cdkl3 | 1.6475011 |
| Cdkl4 | 1.3872944 |
| Cdkn1a | 0.4485712 |
| Cdkn1a | 1.4231842 |
| Cdkn1a | -0.4844313 |
| Cdkn1b | -0.329589 |
| Cdkn1b | -0.5126918 |
| Cdkn1c | -0.0605125 |
| Cdkn2a | 1.5631189 |
| Cdkn2a | 0.1725556 |
| Cdkn2aipnl | 0.2644755 |
| Cdkn2b | -0.5225487 |
| Cdkn2c | -0.0802134 |
| Cdkn2c | -0.0588195 |
| Cdkn2d | 1.0723739 |
| Cdo1 | 0.7891809 |
| Cdr2 | 0.6221934 |
| Cdrt4 | -0.448515 |
| Cds1 | 0.9936122 |
| Cds2 | -0.4027534 |
| Cdsn | 0.4500135 |
| Cdt1 | 1.5076239 |
| Cdv3 | -0.3801123 |
| Cdv3 | 2.9379169 |
| Cdyl | -0.0042705 |
| Cdyl | 0.8599718 |
| Cdyl | -0.2114414 |
| Cdyl2 | -0.5962823 |
| Ceacam1 | 0.4008976 |
| Ceacam13 | -0.0090587 |
| Ceacam2 | -0.6251716 |
| Cebpa | 0.4961304 |
| Cebpb | -0.5653173 |
| Cecr5 | 0.6792593 |
| Cecr6 | 0.1193543 |
| Celsr1 | -0.467625 |
| Celsr2 | 0.0411259 |
| Celsr3 | 1.6876582 |
| Cend1 | -0.0329787 |
| Cenpa | 1.7825691 |
| Cenpb | -0.2245624 |
| Cenph | 0.8175625 |
| Cenpi | -0.8361264 |
| Cenpj | -0.4803691 |
| Cenpk | 0.3777722 |
| Cenpl | 1.7735105 |
| Cenpl | -0.477671 |
| Cenpm | -0.0583968 |
| Cenpm | -0.5787807 |
| Cenpm | -0.341088 |
| Cenpn | 1.5790802 |
| Cenpp | -0.6149153 |
| Cenpq | 0.9486608 |
| Cenpt | -0.6157001 |
| Centa1 | 0.0639159 |
| Centa1 | 1.3455737 |
| Centa2 | -0.6665762 |
| Centb5 | 2.2169638 |
| Centb5 | 0.070532 |
| Centd2 | 1.7753846 |
| Centd2 | 0.2178253 |
| Centd3 | 0.3026223 |
| Centg1 | 2.1251309 |
| Centg1 | -0.0504024 |
| Centg2 | -0.6419904 |
| Centg2 | 0.3161025 |
| Centg3 | 0.4538485 |
| Centg3 | -0.381927 |
| Cep135 | 2.1999159 |
| Cep164 | -0.5947468 |
| Cep170 | 2.2032077 |
| Cep55 | -0.4027534 |
| Cep57 | -0.5993611 |
| Cep57 | 0.0196833 |
| Cep63 | 0.4753545 |
| Cep68 | -0.4452608 |
| Cep70 | 2.4373099 |
| Cep72 | -0.0474749 |
| Cep78 | 1.8730724 |
| Cep97 | 0.4134927 |
| Cept1 | -0.2822943 |
| Cercam | -0.5513212 |
| Ces3 | 0.1259208 |
| Cetn2 | -0.4247081 |
| Cetn3 | 2.6298152 |
| Cetn4 | -0.4171203 |
| Cetn4 | 0.7369459 |
| Cfd | 1.1321644 |
| Cfdp1 | 1.2562302 |
| Cfh | 1.1026494 |
| Cfhr1 | -0.6892929 |
| Cfi | -0.4394327 |
| Cfl1 | 0.1043138 |
| Cfl2 | -0.7788083 |
| Cfl2 | 1.3263714 |
| Cfp | 0.675715 |
| Cftr | 0.192602 |
| Cga | 0.8683095 |
| Cggbp1 | 0.103255 |
| Cgnl1 | 2.2620289 |
| Cgrrf1 | 2.344912 |
| Ch25h | 0.599901 |
| Chac1 | 1.7598894 |
| Chad | 1.2228715 |
| Chaf1a | 0.4347523 |
| Chaf1b | 0.8405745 |
| Chat | 1.6225958 |
| Chchd1 | 0.7840587 |
| Chchd2 | -0.2154583 |
| Chchd4 | -0.4830752 |
| Chchd5 | 0.9250306 |
| Chchd6 | 2.3735164 |
| Chchd7 | -0.3370466 |
| Chchd8 | 0.1262651 |
| Chd1 | 0.7205585 |
| Chd1 | -0.4709612 |
| Chd1l | -0.3480586 |
| Chd1l | -0.078483 |
| Chd4 | -0.6411824 |
| Chd4 | 0.7025982 |
| Chd4 | 2.4270402 |
| Chd5 | -0.6935633 |
| Chd8 | 0.5215541 |
| Chdh | 0.2940935 |
| Chdh | 2.8236671 |
| Chek1 | -0.623586 |
| Chek1 | -0.5955143 |
| Chek2 | -0.4202741 |
| Chek2 | -0.0968199 |
| Cherp | -0.3381994 |
| Chfr | -0.6291476 |
| Chfr | -0.7949526 |
| Chfr | -0.6347438 |
| Chga | 1.2802233 |
| Chga | 0.6104131 |
| Chga | 0.2172032 |
| Chgb | -0.6355461 |
| Chi3l1 | 3.2625487 |
| Chi3l3 | 2.0758764 |
| Chi3l3 | 1.5977197 |
| Chia | 2.1595894 |
| Chia | 0.7747309 |
| Chic1 | 4.274519 |
| Chic2 | -0.2271078 |
| Chic2 | 0.0668624 |
| Chit1 | 1.0142471 |
| Chka | 1.3358443 |
| Chka | -0.6649161 |
| Chka | 2.6745716 |
| Chkb | -0.524675 |
| Chl1 | -0.6961353 |
| Chl1 | -0.470293 |
| Chl1 | 0.7865408 |
| Chm | -0.6766018 |
| Chmp1b | -0.573521 |
| Chmp2a | 2.2623495 |
| Chmp2a | 0.941734 |
| Chmp2b | -0.8143244 |
| Chmp4b | 0.140946 |
| Chmp5 | 0.268011 |
| Chmp7 | 2.4058418 |
| Chmp7 | -0.7537483 |
| Chmp7 | -0.4926112 |
| Chn2 | 0.4972706 |
| Chodl | 1.6938285 |
| Chordc1 | 1.7551714 |
| Chpf | 1.8289322 |
| Chpt1 | -0.6674074 |
| Chpt1 | -0.2014769 |
| Chpt1 | -0.4817211 |
| Chrac1 | 1.5699941 |
| Chrd | 1.9061941 |
| Chrm1 | -0.3318771 |
| Chrm2 | -0.2094396 |
| Chrm3 | 0.7760694 |
| Chrna1 | -0.2378776 |
| Chrna4 | -0.5462192 |
| Chrna4 | 1.1966483 |
| Chrna4 | -0.3837453 |
| Chrna5 | -0.5204274 |
| Chrna6 | -0.7574171 |
| Chrna6 | 0.5273035 |
| Chrna7 | -0.6533803 |
| Chrna9 | -0.1337346 |
| Chrnb1 | 0.0392321 |
| Chrnb1 | 1.5443104 |
| Chrnb2 | -0.8172665 |
| Chrnb2 | -0.5425925 |
| Chrnb3 | -0.5549834 |
| Chrnb3 | -0.6427992 |
| Chrnb3 | 1.0971668 |
| Chrne | 0.191322 |
| Chst1 | 0.3777722 |
| Chst10 | 1.4088176 |
| Chst12 | 0.9250306 |
| Chst14 | -0.5549834 |
| Chst2 | 1.5301041 |
| Chst3 | 0.5909092 |
| Chst3 | -0.2871859 |
| Chst7 | 1.7270875 |
| Chst8 | 1.6938888 |
| Chtf18 | -0.6699057 |
| Chuk | -0.2904618 |
| Churc1 | 1.3204568 |
| Ciao1 | 3.1483302 |
| Ciapin1 | 1.8497534 |
| Cib1 | 0.103255 |
| Cib2 | 2.4459753 |
| Cib2 | -0.4643011 |
| Cic | 0.5797496 |
| Cidea | -0.1383418 |
| Cideb | -0.3904443 |
| Cidec | 2.3019436 |
| Ciita | 0.9849059 |
| Cirbp | 3.3635579 |
| Cirh1a | -0.4817211 |
| Cisd1 | -0.5176068 |
| Cish | -0.6024505 |
| Cit | 2.5807828 |
| Cited1 | 3.5168967 |
| Cited2 | -0.3527358 |
| Cited4 | -0.3789045 |
| Ciz1 | 0.668961 |
| Ciz1 | -0.8036184 |
| Ckap2l | -0.2667109 |
| Ckap5 | -0.2054495 |
| Ckb | 1.476928 |
| Cklf | -0.628351 |
| Cklf | -0.2189878 |
| Ckmt1 | 0.4159907 |
| Ckmt2 | 0.0036537 |
| Ckmt2 | -0.5742705 |
| Ckt2 | -0.5282301 |
| Ckt2 | 0.850941 |
| Clca5 | 1.9412589 |
| Clcc1 | -0.0802134 |
| Clcf1 | 0.9972805 |
| Clcf1 | -0.1164393 |
| Clcf1 | 0.4631284 |
| Clcn1 | -0.5462192 |
| Clcn2 | -0.4960416 |
| Clcn3 | 1.5220407 |
| Clcn3 | -0.4550589 |
| Clcn3 | 2.4150035 |
| Clcn4-2 | -0.5535167 |
| Clcn7 | -0.6032245 |
| Clcnka | -0.4114711 |
| Cldn1 | -0.5382596 |
| Cldn10 | 1.1706089 |
| Cldn10 | 0.9230449 |
| Cldn10 | 0.5587109 |
| Cldn11 | 2.1423237 |
| Cldn12 | -0.536101 |
| Cldn15 | 0.0192957 |
| Cldn15 | -0.6227943 |
| Cldn17 | -0.5840713 |
| Cldn2 | -0.4939818 |
| Cldn23 | 0.8997436 |
| Cldn3 | -0.4557159 |
| Cldn4 | 1.5485714 |
| Cldn5 | -0.5119919 |
| Cldn6 | -0.2230386 |
| Cldn9 | 0.8153246 |
| Cldnd1 | -0.5022494 |
| Cldnd2 | 0.0150212 |
| Clec11a | 0.2477903 |
| Clec16a | -0.7997566 |
| Clec1a | -0.6172717 |
| Clec2d | 1.2665637 |
| Clec2e | -0.5317993 |
| Clec3b | 0.3960648 |
| Clec4a1 | 2.0257302 |
| Clec4a3 | 2.0068993 |
| Clec4b1 | 0.2227862 |
| Clec4b1 | 1.7208493 |
| Clec4d | -0.5878695 |
| Clec4g | 0.2929502 |
| Clec4n | 0.7691886 |
| Clec7a | 1.5298871 |
| Clecsf12 | -0.5675473 |
| Clgn | 0.3894092 |
| Clic1 | 1.5778465 |
| Clic3 | -0.6188462 |
| Clic4 | 0.5332355 |
| Clic6 | 2.2819724 |
| Clip1 | 0.6132224 |
| Clip1 | 3.0334232 |
| Clip4 | -0.6961353 |
| Clk1 | 0.8722242 |
| Clk2 | 1.6395959 |
| Clk2 | -0.7648 |
| Clk2 | -0.7629486 |
| Clk3 | 1.4675191 |
| Clk4 | 1.221242 |
| Clk4 | -0.6188462 |
| Clk4 | -0.7592572 |
| Clm3 | 0.5312625 |
| Clm3 | 1.3075841 |
| Clmn | -0.5705292 |
| Clmn | -0.6850426 |
| Cln3 | -0.4871497 |
| Cln5 | 0.3814087 |
| Cln6 | 1.0414177 |
| Cln6 | -0.3831388 |
| Cln8 | -0.8636262 |
| Clns1a | -0.1955507 |
| Clns1a | -0.1584261 |
| Clns1a | 1.4708466 |
| Clock | -0.2265981 |
| Clock | -0.1030162 |
| Clp1 | -0.1523067 |
| Clpb | 1.8918441 |
| Clpp | 0.1392512 |
| Clptm1 | 1.6057339 |
| Clptm1l | -0.7311508 |
| Clpx | 1.8702028 |
| Clpx | -0.3603886 |
| Clrn1 | 0.4413246 |
| Clspn | -0.7302586 |
| Clspn | 0.8182009 |
| Clspn | 1.3183546 |
| Clstn1 | 2.9416756 |
| Clstn3 | -0.7338327 |
| Clstn3 | 0.376991 |
| Clta | -0.0741717 |
| Cltb | 0.3678268 |
| Cluap1 | 0.7870364 |
| Cma1 | 1.8404281 |
| Cma1 | 0.478618 |
| Cmas | 0.6476182 |
| Cmbl | -0.4967292 |
| Cml1 | -0.5339476 |
| Cml3 | -0.56606 |
| Cml4 | 1.7210833 |
| Cmpk | -0.6766018 |
| Cmtm2a | 3.0022265 |
| Cmtm2b | 0.7475348 |
| Cmtm4 | -0.7143485 |
| Cmtm4 | -0.8094422 |
| Cmtm5 | -0.5542498 |
| Cmtm6 | -0.4636377 |
| Cmtm7 | 4.2543146 |
| Cmtm7 | 0.6901771 |
| Cmtm7 | 3.2905285 |
| Cmtm8 | -0.8291319 |
| Cmtm8 | 1.5085132 |
| Cmya1 | -0.621213 |
| CN716893 | 1.5433132 |
| Cnbp2 | 1.7099013 |
| Cnbp2 | -0.3267371 |
| Cndp1 | 1.5242999 |
| Cnga4 | -0.4629749 |
| Cnih | -0.2414964 |
| Cnih3 | 0.1217791 |
| Cnih4 | 1.424161 |
| Cnksr2 | 1.5514715 |
| Cnksr2 | -0.1645874 |
| Cnksr3 | -0.0609362 |
| Cnn1 | 1.6879616 |
| Cnn2 | -0.67074 |
| Cnn2 | 0.2992205 |
| Cnnm1 | 2.1481644 |
| Cnnm1 | -0.3273067 |
| Cnnm2 | -0.2915564 |
| Cnnm3 | -0.6640872 |
| Cno | -0.5382596 |
| Cnot10 | 0.1443261 |
| Cnot2 | -0.1703124 |
| Cnot2 | 0.889338 |
| Cnot2 | 1.005739 |
| Cnot2 | -0.5757714 |
| Cnot3 | 1.2063386 |
| Cnot3 | 1.3288168 |
| Cnot4 | -0.7675843 |
| Cnot4 | 1.5882109 |
| Cnot4 | -0.3364707 |
| Cnot6l | -0.3284471 |
| Cnot6l | -0.5447667 |
| Cnot7 | -0.8667342 |
| Cnp | 0.1279845 |
| Cnp | -0.1227727 |
| Cnpy2 | 2.3624875 |
| Cnpy3 | -0.6884412 |
| Cnr1 | -0.2240542 |
| Cnr1 | -0.7249239 |
| Cntd1 | 0.1193543 |
| Cntd1 | 3.6193363 |
| Cntfr | -0.2871859 |
| Cntln | -0.5535167 |
| Cntn1 | 1.8330216 |
| Cntn4 | 0.6848273 |
| Cntn6 | 1.0302697 |
| Cntnap1 | -0.547674 |
| Cntnap2 | 0.7742285 |
| Cntnap4 | 2.8050122 |
| Cntnap4 | 0.9030888 |
| Coasy | 0.300923 |
| Cobl | 1.4960569 |
| Cobll1 | -0.8065256 |
| Cobll1 | -0.5332309 |
| Coch | -0.2317076 |
| Cog1 | 2.286071 |
| Cog2 | -0.4810448 |
| Cog3 | -0.6110014 |
| Cog4 | -0.0146774 |
| Cog6 | 1.8572251 |
| Cog6 | -0.5623526 |
| Cog8 | -0.1881975 |
| Coil | 2.9778159 |
| Coil | -0.1598442 |
| Col11a1 | 2.1996067 |
| Col12a1 | 1.295933 |
| Col14a1 | 3.0161731 |
| Col15a1 | -0.5757714 |
| Col16a1 | 2.308209 |
| Col17a1 | -0.2910089 |
| Col17a1 | -0.5183111 |
| Col17a1 | 3.1766267 |
| Col18a1 | 0.4227009 |
| Col18a1 | -0.4629749 |
| Col1a1 | -0.6782836 |
| Col1a2 | 0.6285188 |
| Col24a1 | 0.4291156 |
| Col24a1 | -0.7356251 |
| Col25a1 | 1.5999976 |
| Col25a1 | 1.7161568 |
| Col25a1 | 1.545804 |
| Col27a1 | -0.7546641 |
| Col28a1 | -0.1896634 |
| Col2a1 | -0.6690721 |
| Col2a1 | -0.3716918 |
| Col3a1 | 1.2960268 |
| Col3a1 | -0.5645752 |
| Col4a1 | 0.3334941 |
| Col4a2 | 0.1351705 |
| Col4a3 | -0.5382596 |
| Col4a3bp | 2.312281 |
| Col4a3bp | -0.7437354 |
| Col4a3bp | -0.0030774 |
| Col4a4 | 1.9881007 |
| Col4a4 | 0.6144237 |
| Col4a5 | 0.0631778 |
| Col4a6 | 1.1393385 |
| Col4a6 | -0.6624316 |
| Col5a1 | -0.2752752 |
| Col5a3 | 0.8008039 |
| Col6a1 | 2.6127607 |
| Col6a1 | 0.9399243 |
| Col6a1 | -0.3837453 |
| Col6a2 | -0.5586605 |
| Col7a1 | -0.5440414 |
| Col8a2 | -0.4783448 |
| Col9a2 | -0.0130685 |
| Colec12 | 0.4709065 |
| Commd1 | 0.6178187 |
| Commd1 | -0.7284769 |
| Commd10 | -0.4381427 |
| Commd2 | -0.4027534 |
| Commd2 | -0.4228051 |
| Commd4 | -0.2114414 |
| Commd5 | -0.644419 |
| Commd6 | 1.1009033 |
| Commd8 | -0.4960416 |
| Commd9 | -0.4272516 |
| Comp | -0.5098953 |
| Comtd1 | -0.0946172 |
| Copa | -0.5346648 |
| Copb2 | -0.4783448 |
| Cope | -0.6331414 |
| Cope | -0.6533803 |
| Cope | -0.7056296 |
| Cope | 0.5751631 |
| Copg | 0.0262468 |
| Copg | -0.7178598 |
| Copg | -0.4400784 |
| Copg | 0.2600365 |
| Cops2 | -0.5001753 |
| Cops3 | -0.8584698 |
| Cops4 | -0.640375 |
| Cops4 | 1.6393398 |
| Cops5 | 1.9125989 |
| Cops5 | 0.7091053 |
| Cops6 | 1.1436824 |
| Cops7a | -0.0858601 |
| Cops7a | -0.5225487 |
| Cops7a | -0.1780029 |
| Cops7b | -0.6799686 |
| Cops7b | 3.6360619 |
| Cops8 | 1.6957557 |
| Copz1 | 0.1986568 |
| Copz2 | -0.5126918 |
| Copz2 | -0.181873 |
| Copz2 | 3.8987037 |
| Copz2 | 0.248692 |
| Coq10b | 0.6773956 |
| Coq10b | 0.5589237 |
| Coq2 | -0.1101503 |
| Coq3 | 2.4371779 |
| Coq3 | -0.6347438 |
| Coq4 | -0.0600889 |
| Coq4 | 1.9794522 |
| Coq5 | 1.042786 |
| Coq5 | -0.4987953 |
| Coq5 | 2.1880553 |
| Coq6 | 0.5281848 |
| Coq6 | 0.2112723 |
| Coq7 | -0.483753 |
| Coq9 | -0.7213849 |
| Corin | 0.2274123 |
| Coro1a | 2.0911628 |
| Coro1a | -0.6164856 |
| Coro1a | -0.3928927 |
| Coro1a | 2.9702304 |
| Coro1b | -0.4722992 |
| Coro1b | -0.7863696 |
| Coro1c | 0.6541509 |
| Coro2a | 1.2668544 |
| Coro2a | -0.3503942 |
| Coro2b | -0.1070221 |
| Coro2b | 1.8675228 |
| Coro6 | 2.5478598 |
| Coro7 | 2.0249795 |
| Cort | -0.5043282 |
| Cort | -0.2144524 |
| Cotl1 | -0.5682918 |
| Cova1 | -0.3238941 |
| Cox10 | -0.4823979 |
| Cox11 | 1.5795594 |
| Cox15 | -0.5727721 |
| Cox17 | -0.5909196 |
| Cox18 | -0.7892215 |
| Cox18 | -0.2850085 |
| Cox19 | 2.1313434 |
| Cox4i1 | 0.9333509 |
| Cox4nb | -0.7240379 |
| Cox4nb | 0.3219371 |
| Cox5a | -0.3922799 |
| Cox5b | 1.9985594 |
| Cox6a1 | -0.2828365 |
| Cox6a1 | -0.7338327 |
| Cox6a2 | 3.291397 |
| Cox6b1 | -0.3434055 |
| Cox6b1 | 2.019161 |
| Cox6b2 | 0.3403035 |
| Cox6b2 | -0.6961353 |
| Cox6c | 0.693665 |
| Cox7a1 | 0.1910018 |
| Cox7a2 | -0.6665762 |
| Cox7a2l | 0.4711412 |
| Cox7b | -0.6566611 |
| Cox7b2 | -0.6649161 |
| Cox8b | -0.5675473 |
| Cox8c | 1.0373002 |
| Cp | -0.7213849 |
| Cpa1 | -0.2409785 |
| Cpa2 | 0.1214331 |
| Cpa2 | 2.4317252 |
| Cpa3 | 0.3622832 |
| Cpa3 | -0.2399437 |
| Cpa3 | -0.7528334 |
| Cpa4 | 1.4826638 |
| Cpa5 | -0.6749231 |
| Cpa6 | -0.6220033 |
| Cpb2 | 0.0821752 |
| Cpeb1 | 2.5102865 |
| Cpeb2 | 0.2703603 |
| Cpeb3 | -0.2451298 |
| Cpeb4 | 1.4502993 |
| Cphx | 2.7488134 |
| Cplx1 | -0.2477341 |
| Cplx2 | 1.0226754 |
| Cpn1 | 0.594394 |
| Cpne2 | -0.5105936 |
| Cpne4 | 1.8873822 |
| Cpne5 | -0.6909986 |
| Cpne6 | 0.5667637 |
| Cpne7 | 2.4001126 |
| Cpne8 | 2.1076032 |
| Cpne8 | 0.1224707 |
| Cpne9 | -0.1341943 |
| Cpsf1 | 1.2920783 |
| Cpsf1 | -0.3698976 |
| Cpsf2 | 0.1560575 |
| Cpsf3l | 1.1219781 |
| Cpsf4 | 1.7323013 |
| Cpsf4l | 0.2215485 |
| Cpsf4l | -0.7410238 |
| Cpsf6 | -0.0215475 |
| Cpsf6 | -0.77975 |
| Cpt1b | 2.0772947 |
| Cpt1b | -0.5757714 |
| Cpt1c | -0.3009149 |
| Cpt2 | -0.2779684 |
| Cpxcr1 | -0.5112925 |
| Cpxm1 | -0.0268368 |
| Cpxm2 | -0.6204233 |
| Cpz | -0.6339422 |
| Crabp2 | -0.524675 |
| Crat | 1.5821568 |
| Crat | -0.1191482 |
| Crat | -0.6024505 |
| Crb1 | 0.9605877 |
| Crbn | -0.3199291 |
| Crbn | 0.0657586 |
| Crbn | 4.0755163 |
| Crcp | -0.3947333 |
| Creb1 | -0.6371529 |
| Creb1 | -0.6501113 |
| Creb3 | 1.8718366 |
| Creb3l1 | -0.6436087 |
| Creb3l2 | 0.0792785 |
| Creb3l2 | 3.3616236 |
| Creb3l3 | -0.7030303 |
| Creb3l4 | 1.6535739 |
| Creb5 | 1.4287054 |
| Crebl1 | -0.1717494 |
| Crebl2 | 0.887715 |
| Creg2 | 2.5666799 |
| Creld1 | -0.6525619 |
| Creld2 | 0.140946 |
| Creld2 | 0.6861215 |
| Crem | 0.8648322 |
| Crem | 2.5041832 |
| Crem | 0.9487988 |
| Crem | 0.9797213 |
| Crhbp | -0.2806696 |
| Crhr1 | -0.6484812 |
| Crim1 | 0.8061554 |
| Crim2 | -0.0605125 |
| Crip2 | -0.7108508 |
| Crip3 | 1.0145037 |
| Crip3 | 1.4187755 |
| Cript | -0.6740849 |
| Cript | -0.2545407 |
| Crisp2 | -0.5310843 |
| Crispld2 | -0.3347454 |
| Crk | -0.3498097 |
| Crk | 3.0881515 |
| Crk | -0.6492959 |
| Crkl | 1.3890726 |
| Crkrs | 0.4454381 |
| Crkrs | 2.1168808 |
| Crlf3 | 1.2561322 |
| Crlf3 | -0.5050222 |
| Crls1 | -0.6063272 |
| Crls1 | 0.9834466 |
| Crls1 | -0.7266986 |
| Crls1 | -0.7284769 |
| Crmp1 | 1.2929259 |
| Crmp1 | 0.2537849 |
| Crnkl1 | -0.6525619 |
| Crocc | 0.2153345 |
| Crry | 2.9461085 |
| Crry | 0.7745635 |
| Crtac1 | 1.4994304 |
| Crtam | 1.4248116 |
| Crtap | -0.090228 |
| Crtap | 0.362548 |
| Crtc1 | -0.6732475 |
| Crtc3 | -0.0647587 |
| Crxos1 | 0.7814035 |
| Cry2 | 1.7360481 |
| Cryab | -0.4046144 |
| Cryba2 | -0.1712702 |
| Cryba4 | -0.3801123 |
| Crybb1 | -0.2024684 |
| Crybb2 | 0.4256672 |
| Crybb3 | -0.7410238 |
| Crygn | -0.7249239 |
| Crygn | -0.7258108 |
| Cryl1 | -0.5818001 |
| Crym | 1.205924 |
| Cryz | 0.5673963 |
| Cryz | 1.7491255 |
| Cryz | 0.228642 |
| Cryzl1 | -0.4898763 |
| Cryzl1 | -0.3722907 |
| Cs | 2.3790791 |
| Csad | -0.7638738 |
| Csda | -0.709107 |
| Csda | 1.1573558 |
| Csda | -0.4190112 |
| Csdc2 | 1.0319067 |
| Csde1 | 1.8126506 |
| Csde1 | 0.0861429 |
| Csde1 | 0.8319746 |
| Cse1l | -0.5727721 |
| Csf1 | 1.6600443 |
| Csf1r | -0.425979 |
| Csf1r | 0.0950961 |
| Csf2ra | -0.5727721 |
| Csgalnact1 | -0.6833481 |
| Csgalnact2 | -0.0789153 |
| Csk | 2.0002765 |
| Csl | -0.4046144 |
| Csl | 0.2543821 |
| Csmd1 | 0.4283778 |
| Csnk1d | -0.3193641 |
| Csnk1d | 1.3205481 |
| Csnk1d | 1.248352 |
| Csnk1d | 1.8747504 |
| Csnk1d | -0.3009149 |
| Csnk1e | 0.9429848 |
| Csnk1e | -0.070309 |
| Csnk1g1 | 0.0605896 |
| Csnk1g1 | -0.4756528 |
| Csnk1g1 | -0.6227943 |
| Csnk1g2 | -0.7143485 |
| Csnk1g2 | 1.3471703 |
| Csnk1g2 | 0.8142036 |
| Csnk1g3 | -0.5462192 |
| Csnk2a1 | -0.2104399 |
| Csnk2a2 | 2.4695748 |
| Csnk2a2 | 0.3351331 |
| Csnrp2 | -0.5833136 |
| Csnrp3 | 0.6404548 |
| Cspg4 | -0.0280618 |
| Cspg5 | 1.4540845 |
| Cspp1 | 0.565497 |
| Csprs | 0.9101743 |
| Csrp1 | 0.9400636 |
| Csrp2 | -0.7038959 |
| Csrp2bp | 0.9486608 |
| Cst11 | 0.0679649 |
| Cst3 | 1.21869 |
| Cst8 | 2.1941607 |
| Cst9 | 0.7207364 |
| Cstad | 3.1839293 |
| Cstb | -0.5720238 |
| Cstf1 | 3.8205512 |
| Cstf2 | 0.4853406 |
| Cstf3 | -0.4722992 |
| Cstf3 | 0.1996092 |
| Cstl1 | 0.2656555 |
| Ctage5 | 0.2234044 |
| Ctcf | 0.7777397 |
| Ctdp1 | -0.7854209 |
| Ctdp1 | -0.6875903 |
| Ctdp1 | 0.2546806 |
| Ctdsp1 | 1.4766978 |
| Ctdsp1 | 2.1550512 |
| Ctdsp1 | -0.7537483 |
| Ctdsp2 | -0.6355461 |
| Ctdspl | -0.5498606 |
| Ctdspl2 | -0.5593977 |
| Ctdspl2 | 0.2995045 |
| Ctf1 | -0.3615717 |
| Ctgf | -0.5098953 |
| Ctla4 | -0.5638338 |
| Ctnna1 | -0.2921042 |
| Ctnna3 | -0.1388039 |
| Ctnnal1 | -0.5001753 |
| Ctnnb1 | -0.6047745 |
| Ctnnb1 | 0.1259208 |
| Ctnnb1 | 0.8966784 |
| Ctnnbip1 | -0.561613 |
| Ctnnbl1 | 0.6414261 |
| Ctnnbl1 | -0.477671 |
| Ctnnd2 | -0.3971935 |
| Ctnnd2 | 0.1916422 |
| Ctnnd2 | -0.5353826 |
| Ctns | -0.4967292 |
| Ctns | -0.0453897 |
| Ctps | -0.6117828 |
| Ctps | -0.2240542 |
| Ctps | 0.5213322 |
| Ctps2 | -0.5425925 |
| Ctps2 | 0.9891376 |
| Ctps2 | 0.3340408 |
| Ctr9 | -0.560874 |
| Ctrb1 | -0.5601355 |
| Ctrl | 1.6800995 |
| Ctsa | -0.0841189 |
| Ctsa | -0.4058572 |
| Ctsa | -0.4851101 |
| Ctsa | -0.0815134 |
| Ctsb | 0.7646285 |
| Ctsc | 2.0158373 |
| Ctsc | 2.748384 |
| Ctsc | -0.6267599 |
| Ctse | -0.4743099 |
| Ctsf | 1.0451445 |
| Ctsh | -0.5126918 |
| Ctsk | 0.1823112 |
| Ctsl | -0.6978541 |
| Ctso | 0.3850306 |
| Ctsr | 0.021232 |
| Ctss | -0.6649161 |
| Ctsw | -0.0346236 |
| Ctsz | -0.1114942 |
| Ctsz | -0.1891745 |
| Cttn | -0.6987148 |
| Cttn | 1.2060276 |
| Cttnbp2nl | -0.4052356 |
| Ctxn1 | -0.0554432 |
| Cuedc1 | 0.3233208 |
| Cuedc2 | 0.3837388 |
| Cugbp1 | 1.3750918 |
| Cugbp1 | -0.4857895 |
| Cugbp1 | 2.0533182 |
| Cugbp1 | -0.4878306 |
| Cugbp2 | -0.287731 |
| Cugbp2 | -0.1684 |
| Cul1 | 1.1414574 |
| Cul2 | 1.2623861 |
| Cul2 | -0.5623526 |
| Cul3 | -0.5469463 |
| Cul4a | 0.6298965 |
| Cul4a | 0.0388528 |
| Cul4a | -0.628351 |
| Cul4b | 1.1886511 |
| Cul5 | -0.0845539 |
| Cul7 | 0.2992205 |
| Cuta | 0.7189567 |
| Cuta | -0.5848297 |
| Cuta | -0.5720238 |
| Cutc | -0.3645364 |
| Cutc | 0.1148336 |
| Cux1 | 0.6794455 |
| Cux1 | -0.278508 |
| Cux1 | -0.5310843 |
| Cux1 | 2.1580073 |
| Cux1 | 1.7791211 |
| Cux2 | 3.3093841 |
| Cux2 | 0.266834 |
| Cuzd1 | -0.2613993 |
| Cwf19l2 | 0.1603762 |
| Cx3cl1 | -0.2477341 |
| Cx3cr1 | 2.6127173 |
| Cxadr | 0.5699227 |
| Cxadr | -0.5218411 |
| Cxadr | -0.3159814 |
| Cxcl1 | 1.5132415 |
| Cxcl12 | -0.6841949 |
| Cxcl12 | -0.6952772 |
| Cxcl12 | 2.0022899 |
| Cxcl13 | -0.345729 |
| Cxcl14 | 0.022778 |
| Cxcl14 | -0.7437354 |
| Cxcl15 | 1.0139905 |
| Cxcl16 | -0.2860966 |
| Cxcl17 | 1.1706089 |
| Cxcl4 | -0.561613 |
| Cxcl9 | -0.5653173 |
| Cxcr3 | -0.7099784 |
| Cxcr4 | 0.3876088 |
| Cxcr5 | -0.682502 |
| Cxcr7 | -0.2608698 |
| Cxx1a | -0.4974174 |
| Cxx1b | -0.3627564 |
| Cxx1c | -0.5043282 |
| Cxxc1 | -0.5653173 |
| Cxxc1 | -0.5630929 |
| Cxxc4 | -0.4609894 |
| Cxxc4 | 3.0317621 |
| Cyb5 | 0.8687621 |
| Cyb561 | -0.1872216 |
| Cyb561d1 | 1.3171642 |
| Cyb561d1 | 1.8596351 |
| Cyb561d2 | 1.0200023 |
| Cyb5b | 0.6608266 |
| Cyb5d1 | 2.2634385 |
| Cyb5d2 | 1.5096976 |
| Cyb5d2 | -0.5978204 |
| Cyb5r1 | 0.7765708 |
| Cyb5r2 | -0.6141311 |
| Cyb5r3 | -0.6251716 |
| Cyb5r3 | 0.6907287 |
| Cyb5r3 | 2.8060286 |
| Cyb5r4 | 0.3598969 |
| Cyba | 0.0373342 |
| Cybasc3 | -0.4064792 |
| Cyc1 | -0.628351 |
| Cycs | 2.3300236 |
| Cycs | -0.597051 |
| Cyfip1 | 0.7598749 |
| Cyfip2 | 1.1678008 |
| Cyfip2 | 0.8443016 |
| Cygb | -0.5571878 |
| Cyhr1 | 0.3299328 |
| Cyhr1 | 3.0572642 |
| Cyhr1 | -0.6009045 |
| Cyhr1 | -0.6243784 |
| Cyld | -0.4932962 |
| Cyln2 | 3.6140505 |
| Cyp11a1 | -0.2451298 |
| Cyp11b2 | 0.1738625 |
| Cyp17a1 | -0.6816567 |
| Cyp19a1 | -0.4583488 |
| Cyp1b1 | -0.4743099 |
| Cyp20a1 | -0.6841949 |
| Cyp24a1 | -0.7258108 |
| Cyp26a1 | 1.2403044 |
| Cyp26b1 | -0.8045864 |
| Cyp27a1 | -0.5183111 |
| Cyp27a1 | -0.1411175 |
| Cyp27a1 | -0.527518 |
| Cyp2a12 | -0.628351 |
| Cyp2a5 | 2.1047821 |
| Cyp2a5 | -0.5029418 |
| Cyp2b10 | 0.0403689 |
| Cyp2c55 | 3.2917135 |
| Cyp2d10 | 1.1011363 |
| Cyp2d22 | -0.5513212 |
| Cyp2d26 | 0.0825366 |
| Cyp2d26 | -0.1693557 |
| Cyp2d26 | 1.3038722 |
| Cyp2e1 | 2.28816 |
| Cyp2f2 | -0.6724109 |
| Cyp2j11 | 1.4726214 |
| Cyp2j9 | -0.2347873 |
| Cyp2s1 | -0.4830752 |
| Cyp2u1 | -0.4736392 |
| Cyp39a1 | -0.2964988 |
| Cyp3a13 | 0.300923 |
| Cyp3a13 | -0.6674074 |
| Cyp46a1 | -0.4021339 |
| Cyp4b1 | 0.4538485 |
| Cyp4f13 | 0.765813 |
| Cyp4f14 | 1.1836697 |
| Cyp4f15 | 1.3628955 |
| Cyp4f16 | -0.6102207 |
| Cyp4f16 | -0.6674074 |
| Cyp4f16 | 1.3996683 |
| Cyp4f18 | 2.4452956 |
| Cyp4f39 | -0.5564524 |
| Cyp4v3 | 0.6092064 |
| Cyp51 | 0.1200478 |
| Cyp7b1 | -0.7108508 |
| Cypt10 | -0.6995762 |
| Cypt2 | -0.1101503 |
| Cypt3 | 0.8239259 |
| Cypt6 | 2.1032535 |
| Cypt8 | 0.1919622 |
| Cypt9 | 0.4536093 |
| Cyr61 | -0.2245624 |
| Cyr61 | -0.6476673 |
| Cysltr1 | -0.4247081 |
| Cytl1 | 0.466908 |
| D030011O10Rik | 1.5213838 |
| D030013I16Rik | 1.91722 |
| D030018L15Rik | 2.7732466 |
| D030028O16Rik | -0.2942988 |
| D030051N19Rik | 0.1245424 |
| D030056L22Rik | -0.7574171 |
| D030070L09Rik | -0.2317076 |
| D030070L09Rik | -0.3009149 |
| D030070L09Rik | -0.2926524 |
| D030070L09Rik | 0.1977035 |
| D030074E01Rik | 1.9708085 |
| D0H4S114 | 0.0774633 |
| D0H8S2298E | -0.0919811 |
| D0HXS9928E | -0.4465611 |
| D0HXS9928E | -0.7004385 |
| D10627 | -0.6039992 |
| D10Bwg1364e | -0.4452608 |
| D10Bwg1379e | 0.1116906 |
| D10Ertd322e | 0.5762075 |
| D10Ertd610e | 0.8329168 |
| D10Ertd610e | -0.640375 |
| D10Ertd610e | 1.0037941 |
| D10Ertd641e | -0.3978095 |
| D10Jhu81e | 3.2916931 |
| D10Wsu102e | 1.0364244 |
| D10Wsu52e | -0.2709829 |
| D11Bwg0517e | 1.9171259 |
| D11Wsu47e | -0.0617842 |
| D11Wsu47e | 0.4497733 |
| D12Ertd551e | 3.3466222 |
| D12Ertd551e | -0.6032245 |
| D12Ertd553e | -0.5382596 |
| D12Ertd647e | -0.2893685 |
| D12Ertd647e | -0.7134728 |
| D130040H23Rik | 0.0430158 |
| D130043K22Rik | -0.6032245 |
| D130059P03Rik | 1.975273 |
| D13Ertd608e | -0.6363491 |
| D14Ertd171e | 0.8984312 |
| D14Ertd231e | -0.4769978 |
| D14Ertd449e | -0.6892929 |
| D14Ertd500e | -0.6892929 |
| D14Ertd668e | -0.7564985 |
| D14Ertd668e | 2.0374105 |
| D15Ertd621e | -0.5353826 |
| D15Ertd682e | -0.6339422 |
| D15Mgi27 | -0.8202182 |
| D15Wsu169e | 0.535641 |
| D15Wsu75e | 0.7967284 |
| D16Ertd472e | -0.5303699 |
| D16H22S680E | 1.2003074 |
| D17H6S56E-5 | 0.7164593 |
| D17Wsu104e | 0.9532034 |
| D17Wsu92e | -0.6927076 |
| D19Bwg1357e | -0.593214 |
| D19Ertd721e | -0.5440414 |
| D19Ertd737e | -0.524675 |
| D19Wsu162e | 0.1422996 |
| D1Bwg0212e | 1.5004774 |
| D1Bwg0212e | 2.0138963 |
| D1Pas1 | -0.381927 |
| D230007K08Rik | 0.0392321 |
| D230014K01Rik | -0.6732475 |
| D2Ertd750e | 0.8846085 |
| D2Ertd750e | 0.1158789 |
| D330014H01Rik | -0.4058572 |
| D330017J20Rik | -0.3633494 |
| D330028D13Rik | 1.0441522 |
| D330045A20Rik | 0.0965204 |
| D330045A20Rik | 0.9298347 |
| D330045A20Rik | 0.5728611 |
| D3Bwg0562e | -0.5993611 |
| D3Ertd300e | -0.593214 |
| D3Ertd751e | 1.6266975 |
| D3Ucla1 | 1.7907799 |
| D430015B01Rik | -0.6315417 |
| D430028G21Rik | -0.64523 |
| D430039N05Rik | -0.5397016 |
| D430041B17Rik | -0.5253849 |
| D430042O09Rik | -0.7205024 |
| D4Bwg0951e | 0.0861429 |
| D4Bwg1540e | -0.1259561 |
| D4Ertd22e | -0.5197214 |
| D4Ertd22e | -0.671575 |
| D4Wsu114e | -0.368107 |
| D4Wsu132e | 3.4978666 |
| D4Wsu132e | -0.1679226 |
| D530004J12Rik | -0.7901742 |
| D530033C11Rik | 1.6925616 |
| D5Ertd135e | 3.5666226 |
| D5Ertd579e | 3.6724748 |
| D5Wsu178e | -0.3387764 |
| D630003M21Rik | -0.7464551 |
| D630003M21Rik | 0.3927431 |
| D630014A15Rik | -0.6841949 |
| D630023F18Rik | 0.1429756 |
| D630042P16Rik | -0.5520525 |
| D630048P19Rik | -0.242533 |
| D6Ertd474e | -0.560874 |
| D6Mm5e | 1.9505072 |
| D6Wsu116e | 0.2267969 |
| D6Wsu163e | -0.5454927 |
| D6Wsu163e | -0.6204233 |
| D6Wsu163e | -0.1476276 |
| D6Wsu176e | 2.3022809 |
| D6Wsu176e | -0.5579238 |
| D730039F16Rik | 0.1964308 |
| D730039F16Rik | 0.5958249 |
| D7Bwg0611e | -0.709107 |
| D830014E11Rik | -0.5513212 |
| D830030K20Rik | 0.0467838 |
| D830030K20Rik | -0.2672438 |
| D830050J10Rik | 0.735373 |
| D8Ertd738e | 1.8958023 |
| D8Ertd82e | 0.3427423 |
| D930001I22Rik | -0.6094407 |
| D930005D10Rik | -0.3722907 |
| D930014E17Rik | 1.5665978 |
| D930015E06Rik | -0.527518 |
| D930020B18Rik | 2.561868 |
| D930028F11Rik | -0.3716918 |
| D930049A15Rik | 0.9527914 |
| D9Ertd280e | 0.268011 |
| D9Ertd392e | 2.019161 |
| D9Ertd402e | -0.6164856 |
| D9Wsu20e | 1.2431939 |
| Daam1 | -0.5795346 |
| Daam2 | 0.8202725 |
| Dab1 | -0.640375 |
| Dab1 | 1.2086158 |
| Dab1 | 1.2497357 |
| Dab2 | -0.6188462 |
| Dab2 | -0.0802134 |
| Dab2ip | 0.5495108 |
| Dact1 | 1.6570348 |
| Dact2 | 0.6583529 |
| Dad1 | 1.5235718 |
| Daf2 | 0.6969569 |
| Dag1 | 1.3054516 |
| Dag1 | -0.628351 |
| Dagla | -0.1584261 |
| Daglb | 1.3759505 |
| Dak | -0.5498606 |
| Dalrd3 | 1.849297 |
| Dap | -0.6892929 |
| Dap3 | 1.2672419 |
| Dap3 | 0.0940264 |
| Dap3 | 0.2969454 |
| Dap3 | 0.0274002 |
| Dapk1 | -0.6927076 |
| Dapk1 | 1.0875208 |
| Dapk2 | -0.4381427 |
| Dapk2 | -0.4272516 |
| Dapl1 | 3.3028638 |
| Dapp1 | -0.6164856 |
| Dapp1 | -0.5705292 |
| Darc | -0.593214 |
| Dars | -0.6227943 |
| Dars | -0.5757714 |
| Daxx | -0.6541994 |
| Dazap1 | 2.0272714 |
| Dazap1 | -0.3651305 |
| Dazap2 | 0.5179977 |
| Dazl | -0.6032245 |
| Dbc1 | 1.7839337 |
| Dbf4 | -0.342246 |
| Dbi | 2.6792015 |
| Dbi | -0.7365227 |
| Dbil5 | -0.5520525 |
| Dbn1 | 0.035813 |
| Dbndd1 | -0.2715183 |
| Dbndd2 | -0.6172717 |
| Dbndd2 | 0.3277342 |
| Dbndd2 | -0.588631 |
| Dbnl | 0.6114174 |
| Dbp | -0.6141311 |
| Dbpht2 | 1.7612888 |
| Dbr1 | 0.8259844 |
| Dbt | 0.0289356 |
| Dcakd | -0.5282301 |
| Dcbld1 | 1.3795483 |
| Dcbld2 | 0.4936167 |
| Dci | 0.1640144 |
| Dck | 0.6636726 |
| Dclk1 | -0.2296603 |
| Dclk2 | 0.8698172 |
| Dclk3 | -0.4394327 |
| Dclre1b | 0.8674037 |
| Dclre1b | -0.64523 |
| Dclre1c | -0.5916838 |
| Dclre1c | -0.4749811 |
| Dclre1c | -0.5140934 |
| Dcn | -0.5653173 |
| Dcn | 0.6822332 |
| Dcp1a | 1.2118147 |
| Dcp1a | 1.2522012 |
| Dcp1a | 1.0702064 |
| Dcp1a | -0.5505906 |
| Dcp1b | 0.5301645 |
| Dcp1b | 1.2692736 |
| Dcpp1 | -0.5433166 |
| Dcpp2 | -0.7455476 |
| Dcpp2 | -0.2184828 |
| Dcpp3 | -0.8301278 |
| Dcps | -0.5571878 |
| Dcps | -0.7108508 |
| Dct | 0.9108942 |
| Dctd | 2.637621 |
| Dctn1 | 0.9884777 |
| Dctn2 | -0.0399905 |
| Dctn2 | 1.2358022 |
| Dctn2 | 0.4569525 |
| Dctn3 | -0.6492959 |
| Dctn3 | -0.6732475 |
| Dctn4 | 0.1679666 |
| Dctn4 | -0.1911316 |
| Dctn5 | 0.6168215 |
| Dctn6 | -0.128237 |
| Dcun1d2 | 0.6449144 |
| Dcun1d3 | -0.7222684 |
| Dcun1d4 | -0.0412336 |
| Dcun1d4 | 1.9309887 |
| Dcun1d5 | 1.9153838 |
| Dcx | 0.4699673 |
| Dcx | 0.4790833 |
| Dcxr | 1.1033469 |
| Dcxr | 0.1751675 |
| Ddah1 | -0.7338327 |
| Ddah2 | 0.0561352 |
| Ddb1 | 0.0531533 |
| Ddb2 | -0.7056296 |
| Ddb2 | 0.3211059 |
| Ddb2 | -0.5909196 |
| Ddc | -0.5833136 |
| Ddc8 | 1.0288822 |
| Ddef2 | -0.5057167 |
| Ddef2 | 0.0668624 |
| Ddefl1 | 0.25766 |
| Ddhd1 | 0.8180413 |
| Ddhd1 | -0.675762 |
| Ddi2 | -0.1541852 |
| Ddit3 | -0.2129456 |
| Ddit3 | 0.8281959 |
| Ddit4 | 1.9070507 |
| Ddit4l | -0.4478632 |
| Ddo | 1.9629753 |
| Ddo | 0.4396253 |
| Ddo | 2.9720381 |
| Ddost | 3.1399827 |
| Ddr1 | -0.3669152 |
| Ddr2 | 1.1498832 |
| Ddt | 0.7698622 |
| Ddx1 | -0.3758922 |
| Ddx1 | 1.5321267 |
| Ddx17 | 0.7397354 |
| Ddx17 | -0.1755926 |
| Ddx17 | 0.382704 |
| Ddx19a | -0.1641119 |
| Ddx19b | -0.4394327 |
| Ddx19b | -0.0441408 |
| Ddx20 | 0.1190073 |
| Ddx21 | -0.4629749 |
| Ddx24 | -0.5064118 |
| Ddx24 | 2.9669116 |
| Ddx24 | 0.3617534 |
| Ddx25 | 0.3824451 |
| Ddx26b | -0.7365227 |
| Ddx27 | -0.368107 |
| Ddx28 | 0.0433933 |
| Ddx3x | -0.1021284 |
| Ddx3y | -0.7240379 |
| Ddx4 | -0.5916838 |
| Ddx4 | 0.7168165 |
| Ddx41 | 0.36809 |
| Ddx46 | 0.6663206 |
| Ddx47 | -0.3428256 |
| Ddx47 | -0.2317076 |
| Ddx47 | 1.4648015 |
| Ddx49 | 3.2165434 |
| Ddx5 | -0.682502 |
| Ddx50 | -0.4152333 |
| Ddx50 | 3.3793198 |
| Ddx51 | 1.0446485 |
| Ddx52 | -0.4636377 |
| Ddx54 | 2.0208812 |
| Ddx58 | -0.6047745 |
| Ddx6 | 1.0813497 |
| Ddx6 | -0.3210602 |
| Ddx6 | -0.429164 |
| Ddx6 | -0.6640872 |
| Deadc1 | -0.6774423 |
| Deb1 | 0.1874713 |
| Decr1 | 0.8012917 |
| Decr2 | 1.8330732 |
| Decr2 | -0.1955507 |
| Dedd | 0.4769878 |
| Dedd | -0.6071046 |
| Dedd2 | -0.5924485 |
| Def6 | 0.8429058 |
| Def8 | 0.6176194 |
| Defb11 | 0.7119823 |
| Defb14 | -0.3527358 |
| Defb15 | -0.2149552 |
| Defb19 | 0.6277305 |
| Defb20 | 1.0440281 |
| Defb21 | -0.2215174 |
| Defb25 | -0.2839218 |
| Defb26 | 0.6631041 |
| Defb30 | -0.3114909 |
| Defb36 | 0.4595705 |
| Defb36 | -0.5303699 |
| Defb4 | -0.6395684 |
| Defcr20 | 2.467587 |
| Defcr6 | -0.6690721 |
| Defcr-rs7 | 1.2371052 |
| Degs1 | 0.8611894 |
| Degs2 | -0.6024505 |
| Dek | 0.2402404 |
| Dek | 0.1071311 |
| Dennd1a | -0.5346648 |
| Dennd1c | -0.141581 |
| Dennd2a | 1.0457642 |
| Dennd3 | -0.4177502 |
| Denr | -0.7510064 |
| Denr | 1.7927804 |
| Depdc1b | -0.1636367 |
| Depdc5 | 1.184838 |
| Depdc5 | -0.6699057 |
| Depdc6 | -0.6492959 |
| Depdc6 | 0.8489389 |
| Dera | -0.5757714 |
| Derl2 | 1.1776981 |
| Derl3 | 1.490255 |
| Des | -0.4439624 |
| Det1 | 1.7831697 |
| Dexi | -0.0889154 |
| Dexi | 2.4620336 |
| Dffa | 1.5126519 |
| Dfna5h | 0.3484073 |
| Dgat1 | 0.7962381 |
| Dgat2 | -0.6740849 |
| Dgcr2 | -0.0660365 |
| Dgcr2 | 2.162817 |
| Dgcr6 | 0.1800444 |
| Dgcr6 | 2.4063881 |
| Dgcr8 | 2.8312435 |
| Dgka | -0.7329379 |
| Dgka | 0.1500433 |
| Dgkb | -0.5750206 |
| Dgke | -0.7213849 |
| Dgkg | -0.4433139 |
| Dgkh | -0.7196206 |
| Dgki | 0.6249662 |
| Dgkk | 1.3586181 |
| Dgkq | 0.2920917 |
| Dgkz | 2.1560978 |
| Dgkz | 0.6152237 |
| Dgkz | 2.9041642 |
| Dguok | 3.35779 |
| Dhcr24 | -0.3109312 |
| Dhcr24 | 0.45337 |
| Dhcr24 | 2.5030067 |
| Dhcr7 | -0.0876048 |
| Dhdds | -0.5454927 |
| Dhdds | 1.052921 |
| Dhdh | 2.9601191 |
| Dhdh | -0.6574832 |
| Dhdh | 2.6220915 |
| Dhh | 0.6980515 |
| Dhodh | -0.3687035 |
| Dhps | 0.6518507 |
| Dhrs1 | 0.5711831 |
| Dhrs4 | 0.3516284 |
| Dhrs7 | -0.6640872 |
| Dhrs7 | 1.6456586 |
| Dhrs7 | -0.3758922 |
| Dhrs7b | -0.1877094 |
| Dhrs7b | -0.5825565 |
| Dhrs7b | 0.6827897 |
| Dhrsx | -0.4926112 |
| Dhrsx | 0.085783 |
| Dhtkd1 | 2.8293682 |
| Dhx15 | 0.5036295 |
| Dhx15 | 1.0375503 |
| Dhx16 | -0.4729689 |
| Dhx29 | -0.3783013 |
| Dhx30 | 0.6526181 |
| Dhx32 | 0.3612234 |
| Dhx33 | -0.1369572 |
| Dhx36 | -0.3568485 |
| Dhx37 | -0.0118637 |
| Dhx38 | -0.3795082 |
| Dhx57 | -0.5411459 |
| Dhx58 | -0.4355682 |
| Dhx8 | -0.661605 |
| Dhx9 | 1.3608903 |
| Diablo | -0.6379573 |
| Diap1 | -0.6978541 |
| Diap3 | 2.7273375 |
| Dicer1 | -0.4247081 |
| Dido1 | -0.3562598 |
| Dido1 | 1.3444188 |
| Dido1 | 1.0513218 |
| Dio1 | -0.5947468 |
| Dio1 | 1.7990773 |
| Dio3 | -0.4052356 |
| Dip2b | 0.069066 |
| Dip2b | 0.7139549 |
| Diras1 | 1.136543 |
| Diras2 | -0.2210109 |
| Dirc2 | -0.5720238 |
| Dis3 | 0.3326735 |
| Dis3l | 0.6562544 |
| Dis3l2 | 0.4956738 |
| Disp1 | 1.172225 |
| Dixdc1 | 1.8398132 |
| Dkk3 | 0.5520878 |
| Dkk3 | -0.4803691 |
| Dkk3 | -0.0313367 |
| Dkkl1 | -0.6251716 |
| Dkkl1 | 1.0409196 |
| Dlat | 1.7012732 |
| Dlc1 | -0.3451476 |
| Dld | -0.6492959 |
| Dleu7 | -0.6690721 |
| Dlg1 | 0.9139115 |
| Dlg2 | 1.4181205 |
| Dlg3 | 2.4576332 |
| Dlg4 | -0.5630929 |
| Dlg7 | -0.5353826 |
| Dlgap1 | -0.4114711 |
| Dlgap2 | -0.516903 |
| Dlgap2 | -0.7320439 |
| Dlgap2 | 0.2492927 |
| Dlgap3 | 0.183281 |
| Dlgap4 | -0.7987938 |
| Dlgap4 | -0.4008963 |
| Dlgap4 | 0.6549164 |
| Dlgap4 | 0.5749541 |
| Dlk1 | -0.53682 |
| Dlk2 | 0.0908097 |
| Dll1 | -0.0042705 |
| Dlst | 0.3876088 |
| Dlx1 | -0.685891 |
| Dlx1as | 0.4961304 |
| Dlx2 | 0.170264 |
| Dlx3 | 3.0528286 |
| Dmap1 | 1.5749615 |
| Dmbt1 | 0.4734844 |
| Dmkn | -0.5268064 |
| Dmkn | 1.1640068 |
| Dmkn | -0.7258108 |
| Dmn | 1.2493406 |
| Dmn | -0.6427992 |
| Dmpk | 1.0006735 |
| Dmrt2 | 0.0052302 |
| Dmrt3 | -0.1994972 |
| Dmrta1 | 0.4744199 |
| Dmrta2 | 0.3394891 |
| Dmrtc1a | 1.5778465 |
| Dmrtc1a | 0.8376129 |
| Dmrtc1a | 0.6379243 |
| Dmrtc1b | -0.0802134 |
| Dmrtc2 | -0.7638738 |
| Dmtf1 | -0.6987148 |
| Dmtf1 | -0.7347285 |
| Dmtf1 | 2.1199272 |
| Dmwd | 0.11239 |
| Dmwd | 2.2479407 |
| Dmwd | 0.3313042 |
| Dnahc11 | 0.2638849 |
| Dnahc2 | -0.5705292 |
| Dnahc5 | 1.0611182 |
| Dnahc8 | -0.1755926 |
| Dnaic1 | 0.2492927 |
| Dnaic1 | 1.3225541 |
| Dnaja1 | -0.5765228 |
| Dnaja2 | 0.2495928 |
| Dnaja2 | -0.7240379 |
| Dnaja3 | -0.5848297 |
| Dnajb1 | -0.4400784 |
| Dnajb10 | -0.6347438 |
| Dnajb12 | 0.1725556 |
| Dnajb13 | 0.6245706 |
| Dnajb3 | 1.2809867 |
| Dnajb4 | -0.287731 |
| Dnajb4 | 3.0295242 |
| Dnajb5 | 1.5214568 |
| Dnajb6 | -0.2693785 |
| Dnajb6 | 0.2065622 |
| Dnajb7 | 1.3156052 |
| Dnajb8 | 4.1834573 |
| Dnajb9 | 0.7287014 |
| Dnajc10 | 1.3678458 |
| Dnajc10 | 0.5690813 |
| Dnajc12 | 0.5919355 |
| Dnajc14 | -0.4374984 |
| Dnajc15 | -0.6331414 |
| Dnajc16 | -0.1717494 |
| Dnajc17 | -0.5939801 |
| Dnajc18 | 1.239106 |
| Dnajc19 | -0.5787807 |
| Dnajc19 | -0.5780274 |
| Dnajc2 | 1.6570976 |
| Dnajc2 | -0.2624591 |
| Dnajc3a | -0.7178598 |
| Dnajc4 | 0.3824451 |
| Dnajc5 | -0.5057167 |
| Dnajc5b | -0.1921118 |
| Dnajc5g | -0.2871859 |
| Dnajc6 | 0.6273361 |
| Dnajc6 | -0.4400784 |
| Dnajc7 | -0.3752909 |
| Dnajc8 | 0.0825366 |
| Dnalc1 | 0.9548496 |
| Dnali1 | 1.5564717 |
| Dnase1l1 | -0.2440902 |
| Dnase1l2 | -0.5339476 |
| Dnase1l2 | -0.1843002 |
| Dnase2a | 3.4050302 |
| Dnd1 | 0.2375069 |
| Dner | -0.5549834 |
| Dnm | 0.2399371 |
| Dnm1 | 0.6516587 |
| Dnm1l | -0.7731788 |
| Dnm1l | 0.7535546 |
| Dnmbp | -0.4304412 |
| Dnmt1 | -0.0191168 |
| Dnmt3b | -0.1264118 |
| Dnmt3l | -0.6204233 |
| Dnmt3l | -0.0470575 |
| Dnttip1 | -0.232733 |
| Doc2a | -0.2124439 |
| Doc2b | 1.1683415 |
| Doc2b | -0.6460417 |
| Dock1 | -0.7374211 |
| Dock3 | -0.24565 |
| Dock4 | -0.630743 |
| Dock6 | 0.025092 |
| Dock8 | 0.1948374 |
| Dock9 | -0.1012414 |
| Dok1 | -0.3550836 |
| Dok1 | -0.4743099 |
| Dok2 | -0.7047623 |
| Dok3 | 1.4520362 |
| Dok4 | -0.536101 |
| Dok5 | 1.8360121 |
| Dok5 | -0.2817524 |
| Dok7 | 0.5548711 |
| Dolk | 0.1797201 |
| Dolpp1 | 0.5211103 |
| Dolpp1 | 2.0515375 |
| Dolpp1 | 1.7774196 |
| Dom3z | -0.3081377 |
| Dom3z | -0.7125979 |
| Donson | -0.7428306 |
| Donson | 2.7760939 |
| Dos | -0.1364961 |
| Dos | -0.4342837 |
| Dot1l | -0.0972611 |
| Dpagt1 | 0.6476182 |
| Dpep1 | 0.7795734 |
| Dpep2 | 0.6901771 |
| Dpep2 | 2.0901543 |
| Dpep3 | 1.9893605 |
| Dpf1 | -0.0512406 |
| Dpf1 | 1.5016724 |
| Dpf3 | 0.6872294 |
| Dph2 | -0.5382596 |
| Dph2 | -0.1490288 |
| Dph2 | -0.5462192 |
| Dph3 | 2.2076121 |
| Dph3 | -0.2250709 |
| Dph3 | -0.3451476 |
| Dph4 | 0.1793957 |
| Dpm1 | -0.6657458 |
| Dpm1 | 1.3346768 |
| Dpm2 | -0.0074598 |
| Dpm3 | -0.1703124 |
| Dpp10 | -0.6699057 |
| Dpp3 | -0.5947468 |
| Dpp3 | -0.7284769 |
| Dpp4 | -0.3807168 |
| Dpp6 | 3.45041 |
| Dpp7 | 0.5103932 |
| Dpp8 | -0.6649161 |
| Dpp9 | -0.588631 |
| Dpp9 | -0.2174735 |
| Dppa3 | 0.5795416 |
| Dppa4 | 0.8675547 |
| Dppa5 | 1.2869761 |
| Dppa5 | 2.0731121 |
| Dpt | 1.4760837 |
| Dpy30 | -0.5289428 |
| Dpyd | -0.5772748 |
| Dpysl2 | -0.3959626 |
| Dpysl2 | 1.1819675 |
| Dpysl2 | 1.2641372 |
| Dpysl3 | -0.1731887 |
| Dpysl4 | 1.2945246 |
| Dpysl5 | 1.1909734 |
| Drbp1 | -0.629945 |
| Drctnnb1a | -0.5955143 |
| Drd1a | -0.5993611 |
| Drd2 | -0.1499641 |
| Drd4 | -0.7021655 |
| Drd5 | 0.9094539 |
| Drg1 | 1.8331249 |
| Drg1 | -0.5535167 |
| Drg2 | -0.1911316 |
| Drp2 | 3.6311468 |
| Dscam | -0.5638338 |
| Dscam | 1.551189 |
| Dscc1 | 0.6079981 |
| Dscr1 | 0.2920917 |
| Dscr1l2 | -0.5491311 |
| Dscr2 | 3.4364813 |
| Dscr3 | -0.5712762 |
| Dscr3 | 1.3662855 |
| Dscr6 | -0.2306834 |
| Dsg2 | 0.116575 |
| Dsg2 | -0.358027 |
| Dsn1 | -0.6978541 |
| Dsp | -0.5557176 |
| Dst | -0.685891 |
| Dst | 0.594803 |
| Dst | 1.003015 |
| Dstn | 0.3894092 |
| Dtd1 | -0.0724529 |
| Dtl | -0.2850085 |
| Dtna | -0.5469463 |
| Dtna | 1.0726144 |
| Dtna | -0.6740849 |
| Dtna | 1.2855536 |
| Dtnb | 2.0718455 |
| Dtnbp1 | -0.3734896 |
| Dtwd1 | 1.5400291 |
| Dtx1 | -0.5871086 |
| Dtx2 | 0.8916934 |
| Dtx2 | -0.5484022 |
| Dtx2 | -0.6117828 |
| Dtx4 | 0.4367047 |
| Dtx4 | -0.2466914 |
| Dtymk | -0.0841189 |
| Dus1l | 1.7444076 |
| Dus1l | -0.4749811 |
| Dus2l | -0.5239657 |
| Dus3l | -0.3740896 |
| Dus3l | 1.7611769 |
| Dus3l | 0.5838989 |
| Dus4l | 0.1715742 |
| Dusp1 | 2.5011406 |
| Dusp11 | 1.2872603 |
| Dusp11 | 0.2140866 |
| Dusp13 | 1.2956515 |
| Dusp13 | 0.1992918 |
| Dusp13 | 0.0697993 |
| Dusp14 | -0.5064118 |
| Dusp15 | 0.0312339 |
| Dusp15 | 3.1322276 |
| Dusp16 | 1.9065749 |
| Dusp16 | 0.845231 |
| Dusp16 | 0.4739523 |
| Dusp18 | 0.2555755 |
| Dusp19 | -0.261929 |
| Dusp2 | 2.4247688 |
| Dusp2 | 0.209705 |
| Dusp21 | -0.3064657 |
| Dusp22 | 0.9753109 |
| Dusp23 | -0.329589 |
| Dusp26 | 0.3593657 |
| Dusp28 | 1.4265172 |
| Dusp3 | 1.5316937 |
| Dusp3 | -0.1518377 |
| Dusp3 | -0.5176068 |
| Dusp4 | -0.640375 |
| Dusp4 | -0.3515642 |
| Dusp4 | -0.4374984 |
| Dusp6 | -0.0849891 |
| Dusp6 | 0.3591 |
| Dusp6 | -0.5593977 |
| Dusp7 | -0.0880414 |
| Dusp8 | 0.8172432 |
| Dut | 0.5501557 |
| Duxbl | 3.1134203 |
| Duxbl | -0.3109312 |
| Dvl1 | -0.3165443 |
| Dvl1 | -0.5757714 |
| Dvl2 | -0.3267371 |
| DXBwg1396e | -0.7125979 |
| Dydc2 | -0.2306834 |
| Dym | -0.7169807 |
| Dync1h1 | 0.7191348 |
| Dync1i1 | -0.6259654 |
| Dync1i1 | -0.1994972 |
| Dync1i2 | 0.737993 |
| Dync1li1 | -0.521134 |
| Dync1li2 | 2.3948448 |
| Dync2li1 | 0.0437707 |
| Dynll1 | -0.1434372 |
| Dynll2 | 0.0028644 |
| Dynlrb1 | -0.5176068 |
| Dynlrb2 | -0.1555966 |
| Dynlt3 | 1.8025033 |
| Dyrk1a | -0.5454927 |
| Dyrk1b | 0.1563904 |
| Dyrk1b | 0.268305 |
| Dyrk2 | 4.1550675 |
| Dyrk2 | 0.1334648 |
| Dyrk3 | -0.6331414 |
| Dyrk4 | -0.1052395 |
| Dysf | 0.8937494 |
| Dysfip1 | -0.5878695 |
| Dzip1 | 2.1191008 |
| E030010A14Rik | -0.5855887 |
| E030034P13Rik | -0.3187994 |
| E130009J12Rik | -0.2915564 |
| E130012A19Rik | -0.2342733 |
| E130014J05Rik | 2.5757068 |
| E130016E03Rik | 0.3870938 |
| E130203B14Rik | -0.448515 |
| E130203B14Rik | 0.1327816 |
| E130303B06Rik | -0.8667342 |
| E130306D19Rik | 0.7096454 |
| E130308A19Rik | -0.7073666 |
| E130309D02Rik | -0.5542498 |
| E130309D02Rik | -0.4304412 |
| E130309D14Rik | 1.260534 |
| E130309F12Rik | -0.6909986 |
| E130309F12Rik | 1.7559594 |
| E130309F12Rik | -0.4394327 |
| E230008N13Rik | 2.6624294 |
| E2f1 | 1.1434601 |
| E2f2 | 0.9014905 |
| E2f2 | -0.6086613 |
| E2f3 | -0.6227943 |
| E2f4 | -0.0999127 |
| E2f5 | 2.9915446 |
| E2f6 | -0.479019 |
| E2f6 | 0.7595344 |
| E2f6 | -0.815304 |
| E330016A19Rik | -0.0690251 |
| E330017A01Rik | 0.882383 |
| E330018D03Rik | -0.6371529 |
| E330021D16Rik | 0.3559055 |
| E330034G19Rik | -0.6196344 |
| E330034G19Rik | -0.7143485 |
| E330036I19Rik | 1.9705869 |
| E430002D04Rik | -0.5840713 |
| E430002G05Rik | -0.6315417 |
| E430018J23Rik | -0.4452608 |
| E430025E21Rik | 1.1023005 |
| E430028B21Rik | -0.7592572 |
| E4f1 | 1.1249327 |
| Eaf1 | -0.0391628 |
| Ear11 | 0.2582547 |
| Ear2 | -0.1119427 |
| Ear4 | -0.0915425 |
| Ears2 | 2.5057252 |
| Ebag9 | 0.3633418 |
| Ebf2 | 0.3839973 |
| Ebf3 | -0.2540153 |
| Ebf4 | 1.5462303 |
| Ebi2 | 0.7683459 |
| Ebi3 | -0.6387625 |
| Ebna1bp2 | 2.585271 |
| Ebna1bp2 | 1.0660977 |
| Ebp | -0.4504732 |
| Ebpl | -0.4885119 |
| Ebpl | 0.9726274 |
| Ecd | 0.2567672 |
| Ecd | 0.0501615 |
| Ece1 | 1.0580683 |
| Ece1 | 1.9055749 |
| Ece2 | -0.6624316 |
| Ecel1 | 1.8740105 |
| Ech1 | -0.7482729 |
| Echdc1 | 0.6640513 |
| Echdc2 | 1.5668755 |
| Echdc2 | -0.6476673 |
| Echdc2 | 0.1715742 |
| Echdc3 | 0.1919622 |
| Echs1 | 1.789263 |
| Ecm1 | 0.0886588 |
| Ecm1 | -0.4960416 |
| Ecsit | 0.2438723 |
| Eda2r | 1.5630492 |
| Edaradd | 0.700964 |
| Edc4 | 0.228642 |
| Edem1 | 2.0686714 |
| Edem2 | -0.5085003 |
| Edem2 | 0.7309894 |
| Edem2 | 1.1950755 |
| Edem2 | -0.7694452 |
| Edf1 | -0.7564985 |
| Edg2 | -0.4609894 |
| Edg5 | 1.2330901 |
| Edg7 | 1.3953106 |
| Edg8 | 0.2932361 |
| Edg8 | -0.0199263 |
| Edil3 | 1.3126632 |
| Edn1 | 0.3375859 |
| Edn2 | -0.3807168 |
| Ednra | -0.4905592 |
| Ednrb | 2.2013918 |
| Ednrb | 1.0298915 |
| Ednrb | -0.7419268 |
| Eed | 0.5490806 |
| Eef1a1 | -0.8094422 |
| Eef1a1 | 1.3716486 |
| Eef1a2 | 0.9265877 |
| Eef1a2 | -0.5440414 |
| Eef1a2 | 1.4061655 |
| Eef1b2 | -0.425979 |
| Eef1b2 | 1.0071627 |
| Eef1b2 | 1.0991538 |
| Eef1d | 2.8736888 |
| Eef1d | 0.7795734 |
| Eef1d | 0.43353 |
| Eef1d | 1.1621582 |
| Eef1e1 | 1.0769328 |
| Eef1g | -0.659954 |
| Eef2 | 0.0304685 |
| Eef2 | 0.7013274 |
| Eef2 | 0.4104859 |
| Eef2k | 0.0568791 |
| Eefsec | 0.1231618 |
| Efcab1 | -0.2378776 |
| Efcab2 | -0.269913 |
| Efcab2 | 2.680189 |
| Efcab3 | -0.6657458 |
| Efcab3 | 1.2386062 |
| Efcab3 | 0.0411259 |
| Efcab4a | 0.9961031 |
| Efcab4a | -0.5564524 |
| Efcab5 | -0.67074 |
| Efemp2 | 0.819317 |
| Efha1 | 1.9346405 |
| Efha1 | -0.671575 |
| Efhc2 | 0.5980691 |
| Efhd1 | 0.6108149 |
| Efna1 | 1.3353057 |
| Efna3 | -0.7997566 |
| Efna4 | -0.7240379 |
| Efna4 | 0.0300855 |
| Efna5 | 1.1948656 |
| Efnb1 | 0.9540269 |
| Efnb2 | -0.1555966 |
| Efnb3 | -0.2555925 |
| Efr3a | -0.7528334 |
| Efs | -0.1985089 |
| Eftud1 | 0.8884531 |
| Eftud2 | 0.6530015 |
| Eftud2 | -0.6016772 |
| Eftud2 | -0.4190112 |
| EG194588 | 0.1964308 |
| EG208426 | -0.675762 |
| EG210155 | 0.1507135 |
| EG212225 | 1.7417822 |
| EG214321 | 0.4758215 |
| EG232599 | 0.5475732 |
| EG237009 | -0.399043 |
| EG237009 | -0.5057167 |
| EG237300 | 0.1954751 |
| EG238829 | 1.3710447 |
| EG240916 | 2.037287 |
| EG245297 | -0.4139775 |
| EG245297 | 2.6819399 |
| EG245376 | -0.7108508 |
| EG245376 | -0.5593977 |
| EG272350 | -0.5353826 |
| EG277333 | 1.175663 |
| EG317677 | -0.1999917 |
| EG328264 | -0.5955143 |
| EG328280 | 0.0936696 |
| EG328644 | -0.8513002 |
| EG328839 | 0.7868713 |
| EG330305 | -0.6427992 |
| EG330503 | -0.6918527 |
| EG330513 | -0.5353826 |
| EG333669 | 0.0486619 |
| EG368203 | -0.3330233 |
| EG380907 | -0.4669592 |
| EG381438 | 1.7841518 |
| EG382109 | 1.8547583 |
| EG382109 | -0.7284769 |
| EG382109 | -0.6427992 |
| EG384639 | 0.1279845 |
| EG384813 | 3.2616519 |
| EG384813 | 1.8070864 |
| EG384814 | -0.7713101 |
| EG385328 | 0.7157445 |
| EG408196 | 0.1487014 |
| EG432825 | 0.4480899 |
| EG432879 | 1.1630286 |
| EG432939 | 0.7018723 |
| EG433016 | 1.9247139 |
| EG433182 | -0.5133923 |
| EG433224 | 2.2404191 |
| EG433923 | 2.5061408 |
| EG434171 | 1.2996778 |
| EG434197 | 0.528405 |
| EG434280 | -0.6525619 |
| EG434459 | 2.4760176 |
| EG434459 | -0.3783013 |
| EG434858 | 0.6126211 |
| EG435391 | -0.6315417 |
| EG436332 | -0.3416668 |
| EG545047 | 1.9719156 |
| EG545056 | 0.1659926 |
| EG545253 | 1.5909863 |
| EG545370 | 1.3021036 |
| EG545758 | 1.7135065 |
| EG545861 | -0.7047623 |
| EG545963 | -0.1707912 |
| EG546166 | 2.758744 |
| EG546282 | 1.2644287 |
| EG546325 | 1.4012553 |
| EG546347 | -0.5757714 |
| EG547109 | -0.7939949 |
| EG574403 | 1.7786823 |
| EG622320 | -0.1833286 |
| EG622339 | -0.6969943 |
| EG624866 | 1.3006116 |
| EG625054 | -0.5645752 |
| EG625424 | 0.3310301 |
| EG630499 | 0.5707632 |
| EG633640 | 0.9854359 |
| EG638695 | 2.2114526 |
| EG654453 | -0.6243784 |
| EG665186 | 0.4461621 |
| EG665378 | -0.2871859 |
| EG667977 | 2.8795507 |
| EG668668 | -0.0042705 |
| Egf | 1.2016628 |
| Egfbp2 | -0.8401478 |
| Egfl7 | 1.7690854 |
| Egfl7 | 0.205302 |
| Egflam | 1.5542215 |
| Egflam | 1.2338945 |
| Egfr | -0.7365227 |
| Egfr | -0.5091975 |
| Egfr | 0.4369484 |
| Egfr | -0.5325148 |
| Egln1 | 0.0399901 |
| Egln1 | -0.4596681 |
| Egln1 | -0.7685143 |
| Egln2 | 0.1826346 |
| Egln3 | 1.048609 |
| Egr1 | 0.2405437 |
| Egr2 | -0.3347454 |
| Egr3 | 2.1974047 |
| Egr4 | -0.6071046 |
| Ehbp1 | 1.7619597 |
| Ehbp1l1 | -0.5962823 |
| Ehd1 | -0.7222684 |
| Ehd2 | 0.3521642 |
| Ehd3 | 0.2995045 |
| Ehd4 | 1.1343002 |
| Ehmt1 | -0.6558398 |
| Ehmt2 | -0.4139775 |
| Ehmt2 | -0.630743 |
| Ei24 | -0.2399437 |
| Eid1 | 1.0805157 |
| Eid2 | 2.7515901 |
| Eid3 | -0.7528334 |
| Eif1a | -0.2019725 |
| Eif1b | 0.4557599 |
| Eif2a | 1.5750991 |
| Eif2ak1 | -0.5780274 |
| Eif2ak2 | -0.5855887 |
| Eif2b1 | 1.7882322 |
| Eif2b1 | 2.6001453 |
| Eif2b2 | 0.3790727 |
| Eif2b4 | 1.6367741 |
| Eif2b5 | -0.5225487 |
| Eif2b5 | 0.8966784 |
| Eif2b5 | -0.5382596 |
| Eif2c2 | 0.5505854 |
| Eif2c3 | -0.6371529 |
| Eif2c3 | -0.3892226 |
| Eif2c3 | -0.6884412 |
| Eif2c3 | 0.6693375 |
| Eif2c4 | 1.3353057 |
| Eif2s2 | 0.206877 |
| Eif2s2 | -0.7311508 |
| Eif2s3x | 0.336769 |
| Eif2s3y | -0.6690721 |
| Eif3b | 0.1460114 |
| Eif3b | -0.470293 |
| Eif3d | 0.4848786 |
| Eif3eip | -0.1268678 |
| Eif3eip | 0.2756239 |
| Eif3f | -0.6961353 |
| Eif3g | 1.007809 |
| Eif3i | 0.103961 |
| Eif3i | 1.2727458 |
| Eif3i | 0.4276394 |
| Eif3k | -0.0134704 |
| Eif3s1 | -0.3764939 |
| Eif3s10 | -0.3347454 |
| Eif3s10 | -0.5232569 |
| Eif3s4 | 1.2131528 |
| Eif3s8 | 2.8057133 |
| Eif4a1 | 0.1406073 |
| Eif4a1 | 0.7898398 |
| Eif4a2 | -0.5317993 |
| Eif4a3 | -0.4803691 |
| Eif4b | 1.8782934 |
| Eif4e | -0.1999917 |
| Eif4e1b | -0.6110014 |
| Eif4e1b | 0.5606247 |
| Eif4e2 | -0.6875903 |
| Eif4e2 | -0.5404234 |
| Eif4e3 | -0.3284471 |
| Eif4e3 | 0.5128605 |
| Eif4e3 | 0.9281421 |
| Eif4ebp1 | -0.3621639 |
| Eif4ebp2 | 2.4282137 |
| Eif4el3 | 1.4829687 |
| Eif4enif1 | -0.2189878 |
| Eif4enif1 | -0.2545407 |
| Eif4g1 | 0.3020562 |
| Eif4g2 | -0.3347454 |
| Eif4g2 | 0.1613699 |
| Eif4h | -0.6492959 |
| Eif5 | -0.0478926 |
| Eif5 | -0.5085003 |
| Eif5 | 0.872074 |
| Eif5a | -0.470293 |
| Eif5a | -0.7863696 |
| Eif6 | 0.2030924 |
| Ela1 | 3.1302265 |
| Ela2 | -0.3521498 |
| Ela2a | 1.3783506 |
| Ela3b | -0.6164856 |
| Elac2 | 2.6929896 |
| Elac2 | 2.7935242 |
| Elac2 | 0.8681586 |
| Elavl1 | -0.429164 |
| Elavl2 | -0.3428256 |
| Elavl2 | -0.5901561 |
| Elavl3 | 2.1511793 |
| Elavl4 | 3.8559529 |
| Elavl4 | -0.0424784 |
| Elf1 | 1.8531436 |
| Elf2 | 0.0553906 |
| Elf2 | 1.0277454 |
| Elf2 | -0.7196206 |
| Elf2 | -0.1780029 |
| Elf2 | 2.411969 |
| Elf3 | -0.4636377 |
| Elf3 | 1.4596545 |
| Elf3 | 1.5067337 |
| Elf5 | -0.8261509 |
| Elfn1 | -0.368107 |
| Elfn2 | -0.4317203 |
| Elk3 | 2.0264386 |
| Elk3 | 1.9057654 |
| Elk3 | 0.6324497 |
| Ell | 0.3040359 |
| Ell3 | 0.0642848 |
| Elmo1 | 0.7855488 |
| Elmo1 | -0.2420145 |
| Elmo1 | 0.4471265 |
| Elmo1 | -0.1164393 |
| Elmo1 | -0.1945668 |
| Elmo1 | 1.3482332 |
| Elmo1 | 2.3809632 |
| Elmo1 | 0.0961645 |
| Elmo1 | -0.5593977 |
| Elmo1 | 0.6394823 |
| Elmo1 | -0.7873192 |
| Elmo1 | -0.329589 |
| Elmo1 | -0.1494963 |
| Elmo2 | -0.4796938 |
| Elmo2 | -0.4400784 |
| Elmo3 | 1.0297654 |
| Elmo3 | -0.5962823 |
| Elmod1 | -0.8341224 |
| Eln | -0.644419 |
| Elof1 | -0.5239657 |
| Elovl1 | -0.7759892 |
| Elovl1 | 0.4432627 |
| Elovl1 | -0.4946678 |
| Elovl1 | -0.3047969 |
| Elovl2 | -0.7178598 |
| Elovl4 | 0.3545711 |
| Elovl5 | 1.0788455 |
| Elovl6 | 0.6487745 |
| Elp2 | 0.94645 |
| Elp3 | -0.6799686 |
| Elp3 | 1.1918164 |
| Eltd1 | 1.1195846 |
| Emb | -0.6766018 |
| Emcn | 1.6231829 |
| Emd | -0.0342121 |
| Emd | -0.412097 |
| Emg1 | 1.8712923 |
| Emg1 | 0.1344886 |
| Emid1 | -0.4330011 |
| Emid2 | -0.0647587 |
| Emilin1 | 1.3155134 |
| Emilin2 | 0.1874713 |
| Eml1 | -0.597051 |
| Eml3 | -0.588631 |
| Eml3 | -0.090666 |
| Eml4 | -0.470293 |
| Eml5 | 1.4417238 |
| Emp1 | -0.644419 |
| Emp2 | -0.6141311 |
| Emp3 | -0.6918527 |
| Emr1 | -0.3928927 |
| Emr1 | 1.991918 |
| Emr1 | 1.2782162 |
| En2 | 3.4665002 |
| Enah | 0.6439468 |
| Enc1 | 0.7242851 |
| Endod1 | 1.5139045 |
| Endogl1 | -0.5645752 |
| Endogl1 | 0.7807384 |
| Eng | 4.0242371 |
| Eno1 | -0.4433139 |
| Eno2 | -0.5727721 |
| Eno3 | 1.668703 |
| Enoph1 | 0.5097191 |
| Enox1 | 0.1897196 |
| Enpp1 | 0.1890778 |
| Enpp1 | 1.9129298 |
| Enpp2 | 2.4191054 |
| Enpp4 | -0.2394267 |
| Enpp5 | -0.3503942 |
| Enpp6 | 0.0620695 |
| Ensa | 2.3079653 |
| Ensa | -0.0911042 |
| ENSMUSG00000043795 | -0.7013016 |
| ENSMUSG00000053178 | -0.8677726 |
| ENSMUSG00000068790 | -0.5871086 |
| Entpd2 | 0.968722 |
| Entpd3 | 2.0817323 |
| Entpd4 | 0.9110381 |
| Entpd5 | -0.0981441 |
| Entpd5 | 1.203536 |
| Entpd5 | 0.0900933 |
| Entpd6 | 0.6400659 |
| Entpd7 | 1.9702766 |
| Ep300 | -0.3675109 |
| Ep400 | -0.4796938 |
| Epas1 | -0.7392207 |
| Epb4.1 | 0.9794546 |
| Epb4.1l1 | 0.9004719 |
| Epb4.1l1 | 0.5550849 |
| Epb4.1l1 | 1.9199437 |
| Epb4.1l2 | 0.8410413 |
| Epb4.1l3 | 0.6396769 |
| Epb4.1l3 | 0.259443 |
| Epb4.1l3 | 0.9071445 |
| Epb4.1l4a | -0.6583061 |
| Epb4.1l5 | -0.2430518 |
| Epb4.1l5 | -0.0086587 |
| Epb4.9 | -0.6550192 |
| Epb4.9 | 0.2510922 |
| Epc1 | 1.0174489 |
| Epc1 | 0.5512296 |
| Epc1 | 1.4876812 |
| Epdr1 | -0.7592572 |
| Epgn | 1.0494731 |
| Epha1 | 1.4263549 |
| Epha1 | -0.5527843 |
| Epha3 | 0.4984094 |
| Epha3 | -0.5232569 |
| Epha3 | 1.412369 |
| Epha4 | -0.5557176 |
| Epha5 | 0.3174951 |
| Epha6 | 0.2368983 |
| Epha7 | 0.0847021 |
| Epha7 | -0.1607908 |
| Epha7 | 2.6649939 |
| Ephb1 | -0.4722992 |
| Ephb2 | 2.1195141 |
| Ephb4 | 1.0772918 |
| Ephb6 | 0.280277 |
| Ephx1 | -0.6039992 |
| Ephx2 | -0.4209062 |
| Epm2a | 0.1580533 |
| Epm2aip1 | 1.3933764 |
| Epm2aip1 | 0.7885216 |
| Epm2aip1 | -0.4974174 |
| Epn1 | -0.6133477 |
| Epn2 | 0.5286251 |
| Epn2 | -0.6995762 |
| Epn2 | 1.1038116 |
| Epn2 | -0.5571878 |
| Epn2 | 0.9180616 |
| Epor | 0.5597746 |
| Eprs | -0.6808123 |
| Eprs | -0.5623526 |
| Eps15 | 0.3598969 |
| Eps15l1 | -0.4939818 |
| Eps8 | 1.399919 |
| Eps8l1 | -0.5535167 |
| Eps8l2 | 0.08362 |
| Eps8l2 | -0.5339476 |
| Eps8l3 | 1.8521835 |
| Epsti1 | -0.6133477 |
| Epsti1 | 1.888694 |
| Eraf | 0.725347 |
| Eral1 | 0.2872117 |
| Ercc1 | 1.6366456 |
| Ercc2 | 0.1067795 |
| Ercc3 | 0.1709194 |
| Ercc3 | 1.8313674 |
| Ercc4 | -0.685891 |
| Ercc4 | 2.1545094 |
| Ercc5 | -0.0453897 |
| Ercc5 | -0.5520525 |
| Ercc8 | -0.4709612 |
| Ercc8 | 0.5803732 |
| Erdr1 | -0.4981061 |
| Erf | -0.7099784 |
| Erf | 0.0008882 |
| Ergic1 | 1.1038116 |
| Ergic2 | 0.8587526 |
| Ergic2 | 0.0646535 |
| Ergic3 | 0.1395904 |
| Ergic3 | 2.1794164 |
| Erh | -0.2981523 |
| Erich1 | -0.6509274 |
| Erlin1 | 1.1366549 |
| Erlin2 | -0.5036347 |
| Erlin2 | 2.3393981 |
| Ermap | -0.5787807 |
| Ermp1 | -0.661605 |
| Ern1 | 1.2645259 |
| Ern2 | 1.4773882 |
| Ero1lb | -0.5469463 |
| Erp29 | -0.4583488 |
| Errfi1 | 1.8790298 |
| Es22 | -0.5978204 |
| Esam1 | 1.6981588 |
| Esco2 | 0.2966606 |
| Esd | -0.1177927 |
| Esd | 1.6664685 |
| Esd | 1.8627385 |
| Esd | -0.7275873 |
| Esm1 | -0.3047969 |
| Espl1 | -0.2513928 |
| Espn | 1.1637895 |
| Espn | 1.8224758 |
| Espn | 0.059478 |
| Espn | -0.6078826 |
| Esr1 | 0.0749159 |
| Esr2 | 0.5604122 |
| Esr2 | -0.3142948 |
| Esr2 | -0.3364707 |
| Esrra | -0.6204233 |
| Esrrb | 0.7602153 |
| Etaa1 | 1.1960195 |
| Etf1 | 2.4172727 |
| Etfa | -0.4127234 |
| Etfa | 1.5835208 |
| Etfa | -0.3428256 |
| Etfb | 0.8084162 |
| Etfb | 1.5046531 |
| Etfdh | 0.6071916 |
| Ethe1 | 0.016189 |
| Etnk2 | -0.0968199 |
| Ets1 | 1.1906571 |
| Ets2 | -0.345729 |
| Etsrp71 | -0.3307323 |
| Etv1 | -0.5916838 |
| Etv1 | 2.977758 |
| Etv4 | -0.3047969 |
| Etv5 | -0.6808123 |
| Etv6 | -0.7004385 |
| Evc | -0.4228051 |
| Evc2 | -0.0763248 |
| Evi1 | 0.9779865 |
| Evi1 | 0.5972537 |
| Evi2a | -0.5282301 |
| Evi2a | -0.47163 |
| Evi5 | -0.0854245 |
| Evi5l | -0.7056296 |
| Evl | -0.5871086 |
| Evx1 | -0.2855524 |
| Exdl1 | -0.671575 |
| Exdl2 | -0.7978319 |
| Exdl2 | 1.1838822 |
| Exdl2 | 1.439727 |
| Exoc2 | 0.0008882 |
| Exoc3 | -0.3795082 |
| Exoc3l | -0.2059473 |
| Exoc4 | 1.7130935 |
| Exoc7 | -0.64523 |
| Exoc8 | -0.569037 |
| Exod1 | -0.4215387 |
| Exosc1 | 0.4384095 |
| Exosc10 | 1.1646583 |
| Exosc2 | 0.0972317 |
| Exosc2 | -0.6808123 |
| Exosc4 | -0.0272449 |
| Exosc5 | -0.4133502 |
| Exosc6 | 1.7562407 |
| Exosc7 | -0.2959483 |
| Exosc8 | -0.2860966 |
| Ext1 | -0.8133458 |
| Ext2 | 0.0628085 |
| Extl1 | 2.1441895 |
| Extl2 | 0.2677169 |
| Extl3 | 1.526263 |
| Extl3 | 2.7832 |
| Eya1 | -0.5353826 |
| Eya1 | 0.1722286 |
| Eya2 | -0.4783448 |
| Eya3 | -0.4253433 |
| Eya3 | 1.4285435 |
| Eya3 | -0.6978541 |
| Eya4 | -0.5985904 |
| Ezh1 | 2.9945182 |
| Ezh2 | 1.0663399 |
| Ezh2 | -0.7302586 |
| F10 | -0.5085003 |
| F11 | -0.6071046 |
| F11 | -0.4362112 |
| F11r | -0.6259654 |
| F13a1 | -0.6850426 |
| F2r | 0.9250306 |
| F2rl1 | 0.0591072 |
| F2rl3 | -0.342246 |
| F430201B04Rik | 0.4758215 |
| F5 | 1.5929442 |
| F630043A04Rik | -0.7455476 |
| F730014I05Rik | 0.0800035 |
| F730047E07Rik | 0.7272906 |
| F730047E07Rik | 0.0107262 |
| F730047E07Rik | -0.3734896 |
| F8 | 0.2147108 |
| F8 | 1.6884469 |
| F8 | -0.6347438 |
| F8a | 1.4974079 |
| Fa2h | -0.0613601 |
| Faah | 0.0752802 |
| Faah | 0.4102349 |
| Fabp3 | -0.715225 |
| Fabp5 | 1.2492417 |
| Fabp9 | 2.0565469 |
| Fadd | 2.135043 |
| Fadd | 1.6144678 |
| Fadd | 1.7831697 |
| Fads1 | 0.6182173 |
| Fads2 | 0.9799879 |
| Fads3 | -0.6363491 |
| Fads6 | 0.2519907 |
| Faf1 | 1.0105186 |
| Faf1 | -0.53682 |
| Fah | -0.685891 |
| Fahd1 | 0.7031423 |
| Fahd2a | 0.2659503 |
| Fahd2a | -0.3307323 |
| Fahd2a | 1.0680335 |
| Fahd2a | 0.880003 |
| Faim | -0.334171 |
| Faim | -0.5469463 |
| Faim2 | -0.2921042 |
| Faim2 | 1.6315561 |
| Faim3 | 2.5384062 |
| Fancc | 1.1439046 |
| Fancc | -0.6987148 |
| Fancd2 | 0.4067132 |
| Fancg | -0.6566611 |
| Fanci | 0.2326267 |
| Fancl | 0.6480038 |
| Fancl | -0.5447667 |
| Fank1 | 0.1000712 |
| Fank1 | -0.2550665 |
| Fap | -0.3399314 |
| Fars2 | -0.0211419 |
| Farsa | -0.6566611 |
| Farsb | -0.4368546 |
| Farsb | -0.3746901 |
| Fas | 0.1412846 |
| Fas | 0.1741889 |
| Fasl | -0.398426 |
| Fastk | 0.8313459 |
| Fastk | -0.6291476 |
| Fastkd1 | 0.5045343 |
| Fastkd5 | 0.6400659 |
| Fat1 | 0.9727618 |
| Fat4 | 0.0861429 |
| Fbf1 | -0.0395766 |
| Fblim1 | -0.6969943 |
| Fbln1 | 0.1228163 |
| Fbln1 | 0.1513833 |
| Fbln1 | -0.5232569 |
| Fbln2 | 0.2606296 |
| Fbln2 | -0.659954 |
| Fbn1 | -0.6355461 |
| Fbp1 | 2.0965351 |
| Fbp2 | 0.4420519 |
| Fbxl10 | 0.3014898 |
| Fbxl12 | 1.0315292 |
| Fbxl12 | 0.7873667 |
| Fbxl12 | 0.5927558 |
| Fbxl12 | 1.7392637 |
| Fbxl13 | 0.1144849 |
| Fbxl13 | -0.2948483 |
| Fbxl14 | 0.0071969 |
| Fbxl15 | 0.8963859 |
| Fbxl18 | -0.2661783 |
| Fbxl18 | 1.1316015 |
| Fbxl21 | 0.8373006 |
| Fbxl22 | 0.3177734 |
| Fbxl4 | -0.7816364 |
| Fbxl5 | -0.5863483 |
| Fbxl6 | -0.6009045 |
| Fbxl6 | -0.4919267 |
| Fbxl8 | -0.1318984 |
| Fbxo15 | 0.3062932 |
| Fbxo16 | -0.6799686 |
| Fbxo18 | -0.1074683 |
| Fbxo18 | 0.2534861 |
| Fbxo2 | 0.9263048 |
| Fbxo21 | 0.7533832 |
| Fbxo25 | -0.1916215 |
| Fbxo27 | -0.1443667 |
| Fbxo28 | 0.4449552 |
| Fbxo3 | -0.5404234 |
| Fbxo3 | 0.8174029 |
| Fbxo30 | 2.0321276 |
| Fbxo31 | 1.3520314 |
| Fbxo31 | 0.4246796 |
| Fbxo32 | 1.2658849 |
| Fbxo32 | -0.4285261 |
| Fbxo32 | -0.4769978 |
| Fbxo33 | 0.0990074 |
| Fbxo34 | -0.685891 |
| Fbxo34 | -0.6331414 |
| Fbxo34 | 0.376991 |
| Fbxo36 | 0.0672301 |
| Fbxo38 | 0.3062932 |
| Fbxo38 | -0.5454927 |
| Fbxo4 | -0.5085003 |
| Fbxo42 | -0.0034749 |
| Fbxo44 | 2.4068795 |
| Fbxo6 | -0.2629895 |
| Fbxo7 | -0.6952772 |
| Fbxo8 | -0.5447667 |
| Fbxo9 | 0.2629982 |
| Fbxo9 | -0.0110613 |
| Fbxw12 | 1.7579827 |
| Fbxw13 | -0.6961353 |
| Fbxw15 | 0.3040359 |
| Fbxw16 | 0.3532347 |
| Fbxw17 | 0.8106713 |
| Fbxw19 | -0.5712762 |
| Fbxw2 | 2.0882895 |
| Fbxw4 | 0.9924299 |
| Fbxw4 | -0.1921118 |
| Fbxw5 | -0.4190112 |
| Fbxw7 | 0.3006395 |
| Fbxw7 | -0.2373618 |
| Fbxw7 | 0.8477046 |
| Fbxw8 | -0.5433166 |
| Fcer1g | 0.8335443 |
| Fcer2a | 0.1053713 |
| Fcf1 | -0.7491831 |
| Fcgr2b | 2.5629013 |
| Fcgr2b | 0.1597131 |
| Fcgr2b | -0.5916838 |
| Fcgr3 | -0.7021655 |
| Fcgr4 | 2.1709422 |
| Fcgrt | -0.5133923 |
| Fcho1 | -0.7473636 |
| Fcho2 | -0.1065761 |
| Fchsd1 | 0.1172706 |
| Fchsd2 | 0.8617976 |
| Fcna | 0.6749671 |
| Fcrla | 2.1093145 |
| Fcrla | 0.4320609 |
| Fcrla | 2.7953871 |
| Fdps | 1.2499332 |
| Fdxr | -0.6016772 |
| Fdxr | 2.0729143 |
| Fem1b | 2.9098003 |
| Fem1c | -0.4152333 |
| Fem1c | 1.6238348 |
| Fen1 | -0.5557176 |
| Fermt2 | 1.5843383 |
| Fermt3 | 0.3247023 |
| Fert2 | 1.4370845 |
| Fert2 | -0.5513212 |
| Fes | 2.1060031 |
| Fetub | -0.4987953 |
| Fev | 0.3596313 |
| Fez1 | 0.3696672 |
| Fez1 | 0.3500193 |
| Fez2 | -0.6952772 |
| Fezf2 | 2.7723379 |
| Ffar2 | -0.4603285 |
| Fgd2 | -0.7258108 |
| Fgd4 | 2.6807126 |
| Fgd4 | -0.7741146 |
| Fgd5 | -0.5939801 |
| Fgd6 | 0.7671647 |
| Fgf1 | -0.4362112 |
| Fgf1 | 3.0586938 |
| Fgf10 | -0.2144524 |
| Fgf10 | -0.7365227 |
| Fgf12 | 1.2174624 |
| Fgf12 | 0.8232916 |
| Fgf13 | 0.2624067 |
| Fgf13 | 1.5990604 |
| Fgf17 | 1.4410853 |
| Fgf21 | 2.4277071 |
| Fgf22 | 1.0271132 |
| Fgf22 | 0.882977 |
| Fgf5 | 0.8696665 |
| Fgf7 | -0.6125649 |
| Fgf8 | 0.6965917 |
| Fgf9 | 0.7970551 |
| Fgfbp3 | -0.0305169 |
| Fgfr1 | -0.3928927 |
| Fgfr1op | -0.5218411 |
| Fgfr1op2 | -0.6833481 |
| Fgfr1op2 | 0.4776868 |
| Fgfr2 | 0.0542727 |
| Fgfr2 | -0.6492959 |
| Fgfr3 | -0.5601355 |
| Fgfrl1 | -0.5780274 |
| Fgl2 | 1.0553758 |
| Fh1 | 0.1241974 |
| Fhad1 | 2.2191857 |
| Fhit | 2.9905464 |
| Fhl1 | 2.0586795 |
| Fhl1 | 0.6336256 |
| Fhl2 | 1.3389799 |
| Fhl4 | -0.7338327 |
| Fhl5 | 1.4944783 |
| Fhod1 | 2.5894695 |
| Fhod3 | -0.4729689 |
| Fibcd1 | 1.6872331 |
| Fibp | 0.3575042 |
| Figf | -0.7997566 |
| Figla | -0.5638338 |
| Fign | -0.6204233 |
| Fignl1 | -0.7178598 |
| Fip1l1 | -0.2311954 |
| Fis1 | 1.7153331 |
| Fis1 | -0.6607792 |
| Fiz1 | -0.3159814 |
| Fjx1 | 0.9604515 |
| Fkbp10 | 2.1023351 |
| Fkbp11 | 0.4480899 |
| Fkbp14 | -0.5404234 |
| Fkbp1a | 1.1217504 |
| Fkbp1b | 0.3580364 |
| Fkbp2 | -0.3639427 |
| Fkbp2 | -0.1731887 |
| Fkbp4 | -0.3813217 |
| Fkbp5 | 0.0281682 |
| Fkbp6 | 1.2009332 |
| Fkbp7 | 1.9733308 |
| Fkbp8 | 0.7787404 |
| Fkbp9 | -0.3710933 |
| Fkbpl | 0.7276435 |
| Fkbpl | -0.3789045 |
| Fkhl18 | -0.0613601 |
| Fkrp | 1.3460175 |
| Fktn | -0.169834 |
| Flad1 | -0.0537598 |
| Flcn | 1.9761104 |
| Flcn | -0.3574375 |
| Fli1 | -0.3047969 |
| Flii | 1.3218252 |
| Flna | 2.722642 |
| Flot1 | 0.2724109 |
| Flot1 | 0.2221676 |
| Flot2 | -0.4960416 |
| Flot2 | 1.7574211 |
| Flot2 | 0.9887418 |
| Flrt1 | 1.5570333 |
| Flrt2 | -0.2693785 |
| Flrt3 | -0.663259 |
| Flt1 | 0.1540572 |
| Flt4 | 1.3260085 |
| Flt4 | -0.6347438 |
| Flvcr2 | 1.7350121 |
| Flywch2 | 0.7358976 |
| Fmn2 | -0.1141882 |
| Fmn2 | -0.261929 |
| Fmnl1 | 0.3224908 |
| Fmnl3 | 1.5524597 |
| Fmnl3 | 0.7624246 |
| Fmo1 | -0.5742705 |
| Fmo2 | 1.2811774 |
| Fmo5 | -0.547674 |
| Fmod | -0.5332309 |
| Fmr1nb | 0.6503139 |
| Fmr1nb | -0.7143485 |
| Fn3k | 1.6139408 |
| Fn3k | -0.0668894 |
| Fnbp1 | 0.9496263 |
| Fnbp1 | 1.7598333 |
| Fnbp1 | 1.2194053 |
| Fnbp1l | 2.4703382 |
| Fnbp4 | -0.6227943 |
| Fndc3 | -0.1527759 |
| Fndc3a | 1.7354152 |
| Fndc3b | 0.2323208 |
| Fndc4 | 3.5933581 |
| Fndc5 | 2.061889 |
| Fndc5 | -0.4796938 |
| Fndc7 | -0.569037 |
| Fndc7 | -0.4127234 |
| Fndc8 | -0.3675109 |
| Fndc8 | -0.3267371 |
| Fnip1 | -0.6533803 |
| Fnip1 | -0.7169807 |
| Fnta | -0.6476673 |
| Fnta | -0.2571724 |
| Fntb | -0.4164908 |
| Folr1 | 0.8919874 |
| Folr1 | 0.9633073 |
| Folr2 | 2.1221396 |
| Fos | 1.4024229 |
| Fosb | -0.6791257 |
| Foxa1 | 2.2287179 |
| Foxa2 | -0.5727721 |
| Foxa3 | -0.165539 |
| Foxc1 | -0.4590082 |
| Foxd2 | 2.1386172 |
| Foxg1 | 0.1813404 |
| Foxh1 | 0.8220216 |
| Foxh1 | 0.6221934 |
| Foxj1 | -0.0106605 |
| Foxj2 | -0.8074967 |
| Foxj3 | 1.5231346 |
| Foxj3 | -0.5947468 |
| Foxj3 | 2.4993446 |
| Foxj3 | 0.8319746 |
| Foxj3 | 0.4645476 |
| Foxk1 | -0.4465611 |
| Foxk1 | 0.0028644 |
| Foxk1 | 1.4155779 |
| Foxl2 | -0.629945 |
| Foxm1 | -0.3109312 |
| Foxm1 | -0.1622127 |
| Foxn2 | 3.0954946 |
| Foxn3 | -0.4960416 |
| Foxo1 | 1.8429345 |
| Foxo6 | -0.5253849 |
| Foxo6 | -0.64523 |
| Foxp1 | -0.451781 |
| Foxp2 | -0.4769978 |
| Foxp2 | -0.6808123 |
| Foxp2 | 0.4598082 |
| Foxp3 | 0.4122411 |
| Foxp4 | 1.506214 |
| Foxq1 | 1.4222879 |
| Foxred1 | -0.7401218 |
| Foxred1 | -0.0090587 |
| Foxred1 | -0.2758132 |
| Foxred1 | 0.2889371 |
| Foxred1 | -0.1209586 |
| Foxred2 | 0.9660187 |
| Fpgs | 0.5724419 |
| Fpgs | 0.4449552 |
| Fpgs | -0.4095959 |
| Fpr2 | -0.5204274 |
| Frag1 | 0.070532 |
| Frag1 | 1.7833335 |
| Frag1 | -0.7374211 |
| Frap1 | 3.3336397 |
| Fras1 | 2.5312087 |
| Fras1 | -0.5015575 |
| Frat1 | 0.8595148 |
| Frat1 | -0.479019 |
| Frat2 | 0.8337011 |
| Freq | 1.8380174 |
| Freq | 0.2977992 |
| Freq | -0.5375395 |
| Frg1 | -0.7629486 |
| Frk | -0.2291492 |
| Frmd3 | -0.64523 |
| Frmd6 | -0.4215387 |
| Frmd8 | 2.2599747 |
| Frmpd4 | -0.5119919 |
| Frrs1 | -0.2603407 |
| Frs2 | 1.2662729 |
| Frs3 | 1.4453863 |
| Fryl | -0.33245 |
| Fryl | 0.1043138 |
| Frzb | 0.3777722 |
| Fscn1 | 3.6997656 |
| Fscn2 | -0.2513928 |
| Fscn3 | -0.7030303 |
| Fsd1 | 0.4590951 |
| Fsd2 | 1.107405 |
| Fshb | 0.2773716 |
| Fshr | 3.3623884 |
| Fshr | -0.5645752 |
| Fshr | -0.3947333 |
| Fsip1 | 0.6855671 |
| Fst | -0.2430518 |
| Fstl1 | -0.89842 |
| Fstl4 | -0.0499836 |
| Fstl5 | -0.6558398 |
| Fth1 | -0.709107 |
| Fth1 | 1.0849151 |
| Ftmt | 0.9340525 |
| Fto | -0.2693785 |
| Ftsj1 | -0.3807168 |
| Ftsj3 | -0.7258108 |
| Fubp1 | -0.7240379 |
| Fubp3 | 0.435729 |
| Fuk | 3.0079543 |
| Fuk | 1.5704091 |
| Fundc1 | -0.3187994 |
| Fundc1 | 2.1799434 |
| Fundc2 | 0.791156 |
| Furin | -0.6850426 |
| Furin | -0.761101 |
| Fus | 1.876868 |
| Fusip1 | 2.4332373 |
| Fusip1 | -0.4763251 |
| Fusip1 | -0.5901561 |
| Fut10 | 1.3858524 |
| Fut10 | 0.5384757 |
| Fut2 | -0.3261678 |
| Fut4 | 0.4702022 |
| Fut8 | 0.0719957 |
| Fut8 | -0.4967292 |
| Fut8 | 0.3521642 |
| Fvt1 | -0.6339422 |
| Fxc1 | -0.2144524 |
| Fxn | 0.0266314 |
| Fxn | -0.4133502 |
| Fxyd1 | 2.3223211 |
| Fxyd2 | 3.5587131 |
| Fxyd2 | 0.0048363 |
| Fxyd2 | -0.7920825 |
| Fxyd3 | -0.6525619 |
| Fxyd4 | -0.4743099 |
| Fxyd4 | -0.5840713 |
| Fxyd5 | 2.0061327 |
| Fxyd5 | 0.0142418 |
| Fxyd5 | 0.0738219 |
| Fxyd6 | 0.5211103 |
| Fxyd7 | -0.569037 |
| Fyb | -0.6395684 |
| Fyco1 | -0.5260954 |
| Fyn | 2.744454 |
| Fyttd1 | 0.0527799 |
| Fzd1 | -0.1804198 |
| Fzd10 | -0.8341224 |
| Fzd10 | -0.5091975 |
| Fzd2 | -0.7222684 |
| Fzd3 | 1.2815588 |
| Fzd4 | -0.7482729 |
| Fzd5 | -0.6574832 |
| Fzd6 | -0.2513928 |
| Fzd7 | -0.560874 |
| Fzd8 | 0.8911052 |
| Fzr1 | 1.5277869 |
| Fzr1 | 0.6172205 |
| Fzr1 | 1.0370501 |
| G0s2 | -0.7428306 |
| G3bp1 | -0.3486419 |
| G3bp1 | -0.709107 |
| G3bp2 | 0.0672301 |
| G3bp2 | -0.1141882 |
| G3bp2 | 1.7127984 |
| G430022H21Rik | 0.9510038 |
| G430055L02Rik | -0.8123683 |
| G6pc | -0.0207365 |
| G6pc2 | 0.3912059 |
| G6pc2 | -0.7410238 |
| G6pc3 | 0.7288776 |
| G6pd2 | -0.3892226 |
| G6pd2 | -0.3971935 |
| G6pdx | -0.3474756 |
| Gaa | 0.0262468 |
| Gab1 | 2.0610877 |
| Gab2 | 1.4686038 |
| Gab2 | 1.6155207 |
| Gab2 | 1.8130731 |
| Gab3 | -0.7258108 |
| Gab3 | -0.2332462 |
| Gab3 | -0.6969943 |
| Gabarap | -0.176556 |
| Gabarapl1 | 1.094941 |
| Gabarapl2 | 0.457429 |
| Gabbr1 | -0.4817211 |
| Gabpa | -0.6850426 |
| Gabpa | -0.7882699 |
| Gabpb1 | 0.0118996 |
| Gabpb1 | 0.2659503 |
| Gabpb2 | -0.4171203 |
| Gabra3 | -0.1551259 |
| Gabra4 | 2.3647499 |
| Gabrb1 | -0.3813217 |
| Gabrb2 | -0.7056296 |
| Gabrb3 | -0.6944198 |
| Gabrb3 | -0.165539 |
| Gabrd | -0.6204233 |
| Gabrd | -0.2225313 |
| Gabrg1 | 2.019077 |
| Gabrg2 | -0.3405095 |
| Gabrg2 | -0.7464551 |
| Gabrg2 | -0.4374984 |
| Gabrg2 | -0.2039577 |
| Gabrp | -0.4221717 |
| Gabrp | -0.569037 |
| Gad1 | -0.6833481 |
| Gad1 | -0.5601355 |
| Gadd45a | 1.5423149 |
| Gadd45a | 0.7632729 |
| Gadd45b | -0.2394267 |
| Gadd45g | 1.5253184 |
| Gadd45g | 1.0484855 |
| Gak | -0.0622086 |
| Gal | 0.4581434 |
| Gal3st1 | -0.2260887 |
| Gal3st2 | 0.1375532 |
| Gal3st3 | 0.7383418 |
| Galc | -0.47163 |
| Galc | 0.2027763 |
| Gale | -0.5705292 |
| Gale | -0.7108508 |
| Galk1 | -0.4472119 |
| Galm | 2.8116856 |
| Galns | 0.7490866 |
| Galnt1 | 0.3291089 |
| Galnt1 | -0.2855524 |
| Galnt1 | 0.9503153 |
| Galnt1 | -0.3801123 |
| Galnt10 | 0.6172205 |
| Galnt10 | 0.2735806 |
| Galnt10 | 0.7334469 |
| Galnt11 | -0.3740896 |
| Galnt12 | -0.5418689 |
| Galnt13 | -0.6363491 |
| Galnt2 | 1.2531856 |
| Galnt2 | 1.7018709 |
| Galnt2 | -0.5557176 |
| Galnt3 | 1.2693702 |
| Galnt9 | -0.5112925 |
| Galntl2 | 0.7978714 |
| Galntl4 | -0.3003617 |
| Galntl5 | 2.880438 |
| Galt | 1.355199 |
| Galt | -0.3064657 |
| Gamt | 2.986471 |
| Gan | -0.7601786 |
| Ganab | -0.5346648 |
| Ganab | 1.0757354 |
| Ganab | 0.9319461 |
| Ganc | 1.5450219 |
| Gap43 | -0.2440902 |
| Gapdh | 1.0376753 |
| Gapdhs | -0.2731265 |
| Gapvd1 | 2.7192151 |
| Gapvd1 | 0.6787006 |
| Garnl3 | -0.1731887 |
| Garnl4 | -0.5893933 |
| Gars | 1.9305254 |
| Gart | -0.573521 |
| Gart | -0.8729827 |
| Gas2l1 | 0.2918054 |
| Gas2l1 | 2.9934238 |
| Gas2l1 | 1.8906822 |
| Gas2l1 | 2.1817329 |
| Gas6 | -0.561613 |
| Gas7 | 0.8204316 |
| Gas7 | -0.4939818 |
| Gas8 | -0.2109405 |
| Gata2 | -0.2524409 |
| Gata4 | -0.5955143 |
| Gata6 | -0.5375395 |
| Gatad1 | -0.5947468 |
| Gatad1 | -0.3064657 |
| Gatad2b | 1.6239 |
| Gats | -0.5675473 |
| Gats | 3.8876536 |
| Gba | -0.0668894 |
| Gba2 | 1.4064145 |
| Gbas | 1.9769911 |
| Gbf1 | -0.675762 |
| Gbl | -0.3855674 |
| Gbl | -0.3941194 |
| Gbl | -0.3556715 |
| Gbp2 | -0.1612645 |
| Gbp3 | -0.7021655 |
| Gbp3 | -0.4563734 |
| Gbp6 | 1.5609552 |
| Gbp6 | 2.5816598 |
| Gbx2 | 2.3087266 |
| Gca | -0.2866411 |
| Gca | -0.7311508 |
| Gcap14 | 0.8068019 |
| Gcap14 | 0.8791089 |
| Gcc1 | -0.6009045 |
| Gcc2 | -0.4609894 |
| Gcdh | 0.5782928 |
| Gch1 | -0.7629486 |
| Gch1 | 2.9231384 |
| Gchfr | 1.1049724 |
| Gck | -0.6395684 |
| Gclm | 0.2271047 |
| Gcn5l2 | 1.2499332 |
| Gcnt1 | 3.744626 |
| Gcnt1 | -0.64523 |
| Gcnt2 | 2.0288099 |
| Gcnt2 | -0.176556 |
| Gcs1 | -0.451781 |
| Gda | 0.3965747 |
| Gdap1 | -0.7537483 |
| Gdap1 | 0.6400659 |
| Gdap1l1 | 1.6423451 |
| Gdap1l1 | -0.7491831 |
| Gde1 | 1.7041386 |
| Gde1 | 1.2773547 |
| Gdf10 | -0.6550192 |
| Gdf15 | 0.9220505 |
| Gdf3 | 0.7456345 |
| Gdf9 | 2.385726 |
| Gdi1 | 0.6013232 |
| Gdi1 | 0.9588155 |
| Gdi1 | 1.0612399 |
| Gdi1 | 0.8111538 |
| Gdi1 | 3.7230033 |
| Gdi2 | -0.3307323 |
| Gdnf | -0.3801123 |
| Gdpd1 | 1.2446849 |
| Gdpd2 | 1.4142628 |
| Gdpd2 | 0.4308349 |
| Gdpd3 | 0.1557244 |
| Gdpd4 | -0.350979 |
| Gdpd5 | -0.3928927 |
| Gemin4 | -0.4544024 |
| Gemin4 | 0.6104131 |
| Gemin4 | 0.2030924 |
| Gemin5 | 1.0952928 |
| Gemin6 | -0.7178598 |
| Gemin6 | -0.661605 |
| Gemin7 | 0.2043557 |
| Gemin7 | -0.7750514 |
| Gfap | 0.0293191 |
| Gfer | -0.5863483 |
| Gfi1 | -0.4803691 |
| Gfm1 | -0.2149552 |
| Gfod1 | -0.6110014 |
| Gfod2 | 0.8373006 |
| Gfpt2 | -0.2286384 |
| Gfra1 | -0.3278767 |
| Gfra4 | -0.4253433 |
| Gga1 | 1.5520363 |
| Gga1 | 0.0965204 |
| Gga1 | -0.4898763 |
| Gga3 | -0.141581 |
| Ggcx | -0.0375095 |
| Ggcx | 0.0270159 |
| Ggcx | -0.8361264 |
| Ggn | -0.6591297 |
| Ggn | 2.3735164 |
| Ggnbp1 | -0.5085003 |
| Ggnbp2 | 1.22114 |
| Ggt5 | 0.9628999 |
| Ggt7 | -0.6094407 |
| Ggta1 | 0.499092 |
| Ggtla1 | 0.1935607 |
| Gh | -0.7108508 |
| Ghitm | -0.6427992 |
| Ghr | 0.1169229 |
| Ghr | -0.3947333 |
| Ghrh | -0.4722992 |
| Ghrhr | 1.1983229 |
| Ghrl | -0.7134728 |
| Ghrl | -0.675762 |
| Ghsr | 0.9243219 |
| Ghsr | -0.2667109 |
| Gif | -0.4769978 |
| Gigyf1 | 3.7637316 |
| Gimap1 | 0.6025405 |
| Gimap4 | -0.5225487 |
| Gimap5 | -0.6875903 |
| Gimap6 | 2.8759766 |
| Gimap8 | -0.4342837 |
| Gimap9 | -0.5601355 |
| Gins3 | 1.4952304 |
| Gins4 | -0.5043282 |
| Gipc1 | 0.1666511 |
| Gipc1 | -0.078051 |
| Gipc1 | 0.9540269 |
| Gipc2 | -0.4133502 |
| Git1 | -0.2704477 |
| Git2 | -0.6379573 |
| Git2 | 0.3046008 |
| Git2 | 0.3958097 |
| Gja1 | 0.2344599 |
| Gja4 | 0.6678303 |
| Gja5 | -0.3204944 |
| Gja5 | 3.1529398 |
| Gja6 | 0.2952353 |
| Gja7 | -0.7082364 |
| Gjb1 | 0.1613699 |
| Gjb2 | -0.6039992 |
| Gjb2 | -0.6492959 |
| Gjb3 | 1.5542215 |
| Gjb6 | 2.0421305 |
| Gjc2 | 1.4089003 |
| Gjc2 | 0.2438723 |
| Gjd2 | 0.6861215 |
| Gjd2 | 0.4340191 |
| Gjd3 | -0.7056296 |
| Gk2 | -0.7117239 |
| Gk5 | -0.5638338 |
| Gkap1 | 1.5289462 |
| Gkap1 | 1.6392117 |
| Glb1 | 1.4186118 |
| Glb1l2 | -0.7258108 |
| Glcci1 | -0.2169692 |
| Gldc | 0.9508661 |
| Gldc | -0.644419 |
| Gldn | -0.1617385 |
| Gle1 | -0.0776191 |
| Glg1 | 0.619213 |
| Glipr2 | 1.6669034 |
| Glis1 | 1.9077164 |
| Glo1 | -0.7500943 |
| Gloxd1 | 2.1894466 |
| Glp2r | 0.1419614 |
| Glra2 | 1.9689898 |
| Glrb | 0.7995017 |
| Glrp1 | -0.6657458 |
| Glrx | -0.640375 |
| Glrx2 | 1.0234378 |
| Glrx2 | -0.5645752 |
| Glrx2 | -0.7004385 |
| Glrx3 | 0.0763724 |
| Glrx5 | -0.7196206 |
| Gls2 | 2.0609273 |
| Gls2 | 1.6577881 |
| Glt25d1 | 0.1980214 |
| Glt25d2 | -0.5078036 |
| Glt25d2 | 0.3738595 |
| Glt6d1 | 1.1582309 |
| Glt8d1 | -0.0272449 |
| Glt8d1 | -0.3740896 |
| Glt8d1 | 0.3141491 |
| Glt8d2 | 1.4816722 |
| Glt8d3 | 0.0115087 |
| Gltp | 0.9280009 |
| Gltscr1 | -0.6969943 |
| Glud1 | 3.0562701 |
| Glud1 | 0.1169229 |
| Glul | -0.5871086 |
| Glyctk | 0.6923816 |
| Glyctk | -0.4967292 |
| Glyctk | -0.7968711 |
| Gm1006 | -0.5498606 |
| Gm1006 | -0.5491311 |
| Gm1019 | -0.6117828 |
| Gm1040 | 0.6398714 |
| Gm1070 | 3.7662113 |
| Gm1082 | -0.0166926 |
| Gm114 | -0.3728899 |
| Gm114 | 0.6090051 |
| Gm1141 | -0.6550192 |
| Gm128 | 2.0021187 |
| Gm129 | -0.2134476 |
| Gm129 | -0.0317469 |
| Gm1322 | -0.9081475 |
| Gm136 | 1.3665458 |
| Gm136 | 0.8633162 |
| Gm1381 | -0.7073666 |
| Gm1527 | -0.5317993 |
| Gm1568 | 1.0002826 |
| Gm1574 | 0.791156 |
| Gm1574 | -0.5204274 |
| Gm1604 | -0.5947468 |
| Gm1604 | -0.7500943 |
| Gm166 | -0.6774423 |
| Gm166 | -0.6918527 |
| Gm1661 | -0.0550221 |
| Gm1673 | -0.2519167 |
| Gm1673 | -0.2850085 |
| Gm1698 | 2.9884753 |
| Gm1821 | -0.6624316 |
| Gm1943 | -0.7741146 |
| Gm1965 | -0.470293 |
| Gm1967 | -0.1906419 |
| Gm1967 | 1.1035793 |
| Gm216 | -0.83713 |
| Gm239 | 2.3741393 |
| Gm249 | 1.316706 |
| Gm266 | 1.8619387 |
| Gm2a | -0.3439858 |
| Gm347 | -0.1916215 |
| Gm382 | -0.4046144 |
| Gm410 | 0.1673091 |
| Gm428 | -0.0845539 |
| Gm443 | 0.1300435 |
| Gm484 | -0.5916838 |
| Gm50 | -0.524675 |
| Gm525 | -0.5057167 |
| Gm525 | -0.0066614 |
| Gm527 | -0.3014685 |
| Gm527 | 0.6468465 |
| Gm561 | -0.5204274 |
| Gm561 | -0.7574171 |
| Gm597 | -0.5454927 |
| Gm603 | 2.1405274 |
| Gm609 | -0.7021655 |
| Gm614 | -0.2545407 |
| Gm614 | -0.7713101 |
| Gm648 | 1.0352971 |
| Gm648 | 1.2229732 |
| Gm71 | -0.3904443 |
| Gm711 | 1.0067746 |
| Gm732 | 1.3034071 |
| Gm732 | -0.5630929 |
| Gm767 | 1.1936051 |
| Gm773 | 1.9074311 |
| Gm773 | -0.7564985 |
| Gm784 | 0.7580005 |
| Gm813 | -0.7143485 |
| Gm813 | -0.3807168 |
| Gm817 | -0.6243784 |
| Gm867 | -0.5924485 |
| Gm884 | -0.4413712 |
| Gm889 | 0.8255098 |
| Gm906 | -0.7169807 |
| Gm93 | -0.1360353 |
| Gm93 | -0.6674074 |
| Gm944 | -0.6419904 |
| Gm949 | 0.1144849 |
| Gm962 | 1.5844064 |
| Gm962 | 2.5430399 |
| Gm97 | -0.381927 |
| Gm97 | 0.9826496 |
| Gmcl1 | 0.6809333 |
| Gmcl1 | -0.6355461 |
| Gmcl1 | 2.0723207 |
| Gmds | -0.3255988 |
| Gmeb2 | 1.6673381 |
| Gmfg | -0.7030303 |
| Gmfg | -0.6558398 |
| Gmfg | 1.3568664 |
| Gmip | 1.2421986 |
| Gmip | -0.7284769 |
| Gmppa | 1.4864674 |
| Gmppa | -0.4413712 |
| Gmppb | 1.6234437 |
| Gmpr | 2.6218551 |
| Gmpr2 | -0.3953477 |
| Gna11 | -0.6001325 |
| Gna13 | 0.0701657 |
| Gna14 | -0.5593977 |
| Gnai2 | 1.514199 |
| Gnao1 | -0.5549834 |
| Gnao1 | -0.5325148 |
| Gnaq | 0.6775822 |
| Gnaq | -0.6833481 |
| Gna-rs1 | -0.5924485 |
| Gnas | -0.6259654 |
| Gnas | -0.2409785 |
| Gnas | -0.2435708 |
| Gnas | -0.2435708 |
| Gnas | -0.0268368 |
| Gnat1 | -0.5557176 |
| Gnat2 | 2.7667932 |
| Gnaz | 2.2611308 |
| Gnb1 | -0.4898763 |
| Gnb1l | 1.9751848 |
| Gnb2 | -0.5239657 |
| Gnb2l1 | -0.5712762 |
| Gnb3 | 0.0471597 |
| Gnb4 | 0.2823051 |
| Gnb4 | 1.8198592 |
| Gnb5 | 0.836363 |
| Gnb5 | 1.3551112 |
| Gne | -0.5325148 |
| Gng10 | 1.2520042 |
| Gng11 | 1.9610063 |
| Gng13 | 2.5178698 |
| Gng2 | -0.3193641 |
| Gng2 | 0.9091655 |
| Gng3 | 0.4564756 |
| Gng4 | -0.6251716 |
| Gng7 | -0.8221914 |
| Gng8 | -0.3204944 |
| Gngt2 | 0.5739082 |
| Gnl2 | -0.7437354 |
| Gnl2 | 0.1116906 |
| Gnl3 | -0.1255007 |
| Gnl3 | -0.4209062 |
| Gnl3 | -0.5668034 |
| Gnl3l | 1.5719982 |
| Gnl3l | -0.6918527 |
| Gnmt | -0.5253849 |
| Gnmt | 0.0335264 |
| Gnpat | -0.4823979 |
| Gnpda1 | -0.185273 |
| Gnpda1 | -0.7555808 |
| Gnpda1 | -0.2926524 |
| Gnpda2 | 1.0618486 |
| Gnptab | -0.6157001 |
| Gnptg | 1.245082 |
| Gnptg | 0.1881142 |
| Gnrhr | -0.3131722 |
| Gns | 1.4700738 |
| Golga1 | 0.8464687 |
| Golga2 | -0.6055506 |
| Golga2 | -0.6607792 |
| Golga2 | -0.4743099 |
| Golga2 | 1.3324275 |
| Golga2 | 1.7180368 |
| Golga3 | 0.7496033 |
| Golga4 | -0.6291476 |
| Golga5 | 0.8322887 |
| Golga7 | 0.8124392 |
| Golim4 | -0.5645752 |
| Golm1 | -0.2009816 |
| Golm1 | 0.3427423 |
| Golph3l | -0.2044547 |
| Golt1a | -0.2487779 |
| Golt1b | 0.7306378 |
| Gopc | -0.6180586 |
| Gorasp1 | -0.5557176 |
| Gorasp2 | 1.1302492 |
| Gorasp2 | 0.1446634 |
| Gorasp2 | -0.5564524 |
| Gorasp2 | 0.3628126 |
| Gosr1 | -0.5742705 |
| Gosr2 | 1.807299 |
| Gosr2 | 2.028893 |
| Gosr2 | 1.2529888 |
| Gosr2 | 2.8099783 |
| Got1l1 | -0.3941194 |
| Got2 | 0.2456827 |
| Gp1bb | -0.7410238 |
| Gp38 | 1.3487641 |
| Gp5 | -0.0449732 |
| Gp9 | -0.5833136 |
| Gpaa1 | 1.3844931 |
| Gpam | -0.3621639 |
| Gpatch1 | 1.8299693 |
| Gpatch2 | 0.0550181 |
| Gpatch3 | -0.4871497 |
| Gpatch4 | -0.7117239 |
| Gpbp1 | 0.6023378 |
| Gpbp1 | -0.7437354 |
| Gpbp1l1 | 3.0605305 |
| Gpc1 | -0.8212043 |
| Gpc3 | 0.5215541 |
| Gpc3 | 1.3320671 |
| Gpc4 | 1.3752636 |
| Gpc5 | 0.5972537 |
| Gpc5 | 3.0156598 |
| Gpc5 | 0.9424291 |
| Gpd1 | -0.7329379 |
| Gpd2 | 2.1442626 |
| Gpd2 | 1.2763006 |
| Gper | -0.8574421 |
| Gpha2 | 0.1176183 |
| Gphb5 | 0.2353751 |
| Gphn | 0.6850123 |
| Gphn | 0.935874 |
| Gphn | -0.4803691 |
| Gpihbp1 | 0.3662464 |
| Gpkow | -0.7143485 |
| Gpld1 | -0.3313045 |
| Gpld1 | 0.4109877 |
| Gpm6a | 0.6200087 |
| Gpm6a | -0.6566611 |
| Gpm6b | 0.5923457 |
| Gpnmb | 0.1172706 |
| Gpnmb | -0.6640872 |
| Gpr1 | -0.3965778 |
| Gpr1 | 2.8997087 |
| Gpr103 | 1.883582 |
| Gpr107 | -0.6419904 |
| Gpr108 | 0.4261607 |
| Gpr108 | -0.4960416 |
| Gpr108 | -0.4317203 |
| Gpr109a | -0.5535167 |
| Gpr113 | 0.0099431 |
| Gpr113 | 1.7843699 |
| Gpr114 | -0.6387625 |
| Gpr12 | 2.5310661 |
| Gpr12 | -0.6935633 |
| Gpr120 | -0.0098592 |
| Gpr123 | -0.7013016 |
| Gpr124 | 0.266834 |
| Gpr128 | 2.7342498 |
| Gpr135 | 0.668961 |
| Gpr137 | 1.4591851 |
| Gpr137b | 0.9692617 |
| Gpr141 | -0.3873931 |
| Gpr146 | -0.2992563 |
| Gpr146 | 0.6324497 |
| Gpr149 | 0.6241748 |
| Gpr151 | -0.4682912 |
| Gpr153 | -0.6220033 |
| Gpr156 | 2.0039571 |
| Gpr156 | -0.7528334 |
| Gpr158 | -0.2981523 |
| Gpr162 | -0.0677431 |
| Gpr162 | -0.5204274 |
| Gpr17 | 2.4440656 |
| Gpr172b | 1.3244648 |
| Gpr172b | 0.1643444 |
| Gpr176 | 0.0843416 |
| Gpr177 | -0.0946172 |
| Gpr18 | 2.9953985 |
| Gpr180 | 1.1494417 |
| Gpr182 | 2.039671 |
| Gpr182 | -0.3971935 |
| Gpr19 | 1.2542671 |
| Gpr20 | -0.4530907 |
| Gpr21 | -0.3313045 |
| Gpr22 | 0.4189792 |
| Gpr23 | 0.8624053 |
| Gpr26 | -0.7356251 |
| Gpr27 | 1.4059165 |
| Gpr27 | 2.4268533 |
| Gpr27 | -0.4021339 |
| Gpr34 | 0.4781525 |
| Gpr34 | 2.5611325 |
| Gpr37l1 | -0.6591297 |
| Gpr45 | -0.4209062 |
| Gpr6 | -0.0026801 |
| Gpr64 | 0.4352408 |
| Gpr65 | 0.8956543 |
| Gpr68 | -0.5071074 |
| Gpr75 | 1.6736489 |
| Gpr81 | 2.4295986 |
| Gpr83 | 0.5036295 |
| Gpr85 | -0.3941194 |
| Gpr85 | -0.8533428 |
| Gpr89 | 0.8523244 |
| Gpr89 | -0.3527358 |
| Gpr97 | -0.6110014 |
| Gprasp1 | -0.6492959 |
| Gprasp1 | 1.3521195 |
| Gprc2a-rs5 | 1.011806 |
| Gprc2a-rs5 | 0.1245424 |
| Gprc5a | -0.685891 |
| Gprc5b | 2.6283013 |
| Gprc5c | -0.4530907 |
| Gprk6 | -0.5253849 |
| Gprk6 | 0.5968457 |
| Gps1 | -0.2255797 |
| Gps1 | -0.5491311 |
| Gps1 | -0.5404234 |
| Gps1 | -0.346893 |
| Gps2 | 0.6831605 |
| Gpsm1 | 1.2610218 |
| Gpsm2 | -0.5668034 |
| Gpsm3 | -0.2240542 |
| Gpsn2 | -0.4557159 |
| Gpsn2 | 0.1053713 |
| Gpsn2 | 0.4315707 |
| Gpt1 | -0.6541994 |
| Gpt2 | 0.9041044 |
| Gpx1 | 1.8616886 |
| Gpx2 | 0.968317 |
| Gpx3 | -0.3255988 |
| Gpx4 | 0.7750657 |
| Gpx4 | 1.7711347 |
| Gpx6 | -0.4557159 |
| Gpx7 | -0.621213 |
| Gramd1a | -0.1780029 |
| Gramd1b | -0.4304412 |
| Gramd2 | -0.5085003 |
| Gramd2 | -0.1268678 |
| Gramd3 | -0.4844313 |
| Gramd4 | -0.2915564 |
| Grap2 | -0.3855674 |
| Grasp | -0.6291476 |
| Grasp | 0.0745514 |
| Grb10 | -0.0758937 |
| Grb14 | -0.6484812 |
| Grb14 | 0.9054086 |
| Grb2 | -0.5712762 |
| Grb7 | 0.5701329 |
| Grcc10 | 1.7673648 |
| Greb1 | 0.2087633 |
| Greb1 | 1.1266328 |
| Grem1 | -0.1155382 |
| Grem2 | -0.4810448 |
| Grhl1 | 0.9652062 |
| Grhl2 | -0.2715183 |
| Grhpr | -0.2571724 |
| Gria1 | -0.5310843 |
| Gria1 | -0.6395684 |
| Gria1 | -0.6833481 |
| Gria2 | 1.8386335 |
| Gria2 | -0.5232569 |
| Gria3 | -0.64523 |
| Gria3 | 0.1774467 |
| Gria3 | -0.3176711 |
| Grid2 | 2.2423873 |
| Grik1 | -0.3221927 |
| Grik2 | 1.2918898 |
| Grik4 | 2.3503341 |
| Grik5 | -0.5623526 |
| Grin1 | 1.9541259 |
| Grin1 | 1.4075754 |
| Grin2a | 2.0882895 |
| Grin2b | 1.3698357 |
| Grin2c | 0.3940221 |
| Grin3b | -0.6468541 |
| Grina | -0.782581 |
| Grip1 | 2.9543747 |
| Grip1 | 0.0850626 |
| Grip1 | -0.5848297 |
| Gripap1 | -0.5491311 |
| Gripap1 | -0.6371529 |
| Grit | -0.6782836 |
| Grk4 | 1.1570274 |
| Grk4 | -0.6094407 |
| Grk4 | 1.959437 |
| Grk5 | -0.5638338 |
| Grlf1 | -0.5112925 |
| Grm1 | 2.4068249 |
| Grm7 | -0.467625 |
| Grm8 | 0.050536 |
| Grn | 1.2708185 |
| Grp | -0.4102205 |
| Grpel1 | 0.3026223 |
| Grpr | -0.7056296 |
| Grsf1 | 1.0780092 |
| Grtp1 | -0.4426659 |
| Grwd1 | -0.3586168 |
| Grwd1 | -0.3669152 |
| Gsbs | 0.2753323 |
| Gsc | -0.7117239 |
| Gsdma1 | -0.5289428 |
| Gsdmdc1 | 0.8097055 |
| Gsdmdc1 | -0.2230386 |
| Gsdmdc1 | -0.0754629 |
| Gse1 | -0.6699057 |
| Gsg1 | -0.6969943 |
| Gsg1 | 0.6539595 |
| Gsg1 | -0.4102205 |
| Gsg2 | -0.358027 |
| Gsk3a | 0.5081443 |
| Gsk3b | -0.1960431 |
| Gsn | 1.430242 |
| Gspt1 | -0.6149153 |
| Gspt2 | -0.3953477 |
| Gss | -0.6331414 |
| Gss | -0.663259 |
| Gss | 1.3099887 |
| Gsta2 | -0.5154971 |
| Gsta3 | 1.9455988 |
| Gsta3 | 0.1460114 |
| Gsta4 | -0.2225313 |
| Gstcd | 0.3793325 |
| Gstk1 | -0.7030303 |
| Gstm1 | 0.2420582 |
| Gstm1 | -0.3434055 |
| Gstm2 | -0.1727087 |
| Gstm2 | 0.3638706 |
| Gstm2 | 3.2718749 |
| Gstm4 | 1.691716 |
| Gstm5 | 0.1890778 |
| Gstm6 | -0.573521 |
| Gstm6 | -0.629945 |
| Gsto1 | 3.6143573 |
| Gsto2 | -0.4823979 |
| Gsto2 | -0.185273 |
| Gstp1 | -0.4387875 |
| Gstp1 | -0.6749231 |
| Gstp2 | -0.1813883 |
| Gstt1 | 0.6487745 |
| Gstt2 | 0.9731647 |
| Gstt3 | -0.3734896 |
| Gstt4 | 0.3288342 |
| Gstz1 | 0.3728133 |
| Gtdc1 | -0.8162847 |
| Gtf2a1 | 1.1337387 |
| Gtf2e1 | 0.3709794 |
| Gtf2e2 | -0.6009045 |
| Gtf2e2 | 1.7932124 |
| Gtf2f1 | 0.7921418 |
| Gtf2f2 | 0.0745514 |
| Gtf2h1 | 0.3478693 |
| Gtf2h1 | 1.0944717 |
| Gtf2h2 | 0.9287066 |
| Gtf2h3 | -0.4472119 |
| Gtf2h4 | 0.1151822 |
| Gtf2h5 | 1.6021363 |
| Gtf2ird1 | -0.5078036 |
| Gtf2ird1 | 1.3047087 |
| Gtf2ird2 | 1.2917014 |
| Gtf2ird2 | 0.4694973 |
| Gtf2ird2 | 0.0087672 |
| Gtf3a | -0.1137386 |
| Gtf3a | 0.534767 |
| Gtf3c1 | 0.543036 |
| Gtf3c2 | 0.6357775 |
| Gtf3c3 | 0.4084758 |
| Gtf3c4 | 1.3450409 |
| Gtf3c4 | -0.345729 |
| Gtf3c5 | -0.5239657 |
| Gtf3c5 | -0.2477341 |
| Gtf3c5 | 0.1590496 |
| Gtl3 | -0.2461706 |
| Gtpbp1 | 1.6258526 |
| Gtpbp2 | -0.0889154 |
| Gtpbp3 | -0.5638338 |
| Gtpbp4 | -0.630743 |
| Gtpbp5 | 0.5941893 |
| Gtpbp6 | -0.7731788 |
| Gtpbp8 | 1.8156568 |
| Gtpbp8 | 0.1348296 |
| Gtrgeo22 | 1.6072618 |
| Gtse1 | -0.4064792 |
| Gtse1 | -0.0789153 |
| Gtse1 | -0.6558398 |
| Gtsf1l | 0.4008976 |
| Guca1a | -0.6063272 |
| Guca1b | -0.448515 |
| Guca2a | 2.8136502 |
| Guca2b | 0.668961 |
| Gucy1a2 | 1.8369896 |
| Gucy1a3 | -0.6172717 |
| Gucy1a3 | 0.3163812 |
| Gucy1a3 | -0.0776191 |
| Gucy1b3 | 1.5964442 |
| Guk1 | 1.8640368 |
| Gulo | 2.3010231 |
| Gulp1 | -0.3307323 |
| Gusb | -0.5916838 |
| Gusb | 0.1500433 |
| Gusb | -0.663259 |
| Gvin1 | 1.3205481 |
| Gyg | 1.2846041 |
| Gyg | 0.6649976 |
| Gykl1 | 0.9886098 |
| Gyltl1b | -0.6024505 |
| Gyltl1b | 0.8912523 |
| Gypa | 0.5518733 |
| Gypc | -0.6259654 |
| Gys1 | -0.0741717 |
| Gys3 | 1.4625436 |
| Gzf1 | -0.6987148 |
| Gzf1 | -0.3103718 |
| Gzmd | 2.8379625 |
| Gzmd | 0.8503254 |
| Gzmk | 0.0008882 |
| Gzmm | -0.6749231 |
| Gzmn | 0.8415078 |
| H13 | -0.4439624 |
| H13 | 2.6049981 |
| H13 | -0.4240733 |
| H13 | -0.6460417 |
| H19 | 1.6986986 |
| H1f0 | -0.644419 |
| H1fnt | 0.0943831 |
| H1foo | -0.0550221 |
| H1fx | -0.7082364 |
| H1fx | -0.5289428 |
| H2-Aa | 0.5667637 |
| H2-Ab1 | 0.9163466 |
| H2-Ab1 | 1.4052521 |
| H2-Ab1 | 0.3422009 |
| H2afj | -0.1052395 |
| H2afx | 2.4858212 |
| H2afy | -0.5955143 |
| H2afz | 0.4860332 |
| H2-Bl | 0.116575 |
| H2-DMa | 1.2247011 |
| H2-DMb1 | -0.7178598 |
| H2-Eb1 | -0.3014685 |
| H2-K1 | -0.7694452 |
| H2-Ke6 | -0.2409785 |
| H2-M10.3 | -0.423439 |
| H2-M2 | 0.7323945 |
| H2-M3 | 0.0274002 |
| H2-Oa | 1.9350095 |
| H2-Ob | 0.7941104 |
| H2-Q1 | 1.0811115 |
| H2-Q2 | -0.2373618 |
| H2-Q7 | 0.1238523 |
| H2-Q8 | -0.5653173 |
| H2-T10 | -0.5780274 |
| H2-T23 | 0.3551051 |
| H2-T23 | -0.6291476 |
| H2-T24 | 3.2798029 |
| H2-Tw3 | -0.1975218 |
| H3f3a | 0.535641 |
| H3f3a | -0.4823979 |
| H3f3b | 0.7490866 |
| H47 | 2.6019929 |
| H47 | -0.6188462 |
| H6pd | -0.1945668 |
| Habp4 | -0.7030303 |
| Habp4 | 2.3043939 |
| Hace1 | -0.6969943 |
| Hacl1 | -0.7401218 |
| Hadhb | -0.6476673 |
| Hagh | -0.5863483 |
| Haghl | 2.9060625 |
| Haghl | 3.9719983 |
| Hal | 2.5165419 |
| Hamp2 | -0.7510064 |
| Hao1 | -0.8553901 |
| Hao3 | -0.5332309 |
| Hap1 | 0.4154917 |
| Hap1 | 0.1372132 |
| Hap1 | 1.0842031 |
| Hapln1 | -0.1137386 |
| Hapln2 | -0.3651305 |
| Hapln4 | 0.9983259 |
| Harbi1 | -0.3070227 |
| Hars | 1.2324863 |
| Has1 | -0.6009045 |
| Hat1 | -0.3922799 |
| Havcr1 | -0.6323412 |
| Havcr2 | 1.7318391 |
| Hax1 | -0.6550192 |
| Hba-a1 | -0.7882699 |
| Hbb-b1 | -0.7258108 |
| Hbegf | -0.6071046 |
| Hbp1 | -0.5697828 |
| Hbp1 | 1.1137452 |
| Hbs1l | -0.560874 |
| Hbs1l | -0.7592572 |
| Hbs1l | 0.0749159 |
| Hbs1l | 0.6940314 |
| Hc | -0.4052356 |
| Hccs | -0.477671 |
| Hcfc1 | -0.4368546 |
| Hcfc1r1 | 1.7290027 |
| Hcfc2 | 3.1822015 |
| Hcls1 | -0.5469463 |
| Hcn2 | 2.0953397 |
| Hcn3 | 1.4168091 |
| Hcn3 | 1.0636721 |
| Hcr | -0.6291476 |
| Hcst | -0.0897903 |
| Hdac10 | -0.4563734 |
| Hdac11 | 0.1266092 |
| Hdac2 | -0.5802891 |
| Hdac3 | -0.6952772 |
| Hdac4 | -0.1707912 |
| Hdac5 | -0.076756 |
| Hdac6 | 0.0115087 |
| Hdac7 | 2.9461385 |
| Hdac8 | -0.0588195 |
| Hdac9 | -0.6674074 |
| Hdc | -0.6125649 |
| Hddc2 | -0.4253433 |
| Hddc2 | -0.5140934 |
| Hdgf | -0.4139775 |
| Hdgf | -0.5924485 |
| Hdgf | -0.514795 |
| Hdgf | 0.1439887 |
| Hdgfl1 | -0.2866411 |
| Hdgfrp2 | -0.5955143 |
| Hdhd1a | 2.3635764 |
| Hdhd2 | -0.3880025 |
| Hdhd2 | 1.2711078 |
| Hdhd2 | 0.102195 |
| Hdlbp | -0.3965778 |
| Hdlbp | 1.9965377 |
| Heatr1 | 2.3780651 |
| Heatr1 | -0.4590082 |
| Heatr3 | -0.2932009 |
| Heatr5a | -0.7284769 |
| Hebp1 | 3.0341711 |
| Hebp2 | -0.6157001 |
| Heca | 1.2395057 |
| Hectd2 | -0.242533 |
| Hectd2 | 2.2428132 |
| Hectd3 | 0.186506 |
| Hecw2 | 0.4069652 |
| Hecw2 | -0.000696 |
| Hecw2 | -0.6961353 |
| Heg1 | 2.593094 |
| Hel308 | 0.9388088 |
| Helb | -0.329589 |
| Helb | -0.1555966 |
| Helz | -0.4939818 |
| Hemgn | 1.1147784 |
| Hemk1 | -0.5848297 |
| Hemt1 | 0.3952993 |
| Hepacam | 1.5532353 |
| Heph | -0.2054495 |
| Heph | 0.3302072 |
| Heph | -0.3233266 |
| Heph | -0.3439858 |
| Heph | 1.923874 |
| Herc2 | -0.4763251 |
| Herc3 | -0.4623126 |
| Herc3 | 0.6606366 |
| Herc4 | -0.5549834 |
| Herc4 | 0.7518388 |
| Herpud1 | 0.3932549 |
| Herpud1 | 0.9915093 |
| Herpud2 | 0.0044423 |
| Herpud2 | -0.5057167 |
| Herpud2 | 1.0481149 |
| Herpud2 | -0.3556715 |
| Hes1 | -0.3855674 |
| Hes5 | -0.5447667 |
| Hes5 | -0.4139775 |
| Hes6 | -0.7638738 |
| Hes6 | 0.7634424 |
| Hes6 | 1.1270857 |
| Hexa | -0.6492959 |
| Hexb | 0.5334544 |
| Hexdc | 1.0830153 |
| Hexdc | 2.5135893 |
| Hexim1 | 3.5804823 |
| Hexim2 | 1.9930427 |
| Hey1 | 3.5270532 |
| Hey2 | 1.2135642 |
| Hey2 | 1.8464013 |
| Heyl | 0.7445963 |
| Hfe | -0.1499641 |
| Hfe | 0.2906592 |
| Hgfac | -0.5126918 |
| Hgs | 0.4514536 |
| Hgs | 0.2037243 |
| Hgsnat | 1.1106383 |
| Hhat | 2.5451021 |
| Hhat | -0.6157001 |
| Hhat | -0.4656292 |
| Hhatl | -0.5353826 |
| Hhex | -0.1494963 |
| Hhip | -0.2964988 |
| Hibadh | 0.8655893 |
| Hic1 | -0.6291476 |
| Hic2 | -0.6492959 |
| Hif1a | 2.2736873 |
| Hif3a | -0.6149153 |
| Hif3a | 2.6367124 |
| Higd1a | -0.0512406 |
| Higd1b | -0.2482558 |
| Higd2a | 1.1574652 |
| Hils1 | -0.4511268 |
| Hint1 | 2.2507371 |
| Hint2 | -0.4452608 |
| Hint3 | 0.8352679 |
| Hint3 | -0.2915564 |
| Hip1 | 0.2794063 |
| Hip1r | -0.4563734 |
| Hip2 | 0.4283778 |
| Hipk1 | 2.0905423 |
| Hipk2 | -0.6850426 |
| Hipk3 | 2.0406146 |
| Hipk4 | 0.1176183 |
| Hira | -0.451781 |
| Hira | 1.9230799 |
| Hirip3 | 0.8544723 |
| Hisppd1 | -0.4433139 |
| Hist1h1b | -0.5183111 |
| Hist1h1c | -0.5668034 |
| Hist1h2aa | -0.7464551 |
| Hist1h2ac | -0.1960431 |
| Hist1h2ad | 2.0924798 |
| Hist1h2ae | -0.5579238 |
| Hist1h2af | 2.2276194 |
| Hist1h2ag | -0.597051 |
| Hist1h2ah | 1.3170726 |
| Hist1h2ak | -0.7004385 |
| Hist1h2an | -0.2059473 |
| Hist1h2ao | -0.3527358 |
| Hist1h2bc | -0.7293673 |
| Hist1h2be | -0.5491311 |
| Hist1h2bf | 1.5839296 |
| Hist1h2bg | 1.2675325 |
| Hist1h2bh | 1.6852268 |
| Hist1h2bj | -0.621213 |
| Hist1h2bk | -0.2210109 |
| Hist1h2bm | 1.2120207 |
| Hist1h2bn | 0.865892 |
| Hist1h3a | 2.250315 |
| Hist1h3d | -0.6024505 |
| Hist1h3e | -0.3393537 |
| Hist1h3f | -0.4912427 |
| Hist1h4c | 1.2231767 |
| Hist1h4d | 0.3998823 |
| Hist1h4f | 0.4500135 |
| Hist1h4h | 1.9181131 |
| Hist1h4i | 2.8820973 |
| Hist1h4j | -0.448515 |
| Hist1h4k | -0.5071074 |
| Hist1h4m | 1.0744163 |
| Hist2h2aa1 | 0.322214 |
| Hist2h2ab | -0.0994702 |
| Hist2h2ac | -0.623586 |
| Hist2h2bb | 0.2118985 |
| Hist2h2be | 0.36809 |
| Hist2h3b | -0.4851101 |
| Hist2h3c1 | -0.4946678 |
| Hist2h3c1 | -0.4374984 |
| Hist2h4 | 0.3149868 |
| Hist3h2a | -0.5825565 |
| Hist3h2bb | -0.4285261 |
| Hist4h4 | 1.5672226 |
| Hk1 | -0.4987953 |
| Hk3 | -0.7601786 |
| Hkdc1 | -0.6901453 |
| Hlcs | 0.9470032 |
| Hlf | 0.5268626 |
| Hltf | 2.5530254 |
| Hlx | 0.6215981 |
| Hmg20a | 2.4543252 |
| Hmg20a | -0.5939801 |
| Hmg20b | -0.3142948 |
| Hmga1 | -0.5682918 |
| Hmgb2 | 0.8085774 |
| Hmgb2l1 | 1.9162788 |
| Hmgb2l1 | 1.0087131 |
| Hmgb4 | -0.2358162 |
| Hmgcl | -0.5527843 |
| Hmgcl | 0.6332338 |
| Hmgcl | 2.6735575 |
| Hmgcll1 | -0.5183111 |
| Hmgcs2 | -0.6875903 |
| Hmgn1 | 1.5758555 |
| Hmgn2 | -0.6110014 |
| Hmgn3 | -0.1088083 |
| Hmgn3 | 1.0780092 |
| Hmha1 | -0.5098953 |
| Hmox1 | 0.4901776 |
| Hmox2 | -0.3318771 |
| Hmx1 | -0.4127234 |
| Hmx1 | -0.0449732 |
| Hn1 | 1.4161527 |
| Hn1 | 1.4729297 |
| Hn1l | -0.1843002 |
| Hnf1b | 0.8963859 |
| Hnf1b | -0.709107 |
| Hnf4a | 1.4501412 |
| Hnrnpc | 1.7992382 |
| Hnrpa1 | 0.8707206 |
| Hnrpa1 | -0.4407246 |
| Hnrpab | 1.8767697 |
| Hnrpc | 3.6482816 |
| Hnrpdl | -0.4981061 |
| Hnrpdl | -0.5317993 |
| Hnrpf | -0.5564524 |
| Hnrph1 | -0.5404234 |
| Hnrph1 | -0.5962823 |
| Hnrph1 | 1.1557128 |
| Hnrph1 | 0.3720278 |
| Hnrph1 | -0.3020223 |
| Hnrph2 | -0.6841949 |
| Hnrph3 | 0.4179842 |
| Hnrpk | 1.5207994 |
| Hnrpl | 0.1169229 |
| Hnrpll | 0.2644755 |
| Hnrpll | -0.3698976 |
| Hnrpm | -0.6774423 |
| Hnrpm | -0.7030303 |
| Hnrpm | 3.0752849 |
| Hnrpul1 | 0.6894411 |
| Hnrpul1 | 0.5354226 |
| Hnrpul1 | -0.4987953 |
| Hnrpul1 | 1.2545618 |
| Hnrpul1 | 0.0064107 |
| Hnt | -0.05166 |
| Homer1 | 1.2356016 |
| Homer1 | -0.6323412 |
| Homer1 | -0.147161 |
| Homer1 | -0.6799686 |
| Homez | -0.4729689 |
| Homez | 1.2237868 |
| Hook1 | -0.2114414 |
| Hook1 | -0.5765228 |
| Hook1 | 3.290876 |
| Hook1 | 0.5159909 |
| Hook2 | -0.2763515 |
| Hormad2 | -0.4994851 |
| Hoxa13 | -0.6047745 |
| Hoxa2 | -0.7249239 |
| Hoxa2 | 0.1696082 |
| Hoxa4 | -0.1341943 |
| Hoxa5 | 0.0631778 |
| Hoxa7 | 1.8540522 |
| Hoxa9 | 1.3295401 |
| Hoxa9 | 1.8876253 |
| Hoxb2 | -0.4729689 |
| Hoxb3 | 2.882966 |
| Hoxb3 | -0.5050222 |
| Hoxb4 | -0.7882699 |
| Hoxb5 | -0.5064118 |
| Hoxb6 | -0.6315417 |
| Hoxb7 | -0.4459107 |
| Hoxc10 | 1.0065158 |
| Hoxc10 | 1.8429856 |
| Hoxc6 | 2.4682246 |
| Hoxc9 | -0.4662939 |
| Hoxd10 | -0.5447667 |
| Hoxd3 | 2.7824452 |
| Hoxd4 | 0.1078341 |
| Hoxd8 | 0.6353866 |
| Hoxd9 | 0.258849 |
| Hp | 1.9212089 |
| Hp | 0.1109906 |
| Hp | 0.6023378 |
| Hp1bp3 | 1.7528596 |
| Hpca | -0.6039992 |
| Hpcal1 | -0.5397016 |
| Hpcal1 | 1.6205707 |
| Hpcal4 | -0.5425925 |
| Hpgd | -0.6102207 |
| Hpn | -0.1155382 |
| Hpn | 0.0915255 |
| Hprt1 | 2.2022146 |
| Hprt1 | 2.1243093 |
| Hprt1 | -0.6371529 |
| Hps1 | 1.3905095 |
| Hps3 | -0.3603886 |
| Hps3 | -0.3776984 |
| Hps4 | -0.4550589 |
| Hps5 | -0.2169692 |
| Hps6 | -0.7968711 |
| Hpse | -0.5871086 |
| Hpx | 2.1491099 |
| Hr | -0.429164 |
| Hr | -0.4491672 |
| Hras1 | 1.8405817 |
| Hrasls | -0.5593977 |
| Hrasls5 | 3.3896154 |
| Hrasls5 | -0.4317203 |
| Hrb | -0.6164856 |
| Hrb | 0.0091594 |
| Hrbl | -0.6732475 |
| Hrbl | 1.4564423 |
| Hrc | 1.3034071 |
| Hrg | -0.5557176 |
| Hrh3 | 0.7812372 |
| Hrk | 1.8920859 |
| Hrmt1l2 | 2.3948448 |
| Hrsp12 | 0.1777718 |
| Hrsp12 | -0.6125649 |
| Hrsp12 | 0.2381151 |
| Hs1bp3 | 2.1960257 |
| Hs2st1 | 2.302097 |
| Hs3st1 | -0.6009045 |
| Hs3st3a1 | 0.5244334 |
| Hs3st3b1 | 2.6712615 |
| Hs3st6 | 1.1220919 |
| Hs6st1 | -0.2477341 |
| Hs6st2 | -0.5571878 |
| Hs6st2 | 0.8103495 |
| Hs6st3 | 2.1844979 |
| Hsbp1 | 0.4867253 |
| Hsd11b1 | -0.597051 |
| Hsd11b1 | -0.5126918 |
| Hsd11b1 | -0.4158618 |
| Hsd11b1 | 0.2112723 |
| Hsd11b2 | -0.025206 |
| Hsd17b1 | -0.7056296 |
| Hsd17b10 | -0.6541994 |
| Hsd17b11 | -0.5727721 |
| Hsd17b11 | -0.7143485 |
| Hsd17b11 | 2.3110067 |
| Hsd17b12 | 0.5206663 |
| Hsd17b2 | -0.6094407 |
| Hsd17b3 | -0.2230386 |
| Hsd17b4 | -0.8016854 |
| Hsd17b7 | 1.833383 |
| Hsd3b1 | -0.573521 |
| Hsd3b2 | -0.675762 |
| Hsd3b2 | -0.5645752 |
| Hsd3b4 | 0.3397606 |
| Hsd3b7 | -0.6243784 |
| Hsd3b7 | -0.2763515 |
| Hsdl1 | -0.6133477 |
| Hsf1 | -0.2353016 |
| Hsf4 | -0.6220033 |
| Hsf5 | -0.6841949 |
| Hsn2 | 1.6273468 |
| Hsp105 | 0.1748414 |
| Hsp90aa1 | -0.536101 |
| Hsp90ab1 | 0.5391286 |
| Hsp90ab1 | 1.6769721 |
| Hsp90ab1 | 1.5418154 |
| Hsp90b1 | -0.7082364 |
| Hspa12b | 1.8219008 |
| Hspa1a | 0.7653055 |
| Hspa1l | 1.5428855 |
| Hspa2 | -0.7187398 |
| Hspa2 | 1.3677592 |
| Hspa4 | -0.5557176 |
| Hspa4l | -0.5303699 |
| Hspa5 | 0.6572088 |
| Hspa9 | -0.4039936 |
| Hspa9 | 0.9511414 |
| Hspb1 | -0.6251716 |
| Hspb2 | 1.4422821 |
| Hspb3 | -0.516903 |
| Hspb6 | -0.5310843 |
| Hspb7 | -0.2220242 |
| Hspb8 | -0.3965778 |
| Hspbap1 | -0.6196344 |
| Hspd1 | 0.2462854 |
| Hspd1 | -0.3261678 |
| Hspe1 | 2.5680499 |
| Hspg2 | -0.2215174 |
| Htatip | 0.4602832 |
| Htatip | 1.3477019 |
| Htatip2 | -0.1056848 |
| Htatip2 | -0.6094407 |
| Htatsf1 | 0.1663219 |
| Htf9c | -0.1693557 |
| Htr1a | -0.7178598 |
| Htr1b | -0.483753 |
| Htr1d | -0.5893933 |
| Htr1f | -0.5901561 |
| Htr1f | -0.5112925 |
| Htr2b | 1.6513646 |
| Htr3a | 0.266834 |
| Htr5b | 0.3803712 |
| Htr7 | -0.5462192 |
| Htra1 | 2.6133684 |
| Htra1 | 1.6192617 |
| Htra2 | -0.2317076 |
| Htra3 | 1.8850455 |
| Htra3 | 0.9116135 |
| Htt | 1.9886222 |
| Hus1 | 1.3601918 |
| Hus1 | 1.5861748 |
| Hus1 | 0.2960908 |
| Huwe1 | 2.1474728 |
| Hvcn1 | 0.2546806 |
| Hvcn1 | 0.4256672 |
| Hyal1 | 0.2444761 |
| Hyal2 | -0.629945 |
| Hyal3 | -0.4342837 |
| Hyal5 | 0.2274123 |
| Hyal6 | -0.7021655 |
| Hydin | -0.2860966 |
| Hyi | -0.4452608 |
| Hyou1 | -0.4994851 |
| Iah1 | 1.9981296 |
| Iap | 0.0028644 |
| Iars | -0.6774423 |
| Iars | 0.5410844 |
| Iars2 | 0.6975044 |
| Ibtk | 2.470745 |
| Ica1 | -0.6117828 |
| Ica1l | -0.3947333 |
| Icam1 | 0.6581623 |
| Icam2 | -0.6841949 |
| Icam4 | 1.7901301 |
| Icam5 | -0.4796938 |
| Ick | 0.0274002 |
| Icmt | -0.6533803 |
| Ict1 | 2.3617417 |
| Id1 | -0.334171 |
| Id2 | -0.7064977 |
| Id2 | 1.4912372 |
| Id3 | 1.6182132 |
| Id4 | -0.2245624 |
| Ide | -0.1318984 |
| Idh1 | 1.7166859 |
| Idh2 | 1.7786823 |
| Idh3a | -0.5029418 |
| Idh3b | -0.2394267 |
| Idh3g | 1.6338789 |
| Idh3g | 2.1820133 |
| Idi2 | -0.5036347 |
| Ids | 0.0749159 |
| Ids | 1.6991182 |
| Ids | 0.9404815 |
| Idua | 0.913194 |
| Ier3 | -0.6347438 |
| Ier3ip1 | -0.6867403 |
| Ier5l | 1.9580897 |
| Ifi202b | -0.7592572 |
| Ifi202b | 0.6813049 |
| Ifi27 | 0.6096088 |
| Ifi30 | -0.4974174 |
| Ifi30 | -0.4323604 |
| Ifi35 | 0.7092854 |
| Ifi47 | 3.6256625 |
| Ifit2 | 1.7150976 |
| Ifit2 | -0.4898763 |
| Ifit3 | -0.4557159 |
| Ifit3 | 1.717391 |
| Ifitm1 | -0.5893933 |
| Ifitm1 | 0.3077011 |
| Ifitm1 | -0.4912427 |
| Ifitm2 | 0.8779153 |
| Ifitm3 | 1.5711004 |
| Ifitm5 | -0.0893528 |
| Ifitm6 | -0.8212043 |
| Ifna1 | -0.0276533 |
| Ifna6 | -0.5810443 |
| Ifnar2 | 1.2022876 |
| Ifnar2 | 2.6496419 |
| Ifnar2 | 3.0977898 |
| Ifngr1 | 0.2399371 |
| Ifngr2 | -0.2866411 |
| Ifngr2 | -0.2822943 |
| Ifngr2 | -0.147161 |
| Ifnz | -0.4662939 |
| Ifrd1 | 0.2671284 |
| Ifrg15 | 0.1389118 |
| Ift122 | -0.3463109 |
| Ift140 | -0.3364707 |
| Ift140 | 0.1553912 |
| Ift140 | -0.53682 |
| Ift172 | -0.2194932 |
| Ift172 | 1.817235 |
| Ift172 | -0.7293673 |
| Ift20 | -0.5154971 |
| Ift20 | 1.6509221 |
| Ift20 | -0.5571878 |
| Ift52 | 1.2345978 |
| Ift80 | 2.3399569 |
| Ift81 | 0.4702022 |
| Ift81 | 1.4144273 |
| Ift81 | -0.3148567 |
| Ift88 | 1.3391587 |
| Igbp1 | 0.6833459 |
| Igbp1b | -0.3216262 |
| Igf1 | -0.3148567 |
| Igf1 | 0.2528882 |
| Igf1r | 0.9243219 |
| Igf2 | -0.5802891 |
| Igf2bp1 | -0.675762 |
| Igf2bp2 | -0.4912427 |
| Igf2bp3 | 2.4809579 |
| Igf2bp3 | 3.4317492 |
| Igf2r | -0.5579238 |
| Igf2r | -0.2779684 |
| Igfals | -0.0231717 |
| Igfbp2 | -0.5601355 |
| Igfbp2 | 0.8106713 |
| Igfbp3 | -0.7038959 |
| Igfbp4 | 0.7132381 |
| Igfbp4 | -0.6690721 |
| Igfbp5 | 1.814445 |
| Igfbp6 | -0.6782836 |
| Igfbp7 | -0.6624316 |
| Igfbpl1 | 0.5061154 |
| Igsf1 | -0.6850426 |
| Igsf1 | -0.6016772 |
| Igsf1 | 2.027313 |
| Igsf1 | 0.1777718 |
| Igsf11 | 0.0111175 |
| Igsf11 | -0.2044547 |
| Igsf11 | -0.5491311 |
| Igsf21 | -0.7601786 |
| Igsf3 | -0.5382596 |
| Igsf4a | 0.332126 |
| Igsf5 | -0.4349258 |
| Igsf8 | -0.2688443 |
| Igsf9 | -0.4596681 |
| Igtp | 1.4669762 |
| Ihh | 1.007292 |
| Ihpk1 | 1.367066 |
| Ihpk2 | 0.3699298 |
| Ihpk2 | 1.0806349 |
| Iigp2 | 0.5380402 |
| Ik | 0.8407301 |
| Ikbkap | -0.3645364 |
| Ikbkb | -0.83713 |
| Ikbke | 0.6361682 |
| Ikbkg | 1.7203812 |
| Ikbkg | -0.7338327 |
| Il10ra | -0.0122651 |
| Il10rb | -0.6125649 |
| Il11 | 0.9950551 |
| Il11ra1 | 0.8404189 |
| Il12a | 0.610011 |
| Il13 | -0.5085003 |
| Il13ra1 | -0.573521 |
| Il13ra2 | -0.6649161 |
| Il15 | -0.0470575 |
| Il15 | 0.9698011 |
| Il15 | -0.4544024 |
| Il15ra | -0.4912427 |
| Il15ra | 0.3643991 |
| Il15ra | 1.2621913 |
| Il16 | 0.5701329 |
| Il17d | 0.9765159 |
| Il17d | -0.6961353 |
| Il17ra | -0.5346648 |
| Il17rb | -0.7419268 |
| Il17rc | -0.64523 |
| Il17rc | -0.6533803 |
| Il17rc | -0.7329379 |
| Il17rd | 2.1146553 |
| Il17rd | 2.0034444 |
| Il17re | 0.6969569 |
| Il17re | 0.1682951 |
| Il17re | 1.9704982 |
| Il18 | 2.5310898 |
| Il18bp | -0.6952772 |
| Il18r1 | 2.4180278 |
| Il1b | -0.5176068 |
| Il1r1 | 1.2145917 |
| Il1rap | -0.7620243 |
| Il1rap | 1.2818448 |
| Il1rap | -0.5795346 |
| Il1rap | 1.4521939 |
| Il1rap | 0.2131495 |
| Il1rl1l | 1.0792037 |
| Il1rl2 | 0.7993388 |
| Il20ra | -0.3376228 |
| Il20rb | -0.5623526 |
| Il20rb | -0.4994851 |
| Il23a | 0.7404314 |
| Il27ra | -0.7383204 |
| Il28ra | -0.6016772 |
| Il28ra | 0.2555755 |
| Il28ra | 1.6188031 |
| Il31ra | 0.4769878 |
| Il33 | -0.2806696 |
| Il3ra | -0.5433166 |
| Il4i1 | 1.8052772 |
| Il6ra | -0.2296603 |
| Il6st | 0.7526971 |
| Il7 | 2.308209 |
| Ildr1 | 1.0017149 |
| Ildr1 | 0.1826346 |
| Ilf2 | 0.1973855 |
| Ilf3 | -0.4058572 |
| Ilf3 | 0.2677169 |
| Ilf3 | 0.4179842 |
| Ilf3 | 1.0406705 |
| Ilk | 1.4098101 |
| Ilkap | -0.4596681 |
| Ilvbl | 0.8570735 |
| Ilvbl | -0.4002781 |
| Ilvbl | -0.287731 |
| Ilvbl | -0.5810443 |
| Immp2l | 0.0697993 |
| Immt | 2.9764546 |
| Imp3 | 0.6420085 |
| Imp4 | -0.2866411 |
| Imp4 | -0.1574819 |
| Impa2 | 3.2468048 |
| Impact | 0.9230449 |
| Impad1 | 0.9739702 |
| Impdh1 | 0.3183296 |
| Impdh2 | 0.4461621 |
| Ina | -0.1584261 |
| Inadl | -0.4407246 |
| Inadl | 0.3291089 |
| Incenp | 0.1640144 |
| Indo | 2.0217191 |
| Ing2 | -0.2667109 |
| Ing3 | 0.534111 |
| Ing3 | 0.8637713 |
| Ing3 | 0.3299328 |
| Ing4 | 2.0458481 |
| Ing4 | -0.7073666 |
| Ing4 | -0.6117828 |
| Ing5 | -0.0946172 |
| Inha | -0.4960416 |
| Inhba | 0.1331232 |
| Inmt | 1.7908881 |
| Inoc1 | -0.4537463 |
| Inpp4a | -0.4039936 |
| Inpp4a | -0.399043 |
| Inpp4a | 0.3973391 |
| Inpp4b | 1.0354224 |
| Inpp5a | -0.468958 |
| Inpp5d | -0.5119919 |
| Inpp5e | 0.3691417 |
| Inpp5f | -0.573521 |
| Inppl1 | 3.7175695 |
| Inppl1 | 0.2986522 |
| Inppl1 | -0.6892929 |
| Ins2 | -0.5197214 |
| Insc | -0.1255007 |
| Insig1 | -0.7374211 |
| Insig1 | 2.3320131 |
| Insig1 | 0.4657286 |
| Insig2 | 0.557859 |
| Insig2 | -0.3722907 |
| Insl3 | 1.493349 |
| Insl5 | -0.0754629 |
| Insl6 | -0.2440902 |
| Insm1 | 0.5834849 |
| Ints1 | -0.3182351 |
| Ints12 | -0.7816364 |
| Ints3 | 0.1623625 |
| Ints3 | 1.4845676 |
| Ints4 | 1.0471259 |
| Ints5 | -0.3474756 |
| Ints7 | 1.1689899 |
| Ints9 | -0.4491672 |
| Ints9 | -0.3801123 |
| Ipmk | -0.6791257 |
| Ipmk | 2.0662835 |
| Ipo13 | 0.1607075 |
| Ipo4 | -0.6640872 |
| Ipo5 | 2.288876 |
| Ipp | -0.56606 |
| Ippk | -0.470293 |
| Iqcb1 | -0.1579539 |
| Iqcb1 | 0.7842244 |
| Iqcd | 0.121087 |
| Iqce | -0.2159616 |
| Iqcf3 | 0.5301645 |
| Iqcf4 | -0.6267599 |
| Iqgap1 | 0.4949886 |
| Iqgap2 | 0.724108 |
| Iqgap3 | 2.1689889 |
| Iqsec2 | 0.6531932 |
| Iqsec3 | -0.4413712 |
| Irak1 | -0.7365227 |
| Irak1bp1 | -0.3216262 |
| Irak2 | 1.1984275 |
| Irak2 | 0.4184818 |
| Irak3 | 0.9173474 |
| Irak4 | 0.3811495 |
| Irak4 | -0.2109405 |
| Irak4 | -0.5057167 |
| Ireb2 | -0.561613 |
| Irf1 | -0.7275873 |
| Irf1 | -0.7564985 |
| Irf1 | -0.5871086 |
| Irf1 | 0.3777722 |
| Irf2 | -0.5225487 |
| Irf2bp1 | -0.6220033 |
| Irf3 | 0.9771846 |
| Irf3 | 1.3559892 |
| Irf3 | 2.2919824 |
| Irf4 | -0.4946678 |
| Irf5 | 1.4659672 |
| Irf6 | -0.7338327 |
| Irf6 | 1.8899553 |
| Irf7 | 0.968722 |
| Irf8 | 0.2147108 |
| Irf9 | -0.1070221 |
| Irf9 | 2.565628 |
| Irgm | 2.7255963 |
| Irgm | -0.5978204 |
| Irgq | 1.0914152 |
| Irgq | -0.5795346 |
| Irgq | -0.3204944 |
| Irs2 | 0.0968761 |
| Irs3 | 0.0479111 |
| Irx2 | -0.6978541 |
| Irx3 | -0.6331414 |
| Irx5 | 0.62259 |
| Isca1 | -0.5727721 |
| Iscu | -0.4413712 |
| Isg20 | -0.0600889 |
| Isg20l1 | -0.2399437 |
| Isg20l2 | -0.7249239 |
| Isl1 | -0.4960416 |
| Islr2 | 2.2222068 |
| Isy1 | -0.5310843 |
| Isyna1 | 1.9712515 |
| Itfg2 | 0.3612234 |
| Itfg3 | 2.0606467 |
| Itga1 | -0.6427992 |
| Itga11 | 0.3102297 |
| Itga11 | 0.8352679 |
| Itga2b | 1.2804142 |
| Itga3 | -0.5601355 |
| Itga5 | -0.4330011 |
| Itga5 | -0.2817524 |
| Itga6 | 1.9421743 |
| Itga7 | -0.3064657 |
| Itgad | 1.3272779 |
| Itgad | -0.7284769 |
| Itgad | -0.4491672 |
| Itgae | 1.4476084 |
| Itgae | -0.5253849 |
| Itgae | 0.8651351 |
| Itgav | 1.724411 |
| Itgb1 | 1.9294125 |
| Itgb1 | 0.2943791 |
| Itgb1bp1 | 0.6623456 |
| Itgb3bp | -0.5425925 |
| Itgb4 | 0.7210919 |
| Itgb4 | -0.5325148 |
| Itgb5 | -0.2608698 |
| Itgb6 | 4.232746 |
| Itgb6 | 0.8940427 |
| Itgb6 | 1.6973785 |
| Itgb7 | -0.5098953 |
| Itgbl1 | -0.1881975 |
| Itih2 | -0.2855524 |
| Itih3 | 1.6879009 |
| Itih5 | 0.0789157 |
| Itih5 | -0.7592572 |
| Itk | -0.5303699 |
| Itlnb | 0.4832597 |
| Itm2a | 0.8088999 |
| Itm2b | -0.1755926 |
| Itm2b | 0.4938455 |
| Itpk1 | -0.7293673 |
| Itpka | -0.4939818 |
| Itpkc | -0.4530907 |
| Itpr1 | -0.7500943 |
| Itpr2 | -0.5462192 |
| Itpr3 | -0.4709612 |
| Itsn1 | 2.1285215 |
| Itsn2 | -0.4374984 |
| Ivd | -0.1950587 |
| Ivns1abp | -0.303686 |
| Ivns1abp | 0.4974985 |
| Iws1 | -0.3597976 |
| Iyd | 1.2190988 |
| Izumo1 | 1.5723433 |
| Jag1 | -0.6110014 |
| Jag2 | -0.4851101 |
| Jagn1 | -0.2768901 |
| Jak1 | -0.4926112 |
| Jak2 | 0.746326 |
| Jak2 | -0.4071017 |
| Jak3 | 1.9548028 |
| Jak3 | 0.1116906 |
| Jakmip1 | 0.0392321 |
| Jam2 | -0.514795 |
| Jam2 | 0.5144271 |
| Jam2 | 2.8588152 |
| Jam2 | 2.958509 |
| Jam3 | 0.0319987 |
| Jam4 | 0.5753721 |
| Jarid1b | 0.968722 |
| Jarid1c | -0.3574375 |
| Jarid1d | 3.2458921 |
| Jazf1 | -0.7275873 |
| Jdp2 | -0.4215387 |
| Jmjd1a | -0.4596681 |
| Jmjd1a | -0.3910558 |
| Jmjd1a | -0.623586 |
| Jmjd2a | -0.312051 |
| Jmjd2b | -0.1593712 |
| Jmjd2b | -0.5433166 |
| Jmjd2c | -0.4570314 |
| Jmjd2d | -0.6227943 |
| Jmjd3 | 0.1060757 |
| Jmjd3 | -0.4504732 |
| Jmjd5 | -0.6149153 |
| Jmjd6 | -0.7284769 |
| Jmy | 2.9510713 |
| Josd1 | -0.169834 |
| Josd2 | -0.5939801 |
| Josd3 | -0.3746901 |
| Josd3 | 2.9679802 |
| Jph1 | -0.6841949 |
| Jph2 | -0.4623126 |
| Jph3 | 2.3853347 |
| Jph4 | 0.489718 |
| Jph4 | -0.5787807 |
| Jtb | 0.7027796 |
| Jtv1 | -0.5772748 |
| Jtv1 | -0.7038959 |
| Jun | 2.2273528 |
| Junb | -0.0146774 |
| Jund1 | 1.9640921 |
| Jup | -0.3109312 |
| Kal1 | 2.900182 |
| Kalrn | -0.4152333 |
| Kank1 | -0.2667109 |
| Kank3 | -0.0972611 |
| Kank4 | 0.254979 |
| Kank4 | 1.5443815 |
| Kank4 | 0.0538997 |
| Kars | 0.7439035 |
| Katna1 | -0.661605 |
| Katnal1 | 1.9441849 |
| Katnb1 | -0.6927076 |
| Kazald1 | 1.1388918 |
| Kbtbd2 | -0.0911042 |
| Kbtbd2 | -0.5571878 |
| Kbtbd4 | -0.4046144 |
| Kbtbd4 | -0.7169807 |
| Kbtbd7 | 0.2840399 |
| Kbtbd7 | -0.4974174 |
| Kbtbd8 | 0.6388984 |
| Kcmf1 | -0.4355682 |
| Kcna1 | -0.4362112 |
| Kcna2 | 3.6401813 |
| Kcna3 | 0.8731252 |
| Kcna5 | -0.3752909 |
| Kcna6 | 1.8690623 |
| Kcnab1 | -0.7205024 |
| Kcnab1 | -0.5757714 |
| Kcnab1 | 1.2832731 |
| Kcnab2 | -0.6674074 |
| Kcnab3 | 1.122547 |
| Kcnb1 | -0.7240379 |
| Kcnc1 | -0.3633494 |
| Kcnc1 | 0.1780968 |
| Kcnc4 | -0.6133477 |
| Kcnc4 | 0.2156463 |
| Kcnd2 | 0.0712642 |
| Kcne1 | -0.6347438 |
| Kcne1l | -0.5008661 |
| Kcne2 | -0.2624591 |
| Kcne4 | -0.7638738 |
| Kcnf1 | -0.3042413 |
| Kcng4 | -0.6918527 |
| Kcnh1 | 2.4435155 |
| Kcnh2 | 1.8030908 |
| Kcnh2 | -0.5527843 |
| Kcnh3 | 0.2866359 |
| Kcnh5 | -0.2245624 |
| Kcnip1 | -0.5310843 |
| Kcnip2 | -0.5772748 |
| Kcnip2 | 1.1129407 |
| Kcnip2 | -0.0977025 |
| Kcnip3 | 2.957947 |
| Kcnip4 | -0.3770959 |
| Kcnj12 | -0.4278887 |
| Kcnj16 | 2.3210009 |
| Kcnj2 | 1.3313458 |
| Kcnj3 | -0.7491831 |
| Kcnj4 | 0.2938078 |
| Kcnj6 | 0.3116315 |
| Kcnj6 | 1.6504161 |
| Kcnj8 | 1.3136756 |
| Kcnj9 | 2.069188 |
| Kcnk1 | 0.3580364 |
| Kcnk10 | 0.7715441 |
| Kcnk12 | -0.640375 |
| Kcnk13 | -0.2014769 |
| Kcnk2 | -0.6102207 |
| Kcnk2 | 0.8088999 |
| Kcnk3 | -0.4064792 |
| Kcnk4 | 0.7888514 |
| Kcnk5 | 0.4952171 |
| Kcnk5 | 0.3876088 |
| Kcnk6 | 0.2274123 |
| Kcnk6 | 0.0095513 |
| Kcnk9 | 1.4265983 |
| Kcnma1 | -0.2915564 |
| Kcnmb1 | -0.358027 |
| Kcnmb4 | 1.7840973 |
| Kcnmb4 | 0.4779197 |
| Kcnn1 | 1.9597062 |
| Kcnn2 | -0.5418689 |
| Kcnn4 | 0.502044 |
| Kcnq1 | 0.435729 |
| Kcnq2 | -0.1092554 |
| Kcnq2 | 1.6379296 |
| Kcnq3 | 0.6736566 |
| Kcnq5 | -0.4039936 |
| Kcnrg | -0.2822943 |
| Kcnrg | -0.1245904 |
| Kcns1 | 0.1666511 |
| Kcns2 | 2.469473 |
| Kcnt1 | -0.3434055 |
| Kcnu1 | 0.2632939 |
| Kcnv1 | 0.6826042 |
| Kcnv2 | -0.0428937 |
| Kctd10 | -0.6665762 |
| Kctd11 | 0.6708422 |
| Kctd12 | -0.5653173 |
| Kctd12b | 1.4876812 |
| Kctd12b | 0.2495928 |
| Kctd14 | -0.6387625 |
| Kctd14 | -0.8311248 |
| Kctd15 | -0.309254 |
| Kctd18 | 1.246768 |
| Kctd19 | 0.8127602 |
| Kctd2 | 0.1587176 |
| Kctd20 | -0.4478632 |
| Kctd20 | -0.7455476 |
| Kctd3 | 0.3537695 |
| Kctd4 | -0.7047623 |
| Kctd5 | 0.0828979 |
| Kctd5 | 1.1587774 |
| Kctd6 | 1.1177567 |
| Kctd6 | 0.9474179 |
| Kctd9 | -0.358027 |
| Kdelc1 | -0.7284769 |
| Kdelc1 | 1.6564694 |
| Kdelr2 | -0.8973456 |
| Kdelr3 | 0.6229865 |
| Kdr | -0.67074 |
| Keap1 | -0.043725 |
| Keap1 | -0.1755926 |
| Keap1 | 0.4381661 |
| Keap1 | -0.6607792 |
| Kera | -0.2752752 |
| Khdc1c | 3.3104146 |
| Khdrbs1 | -0.4240733 |
| Khdrbs2 | -0.2378776 |
| Khdrbs3 | 0.4234434 |
| Khk | -0.1975218 |
| Khk | 0.7750657 |
| Khsrp | 0.7637815 |
| Kif11 | -0.6640872 |
| Kif13a | 2.1406742 |
| Kif14 | -0.3698976 |
| Kif15 | -0.1223188 |
| Kif17 | 0.5830706 |
| Kif17 | -0.3364707 |
| Kif18a | -0.5008661 |
| Kif1a | 0.8357374 |
| Kif1b | -0.6039992 |
| Kif1b | 2.7986069 |
| Kif1b | 0.807448 |
| Kif1b | 0.4468855 |
| Kif1b | -0.5498606 |
| Kif1b | 1.3165226 |
| Kif1c | 2.0971899 |
| Kif21a | -0.5317993 |
| Kif21b | -0.7694452 |
| Kif22 | 0.0365739 |
| Kif23 | 0.6279277 |
| Kif24 | 3.4287122 |
| Kif27 | 0.1235071 |
| Kif2a | -0.5579238 |
| Kif2b | 1.8458923 |
| Kif2c | -0.4563734 |
| Kif3a | -0.5133923 |
| Kif3a | 3.6867898 |
| Kif3b | 0.6680189 |
| Kif3c | -0.1703124 |
| Kif3c | -0.7685143 |
| Kif4 | 0.0258621 |
| Kif4 | 0.3034708 |
| Kif4 | 0.7371205 |
| Kif5a | -0.7169807 |
| Kif5a | -0.2672438 |
| Kif5a | 2.746627 |
| Kif5b | 1.5661116 |
| Kif5c | 0.5231057 |
| Kif9 | -0.6724109 |
| Kifap3 | 1.5293082 |
| Kifap3 | 0.6547251 |
| Kifap3 | 0.5477887 |
| Kifc2 | -0.7592572 |
| Kifc2 | 0.4490522 |
| Kifc3 | 0.3516284 |
| Kin | -0.7383204 |
| Kirrel3 | -0.6016772 |
| Kiss1 | -0.5140934 |
| Kiss1r | 0.3023393 |
| Kitl | -0.7320439 |
| Kl | -0.4304412 |
| Klb | -0.6331414 |
| Klc1 | 1.9354705 |
| Klc1 | 0.6717813 |
| Klc1 | -0.7021655 |
| Klc2 | -0.1355747 |
| Klc2 | 0.2150227 |
| Klc4 | 2.4707959 |
| Klf1 | 0.5962334 |
| Klf10 | -0.3273067 |
| Klf12 | -0.3831388 |
| Klf12 | 0.157056 |
| Klf12 | 0.8717734 |
| Klf13 | -0.682502 |
| Klf16 | -0.5296561 |
| Klf17 | 0.0407475 |
| Klf17 | 0.2118985 |
| Klf2 | 0.3026223 |
| Klf4 | 0.0741867 |
| Klf4 | 0.6416203 |
| Klf4 | -0.4247081 |
| Klf5 | -0.4491672 |
| Klf6 | 0.3457141 |
| Klf6 | -0.3405095 |
| Klf7 | -0.6492959 |
| Klf9 | 0.9639859 |
| Klhdc1 | 1.6063321 |
| Klhdc1 | 0.4325509 |
| Klhdc1 | -0.8502806 |
| Klhdc2 | -0.6180586 |
| Klhdc3 | -0.7143485 |
| Klhdc4 | 2.2044048 |
| Klhdc4 | 1.2335929 |
| Klhdc4 | 0.2156463 |
| Klhdc8b | -0.6550192 |
| Klhdc9 | 0.1916422 |
| Klhl10 | -0.6909986 |
| Klhl11 | -0.5780274 |
| Klhl12 | -0.7620243 |
| Klhl14 | -0.3533222 |
| Klhl15 | 0.116575 |
| Klhl15 | 0.7957475 |
| Klhl17 | 0.2767894 |
| Klhl2 | -0.6808123 |
| Klhl21 | 3.0944402 |
| Klhl22 | -0.2301717 |
| Klhl22 | 0.7098254 |
| Klhl23 | 1.284129 |
| Klhl24 | 0.937133 |
| Klhl24 | 0.9119011 |
| Klhl25 | -0.4394327 |
| Klhl26 | -0.5462192 |
| Klhl26 | 0.3000721 |
| Klhl30 | 2.0863819 |
| Klhl31 | -0.6624316 |
| Klhl32 | 0.9998916 |
| Klhl5 | 0.8034025 |
| Klhl5 | -0.5183111 |
| Klhl6 | 3.6140077 |
| Klhl6 | -0.6419904 |
| Klhl7 | -0.7997566 |
| Klhl8 | 0.3806307 |
| Klhl9 | 0.812118 |
| Klhl9 | -0.5557176 |
| Klk1 | -0.7930382 |
| Klk11 | 1.1666101 |
| Klk11 | -0.6816567 |
| Klk15 | -0.1736689 |
| Klk1b1 | -0.5375395 |
| Klk1b11 | 0.1324398 |
| Klk1b16 | 0.243268 |
| Klk1b21 | 1.4028395 |
| Klk1b21 | -0.2049519 |
| Klk1b22 | -0.4133502 |
| Klk1b24 | -0.560874 |
| Klk1b26 | 1.5203609 |
| Klk1b27 | -0.5705292 |
| Klk1b27 | -0.7099784 |
| Klk1b4 | -0.7620243 |
| Klk1b4 | 1.8697567 |
| Klk1b5 | -0.7657271 |
| Klk1b8 | -0.1799359 |
| Klk1b9 | 0.993087 |
| Klk6 | -0.1955507 |
| Klk8 | -0.623586 |
| Klkb1 | -0.185273 |
| Klra17 | -0.5140934 |
| Klra4 | -0.6961353 |
| Klrd1 | -0.4743099 |
| Kndc1 | 1.048362 |
| Kng1 | 0.1426377 |
| Kng1 | -0.3978095 |
| Kng2 | 1.073696 |
| Kns2 | 0.8636196 |
| Kns2 | -0.5593977 |
| Kntc1 | 1.3001448 |
| Kpna1 | 0.4968147 |
| Kpna2 | -0.7731788 |
| Kpna3 | -0.6419904 |
| Kpna6 | 0.4246796 |
| Kpnb1 | -0.111046 |
| Kptn | -0.2683105 |
| Kras | 1.8291916 |
| Kras | -0.6094407 |
| Krba1 | -0.5765228 |
| Krba1 | 2.0247708 |
| Krba1 | -0.6987148 |
| Krcc1 | -0.5978204 |
| Kremen | 3.3671231 |
| Kremen1 | -0.8887966 |
| Kremen2 | -0.7446411 |
| Krit1 | -0.4609894 |
| Krit1 | -0.4196424 |
| Krr1 | -0.7491831 |
| Krr1 | -0.1034604 |
| Krr1 | -0.4729689 |
| Krt10 | 3.2161327 |
| Krt12 | -0.2953981 |
| Krt13 | 0.2764982 |
| Krt14 | -0.2347873 |
| Krt17 | -0.7519194 |
| Krt18 | -0.5183111 |
| Krt19 | 0.4039365 |
| Krt2 | -0.6649161 |
| Krt20 | 1.2697566 |
| Krt222 | -0.7806927 |
| Krt23 | -0.1518377 |
| Krt27 | -0.2358162 |
| Krt32 | 2.1811018 |
| Krt4 | 2.0428671 |
| Krt7 | -0.2860966 |
| Krt73 | 2.3236992 |
| Krt75 | 0.2712397 |
| Krt78 | -0.7500943 |
| Krt79 | -0.5549834 |
| Krt8 | -0.8074967 |
| Krt84 | -0.6180586 |
| Krt86 | -0.524675 |
| Krt86 | 1.1024168 |
| Krt9 | -0.6591297 |
| Krtap16-4 | 0.0900933 |
| Krtap8-2 | -0.6833481 |
| Krtcap2 | 0.1228163 |
| Krtcap3 | 1.1961243 |
| Ksr2 | -0.5140934 |
| Ktelc1 | 0.6661318 |
| Kti12 | 1.4058335 |
| Ktn1 | 0.9094539 |
| Ktn1 | -0.7703772 |
| Ky | -0.3416668 |
| L1cam | 2.5288284 |
| L2hgdh | 0.8634679 |
| L3mbtl2 | 1.203536 |
| L3mbtl3 | -0.6468541 |
| l7Rn6 | 1.3256455 |
| Lace1 | 1.4491131 |
| Lad1 | -0.7365227 |
| Lag3 | 2.6317092 |
| Lage3 | 0.4869558 |
| Lama1 | -0.6243784 |
| Lama2 | 0.3834802 |
| Lamb1-1 | -0.6987148 |
| Lamb2 | 2.203105 |
| Lamb3 | -0.7491831 |
| Lamc1 | 1.9154309 |
| Lamc2 | -0.4736392 |
| Lamc2 | 1.9685901 |
| Lamc2 | 0.4869558 |
| Lamc3 | 0.2546806 |
| Lamp1 | 0.5552986 |
| Lamp2 | -0.1420447 |
| Lamp2 | -0.7284769 |
| Lamp2 | -0.5795346 |
| Lamp2 | -0.4355682 |
| Lancl1 | -0.6251716 |
| Lancl1 | -0.1684 |
| Lancl1 | 0.4718448 |
| Laptm4a | -0.7446411 |
| Laptm4b | -0.7392207 |
| Laptm4b | -0.4590082 |
| Laptm5 | 0.7705354 |
| Large | -0.5098953 |
| Larp2 | 1.8345181 |
| Larp5 | 1.0905905 |
| Larp6 | 0.0403689 |
| Larp6 | -0.5078036 |
| Lars | 0.6355821 |
| Lars2 | -0.5382596 |
| Las1l | -0.6196344 |
| Lasp1 | 0.9454811 |
| Lasp1 | -0.1574819 |
| Lasp1 | -0.6558398 |
| Lass1 | 0.8918405 |
| Lass2 | -0.2519167 |
| Lass2 | -0.5535167 |
| Lass2 | -0.5593977 |
| Lass4 | 0.1767961 |
| Lass4 | -0.7240379 |
| Lass5 | -0.2119425 |
| Lass6 | -0.1296082 |
| Lat2 | -0.5078036 |
| Lat2 | -0.5772748 |
| Lats2 | -0.671575 |
| Lbh | -0.2888223 |
| Lbh | -0.3216262 |
| Lbp | -0.7648 |
| Lbr | -0.7266986 |
| Lbx2 | -0.6315417 |
| Lca5 | 1.2751492 |
| Lca5 | -0.5962823 |
| Lca5 | 0.7098254 |
| Lcat | -0.0963789 |
| Lcat | 0.4079725 |
| Lce1c | 0.2230954 |
| Lck | -0.5253849 |
| Lcmt1 | -0.0720237 |
| Lcmt1 | 1.3729413 |
| Lcmt1 | -0.6347438 |
| Lcn10 | -0.5818001 |
| Lcn12 | 1.5908511 |
| Lcn13 | 1.1010198 |
| Lcn2 | -0.2440902 |
| Lcn3 | -0.6640872 |
| Lcn8 | -0.0387492 |
| Lcor | 0.7605555 |
| Lcp1 | -0.4570314 |
| Ldb1 | -0.5863483 |
| Ldb2 | 0.968857 |
| Ldb2 | 0.5923457 |
| Ldha | -0.4433139 |
| Ldhc | -0.6468541 |
| Ldhc | -0.1641119 |
| Ldhc | -0.6724109 |
| Ldhd | 0.8113146 |
| Ldhd | -0.675762 |
| Ldoc1 | 2.6340023 |
| Ldoc1l | 3.0189283 |
| Lef1 | -0.6110014 |
| Lef1 | 0.1463481 |
| Lefty1 | 0.5818267 |
| Lefty2 | -0.5071074 |
| Lemd2 | 2.9490267 |
| Leng1 | 3.0385777 |
| Leng8 | 2.039671 |
| Leng9 | -0.7266986 |
| Leo1 | 0.0572508 |
| Lep | 1.556261 |
| Lepre1 | -0.2888223 |
| Lepre1 | -0.5411459 |
| Lepre1 | 2.1159762 |
| Leprel1 | -0.4089717 |
| Leprel2 | -0.1570102 |
| Leprotl1 | 1.4529034 |
| Letm1 | 1.1502142 |
| Letm1 | 2.9948165 |
| Letm1 | 1.7055679 |
| Letmd1 | 0.997019 |
| Lgals1 | -0.0963789 |
| Lgals2 | 2.9448935 |
| Lgals3 | -0.6995762 |
| Lgals3bp | 0.9470032 |
| Lgals4 | -0.4994851 |
| Lgals4 | 1.4773116 |
| Lgals6 | -0.7437354 |
| Lgals7 | -0.5802891 |
| Lgals8 | -0.4960416 |
| Lgals8 | 0.3795923 |
| Lgals9 | 3.7163795 |
| Lgals9 | 0.2920917 |
| Lgi1 | 1.5396714 |
| Lgi1 | 1.2357019 |
| Lgi2 | -0.6533803 |
| Lgi3 | -0.621213 |
| Lgi4 | -0.5924485 |
| Lgmn | 1.9381856 |
| Lgr5 | 2.2835703 |
| Lgtn | 0.2779534 |
| Lgtn | -0.4656292 |
| Lhb | -0.3904443 |
| Lhcgr | 1.1801555 |
| Lhcgr | -0.1886859 |
| Lhfp | 2.6711394 |
| Lhfp | -0.3728899 |
| Lhfp | 0.2647706 |
| Lhfpl1 | 1.4262737 |
| Lhfpl2 | -0.8281371 |
| Lhx1 | 2.2733707 |
| Lhx2 | -0.64523 |
| Lhx3 | -0.425979 |
| Lhx4 | -0.682502 |
| Lhx9 | -0.6952772 |
| Lhx9 | 0.1307288 |
| Lias | 1.9539453 |
| Lias | 1.7089533 |
| Lias | 3.1511275 |
| Lifr | -0.5050222 |
| Lig1 | -0.9357335 |
| Lig1 | 0.1141361 |
| Lig1 | 1.0513218 |
| Lig3 | -0.6901453 |
| Lig4 | -0.0195214 |
| Lima1 | -0.368107 |
| Lima1 | 1.0125776 |
| Limd1 | -0.7222684 |
| Limd2 | -0.8533428 |
| Lime1 | 0.0369541 |
| Limk1 | -0.8845523 |
| Limk2 | -0.5043282 |
| Limk2 | 1.6332988 |
| Lims2 | 2.0016906 |
| Lims2 | -0.2363311 |
| Lims2 | -0.5586605 |
| Lin54 | -0.6665762 |
| Lin7b | 1.5976526 |
| Lin7b | 1.2632621 |
| Lin7b | -0.1901525 |
| Lin7b | -0.5549834 |
| Lin7c | -0.560874 |
| Lincr | 1.9176902 |
| Lincr | -0.7455476 |
| Lingo1 | -0.5955143 |
| Lingo2 | -0.893061 |
| Lingo2 | 1.2112995 |
| Lingo4 | -0.659954 |
| Lip1 | 0.0231641 |
| Lipe | -0.3486419 |
| Lipf | 0.6023378 |
| Liph | 1.4316147 |
| Liph | -0.3199291 |
| Litaf | 1.8690126 |
| Lix1 | -0.4183805 |
| Lix1 | -0.7108508 |
| Lix1 | -0.3527358 |
| Lix1 | 1.547508 |
| Llgl1 | 0.4702022 |
| Llgl2 | -0.0022829 |
| Lman1 | 0.7355479 |
| Lman2 | 0.36809 |
| Lman2l | -0.6892929 |
| Lman2l | 1.3139515 |
| Lmbr1 | -0.6816567 |
| Lmbr1l | -0.7073666 |
| Lmcd1 | -0.6339422 |
| Lmcd1 | -0.6251716 |
| Lmcd1 | -0.6323412 |
| Lmf1 | -0.0474749 |
| Lmf2 | -0.671575 |
| Lmna | 3.1760928 |
| Lmna | 0.6721567 |
| Lmna | -0.1975218 |
| Lmna | -0.6063272 |
| Lmna | -0.6699057 |
| Lmnb1 | -0.5962823 |
| Lmnb1 | -0.7266986 |
| Lmnb2 | -0.6243784 |
| Lmo2 | 1.1510962 |
| Lmo3 | -0.0828152 |
| Lmo3 | -0.3922799 |
| Lmo4 | -0.6196344 |
| Lmod1 | 1.8136538 |
| Lmtk2 | 0.3315782 |
| Lmtk3 | 0.8021042 |
| Lmx1a | -0.573521 |
| Lmx1b | -0.303686 |
| Lnx1 | -0.3159814 |
| Lnx2 | 0.5022707 |
| Lnx2 | -0.5513212 |
| LOC100038882 | -0.5375395 |
| LOC100038984 | -0.269913 |
| LOC100039087 | -0.2044547 |
| LOC100039227 | 2.6217691 |
| LOC100039479 | -0.7161024 |
| LOC100039532 | -0.1466947 |
| LOC100039571 | -0.6363491 |
| LOC100039728 | -0.6724109 |
| LOC100040573 | -0.5593977 |
| LOC100040592 | 1.8681687 |
| LOC100040601 | -0.5260954 |
| LOC100040799 | -0.2009816 |
| LOC100040899 | 0.6039586 |
| LOC100040919 | -0.128237 |
| LOC100041103 | -0.7266986 |
| LOC100041290 | 0.1767961 |
| LOC100041463 | -0.0959382 |
| LOC100041483 | 1.3350362 |
| LOC100041500 | -0.6468541 |
| LOC100041504 | 0.6094076 |
| LOC100041703 | 0.0411259 |
| LOC100041722 | 1.7550024 |
| LOC100041725 | -0.7021655 |
| LOC100041835 | 2.6382333 |
| LOC100042179 | 0.2426633 |
| LOC100042427 | -0.4905592 |
| LOC100042777 | -0.6371529 |
| LOC100043257 | -0.6180586 |
| LOC100043391 | -0.425979 |
| LOC100043671 | 3.2616413 |
| LOC100043822 | 3.0938298 |
| LOC100043986 | -0.0558646 |
| LOC100044087 | 0.3891522 |
| LOC100044089 | 3.3944371 |
| LOC100044101 | -0.0733119 |
| LOC100044103 | -0.6591297 |
| LOC100044103 | -0.7419268 |
| LOC100044122 | -0.4729689 |
| LOC100044133 | 1.1748047 |
| LOC100044159 | -0.1813883 |
| LOC100044170 | 2.7189643 |
| LOC100044172 | -0.3020223 |
| LOC100044177 | -0.7021655 |
| LOC100044190 | -0.4046144 |
| LOC100044204 | -0.082381 |
| LOC100044210 | 0.1874713 |
| LOC100044221 | -0.7047623 |
| LOC100044257 | 1.9264846 |
| LOC100044275 | -0.6799686 |
| LOC100044289 | 3.0164365 |
| LOC100044292 | -0.1513689 |
| LOC100044294 | 0.3329471 |
| LOC100044298 | 0.7418219 |
| LOC100044322 | -0.5871086 |
| LOC100044324 | 1.0416666 |
| LOC100044324 | -0.782581 |
| LOC100044363 | -0.0579743 |
| LOC100044363 | -0.2970496 |
| LOC100044439 | 1.4985321 |
| LOC100044468 | 0.2564694 |
| LOC100044475 | 1.4506943 |
| LOC100044538 | 1.2939607 |
| LOC100044566 | -0.1074683 |
| LOC100044576 | -0.5268064 |
| LOC100044636 | 1.0826586 |
| LOC100044692 | -0.0223592 |
| LOC100044708 | 2.2361727 |
| LOC100044736 | 0.3588342 |
| LOC100044756 | -0.3831388 |
| LOC100044776 | 0.6456878 |
| LOC100044779 | 1.7149799 |
| LOC100044812 | -0.5623526 |
| LOC100044829 | -0.2194932 |
| LOC100044862 | 1.1147784 |
| LOC100044873 | 0.6328418 |
| LOC100044934 | 1.582498 |
| LOC100045005 | 0.0277843 |
| LOC100045019 | -0.8719383 |
| LOC100045040 | -0.114638 |
| LOC100045098 | 2.941088 |
| LOC100045228 | 1.8893734 |
| LOC100045241 | 0.425914 |
| LOC100045250 | -0.7064977 |
| LOC100045280 | 1.0089713 |
| LOC100045300 | -0.1588985 |
| LOC100045304 | -0.4898763 |
| LOC100045343 | -0.5404234 |
| LOC100045359 | 0.5004557 |
| LOC100045359 | -0.661605 |
| LOC100045403 | 0.1395904 |
| LOC100045413 | 2.5447275 |
| LOC100045419 | 1.5119142 |
| LOC100045430 | -0.5015575 |
| LOC100045439 | 1.8463504 |
| LOC100045491 | 0.1510485 |
| LOC100045505 | -0.2779684 |
| LOC100045522 | 0.1385724 |
| LOC100045542 | 0.7423427 |
| LOC100045551 | -0.4643011 |
| LOC100045567 | -0.451781 |
| LOC100045607 | 1.7284808 |
| LOC100045617 | -0.4171203 |
| LOC100045644 | 0.5869984 |
| LOC100045697 | 0.3023393 |
| LOC100045780 | -0.0301072 |
| LOC100045782 | 1.9766829 |
| LOC100045877 | 0.2314025 |
| LOC100045882 | -0.5593977 |
| LOC100045887 | -0.6640872 |
| LOC100045950 | 0.368353 |
| LOC100045963 | 1.1617226 |
| LOC100045981 | 0.4227009 |
| LOC100046003 | 0.8315031 |
| LOC100046035 | -0.1434372 |
| LOC100046035 | 2.2279857 |
| LOC100046039 | 0.2997883 |
| LOC100046044 | -0.675762 |
| LOC100046049 | 0.804861 |
| LOC100046056 | 0.9949241 |
| LOC100046068 | 0.9925614 |
| LOC100046080 | -0.5893933 |
| LOC100046081 | 0.0968761 |
| LOC100046120 | -0.4387875 |
| LOC100046128 | 0.853399 |
| LOC100046129 | 0.848322 |
| LOC100046163 | -0.4407246 |
| LOC100046163 | -0.6558398 |
| LOC100046207 | -0.5878695 |
| LOC100046207 | -0.7064977 |
| LOC100046207 | 0.1255764 |
| LOC100046232 | -0.5901561 |
| LOC100046254 | 1.1983229 |
| LOC100046320 | 1.0682752 |
| LOC100046393 | -0.3527358 |
| LOC100046406 | -0.5197214 |
| LOC100046457 | 0.5880292 |
| LOC100046518 | -0.6640872 |
| LOC100046568 | 1.107405 |
| LOC100046586 | 2.9678339 |
| LOC100046608 | 1.5045787 |
| LOC100046608 | 0.1200478 |
| LOC100046608 | 0.0482866 |
| LOC100046608 | 1.8457396 |
| LOC100046616 | -0.2430518 |
| LOC100046641 | 0.5288452 |
| LOC100046650 | 0.9152015 |
| LOC100046690 | 3.1537018 |
| LOC100046690 | 0.1823112 |
| LOC100046741 | 0.4612326 |
| LOC100046744 | -0.6387625 |
| LOC100046744 | -0.7546641 |
| LOC100046746 | 1.4754692 |
| LOC100046775 | 1.2034321 |
| LOC100046781 | -0.6363491 |
| LOC100046781 | -0.2487779 |
| LOC100046792 | -0.5742705 |
| LOC100046796 | 0.2134619 |
| LOC100046802 | 1.4531397 |
| LOC100046841 | -0.6699057 |
| LOC100046855 | -0.782581 |
| LOC100046883 | 0.5024973 |
| LOC100046891 | 0.8480134 |
| LOC100046898 | -0.6172717 |
| LOC100046918 | -0.6196344 |
| LOC100046953 | -0.5727721 |
| LOC100046959 | -0.879275 |
| LOC100046996 | 0.9897971 |
| LOC100047009 | -0.7930382 |
| LOC100047009 | 0.522441 |
| LOC100047012 | -0.6468541 |
| LOC100047028 | -0.5462192 |
| LOC100047046 | 1.7699168 |
| LOC100047052 | 1.2628729 |
| LOC100047052 | 1.4328241 |
| LOC100047155 | -0.5939801 |
| LOC100047155 | -0.6188462 |
| LOC100047167 | -0.3387764 |
| LOC100047173 | -0.2817524 |
| LOC100047184 | 1.9114636 |
| LOC100047200 | 0.0433933 |
| LOC100047214 | 1.0635507 |
| LOC100047214 | -0.1867341 |
| LOC100047238 | 2.4241532 |
| LOC100047261 | 2.0903095 |
| LOC100047323 | -0.8172665 |
| LOC100047323 | 1.8182856 |
| LOC100047339 | 0.3208287 |
| LOC100047353 | 3.3821864 |
| LOC100047368 | 1.1192422 |
| LOC100047369 | 1.0568454 |
| LOC100047369 | -0.4196424 |
| LOC100047391 | 0.4006438 |
| LOC100047393 | -0.1411175 |
| LOC100047427 | -0.6724109 |
| LOC100047480 | 1.7516166 |
| LOC100047490 | 2.0685919 |
| LOC100047579 | 0.7528687 |
| LOC100047583 | -0.6517443 |
| LOC100047606 | -0.6164856 |
| LOC100047615 | -0.1476276 |
| LOC100047619 | -0.3070227 |
| LOC100047634 | 1.6165724 |
| LOC100047651 | -0.8172665 |
| LOC100047653 | 1.7809288 |
| LOC100047659 | -0.5218411 |
| LOC100047674 | 0.2426633 |
| LOC100047707 | -0.2932009 |
| LOC100047717 | 1.6354885 |
| LOC100047749 | 0.3107907 |
| LOC100047762 | -0.6331414 |
| LOC100047776 | -0.7117239 |
| LOC100047794 | -0.0819471 |
| LOC100047800 | 0.9176331 |
| LOC100047810 | -0.5382596 |
| LOC100047815 | -0.8341224 |
| LOC100047816 | -0.6850426 |
| LOC100047834 | -0.4939818 |
| LOC100047837 | 1.1455693 |
| LOC100047837 | 0.1557244 |
| LOC100047841 | -0.6180586 |
| LOC100047856 | 0.5824488 |
| LOC100047870 | -0.4511268 |
| LOC100047911 | -0.3109312 |
| LOC100047934 | 1.9884484 |
| LOC100047935 | -0.7583367 |
| LOC100047937 | 2.2794917 |
| LOC100047937 | 0.4504938 |
| LOC100047958 | 0.3299328 |
| LOC100047963 | -0.4355682 |
| LOC100047963 | 0.5027239 |
| LOC100047998 | -0.5750206 |
| LOC100048046 | -0.0579743 |
| LOC100048046 | -0.1828431 |
| LOC100048049 | -0.4623126 |
| LOC100048049 | -0.865697 |
| LOC100048049 | 0.8838673 |
| LOC100048056 | 2.4803544 |
| LOC100048071 | -0.4939818 |
| LOC100048076 | -0.5161998 |
| LOC100048083 | -0.1296082 |
| LOC100048105 | 2.5179181 |
| LOC100048105 | 0.4320609 |
| LOC100048187 | 1.8645855 |
| LOC100048251 | -0.5105936 |
| LOC100048295 | 0.2106457 |
| LOC100048299 | -0.334171 |
| LOC100048301 | 1.4413248 |
| LOC100048313 | -0.7592572 |
| LOC100048331 | 0.5711831 |
| LOC100048332 | 1.9638242 |
| LOC100048346 | 0.8886006 |
| LOC100048372 | 2.3388095 |
| LOC100048384 | -0.1291509 |
| LOC100048413 | -0.1296082 |
| LOC100048436 | -0.4433139 |
| LOC100048445 | 1.2728421 |
| LOC100048461 | -0.4857895 |
| LOC100048480 | 2.3574844 |
| LOC100048483 | -0.7555808 |
| LOC100048504 | 0.0825366 |
| LOC100048530 | -0.076756 |
| LOC100048554 | 2.8582039 |
| LOC100048556 | -0.6533803 |
| LOC100048589 | 1.2586781 |
| LOC100048613 | 0.7632729 |
| LOC100048622 | 2.5290906 |
| LOC100048645 | 0.1874713 |
| LOC100048684 | 0.5518733 |
| LOC100048703 | 2.2637905 |
| LOC100048710 | 2.7583378 |
| LOC100048721 | -0.6740849 |
| LOC100048721 | -0.5036347 |
| LOC100048726 | 0.6390931 |
| LOC100048733 | -0.8482449 |
| LOC100048816 | -0.5176068 |
| LOC100048832 | 0.862709 |
| LOC100048832 | -0.1940753 |
| LOC100048858 | 0.0192957 |
| LOC192758 | 0.6685843 |
| LOC194227 | -0.5491311 |
| LOC212390 | -0.8401478 |
| LOC216443 | -0.3126114 |
| LOC216963 | -0.5993611 |
| LOC217341 | 2.7619319 |
| LOC218963 | -0.569037 |
| LOC225922 | -0.7901742 |
| LOC233529 | -0.715225 |
| LOC234374 | -0.7685143 |
| LOC235497 | -0.5078036 |
| LOC236749 | -0.4609894 |
| LOC237408 | 1.0709295 |
| LOC237877 | 0.9806541 |
| LOC238829 | -0.5623526 |
| LOC241593 | -0.7125979 |
| LOC244595 | 1.2865022 |
| LOC245263 | -0.3867841 |
| LOC329575 | 0.3213831 |
| LOC329575 | 0.9913777 |
| LOC333331 | 2.9809938 |
| LOC381066 | -0.7240379 |
| LOC381302 | 0.7779066 |
| LOC381375 | -0.272054 |
| LOC381483 | 0.3213831 |
| LOC381629 | 2.2419612 |
| LOC381801 | 0.1983392 |
| LOC381936 | -0.1641119 |
| LOC382010 | 1.1196987 |
| LOC382044 | 1.4593416 |
| LOC382106 | -0.3463109 |
| LOC382106 | -0.5985904 |
| LOC382133 | -0.1809039 |
| LOC384806 | -0.5871086 |
| LOC384806 | -0.7759892 |
| LOC386486 | 1.4508523 |
| LOC433479 | -0.663259 |
| LOC433801 | 3.2103291 |
| LOC433886 | -0.6875903 |
| LOC434172 | 0.8278803 |
| LOC434172 | -0.7117239 |
| LOC434864 | -0.782581 |
| LOC434960 | 3.1147262 |
| LOC435023 | -0.5947468 |
| LOC435337 | -0.7500943 |
| LOC435565 | -0.5787807 |
| LOC436177 | -0.8523209 |
| LOC544988 | -0.2608698 |
| LOC544988 | -0.0690251 |
| LOC545013 | 0.9767834 |
| LOC545013 | -0.7564985 |
| LOC545056 | -0.3574375 |
| LOC545238 | -0.3307323 |
| LOC545703 | 1.0291346 |
| LOC545732 | 2.6433683 |
| LOC545952 | 0.1327816 |
| LOC546015 | -0.0664629 |
| LOC546015 | -0.2970496 |
| LOC546723 | 1.6426643 |
| LOC546840 | 1.6841915 |
| LOC547323 | 0.4543267 |
| LOC547343 | -0.7564985 |
| LOC620807 | -0.7056296 |
| LOC621823 | 0.1527212 |
| LOC621824 | 0.2314025 |
| LOC622404 | -0.9334952 |
| LOC622665 | 2.1213528 |
| LOC624855 | -0.7302586 |
| LOC625480 | 0.5043082 |
| LOC626152 | -0.7374211 |
| LOC628053 | 1.6033371 |
| LOC631002 | 0.0792785 |
| LOC631784 | -0.6517443 |
| LOC632431 | 2.0581971 |
| LOC639396 | -0.3892226 |
| LOC640963 | -0.4810448 |
| LOC641240 | -0.6501113 |
| LOC654426 | 1.0287559 |
| LOC654467 | 1.0207668 |
| LOC654494 | -0.5765228 |
| LOC654842 | -0.3910558 |
| LOC665250 | -0.4810448 |
| LOC666676 | -0.6047745 |
| LOC666904 | 2.3720991 |
| LOC667250 | 1.1822869 |
| LOC667370 | 1.1894963 |
| LOC667609 | 0.5462792 |
| LOC668492 | 0.2212389 |
| LOC668837 | 2.5958194 |
| LOC669168 | -0.2451298 |
| LOC669660 | 0.3443645 |
| LOC670044 | -0.368107 |
| LOC671564 | 1.3250099 |
| LOC672705 | 3.0718503 |
| LOC674706 | 0.7049534 |
| LOC675228 | 0.644721 |
| LOC675567 | -0.3358952 |
| LOC675567 | 2.4601063 |
| LOC675813 | 3.0991691 |
| LOC676420 | 0.6855671 |
| LOC676420 | -0.4272516 |
| LOC677144 | -0.1784858 |
| LOC677205 | 1.8973423 |
| LOC677317 | -0.4524356 |
| LOC677319 | 1.2021835 |
| LOC677528 | 1.0169374 |
| LOC732482 | 0.6381192 |
| Loh11cr2a | 0.4807097 |
| Loh12cr1 | 1.8475201 |
| Loh12cr1 | 0.0270159 |
| Lonp2 | -0.4330011 |
| Lonrf1 | 2.0253132 |
| Lonrf2 | -0.4550589 |
| Lor | -0.4323604 |
| Lox | 0.5807887 |
| Loxl1 | -0.6055506 |
| Lpar2 | 0.4694973 |
| Lpar2 | 1.4321793 |
| Lpar3 | -0.23891 |
| Lpcat2 | -0.5682918 |
| Lpcat2 | 3.0690304 |
| Lpcat2 | -0.7311508 |
| Lpgat1 | 1.8945492 |
| Lphn1 | -0.6808123 |
| Lphn3 | -0.2571724 |
| Lpin1 | -0.4095959 |
| Lpin1 | -0.7275873 |
| Lpin2 | 2.3165157 |
| Lpin3 | 1.5303933 |
| Lpl | -0.7338327 |
| Lpxn | -0.7987938 |
| Lrat | -0.7374211 |
| Lrba | 2.4161118 |
| Lrba | 1.188334 |
| Lrch1 | 0.5162141 |
| Lrch4 | 2.443332 |
| Lrch4 | 1.8827027 |
| Lrdd | -0.7713101 |
| Lrfn1 | -0.8094422 |
| Lrfn2 | -0.1504321 |
| Lrfn3 | -0.3898332 |
| Lrfn4 | 2.3045468 |
| Lrg1 | -0.7731788 |
| Lrig1 | 0.1310713 |
| Lrig3 | 1.9450061 |
| Lrmp | -0.8182493 |
| Lrp1 | -0.3996603 |
| Lrp10 | -0.5645752 |
| Lrp11 | -0.5675473 |
| Lrp12 | 0.5945985 |
| Lrp12 | -0.8740284 |
| Lrp4 | -0.6808123 |
| Lrp5 | 1.0150166 |
| Lrp8 | -0.1250454 |
| Lrp8 | -0.4590082 |
| Lrpap1 | -0.9027304 |
| Lrpprc | -0.6607792 |
| Lrpprc | -0.6024505 |
| Lrpprc | -0.2998089 |
| Lrrc1 | -0.2817524 |
| Lrrc1 | -0.6732475 |
| Lrrc1 | 1.1644412 |
| Lrrc10 | -0.7108508 |
| Lrrc15 | 1.0455164 |
| Lrrc16b | 1.3972406 |
| Lrrc16b | -0.44982 |
| Lrrc18 | 2.5027613 |
| Lrrc2 | 1.9290413 |
| Lrrc20 | 1.6352312 |
| Lrrc20 | 0.0181319 |
| Lrrc23 | -0.6816567 |
| Lrrc23 | 0.2918054 |
| Lrrc24 | 0.6790731 |
| Lrrc26 | 0.9530661 |
| Lrrc28 | -0.3064657 |
| Lrrc29 | 0.6659428 |
| Lrrc3 | 0.9284244 |
| Lrrc30 | 1.8364238 |
| Lrrc33 | -0.5840713 |
| Lrrc34 | 1.4373251 |
| Lrrc34 | 1.255936 |
| Lrrc36 | 0.5177749 |
| Lrrc39 | -0.7056296 |
| Lrrc39 | 0.6678303 |
| Lrrc4 | -0.3290179 |
| Lrrc40 | -0.5015575 |
| Lrrc41 | -0.8094422 |
| Lrrc43 | 1.2170529 |
| Lrrc44 | -0.782581 |
| Lrrc45 | -0.5411459 |
| Lrrc46 | 0.0972317 |
| Lrrc48 | -0.7082364 |
| Lrrc48 | 2.1428363 |
| Lrrc48 | 0.5393461 |
| Lrrc49 | 1.6386349 |
| Lrrc49 | 3.2164435 |
| Lrrc49 | 2.9217534 |
| Lrrc4c | 0.8690637 |
| Lrrc50 | 3.431557 |
| Lrrc50 | -0.5225487 |
| Lrrc52 | -0.5924485 |
| Lrrc52 | 0.7593641 |
| Lrrc56 | 0.2310962 |
| Lrrc57 | -0.0239849 |
| Lrrc59 | -0.2871859 |
| Lrrc6 | -0.715225 |
| Lrrc7 | 1.233794 |
| Lrrc8 | -0.547674 |
| Lrrc8a | 2.2514511 |
| Lrrc8b | -0.1809039 |
| Lrrc8c | 0.4725478 |
| Lrrc8d | -0.2651141 |
| Lrrc9 | -0.0596656 |
| Lrrfip1 | 0.5994942 |
| Lrrfip1 | 1.471001 |
| Lrriq2 | -0.5601355 |
| Lrriq2 | 2.0270216 |
| Lrrk1 | 0.879407 |
| Lrrk2 | -0.3633494 |
| Lrrn1 | 1.2434923 |
| Lrrn2 | -0.5133923 |
| Lrrn3 | 0.9445111 |
| Lrrtm1 | -0.53682 |
| Lrrtm2 | -0.5433166 |
| Lrrtm3 | 1.1594327 |
| Lrsam1 | -0.2139498 |
| Lrsam1 | -0.5993611 |
| Lrtm1 | 0.3249783 |
| Lrtm1 | 0.3793325 |
| Lsamp | -0.240461 |
| Lsamp | -0.1462286 |
| Lsg1 | -0.5893933 |
| Lsg1 | 0.0501615 |
| Lsm10 | 1.0668242 |
| Lsm11 | 0.5413014 |
| Lsm12 | 3.5190324 |
| Lsm12 | -0.6682394 |
| Lsm12 | 0.6422025 |
| Lsm14a | 1.3376374 |
| Lsm14b | -0.7004385 |
| Lsm14b | -0.1847865 |
| Lsm14b | 0.6420085 |
| Lsm2 | 1.7653626 |
| Lsm2 | 1.3267341 |
| Lsm3 | -0.5397016 |
| Lsm4 | 2.1691311 |
| Lsm5 | -0.2932009 |
| Lsm6 | 1.3321572 |
| Lsm6 | 1.2284504 |
| Lsm8 | -0.2981523 |
| Lsr | -0.8502806 |
| Lst1 | -0.6649161 |
| Ltb4dh | -0.359207 |
| Ltb4r1 | -0.7473636 |
| Ltb4r2 | 1.5746863 |
| Ltbp1 | 0.3712416 |
| Ltbp1 | 0.6098099 |
| Ltbp2 | -0.3255988 |
| Ltbp3 | 2.3737146 |
| Ltbp3 | -0.4810448 |
| Ltbp4 | -0.6110014 |
| Ltbp4 | -0.7583367 |
| Ltbp4 | 2.1778688 |
| Ltbr | 2.3191674 |
| Ltc4s | -0.7419268 |
| Ltf | 0.3147077 |
| Ltf | -0.4743099 |
| Ltk | 0.2156463 |
| Ltk | -0.1277803 |
| Ltv1 | -0.0876048 |
| Luc7l | -0.0932982 |
| Luc7l | -0.1388039 |
| Luc7l2 | 0.1751675 |
| Luc7l2 | -0.1588985 |
| Lum | 1.8728254 |
| Luzp1 | 2.1941262 |
| Luzp1 | -0.7987938 |
| Luzp1 | -0.7293673 |
| Luzp2 | 0.1279845 |
| Lxn | -0.4039936 |
| Ly6a | 1.7438375 |
| Ly6c1 | -0.7788083 |
| Ly6d | 1.1421254 |
| Ly6e | -0.4912427 |
| Ly6f | 0.3020562 |
| Ly6g6c | 0.2252564 |
| Ly6g6d | -0.0416484 |
| Ly6g6e | 0.3006395 |
| Ly6k | -0.6001325 |
| Ly86 | 0.3521642 |
| Ly9 | -0.6094407 |
| Ly9 | 2.1259518 |
| Ly96 | 0.8143639 |
| Lyar | -0.4596681 |
| Lyl1 | 2.2462783 |
| Lynx1 | 0.4006438 |
| Lynx1 | 1.592202 |
| Lypd1 | -0.0058637 |
| Lypd3 | -0.7510064 |
| Lypd4 | 0.2175143 |
| Lypd5 | -0.479019 |
| Lypd6 | 0.9791879 |
| Lypla1 | -0.6901453 |
| Lypla1 | 0.1890778 |
| Lypla2 | 0.4790833 |
| Lypla3 | -0.6541994 |
| Lyplal1 | -0.5043282 |
| Lyrm1 | -0.5848297 |
| Lyrm2 | -0.3776984 |
| Lyrm2 | 0.8084162 |
| Lyrm5 | -0.4590082 |
| Lysmd1 | -0.5682918 |
| Lysmd1 | -0.2009816 |
| Lysmd2 | 0.0868625 |
| Lysmd4 | 0.2399371 |
| Lyst | -0.67074 |
| Lyve1 | -0.2493003 |
| Lyz | 0.2128369 |
| Lyz1 | 1.0783677 |
| Lyz1 | -0.4729689 |
| Lyz2 | 3.7058619 |
| Lyzl1 | 2.8905396 |
| Lyzl4 | 2.1294136 |
| Lyzl6 | 3.7686662 |
| Lyzs | -0.2276177 |
| Lzic | -0.4696252 |
| Lztr1 | -0.6649161 |
| Lzts2 | -0.4756528 |
| M6pr | 0.5618982 |
| M6pr | -0.4478632 |
| M6prbp1 | 0.8569207 |
| Mab21l2 | 0.6017292 |
| Macrod1 | -0.561613 |
| Mad1l1 | -0.5727721 |
| Mad2l1 | 1.1432378 |
| Mad2l1bp | -0.8482449 |
| Mad2l1bp | -0.6141311 |
| Mad2l1bp | 2.8953896 |
| Mad2l2 | -0.772244 |
| Madcam1 | 0.6114174 |
| Madd | 0.1172706 |
| Maea | 1.1889681 |
| Maf | 0.085783 |
| Maf1 | -0.3807168 |
| Mafg | 1.5897012 |
| Mag | -0.573521 |
| Magea2 | -0.2747375 |
| Magea5 | -0.640375 |
| Mageb3 | -0.6024505 |
| Mageb3 | -0.3020223 |
| Maged1 | 1.1285558 |
| Maged2 | 0.1593814 |
| Magee1 | -0.1092554 |
| Mageh1 | -0.3922799 |
| Magi1 | -0.4609894 |
| Magi1 | 1.0842031 |
| Magi1 | -0.593214 |
| Magi2 | -0.5382596 |
| Magi3 | -0.2937497 |
| Magi3 | -0.7056296 |
| Magix | -0.5505906 |
| Magmas | 3.1157199 |
| Magoh | 0.8357374 |
| Magoh | -0.4400784 |
| Magohb | 1.4767746 |
| Mak | -0.4682912 |
| Mak10 | 0.9834466 |
| Mak10 | -0.3533222 |
| Mal | 2.1405274 |
| Mal | 0.7062191 |
| Mall | -0.6141311 |
| Malt1 | 0.6572088 |
| Mamdc2 | 0.4352408 |
| Maml1 | -0.7703772 |
| Maml3 | 0.9895334 |
| Man1a | 1.0110338 |
| Man1a2 | -0.6665762 |
| Man1a2 | 1.6028036 |
| Man1b1 | 2.726783 |
| Man1c1 | 0.2143987 |
| Man1c1 | -0.3758922 |
| Man2a1 | 2.5473697 |
| Man2a2 | 1.5882109 |
| Man2a2 | 1.8518801 |
| Man2a2 | 0.498637 |
| Man2b1 | -0.1843002 |
| Man2b2 | 0.9219083 |
| Man2c1 | -0.7222684 |
| Manba | -0.6164856 |
| Manba | 2.0706169 |
| Manbal | -0.0002997 |
| Manea | -0.4524356 |
| Mansc1 | 0.6351911 |
| Maob | 1.7895883 |
| Map1lc3a | -0.3137334 |
| Map1lc3a | -0.1434372 |
| Map1lc3b | 1.7399513 |
| Map2k1 | 1.5211647 |
| Map2k2 | 0.293522 |
| Map2k2 | -0.5505906 |
| Map2k3 | 1.8808926 |
| Map2k4 | -0.8084689 |
| Map2k6 | 0.7025982 |
| Map2k6 | 0.1751675 |
| Map2k6 | -0.2882765 |
| Map2k7 | 0.4636017 |
| Map2k7 | 0.2043557 |
| Map2k7 | -0.64523 |
| Map3k1 | 0.9204855 |
| Map3k11 | 0.4741861 |
| Map3k12 | 3.54709 |
| Map3k12 | 0.668961 |
| Map3k12 | 0.6118188 |
| Map3k14 | 2.6805918 |
| Map3k3 | 0.2267969 |
| Map3k6 | 2.6399833 |
| Map3k7 | 0.5853467 |
| Map3k7ip1 | 0.6349956 |
| Map3k7ip1 | -0.5863483 |
| Map3k7ip1 | -0.6892929 |
| Map3k7ip2 | -0.5353826 |
| Map3k7ip3 | -0.0941773 |
| Map3k8 | 2.0496721 |
| Map3k9 | 0.1141361 |
| Map4k2 | 3.0090312 |
| Map4k4 | -0.6782836 |
| Map4k5 | 1.0026252 |
| Map4k5 | 0.7316922 |
| Mapbpip | -0.2598119 |
| Mapk1 | 1.7043174 |
| Mapk1 | -0.3273067 |
| Mapk1 | -0.6740849 |
| Mapk10 | 0.882977 |
| Mapk10 | 0.2307898 |
| Mapk11 | -0.0977025 |
| Mapk12 | -0.132816 |
| Mapk12 | -0.5527843 |
| Mapk12 | 0.2759154 |
| Mapk13 | -0.7437354 |
| Mapk13 | -0.7258108 |
| Mapk14 | 0.1085365 |
| Mapk15 | 0.2860597 |
| Mapk15 | -0.1466947 |
| Mapk15 | -0.412097 |
| Mapk1ip1 | 1.9060036 |
| Mapk1ip1 | -0.7583367 |
| Mapk1ip1 | 2.3029552 |
| Mapk3 | -0.659954 |
| Mapk3 | 1.2092358 |
| Mapk4 | 1.1067106 |
| Mapk6 | 1.167909 |
| Mapk6 | 0.4386528 |
| Mapk6 | -0.5985904 |
| Mapk8 | -0.6196344 |
| Mapk8ip1 | -0.4524356 |
| Mapk8ip1 | 2.6108263 |
| Mapk8ip2 | -0.1906419 |
| Mapk8ip3 | 2.4895635 |
| Mapk8ip3 | 0.0803658 |
| Mapk9 | 0.7559513 |
| Mapk9 | -0.0179038 |
| Mapk9 | 1.0300176 |
| Mapkap1 | 0.9439564 |
| Mapkap1 | -0.4590082 |
| Mapkapk2 | 0.2624067 |
| Mapkapk3 | 0.2396337 |
| Mapkbp1 | 0.0467838 |
| Mapre1 | -0.6774423 |
| Mapre2 | -0.5645752 |
| Mapre2 | -0.56606 |
| Mapre3 | -0.6740849 |
| Mapt | -0.4783448 |
| Mapt | 0.3906929 |
| Mapt | -0.7169807 |
| Mapt | -0.5668034 |
| March1 | -0.3633494 |
| March2 | -0.7882699 |
| March2 | 0.6964091 |
| March4 | -0.4021339 |
| March4 | -0.2260887 |
| March6 | -0.4967292 |
| March7 | -0.6674074 |
| March8 | 3.0972451 |
| March10 | -0.3615717 |
| Marcks | 0.3870938 |
| Marcksl1 | 0.0142418 |
| Marcksl1 | -0.2079412 |
| Marcksl1 | -0.3544961 |
| Mare | -0.0529193 |
| Mark1 | 2.3299047 |
| Mark2 | -0.0086587 |
| Mark2 | 0.9180616 |
| Mark3 | 0.1493726 |
| Mark3 | -0.4071017 |
| Mark4 | -0.2435708 |
| Mars | 0.7848868 |
| Mars2 | -0.477671 |
| Marveld3 | -0.6032245 |
| Marveld3 | 2.4805556 |
| Mas1 | -0.4298024 |
| Masp1 | -0.6550192 |
| Masp2 | -0.5802891 |
| Mast1 | -0.1722289 |
| Mast1 | -0.1722289 |
| Mast2 | -0.2571724 |
| Mast2 | 0.2353751 |
| Mat1a | -0.1551259 |
| Mat2a | 0.1600447 |
| Mat2a | 1.6246164 |
| Mat2a | 1.0875208 |
| Mat2a | -0.5036347 |
| Mat2b | -0.4696252 |
| Mat2b | -0.7769279 |
| Matk | 0.2230954 |
| Matk | 0.4134927 |
| Matn4 | -0.4550589 |
| Matn4 | -0.7161024 |
| Matn4 | 2.1332691 |
| Matr3 | 0.6170211 |
| Max | -0.4682912 |
| Maz | -0.7320439 |
| Mb | 0.5512296 |
| Mbc2 | -0.6141311 |
| Mbd1 | -0.5653173 |
| Mbd1 | -0.4729689 |
| Mbd3 | 1.1358707 |
| Mbd3l2 | -0.1236811 |
| Mbd4 | 0.5669746 |
| Mbd5 | -0.5015575 |
| Mbd6 | 2.432256 |
| Mbip | -0.345729 |
| Mbl2 | 0.2656555 |
| Mbnl1 | -0.7901742 |
| Mbnl2 | 0.049037 |
| Mbnl2 | -0.4851101 |
| Mboat1 | 0.2277199 |
| Mboat2 | -0.267777 |
| Mboat2 | 1.717156 |
| Mboat2 | 1.7664199 |
| Mboat2 | -0.6371529 |
| Mboat5 | -0.7169807 |
| Mboat5 | 1.6461672 |
| Mbp | -0.6657458 |
| Mbp | -0.5112925 |
| Mbp | -0.3480586 |
| Mbtd1 | 1.0554983 |
| Mbtps1 | -0.7143485 |
| Mbtps1 | -0.6024505 |
| Mbtps1 | 1.5456619 |
| Mbtps2 | 0.6687726 |
| Mc3r | -0.1047944 |
| Mc4r | 0.3205513 |
| Mc5r | 0.350556 |
| Mcam | -0.6674074 |
| Mcam | 1.9702322 |
| Mcam | 0.3060113 |
| Mcat | 0.7732228 |
| Mcc | -0.6476673 |
| Mccc1 | -0.4171203 |
| Mcee | -0.3849596 |
| Mcf2l | -0.1236811 |
| Mcf2l | 3.769555 |
| Mcf2l | -0.0836841 |
| Mcfd2 | -0.659954 |
| Mchr1 | -0.4616507 |
| Mcl1 | -0.644419 |
| Mcm10 | -0.4002781 |
| Mcm10 | -0.6799686 |
| Mcm2 | 2.4631881 |
| Mcm3 | 3.1963411 |
| Mcm4 | 1.4946288 |
| Mcm5 | -0.6867403 |
| Mcm6 | -0.8261509 |
| Mcm6 | 0.2109591 |
| Mcm6 | 0.7033235 |
| Mcm7 | 0.5857599 |
| Mcoln1 | 0.325806 |
| Mcoln2 | 1.8685659 |
| Mcoln3 | 0.4349966 |
| Mcph1 | -0.4736392 |
| Mcph1 | -0.5411459 |
| Mcph1 | 1.4648793 |
| Mcph1 | 0.7166379 |
| Mcpt4 | 0.2612224 |
| Mcrs1 | -0.6243784 |
| Mcrs1 | -0.5878695 |
| Mcrs1 | 3.1753031 |
| Mcts1 | -0.6935633 |
| Mdfic | -0.521134 |
| Mdga1 | 1.5593465 |
| Mdga2 | -0.5462192 |
| Mdga2 | 0.6394823 |
| Mdh1 | -0.4478632 |
| Mdh1 | 0.337858 |
| Mdh1b | -0.7546641 |
| Mdh2 | -0.7347285 |
| Mdk | 1.2763964 |
| Mdk | 3.8543591 |
| Mdm1 | 0.3526996 |
| Mdm1 | -0.6574832 |
| Mdm1 | 2.3281786 |
| Mdm1 | -0.628351 |
| Mdm1 | -0.5855887 |
| Mdm1 | -0.5505906 |
| Mdm1 | -0.7564985 |
| Mdm1 | 1.3266434 |
| Mdm1 | -0.4002781 |
| Mdm2 | -0.240461 |
| Mdm2 | -0.4550589 |
| Mdm4 | 0.0789157 |
| Mdn1 | 0.5334544 |
| Me2 | -0.4221717 |
| Me3 | 2.4022825 |
| Mea1 | -0.6251716 |
| Mecp2 | 0.2221676 |
| Mecr | 0.4924718 |
| Med1 | 0.1712469 |
| Med1 | -0.6690721 |
| Med10 | 1.4837304 |
| Med11 | 0.8114753 |
| Med11 | 0.303188 |
| Med12 | 0.7269375 |
| Med15 | 1.1142045 |
| Med15 | 1.6231829 |
| Med15 | 0.6158232 |
| Med16 | 1.0721333 |
| Med18 | -0.4374984 |
| Med18 | 1.5875327 |
| Med19 | -0.5282301 |
| Med21 | -0.6640872 |
| Med22 | -0.2855524 |
| Med22 | 0.2897986 |
| Med23 | -0.3953477 |
| Med23 | 2.2286514 |
| Med24 | 1.8027169 |
| Med25 | -0.4709612 |
| Med25 | -0.3621639 |
| Med26 | 0.0304685 |
| Med27 | -0.2812108 |
| Med28 | -0.644419 |
| Med30 | 1.3371895 |
| Med31 | 2.029599 |
| Med4 | -0.2059473 |
| Med6 | 0.2206192 |
| Med7 | 1.4994304 |
| Med8 | -0.1016848 |
| Med8 | 2.0722811 |
| Med9 | -0.7213849 |
| Mef2b | 1.3587931 |
| Mef2b | -0.6149153 |
| Mef2c | -0.6419904 |
| Mef2c | 1.8578785 |
| Mef2c | -0.4756528 |
| Megf10 | -0.5050222 |
| Megf11 | -0.8065256 |
| Megf11 | -0.483753 |
| Megf11 | -0.6566611 |
| Megf11 | 2.5386892 |
| Megf6 | -0.514795 |
| Megf9 | -0.5260954 |
| Mei1 | 2.6104999 |
| Meis1 | 1.5516833 |
| Meis2 | -0.7231527 |
| Meis3 | 1.6314915 |
| Melk | 2.1131434 |
| Memo1 | -0.284465 |
| Men1 | 2.7455037 |
| Men1 | -0.6927076 |
| Meox1 | -0.6436087 |
| Meox2 | 1.3575675 |
| Mep1b | -0.6766018 |
| Mepce | -0.1560675 |
| Mertk | -0.7099784 |
| Mesdc1 | -0.2240542 |
| Mesp1 | -0.7873192 |
| Mest | -0.3233266 |
| Mest | -0.7383204 |
| Mest | -0.2910089 |
| Metap1 | 2.2227428 |
| Metap2 | -0.6901453 |
| Metap2 | 0.7661511 |
| Metapl1 | -0.4253433 |
| Metrn | -0.5535167 |
| Metrnl | 0.0118996 |
| Metrnl | -0.3562598 |
| Mett11d1 | -0.3463109 |
| Mettl1 | -0.7713101 |
| Mettl1 | 0.4767546 |
| Mettl2 | -0.761101 |
| Mettl3 | -0.5825565 |
| Mettl4 | -0.6063272 |
| Mettl6 | -0.7620243 |
| Mettl8 | 1.0306477 |
| Mettl9 | -0.3693004 |
| Mettl9 | -0.1439018 |
| Mex3a | 3.2791406 |
| Mex3a | 0.2240222 |
| Mex3c | 0.3981028 |
| Mfap1b | -0.5022494 |
| Mfap2 | 2.0722811 |
| Mfap2 | 0.6591148 |
| Mfap2 | -0.2094396 |
| Mfap3 | 3.2159883 |
| Mfap3 | 2.0698233 |
| Mfap3l | -0.3550836 |
| Mfap3l | -0.1607908 |
| Mfap4 | 0.7576593 |
| Mfap5 | 3.1411903 |
| Mfge8 | 1.7591048 |
| Mfge8 | 0.0854228 |
| Mfi2 | -0.1741495 |
| Mfn1 | -0.5579238 |
| Mfn1 | 1.1031145 |
| Mfn2 | -0.5542498 |
| Mfng | -0.547674 |
| Mfrp | -0.4810448 |
| Mfsd1 | -0.6180586 |
| Mfsd10 | -0.621213 |
| Mfsd10 | 2.6274474 |
| Mfsd11 | 1.4435569 |
| Mfsd2 | 4.6001895 |
| Mfsd3 | -0.5126918 |
| Mfsd4 | 1.1056681 |
| Mfsd7 | -0.6640872 |
| Mfsd8 | 1.2164382 |
| Mfsd9 | 0.594394 |
| Mga | 0.1341475 |
| Mgat1 | 0.3762092 |
| Mgat3 | 1.5323432 |
| Mgat4b | 0.5510149 |
| Mgat4b | -0.4228051 |
| Mgat5 | -0.6267599 |
| MGC107098 | 1.1251596 |
| MGC117608 | -0.7073666 |
| MGC118210 | 0.9642573 |
| MGC18837 | 0.77974 |
| MGC41689 | -0.6188462 |
| MGC67181 | -0.7302586 |
| Mgea6 | 0.6951296 |
| Mgl1 | -0.6624316 |
| Mgl2 | 0.4308349 |
| Mgll | 2.0141075 |
| Mgll | -0.5772748 |
| Mgll | -0.629945 |
| Mgmt | 0.3912059 |
| Mgp | 0.8270908 |
| Mgrn1 | 3.2740639 |
| Mgst1 | -0.6732475 |
| Mgst1 | 0.0479111 |
| Mgst2 | 1.3028487 |
| Mgst3 | 1.0594115 |
| Mgst3 | -0.715225 |
| Mia1 | -0.2693785 |
| Mia3 | -0.0690251 |
| Mib1 | 2.2290173 |
| Mib2 | 0.7673336 |
| Mic2l1 | -0.114638 |
| Mical1 | 1.1644412 |
| Mical1 | -0.0897903 |
| Mical1 | -0.5878695 |
| Mical2 | 0.8267749 |
| Mical3 | 1.5582257 |
| Micall2 | -0.5833136 |
| Micall2 | -0.0639079 |
| Mid1 | 1.544595 |
| Mid1 | 0.157056 |
| Mid1ip1 | -0.1264118 |
| Midn | -0.3586168 |
| Midn | 1.2522997 |
| Mier1 | 0.8376129 |
| Mier1 | -0.2672438 |
| Mif | -0.1388039 |
| Mif4gd | -0.3935058 |
| Mif4gd | -0.468958 |
| Mill2 | 2.1308243 |
| Mill2 | -0.3399314 |
| Mina | -0.1016848 |
| Mink1 | -0.3728899 |
| Mink1 | -0.7249239 |
| Minpp1 | 1.1329519 |
| Minpp1 | -0.7428306 |
| Miox | -0.4844313 |
| Mipep | 3.5918601 |
| Mir16 | -0.7392207 |
| Mist1 | -0.5133923 |
| Mitf | -0.5296561 |
| Mizf | -0.2812108 |
| Mki67ip | -0.5289428 |
| Mkks | -0.115088 |
| Mkks | 2.0549743 |
| Mkl1 | 1.932146 |
| Mkl2 | 0.9207702 |
| Mkl2 | -0.3451476 |
| Mkl2 | -0.6732475 |
| Mkln1 | 1.0023652 |
| Mknk1 | -0.8172665 |
| Mknk2 | 1.0092293 |
| Mkrn1 | 1.5278594 |
| Mkrn2 | -0.5303699 |
| Mkrn3 | 0.5606247 |
| Mkx | 0.9353139 |
| Mlana | 0.2062473 |
| Mlf1 | -0.5586605 |
| Mlf1 | -0.3568485 |
| Mlf2 | 2.3633473 |
| Mlf2 | -0.5787807 |
| Mlh1 | -0.6196344 |
| Mlh3 | 1.1501039 |
| Mlkl | -0.4183805 |
| Mllt1 | 0.1450006 |
| Mllt1 | 0.3219371 |
| Mllt1 | -0.4967292 |
| Mllt10 | -0.0919811 |
| Mllt11 | 0.0216187 |
| Mllt3 | -0.2019725 |
| Mllt3 | -0.5001753 |
| Mllt3 | -0.56606 |
| Mlp | -0.6460417 |
| Mlstd2 | 1.2481542 |
| Mlx | 0.576625 |
| Mlx | -0.7222684 |
| Mlxipl | -0.6009045 |
| Mlycd | -0.2260887 |
| Mmab | -0.3783013 |
| Mmab | 0.2365939 |
| Mmd | 0.5847265 |
| Mmd2 | 1.3112809 |
| Mme | -0.1975218 |
| Mme | 0.8931625 |
| Mmel1 | 0.836363 |
| Mmp10 | -0.5712762 |
| Mmp11 | -0.0994702 |
| Mmp12 | 3.0914225 |
| Mmp13 | 1.2094424 |
| Mmp13 | 1.2498344 |
| Mmp14 | 2.0861871 |
| Mmp15 | -0.7741146 |
| Mmp16 | -0.8271434 |
| Mmp17 | -0.5325148 |
| Mmp1a | 0.3048831 |
| Mmp1a | 0.1580533 |
| Mmp2 | 0.5795416 |
| Mmp23 | -0.663259 |
| Mmp24 | 1.3510613 |
| Mmp3 | 2.2303467 |
| Mmp3 | -0.7528334 |
| Mmp3 | 0.8593624 |
| Mmp9 | -0.6492959 |
| Mmrn2 | -0.0828152 |
| Mnat1 | 0.8349548 |
| Mnat1 | -0.7365227 |
| Mnd1 | 0.2372027 |
| Mns1 | -0.6102207 |
| Mnt | 0.208135 |
| Mobk1b | 1.451326 |
| Mobkl2a | -0.3003617 |
| Mobkl2b | -0.6740849 |
| Mobkl2c | -0.6640872 |
| Mobkl3 | -0.644419 |
| Mobp | 0.3392174 |
| Mobp | -0.6395684 |
| Mobp | 2.726936 |
| Mocos | -0.4374984 |
| Mocs1 | 0.7583416 |
| Mocs2 | -0.272054 |
| Mod1 | -0.7806927 |
| Mog | 2.0442159 |
| Mog | -0.1462286 |
| Mon1a | 0.6128216 |
| Mon1a | 1.6191307 |
| Mon1b | 0.063547 |
| Mon1b | -0.709107 |
| Mon1b | 0.2353751 |
| Morc2a | 0.1395904 |
| Morc2b | 0.4283778 |
| Morc3 | -0.8909262 |
| Morf4l1 | -0.7939949 |
| Morf4l1 | 0.4371921 |
| Morf4l2 | -0.5098953 |
| Morn2 | 1.9076213 |
| Morn3 | 0.8258262 |
| Morn3 | 0.0071969 |
| Mos | -0.5057167 |
| Mosc2 | -0.6387625 |
| Mospd1 | -0.560874 |
| Mospd1 | 1.5615839 |
| Mospd1 | -0.6339422 |
| Mospd3 | -0.6995762 |
| Mospd3 | -0.5346648 |
| Mov10 | 1.5217488 |
| Mov10l1 | 1.5145669 |
| Moxd1 | 0.7619152 |
| Moxd1 | -0.5204274 |
| Mpdu1 | 0.5365142 |
| Mpdz | -0.5638338 |
| Mpdz | -0.6952772 |
| Mpg | -0.4563734 |
| Mpg | 2.2001907 |
| Mphosph6 | -0.6749231 |
| Mpnd | 0.3806307 |
| Mpo | 0.7404314 |
| Mpo | 0.9351739 |
| Mpp1 | -0.6952772 |
| Mpp2 | 0.2647706 |
| Mpp3 | 0.1450006 |
| Mpp3 | 2.6166387 |
| Mpp5 | 2.0924411 |
| Mpp6 | 2.3200094 |
| Mppe1 | -0.3758922 |
| Mpped1 | -0.1092554 |
| Mpst | 1.1107536 |
| Mpv17 | 0.0091594 |
| Mpv17 | -0.7222684 |
| Mpv17 | 0.9017813 |
| Mpv17 | -0.7231527 |
| Mpv17l | 0.1630236 |
| Mpv17l | 0.457429 |
| Mpv17l | 2.9899612 |
| Mpz | 0.9783871 |
| Mr1 | 0.7390389 |
| Mrap | 1.906908 |
| Mras | -0.36098 |
| Mrc1 | -0.8472287 |
| Mrc2 | -0.2139498 |
| Mrc2 | 1.10833 |
| Mre11a | 0.1314136 |
| Mre11a | -0.5705292 |
| Mre11a | -0.5091975 |
| Mreg | 0.6648084 |
| Mrgpra2 | -0.4722992 |
| Mrgpra2 | 2.0569899 |
| Mrgprb2 | 0.0947397 |
| Mrgpre | -0.5579238 |
| Mrgprf | -0.761101 |
| Mrgprg | 0.1046665 |
| Mrm1 | -0.661605 |
| Mro | -0.6749231 |
| Mrp63 | 0.0818136 |
| Mrpl1 | 0.8463141 |
| Mrpl1 | -0.7648 |
| Mrpl10 | -0.597051 |
| Mrpl10 | 2.6913796 |
| Mrpl11 | -0.6094407 |
| Mrpl12 | -0.67074 |
| Mrpl13 | 0.2597398 |
| Mrpl13 | 0.9085885 |
| Mrpl14 | -0.5447667 |
| Mrpl15 | 0.847859 |
| Mrpl16 | -0.6204233 |
| Mrpl17 | 1.2588736 |
| Mrpl18 | 0.4352408 |
| Mrpl2 | -0.1532454 |
| Mrpl20 | -0.524675 |
| Mrpl22 | -0.3405095 |
| Mrpl23 | 0.5031768 |
| Mrpl24 | -0.5727721 |
| Mrpl27 | -0.623586 |
| Mrpl28 | -0.7583367 |
| Mrpl3 | 0.4628916 |
| Mrpl3 | -0.6355461 |
| Mrpl30 | 0.3842558 |
| Mrpl33 | -0.4563734 |
| Mrpl33 | -0.547674 |
| Mrpl34 | 1.4527457 |
| Mrpl36 | -0.6884412 |
| Mrpl37 | 0.2585519 |
| Mrpl37 | 0.0825366 |
| Mrpl38 | 0.9194882 |
| Mrpl39 | -0.4108456 |
| Mrpl40 | -0.3387764 |
| Mrpl43 | 1.6257226 |
| Mrpl43 | 1.0416666 |
| Mrpl45 | -0.5601355 |
| Mrpl46 | 0.1945184 |
| Mrpl47 | -0.6884412 |
| Mrpl47 | 0.7281726 |
| Mrpl48 | 0.025092 |
| Mrpl48 | 0.3575042 |
| Mrpl48 | -0.1214118 |
| Mrpl49 | 2.1862784 |
| Mrpl49 | -0.4905592 |
| Mrpl49 | 2.1654622 |
| Mrpl49 | 1.1219781 |
| Mrpl50 | -0.6259654 |
| Mrpl51 | 0.2756239 |
| Mrpl51 | -0.4649649 |
| Mrpl51 | -0.630743 |
| Mrpl52 | -0.2893685 |
| Mrpl53 | 0.2368983 |
| Mrpl54 | 0.0471597 |
| Mrpl55 | -0.3770959 |
| Mrpl9 | -0.3273067 |
| Mrps10 | 0.9061323 |
| Mrps10 | 2.1224392 |
| Mrps11 | -0.4058572 |
| Mrps11 | 0.710545 |
| Mrps12 | -0.5397016 |
| Mrps14 | 0.8341715 |
| Mrps15 | -0.6892929 |
| Mrps16 | -0.3651305 |
| Mrps17 | -0.5353826 |
| Mrps18a | -0.4027534 |
| Mrps18b | 0.4019117 |
| Mrps18b | 1.2114026 |
| Mrps18c | -0.5382596 |
| Mrps2 | -0.6379573 |
| Mrps21 | -0.2039577 |
| Mrps22 | 1.0689999 |
| Mrps23 | 2.1649623 |
| Mrps24 | 1.4320986 |
| Mrps25 | -0.5909196 |
| Mrps25 | 0.2866359 |
| Mrps25 | 1.2090292 |
| Mrps26 | 0.3166598 |
| Mrps26 | -0.3621639 |
| Mrps27 | 2.6267422 |
| Mrps28 | -0.6094407 |
| Mrps30 | 0.5555122 |
| Mrps31 | 1.8631881 |
| Mrps31 | -0.4596681 |
| Mrps31 | -0.6766018 |
| Mrps33 | 0.5308235 |
| Mrps33 | -0.7930382 |
| Mrps34 | 0.7187786 |
| Mrps35 | 1.5848828 |
| Mrps5 | 3.1610033 |
| Mrps6 | -0.6969943 |
| Mrps7 | -0.7047623 |
| Mrps7 | 1.1799421 |
| Mrps9 | -0.2839218 |
| Mrrf | 0.1823112 |
| Mrrf | 0.1429756 |
| Mrto4 | -0.6172717 |
| Mrvi1 | -0.4994851 |
| Ms4a1 | 3.7945478 |
| Ms4a10 | -0.4709612 |
| Ms4a13 | 1.8371952 |
| Ms4a5 | 1.0912974 |
| Ms4a6d | -0.4749811 |
| Ms4a6d | 0.5314819 |
| Ms4a7 | -0.6363491 |
| Ms4a8a | 1.9007498 |
| Msc | -0.3886123 |
| Msh2 | 0.0875815 |
| Msh3 | 1.4251367 |
| Msh3 | -0.5036347 |
| Msh4 | 1.1340756 |
| Msh5 | 1.0611182 |
| Msh5 | -0.7437354 |
| Msh6 | 0.5736989 |
| Msh6 | 0.6665095 |
| Msi2 | -0.0362716 |
| Msi2h | 0.4832597 |
| Msln | -0.193093 |
| Msn | -0.5712762 |
| Msr2 | -0.6387625 |
| Msrb2 | -0.5675473 |
| Msrb2 | 3.7688524 |
| Msrb3 | 1.4747004 |
| Mst1r | -0.7222684 |
| Msto1 | -0.7073666 |
| Msx1 | 2.690444 |
| Mt1 | -0.269913 |
| Mt3 | 0.9917724 |
| Mta1 | 0.2721182 |
| Mta2 | 0.8931625 |
| Mta3 | 1.2536773 |
| Mta3 | 0.6369491 |
| Mtag2 | 2.1983343 |
| Mtap | -0.6460417 |
| Mtap | -0.2466914 |
| Mtap1b | -0.0370967 |
| Mtap1b | -0.3764939 |
| Mtap1b | 0.2647706 |
| Mtap1b | 1.2033281 |
| Mtap1s | -0.5955143 |
| Mtap2 | 1.2232784 |
| Mtap2 | -0.1688778 |
| Mtap2 | -0.2910089 |
| Mtap4 | 0.9369932 |
| Mtap6 | 1.5608154 |
| Mtap6 | -0.6808123 |
| Mtap7 | 2.98485 |
| Mtap7d1 | -0.7196206 |
| Mtap7d1 | -0.5978204 |
| Mtap7d2 | -0.6220033 |
| Mtap7d3 | 1.8478249 |
| Mtbp | 0.4694973 |
| Mtbp | 2.0697836 |
| Mtbp | 0.0399901 |
| Mtch1 | 2.4196438 |
| Mtch1 | 0.1992918 |
| Mtch2 | -0.7205024 |
| Mtch2 | -0.0968199 |
| Mtch2 | -0.3109312 |
| Mtcp1 | 0.4654925 |
| Mtdh | 1.1954952 |
| Mterf | 4.000382 |
| Mterf | -0.7178598 |
| Mterfd1 | -0.5105936 |
| Mterfd2 | -0.3165443 |
| Mterfd2 | 2.1217275 |
| Mterfd2 | 3.5205299 |
| Mterfd3 | -0.3867841 |
| Mterfd3 | -0.176556 |
| Mtf1 | 0.1088875 |
| Mtf1 | 1.7207323 |
| Mtf2 | -0.6149153 |
| Mtfmt | 0.3735981 |
| Mtfmt | -0.7258108 |
| Mtg1 | 0.0639159 |
| Mthfd1 | 0.6631041 |
| Mthfd2 | -0.1323571 |
| Mthfs | -0.2169692 |
| Mthfsd | -0.6774423 |
| Mthfsd | -0.2059473 |
| Mtif3 | -0.5078036 |
| Mtl5 | 0.5417354 |
| Mtl5 | 1.7412676 |
| Mtmr1 | -0.4583488 |
| Mtmr11 | -0.7249239 |
| Mtmr11 | -0.772244 |
| Mtmr12 | 0.2785349 |
| Mtmr14 | 0.7473622 |
| Mtmr14 | 1.7846424 |
| Mtmr14 | -0.4649649 |
| Mtmr14 | -0.6732475 |
| Mtmr2 | -0.6427992 |
| Mtmr2 | -0.6157001 |
| Mtmr2 | 0.2209291 |
| Mtmr3 | 0.1046665 |
| Mtmr4 | 0.1653337 |
| Mtmr9 | 0.7367713 |
| Mto1 | 0.3978483 |
| Mtpn | -0.5112925 |
| Mtrf1 | -0.7338327 |
| Mtrf1 | 0.3356787 |
| Mtrf1l | -0.6196344 |
| Mtrr | -0.536101 |
| Mtss1 | -0.7347285 |
| Mttp | 2.0653664 |
| Mtus1 | -0.1137386 |
| Mtus1 | -0.6267599 |
| Mtvr2 | 0.4315707 |
| Mtvr2 | -0.5043282 |
| Mtx1 | 0.2561715 |
| Mtx1 | 0.0961645 |
| Mtx1 | 3.362379 |
| Mtx2 | 0.0384734 |
| Muc1 | 3.2634762 |
| Muc1 | -0.2774291 |
| Muc1 | -0.3669152 |
| Muc1 | 0.3362241 |
| Muc13 | 0.0763724 |
| Muc5ac | 3.5621287 |
| Mug2 | 0.1741889 |
| Mug2 | 0.9257387 |
| Mug4 | -0.7759892 |
| Mum1 | -0.078051 |
| Mup1 | 2.5448445 |
| Mup2 | -0.4871497 |
| Mup2 | 1.6658468 |
| Mup3 | 0.4264073 |
| Mupcdh | 0.4461621 |
| Mus81 | -0.5375395 |
| Mus81 | 2.0538841 |
| Mustn1 | -0.6476673 |
| Mustn1 | 3.4651542 |
| Muted | 2.0029315 |
| Muted | 0.5682392 |
| Mutyh | 0.8335443 |
| Mvd | -0.7161024 |
| Mvp | -0.7187398 |
| Mx2 | -0.6251716 |
| Mxd1 | 2.6574023 |
| Mxd4 | -0.7510064 |
| Mxi1 | -0.2640512 |
| Mxra7 | 1.5964442 |
| Mxra8 | 0.9830482 |
| Mxra8 | -0.4355682 |
| Myadm | 1.5455908 |
| Mybbp1a | -0.2109405 |
| Mybbp1a | 0.5898817 |
| Mybbp1a | 1.7943992 |
| Mybl2 | 0.0441479 |
| Mybpc2 | -0.3081377 |
| Mybpc3 | -0.7082364 |
| Myc | -0.6259654 |
| Mycbp | 0.6921981 |
| Mycbp2 | -0.6987148 |
| Mycbp2 | -0.3722907 |
| Mycbpap | -0.56606 |
| Mycl1 | -0.5105936 |
| Myd116 | 0.8077708 |
| Myd88 | -0.7731788 |
| Myef2 | -0.1794523 |
| Myg1 | 2.4020356 |
| Myg1 | -0.2265981 |
| Myg1 | 2.4869455 |
| Myh10 | -0.2540153 |
| Myh11 | 1.9243874 |
| Myh8 | 1.9018987 |
| Myh9 | 1.6797933 |
| Myl1 | 0.7789071 |
| Myl2 | -0.6149153 |
| Myl4 | -0.4817211 |
| Myl6 | -0.2363311 |
| Myl6b | -0.6665762 |
| Mylc2b | -0.5564524 |
| Mylc2b | -0.6492959 |
| Mylc2b | -0.6227943 |
| Mylc2pl | -0.1650631 |
| Mylk | 1.5953687 |
| Mylk | 1.0782482 |
| Mynn | -0.412097 |
| Myo10 | -0.7949526 |
| Myo10 | 1.2380061 |
| Myo15 | -0.1494963 |
| Myo18a | 1.4481631 |
| Myo1b | -0.7629486 |
| Myo1c | -0.3645364 |
| Myo1c | 0.5661305 |
| Myo1f | 0.1092384 |
| Myo1g | -0.4021339 |
| Myo3b | -0.5022494 |
| Myo5a | 0.1597131 |
| Myo5b | -0.7537483 |
| Myo6 | -0.4478632 |
| Myo7a | 1.8804518 |
| Myo9a | -0.0478926 |
| Myo9b | -0.5091975 |
| Myo9b | 0.6608266 |
| Myoc | -0.4285261 |
| Myocd | -0.2089399 |
| Myocd | 0.9263048 |
| Myom1 | -0.5098953 |
| Myom1 | 4.6391158 |
| Myrip | -0.4459107 |
| Mysm1 | 1.8676719 |
| Myst1 | 2.2381502 |
| Myst2 | -0.2964988 |
| Myst4 | -0.6078826 |
| Myst4 | -0.561613 |
| Myst4 | 0.3785527 |
| Myt1 | 0.8308741 |
| Myt1l | -0.1736689 |
| Myt1l | -0.0022829 |
| Mzf1 | -0.1323571 |
| Mzf1 | -0.628351 |
| N28178 | -0.309254 |
| N4bp2 | 0.6485819 |
| N6amt1 | -0.6884412 |
| N6amt1 | -0.3221927 |
| N6amt2 | -0.2948483 |
| Naalad2 | 0.5142034 |
| Nab1 | -0.4253433 |
| Nab1 | -0.4349258 |
| Nab1 | -0.6055506 |
| Nab1 | -0.4330011 |
| Nab2 | 2.2785164 |
| Naca | -0.0841189 |
| Naca | -0.7108508 |
| Naca | -0.3722907 |
| Nadk | 0.6349956 |
| Nadsyn1 | 0.3188856 |
| Nadsyn1 | -0.1351143 |
| Naga | -0.6690721 |
| Nagk | 1.6128201 |
| Nagk | 0.4908664 |
| Nagk | -0.4844313 |
| Nagk | -0.6574832 |
| Naglu | -0.1186961 |
| Nagpa | 2.3625162 |
| Nags | -0.6251716 |
| Nags | 0.7024168 |
| Naif1 | 1.8255015 |
| Naif1 | -0.6665762 |
| Naip5 | -0.5712762 |
| Nalp10 | -0.5962823 |
| Nampt | 0.0587363 |
| Nanos1 | -0.7249239 |
| Nanos2 | -0.621213 |
| Nans | 2.2622533 |
| Nap1l1 | -0.521134 |
| Nap1l1 | -0.47163 |
| Nap1l3 | 0.0821752 |
| Nap1l4 | -0.3474756 |
| Nap1l5 | -0.4576898 |
| Napa | 3.4646236 |
| Napa | -0.2004865 |
| Napa | 1.8905853 |
| Napb | -0.2540153 |
| Napepld | 0.0083749 |
| Napg | 0.952654 |
| Napg | 0.1029018 |
| Naprt1 | 0.2323208 |
| Naprt1 | -0.4729689 |
| Napsa | -0.1411175 |
| Napsa | -0.3148567 |
| Napsa | 1.1523076 |
| Narf | -0.3928927 |
| Narfl | 2.6135637 |
| Narg1 | 0.0169666 |
| Narg1 | -0.5985904 |
| Narg1l | 1.5837934 |
| Narg1l | -0.468958 |
| Narg1l | 2.1659617 |
| Narg2 | -0.3318771 |
| Nars | 0.259146 |
| Nars2 | 0.1399295 |
| Nasp | -0.4400784 |
| Nasp | -0.1684 |
| Nasp | -0.7455476 |
| Nasp | -0.6774423 |
| Nat1 | -0.6749231 |
| Nat10 | -0.3047969 |
| Nat14 | 1.0662189 |
| Nat2 | -0.6387625 |
| Nat5 | -0.5382596 |
| Nat5 | -0.5542498 |
| Nat6 | 1.5209455 |
| Nat6 | 2.8501852 |
| Nav1 | 1.3432625 |
| Nav1 | -0.7258108 |
| Nbl1 | 0.3824451 |
| Nbr1 | 0.96656 |
| Nbr1 | 0.5859665 |
| Ncald | -0.2534902 |
| Ncam1 | -0.2337596 |
| Ncam1 | 0.4036837 |
| Ncam2 | 0.3942777 |
| Ncan | 1.1525277 |
| Ncapd2 | 2.0348554 |
| Ncapd2 | 0.1064276 |
| Ncapd2 | 0.5436855 |
| Ncapd3 | 1.8069801 |
| Ncapg2 | -0.3434055 |
| Ncapg2 | 2.8927372 |
| Ncaph | 0.8207499 |
| Ncaph2 | 1.5012245 |
| Ncaph2 | -0.5260954 |
| Ncaph2 | -0.5535167 |
| Ncbp2 | 0.7985239 |
| Ncbp2 | 0.0107262 |
| Ncdn | 1.2833682 |
| Ncdn | -0.3770959 |
| Ncdn | -0.7266986 |
| Ncdn | -0.7082364 |
| Ncf4 | -0.2265981 |
| Nck1 | 0.1754934 |
| Nckap1 | 0.4922426 |
| Nckap1l | 0.7569765 |
| Nckipsd | 0.823133 |
| Ncl | -0.6188462 |
| Ncl | -0.53682 |
| Ncoa2 | 1.1168414 |
| Ncoa2 | 3.2866151 |
| Ncoa4 | 0.1826346 |
| Ncoa5 | 0.5996976 |
| Ncoa6 | -0.4885119 |
| Ncoa6 | -0.7583367 |
| Ncoa6 | -0.6492959 |
| Ncor1 | -0.5623526 |
| Ncstn | 2.4533148 |
| Ncstn | 0.9395061 |
| Ncstn | 2.099651 |
| Ndc80 | -0.623586 |
| Nde1 | 1.6577881 |
| Nde1 | 0.543902 |
| Ndel1 | 2.3800919 |
| Ndfip1 | 1.797252 |
| Ndfip2 | -0.6566611 |
| Ndg2 | -0.6001325 |
| Ndn | -0.4015149 |
| Ndor1 | -0.7778676 |
| Ndor1 | 2.3993699 |
| Ndor1 | -0.44982 |
| Ndrg1 | -0.5404234 |
| Ndrg2 | -0.1693557 |
| Ndrg3 | -0.1092554 |
| Ndrg4 | -0.5303699 |
| Ndrl | 2.2279191 |
| Ndst1 | -0.3439858 |
| Ndst1 | -0.5810443 |
| Ndst4 | -0.309254 |
| Ndufa1 | -0.4629749 |
| Ndufa10 | 1.5462303 |
| Ndufa12 | 1.2958392 |
| Ndufa13 | -0.7064977 |
| Ndufa2 | 2.0778457 |
| Ndufa3 | 0.1573885 |
| Ndufa4 | -0.3645364 |
| Ndufa5 | 2.3931559 |
| Ndufa5 | 0.6296999 |
| Ndufa6 | -0.2534902 |
| Ndufa7 | 0.2065622 |
| Ndufa8 | -0.6952772 |
| Ndufa8 | -0.6157001 |
| Ndufa9 | 1.043283 |
| Ndufaf1 | 1.8478757 |
| Ndufb10 | -0.2296603 |
| Ndufb11 | -0.6323412 |
| Ndufb2 | 0.0975871 |
| Ndufb3 | -0.6987148 |
| Ndufb4 | 0.4107369 |
| Ndufb5 | -0.7356251 |
| Ndufb6 | 0.9543012 |
| Ndufb6 | 1.7975207 |
| Ndufb7 | 0.9555347 |
| Ndufb8 | 1.0552532 |
| Ndufb9 | -0.3416668 |
| Ndufc1 | -0.1877094 |
| Ndufc1 | -0.3586168 |
| Ndufc2 | 1.1234566 |
| Ndufs2 | 0.3244261 |
| Ndufs2 | 1.1778051 |
| Ndufs3 | 2.7682374 |
| Ndufs4 | 1.5764052 |
| Ndufs4 | -0.6665762 |
| Ndufs4 | -0.7464551 |
| Ndufs4 | 1.020512 |
| Ndufs6 | 1.9443218 |
| Ndufs7 | 1.0950583 |
| Ndufs8 | -0.6699057 |
| Ndufv1 | -0.3544961 |
| Ndufv2 | 0.8921344 |
| Necab1 | 2.9786114 |
| Necab2 | 1.0193646 |
| Necab3 | 2.3921855 |
| Necap1 | 0.3667735 |
| Necap1 | 0.0947397 |
| Necap1 | 1.8547583 |
| Necap2 | -0.561613 |
| Nedd1 | 2.5878416 |
| Nedd4 | 0.7135966 |
| Nedd4l | 0.2034084 |
| Nedd8 | -0.6791257 |
| Nedd9 | -0.2981523 |
| Nedd9 | -0.6841949 |
| Nefm | -0.6172717 |
| Negr1 | -0.527518 |
| Negr1 | 1.3441521 |
| Neil1 | -0.6039992 |
| Neil2 | 1.6097805 |
| Nek11 | 0.9369932 |
| Nek3 | -0.5346648 |
| Nek3 | -0.6164856 |
| Nek3 | 2.6835067 |
| Nek4 | 0.7824001 |
| Nek5 | 1.5182383 |
| Nek6 | -0.597051 |
| Nek8 | -0.4478632 |
| Nek9 | 1.2220571 |
| Nek9 | 1.0056095 |
| Nelf | -0.6476673 |
| Nelf | -0.0609362 |
| Nell2 | -0.4253433 |
| Nell2 | -0.6517443 |
| Nell2 | 0.02355 |
| Nell2 | 1.17459 |
| Nenf | -0.7302586 |
| Neo1 | -0.2779684 |
| Nes | 1.5022693 |
| Net1 | -0.5296561 |
| Net1 | -0.6039992 |
| Neto1 | 0.3260817 |
| Neto1 | -0.6927076 |
| Neto1 | 2.0469888 |
| Neu1 | -0.4046144 |
| Neu2 | -0.3301605 |
| Neu3 | -0.4039936 |
| Neu4 | 1.1134005 |
| Neu4 | -0.6978541 |
| Neu4 | 1.2077884 |
| Neurl | -0.7510064 |
| Neurod1 | 0.5000014 |
| Neurod2 | 1.7379444 |
| Neurod4 | -0.6901453 |
| Neurog1 | 1.0173211 |
| Neurog2 | 2.2214022 |
| Nf1 | -0.5939801 |
| Nfasc | -0.6665762 |
| Nfasc | 2.4749048 |
| Nfasc | -0.6799686 |
| Nfat5 | 0.5508002 |
| Nfat5 | -0.1588985 |
| Nfatc1 | -0.5787807 |
| Nfatc1 | 0.6822332 |
| Nfatc2 | 0.3470617 |
| Nfatc2 | 1.378693 |
| Nfatc2ip | -0.7528334 |
| Nfatc3 | -0.7901742 |
| Nfatc4 | -0.5296561 |
| Nfe2 | 1.4937256 |
| Nfe2l1 | -0.3722907 |
| Nfe2l2 | -0.2508692 |
| Nfe2l3 | -0.3210602 |
| Nfib | 1.2266284 |
| Nfic | 0.2860597 |
| Nfic | 0.8530921 |
| Nfic | -0.5564524 |
| Nfix | -0.1945668 |
| Nfix | 0.3585684 |
| Nfkb1 | -0.3873931 |
| Nfkb2 | -0.3783013 |
| Nfkbia | -0.5750206 |
| Nfkbib | 0.7733905 |
| Nfkbid | 1.1050883 |
| Nfkbie | 1.9939933 |
| Nfkbil1 | -0.536101 |
| Nfkbil2 | 1.91378 |
| Nfkbiz | 0.4654925 |
| Nfrkb | -0.4769978 |
| Nfrkb | 1.1681253 |
| Nfs1 | 0.9908511 |
| Nfs1 | 2.4000026 |
| Nfs1 | 0.36809 |
| Nfu1 | -0.0715947 |
| Nfx1 | -0.7013016 |
| Nfya | 1.3406775 |
| Nfyb | -0.1999917 |
| Nfyc | 1.2187922 |
| Ngb | 0.9045394 |
| Ngb | 0.4254204 |
| Ngef | 1.4461012 |
| Ngfb | -0.4491672 |
| Ngfb | -0.1664917 |
| Ngfr | -0.7187398 |
| Ngfrap1 | -0.6665762 |
| Ngp | -0.6690721 |
| Nhedc1 | -0.1232268 |
| Nhej1 | -0.1565388 |
| Nhlrc1 | 1.018088 |
| Nhlrc2 | -0.4609894 |
| Nhlrc2 | -0.8813822 |
| Nhp2l1 | 1.9724908 |
| Nhsl1 | -0.7169807 |
| Niban | -0.5985904 |
| Nicn1 | -0.5140934 |
| Nicn1 | 3.0662672 |
| Nid1 | -0.1223188 |
| Nid2 | 0.8605808 |
| Nif3l1 | -0.6927076 |
| Nin | 0.2597398 |
| Ninj1 | -0.3210602 |
| Ninj2 | -0.8123683 |
| Nip7 | -0.1218652 |
| Nipa1 | 1.3165226 |
| Nipbl | -0.536101 |
| Nipbl | 3.326675 |
| Nipsnap1 | -0.23891 |
| Nipsnap3a | -0.77975 |
| Nipsnap3a | 0.1064276 |
| Nisch | 0.2932361 |
| Nit1 | 0.1018414 |
| Nit2 | -0.1411175 |
| Nit2 | -0.6816567 |
| Nkain3 | -0.2812108 |
| Nkain4 | -0.815304 |
| Nkd2 | -0.6331414 |
| Nkg7 | -0.0030774 |
| Nkiras1 | -0.7249239 |
| Nkiras2 | 1.958539 |
| Nkrf | 0.5162141 |
| Nktr | -0.3042413 |
| Nkx1-2 | 0.7590234 |
| Nkx2-1 | -0.4407246 |
| Nkx2-2 | -0.8321229 |
| Nkx2-2 | -0.2024684 |
| Nkx2-3 | -0.7806927 |
| Nkx2-4 | 1.0330382 |
| Nkx2-6 | 2.8100306 |
| Nkx3-1 | -0.1360353 |
| Nkx6-1 | 2.0080055 |
| Nle1 | 0.821068 |
| Nlgn1 | 1.4248929 |
| Nlgn2 | 0.8572262 |
| Nlk | -0.6566611 |
| Nln | -0.2839218 |
| Nln | -0.6799686 |
| Nln | -0.9016508 |
| Nlrp14 | 0.5406501 |
| Nlrp4a | -0.3307323 |
| Nlrp4c | 2.4292526 |
| Nlrp5 | -0.115088 |
| Nlrp5 | 1.7438375 |
| Nlrp5 | -0.4152333 |
| Nlrp9a | -0.4139775 |
| Nlrp9c | 0.7109045 |
| Nlrx1 | -0.3227595 |
| Nlrx1 | -0.1693557 |
| Nmb | 0.4107369 |
| Nmbr | -0.5161998 |
| Nmd3 | -0.9368547 |
| Nme1 | 0.807448 |
| Nme2 | 1.9183011 |
| Nme2 | -0.5484022 |
| Nme2 | -0.2959483 |
| Nme3 | 1.0890569 |
| Nme4 | 2.3970274 |
| Nme4 | 0.5110667 |
| Nme4 | -0.8311248 |
| Nme5 | 0.5270831 |
| Nme7 | -0.312051 |
| Nme7 | 0.6604465 |
| Nme7 | -0.6961353 |
| Nme7 | 2.5339619 |
| Nme7 | 1.0410441 |
| Nmnat1 | 0.5323591 |
| Nmnat2 | -0.01508 |
| Nmnat3 | 1.4726985 |
| Nmral1 | 0.1813404 |
| Nmt1 | -0.2435708 |
| Nmu | -0.6944198 |
| Nmur2 | 0.1245424 |
| Nnat | -0.3370466 |
| Nnat | -0.7392207 |
| Nnmt | -0.1119427 |
| Nnmt | -0.685891 |
| Nob1 | -0.4796938 |
| Nob1 | 1.2089258 |
| Nobox | 0.657018 |
| Noc3l | -0.2225313 |
| Noc4l | -0.2009816 |
| Noc4l | -0.6833481 |
| Nod1 | 0.6221934 |
| Nog | -0.6541994 |
| Nol1 | 1.8132315 |
| Nol1 | -0.6009045 |
| Nol1 | 2.2771618 |
| Nol10 | 0.2597398 |
| Nol11 | -0.5197214 |
| Nol12 | 2.0388086 |
| Nol14 | 3.2457954 |
| Nol3 | -0.5484022 |
| Nol4 | 1.5179451 |
| Nol4 | 0.5814116 |
| Nol4 | 0.7187786 |
| Nol5 | 1.8082551 |
| Nol5a | 2.9727078 |
| Nol6 | 0.0396111 |
| Nol7 | -0.7788083 |
| Nol9 | -0.6657458 |
| Nola1 | 3.1570517 |
| Nola2 | -0.2104399 |
| Nola3 | -0.6331414 |
| Nola3 | 0.5222194 |
| Nolc1 | 0.9799879 |
| Nomo1 | -0.4662939 |
| Nono | 3.9064546 |
| Nope | -0.5404234 |
| Nos1 | 1.3011714 |
| Nos1ap | -0.53682 |
| Nos2 | 1.9083816 |
| Nos3 | 0.102195 |
| Nos3 | -0.5825565 |
| Nos3 | -0.6978541 |
| Nosip | 0.4167388 |
| Nosip | -0.4576898 |
| Nosip | -0.2779684 |
| Nostrin | 0.9364338 |
| Notch1 | -0.67074 |
| Notch3 | 0.7134174 |
| Notch4 | -0.4898763 |
| Notch4 | 1.3097116 |
| Notum | -0.6566611 |
| Nova2 | 0.9950551 |
| Nox4 | 1.3865313 |
| Noxo1 | -0.3710933 |
| Noxo1 | 2.4989751 |
| Npal2 | 0.3730749 |
| Npas1 | 0.7492589 |
| Npas2 | -0.33245 |
| Npas4 | -0.1168902 |
| Npb | -0.3669152 |
| Npc2 | 1.4033392 |
| Npcd | 1.5442392 |
| Npcd | -0.7574171 |
| Npdc1 | 0.3476002 |
| Npepl1 | 2.5931827 |
| Npff | -0.4662939 |
| Nphp1 | -0.6363491 |
| Nphp1 | -0.8740284 |
| Nphp3 | -0.1862468 |
| Nphp4 | -0.8698531 |
| Nphs2 | -0.6969943 |
| Nploc4 | 0.8427505 |
| Npm1 | -0.5916838 |
| Npm2 | -0.53682 |
| Npm3 | -0.1862468 |
| Npm3 | 0.52598 |
| Npm3-ps1 | 0.9734333 |
| Npr1 | 2.3323393 |
| Npr2 | -0.4987953 |
| Npr3 | 0.0479111 |
| Npsr1 | -0.6071046 |
| Nptn | -0.6204233 |
| Nptx1 | -0.6016772 |
| Nptx2 | 0.466908 |
| Nptxr | 2.3466063 |
| Npy | 0.1754934 |
| Npy1r | -0.5232569 |
| Npy2r | -0.0776191 |
| Npy5r | -0.6291476 |
| Nqo1 | -0.468958 |
| Nqo2 | 1.2747651 |
| Nqo2 | -0.7592572 |
| Nqo2 | -0.6078826 |
| Nr1d1 | 0.52598 |
| Nr1d2 | -0.5520525 |
| Nr1h2 | -0.685891 |
| Nr1h2 | 0.0316164 |
| Nr1h3 | -0.6251716 |
| Nr1h4 | -0.6808123 |
| Nr1h5 | 0.7218024 |
| Nr1i2 | -0.4413712 |
| Nr2c1 | 2.3585218 |
| Nr2c2ap | 1.3628084 |
| Nr2e1 | 3.5269431 |
| Nr2f2 | -0.6355461 |
| Nr2f2 | -0.2529654 |
| Nr2f6 | -0.0370967 |
| Nr2f6 | 0.5972537 |
| Nr4a2 | 0.4927009 |
| Nr4a3 | 0.9501776 |
| Nr4a3 | -0.1693557 |
| Nr5a1 | -0.6833481 |
| Nr5a2 | 1.285174 |
| Nr5a2 | 2.3130993 |
| Nr5a2 | -0.1056848 |
| Nr6a1 | -0.1397286 |
| Nradd | 0.2615186 |
| Nrap | -0.4851101 |
| Nrarp | 0.4945316 |
| Nras | -0.3849596 |
| Nrbf2 | -0.6749231 |
| Nrbp1 | -0.1679226 |
| Nrbp2 | 3.3701411 |
| Nrcam | 1.361501 |
| Nrd1 | -0.4743099 |
| Nrf1 | -0.7311508 |
| Nrg1 | -0.3746901 |
| Nrg3 | -0.7196206 |
| Nrg4 | 0.1361924 |
| Nrgn | 1.9910085 |
| Nrip2 | -0.47163 |
| Nrip3 | 0.8408857 |
| Nrl | -0.2747375 |
| Nrm | -0.2493003 |
| Nrn1 | -0.6039992 |
| Nrn1l | -0.6259654 |
| Nrp1 | -0.4511268 |
| Nrp1 | 1.2492417 |
| Nrp2 | 0.6665095 |
| Nrp2 | -0.4058572 |
| Nrsn2 | -0.2220242 |
| Nrsn2 | -0.0191168 |
| Nrtn | 0.0189079 |
| Nrxn1 | 0.5162141 |
| Nrxn1 | 3.0464691 |
| Nrxn1 | 1.202704 |
| Nrxn2 | 1.0331639 |
| Nrxn3 | -0.3148567 |
| Nrxn3 | -0.284465 |
| Nsbp1 | 0.4892583 |
| Nsd1 | -0.5498606 |
| Nsd1 | -0.4796938 |
| Nsdhl | -0.7491831 |
| Nsdhl | 4.3202639 |
| Nsdhl | -0.7056296 |
| Nsf | -0.5491311 |
| Nsfl1c | -0.5204274 |
| Nsg1 | 0.7483973 |
| Nsg2 | 0.1823112 |
| Nsl1 | 0.0377141 |
| Nsmaf | -0.7574171 |
| Nsmaf | -0.5425925 |
| Nsmce1 | -0.7196206 |
| Nsmce2 | -0.5397016 |
| Nsmce2 | 2.6331961 |
| Nsmce2 | -0.0832496 |
| Nsun2 | -0.4102205 |
| Nsun4 | -0.6086613 |
| Nsun5 | 2.3972206 |
| Nsun5 | 0.5799575 |
| Nsun5 | -0.5962823 |
| Nsun5 | -0.5863483 |
| Nt5c | -0.64523 |
| Nt5c1b | -0.5962823 |
| Nt5c2 | 1.0362992 |
| Nt5c2 | -0.4864693 |
| Nt5c2 | -0.6227943 |
| Nt5c3 | 0.5713931 |
| Nt5c3l | -0.5491311 |
| Nt5c3l | -0.7410238 |
| Nt5dc3 | -0.1241356 |
| Nt5dc3 | -0.661605 |
| Nt5dc3 | 1.7965531 |
| Nt5e | -0.5833136 |
| Nt5e | -0.67074 |
| Nt5m | -0.5878695 |
| Nt5m | 0.5369504 |
| Ntan1 | -0.4253433 |
| Ntf3 | -0.090666 |
| Nthl1 | 0.0954524 |
| Ntn2l | -0.547674 |
| Ntn2l | -0.1448318 |
| Ntn4 | -0.1921118 |
| Ntng1 | -0.4064792 |
| Ntng1 | -0.470293 |
| Ntng1 | 0.5787093 |
| Ntrk2 | 0.2320148 |
| Ntrk2 | 0.0165779 |
| Ntrk2 | -0.2430518 |
| Ntrk3 | 1.3621114 |
| Ntrk3 | 1.2644287 |
| Ntrk3 | 0.5159909 |
| Nts | 0.4107369 |
| Ntsr1 | -0.3025766 |
| Ntsr2 | 2.4964337 |
| Nuak1 | -0.6157001 |
| Nuak1 | -0.5411459 |
| Nuak2 | 1.6094495 |
| Nub1 | 0.0189079 |
| Nubp1 | -0.0617842 |
| Nubp2 | -0.0491466 |
| Nubp2 | 1.0515681 |
| Nubp2 | 1.4754692 |
| Nucb1 | 0.3819271 |
| Nucb1 | 0.3163812 |
| Nucks1 | 0.0471597 |
| Nudc | -0.7082364 |
| Nudc | -0.3515642 |
| Nudcd2 | -0.2513928 |
| Nudcd3 | 1.1231156 |
| Nudc-ps1 | 0.9571766 |
| Nudt1 | 0.4993195 |
| Nudt10 | 0.8803008 |
| Nudt11 | 0.9200582 |
| Nudt12 | 0.5550849 |
| Nudt13 | 1.2216496 |
| Nudt14 | 0.7977082 |
| Nudt16l1 | -0.5218411 |
| Nudt18 | 1.3612393 |
| Nudt19 | -0.5901561 |
| Nudt19 | 0.5013637 |
| Nudt2 | -0.1462286 |
| Nudt21 | -0.4387875 |
| Nudt22 | 0.5034032 |
| Nudt4 | 1.4773116 |
| Nudt5 | 1.0472496 |
| Nudt5 | -0.0433093 |
| Nudt6 | 0.3202739 |
| Nudt7 | -0.5433166 |
| Nudt8 | -0.1886859 |
| Nudt9 | -0.4864693 |
| Nufip1 | -0.6833481 |
| Numa1 | -0.2353016 |
| Numb | 0.5126365 |
| Numb | 0.0079824 |
| Numb | -0.5795346 |
| Numb | 1.1355344 |
| Numbl | -0.1012414 |
| Nup107 | -0.342246 |
| Nup133 | -0.5325148 |
| Nup133 | 1.6365814 |
| Nup155 | 0.7898398 |
| Nup155 | 0.1310713 |
| Nup160 | -0.5825565 |
| Nup210 | -0.0789153 |
| Nup37 | 2.1852315 |
| Nup43 | 1.9548479 |
| Nup50 | -0.5978204 |
| Nup50 | -0.1570102 |
| Nup50 | 1.1158105 |
| Nup62 | 1.1375504 |
| Nup85 | -0.4504732 |
| Nup88 | 1.8690126 |
| Nup88 | -0.6125649 |
| Nup88 | -0.0082589 |
| Nup93 | -0.519016 |
| Nup98 | 0.9236127 |
| Nup98 | 0.1081854 |
| Nupl1 | -0.1760741 |
| Nupl1 | -0.597051 |
| Nupl1 | -0.7021655 |
| Nupl2 | -0.5787807 |
| Nupr1 | -0.0990279 |
| Nus1 | 2.0441342 |
| Nusap1 | -0.2561188 |
| Nut | 0.3865784 |
| Nutf2 | -0.365725 |
| Nvl | -0.4342837 |
| Nwd1 | -0.6995762 |
| Nxf1 | -0.7161024 |
| Nxf2 | -0.7419268 |
| Nxf3 | -0.4407246 |
| Nxph1 | 1.5474371 |
| Nxph2 | -0.2099396 |
| Nxph3 | 1.471001 |
| Nxt1 | 0.7053152 |
| Nxt1 | 1.0207668 |
| Nxt2 | -0.1043495 |
| Nyx | -0.5008661 |
| Oaf | -0.4857895 |
| Oas1b | 0.8866807 |
| Oas1b | -0.547674 |
| Oas1c | -0.3250302 |
| Oas1d | 0.6166219 |
| Oas1d | -0.0106605 |
| Oas1e | 0.5217759 |
| Oas1g | -0.6275551 |
| Oas1g | 1.1085611 |
| Oas2 | 0.1439887 |
| Oasl1 | 0.9189178 |
| Oasl2 | -0.0391628 |
| Oasl2 | -0.5469463 |
| Oat | 0.3121916 |
| Oaz2 | -0.7013016 |
| Oaz3 | -0.7117239 |
| Obfc1 | 0.1190073 |
| Obfc2a | -0.5848297 |
| Obfc2b | -0.3486419 |
| Obfc2b | 0.9482467 |
| Obox1 | -0.5303699 |
| Obox5 | -0.6267599 |
| Obrgrp | 0.5516588 |
| Obsl1 | -0.1268678 |
| Oc90 | -0.5705292 |
| Oca2 | -0.1021284 |
| Ociad1 | -0.2768901 |
| Ociad1 | 0.4057045 |
| Ociad2 | -0.6468541 |
| Ocln | 1.4219617 |
| Ocrl | 0.1279845 |
| Odc1 | 2.4565491 |
| Odf1 | -0.4368546 |
| Odf2 | -0.7021655 |
| Odf2 | -0.2363311 |
| Odf2 | -0.6550192 |
| Odf2 | 0.0056239 |
| Odf3 | -0.5008661 |
| Odf3l1 | -0.470293 |
| Odf4 | 2.902435 |
| Odf4 | -0.4355682 |
| Odz1 | -0.7275873 |
| Odz3 | -0.0737417 |
| Odz3 | -0.0567079 |
| Odz4 | 0.0044423 |
| Og9x | -0.0613601 |
| Ogdh | -0.5397016 |
| Ogfod1 | -0.6558398 |
| Ogfod2 | 1.3080471 |
| Ogfr | 1.2671451 |
| Ogfrl1 | -0.6363491 |
| Ogg1 | -0.0030774 |
| Ogn | 2.5896923 |
| Ogn | 0.5376044 |
| Ogn | -0.6525619 |
| Ogt | -0.36098 |
| Ogt | 0.3147077 |
| Ogt | 1.1951805 |
| Oit1 | 0.9552607 |
| Oit3 | -0.6188462 |
| Ola1 | 0.9485228 |
| Olfm1 | 1.149 |
| Olfm1 | -0.5454927 |
| Olfm2 | -0.629945 |
| Olfm2 | 0.8444566 |
| Olfm3 | 0.3775119 |
| Olfm3 | -0.0317469 |
| Olfm4 | 2.1252802 |
| Olfml1 | -0.4570314 |
| Olfml2b | -0.5787807 |
| Olfml3 | 1.4983823 |
| Olfr100 | -0.2860966 |
| Olfr1006 | 2.0371635 |
| Olfr1012 | -0.3776984 |
| Olfr1020 | -0.4596681 |
| Olfr1065 | 1.4392472 |
| Olfr1094 | 0.8167639 |
| Olfr1099 | -0.5491311 |
| Olfr1105 | 3.4044179 |
| Olfr1112 | 1.8973423 |
| Olfr1113 | -0.1970286 |
| Olfr1189 | 0.2043557 |
| Olfr1197 | -0.312051 |
| Olfr1198 | 1.7818587 |
| Olfr1249 | -0.429164 |
| Olfr1280 | -0.399043 |
| Olfr133 | -0.0841189 |
| Olfr1330 | -0.7134728 |
| Olfr134 | -0.1177927 |
| Olfr1340 | -0.4932962 |
| Olfr1384 | 3.2547324 |
| Olfr1386 | -0.7741146 |
| Olfr1390 | -0.1838143 |
| Olfr1394 | -0.7161024 |
| Olfr1412 | 2.0976134 |
| Olfr1437 | 0.4192278 |
| Olfr144 | 1.9636456 |
| Olfr1443 | 1.3965698 |
| Olfr1443 | -0.6665762 |
| Olfr1459 | -0.5491311 |
| Olfr1500 | 3.7931923 |
| Olfr1508 | -0.5126918 |
| Olfr166 | -0.6517443 |
| Olfr168 | 1.5209455 |
| Olfr206 | 0.9043944 |
| Olfr213 | -0.3103718 |
| Olfr215 | 0.0613298 |
| Olfr232 | 0.2435702 |
| Olfr257 | -0.1891745 |
| Olfr267 | -0.5425925 |
| Olfr272 | -0.3795082 |
| Olfr303 | 2.3204903 |
| Olfr310 | 3.8219568 |
| Olfr352 | -0.7555808 |
| Olfr371 | 0.8634679 |
| Olfr378 | 1.0995039 |
| Olfr419 | 1.1712557 |
| Olfr437 | -0.7528334 |
| Olfr464 | -0.6574832 |
| Olfr47 | 0.7216249 |
| Olfr49 | -0.8271434 |
| Olfr503 | -0.8261509 |
| Olfr51 | 2.4980383 |
| Olfr536 | 1.8530426 |
| Olfr538 | -0.7939949 |
| Olfr54 | -0.880328 |
| Olfr550 | 0.4286238 |
| Olfr574 | -0.6078826 |
| Olfr591 | 3.26049 |
| Olfr593 | -0.2154583 |
| Olfr608 | 1.9077164 |
| Olfr638 | -0.6125649 |
| Olfr65 | 2.2040629 |
| Olfr655 | 0.7685145 |
| Olfr658 | -0.524675 |
| Olfr665 | -0.1921118 |
| Olfr675 | 1.3296305 |
| Olfr691 | 0.7586826 |
| Olfr692 | 0.7407793 |
| Olfr702 | 0.3909495 |
| Olfr724 | -0.1799359 |
| Olfr725 | -0.242533 |
| Olfr748 | 0.0997168 |
| Olfr786 | 0.4122411 |
| Olfr802 | 1.3559014 |
| Olfr824 | 0.1473574 |
| Olfr91 | 1.6816896 |
| Olfr910 | 2.6489122 |
| Olfr920 | -0.7258108 |
| Olfr924 | 1.7460589 |
| Olfr952 | 1.6016021 |
| Olfr965 | -0.4285261 |
| Olfr982 | -0.3515642 |
| Olfr983 | 0.5110667 |
| Olfr984 | -0.5411459 |
| Olig1 | -0.4981061 |
| Olig3 | -0.6674074 |
| Oma1 | -0.4102205 |
| Omd | 0.4435047 |
| Omg | -0.1155382 |
| Omt2b | -0.7759892 |
| Onecut2 | -0.6799686 |
| Onecut3 | -0.8856115 |
| Onecut3 | 1.7805457 |
| Oog4 | -0.232733 |
| Oosp1 | -0.3922799 |
| Opa1 | -0.1364961 |
| Opa1 | 1.0844405 |
| Ophn1 | 1.2918898 |
| Oplah | 1.5624913 |
| Opn1mw | -0.0562861 |
| Opn3 | -0.4878306 |
| Oprl1 | 2.869308 |
| Oprl1 | -0.7482729 |
| Oprl1 | 0.9894015 |
| Oprs1 | -0.4077246 |
| Optc | -0.1092554 |
| Optn | -0.5382596 |
| Optn | 0.2903724 |
| Orai1 | -0.5433166 |
| Orai3 | -0.5239657 |
| Oraov1 | 0.2489924 |
| Orc2l | 1.3734579 |
| Orc3l | -0.685891 |
| Orc5l | -0.6732475 |
| Orc5l | -0.7537483 |
| Orc6l | 1.3282739 |
| ORF19 | 2.0269384 |
| ORF28 | -0.6850426 |
| ORF34 | -0.1617385 |
| ORF61 | 0.6960437 |
| ORF63 | 0.4349966 |
| ORF9 | -0.2004865 |
| ORF9 | -0.0524993 |
| ORF9 | -0.6682394 |
| Orm1 | 1.391016 |
| Ormdl2 | 0.209705 |
| Ormdl3 | 0.6781416 |
| Os9 | -0.6355461 |
| Osbp2 | -0.0571298 |
| Osbpl10 | 2.5782597 |
| Osbpl11 | 0.9213395 |
| Osbpl2 | -0.2932009 |
| Osbpl3 | 0.0792785 |
| Osbpl3 | 0.2635894 |
| Osbpl6 | 2.5505161 |
| Osbpl8 | 0.0868625 |
| Osbpl9 | -0.5001753 |
| Oscar | -0.5520525 |
| Osgep | 0.6954953 |
| Osgepl1 | 1.4809087 |
| Osgin1 | 1.0706885 |
| Osm | -0.7099784 |
| Osmr | 1.392197 |
| Osr1 | 1.8761299 |
| Osr2 | -0.3009149 |
| Ostf1 | 2.9165328 |
| Ostm1 | -0.7240379 |
| Otof | 0.7205585 |
| Otop1 | -0.1341943 |
| Otop2 | -0.4089717 |
| Ott | 0.7812372 |
| Ott | 2.0868105 |
| OTTMUSG00000000421 | 1.585291 |
| OTTMUSG00000000971 | -0.0199263 |
| OTTMUSG00000000990 | 0.6613965 |
| OTTMUSG00000001305 | -0.3126114 |
| OTTMUSG00000002038 | 1.2437906 |
| OTTMUSG00000004461 | -0.6995762 |
| OTTMUSG00000004551 | -0.6550192 |
| OTTMUSG00000005148 | 2.2964678 |
| OTTMUSG00000005491 | -0.4756528 |
| OTTMUSG00000008540 | 1.8093162 |
| OTTMUSG00000010009 | 2.6174598 |
| OTTMUSG00000010328 | 1.8773106 |
| OTTMUSG00000015762 | -0.6640872 |
| OTTMUSG00000015859 | 1.0754957 |
| OTTMUSG00000016293 | 0.020845 |
| OTTMUSG00000016790 | -0.7455476 |
| OTTMUSG00000016790 | 1.2134614 |
| OTTMUSG00000017677 | -0.3996603 |
| OTTMUSG00000020946 | 0.5244334 |
| Otub1 | -0.4304412 |
| Otub2 | -0.1911316 |
| Otub2 | -0.7500943 |
| Otub2 | -0.5389803 |
| Otud5 | -0.309254 |
| Otud6b | 0.7033235 |
| Otud7b | 0.2712397 |
| Otud7b | 1.7285388 |
| Otx1 | -0.5924485 |
| Otx2 | -0.4240733 |
| Ovca2 | -0.8036184 |
| Ovgp1 | 1.7164508 |
| Ovgp1 | 0.2075062 |
| Ovol1 | -0.3928927 |
| Ovol2 | 0.9604515 |
| Ovol2 | 0.0958085 |
| Oxct1 | -0.5750206 |
| Oxct2a | -0.630743 |
| Oxct2b | 2.0730725 |
| Oxgr1 | 1.7352424 |
| Oxnad1 | -0.7365227 |
| Oxr1 | 0.1543909 |
| Oxr1 | -0.7911278 |
| Oxt | -0.6649161 |
| P140 | -0.0203313 |
| P2rx2 | -0.4796938 |
| P2rx3 | 2.8926418 |
| P2rx4 | 0.3194412 |
| P2rx4 | -0.6196344 |
| P2rx5 | 0.3620184 |
| P2rx7 | -0.8574421 |
| P2rx7 | 2.9516525 |
| P2ry1 | 1.3166143 |
| P2ry12 | -0.659954 |
| P2ry13 | 2.8814533 |
| P2ry14 | -0.4465611 |
| P2ry14 | -0.6024505 |
| P2ry5 | -0.6291476 |
| P2ry6 | 0.8090611 |
| P42pop | 0.0460315 |
| P4ha1 | 0.3348601 |
| P4ha2 | -0.1641119 |
| P4hb | 1.0500898 |
| Pa2g4 | -0.5140934 |
| Pabpc1 | 1.4615296 |
| Pabpc1 | -0.6315417 |
| Pabpc1 | 0.0079824 |
| Pabpc2 | 0.0247068 |
| Pabpc4 | -0.3335969 |
| Pabpn1 | 0.0361935 |
| Pabpnl1 | 0.2471886 |
| Pacrg | 2.0759158 |
| Pacs1 | -0.2693785 |
| Pacs2 | 1.5079945 |
| Pacsin1 | -0.2094396 |
| Pacsin1 | 2.4427028 |
| Pacsin1 | -0.2624591 |
| Pacsin2 | 2.1169561 |
| Pacsin2 | -0.7769279 |
| Pacsin3 | 0.3502877 |
| Pacsin3 | 0.8255098 |
| Padi1 | 1.6433022 |
| Padi2 | -0.2758132 |
| Padi6 | 0.2005605 |
| Paf1 | -0.3704953 |
| Paf1 | 0.6196109 |
| Pafah1b1 | -0.0512406 |
| Pafah1b1 | 0.2471886 |
| Pafah1b3 | -0.3801123 |
| Pafah2 | 0.0392321 |
| Pafah2 | -0.8421652 |
| Paip1 | -0.6078826 |
| Paip2 | 2.0917441 |
| Paip2b | -0.7213849 |
| Paip2b | 0.9945307 |
| Pak1 | -0.6492959 |
| Pak3 | 1.5847467 |
| Pak4 | 0.1266092 |
| Pak6 | 2.6134986 |
| Pak7 | -0.77975 |
| Palld | 0.8677057 |
| Palm | 1.6361315 |
| Palm | -0.6468541 |
| Palm2 | -0.5375395 |
| Palmd | -0.4857895 |
| Palmd | 1.3110041 |
| Palmd | 1.6340722 |
| Pam | 2.8630993 |
| Pank1 | 0.0850626 |
| Pank1 | -0.6566611 |
| Pank2 | 1.9397006 |
| Pank3 | 1.6642906 |
| Pank4 | 0.6683958 |
| Panx1 | 1.5990604 |
| Panx3 | -0.6509274 |
| Paox | -0.7117239 |
| Paox | 1.0955273 |
| Paox | 1.5432419 |
| Paox | 3.3479946 |
| Papd1 | -0.6624316 |
| Papd1 | -0.4046144 |
| Papd4 | -0.3353201 |
| Papln | 1.1332892 |
| Papola | 1.1040439 |
| Papolb | -0.6766018 |
| Papolg | -0.3199291 |
| Papss1 | -0.573521 |
| Papss1 | -0.7169807 |
| Papss2 | 0.3524319 |
| Paqr3 | 0.5097191 |
| Paqr6 | -0.2420145 |
| Paqr7 | -0.4796938 |
| Paqr9 | -0.7930382 |
| Paqr9 | -0.5353826 |
| Paqr9 | 1.8063951 |
| Pard3 | -0.2503459 |
| Pard3 | -0.4817211 |
| Pard6a | -0.6791257 |
| Pard6a | 1.0468785 |
| Pard6b | -0.2119425 |
| Pard6g | -0.4095959 |
| Park7 | -0.5303699 |
| Parl | 1.1843071 |
| Parp1 | 0.7406054 |
| Parp1 | -0.1980152 |
| Parp1 | 1.6548969 |
| Parp12 | 1.1592143 |
| Parp14 | -0.0954976 |
| Parp16 | -0.5675473 |
| Parp2 | -0.4008963 |
| Parp2 | -0.8133458 |
| Parp3 | 0.4588573 |
| Parp6 | -0.7769279 |
| Parp8 | 0.3012064 |
| Pars2 | -0.0074598 |
| Pars2 | -0.6657458 |
| Parva | -0.547674 |
| Parvg | -0.7383204 |
| Pask | 0.2900855 |
| Patl1 | -0.7240379 |
| Patz1 | -0.4926112 |
| Pawr | -0.675762 |
| Pax8 | -0.5404234 |
| Paxip1 | -0.8813822 |
| Pbk | 2.140454 |
| Pbld | -0.2667109 |
| Pbp2 | 0.3394891 |
| Pbrm1 | 0.2267969 |
| Pbrm1 | -0.6047745 |
| Pbx1 | -0.6674074 |
| Pbx1 | -0.309254 |
| Pbx2 | 0.6575903 |
| Pbx2 | -0.2194932 |
| Pbx2 | -0.5985904 |
| Pbx3 | -0.4478632 |
| Pbx3 | 1.1698538 |
| Pbx4 | 1.5487131 |
| Pcaf | -0.3996603 |
| Pcaf | 0.4588573 |
| Pcbd1 | 2.4582521 |
| Pcbd2 | -0.5268064 |
| Pcbp1 | -0.761101 |
| Pcbp2 | -0.7446411 |
| Pcbp3 | -0.5126918 |
| Pcca | 0.9224768 |
| Pcca | -0.4570314 |
| Pccb | -0.2992563 |
| Pccb | 0.1120403 |
| Pcdh1 | 0.7728873 |
| Pcdh10 | -0.8411559 |
| Pcdh10 | 2.2112152 |
| Pcdh10 | 3.5947515 |
| Pcdh15 | 1.3402311 |
| Pcdh17 | -0.3764939 |
| Pcdh20 | 1.5564717 |
| Pcdh21 | -0.8472287 |
| Pcdh7 | 3.8959278 |
| Pcdh7 | 1.7380592 |
| Pcdh8 | -0.7222684 |
| Pcdh9 | 0.1673091 |
| Pcdha10 | 0.3155448 |
| Pcdha11 | 0.8125997 |
| Pcdha2 | 0.2396337 |
| Pcdha6 | 4.6284336 |
| Pcdhac1 | 2.0804778 |
| Pcdhac2 | 1.2722644 |
| Pcdhb1 | -0.5757714 |
| Pcdhb10 | 2.2474847 |
| Pcdhb12 | 1.5283668 |
| Pcdhb14 | 0.8804497 |
| Pcdhb19 | 0.4327957 |
| Pcdhb20 | -0.6533803 |
| Pcdhb22 | -0.8533428 |
| Pcdhb3 | -0.5765228 |
| Pcdhb7 | -0.3996603 |
| Pcdhb8 | -0.0677431 |
| Pcdhga10 | -0.1378801 |
| Pcdhga12 | -0.7284769 |
| Pcdhga4 | -0.1603174 |
| Pcdhga7 | -0.0954976 |
| Pcdhga8 | 2.1410043 |
| Pcdhgb2 | -0.0789153 |
| Pcdhgb6 | 1.2629702 |
| Pcdhgc3 | 1.8262303 |
| Pcdhgc4 | -0.3770959 |
| Pcdhgc5 | 2.6584141 |
| Pcf11 | 1.6009339 |
| Pcgf1 | -0.8123683 |
| Pcgf2 | -0.0588195 |
| Pcgf3 | 1.141346 |
| Pcgf5 | -0.8221914 |
| Pcgf6 | -0.5204274 |
| Pck1 | 0.5067922 |
| Pclo | 0.2510922 |
| Pclo | 3.3706088 |
| Pcmt1 | 0.2843287 |
| Pcmt1 | -0.359207 |
| Pcmt1 | -0.4878306 |
| Pcmtd1 | -0.3615717 |
| Pcmtd2 | 0.2886498 |
| Pcna | -0.309254 |
| Pcnt | 0.8654379 |
| Pcnx | -0.5253849 |
| Pcnxl2 | -0.4743099 |
| Pcnxl2 | 0.1613699 |
| Pcolce | 2.2274528 |
| Pcolce2 | 0.3062932 |
| Pcolce2 | 0.4605207 |
| Pcsk1n | 2.753913 |
| Pcsk2 | 1.0910618 |
| Pcsk2 | -0.7240379 |
| Pcsk2 | 1.4885904 |
| Pcsk2 | 1.5957722 |
| Pcsk4 | -0.2503459 |
| Pcsk4 | -0.6558398 |
| Pcsk4 | 0.1790711 |
| Pcsk4 | -0.283379 |
| Pcsk4 | 2.1594457 |
| Pcsk9 | 1.4960569 |
| Pctk1 | -0.4133502 |
| Pcx | -0.1622127 |
| Pcyox1 | -0.3364707 |
| Pcyox1l | -0.6525619 |
| Pcyt1a | -0.7258108 |
| Pcyt1b | -0.4583488 |
| Pcyt1b | 0.6945806 |
| Pcyt2 | 0.1989744 |
| Pcyt2 | -0.1626871 |
| Pcyt2 | 0.2828838 |
| Pdcd10 | -0.261929 |
| Pdcd2 | -0.4803691 |
| Pdcd2l | 1.4352381 |
| Pdcd4 | 1.7767051 |
| Pdcd5 | 0.0868625 |
| Pdcd5 | -0.4729689 |
| Pdcd6ip | 1.3710447 |
| Pdcl | -0.5015575 |
| Pdcl3 | 1.0043132 |
| Pddc1 | 0.1583855 |
| Pde10a | -0.4682912 |
| Pde1a | -0.6782836 |
| Pde1a | 0.3105103 |
| Pde1a | 1.1779121 |
| Pde1b | 1.3476133 |
| Pde1c | 0.0079824 |
| Pde2a | -0.1833286 |
| Pde3a | -0.8084689 |
| Pde4a | 2.2934078 |
| Pde4a | -0.2487779 |
| Pde4a | -0.7437354 |
| Pde4a | -0.3916676 |
| Pde4b | 0.5697124 |
| Pde4d | -0.47163 |
| Pde4dip | 1.0008037 |
| Pde4dip | -0.5339476 |
| Pde4dip | -0.4190112 |
| Pde4dip | 0.4194763 |
| Pde4dip | -0.2414964 |
| Pde5a | 1.0287559 |
| Pde6a | -0.5645752 |
| Pde6d | -0.4709612 |
| Pde6d | -0.4769978 |
| Pde6d | -0.6323412 |
| Pde6d | 2.5195089 |
| Pde6h | -0.2383937 |
| Pde7a | -0.3843523 |
| Pdgfa | -0.6978541 |
| Pdgfb | 2.6506 |
| Pdgfc | -0.341088 |
| Pdgfra | 3.0187486 |
| Pdgfrb | -0.6315417 |
| Pdgfrl | 1.234397 |
| Pdha1 | 1.5122093 |
| Pdhb | -0.1074683 |
| Pdhb | -0.5871086 |
| Pdia3 | -0.5064118 |
| Pdia4 | 0.2659503 |
| Pdia5 | -0.6110014 |
| Pdia5 | -0.334171 |
| Pdia6 | -0.0711659 |
| Pdik1l | 0.2075062 |
| Pdk1 | 2.0262303 |
| Pdk2 | -0.7169807 |
| Pdk3 | -0.3047969 |
| Pdk4 | 0.1406073 |
| Pdlim1 | 0.6512747 |
| Pdlim2 | 0.491096 |
| Pdlim2 | -0.7117239 |
| Pdlim2 | 2.6433683 |
| Pdlim2 | -0.6774423 |
| Pdlim3 | 1.1319393 |
| Pdlim3 | -0.078483 |
| Pdlim4 | -0.2959483 |
| Pdlim5 | 2.5037422 |
| Pdlim5 | -0.4803691 |
| Pdlim7 | -0.7196206 |
| Pdpk1 | -0.4183805 |
| Pdpk1 | 2.6046476 |
| Pdrg1 | -0.7769279 |
| Pdss1 | -0.2184828 |
| Pdxdc1 | 1.2508214 |
| Pdxdc1 | 2.2312761 |
| Pdxk | -0.2948483 |
| Pdxp | -0.7806927 |
| Pdyn | -0.4609894 |
| Pdyn | 1.2848891 |
| Pdzd11 | -0.2725901 |
| Pdzd11 | -0.0499836 |
| Pdzd11 | -0.1809039 |
| Pdzd8 | 1.4063315 |
| Pdzk1 | 0.035813 |
| Pdzk1ip1 | -0.2291492 |
| Pdzrn3 | -0.4102205 |
| Pdzrn3 | 1.1797286 |
| Pea15 | -0.6987148 |
| Pear1 | 0.5277443 |
| Pear1 | 0.6172205 |
| Pebp1 | -0.5433166 |
| Pecam1 | -0.2959483 |
| Pecam1 | -0.7528334 |
| Peci | 0.2274123 |
| Pef1 | 0.8903693 |
| Peg10 | -0.527518 |
| Peg3 | 0.1550579 |
| Peli1 | -0.7455476 |
| Peli1 | 0.0056239 |
| Peli2 | -0.4374984 |
| Peli3 | 0.1385724 |
| Pelo | -0.0720237 |
| Pelp1 | -0.6918527 |
| Pemt | 1.4638679 |
| Peo1 | 2.6264857 |
| Pepd | 1.3130315 |
| Per1 | -0.675762 |
| Per1 | -0.7383204 |
| Per2 | 0.8634679 |
| Per2 | -0.6918527 |
| Per2 | 0.7814035 |
| Per3 | 0.4318159 |
| Perld1 | 0.8264588 |
| Pes1 | -0.2301717 |
| Pet112l | -0.265646 |
| Pet112l | 0.4963585 |
| Pet112l | 0.5697124 |
| Pet112l | 0.6148238 |
| Pex11a | 0.368616 |
| Pex11a | -0.6816567 |
| Pex11a | -0.5469463 |
| Pex11b | -0.6387625 |
| Pex11c | -0.3416668 |
| Pex14 | -0.7196206 |
| Pex16 | 1.6581645 |
| Pex19 | -0.1291509 |
| Pex19 | -0.6674074 |
| Pex2 | 1.0534125 |
| Pex26 | 0.9483847 |
| Pex3 | 0.762085 |
| Pex3 | 2.3664929 |
| Pex5 | -0.0424784 |
| Pex5 | -0.114638 |
| Pex5 | -0.6766018 |
| Pex5l | -0.3193641 |
| Pex6 | 0.2062473 |
| Pex6 | 0.6257569 |
| Pex6 | 0.0134618 |
| Pex7 | -0.6055506 |
| Pfdn1 | -0.5780274 |
| Pfdn2 | 0.6200087 |
| Pfdn4 | 0.2495928 |
| Pfdn5 | -0.6665762 |
| Pfdn5 | 1.5900396 |
| Pfkfb2 | 2.603025 |
| Pfkfb2 | -0.5947468 |
| Pfkfb4 | 0.2629982 |
| Pfkl | 1.365678 |
| Pfkl | -0.269913 |
| Pfkm | -0.0592425 |
| Pfkm | 3.3168607 |
| Pfkm | 0.2178253 |
| Pfkp | -0.6909986 |
| Pfkp | 0.1780968 |
| Pfkp | 1.0674291 |
| Pfn1 | 3.3455651 |
| Pfn1 | -0.7657271 |
| Pfn2 | 0.6035537 |
| Pfn2 | 1.8872364 |
| Pfn2 | -0.0138725 |
| Pfn3 | 1.1142045 |
| Pfn4 | -0.1955507 |
| Pftk1 | -0.467625 |
| Pga5 | 1.3857675 |
| Pgam1 | -0.1560675 |
| Pgam2 | 1.3060084 |
| Pgam5 | 1.2697566 |
| Pgbd5 | 0.2221676 |
| Pgcp | -0.6339422 |
| Pgd | 0.7022353 |
| Pgk1 | -0.1650631 |
| Pgk2 | -0.5571878 |
| Pgls | 1.9606927 |
| Pglyrp1 | -0.2054495 |
| Pglyrp4 | -0.2645825 |
| Pgm2 | -0.4803691 |
| Pgm2l1 | -0.1374185 |
| Pgm3 | -0.5454927 |
| Pgm5 | 0.4189792 |
| Pgm5 | -0.1838143 |
| Pgpep1 | -0.8301278 |
| Pgpep1 | 0.1007797 |
| Pgr | -0.0203313 |
| Pgrmc1 | -0.7205024 |
| Pgs1 | -0.1527759 |
| Phactr1 | -0.593214 |
| Phactr1 | -0.514795 |
| Phactr1 | -0.4596681 |
| Phactr4 | -0.7778676 |
| Phb2 | 0.8753738 |
| Phc1 | -0.7117239 |
| Phc1 | -0.6204233 |
| Phc2 | 1.6132818 |
| Phca | -0.0342121 |
| Phca | 2.9171986 |
| Phex | 1.3706131 |
| Phf1 | 1.2928317 |
| Phf11 | 1.9358852 |
| Phf11 | -0.5653173 |
| Phf12 | 1.2465699 |
| Phf12 | 0.4011512 |
| Phf13 | 0.0220053 |
| Phf13 | -0.5418689 |
| Phf14 | 0.3427423 |
| Phf16 | -0.597051 |
| Phf17 | -0.4253433 |
| Phf19 | 1.6354885 |
| Phf2 | 0.7942742 |
| Phf20l1 | 1.9943387 |
| Phf21a | 0.1922821 |
| Phf5a | 1.651491 |
| Phf6 | 1.2072709 |
| Phf7 | 2.6007175 |
| Phgdh | 2.0972284 |
| Phka1 | 2.0207554 |
| Phka1 | -0.0810798 |
| Phka2 | -0.2271078 |
| Phka2 | -0.5204274 |
| Phka2 | -0.3165443 |
| Phkb | -0.3825327 |
| Phkg1 | 0.8218628 |
| Phkg2 | 0.3183296 |
| Phlda1 | 1.2188944 |
| Phlda2 | 1.2658849 |
| Phlda3 | 0.6759019 |
| Phlda3 | 0.3329471 |
| Phldb1 | -0.4253433 |
| Phldb1 | -0.1970286 |
| Phlpp | -0.5978204 |
| Phospho1 | 1.5094017 |
| Phospho2 | -0.2245624 |
| Phox2a | 0.1884355 |
| Phox2b | 2.3209408 |
| Phpt1 | 0.9124759 |
| Phtf1 | -0.7546641 |
| Phtf2 | -0.4974174 |
| Phyhip | -0.629945 |
| Pi16 | -0.6583061 |
| Pi4k2b | 0.192602 |
| Pi4k2b | 2.8282582 |
| Pi4ka | 1.7075294 |
| Pi4kb | -0.1007983 |
| Pi4kb | 1.76903 |
| Pias1 | 0.575581 |
| Pias2 | 0.0587363 |
| Pias3 | 0.7755677 |
| Pias3 | -0.0038726 |
| Pias4 | 0.3935108 |
| Pibf1 | -0.4596681 |
| Pibf1 | -0.6110014 |
| Picalm | -0.3439858 |
| Pick1 | 1.4635564 |
| Pick1 | 1.0306477 |
| Pif1 | -0.0600889 |
| Piga | 1.2425969 |
| Piga | 2.6645841 |
| Pigb | 1.8152882 |
| Pigc | -0.6063272 |
| Pigf | -0.6782836 |
| Pigh | 0.1088875 |
| Pigk | -0.4426659 |
| Pigk | 0.9874209 |
| Pigl | 0.2159579 |
| Pign | -0.6180586 |
| Pigo | 2.2488847 |
| Pigo | -0.6657458 |
| Pigo | 0.527524 |
| Pigp | -0.6657458 |
| Pigp | 2.9330633 |
| Pigq | -0.640375 |
| Pigt | 1.2701429 |
| Pigx | -0.4221717 |
| Pigy | -0.7666552 |
| Pigyl | 1.4676741 |
| Pigz | -0.2608698 |
| Pih1d1 | 0.6809333 |
| Pik3ap1 | 1.0536581 |
| Pik3c2a | -0.467625 |
| Pik3c3 | -0.7410238 |
| Pik3ca | 0.6420085 |
| Pik3cb | -0.165539 |
| Pik3cd | 1.7930504 |
| Pik3cg | -0.4355682 |
| Pik3ip1 | -0.5008661 |
| Pik3r1 | -0.7125979 |
| Pik3r1 | 0.2402404 |
| Pik3r1 | 0.1868279 |
| Pik3r2 | 0.6589243 |
| Pik3r2 | 0.9516918 |
| Pik3r3 | -0.521134 |
| Pik3r4 | 1.7735657 |
| Pik3r6 | 2.1464526 |
| Pik3r6 | -0.7082364 |
| Pim1 | -0.1872216 |
| Pim2 | -0.0858601 |
| Pim2 | 1.4979327 |
| Pim3 | 0.0032591 |
| Pip4k2a | 2.0960725 |
| Pip4k2b | 1.6927427 |
| Pip4k2b | 0.8791089 |
| Pip4k2c | -0.1886859 |
| Pip5k1a | -0.782581 |
| Pip5k1a | -0.4649649 |
| Pip5k1b | -0.5289428 |
| Pip5kl1 | -0.4491672 |
| Pipox | -0.3278767 |
| Pir | 2.0845484 |
| Pira11 | 1.4003368 |
| Pira4 | 2.0683534 |
| Pisd | -0.593214 |
| Pit1 | -0.0707374 |
| Pitpna | 1.2621913 |
| Pitpnb | 0.9447884 |
| Pitpnc1 | 3.6026481 |
| Pitpnm1 | -0.3064657 |
| Pitpnm2 | 2.498211 |
| Pitpnm3 | 1.0548853 |
| Pitx1 | 0.4609954 |
| Pitx2 | -0.7500943 |
| Pitx2 | -0.3210602 |
| Pitx2 | -0.628351 |
| Pitx2 | 3.8450022 |
| Piwil1 | -0.630743 |
| Piwil2 | -0.4649649 |
| Piwil4 | 1.3192692 |
| Pja1 | -0.5022494 |
| Pja2 | 0.7544113 |
| Pja2 | 2.0891058 |
| Pkd1 | -0.0466402 |
| Pkd1l3 | 2.578034 |
| Pkd2 | -0.3698976 |
| Pkd2l1 | -0.7365227 |
| Pkd2l2 | -0.8202182 |
| Pkdrej | 0.0631778 |
| Pkhd1l1 | 1.5949651 |
| Pkhd1l1 | 1.4563638 |
| Pkia | 0.4887982 |
| Pkib | 2.2694364 |
| Pkib | -0.8172665 |
| Pkig | -0.4027534 |
| Pkig | -0.5871086 |
| Pkig | -0.3935058 |
| Pkm2 | 0.5728611 |
| Pkmyt1 | -0.2926524 |
| Pkn1 | -0.6541994 |
| Pkn1 | 1.4763908 |
| Pkn2 | 1.4459424 |
| Pkn2 | -0.0034749 |
| Pkn2 | 1.6502263 |
| Pkn3 | 0.0697993 |
| Pknox1 | 2.9535425 |
| Pknox1 | 2.6105652 |
| Pknox2 | 0.0418824 |
| Pknox2 | 1.3061012 |
| Pknox2 | 1.133851 |
| Pknox2 | -0.4939818 |
| Pknox2 | 1.7111441 |
| Pkp1 | 1.0019751 |
| Pkp2 | -0.6884412 |
| Pkp2 | -0.2466914 |
| Pkp2 | 1.29302 |
| Pkp3 | -0.7741146 |
| Pla1a | -0.4083479 |
| Pla1a | -0.7629486 |
| Pla1a | -0.6204233 |
| Pla2g10 | -0.6808123 |
| Pla2g12a | -0.7266986 |
| Pla2g1b | -0.5078036 |
| Pla2g2c | -0.6275551 |
| Pla2g2d | 0.1813404 |
| Pla2g2f | -0.5542498 |
| Pla2g4c | -0.3020223 |
| Pla2g4d | -0.4981061 |
| Pla2g4e | -0.6387625 |
| Pla2g4f | 2.091279 |
| Pla2g5 | 2.1117047 |
| Pla2g6 | 0.6158232 |
| Pla2g7 | 0.1176183 |
| Plaa | 0.6306829 |
| Plac1 | -0.6117828 |
| Plac1l | -0.3764939 |
| Plac8 | 0.5384757 |
| Plag1 | 0.7845556 |
| Plag1 | 3.1566722 |
| Plagl2 | -0.6674074 |
| Plau | -0.0797805 |
| Plb1 | 0.6827897 |
| Plcb2 | 1.7461157 |
| Plcb3 | 1.2884909 |
| Plcb4 | 0.1543909 |
| Plcd1 | -0.6047745 |
| Plcd3 | -0.573521 |
| Plcd4 | -0.8553901 |
| Plcd4 | -0.3831388 |
| Plcd4 | -0.6063272 |
| Plce1 | 2.3198591 |
| Plcg2 | 0.644334 |
| Plch2 | 0.3397606 |
| Plcl2 | -0.0301072 |
| Plcxd1 | 0.1874713 |
| Plcxd1 | -0.3627564 |
| Plcz1 | 2.8455373 |
| Pld1 | 1.2282482 |
| Pld2 | -0.671575 |
| Pld3 | 0.2483915 |
| Pld3 | 0.4590951 |
| Pld4 | 0.9153447 |
| Pld5 | -0.470293 |
| Pldn | -0.0416484 |
| Plec1 | 0.5726515 |
| Plec1 | -0.2094396 |
| Plec1 | 0.1361924 |
| Plec1 | 0.0918832 |
| Plek | 2.3704806 |
| Plek2 | -0.6172717 |
| Plekha1 | 1.340499 |
| Plekha1 | 0.5451994 |
| Plekha2 | 1.3288168 |
| Plekha3 | -0.6047745 |
| Plekha4 | 2.4172996 |
| Plekha4 | -0.6427992 |
| Plekha5 | -0.5022494 |
| Plekha6 | -0.7222684 |
| Plekha7 | -0.5382596 |
| Plekha8 | -0.7134728 |
| Plekhb1 | 0.268305 |
| Plekhb2 | 0.0095513 |
| Plekhf1 | -0.1168902 |
| Plekhf1 | -0.2271078 |
| Plekhf2 | -0.628351 |
| Plekhf2 | 2.3702816 |
| Plekhg2 | 0.9829153 |
| Plekhg2 | -0.1523067 |
| Plekhg2 | 1.3225541 |
| Plekhg3 | 1.9265777 |
| Plekhg3 | 0.7350231 |
| Plekhg4 | -0.1794523 |
| Plekhg5 | -0.56606 |
| Plekhg6 | -0.7187398 |
| Plekhj1 | 2.2479082 |
| Plekhm1 | 0.3079824 |
| Plekhm3 | -0.5871086 |
| Plekho2 | 1.3443299 |
| Plin | -0.6541994 |
| Plk1 | 0.1819878 |
| Plk2 | -0.2159616 |
| Plk3 | 0.9487988 |
| Plk4 | 0.337858 |
| Pllp | -0.6275551 |
| Plod1 | 2.2618686 |
| Plod2 | -0.1259561 |
| Plod3 | 0.0767362 |
| Plp1 | -0.6901453 |
| Plp2 | 0.8874196 |
| Plrg1 | -0.2159616 |
| Pls3 | -0.6665762 |
| Plscr1 | 1.6885682 |
| Plscr1 | 0.6110158 |
| Plscr3 | -0.7519194 |
| Plscr4 | 0.4234434 |
| Pltp | -0.524675 |
| Plunc | 1.0956445 |
| Plvap | -0.3801123 |
| Plxdc1 | 1.0327869 |
| Plxdc1 | 1.811276 |
| Plxna2 | -0.6884412 |
| Plxnb1 | -0.5440414 |
| Plxnb3 | -0.6001325 |
| Plxnc1 | 0.6257569 |
| Plxnd1 | 0.266245 |
| Pmaip1 | -0.3047969 |
| Pmch | -0.3142948 |
| Pmepa1 | 0.157721 |
| Pmf1 | 2.2195891 |
| Pmfbp1 | -0.5535167 |
| Pml | 1.2854587 |
| Pml | -0.3586168 |
| Pmm1 | -0.5310843 |
| Pmm1 | -0.0074598 |
| Pmm2 | -0.2451298 |
| Pmm2 | -0.169834 |
| Pmm2 | 0.5015905 |
| Pmm2 | 1.1624847 |
| Pmp2 | 0.1932412 |
| Pmp22 | -0.5015575 |
| Pmp22 | -0.8231796 |
| Pmpca | 1.8163411 |
| Pmpcb | 0.4483306 |
| Pms2 | -0.7143485 |
| Pmvk | -0.4491672 |
| Pnck | 2.0696645 |
| Pnkd | -0.644419 |
| Pnkd | 0.6503139 |
| Pnkp | 0.4114893 |
| Pnkp | 3.1147013 |
| Pnliprp1 | -0.4830752 |
| Pnma1 | -0.5239657 |
| Pnma2 | 0.3497508 |
| Pno1 | 1.4866951 |
| Pnpla2 | 0.935874 |
| Pnpla6 | 1.0476205 |
| Pnpla7 | -0.5818001 |
| Pnpla8 | -0.3978095 |
| Pnpla8 | -0.3047969 |
| Pnpo | 1.4222064 |
| Pnpo | -0.090228 |
| Pnrc2 | 0.9422901 |
| Podn | -0.5586605 |
| Podxl | -0.5289428 |
| Podxl2 | -0.0724529 |
| Pof1b | 0.7069415 |
| Pofut1 | 0.890958 |
| Pofut1 | -0.6371529 |
| Pofut2 | -0.621213 |
| Pogz | -0.8074967 |
| Pogz | 0.6296999 |
| Pola2 | -0.1047944 |
| Polb | 1.029387 |
| Pold1 | -0.7222684 |
| Pold2 | -0.7013016 |
| Pold3 | 1.3528244 |
| Pold4 | -0.6110014 |
| Poldip2 | -0.6411824 |
| Poldip3 | -0.685891 |
| Poldip3 | 0.2585519 |
| Pole | -0.0685976 |
| Pole3 | -0.4583488 |
| Pole3 | -0.8381348 |
| Pole4 | -0.4851101 |
| Polg | 1.589295 |
| Polg2 | 2.4788187 |
| Polh | -0.7564985 |
| Poli | -0.4722992 |
| Polk | -0.5962823 |
| Poll | 2.3894347 |
| Poll | 1.7464001 |
| Polm | 2.9094568 |
| Polm | -0.36098 |
| Poln | 2.1813823 |
| Polq | -0.7911278 |
| Polr1e | 0.1560575 |
| Polr2d | 1.4449094 |
| Polr2d | -0.3704953 |
| Polr2d | -0.6682394 |
| Polr2d | -0.8361264 |
| Polr2e | -0.7601786 |
| Polr2e | 0.0865028 |
| Polr2f | 1.1665018 |
| Polr2g | -0.7196206 |
| Polr2h | 0.7269375 |
| Polr2j | -0.7365227 |
| Polr3a | -0.4102205 |
| Polr3a | -0.7134728 |
| Polr3b | -0.3722907 |
| Polr3d | 2.3724394 |
| Polr3e | 3.1106405 |
| Polr3g | 0.3113513 |
| Polr3h | -0.5712762 |
| Polr3h | 0.1620317 |
| Polr3h | -0.7741146 |
| Polr3k | -0.7047623 |
| Polr3k | 1.5943593 |
| Polrmt | 0.0908097 |
| Pols | -0.3233266 |
| Pom121 | -0.7030303 |
| Pomc | -0.6550192 |
| Pomgnt1 | -0.2271078 |
| Pomgnt1 | 2.8082503 |
| Pomp | 1.9076213 |
| Pomt1 | 2.5347436 |
| Pomt1 | -0.4803691 |
| Pomt2 | -0.7161024 |
| Pon1 | 0.8162844 |
| Pon2 | 0.8346415 |
| Pon3 | 0.3174951 |
| Pon3 | -0.1823579 |
| Pop4 | 0.9365737 |
| Pop5 | 0.2049867 |
| Pop5 | -0.6032245 |
| Pop7 | 0.6700902 |
| Popdc2 | -0.7047623 |
| Popdc2 | 1.3775798 |
| Popdc3 | -0.6509274 |
| Por | 0.4609954 |
| Por | -0.3693004 |
| Porcn | 0.4741861 |
| Porcn | -0.3227595 |
| Pou2af1 | 1.1054362 |
| Pou2f1 | -0.2970496 |
| Pou2f1 | -0.448515 |
| Pou2f2 | -0.4400784 |
| Pou3f1 | -0.623586 |
| Pou3f3 | 0.2165808 |
| Pou5f1 | 1.493575 |
| Pou5f1 | 0.105019 |
| Pou6f1 | 0.7011458 |
| Ppa1 | 1.4282196 |
| Ppa2 | 0.9879495 |
| Ppa2 | 1.9961069 |
| Ppa2 | -0.8084689 |
| Ppap2a | -0.640375 |
| Ppap2a | 2.3106423 |
| Ppap2a | -0.8172665 |
| Ppap2b | -0.7064977 |
| Ppap2b | 1.4468154 |
| Ppap2c | -0.5985904 |
| Ppapdc1 | 1.5253911 |
| Ppapdc1a | 1.4414844 |
| Ppapdc2 | -0.6363491 |
| Ppapdc3 | -0.0698808 |
| Ppapdc3 | 2.8105535 |
| Pparbp | -0.6774423 |
| Pparg | -0.7205024 |
| Ppargc1a | 3.863609 |
| Ppargc1b | 1.5307547 |
| Ppat | 1.1148931 |
| Ppcs | -0.2555925 |
| Ppef2 | 0.2115854 |
| Ppfia3 | 2.6048229 |
| Ppfia3 | -0.2383937 |
| Ppfibp1 | -0.7464551 |
| Ppfibp2 | 1.7024683 |
| Ppfibp2 | 1.9046215 |
| Ppia | 0.4109877 |
| Ppic | -0.0990279 |
| Ppic | -0.7004385 |
| Ppid | 0.2621107 |
| Ppie | -0.2582273 |
| Ppie | -0.4298024 |
| Ppif | -0.8104165 |
| Ppil2 | -0.5022494 |
| Ppil3 | -0.4629749 |
| Ppl | 0.9549867 |
| Ppm1a | -0.7638738 |
| Ppm1a | 0.0723612 |
| Ppm1a | -0.451781 |
| Ppm1b | 0.4097326 |
| Ppm1b | -0.4524356 |
| Ppm1b | 0.3942777 |
| Ppm1e | -0.7873192 |
| Ppm1e | -0.7528334 |
| Ppm1f | 2.2156145 |
| Ppm1f | -0.715225 |
| Ppm1f | 4.223642 |
| Ppm1g | 0.0231641 |
| Ppm1h | 0.7470169 |
| Ppm1j | -0.5985904 |
| Ppm1k | 0.8461594 |
| Ppm1l | -0.5557176 |
| Ppm1l | -0.7987938 |
| Ppm1m | -0.3486419 |
| Ppm1m | 0.4369484 |
| Ppm2c | 0.7207364 |
| Ppme1 | -0.6649161 |
| Ppnr | 1.3447743 |
| Ppox | 0.6971394 |
| Ppox | -0.4021339 |
| Ppp1ca | -0.6063272 |
| Ppp1ca | 0.2799868 |
| Ppp1ca | 0.1826346 |
| Ppp1cb | 0.0426382 |
| Ppp1cb | 0.2193785 |
| Ppp1cc | -0.6419904 |
| Ppp1r10 | 1.2243964 |
| Ppp1r10 | 0.5625344 |
| Ppp1r11 | 1.1910788 |
| Ppp1r12c | -0.1693557 |
| Ppp1r13b | 2.0344427 |
| Ppp1r13b | -0.09242 |
| Ppp1r13b | 2.466336 |
| Ppp1r14a | 1.2752452 |
| Ppp1r14a | -0.6509274 |
| Ppp1r14c | 2.0499156 |
| Ppp1r14d | 2.5386892 |
| Ppp1r15b | -0.2461706 |
| Ppp1r16a | -0.64523 |
| Ppp1r1a | -0.7169807 |
| Ppp1r1b | 1.2719754 |
| Ppp1r1b | 0.7523539 |
| Ppp1r2 | -0.8492621 |
| Ppp1r3c | -0.3687035 |
| Ppp1r3f | -0.3898332 |
| Ppp1r8 | -0.4374984 |
| Ppp1r9a | -0.4015149 |
| Ppp1r9b | 0.1169229 |
| Ppp2ca | -0.423439 |
| Ppp2cb | -0.628351 |
| Ppp2cz | -0.5133923 |
| Ppp2r1a | -0.4133502 |
| Ppp2r1b | -0.3965778 |
| Ppp2r2b | 0.7593641 |
| Ppp2r2b | 3.3716649 |
| Ppp2r2b | 1.2167456 |
| Ppp2r2c | -0.2446098 |
| Ppp2r2c | 0.8244014 |
| Ppp2r2d | 0.3788127 |
| Ppp2r3c | 0.1000712 |
| Ppp2r4 | -0.7583367 |
| Ppp2r5b | 1.70747 |
| Ppp2r5c | -0.303686 |
| Ppp2r5c | 0.7062191 |
| Ppp2r5c | -0.4064792 |
| Ppp2r5d | 0.2084492 |
| Ppp2r5d | -0.8045864 |
| Ppp2r5d | -0.1838143 |
| Ppp2r5d | 0.9303982 |
| Ppp2r5e | -0.5638338 |
| Ppp2r5e | 0.173536 |
| Ppp3ca | -0.3070227 |
| Ppp3ca | -0.8677726 |
| Ppp3ca | -0.5036347 |
| Ppp3cb | -1.0631152 |
| Ppp3cc | -0.6460417 |
| Ppp3r1 | -0.621213 |
| Ppp4c | -0.6267599 |
| Ppp4r1 | -0.8845523 |
| Ppp4r1 | 0.7900044 |
| Ppp4r1 | -0.7258108 |
| Ppp4r1l | 0.0701657 |
| Ppp5c | -0.4413712 |
| Ppp5c | -0.7741146 |
| Ppp6c | 0.570343 |
| Pps | 0.5117398 |
| Ppt1 | 0.1884355 |
| Ppt2 | -0.644419 |
| Pptc7 | -0.4557159 |
| Pqbp1 | 0.1493726 |
| Pqlc1 | -0.2024684 |
| Pqlc2 | -0.8451999 |
| Pqlc3 | 0.538911 |
| Pqlc3 | 0.4413246 |
| Pqlc3 | 1.5423863 |
| Pqlc3 | -0.5289428 |
| Pramef12 | 1.010905 |
| Prap1 | 1.2219553 |
| Prc1 | -0.979354 |
| Prcp | -0.3562598 |
| Prdm10 | 1.2621913 |
| Prdm4 | -0.4583488 |
| Prdm8 | -0.3752909 |
| Prdm9 | -0.2435708 |
| Prdx1 | 1.0989202 |
| Prdx2 | -0.3126114 |
| Prdx2 | 2.6380644 |
| Prdx2 | -0.2029645 |
| Prdx3 | 0.1935607 |
| Prdx4 | -0.6275551 |
| Prdx5 | 0.3945332 |
| Prdx5 | -0.8594987 |
| Prdx6 | -0.5064118 |
| Prdx6-rs1 | -0.2225313 |
| Prdx6-rs1 | 1.0928271 |
| Preb | -0.1291509 |
| Preb | 2.0750085 |
| Prei3 | 0.392231 |
| Prei4 | 1.0883482 |
| Prei4 | 0.0818136 |
| Prelp | 1.8380174 |
| Prep | -0.4336422 |
| Prep | -0.6517443 |
| Prf1 | -0.2159616 |
| Prickle1 | -0.0793478 |
| Prickle2 | -0.2855524 |
| Prickle3 | -0.4190112 |
| Prickle4 | -0.2742002 |
| Prim2 | -0.1579539 |
| Prim2 | -0.451781 |
| Prima1 | 2.2013575 |
| Prkaa2 | 0.9992396 |
| Prkaa2 | -0.1504321 |
| Prkab1 | 0.499092 |
| Prkab2 | -0.1485615 |
| Prkaca | 1.9897945 |
| Prkaca | -0.8104165 |
| Prkacb | 0.9223347 |
| Prkag1 | -0.5549834 |
| Prkag2 | -0.7901742 |
| Prkar1a | 0.2414527 |
| Prkar1b | -0.4228051 |
| Prkar2b | 0.3419301 |
| Prkca | 0.8708711 |
| Prkcb1 | -0.9481445 |
| Prkcbp1 | 1.2040557 |
| Prkcbp1 | -0.9504195 |
| Prkcc | -0.1965357 |
| Prkcd | 1.0964645 |
| Prkcdbp | 3.257462 |
| Prkcdbp | -0.8182493 |
| Prkce | -0.7222684 |
| Prkch | -0.4052356 |
| Prkcq | -0.8221914 |
| Prkcsh | -0.3370466 |
| Prkcz | 0.1777718 |
| Prkcz | -0.1383418 |
| Prkcz | -0.4108456 |
| Prkd1 | -0.5993611 |
| Prkd1 | -0.6944198 |
| Prkd2 | 0.5085945 |
| Prkd3 | -0.3710933 |
| Prkd3 | 3.8629058 |
| Prkd3 | 0.7868713 |
| Prkg1 | -0.659954 |
| Prkg2 | -0.0074598 |
| Prkra | 0.2274123 |
| Prkrip1 | -0.8594987 |
| Prkrip1 | -0.4407246 |
| Prkrir | 1.5830437 |
| Prkx | 1.0141188 |
| Prkx | 0.9843755 |
| Prl | -0.3142948 |
| Prlr | -0.5043282 |
| Prlr | -0.0554432 |
| Prm1 | 2.2407474 |
| Prm3 | 2.574167 |
| Prmt2 | -0.6509274 |
| Prmt2 | -0.2932009 |
| Prmt3 | -0.4649649 |
| Prmt5 | -0.5772748 |
| Prmt5 | -0.0575519 |
| Prmt5 | -0.470293 |
| Prmt7 | 0.5321399 |
| Prmt7 | 0.1663219 |
| Prmt7 | -0.6766018 |
| Prmt7 | -0.5389803 |
| Prmt8 | -0.3301605 |
| Prnp | -0.111046 |
| Prnpip1 | -0.6149153 |
| Prnpip1 | -0.4240733 |
| Prnpip1 | -0.6339422 |
| Proc | 1.0871659 |
| Proc | 2.5232579 |
| Procr | 2.0747717 |
| Prodh | 0.8791089 |
| Prok2 | -0.9424819 |
| Prok2 | -0.7816364 |
| Prokr1 | 0.7371205 |
| Prokr2 | -0.3776984 |
| Prom1 | -0.7302586 |
| Prom2 | 0.6152237 |
| Prom2 | 2.1203401 |
| Pros1 | -0.4317203 |
| Prosapip1 | 0.5669746 |
| Prosapip1 | -0.7939949 |
| Prosapip1 | -0.573521 |
| Prosc | 1.0673081 |
| Prosc | 0.0418824 |
| Prosc | -0.0806465 |
| Prox1 | -0.4537463 |
| Prox1 | 2.072756 |
| Prpf18 | -0.3953477 |
| Prpf19 | 0.1018414 |
| Prpf19 | -0.7657271 |
| Prpf19 | 3.3264294 |
| Prpf3 | -0.6427992 |
| Prpf3 | 0.3965747 |
| Prpf3 | 2.4663104 |
| Prpf3 | -0.086296 |
| Prpf3 | -0.7004385 |
| Prpf31 | -0.2461706 |
| Prpf38a | -0.7258108 |
| Prpf38a | -0.864661 |
| Prpf38a | -0.036684 |
| Prpf38b | 3.4067208 |
| Prpf39 | 0.9209126 |
| Prpf40a | -0.5029418 |
| Prpf40a | 1.5114713 |
| Prpf40b | -0.3376228 |
| Prpf40b | -0.6808123 |
| Prpf40b | -0.2255797 |
| Prpf6 | 0.3282843 |
| Prpf8 | -0.4336422 |
| Prpf8 | -0.2534902 |
| Prph2 | 0.266834 |
| Prps1 | -0.6102207 |
| Prps1l1 | -0.8351238 |
| Prps2 | -0.2353016 |
| Prpsap2 | -0.5962823 |
| Prpsap2 | 1.2488464 |
| Prr11 | -0.8411559 |
| Prr13 | -0.6558398 |
| Prr14 | -0.1541852 |
| Prr15 | 2.2379856 |
| Prr16 | -0.2981523 |
| Prr18 | 0.6334297 |
| Prr19 | 1.5009257 |
| Prr3 | 1.0465072 |
| Prr7 | 0.4142428 |
| Prrc1 | 2.5122551 |
| Prrg2 | 0.2874995 |
| Prrt1 | -0.7863696 |
| Prrt1 | -0.3031311 |
| Prrt1 | -0.5513212 |
| Prrt3 | -0.7108508 |
| Prrx1 | 0.0319987 |
| Prrx1 | -0.0199263 |
| Prrx1 | -0.2144524 |
| Prss21 | -0.7510064 |
| Prss23 | 0.4367047 |
| Prss27 | -0.0235782 |
| Prss28 | -0.1177927 |
| Prss29 | 1.8342086 |
| Prss29 | 0.3513605 |
| Prss34 | -0.4310805 |
| Prss35 | 1.8069269 |
| Prss36 | -0.6533803 |
| Prss7 | -0.470293 |
| Prss8 | 0.20404 |
| Prss8 | 1.5878719 |
| Prtn3 | 1.2133585 |
| Prune | 0.7586826 |
| Prune | 0.085783 |
| Prune | 1.6844352 |
| Prune | 2.3851949 |
| Psap | -0.7047623 |
| Psat1 | -0.1940753 |
| Psca | 0.7506357 |
| Pscd1 | -0.8094422 |
| Pscd2 | 2.5937151 |
| Pscd3 | 0.3532347 |
| Pscdbp | -0.3813217 |
| Psd2 | 0.0818136 |
| Psd3 | 1.0269867 |
| Psd4 | 0.2453812 |
| Psen1 | -0.7038959 |
| Psen2 | 1.224498 |
| Psenen | -0.2932009 |
| Psg19 | 1.9254133 |
| Psg23 | -0.7038959 |
| Psg27 | 0.2504928 |
| Psip1 | -0.7161024 |
| Psip1 | 2.8918142 |
| Pskh1 | 2.6723594 |
| Psma2 | 0.734498 |
| Psma4 | 0.2615186 |
| Psma5 | 0.4442302 |
| Psma7 | 3.0087656 |
| Psma7 | -0.1255007 |
| Psmb1 | -0.561613 |
| Psmb10 | 1.2477583 |
| Psmb10 | -0.4629749 |
| Psmb2 | -0.8856115 |
| Psmb2 | 0.1466847 |
| Psmb3 | -0.0276533 |
| Psmb4 | 0.4974985 |
| Psmb5 | -0.0950573 |
| Psmb6 | -0.6172717 |
| Psmb7 | -0.4459107 |
| Psmb7 | -0.7911278 |
| Psmb7 | -0.4926112 |
| Psmb8 | -0.1043495 |
| Psmb9 | 1.5145669 |
| Psmc1 | 0.9855684 |
| Psmc2 | -0.8065256 |
| Psmc3 | -0.893061 |
| Psmc3ip | -0.6460417 |
| Psmc4 | 1.6564065 |
| Psmc5 | -0.7601786 |
| Psmc5 | 0.7554382 |
| Psmd1 | 0.2507926 |
| Psmd10 | -0.8677726 |
| Psmd10 | -0.3996603 |
| Psmd10 | -0.6657458 |
| Psmd11 | -0.5268064 |
| Psmd11 | 1.8086797 |
| Psmd12 | 1.9066701 |
| Psmd12 | 1.6417065 |
| Psmd13 | 0.8994521 |
| Psmd14 | 1.8946938 |
| Psmd2 | 1.1871702 |
| Psmd2 | 0.2501929 |
| Psmd4 | -0.0354472 |
| Psmd5 | -0.6259654 |
| Psmd6 | -0.2561188 |
| Psmd6 | 0.61742 |
| Psmd7 | -0.6071046 |
| Psmd7 | 1.5744109 |
| Psmd8 | 0.3476002 |
| Psmd9 | 1.3659384 |
| Psmd9 | 0.1476936 |
| Psme1 | -0.217978 |
| Psme1 | 0.3759484 |
| Psme2 | 1.1129407 |
| Psme3 | 0.3332207 |
| Psme4 | -0.8192332 |
| Psme4 | -0.7500943 |
| Psmf1 | 0.6112166 |
| Psmf1 | -0.5447667 |
| Psmg2 | 2.4355932 |
| Psmg2 | 2.1234496 |
| Psmg3 | 2.3734597 |
| Psors1c2 | 2.1061175 |
| Psp | 0.8425953 |
| Pspc1 | -0.5765228 |
| Pstpip1 | 0.4807097 |
| Pstpip2 | 0.8905165 |
| Ptbp1 | -0.7939949 |
| Ptbp1 | 0.0064107 |
| Ptcd3 | 2.6597339 |
| Ptch1 | 1.53702 |
| Ptch2 | -0.7835267 |
| Ptdss2 | 0.8354244 |
| Pten | 3.4510944 |
| Pter | -0.5197214 |
| Ptgds | 1.3283644 |
| Ptgds2 | 2.3845516 |
| Ptgds2 | 0.724108 |
| Ptger2 | -0.6833481 |
| Ptger2 | -0.593214 |
| Ptger3 | -0.1975218 |
| Ptges | -0.519016 |
| Ptges | 0.0157999 |
| Ptges2 | 0.3896661 |
| Ptges3 | 0.8461594 |
| Ptgfr | -0.2774291 |
| Ptgir | -0.6749231 |
| Ptgis | -0.7491831 |
| Ptgs1 | -0.964192 |
| Ptgs1 | -0.7731788 |
| Pth2 | -0.4209062 |
| Pth2r | -0.5579238 |
| Pthlh | 0.283462 |
| Pthr1 | -0.864661 |
| Pthr1 | -0.9048933 |
| Pthr1 | 0.6905449 |
| Ptk2 | 1.3021968 |
| Ptk2b | 2.4582263 |
| Ptk7 | -0.6157001 |
| Ptms | 0.5762075 |
| Ptn | -0.6492959 |
| Ptp4a2 | -0.1916215 |
| Ptp4a2 | 2.030014 |
| Ptp4a2 | -0.8482449 |
| Ptp4a2 | -0.7143485 |
| Ptp4a3 | -0.4478632 |
| Ptp4a3 | -0.0030774 |
| Ptpdc1 | 1.2106809 |
| Ptpdc1 | 0.534111 |
| Ptpdc1 | 0.3141491 |
| Ptpla | -0.6875903 |
| Ptpla | -0.0301072 |
| Ptplad1 | -0.4058572 |
| Ptplad2 | 0.5484349 |
| Ptplb | -0.3873931 |
| Ptpn1 | -0.8045864 |
| Ptpn11 | -0.2276177 |
| Ptpn11 | 0.0300855 |
| Ptpn11 | -0.4810448 |
| Ptpn12 | -0.6039992 |
| Ptpn12 | -0.6227943 |
| Ptpn12 | -0.4682912 |
| Ptpn12 | -0.6558398 |
| Ptpn12 | 0.2426633 |
| Ptpn14 | 1.1993679 |
| Ptpn18 | 2.5581384 |
| Ptpn20 | 1.797037 |
| Ptpn21 | -0.4089717 |
| Ptpn22 | -0.9114135 |
| Ptpn5 | 1.9668114 |
| Ptpn6 | -0.9346136 |
| Ptpn6 | -0.324462 |
| Ptpra | -0.2164653 |
| Ptprb | -0.114638 |
| Ptprd | 1.0924744 |
| Ptprd | -0.4919267 |
| Ptpre | -0.1872216 |
| Ptpre | -0.147161 |
| Ptprf | 0.7425163 |
| Ptprf | -0.8677726 |
| Ptprg | 0.4524123 |
| Ptprj | -0.7657271 |
| Ptprk | 1.0985698 |
| Ptprk | -0.0919811 |
| Ptprn | 1.4989065 |
| Ptprn2 | -0.267777 |
| Ptpro | 1.0411687 |
| Ptpro | -0.8543659 |
| Ptprq | 1.2270336 |
| Ptprr | 1.7610091 |
| Ptprr | 1.5392419 |
| Ptprs | 0.3502877 |
| Ptprs | -0.6740849 |
| Ptprt | -0.1940753 |
| Ptpru | 0.8460047 |
| Ptprv | -0.6363491 |
| Ptrf | -0.6078826 |
| Ptrh1 | 3.1872146 |
| Ptrh1 | -0.6607792 |
| Ptrh2 | 1.5737222 |
| Pts | -0.6419904 |
| Pts | -0.4722992 |
| Pttg1 | 0.6427843 |
| Pttg1ip | 1.8296064 |
| Ptx3 | 1.0435315 |
| Ptx3 | -0.3387764 |
| Puf60 | 1.6952141 |
| Puf60 | -0.8636262 |
| Pum1 | -0.1727087 |
| Pum2 | -0.4285261 |
| Pum2 | 0.0968761 |
| Punc | 2.1343043 |
| Purb | -0.7410238 |
| Purg | 1.3501784 |
| Pus1 | -0.6591297 |
| Pus1 | -0.83713 |
| Pus10 | 0.0792785 |
| Pus10 | 0.7272906 |
| Pus10 | 0.6985983 |
| Pus3 | -0.8133458 |
| Pus7l | -0.1722289 |
| Pvalb | -0.5535167 |
| Pvr | 0.7399095 |
| Pvr | -0.8431756 |
| Pvrl1 | 1.9235004 |
| Pvrl2 | 0.1011337 |
| Pvrl2 | 2.665424 |
| Pvrl2 | 1.0474969 |
| Pvrl3 | -0.5947468 |
| Pvrl3 | 1.1451257 |
| Pvrl3 | -0.3698976 |
| Pwp1 | 2.517194 |
| Pwp2 | 0.0568791 |
| Pwwp2b | -0.6492959 |
| Pxdn | 1.5277144 |
| Pxmp2 | 1.0665821 |
| Pxmp4 | -0.2582273 |
| Pxn | -0.6978541 |
| Pxt1 | 0.3235972 |
| Pycr2 | 1.5153759 |
| Pycrl | 2.4466545 |
| Pygb | -0.477671 |
| Pygl | -0.2871859 |
| Pygl | 0.063547 |
| Pygm | -0.3568485 |
| Pygo1 | -0.5593977 |
| Pygo2 | 0.5894704 |
| Pyroxd1 | -0.7284769 |
| Pyy | -0.6995762 |
| Qars | 0.157721 |
| Qdpr | 0.4509738 |
| Qpct | 0.6861215 |
| Qprt | -0.1911316 |
| Qrich2 | 1.5489964 |
| Qrich2 | 1.6593555 |
| Qrsl1 | 1.3366516 |
| Qsox1 | -0.3221927 |
| Qtrt1 | 0.3720278 |
| R3hdm1 | 3.2956761 |
| R3hdm1 | -0.4656292 |
| R3hdm2 | 0.0346704 |
| R74862 | -0.1227727 |
| Rab1 | 0.1916422 |
| Rab10 | 1.3403204 |
| Rab10 | -0.8411559 |
| Rab11a | 0.0947397 |
| Rab11b | 0.6348 |
| Rab11fip2 | 1.196334 |
| Rab11fip3 | -0.623586 |
| Rab11fip5 | 0.977452 |
| Rab11fip5 | 1.1540667 |
| Rab11fip5 | 1.0043132 |
| Rab12 | 0.6422025 |
| Rab13 | 0.7745635 |
| Rab13 | -0.0090587 |
| Rab14 | -0.6149153 |
| Rab15 | 0.2872117 |
| Rab17 | 3.0376711 |
| Rab17 | -0.7329379 |
| Rab18 | 0.691831 |
| Rab19 | -0.5623526 |
| Rab19 | 1.3074915 |
| Rab1b | 1.0094873 |
| Rab1b | -0.64523 |
| Rab1b | 0.2770806 |
| Rab21 | 0.7110842 |
| Rab22a | 1.6554633 |
| Rab22a | -0.3873931 |
| Rab23 | 1.2608267 |
| Rab24 | -0.6331414 |
| Rab24 | -0.1612645 |
| Rab24 | -0.1065761 |
| Rab25 | 1.585699 |
| Rab27a | 2.3162441 |
| Rab27b | 1.7015124 |
| Rab28 | -0.4960416 |
| Rab28 | 1.0215308 |
| Rab2a | 2.1352645 |
| Rab31 | 2.4089514 |
| Rab32 | -0.3131722 |
| Rab33a | -0.5955143 |
| Rab33b | -0.4272516 |
| Rab34 | -0.0876048 |
| Rab34 | 2.8824191 |
| Rab34 | 0.0868625 |
| Rab35 | 0.224948 |
| Rab36 | -0.6055506 |
| Rab38 | -0.1200529 |
| Rab38 | -0.5289428 |
| Rab39 | -0.630743 |
| Rab39b | -0.6172717 |
| Rab3a | -0.6016772 |
| Rab3b | -0.1476276 |
| Rab3d | -0.6016772 |
| Rab3gap1 | -0.6243784 |
| Rab3gap2 | -0.5078036 |
| Rab3il1 | -0.5418689 |
| Rab3il1 | -0.203461 |
| Rab3ip | -0.5909196 |
| Rab40b | -0.6509274 |
| Rab40c | -0.0698808 |
| Rab43 | -0.77975 |
| Rab4a | 0.4732503 |
| Rab4b | 1.0121919 |
| Rab5a | 2.0665624 |
| Rab5a | -0.5955143 |
| Rab5b | -0.67074 |
| Rab5c | 0.0196833 |
| Rab6 | -0.4164908 |
| Rab6 | 0.0646535 |
| Rab6 | -0.398426 |
| Rab6b | -0.6665762 |
| Rab6ip1 | 0.8723745 |
| Rab7 | 0.5264214 |
| Rab7 | 2.1172951 |
| Rab7 | -0.7284769 |
| Rab7l1 | -0.5260954 |
| Rab8a | -0.1833286 |
| Rab8a | -0.6501113 |
| Rab8b | -0.1828431 |
| Rab8b | -0.1056848 |
| Rab9 | -0.5579238 |
| Rab9 | 0.4192278 |
| Rab9b | 1.7198542 |
| Rabac1 | 0.6158232 |
| Rabac1 | 2.8594921 |
| Rabep1 | -0.5593977 |
| Rabep1 | -0.36098 |
| Rabep2 | -0.4209062 |
| Rabgap1 | 0.7242851 |
| Rabgap1 | -0.1155382 |
| Rabgap1l | 0.0369541 |
| Rabgap1l | -0.0946172 |
| Rabgef1 | -0.1164393 |
| Rabggta | 0.6953125 |
| Rabggtb | -0.5498606 |
| Rabif | 0.5734895 |
| Rabif | -0.5253849 |
| Rabl2a | 2.3709636 |
| Rabl2a | -0.6987148 |
| Rabl3 | -0.2296603 |
| Rabl4 | -0.5542498 |
| Rabl4 | 0.3715037 |
| Rabl5 | 0.6140234 |
| Rac1 | 0.9193456 |
| Rac2 | -0.6220033 |
| Rac3 | 1.0699652 |
| Rac3 | -0.0776191 |
| Rad1 | -0.67074 |
| Rad17 | 1.2249042 |
| Rad18 | -0.3704953 |
| Rad23a | -0.468958 |
| Rad23a | -0.169834 |
| Rad23a | -0.4133502 |
| Rad23b | 0.2090773 |
| Rad50 | 2.0979213 |
| Rad51 | -0.4272516 |
| Rad51 | -0.6476673 |
| Rad51ap1 | 0.5732801 |
| Rad51c | 0.7053152 |
| Rad51l3 | 0.2727035 |
| Rad51l3 | 1.9629753 |
| Rad51l3 | -0.5909196 |
| Rad51l3 | 2.5586689 |
| Rad52 | -0.4374984 |
| Rad54l | -0.0174999 |
| Rad9 | -0.217978 |
| Rad9 | -0.5425925 |
| Rad9 | -0.399043 |
| Rad9b | -0.4803691 |
| Rae1 | -0.5036347 |
| Raet1b | -0.7108508 |
| Raet1b | 0.4480899 |
| Raf1 | -0.2394267 |
| Rag1 | 0.4109877 |
| Rag1ap1 | -0.8026514 |
| Rage | -0.7329379 |
| Rage | -0.6841949 |
| Rai1 | -0.1994972 |
| Rai12 | -0.1097027 |
| Rai12 | 0.7759022 |
| Rai12 | -0.0042705 |
| Rai12 | 2.835864 |
| Rai14 | -0.3103718 |
| Rala | 2.1145798 |
| Ralb | 0.2122114 |
| Ralbp1 | -0.6476673 |
| Ralgds | 1.5564015 |
| Ralgps1 | 1.9066701 |
| Ralgps1 | 0.519333 |
| Ralgps1 | -0.3064657 |
| Ralgps2 | 0.350556 |
| Ralgps2 | 0.0008882 |
| Raly | 1.2685002 |
| Raly | -0.6243784 |
| Ralyl | 2.047111 |
| Ramp1 | -0.3227595 |
| Ramp2 | -0.6086613 |
| Ramp3 | 1.8754897 |
| Ramp3 | -0.0002997 |
| Ran | -0.6918527 |
| Ranbp1 | -0.0562861 |
| Ranbp10 | 0.42171 |
| Ranbp17 | -0.4623126 |
| Ranbp2 | -0.6884412 |
| Ranbp3l | 0.6168215 |
| Ranbp6 | 1.8203831 |
| Ranbp9 | 2.1877768 |
| Rangap1 | -0.6427992 |
| Rangrf | 1.2948065 |
| Rap1b | -0.5454927 |
| Rap1gap | 3.2046565 |
| Rap2a | -0.7968711 |
| Rap2b | -0.7117239 |
| Rap2c | 0.1439887 |
| Rap2c | -0.7778676 |
| Rap2c | 0.1176183 |
| Rap2ip | 1.6691369 |
| Rapgef1 | 0.3233208 |
| Rapgef1 | -0.4709612 |
| Rapgef1 | -0.1707912 |
| Rapgef1 | -0.5112925 |
| Rapgef3 | -0.5720238 |
| Rapgef4 | 0.9754449 |
| Rapgef5 | 2.4767755 |
| Rapgef6 | -0.6935633 |
| Rapgefl1 | 0.8117967 |
| Rapgefl1 | -0.3313045 |
| Rapsn | -0.7143485 |
| Rapsn | -0.0520796 |
| Rara | 0.8521708 |
| Rarb | -0.772244 |
| Rarb | 0.1793957 |
| Rarg | -0.6172717 |
| Rarg | -0.0889154 |
| Rarg | -0.5289428 |
| Rarres2 | 2.212029 |
| Rars | -0.7108508 |
| Rars | -0.6799686 |
| Rars2 | -0.6243784 |
| Rars2 | 1.6058003 |
| Rars2 | -0.3131722 |
| Rasa1 | -0.5064118 |
| Rasa2 | -0.623586 |
| Rasal1 | -0.3451476 |
| Rasd1 | 1.6748196 |
| Rasef | -0.4878306 |
| Rasgef1b | -0.2970496 |
| Rasgef1b | -0.5564524 |
| Rasgef1c | -0.2768901 |
| Rasgef1c | -0.573521 |
| Rasgrf1 | -0.7013016 |
| Rasgrf1 | 0.6594955 |
| Rasgrf1 | -0.7213849 |
| Rasgrf1 | 0.0297024 |
| Rasgrp1 | -0.4662939 |
| Rasgrp1 | -0.7021655 |
| Rasgrp1 | -0.3734896 |
| Rasgrp3 | 0.325806 |
| Rasip1 | 0.4885681 |
| Rasl10a | -0.2144524 |
| Rasl10b | 1.1156959 |
| Rasl10b | -0.6740849 |
| Rasl11a | 0.4415671 |
| Rasl11b | 0.7485696 |
| Rasl11b | 1.6273468 |
| Rasl12 | 1.1554935 |
| Rasl2-9 | -0.5382596 |
| Rassf1 | 2.0083031 |
| Rassf1 | -0.5955143 |
| Rassf10 | -0.3387764 |
| Rassf2 | -0.4912427 |
| Rassf2 | -0.5878695 |
| Rassf3 | 2.3261806 |
| Rassf4 | -0.0034749 |
| Rassf4 | 0.4072172 |
| Rassf4 | -0.2503459 |
| Rassf4 | -0.5520525 |
| Rassf5 | 0.4876472 |
| Rassf7 | -0.5825565 |
| Rassf7 | 1.398246 |
| Raver1 | -0.0280618 |
| Raver2 | 0.2423608 |
| Rb1 | 0.6186157 |
| Rb1 | 0.8399518 |
| Rbak | -0.2059473 |
| Rbak | -0.4974174 |
| Rbbp5 | -0.2736632 |
| Rbbp5 | -0.2368463 |
| Rbbp6 | -0.6243784 |
| Rbbp7 | -0.365725 |
| Rbbp7 | 1.391438 |
| Rbbp9 | -0.663259 |
| Rbck1 | -0.2709829 |
| Rbed1 | -0.7311508 |
| Rbed1 | -0.4221717 |
| Rbj | -0.3053528 |
| Rbks | -0.2271078 |
| Rbl1 | 0.5301645 |
| Rbl2 | 0.4765214 |
| Rbm10 | 1.7826237 |
| Rbm10 | -0.5863483 |
| Rbm10 | 2.2205968 |
| Rbm11 | -0.7347285 |
| Rbm12 | 3.1815209 |
| Rbm12 | 2.14258 |
| Rbm12 | 1.9161847 |
| Rbm12 | 0.0579939 |
| Rbm13 | -0.5310843 |
| Rbm13 | 1.2966832 |
| Rbm14 | -0.4400784 |
| Rbm14 | 0.4300985 |
| Rbm15 | 1.1926586 |
| Rbm16 | 1.0021051 |
| Rbm18 | -0.6901453 |
| Rbm18 | -0.1975218 |
| Rbm19 | 0.1682951 |
| Rbm24 | 1.2554455 |
| Rbm26 | -0.5411459 |
| Rbm28 | 1.8218485 |
| Rbm28 | 0.7552672 |
| Rbm3 | -0.3807168 |
| Rbm35a | -0.5268064 |
| Rbm38 | 0.0628085 |
| Rbm38 | -0.2291492 |
| Rbm38 | -0.3480586 |
| Rbm4 | 0.968857 |
| Rbm41 | 0.5043082 |
| Rbm42 | 0.2304833 |
| Rbm42 | 0.1279845 |
| Rbm43 | -0.5712762 |
| Rbm45 | -0.5855887 |
| Rbm47 | 0.4164895 |
| Rbm47 | -0.5339476 |
| Rbm47 | 0.8537058 |
| Rbm47 | 2.5736683 |
| Rbm4b | -0.7759892 |
| Rbm5 | -0.1707912 |
| Rbm5 | 2.1521941 |
| Rbm6 | 0.1057236 |
| Rbm6 | 2.3748749 |
| Rbm6 | 2.4521217 |
| Rbm8a | -0.4240733 |
| Rbm9 | -0.3675109 |
| Rbm9 | -0.4374984 |
| Rbms1 | 0.5492957 |
| Rbms1 | -0.6533803 |
| Rbms2 | 1.9266242 |
| Rbms2 | -0.6141311 |
| Rbms3 | 0.9612684 |
| Rbmx | 1.2312774 |
| Rbmy1a1 | 0.9875531 |
| Rbp1 | -0.2866411 |
| Rbp7 | 2.7414293 |
| Rbpms | 2.553327 |
| Rbpms | 1.089175 |
| Rbpms | -0.6509274 |
| Rbpms2 | 2.1237861 |
| Rbx1 | -0.0567079 |
| Rbx1 | -0.5557176 |
| Rc3h1 | 1.3795483 |
| Rcan1 | 0.0471597 |
| Rcan2 | -0.5397016 |
| Rcan2 | 1.545804 |
| Rcan2 | -0.6315417 |
| Rcan3 | -0.6978541 |
| Rcbtb2 | -0.7844733 |
| Rcbtb2 | -0.7213849 |
| Rcc1 | 0.0854228 |
| Rccd1 | 0.9753109 |
| Rce1 | -0.0626331 |
| Rchy1 | 0.1279845 |
| Rcl1 | 1.8703019 |
| Rcl1 | -0.6141311 |
| Rcl1 | 0.7825661 |
| Rcn3 | -0.1794523 |
| Rcor1 | 0.2712397 |
| Rcor2 | 0.3113513 |
| Rcsd1 | 2.133676 |
| Rdbp | -0.2704477 |
| Rdbp | -0.0639079 |
| Rdh11 | -0.5795346 |
| Rdh12 | 1.4200023 |
| Rdh14 | -0.6009045 |
| Rdh14 | -0.3210602 |
| Rdh5 | 0.3852888 |
| Rdh7 | 0.2638849 |
| Rdh9 | 2.0706962 |
| Rdm1 | -0.6172717 |
| Rec8 | 1.7347817 |
| Reck | 0.4720792 |
| Recql | 1.2408033 |
| Recql4 | -0.7161024 |
| Recql5 | 1.1493313 |
| Reep1 | 2.1245335 |
| Reep2 | -0.3783013 |
| Reep3 | 3.1650551 |
| Reep4 | -0.4177502 |
| Reep5 | -0.078483 |
| Reep6 | 1.4801446 |
| Reep6 | 1.84691 |
| Refbp2 | -0.4994851 |
| Reg1 | -0.4736392 |
| Rela | -0.7125979 |
| Relb | 0.5417354 |
| Rell1 | 1.2933964 |
| Rell2 | 2.3864522 |
| Rell2 | 1.4001697 |
| Reln | 2.4001126 |
| Relt | 0.266245 |
| Relt | 0.8966784 |
| Rem1 | -0.1173414 |
| Rem2 | 2.4165709 |
| Renbp | 1.2441882 |
| Repin1 | -0.7356251 |
| Repin1 | 0.0639159 |
| Reps1 | -0.2144524 |
| Reps1 | -0.01508 |
| Rer1 | 0.5954163 |
| Rer1 | 0.3356787 |
| Rerg | 0.2709467 |
| Resp18 | 1.4361216 |
| Rest | -0.3597976 |
| Ret | 1.8968132 |
| Retnla | -0.9436115 |
| Retnlg | -0.6833481 |
| Retsat | -0.365725 |
| Retsat | -0.5375395 |
| Rev1 | 1.5871934 |
| Rev3l | 0.9523792 |
| Rex3 | 2.522034 |
| Rexo1 | -0.1457628 |
| Rexo1 | -0.5382596 |
| Rexo2 | 3.2603631 |
| Rfc1 | 2.5534197 |
| Rfc2 | 2.0240193 |
| Rfc3 | -0.6251716 |
| Rfc4 | -0.2059473 |
| Rfc4 | -0.6509274 |
| Rfc5 | 0.6412319 |
| Rfesd | 1.1065948 |
| Rffl | 1.3940496 |
| Rffl | -0.6841949 |
| Rffl | 3.0252102 |
| Rffl | -0.7769279 |
| Rffl | -0.4926112 |
| Rfng | 3.4739761 |
| Rfpl4 | 2.3433902 |
| Rfpl4 | -0.4472119 |
| Rft1 | 0.0950961 |
| Rftn1 | 0.5237698 |
| Rftn2 | 1.0345448 |
| Rftn2 | 2.3707079 |
| Rfwd2 | -0.6180586 |
| Rfwd3 | 0.5462792 |
| Rfwd3 | 1.272168 |
| Rfx1 | 1.5113237 |
| Rfx1 | 1.1653094 |
| Rfx2 | 1.2649144 |
| Rfx3 | -0.8431756 |
| Rfx4 | -0.5239657 |
| Rfx4 | 0.4595705 |
| Rfx4 | -0.1896634 |
| Rfxank | 2.5466689 |
| Rfxap | 2.8283265 |
| Rfxdc1 | 1.0885845 |
| Rg9mtd1 | -0.7648 |
| Rg9mtd2 | 0.8441466 |
| Rgag4 | -0.2430518 |
| Rgl1 | 1.6352955 |
| Rgl2 | -0.3204944 |
| Rgl2 | -0.6558398 |
| Rgl3 | 1.4063315 |
| Rgma | 0.7985239 |
| Rgmb | 2.8291121 |
| Rgmb | 2.5675478 |
| Rgnef | 2.091279 |
| Rgs1 | -0.7082364 |
| Rgs1 | -0.3996603 |
| Rgs1 | -0.7769279 |
| Rgs10 | -0.5520525 |
| Rgs12 | 2.8727623 |
| Rgs14 | 2.8381822 |
| Rgs16 | 0.4342636 |
| Rgs17 | 1.5291635 |
| Rgs2 | 1.5342888 |
| Rgs3 | 0.4236908 |
| Rgs3 | 0.9660187 |
| Rgs3 | -0.2855524 |
| Rgs3 | 1.2295621 |
| Rgs3 | -0.7401218 |
| Rgs3 | -0.8104165 |
| Rgs3 | -0.5765228 |
| Rgs4 | 3.7589886 |
| Rgs5 | -0.0981441 |
| Rgs6 | -0.4919267 |
| Rgs7 | 0.9038144 |
| Rgs7bp | 1.5329922 |
| Rgs8 | 0.0882998 |
| Rgs9 | 2.1902803 |
| Rgs9bp | -0.8231796 |
| Rhag | 3.1778324 |
| Rhbdd1 | 1.2333918 |
| Rhbdd3 | -0.6484812 |
| Rhbdf1 | 2.6837274 |
| Rhbdf2 | -0.2210109 |
| Rhbdl1 | -0.425979 |
| Rhbdl2 | 2.4576074 |
| Rhbdl3 | 0.1607075 |
| Rhbdl7 | 0.5824488 |
| Rhcg | 1.7649727 |
| Rheb | 0.6217966 |
| Rhebl1 | -0.7302586 |
| Rhob | -0.2693785 |
| Rhobtb2 | 1.2214458 |
| Rhobtb2 | 1.2009332 |
| Rhobtb2 | -0.8143244 |
| Rhobtb2 | 1.5143462 |
| Rhobtb3 | 0.8138831 |
| Rhoc | -0.77975 |
| Rhoc | 0.4189792 |
| Rhod | 3.590052 |
| Rhof | 0.478618 |
| Rhog | -0.1429727 |
| Rhoj | -0.7939949 |
| Rhot1 | 1.142793 |
| Rhot2 | 1.8231548 |
| Rhot2 | 0.2122114 |
| Rhot2 | 0.1228163 |
| Rhou | -0.5901561 |
| Rhov | -0.1727087 |
| Rhox11 | -0.8482449 |
| Rhox12 | 0.8053466 |
| Rhox3a | -0.6460417 |
| Rhox3f | 1.8961876 |
| Rhox4d | -0.3764939 |
| Rhox5 | -0.8441872 |
| Rhox8 | -0.7675843 |
| Rhpn1 | 0.3293836 |
| Rhpn1 | -0.0554432 |
| Rhpn1 | 0.3612234 |
| Ribc1 | 0.1095891 |
| Ric3 | 1.5713767 |
| Ric3 | -0.3238941 |
| Ric3 | 1.9641368 |
| Ric8b | -0.8045864 |
| Ric8b | -0.185273 |
| Rif1 | 0.4320609 |
| Rilp | -0.7302586 |
| Rilpl1 | 0.0940264 |
| Rilpl1 | -0.5818001 |
| Rilpl2 | 0.4864946 |
| Rims1 | 0.192602 |
| Rims2 | -0.5513212 |
| Rims3 | 2.2648778 |
| Rin1 | -0.4077246 |
| Rin2 | -0.5535167 |
| Rin2 | -0.7741146 |
| Rin2 | 0.9614045 |
| Rin3 | -0.6927076 |
| Riok1 | 0.2552773 |
| Riok1 | 1.5338568 |
| Riok3 | -0.4426659 |
| Riok3 | 0.3191634 |
| Ripk1 | -0.0301072 |
| Ripk2 | 0.0936696 |
| Ripk4 | 2.2709297 |
| Ripk5 | 1.3546719 |
| Rit1 | -0.6204233 |
| Rit2 | -0.448515 |
| Rlbp1 | -0.3764939 |
| Rlbp1l1 | 1.5915268 |
| Rlbp1l1 | -0.629945 |
| Rln1 | 1.2678229 |
| Rltpr | 1.0212762 |
| Rmnd1 | 1.5155964 |
| Rmnd5b | -0.6063272 |
| Rmnd5b | 0.3051653 |
| Rnase1 | 1.4394871 |
| Rnase4 | -0.6331414 |
| Rnase4 | 1.6372236 |
| Rnase6 | -0.6157001 |
| Rnaseh1 | 0.2393302 |
| Rnaseh1 | -0.5325148 |
| Rnaseh2c | 0.3524319 |
| Rnasek | -0.4783448 |
| Rnasen | 1.1178711 |
| Rnaset2 | -0.0070605 |
| Rnaset2b | -0.5008661 |
| Rnd2 | 0.291519 |
| Rnd3 | 1.5885499 |
| Rnf10 | -0.0346236 |
| Rnf103 | -0.7428306 |
| Rnf11 | -0.7275873 |
| Rnf11 | -0.4146052 |
| Rnf11 | -0.5126918 |
| Rnf111 | -0.6419904 |
| Rnf113a1 | 1.759329 |
| Rnf113a2 | 3.529008 |
| Rnf12 | -0.5878695 |
| Rnf121 | -0.6927076 |
| Rnf123 | -0.7537483 |
| Rnf125 | 0.0304685 |
| Rnf125 | -0.4407246 |
| Rnf128 | -0.4071017 |
| Rnf133 | 2.8869929 |
| Rnf135 | 2.4553344 |
| Rnf135 | 0.7164593 |
| Rnf135 | -0.621213 |
| Rnf138 | 1.9120314 |
| Rnf138 | 0.0060174 |
| Rnf138 | -0.5389803 |
| Rnf138 | 1.3564279 |
| Rnf139 | 0.1200478 |
| Rnf139 | -0.4304412 |
| Rnf14 | -0.5105936 |
| Rnf14 | -0.7491831 |
| Rnf14 | -0.0915425 |
| Rnf141 | 3.9664932 |
| Rnf141 | 1.9895775 |
| Rnf144a | -0.2446098 |
| Rnf145 | -0.6484812 |
| Rnf150 | -0.6509274 |
| Rnf166 | 0.0449018 |
| Rnf167 | -0.6188462 |
| Rnf167 | -0.4108456 |
| Rnf167 | -0.1191482 |
| Rnf167 | -0.661605 |
| Rnf167 | -0.026021 |
| Rnf168 | -0.1970286 |
| Rnf17 | 0.6760887 |
| Rnf170 | -0.8261509 |
| Rnf181 | 2.7010559 |
| Rnf182 | 0.5428193 |
| Rnf182 | 1.6026035 |
| Rnf183 | -0.4407246 |
| Rnf185 | 0.2975147 |
| Rnf185 | -0.2306834 |
| Rnf185 | 1.9486015 |
| Rnf185 | 0.1852174 |
| Rnf186 | 2.5084598 |
| Rnf19a | -0.8351238 |
| Rnf2 | 0.9864951 |
| Rnf20 | 1.1916057 |
| Rnf207 | 1.5481462 |
| Rnf208 | 3.2563691 |
| Rnf214 | 0.5574328 |
| Rnf215 | -0.3904443 |
| Rnf216 | 2.2024201 |
| Rnf216 | -0.6509274 |
| Rnf219 | 0.0639159 |
| Rnf24 | -0.7038959 |
| Rnf24 | 0.6625353 |
| Rnf25 | -0.4905592 |
| Rnf25 | -0.7537483 |
| Rnf25 | -0.1688778 |
| Rnf26 | -0.7428306 |
| Rnf31 | 1.6591676 |
| Rnf32 | 1.2320835 |
| Rnf34 | -0.1196004 |
| Rnf34 | -0.165539 |
| Rnf34 | 3.7290891 |
| Rnf38 | 1.5675695 |
| Rnf38 | 2.2844463 |
| Rnf38 | 3.5332408 |
| Rnf4 | -0.7750514 |
| Rnf4 | -0.2850085 |
| Rnf40 | -0.7788083 |
| Rnf41 | -0.3935058 |
| Rnf41 | -0.4008963 |
| Rnf41 | -0.5282301 |
| Rnf44 | -0.7778676 |
| Rnf5 | -0.659954 |
| Rnf6 | -0.2893685 |
| Rnf6 | -0.663259 |
| Rnf8 | -0.2953981 |
| Rnf8 | -0.7161024 |
| Rngtt | -0.0758937 |
| Rnmt | 1.6504161 |
| Rnmtl1 | 0.5286251 |
| Rnpc2 | 0.1067795 |
| Rnpep | -0.6607792 |
| Rnpepl1 | 0.3188856 |
| Rnps1 | -0.5126918 |
| Rnps1 | 0.3816679 |
| Rnu6 | -0.5780274 |
| Rnuxa | -0.0466402 |
| Rnuxa | -0.6674074 |
| Robo1 | -0.7099784 |
| Robo2 | -0.5425925 |
| Robo4 | 0.0445249 |
| Rock1 | -0.3434055 |
| Rock2 | 0.9879495 |
| Rock2 | 0.020845 |
| Rod1 | 0.5423859 |
| Rogdi | -0.2159616 |
| Rom1 | 0.6363635 |
| Ropn1 | 0.5290652 |
| Ror1 | -0.5036347 |
| Ror2 | 1.83266 |
| Rora | 1.3905939 |
| Rorb | -0.7293673 |
| Rorb | -0.6133477 |
| Rorc | -0.5939801 |
| Rorc | 2.8289756 |
| Ros1 | -0.1341943 |
| Rp23-297j14.5 | 2.1456137 |
| Rp23-438h3.2 | 0.7520105 |
| rp9 | -0.0022829 |
| rp9 | 1.072013 |
| Rpa1 | -0.3633494 |
| Rpa2 | -0.5447667 |
| Rpa2 | -0.6315417 |
| Rpa3 | 0.8785123 |
| Rpain | 0.9751769 |
| Rpap1 | -0.6682394 |
| Rpap1 | 1.178019 |
| Rpap2 | -0.6987148 |
| Rpap2 | 0.3315782 |
| Rpap3 | 1.0993872 |
| Rpe | -0.3439858 |
| Rpgr | -0.4400784 |
| Rpgrip1 | 1.4614515 |
| Rpgrip1l | 3.4096627 |
| Rph3a | -0.1626871 |
| Rph3al | 1.1806888 |
| Rpia | 0.1193543 |
| Rpl12 | -0.2440902 |
| Rpl13a | -0.547674 |
| Rpl18 | 0.2159579 |
| Rpl19 | 1.0389245 |
| Rpl21 | -0.7939949 |
| Rpl21 | 1.1915004 |
| Rpl22 | 0.5778761 |
| Rpl22l1 | -0.5375395 |
| Rpl23 | 0.9765159 |
| Rpl23 | 0.5031768 |
| Rpl23 | 0.283751 |
| Rpl24 | 0.8092222 |
| Rpl27 | -0.6323412 |
| Rpl27a | -0.5787807 |
| Rpl28 | -0.5505906 |
| Rpl29 | -0.53682 |
| Rpl29 | -0.0746019 |
| Rpl3 | -0.7073666 |
| Rpl30 | 0.8022666 |
| Rpl31 | -0.6724109 |
| Rpl31 | -0.4696252 |
| Rpl34 | 2.6029152 |
| Rpl35 | -0.3142948 |
| Rpl36a | -0.1218652 |
| Rpl36al | -0.1990029 |
| Rpl37 | -0.6649161 |
| Rpl38 | 0.0304685 |
| Rpl38 | 0.0968761 |
| Rpl39 | 0.1823112 |
| Rpl39l | 1.9105638 |
| Rpl3l | 1.7031248 |
| Rpl4 | -0.5848297 |
| Rpl41 | 0.6921981 |
| Rpl7a | -0.5071074 |
| Rpl7l1 | 0.6663206 |
| Rpl7l1 | 1.1038116 |
| Rpl9 | -0.5985904 |
| Rplp1 | -0.7939949 |
| Rpn2 | 0.6508905 |
| Rpo1-1 | 0.1456746 |
| Rpo1-3 | 0.4807097 |
| Rpo1-3 | 1.9330245 |
| Rpo1-4 | 2.7487947 |
| Rpo1-4 | 0.4077208 |
| Rpo1-4 | -0.64523 |
| Rpo2tc1 | 2.072479 |
| Rpp21 | 0.1011337 |
| Rpp25 | -0.1906419 |
| Rpp30 | -0.283379 |
| Rpp38 | 1.9636456 |
| Rpp40 | -0.0677431 |
| Rprm | 2.973406 |
| Rprml | -0.7392207 |
| Rps10 | -0.9504195 |
| Rps11 | 1.2373055 |
| Rps12 | 0.2199991 |
| Rps13 | -0.0634827 |
| Rps14 | 1.6135455 |
| Rps15 | -0.6243784 |
| Rps15a | -0.8016854 |
| Rps16 | -0.7759892 |
| Rps19 | -0.4196424 |
| Rps19 | -0.3498097 |
| Rps19bp1 | -0.6649161 |
| Rps2 | 1.3902561 |
| Rps21 | 0.2753323 |
| Rps24 | 3.7246391 |
| Rps24 | 0.4509738 |
| Rps25 | 0.4837225 |
| Rps26 | -0.3831388 |
| Rps27a | 0.5159909 |
| Rps27a | 0.7735582 |
| Rps27l | 0.1617009 |
| Rps3 | 0.6152237 |
| Rps3 | 2.0647279 |
| Rps3a | -0.6395684 |
| Rps4x | -0.4844313 |
| Rps4y2 | -0.0483104 |
| Rps5 | -0.115088 |
| Rps6 | 0.9209126 |
| Rps6ka1 | 1.597988 |
| Rps6ka3 | 3.4075021 |
| Rps6ka4 | -0.6149153 |
| Rps6ka5 | -0.2281279 |
| Rps6kb1 | 2.162638 |
| Rps6kl1 | 0.0620695 |
| Rps7 | 1.3590555 |
| Rps8 | 0.8489389 |
| Rps9 | 0.1088875 |
| Rps9 | -0.2812108 |
| Rpsa | -0.8719383 |
| Rpusd1 | 0.9048292 |
| Rpusd4 | 0.5818267 |
| Rqcd1 | -0.7082364 |
| Rrad | 2.2128082 |
| Rragb | 3.0855877 |
| Rragc | -0.7231527 |
| Rragd | -0.640375 |
| Rras | -0.5353826 |
| Rras2 | -0.5057167 |
| Rrbp1 | -0.1476276 |
| Rrbp1 | -0.0656104 |
| Rreb1 | -0.3776984 |
| Rreb1 | -0.5091975 |
| Rrh | 0.7619152 |
| Rrm1 | -0.3645364 |
| Rrm2 | 1.846045 |
| Rrm2 | -0.6867403 |
| Rrm2b | 0.7301101 |
| Rrn3 | 1.5419582 |
| Rrp1 | -0.6323412 |
| Rrp12 | 0.1422996 |
| Rrp15 | 1.2313782 |
| Rrp1b | -0.2774291 |
| Rrp1b | 0.1861841 |
| Rrp1b | 1.1208391 |
| Rrp9 | -0.4623126 |
| Rs1 | -0.6419904 |
| Rsad1 | -0.630743 |
| Rsad2 | 1.2105778 |
| Rsf1 | 2.3868151 |
| Rshl1 | -0.5197214 |
| Rshl2a | 0.035813 |
| Rsl1d1 | 0.0774633 |
| Rsl1d1 | 0.0297024 |
| Rsn | 0.1389118 |
| Rsph1 | 0.0664946 |
| Rspo1 | -0.2477341 |
| Rspo2 | -0.685891 |
| Rspo3 | 1.7504285 |
| Rspo4 | -0.3621639 |
| Rspry1 | -0.5375395 |
| Rsrc1 | 1.6143361 |
| Rsrc1 | -0.3704953 |
| Rsrc2 | 3.4943607 |
| Rsu1 | -0.6339422 |
| Rtbdn | -0.2774291 |
| Rtel1 | -0.3399314 |
| Rtel1 | -0.5303699 |
| Rtkn | 0.7121618 |
| Rtn1 | 0.5090445 |
| Rtn1 | -0.4407246 |
| Rtn1 | 0.0620695 |
| Rtn1 | 2.0599245 |
| Rtn2 | 0.9216239 |
| Rtn3 | 0.1266092 |
| Rtn4 | -0.9402268 |
| Rtn4 | -0.8341224 |
| Rtn4 | -0.2109405 |
| Rtn4ip1 | 0.1748414 |
| Rtn4r | 0.4920134 |
| Rtn4rl1 | -0.5325148 |
| Rtn4rl2 | 3.0084859 |
| Rtp1 | -0.4478632 |
| Rtp4 | -0.7601786 |
| Rttn | -0.5947468 |
| Rufy1 | 0.6885202 |
| Rufy2 | 1.4632449 |
| Rufy3 | 2.0053228 |
| Rufy3 | 0.9140549 |
| Rufy3 | 0.5716029 |
| Rufy4 | -0.5705292 |
| Rundc1 | -0.6086613 |
| Rundc3b | -0.4550589 |
| Runx1 | -0.4987953 |
| Runx1t1 | 0.4654925 |
| Runx1t1 | -0.2322202 |
| Runx2 | 2.5113078 |
| Rusc1 | 0.8209089 |
| Rusc2 | -0.7601786 |
| Rusc2 | -0.5425925 |
| Ruvbl1 | 0.4305895 |
| Ruvbl1 | -0.6188462 |
| Ruvbl1 | 1.6388272 |
| Ruvbl2 | 1.380659 |
| Rwdd1 | -0.597051 |
| Rwdd1 | 2.1919107 |
| Rwdd3 | 0.1823112 |
| Rwdd3 | 1.5599064 |
| Rwdd4a | -0.6133477 |
| Rxfp1 | 1.3211869 |
| Rxfp3 | 0.2347651 |
| Rxra | -0.0724529 |
| Rxrb | -0.1214118 |
| Rxrb | 0.0024695 |
| Rxrg | -0.6749231 |
| Rya3 | 0.6914637 |
| Ryk | -0.3109312 |
| Ryk | -0.536101 |
| Ryr1 | -0.176556 |
| Ryr1 | 0.4291156 |
| Ryr1 | 1.0026252 |
| Ryr3 | -0.6927076 |
| S100a1 | 0.1344886 |
| S100a10 | -0.659954 |
| S100a11 | -0.01508 |
| S100a13 | 0.4069652 |
| S100a16 | -0.5705292 |
| S100a4 | -0.6724109 |
| S100a6 | 0.5426026 |
| S100a8 | -0.4368546 |
| S100a9 | 0.2489924 |
| S100b | 1.7694735 |
| S100pbp | 0.5217759 |
| S100pbp | -0.3445665 |
| S3-12 | 1.9448237 |
| Saa3 | -0.4433139 |
| Sac3d1 | -0.0994702 |
| Sacm1l | -0.2099396 |
| Sacs | -0.5623526 |
| Sae1 | -0.0741717 |
| Sae1 | 0.4405967 |
| Sae1 | -0.6624316 |
| Sag | -0.0296978 |
| Sall1 | -0.7648 |
| Sall1 | -0.4355682 |
| Sall2 | -0.8381348 |
| Sall4 | 0.6296999 |
| Sall4 | 2.0917441 |
| Samd10 | -0.6024505 |
| Samd11 | 0.3917186 |
| Samd12 | -0.6517443 |
| Samd14 | 0.7586826 |
| Samd4 | -0.3722907 |
| Samd4 | 3.3447666 |
| Samd4b | 0.7962381 |
| Samd5 | -0.6172717 |
| Samd7 | -0.7939949 |
| Samd8 | 0.025092 |
| Samd9l | -0.7854209 |
| Samhd1 | 0.2267969 |
| Samm50 | 1.1439046 |
| Samsn1 | -0.4987953 |
| Sap130 | -0.6347438 |
| Sap30 | 1.4820537 |
| Sap30bp | 2.8445814 |
| Sap30l | -0.7338327 |
| Saps1 | 0.0111175 |
| Saps2 | 1.827894 |
| Saps3 | -0.5029418 |
| Saps3 | 1.4842633 |
| Sar1a | -0.6436087 |
| Sar1b | 0.7031423 |
| Sar1b | -0.4636377 |
| Sarm1 | -0.6355461 |
| Sars | 0.4192278 |
| Sars | 2.6440186 |
| Sars2 | -0.7004385 |
| Sart1 | 1.1499935 |
| Sart3 | -0.4298024 |
| Sas | -0.3533222 |
| Sass6 | -0.3663199 |
| Sat2 | -0.6850426 |
| Satb1 | 1.272457 |
| Sav1 | 3.3028335 |
| Sbds | 1.0290084 |
| Sbf1 | -0.5878695 |
| Sbk | 3.1178402 |
| Sbno1 | -0.0034749 |
| Sbno2 | 0.0091594 |
| Sc4mol | -0.3290179 |
| Sc5d | -0.7143485 |
| Scaf1 | -0.7047623 |
| Scamp1 | 1.9858374 |
| Scamp2 | -0.6525619 |
| Scamp3 | 1.6048691 |
| Scamp4 | -0.3764939 |
| Scamp5 | 2.3269266 |
| Scamp5 | 3.9405432 |
| Scand1 | 0.5406501 |
| Scap | 0.5673963 |
| Scaper | 0.3158237 |
| Scara3 | -0.3947333 |
| Scara5 | 2.067717 |
| Scarb1 | 1.505471 |
| Scarb2 | -0.6227943 |
| Scarf1 | -0.2189878 |
| Scarf2 | 0.165004 |
| Sccpdh | -0.6541994 |
| Scd1 | -0.2715183 |
| Scd2 | 0.3432833 |
| Scd4 | -0.4616507 |
| Scel | -0.7592572 |
| Scg2 | 0.0760085 |
| Scg3 | -0.3137334 |
| Scg5 | -0.7911278 |
| Scgn | 0.2986522 |
| Scgn | -0.7685143 |
| Scgn | 1.6476281 |
| Schip1 | 1.4662001 |
| Schip1 | 0.9204855 |
| Scin | -0.2451298 |
| scl0003251.1_21 | 1.7164508 |
| Sclt1 | -0.477671 |
| Scly | -0.6180586 |
| Scmh1 | -0.5871086 |
| Scmh1 | -0.0994702 |
| Scml2 | 1.5099935 |
| Scml4 | -0.4133502 |
| Scn10a | 1.2449828 |
| Scn11a | -0.7873192 |
| Scn1a | 0.0087672 |
| Scn2b | 1.1995768 |
| Scn3b | -0.4002781 |
| Scn3b | 1.4580106 |
| Scn3b | -0.47163 |
| Scn3b | -0.7258108 |
| Scn4b | -0.7694452 |
| Scn5a | 0.6200087 |
| Scn7a | -0.6987148 |
| Scn8a | 0.0122904 |
| Scnm1 | -0.2440902 |
| Scnm1 | 0.9731647 |
| Scnn1a | -0.7583367 |
| Scnn1b | 0.0024695 |
| Scnn1g | -0.3335969 |
| Scoc | 2.5939369 |
| Scoc | 1.3463724 |
| Scotin | -0.4139775 |
| Scotin | -0.6133477 |
| Scotin | -0.3669152 |
| Scp2 | 0.3282843 |
| Scp2 | 1.6086546 |
| Scp2 | -0.6541994 |
| Scrg1 | -0.6501113 |
| Scrib | 2.1601641 |
| Scrib | 1.3532646 |
| Scrn1 | -0.1555966 |
| Scrn1 | -0.2932009 |
| Scrn1 | -0.3031311 |
| Scrn2 | -0.6875903 |
| Sct | -0.6055506 |
| Sctr | 0.7881918 |
| Scube1 | 1.3660252 |
| Scube2 | 1.132727 |
| Scube3 | 0.3000721 |
| Scube3 | -0.7169807 |
| Scx | 0.2255647 |
| Scye1 | -0.5389803 |
| Scyl1 | 1.4095621 |
| Scyl1bp1 | 0.890222 |
| Scyl3 | 3.3526624 |
| Scyl3 | -0.6732475 |
| Sdad1 | -0.3307323 |
| Sdc1 | 1.7843154 |
| Sdc1 | 0.1980214 |
| Sdc2 | 0.1000712 |
| Sdc3 | -0.4420183 |
| Sdc4 | 4.3835482 |
| Sdcbp | 2.1120078 |
| Sdcbp | 3.2129737 |
| Sdcbp2 | -0.0990279 |
| Sdcbp2 | 0.2796966 |
| Sdccag1 | 0.3107907 |
| Sdccag1 | -0.7240379 |
| Sdccag3 | -0.5720238 |
| Sdccag3 | 0.5686604 |
| Sdf2 | -0.6892929 |
| Sdf2 | -0.77975 |
| Sdf4 | -0.5001753 |
| Sdhb | -0.5296561 |
| Sdhb | 2.680048 |
| Sdhb | -0.5440414 |
| Sdhc | -0.675762 |
| Sdhd | 0.6987805 |
| Sdk1 | 0.7673336 |
| Sdpr | -0.2752752 |
| Sds | -0.6243784 |
| Sdsl | -0.7178598 |
| Sec1 | -0.7038959 |
| Sec11a | -0.6476673 |
| Sec11c | 1.4019226 |
| Sec13 | 2.6091494 |
| Sec14l1 | 0.8722242 |
| Sec14l1 | 1.060509 |
| Sec14l1 | 0.1906814 |
| Sec14l2 | 2.8458893 |
| Sec14l3 | 0.2453812 |
| Sec14l3 | -0.7169807 |
| Sec14l4 | 0.1422996 |
| Sec15l1 | -0.1833286 |
| Sec16a | 0.2122114 |
| Sec16b | -0.1443667 |
| Sec22a | -0.5064118 |
| Sec22b | -0.4039936 |
| Sec22c | 1.5987254 |
| Sec22c | -0.671575 |
| Sec23a | 2.7026684 |
| Sec23ip | 0.2147108 |
| Sec24b | -0.0138725 |
| Sec24c | 0.3780324 |
| Sec24d | 2.8959603 |
| Sec61a1 | -0.7428306 |
| Sec61a2 | -0.3335969 |
| Sec61a2 | 1.1640068 |
| Sec61b | 0.4671437 |
| Sec61b | 2.8930871 |
| Sec61g | -0.2921042 |
| Sec63 | 0.4039365 |
| Sec63 | -0.5404234 |
| Secisbp2 | -0.6204233 |
| Sectm1b | -0.5765228 |
| Seh1l | -0.0884783 |
| Seh1l | 1.4030894 |
| Sel1l | -0.6566611 |
| Sel1l | -0.4083479 |
| Sel1l | 0.3630772 |
| Sel1l2 | 1.2786945 |
| Sel1l2 | 1.4757765 |
| Selenbp2 | 0.69128 |
| Selk | 2.2379527 |
| Sell | 3.4652636 |
| Selp | -0.4656292 |
| Selplg | -0.6640872 |
| Sema3a | -0.6001325 |
| Sema3b | -0.1374185 |
| Sema3b | 0.184895 |
| Sema3b | 2.2589458 |
| Sema3e | -0.6009045 |
| Sema3f | 1.2393059 |
| Sema3f | 0.594394 |
| Sema3g | -0.4656292 |
| Sema4a | -0.1891745 |
| Sema4b | -0.6371529 |
| Sema4d | 1.3129395 |
| Sema4f | -0.7311508 |
| Sema4g | 1.8240421 |
| Sema5a | -0.6995762 |
| Sema5a | -0.5675473 |
| Sema5b | 2.8141016 |
| Sema6b | -0.7769279 |
| Sema6b | 0.944927 |
| Sema6b | -0.025206 |
| Sema6d | 0.5621103 |
| Sema6d | -0.628351 |
| Sema7a | -0.7196206 |
| Sema7a | -0.6927076 |
| Senp1 | -0.621213 |
| Senp2 | -0.3752909 |
| Senp3 | 0.2756239 |
| Senp5 | -0.0715947 |
| Senp5 | -0.4696252 |
| Senp5 | 0.3516284 |
| Senp6 | -0.451781 |
| Senp6 | 0.8180413 |
| Senp7 | 0.9684521 |
| Senp7 | -0.6649161 |
| Senp7 | 1.9527254 |
| Senp8 | 2.3577438 |
| Sephs1 | -0.8221914 |
| Sephs2 | 0.0825366 |
| Sepm | -0.685891 |
| Sepn1 | -0.0276533 |
| Sepp1 | 0.3238736 |
| Sepp1 | 0.327459 |
| Sepsecs | -0.623586 |
| Sept2 | -0.2322202 |
| Sept3 | 3.2212038 |
| Sept3 | -0.3250302 |
| Sept5 | -0.0082589 |
| Sept6 | 1.1131706 |
| Sept6 | -0.6220033 |
| Sept7 | 0.2653607 |
| Sept8 | -0.685891 |
| Sept9 | 0.3082636 |
| Sept10 | 0.8732753 |
| Sept10 | -0.6267599 |
| Sept12 | -0.1760741 |
| Sept15 | 1.3867009 |
| Sepw1 | -0.514795 |
| Sepx1 | -0.5325148 |
| Sepx1 | 0.3085447 |
| Serac1 | 0.0300855 |
| Serbp1 | 0.0785528 |
| Serbp1 | 1.4559712 |
| Serbp1 | -0.1420447 |
| Serf1 | -0.7266986 |
| Serf2 | 1.4263549 |
| Sergef | -0.2493003 |
| Serhl | -0.3171076 |
| Serhl | 0.9553977 |
| Serinc1 | 1.5670838 |
| Serinc2 | 1.8002029 |
| Serinc3 | -0.3290179 |
| Serpina1a | 1.9643153 |
| Serpina1a | 0.4405967 |
| Serpina1b | -0.0317469 |
| Serpina1b | -0.1245904 |
| Serpina1d | 0.8899274 |
| Serpina1e | -0.1273239 |
| Serpina3b | -0.425979 |
| Serpina3c | 0.6610166 |
| Serpina3g | 0.4473675 |
| Serpina3h | 1.5890919 |
| Serpina3k | 1.4025062 |
| Serpina3n | -0.7750514 |
| Serpina5 | -0.593214 |
| Serpina5 | 2.9206747 |
| Serpina7 | -0.6749231 |
| Serpina9 | 0.1025485 |
| Serpinb11 | 1.3451297 |
| Serpinb12 | 1.0283771 |
| Serpinb1a | 0.935734 |
| Serpinb3a | -0.1940753 |
| Serpinb6a | -0.5601355 |
| Serpinb6b | 0.1948374 |
| Serpinb6c | -0.4215387 |
| Serpind1 | -0.5375395 |
| Serpine2 | 0.4069652 |
| Serpinf1 | -0.4967292 |
| Serping1 | -0.6892929 |
| Serpinh1 | -0.7231527 |
| Serpini1 | 1.2750532 |
| Sertad1 | -0.1494963 |
| Sertad2 | 0.6646192 |
| Sertad2 | -0.4563734 |
| Sertad3 | -0.6275551 |
| Sertad4 | 3.7376937 |
| Sesn1 | 0.1286714 |
| Sesn1 | 0.2825945 |
| Sesn2 | -0.6331414 |
| Sesn3 | -0.0828152 |
| Setd1b | -0.5050222 |
| Setd2 | -0.5282301 |
| Setd3 | 1.1727631 |
| Setd4 | 1.2095457 |
| Setd4 | 1.1332892 |
| Setd5 | 2.5380053 |
| Setd6 | -0.7428306 |
| Setd6 | 0.2750406 |
| Setd7 | 1.7674204 |
| Setd7 | -0.6517443 |
| Setd8 | 1.1641154 |
| Setd8 | -0.5105936 |
| Setd8 | -0.67074 |
| Setdb1 | 0.5029504 |
| Setdb2 | -0.30758 |
| Setmar | -0.6339422 |
| Setx | -0.4452608 |
| Sez6 | -0.5296561 |
| Sez6l | 0.9016359 |
| Sez6l2 | -0.6149153 |
| Sez6l2 | -0.4609894 |
| Sf1 | 2.9102839 |
| Sf1 | -0.5795346 |
| Sf3a1 | -0.1378801 |
| Sf3a2 | 1.5502701 |
| Sf3a3 | 1.1800488 |
| Sf3b1 | -0.7383204 |
| Sf3b2 | -0.6558398 |
| Sf3b3 | 0.9794546 |
| Sf3b4 | 0.0968761 |
| Sf3b4 | -0.5586605 |
| Sf3b5 | -0.2822943 |
| Sf4 | 0.4384095 |
| Sfi1 | 1.1593235 |
| Sfi1 | -0.6102207 |
| Sfmbt1 | 0.6497369 |
| Sfmbt1 | 0.2353751 |
| Sfmbt1 | 1.3988319 |
| Sfmbt2 | -0.5742705 |
| Sfrp1 | -0.597051 |
| Sfrp2 | 0.1543909 |
| Sfrp4 | 1.8638372 |
| Sfrp5 | -0.5623526 |
| Sfrs1 | 2.9139266 |
| Sfrs1 | -0.7657271 |
| Sfrs11 | 1.4293527 |
| Sfrs12 | 0.1560575 |
| Sfrs14 | 0.7399095 |
| Sfrs16 | 0.3569716 |
| Sfrs2 | 0.9897971 |
| Sfrs2 | 2.3264492 |
| Sfrs2 | -0.7347285 |
| Sfrs3 | 0.5054382 |
| Sfrs3 | -0.675762 |
| Sfrs4 | 2.1671738 |
| Sfrs5 | -0.4864693 |
| Sfrs5 | -0.5491311 |
| Sfrs6 | 3.6659452 |
| Sfrs7 | -0.6492959 |
| Sfrs8 | 2.0978058 |
| Sfrs9 | -0.6180586 |
| Sft2d2 | 0.4730162 |
| Sft2d2 | -0.3704953 |
| Sft2d2 | -0.6624316 |
| Sftpc | 0.1861841 |
| Sftpd | -0.6724109 |
| Sfxn1 | 1.939563 |
| Sfxn1 | 0.012681 |
| Sfxn3 | -0.7500943 |
| Sfxn3 | -0.7222684 |
| Sfxn4 | 1.0895291 |
| Sfxn4 | -0.6149153 |
| Sfxn5 | 0.8804497 |
| Sgca | -0.3758922 |
| Sgca | 0.7921418 |
| Sgcb | 1.7857318 |
| Sgcb | -0.7473636 |
| Sgcz | -0.8431756 |
| Sgip1 | 1.5436695 |
| Sgip1 | -0.1833286 |
| Sgip1 | 0.4904073 |
| Sgip1 | -0.7365227 |
| Sgip1 | -0.3498097 |
| Sgk1 | -0.7968711 |
| Sgk2 | 1.7051512 |
| Sgk2 | -0.6371529 |
| Sgk3 | 1.688932 |
| Sgk3 | 0.5153211 |
| Sgk3 | -0.6969943 |
| Sgk3 | -0.7143485 |
| Sgk3 | 0.6279277 |
| Sgms2 | 0.0598487 |
| Sgol1 | -0.6355461 |
| Sgpl1 | 1.7679202 |
| Sgpl1 | -0.761101 |
| Sgpp1 | 1.4864674 |
| Sgpp2 | -0.6094407 |
| Sgsm1 | -0.8074967 |
| Sgsm2 | 0.3883808 |
| Sgsm3 | 1.2463716 |
| Sgta | -0.1012414 |
| Sgtb | -0.4524356 |
| Sgtb | 1.1227745 |
| Sh2b1 | 1.8623387 |
| Sh2b1 | 0.6385089 |
| Sh2b3 | -0.7666552 |
| Sh2d2a | -0.5901561 |
| Sh3bgrl | -0.7844733 |
| Sh3bgrl3 | 1.4766978 |
| Sh3bp2 | 0.0668624 |
| Sh3bp5 | 1.5744798 |
| Sh3bp5l | -0.7510064 |
| Sh3gl1 | 2.1705873 |
| Sh3gl2 | -0.6952772 |
| Sh3gl2 | -0.4885119 |
| Sh3gl2 | -0.7657271 |
| Sh3gl3 | 0.4369484 |
| Sh3glb1 | -0.5818001 |
| Sh3glb2 | -0.2921042 |
| Sh3kbp1 | -0.2779684 |
| Sh3kbp1 | 0.2059323 |
| Sh3kbp1 | -0.6901453 |
| Sh3kbp1 | 1.6137432 |
| Sh3md4 | 0.9928242 |
| Sh3md4 | -0.5630929 |
| Sh3pxd2a | 0.6267442 |
| Sh3pxd2b | -0.2976008 |
| Sh3rf1 | -0.5772748 |
| Sh3rf2 | -0.358027 |
| Sh3tc1 | -0.6892929 |
| Sh3tc2 | -0.5668034 |
| Sh3yl1 | -0.5268064 |
| Shank2 | -0.6395684 |
| Sharpin | -0.7266986 |
| Shbg | -0.4400784 |
| Shc1 | 1.1082144 |
| Shc1 | 1.1449038 |
| Shc3 | -0.0375095 |
| Shcbp1 | -0.4576898 |
| Shd | -0.3171076 |
| She | -0.2124439 |
| Shf | -0.4209062 |
| Shf | 0.5589237 |
| Shf | -0.078483 |
| Shfm1 | -0.4452608 |
| Shh | 0.8617976 |
| Shisa2 | -0.1218652 |
| Shisa3 | 0.5462792 |
| Shisa4 | -0.1137386 |
| Shkbp1 | 2.183239 |
| Shkbp1 | -0.6918527 |
| Shmt1 | -0.329589 |
| Shmt2 | -0.3313045 |
| Shoc2 | 1.9245273 |
| Shox2 | -0.3053528 |
| Shprh | 0.8497095 |
| Shq1 | -0.36098 |
| Shroom3 | -0.5064118 |
| Shroom3 | -0.2998089 |
| Si | -0.423439 |
| Si | 0.5692917 |
| Siah1a | 2.8149171 |
| Siah1b | 0.531043 |
| Siah1b | -0.5682918 |
| Siah1b | -0.8962726 |
| Siah1b | 0.9490747 |
| Siah2 | 0.7760694 |
| Siat7f | -0.232733 |
| Siat9 | 2.0546918 |
| Sidt1 | 0.6379243 |
| Sidt2 | -0.4083479 |
| Sidt2 | -0.2725901 |
| Sidt2 | -0.0082589 |
| Sigirr | -0.3165443 |
| Siglec1 | -0.2850085 |
| Siglech | 0.2100187 |
| Siglech | -0.177038 |
| Sil1 | -0.3556715 |
| Sin3a | -0.7437354 |
| Sin3b | 0.0064107 |
| Sip1 | 1.0416666 |
| Sip1 | 0.1507135 |
| Sipa1 | -0.2104399 |
| Sipa1l1 | 0.184895 |
| Sirpa | -0.6024505 |
| Sirpa | -0.1881975 |
| Sirpb1 | -0.3843523 |
| Sirt1 | -0.4891939 |
| Sirt2 | -0.5527843 |
| Sirt3 | 0.4349966 |
| Sirt5 | -0.6682394 |
| Sirt5 | 1.537307 |
| Sirt6 | -0.7082364 |
| Sirt7 | -0.329589 |
| Siva1 | -0.0441408 |
| Six1 | 0.5720225 |
| Six2 | 0.1520525 |
| Six3 | 2.359558 |
| Six5 | -0.5071074 |
| Six6os1 | 2.1452853 |
| Skap2 | -0.6387625 |
| Skil | -0.4576898 |
| Skiv2l | 0.4325509 |
| Skiv2l | 0.2441743 |
| Skiv2l2 | -0.2926524 |
| Skp1a | 2.0121204 |
| Skp2 | 1.2960268 |
| Skp2 | 0.0672301 |
| Skp2 | 4.1220726 |
| Sla | 0.5090445 |
| Slain1 | -0.4278887 |
| Slain2 | -0.2806696 |
| Slamf7 | 1.3197262 |
| Slamf8 | 1.870401 |
| Slamf9 | 0.063547 |
| Slc10a3 | 4.042127 |
| Slc10a4 | 0.9116135 |
| Slc10a6 | -0.5893933 |
| Slc10a6 | -0.2383937 |
| Slc10a7 | 1.1843071 |
| Slc10a7 | -0.3746901 |
| Slc10a7 | -0.7169807 |
| Slc11a1 | -0.623586 |
| Slc11a1 | 1.9133078 |
| Slc11a1 | 1.5095497 |
| Slc11a1 | 1.154945 |
| Slc11a2 | 1.4186936 |
| Slc12a2 | 0.4147426 |
| Slc12a2 | 2.2028311 |
| Slc12a3 | -0.630743 |
| Slc12a5 | -0.6690721 |
| Slc12a6 | -0.5893933 |
| Slc12a7 | -0.2953981 |
| Slc12a8 | -0.8016854 |
| Slc12a9 | 0.0602192 |
| Slc12a9 | -0.7284769 |
| Slc13a2 | -0.6251716 |
| Slc13a3 | -0.6550192 |
| Slc13a3 | 0.1999264 |
| Slc13a4 | -0.5675473 |
| Slc14a1 | 2.6462596 |
| Slc14a1 | 0.4079725 |
| Slc14a1 | -0.5645752 |
| Slc14a2 | -0.7666552 |
| Slc14a2 | -0.7574171 |
| Slc15a2 | -0.7666552 |
| Slc15a3 | -0.2014769 |
| Slc15a4 | -0.3353201 |
| Slc15a4 | -0.7231527 |
| Slc16a10 | -0.6251716 |
| Slc16a11 | 1.9440936 |
| Slc16a4 | 2.1562781 |
| Slc16a6 | -0.3428256 |
| Slc16a6 | -0.7302586 |
| Slc16a6 | 1.6400441 |
| Slc16a8 | 0.6092064 |
| Slc16a9 | -0.7666552 |
| Slc17a2 | 0.0825366 |
| Slc17a5 | -0.6204233 |
| Slc17a6 | -0.4247081 |
| Slc17a7 | 0.0512846 |
| Slc17a8 | -0.6149153 |
| Slc18a2 | 1.2473624 |
| Slc19a1 | -0.2550665 |
| Slc19a2 | -0.6323412 |
| Slc1a1 | 1.6147969 |
| Slc1a1 | -0.070309 |
| Slc1a2 | 1.8335895 |
| Slc1a2 | -0.0122651 |
| Slc1a2 | 1.3962341 |
| Slc1a2 | 0.6625353 |
| Slc1a3 | 1.062092 |
| Slc1a4 | -0.4871497 |
| Slc1a6 | -0.0537598 |
| Slc1a7 | 0.3413882 |
| Slc20a1 | -0.5878695 |
| Slc22a1 | 0.2362893 |
| Slc22a12 | -0.6086613 |
| Slc22a13 | -0.078483 |
| Slc22a14 | 0.8613415 |
| Slc22a17 | -0.6566611 |
| Slc22a17 | 1.0138621 |
| Slc22a18 | -0.3651305 |
| Slc22a21 | -0.1209586 |
| Slc22a3 | 2.5070935 |
| Slc22a4 | -0.6816567 |
| Slc22a5 | 0.5072431 |
| Slc22a5 | -0.283379 |
| Slc22a5 | -0.8421652 |
| Slc22a6 | 0.1728825 |
| Slc22a7 | 0.2946646 |
| Slc22a8 | -0.0453897 |
| Slc23a2 | 0.6591148 |
| Slc23a3 | 2.1927768 |
| Slc24a1 | 1.4741618 |
| Slc24a3 | -0.3910558 |
| Slc24a4 | -0.6371529 |
| Slc24a5 | 0.4821015 |
| Slc24a6 | 1.5706166 |
| Slc25a1 | -0.1518377 |
| Slc25a1 | -0.5527843 |
| Slc25a10 | 2.8369815 |
| Slc25a11 | -0.4939818 |
| Slc25a11 | -0.076756 |
| Slc25a11 | 0.8153246 |
| Slc25a12 | 1.5636764 |
| Slc25a14 | -0.6016772 |
| Slc25a15 | 0.7649671 |
| Slc25a15 | 0.777072 |
| Slc25a16 | 0.07163 |
| Slc25a17 | -0.4669592 |
| Slc25a18 | 1.0404212 |
| Slc25a19 | -0.4095959 |
| Slc25a19 | 3.219181 |
| Slc25a20 | 0.9496263 |
| Slc25a20 | -0.5795346 |
| Slc25a23 | -0.6969943 |
| Slc25a24 | -0.6674074 |
| Slc25a24 | 0.1018414 |
| Slc25a25 | 0.3280093 |
| Slc25a26 | -0.4844313 |
| Slc25a27 | 1.2461733 |
| Slc25a28 | -0.6884412 |
| Slc25a29 | 1.5599763 |
| Slc25a3 | -0.1537152 |
| Slc25a3 | -0.169834 |
| Slc25a30 | -0.7205024 |
| Slc25a32 | -0.6884412 |
| Slc25a33 | -0.8094422 |
| Slc25a34 | -0.0664629 |
| Slc25a35 | -0.3492256 |
| Slc25a36 | 1.0381752 |
| Slc25a37 | -0.5564524 |
| Slc25a38 | -0.7196206 |
| Slc25a38 | 3.0160621 |
| Slc25a38 | 1.7414963 |
| Slc25a39 | -0.2921042 |
| Slc25a41 | -0.2888223 |
| Slc25a42 | 0.466908 |
| Slc25a44 | 1.1550547 |
| Slc25a45 | 1.8245114 |
| Slc25a46 | 0.4480899 |
| Slc25a5 | -0.4108456 |
| Slc26a4 | -0.4046144 |
| Slc26a6 | -0.5232569 |
| Slc26a7 | -0.2220242 |
| Slc26a8 | -0.6841949 |
| Slc27a1 | -0.7338327 |
| Slc27a2 | 0.6422025 |
| Slc27a3 | -0.5802891 |
| Slc27a4 | 0.2903724 |
| Slc27a6 | -0.33245 |
| Slc29a1 | -0.8553901 |
| Slc29a2 | -0.4083479 |
| Slc29a3 | -0.6766018 |
| Slc29a4 | 1.3635919 |
| Slc2a1 | 0.9963649 |
| Slc2a10 | 0.0854228 |
| Slc2a10 | -0.6032245 |
| Slc2a13 | -0.1886859 |
| Slc2a3 | 1.4040882 |
| Slc2a4 | -0.6978541 |
| Slc2a5 | -0.6566611 |
| Slc2a6 | -0.7694452 |
| Slc2a8 | 0.1046665 |
| Slc2a8 | -0.3783013 |
| Slc2a8 | 1.1314889 |
| Slc2a9 | -0.5050222 |
| Slc30a1 | 0.703686 |
| Slc30a10 | 0.2600365 |
| Slc30a3 | -0.1731887 |
| Slc30a5 | 1.0283771 |
| Slc30a5 | -0.6071046 |
| Slc30a6 | -0.7854209 |
| Slc30a7 | 3.0306575 |
| Slc30a7 | 1.6075936 |
| Slc30a9 | -0.5346648 |
| Slc31a1 | -0.1466947 |
| Slc31a2 | 1.2685002 |
| Slc32a1 | 0.1071311 |
| Slc34a2 | -0.4851101 |
| Slc35a1 | -0.1537152 |
| Slc35a2 | 0.8521708 |
| Slc35a3 | -0.4851101 |
| Slc35a4 | -0.3387764 |
| Slc35a5 | 2.1281123 |
| Slc35a5 | 2.2322708 |
| Slc35b1 | 0.1433134 |
| Slc35b2 | 0.7683459 |
| Slc35b3 | 2.3294883 |
| Slc35b3 | 1.8981113 |
| Slc35b3 | -0.3498097 |
| Slc35b4 | -0.6047745 |
| Slc35c1 | -0.4511268 |
| Slc35c1 | 1.7020502 |
| Slc35c2 | -0.7213849 |
| Slc35c2 | -0.5623526 |
| Slc35c2 | -0.3710933 |
| Slc35e1 | -0.6157001 |
| Slc35e3 | -0.6251716 |
| Slc35e4 | -0.1401913 |
| Slc35f1 | -0.3445665 |
| Slc35f2 | 1.2978073 |
| Slc35f3 | -0.3789045 |
| Slc35f4 | -0.1867341 |
| Slc35f4 | -0.6476673 |
| Slc36a1 | 0.5544435 |
| Slc36a3 | 1.2150023 |
| Slc37a1 | -0.527518 |
| Slc37a3 | -0.467625 |
| Slc38a1 | 0.4581434 |
| Slc38a10 | 2.2355456 |
| Slc38a11 | -0.1641119 |
| Slc38a2 | 0.5358594 |
| Slc38a2 | -0.6078826 |
| Slc38a2 | -0.5947468 |
| Slc38a3 | -0.6315417 |
| Slc38a4 | 1.1585588 |
| Slc38a5 | -0.2725901 |
| Slc38a8 | 1.0531668 |
| Slc38a9 | 1.1222057 |
| Slc38a9 | -0.7013016 |
| Slc39a10 | -0.7320439 |
| Slc39a11 | 0.1550579 |
| Slc39a12 | 0.4059568 |
| Slc39a13 | -0.1760741 |
| Slc39a13 | -0.3216262 |
| Slc39a14 | 0.5962334 |
| Slc39a3 | 0.055763 |
| Slc39a3 | -0.6204233 |
| Slc39a4 | -0.8094422 |
| Slc39a5 | 0.0380938 |
| Slc39a5 | -0.0728823 |
| Slc39a5 | 2.0930603 |
| Slc39a6 | -0.8143244 |
| Slc39a7 | -0.7030303 |
| Slc39a7 | -0.2466914 |
| Slc39a7 | -0.5840713 |
| Slc39a8 | -0.3746901 |
| Slc3a2 | 0.6096088 |
| Slc40a1 | -0.2149552 |
| Slc41a2 | -0.0276533 |
| Slc41a3 | 3.1549506 |
| Slc41a3 | -0.6978541 |
| Slc41a3 | -0.671575 |
| Slc43a2 | 1.2253102 |
| Slc43a2 | -0.5339476 |
| Slc44a1 | -0.2550665 |
| Slc44a1 | 3.2766525 |
| Slc44a2 | -0.5924485 |
| Slc44a2 | 1.0207668 |
| Slc44a2 | -0.1323571 |
| Slc44a3 | -0.7500943 |
| Slc44a4 | 2.6772634 |
| Slc45a1 | -0.772244 |
| Slc45a2 | -0.3250302 |
| Slc45a3 | 0.2456827 |
| Slc45a4 | -0.2519167 |
| Slc46a1 | 0.8021042 |
| Slc46a3 | -0.4898763 |
| Slc47a1 | -0.3233266 |
| Slc4a1 | -0.7648 |
| Slc4a1ap | -0.4330011 |
| Slc4a2 | 1.8838261 |
| Slc4a3 | -0.3131722 |
| Slc4a3 | 0.1480296 |
| Slc4a3 | 0.0064107 |
| Slc4a3 | 0.945758 |
| Slc4a8 | -0.4803691 |
| Slc4a8 | 0.2546806 |
| Slc5a4a | -0.5140934 |
| Slc5a4b | -0.5863483 |
| Slc5a5 | 0.3508242 |
| Slc5a6 | -0.4478632 |
| Slc5a7 | 0.876122 |
| Slc5a8 | 1.6030704 |
| Slc5a9 | 0.7334469 |
| Slc6a1 | -0.5183111 |
| Slc6a11 | 0.1961124 |
| Slc6a12 | -0.3795082 |
| Slc6a13 | -0.7583367 |
| Slc6a15 | 1.3174391 |
| Slc6a15 | 0.4184818 |
| Slc6a17 | -0.5447667 |
| Slc6a2 | 0.7011458 |
| Slc6a4 | 0.0270159 |
| Slc6a6 | -0.1674454 |
| Slc6a7 | -0.7213849 |
| Slc6a8 | -0.451781 |
| Slc6a9 | -0.0737417 |
| Slc7a1 | 0.5358594 |
| Slc7a10 | -0.2768901 |
| Slc7a11 | -0.8492621 |
| Slc7a14 | 2.4860462 |
| Slc7a14 | 0.505664 |
| Slc7a14 | 0.9552607 |
| Slc7a2 | 2.0564663 |
| Slc7a3 | -0.4400784 |
| Slc7a4 | -0.7473636 |
| Slc7a6 | 0.3743822 |
| Slc7a6os | -0.761101 |
| Slc7a7 | 1.1216366 |
| Slc7a7 | 2.030802 |
| Slc7a8 | 0.1916422 |
| Slc7a8 | 2.8682299 |
| Slc8a1 | 0.2049867 |
| Slc8a2 | -0.6850426 |
| Slc8a2 | 0.7346731 |
| Slc9a1 | 1.8459941 |
| Slc9a10 | -0.4912427 |
| Slc9a3r1 | -0.3278767 |
| Slc9a3r2 | 0.9596339 |
| Slc9a4 | -0.815304 |
| Slc9a6 | -0.9114135 |
| Slc9a6 | 2.210808 |
| Slc9a8 | -0.8143244 |
| Slc9a8 | 1.4946288 |
| Slc9a8 | -0.7769279 |
| Slc9a8 | 3.0663328 |
| Slc9a9 | 0.0150212 |
| Slco1a4 | -0.3393537 |
| Slco1c1 | 0.9080112 |
| Slco2a1 | -0.7778676 |
| Slco2b1 | -0.7178598 |
| Slco3a1 | -0.3928927 |
| Slco3a1 | -0.0191168 |
| Slco3a1 | -0.7196206 |
| Slco4a1 | 0.9421511 |
| Slfnl1 | -1.0528465 |
| Slit1 | 0.5720225 |
| Slit2 | -0.644419 |
| Slit3 | 0.3952993 |
| Slitrk1 | 0.6251639 |
| Slitrk3 | -0.329589 |
| Slitrk4 | 1.9980437 |
| Slitrk5 | 0.3105103 |
| Slitrk5 | -0.7365227 |
| Slitrk6 | 0.3917186 |
| Slk | -0.4629749 |
| Slk | 1.5098456 |
| Slmap | 1.3338677 |
| Slmap | -0.2286384 |
| Slmap | -0.6468541 |
| Slmo1 | -0.6509274 |
| Slmo2 | -0.2970496 |
| Sln | -0.9146914 |
| Slpi | 0.1777718 |
| Sltm | 0.2341546 |
| Sltm | 2.7727015 |
| Slu7 | 0.8941894 |
| Slu7 | 1.4522728 |
| Sly | 3.1558771 |
| Smad1 | -0.6909986 |
| Smad1 | -0.7978319 |
| Smad2 | 1.3549355 |
| Smad3 | 1.8649843 |
| Smad5 | 0.4381661 |
| Smad5 | 1.6154549 |
| Smad6 | -0.6944198 |
| Smap2 | -0.4623126 |
| Smap2 | -0.5447667 |
| Smap2 | 1.851526 |
| Smarca1 | -0.5840713 |
| Smarca2 | -0.8411559 |
| Smarca2 | -0.8261509 |
| Smarca4 | 1.9954173 |
| Smarcad1 | 0.6128216 |
| Smarcal1 | 0.0631778 |
| Smarcb1 | -0.1901525 |
| Smarcc1 | 0.2546806 |
| Smarcc1 | -0.6460417 |
| Smarcc2 | 0.8938961 |
| Smarcd1 | 2.0307191 |
| Smarcd1 | 0.5408673 |
| Smarcd1 | 3.5143097 |
| Smarcd2 | 1.3115575 |
| Smarcd3 | -0.4871497 |
| Smarce1 | 0.5621103 |
| Smarce1 | 0.9093097 |
| Smc1a | 1.083847 |
| Smc1a | -0.3978095 |
| Smc1a | 0.3569716 |
| Smc1a | -0.6909986 |
| Smc1b | -0.5833136 |
| Smc3 | 1.9702322 |
| Smc5l1 | 0.6839017 |
| Smc6 | 0.8741752 |
| Smchd1 | 0.2125242 |
| Smcp | -0.5705292 |
| Smcr7 | 1.9720926 |
| Smcr7l | 1.3075841 |
| Smek1 | 0.5567931 |
| Smek2 | -0.2774291 |
| Smek2 | 2.643704 |
| Smg5 | -0.0977025 |
| Smg6 | -0.3014685 |
| Smn1 | 0.1004255 |
| Smo | 0.8435264 |
| Smo | -0.7491831 |
| Smo | -0.5454927 |
| Smoc1 | 1.7365657 |
| Smox | -0.448515 |
| Smox | 0.3537695 |
| Smox | 2.2251844 |
| Smox | 1.8984956 |
| Smox | 2.3953703 |
| Smpd1 | 3.4025335 |
| Smpd1 | 2.3926015 |
| Smpd1 | 0.6493521 |
| Smpd2 | 1.9874484 |
| Smpd3 | 0.3532347 |
| Smpd4 | 0.6116181 |
| Smpdl3a | 0.8710215 |
| Smpdl3b | -0.2540153 |
| Smpx | 1.6560294 |
| Smtn | 2.1962327 |
| Smtn | -0.4278887 |
| Smtn | 1.3358443 |
| Smtnl1 | 2.2228768 |
| Smtnl2 | 0.2405437 |
| Smu1 | -0.4898763 |
| Smu1 | 0.8773179 |
| Smug1 | 1.1043922 |
| Smurf1 | 0.1560575 |
| Smyd1 | 0.5527308 |
| Smyd2 | -0.6460417 |
| Smyd2 | 1.8158148 |
| Smyd3 | 1.9753612 |
| Smyd4 | 0.3513605 |
| Smyd5 | -0.483753 |
| Smyd5 | 0.5248756 |
| Snai1 | -0.0478926 |
| Snai3 | 1.4263549 |
| Snap23 | -0.2342733 |
| Snap29 | 0.0181319 |
| Snap91 | -0.4478632 |
| Snap91 | -0.4304412 |
| Snapc1 | -0.4656292 |
| Snapc3 | -0.6379573 |
| Snapc3 | 0.6785143 |
| Snapc3 | 0.0411259 |
| Snapc3 | -0.2953981 |
| Snapc4 | 1.5158903 |
| Snapin | 0.3968296 |
| Snca | 0.6987805 |
| Snca | 0.6094076 |
| Snca | 0.1172706 |
| Sncaip | -0.5653173 |
| Sncg | 0.5884412 |
| Sncg | 4.3489302 |
| Snd1 | -0.3393537 |
| Snf1lk | 0.967101 |
| Snf1lk2 | -0.3533222 |
| Snf8 | 0.4818697 |
| Snip1 | -0.6782836 |
| Snip1 | -0.6157001 |
| Snn | 2.0903871 |
| Snora65 | -0.3165443 |
| Snph | -0.7500943 |
| Snrk | -0.6071046 |
| Snrk | -0.324462 |
| Snrk | -0.2817524 |
| Snrp70 | -0.1532454 |
| Snrpa | -0.3663199 |
| Snrpb | -0.5682918 |
| Snrpb | -0.6995762 |
| Snrpd1 | -0.659954 |
| Snrpd1 | -0.7108508 |
| Snrpd2 | -0.7437354 |
| Snrpd3 | -0.0042705 |
| Snrpg | 0.0083749 |
| Snta1 | -0.6379573 |
| Sntb2 | -0.7196206 |
| Sntb2 | 1.1125957 |
| Sntg2 | -0.2225313 |
| Snupn | 2.5725792 |
| Snurf | 2.5908721 |
| Snurf | 1.7745031 |
| Snx1 | -0.7455476 |
| Snx10 | -0.6927076 |
| Snx11 | -0.3009149 |
| Snx12 | -0.3031311 |
| Snx13 | 1.0123205 |
| Snx14 | 0.7742285 |
| Snx14 | -0.6749231 |
| Snx14 | -0.5375395 |
| Snx15 | -0.6816567 |
| Snx16 | -0.2790479 |
| Snx17 | -0.4285261 |
| Snx17 | 0.1673091 |
| Snx18 | 0.0004924 |
| Snx19 | 1.3083247 |
| Snx19 | -0.644419 |
| Snx2 | 1.4795328 |
| Snx2 | -0.7546641 |
| Snx20 | -0.4763251 |
| Snx21 | -0.519016 |
| Snx22 | 2.6106523 |
| Snx24 | -0.6379573 |
| Snx25 | 1.0935323 |
| Snx25 | -0.5440414 |
| Snx26 | 2.0356804 |
| Snx29 | -0.5818001 |
| Snx3 | -0.1722289 |
| Snx30 | -0.7939949 |
| Snx33 | 1.4112142 |
| Snx4 | 1.259753 |
| Snx6 | -0.3070227 |
| Snx7 | -0.1847865 |
| Snx8 | -0.5579238 |
| Snx9 | -0.6339422 |
| Soat1 | 1.0247068 |
| Socs2 | 1.7590487 |
| Socs3 | -0.6251716 |
| Socs4 | -0.1309816 |
| Socs5 | -0.4926112 |
| Socs6 | -0.3597976 |
| Socs7 | -0.3533222 |
| Socs7 | -0.0622086 |
| Sod1 | -0.1030162 |
| Sod2 | 0.8837189 |
| Sod3 | 0.7406054 |
| Sohlh1 | 0.8610373 |
| Sohlh1 | -0.6157001 |
| Son | -0.0362716 |
| Sorbs1 | -0.1388039 |
| Sorbs2 | 3.136617 |
| Sorbs3 | 0.6466535 |
| Sorcs1 | 1.5166247 |
| Sorcs1 | -0.628351 |
| Sorcs2 | 1.9038103 |
| Sorcs3 | 2.6542357 |
| Sorcs3 | -0.6227943 |
| Sord | 1.3588806 |
| Sorl1 | 0.2206192 |
| Sort1 | -0.5260954 |
| Sort1 | -0.7134728 |
| Sort1 | -0.623586 |
| Sort1 | 1.1523076 |
| Sostdc1 | 0.3759484 |
| Sox12 | 1.0733356 |
| Sox13 | -0.7464551 |
| Sox17 | 1.4106363 |
| Sox17 | -0.6918527 |
| Sox18 | 0.5695021 |
| Sox2 | 1.1839885 |
| Sox2 | 0.4137428 |
| Sox21 | 0.4401111 |
| Sox21 | 0.0825366 |
| Sox30 | 1.603737 |
| Sox5 | -0.4152333 |
| Sox5 | -0.6690721 |
| Sox5 | -0.381927 |
| Sox5 | 0.049037 |
| Sox7 | -0.7329379 |
| Sox7 | 0.3277342 |
| Sox8 | -0.6649161 |
| Sox9 | 0.8750743 |
| Sp100 | 2.0876282 |
| Sp140 | 0.2986522 |
| Sp2 | 2.744604 |
| Sp4 | -0.3740896 |
| Sp5 | -0.7125979 |
| Sp6 | 0.7423427 |
| Sp7 | 2.7149228 |
| Sp8 | -0.5353826 |
| Sp9 | 0.2071916 |
| Spaca3 | 1.4723901 |
| Spaca4 | 1.048609 |
| Spag1 | 3.4933659 |
| Spag1 | 0.8972631 |
| Spag16 | -0.169834 |
| Spag16 | 0.7876968 |
| Spag4 | 1.5566121 |
| Spag4 | -0.7638738 |
| Spag4l | 0.3559055 |
| Spag4l | -0.6525619 |
| Spag5 | -0.078483 |
| Spag5 | 0.0512846 |
| Spag6 | -0.7930382 |
| Spag6 | -0.6782836 |
| Spag7 | 0.3730749 |
| Spag8 | 1.6781385 |
| Spag9 | 0.9306799 |
| Spag9 | -0.3176711 |
| Spag9 | -0.176556 |
| Spag9 | -0.309254 |
| Spam1 | -0.2566455 |
| Spam1 | -0.070309 |
| Sparc | -0.2220242 |
| Sparc | -0.6740849 |
| Sparcl1 | -0.1485615 |
| Spast | -0.4926112 |
| Spast | -0.6674074 |
| Spast | -0.3335969 |
| Spata16 | -0.3904443 |
| Spata16 | 2.5266091 |
| Spata17 | -0.5840713 |
| Spata17 | -0.2624591 |
| Spata19 | 0.0929555 |
| Spata2 | 0.9173474 |
| Spata20 | -0.3758922 |
| Spata20 | -0.6086613 |
| Spata21 | -0.6275551 |
| Spata22 | 0.8456953 |
| Spata2L | 1.624356 |
| Spata3 | 1.9871874 |
| Spata3 | -0.3904443 |
| Spata3 | -0.0276533 |
| Spata3 | 3.6559146 |
| Spata4 | -0.7491831 |
| Spata6 | 0.5828634 |
| Spata7 | -0.132816 |
| Spata7 | 0.8146842 |
| Spatc1 | -0.44982 |
| Spatc1 | -0.309254 |
| Spats1 | -0.7873192 |
| Spc24 | -0.644419 |
| Spc24 | 0.681862 |
| Spc25 | -0.6791257 |
| Spc25 | -0.3539089 |
| Spcs1 | -0.5985904 |
| Spcs1 | 0.0118996 |
| Spcs3 | 0.0331447 |
| Spdef | 0.1338062 |
| Specc1l | 0.2078206 |
| Speer1-ps1 | 0.6061826 |
| Speer1-ps1 | -0.3290179 |
| Speer2 | 0.3260817 |
| Speer2 | 3.3737083 |
| Speer3 | 0.5139798 |
| Speer4b | 0.0591072 |
| Speer4d | -0.6665762 |
| Speer4f | 1.0494731 |
| Speer6-ps1 | 0.0380938 |
| Spef1 | -0.4368546 |
| Spen | 0.9649352 |
| Spert | -0.3364707 |
| Spesp1 | -0.7675843 |
| Spg20 | -0.3064657 |
| Spg21 | 0.3909495 |
| Spg7 | 2.7571735 |
| Spg7 | 0.2969454 |
| Sphk1 | -0.4240733 |
| Sphk2 | 0.4654925 |
| Sphk2 | -0.597051 |
| Spic | 1.3362929 |
| Spin | -0.7347285 |
| Spin2 | 0.3040359 |
| Spink10 | -0.761101 |
| Spink2 | 2.283539 |
| Spink4 | 0.0509104 |
| Spink8 | 1.2530872 |
| Spinlw1 | -0.0634827 |
| Spinlw1 | -0.7666552 |
| Spinlw1 | 1.6406838 |
| Spint1 | -0.7854209 |
| Spint2 | 0.7732228 |
| Spire1 | -0.4905592 |
| Spire1 | -0.7537483 |
| Spire1 | -0.5105936 |
| Spire2 | 1.5719292 |
| Spna1 | -0.5440414 |
| Spnb1 | -0.6125649 |
| Spnb2 | 1.4395671 |
| Spnb2 | 0.663862 |
| Spnb2 | -0.8007205 |
| Spnb2 | 1.3592304 |
| Spnb3 | -0.4537463 |
| Spnb4 | -0.6791257 |
| Spns1 | -0.5653173 |
| Spo11 | 0.9187752 |
| Spo11 | 1.1243652 |
| Spock1 | 1.0518142 |
| Spock2 | 0.5785011 |
| Spock3 | 1.412781 |
| Spon1 | 0.2767894 |
| Spon2 | -0.6157001 |
| Spop | 2.245201 |
| Spp1 | -0.5112925 |
| Spp2 | -0.2893685 |
| Sppl3 | -0.6323412 |
| Spr | 1.7591048 |
| Spred1 | 1.8263344 |
| Spred2 | 1.1728706 |
| Spred2 | 2.5519807 |
| Sprn | 0.0146316 |
| Sprn | 0.4011512 |
| Sprr2a | 0.1317558 |
| Sprr2a | 0.602135 |
| Sprr2f | -0.6749231 |
| Spry3 | 0.4846475 |
| Spry4 | 1.6722298 |
| Spryd4 | 1.3060084 |
| Spsb1 | -0.7249239 |
| Spsb2 | -0.0387492 |
| Spsb3 | -0.1030162 |
| Spsb3 | -0.2079412 |
| Spsb4 | 0.9277184 |
| Sptlc1 | -0.6816567 |
| Sptlc2 | -0.5697828 |
| Spz1 | 0.4860332 |
| Sqle | -0.1736689 |
| Sqle | 1.2671451 |
| Sqrdl | -0.5623526 |
| Sqstm1 | -0.7939949 |
| Sra1 | -0.5204274 |
| Sra1 | 0.9220505 |
| Src | 1.8733688 |
| Srd5a1 | -0.6927076 |
| Srd5a2 | 0.1310713 |
| Srd5a3 | 0.5177749 |
| Srd5a3 | 0.8371444 |
| Srd5a3 | -0.675762 |
| Srebf1 | -0.7500943 |
| Srebf2 | 1.6613578 |
| Srf | 0.5447671 |
| Srf | 2.4931664 |
| Srfbp1 | -0.4682912 |
| Srgn | -0.2409785 |
| Srgn | 0.6480038 |
| Sri | -0.7464551 |
| Sri | -0.6141311 |
| Sri | -0.1809039 |
| Srm | -0.6102207 |
| Srm | 0.9223347 |
| Srm | 1.6758658 |
| Srp14 | 3.5151065 |
| Srp19 | -0.1504321 |
| Srp19 | -0.7347285 |
| Srp54 | 0.4995468 |
| Srp68 | 2.1374765 |
| Srp72 | 0.7654747 |
| Srp9 | -0.6740849 |
| Srp9 | -0.193584 |
| Srpk1 | 1.0521833 |
| Srpk2 | 2.3144311 |
| Srpk3 | 0.6831605 |
| Srpr | 1.4626215 |
| Srprb | 0.8911052 |
| Srprb | -0.6566611 |
| Srpx | -0.4891939 |
| Srpx | 0.5520878 |
| Srpx2 | 0.7735582 |
| Srpx2 | 1.6479453 |
| Srpx2 | -0.3867841 |
| Srr | 0.7168165 |
| Srrd | -0.671575 |
| Srrm1 | 0.0657586 |
| Srrm2 | -0.6690721 |
| Srrm2 | 0.9660187 |
| Srrp | 1.3738882 |
| Srxn1 | -0.4420183 |
| Srxn1 | 3.4231984 |
| Srxn1 | 0.3172167 |
| Ss18 | 2.3148242 |
| Ss18 | -0.1443667 |
| Ss18l1 | -0.4636377 |
| Ss18l1 | 2.1412244 |
| Ss18l1 | 1.167368 |
| Ssbp1 | 2.1312693 |
| Ssbp2 | -0.6001325 |
| Ssbp2 | 0.3152658 |
| Ssbp3 | -0.5564524 |
| Ssbp3 | 1.0827775 |
| Ssbp4 | 1.3752636 |
| Ssfa2 | -0.629945 |
| Ssh2 | 2.390825 |
| Ssh3 | 0.2140866 |
| Ssh3 | 0.7464988 |
| Ssna1 | -0.1570102 |
| Sspn | 2.2299481 |
| Ssr2 | -0.7648 |
| Ssr3 | 0.059478 |
| Ssr4 | 0.1172706 |
| Ssrp1 | 0.8527851 |
| Sssca1 | 1.248352 |
| Sssca1 | -0.1155382 |
| Sst | 0.5146507 |
| Sstr1 | 0.4464033 |
| Sstr5 | 2.1284099 |
| Ssty2 | -0.6850426 |
| Ssu72 | -0.5498606 |
| Ssx2ip | 1.5069563 |
| Ssxb2 | -0.7004385 |
| Ssxb5 | -0.1186961 |
| St13 | 1.3974083 |
| St14 | 0.0821752 |
| St14 | 3.3157382 |
| St18 | -0.5795346 |
| St3gal1 | -0.2987041 |
| St3gal2 | 0.1967492 |
| St3gal2 | -0.2508692 |
| St3gal3 | 0.5327974 |
| St3gal3 | -0.6094407 |
| St3gal4 | 1.0198748 |
| St3gal5 | -0.1960431 |
| St3gal6 | -0.7778676 |
| St5 | 0.4988646 |
| St5 | -0.7038959 |
| St6gal1 | 0.9042494 |
| St6galnac2 | -0.2049519 |
| St6galnac3 | 0.5327974 |
| St6galnac4 | -0.2613993 |
| St6galnac4 | 2.2049174 |
| St6galnac4 | -0.1337346 |
| St6galnac5 | -0.181873 |
| St6galnac6 | 0.1141361 |
| St6galnac6 | -0.7275873 |
| St7 | 0.6560633 |
| St7l | 1.0640364 |
| St7l | 0.6872294 |
| St8sia1 | -0.8065256 |
| St8sia1 | 1.0509524 |
| St8sia2 | -0.3159814 |
| St8sia3 | -0.7222684 |
| St8sia4 | 1.132277 |
| St8sia4 | -0.560874 |
| St8sia5 | -0.1236811 |
| Stab1 | -0.3381994 |
| Stab2 | -0.0664629 |
| Stac | -0.4817211 |
| Stac2 | -0.5346648 |
| Stac3 | -0.0858601 |
| Stag1 | -0.4583488 |
| Stag2 | 1.2504267 |
| Stag2 | -0.4783448 |
| Stag3 | 3.5885044 |
| Stam | -0.5863483 |
| Stam | 2.2880665 |
| Stam | -0.0954976 |
| Stam2 | -0.0142749 |
| Stam2 | -0.381927 |
| Stambp | -0.2009816 |
| Stambpl1 | 0.8305594 |
| Stap1 | 3.0389293 |
| Stap2 | -0.7021655 |
| Star | 1.5353679 |
| Stard10 | -0.6411824 |
| Stard10 | 3.2695968 |
| Stard13 | -0.3335969 |
| Stard3 | -0.6867403 |
| Stard3nl | -0.5978204 |
| Stard4 | 0.4993195 |
| Stard5 | 0.3435538 |
| Stard5 | -0.3710933 |
| Stard6 | 2.6374943 |
| Stard7 | -0.6640872 |
| Stard7 | 0.1961124 |
| Stard8 | 2.5890684 |
| Stard8 | -0.630743 |
| Stat1 | -0.2378776 |
| Stat1 | 0.3991201 |
| Stat2 | 0.528405 |
| Stat3 | 0.0091594 |
| Stat3 | -0.2904618 |
| Stat3 | 0.0040481 |
| Stat4 | -0.5916838 |
| Stat4 | 1.9817754 |
| Stat5a | 0.6967743 |
| Stat5a | 0.1989744 |
| Stat6 | -0.5505906 |
| Statip1 | -0.5454927 |
| Stau1 | 2.7823013 |
| Stau2 | 0.8196356 |
| Stbd1 | -0.1434372 |
| Stc2 | 1.3437963 |
| Steap1 | 2.1284843 |
| Steap2 | -0.5204274 |
| Steap3 | -0.640375 |
| Stfa1 | -0.6816567 |
| Stfa2 | -0.6841949 |
| Stim1 | -0.8182493 |
| Stip1 | -0.4783448 |
| Stk11 | 0.347331 |
| Stk11ip | -0.4336422 |
| Stk17b | 1.2219553 |
| Stk19 | 0.8379251 |
| Stk19 | 1.8488404 |
| Stk19 | -0.3148567 |
| Stk22b | 1.360017 |
| Stk22s1 | -0.7082364 |
| Stk24 | -0.4603285 |
| Stk25 | 0.9255971 |
| Stk25 | 2.9122161 |
| Stk3 | 0.0605896 |
| Stk31 | -0.5389803 |
| Stk32a | 2.1762832 |
| Stk32b | 0.6400659 |
| Stk32c | 0.1957938 |
| Stk35 | 2.1159762 |
| Stk35 | 0.7715441 |
| Stk36 | -0.3227595 |
| Stk38l | 0.9837121 |
| Stk39 | -0.6009045 |
| Stk4 | 0.2600365 |
| Stk40 | 0.2093912 |
| Stmn1 | -0.7939949 |
| Stmn2 | -0.6583061 |
| Stmn3 | 0.3513605 |
| Stmn4 | -0.516903 |
| Stmn4 | -0.2368463 |
| Stoml1 | 1.1492209 |
| Stoml1 | -0.2667109 |
| Stoml1 | 0.1392512 |
| Stoml2 | 0.2071916 |
| Stoml3 | -0.3031311 |
| Stra13 | 0.3596313 |
| Stra13 | 0.4881078 |
| Stra13 | 0.8217039 |
| Stra6 | -0.6411824 |
| Strap | 1.0463834 |
| Strbp | -0.5802891 |
| Strn4 | 0.1169229 |
| Strn4 | -0.7293673 |
| Stt3a | 1.0162976 |
| Stt3b | -0.6125649 |
| Stx12 | -0.7329379 |
| Stx17 | 2.6755643 |
| Stx18 | 0.8177222 |
| Stx1a | 0.1640144 |
| Stx2 | 0.4067132 |
| Stx3 | -0.0030774 |
| Stx3 | -0.5593977 |
| Stx4a | -0.0276533 |
| Stx5a | -0.0630578 |
| Stx5a | 0.0950961 |
| Stx6 | -0.5772748 |
| Stx7 | -0.4891939 |
| Stx8 | -0.6799686 |
| Stx8 | 0.0323809 |
| Stx8 | -0.6039992 |
| Stx8 | 1.0741763 |
| Stxbp1 | 2.6009156 |
| Stxbp1 | -0.217978 |
| Stxbp1 | 0.4219578 |
| Stxbp2 | 1.1424593 |
| Stxbp4 | -0.6649161 |
| Stxbp4 | 1.8224758 |
| Stxbp5l | -0.573521 |
| Stxbp5l | -0.6509274 |
| Styx | -0.7464551 |
| Styxl1 | -0.6657458 |
| Styxl1 | 0.1996092 |
| Styxl1 | 1.926019 |
| Suclg1 | 0.2938078 |
| Suclg1 | 0.1385724 |
| Suclg2 | 2.5253658 |
| Suclg2 | -0.7657271 |
| Suds3 | -0.5893933 |
| Sufu | -0.6071046 |
| Suhw2 | 0.3772515 |
| Suhw4 | 1.8460959 |
| Sulf1 | 1.3122948 |
| Sult1a1 | 0.0384734 |
| Sult1c2 | 2.4787935 |
| Sult1d1 | 0.4036837 |
| Sult1d1 | -0.6867403 |
| Sult2b1 | -0.4871497 |
| Sult2b1 | 0.1217791 |
| Sult4a1 | -0.0639079 |
| Sult5a1 | 0.6800037 |
| Sult5a1 | 2.1163532 |
| Sumf2 | 1.1854746 |
| Sumo1 | 0.1141361 |
| Sumo1 | 0.0373342 |
| Sumo1 | 1.0562333 |
| Sumo2 | 0.063547 |
| Sumo3 | -0.1985089 |
| Sumo3 | 0.7471896 |
| Sunc1 | 1.4928215 |
| Suox | -0.4133502 |
| Supt3h | -0.4864693 |
| Supt4h1 | 1.2215477 |
| Supt4h2 | 0.8146842 |
| Supt5h | -0.1250454 |
| Supt5h | 0.1014876 |
| Supt6h | -0.4810448 |
| Supv3l1 | 2.0055786 |
| Surf1 | -0.4008963 |
| Surf1 | 0.0893764 |
| Surf2 | 2.2752051 |
| Surf4 | -0.7437354 |
| Susd2 | -0.3687035 |
| Susd3 | 1.8395569 |
| Susd4 | 2.0022899 |
| Suv39h1 | -0.5955143 |
| Suv420h1 | 0.2059323 |
| Suv420h1 | 0.9458965 |
| Suv420h1 | 2.9513992 |
| Suv420h2 | 2.322411 |
| Suz12 | 1.4135222 |
| Sv2a | -0.83713 |
| Sv2a | 4.4531931 |
| Sv2b | 0.2814365 |
| Sval2 | 0.5966416 |
| Svep1 | 0.1520525 |
| Svil | 0.4485712 |
| Svil | 1.7467981 |
| Svop | -0.7347285 |
| Svopl | 0.08362 |
| Svs4 | 0.6025405 |
| Svs5 | -0.7196206 |
| Svs6 | 2.4044745 |
| Swap70 | -0.448515 |
| Swap70 | -0.3480586 |
| Swap70 | 0.9049741 |
| Syap1 | 2.1887164 |
| Syap1 | -0.6001325 |
| Sybl1 | -0.3675109 |
| Syce2 | -0.6833481 |
| Sycp1 | -0.6699057 |
| Syf2 | -0.6808123 |
| Syf2 | 0.8614935 |
| Syn1 | -0.2493003 |
| Syn1 | -0.2598119 |
| Syn2 | -0.3148567 |
| Sync | -0.5645752 |
| Syncrip | -0.7178598 |
| Syne1 | 1.7263319 |
| Syne1 | -0.5542498 |
| Syngr1 | -0.6117828 |
| Syngr1 | 2.6348924 |
| Syngr1 | 1.4195936 |
| Syngr1 | 2.6800279 |
| Syngr2 | -0.7134728 |
| Syngr2 | 0.1874713 |
| Syngr2 | -0.2368463 |
| Syngr3 | 0.9553977 |
| Syngr4 | 3.138483 |
| Syngr4 | -0.5985904 |
| Syngr4 | -0.6387625 |
| Synj2 | -0.524675 |
| Synj2bp | 2.2132145 |
| Synj2bp | 1.2867866 |
| Synpo2l | -0.7311508 |
| Syp | -0.3615717 |
| Sypl | -0.5705292 |
| Sypl | 1.4308075 |
| Sypl2 | 1.9563352 |
| Sys1 | -0.5742705 |
| Syt1 | 0.9992396 |
| Syt11 | -0.6649161 |
| Syt11 | -0.5586605 |
| Syt12 | -0.2094396 |
| Syt13 | 0.2779534 |
| Syt15 | -0.0977025 |
| Syt16 | -0.7482729 |
| Syt3 | 0.5195553 |
| Syt4 | 1.0273661 |
| Syt5 | 1.1379977 |
| Syt9 | 0.3577703 |
| Sytl2 | -0.6001325 |
| Sytl3 | 0.0828979 |
| Sytl3 | 0.4202215 |
| Sytl3 | 1.0511987 |
| Sytl4 | 0.1557244 |
| Sytl5 | 0.7693571 |
| Syvn1 | -0.5015575 |
| Taar2 | 0.0342892 |
| Taar3 | 1.6113016 |
| Taar4 | 1.0070333 |
| Tac1 | 0.0661267 |
| Tacc1 | -0.2860966 |
| Tacc1 | 0.436217 |
| Tacr3 | 0.9278597 |
| Tacr3 | -0.4209062 |
| Tacstd2 | -0.7601786 |
| Tada1l | 1.3631567 |
| Tada2l | -0.1268678 |
| Tada2l | -0.398426 |
| Tada3l | 0.0965204 |
| Tada3l | 2.4419156 |
| Taf10 | -0.2094396 |
| Taf11 | 1.407161 |
| Taf12 | -0.5105936 |
| Taf12 | -0.5833136 |
| Taf12 | 2.5878639 |
| Taf13 | -0.2731265 |
| Taf1c | 0.5393461 |
| Taf3 | 0.2582547 |
| Taf5 | -0.4803691 |
| Taf5 | 0.2468876 |
| Taf5 | -0.5078036 |
| Taf5l | -0.3716918 |
| Taf6 | 0.2788255 |
| Taf6 | 0.4094814 |
| Taf6l | -0.4342837 |
| Taf7l | 0.7265843 |
| Taf9 | -0.1780029 |
| Taf9 | -0.5750206 |
| Taf9 | 0.0850626 |
| Taf9b | -0.6740849 |
| Tagap | 2.8021847 |
| Tagap | 2.0727165 |
| Tagln | 2.7023147 |
| Tagln3 | 0.3166598 |
| Tal1 | -0.521134 |
| Tal2 | 0.6346044 |
| Taldo1 | 3.3174859 |
| Tank | -0.1196004 |
| Tank | -0.621213 |
| Tank | 0.6539595 |
| Tap1 | 0.6298965 |
| Tap2 | -0.7056296 |
| Tapbp | 1.8814801 |
| Tapbp | -0.3971935 |
| Tapbpl | 2.0676374 |
| Tapbpl | -0.5078036 |
| Tapt1 | -0.2265981 |
| Tarbp2 | -0.5022494 |
| Tarbp2 | -0.4960416 |
| Tardbp | -0.640375 |
| Tardbp | 2.6022565 |
| Tardbp | 0.9039594 |
| Tars | -0.682502 |
| Tarsl2 | 0.1951563 |
| Tarsl2 | 1.2118147 |
| Tas1r1 | 0.8933092 |
| Tasp1 | -0.5668034 |
| Tatdn2 | 1.0658555 |
| Tax1bp1 | 0.1259208 |
| Tax1bp3 | 1.3820241 |
| Tax1bp3 | 0.8101885 |
| Taz | -0.5346648 |
| Taz | -0.4459107 |
| Tbc1d1 | 0.7857142 |
| Tbc1d10a | -0.640375 |
| Tbc1d10a | 0.4435047 |
| Tbc1d10c | 2.1810667 |
| Tbc1d12 | 3.7143841 |
| Tbc1d13 | -0.5623526 |
| Tbc1d14 | 0.1365328 |
| Tbc1d15 | -0.5204274 |
| Tbc1d16 | -0.661605 |
| Tbc1d17 | 0.5204442 |
| Tbc1d19 | -0.6799686 |
| Tbc1d2 | 1.7615125 |
| Tbc1d20 | -0.5840713 |
| Tbc1d22a | -0.5630929 |
| Tbc1d22b | -0.5564524 |
| Tbc1d23 | 2.2007057 |
| Tbc1d24 | -0.7788083 |
| Tbc1d25 | 0.6651867 |
| Tbc1d2b | -0.7410238 |
| Tbc1d5 | -0.4420183 |
| Tbc1d5 | -0.6574832 |
| Tbc1d7 | -0.4669592 |
| Tbc1d7 | -0.6591297 |
| Tbc1d8 | -0.4146052 |
| Tbc1d9 | -0.2801287 |
| Tbc1d9 | -0.573521 |
| Tbc1d9b | -0.2466914 |
| Tbca | 4.5162867 |
| Tbcb | 0.7203807 |
| Tbcc | -0.358027 |
| Tbcd | 0.5237698 |
| Tbcel | -0.2904618 |
| Tbk1 | 1.8281017 |
| Tbkbp1 | -0.1823579 |
| Tbl1x | 2.0107226 |
| Tbl1xr1 | -0.6740849 |
| Tbl2 | 2.8250057 |
| Tbl3 | -0.685891 |
| Tbp | -0.0138725 |
| Tbpl1 | -0.6566611 |
| Tbr1 | -0.5411459 |
| Tbrg1 | -0.4643011 |
| Tbrg1 | -0.3307323 |
| Tbrg1 | -0.4891939 |
| Tbrg4 | 1.777035 |
| Tbrg4 | -0.5675473 |
| Tbx1 | 0.8114753 |
| Tbx19 | -0.7178598 |
| Tbx2 | 1.3008915 |
| Tbx22 | -0.5623526 |
| Tbx3 | 2.3478312 |
| Tbx3 | 1.0019751 |
| Tbx5 | 0.4844164 |
| Tbx6 | -0.5346648 |
| Tbxa2r | 1.3235554 |
| Tbxa2r | -0.7143485 |
| Tcam1 | 1.0623352 |
| Tcam1 | -0.7500943 |
| Tcam1 | -0.6188462 |
| Tcap | 1.0290084 |
| Tcea2 | 0.4384095 |
| Tcea3 | 0.1852174 |
| Tceal1 | -0.2910089 |
| Tceal1 | 0.1600447 |
| Tceal3 | -0.6436087 |
| Tceal5 | -0.5520525 |
| Tceal6 | 0.2579574 |
| Tceal6 | -0.3941194 |
| Tceal8 | -0.3861756 |
| Tceb1 | 0.4425364 |
| Tceb2 | -0.110598 |
| Tceb2 | 1.5611648 |
| Tceb3 | 0.0323809 |
| Tcerg1 | -0.2306834 |
| Tcf12 | -0.272054 |
| Tcf12 | 1.9788815 |
| Tcf15 | 1.5971829 |
| Tcf19 | -0.3318771 |
| Tcf20 | -0.2129456 |
| Tcf20 | 0.0350514 |
| Tcf20 | 0.7357228 |
| Tcf20 | -0.5112925 |
| Tcf20 | -0.7930382 |
| Tcf21 | -0.7082364 |
| Tcf23 | -0.7750514 |
| Tcf25 | -0.8192332 |
| Tcf25 | 0.0538997 |
| Tcf25 | -0.265646 |
| Tcf3 | 0.6124206 |
| Tcf3 | 0.8743251 |
| Tcf4 | -0.772244 |
| Tcf7 | -0.6484812 |
| Tcf7 | 0.1593814 |
| Tcf7l2 | 2.9228155 |
| Tcf7l2 | 1.7701383 |
| Tcfap2b | -0.4058572 |
| Tcfap2b | 0.8523244 |
| Tcfap2c | -0.560874 |
| Tcfap2c | 1.0208942 |
| Tcfcp2 | -0.6436087 |
| Tcfe3 | 0.9692617 |
| Tcfl5 | 1.2920783 |
| Tchp | 2.014403 |
| Tcirg1 | -0.4095959 |
| Tcirg1 | 2.6435781 |
| Tcirg1 | -0.7854209 |
| Tcirg1 | -0.4912427 |
| Tcl1 | 3.0122123 |
| Tcl1 | 0.3873513 |
| Tcl1b2 | 0.5085945 |
| Tcl1b2 | -0.5332309 |
| Tcl1b3 | 0.1719015 |
| Tcl1b4 | 0.406209 |
| Tcn2 | -0.2715183 |
| Tcof1 | -0.6086613 |
| Tcp1 | 0.7921418 |
| Tcp1 | -0.4330011 |
| Tcp10a | 0.7272906 |
| Tcp10a | 1.3766369 |
| Tcp11 | 0.049037 |
| Tcstv1 | -0.7519194 |
| Tcte3 | -0.644419 |
| Tctex1d1 | 0.9601791 |
| Tctex1d2 | -0.5133923 |
| Tctex1d4 | -0.4857895 |
| Tctn3 | -0.4310805 |
| Tctn3 | -0.6724109 |
| Tctn3 | 1.3165226 |
| Tdg | -0.4864693 |
| Tdp1 | -0.2311954 |
| Tdpoz1 | 1.6490232 |
| Tdpoz2 | 1.1437935 |
| Tdrd1 | -0.7082364 |
| Tdrd12 | 0.8051848 |
| Tdrd3 | 0.5878232 |
| Tdrd3 | -0.0276533 |
| Tdrd3 | 0.4811739 |
| Tdrd6 | -0.4810448 |
| Tdrd7 | 1.9711629 |
| Tdrkh | 0.3878662 |
| Tead2 | -0.1877094 |
| Tec | 0.5789174 |
| Tecta | 2.2733391 |
| Tef | 2.1750127 |
| Tef | -0.7419268 |
| Tegt | 0.6110158 |
| Tek | 1.4972579 |
| Tekt1 | -0.8472287 |
| Tekt1 | -0.1523067 |
| Tekt3 | -0.7788083 |
| Tenc1 | 3.2866767 |
| Tenc1 | -0.2353016 |
| Terf2 | -0.6339422 |
| Terf2 | -0.5029418 |
| Terf2ip | 1.3854278 |
| Terf2ip | 0.5162141 |
| Terf2ip | 1.2402046 |
| Terf2ip | -0.0763248 |
| Tes | -0.6952772 |
| Tes | -0.663259 |
| Tes | -0.8251594 |
| Tesc | 0.4626548 |
| Tesk1 | -0.6952772 |
| Tesp1 | 1.1695299 |
| Tesp2 | 0.6025405 |
| Tessp2 | 1.7168035 |
| Tessp3 | -0.3376228 |
| Tex10 | -0.5303699 |
| Tex10 | 0.5233271 |
| Tex101 | -0.1273239 |
| Tex11 | 2.6440815 |
| Tex13 | -0.6339422 |
| Tex14 | -0.3521498 |
| Tex16 | -0.1314399 |
| Tex19 | 0.7752331 |
| Tex2 | -0.8084689 |
| Tex2 | -0.6808123 |
| Tex22 | 1.4904062 |
| Tex24 | -0.6331414 |
| Tex261 | -0.524675 |
| Tex261 | 0.872825 |
| Tex264 | 1.5968472 |
| Tex264 | -0.6816567 |
| Tex9 | 0.0843416 |
| Tfam | -0.4603285 |
| Tfam | 0.5941893 |
| Tfb1m | -0.7082364 |
| Tfb2m | 2.6197894 |
| Tfdp1 | 1.9970543 |
| Tfdp2 | 0.935874 |
| Tfdp2 | 2.7733374 |
| Tfg | 3.4112642 |
| Tfip11 | 0.8517098 |
| Tfpi | 0.360693 |
| Tfpt | 1.3336878 |
| Tfpt | -0.2286384 |
| Tfpt | 1.8733688 |
| Tfrc | 0.012681 |
| Tfrc | -0.7347285 |
| Tg | 0.0512846 |
| Tgfb1 | -0.7240379 |
| Tgfb1i1 | 1.4575404 |
| Tgfb1i1 | -0.6927076 |
| Tgfb2 | -0.1429727 |
| Tgfb3 | -0.3109312 |
| Tgfbi | -0.6086613 |
| Tgfbi | 0.3622832 |
| Tgfbr1 | -0.240461 |
| Tgfbr1 | -0.4662939 |
| Tgfbr1 | -0.6995762 |
| Tgfbr2 | 2.3643494 |
| Tgfbr2 | -0.8482449 |
| Tgfbr3 | 1.1270857 |
| Tgfbr3 | 1.4651902 |
| Tgfbrap1 | -0.4133502 |
| Tgif1 | -0.6995762 |
| Tgif1 | -0.6533803 |
| Tgif1 | 0.5188881 |
| Tgif2 | 0.4456795 |
| Tgif2lx | 2.2348519 |
| Tgm2 | -0.7844733 |
| Tgm4 | 0.3788127 |
| Tgoln1 | -0.2109405 |
| Tgs1 | -0.5346648 |
| Tgs1 | -0.1926022 |
| Th1l | 1.2174624 |
| Tha1 | -0.6323412 |
| Thada | 2.8138933 |
| Thada | 0.1560575 |
| Thap1 | -0.3831388 |
| Thap1 | -0.4071017 |
| Thap1 | -0.7564985 |
| Thap11 | 0.9809204 |
| Thap3 | -0.4946678 |
| Thap4 | -0.6740849 |
| Thap7 | -0.7013016 |
| Thbd | 0.5608371 |
| Thbs1 | -0.7491831 |
| Thbs2 | 0.6312722 |
| Thbs3 | -0.1101503 |
| Thbs3 | -0.2839218 |
| Theg | -0.2205047 |
| Theg | 0.228642 |
| Theg | 0.4988646 |
| Them2 | 0.5954163 |
| Them4 | -0.663259 |
| Them5 | -0.5389803 |
| Thg1l | 0.489718 |
| Thnsl1 | 0.5705531 |
| Thnsl2 | -0.6492959 |
| Thnsl2 | 0.6589243 |
| Thoc1 | 1.2906639 |
| Thoc3 | -0.6032245 |
| Thoc4 | -0.2353016 |
| Thoc4 | 0.3654552 |
| Thoc5 | -0.1684 |
| Thoc6 | 2.3541624 |
| Thop1 | 0.5917304 |
| Thra | 0.5175521 |
| Thra | 1.1116751 |
| Thrap2 | 1.4278956 |
| Thrap3 | 0.6152237 |
| Thrsp | -0.1950587 |
| Thsd1 | 0.1692801 |
| Thsd4 | 2.8552073 |
| Thsd4 | -0.7099784 |
| Thsd7b | 0.9604515 |
| Thsd7b | 0.0445249 |
| Thsd7b | -0.5411459 |
| Thtpa | -0.597051 |
| Thumpd1 | 2.5902045 |
| Thumpd3 | -0.8636262 |
| Thy1 | 0.0990074 |
| Thyn1 | -0.6427992 |
| Tia1 | -0.623586 |
| Tial1 | -0.8065256 |
| Tial1 | 0.270067 |
| Tiam1 | -0.0815134 |
| Tiam2 | -0.2373618 |
| Ticam1 | 0.5415184 |
| Tie1 | 2.0116124 |
| Tigd2 | 1.4376457 |
| Tigd3 | 1.9494187 |
| Timeless | -0.9538429 |
| Timm10 | 0.1245424 |
| Timm13 | 2.72835 |
| Timm17a | -0.4946678 |
| Timm17b | 0.3855469 |
| Timm22 | 0.0697993 |
| Timm44 | 1.7416107 |
| Timm44 | 3.5482422 |
| Timm44 | -0.621213 |
| Timm50 | 3.4042108 |
| Timm8a1 | -0.6411824 |
| Timm8a2 | 1.693708 |
| Timm8a2 | -0.4590082 |
| Timm8b | -0.6267599 |
| Timm8b | -0.2189878 |
| Timm9 | 1.5199953 |
| Timm9 | 1.2274387 |
| Timm9 | -0.3261678 |
| Timp1 | 0.1169229 |
| Timp1 | 0.3074197 |
| Timp2 | -0.5204274 |
| Timp3 | 0.8466233 |
| Timp3 | -0.3663199 |
| Timp4 | -0.5425925 |
| Tinagl | -0.6016772 |
| Tipin | -0.1364961 |
| Tipin | -0.6125649 |
| Tirap | -0.4285261 |
| Tirap | 2.7249257 |
| Tirap | -0.5397016 |
| Tjap1 | -0.6039992 |
| Tjap1 | 1.0710499 |
| Tjap1 | -0.3539089 |
| Tjp1 | 3.9883425 |
| Tjp2 | -0.5653173 |
| Tjp3 | 0.7708717 |
| Tjp3 | 0.3960648 |
| Tk1 | -0.6476673 |
| Tk2 | 3.7894013 |
| Tkt | 2.1725726 |
| Tkt | 2.1247576 |
| Tkt | 2.9877886 |
| Tktl1 | -0.4851101 |
| Tktl1 | -0.1332752 |
| Tktl2 | 1.000934 |
| Tlcd1 | 1.0618486 |
| Tle1 | -0.7338327 |
| Tle1 | -0.6995762 |
| Tle2 | 1.3083247 |
| Tle3 | -0.3996603 |
| Tle3 | -0.4817211 |
| Tle3 | 0.3728133 |
| Tle4 | 0.2688927 |
| Tle4 | 1.5321989 |
| Tle4 | 1.7375425 |
| Tle6 | 2.8172899 |
| Tlk1 | -0.4272516 |
| Tlk2 | 0.9413167 |
| Tln1 | -0.3898332 |
| Tln2 | -0.1388039 |
| Tlr13 | 3.3657232 |
| Tlr2 | 0.1983392 |
| Tlr3 | -0.6816567 |
| Tlr4 | 0.5222194 |
| Tlr6 | 0.1172706 |
| Tlr7 | 2.6445636 |
| Tm2d2 | -0.4008963 |
| Tm2d3 | 1.6115659 |
| Tm2d3 | -0.477671 |
| Tm4sf4 | -0.7741146 |
| Tm4sf5 | 1.7491255 |
| Tm6sf1 | 0.2900855 |
| Tm6sf2 | 0.2426633 |
| Tm9sf2 | 1.70646 |
| Tm9sf2 | -0.6133477 |
| Tm9sf2 | 2.5157684 |
| Tm9sf3 | -0.5571878 |
| Tm9sf4 | 0.8357374 |
| Tm9sf4 | 2.2665701 |
| Tmbim1 | -0.671575 |
| Tmbim4 | -0.6188462 |
| Tmc1 | -0.597051 |
| Tmc4 | 0.2846175 |
| Tmc5 | -0.2937497 |
| Tmc6 | 1.6471837 |
| Tmc6 | -0.1631618 |
| Tmc6 | 3.0194949 |
| Tmc7 | 1.3115575 |
| Tmcc2 | 3.0351628 |
| Tmcc3 | 1.5147141 |
| Tmco3 | -0.3904443 |
| Tmco3 | -0.1901525 |
| Tmco4 | -0.5623526 |
| Tmco4 | -0.7030303 |
| Tmco5 | -0.5161998 |
| Tmco6 | 0.907 |
| Tmco6 | -0.5893933 |
| Tmco7 | -0.7930382 |
| Tmed1 | 0.839017 |
| Tmed1 | -0.6063272 |
| Tmed10 | -0.6110014 |
| Tmed2 | -0.2704477 |
| Tmed3 | -0.7178598 |
| Tmed3 | 2.2051906 |
| Tmed4 | 1.0375503 |
| Tmed4 | -0.7901742 |
| Tmed4 | 3.0211513 |
| Tmed4 | -0.6164856 |
| Tmed8 | 1.2931141 |
| Tmed8 | 2.1032152 |
| Tmed9 | -0.3358952 |
| Tmed9 | -0.5645752 |
| Tmeff2 | -0.4939818 |
| Tmem10 | -0.2801287 |
| Tmem100 | 1.4329853 |
| Tmem101 | -0.7320439 |
| Tmem102 | -0.5098953 |
| Tmem102 | -0.5527843 |
| Tmem106a | -0.6558398 |
| Tmem106c | 1.518165 |
| Tmem107 | 2.4494183 |
| Tmem107 | -0.4939818 |
| Tmem107 | -0.2024684 |
| Tmem108 | 0.5002286 |
| Tmem109 | 3.8637766 |
| Tmem11 | 1.2417005 |
| Tmem110 | -0.6204233 |
| Tmem111 | 0.9643929 |
| Tmem111 | -0.2296603 |
| Tmem111 | 1.8682184 |
| Tmem115 | -0.1369572 |
| Tmem116 | -0.2220242 |
| Tmem116 | -0.8351238 |
| Tmem117 | -0.3047969 |
| Tmem118 | -0.7959114 |
| Tmem119 | 1.3198175 |
| Tmem120a | 1.3252824 |
| Tmem120b | 1.5741355 |
| Tmem121 | 0.5646515 |
| Tmem125 | -0.4202741 |
| Tmem126a | 1.581474 |
| Tmem126b | 0.1429756 |
| Tmem127 | -0.5802891 |
| Tmem128 | 0.3601623 |
| Tmem130 | 0.2118985 |
| Tmem131 | 0.7397354 |
| Tmem132a | 0.7323945 |
| Tmem132a | 0.2190681 |
| Tmem132c | 0.5843128 |
| Tmem132d | 1.1475629 |
| Tmem132e | -0.6944198 |
| Tmem134 | -0.1360353 |
| Tmem134 | 1.3927869 |
| Tmem135 | -0.6901453 |
| Tmem136 | 1.5612347 |
| Tmem138 | 1.6233133 |
| Tmem139 | 0.1081854 |
| Tmem140 | -0.3861756 |
| Tmem141 | -0.1155382 |
| Tmem143 | -0.0673161 |
| Tmem143 | 0.8696665 |
| Tmem143 | -0.6196344 |
| Tmem143 | -0.2461706 |
| Tmem144 | 0.903234 |
| Tmem146 | 2.5065318 |
| Tmem147 | -0.2524409 |
| Tmem14c | 0.5148742 |
| Tmem14c | 0.1771214 |
| Tmem14c | 1.5676389 |
| Tmem150 | 1.4881359 |
| Tmem150 | 0.5303842 |
| Tmem151a | -0.1579539 |
| Tmem154 | -0.5325148 |
| Tmem158 | -0.7169807 |
| Tmem159 | 1.7278425 |
| Tmem159 | -0.3492256 |
| Tmem160 | -0.5825565 |
| Tmem160 | -0.4439624 |
| Tmem161a | -0.8311248 |
| Tmem162 | -0.2693785 |
| Tmem164 | 1.6405559 |
| Tmem166 | -0.1268678 |
| Tmem167 | -0.09242 |
| Tmem168 | -0.0333896 |
| Tmem168 | -0.7082364 |
| Tmem168 | -0.4967292 |
| Tmem169 | 2.6029591 |
| Tmem16a | 1.337727 |
| Tmem16b | 1.5516127 |
| Tmem16d | -0.0596656 |
| Tmem16e | -0.0588195 |
| Tmem16g | -0.3086957 |
| Tmem16j | 1.8702523 |
| Tmem16k | -0.1679226 |
| Tmem17 | 1.5113975 |
| Tmem17 | -0.3898332 |
| Tmem170 | 2.1061938 |
| Tmem176a | -0.6204233 |
| Tmem176a | 2.2304795 |
| Tmem176b | -0.5303699 |
| Tmem177 | 0.668961 |
| Tmem178 | 1.7061627 |
| Tmem179b | -0.5623526 |
| Tmem180 | 0.2021438 |
| Tmem183a | 1.2179741 |
| Tmem183a | -0.5513212 |
| Tmem184a | -0.64523 |
| Tmem184a | 0.5996976 |
| Tmem184b | 0.5867921 |
| Tmem185b | 0.1446634 |
| Tmem186 | -0.7284769 |
| Tmem19 | 0.2943791 |
| Tmem192 | -0.4285261 |
| Tmem198 | 0.9666953 |
| Tmem199 | 1.7863847 |
| Tmem2 | 2.0035726 |
| Tmem2 | 0.6208038 |
| Tmem2 | 0.7802393 |
| Tmem201 | -0.547674 |
| Tmem204 | -0.6699057 |
| Tmem205 | -0.0941773 |
| Tmem208 | -0.6251716 |
| Tmem209 | -0.5382596 |
| Tmem209 | -0.2451298 |
| Tmem23 | 0.2347651 |
| Tmem25 | 0.0968761 |
| Tmem25 | -0.2545407 |
| Tmem25 | -0.4953544 |
| Tmem27 | 0.2046712 |
| Tmem32 | 0.2267969 |
| Tmem33 | -0.470293 |
| Tmem33 | 0.5950075 |
| Tmem34 | 0.8788106 |
| Tmem35 | 0.8734254 |
| Tmem38a | -0.5947468 |
| Tmem38b | 0.9575866 |
| Tmem38b | 0.0631778 |
| Tmem39a | 1.0444004 |
| Tmem40 | 0.5279646 |
| Tmem41a | 0.3133107 |
| Tmem41b | 1.7541008 |
| Tmem42 | -0.4763251 |
| Tmem43 | -0.6867403 |
| Tmem43 | 2.6985133 |
| Tmem44 | -0.3428256 |
| Tmem45a | 1.1058999 |
| Tmem45b | -0.7374211 |
| Tmem47 | 1.7452052 |
| Tmem47 | 3.0430571 |
| Tmem48 | -0.272054 |
| Tmem49 | -0.2225313 |
| Tmem49 | -0.6782836 |
| Tmem49 | -0.3182351 |
| Tmem49 | 0.5443347 |
| Tmem5 | -0.4285261 |
| Tmem50a | -0.3813217 |
| Tmem50b | -0.7311508 |
| Tmem50b | -0.6355461 |
| Tmem51 | 0.6594955 |
| Tmem53 | 0.2892244 |
| Tmem53 | -0.0673161 |
| Tmem53 | 0.1676379 |
| Tmem54 | 0.3614885 |
| Tmem55a | -0.2044547 |
| Tmem55b | -0.7428306 |
| Tmem56 | 2.2241484 |
| Tmem57 | -0.2932009 |
| Tmem59 | 1.1812219 |
| Tmem59l | -0.4272516 |
| Tmem60 | 0.9436789 |
| Tmem62 | -0.5750206 |
| Tmem63a | 0.2564694 |
| Tmem63b | -0.1273239 |
| Tmem63b | 0.0040481 |
| Tmem63c | -0.5498606 |
| Tmem65 | 3.2641396 |
| Tmem66 | 0.0016792 |
| Tmem66 | -0.5893933 |
| Tmem66 | -0.3154189 |
| Tmem68 | 1.1437935 |
| Tmem69 | -0.0256134 |
| Tmem70 | 0.4254204 |
| Tmem71 | 2.7131185 |
| Tmem74 | -0.3330233 |
| Tmem74 | -0.6387625 |
| Tmem74 | 2.1747301 |
| Tmem77 | 0.0350514 |
| Tmem77 | 2.5166385 |
| Tmem77 | -0.8261509 |
| Tmem79 | -0.4783448 |
| Tmem8 | 0.2796966 |
| Tmem8 | -0.6476673 |
| Tmem8 | 0.9170616 |
| Tmem81 | -0.2124439 |
| Tmem82 | 0.3839973 |
| Tmem85 | 0.2109591 |
| Tmem85 | 2.3657504 |
| Tmem86a | 0.8088999 |
| Tmem86b | -0.7383204 |
| Tmem87a | -0.5339476 |
| Tmem88 | -0.6133477 |
| Tmem9 | -0.7222684 |
| Tmem90a | 0.0103348 |
| Tmem91 | -0.569037 |
| Tmem93 | 0.7914847 |
| Tmem97 | 1.493575 |
| Tmem98 | 3.8124208 |
| Tmem98 | 0.6196109 |
| Tmem9b | 2.6594866 |
| Tmem9b | 1.8205926 |
| Tmem9b | -0.4027534 |
| Tmie | 2.8979392 |
| Tmod1 | -0.5513212 |
| Tmod2 | -0.3801123 |
| Tmod2 | -0.1119427 |
| Tmod3 | -0.6016772 |
| Tmod4 | 2.9299402 |
| Tmod4 | 0.968317 |
| Tmod4 | 2.4387599 |
| Tmpo | -0.4871497 |
| Tmpo | 2.2772249 |
| Tmpo | 2.6508082 |
| Tmprss11a | -0.7685143 |
| Tmprss11c | -0.4629749 |
| Tmprss13 | -0.5183111 |
| Tmprss13 | -0.4550589 |
| Tmprss2 | 0.0403689 |
| Tmprss4 | -0.6259654 |
| Tmprss4 | -0.483753 |
| Tmprss4 | 0.3348601 |
| Tmprss7 | -0.4857895 |
| Tmprss9 | 0.5559394 |
| Tmsb10 | -0.5105936 |
| Tmsb10 | -0.070309 |
| Tmsb10 | 0.9033791 |
| Tmsb4x | 0.2540835 |
| Tmtc2 | -0.5697828 |
| Tmtc4 | -0.5425925 |
| Tmub1 | 0.1964308 |
| Tmub1 | 0.671218 |
| Tnc | 0.1269533 |
| Tnc | 1.4564423 |
| Tnc | 1.9944682 |
| Tnc | -0.2301717 |
| Tnfaip1 | 1.1360949 |
| Tnfaip2 | -0.0977025 |
| Tnfaip2 | -0.4209062 |
| Tnfaip2 | 1.250328 |
| Tnfaip3 | -0.6323412 |
| Tnfaip8 | 1.1319393 |
| Tnfaip8l2 | 2.5392548 |
| Tnfaip8l2 | 1.6562808 |
| Tnfaip8l3 | -0.7004385 |
| Tnfrsf10b | 0.9200582 |
| Tnfrsf10b | -0.4932962 |
| Tnfrsf10b | 1.9602893 |
| Tnfrsf11a | 1.7686972 |
| Tnfrsf11b | 0.4993195 |
| Tnfrsf12a | -0.7778676 |
| Tnfrsf13c | -0.7222684 |
| Tnfrsf19 | 0.7308136 |
| Tnfrsf19 | 1.7914833 |
| Tnfrsf1a | -0.2812108 |
| Tnfrsf1a | -0.5527843 |
| Tnfrsf1a | -0.5411459 |
| Tnfrsf1b | 1.5163311 |
| Tnfrsf21 | 0.7413007 |
| Tnfrsf21 | -0.6850426 |
| Tnfrsf22 | -0.1760741 |
| Tnfrsf22 | -0.5818001 |
| Tnfrsf25 | 1.5724123 |
| Tnfrsf4 | -0.630743 |
| Tnfsf13b | 0.2353751 |
| Tnfsf13b | 0.5625344 |
| Tnip1 | -0.078483 |
| Tnip1 | -0.7169807 |
| Tnip1 | -0.1804198 |
| Tnip1 | 2.7733374 |
| Tnip2 | 3.0188039 |
| Tnks | -0.5901561 |
| Tnks1bp1 | -0.4127234 |
| Tnnc1 | 0.055763 |
| Tnnc2 | 0.1338062 |
| Tnni2 | 1.619851 |
| Tnni3 | 1.1319393 |
| Tnnt1 | 1.6804667 |
| Tnp1 | 0.7653055 |
| Tnp2 | 3.5791036 |
| Tnpo1 | 0.5386934 |
| Tnpo2 | -0.4932962 |
| Tnr | 0.5711831 |
| Tnrc15 | -0.6583061 |
| Tnrc18 | -0.6102207 |
| Tnrc18 | -0.675762 |
| Tnrc18 | -0.7401218 |
| Tnrc6a | 1.9800226 |
| Tnrc6a | 0.3638706 |
| Tnrc6b | -0.7437354 |
| Tnrc6b | -0.7161024 |
| Tnrc6c | -0.1645874 |
| Tns4 | 2.7872688 |
| Tnxb | 0.7941104 |
| Tnxb | -0.6657458 |
| Tnxb | 4.0182334 |
| Tob1 | 0.0900933 |
| Tob2 | 0.035813 |
| Toe1 | -0.4058572 |
| Toe1 | -0.4696252 |
| Tollip | 2.9665747 |
| Tollip | 1.0259741 |
| Tom1 | 1.7053893 |
| Tom1l2 | 1.6935874 |
| Tom1l2 | -0.3358952 |
| Tom1l2 | -0.44982 |
| Tomm22 | 2.4903101 |
| Tomm34 | -0.3364707 |
| Tomm34 | 1.4588721 |
| Tomm7 | 0.6308794 |
| Tomm70a | -0.519016 |
| Top1 | -0.5353826 |
| Top1mt | -0.5268064 |
| Top2a | -0.6690721 |
| Top3a | 1.0397979 |
| Top3b | -0.3825327 |
| Topors | 2.1984031 |
| Tor1a | -0.4563734 |
| Tor1a | -0.4446114 |
| Tor1aip1 | -0.0707374 |
| Tor1aip2 | -0.6331414 |
| Tor1aip2 | -0.3003617 |
| Tor1b | 2.3392216 |
| Tor3a | 0.1193543 |
| Tox3 | 0.8520172 |
| Tpbg | 1.8120164 |
| Tpcn1 | 2.0350618 |
| Tpcn1 | -0.5282301 |
| Tpcn2 | 1.1319393 |
| Tpd52 | -0.6740849 |
| Tpd52l1 | -0.4709612 |
| Tpd52l1 | -0.7675843 |
| Tpd52l2 | -0.5418689 |
| Tph2 | -0.1141882 |
| Tpi1 | 1.5652774 |
| Tpi1 | 1.8116463 |
| Tpi1 | 0.1228163 |
| Tpk1 | -0.3928927 |
| Tpk1 | -0.0677431 |
| Tpk1 | -0.6436087 |
| Tpm1 | 1.5341448 |
| Tpm2 | -0.4368546 |
| Tpm2 | 1.6309744 |
| Tpm3 | -0.8094422 |
| Tpm3 | -0.4452608 |
| Tpm4 | 0.0407475 |
| Tpo | 1.4760837 |
| Tpp1 | 0.2715326 |
| Tpp2 | 1.3103581 |
| Tppp | 0.2546806 |
| Tppp | -0.7117239 |
| Tppp3 | -0.6791257 |
| Tpr | -0.6682394 |
| Tpra40 | -0.5705292 |
| Tprgl | -0.6196344 |
| Tprgl | 1.6875367 |
| Tprkb | -0.5833136 |
| Tprkb | 2.8877609 |
| Tpsab1 | 2.168064 |
| Tpst1 | 1.9009414 |
| Tpst2 | -0.6944198 |
| Tpte | -0.5397016 |
| Tpte | -0.1452972 |
| Tpx2 | -0.5848297 |
| Tra2a | -0.4027534 |
| Trabd | 0.184895 |
| Trabd | -0.7073666 |
| Traf1 | -0.7187398 |
| Traf2 | -0.7657271 |
| Traf3 | 1.1565894 |
| Traf3 | -0.2311954 |
| Traf3ip1 | -0.5339476 |
| Traf3ip2 | 1.7216095 |
| Traf4 | -0.0724529 |
| Traf4 | 2.4053771 |
| Traf4 | 0.3808901 |
| Traf4 | 3.0125326 |
| Traf5 | 0.9253139 |
| Traf6 | -0.7356251 |
| Traf6 | -0.6355461 |
| Traf6 | -0.5924485 |
| Traf7 | 1.903858 |
| Trafd1 | -0.7329379 |
| Trafd1 | -0.6094407 |
| Trafd1 | -0.8605288 |
| Traip | 1.0087131 |
| Trak1 | -0.4064792 |
| Trak2 | -0.6918527 |
| Trak2 | 0.4087273 |
| Trak2 | 0.9117573 |
| Tram1 | -0.6791257 |
| Tram1 | 0.0796411 |
| Tram1 | -0.6927076 |
| Tram2 | -0.4336422 |
| Tram2 | -0.4349258 |
| Trap1 | -0.4349258 |
| Trappc1 | 0.4046946 |
| Trappc1 | 0.9048292 |
| Trappc2 | 1.0500898 |
| Trappc2l | 0.4645476 |
| Trappc3 | 2.6229291 |
| Trappc3 | -0.7844733 |
| Trappc3 | -0.4215387 |
| Trappc3 | 1.0745363 |
| Trappc4 | -0.5043282 |
| Trappc4 | -0.3255988 |
| Trappc5 | -0.6649161 |
| Trappc6a | 0.7837272 |
| Trat1 | 2.1813823 |
| Trcg1 | -0.2134476 |
| Trdmt1 | -0.6961353 |
| Trdmt1 | 1.6893562 |
| Trem2 | -0.5742705 |
| Treml1 | 0.7639509 |
| Treml2 | 1.756072 |
| Treml4 | -0.6172717 |
| Trerf1 | 3.7630305 |
| Trerf1 | 1.0589233 |
| Trex1 | -0.6799686 |
| Trex1 | 0.3144284 |
| Trex2 | -0.3053528 |
| Trf | 0.4202215 |
| Trfp | -0.5346648 |
| Trfr2 | -0.6149153 |
| Trfr2 | 0.2920917 |
| Trh | 1.3316164 |
| Trhde | -0.1843002 |
| Trhr | -0.468958 |
| Triap1 | -0.1141882 |
| Trib2 | 0.9548496 |
| Trib3 | -0.2383937 |
| Trib3 | -0.6379573 |
| Trim11 | 3.4938634 |
| Trim16 | 1.5991273 |
| Trim17 | -0.4844313 |
| Trim2 | -0.6690721 |
| Trim2 | -0.7675843 |
| Trim21 | 0.1893987 |
| Trim21 | 1.7476503 |
| Trim23 | 0.3725515 |
| Trim23 | -0.6427992 |
| Trim23 | -0.4530907 |
| Trim25 | -0.6363491 |
| Trim26 | -0.7555808 |
| Trim28 | -0.7338327 |
| Trim3 | 2.2880665 |
| Trim3 | -0.3261678 |
| Trim30 | -0.7161024 |
| Trim32 | -0.6657458 |
| Trim32 | 0.8553912 |
| Trim33 | 1.5624913 |
| Trim33 | 0.5166603 |
| Trim33 | 0.065022 |
| Trim35 | -0.0491466 |
| Trim36 | -0.4459107 |
| Trim36 | -0.6164856 |
| Trim36 | 0.2005605 |
| Trim37 | 0.4239381 |
| Trim38 | 1.1329519 |
| Trim38 | -0.6094407 |
| Trim39 | 1.9699218 |
| Trim40 | 0.6416203 |
| Trim41 | 1.1579028 |
| Trim42 | -0.5469463 |
| Trim44 | 0.7143131 |
| Trim45 | -0.6039992 |
| Trim45 | 1.4350774 |
| Trim46 | 1.8033577 |
| Trim47 | 0.2408468 |
| Trim54 | -0.4439624 |
| Trim56 | -0.6740849 |
| Trim59 | 0.1120403 |
| Trim61 | 0.2087633 |
| Trim62 | 0.5778761 |
| Trim67 | -0.5682918 |
| Trim68 | 2.1816979 |
| Trim69 | 0.0579939 |
| Trim72 | -0.4511268 |
| Trim8 | -0.0179038 |
| Trim9 | -0.5078036 |
| Trim9 | -0.6243784 |
| Trim9 | 0.7521822 |
| Trim9 | -0.5557176 |
| Triml1 | -0.1420447 |
| Triobp | -0.0054652 |
| Triobp | -0.7205024 |
| Trip10 | 0.1800444 |
| Trip12 | -0.4368546 |
| Trip12 | 3.2519198 |
| Trip12 | 1.402006 |
| Trip6 | -0.6227943 |
| Trip6 | 0.0774633 |
| Trit1 | -0.5176068 |
| Trmt1 | 1.7891545 |
| Trmt12 | 1.5087354 |
| Trmu | -0.6649161 |
| Tro | -0.6533803 |
| Tro | 0.418233 |
| Tro | 0.0639159 |
| Trove2 | 0.1580533 |
| Trp53 | 0.1715742 |
| Trp53bp1 | -0.7082364 |
| Trp53bp1 | -0.4304412 |
| Trp53bp2 | -0.5057167 |
| Trp53i11 | -0.1857597 |
| Trp53i11 | -0.44982 |
| Trp53inp1 | -0.4349258 |
| Trp53inp1 | 1.6045363 |
| Trp53inp2 | 0.6549164 |
| Trp53rk | -0.1664917 |
| Trp63 | -0.815304 |
| Trpc1 | -0.1999917 |
| Trpc1 | -0.5542498 |
| Trpc2 | 0.7049534 |
| Trpc3 | -0.4643011 |
| Trpc4 | -0.0333896 |
| Trpc4ap | -0.5742705 |
| Trpc4ap | -0.6850426 |
| Trpc5 | -0.5225487 |
| Trpc6 | 0.022778 |
| Trpc7 | 0.94076 |
| Trpd52l3 | -0.7510064 |
| Trpm1 | -0.1645874 |
| Trpm2 | -0.6782836 |
| Trpm3 | -0.0746019 |
| Trpm3 | -0.2029645 |
| Trpm3 | -0.64523 |
| Trpm3 | -0.7455476 |
| Trpm3 | 2.0806347 |
| Trpm4 | -0.7844733 |
| Trpm6 | -0.569037 |
| Trpm6 | -0.1551259 |
| Trpm6 | 1.2916071 |
| Trpm8 | 0.4286238 |
| Trps1 | -0.7125979 |
| Trpt1 | 2.0220959 |
| Trpt1 | 0.3772515 |
| Trpt1 | -0.5593977 |
| Trpv2 | 0.8129207 |
| Trpv4 | -0.1007983 |
| Trpv6 | 0.3855469 |
| Trrap | 1.7455467 |
| Trub1 | 3.3018448 |
| Trub1 | 4.1990324 |
| Trub1 | -0.5389803 |
| Trub2 | 1.9542162 |
| Tsc1 | -0.2184828 |
| Tsc2 | -0.5404234 |
| Tsc2 | 0.2966606 |
| Tsc22d1 | 0.8387052 |
| Tsc22d1 | 0.6239769 |
| Tsc22d2 | -0.6875903 |
| Tsc22d3 | 0.7062191 |
| Tsc22d3 | -0.6909986 |
| Tsc22d4 | -0.3675109 |
| Tsen2 | 1.315697 |
| Tsen34 | -0.1741495 |
| Tsen54 | -0.5126918 |
| Tsfm | -0.6016772 |
| Tsg101 | -0.7178598 |
| Tsga10 | 2.7576911 |
| Tsga10 | -0.7731788 |
| Tsga14 | -0.761101 |
| Tsga8 | -0.8172665 |
| Tshb | 1.3302628 |
| Tshr | 0.3034708 |
| Tshz3 | -0.5878695 |
| Tsku | -0.3182351 |
| Tsn | -0.7347285 |
| Tsnaxip1 | 1.4617637 |
| Tsnaxip1 | -0.6484812 |
| Tspan1 | -0.0211419 |
| Tspan11 | -0.7901742 |
| Tspan12 | -0.5036347 |
| Tspan13 | -0.8492621 |
| Tspan14 | -0.092859 |
| Tspan17 | 1.253874 |
| Tspan17 | -0.0142749 |
| Tspan18 | 0.7233992 |
| Tspan18 | 1.2446849 |
| Tspan2 | -0.0130685 |
| Tspan3 | -0.1374185 |
| Tspan31 | 0.4124916 |
| Tspan33 | -0.4817211 |
| Tspan6 | 0.5972537 |
| Tspan6 | 1.5448085 |
| Tspan7 | -0.1631618 |
| Tspan8 | -0.7446411 |
| Tspo | -0.1052395 |
| Tspo | 2.7264769 |
| Tspo | -0.7030303 |
| Tspyl1 | 3.0455435 |
| Tspyl3 | -0.0884783 |
| Tsr1 | 1.3163392 |
| Tsr2 | -0.2435708 |
| Tsr2 | 1.5385258 |
| Tsr2 | 0.141623 |
| Tssc1 | -0.4368546 |
| Tssc1 | -0.6867403 |
| Tssc1 | -0.2399437 |
| Tssc1 | 1.6512382 |
| Tssc1 | 0.3572379 |
| Tssc4 | 1.3694035 |
| Tssc4 | -0.6001325 |
| Tssk4 | -0.4139775 |
| Tssk5 | 1.3445077 |
| Tssk6 | -0.5638338 |
| Tst | -0.0211419 |
| Tsta3 | -0.3255988 |
| Tsx | -0.1950587 |
| Ttbk2 | -0.629945 |
| Ttc1 | -0.7038959 |
| Ttc12 | -0.6149153 |
| Ttc13 | 0.6229865 |
| Ttc14 | -0.5375395 |
| Ttc16 | -0.2893685 |
| Ttc17 | 0.4097326 |
| Ttc19 | 3.8074219 |
| Ttc19 | 0.8504794 |
| Ttc21a | 1.2129471 |
| Ttc22 | 0.574745 |
| Ttc23 | -0.1799359 |
| Ttc24 | 0.774396 |
| Ttc24 | 1.4395671 |
| Ttc26 | 0.7824001 |
| Ttc27 | 2.4902106 |
| Ttc29 | 1.4760069 |
| Ttc29 | -0.4769978 |
| Ttc3 | 2.1138619 |
| Ttc3 | -0.5029418 |
| Ttc3 | -0.2953981 |
| Ttc3 | -0.7205024 |
| Ttc3 | 0.1361924 |
| Ttc3 | 1.7212587 |
| Ttc30a1 | 0.1813404 |
| Ttc30a2 | -0.2731265 |
| Ttc33 | -0.4511268 |
| Ttc33 | -0.0070605 |
| Ttc33 | 1.6860177 |
| Ttc35 | 2.0467038 |
| Ttc4 | 3.1894363 |
| Ttc5 | -0.6141311 |
| Ttc5 | 0.7454616 |
| Ttc5 | 0.4214621 |
| Ttc7 | -0.2265981 |
| Ttc8 | -0.6724109 |
| Ttc8 | 1.3220074 |
| Ttf2 | -0.0990279 |
| Ttk | -0.7240379 |
| Ttl | -0.01508 |
| Ttll1 | 0.8501715 |
| Ttll1 | 0.3462534 |
| Ttll1 | 0.7433836 |
| Ttll10 | 0.1973855 |
| Ttll11 | -0.3358952 |
| Ttll13 | -0.4844313 |
| Ttll4 | 3.3682021 |
| Ttll5 | -0.6204233 |
| Ttll5 | 1.1613959 |
| Ttll6 | 2.7482906 |
| Ttll8 | 2.8140843 |
| Ttll9 | 0.7675024 |
| Ttn | 1.6530064 |
| Ttn | -0.2667109 |
| Ttr | -0.0441408 |
| Ttyh1 | 0.388638 |
| Ttyh1 | 2.8600861 |
| Ttyh2 | -0.3210602 |
| Ttyh2 | 0.0661267 |
| Ttyh3 | -0.7013016 |
| Tub | -0.5520525 |
| Tub | -0.6533803 |
| Tuba1a | -0.36098 |
| Tuba1b | -0.623586 |
| Tuba3a | -0.6909986 |
| Tuba3a | 1.5129467 |
| Tuba3a | -0.7638738 |
| Tuba3a | -0.2942988 |
| Tuba3b | 3.4316881 |
| Tuba4a | 0.4273931 |
| Tuba6 | -0.4905592 |
| Tuba8 | 3.2656647 |
| Tubb2b | -0.4387875 |
| Tubb2b | 0.0800035 |
| Tubb2b | -0.3758922 |
| Tubb2b | 1.8761791 |
| Tubb2b | 2.7100644 |
| Tubb2c | -0.6774423 |
| Tubb4 | -0.7816364 |
| Tubb5 | -0.6125649 |
| Tubb6 | 1.7452621 |
| Tubd1 | 2.2142969 |
| Tubd1 | -0.4939818 |
| Tube1 | -0.8084689 |
| Tubg1 | -0.6884412 |
| Tubgcp2 | 1.3349464 |
| Tubgcp4 | 1.2847941 |
| Tufm | -0.6541994 |
| Tuft1 | -0.3789045 |
| Tug1 | 1.1581215 |
| Tulp1 | -0.5008661 |
| Tulp2 | 2.122589 |
| Tulp3 | 2.55033 |
| Tulp4 | 1.4689135 |
| Tusc2 | -0.6347438 |
| Tusc2 | 1.0545174 |
| Tusc4 | 0.6017292 |
| Tut1 | -0.7713101 |
| Twf2 | 0.9183471 |
| Twist1 | -0.7620243 |
| Twist2 | -0.5296561 |
| Twistnb | 1.2694668 |
| Twistnb | 1.1947606 |
| Twsg1 | -0.628351 |
| Txk | -0.6541994 |
| Txlna | -0.6841949 |
| Txn1 | 1.8106938 |
| Txn2 | 0.4593328 |
| Txndc1 | -0.5057167 |
| Txndc11 | 1.299958 |
| Txndc11 | -0.3586168 |
| Txndc11 | 3.6184137 |
| Txndc12 | 1.7900218 |
| Txndc13 | -0.0002997 |
| Txndc13 | 2.769661 |
| Txndc15 | 1.7557906 |
| Txndc2 | -0.7125979 |
| Txndc5 | -0.7284769 |
| Txndc8 | -0.5962823 |
| Txndc9 | 0.0781898 |
| Txnip | 1.6970782 |
| Txnl2 | 0.402165 |
| Txnl4 | -0.6509274 |
| Txnl4a | 2.2733707 |
| Txnl4a | -0.5978204 |
| Txnl4a | 1.2332913 |
| Txnl4b | -0.0959382 |
| Txnrd2 | -0.7939949 |
| Txnrd3 | -0.7004385 |
| Tyk2 | 1.3983297 |
| Tyk2 | 1.5933487 |
| Tyk2 | 1.5348645 |
| Tyki | 0.379852 |
| Tyms | -0.0358593 |
| Tyms | 0.07163 |
| Tyms | -0.6347438 |
| Tyms-ps | 0.3795923 |
| Tyro3 | -0.6063272 |
| Tyrobp | 1.5657641 |
| Tyw1 | -0.2571724 |
| Tyw1 | 0.3955546 |
| U2af1l4 | 2.8963881 |
| U2af2 | 0.3633418 |
| U2af2 | -0.5176068 |
| Uaca | 0.049412 |
| Uap1 | 0.1252319 |
| Uap1l1 | -0.4537463 |
| Uba1 | 0.102195 |
| Uba1 | -0.6657458 |
| Uba2 | -0.7920825 |
| Uba5 | 0.1890778 |
| Ubac1 | -0.7347285 |
| Ubac1 | 1.6411953 |
| Ubac1 | -0.6699057 |
| Ubac2 | 2.4354874 |
| Ubap1 | 1.9738168 |
| Ubap2 | -0.6607792 |
| Ubap2l | -0.1916215 |
| Ubap2l | 0.8979933 |
| Ubap2l | -0.7601786 |
| Ubb | 3.5552313 |
| Ubd | -0.2215174 |
| Ube1c | -0.7592572 |
| Ube1l | -0.2603407 |
| Ube1x | -0.3474756 |
| Ube2a | 2.0060475 |
| Ube2b | 1.5158903 |
| Ube2d1 | -0.6699057 |
| Ube2d2 | -0.6071046 |
| Ube2d3 | -0.7930382 |
| Ube2e1 | -0.3014685 |
| Ube2e2 | -0.3284471 |
| Ube2e3 | 3.2585113 |
| Ube2f | -0.6387625 |
| Ube2f | -0.2555925 |
| Ube2f | 0.6685843 |
| Ube2g1 | 1.2804142 |
| Ube2g1 | -0.5140934 |
| Ube2g1 | -0.4763251 |
| Ube2g2 | -0.5154971 |
| Ube2i | 0.3163812 |
| Ube2j1 | -0.2513928 |
| Ube2j2 | 2.4839442 |
| Ube2j2 | 0.1158789 |
| Ube2j2 | 0.7334469 |
| Ube2k | 2.1473271 |
| Ube2k | 1.050213 |
| Ube2l3 | 2.0205039 |
| Ube2l3 | 0.4396253 |
| Ube2l6 | 0.7656438 |
| Ube2m | 0.0745514 |
| Ube2n | -0.5848297 |
| Ube2n | -0.7873192 |
| Ube2o | -0.2582273 |
| Ube2q1 | -0.5183111 |
| Ube2q1 | -0.6833481 |
| Ube2q2 | -0.4355682 |
| Ube2q2 | 1.693708 |
| Ube2q2 | -0.5750206 |
| Ube2r2 | 1.775825 |
| Ube2s | 1.8861659 |
| Ube2t | 0.2501929 |
| Ube2u | 1.6084558 |
| Ube2u | -0.8553901 |
| Ube3a | 0.0800035 |
| Ube3a | -0.3722907 |
| Ube3b | -0.661605 |
| Ube3b | -0.3261678 |
| Ube3c | -0.6640872 |
| Ube4a | -0.3675109 |
| Ube4b | 0.4046946 |
| Ubfd1 | 1.1372147 |
| Ubiad1 | 1.4021728 |
| Ubie | -0.7863696 |
| Ubl3 | 1.5332805 |
| Ubl4 | -0.4729689 |
| Ubl4b | -0.6419904 |
| Ubl5 | -0.3498097 |
| Ubl7 | -0.7056296 |
| Ublcp1 | -0.7099784 |
| Ubox5 | -0.3698976 |
| Ubox5 | -0.8441872 |
| Ubp1 | -0.4851101 |
| Ubp1 | -0.6355461 |
| Ubqln1 | -0.5586605 |
| Ubqln1 | -0.3486419 |
| Ubqln1 | 1.0924744 |
| Ubqln3 | -0.6291476 |
| Ubqln4 | -0.6157001 |
| Ubqlnl | -0.6987148 |
| Ubr1 | 1.8979191 |
| Ubr5 | -0.283379 |
| Ubtd1 | -0.6766018 |
| Ubtd2 | 1.1527477 |
| Ubtd2 | -0.6766018 |
| Ubtd2 | 0.7164593 |
| Ubtf | -0.2576997 |
| Ubxd1 | -0.2774291 |
| Ubxd1 | -0.7004385 |
| Ubxd1 | -0.4139775 |
| Ubxd3 | 0.7906626 |
| Ubxd4 | -0.6243784 |
| Ubxd5 | 0.8884531 |
| Ubxd8 | -0.6188462 |
| Uchl1 | -0.0871683 |
| Uchl3 | -0.5389803 |
| Uchl5 | -0.3492256 |
| Uchl5 | 0.3548381 |
| Uchl5 | -0.2672438 |
| Uck1 | 0.1060757 |
| Uck1 | 1.3327878 |
| Uck1 | 0.8686113 |
| Uck1 | 0.1597131 |
| Uck2 | -0.1411175 |
| Uck2 | -0.4544024 |
| Uck2 | 0.0377141 |
| Uevld | 0.1823112 |
| Ufc1 | -0.5310843 |
| Ufsp1 | 1.2305715 |
| Ufsp2 | 0.6239769 |
| Ugcg | -0.1043495 |
| Ugp2 | 1.0125776 |
| Ugt1a10 | -0.451781 |
| Ugt1a6a | 2.1600563 |
| Ugt1a6a | 0.7790737 |
| Ugt1a6a | 0.4471265 |
| Ugt3a2 | -0.0158859 |
| Ugt8a | 0.8683095 |
| Uhmk1 | -0.3059091 |
| Uhrf1 | 1.2282482 |
| Uhrf1bp1 | -0.5454927 |
| Uhrf1bp1l | -0.7073666 |
| Uhrf2 | -0.5397016 |
| Uhrf2 | -0.0802134 |
| Uhrf2 | -0.4413712 |
| Uhrf2 | 0.8786615 |
| Uimc1 | 1.9716943 |
| Ulk1 | -0.6566611 |
| Ulk1 | 0.7634424 |
| Ulk2 | 1.1281037 |
| Ulk2 | -0.6117828 |
| Umodl1 | -0.5682918 |
| Umps | -0.6001325 |
| Umps | -0.6227943 |
| Umps | -0.6196344 |
| Unc119 | 0.9763821 |
| Unc119 | -0.3474756 |
| Unc119b | -0.7143485 |
| Unc119b | 0.1141361 |
| Unc119b | -0.193093 |
| Unc13b | 1.5571737 |
| Unc13c | -0.5462192 |
| Unc13d | -0.5346648 |
| Unc45a | -0.2987041 |
| Unc45a | 2.3496072 |
| Unc45b | -0.4871497 |
| Unc5a | -0.342246 |
| Unc5a | 1.4299996 |
| Unc5b | 1.7934283 |
| Unc5c | 1.4134399 |
| Unc84a | -0.7987938 |
| Unc84a | 0.8515561 |
| Unc84b | -0.6267599 |
| Unc93a | -0.4064792 |
| Unc93b1 | -0.5564524 |
| Unc93b1 | -0.4530907 |
| Ung | -0.4583488 |
| Ung | 1.8302283 |
| Unk | 0.0254771 |
| Unkl | -0.1862468 |
| Uox | 0.5853467 |
| Upb1 | -0.2134476 |
| Upb1 | -0.4015149 |
| Upb1 | 0.0036537 |
| Upf1 | -0.6339422 |
| Upf1 | -0.6125649 |
| Upf1 | -0.0346236 |
| Upf2 | 0.9967574 |
| Upf3a | 0.9127632 |
| Upk1a | -0.6875903 |
| Upk1a | -0.682502 |
| Upk1b | -0.547674 |
| Upk2 | -0.5947468 |
| Upk3a | 0.5345484 |
| Upk3b | -0.303686 |
| Upk3b | 1.8476725 |
| Upk3b | 1.1143193 |
| Upp1 | 2.7296475 |
| Upp1 | -0.3527358 |
| Upp1 | -0.1886859 |
| Upp2 | 1.270336 |
| Upp2 | 2.8111459 |
| Upp2 | 1.2021835 |
| Upp2 | 0.4204697 |
| Uqcc | 2.3142496 |
| Uqcr | 0.907 |
| Uqcrc1 | -0.6267599 |
| Uqcrc2 | 0.2474895 |
| Uqcrh | 0.6423965 |
| Urm1 | -0.6766018 |
| Urod | 1.5050993 |
| Uros | -0.3370466 |
| Uros | -0.1896634 |
| Use1 | 0.5471421 |
| Use1 | -0.685891 |
| Use1 | -0.524675 |
| Usf1 | 1.2177695 |
| Usf1 | -0.5154971 |
| Usf2 | 0.8307168 |
| Usf2 | -0.5505906 |
| Ush1c | -0.5225487 |
| Ush1g | 0.8707206 |
| Ush2a | -0.8026514 |
| Ush3a | -0.4563734 |
| Ushbp1 | 1.3714761 |
| Usmg5 | -0.5397016 |
| Uso1 | 0.8338579 |
| Usp1 | 1.6284496 |
| Usp1 | -0.6740849 |
| Usp1 | -0.5154971 |
| Usp10 | 0.2323208 |
| Usp10 | 0.9559454 |
| Usp10 | -0.3953477 |
| Usp11 | 0.6410377 |
| Usp11 | 1.237806 |
| Usp12 | -0.7629486 |
| Usp12 | -0.1097027 |
| Usp13 | 0.1806926 |
| Usp14 | -0.3313045 |
| Usp15 | -0.8391407 |
| Usp16 | 0.8255098 |
| Usp18 | -0.514795 |
| Usp18 | 3.060966 |
| Usp19 | 1.134637 |
| Usp2 | -0.8431756 |
| Usp2 | 1.0444004 |
| Usp2 | 1.3855128 |
| Usp20 | -0.2779684 |
| Usp21 | 1.089175 |
| Usp22 | -0.3996603 |
| Usp25 | -0.1255007 |
| Usp28 | 2.1136351 |
| Usp29 | 0.0843416 |
| Usp3 | -0.3978095 |
| Usp3 | -0.5825565 |
| Usp33 | 1.275821 |
| Usp37 | -0.7205024 |
| Usp38 | -0.4769978 |
| Usp38 | 1.2763964 |
| Usp39 | -0.2860966 |
| Usp39 | -0.1364961 |
| Usp39 | -0.6995762 |
| Usp39 | -0.5712762 |
| Usp4 | 2.6274046 |
| Usp4 | -0.6339422 |
| Usp43 | 2.0128819 |
| Usp45 | 0.5667637 |
| Usp46 | 1.3645487 |
| Usp47 | 1.6675864 |
| Usp48 | -0.3795082 |
| Usp49 | 0.1890778 |
| Usp5 | 0.6816763 |
| Usp50 | 1.4908596 |
| Usp52 | 0.3909495 |
| Usp52 | -0.0166926 |
| Usp52 | -0.0050668 |
| Usp53 | 2.1708712 |
| Usp6nl | 2.7458221 |
| Usp6nl | -0.5520525 |
| Usp7 | 0.2314025 |
| Usp8 | 0.5950075 |
| Usp8 | 0.5557258 |
| Usp9x | 2.6514323 |
| Usp9y | 0.6302898 |
| Uspl1 | -0.7428306 |
| Utp11l | 1.2482531 |
| Utp15 | -0.6841949 |
| Utp6 | 0.065022 |
| Utx | -0.0533395 |
| Utx | -0.284465 |
| Uty | 1.3724245 |
| Uvrag | -0.5008661 |
| Uxt | 1.0563558 |
| Uxt | 2.4635469 |
| V1ra2 | 1.0801581 |
| V1rd2 | -0.086296 |
| V1rd20 | 1.7624067 |
| V1rd21 | -0.7064977 |
| V2r8 | 1.3946383 |
| Vac14 | 3.5905062 |
| Vamp1 | -0.6927076 |
| Vamp1 | -0.5955143 |
| Vamp2 | -0.3746901 |
| Vamp3 | 0.5058897 |
| Vamp3 | -0.0313367 |
| Vamp4 | -0.663259 |
| Vamp5 | -0.5454927 |
| Vamp8 | 0.3839973 |
| Vamp8 | -0.483753 |
| Vangl2 | -0.5397016 |
| Vapa | -0.44982 |
| Vapb | 3.1642321 |
| Vars | -0.7082364 |
| Vars | 0.8530921 |
| Vars | 1.637352 |
| Vars2 | 1.001845 |
| Vash1 | -0.2477341 |
| Vash1 | -0.7196206 |
| Vasn | -0.4504732 |
| Vasp | 0.3780324 |
| Vat1 | 0.1106404 |
| Vav1 | 0.491096 |
| Vav2 | 2.5257007 |
| Vav3 | 0.7121618 |
| Vax1 | -0.4190112 |
| Vbp1 | -0.0815134 |
| Vcam1 | -0.0082589 |
| Vcl | -0.621213 |
| Vcp | -0.6690721 |
| Vcpip1 | 2.2044048 |
| Vdac1 | -0.7125979 |
| Vdac2 | -0.4146052 |
| Vdac3 | 0.6237789 |
| Vdr | -0.2144524 |
| Vdr | 0.6916474 |
| Vdr | 0.6522345 |
| Vegfa | 0.5890589 |
| Vegfa | -0.4729689 |
| Vegfb | 2.6055235 |
| Vegfc | -0.0026801 |
| Vezf1 | -0.7482729 |
| Vezt | 0.2579574 |
| Vgf | 1.9574153 |
| Vgll2 | -0.1901525 |
| Vgll4 | 2.0053655 |
| Vhlh | 0.2399371 |
| Vil1 | 1.2196095 |
| Vill | -0.5404234 |
| Vim | -0.7013016 |
| Vip | 2.0457665 |
| Vit | -0.4164908 |
| Vkorc1 | -0.7519194 |
| Vkorc1 | 0.2555755 |
| Vkorc1 | -0.0325679 |
| Vkorc1l1 | 2.1101119 |
| Vkorc1l1 | 0.2706535 |
| Vkorc1l1 | 0.5520878 |
| Vldlr | -0.5742705 |
| Vldlr | -0.0321573 |
| Vldlr | 0.0628085 |
| Vldlr | -0.6509274 |
| Vldlr | 1.2986495 |
| Vmn2r81 | 0.5492957 |
| Vmo1 | -0.2904618 |
| Vnn3 | -0.177038 |
| Vnn3 | 0.2468876 |
| Vpreb1 | -0.5579238 |
| Vpreb3 | 0.3824451 |
| Vps11 | -0.1994972 |
| Vps13a | -0.6331414 |
| Vps16 | 1.0518142 |
| Vps16 | -0.561613 |
| Vps16 | -0.468958 |
| Vps16 | -0.6952772 |
| Vps18 | 0.8251932 |
| Vps24 | 1.1863228 |
| Vps25 | -0.0758937 |
| Vps25 | -0.6833481 |
| Vps26 | -0.4202741 |
| Vps26a | -0.5260954 |
| Vps26b | -0.4537463 |
| Vps28 | 1.4318567 |
| Vps29 | 0.5031768 |
| Vps29 | -0.7675843 |
| Vps29 | -0.5296561 |
| Vps33a | 2.8235125 |
| Vps33a | 0.1751675 |
| Vps33b | 0.7985239 |
| Vps33b | 2.4995416 |
| Vps35 | 0.4807097 |
| Vps36 | 0.6011202 |
| Vps37b | -0.7835267 |
| Vps37c | -0.4557159 |
| Vps37d | 1.3832169 |
| Vps39 | -0.3313045 |
| Vps39 | -0.6476673 |
| Vps41 | 1.3041511 |
| Vps41 | -0.0741717 |
| Vps45 | -0.5593977 |
| Vps4a | -0.6379573 |
| Vps4b | 0.4988646 |
| Vps53 | -0.7844733 |
| Vps53 | 0.7502917 |
| Vps53 | 0.7959111 |
| Vps54 | -0.1223188 |
| Vps72 | 0.5673963 |
| Vrk1 | 1.3241011 |
| Vrk1 | -0.6172717 |
| Vrk2 | 2.5955981 |
| Vrk3 | -0.8401478 |
| Vsig2 | -0.2763515 |
| Vsig4 | -0.6850426 |
| Vsnl1 | 3.6955979 |
| Vstm2a | 1.5860389 |
| Vstm2a | -0.3734896 |
| Vstm2a | 0.3785527 |
| Vstm2b | 0.2638849 |
| Vsx2 | -0.6935633 |
| Vti1a | -0.4033733 |
| Vti1a | 2.450303 |
| Vti1a | -0.1236811 |
| Vti1b | 1.3809151 |
| Vti1b | -0.640375 |
| Vtn | -0.284465 |
| Vwa2 | 2.3415138 |
| Vwa3a | -0.7968711 |
| Vwf | -0.6363491 |
| Wars | -0.8036184 |
| Wars | -0.6850426 |
| Wars | -0.5332309 |
| Wars | -0.1088083 |
| Wars2 | 2.4636238 |
| Wars2 | -0.5520525 |
| Was | -0.8301278 |
| Wasf1 | -0.4400784 |
| Wasf2 | 0.0111175 |
| Wasf3 | -0.0449732 |
| Wasl | 1.8989757 |
| Wbp1 | -0.3284471 |
| Wbp1 | -0.2363311 |
| Wbp11 | 0.2685989 |
| Wbp2 | 2.4175154 |
| Wbp2nl | 0.3896661 |
| Wbp4 | 0.5288452 |
| Wbp5 | 0.0918832 |
| Wbp7 | -0.7108508 |
| Wbscr16 | 1.4148384 |
| Wbscr18 | -0.5404234 |
| Wbscr22 | 0.069066 |
| Wbscr22 | 0.1938801 |
| Wbscr22 | -0.621213 |
| Wbscr27 | 1.5371635 |
| Wbscr28 | -0.1052395 |
| Wbscr28 | 0.1719015 |
| Wdfy1 | 0.191322 |
| Wdfy3 | -0.0849891 |
| Wdhd1 | 0.2938078 |
| Wdr1 | -0.5091975 |
| Wdr1 | 1.6048025 |
| Wdr13 | -0.5787807 |
| Wdr13 | -0.7108508 |
| Wdr16 | -0.6227943 |
| Wdr17 | 1.5744109 |
| Wdr18 | -0.5978204 |
| Wdr20a | 0.9038144 |
| Wdr20b | 0.7696939 |
| Wdr21 | -0.2608698 |
| Wdr22 | -0.5126918 |
| Wdr23 | 1.6925012 |
| Wdr24 | -0.2624591 |
| Wdr27 | 1.2431939 |
| Wdr3 | 0.6172205 |
| Wdr31 | -0.6039992 |
| Wdr31 | -0.2059473 |
| Wdr31 | 0.2691864 |
| Wdr32 | 0.6420085 |
| Wdr33 | 1.5652079 |
| Wdr34 | -0.3959626 |
| Wdr37 | 0.4881078 |
| Wdr37 | -0.5239657 |
| Wdr37 | -0.7374211 |
| Wdr37 | 0.2759154 |
| Wdr38 | 0.1666511 |
| Wdr41 | 0.1613699 |
| Wdr45 | 0.498637 |
| Wdr45l | -0.536101 |
| Wdr45l | 1.3933764 |
| Wdr46 | -0.1434372 |
| Wdr46 | -0.5484022 |
| Wdr47 | -0.2079412 |
| Wdr5 | -0.7500943 |
| Wdr51b | -0.3978095 |
| Wdr53 | -0.6339422 |
| Wdr54 | -0.1612645 |
| Wdr55 | -0.4046144 |
| Wdr59 | -0.64523 |
| Wdr5b | 0.6172205 |
| Wdr6 | 0.0501615 |
| Wdr60 | -0.3064657 |
| Wdr61 | -0.6566611 |
| Wdr62 | 0.1116906 |
| Wdr63 | -0.0062625 |
| Wdr66 | -0.1843002 |
| Wdr67 | 1.0228025 |
| Wdr68 | 0.4725478 |
| Wdr69 | 1.7909422 |
| Wdr70 | -0.5440414 |
| Wdr70 | -0.527518 |
| Wdr74 | 0.3759484 |
| Wdr75 | -0.8472287 |
| Wdr75 | 1.8785881 |
| Wdr76 | -0.5078036 |
| Wdr77 | -0.2998089 |
| Wdr78 | 0.4114893 |
| Wdr8 | 1.4575404 |
| Wdr8 | -0.6657458 |
| Wdr9 | 0.4480899 |
| Wdr93 | -0.1070221 |
| Wdsof1 | -0.5454927 |
| Wfdc1 | -0.7196206 |
| Wfdc10 | 1.6379937 |
| Wfdc10 | 0.5952119 |
| Wfdc12 | 0.7983608 |
| Wfdc15b | -0.6640872 |
| Wfdc15b | -0.7455476 |
| Wfdc2 | -0.2937497 |
| Wfdc3 | -0.623586 |
| Wfdc6a | -0.7401218 |
| Wfdc6b | 0.4680859 |
| Wfdc6b | -0.7648 |
| Wfikkn2 | -0.1196004 |
| Wfs1 | -0.3978095 |
| Whdc1 | 0.7727195 |
| Whrn | -0.6833481 |
| Whrn | -0.4439624 |
| Whsc2 | -0.3376228 |
| Wif1 | -0.342246 |
| Wipf1 | 1.741382 |
| Wipf2 | -0.6275551 |
| Wipi1 | 1.5971158 |
| Wipi1 | 0.1120403 |
| Wipi1 | -0.6591297 |
| Wipi2 | -0.1707912 |
| Wipi2 | -0.6952772 |
| Wipi2 | 0.4894882 |
| Wisp1 | 0.7083846 |
| Wisp1 | 1.5471533 |
| Wisp2 | 1.4662778 |
| Wiz | 4.5349318 |
| Wiz | 0.2423608 |
| Wnk1 | -0.7816364 |
| Wnk1 | 0.338402 |
| Wnk2 | -0.2582273 |
| Wnk4 | 0.5610494 |
| Wnt10a | -0.3789045 |
| Wnt10b | 1.0341684 |
| Wnt16 | -0.1537152 |
| Wnt16 | -0.4709612 |
| Wnt16 | 1.5001784 |
| Wnt2 | -0.2566455 |
| Wnt3a | -0.0533395 |
| Wnt4 | -0.6699057 |
| Wnt4 | 0.3654552 |
| Wnt5a | -0.4857895 |
| Wnt5a | 0.8860891 |
| Wnt5b | 2.1837288 |
| Wnt6 | 0.5614739 |
| Wnt7a | 0.9890057 |
| Wnt7b | 0.5931656 |
| Wnt7b | 0.1419614 |
| Wrb | 0.0579939 |
| Wrn | -0.6574832 |
| Wsb1 | 0.6406491 |
| Wsb2 | -0.2576997 |
| Wscd1 | 1.6548969 |
| Wscd2 | 2.0673986 |
| Wtap | 2.7898047 |
| Wtip | -0.0387492 |
| Wwc1 | -0.4247081 |
| Wwc1 | -0.6024505 |
| Wwc2 | 3.8526258 |
| Wwc2 | -0.7675843 |
| Wwox | -0.423439 |
| Wwox | -0.6791257 |
| Wwp1 | 2.2307119 |
| Wwp2 | 1.3507965 |
| Wwp2 | -0.0211419 |
| Wwp2 | 0.1419614 |
| Wwtr1 | 1.3275496 |
| X99384 | 0.2869239 |
| Xab1 | -0.6799686 |
| Xab1 | 0.502044 |
| Xbp1 | -0.597051 |
| Xdh | 0.7569765 |
| Xist | 0.6069899 |
| Xist | 0.2071916 |
| Xk | -0.5154971 |
| Xkr4 | 1.7042578 |
| Xkr5 | -0.6558398 |
| Xkr6 | 0.6437532 |
| Xkr8 | 0.3293836 |
| Xkrx | 0.3960648 |
| Xlr4a | 2.2993637 |
| Xpc | 3.0386454 |
| Xpnpep1 | 3.0140628 |
| Xpnpep1 | 0.6907287 |
| Xpnpep1 | 0.4468855 |
| Xpnpep2 | 1.1698538 |
| Xpnpep2 | 1.7046748 |
| Xpnpep3 | -0.7401218 |
| Xpo4 | -0.7187398 |
| Xpo4 | -0.8834944 |
| Xpo5 | -0.4127234 |
| Xpo6 | -0.4171203 |
| Xpo7 | 1.9386909 |
| Xpo7 | -0.6901453 |
| Xpr1 | -0.3645364 |
| Xrcc1 | 1.6352312 |
| Xrcc2 | 1.2366043 |
| Xrcc2 | -0.623586 |
| Xrcc3 | 0.8085774 |
| Xrcc6 | 2.9530221 |
| Xrcc6 | -0.3971935 |
| Xrcc6 | 2.0741398 |
| Xrn1 | 1.980505 |
| Xrn2 | 1.5103632 |
| Xylb | -0.5901561 |
| Xylt2 | -0.8074967 |
| Yaf2 | 0.4003901 |
| Yap1 | 0.5843128 |
| Ybx2 | 0.9846407 |
| Ybx2 | -0.7873192 |
| Yeats2 | 2.9795652 |
| Yeats4 | 0.8019417 |
| Yeats4 | 0.0475355 |
| Yif1a | -0.3698976 |
| Yif1a | -0.7231527 |
| Yif1a | 1.4604363 |
| Yif1b | 1.373544 |
| Yipf1 | 2.5607416 |
| Yipf2 | 1.8683673 |
| Yipf3 | 0.163354 |
| Yipf3 | -0.2322202 |
| Yipf3 | 3.3004911 |
| Yipf4 | 1.7122079 |
| Yipf6 | -0.7713101 |
| Yipf6 | -0.2235463 |
| Yipf6 | -0.1277803 |
| Yipf6 | -0.560874 |
| Yipf7 | -0.2992563 |
| Ykt6 | 0.3012064 |
| Ykt6 | 2.4464978 |
| Ylpm1 | 1.8445166 |
| Yme1l1 | -0.4596681 |
| Yme1l1 | -0.1123913 |
| Yme1l1 | -0.7178598 |
| Yod1 | 1.1845195 |
| Ypel1 | -0.7320439 |
| Ypel3 | -0.8074967 |
| Ypel3 | -0.5197214 |
| Ypel4 | 2.8716883 |
| Ypel4 | -0.3451476 |
| Ypel5 | 0.0843416 |
| Yrdc | -0.2860966 |
| Ythdf1 | 1.5657641 |
| Ythdf2 | -0.9257048 |
| Ythdf2 | -0.1823579 |
| Ythdf3 | 0.2504928 |
| Ythdf3 | -0.4215387 |
| Ywhab | -0.2566455 |
| Ywhab | -0.6892929 |
| Ywhae | -0.6339422 |
| Ywhag | 1.240504 |
| Ywhah | 0.9739702 |
| Ywhaq | -0.5140934 |
| Ywhaz | -0.3533222 |
| Yy2 | 0.063547 |
| Zadh1 | -0.7117239 |
| Zadh2 | 1.3069357 |
| Zan | -0.573521 |
| Zap70 | 0.5501557 |
| Zap70 | 2.5249351 |
| Zbbx | -0.2281279 |
| Zbed4 | 0.0415042 |
| Zbp1 | 1.1884397 |
| Zbtb17 | 0.460758 |
| Zbtb2 | 1.7063411 |
| Zbtb22 | -0.7338327 |
| Zbtb24 | -0.6180586 |
| Zbtb3 | -0.9179813 |
| Zbtb32 | 0.8604286 |
| Zbtb33 | -0.8055555 |
| Zbtb38 | 0.404442 |
| Zbtb39 | -0.7863696 |
| Zbtb40 | -0.0191168 |
| Zbtb41 | 1.0001523 |
| Zbtb43 | 0.4077208 |
| Zbtb45 | -0.4729689 |
| Zbtb46 | 0.5308235 |
| Zbtb5 | -0.6892929 |
| Zbtb7a | 0.6764623 |
| Zbtb7c | -0.8251594 |
| Zbtb8 | 1.2310758 |
| Zbtb9 | 0.2621107 |
| Zbtb9 | -0.772244 |
| Zc3h10 | -0.5253849 |
| Zc3h11a | -0.569037 |
| Zc3h12a | 1.8929077 |
| Zc3h12b | -0.6039992 |
| Zc3h13 | -0.5176068 |
| Zc3h14 | -0.4609894 |
| Zc3h14 | 2.7539873 |
| Zc3h15 | -0.1214118 |
| Zc3h18 | -0.7601786 |
| Zc3h3 | 2.2048832 |
| Zc3h3 | -0.6484812 |
| Zc3h3 | 0.025092 |
| Zc3h6 | 1.9124571 |
| Zc3h7a | -0.6533803 |
| Zc3h7a | 0.8164443 |
| Zc3h8 | 3.129004 |
| Zc3hc1 | -0.0613601 |
| Zc3hc1 | -0.3947333 |
| Zc3hc1 | -0.4164908 |
| Zc3hc1 | 0.065022 |
| Zcchc12 | -0.7266986 |
| Zcchc14 | 2.8407136 |
| Zcchc16 | -0.4089717 |
| Zcchc17 | -0.6533803 |
| Zcchc17 | 2.6830851 |
| Zcchc18 | -0.7638738 |
| Zcchc18 | 0.9871565 |
| Zcchc3 | 2.2198579 |
| Zcchc3 | 2.874452 |
| Zcchc3 | -0.8182493 |
| Zcchc5 | -0.4898763 |
| Zcchc6 | -0.5154971 |
| Zcchc8 | -0.560874 |
| Zcchc8 | 0.7053152 |
| Zcchc8 | 0.8053466 |
| Zcchc9 | -0.8331221 |
| Zcrb1 | -0.2049519 |
| Zcwpw1 | 0.9884777 |
| Zdhhc1 | 0.0828979 |
| Zdhhc12 | 0.3233208 |
| Zdhhc12 | -0.1097027 |
| Zdhhc13 | 1.1003205 |
| Zdhhc13 | -0.7816364 |
| Zdhhc13 | -0.4709612 |
| Zdhhc14 | -0.6987148 |
| Zdhhc15 | -0.67074 |
| Zdhhc16 | -0.8055555 |
| Zdhhc16 | -0.7731788 |
| Zdhhc17 | -0.6774423 |
| Zdhhc19 | 0.8425953 |
| Zdhhc19 | -0.6808123 |
| Zdhhc21 | 2.5634519 |
| Zdhhc24 | 1.1183283 |
| Zdhhc3 | 0.6472325 |
| Zdhhc3 | 0.4671437 |
| Zdhhc4 | -0.2099396 |
| Zdhhc6 | 1.485328 |
| Zdhhc7 | 1.2685969 |
| Zdhhc9 | -0.5310843 |
| Zeb1 | -0.3154189 |
| Zeb2 | -0.6063272 |
| Zer1 | 0.5759988 |
| Zf | 0.5874109 |
| Zfa | -0.644419 |
| Zfand2a | -0.7892215 |
| Zfand2b | 0.3348601 |
| Zfand2b | 2.1388378 |
| Zfand2b | 2.5187138 |
| Zfand2b | 0.2653607 |
| Zfand3 | -0.7997566 |
| Zfand6 | 1.5751679 |
| Zfand6 | -0.6039992 |
| Zfat | 3.2023368 |
| Zfh4 | 2.3441222 |
| Zfhx2 | 0.907289 |
| Zfhx2 | -0.2550665 |
| Zfhx3 | 0.2555755 |
| Zfhx3 | 2.0794966 |
| Zfml | 1.5472952 |
| Zfp1 | -0.2519167 |
| Zfp101 | 2.4476463 |
| Zfp105 | -0.7161024 |
| Zfp106 | 0.7425163 |
| Zfp106 | 0.8480134 |
| Zfp109 | 0.4720792 |
| Zfp11 | 0.425914 |
| Zfp119 | -0.6833481 |
| Zfp12 | -0.7510064 |
| Zfp128 | -0.8898608 |
| Zfp13 | -0.5513212 |
| Zfp131 | 0.0154106 |
| Zfp142 | 0.1910018 |
| Zfp143 | 1.3690576 |
| Zfp148 | -0.6251716 |
| Zfp160 | 0.2298699 |
| Zfp161 | -0.4478632 |
| Zfp161 | -0.2435708 |
| Zfp169 | -0.0219533 |
| Zfp179 | 0.2938078 |
| Zfp182 | -0.6476673 |
| Zfp184 | -0.181873 |
| Zfp185 | 0.0975871 |
| Zfp187 | 0.0579939 |
| Zfp187 | -0.1843002 |
| Zfp189 | -0.2317076 |
| Zfp191 | 1.3713036 |
| Zfp2 | -0.2790479 |
| Zfp2 | -0.1191482 |
| Zfp202 | -0.2004865 |
| Zfp207 | 2.4734612 |
| Zfp212 | -0.5513212 |
| Zfp212 | -0.267777 |
| Zfp212 | 1.7943453 |
| Zfp212 | -0.5720238 |
| Zfp213 | -0.2149552 |
| Zfp217 | 0.3641349 |
| Zfp219 | -0.5993611 |
| Zfp235 | -0.0977025 |
| Zfp236 | -0.3025766 |
| Zfp236 | 0.6653758 |
| Zfp238 | -0.2645825 |
| Zfp238 | 0.6932985 |
| Zfp238 | -0.3330233 |
| Zfp238 | -0.4190112 |
| Zfp238 | -0.663259 |
| Zfp239 | -0.661605 |
| Zfp251 | -0.9447426 |
| Zfp259 | -0.5993611 |
| Zfp26 | -0.3770959 |
| Zfp263 | 0.7177089 |
| Zfp27 | -0.8104165 |
| Zfp27 | -0.4433139 |
| Zfp275 | 1.4870745 |
| Zfp276 | -0.5239657 |
| Zfp277 | -0.4932962 |
| Zfp277 | 0.7483973 |
| Zfp280c | 1.5013738 |
| Zfp281 | 2.2718181 |
| Zfp282 | -0.3698976 |
| Zfp286 | -0.8677726 |
| Zfp288 | -0.3025766 |
| Zfp292 | 1.28868 |
| Zfp292 | -0.6492959 |
| Zfp295 | -0.0626331 |
| Zfp296 | -0.7759892 |
| Zfp296 | 2.4076707 |
| Zfp3 | 3.1304952 |
| Zfp30 | 3.4119076 |
| Zfp313 | 0.1767961 |
| Zfp313 | -0.4557159 |
| Zfp316 | -0.2399437 |
| Zfp316 | -0.7620243 |
| Zfp318 | -0.4420183 |
| Zfp322a | -0.0711659 |
| Zfp324 | 2.9698071 |
| Zfp326 | 2.358493 |
| Zfp326 | 0.6279277 |
| Zfp326 | 0.5150976 |
| Zfp329 | -0.2932009 |
| Zfp329 | 2.8771264 |
| Zfp330 | -0.5154971 |
| Zfp330 | 2.2658999 |
| Zfp334 | 2.1895856 |
| Zfp335 | 0.116575 |
| Zfp339 | -0.303686 |
| Zfp346 | 0.9879495 |
| Zfp346 | -0.365725 |
| Zfp35 | 0.2712397 |
| Zfp354a | -0.6102207 |
| Zfp36 | 0.576625 |
| Zfp365 | -0.9157867 |
| Zfp367 | 0.9434013 |
| Zfp367 | -0.1137386 |
| Zfp36l1 | -0.7519194 |
| Zfp36l1 | -0.2624591 |
| Zfp36l1 | -0.8421652 |
| Zfp36l2 | 0.3767305 |
| Zfp37 | 1.1033469 |
| Zfp371 | -0.2508692 |
| Zfp382 | 0.9190604 |
| Zfp385a | -0.9257048 |
| Zfp385a | 0.0661267 |
| Zfp385c | -0.7601786 |
| Zfp386 | 0.3216601 |
| Zfp386 | -0.6901453 |
| Zfp39 | 0.1255764 |
| Zfp393 | 1.5074756 |
| Zfp395 | -0.7073666 |
| Zfp397 | 1.214489 |
| Zfp398 | 0.6777687 |
| Zfp398 | -0.6944198 |
| Zfp398 | -0.7510064 |
| Zfp41 | 1.6734023 |
| Zfp41 | -0.2640512 |
| Zfp410 | -0.9103235 |
| Zfp410 | -0.7356251 |
| Zfp410 | -0.4796938 |
| Zfp414 | -0.3978095 |
| Zfp418 | 0.1443261 |
| Zfp42 | 0.2453812 |
| Zfp423 | 0.2209291 |
| Zfp423 | 0.5054382 |
| Zfp428 | 0.7732228 |
| Zfp428 | -0.4576898 |
| Zfp446 | 1.0431588 |
| Zfp446 | -0.1168902 |
| Zfp449 | -0.7546641 |
| Zfp451 | 1.4265983 |
| Zfp457 | 0.5451994 |
| Zfp46 | 1.3016376 |
| Zfp467 | 2.8938023 |
| Zfp472 | -0.5126918 |
| Zfp473 | -0.3137334 |
| Zfp473 | 2.1262501 |
| Zfp474 | -0.7013016 |
| Zfp474 | -0.0828152 |
| Zfp512 | 1.9152424 |
| Zfp513 | 0.8017793 |
| Zfp516 | 0.0426382 |
| Zfp52 | 1.6106406 |
| Zfp521 | -0.4623126 |
| Zfp523 | -0.0296978 |
| Zfp523 | 0.7004187 |
| Zfp524 | 0.1169229 |
| Zfp526 | 1.1966483 |
| Zfp532 | -0.1182443 |
| Zfp532 | 2.1761774 |
| Zfp536 | 0.7718801 |
| Zfp551 | 1.237806 |
| Zfp553 | 0.1453377 |
| Zfp553 | 0.0312339 |
| Zfp560 | 0.6807475 |
| Zfp566 | 1.5477917 |
| Zfp57 | -0.5682918 |
| Zfp574 | 0.5128605 |
| Zfp579 | 1.0852708 |
| Zfp592 | -0.7854209 |
| Zfp593 | 1.5301041 |
| Zfp595 | 0.2093912 |
| Zfp598 | 1.9521826 |
| Zfp60 | 0.7703671 |
| Zfp606 | 0.6773956 |
| Zfp607 | 1.6872331 |
| Zfp608 | 1.3023831 |
| Zfp609 | -0.4939818 |
| Zfp61 | 0.3780324 |
| Zfp612 | -0.7222684 |
| Zfp617 | 1.5804489 |
| Zfp619 | 1.3961502 |
| Zfp62 | -0.0605125 |
| Zfp622 | 0.1099397 |
| Zfp623 | -0.3255988 |
| Zfp629 | 1.9309424 |
| Zfp637 | -0.2019725 |
| Zfp639 | -0.4355682 |
| Zfp64 | 0.1172706 |
| Zfp64 | -0.2513928 |
| Zfp64 | -0.3639427 |
| Zfp641 | 0.638314 |
| Zfp644 | 0.049412 |
| Zfp646 | 0.7440768 |
| Zfp653 | 3.8299233 |
| Zfp654 | 2.2591388 |
| Zfp654 | 0.6073933 |
| Zfp655 | -0.8212043 |
| Zfp661 | -0.7117239 |
| Zfp667 | -0.6071046 |
| Zfp668 | 2.5153572 |
| Zfp67 | -0.5140934 |
| Zfp672 | 2.0051522 |
| Zfp68 | 2.5968365 |
| Zfp68 | 0.7346731 |
| Zfp68 | 2.1085923 |
| Zfp687 | -0.2752752 |
| Zfp688 | -0.4603285 |
| Zfp689 | -0.954987 |
| Zfp69 | -0.8941302 |
| Zfp691 | 0.6870449 |
| Zfp692 | -0.782581 |
| Zfp692 | 2.0202103 |
| Zfp704 | 2.8462746 |
| Zfp704 | 1.0006735 |
| Zfp706 | 1.6232481 |
| Zfp709 | 1.1099464 |
| Zfp709 | -0.5586605 |
| Zfp710 | -0.2322202 |
| Zfp711 | -0.0541804 |
| Zfp715 | -0.7169807 |
| Zfp71-rs1 | -0.4939818 |
| Zfp74 | 1.3731997 |
| Zfp75 | 2.1924997 |
| Zfp75 | 1.9417168 |
| Zfp75 | 2.734098 |
| Zfp750 | -0.7901742 |
| Zfp758 | 1.3700085 |
| Zfp760 | -0.4426659 |
| Zfp764 | -0.7694452 |
| Zfp768 | -0.115088 |
| Zfp770 | 2.8011463 |
| Zfp771 | -0.8261509 |
| Zfp771 | 0.8849048 |
| Zfp78 | -0.7240379 |
| Zfp78 | 0.9485228 |
| Zfp780b | -0.5454927 |
| Zfp786 | 1.8918441 |
| Zfp787 | -0.5765228 |
| Zfp787 | -0.7694452 |
| Zfp790 | -0.2271078 |
| Zfp804a | 0.2958057 |
| Zfp809 | -0.9911934 |
| Zfp810 | 0.5970497 |
| Zfp811 | 1.5006269 |
| Zfp817 | 0.756464 |
| Zfp819 | 0.226181 |
| Zfp82 | 1.2079953 |
| Zfp825 | 1.6208323 |
| Zfp828 | 1.4112142 |
| Zfp85-rs1 | -0.0146774 |
| Zfp87 | 2.2132821 |
| Zfp87 | -0.5091975 |
| Zfp90 | -0.4108456 |
| Zfp91 | -0.5727721 |
| Zfp91-cntf | 0.6691493 |
| Zfp92 | -0.1513689 |
| Zfp93 | 1.050213 |
| Zfp93 | 1.6218778 |
| Zfp94 | 2.4830418 |
| Zfp97 | 2.4611861 |
| Zfpl1 | 2.7365043 |
| Zfpm2 | -0.3492256 |
| Zfr | -0.4465611 |
| Zfx | -0.5078036 |
| Zfx | 0.3169383 |
| Zfy1 | 1.6026035 |
| Zfyve1 | -0.5112925 |
| Zfyve16 | -0.7347285 |
| Zfyve19 | 0.9207702 |
| Zfyve20 | -0.4008963 |
| Zfyve21 | 0.8846085 |
| Zfyve26 | 0.9160604 |
| Zfyve27 | -0.0841189 |
| Zgpat | 0.6281247 |
| Zgpat | 1.3947223 |
| Zhx1 | -0.8065256 |
| Zhx1 | -0.7275873 |
| Zhx1 | -0.519016 |
| Zhx2 | -0.6275551 |
| Zhx3 | -0.7666552 |
| Zic1 | -0.358027 |
| Zic2 | 3.8020304 |
| Zic3 | -0.3330233 |
| Zic4 | -0.6987148 |
| Zkscan1 | -0.7666552 |
| Zkscan14 | 0.3743822 |
| Zkscan17 | -0.9201813 |
| Zkscan3 | 0.7098254 |
| Zkscan6 | -0.8192332 |
| Zkscan6 | 1.3123869 |
| Zmat1 | -0.7537483 |
| Zmat2 | -0.6460417 |
| Zmat3 | 0.9946618 |
| Zmat4 | 1.0279981 |
| Zmat5 | 1.7214342 |
| Zmiz1 | -0.7178598 |
| Zmiz1 | -0.7169807 |
| Zmiz2 | 0.6953125 |
| Zmiz2 | -0.630743 |
| Zmpste24 | 0.1361924 |
| Zmpste24 | 0.8108321 |
| Zmpste24 | 0.206877 |
| Zmym3 | -0.0301072 |
| Zmym3 | 0.0079824 |
| Zmym5 | 0.049037 |
| Zmym6 | 3.5963466 |
| Zmynd10 | -0.4709612 |
| Zmynd11 | 0.8526316 |
| Zmynd11 | 2.8830464 |
| Zmynd11 | -0.6682394 |
| Zmynd12 | -0.3273067 |
| Zmynd15 | -0.6484812 |
| Znhit2 | -0.1560675 |
| Znhit3 | -0.6791257 |
| Znhit4 | -0.1579539 |
| Znrd1 | 0.0016792 |
| Znrd1 | -0.5433166 |
| Znrd1 | 0.1355113 |
| Znrf1 | 1.2891528 |
| Znrf2 | -0.4285261 |
| Znrf3 | -0.4253433 |
| Znrf4 | -0.2164653 |
| Zp1 | 3.9495568 |
| Zp3 | -0.4885119 |
| Zp3r | -0.7510064 |
| Zpbp | -0.4504732 |
| Zpbp2 | -0.5260954 |
| Zpbp2 | 3.4196188 |
| Zranb1 | 1.8950796 |
| Zranb2 | -0.5909196 |
| Zranb3 | 1.4122865 |
| Zrsr2 | -0.0698808 |
| Zscan10 | -0.3783013 |
| Zscan12 | -0.0187123 |
| Zscan2 | -0.5008661 |
| Zscan2 | -0.3405095 |
| Zscan2 | 0.083259 |
| Zscan20 | 1.695876 |
| Zscan20 | 0.9332105 |
| Zscan21 | 0.1533895 |
| Zscan21 | -0.4240733 |
| Zswim1 | 0.1663219 |
| Zswim2 | -0.0643332 |
| Zswim3 | -0.6476673 |
| Zswim4 | -0.3764939 |
| Zswim5 | 2.4127286 |
| Zw10 | -0.0223592 |
| Zw10 | 0.513756 |
| Zw10 | -0.7021655 |
| Zwilch | 0.6196109 |
| Zxda | 0.1011337 |
| Zxdc | 2.0005766 |
| Zxdc | 0.663862 |
| Zxdc | -0.1012414 |
| Zyg11b | 0.9596339 |
| Zyx | -0.0771875 |
| Zzz3 | 1.5494212 |
| Zzz3 | -0.7997566 |
| Zzz3 | 1.6612953 |
| Zzz3 | 0.0403689 |

| **Gene Symbol** | **WT3** |
| --- | --- |
| 0610006I08Rik | -0.4435225 |
| 0610006I08Rik | -0.2641852 |
| 0610007C21Rik | -0.350166 |
| 0610007C21Rik | 0.0260023 |
| 0610007P08Rik | 3.4927042 |
| 0610007P14Rik | -0.4898832 |
| 0610007P22Rik | -0.4802768 |
| 0610009B22Rik | 0.2379507 |
| 0610009D07Rik | -0.4477717 |
| 0610009O20Rik | -0.2641852 |
| 0610010E21Rik | 1.7076192 |
| 0610010K06Rik | -0.5363373 |
| 0610010K14Rik | 0.5652882 |
| 0610012A05Rik | -0.2148526 |
| 0610012D14Rik | -0.5893657 |
| 0610012G03Rik | -0.464966 |
| 0610012H03Rik | 0.4394635 |
| 0610025P10Rik | -0.7417971 |
| 0610031J06Rik | -0.4407005 |
| 0610031J06Rik | 1.0072675 |
| 0610031J06Rik | 1.547673 |
| 0610037D15Rik | -0.464966 |
| 0610037L13Rik | -0.0751594 |
| 0610037L13Rik | 0.1733859 |
| 0610038F07Rik | 0.1686497 |
| 0610040J01Rik | 1.4059401 |
| 0710008K08Rik | 0.51261 |
| 0910001A06Rik | 1.681458 |
| 0910001A06Rik | -0.5316635 |
| 0910001L09Rik | 0.5513694 |
| 1110001A07Rik | 2.1841459 |
| 1110001J03Rik | 1.2695291 |
| 1110002B05Rik | -0.6878487 |
| 1110002N22Rik | 1.3248219 |
| 1110003E01Rik | 1.351841 |
| 1110006G06Rik | -0.2259635 |
| 1110007A13Rik | -0.7036129 |
| 1110007A13Rik | 0.2550788 |
| 1110007C09Rik | 2.1989208 |
| 1110007L15Rik | 1.4740263 |
| 1110007L15Rik | 2.3217643 |
| 1110007L15Rik | -0.5147249 |
| 1110007L15Rik | -0.3973496 |
| 1110007M04Rik | 0.0420493 |
| 1110008F13Rik | 0.3239985 |
| 1110008J03Rik | 1.7234066 |
| 1110008J03Rik | 0.0849986 |
| 1110008J03Rik | -0.2883758 |
| 1110008P14Rik | -0.6714441 |
| 1110012D08Rik | -0.6377365 |
| 1110012J17Rik | -0.6642453 |
| 1110012L19Rik | -0.1299499 |
| 1110012L19Rik | -0.2877783 |
| 1110012M11Rik | 3.1968795 |
| 1110013L07Rik | -0.5910209 |
| 1110017D15Rik | -0.7282531 |
| 1110017D15Rik | 0.6119697 |
| 1110017D15Rik | -0.6750646 |
| 1110018G07Rik | -0.5926791 |
| 1110018J18Rik | -0.3767396 |
| 1110018J18Rik | 0.1415955 |
| 1110019N10Rik | 0.6971755 |
| 1110019N10Rik | 0.4623653 |
| 1110020C03Rik | 2.4308018 |
| 1110020P15Rik | -0.5254682 |
| 1110020P15Rik | -0.6500131 |
| 1110031B06Rik | -0.4343821 |
| 1110031I02Rik | 0.9617955 |
| 1110032A13Rik | -0.4211829 |
| 1110032A13Rik | -0.6850944 |
| 1110032E23Rik | 0.0953335 |
| 1110034A24Rik | 1.0115472 |
| 1110036O03Rik | 0.736617 |
| 1110038D17Rik | -0.6850944 |
| 1110038F14Rik | 0.158735 |
| 1110038F14Rik | -0.0498557 |
| 1110039B18Rik | -0.6136602 |
| 1110039B18Rik | -0.4950974 |
| 1110039B18Rik | -0.0732612 |
| 1110048D14Rik | 3.6784499 |
| 1110049B09Rik | 0.1531808 |
| 1110049B09Rik | -0.1036186 |
| 1110049F12Rik | 0.1770127 |
| 1110049F12Rik | -0.5811337 |
| 1110057K04Rik | -0.5457561 |
| 1110057K04Rik | -0.0171564 |
| 1110057K04Rik | 2.2264324 |
| 1110058L19Rik | 0.8030902 |
| 1110059E24Rik | -0.3144429 |
| 1110059G10Rik | -0.2776778 |
| 1110067D22Rik | -0.7715033 |
| 1110067I12Rik | -0.1395744 |
| 1190002A17Rik | 0.7807023 |
| 1190002H23Rik | 1.4455088 |
| 1190002N15Rik | -0.8568128 |
| 1190003J15Rik | -0.520081 |
| 1190003M12Rik | -0.0605489 |
| 1190005F20Rik | -0.4527539 |
| 1190005I06Rik | 1.7157877 |
| 1190005P17Rik | 0.1047723 |
| 1190005P17Rik | -0.1095153 |
| 1190007F08Rik | 0.6173899 |
| 1190017O12Rik | 3.3658971 |
| 1190020J12Rik | -0.0198378 |
| 1200002N14Rik | -0.5697302 |
| 1200003C05Rik | -0.5473352 |
| 1200003C05Rik | 0.0217329 |
| 1200003C05Rik | -0.5664976 |
| 1200003I07Rik | -0.7408231 |
| 1200009F10Rik | -0.2676862 |
| 1200009I06Rik | 3.5820714 |
| 1200009I06Rik | -0.549709 |
| 1200009O22Rik | -0.0480085 |
| 1200011O22Rik | 2.601633 |
| 1200013B08Rik | 0.0569998 |
| 1200013P24Rik | -0.6403535 |
| 1200013P24Rik | 1.0053425 |
| 1200014J11Rik | 2.5562422 |
| 1200015F23Rik | -0.5316635 |
| 1200015F23Rik | 0.5004293 |
| 1200015N20Rik | 0.730059 |
| 1200016B10Rik | -0.6696392 |
| 1200016B10Rik | -0.6136602 |
| 1200016D23Rik | 3.4046257 |
| 1300001I01Rik | 1.7863018 |
| 1300007L22Rik | 2.7470941 |
| 1300010M03Rik | 0.3579701 |
| 1300013J15Rik | -0.0265752 |
| 1300013J15Rik | -0.6553246 |
| 1300014I06Rik | -0.064767 |
| 1300017J02Rik | -0.0890387 |
| 1300018I05Rik | -0.3643303 |
| 1300018J18Rik | -0.5025974 |
| 1500001L15Rik | -0.1472439 |
| 1500001M20Rik | -0.1100084 |
| 1500001M20Rik | 1.9076743 |
| 1500001M20Rik | -0.6094258 |
| 1500002O20Rik | -0.3873199 |
| 1500003O03Rik | 1.5346112 |
| 1500005A01Rik | 0.1329055 |
| 1500010J02Rik | 1.4958587 |
| 1500010J02Rik | 0.2954502 |
| 1500011H22Rik | -0.3224701 |
| 1500012F01Rik | -0.40342 |
| 1500015O10Rik | 0.0191618 |
| 1500019C06Rik | 1.0084501 |
| 1500019G21Rik | 1.1551696 |
| 1500031L02Rik | 2.3179187 |
| 1500032D16Rik | -0.4950974 |
| 1500032D16Rik | 0.145723 |
| 1500032D16Rik | 0.552336 |
| 1500032L24Rik | -0.5262403 |
| 1500041J02Rik | 2.3348346 |
| 1500041N16Rik | -0.6814346 |
| 1520401A03Rik | -0.4642431 |
| 1600002K03Rik | 0.0668347 |
| 1600012H06Rik | -0.7234635 |
| 1600012H06Rik | 0.958995 |
| 1600014C10Rik | -0.693382 |
| 1600016N20Rik | -0.5877134 |
| 1600021P15Rik | 0.3374932 |
| 1600027N09Rik | -0.0723135 |
| 1600029D21Rik | 2.4064078 |
| 1700001C02Rik | -0.0338162 |
| 1700001C19Rik | -0.3604458 |
| 1700001E04Rik | -0.4693148 |
| 1700001J03Rik | 1.6765819 |
| 1700001O22Rik | 1.7542637 |
| 1700001O22Rik | -0.5193139 |
| 1700001P01Rik | 0.2111274 |
| 1700003F12Rik | 0.9528981 |
| 1700003M02Rik | 0.6274681 |
| 1700007G11Rik | 2.147531 |
| 1700007I06Rik | 0.1145033 |
| 1700007K09Rik | 0.4259998 |
| 1700008F21Rik | -0.1219218 |
| 1700008I05Rik | -0.1436569 |
| 1700008P20Rik | 0.8677 |
| 1700009P17Rik | 0.391029 |
| 1700010A17Rik | -0.5301108 |
| 1700010A17Rik | 0.5600332 |
| 1700010D01Rik | -0.0360906 |
| 1700010M22Rik | -0.3132141 |
| 1700010M22Rik | -0.2572228 |
| 1700011A15Rik | -0.6213311 |
| 1700011F03Rik | 0.4888629 |
| 1700011F14Rik | 2.6915866 |
| 1700011H14Rik | 0.3190422 |
| 1700011K15Rik | -0.0619528 |
| 1700012A03Rik | 0.405855 |
| 1700012B09Rik | 0.6851132 |
| 1700012G19Rik | -0.3224701 |
| 1700012H05Rik | -0.6010147 |
| 1700012H17Rik | -0.3973496 |
| 1700012L04Rik | -0.4301936 |
| 1700013B16Rik | 0.6564145 |
| 1700013G24Rik | -0.5254682 |
| 1700013N18Rik | -0.7674871 |
| 1700013N18Rik | 0.415982 |
| 1700015E13Rik | -0.6221873 |
| 1700016C15Rik | -0.4788077 |
| 1700016D06Rik | -0.3230904 |
| 1700016G05Rik | 1.3193435 |
| 1700016K19Rik | 0.3887269 |
| 1700016K19Rik | -0.6342585 |
| 1700017N19Rik | 2.6186385 |
| 1700018B24Rik | -0.7263343 |
| 1700018C11Rik | 1.4006312 |
| 1700018F24Rik | -0.1995176 |
| 1700019D03Rik | -0.5473352 |
| 1700019E19Rik | -0.5410347 |
| 1700019G17Rik | -0.2270819 |
| 1700019H03Rik | -0.6394804 |
| 1700019L03Rik | 0.1838649 |
| 1700019M22Rik | 2.6398036 |
| 1700019N19Rik | -0.5632762 |
| 1700020C11Rik | -0.2659341 |
| 1700020D05Rik | -0.6307936 |
| 1700020L24Rik | -0.2044192 |
| 1700020N01Rik | 0.0829979 |
| 1700020O03Rik | 2.5310558 |
| 1700021C14Rik | -0.1041085 |
| 1700021F05Rik | 2.1493468 |
| 1700021K02Rik | -0.2265225 |
| 1700021K02Rik | 2.3404406 |
| 1700021K19Rik | 0.4847154 |
| 1700022C21Rik | -0.1838978 |
| 1700023B02Rik | 1.1199524 |
| 1700023B02Rik | 3.3466699 |
| 1700023E05Rik | -0.3482511 |
| 1700023E05Rik | -0.3787143 |
| 1700023I07Rik | -0.0220782 |
| 1700023M03Rik | -0.411576 |
| 1700024G13Rik | -0.7575219 |
| 1700025D03Rik | -0.7092413 |
| 1700025E21Rik | 0.8754048 |
| 1700025F22Rik | 0.0885892 |
| 1700025G04Rik | -0.456329 |
| 1700025K23Rik | -0.5935093 |
| 1700026D08Rik | -0.4751451 |
| 1700026L06Rik | -0.4477717 |
| 1700027A23Rik | 1.0060834 |
| 1700027D21Rik | -0.7311388 |
| 1700027J05Rik | -0.7206016 |
| 1700027N10Rik | -0.7244194 |
| 1700028K03Rik | -0.6307936 |
| 1700029F12Rik | 0.4586319 |
| 1700029F12Rik | -0.5770449 |
| 1700029G01Rik | 2.4024379 |
| 1700029H14Rik | 0.1730224 |
| 1700029H14Rik | -0.7486439 |
| 1700029I01Rik | 0.8137204 |
| 1700029I08Rik | 2.5051077 |
| 1700029I15Rik | -0.5139623 |
| 1700029J07Rik | 0.2104294 |
| 1700029P11Rik | -0.063828 |
| 1700029P11Rik | 1.5879282 |
| 1700029P11Rik | -0.5827742 |
| 1700030J22Rik | 1.7858545 |
| 1700030K09Rik | -0.7167993 |
| 1700034E13Rik | 0.2463863 |
| 1700034H14Rik | -0.4054523 |
| 1700034H14Rik | -0.3034419 |
| 1700034H14Rik | 1.8409364 |
| 1700034H14Rik | 1.9282369 |
| 1700034I23Rik | 2.254271 |
| 1700034I23Rik | 1.4293203 |
| 1700034O15Rik | 0.6499146 |
| 1700036D21Rik | 2.4515822 |
| 1700037C18Rik | 0.91447 |
| 1700037C18Rik | 1.6005775 |
| 1700037H04Rik | 3.7485834 |
| 1700039E15Rik | -0.6473687 |
| 1700040I03Rik | -0.4322855 |
| 1700042B14Rik | 1.3570299 |
| 1700045I19Rik | -0.5193139 |
| 1700047I17Rik1 | -0.1674864 |
| 1700047I17Rik1 | -0.037002 |
| 1700049G17Rik | -0.5208487 |
| 1700049K14Rik | -0.5860641 |
| 1700049L16Rik | 1.8717357 |
| 1700052N19Rik | 0.4295909 |
| 1700052N19Rik | 0.5470069 |
| 1700054O13Rik | 0.0829979 |
| 1700055M20Rik | 2.2798362 |
| 1700057G04Rik | 1.2887291 |
| 1700057K13Rik | 0.5657645 |
| 1700058C13Rik | -0.6814346 |
| 1700060H10Rik | 2.7812 |
| 1700061G19Rik | -0.7644863 |
| 1700063I17Rik | 2.8063215 |
| 1700065I17Rik | -0.3064847 |
| 1700065O13Rik | -0.7311388 |
| 1700065O13Rik | 0.1434739 |
| 1700067C01Rik | -0.4047743 |
| 1700067K01Rik | -0.3418967 |
| 1700067P10Rik | 0.1415955 |
| 1700071K01Rik | -0.7234635 |
| 1700072E05Rik | 0.0761629 |
| 1700072E05Rik | 1.2613541 |
| 1700072E05Rik | 2.8972192 |
| 1700073E17Rik | 0.2570732 |
| 1700080E11Rik | -0.7064228 |
| 1700081D17Rik | -0.4371849 |
| 1700081D17Rik | 0.7893483 |
| 1700084C01Rik | 0.5173876 |
| 1700084C01Rik | 2.0736972 |
| 1700088E04Rik | -0.1105017 |
| 1700093K21Rik | 2.1312911 |
| 1700094C09Rik | 0.6590018 |
| 1700095K08Rik | 1.8689352 |
| 1700102P08Rik | -0.0429476 |
| 1700108M19Rik | 0.2527465 |
| 1700109H08Rik | -0.8168688 |
| 1700112C13Rik | -0.4548973 |
| 1700113I22Rik | -0.3552917 |
| 1700113I22Rik | 0.892301 |
| 1700113O17Rik | 0.1693799 |
| 1700120B06Rik | -0.6491308 |
| 1700123D08Rik | -0.0943581 |
| 1700123K08Rik | -0.2282017 |
| 1700123L14Rik | 1.8107607 |
| 1700123O20Rik | -0.4788077 |
| 1700123O20Rik | -0.7457034 |
| 1700127D06Rik | -0.3973496 |
| 1700129C05Rik | -0.3747692 |
| 1700129I15Rik | -0.6247608 |
| 1810007M14Rik | 0.7145745 |
| 1810007P19Rik | -0.6887686 |
| 1810007P19Rik | 3.5898202 |
| 1810008A18Rik | 0.2020112 |
| 1810008A18Rik | -0.2889738 |
| 1810009A15Rik | -0.0582138 |
| 1810009A15Rik | -0.1070538 |
| 1810009N02Rik | 1.9308643 |
| 1810009O10Rik | -0.4149957 |
| 1810009O10Rik | 1.0085978 |
| 1810010M01Rik | 1.9823732 |
| 1810011O10Rik | 0.463695 |
| 1810014F10Rik | -0.5616698 |
| 1810015A11Rik | 1.1328316 |
| 1810015C04Rik | -0.6615601 |
| 1810019J16Rik | -0.366929 |
| 1810020D17Rik | -0.5592652 |
| 1810020G14Rik | -0.6094258 |
| 1810020G14Rik | 3.6249325 |
| 1810021J13Rik | -0.824295 |
| 1810021J13Rik | -0.1769802 |
| 1810022C23Rik | -0.2293228 |
| 1810026J23Rik | 2.3697799 |
| 1810027O10Rik | 1.7998716 |
| 1810030J14Rik | -0.1919442 |
| 1810030N24Rik | -0.5819536 |
| 1810030N24Rik | -0.7130126 |
| 1810030N24Rik | -0.7476627 |
| 1810031K17Rik | 2.6496789 |
| 1810035L17Rik | 2.8653534 |
| 1810035L17Rik | 0.0332155 |
| 1810037C20Rik | -0.4477717 |
| 1810043G02Rik | 0.3423521 |
| 1810044A24Rik | 0.3823665 |
| 1810046J19Rik | 1.469903 |
| 1810048J11Rik | 0.0594683 |
| 1810049H13Rik | 1.5775071 |
| 1810049H19Rik | 0.8171763 |
| 1810055E12Rik | 0.0757593 |
| 1810055E12Rik | 1.1880037 |
| 1810055E12Rik | -0.5246966 |
| 1810055G02Rik | 0.6777576 |
| 1810063B05Rik | -0.6077375 |
| 1810064F22Rik | 0.2967237 |
| 1810065E05Rik | -0.0642974 |
| 1810073G14Rik | -0.7244194 |
| 1810074P20Rik | 1.7436235 |
| 1810074P20Rik | -0.8021923 |
| 2010001J22Rik | -0.2700275 |
| 2010001J22Rik | -0.233821 |
| 2010001M09Rik | 0.4086791 |
| 2010003J03Rik | 0.1942269 |
| 2010003O18Rik | 0.9851131 |
| 2010004A03Rik | -0.5624727 |
| 2010005H15Rik | -0.3362147 |
| 2010005J08Rik | -0.5033507 |
| 2010005J08Rik | -0.7225085 |
| 2010005J08Rik | -0.1716939 |
| 2010007H12Rik | -0.0211813 |
| 2010007H12Rik | -0.2961796 |
| 2010011I20Rik | 0.7025323 |
| 2010012O05Rik | 0.5062858 |
| 2010100O12Rik | -0.4047743 |
| 2010100O12Rik | 0.3081072 |
| 2010106G01Rik | 2.9099773 |
| 2010106G01Rik | 0.8966441 |
| 2010107E04Rik | 0.7252603 |
| 2010107G12Rik | -0.5770449 |
| 2010109I03Rik | -0.6403535 |
| 2010110K16Rik | 0.2722231 |
| 2010111I01Rik | -0.5093996 |
| 2010200O16Rik | -0.3527253 |
| 2010301N04Rik | 0.9583715 |
| 2010305A19Rik | 1.5895879 |
| 2010305C02Rik | -0.3960061 |
| 2010305C02Rik | 1.6849558 |
| 2010308M01Rik | 0.8714748 |
| 2010311D03Rik | -0.6187671 |
| 2010316F05Rik | 0.9684516 |
| 2010321M09Rik | 0.0289793 |
| 2010321M09Rik | -0.4020676 |
| 2200001I15Rik | 1.3539811 |
| 2210009G21Rik | -0.5332188 |
| 2210009G21Rik | -0.6961611 |
| 2210009G21Rik | 0.7431289 |
| 2210010B09Rik | -0.0096011 |
| 2210010L05Rik | 3.8500888 |
| 2210012G02Rik | -0.5056144 |
| 2210012G02Rik | -0.0671187 |
| 2210012G02Rik | -0.6377365 |
| 2210012G02Rik | 0.2725497 |
| 2210016L21Rik | 0.221881 |
| 2210016L21Rik | 4.4097191 |
| 2210016L21Rik | -0.350166 |
| 2210021J22Rik | 2.8180121 |
| 2210023G05Rik | -0.5520888 |
| 2210023G05Rik | 0.7519357 |
| 2210407C18Rik | -0.5576657 |
| 2210412D01Rik | 0.391029 |
| 2210417D09Rik | 0.7884124 |
| 2210418O10Rik | 1.5276309 |
| 2300002D11Rik | 0.5014505 |
| 2300002D11Rik | 0.1690149 |
| 2300002M23Rik | -0.5803145 |
| 2300005B03Rik | -0.7918494 |
| 2310001A20Rik | -0.4928591 |
| 2310002B06Rik | 0.1910235 |
| 2310003C23Rik | 0.5470069 |
| 2310003F16Rik | -0.1184312 |
| 2310003H01Rik | 0.4229503 |
| 2310003L22Rik | 1.2960175 |
| 2310003L22Rik | -0.6230444 |
| 2310004I24Rik | -0.6814346 |
| 2310005E10Rik | 0.0893852 |
| 2310005E10Rik | -0.6777893 |
| 2310005G13Rik | 0.7577608 |
| 2310005N01Rik | 0.3869965 |
| 2310005N03Rik | 2.1980196 |
| 2310005P05Rik | 0.0909751 |
| 2310006J04Rik | -0.2332575 |
| 2310007A19Rik | -0.4693148 |
| 2310007B03Rik | -0.3933247 |
| 2310007F21Rik | -0.2531853 |
| 2310007F21Rik | -0.2360783 |
| 2310008H04Rik | -0.5960043 |
| 2310008H09Rik | -0.8780129 |
| 2310008H09Rik | -0.668738 |
| 2310008M10Rik | 2.0048502 |
| 2310009E04Rik | 0.114116 |
| 2310010B21Rik | -0.2601175 |
| 2310010M24Rik | 2.2570441 |
| 2310011J03Rik | -0.0189431 |
| 2310014L17Rik | -0.6924575 |
| 2310016C08Rik | -0.7177484 |
| 2310016C08Rik | -0.7369372 |
| 2310016C16Rik | 1.6268126 |
| 2310016C16Rik | 2.5551273 |
| 2310016C16Rik | 1.5336053 |
| 2310016E02Rik | 1.5304967 |
| 2310016M24Rik | 1.5287277 |
| 2310021P13Rik | 0.6232262 |
| 2310022A10Rik | -0.7244194 |
| 2310022B05Rik | 1.7736319 |
| 2310031L18Rik | 0.8976435 |
| 2310031L18Rik | 0.8235091 |
| 2310033E01Rik | -0.5778612 |
| 2310033K02Rik | -0.5285606 |
| 2310033P09Rik | -0.4950974 |
| 2310035C23Rik | -0.4308904 |
| 2310035C23Rik | -0.2209471 |
| 2310035K24Rik | -0.479542 |
| 2310036D22Rik | 1.6635495 |
| 2310036O22Rik | -0.4729544 |
| 2310037I24Rik | 1.188977 |
| 2310037I24Rik | 1.3080747 |
| 2310038H17Rik | -0.3034419 |
| 2310039E09Rik | 0.3677468 |
| 2310039H08Rik | 2.664496 |
| 2310040C09Rik | 0.3552857 |
| 2310042D19Rik | 0.2030677 |
| 2310044G17Rik | 0.1199084 |
| 2310044H10Rik | 1.558351 |
| 2310045A20Rik | -0.6377365 |
| 2310046K01Rik | 0.2640202 |
| 2310046O06Rik | -0.459918 |
| 2310047C04Rik | -0.5078837 |
| 2310047D13Rik | -0.3450685 |
| 2310047D13Rik | 0.7037641 |
| 2310047M10Rik | 1.6647154 |
| 2310047M10Rik | -0.252035 |
| 2310047O13Rik | -0.3206115 |
| 2310056P07Rik | 0.7744498 |
| 2310057J16Rik | 0.9206615 |
| 2310058J06Rik | -0.4499037 |
| 2310061C15Rik | -0.5852406 |
| 2310061F22Rik | -0.7775604 |
| 2310061F22Rik | -0.6491308 |
| 2310065K24Rik | 3.3503935 |
| 2310065K24Rik | -0.1254255 |
| 2310066E14Rik | -0.6060522 |
| 2310067B10Rik | -0.5835956 |
| 2310076L09Rik | 0.71356 |
| 2310076L09Rik | -0.7158512 |
| 2310079F23Rik | -0.5697302 |
| 2310079N02Rik | 1.4214975 |
| 2400001E08Rik | 1.7404746 |
| 2400003C14Rik | 1.606179 |
| 2400003C14Rik | -0.2991987 |
| 2400003C14Rik | -0.6447318 |
| 2400006N03Rik | 0.3970449 |
| 2400010D15Rik | 1.8168377 |
| 2410001C21Rik | -0.4343821 |
| 2410001C21Rik | 1.0632332 |
| 2410001C21Rik | 1.8144241 |
| 2410002F23Rik | -0.4824849 |
| 2410002F23Rik | 3.578404 |
| 2410002F23Rik | -0.5262403 |
| 2410002I01Rik | 2.5419257 |
| 2410002O22Rik | 0.121447 |
| 2410003P15Rik | 0.0643857 |
| 2410004A20Rik | 1.6178179 |
| 2410004B18Rik | 1.3171218 |
| 2410004L22Rik | -0.4541823 |
| 2410005O16Rik | 1.2363878 |
| 2410008K03Rik | 0.4176592 |
| 2410014A08Rik | 1.0236882 |
| 2410014A08Rik | -0.5308868 |
| 2410014A08Rik | 1.0315026 |
| 2410014A08Rik | 0.5721701 |
| 2410015M20Rik | 0.6982081 |
| 2410015N17Rik | 2.1748641 |
| 2410016F19Rik | 0.8822003 |
| 2410016F19Rik | 1.5139392 |
| 2410016O06Rik | -0.3387357 |
| 2410017P07Rik | 1.3776986 |
| 2410018C17Rik | -0.7959727 |
| 2410018G20Rik | 0.474002 |
| 2410018M08Rik | 0.207982 |
| 2410019A14Rik | -0.5048592 |
| 2410019G02Rik | 2.0126403 |
| 2410022L05Rik | 0.6652247 |
| 2410025L10Rik | 0.6209858 |
| 2410042D21Rik | -0.6473687 |
| 2410042D21Rik | 0.1924486 |
| 2410066E13Rik | -0.6018524 |
| 2410081M15Rik | 0.8796578 |
| 2410091C18Rik | -0.5926791 |
| 2410116G06Rik | 2.5217643 |
| 2410127E18Rik | -0.6464889 |
| 2410127L17Rik | 1.6279498 |
| 2410127L17Rik | -0.7836573 |
| 2410129H14Rik | 0.9276182 |
| 2410137M14Rik | 2.2483894 |
| 2410146L05Rik | -0.497341 |
| 2410166I05Rik | -0.2630212 |
| 2500002L14Rik | 0.5050158 |
| 2500003M10Rik | -0.2176179 |
| 2500003M10Rik | -0.8189845 |
| 2500003M10Rik | -0.7398501 |
| 2510002A14Rik | 0.2590634 |
| 2510003E04Rik | -0.714904 |
| 2510006D16Rik | -0.638608 |
| 2510009E07Rik | -0.8480293 |
| 2510010F15Rik | -0.1855015 |
| 2510012J08Rik | 0.8091533 |
| 2510039O18Rik | 0.0378532 |
| 2510040D07Rik | -0.5877134 |
| 2510048L02Rik | -0.520081 |
| 2510049I19Rik | -0.9350334 |
| 2600001B17Rik | -0.5324408 |
| 2600009E05Rik | 0.0749515 |
| 2600009E05Rik | -0.7555417 |
| 2600010E01Rik | -0.6394804 |
| 2600011E07Rik | -0.1738048 |
| 2600011E07Rik | 1.9347915 |
| 2610002J02Rik | -0.5301108 |
| 2610002M06Rik | -0.4428162 |
| 2610003J06Rik | -0.4149957 |
| 2610016F04Rik | -0.7506095 |
| 2610019A05Rik | 1.6721136 |
| 2610019P18Rik | -0.4958446 |
| 2610020C11Rik | 3.1968795 |
| 2610020H08Rik | -0.6068945 |
| 2610020H08Rik | 4.0975994 |
| 2610020H08Rik | -0.5433924 |
| 2610020O08Rik | 2.2361591 |
| 2610021K21Rik | 2.2531903 |
| 2610021K21Rik | 0.9577476 |
| 2610024G14Rik | 0.1442242 |
| 2610024G14Rik | 1.4346546 |
| 2610024G14Rik | -0.8360863 |
| 2610027C15Rik | 1.8999644 |
| 2610027C15Rik | -0.7816206 |
| 2610027L16Rik | 1.0155142 |
| 2610028A01Rik | 1.2332627 |
| 2610028H24Rik | -0.3886509 |
| 2610029G23Rik | -0.9231085 |
| 2610029G23Rik | 1.5507247 |
| 2610034E13Rik | -0.4883988 |
| 2610034E13Rik | 1.6485547 |
| 2610034M16Rik | 2.1724238 |
| 2610036D13Rik | 0.867528 |
| 2610039C10Rik | 0.3134346 |
| 2610042L04Rik | -0.638608 |
| 2610044O15Rik | -0.4007171 |
| 2610101N10Rik | 0.7064274 |
| 2610101N10Rik | -0.9008649 |
| 2610103J23Rik | -0.4707689 |
| 2610110G12Rik | 1.988917 |
| 2610200G18Rik | -0.5223859 |
| 2610204K14Rik | 2.6566297 |
| 2610204L23Rik | -0.7806039 |
| 2610204M08Rik | -0.9043418 |
| 2610205E22Rik | 3.4313408 |
| 2610207I05Rik | -0.1544597 |
| 2610208M17Rik | -0.2630212 |
| 2610209A20Rik | -0.4315877 |
| 2610209A20Rik | 2.0431142 |
| 2610209M04Rik | -0.4943507 |
| 2610301B20Rik | 0.1748383 |
| 2610301F02Rik | 3.3323003 |
| 2610304G08Rik | -0.4329838 |
| 2610305D13Rik | -0.8168688 |
| 2610318N02Rik | -0.7045486 |
| 2610507B11Rik | -0.4198044 |
| 2610507B11Rik | -0.7120683 |
| 2610510B01Rik | -0.2355135 |
| 2610510J17Rik | -0.3028345 |
| 2610524A10Rik | -0.5124389 |
| 2610524H06Rik | 1.6447678 |
| 2610528E23Rik | -0.5048592 |
| 2610528J11Rik | 1.0769184 |
| 2610528O22Rik | 2.7297671 |
| 2700007P21Rik | -0.2806371 |
| 2700007P21Rik | -0.3089261 |
| 2700038C09Rik | 2.5633461 |
| 2700038C09Rik | 1.241573 |
| 2700050L05Rik | -0.7369372 |
| 2700055A20Rik | 0.7717844 |
| 2700060E02Rik | 1.3104248 |
| 2700062C07Rik | -0.3721484 |
| 2700062C07Rik | 1.681601 |
| 2700078K21Rik | -0.5262403 |
| 2700081O15Rik | 2.1648517 |
| 2700087H15Rik | 0.7302584 |
| 2700094K13Rik | -0.5608676 |
| 2700097O09Rik | 1.1963656 |
| 2810002N01Rik | 0.0075027 |
| 2810002O09Rik | -0.6060522 |
| 2810003C17Rik | -0.3899839 |
| 2810004N20Rik | -0.7466825 |
| 2810004N23Rik | 0.3258503 |
| 2810008M24Rik | 0.7567925 |
| 2810021B07Rik | 1.3305813 |
| 2810021J22Rik | 1.0109581 |
| 2810021J22Rik | -0.7846774 |
| 2810022L02Rik | -0.5640805 |
| 2810022L02Rik | -0.6010147 |
| 2810022L02Rik | -0.6571019 |
| 2810030E01Rik | 2.1163745 |
| 2810046L04Rik | -0.5819536 |
| 2810046M22Rik | -0.5713507 |
| 2810403A07Rik | 0.6828078 |
| 2810405J04Rik | -0.7045486 |
| 2810405K02Rik | -0.3206115 |
| 2810408A11Rik | -0.3362147 |
| 2810408M09Rik | 1.927524 |
| 2810410C14Rik | -0.7887689 |
| 2810410P22Rik | 1.3949997 |
| 2810410P22Rik | -0.9020225 |
| 2810417H13Rik | -0.8328556 |
| 2810422O20Rik | 2.4055234 |
| 2810428I15Rik | -0.3463402 |
| 2810432D09Rik | 0.0841989 |
| 2810432L12Rik | 0.8658068 |
| 2810439F02Rik | 0.6490445 |
| 2810439F02Rik | -0.4527539 |
| 2810441K11Rik | -0.6998795 |
| 2810452K22Rik | -0.8590217 |
| 2810453I06Rik | -0.8032329 |
| 2810453I06Rik | 0.7940137 |
| 2810470D21Rik | -0.6970893 |
| 2810485I05Rik | -0.4802768 |
| 2900006K08Rik | 1.5868201 |
| 2900010M23Rik | 0.6171647 |
| 2900011O08Rik | 2.6713124 |
| 2900011O08Rik | 2.0399608 |
| 2900024C23Rik | -0.7215546 |
| 2900024O10Rik | -0.6001777 |
| 2900026A02Rik | -0.1194272 |
| 2900046G09Rik | -0.6606667 |
| 2900062L11Rik | -0.3437985 |
| 2900064A13Rik | 0.8137204 |
| 2900073G15Rik | -0.037458 |
| 2900092C05Rik | -0.933834 |
| 3000004C01Rik | -0.8712657 |
| 3000004C01Rik | -0.8021923 |
| 3000004C01Rik | -0.5568669 |
| 3010003L21Rik | -0.7177484 |
| 3010026O09Rik | 1.4492825 |
| 3110001A13Rik | -0.6943074 |
| 3110001D03Rik | -0.8601281 |
| 3110004L20Rik | -0.7735179 |
| 3110007F17Rik | -0.8021923 |
| 3110009E18Rik | 2.180436 |
| 3110023B02Rik | -0.2434527 |
| 3110035E14Rik | 0.566954 |
| 3110035E14Rik | 1.5805421 |
| 3110037I16Rik | 0.0833984 |
| 3110040N11Rik | 0.0949382 |
| 3110040N11Rik | 0.359458 |
| 3110043J09Rik | -0.7408231 |
| 3110043J09Rik | 1.8373159 |
| 3110043J09Rik | -0.7417971 |
| 3110048E14Rik | -0.6473687 |
| 3110049J23Rik | -0.6535507 |
| 3110050N22Rik | -0.4883988 |
| 3110050N22Rik | 1.180916 |
| 3110056O03Rik | -0.3840006 |
| 3110057O12Rik | 1.6204949 |
| 3110057O12Rik | -0.1482713 |
| 3110070M22Rik | -0.0997082 |
| 3110082I17Rik | 0.4459874 |
| 3200001I04Rik | 0.6407376 |
| 3200002M19Rik | -0.1978895 |
| 3200002M19Rik | 0.3528931 |
| 3300001G02Rik | -0.5951719 |
| 3300001P08Rik | 0.6216585 |
| 3300001P08Rik | 0.0627495 |
| 3300001P08Rik | 0.2450418 |
| 3300002I08Rik | 0.0449753 |
| 3321401G04Rik | -0.1643433 |
| 3322402L07Rik | -0.5270131 |
| 3632451O06Rik | 1.1940706 |
| 3830402I07Rik | 0.6319124 |
| 3830406C13Rik | 0.8191713 |
| 3830406C13Rik | -0.8546091 |
| 3830406C13Rik | 2.8865493 |
| 3830408P04Rik | 2.4985797 |
| 3930401K13Rik | -0.5147249 |
| 4121402D02Rik | 2.2718846 |
| 4121402D02Rik | -0.4758764 |
| 4430402I18Rik | 1.001034 |
| 4432405B04Rik | 0.3396221 |
| 4432416J03Rik | 0.0761629 |
| 4631426J05Rik | -0.5223859 |
| 4631427C17Rik | -0.2270819 |
| 4632404H12Rik | 1.2828129 |
| 4632404H12Rik | 3.3487589 |
| 4632404H12Rik | -0.6342585 |
| 4632404H22Rik | -0.4988398 |
| 4632411B12Rik | -0.8436686 |
| 4632411B12Rik | 0.0536983 |
| 4632412N22Rik | 2.8197122 |
| 4632415K11Rik | 1.4446783 |
| 4632417K18Rik | 1.4948098 |
| 4632417N05Rik | 0.5342878 |
| 4632419K20Rik | 1.4166578 |
| 4632434I11Rik | 1.7228595 |
| 4732415M23Rik | 0.7049942 |
| 4732418C07Rik | -0.1685365 |
| 4732418C07Rik | 0.3319963 |
| 4732418C07Rik | -0.6239022 |
| 4732429D16Rik | 1.9805616 |
| 4732456N10Rik | -0.4693148 |
| 4732471D19Rik | -0.3540076 |
| 4732473B16Rik | -0.638608 |
| 4732479N06Rik | -0.8021923 |
| 4732479N06Rik | -0.0549545 |
| 4732496O08Rik | 1.3020657 |
| 4733401H18Rik | 0.3246162 |
| 4831426I19Rik | 0.4753151 |
| 4833418A01Rik | -0.0535611 |
| 4833420G17Rik | 0.294175 |
| 4833421E05Rik | -0.6077375 |
| 4833424O15Rik | 1.537372 |
| 4833424O15Rik | -0.8021923 |
| 4833426J09Rik | -0.5025974 |
| 4833426J09Rik | -0.5465453 |
| 4833427G06Rik | 1.1736502 |
| 4833436C18Rik | -0.362386 |
| 4833439L19Rik | -0.2165108 |
| 4833439L19Rik | 1.072517 |
| 4833439L19Rik | 1.811942 |
| 4833442J19Rik | 0.013998 |
| 4833446K15Rik | -0.8535092 |
| 4921501E09Rik | -0.3617388 |
| 4921504E06Rik | -0.8189845 |
| 4921504I05Rik | 2.4774733 |
| 4921505C17Rik | -0.4846982 |
| 4921505C17Rik | -0.5552715 |
| 4921507P07Rik | 1.6861663 |
| 4921511H03Rik | 0.731454 |
| 4921511H03Rik | 0.1175958 |
| 4921511K06Rik | 0.2741811 |
| 4921511K06Rik | 0.172295 |
| 4921515J06Rik | -0.1529088 |
| 4921517D21Rik | -0.5713507 |
| 4921517D22Rik | 1.9152651 |
| 4921520G13Rik | 2.031172 |
| 4921521F21Rik | 1.8177027 |
| 4921521F21Rik | -0.1239223 |
| 4921523A10Rik | 0.5686169 |
| 4921523A10Rik | -0.5852406 |
| 4921523A10Rik | -0.4364834 |
| 4921525H12Rik | -0.3754255 |
| 4921528G01Rik | -0.6273415 |
| 4921528I01Rik | -0.6162101 |
| 4921530G04Rik | -0.1174363 |
| 4921530L21Rik | -0.5877134 |
| 4921530L21Rik | 1.4598771 |
| 4921536K21Rik | -0.4936046 |
| 4921537P18Rik | 0.6965555 |
| 4922502D21Rik | 0.0725239 |
| 4922503N01Rik | -0.6213311 |
| 4922503N01Rik | 0.6037797 |
| 4922505E12Rik | -0.5132003 |
| 4922505G16Rik | 4.0159528 |
| 4922505G16Rik | -0.4869168 |
| 4930401F20Rik | -0.6669383 |
| 4930402H24Rik | 2.4858654 |
| 4930403C10Rik | 0.4013183 |
| 4930403L05Rik | 0.2467222 |
| 4930403O06Rik | 0.0574117 |
| 4930404H21Rik | 1.2622535 |
| 4930404N11Rik | -0.4534678 |
| 4930408O21Rik | -0.6597742 |
| 4930408O21Rik | -0.4861767 |
| 4930412F15Rik | -0.1400838 |
| 4930415L06Rik | -0.7535657 |
| 4930418G15Rik | 0.1202933 |
| 4930427A07Rik | -0.1753912 |
| 4930428D18Rik | 0.0344827 |
| 4930428E23Rik | 2.0267955 |
| 4930430D24Rik | -0.5860641 |
| 4930430E16Rik | -0.7398501 |
| 4930430E16Rik | -0.2694416 |
| 4930431L04Rik | -0.5139623 |
| 4930432H15Rik | -0.2332575 |
| 4930432K21Rik | -0.538683 |
| 4930432O21Rik | 0.2900183 |
| 4930432O21Rik | 0.3465829 |
| 4930438O05Rik | -0.2812301 |
| 4930438O05Rik | -0.7476627 |
| 4930449I24Rik | 0.4004652 |
| 4930451C15Rik | -0.5154882 |
| 4930451I11Rik | 0.1744754 |
| 4930452B06Rik | 0.2430213 |
| 4930453N24Rik | 0.5782969 |
| 4930455C21Rik | -0.7045486 |
| 4930455F23Rik | 0.0285546 |
| 4930467M19Rik | 3.9777506 |
| 4930470P17Rik | -0.6869297 |
| 4930473A06Rik | 2.0402523 |
| 4930473A06Rik | -0.7684895 |
| 4930473A06Rik | -0.6814346 |
| 4930474N05Rik | 0.7855991 |
| 4930478A21Rik | 2.3327812 |
| 4930481F22Rik | -0.0225269 |
| 4930481F22Rik | 0.3387103 |
| 4930481M05 | -0.5339974 |
| 4930486L24Rik | 1.2667376 |
| 4930502E18Rik | 1.2290795 |
| 4930503B20Rik | 2.4087293 |
| 4930503L19Rik | -0.6980184 |
| 4930504E06Rik | -0.2961796 |
| 4930504E06Rik | 3.3031122 |
| 4930504O13Rik | -0.4061307 |
| 4930506M07Rik | 0.1646232 |
| 4930507C10Rik | -0.6841781 |
| 4930511I11Rik | 1.8550414 |
| 4930511J11Rik | -0.5926791 |
| 4930515G01Rik | 1.2963425 |
| 4930518C23Rik | -0.3425302 |
| 4930519F16Rik | 3.8353939 |
| 4930519G04Rik | -0.2925697 |
| 4930519N13Rik | 0.9768962 |
| 4930519N16Rik | -0.0582138 |
| 4930522H14Rik | -0.411576 |
| 4930524B15Rik | 2.4517692 |
| 4930524B15Rik | 0.894141 |
| 4930524E20Rik | -0.1759205 |
| 4930526D03Rik | -0.6615601 |
| 4930535B03Rik | 1.6873751 |
| 4930535E21Rik | -0.5512949 |
| 4930538K18Rik | -0.2560674 |
| 4930539E08Rik | -0.5827742 |
| 4930540L03Rik | -0.5877134 |
| 4930544G11Rik | 0.1494594 |
| 4930544G21Rik | 2.1621719 |
| 4930547C10Rik | 2.2812235 |
| 4930547N16Rik | -0.4329838 |
| 4930549C01Rik | -0.3760823 |
| 4930550C14Rik | -0.4392927 |
| 4930550L24Rik | 1.8129977 |
| 4930555G01Rik | 0.6546857 |
| 4930555I21Rik | 0.3438653 |
| 4930562D19Rik | -0.3437985 |
| 4930562D19Rik | 0.0044559 |
| 4930563D23Rik | -0.8317812 |
| 4930563P21Rik | 0.7984704 |
| 4930570C03Rik | -0.4534678 |
| 4930572J05Rik | 2.5699569 |
| 4930572J05Rik | 0.4419154 |
| 4930572J05Rik | 0.3273906 |
| 4930573I19Rik | -0.2254048 |
| 4930578I06Rik | 2.5754575 |
| 4930579C15Rik | 2.440572 |
| 4930579E17Rik | 0.4620992 |
| 4930579E17Rik | -0.5918496 |
| 4930579J09Rik | -0.3495273 |
| 4930583H14Rik | 0.2530801 |
| 4930590J08Rik | -0.0365462 |
| 4930595M18Rik | 0.5208923 |
| 4930596D02Rik | -0.6421024 |
| 4930599N23Rik | 0.4035894 |
| 4931406B18Rik | -0.60437 |
| 4931406C07Rik | -0.5124389 |
| 4931406H21Rik | -0.3853269 |
| 4931406I20Rik | 1.352351 |
| 4931406I20Rik | 0.4615666 |
| 4931406P16Rik | 2.4494906 |
| 4931407G18Rik | -0.3565775 |
| 4931408A02Rik | -0.6588826 |
| 4931408C20Rik | 2.3399835 |
| 4931408C20Rik | 0.0101063 |
| 4931409K22Rik | 1.5047267 |
| 4931417E11Rik | -0.7120683 |
| 4931417G12Rik | -0.6696392 |
| 4931428L18Rik | 1.627874 |
| 4931432M23Rik | -0.2913695 |
| 4931433A01Rik | -0.5844177 |
| 4931440B09Rik | 2.3212621 |
| 4931440F15Rik | -0.6403535 |
| 4931440F15Rik | -0.49137 |
| 4931440L10Rik | 0.4878277 |
| 4932409I22Rik | 2.5425761 |
| 4932411E22Rik | -0.6111173 |
| 4932411G14Rik | 0.0349047 |
| 4932412H11Rik | 2.4097737 |
| 4932413O14Rik | 2.3131887 |
| 4932414J04Rik | 0.6503494 |
| 4932414N04Rik | 0.5862484 |
| 4932418E24Rik | -0.3274446 |
| 4932431H17Rik | 0.7679634 |
| 4932431H17Rik | -0.6342585 |
| 4932441K18Rik | 3.086849 |
| 4932441K18Rik | 0.2564089 |
| 4932442K08Rik | 0.5808769 |
| 4932702K14Rik | 0.5365017 |
| 4933400A11Rik | -0.4392927 |
| 4933400C05Rik | -0.1833638 |
| 4933400E14Rik | 1.2864328 |
| 4933402J07Rik | 0.3890149 |
| 4933402N03Rik | -0.5332188 |
| 4933402P03Rik | 0.7383975 |
| 4933403G14Rik | -0.7301759 |
| 4933404M02Rik | -0.462078 |
| 4933405O20Rik | -0.3540076 |
| 4933406M09Rik | 0.8522704 |
| 4933407N01Rik | 0.9412183 |
| 4933407N01Rik | -0.4876575 |
| 4933407N01Rik | 0.6731041 |
| 4933407P14Rik | -0.0614846 |
| 4933407P14Rik | 2.8054488 |
| 4933407P14Rik | 1.0215058 |
| 4933409N07Rik | 1.582613 |
| 4933411G06Rik | -0.3274446 |
| 4933411K20Rik | -0.0963 |
| 4933412E14Rik | -0.1643433 |
| 4933413G11Rik | -0.037002 |
| 4933415F23Rik | 3.4732849 |
| 4933416C03Rik | -0.7196495 |
| 4933417A18Rik | -0.6989485 |
| 4933417E01Rik | -0.4456447 |
| 4933421E11Rik | -0.4385896 |
| 4933421E11Rik | 1.0931056 |
| 4933421E11Rik | 1.4108358 |
| 4933422H20Rik | -0.6571019 |
| 4933424B01Rik | -0.2143006 |
| 4933424B01Rik | -0.3682311 |
| 4933424B01Rik | -0.6264805 |
| 4933425O20Rik | 1.5524522 |
| 4933426M11Rik | -0.3833381 |
| 4933427D14Rik | 0.4615666 |
| 4933428G09Rik | 3.6751784 |
| 4933428G20Rik | -0.60437 |
| 4933429E10Rik | 2.3852818 |
| 4933429E10Rik | -0.5147249 |
| 4933430I17Rik | 0.5062858 |
| 4933434E20Rik | -0.5835956 |
| 4933434E20Rik | -0.7036129 |
| 4933434E20Rik | -0.4385896 |
| 4933434I06Rik | 0.3153076 |
| 4933436I01Rik | 0.183146 |
| 4933437F05Rik | 1.4278174 |
| 4933439C20Rik | 0.5305862 |
| 4933440H19Rik | 0.8440175 |
| 5031414D18Rik | -0.459918 |
| 5031414D18Rik | -0.2265225 |
| 5033411D12Rik | -0.6170616 |
| 5033414D02Rik | -0.6094258 |
| 5033414K04Rik | -0.3946644 |
| 5133400G04Rik | 1.5943923 |
| 5133400G04Rik | -0.6068945 |
| 5133400G04Rik | 1.8923608 |
| 5133401N09Rik | -0.4839598 |
| 5230400G24Rik | -0.5852406 |
| 5230400G24Rik | 1.4751883 |
| 5330401P04Rik | 1.0181494 |
| 5330417C22Rik | 0.8195335 |
| 5330431N19Rik | -0.6562128 |
| 5330431N19Rik | 0.3081072 |
| 5330431N19Rik | -0.6553246 |
| 5330437I02Rik | 2.4537935 |
| 5330439C02Rik | 0.3630194 |
| 5330439J01Rik | -0.5426058 |
| 5330439J01Rik | -0.131463 |
| 5430407P10Rik | -0.3682311 |
| 5430432M24Rik | -0.5465453 |
| 5430432M24Rik | -0.7980413 |
| 5430432M24Rik | 1.0802059 |
| 5430435G22Rik | 3.5904143 |
| 5430437P03Rik | 1.2235895 |
| 5530601I19Rik | -0.6119641 |
| 5630401D24Rik | -0.2618586 |
| 5630401D24Rik | -0.617914 |
| 5630401D24Rik | 1.8325273 |
| 5730403M16Rik | -0.2282017 |
| 5730409E04Rik | 1.428757 |
| 5730410E15Rik | 0.607201 |
| 5730410E15Rik | 0.7383975 |
| 5730410I19Rik | -0.7417971 |
| 5730410I19Rik | 0.828365 |
| 5730410I19Rik | -0.3591546 |
| 5730419I09Rik | -0.435082 |
| 5730437N04Rik | -0.5868884 |
| 5730446C15Rik | -0.4936046 |
| 5730449L18Rik | 1.7756372 |
| 5730449L18Rik | -0.4627992 |
| 5730449L18Rik | -0.4766084 |
| 5730453I16Rik | 0.7186214 |
| 5730453I16Rik | -0.2044192 |
| 5730469M10Rik | 1.6555677 |
| 5730472N09Rik | 0.3074785 |
| 5730494M16Rik | -0.7186985 |
| 5730494N06Rik | -0.2104453 |
| 5730502D15Rik | 2.457612 |
| 5730507A09Rik | 2.3323913 |
| 5730507A09Rik | -0.6394804 |
| 5730528L13Rik | 1.1545388 |
| 5730589K01Rik | 0.3733295 |
| 5730593F17Rik | 0.1468455 |
| 5730596K20Rik | -0.6553246 |
| 5730596K20Rik | 0.1468455 |
| 5730596K20Rik | -0.380033 |
| 5730596K20Rik | 0.0174437 |
| 5830403L16Rik | 1.2193623 |
| 5830404H04Rik | 0.8179023 |
| 5830405N20Rik | -0.6230444 |
| 5830415F09Rik | -0.5552715 |
| 5830415L20Rik | -0.4980901 |
| 5830417C01Rik | -0.416367 |
| 5830426I05Rik | -0.3349567 |
| 5830427D03Rik | -0.3431641 |
| 5830434P21Rik | -0.2566449 |
| 5830435K17Rik | -0.6447318 |
| 5830457O10Rik | -0.6325244 |
| 5830457O10Rik | -0.711125 |
| 5830457O10Rik | -0.4958446 |
| 5830467P10Rik | -0.3806931 |
| 5830468K18Rik | 3.5813981 |
| 5830472M02Rik | -0.3268213 |
| 5830472M02Rik | 0.5740596 |
| 5830472M02Rik | 0.4572949 |
| 5930416I19Rik | -0.6204756 |
| 5930431H10 | -0.6980184 |
| 5930434B04Rik | -0.6325244 |
| 5930434B04Rik | 0.1306251 |
| 5930434B04Rik | -0.1796347 |
| 6030405A18Rik | -0.4357825 |
| 6030408B16Rik | -0.4392927 |
| 6030408B16Rik | 0.1393364 |
| 6030419C18Rik | -0.7186985 |
| 6030429G01Rik | 0.5662405 |
| 6030443O07Rik | 0.6398589 |
| 6030446N20Rik | 0.1704742 |
| 6030458C11Rik | 0.5850834 |
| 6230409E13Rik | 2.3293725 |
| 6230409E13Rik | 0.3872851 |
| 6230416J20Rik | -0.7644863 |
| 6230427J02Rik | 0.6400786 |
| 6330403K07Rik | 0.7708306 |
| 6330406I15Rik | -0.0765856 |
| 6330406I15Rik | 0.6863684 |
| 6330407J23Rik | 0.0268538 |
| 6330408A02Rik | -0.5632762 |
| 6330409N04Rik | -0.5071266 |
| 6330416G13Rik | -0.3760823 |
| 6330439K17Rik | 1.2737563 |
| 6330442E10Rik | 0.1513219 |
| 6330442E10Rik | -0.6998795 |
| 6330500D04Rik | 2.931974 |
| 6330503C03Rik | -0.7167993 |
| 6330503K22Rik | -0.5208487 |
| 6330503K22Rik | -0.6256202 |
| 6330503K22Rik | -0.6316586 |
| 6330505N24Rik | 3.2102858 |
| 6330512M04Rik | -0.300409 |
| 6330534C20Rik | -0.2665177 |
| 6330548G22Rik | -0.6239022 |
| 6330569M22Rik | -0.5339974 |
| 6330577E15Rik | -0.7795883 |
| 6330578E17Rik | 0.6407376 |
| 6332401O19Rik | -0.7158512 |
| 6430411K18Rik | 2.3188906 |
| 6430502M16Rik | 0.8106172 |
| 6430510M02Rik | 1.8161576 |
| 6430517E21Rik | -0.7506095 |
| 6430526N21Rik | -0.4824849 |
| 6430527G18Rik | 1.1796885 |
| 6430527G18Rik | 0.5141214 |
| 6430531B16Rik | -0.7939088 |
| 6430537H07Rik | -0.5844177 |
| 6430537H07Rik | 0.9448643 |
| 6430548M08Rik | 4.5422401 |
| 6430550H21Rik | 2.044179 |
| 6430571L13Rik | -0.0789675 |
| 6430573F11Rik | 0.6518694 |
| 6430598A04Rik | 0.4041563 |
| 6430604K15Rik | 2.4206376 |
| 6430706D22Rik | -0.3866551 |
| 6530404N21Rik | -0.2287621 |
| 6530406M24Rik | -0.6805219 |
| 6530418L21Rik | 2.125238 |
| 6530418L21Rik | 1.8765006 |
| 6530418L21Rik | 3.1422067 |
| 6530421E24Rik | -0.60437 |
| 6620401K05Rik | -0.5116781 |
| 6720456B07Rik | -0.6204756 |
| 6720456B07Rik | -0.4664133 |
| 6720456H20Rik | -0.6052107 |
| 6720456H20Rik | 0.364795 |
| 6720458F09Rik | 0.4713701 |
| 6720463M24Rik | -0.1174363 |
| 6720467C03Rik | 2.3075973 |
| 6720467C03Rik | 0.2009535 |
| 6820408C15Rik | -0.0069491 |
| 7420416P09Rik | -0.5124389 |
| 7530404M11Rik | -0.3919869 |
| 8030411F24Rik | -0.0420304 |
| 8030451F13Rik | -0.3218501 |
| 8030451F13Rik | 1.4194129 |
| 8030451F13Rik | 1.304431 |
| 8430408G22Rik | 0.3498936 |
| 8430410A17Rik | -0.2764967 |
| 8430410K20Rik | 0.038694 |
| 8430415E04Rik | -0.6230444 |
| 8430415E04Rik | -0.5147249 |
| 8430419L09Rik | -0.6456099 |
| 8430427H17Rik | 1.2718676 |
| 8430432M10Rik | -0.1149537 |
| 9030025P20Rik | -0.0270261 |
| 9030025P20Rik | -0.411576 |
| 9030025P20Rik | 0.108677 |
| 9030205A07Rik | -0.4839598 |
| 9030221M09Rik | 1.0048978 |
| 9030224M15Rik | 0.8369354 |
| 9030227G01Rik | -0.568921 |
| 9030409G11Rik | -0.0252238 |
| 9030418K01Rik | 0.736617 |
| 9030425E11Rik | 0.0200196 |
| 9030607L17Rik | 3.2236912 |
| 9030607L17Rik | -0.7515938 |
| 9030611O19Rik | 0.1967108 |
| 9030612M13Rik | -0.5877134 |
| 9030617O03Rik | 0.7456817 |
| 9030624J02Rik | 0.2006007 |
| 9030625A04Rik | 0.8151771 |
| 9130005N14Rik | 0.1090665 |
| 9130011E15Rik | 0.4246149 |
| 9130011J15Rik | -0.0216296 |
| 9130014G24Rik | 0.4546151 |
| 9130017N09Rik | -0.1395744 |
| 9130206N08Rik | 1.5714072 |
| 9130211I03Rik | 3.9144111 |
| 9130213B05Rik | -0.4758764 |
| 9130213B05Rik | 0.9979015 |
| 9130218O11Rik | 0.5636194 |
| 9130221H12Rik | 3.2522657 |
| 9130227C08Rik | -0.7939088 |
| 9130401M01Rik | -0.4108936 |
| 9130404D08Rik | -0.5339974 |
| 9130404D08Rik | 1.4462463 |
| 9130404D14Rik | -0.5324408 |
| 9130422G05Rik | -0.3913188 |
| 9230104L09Rik | -0.5481258 |
| 9330129D05Rik | 0.9872316 |
| 9330134C04Rik | -0.215405 |
| 9330176C04Rik | -0.6351268 |
| 9330186A19Rik | 0.6473019 |
| 9430015G10Rik | 0.7565987 |
| 9430015G10Rik | 3.6828768 |
| 9430023L20Rik | 0.7567925 |
| 9430025C20Rik | -0.3274446 |
| 9430028L06Rik | 0.038694 |
| 9430029K10Rik | 0.2177331 |
| 9430031J16Rik | 0.3435629 |
| 9430034D17Rik | 0.1646232 |
| 9430057O19Rik | 0.8380012 |
| 9430069J07Rik | 2.3306878 |
| 9430077A04Rik | 0.2877723 |
| 9430079M16Rik | -0.411576 |
| 9530015I07Rik | 0.7421452 |
| 9530027K23Rik | 4.0064028 |
| 9530058B02Rik | 0.8395975 |
| 9530058B02Rik | 0.3374932 |
| 9530068E07Rik | -0.6111173 |
| 9530077C05Rik | 0.713357 |
| 9530096D07Rik | 1.2506064 |
| 9530096D07Rik | -0.8535092 |
| 9630015D15Rik | 0.5223903 |
| 9630020C08Rik | 0.9029553 |
| 9630028B13Rik | -0.4020676 |
| 9630031F12Rik | -0.49137 |
| 9630050M13Rik | -0.4499037 |
| 9630058J23Rik | 0.1546652 |
| 9830163H01Rik | -0.5754144 |
| 9830169C18Rik | -0.5868884 |
| 9930013L23Rik | -0.5552715 |
| 9930021D14Rik | -0.5778612 |
| 9930023K05Rik | -0.5648855 |
| 9930023K05Rik | 0.6869953 |
| 9930104L06Rik | -0.3156734 |
| a | 1.7349551 |
| A030003K21Rik | -0.5277866 |
| A030007L17Rik | 0.3096773 |
| A030007L17Rik | -0.6606667 |
| A030007L17Rik | 0.2232595 |
| A030007L17Rik | 1.3477508 |
| A030009H04Rik | 1.9716597 |
| A030010B05Rik | 0.4940213 |
| A030012M09Rik | 3.0377264 |
| A130010J15Rik | 0.345376 |
| A130038H09Rik | 1.6763662 |
| A130042E20Rik | -0.7101827 |
| A130090K04Rik | 0.6878305 |
| A130092J06Rik | 0.2345549 |
| A230050P20Rik | -0.5754144 |
| A230051G13Rik | -0.4541823 |
| A230054D04Rik | 1.8263689 |
| A230067G21Rik | -0.6473687 |
| A230069A22Rik | 1.1904352 |
| A230074B11Rik | -0.2242883 |
| A230074B11Rik | -0.2653508 |
| A230074B11Rik | -0.3826762 |
| A230106M15Rik | -0.1329785 |
| A230106N23Rik | 2.0830223 |
| A230107C01Rik | 1.1818968 |
| A2bp1 | -0.5893657 |
| A2bp1 | 0.9226082 |
| A330021E22Rik | -0.6961611 |
| A330021E22Rik | 2.1581162 |
| A330049M08Rik | 1.1882471 |
| A330080J22Rik | 0.8075037 |
| A3galt2 | 2.42257 |
| A430005L14Rik | -0.8189845 |
| A430005L14Rik | 1.7319121 |
| A430005L14Rik | 1.9653087 |
| A430005L14Rik | -0.4988398 |
| A430033K04Rik | -0.4449368 |
| A430079E08 | -0.1903293 |
| A430083B19Rik | -0.2665177 |
| A430083B19Rik | 1.3703268 |
| A430107D22Rik | -0.2665177 |
| A430107P09Rik | 1.424427 |
| A430110N23Rik | -0.063828 |
| A4galt | 0.063568 |
| A4gnt | 0.6254612 |
| A530050D06Rik | -0.6723479 |
| A530082C11Rik | 0.8434882 |
| A530082C11Rik | 0.7693025 |
| A530088H08Rik | -0.6615601 |
| A530088H08Rik | 1.6107441 |
| A530098C11Rik | 0.8874327 |
| A630042L21Rik | -0.6247608 |
| A630054L15Rik | -0.3268213 |
| A630077B13Rik | -0.5324408 |
| A630095E13Rik | 1.1763661 |
| A730008H23Rik | -0.0060668 |
| A730008L03Rik | 1.9599739 |
| A730008L03Rik | -0.416367 |
| A730017C20Rik | 0.120678 |
| A730024F05Rik | -0.6316586 |
| A730037C10Rik | -0.0837497 |
| A730042J05Rik | 0.4605005 |
| A730098D12Rik | 0.9776601 |
| A830006F12Rik | 0.0789834 |
| A830007P12Rik | -0.5473352 |
| A830053O21Rik | -0.5301108 |
| A830059I20Rik | -0.6998795 |
| A830073O21Rik | -0.3919869 |
| A830080D01Rik | 2.331327 |
| A830093I24Rik | -0.4499037 |
| A930001N09Rik | -0.253761 |
| A930005I04Rik | -0.3089261 |
| A930008G19Rik | 0.0040199 |
| A930010I20Rik | 0.6583556 |
| A930016P21Rik | 1.2769693 |
| A930018P22Rik | 1.4544123 |
| A930024E05Rik | 1.243295 |
| A930025D01Rik | -0.456329 |
| A930025D01Rik | 2.2493205 |
| A930025D01Rik | -0.5844177 |
| A930034L06Rik | -0.5528835 |
| A930038C07Rik | 0.4834155 |
| A930041I02Rik | -0.5713507 |
| AA388235 | -0.2595378 |
| AA407270 | 0.8891143 |
| AA407659 | 0.6915798 |
| AA407659 | 0.5973593 |
| AA408296 | 0.4836756 |
| AA408296 | -0.7447253 |
| AA408296 | -0.5246966 |
| AA409316 | -0.6377365 |
| AA415398 | -0.6660398 |
| AA467197 | 1.1938286 |
| AA536717 | -0.6786993 |
| AA536717 | -0.6869297 |
| AA536749 | -0.3886509 |
| AA536749 | -0.1759205 |
| AA536749 | -0.4357825 |
| AA536749 | 0.8180837 |
| AA673488 | -0.1769802 |
| AA691260 | 0.6709811 |
| AA792892 | -0.6010147 |
| AA881470 | -0.5101585 |
| AA881470 | 1.2706434 |
| AA960436 | 2.1719603 |
| AA960436 | -0.2503122 |
| Aaas | 1.6394701 |
| Aaas | -0.3552917 |
| Aacs | 0.5511275 |
| Aadacl1 | -0.6052107 |
| Aak1 | 0.0412116 |
| Aak1 | 0.8186276 |
| Aamp | -0.395335 |
| Aard | -0.1554952 |
| Aars | -0.5943402 |
| Aars2 | -0.5803145 |
| Aarsd1 | 0.2253235 |
| Aasdh | -0.4204934 |
| Aatf | -0.7130126 |
| Aatf | 1.1623308 |
| Aatk | -0.4414052 |
| AB112350 | 2.0945026 |
| AB182283 | -0.1674864 |
| AB182283 | 1.1117946 |
| Abat | 3.1141929 |
| Abat | -0.333073 |
| Abat | -0.4591991 |
| Abca14 | -0.4577629 |
| Abca14 | 1.4977786 |
| Abca15 | -0.6642453 |
| Abca15 | -0.5177817 |
| Abca15 | -0.2723747 |
| Abca15 | 1.466573 |
| Abca17 | -0.1329785 |
| Abca2 | -0.0411141 |
| Abca3 | -0.6456099 |
| Abca4 | -0.7715033 |
| Abca4 | -0.3268213 |
| Abca5 | 0.6164887 |
| Abca6 | -0.5223859 |
| Abca6 | -0.0628899 |
| Abca7 | -0.6805219 |
| Abca7 | 0.8358685 |
| Abca8a | 0.5602727 |
| Abca8b | -0.7369372 |
| Abca9 | -0.3754255 |
| Abca9 | -0.5976714 |
| Abca9 | 1.5771071 |
| Abcb11 | -0.7130126 |
| Abcb1b | 1.080616 |
| Abcb4 | 1.1540338 |
| Abcb6 | -0.8296361 |
| Abcb8 | 0.9084015 |
| Abcb8 | -0.7715033 |
| Abcb8 | -0.4758764 |
| Abcb9 | -0.7949402 |
| Abcb9 | 0.101244 |
| Abcc1 | -0.4869168 |
| Abcc10 | -0.6535507 |
| Abcc10 | 1.5960405 |
| Abcc12 | -0.2276416 |
| Abcc3 | 0.5922833 |
| Abcc5 | -0.7867209 |
| Abcc6 | 2.0962422 |
| Abcc8 | -0.5568669 |
| Abcc9 | -0.2853918 |
| Abcc9 | 0.1704742 |
| Abcd1 | -0.5632762 |
| Abcd2 | -0.6102712 |
| Abcd2 | -0.3773974 |
| Abcd3 | 0.8346221 |
| Abcd4 | -0.3591546 |
| Abcd4 | 0.1712029 |
| Abce1 | -0.2985941 |
| Abcf1 | -0.8074073 |
| Abcf1 | 0.3820763 |
| Abcf1 | 2.972472 |
| Abcf2 | 1.9499614 |
| Abcf2 | -0.6204756 |
| Abcf2 | -0.1431456 |
| Abcf2 | -0.300409 |
| Abcf3 | -0.6878487 |
| Abcg1 | 1.205131 |
| Abcg4 | 2.3926876 |
| Abcg5 | -0.572162 |
| Abhd1 | 2.2282987 |
| Abhd1 | 4.0792025 |
| Abhd10 | -0.2919694 |
| Abhd11 | 0.8084205 |
| Abhd12 | -0.5086413 |
| Abhd14a | 0.9440729 |
| Abhd14b | 0.121447 |
| Abhd2 | 3.3861698 |
| Abhd2 | 1.7216276 |
| Abhd2 | 1.3163801 |
| Abhd3 | 0.2916192 |
| Abhd4 | 0.7276628 |
| Abhd5 | -0.4301936 |
| Abhd6 | -0.5301108 |
| Abhd7 | -0.0176028 |
| Abhd7 | 0.1605791 |
| Abhd8 | -0.184432 |
| Abi1 | -0.4534678 |
| Abi1 | -0.6351268 |
| Abi2 | 0.1047723 |
| Abi2 | -0.1539425 |
| Abi2 | -0.693382 |
| Abi3 | 0.9558734 |
| Abi3bp | 3.8482084 |
| Abi3bp | 0.2009535 |
| Abl1 | 1.2088284 |
| Ablim1 | 0.7033537 |
| Ablim2 | 0.1404666 |
| Ablim2 | -0.6394804 |
| Ablim3 | -0.7765481 |
| Ablim3 | 0.8403062 |
| Abp1 | 0.2301216 |
| Abr | -0.6624543 |
| Abr | -0.6723479 |
| Abtb1 | 0.1145033 |
| Acaa1a | 1.7668076 |
| Acaa1b | -0.6359959 |
| Acaa2 | 0.8597572 |
| Acaa2 | -0.6394804 |
| Acaa2 | 1.1620804 |
| Acaa2 | 1.1927392 |
| Acad10 | 0.6164887 |
| Acad10 | -0.0694764 |
| Acad8 | -0.5520888 |
| Acad8 | 0.48938 |
| Acad9 | -0.2794523 |
| Acad9 | 0.2177331 |
| Acadl | 1.2175953 |
| Acadl | 1.7502879 |
| Acadm | -0.6796102 |
| Acads | -0.521617 |
| Acads | 0.6752222 |
| Acadsb | 3.1618312 |
| Acadvl | -0.6068945 |
| Acan | 4.0366342 |
| Acat2 | -0.6447318 |
| Acbd3 | -0.5976714 |
| Acbd4 | 1.7303529 |
| Acbd4 | 0.6518694 |
| Acbd5 | -0.6943074 |
| Acbd6 | 1.765698 |
| Accn1 | 1.7287232 |
| Accn1 | -0.5968375 |
| Accn2 | 0.9922061 |
| Accn3 | 1.1272596 |
| Accn3 | 0.8475384 |
| Accs | -0.6823482 |
| Acd | 0.0996716 |
| Ace | 1.0949915 |
| Ace | 2.2402799 |
| Ache | -0.5536788 |
| Acin1 | -0.6085813 |
| Acly | 4.5945891 |
| Acn9 | -0.711125 |
| Aco1 | -0.4541823 |
| Aco2 | -0.5308868 |
| Acot1 | -0.6491308 |
| Acot1 | -0.6491308 |
| Acot10 | -0.8011528 |
| Acot11 | -0.3514448 |
| Acot11 | -0.3058753 |
| Acot2 | -0.5918496 |
| Acot2 | -0.1962643 |
| Acot3 | 0.8628733 |
| Acot5 | -0.5465453 |
| Acot7 | -0.5993416 |
| Acot7 | -0.1508448 |
| Acot7 | -0.6412275 |
| Acot7 | -0.6517802 |
| Acot8 | -0.0073906 |
| Acot8 | -0.5844177 |
| Acot8 | -0.6060522 |
| Acot9 | 2.825711 |
| Acox2 | -0.1822968 |
| Acox3 | 1.0818453 |
| Acox3 | -0.4149957 |
| Acoxl | -0.6906112 |
| Acp2 | -0.2298839 |
| Acp6 | -0.6170616 |
| Acpl2 | -0.6924575 |
| Acpp | 2.2977119 |
| Acr | 1.1213923 |
| Acr | -0.6290659 |
| Acrbp | -0.1264291 |
| Acrbp | -0.5852406 |
| Acrv1 | -0.4773409 |
| Acsbg1 | -0.7535657 |
| Acsbg1 | -0.1606897 |
| Acsbg2 | -0.4477717 |
| Acsf3 | -0.6447318 |
| Acsl1 | -0.4965925 |
| Acsl3 | 1.8928165 |
| Acsl3 | 0.0357481 |
| Acsl4 | -0.4950974 |
| Acsl4 | 0.6241208 |
| Acsl5 | -0.2366435 |
| Acsl6 | 2.2131415 |
| Acsl6 | 0.6762795 |
| Acsl6 | -0.3598 |
| Acsm3 | 1.2746438 |
| Acss1 | 4.2316765 |
| Acss2 | -0.2120956 |
| Acss2 | -0.6026908 |
| Acta1 | -0.5426058 |
| Acta2 | -0.7704976 |
| Acta2 | 0.7060181 |
| Acta2 | 0.294175 |
| Acta2 | -0.4288016 |
| Actb | 0.6982081 |
| Actb | -0.1791032 |
| Actb | 0.5305862 |
| Actb | 0.9662898 |
| Actb | 0.6103829 |
| Actg2 | -0.6915339 |
| Actl6a | 0.0532847 |
| Actl6b | -0.4246382 |
| Actl6b | 1.7791842 |
| Actl6b | -0.2865843 |
| Actl6b | 1.1979326 |
| Actl7a | -0.7816206 |
| Actl7b | 0.2041229 |
| Actn1 | -0.6247608 |
| Actn2 | 2.209627 |
| Actn3 | -0.5041047 |
| Actn3 | -0.5147249 |
| Actn4 | 0.8618358 |
| Actn4 | 1.281383 |
| Actn4 | 1.3785911 |
| Actr10 | -0.3591546 |
| Actr10 | -0.5926791 |
| Actr1a | -0.4184279 |
| Actr1b | -0.3495273 |
| Actr3 | 0.2813251 |
| Actr3b | -0.5592652 |
| Actr5 | 0.5231384 |
| Actr5 | -0.5918496 |
| Actr6 | 0.1125653 |
| Actrt1 | 3.0708314 |
| Actrt2 | 0.4083971 |
| Acvr1 | 0.1693799 |
| Acvr1c | -0.0012291 |
| Acvr2a | 1.6113613 |
| Acvr2b | -0.407489 |
| Acvrl1 | 0.6853225 |
| Acy1 | -0.3773974 |
| Acy1l2 | -0.6145094 |
| Acy3 | 2.2265119 |
| Ada | -0.4869168 |
| Adal | 2.2552733 |
| Adal | 1.7762831 |
| Adam11 | -0.4788077 |
| Adam11 | 0.1225992 |
| Adam12 | 0.8697609 |
| Adam15 | -0.5101585 |
| Adam17 | 0.6971755 |
| Adam1b | -0.7674871 |
| Adam1b | -0.5568669 |
| Adam21 | -0.6980184 |
| Adam22 | 2.0067157 |
| Adam22 | -0.2226162 |
| Adam22 | 0.5384652 |
| Adam23 | 0.5211422 |
| Adam24 | 1.5267864 |
| Adam26a | 0.5771218 |
| Adam28 | 1.6880855 |
| Adam29 | 0.9211486 |
| Adam29 | 1.6632578 |
| Adam3 | -0.1648664 |
| Adam30 | -0.0948432 |
| Adam32 | -0.5285606 |
| Adam33 | -0.6500131 |
| Adam39 | 0.2932174 |
| Adam4 | 0.1906668 |
| Adam4 | 0.8418985 |
| Adam4 | -0.6018524 |
| Adam5 | -0.6230444 |
| Adam6 | -0.714904 |
| Adam8 | -0.4766084 |
| Adam8 | -0.6342585 |
| Adam9 | 2.6990199 |
| Adamdec1 | 0.8069531 |
| Adamts1 | 0.3131221 |
| Adamts13 | -0.3747692 |
| Adamts15 | -0.549709 |
| Adamts16 | -0.3280683 |
| Adamts17 | 0.2365939 |
| Adamts18 | -0.2806371 |
| Adamts19 | 0.91267 |
| Adamts2 | 0.274507 |
| Adamts2 | -0.2242883 |
| Adamts2 | 1.3224027 |
| Adamts20 | -0.2717874 |
| Adamts20 | 0.078581 |
| Adamts4 | 0.8818617 |
| Adamts7 | 0.290659 |
| Adamtsl2 | -0.4506154 |
| Adamtsl4 | 0.8076871 |
| Adamtsl5 | -0.5505016 |
| Adar | -0.3495273 |
| Adar | -0.6732526 |
| Adar | 1.3884514 |
| Adarb1 | -0.4810123 |
| Adarb1 | 0.5966687 |
| Adarb1 | -0.7026781 |
| Adat1 | 0.4888629 |
| Adc | -0.1513604 |
| Adc | -0.6705412 |
| Adck1 | -0.6906112 |
| Adck2 | 2.3191424 |
| Adck4 | -0.5762293 |
| Adck5 | 0.4165414 |
| Adcy1 | 0.5315748 |
| Adcy10 | 0.9375579 |
| Adcy2 | 0.9407417 |
| Adcy4 | 1.3571313 |
| Adcy4 | 0.3243074 |
| Adcy6 | 1.1425988 |
| Adcy7 | 1.4346546 |
| Adcy8 | -0.1722212 |
| Adcy9 | 1.9440525 |
| Adcyap1 | 2.6360794 |
| Adcyap1r1 | 1.7779604 |
| Add1 | -0.60437 |
| Add1 | 0.0594683 |
| Add1 | -0.2321316 |
| Add1 | 1.9049241 |
| Add3 | 2.1203936 |
| Add3 | 3.8539139 |
| Add3 | 0.4750526 |
| Adfp | -0.6412275 |
| Adh1 | -0.3919869 |
| Adh1 | 0.6427118 |
| Adh4 | 0.8047477 |
| Adh5 | 0.2758096 |
| Adh7 | -0.028832 |
| Adhfe1 | 0.3782961 |
| Adi1 | 0.6212101 |
| Adipoq | -0.6508962 |
| Adipor1 | -0.6696392 |
| Adipor2 | -0.2343848 |
| Adk | 0.5163838 |
| Adk | -0.4484818 |
| Adm | -0.3591546 |
| Adm2 | -0.500341 |
| Adnp | 0.5100855 |
| Ado | -0.1319679 |
| Adora1 | -0.668738 |
| Adora1 | 0.9588392 |
| Adora1 | 1.9266459 |
| Adora2b | 2.189166 |
| Adora3 | -0.4556129 |
| Adpgk | 1.7484267 |
| Adprh | -0.1759205 |
| Adprhl1 | -0.3169054 |
| Adprhl1 | -0.1930223 |
| Adprhl1 | 2.1139995 |
| Adprhl2 | 1.3400328 |
| Adra1a | 0.2784092 |
| Adra1a | 0.3771299 |
| Adra1d | -0.5347767 |
| Adra2a | -0.6915339 |
| Adra2c | 0.2457143 |
| Adra2c | -0.4527539 |
| Adrb1 | 1.0828002 |
| Adrb2 | -0.6230444 |
| Adrbk1 | -0.2647678 |
| Adrbk2 | -0.3636818 |
| Adrm1 | 0.4831553 |
| Adsl | -0.6438545 |
| Adss | 1.4612383 |
| Adssl1 | 0.1210626 |
| Adssl1 | 1.8808373 |
| Adssl1 | -0.4556129 |
| Aebp1 | -0.6052107 |
| Aebp2 | -0.6264805 |
| Aes | -0.2818235 |
| Afap1 | 0.4459874 |
| Afap1 | 0.2712426 |
| Afap1 | -0.2332575 |
| Afap1l1 | 0.6878305 |
| Aff1 | 0.5716971 |
| Aff4 | -0.5811337 |
| Afg3l1 | -0.3224701 |
| Afg3l1 | -0.6026908 |
| Afg3l1 | 2.4236953 |
| Afg3l1 | -0.7605003 |
| Afg3l2 | 2.5483533 |
| Afmid | -0.6145094 |
| Aftph | 1.1952792 |
| Agbl1 | 1.4192232 |
| Ager | 0.8906252 |
| Ager | -0.362386 |
| Aggf1 | -0.4891407 |
| Aggf1 | -0.3193745 |
| Aggf1 | -0.6482493 |
| Agpat1 | -0.6128118 |
| Agpat2 | 1.7609836 |
| Agpat3 | -0.7158512 |
| Agpat4 | -0.5418199 |
| Agpat5 | -0.4061307 |
| Agpat6 | -0.5101585 |
| Agpat6 | 0.7717844 |
| Agpat7 | 0.5472498 |
| Agpat9 | -0.5223859 |
| Agpat9 | 0.7021213 |
| Agps | -0.6204756 |
| Agps | 0.1974193 |
| Agrn | -0.4707689 |
| Agrp | -0.4204934 |
| Agt | 1.3119172 |
| Agtpbp1 | -0.2187263 |
| Agtr1a | -0.4635209 |
| Agtrap | -0.5185475 |
| Agtrl1 | 2.0502544 |
| Agxt2l1 | -0.0360906 |
| Agxt2l1 | 0.2718964 |
| Agxt2l2 | 1.057646 |
| Agxt2l2 | -0.3144429 |
| Agxt2l2 | 2.4427467 |
| Agxt2l2 | -0.065707 |
| Ahctf1 | -0.3132141 |
| Ahcy | -0.1179336 |
| Ahcyl1 | -0.4301936 |
| Ahdc1 | 1.0601644 |
| Ahi1 | -0.3617388 |
| Ahnak | -0.7836573 |
| Ahnak | 0.0013991 |
| Ahnak2 | -0.4980901 |
| Ahrr | -0.5528835 |
| Ahsa1 | -0.6001777 |
| Ahsa2 | 1.1973303 |
| AI118078 | -0.1769802 |
| AI132487 | 0.2020112 |
| AI314180 | -0.6750646 |
| AI314976 | -0.6633494 |
| AI316807 | -0.3374743 |
| AI317395 | -0.40342 |
| AI413582 | 1.3224027 |
| AI413782 | -0.1436569 |
| AI427515 | -0.3578652 |
| AI428936 | 1.1317975 |
| AI449175 | 0.5829825 |
| AI449175 | -0.5301108 |
| AI450540 | 1.4639547 |
| AI451557 | -0.3412636 |
| AI451617 | 0.4513891 |
| AI467606 | 0.8360464 |
| AI480653 | -0.5262403 |
| AI504432 | -0.4958446 |
| AI553587 | -0.1738048 |
| AI593442 | -0.7506095 |
| AI593442 | -0.6456099 |
| AI593442 | -0.7008114 |
| AI595366 | 0.1464714 |
| AI595406 | 0.8621818 |
| AI597468 | -0.6230444 |
| AI597479 | -0.5441796 |
| AI606181 | 0.6149096 |
| AI646023 | 1.6960751 |
| AI661453 | 1.0081546 |
| AI662250 | 2.25562 |
| AI747699 | 0.5113486 |
| AI836003 | -0.4274118 |
| AI837181 | -0.617914 |
| AI842396 | -0.4421104 |
| AI846148 | -0.510918 |
| AI847670 | -0.6915339 |
| AI851790 | -0.4751451 |
| AI894139 | 0.5462778 |
| AI987692 | 2.8389088 |
| AI987692 | -0.3675799 |
| AI987944 | -0.2066059 |
| Aicda | 1.8730165 |
| Aif1 | -0.1780411 |
| Aif1 | -0.6060522 |
| Aif1 | 0.85262 |
| Aifm1 | -0.6136602 |
| Aifm1 | 4.3764726 |
| Aifm2 | -0.6579918 |
| Aifm2 | -0.3773974 |
| Aifm2 | 0.8644275 |
| Aifm3 | 3.0780973 |
| Aig1 | -0.0827913 |
| Aig1-pending | 0.5897349 |
| Aim1 | 0.1051635 |
| Aim2 | -0.500341 |
| Aip | -0.5576657 |
| Ak1 | -0.5048592 |
| AK122209 | -0.6508962 |
| AK122525 | 0.9890436 |
| AK129128 | -0.210995 |
| AK129302 | -0.7437482 |
| AK129302 | -0.2688561 |
| AK129302 | 0.374793 |
| AK129341 | -0.4921142 |
| AK162044 | -0.693382 |
| Ak2 | -0.7408231 |
| Ak2 | 0.748423 |
| Ak2 | 0.5491907 |
| Ak3 | 1.5472598 |
| Ak3 | 4.2263377 |
| Ak3l1 | 0.2979955 |
| Ak5 | 0.0627495 |
| Akap1 | 0.871646 |
| Akap1 | -0.572162 |
| Akap1 | -0.0003522 |
| Akap10 | -0.7476627 |
| Akap10 | 0.0503849 |
| Akap12 | -0.411576 |
| Akap14 | -0.8723868 |
| Akap2 | 0.8385336 |
| Akap2 | -0.5410347 |
| Akap3 | 2.0235861 |
| Akap4 | 0.3423521 |
| Akap4 | -0.4906263 |
| Akap4 | -0.5093996 |
| Akap4 | 0.1686497 |
| Akap6 | -0.5713507 |
| Akap7 | 3.2109738 |
| Akap8 | 2.0824655 |
| Akap8l | 2.9261403 |
| Akap9 | -0.7350004 |
| Akap9 | -0.5347767 |
| Akap9 | 2.146795 |
| Akna | -0.6980184 |
| Akp2 | -0.7263343 |
| Akp5 | 1.1405511 |
| Akr1a4 | 1.3804727 |
| Akr1a4 | -0.2595378 |
| Akr1b3 | -0.6651421 |
| Akr1b8 | 0.8585425 |
| Akr1c12 | -0.0842292 |
| Akr1c14 | 0.0660191 |
| Akr1c14 | -0.2389077 |
| Akr1c18 | 0.9652069 |
| Akr1c19 | 1.2760841 |
| Akr1e1 | -0.340631 |
| Akr7a5 | -0.3150579 |
| Akr7a5 | 0.8601039 |
| Akr7a5 | 0.8308729 |
| Akt1s1 | -0.6325244 |
| Akt3 | 1.2171235 |
| Akt3 | 3.644247 |
| Aktip | -0.6464889 |
| Alad | -0.4322855 |
| Alas1 | -0.6094258 |
| Alas2 | -0.5640805 |
| Alcam | -0.2389077 |
| Aldh16a1 | 0.8719883 |
| Aldh18a1 | -0.4606374 |
| Aldh1a1 | -0.3293169 |
| Aldh1a2 | 0.2236038 |
| Aldh1a2 | -0.520081 |
| Aldh1a7 | -0.1973475 |
| Aldh1b1 | 1.6222499 |
| Aldh1l1 | -0.4736841 |
| Aldh1l2 | -0.7177484 |
| Aldh1l2 | -0.3040496 |
| Aldh2 | 1.156178 |
| Aldh3a1 | -0.6438545 |
| Aldh3a2 | -0.5231555 |
| Aldh3b1 | -0.1179336 |
| Aldh3b1 | -0.4281064 |
| Aldh3b1 | 0.2803542 |
| Aldh4a1 | 0.8166315 |
| Aldh5a1 | 0.8349784 |
| Aldh5a1 | 2.1340615 |
| Aldh6a1 | 0.5325622 |
| Aldh8a1 | 0.1773746 |
| Aldh8a1 | 2.208493 |
| Aldoa | -0.3886509 |
| Aldoc | 2.8120478 |
| Alg1 | 1.2565048 |
| Alg10b | 2.0580076 |
| Alg11 | 2.1923878 |
| Alg11 | 1.8613076 |
| Alg12 | 0.3832364 |
| Alg12 | 0.3677468 |
| Alg14 | -0.3299419 |
| Alg2 | -0.1764502 |
| Alg3 | -0.2033277 |
| Alg3 | -0.4744143 |
| Alg3 | -0.1711669 |
| Alg5 | 1.3934401 |
| Alg5 | -0.4980901 |
| Alg5 | 0.9904003 |
| Alg8 | -0.6924575 |
| Alg8 | -0.4898832 |
| Alg8 | -0.714904 |
| Alg8 | 0.1195234 |
| Alg9 | 0.4841956 |
| Alg9 | 1.4272532 |
| Alg9 | 0.9704546 |
| Alk | -0.6119641 |
| Alkbh2 | -0.4548973 |
| Alkbh3 | -0.6273415 |
| Alkbh4 | -0.5262403 |
| Alkbh5 | -0.5489171 |
| Alkbh7 | -0.7388781 |
| Alkbh8 | 1.6091218 |
| Allc | -0.0929043 |
| Allc | 1.220891 |
| Alox12 | -0.520081 |
| Alox12b | 0.8505206 |
| Alox12e | 1.5815781 |
| Alox15 | 0.7556292 |
| Alox5ap | 1.3912914 |
| Alox8 | -0.6351268 |
| Aloxe3 | -0.5410347 |
| Alpk3 | 0.2995827 |
| Als2 | 2.7049715 |
| Als2 | -0.6961611 |
| Als2 | -0.6421024 |
| Als2cl | -0.6145094 |
| Als2cr13 | 1.1791971 |
| Als2cr2 | -0.3708408 |
| Als2cr2 | -0.4534678 |
| Als2cr4 | 0.6218826 |
| Als2cr4 | 0.3858412 |
| Als2cr4 | -0.350166 |
| Alx3 | 1.1034311 |
| Amac1 | -0.5426058 |
| Ambp | 1.4700826 |
| Ambra1 | -0.2287621 |
| Ambra1 | -0.7206016 |
| Amd2 | 0.8367577 |
| Amdhd2 | 0.5857826 |
| Amdhd2 | -0.711125 |
| Amfr | -0.2764967 |
| Amh | 0.0170137 |
| Amhr2 | 2.8683032 |
| Amhr2 | 2.4622783 |
| Amhr2 | 0.2754841 |
| Amigo1 | 1.4439395 |
| Amigo1 | 0.0841989 |
| Amigo2 | -0.6850944 |
| Amigo3 | 0.9781181 |
| Ammecr1l | -0.5101585 |
| Amn | -0.6952338 |
| Amotl2 | 2.4402564 |
| Ampd1 | 2.360917 |
| Ampd1 | 1.6769413 |
| Ampd2 | 1.9247771 |
| Amph | -0.7311388 |
| Amph | 0.7622021 |
| Amy1 | -0.7225085 |
| Amz2 | -0.4995901 |
| Anapc10 | 2.0628088 |
| Anapc11 | -0.6517802 |
| Anapc13 | -0.6714441 |
| Anapc2 | 0.4137411 |
| Anapc4 | -0.2753172 |
| Anapc5 | 0.7330457 |
| Anapc5 | -0.6796102 |
| Anapc5 | 0.0680567 |
| Anapc7 | 0.3112447 |
| Ang | 0.4940213 |
| Angel1 | -0.6136602 |
| Angel2 | -0.2457335 |
| Angpt1 | 2.0993926 |
| Angpt2 | -0.3224701 |
| Angpt2 | 0.2044744 |
| Angptl1 | 0.1744754 |
| Angptl2 | 1.4654002 |
| Angptl3 | 0.7538815 |
| Angptl4 | -0.6508962 |
| Angptl7 | -0.6282033 |
| Ank | 1.487346 |
| Ank1 | -0.3866551 |
| Ank2 | 0.7184194 |
| Ank2 | 0.0929588 |
| Ank2 | -0.1908673 |
| Ank3 | -0.6952338 |
| Ank3 | -0.5885392 |
| Ank3 | 1.6731244 |
| Ankar | -0.5410347 |
| Ankar | -0.0535611 |
| Ankfy1 | 0.0769696 |
| Ankle2 | -0.7321027 |
| Ankle2 | 2.7752795 |
| Ankle2 | 0.0977022 |
| Ankmy1 | 0.3459796 |
| Ankmy1 | -0.6473687 |
| Ankmy1 | -0.6060522 |
| Ankmy2 | 1.2660663 |
| Ankmy2 | 1.1840999 |
| Ankrd10 | -0.5893657 |
| Ankrd12 | -0.459918 |
| Ankrd12 | -0.5926791 |
| Ankrd13a | 0.7025323 |
| Ankrd13b | -0.0401988 |
| Ankrd13d | -0.5960043 |
| Ankrd17 | 0.4370052 |
| Ankrd17 | -0.497341 |
| Ankrd17 | -0.5285606 |
| Ankrd22 | -0.5697302 |
| Ankrd23 | 1.1260891 |
| Ankrd24 | 0.6662934 |
| Ankrd25 | 1.0419631 |
| Ankrd27 | 3.0304868 |
| Ankrd34a | 0.0961238 |
| Ankrd35 | -0.6213311 |
| Ankrd36 | -0.6204756 |
| Ankrd37 | 0.2494045 |
| Ankrd39 | 0.7880378 |
| Ankrd40 | -0.4534678 |
| Ankrd40 | -0.7282531 |
| Ankrd40 | -0.3760823 |
| Ankrd46 | 1.9916342 |
| Ankrd47 | 0.2607186 |
| Ankrd49 | -0.2889738 |
| Ankrd5 | 3.036283 |
| Ankrd50 | -0.2961796 |
| Ankrd53 | 0.619639 |
| Ankrd54 | 0.0079372 |
| Ankrd54 | 0.1842241 |
| Ankrd54 | 0.038694 |
| Ankrd56 | -0.3156734 |
| Ankrd58 | -0.6544372 |
| Ankrd6 | -0.4736841 |
| Ankrd6 | 1.5997188 |
| Ankrd6 | -0.2847961 |
| Ankrd7 | 1.8587116 |
| Ankrd9 | 0.8441939 |
| Anks1 | 2.1588433 |
| Anks3 | 4.4378983 |
| Anks3 | 1.2619164 |
| Anks6 | 1.9753662 |
| Anks6 | 1.482307 |
| Ankzf1 | -0.729214 |
| Ankzf1 | 0.2925784 |
| Anln | -0.5231555 |
| Anln | -0.4428162 |
| Anp32a | -0.5584651 |
| Anp32a | -0.1706402 |
| Anp32a | 0.1988346 |
| Anp32e | -0.268271 |
| Anpep | 1.8612486 |
| Antxr1 | 1.7294705 |
| Antxr1 | 0.0349047 |
| Antxrl | 2.7802815 |
| Anxa1 | -0.6943074 |
| Anxa11 | 3.2705413 |
| Anxa11 | -0.4744143 |
| Anxa2 | -0.3714944 |
| Anxa3 | 0.1576268 |
| Anxa3 | 3.4188273 |
| Anxa4 | -0.078014 |
| Anxa5 | -0.6102712 |
| Anxa6 | -0.7234635 |
| Anxa8 | -0.7535657 |
| Anxa8 | 0.4061378 |
| Anxa9 | -0.5177817 |
| Aoah | 0.3918908 |
| Aoc2 | 0.4128994 |
| Aoc2 | 1.7258642 |
| Aoc3 | -0.3437985 |
| Aof1 | -0.7139578 |
| Aof1 | -0.7244194 |
| Aof2 | -0.7379071 |
| Aox3 | -0.4591991 |
| Aox3l1 | -0.2011486 |
| Ap1b1 | -0.0904864 |
| Ap1g1 | -0.3132141 |
| Ap1g1 | -0.7026781 |
| Ap1g2 | 1.2592145 |
| Ap1gbp1 | -0.5139623 |
| Ap1gbp1 | 1.2937393 |
| Ap1m1 | -0.0429476 |
| Ap1m2 | 0.1870931 |
| Ap1m2 | -0.6943074 |
| Ap1m2 | 1.0968736 |
| Ap1m2 | 0.4243377 |
| Ap1s1 | -0.4788077 |
| Ap1s1 | 1.120869 |
| Ap1s1 | 1.5750239 |
| Ap1s3 | 0.6529536 |
| Ap2a1 | -0.7595064 |
| Ap2a1 | -0.5926791 |
| Ap2a2 | -0.60437 |
| Ap2b1 | 1.3690257 |
| Ap2b1 | -0.0016678 |
| Ap2b1 | -0.4336827 |
| Ap2b1 | -0.3552917 |
| Ap2m1 | 1.9089624 |
| Ap2s1 | -0.6447318 |
| Ap2s1 | 0.9920558 |
| Ap2s1 | -0.6553246 |
| Ap3b1 | -0.7437482 |
| Ap3b1 | 2.0613853 |
| Ap3b2 | 2.524795 |
| Ap3d1 | 1.320294 |
| Ap3m1 | 0.7925233 |
| Ap3m2 | 3.1842256 |
| Ap3s2 | -0.4824849 |
| Ap3s2 | -0.0131489 |
| Ap4b1 | 0.908895 |
| Ap4m1 | 0.2912992 |
| Ap4m1 | -0.8436686 |
| Ap4m1 | -0.617914 |
| Ap4s1 | 1.2005785 |
| Apaf1 | -0.2355135 |
| Apaf1 | 0.4906716 |
| Apba1 | -0.4751451 |
| Apba2 | -0.1544597 |
| Apba2 | 0.052043 |
| Apba3 | -0.7340335 |
| Apbb1 | -0.5993416 |
| Apbb1ip | 0.393612 |
| Apbb1ip | -0.0189431 |
| Apbb1ip | -0.4950974 |
| Apbb2 | 0.140843 |
| Apbb3 | -0.3162892 |
| Apc | -0.7575219 |
| Apc2 | -0.6741582 |
| Apcdd1 | 0.3335265 |
| Apeh | -0.6230444 |
| Apex1 | -0.5185475 |
| Aph1a | 0.0365907 |
| Aph1b | 2.8928691 |
| Apip | 1.6732688 |
| Apip | 0.1355589 |
| Apitd1 | -0.5520888 |
| Aplp1 | -0.4591991 |
| Aplp2 | 1.2910197 |
| Apoa1 | 1.529402 |
| Apoa1bp | -0.1903293 |
| Apoa2 | -0.2440223 |
| Apoa4 | 1.7652407 |
| Apob48r | -0.5935093 |
| Apobec1 | 0.1520659 |
| Apobec3 | -0.3299419 |
| Apoc1 | -0.60437 |
| Apoc1 | -0.2630212 |
| Apoc2 | 1.5402079 |
| Apoc3 | -0.2853918 |
| Apoe | 2.4966807 |
| Apof | -0.6878487 |
| Apoh | -0.5893657 |
| Apol7c | 0.7393852 |
| Apol9b | -0.1462176 |
| App | -0.4766084 |
| Appbp1 | -0.3355855 |
| Appbp1 | 0.6330201 |
| Appbp2 | 0.7060181 |
| Appl1 | -0.3324459 |
| Appl2 | 1.5849173 |
| Aprin | 0.4145821 |
| Aprt | -0.5600661 |
| Aptx | -0.638608 |
| Aqp1 | -0.7664857 |
| Aqp1 | -0.6196209 |
| Aqp1 | -0.6412275 |
| Aqp11 | 1.0489189 |
| Aqp11 | -0.6316586 |
| Aqp3 | -0.3826762 |
| Aqp4 | -0.830708 |
| Aqp5 | 1.0979474 |
| Aqp7 | 2.2267504 |
| Aqp7 | 1.2419177 |
| Aqp8 | -0.6642453 |
| Aqp8 | 1.5240786 |
| Aqp9 | 0.3893029 |
| Aqr | -0.6571019 |
| Aqr | -0.253761 |
| Ar | -0.3224701 |
| Araf | 0.2222258 |
| Araf | 1.6492954 |
| Arbp | -0.2800445 |
| Arc | 0.9802526 |
| Arcn1 | 0.2663243 |
| Ard1 | -0.1105017 |
| Ard1 | -0.5339974 |
| Areg | -0.5985061 |
| Arf2 | -0.1946419 |
| Arf3 | 0.3582678 |
| Arf5 | -0.1513604 |
| Arf6 | -0.7496262 |
| Arfgap1 | 1.2068026 |
| Arfgap1 | 0.0319467 |
| Arfgap1 | -0.8147579 |
| Arfgap2 | -0.395335 |
| Arfgap2 | 1.8304587 |
| Arfgap2 | -0.4088494 |
| Arfgap2 | -0.4613574 |
| Arfgap3 | -0.617914 |
| Arfip2 | -0.5025974 |
| Arfip2 | -0.0530971 |
| Arfip2 | -0.8074073 |
| Arfrp1 | -0.0356353 |
| Arfrp1 | -0.10558 |
| Arg1 | 1.5561434 |
| Arg2 | 0.3794609 |
| Arglu1 | 2.76078 |
| Arhgap1 | -0.7836573 |
| Arhgap1 | 3.5617707 |
| Arhgap1 | 2.4352873 |
| Arhgap10 | -0.0933887 |
| Arhgap12 | -0.0699486 |
| Arhgap15 | -0.7301759 |
| Arhgap15 | -0.2937715 |
| Arhgap17 | -0.2606975 |
| Arhgap18 | -0.1606897 |
| Arhgap19 | 0.50527 |
| Arhgap20 | 0.3128095 |
| Arhgap22 | -0.3138283 |
| Arhgap24 | 0.7654712 |
| Arhgap24 | -0.3734579 |
| Arhgap25 | -0.3077046 |
| Arhgap26 | 0.8323029 |
| Arhgap27 | -0.3508052 |
| Arhgap28 | 0.1679189 |
| Arhgap29 | 0.2900183 |
| Arhgap30 | 1.3130881 |
| Arhgap4 | 0.7629724 |
| Arhgap6 | -0.032908 |
| Arhgap8 | 2.6195756 |
| Arhgap9 | -0.5449675 |
| Arhgdib | 1.204892 |
| Arhgdig | 0.9044438 |
| Arhgef1 | 0.9885909 |
| Arhgef1 | 0.8290822 |
| Arhgef10 | 1.9799397 |
| Arhgef10l | -0.5786782 |
| Arhgef10l | 1.6909216 |
| Arhgef15 | -0.3773974 |
| Arhgef18 | 1.4499253 |
| Arhgef19 | 0.0462264 |
| Arhgef2 | 2.981164 |
| Arhgef3 | -0.5746002 |
| Arhgef4 | -0.5025974 |
| Arhgef6 | 1.1092802 |
| Arid2 | 3.6237345 |
| Arid3a | 0.0536983 |
| Arid3b | 1.8987769 |
| Arid4b | -0.711125 |
| Arid5a | -0.5640805 |
| Arid5b | -0.5910209 |
| Arih1 | -0.0365462 |
| Arih2 | -0.5041047 |
| Arl1 | 3.1717841 |
| Arl11 | -0.4020676 |
| Arl16 | 2.8350393 |
| Arl2 | -0.5254682 |
| Arl2bp | -0.6438545 |
| Arl2bp | 1.3187092 |
| Arl2bp | 0.1535521 |
| Arl3 | -0.0007905 |
| Arl3 | -0.1259272 |
| Arl4a | -0.5943402 |
| Arl4a | 0.761624 |
| Arl4c | -0.6832627 |
| Arl5a | -0.7437482 |
| Arl5b | 0.8750637 |
| Arl6 | 8.59E-05 |
| Arl6ip1 | 0.6869953 |
| Arl6ip2 | 0.5303389 |
| Arl6ip2 | 1.920365 |
| Arl6ip2 | -0.6615601 |
| Arl6ip2 | 0.6735281 |
| Arl6ip4 | -0.340631 |
| Arl6ip4 | -0.4534678 |
| Arl6ip5 | 1.1429823 |
| Arl6ip5 | -0.064767 |
| Arl8a | 0.7458778 |
| Arl8b | -0.5960043 |
| Armc10 | 2.3672221 |
| Armc3 | 2.0128905 |
| Armc6 | 2.6503336 |
| Armc6 | -0.7092413 |
| Armc8 | 0.7532982 |
| Armc8 | -0.5324408 |
| Armc8 | 0.8599306 |
| Armc9 | -0.1254255 |
| Armc9 | -0.5985061 |
| Armcx1 | -0.5355567 |
| Armcx2 | -0.2865843 |
| Armcx3 | 0.0865961 |
| Armcx5 | 3.1792718 |
| Armcx6 | 1.0074154 |
| Armcx6 | 0.0941469 |
| Armetl1 | 0.7784336 |
| Arnt | 2.4227308 |
| Arnt | -0.5770449 |
| Arnt2 | -0.1962643 |
| Arntl | 0.2376117 |
| Arpc1a | 0.0565878 |
| Arpc1a | -0.714904 |
| Arpc1a | -0.4744143 |
| Arpc1b | -0.3598 |
| Arpc1b | 2.7974934 |
| Arpc1b | -0.4980901 |
| Arpc3 | 0.1762885 |
| Arpc4 | -0.6111173 |
| Arpc5 | -0.5193139 |
| Arpc5 | -0.5177817 |
| Arpc5l | -0.1664375 |
| Arpp19 | 0.5404245 |
| Arpp21 | -0.2360783 |
| Arpp21 | -0.6678377 |
| Arrb1 | -0.028832 |
| Arrb1 | -0.6239022 |
| Arrb1 | -0.187108 |
| Arrb1 | -0.3299419 |
| Arrb2 | -0.5926791 |
| Arrdc1 | -0.0929043 |
| Arrdc2 | -0.0434066 |
| Arrdc3 | 0.5355184 |
| Arrdc4 | 1.0963363 |
| Ars2 | 1.8373764 |
| Ars2 | 2.0644668 |
| Ars2 | -0.5063702 |
| Arsa | -0.4729544 |
| Arsb | -0.7321027 |
| Arsb | -0.3261984 |
| Arsg | -0.0914528 |
| Arsi | 1.783551 |
| Arsj | -0.5449675 |
| Arsj | 2.4633257 |
| Art1 | -0.8053177 |
| Art2b | 1.8644836 |
| Art3 | -0.4921142 |
| Art3 | -0.6869297 |
| Art4 | 0.2072815 |
| Art5 | -0.0047449 |
| Artn | 0.4602338 |
| Artn | 0.6660797 |
| Arv1 | -0.5835956 |
| As3mt | -0.4371849 |
| As3mt | 1.30893 |
| Asah1 | -0.0775376 |
| Asah1 | 1.6485547 |
| Asah2 | -0.0784906 |
| Asah3 | -0.0438657 |
| Asah3l | 0.9024586 |
| Asb1 | -0.3374743 |
| Asb1 | -0.5935093 |
| Asb1 | -0.3150579 |
| Asb12 | -0.6535507 |
| Asb13 | -0.1154497 |
| Asb15 | -0.6943074 |
| Asb17 | 0.4763643 |
| Asb2 | -0.0087163 |
| Asb3 | -0.5048592 |
| Asb3 | -0.5033507 |
| Asb4 | 1.4054587 |
| Asb4 | 0.4564918 |
| Asb6 | -0.6615601 |
| Asb6 | -0.7515938 |
| Asb7 | -0.7595064 |
| Asb7 | -0.6750646 |
| Asb8 | -0.4788077 |
| Asb9 | -0.5893657 |
| Ascc1 | 0.7306571 |
| Ascc1 | -0.4456447 |
| Ascc2 | 0.133285 |
| Ascc2 | -0.4817483 |
| Ascc2 | 1.9261516 |
| Ascc2 | -0.333073 |
| Ascc3l1 | 2.7848649 |
| Ascl1 | 2.1213289 |
| Ascl2 | -0.6980184 |
| Asf1a | -0.7282531 |
| Asf1b | -0.7515938 |
| Asgr1 | -0.5616698 |
| Ash1l | -0.2931704 |
| Ash1l | 1.8370136 |
| Ash1l | 0.657062 |
| Ash2l | -0.6010147 |
| Ash2l | -0.6579918 |
| Asl | -0.7321027 |
| Asl | 1.0396805 |
| Asna1 | -0.8105504 |
| Asns | 1.7556512 |
| Asns | 0.0428862 |
| Asns | 1.6258259 |
| Asnsd1 | -0.8404114 |
| Asnsd1 | -0.4407005 |
| Asph | -0.4421104 |
| Asph | -0.7369372 |
| Asph | -0.4541823 |
| Asph | 2.783187 |
| Asph | -0.3866551 |
| Asphd1 | 1.243639 |
| Asphd2 | -0.5332188 |
| Aspm | -0.6213311 |
| Asprv1 | 2.7268514 |
| Aspscr1 | 2.0858017 |
| Aspscr1 | 0.4154223 |
| Aspscr1 | 0.3609436 |
| Asrgl1 | -0.7206016 |
| Ass1 | -0.3425302 |
| Aste1 | -0.1957232 |
| Astn1 | 0.0127026 |
| Astn2 | 3.6990197 |
| Asz1 | 0.1487133 |
| Atad1 | 1.7796347 |
| Atad2 | -0.6571019 |
| Atad3a | -0.0320007 |
| Atad4 | 2.4864362 |
| Atad4 | 0.6514353 |
| Atcay | 1.7630819 |
| Ate1 | 1.1539075 |
| Ate1 | 0.21461 |
| Atf2 | -0.3175221 |
| Atf2 | 0.0660191 |
| Atf2 | 0.1260474 |
| Atf3 | -0.6924575 |
| Atf4 | -0.5193139 |
| Atf4 | 2.342442 |
| Atf5 | -0.5786782 |
| Atf7 | -0.7506095 |
| Atf7ip | 0.2735289 |
| Atg10 | -0.380033 |
| Atg10 | -0.5363373 |
| Atg12 | -0.0953285 |
| Atg16l1 | 0.7927097 |
| Atg2a | 0.2153049 |
| Atg2a | 1.3864877 |
| Atg2b | -0.2665177 |
| Atg2b | -0.5010925 |
| Atg3 | -0.4846982 |
| Atg3 | -0.6786993 |
| Atg4b | -0.4832221 |
| Atg4c | -0.0184961 |
| Atg4c | 2.0099839 |
| Atg4d | -0.7282531 |
| Atg5 | 0.0092393 |
| Atg5 | 1.0941837 |
| Atg7 | 0.0980964 |
| Atg9b | -0.2137488 |
| Athl1 | -0.4322855 |
| Atic | 3.1801964 |
| Atic | -0.1011726 |
| Atic | -0.617914 |
| Atm | -0.572974 |
| Atm | 1.3172277 |
| Atmin | 0.1863767 |
| Atmin | -0.4027435 |
| Atmin | -0.7359683 |
| Atn1 | 0.023443 |
| Atoh8 | 0.8378237 |
| Atox1 | -0.7017443 |
| Atp10a | 0.8736982 |
| Atp10d | -0.7545532 |
| Atp11c | -0.7515938 |
| Atp11c | -0.7350004 |
| Atp12a | -0.3873199 |
| Atp13a1 | -0.6841781 |
| Atp13a1 | -0.0207333 |
| Atp13a2 | -0.6615601 |
| Atp13a4 | -0.5528835 |
| Atp13a5 | -0.1299499 |
| Atp1a1 | -0.6562128 |
| Atp1a2 | 1.7703885 |
| Atp1b1 | -0.4007171 |
| Atp1b2 | -0.4371849 |
| Atp1b3 | -0.4606374 |
| Atp2a1 | 1.4081512 |
| Atp2a2 | 0.2006007 |
| Atp2a2 | 2.2003118 |
| Atp2a3 | 1.0902695 |
| Atp2a3 | 1.320294 |
| Atp2a3 | 4.0053402 |
| Atp2b1 | -0.7263343 |
| Atp2b2 | -0.6633494 |
| Atp2b2 | -0.1529088 |
| Atp2b2 | -0.2671018 |
| Atp2b3 | -0.7301759 |
| Atp2b3 | 1.9504432 |
| Atp2b4 | 1.4699928 |
| Atp2b4 | 0.1620517 |
| Atp2c1 | 0.471897 |
| Atp2c1 | 3.0983184 |
| Atp5a1 | 0.2709155 |
| Atp5b | 2.2621831 |
| Atp5c1 | 0.3774216 |
| Atp5d | 1.0781526 |
| Atp5e | 0.4815925 |
| Atp5f1 | -0.7282531 |
| Atp5g2 | 1.2460435 |
| Atp5g3 | 0.3087356 |
| Atp5h | -0.8137042 |
| Atp5j | -0.5528835 |
| Atp5j | -0.3089261 |
| Atp5j2 | 0.0607001 |
| Atp5l | 1.9251072 |
| Atp5o | -0.6741582 |
| Atp5s | 1.0035621 |
| Atp5sl | 0.1546652 |
| Atp6ap1 | 0.1602106 |
| Atp6ap2 | -0.6961611 |
| Atp6ap2 | 2.3368129 |
| Atp6ap2 | -0.1179336 |
| Atp6ap2 | -0.4371849 |
| Atp6v0a1 | -0.8253608 |
| Atp6v0a1 | 0.8599306 |
| Atp6v0a2 | -0.6723479 |
| Atp6v0b | 1.9471728 |
| Atp6v0d1 | -0.311374 |
| Atp6v0d1 | -0.0065079 |
| Atp6v0d1 | -0.693382 |
| Atp6v0d1 | -0.2717874 |
| Atp6v0e | -0.2485925 |
| Atp6v0e2 | -0.6325244 |
| Atp6v1a | 0.5839168 |
| Atp6v1b1 | -0.3598 |
| Atp6v1b2 | 0.9346831 |
| Atp6v1c1 | -0.8350081 |
| Atp6v1c2 | -0.8168688 |
| Atp6v1d | -0.2497386 |
| Atp6v1e1 | -0.6290659 |
| Atp6v1e2 | 1.7346175 |
| Atp6v1g1 | 0.5540252 |
| Atp6v1g2 | 0.7964304 |
| Atp6v1g2 | 0.225667 |
| Atp6v1h | 2.2046759 |
| Atp7a | -0.4928591 |
| Atp8a1 | 0.7664305 |
| Atp8a2 | -0.2647678 |
| Atp8b1 | 1.9901484 |
| Atp8b3 | -0.7036129 |
| Atp8b3 | -0.7263343 |
| Atp8b3 | 0.221881 |
| Atp8b3 | 0.4151423 |
| Atp9a | 0.3426549 |
| Atp9a | 3.4706169 |
| Atp9b | 2.6954785 |
| Atp9b | 1.2246433 |
| Atpaf1 | -0.5371185 |
| Atpaf2 | -0.668738 |
| Atpaf2 | 0.0161531 |
| Atpbd1b | 0.5619475 |
| Atpbd1c | 0.5105909 |
| Atpbd3 | -0.5132003 |
| Atpif1 | 1.2395024 |
| Atrx | 0.0105395 |
| Atxn1 | 2.5122036 |
| Atxn10 | 0.65252 |
| Atxn2 | 1.3140451 |
| Atxn2l | -0.4854371 |
| Atxn3 | -0.7867209 |
| Atxn3 | 0.2636906 |
| Atxn3 | 0.273855 |
| Atxn7l1 | 2.8133155 |
| Atxn7l2 | -0.5993416 |
| AU014645 | -0.7867209 |
| AU017455 | 2.1895383 |
| AU017455 | -0.459918 |
| AU018778 | 1.3687251 |
| AU018829 | -0.4499037 |
| AU019823 | 2.5346268 |
| AU021034 | -0.0554194 |
| AU021092 | 1.7085921 |
| AU021838 | -0.6162101 |
| AU022252 | 1.1428545 |
| AU022751 | -0.8253608 |
| AU022870 | 1.6386466 |
| AU023871 | 0.205177 |
| AU040320 | 2.7902704 |
| AU040320 | -0.5193139 |
| AU040320 | -0.6896895 |
| AU040829 | 0.7567925 |
| AU042651 | -0.6980184 |
| Aup1 | 0.7226507 |
| Aup1 | 0.0336381 |
| Aurka | -0.037002 |
| Aurkaip1 | -0.5332188 |
| Aurkc | -0.3886509 |
| Auts2 | -0.3993687 |
| Auts2 | 0.2266969 |
| AV028368 | 0.5318217 |
| AV249152 | -0.1716939 |
| AV249152 | 1.8072076 |
| Aven | 0.8892823 |
| Avil | 1.3228239 |
| Avp | -0.3946644 |
| Avpi1 | 0.5022157 |
| Avpi1 | 0.4486922 |
| Avpr1a | -0.5885392 |
| AW049604 | -0.4995901 |
| AW061290 | 0.4013183 |
| AW111922 | -0.5426058 |
| AW121567 | 0.5330556 |
| AW125753 | -0.3010148 |
| AW146242 | 0.6996517 |
| AW146299 | 0.6654385 |
| AW146299 | 1.6423839 |
| AW540478 | -0.5301108 |
| AW544981 | -0.4281064 |
| AW548124 | -0.568921 |
| AW549877 | -0.464966 |
| AW551984 | -0.1518762 |
| AW551984 | -0.325576 |
| AW551984 | -0.4463532 |
| AW555464 | 0.8728436 |
| Axin2 | -0.2889738 |
| Axl | 1.1043641 |
| Axud1 | 0.2696061 |
| Azgp1 | -0.2497386 |
| Azgp1 | 2.0417086 |
| Azi1 | 3.4687749 |
| Azi2 | 1.3588543 |
| Azin1 | -0.3218501 |
| B020018G12Rik | -0.0144828 |
| B230118H07Rik | 0.7360227 |
| B230120H23Rik | 0.6250146 |
| B230206H07Rik | 2.5757576 |
| B230209C24Rik | 0.4711065 |
| B230217C12Rik | -0.3343284 |
| B230219D22Rik | -0.6299293 |
| B230312A22Rik | -0.5778612 |
| B230312I18Rik | -0.5355567 |
| B230317C12Rik | -0.7775604 |
| B230317C12Rik | -0.3089261 |
| B230339H12Rik | 1.1907994 |
| B230339M05Rik | 1.5766267 |
| B230339M05Rik | 2.3239138 |
| B230340J04Rik | -0.6170616 |
| B230342M21Rik | 1.6493695 |
| B230373P09Rik | -0.6264805 |
| B230378H13Rik | 0.4383717 |
| B230380D07Rik | 0.5561926 |
| B230396O12Rik | -0.1149537 |
| B230399E16Rik | -0.2082494 |
| B2m | 1.6543167 |
| B2m | 0.8825387 |
| B3galnt1 | -0.6290659 |
| B3galnt2 | -0.5910209 |
| B3galt1 | 1.303894 |
| B3galt4 | -0.4047743 |
| B3galt4 | -0.7139578 |
| B3galt5 | -0.6732526 |
| B3galt6 | -0.2485925 |
| B3gat1 | 1.2971005 |
| B3gat1 | 1.4951595 |
| B3gat1 | 0.3644993 |
| B3gat2 | -0.5101585 |
| B3gat3 | 0.925359 |
| B3gat3 | 1.7216276 |
| B3gnt1 | 0.3128095 |
| B3gnt3 | -0.1329785 |
| B3gnt5 | -0.219281 |
| B3gnt7 | -0.6299293 |
| B3gnt8 | 0.2121736 |
| B3gnt8 | 0.3644993 |
| B430119L13Rik | 0.605834 |
| B4galnt3 | -0.215405 |
| B4galnt4 | 0.078581 |
| B4galt1 | -0.5827742 |
| B4galt3 | -0.4301936 |
| B4galt3 | -0.6264805 |
| B4galt4 | 0.2780846 |
| B4galt7 | 2.5043715 |
| B630005N14Rik | 1.1236132 |
| B830045N13Rik | 2.1994529 |
| B930006L02Rik | -0.4470622 |
| B930011P16Rik | -0.1822968 |
| B930037P14Rik | -0.497341 |
| B930041F14Rik | -0.0929043 |
| B930041G04 | 1.0658754 |
| B930076A02 | -0.6961611 |
| B9d1 | 1.8648945 |
| B9d2 | 1.6159777 |
| Baalc | 0.4337173 |
| Baalc | -0.4751451 |
| Bace1 | 1.4953344 |
| Bace2 | -0.7017443 |
| Bace2 | -0.7253764 |
| Bace2 | 2.2922853 |
| Bach1 | -0.0109299 |
| Bach2 | -0.5544748 |
| Bad | 1.9276337 |
| Bag1 | -0.4013921 |
| Bag2 | 0.2682948 |
| Bag3 | 0.7620095 |
| Bag4 | -0.7398501 |
| Bag5 | 2.0359674 |
| Bahcc1 | 1.561612 |
| Bahd1 | 0.2793822 |
| Bai1 | 0.3721569 |
| Bai2 | 0.4666137 |
| Bai2 | 2.8311535 |
| Bai3 | -0.3767396 |
| Baiap2 | -0.4301936 |
| Baiap2 | 1.3433337 |
| Banf1 | -0.2508861 |
| Banf1 | -0.4506154 |
| Banf1 | -0.6018524 |
| Banf1 | -0.5584651 |
| Banf2 | -0.002546 |
| Bap1 | 1.3867826 |
| Bard1 | 2.186846 |
| Barx2 | 0.7902833 |
| Basp1 | 1.2269808 |
| Bat1a | -0.1319679 |
| Bat1a | -0.6077375 |
| Bat2 | -0.3431641 |
| Bat3 | -0.5960043 |
| Bat4 | 3.6128375 |
| Bat4 | -0.6878487 |
| Bat5 | -0.6351268 |
| Batf | 0.9902496 |
| Bax | -0.6651421 |
| Baz1a | -0.4700416 |
| Baz1b | 2.7461412 |
| Baz2a | -0.3899839 |
| BB014433 | -0.4184279 |
| BB128963 | 0.3730365 |
| BB146404 | -0.6325244 |
| Bbc3 | 0.4679374 |
| Bbc3 | 0.5545073 |
| Bbox1 | -0.5608676 |
| Bbs1 | 0.1294828 |
| Bbs12 | 1.07417 |
| Bbs2 | 2.7960318 |
| Bbs4 | -0.3966776 |
| Bbs5 | -0.4988398 |
| Bbs7 | -0.3565775 |
| Bbs9 | -0.4685885 |
| Bbs9 | -0.0207333 |
| Bbx | -0.3337005 |
| BC002163 | 0.2577371 |
| BC002199 | -0.3734579 |
| BC002216 | 0.1016367 |
| BC003236 | 0.0789834 |
| BC003266 | -0.3527253 |
| BC003885 | 0.7947579 |
| BC003885 | 0.4328935 |
| BC003993 | 1.3591581 |
| BC003993 | -0.4606374 |
| BC004004 | -0.6750646 |
| BC004044 | -0.60437 |
| BC004044 | -0.0919364 |
| BC004728 | 0.1605791 |
| BC004728 | -0.5835956 |
| BC005537 | 1.6791665 |
| BC005682 | -0.4606374 |
| BC005764 | -0.5985061 |
| BC006662 | 1.6815295 |
| BC006779 | -0.6325244 |
| BC006779 | 1.7736967 |
| BC008155 | 1.8405147 |
| BC008163 | -0.4281064 |
| BC008163 | 0.6288036 |
| BC010462 | 0.2166932 |
| BC011248 | -0.5086413 |
| BC011248 | -0.6162101 |
| BC011487 | -0.6500131 |
| BC013491 | -0.5185475 |
| BC013491 | -0.024324 |
| BC013529 | 0.4176592 |
| BC013712 | -0.2406094 |
| BC016201 | -0.362386 |
| BC016423 | -0.7408231 |
| BC016495 | 0.6645828 |
| BC017158 | 1.8072701 |
| BC017612 | 1.5097318 |
| BC017643 | 0.4193333 |
| BC017647 | 1.1893418 |
| BC018242 | 2.9502241 |
| BC018242 | -0.7704976 |
| BC018465 | 0.6464293 |
| BC020002 | -0.2270819 |
| BC020535 | 0.254413 |
| BC021381 | -0.1119833 |
| BC021395 | -0.6333911 |
| BC021614 | -0.7083008 |
| BC021790 | 3.078621 |
| BC021891 | -0.3181391 |
| BC022224 | -0.1229215 |
| BC022687 | 2.6547027 |
| BC023055 | -0.5664976 |
| BC023151 | 1.3639036 |
| BC023744 | 2.714744 |
| BC023814 | 1.0490603 |
| BC023829 | 2.5204335 |
| BC024561 | 0.3636117 |
| BC024814 | -0.6642453 |
| BC024868 | -0.5713507 |
| BC024997 | -0.1065622 |
| BC025076 | 2.1325674 |
| BC025546 | -0.4491925 |
| BC025575 | 0.6216585 |
| BC025920 | -0.3343284 |
| BC026439 | -0.2931704 |
| BC026585 | 3.5640766 |
| BC026590 | -0.5355567 |
| BC026590 | -0.5086413 |
| BC027072 | 0.1712029 |
| BC027231 | -0.2411774 |
| BC027231 | -0.6170616 |
| BC027231 | 0.5878771 |
| BC027344 | -0.5528835 |
| BC028528 | 1.0004382 |
| BC028528 | 1.3730233 |
| BC029169 | 0.9481807 |
| BC029169 | 4.3202291 |
| BC029214 | 1.9193141 |
| BC030183 | 0.8524452 |
| BC030307 | -0.2259635 |
| BC030476 | -0.7486439 |
| BC030499 | -0.131463 |
| BC030500 | 0.8568045 |
| BC030867 | -0.3993687 |
| BC031181 | -0.7092413 |
| BC031353 | 0.513114 |
| BC031575 | -0.6136602 |
| BC031748 | -0.7715033 |
| BC031781 | 0.0289793 |
| BC031853 | 8.59E-05 |
| BC032265 | 1.6168216 |
| BC034076 | -0.3387357 |
| BC037034 | 0.5178891 |
| BC037594 | -0.6394804 |
| BC038156 | -0.5697302 |
| BC038286 | -0.5528835 |
| BC038613 | -0.4693148 |
| BC038822 | 0.3246162 |
| BC038822 | -0.3826762 |
| BC038822 | -0.4936046 |
| BC038925 | -0.6980184 |
| BC039093 | -0.1259272 |
| BC040758 | 0.2163464 |
| BC043098 | -0.4714968 |
| BC043118 | 0.7656632 |
| BC043301 | -0.3565775 |
| BC046331 | 0.8281856 |
| BC046404 | 1.0518841 |
| BC048355 | -0.4693148 |
| BC048502 | -0.2044192 |
| BC048546 | 0.4052891 |
| BC048546 | 1.7024596 |
| BC048562 | 2.1152997 |
| BC048644 | -0.4499037 |
| BC048651 | -0.4095303 |
| BC048671 | -0.2265225 |
| BC049635 | 0.2232595 |
| BC049635 | 0.7161953 |
| BC049730 | -0.3514448 |
| BC049762 | 3.7763929 |
| BC049806 | 0.0170137 |
| BC049806 | -0.3636818 |
| BC049806 | -0.4671378 |
| BC050196 | 3.2541056 |
| BC050210 | -0.6544372 |
| BC050777 | 0.5333022 |
| BC050811 | -0.1560133 |
| BC050811 | 0.0285546 |
| BC051019 | -0.2676862 |
| BC051070 | 1.7963398 |
| BC051142 | 1.4221598 |
| BC051227 | 1.55056 |
| BC051628 | 2.7966339 |
| BC051665 | 1.5772671 |
| BC052484 | -0.5770449 |
| BC053749 | 1.0994218 |
| BC053749 | 2.4202183 |
| BC054059 | -0.6316586 |
| BC055107 | 0.619639 |
| BC055111 | -0.163298 |
| BC055324 | 0.5759452 |
| BC055368 | 0.989496 |
| BC056474 | -0.49137 |
| BC056923 | 0.1344226 |
| BC057022 | 0.7814574 |
| BC057371 | 2.2586963 |
| BC057371 | -0.7565313 |
| BC057371 | 1.5289806 |
| BC057552 | -0.6998795 |
| BC057627 | 0.0893852 |
| BC057893 | -0.6282033 |
| BC060267 | 0.5991982 |
| BC060615 | 3.04097 |
| BC060632 | 1.0155142 |
| BC061039 | 1.1259589 |
| BC061237 | 0.0217329 |
| BC062115 | 1.3694262 |
| BC062650 | -0.6491308 |
| BC063749 | 0.3913163 |
| BC064033 | -0.2006046 |
| BC065085 | 0.7656632 |
| BC065397 | -0.2242883 |
| BC066028 | -0.7980413 |
| BC066107 | 1.7692832 |
| BC067047 | 0.6402983 |
| BC067068 | 0.0144295 |
| BC068157 | 1.5441968 |
| BC068281 | -0.1421239 |
| BC072620 | 0.1498322 |
| BC085271 | 2.4054251 |
| BC087945 | -0.2618586 |
| BC088983 | 1.9032926 |
| BC089491 | -0.6732526 |
| BC099439 | 1.3484679 |
| BC107230 | -0.324954 |
| Bcam | 1.7428875 |
| Bcan | 1.4302583 |
| Bcan | -0.4693148 |
| Bcap29 | -0.6980184 |
| Bcap29 | -0.711125 |
| Bcap31 | -0.4385896 |
| Bcap31 | -0.5681125 |
| Bcar1 | -0.4685885 |
| Bcar3 | -0.7234635 |
| Bcas1 | 0.5982792 |
| Bcas2 | -0.7887689 |
| Bcas3 | 3.1076974 |
| Bcat1 | 0.7264623 |
| Bcat2 | -0.6247608 |
| Bccip | -0.459918 |
| Bcdin3d | -0.1711669 |
| Bcdo2 | -0.572162 |
| Bckdha | -0.6473687 |
| Bckdhb | -0.1289426 |
| Bckdk | 0.3103046 |
| Bckdk | -0.7654855 |
| Bcl10 | 2.5002974 |
| Bcl11a | 0.5045073 |
| Bcl11b | 2.8734033 |
| Bcl2 | 0.2656666 |
| Bcl2 | -0.5560689 |
| Bcl2l1 | 1.1209998 |
| Bcl2l1 | 0.6639406 |
| Bcl2l1 | -0.6887686 |
| Bcl2l10 | -0.7785738 |
| Bcl2l11 | -0.4943507 |
| Bcl2l11 | -0.7545532 |
| Bcl2l11 | 0.3059047 |
| Bcl2l12 | 3.3531824 |
| Bcl2l13 | -0.2242883 |
| Bcl2l14 | 0.5690914 |
| Bcl2l14 | 0.390454 |
| Bcl2l2 | -0.7555417 |
| Bcl2l2 | 0.5421356 |
| Bcl3 | -0.3859907 |
| Bcl6 | -0.1833638 |
| Bcl6b | 0.8188089 |
| Bcl7a | 0.6656523 |
| Bcl7b | -0.0517065 |
| Bcl9l | -0.5293354 |
| Bclaf1 | -0.2606975 |
| Bclaf1 | -0.6299293 |
| Bclp2 | -0.268271 |
| Bcmo1 | -0.5449675 |
| Bcor | -0.0987331 |
| Bcor | 0.4356366 |
| Bcorl1 | -0.0832703 |
| Bcs1l | 1.2648345 |
| Bdh1 | 1.8516543 |
| Bdh2 | -0.0866309 |
| Bdkrb1 | -0.572162 |
| Bdnf | 0.4454455 |
| Bdnf | -0.5778612 |
| Becn1 | -0.5778612 |
| Becn1 | -0.5600661 |
| Bet1 | -0.050318 |
| Bet1l | 1.567453 |
| Bex2 | 3.4394398 |
| Bex2 | -0.408169 |
| Bex2 | 1.2241751 |
| Bex4 | -0.078014 |
| Bex4 | 2.2538852 |
| Bex6 | -0.7555417 |
| Bfar | -0.6980184 |
| Bfar | 1.0771928 |
| Bglap2 | 1.4485474 |
| Bgn | 1.4972553 |
| Bhlhb2 | 3.6242808 |
| Bhlhb5 | -0.4156811 |
| Bhlhb8 | 0.0615204 |
| Bhlhb8 | 1.2166515 |
| Bhlhb9 | 1.4818637 |
| Bhmt | -0.2293228 |
| Bhmt2 | -0.4274118 |
| Bicc1 | -0.1390654 |
| Bicd1 | -0.4773409 |
| Bicd2 | 2.1812292 |
| Bicd2 | 0.9794909 |
| Bid | -0.5101585 |
| Bid | -0.3559344 |
| Bik | 1.3854059 |
| Bin1 | 1.635721 |
| Bin1 | -0.1775105 |
| Bin1 | 0.279058 |
| Bin3 | 0.5996574 |
| Birc2 | -0.5536788 |
| Birc2 | -0.1045988 |
| Birc2 | -0.2082494 |
| Birc4 | 1.589509 |
| Birc5 | 0.1370717 |
| Birc5 | 2.552446 |
| Birc5 | 0.7196303 |
| Blcap | 1.4730419 |
| Blm | -0.478074 |
| Blm | 3.4913123 |
| Blmh | -0.0100438 |
| Blnk | -0.6669383 |
| Bloc1s1 | -0.4700416 |
| Bloc1s3 | -0.7036129 |
| Blvra | -0.028832 |
| Blvrb | 0.7643187 |
| Bmf | 2.0772051 |
| Bmi1 | 1.3254519 |
| Bmp1 | -0.5293354 |
| Bmp1 | 0.0070681 |
| Bmp1 | -0.0666479 |
| Bmp1 | 1.6860951 |
| Bmp15 | 1.6776597 |
| Bmp2k | 1.103031 |
| Bmp2k | -0.0723135 |
| Bmp3 | 0.8531441 |
| Bmp4 | 1.3675221 |
| Bmp6 | -0.4184279 |
| Bmp7 | 2.3835386 |
| Bmp8a | 1.2377734 |
| Bmper | -0.6970893 |
| Bmpr1a | 2.0720087 |
| Bmpr1a | 1.1357984 |
| Bmpr1b | 1.1685673 |
| Bmpr1b | 1.7197765 |
| Bmpr2 | -0.7036129 |
| Bms1 | -0.8158127 |
| Bnc1 | 1.7348201 |
| Bnc2 | 0.0433044 |
| Bnc2 | 0.425723 |
| Bnip1 | -0.4751451 |
| Bnip2 | 1.0046011 |
| Bnip3l | 1.8801448 |
| Bnipl | 2.0856166 |
| Bnipl | -0.6111173 |
| Bok | 0.3456779 |
| Bok | -0.5935093 |
| Bola1 | 1.5370378 |
| Bola2 | 1.9961284 |
| Bola3 | -0.7535657 |
| Boll | 1.2188915 |
| Bop1 | -0.0832703 |
| Bpi | -0.1876441 |
| Bpil1 | 1.9009246 |
| Bpil3 | -0.6394804 |
| Bpnt1 | -0.579496 |
| Bpnt1 | -0.5985061 |
| Bpnt1 | -0.3508052 |
| Bpnt1 | -0.6796102 |
| Brap | -0.7980413 |
| Brca2 | 0.3276984 |
| Brca2 | -0.6998795 |
| Brcc3 | -0.3919869 |
| Brcc3 | -0.7036129 |
| Brcc3 | 1.6880145 |
| Brd2 | 2.2954639 |
| Brd2 | -0.6553246 |
| Brd2 | 0.2610493 |
| Brd4 | 2.0905996 |
| Brd4 | 1.1668253 |
| Brd8 | -0.6660398 |
| Brd8 | -0.6060522 |
| Brd9 | 2.1423234 |
| Brd9 | -0.4000427 |
| Brdt | -0.3280683 |
| Brdt | -0.7427721 |
| Bre | 0.3849737 |
| Bre | -0.1100084 |
| Bre | -0.5132003 |
| Brf1 | 1.8152912 |
| Brf2 | 1.6938901 |
| Bri3 | 2.6750696 |
| Brip1 | -0.4928591 |
| Brms1 | 1.5011723 |
| Brp16 | 0.6735281 |
| Brp16 | -0.0751594 |
| Brp17 | 2.1518055 |
| Brp44 | -0.5512949 |
| Brpf1 | -0.089521 |
| Brpf1 | -0.3919869 |
| Brs3 | -0.3636818 |
| Brsk1 | -0.4613574 |
| Brsk2 | -0.7196495 |
| Brsk2 | -0.7282531 |
| Brunol4 | -0.8042747 |
| Brunol4 | -0.6669383 |
| Brwd1 | -0.6989485 |
| Brwd1 | -0.4343821 |
| Brwd2 | 0.7507663 |
| Bscl2 | -0.0392843 |
| Bsdc1 | -0.5762293 |
| Bsg | 0.5815793 |
| Bsg | 1.3063618 |
| Bspry | 0.4467997 |
| Bst2 | -0.7064228 |
| Btbd1 | 2.0515991 |
| Btbd10 | -0.5208487 |
| Btbd11 | 0.8251305 |
| Btbd12 | -0.023425 |
| Btbd14a | -0.714904 |
| Btbd14b | -0.638608 |
| Btbd16 | -0.0880748 |
| Btbd3 | 0.1561472 |
| Btbd3 | -0.3193745 |
| Btbd6 | 2.7324433 |
| Btbd9 | 0.9839004 |
| Btc | -0.5935093 |
| Btd | 2.4936758 |
| Btf3 | -0.638608 |
| Btg1 | 1.2034564 |
| Btg1 | 0.0660191 |
| Btg3 | -0.5608676 |
| Btg3 | -0.5885392 |
| Btg4 | 0.7652792 |
| Btk | -0.4810123 |
| Btla | -0.729214 |
| Btrc | -0.1451925 |
| Btrc | -0.4685885 |
| Btrc | 1.9559925 |
| Bub1b | -0.3913188 |
| Bub3 | 1.315001 |
| Bud13 | 0.9165928 |
| Bud31 | -0.1477575 |
| Bves | 1.0704466 |
| Bxdc1 | 1.048636 |
| Bxdc1 | -0.1680113 |
| Bxdc2 | 0.391029 |
| Bxdc2 | 2.4447279 |
| Bxdc5 | 1.5122242 |
| Bxdc5 | 2.3336669 |
| Bxdc5 | -0.49137 |
| Bzrap1 | 0.4218389 |
| Bzrpl1 | -0.5078837 |
| Bzw1 | -0.5681125 |
| Bzw2 | 2.7952355 |
| Bzw2 | 1.0883739 |
| C030006K11Rik | 0.6888735 |
| C030011O14Rik | -0.5063702 |
| C030014K22Rik | 0.1047723 |
| C030018G13Rik | 2.0059095 |
| C030030A07Rik | -0.4876575 |
| C030039L03Rik | 0.5283581 |
| C030045D06Rik | 0.2467222 |
| C030045D06Rik | -0.7282531 |
| C030046I01Rik | -0.549709 |
| C030048B08Rik | -0.4184279 |
| C030048B08Rik | -0.6102712 |
| C130022K22Rik | 1.7576956 |
| C130032J12Rik | 2.0418056 |
| C130034I18Rik | -0.478074 |
| C130038G02Rik | -0.6299293 |
| C130060K24Rik | 3.9608605 |
| C130073D16Rik | 1.8458017 |
| C130074G19Rik | -0.729214 |
| C130090K23Rik | -0.8232305 |
| C130090K23Rik | 2.9750651 |
| C1galt1 | 0.1579964 |
| C1galt1c1 | -0.7715033 |
| C1qa | -0.1229215 |
| C1qb | 0.7374087 |
| C1qbp | 1.3889417 |
| C1qc | -0.7466825 |
| C1qdc2 | 0.6299151 |
| C1ql2 | -0.4832221 |
| C1ql3 | 0.5138696 |
| C1qtnf1 | 0.3861302 |
| C1qtnf2 | 1.220656 |
| C1qtnf3 | 2.2803239 |
| C1qtnf3 | -0.6998795 |
| C1qtnf3 | 1.5047267 |
| C1qtnf4 | 1.06851 |
| C1qtnf6 | -0.4700416 |
| C1qtnf7 | -0.5301108 |
| C1qtnf9 | 0.5426239 |
| C1rl | 0.7240568 |
| C1s | 4.167291 |
| C1s | 0.1748383 |
| C2 | -0.1436569 |
| C230021P08Rik | 1.0134594 |
| C230029F24Rik | -0.7120683 |
| C230052I12Rik | -0.4832221 |
| C230052I12Rik | 0.8939739 |
| C230055K05Rik | 3.2251958 |
| C230071H18Rik | -0.3873199 |
| C230078M08Rik | -0.6768802 |
| C230078M14Rik | -0.6052107 |
| C230090D14 | 0.0925624 |
| C230093N12Rik | 0.5855496 |
| C230096C10Rik | -0.411576 |
| C2cd3 | 0.8411912 |
| C3 | -0.6230444 |
| C330001K17Rik | 0.7899094 |
| C330005M16Rik | -0.7535657 |
| C330016O10Rik | -0.6732526 |
| C330019G07Rik | -0.3064847 |
| C330023M02Rik | -0.6961611 |
| C330027C09Rik | -0.479542 |
| C430003P19Rik | -0.7130126 |
| C430004E15Rik | -0.089521 |
| C4b | 0.0018364 |
| C530028I08Rik | 2.6962933 |
| C530028I08Rik | -0.2440223 |
| C530028O21Rik | 0.2780846 |
| C530043G21Rik | -0.5285606 |
| C530044N13Rik | 3.678159 |
| C6 | 0.4356366 |
| C630004H02Rik | -0.3457041 |
| C630004H02Rik | 0.1063361 |
| C630007B19Rik | -0.3318193 |
| C630028N24Rik | 0.9870804 |
| C630035N08Rik | -0.4685885 |
| C630035N08Rik | -0.7092413 |
| C730025P13Rik | -0.6553246 |
| C730027P07Rik | -0.0746845 |
| C78339 | 0.0225883 |
| C79127 | 3.3617444 |
| C79267 | 0.2426841 |
| C79407 | -0.7634882 |
| C79407 | 3.3548092 |
| C80008 | 1.6843143 |
| C80913 | 0.1468455 |
| C85492 | 1.1708022 |
| C85492 | -0.5762293 |
| C85492 | -0.5324408 |
| C85627 | -0.1264291 |
| C87436 | 3.313022 |
| C87436 | 1.7550568 |
| C87499 | -0.7196495 |
| C8b | 1.8930443 |
| C8g | 1.9508177 |
| C9 | 2.4238881 |
| C9 | 1.0852513 |
| C920005C14Rik | 3.9648358 |
| Cab39l | 0.1620517 |
| Cabc1 | 0.9195241 |
| Cables2 | -0.5433924 |
| Cabp2 | -0.4054523 |
| Cabp7 | 0.7454856 |
| Cabyr | 2.2747188 |
| Cabyr | 1.7800206 |
| Cachd1 | 1.3948049 |
| Cacna1a | -0.5347767 |
| Cacna1b | -0.549709 |
| Cacna1b | 0.2325114 |
| Cacna1b | 0.33169 |
| Cacna1d | -0.479542 |
| Cacna1g | -0.5078837 |
| Cacna1h | 2.4379492 |
| Cacna2d1 | 0.6926186 |
| Cacna2d3 | -0.3873199 |
| Cacnb1 | -0.6394804 |
| Cacnb1 | -0.4392927 |
| Cacnb2 | 2.8314227 |
| Cacnb2 | 0.0598791 |
| Cacnb3 | -0.3946644 |
| Cacnb3 | -0.4817483 |
| Cacnb3 | 2.4338581 |
| Cacnb3 | -0.7545532 |
| Cacnb4 | 1.2799509 |
| Cacnb4 | 2.4208954 |
| Cacng2 | 0.9658258 |
| Cacng3 | -0.5819536 |
| Cacng3 | -0.2913695 |
| Cacng4 | 0.9001372 |
| Cacng5 | -0.572974 |
| Cacng5 | -0.0727872 |
| Cacng5 | -0.500341 |
| Cacng7 | 1.9221324 |
| Cacng8 | -0.4136263 |
| Cacybp | -0.2806371 |
| Cad | -0.2700275 |
| Cadm2 | -0.6247608 |
| Cadm3 | -0.2383411 |
| Cadps | -0.4170535 |
| Cadps2 | 1.9724963 |
| Cadps2 | 0.3855521 |
| Cage1 | -0.3181391 |
| Calb2 | 0.9797956 |
| Calb2 | -0.6068945 |
| Calca | 1.0433869 |
| Calca | 1.2119181 |
| Calcoco1 | -0.6759719 |
| Cald1 | 2.0826511 |
| Cald1 | -0.5835956 |
| Calm2 | 2.1725081 |
| Calm3 | -0.4758764 |
| Calml4 | -0.6723479 |
| Caln1 | 0.0416305 |
| Calr | -0.3425302 |
| Calr3 | -0.5560689 |
| Calr3 | -0.4040969 |
| Calr3 | -0.5616698 |
| Calr4 | 1.6318058 |
| Calu | -0.4671378 |
| Calu | -0.0207333 |
| Calu | 1.0490603 |
| Caly | -0.6351268 |
| Camk1 | 2.1734765 |
| Camk1d | 1.1256986 |
| Camk1g | 0.4771504 |
| Camk1g | 1.3780954 |
| Camk2a | -0.5885392 |
| Camk2a | 0.7666223 |
| Camk2a | 0.233193 |
| Camk2b | -0.2618586 |
| Camk2d | -0.0948432 |
| Camk2d | 1.2132222 |
| Camk2d | -0.5449675 |
| Camk2g | 0.6771244 |
| Camk2g | 1.1576886 |
| Camk2n1 | 0.67374 |
| Camk4 | 0.4055721 |
| Camkk1 | 1.1897064 |
| Camkk2 | 2.035235 |
| Camkk2 | 1.6099719 |
| Camkv | -0.0225269 |
| Caml | -0.3286924 |
| Camp | 2.2663805 |
| Camsap1l1 | 2.0479447 |
| Camta2 | -0.0265752 |
| Cant1 | -0.2543371 |
| Cant1 | -0.5786782 |
| Cap1 | -0.4095303 |
| Capg | -0.4898832 |
| Capg | -0.6403535 |
| Capn1 | 0.0395339 |
| Capn10 | -0.7525792 |
| Capn11 | 0.0035836 |
| Capn2 | 2.4585719 |
| Capn2 | -0.5093996 |
| Capn2 | 0.254746 |
| Capn3 | 0.2884145 |
| Capn5 | 0.2650084 |
| Capn6 | -0.464966 |
| Capn7 | -0.6412275 |
| Capn8 | 1.3462123 |
| Capns1 | -0.5893657 |
| Caprin1 | -0.6980184 |
| Caprin1 | -0.5154882 |
| Capsl | -0.49137 |
| Capza1 | 1.6069543 |
| Capza3 | 0.0027104 |
| Capzb | -0.500341 |
| Capzb | 1.51042 |
| Capzb | 1.6901425 |
| Car11 | -0.3572211 |
| Car12 | 0.384395 |
| Car13 | 1.3001259 |
| Car14 | -0.0972725 |
| Car15 | -0.3444333 |
| Car2 | -0.4129424 |
| Car3 | 0.721645 |
| Car4 | 0.2372725 |
| Car5b | -0.4729544 |
| Car5b | -0.3199928 |
| Car5b | -0.4407005 |
| Car7 | -0.5101585 |
| Car9 | 2.1074254 |
| Card10 | 0.3718636 |
| Card11 | -0.4802768 |
| Carhsp1 | 2.8521849 |
| Carm1 | 0.0941469 |
| Cart | 2.8764657 |
| Cartpt | 0.9004692 |
| Casc1 | 1.7195706 |
| Casc1 | 2.3668464 |
| Casc1 | 4.3062231 |
| Casc3 | -0.2583796 |
| Casc4 | -0.0193903 |
| Casc4 | -0.5935093 |
| Casc4 | -0.5544748 |
| Cask | -0.6714441 |
| Caskin1 | -0.5770449 |
| Caskin1 | 1.5865825 |
| Caskin1 | -0.5048592 |
| Caskin2 | -0.6814346 |
| Casp1 | -0.380033 |
| Casp2 | 1.9528484 |
| Casp4 | -0.5819536 |
| Casp4 | -0.4685885 |
| Casp4 | 0.5108436 |
| Casp6 | -0.6606667 |
| Casp6 | -0.4020676 |
| Casp7 | 0.4072681 |
| Casp8 | 1.3241914 |
| Casp8 | -0.5737868 |
| Casp9 | 0.7248593 |
| Casq2 | -0.3747692 |
| Cass4 | 0.390454 |
| Cast | -0.7234635 |
| Cat | 0.0285546 |
| Catsper1 | 3.8386928 |
| Catsper2 | -0.5935093 |
| Catsper3 | -0.6750646 |
| Catsper4 | 0.9131613 |
| Cav1 | -0.6615601 |
| Cav2 | 1.0396805 |
| Cbara1 | 1.8507013 |
| Cbfa2t3h | -0.478074 |
| Cbfa2t3h | -0.1812311 |
| Cbfb | -0.6615601 |
| Cbfb | -0.234949 |
| Cbfb | 0.3477881 |
| Cbl | 0.3698075 |
| Cblb | -0.6010147 |
| Cblc | -0.3721484 |
| Cbln1 | 0.8180837 |
| Cbln2 | 0.3895908 |
| Cbln4 | 2.3765326 |
| Cbr1 | 0.1520659 |
| Cbr2 | -0.5520888 |
| Cbr3 | 0.9744475 |
| Cbr3 | -0.4108936 |
| Cbs | -0.6403535 |
| Cbs | 1.5217878 |
| Cbwd1 | 1.3207161 |
| Cbx2 | 1.2775222 |
| Cbx5 | 0.6001163 |
| Cbx7 | 0.3465829 |
| Cbx8 | 0.4968463 |
| Cby1 | -0.3946644 |
| Cc2d1a | -0.2326944 |
| Cc2d1b | -0.6307936 |
| Cc2d2a | 1.6402181 |
| Ccar1 | -0.4685885 |
| Ccbe1 | -0.6282033 |
| Ccbl1 | 1.2893841 |
| Ccbl1 | 1.1900709 |
| Ccbl2 | -0.1828302 |
| Ccbp2 | 0.4847154 |
| Ccdc100 | -0.301621 |
| Ccdc101 | -0.2973861 |
| Ccdc108 | 0.4162618 |
| Ccdc109a | 0.1370717 |
| Ccdc109a | 1.4048807 |
| Ccdc109b | 1.6766538 |
| Ccdc11 | -0.6860116 |
| Ccdc110 | 0.0374326 |
| Ccdc111 | -0.5568669 |
| Ccdc113 | -0.6906112 |
| Ccdc114 | 0.6944855 |
| Ccdc115 | 0.2916192 |
| Ccdc116 | -0.3150579 |
| Ccdc117 | -0.1812311 |
| Ccdc12 | -0.7525792 |
| Ccdc120 | 2.289133 |
| Ccdc123 | -0.4722253 |
| Ccdc124 | 0.5906624 |
| Ccdc125 | 0.4361843 |
| Ccdc126 | -0.3546494 |
| Ccdc127 | 0.4075505 |
| Ccdc127 | 2.8212417 |
| Ccdc13 | -0.4407005 |
| Ccdc130 | 0.8855794 |
| Ccdc131 | 0.3435629 |
| Ccdc132 | 1.4011151 |
| Ccdc134 | -0.2925697 |
| Ccdc137 | 2.6842631 |
| Ccdc137 | -0.6162101 |
| Ccdc18 | -0.4428162 |
| Ccdc21 | 2.6176744 |
| Ccdc23 | -0.3520848 |
| Ccdc25 | 0.1977733 |
| Ccdc27 | -0.3993687 |
| Ccdc27 | 0.1016367 |
| Ccdc27 | -0.7585137 |
| Ccdc28a | -0.3119869 |
| Ccdc28b | -0.4315877 |
| Ccdc28b | -0.8116005 |
| Ccdc3 | 1.0859311 |
| Ccdc32 | 0.2184257 |
| Ccdc38 | -0.6491308 |
| Ccdc39 | 0.1294828 |
| Ccdc40 | -0.4584807 |
| Ccdc41 | 2.739146 |
| Ccdc42 | -0.6333911 |
| Ccdc43 | 0.33169 |
| Ccdc45 | -0.456329 |
| Ccdc46 | 0.5978194 |
| Ccdc46 | -0.5185475 |
| Ccdc47 | 2.1098173 |
| Ccdc49 | -0.4435225 |
| Ccdc5 | -0.7225085 |
| Ccdc50 | -0.3973496 |
| Ccdc51 | 2.5809799 |
| Ccdc53 | 2.1017143 |
| Ccdc53 | -0.4198044 |
| Ccdc53 | 0.3438653 |
| Ccdc54 | 0.898808 |
| Ccdc56 | 0.2386284 |
| Ccdc57 | 0.2470579 |
| Ccdc58 | -0.5285606 |
| Ccdc59 | -0.7908214 |
| Ccdc59 | -0.4149957 |
| Ccdc64 | -0.4013921 |
| Ccdc65 | 2.0702231 |
| Ccdc65 | 0.7962447 |
| Ccdc66 | 0.7062228 |
| Ccdc67 | -0.6961611 |
| Ccdc68 | 0.0684637 |
| Ccdc70 | -0.7026781 |
| Ccdc71 | -0.4744143 |
| Ccdc73 | -0.459918 |
| Ccdc77 | 1.6475167 |
| Ccdc79 | 2.3362128 |
| Ccdc80 | -0.8414959 |
| Ccdc82 | 0.6673608 |
| Ccdc84 | 2.0532294 |
| Ccdc85a | -0.8084538 |
| Ccdc85b | 0.5759452 |
| Ccdc86 | 0.9154504 |
| Ccdc87 | -0.3101492 |
| Ccdc88a | -0.8667946 |
| Ccdc88b | 0.822968 |
| Ccdc88c | -0.4685885 |
| Ccdc90b | 0.5932083 |
| Ccdc91 | 0.790657 |
| Ccdc92 | -0.9326361 |
| Ccdc93 | -0.4577629 |
| Ccdc94 | 2.795709 |
| Ccdc95 | 1.1671989 |
| Ccdc96 | 3.2251823 |
| Ccdc97 | 1.4740263 |
| Ccdc98 | 0.669705 |
| Ccdc99 | 0.2550788 |
| Cchcr1 | -0.7506095 |
| Ccin | -0.1026394 |
| Cck | 1.0319349 |
| Cckbr | -0.5301108 |
| Ccl11 | -0.4253308 |
| Ccl17 | -0.7614952 |
| Ccl19 | -0.897401 |
| Ccl21b | -0.5552715 |
| Ccl21c | 2.0796295 |
| Ccl25 | -0.5985061 |
| Ccl25 | -0.1234218 |
| Ccl27 | -0.1219218 |
| Ccl27 | 0.9480231 |
| Ccl28 | 1.07582 |
| Ccl3 | -0.7244194 |
| Ccl4 | 1.1826318 |
| Ccl4 | 0.1616838 |
| Ccl5 | 2.6284829 |
| Ccl6 | -0.7877443 |
| Ccl7 | -0.5410347 |
| Ccl9 | -0.4191159 |
| Ccm2 | -0.5664976 |
| Ccna1 | 1.9682534 |
| Ccnb1 | -0.7745269 |
| Ccnb1 | -0.775537 |
| Ccnb3 | 0.5024706 |
| Ccnc | -0.2694416 |
| Ccnd1 | -0.5301108 |
| Ccnd1 | -0.3126003 |
| Ccnd1 | 1.0756826 |
| Ccnd2 | -0.4239461 |
| Ccnd3 | -0.4548973 |
| Ccnd3 | -0.7867209 |
| Ccndbp1 | 1.7219015 |
| Ccne1 | -0.2423143 |
| Ccne2 | 1.5695533 |
| Ccnf | -0.5071266 |
| Ccng1 | -0.7476627 |
| Ccng1 | -0.4693148 |
| Ccng1 | -0.4936046 |
| Ccng2 | 0.9983496 |
| Ccnk | 0.2473935 |
| Ccnl1 | -0.5262403 |
| Ccno | -0.0078323 |
| Ccr4 | 1.4369845 |
| Ccr5 | -0.6500131 |
| Ccr5 | -0.6316586 |
| Ccr5 | 0.9180596 |
| Ccr6 | 1.4189385 |
| Ccr6 | 2.1501671 |
| Ccrk | 1.3811649 |
| Ccrk | 2.3662654 |
| Ccrk | -0.089521 |
| Ccrk | 2.1703149 |
| Ccrl2 | -0.7990773 |
| Ccrn4l | 0.2497392 |
| Ccrn4l | 0.7058134 |
| Ccs | 2.0358697 |
| Cct2 | -0.6230444 |
| Cct3 | 1.3126625 |
| Cct3 | -0.7654855 |
| Cct4 | -0.1400838 |
| Cct5 | -0.6669383 |
| Cct6a | -0.4906263 |
| Cct6b | -0.8502174 |
| Cct7 | -0.1006842 |
| Cct8 | -0.3463402 |
| Cd109 | 0.1024216 |
| Cd109 | 3.1442781 |
| Cd14 | 0.0152917 |
| Cd151 | -0.1109953 |
| Cd151 | -0.6500131 |
| Cd151 | -0.6145094 |
| Cd160 | -0.0680611 |
| Cd163 | -0.8870863 |
| Cd164 | -0.6333911 |
| Cd177 | 0.7232535 |
| Cd180 | 1.9344104 |
| Cd1d1 | -0.6777893 |
| Cd2 | 0.9521135 |
| Cd200 | -0.7101827 |
| Cd200 | -0.4664133 |
| Cd226 | -0.7496262 |
| Cd247 | -0.1244231 |
| Cd248 | 1.1907994 |
| Cd248 | -0.6678377 |
| Cd27 | -0.5339974 |
| Cd274 | -0.8502174 |
| Cd276 | -0.6952338 |
| Cd2bp2 | 1.0549803 |
| Cd300lg | -0.2729625 |
| Cd320 | -0.4198044 |
| Cd34 | -0.6438545 |
| Cd3d | -0.6342585 |
| Cd3eap | 0.7863502 |
| Cd3g | -0.6714441 |
| Cd4 | 0.615361 |
| Cd40 | 0.1051635 |
| Cd40 | -0.115946 |
| Cd44 | 2.5409351 |
| Cd44 | -0.7846774 |
| Cd44 | -0.5600661 |
| Cd44 | -0.8656802 |
| Cd47 | -0.0193903 |
| Cd52 | 0.1708386 |
| Cd53 | -0.6001777 |
| Cd55 | 2.6552787 |
| Cd59a | -0.6723479 |
| Cd59b | 1.1588199 |
| Cd59b | -0.5048592 |
| Cd6 | 0.1598419 |
| Cd6 | -0.4693148 |
| Cd6 | -0.0765856 |
| Cd63 | -0.5681125 |
| Cd63 | -0.0252238 |
| Cd63 | -0.7795883 |
| Cd68 | -0.3230904 |
| Cd74 | -0.2120956 |
| Cd74 | -0.5860641 |
| Cd79b | -0.7340335 |
| Cd79b | 0.2447053 |
| Cd81 | -0.5576657 |
| Cd82 | -0.7949402 |
| Cd83 | -0.8905118 |
| Cd84 | -0.7856986 |
| Cd86 | -0.2913695 |
| Cd86 | -0.7908214 |
| Cd86 | -0.572974 |
| Cd8b1 | 2.3293014 |
| Cd9 | 0.3750854 |
| Cd93 | -0.6187671 |
| Cd96 | 0.3806242 |
| Cd97 | 2.0004996 |
| Cd97 | -0.5418199 |
| Cd97 | -0.7427721 |
| Cd99l2 | -0.3318193 |
| Cdan1 | -0.6924575 |
| Cdc123 | 1.6924777 |
| Cdc14b | -0.5835956 |
| Cdc14b | -0.6896895 |
| Cdc14b | 0.8244102 |
| Cdc16 | -0.2741391 |
| Cdc20 | -0.8436686 |
| Cdc20 | 1.7062972 |
| Cdc20 | 1.0494844 |
| Cdc20 | 0.179904 |
| Cdc23 | -0.5673047 |
| Cdc25a | -0.2485925 |
| Cdc25b | -0.7379071 |
| Cdc25c | -0.2394746 |
| Cdc25c | -0.7427721 |
| Cdc27 | 1.2046529 |
| Cdc2a | 0.7470534 |
| Cdc2l1 | -0.8074073 |
| Cdc2l1 | -0.7282531 |
| Cdc2l5 | 3.6928221 |
| Cdc2l6 | 2.4802589 |
| Cdc34 | -0.3230904 |
| Cdc37 | 0.6802861 |
| Cdc37l1 | 1.5267019 |
| Cdc37l1 | 1.9084586 |
| Cdc37l1 | -0.4936046 |
| Cdc37l1 | -0.2071534 |
| Cdc42 | 1.9228494 |
| Cdc42bpb | 1.2677435 |
| Cdc42ep1 | 0.1602106 |
| Cdc42ep2 | -0.5568669 |
| Cdc42ep3 | -0.5441796 |
| Cdc42ep4 | 3.0237459 |
| Cdc42ep5 | -0.8848096 |
| Cdc42se1 | 0.0615204 |
| Cdc42se1 | -0.3853269 |
| Cdc45l | 0.1613157 |
| Cdc5l | 0.3855521 |
| Cdc5l | 1.8598335 |
| Cdc5l | -0.4928591 |
| Cdc5l | 0.9407417 |
| Cdc6 | 0.56505 |
| Cdc7 | 0.4610337 |
| Cdc73 | 0.98844 |
| Cdca2 | -0.642978 |
| Cdca3 | -0.0270261 |
| Cdca3 | -0.1259272 |
| Cdca4 | -0.572974 |
| Cdca4 | 1.1973303 |
| Cdca4 | 0.9113587 |
| Cdca5 | 3.9630582 |
| Cdca7 | 0.0607001 |
| Cdca8 | 0.0725239 |
| Cdcp1 | -0.3083151 |
| Cdgap | -0.7167993 |
| Cdh1 | -0.711125 |
| Cdh11 | -0.7959727 |
| Cdh11 | -0.2491654 |
| Cdh12 | 1.7904445 |
| Cdh13 | 1.2774117 |
| Cdh13 | -0.0434066 |
| Cdh16 | -0.3101492 |
| Cdh22 | -0.8147579 |
| Cdh23 | -0.5347767 |
| Cdh23 | 0.3933254 |
| Cdh23 | -0.6119641 |
| Cdh24 | 0.9502279 |
| Cdh26 | 0.2780846 |
| Cdh3 | 0.9107024 |
| Cdh5 | -0.1411033 |
| Cdh6 | -0.4364834 |
| Cdh7 | -0.7206016 |
| Cdh8 | 0.4179384 |
| Cdh8 | -0.6239022 |
| Cdh8 | -0.4336827 |
| Cdipt | -0.219281 |
| Cdipt | -0.3926556 |
| Cdk10 | -0.7506095 |
| Cdk10 | 0.3588631 |
| Cdk10 | -0.7908214 |
| Cdk2 | 0.8029059 |
| Cdk2 | 0.6523032 |
| Cdk2 | 0.5065396 |
| Cdk2 | -0.3418967 |
| Cdk2ap2 | -0.4876575 |
| Cdk3 | -0.0929043 |
| Cdk5 | -0.6860116 |
| Cdk5r1 | -0.3431641 |
| Cdk5rap1 | -0.6887686 |
| Cdk5rap2 | 2.0364553 |
| Cdk5rap3 | 1.8560492 |
| Cdk6 | -0.6860116 |
| Cdk8 | -0.6421024 |
| Cdk9 | -0.6678377 |
| Cdkal1 | -0.3688829 |
| Cdkl1 | -0.5697302 |
| Cdkl2 | -0.1011726 |
| Cdkl2 | -0.4832221 |
| Cdkl2 | -0.6723479 |
| Cdkl2 | 0.9644327 |
| Cdkl3 | 1.6722581 |
| Cdkl4 | 1.2880736 |
| Cdkn1a | 0.5888064 |
| Cdkn1a | 1.529739 |
| Cdkn1a | -0.333073 |
| Cdkn1b | -0.1989746 |
| Cdkn1b | -0.60353 |
| Cdkn1c | -0.0675898 |
| Cdkn2a | 1.4171334 |
| Cdkn2a | 0.1264297 |
| Cdkn2aipnl | 0.1583658 |
| Cdkn2b | -0.426024 |
| Cdkn2c | 0.0277046 |
| Cdkn2c | -0.1100084 |
| Cdkn2d | 1.0398233 |
| Cdo1 | 0.7725467 |
| Cdr2 | 0.6594323 |
| Cdrt4 | -0.5576657 |
| Cds1 | 0.991755 |
| Cds2 | -0.2688561 |
| Cdsn | 0.6977952 |
| Cdt1 | 1.3741196 |
| Cdv3 | -0.5170166 |
| Cdv3 | 2.9620985 |
| Cdyl | 0.0378532 |
| Cdyl | 0.829978 |
| Cdyl | -0.6256202 |
| Cdyl2 | -0.568921 |
| Ceacam1 | 0.4768885 |
| Ceacam13 | -0.2055119 |
| Ceacam2 | -0.6535507 |
| Cebpa | 0.4413711 |
| Cebpb | -0.6187671 |
| Cecr5 | 0.6901236 |
| Cecr6 | 0.1106232 |
| Celsr1 | -0.3604458 |
| Celsr2 | 0.0428862 |
| Celsr3 | 1.3220867 |
| Cend1 | -0.0047449 |
| Cenpa | 1.6832442 |
| Cenpb | -0.2818235 |
| Cenph | 0.7999512 |
| Cenpi | -0.6887686 |
| Cenpj | -0.4322855 |
| Cenpk | 0.6040082 |
| Cenpl | 1.7775091 |
| Cenpl | -0.5481258 |
| Cenpm | -0.2093467 |
| Cenpm | -0.5993416 |
| Cenpm | -0.5093996 |
| Cenpn | 1.602682 |
| Cenpp | -0.7959727 |
| Cenpq | 0.9492836 |
| Cenpt | -0.6651421 |
| Centa1 | 0.0217329 |
| Centa1 | 1.3454935 |
| Centa2 | -0.6553246 |
| Centb5 | 2.2511413 |
| Centb5 | 0.0877925 |
| Centd2 | 1.6549056 |
| Centd2 | 0.1000649 |
| Centd3 | 0.0545247 |
| Centg1 | 1.9747408 |
| Centg1 | 0.0672422 |
| Centg2 | -0.4577629 |
| Centg2 | 0.3411398 |
| Centg3 | 0.4226725 |
| Centg3 | -0.3643303 |
| Cep135 | 2.0763177 |
| Cep164 | -0.5025974 |
| Cep170 | 2.4726696 |
| Cep55 | -0.3946644 |
| Cep57 | -0.5449675 |
| Cep57 | 0.1363156 |
| Cep63 | 0.4443607 |
| Cep68 | -0.4556129 |
| Cep70 | 2.6070814 |
| Cep72 | -0.1179336 |
| Cep78 | 1.6083483 |
| Cep97 | 0.2473935 |
| Cept1 | -0.1674864 |
| Cercam | -0.4766084 |
| Ces3 | 0.1004581 |
| Cetn2 | -0.3126003 |
| Cetn3 | 2.6710909 |
| Cetn4 | -0.3040496 |
| Cetn4 | 0.7202352 |
| Cfd | 1.2532199 |
| Cfdp1 | 1.2574089 |
| Cfh | 1.1639563 |
| Cfhr1 | -0.5737868 |
| Cfi | -0.510918 |
| Cfl1 | 0.1642562 |
| Cfl2 | -0.6187671 |
| Cfl2 | 1.3293278 |
| Cfp | 0.9961067 |
| Cftr | 0.3162427 |
| Cga | 0.8564565 |
| Cggbp1 | 0.2460504 |
| Cgnl1 | 2.1021688 |
| Cgrrf1 | 2.4054906 |
| Ch25h | 0.6042366 |
| Chac1 | 1.7851513 |
| Chad | 1.3137262 |
| Chaf1a | 0.5764161 |
| Chaf1b | 0.8642549 |
| Chat | 1.5693112 |
| Chchd1 | 0.9823822 |
| Chchd2 | -0.1791032 |
| Chchd4 | -0.5147249 |
| Chchd5 | 0.8468353 |
| Chchd6 | 2.3884916 |
| Chchd7 | -0.2326944 |
| Chchd8 | 0.0909751 |
| Chd1 | 0.7370129 |
| Chd1 | -0.5056144 |
| Chd1l | -0.3760823 |
| Chd1l | -0.1279364 |
| Chd4 | -0.5811337 |
| Chd4 | 0.5333022 |
| Chd4 | 2.6741624 |
| Chd5 | -0.5410347 |
| Chd8 | 0.587412 |
| Chdh | 0.1539233 |
| Chdh | 2.8573539 |
| Chek1 | -0.7177484 |
| Chek1 | -0.4693148 |
| Chek2 | -0.295577 |
| Chek2 | -0.1204242 |
| Cherp | -0.3682311 |
| Chfr | -0.6805219 |
| Chfr | -0.714904 |
| Chfr | -0.5770449 |
| Chga | 1.3732227 |
| Chga | 0.6279135 |
| Chga | 0.1419715 |
| Chgb | -0.6732526 |
| Chi3l1 | 3.4262727 |
| Chi3l3 | 1.9894816 |
| Chi3l3 | 1.6353452 |
| Chia | 2.3411082 |
| Chia | 0.6470838 |
| Chic1 | 4.3596428 |
| Chic2 | -0.0633588 |
| Chic2 | 0.2177331 |
| Chit1 | 1.026735 |
| Chka | 1.2549204 |
| Chka | -0.6777893 |
| Chka | 2.7839936 |
| Chkb | -0.521617 |
| Chl1 | -0.6741582 |
| Chl1 | -0.5246966 |
| Chl1 | 0.6919955 |
| Chm | -0.6651421 |
| Chmp1b | -0.6464889 |
| Chmp2a | 2.2236858 |
| Chmp2a | 1.0748579 |
| Chmp2b | -0.7330676 |
| Chmp4b | -0.0756345 |
| Chmp5 | 0.2342146 |
| Chmp7 | 2.455596 |
| Chmp7 | -0.7575219 |
| Chmp7 | -0.2901709 |
| Chn2 | 0.3344434 |
| Chodl | 1.5267019 |
| Chordc1 | 1.7456279 |
| Chpf | 2.1889177 |
| Chpt1 | -0.7856986 |
| Chpt1 | -0.3636818 |
| Chpt1 | -0.6230444 |
| Chrac1 | 1.6638411 |
| Chrd | 1.7532713 |
| Chrm1 | -0.1855015 |
| Chrm2 | -0.4958446 |
| Chrm3 | 1.07582 |
| Chrna1 | -0.234949 |
| Chrna4 | -0.6085813 |
| Chrna4 | 0.7654712 |
| Chrna4 | -0.4693148 |
| Chrna5 | -0.4627992 |
| Chrna6 | -0.6759719 |
| Chrna6 | 0.4243377 |
| Chrna7 | -0.6517802 |
| Chrna9 | -0.2770871 |
| Chrnb1 | 0.2689506 |
| Chrnb1 | 1.6398441 |
| Chrnb2 | -0.6456099 |
| Chrnb2 | -0.6239022 |
| Chrnb3 | -0.6403535 |
| Chrnb3 | -0.6213311 |
| Chrnb3 | 0.8730146 |
| Chrne | -0.1695878 |
| Chst1 | 0.2868081 |
| Chst10 | 1.4467991 |
| Chst12 | 0.7882252 |
| Chst14 | -0.6473687 |
| Chst2 | 1.6088898 |
| Chst3 | 0.5024706 |
| Chst3 | -0.3708408 |
| Chst7 | 1.7657633 |
| Chst8 | 1.6560824 |
| Chtf18 | -0.7225085 |
| Chuk | -0.2967827 |
| Churc1 | 1.4031446 |
| Ciao1 | 3.069807 |
| Ciapin1 | 1.758749 |
| Cib1 | 0.1679189 |
| Cib2 | 2.5673458 |
| Cib2 | -0.4239461 |
| Cic | 1.1272596 |
| Cidea | -0.2961796 |
| Cideb | -0.4392927 |
| Cidec | 2.470564 |
| Ciita | 1.1596988 |
| Cirbp | 3.6088332 |
| Cirh1a | -0.5481258 |
| Cisd1 | -0.4846982 |
| Cish | -0.5697302 |
| Cit | 2.600412 |
| Cited1 | 3.6653445 |
| Cited2 | -0.3508052 |
| Cited4 | -0.3540076 |
| Ciz1 | 1.0191722 |
| Ciz1 | -0.6018524 |
| Ckap2l | -0.3604458 |
| Ckap5 | -0.063828 |
| Ckb | 1.490778 |
| Cklf | -0.6633494 |
| Cklf | -0.3899839 |
| Ckmt1 | 0.1156642 |
| Ckmt2 | -0.132473 |
| Ckmt2 | -0.49137 |
| Ckt2 | -0.5418199 |
| Ckt2 | 0.9899483 |
| Clca5 | 1.8698696 |
| Clcc1 | -0.037002 |
| Clcf1 | 1.0231067 |
| Clcf1 | -0.0521698 |
| Clcf1 | 0.3068493 |
| Clcn1 | -0.4288016 |
| Clcn2 | -0.6128118 |
| Clcn3 | 1.7280433 |
| Clcn3 | -0.5056144 |
| Clcn3 | 2.6220181 |
| Clcn4-2 | -0.5803145 |
| Clcn7 | -0.5762293 |
| Clcnka | -0.3617388 |
| Cldn1 | -0.5426058 |
| Cldn10 | 1.1845888 |
| Cldn10 | 0.7956875 |
| Cldn10 | 0.4642264 |
| Cldn11 | 2.3467114 |
| Cldn12 | -0.652665 |
| Cldn15 | 0.0101063 |
| Cldn15 | -0.5885392 |
| Cldn17 | -0.6102712 |
| Cldn2 | -0.3754255 |
| Cldn23 | 0.6851132 |
| Cldn3 | -0.4642431 |
| Cldn4 | 1.7004998 |
| Cldn5 | -0.6196209 |
| Cldn6 | -0.2497386 |
| Cldn9 | 0.6407376 |
| Cldnd1 | -0.3643303 |
| Cldnd2 | -0.4040969 |
| Clec11a | 0.2208458 |
| Clec16a | -0.7379071 |
| Clec1a | -0.6239022 |
| Clec2d | 1.4637739 |
| Clec2e | -0.497341 |
| Clec3b | 0.1755637 |
| Clec4a1 | 2.0971564 |
| Clec4a3 | 2.2339933 |
| Clec4b1 | 0.2524129 |
| Clec4b1 | 1.8933858 |
| Clec4d | -0.6642453 |
| Clec4g | 0.2055281 |
| Clec4n | 0.6391992 |
| Clec7a | 1.5160786 |
| Clecsf12 | -0.5347767 |
| Clgn | 0.2715695 |
| Clic1 | 1.6731244 |
| Clic3 | -0.4606374 |
| Clic4 | 0.5504017 |
| Clic6 | 2.4761684 |
| Clip1 | 0.5836833 |
| Clip1 | 2.9614327 |
| Clip4 | -0.7565313 |
| Clk1 | 0.9345232 |
| Clk2 | 1.4341879 |
| Clk2 | -0.7398501 |
| Clk2 | -0.6196209 |
| Clk3 | 1.0686485 |
| Clk4 | 1.1516314 |
| Clk4 | -0.6508962 |
| Clk4 | -0.7215546 |
| Clm3 | 0.6564145 |
| Clm3 | 1.3391024 |
| Clmn | -0.4449368 |
| Clmn | -0.6544372 |
| Cln3 | -0.5926791 |
| Cln5 | 0.3423521 |
| Cln6 | 1.2153522 |
| Cln6 | -0.2943729 |
| Cln8 | -0.8546091 |
| Clns1a | -0.4385896 |
| Clns1a | -0.1174363 |
| Clns1a | 1.6875883 |
| Clock | -0.1451925 |
| Clock | -0.2776778 |
| Clp1 | -0.3787143 |
| Clpb | 1.8170232 |
| Clpp | 0.0809928 |
| Clptm1 | 1.3187092 |
| Clptm1l | -0.6290659 |
| Clpx | 1.9185943 |
| Clpx | -0.3156734 |
| Clrn1 | 0.4852348 |
| Clspn | -0.8158127 |
| Clspn | 0.9074137 |
| Clspn | 1.2925436 |
| Clstn1 | 2.9373464 |
| Clstn3 | -0.6651421 |
| Clstn3 | 0.4072681 |
| Clta | -0.1946419 |
| Cltb | 0.3077929 |
| Cluap1 | 0.5667162 |
| Cma1 | 1.8849816 |
| Cma1 | 0.5520944 |
| Cmas | 0.4823742 |
| Cmbl | -0.479542 |
| Cml1 | -0.4846982 |
| Cml3 | -0.6052107 |
| Cml4 | 1.7696084 |
| Cmpk | -0.7535657 |
| Cmtm2a | 2.8620737 |
| Cmtm2b | 0.6503494 |
| Cmtm4 | -0.6562128 |
| Cmtm4 | -0.7614952 |
| Cmtm5 | -0.4143107 |
| Cmtm6 | -0.5371185 |
| Cmtm7 | 4.265568 |
| Cmtm7 | 0.3510945 |
| Cmtm7 | 3.468827 |
| Cmtm8 | -0.7806039 |
| Cmtm8 | 1.6113613 |
| Cmya1 | -0.638608 |
| CN716893 | 1.4281933 |
| Cnbp2 | 1.7892354 |
| Cnbp2 | -0.4371849 |
| Cndp1 | 1.4929714 |
| Cnga4 | -0.4442294 |
| Cnih | -0.2220595 |
| Cnih3 | 0.2191178 |
| Cnih4 | 1.2810527 |
| Cnksr2 | 1.6004995 |
| Cnksr2 | -0.163298 |
| Cnksr3 | -0.0283802 |
| Cnn1 | 1.5637255 |
| Cnn2 | -0.7350004 |
| Cnn2 | 0.4878277 |
| Cnnm1 | 2.1900758 |
| Cnnm1 | -0.3734579 |
| Cnnm2 | -0.1748621 |
| Cnnm3 | -0.6571019 |
| Cno | -0.479542 |
| Cnot10 | -0.0279286 |
| Cnot2 | -0.1184312 |
| Cnot2 | 0.8786389 |
| Cnot2 | 0.8830462 |
| Cnot2 | -0.4198044 |
| Cnot3 | 1.2591018 |
| Cnot3 | 0.6083386 |
| Cnot4 | -0.7330676 |
| Cnot4 | 1.7025295 |
| Cnot4 | -0.3034419 |
| Cnot6l | -0.3193745 |
| Cnot6l | -0.437887 |
| Cnot7 | -0.7139578 |
| Cnp | 0.1156642 |
| Cnp | 0.0053275 |
| Cnpy2 | 2.5193037 |
| Cnpy3 | -0.6264805 |
| Cnr1 | -0.1284393 |
| Cnr1 | -0.7908214 |
| Cntd1 | 0.0845988 |
| Cntd1 | 3.8427809 |
| Cntfr | -0.1199256 |
| Cntln | -0.6077375 |
| Cntn1 | 1.445601 |
| Cntn4 | 0.5073006 |
| Cntn6 | 0.9695307 |
| Cntnap1 | -0.5355567 |
| Cntnap2 | 0.8651174 |
| Cntnap4 | 2.7891441 |
| Cntnap4 | 0.7671973 |
| Coasy | 0.3402295 |
| Cobl | 1.4479037 |
| Cobll1 | -0.8590217 |
| Cobll1 | -0.3859907 |
| Coch | -0.1984319 |
| Cog1 | 2.1118882 |
| Cog2 | -0.4707689 |
| Cog3 | -0.4399963 |
| Cog4 | -0.0140379 |
| Cog6 | 1.8641899 |
| Cog6 | -0.5520888 |
| Cog8 | -0.1924831 |
| Coil | 3.0728128 |
| Coil | -0.2181719 |
| Col11a1 | 1.9969941 |
| Col12a1 | 1.2121554 |
| Col14a1 | 3.1358304 |
| Col15a1 | -0.642978 |
| Col16a1 | 2.1953518 |
| Col17a1 | -0.3355855 |
| Col17a1 | -0.5576657 |
| Col17a1 | 3.2827275 |
| Col18a1 | 0.4758399 |
| Col18a1 | -0.4928591 |
| Col1a1 | -0.6438545 |
| Col1a2 | 0.4564918 |
| Col24a1 | 0.6699178 |
| Col24a1 | -0.6777893 |
| Col25a1 | 1.542786 |
| Col25a1 | 1.8958299 |
| Col25a1 | 1.790826 |
| Col27a1 | -0.6832627 |
| Col28a1 | -0.2497386 |
| Col2a1 | -0.6832627 |
| Col2a1 | -0.5576657 |
| Col3a1 | 1.1266095 |
| Col3a1 | -0.6026908 |
| Col4a1 | 0.1899532 |
| Col4a2 | 0.2318292 |
| Col4a3 | -0.4456447 |
| Col4a3bp | 1.9770319 |
| Col4a3bp | -0.6403535 |
| Col4a3bp | 0.038694 |
| Col4a4 | 1.8003122 |
| Col4a4 | 0.7820232 |
| Col4a5 | -0.029284 |
| Col4a6 | 1.1107368 |
| Col4a6 | -0.6256202 |
| Col5a1 | -0.4399963 |
| Col5a3 | 0.6171647 |
| Col6a1 | 2.504401 |
| Col6a1 | 0.8827079 |
| Col6a1 | 0.8536679 |
| Col6a2 | -0.5124389 |
| Col7a1 | -0.5465453 |
| Col8a2 | -0.4211829 |
| Col9a2 | -0.0122607 |
| Colec12 | 0.3483902 |
| Commd1 | 0.7332444 |
| Commd1 | -0.6597742 |
| Commd10 | -0.3913188 |
| Commd2 | -0.2209471 |
| Commd2 | -0.4013921 |
| Commd4 | -0.1264291 |
| Commd5 | -0.6500131 |
| Commd6 | 1.1490959 |
| Commd8 | -0.5231555 |
| Commd9 | -0.5465453 |
| Comp | -0.3833381 |
| Comtd1 | 0.0466431 |
| Copa | -0.6213311 |
| Copb2 | -0.2759068 |
| Cope | -0.7585137 |
| Cope | -0.7045486 |
| Cope | -0.5835956 |
| Cope | 0.6680007 |
| Copg | -0.0149279 |
| Copg | -0.7008114 |
| Copg | -0.4156811 |
| Copg | 0.5724065 |
| Cops2 | -0.4906263 |
| Cops3 | -0.8404114 |
| Cops4 | -0.6316586 |
| Cops4 | 1.644991 |
| Cops5 | 1.7870041 |
| Cops5 | 0.829978 |
| Cops6 | 1.3287004 |
| Cops7a | -0.0016678 |
| Cops7a | -0.6333911 |
| Cops7a | -0.1722212 |
| Cops7b | -0.668738 |
| Cops7b | 3.6977347 |
| Cops8 | 1.4272532 |
| Copz1 | 0.1770127 |
| Copz2 | -0.479542 |
| Copz2 | -0.3846635 |
| Copz2 | 3.9003022 |
| Copz2 | 0.1287204 |
| Coq10b | 0.6429309 |
| Coq10b | 0.5300915 |
| Coq2 | -0.2937715 |
| Coq3 | 2.3909249 |
| Coq3 | -0.6696392 |
| Coq4 | -0.1727488 |
| Coq4 | 2.1180735 |
| Coq5 | 1.0007361 |
| Coq5 | -0.4678629 |
| Coq5 | 2.1077869 |
| Coq6 | 0.5693286 |
| Coq6 | 0.1565173 |
| Coq7 | -0.4281064 |
| Coq9 | -0.7054852 |
| Corin | -0.0127047 |
| Coro1a | 2.0697996 |
| Coro1a | -0.435082 |
| Coro1a | -0.2937715 |
| Coro1a | 3.0106617 |
| Coro1b | -0.5010925 |
| Coro1b | -0.7311388 |
| Coro1c | 0.8607971 |
| Coro2a | 1.2759734 |
| Coro2a | -0.1701139 |
| Coro2b | -0.4456447 |
| Coro2b | 1.9879409 |
| Coro6 | 2.4438165 |
| Coro7 | 2.1263898 |
| Cort | -0.6282033 |
| Cort | -0.421873 |
| Cotl1 | -0.7301759 |
| Cova1 | -0.1194272 |
| Cox10 | -0.5803145 |
| Cox11 | 1.8960002 |
| Cox15 | -0.6624543 |
| Cox17 | -0.6535507 |
| Cox18 | -0.714904 |
| Cox18 | -0.416367 |
| Cox19 | 2.0598169 |
| Cox4i1 | 0.7882252 |
| Cox4nb | -0.7664857 |
| Cox4nb | 0.5525775 |
| Cox5a | -0.4744143 |
| Cox5b | 2.0806068 |
| Cox6a1 | -0.2242883 |
| Cox6a1 | -0.7565313 |
| Cox6a2 | 3.3368148 |
| Cox6b1 | -0.3960061 |
| Cox6b1 | 2.33487 |
| Cox6b2 | 0.2806779 |
| Cox6b2 | -0.7595064 |
| Cox6c | 0.688665 |
| Cox7a1 | -0.1119833 |
| Cox7a2 | -0.5951719 |
| Cox7a2l | 0.2630309 |
| Cox7b | -0.7036129 |
| Cox7b2 | -0.6128118 |
| Cox8b | -0.5170166 |
| Cox8c | 0.9672169 |
| Cp | -0.6562128 |
| Cpa1 | -0.4308904 |
| Cpa2 | 0.2159993 |
| Cpa2 | 2.3631493 |
| Cpa3 | 0.482895 |
| Cpa3 | -0.1199256 |
| Cpa3 | -0.5935093 |
| Cpa4 | 1.3555067 |
| Cpa5 | -0.6456099 |
| Cpa6 | -0.6421024 |
| Cpb2 | 0.0619303 |
| Cpeb1 | 2.5272986 |
| Cpeb2 | 0.0984905 |
| Cpeb3 | -0.3533662 |
| Cpeb4 | 1.4908658 |
| Cphx | 2.8501578 |
| Cplx1 | -0.3418967 |
| Cplx2 | 1.0647638 |
| Cpn1 | 0.5918205 |
| Cpne2 | -0.572974 |
| Cpne4 | 1.7982335 |
| Cpne5 | -0.5697302 |
| Cpne6 | 0.5629032 |
| Cpne7 | 2.4303553 |
| Cpne8 | 1.9366403 |
| Cpne8 | 0.205879 |
| Cpne9 | -0.3156734 |
| Cpsf1 | 1.3136199 |
| Cpsf1 | -0.4577629 |
| Cpsf2 | 0.0841989 |
| Cpsf3l | 1.2499234 |
| Cpsf4 | 1.7697385 |
| Cpsf4l | 0.093751 |
| Cpsf4l | -0.6989485 |
| Cpsf6 | -0.0184961 |
| Cpsf6 | -0.7272932 |
| Cpt1b | 2.1379175 |
| Cpt1b | -0.5910209 |
| Cpt1c | -0.4407005 |
| Cpt2 | -0.0761099 |
| Cpxcr1 | -0.638608 |
| Cpxm1 | 0.1744754 |
| Cpxm2 | -0.6377365 |
| Cpz | -0.4921142 |
| Crabp2 | -0.5239258 |
| Crat | 1.9558861 |
| Crat | 0.0441402 |
| Crat | -0.6342585 |
| Crb1 | 0.9491261 |
| Crbn | -0.2022375 |
| Crbn | 0.1664556 |
| Crbn | 4.2405825 |
| Crcp | -0.380033 |
| Creb1 | -0.6705412 |
| Creb1 | -0.6732526 |
| Creb3 | 1.9138187 |
| Creb3l1 | -0.5926791 |
| Creb3l2 | -0.0526333 |
| Creb3l2 | 3.4930797 |
| Creb3l3 | -0.6412275 |
| Creb3l4 | 1.6821014 |
| Creb5 | 1.4899871 |
| Crebl1 | -0.2818235 |
| Crebl2 | 0.7290614 |
| Creg2 | 2.5247085 |
| Creld1 | -0.7379071 |
| Creld2 | 0.1802648 |
| Creld2 | 0.6299151 |
| Crem | 0.9402648 |
| Crem | 2.4990835 |
| Crem | 1.0840266 |
| Crem | 0.9072489 |
| Crhbp | -0.2531853 |
| Crhr1 | -0.6759719 |
| Crim1 | 0.8794881 |
| Crim2 | -0.2688561 |
| Crip2 | -0.6597742 |
| Crip3 | 1.0651808 |
| Crip3 | 1.4178939 |
| Cript | -0.5402502 |
| Cript | -0.4020676 |
| Crisp2 | -0.479542 |
| Crispld2 | -0.3578652 |
| Crk | -0.407489 |
| Crk | 3.1185336 |
| Crk | -0.7244194 |
| Crkl | 1.3057187 |
| Crkrs | 0.1977733 |
| Crkrs | 1.5141105 |
| Crlf3 | 1.132444 |
| Crlf3 | -0.5713507 |
| Crls1 | -0.6438545 |
| Crls1 | 0.8946421 |
| Crls1 | -0.638608 |
| Crls1 | -0.7624911 |
| Crmp1 | 1.1904352 |
| Crmp1 | 0.2480643 |
| Crnkl1 | -0.6187671 |
| Crocc | 0.1156642 |
| Crry | 3.0512379 |
| Crry | 0.6942783 |
| Crtac1 | 1.5667249 |
| Crtam | 1.2624783 |
| Crtap | -0.1801665 |
| Crtap | 0.5411582 |
| Crtc1 | -0.6377365 |
| Crtc3 | 0.0066332 |
| Crxos1 | 0.6863684 |
| Cry2 | 1.6242299 |
| Cryab | -0.2203914 |
| Cryba2 | -0.0746845 |
| Cryba4 | -0.4499037 |
| Crybb1 | -0.0577475 |
| Crybb2 | 0.5176384 |
| Crybb3 | -0.7506095 |
| Crygn | -0.6196209 |
| Crygn | -0.7158512 |
| Cryl1 | -0.7008114 |
| Crym | 1.2605664 |
| Cryz | 0.5600332 |
| Cryz | 1.420361 |
| Cryz | 0.1505773 |
| Cryzl1 | -0.5935093 |
| Cryzl1 | -0.1617321 |
| Cs | 2.5697099 |
| Csad | -0.6394804 |
| Csda | -0.5640805 |
| Csda | 1.1255684 |
| Csda | -0.1627757 |
| Csdc2 | 0.918548 |
| Csde1 | 1.9789022 |
| Csde1 | -0.0452446 |
| Csde1 | 0.612649 |
| Cse1l | -0.5600661 |
| Csf1 | 1.604471 |
| Csf1r | -0.2665177 |
| Csf1r | 0.0127026 |
| Csf2ra | -0.568921 |
| Csgalnact1 | -0.6077375 |
| Csgalnact2 | -0.0827913 |
| Csk | 2.1284683 |
| Csl | -0.3973496 |
| Csl | 0.4367316 |
| Csmd1 | 0.2998998 |
| Csnk1d | -0.3040496 |
| Csnk1d | 1.3410656 |
| Csnk1d | 1.3377567 |
| Csnk1d | 1.7991159 |
| Csnk1d | -0.0742098 |
| Csnk1e | 0.9069193 |
| Csnk1e | -0.1259272 |
| Csnk1g1 | 0.1668216 |
| Csnk1g1 | -0.4136263 |
| Csnk1g1 | -0.6325244 |
| Csnk1g2 | -0.7684895 |
| Csnk1g2 | 1.4190334 |
| Csnk1g2 | 0.7930825 |
| Csnk1g3 | -0.4671378 |
| Csnk2a1 | -0.219281 |
| Csnk2a2 | 2.4734009 |
| Csnk2a2 | 0.4001807 |
| Csnrp2 | -0.407489 |
| Csnrp3 | 0.7869131 |
| Cspg4 | -0.200061 |
| Cspg5 | 1.4652196 |
| Cspp1 | 0.713357 |
| Csprs | 0.9239037 |
| Csrp1 | 0.7027377 |
| Csrp2 | -0.638608 |
| Csrp2bp | 0.9539954 |
| Cst11 | -0.0149279 |
| Cst3 | 1.0254304 |
| Cst8 | 2.2738506 |
| Cst9 | 0.6876218 |
| Cstad | 3.188195 |
| Cstb | -0.49137 |
| Cstf1 | 3.9743017 |
| Cstf2 | 0.4538097 |
| Cstf3 | -0.311374 |
| Cstf3 | 0.113341 |
| Cstl1 | 0.2800303 |
| Ctage5 | 0.1039894 |
| Ctcf | 0.3534918 |
| Ctdp1 | -0.7350004 |
| Ctdp1 | -0.5041047 |
| Ctdp1 | 0.2447053 |
| Ctdsp1 | 1.319766 |
| Ctdsp1 | 2.0546654 |
| Ctdsp1 | -0.7253764 |
| Ctdsp2 | -0.6651421 |
| Ctdspl | -0.5918496 |
| Ctdspl2 | -0.4232546 |
| Ctdspl2 | 0.1370717 |
| Ctf1 | -0.3846635 |
| Ctgf | -0.4336827 |
| Ctla4 | -0.5177817 |
| Ctnna1 | -0.301621 |
| Ctnna3 | -0.1355098 |
| Ctnnal1 | -0.5600661 |
| Ctnnb1 | -0.8032329 |
| Ctnnb1 | -0.1001961 |
| Ctnnb1 | 0.7746399 |
| Ctnnbip1 | -0.4656893 |
| Ctnnbl1 | 0.6735281 |
| Ctnnbl1 | -0.4253308 |
| Ctnnd2 | -0.572162 |
| Ctnnd2 | 0.2393056 |
| Ctnnd2 | -0.4246382 |
| Ctns | -0.5018446 |
| Ctns | -0.0351802 |
| Ctps | -0.5803145 |
| Ctps | -0.2400418 |
| Ctps | 0.5118533 |
| Ctps2 | -0.5101585 |
| Ctps2 | 0.7623948 |
| Ctps2 | 0.317488 |
| Ctr9 | -0.5449675 |
| Ctrb1 | -0.5762293 |
| Ctrl | 1.8102006 |
| Ctsa | -0.1833638 |
| Ctsa | -0.257801 |
| Ctsa | -0.4088494 |
| Ctsa | -0.2925697 |
| Ctsb | 0.7258615 |
| Ctsc | 1.8470589 |
| Ctsc | 2.8185792 |
| Ctsc | -0.6196209 |
| Ctse | -0.6579918 |
| Ctsf | 1.0463698 |
| Ctsh | -0.6500131 |
| Ctsk | 0.0013991 |
| Ctsl | -0.5426058 |
| Ctso | 0.1016367 |
| Ctsr | 0.0370117 |
| Ctss | -0.6482493 |
| Ctsw | -0.3187566 |
| Ctsz | -0.1989746 |
| Ctsz | -0.1375399 |
| Cttn | -0.652665 |
| Cttn | 1.4242383 |
| Cttnbp2nl | -0.3846635 |
| Ctxn1 | -0.1045988 |
| Cuedc1 | 0.189239 |
| Cuedc2 | 0.3771299 |
| Cugbp1 | 1.5317582 |
| Cugbp1 | -0.4671378 |
| Cugbp1 | 1.9871696 |
| Cugbp1 | -0.4832221 |
| Cugbp2 | -0.1817638 |
| Cugbp2 | -0.0746845 |
| Cul1 | 0.9596181 |
| Cul2 | 1.1511249 |
| Cul2 | -0.6264805 |
| Cul3 | -0.5835956 |
| Cul4a | 0.609702 |
| Cul4a | 0.0127026 |
| Cul4a | -0.4965925 |
| Cul4b | 1.1260891 |
| Cul5 | -0.0619528 |
| Cul7 | 0.3323026 |
| Cuta | 0.8142669 |
| Cuta | -0.5139623 |
| Cuta | -0.5332188 |
| Cutc | -0.3064847 |
| Cutc | 0.0122704 |
| Cux1 | 0.7825888 |
| Cux1 | -0.4758764 |
| Cux1 | -0.6119641 |
| Cux1 | 2.0382097 |
| Cux1 | 1.7249092 |
| Cux2 | 3.483594 |
| Cux2 | 0.2800303 |
| Cuzd1 | -0.333073 |
| Cwf19l2 | 0.093355 |
| Cx3cl1 | -0.1279364 |
| Cx3cr1 | 2.5620182 |
| Cxadr | 0.6205371 |
| Cxadr | -0.5426058 |
| Cxadr | -0.3833381 |
| Cxcl1 | 1.365413 |
| Cxcl12 | -0.6970893 |
| Cxcl12 | -0.6896895 |
| Cxcl12 | 1.5365363 |
| Cxcl13 | -0.2531853 |
| Cxcl14 | 0.1546652 |
| Cxcl14 | -0.60437 |
| Cxcl15 | 1.0141937 |
| Cxcl16 | -0.2310071 |
| Cxcl17 | 1.1900709 |
| Cxcl4 | -0.4898832 |
| Cxcl9 | -0.6085813 |
| Cxcr3 | -0.7083008 |
| Cxcr4 | 0.4516584 |
| Cxcr5 | -0.6351268 |
| Cxcr7 | -0.2572228 |
| Cxx1a | -0.5033507 |
| Cxx1b | -0.426024 |
| Cxx1c | -0.3773974 |
| Cxxc1 | -0.4824849 |
| Cxxc1 | -0.462078 |
| Cxxc4 | -0.3132141 |
| Cxxc4 | 3.0421586 |
| Cyb5 | 0.6982081 |
| Cyb561 | -0.033362 |
| Cyb561d1 | 1.2269808 |
| Cyb561d1 | 1.9349548 |
| Cyb561d2 | 1.0174181 |
| Cyb5b | 0.6783904 |
| Cyb5d1 | 2.3666756 |
| Cyb5d2 | 1.618507 |
| Cyb5d2 | -0.6403535 |
| Cyb5r1 | 0.617615 |
| Cyb5r2 | -0.6247608 |
| Cyb5r3 | -0.538683 |
| Cyb5r3 | 0.6523032 |
| Cyb5r3 | 2.9777218 |
| Cyb5r4 | 0.3390143 |
| Cyba | -0.0104868 |
| Cybasc3 | -0.4308904 |
| Cyc1 | -0.6887686 |
| Cycs | 2.5036348 |
| Cycs | -0.5449675 |
| Cyfip1 | 0.848592 |
| Cyfip2 | 1.2730901 |
| Cyfip2 | 0.8019835 |
| Cygb | -0.5893657 |
| Cyhr1 | 0.2314879 |
| Cyhr1 | 3.1207191 |
| Cyhr1 | -0.5819536 |
| Cyhr1 | -0.5926791 |
| Cyld | -0.5308868 |
| Cyln2 | 3.7711515 |
| Cyp11a1 | -0.2514604 |
| Cyp11b2 | 0.1389593 |
| Cyp17a1 | -0.6094258 |
| Cyp19a1 | -0.5673047 |
| Cyp1b1 | -0.4274118 |
| Cyp20a1 | -0.6705412 |
| Cyp24a1 | -0.7272932 |
| Cyp26a1 | 1.2537871 |
| Cyp26b1 | -0.6651421 |
| Cyp27a1 | -0.4627992 |
| Cyp27a1 | -0.1269312 |
| Cyp27a1 | -0.4225635 |
| Cyp2a12 | -0.6624543 |
| Cyp2a5 | 2.1846034 |
| Cyp2a5 | -0.6068945 |
| Cyp2b10 | -0.1001961 |
| Cyp2c55 | 3.5989002 |
| Cyp2d10 | 0.9873827 |
| Cyp2d22 | -0.408169 |
| Cyp2d26 | 0.1977733 |
| Cyp2d26 | -0.2800445 |
| Cyp2d26 | 1.5141105 |
| Cyp2e1 | 2.3200771 |
| Cyp2f2 | -0.6915339 |
| Cyp2j11 | 1.3720255 |
| Cyp2j9 | -0.3337005 |
| Cyp2s1 | -0.4170535 |
| Cyp2u1 | -0.5301108 |
| Cyp39a1 | -0.3077046 |
| Cyp3a13 | 0.25408 |
| Cyp3a13 | -0.6896895 |
| Cyp46a1 | -0.4136263 |
| Cyp4b1 | 0.4813318 |
| Cyp4f13 | 0.7837188 |
| Cyp4f14 | 1.13309 |
| Cyp4f15 | 1.2890567 |
| Cyp4f16 | -0.3598 |
| Cyp4f16 | -0.6887686 |
| Cyp4f16 | 1.4449552 |
| Cyp4f18 | 2.5880052 |
| Cyp4f39 | -0.5071266 |
| Cyp4v3 | 1.0500496 |
| Cyp51 | 0.1487133 |
| Cyp7b1 | -0.7704976 |
| Cypt10 | -0.7120683 |
| Cypt2 | -0.3010148 |
| Cypt3 | 0.9989468 |
| Cypt6 | 2.3517255 |
| Cypt8 | 0.2607186 |
| Cypt9 | 0.3618338 |
| Cyr61 | -0.3437985 |
| Cyr61 | -0.510918 |
| Cysltr1 | -0.3598 |
| Cytl1 | 0.4293151 |
| D030011O10Rik | 1.6412642 |
| D030013I16Rik | 2.0424844 |
| D030018L15Rik | 2.7998101 |
| D030028O16Rik | -0.3656288 |
| D030051N19Rik | 0.3650906 |
| D030056L22Rik | -0.6359959 |
| D030070L09Rik | -0.1554952 |
| D030070L09Rik | -0.3973496 |
| D030070L09Rik | -0.1189291 |
| D030070L09Rik | 0.1430985 |
| D030074E01Rik | 1.9984182 |
| D0H4S114 | 0.0013991 |
| D0H8S2298E | -0.1119833 |
| D0HXS9928E | -0.4729544 |
| D0HXS9928E | -0.7054852 |
| D10627 | -0.6153594 |
| D10Bwg1364e | -0.6517802 |
| D10Bwg1379e | 0.2194637 |
| D10Ertd322e | 0.6103829 |
| D10Ertd610e | 0.8365799 |
| D10Ertd610e | -0.6018524 |
| D10Ertd610e | 1.0826639 |
| D10Ertd641e | -0.3040496 |
| D10Jhu81e | 3.4543185 |
| D10Wsu102e | 1.1612037 |
| D10Wsu52e | -0.1817638 |
| D11Bwg0517e | 2.0512151 |
| D11Wsu47e | 0.1067267 |
| D11Wsu47e | 0.424892 |
| D12Ertd551e | 3.4806882 |
| D12Ertd551e | -0.4477717 |
| D12Ertd553e | -0.6111173 |
| D12Ertd647e | -0.2231733 |
| D12Ertd647e | -0.7036129 |
| D130040H23Rik | 0.1445991 |
| D130043K22Rik | -0.7447253 |
| D130059P03Rik | 1.9563648 |
| D13Ertd608e | -0.7476627 |
| D14Ertd171e | 0.8911283 |
| D14Ertd231e | -0.4906263 |
| D14Ertd449e | -0.6136602 |
| D14Ertd500e | -0.5608676 |
| D14Ertd668e | -0.769493 |
| D14Ertd668e | 1.9393523 |
| D15Ertd621e | -0.5910209 |
| D15Ertd682e | -0.5410347 |
| D15Mgi27 | -0.7330676 |
| D15Wsu169e | 0.4481519 |
| D15Wsu75e | 0.9897976 |
| D16Ertd472e | -0.5262403 |
| D16H22S680E | 0.9794909 |
| D17H6S56E-5 | 0.7859747 |
| D17Wsu104e | 0.6566304 |
| D17Wsu92e | -0.827496 |
| D19Bwg1357e | -0.7073613 |
| D19Ertd721e | -0.5193139 |
| D19Ertd737e | -0.6221873 |
| D19Wsu162e | 0.2916192 |
| D1Bwg0212e | 1.6386466 |
| D1Bwg0212e | 1.7470283 |
| D1Pas1 | -0.4232546 |
| D230007K08Rik | 0.0865961 |
| D230014K01Rik | -0.4343821 |
| D2Ertd750e | 0.917082 |
| D2Ertd750e | 0.078581 |
| D330014H01Rik | -0.5512949 |
| D330017J20Rik | -0.1935619 |
| D330028D13Rik | 1.0300605 |
| D330045A20Rik | 0.1442242 |
| D330045A20Rik | 0.9647425 |
| D330045A20Rik | 0.5660025 |
| D3Bwg0562e | -0.5441796 |
| D3Ertd300e | -0.6464889 |
| D3Ertd751e | 1.6967789 |
| D3Ucla1 | 1.8966242 |
| D430015B01Rik | -0.5852406 |
| D430028G21Rik | -0.521617 |
| D430039N05Rik | -0.6196209 |
| D430041B17Rik | -0.5379004 |
| D430042O09Rik | -0.6264805 |
| D4Bwg0951e | -0.1544597 |
| D4Bwg1540e | -0.2985941 |
| D4Ertd22e | -0.5262403 |
| D4Ertd22e | -0.497341 |
| D4Wsu114e | -0.2812301 |
| D4Wsu132e | 3.4200687 |
| D4Wsu132e | -0.2248464 |
| D530004J12Rik | -0.5844177 |
| D530033C11Rik | 1.6417869 |
| D5Ertd135e | 3.6447297 |
| D5Ertd579e | 3.6049735 |
| D5Wsu178e | -0.2830114 |
| D630003M21Rik | -0.7486439 |
| D630003M21Rik | 0.4215608 |
| D630014A15Rik | -0.6273415 |
| D630023F18Rik | 0.0260023 |
| D630042P16Rik | -0.6010147 |
| D630048P19Rik | -0.0904864 |
| D6Ertd474e | -0.6102712 |
| D6Mm5e | 2.0002967 |
| D6Wsu116e | 0.2198094 |
| D6Wsu163e | -0.5033507 |
| D6Wsu163e | -0.568921 |
| D6Wsu163e | -0.1796347 |
| D6Wsu176e | 2.4442566 |
| D6Wsu176e | -0.4810123 |
| D730039F16Rik | 0.1106232 |
| D730039F16Rik | 0.7612384 |
| D7Bwg0611e | -0.6342585 |
| D830014E11Rik | -0.6359959 |
| D830030K20Rik | 0.0627495 |
| D830030K20Rik | -0.4122589 |
| D830050J10Rik | 0.7575673 |
| D8Ertd738e | 1.7750554 |
| D8Ertd82e | 0.0148607 |
| D930001I22Rik | -0.6111173 |
| D930005D10Rik | -0.3343284 |
| D930014E17Rik | 1.5726143 |
| D930015E06Rik | -0.5536788 |
| D930020B18Rik | 2.892559 |
| D930028F11Rik | -0.407489 |
| D930049A15Rik | 1.0506144 |
| D9Ertd280e | 0.2041229 |
| D9Ertd392e | 2.1334466 |
| D9Ertd402e | -0.6500131 |
| D9Wsu20e | 1.2950418 |
| Daam1 | -0.5993416 |
| Daam2 | 0.7824003 |
| Dab1 | -0.6562128 |
| Dab1 | 1.2715339 |
| Dab1 | 1.3178629 |
| Dab2 | -0.520081 |
| Dab2 | 0.0285546 |
| Dab2ip | 0.5090738 |
| Dact1 | 1.5909294 |
| Dact2 | 0.5671918 |
| Dad1 | 1.590535 |
| Daf2 | 0.5537841 |
| Dag1 | 1.3271299 |
| Dag1 | -0.5713507 |
| Dagla | -0.348889 |
| Daglb | 1.1302441 |
| Dak | -0.5093996 |
| Dalrd3 | 1.9110308 |
| Dap | -0.6841781 |
| Dap3 | 1.1281689 |
| Dap3 | 0.1264297 |
| Dap3 | 0.4072681 |
| Dap3 | 0.3742079 |
| Dapk1 | -0.5893657 |
| Dapk1 | 0.9860216 |
| Dapk2 | -0.3591546 |
| Dapk2 | -0.4143107 |
| Dapl1 | 3.3434442 |
| Dapp1 | -0.7565313 |
| Dapp1 | -0.5778612 |
| Darc | -0.5918496 |
| Dars | -0.5101585 |
| Dars | -0.49137 |
| Daxx | -0.7120683 |
| Dazap1 | 1.9314654 |
| Dazap1 | -0.4047743 |
| Dazap2 | 0.657062 |
| Dazl | -0.7244194 |
| Dbc1 | 1.9181511 |
| Dbf4 | -0.2883758 |
| Dbi | 2.7014766 |
| Dbi | -0.6221873 |
| Dbil5 | -0.6162101 |
| Dbn1 | -0.1822968 |
| Dbndd1 | -0.4294974 |
| Dbndd2 | -0.5976714 |
| Dbndd2 | 0.1860183 |
| Dbndd2 | -0.7167993 |
| Dbnl | 0.7683462 |
| Dbp | -0.6377365 |
| Dbpht2 | 1.190678 |
| Dbr1 | 0.8735273 |
| Dbt | -0.0438657 |
| Dcakd | -0.2165108 |
| Dcbld1 | 1.3906067 |
| Dcbld2 | 0.4451744 |
| Dci | -0.0320007 |
| Dck | 0.7738793 |
| Dclk1 | -0.2925697 |
| Dclk2 | 0.8353345 |
| Dclk3 | -0.2220595 |
| Dclre1b | 0.9519566 |
| Dclre1b | -0.5968375 |
| Dclre1c | -0.6906112 |
| Dclre1c | -0.4584807 |
| Dclre1c | -0.4506154 |
| Dcn | -0.549709 |
| Dcn | 0.4971027 |
| Dcp1a | 1.1952792 |
| Dcp1a | 1.0538556 |
| Dcp1a | 1.1195593 |
| Dcp1a | -0.4198044 |
| Dcp1b | 0.4221168 |
| Dcp1b | 1.2834721 |
| Dcpp1 | -0.4965925 |
| Dcpp2 | -0.7369372 |
| Dcpp2 | -0.184432 |
| Dcpp3 | -0.6850944 |
| Dcps | -0.6723479 |
| Dcps | -0.6878487 |
| Dct | 0.8168131 |
| Dctd | 2.5732448 |
| Dctn1 | 1.0226704 |
| Dctn2 | -0.2423143 |
| Dctn2 | 1.2096613 |
| Dctn2 | 0.4223947 |
| Dctn3 | -0.7215546 |
| Dctn3 | -0.7244194 |
| Dctn4 | 0.2517452 |
| Dctn4 | -0.2694416 |
| Dctn5 | 0.8767679 |
| Dctn6 | -0.1865721 |
| Dcun1d2 | 0.5597936 |
| Dcun1d3 | -0.7064228 |
| Dcun1d4 | -0.2104453 |
| Dcun1d4 | 1.6722581 |
| Dcun1d5 | 1.893272 |
| Dcx | 0.4813318 |
| Dcx | 0.4001807 |
| Dcxr | 1.1509982 |
| Dcxr | 0.1809859 |
| Ddah1 | -0.6651421 |
| Ddah2 | 0.0407925 |
| Ddb1 | 0.14009 |
| Ddb2 | -0.6642453 |
| Ddb2 | 0.0586462 |
| Ddb2 | -0.4846982 |
| Ddc | -0.6878487 |
| Ddc8 | 1.1212615 |
| Ddef2 | -0.4664133 |
| Ddef2 | 0.02515 |
| Ddefl1 | 0.0574117 |
| Ddhd1 | 0.7472491 |
| Ddhd1 | -0.6412275 |
| Ddi2 | -0.1204242 |
| Ddit3 | -0.3077046 |
| Ddit3 | 0.8561084 |
| Ddit4 | 2.0336217 |
| Ddit4l | -0.4170535 |
| Ddo | 1.9179294 |
| Ddo | 0.4221168 |
| Ddo | 2.7868006 |
| Ddost | 3.265925 |
| Ddr1 | -0.4906263 |
| Ddr2 | 1.2314058 |
| Ddt | 0.9596181 |
| Ddx1 | -0.4274118 |
| Ddx1 | 1.8224453 |
| Ddx17 | 0.945655 |
| Ddx17 | -0.362386 |
| Ddx17 | 0.446529 |
| Ddx19a | -0.210995 |
| Ddx19b | -0.5071266 |
| Ddx19b | -0.2248464 |
| Ddx20 | 0.1415955 |
| Ddx21 | -0.4499037 |
| Ddx24 | -0.4891407 |
| Ddx24 | 3.1700177 |
| Ddx24 | 0.9319599 |
| Ddx25 | 0.1913799 |
| Ddx26b | -0.6299293 |
| Ddx27 | -0.3899839 |
| Ddx28 | 0.1576268 |
| Ddx3x | -0.0140379 |
| Ddx3y | -0.6998795 |
| Ddx4 | -0.4832221 |
| Ddx4 | 0.7391877 |
| Ddx41 | 0.6971755 |
| Ddx46 | 0.6733161 |
| Ddx47 | -0.2549135 |
| Ddx47 | -0.1385566 |
| Ddx47 | 1.5494887 |
| Ddx49 | 3.1757498 |
| Ddx5 | -0.7206016 |
| Ddx50 | -0.4758764 |
| Ddx50 | 3.2849399 |
| Ddx51 | 0.9893453 |
| Ddx52 | -0.5536788 |
| Ddx54 | 2.0101345 |
| Ddx58 | -0.6368658 |
| Ddx6 | 0.8938067 |
| Ddx6 | -0.3636818 |
| Ddx6 | -0.4407005 |
| Ddx6 | -0.6660398 |
| Deadc1 | -0.7644863 |
| Deb1 | 0.0353265 |
| Decr1 | 0.9599295 |
| Decr2 | 1.4600587 |
| Decr2 | -0.135003 |
| Dedd | 0.6507839 |
| Dedd | -0.5078837 |
| Dedd2 | -0.4729544 |
| Def6 | 0.5365017 |
| Def8 | 0.596899 |
| Defb11 | 0.689082 |
| Defb14 | -0.4204934 |
| Defb15 | 0.0009616 |
| Defb19 | 0.7744498 |
| Defb20 | 1.0654587 |
| Defb21 | -0.4149957 |
| Defb25 | -0.4921142 |
| Defb26 | 0.6796547 |
| Defb30 | -0.4020676 |
| Defb36 | 0.4193333 |
| Defb36 | -0.4891407 |
| Defb4 | -0.6723479 |
| Defcr20 | 2.7203858 |
| Defcr6 | -0.6669383 |
| Defcr-rs7 | 1.1288179 |
| Degs1 | 0.7969872 |
| Degs2 | -0.5632762 |
| Dek | 0.3573742 |
| Dek | -0.0082742 |
| Dennd1a | -0.6342585 |
| Dennd1c | -0.1090224 |
| Dennd2a | 1.0277483 |
| Dennd3 | -0.3813536 |
| Denr | -0.6786993 |
| Denr | 1.750819 |
| Depdc1b | -0.0577475 |
| Depdc5 | 1.3488774 |
| Depdc5 | -0.6128118 |
| Depdc6 | -0.6553246 |
| Depdc6 | 0.8613166 |
| Dera | -0.6085813 |
| Derl2 | 1.226397 |
| Derl3 | 1.5753447 |
| Des | -0.2572228 |
| Det1 | 1.8668292 |
| Dexi | -0.178572 |
| Dexi | 2.509571 |
| Dffa | 1.6345181 |
| Dfna5h | 0.3782961 |
| Dgat1 | 0.6869953 |
| Dgat2 | -0.6588826 |
| Dgcr2 | -0.2027824 |
| Dgcr2 | 2.2361591 |
| Dgcr6 | 0.2944939 |
| Dgcr6 | 2.3768369 |
| Dgcr8 | 2.6691692 |
| Dgka | -0.6633494 |
| Dgka | -0.0060668 |
| Dgkb | -0.6077375 |
| Dgke | -0.6860116 |
| Dgkg | -0.4371849 |
| Dgkh | -0.6741582 |
| Dgki | 0.2044744 |
| Dgkk | 1.5689883 |
| Dgkq | 0.3450741 |
| Dgkz | 2.0285191 |
| Dgkz | 0.345376 |
| Dgkz | 2.9148161 |
| Dguok | 3.3316846 |
| Dhcr24 | -0.2411774 |
| Dhcr24 | 0.0557631 |
| Dhcr24 | 2.6752166 |
| Dhcr7 | -0.1743333 |
| Dhdds | -0.4861767 |
| Dhdds | 0.933883 |
| Dhdh | 3.0302696 |
| Dhdh | -0.6553246 |
| Dhdh | 2.6054449 |
| Dhh | 0.6046933 |
| Dhodh | -0.4477717 |
| Dhps | 0.5095798 |
| Dhrs1 | 0.4826346 |
| Dhrs4 | 0.3987566 |
| Dhrs7 | -0.7340335 |
| Dhrs7 | 1.6151331 |
| Dhrs7 | -0.4191159 |
| Dhrs7b | -0.5394663 |
| Dhrs7b | -0.617914 |
| Dhrs7b | 0.6162633 |
| Dhrsx | -0.5893657 |
| Dhrsx | 0.0428862 |
| Dhtkd1 | 3.1176823 |
| Dhx15 | 0.3008506 |
| Dhx15 | 0.9752134 |
| Dhx16 | -0.6085813 |
| Dhx29 | -0.3826762 |
| Dhx30 | 0.6354526 |
| Dhx32 | 0.3396221 |
| Dhx33 | -0.132473 |
| Dhx36 | -0.5162521 |
| Dhx37 | -0.0680611 |
| Dhx38 | -0.4577629 |
| Dhx57 | -0.638608 |
| Dhx58 | -0.4449368 |
| Dhx8 | -0.6162101 |
| Dhx9 | 1.2726457 |
| Diablo | -0.5154882 |
| Diap1 | -0.6998795 |
| Diap3 | 2.7475929 |
| Dicer1 | -0.3482511 |
| Dido1 | -0.3721484 |
| Dido1 | 1.5492413 |
| Dido1 | 1.0131654 |
| Dio1 | -0.5568669 |
| Dio1 | 1.8992295 |
| Dio3 | -0.479542 |
| Dip2b | 0.1279575 |
| Dip2b | 0.4157022 |
| Diras1 | 1.1849553 |
| Diras2 | -0.4088494 |
| Dirc2 | -0.510918 |
| Dis3 | 0.1627871 |
| Dis3l | 0.470579 |
| Dis3l2 | 0.3668623 |
| Disp1 | 1.2910197 |
| Dixdc1 | 1.8049566 |
| Dkk3 | 0.4187756 |
| Dkk3 | -0.4301936 |
| Dkk3 | -0.0149279 |
| Dkkl1 | -0.6077375 |
| Dkkl1 | 0.8209812 |
| Dlat | 1.934356 |
| Dlc1 | -0.3833381 |
| Dld | -0.5124389 |
| Dleu7 | -0.6325244 |
| Dlg1 | 0.9012986 |
| Dlg2 | 1.243639 |
| Dlg3 | 2.4999127 |
| Dlg4 | -0.6010147 |
| Dlg7 | -0.49137 |
| Dlgap1 | -0.4736841 |
| Dlgap2 | -0.4506154 |
| Dlgap2 | -0.6325244 |
| Dlgap2 | 0.0984905 |
| Dlgap3 | 0.0598791 |
| Dlgap4 | -0.6642453 |
| Dlgap4 | -0.3089261 |
| Dlgap4 | 0.4337173 |
| Dlgap4 | 0.612649 |
| Dlk1 | -0.7379071 |
| Dlk2 | 0.06234 |
| Dll1 | 0.065611 |
| Dlst | 0.424892 |
| Dlx1 | -0.7167993 |
| Dlx1as | 0.3895908 |
| Dlx2 | -0.0424889 |
| Dlx3 | 2.9544047 |
| Dmap1 | 1.534276 |
| Dmbt1 | 0.580174 |
| Dmkn | -0.4088494 |
| Dmkn | 1.1071575 |
| Dmkn | -0.6562128 |
| Dmn | 1.1479527 |
| Dmn | -0.5489171 |
| Dmpk | 0.8936395 |
| Dmrt2 | 0.1867349 |
| Dmrt3 | -0.4061307 |
| Dmrta1 | 0.3800428 |
| Dmrta2 | 0.4033058 |
| Dmrtc1a | 1.3957785 |
| Dmrtc1a | 0.803643 |
| Dmrtc1a | 0.6283587 |
| Dmrtc1b | -0.0521698 |
| Dmrtc2 | -0.6491308 |
| Dmtf1 | -0.652665 |
| Dmtf1 | -0.6239022 |
| Dmtf1 | 2.1461452 |
| Dmwd | 0.0980964 |
| Dmwd | 2.0462086 |
| Dmwd | 0.0412116 |
| Dnahc11 | 0.254746 |
| Dnahc2 | -0.5101585 |
| Dnahc5 | 0.9937083 |
| Dnahc8 | -0.1881805 |
| Dnaic1 | 0.5014505 |
| Dnaic1 | 1.3262913 |
| Dnaja1 | -0.5762293 |
| Dnaja2 | 0.3239985 |
| Dnaja2 | -0.7272932 |
| Dnaja3 | -0.6989485 |
| Dnajb1 | -0.3960061 |
| Dnajb10 | -0.6221873 |
| Dnajb12 | 0.41318 |
| Dnajb13 | 0.6913719 |
| Dnajb3 | 1.1471897 |
| Dnajb4 | -0.3708408 |
| Dnajb4 | 3.1975216 |
| Dnajb5 | 1.620724 |
| Dnajb6 | -0.263603 |
| Dnajb6 | 0.2580688 |
| Dnajb7 | 1.2845698 |
| Dnajb8 | 4.2134874 |
| Dnajb9 | 0.6416155 |
| Dnajc10 | 1.4978657 |
| Dnajc10 | 0.1423473 |
| Dnajc12 | 0.2517452 |
| Dnajc14 | -0.4869168 |
| Dnajc15 | -0.572974 |
| Dnajc16 | 0.4955633 |
| Dnajc17 | -0.5185475 |
| Dnajc18 | 1.3703268 |
| Dnajc19 | -0.5893657 |
| Dnajc19 | -0.5960043 |
| Dnajc2 | 1.4668435 |
| Dnajc2 | -0.1701139 |
| Dnajc3a | -0.6896895 |
| Dnajc4 | 0.3519942 |
| Dnajc5 | -0.4832221 |
| Dnajc5b | -0.2077013 |
| Dnajc5g | -0.4591991 |
| Dnajc6 | 0.6209858 |
| Dnajc6 | -0.5681125 |
| Dnajc7 | -0.3939943 |
| Dnajc8 | 0.0672422 |
| Dnalc1 | 0.7979145 |
| Dnali1 | 1.566644 |
| Dnase1l1 | -0.3052663 |
| Dnase1l2 | -0.4108936 |
| Dnase1l2 | -0.2377749 |
| Dnase2a | 3.5690734 |
| Dnd1 | 0.1256649 |
| Dner | -0.5147249 |
| Dnm | 0.12375 |
| Dnm1 | 0.4813318 |
| Dnm1l | -0.7939088 |
| Dnm1l | 0.7268627 |
| Dnmbp | -0.3682311 |
| Dnmt1 | -0.065707 |
| Dnmt3b | -0.4129424 |
| Dnmt3l | -0.6077375 |
| Dnmt3l | -0.0784906 |
| Dnttip1 | -0.3444333 |
| Doc2a | -0.2717874 |
| Doc2b | 1.1280391 |
| Doc2b | -0.711125 |
| Dock1 | -0.6786993 |
| Dock3 | -0.3224701 |
| Dock4 | -0.6473687 |
| Dock6 | 0.0268538 |
| Dock8 | 0.0310998 |
| Dock9 | -0.2800445 |
| Dok1 | -0.2735506 |
| Dok1 | -0.380033 |
| Dok2 | -0.7036129 |
| Dok3 | 1.3065761 |
| Dok4 | -0.5877134 |
| Dok5 | 1.724841 |
| Dok5 | 0.0247235 |
| Dok7 | 0.3855521 |
| Dolk | 0.2871296 |
| Dolpp1 | 0.608566 |
| Dolpp1 | 1.5189802 |
| Dolpp1 | 1.868526 |
| Dom3z | -0.408169 |
| Dom3z | -0.6052107 |
| Donson | -0.7816206 |
| Donson | 2.7193583 |
| Dos | -0.1941017 |
| Dos | -0.4470622 |
| Dot1l | -0.1989746 |
| Dpagt1 | 0.882031 |
| Dpep1 | 0.8213427 |
| Dpep2 | 0.5904306 |
| Dpep2 | 2.3338439 |
| Dpep3 | 2.0033854 |
| Dpf1 | 0.2359147 |
| Dpf1 | 1.5215329 |
| Dpf3 | 0.6932413 |
| Dph2 | -0.6256202 |
| Dph2 | -0.2321316 |
| Dph2 | -0.5285606 |
| Dph3 | 1.9567901 |
| Dph3 | -0.2170642 |
| Dph3 | -0.3299419 |
| Dph4 | 0.078581 |
| Dpm1 | -0.579496 |
| Dpm1 | 1.30893 |
| Dpm2 | 0.0285546 |
| Dpm3 | -0.1627757 |
| Dpp10 | -0.7545532 |
| Dpp3 | -0.5901929 |
| Dpp3 | -0.6980184 |
| Dpp4 | -0.3546494 |
| Dpp6 | 3.460309 |
| Dpp7 | 0.5248816 |
| Dpp8 | -0.6153594 |
| Dpp9 | -0.538683 |
| Dpp9 | -0.3181391 |
| Dppa3 | 0.3477881 |
| Dppa4 | 0.847714 |
| Dppa5 | 1.4160868 |
| Dppa5 | 2.2374955 |
| Dpt | 1.553438 |
| Dpy30 | -0.6403535 |
| Dpyd | -0.6482493 |
| Dpysl2 | -0.3980222 |
| Dpysl2 | 1.1949168 |
| Dpysl2 | 1.3201884 |
| Dpysl3 | -0.2589585 |
| Dpysl4 | 1.2620288 |
| Dpysl5 | 1.2316381 |
| Drbp1 | -0.4407005 |
| Drctnnb1a | -0.5332188 |
| Drd1a | -0.5324408 |
| Drd2 | -0.2049654 |
| Drd4 | -0.7595064 |
| Drd5 | 0.7000638 |
| Drg1 | 1.7882798 |
| Drg1 | -0.5246966 |
| Drg2 | -0.2770871 |
| Drp2 | 3.7445063 |
| Dscam | -0.5010925 |
| Dscam | 1.2153522 |
| Dscc1 | 0.5554707 |
| Dscr1 | 0.5073006 |
| Dscr1l2 | -0.5852406 |
| Dscr2 | 3.4636669 |
| Dscr3 | -0.5063702 |
| Dscr3 | 1.3307901 |
| Dscr6 | -0.2377749 |
| Dsg2 | 0.0516288 |
| Dsg2 | -0.1244231 |
| Dsn1 | -0.6333911 |
| Dsp | -0.5355567 |
| Dst | -0.6273415 |
| Dst | 0.5578748 |
| Dst | 1.1378566 |
| Dstn | 0.3168655 |
| Dtd1 | -0.0680611 |
| Dtl | 0.0165835 |
| Dtna | -0.4054523 |
| Dtna | 1.0811625 |
| Dtna | -0.572162 |
| Dtna | 0.828365 |
| Dtnb | 2.1371738 |
| Dtnbp1 | -0.4054523 |
| Dtwd1 | 1.5689075 |
| Dtx1 | -0.6316586 |
| Dtx2 | 0.6566304 |
| Dtx2 | -0.5943402 |
| Dtx2 | -0.4225635 |
| Dtx4 | 0.3030651 |
| Dtx4 | -0.2115451 |
| Dtymk | -0.0742098 |
| Dus1l | 1.7096334 |
| Dus1l | -0.5056144 |
| Dus2l | -0.462078 |
| Dus3l | -0.4810123 |
| Dus3l | 1.8025745 |
| Dus3l | 0.8760866 |
| Dus4l | 0.066427 |
| Dusp1 | 2.3721948 |
| Dusp11 | 1.2586506 |
| Dusp11 | 0.163522 |
| Dusp13 | 1.154665 |
| Dusp13 | 0.364795 |
| Dusp13 | -0.0383707 |
| Dusp14 | -0.3337005 |
| Dusp15 | -0.233821 |
| Dusp15 | 3.107236 |
| Dusp16 | 1.8210312 |
| Dusp16 | 0.6033226 |
| Dusp16 | 0.5576347 |
| Dusp18 | 0.4212827 |
| Dusp19 | -0.2531853 |
| Dusp2 | 2.5323143 |
| Dusp2 | 0.2086819 |
| Dusp21 | -0.2723747 |
| Dusp22 | 0.9579037 |
| Dusp23 | -0.407489 |
| Dusp26 | 0.8954768 |
| Dusp28 | 1.3936352 |
| Dusp3 | 1.565429 |
| Dusp3 | -0.2700275 |
| Dusp3 | -0.4294974 |
| Dusp4 | -0.5746002 |
| Dusp4 | -0.2830114 |
| Dusp4 | -0.3708408 |
| Dusp6 | -0.1016613 |
| Dusp6 | 0.2954502 |
| Dusp6 | -0.5489171 |
| Dusp7 | -0.2027824 |
| Dusp8 | 0.7284622 |
| Dut | 0.2355749 |
| Duxbl | 3.2477425 |
| Duxbl | -0.4329838 |
| Dvl1 | -0.3318193 |
| Dvl1 | -0.5746002 |
| Dvl2 | -0.1472439 |
| DXBwg1396e | -0.7026781 |
| Dydc2 | -0.2131974 |
| Dym | -0.7120683 |
| Dync1h1 | 0.8109828 |
| Dync1i1 | -0.5536788 |
| Dync1i1 | -0.064767 |
| Dync1i2 | 0.50527 |
| Dync1li1 | -0.5754144 |
| Dync1li2 | 2.4851739 |
| Dync2li1 | 0.093355 |
| Dynll1 | -0.1105017 |
| Dynll2 | 0.0680567 |
| Dynlrb1 | -0.4357825 |
| Dynlrb2 | -0.0360906 |
| Dynlt3 | 1.6307489 |
| Dyrk1a | -0.5893657 |
| Dyrk1b | -0.1580888 |
| Dyrk1b | -0.033362 |
| Dyrk2 | 4.2871385 |
| Dyrk2 | 0.1370717 |
| Dyrk3 | -0.6606667 |
| Dyrk4 | 0.1423473 |
| Dysf | 0.8139026 |
| Dysfip1 | -0.6500131 |
| Dzip1 | 2.2379274 |
| E030010A14Rik | -0.5568669 |
| E030034P13Rik | -0.2782689 |
| E130009J12Rik | -0.3773974 |
| E130012A19Rik | -0.2694416 |
| E130014J05Rik | 2.5339422 |
| E130016E03Rik | 0.1438491 |
| E130203B14Rik | -0.4802768 |
| E130203B14Rik | 0.638319 |
| E130303B06Rik | -0.7634882 |
| E130306D19Rik | 0.4454455 |
| E130308A19Rik | -0.7120683 |
| E130309D02Rik | -0.5737868 |
| E130309D02Rik | -0.2842008 |
| E130309D14Rik | 1.3574356 |
| E130309F12Rik | -0.6473687 |
| E130309F12Rik | 1.6965678 |
| E130309F12Rik | -0.437887 |
| E230008N13Rik | 2.7787925 |
| E2f1 | 1.2451282 |
| E2f2 | 0.8478897 |
| E2f2 | -0.6491308 |
| E2f3 | -0.6264805 |
| E2f4 | -0.2237306 |
| E2f5 | 3.1763925 |
| E2f6 | -0.6052107 |
| E2f6 | 0.5860155 |
| E2f6 | -0.7369372 |
| E330016A19Rik | -0.089521 |
| E330017A01Rik | 0.695728 |
| E330018D03Rik | -0.5819536 |
| E330021D16Rik | 0.3190422 |
| E330034G19Rik | -0.5673047 |
| E330034G19Rik | -0.6239022 |
| E330036I19Rik | 1.8664191 |
| E430002D04Rik | -0.4773409 |
| E430002G05Rik | -0.6651421 |
| E430018J23Rik | -0.6145094 |
| E430025E21Rik | 1.1564299 |
| E430028B21Rik | -0.6777893 |
| E4f1 | 1.1341227 |
| Eaf1 | -0.210995 |
| Ear11 | -0.0283802 |
| Ear2 | -0.295577 |
| Ear4 | -0.1780411 |
| Ears2 | 2.5273849 |
| Ebag9 | 0.5186408 |
| Ebf2 | 0.3621304 |
| Ebf3 | -0.3946644 |
| Ebf4 | 1.7520128 |
| Ebi2 | 0.8765976 |
| Ebi3 | -0.5819536 |
| Ebna1bp2 | 2.4508335 |
| Ebna1bp2 | 0.9819263 |
| Ebp | -0.4700416 |
| Ebpl | -0.2215032 |
| Ebpl | 1.0445243 |
| Ecd | -0.0051853 |
| Ecd | -0.1395744 |
| Ece1 | 1.0687869 |
| Ece1 | 2.2137864 |
| Ece2 | -0.6642453 |
| Ecel1 | 2.0057078 |
| Ech1 | -0.7026781 |
| Echdc1 | 0.6510011 |
| Echdc2 | 1.6324846 |
| Echdc2 | -0.6869297 |
| Echdc2 | 0.1576268 |
| Echdc3 | 0.0897829 |
| Echs1 | 1.7845115 |
| Ecm1 | 0.1967108 |
| Ecm1 | -0.5536788 |
| Ecsit | 0.3543892 |
| Eda2r | 1.6145184 |
| Edaradd | 0.5039986 |
| Edc4 | 0.2284108 |
| Edem1 | 2.0568631 |
| Edem2 | -0.5086413 |
| Edem2 | 0.7322502 |
| Edem2 | 1.2554866 |
| Edem2 | -0.6571019 |
| Edf1 | -0.7158512 |
| Edg2 | -0.4322855 |
| Edg5 | 1.1004927 |
| Edg7 | 1.5554882 |
| Edg8 | 0.2537468 |
| Edg8 | -0.0383707 |
| Edil3 | 1.4792882 |
| Edn1 | 0.2797063 |
| Edn2 | -0.3132141 |
| Ednra | -0.4678629 |
| Ednrb | 2.1157925 |
| Ednrb | 0.867012 |
| Ednrb | -0.9692624 |
| Eed | 0.7190251 |
| Eef1a1 | -0.652665 |
| Eef1a1 | 1.165579 |
| Eef1a2 | 0.8173579 |
| Eef1a2 | -0.6544372 |
| Eef1a2 | 1.1873948 |
| Eef1b2 | -0.5025974 |
| Eef1b2 | 0.8661513 |
| Eef1b2 | 1.1757496 |
| Eef1d | 2.626574 |
| Eef1d | 0.9319599 |
| Eef1d | 0.3967593 |
| Eef1d | 1.0947223 |
| Eef1e1 | 1.1620804 |
| Eef1g | -0.8158127 |
| Eef2 | 0.179904 |
| Eef2 | 0.6499146 |
| Eef2 | 0.5080609 |
| Eef2k | 0.1051635 |
| Eefsec | 0.0696837 |
| Efcab1 | -0.1080376 |
| Efcab2 | -0.3450685 |
| Efcab2 | 2.7398548 |
| Efcab3 | -0.6010147 |
| Efcab3 | 1.2624783 |
| Efcab3 | -0.0153731 |
| Efcab4a | 0.7623948 |
| Efcab4a | -0.6473687 |
| Efcab5 | -0.6359959 |
| Efemp2 | 0.9239037 |
| Efha1 | 1.866712 |
| Efha1 | -0.6759719 |
| Efhc2 | 0.3615372 |
| Efhd1 | 0.6825979 |
| Efna1 | 1.252993 |
| Efna3 | -0.7139578 |
| Efna4 | -0.6759719 |
| Efna4 | -0.1129724 |
| Efna5 | 1.3717259 |
| Efnb1 | 0.742342 |
| Efnb2 | 0.0370117 |
| Efnb3 | -0.3324459 |
| Efr3a | -0.6342585 |
| Efs | -0.2389077 |
| Eftud1 | 0.8371131 |
| Eftud2 | 0.6146838 |
| Eftud2 | -0.5277866 |
| Eftud2 | -0.4020676 |
| EG194588 | 0.114116 |
| EG208426 | -0.7427721 |
| EG210155 | 0.6014918 |
| EG212225 | 1.7771866 |
| EG214321 | 0.4196121 |
| EG232599 | 0.7958732 |
| EG237009 | -0.2812301 |
| EG237009 | -0.4788077 |
| EG237300 | 0.2480643 |
| EG238829 | 1.3951944 |
| EG240916 | 2.1471415 |
| EG245297 | -0.426024 |
| EG245297 | 2.8480043 |
| EG245376 | -0.7120683 |
| EG245376 | -0.652665 |
| EG272350 | -0.5885392 |
| EG277333 | 1.2272142 |
| EG317677 | -0.0388274 |
| EG328264 | -0.711125 |
| EG328280 | 0.0619303 |
| EG328644 | -0.714904 |
| EG328839 | 0.4451744 |
| EG330305 | -0.6750646 |
| EG330503 | -0.6239022 |
| EG330513 | -0.4584807 |
| EG333669 | -0.1534255 |
| EG368203 | -0.3418967 |
| EG380907 | -0.5033507 |
| EG381438 | 1.9174305 |
| EG382109 | 1.9137074 |
| EG382109 | -0.6805219 |
| EG382109 | -0.5010925 |
| EG384639 | 0.0217329 |
| EG384813 | 3.4435058 |
| EG384813 | 1.7036475 |
| EG384814 | -0.6850944 |
| EG385328 | 0.5352724 |
| EG408196 | 0.2413342 |
| EG432825 | 0.5288537 |
| EG432879 | 1.3826465 |
| EG432939 | 0.5547482 |
| EG433016 | 1.9647291 |
| EG433182 | -0.3656288 |
| EG433224 | 2.1818133 |
| EG433923 | 2.4174403 |
| EG434171 | 1.1143022 |
| EG434197 | 0.6680007 |
| EG434280 | -0.6588826 |
| EG434459 | 2.4440994 |
| EG434459 | -0.3138283 |
| EG434858 | 0.5705136 |
| EG435391 | -0.6403535 |
| EG436332 | -0.4707689 |
| EG545047 | 2.0054556 |
| EG545056 | 0.2463863 |
| EG545253 | 1.6655888 |
| EG545370 | 1.2637132 |
| EG545758 | 1.8089547 |
| EG545861 | -0.8105504 |
| EG545963 | -0.2712004 |
| EG546166 | 2.7013813 |
| EG546282 | 1.0147808 |
| EG546325 | 1.6078839 |
| EG546347 | -0.5762293 |
| EG547109 | -0.7466825 |
| EG574403 | 1.7294026 |
| EG622320 | -0.3349567 |
| EG622339 | -0.6796102 |
| EG624866 | 1.4824843 |
| EG625054 | -0.5835956 |
| EG625424 | 0.2610493 |
| EG630499 | 0.5929771 |
| EG633640 | 0.867356 |
| EG638695 | 2.2719225 |
| EG654453 | -0.6615601 |
| EG665186 | 0.4527347 |
| EG665378 | -0.4246382 |
| EG667977 | 2.995848 |
| EG668668 | -0.3305672 |
| Egf | 1.4797328 |
| Egfbp2 | -0.6705412 |
| Egfl7 | 1.8469393 |
| Egfl7 | 0.3507944 |
| Egflam | 1.4759026 |
| Egflam | 1.3543882 |
| Egfr | -0.7054852 |
| Egfr | -0.3559344 |
| Egfr | 0.464492 |
| Egfr | -0.5457561 |
| Egln1 | -0.2022375 |
| Egln1 | -0.5568669 |
| Egln1 | -0.8859473 |
| Egln2 | 0.3516944 |
| Egln3 | 1.0503321 |
| Egr1 | 0.0217329 |
| Egr2 | -0.0770615 |
| Egr3 | 2.2785971 |
| Egr4 | -0.7775604 |
| Ehbp1 | 1.6294639 |
| Ehbp1l1 | -0.5426058 |
| Ehd1 | -0.5010925 |
| Ehd2 | 0.283264 |
| Ehd3 | 0.2735289 |
| Ehd4 | 1.3029265 |
| Ehmt1 | -0.5901929 |
| Ehmt2 | -0.3064847 |
| Ehmt2 | -0.6438545 |
| Ei24 | -0.3960061 |
| Eid1 | 0.8080539 |
| Eid2 | 2.9651869 |
| Eid3 | -0.7427721 |
| Eif1a | -0.3343284 |
| Eif1b | 0.6003457 |
| Eif2a | 1.5762263 |
| Eif2ak1 | -0.6282033 |
| Eif2ak2 | -0.579496 |
| Eif2b1 | 1.7338743 |
| Eif2b1 | 2.8252942 |
| Eif2b2 | 0.5640965 |
| Eif2b4 | 1.7871956 |
| Eif2b5 | -0.4061307 |
| Eif2b5 | 0.9738343 |
| Eif2b5 | -0.7272932 |
| Eif2c2 | 0.4724237 |
| Eif2c3 | -0.5827742 |
| Eif2c3 | -0.4729544 |
| Eif2c3 | -0.8032329 |
| Eif2c3 | 0.7272628 |
| Eif2c4 | 1.3455962 |
| Eif2s2 | 0.2097308 |
| Eif2s2 | -0.7785738 |
| Eif2s3x | 0.3522939 |
| Eif2s3y | -0.6669383 |
| Eif3b | -0.135003 |
| Eif3b | -0.4421104 |
| Eif3d | 0.4753151 |
| Eif3eip | -0.1031289 |
| Eif3eip | 0.1035977 |
| Eif3f | -0.4491925 |
| Eif3g | 0.9681431 |
| Eif3i | -0.1279364 |
| Eif3i | 1.3247168 |
| Eif3i | 0.7874756 |
| Eif3k | 0.1568873 |
| Eif3s1 | -0.5632762 |
| Eif3s10 | -0.3230904 |
| Eif3s10 | -0.4308904 |
| Eif3s4 | 1.038823 |
| Eif3s8 | 2.8366261 |
| Eif4a1 | 0.2287532 |
| Eif4a1 | 0.6758567 |
| Eif4a2 | -0.5139623 |
| Eif4a3 | -0.4463532 |
| Eif4b | 1.9658352 |
| Eif4e | -0.3181391 |
| Eif4e1b | -0.5379004 |
| Eif4e1b | 0.6483914 |
| Eif4e2 | -0.7515938 |
| Eif4e2 | -0.5976714 |
| Eif4e3 | -0.3343284 |
| Eif4e3 | 0.5957472 |
| Eif4e3 | 1.029483 |
| Eif4ebp1 | -0.2991987 |
| Eif4ebp2 | 2.6241694 |
| Eif4el3 | 1.4619634 |
| Eif4enif1 | -0.1806986 |
| Eif4enif1 | 0.02515 |
| Eif4g1 | 0.2457143 |
| Eif4g2 | -0.2943729 |
| Eif4g2 | 0.2909792 |
| Eif4h | -0.6136602 |
| Eif5 | -0.1822968 |
| Eif5 | -0.5993416 |
| Eif5 | 0.7577608 |
| Eif5a | -0.5481258 |
| Eif5a | -0.7158512 |
| Eif6 | 0.145723 |
| Ela1 | 3.2126365 |
| Ela2 | -0.3565775 |
| Ela2a | 1.4123665 |
| Ela3b | -0.6060522 |
| Elac2 | 2.7692766 |
| Elac2 | 2.8441726 |
| Elac2 | 0.5936704 |
| Elavl1 | -0.3349567 |
| Elavl2 | -0.4788077 |
| Elavl2 | -0.6456099 |
| Elavl3 | 2.2174459 |
| Elavl4 | 3.8368381 |
| Elavl4 | -0.0549545 |
| Elf1 | 1.910584 |
| Elf2 | 0.1114005 |
| Elf2 | 0.5860155 |
| Elf2 | -0.8780129 |
| Elf2 | -0.1716939 |
| Elf2 | 2.8472922 |
| Elf3 | -0.4520405 |
| Elf3 | 1.4898113 |
| Elf3 | 1.3686249 |
| Elf5 | -0.7605003 |
| Elfn1 | -0.3437985 |
| Elfn2 | -0.5262403 |
| Elk3 | 1.9702987 |
| Elk3 | 1.8675317 |
| Elk3 | 0.4421874 |
| Ell | 0.1820666 |
| Ell3 | 0.0524571 |
| Elmo1 | 0.8346221 |
| Elmo1 | -0.3585097 |
| Elmo1 | 0.7280626 |
| Elmo1 | -0.3482511 |
| Elmo1 | -0.3463402 |
| Elmo1 | 1.5433671 |
| Elmo1 | 2.418378 |
| Elmo1 | -0.0761099 |
| Elmo1 | -0.5632762 |
| Elmo1 | 0.8680438 |
| Elmo1 | -0.6588826 |
| Elmo1 | -0.4700416 |
| Elmo1 | -0.3813536 |
| Elmo2 | -0.3040496 |
| Elmo2 | -0.3362147 |
| Elmo3 | 0.9178968 |
| Elmo3 | -0.6128118 |
| Elmod1 | -0.8232305 |
| Eln | -0.6001777 |
| Elof1 | -0.5910209 |
| Elovl1 | -0.7196495 |
| Elovl1 | 0.2159993 |
| Elovl1 | -0.5811337 |
| Elovl1 | -0.263603 |
| Elovl2 | -0.7856986 |
| Elovl4 | 0.1039894 |
| Elovl5 | 1.038251 |
| Elovl6 | 0.7380021 |
| Elp2 | 0.7859747 |
| Elp3 | -0.617914 |
| Elp3 | 1.4232941 |
| Eltd1 | 1.0555422 |
| Emb | -0.7101827 |
| Emcn | 1.6721858 |
| Emd | 0.1520659 |
| Emd | -0.464966 |
| Emg1 | 1.9659405 |
| Emg1 | 0.0881909 |
| Emid1 | -0.5231555 |
| Emid2 | -0.0297362 |
| Emilin1 | 1.2138144 |
| Emilin2 | -0.037458 |
| Eml1 | -0.5632762 |
| Eml3 | -0.5324408 |
| Eml3 | -0.0671187 |
| Eml4 | -0.3230904 |
| Eml5 | 1.5295705 |
| Emp1 | -0.49137 |
| Emp2 | -0.6571019 |
| Emp3 | -0.7120683 |
| Emr1 | -0.3046578 |
| Emr1 | 1.9180957 |
| Emr1 | 1.1319268 |
| En2 | 3.5915923 |
| Enah | 0.5600332 |
| Enc1 | 0.790657 |
| Endod1 | 1.1425988 |
| Endogl1 | -0.5512949 |
| Endogl1 | 0.4532723 |
| Eng | 4.1220537 |
| Eno1 | -0.3305672 |
| Eno2 | -0.3993687 |
| Eno3 | 1.744693 |
| Enoph1 | 0.3202838 |
| Enox1 | 0.2027156 |
| Enpp1 | -0.0447847 |
| Enpp1 | 2.1747381 |
| Enpp2 | 2.5294516 |
| Enpp4 | -0.2806371 |
| Enpp5 | -0.416367 |
| Enpp6 | -0.1591283 |
| Ensa | 2.5111514 |
| Ensa | -0.1989746 |
| ENSMUSG00000043795 | -0.6473687 |
| ENSMUSG00000053178 | -0.7836573 |
| ENSMUSG00000068790 | -0.5860641 |
| Entpd2 | 1.0392518 |
| Entpd3 | 2.1385731 |
| Entpd4 | 0.8470111 |
| Entpd5 | -0.3457041 |
| Entpd5 | 1.2419177 |
| Entpd5 | 0.208332 |
| Entpd6 | 0.5740596 |
| Entpd7 | 2.2125365 |
| Ep300 | -0.3070944 |
| Ep400 | -0.40342 |
| Epas1 | -0.7644863 |
| Epb4.1 | 0.8233287 |
| Epb4.1l1 | 0.849995 |
| Epb4.1l1 | 0.6270225 |
| Epb4.1l1 | 1.6577714 |
| Epb4.1l2 | 0.7587282 |
| Epb4.1l3 | 0.5927459 |
| Epb4.1l3 | 0.2191178 |
| Epb4.1l3 | 1.0182956 |
| Epb4.1l4a | -0.5277866 |
| Epb4.1l5 | -0.1716939 |
| Epb4.1l5 | 0.02387 |
| Epb4.9 | -0.6438545 |
| Epb4.9 | 0.1967108 |
| Epc1 | 1.1623308 |
| Epc1 | 0.5024706 |
| Epc1 | 1.506542 |
| Epdr1 | -0.8535092 |
| Epgn | 1.1772286 |
| Epha1 | 1.4761703 |
| Epha1 | -0.5993416 |
| Epha3 | 0.2580688 |
| Epha3 | -0.4371849 |
| Epha3 | 1.4453243 |
| Epha4 | -0.5433924 |
| Epha5 | 0.142723 |
| Epha6 | 0.3146837 |
| Epha7 | -0.0193903 |
| Epha7 | -0.2411774 |
| Epha7 | 2.7254598 |
| Ephb1 | -0.2612779 |
| Ephb2 | 2.0765513 |
| Ephb4 | 1.1395255 |
| Ephb6 | 0.7900964 |
| Ephx1 | -0.6412275 |
| Ephx2 | -0.4788077 |
| Epm2a | 0.1860183 |
| Epm2aip1 | 1.5767068 |
| Epm2aip1 | 0.6998578 |
| Epm2aip1 | -0.4170535 |
| Epn1 | -0.5552715 |
| Epn2 | 0.4708428 |
| Epn2 | -0.6018524 |
| Epn2 | 1.2214783 |
| Epn2 | -0.49137 |
| Epn2 | 0.8217041 |
| Epor | 0.6861593 |
| Eprs | -0.5844177 |
| Eprs | -0.6588826 |
| Eps15 | 0.3489918 |
| Eps15l1 | -0.4958446 |
| Eps8 | 1.4411638 |
| Eps8l1 | -0.5835956 |
| Eps8l2 | 0.1145033 |
| Eps8l2 | -0.5697302 |
| Eps8l3 | 1.7496901 |
| Epsti1 | -0.6119641 |
| Epsti1 | 1.8406955 |
| Eraf | 0.8616627 |
| Eral1 | 0.408115 |
| Ercc1 | 1.6500356 |
| Ercc2 | 0.1653566 |
| Ercc3 | -0.0021068 |
| Ercc3 | 1.7325215 |
| Ercc4 | -0.7486439 |
| Ercc4 | 1.73455 |
| Ercc5 | -0.1169393 |
| Ercc5 | -0.459918 |
| Ercc8 | -0.4928591 |
| Ercc8 | 0.5709872 |
| Erdr1 | -0.464966 |
| Erf | -0.5993416 |
| Erf | -0.023425 |
| Ergic1 | 1.0837542 |
| Ergic2 | 1.0268798 |
| Ergic2 | 0.0083714 |
| Ergic3 | 0.0433044 |
| Ergic3 | 2.0745403 |
| Erh | -0.2776778 |
| Erich1 | -0.6342585 |
| Erlin1 | 0.6667205 |
| Erlin2 | -0.2653508 |
| Erlin2 | 2.3805137 |
| Ermap | -0.4980901 |
| Ermp1 | -0.6351268 |
| Ern1 | 1.42471 |
| Ern2 | 2.0983436 |
| Ero1lb | -0.617914 |
| Erp29 | -0.4329838 |
| Errfi1 | 1.8937272 |
| Es22 | -0.5402502 |
| Esam1 | 1.6810287 |
| Esco2 | 0.3362745 |
| Esd | -0.301621 |
| Esd | 1.8327096 |
| Esd | 1.720051 |
| Esd | -0.7120683 |
| Esm1 | -0.2706137 |
| Espl1 | -0.3444333 |
| Espn | 1.0647638 |
| Espn | 1.7379209 |
| Espn | 0.2450418 |
| Espn | -0.5811337 |
| Esr1 | 0.0403731 |
| Esr2 | 0.9377173 |
| Esr2 | -0.362386 |
| Esr2 | -0.4184279 |
| Esrra | -0.6307936 |
| Esrrb | 0.8076871 |
| Etaa1 | 1.0951261 |
| Etf1 | 2.3805473 |
| Etfa | -0.5033507 |
| Etfa | 1.7622956 |
| Etfa | -0.3299419 |
| Etfb | 0.6372176 |
| Etfb | 1.4198871 |
| Etfdh | 0.5203925 |
| Ethe1 | -0.1539425 |
| Etnk2 | -0.0718401 |
| Ets1 | 1.0687869 |
| Ets2 | -0.3224701 |
| Etsrp71 | -0.324954 |
| Etv1 | -0.5901929 |
| Etv1 | 3.1078972 |
| Etv4 | -0.3230904 |
| Etv5 | -0.6588826 |
| Etv6 | -0.5746002 |
| Evc | -0.3052663 |
| Evc2 | -0.1269312 |
| Evi1 | 1.0134594 |
| Evi1 | 0.5399351 |
| Evi2a | -0.5746002 |
| Evi2a | -0.4707689 |
| Evi5 | 0.2813251 |
| Evi5l | -0.60353 |
| Evl | -0.5648855 |
| Evx1 | -0.2764967 |
| Exdl1 | -0.5277866 |
| Exdl2 | -0.7725101 |
| Exdl2 | 1.1950376 |
| Exdl2 | 1.519917 |
| Exoc2 | -0.0909695 |
| Exoc3 | -0.29077 |
| Exoc3l | -0.4136263 |
| Exoc4 | 1.6358712 |
| Exoc7 | -0.4570457 |
| Exoc8 | -0.5262403 |
| Exod1 | -0.5993416 |
| Exosc1 | 0.3014838 |
| Exosc10 | 1.1521377 |
| Exosc2 | -0.0992205 |
| Exosc2 | -0.6153594 |
| Exosc4 | -0.1070538 |
| Exosc5 | -0.3741133 |
| Exosc6 | 1.8871614 |
| Exosc7 | -0.3806931 |
| Exosc8 | -0.2423143 |
| Ext1 | -0.6998795 |
| Ext2 | 0.1748383 |
| Extl1 | 2.1182075 |
| Extl2 | 0.5330556 |
| Extl3 | 1.3963621 |
| Extl3 | 2.8283947 |
| Eya1 | -0.4736841 |
| Eya1 | 0.0773728 |
| Eya2 | -0.4068096 |
| Eya3 | -0.4854371 |
| Eya3 | 1.4143716 |
| Eya3 | -0.6377365 |
| Eya4 | -0.60353 |
| Ezh1 | 3.0030693 |
| Ezh2 | 1.2221826 |
| Ezh2 | -0.6273415 |
| F10 | -0.3734579 |
| F11 | -0.6571019 |
| F11 | -0.4635209 |
| F11r | -0.6805219 |
| F13a1 | -0.5208487 |
| F2r | 0.9971541 |
| F2rl1 | -0.0526333 |
| F2rl3 | -0.3598 |
| F430201B04Rik | 0.3143716 |
| F5 | 1.596981 |
| F630043A04Rik | -0.7535657 |
| F730014I05Rik | 0.0088054 |
| F730047E07Rik | 0.8265695 |
| F730047E07Rik | -0.037458 |
| F730047E07Rik | -0.2417457 |
| F8 | 0.1860183 |
| F8 | 1.9542886 |
| F8 | -0.7036129 |
| F8a | 1.7067844 |
| Fa2h | -0.0943581 |
| Faah | -0.0131489 |
| Faah | 0.3283136 |
| Fabp3 | -0.7177484 |
| Fabp5 | 1.3416847 |
| Fabp9 | 2.0683868 |
| Fadd | 2.1389226 |
| Fadd | 1.7266819 |
| Fadd | 1.6478134 |
| Fads1 | 0.4470703 |
| Fads2 | 0.9416947 |
| Fads3 | -0.5811337 |
| Fads6 | 0.2570732 |
| Faf1 | 0.8680438 |
| Faf1 | -0.5254682 |
| Fah | -0.6777893 |
| Fahd1 | 0.8217041 |
| Fahd2a | 0.2864864 |
| Fahd2a | -0.3773974 |
| Fahd2a | 0.8436647 |
| Fahd2a | 0.9410594 |
| Faim | -0.2836059 |
| Faim | -0.6230444 |
| Faim2 | -0.163298 |
| Faim2 | 1.7907624 |
| Faim3 | 2.6467273 |
| Fancc | 1.0359592 |
| Fancc | -0.6869297 |
| Fancd2 | 0.2114763 |
| Fancg | -0.668738 |
| Fanci | 0.3149957 |
| Fancl | 0.3027491 |
| Fancl | -0.5347767 |
| Fank1 | 0.2938559 |
| Fank1 | -0.2406094 |
| Fap | -0.2842008 |
| Fars2 | -0.0411141 |
| Farsa | -0.4570457 |
| Farsb | -0.3305672 |
| Farsb | -0.3833381 |
| Fas | 0.0187325 |
| Fas | 0.0122704 |
| Fasl | -0.2688561 |
| Fastk | 0.690956 |
| Fastk | -0.5339974 |
| Fastkd1 | 0.2696061 |
| Fastkd5 | 0.659217 |
| Fat1 | 1.1932236 |
| Fat4 | -0.0180493 |
| Fbf1 | 0.14834 |
| Fblim1 | -0.6597742 |
| Fbln1 | 0.1928045 |
| Fbln1 | 0.2290955 |
| Fbln1 | -0.6482493 |
| Fbln2 | 0.1741124 |
| Fbln2 | -0.5584651 |
| Fbn1 | -0.6768802 |
| Fbp1 | 2.0392802 |
| Fbp2 | 0.1256649 |
| Fbxl10 | 0.0195908 |
| Fbxl12 | 0.5982792 |
| Fbxl12 | 0.844899 |
| Fbxl12 | 0.5564331 |
| Fbxl12 | 1.5691497 |
| Fbxl13 | 0.0230158 |
| Fbxl13 | -0.2949748 |
| Fbxl14 | 0.0315234 |
| Fbxl15 | 0.8010603 |
| Fbxl18 | -0.2104453 |
| Fbxl18 | 1.4284752 |
| Fbxl21 | 0.6602927 |
| Fbxl22 | 0.3438653 |
| Fbxl4 | -0.6579918 |
| Fbxl5 | -0.5048592 |
| Fbxl6 | -0.4714968 |
| Fbxl6 | -0.4548973 |
| Fbxl8 | -0.0823124 |
| Fbxo15 | 0.3273906 |
| Fbxo16 | -0.60353 |
| Fbxo18 | -0.1041085 |
| Fbxo18 | 0.2273829 |
| Fbxo2 | 0.8618358 |
| Fbxo21 | 0.8590633 |
| Fbxo25 | -0.380033 |
| Fbxo27 | -0.0837497 |
| Fbxo28 | 0.5022157 |
| Fbxo3 | -0.4371849 |
| Fbxo3 | 0.9784233 |
| Fbxo30 | 1.8555751 |
| Fbxo31 | 1.0517431 |
| Fbxo31 | 0.50527 |
| Fbxo32 | 1.6078839 |
| Fbxo32 | -0.3760823 |
| Fbxo32 | -0.4678629 |
| Fbxo33 | 0.2433583 |
| Fbxo34 | -0.6377365 |
| Fbxo34 | -0.8147579 |
| Fbxo34 | 0.3692192 |
| Fbxo36 | -0.1194272 |
| Fbxo38 | 0.1565173 |
| Fbxo38 | -0.4322855 |
| Fbxo4 | -0.5231555 |
| Fbxo42 | 0.0183031 |
| Fbxo44 | 2.374434 |
| Fbxo6 | -0.1968057 |
| Fbxo7 | -0.6915339 |
| Fbxo8 | -0.5673047 |
| Fbxo9 | 0.2979955 |
| Fbxo9 | -0.0184961 |
| Fbxw12 | 1.6780187 |
| Fbxw13 | -0.6624543 |
| Fbxw15 | 0.2016588 |
| Fbxw16 | 0.1039894 |
| Fbxw17 | 0.7525199 |
| Fbxw19 | -0.5086413 |
| Fbxw2 | 2.4512079 |
| Fbxw4 | 0.8627005 |
| Fbxw4 | -0.3181391 |
| Fbxw5 | -0.5489171 |
| Fbxw7 | 0.254746 |
| Fbxw7 | 0.0122704 |
| Fbxw7 | 0.7066319 |
| Fbxw8 | -0.5078837 |
| Fcer1g | 0.8755753 |
| Fcer2a | -0.1189291 |
| Fcf1 | -0.7674871 |
| Fcgr2b | 2.3754839 |
| Fcgr2b | 0.1067267 |
| Fcgr2b | -0.6669383 |
| Fcgr3 | -0.6111173 |
| Fcgr4 | 2.4790185 |
| Fcgrt | -0.5093996 |
| Fcho1 | -0.6359959 |
| Fcho2 | -0.0379142 |
| Fchsd1 | -0.2830114 |
| Fchsd2 | 0.4298665 |
| Fcna | 0.6444631 |
| Fcrla | 2.2765282 |
| Fcrla | 0.4140215 |
| Fcrla | 2.7822268 |
| Fdps | 1.0242693 |
| Fdxr | -0.5762293 |
| Fdxr | 1.9850065 |
| Fem1b | 2.9049984 |
| Fem1c | -0.2423143 |
| Fem1c | 1.775831 |
| Fen1 | -0.6026908 |
| Fermt2 | 1.6332383 |
| Fermt3 | 0.2819718 |
| Fert2 | 1.3980139 |
| Fert2 | -0.4876575 |
| Fes | 2.148958 |
| Fetub | -0.5512949 |
| Fev | 0.2986307 |
| Fez1 | 0.2861647 |
| Fez1 | 0.0399536 |
| Fez2 | -0.60437 |
| Fezf2 | 2.8095283 |
| Ffar2 | -0.3913188 |
| Fgd2 | -0.8328556 |
| Fgd4 | 2.8977591 |
| Fgd4 | -0.7026781 |
| Fgd5 | -0.5926791 |
| Fgd6 | 0.7105099 |
| Fgf1 | -0.4810123 |
| Fgf1 | 3.2282519 |
| Fgf10 | -0.1011726 |
| Fgf10 | -0.7101827 |
| Fgf12 | 1.1297258 |
| Fgf12 | 0.848943 |
| Fgf13 | 0.3872851 |
| Fgf13 | 1.7995568 |
| Fgf17 | 1.4288509 |
| Fgf21 | 2.4877267 |
| Fgf22 | 1.0789745 |
| Fgf22 | 0.6899153 |
| Fgf5 | 0.7641265 |
| Fgf7 | -0.5885392 |
| Fgf8 | 0.6308033 |
| Fgf9 | 0.7943859 |
| Fgfbp3 | -0.114458 |
| Fgfr1 | -0.459918 |
| Fgfr1op | -0.3893172 |
| Fgfr1op2 | -0.5101585 |
| Fgfr1op2 | 0.578062 |
| Fgfr2 | 0.1256649 |
| Fgfr2 | -0.568921 |
| Fgfr3 | -0.5056144 |
| Fgfrl1 | -0.6750646 |
| Fgl2 | 1.0771928 |
| Fh1 | 0.3008506 |
| Fhad1 | 2.3862526 |
| Fhit | 3.0008467 |
| Fhl1 | 2.1467517 |
| Fhl1 | 0.8032745 |
| Fhl2 | 0.9695307 |
| Fhl4 | -0.7506095 |
| Fhl5 | 1.6145952 |
| Fhod1 | 2.7516184 |
| Fhod3 | -0.3714944 |
| Fibcd1 | 1.5529452 |
| Fibp | 0.2086819 |
| Figf | -0.7486439 |
| Figla | -0.549709 |
| Fign | -0.6119641 |
| Fignl1 | -0.7045486 |
| Fip1l1 | -0.3212306 |
| Fis1 | 1.5921901 |
| Fis1 | -0.6273415 |
| Fiz1 | -0.3630337 |
| Fjx1 | 0.8276472 |
| Fkbp10 | 2.0916113 |
| Fkbp11 | 0.3387103 |
| Fkbp14 | -0.4685885 |
| Fkbp1a | 0.9690684 |
| Fkbp1b | 0.4210044 |
| Fkbp2 | -0.3688829 |
| Fkbp2 | -0.4088494 |
| Fkbp4 | -0.456329 |
| Fkbp5 | 0.0749515 |
| Fkbp6 | 1.3176513 |
| Fkbp7 | 2.1191897 |
| Fkbp8 | 0.598509 |
| Fkbp9 | -0.4315877 |
| Fkbpl | 0.5183903 |
| Fkbpl | -0.4928591 |
| Fkhl18 | -0.0283802 |
| Fkrp | 1.0902695 |
| Fktn | -0.219836 |
| Flad1 | -0.1254255 |
| Flcn | 1.9011504 |
| Flcn | -0.3701877 |
| Fli1 | -0.3520848 |
| Flii | 1.2665139 |
| Flna | 2.8145394 |
| Flot1 | 0.4047229 |
| Flot1 | 0.1824265 |
| Flot2 | -0.5632762 |
| Flot2 | 1.7876422 |
| Flot2 | 0.9631926 |
| Flrt1 | 1.6407413 |
| Flrt2 | -0.4606374 |
| Flrt3 | -0.6705412 |
| Flt1 | 0.1686497 |
| Flt4 | 1.3488774 |
| Flt4 | -0.5985061 |
| Flvcr2 | 1.7478942 |
| Flywch2 | 0.482895 |
| Fmn2 | -0.2895722 |
| Fmn2 | -0.3324459 |
| Fmnl1 | 0.2409964 |
| Fmnl3 | 1.7121969 |
| Fmnl3 | 0.7897224 |
| Fmo1 | -0.6351268 |
| Fmo2 | 1.258425 |
| Fmo5 | -0.4499037 |
| Fmod | -0.435082 |
| Fmr1nb | 0.7521305 |
| Fmr1nb | -0.7486439 |
| Fn3k | 1.4749202 |
| Fn3k | -0.0880748 |
| Fnbp1 | 0.8378237 |
| Fnbp1 | 1.5921113 |
| Fnbp1 | 1.2311735 |
| Fnbp1l | 2.5083398 |
| Fnbp4 | -0.693382 |
| Fndc3 | -0.0544898 |
| Fndc3a | 1.5900616 |
| Fndc3b | 0.205879 |
| Fndc4 | 3.5182071 |
| Fndc5 | 1.8638375 |
| Fndc5 | -0.5505016 |
| Fndc7 | -0.4102117 |
| Fndc7 | -0.4149957 |
| Fndc8 | -0.5877134 |
| Fndc8 | -0.4950974 |
| Fnip1 | -0.6579918 |
| Fnip1 | -0.7476627 |
| Fnta | -0.6438545 |
| Fnta | -0.257801 |
| Fntb | -0.4232546 |
| Folr1 | 0.9356424 |
| Folr1 | 0.4543467 |
| Folr2 | 1.9772919 |
| Fos | 1.2921085 |
| Fosb | -0.6841781 |
| Foxa1 | 2.4134525 |
| Foxa2 | -0.6119641 |
| Foxa3 | -0.3533662 |
| Foxc1 | -0.5239258 |
| Foxd2 | 2.0419996 |
| Foxg1 | 0.2440321 |
| Foxh1 | 0.8882739 |
| Foxh1 | 0.7625873 |
| Foxj1 | -0.187108 |
| Foxj2 | -0.7177484 |
| Foxj3 | 1.4366121 |
| Foxj3 | -0.6517802 |
| Foxj3 | 2.4960567 |
| Foxj3 | 0.5778271 |
| Foxj3 | 0.5226397 |
| Foxk1 | -0.4906263 |
| Foxk1 | -0.0060668 |
| Foxk1 | 1.4045916 |
| Foxl2 | -0.6860116 |
| Foxm1 | -0.2712004 |
| Foxm1 | -0.2979899 |
| Foxn2 | 3.1732607 |
| Foxn3 | -0.4921142 |
| Foxo1 | 1.9519939 |
| Foxo6 | -0.7130126 |
| Foxo6 | -0.7186985 |
| Foxp1 | -0.4722253 |
| Foxp2 | -0.5505016 |
| Foxp2 | -0.6878487 |
| Foxp2 | 0.4776741 |
| Foxp3 | 0.410651 |
| Foxp4 | 1.7728545 |
| Foxq1 | 1.6538747 |
| Foxred1 | -0.6705412 |
| Foxred1 | 0.0225883 |
| Foxred1 | -0.2818235 |
| Foxred1 | 0.2938559 |
| Foxred1 | -0.1416134 |
| Foxred2 | 0.848592 |
| Fpgs | 0.6234499 |
| Fpgs | 0.2816485 |
| Fpgs | -0.2979899 |
| Fpr2 | -0.5656912 |
| Frag1 | 0.0191618 |
| Frag1 | 1.6627471 |
| Frag1 | -0.6421024 |
| Frap1 | 3.4490237 |
| Fras1 | 2.5596622 |
| Fras1 | -0.4315877 |
| Frat1 | 0.8850733 |
| Frat1 | -0.4399963 |
| Frat2 | 0.7861625 |
| Freq | 1.9125373 |
| Freq | 0.2880935 |
| Freq | -0.5363373 |
| Frg1 | -0.8882267 |
| Frk | -0.2016929 |
| Frmd3 | -0.7064228 |
| Frmd6 | -0.5063702 |
| Frmd8 | 2.1642994 |
| Frmpd4 | -0.5811337 |
| Frrs1 | -0.3873199 |
| Frs2 | 1.2956924 |
| Frs3 | 1.469184 |
| Fryl | -0.0842292 |
| Fryl | 0.2797063 |
| Frzb | 0.395902 |
| Fscn1 | 3.9161171 |
| Fscn2 | -0.3840006 |
| Fscn3 | -0.6606667 |
| Fsd1 | 0.4508504 |
| Fsd2 | 1.3052897 |
| Fshb | 0.2806779 |
| Fshr | 3.3834214 |
| Fshr | -0.4364834 |
| Fshr | -0.4656893 |
| Fsip1 | 0.6341266 |
| Fst | 0.1682843 |
| Fstl1 | -0.7664857 |
| Fstl4 | 0.1175958 |
| Fstl5 | -0.4832221 |
| Fth1 | -0.7535657 |
| Fth1 | 0.8860852 |
| Ftmt | 0.800506 |
| Fto | -0.3643303 |
| Ftsj1 | -0.4301936 |
| Ftsj3 | -0.568921 |
| Fubp1 | -0.7253764 |
| Fubp3 | 0.3993265 |
| Fuk | 3.3197129 |
| Fuk | 1.6061015 |
| Fundc1 | -0.2943729 |
| Fundc1 | 2.3071966 |
| Fundc2 | 0.8151771 |
| Furin | -0.6768802 |
| Furin | -0.7939088 |
| Fus | 2.0465464 |
| Fusip1 | 2.4154323 |
| Fusip1 | -0.6562128 |
| Fusip1 | -0.6588826 |
| Fut10 | 1.3633999 |
| Fut10 | 0.695728 |
| Fut2 | -0.3374743 |
| Fut4 | 0.4508504 |
| Fut8 | 0.0647943 |
| Fut8 | -0.1380481 |
| Fut8 | 0.3820763 |
| Fvt1 | -0.6213311 |
| Fxc1 | -0.0933887 |
| Fxn | 0.0881909 |
| Fxn | -0.4392927 |
| Fxyd1 | 2.3537374 |
| Fxyd2 | 3.7054427 |
| Fxyd2 | 0.1106232 |
| Fxyd2 | -0.5584651 |
| Fxyd3 | -0.8084538 |
| Fxyd4 | -0.3913188 |
| Fxyd4 | -0.5208487 |
| Fxyd5 | 1.9870667 |
| Fxyd5 | -0.0082742 |
| Fxyd5 | -0.1472439 |
| Fxyd6 | 0.3090496 |
| Fxyd7 | -0.6544372 |
| Fyb | -0.642978 |
| Fyco1 | -0.5078837 |
| Fyn | 2.6455137 |
| Fyttd1 | -0.1570505 |
| Fzd1 | 0.0225883 |
| Fzd10 | -0.7624911 |
| Fzd10 | -0.4832221 |
| Fzd2 | -0.7139578 |
| Fzd3 | 1.21948 |
| Fzd4 | -0.7427721 |
| Fzd5 | -0.6878487 |
| Fzd6 | -0.3578652 |
| Fzd7 | -0.4513277 |
| Fzd8 | 0.9477077 |
| Fzr1 | 1.4920069 |
| Fzr1 | 0.6671474 |
| Fzr1 | 0.6711936 |
| G0s2 | -0.5893657 |
| G3bp1 | -0.4000427 |
| G3bp1 | -0.6325244 |
| G3bp2 | 0.02515 |
| G3bp2 | -0.0429476 |
| G3bp2 | 1.8492102 |
| G430022H21Rik | 1.0780156 |
| G430055L02Rik | -0.7585137 |
| G6pc | -0.0252238 |
| G6pc2 | 0.1730224 |
| G6pc2 | -0.6094258 |
| G6pc3 | 0.7915906 |
| G6pd2 | -0.4343821 |
| G6pd2 | -0.5262403 |
| G6pdx | -0.4744143 |
| Gaa | -0.2209471 |
| Gab1 | 2.1691741 |
| Gab2 | 1.5917963 |
| Gab2 | 1.6639869 |
| Gab2 | 1.6986061 |
| Gab3 | -0.711125 |
| Gab3 | -0.178572 |
| Gab3 | -0.7447253 |
| Gabarap | -0.1109953 |
| Gabarapl1 | 1.0333741 |
| Gabarapl2 | 0.5888064 |
| Gabbr1 | -0.2782689 |
| Gabpa | -0.5811337 |
| Gabpa | -0.6438545 |
| Gabpb1 | -0.3089261 |
| Gabpb1 | 0.2013062 |
| Gabpb2 | -0.5844177 |
| Gabra3 | -0.2554903 |
| Gabra4 | 1.8548634 |
| Gabrb1 | -0.237209 |
| Gabrb2 | -0.6482493 |
| Gabrb3 | -0.6624543 |
| Gabrb3 | -0.4294974 |
| Gabrd | -0.6094258 |
| Gabrd | -0.2055119 |
| Gabrg1 | 1.8811834 |
| Gabrg2 | -0.4584807 |
| Gabrg2 | -0.693382 |
| Gabrg2 | -0.5193139 |
| Gabrg2 | -0.3431641 |
| Gabrp | -0.4315877 |
| Gabrp | -0.5673047 |
| Gad1 | -0.6651421 |
| Gad1 | -0.6351268 |
| Gadd45a | 1.7458948 |
| Gadd45a | 0.7529091 |
| Gadd45b | 0.047892 |
| Gadd45g | 1.509043 |
| Gadd45g | 1.0159539 |
| Gak | 0.0200196 |
| Gal | 0.3924649 |
| Gal3st1 | -0.1189291 |
| Gal3st2 | 0.1449739 |
| Gal3st3 | 0.4282113 |
| Galc | -0.5860641 |
| Galc | 0.205879 |
| Gale | -0.5616698 |
| Gale | -0.6768802 |
| Galk1 | -0.4456447 |
| Galm | 2.7549972 |
| Galns | 0.7589215 |
| Galnt1 | 0.4016025 |
| Galnt1 | -0.2293228 |
| Galnt1 | 0.8894503 |
| Galnt1 | -0.3604458 |
| Galnt10 | 0.6777576 |
| Galnt10 | 0.1164373 |
| Galnt10 | 0.5899669 |
| Galnt11 | -0.3591546 |
| Galnt12 | -0.3820147 |
| Galnt13 | -0.7282531 |
| Galnt2 | 1.3734221 |
| Galnt2 | 1.6467003 |
| Galnt2 | -0.5844177 |
| Galnt3 | 1.3276537 |
| Galnt9 | -0.4047743 |
| Galntl2 | 0.7268627 |
| Galntl4 | -0.5063702 |
| Galntl5 | 2.9177073 |
| Galt | 1.2852278 |
| Galt | -0.2973861 |
| Gamt | 3.0381242 |
| Gan | -0.6553246 |
| Ganab | -0.5819536 |
| Ganab | 1.0605835 |
| Ganab | 1.1755028 |
| Ganc | 1.4869051 |
| Gap43 | -0.362386 |
| Gapdh | 1.0752703 |
| Gapdhs | -0.3022275 |
| Gapvd1 | 2.6878717 |
| Gapvd1 | 0.6731041 |
| Garnl3 | 0.0536983 |
| Garnl4 | -0.5301108 |
| Gars | 1.9340292 |
| Gart | -0.6077375 |
| Gart | -0.8317812 |
| Gas2l1 | 0.2263537 |
| Gas2l1 | 3.2177844 |
| Gas2l1 | 1.9638855 |
| Gas2l1 | 2.4324267 |
| Gas6 | -0.4656893 |
| Gas7 | 0.7222485 |
| Gas7 | -0.4883988 |
| Gas8 | -0.1050892 |
| Gata2 | -0.1329785 |
| Gata4 | -0.5656912 |
| Gata6 | -0.4371849 |
| Gatad1 | -0.572162 |
| Gatad1 | -0.1031289 |
| Gatad2b | 1.1738974 |
| Gats | -0.3960061 |
| Gats | 4.0144268 |
| Gba | -0.1194272 |
| Gba2 | 1.1295961 |
| Gbas | 1.8234278 |
| Gbf1 | -0.6517802 |
| Gbl | -0.4047743 |
| Gbl | -0.3886509 |
| Gbl | -0.2143006 |
| Gbp2 | -0.4281064 |
| Gbp3 | -0.7321027 |
| Gbp3 | -0.4428162 |
| Gbp6 | 1.5592489 |
| Gbp6 | 2.694183 |
| Gbx2 | 2.2809612 |
| Gca | -0.3293169 |
| Gca | -0.3469768 |
| Gcap14 | 0.5208923 |
| Gcap14 | 0.7560171 |
| Gcc1 | -0.5852406 |
| Gcc2 | -0.3355855 |
| Gcdh | 0.1520659 |
| Gch1 | -0.7167993 |
| Gch1 | 3.000726 |
| Gchfr | 1.2985064 |
| Gck | -0.6696392 |
| Gclm | 0.1682843 |
| Gcn5l2 | 1.2765268 |
| Gcnt1 | 3.8660358 |
| Gcnt1 | -0.7263343 |
| Gcnt2 | 2.0310249 |
| Gcnt2 | -0.3355855 |
| Gcs1 | -0.5208487 |
| Gda | 0.294175 |
| Gdap1 | -0.7887689 |
| Gdap1 | 0.6684271 |
| Gdap1l1 | 1.834955 |
| Gdap1l1 | -0.6832627 |
| Gde1 | 1.8452024 |
| Gde1 | 1.239272 |
| Gdf10 | -0.6832627 |
| Gdf15 | 0.9232562 |
| Gdf3 | 0.4232279 |
| Gdf9 | 2.3189626 |
| Gdi1 | 0.8113483 |
| Gdi1 | 1.0732061 |
| Gdi1 | 1.0552613 |
| Gdi1 | 0.6641547 |
| Gdi1 | 3.8515725 |
| Gdi2 | -0.4088494 |
| Gdnf | -0.3355855 |
| Gdpd1 | 0.8633917 |
| Gdpd2 | 1.4098779 |
| Gdpd2 | 0.4497719 |
| Gdpd3 | -0.0568155 |
| Gdpd4 | -0.4407005 |
| Gdpd5 | -0.5093996 |
| Gemin4 | -0.3643303 |
| Gemin4 | 0.5605121 |
| Gemin4 | 0.1241333 |
| Gemin5 | 1.2222999 |
| Gemin6 | -0.8189845 |
| Gemin6 | -0.6915339 |
| Gemin7 | 0.1773746 |
| Gemin7 | -0.7263343 |
| Gfap | -0.0087163 |
| Gfer | -0.7120683 |
| Gfi1 | -0.6153594 |
| Gfm1 | -0.0909695 |
| Gfod1 | -0.4435225 |
| Gfod2 | 0.8157227 |
| Gfpt2 | -0.1586084 |
| Gfra1 | -0.0586803 |
| Gfra4 | -0.5819536 |
| Gga1 | 1.4786655 |
| Gga1 | 0.0941469 |
| Gga1 | -0.5324408 |
| Gga3 | -0.234949 |
| Ggcx | -0.2782689 |
| Ggcx | 0.2696061 |
| Ggcx | -0.7408231 |
| Ggn | -0.5656912 |
| Ggn | 2.1687935 |
| Ggnbp1 | -0.4491925 |
| Ggnbp2 | 1.2851181 |
| Ggt5 | 1.0732061 |
| Ggt7 | -0.6500131 |
| Ggta1 | 0.2253235 |
| Ggtla1 | -0.0429476 |
| Gh | -0.5885392 |
| Ghitm | -0.6906112 |
| Ghr | -0.0498557 |
| Ghr | -0.4744143 |
| Ghrh | -0.3533662 |
| Ghrhr | 1.154665 |
| Ghrl | -0.7887689 |
| Ghrl | -0.6145094 |
| Ghsr | 0.8796578 |
| Ghsr | -0.1812311 |
| Gif | -0.5918496 |
| Gigyf1 | 3.8624785 |
| Gimap1 | 0.4143019 |
| Gimap4 | -0.572162 |
| Gimap5 | -0.7959727 |
| Gimap6 | 2.820383 |
| Gimap8 | -0.4627992 |
| Gimap9 | -0.5552715 |
| Gins3 | 1.4373568 |
| Gins4 | -0.4477717 |
| Gipc1 | 0.1442242 |
| Gipc1 | -0.1812311 |
| Gipc1 | 0.9543086 |
| Gipc2 | -0.3469768 |
| Git1 | -0.4007171 |
| Git2 | -0.6633494 |
| Git2 | 0.208332 |
| Git2 | 0.4554199 |
| Gja1 | 0.3534918 |
| Gja4 | 0.9707623 |
| Gja5 | -0.4442294 |
| Gja5 | 3.1531767 |
| Gja6 | 0.2430213 |
| Gja7 | -0.6860116 |
| Gjb1 | 0.1938715 |
| Gjb2 | -0.6438545 |
| Gjb2 | -0.6814346 |
| Gjb3 | 1.553438 |
| Gjb6 | 1.9149871 |
| Gjc2 | 1.138884 |
| Gjc2 | 0.2215361 |
| Gjd2 | 0.6316907 |
| Gjd2 | 0.354987 |
| Gjd3 | -0.6128118 |
| Gk2 | -0.5910209 |
| Gk5 | -0.638608 |
| Gkap1 | 1.4833701 |
| Gkap1 | 1.6973415 |
| Glb1 | 1.3770037 |
| Glb1l2 | -0.7130126 |
| Glcci1 | -0.3728029 |
| Gldc | 0.9322807 |
| Gldc | -0.6333911 |
| Gldn | -0.1680113 |
| Gle1 | 0.0332155 |
| Glg1 | 0.5293491 |
| Glipr2 | 1.8083935 |
| Glis1 | 1.7934285 |
| Glo1 | -0.6119641 |
| Gloxd1 | 2.2290521 |
| Glp2r | -0.2226162 |
| Glra2 | 2.0154388 |
| Glrb | 1.0316467 |
| Glrp1 | -0.7595064 |
| Glrx | -0.6403535 |
| Glrx2 | 1.1202144 |
| Glrx2 | -0.6473687 |
| Glrx2 | -0.6421024 |
| Glrx3 | 0.1222153 |
| Glrx5 | -0.6896895 |
| Gls2 | 1.7878335 |
| Gls2 | 1.7232015 |
| Glt25d1 | 0.0437224 |
| Glt25d2 | -0.6464889 |
| Glt25d2 | 0.3090496 |
| Glt6d1 | 1.0196102 |
| Glt8d1 | 0.039114 |
| Glt8d1 | -0.4407005 |
| Glt8d1 | 0.2728762 |
| Glt8d2 | 1.4574219 |
| Glt8d3 | -0.3425302 |
| Gltp | 1.0261554 |
| Gltscr1 | -0.8032329 |
| Glud1 | 3.2041682 |
| Glud1 | 0.0247235 |
| Glul | -0.579496 |
| Glyctk | 0.2314879 |
| Glyctk | -0.5293354 |
| Glyctk | -0.6500131 |
| Gm1006 | -0.4232546 |
| Gm1006 | -0.579496 |
| Gm1019 | -0.4267176 |
| Gm1040 | 0.6424926 |
| Gm1070 | 4.0350513 |
| Gm1082 | -0.2468761 |
| Gm114 | -0.3886509 |
| Gm114 | 0.6030939 |
| Gm1141 | -0.579496 |
| Gm128 | 2.0393288 |
| Gm129 | -0.2653508 |
| Gm129 | -0.0563499 |
| Gm1322 | -0.7595064 |
| Gm136 | 1.1207381 |
| Gm136 | 0.9480231 |
| Gm1381 | -0.7785738 |
| Gm1527 | -0.6026908 |
| Gm1568 | 0.9655164 |
| Gm1574 | 0.8916311 |
| Gm1574 | -0.4869168 |
| Gm1604 | -0.7301759 |
| Gm1604 | -0.7379071 |
| Gm166 | -0.6814346 |
| Gm166 | -0.5489171 |
| Gm1661 | 0.0495547 |
| Gm1673 | -0.464966 |
| Gm1673 | -0.1575695 |
| Gm1698 | 2.9172355 |
| Gm1821 | -0.5951719 |
| Gm1943 | -0.6464889 |
| Gm1965 | -0.4232546 |
| Gm1967 | -0.3144429 |
| Gm1967 | 1.0332303 |
| Gm216 | -0.8360863 |
| Gm239 | 2.3511697 |
| Gm249 | 1.2950418 |
| Gm266 | 2.117582 |
| Gm2a | -0.0610166 |
| Gm347 | -0.1930223 |
| Gm382 | -0.408169 |
| Gm410 | 0.2893771 |
| Gm428 | -0.3546494 |
| Gm443 | 0.0315234 |
| Gm484 | -0.5394663 |
| Gm50 | -0.6299293 |
| Gm525 | -0.6553246 |
| Gm525 | -0.0424889 |
| Gm527 | -0.1664375 |
| Gm527 | 0.5095798 |
| Gm561 | -0.5170166 |
| Gm561 | -0.7785738 |
| Gm597 | -0.6642453 |
| Gm603 | 2.1283358 |
| Gm609 | -0.7036129 |
| Gm614 | -0.1194272 |
| Gm614 | -0.7674871 |
| Gm648 | 0.9152871 |
| Gm648 | 1.0405371 |
| Gm71 | -0.4707689 |
| Gm711 | 0.8772785 |
| Gm732 | 1.4512095 |
| Gm732 | -0.4442294 |
| Gm767 | 1.0251403 |
| Gm773 | 1.7855989 |
| Gm773 | -0.729214 |
| Gm784 | 0.598509 |
| Gm813 | -0.6597742 |
| Gm813 | -0.4068096 |
| Gm817 | -0.6588826 |
| Gm867 | -0.3813536 |
| Gm884 | -0.4343821 |
| Gm889 | 0.7683462 |
| Gm906 | -0.8328556 |
| Gm93 | -0.2406094 |
| Gm93 | -0.5056144 |
| Gm944 | -0.6145094 |
| Gm949 | 0.3624268 |
| Gm962 | 1.5639691 |
| Gm962 | 2.7083823 |
| Gm97 | -0.4788077 |
| Gm97 | 1.0974107 |
| Gmcl1 | 0.6422734 |
| Gmcl1 | -0.8480293 |
| Gmcl1 | 2.0697526 |
| Gmds | -0.2434527 |
| Gmeb2 | 1.6736294 |
| Gmfg | -0.6571019 |
| Gmfg | -0.6768802 |
| Gmfg | 1.3057187 |
| Gmip | 1.1580658 |
| Gmip | -0.6464889 |
| Gmppa | 1.4705316 |
| Gmppa | -0.333073 |
| Gmppb | 1.62848 |
| Gmpr | 2.7041405 |
| Gmpr2 | -0.4364834 |
| Gna11 | -0.4736841 |
| Gna13 | 0.2584005 |
| Gna14 | -0.4329838 |
| Gnai2 | 1.6616518 |
| Gnao1 | -0.6351268 |
| Gnao1 | -0.5324408 |
| Gnaq | 0.5499175 |
| Gnaq | -0.7928785 |
| Gna-rs1 | -0.500341 |
| Gnas | -0.4591991 |
| Gnas | -0.1680113 |
| Gnas | -0.3913188 |
| Gnas | -0.2315692 |
| Gnas | 0.0340605 |
| Gnat1 | -0.6196209 |
| Gnat2 | 2.9336446 |
| Gnaz | 2.2120926 |
| Gnb1 | -0.4534678 |
| Gnb1l | 2.0862641 |
| Gnb2 | -0.5505016 |
| Gnb2l1 | -0.5489171 |
| Gnb3 | 0.0474759 |
| Gnb4 | 0.140843 |
| Gnb4 | 1.6790231 |
| Gnb5 | 0.9899483 |
| Gnb5 | 1.4782204 |
| Gne | -0.462078 |
| Gng10 | 0.7583414 |
| Gng11 | 1.801444 |
| Gng13 | 2.4785643 |
| Gng2 | -0.4143107 |
| Gng2 | 0.8505206 |
| Gng3 | 0.5406692 |
| Gng4 | -0.668738 |
| Gng7 | -0.8480293 |
| Gng8 | -0.4149957 |
| Gngt2 | 0.482895 |
| Gnl2 | -0.6651421 |
| Gnl2 | 0.0213049 |
| Gnl3 | 0.0817954 |
| Gnl3 | -0.4788077 |
| Gnl3 | -0.5926791 |
| Gnl3l | 1.3053969 |
| Gnl3l | -0.6624543 |
| Gnmt | -0.5528835 |
| Gnmt | 0.0079372 |
| Gnpat | -0.4477717 |
| Gnpda1 | -0.1375399 |
| Gnpda1 | -0.6732526 |
| Gnpda1 | -0.2877783 |
| Gnpda2 | 1.2836918 |
| Gnptab | -0.5433924 |
| Gnptg | 1.1584429 |
| Gnptg | 0.3504942 |
| Gnrhr | -0.3144429 |
| Gns | 1.3540829 |
| Golga1 | 0.7035589 |
| Golga2 | -0.5231555 |
| Golga2 | -0.6213311 |
| Golga2 | -0.6001777 |
| Golga2 | 1.2913465 |
| Golga2 | 1.6422346 |
| Golga3 | 0.7633572 |
| Golga4 | -0.6273415 |
| Golga5 | 0.8557602 |
| Golga7 | 0.8478897 |
| Golim4 | -0.4876575 |
| Golm1 | -0.2343848 |
| Golm1 | 0.2198094 |
| Golph3l | -0.2082494 |
| Golt1a | -0.2753172 |
| Golt1b | 0.4802882 |
| Gopc | -0.5339974 |
| Gorasp1 | -0.4773409 |
| Gorasp2 | 1.2242922 |
| Gorasp2 | -0.0315473 |
| Gorasp2 | -0.3806931 |
| Gorasp2 | 0.2055281 |
| Gosr1 | -0.5786782 |
| Gosr2 | 1.7754433 |
| Gosr2 | 2.0560037 |
| Gosr2 | 1.1161456 |
| Gosr2 | 2.874748 |
| Got1l1 | -0.5347767 |
| Got2 | 0.1082872 |
| Gp1bb | -0.7083008 |
| Gp38 | 1.4019854 |
| Gp5 | -0.0184961 |
| Gp9 | -0.4591991 |
| Gpaa1 | 1.4194129 |
| Gpam | -0.3089261 |
| Gpatch1 | 1.7922234 |
| Gpatch2 | -0.0851892 |
| Gpatch3 | -0.5465453 |
| Gpatch4 | -0.8350081 |
| Gpbp1 | 0.4886042 |
| Gpbp1 | -0.6970893 |
| Gpbp1l1 | 3.2437814 |
| Gpc1 | -0.7525792 |
| Gpc3 | 0.1773746 |
| Gpc3 | 1.3928546 |
| Gpc4 | 1.4700826 |
| Gpc5 | 0.6081112 |
| Gpc5 | 2.8846743 |
| Gpc5 | 0.4922191 |
| Gpd1 | -0.7340335 |
| Gpd2 | 1.9793692 |
| Gpd2 | 1.2904748 |
| Gper | -0.7398501 |
| Gpha2 | 0.1241333 |
| Gphb5 | 0.221881 |
| Gphn | 0.7129508 |
| Gphn | 0.9332424 |
| Gphn | -0.5223859 |
| Gpihbp1 | -0.1838978 |
| Gpkow | -0.7073613 |
| Gpld1 | -0.5139623 |
| Gpld1 | 0.1445991 |
| Gpm6a | 0.5511275 |
| Gpm6a | -0.7836573 |
| Gpm6b | 0.5934394 |
| Gpnmb | -0.0003522 |
| Gpnmb | -0.711125 |
| Gpr1 | -0.2883758 |
| Gpr1 | 2.8692976 |
| Gpr103 | 1.956737 |
| Gpr107 | -0.6562128 |
| Gpr108 | 0.5426239 |
| Gpr108 | -0.5852406 |
| Gpr108 | -0.4143107 |
| Gpr109a | -0.6421024 |
| Gpr113 | 0.1397133 |
| Gpr113 | 1.8165286 |
| Gpr114 | -0.6633494 |
| Gpr12 | 2.516662 |
| Gpr12 | -0.6119641 |
| Gpr120 | -0.0953285 |
| Gpr123 | -0.5433924 |
| Gpr124 | 0.2764602 |
| Gpr128 | 2.8436006 |
| Gpr135 | 0.6971755 |
| Gpr137 | 1.25163 |
| Gpr137b | 0.9773546 |
| Gpr141 | -0.3058753 |
| Gpr146 | -0.3846635 |
| Gpr146 | 0.3832364 |
| Gpr149 | 0.7521305 |
| Gpr151 | -0.4936046 |
| Gpr153 | -0.579496 |
| Gpr156 | 1.7213536 |
| Gpr156 | -0.7036129 |
| Gpr158 | -0.3175221 |
| Gpr162 | -0.1617321 |
| Gpr162 | -0.4869168 |
| Gpr17 | 2.4315987 |
| Gpr172b | 0.9046091 |
| Gpr172b | 0.2149575 |
| Gpr176 | -0.1209231 |
| Gpr177 | -0.3374743 |
| Gpr18 | 3.0498972 |
| Gpr180 | 0.847714 |
| Gpr182 | 2.3019352 |
| Gpr182 | -0.3349567 |
| Gpr19 | 1.3121302 |
| Gpr20 | -0.4246382 |
| Gpr21 | -0.408169 |
| Gpr22 | 0.3408364 |
| Gpr23 | 1.0722412 |
| Gpr26 | -0.7565313 |
| Gpr27 | 1.3889417 |
| Gpr27 | 2.5275573 |
| Gpr27 | -0.4766084 |
| Gpr34 | 0.3146837 |
| Gpr34 | 2.5565207 |
| Gpr37l1 | -0.5277866 |
| Gpr45 | -0.4556129 |
| Gpr6 | -0.0498557 |
| Gpr64 | 0.4013183 |
| Gpr65 | 0.7955016 |
| Gpr68 | -0.5162521 |
| Gpr75 | 1.7850873 |
| Gpr81 | 2.5050782 |
| Gpr83 | 0.3402295 |
| Gpr85 | -0.4810123 |
| Gpr85 | -0.7644863 |
| Gpr89 | 0.7212424 |
| Gpr89 | -0.4149957 |
| Gpr97 | -0.6989485 |
| Gprasp1 | -0.6544372 |
| Gprasp1 | 1.0887804 |
| Gprc2a-rs5 | 1.1725368 |
| Gprc2a-rs5 | 0.0865961 |
| Gprc5a | -0.5976714 |
| Gprc5b | 2.6337506 |
| Gprc5c | -0.3520848 |
| Gprk6 | -0.4407005 |
| Gprk6 | 0.7938275 |
| Gps1 | -0.2659341 |
| Gps1 | -0.6394804 |
| Gps1 | -0.5332188 |
| Gps1 | -0.3913188 |
| Gps2 | 0.609929 |
| Gpsm1 | 0.9820783 |
| Gpsm2 | -0.5985061 |
| Gpsm3 | -0.1344965 |
| Gpsn2 | -0.3495273 |
| Gpsn2 | 0.0310998 |
| Gpsn2 | 0.3121839 |
| Gpt1 | -0.6010147 |
| Gpt2 | 0.8324815 |
| Gpx1 | 2.0837179 |
| Gpx2 | 1.3245067 |
| Gpx3 | -0.2961796 |
| Gpx4 | 0.5433558 |
| Gpx4 | 1.6899299 |
| Gpx6 | -0.4744143 |
| Gpx7 | -0.5170166 |
| Gramd1a | -0.2694416 |
| Gramd1b | -0.510918 |
| Gramd2 | -0.3268213 |
| Gramd2 | -0.1575695 |
| Gramd3 | -0.4642431 |
| Gramd4 | -0.3826762 |
| Grap2 | -0.3293169 |
| Grasp | -0.5363373 |
| Grasp | 0.0152917 |
| Grb10 | 0.023443 |
| Grb14 | -0.7545532 |
| Grb14 | 0.848943 |
| Grb2 | -0.5139623 |
| Grb7 | 0.4572949 |
| Grcc10 | 1.5561434 |
| Greb1 | -0.0279286 |
| Greb1 | 1.2534468 |
| Grem1 | -0.2104453 |
| Grem2 | -0.4802768 |
| Grhl1 | 0.9779655 |
| Grhl2 | -0.4456447 |
| Grhpr | -0.2428833 |
| Gria1 | -0.4156811 |
| Gria1 | -0.500341 |
| Gria1 | -0.5746002 |
| Gria2 | 1.8921329 |
| Gria2 | -0.3187566 |
| Gria3 | -0.5835956 |
| Gria3 | 0.0035836 |
| Gria3 | -0.2688561 |
| Grid2 | 2.3180268 |
| Grik1 | -0.2676862 |
| Grik2 | 1.3705268 |
| Grik4 | 2.245084 |
| Grik5 | -0.5147249 |
| Grin1 | 1.8708615 |
| Grin1 | 1.4402367 |
| Grin2a | 2.1284241 |
| Grin2b | 1.5587593 |
| Grin2c | 0.6128753 |
| Grin3b | -0.6624543 |
| Grina | -0.6119641 |
| Grip1 | 2.9526075 |
| Grip1 | -0.1664375 |
| Grip1 | -0.6060522 |
| Gripap1 | -0.5254682 |
| Gripap1 | -0.6732526 |
| Grit | -0.5827742 |
| Grk4 | 1.2245263 |
| Grk4 | -0.6544372 |
| Grk4 | 1.4666632 |
| Grk5 | -0.5473352 |
| Grlf1 | -0.5418199 |
| Grm1 | 2.265695 |
| Grm7 | -0.462078 |
| Grm8 | 0.258732 |
| Grn | 0.9335628 |
| Grp | -0.3643303 |
| Grpel1 | 0.3612404 |
| Grpr | -0.6832627 |
| Grsf1 | 1.0787006 |
| Grtp1 | -0.4541823 |
| Grwd1 | -0.3741133 |
| Grwd1 | -0.1643433 |
| Gsbs | 0.3332207 |
| Gsc | -0.775537 |
| Gsdma1 | -0.5093996 |
| Gsdmdc1 | 0.7956875 |
| Gsdmdc1 | -0.0153731 |
| Gsdmdc1 | -0.1674864 |
| Gse1 | -0.7887689 |
| Gsg1 | -0.8095015 |
| Gsg1 | 0.6299151 |
| Gsg1 | -0.3708408 |
| Gsg2 | -0.3721484 |
| Gsk3a | 0.2514112 |
| Gsk3b | -0.135003 |
| Gsn | 1.1354119 |
| Gspt1 | -0.4336827 |
| Gspt2 | -0.4591991 |
| Gss | -0.7525792 |
| Gss | -0.6026908 |
| Gss | 1.2775222 |
| Gsta2 | -0.5457561 |
| Gsta3 | 1.6162847 |
| Gsta3 | 0.0255762 |
| Gsta4 | -0.1264291 |
| Gstcd | 0.4027384 |
| Gstk1 | -0.6768802 |
| Gstm1 | 0.0869951 |
| Gstm1 | -0.3993687 |
| Gstm2 | -0.2400418 |
| Gstm2 | 0.0801896 |
| Gstm2 | 3.3054754 |
| Gstm4 | 1.6093537 |
| Gstm5 | 0.133285 |
| Gstm6 | -0.6094258 |
| Gstm6 | -0.5943402 |
| Gsto1 | 3.6946122 |
| Gsto2 | -0.4421104 |
| Gsto2 | -0.0526333 |
| Gstp1 | -0.4170535 |
| Gstp1 | -0.6571019 |
| Gstp2 | -0.2871811 |
| Gstt1 | 0.6564145 |
| Gstt2 | 0.9125062 |
| Gstt3 | -0.4484818 |
| Gstt4 | 0.2020112 |
| Gstz1 | 0.3543892 |
| Gtdc1 | -0.9125056 |
| Gtf2a1 | 1.1455345 |
| Gtf2e1 | 0.2297797 |
| Gtf2e2 | -0.7101827 |
| Gtf2e2 | 1.8356215 |
| Gtf2f1 | 0.4942785 |
| Gtf2f2 | 0.0027104 |
| Gtf2h1 | 0.1415955 |
| Gtf2h1 | 1.0355288 |
| Gtf2h2 | 1.0307818 |
| Gtf2h3 | -0.3879852 |
| Gtf2h4 | -0.0003522 |
| Gtf2h5 | 1.5484162 |
| Gtf2ird1 | -0.4883988 |
| Gtf2ird1 | 1.4805324 |
| Gtf2ird2 | 1.4381938 |
| Gtf2ird2 | 0.504253 |
| Gtf2ird2 | 0.02515 |
| Gtf3a | 0.0729289 |
| Gtf3a | 0.6783904 |
| Gtf3c1 | 0.7427355 |
| Gtf3c2 | 0.6628692 |
| Gtf3c3 | 0.4198907 |
| Gtf3c4 | 1.3298503 |
| Gtf3c4 | -0.3476137 |
| Gtf3c5 | -0.5713507 |
| Gtf3c5 | -0.2583796 |
| Gtf3c5 | 0.1404666 |
| Gtl3 | -0.331193 |
| Gtpbp1 | 1.5544227 |
| Gtpbp2 | -0.2237306 |
| Gtpbp3 | -0.4584807 |
| Gtpbp4 | -0.6535507 |
| Gtpbp5 | 0.4497719 |
| Gtpbp6 | -0.652665 |
| Gtpbp8 | 1.9966378 |
| Gtpbp8 | 0.2273829 |
| Gtrgeo22 | 1.5664821 |
| Gtse1 | -0.4832221 |
| Gtse1 | -0.1400838 |
| Gtse1 | -0.6145094 |
| Gtsf1l | 0.093751 |
| Guca1a | -0.4385896 |
| Guca1b | -0.4817483 |
| Guca2a | 2.9376604 |
| Guca2b | 0.5655264 |
| Gucy1a2 | 1.7520791 |
| Gucy1a3 | -0.5762293 |
| Gucy1a3 | 0.2100801 |
| Gucy1a3 | -0.3077046 |
| Gucy1b3 | 1.3870773 |
| Guk1 | 2.0442274 |
| Gulo | 2.493795 |
| Gulp1 | -0.4456447 |
| Gusb | -0.5465453 |
| Gusb | 0.2156522 |
| Gusb | -0.6145094 |
| Gvin1 | 1.2101368 |
| Gyg | 1.199016 |
| Gyg | 0.7103062 |
| Gykl1 | 0.6869953 |
| Gyltl1b | -0.5713507 |
| Gyltl1b | 0.8877693 |
| Gypa | 0.2944939 |
| Gypc | -0.693382 |
| Gys1 | -0.033362 |
| Gys3 | 1.6417869 |
| Gzf1 | -0.7398501 |
| Gzf1 | -0.3741133 |
| Gzmd | 2.7894475 |
| Gzmd | 0.8774487 |
| Gzmk | -0.2717874 |
| Gzmm | -0.6651421 |
| Gzmn | 1.2125111 |
| H13 | -0.6579918 |
| H13 | 2.5474539 |
| H13 | -0.4477717 |
| H13 | -0.6841781 |
| H19 | 1.4043024 |
| H1f0 | -0.6571019 |
| H1fnt | -0.0549545 |
| H1foo | 0.1602106 |
| H1fx | -0.7196495 |
| H1fx | -0.5746002 |
| H2-Aa | 0.3585655 |
| H2-Ab1 | 1.1054293 |
| H2-Ab1 | 1.3319375 |
| H2-Ab1 | 0.3615372 |
| H2afj | -0.3662787 |
| H2afx | 2.5853904 |
| H2afy | -0.568921 |
| H2afz | 0.6775466 |
| H2-Bl | 0.2121736 |
| H2-DMa | 0.9811659 |
| H2-DMb1 | -0.7826384 |
| H2-Eb1 | -0.078014 |
| H2-K1 | -0.7359683 |
| H2-Ke6 | -0.295577 |
| H2-M10.3 | 0.0733338 |
| H2-M2 | 0.6798652 |
| H2-M3 | 0.2290955 |
| H2-Oa | 1.9018274 |
| H2-Ob | 1.0476452 |
| H2-Q1 | 1.0651808 |
| H2-Q2 | -0.1498146 |
| H2-Q7 | 0.0315234 |
| H2-Q8 | -0.5697302 |
| H2-T10 | -0.5193139 |
| H2-T23 | 0.3715701 |
| H2-T23 | -0.7417971 |
| H2-T24 | 3.3921288 |
| H2-Tw3 | -0.1775105 |
| H3f3a | 0.5911258 |
| H3f3a | -0.4013921 |
| H3f3b | 0.6936563 |
| H47 | 2.6235997 |
| H47 | -0.6052107 |
| H6pd | -0.1935619 |
| Habp4 | -0.5910209 |
| Habp4 | 2.4444137 |
| Hace1 | -0.7565313 |
| Hacl1 | -0.7120683 |
| Hadhb | -0.6290659 |
| Hagh | -0.5332188 |
| Haghl | 2.7996172 |
| Haghl | 4.1819476 |
| Hal | 2.6647438 |
| Hamp2 | -0.5457561 |
| Hao1 | -0.7836573 |
| Hao3 | -0.4392927 |
| Hap1 | 0.3582678 |
| Hap1 | -0.1204242 |
| Hap1 | 1.0650418 |
| Hapln1 | -0.1924831 |
| Hapln2 | -0.4329838 |
| Hapln4 | 1.0957986 |
| Harbi1 | -0.3591546 |
| Hars | 0.9165928 |
| Has1 | -0.5520888 |
| Hat1 | -0.459918 |
| Havcr1 | -0.4613574 |
| Havcr2 | 1.6587247 |
| Hax1 | -0.5418199 |
| Hba-a1 | -0.6500131 |
| Hbb-b1 | -0.6961611 |
| Hbegf | -0.5786782 |
| Hbp1 | -0.638608 |
| Hbp1 | 1.0554017 |
| Hbs1l | -0.6696392 |
| Hbs1l | -0.8021923 |
| Hbs1l | -0.0880748 |
| Hbs1l | 0.6014918 |
| Hc | -0.4108936 |
| Hccs | -0.5746002 |
| Hcfc1 | -0.3846635 |
| Hcfc1r1 | 1.6198073 |
| Hcfc2 | 3.3097264 |
| Hcls1 | -0.5868884 |
| Hcn2 | 2.0774385 |
| Hcn3 | 1.4872578 |
| Hcn3 | 1.07582 |
| Hcr | -0.6579918 |
| Hcst | -0.1903293 |
| Hdac10 | -0.5041047 |
| Hdac11 | -0.08615 |
| Hdac2 | -0.4758764 |
| Hdac3 | -0.6860116 |
| Hdac4 | -0.29077 |
| Hdac5 | -0.2572228 |
| Hdac6 | -0.0924202 |
| Hdac7 | 2.8499549 |
| Hdac8 | -0.0043047 |
| Hdac9 | -0.6869297 |
| Hdc | -0.6052107 |
| Hddc2 | -0.4294974 |
| Hddc2 | -0.5505016 |
| Hdgf | -0.5489171 |
| Hdgf | -0.5632762 |
| Hdgf | -0.5568669 |
| Hdgf | -0.0238744 |
| Hdgfl1 | -0.2126463 |
| Hdgfrp2 | -0.5985061 |
| Hdhd1a | 2.5683086 |
| Hdhd2 | -0.3046578 |
| Hdhd2 | 1.2103745 |
| Hdhd2 | 0.2153049 |
| Hdlbp | -0.3022275 |
| Hdlbp | 1.9841812 |
| Heatr1 | 2.4524549 |
| Heatr1 | -0.5473352 |
| Heatr3 | -0.1487854 |
| Heatr5a | -0.7139578 |
| Hebp1 | 3.1874335 |
| Hebp2 | -0.6860116 |
| Heca | 1.3547951 |
| Hectd2 | -0.3243324 |
| Hectd2 | 2.3143464 |
| Hectd3 | 0.3350542 |
| Hecw2 | 0.2623709 |
| Hecw2 | -0.0180493 |
| Hecw2 | -0.6544372 |
| Heg1 | 2.594764 |
| Hel308 | 0.865979 |
| Helb | -0.479542 |
| Helb | -0.130454 |
| Helz | -0.4995901 |
| Hemgn | 1.0885094 |
| Hemk1 | -0.6403535 |
| Hemt1 | 0.5394454 |
| Hepacam | 1.5881655 |
| Heph | -0.0526333 |
| Heph | 0.4734762 |
| Heph | -0.4421104 |
| Heph | -0.24802 |
| Heph | 1.6713186 |
| Herc2 | -0.4020676 |
| Herc3 | -0.3960061 |
| Herc3 | 0.8184463 |
| Herc4 | -0.5426058 |
| Herc4 | 0.7234544 |
| Herpud1 | 0.425723 |
| Herpud1 | 1.0193182 |
| Herpud2 | -0.1451925 |
| Herpud2 | -0.5093996 |
| Herpud2 | 1.1207381 |
| Herpud2 | -0.4102117 |
| Hes1 | -0.5078837 |
| Hes5 | -0.5449675 |
| Hes5 | -0.4246382 |
| Hes6 | -0.7624911 |
| Hes6 | 0.8310518 |
| Hes6 | 1.1391407 |
| Hexa | -0.6256202 |
| Hexb | 0.5293491 |
| Hexdc | 1.0376785 |
| Hexdc | 2.3985501 |
| Hexim1 | 3.6225702 |
| Hexim2 | 2.0260065 |
| Hey1 | 3.4998237 |
| Hey2 | 1.1384989 |
| Hey2 | 1.9098574 |
| Heyl | 0.678812 |
| Hfe | -0.1508448 |
| Hfe | 0.0044559 |
| Hgfac | -0.4876575 |
| Hgs | 0.4435463 |
| Hgs | 0.347487 |
| Hgsnat | 1.1937077 |
| Hhat | 2.2823843 |
| Hhat | -0.5293354 |
| Hhat | -0.3286924 |
| Hhatl | -0.538683 |
| Hhex | -0.3393671 |
| Hhip | -0.1968057 |
| Hibadh | 0.974141 |
| Hic1 | -0.5481258 |
| Hic2 | -0.5960043 |
| Hif1a | 2.6131007 |
| Hif3a | -0.6473687 |
| Hif3a | 2.7935546 |
| Higd1a | -0.1139625 |
| Higd1b | -0.3343284 |
| Higd2a | 1.0493431 |
| Hils1 | -0.4191159 |
| Hint1 | 2.3467463 |
| Hint2 | -0.5116781 |
| Hint3 | 0.7354281 |
| Hint3 | -0.2865843 |
| Hip1 | 0.3867078 |
| Hip1r | -0.3418967 |
| Hip2 | 0.2887355 |
| Hipk1 | 2.0288634 |
| Hipk2 | -0.652665 |
| Hipk3 | 2.0987998 |
| Hipk4 | 0.1313858 |
| Hira | -0.3463402 |
| Hira | 1.6676237 |
| Hirip3 | 0.6243444 |
| Hisppd1 | -0.5835956 |
| Hist1h1b | -0.5018446 |
| Hist1h1c | -0.5056144 |
| Hist1h2aa | -0.6351268 |
| Hist1h2ac | -0.3966776 |
| Hist1h2ad | 1.8727256 |
| Hist1h2ae | -0.5481258 |
| Hist1h2af | 2.3520033 |
| Hist1h2ag | -0.6299293 |
| Hist1h2ah | 1.0898636 |
| Hist1h2ak | -0.5705401 |
| Hist1h2an | -0.2847961 |
| Hist1h2ao | -0.4281064 |
| Hist1h2bc | -0.6651421 |
| Hist1h2be | -0.4928591 |
| Hist1h2bf | 2.1221743 |
| Hist1h2bg | 1.1691887 |
| Hist1h2bh | 1.582613 |
| Hist1h2bj | -0.6562128 |
| Hist1h2bk | -0.3101492 |
| Hist1h2bm | 0.9807094 |
| Hist1h2bn | 1.002522 |
| Hist1h3a | 2.1783034 |
| Hist1h3d | -0.4506154 |
| Hist1h3e | -0.3064847 |
| Hist1h3f | -0.4399963 |
| Hist1h4c | 1.3312075 |
| Hist1h4d | 0.3239985 |
| Hist1h4f | 0.5655264 |
| Hist1h4h | 1.8857853 |
| Hist1h4i | 2.7377269 |
| Hist1h4j | -0.3993687 |
| Hist1h4k | -0.5332188 |
| Hist1h4m | 1.0847072 |
| Hist2h2aa1 | 0.2689506 |
| Hist2h2ab | -0.0434066 |
| Hist2h2ac | -0.3721484 |
| Hist2h2bb | 0.1423473 |
| Hist2h2be | 0.2758096 |
| Hist2h3b | -0.5116781 |
| Hist2h3c1 | -0.5270131 |
| Hist2h3c1 | -0.464966 |
| Hist2h4 | 0.2780846 |
| Hist3h2a | -0.6943074 |
| Hist3h2bb | -0.3237112 |
| Hist4h4 | 1.5592489 |
| Hk1 | -0.4428162 |
| Hk3 | -0.7908214 |
| Hkdc1 | -0.7408231 |
| Hlcs | 0.945655 |
| Hlf | 0.5927459 |
| Hltf | 2.6436142 |
| Hlx | 0.4210044 |
| Hmg20a | 2.5230355 |
| Hmg20a | -0.6535507 |
| Hmg20b | -0.2973861 |
| Hmga1 | -0.5544748 |
| Hmgb2 | 0.766814 |
| Hmgb2l1 | 1.8918479 |
| Hmgb2l1 | 0.9107024 |
| Hmgb4 | -0.2800445 |
| Hmgcl | -0.549709 |
| Hmgcl | 0.6065177 |
| Hmgcl | 2.6697609 |
| Hmgcll1 | -0.4570457 |
| Hmgcs2 | -0.7745269 |
| Hmgn1 | 1.9012068 |
| Hmgn2 | -0.6359959 |
| Hmgn3 | -0.2389077 |
| Hmgn3 | 1.0870176 |
| Hmha1 | -0.5208487 |
| Hmox1 | 0.7742596 |
| Hmox2 | -0.3933247 |
| Hmx1 | -0.4556129 |
| Hmx1 | 0.1434739 |
| Hn1 | 1.4142762 |
| Hn1 | 1.1106044 |
| Hn1l | -0.1219218 |
| Hnf1b | 0.898808 |
| Hnf1b | -0.7350004 |
| Hnf4a | 1.2677435 |
| Hnrnpc | 1.7003597 |
| Hnrpa1 | 0.8685592 |
| Hnrpa1 | -0.4635209 |
| Hnrpab | 1.8477168 |
| Hnrpc | 3.8844725 |
| Hnrpdl | -0.3754255 |
| Hnrpdl | -0.4671378 |
| Hnrpf | -0.5592652 |
| Hnrph1 | -0.4758764 |
| Hnrph1 | -0.5379004 |
| Hnrph1 | 1.110075 |
| Hnrph1 | 0.3875737 |
| Hnrph1 | -0.456329 |
| Hnrph2 | -0.6187671 |
| Hnrph3 | 0.290659 |
| Hnrpk | 1.3814614 |
| Hnrpl | -0.1446804 |
| Hnrpll | 0.4459874 |
| Hnrpll | -0.3175221 |
| Hnrpm | -0.6307936 |
| Hnrpm | -0.6136602 |
| Hnrpm | 3.2124443 |
| Hnrpul1 | 0.7246588 |
| Hnrpul1 | 0.4524657 |
| Hnrpul1 | -0.4988398 |
| Hnrpul1 | 1.2488979 |
| Hnrpul1 | -0.0813555 |
| Hnt | 0.0327928 |
| Homer1 | 1.315001 |
| Homer1 | -0.6669383 |
| Homer1 | -0.3299419 |
| Homer1 | -0.7092413 |
| Homez | -0.3833381 |
| Homez | 1.1816517 |
| Hook1 | -0.028832 |
| Hook1 | -0.5918496 |
| Hook1 | 3.1673401 |
| Hook1 | 0.3730365 |
| Hook2 | -0.1903293 |
| Hormad2 | -0.4824849 |
| Hoxa13 | -0.4527539 |
| Hoxa2 | -0.711125 |
| Hoxa2 | 0.0765664 |
| Hoxa4 | -0.1477575 |
| Hoxa5 | -0.0047449 |
| Hoxa7 | 1.5538484 |
| Hoxa9 | 1.4190334 |
| Hoxa9 | 2.050975 |
| Hoxb2 | -0.5426058 |
| Hoxb3 | 2.9629075 |
| Hoxb3 | -0.5608676 |
| Hoxb4 | -0.6491308 |
| Hoxb5 | -0.4995901 |
| Hoxb6 | -0.5901929 |
| Hoxb7 | -0.4958446 |
| Hoxc10 | 0.9986483 |
| Hoxc10 | 1.7223806 |
| Hoxc6 | 2.5300248 |
| Hoxc9 | -0.5139623 |
| Hoxd10 | -0.4685885 |
| Hoxd3 | 2.6922605 |
| Hoxd4 | 0.221881 |
| Hoxd8 | 0.732648 |
| Hoxd9 | 0.5491907 |
| Hp | 1.9251072 |
| Hp | -0.0475473 |
| Hp | 0.7945719 |
| Hp1bp3 | 1.2397327 |
| Hpca | -0.4802768 |
| Hpcal1 | -0.500341 |
| Hpcal1 | 1.7003597 |
| Hpcal4 | -0.4239461 |
| Hpgd | -0.5770449 |
| Hpn | -0.0466255 |
| Hpn | 0.0574117 |
| Hprt1 | 2.2963122 |
| Hprt1 | 2.2270285 |
| Hprt1 | -0.500341 |
| Hps1 | 1.3037865 |
| Hps3 | -0.4442294 |
| Hps3 | -0.3495273 |
| Hps4 | -0.5648855 |
| Hps5 | -0.2176179 |
| Hps6 | -0.7826384 |
| Hpse | -0.5819536 |
| Hpx | 2.3244146 |
| Hr | -0.426024 |
| Hr | -0.3886509 |
| Hras1 | 1.8129977 |
| Hrasls | -0.5608676 |
| Hrasls5 | 3.5202296 |
| Hrasls5 | -0.4184279 |
| Hrb | -0.5170166 |
| Hrb | 0.1098452 |
| Hrbl | -0.7225085 |
| Hrbl | 1.3261864 |
| Hrc | 1.3247168 |
| Hrg | -0.5877134 |
| Hrh3 | 0.6216585 |
| Hrk | 1.9480317 |
| Hrmt1l2 | 2.5459623 |
| Hrsp12 | 0.133285 |
| Hrsp12 | -0.5600661 |
| Hrsp12 | 0.2034195 |
| Hs1bp3 | 2.2905807 |
| Hs2st1 | 2.250793 |
| Hs3st1 | -0.5877134 |
| Hs3st3a1 | 0.5888064 |
| Hs3st3b1 | 2.9479258 |
| Hs3st6 | 1.0442401 |
| Hs6st1 | -0.4149957 |
| Hs6st2 | -0.4883988 |
| Hs6st2 | 0.9191989 |
| Hs6st3 | 2.2585811 |
| Hsbp1 | 0.4064205 |
| Hsd11b1 | -0.579496 |
| Hsd11b1 | -0.5262403 |
| Hsd11b1 | -0.3773974 |
| Hsd11b1 | 0.3703954 |
| Hsd11b2 | -0.2806371 |
| Hsd17b1 | -0.6678377 |
| Hsd17b10 | -0.6887686 |
| Hsd17b11 | -0.4751451 |
| Hsd17b11 | -0.6447318 |
| Hsd17b11 | 2.2806988 |
| Hsd17b12 | 0.695728 |
| Hsd17b2 | -0.5402502 |
| Hsd17b3 | -0.3101492 |
| Hsd17b4 | -0.7379071 |
| Hsd17b7 | 1.9819077 |
| Hsd3b1 | -0.6094258 |
| Hsd3b2 | -0.7321027 |
| Hsd3b2 | -0.5332188 |
| Hsd3b4 | 0.3233804 |
| Hsd3b7 | -0.7634882 |
| Hsd3b7 | -0.3374743 |
| Hsdl1 | -0.6579918 |
| Hsf1 | -0.2508861 |
| Hsf4 | -0.6403535 |
| Hsf5 | -0.6961611 |
| Hsn2 | 1.5490764 |
| Hsp105 | 0.0255762 |
| Hsp90aa1 | -0.6290659 |
| Hsp90ab1 | 0.5782969 |
| Hsp90ab1 | 1.7341446 |
| Hsp90ab1 | 1.4476276 |
| Hsp90b1 | -0.7321027 |
| Hspa12b | 1.8620732 |
| Hspa1a | 0.7899094 |
| Hspa1l | 1.6036159 |
| Hspa2 | -0.7765481 |
| Hspa2 | 1.2641619 |
| Hspa4 | -0.6162101 |
| Hspa4l | -0.4700416 |
| Hspa5 | 0.8623547 |
| Hspa9 | -0.4061307 |
| Hspa9 | 0.9514854 |
| Hspb1 | -0.4442294 |
| Hspb2 | 1.3422004 |
| Hspb3 | -0.4876575 |
| Hspb6 | -0.5835956 |
| Hspb7 | -0.1518762 |
| Hspb8 | -0.3064847 |
| Hspbap1 | -0.6111173 |
| Hspd1 | 0.3374932 |
| Hspd1 | -0.2485925 |
| Hspe1 | 2.6088728 |
| Hspg2 | -0.1669618 |
| Htatip | 0.6341266 |
| Htatip | 1.270532 |
| Htatip2 | -0.1264291 |
| Htatip2 | -0.6213311 |
| Htatsf1 | -0.2049654 |
| Htf9c | -0.2583796 |
| Htr1a | -0.714904 |
| Htr1b | -0.60353 |
| Htr1d | -0.6026908 |
| Htr1f | -0.5481258 |
| Htr1f | -0.3431641 |
| Htr2b | 1.699869 |
| Htr3a | 0.1583658 |
| Htr5b | 0.3501939 |
| Htr7 | -0.6068945 |
| Htra1 | 2.7803909 |
| Htra1 | 1.7166144 |
| Htra2 | -0.2326944 |
| Htra3 | 1.8670049 |
| Htra3 | 0.7904702 |
| Htt | 2.0533731 |
| Hus1 | 1.2231208 |
| Hus1 | 1.3151072 |
| Hus1 | 0.0973079 |
| Huwe1 | 2.1276728 |
| Hvcn1 | 0.0865961 |
| Hvcn1 | 0.4761021 |
| Hyal1 | 0.4337173 |
| Hyal2 | -0.6247608 |
| Hyal3 | -0.3527253 |
| Hyal5 | 0.2013062 |
| Hyal6 | -0.6316586 |
| Hydin | -0.1806986 |
| Hyi | -0.4129424 |
| Hyou1 | -0.5402502 |
| Iah1 | 1.9025039 |
| Iap | -0.1855015 |
| Iars | -0.7437482 |
| Iars | 0.4521967 |
| Iars2 | 0.4359105 |
| Ibtk | 2.5021595 |
| Ica1 | -0.6368658 |
| Ica1l | -0.3747692 |
| Icam1 | 0.6350108 |
| Icam2 | -0.6750646 |
| Icam4 | 1.3674218 |
| Icam5 | -0.4883988 |
| Ick | 0.1032058 |
| Icmt | -0.7158512 |
| Ict1 | 2.2490102 |
| Id1 | -0.2215032 |
| Id2 | -0.6473687 |
| Id2 | 1.3538793 |
| Id3 | 1.7602613 |
| Id4 | -0.3913188 |
| Ide | -0.1812311 |
| Idh1 | 1.6211821 |
| Idh2 | 1.9400562 |
| Idh3a | -0.4170535 |
| Idh3b | -0.1503295 |
| Idh3g | 1.4545036 |
| Idh3g | 2.1754522 |
| Idi2 | -0.5592652 |
| Ids | 0.2527465 |
| Ids | 1.7072019 |
| Ids | 0.8380012 |
| Idua | 0.8213427 |
| Ier3 | -0.5355567 |
| Ier3ip1 | -0.6299293 |
| Ier5l | 1.7682416 |
| Ifi202b | -0.6989485 |
| Ifi202b | 0.463695 |
| Ifi27 | 0.5839168 |
| Ifi30 | -0.4846982 |
| Ifi30 | -0.2871811 |
| Ifi35 | 0.515379 |
| Ifi47 | 3.8096108 |
| Ifit2 | 1.5282217 |
| Ifit2 | -0.2901709 |
| Ifit3 | -0.5402502 |
| Ifit3 | 1.7686323 |
| Ifitm1 | -0.5170166 |
| Ifitm1 | 0.198481 |
| Ifitm1 | -0.4336827 |
| Ifitm2 | 1.0675402 |
| Ifitm3 | 1.6925484 |
| Ifitm5 | 0.1268119 |
| Ifitm6 | -0.7555417 |
| Ifna1 | -0.050318 |
| Ifna6 | -0.5811337 |
| Ifnar2 | 0.9421708 |
| Ifnar2 | 2.6782257 |
| Ifnar2 | 3.1129708 |
| Ifngr1 | 0.21461 |
| Ifngr2 | -0.0900036 |
| Ifngr2 | -0.4513277 |
| Ifngr2 | 0.1047723 |
| Ifnz | -0.3773974 |
| Ifrd1 | 0.1924486 |
| Ifrg15 | 0.1419715 |
| Ift122 | -0.2022375 |
| Ift140 | -0.4921142 |
| Ift140 | 0.0969133 |
| Ift140 | -0.4906263 |
| Ift172 | -0.1174363 |
| Ift172 | 1.8376181 |
| Ift172 | -0.6624543 |
| Ift20 | -0.5116781 |
| Ift20 | 1.5522056 |
| Ift20 | -0.6544372 |
| Ift52 | 1.506801 |
| Ift80 | 2.4378859 |
| Ift81 | 0.2560766 |
| Ift81 | 1.5031679 |
| Ift81 | -0.2016929 |
| Ift88 | 1.2866518 |
| Igbp1 | 0.5211422 |
| Igbp1b | -0.6368658 |
| Igf1 | -0.2800445 |
| Igf1 | 0.1106232 |
| Igf1r | 0.8296198 |
| Igf2 | -0.497341 |
| Igf2bp1 | -0.5746002 |
| Igf2bp2 | -0.5018446 |
| Igf2bp3 | 2.9537334 |
| Igf2bp3 | 3.3674205 |
| Igf2r | -0.3780556 |
| Igf2r | -0.3349567 |
| Igfals | 0.074143 |
| Igfbp2 | -0.5951719 |
| Igfbp2 | 0.6701306 |
| Igfbp3 | -0.6464889 |
| Igfbp4 | 0.6828078 |
| Igfbp4 | -0.6145094 |
| Igfbp5 | 1.1837331 |
| Igfbp6 | -0.6750646 |
| Igfbp7 | -0.7206016 |
| Igfbpl1 | 0.6296929 |
| Igsf1 | -0.7167993 |
| Igsf1 | -0.6145094 |
| Igsf1 | 2.2504057 |
| Igsf1 | -0.0047449 |
| Igsf11 | -0.0360906 |
| Igsf11 | -0.3058753 |
| Igsf11 | -0.6230444 |
| Igsf21 | -0.7806039 |
| Igsf3 | -0.5985061 |
| Igsf4a | 0.4647574 |
| Igsf5 | -0.521617 |
| Igsf8 | -0.311374 |
| Igsf9 | -0.4315877 |
| Igtp | 1.6543903 |
| Ihh | 0.8867592 |
| Ihpk1 | 1.4607848 |
| Ihpk2 | 0.3068493 |
| Ihpk2 | 0.9872316 |
| Iigp2 | 0.6128753 |
| Ik | 0.7529091 |
| Ikbkap | -0.421873 |
| Ikbkb | -0.7263343 |
| Ikbke | 0.5782969 |
| Ikbkg | 1.7302172 |
| Ikbkg | -0.6924575 |
| Il10ra | 0.0652028 |
| Il10rb | -0.6001777 |
| Il11 | 0.990099 |
| Il11ra1 | 0.828903 |
| Il12a | 0.7320512 |
| Il13 | -0.5124389 |
| Il13ra1 | -0.4442294 |
| Il13ra2 | -0.5086413 |
| Il15 | -0.1518762 |
| Il15 | 1.0936448 |
| Il15 | -0.3546494 |
| Il15ra | -0.6102712 |
| Il15ra | 0.3727434 |
| Il15ra | 1.139269 |
| Il16 | 0.5957472 |
| Il17d | 1.1196904 |
| Il17d | -0.6952338 |
| Il17ra | -0.456329 |
| Il17rb | -0.7158512 |
| Il17rc | -0.7139578 |
| Il17rc | -0.4936046 |
| Il17rc | -0.5746002 |
| Il17rd | 2.1090959 |
| Il17rd | 1.9905073 |
| Il17re | 0.7738793 |
| Il17re | 0.1849422 |
| Il17re | 2.0608153 |
| Il18 | 2.2638643 |
| Il18bp | -0.668738 |
| Il18r1 | 2.4702278 |
| Il1b | -0.4729544 |
| Il1r1 | 1.4623258 |
| Il1rap | -0.7083008 |
| Il1rap | 1.3110647 |
| Il1rap | -0.5324408 |
| Il1rap | 1.2091854 |
| Il1rap | 0.2423468 |
| Il1rl1l | 1.2375426 |
| Il1rl2 | 0.7288617 |
| Il20ra | -0.4253308 |
| Il20rb | -0.5960043 |
| Il20rb | -0.4906263 |
| Il23a | 0.7068364 |
| Il27ra | -0.6491308 |
| Il28ra | -0.6615601 |
| Il28ra | 0.0729289 |
| Il28ra | 1.5623441 |
| Il31ra | 0.5728791 |
| Il33 | -0.2332575 |
| Il3ra | -0.5010925 |
| Il4i1 | 1.8796251 |
| Il6ra | -0.2776778 |
| Il6st | 0.7188232 |
| Il7 | 2.1113034 |
| Ildr1 | 0.9187108 |
| Ildr1 | 0.1063361 |
| Ilf2 | 0.3074785 |
| Ilf3 | -0.4232546 |
| Ilf3 | 0.2111274 |
| Ilf3 | 0.5315748 |
| Ilf3 | 0.7204367 |
| Ilk | 1.2909108 |
| Ilkap | -0.520081 |
| Ilvbl | 0.7993962 |
| Ilvbl | -0.3899839 |
| Ilvbl | -0.1643433 |
| Ilvbl | -0.5505016 |
| Immp2l | -0.0457047 |
| Immt | 2.9734675 |
| Imp3 | 0.5075541 |
| Imp4 | -0.2937715 |
| Imp4 | -0.1334842 |
| Impa2 | 3.3073616 |
| Impact | 0.8417218 |
| Impad1 | 0.8280062 |
| Impdh1 | 0.4353627 |
| Impdh2 | 0.3341379 |
| Ina | -0.2242883 |
| Inadl | -0.5568669 |
| Inadl | 0.3522939 |
| Incenp | -0.0034249 |
| Indo | 2.0936316 |
| Ing2 | -0.3193745 |
| Ing3 | 0.4478816 |
| Ing3 | 0.6516524 |
| Ing3 | -0.2508861 |
| Ing4 | 2.1836049 |
| Ing4 | -0.8168688 |
| Ing4 | -0.6119641 |
| Ing5 | 0.0022735 |
| Inha | -0.5640805 |
| Inhba | 0.1438491 |
| Inmt | 1.6692194 |
| Inoc1 | -0.4722253 |
| Inpp4a | -0.4301936 |
| Inpp4a | -0.4664133 |
| Inpp4a | 0.3043282 |
| Inpp4b | 0.9279406 |
| Inpp5a | -0.6447318 |
| Inpp5d | -0.4371849 |
| Inpp5e | 0.3377976 |
| Inpp5f | -0.5885392 |
| Inppl1 | 4.0958788 |
| Inppl1 | 0.2450418 |
| Inppl1 | -0.6544372 |
| Ins2 | -0.5185475 |
| Insc | -0.08615 |
| Insig1 | -0.8317812 |
| Insig1 | 2.210153 |
| Insig1 | 0.2359147 |
| Insig2 | 0.4223947 |
| Insig2 | -0.3826762 |
| Insl3 | 1.6467003 |
| Insl5 | 0.0549377 |
| Insl6 | -0.2115451 |
| Insm1 | 0.4575625 |
| Ints1 | -0.2366435 |
| Ints12 | -0.8189845 |
| Ints3 | 0.0917691 |
| Ints3 | 1.4350278 |
| Ints4 | 0.8912959 |
| Ints5 | -0.4385896 |
| Ints7 | 1.2468436 |
| Ints9 | -0.4294974 |
| Ints9 | -0.4664133 |
| Ipmk | -0.7330676 |
| Ipmk | 2.1483097 |
| Ipo13 | 0.1179816 |
| Ipo4 | -0.6562128 |
| Ipo5 | 2.2032914 |
| Ipp | -0.6221873 |
| Ippk | -0.5786782 |
| Iqcb1 | 0.0332155 |
| Iqcb1 | 0.6675742 |
| Iqcd | 0.1051635 |
| Iqce | -0.1968057 |
| Iqcf3 | 0.4033058 |
| Iqcf4 | -0.6456099 |
| Iqgap1 | 0.5389554 |
| Iqgap2 | 0.8086038 |
| Iqgap3 | 2.344999 |
| Iqsec2 | 0.586714 |
| Iqsec3 | -0.4020676 |
| Irak1 | -0.7130126 |
| Irak1bp1 | -0.2474479 |
| Irak2 | 1.1450246 |
| Irak2 | 0.3332207 |
| Irak3 | 0.7308564 |
| Irak4 | 0.3627232 |
| Irak4 | -0.1477575 |
| Irak4 | -0.6153594 |
| Ireb2 | -0.6001777 |
| Irf1 | -0.5520888 |
| Irf1 | -0.7101827 |
| Irf1 | -0.6018524 |
| Irf1 | 0.1051635 |
| Irf2 | -0.5976714 |
| Irf2bp1 | -0.6896895 |
| Irf3 | 0.9722996 |
| Irf3 | 1.0274589 |
| Irf3 | 2.3028508 |
| Irf4 | -0.4336827 |
| Irf5 | 1.4976042 |
| Irf6 | -0.7359683 |
| Irf6 | 1.8726674 |
| Irf7 | 0.974141 |
| Irf8 | 0.2447053 |
| Irf9 | -0.0517065 |
| Irf9 | 2.5318283 |
| Irgm | 2.9787999 |
| Irgm | -0.6678377 |
| Irgq | 1.1314094 |
| Irgq | -0.5608676 |
| Irgq | -0.1239223 |
| Irs2 | 0.0672422 |
| Irs3 | 0.0277046 |
| Irx2 | -0.5648855 |
| Irx3 | -0.7206016 |
| Irx5 | 0.6245678 |
| Isca1 | -0.4577629 |
| Iscu | -0.5746002 |
| Isg20 | -0.0813555 |
| Isg20l1 | -0.1596485 |
| Isg20l2 | -0.7836573 |
| Isl1 | -0.2287621 |
| Islr2 | 2.0001953 |
| Isy1 | -0.5270131 |
| Isyna1 | 1.8038917 |
| Itfg2 | 0.238967 |
| Itfg3 | 1.9827867 |
| Itga1 | -0.6221873 |
| Itga11 | 0.4157022 |
| Itga11 | 0.7631648 |
| Itga2b | 1.3511265 |
| Itga3 | -0.5293354 |
| Itga5 | -0.4506154 |
| Itga5 | -0.1549773 |
| Itga6 | 1.8703365 |
| Itga7 | -0.2706137 |
| Itgad | 1.0091884 |
| Itgad | -0.769493 |
| Itgad | -0.4315877 |
| Itgae | 1.3823504 |
| Itgae | -0.6060522 |
| Itgae | 0.7885997 |
| Itgav | 1.6871619 |
| Itgb1 | 1.9774998 |
| Itgb1 | 0.5640965 |
| Itgb1bp1 | 0.50527 |
| Itgb3bp | -0.4627992 |
| Itgb4 | 0.9802526 |
| Itgb4 | -0.49137 |
| Itgb5 | -0.2503122 |
| Itgb6 | 4.3115889 |
| Itgb6 | 0.8943081 |
| Itgb6 | 1.8040797 |
| Itgb7 | -0.4671378 |
| Itgbl1 | -0.2998036 |
| Itih2 | -0.4108936 |
| Itih3 | 1.6211057 |
| Itih5 | 0.0420493 |
| Itih5 | -0.7585137 |
| Itk | -0.4958446 |
| Itlnb | 0.5198923 |
| Itm2a | 0.917082 |
| Itm2b | -0.2066059 |
| Itm2b | 0.4226725 |
| Itpk1 | -0.6696392 |
| Itpka | -0.5308868 |
| Itpkc | -0.5048592 |
| Itpr1 | -0.6500131 |
| Itpr2 | -0.642978 |
| Itpr3 | -0.3591546 |
| Itsn1 | 2.0738847 |
| Itsn2 | -0.5093996 |
| Ivd | -0.1680113 |
| Ivns1abp | -0.2360783 |
| Ivns1abp | 0.5433558 |
| Iws1 | -0.4129424 |
| Iyd | 1.1327024 |
| Izumo1 | 1.715512 |
| Jag1 | -0.652665 |
| Jag2 | -0.411576 |
| Jagn1 | -0.3324459 |
| Jak1 | -0.500341 |
| Jak2 | 0.7700669 |
| Jak2 | -0.459918 |
| Jak3 | 2.2425874 |
| Jak3 | 0.163522 |
| Jakmip1 | 0.0713077 |
| Jam2 | -0.4442294 |
| Jam2 | 0.7652792 |
| Jam2 | 2.7740468 |
| Jam2 | 3.2177298 |
| Jam3 | 0.0761629 |
| Jam4 | 0.3869965 |
| Jarid1b | 0.8973105 |
| Jarid1c | -0.5086413 |
| Jarid1d | 3.2916088 |
| Jazf1 | -0.6068945 |
| Jdp2 | -0.5116781 |
| Jmjd1a | -0.2115451 |
| Jmjd1a | -0.3274446 |
| Jmjd1a | -0.6204756 |
| Jmjd2a | -0.2170642 |
| Jmjd2b | 0.0453925 |
| Jmjd2b | -0.6094258 |
| Jmjd2c | -0.5347767 |
| Jmjd2d | -0.7026781 |
| Jmjd3 | 0.047892 |
| Jmjd3 | -0.1612108 |
| Jmjd5 | -0.6714441 |
| Jmjd6 | -0.5993416 |
| Jmy | 2.8667896 |
| Josd1 | -0.3193745 |
| Josd2 | -0.6162101 |
| Josd3 | -0.2400418 |
| Josd3 | 2.9485466 |
| Jph1 | -0.7139578 |
| Jph2 | -0.4891407 |
| Jph3 | 2.4032595 |
| Jph4 | 0.578062 |
| Jph4 | -0.4463532 |
| Jtb | 0.6350108 |
| Jtv1 | -0.6696392 |
| Jtv1 | -0.693382 |
| Jun | 2.0736035 |
| Junb | 0.0204482 |
| Jund1 | 2.04833 |
| Jup | -0.3052663 |
| Kal1 | 2.7458234 |
| Kalrn | -0.3387357 |
| Kank1 | -0.3349567 |
| Kank3 | -0.1400838 |
| Kank4 | 0.1055546 |
| Kank4 | 1.6369974 |
| Kank4 | -0.1822968 |
| Kars | 0.6602927 |
| Katna1 | -0.7073613 |
| Katnal1 | 1.9854705 |
| Katnb1 | -0.6010147 |
| Kazald1 | 1.0462279 |
| Kbtbd2 | -0.2440223 |
| Kbtbd2 | -0.4225635 |
| Kbtbd4 | -0.350166 |
| Kbtbd4 | -0.6624543 |
| Kbtbd7 | 0.1928045 |
| Kbtbd7 | -0.4995901 |
| Kbtbd8 | 0.4240603 |
| Kcmf1 | -0.4143107 |
| Kcna1 | -0.3643303 |
| Kcna2 | 3.9213955 |
| Kcna3 | 0.7153853 |
| Kcna5 | -0.3064847 |
| Kcna6 | 2.0569108 |
| Kcnab1 | -0.7644863 |
| Kcnab1 | -0.7684895 |
| Kcnab1 | 1.2805019 |
| Kcnab2 | -0.6290659 |
| Kcnab3 | 1.1369567 |
| Kcnb1 | -0.6832627 |
| Kcnc1 | -0.3393671 |
| Kcnc1 | 0.0378532 |
| Kcnc4 | -0.5528835 |
| Kcnc4 | 0.1806254 |
| Kcnd2 | -0.0470863 |
| Kcne1 | -0.6714441 |
| Kcne1l | -0.5147249 |
| Kcne2 | -0.3960061 |
| Kcne4 | -0.6980184 |
| Kcnf1 | -0.3150579 |
| Kcng4 | -0.6438545 |
| Kcnh1 | 2.6503084 |
| Kcnh2 | 1.8293618 |
| Kcnh2 | -0.5560689 |
| Kcnh3 | 0.2718964 |
| Kcnh5 | -0.1812311 |
| Kcnip1 | -0.5041047 |
| Kcnip2 | -0.642978 |
| Kcnip2 | 1.2351159 |
| Kcnip2 | -0.1431456 |
| Kcnip3 | 2.9162534 |
| Kcnip4 | -0.4788077 |
| Kcnj12 | -0.4883988 |
| Kcnj16 | 2.214592 |
| Kcnj2 | 1.5172744 |
| Kcnj3 | -0.8612359 |
| Kcnj4 | 0.1744754 |
| Kcnj6 | 0.3093635 |
| Kcnj6 | 1.7572344 |
| Kcnj8 | 1.0874247 |
| Kcnj9 | 1.9814937 |
| Kcnk1 | 0.4655535 |
| Kcnk10 | 0.689082 |
| Kcnk12 | -0.6588826 |
| Kcnk13 | -0.350166 |
| Kcnk2 | -0.6850944 |
| Kcnk2 | 0.9613294 |
| Kcnk3 | -0.4315877 |
| Kcnk4 | 1.274422 |
| Kcnk5 | 0.5890386 |
| Kcnk5 | 0.3823665 |
| Kcnk6 | 0.172295 |
| Kcnk6 | 0.0281297 |
| Kcnk9 | 1.7268863 |
| Kcnma1 | -0.1229215 |
| Kcnmb1 | -0.2131974 |
| Kcnmb4 | 1.6870197 |
| Kcnmb4 | 0.564335 |
| Kcnn1 | 2.00732 |
| Kcnn2 | -0.5433924 |
| Kcnn4 | 0.3927518 |
| Kcnq1 | 0.3444699 |
| Kcnq2 | -0.2554903 |
| Kcnq2 | 1.5796646 |
| Kcnq3 | 0.684066 |
| Kcnq5 | -0.5063702 |
| Kcnrg | -0.2665177 |
| Kcnrg | -0.0388274 |
| Kcns1 | 0.0152917 |
| Kcns2 | 2.7381391 |
| Kcnt1 | -0.2016929 |
| Kcnu1 | 0.2369333 |
| Kcnv1 | 0.6420542 |
| Kcnv2 | 0.0545247 |
| Kctd10 | -0.5656912 |
| Kctd11 | 0.6982081 |
| Kctd12 | -0.6102712 |
| Kctd12b | 1.5226369 |
| Kctd12b | 0.4271062 |
| Kctd14 | -0.6085813 |
| Kctd14 | -0.8021923 |
| Kctd15 | -0.4921142 |
| Kctd18 | 1.1132472 |
| Kctd19 | 0.7206382 |
| Kctd2 | 0.0148607 |
| Kctd20 | -0.4824849 |
| Kctd20 | -0.6768802 |
| Kctd3 | 0.3585655 |
| Kctd4 | -0.5616698 |
| Kctd5 | -0.0065079 |
| Kctd5 | 1.0891868 |
| Kctd6 | 1.1887338 |
| Kctd6 | 1.1139067 |
| Kctd9 | -0.4499037 |
| Kdelc1 | -0.579496 |
| Kdelc1 | 1.8299714 |
| Kdelr2 | -0.7684895 |
| Kdelr3 | 0.5547482 |
| Kdr | -0.7846774 |
| Keap1 | -0.3324459 |
| Keap1 | -0.1908673 |
| Keap1 | 0.3973304 |
| Keap1 | -0.7466825 |
| Kera | -0.1544597 |
| Khdc1c | 3.2482183 |
| Khdrbs1 | -0.3604458 |
| Khdrbs2 | -0.3437985 |
| Khdrbs3 | 0.4800272 |
| Khk | -0.3469768 |
| Khk | 0.5936704 |
| Khsrp | 0.7517409 |
| Kif11 | -0.6606667 |
| Kif13a | 2.2729438 |
| Kif14 | -0.3688829 |
| Kif15 | -0.106071 |
| Kif17 | 0.4836756 |
| Kif17 | -0.4156811 |
| Kif18a | -0.5379004 |
| Kif1a | 0.9418534 |
| Kif1b | -0.5901929 |
| Kif1b | 2.7464816 |
| Kif1b | 0.8312306 |
| Kif1b | 0.3024329 |
| Kif1b | -0.6204756 |
| Kif1b | 1.3848153 |
| Kif1c | 2.0047493 |
| Kif21a | -0.6111173 |
| Kif21b | -0.7282531 |
| Kif22 | 0.1264297 |
| Kif23 | 0.3653861 |
| Kif24 | 3.4646406 |
| Kif27 | -0.1385566 |
| Kif2a | -0.6915339 |
| Kif2b | 1.9649926 |
| Kif2c | -0.6282033 |
| Kif3a | -0.5505016 |
| Kif3a | 3.8625467 |
| Kif3b | 0.830336 |
| Kif3c | -0.4766084 |
| Kif3c | -0.7092413 |
| Kif4 | 0.0491393 |
| Kif4 | 0.2290955 |
| Kif4 | 1.0137532 |
| Kif5a | -0.8579166 |
| Kif5a | -0.2812301 |
| Kif5a | 2.6858875 |
| Kif5b | 1.6281771 |
| Kif5c | 0.6407376 |
| Kif9 | -0.7026781 |
| Kifap3 | 1.5930559 |
| Kifap3 | 0.6354526 |
| Kifap3 | 0.4016025 |
| Kifc2 | -0.7045486 |
| Kifc2 | 0.1766507 |
| Kifc3 | 0.070902 |
| Kin | -0.6869297 |
| Kirrel3 | -0.6333911 |
| Kiss1 | -0.4470622 |
| Kiss1r | 0.2335337 |
| Kitl | -0.7073613 |
| Kl | -0.3150579 |
| Klb | -0.5664976 |
| Klc1 | 1.9874268 |
| Klc1 | 0.8998051 |
| Klc1 | -0.7476627 |
| Klc2 | -0.0452446 |
| Klc2 | 0.3785875 |
| Klc4 | 2.6881375 |
| Klf1 | 1.057646 |
| Klf10 | -0.263603 |
| Klf12 | -0.5193139 |
| Klf12 | 0.0578234 |
| Klf12 | 0.7224496 |
| Klf13 | -0.8032329 |
| Klf16 | -0.366929 |
| Klf17 | -0.0021068 |
| Klf17 | 0.2909792 |
| Klf2 | 0.4567596 |
| Klf4 | -0.0808774 |
| Klf4 | 0.4983836 |
| Klf4 | -0.5270131 |
| Klf5 | -0.6660398 |
| Klf6 | 0.4162618 |
| Klf6 | -0.187108 |
| Klf7 | -0.6204756 |
| Klf9 | 1.0803426 |
| Klhdc1 | 1.5767068 |
| Klhdc1 | 0.2998998 |
| Klhdc1 | -0.7555417 |
| Klhdc2 | -0.8001144 |
| Klhdc3 | -0.7008114 |
| Klhdc4 | 2.2589266 |
| Klhdc4 | 1.1759962 |
| Klhdc4 | 0.1152774 |
| Klhdc8b | -0.8200442 |
| Klhdc9 | 0.0315234 |
| Klhl10 | -0.6491308 |
| Klhl11 | -0.6299293 |
| Klhl12 | -0.7206016 |
| Klhl14 | -0.2434527 |
| Klhl15 | 0.3591606 |
| Klhl15 | 0.7415544 |
| Klhl17 | 0.078581 |
| Klhl2 | -0.775537 |
| Klhl21 | 3.2400833 |
| Klhl22 | -0.407489 |
| Klhl22 | 0.7863502 |
| Klhl23 | 1.2002182 |
| Klhl24 | 0.9904003 |
| Klhl24 | 0.9412183 |
| Klhl25 | -0.5819536 |
| Klhl26 | -0.3476137 |
| Klhl26 | 0.4293151 |
| Klhl30 | 2.1981425 |
| Klhl31 | -0.5600661 |
| Klhl32 | 1.1951584 |
| Klhl5 | 0.4747901 |
| Klhl5 | -0.4824849 |
| Klhl6 | 3.5321853 |
| Klhl6 | -0.638608 |
| Klhl7 | -0.6869297 |
| Klhl8 | 0.3501939 |
| Klhl9 | 0.9785759 |
| Klhl9 | -0.6136602 |
| Klk1 | -0.8634554 |
| Klk11 | 1.2439829 |
| Klk11 | -0.6438545 |
| Klk15 | -0.2406094 |
| Klk1b1 | -0.6447318 |
| Klk1b11 | 0.0399536 |
| Klk1b16 | 0.328006 |
| Klk1b21 | 1.3923664 |
| Klk1b21 | -0.3773974 |
| Klk1b22 | -0.3793734 |
| Klk1b24 | -0.3181391 |
| Klk1b26 | 1.6381221 |
| Klk1b27 | -0.6906112 |
| Klk1b27 | -0.7008114 |
| Klk1b4 | -0.8084538 |
| Klk1b4 | 1.7729842 |
| Klk1b5 | -0.6860116 |
| Klk1b8 | -0.1539425 |
| Klk1b9 | 0.6762795 |
| Klk6 | -0.2895722 |
| Klk8 | -0.549709 |
| Klkb1 | -0.268271 |
| Klra17 | -0.4534678 |
| Klra4 | -0.6924575 |
| Klrd1 | -0.4980901 |
| Kndc1 | 1.0538556 |
| Kng1 | -0.0158186 |
| Kng1 | -0.4315877 |
| Kng2 | 0.9765905 |
| Kns2 | 0.7113242 |
| Kns2 | -0.5584651 |
| Kntc1 | 1.3567254 |
| Kpna1 | 0.6092478 |
| Kpna2 | -0.6814346 |
| Kpna3 | -0.711125 |
| Kpna6 | 0.3149957 |
| Kpnb1 | 0.0061982 |
| Kptn | -0.2630212 |
| Kras | 1.8168996 |
| Kras | -0.7008114 |
| Krba1 | -0.5324408 |
| Krba1 | 1.8442428 |
| Krba1 | -0.5943402 |
| Krcc1 | -0.7139578 |
| Kremen | 3.4265562 |
| Kremen1 | -0.7684895 |
| Kremen2 | -0.7045486 |
| Krit1 | -0.5926791 |
| Krit1 | -0.3585097 |
| Krr1 | 0.0725239 |
| Krr1 | 0.0428862 |
| Krr1 | -0.3212306 |
| Krt10 | 3.2769042 |
| Krt12 | -0.4606374 |
| Krt13 | 1.0097787 |
| Krt14 | -0.3064847 |
| Krt17 | -0.6579918 |
| Krt18 | -0.5010925 |
| Krt19 | 0.5477354 |
| Krt2 | -0.6723479 |
| Krt20 | 0.7094909 |
| Krt222 | -0.7321027 |
| Krt23 | -0.1743333 |
| Krt27 | -0.1544597 |
| Krt32 | 2.1290424 |
| Krt4 | 2.0639934 |
| Krt7 | -0.3946644 |
| Krt73 | 2.3774788 |
| Krt75 | 0.2263537 |
| Krt78 | -0.6906112 |
| Krt79 | -0.5489171 |
| Krt8 | -0.6777893 |
| Krt84 | -0.6128118 |
| Krt86 | -0.5951719 |
| Krt86 | 1.4890196 |
| Krt9 | -0.4577629 |
| Krtap16-4 | 0.0361695 |
| Krtap8-2 | -0.6732526 |
| Krtcap2 | 0.0407925 |
| Krtcap3 | 1.1712982 |
| Ksr2 | -0.4928591 |
| Ktelc1 | 0.4610337 |
| Kti12 | 1.3900194 |
| Ktn1 | 0.8227875 |
| Ktn1 | -0.7644863 |
| Ky | -0.3040496 |
| L1cam | 2.562129 |
| L2hgdh | 0.848592 |
| L3mbtl2 | 1.0957986 |
| L3mbtl3 | -0.8285654 |
| l7Rn6 | 1.3616853 |
| Lace1 | 1.3299548 |
| Lad1 | -0.7496262 |
| Lag3 | 2.8224133 |
| Lage3 | 0.482895 |
| Lama1 | -0.5811337 |
| Lama2 | 0.6037797 |
| Lamb1-1 | -0.6759719 |
| Lamb2 | 2.1716652 |
| Lamb3 | -0.6850944 |
| Lamc1 | 1.9321206 |
| Lamc2 | -0.462078 |
| Lamc2 | 1.7934919 |
| Lamc2 | 0.5450614 |
| Lamc3 | 0.2173866 |
| Lamp1 | 0.5597936 |
| Lamp2 | -0.1648664 |
| Lamp2 | -0.7215546 |
| Lamp2 | -0.1978895 |
| Lamp2 | -0.3853269 |
| Lancl1 | -0.5844177 |
| Lancl1 | -0.1249242 |
| Lancl1 | 0.4981275 |
| Laptm4a | -0.6878487 |
| Laptm4b | -0.6980184 |
| Laptm4b | -0.4267176 |
| Laptm5 | 0.8038272 |
| Large | -0.5844177 |
| Larp2 | 1.9631996 |
| Larp5 | 1.0047495 |
| Larp6 | 0.1004581 |
| Larp6 | -0.3893172 |
| Lars | 0.5989686 |
| Lars2 | -0.5528835 |
| Las1l | -0.5960043 |
| Lasp1 | 0.7066319 |
| Lasp1 | -0.2572228 |
| Lasp1 | -0.6723479 |
| Lass1 | 0.8394203 |
| Lass2 | -0.1817638 |
| Lass2 | -0.5426058 |
| Lass2 | -0.5860641 |
| Lass4 | 0.0781784 |
| Lass4 | -0.5673047 |
| Lass5 | -0.0073906 |
| Lass6 | -0.1229215 |
| Lat2 | -0.6943074 |
| Lat2 | -0.4613574 |
| Lats2 | -0.6970893 |
| Lbh | -0.1209231 |
| Lbh | -0.3305672 |
| Lbp | -0.7535657 |
| Lbr | -0.7225085 |
| Lbx2 | -0.5512949 |
| Lca5 | 1.0879672 |
| Lca5 | -0.4198044 |
| Lca5 | 0.5263731 |
| Lcat | -0.0938733 |
| Lcat | 0.3319963 |
| Lce1c | 0.0470596 |
| Lck | -0.5512949 |
| Lcmt1 | -0.0929043 |
| Lcmt1 | 1.5658342 |
| Lcmt1 | -0.7253764 |
| Lcn10 | -0.6651421 |
| Lcn12 | 1.4579681 |
| Lcn13 | 1.1941915 |
| Lcn2 | -0.184432 |
| Lcn3 | -0.5673047 |
| Lcn8 | -0.0038647 |
| Lcor | 0.6967622 |
| Lcp1 | -0.4700416 |
| Ldb1 | -0.5560689 |
| Ldb2 | 0.8487675 |
| Ldb2 | 0.6110633 |
| Ldha | -0.568921 |
| Ldhc | -0.6282033 |
| Ldhc | -0.2747279 |
| Ldhc | -0.7130126 |
| Ldhd | 1.2081139 |
| Ldhd | -0.6204756 |
| Ldoc1 | 2.6233924 |
| Ldoc1l | 3.1430654 |
| Lef1 | -0.6544372 |
| Lef1 | 0.165723 |
| Lefty1 | 0.5876446 |
| Lefty2 | -0.5293354 |
| Lemd2 | 2.8751629 |
| Leng1 | 2.949331 |
| Leng8 | 2.0433079 |
| Leng9 | -0.6669383 |
| Leo1 | 0.0627495 |
| Lep | 1.4979529 |
| Lepre1 | -0.2735506 |
| Lepre1 | -0.4802768 |
| Lepre1 | 2.100759 |
| Leprel1 | -0.380033 |
| Leprel2 | -0.1780411 |
| Leprotl1 | 1.3246118 |
| Letm1 | 1.0763694 |
| Letm1 | 3.0281951 |
| Letm1 | 1.5390412 |
| Letmd1 | 0.9893453 |
| Lgals1 | -0.1365243 |
| Lgals2 | 2.8008165 |
| Lgals3 | -0.7139578 |
| Lgals3bp | 0.7054039 |
| Lgals4 | -0.5433924 |
| Lgals4 | 2.1889591 |
| Lgals6 | -0.6001777 |
| Lgals7 | -0.5608676 |
| Lgals8 | -0.4693148 |
| Lgals8 | 0.4443607 |
| Lgals9 | 3.7638816 |
| Lgals9 | 0.279058 |
| Lgi1 | 1.5822151 |
| Lgi1 | 1.0944531 |
| Lgi2 | -0.6660398 |
| Lgi3 | -0.6805219 |
| Lgi4 | -0.4707689 |
| Lgmn | 1.9203098 |
| Lgr5 | 2.2962016 |
| Lgtn | 0.221881 |
| Lgtn | -0.4627992 |
| Lhb | -0.4456447 |
| Lhcgr | 1.0284715 |
| Lhcgr | -0.3126003 |
| Lhfp | 2.8135689 |
| Lhfp | -0.4007171 |
| Lhfp | 0.1202933 |
| Lhfpl1 | 1.5031679 |
| Lhfpl2 | -0.7036129 |
| Lhx1 | 2.3539453 |
| Lhx2 | -0.7244194 |
| Lhx3 | -0.4591991 |
| Lhx4 | -0.8469372 |
| Lhx9 | -0.6998795 |
| Lhx9 | 0.1598419 |
| Lias | 1.9576933 |
| Lias | 1.7634093 |
| Lias | 3.1191109 |
| Lifr | -0.4898832 |
| Lig1 | -0.8095015 |
| Lig1 | -0.1627757 |
| Lig1 | 1.1610784 |
| Lig3 | -0.6535507 |
| Lig4 | -0.0131489 |
| Lima1 | -0.4253308 |
| Lima1 | 0.7395826 |
| Limd1 | -0.8063619 |
| Limd2 | -0.7017443 |
| Lime1 | 0.0627495 |
| Limk1 | -0.8074073 |
| Limk2 | -0.5819536 |
| Limk2 | 1.9267557 |
| Lims2 | 1.8563454 |
| Lims2 | -0.0977591 |
| Lims2 | -0.3701877 |
| Lin54 | -0.6482493 |
| Lin7b | 1.5099039 |
| Lin7b | 1.0379648 |
| Lin7b | -0.1539425 |
| Lin7b | -0.3899839 |
| Lin7c | -0.6732526 |
| Lincr | 1.940002 |
| Lincr | -0.7535657 |
| Lingo1 | -0.5943402 |
| Lingo2 | -0.8735093 |
| Lingo2 | 1.0443822 |
| Lingo4 | -0.7083008 |
| Lip1 | -0.0038647 |
| Lipe | -0.497341 |
| Lipf | 0.4513891 |
| Liph | 1.4456932 |
| Liph | -0.4253308 |
| Litaf | 1.8869322 |
| Lix1 | -0.3754255 |
| Lix1 | -0.652665 |
| Lix1 | -0.2445924 |
| Lix1 | 1.6883695 |
| Llgl1 | 0.3967593 |
| Llgl2 | 0.0676496 |
| Lman1 | 0.8561084 |
| Lman2 | 0.3307704 |
| Lman2l | -0.6705412 |
| Lman2l | 1.3102114 |
| Lmbr1 | -0.6906112 |
| Lmbr1l | -0.7017443 |
| Lmcd1 | -0.6102712 |
| Lmcd1 | -0.638608 |
| Lmcd1 | -0.5308868 |
| Lmf1 | -0.1189291 |
| Lmf2 | -0.6796102 |
| Lmna | 3.0658108 |
| Lmna | 0.6849039 |
| Lmna | -0.3714944 |
| Lmna | -0.5371185 |
| Lmna | -0.6447318 |
| Lmnb1 | -0.7026781 |
| Lmnb1 | -0.7092413 |
| Lmnb2 | -0.7634882 |
| Lmo2 | 0.9555607 |
| Lmo3 | -0.1006842 |
| Lmo3 | -0.4371849 |
| Lmo4 | -0.6998795 |
| Lmod1 | 1.9656246 |
| Lmtk2 | 0.2372725 |
| Lmtk3 | 0.7131539 |
| Lmx1a | -0.4995901 |
| Lmx1b | -0.2560674 |
| Lnx1 | -0.219836 |
| Lnx2 | 0.5198923 |
| Lnx2 | -0.4329838 |
| LOC100038882 | -0.4995901 |
| LOC100038984 | -0.1539425 |
| LOC100039087 | -0.1680113 |
| LOC100039227 | 2.7045205 |
| LOC100039479 | -0.6732526 |
| LOC100039532 | -0.219281 |
| LOC100039571 | -0.6377365 |
| LOC100039728 | -0.7398501 |
| LOC100040573 | -0.4184279 |
| LOC100040592 | 2.087973 |
| LOC100040601 | -0.5868884 |
| LOC100040799 | -0.2583796 |
| LOC100040899 | 0.5987388 |
| LOC100040919 | -0.1780411 |
| LOC100041103 | -0.6759719 |
| LOC100041290 | 0.3895908 |
| LOC100041463 | 0.048308 |
| LOC100041483 | 1.3236657 |
| LOC100041500 | -0.520081 |
| LOC100041504 | 0.5768866 |
| LOC100041703 | -0.1259272 |
| LOC100041722 | 1.9694077 |
| LOC100041725 | -0.6832627 |
| LOC100041835 | 2.6333918 |
| LOC100042179 | 0.2567411 |
| LOC100042427 | -0.5624727 |
| LOC100042777 | -0.7330676 |
| LOC100043257 | -0.5056144 |
| LOC100043391 | -0.407489 |
| LOC100043671 | 3.4100351 |
| LOC100043822 | 3.0820411 |
| LOC100043986 | 0.0412116 |
| LOC100044087 | 0.3002169 |
| LOC100044089 | 3.5536024 |
| LOC100044101 | 0.1568873 |
| LOC100044103 | -0.6316586 |
| LOC100044103 | -0.5640805 |
| LOC100044122 | -0.4707689 |
| LOC100044133 | 1.1460441 |
| LOC100044159 | -0.1259272 |
| LOC100044170 | 2.6498552 |
| LOC100044172 | -0.2919694 |
| LOC100044177 | -0.5844177 |
| LOC100044190 | -0.4122589 |
| LOC100044204 | -0.1508448 |
| LOC100044210 | -0.0270261 |
| LOC100044221 | -0.6714441 |
| LOC100044257 | 1.9994849 |
| LOC100044275 | -0.6359959 |
| LOC100044289 | 3.1453859 |
| LOC100044292 | -0.1254255 |
| LOC100044294 | 0.3832364 |
| LOC100044298 | 0.7852233 |
| LOC100044322 | -0.6615601 |
| LOC100044324 | 1.0371058 |
| LOC100044324 | -0.7073613 |
| LOC100044363 | -0.1643433 |
| LOC100044363 | -0.3741133 |
| LOC100044439 | 1.4184638 |
| LOC100044468 | 0.4548835 |
| LOC100044475 | 1.5741411 |
| LOC100044538 | 1.1688159 |
| LOC100044566 | 0.0378532 |
| LOC100044576 | -0.5951719 |
| LOC100044636 | 0.8993067 |
| LOC100044692 | -0.2901709 |
| LOC100044708 | 2.1614475 |
| LOC100044736 | 0.4588991 |
| LOC100044756 | -0.3565775 |
| LOC100044776 | 0.5148761 |
| LOC100044779 | 1.7062275 |
| LOC100044812 | -0.6077375 |
| LOC100044829 | -0.0225269 |
| LOC100044862 | 0.9950582 |
| LOC100044873 | 0.446529 |
| LOC100044934 | 1.5887189 |
| LOC100045005 | -0.0958141 |
| LOC100045019 | -0.8404114 |
| LOC100045040 | -0.0424889 |
| LOC100045098 | 2.8448872 |
| LOC100045228 | 1.7588806 |
| LOC100045241 | 0.391029 |
| LOC100045250 | -0.6823482 |
| LOC100045280 | 1.1696854 |
| LOC100045300 | -0.3381048 |
| LOC100045304 | -0.5223859 |
| LOC100045343 | -0.4520405 |
| LOC100045359 | 0.6021787 |
| LOC100045359 | -0.5673047 |
| LOC100045403 | -0.0475473 |
| LOC100045413 | 2.647182 |
| LOC100045419 | 1.5009117 |
| LOC100045430 | -0.4714968 |
| LOC100045439 | 1.7977289 |
| LOC100045491 | 0.208332 |
| LOC100045505 | -0.5324408 |
| LOC100045522 | 0.0733338 |
| LOC100045542 | 0.6223307 |
| LOC100045551 | -0.5124389 |
| LOC100045567 | -0.331193 |
| LOC100045607 | 1.7693482 |
| LOC100045617 | -0.6052107 |
| LOC100045644 | 0.4210044 |
| LOC100045697 | 0.3292356 |
| LOC100045780 | 0.0361695 |
| LOC100045782 | 1.8194917 |
| LOC100045877 | 0.2780846 |
| LOC100045882 | -0.6651421 |
| LOC100045887 | -0.7064228 |
| LOC100045950 | 0.1903101 |
| LOC100045963 | 1.0525887 |
| LOC100045981 | 0.2793822 |
| LOC100046003 | 0.8117136 |
| LOC100046035 | -0.1876441 |
| LOC100046035 | 2.1033496 |
| LOC100046039 | 0.1256649 |
| LOC100046044 | -0.6394804 |
| LOC100046049 | 0.5664784 |
| LOC100046056 | 0.867356 |
| LOC100046068 | 1.0541369 |
| LOC100046080 | -0.6678377 |
| LOC100046081 | 0.0849986 |
| LOC100046120 | -0.4810123 |
| LOC100046128 | 0.8892823 |
| LOC100046129 | 0.9074137 |
| LOC100046163 | -0.416367 |
| LOC100046163 | -0.6026908 |
| LOC100046207 | -0.7272932 |
| LOC100046207 | -0.6482493 |
| LOC100046207 | -0.1179336 |
| LOC100046232 | -0.5270131 |
| LOC100046254 | 1.2166515 |
| LOC100046320 | 1.1720415 |
| LOC100046393 | -0.2770871 |
| LOC100046406 | -0.2137488 |
| LOC100046457 | 0.1878089 |
| LOC100046518 | -0.6915339 |
| LOC100046568 | 1.0463698 |
| LOC100046586 | 3.0951605 |
| LOC100046608 | 1.506542 |
| LOC100046608 | 0.1708386 |
| LOC100046608 | 0.2273829 |
| LOC100046608 | 1.4742051 |
| LOC100046616 | -0.4357825 |
| LOC100046641 | 0.6510011 |
| LOC100046650 | 0.8837224 |
| LOC100046690 | 3.4716975 |
| LOC100046690 | 0.0897829 |
| LOC100046741 | 0.6090206 |
| LOC100046744 | -0.6077375 |
| LOC100046744 | -0.6018524 |
| LOC100046746 | 1.4490069 |
| LOC100046775 | 1.4894595 |
| LOC100046781 | -0.7177484 |
| LOC100046781 | -0.1105017 |
| LOC100046792 | -0.5893657 |
| LOC100046796 | 0.0692772 |
| LOC100046802 | 1.3249269 |
| LOC100046841 | -0.6651421 |
| LOC100046855 | -0.7939088 |
| LOC100046883 | 0.3727434 |
| LOC100046891 | 1.0871533 |
| LOC100046898 | -0.60353 |
| LOC100046918 | -0.7330676 |
| LOC100046953 | -0.5208487 |
| LOC100046959 | -0.876885 |
| LOC100046996 | 0.9281017 |
| LOC100047009 | -0.7244194 |
| LOC100047009 | 0.2603878 |
| LOC100047012 | -0.7263343 |
| LOC100047028 | -0.6796102 |
| LOC100047046 | 1.7124736 |
| LOC100047052 | 1.4175137 |
| LOC100047052 | 1.2758626 |
| LOC100047155 | -0.7253764 |
| LOC100047155 | -0.572162 |
| LOC100047167 | -0.4627992 |
| LOC100047173 | -0.3643303 |
| LOC100047184 | 1.6882275 |
| LOC100047200 | 0.1359373 |
| LOC100047214 | 1.0614211 |
| LOC100047214 | -0.3280683 |
| LOC100047238 | 2.3220512 |
| LOC100047261 | 2.2434854 |
| LOC100047323 | -0.8232305 |
| LOC100047323 | 1.8438226 |
| LOC100047339 | 0.013998 |
| LOC100047353 | 3.5707641 |
| LOC100047368 | 1.1272596 |
| LOC100047369 | 1.0895929 |
| LOC100047369 | -0.4936046 |
| LOC100047391 | 0.4378254 |
| LOC100047393 | -0.1375399 |
| LOC100047427 | -0.6500131 |
| LOC100047480 | 1.7858545 |
| LOC100047490 | 1.9025039 |
| LOC100047579 | 0.8843981 |
| LOC100047583 | -0.6316586 |
| LOC100047606 | -0.7795883 |
| LOC100047615 | -0.0770615 |
| LOC100047619 | -0.3381048 |
| LOC100047634 | 1.7564431 |
| LOC100047651 | -0.7634882 |
| LOC100047653 | 1.744693 |
| LOC100047659 | -0.5656912 |
| LOC100047674 | 0.283264 |
| LOC100047707 | -0.4642431 |
| LOC100047717 | 1.6897173 |
| LOC100047749 | 0.3916036 |
| LOC100047762 | -0.6943074 |
| LOC100047776 | -0.775537 |
| LOC100047794 | -0.0558845 |
| LOC100047800 | 0.8378237 |
| LOC100047810 | -0.6412275 |
| LOC100047815 | -0.8221672 |
| LOC100047816 | -0.7664857 |
| LOC100047834 | -0.3218501 |
| LOC100047837 | 1.0819817 |
| LOC100047837 | 0.1351803 |
| LOC100047841 | -0.3305672 |
| LOC100047856 | 0.9391511 |
| LOC100047870 | -0.6001777 |
| LOC100047911 | -0.2865843 |
| LOC100047934 | 2.1396649 |
| LOC100047935 | -0.7408231 |
| LOC100047937 | 2.3123558 |
| LOC100047937 | 0.0821964 |
| LOC100047958 | 0.399896 |
| LOC100047963 | -0.5270131 |
| LOC100047963 | 0.4326187 |
| LOC100047998 | -0.6508962 |
| LOC100048046 | 0.0148607 |
| LOC100048046 | -0.2276416 |
| LOC100048049 | -0.6394804 |
| LOC100048049 | -0.8712657 |
| LOC100048049 | 0.8191713 |
| LOC100048056 | 2.5612705 |
| LOC100048071 | -0.4707689 |
| LOC100048076 | -0.5713507 |
| LOC100048083 | -0.1070538 |
| LOC100048105 | 2.402405 |
| LOC100048105 | 0.4724237 |
| LOC100048187 | 1.8355609 |
| LOC100048251 | -0.456329 |
| LOC100048295 | 0.2633608 |
| LOC100048299 | -0.2671018 |
| LOC100048301 | 1.0838904 |
| LOC100048313 | -0.693382 |
| LOC100048331 | 0.5991982 |
| LOC100048332 | 1.9443219 |
| LOC100048346 | 0.9647425 |
| LOC100048372 | 2.1803524 |
| LOC100048384 | -0.2753172 |
| LOC100048413 | -0.1380481 |
| LOC100048436 | -0.4225635 |
| LOC100048445 | 1.2023782 |
| LOC100048461 | -0.4068096 |
| LOC100048480 | 2.4001994 |
| LOC100048483 | -0.6221873 |
| LOC100048504 | 0.1415955 |
| LOC100048530 | -0.1085299 |
| LOC100048554 | 2.9295555 |
| LOC100048556 | -0.380033 |
| LOC100048589 | 1.5296547 |
| LOC100048613 | 0.8748932 |
| LOC100048622 | 2.4294935 |
| LOC100048645 | 0.3771299 |
| LOC100048684 | 0.4315188 |
| LOC100048703 | 2.1976096 |
| LOC100048710 | 2.7755874 |
| LOC100048721 | -0.6588826 |
| LOC100048721 | -0.5592652 |
| LOC100048726 | 0.6804965 |
| LOC100048733 | -0.8524106 |
| LOC100048816 | -0.4685885 |
| LOC100048832 | 0.7272628 |
| LOC100048832 | -0.2606975 |
| LOC100048858 | 0.0977022 |
| LOC192758 | 0.7715937 |
| LOC194227 | -0.5293354 |
| LOC212390 | -0.7970064 |
| LOC216443 | -0.2653508 |
| LOC216963 | -0.6377365 |
| LOC217341 | 2.814961 |
| LOC218963 | -0.4239461 |
| LOC225922 | -0.7282531 |
| LOC233529 | -0.7725101 |
| LOC234374 | -0.6579918 |
| LOC235497 | -0.464966 |
| LOC236749 | -0.5616698 |
| LOC237408 | 0.8838914 |
| LOC237877 | 0.9353228 |
| LOC238829 | -0.6111173 |
| LOC241593 | -0.5819536 |
| LOC244595 | 1.1961243 |
| LOC245263 | -0.5568669 |
| LOC329575 | 0.2277256 |
| LOC329575 | 0.8041955 |
| LOC333331 | 2.9895135 |
| LOC381066 | -0.7525792 |
| LOC381302 | 0.9630375 |
| LOC381375 | -0.3144429 |
| LOC381483 | 0.3264667 |
| LOC381629 | 2.2641315 |
| LOC381801 | 0.6505667 |
| LOC381936 | -0.3741133 |
| LOC382010 | 0.9987976 |
| LOC382044 | 1.4572398 |
| LOC382106 | -0.3355855 |
| LOC382106 | -0.6239022 |
| LOC382133 | -0.263603 |
| LOC384806 | -0.6316586 |
| LOC384806 | -0.7765481 |
| LOC386486 | 1.5648615 |
| LOC433479 | -0.6823482 |
| LOC433801 | 3.2083982 |
| LOC433886 | -0.729214 |
| LOC434172 | 0.7066319 |
| LOC434172 | -0.6624543 |
| LOC434864 | -0.7785738 |
| LOC434960 | 3.2096938 |
| LOC435023 | -0.4995901 |
| LOC435337 | -0.8063619 |
| LOC435565 | -0.5512949 |
| LOC436177 | -0.8296361 |
| LOC544988 | -0.3243324 |
| LOC544988 | -0.1436569 |
| LOC545013 | 0.8058511 |
| LOC545013 | -0.729214 |
| LOC545056 | -0.5544748 |
| LOC545238 | -0.3393671 |
| LOC545703 | 0.8835534 |
| LOC545732 | 2.7302522 |
| LOC545952 | 0.0264282 |
| LOC546015 | -0.005626 |
| LOC546015 | -0.2776778 |
| LOC546723 | 1.558596 |
| LOC546840 | 1.6547584 |
| LOC547323 | 0.4519276 |
| LOC547343 | -0.8404114 |
| LOC620807 | -0.572974 |
| LOC621823 | -0.0799219 |
| LOC621824 | 0.2409964 |
| LOC622404 | -0.89395 |
| LOC622665 | 2.2236459 |
| LOC624855 | -0.6571019 |
| LOC625480 | 0.4284874 |
| LOC626152 | -0.7311388 |
| LOC628053 | 1.6658071 |
| LOC631002 | -0.1680113 |
| LOC631784 | -0.6714441 |
| LOC632431 | 1.7736967 |
| LOC639396 | -0.3598 |
| LOC640963 | -0.4392927 |
| LOC641240 | -0.4861767 |
| LOC654426 | 1.2448993 |
| LOC654467 | 1.0140469 |
| LOC654494 | -0.5010925 |
| LOC654842 | -0.2503122 |
| LOC665250 | -0.4591991 |
| LOC666676 | -0.4810123 |
| LOC666904 | 2.3460827 |
| LOC667250 | 0.9156137 |
| LOC667370 | 1.2411133 |
| LOC667609 | 0.3359695 |
| LOC668492 | 0.3961879 |
| LOC668837 | 2.6061843 |
| LOC669168 | -0.2468761 |
| LOC669660 | 0.2725497 |
| LOC670044 | -0.3734579 |
| LOC671564 | 1.4371707 |
| LOC672705 | 3.2298705 |
| LOC674706 | 0.6654385 |
| LOC675228 | 0.4052891 |
| LOC675567 | -0.3305672 |
| LOC675567 | 2.5134296 |
| LOC675813 | 3.1993201 |
| LOC676420 | 0.5557114 |
| LOC676420 | -0.4399963 |
| LOC677144 | -0.1680113 |
| LOC677205 | 1.6113613 |
| LOC677317 | -0.3893172 |
| LOC677319 | 1.1569336 |
| LOC677528 | 0.884567 |
| LOC732482 | 0.3027491 |
| Loh11cr2a | 0.3365793 |
| Loh12cr1 | 1.8327096 |
| Loh12cr1 | -0.0324542 |
| Lonp2 | -0.4267176 |
| Lonrf1 | 1.8773116 |
| Lonrf2 | -0.5162521 |
| Lor | -0.3450685 |
| Lox | 0.4602338 |
| Loxl1 | -0.5223859 |
| Lpar2 | 0.5453049 |
| Lpar2 | 1.2063253 |
| Lpar3 | -0.3028345 |
| Lpcat2 | -0.5592652 |
| Lpcat2 | 2.9870305 |
| Lpcat2 | -0.7215546 |
| Lpgat1 | 1.8698696 |
| Lphn1 | -0.7359683 |
| Lphn3 | -0.233821 |
| Lpin1 | -0.4000427 |
| Lpin1 | -0.6464889 |
| Lpin2 | 2.1832302 |
| Lpin3 | 1.5906139 |
| Lpl | -0.7054852 |
| Lpxn | -0.6759719 |
| Lrat | -0.6796102 |
| Lrba | 2.2548879 |
| Lrba | 1.1303737 |
| Lrch1 | 0.5186408 |
| Lrch4 | 2.4413921 |
| Lrch4 | 1.6108984 |
| Lrdd | -0.714904 |
| Lrfn1 | -0.6814346 |
| Lrfn2 | -0.1806986 |
| Lrfn3 | -0.3425302 |
| Lrfn4 | 2.3352943 |
| Lrg1 | -0.6970893 |
| Lrig1 | 0.2761349 |
| Lrig3 | 1.946098 |
| Lrmp | -0.7555417 |
| Lrp1 | -0.3846635 |
| Lrp10 | -0.6660398 |
| Lrp11 | -0.5528835 |
| Lrp12 | 0.579236 |
| Lrp12 | -0.7735179 |
| Lrp4 | -0.7083008 |
| Lrp5 | 0.7997663 |
| Lrp8 | -0.1482713 |
| Lrp8 | -0.4584807 |
| Lrpap1 | -0.6553246 |
| Lrpprc | -0.4846982 |
| Lrpprc | -0.5285606 |
| Lrpprc | -0.4315877 |
| Lrrc1 | -0.2531853 |
| Lrrc1 | -0.693382 |
| Lrrc1 | 1.1637064 |
| Lrrc10 | -0.7036129 |
| Lrrc15 | 0.9157769 |
| Lrrc16b | 1.5462675 |
| Lrrc16b | -0.3399988 |
| Lrrc18 | 2.4600253 |
| Lrrc2 | 1.6797399 |
| Lrrc20 | 1.6799548 |
| Lrrc20 | -0.0948432 |
| Lrrc23 | -0.6368658 |
| Lrrc23 | 0.3712766 |
| Lrrc24 | 0.6587864 |
| Lrrc26 | 0.987836 |
| Lrrc28 | -0.1973475 |
| Lrrc29 | 0.7234544 |
| Lrrc3 | 0.9032863 |
| Lrrc30 | 1.8175174 |
| Lrrc33 | -0.6102712 |
| Lrrc34 | 1.4070943 |
| Lrrc34 | 0.9873827 |
| Lrrc36 | 0.4652882 |
| Lrrc39 | -0.5926791 |
| Lrrc39 | 0.6656523 |
| Lrrc4 | -0.2595378 |
| Lrrc40 | -0.4414052 |
| Lrrc41 | -0.6517802 |
| Lrrc43 | 0.8742105 |
| Lrrc44 | -0.7350004 |
| Lrrc45 | -0.4854371 |
| Lrrc46 | 0.1098452 |
| Lrrc48 | -0.7715033 |
| Lrrc48 | 2.4320128 |
| Lrrc48 | 0.6635122 |
| Lrrc49 | 1.5934491 |
| Lrrc49 | 3.1928911 |
| Lrrc49 | 2.9483823 |
| Lrrc4c | 0.9321203 |
| Lrrc50 | 3.4829377 |
| Lrrc50 | -0.5754144 |
| Lrrc52 | -0.5481258 |
| Lrrc52 | 0.9750603 |
| Lrrc56 | 0.3402295 |
| Lrrc57 | 0.0532847 |
| Lrrc59 | -0.3077046 |
| Lrrc6 | -0.5254682 |
| Lrrc7 | 1.0803426 |
| Lrrc8 | -0.6085813 |
| Lrrc8a | 2.2925815 |
| Lrrc8b | -0.1764502 |
| Lrrc8c | 0.3489918 |
| Lrrc8d | -0.3946644 |
| Lrrc9 | 0.2970418 |
| Lrrfip1 | 0.507047 |
| Lrrfip1 | 1.6015135 |
| Lrriq2 | -0.3980222 |
| Lrriq2 | 2.0523187 |
| Lrrk1 | 0.734833 |
| Lrrk2 | -0.2836059 |
| Lrrn1 | 1.4455088 |
| Lrrn2 | -0.421873 |
| Lrrn3 | 1.0754078 |
| Lrrtm1 | -0.4627992 |
| Lrrtm2 | -0.5624727 |
| Lrrtm3 | 1.1586943 |
| Lrsam1 | -0.1941017 |
| Lrsam1 | -0.4627992 |
| Lrtm1 | 0.4449033 |
| Lrtm1 | 0.3698075 |
| Lsamp | -0.2209471 |
| Lsamp | -0.3187566 |
| Lsg1 | -0.6256202 |
| Lsg1 | 0.0306761 |
| Lsm10 | 0.9546218 |
| Lsm11 | 0.5545073 |
| Lsm12 | 3.5602617 |
| Lsm12 | -0.6705412 |
| Lsm12 | 0.3507944 |
| Lsm14a | 1.2839114 |
| Lsm14b | -0.6732526 |
| Lsm14b | -0.1881805 |
| Lsm14b | 0.3970449 |
| Lsm2 | 1.7176467 |
| Lsm2 | 1.2234723 |
| Lsm3 | -0.5811337 |
| Lsm4 | 2.5256879 |
| Lsm5 | -0.3780556 |
| Lsm6 | 1.2845698 |
| Lsm6 | 1.1506181 |
| Lsm8 | -0.3169054 |
| Lsr | -0.8656802 |
| Lst1 | -0.6805219 |
| Ltb4dh | -0.4274118 |
| Ltb4r1 | -0.6924575 |
| Ltb4r2 | 1.4536812 |
| Ltbp1 | 0.4356366 |
| Ltbp1 | 0.7064274 |
| Ltbp2 | -0.5379004 |
| Ltbp3 | 2.3883581 |
| Ltbp3 | -0.4722253 |
| Ltbp4 | -0.5584651 |
| Ltbp4 | -0.5835956 |
| Ltbp4 | 2.3825314 |
| Ltbr | 2.3134783 |
| Ltc4s | -0.5968375 |
| Ltf | 0.2630309 |
| Ltf | -0.4714968 |
| Ltk | 0.1715671 |
| Ltk | -0.3859907 |
| Ltv1 | -0.0279286 |
| Luc7l | -0.2706137 |
| Luc7l | 0.0797877 |
| Luc7l2 | 0.1363156 |
| Luc7l2 | -0.1421239 |
| Lum | 1.6784493 |
| Luzp1 | 2.0810253 |
| Luzp1 | -0.8105504 |
| Luzp1 | -0.6786993 |
| Luzp2 | 0.0183031 |
| Lxn | -0.4577629 |
| Ly6a | 1.7805992 |
| Ly6c1 | -0.8296361 |
| Ly6d | 1.0642075 |
| Ly6e | -0.5262403 |
| Ly6f | 0.247729 |
| Ly6g6c | 0.3062197 |
| Ly6g6d | -0.1234218 |
| Ly6g6e | 0.262701 |
| Ly6k | -0.6805219 |
| Ly86 | 0.2447053 |
| Ly9 | -0.572162 |
| Ly9 | 2.2136656 |
| Ly96 | 0.7494001 |
| Lyar | -0.5868884 |
| Lyl1 | 2.383505 |
| Lynx1 | 0.443003 |
| Lynx1 | 1.5477556 |
| Lypd1 | -0.1390654 |
| Lypd3 | -0.6333911 |
| Lypd4 | 0.1895962 |
| Lypd5 | -0.5208487 |
| Lypd6 | 1.0593257 |
| Lypla1 | -0.7321027 |
| Lypla1 | 0.1059454 |
| Lypla2 | 0.3267748 |
| Lypla3 | -0.7282531 |
| Lyplal1 | -0.5208487 |
| Lyrm1 | -0.6128118 |
| Lyrm2 | -0.2949748 |
| Lyrm2 | 0.7964304 |
| Lyrm5 | -0.408169 |
| Lysmd1 | -0.6579918 |
| Lysmd1 | -0.1638205 |
| Lysmd2 | 0.0079372 |
| Lysmd4 | 0.3199735 |
| Lyst | -0.6814346 |
| Lyve1 | -0.29077 |
| Lyz | 0.2187718 |
| Lyz1 | 1.0815722 |
| Lyz1 | -0.5640805 |
| Lyz2 | 3.641406 |
| Lyzl1 | 2.8946116 |
| Lyzl4 | 2.1428019 |
| Lyzl6 | 4.0201537 |
| Lyzs | -0.1924831 |
| Lzic | -0.5951719 |
| Lztr1 | -0.6316586 |
| Lzts2 | -0.497341 |
| M6pr | 0.5578748 |
| M6pr | -0.4642431 |
| M6prbp1 | 0.5602727 |
| Mab21l2 | 0.513366 |
| Macrod1 | -0.5418199 |
| Mad1l1 | -0.4832221 |
| Mad2l1 | 1.2109685 |
| Mad2l1bp | -0.8001144 |
| Mad2l1bp | -0.7026781 |
| Mad2l1bp | 2.82619 |
| Mad2l2 | -0.6814346 |
| Madcam1 | 0.5542663 |
| Madd | 0.1788208 |
| Maea | 0.7975437 |
| Maf | -0.0351802 |
| Maf1 | -0.5162521 |
| Mafg | 1.4414417 |
| Mag | -0.6500131 |
| Magea2 | -0.5285606 |
| Magea5 | -0.5697302 |
| Mageb3 | -0.6068945 |
| Mageb3 | -0.2531853 |
| Maged1 | 0.9547784 |
| Maged2 | -0.0198378 |
| Magee1 | -0.002546 |
| Mageh1 | -0.4556129 |
| Magi1 | -0.4988398 |
| Magi1 | 1.1000913 |
| Magi1 | -0.6204756 |
| Magi2 | -0.6705412 |
| Magi3 | -0.3833381 |
| Magi3 | -0.7282531 |
| Magix | -0.6094258 |
| Magmas | 3.0730842 |
| Magoh | 0.8628733 |
| Magoh | -0.4274118 |
| Magohb | 1.5709239 |
| Mak | -0.4861767 |
| Mak10 | 0.6718309 |
| Mak10 | -0.3393671 |
| Mal | 2.1236852 |
| Mal | 0.7236552 |
| Mall | -0.6333911 |
| Malt1 | 0.4218389 |
| Mamdc2 | 0.3084215 |
| Maml1 | -0.7026781 |
| Maml3 | 0.8464835 |
| Man1a | 1.1773518 |
| Man1a2 | -0.7073613 |
| Man1a2 | 1.7470283 |
| Man1b1 | 2.9170278 |
| Man1c1 | 0.3344434 |
| Man1c1 | -0.4308904 |
| Man2a1 | 2.9755084 |
| Man2a2 | 1.5200021 |
| Man2a2 | 1.7304208 |
| Man2a2 | 0.6281361 |
| Man2b1 | -0.2463046 |
| Man2b2 | 0.9279406 |
| Man2c1 | -0.5277866 |
| Manba | -0.7506095 |
| Manba | 2.1700615 |
| Manbal | -0.0207333 |
| Manea | -0.4329838 |
| Mansc1 | 0.5123578 |
| Maob | 1.731099 |
| Map1lc3a | -0.3412636 |
| Map1lc3a | -0.333073 |
| Map1lc3b | 1.9145422 |
| Map2k1 | 1.6250662 |
| Map2k2 | 0.3438653 |
| Map2k2 | -0.5681125 |
| Map2k3 | 1.8816446 |
| Map2k4 | -0.7908214 |
| Map2k6 | 0.7115277 |
| Map2k6 | 0.1802648 |
| Map2k6 | -0.2434527 |
| Map2k7 | 0.7119345 |
| Map2k7 | 0.1039894 |
| Map2k7 | -0.5976714 |
| Map3k1 | 0.6442443 |
| Map3k11 | 0.5281102 |
| Map3k12 | 3.5882097 |
| Map3k12 | 0.8188089 |
| Map3k12 | 0.8023526 |
| Map3k14 | 2.7507605 |
| Map3k3 | 0.158735 |
| Map3k6 | 2.8002385 |
| Map3k7 | 0.3953301 |
| Map3k7ip1 | 0.3724502 |
| Map3k7ip1 | -0.4308904 |
| Map3k7ip1 | -0.7244194 |
| Map3k7ip2 | -0.5926791 |
| Map3k7ip3 | -0.2753172 |
| Map3k8 | 2.1597834 |
| Map3k9 | 0.113341 |
| Map4k2 | 3.1289744 |
| Map4k4 | -0.497341 |
| Map4k5 | 0.7407662 |
| Map4k5 | 0.689082 |
| Mapbpip | -0.1664375 |
| Mapk1 | 1.6608478 |
| Mapk1 | -0.2428833 |
| Mapk1 | -0.617914 |
| Mapk10 | 0.8290822 |
| Mapk10 | 0.1302445 |
| Mapk11 | 0.1981272 |
| Mapk12 | -0.0131489 |
| Mapk12 | -0.5441796 |
| Mapk12 | 0.1977733 |
| Mapk13 | -0.6403535 |
| Mapk13 | -0.6482493 |
| Mapk14 | 0.0925624 |
| Mapk15 | 0.4271062 |
| Mapk15 | -0.2812301 |
| Mapk15 | -0.5664976 |
| Mapk1ip1 | 1.7367088 |
| Mapk1ip1 | -0.6394804 |
| Mapk1ip1 | 2.260652 |
| Mapk3 | -0.6906112 |
| Mapk3 | 1.2632643 |
| Mapk4 | 1.0496258 |
| Mapk6 | 1.1031644 |
| Mapk6 | 0.4184966 |
| Mapk6 | -0.5737868 |
| Mapk8 | -0.6282033 |
| Mapk8ip1 | -0.4204934 |
| Mapk8ip1 | 2.8665703 |
| Mapk8ip2 | -0.1951824 |
| Mapk8ip3 | 2.426679 |
| Mapk8ip3 | 0.3215237 |
| Mapk9 | 0.9514854 |
| Mapk9 | -0.1769802 |
| Mapk9 | 1.0689253 |
| Mapkap1 | 1.094049 |
| Mapkap1 | -0.5713507 |
| Mapkapk2 | 0.0849986 |
| Mapkapk3 | 0.2460504 |
| Mapkbp1 | 0.2325114 |
| Mapre1 | -0.6796102 |
| Mapre2 | -0.6633494 |
| Mapre2 | -0.6562128 |
| Mapre3 | -0.6989485 |
| Mapt | -0.3966776 |
| Mapt | 0.354987 |
| Mapt | -0.6850944 |
| Mapt | -0.6796102 |
| March1 | -0.5293354 |
| March2 | -0.6714441 |
| March2 | 0.7534927 |
| March4 | -0.4294974 |
| March4 | -0.0180493 |
| March6 | -0.4068096 |
| March7 | -0.7120683 |
| March8 | 3.0663897 |
| March10 | -0.4751451 |
| Marcks | 0.4339917 |
| Marcksl1 | 0.0361695 |
| Marcksl1 | -0.2131974 |
| Marcksl1 | -0.2973861 |
| Mare | 0.0453925 |
| Mark1 | 1.9421645 |
| Mark2 | -0.1129724 |
| Mark2 | 0.8111656 |
| Mark3 | 0.2497392 |
| Mark3 | -0.5147249 |
| Mark4 | 0.1397133 |
| Mars | 0.6259075 |
| Mars2 | -0.5025974 |
| Marveld3 | -0.5576657 |
| Marveld3 | 2.5051665 |
| Mas1 | -0.4824849 |
| Masp1 | -0.5918496 |
| Masp2 | -0.5673047 |
| Mast1 | -0.1711669 |
| Mast1 | -0.0732612 |
| Mast2 | -0.3299419 |
| Mast2 | 0.0230158 |
| Mat1a | -0.0851892 |
| Mat2a | 0.1820666 |
| Mat2a | 1.6290856 |
| Mat2a | 0.802537 |
| Mat2a | -0.5293354 |
| Mat2b | -0.5025974 |
| Mat2b | -0.7008114 |
| Matk | 0.1359373 |
| Matk | 0.3384062 |
| Matn4 | -0.4385896 |
| Matn4 | -0.7206016 |
| Matn4 | 2.1380486 |
| Matr3 | 0.370983 |
| Max | -0.3324459 |
| Maz | -0.6989485 |
| Mb | 0.6457744 |
| Mbc2 | -0.6264805 |
| Mbd1 | -0.5193139 |
| Mbd1 | -0.5489171 |
| Mbd3 | 1.1135111 |
| Mbd3l2 | 0.0242969 |
| Mbd4 | 0.6731041 |
| Mbd5 | -0.5713507 |
| Mbd6 | 2.39703 |
| Mbip | -0.210995 |
| Mbl2 | 0.0395339 |
| Mbnl1 | -0.6850944 |
| Mbnl2 | 0.1370717 |
| Mbnl2 | -0.6128118 |
| Mboat1 | 0.2893771 |
| Mboat2 | -0.4343821 |
| Mboat2 | 1.9006423 |
| Mboat2 | 1.8532008 |
| Mboat2 | -0.6786993 |
| Mboat5 | -0.7054852 |
| Mboat5 | 1.6966382 |
| Mbp | -0.6247608 |
| Mbp | -0.6359959 |
| Mbp | -0.4122589 |
| Mbtd1 | 0.9343632 |
| Mbtps1 | -0.5893657 |
| Mbtps1 | -0.7447253 |
| Mbtps1 | 1.537372 |
| Mbtps2 | 0.736815 |
| Mc3r | -0.0207333 |
| Mc4r | 0.274507 |
| Mc5r | 0.1325258 |
| Mcam | -0.6961611 |
| Mcam | 2.1393593 |
| Mcam | 0.591589 |
| Mcat | 0.6350108 |
| Mcc | -0.5086413 |
| Mccc1 | -0.568921 |
| Mcee | -0.4751451 |
| Mcf2l | 0.0191618 |
| Mcf2l | 3.9760338 |
| Mcf2l | -0.1472439 |
| Mcfd2 | -0.7644863 |
| Mchr1 | -0.3939943 |
| Mcl1 | -0.6204756 |
| Mcm10 | -0.3546494 |
| Mcm10 | -0.7486439 |
| Mcm2 | 2.5561586 |
| Mcm3 | 3.087352 |
| Mcm4 | 1.5679381 |
| Mcm5 | -0.5993416 |
| Mcm6 | -0.8084538 |
| Mcm6 | 0.1520659 |
| Mcm6 | 0.7256612 |
| Mcm7 | 0.6635122 |
| Mcoln1 | 0.3124967 |
| Mcoln2 | 1.7522779 |
| Mcoln3 | 0.292898 |
| Mcph1 | -0.462078 |
| Mcph1 | -0.6553246 |
| Mcph1 | 1.4783984 |
| Mcph1 | 0.6579246 |
| Mcpt4 | 0.4173799 |
| Mcrs1 | -0.6571019 |
| Mcrs1 | -0.4965925 |
| Mcrs1 | 3.3127879 |
| Mcts1 | -0.6204756 |
| Mdfic | -0.5520888 |
| Mdga1 | 1.4454165 |
| Mdga2 | -0.6351268 |
| Mdga2 | 0.3633156 |
| Mdh1 | -0.4040969 |
| Mdh1 | 0.3543892 |
| Mdh1b | -0.7073613 |
| Mdh2 | -0.4817483 |
| Mdk | 1.1171973 |
| Mdk | 3.8662666 |
| Mdm1 | 0.3964737 |
| Mdm1 | -0.6325244 |
| Mdm1 | 2.4559685 |
| Mdm1 | -0.572162 |
| Mdm1 | -0.5568669 |
| Mdm1 | -0.6196209 |
| Mdm1 | -0.7585137 |
| Mdm1 | 1.4818637 |
| Mdm1 | -0.3495273 |
| Mdm2 | -0.3064847 |
| Mdm2 | -0.3324459 |
| Mdm4 | -0.1523923 |
| Mdn1 | 0.3153076 |
| Me2 | -0.2895722 |
| Me3 | 2.3170179 |
| Mea1 | -0.4928591 |
| Mecp2 | 0.1351803 |
| Mecr | 0.5462778 |
| Med1 | -0.0383707 |
| Med1 | -0.60437 |
| Med10 | 1.3759108 |
| Med11 | 0.9709162 |
| Med11 | 0.205879 |
| Med12 | 0.65252 |
| Med15 | 1.156304 |
| Med15 | 1.6876593 |
| Med15 | 0.6400786 |
| Med16 | 1.1580658 |
| Med18 | -0.4456447 |
| Med18 | 1.543782 |
| Med19 | -0.4329838 |
| Med21 | -0.7215546 |
| Med22 | -0.3393671 |
| Med22 | 0.2069311 |
| Med23 | -0.5544748 |
| Med23 | 2.2786722 |
| Med24 | 1.8308241 |
| Med25 | -0.4839598 |
| Med25 | -0.4364834 |
| Med26 | -0.0202854 |
| Med27 | -0.1179336 |
| Med28 | -0.5394663 |
| Med30 | 1.3334998 |
| Med31 | 2.0110374 |
| Med4 | -0.3156734 |
| Med6 | 0.1748383 |
| Med7 | 1.4723254 |
| Med8 | -0.2332575 |
| Med8 | 1.6588713 |
| Med9 | -0.693382 |
| Mef2b | 1.2657306 |
| Mef2b | -0.6732526 |
| Mef2c | -0.6642453 |
| Mef2c | 1.9694601 |
| Mef2c | -0.4664133 |
| Megf10 | -0.4656893 |
| Megf11 | -0.6768802 |
| Megf11 | -0.4470622 |
| Megf11 | -0.6535507 |
| Megf11 | 2.4762899 |
| Megf6 | -0.421873 |
| Megf9 | -0.5528835 |
| Mei1 | 2.5502618 |
| Meis1 | 1.4357737 |
| Meis2 | -0.6786993 |
| Meis3 | 1.6078064 |
| Melk | 2.2525722 |
| Memo1 | -0.1833638 |
| Men1 | 2.58201 |
| Men1 | -0.5852406 |
| Meox1 | -0.5910209 |
| Meox2 | 1.3269204 |
| Mep1b | -0.638608 |
| Mepce | -0.0746845 |
| Mertk | -0.7826384 |
| Mesdc1 | -0.2400418 |
| Mesp1 | -0.7515938 |
| Mest | -0.4758764 |
| Mest | -0.9183811 |
| Mest | -0.3520848 |
| Metap1 | 2.3851814 |
| Metap2 | -0.6869297 |
| Metap2 | 0.913325 |
| Metapl1 | -0.4399963 |
| Metrn | -0.6805219 |
| Metrnl | -0.1139625 |
| Metrnl | -0.4891407 |
| Mett11d1 | -0.4513277 |
| Mettl1 | -0.668738 |
| Mettl1 | 0.5027254 |
| Mettl2 | -0.7054852 |
| Mettl3 | -0.6438545 |
| Mettl4 | -0.4876575 |
| Mettl6 | -0.6998795 |
| Mettl8 | 0.8981427 |
| Mettl9 | -0.4642431 |
| Mettl9 | -0.1941017 |
| Mex3a | 3.2739917 |
| Mex3a | 0.0647943 |
| Mex3c | 0.3718636 |
| Mfap1b | -0.5041047 |
| Mfap2 | 1.6718246 |
| Mfap2 | 0.408115 |
| Mfap2 | -0.2474479 |
| Mfap3 | 3.3976676 |
| Mfap3 | 2.1535269 |
| Mfap3l | -0.5018446 |
| Mfap3l | -0.0842292 |
| Mfap4 | 0.6673608 |
| Mfap5 | 3.0300858 |
| Mfge8 | 1.6805277 |
| Mfge8 | 0.1268119 |
| Mfi2 | 0.0105395 |
| Mfn1 | -0.6952338 |
| Mfn1 | 0.7182175 |
| Mfn2 | -0.5835956 |
| Mfng | -0.4802768 |
| Mfrp | -0.4788077 |
| Mfsd1 | -0.6517802 |
| Mfsd10 | -0.4729544 |
| Mfsd10 | 2.6285087 |
| Mfsd11 | 1.3840273 |
| Mfsd2 | 4.6596057 |
| Mfsd3 | -0.5705401 |
| Mfsd4 | 0.8180837 |
| Mfsd7 | -0.6517802 |
| Mfsd8 | 0.9293895 |
| Mfsd9 | 0.6252379 |
| Mga | -0.1674864 |
| Mgat1 | 0.4543467 |
| Mgat3 | 1.4783094 |
| Mgat4b | 0.5864813 |
| Mgat4b | -0.4758764 |
| Mgat5 | -0.6723479 |
| MGC107098 | 0.9823822 |
| MGC117608 | -0.7026781 |
| MGC118210 | 1.0538556 |
| MGC18837 | 0.7393852 |
| MGC41689 | -0.6145094 |
| MGC67181 | -0.7054852 |
| Mgea6 | 0.552336 |
| Mgl1 | -0.5592652 |
| Mgl2 | 0.6305813 |
| Mgll | 2.34957 |
| Mgll | -0.5465453 |
| Mgll | -0.5705401 |
| Mgmt | 0.4323438 |
| Mgp | 0.5396903 |
| Mgrn1 | 3.250778 |
| Mgst1 | -0.6606667 |
| Mgst1 | -0.3083151 |
| Mgst2 | 1.1909207 |
| Mgst3 | 0.9102098 |
| Mgst3 | -0.7321027 |
| Mia1 | -0.350166 |
| Mia3 | -0.1426346 |
| Mib1 | 2.3702226 |
| Mib2 | 0.6859502 |
| Mic2l1 | 0.0495547 |
| Mical1 | 1.2311735 |
| Mical1 | -0.0507806 |
| Mical1 | -0.6111173 |
| Mical2 | 0.6272453 |
| Mical3 | 1.4301646 |
| Micall2 | -0.5885392 |
| Micall2 | -0.1355098 |
| Mid1 | 1.7962134 |
| Mid1 | 0.2861647 |
| Mid1ip1 | -0.0953285 |
| Midn | -0.2457335 |
| Midn | 1.2321026 |
| Mier1 | 0.7921503 |
| Mier1 | -0.2717874 |
| Mif | -0.1801665 |
| Mif4gd | -0.4928591 |
| Mif4gd | -0.4007171 |
| Mill2 | 2.0421935 |
| Mill2 | -0.3444333 |
| Mina | -0.2794523 |
| Mink1 | -0.4156811 |
| Mink1 | -0.6456099 |
| Minpp1 | 1.2933047 |
| Minpp1 | -0.6860116 |
| Miox | -0.5433924 |
| Mipep | 3.6381312 |
| Mir16 | -0.7634882 |
| Mist1 | -0.4414052 |
| Mitf | -0.5324408 |
| Mizf | -0.3546494 |
| Mki67ip | -0.5762293 |
| Mkks | 0.0255762 |
| Mkks | 1.6957935 |
| Mkl1 | 2.0477519 |
| Mkl2 | 0.7863502 |
| Mkl2 | -0.3853269 |
| Mkl2 | -0.7437482 |
| Mkln1 | 0.9650521 |
| Mknk1 | -0.7684895 |
| Mknk2 | 0.7476406 |
| Mkrn1 | 1.5484988 |
| Mkrn2 | -0.7856986 |
| Mkrn3 | 0.650132 |
| Mkx | 0.6998578 |
| Mlana | 0.1646232 |
| Mlf1 | -0.7215546 |
| Mlf1 | -0.5086413 |
| Mlf2 | 2.2697628 |
| Mlf2 | -0.4714968 |
| Mlh1 | -0.6633494 |
| Mlh3 | 1.2708661 |
| Mlkl | -0.5147249 |
| Mllt1 | 0.5136178 |
| Mllt1 | 0.5379747 |
| Mllt1 | -0.5592652 |
| Mllt10 | -0.0568155 |
| Mllt11 | -0.0600814 |
| Mllt3 | -0.2901709 |
| Mllt3 | -0.6624543 |
| Mllt3 | -0.5489171 |
| Mlp | -0.6421024 |
| Mlstd2 | 1.1512516 |
| Mlx | 0.4671434 |
| Mlx | -0.7496262 |
| Mlxipl | -0.6850944 |
| Mlycd | -0.1822968 |
| Mmab | -0.4435225 |
| Mmab | -0.0452446 |
| Mmd | 0.3319963 |
| Mmd2 | 1.3988872 |
| Mme | -0.1234218 |
| Mme | 0.8281856 |
| Mmel1 | 0.6961418 |
| Mmp10 | -0.3933247 |
| Mmp11 | -0.08615 |
| Mmp12 | 2.7052325 |
| Mmp13 | 1.0864745 |
| Mmp13 | 1.2244092 |
| Mmp14 | 1.9308096 |
| Mmp15 | -0.6204756 |
| Mmp16 | -0.7350004 |
| Mmp17 | -0.4294974 |
| Mmp1a | 0.3800428 |
| Mmp1a | 0.1690149 |
| Mmp2 | 0.4986395 |
| Mmp23 | -0.7795883 |
| Mmp24 | 1.4079591 |
| Mmp3 | 2.270559 |
| Mmp3 | -0.6464889 |
| Mmp3 | 0.8168131 |
| Mmp9 | -0.7535657 |
| Mmrn2 | 0.0729289 |
| Mnat1 | 0.6773356 |
| Mnat1 | -0.6588826 |
| Mnd1 | 0.1960018 |
| Mns1 | -0.5347767 |
| Mnt | -0.1596485 |
| Mobk1b | 1.5820559 |
| Mobkl2a | -0.4329838 |
| Mobkl2b | -0.7130126 |
| Mobkl2c | -0.7301759 |
| Mobkl3 | -0.6290659 |
| Mobp | 0.2135666 |
| Mobp | -0.5943402 |
| Mobp | 2.7974504 |
| Mocos | -0.5132003 |
| Mocs1 | 0.4761021 |
| Mocs2 | -0.3813536 |
| Mod1 | -0.6273415 |
| Mog | 2.1798928 |
| Mog | -0.1244231 |
| Mon1a | 0.6739519 |
| Mon1a | 1.5412899 |
| Mon1b | 0.3084215 |
| Mon1b | -0.6213311 |
| Mon1b | 0.1513219 |
| Morc2a | -0.089521 |
| Morc2b | 0.6135539 |
| Morc3 | -0.8371657 |
| Morf4l1 | -0.8480293 |
| Morf4l1 | 0.6758567 |
| Morf4l2 | -0.579496 |
| Morn2 | 2.0172832 |
| Morn3 | 0.750181 |
| Morn3 | 0.048308 |
| Mos | -0.3387357 |
| Mosc2 | -0.5363373 |
| Mospd1 | -0.3181391 |
| Mospd1 | 1.5028211 |
| Mospd1 | -0.6412275 |
| Mospd3 | -0.7836573 |
| Mospd3 | -0.5951719 |
| Mov10 | 1.4653099 |
| Mov10l1 | 1.6191956 |
| Moxd1 | 0.7912173 |
| Moxd1 | -0.5600661 |
| Mpdu1 | 0.5340415 |
| Mpdz | -0.4520405 |
| Mpdz | -0.6325244 |
| Mpg | -0.2428833 |
| Mpg | 1.925657 |
| Mphosph6 | -0.5993416 |
| Mpnd | 0.4273826 |
| Mpo | 0.7260619 |
| Mpo | 1.2426066 |
| Mpp1 | -0.6624543 |
| Mpp2 | 0.2139145 |
| Mpp3 | 0.0416305 |
| Mpp3 | 2.6617901 |
| Mpp5 | 2.1132813 |
| Mpp6 | 2.4515822 |
| Mppe1 | -0.6500131 |
| Mpped1 | -0.1095153 |
| Mpst | 1.1455345 |
| Mpv17 | 0.086197 |
| Mpv17 | -0.7206016 |
| Mpv17 | 1.0203397 |
| Mpv17 | -0.6597742 |
| Mpv17l | 0.1382047 |
| Mpv17l | 0.3106181 |
| Mpv17l | 3.1350546 |
| Mpz | 0.9713775 |
| Mr1 | 0.7157904 |
| Mrap | 2.2648945 |
| Mras | -0.3199928 |
| Mrc1 | -0.8425816 |
| Mrc2 | -0.002546 |
| Mrc2 | 1.1074231 |
| Mre11a | 0.1967108 |
| Mre11a | -0.5656912 |
| Mre11a | -0.5520888 |
| Mreg | 0.5640965 |
| Mrgpra2 | -0.4556129 |
| Mrgpra2 | 2.2691556 |
| Mrgprb2 | -0.0180493 |
| Mrgpre | -0.5918496 |
| Mrgprf | -0.7225085 |
| Mrgprg | 0.2948128 |
| Mrm1 | -0.6290659 |
| Mro | -0.7330676 |
| Mrp63 | 0.1195234 |
| Mrpl1 | 1.0155142 |
| Mrpl1 | -0.7244194 |
| Mrpl10 | -0.4281064 |
| Mrpl10 | 2.7919572 |
| Mrpl11 | -0.4736841 |
| Mrpl12 | -0.7939088 |
| Mrpl13 | 0.1849422 |
| Mrpl13 | 0.8728436 |
| Mrpl14 | -0.5754144 |
| Mrpl15 | 1.1245261 |
| Mrpl16 | -0.6924575 |
| Mrpl17 | 1.1961243 |
| Mrpl18 | 0.2009535 |
| Mrpl2 | -0.1405934 |
| Mrpl20 | -0.5457561 |
| Mrpl22 | -0.325576 |
| Mrpl23 | 0.3812053 |
| Mrpl24 | -0.5324408 |
| Mrpl27 | -0.6714441 |
| Mrpl28 | -0.6464889 |
| Mrpl3 | 0.2650084 |
| Mrpl3 | -0.521617 |
| Mrpl30 | 0.3283136 |
| Mrpl33 | -0.4149957 |
| Mrpl33 | -0.5093996 |
| Mrpl34 | 1.8100138 |
| Mrpl36 | -0.6351268 |
| Mrpl37 | 0.3872851 |
| Mrpl37 | 0.0619303 |
| Mrpl38 | 0.9684516 |
| Mrpl39 | -0.3362147 |
| Mrpl40 | -0.3052663 |
| Mrpl43 | 1.6004995 |
| Mrpl43 | 0.94755 |
| Mrpl45 | -0.6102712 |
| Mrpl46 | 0.0897829 |
| Mrpl47 | -0.6777893 |
| Mrpl47 | 0.3895908 |
| Mrpl48 | -0.0356353 |
| Mrpl48 | 0.2270399 |
| Mrpl48 | -0.2120956 |
| Mrpl49 | 2.0904155 |
| Mrpl49 | -0.497341 |
| Mrpl49 | 2.239222 |
| Mrpl49 | 1.0891868 |
| Mrpl50 | -0.6213311 |
| Mrpl51 | 0.3399258 |
| Mrpl51 | -0.4839598 |
| Mrpl51 | -0.5465453 |
| Mrpl52 | -0.3337005 |
| Mrpl53 | 0.3236895 |
| Mrpl54 | 0.1445991 |
| Mrpl55 | -0.0992205 |
| Mrpl9 | -0.4027435 |
| Mrps10 | 0.9773546 |
| Mrps10 | 2.1852269 |
| Mrps11 | -0.3649793 |
| Mrps11 | 0.803643 |
| Mrps12 | -0.5270131 |
| Mrps14 | 0.5323155 |
| Mrps15 | -0.6094258 |
| Mrps16 | -0.416367 |
| Mrps17 | -0.5835956 |
| Mrps18a | -0.3610921 |
| Mrps18b | 0.7082667 |
| Mrps18b | 1.0589061 |
| Mrps18c | -0.4584807 |
| Mrps2 | -0.7350004 |
| Mrps21 | -0.257801 |
| Mrps22 | 1.1329608 |
| Mrps23 | 2.3568853 |
| Mrps24 | 1.14311 |
| Mrps25 | -0.5737868 |
| Mrps25 | 0.2893771 |
| Mrps25 | 1.4926208 |
| Mrps26 | 0.1809859 |
| Mrps26 | -0.1622538 |
| Mrps27 | 2.771002 |
| Mrps28 | -0.6606667 |
| Mrps30 | 0.5612301 |
| Mrps31 | 1.8624263 |
| Mrps31 | -0.4336827 |
| Mrps31 | -0.5010925 |
| Mrps33 | 0.2780846 |
| Mrps33 | -0.821105 |
| Mrps34 | 0.8131735 |
| Mrps35 | 1.6751428 |
| Mrps5 | 3.0253434 |
| Mrps6 | -0.6018524 |
| Mrps7 | -0.5935093 |
| Mrps7 | 0.9662898 |
| Mrps9 | -0.3457041 |
| Mrrf | 0.4524657 |
| Mrrf | 0.1524377 |
| Mrto4 | -0.5918496 |
| Mrvi1 | -0.5803145 |
| Ms4a1 | 3.9288544 |
| Ms4a10 | -0.4883988 |
| Ms4a13 | 1.8835447 |
| Ms4a5 | 0.9359619 |
| Ms4a6d | -0.3879852 |
| Ms4a6d | 0.6587864 |
| Ms4a7 | -0.6669383 |
| Ms4a8a | 1.8221996 |
| Msc | -0.3318193 |
| Msh2 | 0.0897829 |
| Msh3 | 1.338792 |
| Msh3 | -0.6001777 |
| Msh4 | 1.219833 |
| Msh5 | 0.9808616 |
| Msh5 | -0.7008114 |
| Msh6 | 0.6128753 |
| Msh6 | 0.7515461 |
| Msi2 | -0.1164425 |
| Msi2h | 0.4927344 |
| Msln | -0.1612108 |
| Msn | -0.4136263 |
| Msr2 | -0.7816206 |
| Msrb2 | -0.5071266 |
| Msrb2 | 3.7912724 |
| Msrb3 | 1.5734182 |
| Mst1r | -0.6221873 |
| Msto1 | -0.7785738 |
| Msx1 | 2.7163394 |
| Mt1 | -0.178572 |
| Mt3 | 0.7105099 |
| Mta1 | -0.0466255 |
| Mta2 | 0.5971292 |
| Mta3 | 1.215234 |
| Mta3 | 0.7270628 |
| Mtag2 | 2.1854346 |
| Mtap | -0.5811337 |
| Mtap | -0.1706402 |
| Mtap1b | 0.0191618 |
| Mtap1b | -0.4232546 |
| Mtap1b | 0.0643857 |
| Mtap1b | 1.4066136 |
| Mtap1s | -0.6094258 |
| Mtap2 | 1.2475289 |
| Mtap2 | -0.0842292 |
| Mtap2 | -0.4477717 |
| Mtap4 | 0.9558734 |
| Mtap6 | 1.5866617 |
| Mtap6 | -0.5910209 |
| Mtap7 | 3.0343217 |
| Mtap7d1 | -0.6998795 |
| Mtap7d1 | -0.5737868 |
| Mtap7d2 | -0.5713507 |
| Mtap7d3 | 1.9091303 |
| Mtbp | 0.5892708 |
| Mtbp | 2.1606371 |
| Mtbp | 0.1210626 |
| Mtch1 | 2.4094801 |
| Mtch1 | 0.1494594 |
| Mtch2 | -0.817926 |
| Mtch2 | -0.2394746 |
| Mtch2 | -0.2170642 |
| Mtcp1 | 0.1445991 |
| Mtdh | 1.3565224 |
| Mterf | 4.132305 |
| Mterf | -0.714904 |
| Mterfd1 | -0.2366435 |
| Mterfd2 | -0.3175221 |
| Mterfd2 | 2.2137462 |
| Mterfd2 | 3.4707313 |
| Mterfd3 | -0.4108936 |
| Mterfd3 | -0.4232546 |
| Mtf1 | 0.1928045 |
| Mtf1 | 1.8033901 |
| Mtf2 | -0.5584651 |
| Mtfmt | 0.2460504 |
| Mtfmt | -0.6351268 |
| Mtg1 | 0.1991881 |
| Mthfd1 | 0.5971292 |
| Mthfd2 | -0.2595378 |
| Mthfs | -0.3349567 |
| Mthfsd | -0.693382 |
| Mthfsd | -0.0847091 |
| Mtif3 | -0.5170166 |
| Mtl5 | 0.6490445 |
| Mtl5 | 1.9168202 |
| Mtmr1 | -0.3337005 |
| Mtmr11 | -0.6669383 |
| Mtmr11 | -0.7476627 |
| Mtmr12 | -0.0078323 |
| Mtmr14 | 0.8058511 |
| Mtmr14 | 1.6984657 |
| Mtmr14 | -0.4470622 |
| Mtmr14 | -0.8200442 |
| Mtmr2 | -0.5754144 |
| Mtmr2 | -0.5656912 |
| Mtmr2 | -0.0411141 |
| Mtmr3 | 0.0893852 |
| Mtmr4 | 0.1160508 |
| Mtmr9 | 0.6998578 |
| Mto1 | 0.2107785 |
| Mtpn | -0.4671378 |
| Mtrf1 | -0.7064228 |
| Mtrf1 | 0.1977733 |
| Mtrf1l | -0.6351268 |
| Mtrr | -0.49137 |
| Mtss1 | -0.6841781 |
| Mttp | 2.322983 |
| Mtus1 | -0.1544597 |
| Mtus1 | -0.5584651 |
| Mtvr2 | 0.4955633 |
| Mtvr2 | -0.5018446 |
| Mtx1 | 0.3510945 |
| Mtx1 | 0.0507997 |
| Mtx1 | 3.478227 |
| Mtx2 | 0.0602897 |
| Muc1 | 3.2761994 |
| Muc1 | -0.1400838 |
| Muc1 | -0.2717874 |
| Muc1 | 0.0353265 |
| Muc13 | 0.0487237 |
| Muc5ac | 3.5416613 |
| Mug2 | 0.2297797 |
| Mug2 | 0.8791485 |
| Mug4 | -0.7614952 |
| Mum1 | -0.0100438 |
| Mup1 | 2.4309294 |
| Mup2 | -0.3986952 |
| Mup2 | 1.7247044 |
| Mup3 | 0.3630194 |
| Mupcdh | 0.3701015 |
| Mus81 | -0.7330676 |
| Mus81 | 2.0497256 |
| Mustn1 | -0.6473687 |
| Mustn1 | 3.1774341 |
| Muted | 2.1735186 |
| Muted | 0.6067456 |
| Mutyh | 0.6214343 |
| Mvd | -0.5778612 |
| Mvp | -0.6998795 |
| Mx2 | -0.6196209 |
| Mxd1 | 2.7482273 |
| Mxd4 | -0.6508962 |
| Mxi1 | -0.3760823 |
| Mxra7 | 1.5405409 |
| Mxra8 | 1.0136063 |
| Mxra8 | -0.4020676 |
| Myadm | 1.5907716 |
| Mybbp1a | -0.1659135 |
| Mybbp1a | 0.5991982 |
| Mybbp1a | 1.8340454 |
| Mybl2 | -0.0723135 |
| Mybpc2 | -0.1722212 |
| Mybpc3 | -0.6768802 |
| Myc | -0.6491308 |
| Mycbp | 0.5545073 |
| Mycbp2 | -0.7379071 |
| Mycbp2 | -0.3899839 |
| Mycbpap | -0.6333911 |
| Mycl1 | -0.4584807 |
| Myd116 | 0.8335524 |
| Myd88 | -0.8116005 |
| Myef2 | -0.2659341 |
| Myg1 | 2.4052612 |
| Myg1 | -0.0799219 |
| Myg1 | 2.6335969 |
| Myh10 | -0.3879852 |
| Myh11 | 2.2050014 |
| Myh8 | 2.039572 |
| Myh9 | 1.6504054 |
| Myl1 | 0.8285443 |
| Myl2 | -0.6651421 |
| Myl4 | -0.572974 |
| Myl6 | -0.0091586 |
| Myl6b | -0.5910209 |
| Mylc2b | -0.5379004 |
| Mylc2b | -0.6403535 |
| Mylc2b | -0.5926791 |
| Mylc2pl | -0.2549135 |
| Mylk | 1.6019031 |
| Mylk | 0.9532117 |
| Mynn | -0.5426058 |
| Myo10 | -0.7427721 |
| Myo10 | 1.1294665 |
| Myo15 | 0.0582349 |
| Myo18a | 1.420361 |
| Myo1b | -0.7545532 |
| Myo1c | -0.510918 |
| Myo1c | 0.5365017 |
| Myo1f | 0.4182176 |
| Myo1g | -0.4301936 |
| Myo3b | -0.4343821 |
| Myo5a | 0.2584005 |
| Myo5b | -0.6588826 |
| Myo6 | -0.5239258 |
| Myo7a | 1.8924178 |
| Myo9a | -0.0452446 |
| Myo9b | -0.5910209 |
| Myo9b | 0.5955167 |
| Myoc | -0.5277866 |
| Myocd | -0.2991987 |
| Myocd | 1.2316381 |
| Myom1 | -0.3649793 |
| Myom1 | 4.7779942 |
| Myrip | -0.4267176 |
| Mysm1 | 1.8523684 |
| Myst1 | 2.2193703 |
| Myst2 | -0.3218501 |
| Myst4 | -0.5394663 |
| Myst4 | -0.5656912 |
| Myst4 | 0.101244 |
| Myt1 | 0.801245 |
| Myt1l | -0.3274446 |
| Myt1l | 0.0365907 |
| Mzf1 | -0.1565318 |
| Mzf1 | -0.5705401 |
| N28178 | -0.3022275 |
| N4bp2 | 0.5966687 |
| N6amt1 | -0.6915339 |
| N6amt1 | -0.1892543 |
| N6amt2 | -0.2434527 |
| Naalad2 | 0.2732026 |
| Nab1 | -0.5154882 |
| Nab1 | -0.5505016 |
| Nab1 | -0.5018446 |
| Nab1 | -0.4577629 |
| Nab2 | 2.295058 |
| Naca | 0.0382737 |
| Naca | -0.6273415 |
| Naca | -0.4301936 |
| Nadk | 0.7080625 |
| Nadsyn1 | 0.2938559 |
| Nadsyn1 | -0.219836 |
| Naga | -0.668738 |
| Nagk | 1.539958 |
| Nagk | 0.2848766 |
| Nagk | -0.5473352 |
| Nagk | -0.6980184 |
| Naglu | -0.1817638 |
| Nagpa | 2.0925302 |
| Nags | -0.652665 |
| Nags | 0.8644275 |
| Naif1 | 1.8802025 |
| Naif1 | -0.8021923 |
| Naip5 | -0.5449675 |
| Nalp10 | -0.6119641 |
| Nampt | -0.311374 |
| Nanos1 | -0.6777893 |
| Nanos2 | -0.6759719 |
| Nans | 2.2443825 |
| Nap1l1 | -0.6491308 |
| Nap1l1 | -0.5371185 |
| Nap1l3 | -0.1643433 |
| Nap1l4 | -0.1426346 |
| Nap1l5 | -0.4442294 |
| Napa | 3.6299019 |
| Napa | -0.1653898 |
| Napa | 1.9041931 |
| Napb | -0.4824849 |
| Napepld | -0.1139625 |
| Napg | 0.8784689 |
| Napg | 0.1199084 |
| Naprt1 | 0.0105395 |
| Naprt1 | -0.4635209 |
| Napsa | -0.1095153 |
| Napsa | -0.3206115 |
| Napsa | 0.8764273 |
| Narf | -0.5254682 |
| Narfl | 2.2659235 |
| Narg1 | 0.0079372 |
| Narg1 | -0.6307936 |
| Narg1l | 1.4070943 |
| Narg1l | -0.4591991 |
| Narg1l | 2.1898278 |
| Narg2 | -0.2332575 |
| Nars | 0.3307704 |
| Nars2 | -0.08615 |
| Nasp | -0.6869297 |
| Nasp | -0.2148526 |
| Nasp | -0.6562128 |
| Nasp | -0.5746002 |
| Nat1 | -0.6989485 |
| Nat10 | -0.3913188 |
| Nat14 | 0.8580215 |
| Nat2 | -0.5394663 |
| Nat5 | -0.5332188 |
| Nat5 | -0.5926791 |
| Nat6 | 1.6015135 |
| Nat6 | 2.8879726 |
| Nav1 | 1.1790742 |
| Nav1 | -0.7083008 |
| Nbl1 | 0.3008506 |
| Nbr1 | 0.9026242 |
| Nbr1 | 0.6590018 |
| Ncald | -0.2049654 |
| Ncam1 | -0.2126463 |
| Ncam1 | 0.3794609 |
| Ncam2 | 0.4808102 |
| Ncan | 1.2115621 |
| Ncapd2 | 2.0577693 |
| Ncapd2 | 0.1487133 |
| Ncapd2 | 0.6692792 |
| Ncapd3 | 1.7146843 |
| Ncapg2 | -0.3083151 |
| Ncapg2 | 2.8164352 |
| Ncaph | 0.8450752 |
| Ncaph2 | 1.55056 |
| Ncaph2 | -0.4191159 |
| Ncaph2 | -0.5993416 |
| Ncbp2 | 0.9342032 |
| Ncbp2 | 0.0877925 |
| Ncdn | 1.0212144 |
| Ncdn | -0.4129424 |
| Ncdn | -0.8371657 |
| Ncdn | -0.6924575 |
| Ncf4 | -0.1416134 |
| Nck1 | 0.1397133 |
| Nckap1 | 0.6151353 |
| Nckap1l | 0.6802861 |
| Nckipsd | 0.7842833 |
| Ncl | -0.5608676 |
| Ncl | -0.5433924 |
| Ncoa2 | 1.3929522 |
| Ncoa2 | 3.2496573 |
| Ncoa4 | 0.0503849 |
| Ncoa5 | 0.598509 |
| Ncoa6 | -0.579496 |
| Ncoa6 | -0.7466825 |
| Ncoa6 | -0.6111173 |
| Ncor1 | -0.7073613 |
| Ncstn | 2.5245644 |
| Ncstn | 1.0715514 |
| Ncstn | 2.1838962 |
| Ndc80 | -0.6553246 |
| Nde1 | 1.7929847 |
| Nde1 | 0.6675742 |
| Ndel1 | 2.8046606 |
| Ndfip1 | 1.4984757 |
| Ndfip2 | -0.5673047 |
| Ndg2 | -0.6553246 |
| Ndn | -0.2723747 |
| Ndor1 | -0.8042747 |
| Ndor1 | 2.1661251 |
| Ndor1 | -0.3585097 |
| Ndrg1 | -0.5592652 |
| Ndrg2 | -0.0866309 |
| Ndrg3 | -0.0360906 |
| Ndrg4 | -0.5993416 |
| Ndrl | 2.3252368 |
| Ndst1 | -0.4758764 |
| Ndst1 | -0.6111173 |
| Ndst4 | -0.3199928 |
| Ndufa1 | -0.4301936 |
| Ndufa10 | 1.4721462 |
| Ndufa12 | 1.2469579 |
| Ndufa13 | -0.5901929 |
| Ndufa2 | 2.02783 |
| Ndufa3 | 0.1464714 |
| Ndufa4 | -0.4239461 |
| Ndufa5 | 2.3571961 |
| Ndufa5 | 0.6205371 |
| Ndufa6 | -0.2794523 |
| Ndufa7 | 0.1393364 |
| Ndufa8 | -0.6823482 |
| Ndufa8 | -0.5786782 |
| Ndufa9 | 1.0001401 |
| Ndufaf1 | 1.7220384 |
| Ndufb10 | -0.1732766 |
| Ndufb11 | -0.6980184 |
| Ndufb2 | 0.0713077 |
| Ndufb3 | -0.7036129 |
| Ndufb4 | 0.5423797 |
| Ndufb5 | -0.6421024 |
| Ndufb6 | 0.6867864 |
| Ndufb6 | 1.7882161 |
| Ndufb7 | 0.8602773 |
| Ndufb8 | 0.9989468 |
| Ndufb9 | -0.5568669 |
| Ndufc1 | -0.3913188 |
| Ndufc1 | -0.2836059 |
| Ndufc2 | 0.9808616 |
| Ndufs2 | 0.3950439 |
| Ndufs2 | 1.2170056 |
| Ndufs3 | 2.6328021 |
| Ndufs4 | 1.400728 |
| Ndufs4 | -0.652665 |
| Ndufs4 | -0.6786993 |
| Ndufs4 | 0.8424287 |
| Ndufs6 | 2.0795364 |
| Ndufs7 | 1.0565246 |
| Ndufs8 | -0.6571019 |
| Ndufv1 | -0.3675799 |
| Ndufv2 | 0.8742105 |
| Necab1 | 2.972561 |
| Necab2 | 0.91447 |
| Necab3 | 2.4618466 |
| Necap1 | 0.5085675 |
| Necap1 | 0.0541116 |
| Necap1 | 1.6702334 |
| Necap2 | -0.6273415 |
| Nedd1 | 2.5351398 |
| Nedd4 | 0.5438435 |
| Nedd4l | 0.1168236 |
| Nedd8 | -0.4253308 |
| Nedd9 | -0.2788604 |
| Nedd9 | -0.6544372 |
| Nefm | -0.711125 |
| Negr1 | -0.5363373 |
| Negr1 | 1.3067903 |
| Neil1 | -0.6204756 |
| Neil2 | 1.6199601 |
| Nek11 | 0.8872644 |
| Nek3 | -0.5877134 |
| Nek3 | -0.5544748 |
| Nek3 | 2.673107 |
| Nek4 | 0.8794881 |
| Nek5 | 1.5395414 |
| Nek6 | -0.5584651 |
| Nek8 | -0.4232546 |
| Nek9 | 1.1807933 |
| Nek9 | 1.0577861 |
| Nelf | -0.5681125 |
| Nelf | -0.2549135 |
| Nell2 | -0.5033507 |
| Nell2 | -0.6759719 |
| Nell2 | -0.3980222 |
| Nell2 | 1.0002891 |
| Nenf | -0.5951719 |
| Neo1 | -0.2366435 |
| Nes | 1.4809764 |
| Net1 | -0.5512949 |
| Net1 | -0.5544748 |
| Neto1 | 0.3071639 |
| Neto1 | -0.714904 |
| Neto1 | 1.9442142 |
| Neu1 | -0.4534678 |
| Neu2 | -0.5616698 |
| Neu3 | -0.4288016 |
| Neu4 | 1.1668253 |
| Neu4 | -0.7263343 |
| Neu4 | 1.168443 |
| Neurl | -0.6464889 |
| Neurod1 | 0.586714 |
| Neurod2 | 1.7094252 |
| Neurod4 | -0.7417971 |
| Neurog1 | 0.979948 |
| Neurog2 | 2.0558604 |
| Nf1 | -0.5993416 |
| Nfasc | -0.5608676 |
| Nfasc | 2.634519 |
| Nfasc | -0.7437482 |
| Nfat5 | 0.6861593 |
| Nfat5 | -0.163298 |
| Nfatc1 | -0.4198044 |
| Nfatc1 | 0.6341266 |
| Nfatc2 | 0.2610493 |
| Nfatc2 | 1.711574 |
| Nfatc2ip | -0.5893657 |
| Nfatc3 | -0.7017443 |
| Nfatc4 | -0.6768802 |
| Nfe2 | 1.5488289 |
| Nfe2l1 | -0.3636818 |
| Nfe2l2 | -0.3741133 |
| Nfe2l3 | -0.4315877 |
| Nfib | 1.2361567 |
| Nfic | 0.207982 |
| Nfic | 0.8993067 |
| Nfic | -0.5901929 |
| Nfix | -0.1513604 |
| Nfix | 0.2335337 |
| Nfkb1 | -0.3101492 |
| Nfkb2 | -0.4315877 |
| Nfkbia | -0.4577629 |
| Nfkbib | 0.7593081 |
| Nfkbid | 0.9614848 |
| Nfkbie | 1.83447 |
| Nfkbil1 | -0.3734579 |
| Nfkbil2 | 1.9590729 |
| Nfkbiz | 0.4695233 |
| Nfrkb | -0.4729544 |
| Nfrkb | 1.0849793 |
| Nfs1 | 0.5688542 |
| Nfs1 | 2.2417668 |
| Nfs1 | 0.0416305 |
| Nfu1 | -0.0799219 |
| Nfx1 | -0.7826384 |
| Nfya | 1.171546 |
| Nfyb | -0.1908673 |
| Nfyc | 1.1811613 |
| Ngb | 0.956186 |
| Ngb | 0.4112136 |
| Ngef | 1.5084399 |
| Ngfb | -0.411576 |
| Ngfb | -0.1334842 |
| Ngfr | -0.6162101 |
| Ngfrap1 | -0.6162101 |
| Ngp | -0.6823482 |
| Nhedc1 | 0.0013991 |
| Nhej1 | 0.0230158 |
| Nhlrc1 | 1.0229613 |
| Nhlrc2 | -0.521617 |
| Nhlrc2 | -0.7506095 |
| Nhp2l1 | 1.6300688 |
| Nhsl1 | -0.6943074 |
| Niban | -0.6669383 |
| Nicn1 | -0.5616698 |
| Nicn1 | 3.0967797 |
| Nid1 | 0.0416305 |
| Nid2 | 0.7131539 |
| Nif3l1 | -0.456329 |
| Nin | 0.0187325 |
| Ninj1 | -0.3866551 |
| Ninj2 | -0.7856986 |
| Nip7 | -0.3387357 |
| Nipa1 | 1.4898992 |
| Nipbl | -0.3230904 |
| Nipbl | 3.314118 |
| Nipsnap1 | -0.1580888 |
| Nipsnap3a | -0.7614952 |
| Nipsnap3a | 0.1733859 |
| Nisch | 0.3218334 |
| Nit1 | 0.1627871 |
| Nit2 | -0.0315473 |
| Nit2 | -0.6832627 |
| Nkain3 | -0.3708408 |
| Nkain4 | -0.7908214 |
| Nkd2 | -0.6145094 |
| Nkg7 | 0.0797877 |
| Nkiras1 | -0.7177484 |
| Nkiras2 | 1.9709271 |
| Nkrf | 0.6069733 |
| Nktr | -0.1711669 |
| Nkx1-2 | 0.6892904 |
| Nkx2-1 | -0.3728029 |
| Nkx2-2 | -0.6768802 |
| Nkx2-2 | -0.1643433 |
| Nkx2-3 | -0.7515938 |
| Nkx2-4 | 1.0929707 |
| Nkx2-6 | 2.7118748 |
| Nkx3-1 | -0.2595378 |
| Nkx6-1 | 2.2010882 |
| Nle1 | 0.689082 |
| Nlgn1 | 0.8588897 |
| Nlgn2 | 0.7799467 |
| Nlk | -0.6952338 |
| Nln | -0.1319679 |
| Nln | -0.5473352 |
| Nln | -0.6832627 |
| Nlrp14 | 0.6053779 |
| Nlrp4a | -0.2735506 |
| Nlrp4c | 2.4613222 |
| Nlrp5 | -0.2641852 |
| Nlrp5 | 1.6990273 |
| Nlrp5 | -0.3374743 |
| Nlrp9a | -0.3444333 |
| Nlrp9c | 0.695728 |
| Nlrx1 | -0.2723747 |
| Nlrx1 | -0.0591471 |
| Nmb | 0.3341379 |
| Nmbr | -0.4758764 |
| Nmd3 | -0.9398466 |
| Nme1 | 0.5428679 |
| Nme2 | 1.8901361 |
| Nme2 | -0.6170616 |
| Nme2 | -0.3212306 |
| Nme3 | 0.9555607 |
| Nme4 | 2.393219 |
| Nme4 | 0.3267748 |
| Nme4 | -0.8011528 |
| Nme5 | 0.4326187 |
| Nme7 | -0.1129724 |
| Nme7 | 0.5489483 |
| Nme7 | -0.6325244 |
| Nme7 | 2.2292106 |
| Nme7 | 0.8217041 |
| Nmnat1 | 0.7107135 |
| Nmnat2 | -0.2187263 |
| Nmnat3 | 1.1359271 |
| Nmral1 | 0.1568873 |
| Nmt1 | -0.324954 |
| Nmu | -0.6256202 |
| Nmur2 | 0.0170137 |
| Nnat | -0.3362147 |
| Nnat | -0.7704976 |
| Nnmt | -0.1395744 |
| Nnmt | -0.6325244 |
| Nob1 | -0.4766084 |
| Nob1 | 1.2974251 |
| Nobox | 0.7499859 |
| Noc3l | -0.3064847 |
| Noc4l | -0.1612108 |
| Noc4l | -0.7664857 |
| Nod1 | 0.51261 |
| Nog | -0.60437 |
| Nol1 | 1.7811133 |
| Nol1 | -0.5124389 |
| Nol1 | 2.4644643 |
| Nol10 | 0.2149575 |
| Nol11 | -0.4088494 |
| Nol12 | 2.0920249 |
| Nol14 | 3.491536 |
| Nol3 | -0.6221873 |
| Nol4 | 1.5588409 |
| Nol4 | 0.7812687 |
| Nol4 | 0.9324411 |
| Nol5 | 2.1180289 |
| Nol5a | 3.0881531 |
| Nol6 | 0.0044559 |
| Nol7 | -0.6814346 |
| Nol9 | -0.6544372 |
| Nola1 | 3.2035029 |
| Nola2 | -0.1968057 |
| Nola3 | -0.6615601 |
| Nola3 | 0.3698075 |
| Nolc1 | 1.072517 |
| Nomo1 | -0.5056144 |
| Nono | 4.1229084 |
| Nope | -0.5301108 |
| Nos1 | 1.3175454 |
| Nos1ap | -0.4766084 |
| Nos2 | 1.8474178 |
| Nos3 | -0.0987331 |
| Nos3 | -0.6777893 |
| Nos3 | -0.6256202 |
| Nosip | 0.2620406 |
| Nosip | -0.4729544 |
| Nosip | -0.1451925 |
| Nostrin | 0.9755196 |
| Notch1 | -0.7054852 |
| Notch3 | 0.4971027 |
| Notch4 | -0.4047743 |
| Notch4 | 1.2226518 |
| Notum | -0.7196495 |
| Nova2 | 0.8999711 |
| Nox4 | 1.1132472 |
| Noxo1 | -0.4685885 |
| Noxo1 | 2.4532956 |
| Npal2 | 0.3712766 |
| Npas1 | 0.5571542 |
| Npas2 | -0.2895722 |
| Npas4 | -0.1209231 |
| Npb | -0.4584807 |
| Npc2 | 1.1771055 |
| Npcd | 1.2117995 |
| Npcd | -0.6732526 |
| Npdc1 | 0.178098 |
| Npepl1 | 2.8050016 |
| Npff | -0.510918 |
| Nphp1 | -0.5852406 |
| Nphp1 | -0.8612359 |
| Nphp3 | -0.2400418 |
| Nphp4 | -0.7282531 |
| Nphs2 | -0.6915339 |
| Nploc4 | 0.8767679 |
| Npm1 | -0.5600661 |
| Npm2 | -0.5363373 |
| Npm3 | -0.3873199 |
| Npm3 | 0.5474926 |
| Npm3-ps1 | 0.773118 |
| Npr1 | 2.3859515 |
| Npr2 | -0.2181719 |
| Npr3 | -0.1513604 |
| Npsr1 | -0.3793734 |
| Nptn | -0.6018524 |
| Nptx1 | -0.5505016 |
| Nptx2 | 0.4942785 |
| Nptxr | 2.3939493 |
| Npy | 0.1298637 |
| Npy1r | -0.4995901 |
| Npy2r | -0.1021502 |
| Npy5r | -0.5754144 |
| Nqo1 | -0.5308868 |
| Nqo2 | 1.1850774 |
| Nqo2 | -0.7928785 |
| Nqo2 | -0.6119641 |
| Nr1d1 | 0.3196632 |
| Nr1d2 | -0.6085813 |
| Nr1h2 | -0.6796102 |
| Nr1h2 | -0.0694764 |
| Nr1h3 | -0.6906112 |
| Nr1h4 | -0.7816206 |
| Nr1h5 | 0.5216416 |
| Nr1i2 | -0.3813536 |
| Nr2c1 | 2.2667992 |
| Nr2c2ap | 1.4052661 |
| Nr2e1 | 3.5742201 |
| Nr2f2 | -0.8011528 |
| Nr2f2 | -0.2723747 |
| Nr2f6 | 0.011838 |
| Nr2f6 | 0.5472498 |
| Nr4a2 | 0.5885742 |
| Nr4a3 | 0.8491184 |
| Nr4a3 | -0.2647678 |
| Nr5a1 | -0.6394804 |
| Nr5a2 | 1.1888554 |
| Nr5a2 | 2.3073059 |
| Nr5a2 | 0.14834 |
| Nr6a1 | -0.033362 |
| Nradd | 0.2208458 |
| Nrap | -0.4642431 |
| Nrarp | 0.3540901 |
| Nras | -0.4122589 |
| Nrbf2 | -0.7083008 |
| Nrbp1 | -0.2411774 |
| Nrbp2 | 3.4968976 |
| Nrcam | 1.2260465 |
| Nrd1 | -0.5465453 |
| Nrf1 | -0.6989485 |
| Nrg1 | -0.3508052 |
| Nrg3 | -0.7614952 |
| Nrg4 | 0.0225883 |
| Nrgn | 1.9216357 |
| Nrip2 | -0.3806931 |
| Nrip3 | 0.7757796 |
| Nrl | -0.2979899 |
| Nrm | -0.0967861 |
| Nrn1 | -0.6136602 |
| Nrn1l | -0.60353 |
| Nrp1 | -0.4839598 |
| Nrp1 | 1.1999779 |
| Nrp2 | 0.4689949 |
| Nrp2 | -0.3617388 |
| Nrsn2 | -0.3064847 |
| Nrsn2 | 0.0285546 |
| Nrtn | -0.002546 |
| Nrxn1 | 0.6442443 |
| Nrxn1 | 3.1451644 |
| Nrxn1 | 1.3277584 |
| Nrxn2 | 0.9483384 |
| Nrxn3 | -0.3747692 |
| Nrxn3 | -0.1085299 |
| Nsbp1 | 0.4779359 |
| Nsd1 | -0.6001777 |
| Nsd1 | -0.5262403 |
| Nsdhl | -0.7215546 |
| Nsdhl | 4.5983857 |
| Nsdhl | -0.6759719 |
| Nsf | -0.714904 |
| Nsfl1c | -0.5418199 |
| Nsg1 | 0.7464657 |
| Nsg2 | -0.0007905 |
| Nsl1 | -0.1179336 |
| Nsmaf | -0.7330676 |
| Nsmaf | -0.6535507 |
| Nsmce1 | -0.6368658 |
| Nsmce2 | -0.3636818 |
| Nsmce2 | 2.6246094 |
| Nsmce2 | -0.132473 |
| Nsun2 | -0.3591546 |
| Nsun4 | -0.4995901 |
| Nsun5 | 2.6100302 |
| Nsun5 | 0.6867864 |
| Nsun5 | -0.6213311 |
| Nsun5 | -0.4802768 |
| Nt5c | -0.775537 |
| Nt5c1b | -0.5746002 |
| Nt5c2 | 1.1277793 |
| Nt5c2 | -0.348889 |
| Nt5c2 | -0.6136602 |
| Nt5c3 | 0.7902833 |
| Nt5c3l | -0.5441796 |
| Nt5c3l | -0.7725101 |
| Nt5dc3 | -0.1498146 |
| Nt5dc3 | -0.7980413 |
| Nt5dc3 | 1.6737016 |
| Nt5e | -0.6282033 |
| Nt5e | -0.6316586 |
| Nt5m | -0.5976714 |
| Nt5m | 0.5731153 |
| Ntan1 | -0.4013921 |
| Ntf3 | -0.0704211 |
| Nthl1 | 0.2355749 |
| Ntn2l | -0.572974 |
| Ntn2l | 0.078581 |
| Ntn4 | -0.2440223 |
| Ntng1 | -0.3966776 |
| Ntng1 | -0.3482511 |
| Ntng1 | 0.4960767 |
| Ntrk2 | 0.1412193 |
| Ntrk2 | -0.1229215 |
| Ntrk2 | -0.331193 |
| Ntrk3 | 1.3996627 |
| Ntrk3 | 1.1583172 |
| Ntrk3 | 0.7153853 |
| Nts | 0.5908941 |
| Ntsr1 | -0.3559344 |
| Ntsr2 | 2.6721978 |
| Nuak1 | -0.5935093 |
| Nuak1 | -0.510918 |
| Nuak2 | 1.5503952 |
| Nub1 | -0.0875933 |
| Nubp1 | -0.0310942 |
| Nubp2 | 0.1415955 |
| Nubp2 | 1.0730683 |
| Nubp2 | 1.5067147 |
| Nucb1 | 0.052043 |
| Nucb1 | 0.393612 |
| Nucks1 | -0.0808774 |
| Nudc | -0.6952338 |
| Nudc | -0.4170535 |
| Nudcd2 | -0.3064847 |
| Nudcd3 | 1.2286135 |
| Nudc-ps1 | 1.3725245 |
| Nudt1 | 0.4304176 |
| Nudt10 | 0.7812687 |
| Nudt11 | 0.5994278 |
| Nudt12 | 0.4495021 |
| Nudt13 | 1.0752703 |
| Nudt14 | 0.8457796 |
| Nudt16l1 | -0.380033 |
| Nudt18 | 1.3112779 |
| Nudt19 | -0.693382 |
| Nudt19 | 0.7419483 |
| Nudt2 | -0.2859878 |
| Nudt21 | -0.5893657 |
| Nudt22 | 0.6090206 |
| Nudt4 | 1.4027584 |
| Nudt5 | 1.3163801 |
| Nudt5 | -0.0180493 |
| Nudt6 | 0.322143 |
| Nudt7 | -0.5576657 |
| Nudt8 | -0.2445924 |
| Nudt9 | -0.5697302 |
| Nufip1 | -0.6714441 |
| Numa1 | -0.1174363 |
| Numb | 0.6389793 |
| Numb | 0.0652028 |
| Numb | -0.638608 |
| Numb | 1.1883688 |
| Numbl | -0.2115451 |
| Nup107 | -0.1026394 |
| Nup133 | -0.5893657 |
| Nup133 | 1.7807278 |
| Nup155 | 0.8576739 |
| Nup155 | 0.1777364 |
| Nup160 | -0.520081 |
| Nup210 | -0.3089261 |
| Nup37 | 2.2826462 |
| Nup43 | 2.0601498 |
| Nup50 | -0.5147249 |
| Nup50 | -0.2126463 |
| Nup50 | 1.430727 |
| Nup62 | 1.0860669 |
| Nup85 | -0.6325244 |
| Nup88 | 2.0848762 |
| Nup88 | -0.6187671 |
| Nup88 | -0.0799219 |
| Nup93 | -0.5770449 |
| Nup98 | 0.9599295 |
| Nup98 | 0.0905779 |
| Nupl1 | -0.0540253 |
| Nupl1 | -0.6204756 |
| Nupl1 | -0.5778612 |
| Nupl2 | -0.4484818 |
| Nupr1 | 0.0306761 |
| Nus1 | 1.90941 |
| Nusap1 | -0.1075455 |
| Nut | 0.0849986 |
| Nutf2 | -0.3187566 |
| Nvl | -0.5926791 |
| Nwd1 | -0.7234635 |
| Nxf1 | -0.6342585 |
| Nxf2 | -0.6850944 |
| Nxf3 | -0.4802768 |
| Nxph1 | 1.6996587 |
| Nxph2 | -0.1184312 |
| Nxph3 | 1.4333472 |
| Nxt1 | 0.3576722 |
| Nxt1 | 0.9849616 |
| Nxt2 | -0.3064847 |
| Nyx | -0.4869168 |
| Oaf | -0.3859907 |
| Oas1b | 0.8129911 |
| Oas1b | -0.5968375 |
| Oas1c | -0.3533662 |
| Oas1d | 0.385263 |
| Oas1d | -0.2060588 |
| Oas1e | 0.5423797 |
| Oas1g | -0.4499037 |
| Oas1g | 1.2540139 |
| Oas2 | 0.1366937 |
| Oasl1 | 0.8692461 |
| Oasl2 | -0.0577475 |
| Oasl2 | -0.3793734 |
| Oat | 0.3456779 |
| Oaz2 | -0.714904 |
| Oaz3 | -0.5901929 |
| Obfc1 | 0.3686305 |
| Obfc2a | -0.5239258 |
| Obfc2b | -0.3318193 |
| Obfc2b | 0.9159402 |
| Obox1 | -0.5041047 |
| Obox5 | -0.6102712 |
| Obrgrp | 0.2922588 |
| Obsl1 | -0.2653508 |
| Oc90 | -0.6869297 |
| Oca2 | -0.0356353 |
| Ociad1 | -0.3034419 |
| Ociad1 | 0.1199084 |
| Ociad2 | -0.6980184 |
| Ocln | 1.0607232 |
| Ocrl | 0.2166932 |
| Odc1 | 2.5738736 |
| Odf1 | -0.510918 |
| Odf2 | -0.7272932 |
| Odf2 | -0.2226162 |
| Odf2 | -0.5910209 |
| Odf2 | -0.1441685 |
| Odf3 | -0.5926791 |
| Odf3l1 | -0.3966776 |
| Odf4 | 3.0404412 |
| Odf4 | -0.3734579 |
| Odz1 | -0.6869297 |
| Odz3 | -0.1775105 |
| Odz3 | -0.1036186 |
| Odz4 | -0.1855015 |
| Og9x | -0.115946 |
| Ogdh | -0.5648855 |
| Ogfod1 | -0.6508962 |
| Ogfod2 | 1.067263 |
| Ogfr | 1.0477868 |
| Ogfrl1 | -0.6111173 |
| Ogg1 | 0.0306761 |
| Ogn | 2.4456699 |
| Ogn | 0.4729501 |
| Ogn | -0.7206016 |
| Ogt | -0.4463532 |
| Ogt | 0.2128703 |
| Ogt | 1.0408225 |
| Oit1 | 1.2312896 |
| Oit3 | -0.6544372 |
| Ola1 | 0.7380021 |
| Olfm1 | 1.09351 |
| Olfm1 | -0.5262403 |
| Olfm2 | -0.6196209 |
| Olfm2 | 0.8195335 |
| Olfm3 | 0.4114948 |
| Olfm3 | -0.1586084 |
| Olfm4 | 2.0040423 |
| Olfml1 | -0.5778612 |
| Olfml2b | -0.6333911 |
| Olfml3 | 1.4717876 |
| Olfr100 | -0.3565775 |
| Olfr1006 | 2.1090056 |
| Olfr1012 | -0.263603 |
| Olfr1020 | -0.456329 |
| Olfr1065 | 1.1694371 |
| Olfr1094 | 0.8166315 |
| Olfr1099 | -0.5246966 |
| Olfr1105 | 3.5611297 |
| Olfr1112 | 1.7846395 |
| Olfr1113 | -0.215405 |
| Olfr1189 | -0.0034249 |
| Olfr1197 | -0.1596485 |
| Olfr1198 | 1.6437256 |
| Olfr1249 | -0.3268213 |
| Olfr1280 | -0.4170535 |
| Olfr133 | -0.1570505 |
| Olfr1330 | -0.693382 |
| Olfr134 | 0.0208766 |
| Olfr1340 | -0.4876575 |
| Olfr1384 | 3.3152249 |
| Olfr1386 | -0.7486439 |
| Olfr1390 | -0.219281 |
| Olfr1394 | -0.7388781 |
| Olfr1412 | 2.1889177 |
| Olfr1437 | 0.4492322 |
| Olfr144 | 1.9234006 |
| Olfr1443 | 1.2790684 |
| Olfr1443 | -0.7101827 |
| Olfr1459 | -0.4714968 |
| Olfr1500 | 3.8041625 |
| Olfr1508 | -0.5640805 |
| Olfr166 | -0.6145094 |
| Olfr168 | 1.5801433 |
| Olfr206 | 0.8678719 |
| Olfr213 | -0.2753172 |
| Olfr215 | 0.1256649 |
| Olfr232 | 0.2925784 |
| Olfr257 | -0.233821 |
| Olfr267 | -0.5339974 |
| Olfr272 | -0.2659341 |
| Olfr303 | 2.4104259 |
| Olfr310 | 3.7608984 |
| Olfr352 | -0.6403535 |
| Olfr371 | 0.9415359 |
| Olfr378 | 0.9242273 |
| Olfr419 | 1.1721654 |
| Olfr437 | -0.5664976 |
| Olfr464 | -0.7167993 |
| Olfr47 | 0.8699324 |
| Olfr49 | -0.8735093 |
| Olfr503 | -0.7379071 |
| Olfr51 | 2.3571961 |
| Olfr536 | 1.7524767 |
| Olfr538 | -0.7101827 |
| Olfr54 | -0.7980413 |
| Olfr550 | 0.3477881 |
| Olfr574 | -0.5976714 |
| Olfr591 | 3.3326743 |
| Olfr593 | -0.2304453 |
| Olfr608 | 1.9909172 |
| Olfr638 | -0.6085813 |
| Olfr65 | 1.9441064 |
| Olfr655 | 0.9506997 |
| Olfr658 | -0.4371849 |
| Olfr665 | -0.0256741 |
| Olfr675 | 1.1027641 |
| Olfr691 | 0.5895029 |
| Olfr692 | 0.8697609 |
| Olfr702 | 0.0797877 |
| Olfr724 | -0.1204242 |
| Olfr725 | -0.2027824 |
| Olfr748 | 0.0757593 |
| Olfr786 | 0.3264667 |
| Olfr802 | 1.3569284 |
| Olfr824 | 0.132146 |
| Olfr91 | 1.6182774 |
| Olfr910 | 2.5699844 |
| Olfr920 | -0.6491308 |
| Olfr924 | 1.8681752 |
| Olfr952 | 1.5195764 |
| Olfr965 | -0.3793734 |
| Olfr982 | -0.4315877 |
| Olfr983 | 0.3159311 |
| Olfr984 | -0.5835956 |
| Olig1 | -0.60437 |
| Olig3 | -0.7388781 |
| Oma1 | -0.3747692 |
| Omd | 0.3841055 |
| Omg | -0.0900036 |
| Omt2b | -0.7684895 |
| Onecut2 | -0.6606667 |
| Onecut3 | -0.821105 |
| Onecut3 | 1.8651292 |
| Oog4 | -0.1722212 |
| Oosp1 | -0.520081 |
| Opa1 | 0.108677 |
| Opa1 | 1.112191 |
| Ophn1 | 1.4856694 |
| Oplah | 1.5747029 |
| Opn1mw | -0.0324542 |
| Opn3 | -0.6482493 |
| Oprl1 | 3.0969819 |
| Oprl1 | -0.7476627 |
| Oprl1 | 0.9499132 |
| Oprs1 | -0.4020676 |
| Optc | -0.1259272 |
| Optn | -0.4392927 |
| Optn | 0.3071639 |
| Orai1 | -0.4635209 |
| Orai3 | -0.5786782 |
| Oraov1 | 0.193516 |
| Orc2l | 1.2200682 |
| Orc3l | -0.7244194 |
| Orc5l | -0.5860641 |
| Orc5l | -0.5819536 |
| Orc6l | 0.8157227 |
| ORF19 | 2.0545219 |
| ORF28 | -0.6153594 |
| ORF34 | -0.1329785 |
| ORF61 | 0.4345403 |
| ORF63 | 0.3118709 |
| ORF9 | -0.2143006 |
| ORF9 | 0.0565878 |
| ORF9 | -0.6896895 |
| Orm1 | 1.3260815 |
| Ormdl2 | 0.1766507 |
| Ormdl3 | 0.6312471 |
| Os9 | -0.5737868 |
| Osbp2 | 0.1059454 |
| Osbpl10 | 2.5182885 |
| Osbpl11 | 0.9650521 |
| Osbpl2 | -0.300409 |
| Osbpl3 | 0.0470596 |
| Osbpl3 | 0.1863767 |
| Osbpl6 | 2.5233819 |
| Osbpl8 | -0.2209471 |
| Osbpl9 | -0.5926791 |
| Oscar | -0.3946644 |
| Osgep | 0.6988271 |
| Osgepl1 | 1.3804727 |
| Osgin1 | 1.1741446 |
| Osm | -0.8137042 |
| Osmr | 1.5526166 |
| Osr1 | 2.028027 |
| Osr2 | -0.3457041 |
| Ostf1 | 3.0208467 |
| Ostm1 | -0.6325244 |
| Otof | 0.933883 |
| Otop1 | -0.0582138 |
| Otop2 | -0.497341 |
| Ott | 1.0274589 |
| Ott | 2.0375278 |
| OTTMUSG00000000421 | 1.6515879 |
| OTTMUSG00000000971 | -0.0306413 |
| OTTMUSG00000000990 | 0.2948128 |
| OTTMUSG00000001305 | -0.2806371 |
| OTTMUSG00000002038 | 1.132444 |
| OTTMUSG00000004461 | -0.5770449 |
| OTTMUSG00000004551 | -0.6544372 |
| OTTMUSG00000005148 | 2.4588194 |
| OTTMUSG00000005491 | -0.4315877 |
| OTTMUSG00000008540 | 1.4670237 |
| OTTMUSG00000010009 | 2.6358749 |
| OTTMUSG00000010328 | 1.7718819 |
| OTTMUSG00000015762 | -0.6052107 |
| OTTMUSG00000015859 | 1.0630939 |
| OTTMUSG00000016293 | 0.0200196 |
| OTTMUSG00000016790 | -0.6980184 |
| OTTMUSG00000016790 | 1.0712753 |
| OTTMUSG00000017677 | -0.642978 |
| OTTMUSG00000020946 | 0.5078076 |
| Otub1 | -0.6052107 |
| Otub2 | -0.1400838 |
| Otub2 | -0.6010147 |
| Otub2 | -0.5418199 |
| Otud5 | -0.5177817 |
| Otud6b | 0.6861593 |
| Otud7b | 0.1741124 |
| Otud7b | 1.6962863 |
| Otx1 | -0.4958446 |
| Otx2 | -0.4802768 |
| Ovca2 | -0.8232305 |
| Ovgp1 | 1.6590911 |
| Ovgp1 | 0.0225883 |
| Ovol1 | -0.5943402 |
| Ovol2 | 1.04296 |
| Ovol2 | 0.2229151 |
| Oxct1 | -0.5860641 |
| Oxct2a | -0.7350004 |
| Oxct2b | 1.989892 |
| Oxgr1 | 1.871794 |
| Oxnad1 | -0.7704976 |
| Oxr1 | 0.0877925 |
| Oxr1 | -0.693382 |
| Oxt | -0.5697302 |
| P140 | 0.0953335 |
| P2rx2 | -0.4129424 |
| P2rx3 | 2.5961525 |
| P2rx4 | 0.2184257 |
| P2rx4 | -0.6535507 |
| P2rx5 | 0.3128095 |
| P2rx7 | -0.6342585 |
| P2rx7 | 2.8769784 |
| P2ry1 | 1.2579735 |
| P2ry12 | -0.60437 |
| P2ry13 | 2.8898021 |
| P2ry14 | -0.4722253 |
| P2ry14 | -0.5363373 |
| P2ry5 | -0.6421024 |
| P2ry6 | 0.6825979 |
| P42pop | -0.2400418 |
| P4ha1 | 0.2709155 |
| P4ha2 | -0.1214223 |
| P4hb | 1.1000913 |
| Pa2g4 | -0.4591991 |
| Pabpc1 | 1.6187366 |
| Pabpc1 | -0.7282531 |
| Pabpc1 | -0.0591471 |
| Pabpc2 | 0.0105395 |
| Pabpc4 | -0.4054523 |
| Pabpn1 | -0.1575695 |
| Pabpnl1 | 0.2935367 |
| Pacrg | 2.1903651 |
| Pacs1 | -0.1508448 |
| Pacs2 | 1.4099738 |
| Pacsin1 | -0.2310071 |
| Pacsin1 | 2.3997049 |
| Pacsin1 | -0.3206115 |
| Pacsin2 | 1.9460443 |
| Pacsin2 | -0.7330676 |
| Pacsin3 | 0.5542663 |
| Pacsin3 | 0.7694937 |
| Padi1 | 1.796782 |
| Padi2 | -0.3540076 |
| Padi6 | 0.552336 |
| Paf1 | -0.3899839 |
| Paf1 | 0.8378237 |
| Pafah1b1 | -0.1622538 |
| Pafah1b1 | 0.1849422 |
| Pafah1b3 | -0.333073 |
| Pafah2 | -0.0727872 |
| Pafah2 | -0.7565313 |
| Paip1 | -0.5347767 |
| Paip2 | 1.9837166 |
| Paip2b | -0.7282531 |
| Paip2b | 0.8073202 |
| Pak1 | -0.7350004 |
| Pak3 | 1.7331982 |
| Pak4 | 0.1202933 |
| Pak6 | 2.9040415 |
| Pak7 | -0.6732526 |
| Palld | 0.8520956 |
| Palm | 1.6728357 |
| Palm | -0.5624727 |
| Palm2 | -0.5063702 |
| Palmd | -0.5885392 |
| Palmd | 1.2600034 |
| Palmd | 1.6424584 |
| Pam | 2.9755971 |
| Pank1 | 0.1505773 |
| Pank1 | -0.6998795 |
| Pank2 | 2.1117983 |
| Pank3 | 1.8408762 |
| Pank4 | 0.5034895 |
| Panx1 | 1.618507 |
| Panx3 | -0.7272932 |
| Paox | -0.8590217 |
| Paox | 1.1405511 |
| Paox | 1.3739204 |
| Paox | 3.7027752 |
| Papd1 | -0.6196209 |
| Papd1 | -0.5170166 |
| Papd4 | -0.2148526 |
| Papln | 0.7364189 |
| Papola | 1.0307818 |
| Papolb | -0.7486439 |
| Papolg | -0.4506154 |
| Papss1 | -0.6553246 |
| Papss1 | -0.7990773 |
| Papss2 | 0.3695134 |
| Paqr3 | 0.5709872 |
| Paqr6 | -0.1149537 |
| Paqr7 | -0.4891407 |
| Paqr9 | -0.6394804 |
| Paqr9 | -0.4685885 |
| Paqr9 | 1.8613076 |
| Pard3 | -0.3873199 |
| Pard3 | -0.3966776 |
| Pard6a | -0.6759719 |
| Pard6a | 0.9750603 |
| Pard6b | -0.3399988 |
| Pard6g | -0.2812301 |
| Park7 | -0.6307936 |
| Parl | 1.3655136 |
| Parp1 | 0.7536871 |
| Parp1 | -0.0480085 |
| Parp1 | 2.4248514 |
| Parp12 | 1.2324508 |
| Parp14 | -0.1299499 |
| Parp16 | -0.5624727 |
| Parp2 | -0.4315877 |
| Parp2 | -0.6741582 |
| Parp3 | 0.5401798 |
| Parp6 | -0.7476627 |
| Parp8 | 0.165723 |
| Pars2 | -0.2985941 |
| Pars2 | -0.5713507 |
| Parva | -0.4876575 |
| Parvg | -0.7036129 |
| Pask | 0.5283581 |
| Patl1 | -0.729214 |
| Patz1 | -0.5347767 |
| Pawr | -0.6153594 |
| Pax8 | -0.5177817 |
| Paxip1 | -0.8032329 |
| Pbk | 2.2323755 |
| Pbld | -0.1780411 |
| Pbp2 | 0.3677468 |
| Pbrm1 | 0.1931603 |
| Pbrm1 | -0.6860116 |
| Pbx1 | -0.6924575 |
| Pbx1 | -0.3058753 |
| Pbx2 | 0.7324491 |
| Pbx2 | -0.0799219 |
| Pbx2 | -0.6705412 |
| Pbx3 | -0.4824849 |
| Pbx3 | 0.8983091 |
| Pbx4 | 1.8415986 |
| Pcaf | -0.3162892 |
| Pcaf | 0.353791 |
| Pcbd1 | 2.5646996 |
| Pcbd2 | -0.5584651 |
| Pcbp1 | -0.7083008 |
| Pcbp2 | -0.572162 |
| Pcbp3 | -0.6128118 |
| Pcca | 0.8280062 |
| Pcca | -0.4232546 |
| Pccb | -0.1174363 |
| Pccb | 0.0208766 |
| Pcdh1 | 0.721645 |
| Pcdh10 | -0.7959727 |
| Pcdh10 | 2.2268298 |
| Pcdh10 | 3.6786742 |
| Pcdh15 | 1.3058259 |
| Pcdh17 | -0.5078837 |
| Pcdh20 | 1.6737016 |
| Pcdh21 | -0.8893686 |
| Pcdh7 | 4.152788 |
| Pcdh7 | 1.6969196 |
| Pcdh8 | -0.6204756 |
| Pcdh9 | 0.2643497 |
| Pcdha10 | 0.1175958 |
| Pcdha11 | 0.7687288 |
| Pcdha2 | 0.1956471 |
| Pcdha6 | 4.7694758 |
| Pcdhac1 | 2.0014119 |
| Pcdhac2 | 1.2894933 |
| Pcdhb1 | -0.6500131 |
| Pcdhb10 | 2.340792 |
| Pcdhb12 | 1.4682845 |
| Pcdhb14 | 0.8209812 |
| Pcdhb19 | -0.3156734 |
| Pcdhb20 | -0.7745269 |
| Pcdhb22 | -0.769493 |
| Pcdhb3 | -0.5371185 |
| Pcdhb7 | -0.3280683 |
| Pcdhb8 | -0.1390654 |
| Pcdhga10 | -0.2066059 |
| Pcdhga12 | -0.7186985 |
| Pcdhga4 | -0.2671018 |
| Pcdhga7 | -0.0274773 |
| Pcdhga8 | 2.0597694 |
| Pcdhgb2 | -0.1426346 |
| Pcdhgb6 | 1.2221826 |
| Pcdhgc3 | 1.8325273 |
| Pcdhgc4 | -0.4267176 |
| Pcdhgc5 | 2.7953647 |
| Pcf11 | 1.6571843 |
| Pcgf1 | -0.8021923 |
| Pcgf2 | -0.1329785 |
| Pcgf3 | 1.5239939 |
| Pcgf5 | -0.6170616 |
| Pcgf6 | -0.5528835 |
| Pck1 | 0.4356366 |
| Pclo | 0.3459796 |
| Pclo | 3.4349671 |
| Pcmt1 | 0.0061982 |
| Pcmt1 | -0.4491925 |
| Pcmt1 | -0.3714944 |
| Pcmtd1 | -0.2554903 |
| Pcmtd2 | 0.0925624 |
| Pcna | -0.4477717 |
| Pcnt | 0.5688542 |
| Pcnx | -0.617914 |
| Pcnxl2 | -0.5124389 |
| Pcnxl2 | 0.273855 |
| Pcolce | 2.056338 |
| Pcolce2 | 0.2754841 |
| Pcolce2 | 0.3872851 |
| Pcsk1n | 2.9871356 |
| Pcsk2 | 1.1518846 |
| Pcsk2 | -0.6571019 |
| Pcsk2 | 1.404206 |
| Pcsk2 | 1.6563764 |
| Pcsk4 | -0.3546494 |
| Pcsk4 | -0.5901929 |
| Pcsk4 | 0.2450418 |
| Pcsk4 | -0.2788604 |
| Pcsk4 | 2.2322965 |
| Pcsk9 | 1.3879609 |
| Pctk1 | -0.3224701 |
| Pcx | -0.2220595 |
| Pcyox1 | -0.1764502 |
| Pcyox1l | -0.6307936 |
| Pcyt1a | -0.5147249 |
| Pcyt1b | -0.6482493 |
| Pcyt1b | 0.7515461 |
| Pcyt2 | 0.1777364 |
| Pcyt2 | -0.1129724 |
| Pcyt2 | 0.1888817 |
| Pdcd10 | -0.2937715 |
| Pdcd2 | -0.4129424 |
| Pdcd2l | 1.5397914 |
| Pdcd4 | 1.734685 |
| Pdcd5 | 0.2301216 |
| Pdcd5 | -0.4095303 |
| Pdcd6ip | 1.1145658 |
| Pdcl | -0.4435225 |
| Pdcl3 | 0.8189901 |
| Pddc1 | 0.3307704 |
| Pde10a | -0.4027435 |
| Pde1a | -0.5713507 |
| Pde1a | 0.2097308 |
| Pde1a | 1.2624783 |
| Pde1b | 1.4672039 |
| Pde1c | -0.0484699 |
| Pde2a | -0.1344965 |
| Pde3a | -0.6571019 |
| Pde4a | 2.3113407 |
| Pde4a | -0.3089261 |
| Pde4a | -0.7496262 |
| Pde4a | -0.4876575 |
| Pde4b | 0.2826181 |
| Pde4d | -0.3591546 |
| Pde4dip | 1.0204855 |
| Pde4dip | -0.520081 |
| Pde4dip | -0.4281064 |
| Pde4dip | 0.5278623 |
| Pde4dip | -0.219281 |
| Pde5a | 0.9036172 |
| Pde6a | -0.5600661 |
| Pde6d | -0.3636818 |
| Pde6d | -0.6001777 |
| Pde6d | -0.6351268 |
| Pde6d | 2.5089849 |
| Pde6h | -0.3101492 |
| Pde7a | -0.3218501 |
| Pdgfa | -0.6850944 |
| Pdgfb | 2.7799533 |
| Pdgfc | -0.3343284 |
| Pdgfra | 2.9463173 |
| Pdgfrb | -0.5536788 |
| Pdgfrl | 1.0116945 |
| Pdha1 | 1.6031491 |
| Pdhb | -0.3040496 |
| Pdhb | -0.5681125 |
| Pdia3 | -0.6128118 |
| Pdia4 | 0.3715701 |
| Pdia5 | -0.5418199 |
| Pdia5 | -0.2531853 |
| Pdia6 | 0.0070681 |
| Pdik1l | 0.2944939 |
| Pdk1 | 1.8175174 |
| Pdk2 | -0.6377365 |
| Pdk3 | -0.3299419 |
| Pdk4 | 0.1910235 |
| Pdlim1 | 0.4524657 |
| Pdlim2 | 0.5804084 |
| Pdlim2 | -0.6544372 |
| Pdlim2 | 2.6117897 |
| Pdlim2 | -0.6832627 |
| Pdlim3 | 1.1090152 |
| Pdlim3 | -0.0818338 |
| Pdlim4 | -0.2842008 |
| Pdlim5 | 2.6501825 |
| Pdlim5 | -0.4470622 |
| Pdlim7 | -0.6860116 |
| Pdpk1 | -0.4191159 |
| Pdpk1 | 2.6667242 |
| Pdrg1 | -0.8360863 |
| Pdss1 | -0.2282017 |
| Pdxdc1 | 1.2671848 |
| Pdxdc1 | 2.1335345 |
| Pdxk | -0.0065079 |
| Pdxp | -0.5910209 |
| Pdyn | -0.4678629 |
| Pdyn | 1.1338646 |
| Pdzd11 | -0.3540076 |
| Pdzd11 | 0.074143 |
| Pdzd11 | 0.0195908 |
| Pdzd8 | 1.2560524 |
| Pdzk1 | 0.0470596 |
| Pdzk1ip1 | -0.1801665 |
| Pdzrn3 | -0.5673047 |
| Pdzrn3 | 1.208233 |
| Pea15 | -0.8360863 |
| Pear1 | 0.4647574 |
| Pear1 | 0.5698028 |
| Pebp1 | -0.6111173 |
| Pecam1 | -0.2842008 |
| Pecam1 | -0.7215546 |
| Peci | 0.3289284 |
| Pef1 | 0.4117758 |
| Peg10 | -0.5520888 |
| Peg3 | 0.2806779 |
| Peli1 | -0.8916565 |
| Peli1 | 0.0873939 |
| Peli2 | -0.3787143 |
| Peli3 | 0.2301216 |
| Pelo | 0.0524571 |
| Pelp1 | -0.7282531 |
| Pemt | 1.5520412 |
| Peo1 | 2.7206658 |
| Pepd | 1.3789876 |
| Per1 | -0.6119641 |
| Per1 | -0.7130126 |
| Per2 | 0.7486185 |
| Per2 | -0.8221672 |
| Per2 | 0.7681548 |
| Per3 | 0.2163464 |
| Perld1 | 1.0054908 |
| Pes1 | -0.4839598 |
| Pet112l | -0.4606374 |
| Pet112l | 0.356778 |
| Pet112l | 1.2017787 |
| Pet112l | 0.4408265 |
| Pex11a | 0.4221168 |
| Pex11a | -0.6615601 |
| Pex11a | -0.7408231 |
| Pex11b | -0.7282531 |
| Pex11c | -0.4122589 |
| Pex14 | -0.5041047 |
| Pex16 | 1.7731137 |
| Pex19 | -0.1289426 |
| Pex19 | -0.7515938 |
| Pex2 | 0.7017102 |
| Pex26 | 0.9431223 |
| Pex3 | 1.5126532 |
| Pex3 | 2.5429153 |
| Pex5 | -0.0360906 |
| Pex5 | -0.0475473 |
| Pex5 | -0.7918494 |
| Pex5l | -0.2812301 |
| Pex6 | 0.1000649 |
| Pex6 | 1.0862028 |
| Pex6 | -0.2998036 |
| Pex7 | -0.5465453 |
| Pfdn1 | -0.5505016 |
| Pfdn2 | 0.5655264 |
| Pfdn4 | 0.2409964 |
| Pfdn5 | -0.6010147 |
| Pfdn5 | 1.490778 |
| Pfkfb2 | 2.6688731 |
| Pfkfb2 | -0.4898832 |
| Pfkfb4 | 0.1649899 |
| Pfkl | 1.303894 |
| Pfkl | -0.2919694 |
| Pfkm | -0.0685326 |
| Pfkm | 3.4807499 |
| Pfkm | -0.2298839 |
| Pfkp | -0.7186985 |
| Pfkp | 0.2416718 |
| Pfkp | 1.0928359 |
| Pfn1 | 3.3086005 |
| Pfn1 | -0.7008114 |
| Pfn2 | 0.5913574 |
| Pfn2 | 1.8434023 |
| Pfn2 | -0.163298 |
| Pfn3 | 1.0524478 |
| Pfn4 | -0.0176028 |
| Pftk1 | -0.3886509 |
| Pga5 | 1.4051698 |
| Pgam1 | -0.2723747 |
| Pgam2 | 1.0019271 |
| Pgam5 | 1.1157509 |
| Pgbd5 | -0.1544597 |
| Pgcp | -0.5379004 |
| Pgd | 0.6731041 |
| Pgk1 | -0.1339902 |
| Pgk2 | -0.5223859 |
| Pgls | 1.9641493 |
| Pglyrp1 | -0.2508861 |
| Pglyrp4 | -0.2098958 |
| Pgm2 | -0.4995901 |
| Pgm2l1 | -0.0784906 |
| Pgm3 | -0.6018524 |
| Pgm5 | 0.374793 |
| Pgm5 | -0.1685365 |
| Pgpep1 | -0.8095015 |
| Pgpep1 | -0.0301887 |
| Pgr | 0.2527465 |
| Pgrmc1 | -0.7026781 |
| Pgs1 | -0.3187566 |
| Phactr1 | -0.6094258 |
| Phactr1 | -0.5363373 |
| Phactr1 | -0.6153594 |
| Phactr4 | -0.6823482 |
| Phb2 | 0.4674082 |
| Phc1 | -0.7486439 |
| Phc1 | -0.5293354 |
| Phc2 | 1.6983955 |
| Phca | -0.2181719 |
| Phca | 2.9267945 |
| Phex | 1.2554866 |
| Phf1 | 1.3923664 |
| Phf11 | 2.0020702 |
| Phf11 | -0.5347767 |
| Phf12 | 1.1429823 |
| Phf12 | 0.7968016 |
| Phf13 | -0.1164425 |
| Phf13 | -0.642978 |
| Phf14 | 0.3887269 |
| Phf16 | -0.5041047 |
| Phf17 | -0.3747692 |
| Phf19 | 1.6189661 |
| Phf2 | 0.7715937 |
| Phf20l1 | 2.1081482 |
| Phf21a | 0.3071639 |
| Phf5a | 1.7363044 |
| Phf6 | 1.0941837 |
| Phf7 | 2.6919236 |
| Phgdh | 2.0743531 |
| Phka1 | 2.0388911 |
| Phka1 | -0.2583796 |
| Phka2 | -0.1001961 |
| Phka2 | -0.4700416 |
| Phka2 | -0.3418967 |
| Phkb | -0.3463402 |
| Phkg1 | 0.7666223 |
| Phkg2 | 0.4103695 |
| Phlda1 | 1.4108358 |
| Phlda2 | 1.1815292 |
| Phlda3 | 0.9919054 |
| Phlda3 | 0.3993265 |
| Phldb1 | -0.4211829 |
| Phldb1 | -0.4143107 |
| Phlpp | -0.500341 |
| Phospho1 | 1.4057476 |
| Phospho2 | -0.4584807 |
| Phox2a | -0.1021502 |
| Phox2b | 2.3002482 |
| Phpt1 | 1.1791971 |
| Phtf1 | -0.6980184 |
| Phtf2 | -0.421873 |
| Phyhip | -0.6447318 |
| Pi16 | -0.6796102 |
| Pi4k2b | -0.0953285 |
| Pi4k2b | 2.9858392 |
| Pi4ka | 1.8993991 |
| Pi4kb | -0.1903293 |
| Pi4kb | 1.9953639 |
| Pias1 | 0.5206424 |
| Pias2 | 0.3399258 |
| Pias3 | 0.6585711 |
| Pias3 | -0.2254048 |
| Pias4 | 0.3585655 |
| Pibf1 | -0.60437 |
| Pibf1 | -0.6456099 |
| Picalm | -0.2931704 |
| Pick1 | 1.5625068 |
| Pick1 | 0.7673888 |
| Pif1 | -0.0842292 |
| Piga | 1.1685673 |
| Piga | 2.8837354 |
| Pigb | 1.5171891 |
| Pigc | -0.6162101 |
| Pigf | -0.693382 |
| Pigh | 0.0433044 |
| Pigk | -0.459918 |
| Pigk | 1.061142 |
| Pigl | -0.0480085 |
| Pign | -0.6094258 |
| Pigo | 2.1291748 |
| Pigo | -0.6290659 |
| Pigo | 0.4348145 |
| Pigp | -0.6377365 |
| Pigp | 3.0228872 |
| Pigq | -0.5301108 |
| Pigt | 1.2689715 |
| Pigx | -0.348889 |
| Pigy | -0.8147579 |
| Pigyl | 1.6375974 |
| Pigz | -0.1806986 |
| Pih1d1 | 0.7123411 |
| Pik3ap1 | 0.9330822 |
| Pik3c2a | -0.5600661 |
| Pik3c3 | -0.5811337 |
| Pik3ca | 0.408115 |
| Pik3cb | -0.2788604 |
| Pik3cd | 1.7484932 |
| Pik3cg | -0.3859907 |
| Pik3ip1 | -0.5116781 |
| Pik3r1 | -0.7867209 |
| Pik3r1 | 0.233193 |
| Pik3r1 | -0.1421239 |
| Pik3r2 | 0.6630835 |
| Pik3r2 | 1.017857 |
| Pik3r3 | -0.6060522 |
| Pik3r4 | 1.8924178 |
| Pik3r6 | 2.0283715 |
| Pik3r6 | -0.7215546 |
| Pim1 | -0.2220595 |
| Pim2 | 0.1225992 |
| Pim2 | 1.6435021 |
| Pim3 | 0.0057629 |
| Pip4k2a | 2.080095 |
| Pip4k2b | 1.8701031 |
| Pip4k2b | 0.8344439 |
| Pip4k2c | -0.257801 |
| Pip5k1a | -0.7684895 |
| Pip5k1a | -0.4484818 |
| Pip5k1b | -0.4758764 |
| Pip5kl1 | -0.2287621 |
| Pipox | -0.1586084 |
| Pir | 2.1319075 |
| Pira11 | 1.6086578 |
| Pira4 | 1.9684634 |
| Pisd | -0.6068945 |
| Pit1 | -0.1472439 |
| Pitpna | 1.1461715 |
| Pitpnb | 0.9309968 |
| Pitpnc1 | 3.7441116 |
| Pitpnm1 | -0.3939943 |
| Pitpnm2 | 2.5004749 |
| Pitpnm3 | 1.0196102 |
| Pitx1 | 0.4721604 |
| Pitx2 | -0.7158512 |
| Pitx2 | -0.3126003 |
| Pitx2 | -0.5819536 |
| Pitx2 | 3.889179 |
| Piwil1 | -0.7167993 |
| Piwil2 | -0.4556129 |
| Piwil4 | 1.3005574 |
| Pja1 | -0.5132003 |
| Pja2 | 0.8731855 |
| Pja2 | 2.2359625 |
| Pkd1 | -0.1446804 |
| Pkd1l3 | 2.6590514 |
| Pkd2 | -0.478074 |
| Pkd2l1 | -0.5648855 |
| Pkd2l2 | -0.7908214 |
| Pkdrej | -0.0342706 |
| Pkhd1l1 | 1.5807016 |
| Pkhd1l1 | 1.3545916 |
| Pkia | 0.3128095 |
| Pkib | 2.189621 |
| Pkib | -0.7816206 |
| Pkig | -0.5576657 |
| Pkig | -0.5951719 |
| Pkig | -0.408169 |
| Pkm2 | 0.5588347 |
| Pkmyt1 | -0.366929 |
| Pkn1 | -0.6256202 |
| Pkn1 | 1.6599699 |
| Pkn2 | 1.4146577 |
| Pkn2 | 0.0809928 |
| Pkn2 | 1.7528741 |
| Pkn3 | 0.193516 |
| Pknox1 | 3.0497827 |
| Pknox1 | 2.5752119 |
| Pknox2 | -0.114458 |
| Pknox2 | 1.3793838 |
| Pknox2 | 1.2303597 |
| Pknox2 | -0.4980901 |
| Pknox2 | 1.720051 |
| Pkp1 | 1.0088932 |
| Pkp2 | -0.6759719 |
| Pkp2 | -0.1239223 |
| Pkp2 | 1.2175953 |
| Pkp3 | -0.8568128 |
| Pla1a | -0.4371849 |
| Pla1a | -0.711125 |
| Pla1a | -0.6162101 |
| Pla2g10 | -0.6500131 |
| Pla2g12a | -0.7017443 |
| Pla2g1b | -0.3933247 |
| Pla2g2c | -0.5754144 |
| Pla2g2d | -0.0087163 |
| Pla2g2f | -0.5786782 |
| Pla2g4c | -0.3993687 |
| Pla2g4d | -0.5528835 |
| Pla2g4e | -0.7045486 |
| Pla2g4f | 2.1481799 |
| Pla2g5 | 2.1927176 |
| Pla2g6 | 0.6236736 |
| Pla2g7 | 0.1464714 |
| Plaa | 0.579236 |
| Plac1 | -0.4846982 |
| Plac1l | -0.3156734 |
| Plac8 | 0.1967108 |
| Plag1 | 0.6274681 |
| Plag1 | 3.1830782 |
| Plagl2 | -0.6085813 |
| Plau | 0.0302522 |
| Plb1 | 0.3408364 |
| Plcb2 | 1.7388626 |
| Plcb3 | 1.1221767 |
| Plcb4 | 0.1363156 |
| Plcd1 | -0.6421024 |
| Plcd3 | -0.6796102 |
| Plcd4 | -0.8200442 |
| Plcd4 | -0.3243324 |
| Plcd4 | -0.5960043 |
| Plce1 | 2.4328087 |
| Plcg2 | 0.6180652 |
| Plch2 | 0.225667 |
| Plcl2 | 0.1179816 |
| Plcxd1 | 0.3335265 |
| Plcxd1 | -0.3980222 |
| Plcz1 | 2.9644516 |
| Pld1 | 1.0653198 |
| Pld2 | -0.6299293 |
| Pld3 | 0.0893852 |
| Pld3 | 0.4276589 |
| Pld4 | 0.7604668 |
| Pld5 | -0.617914 |
| Pldn | -0.4191159 |
| Plec1 | 0.4906716 |
| Plec1 | -0.1405934 |
| Plec1 | 0.0553505 |
| Plec1 | 0.0503849 |
| Plek | 2.5479037 |
| Plek2 | -0.6282033 |
| Plekha1 | 1.4687343 |
| Plekha1 | 0.3861302 |
| Plekha2 | 1.2499234 |
| Plekha3 | -0.5943402 |
| Plekha4 | 2.3881911 |
| Plekha4 | -0.6500131 |
| Plekha5 | -0.4832221 |
| Plekha6 | -0.5746002 |
| Plekha7 | -0.5139623 |
| Plekha8 | -0.7311388 |
| Plekhb1 | 0.4421874 |
| Plekhb2 | -0.0746845 |
| Plekhf1 | -0.0746845 |
| Plekhf1 | -0.1360169 |
| Plekhf2 | -0.6001777 |
| Plekhf2 | 2.3558833 |
| Plekhg2 | 1.0161004 |
| Plekhg2 | -0.0256741 |
| Plekhg2 | 1.4839897 |
| Plekhg3 | 1.7945055 |
| Plekhg3 | 0.7101024 |
| Plekhg4 | -0.1897916 |
| Plekhg5 | -0.5010925 |
| Plekhg6 | -0.6001777 |
| Plekhj1 | 2.3585415 |
| Plekhm1 | 0.2184257 |
| Plekhm3 | -0.4671378 |
| Plekho2 | 1.4009216 |
| Plin | -0.711125 |
| Plk1 | 0.2580688 |
| Plk2 | -0.2143006 |
| Plk3 | 1.0847072 |
| Plk4 | 0.3332207 |
| Pllp | -0.6316586 |
| Plod1 | 2.3779178 |
| Plod2 | -0.1244231 |
| Plod3 | -0.0977591 |
| Plp1 | -0.6741582 |
| Plp2 | 0.8292614 |
| Plrg1 | -0.2800445 |
| Pls3 | -0.6615601 |
| Plscr1 | 1.7317766 |
| Plscr1 | 0.6236736 |
| Plscr3 | -0.8557103 |
| Plscr4 | 0.2839094 |
| Pltp | -0.4527539 |
| Plunc | 0.9805571 |
| Plvap | -0.4506154 |
| Plxdc1 | 0.9282627 |
| Plxdc1 | 1.7692832 |
| Plxna2 | -0.6421024 |
| Plxnb1 | -0.4484818 |
| Plxnb3 | -0.5254682 |
| Plxnc1 | 0.5178891 |
| Plxnd1 | -0.0051853 |
| Pmaip1 | -0.4707689 |
| Pmch | -0.3187566 |
| Pmepa1 | 0.0079372 |
| Pmf1 | 2.1326554 |
| Pmfbp1 | -0.5754144 |
| Pml | 1.2691946 |
| Pml | -0.4921142 |
| Pmm1 | -0.5355567 |
| Pmm1 | -0.0140379 |
| Pmm2 | -0.3754255 |
| Pmm2 | -0.263603 |
| Pmm2 | 0.5943632 |
| Pmm2 | 1.1120589 |
| Pmp2 | 0.0399536 |
| Pmp22 | -0.5457561 |
| Pmp22 | -0.7417971 |
| Pmpca | 1.5966676 |
| Pmpcb | 0.399896 |
| Pms2 | -0.8985542 |
| Pmvk | -0.4470622 |
| Pnck | 2.1238628 |
| Pnkd | -0.6553246 |
| Pnkd | 0.5211422 |
| Pnkp | 0.2890564 |
| Pnkp | 3.5585517 |
| Pnliprp1 | -0.4839598 |
| Pnma1 | -0.4635209 |
| Pnma2 | 0.4334427 |
| Pno1 | 1.5480034 |
| Pnpla2 | 1.0751329 |
| Pnpla6 | 1.0463698 |
| Pnpla7 | -0.6290659 |
| Pnpla8 | -0.3960061 |
| Pnpla8 | -0.1791032 |
| Pnpo | 1.6078064 |
| Pnpo | -0.1016613 |
| Pnrc2 | 0.6741638 |
| Podn | -0.5656912 |
| Podxl | -0.5465453 |
| Podxl2 | -0.1105017 |
| Pof1b | 0.8552375 |
| Pofut1 | 0.3270827 |
| Pofut1 | -0.7545532 |
| Pofut2 | -0.5926791 |
| Pogz | -0.7515938 |
| Pogz | 0.6158123 |
| Pola2 | -0.0365462 |
| Polb | 1.0625367 |
| Pold1 | -0.6394804 |
| Pold2 | -0.7408231 |
| Pold3 | 1.300773 |
| Pold4 | -0.638608 |
| Poldip2 | -0.5162521 |
| Poldip3 | -0.642978 |
| Poldip3 | 0.1784595 |
| Pole | -0.1011726 |
| Pole3 | -0.3721484 |
| Pole3 | -0.8645671 |
| Pole4 | -0.3299419 |
| Polg | 1.4974298 |
| Polg2 | 2.5155276 |
| Polh | -0.6299293 |
| Poli | -0.4642431 |
| Polk | -0.6579918 |
| Poll | 2.3430381 |
| Poll | 1.7872594 |
| Polm | 2.8894326 |
| Polm | -0.2321316 |
| Poln | 2.2153565 |
| Polq | -0.6316586 |
| Polr1e | 0.0545247 |
| Polr2d | 1.5160786 |
| Polr2d | -0.5324408 |
| Polr2d | -0.711125 |
| Polr2d | -0.7301759 |
| Polr2e | -0.6860116 |
| Polr2e | 0.0777757 |
| Polr2f | 1.1016959 |
| Polr2g | -0.7234635 |
| Polr2h | 0.7544644 |
| Polr2j | -0.7408231 |
| Polr3a | -0.3181391 |
| Polr3a | -0.7054852 |
| Polr3b | -0.3980222 |
| Polr3d | 2.3486297 |
| Polr3e | 3.3463134 |
| Polr3g | 0.3878621 |
| Polr3h | -0.3540076 |
| Polr3h | 0.1913799 |
| Polr3h | -0.8001144 |
| Polr3k | -0.7101827 |
| Polr3k | 1.4279114 |
| Polrmt | 0.2198094 |
| Pols | -0.3132141 |
| Pom121 | -0.7158512 |
| Pomc | -0.7083008 |
| Pomgnt1 | -0.4239461 |
| Pomgnt1 | 2.6245059 |
| Pomp | 1.954022 |
| Pomt1 | 2.6936067 |
| Pomt1 | -0.5093996 |
| Pomt2 | -0.7045486 |
| Pon1 | 0.7923368 |
| Pon2 | 0.9814701 |
| Pon3 | 0.239644 |
| Pon3 | -0.1828302 |
| Pop4 | 1.0111054 |
| Pop5 | 0.1795431 |
| Pop5 | -0.5778612 |
| Pop7 | 0.7685375 |
| Popdc2 | -0.8425816 |
| Popdc2 | 1.4695435 |
| Popdc3 | -0.6094258 |
| Por | 0.4293151 |
| Por | -0.4407005 |
| Porcn | 0.2139145 |
| Porcn | -0.3767396 |
| Pou2af1 | 0.9389919 |
| Pou2f1 | -0.2836059 |
| Pou2f1 | -0.4371849 |
| Pou2f2 | -0.3662787 |
| Pou3f1 | -0.4642431 |
| Pou3f3 | 0.2979955 |
| Pou5f1 | 1.5419551 |
| Pou5f1 | 0.0365907 |
| Pou6f1 | 0.7208396 |
| Ppa1 | 1.3774008 |
| Ppa2 | 1.0338055 |
| Ppa2 | 1.9795767 |
| Ppa2 | -0.8053177 |
| Ppap2a | -0.6860116 |
| Ppap2a | 2.3651708 |
| Ppap2a | -0.8870863 |
| Ppap2b | -0.7120683 |
| Ppap2b | 1.2805019 |
| Ppap2c | -0.5664976 |
| Ppapdc1 | 1.8035782 |
| Ppapdc1a | 1.4502924 |
| Ppapdc2 | -0.6998795 |
| Ppapdc3 | 0.0035836 |
| Ppapdc3 | 2.9378266 |
| Pparbp | -0.7427721 |
| Pparg | -0.6961611 |
| Ppargc1a | 3.9826727 |
| Ppargc1b | 1.5854727 |
| Ppat | 1.2352316 |
| Ppcs | -0.2871811 |
| Ppef2 | -0.0047449 |
| Ppfia3 | 2.6857179 |
| Ppfia3 | -0.1919442 |
| Ppfibp1 | -0.7654855 |
| Ppfibp2 | 1.8827391 |
| Ppfibp2 | 1.8380411 |
| Ppia | 0.4004652 |
| Ppic | -0.1539425 |
| Ppic | -0.6705412 |
| Ppid | 0.131766 |
| Ppie | 0.0105395 |
| Ppie | -0.4407005 |
| Ppif | -0.7614952 |
| Ppil2 | -0.4491925 |
| Ppil3 | -0.4204934 |
| Ppl | 1.0002891 |
| Ppm1a | -0.7466825 |
| Ppm1a | -0.2165108 |
| Ppm1a | -0.3806931 |
| Ppm1b | 0.328621 |
| Ppm1b | -0.6060522 |
| Ppm1b | 0.254746 |
| Ppm1e | -0.7877443 |
| Ppm1e | -0.7263343 |
| Ppm1f | 2.0397178 |
| Ppm1f | -0.7092413 |
| Ppm1f | 4.4526495 |
| Ppm1g | -0.1299499 |
| Ppm1h | 0.8774487 |
| Ppm1j | -0.5449675 |
| Ppm1k | 1.0007361 |
| Ppm1l | -0.3675799 |
| Ppm1l | -0.6651421 |
| Ppm1m | -0.5101585 |
| Ppm1m | 0.4924768 |
| Ppm2c | 0.3771299 |
| Ppme1 | -0.7186985 |
| Ppnr | 1.4756348 |
| Ppox | 0.5333022 |
| Ppox | -0.3682311 |
| Ppp1ca | -0.5520888 |
| Ppp1ca | 0.3961879 |
| Ppp1ca | 0.1449739 |
| Ppp1cb | 0.0721187 |
| Ppp1cb | 0.1572571 |
| Ppp1cc | -0.5254682 |
| Ppp1r10 | 1.3439513 |
| Ppp1r10 | 0.4779359 |
| Ppp1r11 | 1.0242693 |
| Ppp1r12c | -0.1638205 |
| Ppp1r13b | 1.8520709 |
| Ppp1r13b | -0.3552917 |
| Ppp1r13b | 2.4352238 |
| Ppp1r14a | 1.3579426 |
| Ppp1r14a | -0.7715033 |
| Ppp1r14c | 2.0562425 |
| Ppp1r14d | 2.4854446 |
| Ppp1r15b | -0.2215032 |
| Ppp1r16a | -0.6597742 |
| Ppp1r1a | -0.7867209 |
| Ppp1r1b | 1.228497 |
| Ppp1r1b | 0.9639679 |
| Ppp1r2 | -0.8893686 |
| Ppp1r3c | -0.3387357 |
| Ppp1r3f | -0.2889738 |
| Ppp1r8 | -0.5223859 |
| Ppp1r9a | -0.407489 |
| Ppp1r9b | 0.101244 |
| Ppp2ca | -0.5505016 |
| Ppp2cb | -0.5332188 |
| Ppp2cz | -0.4513277 |
| Ppp2r1a | -0.3662787 |
| Ppp2r1b | -0.3643303 |
| Ppp2r2b | 0.7660469 |
| Ppp2r2b | 3.5779225 |
| Ppp2r2b | 0.9095526 |
| Ppp2r2c | -0.6759719 |
| Ppp2r2c | 0.7757796 |
| Ppp2r2d | 0.1453485 |
| Ppp2r3c | 0.093355 |
| Ppp2r4 | -0.7908214 |
| Ppp2r5b | 1.7724008 |
| Ppp2r5c | -0.1764502 |
| Ppp2r5c | 0.5733515 |
| Ppp2r5c | -0.5418199 |
| Ppp2r5d | 0.158735 |
| Ppp2r5d | -0.8200442 |
| Ppp2r5d | -0.1919442 |
| Ppp2r5d | 0.9979015 |
| Ppp2r5e | -0.4211829 |
| Ppp2r5e | 0.2650084 |
| Ppp3ca | -0.3144429 |
| Ppp3ca | -0.7282531 |
| Ppp3ca | -0.4883988 |
| Ppp3cb | -0.938641 |
| Ppp3cc | -0.4556129 |
| Ppp3r1 | -0.5177817 |
| Ppp4c | -0.817926 |
| Ppp4r1 | -0.8997089 |
| Ppp4r1 | 0.8548889 |
| Ppp4r1 | -0.7555417 |
| Ppp4r1l | 0.2321703 |
| Ppp5c | -0.3886509 |
| Ppp5c | -0.7206016 |
| Ppp6c | 0.5323155 |
| Pps | 0.415982 |
| Ppt1 | 0.1160508 |
| Ppt2 | -0.6624543 |
| Pptc7 | -0.4054523 |
| Pqbp1 | 0.1071171 |
| Pqlc1 | -0.1299499 |
| Pqlc2 | -0.8712657 |
| Pqlc3 | 0.7084709 |
| Pqlc3 | 0.4004652 |
| Pqlc3 | 1.4809764 |
| Pqlc3 | -0.6273415 |
| Pramef12 | 0.744308 |
| Prap1 | 1.242377 |
| Prc1 | -0.9160264 |
| Prcp | -0.3193745 |
| Prdm10 | 1.2969922 |
| Prdm4 | -0.538683 |
| Prdm8 | -0.4988398 |
| Prdm9 | -0.5640805 |
| Prdx1 | 1.0814357 |
| Prdx2 | -0.3859907 |
| Prdx2 | 2.2328098 |
| Prdx2 | -0.4642431 |
| Prdx3 | 0.5065396 |
| Prdx4 | -0.6145094 |
| Prdx5 | 0.2453781 |
| Prdx5 | -0.9422626 |
| Prdx6 | -0.6273415 |
| Prdx6-rs1 | -0.3514448 |
| Prdx6-rs1 | 0.9125062 |
| Preb | 0.2590634 |
| Preb | 2.010586 |
| Prei3 | 0.4508504 |
| Prei4 | 1.1673233 |
| Prei4 | 0.0264282 |
| Prelp | 1.5255181 |
| Prep | -0.3879852 |
| Prep | -0.5124389 |
| Prf1 | -0.1695878 |
| Prickle1 | -0.1016613 |
| Prickle2 | -0.4729544 |
| Prickle3 | -0.3946644 |
| Prickle4 | -0.4143107 |
| Prim2 | -0.2423143 |
| Prim2 | -0.3299419 |
| Prima1 | 2.2291313 |
| Prkaa2 | 1.0140469 |
| Prkaa2 | -0.0685326 |
| Prkab1 | 0.5001739 |
| Prkab2 | -0.2700275 |
| Prkaca | 1.9273595 |
| Prkaca | -0.8317812 |
| Prkacb | 0.7915906 |
| Prkag1 | -0.5033507 |
| Prkag2 | -0.7816206 |
| Prkar1a | 0.2732026 |
| Prkar1b | -0.4095303 |
| Prkar2b | 0.3307704 |
| Prkca | 0.6067456 |
| Prkcb1 | -0.9422626 |
| Prkcbp1 | 1.1004927 |
| Prkcbp1 | -0.9446849 |
| Prkcc | -0.2126463 |
| Prkcd | 0.9977521 |
| Prkcdbp | 3.3140564 |
| Prkcdbp | -0.9362344 |
| Prkce | -0.4980901 |
| Prkch | -0.2451628 |
| Prkcq | -0.8382463 |
| Prkcsh | -0.2641852 |
| Prkcz | 0.0048918 |
| Prkcz | -0.1129724 |
| Prkcz | -0.5910209 |
| Prkd1 | -0.5713507 |
| Prkd1 | -0.6777893 |
| Prkd2 | 0.0445578 |
| Prkd3 | -0.464966 |
| Prkd3 | 3.8568942 |
| Prkd3 | 1.0146341 |
| Prkg1 | -0.6162101 |
| Prkg2 | -0.0929043 |
| Prkra | 0.0035836 |
| Prkrip1 | -0.8746332 |
| Prkrip1 | -0.4047743 |
| Prkrir | 1.4734 |
| Prkx | 0.9995437 |
| Prkx | 0.7915906 |
| Prl | -0.295577 |
| Prlr | -0.4308904 |
| Prlr | -0.1968057 |
| Prm1 | 2.2746056 |
| Prm3 | 2.7424109 |
| Prmt2 | -0.5426058 |
| Prmt2 | -0.1070538 |
| Prmt3 | -0.6473687 |
| Prmt5 | -0.3787143 |
| Prmt5 | -0.178572 |
| Prmt5 | -0.4061307 |
| Prmt7 | 0.239644 |
| Prmt7 | 0.0821964 |
| Prmt7 | -0.60437 |
| Prmt7 | -0.3946644 |
| Prmt8 | 0.1183673 |
| Prnp | 0.0717133 |
| Prnpip1 | -0.6359959 |
| Prnpip1 | -0.3643303 |
| Prnpip1 | -0.6562128 |
| Proc | 1.2822632 |
| Proc | 2.4931393 |
| Procr | 2.1226189 |
| Prodh | 0.899639 |
| Prok2 | -0.9932299 |
| Prok2 | -0.830708 |
| Prokr1 | 0.7464657 |
| Prokr2 | 0.0781784 |
| Prom1 | -0.7379071 |
| Prom2 | 0.4732132 |
| Prom2 | 2.2058552 |
| Pros1 | -0.6068945 |
| Prosapip1 | 0.3835262 |
| Prosapip1 | -0.881405 |
| Prosapip1 | -0.7026781 |
| Prosc | 1.2736453 |
| Prosc | -0.1026394 |
| Prosc | -0.1849666 |
| Prox1 | -0.3662787 |
| Prox1 | 2.2308735 |
| Prpf18 | -0.3349567 |
| Prpf19 | 0.0044559 |
| Prpf19 | -0.6517802 |
| Prpf19 | 3.3608672 |
| Prpf3 | -0.7186985 |
| Prpf3 | 0.1602106 |
| Prpf3 | 2.500682 |
| Prpf3 | 0.1557769 |
| Prpf3 | -0.5632762 |
| Prpf31 | -0.2343848 |
| Prpf38a | -0.7897946 |
| Prpf38a | -0.9078318 |
| Prpf38a | -0.0007905 |
| Prpf38b | 3.5364465 |
| Prpf39 | 0.9309968 |
| Prpf40a | -0.5827742 |
| Prpf40a | 1.4542295 |
| Prpf40b | -0.2806371 |
| Prpf40b | -0.7186985 |
| Prpf40b | -0.2304453 |
| Prpf6 | 0.1145033 |
| Prpf8 | -0.6052107 |
| Prpf8 | -0.4007171 |
| Prph2 | -0.0238744 |
| Prps1 | -0.49137 |
| Prps1l1 | -0.7101827 |
| Prps2 | -0.0997082 |
| Prpsap2 | -0.5363373 |
| Prpsap2 | 1.3867826 |
| Prr11 | -0.7321027 |
| Prr13 | -0.6860116 |
| Prr14 | -0.1080376 |
| Prr15 | 2.343879 |
| Prr16 | -0.2806371 |
| Prr18 | 0.5537841 |
| Prr19 | 1.5280529 |
| Prr3 | 1.0448085 |
| Prr7 | 0.3621304 |
| Prrc1 | 2.5604667 |
| Prrg2 | 0.0672422 |
| Prrt1 | -0.8350081 |
| Prrt1 | -0.3412636 |
| Prrt1 | -0.4736841 |
| Prrt3 | -0.7359683 |
| Prrx1 | 0.070902 |
| Prrx1 | -0.2389077 |
| Prrx1 | -0.4225635 |
| Prss21 | -0.6860116 |
| Prss23 | 0.6012627 |
| Prss27 | -0.311374 |
| Prss28 | 0.0040199 |
| Prss29 | 1.7669381 |
| Prss29 | 0.3872851 |
| Prss34 | -0.5086413 |
| Prss35 | 1.8095155 |
| Prss36 | -0.4707689 |
| Prss7 | -0.5778612 |
| Prss8 | 0.1938715 |
| Prss8 | 1.1954 |
| Prtn3 | 1.1694371 |
| Prune | 0.8512209 |
| Prune | -0.0713669 |
| Prune | 1.6396945 |
| Prune | 2.407095 |
| Psap | -0.7263343 |
| Psat1 | -0.3337005 |
| Psca | 0.4342661 |
| Pscd1 | -0.7130126 |
| Pscd2 | 2.6155602 |
| Pscd3 | 0.1032058 |
| Pscdbp | -0.5656912 |
| Psd2 | -0.0324542 |
| Psd3 | 1.3949997 |
| Psd4 | 0.2620406 |
| Psen1 | -0.6394804 |
| Psen2 | 0.8367577 |
| Psenen | -0.0958141 |
| Psg19 | 1.8658916 |
| Psg23 | -0.729214 |
| Psg27 | 0.2787336 |
| Psip1 | -0.7515938 |
| Psip1 | 2.9445594 |
| Pskh1 | 2.6902133 |
| Psma2 | 0.6464293 |
| Psma4 | 0.2758096 |
| Psma5 | 0.6356734 |
| Psma7 | 2.9353123 |
| Psma7 | -0.4421104 |
| Psmb1 | -0.7101827 |
| Psmb10 | 1.309998 |
| Psmb10 | -0.5465453 |
| Psmb2 | -0.8350081 |
| Psmb2 | -0.1968057 |
| Psmb3 | -0.1596485 |
| Psmb4 | 0.5431119 |
| Psmb5 | -0.0943581 |
| Psmb6 | -0.187108 |
| Psmb7 | -0.5147249 |
| Psmb7 | -0.8928025 |
| Psmb7 | -0.5308868 |
| Psmb8 | -0.1239223 |
| Psmb9 | 1.7036475 |
| Psmc1 | 1.0066756 |
| Psmc2 | -0.7476627 |
| Psmc3 | -1.0060976 |
| Psmc3ip | -0.6213311 |
| Psmc4 | 1.6458832 |
| Psmc5 | -0.7466825 |
| Psmc5 | 0.6975887 |
| Psmd1 | -0.0167103 |
| Psmd10 | -0.7634882 |
| Psmd10 | -0.340631 |
| Psmd10 | -0.6273415 |
| Psmd11 | -0.7806039 |
| Psmd11 | 1.6337655 |
| Psmd12 | 1.934356 |
| Psmd12 | 1.6392455 |
| Psmd13 | 0.9224461 |
| Psmd14 | 1.8900218 |
| Psmd2 | 1.2167695 |
| Psmd2 | 0.405006 |
| Psmd4 | -0.1575695 |
| Psmd5 | -0.6544372 |
| Psmd6 | -0.4322855 |
| Psmd6 | 0.5540252 |
| Psmd7 | -0.5811337 |
| Psmd7 | 1.7574321 |
| Psmd8 | 0.3140594 |
| Psmd9 | 1.3671207 |
| Psmd9 | 0.2912992 |
| Psme1 | -0.2630212 |
| Psme1 | 0.354987 |
| Psme2 | 0.8959773 |
| Psme3 | 0.3313836 |
| Psme4 | -0.906667 |
| Psme4 | -0.7350004 |
| Psmf1 | 0.6200881 |
| Psmf1 | -0.5713507 |
| Psmg2 | 2.4021748 |
| Psmg2 | 2.1444534 |
| Psmg3 | 2.2603072 |
| Psors1c2 | 2.1348515 |
| Psp | 0.7043794 |
| Pspc1 | -0.7225085 |
| Pstpip1 | 0.436458 |
| Pstpip2 | 0.8907929 |
| Ptbp1 | -0.6786993 |
| Ptbp1 | 0.0349047 |
| Ptcd3 | 2.7016671 |
| Ptch1 | 1.586424 |
| Ptch2 | -0.8084538 |
| Ptdss2 | 0.884567 |
| Pten | 3.4555874 |
| Pter | -0.5656912 |
| Ptgds | 1.5924263 |
| Ptgds2 | 2.4717242 |
| Ptgds2 | 0.6786012 |
| Ptger2 | -0.7369372 |
| Ptger2 | -0.711125 |
| Ptger3 | -0.0997082 |
| Ptges | -0.5116781 |
| Ptges | -0.2270819 |
| Ptges2 | 0.3362745 |
| Ptges3 | 1.1132472 |
| Ptgfr | -0.2423143 |
| Ptgir | -0.8404114 |
| Ptgis | -0.5656912 |
| Ptgs1 | -0.8859473 |
| Ptgs1 | -0.8701459 |
| Pth2 | -0.5544748 |
| Pth2r | -0.5737868 |
| Pthlh | 0.2236038 |
| Pthr1 | -0.9680179 |
| Pthr1 | -0.7359683 |
| Pthr1 | 0.2887355 |
| Ptk2 | 1.1617048 |
| Ptk2b | 2.3488039 |
| Ptk7 | -0.7990773 |
| Ptms | 0.27776 |
| Ptn | -0.7735179 |
| Ptp4a2 | -0.0818338 |
| Ptp4a2 | 1.7665466 |
| Ptp4a2 | -0.8137042 |
| Ptp4a2 | -0.7177484 |
| Ptp4a3 | -0.3598 |
| Ptp4a3 | -0.1759205 |
| Ptpdc1 | 1.0510378 |
| Ptpdc1 | 0.6341266 |
| Ptpdc1 | -0.005626 |
| Ptpla | -0.642978 |
| Ptpla | 0.1121772 |
| Ptplad1 | -0.2451628 |
| Ptplad2 | 0.3081072 |
| Ptplb | -0.4013921 |
| Ptpn1 | -0.7437482 |
| Ptpn11 | -0.49137 |
| Ptpn11 | -0.1978895 |
| Ptpn11 | -0.3150579 |
| Ptpn12 | -0.6464889 |
| Ptpn12 | -0.4854371 |
| Ptpn12 | -0.3187566 |
| Ptpn12 | -0.6438545 |
| Ptpn12 | 0.1638892 |
| Ptpn14 | 1.1566818 |
| Ptpn18 | 2.5679786 |
| Ptpn20 | 1.8873333 |
| Ptpn21 | -0.3187566 |
| Ptpn22 | -0.9020225 |
| Ptpn5 | 1.9609798 |
| Ptpn6 | -0.8836734 |
| Ptpn6 | -0.3846635 |
| Ptpra | -0.5363373 |
| Ptprb | -0.0708939 |
| Ptprd | 1.1263493 |
| Ptprd | -0.4943507 |
| Ptpre | -0.1472439 |
| Ptpre | -0.2143006 |
| Ptprf | 0.6564145 |
| Ptprf | -0.7457034 |
| Ptprg | 0.5146246 |
| Ptprj | -0.6660398 |
| Ptprk | 0.9255206 |
| Ptprk | -0.1036186 |
| Ptprn | 1.4777751 |
| Ptprn2 | -0.3237112 |
| Ptpro | 0.9277794 |
| Ptpro | -0.7644863 |
| Ptprq | 1.0472203 |
| Ptprr | 1.7915884 |
| Ptprr | 1.4734895 |
| Ptprs | 0.3510945 |
| Ptprs | -0.7877443 |
| Ptprt | -0.1951824 |
| Ptpru | 0.9017959 |
| Ptprv | -0.5737868 |
| Ptrf | -0.7064228 |
| Ptrh1 | 3.2710055 |
| Ptrh1 | -0.5473352 |
| Ptrh2 | 1.7090782 |
| Pts | -0.5239258 |
| Pts | -0.7785738 |
| Pttg1 | 0.5716971 |
| Pttg1ip | 1.9012632 |
| Ptx3 | 1.2282639 |
| Ptx3 | -0.3425302 |
| Puf60 | 1.7892354 |
| Puf60 | -0.8328556 |
| Pum1 | 0.0357481 |
| Pum2 | -0.4336827 |
| Pum2 | 0.0264282 |
| Punc | 1.9963831 |
| Purb | -0.6136602 |
| Purg | 1.2681902 |
| Pus1 | -0.5811337 |
| Pus1 | -0.9043418 |
| Pus10 | -0.0765856 |
| Pus10 | 0.9837487 |
| Pus10 | 0.7859747 |
| Pus3 | -0.7457034 |
| Pus7l | -0.1638205 |
| Pvalb | -0.4392927 |
| Pvr | 0.71498 |
| Pvr | -0.8425816 |
| Pvrl1 | 1.9291683 |
| Pvrl2 | 0.052043 |
| Pvrl2 | 2.7128401 |
| Pvrl2 | 1.0497671 |
| Pvrl3 | -0.5943402 |
| Pvrl3 | 0.9473922 |
| Pvrl3 | -0.5868884 |
| Pwp1 | 2.4528909 |
| Pwp2 | -0.0761099 |
| Pwwp2b | -0.6119641 |
| Pxdn | 1.4920946 |
| Pxmp2 | 0.8798275 |
| Pxmp4 | -0.2877783 |
| Pxn | -0.6952338 |
| Pxt1 | 0.4128994 |
| Pycr2 | 1.7472282 |
| Pycrl | 2.5002382 |
| Pygb | -0.2901709 |
| Pygl | -0.4108936 |
| Pygl | -0.0301887 |
| Pygm | -0.5056144 |
| Pygo1 | -0.6841781 |
| Pygo2 | 0.3084215 |
| Pyroxd1 | -0.7083008 |
| Pyy | -0.7457034 |
| Qars | 0.0221607 |
| Qdpr | 0.4438179 |
| Qpct | 0.7049942 |
| Qprt | -0.1753912 |
| Qrich2 | 1.6260537 |
| Qrich2 | 1.7117817 |
| Qrsl1 | 1.3057187 |
| Qsox1 | -0.5078837 |
| Qtrt1 | 0.238967 |
| R3hdm1 | 3.3481185 |
| R3hdm1 | -0.5608676 |
| R3hdm2 | 0.0281297 |
| R74862 | -0.4308904 |
| Rab1 | 0.1329055 |
| Rab10 | 1.3516369 |
| Rab10 | -0.8447568 |
| Rab11a | 0.2656666 |
| Rab11b | 0.4519276 |
| Rab11fip2 | 1.0290496 |
| Rab11fip3 | -0.5568669 |
| Rab11fip5 | 0.8362242 |
| Rab11fip5 | 1.2544673 |
| Rab11fip5 | 1.0694788 |
| Rab12 | 0.2826181 |
| Rab13 | 0.8855794 |
| Rab13 | -0.0967861 |
| Rab14 | -0.479542 |
| Rab15 | 0.3209039 |
| Rab17 | 3.2026151 |
| Rab17 | -0.7765481 |
| Rab18 | 0.3653861 |
| Rab19 | -0.5737868 |
| Rab19 | 1.112191 |
| Rab1b | 1.0359592 |
| Rab1b | -0.7244194 |
| Rab1b | -0.1219218 |
| Rab21 | 0.6499146 |
| Rab22a | 1.5137679 |
| Rab22a | -0.4591991 |
| Rab23 | 1.0165399 |
| Rab24 | -0.6102712 |
| Rab24 | -0.1892543 |
| Rab24 | 0.0757593 |
| Rab25 | 1.6128257 |
| Rab27a | 2.233875 |
| Rab27b | 1.3814614 |
| Rab28 | -0.4928591 |
| Rab28 | 0.9764376 |
| Rab2a | 2.0249206 |
| Rab31 | 2.407651 |
| Rab32 | -0.3476137 |
| Rab33a | -0.7026781 |
| Rab33b | -0.3565775 |
| Rab34 | -0.1833638 |
| Rab34 | 2.9372725 |
| Rab34 | 0.0647943 |
| Rab35 | 0.1675533 |
| Rab36 | -0.7008114 |
| Rab38 | -0.1031289 |
| Rab38 | -0.5926791 |
| Rab39 | -0.6136602 |
| Rab39b | -0.6094258 |
| Rab3a | -0.435082 |
| Rab3b | -0.2853918 |
| Rab3d | -0.6342585 |
| Rab3gap1 | -0.6299293 |
| Rab3gap2 | -0.5355567 |
| Rab3il1 | -0.4707689 |
| Rab3il1 | -0.1892543 |
| Rab3ip | -0.4758764 |
| Rab40b | -0.6562128 |
| Rab40c | -0.137032 |
| Rab43 | -0.6085813 |
| Rab4a | 0.3059047 |
| Rab4b | 1.0612815 |
| Rab5a | 2.1579879 |
| Rab5a | -0.49137 |
| Rab5b | -0.6351268 |
| Rab5c | 0.0277046 |
| Rab6 | -0.4308904 |
| Rab6 | -0.0415722 |
| Rab6 | -0.421873 |
| Rab6b | -0.4449368 |
| Rab6ip1 | 1.0111054 |
| Rab7 | 0.5561926 |
| Rab7 | 2.3228397 |
| Rab7 | -0.6579918 |
| Rab7l1 | -0.4322855 |
| Rab8a | -0.237209 |
| Rab8a | -0.6136602 |
| Rab8b | -0.3040496 |
| Rab8b | 0.1378272 |
| Rab9 | -0.4736841 |
| Rab9 | 0.3996113 |
| Rab9b | 1.9204203 |
| Rabac1 | 0.5484633 |
| Rabac1 | 2.7798 |
| Rabep1 | -0.5648855 |
| Rabep1 | -0.4729544 |
| Rabep2 | -0.5116781 |
| Rabgap1 | 0.4078328 |
| Rabgap1 | -0.2033277 |
| Rabgap1l | 0.0315234 |
| Rabgap1l | -0.2022375 |
| Rabgef1 | -0.2717874 |
| Rabggta | 0.4909297 |
| Rabggtb | -0.5656912 |
| Rabif | 0.6559826 |
| Rabif | -0.510918 |
| Rabl2a | 2.2014558 |
| Rabl2a | -0.5239258 |
| Rabl3 | -0.3708408 |
| Rabl4 | -0.5985061 |
| Rabl4 | 0.4922191 |
| Rabl5 | 0.7288617 |
| Rac1 | 0.6984145 |
| Rac2 | -0.6447318 |
| Rac3 | 1.2003383 |
| Rac3 | 0.0294038 |
| Rad1 | -0.7282531 |
| Rad17 | 1.2060866 |
| Rad18 | -0.4102117 |
| Rad23a | -0.4061307 |
| Rad23a | -0.1219218 |
| Rad23a | -0.4758764 |
| Rad23b | 0.0361695 |
| Rad50 | 1.9253821 |
| Rad51 | -0.3787143 |
| Rad51 | -0.7816206 |
| Rad51ap1 | 0.6189648 |
| Rad51c | 0.6424926 |
| Rad51l3 | 0.4413711 |
| Rad51l3 | 1.9875296 |
| Rad51l3 | -0.6368658 |
| Rad51l3 | 2.217205 |
| Rad52 | -0.4253308 |
| Rad54l | 0.0105395 |
| Rad9 | -0.3095375 |
| Rad9 | -0.6196209 |
| Rad9 | -0.421873 |
| Rad9b | -0.4664133 |
| Rae1 | -0.5910209 |
| Raet1b | -0.6841781 |
| Raet1b | 0.0445578 |
| Raf1 | -0.163298 |
| Rag1 | 0.2960872 |
| Rag1ap1 | -0.7073613 |
| Rage | -0.6588826 |
| Rage | -0.6814346 |
| Rai1 | -0.3119869 |
| Rai12 | -0.0198378 |
| Rai12 | 0.555952 |
| Rai12 | 0.0536983 |
| Rai12 | 2.7414532 |
| Rai14 | -0.4385896 |
| Rala | 1.9957208 |
| Ralb | -0.0512435 |
| Ralbp1 | -0.7054852 |
| Ralgds | 1.6387214 |
| Ralgps1 | 1.9963831 |
| Ralgps1 | 0.4092429 |
| Ralgps1 | -0.163298 |
| Ralgps2 | 0.5186408 |
| Ralgps2 | 0.1977733 |
| Raly | 1.8277749 |
| Raly | -0.6553246 |
| Ralyl | 2.1540428 |
| Ramp1 | -0.2735506 |
| Ramp2 | -0.6368658 |
| Ramp3 | 1.7023897 |
| Ramp3 | -0.0842292 |
| Ran | -0.6732526 |
| Ranbp1 | -0.1365243 |
| Ranbp10 | 0.5566735 |
| Ranbp17 | -0.3721484 |
| Ranbp2 | -0.7272932 |
| Ranbp3l | 0.5278623 |
| Ranbp6 | 1.7618366 |
| Ranbp9 | 2.1747801 |
| Rangap1 | -0.5935093 |
| Rangrf | 1.1734029 |
| Rap1b | -0.6777893 |
| Rap1gap | 3.2798715 |
| Rap2a | -0.7476627 |
| Rap2b | -0.6421024 |
| Rap2c | 0.1998947 |
| Rap2c | -0.7605003 |
| Rap2c | 0.1693799 |
| Rap2ip | 1.4517593 |
| Rapgef1 | 0.3215237 |
| Rapgef1 | -0.4211829 |
| Rapgef1 | -0.2071534 |
| Rapgef1 | -0.4336827 |
| Rapgef3 | -0.6924575 |
| Rapgef4 | 1.124135 |
| Rapgef5 | 2.4246589 |
| Rapgef6 | -0.729214 |
| Rapgefl1 | 0.8554118 |
| Rapgefl1 | -0.1560133 |
| Rapsn | -0.6741582 |
| Rapsn | -0.2093467 |
| Rara | 0.6520863 |
| Rarb | -0.6299293 |
| Rarb | -0.1075455 |
| Rarg | -0.6085813 |
| Rarg | -0.2428833 |
| Rarg | -0.4463532 |
| Rarres2 | 2.3327812 |
| Rars | -0.7457034 |
| Rars | -0.6786993 |
| Rars2 | -0.4928591 |
| Rars2 | 1.1843444 |
| Rars2 | -0.263603 |
| Rasa1 | -0.5473352 |
| Rasa2 | -0.6221873 |
| Rasal1 | -0.2913695 |
| Rasd1 | 1.8062703 |
| Rasef | -0.5418199 |
| Rasgef1b | -0.3754255 |
| Rasgef1b | -0.5208487 |
| Rasgef1c | -0.1031289 |
| Rasgef1c | -0.6456099 |
| Rasgrf1 | -0.6732526 |
| Rasgrf1 | 0.3656815 |
| Rasgrf1 | -0.6998795 |
| Rasgrf1 | -0.0247738 |
| Rasgrp1 | -0.4773409 |
| Rasgrp1 | -0.5616698 |
| Rasgrp1 | -0.3747692 |
| Rasgrp3 | 0.0869951 |
| Rasip1 | 0.5614693 |
| Rasl10a | -0.4484818 |
| Rasl10b | 1.0293386 |
| Rasl10b | -0.7765481 |
| Rasl11a | 0.6252379 |
| Rasl11b | 0.8010603 |
| Rasl11b | 1.635721 |
| Rasl12 | 1.2408833 |
| Rasl2-9 | -0.5465453 |
| Rassf1 | 1.7933017 |
| Rassf1 | -0.5033507 |
| Rassf10 | -0.3714944 |
| Rassf2 | -0.5293354 |
| Rassf2 | -0.4958446 |
| Rassf3 | 2.3081798 |
| Rassf4 | 0.1557769 |
| Rassf4 | 0.6303593 |
| Rassf4 | -0.1139625 |
| Rassf4 | -0.4906263 |
| Rassf5 | 0.3887269 |
| Rassf7 | -0.5223859 |
| Rassf7 | 2.1218629 |
| Raver1 | 0.0980964 |
| Raver2 | 0.233193 |
| Rb1 | 0.4137411 |
| Rb1 | 0.8623547 |
| Rbak | -0.2549135 |
| Rbak | -0.462078 |
| Rbbp5 | -0.3070944 |
| Rbbp5 | -0.2700275 |
| Rbbp6 | -0.5852406 |
| Rbbp7 | -0.4421104 |
| Rbbp7 | 1.2039352 |
| Rbbp9 | -0.5441796 |
| Rbck1 | -0.257801 |
| Rbed1 | -0.6678377 |
| Rbed1 | -0.3457041 |
| Rbj | -0.3840006 |
| Rbks | -0.2618586 |
| Rbl1 | 0.2399823 |
| Rbl2 | 0.3627232 |
| Rbm10 | 1.8412976 |
| Rbm10 | -0.6669383 |
| Rbm10 | 2.3499528 |
| Rbm11 | -0.8757584 |
| Rbm12 | 3.3109003 |
| Rbm12 | 2.3084345 |
| Rbm12 | 1.9801989 |
| Rbm12 | 0.1260474 |
| Rbm13 | -0.5170166 |
| Rbm13 | 1.3108514 |
| Rbm14 | -0.3682311 |
| Rbm14 | 0.6323556 |
| Rbm15 | 1.1339937 |
| Rbm16 | 0.7645109 |
| Rbm18 | -0.6961611 |
| Rbm18 | -0.3610921 |
| Rbm19 | 0.2266969 |
| Rbm24 | 1.7899356 |
| Rbm26 | -0.4456447 |
| Rbm28 | 1.626585 |
| Rbm28 | 0.6645828 |
| Rbm3 | -0.3591546 |
| Rbm35a | -0.4513277 |
| Rbm38 | 0.0298281 |
| Rbm38 | -0.3859907 |
| Rbm38 | -0.3572211 |
| Rbm4 | 0.8752343 |
| Rbm41 | 0.4618329 |
| Rbm42 | 0.1129532 |
| Rbm42 | 0.0873939 |
| Rbm43 | -0.5371185 |
| Rbm45 | -0.6010147 |
| Rbm47 | 0.3504942 |
| Rbm47 | -0.4846982 |
| Rbm47 | 1.0342366 |
| Rbm47 | 2.6299506 |
| Rbm4b | -0.7206016 |
| Rbm5 | -0.1903293 |
| Rbm5 | 2.0309267 |
| Rbm6 | 0.2650084 |
| Rbm6 | 2.4588504 |
| Rbm6 | 2.5290787 |
| Rbm8a | -0.4054523 |
| Rbm9 | -0.4143107 |
| Rbm9 | -0.5544748 |
| Rbms1 | 0.6615818 |
| Rbms1 | -0.6247608 |
| Rbms2 | 2.0511671 |
| Rbms2 | -0.6068945 |
| Rbms3 | 1.0190261 |
| Rbmx | 1.0411078 |
| Rbmy1a1 | 0.9469187 |
| Rbp1 | -0.4108936 |
| Rbp7 | 2.845336 |
| Rbpms | 2.3915239 |
| Rbpms | 0.9290677 |
| Rbpms | -0.5147249 |
| Rbpms2 | 2.1279823 |
| Rbx1 | 0.2600569 |
| Rbx1 | -0.3840006 |
| Rc3h1 | 1.1748857 |
| Rcan1 | 0.0602897 |
| Rcan2 | -0.5101585 |
| Rcan2 | 1.493059 |
| Rcan2 | -0.7545532 |
| Rcan3 | -0.568921 |
| Rcbtb2 | -0.8404114 |
| Rcbtb2 | -0.6823482 |
| Rcc1 | 0.1809859 |
| Rccd1 | 0.991755 |
| Rce1 | -0.0624213 |
| Rchy1 | 0.1370717 |
| Rcl1 | 1.6681317 |
| Rcl1 | -0.5608676 |
| Rcl1 | 0.7679634 |
| Rcn3 | -0.2485925 |
| Rcor1 | 0.0777757 |
| Rcor2 | 0.0289793 |
| Rcsd1 | 2.1959681 |
| Rdbp | -0.3533662 |
| Rdbp | 0.0361695 |
| Rdh11 | -0.426024 |
| Rdh12 | 1.2269808 |
| Rdh14 | -0.4371849 |
| Rdh14 | -0.1903293 |
| Rdh5 | 0.4802882 |
| Rdh7 | 0.1952922 |
| Rdh9 | 2.3743663 |
| Rdm1 | -0.6887686 |
| Rec8 | 1.7246362 |
| Reck | 0.3644993 |
| Recql | 1.1978122 |
| Recql4 | -0.6696392 |
| Recql5 | 0.9269733 |
| Reep1 | 2.2133431 |
| Reep2 | -0.3431641 |
| Reep3 | 3.2530416 |
| Reep4 | -0.3546494 |
| Reep5 | -0.1612108 |
| Reep6 | 1.4059401 |
| Reep6 | 1.9070576 |
| Refbp2 | -0.3206115 |
| Reg1 | -0.3546494 |
| Rela | -0.7017443 |
| Relb | 0.8597572 |
| Rell1 | 1.2091854 |
| Rell2 | 2.2973438 |
| Rell2 | 1.4600587 |
| Reln | 2.2845896 |
| Relt | 0.2715695 |
| Relt | 0.6913719 |
| Rem1 | -0.1090224 |
| Rem2 | 2.4712053 |
| Renbp | 1.0996897 |
| Repin1 | -0.7457034 |
| Repin1 | 0.1528093 |
| Reps1 | -0.2700275 |
| Reps1 | -0.0144828 |
| Rer1 | 0.4639608 |
| Rer1 | 0.4800272 |
| Rerg | 0.3249248 |
| Resp18 | 1.4292264 |
| Rest | -0.3324459 |
| Ret | 2.0079741 |
| Retnla | -0.7486439 |
| Retnlg | -0.7130126 |
| Retsat | -0.478074 |
| Retsat | -0.4343821 |
| Rev1 | 1.71627 |
| Rev3l | 0.6967622 |
| Rex3 | 2.6434875 |
| Rexo1 | -0.1179336 |
| Rexo1 | -0.7225085 |
| Rexo2 | 3.4747041 |
| Rfc1 | 2.8013299 |
| Rfc2 | 1.9311376 |
| Rfc3 | -0.6026908 |
| Rfc4 | 0.0272793 |
| Rfc4 | -0.6204756 |
| Rfc5 | 0.6637264 |
| Rfesd | 1.198655 |
| Rffl | 1.1022302 |
| Rffl | -0.5426058 |
| Rffl | 2.9780224 |
| Rffl | -0.7466825 |
| Rffl | -0.4463532 |
| Rfng | 3.4483744 |
| Rfpl4 | 2.6806161 |
| Rfpl4 | -0.3853269 |
| Rft1 | 0.0805913 |
| Rftn1 | 0.2436953 |
| Rftn2 | 0.9673713 |
| Rftn2 | 2.3229472 |
| Rfwd2 | -0.7253764 |
| Rfwd3 | 0.4229503 |
| Rfwd3 | 1.3995658 |
| Rfx1 | 1.4030481 |
| Rfx1 | 1.13128 |
| Rfx2 | 1.2205385 |
| Rfx3 | -0.7330676 |
| Rfx4 | -0.3767396 |
| Rfx4 | 0.6308033 |
| Rfx4 | -0.3181391 |
| Rfxank | 2.6691692 |
| Rfxap | 2.7765325 |
| Rfxdc1 | 1.1766126 |
| Rg9mtd1 | -0.7725101 |
| Rg9mtd2 | 0.5525775 |
| Rgag4 | -0.2741391 |
| Rgl1 | 1.6390958 |
| Rgl2 | -0.3437985 |
| Rgl2 | -0.617914 |
| Rgl3 | 1.3296413 |
| Rgma | 0.98844 |
| Rgmb | 2.8178441 |
| Rgmb | 2.636335 |
| Rgnef | 2.2826837 |
| Rgs1 | -0.7545532 |
| Rgs1 | -0.4442294 |
| Rgs1 | -0.7447253 |
| Rgs10 | -0.7496262 |
| Rgs12 | 2.9855937 |
| Rgs14 | 3.01804 |
| Rgs16 | 0.2520791 |
| Rgs17 | 1.3433337 |
| Rgs2 | 1.4694537 |
| Rgs3 | 0.415982 |
| Rgs3 | 1.2701979 |
| Rgs3 | -0.2889738 |
| Rgs3 | 1.3058259 |
| Rgs3 | -0.8371657 |
| Rgs3 | -0.7867209 |
| Rgs3 | -0.6535507 |
| Rgs4 | 3.7040129 |
| Rgs5 | -0.2589585 |
| Rgs6 | -0.6544372 |
| Rgs7 | 0.815359 |
| Rgs7bp | 1.6449166 |
| Rgs8 | -0.0131489 |
| Rgs9 | 2.1675662 |
| Rgs9bp | -0.7816206 |
| Rhag | 3.1744918 |
| Rhbdd1 | 1.1935867 |
| Rhbdd3 | -0.5910209 |
| Rhbdf1 | 2.8468239 |
| Rhbdf2 | -0.263603 |
| Rhbdl1 | -0.2979899 |
| Rhbdl2 | 2.4566511 |
| Rhbdl3 | 0.128339 |
| Rhbdl7 | 0.4226725 |
| Rhcg | 1.7632783 |
| Rheb | 0.6232262 |
| Rhebl1 | -0.5968375 |
| Rhob | -0.3089261 |
| Rhobtb2 | 1.1535285 |
| Rhobtb2 | 1.1662023 |
| Rhobtb2 | -0.7775604 |
| Rhobtb2 | 1.4544123 |
| Rhobtb3 | 0.7893483 |
| Rhoc | -0.8667946 |
| Rhoc | 0.5323155 |
| Rhod | 3.6961136 |
| Rhof | 0.3803335 |
| Rhog | -0.0558845 |
| Rhoj | -0.8063619 |
| Rhot1 | 1.2083521 |
| Rhot2 | 1.9509782 |
| Rhot2 | 0.1870931 |
| Rhot2 | -0.0118169 |
| Rhou | -0.6377365 |
| Rhov | -0.3199928 |
| Rhox11 | -0.6368658 |
| Rhox12 | 0.7546587 |
| Rhox3a | -0.6508962 |
| Rhox3f | 1.8109473 |
| Rhox4d | -0.5600661 |
| Rhox5 | -0.8656802 |
| Rhox8 | -0.8645671 |
| Rhpn1 | 0.6303593 |
| Rhpn1 | -0.1518762 |
| Rhpn1 | 0.2450418 |
| Ribc1 | 0.1609475 |
| Ric3 | 1.2191269 |
| Ric3 | -0.4027435 |
| Ric3 | 1.949265 |
| Ric8b | -0.6615601 |
| Ric8b | -0.1919442 |
| Rif1 | 0.234895 |
| Rilp | -0.7272932 |
| Rilpl1 | 0.4182176 |
| Rilpl1 | -0.579496 |
| Rilpl2 | 0.2819718 |
| Rims1 | 0.1430985 |
| Rims2 | -0.652665 |
| Rims3 | 2.3772762 |
| Rin1 | -0.4421104 |
| Rin2 | -0.5018446 |
| Rin2 | -0.6943074 |
| Rin2 | 0.9982003 |
| Rin3 | -0.7765481 |
| Riok1 | 0.3062197 |
| Riok1 | 1.6621631 |
| Riok3 | -0.5208487 |
| Riok3 | 0.0647943 |
| Ripk1 | -0.2463046 |
| Ripk2 | 0.1160508 |
| Ripk4 | 2.1700193 |
| Ripk5 | 1.156304 |
| Rit1 | -0.2406094 |
| Rit2 | -0.5473352 |
| Rlbp1 | -0.4766084 |
| Rlbp1l1 | 1.8046435 |
| Rlbp1l1 | -0.6464889 |
| Rln1 | 1.1187727 |
| Rltpr | 1.1690644 |
| Rmnd1 | 1.6280256 |
| Rmnd5b | -0.4758764 |
| Rmnd5b | 0.3018003 |
| Rnase1 | 1.4329734 |
| Rnase4 | -0.6696392 |
| Rnase4 | 1.4474436 |
| Rnase6 | -0.6136602 |
| Rnaseh1 | -0.0113733 |
| Rnaseh1 | -0.4832221 |
| Rnaseh2c | 0.1415955 |
| Rnasek | -0.4766084 |
| Rnasen | 1.0249952 |
| Rnaset2 | 0.1183673 |
| Rnaset2b | -0.500341 |
| Rnd2 | 0.3018003 |
| Rnd3 | 1.4727733 |
| Rnf10 | -0.0568155 |
| Rnf103 | -0.8371657 |
| Rnf11 | -0.7234635 |
| Rnf11 | -0.4143107 |
| Rnf11 | -0.5246966 |
| Rnf111 | -0.6153594 |
| Rnf113a1 | 1.7341446 |
| Rnf113a2 | 3.6597229 |
| Rnf12 | -0.6579918 |
| Rnf121 | -0.7765481 |
| Rnf123 | -0.8723868 |
| Rnf125 | -0.0577475 |
| Rnf125 | -0.510918 |
| Rnf128 | -0.3070944 |
| Rnf133 | 3.0236281 |
| Rnf135 | 2.3668806 |
| Rnf135 | 0.4896385 |
| Rnf135 | -0.6333911 |
| Rnf138 | 1.9065528 |
| Rnf138 | -0.1887172 |
| Rnf138 | -0.5025974 |
| Rnf138 | 1.4990853 |
| Rnf139 | 0.2420094 |
| Rnf139 | -0.3418967 |
| Rnf14 | -0.6256202 |
| Rnf14 | -0.6562128 |
| Rnf14 | -0.3649793 |
| Rnf141 | 4.0052759 |
| Rnf141 | 1.9292231 |
| Rnf144a | -0.3230904 |
| Rnf145 | -0.7867209 |
| Rnf150 | -0.5835956 |
| Rnf166 | 0.0688706 |
| Rnf167 | -0.5339974 |
| Rnf167 | -0.5270131 |
| Rnf167 | -0.1722212 |
| Rnf167 | -0.6464889 |
| Rnf167 | 0.0161531 |
| Rnf168 | -0.1045988 |
| Rnf17 | 0.6600777 |
| Rnf170 | -0.6777893 |
| Rnf181 | 2.8879921 |
| Rnf182 | 0.8788088 |
| Rnf182 | 1.4689142 |
| Rnf183 | -0.5071266 |
| Rnf185 | 0.4689949 |
| Rnf185 | -0.064767 |
| Rnf185 | 1.8976445 |
| Rnf185 | 0.1576268 |
| Rnf186 | 2.5616583 |
| Rnf19a | -0.7417971 |
| Rnf2 | 1.1293368 |
| Rnf20 | 0.9466029 |
| Rnf207 | 1.4072866 |
| Rnf208 | 3.4539376 |
| Rnf214 | 0.3307704 |
| Rnf215 | -0.464966 |
| Rnf216 | 2.2306361 |
| Rnf216 | -0.5162521 |
| Rnf219 | 0.1770127 |
| Rnf24 | -0.7036129 |
| Rnf24 | 0.5724065 |
| Rnf25 | -0.4198044 |
| Rnf25 | -0.6128118 |
| Rnf25 | -0.2087979 |
| Rnf26 | -0.7826384 |
| Rnf31 | 1.5937636 |
| Rnf32 | 0.988138 |
| Rnf34 | -0.3993687 |
| Rnf34 | -0.1309583 |
| Rnf34 | 3.8534461 |
| Rnf38 | 1.4267827 |
| Rnf38 | 2.2967913 |
| Rnf38 | 3.4877164 |
| Rnf4 | -0.7735179 |
| Rnf4 | -0.2606975 |
| Rnf40 | -0.7158512 |
| Rnf41 | -0.4020676 |
| Rnf41 | -0.380033 |
| Rnf41 | -0.6351268 |
| Rnf44 | -0.8579166 |
| Rnf5 | -0.5877134 |
| Rnf6 | -0.2782689 |
| Rnf6 | -0.5786782 |
| Rnf8 | -0.3193745 |
| Rnf8 | -0.7496262 |
| Rngtt | -0.1194272 |
| Rnmt | 1.5730967 |
| Rnmtl1 | 0.6247913 |
| Rnpc2 | 0.0921658 |
| Rnpep | -0.6170616 |
| Rnpepl1 | 0.2643497 |
| Rnps1 | -0.4773409 |
| Rnps1 | 0.2365939 |
| Rnu6 | -0.5308868 |
| Rnuxa | 0.1082872 |
| Rnuxa | -0.6077375 |
| Robo1 | -0.8221672 |
| Robo2 | -0.6119641 |
| Robo4 | -0.0021068 |
| Rock1 | -0.3349567 |
| Rock2 | 0.8652898 |
| Rock2 | 0.0061982 |
| Rod1 | 0.6566304 |
| Rogdi | -0.3873199 |
| Rom1 | 0.801799 |
| Ropn1 | 0.4350886 |
| Ror1 | -0.4281064 |
| Ror2 | 1.5147099 |
| Rora | 1.2616915 |
| Rorb | -0.7398501 |
| Rorb | -0.4950974 |
| Rorc | -0.5308868 |
| Rorc | 3.1487307 |
| Ros1 | -0.1727488 |
| Rp23-297j14.5 | 2.1172691 |
| Rp23-438h3.2 | 0.8583689 |
| rp9 | -0.2973861 |
| rp9 | 1.0102212 |
| Rpa1 | -0.3906511 |
| Rpa2 | -0.60437 |
| Rpa2 | -0.6615601 |
| Rpa3 | 0.8627005 |
| Rpain | 1.1818968 |
| Rpap1 | -0.5976714 |
| Rpap1 | 1.0643466 |
| Rpap2 | -0.7575219 |
| Rpap2 | 0.3603497 |
| Rpap3 | 1.1273895 |
| Rpe | -0.187108 |
| Rpgr | -0.3187566 |
| Rpgrip1 | 1.4740263 |
| Rpgrip1l | 3.4500128 |
| Rph3a | -0.2985941 |
| Rph3al | 1.2247603 |
| Rpia | -0.0933887 |
| Rpl12 | -0.2503122 |
| Rpl13a | -0.5262403 |
| Rpl18 | 0.0487237 |
| Rpl19 | 0.3230712 |
| Rpl21 | -0.7234635 |
| Rpl21 | 1.0823911 |
| Rpl22 | 0.5168859 |
| Rpl22l1 | -0.568921 |
| Rpl23 | 0.9415359 |
| Rpl23 | 0.3021167 |
| Rpl23 | 0.1094559 |
| Rpl24 | 0.7129508 |
| Rpl27 | -0.5308868 |
| Rpl27a | -0.6153594 |
| Rpl28 | -0.4744143 |
| Rpl29 | -0.5505016 |
| Rpl29 | 0.0174437 |
| Rpl3 | -0.7186985 |
| Rpl30 | 1.1339937 |
| Rpl31 | -0.6759719 |
| Rpl31 | -0.4456447 |
| Rpl34 | 2.6314673 |
| Rpl35 | -0.2741391 |
| Rpl36a | -0.2231733 |
| Rpl36al | -0.4315877 |
| Rpl37 | -0.5943402 |
| Rpl38 | 0.0412116 |
| Rpl38 | 0.1225992 |
| Rpl39 | 0.399896 |
| Rpl39l | 1.4078631 |
| Rpl3l | 1.4384726 |
| Rpl4 | -0.6924575 |
| Rpl41 | 0.7129508 |
| Rpl7a | -0.6359959 |
| Rpl7l1 | 0.7951299 |
| Rpl7l1 | 1.0207772 |
| Rpl9 | -0.6299293 |
| Rplp1 | -0.8723868 |
| Rpn2 | 0.579236 |
| Rpo1-1 | -0.0007905 |
| Rpo1-3 | 0.4540782 |
| Rpo1-3 | 2.0066654 |
| Rpo1-4 | 2.4480205 |
| Rpo1-4 | 0.270261 |
| Rpo1-4 | -0.4714968 |
| Rpo2tc1 | 2.102396 |
| Rpp21 | -0.1380481 |
| Rpp25 | -0.2589585 |
| Rpp30 | -0.5041047 |
| Rpp38 | 1.8539137 |
| Rpp40 | -0.2159577 |
| Rprm | 3.221885 |
| Rprml | -0.6535507 |
| Rps10 | -0.8836734 |
| Rps11 | 1.3652119 |
| Rps12 | 0.3109314 |
| Rps13 | -0.3052663 |
| Rps14 | 1.6238494 |
| Rps15 | -0.638608 |
| Rps15a | -0.6010147 |
| Rps16 | -0.5193139 |
| Rps19 | -0.549709 |
| Rps19 | -0.0784906 |
| Rps19bp1 | -0.7715033 |
| Rps2 | 1.1076887 |
| Rps21 | 0.3184208 |
| Rps24 | 3.7332402 |
| Rps24 | 0.2338742 |
| Rps25 | 0.5188913 |
| Rps26 | -0.5285606 |
| Rps27a | 0.3498936 |
| Rps27a | 0.9232562 |
| Rps27l | 0.2294377 |
| Rps3 | 0.5679046 |
| Rps3 | 2.0035876 |
| Rps3a | -0.6213311 |
| Rps4x | -0.6952338 |
| Rps4y2 | -0.064767 |
| Rps5 | -0.0397414 |
| Rps6 | 0.6626547 |
| Rps6ka1 | 1.4170383 |
| Rps6ka3 | 3.5351285 |
| Rps6ka4 | -0.6615601 |
| Rps6ka5 | -0.301621 |
| Rps6kb1 | 2.1092312 |
| Rps6kl1 | -0.2764967 |
| Rps7 | 1.416182 |
| Rps8 | 0.7344361 |
| Rps9 | 0.070496 |
| Rps9 | -0.4958446 |
| Rpsa | -0.821105 |
| Rpusd1 | 0.7564049 |
| Rpusd4 | 0.3350542 |
| Rqcd1 | -0.6961611 |
| Rrad | 2.389959 |
| Rragb | 3.1877297 |
| Rragc | -0.824295 |
| Rragd | -0.6010147 |
| Rras | -0.4281064 |
| Rras2 | -0.5473352 |
| Rrbp1 | -0.1199256 |
| Rrbp1 | 0.0437224 |
| Rreb1 | -0.2700275 |
| Rreb1 | -0.49137 |
| Rrh | 0.6389793 |
| Rrm1 | -0.3767396 |
| Rrm2 | 1.8653052 |
| Rrm2 | -0.6823482 |
| Rrm2b | 0.4047229 |
| Rrn3 | 1.5667249 |
| Rrp1 | -0.6060522 |
| Rrp12 | -0.2016929 |
| Rrp15 | 1.2707548 |
| Rrp1b | -0.1503295 |
| Rrp1b | 0.14009 |
| Rrp1b | 0.9300328 |
| Rrp9 | -0.5048592 |
| Rs1 | -0.6264805 |
| Rsad1 | -0.6290659 |
| Rsad2 | 1.0034136 |
| Rsf1 | 2.3856837 |
| Rshl1 | -0.5803145 |
| Rshl2a | -0.1279364 |
| Rsl1d1 | 0.2728762 |
| Rsl1d1 | -0.0765856 |
| Rsn | 0.1145033 |
| Rsph1 | 0.0797877 |
| Rspo1 | -0.3046578 |
| Rspo2 | -0.6535507 |
| Rspo3 | 1.6236972 |
| Rspo4 | -0.3688829 |
| Rspry1 | -0.4664133 |
| Rsrc1 | 1.685668 |
| Rsrc1 | -0.3212306 |
| Rsrc2 | 3.5268874 |
| Rsu1 | -0.638608 |
| Rtbdn | -0.0992205 |
| Rtel1 | -0.5608676 |
| Rtel1 | -0.5705401 |
| Rtkn | 0.7356263 |
| Rtn1 | 0.4942785 |
| Rtn1 | -0.4861767 |
| Rtn1 | 0.1479666 |
| Rtn1 | 2.1055709 |
| Rtn2 | 0.7272628 |
| Rtn3 | 0.0737385 |
| Rtn4 | -0.8350081 |
| Rtn4 | -0.7408231 |
| Rtn4 | -0.3721484 |
| Rtn4ip1 | 0.0765664 |
| Rtn4r | 0.3739152 |
| Rtn4rl1 | -0.4810123 |
| Rtn4rl2 | 3.1799547 |
| Rtp1 | -0.6359959 |
| Rtp4 | -0.7704976 |
| Rttn | -0.5086413 |
| Rufy1 | 0.7887869 |
| Rufy2 | 1.5328501 |
| Rufy3 | 2.1981425 |
| Rufy3 | 0.932922 |
| Rufy3 | 0.4486922 |
| Rufy4 | -0.5355567 |
| Rundc1 | -0.6018524 |
| Rundc3b | -0.4211829 |
| Runx1 | -0.4988398 |
| Runx1t1 | 0.179904 |
| Runx1t1 | 0.0399536 |
| Runx2 | 2.7297209 |
| Rusc1 | 0.8362242 |
| Rusc2 | -0.7225085 |
| Rusc2 | -0.4958446 |
| Ruvbl1 | 0.4284874 |
| Ruvbl1 | -0.6491308 |
| Ruvbl1 | 1.564456 |
| Ruvbl2 | 1.4008248 |
| Rwdd1 | -0.6491308 |
| Rwdd1 | 2.1671426 |
| Rwdd3 | 0.0420493 |
| Rwdd3 | 1.5324303 |
| Rwdd4a | -0.5935093 |
| Rxfp1 | 1.1496036 |
| Rxfp3 | 0.0660191 |
| Rxra | -0.132473 |
| Rxrb | -0.1189291 |
| Rxrb | 0.0412116 |
| Rxrg | -0.7476627 |
| Rya3 | 0.183146 |
| Ryk | -0.3476137 |
| Ryk | -0.5592652 |
| Ryr1 | -0.2120956 |
| Ryr1 | 0.6650108 |
| Ryr1 | 0.931639 |
| Ryr3 | -0.6187671 |
| S100a1 | -0.0756345 |
| S100a10 | -0.7177484 |
| S100a11 | 0.0873939 |
| S100a13 | 0.4800272 |
| S100a16 | -0.497341 |
| S100a4 | -0.7515938 |
| S100a6 | 0.5855496 |
| S100a8 | -0.4364834 |
| S100a9 | 0.0769696 |
| S100b | 1.6400685 |
| S100pbp | 0.4668786 |
| S100pbp | -0.4744143 |
| S3-12 | 2.0086278 |
| Saa3 | -0.411576 |
| Sac3d1 | -0.1855015 |
| Sacm1l | -0.1664375 |
| Sacs | -0.6915339 |
| Sae1 | -0.1462176 |
| Sae1 | 0.2954502 |
| Sae1 | -0.6359959 |
| Sag | -0.2717874 |
| Sall1 | -0.821105 |
| Sall1 | -0.5170166 |
| Sall2 | -0.6832627 |
| Sall4 | 0.6216585 |
| Sall4 | 1.9269204 |
| Samd10 | -0.6213311 |
| Samd11 | 0.3264667 |
| Samd12 | -0.5473352 |
| Samd14 | 0.7431289 |
| Samd4 | -0.4191159 |
| Samd4 | 3.4936174 |
| Samd4b | 0.4986395 |
| Samd5 | -0.5910209 |
| Samd7 | -0.7369372 |
| Samd8 | 0.1445991 |
| Samd9l | -0.7045486 |
| Samhd1 | -0.0890387 |
| Samm50 | 1.2347687 |
| Samsn1 | -0.5347767 |
| Sap130 | -0.6869297 |
| Sap30 | 1.6519571 |
| Sap30bp | 2.8926559 |
| Sap30l | -0.6333911 |
| Saps1 | -0.2203914 |
| Saps2 | 1.874644 |
| Saps3 | -0.4020676 |
| Saps3 | 1.3690257 |
| Sar1a | -0.6368658 |
| Sar1b | 0.6973821 |
| Sar1b | -0.572974 |
| Sarm1 | -0.5544748 |
| Sars | 0.3426549 |
| Sars | 2.6720011 |
| Sars2 | -0.6998795 |
| Sart1 | 1.472415 |
| Sart3 | -0.5449675 |
| Sas | -0.4343821 |
| Sass6 | -0.5885392 |
| Sat2 | -0.6906112 |
| Satb1 | 1.3250319 |
| Sav1 | 3.2285489 |
| Sbds | 1.1379851 |
| Sbf1 | -0.5960043 |
| Sbk | 2.9232753 |
| Sbno1 | -0.1021502 |
| Sbno2 | 0.0602897 |
| Sc4mol | -0.3866551 |
| Sc5d | -0.6896895 |
| Scaf1 | -0.5943402 |
| Scamp1 | 1.9682534 |
| Scamp2 | -0.6230444 |
| Scamp3 | 1.5377061 |
| Scamp4 | -0.5154882 |
| Scamp5 | 2.3625316 |
| Scamp5 | 4.1852259 |
| Scand1 | 0.5440872 |
| Scap | 0.283264 |
| Scaper | 0.3106181 |
| Scara3 | -0.4274118 |
| Scara5 | 2.0791172 |
| Scarb1 | 1.5163349 |
| Scarb2 | -0.6102712 |
| Scarf1 | -0.4308904 |
| Scarf2 | 0.1306251 |
| Sccpdh | -0.6777893 |
| Scd1 | -0.0977591 |
| Scd2 | 0.2023635 |
| Scd4 | -0.4965925 |
| Scel | -0.5592652 |
| Scg2 | 0.0913722 |
| Scg3 | -0.3083151 |
| Scg5 | -0.6624543 |
| Scgn | 0.1000649 |
| Scgn | -0.7684895 |
| Scgn | 1.7093558 |
| Schip1 | 1.4704418 |
| Schip1 | 0.7748299 |
| Scin | -0.3853269 |
| scl0003251.1_21 | 1.8428014 |
| Sclt1 | -0.4802768 |
| Scly | -0.5811337 |
| Scmh1 | -0.5860641 |
| Scmh1 | -0.1612108 |
| Scml2 | 1.4419974 |
| Scml4 | -0.4294974 |
| Scn10a | 1.3525549 |
| Scn11a | -0.6282033 |
| Scn1a | -0.2298839 |
| Scn2b | 1.1647056 |
| Scn3b | -0.4606374 |
| Scn3b | 1.402855 |
| Scn3b | -0.5262403 |
| Scn3b | -0.6588826 |
| Scn4b | -0.6786993 |
| Scn5a | 0.4650229 |
| Scn7a | -0.7272932 |
| Scn8a | 0.0242969 |
| Scnm1 | -0.311374 |
| Scnm1 | 0.8854108 |
| Scnn1a | -0.7253764 |
| Scnn1b | 0.0208766 |
| Scnn1g | -0.4013921 |
| Scoc | 2.7304831 |
| Scoc | 1.2572959 |
| Scotin | -0.4513277 |
| Scotin | -0.6896895 |
| Scotin | -0.3662787 |
| Scp2 | 0.2983131 |
| Scp2 | 1.539958 |
| Scp2 | -0.7585137 |
| Scrg1 | -0.6256202 |
| Scrib | 2.1480934 |
| Scrib | 1.3209271 |
| Scrn1 | -0.1549773 |
| Scrn1 | -0.2595378 |
| Scrn1 | -0.4484818 |
| Scrn2 | -0.6642453 |
| Sct | -0.5713507 |
| Sctr | 0.5629032 |
| Scube1 | 1.4897233 |
| Scube2 | 1.3042163 |
| Scube3 | 0.0652028 |
| Scube3 | -0.7959727 |
| Scx | 0.367452 |
| Scye1 | -0.5154882 |
| Scyl1 | 1.129985 |
| Scyl1bp1 | 0.7738793 |
| Scyl3 | 3.3682916 |
| Scyl3 | -0.4744143 |
| Sdad1 | -0.3813536 |
| Sdc1 | 1.73819 |
| Sdc1 | 0.2335337 |
| Sdc2 | 0.0833984 |
| Sdc3 | -0.5681125 |
| Sdc4 | 4.5995349 |
| Sdcbp | 2.0750552 |
| Sdcbp | 3.4797724 |
| Sdcbp2 | -0.0568155 |
| Sdcbp2 | 0.1035977 |
| Sdccag1 | 0.0101063 |
| Sdccag1 | -0.7330676 |
| Sdccag3 | -0.5960043 |
| Sdccag3 | 0.598509 |
| Sdf2 | -0.6473687 |
| Sdf2 | -0.7745269 |
| Sdf4 | -0.5177817 |
| Sdhb | -0.60437 |
| Sdhb | 2.7878429 |
| Sdhb | -0.5473352 |
| Sdhc | -0.5285606 |
| Sdhd | 0.7376066 |
| Sdk1 | 0.7993962 |
| Sdpr | -0.4315877 |
| Sds | -0.572974 |
| Sdsl | -0.6368658 |
| Sec1 | -0.7206016 |
| Sec11a | -0.6068945 |
| Sec11c | 1.2927611 |
| Sec13 | 2.5593291 |
| Sec14l1 | 0.8855794 |
| Sec14l1 | 1.1262192 |
| Sec14l1 | 0.1468455 |
| Sec14l2 | 2.810376 |
| Sec14l3 | 0.1252822 |
| Sec14l3 | -0.7092413 |
| Sec14l4 | 0.2090316 |
| Sec15l1 | -0.0429476 |
| Sec16a | 0.1359373 |
| Sec16b | -0.0900036 |
| Sec22a | -0.3482511 |
| Sec22b | -0.5616698 |
| Sec22c | 1.4849625 |
| Sec22c | -0.6989485 |
| Sec23a | 2.6521189 |
| Sec23ip | 0.2463863 |
| Sec24b | 0.1102342 |
| Sec24c | 0.4295909 |
| Sec24d | 3.0080294 |
| Sec61a1 | -0.7244194 |
| Sec61a2 | -0.2913695 |
| Sec61a2 | 1.2845698 |
| Sec61b | 0.2416718 |
| Sec61b | 3.0172777 |
| Sec61g | 0.0183031 |
| Sec63 | 0.3656815 |
| Sec63 | -0.49137 |
| Secisbp2 | -0.6196209 |
| Sectm1b | -0.6359959 |
| Seh1l | -0.1930223 |
| Seh1l | 1.3336038 |
| Sel1l | -0.5640805 |
| Sel1l | -0.4477717 |
| Sel1l | 0.4750526 |
| Sel1l2 | 1.0891868 |
| Sel1l2 | 1.5908505 |
| Selenbp2 | 0.6581401 |
| Selk | 2.1787637 |
| Sell | 3.4828761 |
| Selp | -0.4184279 |
| Selplg | -0.6732526 |
| Sema3a | -0.5528835 |
| Sema3b | -0.2181719 |
| Sema3b | 0.1199084 |
| Sema3b | 2.2949103 |
| Sema3e | -0.7017443 |
| Sema3f | 1.1820194 |
| Sema3f | 0.4854944 |
| Sema3g | -0.4336827 |
| Sema4a | -0.2712004 |
| Sema4b | -0.7806039 |
| Sema4d | 1.2646104 |
| Sema4f | -0.7506095 |
| Sema4g | 1.8170232 |
| Sema5a | -0.7575219 |
| Sema5a | -0.4102117 |
| Sema5b | 2.8619736 |
| Sema6b | -0.7447253 |
| Sema6b | 0.8632189 |
| Sema6b | 0.0495547 |
| Sema6d | 0.374793 |
| Sema6d | -0.7177484 |
| Sema7a | -0.6961611 |
| Sema7a | -0.7369372 |
| Senp1 | -0.6500131 |
| Senp2 | -0.3933247 |
| Senp3 | 0.2433583 |
| Senp5 | -0.1775105 |
| Senp5 | -0.4891407 |
| Senp5 | 0.5477354 |
| Senp6 | -0.6026908 |
| Senp6 | 0.9858703 |
| Senp7 | 1.0368193 |
| Senp7 | -0.6961611 |
| Senp7 | 2.0514071 |
| Senp8 | 2.3090165 |
| Sephs1 | -0.7476627 |
| Sephs2 | 0.1179816 |
| Sepm | -0.6018524 |
| Sepn1 | 0.122983 |
| Sepp1 | 0.0524571 |
| Sepp1 | 0.2055281 |
| Sepsecs | -0.579496 |
| Sept2 | -0.3230904 |
| Sept3 | 3.3734662 |
| Sept3 | -0.3119869 |
| Sept5 | -0.1962643 |
| Sept6 | 1.0991539 |
| Sept6 | -0.6660398 |
| Sept7 | 0.0627495 |
| Sept8 | -0.6841781 |
| Sept9 | 0.3624268 |
| Sept10 | 0.5989686 |
| Sept10 | -0.7595064 |
| Sept12 | -0.1523923 |
| Sept15 | 1.3733224 |
| Sepw1 | -0.4308904 |
| Sepx1 | -0.6239022 |
| Sepx1 | 0.4538097 |
| Serac1 | 0.0829979 |
| Serbp1 | 0.1102342 |
| Serbp1 | 1.524841 |
| Serbp1 | 0.0127026 |
| Serf1 | -0.8084538 |
| Serf2 | 1.3859962 |
| Sergef | 0.02387 |
| Serhl | -0.3362147 |
| Serhl | 0.685741 |
| Serinc1 | 1.3700267 |
| Serinc2 | 1.7634093 |
| Serinc3 | -0.3077046 |
| Serpina1a | 2.0743062 |
| Serpina1a | 0.4432747 |
| Serpina1b | -0.2137488 |
| Serpina1b | -0.3533662 |
| Serpina1d | 0.7908438 |
| Serpina1e | -0.0434066 |
| Serpina3b | -0.3980222 |
| Serpina3c | 0.393612 |
| Serpina3g | 0.1942269 |
| Serpina3h | 1.6775879 |
| Serpina3k | 1.3118107 |
| Serpina3n | -0.8667946 |
| Serpina5 | -0.4336827 |
| Serpina5 | 2.995449 |
| Serpina7 | -0.6943074 |
| Serpina9 | 0.1344226 |
| Serpinb11 | 0.9922061 |
| Serpinb12 | 1.0099262 |
| Serpinb1a | 0.6684271 |
| Serpinb3a | -0.3095375 |
| Serpinb6a | -0.5844177 |
| Serpinb6b | 0.2440321 |
| Serpinb6c | -0.5025974 |
| Serpind1 | -0.5418199 |
| Serpine2 | 0.3861302 |
| Serpinf1 | -0.464966 |
| Serping1 | -0.7008114 |
| Serpinh1 | -0.7959727 |
| Serpini1 | 1.2411133 |
| Sertad1 | -0.1855015 |
| Sertad2 | 0.5860155 |
| Sertad2 | -0.1711669 |
| Sertad3 | -0.6588826 |
| Sertad4 | 3.8625467 |
| Sesn1 | -0.0808774 |
| Sesn1 | 0.0221607 |
| Sesn2 | -0.7437482 |
| Sesn3 | -0.2560674 |
| Setd1b | -0.521617 |
| Setd2 | -0.568921 |
| Setd3 | 1.1466808 |
| Setd4 | 1.2829228 |
| Setd4 | 1.0496258 |
| Setd5 | 2.5483533 |
| Setd6 | -0.8502174 |
| Setd6 | 0.2379507 |
| Setd7 | 1.8184434 |
| Setd7 | -0.6196209 |
| Setd8 | 1.0654587 |
| Setd8 | -0.5512949 |
| Setd8 | -0.6136602 |
| Setdb1 | 0.4761021 |
| Setdb2 | -0.3469768 |
| Setmar | -0.6290659 |
| Setx | -0.3299419 |
| Sez6 | -0.6359959 |
| Sez6l | 0.8346221 |
| Sez6l2 | -0.6001777 |
| Sez6l2 | -0.5481258 |
| Sf1 | 2.8028473 |
| Sf1 | -0.5918496 |
| Sf3a1 | -0.1544597 |
| Sf3a2 | 1.6455116 |
| Sf3a3 | 1.0096312 |
| Sf3b1 | -0.6860116 |
| Sf3b2 | -0.568921 |
| Sf3b3 | 0.9123424 |
| Sf3b4 | 0.2249798 |
| Sf3b4 | -0.5901929 |
| Sf3b5 | -0.2187263 |
| Sf4 | 0.5808769 |
| Sfi1 | 0.8440175 |
| Sfi1 | -0.6421024 |
| Sfmbt1 | 0.5862484 |
| Sfmbt1 | 0.4443607 |
| Sfmbt1 | 1.153023 |
| Sfmbt2 | -0.5086413 |
| Sfrp1 | -0.6447318 |
| Sfrp2 | 0.1860183 |
| Sfrp4 | 1.8869895 |
| Sfrp5 | -0.5520888 |
| Sfrs1 | 3.1571351 |
| Sfrs1 | -0.714904 |
| Sfrs11 | 1.2546939 |
| Sfrs12 | 0.0805913 |
| Sfrs14 | 0.8470111 |
| Sfrs16 | 0.0424678 |
| Sfrs2 | 0.9358022 |
| Sfrs2 | 2.3031802 |
| Sfrs2 | -0.7664857 |
| Sfrs3 | 0.4251691 |
| Sfrs3 | -0.60353 |
| Sfrs4 | 2.081676 |
| Sfrs5 | -0.5656912 |
| Sfrs5 | -0.6500131 |
| Sfrs6 | 3.7805261 |
| Sfrs7 | -0.7282531 |
| Sfrs8 | 2.0972935 |
| Sfrs9 | -0.6841781 |
| Sft2d2 | 0.4660837 |
| Sft2d2 | -0.4898832 |
| Sft2d2 | -0.6642453 |
| Sftpc | 0.1744754 |
| Sftpd | -0.7565313 |
| Sfxn1 | 1.6896464 |
| Sfxn1 | -0.2497386 |
| Sfxn3 | -0.7836573 |
| Sfxn3 | -0.5770449 |
| Sfxn4 | 1.4431077 |
| Sfxn4 | -0.462078 |
| Sfxn5 | 0.6076562 |
| Sgca | -0.462078 |
| Sgca | 0.7812687 |
| Sgcb | 1.85427 |
| Sgcb | -0.7565313 |
| Sgcz | -0.6850944 |
| Sgip1 | 1.830276 |
| Sgip1 | -0.215405 |
| Sgip1 | 0.7002697 |
| Sgip1 | -0.6850944 |
| Sgip1 | -0.3578652 |
| Sgk1 | -0.6896895 |
| Sgk2 | 1.7661551 |
| Sgk2 | -0.7234635 |
| Sgk3 | 1.6142877 |
| Sgk3 | 0.5496753 |
| Sgk3 | -0.6077375 |
| Sgk3 | -0.6579918 |
| Sgk3 | 1.1520112 |
| Sgms2 | 0.2062298 |
| Sgol1 | -0.7466825 |
| Sgpl1 | 1.8243479 |
| Sgpl1 | -0.729214 |
| Sgpp1 | 1.4810652 |
| Sgpp2 | -0.5347767 |
| Sgsm1 | -0.7575219 |
| Sgsm2 | 0.390454 |
| Sgsm3 | 1.421876 |
| Sgta | -0.2170642 |
| Sgtb | -0.3425302 |
| Sgtb | 0.9123424 |
| Sh2b1 | 1.943082 |
| Sh2b1 | 0.394185 |
| Sh2b3 | -0.5786782 |
| Sh2d2a | -0.4095303 |
| Sh3bgrl | -0.8042747 |
| Sh3bgrl3 | 1.487346 |
| Sh3bp2 | 0.1981272 |
| Sh3bp5 | 1.5909294 |
| Sh3bp5l | -0.7918494 |
| Sh3gl1 | 2.1444534 |
| Sh3gl2 | -0.7158512 |
| Sh3gl2 | -0.1822968 |
| Sh3gl2 | -0.7826384 |
| Sh3gl3 | 0.4588991 |
| Sh3glb1 | -0.6606667 |
| Sh3glb2 | -0.4463532 |
| Sh3kbp1 | -0.2712004 |
| Sh3kbp1 | 0.3546881 |
| Sh3kbp1 | -0.824295 |
| Sh3kbp1 | 1.7607867 |
| Sh3md4 | 0.9982003 |
| Sh3md4 | -0.6247608 |
| Sh3pxd2a | 0.5975893 |
| Sh3pxd2b | -0.3318193 |
| Sh3rf1 | -0.5254682 |
| Sh3rf2 | -0.3913188 |
| Sh3tc1 | -0.7398501 |
| Sh3tc2 | -0.6001777 |
| Sh3yl1 | -0.5918496 |
| Shank2 | -0.4988398 |
| Sharpin | -0.6768802 |
| Shbg | -0.3980222 |
| Shc1 | 1.0601644 |
| Shc1 | 1.2639376 |
| Shc3 | -0.0661773 |
| Shcbp1 | -0.3040496 |
| Shd | -0.4788077 |
| She | -0.2830114 |
| Shf | -0.4225635 |
| Shf | 0.4823742 |
| Shf | -0.3546494 |
| Shfm1 | -0.4047743 |
| Shh | 1.0551208 |
| Shisa2 | -0.2209471 |
| Shisa3 | 0.4978714 |
| Shisa4 | -0.1743333 |
| Shkbp1 | 2.307488 |
| Shkbp1 | -0.7466825 |
| Shmt1 | -0.2913695 |
| Shmt2 | -0.4225635 |
| Shoc2 | 1.9821663 |
| Shox2 | -0.2853918 |
| Shprh | 0.9420121 |
| Shq1 | -0.4170535 |
| Shroom3 | -0.5086413 |
| Shroom3 | -0.2027824 |
| Si | -0.4965925 |
| Si | 0.4901552 |
| Siah1a | 2.9683901 |
| Siah1b | 0.8091533 |
| Siah1b | -0.5355567 |
| Siah1b | -0.7359683 |
| Siah1b | 1.0879672 |
| Siah2 | 0.6620111 |
| Siat7f | -0.1989746 |
| Siat9 | 1.9091303 |
| Sidt1 | 0.7618168 |
| Sidt2 | -0.4421104 |
| Sidt2 | -0.3966776 |
| Sidt2 | 0.1554065 |
| Sigirr | -0.3793734 |
| Siglec1 | -0.2474479 |
| Siglech | -0.0216296 |
| Siglech | -0.1041085 |
| Sil1 | -0.3064847 |
| Sin3a | -0.8063619 |
| Sin3b | 0.0495547 |
| Sip1 | 0.9676801 |
| Sip1 | 0.070496 |
| Sipa1 | -0.1036186 |
| Sipa1l1 | 0.1121772 |
| Sirpa | -0.5544748 |
| Sirpa | -0.3374743 |
| Sirpb1 | -0.0784906 |
| Sirt1 | -0.549709 |
| Sirt2 | -0.4898832 |
| Sirt3 | 0.5525775 |
| Sirt5 | -0.6351268 |
| Sirt5 | 1.4391229 |
| Sirt6 | -0.5819536 |
| Sirt7 | -0.1544597 |
| Siva1 | 0.0474759 |
| Six1 | 0.6422734 |
| Six2 | 0.1974193 |
| Six3 | 2.3661287 |
| Six5 | -0.5162521 |
| Six6os1 | 2.2371419 |
| Skap2 | -0.6438545 |
| Skil | -0.5071266 |
| Skiv2l | 0.3753778 |
| Skiv2l | -0.2131974 |
| Skiv2l2 | -0.3052663 |
| Skp1a | 2.0242289 |
| Skp2 | 1.0949915 |
| Skp2 | 0.128339 |
| Skp2 | 4.2334143 |
| Sla | 0.3973304 |
| Slain1 | -0.348889 |
| Slain2 | -0.3150579 |
| Slamf7 | 1.3787894 |
| Slamf8 | 2.0884344 |
| Slamf9 | 0.2065805 |
| Slc10a3 | 4.2785573 |
| Slc10a4 | 0.9295504 |
| Slc10a6 | -0.7379071 |
| Slc10a6 | -0.2120956 |
| Slc10a7 | 1.1725368 |
| Slc10a7 | -0.3431641 |
| Slc10a7 | -0.7545532 |
| Slc11a1 | -0.5835956 |
| Slc11a1 | 1.954555 |
| Slc11a1 | 1.6971305 |
| Slc11a1 | 1.0575059 |
| Slc11a2 | 1.4618728 |
| Slc12a2 | 0.2270399 |
| Slc12a2 | 2.3005051 |
| Slc12a3 | -0.568921 |
| Slc12a5 | -0.6832627 |
| Slc12a6 | -0.5943402 |
| Slc12a7 | -0.2055119 |
| Slc12a8 | -0.7388781 |
| Slc12a9 | 0.0340605 |
| Slc12a9 | -0.6723479 |
| Slc13a2 | -0.7457034 |
| Slc13a3 | -0.6307936 |
| Slc13a3 | 0.0627495 |
| Slc13a4 | -0.6438545 |
| Slc14a1 | 2.5947105 |
| Slc14a1 | 0.410088 |
| Slc14a1 | -0.7008114 |
| Slc14a2 | -0.7340335 |
| Slc14a2 | -0.7595064 |
| Slc15a2 | -0.7253764 |
| Slc15a3 | -0.3181391 |
| Slc15a4 | -0.4013921 |
| Slc15a4 | -0.8158127 |
| Slc16a10 | -0.6299293 |
| Slc16a11 | 1.8716192 |
| Slc16a4 | 1.9995864 |
| Slc16a6 | -0.3150579 |
| Slc16a6 | -0.4869168 |
| Slc16a6 | 1.5985464 |
| Slc16a8 | 0.586714 |
| Slc16a9 | -0.7806039 |
| Slc17a2 | 0.1546652 |
| Slc17a5 | -0.7130126 |
| Slc17a6 | -0.5116781 |
| Slc17a7 | 0.147593 |
| Slc17a8 | -0.6018524 |
| Slc18a2 | 1.184711 |
| Slc19a1 | -0.2254048 |
| Slc19a2 | -0.711125 |
| Slc1a1 | 1.3667192 |
| Slc1a1 | -0.0082742 |
| Slc1a2 | 1.1354119 |
| Slc1a2 | 0.0753555 |
| Slc1a2 | 1.5098178 |
| Slc1a2 | 0.6279135 |
| Slc1a3 | 0.9834452 |
| Slc1a4 | -0.3826762 |
| Slc1a6 | -0.2321316 |
| Slc1a7 | 0.4623653 |
| Slc20a1 | -0.6368658 |
| Slc22a1 | 0.0088054 |
| Slc22a12 | -0.4491925 |
| Slc22a13 | -0.0987331 |
| Slc22a14 | 0.7415544 |
| Slc22a17 | -0.5762293 |
| Slc22a17 | 0.9324411 |
| Slc22a18 | -0.4281064 |
| Slc22a21 | -0.0247738 |
| Slc22a3 | 2.5428588 |
| Slc22a4 | -0.6290659 |
| Slc22a5 | 0.3528931 |
| Slc22a5 | -0.4239461 |
| Slc22a5 | -0.7398501 |
| Slc22a6 | 0.0957287 |
| Slc22a7 | 0.4154223 |
| Slc22a8 | 0.0378532 |
| Slc23a2 | 0.6587864 |
| Slc23a3 | 2.092622 |
| Slc24a1 | 1.3430247 |
| Slc24a3 | -0.435082 |
| Slc24a4 | -0.5993416 |
| Slc24a5 | 0.1949372 |
| Slc24a6 | 1.6217162 |
| Slc25a1 | -0.219836 |
| Slc25a1 | -0.4054523 |
| Slc25a10 | 2.8885179 |
| Slc25a11 | -0.5811337 |
| Slc25a11 | -0.1169393 |
| Slc25a11 | 0.8891143 |
| Slc25a12 | 1.6500356 |
| Slc25a14 | -0.6869297 |
| Slc25a15 | 0.8887783 |
| Slc25a15 | 0.6611523 |
| Slc25a16 | -0.1184312 |
| Slc25a17 | -0.4477717 |
| Slc25a18 | 0.922284 |
| Slc25a19 | -0.5071266 |
| Slc25a19 | 3.3745507 |
| Slc25a20 | 1.0184417 |
| Slc25a20 | -0.5901929 |
| Slc25a23 | -0.7350004 |
| Slc25a24 | -0.6508962 |
| Slc25a24 | 0.1513219 |
| Slc25a25 | 0.1627871 |
| Slc25a26 | -0.5371185 |
| Slc25a27 | 1.2158249 |
| Slc25a28 | -0.6750646 |
| Slc25a29 | 1.4902508 |
| Slc25a3 | -0.0690044 |
| Slc25a3 | -0.1769802 |
| Slc25a30 | -0.6077375 |
| Slc25a32 | -0.6606667 |
| Slc25a33 | -0.7311388 |
| Slc25a34 | -0.1360169 |
| Slc25a35 | -0.3034419 |
| Slc25a36 | 0.9744475 |
| Slc25a37 | -0.6264805 |
| Slc25a38 | -0.6841781 |
| Slc25a38 | 3.1056042 |
| Slc25a38 | 1.6308999 |
| Slc25a39 | -0.2389077 |
| Slc25a41 | -0.1674864 |
| Slc25a42 | 0.3742079 |
| Slc25a44 | 1.0528704 |
| Slc25a45 | 1.7805992 |
| Slc25a46 | 0.4140215 |
| Slc25a5 | -0.1596485 |
| Slc26a4 | -0.3741133 |
| Slc26a6 | -0.4463532 |
| Slc26a7 | -0.4007171 |
| Slc26a8 | -0.7826384 |
| Slc27a1 | -0.7379071 |
| Slc27a2 | 0.7639343 |
| Slc27a3 | -0.5048592 |
| Slc27a4 | 0.2570732 |
| Slc27a6 | -0.4143107 |
| Slc29a1 | -0.7301759 |
| Slc29a2 | -0.6438545 |
| Slc29a3 | -0.7073613 |
| Slc29a4 | 1.0785636 |
| Slc2a1 | 0.9200117 |
| Slc2a10 | -0.130454 |
| Slc2a10 | -0.5935093 |
| Slc2a13 | -0.3028345 |
| Slc2a3 | 1.1015623 |
| Slc2a4 | -0.5960043 |
| Slc2a5 | -0.6153594 |
| Slc2a6 | -0.7545532 |
| Slc2a8 | 0.0602897 |
| Slc2a8 | -0.2824173 |
| Slc2a8 | 0.9198492 |
| Slc2a9 | -0.4839598 |
| Slc30a1 | 0.5335487 |
| Slc30a10 | -0.1472439 |
| Slc30a3 | -0.3132141 |
| Slc30a5 | 1.0680945 |
| Slc30a5 | -0.521617 |
| Slc30a6 | -0.711125 |
| Slc30a7 | 3.1029811 |
| Slc30a7 | 1.5367035 |
| Slc30a9 | -0.4054523 |
| Slc31a1 | 0.0688706 |
| Slc31a2 | 1.191406 |
| Slc32a1 | 0.0574117 |
| Slc34a2 | -0.49137 |
| Slc35a1 | 0.0281297 |
| Slc35a2 | 0.7194286 |
| Slc35a3 | -0.3649793 |
| Slc35a4 | -0.1935619 |
| Slc35a5 | 1.8939548 |
| Slc35a5 | 2.1226634 |
| Slc35b1 | 0.1389593 |
| Slc35b2 | 0.4391907 |
| Slc35b3 | 2.3391391 |
| Slc35b3 | 1.810076 |
| Slc35b3 | -0.4810123 |
| Slc35b4 | -0.7654855 |
| Slc35c1 | -0.2812301 |
| Slc35c1 | 1.7037871 |
| Slc35c2 | -0.6823482 |
| Slc35c2 | -0.5560689 |
| Slc35c2 | -0.5418199 |
| Slc35e1 | -0.5576657 |
| Slc35e3 | -0.6535507 |
| Slc35e4 | -0.0875933 |
| Slc35f1 | -0.2497386 |
| Slc35f2 | 1.4894595 |
| Slc35f3 | -0.3973496 |
| Slc35f4 | -0.0997082 |
| Slc35f4 | -0.6102712 |
| Slc36a1 | 0.4774123 |
| Slc36a3 | 1.0329426 |
| Slc37a1 | -0.5162521 |
| Slc37a3 | -0.2700275 |
| Slc38a1 | 0.2842319 |
| Slc38a10 | 2.3324622 |
| Slc38a11 | -0.1139625 |
| Slc38a2 | 0.2338742 |
| Slc38a2 | -0.4700416 |
| Slc38a2 | -0.5868884 |
| Slc38a3 | -0.6980184 |
| Slc38a4 | 1.1375996 |
| Slc38a5 | -0.2595378 |
| Slc38a8 | 0.986173 |
| Slc38a9 | 1.0953952 |
| Slc38a9 | -0.6500131 |
| Slc39a10 | -0.7311388 |
| Slc39a11 | 0.1366937 |
| Slc39a12 | 0.3432603 |
| Slc39a13 | -0.0699486 |
| Slc39a13 | -0.4484818 |
| Slc39a14 | 0.6733161 |
| Slc39a3 | 0.0582349 |
| Slc39a3 | -0.5868884 |
| Slc39a4 | -0.7466825 |
| Slc39a5 | -0.1612108 |
| Slc39a5 | -0.3598 |
| Slc39a5 | 2.0877423 |
| Slc39a6 | -0.7340335 |
| Slc39a7 | -0.6669383 |
| Slc39a7 | -0.3986952 |
| Slc39a7 | -0.5324408 |
| Slc39a8 | -0.3206115 |
| Slc3a2 | 0.6341266 |
| Slc40a1 | -0.0770615 |
| Slc41a2 | 0.0749515 |
| Slc41a3 | 3.3785318 |
| Slc41a3 | -0.5960043 |
| Slc41a3 | -0.6562128 |
| Slc43a2 | 1.5305808 |
| Slc43a2 | -0.4988398 |
| Slc44a1 | -0.2741391 |
| Slc44a1 | 3.4538741 |
| Slc44a2 | -0.6588826 |
| Slc44a2 | 1.0399661 |
| Slc44a2 | -0.2865843 |
| Slc44a3 | -0.7369372 |
| Slc44a4 | 2.7634575 |
| Slc45a1 | -0.8656802 |
| Slc45a2 | -0.3293169 |
| Slc45a3 | 0.0453925 |
| Slc45a4 | -0.3833381 |
| Slc46a1 | 0.7196303 |
| Slc46a3 | -0.4184279 |
| Slc47a1 | -0.3565775 |
| Slc4a1 | -0.7897946 |
| Slc4a1ap | -0.4685885 |
| Slc4a2 | 1.9170422 |
| Slc4a3 | -0.2717874 |
| Slc4a3 | 0.1741124 |
| Slc4a3 | -0.1204242 |
| Slc4a3 | 0.5176384 |
| Slc4a8 | -0.4729544 |
| Slc4a8 | 0.2983131 |
| Slc5a4a | -0.5943402 |
| Slc5a4b | -0.5093996 |
| Slc5a5 | 0.1817065 |
| Slc5a6 | -0.4336827 |
| Slc5a7 | 1.1685673 |
| Slc5a8 | 1.5948635 |
| Slc5a9 | 0.6605077 |
| Slc6a1 | -0.5827742 |
| Slc6a11 | 0.0949382 |
| Slc6a12 | -0.2991987 |
| Slc6a13 | -0.6606667 |
| Slc6a15 | 1.5200021 |
| Slc6a15 | 0.4839356 |
| Slc6a17 | -0.324954 |
| Slc6a2 | 0.6773356 |
| Slc6a4 | -0.2706137 |
| Slc6a6 | -0.3144429 |
| Slc6a7 | -0.817926 |
| Slc6a8 | -0.3305672 |
| Slc6a9 | -0.1339902 |
| Slc7a1 | 0.6942783 |
| Slc7a10 | -0.3540076 |
| Slc7a11 | -0.7330676 |
| Slc7a14 | 2.5124373 |
| Slc7a14 | 0.3820763 |
| Slc7a14 | 0.9034517 |
| Slc7a2 | 2.0204152 |
| Slc7a3 | -0.5681125 |
| Slc7a4 | -0.7408231 |
| Slc7a6 | 0.2574052 |
| Slc7a6os | -0.5968375 |
| Slc7a7 | 1.0614211 |
| Slc7a7 | 2.1521931 |
| Slc7a8 | 0.3656815 |
| Slc7a8 | 2.8363172 |
| Slc8a1 | 0.1256649 |
| Slc8a2 | -0.6660398 |
| Slc8a2 | 0.5146246 |
| Slc9a1 | 1.7314379 |
| Slc9a10 | -0.366929 |
| Slc9a3r1 | -0.187108 |
| Slc9a3r2 | 0.9217976 |
| Slc9a4 | -0.862345 |
| Slc9a6 | -0.8667946 |
| Slc9a6 | 2.370665 |
| Slc9a8 | -0.7388781 |
| Slc9a8 | 1.4141808 |
| Slc9a8 | -0.5868884 |
| Slc9a8 | 3.1621212 |
| Slc9a9 | -0.063828 |
| Slco1a4 | -0.3656288 |
| Slco1c1 | 0.8217041 |
| Slco2a1 | -0.6989485 |
| Slco2b1 | -0.7177484 |
| Slco3a1 | -0.3846635 |
| Slco3a1 | 0.0821964 |
| Slco3a1 | -0.7321027 |
| Slco4a1 | 1.157437 |
| Slfnl1 | -0.9680179 |
| Slit1 | 0.5014505 |
| Slit2 | -0.4040969 |
| Slit3 | 0.53822 |
| Slitrk1 | 0.5998869 |
| Slitrk3 | -0.4832221 |
| Slitrk4 | 1.9384853 |
| Slitrk5 | 0.3350542 |
| Slitrk5 | -0.6860116 |
| Slitrk6 | 0.3014838 |
| Slk | -0.1716939 |
| Slk | 1.3384815 |
| Slmap | 1.4166578 |
| Slmap | -0.2434527 |
| Slmap | -0.7158512 |
| Slmo1 | -0.6850944 |
| Slmo2 | -0.3425302 |
| Sln | -0.7959727 |
| Slpi | 0.2376117 |
| Sltm | 0.1795431 |
| Sltm | 2.8994925 |
| Slu7 | 1.0667082 |
| Slu7 | 1.45815 |
| Sly | 3.1906022 |
| Smad1 | -0.7437482 |
| Smad1 | -0.7054852 |
| Smad2 | 1.187273 |
| Smad3 | 1.8297276 |
| Smad5 | 0.5014505 |
| Smad5 | 1.7144083 |
| Smad6 | -0.7970064 |
| Smap2 | -0.4700416 |
| Smap2 | -0.5648855 |
| Smap2 | 1.485846 |
| Smarca1 | -0.7282531 |
| Smarca2 | -0.7775604 |
| Smarca2 | -0.729214 |
| Smarca4 | 2.0973849 |
| Smarcad1 | 0.5387103 |
| Smarcal1 | 0.3246162 |
| Smarcb1 | -0.2468761 |
| Smarcc1 | 0.4642264 |
| Smarcc1 | -0.4891407 |
| Smarcc2 | 0.9164297 |
| Smarcd1 | 2.0682454 |
| Smarcd1 | 0.5566735 |
| Smarcd1 | 3.5140199 |
| Smarcd2 | 1.3878628 |
| Smarcd3 | -0.4988398 |
| Smarce1 | 0.4055721 |
| Smarce1 | 0.9380362 |
| Smc1a | 1.0313585 |
| Smc1a | -0.3780556 |
| Smc1a | 0.0277046 |
| Smc1a | -0.7167993 |
| Smc1b | -0.6915339 |
| Smc3 | 1.6825301 |
| Smc5l1 | 0.6507839 |
| Smc6 | 0.7094909 |
| Smchd1 | 0.1094559 |
| Smcp | -0.4442294 |
| Smcr7 | 2.0741657 |
| Smcr7l | 1.3735218 |
| Smek1 | 0.4792435 |
| Smek2 | -0.1090224 |
| Smek2 | 2.5079291 |
| Smg5 | -0.0207333 |
| Smg6 | -0.2985941 |
| Smn1 | 0.3423521 |
| Smo | 0.6173899 |
| Smo | -0.7897946 |
| Smo | -0.6153594 |
| Smoc1 | 1.7158566 |
| Smox | -0.3119869 |
| Smox | 0.2460504 |
| Smox | 2.1035765 |
| Smox | 2.0997571 |
| Smox | 2.3198255 |
| Smpd1 | 3.444319 |
| Smpd1 | 2.5821183 |
| Smpd1 | 0.5927459 |
| Smpd2 | 1.7701286 |
| Smpd3 | 0.3118709 |
| Smpd4 | 0.6435878 |
| Smpdl3a | 0.8534933 |
| Smpdl3b | -0.3028345 |
| Smpx | 1.8761527 |
| Smtn | 2.3274139 |
| Smtn | -0.5101585 |
| Smtn | 0.9872316 |
| Smtnl1 | 1.9663088 |
| Smtnl2 | 0.3156194 |
| Smu1 | -0.4000427 |
| Smu1 | 0.9732207 |
| Smug1 | 1.304431 |
| Smurf1 | 0.120678 |
| Smyd1 | 0.4459874 |
| Smyd2 | -0.6705412 |
| Smyd2 | 1.802951 |
| Smyd3 | 1.9649399 |
| Smyd4 | 0.25408 |
| Smyd5 | -0.3591546 |
| Smyd5 | 0.6131016 |
| Snai1 | 0.2839094 |
| Snai3 | 1.5744622 |
| Snap23 | -0.3966776 |
| Snap29 | -0.187108 |
| Snap91 | -0.3859907 |
| Snap91 | -0.5673047 |
| Snapc1 | -0.5379004 |
| Snapc3 | -0.5943402 |
| Snapc3 | 0.9470766 |
| Snapc3 | 0.1609475 |
| Snapc3 | -0.2033277 |
| Snapc4 | 1.4191283 |
| Snapin | 0.4758399 |
| Snca | 0.4870506 |
| Snca | 0.4086791 |
| Snca | 0.0323698 |
| Sncaip | -0.5162521 |
| Sncg | 0.5738236 |
| Sncg | 4.3666426 |
| Snd1 | -0.3224701 |
| Snf1lk | 0.7818347 |
| Snf1lk2 | -0.3833381 |
| Snf8 | 0.254413 |
| Snip1 | -0.6018524 |
| Snip1 | -0.6377365 |
| Snn | 2.0829295 |
| Snora65 | -0.4061307 |
| Snph | -0.7959727 |
| Snrk | -0.5560689 |
| Snrk | -0.1924831 |
| Snrk | 0.0096729 |
| Snrp70 | -0.046165 |
| Snrpa | -0.1244231 |
| Snrpb | -0.6052107 |
| Snrpb | -0.8116005 |
| Snrpd1 | -0.572162 |
| Snrpd1 | -0.6642453 |
| Snrpd2 | -0.6970893 |
| Snrpd3 | 0.074143 |
| Snrpg | -0.0732612 |
| Snta1 | -0.5835956 |
| Sntb2 | -0.6052107 |
| Sntb2 | 1.0788375 |
| Sntg2 | -0.2071534 |
| Snupn | 2.4751353 |
| Snurf | 2.4892849 |
| Snurf | 1.8011298 |
| Snx1 | -0.6579918 |
| Snx10 | -0.6412275 |
| Snx11 | -0.215405 |
| Snx12 | -0.2549135 |
| Snx13 | 0.8129911 |
| Snx14 | 0.7274628 |
| Snx14 | -0.7167993 |
| Snx14 | -0.5632762 |
| Snx15 | -0.6403535 |
| Snx16 | -0.2735506 |
| Snx17 | -0.3286924 |
| Snx17 | 0.254746 |
| Snx18 | -0.1523923 |
| Snx19 | 1.5713267 |
| Snx19 | -0.6464889 |
| Snx2 | 1.3648095 |
| Snx2 | -0.7054852 |
| Snx20 | -0.5301108 |
| Snx21 | -0.4988398 |
| Snx22 | 2.7781787 |
| Snx24 | -0.6368658 |
| Snx25 | 1.0182956 |
| Snx25 | -0.4364834 |
| Snx26 | 1.9052051 |
| Snx29 | -0.6473687 |
| Snx3 | -0.3813536 |
| Snx30 | -0.6606667 |
| Snx33 | 1.4560551 |
| Snx4 | 1.2132222 |
| Snx6 | -0.311374 |
| Snx7 | -0.0167103 |
| Snx8 | -0.538683 |
| Snx9 | -0.5985061 |
| Soat1 | 0.8411912 |
| Socs2 | 1.8073325 |
| Socs3 | -0.6759719 |
| Socs4 | -0.2231733 |
| Socs5 | -0.5528835 |
| Socs6 | -0.1648664 |
| Socs7 | -0.2377749 |
| Socs7 | -0.1833638 |
| Sod1 | -0.1554952 |
| Sod2 | 1.0660142 |
| Sod3 | 0.8519207 |
| Sohlh1 | 0.9149603 |
| Sohlh1 | -0.4149957 |
| Son | 0.0005239 |
| Sorbs1 | -0.2394746 |
| Sorbs2 | 3.5467608 |
| Sorbs3 | 0.6786012 |
| Sorcs1 | 1.3548968 |
| Sorcs1 | -0.7846774 |
| Sorcs2 | 1.8102006 |
| Sorcs3 | 2.5459623 |
| Sorcs3 | -0.6196209 |
| Sord | 1.3568269 |
| Sorl1 | 0.347487 |
| Sort1 | -0.435082 |
| Sort1 | -0.7605003 |
| Sort1 | -0.4906263 |
| Sort1 | 1.0112527 |
| Sostdc1 | 0.4927344 |
| Sox12 | 0.882031 |
| Sox13 | -0.7311388 |
| Sox17 | 1.3270252 |
| Sox17 | -0.7795883 |
| Sox18 | 0.6572777 |
| Sox2 | 0.8748932 |
| Sox2 | 0.3441676 |
| Sox21 | 0.4069857 |
| Sox21 | 0.1795431 |
| Sox30 | 1.5587593 |
| Sox5 | -0.3721484 |
| Sox5 | -0.7073613 |
| Sox5 | -0.4802768 |
| Sox5 | 0.2260105 |
| Sox7 | -0.6998795 |
| Sox7 | 0.2656666 |
| Sox8 | -0.6290659 |
| Sox9 | 0.849995 |
| Sp100 | 2.2311899 |
| Sp140 | 0.0336381 |
| Sp2 | 2.5907468 |
| Sp4 | -0.4288016 |
| Sp5 | -0.729214 |
| Sp6 | 0.7157904 |
| Sp7 | 2.4934672 |
| Sp8 | -0.6239022 |
| Sp9 | -0.0880748 |
| Spaca3 | 1.2924349 |
| Spaca4 | 1.2769693 |
| Spag1 | 3.6509393 |
| Spag1 | 0.9560297 |
| Spag16 | 0.0079372 |
| Spag16 | 0.6254612 |
| Spag4 | 1.5237396 |
| Spag4 | -0.6651421 |
| Spag4l | -0.1508448 |
| Spag4l | -0.5968375 |
| Spag5 | 0.0079372 |
| Spag5 | -0.1523923 |
| Spag6 | -0.7282531 |
| Spag6 | -0.510918 |
| Spag7 | 0.3362745 |
| Spag8 | 1.878122 |
| Spag9 | 0.9375579 |
| Spag9 | -0.2712004 |
| Spag9 | -0.0530971 |
| Spag9 | -0.2618586 |
| Spam1 | -0.1339902 |
| Spam1 | 0.0079372 |
| Sparc | -0.1400838 |
| Sparc | -0.638608 |
| Sparcl1 | -0.1653898 |
| Spast | -0.6273415 |
| Spast | -0.6624543 |
| Spast | -0.5177817 |
| Spata16 | -0.4007171 |
| Spata16 | 2.5432826 |
| Spata17 | -0.5078837 |
| Spata17 | -0.2913695 |
| Spata19 | 0.0809928 |
| Spata2 | 0.9986483 |
| Spata20 | -0.5048592 |
| Spata20 | -0.5835956 |
| Spata21 | -0.4685885 |
| Spata22 | 0.9934081 |
| Spata2L | 1.5331858 |
| Spata3 | 2.0502064 |
| Spata3 | -0.456329 |
| Spata3 | -0.0401988 |
| Spata3 | 3.3708651 |
| Spata4 | -0.6508962 |
| Spata6 | 0.4721604 |
| Spata7 | -0.1903293 |
| Spata7 | 0.828365 |
| Spatc1 | -0.6597742 |
| Spatc1 | -0.065707 |
| Spats1 | -0.6723479 |
| Spc24 | -0.5132003 |
| Spc24 | 0.5962081 |
| Spc25 | -0.5951719 |
| Spc25 | -0.4177405 |
| Spcs1 | -0.5926791 |
| Spcs1 | 0.0717133 |
| Spcs3 | 0.0349047 |
| Spdef | 0.0449753 |
| Specc1l | 0.0109726 |
| Speer1-ps1 | 0.5927459 |
| Speer1-ps1 | -0.1951824 |
| Speer2 | 0.1895962 |
| Speer2 | 3.2576961 |
| Speer3 | 0.3832364 |
| Speer4b | -0.1876441 |
| Speer4d | -0.7054852 |
| Speer4f | 1.4678344 |
| Speer6-ps1 | 0.0247235 |
| Spef1 | -0.2889738 |
| Spen | 0.9477077 |
| Spert | -0.3840006 |
| Spesp1 | -0.7253764 |
| Spg20 | -0.2641852 |
| Spg21 | 0.4193333 |
| Spg7 | 2.8122169 |
| Spg7 | 0.3341379 |
| Sphk1 | -0.4846982 |
| Sphk2 | 0.5576347 |
| Sphk2 | -0.5449675 |
| Spic | 1.2337264 |
| Spin | -0.7555417 |
| Spin2 | 0.4176592 |
| Spink10 | -0.7496262 |
| Spink2 | 2.5053137 |
| Spink4 | 0.1351803 |
| Spink8 | 1.0610024 |
| Spinlw1 | -0.0751594 |
| Spinlw1 | -0.6678377 |
| Spinlw1 | 1.7133723 |
| Spint1 | -0.8264278 |
| Spint2 | 0.5733515 |
| Spire1 | -0.411576 |
| Spire1 | -0.7856986 |
| Spire1 | -0.3324459 |
| Spire2 | 1.4004376 |
| Spna1 | -0.572162 |
| Spnb1 | -0.638608 |
| Spnb2 | 1.3199772 |
| Spnb2 | 0.8351564 |
| Spnb2 | -0.7908214 |
| Spnb2 | 1.2557129 |
| Spnb3 | -0.4499037 |
| Spnb4 | -0.7388781 |
| Spns1 | -0.5600661 |
| Spo11 | 0.7997663 |
| Spo11 | 1.1731555 |
| Spock1 | 1.1271296 |
| Spock2 | 0.5991982 |
| Spock3 | 1.2528794 |
| Spon1 | 0.3806242 |
| Spon2 | -0.572974 |
| Spop | 2.3149246 |
| Spp1 | -0.7167993 |
| Spp2 | -0.3077046 |
| Sppl3 | -0.7026781 |
| Spr | 1.8726674 |
| Spred1 | 1.9705082 |
| Spred2 | 1.0220882 |
| Spred2 | 2.6290754 |
| Sprn | 0.0005239 |
| Sprn | 0.4235055 |
| Sprr2a | -0.0866309 |
| Sprr2a | 0.5431119 |
| Sprr2f | -0.5457561 |
| Spry3 | 0.4229503 |
| Spry4 | 1.4272532 |
| Spryd4 | 1.2911286 |
| Spsb1 | -0.6989485 |
| Spsb2 | 0.011838 |
| Spsb3 | -0.1638205 |
| Spsb3 | -0.1441685 |
| Spsb4 | 0.9973036 |
| Sptlc1 | -0.6615601 |
| Sptlc2 | -0.6001777 |
| Spz1 | 0.3841055 |
| Sqle | -0.1855015 |
| Sqle | 1.3667192 |
| Sqrdl | -0.5640805 |
| Sqstm1 | -0.7427721 |
| Sra1 | -0.4664133 |
| Sra1 | 0.7391877 |
| Src | 1.8525468 |
| Srd5a1 | -0.7466825 |
| Srd5a2 | 0.086197 |
| Srd5a3 | 0.470579 |
| Srd5a3 | 0.7771075 |
| Srd5a3 | -0.6535507 |
| Srebf1 | -0.4788077 |
| Srebf2 | 1.6877304 |
| Srf | 0.6227785 |
| Srf | 2.6246094 |
| Srfbp1 | -0.5048592 |
| Srgn | 0.0611104 |
| Srgn | 0.7429322 |
| Sri | -0.6456099 |
| Sri | -0.5844177 |
| Sri | -0.3199928 |
| Srm | -0.6438545 |
| Srm | 0.6398589 |
| Srm | 1.3646083 |
| Srp14 | 3.6356718 |
| Srp19 | -0.1695878 |
| Srp19 | -0.7525792 |
| Srp54 | 0.4151423 |
| Srp68 | 2.3304746 |
| Srp72 | 0.6984145 |
| Srp9 | -0.7225085 |
| Srp9 | -0.0104868 |
| Srpk1 | 0.8247705 |
| Srpk2 | 2.2602305 |
| Srpk3 | 0.5047616 |
| Srpr | 1.4348412 |
| Srprb | 0.6743756 |
| Srprb | -0.6359959 |
| Srpx | -0.5162521 |
| Srpx | 0.6934488 |
| Srpx2 | 0.7958732 |
| Srpx2 | 1.7969715 |
| Srpx2 | -0.4061307 |
| Srr | 0.719832 |
| Srrd | -0.572974 |
| Srrm1 | -0.0073906 |
| Srrm2 | -0.6696392 |
| Srrm2 | 0.8067695 |
| Srrp | 1.7023897 |
| Srxn1 | -0.6456099 |
| Srxn1 | 3.4938304 |
| Srxn1 | 0.2617103 |
| Ss18 | 2.4013852 |
| Ss18 | -0.1801665 |
| Ss18l1 | -0.5600661 |
| Ss18l1 | 2.1971175 |
| Ss18l1 | 1.0421056 |
| Ssbp1 | 2.1762498 |
| Ssbp2 | -0.4463532 |
| Ssbp2 | 0.4212827 |
| Ssbp3 | -0.5976714 |
| Ssbp3 | 0.828903 |
| Ssbp4 | 1.4354008 |
| Ssfa2 | -0.6482493 |
| Ssh2 | 2.6430056 |
| Ssh3 | 0.1542943 |
| Ssh3 | 0.6750106 |
| Ssna1 | -0.2543371 |
| Sspn | 2.4577668 |
| Ssr2 | -0.8001144 |
| Ssr3 | 0.1737492 |
| Ssr4 | 0.2487346 |
| Ssrp1 | 0.9701467 |
| Sssca1 | 1.0622579 |
| Sssca1 | -0.0948432 |
| Sst | 0.3788787 |
| Sstr1 | 0.2787336 |
| Sstr5 | 2.2279416 |
| Ssty2 | -0.7816206 |
| Ssu72 | -0.6642453 |
| Ssx2ip | 1.4328799 |
| Ssxb2 | -0.6010147 |
| Ssxb5 | -0.2440223 |
| St13 | 1.4448629 |
| St14 | -0.0365462 |
| St14 | 2.9157998 |
| St18 | -0.5025974 |
| St3gal1 | -0.4527539 |
| St3gal2 | 0.4243377 |
| St3gal2 | -0.300409 |
| St3gal3 | 0.5850834 |
| St3gal3 | -0.5640805 |
| St3gal4 | 1.1629563 |
| St3gal5 | -0.325576 |
| St3gal6 | -0.7664857 |
| St5 | 0.4729501 |
| St5 | -0.7244194 |
| St6gal1 | 0.5404245 |
| St6galnac2 | -0.3286924 |
| St6galnac3 | 0.7372109 |
| St6galnac4 | -0.3813536 |
| St6galnac4 | 2.1441059 |
| St6galnac4 | -0.0813555 |
| St6galnac5 | -0.2842008 |
| St6galnac6 | 0.038694 |
| St6galnac6 | -0.7634882 |
| St7 | 0.6855318 |
| St7l | 1.190678 |
| St7l | 0.8478897 |
| St8sia1 | -0.7398501 |
| St8sia1 | 1.1168031 |
| St8sia2 | -0.5993416 |
| St8sia3 | -0.7674871 |
| St8sia4 | 1.0712753 |
| St8sia4 | -0.6187671 |
| St8sia5 | -0.1503295 |
| Stab1 | -0.1560133 |
| Stab2 | 0.013998 |
| Stac | -0.5489171 |
| Stac2 | -0.4606374 |
| Stac3 | -0.2071534 |
| Stag1 | -0.1539425 |
| Stag2 | 1.3146825 |
| Stag2 | -0.4950974 |
| Stag3 | 3.6547918 |
| Stam | -0.4693148 |
| Stam | 2.3151775 |
| Stam | 0.0507997 |
| Stam2 | -0.0424889 |
| Stam2 | -0.3649793 |
| Stambp | -0.2800445 |
| Stambpl1 | 0.773118 |
| Stap1 | 2.9745329 |
| Stap2 | -0.6544372 |
| Star | 1.5913235 |
| Stard10 | -0.5608676 |
| Stard10 | 3.3620484 |
| Stard13 | -0.3482511 |
| Stard3 | -0.6421024 |
| Stard3nl | -0.5332188 |
| Stard4 | 0.3736223 |
| Stard5 | 0.3501939 |
| Stard5 | -0.3206115 |
| Stard6 | 2.5666574 |
| Stard7 | -0.5154882 |
| Stard7 | 0.2311465 |
| Stard8 | 2.6414314 |
| Stard8 | -0.7101827 |
| Stat1 | -0.1334842 |
| Stat1 | 0.5564331 |
| Stat2 | 0.5688542 |
| Stat3 | 0.0769696 |
| Stat3 | -0.3175221 |
| Stat3 | -0.0466255 |
| Stat4 | -0.5457561 |
| Stat4 | 1.959285 |
| Stat5a | 0.515379 |
| Stat5a | 0.2728762 |
| Stat6 | -0.3444333 |
| Statip1 | -0.4950974 |
| Stau1 | 2.9752425 |
| Stau2 | 0.6380988 |
| Stbd1 | -0.2400418 |
| Stc2 | 1.3103181 |
| Steap1 | 2.1567461 |
| Steap2 | -0.572974 |
| Steap3 | -0.5827742 |
| Stfa1 | -0.5101585 |
| Stfa2 | -0.693382 |
| Stim1 | -0.7045486 |
| Stip1 | -0.3206115 |
| Stk11 | 0.3236895 |
| Stk11ip | -0.3399988 |
| Stk17b | 1.0583463 |
| Stk19 | 1.0421056 |
| Stk19 | 2.00908 |
| Stk19 | -0.4527539 |
| Stk22b | 1.416753 |
| Stk22s1 | -0.7092413 |
| Stk24 | -0.5010925 |
| Stk25 | 0.672892 |
| Stk25 | 2.9363669 |
| Stk3 | -0.0751594 |
| Stk31 | -0.5505016 |
| Stk32a | 2.1990436 |
| Stk32b | 0.4763643 |
| Stk32c | 0.2826181 |
| Stk35 | 1.9276886 |
| Stk35 | 0.7376066 |
| Stk36 | -0.2764967 |
| Stk38l | 0.9950582 |
| Stk39 | -0.5960043 |
| Stk4 | 0.0361695 |
| Stk40 | 0.099278 |
| Stmn1 | -0.6562128 |
| Stmn2 | -0.5592652 |
| Stmn3 | 0.4148623 |
| Stmn4 | -0.4040969 |
| Stmn4 | -0.1482713 |
| Stoml1 | 0.9477077 |
| Stoml1 | -0.5162521 |
| Stoml1 | 0.2534135 |
| Stoml2 | 0.2957688 |
| Stoml3 | -0.1764502 |
| Stra13 | 0.2194637 |
| Stra13 | 0.4591662 |
| Stra13 | 0.6065177 |
| Stra6 | -0.6860116 |
| Strap | 0.7230526 |
| Strbp | -0.5033507 |
| Strn4 | 0.2215361 |
| Strn4 | -0.6915339 |
| Stt3a | 0.9977521 |
| Stt3b | -0.6412275 |
| Stx12 | -0.7158512 |
| Stx17 | 2.8781999 |
| Stx18 | 0.8240499 |
| Stx1a | 0.1719312 |
| Stx2 | 0.3938986 |
| Stx3 | -0.2282017 |
| Stx3 | -0.5512949 |
| Stx4a | -0.1591283 |
| Stx5a | -0.1653898 |
| Stx5a | 0.0817954 |
| Stx6 | -0.4965925 |
| Stx7 | -0.3412636 |
| Stx8 | -0.7826384 |
| Stx8 | 0.0845988 |
| Stx8 | -0.3820147 |
| Stx8 | 1.0054908 |
| Stxbp1 | 2.7731873 |
| Stxbp1 | -0.2943729 |
| Stxbp1 | 0.4165414 |
| Stxbp2 | 1.1764894 |
| Stxbp4 | -0.5656912 |
| Stxbp4 | 1.8585935 |
| Stxbp5l | -0.693382 |
| Stxbp5l | -0.5976714 |
| Styx | -0.7949402 |
| Styxl1 | -0.7092413 |
| Styxl1 | 0.0913722 |
| Styxl1 | 1.8868176 |
| Suclg1 | 0.3930386 |
| Suclg1 | 0.0255762 |
| Suclg2 | 2.5742835 |
| Suclg2 | -0.6786993 |
| Suds3 | -0.4435225 |
| Sufu | -0.6571019 |
| Suhw2 | 0.4210044 |
| Suhw4 | 1.9215805 |
| Sulf1 | 1.07733 |
| Sult1a1 | -0.2440223 |
| Sult1c2 | 2.3787277 |
| Sult1d1 | 0.5085675 |
| Sult1d1 | -0.6669383 |
| Sult2b1 | -0.4685885 |
| Sult2b1 | 0.0512143 |
| Sult4a1 | -0.1021502 |
| Sult5a1 | 0.8064023 |
| Sult5a1 | 2.1001671 |
| Sumf2 | 1.1051631 |
| Sumo1 | 0.114116 |
| Sumo1 | -0.3010148 |
| Sumo1 | 0.956811 |
| Sumo2 | -0.1011726 |
| Sumo3 | -0.0577475 |
| Sumo3 | 0.9138159 |
| Sunc1 | 1.4400512 |
| Suox | -0.4020676 |
| Supt3h | -0.5951719 |
| Supt4h1 | 1.1454071 |
| Supt4h2 | 0.7554352 |
| Supt5h | -0.0813555 |
| Supt5h | -0.0401988 |
| Supt6h | -0.4898832 |
| Supv3l1 | 2.0527502 |
| Surf1 | -0.5308868 |
| Surf1 | 0.1719312 |
| Surf2 | 2.2212109 |
| Surf4 | -0.6162101 |
| Susd2 | -0.4491925 |
| Susd3 | 1.8375577 |
| Susd4 | 1.7896174 |
| Suv39h1 | -0.6342585 |
| Suv420h1 | 0.341443 |
| Suv420h1 | 0.8378237 |
| Suv420h1 | 3.1109662 |
| Suv420h2 | 2.2486611 |
| Suz12 | 1.3448769 |
| Sv2a | -0.8168688 |
| Sv2a | 4.6385725 |
| Sv2b | 0.2128703 |
| Sval2 | 0.7322502 |
| Svep1 | 0.1513219 |
| Svil | 0.3124967 |
| Svil | 1.9507107 |
| Svop | -0.7282531 |
| Svopl | -0.0310942 |
| Svs4 | 0.4597002 |
| Svs5 | -0.6597742 |
| Svs6 | 2.3814558 |
| Swap70 | -0.3362147 |
| Swap70 | -0.5528835 |
| Swap70 | 0.9245508 |
| Syap1 | 2.1502966 |
| Syap1 | -0.6377365 |
| Sybl1 | -0.2985941 |
| Syce2 | -0.6850944 |
| Sycp1 | -0.693382 |
| Syf2 | -0.6299293 |
| Syf2 | 0.9435977 |
| Syn1 | -0.3324459 |
| Syn1 | -0.4414052 |
| Syn2 | -0.5576657 |
| Sync | -0.6473687 |
| Syncrip | -0.5697302 |
| Syne1 | 1.6904967 |
| Syne1 | -0.638608 |
| Syngr1 | -0.5544748 |
| Syngr1 | 2.7597293 |
| Syngr1 | 1.4830159 |
| Syngr1 | 2.8838528 |
| Syngr2 | -0.6196209 |
| Syngr2 | 0.0725239 |
| Syngr2 | -0.29077 |
| Syngr3 | 0.8541914 |
| Syngr4 | 3.1567415 |
| Syngr4 | -0.5584651 |
| Syngr4 | -0.6307936 |
| Synj2 | -0.4061307 |
| Synj2bp | 2.1549018 |
| Synj2bp | 1.1998577 |
| Synpo2l | -0.6896895 |
| Syp | -0.60437 |
| Sypl | -0.6915339 |
| Sypl | 1.0469368 |
| Sypl2 | 2.0359674 |
| Sys1 | -0.6128118 |
| Syt1 | 1.096605 |
| Syt11 | -0.5901929 |
| Syt11 | -0.4570457 |
| Syt12 | -0.1984319 |
| Syt13 | 0.2450418 |
| Syt15 | 0.2236038 |
| Syt16 | -0.7167993 |
| Syt3 | 0.750181 |
| Syt4 | 1.3103181 |
| Syt5 | 0.6003457 |
| Syt9 | 0.3639076 |
| Sytl2 | -0.6085813 |
| Sytl3 | -0.0021068 |
| Sytl3 | 0.2409964 |
| Sytl3 | 1.0978133 |
| Sytl4 | 0.179182 |
| Sytl5 | 0.7548529 |
| Syvn1 | -0.4570457 |
| Taar2 | -0.1738048 |
| Taar3 | 1.7966557 |
| Taar4 | 0.7186214 |
| Tac1 | -0.0493935 |
| Tacc1 | -0.1045988 |
| Tacc1 | 0.5621866 |
| Tacr3 | 0.7641265 |
| Tacr3 | -0.3966776 |
| Tacstd2 | -0.6068945 |
| Tada1l | 1.2805019 |
| Tada2l | -0.1903293 |
| Tada2l | -0.3169054 |
| Tada3l | 0.3371887 |
| Tada3l | 2.4269352 |
| Taf10 | -0.0851892 |
| Taf11 | 1.0309261 |
| Taf12 | -0.5465453 |
| Taf12 | -0.579496 |
| Taf12 | 2.5100102 |
| Taf13 | -0.3181391 |
| Taf1c | 0.6815478 |
| Taf3 | 0.0565878 |
| Taf5 | -0.5528835 |
| Taf5 | 0.2280683 |
| Taf5 | -0.5041047 |
| Taf5l | -0.2055119 |
| Taf6 | 0.5876446 |
| Taf6 | 0.0148607 |
| Taf6l | -0.4921142 |
| Taf7l | 0.7729276 |
| Taf9 | -0.2326944 |
| Taf9 | -0.60353 |
| Taf9 | -0.3793734 |
| Taf9b | -0.4449368 |
| Tagap | 2.7684349 |
| Tagap | 2.0068668 |
| Tagln | 2.7227866 |
| Tagln3 | 0.1795431 |
| Tal1 | -0.5301108 |
| Tal2 | 0.5683795 |
| Taldo1 | 3.5753166 |
| Tank | -0.2688561 |
| Tank | -0.456329 |
| Tank | 0.7096948 |
| Tap1 | 0.7780549 |
| Tap2 | -0.5505016 |
| Tapbp | 1.7908895 |
| Tapbp | -0.2554903 |
| Tapbpl | 2.1048462 |
| Tapbpl | -0.5355567 |
| Tapt1 | -0.2770871 |
| Tarbp2 | -0.4635209 |
| Tarbp2 | -0.4943507 |
| Tardbp | -0.5926791 |
| Tardbp | 2.7320977 |
| Tardbp | 1.1043641 |
| Tars | -0.7624911 |
| Tarsl2 | 0.5083143 |
| Tarsl2 | 1.3583479 |
| Tas1r1 | 0.8339091 |
| Tasp1 | -0.5592652 |
| Tatdn2 | 1.0508967 |
| Tax1bp1 | 0.1686497 |
| Tax1bp3 | 1.2687483 |
| Tax1bp3 | 0.9006351 |
| Taz | -0.6952338 |
| Taz | -0.40342 |
| Tbc1d1 | 0.6214343 |
| Tbc1d10a | -0.5951719 |
| Tbc1d10a | 0.5698028 |
| Tbc1d10c | 2.2083309 |
| Tbc1d12 | 3.7628655 |
| Tbc1d13 | -0.3866551 |
| Tbc1d14 | 0.3109314 |
| Tbc1d15 | -0.5803145 |
| Tbc1d16 | -0.6832627 |
| Tbc1d17 | 0.5088207 |
| Tbc1d19 | -0.7253764 |
| Tbc1d2 | 1.8503436 |
| Tbc1d20 | -0.6732526 |
| Tbc1d22a | -0.5473352 |
| Tbc1d22b | -0.4506154 |
| Tbc1d23 | 2.2756616 |
| Tbc1d24 | -0.5246966 |
| Tbc1d25 | 0.5062858 |
| Tbc1d2b | -0.7301759 |
| Tbc1d5 | -0.3305672 |
| Tbc1d5 | -0.6018524 |
| Tbc1d7 | -0.4846982 |
| Tbc1d7 | -0.7340335 |
| Tbc1d8 | -0.5093996 |
| Tbc1d9 | -0.29077 |
| Tbc1d9 | -0.6001777 |
| Tbc1d9b | -0.3859907 |
| Tbca | 4.6716939 |
| Tbcb | 0.6446817 |
| Tbcc | -0.6153594 |
| Tbcd | 0.463695 |
| Tbcel | -0.2871811 |
| Tbk1 | 1.9876839 |
| Tbkbp1 | -0.3144429 |
| Tbl1x | 2.1277612 |
| Tbl1xr1 | -0.6777893 |
| Tbl2 | 2.877412 |
| Tbl3 | -0.6850944 |
| Tbp | 0.0428862 |
| Tbpl1 | -0.5713507 |
| Tbr1 | -0.5893657 |
| Tbrg1 | -0.4980901 |
| Tbrg1 | -0.3893172 |
| Tbrg1 | -0.6342585 |
| Tbrg4 | 1.6474425 |
| Tbrg4 | -0.4788077 |
| Tbx1 | 0.9036172 |
| Tbx19 | -0.6705412 |
| Tbx2 | 1.399275 |
| Tbx22 | -0.6544372 |
| Tbx3 | 2.5446093 |
| Tbx3 | 0.9958073 |
| Tbx5 | 0.4134606 |
| Tbx6 | -0.6128118 |
| Tbxa2r | 1.377103 |
| Tbxa2r | -0.7674871 |
| Tcam1 | 1.1717938 |
| Tcam1 | -0.6669383 |
| Tcam1 | -0.5926791 |
| Tcap | 1.0532927 |
| Tcea2 | 0.8317669 |
| Tcea3 | 0.140843 |
| Tceal1 | -0.1946419 |
| Tceal1 | 0.1524377 |
| Tceal3 | -0.6924575 |
| Tceal5 | -0.4274118 |
| Tceal6 | 0.3768381 |
| Tceal6 | -0.3933247 |
| Tceal8 | -0.219836 |
| Tceb1 | 0.5731153 |
| Tceb2 | -0.0140379 |
| Tceb2 | 1.5985464 |
| Tceb3 | -0.1881805 |
| Tcerg1 | -0.3095375 |
| Tcf12 | -0.4499037 |
| Tcf12 | 1.987221 |
| Tcf15 | 1.4758133 |
| Tcf19 | -0.49137 |
| Tcf20 | -0.2859878 |
| Tcf20 | 0.0260023 |
| Tcf20 | 0.607201 |
| Tcf20 | -0.3926556 |
| Tcf20 | -0.7836573 |
| Tcf21 | -0.5993416 |
| Tcf23 | -0.7867209 |
| Tcf25 | -0.6924575 |
| Tcf25 | 0.1609475 |
| Tcf25 | -0.2985941 |
| Tcf3 | 0.4888629 |
| Tcf3 | 0.7256612 |
| Tcf4 | -0.5705401 |
| Tcf7 | -0.6001777 |
| Tcf7 | 0.2149575 |
| Tcf7l2 | 2.9892166 |
| Tcf7l2 | 1.5879282 |
| Tcfap2b | -0.5316635 |
| Tcfap2b | 0.9239037 |
| Tcfap2c | -0.5624727 |
| Tcfap2c | 0.9451806 |
| Tcfcp2 | -0.7045486 |
| Tcfe3 | 1.0175644 |
| Tcfl5 | 1.3346438 |
| Tchp | 1.8561677 |
| Tcirg1 | -0.4642431 |
| Tcirg1 | 2.5276147 |
| Tcirg1 | -0.6153594 |
| Tcirg1 | -0.462078 |
| Tcl1 | 2.9067183 |
| Tcl1 | 0.3759622 |
| Tcl1b2 | 0.4033058 |
| Tcl1b2 | -0.3933247 |
| Tcl1b3 | 0.1715671 |
| Tcl1b4 | 0.3642035 |
| Tcn2 | -0.1570505 |
| Tcof1 | -0.5520888 |
| Tcp1 | 0.7973582 |
| Tcp1 | -0.2729625 |
| Tcp10a | 0.684485 |
| Tcp10a | 1.5035145 |
| Tcp11 | 0.0466431 |
| Tcstv1 | -0.7959727 |
| Tcte3 | -0.5762293 |
| Tctex1d1 | 1.0945877 |
| Tctex1d2 | -0.4294974 |
| Tctex1d4 | -0.5624727 |
| Tctn3 | -0.3156734 |
| Tctn3 | -0.520081 |
| Tctn3 | 1.4778642 |
| Tdg | -0.6085813 |
| Tdp1 | -0.2417457 |
| Tdpoz1 | 1.4925331 |
| Tdpoz2 | 1.2181847 |
| Tdrd1 | -0.8350081 |
| Tdrd12 | 0.7360227 |
| Tdrd3 | 0.5738236 |
| Tdrd3 | -0.1129724 |
| Tdrd3 | 0.1719312 |
| Tdrd6 | -0.5063702 |
| Tdrd7 | 1.642533 |
| Tdrkh | 0.3826565 |
| Tead2 | -0.0827913 |
| Tec | 0.7288617 |
| Tecta | 2.1792657 |
| Tef | 2.3481418 |
| Tef | -0.8200442 |
| Tegt | 0.6330201 |
| Tek | 1.3619881 |
| Tekt1 | -0.7177484 |
| Tekt1 | -0.2343848 |
| Tekt3 | -0.8656802 |
| Tenc1 | 3.3272179 |
| Tenc1 | -0.3656288 |
| Terf2 | -0.5308868 |
| Terf2 | -0.538683 |
| Terf2ip | 1.3612814 |
| Terf2ip | 0.4443607 |
| Terf2ip | 1.2428361 |
| Terf2ip | -0.1503295 |
| Tes | -0.5681125 |
| Tes | -0.7177484 |
| Tes | -0.714904 |
| Tesc | 0.4489622 |
| Tesk1 | -0.6368658 |
| Tesp1 | 1.1285584 |
| Tesp2 | 0.532809 |
| Tessp2 | 1.8939548 |
| Tessp3 | -0.4513277 |
| Tex10 | -0.5568669 |
| Tex10 | 0.4473408 |
| Tex101 | -0.1627757 |
| Tex11 | 2.2964596 |
| Tex13 | -0.5835956 |
| Tex14 | -0.4802768 |
| Tex16 | -0.3119869 |
| Tex19 | 0.7687288 |
| Tex2 | -0.7139578 |
| Tex2 | -0.6377365 |
| Tex22 | 1.7038569 |
| Tex24 | -0.7196495 |
| Tex261 | -0.572162 |
| Tex261 | 0.7589215 |
| Tex264 | 1.473221 |
| Tex264 | -0.7340335 |
| Tex9 | 0.354987 |
| Tfam | -0.5656912 |
| Tfam | 0.513366 |
| Tfb1m | -0.7008114 |
| Tfb2m | 2.5586903 |
| Tfdp1 | 2.1034404 |
| Tfdp2 | 0.8726726 |
| Tfdp2 | 2.7963329 |
| Tfg | 3.4514361 |
| Tfip11 | 0.7565987 |
| Tfpi | 0.0793857 |
| Tfpt | 1.16183 |
| Tfpt | -0.3132141 |
| Tfpt | 1.9014325 |
| Tfrc | 0.2524129 |
| Tfrc | -0.7634882 |
| Tg | -0.0211813 |
| Tgfb1 | -0.6421024 |
| Tgfb1i1 | 1.1254382 |
| Tgfb1i1 | -0.6136602 |
| Tgfb2 | 0.0470596 |
| Tgfb3 | -0.3095375 |
| Tgfbi | -0.5681125 |
| Tgfbi | 0.5612301 |
| Tgfbr1 | -0.2383411 |
| Tgfbr1 | -0.5592652 |
| Tgfbr1 | -0.7263343 |
| Tgfbr2 | 2.3690986 |
| Tgfbr2 | -0.8221672 |
| Tgfbr3 | 1.0406798 |
| Tgfbr3 | 1.5499009 |
| Tgfbrap1 | -0.6094258 |
| Tgif1 | -0.7664857 |
| Tgif1 | -0.6421024 |
| Tgif1 | 0.5367474 |
| Tgif2 | 0.3143716 |
| Tgif2lx | 2.2934324 |
| Tgm2 | -0.7017443 |
| Tgm4 | 0.1991881 |
| Tgoln1 | -0.0718401 |
| Tgs1 | -0.5560689 |
| Tgs1 | -0.0948432 |
| Th1l | 1.3247168 |
| Tha1 | -0.7158512 |
| Thada | 2.4742533 |
| Thada | 0.1306251 |
| Thap1 | -0.3833381 |
| Thap1 | -0.2148526 |
| Thap1 | -0.8458464 |
| Thap11 | 0.8917986 |
| Thap3 | -0.5673047 |
| Thap4 | -0.6878487 |
| Thap7 | -0.6660398 |
| Thbd | 0.6227785 |
| Thbs1 | -0.7301759 |
| Thbs2 | 0.6325772 |
| Thbs3 | -0.1930223 |
| Thbs3 | -0.5124389 |
| Theg | -0.2943729 |
| Theg | 0.2090316 |
| Theg | 0.4668786 |
| Them2 | 0.7080625 |
| Them4 | -0.7321027 |
| Them5 | -0.4421104 |
| Thg1l | 0.4064205 |
| Thnsl1 | 0.6632979 |
| Thnsl2 | -0.6290659 |
| Thnsl2 | 0.8175394 |
| Thoc1 | 1.4309144 |
| Thoc3 | -0.6264805 |
| Thoc4 | 0.1137286 |
| Thoc4 | 0.1423473 |
| Thoc5 | -0.2276416 |
| Thoc6 | 2.1581162 |
| Thop1 | 0.5890386 |
| Thra | 0.4143019 |
| Thra | 0.9701467 |
| Thrap2 | 1.6274193 |
| Thrap3 | 0.7608527 |
| Thrsp | -0.2176179 |
| Thsd1 | 0.1827863 |
| Thsd4 | 2.8923845 |
| Thsd4 | -0.6961611 |
| Thsd7b | 0.7047893 |
| Thsd7b | -0.0356353 |
| Thsd7b | -0.4442294 |
| Thtpa | -0.638608 |
| Thumpd1 | 2.743527 |
| Thumpd3 | -0.5860641 |
| Thy1 | 0.0841989 |
| Thyn1 | -0.711125 |
| Tia1 | -0.7282531 |
| Tial1 | -0.6571019 |
| Tial1 | 0.2922588 |
| Tiam1 | -0.0073906 |
| Tiam2 | -0.2126463 |
| Ticam1 | 0.6270225 |
| Tie1 | 1.7271586 |
| Tigd2 | 1.3752146 |
| Tigd3 | 1.9623017 |
| Timeless | -0.8042747 |
| Timm10 | 0.0961238 |
| Timm13 | 2.6986616 |
| Timm17a | -0.4122589 |
| Timm17b | 0.4484221 |
| Timm22 | 0.0598791 |
| Timm44 | 1.8011298 |
| Timm44 | 3.6804421 |
| Timm44 | -0.6333911 |
| Timm50 | 3.5216575 |
| Timm8a1 | -0.6085813 |
| Timm8a2 | 1.6149795 |
| Timm8a2 | -0.4274118 |
| Timm8b | -0.6359959 |
| Timm8b | -0.3986952 |
| Timm9 | 1.6585781 |
| Timm9 | 1.0984838 |
| Timm9 | -0.5441796 |
| Timp1 | -0.187108 |
| Timp1 | 0.1838649 |
| Timp2 | -0.5223859 |
| Timp3 | 0.8651174 |
| Timp3 | -0.4364834 |
| Timp4 | -0.497341 |
| Tinagl | -0.5170166 |
| Tipin | -0.1978895 |
| Tipin | -0.5576657 |
| Tirap | -0.2595378 |
| Tirap | 2.7081931 |
| Tirap | -0.520081 |
| Tjap1 | -0.5910209 |
| Tjap1 | 1.137471 |
| Tjap1 | -0.4513277 |
| Tjp1 | 4.1136289 |
| Tjp2 | -0.693382 |
| Tjp3 | 0.7769179 |
| Tjp3 | 0.3552857 |
| Tk1 | -0.5844177 |
| Tk2 | 3.7464853 |
| Tkt | 2.3469907 |
| Tkt | 2.0710223 |
| Tkt | 3.1988185 |
| Tktl1 | -0.4541823 |
| Tktl1 | -0.3767396 |
| Tktl2 | 0.9206615 |
| Tlcd1 | 0.7814574 |
| Tle1 | -0.6517802 |
| Tle1 | -0.7908214 |
| Tle2 | 1.1881254 |
| Tle3 | -0.3368443 |
| Tle3 | -0.5418199 |
| Tle3 | 0.4072681 |
| Tle4 | 0.3701015 |
| Tle4 | 1.7534699 |
| Tle4 | 1.7448266 |
| Tle6 | 2.935738 |
| Tlk1 | -0.3813536 |
| Tlk2 | 0.9029553 |
| Tln1 | -0.4357825 |
| Tln2 | -0.0851892 |
| Tlr13 | 3.0732756 |
| Tlr2 | 0.5747672 |
| Tlr3 | -0.7311388 |
| Tlr4 | 0.6369971 |
| Tlr6 | 0.2842319 |
| Tlr7 | 2.6971313 |
| Tm2d2 | -0.1643433 |
| Tm2d3 | 1.5465157 |
| Tm2d3 | -0.6162101 |
| Tm4sf4 | -0.5616698 |
| Tm4sf5 | 1.82398 |
| Tm6sf1 | 0.2669816 |
| Tm6sf2 | 0.2715695 |
| Tm9sf2 | 1.9326118 |
| Tm9sf2 | -0.5608676 |
| Tm9sf2 | 2.5627654 |
| Tm9sf3 | -0.4950974 |
| Tm9sf4 | 1.0760948 |
| Tm9sf4 | 2.2563516 |
| Tmbim1 | -0.4054523 |
| Tmbim4 | -0.6204756 |
| Tmc1 | -0.5681125 |
| Tmc4 | 0.3878621 |
| Tmc5 | -0.2723747 |
| Tmc6 | 1.4748309 |
| Tmc6 | -0.0429476 |
| Tmc6 | 2.96019 |
| Tmc7 | 1.219833 |
| Tmcc2 | 3.1698595 |
| Tmcc3 | 1.4522173 |
| Tmco3 | -0.4421104 |
| Tmco3 | -0.2729625 |
| Tmco4 | -0.5239258 |
| Tmco4 | -0.6026908 |
| Tmco5 | -0.4267176 |
| Tmco6 | 0.9424881 |
| Tmco6 | -0.3636818 |
| Tmco7 | -0.7806039 |
| Tmed1 | 0.761624 |
| Tmed1 | -0.6482493 |
| Tmed10 | -0.6579918 |
| Tmed2 | -0.25261 |
| Tmed3 | -0.5457561 |
| Tmed3 | 2.1742336 |
| Tmed4 | 1.1058284 |
| Tmed4 | -0.7369372 |
| Tmed4 | 2.8598273 |
| Tmed4 | -0.6230444 |
| Tmed8 | 1.2621412 |
| Tmed8 | 1.9117007 |
| Tmed9 | -0.3058753 |
| Tmed9 | -0.5473352 |
| Tmeff2 | -0.3853269 |
| Tmem10 | -0.1989746 |
| Tmem100 | 1.3004495 |
| Tmem101 | -0.7545532 |
| Tmem102 | -0.5544748 |
| Tmem102 | -0.6111173 |
| Tmem106a | -0.7437482 |
| Tmem106c | 1.1811613 |
| Tmem107 | 2.5587459 |
| Tmem107 | -0.4693148 |
| Tmem107 | -0.1775105 |
| Tmem108 | 0.7505712 |
| Tmem109 | 3.9153192 |
| Tmem11 | 1.3439513 |
| Tmem110 | -0.60353 |
| Tmem111 | 0.9477077 |
| Tmem111 | -0.2806371 |
| Tmem111 | 2.0671135 |
| Tmem115 | -0.2265225 |
| Tmem116 | -0.3343284 |
| Tmem116 | -0.7575219 |
| Tmem117 | -0.4027435 |
| Tmem118 | -0.8116005 |
| Tmem119 | 1.3062547 |
| Tmem120a | 1.1603262 |
| Tmem120b | 1.4625069 |
| Tmem121 | 0.5750029 |
| Tmem125 | -0.5147249 |
| Tmem126a | 1.3769044 |
| Tmem126b | 0.1931603 |
| Tmem127 | -0.5441796 |
| Tmem128 | 0.2507428 |
| Tmem130 | 0.0170137 |
| Tmem131 | 0.8573263 |
| Tmem132a | 0.7859747 |
| Tmem132a | 0.3546881 |
| Tmem132c | 0.7248593 |
| Tmem132d | 1.31691 |
| Tmem132e | -0.8001144 |
| Tmem134 | -0.3833381 |
| Tmem134 | 1.1254382 |
| Tmem135 | -0.6145094 |
| Tmem136 | 1.5217878 |
| Tmem138 | 1.3273395 |
| Tmem139 | -0.0808774 |
| Tmem140 | -0.5056144 |
| Tmem141 | -0.0149279 |
| Tmem143 | -0.1951824 |
| Tmem143 | 0.8550633 |
| Tmem143 | -0.7101827 |
| Tmem143 | -0.2049654 |
| Tmem144 | 0.687413 |
| Tmem146 | 2.5760303 |
| Tmem147 | -0.4204934 |
| Tmem14c | 0.3872851 |
| Tmem14c | 0.0569998 |
| Tmem14c | 1.7142702 |
| Tmem150 | 1.8797983 |
| Tmem150 | 0.4650229 |
| Tmem151a | -0.1659135 |
| Tmem154 | -0.3966776 |
| Tmem158 | -0.5885392 |
| Tmem159 | 1.484432 |
| Tmem159 | -0.4211829 |
| Tmem160 | -0.4883988 |
| Tmem160 | -0.426024 |
| Tmem161a | -0.7321027 |
| Tmem162 | -0.1400838 |
| Tmem164 | 1.6808141 |
| Tmem166 | -0.2248464 |
| Tmem167 | -0.1431456 |
| Tmem168 | 0.0503849 |
| Tmem168 | -0.7206016 |
| Tmem168 | -0.3926556 |
| Tmem169 | 2.5666574 |
| Tmem16a | 1.2756411 |
| Tmem16b | 1.4876104 |
| Tmem16d | -0.2282017 |
| Tmem16e | 0.0941469 |
| Tmem16g | -0.25261 |
| Tmem16j | 1.9361512 |
| Tmem16k | -0.3199928 |
| Tmem17 | 1.470352 |
| Tmem17 | -0.3866551 |
| Tmem170 | 2.3398076 |
| Tmem176a | -0.5697302 |
| Tmem176a | 2.3319657 |
| Tmem176b | -0.6170616 |
| Tmem177 | 0.582515 |
| Tmem178 | 1.7705834 |
| Tmem179b | -0.6170616 |
| Tmem180 | 0.2413342 |
| Tmem183a | 0.9128338 |
| Tmem183a | -0.5935093 |
| Tmem184a | -0.7130126 |
| Tmem184a | 0.6607226 |
| Tmem184b | 0.4527347 |
| Tmem185b | 0.0195908 |
| Tmem186 | -0.6615601 |
| Tmem19 | 0.3393183 |
| Tmem192 | -0.4329838 |
| Tmem198 | 0.9402648 |
| Tmem199 | 1.8507013 |
| Tmem2 | 1.6455116 |
| Tmem2 | 0.7593081 |
| Tmem2 | 0.9905509 |
| Tmem201 | -0.4627992 |
| Tmem204 | -0.6273415 |
| Tmem205 | -0.3318193 |
| Tmem208 | -0.5465453 |
| Tmem209 | -0.5877134 |
| Tmem209 | -0.380033 |
| Tmem23 | 0.2835867 |
| Tmem25 | 0.1554065 |
| Tmem25 | -0.4876575 |
| Tmem25 | -0.5018446 |
| Tmem27 | 0.1287204 |
| Tmem32 | 0.2483995 |
| Tmem33 | -0.6412275 |
| Tmem33 | 0.7704488 |
| Tmem34 | 0.763742 |
| Tmem35 | 0.5948248 |
| Tmem38a | -0.4392927 |
| Tmem38b | 0.9108665 |
| Tmem38b | -0.0885567 |
| Tmem39a | 0.9887418 |
| Tmem40 | 0.6643688 |
| Tmem41a | 0.1256649 |
| Tmem41b | 1.6844569 |
| Tmem42 | -0.411576 |
| Tmem43 | -0.7506095 |
| Tmem43 | 2.6628834 |
| Tmem44 | -0.333073 |
| Tmem45a | 1.215943 |
| Tmem45b | -0.6221873 |
| Tmem47 | 1.7446262 |
| Tmem47 | 3.1506261 |
| Tmem48 | -0.3138283 |
| Tmem49 | -0.2445924 |
| Tmem49 | -0.5926791 |
| Tmem49 | -0.3399988 |
| Tmem49 | 0.639639 |
| Tmem5 | -0.4184279 |
| Tmem50a | -0.263603 |
| Tmem50b | -0.6989485 |
| Tmem50b | -0.5193139 |
| Tmem51 | 0.5198923 |
| Tmem53 | 0.5191416 |
| Tmem53 | -0.0324542 |
| Tmem53 | 0.359458 |
| Tmem54 | 0.4232279 |
| Tmem55a | -0.2237306 |
| Tmem55b | -0.7664857 |
| Tmem56 | 2.2932105 |
| Tmem57 | -0.2485925 |
| Tmem59 | 1.2052505 |
| Tmem59l | -0.4156811 |
| Tmem60 | 1.243295 |
| Tmem62 | -0.6878487 |
| Tmem63a | 0.099278 |
| Tmem63b | -0.366929 |
| Tmem63b | -0.0144828 |
| Tmem63c | -0.5208487 |
| Tmem65 | 3.350855 |
| Tmem66 | -0.0526333 |
| Tmem66 | -0.572974 |
| Tmem66 | -0.437887 |
| Tmem68 | 1.4741157 |
| Tmem69 | -0.0113733 |
| Tmem70 | 0.5295966 |
| Tmem71 | 2.5241897 |
| Tmem74 | -0.1775105 |
| Tmem74 | -0.6473687 |
| Tmem74 | 2.0260065 |
| Tmem77 | 0.0101063 |
| Tmem77 | 1.9329936 |
| Tmem77 | -0.6196209 |
| Tmem79 | -0.4484818 |
| Tmem8 | 0.0195908 |
| Tmem8 | -0.5935093 |
| Tmem8 | 0.8949761 |
| Tmem81 | -0.2137488 |
| Tmem82 | 0.4103695 |
| Tmem85 | 0.1795431 |
| Tmem85 | 1.9063283 |
| Tmem86a | 0.7113242 |
| Tmem86b | -0.7139578 |
| Tmem87a | -0.4520405 |
| Tmem88 | -0.5778612 |
| Tmem9 | -0.6491308 |
| Tmem90a | 0.12375 |
| Tmem91 | -0.4664133 |
| Tmem93 | 0.8534933 |
| Tmem97 | 1.3586518 |
| Tmem98 | 3.9444123 |
| Tmem98 | 0.7360227 |
| Tmem9b | 2.4382656 |
| Tmem9b | 1.7927944 |
| Tmem9b | -0.4980901 |
| Tmie | 2.9270934 |
| Tmod1 | -0.5746002 |
| Tmod2 | -0.2913695 |
| Tmod2 | 0.0869951 |
| Tmod3 | -0.6264805 |
| Tmod4 | 3.0229209 |
| Tmod4 | 0.8536679 |
| Tmod4 | 2.5572166 |
| Tmpo | -0.5355567 |
| Tmpo | 2.1443231 |
| Tmpo | 2.8725519 |
| Tmprss11a | -0.6970893 |
| Tmprss11c | -0.5885392 |
| Tmprss13 | -0.4456447 |
| Tmprss13 | -0.4891407 |
| Tmprss2 | -0.0324542 |
| Tmprss4 | -0.5208487 |
| Tmprss4 | -0.3688829 |
| Tmprss4 | -0.2104453 |
| Tmprss7 | -0.5457561 |
| Tmprss9 | 0.6049215 |
| Tmsb10 | -0.1706402 |
| Tmsb10 | -0.1984319 |
| Tmsb10 | 1.0172718 |
| Tmsb4x | 0.1835055 |
| Tmtc2 | -0.4861767 |
| Tmtc4 | -0.5410347 |
| Tmub1 | 0.099278 |
| Tmub1 | 0.6270225 |
| Tnc | 0.038694 |
| Tnc | 1.3714263 |
| Tnc | 1.7545942 |
| Tnc | -0.4040969 |
| Tnfaip1 | 1.148969 |
| Tnfaip2 | -0.2215032 |
| Tnfaip2 | -0.3714944 |
| Tnfaip2 | 1.2551469 |
| Tnfaip3 | -0.6723479 |
| Tnfaip8 | 0.8979764 |
| Tnfaip8l2 | 2.6004917 |
| Tnfaip8l2 | 1.6007336 |
| Tnfaip8l3 | -0.5868884 |
| Tnfrsf10b | 0.8450752 |
| Tnfrsf10b | -0.6438545 |
| Tnfrsf10b | 2.1208391 |
| Tnfrsf11a | 1.7159945 |
| Tnfrsf11b | 0.5547482 |
| Tnfrsf12a | -0.7654855 |
| Tnfrsf13c | -0.617914 |
| Tnfrsf19 | 0.9287458 |
| Tnfrsf19 | 1.769153 |
| Tnfrsf1a | -0.1596485 |
| Tnfrsf1a | -0.4225635 |
| Tnfrsf1a | -0.6001777 |
| Tnfrsf1b | 1.6714632 |
| Tnfrsf21 | 0.7155878 |
| Tnfrsf21 | -0.6077375 |
| Tnfrsf22 | -0.2497386 |
| Tnfrsf22 | -0.5162521 |
| Tnfrsf25 | 1.6809572 |
| Tnfrsf4 | -0.6247608 |
| Tnfsf13b | -0.0306413 |
| Tnfsf13b | 0.7776761 |
| Tnip1 | -0.0909695 |
| Tnip1 | -0.6998795 |
| Tnip1 | -0.2027824 |
| Tnip1 | 2.8742736 |
| Tnip2 | 3.2334484 |
| Tnks | -0.6508962 |
| Tnks1bp1 | -0.4693148 |
| Tnnc1 | 0.1438491 |
| Tnnc2 | -0.0043047 |
| Tnni2 | 1.5684229 |
| Tnni3 | 1.087289 |
| Tnnt1 | 1.4927961 |
| Tnp1 | 0.7155878 |
| Tnp2 | 3.7295906 |
| Tnpo1 | 0.578062 |
| Tnpo2 | -0.7369372 |
| Tnr | 0.6859502 |
| Tnrc15 | -0.6196209 |
| Tnrc18 | -0.668738 |
| Tnrc18 | -0.5673047 |
| Tnrc18 | -0.817926 |
| Tnrc6a | 1.9926064 |
| Tnrc6a | 0.4440893 |
| Tnrc6b | -0.7457034 |
| Tnrc6b | -0.7225085 |
| Tnrc6c | -0.325576 |
| Tns4 | 2.7295129 |
| Tnxb | 0.8805061 |
| Tnxb | -0.6196209 |
| Tnxb | 4.0122838 |
| Tob1 | -0.5239258 |
| Tob2 | -0.1365243 |
| Toe1 | -0.668738 |
| Toe1 | -0.456329 |
| Tollip | 3.1029966 |
| Tollip | 1.1408073 |
| Tom1 | 1.8122526 |
| Tom1l2 | 1.5477556 |
| Tom1l2 | -0.3754255 |
| Tom1l2 | -0.7215546 |
| Tomm22 | 2.5806543 |
| Tomm34 | -0.3077046 |
| Tomm34 | 1.3578412 |
| Tomm7 | 0.7660469 |
| Tomm70a | -0.4861767 |
| Top1 | -0.6714441 |
| Top1mt | -0.3617388 |
| Top2a | -0.6777893 |
| Top3a | 1.4308207 |
| Top3b | -0.5426058 |
| Topors | 2.0784647 |
| Tor1a | -0.5262403 |
| Tor1a | -0.4225635 |
| Tor1aip1 | -0.0443251 |
| Tor1aip2 | -0.5893657 |
| Tor1aip2 | -0.3591546 |
| Tor1b | 2.1413654 |
| Tor3a | 0.1594731 |
| Tox3 | 0.9256821 |
| Tpbg | 1.7820763 |
| Tpcn1 | 2.3138039 |
| Tpcn1 | -0.5208487 |
| Tpcn2 | 1.1668253 |
| Tpd52 | -0.7263343 |
| Tpd52l1 | -0.4520405 |
| Tpd52l1 | -0.6814346 |
| Tpd52l2 | -0.6325244 |
| Tph2 | -0.1544597 |
| Tpi1 | 1.7715575 |
| Tpi1 | 1.9688832 |
| Tpi1 | 0.108677 |
| Tpk1 | -0.3773974 |
| Tpk1 | -0.0507806 |
| Tpk1 | -0.6464889 |
| Tpm1 | 1.5871368 |
| Tpm2 | -0.5293354 |
| Tpm2 | 1.3659156 |
| Tpm3 | -0.8612359 |
| Tpm3 | -0.3993687 |
| Tpm4 | 0.1653566 |
| Tpo | 1.482307 |
| Tpp1 | 0.1977733 |
| Tpp2 | 1.2311735 |
| Tppp | 0.384395 |
| Tppp | -0.6351268 |
| Tppp3 | -0.638608 |
| Tpr | -0.7206016 |
| Tpra40 | -0.7045486 |
| Tprgl | -0.6299293 |
| Tprgl | 1.6952299 |
| Tprkb | -0.5410347 |
| Tprkb | 2.9518986 |
| Tpsab1 | 2.0238334 |
| Tpst1 | 1.7601299 |
| Tpst2 | -0.6961611 |
| Tpte | -0.5371185 |
| Tpte | -0.1194272 |
| Tpx2 | -0.617914 |
| Tra2a | -0.3058753 |
| Trabd | 0.3411398 |
| Trabd | -0.6482493 |
| Traf1 | -0.6325244 |
| Traf2 | -0.6239022 |
| Traf3 | 1.4394015 |
| Traf3 | -0.0270261 |
| Traf3ip1 | -0.4506154 |
| Traf3ip2 | 1.5624254 |
| Traf4 | -0.0512435 |
| Traf4 | 2.5306836 |
| Traf4 | 0.2597258 |
| Traf4 | 3.2490503 |
| Traf5 | 0.9389919 |
| Traf6 | -0.5231555 |
| Traf6 | -0.6924575 |
| Traf6 | -0.5208487 |
| Traf7 | 1.9057669 |
| Trafd1 | -0.8074073 |
| Trafd1 | -0.4635209 |
| Trafd1 | -0.7806039 |
| Traip | 1.1907994 |
| Trak1 | -0.2265225 |
| Trak2 | -0.6544372 |
| Trak2 | 0.3323026 |
| Trak2 | 0.8543658 |
| Tram1 | -0.5705401 |
| Tram1 | -0.1796347 |
| Tram1 | -0.60353 |
| Tram2 | -0.4308904 |
| Tram2 | -0.3299419 |
| Trap1 | -0.5528835 |
| Trappc1 | 0.5163838 |
| Trappc1 | 0.7780549 |
| Trappc2 | 0.9521135 |
| Trappc2l | 0.5716971 |
| Trappc3 | 2.7026906 |
| Trappc3 | -0.7575219 |
| Trappc3 | -0.3675799 |
| Trappc3 | 1.0607232 |
| Trappc4 | -0.5592652 |
| Trappc4 | -0.3780556 |
| Trappc5 | -0.5285606 |
| Trappc6a | 0.3750854 |
| Trat1 | 2.3455585 |
| Trcg1 | -0.2967827 |
| Trdmt1 | -0.6403535 |
| Trdmt1 | 1.6473683 |
| Trem2 | -0.4294974 |
| Treml1 | 0.7356263 |
| Treml2 | 1.6217924 |
| Treml4 | -0.6153594 |
| Trerf1 | 3.8058952 |
| Trerf1 | 0.6312471 |
| Trex1 | -0.6491308 |
| Trex1 | 0.1766507 |
| Trex2 | -0.2931704 |
| Trf | 0.4348145 |
| Trfp | -0.4527539 |
| Trfr2 | -0.6204756 |
| Trfr2 | 0.2787336 |
| Trh | 1.2174774 |
| Trhde | -0.3218501 |
| Trhr | -0.5664976 |
| Triap1 | -0.4061307 |
| Trib2 | 0.818265 |
| Trib3 | -0.1269312 |
| Trib3 | -0.6786993 |
| Trim11 | 3.545419 |
| Trim16 | 1.5943137 |
| Trim17 | -0.4336827 |
| Trim2 | -0.6850944 |
| Trim2 | -0.6553246 |
| Trim21 | 0.0713077 |
| Trim21 | 1.7196393 |
| Trim23 | 0.4348145 |
| Trim23 | -0.642978 |
| Trim23 | -0.4729544 |
| Trim25 | -0.5885392 |
| Trim26 | -0.6571019 |
| Trim28 | -0.6869297 |
| Trim3 | 2.1069734 |
| Trim3 | -0.3552917 |
| Trim30 | -0.7359683 |
| Trim32 | -0.5926791 |
| Trim32 | 0.8369354 |
| Trim33 | 1.4013086 |
| Trim33 | 0.7051991 |
| Trim33 | 0.1824265 |
| Trim35 | -0.215405 |
| Trim36 | -0.4149957 |
| Trim36 | -0.7388781 |
| Trim36 | 0.2277256 |
| Trim37 | 0.3579701 |
| Trim38 | 1.3067903 |
| Trim38 | -0.6145094 |
| Trim39 | 1.8299714 |
| Trim40 | 0.6694921 |
| Trim41 | 1.2114434 |
| Trim42 | -0.5951719 |
| Trim44 | 0.6323556 |
| Trim45 | -0.5465453 |
| Trim45 | 1.3786903 |
| Trim46 | 1.6751428 |
| Trim47 | 0.1920925 |
| Trim54 | -0.5262403 |
| Trim56 | -0.7045486 |
| Trim59 | 0.3292356 |
| Trim61 | 0.2153049 |
| Trim62 | 0.5804084 |
| Trim67 | -0.5664976 |
| Trim68 | 2.0296008 |
| Trim69 | -0.1375399 |
| Trim72 | -0.6952338 |
| Trim8 | 0.2851988 |
| Trim9 | -0.4177405 |
| Trim9 | -0.6290659 |
| Trim9 | 0.6308033 |
| Trim9 | -0.4995901 |
| Triml1 | -0.2027824 |
| Triobp | 0.12375 |
| Triobp | -0.7908214 |
| Trip10 | 0.0725239 |
| Trip12 | -0.2400418 |
| Trip12 | 3.3496358 |
| Trip12 | 1.3063618 |
| Trip6 | -0.6256202 |
| Trip6 | 0.0213049 |
| Trit1 | -0.4729544 |
| Trmt1 | 1.9048679 |
| Trmt12 | 1.6214873 |
| Trmu | -0.6473687 |
| Tro | -0.6394804 |
| Tro | 0.4530035 |
| Tro | 0.0881909 |
| Trove2 | 0.0013991 |
| Trp53 | 0.1355589 |
| Trp53bp1 | -0.7506095 |
| Trp53bp1 | -0.3853269 |
| Trp53bp2 | -0.6544372 |
| Trp53i11 | -0.0871119 |
| Trp53i11 | -0.3939943 |
| Trp53inp1 | -0.6678377 |
| Trp53inp1 | 1.3969454 |
| Trp53inp2 | 0.5141214 |
| Trp53rk | -0.0652369 |
| Trp63 | -0.6887686 |
| Trpc1 | -0.24802 |
| Trpc1 | -0.5449675 |
| Trpc2 | 0.6596474 |
| Trpc3 | -0.464966 |
| Trpc4 | -0.0162644 |
| Trpc4ap | -0.5552715 |
| Trpc4ap | -0.6094258 |
| Trpc5 | -0.4656893 |
| Trpc6 | 0.113341 |
| Trpc7 | 1.3295368 |
| Trpd52l3 | -0.7092413 |
| Trpm1 | -0.1796347 |
| Trpm2 | -0.6204756 |
| Trpm3 | 0.0157225 |
| Trpm3 | -0.1319679 |
| Trpm3 | -0.5379004 |
| Trpm3 | -0.6998795 |
| Trpm3 | 1.8679413 |
| Trpm4 | -0.6768802 |
| Trpm6 | -0.7447253 |
| Trpm6 | -0.3476137 |
| Trpm6 | 1.0284715 |
| Trpm8 | 0.3528931 |
| Trps1 | -0.5976714 |
| Trpt1 | 2.291915 |
| Trpt1 | 0.3261585 |
| Trpt1 | -0.4548973 |
| Trpv2 | 1.0286161 |
| Trpv4 | -0.2259635 |
| Trpv6 | 0.1591041 |
| Trrap | 1.6976227 |
| Trub1 | 3.5202986 |
| Trub1 | 4.2891198 |
| Trub1 | -0.2360783 |
| Trub2 | 1.8151674 |
| Tsc1 | -0.2943729 |
| Tsc2 | -0.3268213 |
| Tsc2 | 0.3933254 |
| Tsc22d1 | 0.8269288 |
| Tsc22d1 | 0.5168859 |
| Tsc22d2 | -0.7215546 |
| Tsc22d3 | 0.6372176 |
| Tsc22d3 | -0.7725101 |
| Tsc22d4 | -0.4364834 |
| Tsen2 | 1.5463503 |
| Tsen34 | -0.350166 |
| Tsen54 | -0.3144429 |
| Tsfm | -0.6544372 |
| Tsg101 | -0.7437482 |
| Tsga10 | 2.8124917 |
| Tsga10 | -0.821105 |
| Tsga14 | -0.7437482 |
| Tsga8 | -0.8339312 |
| Tshb | 1.2934133 |
| Tshr | 0.2617103 |
| Tshz3 | -0.1727488 |
| Tsku | -0.2937715 |
| Tsn | -0.7457034 |
| Tsnaxip1 | 1.3464176 |
| Tsnaxip1 | -0.6145094 |
| Tspan1 | 0.0619303 |
| Tspan11 | -0.6230444 |
| Tspan12 | -0.4965925 |
| Tspan13 | -0.817926 |
| Tspan14 | 0.0833984 |
| Tspan17 | 1.3227186 |
| Tspan17 | 0.0148607 |
| Tspan18 | 0.8381787 |
| Tspan18 | 1.2647224 |
| Tspan2 | 0.0428862 |
| Tspan3 | -0.2877783 |
| Tspan31 | 0.7101024 |
| Tspan33 | -0.4700416 |
| Tspan6 | 0.4215608 |
| Tspan6 | 1.7398704 |
| Tspan7 | -0.1319679 |
| Tspan8 | -0.6651421 |
| Tspo | -0.3052663 |
| Tspo | 2.810736 |
| Tspo | -0.6464889 |
| Tspyl1 | 3.0606174 |
| Tspyl3 | -0.1239223 |
| Tsr1 | 1.3076468 |
| Tsr2 | -0.2723747 |
| Tsr2 | 1.5221275 |
| Tsr2 | 0.2016588 |
| Tssc1 | -0.3946644 |
| Tssc1 | -0.7806039 |
| Tssc1 | -0.3083151 |
| Tssc1 | 1.5278841 |
| Tssc1 | 0.3307704 |
| Tssc4 | 1.4885794 |
| Tssc4 | -0.5860641 |
| Tssk4 | -0.5018446 |
| Tssk5 | 1.0916211 |
| Tssk6 | -0.3610921 |
| Tst | 0.0797877 |
| Tsta3 | -0.325576 |
| Tsx | -0.2304453 |
| Ttbk2 | -0.6998795 |
| Ttc1 | -0.6162101 |
| Ttc12 | -0.7196495 |
| Ttc13 | 0.7117311 |
| Ttc14 | -0.6660398 |
| Ttc16 | -0.2770871 |
| Ttc17 | 0.5203925 |
| Ttc19 | 3.7757122 |
| Ttc19 | 0.7780549 |
| Ttc21a | 1.018734 |
| Ttc22 | 0.5936704 |
| Ttc23 | -0.1309583 |
| Ttc24 | 0.6261306 |
| Ttc24 | 1.274311 |
| Ttc26 | 0.8109828 |
| Ttc27 | 2.6134412 |
| Ttc29 | 1.4660319 |
| Ttc29 | -0.6412275 |
| Ttc3 | 2.0021208 |
| Ttc3 | -0.5301108 |
| Ttc3 | -0.0919364 |
| Ttc3 | -0.5926791 |
| Ttc3 | 0.2280683 |
| Ttc3 | 1.5732575 |
| Ttc30a1 | 0.1294828 |
| Ttc30a2 | -0.2618586 |
| Ttc33 | -0.5616698 |
| Ttc33 | -0.0666479 |
| Ttc33 | 1.6530639 |
| Ttc35 | 2.1126974 |
| Ttc4 | 3.2790667 |
| Ttc5 | -0.6239022 |
| Ttc5 | 1.2386959 |
| Ttc5 | 0.3146837 |
| Ttc7 | -0.2937715 |
| Ttc8 | -0.4817483 |
| Ttc8 | 1.3035716 |
| Ttf2 | -0.0568155 |
| Ttk | -0.5935093 |
| Ttl | -0.3150579 |
| Ttll1 | 0.8395975 |
| Ttll1 | 0.5320686 |
| Ttll1 | 0.6261306 |
| Ttll10 | 0.3456779 |
| Ttll11 | -0.1978895 |
| Ttll13 | -0.4627992 |
| Ttll4 | 3.3810428 |
| Ttll5 | -0.7970064 |
| Ttll5 | 1.0743076 |
| Ttll6 | 2.8129565 |
| Ttll8 | 3.0182432 |
| Ttll9 | 0.8999711 |
| Ttn | 1.3483655 |
| Ttn | -0.4143107 |
| Ttr | -0.2659341 |
| Ttyh1 | 0.536993 |
| Ttyh1 | 2.878062 |
| Ttyh2 | -0.1984319 |
| Ttyh2 | -0.1462176 |
| Ttyh3 | -0.5770449 |
| Tub | -0.4534678 |
| Tub | -0.7064228 |
| Tuba1a | -0.5901929 |
| Tuba1b | -0.5778612 |
| Tuba3a | -0.7398501 |
| Tuba3a | 1.260679 |
| Tuba3a | -0.6832627 |
| Tuba3a | -0.3412636 |
| Tuba3b | 3.6336333 |
| Tuba4a | 0.8342657 |
| Tuba6 | -0.4839598 |
| Tuba8 | 3.2675181 |
| Tubb2b | -0.4758764 |
| Tubb2b | 0.0996716 |
| Tubb2b | -0.395335 |
| Tubb2b | 1.5322623 |
| Tubb2b | 2.7489065 |
| Tubb2c | -0.6651421 |
| Tubb4 | -0.6342585 |
| Tubb5 | -0.6204756 |
| Tubb6 | 1.6228596 |
| Tubd1 | 2.1546442 |
| Tubd1 | -0.437887 |
| Tube1 | -0.6970893 |
| Tubg1 | -0.7496262 |
| Tubgcp2 | 1.3624926 |
| Tubgcp4 | 1.4713392 |
| Tufm | -0.6052107 |
| Tuft1 | -0.3899839 |
| Tug1 | 1.2477572 |
| Tulp1 | -0.4883988 |
| Tulp2 | 1.9815455 |
| Tulp3 | 2.7614278 |
| Tulp4 | 1.3958758 |
| Tusc2 | -0.5316635 |
| Tusc2 | 0.985416 |
| Tusc4 | 0.4784591 |
| Tut1 | -0.7427721 |
| Twf2 | 0.5808769 |
| Twist1 | -0.7311388 |
| Twist2 | -0.3533662 |
| Twistnb | 1.0721033 |
| Twistnb | 1.2944993 |
| Twsg1 | -0.6239022 |
| Txk | -0.6832627 |
| Txlna | -0.7167993 |
| Txn1 | 1.5868201 |
| Txn2 | 0.527118 |
| Txndc1 | -0.3879852 |
| Txndc11 | 1.0065276 |
| Txndc11 | -0.3886509 |
| Txndc11 | 3.6809395 |
| Txndc12 | 1.7379209 |
| Txndc13 | -0.132473 |
| Txndc13 | 2.8936439 |
| Txndc15 | 1.760327 |
| Txndc2 | -0.6869297 |
| Txndc5 | -0.6961611 |
| Txndc8 | -0.5835956 |
| Txndc9 | -0.0247738 |
| Txnip | 1.8010669 |
| Txnl2 | 0.4826346 |
| Txnl4 | -0.5835956 |
| Txnl4a | 2.4394985 |
| Txnl4a | -0.7008114 |
| Txnl4a | 1.1008939 |
| Txnl4b | -0.0904864 |
| Txnrd2 | -0.7595064 |
| Txnrd3 | -0.7225085 |
| Tyk2 | 1.1671989 |
| Tyk2 | 1.6895755 |
| Tyk2 | 1.6808141 |
| Tyki | 0.2345549 |
| Tyms | 0.0487237 |
| Tyms | 0.1106232 |
| Tyms | -0.6887686 |
| Tyms-ps | 0.5223903 |
| Tyro3 | -0.5918496 |
| Tyrobp | 1.6231643 |
| Tyw1 | -0.1722212 |
| Tyw1 | 0.5911258 |
| U2af1l4 | 2.912637 |
| U2af2 | 0.2689506 |
| U2af2 | -0.5536788 |
| Uaca | -0.1924831 |
| Uap1 | 0.2132185 |
| Uap1l1 | -0.5339974 |
| Uba1 | 0.1845832 |
| Uba1 | -0.7008114 |
| Uba2 | -0.6860116 |
| Uba5 | 0.1329055 |
| Ubac1 | -0.7206016 |
| Ubac1 | 1.4394943 |
| Ubac1 | -0.7496262 |
| Ubac2 | 1.9765117 |
| Ubap1 | 2.0145402 |
| Ubap2 | -0.6805219 |
| Ubap2l | -0.1806986 |
| Ubap2l | 0.8434882 |
| Ubap2l | -0.7525792 |
| Ubb | 3.6281577 |
| Ubd | -0.0972725 |
| Ube1c | -0.7130126 |
| Ube1l | -0.2293228 |
| Ube1x | -0.137032 |
| Ube2a | 1.9755225 |
| Ube2b | 1.6250662 |
| Ube2d1 | -0.6750646 |
| Ube2d2 | -0.4627992 |
| Ube2d3 | -0.5926791 |
| Ube2e1 | -0.25261 |
| Ube2e2 | -0.3150579 |
| Ube2e3 | 3.3640447 |
| Ube2f | -0.6264805 |
| Ube2f | -0.380033 |
| Ube2f | 0.3435629 |
| Ube2g1 | 1.3677227 |
| Ube2g1 | -0.5985061 |
| Ube2g1 | -0.4714968 |
| Ube2g2 | -0.4225635 |
| Ube2i | 0.2253235 |
| Ube2j1 | -0.2203914 |
| Ube2j2 | 2.3518644 |
| Ube2j2 | 0.3573742 |
| Ube2j2 | 0.7652792 |
| Ube2k | 2.1805613 |
| Ube2k | 0.8109828 |
| Ube2l3 | 2.0448561 |
| Ube2l3 | 0.1863767 |
| Ube2l6 | 0.7752099 |
| Ube2m | 0.0127026 |
| Ube2n | -0.5713507 |
| Ube2n | -0.7725101 |
| Ube2o | -0.252035 |
| Ube2q1 | -0.5968375 |
| Ube2q1 | -0.775537 |
| Ube2q2 | -0.5041047 |
| Ube2q2 | 1.7644562 |
| Ube2q2 | -0.6230444 |
| Ube2r2 | 1.9881465 |
| Ube2s | 1.6423093 |
| Ube2t | 0.2480643 |
| Ube2u | 1.4481796 |
| Ube2u | -0.6342585 |
| Ube3a | 0.14009 |
| Ube3a | -0.2871811 |
| Ube3b | -0.6438545 |
| Ube3b | -0.2961796 |
| Ube3c | -0.6768802 |
| Ube4a | -0.3552917 |
| Ube4b | 0.5409137 |
| Ubfd1 | 1.017857 |
| Ubiad1 | 1.153023 |
| Ubie | -0.8021923 |
| Ubl3 | 1.4378219 |
| Ubl4 | -0.4943507 |
| Ubl4b | -0.7167993 |
| Ubl5 | -0.3793734 |
| Ubl7 | -0.7083008 |
| Ublcp1 | -0.7555417 |
| Ubox5 | -0.263603 |
| Ubox5 | -0.7725101 |
| Ubp1 | -0.5246966 |
| Ubp1 | -0.6307936 |
| Ubqln1 | -0.3520848 |
| Ubqln1 | -0.4027435 |
| Ubqln1 | 1.0273142 |
| Ubqln3 | -0.5410347 |
| Ubqln4 | -0.60437 |
| Ubqlnl | -0.6796102 |
| Ubr1 | 1.9243919 |
| Ubr5 | -0.3437985 |
| Ubtd1 | -0.8137042 |
| Ubtd2 | 1.1967275 |
| Ubtd2 | -0.5770449 |
| Ubtd2 | 0.1366937 |
| Ubtf | -0.1580888 |
| Ubxd1 | -0.2895722 |
| Ubxd1 | -0.7167993 |
| Ubxd1 | -0.3787143 |
| Ubxd3 | 0.7928961 |
| Ubxd4 | -0.4184279 |
| Ubxd5 | 0.9610185 |
| Ubxd8 | -0.7644863 |
| Uchl1 | -0.1462176 |
| Uchl3 | -0.3028345 |
| Uchl5 | -0.3444333 |
| Uchl5 | 0.1329055 |
| Uchl5 | 0.0536983 |
| Uck1 | 0.1795431 |
| Uck1 | 1.2900387 |
| Uck1 | 0.8656345 |
| Uck1 | 0.1291017 |
| Uck2 | -0.2457335 |
| Uck2 | -0.3237112 |
| Uck2 | 0.1881667 |
| Uevld | 0.2037713 |
| Ufc1 | -0.497341 |
| Ufsp1 | 1.1943124 |
| Ufsp2 | 0.8010603 |
| Ugcg | -0.0938733 |
| Ugp2 | 0.8104344 |
| Ugt1a10 | -0.5860641 |
| Ugt1a6a | 2.2713546 |
| Ugt1a6a | 0.6656523 |
| Ugt1a6a | 0.1970651 |
| Ugt3a2 | -0.0338162 |
| Ugt8a | 0.8436647 |
| Uhmk1 | -0.3393671 |
| Uhrf1 | 1.2435243 |
| Uhrf1bp1 | -0.5033507 |
| Uhrf1bp1l | -0.6660398 |
| Uhrf2 | -0.6060522 |
| Uhrf2 | 0.0769696 |
| Uhrf2 | -0.416367 |
| Uhrf2 | 1.0982157 |
| Uimc1 | 1.7968452 |
| Ulk1 | -0.4802768 |
| Ulk1 | 0.5065396 |
| Ulk2 | 1.182999 |
| Ulk2 | -0.6456099 |
| Umodl1 | -0.5512949 |
| Umps | -0.5520888 |
| Umps | -0.7476627 |
| Umps | -0.6741582 |
| Unc119 | 0.8512209 |
| Unc119 | -0.3721484 |
| Unc119b | -0.6860116 |
| Unc119b | 0.1509497 |
| Unc119b | -0.0727872 |
| Unc13b | 1.5163349 |
| Unc13c | -0.572162 |
| Unc13d | -0.5528835 |
| Unc45a | -0.3649793 |
| Unc45a | 2.3121384 |
| Unc45b | -0.3274446 |
| Unc5a | -0.324954 |
| Unc5a | 1.4289448 |
| Unc5b | 1.9538088 |
| Unc5c | 1.4551428 |
| Unc84a | -0.7427721 |
| Unc84a | 0.9998419 |
| Unc84b | -0.5985061 |
| Unc93a | -0.2925697 |
| Unc93b1 | -0.6010147 |
| Unc93b1 | -0.426024 |
| Ung | -0.3230904 |
| Ung | 1.8391882 |
| Unk | 0.0277046 |
| Unkl | -0.2016929 |
| Uox | 0.6998578 |
| Upb1 | -0.2606975 |
| Upb1 | -0.5552715 |
| Upb1 | -0.1946419 |
| Upf1 | -0.6980184 |
| Upf1 | -0.4591991 |
| Upf1 | 0.0984905 |
| Upf2 | 1.0168327 |
| Upf3a | 0.9319599 |
| Upk1a | -0.8590217 |
| Upk1a | -0.668738 |
| Upk1b | -0.6001777 |
| Upk2 | -0.3546494 |
| Upk3a | 0.7988408 |
| Upk3b | -0.3886509 |
| Upk3b | 1.4823956 |
| Upk3b | 1.0640684 |
| Upp1 | 2.7615171 |
| Upp1 | -0.4685885 |
| Upp1 | -0.1134673 |
| Upp2 | 1.2612416 |
| Upp2 | 3.0188863 |
| Upp2 | 1.3734221 |
| Upp2 | 0.4301421 |
| Uqcc | 2.4932287 |
| Uqcr | 0.9258436 |
| Uqcrc1 | -0.5162521 |
| Uqcrc2 | 0.3618338 |
| Uqcrh | 0.684066 |
| Urm1 | -0.8105504 |
| Urod | 1.1196904 |
| Uros | -0.4095303 |
| Uros | -0.2060588 |
| Use1 | 0.3483902 |
| Use1 | -0.6759719 |
| Use1 | -0.4898832 |
| Usf1 | 1.3015273 |
| Usf1 | -0.4232546 |
| Usf2 | 0.822968 |
| Usf2 | -0.5162521 |
| Ush1c | -0.6153594 |
| Ush1g | 0.9290677 |
| Ush2a | -0.8601281 |
| Ush3a | -0.5208487 |
| Ushbp1 | 1.1956415 |
| Usmg5 | -0.4928591 |
| Uso1 | 0.8748932 |
| Usp1 | 1.6838864 |
| Usp1 | -0.6961611 |
| Usp1 | -0.5101585 |
| Usp10 | 0.0777757 |
| Usp10 | 0.8093364 |
| Usp10 | -0.2087979 |
| Usp11 | 0.6559826 |
| Usp11 | 1.2882922 |
| Usp12 | -0.7457034 |
| Usp12 | -0.263603 |
| Usp13 | 0.0458096 |
| Usp14 | -0.0279286 |
| Usp15 | -0.7120683 |
| Usp16 | 0.9157769 |
| Usp18 | -0.5185475 |
| Usp18 | 3.187772 |
| Usp19 | 1.1526437 |
| Usp2 | -0.7856986 |
| Usp2 | 0.8395975 |
| Usp2 | 1.2520845 |
| Usp20 | -0.3175221 |
| Usp21 | 0.9404238 |
| Usp22 | -0.5664976 |
| Usp25 | -0.2572228 |
| Usp28 | 1.8175174 |
| Usp29 | 0.1835055 |
| Usp3 | -0.4506154 |
| Usp3 | -0.6544372 |
| Usp33 | 1.3367202 |
| Usp37 | -0.6952338 |
| Usp38 | -0.3546494 |
| Usp38 | 1.114434 |
| Usp39 | -0.4513277 |
| Usp39 | -0.1828302 |
| Usp39 | -0.693382 |
| Usp39 | -0.5803145 |
| Usp4 | 2.4039163 |
| Usp4 | -0.5943402 |
| Usp43 | 2.2073171 |
| Usp45 | 0.5640965 |
| Usp46 | 1.1502378 |
| Usp47 | 1.4384726 |
| Usp48 | -0.2022375 |
| Usp49 | 0.047892 |
| Usp5 | 0.7519357 |
| Usp50 | 1.4609662 |
| Usp52 | 0.5090738 |
| Usp52 | 0.0524571 |
| Usp52 | 0.0165835 |
| Usp53 | 2.2024353 |
| Usp6nl | 2.8227687 |
| Usp6nl | -0.5552715 |
| Usp7 | 0.4367316 |
| Usp8 | 0.5813453 |
| Usp8 | -0.0047449 |
| Usp9x | 3.0981631 |
| Usp9y | 0.5843836 |
| Uspl1 | -0.6438545 |
| Utp11l | 0.9846585 |
| Utp15 | -0.5877134 |
| Utp6 | -0.0953285 |
| Utx | -0.0775376 |
| Utx | -0.3286924 |
| Uty | 1.2457004 |
| Uvrag | -0.4773409 |
| Uxt | 1.2077565 |
| Uxt | 2.3895924 |
| V1ra2 | 0.9348431 |
| V1rd2 | -0.1508448 |
| V1rd20 | 1.8984373 |
| V1rd21 | -0.5976714 |
| V2r8 | 1.3825478 |
| Vac14 | 3.7618711 |
| Vamp1 | -0.6841781 |
| Vamp1 | -0.6162101 |
| Vamp2 | -0.4108936 |
| Vamp3 | 0.471897 |
| Vamp3 | 0.0105395 |
| Vamp4 | -0.6282033 |
| Vamp5 | -0.5162521 |
| Vamp8 | 0.4971027 |
| Vamp8 | -0.5713507 |
| Vangl2 | -0.4928591 |
| Vapa | -0.5162521 |
| Vapb | 3.2789005 |
| Vars | -0.6924575 |
| Vars | 1.4035307 |
| Vars | 1.4363327 |
| Vars2 | 1.0156608 |
| Vash1 | -0.4980901 |
| Vash1 | -0.6906112 |
| Vasn | -0.4854371 |
| Vasp | 0.1199084 |
| Vat1 | 0.0696837 |
| Vav1 | 0.359458 |
| Vav2 | 2.3197896 |
| Vav3 | 0.7790013 |
| Vax1 | -0.29077 |
| Vbp1 | -0.2265225 |
| Vcam1 | 0.1513219 |
| Vcl | -0.6696392 |
| Vcp | -0.5985061 |
| Vcpip1 | 2.3013855 |
| Vdac1 | -0.729214 |
| Vdac2 | -0.257801 |
| Vdac3 | 1.0649028 |
| Vdr | -0.1544597 |
| Vdr | 0.5707504 |
| Vdr | 0.692411 |
| Vegfa | 0.5754742 |
| Vegfa | -0.5868884 |
| Vegfb | 2.8038504 |
| Vegfc | -0.0082742 |
| Vezf1 | -0.7017443 |
| Vezt | 0.266653 |
| Vgf | 1.9498007 |
| Vgll2 | -0.3820147 |
| Vgll4 | 1.8762687 |
| Vhlh | 0.9851131 |
| Vil1 | 1.1614543 |
| Vill | -0.4943507 |
| Vim | -0.6553246 |
| Vip | 1.9925553 |
| Vit | -0.5656912 |
| Vkorc1 | -0.6786993 |
| Vkorc1 | 0.2848766 |
| Vkorc1 | -0.0189431 |
| Vkorc1l1 | 1.9500149 |
| Vkorc1l1 | 0.6777576 |
| Vkorc1l1 | 0.282295 |
| Vldlr | -0.6412275 |
| Vldlr | -0.2044192 |
| Vldlr | -0.0180493 |
| Vldlr | -0.4527539 |
| Vldlr | 0.8588897 |
| Vmn2r81 | 0.4924768 |
| Vmo1 | -0.4274118 |
| Vnn3 | -0.2366435 |
| Vnn3 | 0.4334427 |
| Vpreb1 | -0.4950974 |
| Vpreb3 | 0.3106181 |
| Vps11 | -0.1209231 |
| Vps13a | -0.6669383 |
| Vps16 | 1.0438136 |
| Vps16 | -0.3926556 |
| Vps16 | -0.5713507 |
| Vps16 | -0.6544372 |
| Vps18 | 0.8456035 |
| Vps24 | 0.7837188 |
| Vps25 | -0.0727872 |
| Vps25 | -0.6998795 |
| Vps26 | -0.5301108 |
| Vps26a | -0.572162 |
| Vps26b | -0.4246382 |
| Vps28 | 1.3777978 |
| Vps29 | 0.5897349 |
| Vps29 | -0.7867209 |
| Vps29 | -0.6412275 |
| Vps33a | 3.1672536 |
| Vps33a | -0.0530971 |
| Vps33b | 0.5952861 |
| Vps33b | 2.516284 |
| Vps35 | 0.5585948 |
| Vps36 | 0.6350108 |
| Vps37b | -0.7036129 |
| Vps37c | -0.520081 |
| Vps37d | 1.2485559 |
| Vps39 | -0.3565775 |
| Vps39 | -0.5713507 |
| Vps41 | 1.0019271 |
| Vps41 | -0.037002 |
| Vps45 | -0.4664133 |
| Vps4a | -0.479542 |
| Vps4b | 0.8034588 |
| Vps53 | -0.7806039 |
| Vps53 | 0.7334432 |
| Vps53 | 0.7387927 |
| Vps54 | -0.2389077 |
| Vps72 | 0.5571542 |
| Vrk1 | 1.4265945 |
| Vrk1 | -0.6359959 |
| Vrk2 | 2.8187681 |
| Vrk3 | -0.830708 |
| Vsig2 | -0.2653508 |
| Vsig4 | -0.7715033 |
| Vsnl1 | 3.6402637 |
| Vstm2a | 1.4970809 |
| Vstm2a | -0.4239461 |
| Vstm2a | 0.3236895 |
| Vstm2b | 0.1653566 |
| Vsx2 | -0.7282531 |
| Vti1a | -0.5379004 |
| Vti1a | 2.3671197 |
| Vti1a | 0.0773728 |
| Vti1b | 1.2321026 |
| Vti1b | -0.7263343 |
| Vtn | -0.2242883 |
| Vwa2 | 2.3770734 |
| Vwa3a | -0.5770449 |
| Vwf | -0.6588826 |
| Wars | -0.7785738 |
| Wars | -0.6273415 |
| Wars | -0.4788077 |
| Wars | -0.0549545 |
| Wars2 | 2.4796238 |
| Wars2 | -0.7092413 |
| Was | -0.7092413 |
| Wasf1 | -0.3495273 |
| Wasf2 | 0.1712029 |
| Wasf3 | -0.0256741 |
| Wasl | 1.9842844 |
| Wbp1 | -0.4088494 |
| Wbp1 | -0.1941017 |
| Wbp11 | 0.7294606 |
| Wbp2 | 2.4619083 |
| Wbp2nl | 0.4293151 |
| Wbp4 | 0.317799 |
| Wbp5 | 0.121447 |
| Wbp7 | -0.5705401 |
| Wbscr16 | 1.2866518 |
| Wbscr18 | -0.60353 |
| Wbscr22 | -0.331193 |
| Wbscr22 | 0.2177331 |
| Wbscr22 | -0.7496262 |
| Wbscr27 | 1.4649487 |
| Wbscr28 | -0.2428833 |
| Wbscr28 | 0.2983131 |
| Wdfy1 | 0.2500739 |
| Wdfy3 | -0.0704211 |
| Wdhd1 | 0.247729 |
| Wdr1 | -0.5877134 |
| Wdr1 | 1.7001494 |
| Wdr13 | -0.5968375 |
| Wdr13 | -0.7120683 |
| Wdr16 | -0.6714441 |
| Wdr17 | 1.4524919 |
| Wdr18 | -0.7017443 |
| Wdr20a | 0.7878505 |
| Wdr20b | 0.5602727 |
| Wdr21 | -0.2764967 |
| Wdr22 | -0.5505016 |
| Wdr23 | 1.7466284 |
| Wdr24 | -0.1149537 |
| Wdr27 | 1.3808683 |
| Wdr3 | 0.8625276 |
| Wdr31 | -0.6325244 |
| Wdr31 | -0.333073 |
| Wdr31 | 0.2159993 |
| Wdr32 | 0.6021787 |
| Wdr33 | 1.6039269 |
| Wdr34 | -0.4357825 |
| Wdr37 | 0.4276589 |
| Wdr37 | -0.5025974 |
| Wdr37 | -0.7745269 |
| Wdr37 | 0.1701095 |
| Wdr38 | 0.0553505 |
| Wdr41 | 0.1464714 |
| Wdr45 | 0.1602106 |
| Wdr45l | -0.4520405 |
| Wdr45l | 1.4988241 |
| Wdr46 | -0.380033 |
| Wdr46 | -0.5827742 |
| Wdr47 | -0.2601175 |
| Wdr5 | -0.7867209 |
| Wdr51b | -0.5170166 |
| Wdr53 | -0.5624727 |
| Wdr54 | -0.2659341 |
| Wdr55 | -0.2985941 |
| Wdr59 | -0.6896895 |
| Wdr5b | 0.5609909 |
| Wdr6 | -0.2543371 |
| Wdr60 | -0.2913695 |
| Wdr61 | -0.5608676 |
| Wdr62 | 0.2597258 |
| Wdr63 | 0.1168236 |
| Wdr66 | -0.5293354 |
| Wdr67 | 1.0349547 |
| Wdr68 | 0.591589 |
| Wdr69 | 1.685312 |
| Wdr70 | -0.6482493 |
| Wdr70 | -0.4980901 |
| Wdr74 | 0.5226397 |
| Wdr75 | -0.729214 |
| Wdr75 | 1.9628828 |
| Wdr76 | -0.4177405 |
| Wdr77 | -0.2389077 |
| Wdr78 | 0.5614693 |
| Wdr8 | 1.3908024 |
| Wdr8 | -0.6998795 |
| Wdr9 | 0.4911877 |
| Wdr93 | -0.2979899 |
| Wdsof1 | -0.668738 |
| Wfdc1 | -0.7427721 |
| Wfdc10 | 1.5937636 |
| Wfdc10 | 0.5453049 |
| Wfdc12 | 0.8200767 |
| Wfdc15b | -0.6264805 |
| Wfdc15b | -0.6145094 |
| Wfdc2 | -0.2624397 |
| Wfdc3 | -0.6256202 |
| Wfdc6a | -0.7244194 |
| Wfdc6b | 0.234895 |
| Wfdc6b | -0.714904 |
| Wfikkn2 | -0.3444333 |
| Wfs1 | -0.4068096 |
| Whdc1 | 0.5291014 |
| Whrn | -0.6221873 |
| Whrn | -0.5246966 |
| Whsc2 | -0.2782689 |
| Wif1 | -0.395335 |
| Wipf1 | 1.5775871 |
| Wipf2 | -0.3431641 |
| Wipi1 | 1.4869933 |
| Wipi1 | -0.0596142 |
| Wipi1 | -0.6256202 |
| Wipi2 | 0.0294038 |
| Wipi2 | -0.7466825 |
| Wipi2 | 0.4999183 |
| Wisp1 | 0.6566304 |
| Wisp1 | 1.572936 |
| Wisp2 | 1.5797444 |
| Wiz | 4.6682035 |
| Wiz | 0.223948 |
| Wnk1 | -0.7605003 |
| Wnk1 | 0.4668786 |
| Wnk2 | -0.1446804 |
| Wnk4 | 0.4947926 |
| Wnt10a | -0.2818235 |
| Wnt10b | 1.2212435 |
| Wnt16 | -0.2231733 |
| Wnt16 | -0.3444333 |
| Wnt16 | 1.5128248 |
| Wnt2 | -0.2572228 |
| Wnt3a | -0.1534255 |
| Wnt4 | -0.5835956 |
| Wnt4 | 0.2682948 |
| Wnt5a | -0.4414052 |
| Wnt5a | 0.9949083 |
| Wnt5b | 2.0883882 |
| Wnt6 | 0.7008874 |
| Wnt7a | 1.199016 |
| Wnt7b | 1.0991539 |
| Wnt7b | 0.1609475 |
| Wrb | -0.0131489 |
| Wrn | -0.5347767 |
| Wsb1 | 0.7941998 |
| Wsb2 | -0.3132141 |
| Wscd1 | 1.460331 |
| Wscd2 | 2.105435 |
| Wtap | 2.9614867 |
| Wtip | -0.0301887 |
| Wwc1 | -0.5246966 |
| Wwc1 | -0.60353 |
| Wwc2 | 3.80701 |
| Wwc2 | -0.6869297 |
| Wwox | -0.4642431 |
| Wwox | -0.5673047 |
| Wwp1 | 2.2440706 |
| Wwp2 | 1.1123231 |
| Wwp2 | -0.0875933 |
| Wwp2 | 0.0524571 |
| Wwtr1 | 1.1976917 |
| X99384 | 0.3444699 |
| Xab1 | -0.5844177 |
| Xab1 | 0.3826565 |
| Xbp1 | -0.5664976 |
| Xdh | 0.8682156 |
| Xist | 0.3591606 |
| Xist | 0.066427 |
| Xk | -0.5868884 |
| Xkr4 | 1.8487923 |
| Xkr5 | -0.6562128 |
| Xkr6 | 0.7019158 |
| Xkr8 | 0.353791 |
| Xkrx | 0.3224525 |
| Xlr4a | 2.1460151 |
| Xpc | 3.044087 |
| Xpnpep1 | 3.0680277 |
| Xpnpep1 | 0.6720432 |
| Xpnpep1 | 0.2725497 |
| Xpnpep2 | 0.9309968 |
| Xpnpep2 | 1.879105 |
| Xpnpep3 | -0.6660398 |
| Xpo4 | -0.7856986 |
| Xpo4 | -0.6777893 |
| Xpo5 | -0.4664133 |
| Xpo6 | -0.5552715 |
| Xpo7 | 1.7801492 |
| Xpo7 | -0.8264278 |
| Xpr1 | -0.4965925 |
| Xrcc1 | 1.5085261 |
| Xrcc2 | 1.2810527 |
| Xrcc2 | -0.6606667 |
| Xrcc3 | 0.8680438 |
| Xrcc6 | 2.9261964 |
| Xrcc6 | -0.3482511 |
| Xrcc6 | 2.3029606 |
| Xrn1 | 2.0487634 |
| Xrn2 | 1.5786264 |
| Xylb | -0.4477717 |
| Xylt2 | -0.817926 |
| Yaf2 | 0.609702 |
| Yap1 | 0.7984704 |
| Ybx2 | 1.1346386 |
| Ybx2 | -0.7486439 |
| Yeats2 | 3.0607953 |
| Yeats4 | 0.7912173 |
| Yeats4 | 0.1389593 |
| Yif1a | -0.4143107 |
| Yif1a | -0.7073613 |
| Yif1a | 1.4721462 |
| Yif1b | 1.0729305 |
| Yipf1 | 2.4405404 |
| Yipf2 | 1.8654812 |
| Yipf3 | 0.208332 |
| Yipf3 | -0.49137 |
| Yipf3 | 3.380367 |
| Yipf4 | 1.8080192 |
| Yipf6 | -0.7311388 |
| Yipf6 | -0.1421239 |
| Yipf6 | 0.0152917 |
| Yipf6 | -0.4513277 |
| Yipf7 | -0.4950974 |
| Ykt6 | 0.4262765 |
| Ykt6 | 2.3453487 |
| Ylpm1 | 1.7634093 |
| Yme1l1 | -0.5951719 |
| Yme1l1 | -0.0225269 |
| Yme1l1 | -0.7496262 |
| Yod1 | 1.2480996 |
| Ypel1 | -0.7215546 |
| Ypel3 | -0.8557103 |
| Ypel3 | -0.5713507 |
| Ypel4 | 2.9241001 |
| Ypel4 | -0.5418199 |
| Ypel5 | 0.2813251 |
| Yrdc | -0.2653508 |
| Ythdf1 | 1.4311018 |
| Ythdf2 | -0.8645671 |
| Ythdf2 | -0.1426346 |
| Ythdf3 | 0.368336 |
| Ythdf3 | -0.6256202 |
| Ywhab | -0.2226162 |
| Ywhab | -0.7158512 |
| Ywhae | -0.6221873 |
| Ywhag | 1.1321854 |
| Ywhah | 1.5079227 |
| Ywhaq | -0.6213311 |
| Ywhaz | -0.4040969 |
| Yy2 | 0.4400089 |
| Zadh1 | -0.6705412 |
| Zadh2 | 1.4344679 |
| Zan | -0.5770449 |
| Zap70 | 0.5004293 |
| Zap70 | 2.4387716 |
| Zbbx | -0.3343284 |
| Zbed4 | 0.0191618 |
| Zbp1 | 1.09351 |
| Zbtb17 | 0.6151353 |
| Zbtb2 | 1.6367722 |
| Zbtb22 | -0.5960043 |
| Zbtb24 | -0.4921142 |
| Zbtb3 | -0.8447568 |
| Zbtb32 | 1.0975449 |
| Zbtb33 | -0.9242941 |
| Zbtb38 | 0.5088207 |
| Zbtb39 | -0.6282033 |
| Zbtb40 | 0.1554065 |
| Zbtb41 | 0.873869 |
| Zbtb43 | 0.0769696 |
| Zbtb45 | -0.416367 |
| Zbtb46 | 0.3043282 |
| Zbtb5 | -0.4232546 |
| Zbtb7a | 0.8189901 |
| Zbtb7c | -0.7186985 |
| Zbtb8 | 1.2764162 |
| Zbtb9 | 0.1824265 |
| Zbtb9 | -0.6438545 |
| Zc3h10 | -0.5078837 |
| Zc3h11a | -0.5976714 |
| Zc3h12a | 1.8731328 |
| Zc3h12b | -0.7215546 |
| Zc3h13 | -0.4685885 |
| Zc3h14 | -0.4129424 |
| Zc3h14 | 2.8640549 |
| Zc3h15 | 0.0353265 |
| Zc3h18 | -0.7918494 |
| Zc3h3 | 2.1517624 |
| Zc3h3 | -0.7225085 |
| Zc3h3 | 0.1114005 |
| Zc3h6 | 2.2493205 |
| Zc3h7a | -0.5910209 |
| Zc3h7a | 0.7552411 |
| Zc3h8 | 3.148819 |
| Zc3hc1 | -0.4322855 |
| Zc3hc1 | -0.3393671 |
| Zc3hc1 | -0.3846635 |
| Zc3hc1 | -0.0742098 |
| Zcchc12 | -0.711125 |
| Zcchc14 | 3.1109508 |
| Zcchc16 | -0.3813536 |
| Zcchc17 | -0.6678377 |
| Zcchc17 | 2.7869092 |
| Zcchc18 | -0.7253764 |
| Zcchc18 | 1.0191722 |
| Zcchc3 | 2.4039819 |
| Zcchc3 | 2.9713687 |
| Zcchc3 | -0.7605003 |
| Zcchc5 | -0.5624727 |
| Zcchc6 | -0.5154882 |
| Zcchc8 | -0.5063702 |
| Zcchc8 | 0.5080609 |
| Zcchc8 | 0.5050158 |
| Zcchc9 | -0.6111173 |
| Zcrb1 | -0.3218501 |
| Zcwpw1 | 0.9391511 |
| Zdhhc1 | 0.2656666 |
| Zdhhc12 | 0.172295 |
| Zdhhc12 | -0.1523923 |
| Zdhhc13 | 1.1196904 |
| Zdhhc13 | -0.6777893 |
| Zdhhc13 | -0.5985061 |
| Zdhhc14 | -0.6624543 |
| Zdhhc15 | -0.6741582 |
| Zdhhc16 | -0.769493 |
| Zdhhc16 | -0.7244194 |
| Zdhhc17 | -0.5640805 |
| Zdhhc19 | 0.9696847 |
| Zdhhc19 | -0.6777893 |
| Zdhhc21 | 2.5449759 |
| Zdhhc24 | 1.3109581 |
| Zdhhc3 | 0.4348145 |
| Zdhhc3 | 0.3510945 |
| Zdhhc4 | 0.0088054 |
| Zdhhc6 | 1.5711656 |
| Zdhhc7 | 1.1302441 |
| Zdhhc9 | -0.5093996 |
| Zeb1 | -0.2659341 |
| Zeb2 | -0.5552715 |
| Zer1 | 0.4971027 |
| Zf | 0.6800757 |
| Zfa | -0.6660398 |
| Zfand2a | -0.7644863 |
| Zfand2b | 0.1160508 |
| Zfand2b | 2.039037 |
| Zfand2b | 2.5803015 |
| Zfand2b | 0.1572571 |
| Zfand3 | -0.6878487 |
| Zfand6 | 1.5167621 |
| Zfand6 | -0.5835956 |
| Zfat | 3.1560267 |
| Zfh4 | 2.2724143 |
| Zfhx2 | 1.2501511 |
| Zfhx2 | -0.3181391 |
| Zfhx3 | 0.1938715 |
| Zfhx3 | 2.1236852 |
| Zfml | 1.4081512 |
| Zfp1 | -0.4047743 |
| Zfp101 | 2.3491522 |
| Zfp105 | -0.7083008 |
| Zfp106 | 0.4713701 |
| Zfp106 | 0.9125062 |
| Zfp109 | 0.5261247 |
| Zfp11 | 0.344772 |
| Zfp119 | -0.6187671 |
| Zfp12 | -0.6796102 |
| Zfp128 | -0.7816206 |
| Zfp13 | -0.437887 |
| Zfp131 | 0.1055546 |
| Zfp142 | 0.1913799 |
| Zfp143 | 1.393245 |
| Zfp148 | -0.6447318 |
| Zfp160 | 0.1867349 |
| Zfp161 | -0.4143107 |
| Zfp161 | -0.1638205 |
| Zfp169 | -0.0685326 |
| Zfp179 | 0.5740596 |
| Zfp182 | -0.6482493 |
| Zfp184 | -0.3010148 |
| Zfp185 | 0.1649899 |
| Zfp187 | 0.0466431 |
| Zfp187 | -0.2011486 |
| Zfp189 | -0.2248464 |
| Zfp191 | 1.2776327 |
| Zfp2 | -0.4627992 |
| Zfp2 | -0.3034419 |
| Zfp202 | -0.3224701 |
| Zfp207 | 2.5974591 |
| Zfp212 | -0.6136602 |
| Zfp212 | -0.4520405 |
| Zfp212 | 1.5876909 |
| Zfp212 | -0.416367 |
| Zfp213 | -0.1309583 |
| Zfp217 | 0.120678 |
| Zfp219 | -0.5893657 |
| Zfp235 | 0.1106232 |
| Zfp236 | -0.5124389 |
| Zfp236 | 0.6033226 |
| Zfp238 | -0.0466255 |
| Zfp238 | 0.7778655 |
| Zfp238 | -0.3181391 |
| Zfp238 | -0.333073 |
| Zfp238 | -0.4958446 |
| Zfp239 | -0.6796102 |
| Zfp251 | -0.9125056 |
| Zfp259 | -0.668738 |
| Zfp26 | -0.3533662 |
| Zfp263 | 0.7639343 |
| Zfp27 | -0.8021923 |
| Zfp27 | -0.5277866 |
| Zfp275 | 1.2524253 |
| Zfp276 | -0.5394663 |
| Zfp277 | -0.5433924 |
| Zfp277 | 0.6238973 |
| Zfp280c | 1.3920733 |
| Zfp281 | 2.1519347 |
| Zfp282 | -0.3610921 |
| Zfp286 | -0.7186985 |
| Zfp288 | -0.2943729 |
| Zfp292 | 1.1685673 |
| Zfp292 | -0.6085813 |
| Zfp295 | 0.1245164 |
| Zfp296 | -0.930245 |
| Zfp296 | 2.2943564 |
| Zfp3 | 3.0538809 |
| Zfp30 | 3.3660018 |
| Zfp313 | -0.0261245 |
| Zfp313 | -0.5877134 |
| Zfp316 | -0.0540253 |
| Zfp316 | -0.6878487 |
| Zfp318 | -0.3083151 |
| Zfp322a | -0.1727488 |
| Zfp324 | 3.0542067 |
| Zfp326 | 2.6599985 |
| Zfp326 | 0.5281102 |
| Zfp326 | 0.708675 |
| Zfp329 | -0.5025974 |
| Zfp329 | 2.7732534 |
| Zfp330 | -0.3806931 |
| Zfp330 | 2.3587483 |
| Zfp334 | 2.1443665 |
| Zfp335 | 0.3724502 |
| Zfp339 | -0.1796347 |
| Zfp346 | 0.9796433 |
| Zfp346 | -0.2055119 |
| Zfp35 | 0.6771244 |
| Zfp354a | -0.6325244 |
| Zfp36 | 0.552819 |
| Zfp365 | -0.8491227 |
| Zfp367 | 0.9664443 |
| Zfp367 | -0.0977591 |
| Zfp36l1 | -0.8371657 |
| Zfp36l1 | -0.1570505 |
| Zfp36l1 | -0.8137042 |
| Zfp36l2 | 0.7190251 |
| Zfp37 | 1.2020186 |
| Zfp371 | -0.1534255 |
| Zfp382 | 0.9782707 |
| Zfp385a | -0.8317812 |
| Zfp385a | -0.1748621 |
| Zfp385c | -0.7083008 |
| Zfp386 | 0.1967108 |
| Zfp386 | -0.7073613 |
| Zfp39 | 0.1218312 |
| Zfp393 | 1.3980139 |
| Zfp395 | -0.7083008 |
| Zfp397 | 1.2108498 |
| Zfp398 | 0.5987388 |
| Zfp398 | -0.456329 |
| Zfp398 | -0.7654855 |
| Zfp41 | 1.6617979 |
| Zfp41 | -0.1727488 |
| Zfp410 | -0.9242941 |
| Zfp410 | -0.9043418 |
| Zfp410 | -0.60437 |
| Zfp414 | -0.3793734 |
| Zfp418 | -0.1254255 |
| Zfp42 | 0.2301216 |
| Zfp423 | 0.0905779 |
| Zfp423 | 0.6449004 |
| Zfp428 | 0.4572949 |
| Zfp428 | -0.3826762 |
| Zfp446 | 0.8931378 |
| Zfp446 | -0.0775376 |
| Zfp449 | -0.7745269 |
| Zfp451 | 1.4269709 |
| Zfp457 | 0.5962081 |
| Zfp46 | 1.2859948 |
| Zfp467 | 2.8827759 |
| Zfp472 | -0.4204934 |
| Zfp473 | -0.3206115 |
| Zfp473 | 2.0353815 |
| Zfp474 | -0.5918496 |
| Zfp474 | -0.3767396 |
| Zfp512 | 1.7398704 |
| Zfp513 | 0.578062 |
| Zfp516 | 0.1378272 |
| Zfp52 | 1.5685845 |
| Zfp521 | -0.3381048 |
| Zfp523 | -0.0229758 |
| Zfp523 | 0.555952 |
| Zfp524 | 0.0536983 |
| Zfp526 | 1.1450246 |
| Zfp532 | -0.1309583 |
| Zfp532 | 2.1813961 |
| Zfp536 | 0.8310518 |
| Zfp551 | 1.0264452 |
| Zfp553 | 0.133285 |
| Zfp553 | -0.0775376 |
| Zfp560 | 0.7109171 |
| Zfp566 | 1.6686394 |
| Zfp57 | -0.7177484 |
| Zfp574 | 0.235235 |
| Zfp579 | 1.4324123 |
| Zfp592 | -0.8469372 |
| Zfp593 | 1.5385408 |
| Zfp595 | 0.039114 |
| Zfp598 | 1.8815293 |
| Zfp60 | 0.5709872 |
| Zfp606 | 0.7413574 |
| Zfp607 | 1.7495572 |
| Zfp608 | 1.1550434 |
| Zfp609 | -0.6850944 |
| Zfp61 | 0.6092478 |
| Zfp612 | -0.7330676 |
| Zfp617 | 1.5540125 |
| Zfp619 | 1.4311954 |
| Zfp62 | -0.1189291 |
| Zfp622 | 0.1195234 |
| Zfp623 | -0.2735506 |
| Zfp629 | 1.8604234 |
| Zfp637 | -0.1492999 |
| Zfp639 | -0.4832221 |
| Zfp64 | 0.2086819 |
| Zfp64 | -0.1523923 |
| Zfp64 | -0.301621 |
| Zfp641 | 0.6194143 |
| Zfp644 | 0.0319467 |
| Zfp646 | 0.8557602 |
| Zfp653 | 3.8076681 |
| Zfp654 | 2.425429 |
| Zfp654 | 0.5883419 |
| Zfp655 | -0.7775604 |
| Zfp661 | -0.7130126 |
| Zfp667 | -0.497341 |
| Zfp668 | 2.4258458 |
| Zfp67 | -0.7101827 |
| Zfp672 | 1.9114775 |
| Zfp68 | 2.4744663 |
| Zfp68 | -0.3218501 |
| Zfp68 | 1.8010669 |
| Zfp687 | -0.3993687 |
| Zfp688 | -0.5656912 |
| Zfp689 | -0.9078318 |
| Zfp69 | -0.7949402 |
| Zfp691 | 0.6913719 |
| Zfp692 | -0.7244194 |
| Zfp692 | 2.2691556 |
| Zfp704 | 2.7976008 |
| Zfp704 | 1.1640812 |
| Zfp706 | 1.5572887 |
| Zfp709 | 1.0358157 |
| Zfp709 | -0.6896895 |
| Zfp710 | -0.2104453 |
| Zfp711 | 0.1063361 |
| Zfp715 | -0.6508962 |
| Zfp71-rs1 | -0.4943507 |
| Zfp74 | 1.0802059 |
| Zfp75 | 2.0651292 |
| Zfp75 | 1.8253283 |
| Zfp75 | 2.6116323 |
| Zfp750 | -0.6906112 |
| Zfp758 | 1.3365128 |
| Zfp760 | -0.1309583 |
| Zfp764 | -0.5993416 |
| Zfp768 | -0.4421104 |
| Zfp770 | 2.7397405 |
| Zfp771 | -0.8200442 |
| Zfp771 | 0.8646 |
| Zfp78 | -0.7506095 |
| Zfp78 | 0.8929705 |
| Zfp780b | -0.5901929 |
| Zfp786 | 1.8071452 |
| Zfp787 | -0.5433924 |
| Zfp787 | -0.7311388 |
| Zfp790 | -0.4664133 |
| Zfp804a | -0.024324 |
| Zfp809 | -0.9266699 |
| Zfp810 | 0.4312436 |
| Zfp811 | 1.4406076 |
| Zfp817 | 0.8989743 |
| Zfp819 | 0.1310055 |
| Zfp82 | 1.1372139 |
| Zfp825 | 1.6801697 |
| Zfp828 | 1.3974313 |
| Zfp85-rs1 | -0.2332575 |
| Zfp87 | 2.1865556 |
| Zfp87 | -0.7856986 |
| Zfp90 | -0.4343821 |
| Zfp91 | -0.5885392 |
| Zfp91-cntf | 0.8233287 |
| Zfp92 | -0.1769802 |
| Zfp93 | 0.8010603 |
| Zfp93 | 1.7052521 |
| Zfp94 | 2.5518305 |
| Zfp97 | 2.4002983 |
| Zfpl1 | 2.7940722 |
| Zfpm2 | -0.3617388 |
| Zfr | -0.4802768 |
| Zfx | -0.6307936 |
| Zfx | 0.443003 |
| Zfy1 | 1.5722121 |
| Zfyve1 | -0.5778612 |
| Zfyve16 | -0.7496262 |
| Zfyve19 | 0.9396286 |
| Zfyve20 | -0.497341 |
| Zfyve21 | 0.7354281 |
| Zfyve26 | 0.9391511 |
| Zfyve27 | -0.1026394 |
| Zgpat | 0.5513694 |
| Zgpat | 1.4297894 |
| Zhx1 | -0.7624911 |
| Zhx1 | -0.6633494 |
| Zhx1 | -0.4143107 |
| Zhx2 | -0.6068945 |
| Zhx3 | -0.7427721 |
| Zic1 | -0.421873 |
| Zic2 | 3.7644271 |
| Zic3 | -0.4442294 |
| Zic4 | -0.729214 |
| Zkscan1 | -0.6906112 |
| Zkscan14 | 0.3159311 |
| Zkscan17 | -0.7206016 |
| Zkscan3 | 0.7712122 |
| Zkscan6 | -0.6750646 |
| Zkscan6 | 1.5005641 |
| Zmat1 | -0.881405 |
| Zmat2 | -0.7614952 |
| Zmat3 | 0.9343632 |
| Zmat4 | 1.2085903 |
| Zmat5 | 1.5790259 |
| Zmiz1 | -0.7045486 |
| Zmiz1 | -0.7263343 |
| Zmiz2 | 0.7096948 |
| Zmiz2 | -0.6247608 |
| Zmpste24 | -0.0109299 |
| Zmpste24 | 0.9200117 |
| Zmpste24 | 0.1995415 |
| Zmym3 | -0.1095153 |
| Zmym3 | -0.1189291 |
| Zmym5 | 0.1291017 |
| Zmym6 | 3.6301558 |
| Zmynd10 | -0.5193139 |
| Zmynd11 | 0.6332415 |
| Zmynd11 | 2.9350345 |
| Zmynd11 | -0.5285606 |
| Zmynd12 | -0.3714944 |
| Zmynd15 | -0.7167993 |
| Znhit2 | -0.3846635 |
| Znhit3 | -0.7139578 |
| Znhit4 | -0.1477575 |
| Znrd1 | 0.0221607 |
| Znrd1 | -0.5185475 |
| Znrd1 | 0.0395339 |
| Znrf1 | 1.0842989 |
| Znrf2 | -0.4414052 |
| Znrf3 | -0.4817483 |
| Znrf4 | -0.2485925 |
| Zp1 | 4.1497079 |
| Zp3 | -0.3495273 |
| Zp3r | -0.6145094 |
| Zpbp | -0.3243324 |
| Zpbp2 | -0.5139623 |
| Zpbp2 | 3.4544772 |
| Zranb1 | 1.9184282 |
| Zranb2 | -0.8491227 |
| Zranb3 | 1.1735265 |
| Zrsr2 | -0.0021068 |
| Zscan10 | -0.4364834 |
| Zscan12 | 0.1210626 |
| Zscan2 | -0.4788077 |
| Zscan2 | -0.3939943 |
| Zscan2 | 0.2497392 |
| Zscan20 | 1.5021272 |
| Zscan20 | 1.0283269 |
| Zscan21 | 0.1404666 |
| Zscan21 | -0.4570457 |
| Zswim1 | 0.1498322 |
| Zswim2 | 0.0586462 |
| Zswim3 | -0.7545532 |
| Zswim4 | -0.3728029 |
| Zswim5 | 2.2588498 |
| Zw10 | 0.1697448 |
| Zw10 | 0.6779686 |
| Zw10 | -0.7654855 |
| Zwilch | 0.4109323 |
| Zxda | 0.0696837 |
| Zxdc | 1.6994482 |
| Zxdc | 0.5226397 |
| Zxdc | -0.2087979 |
| Zyg11b | 0.9017959 |
| Zyx | -0.0808774 |
| Zzz3 | 1.5718098 |
| Zzz3 | -0.6924575 |
| Zzz3 | 1.7191587 |
| Zzz3 | 0.108677 |

| **Gene Symbol** | **WT4** |
| --- | --- |
| 0610006I08Rik | -0.3662917 |
| 0610006I08Rik | -0.0595064 |
| 0610007C21Rik | -0.6410056 |
| 0610007C21Rik | 0.0742993 |
| 0610007P08Rik | 1.005765 |
| 0610007P14Rik | -0.55776 |
| 0610007P22Rik | -0.5946796 |
| 0610009B22Rik | -0.0063166 |
| 0610009D07Rik | -0.3086194 |
| 0610009O20Rik | 0.2269732 |
| 0610010E21Rik | 1.7328224 |
| 0610010K06Rik | -0.0402813 |
| 0610010K14Rik | -0.4679241 |
| 0610012A05Rik | -0.3879385 |
| 0610012D14Rik | -0.3595833 |
| 0610012G03Rik | -0.6016101 |
| 0610012H03Rik | 0.8548615 |
| 0610025P10Rik | -0.6687015 |
| 0610031J06Rik | -0.0301466 |
| 0610031J06Rik | 1.4618013 |
| 0610031J06Rik | 1.9287744 |
| 0610037D15Rik | 0.0450219 |
| 0610037L13Rik | -0.4726727 |
| 0610037L13Rik | 0.7261289 |
| 0610038F07Rik | 0.1062033 |
| 0610040J01Rik | 1.8031217 |
| 0710008K08Rik | 0.3580518 |
| 0910001A06Rik | 1.1513638 |
| 0910001A06Rik | -0.6596576 |
| 0910001L09Rik | 1.0096811 |
| 1110001A07Rik | -0.1127995 |
| 1110001J03Rik | 1.0556745 |
| 1110002B05Rik | -0.7924408 |
| 1110002N22Rik | 0.3433261 |
| 1110003E01Rik | 1.9969253 |
| 1110006G06Rik | -0.1725955 |
| 1110007A13Rik | -0.6078109 |
| 1110007A13Rik | -0.3208285 |
| 1110007C09Rik | 1.2841805 |
| 1110007L15Rik | 3.138831 |
| 1110007L15Rik | 0.7951624 |
| 1110007L15Rik | -0.4478225 |
| 1110007L15Rik | -0.1344449 |
| 1110007M04Rik | -0.5622088 |
| 1110008F13Rik | 0.9511044 |
| 1110008J03Rik | 2.0809012 |
| 1110008J03Rik | -0.5908497 |
| 1110008J03Rik | -0.2645234 |
| 1110008P14Rik | -0.2399339 |
| 1110012D08Rik | 0.1617116 |
| 1110012J17Rik | -0.5065541 |
| 1110012L19Rik | -0.3583683 |
| 1110012L19Rik | 0.2387951 |
| 1110012M11Rik | 2.2414744 |
| 1110013L07Rik | -0.2491529 |
| 1110017D15Rik | -0.7361521 |
| 1110017D15Rik | 0.2531319 |
| 1110017D15Rik | -0.0482845 |
| 1110018G07Rik | -0.6490728 |
| 1110018J18Rik | -0.4235701 |
| 1110018J18Rik | 0.6064247 |
| 1110019N10Rik | 0.0587637 |
| 1110019N10Rik | 0.5849762 |
| 1110020C03Rik | 1.2790469 |
| 1110020P15Rik | 1.8814299 |
| 1110020P15Rik | -0.7100039 |
| 1110031B06Rik | -0.4049609 |
| 1110031I02Rik | 1.2546975 |
| 1110032A13Rik | -0.6306138 |
| 1110032A13Rik | -0.5365298 |
| 1110032E23Rik | -0.0398385 |
| 1110034A24Rik | -0.16005 |
| 1110036O03Rik | 0.3378621 |
| 1110038D17Rik | -0.1137517 |
| 1110038F14Rik | 0.8973378 |
| 1110038F14Rik | 0.2815301 |
| 1110039B18Rik | -0.5992946 |
| 1110039B18Rik | -0.826627 |
| 1110039B18Rik | -0.0694861 |
| 1110048D14Rik | 1.9586173 |
| 1110049B09Rik | 1.2011908 |
| 1110049B09Rik | 0.5837844 |
| 1110049F12Rik | 0.3909244 |
| 1110049F12Rik | -0.6393999 |
| 1110057K04Rik | -0.5423419 |
| 1110057K04Rik | 0.7565249 |
| 1110057K04Rik | 2.613119 |
| 1110058L19Rik | -0.433332 |
| 1110059E24Rik | -0.7100039 |
| 1110059G10Rik | 1.5050926 |
| 1110067D22Rik | -0.7943099 |
| 1110067I12Rik | -0.4184021 |
| 1190002A17Rik | 0.1168702 |
| 1190002H23Rik | 1.7792818 |
| 1190002N15Rik | -0.8976489 |
| 1190003J15Rik | -0.5533307 |
| 1190003M12Rik | 0.7970946 |
| 1190005F20Rik | -0.6670511 |
| 1190005I06Rik | 1.3277621 |
| 1190005P17Rik | 0.0619701 |
| 1190005P17Rik | 0.9877122 |
| 1190007F08Rik | -0.5525944 |
| 1190017O12Rik | 3.3590174 |
| 1190020J12Rik | -0.6654034 |
| 1200002N14Rik | -0.6330024 |
| 1200003C05Rik | 0.3613282 |
| 1200003C05Rik | 1.0276554 |
| 1200003C05Rik | 0.1718101 |
| 1200003I07Rik | -0.6753303 |
| 1200009F10Rik | -0.4578232 |
| 1200009I06Rik | 2.2444196 |
| 1200009I06Rik | -0.4733529 |
| 1200009O22Rik | 0.0490831 |
| 1200011O22Rik | 3.1448697 |
| 1200013B08Rik | 0.4308408 |
| 1200013P24Rik | 0.1145939 |
| 1200013P24Rik | -0.4870541 |
| 1200014J11Rik | 3.0908428 |
| 1200015F23Rik | -0.2243992 |
| 1200015F23Rik | 0.6054915 |
| 1200015N20Rik | 1.9684525 |
| 1200016B10Rik | -0.5051487 |
| 1200016B10Rik | -0.2965565 |
| 1200016D23Rik | 1.7807384 |
| 1300001I01Rik | 1.1851604 |
| 1300007L22Rik | 3.8317412 |
| 1300010M03Rik | 0.5364195 |
| 1300013J15Rik | -0.1610479 |
| 1300013J15Rik | -0.5992946 |
| 1300014I06Rik | 0.3839699 |
| 1300017J02Rik | -0.2372383 |
| 1300018I05Rik | -0.5365298 |
| 1300018J18Rik | -0.6531307 |
| 1500001L15Rik | -0.6720105 |
| 1500001M20Rik | -0.294275 |
| 1500001M20Rik | 1.9333513 |
| 1500001M20Rik | -0.5636961 |
| 1500002O20Rik | -0.5242892 |
| 1500003O03Rik | 1.3719422 |
| 1500005A01Rik | 0.5984643 |
| 1500010J02Rik | -0.7620901 |
| 1500010J02Rik | -0.0020437 |
| 1500011H22Rik | -0.220685 |
| 1500012F01Rik | -0.4692786 |
| 1500015O10Rik | 0.0996715 |
| 1500019C06Rik | -0.0717681 |
| 1500019G21Rik | 2.3044903 |
| 1500031L02Rik | 0.1142141 |
| 1500032D16Rik | -0.3910692 |
| 1500032D16Rik | 0.1206527 |
| 1500032D16Rik | 0.877928 |
| 1500032L24Rik | 0.0013616 |
| 1500041J02Rik | 2.1453733 |
| 1500041N16Rik | -0.3255187 |
| 1520401A03Rik | -0.5749214 |
| 1600002K03Rik | 0.6549135 |
| 1600012H06Rik | -0.5107822 |
| 1600012H06Rik | 1.3189421 |
| 1600014C10Rik | -0.420337 |
| 1600016N20Rik | -0.5365298 |
| 1600021P15Rik | 2.0999053 |
| 1600027N09Rik | -0.0393959 |
| 1600029D21Rik | 2.7268911 |
| 1700001C02Rik | 0.2279919 |
| 1700001C19Rik | -0.4106995 |
| 1700001E04Rik | -0.6179692 |
| 1700001J03Rik | 1.7651166 |
| 1700001O22Rik | 1.7355661 |
| 1700001O22Rik | 0.1035189 |
| 1700001P01Rik | -0.5954473 |
| 1700003F12Rik | 1.155972 |
| 1700003M02Rik | 0.953256 |
| 1700007G11Rik | 1.581427 |
| 1700007I06Rik | -0.1238046 |
| 1700007K09Rik | 0.7543119 |
| 1700008F21Rik | 2.1518413 |
| 1700008I05Rik | 0.5451308 |
| 1700008P20Rik | 0.1130736 |
| 1700009P17Rik | 0.5503217 |
| 1700010A17Rik | -0.5900855 |
| 1700010A17Rik | -0.126213 |
| 1700010D01Rik | -0.276207 |
| 1700010M22Rik | 1.1597513 |
| 1700010M22Rik | 0.0486777 |
| 1700011A15Rik | -0.7575702 |
| 1700011F03Rik | -0.5114886 |
| 1700011F14Rik | 3.8448419 |
| 1700011H14Rik | -0.4005201 |
| 1700011K15Rik | -0.3742778 |
| 1700012A03Rik | 0.3801828 |
| 1700012B09Rik | 0.5235895 |
| 1700012G19Rik | -0.2307983 |
| 1700012H05Rik | -0.4544786 |
| 1700012H17Rik | -0.703999 |
| 1700012L04Rik | 0.2136349 |
| 1700013B16Rik | 0.7376606 |
| 1700013G24Rik | -0.2033573 |
| 1700013N18Rik | -0.6490728 |
| 1700013N18Rik | 1.1731343 |
| 1700015E13Rik | -0.5592407 |
| 1700016C15Rik | -0.5511233 |
| 1700016D06Rik | -0.2595573 |
| 1700016G05Rik | 0.0958094 |
| 1700016K19Rik | 0.0223881 |
| 1700016K19Rik | 0.1853554 |
| 1700017N19Rik | 3.8389227 |
| 1700018B24Rik | -0.5207172 |
| 1700018C11Rik | 2.2652035 |
| 1700018F24Rik | -0.1223624 |
| 1700019D03Rik | -0.4974536 |
| 1700019E19Rik | -0.5939124 |
| 1700019G17Rik | 0.3019217 |
| 1700019H03Rik | -0.5481877 |
| 1700019L03Rik | -0.5321926 |
| 1700019M22Rik | 2.385205 |
| 1700019N19Rik | -0.3051581 |
| 1700020C11Rik | -0.5607237 |
| 1700020D05Rik | -0.6870365 |
| 1700020L24Rik | 0.2474882 |
| 1700020N01Rik | 2.2551607 |
| 1700020O03Rik | 2.7198325 |
| 1700021C14Rik | 0.0671588 |
| 1700021F05Rik | 2.3621238 |
| 1700021K02Rik | 1.0110872 |
| 1700021K02Rik | 1.4351925 |
| 1700021K19Rik | -0.6466458 |
| 1700022C21Rik | 0.9117399 |
| 1700023B02Rik | 1.6704097 |
| 1700023B02Rik | 2.4277355 |
| 1700023E05Rik | -0.4808655 |
| 1700023E05Rik | -0.5423419 |
| 1700023I07Rik | -0.1325019 |
| 1700023M03Rik | -0.342706 |
| 1700024G13Rik | -0.0402813 |
| 1700025D03Rik | -0.2007577 |
| 1700025E21Rik | 1.2634874 |
| 1700025F22Rik | -0.2661842 |
| 1700025G04Rik | -0.4132605 |
| 1700025K23Rik | -0.6047057 |
| 1700026D08Rik | -0.7326263 |
| 1700026L06Rik | 0.0248821 |
| 1700027A23Rik | -0.3885639 |
| 1700027D21Rik | -0.5065541 |
| 1700027J05Rik | -0.408145 |
| 1700027N10Rik | -0.6803306 |
| 1700028K03Rik | -0.6604764 |
| 1700029F12Rik | 1.4245772 |
| 1700029F12Rik | -0.5992946 |
| 1700029G01Rik | 2.9186857 |
| 1700029H14Rik | 0.2367783 |
| 1700029H14Rik | 0.1969726 |
| 1700029I01Rik | -0.5855123 |
| 1700029I08Rik | 2.7761316 |
| 1700029I15Rik | -0.4190467 |
| 1700029J07Rik | -0.5764276 |
| 1700029P11Rik | -0.6442247 |
| 1700029P11Rik | 1.5573759 |
| 1700029P11Rik | -0.4145435 |
| 1700030J22Rik | 2.1665919 |
| 1700030K09Rik | -0.7134514 |
| 1700034E13Rik | 0.4492683 |
| 1700034H14Rik | 0.0543383 |
| 1700034H14Rik | -0.0451646 |
| 1700034H14Rik | 2.7975979 |
| 1700034H14Rik | -0.5030442 |
| 1700034I23Rik | 2.3125539 |
| 1700034I23Rik | 1.8858329 |
| 1700034O15Rik | 1.0060788 |
| 1700036D21Rik | -0.5293115 |
| 1700037C18Rik | 0.8793523 |
| 1700037C18Rik | 1.1486432 |
| 1700037H04Rik | 3.7218251 |
| 1700039E15Rik | -0.5726663 |
| 1700040I03Rik | 1.9852285 |
| 1700042B14Rik | 1.0983736 |
| 1700045I19Rik | -0.6954821 |
| 1700047I17Rik1 | 1.0210298 |
| 1700047I17Rik1 | -0.0398385 |
| 1700049G17Rik | 0.7281979 |
| 1700049K14Rik | -0.7620901 |
| 1700049L16Rik | 1.0180883 |
| 1700052N19Rik | -0.0451646 |
| 1700052N19Rik | 0.5466167 |
| 1700054O13Rik | -0.7125884 |
| 1700055M20Rik | 1.648827 |
| 1700057G04Rik | 1.7554618 |
| 1700057K13Rik | -0.5093708 |
| 1700058C13Rik | -0.6547585 |
| 1700060H10Rik | 2.5533971 |
| 1700061G19Rik | -0.6828401 |
| 1700063I17Rik | 3.3185042 |
| 1700065I17Rik | -0.0635771 |
| 1700065O13Rik | -0.703999 |
| 1700065O13Rik | 0.605258 |
| 1700067C01Rik | -0.7684522 |
| 1700067K01Rik | -0.1009721 |
| 1700067P10Rik | 0.1404661 |
| 1700071K01Rik | -0.7125884 |
| 1700072E05Rik | -0.6539443 |
| 1700072E05Rik | 1.2628794 |
| 1700072E05Rik | 1.2442208 |
| 1700073E17Rik | 0.4432545 |
| 1700080E11Rik | -0.6588394 |
| 1700081D17Rik | -0.2437197 |
| 1700081D17Rik | 0.5758829 |
| 1700084C01Rik | -0.0358624 |
| 1700084C01Rik | 1.6955868 |
| 1700088E04Rik | -0.4953651 |
| 1700093K21Rik | 2.199745 |
| 1700094C09Rik | 1.8774043 |
| 1700095K08Rik | 1.8437197 |
| 1700102P08Rik | 1.0375889 |
| 1700108M19Rik | -0.4062334 |
| 1700109H08Rik | 0.3357293 |
| 1700112C13Rik | -0.125249 |
| 1700113I22Rik | -0.6179692 |
| 1700113I22Rik | 1.0744682 |
| 1700113O17Rik | 0.544635 |
| 1700120B06Rik | -0.4405512 |
| 1700123D08Rik | -0.6442247 |
| 1700123K08Rik | -0.6000659 |
| 1700123L14Rik | 1.6634847 |
| 1700123O20Rik | 0.0679546 |
| 1700123O20Rik | -0.8160577 |
| 1700127D06Rik | -0.0832559 |
| 1700129C05Rik | -0.2925673 |
| 1700129I15Rik | -0.3320036 |
| 1810007M14Rik | 1.3985499 |
| 1810007P19Rik | -0.8037079 |
| 1810007P19Rik | 3.6239189 |
| 1810008A18Rik | 0.0240514 |
| 1810008A18Rik | -0.634598 |
| 1810009A15Rik | -0.5079615 |
| 1810009A15Rik | 0.5381678 |
| 1810009N02Rik | 1.5224472 |
| 1810009O10Rik | -0.4605069 |
| 1810009O10Rik | -0.2801318 |
| 1810010M01Rik | 3.8557499 |
| 1810011O10Rik | -0.3873136 |
| 1810014F10Rik | -0.4981507 |
| 1810015A11Rik | 2.0101124 |
| 1810015C04Rik | -0.6588394 |
| 1810019J16Rik | -0.6563889 |
| 1810020D17Rik | -0.311513 |
| 1810020G14Rik | -0.0934747 |
| 1810020G14Rik | 2.676141 |
| 1810021J13Rik | 0.4252742 |
| 1810021J13Rik | -0.0653916 |
| 1810022C23Rik | 0.2978129 |
| 1810026J23Rik | 1.2236943 |
| 1810027O10Rik | 1.4126964 |
| 1810030J14Rik | -0.2745296 |
| 1810030N24Rik | -0.6490728 |
| 1810030N24Rik | -0.3711987 |
| 1810030N24Rik | -0.0832559 |
| 1810031K17Rik | 2.2595158 |
| 1810035L17Rik | 2.5141515 |
| 1810035L17Rik | 1.0659287 |
| 1810037C20Rik | -0.5438002 |
| 1810043G02Rik | 0.7579306 |
| 1810044A24Rik | -0.8131943 |
| 1810046J19Rik | 1.3276481 |
| 1810048J11Rik | 0.5172402 |
| 1810049H13Rik | 0.7194797 |
| 1810049H19Rik | -0.4017869 |
| 1810055E12Rik | -0.6322056 |
| 1810055E12Rik | 1.8491745 |
| 1810055E12Rik | -0.6515056 |
| 1810055G02Rik | -0.5832334 |
| 1810063B05Rik | -0.311513 |
| 1810064F22Rik | 0.7760287 |
| 1810065E05Rik | -0.0676643 |
| 1810073G14Rik | -0.5794467 |
| 1810074P20Rik | -0.4706349 |
| 1810074P20Rik | 0.014869 |
| 2010001J22Rik | -0.4598353 |
| 2010001J22Rik | 0.1337805 |
| 2010001M09Rik | 0.0360292 |
| 2010003J03Rik | 0.6497885 |
| 2010003O18Rik | 1.3687717 |
| 2010004A03Rik | -0.4905087 |
| 2010005H15Rik | -0.1028552 |
| 2010005J08Rik | -0.4829242 |
| 2010005J08Rik | 0.4429803 |
| 2010005J08Rik | -0.2270605 |
| 2010007H12Rik | -0.042942 |
| 2010007H12Rik | 0.2590733 |
| 2010011I20Rik | -0.8027642 |
| 2010012O05Rik | 0.7113162 |
| 2010100O12Rik | -0.703999 |
| 2010100O12Rik | -0.3954686 |
| 2010106G01Rik | -0.6482631 |
| 2010106G01Rik | 1.0872882 |
| 2010107E04Rik | 1.9729133 |
| 2010107G12Rik | 0.5424011 |
| 2010109I03Rik | -0.6179692 |
| 2010110K16Rik | -0.6912504 |
| 2010111I01Rik | -0.6482631 |
| 2010200O16Rik | -0.5794467 |
| 2010301N04Rik | -0.3601913 |
| 2010305A19Rik | 1.4228143 |
| 2010305C02Rik | -0.7450211 |
| 2010305C02Rik | 0.5118757 |
| 2010308M01Rik | 0.6892182 |
| 2010311D03Rik | -0.2678478 |
| 2010316F05Rik | 1.2948415 |
| 2010321M09Rik | 1.1578635 |
| 2010321M09Rik | -0.4287647 |
| 2200001I15Rik | -0.5629522 |
| 2210009G21Rik | -0.3979912 |
| 2210009G21Rik | -0.7151796 |
| 2210009G21Rik | 1.2519963 |
| 2210010B09Rik | -0.119484 |
| 2210010L05Rik | 0.0798183 |
| 2210012G02Rik | 0.6036223 |
| 2210012G02Rik | 1.6046579 |
| 2210012G02Rik | -0.6946343 |
| 2210012G02Rik | 0.3069558 |
| 2210016L21Rik | 0.0298516 |
| 2210016L21Rik | 4.4273894 |
| 2210016L21Rik | -0.5178686 |
| 2210021J22Rik | 3.599381 |
| 2210023G05Rik | -0.2925673 |
| 2210023G05Rik | 1.7036319 |
| 2210407C18Rik | -0.5908497 |
| 2210412D01Rik | 0.7825123 |
| 2210417D09Rik | 0.9322053 |
| 2210418O10Rik | 1.5437518 |
| 2300002D11Rik | 0.902741 |
| 2300002D11Rik | -0.4068702 |
| 2300002M23Rik | -0.6377967 |
| 2300005B03Rik | -0.1570622 |
| 2310001A20Rik | -0.5946796 |
| 2310002B06Rik | 0.7327346 |
| 2310003C23Rik | 1.0469736 |
| 2310003F16Rik | -0.0823321 |
| 2310003H01Rik | 1.2822734 |
| 2310003L22Rik | 1.4677746 |
| 2310003L22Rik | -0.0622184 |
| 2310004I24Rik | -0.4760783 |
| 2310005E10Rik | -0.5651856 |
| 2310005E10Rik | -0.2318688 |
| 2310005G13Rik | 1.1245194 |
| 2310005N01Rik | 1.4595021 |
| 2310005N03Rik | -0.6234817 |
| 2310005P05Rik | -0.1099482 |
| 2310006J04Rik | 0.7188541 |
| 2310007A19Rik | -0.7352695 |
| 2310007B03Rik | -0.3742778 |
| 2310007F21Rik | -0.61405 |
| 2310007F21Rik | -0.5900855 |
| 2310008H04Rik | -0.7151796 |
| 2310008H09Rik | -0.5607237 |
| 2310008H09Rik | 3.2500436 |
| 2310008M10Rik | 2.3846053 |
| 2310009E04Rik | 0.7380701 |
| 2310010B21Rik | 0.1307949 |
| 2310010M24Rik | 2.4343039 |
| 2310011J03Rik | 1.5932409 |
| 2310014L17Rik | -0.5221445 |
| 2310016C08Rik | -0.6929409 |
| 2310016C08Rik | -0.311513 |
| 2310016C16Rik | -0.5636961 |
| 2310016C16Rik | 2.3856844 |
| 2310016C16Rik | 0.4168662 |
| 2310016E02Rik | 1.3210108 |
| 2310016M24Rik | 1.8010554 |
| 2310021P13Rik | -0.2885939 |
| 2310022A10Rik | 0.0064481 |
| 2310022B05Rik | 2.4443031 |
| 2310031L18Rik | -0.0262084 |
| 2310031L18Rik | 0.3332861 |
| 2310033E01Rik | -0.1655508 |
| 2310033K02Rik | 1.7507495 |
| 2310033P09Rik | -0.4578232 |
| 2310035C23Rik | -0.408783 |
| 2310035C23Rik | -0.2064857 |
| 2310035K24Rik | -0.7459123 |
| 2310036D22Rik | 2.3158983 |
| 2310036O22Rik | -0.276207 |
| 2310037I24Rik | 0.6816711 |
| 2310037I24Rik | 0.231042 |
| 2310038H17Rik | 1.0855496 |
| 2310039E09Rik | 1.1368601 |
| 2310039H08Rik | 2.8283462 |
| 2310040C09Rik | 0.0563523 |
| 2310042D19Rik | 0.8637541 |
| 2310044G17Rik | -0.1878591 |
| 2310044H10Rik | 1.8618785 |
| 2310045A20Rik | -0.5622088 |
| 2310046K01Rik | 0.5705806 |
| 2310046O06Rik | -0.6418094 |
| 2310047C04Rik | -0.6621161 |
| 2310047D13Rik | 0.4336127 |
| 2310047D13Rik | 1.3067891 |
| 2310047M10Rik | 2.0926671 |
| 2310047M10Rik | 1.0653371 |
| 2310047O13Rik | -0.7143151 |
| 2310056P07Rik | 0.2327325 |
| 2310057J16Rik | 0.9337236 |
| 2310058J06Rik | -0.6418094 |
| 2310061C15Rik | 0.01403 |
| 2310061F22Rik | -0.6547585 |
| 2310061F22Rik | -0.5365298 |
| 2310065K24Rik | 0.6054915 |
| 2310065K24Rik | -0.4767608 |
| 2310066E14Rik | -0.5387054 |
| 2310067B10Rik | 0.5123878 |
| 2310076L09Rik | 0.7965153 |
| 2310076L09Rik | -0.3935808 |
| 2310079F23Rik | -0.5592407 |
| 2310079N02Rik | -0.5651856 |
| 2400001E08Rik | 2.2077824 |
| 2400003C14Rik | 2.8796799 |
| 2400003C14Rik | -0.5164473 |
| 2400003C14Rik | 0.0182177 |
| 2400006N03Rik | 0.2834594 |
| 2400010D15Rik | 2.2986287 |
| 2410001C21Rik | 0.4969095 |
| 2410001C21Rik | 1.169405 |
| 2410001C21Rik | -0.0939416 |
| 2410002F23Rik | -0.6070337 |
| 2410002F23Rik | 3.6856813 |
| 2410002F23Rik | -0.6861958 |
| 2410002I01Rik | 2.1066179 |
| 2410002O22Rik | -0.0473921 |
| 2410003P15Rik | -0.007602 |
| 2410004A20Rik | 0.5875933 |
| 2410004B18Rik | 1.348978 |
| 2410004L22Rik | -0.0976851 |
| 2410005O16Rik | 0.6244529 |
| 2410008K03Rik | 1.0650412 |
| 2410014A08Rik | 1.1831879 |
| 2410014A08Rik | 0.2551163 |
| 2410014A08Rik | 0.939774 |
| 2410014A08Rik | 1.3414681 |
| 2410015M20Rik | 0.0376702 |
| 2410015N17Rik | 0.6255976 |
| 2410016F19Rik | 0.8512092 |
| 2410016F19Rik | 1.3603047 |
| 2410016O06Rik | -0.4740336 |
| 2410017P07Rik | 1.0663722 |
| 2410018C17Rik | -0.6330024 |
| 2410018G20Rik | 1.2310015 |
| 2410018M08Rik | 0.3487605 |
| 2410019A14Rik | -0.6523178 |
| 2410019G02Rik | 2.3748817 |
| 2410022L05Rik | -0.3433039 |
| 2410025L10Rik | 0.646432 |
| 2410042D21Rik | -0.6580219 |
| 2410042D21Rik | -0.7405769 |
| 2410066E13Rik | -0.5178686 |
| 2410081M15Rik | 1.3740141 |
| 2410091C18Rik | -0.6794955 |
| 2410116G06Rik | 2.6155389 |
| 2410127E18Rik | -0.7414642 |
| 2410127L17Rik | 1.2421124 |
| 2410127L17Rik | -0.824697 |
| 2410129H14Rik | 1.2914186 |
| 2410137M14Rik | -0.3028572 |
| 2410146L05Rik | -0.6637584 |
| 2410166I05Rik | -0.2684029 |
| 2500002L14Rik | 0.5213084 |
| 2500003M10Rik | -0.2054418 |
| 2500003M10Rik | -0.7521729 |
| 2500003M10Rik | -0.7961825 |
| 2510002A14Rik | 0.1825188 |
| 2510003E04Rik | -0.705711 |
| 2510006D16Rik | -0.5503886 |
| 2510009E07Rik | -0.6937873 |
| 2510010F15Rik | -0.3577614 |
| 2510012J08Rik | -0.3243441 |
| 2510039O18Rik | 0.1750347 |
| 2510040D07Rik | -0.2914305 |
| 2510048L02Rik | 0.5749209 |
| 2510049I19Rik | -0.4625244 |
| 2600001B17Rik | -0.5365298 |
| 2600009E05Rik | -0.719513 |
| 2600009E05Rik | -0.3487012 |
| 2600010E01Rik | -0.5474551 |
| 2600011E07Rik | -0.2469761 |
| 2600011E07Rik | 1.9348515 |
| 2610002J02Rik | -0.6402024 |
| 2610002M06Rik | -0.8660224 |
| 2610003J06Rik | 0.3320624 |
| 2610016F04Rik | 1.1156916 |
| 2610019A05Rik | 1.5075766 |
| 2610019P18Rik | -0.4558152 |
| 2610020C11Rik | 2.3281096 |
| 2610020H08Rik | -0.6711822 |
| 2610020H08Rik | 3.9934528 |
| 2610020H08Rik | -0.5570204 |
| 2610020O08Rik | 2.2112074 |
| 2610021K21Rik | 2.148562 |
| 2610021K21Rik | 0.9323741 |
| 2610024G14Rik | -0.3786044 |
| 2610024G14Rik | 2.0911169 |
| 2610024G14Rik | -0.3823277 |
| 2610027C15Rik | 2.1320034 |
| 2610027C15Rik | 0.1004421 |
| 2610027L16Rik | 0.6306191 |
| 2610028A01Rik | 0.9437875 |
| 2610028H24Rik | 0.2646528 |
| 2610029G23Rik | -0.8037079 |
| 2610029G23Rik | 1.1194844 |
| 2610034E13Rik | -0.581717 |
| 2610034E13Rik | 1.0118675 |
| 2610034M16Rik | 0.6692579 |
| 2610036D13Rik | 1.1455053 |
| 2610039C10Rik | -0.7238652 |
| 2610042L04Rik | -0.3786044 |
| 2610044O15Rik | -0.2175123 |
| 2610101N10Rik | -0.719513 |
| 2610101N10Rik | -0.9421442 |
| 2610103J23Rik | -0.7803752 |
| 2610110G12Rik | -0.8852885 |
| 2610200G18Rik | 0.6679423 |
| 2610204K14Rik | 3.2234039 |
| 2610204L23Rik | -0.6290245 |
| 2610204M08Rik | -0.3367462 |
| 2610205E22Rik | 0.6971356 |
| 2610207I05Rik | -0.7326263 |
| 2610208M17Rik | -0.7326263 |
| 2610209A20Rik | -0.5585001 |
| 2610209A20Rik | -0.3687422 |
| 2610209M04Rik | -0.5264386 |
| 2610301B20Rik | 0.6984137 |
| 2610301F02Rik | 3.0482306 |
| 2610304G08Rik | -0.6078109 |
| 2610305D13Rik | -0.7100039 |
| 2610318N02Rik | -0.6361961 |
| 2610507B11Rik | 0.165691 |
| 2610507B11Rik | -0.7022898 |
| 2610510B01Rik | 0.4611893 |
| 2610510J17Rik | 0.5008014 |
| 2610524A10Rik | -0.6000659 |
| 2610524H06Rik | 1.2039024 |
| 2610528E23Rik | 0.3100894 |
| 2610528J11Rik | 0.5525383 |
| 2610528O22Rik | 3.3101386 |
| 2700007P21Rik | -0.0007654 |
| 2700007P21Rik | -0.6070337 |
| 2700038C09Rik | 2.4133814 |
| 2700038C09Rik | 3.2737484 |
| 2700050L05Rik | -0.3662917 |
| 2700055A20Rik | 1.1585381 |
| 2700060E02Rik | -0.0581532 |
| 2700062C07Rik | -0.7352695 |
| 2700062C07Rik | 1.3650418 |
| 2700078K21Rik | -0.4665715 |
| 2700081O15Rik | 0.1966225 |
| 2700087H15Rik | 0.4021241 |
| 2700094K13Rik | 0.4644161 |
| 2700097O09Rik | 1.4947086 |
| 2810002N01Rik | 1.4584006 |
| 2810002O09Rik | -0.6314094 |
| 2810003C17Rik | -0.4139018 |
| 2810004N20Rik | -0.55776 |
| 2810004N23Rik | 0.4533481 |
| 2810008M24Rik | 0.5751615 |
| 2810021B07Rik | 1.3109724 |
| 2810021J22Rik | 0.8723898 |
| 2810021J22Rik | -0.8179712 |
| 2810022L02Rik | -0.779453 |
| 2810022L02Rik | -0.657205 |
| 2810022L02Rik | -0.3214136 |
| 2810030E01Rik | 1.8145873 |
| 2810046L04Rik | -0.4385772 |
| 2810046M22Rik | -0.5257216 |
| 2810403A07Rik | 0.093485 |
| 2810405J04Rik | -0.5452606 |
| 2810405K02Rik | 2.294833 |
| 2810408A11Rik | 0.0405356 |
| 2810408M09Rik | -0.6442247 |
| 2810410C14Rik | -0.3149962 |
| 2810410P22Rik | 2.5801655 |
| 2810410P22Rik | 0.0244668 |
| 2810417H13Rik | -0.821809 |
| 2810422O20Rik | 1.6304959 |
| 2810428I15Rik | 0.5411578 |
| 2810432D09Rik | -0.2091002 |
| 2810432L12Rik | 0.4115046 |
| 2810439F02Rik | 0.6747211 |
| 2810439F02Rik | -0.6744993 |
| 2810441K11Rik | -0.4451724 |
| 2810452K22Rik | -0.9324428 |
| 2810453I06Rik | -0.8771315 |
| 2810453I06Rik | 1.4128012 |
| 2810470D21Rik | -0.7343877 |
| 2810485I05Rik | 0.046242 |
| 2900006K08Rik | 1.647499 |
| 2900010M23Rik | 0.6115423 |
| 2900011O08Rik | 2.1764709 |
| 2900011O08Rik | 2.2985416 |
| 2900024C23Rik | -0.5379797 |
| 2900024O10Rik | -0.5839925 |
| 2900026A02Rik | 0.7764228 |
| 2900046G09Rik | -0.5870344 |
| 2900062L11Rik | -0.6769943 |
| 2900064A13Rik | 0.9151798 |
| 2900073G15Rik | 0.186417 |
| 2900092C05Rik | -0.8343839 |
| 3000004C01Rik | -0.7803752 |
| 3000004C01Rik | 0.8999559 |
| 3000004C01Rik | 0.0425773 |
| 3010003L21Rik | 0.5979941 |
| 3010026O09Rik | 1.7923879 |
| 3110001A13Rik | -0.6458381 |
| 3110001D03Rik | -0.5178686 |
| 3110004L20Rik | -0.667876 |
| 3110007F17Rik | -0.7512761 |
| 3110009E18Rik | -0.7100039 |
| 3110023B02Rik | 0.9044778 |
| 3110035E14Rik | -0.0541043 |
| 3110035E14Rik | -0.6596576 |
| 3110037I16Rik | 0.2962281 |
| 3110040N11Rik | 0.3772599 |
| 3110040N11Rik | 0.2441535 |
| 3110043J09Rik | -0.8237334 |
| 3110043J09Rik | 1.9474524 |
| 3110043J09Rik | -0.3086194 |
| 3110048E14Rik | -0.8295288 |
| 3110049J23Rik | -0.598524 |
| 3110050N22Rik | -0.3693558 |
| 3110050N22Rik | 2.1878593 |
| 3110056O03Rik | -0.229729 |
| 3110057O12Rik | 1.7460904 |
| 3110057O12Rik | 0.8996072 |
| 3110070M22Rik | 0.250148 |
| 3110082I17Rik | 0.6549135 |
| 3200001I04Rik | 1.2581249 |
| 3200002M19Rik | -0.8160577 |
| 3200002M19Rik | 0.3390789 |
| 3300001G02Rik | -0.749485 |
| 3300001P08Rik | 0.8813074 |
| 3300001P08Rik | -0.4618515 |
| 3300001P08Rik | -0.2835081 |
| 3300002I08Rik | -0.1315318 |
| 3321401G04Rik | -0.6703546 |
| 3322402L07Rik | -0.4891255 |
| 3632451O06Rik | 1.1116019 |
| 3830402I07Rik | -0.8431824 |
| 3830406C13Rik | 1.5181129 |
| 3830406C13Rik | 1.5047099 |
| 3830406C13Rik | 2.6176357 |
| 3830408P04Rik | 1.9426293 |
| 3930401K13Rik | -0.4856756 |
| 4121402D02Rik | 2.1199106 |
| 4121402D02Rik | -0.6000659 |
| 4430402I18Rik | 0.5404111 |
| 4432405B04Rik | 0.2971793 |
| 4432416J03Rik | 0.2600602 |
| 4631426J05Rik | 2.0804147 |
| 4631427C17Rik | -0.1471662 |
| 4632404H12Rik | 0.5958753 |
| 4632404H12Rik | 3.2209119 |
| 4632404H12Rik | -0.4464966 |
| 4632404H22Rik | -0.6711822 |
| 4632411B12Rik | -0.7396904 |
| 4632411B12Rik | 1.5944672 |
| 4632412N22Rik | 2.9267295 |
| 4632415K11Rik | 1.3585363 |
| 4632417K18Rik | 1.8874055 |
| 4632417N05Rik | -0.0640304 |
| 4632419K20Rik | 1.2592241 |
| 4632434I11Rik | 1.5710872 |
| 4732415M23Rik | 0.7821205 |
| 4732418C07Rik | 0.1496786 |
| 4732418C07Rik | 0.7056256 |
| 4732418C07Rik | 0.4893422 |
| 4732429D16Rik | -0.0658457 |
| 4732456N10Rik | -0.6274377 |
| 4732471D19Rik | -0.3284612 |
| 4732473B16Rik | -0.6442247 |
| 4732479N06Rik | -0.8620129 |
| 4732479N06Rik | -0.3302308 |
| 4732496O08Rik | 0.785055 |
| 4733401H18Rik | -0.2806938 |
| 4831426I19Rik | 1.2603221 |
| 4833418A01Rik | 0.2163854 |
| 4833420G17Rik | 0.7520941 |
| 4833421E05Rik | -0.5474551 |
| 4833424O15Rik | 1.2151867 |
| 4833424O15Rik | -0.6711822 |
| 4833426J09Rik | -0.2464327 |
| 4833426J09Rik | 0.1285499 |
| 4833427G06Rik | 0.7136254 |
| 4833436C18Rik | -0.4524772 |
| 4833439L19Rik | -0.7432411 |
| 4833439L19Rik | 1.0196376 |
| 4833439L19Rik | 1.7991278 |
| 4833442J19Rik | -0.1393187 |
| 4833446K15Rik | -0.7915076 |
| 4921501E09Rik | -0.6498831 |
| 4921504E06Rik | 0.6432892 |
| 4921504I05Rik | 0.8793523 |
| 4921505C17Rik | -0.3948389 |
| 4921505C17Rik | -0.1447073 |
| 4921507P07Rik | 1.5873513 |
| 4921511H03Rik | -0.5585001 |
| 4921511H03Rik | -0.0599579 |
| 4921511K06Rik | 0.6657459 |
| 4921511K06Rik | -0.0948762 |
| 4921515J06Rik | 2.5745173 |
| 4921517D21Rik | -0.5847521 |
| 4921517D22Rik | 1.2217958 |
| 4921520G13Rik | 1.3959951 |
| 4921521F21Rik | 1.9322873 |
| 4921521F21Rik | 3.2717927 |
| 4921523A10Rik | 0.9616532 |
| 4921523A10Rik | -0.4294159 |
| 4921523A10Rik | 1.4886015 |
| 4921525H12Rik | -0.3456991 |
| 4921528G01Rik | -0.5659312 |
| 4921528I01Rik | -0.6637584 |
| 4921530G04Rik | -0.243178 |
| 4921530L21Rik | -0.342706 |
| 4921530L21Rik | 1.7122367 |
| 4921536K21Rik | -0.6720105 |
| 4921537P18Rik | 0.4538909 |
| 4922502D21Rik | 0.6655259 |
| 4922503N01Rik | -0.3935808 |
| 4922503N01Rik | -0.0438304 |
| 4922505E12Rik | 0.8070825 |
| 4922505G16Rik | 2.846871 |
| 4922505G16Rik | -0.5214306 |
| 4930401F20Rik | -0.6861958 |
| 4930402H24Rik | 2.7399909 |
| 4930403C10Rik | 0.5490882 |
| 4930403L05Rik | -0.1447073 |
| 4930403O06Rik | 0.3219091 |
| 4930404H21Rik | 1.518868 |
| 4930404N11Rik | -0.421629 |
| 4930408O21Rik | -0.603931 |
| 4930408O21Rik | -0.4788111 |
| 4930412F15Rik | 0.3075833 |
| 4930415L06Rik | -0.6845165 |
| 4930418G15Rik | 0.029025 |
| 4930427A07Rik | -0.2212148 |
| 4930428D18Rik | 0.0169633 |
| 4930428E23Rik | 1.9267615 |
| 4930430D24Rik | -0.6450311 |
| 4930430E16Rik | -0.636996 |
| 4930430E16Rik | -0.5467231 |
| 4930431L04Rik | -0.530031 |
| 4930432H15Rik | 0.1217846 |
| 4930432K21Rik | 0.0762738 |
| 4930432O21Rik | -0.3559429 |
| 4930432O21Rik | 0.5468641 |
| 4930438O05Rik | -0.2988431 |
| 4930438O05Rik | -0.6070337 |
| 4930449I24Rik | 0.7924511 |
| 4930451C15Rik | -0.2383157 |
| 4930451I11Rik | -0.7221221 |
| 4930452B06Rik | 1.0474255 |
| 4930453N24Rik | 2.1444097 |
| 4930455C21Rik | -0.7125884 |
| 4930455F23Rik | -0.4939752 |
| 4930467M19Rik | -0.5562814 |
| 4930470P17Rik | -0.1899118 |
| 4930473A06Rik | 2.0800902 |
| 4930473A06Rik | -0.5503886 |
| 4930473A06Rik | -0.5228589 |
| 4930474N05Rik | -0.3577614 |
| 4930478A21Rik | 1.3371838 |
| 4930481F22Rik | 0.5686456 |
| 4930481F22Rik | 0.5766038 |
| 4930481M05 | -0.7186448 |
| 4930486L24Rik | 0.4333358 |
| 4930502E18Rik | -0.087888 |
| 4930503B20Rik | 3.5681288 |
| 4930503L19Rik | -0.3045824 |
| 4930504E06Rik | -0.5525944 |
| 4930504E06Rik | 4.3708472 |
| 4930504O13Rik | -0.1847879 |
| 4930506M07Rik | -0.6314094 |
| 4930507C10Rik | -0.7048546 |
| 4930511I11Rik | 1.6439212 |
| 4930511J11Rik | -0.61405 |
| 4930515G01Rik | -0.581717 |
| 4930518C23Rik | -0.1266954 |
| 4930519F16Rik | 1.2980179 |
| 4930519G04Rik | -0.1108978 |
| 4930519N13Rik | -0.530031 |
| 4930519N16Rik | -0.0041779 |
| 4930522H14Rik | -0.6353967 |
| 4930524B15Rik | 2.2743001 |
| 4930524B15Rik | 0.8280777 |
| 4930524E20Rik | -0.385441 |
| 4930526D03Rik | -0.7143151 |
| 4930535B03Rik | 2.8592092 |
| 4930535E21Rik | -0.6093671 |
| 4930538K18Rik | -0.4891255 |
| 4930539E08Rik | -0.500245 |
| 4930540L03Rik | -0.6861958 |
| 4930544G11Rik | -0.4346407 |
| 4930544G21Rik | 1.1896171 |
| 4930547C10Rik | 2.18157 |
| 4930547N16Rik | -0.535082 |
| 4930549C01Rik | -0.683678 |
| 4930550C14Rik | -0.5824749 |
| 4930550L24Rik | -0.5570204 |
| 4930555G01Rik | -0.5336363 |
| 4930555I21Rik | 0.5977589 |
| 4930562D19Rik | -0.3493027 |
| 4930562D19Rik | -0.011468 |
| 4930563D23Rik | -0.4458343 |
| 4930563P21Rik | 2.0750478 |
| 4930570C03Rik | -0.5570204 |
| 4930572J05Rik | 2.453308 |
| 4930572J05Rik | 1.1293905 |
| 4930572J05Rik | -0.4132605 |
| 4930573I19Rik | 0.6990521 |
| 4930578I06Rik | 2.8719657 |
| 4930579C15Rik | 0.9016975 |
| 4930579E17Rik | -0.0218506 |
| 4930579E17Rik | -0.4372633 |
| 4930579J09Rik | -0.3249312 |
| 4930583H14Rik | 0.9570517 |
| 4930590J08Rik | -0.2464327 |
| 4930595M18Rik | 1.4411213 |
| 4930596D02Rik | -0.821809 |
| 4930599N23Rik | 0.8910265 |
| 4931406B18Rik | -0.6402024 |
| 4931406C07Rik | -0.4692786 |
| 4931406H21Rik | -0.3445008 |
| 4931406I20Rik | 1.9319115 |
| 4931406I20Rik | 0.2541246 |
| 4931406P16Rik | 0.8154573 |
| 4931407G18Rik | -0.1238046 |
| 4931408A02Rik | -0.7459123 |
| 4931408C20Rik | 2.9365851 |
| 4931408C20Rik | -0.7432411 |
| 4931409K22Rik | 1.2331333 |
| 4931417E11Rik | -0.6769943 |
| 4931417G12Rik | -0.7423522 |
| 4931428L18Rik | 1.4514639 |
| 4931432M23Rik | -0.4196916 |
| 4931433A01Rik | -0.057252 |
| 4931440B09Rik | 0.3705049 |
| 4931440F15Rik | 0.2997113 |
| 4931440F15Rik | -0.6023831 |
| 4931440L10Rik | 1.1557015 |
| 4932409I22Rik | -0.3231709 |
| 4932411E22Rik | -0.2639704 |
| 4932411G14Rik | 0.4213592 |
| 4932412H11Rik | 0.7599353 |
| 4932413O14Rik | 2.1336459 |
| 4932414J04Rik | 0.5169854 |
| 4932414N04Rik | 0.7518923 |
| 4932418E24Rik | -0.3675162 |
| 4932431H17Rik | 0.7778011 |
| 4932431H17Rik | -0.7693644 |
| 4932441K18Rik | 3.6211892 |
| 4932441K18Rik | 0.0938728 |
| 4932442K08Rik | 1.0354576 |
| 4932702K14Rik | 0.4261111 |
| 4933400A11Rik | -0.5221445 |
| 4933400C05Rik | 0.8996072 |
| 4933400E14Rik | 1.9871874 |
| 4933402J07Rik | -0.6588394 |
| 4933402N03Rik | 0.5902035 |
| 4933402P03Rik | -0.5100763 |
| 4933403G14Rik | -0.7459123 |
| 4933404M02Rik | -0.6078109 |
| 4933405O20Rik | 0.6159412 |
| 4933406M09Rik | 1.208534 |
| 4933407N01Rik | 1.6019678 |
| 4933407N01Rik | -0.4484862 |
| 4933407N01Rik | 0.6699151 |
| 4933407P14Rik | -0.220685 |
| 4933407P14Rik | 0.6945746 |
| 4933407P14Rik | 0.8005631 |
| 4933409N07Rik | 2.0920259 |
| 4933411G06Rik | -0.3439022 |
| 4933411K20Rik | 0.3085238 |
| 4933412E14Rik | -0.8112899 |
| 4933413G11Rik | -0.294275 |
| 4933415F23Rik | -0.598524 |
| 4933416C03Rik | -0.6954821 |
| 4933417A18Rik | -0.6337999 |
| 4933417E01Rik | -0.3891897 |
| 4933421E11Rik | -0.2994156 |
| 4933421E11Rik | 1.3826913 |
| 4933421E11Rik | -0.0874238 |
| 4933422H20Rik | -0.4781272 |
| 4933424B01Rik | 0.5306536 |
| 4933424B01Rik | -0.0433861 |
| 4933424B01Rik | -0.6337999 |
| 4933425O20Rik | 1.3866794 |
| 4933426M11Rik | -0.4248662 |
| 4933427D14Rik | 0.3819325 |
| 4933428G09Rik | 4.2579591 |
| 4933428G20Rik | -0.6629369 |
| 4933429E10Rik | 1.7774581 |
| 4933429E10Rik | -0.2925673 |
| 4933430I17Rik | -0.643419 |
| 4933434E20Rik | -0.7822221 |
| 4933434E20Rik | -0.4106995 |
| 4933434E20Rik | -0.3948389 |
| 4933434I06Rik | 0.762935 |
| 4933436I01Rik | 0.9893088 |
| 4933437F05Rik | 1.7194548 |
| 4933439C20Rik | 0.5756425 |
| 4933440H19Rik | -0.8600141 |
| 5031414D18Rik | -0.5408857 |
| 5031414D18Rik | -0.6555733 |
| 5033411D12Rik | -0.4524772 |
| 5033414D02Rik | -0.1560682 |
| 5033414K04Rik | -0.1940298 |
| 5133400G04Rik | -0.6442247 |
| 5133400G04Rik | -0.6929409 |
| 5133400G04Rik | 3.243155 |
| 5133401N09Rik | 0.6184792 |
| 5230400G24Rik | -0.6980296 |
| 5230400G24Rik | 1.5264821 |
| 5330401P04Rik | 0.4338894 |
| 5330417C22Rik | -0.4119792 |
| 5330431N19Rik | -0.6377967 |
| 5330431N19Rik | 0.2394664 |
| 5330431N19Rik | -0.8008793 |
| 5330437I02Rik | 1.7454877 |
| 5330439C02Rik | 0.0397177 |
| 5330439J01Rik | -0.1940298 |
| 5330439J01Rik | 1.028423 |
| 5430407P10Rik | -0.1832557 |
| 5430432M24Rik | -0.5666773 |
| 5430432M24Rik | -0.720382 |
| 5430432M24Rik | 0.2728021 |
| 5430435G22Rik | 2.566169 |
| 5430437P03Rik | 1.3250213 |
| 5530601I19Rik | -0.408145 |
| 5630401D24Rik | -0.6337999 |
| 5630401D24Rik | -0.8285606 |
| 5630401D24Rik | 2.2443277 |
| 5730403M16Rik | -0.4229226 |
| 5730409E04Rik | 1.5654268 |
| 5730410E15Rik | 0.726957 |
| 5730410E15Rik | -0.4753963 |
| 5730410I19Rik | -0.5214306 |
| 5730410I19Rik | 0.341508 |
| 5730410I19Rik | -0.4981507 |
| 5730419I09Rik | -0.0376276 |
| 5730437N04Rik | -0.6031568 |
| 5730446C15Rik | -0.0420543 |
| 5730449L18Rik | 1.1898787 |
| 5730449L18Rik | -0.6637584 |
| 5730449L18Rik | -0.4578232 |
| 5730453I16Rik | 0.5384173 |
| 5730453I16Rik | -0.6490728 |
| 5730469M10Rik | 1.6401634 |
| 5730472N09Rik | 0.1860633 |
| 5730494M16Rik | -0.6695277 |
| 5730494N06Rik | -0.2801318 |
| 5730502D15Rik | 2.817446 |
| 5730507A09Rik | 1.8541265 |
| 5730507A09Rik | -0.6393999 |
| 5730528L13Rik | 1.1573234 |
| 5730589K01Rik | 0.6814546 |
| 5730593F17Rik | 0.0110883 |
| 5730596K20Rik | -0.5764276 |
| 5730596K20Rik | 0.1649686 |
| 5730596K20Rik | -0.4158281 |
| 5730596K20Rik | -0.3462988 |
| 5830403L16Rik | 0.6338016 |
| 5830404H04Rik | 0.9228769 |
| 5830405N20Rik | -0.4113392 |
| 5830415F09Rik | -0.4891255 |
| 5830415L20Rik | -0.2891606 |
| 5830417C01Rik | 0.292096 |
| 5830426I05Rik | 0.4854058 |
| 5830427D03Rik | -0.6306138 |
| 5830434P21Rik | 0.1251729 |
| 5830435K17Rik | -0.2959856 |
| 5830457O10Rik | -0.1315318 |
| 5830457O10Rik | -0.1832557 |
| 5830457O10Rik | -0.6171841 |
| 5830467P10Rik | -0.7575702 |
| 5830468K18Rik | 4.3360313 |
| 5830472M02Rik | 0.6073571 |
| 5830472M02Rik | 0.8597712 |
| 5830472M02Rik | -0.4692786 |
| 5930416I19Rik | -0.5178686 |
| 5930431H10 | -0.4767608 |
| 5930434B04Rik | -0.7134514 |
| 5930434B04Rik | -0.4638716 |
| 5930434B04Rik | -0.2243992 |
| 6030405A18Rik | -0.5459915 |
| 6030408B16Rik | -0.2491529 |
| 6030408B16Rik | 0.9271278 |
| 6030419C18Rik | -0.6604764 |
| 6030429G01Rik | 0.8074647 |
| 6030443O07Rik | 0.4681678 |
| 6030446N20Rik | -0.7485906 |
| 6030458C11Rik | 1.3598629 |
| 6230409E13Rik | 1.8585154 |
| 6230409E13Rik | 0.8839674 |
| 6230416J20Rik | -0.6828401 |
| 6230427J02Rik | 1.1605593 |
| 6330403K07Rik | 0.2279919 |
| 6330406I15Rik | 1.1413978 |
| 6330406I15Rik | 0.8409119 |
| 6330407J23Rik | 0.5645213 |
| 6330408A02Rik | -0.5264386 |
| 6330409N04Rik | -0.5257216 |
| 6330416G13Rik | -0.8314681 |
| 6330439K17Rik | 2.5128495 |
| 6330442E10Rik | 0.6096844 |
| 6330442E10Rik | 0.4932634 |
| 6330500D04Rik | 1.8976973 |
| 6330503C03Rik | -0.0953438 |
| 6330503K22Rik | -0.4405512 |
| 6330503K22Rik | -0.4774438 |
| 6330503K22Rik | -0.6861958 |
| 6330505N24Rik | 3.234826 |
| 6330512M04Rik | 0.4609199 |
| 6330534C20Rik | -0.16005 |
| 6330548G22Rik | -0.4774438 |
| 6330569M22Rik | -0.6298188 |
| 6330577E15Rik | -0.6515056 |
| 6330578E17Rik | 0.5064825 |
| 6332401O19Rik | -0.6306138 |
| 6430411K18Rik | 2.4765338 |
| 6430502M16Rik | 1.561726 |
| 6430510M02Rik | 0.6712281 |
| 6430517E21Rik | -0.6811664 |
| 6430526N21Rik | -0.530031 |
| 6430527G18Rik | 0.5559765 |
| 6430527G18Rik | 0.4092385 |
| 6430531B16Rik | -0.7557679 |
| 6430537H07Rik | -0.3687422 |
| 6430537H07Rik | 1.0359147 |
| 6430548M08Rik | 2.0391396 |
| 6430550H21Rik | -0.6000659 |
| 6430571L13Rik | 0.1710921 |
| 6430573F11Rik | 1.2216691 |
| 6430598A04Rik | -0.0283944 |
| 6430604K15Rik | -0.4960608 |
| 6430706D22Rik | -0.6156158 |
| 6530404N21Rik | 1.233008 |
| 6530406M24Rik | -0.5489208 |
| 6530418L21Rik | -0.5802029 |
| 6530418L21Rik | 1.8019822 |
| 6530418L21Rik | 4.5443811 |
| 6530421E24Rik | -0.7160448 |
| 6620401K05Rik | -0.4425291 |
| 6720456B07Rik | -0.6226923 |
| 6720456B07Rik | -0.5533307 |
| 6720456H20Rik | -0.4829242 |
| 6720456H20Rik | 1.8838638 |
| 6720458F09Rik | 1.5735927 |
| 6720463M24Rik | 0.1145939 |
| 6720467C03Rik | 1.4486353 |
| 6720467C03Rik | 0.4969095 |
| 6820408C15Rik | 0.5301507 |
| 7420416P09Rik | -0.6744993 |
| 7530404M11Rik | -0.4686011 |
| 8030411F24Rik | 0.2744241 |
| 8030451F13Rik | -0.2243992 |
| 8030451F13Rik | 2.1736652 |
| 8030451F13Rik | 1.581161 |
| 8430408G22Rik | 0.2763671 |
| 8430410A17Rik | -0.436607 |
| 8430410K20Rik | 1.2689424 |
| 8430415E04Rik | -0.6937873 |
| 8430415E04Rik | -0.4438499 |
| 8430419L09Rik | -0.6093671 |
| 8430427H17Rik | 1.8348611 |
| 8430432M10Rik | 0.1039028 |
| 9030025P20Rik | -0.5607237 |
| 9030025P20Rik | -0.4760783 |
| 9030025P20Rik | -0.3379353 |
| 9030205A07Rik | -0.4981507 |
| 9030221M09Rik | 1.5616355 |
| 9030224M15Rik | 2.0563403 |
| 9030227G01Rik | -0.6054811 |
| 9030409G11Rik | -0.5121955 |
| 9030418K01Rik | 0.8922568 |
| 9030425E11Rik | 0.155166 |
| 9030607L17Rik | 2.1416659 |
| 9030607L17Rik | -0.4055969 |
| 9030611O19Rik | -0.3325952 |
| 9030612M13Rik | -0.5802029 |
| 9030617O03Rik | 0.4468125 |
| 9030624J02Rik | 1.0813355 |
| 9030625A04Rik | -0.6887199 |
| 9130005N14Rik | -0.2175123 |
| 9130011E15Rik | 2.1953163 |
| 9130011J15Rik | -0.0722251 |
| 9130014G24Rik | 0.7498714 |
| 9130017N09Rik | 0.0265413 |
| 9130206N08Rik | 1.1833196 |
| 9130211I03Rik | 0.5268754 |
| 9130213B05Rik | -0.6156158 |
| 9130213B05Rik | 1.7208459 |
| 9130218O11Rik | 1.027809 |
| 9130221H12Rik | -0.3249312 |
| 9130227C08Rik | -0.6274377 |
| 9130401M01Rik | 0.0165447 |
| 9130404D08Rik | -0.2437197 |
| 9130404D08Rik | 0.3019217 |
| 9130404D14Rik | -0.3080417 |
| 9130422G05Rik | -0.327872 |
| 9230104L09Rik | -0.125249 |
| 9330129D05Rik | 1.3317458 |
| 9330134C04Rik | 0.2320567 |
| 9330176C04Rik | -0.6337999 |
| 9330186A19Rik | 1.0469736 |
| 9430015G10Rik | 1.1035148 |
| 9430015G10Rik | 4.8740262 |
| 9430023L20Rik | 1.069029 |
| 9430025C20Rik | -0.5802029 |
| 9430028L06Rik | 1.2110979 |
| 9430029K10Rik | 0.4083875 |
| 9430031J16Rik | 0.4075357 |
| 9430034D17Rik | 0.4140478 |
| 9430057O19Rik | 1.059253 |
| 9430069J07Rik | 2.7001406 |
| 9430077A04Rik | -0.244804 |
| 9430079M16Rik | -0.4960608 |
| 9530015I07Rik | 0.864296 |
| 9530027K23Rik | 2.0539009 |
| 9530058B02Rik | 0.3949591 |
| 9530058B02Rik | 0.9644368 |
| 9530068E07Rik | -0.4209828 |
| 9530077C05Rik | 0.2737756 |
| 9530096D07Rik | 1.076226 |
| 9530096D07Rik | -0.6588394 |
| 9630015D15Rik | 0.2242515 |
| 9630020C08Rik | 0.0421693 |
| 9630028B13Rik | -0.4268136 |
| 9630031F12Rik | -0.3361521 |
| 9630050M13Rik | -0.5962156 |
| 9630058J23Rik | 0.0707353 |
| 9830163H01Rik | 0.62948 |
| 9830169C18Rik | -0.1066319 |
| 9930013L23Rik | -0.6418094 |
| 9930021D14Rik | -0.5931459 |
| 9930023K05Rik | -0.7812982 |
| 9930023K05Rik | 0.3929438 |
| 9930104L06Rik | -0.6621161 |
| a | -0.3196593 |
| A030003K21Rik | 0.955898 |
| A030007L17Rik | -0.0934747 |
| A030007L17Rik | -0.6385979 |
| A030007L17Rik | 1.4123819 |
| A030007L17Rik | 2.5453146 |
| A030009H04Rik | 2.0166419 |
| A030010B05Rik | -0.5839925 |
| A030012M09Rik | 1.2831082 |
| A130010J15Rik | 1.0118675 |
| A130038H09Rik | 1.3286741 |
| A130042E20Rik | -0.5939124 |
| A130090K04Rik | -0.9028442 |
| A130092J06Rik | -0.1852991 |
| A230050P20Rik | -0.5372545 |
| A230051G13Rik | -0.6870365 |
| A230054D04Rik | 2.2499187 |
| A230067G21Rik | -0.6179692 |
| A230069A22Rik | 1.7551633 |
| A230074B11Rik | 0.5510611 |
| A230074B11Rik | 0.7259218 |
| A230074B11Rik | -0.1695704 |
| A230106M15Rik | -0.2491529 |
| A230106N23Rik | 1.5200939 |
| A230107C01Rik | 1.1992495 |
| A2bp1 | -0.3022828 |
| A2bp1 | 1.0962236 |
| A330021E22Rik | -0.51858 |
| A330021E22Rik | 1.7653383 |
| A330049M08Rik | 2.8797778 |
| A330080J22Rik | 1.3872171 |
| A3galt2 | -0.2259952 |
| A430005L14Rik | -0.9143688 |
| A430005L14Rik | 1.1748612 |
| A430005L14Rik | 1.9793886 |
| A430005L14Rik | -0.4829242 |
| A430033K04Rik | -0.5622088 |
| A430079E08 | 0.4737693 |
| A430083B19Rik | -0.2524269 |
| A430083B19Rik | 1.4741074 |
| A430107D22Rik | -0.1565651 |
| A430107P09Rik | 1.675027 |
| A430110N23Rik | -0.3860648 |
| A4galt | -0.5107822 |
| A4gnt | -0.1858106 |
| A530050D06Rik | -0.613268 |
| A530082C11Rik | 0.9227065 |
| A530082C11Rik | 0.6681617 |
| A530088H08Rik | -0.4418694 |
| A530088H08Rik | 1.1917076 |
| A530098C11Rik | 2.0990564 |
| A630042L21Rik | -0.496757 |
| A630054L15Rik | 0.4266687 |
| A630077B13Rik | -0.4605069 |
| A630095E13Rik | 1.7011148 |
| A730008H23Rik | -0.5636961 |
| A730008L03Rik | -0.0398385 |
| A730008L03Rik | -0.567424 |
| A730017C20Rik | -0.4665715 |
| A730024F05Rik | -0.7849988 |
| A730037C10Rik | 0.2587442 |
| A730042J05Rik | 0.9469536 |
| A730098D12Rik | 1.8683711 |
| A830006F12Rik | 0.2039487 |
| A830007P12Rik | -0.6753303 |
| A830053O21Rik | -0.4713137 |
| A830059I20Rik | -0.530031 |
| A830073O21Rik | 1.2659155 |
| A830080D01Rik | 2.1799548 |
| A830093I24Rik | -0.5749214 |
| A930001N09Rik | -0.1204425 |
| A930005I04Rik | -0.3638471 |
| A930008G19Rik | -0.4024209 |
| A930010I20Rik | 1.4493431 |
| A930016P21Rik | 0.0946479 |
| A930018P22Rik | -0.4062334 |
| A930024E05Rik | 0.2518068 |
| A930025D01Rik | 2.0619175 |
| A930025D01Rik | 2.7060596 |
| A930025D01Rik | -0.5977539 |
| A930034L06Rik | -0.5719157 |
| A930038C07Rik | 0.7018139 |
| A930041I02Rik | -0.5939124 |
| AA388235 | -0.3535232 |
| AA407270 | 0.2039487 |
| AA407659 | -0.0967479 |
| AA407659 | 0.5195306 |
| AA408296 | 1.0070198 |
| AA408296 | -0.5855123 |
| AA408296 | -0.6786611 |
| AA409316 | -0.408145 |
| AA415398 | -0.4287647 |
| AA467197 | 0.4536195 |
| AA536717 | -0.4425291 |
| AA536717 | -0.4132605 |
| AA536749 | -0.6887199 |
| AA536749 | -0.0986232 |
| AA536749 | -0.4760783 |
| AA536749 | -0.8560283 |
| AA673488 | 0.1255487 |
| AA691260 | 0.7312933 |
| AA792892 | 0.1969726 |
| AA881470 | -0.5644406 |
| AA881470 | 1.0914775 |
| AA960436 | 1.4835327 |
| AA960436 | -0.2628653 |
| Aaas | -0.1776578 |
| Aaas | -0.2689584 |
| Aacs | 1.8102146 |
| Aadacl1 | -0.484987 |
| Aak1 | -0.4753963 |
| Aak1 | 0.8732851 |
| Aamp | 0.1507784 |
| Aard | -0.5540676 |
| Aars | -0.2891606 |
| Aars2 | 1.0081165 |
| Aarsd1 | 1.0196376 |
| Aasdh | -0.5847521 |
| Aatf | -0.581717 |
| Aatf | 1.5824907 |
| Aatk | -0.5394317 |
| AB112350 | 2.7411989 |
| AB182283 | 0.0841335 |
| AB182283 | 1.2433532 |
| Abat | 3.3669132 |
| Abat | -0.4863646 |
| Abat | -0.5372545 |
| Abca14 | 1.225463 |
| Abca14 | -0.5489208 |
| Abca15 | -0.7629965 |
| Abca15 | -0.4605069 |
| Abca15 | -0.3718138 |
| Abca15 | 2.2646632 |
| Abca17 | 0.2521382 |
| Abca2 | 0.7637334 |
| Abca3 | -0.3468989 |
| Abca4 | -0.6290245 |
| Abca4 | 0.3651867 |
| Abca5 | 1.1684705 |
| Abca6 | -0.5171577 |
| Abca6 | -0.2281271 |
| Abca7 | -0.6515056 |
| Abca7 | 0.5883058 |
| Abca8a | -0.6498831 |
| Abca8b | -0.2639704 |
| Abca9 | -0.6282308 |
| Abca9 | -0.7396904 |
| Abca9 | 0.8462575 |
| Abcb11 | -0.7468043 |
| Abcb1b | 1.678174 |
| Abcb4 | -0.5121955 |
| Abcb6 | -0.7238652 |
| Abcb8 | -0.7476971 |
| Abcb8 | -0.1238046 |
| Abcb8 | -0.5511233 |
| Abcb9 | -0.7229933 |
| Abcb9 | -0.6887199 |
| Abcc1 | -0.5416136 |
| Abcc10 | -0.6298188 |
| Abcc10 | 1.3427049 |
| Abcc12 | -0.3017087 |
| Abcc3 | 0.6117743 |
| Abcc5 | -0.6187548 |
| Abcc6 | 2.0364323 |
| Abcc8 | -0.6450311 |
| Abcc9 | -0.1940298 |
| Abcc9 | -0.7229933 |
| Abcd1 | -0.2931363 |
| Abcd2 | -0.5749214 |
| Abcd2 | -0.6093671 |
| Abcd3 | 0.1240448 |
| Abcd4 | -0.1827456 |
| Abcd4 | 1.1796277 |
| Abce1 | 0.0587637 |
| Abcf1 | -0.7238652 |
| Abcf1 | 0.4601114 |
| Abcf1 | 2.8708059 |
| Abcf2 | 1.9110878 |
| Abcf2 | -0.6811664 |
| Abcf2 | -0.3068873 |
| Abcf2 | -0.1349312 |
| Abcf3 | -0.6988802 |
| Abcg1 | 0.7603358 |
| Abcg4 | 1.7154252 |
| Abcg5 | -0.6878779 |
| Abhd1 | 2.3369519 |
| Abhd1 | 0.1945195 |
| Abhd10 | -0.2734129 |
| Abhd11 | 0.8561367 |
| Abhd12 | -0.4618515 |
| Abhd14a | -0.0478382 |
| Abhd14b | 0.1533401 |
| Abhd2 | 3.9907973 |
| Abhd2 | 2.3046202 |
| Abhd2 | 2.1606676 |
| Abhd3 | 0.5228297 |
| Abhd4 | 0.2367783 |
| Abhd5 | 0.195922 |
| Abhd6 | -0.554805 |
| Abhd7 | 0.3839699 |
| Abhd7 | 0.462804 |
| Abhd8 | 1.2071211 |
| Abi1 | -0.4484862 |
| Abi1 | -0.5607237 |
| Abi2 | 0.1825188 |
| Abi2 | -0.1257309 |
| Abi2 | -0.6195411 |
| Abi3 | -0.408783 |
| Abi3bp | 1.4994331 |
| Abi3bp | -0.4564841 |
| Abl1 | 1.1118844 |
| Ablim1 | -0.5696674 |
| Ablim2 | 0.4383074 |
| Ablim2 | -0.5908497 |
| Ablim3 | -0.4558152 |
| Ablim3 | 0.5963465 |
| Abp1 | -0.1305627 |
| Abr | -0.7229933 |
| Abr | -0.6555733 |
| Abtb1 | 1.810144 |
| Acaa1a | 2.3633088 |
| Acaa1b | 0.2860261 |
| Acaa2 | 1.4617015 |
| Acaa2 | -0.5093708 |
| Acaa2 | 1.1032299 |
| Acaa2 | 1.1183621 |
| Acad10 | 0.7569267 |
| Acad10 | 1.3274199 |
| Acad8 | -0.4544786 |
| Acad8 | -0.1329873 |
| Acad9 | -0.2784479 |
| Acad9 | 2.8118859 |
| Acadl | -0.2023166 |
| Acadl | 1.8983452 |
| Acadm | -0.568919 |
| Acads | -0.7924408 |
| Acads | 0.2215224 |
| Acadsb | -0.3620175 |
| Acadvl | -0.6124866 |
| Acan | 4.8497585 |
| Acat2 | -0.5459915 |
| Acbd3 | -0.6703546 |
| Acbd4 | -0.1075783 |
| Acbd4 | -0.6078109 |
| Acbd5 | -0.6937873 |
| Acbd6 | 2.4882198 |
| Accn1 | 1.3998249 |
| Accn1 | -0.5839925 |
| Accn2 | 1.3524339 |
| Accn3 | -0.7629965 |
| Accn3 | 1.4219837 |
| Accs | -0.6523178 |
| Acd | 1.2671274 |
| Ace | 0.6937195 |
| Ace | 1.3782536 |
| Ache | -0.1180478 |
| Acin1 | -0.4094215 |
| Acly | 4.591265 |
| Acn9 | -0.7143151 |
| Aco1 | -0.0892818 |
| Aco2 | -0.5962156 |
| Acot1 | -0.577936 |
| Acot1 | -0.7648118 |
| Acot10 | -0.817014 |
| Acot11 | 0.1482102 |
| Acot11 | -0.4222756 |
| Acot2 | -0.258457 |
| Acot2 | -0.6266453 |
| Acot3 | 1.3227316 |
| Acot5 | -0.535082 |
| Acot7 | 0.0339742 |
| Acot7 | -0.2937055 |
| Acot7 | 0.6922213 |
| Acot7 | -0.7151796 |
| Acot8 | -0.5946796 |
| Acot8 | -0.6637584 |
| Acot8 | -0.2064857 |
| Acot9 | -0.3308214 |
| Acox2 | -0.2426366 |
| Acox3 | 1.2464485 |
| Acox3 | -0.1300785 |
| Acoxl | -0.6314094 |
| Acp2 | -0.3302308 |
| Acp6 | -0.5394317 |
| Acpl2 | -0.7629965 |
| Acpp | 2.332372 |
| Acr | 1.2794059 |
| Acr | 1.2283619 |
| Acrbp | -0.3314123 |
| Acrbp | -0.5121955 |
| Acrv1 | -0.4209828 |
| Acsbg1 | -0.6769943 |
| Acsbg1 | -0.5562814 |
| Acsbg2 | 0.7138351 |
| Acsf3 | -0.6920953 |
| Acsl1 | -0.6195411 |
| Acsl3 | 3.0311065 |
| Acsl3 | 0.6874981 |
| Acsl4 | -0.4981507 |
| Acsl4 | -0.6695277 |
| Acsl5 | 0.0490831 |
| Acsl6 | 2.3408431 |
| Acsl6 | 0.8948881 |
| Acsl6 | -0.0301466 |
| Acsm3 | -0.6604764 |
| Acss1 | 3.621107 |
| Acss2 | -0.4981507 |
| Acss2 | -0.4524772 |
| Acta1 | -0.3028572 |
| Acta2 | -0.6946343 |
| Acta2 | 0.4896041 |
| Acta2 | 0.4630729 |
| Acta2 | -0.4339861 |
| Actb | 0.7736606 |
| Actb | -0.1218821 |
| Actb | 0.5396639 |
| Actb | 1.0298032 |
| Actb | 0.2481538 |
| Actg2 | 0.0860889 |
| Actl6a | -0.7177774 |
| Actl6b | -0.5756742 |
| Actl6b | 2.0923466 |
| Actl6b | -0.4431893 |
| Actl6b | 1.5277924 |
| Actl7a | -0.8046525 |
| Actl7b | -0.2518805 |
| Actn1 | 0.2354315 |
| Actn2 | 0.6582419 |
| Actn3 | -0.5719157 |
| Actn3 | 1.1917076 |
| Actn4 | 0.8019088 |
| Actn4 | 0.2617028 |
| Actn4 | 1.4696588 |
| Actr10 | -0.272855 |
| Actr10 | -0.4346407 |
| Actr1a | -0.2880276 |
| Actr1b | -0.4842989 |
| Actr3 | 0.9303465 |
| Actr3b | -0.6920953 |
| Actr5 | 0.5525383 |
| Actr5 | -0.530031 |
| Actr6 | 0.8082287 |
| Actrt1 | 1.8945818 |
| Actrt2 | 0.2636704 |
| Acvr1 | -0.7476971 |
| Acvr1c | 0.3372532 |
| Acvr2a | 1.7354139 |
| Acvr2b | -0.4891255 |
| Acvrl1 | -0.1620469 |
| Acy1 | -0.5416136 |
| Acy1l2 | -0.6980296 |
| Acy3 | 1.9980993 |
| Ada | -0.3718138 |
| Adal | 3.1532635 |
| Adal | 0.4366529 |
| Adam11 | -0.321999 |
| Adam11 | -0.6963305 |
| Adam12 | 1.1259135 |
| Adam15 | -0.4544786 |
| Adam17 | 0.3696205 |
| Adam1b | -0.7273605 |
| Adam1b | -0.5023437 |
| Adam21 | -0.7657207 |
| Adam22 | 2.6126726 |
| Adam22 | -0.810339 |
| Adam22 | -0.703999 |
| Adam23 | -0.127178 |
| Adam24 | 1.4097568 |
| Adam26a | 0.4361008 |
| Adam28 | 1.324106 |
| Adam29 | 0.8226346 |
| Adam29 | 1.9958675 |
| Adam3 | -0.1432348 |
| Adam30 | -0.705711 |
| Adam32 | -0.6109256 |
| Adam33 | -0.5221445 |
| Adam39 | -0.3644577 |
| Adam4 | 0.4943065 |
| Adam4 | 0.081782 |
| Adam4 | -0.55776 |
| Adam5 | -0.1061591 |
| Adam6 | -0.3823277 |
| Adam8 | -0.5622088 |
| Adam8 | -0.5741691 |
| Adam9 | 2.3040573 |
| Adamdec1 | 1.8166261 |
| Adamts1 | 0.5495818 |
| Adamts13 | -0.5644406 |
| Adamts15 | -0.703144 |
| Adamts16 | 0.2204971 |
| Adamts17 | 0.7242633 |
| Adamts18 | -0.4665715 |
| Adamts19 | 1.3732513 |
| Adamts2 | 0.9431197 |
| Adamts2 | -0.6612959 |
| Adamts2 | 1.3758643 |
| Adamts20 | -0.6171841 |
| Adamts20 | 0.8535847 |
| Adamts4 | -0.4788111 |
| Adamts7 | 0.4566001 |
| Adamtsl2 | -0.2700701 |
| Adamtsl4 | 0.440784 |
| Adamtsl5 | -0.2075307 |
| Adar | 0.4827729 |
| Adar | -0.2518805 |
| Adar | 0.7720788 |
| Adarb1 | -0.5893219 |
| Adarb1 | 0.7718809 |
| Adarb1 | -0.6820029 |
| Adat1 | -0.0892818 |
| Adc | -0.6163997 |
| Adc | -0.5051487 |
| Adck1 | 0.1104074 |
| Adck2 | 3.3982876 |
| Adck4 | -0.2307983 |
| Adck5 | 1.5824021 |
| Adcy1 | -0.1940298 |
| Adcy10 | 1.5653367 |
| Adcy2 | 1.2910638 |
| Adcy4 | 1.7969101 |
| Adcy4 | 1.0973708 |
| Adcy6 | 0.8034445 |
| Adcy7 | 2.2342129 |
| Adcy8 | -0.3547323 |
| Adcy9 | 1.2247053 |
| Adcyap1 | 2.3971628 |
| Adcyap1r1 | 1.3888286 |
| Add1 | -0.6920953 |
| Add1 | -0.6539443 |
| Add1 | -0.1354178 |
| Add1 | 2.1390677 |
| Add3 | -0.4584935 |
| Add3 | 3.610919 |
| Add3 | 0.0833503 |
| Adfp | -0.6171841 |
| Adh1 | -0.1630468 |
| Adh1 | 1.9393388 |
| Adh4 | 0.8962886 |
| Adh5 | 0.2882666 |
| Adh7 | 0.2892253 |
| Adhfe1 | 0.2481538 |
| Adi1 | 1.2904723 |
| Adipoq | -0.2196263 |
| Adipor1 | -0.4043253 |
| Adipor2 | -0.1955784 |
| Adk | -0.3960986 |
| Adk | -0.1344449 |
| Adm | -0.6498831 |
| Adm2 | -0.6377967 |
| Adnp | 0.4089549 |
| Ado | -0.259007 |
| Adora1 | -0.7256113 |
| Adora1 | 1.4852901 |
| Adora1 | -0.4339861 |
| Adora2b | -0.567424 |
| Adora3 | -0.4471593 |
| Adpgk | 1.9058301 |
| Adprh | 0.3772599 |
| Adprhl1 | -0.0420543 |
| Adprhl1 | 0.174319 |
| Adprhl1 | 1.0897461 |
| Adprhl2 | 0.9067312 |
| Adra1a | 1.0460692 |
| Adra1a | 0.0742993 |
| Adra1d | -0.2122465 |
| Adra2a | -0.5939124 |
| Adra2c | 0.3583501 |
| Adra2c | -0.5459915 |
| Adrb1 | 0.3003433 |
| Adrb2 | -0.6588394 |
| Adrbk1 | 0.3807664 |
| Adrbk2 | -0.4571534 |
| Adrm1 | 1.1594818 |
| Adsl | -0.6811664 |
| Adss | 0.6303914 |
| Adssl1 | 0.6637649 |
| Adssl1 | 1.4375477 |
| Adssl1 | 0.0950352 |
| Aebp1 | -0.1276608 |
| Aebp2 | -0.1873466 |
| Aes | 0.0547414 |
| Afap1 | -0.6474542 |
| Afap1 | 0.1721689 |
| Afap1 | 0.2531319 |
| Afap1l1 | 0.6917928 |
| Aff1 | 0.9506072 |
| Aff4 | -0.5659312 |
| Afg3l1 | -0.8924803 |
| Afg3l1 | -0.5445301 |
| Afg3l1 | 1.4283001 |
| Afg3l1 | -0.5379797 |
| Afg3l2 | 3.0055972 |
| Afmid | -0.5416136 |
| Aftph | 0.8295742 |
| Agbl1 | 1.5395083 |
| Ager | 0.545874 |
| Ager | 1.2036445 |
| Aggf1 | -0.5696674 |
| Aggf1 | -0.6450311 |
| Aggf1 | -0.3474993 |
| Agpat1 | -0.4794954 |
| Agpat2 | 1.4584006 |
| Agpat3 | -0.4822375 |
| Agpat4 | -0.6711822 |
| Agpat5 | 0.033151 |
| Agpat6 | -0.4726727 |
| Agpat6 | -0.6171841 |
| Agpat7 | 1.2390037 |
| Agpat9 | -0.6563889 |
| Agpat9 | 2.2666429 |
| Agps | -0.6322056 |
| Agps | -0.5293115 |
| Agrn | -0.3421084 |
| Agrp | -0.4558152 |
| Agt | 2.232215 |
| Agtpbp1 | 1.7500745 |
| Agtr1a | -0.6912504 |
| Agtrap | -0.281256 |
| Agtrl1 | 1.8812323 |
| Agxt2l1 | 0.0315027 |
| Agxt2l1 | 0.6214705 |
| Agxt2l2 | 0.6417141 |
| Agxt2l2 | -0.4905087 |
| Agxt2l2 | 1.3324272 |
| Agxt2l2 | -0.4139018 |
| Ahctf1 | -0.2383157 |
| Ahcy | 0.0872603 |
| Ahcyl1 | -0.2002386 |
| Ahdc1 | -0.7639038 |
| Ahi1 | -0.276207 |
| Ahnak | -0.7388046 |
| Ahnak | -0.0420543 |
| Ahnak2 | -0.4842989 |
| Ahrr | -0.4445109 |
| Ahsa1 | -0.4740336 |
| Ahsa2 | 1.7840081 |
| AI118078 | -0.3829496 |
| AI132487 | -0.3693558 |
| AI314180 | -0.2496978 |
| AI314976 | -0.6769943 |
| AI316807 | 0.7873965 |
| AI317395 | -0.3045824 |
| AI413582 | 1.7138711 |
| AI413782 | -0.5614659 |
| AI427515 | -0.5293115 |
| AI428936 | 1.3167538 |
| AI449175 | 1.8614755 |
| AI449175 | -0.6361961 |
| AI450540 | -0.6761619 |
| AI451557 | -0.0222855 |
| AI451617 | 0.381058 |
| AI467606 | 0.8641154 |
| AI480653 | -0.3011349 |
| AI504432 | -0.6078109 |
| AI553587 | -0.2568089 |
| AI593442 | -0.2711832 |
| AI593442 | -0.5893219 |
| AI593442 | -0.5900855 |
| AI595366 | -0.4774438 |
| AI595406 | 2.0537344 |
| AI597468 | -0.6971797 |
| AI597479 | -0.5681712 |
| AI606181 | 0.987872 |
| AI646023 | 1.9777138 |
| AI661453 | 1.4323178 |
| AI662250 | -0.3998872 |
| AI747699 | 1.6012725 |
| AI836003 | 0.3141486 |
| AI837181 | -0.5143192 |
| AI842396 | -0.5016436 |
| AI846148 | 0.6622215 |
| AI847670 | 0.4024097 |
| AI851790 | -0.7405769 |
| AI894139 | 1.0259646 |
| AI987692 | 3.0367352 |
| AI987692 | 0.106969 |
| AI987944 | -0.4912011 |
| Aicda | 1.9069866 |
| Aif1 | -0.2154027 |
| Aif1 | -0.4418694 |
| Aif1 | 1.2508892 |
| Aifm1 | -0.5741691 |
| Aifm1 | 4.5745039 |
| Aifm2 | -0.6282308 |
| Aifm2 | -0.3231709 |
| Aifm2 | 1.1331322 |
| Aifm3 | 0.9148363 |
| Aig1 | 0.0030599 |
| Aig1-pending | -0.1837662 |
| Aim1 | 0.0841335 |
| Aim2 | -0.3373406 |
| Aip | -0.5336363 |
| Ak1 | 0.1322888 |
| AK122209 | -0.673669 |
| AK122525 | 0.356559 |
| AK129128 | 0.0699416 |
| AK129302 | -0.6820029 |
| AK129302 | -0.4665715 |
| AK129302 | -0.55776 |
| AK129341 | 0.0895991 |
| AK162044 | -0.51858 |
| Ak2 | -0.5121955 |
| Ak2 | 1.2032575 |
| Ak2 | 0.7248855 |
| Ak3 | 2.388397 |
| Ak3 | 4.6055931 |
| Ak3l1 | -0.5977539 |
| Ak5 | 0.7980592 |
| Akap1 | 3.2982185 |
| Akap1 | 0.2018609 |
| Akap1 | 0.4266687 |
| Akap10 | -0.7326263 |
| Akap10 | -0.2415546 |
| Akap12 | -0.5079615 |
| Akap14 | 0.0679546 |
| Akap2 | 0.5202929 |
| Akap2 | -0.4658958 |
| Akap3 | 1.7362509 |
| Akap4 | -0.3873136 |
| Akap4 | -0.4788111 |
| Akap4 | 0.5785236 |
| Akap4 | -0.5771815 |
| Akap6 | -0.1625467 |
| Akap7 | 1.7954767 |
| Akap8 | 1.1036572 |
| Akap8l | 2.8393338 |
| Akap9 | -0.4995464 |
| Akap9 | -0.3979912 |
| Akap9 | 1.9976885 |
| Akna | -0.4464966 |
| Akp2 | -0.4884345 |
| Akp5 | 1.2891697 |
| Akr1a4 | -0.6596576 |
| Akr1a4 | -0.7151796 |
| Akr1b3 | -0.3848175 |
| Akr1b8 | -0.2971276 |
| Akr1c12 | 0.3547647 |
| Akr1c14 | 0.1119318 |
| Akr1c14 | -0.6337999 |
| Akr1c18 | 1.1858171 |
| Akr1c19 | 1.4428524 |
| Akr1e1 | -0.5741691 |
| Akr7a5 | -0.5622088 |
| Akr7a5 | 0.0802113 |
| Akr7a5 | 0.7961289 |
| Akt1s1 | 1.8952966 |
| Akt3 | 1.1922296 |
| Akt3 | 2.6679018 |
| Aktip | -0.5839925 |
| Alad | -0.381085 |
| Alas1 | -0.5946796 |
| Alas2 | -0.2313334 |
| Alcam | -0.6093671 |
| Aldh16a1 | 0.4280613 |
| Aldh18a1 | -0.6637584 |
| Aldh1a1 | 0.0215554 |
| Aldh1a2 | -0.2948449 |
| Aldh1a2 | 2.6813385 |
| Aldh1a7 | -0.0953438 |
| Aldh1b1 | 2.5297496 |
| Aldh1l1 | -0.2540679 |
| Aldh1l2 | -0.8074918 |
| Aldh1l2 | -0.6563889 |
| Aldh2 | 1.0637084 |
| Aldh3a1 | -0.603931 |
| Aldh3a2 | 0.3442338 |
| Aldh3b1 | 0.3050709 |
| Aldh3b1 | -0.0157811 |
| Aldh3b1 | 2.9860326 |
| Aldh4a1 | -0.0209813 |
| Aldh5a1 | 0.8190523 |
| Aldh5a1 | 3.1554628 |
| Aldh6a1 | 0.4227591 |
| Aldh8a1 | 1.714493 |
| Aldh8a1 | -0.5855123 |
| Aldoa | -0.500245 |
| Aldoc | -0.3091975 |
| Alg1 | 1.5349683 |
| Alg10b | -0.6195411 |
| Alg11 | 2.2431781 |
| Alg11 | 2.2154252 |
| Alg12 | 0.0413528 |
| Alg12 | -0.0253353 |
| Alg14 | 0.2428166 |
| Alg2 | -0.4405512 |
| Alg3 | 0.0703386 |
| Alg3 | -0.1832557 |
| Alg3 | 0.4148942 |
| Alg5 | 1.528914 |
| Alg5 | -0.5614659 |
| Alg5 | -0.4491503 |
| Alg8 | -0.6402024 |
| Alg8 | 0.1088806 |
| Alg8 | -0.6148326 |
| Alg8 | -0.3214136 |
| Alg9 | 0.1533401 |
| Alg9 | 1.331064 |
| Alg9 | 1.0607403 |
| Alk | -0.5900855 |
| Alkbh2 | -0.2897276 |
| Alkbh3 | -0.4774438 |
| Alkbh4 | -0.6219036 |
| Alkbh5 | -0.4235701 |
| Alkbh7 | -0.2286608 |
| Alkbh8 | 1.525264 |
| Allc | -0.4464966 |
| Allc | 1.1719371 |
| Alox12 | -0.1019132 |
| Alox12b | -0.3841945 |
| Alox12e | 1.219386 |
| Alox15 | 1.4810867 |
| Alox5ap | 1.3456223 |
| Alox8 | -0.5962156 |
| Aloxe3 | -0.1720907 |
| Alpk3 | 0.2350945 |
| Als2 | 1.6445878 |
| Als2 | -0.613268 |
| Als2 | -0.7971202 |
| Als2cl | -0.6794955 |
| Als2cr13 | 0.0907664 |
| Als2cr2 | -0.4918939 |
| Als2cr2 | -0.2404738 |
| Als2cr4 | 1.2356355 |
| Als2cr4 | 0.1478428 |
| Als2cr4 | -0.515028 |
| Alx3 | 0.6196308 |
| Amac1 | -0.0518619 |
| Ambp | 0.7085803 |
| Ambra1 | 1.4624002 |
| Ambra1 | -0.581717 |
| Amd2 | 0.8614025 |
| Amdhd2 | -0.2238677 |
| Amdhd2 | -0.2756476 |
| Amfr | -0.7343877 |
| Amh | -0.1675586 |
| Amhr2 | 2.1251479 |
| Amhr2 | 2.2114923 |
| Amhr2 | 1.3860338 |
| Amigo1 | 1.6459197 |
| Amigo1 | 0.1714512 |
| Amigo2 | 0.5856906 |
| Amigo3 | 1.0967974 |
| Ammecr1l | -0.7229933 |
| Amn | -0.3468989 |
| Amotl2 | 1.6271962 |
| Ampd1 | -0.2054418 |
| Ampd1 | 1.8802437 |
| Ampd2 | 2.8538753 |
| Amph | -0.6219036 |
| Amph | 0.4349957 |
| Amy1 | -0.634598 |
| Amz2 | -0.5916145 |
| Anapc10 | 2.4227425 |
| Anapc11 | -0.4126197 |
| Anapc13 | -0.4478225 |
| Anapc2 | 0.9392711 |
| Anapc4 | 0.8675413 |
| Anapc5 | -0.0823321 |
| Anapc5 | 2.2369942 |
| Anapc5 | -0.1432348 |
| Anapc7 | 0.388901 |
| Ang | -0.2154027 |
| Angel1 | -0.5636961 |
| Angel2 | -0.1407855 |
| Angpt1 | -0.3208285 |
| Angpt2 | -0.7335066 |
| Angpt2 | 0.186417 |
| Angptl1 | -0.259007 |
| Angptl2 | 1.3969539 |
| Angptl3 | 1.1548896 |
| Angptl4 | -0.5016436 |
| Angptl7 | -0.7169107 |
| Ank | 1.9430631 |
| Ank1 | -0.5445301 |
| Ank2 | 0.4689699 |
| Ank2 | 0.5930434 |
| Ank2 | 0.6877133 |
| Ank3 | -0.6078109 |
| Ank3 | -0.5900855 |
| Ank3 | 2.5521448 |
| Ankar | -0.4808655 |
| Ankar | -0.2238677 |
| Ankfy1 | 1.8900862 |
| Ankle2 | 0.280564 |
| Ankle2 | 3.9241958 |
| Ankle2 | 0.9596837 |
| Ankmy1 | 0.9742003 |
| Ankmy1 | -0.5129029 |
| Ankmy1 | -0.6555733 |
| Ankmy2 | 1.0110872 |
| Ankmy2 | 1.8699045 |
| Ankrd10 | -0.1807074 |
| Ankrd12 | -0.4017869 |
| Ankrd12 | -0.6466458 |
| Ankrd13a | -0.5622088 |
| Ankrd13b | 0.2444875 |
| Ankrd13d | 0.174319 |
| Ankrd17 | 1.1885703 |
| Ankrd17 | -0.6853558 |
| Ankrd17 | -0.6211155 |
| Ankrd22 | 0.3212905 |
| Ankrd23 | 0.3529672 |
| Ankrd24 | 0.5518 |
| Ankrd25 | 1.5537365 |
| Ankrd27 | 2.5695366 |
| Ankrd34a | -0.2880276 |
| Ankrd35 | -0.5207172 |
| Ankrd36 | -0.6687015 |
| Ankrd37 | -0.0433861 |
| Ankrd39 | 1.2049335 |
| Ankrd40 | -0.5093708 |
| Ankrd40 | -0.4808655 |
| Ankrd40 | 0.6278832 |
| Ankrd46 | 0.5849762 |
| Ankrd47 | 0.0802113 |
| Ankrd49 | 0.2001178 |
| Ankrd5 | 2.0477766 |
| Ankrd50 | -0.4753963 |
| Ankrd53 | 0.9261092 |
| Ankrd54 | 1.1960056 |
| Ankrd54 | 0.2776603 |
| Ankrd54 | -0.620328 |
| Ankrd56 | -0.7521729 |
| Ankrd58 | 0.124797 |
| Ankrd6 | -0.6402024 |
| Ankrd6 | 1.9075001 |
| Ankrd6 | -0.5636961 |
| Ankrd7 | 1.9290886 |
| Ankrd9 | -0.2925673 |
| Anks1 | 2.3228093 |
| Anks3 | 4.9797671 |
| Anks3 | 1.2873907 |
| Anks6 | 3.3906641 |
| Anks6 | 1.0182434 |
| Ankzf1 | -0.5051487 |
| Ankzf1 | 0.6932916 |
| Anln | -0.6298188 |
| Anln | -0.4274635 |
| Anp32a | -0.6853558 |
| Anp32a | -0.0496245 |
| Anp32a | -0.2377769 |
| Anp32e | -0.1545791 |
| Anpep | 1.1067858 |
| Antxr1 | 2.7522285 |
| Antxr1 | 0.1441612 |
| Antxrl | 2.5423439 |
| Anxa1 | -0.4062334 |
| Anxa11 | 4.6293798 |
| Anxa11 | -0.4578232 |
| Anxa2 | 0.2541246 |
| Anxa3 | 3.038493 |
| Anxa3 | 3.335139 |
| Anxa4 | 1.6341215 |
| Anxa5 | -0.3910692 |
| Anxa6 | -0.6474542 |
| Anxa8 | -0.4584935 |
| Anxa8 | 0.3862934 |
| Anxa9 | -0.420337 |
| Aoah | 1.273887 |
| Aoc2 | 1.9893201 |
| Aoc2 | 0.6258264 |
| Aoc3 | -0.6728394 |
| Aof1 | -0.6179692 |
| Aof1 | -0.7005836 |
| Aof2 | 0.0294384 |
| Aox3 | -0.7776112 |
| Aox3l1 | -0.4672476 |
| Ap1b1 | -0.4209828 |
| Ap1g1 | -0.6523178 |
| Ap1g1 | -0.7849988 |
| Ap1g2 | 1.2230619 |
| Ap1gbp1 | 0.1019817 |
| Ap1gbp1 | 1.1334089 |
| Ap1m1 | -0.6621161 |
| Ap1m2 | 0.0703386 |
| Ap1m2 | -0.5651856 |
| Ap1m2 | 0.8425739 |
| Ap1m2 | 1.2771301 |
| Ap1s1 | -0.634598 |
| Ap1s1 | 0.3883221 |
| Ap1s1 | 1.1156916 |
| Ap1s3 | 0.6010466 |
| Ap2a1 | -0.6621161 |
| Ap2a1 | -0.5058511 |
| Ap2a2 | -0.5343589 |
| Ap2b1 | 3.0703697 |
| Ap2b1 | -0.4863646 |
| Ap2b1 | -0.1218821 |
| Ap2b1 | -0.3767479 |
| Ap2m1 | 1.9275797 |
| Ap2s1 | -0.6402024 |
| Ap2s1 | 1.5497179 |
| Ap2s1 | 2.0618074 |
| Ap3b1 | -0.7684522 |
| Ap3b1 | 3.0217754 |
| Ap3b2 | 2.7442266 |
| Ap3d1 | 1.6155186 |
| Ap3m1 | -0.0222855 |
| Ap3m2 | 3.0841911 |
| Ap3s2 | -0.2568089 |
| Ap3s2 | 0.211222 |
| Ap4b1 | -0.3231709 |
| Ap4m1 | 1.8178194 |
| Ap4m1 | -0.3547323 |
| Ap4m1 | -0.5207172 |
| Ap4s1 | 0.8215047 |
| Apaf1 | 1.2455828 |
| Apaf1 | 0.9964622 |
| Apba1 | -0.7014363 |
| Apba2 | -0.2159297 |
| Apba2 | 0.1602607 |
| Apba3 | -0.530751 |
| Apbb1 | -0.5178686 |
| Apbb1ip | 1.9567855 |
| Apbb1ip | -0.610146 |
| Apbb1ip | -0.6596576 |
| Apbb2 | 0.1577166 |
| Apbb3 | -0.267293 |
| Apc | -0.6171841 |
| Apc2 | 0.3314499 |
| Apcdd1 | -0.4808655 |
| Apeh | -0.5839925 |
| Apex1 | -0.4870541 |
| Aph1a | 0.732323 |
| Aph1b | 2.710411 |
| Apip | 1.68963 |
| Apip | -0.5847521 |
| Apitd1 | -0.4863646 |
| Aplp1 | -0.281256 |
| Aplp2 | 1.4298473 |
| Apoa1 | 1.1540771 |
| Apoa1bp | -0.3481001 |
| Apoa2 | -0.6258534 |
| Apoa4 | -0.3553374 |
| Apob48r | -0.6621161 |
| Apobec1 | 0.2414779 |
| Apobec3 | -0.4139018 |
| Apoc1 | -0.0906775 |
| Apoc1 | -0.535082 |
| Apoc2 | 1.6852393 |
| Apoc3 | -0.0402813 |
| Apoe | 0.079425 |
| Apof | -0.3149962 |
| Apoh | -0.5293115 |
| Apol7c | 0.8479108 |
| Apol9b | -0.2190974 |
| App | -0.3290507 |
| Appbp1 | -0.2535206 |
| Appbp1 | 0.5963465 |
| Appbp2 | -0.169067 |
| Appl1 | -0.527156 |
| Appl2 | -0.6474542 |
| Aprin | 1.1207455 |
| Aprt | -0.1393187 |
| Aptx | -0.785926 |
| Aqp1 | -0.6828401 |
| Aqp1 | -0.7648118 |
| Aqp1 | -0.7611845 |
| Aqp11 | 0.9364171 |
| Aqp11 | -0.613268 |
| Aqp3 | -0.7065681 |
| Aqp4 | -0.730868 |
| Aqp5 | 1.6591426 |
| Aqp7 | 1.3044576 |
| Aqp7 | 0.6734126 |
| Aqp8 | -0.6980296 |
| Aqp8 | 0.0742993 |
| Aqp9 | 1.6677268 |
| Aqr | -0.1175696 |
| Aqr | 0.3926555 |
| Ar | 0.3153943 |
| Araf | 0.1225386 |
| Araf | 2.3010209 |
| Arbp | 1.8601311 |
| Arc | 1.2924821 |
| Arcn1 | -0.3190753 |
| Ard1 | 0.8499278 |
| Ard1 | 0.0081379 |
| Areg | -0.6588394 |
| Arf2 | -0.5954473 |
| Arf3 | 1.662585 |
| Arf5 | -0.6580219 |
| Arf6 | -0.4464966 |
| Arfgap1 | -0.5726663 |
| Arfgap1 | 0.0623702 |
| Arfgap1 | -0.720382 |
| Arfgap2 | 0.0711319 |
| Arfgap2 | 2.0793328 |
| Arfgap2 | -0.2334767 |
| Arfgap2 | 0.1375002 |
| Arfgap3 | -0.2243992 |
| Arfip2 | -0.4836113 |
| Arfip2 | -0.4379201 |
| Arfip2 | -0.7766916 |
| Arfrp1 | 0.8595898 |
| Arfrp1 | -0.7282362 |
| Arg1 | 0.6439635 |
| Arg2 | 0.2882666 |
| Arglu1 | 2.6094142 |
| Arhgap1 | -0.7423522 |
| Arhgap1 | 2.8694223 |
| Arhgap1 | 2.4033441 |
| Arhgap10 | 1.1744629 |
| Arhgap12 | -0.3091975 |
| Arhgap15 | -0.7361521 |
| Arhgap15 | 0.3160166 |
| Arhgap17 | 0.3268447 |
| Arhgap18 | 0.6952154 |
| Arhgap19 | -0.4119792 |
| Arhgap20 | 0.5116195 |
| Arhgap22 | 0.7125764 |
| Arhgap24 | -0.3711987 |
| Arhgap24 | -0.4918939 |
| Arhgap25 | -0.5622088 |
| Arhgap26 | -0.125249 |
| Arhgap27 | 0.2705269 |
| Arhgap28 | 1.6238857 |
| Arhgap29 | -0.2645234 |
| Arhgap30 | 1.9225972 |
| Arhgap4 | 1.3737962 |
| Arhgap6 | 0.5562217 |
| Arhgap8 | 2.4149347 |
| Arhgap9 | 0.4188343 |
| Arhgdib | 0.2993952 |
| Arhgdig | -0.603931 |
| Arhgef1 | -0.4692786 |
| Arhgef1 | 1.7271589 |
| Arhgef10 | 2.6336615 |
| Arhgef10l | -0.5200043 |
| Arhgef10l | -0.6314094 |
| Arhgef15 | -0.3272832 |
| Arhgef18 | -0.1383421 |
| Arhgef19 | 0.6378786 |
| Arhgef2 | 1.7303007 |
| Arhgef3 | -0.6547585 |
| Arhgef4 | -0.5438002 |
| Arhgef6 | 0.1718101 |
| Arid2 | 3.5411488 |
| Arid3a | -0.5916145 |
| Arid3b | 1.592364 |
| Arid4b | -0.5250052 |
| Arid5a | -0.5533307 |
| Arid5b | -0.5946796 |
| Arih1 | -0.4774438 |
| Arih2 | -0.810339 |
| Arl1 | 0.3868735 |
| Arl11 | -0.5474551 |
| Arl16 | 2.69501 |
| Arl2 | -0.586273 |
| Arl2bp | 0.0829585 |
| Arl2bp | 0.4137656 |
| Arl2bp | -0.3583683 |
| Arl3 | -0.0972164 |
| Arl3 | -0.6078109 |
| Arl4a | -0.3314123 |
| Arl4a | 0.1426848 |
| Arl4c | -0.6645806 |
| Arl5a | -0.8065445 |
| Arl5b | 1.4844118 |
| Arl6 | -0.0667546 |
| Arl6ip1 | 0.8745372 |
| Arl6ip2 | 0.2869869 |
| Arl6ip2 | 2.9069867 |
| Arl6ip2 | -0.730868 |
| Arl6ip2 | 1.2628794 |
| Arl6ip4 | 0.7819246 |
| Arl6ip4 | -0.4571534 |
| Arl6ip5 | 1.2128888 |
| Arl6ip5 | 0.5562217 |
| Arl8a | 1.7002481 |
| Arl8b | -0.5372545 |
| Armc10 | 1.0571671 |
| Armc3 | 0.830695 |
| Armc6 | 1.5943797 |
| Armc6 | 1.0132705 |
| Armc8 | 0.8297611 |
| Armc8 | -0.3261065 |
| Armc8 | 1.1030874 |
| Armc9 | -0.2111966 |
| Armc9 | -0.4551467 |
| Armcx1 | -0.5496544 |
| Armcx2 | -0.0505189 |
| Armcx3 | -0.1665542 |
| Armcx5 | 3.4827976 |
| Armcx6 | 1.4104925 |
| Armcx6 | 0.723433 |
| Armetl1 | 1.4345772 |
| Arnt | 2.4663714 |
| Arnt | -0.1710818 |
| Arnt2 | -0.1976469 |
| Arntl | 0.1232919 |
| Arpc1a | 0.5923342 |
| Arpc1a | -0.1610479 |
| Arpc1a | -0.4631978 |
| Arpc1b | 0.0699416 |
| Arpc1b | 4.0624998 |
| Arpc1b | -0.4196916 |
| Arpc3 | -0.3373406 |
| Arpc4 | -0.535082 |
| Arpc5 | -0.4856756 |
| Arpc5 | -0.581717 |
| Arpc5l | 1.2617842 |
| Arpp19 | 0.8655593 |
| Arpp21 | 0.0663622 |
| Arpp21 | -0.5093708 |
| Arrb1 | 1.0076466 |
| Arrb1 | -0.3063106 |
| Arrb1 | -0.2656303 |
| Arrb1 | -0.36568 |
| Arrb2 | -0.5802029 |
| Arrdc1 | -0.2169845 |
| Arrdc2 | -0.1481514 |
| Arrdc3 | 0.0798183 |
| Arrdc4 | -0.6929409 |
| Ars2 | 2.6611461 |
| Ars2 | 2.7185412 |
| Ars2 | -0.5992946 |
| Arsa | -0.5445301 |
| Arsb | 1.0123354 |
| Arsb | 0.1202751 |
| Arsg | 0.2222053 |
| Arsi | 0.2924144 |
| Arsj | -0.5394317 |
| Arsj | 1.7926757 |
| Art1 | -0.6971797 |
| Art2b | -0.4094215 |
| Art3 | -0.568919 |
| Art3 | -0.643419 |
| Art4 | -0.0179446 |
| Art5 | 0.1906525 |
| Artn | -0.0713113 |
| Artn | 0.9206593 |
| Arv1 | -0.5794467 |
| As3mt | -0.6008377 |
| As3mt | 1.834999 |
| Asah1 | 0.029025 |
| Asah1 | 1.0085861 |
| Asah2 | -0.1610479 |
| Asah3 | -0.386689 |
| Asah3l | 0.4163031 |
| Asb1 | -0.4672476 |
| Asb1 | -0.703999 |
| Asb1 | -0.4760783 |
| Asb12 | -0.577936 |
| Asb13 | -0.1000319 |
| Asb15 | -0.7100039 |
| Asb17 | 0.2924144 |
| Asb2 | 0.4075357 |
| Asb3 | -0.6179692 |
| Asb3 | -0.4036901 |
| Asb4 | -0.5786911 |
| Asb4 | 1.8526367 |
| Asb6 | -0.6980296 |
| Asb6 | -0.5704163 |
| Asb7 | -0.6418094 |
| Asb7 | -0.6580219 |
| Asb8 | -0.4307195 |
| Asb9 | -0.6637584 |
| Ascc1 | 0.4038362 |
| Ascc1 | -0.7238652 |
| Ascc2 | 2.3897903 |
| Ascc2 | -0.6353967 |
| Ascc2 | -0.2190974 |
| Ascc2 | -0.0855693 |
| Ascc3l1 | 1.4848023 |
| Ascl1 | 1.6841186 |
| Ascl2 | -0.6258534 |
| Asf1a | -0.6490728 |
| Asf1b | -0.8560283 |
| Asgr1 | -0.5696674 |
| Ash1l | -0.6904062 |
| Ash1l | 0.6219299 |
| Ash1l | 1.8802437 |
| Ash2l | -0.4445109 |
| Ash2l | -0.4471593 |
| Asl | -0.2982709 |
| Asl | 1.5857631 |
| Asna1 | -0.8441647 |
| Asns | 0.3323684 |
| Asns | 0.1500453 |
| Asns | 0.5584253 |
| Asnsd1 | -0.7282362 |
| Asnsd1 | -0.4836113 |
| Asph | -0.0672093 |
| Asph | -0.5423419 |
| Asph | -0.2367001 |
| Asph | -0.7776112 |
| Asph | -0.7933749 |
| Asphd1 | -0.0590551 |
| Asphd2 | -0.5051487 |
| Aspm | -0.2700701 |
| Asprv1 | 2.4846051 |
| Aspscr1 | -0.2880276 |
| Aspscr1 | 0.9367533 |
| Aspscr1 | 0.0102462 |
| Asrgl1 | -0.650694 |
| Ass1 | -0.3767479 |
| Aste1 | 0.4719056 |
| Astn1 | -0.705711 |
| Astn2 | 1.2959014 |
| Asz1 | 0.2850643 |
| Atad1 | 0.9840305 |
| Atad2 | 2.1331842 |
| Atad3a | -0.0577025 |
| Atad4 | 2.2824308 |
| Atad4 | -0.188885 |
| Atcay | 2.1640572 |
| Ate1 | 0.908634 |
| Ate1 | 1.0983736 |
| Atf2 | -0.3742778 |
| Atf2 | 0.1073516 |
| Atf2 | 0.810517 |
| Atf3 | -0.7730213 |
| Atf4 | -0.673669 |
| Atf4 | 2.6942455 |
| Atf5 | -0.2190974 |
| Atf7 | -0.2885939 |
| Atf7ip | 0.2692245 |
| Atg10 | -0.4524772 |
| Atg10 | -0.719513 |
| Atg12 | 0.6564681 |
| Atg16l1 | 1.1536705 |
| Atg2a | -0.2286608 |
| Atg2a | 1.6277045 |
| Atg2b | -0.4518109 |
| Atg2b | -0.6242717 |
| Atg3 | -0.7602797 |
| Atg3 | -0.5794467 |
| Atg4b | -0.5562814 |
| Atg4c | -0.3559429 |
| Atg4c | 1.874358 |
| Atg4d | -0.720382 |
| Atg5 | -0.1665542 |
| Atg5 | -0.1812165 |
| Atg7 | -0.3086194 |
| Atg9b | -0.0301466 |
| Athl1 | -0.5058511 |
| Atic | 2.7352008 |
| Atic | 0.0494883 |
| Atic | -0.6078109 |
| Atm | -0.61405 |
| Atm | 1.3106244 |
| Atmin | -0.7238652 |
| Atmin | 0.4893422 |
| Atmin | -0.6242717 |
| Atn1 | 1.4415288 |
| Atoh8 | 0.7364314 |
| Atox1 | 0.053128 |
| Atp10a | 1.0628189 |
| Atp10d | -0.5086659 |
| Atp11c | -0.4119792 |
| Atp11c | -0.5511233 |
| Atp12a | -0.4478225 |
| Atp13a1 | -0.6290245 |
| Atp13a1 | 0.3463488 |
| Atp13a2 | -0.5855123 |
| Atp13a4 | -0.5923799 |
| Atp13a5 | -0.7151796 |
| Atp1a1 | -0.5885588 |
| Atp1a2 | 1.1140013 |
| Atp1b1 | -0.6523178 |
| Atp1b2 | 0.6644257 |
| Atp1b3 | -0.4385772 |
| Atp2a1 | 1.1194844 |
| Atp2a2 | 2.4731333 |
| Atp2a2 | 2.7725725 |
| Atp2a3 | 1.5792964 |
| Atp2a3 | 1.6252451 |
| Atp2a3 | 3.4458764 |
| Atp2b1 | -0.7785317 |
| Atp2b2 | -0.484987 |
| Atp2b2 | -0.7575702 |
| Atp2b2 | -0.5651856 |
| Atp2b3 | 1.4728243 |
| Atp2b3 | 2.2503299 |
| Atp2b4 | -0.3415112 |
| Atp2b4 | 0.2799195 |
| Atp2c1 | 0.3795989 |
| Atp2c1 | 2.5606431 |
| Atp5a1 | 0.3360342 |
| Atp5b | 2.530338 |
| Atp5c1 | 0.7569267 |
| Atp5d | 0.393808 |
| Atp5e | 0.5174949 |
| Atp5f1 | -0.7151796 |
| Atp5g2 | 0.6210109 |
| Atp5g3 | 0.4743011 |
| Atp5h | -0.6861958 |
| Atp5j | 1.1533994 |
| Atp5j | -0.3474993 |
| Atp5j2 | -0.7877831 |
| Atp5l | -0.4891255 |
| Atp5o | -0.657205 |
| Atp5s | 0.9642733 |
| Atp5sl | 0.2789519 |
| Atp6ap1 | 0.5098246 |
| Atp6ap2 | -0.6887199 |
| Atp6ap2 | 2.6596252 |
| Atp6ap2 | -0.4068702 |
| Atp6ap2 | -0.2023166 |
| Atp6v0a1 | -0.2650767 |
| Atp6v0a1 | 0.9022194 |
| Atp6v0a2 | -0.6937873 |
| Atp6v0b | 1.4889903 |
| Atp6v0d1 | -0.6728394 |
| Atp6v0d1 | 0.4937851 |
| Atp6v0d1 | -0.7593757 |
| Atp6v0d1 | -0.2645234 |
| Atp6v0e | -0.636996 |
| Atp6v0e2 | 1.4852901 |
| Atp6v1a | 0.2357684 |
| Atp6v1b1 | 0.2917774 |
| Atp6v1b2 | 1.0996613 |
| Atp6v1c1 | 0.1703736 |
| Atp6v1c2 | -0.2756476 |
| Atp6v1d | -0.6596576 |
| Atp6v1e1 | 0.5564667 |
| Atp6v1e2 | 1.3593104 |
| Atp6v1g1 | 1.7276193 |
| Atp6v1g2 | 0.8992584 |
| Atp6v1g2 | 1.5542832 |
| Atp6v1h | 0.9003044 |
| Atp7a | -0.4431893 |
| Atp8a1 | 0.5326629 |
| Atp8a2 | 0.0806042 |
| Atp8b1 | 3.0102737 |
| Atp8b3 | 2.6502973 |
| Atp8b3 | -0.7602797 |
| Atp8b3 | 0.4687026 |
| Atp8b3 | 0.2474882 |
| Atp9a | 0.8425739 |
| Atp9a | 3.6422829 |
| Atp9b | 3.8213387 |
| Atp9b | 0.9923353 |
| Atpaf1 | -0.6226923 |
| Atpaf2 | -0.7273605 |
| Atpaf2 | -0.2075307 |
| Atpbd1b | 2.0144509 |
| Atpbd1c | 0.2965452 |
| Atpbd3 | 0.0735084 |
| Atpif1 | 1.4012043 |
| Atrx | 0.6387824 |
| Atxn1 | 2.570069 |
| Atxn10 | 1.9364743 |
| Atxn2 | 1.2838232 |
| Atxn2l | -0.4591642 |
| Atxn3 | -0.7134514 |
| Atxn3 | 1.2583692 |
| Atxn3 | 0.7047798 |
| Atxn7l1 | 2.6159204 |
| Atxn7l2 | -0.4912011 |
| AU014645 | -0.6803306 |
| AU017455 | 1.8957512 |
| AU017455 | -0.3879385 |
| AU018778 | 1.8056809 |
| AU018829 | -0.3144148 |
| AU019823 | 3.2977821 |
| AU021034 | 0.4830365 |
| AU021092 | 0.892608 |
| AU021838 | -0.5079615 |
| AU022252 | 0.9288231 |
| AU022751 | -0.7065681 |
| AU022870 | 1.7233142 |
| AU023871 | 0.4377562 |
| AU040320 | 2.5938404 |
| AU040320 | -0.6490728 |
| AU040320 | -0.5741691 |
| AU040829 | 0.2387951 |
| AU042651 | -0.6604764 |
| Aup1 | 0.540909 |
| Aup1 | 0.2067257 |
| Aurka | -0.2562601 |
| Aurkaip1 | -0.1771504 |
| Aurkc | -0.4145435 |
| Auts2 | 1.2228088 |
| Auts2 | 0.7117365 |
| AV028368 | 0.9889897 |
| AV249152 | 1.3776025 |
| AV249152 | 0.771287 |
| Aven | 0.2757198 |
| Avil | 2.1715435 |
| Avp | 0.1176279 |
| Avpi1 | 1.113014 |
| Avpi1 | 0.7425627 |
| Avpr1a | -0.4352957 |
| AW049604 | -0.7639038 |
| AW061290 | 0.6217002 |
| AW111922 | -0.3086194 |
| AW121567 | 1.1615012 |
| AW125753 | -0.5086659 |
| AW146242 | 0.837765 |
| AW146299 | -0.5235738 |
| AW146299 | 2.2752811 |
| AW540478 | -0.5336363 |
| AW544981 | -0.5107822 |
| AW548124 | -0.4346407 |
| AW549877 | -0.4255149 |
| AW551984 | 1.1517712 |
| AW551984 | 0.2993952 |
| AW551984 | -0.4209828 |
| AW555464 | 1.2740073 |
| Axin2 | -0.6997315 |
| Axl | -0.5659312 |
| Axud1 | -0.2111966 |
| Azgp1 | -0.4248662 |
| Azgp1 | 1.9880766 |
| Azi1 | 3.8753782 |
| Azi2 | 1.2503967 |
| Azin1 | 0.079425 |
| B020018G12Rik | -0.417758 |
| B230118H07Rik | 0.3274599 |
| B230120H23Rik | 0.7155109 |
| B230206H07Rik | 2.7252951 |
| B230209C24Rik | 0.4822455 |
| B230217C12Rik | 0.1198973 |
| B230219D22Rik | -0.6195411 |
| B230312A22Rik | -0.5570204 |
| B230312I18Rik | -0.6687015 |
| B230317C12Rik | -0.4822375 |
| B230317C12Rik | -0.5681712 |
| B230339H12Rik | 2.9519194 |
| B230339M05Rik | 1.3333349 |
| B230339M05Rik | 2.0377305 |
| B230340J04Rik | -0.6442247 |
| B230342M21Rik | 2.0200344 |
| B230373P09Rik | 1.0207206 |
| B230378H13Rik | -0.0897468 |
| B230380D07Rik | 0.4157398 |
| B230396O12Rik | 0.1382425 |
| B230399E16Rik | -0.0518619 |
| B2m | 0.3535667 |
| B2m | 0.9953528 |
| B3galnt1 | -0.385441 |
| B3galnt2 | -0.5962156 |
| B3galt1 | 2.619443 |
| B3galt4 | -0.6085887 |
| B3galt4 | -0.7666304 |
| B3galt5 | -0.6845165 |
| B3galt6 | -0.567424 |
| B3gat1 | 1.5392309 |
| B3gat1 | -0.5372545 |
| B3gat1 | 0.6509049 |
| B3gat2 | -0.3736612 |
| B3gat3 | 1.0564211 |
| B3gat3 | 0.8493781 |
| B3gnt1 | -0.2404738 |
| B3gnt3 | -0.3331871 |
| B3gnt5 | 0.4081036 |
| B3gnt7 | -0.7335066 |
| B3gnt8 | 0.1899478 |
| B3gnt8 | 1.6767228 |
| B430119L13Rik | 0.2377872 |
| B4galnt3 | -0.3711987 |
| B4galnt4 | -0.4808655 |
| B4galt1 | -0.6330024 |
| B4galt3 | -0.6637584 |
| B4galt3 | -0.4740336 |
| B4galt4 | -0.0967479 |
| B4galt7 | 2.146893 |
| B630005N14Rik | 1.5907837 |
| B830045N13Rik | 2.2912424 |
| B930006L02Rik | 0.4438027 |
| B930011P16Rik | -0.3214136 |
| B930037P14Rik | -0.5467231 |
| B930041F14Rik | 1.0972275 |
| B930041G04 | 0.7637334 |
| B930076A02 | -0.6195411 |
| B9d1 | 1.6409161 |
| B9d2 | 3.2691682 |
| Baalc | 0.6421644 |
| Baalc | -0.4960608 |
| Bace1 | 2.5837821 |
| Bace2 | -0.4235701 |
| Bace2 | 0.2371147 |
| Bace2 | 2.285303 |
| Bach1 | -0.304007 |
| Bach2 | -0.4458343 |
| Bad | 2.7824579 |
| Bag1 | -0.2650767 |
| Bag2 | 0.8449698 |
| Bag3 | 0.5601359 |
| Bag4 | -0.6274377 |
| Bag5 | 1.714493 |
| Bahcc1 | 2.0145086 |
| Bahd1 | 0.629024 |
| Bai1 | 2.292689 |
| Bai2 | 0.5238427 |
| Bai2 | 3.0843509 |
| Bai3 | -0.4713137 |
| Baiap2 | 0.4901277 |
| Baiap2 | 1.0143604 |
| Banf1 | -0.5221445 |
| Banf1 | 0.4811898 |
| Banf1 | -0.5525944 |
| Banf1 | -0.4544786 |
| Banf2 | 1.7810295 |
| Bap1 | 1.199897 |
| Bard1 | -0.1033265 |
| Barx2 | 0.8809522 |
| Basp1 | 0.7079479 |
| Bat1a | 1.0769576 |
| Bat1a | -0.6474542 |
| Bat2 | -0.281256 |
| Bat3 | -0.2546155 |
| Bat4 | 3.2162075 |
| Bat4 | -0.7379197 |
| Bat5 | -0.3325952 |
| Batf | 1.5113861 |
| Bax | -0.719513 |
| Baz1a | -0.6282308 |
| Baz1b | 2.2388903 |
| Baz2a | -0.461179 |
| BB014433 | -0.5681712 |
| BB128963 | 0.7304688 |
| BB146404 | -0.2340132 |
| Bbc3 | -0.3523155 |
| Bbc3 | 0.9261092 |
| Bbox1 | -0.6171841 |
| Bbs1 | 0.8989094 |
| Bbs12 | 1.3486429 |
| Bbs2 | 2.7431343 |
| Bbs4 | -0.5423419 |
| Bbs5 | -0.3063106 |
| Bbs7 | -0.2931363 |
| Bbs9 | -0.5387054 |
| Bbs9 | -0.6070337 |
| Bbx | 1.1954856 |
| BC002163 | 1.5132855 |
| BC002199 | 1.3149074 |
| BC002216 | 0.0298516 |
| BC003236 | -0.3349651 |
| BC003266 | -0.4511451 |
| BC003885 | -0.2874615 |
| BC003885 | 0.7840778 |
| BC003993 | 1.5639842 |
| BC003993 | -0.4359512 |
| BC004004 | -0.7803752 |
| BC004044 | -0.643419 |
| BC004044 | -0.0934747 |
| BC004728 | 0.2394664 |
| BC004728 | -0.6711822 |
| BC005537 | 2.4633063 |
| BC005682 | -0.5379797 |
| BC005764 | -0.610146 |
| BC006662 | 1.3765164 |
| BC006779 | -0.4145435 |
| BC006779 | 0.6120063 |
| BC008155 | 2.9805115 |
| BC008163 | -0.0205469 |
| BC008163 | 0.6143228 |
| BC010462 | 0.4522618 |
| BC011248 | -0.5847521 |
| BC011248 | -0.6954821 |
| BC011487 | -0.6258534 |
| BC013491 | -0.0939416 |
| BC013491 | -0.6124866 |
| BC013529 | -0.5704163 |
| BC013712 | 0.474567 |
| BC016201 | -0.2180404 |
| BC016423 | 2.2237143 |
| BC016495 | 0.0005113 |
| BC017158 | 1.6186003 |
| BC017612 | 1.6073409 |
| BC017643 | 0.5321609 |
| BC017647 | 1.9374093 |
| BC018242 | 2.6695309 |
| BC018242 | -0.8037079 |
| BC018465 | -0.2313334 |
| BC020002 | 0.2215224 |
| BC020535 | 0.124421 |
| BC021381 | -0.119484 |
| BC021395 | -0.7151796 |
| BC021614 | -0.55776 |
| BC021790 | 0.5909143 |
| BC021891 | -0.1924836 |
| BC022224 | -0.7169107 |
| BC022687 | 1.9261317 |
| BC023055 | -0.5394317 |
| BC023151 | 2.044028 |
| BC023744 | 0.5341672 |
| BC023814 | 1.338313 |
| BC023829 | 1.3886139 |
| BC024561 | 1.2396262 |
| BC024814 | -0.55776 |
| BC024868 | -0.3296406 |
| BC024997 | -0.0703983 |
| BC025076 | -0.6258534 |
| BC025546 | -0.7739376 |
| BC025575 | -0.8037079 |
| BC025920 | -0.8314681 |
| BC026439 | -0.1354178 |
| BC026585 | 4.0943375 |
| BC026590 | -0.4747147 |
| BC026590 | -0.5474551 |
| BC027072 | 0.4961293 |
| BC027231 | 0.1682151 |
| BC027231 | -0.4925872 |
| BC027231 | 0.2580855 |
| BC027344 | -0.4100603 |
| BC028528 | 1.8001278 |
| BC028528 | 1.2995437 |
| BC029169 | 2.6619665 |
| BC029169 | 4.4689449 |
| BC029214 | 2.6063379 |
| BC030183 | -0.8500791 |
| BC030307 | -0.2925673 |
| BC030476 | -0.6878779 |
| BC030499 | 0.0310902 |
| BC030500 | 0.133035 |
| BC030867 | -0.5533307 |
| BC031181 | -0.7177774 |
| BC031353 | 0.5401621 |
| BC031575 | -0.4747147 |
| BC031748 | -0.6954821 |
| BC031781 | 0.6569119 |
| BC031853 | 0.0821743 |
| BC032265 | 1.7211547 |
| BC034076 | -0.0380694 |
| BC037034 | -0.2233366 |
| BC037594 | -0.577936 |
| BC038156 | -0.7100039 |
| BC038286 | -0.6117058 |
| BC038613 | -0.4781272 |
| BC038822 | 0.1653299 |
| BC038822 | -0.3314123 |
| BC038822 | -0.6703546 |
| BC038925 | -0.500245 |
| BC039093 | -0.4036901 |
| BC040758 | -0.5969845 |
| BC043098 | -0.577936 |
| BC043118 | 0.1518771 |
| BC043301 | -0.1506188 |
| BC046331 | -0.6298188 |
| BC046404 | 1.6174885 |
| BC048355 | -0.6393999 |
| BC048502 | 0.0930971 |
| BC048546 | -0.3068873 |
| BC048546 | 1.5137598 |
| BC048562 | 0.7188541 |
| BC048644 | -0.5533307 |
| BC048651 | -0.535082 |
| BC048671 | -0.3468989 |
| BC049635 | -0.4242179 |
| BC049635 | 1.1934029 |
| BC049730 | -0.6047057 |
| BC049762 | 0.4165847 |
| BC049806 | 0.5913878 |
| BC049806 | -0.4287647 |
| BC049806 | -0.6117058 |
| BC050196 | 3.5170367 |
| BC050210 | -0.0958116 |
| BC050777 | 1.3513204 |
| BC050811 | 1.3239915 |
| BC050811 | -0.2420955 |
| BC051019 | -0.5562814 |
| BC051070 | 2.5937754 |
| BC051142 | 1.3541019 |
| BC051227 | 1.5841724 |
| BC051628 | 2.3144847 |
| BC051665 | 1.6988283 |
| BC052484 | -0.6778273 |
| BC053749 | 1.2777295 |
| BC053749 | 1.9409544 |
| BC054059 | -0.4287647 |
| BC055107 | -0.1132755 |
| BC055111 | -0.6442247 |
| BC055324 | 0.8782843 |
| BC055368 | -0.6980296 |
| BC056474 | -0.3823277 |
| BC056923 | 0.3147716 |
| BC057022 | 2.6036384 |
| BC057371 | 2.3433457 |
| BC057371 | -0.6402024 |
| BC057371 | 1.3622903 |
| BC057552 | -0.0218506 |
| BC057627 | -0.3898158 |
| BC057893 | -0.5065541 |
| BC060267 | 0.8011401 |
| BC060615 | 2.2327729 |
| BC060632 | 0.573958 |
| BC061039 | 0.8293872 |
| BC061237 | 0.1803862 |
| BC062115 | -0.1710818 |
| BC062650 | -0.7548679 |
| BC063749 | 1.1303619 |
| BC064033 | -0.1104229 |
| BC065085 | 1.0986599 |
| BC065397 | 0.2286705 |
| BC066028 | -0.6786611 |
| BC066107 | 0.1397254 |
| BC067047 | 0.2350945 |
| BC067068 | -0.610146 |
| BC068157 | 3.0997753 |
| BC068281 | 0.2885863 |
| BC072620 | -0.6786611 |
| BC085271 | 1.5532806 |
| BC087945 | 0.4735033 |
| BC088983 | 2.2584741 |
| BC089491 | -0.1295945 |
| BC099439 | -0.4686011 |
| BC107230 | -0.4294159 |
| Bcam | 1.4465086 |
| Bcan | 1.5746646 |
| Bcan | 0.1500453 |
| Bcap29 | -0.5314716 |
| Bcap29 | 1.5082443 |
| Bcap31 | 0.374622 |
| Bcap31 | -0.5946796 |
| Bcar1 | -0.4767608 |
| Bcar3 | -0.4726727 |
| Bcas1 | -0.3367462 |
| Bcas2 | -0.4242179 |
| Bcas3 | 0.0466483 |
| Bcat1 | 0.7173928 |
| Bcat2 | -0.6555733 |
| Bccip | 0.0490831 |
| Bcdin3d | -0.1329873 |
| Bcdo2 | -0.6515056 |
| Bckdha | -0.2286608 |
| Bckdhb | -0.5644406 |
| Bckdk | -0.0354215 |
| Bckdk | -0.6803306 |
| Bcl10 | -0.4692786 |
| Bcl11a | 0.7860313 |
| Bcl11b | 2.7499766 |
| Bcl2 | 0.6152479 |
| Bcl2 | 0.053128 |
| Bcl2l1 | 0.5913878 |
| Bcl2l1 | 1.2703922 |
| Bcl2l1 | -0.634598 |
| Bcl2l10 | 0.6459837 |
| Bcl2l11 | -0.5343589 |
| Bcl2l11 | -0.2169845 |
| Bcl2l11 | 0.7329404 |
| Bcl2l12 | 2.4488906 |
| Bcl2l13 | -0.3272832 |
| Bcl2l14 | -0.3051581 |
| Bcl2l14 | 1.104796 |
| Bcl2l2 | -0.7657207 |
| Bcl2l2 | -0.3879385 |
| Bcl3 | -0.4151856 |
| Bcl6 | -0.7721059 |
| Bcl6b | 0.2001178 |
| Bcl7a | 1.0060788 |
| Bcl7b | -0.6870365 |
| Bcl9l | -0.6330024 |
| Bclaf1 | -0.7539688 |
| Bclaf1 | -0.4953651 |
| Bclp2 | -0.2023166 |
| Bcmo1 | -0.6853558 |
| Bcor | -0.4856756 |
| Bcor | -0.6482631 |
| Bcorl1 | 0.4429803 |
| Bcs1l | 1.2894066 |
| Bdh1 | 1.7484978 |
| Bdh2 | 0.1671341 |
| Bdkrb1 | -0.1511129 |
| Bdnf | -0.6929409 |
| Bdnf | -0.294275 |
| Becn1 | -0.6654034 |
| Becn1 | 0.1573526 |
| Bet1 | -0.3487012 |
| Bet1l | 1.6771261 |
| Bex2 | 3.0073669 |
| Bex2 | -0.5533307 |
| Bex2 | 0.674939 |
| Bex4 | -0.5636961 |
| Bex4 | 2.4349888 |
| Bex6 | -0.810339 |
| Bfar | -0.2291947 |
| Bfar | 1.0486296 |
| Bglap2 | 1.2174794 |
| Bgn | 0.8034445 |
| Bhlhb2 | 0.1478428 |
| Bhlhb5 | -0.6402024 |
| Bhlhb8 | 0.3194322 |
| Bhlhb8 | -0.1858106 |
| Bhlhb9 | 1.4557924 |
| Bhmt | -0.6954821 |
| Bhmt2 | -0.6769943 |
| Bicc1 | -0.1720907 |
| Bicd1 | -0.6031568 |
| Bicd2 | 2.1428355 |
| Bicd2 | 0.4854058 |
| Bid | -0.1393187 |
| Bid | -0.1909398 |
| Bik | 1.8371347 |
| Bin1 | 1.8646279 |
| Bin1 | -0.2601079 |
| Bin1 | 0.5522922 |
| Bin3 | 0.8394322 |
| Birc2 | -0.6016101 |
| Birc2 | 0.3842607 |
| Birc2 | 1.0864193 |
| Birc4 | 0.3568577 |
| Birc5 | -0.1080519 |
| Birc5 | 1.7508245 |
| Birc5 | 0.7617362 |
| Blcap | 1.2391282 |
| Blm | 1.6684592 |
| Blm | 4.5031575 |
| Blmh | 0.174319 |
| Blnk | -0.8620129 |
| Bloc1s1 | -0.6234817 |
| Bloc1s3 | -0.8955783 |
| Blvra | -0.4571534 |
| Blvrb | 1.2873907 |
| Bmf | 1.2802431 |
| Bmi1 | 1.6784963 |
| Bmp1 | -0.4392348 |
| Bmp1 | 1.4708471 |
| Bmp1 | 0.2053381 |
| Bmp1 | 0.0286115 |
| Bmp15 | 0.6782021 |
| Bmp2k | 1.1776444 |
| Bmp2k | 0.5996389 |
| Bmp3 | 0.695429 |
| Bmp4 | 0.8201849 |
| Bmp6 | -0.5250052 |
| Bmp7 | 1.9640325 |
| Bmp8a | 1.9970427 |
| Bmper | -0.5756742 |
| Bmpr1a | 1.3087667 |
| Bmpr1a | 1.1843719 |
| Bmpr1b | 3.7417361 |
| Bmpr1b | 1.469956 |
| Bmpr2 | -0.6226923 |
| Bms1 | 1.2293683 |
| Bnc1 | 1.7496993 |
| Bnc2 | -0.436607 |
| Bnc2 | -0.8112899 |
| Bnip1 | 0.093485 |
| Bnip2 | -0.6458381 |
| Bnip3l | 1.3621801 |
| Bnipl | 2.8715464 |
| Bnipl | -0.5726663 |
| Bok | -0.5065541 |
| Bok | -0.5358057 |
| Bola1 | 1.3559889 |
| Bola2 | 1.9799263 |
| Bola3 | -0.7186448 |
| Boll | 1.8287731 |
| Bop1 | 0.5861666 |
| Bpi | 0.1642458 |
| Bpil1 | 2.468655 |
| Bpil3 | -0.5235738 |
| Bpnt1 | -0.1276608 |
| Bpnt1 | -0.5423419 |
| Bpnt1 | -0.4618515 |
| Bpnt1 | -0.2891606 |
| Brap | -0.7887129 |
| Brca2 | 0.6175571 |
| Brca2 | 0.1345256 |
| Brcc3 | -0.0804869 |
| Brcc3 | -0.6306138 |
| Brcc3 | 2.3601182 |
| Brd2 | 1.6886737 |
| Brd2 | -0.4268136 |
| Brd2 | -0.4652206 |
| Brd4 | 1.2742479 |
| Brd4 | -0.7091438 |
| Brd8 | -0.5143192 |
| Brd8 | -0.3718138 |
| Brd9 | 2.5690371 |
| Brd9 | -0.2249309 |
| Brdt | -0.3202437 |
| Brdt | -0.6148326 |
| Bre | 0.2170718 |
| Bre | 0.4956088 |
| Bre | -0.0906775 |
| Brf1 | 3.0732421 |
| Brf2 | 1.7457891 |
| Bri3 | 0.6964959 |
| Brip1 | -0.4981507 |
| Brms1 | 1.9060872 |
| Brp16 | 0.5591587 |
| Brp16 | 0.1452671 |
| Brp17 | 2.5383006 |
| Brp44 | 0.6699151 |
| Brpf1 | -0.1852991 |
| Brpf1 | -0.6720105 |
| Brs3 | -0.195062 |
| Brsk1 | -0.1447073 |
| Brsk2 | -0.6828401 |
| Brsk2 | -0.7803752 |
| Brunol4 | 0.6001083 |
| Brunol4 | -0.5051487 |
| Brwd1 | -0.5321926 |
| Brwd1 | -0.2529736 |
| Brwd2 | -0.4300675 |
| Bscl2 | -0.6482631 |
| Bsdc1 | -0.705711 |
| Bsg | 0.7345847 |
| Bsg | 1.1700719 |
| Bspry | -0.7739376 |
| Bst2 | -0.6654034 |
| Btbd1 | 1.8809687 |
| Btbd10 | -0.6870365 |
| Btbd11 | -0.4891255 |
| Btbd12 | -0.0722251 |
| Btbd14a | -0.5207172 |
| Btbd14b | -0.6803306 |
| Btbd16 | 0.0806042 |
| Btbd3 | 0.7661249 |
| Btbd3 | 0.233408 |
| Btbd6 | 2.4267389 |
| Btbd9 | -0.1005019 |
| Btc | -0.4774438 |
| Btd | 0.2590733 |
| Btf3 | -0.6298188 |
| Btg1 | 1.9351014 |
| Btg1 | 0.6235362 |
| Btg3 | -0.2475199 |
| Btg3 | -0.1180478 |
| Btg4 | 1.4538821 |
| Btk | 0.3801828 |
| Btla | 0.1004421 |
| Btrc | 0.5414066 |
| Btrc | 1.2689424 |
| Btrc | 0.9825861 |
| Bub1b | -0.1151817 |
| Bub3 | 1.3227316 |
| Bud13 | 1.1480982 |
| Bud31 | 0.0194707 |
| Bves | 1.3405677 |
| Bxdc1 | -0.4877441 |
| Bxdc1 | -0.2750884 |
| Bxdc2 | 0.1987211 |
| Bxdc2 | 0.2708523 |
| Bxdc5 | 2.8005935 |
| Bxdc5 | 2.66935 |
| Bxdc5 | -0.436607 |
| Bzrap1 | 1.5016431 |
| Bzrpl1 | -0.5474551 |
| Bzw1 | -0.577936 |
| Bzw2 | 3.2674536 |
| Bzw2 | 3.1503111 |
| C030006K11Rik | 3.1249761 |
| C030011O14Rik | -0.5379797 |
| C030014K22Rik | -0.6695277 |
| C030018G13Rik | 2.5857167 |
| C030030A07Rik | -0.4760783 |
| C030039L03Rik | 0.6849125 |
| C030045D06Rik | 0.1642458 |
| C030045D06Rik | -0.6946343 |
| C030046I01Rik | -0.6588394 |
| C030048B08Rik | -0.4405512 |
| C030048B08Rik | -0.4658958 |
| C130022K22Rik | 1.2376329 |
| C130032J12Rik | -0.567424 |
| C130034I18Rik | -0.5636961 |
| C130038G02Rik | -0.6187548 |
| C130060K24Rik | 1.9703839 |
| C130073D16Rik | 1.1483707 |
| C130074G19Rik | -0.7005836 |
| C130090K23Rik | -0.1089996 |
| C130090K23Rik | 2.5603408 |
| C1galt1 | 0.8181075 |
| C1galt1c1 | -0.7423522 |
| C1qa | 0.6943609 |
| C1qb | 0.8427584 |
| C1qbp | 1.5619972 |
| C1qc | -0.6728394 |
| C1qdc2 | 1.3839865 |
| C1ql2 | -0.4713137 |
| C1ql3 | 2.0675736 |
| C1qtnf1 | 0.5433946 |
| C1qtnf2 | 1.4848999 |
| C1qtnf3 | 1.7522479 |
| C1qtnf3 | -0.6937873 |
| C1qtnf3 | 1.835137 |
| C1qtnf4 | 1.4437677 |
| C1qtnf6 | -0.577936 |
| C1qtnf7 | -0.3804643 |
| C1qtnf9 | 0.5098246 |
| C1rl | 0.6459837 |
| C1s | 3.4329941 |
| C1s | -0.6117058 |
| C2 | 0.1168702 |
| C230021P08Rik | 0.3577534 |
| C230029F24Rik | -0.5401584 |
| C230052I12Rik | 0.8235751 |
| C230052I12Rik | -0.5749214 |
| C230055K05Rik | 3.5186484 |
| C230071H18Rik | -0.2180404 |
| C230078M08Rik | 0.9709563 |
| C230078M14Rik | -0.4863646 |
| C230090D14 | 0.8059351 |
| C230093N12Rik | 0.7852504 |
| C230096C10Rik | -0.5423419 |
| C2cd3 | -0.3144148 |
| C3 | -0.421629 |
| C330001K17Rik | -0.4235701 |
| C330005M16Rik | -0.5962156 |
| C330016O10Rik | -0.6523178 |
| C330019G07Rik | -0.55776 |
| C330023M02Rik | 0.3314499 |
| C330027C09Rik | -0.3138338 |
| C430003P19Rik | 1.3675665 |
| C430004E15Rik | 0.3280748 |
| C4b | -0.6954821 |
| C530028I08Rik | 2.6562099 |
| C530028I08Rik | 0.2211807 |
| C530028O21Rik | 1.5766269 |
| C530043G21Rik | -0.7186448 |
| C530044N13Rik | 3.7911876 |
| C6 | -0.598524 |
| C630004H02Rik | -0.6385979 |
| C630004H02Rik | 0.5002833 |
| C630007B19Rik | 0.0813896 |
| C630028N24Rik | 0.0802113 |
| C630035N08Rik | 0.4547044 |
| C630035N08Rik | -0.785926 |
| C730025P13Rik | -0.7091438 |
| C730027P07Rik | 0.2084574 |
| C78339 | 0.4171476 |
| C79127 | 3.6137357 |
| C79267 | 0.0735084 |
| C79407 | -0.7675409 |
| C79407 | 2.6762608 |
| C80008 | 1.6966948 |
| C80913 | -0.3167424 |
| C85492 | 0.4609199 |
| C85492 | -0.1042698 |
| C85492 | -0.6148326 |
| C85627 | -0.3541276 |
| C87436 | 2.0099965 |
| C87436 | 1.822299 |
| C87499 | -0.7629965 |
| C8b | 1.9396497 |
| C8g | 0.7518923 |
| C9 | 2.9748487 |
| C9 | 1.8292587 |
| C920005C14Rik | 2.4632694 |
| Cab39l | -0.5802029 |
| Cabc1 | 1.1706052 |
| Cables2 | -0.7186448 |
| Cabp2 | -0.3450998 |
| Cabp7 | 0.4737693 |
| Cabyr | 1.9534796 |
| Cabyr | 2.0681757 |
| Cachd1 | 1.9931004 |
| Cacna1a | -0.5540676 |
| Cacna1b | -0.6474542 |
| Cacna1b | -0.1315318 |
| Cacna1b | 0.8791744 |
| Cacna1d | -0.3499045 |
| Cacna1g | -0.4981507 |
| Cacna1h | 2.1635594 |
| Cacna2d1 | 0.4974293 |
| Cacna2d3 | -0.5086659 |
| Cacnb1 | -0.5293115 |
| Cacnb1 | -0.496757 |
| Cacnb2 | 1.7756311 |
| Cacnb2 | -0.4119792 |
| Cacnb3 | -0.5741691 |
| Cacnb3 | -0.098154 |
| Cacnb3 | 1.0656329 |
| Cacnb3 | -0.6187548 |
| Cacnb4 | 1.7494742 |
| Cacnb4 | 1.7344238 |
| Cacng2 | -0.2612099 |
| Cacng3 | -0.5136108 |
| Cacng3 | -0.436607 |
| Cacng4 | 1.1539416 |
| Cacng5 | -0.304007 |
| Cacng5 | -0.5214306 |
| Cacng5 | 0.0269557 |
| Cacng7 | 0.9506072 |
| Cacng8 | -0.5264386 |
| Cacybp | -0.0314627 |
| Cad | 0.0098249 |
| Cadm2 | -0.1286272 |
| Cadm3 | 1.5609118 |
| Cadps | -0.5285925 |
| Cadps2 | 2.4613813 |
| Cadps2 | 1.0294967 |
| Cage1 | -0.4706349 |
| Calb2 | 0.1685752 |
| Calb2 | -0.5607237 |
| Calca | 1.132302 |
| Calca | 1.0374368 |
| Calcoco1 | -0.6870365 |
| Cald1 | 1.9894976 |
| Cald1 | -0.6820029 |
| Calm2 | 2.2987593 |
| Calm3 | -0.5908497 |
| Calml4 | -0.6242717 |
| Caln1 | -0.0735974 |
| Calr | 0.1577166 |
| Calr3 | -0.6878779 |
| Calr3 | 1.2018371 |
| Calr3 | 0.0064481 |
| Calr4 | 2.129071 |
| Calu | -0.0690303 |
| Calu | 0.033151 |
| Calu | 1.6936051 |
| Caly | -0.5489208 |
| Camk1 | 3.203631 |
| Camk1d | 1.0768113 |
| Camk1g | 1.3887212 |
| Camk1g | 1.4470153 |
| Camk2a | 0.8120397 |
| Camk2a | 0.4196766 |
| Camk2a | 0.5306536 |
| Camk2b | -0.2891606 |
| Camk2d | -0.5481877 |
| Camk2d | -0.6828401 |
| Camk2d | -0.2756476 |
| Camk2g | 0.7194797 |
| Camk2g | 1.1390628 |
| Camk2n1 | 0.8968133 |
| Camk4 | -0.0685748 |
| Camkk1 | 1.021339 |
| Camkk2 | 1.6096718 |
| Camkk2 | 2.0975692 |
| Camkv | -0.6498831 |
| Caml | 0.8773934 |
| Camp | 1.3895797 |
| Camsap1l1 | 0.4199573 |
| Camta2 | -0.5257216 |
| Cant1 | -0.7803752 |
| Cant1 | -0.4524772 |
| Cap1 | -0.3132531 |
| Capg | -0.3979912 |
| Capg | 0.186417 |
| Capn1 | -0.2164569 |
| Capn10 | -0.5336363 |
| Capn11 | 0.382515 |
| Capn2 | -0.2829446 |
| Capn2 | -0.4418694 |
| Capn2 | 0.053935 |
| Capn3 | 0.3110276 |
| Capn5 | -0.4605069 |
| Capn6 | -0.484987 |
| Capn7 | -0.5136108 |
| Capn8 | 1.9444871 |
| Capns1 | -0.5079615 |
| Caprin1 | -0.7450211 |
| Caprin1 | -0.6878779 |
| Capsl | -0.6920953 |
| Capza1 | 1.2575137 |
| Capza3 | -0.0349809 |
| Capzb | -0.3474993 |
| Capzb | -0.6442247 |
| Capzb | 1.2151867 |
| Car11 | -0.5178686 |
| Car12 | -0.461179 |
| Car13 | 0.7460207 |
| Car14 | 2.3588063 |
| Car15 | 0.1725276 |
| Car2 | -0.3835718 |
| Car3 | 1.6613569 |
| Car4 | -0.2706265 |
| Car5b | -0.4524772 |
| Car5b | -0.6163997 |
| Car5b | -0.2513344 |
| Car7 | -0.4679241 |
| Car9 | 2.0772739 |
| Card10 | -0.3149962 |
| Card11 | -0.3167424 |
| Carhsp1 | 3.1924402 |
| Carm1 | 0.4399591 |
| Cart | 2.0185408 |
| Cartpt | 2.6861239 |
| Casc1 | 0.1924121 |
| Casc1 | 2.3996083 |
| Casc1 | 4.2067312 |
| Casc3 | -0.6402024 |
| Casc4 | -0.4011533 |
| Casc4 | -0.705711 |
| Casc4 | -0.6963305 |
| Cask | -0.6274377 |
| Caskin1 | -0.7352695 |
| Caskin1 | 2.5407011 |
| Caskin1 | -0.2976991 |
| Caskin2 | -0.603931 |
| Casp1 | 0.7760287 |
| Casp2 | 0.4665617 |
| Casp4 | -0.5794467 |
| Casp4 | -0.6290245 |
| Casp4 | 0.2039487 |
| Casp6 | -0.0827939 |
| Casp6 | -0.1786733 |
| Casp7 | -0.0170786 |
| Casp8 | 1.4445806 |
| Casp8 | -0.3462988 |
| Casp9 | 1.2009322 |
| Casq2 | -0.3879385 |
| Cass4 | 1.1789671 |
| Cast | -0.6654034 |
| Cat | -0.4822375 |
| Catsper1 | 3.2444853 |
| Catsper2 | -0.4591642 |
| Catsper3 | -0.613268 |
| Catsper4 | 2.08758 |
| Cav1 | -0.5764276 |
| Cav2 | -0.7247379 |
| Cbara1 | 1.2655517 |
| Cbfa2t3h | 0.5567118 |
| Cbfa2t3h | -0.3662917 |
| Cbfb | -0.6266453 |
| Cbfb | -0.1398074 |
| Cbfb | 0.5669494 |
| Cbl | 0.334203 |
| Cblb | -0.6016101 |
| Cblc | -0.4196916 |
| Cbln1 | -0.6266453 |
| Cbln2 | 0.3072696 |
| Cbln4 | 2.3864032 |
| Cbr1 | 0.4207986 |
| Cbr2 | 0.3053853 |
| Cbr3 | 0.9075966 |
| Cbr3 | -0.2852006 |
| Cbs | -0.7379197 |
| Cbs | 0.8837903 |
| Cbwd1 | 2.2924699 |
| Cbx2 | 1.5393234 |
| Cbx5 | 2.0972502 |
| Cbx7 | -0.0690303 |
| Cbx8 | -0.7361521 |
| Cby1 | 1.2546975 |
| Cc2d1a | 2.2708146 |
| Cc2d1b | -0.7247379 |
| Cc2d2a | 1.4522706 |
| Ccar1 | -0.4578232 |
| Ccbe1 | -0.4652206 |
| Ccbl1 | 2.0417834 |
| Ccbl1 | 1.1490517 |
| Ccbl2 | 0.3490616 |
| Ccbp2 | -0.119484 |
| Ccdc100 | 0.4919581 |
| Ccdc101 | -0.2138234 |
| Ccdc108 | 0.7809443 |
| Ccdc109a | -0.61405 |
| Ccdc109a | 0.2088034 |
| Ccdc109b | 0.2218639 |
| Ccdc11 | -0.6612959 |
| Ccdc110 | 0.08687 |
| Ccdc111 | -0.2367001 |
| Ccdc113 | -0.6612959 |
| Ccdc114 | 1.3009501 |
| Ccdc115 | 0.2978129 |
| Ccdc116 | -0.2329404 |
| Ccdc117 | -0.4645459 |
| Ccdc12 | -0.7785317 |
| Ccdc120 | 2.0157776 |
| Ccdc123 | -0.31616 |
| Ccdc124 | -0.6282308 |
| Ccdc125 | 1.4471167 |
| Ccdc126 | -0.311513 |
| Ccdc127 | -0.5058511 |
| Ccdc127 | 1.2528565 |
| Ccdc13 | -0.5592407 |
| Ccdc130 | 1.3144452 |
| Ccdc131 | 0.9785632 |
| Ccdc132 | 1.1743301 |
| Ccdc134 | -0.3132531 |
| Ccdc137 | 4.0400776 |
| Ccdc137 | -0.6523178 |
| Ccdc18 | -0.3577614 |
| Ccdc21 | 1.8894984 |
| Ccdc23 | 0.0306775 |
| Ccdc25 | 0.666625 |
| Ccdc27 | -0.5533307 |
| Ccdc27 | 1.220655 |
| Ccdc27 | -0.7485906 |
| Ccdc28a | -0.5681712 |
| Ccdc28b | -0.0694861 |
| Ccdc28b | 0.3056996 |
| Ccdc3 | 1.5222592 |
| Ccdc32 | -0.0257717 |
| Ccdc38 | 0.0954224 |
| Ccdc39 | 0.3249967 |
| Ccdc40 | -0.6163997 |
| Ccdc41 | 1.8059649 |
| Ccdc42 | -0.515028 |
| Ccdc43 | 0.4188343 |
| Ccdc45 | -0.0860326 |
| Ccdc46 | -0.2185688 |
| Ccdc46 | -0.1992011 |
| Ccdc47 | -0.0586041 |
| Ccdc49 | -0.3705841 |
| Ccdc5 | -0.7602797 |
| Ccdc50 | -0.5824749 |
| Ccdc51 | 3.7589719 |
| Ccdc53 | 2.7425176 |
| Ccdc53 | -0.3517122 |
| Ccdc53 | 0.1471075 |
| Ccdc54 | 1.6581568 |
| Ccdc56 | 0.1606236 |
| Ccdc57 | 1.7339665 |
| Ccdc58 | -0.8037079 |
| Ccdc59 | -0.3462988 |
| Ccdc59 | -0.8490909 |
| Ccdc64 | -0.7485906 |
| Ccdc65 | 2.2881671 |
| Ccdc65 | 0.668381 |
| Ccdc66 | 0.2401374 |
| Ccdc67 | -0.826627 |
| Ccdc68 | 0.1696546 |
| Ccdc70 | -0.4151856 |
| Ccdc71 | -0.720382 |
| Ccdc73 | 0.3091503 |
| Ccdc77 | -0.2568089 |
| Ccdc79 | 2.7262644 |
| Ccdc80 | -0.5802029 |
| Ccdc82 | 1.443666 |
| Ccdc84 | 2.1103513 |
| Ccdc85a | -0.8227708 |
| Ccdc85b | 0.8282649 |
| Ccdc86 | 1.9227236 |
| Ccdc87 | -0.6845165 |
| Ccdc88a | -0.7143151 |
| Ccdc88b | 1.2063495 |
| Ccdc88c | -0.8771315 |
| Ccdc90b | 0.0429851 |
| Ccdc91 | 1.3924717 |
| Ccdc92 | -0.3011349 |
| Ccdc93 | -0.5809597 |
| Ccdc94 | 2.7499209 |
| Ccdc95 | 1.1162544 |
| Ccdc96 | 3.0494515 |
| Ccdc97 | 0.6033884 |
| Ccdc98 | 1.152857 |
| Ccdc99 | 0.5659788 |
| Cchcr1 | -0.7961825 |
| Ccin | 0.0627701 |
| Cck | 1.0893128 |
| Cckbr | -0.6054811 |
| Ccl11 | -0.7125884 |
| Ccl17 | -0.5931459 |
| Ccl19 | -0.4856756 |
| Ccl21b | -0.01276 |
| Ccl21c | 1.3439402 |
| Ccl25 | -0.5503886 |
| Ccl25 | 0.5008014 |
| Ccl27 | 0.2626871 |
| Ccl27 | 0.0376702 |
| Ccl28 | 1.1210255 |
| Ccl3 | 0.9801743 |
| Ccl4 | 1.4534794 |
| Ccl4 | 0.0215554 |
| Ccl5 | 2.2679007 |
| Ccl6 | -0.8730777 |
| Ccl7 | -0.5885588 |
| Ccl9 | -0.3255187 |
| Ccm2 | -0.408145 |
| Ccna1 | 1.0953623 |
| Ccnb1 | -0.72999 |
| Ccnb1 | -0.3761298 |
| Ccnb3 | 0.371683 |
| Ccnc | -0.72999 |
| Ccnd1 | -0.7999382 |
| Ccnd1 | 0.1878308 |
| Ccnd1 | -0.5719157 |
| Ccnd2 | -0.5329142 |
| Ccnd3 | -0.6258534 |
| Ccnd3 | 1.5503583 |
| Ccndbp1 | 0.3215998 |
| Ccne1 | -0.6621161 |
| Ccne2 | 0.8823722 |
| Ccnf | -0.8690401 |
| Ccng1 | -0.6054811 |
| Ccng1 | -0.5794467 |
| Ccng1 | -0.6117058 |
| Ccng2 | 1.1779091 |
| Ccnk | -0.705711 |
| Ccnl1 | -0.61405 |
| Ccno | 0.9022194 |
| Ccr4 | 0.2098407 |
| Ccr5 | 0.6979878 |
| Ccr5 | -0.2404738 |
| Ccr5 | 1.5924517 |
| Ccr6 | 1.7095067 |
| Ccr6 | 0.9994674 |
| Ccrk | -0.7575702 |
| Ccrk | 1.9708059 |
| Ccrk | -0.5931459 |
| Ccrk | 2.0267853 |
| Ccrl2 | -0.7048546 |
| Ccrn4l | -0.6728394 |
| Ccrn4l | 1.0095247 |
| Ccs | 1.6438379 |
| Cct2 | 0.2659611 |
| Cct3 | 2.3290818 |
| Cct3 | -0.9541295 |
| Cct4 | -0.4145435 |
| Cct5 | -0.7822221 |
| Cct6a | -0.51858 |
| Cct6b | -0.8189293 |
| Cct7 | 0.3286893 |
| Cct8 | -0.6226923 |
| Cd109 | 0.3842607 |
| Cd109 | 3.4471459 |
| Cd14 | -0.7757728 |
| Cd151 | -0.7739376 |
| Cd151 | 0.4058299 |
| Cd151 | -0.703144 |
| Cd160 | -0.3103546 |
| Cd163 | -0.2745296 |
| Cd164 | 0.2300264 |
| Cd177 | -0.6385979 |
| Cd180 | 2.3862036 |
| Cd1d1 | -0.6410056 |
| Cd2 | 1.1489155 |
| Cd200 | -0.6761619 |
| Cd200 | -0.6515056 |
| Cd226 | -0.6997315 |
| Cd247 | -0.4524772 |
| Cd248 | 0.9609971 |
| Cd248 | 0.2235699 |
| Cd27 | -0.6929409 |
| Cd274 | -0.8373082 |
| Cd276 | -0.821809 |
| Cd2bp2 | 1.6889926 |
| Cd300lg | -0.8660224 |
| Cd320 | -0.3595833 |
| Cd34 | 0.2679204 |
| Cd3d | -0.7108646 |
| Cd3eap | 0.9665602 |
| Cd3g | -0.6794955 |
| Cd4 | 0.2132905 |
| Cd40 | -0.2033573 |
| Cd40 | -0.2404738 |
| Cd44 | 2.1680303 |
| Cd44 | -0.7611845 |
| Cd44 | -0.7566686 |
| Cd44 | -0.8600141 |
| Cd47 | -0.0385114 |
| Cd52 | -0.088817 |
| Cd53 | -0.720382 |
| Cd55 | 2.106776 |
| Cd59a | -0.6322056 |
| Cd59b | 1.1077792 |
| Cd59b | -0.7335066 |
| Cd6 | 0.8133702 |
| Cd6 | 0.4038362 |
| Cd6 | -0.7459123 |
| Cd63 | -0.3547323 |
| Cd63 | 1.4753889 |
| Cd63 | -0.9552262 |
| Cd68 | 0.4052607 |
| Cd74 | -0.3724292 |
| Cd74 | -0.1535875 |
| Cd79b | -0.6971797 |
| Cd79b | 0.3595424 |
| Cd81 | 0.1496786 |
| Cd82 | -0.1247673 |
| Cd83 | -0.8227708 |
| Cd84 | -0.703144 |
| Cd86 | -0.6612959 |
| Cd86 | -0.6523178 |
| Cd86 | -0.0482845 |
| Cd8b1 | 2.0126599 |
| Cd9 | -0.1640476 |
| Cd93 | 2.7810015 |
| Cd96 | 1.2343852 |
| Cd97 | -0.9080663 |
| Cd97 | -0.6337999 |
| Cd97 | -0.5365298 |
| Cd99l2 | -0.3355584 |
| Cdan1 | -0.6393999 |
| Cdc123 | 1.6520568 |
| Cdc14b | -0.9154231 |
| Cdc14b | -0.1761364 |
| Cdc14b | 1.3156002 |
| Cdc16 | -0.8570233 |
| Cdc20 | -0.6711822 |
| Cdc20 | 1.6264332 |
| Cdc20 | 0.5849762 |
| Cdc20 | 0.1566242 |
| Cdc23 | -0.7151796 |
| Cdc25a | -0.3462988 |
| Cdc25b | -0.7221221 |
| Cdc25c | -0.0179446 |
| Cdc25c | -0.7840723 |
| Cdc27 | 1.3582044 |
| Cdc2a | 1.7161238 |
| Cdc2l1 | -0.8986858 |
| Cdc2l1 | -0.4464966 |
| Cdc2l5 | 3.9424709 |
| Cdc2l6 | 3.1174837 |
| Cdc34 | 0.0679546 |
| Cdc37 | 1.1247984 |
| Cdc37l1 | 1.1759223 |
| Cdc37l1 | 2.1140185 |
| Cdc37l1 | -0.3308214 |
| Cdc37l1 | -0.0385114 |
| Cdc42 | 2.2927766 |
| Cdc42bpb | 1.6926525 |
| Cdc42ep1 | -0.1310472 |
| Cdc42ep2 | 0.4018385 |
| Cdc42ep3 | -0.6954821 |
| Cdc42ep4 | 2.2843318 |
| Cdc42ep5 | -0.7468043 |
| Cdc42se1 | 2.0252446 |
| Cdc42se1 | -0.7326263 |
| Cdc45l | -0.2012771 |
| Cdc5l | 0.6571337 |
| Cdc5l | 2.5203917 |
| Cdc5l | -0.567424 |
| Cdc5l | 1.0846792 |
| Cdc6 | 0.7754372 |
| Cdc7 | 0.1096443 |
| Cdc73 | 1.3308367 |
| Cdca2 | 0.8563187 |
| Cdca3 | -0.7326263 |
| Cdca3 | -0.1432348 |
| Cdca4 | -0.4932809 |
| Cdca4 | 1.6556059 |
| Cdca4 | 1.2463249 |
| Cdca5 | 0.2049909 |
| Cdca7 | -0.7432411 |
| Cdca8 | -0.6920953 |
| Cdcp1 | -0.5336363 |
| Cdgap | -0.6385979 |
| Cdh1 | 1.0776885 |
| Cdh11 | -0.4836113 |
| Cdh11 | -0.785926 |
| Cdh12 | -0.1899118 |
| Cdh13 | 1.9823723 |
| Cdh13 | 0.0813896 |
| Cdh16 | -0.0532067 |
| Cdh22 | -0.6555733 |
| Cdh23 | -0.5207172 |
| Cdh23 | 0.4622661 |
| Cdh23 | -0.4733529 |
| Cdh24 | 0.3478568 |
| Cdh26 | 0.52738 |
| Cdh3 | 0.505452 |
| Cdh5 | -0.5242892 |
| Cdh6 | -0.6377967 |
| Cdh7 | -0.0253353 |
| Cdh8 | 0.4459925 |
| Cdh8 | -0.1066319 |
| Cdh8 | -0.2540679 |
| Cdipt | -0.0851062 |
| Cdipt | -0.6604764 |
| Cdk10 | -0.6498831 |
| Cdk10 | 0.3819325 |
| Cdk10 | -0.6314094 |
| Cdk2 | 1.4942252 |
| Cdk2 | 0.5859287 |
| Cdk2 | 0.5077692 |
| Cdk2 | -0.4043253 |
| Cdk2ap2 | -0.6298188 |
| Cdk3 | 1.1281402 |
| Cdk5 | -0.6474542 |
| Cdk5r1 | 0.8763233 |
| Cdk5rap1 | -0.5555429 |
| Cdk5rap2 | 4.004222 |
| Cdk5rap3 | 0.9396064 |
| Cdk6 | -0.6393999 |
| Cdk8 | -0.7221221 |
| Cdk9 | -0.7108646 |
| Cdkal1 | -0.0613136 |
| Cdkl1 | -0.5496544 |
| Cdkl2 | -0.4412101 |
| Cdkl2 | -0.1089996 |
| Cdkl2 | -0.5503886 |
| Cdkl2 | 0.8650181 |
| Cdkl3 | 0.9020454 |
| Cdkl4 | 0.3044419 |
| Cdkn1a | 0.025712 |
| Cdkn1a | 1.4582003 |
| Cdkn1a | -0.381085 |
| Cdkn1b | 2.2335164 |
| Cdkn1b | 0.5416553 |
| Cdkn1c | 0.3946715 |
| Cdkn2a | 1.0570179 |
| Cdkn2a | -0.2948449 |
| Cdkn2aipnl | 0.9204885 |
| Cdkn2b | 0.0203051 |
| Cdkn2c | 0.113834 |
| Cdkn2c | 3.6396256 |
| Cdkn2d | -0.203878 |
| Cdo1 | 0.3830972 |
| Cdr2 | -0.5659312 |
| Cdrt4 | -0.8065445 |
| Cds1 | 1.3210108 |
| Cds2 | -0.4870541 |
| Cdsn | 0.8373941 |
| Cdt1 | 1.9128769 |
| Cdv3 | 0.2132905 |
| Cdv3 | 2.0174479 |
| Cdyl | -0.5977539 |
| Cdyl | 0.9179233 |
| Cdyl | 0.8211278 |
| Cdyl2 | -0.5741691 |
| Ceacam1 | -0.4960608 |
| Ceacam13 | -0.1873466 |
| Ceacam2 | -0.6744993 |
| Cebpa | 0.1482102 |
| Cebpb | -0.527874 |
| Cecr5 | 0.7127863 |
| Cecr6 | 0.440784 |
| Celsr1 | -0.385441 |
| Celsr2 | 0.0845249 |
| Celsr3 | 0.6551357 |
| Cend1 | -0.2007577 |
| Cenpa | 1.0271946 |
| Cenpb | -0.4229226 |
| Cenph | 0.1471075 |
| Cenpi | -0.3910692 |
| Cenpj | -0.7593757 |
| Cenpk | -0.4891255 |
| Cenpl | -0.6563889 |
| Cenpl | -0.3225848 |
| Cenpm | -0.1899118 |
| Cenpm | -0.657205 |
| Cenpm | 1.4225029 |
| Cenpn | -0.1402963 |
| Cenpp | -0.7657207 |
| Cenpq | 1.2578804 |
| Cenpt | -0.6418094 |
| Centa1 | -0.0855693 |
| Centa1 | 1.8461098 |
| Centa2 | -0.5562814 |
| Centb5 | 1.4861677 |
| Centb5 | 0.5069974 |
| Centd2 | 2.0940016 |
| Centd2 | 0.1412062 |
| Centd3 | 0.220839 |
| Centg1 | 1.8241131 |
| Centg1 | -0.0201127 |
| Centg2 | -0.0869599 |
| Centg2 | 0.7953558 |
| Centg3 | -0.3929523 |
| Centg3 | -0.4235701 |
| Cep135 | 0.7413394 |
| Cep164 | -0.5977539 |
| Cep170 | 2.6476207 |
| Cep55 | -0.6219036 |
| Cep57 | -0.5704163 |
| Cep57 | -0.5839925 |
| Cep63 | 0.1319156 |
| Cep68 | -0.6803306 |
| Cep70 | 0.4660257 |
| Cep72 | -0.0054606 |
| Cep78 | 1.7648948 |
| Cep97 | 2.4616036 |
| Cept1 | -0.2954151 |
| Cercam | 0.1753923 |
| Ces3 | 0.2283313 |
| Cetn2 | -0.1442162 |
| Cetn3 | 2.7605532 |
| Cetn4 | -0.4478225 |
| Cetn4 | 0.2847436 |
| Cfd | 1.8422144 |
| Cfdp1 | 1.8540588 |
| Cfh | 0.4940458 |
| Cfhr1 | -0.6937873 |
| Cfi | -0.2650767 |
| Cfl1 | -0.1123237 |
| Cfl2 | -0.6820029 |
| Cfl2 | 1.242733 |
| Cfp | 1.7712328 |
| Cftr | 0.2792745 |
| Cga | -0.1530921 |
| Cggbp1 | 0.6417141 |
| Cgnl1 | 2.5265936 |
| Cgrrf1 | 2.6775476 |
| Ch25h | 0.1540709 |
| Chac1 | 1.881825 |
| Chad | 0.188537 |
| Chaf1a | -0.6250622 |
| Chaf1b | 2.2786624 |
| Chat | 2.4244352 |
| Chchd1 | 0.6421644 |
| Chchd2 | 0.0758792 |
| Chchd4 | -0.5847521 |
| Chchd5 | 0.2514752 |
| Chchd6 | 0.7529012 |
| Chchd7 | -0.4132605 |
| Chchd8 | 0.5039042 |
| Chd1 | -0.4699565 |
| Chd1 | -0.6234817 |
| Chd1l | -0.4055969 |
| Chd1l | 0.0961963 |
| Chd4 | -0.5379797 |
| Chd4 | 1.1033723 |
| Chd4 | -0.7766916 |
| Chd5 | -0.6322056 |
| Chd8 | 0.9913806 |
| Chdh | 0.4177101 |
| Chdh | -0.6761619 |
| Chek1 | -0.3284612 |
| Chek1 | -0.720382 |
| Chek2 | 0.4856687 |
| Chek2 | 0.6491181 |
| Cherp | -0.3916965 |
| Chfr | -0.5900855 |
| Chfr | 0.2218639 |
| Chfr | -0.0266452 |
| Chga | 2.5713657 |
| Chga | -0.5809597 |
| Chga | 0.2053381 |
| Chgb | -0.6385979 |
| Chi3l1 | 4.4857086 |
| Chi3l3 | 1.375538 |
| Chi3l3 | 2.2838016 |
| Chia | 1.8989926 |
| Chia | 0.7756344 |
| Chic1 | 4.5222941 |
| Chic2 | -0.1822356 |
| Chic2 | 0.3613282 |
| Chit1 | -0.6008377 |
| Chka | 2.1684764 |
| Chka | -0.577936 |
| Chka | -0.2190974 |
| Chkb | 0.1039028 |
| Chl1 | 4.6021488 |
| Chl1 | -0.2329404 |
| Chl1 | 1.019173 |
| Chm | -0.7238652 |
| Chmp1b | -0.3823277 |
| Chmp2a | 2.138098 |
| Chmp2a | 0.8175402 |
| Chmp2b | -0.7065681 |
| Chmp4b | 0.3246883 |
| Chmp5 | 0.7868116 |
| Chmp7 | 1.8211809 |
| Chmp7 | -0.703999 |
| Chmp7 | -0.1771504 |
| Chn2 | 0.5240957 |
| Chodl | 2.695539 |
| Chordc1 | 1.4756844 |
| Chpf | 0.6358422 |
| Chpt1 | -0.6912504 |
| Chpt1 | 0.4825092 |
| Chpt1 | -0.5614659 |
| Chrac1 | -0.6853558 |
| Chrd | 1.4660858 |
| Chrm1 | 0.7190627 |
| Chrm2 | -0.214876 |
| Chrm3 | 1.3306092 |
| Chrna1 | 0.2070723 |
| Chrna4 | -0.4801802 |
| Chrna4 | 1.02919 |
| Chrna4 | -0.1575596 |
| Chrna5 | -0.4808655 |
| Chrna6 | -0.7125884 |
| Chrna6 | -0.2919988 |
| Chrna7 | -0.6195411 |
| Chrna9 | -0.3517122 |
| Chrnb1 | 0.3526673 |
| Chrnb1 | 0.7920632 |
| Chrnb2 | -0.4842989 |
| Chrnb2 | 0.2927328 |
| Chrnb3 | -0.6654034 |
| Chrnb3 | -0.4062334 |
| Chrnb3 | 0.9153515 |
| Chrne | 0.1703736 |
| Chst1 | 0.940109 |
| Chst10 | 1.8318217 |
| Chst12 | 0.9909028 |
| Chst14 | -0.6474542 |
| Chst2 | 0.5057097 |
| Chst3 | 0.573958 |
| Chst3 | -0.3456991 |
| Chst7 | 1.6560178 |
| Chst8 | 1.775119 |
| Chtf18 | -0.5870344 |
| Chuk | -0.3474993 |
| Churc1 | 3.7360992 |
| Ciao1 | 2.6707059 |
| Ciapin1 | 2.4559566 |
| Cib1 | 0.1721689 |
| Cib2 | 2.4040503 |
| Cib2 | -0.3237573 |
| Cic | 0.9446216 |
| Cidea | -0.1645485 |
| Cideb | 0.2377872 |
| Cidec | 1.8347232 |
| Ciita | -0.0218506 |
| Cirbp | 0.3493626 |
| Cirh1a | -0.2291947 |
| Cisd1 | -0.6047057 |
| Cish | -0.3687422 |
| Cit | 2.2378733 |
| Cited1 | 3.3532039 |
| Cited2 | 0.1718101 |
| Cited4 | -0.426164 |
| Ciz1 | 1.4467113 |
| Ciz1 | -0.6322056 |
| Ckap2l | -0.1899118 |
| Ckap5 | 0.2262934 |
| Ckb | 1.4545863 |
| Cklf | 1.3868945 |
| Cklf | -0.5992946 |
| Ckmt1 | 0.960833 |
| Ckmt2 | -0.3736612 |
| Ckmt2 | -0.5416136 |
| Ckt2 | -0.1238046 |
| Ckt2 | 1.1491879 |
| Clca5 | 2.0978881 |
| Clcc1 | -0.7256113 |
| Clcf1 | 1.0023062 |
| Clcf1 | -0.1565651 |
| Clcf1 | 0.7502759 |
| Clcn1 | 0.237451 |
| Clcn2 | -0.5644406 |
| Clcn3 | -0.673669 |
| Clcn3 | 0.2698759 |
| Clcn3 | 3.7302832 |
| Clcn4-2 | -0.6498831 |
| Clcn7 | -0.2070081 |
| Clcnka | 0.0466483 |
| Cldn1 | -0.5651856 |
| Cldn10 | 1.1422207 |
| Cldn10 | 1.4810867 |
| Cldn10 | 0.6237654 |
| Cldn11 | -0.567424 |
| Cldn12 | 0.1111698 |
| Cldn15 | -0.0703983 |
| Cldn15 | -0.5107822 |
| Cldn17 | -0.5445301 |
| Cldn2 | -0.386689 |
| Cldn23 | 0.5911511 |
| Cldn3 | -0.527156 |
| Cldn4 | 1.5727881 |
| Cldn5 | -0.125249 |
| Cldn6 | 0.0643682 |
| Cldn9 | 0.0643682 |
| Cldnd1 | -0.6211155 |
| Cldnd2 | -0.0093184 |
| Clec11a | 0.9711187 |
| Clec16a | -0.7721059 |
| Clec1a | -0.6482631 |
| Clec2d | 3.3435632 |
| Clec2e | 0.4846166 |
| Clec3b | 0.3891903 |
| Clec4a1 | 1.8138833 |
| Clec4a3 | -0.1550752 |
| Clec4b1 | 0.5698554 |
| Clec4b1 | 1.2438491 |
| Clec4d | -0.4478225 |
| Clec4g | -0.5764276 |
| Clec4n | 0.5508147 |
| Clec7a | 0.9155231 |
| Clecsf12 | -0.0622184 |
| Clgn | -0.5832334 |
| Clic1 | 1.862147 |
| Clic3 | -0.3779852 |
| Clic4 | 0.9680277 |
| Clic6 | 2.08333 |
| Clip1 | 0.2074187 |
| Clip1 | -0.4939752 |
| Clip4 | -0.6778273 |
| Clk1 | 0.5594031 |
| Clk2 | 2.1764218 |
| Clk2 | -0.5870344 |
| Clk2 | -0.6442247 |
| Clk3 | 1.6877164 |
| Clk4 | 1.3254786 |
| Clk4 | -0.6687015 |
| Clk4 | -0.7065681 |
| Clm3 | 0.3940959 |
| Clm3 | 1.1629795 |
| Clmn | -0.55776 |
| Clmn | -0.5931459 |
| Cln3 | -0.4699565 |
| Cln5 | 0.1485775 |
| Cln6 | 2.1308212 |
| Cln6 | 1.7523227 |
| Cln8 | -0.8392624 |
| Clns1a | -0.4498148 |
| Clns1a | -0.4372633 |
| Clns1a | 1.4870445 |
| Clock | -0.4981507 |
| Clock | 0.1149737 |
| Clp1 | -0.6811664 |
| Clpb | 1.5151814 |
| Clpp | 0.5902035 |
| Clptm1 | 1.8947118 |
| Clptm1l | -0.4281139 |
| Clpx | 1.076665 |
| Clpx | 0.1537056 |
| Clrn1 | 1.7661508 |
| Clspn | -0.6337999 |
| Clspn | -0.6920953 |
| Clspn | 1.6198816 |
| Clstn1 | -0.6971797 |
| Clstn3 | 1.9577018 |
| Clstn3 | 0.3610308 |
| Clta | 0.9386003 |
| Cltb | -0.4767608 |
| Cluap1 | 1.6429205 |
| Cma1 | 2.5228701 |
| Cma1 | 0.5371691 |
| Cmas | 0.5821135 |
| Cmbl | -0.5651856 |
| Cml1 | -0.4713137 |
| Cml3 | -0.6282308 |
| Cml4 | 1.8522978 |
| Cmpk | -0.667876 |
| Cmtm2a | 2.963787 |
| Cmtm2b | -0.567424 |
| Cmtm4 | -0.5855123 |
| Cmtm4 | -0.810339 |
| Cmtm5 | -0.3045824 |
| Cmtm6 | -0.3885639 |
| Cmtm7 | 4.8304809 |
| Cmtm7 | 0.8379504 |
| Cmtm7 | 3.0617848 |
| Cmtm8 | -0.8237334 |
| Cmtm8 | -0.4571534 |
| Cmya1 | -0.3638471 |
| CN716893 | 3.3291858 |
| Cnbp2 | 1.7794276 |
| Cnbp2 | -0.2954151 |
| Cndp1 | 0.9053451 |
| Cnga4 | -0.5372545 |
| Cnih | -0.1175696 |
| Cnih3 | 2.0501194 |
| Cnih4 | 0.5647643 |
| Cnksr2 | 1.667971 |
| Cnksr2 | -0.1422544 |
| Cnksr3 | 0.0380801 |
| Cnn1 | 1.3106244 |
| Cnn2 | -0.4801802 |
| Cnn2 | -0.007602 |
| Cnnm1 | 1.2726833 |
| Cnnm1 | -0.0699421 |
| Cnnm2 | -0.5481877 |
| Cnnm3 | -0.6498831 |
| Cno | -0.2914305 |
| Cnot10 | 1.1706052 |
| Cnot2 | -0.5847521 |
| Cnot2 | 0.8745372 |
| Cnot2 | 0.5552407 |
| Cnot2 | 0.3060138 |
| Cnot3 | 1.3934339 |
| Cnot3 | -0.5734174 |
| Cnot4 | -0.6515056 |
| Cnot4 | 1.6317621 |
| Cnot4 | -0.4891255 |
| Cnot6l | -0.2661842 |
| Cnot6l | -0.368129 |
| Cnot7 | -0.530031 |
| Cnp | 0.42639 |
| Cnp | 0.8961137 |
| Cnpy2 | 0.8207508 |
| Cnpy3 | -0.4925872 |
| Cnr1 | -0.119484 |
| Cnr1 | -0.6458381 |
| Cntd1 | -0.6054811 |
| Cntd1 | 3.5820178 |
| Cntfr | 0.3019217 |
| Cntln | -0.5009441 |
| Cntn1 | 0.4015528 |
| Cntn4 | 1.2765303 |
| Cntn6 | 0.3405978 |
| Cntnap1 | -0.5511233 |
| Cntnap2 | 1.6152614 |
| Cntnap4 | 2.3828043 |
| Cntnap4 | 0.9529253 |
| Coasy | -0.1899118 |
| Cobl | -0.683678 |
| Cobll1 | -0.7812982 |
| Cobll1 | 2.3823235 |
| Coch | 0.1642458 |
| Cog1 | 2.0669162 |
| Cog2 | -0.5900855 |
| Cog3 | -0.5423419 |
| Cog4 | 0.0849161 |
| Cog6 | 0.7401147 |
| Cog6 | -0.6778273 |
| Cog8 | -0.6580219 |
| Coil | 3.9660281 |
| Coil | -0.0011913 |
| Col11a1 | 2.8157525 |
| Col12a1 | 1.1555663 |
| Col14a1 | -0.0851062 |
| Col15a1 | -0.4753963 |
| Col16a1 | 0.9784019 |
| Col17a1 | 0.263015 |
| Col17a1 | -0.5214306 |
| Col17a1 | 2.5756098 |
| Col18a1 | 0.9649272 |
| Col18a1 | -0.1530921 |
| Col1a1 | -0.5741691 |
| Col1a2 | 0.3141486 |
| Col24a1 | -0.5408857 |
| Col24a1 | -0.7757728 |
| Col25a1 | 1.4838258 |
| Col25a1 | 1.7713797 |
| Col25a1 | 1.9208886 |
| Col27a1 | -0.7441307 |
| Col28a1 | -0.0986232 |
| Col2a1 | -0.5832334 |
| Col2a1 | 0.1807419 |
| Col3a1 | 0.9502756 |
| Col3a1 | -0.6753303 |
| Col4a1 | -0.0451646 |
| Col4a2 | 1.0656329 |
| Col4a3 | -0.8018213 |
| Col4a3bp | 1.2819154 |
| Col4a3bp | -0.6195411 |
| Col4a3bp | -0.1388303 |
| Col4a4 | 2.0968248 |
| Col4a4 | 0.6538016 |
| Col4a5 | -0.4713137 |
| Col4a6 | 1.2066068 |
| Col4a6 | 1.7283095 |
| Col5a1 | -0.7022898 |
| Col5a3 | 1.0293434 |
| Col6a1 | -0.4132605 |
| Col6a1 | 0.9227065 |
| Col6a1 | -0.4106995 |
| Col6a2 | -0.6187548 |
| Col7a1 | -0.2689584 |
| Col8a2 | -0.6171841 |
| Col9a2 | 0.2132905 |
| Colec12 | 0.4377562 |
| Commd1 | -0.4760783 |
| Commd1 | -0.7822221 |
| Commd10 | -0.2012771 |
| Commd2 | -0.527874 |
| Commd2 | -0.4268136 |
| Commd4 | 0.020722 |
| Commd5 | -0.5416136 |
| Commd6 | -0.6211155 |
| Commd8 | -0.581717 |
| Commd9 | -0.3998872 |
| Comp | -0.3998872 |
| Comtd1 | -0.5438002 |
| Copa | -0.5592407 |
| Copb2 | -0.3935808 |
| Cope | -0.3481001 |
| Cope | -0.6031568 |
| Cope | -0.7748548 |
| Cope | 1.4940318 |
| Copg | 0.817351 |
| Copg | -0.6109256 |
| Copg | -0.2164569 |
| Copg | -0.6920953 |
| Cops2 | -0.6070337 |
| Cops3 | -0.8227708 |
| Cops4 | -0.5931459 |
| Cops4 | 0.711106 |
| Cops5 | -0.6929409 |
| Cops5 | 0.2269732 |
| Cops6 | -0.2919988 |
| Cops7a | -0.6450311 |
| Cops7a | -0.5809597 |
| Cops7a | -0.500245 |
| Cops7b | -0.6988802 |
| Cops7b | 4.5042674 |
| Cops8 | 2.224652 |
| Copz1 | -0.4242179 |
| Copz2 | -0.4981507 |
| Copz2 | -0.3860648 |
| Copz2 | 2.3506508 |
| Copz2 | 1.0271946 |
| Coq10b | -0.7530704 |
| Coq10b | 1.6235455 |
| Coq2 | 0.0252971 |
| Coq3 | -0.6820029 |
| Coq3 | -0.6124866 |
| Coq4 | 0.3100894 |
| Coq4 | -0.5659312 |
| Coq5 | 1.4399995 |
| Coq5 | -0.5555429 |
| Coq5 | 2.8673186 |
| Coq6 | 0.9216834 |
| Coq6 | 0.8502941 |
| Coq7 | -0.4405512 |
| Coq9 | -0.1388303 |
| Corin | 0.4021241 |
| Coro1a | 1.7165892 |
| Coro1a | -0.0640304 |
| Coro1a | 0.1345256 |
| Coro1a | 2.9841147 |
| Coro1b | -0.5079615 |
| Coro1b | -0.5607237 |
| Coro1c | 1.5454994 |
| Coro2a | 1.7260071 |
| Coro2a | -0.6466458 |
| Coro2b | -0.530031 |
| Coro2b | 2.6985895 |
| Coro6 | -0.7620901 |
| Coro7 | 0.366075 |
| Cort | -0.0270822 |
| Cort | -0.6093671 |
| Cotl1 | -0.152102 |
| Cova1 | -0.6306138 |
| Cox10 | -0.3173251 |
| Cox11 | 2.1657974 |
| Cox15 | -0.6314094 |
| Cox17 | -0.5839925 |
| Cox18 | -0.3705841 |
| Cox18 | -0.1847879 |
| Cox19 | 1.3579831 |
| Cox4i1 | 1.2403727 |
| Cox4nb | -0.6794955 |
| Cox4nb | -0.2383157 |
| Cox5a | 0.5220693 |
| Cox5b | 2.4365851 |
| Cox6a1 | -0.3829496 |
| Cox6a1 | -0.5293115 |
| Cox6a2 | -0.5051487 |
| Cox6b1 | -0.2138234 |
| Cox6b1 | 1.2969602 |
| Cox6b2 | -0.2868958 |
| Cox6b2 | -0.6687015 |
| Cox6c | 1.317791 |
| Cox7a1 | 0.670134 |
| Cox7a2 | -0.3337794 |
| Cox7a2l | 0.6937195 |
| Cox7b | -0.4686011 |
| Cox7b2 | -0.5481877 |
| Cox8b | -0.3290507 |
| Cox8c | 1.1132962 |
| Cp | -0.5336363 |
| Cpa1 | -0.3022828 |
| Cpa2 | -0.4822375 |
| Cpa2 | -0.5401584 |
| Cpa3 | -0.2756476 |
| Cpa3 | -0.7396904 |
| Cpa3 | -0.5794467 |
| Cpa4 | 1.0251952 |
| Cpa5 | -0.7134514 |
| Cpa6 | -0.577936 |
| Cpb2 | -0.6937873 |
| Cpeb1 | 1.4716384 |
| Cpeb2 | -0.1315318 |
| Cpeb3 | -0.4753963 |
| Cpeb4 | 0.1899478 |
| Cphx | -0.5839925 |
| Cplx1 | -0.0505189 |
| Cplx2 | 0.427226 |
| Cpn1 | -0.7014363 |
| Cpne2 | -0.3017087 |
| Cpne4 | 2.1778954 |
| Cpne5 | -0.5786911 |
| Cpne6 | 0.8049779 |
| Cpne7 | 3.0596782 |
| Cpne8 | 3.6402474 |
| Cpne8 | -0.3817062 |
| Cpne9 | -0.5100763 |
| Cpsf1 | 0.7772106 |
| Cpsf1 | -0.6163997 |
| Cpsf2 | 0.0072933 |
| Cpsf3l | 0.6580203 |
| Cpsf4 | 2.2481805 |
| Cpsf4l | 1.3352612 |
| Cpsf4l | -0.433332 |
| Cpsf6 | -0.1501248 |
| Cpsf6 | -0.4464966 |
| Cpt1b | 2.3148276 |
| Cpt1b | -0.1373664 |
| Cpt1c | -0.3499045 |
| Cpt2 | -0.4767608 |
| Cpxcr1 | -0.4294159 |
| Cpxm1 | 0.7852504 |
| Cpxm2 | 0.0372602 |
| Cpz | -0.6845165 |
| Crabp2 | -0.6070337 |
| Crat | 0.8743585 |
| Crat | -0.1511129 |
| Crat | 0.4061144 |
| Crb1 | -0.636996 |
| Crbn | -0.3755121 |
| Crbn | 1.2850138 |
| Crbn | 3.8222137 |
| Crcp | 0.1555307 |
| Creb1 | -0.6274377 |
| Creb1 | -0.6226923 |
| Creb3 | 1.9240504 |
| Creb3l1 | -0.4774438 |
| Creb3l2 | -0.1635471 |
| Creb3l2 | 3.1947707 |
| Creb3l3 | -0.5923799 |
| Creb3l4 | 1.4473193 |
| Creb5 | 1.0103063 |
| Crebl1 | -0.1650495 |
| Crebl2 | 1.253716 |
| Creg2 | 2.3688885 |
| Creld1 | -0.51858 |
| Creld2 | 0.1471075 |
| Creld2 | 1.5857631 |
| Crem | -0.6887199 |
| Crem | -0.5228589 |
| Crem | 1.028116 |
| Crem | 0.88556 |
| Crhbp | -0.2739711 |
| Crhr1 | 0.1496786 |
| Crim1 | 0.3778452 |
| Crim2 | -0.4484862 |
| Crip2 | -0.4905087 |
| Crip3 | 0.8460737 |
| Crip3 | 0.6414889 |
| Cript | -0.6728394 |
| Cript | -0.673669 |
| Crisp2 | -0.5293115 |
| Crispld2 | -0.1161362 |
| Crk | 0.1471075 |
| Crk | 2.8518395 |
| Crk | -0.5885588 |
| Crkl | 1.331405 |
| Crkrs | 2.0193453 |
| Crkrs | -0.6211155 |
| Crlf3 | 1.6226946 |
| Crlf3 | -0.4713137 |
| Crls1 | -0.5636961 |
| Crls1 | 0.3877429 |
| Crls1 | -0.6753303 |
| Crls1 | -0.7065681 |
| Crmp1 | 1.4536808 |
| Crmp1 | 0.4081036 |
| Crnkl1 | -0.0550026 |
| Crocc | 0.7819246 |
| Crry | 2.0642826 |
| Crry | 0.8969882 |
| Crtac1 | 1.6563472 |
| Crtam | 1.8023385 |
| Crtap | -0.2228057 |
| Crtap | -0.2623132 |
| Crtc1 | -0.6794955 |
| Crtc3 | 0.366075 |
| Crxos1 | -0.6148326 |
| Cry2 | -0.0402813 |
| Cryab | 0.9131173 |
| Cryba2 | -0.6547585 |
| Cryba4 | -0.0874238 |
| Crybb1 | -0.6786611 |
| Crybb2 | 0.7661249 |
| Crybb3 | 0.4241572 |
| Crygn | -0.5562814 |
| Crygn | 0.4123531 |
| Cryl1 | -0.5540676 |
| Crym | 1.5324578 |
| Cryz | 0.7205215 |
| Cryz | 2.8855366 |
| Cryz | 0.3484594 |
| Cryzl1 | -0.6555733 |
| Cryzl1 | -0.1786733 |
| Cs | -0.7584725 |
| Csad | -0.6242717 |
| Csda | -0.5696674 |
| Csda | 1.2325068 |
| Csda | -0.2404738 |
| Csdc2 | -0.6904062 |
| Csde1 | 2.2045847 |
| Csde1 | 0.0587637 |
| Csde1 | 0.8001783 |
| Cse1l | -0.7100039 |
| Csf1 | 2.8029202 |
| Csf1r | 0.1689351 |
| Csf1r | -0.4953651 |
| Csf2ra | -0.3848175 |
| Csgalnact1 | -0.6670511 |
| Csgalnact2 | -0.5651856 |
| Csk | 3.5527912 |
| Csl | 0.3926555 |
| Csl | -0.5394317 |
| Csmd1 | 0.4174289 |
| Csnk1d | -0.1014425 |
| Csnk1d | 1.04878 |
| Csnk1d | 0.3903467 |
| Csnk1d | 1.2073781 |
| Csnk1d | -0.0093184 |
| Csnk1e | 1.3531014 |
| Csnk1e | 0.1262998 |
| Csnk1g1 | 4.0254371 |
| Csnk1g1 | -0.5704163 |
| Csnk1g1 | -0.554805 |
| Csnk1g2 | -0.707426 |
| Csnk1g2 | 0.5438909 |
| Csnk1g2 | 2.689735 |
| Csnk1g3 | -0.5423419 |
| Csnk2a1 | 0.0770625 |
| Csnk2a2 | 2.2089254 |
| Csnk2a2 | 0.9187791 |
| Csnrp2 | -0.4988484 |
| Csnrp3 | 1.9224707 |
| Cspg4 | -0.6937873 |
| Cspg5 | 1.5896409 |
| Cspp1 | 0.8196188 |
| Csprs | 1.2819154 |
| Csrp1 | 1.2989571 |
| Csrp2 | -0.6353967 |
| Csrp2bp | 0.6664053 |
| Cst11 | 0.4046911 |
| Cst3 | -0.1530921 |
| Cst8 | 4.0036522 |
| Cst9 | 0.1054371 |
| Cstad | 2.5671037 |
| Cstb | -0.5636961 |
| Cstf1 | 4.5171495 |
| Cstf2 | 0.1913567 |
| Cstf3 | -0.5562814 |
| Cstf3 | -0.515028 |
| Cstl1 | 0.0833503 |
| Ctage5 | 0.4179913 |
| Ctcf | -0.6811664 |
| Ctdp1 | -0.3986228 |
| Ctdp1 | 0.4098055 |
| Ctdp1 | 0.1356421 |
| Ctdsp1 | 1.6428371 |
| Ctdsp1 | 2.0571152 |
| Ctdsp1 | -0.6474542 |
| Ctdsp2 | -0.5877963 |
| Ctdspl | -0.6171841 |
| Ctdspl2 | -0.5681712 |
| Ctdspl2 | -0.0500716 |
| Ctf1 | -0.4190467 |
| Ctgf | -0.2350871 |
| Ctla4 | -0.4870541 |
| Ctnna1 | -0.4209828 |
| Ctnna3 | 0.4394088 |
| Ctnnal1 | -0.5321926 |
| Ctnnb1 | -0.826627 |
| Ctnnb1 | -0.4307195 |
| Ctnnb1 | 0.6542465 |
| Ctnnbip1 | 0.4308408 |
| Ctnnbl1 | 0.9407788 |
| Ctnnbl1 | -0.214876 |
| Ctnnd2 | -0.4036901 |
| Ctnnd2 | -0.5372545 |
| Ctnnd2 | -0.5908497 |
| Ctns | 1.3112043 |
| Ctns | -0.1175696 |
| Ctps | -0.6195411 |
| Ctps | 0.1452671 |
| Ctps | 1.3452861 |
| Ctps2 | -0.581717 |
| Ctps2 | 1.6859591 |
| Ctps2 | 1.1940542 |
| Ctr9 | -0.5192919 |
| Ctrb1 | -0.5030442 |
| Ctrl | -0.2464327 |
| Ctsa | -0.0791052 |
| Ctsa | -0.386689 |
| Ctsa | -0.4665715 |
| Ctsa | -0.5459915 |
| Ctsb | 0.8186745 |
| Ctsc | 1.1760549 |
| Ctsc | 2.0844615 |
| Ctsc | -0.568919 |
| Ctse | -0.5741691 |
| Ctsf | -0.5336363 |
| Ctsh | -0.568919 |
| Ctsk | -0.6912504 |
| Ctsl | 0.5468641 |
| Ctso | -0.4788111 |
| Ctsr | -0.3138338 |
| Ctss | -0.6266453 |
| Ctsw | 0.7142543 |
| Ctsz | 0.6170957 |
| Ctsz | -0.3553374 |
| Cttn | -0.5030442 |
| Cttn | 1.8071708 |
| Cttnbp2nl | -0.4287647 |
| Ctxn1 | 0.6593489 |
| Cuedc1 | 0.8940118 |
| Cuedc2 | 0.1115509 |
| Cugbp1 | 2.1897044 |
| Cugbp1 | -0.5459915 |
| Cugbp1 | 1.6144893 |
| Cugbp1 | -0.4870541 |
| Cugbp2 | 2.8682844 |
| Cugbp2 | 0.3412047 |
| Cul1 | 1.3559889 |
| Cul2 | -0.5908497 |
| Cul2 | -0.5802029 |
| Cul3 | 0.0115091 |
| Cul4a | 0.8340501 |
| Cul4a | 1.2164611 |
| Cul4a | -0.3632369 |
| Cul4b | -0.4196916 |
| Cul5 | 0.2398019 |
| Cul7 | 0.003908 |
| Cuta | 0.9618172 |
| Cuta | -0.4884345 |
| Cuta | -0.620328 |
| Cutc | -0.3361521 |
| Cutc | 0.1803862 |
| Cux1 | -0.6458381 |
| Cux1 | -0.7441307 |
| Cux1 | 0.2418127 |
| Cux1 | 0.4803974 |
| Cux1 | 1.7762161 |
| Cux2 | 4.2137173 |
| Cux2 | 0.2474882 |
| Cuzd1 | 0.0043318 |
| Cwf19l2 | 0.6239946 |
| Cx3cl1 | -0.6878779 |
| Cx3cr1 | 2.8211876 |
| Cxadr | -0.2442617 |
| Cxadr | -0.3415112 |
| Cxadr | 1.4138489 |
| Cxcl1 | 0.5625746 |
| Cxcl12 | -0.7980586 |
| Cxcl12 | -0.6853558 |
| Cxcl12 | 2.1535524 |
| Cxcl13 | -0.4918939 |
| Cxcl14 | -0.6621161 |
| Cxcl14 | -0.5336363 |
| Cxcl15 | 0.9833888 |
| Cxcl16 | -0.294275 |
| Cxcl17 | -0.5503886 |
| Cxcl4 | -0.2645234 |
| Cxcl9 | -0.5832334 |
| Cxcr3 | -0.7657207 |
| Cxcr4 | 0.7409313 |
| Cxcr5 | -0.5009441 |
| Cxcr7 | -0.0644839 |
| Cxx1a | -0.0487309 |
| Cxx1b | -0.7459123 |
| Cxx1c | -0.5086659 |
| Cxxc1 | -0.6266453 |
| Cxxc1 | -0.1104229 |
| Cxxc4 | -0.0505189 |
| Cxxc4 | 3.2237171 |
| Cyb5 | 1.5032735 |
| Cyb561 | -0.1349312 |
| Cyb561d1 | 1.7780421 |
| Cyb561d1 | 2.1697637 |
| Cyb561d2 | 1.0275018 |
| Cyb5b | 1.1185024 |
| Cyb5d1 | 2.2666879 |
| Cyb5d2 | 1.6580746 |
| Cyb5d2 | -0.6744993 |
| Cyb5r1 | -0.5585001 |
| Cyb5r2 | 0.5904405 |
| Cyb5r3 | -0.6330024 |
| Cyb5r3 | 2.0909029 |
| Cyb5r3 | -0.6458381 |
| Cyb5r4 | 0.0511076 |
| Cyba | 0.9577103 |
| Cybasc3 | -0.3923242 |
| Cyc1 | -0.7868541 |
| Cycs | -0.620328 |
| Cycs | -0.7343877 |
| Cyfip1 | 0.9966206 |
| Cyfip2 | 1.8220196 |
| Cyfip2 | -0.1151817 |
| Cygb | -0.5885588 |
| Cyhr1 | 0.9997832 |
| Cyhr1 | 3.3221404 |
| Cyhr1 | -0.3779852 |
| Cyhr1 | -0.4713137 |
| Cyld | -0.5704163 |
| Cyln2 | 4.509371 |
| Cyp11a1 | -0.2143495 |
| Cyp11b2 | 0.1994197 |
| Cyp17a1 | -0.6580219 |
| Cyp19a1 | -0.6337999 |
| Cyp1b1 | -0.4036901 |
| Cyp20a1 | -0.6563889 |
| Cyp24a1 | -0.6163997 |
| Cyp26a1 | 1.5358964 |
| Cyp26b1 | -0.7971202 |
| Cyp27a1 | 0.133035 |
| Cyp27a1 | -0.3904423 |
| Cyp27a1 | -0.4412101 |
| Cyp2a12 | 0.7708908 |
| Cyp2a5 | 1.7112235 |
| Cyp2a5 | -0.4242179 |
| Cyp2b10 | 0.2039487 |
| Cyp2c55 | 4.25209 |
| Cyp2d10 | 0.4168662 |
| Cyp2d22 | -0.0604096 |
| Cyp2d26 | 0.2398019 |
| Cyp2d26 | -0.2639704 |
| Cyp2d26 | 1.1755245 |
| Cyp2e1 | 2.3254841 |
| Cyp2f2 | -0.6474542 |
| Cyp2j11 | 2.4339612 |
| Cyp2j9 | -0.2291947 |
| Cyp2s1 | -0.8037079 |
| Cyp2u1 | -0.5977539 |
| Cyp39a1 | -0.1388303 |
| Cyp3a13 | 0.3424175 |
| Cyp3a13 | -0.6645806 |
| Cyp46a1 | -0.7684522 |
| Cyp4b1 | 0.8310684 |
| Cyp4f13 | 0.8885614 |
| Cyp4f14 | 1.6391589 |
| Cyp4f15 | 1.253225 |
| Cyp4f16 | -0.2388548 |
| Cyp4f16 | -0.6820029 |
| Cyp4f16 | 2.2389365 |
| Cyp4f18 | 2.6926264 |
| Cyp4f39 | 0.2344202 |
| Cyp4v3 | 3.4155528 |
| Cyp51 | -0.0749715 |
| Cyp7b1 | -0.6531307 |
| Cypt10 | -0.008889 |
| Cypt2 | -0.4932809 |
| Cypt3 | 1.1413978 |
| Cypt6 | -0.7396904 |
| Cypt8 | -0.2238677 |
| Cypt9 | 0.9305156 |
| Cyr61 | -0.0179446 |
| Cyr61 | -0.3547323 |
| Cysltr1 | -0.496757 |
| Cytl1 | 0.9379291 |
| D030011O10Rik | -0.3355584 |
| D030013I16Rik | 2.0219842 |
| D030018L15Rik | 1.9588004 |
| D030028O16Rik | -0.3942097 |
| D030051N19Rik | -0.7117262 |
| D030056L22Rik | -0.6703546 |
| D030070L09Rik | -0.7405769 |
| D030070L09Rik | -0.1660524 |
| D030070L09Rik | -0.6474542 |
| D030070L09Rik | 0.4063988 |
| D030074E01Rik | 2.5058179 |
| D0H4S114 | 0.2039487 |
| D0H8S2298E | 0.1906525 |
| D0HXS9928E | 0.6788534 |
| D0HXS9928E | -0.5474551 |
| D10627 | -0.667876 |
| D10Bwg1364e | -0.6078109 |
| D10Bwg1379e | -0.707426 |
| D10Ertd322e | 1.6235455 |
| D10Ertd610e | 0.4427061 |
| D10Ertd610e | -0.61405 |
| D10Ertd610e | 0.6642055 |
| D10Ertd641e | -0.5518586 |
| D10Jhu81e | 3.6180402 |
| D10Wsu102e | 0.7877862 |
| D10Wsu52e | -0.535082 |
| D11Bwg0517e | 2.416718 |
| D11Wsu47e | 0.2924144 |
| D11Wsu47e | 1.07725 |
| D12Ertd551e | -0.484987 |
| D12Ertd551e | -0.4932809 |
| D12Ertd553e | -0.2033573 |
| D12Ertd647e | 0.2662879 |
| D12Ertd647e | -0.7134514 |
| D130040H23Rik | 1.3202068 |
| D130043K22Rik | -0.6266453 |
| D130059P03Rik | 1.0117115 |
| D13Ertd608e | -0.6803306 |
| D14Ertd171e | 1.1610976 |
| D14Ertd231e | -0.5939124 |
| D14Ertd449e | -0.5908497 |
| D14Ertd500e | 1.0132705 |
| D14Ertd668e | -0.7530704 |
| D14Ertd668e | -0.6670511 |
| D15Ertd621e | -0.5496544 |
| D15Ertd682e | -0.6498831 |
| D15Mgi27 | 0.29114 |
| D15Wsu169e | 0.9272974 |
| D15Wsu75e | 0.4922193 |
| D16Ertd472e | -0.5481877 |
| D16H22S680E | 1.9028686 |
| D17H6S56E-5 | 1.3227316 |
| D17Wsu104e | 1.5267631 |
| D17Wsu92e | -0.3929523 |
| D19Bwg1357e | -0.5044467 |
| D19Ertd721e | -0.6498831 |
| D19Ertd737e | -0.1286272 |
| D19Wsu162e | 0.1775356 |
| D1Bwg0212e | 1.3387643 |
| D1Bwg0212e | 1.6798646 |
| D1Pas1 | -0.3541276 |
| D230007K08Rik | 0.6223891 |
| D230014K01Rik | -0.554805 |
| D2Ertd750e | 2.5414544 |
| D2Ertd750e | 0.8757878 |
| D330014H01Rik | -0.1909398 |
| D330017J20Rik | -0.6711822 |
| D330028D13Rik | -0.496757 |
| D330045A20Rik | -0.0869599 |
| D330045A20Rik | 1.2564126 |
| D330045A20Rik | 2.1918852 |
| D3Bwg0562e | -0.4898169 |
| D3Ertd300e | -0.6298188 |
| D3Ertd751e | -0.0473921 |
| D3Ucla1 | 0.5141783 |
| D430015B01Rik | -0.5771815 |
| D430028G21Rik | -0.5114886 |
| D430039N05Rik | -0.4658958 |
| D430041B17Rik | -0.527874 |
| D430042O09Rik | -0.4856756 |
| D4Bwg0951e | -0.7980586 |
| D4Bwg1540e | 0.9308538 |
| D4Ertd22e | -0.4346407 |
| D4Ertd22e | -0.4287647 |
| D4Wsu114e | -0.3935808 |
| D4Wsu132e | 3.4736457 |
| D4Wsu132e | 0.2434853 |
| D530004J12Rik | -0.6031568 |
| D530033C11Rik | 1.6688658 |
| D5Ertd135e | 0.6538016 |
| D5Ertd579e | -0.4100603 |
| D5Wsu178e | -0.321999 |
| D630003M21Rik | -0.6778273 |
| D630003M21Rik | 1.2459539 |
| D630014A15Rik | -0.5235738 |
| D630023F18Rik | -0.0131911 |
| D630042P16Rik | -0.3910692 |
| D630048P19Rik | -0.3415112 |
| D6Ertd474e | -0.5946796 |
| D6Mm5e | 1.8682376 |
| D6Wsu116e | 0.3681447 |
| D6Wsu163e | -0.5058511 |
| D6Wsu163e | -0.5257216 |
| D6Wsu163e | -0.0590551 |
| D6Wsu176e | 1.9555623 |
| D6Wsu176e | 0.1039028 |
| D730039F16Rik | 0.1445299 |
| D730039F16Rik | -0.1276608 |
| D7Bwg0611e | -0.5171577 |
| D830014E11Rik | -0.420337 |
| D830030K20Rik | 1.5294744 |
| D830030K20Rik | -0.0473921 |
| D830050J10Rik | 0.9835493 |
| D8Ertd738e | 2.5750802 |
| D8Ertd82e | 2.2372719 |
| D930001I22Rik | -0.3266947 |
| D930005D10Rik | 0.3577534 |
| D930014E17Rik | 1.2625145 |
| D930015E06Rik | -0.3798439 |
| D930020B18Rik | 2.5688373 |
| D930028F11Rik | -0.5977539 |
| D930049A15Rik | 1.2807212 |
| D9Ertd280e | 0.8646571 |
| D9Ertd392e | 1.1808159 |
| D9Ertd402e | -0.3823277 |
| D9Wsu20e | 0.9291618 |
| Daam1 | -0.0438304 |
| Daam2 | 0.9894683 |
| Dab1 | -0.6490728 |
| Dab1 | 0.0269557 |
| Dab1 | 0.6712281 |
| Dab2 | -0.4139018 |
| Dab2 | 0.050703 |
| Dab2ip | 1.1343764 |
| Dact1 | 1.6953492 |
| Dact2 | -0.0080308 |
| Dad1 | 1.6581568 |
| Daf2 | 1.6509814 |
| Dag1 | 1.2593461 |
| Dag1 | -0.5756742 |
| Dagla | 0.1460037 |
| Daglb | 0.7547146 |
| Dak | -0.5893219 |
| Dalrd3 | -0.6148326 |
| Dap | -0.610146 |
| Dap3 | -0.5235738 |
| Dap3 | 0.1973225 |
| Dap3 | -0.2138234 |
| Dap3 | -0.7317467 |
| Dapk1 | -0.5585001 |
| Dapk1 | 2.4589326 |
| Dapk2 | 0.1810975 |
| Dapk2 | -0.5044467 |
| Dapl1 | 1.2862029 |
| Dapp1 | -0.657205 |
| Dapp1 | -0.4884345 |
| Darc | -0.6393999 |
| Dars | -0.3337794 |
| Dars | -0.6117058 |
| Daxx | -0.6117058 |
| Dazap1 | 2.2670025 |
| Dazap1 | -0.3034319 |
| Dazap2 | 0.184647 |
| Dazl | -0.7177774 |
| Dbc1 | 1.2403727 |
| Dbf4 | -0.2486083 |
| Dbi | -0.2908625 |
| Dbi | -0.6753303 |
| Dbil5 | 1.075201 |
| Dbn1 | 0.7533044 |
| Dbndd1 | -0.4692786 |
| Dbndd2 | -0.5855123 |
| Dbndd2 | 0.3317562 |
| Dbndd2 | -0.6753303 |
| Dbnl | 0.025712 |
| Dbp | -0.5870344 |
| Dbpht2 | -0.2628653 |
| Dbr1 | 0.9416155 |
| Dbt | 0.1871242 |
| Dcakd | -0.5824749 |
| Dcbld1 | 1.3818269 |
| Dcbld2 | 0.4843534 |
| Dci | 0.3387748 |
| Dck | 0.9129452 |
| Dclk1 | -0.1873466 |
| Dclk2 | 0.9529253 |
| Dclk3 | 0.0790317 |
| Dclre1b | 1.6332795 |
| Dclre1b | -0.4672476 |
| Dclre1c | -0.5719157 |
| Dclre1c | 0.3399905 |
| Dclre1c | -0.1501248 |
| Dcn | -0.0613136 |
| Dcn | -0.7169107 |
| Dcp1a | 1.0098374 |
| Dcp1a | 1.069029 |
| Dcp1a | 1.1371357 |
| Dcp1a | -0.673669 |
| Dcp1b | 0.3012906 |
| Dcp1b | 0.6810216 |
| Dcpp1 | -0.2711832 |
| Dcpp2 | -0.4808655 |
| Dcpp2 | 1.003408 |
| Dcpp3 | 0.0236359 |
| Dcps | -0.5496544 |
| Dcps | -0.6971797 |
| Dct | 0.4188343 |
| Dctd | 1.937783 |
| Dctn1 | -0.4672476 |
| Dctn2 | 0.2350945 |
| Dctn2 | 1.5079582 |
| Dctn2 | 1.122005 |
| Dctn3 | -0.7521729 |
| Dctn3 | -0.5939124 |
| Dctn4 | -0.284072 |
| Dctn4 | -0.3138338 |
| Dctn5 | 0.3348138 |
| Dctn6 | 0.7531028 |
| Dcun1d2 | 0.9596837 |
| Dcun1d3 | -0.5916145 |
| Dcun1d4 | -0.088817 |
| Dcun1d4 | 2.4169117 |
| Dcun1d5 | 1.7897226 |
| Dcx | 0.0177997 |
| Dcx | 0.8184856 |
| Dcxr | 1.8394031 |
| Dcxr | 0.3766743 |
| Ddah1 | -0.6226923 |
| Ddah2 | -0.7264855 |
| Ddb1 | 1.5901685 |
| Ddb2 | -0.7238652 |
| Ddb2 | 0.3595424 |
| Ddb2 | -0.4578232 |
| Ddc | -0.6124866 |
| Ddc8 | 1.0963671 |
| Ddef2 | -0.4815513 |
| Ddef2 | 0.0177997 |
| Ddefl1 | -0.3179081 |
| Ddhd1 | 0.8082287 |
| Ddhd1 | -0.4471593 |
| Ddi2 | -0.4925872 |
| Ddit3 | 0.2070723 |
| Ddit3 | 0.9327116 |
| Ddit4 | 1.5350611 |
| Ddit4l | -0.6645806 |
| Ddo | 1.9370354 |
| Ddo | 0.1142141 |
| Ddo | 2.8574594 |
| Ddost | -0.5908497 |
| Ddr1 | -0.3255187 |
| Ddr2 | 1.4724292 |
| Ddt | 1.3851722 |
| Ddx1 | 0.186417 |
| Ddx1 | -0.5343589 |
| Ddx17 | 0.8738219 |
| Ddx17 | -0.6555733 |
| Ddx17 | 0.3405978 |
| Ddx19a | -0.4774438 |
| Ddx19b | -0.2695141 |
| Ddx19b | 0.1493117 |
| Ddx20 | -0.5207172 |
| Ddx21 | -0.3184915 |
| Ddx24 | -0.4043253 |
| Ddx24 | 1.9329133 |
| Ddx24 | 0.7246782 |
| Ddx25 | -0.6163997 |
| Ddx26b | -0.6211155 |
| Ddx27 | -0.6195411 |
| Ddx28 | -0.4132605 |
| Ddx3x | -0.0067449 |
| Ddx3y | -0.6361961 |
| Ddx4 | -0.5285925 |
| Ddx4 | 0.0607689 |
| Ddx41 | 0.2990789 |
| Ddx46 | 0.8479108 |
| Ddx47 | -0.3017087 |
| Ddx47 | -0.0020437 |
| Ddx47 | -0.5622088 |
| Ddx49 | 2.3354836 |
| Ddx5 | -0.5430708 |
| Ddx50 | -0.4645459 |
| Ddx50 | 3.4092469 |
| Ddx51 | 1.1300845 |
| Ddx52 | 1.5794742 |
| Ddx54 | 1.7852405 |
| Ddx58 | -0.6274377 |
| Ddx6 | 1.2156967 |
| Ddx6 | 0.9364171 |
| Ddx6 | -0.042942 |
| Ddx6 | -0.3798439 |
| Deadc1 | -0.7221221 |
| Deb1 | 0.9077695 |
| Decr1 | 1.5290075 |
| Decr2 | 2.7363581 |
| Decr2 | 0.5956396 |
| Dedd | 0.9138053 |
| Dedd | -0.4767608 |
| Dedd2 | -0.5157374 |
| Def6 | 1.3507632 |
| Def8 | 1.2633658 |
| Defb11 | 0.9701436 |
| Defb14 | 0.1635224 |
| Defb15 | 0.5406601 |
| Defb19 | -0.0911431 |
| Defb20 | 0.1746769 |
| Defb21 | -0.1295945 |
| Defb25 | -0.5607237 |
| Defb26 | 0.3868735 |
| Defb30 | -0.5192919 |
| Defb36 | 0.7003277 |
| Defb36 | -0.2496978 |
| Defb4 | -0.4011533 |
| Defcr20 | 2.7344096 |
| Defcr6 | -0.610146 |
| Defcr-rs7 | 1.6198816 |
| Degs1 | 2.2353727 |
| Degs2 | -0.4631978 |
| Dek | -0.2639704 |
| Dek | -0.7160448 |
| Dennd1a | -0.6322056 |
| Dennd1c | 0.9983613 |
| Dennd2a | -0.4974536 |
| Dennd3 | -0.3669038 |
| Denr | -0.6920953 |
| Denr | 1.6722753 |
| Depdc1b | 0.2428166 |
| Depdc5 | 1.9997991 |
| Depdc5 | -0.5666773 |
| Depdc6 | -0.1511129 |
| Depdc6 | 1.0759333 |
| Dera | 0.2025573 |
| Derl2 | 1.520565 |
| Derl3 | 0.5722707 |
| Des | 0.2401374 |
| Det1 | 0.5316587 |
| Dexi | -0.0123292 |
| Dexi | -0.5540676 |
| Dffa | 1.9453528 |
| Dfna5h | 0.0559498 |
| Dgat1 | 0.2070723 |
| Dgat2 | -0.4113392 |
| Dgcr2 | 0.4157398 |
| Dgcr2 | -0.5756742 |
| Dgcr6 | 0.6947883 |
| Dgcr6 | 2.6367492 |
| Dgcr8 | 1.2341349 |
| Dgka | -0.1325019 |
| Dgka | 0.0996715 |
| Dgkb | -0.3817062 |
| Dgke | -0.7485906 |
| Dgkg | -0.7048546 |
| Dgkh | -0.4808655 |
| Dgki | 0.6905063 |
| Dgkk | 2.1703573 |
| Dgkq | 0.0136103 |
| Dgkz | 2.9144623 |
| Dgkz | 0.6973487 |
| Dgkz | 2.4097217 |
| Dguok | 2.0225569 |
| Dhcr24 | -0.1976469 |
| Dhcr24 | -0.3823277 |
| Dhcr24 | 0.1434232 |
| Dhcr7 | 0.2018609 |
| Dhdds | -0.6588394 |
| Dhdds | 1.3675665 |
| Dhdh | 3.7721577 |
| Dhdh | 0.7961289 |
| Dhdh | 2.6000601 |
| Dhh | 0.237451 |
| Dhodh | -0.7521729 |
| Dhps | 1.4946119 |
| Dhrs1 | 0.6180183 |
| Dhrs4 | 1.2413672 |
| Dhrs7 | -0.2976991 |
| Dhrs7 | 1.1099047 |
| Dhrs7 | -0.0967479 |
| Dhrs7b | -0.4591642 |
| Dhrs7b | -0.1247673 |
| Dhrs7b | -0.0469462 |
| Dhrsx | -0.5946796 |
| Dhrsx | -0.3120927 |
| Dhtkd1 | 1.7892897 |
| Dhx15 | 1.0884456 |
| Dhx15 | 1.1440012 |
| Dhx16 | -0.7014363 |
| Dhx29 | -0.0795656 |
| Dhx30 | 1.1533994 |
| Dhx32 | -0.0869599 |
| Dhx33 | 0.1839381 |
| Dhx36 | 1.6680524 |
| Dhx37 | 0.1493117 |
| Dhx38 | -0.6637584 |
| Dhx57 | -0.4822375 |
| Dhx58 | -0.6670511 |
| Dhx8 | -0.1899118 |
| Dhx9 | 1.7019021 |
| Diablo | -0.3929523 |
| Diap1 | -0.3343721 |
| Diap3 | 3.0139866 |
| Dicer1 | -0.5100763 |
| Dido1 | -0.4145435 |
| Dido1 | 1.4092309 |
| Dido1 | 0.1913567 |
| Dio1 | -0.5207172 |
| Dio1 | 2.4588212 |
| Dio3 | -0.55776 |
| Dip2b | -0.2689584 |
| Dip2b | 1.2517504 |
| Diras1 | 0.7967084 |
| Diras2 | 0.5374189 |
| Dirc2 | 0.3828062 |
| Dis3 | 0.1533401 |
| Dis3l | -0.0080308 |
| Dis3l2 | 0.646432 |
| Disp1 | 1.1182217 |
| Dixdc1 | 1.8112743 |
| Dkk3 | 0.3345084 |
| Dkk3 | -0.5599819 |
| Dkk3 | 0.1613491 |
| Dkkl1 | -0.5445301 |
| Dkkl1 | 1.6362235 |
| Dlat | 2.1674354 |
| Dlc1 | -0.2695141 |
| Dld | -0.6211155 |
| Dleu7 | -0.5365298 |
| Dlg1 | 0.7941949 |
| Dlg2 | 1.5881444 |
| Dlg3 | 1.5106254 |
| Dlg4 | -0.2458895 |
| Dlg7 | -0.0545534 |
| Dlgap1 | -0.0509664 |
| Dlgap2 | -0.0595064 |
| Dlgap2 | -0.6124866 |
| Dlgap2 | -0.2999883 |
| Dlgap3 | 0.7704945 |
| Dlgap4 | -0.6612959 |
| Dlgap4 | 0.2387951 |
| Dlgap4 | 0.4961293 |
| Dlgap4 | -0.3523155 |
| Dlk1 | 0.0089817 |
| Dlk2 | 0.6045573 |
| Dll1 | 0.0782445 |
| Dlst | -0.103798 |
| Dlx1 | -0.4912011 |
| Dlx1as | 0.2528008 |
| Dlx2 | 0.2567669 |
| Dlx3 | -0.3860648 |
| Dmap1 | 2.4777741 |
| Dmbt1 | 0.4492683 |
| Dmkn | 0.0277839 |
| Dmkn | 2.9768954 |
| Dmkn | -0.4425291 |
| Dmn | 1.0985167 |
| Dmn | -0.5771815 |
| Dmpk | 0.3442338 |
| Dmrt2 | 0.3654829 |
| Dmrt3 | -0.5365298 |
| Dmrta1 | 1.2503967 |
| Dmrta2 | 0.958533 |
| Dmrtc1a | 0.7541105 |
| Dmrtc1a | 0.7639329 |
| Dmrtc1a | 0.2685727 |
| Dmrtc1b | -0.4471593 |
| Dmrtc2 | -0.6904062 |
| Dmtf1 | -0.6695277 |
| Dmtf1 | -0.6878779 |
| Dmtf1 | 2.2164178 |
| Dmwd | -0.1383421 |
| Dmwd | -0.6539443 |
| Dmwd | 1.3414681 |
| Dnahc11 | -0.3547323 |
| Dnahc2 | -0.5809597 |
| Dnahc5 | 2.1740595 |
| Dnahc8 | -0.0563517 |
| Dnaic1 | 1.7397435 |
| Dnaic1 | 0.9437875 |
| Dnaja1 | -0.2557116 |
| Dnaja2 | 2.1833291 |
| Dnaja2 | -0.6353967 |
| Dnaja3 | -0.61405 |
| Dnajb1 | -0.4075074 |
| Dnajb10 | -0.4451724 |
| Dnajb12 | 0.3323684 |
| Dnajb13 | -0.127178 |
| Dnajb3 | 1.1360329 |
| Dnajb4 | -0.7100039 |
| Dnajb4 | 3.5312576 |
| Dnajb5 | 1.5144235 |
| Dnajb6 | -0.5824749 |
| Dnajb6 | 0.4182724 |
| Dnajb7 | 1.1059335 |
| Dnajb8 | 4.5181333 |
| Dnajb9 | -0.1827456 |
| Dnajc10 | 2.0103443 |
| Dnajc10 | 1.9000923 |
| Dnajc12 | 0.9474526 |
| Dnajc14 | -0.3632369 |
| Dnajc15 | -0.6547585 |
| Dnajc16 | 0.151511 |
| Dnajc17 | -0.6474542 |
| Dnajc18 | 0.8333055 |
| Dnajc19 | -0.4425291 |
| Dnajc19 | -0.6047057 |
| Dnajc2 | 1.9508395 |
| Dnajc2 | -0.2496978 |
| Dnajc3a | -0.1924836 |
| Dnajc4 | -0.2212148 |
| Dnajc5 | -0.5599819 |
| Dnajc5b | 0.881485 |
| Dnajc5g | 0.7752399 |
| Dnajc6 | 2.0477766 |
| Dnajc6 | -0.6769943 |
| Dnajc7 | -0.6322056 |
| Dnajc8 | -0.1550752 |
| Dnalc1 | 1.6147467 |
| Dnali1 | 1.7722608 |
| Dnase1l1 | -0.0731397 |
| Dnase1l2 | -0.6361961 |
| Dnase1l2 | 0.4385829 |
| Dnase2a | 4.0278463 |
| Dnd1 | 0.2163854 |
| Dner | -0.3620175 |
| Dnm | 0.6029205 |
| Dnm1 | 1.153535 |
| Dnm1l | -0.6662269 |
| Dnm1l | 0.9330491 |
| Dnmbp | 0.7854457 |
| Dnmt1 | 1.5561945 |
| Dnmt3b | 1.8265501 |
| Dnmt3l | -0.6895627 |
| Dnmt3l | -0.719513 |
| Dnttip1 | 0.1853554 |
| Doc2a | -0.5555429 |
| Doc2b | 1.2535933 |
| Doc2b | -0.5741691 |
| Dock1 | -0.6596576 |
| Dock3 | -0.5908497 |
| Dock4 | -0.6637584 |
| Dock6 | 0.9660706 |
| Dock8 | 1.1423577 |
| Dock9 | -0.5503886 |
| Dok1 | -0.3736612 |
| Dok1 | -0.3149962 |
| Dok2 | -0.6828401 |
| Dok3 | -0.3607997 |
| Dok4 | -0.6250622 |
| Dok5 | 2.645184 |
| Dok5 | -0.6458381 |
| Dok7 | 0.9441212 |
| Dolk | 0.2834594 |
| Dolpp1 | -0.1893983 |
| Dolpp1 | -0.6031568 |
| Dolpp1 | 0.4681678 |
| Dom3z | 0.0746945 |
| Dom3z | -0.6820029 |
| Donson | -0.6187548 |
| Donson | -0.5599819 |
| Dos | -0.257358 |
| Dos | -0.3184915 |
| Dot1l | 0.1671341 |
| Dpagt1 | 0.1363857 |
| Dpep1 | 0.6626628 |
| Dpep2 | 1.3060902 |
| Dpep2 | 3.6394297 |
| Dpep3 | 1.9376585 |
| Dpf1 | -0.4564841 |
| Dpf1 | 0.6196308 |
| Dpf3 | 0.3194322 |
| Dph2 | -0.2259952 |
| Dph2 | 2.7840737 |
| Dph2 | -0.4598353 |
| Dph3 | 2.5215095 |
| Dph3 | 0.0746945 |
| Dph3 | 0.3308371 |
| Dph4 | 0.6417141 |
| Dpm1 | -0.5214306 |
| Dpm1 | 1.7802289 |
| Dpm2 | 0.0056021 |
| Dpm3 | -0.7326263 |
| Dpp10 | -0.581717 |
| Dpp3 | -0.0054606 |
| Dpp3 | -0.7134514 |
| Dpp4 | -0.6828401 |
| Dpp6 | 0.2276525 |
| Dpp7 | -0.5394317 |
| Dpp8 | -0.7221221 |
| Dpp9 | -0.4551467 |
| Dpp9 | -0.0653916 |
| Dppa3 | 0.8823722 |
| Dppa4 | 0.9120845 |
| Dppa5 | 1.3888286 |
| Dppa5 | 3.3481875 |
| Dpt | 2.0500637 |
| Dpy30 | -0.51858 |
| Dpyd | -0.673669 |
| Dpysl2 | -0.3675162 |
| Dpysl2 | 0.2518068 |
| Dpysl2 | 1.2328827 |
| Dpysl3 | 0.8506602 |
| Dpysl4 | 0.5116195 |
| Dpysl5 | 1.3196322 |
| Drbp1 | -0.8510682 |
| Drctnnb1a | -0.2795702 |
| Drd1a | -0.5221445 |
| Drd2 | -0.5329142 |
| Drd4 | 2.0487255 |
| Drd5 | 1.0498322 |
| Drg1 | 1.4551895 |
| Drg1 | -0.6761619 |
| Drg2 | -0.015349 |
| Drp2 | 2.922094 |
| Dscam | -0.6895627 |
| Dscam | 1.4244736 |
| Dscc1 | -0.0740552 |
| Dscr1 | -0.0860326 |
| Dscr1l2 | -0.1955784 |
| Dscr2 | 3.0976285 |
| Dscr3 | -0.5192919 |
| Dscr3 | -0.3138338 |
| Dscr6 | 0.8453379 |
| Dsg2 | -0.2012771 |
| Dsg2 | -0.1349312 |
| Dsn1 | -0.6047057 |
| Dsp | -0.3650687 |
| Dst | 0.2354315 |
| Dst | 0.8013323 |
| Dst | 1.0477267 |
| Dstn | 0.3839699 |
| Dtd1 | 0.6143228 |
| Dtl | 2.0149702 |
| Dtna | -0.6171841 |
| Dtna | -0.3261065 |
| Dtna | -0.6290245 |
| Dtna | 2.8947407 |
| Dtnb | 1.5705495 |
| Dtnbp1 | 0.0203051 |
| Dtwd1 | 1.9300932 |
| Dtx1 | -0.3367462 |
| Dtx2 | 1.7779691 |
| Dtx2 | -0.6234817 |
| Dtx2 | -0.3923242 |
| Dtx4 | 0.9284843 |
| Dtx4 | -0.4960608 |
| Dtymk | 0.1042866 |
| Dus1l | 1.8457005 |
| Dus1l | -0.4753963 |
| Dus2l | -0.1796898 |
| Dus3l | -0.4995464 |
| Dus3l | 1.8916522 |
| Dus3l | 0.9070774 |
| Dus4l | 0.8599526 |
| Dusp1 | 3.4399263 |
| Dusp11 | 2.2256356 |
| Dusp11 | 0.0043318 |
| Dusp13 | -0.158555 |
| Dusp13 | 1.5910473 |
| Dusp13 | -0.431372 |
| Dusp14 | -0.6490728 |
| Dusp15 | 0.0942604 |
| Dusp15 | 2.2612797 |
| Dusp16 | 0.7704945 |
| Dusp16 | 1.065485 |
| Dusp16 | 0.7781945 |
| Dusp18 | -0.0279568 |
| Dusp19 | 1.4608023 |
| Dusp2 | 2.4660395 |
| Dusp2 | 0.0627701 |
| Dusp21 | -0.2891606 |
| Dusp22 | 1.1141423 |
| Dusp23 | 0.2914588 |
| Dusp26 | 0.817351 |
| Dusp28 | 1.1885703 |
| Dusp3 | 1.6282973 |
| Dusp3 | -0.4307195 |
| Dusp3 | -0.3138338 |
| Dusp4 | -0.4017869 |
| Dusp4 | -0.2415546 |
| Dusp4 | -0.3439022 |
| Dusp6 | 0.4083875 |
| Dusp6 | 1.3004815 |
| Dusp6 | -0.4870541 |
| Dusp7 | 0.201164 |
| Dusp8 | 1.463298 |
| Dut | 0.2715026 |
| Duxbl | 1.181739 |
| Duxbl | -0.1005019 |
| Dvl1 | -0.0144852 |
| Dvl1 | -0.4988484 |
| Dvl2 | -0.3138338 |
| DXBwg1396e | -0.3511093 |
| Dydc2 | -0.4005201 |
| Dym | -0.4625244 |
| Dync1h1 | 0.9143209 |
| Dync1i1 | -0.6426139 |
| Dync1i1 | -0.2302635 |
| Dync1i2 | -0.6117058 |
| Dync1li1 | 0.2498159 |
| Dync1li2 | 2.5378885 |
| Dync2li1 | 0.1635224 |
| Dynll1 | 1.9320368 |
| Dynll2 | 0.4919581 |
| Dynlrb1 | -0.0380694 |
| Dynlrb2 | 0.1537056 |
| Dynlt3 | 1.6693536 |
| Dyrk1a | -0.5474551 |
| Dyrk1b | 1.1127317 |
| Dyrk1b | -0.362627 |
| Dyrk2 | 1.7828468 |
| Dyrk2 | 0.029025 |
| Dyrk3 | -0.3904423 |
| Dyrk4 | 0.2666146 |
| Dysf | 0.8126101 |
| Dysfip1 | -0.5438002 |
| Dzip1 | 2.0643376 |
| E030010A14Rik | 0.2347574 |
| E030034P13Rik | -0.5756742 |
| E130009J12Rik | -0.4359512 |
| E130012A19Rik | -0.5711657 |
| E130014J05Rik | 2.1688231 |
| E130016E03Rik | 0.5498285 |
| E130203B14Rik | -0.6645806 |
| E130203B14Rik | -0.7557679 |
| E130303B06Rik | -0.8027642 |
| E130306D19Rik | 1.2705129 |
| E130308A19Rik | -0.420337 |
| E130309D02Rik | -0.6117058 |
| E130309D02Rik | -0.408783 |
| E130309D14Rik | 1.9778934 |
| E130309F12Rik | -0.0681194 |
| E130309F12Rik | 1.8345162 |
| E130309F12Rik | -0.7022898 |
| E230008N13Rik | 2.140495 |
| E2f1 | -0.7221221 |
| E2f2 | -0.3255187 |
| E2f2 | -0.5100763 |
| E2f3 | -0.5832334 |
| E2f4 | -0.6878779 |
| E2f5 | 3.4221627 |
| E2f6 | 0.1478428 |
| E2f6 | 2.9545132 |
| E2f6 | -0.7343877 |
| E330016A19Rik | 0.1363857 |
| E330017A01Rik | 1.0463708 |
| E330018D03Rik | -0.3450998 |
| E330021D16Rik | 0.3311436 |
| E330034G19Rik | -0.1868343 |
| E330034G19Rik | 0.3631109 |
| E330036I19Rik | -0.6353967 |
| E430002D04Rik | -0.5072575 |
| E430002G05Rik | -0.6047057 |
| E430018J23Rik | -0.5607237 |
| E430025E21Rik | 0.9235582 |
| E430028B21Rik | -0.0536554 |
| E4f1 | 1.0258108 |
| Eaf1 | 0.1289244 |
| Ear11 | -0.188885 |
| Ear2 | 1.0884456 |
| Ear4 | -0.0827939 |
| Ears2 | -0.7620901 |
| Ebag9 | 0.9514357 |
| Ebf2 | -0.5908497 |
| Ebf3 | -0.037186 |
| Ebf4 | -0.0694861 |
| Ebi2 | 1.5867339 |
| Ebi3 | -0.6195411 |
| Ebna1bp2 | 2.7528116 |
| Ebna1bp2 | 1.8671026 |
| Ebp | -0.7803752 |
| Ebpl | 2.0688322 |
| Ebpl | 2.2820325 |
| Ecd | 1.6470005 |
| Ecd | 1.1280012 |
| Ece1 | -0.643419 |
| Ece1 | 2.0629082 |
| Ece2 | -0.6482631 |
| Ecel1 | 2.8240623 |
| Ech1 | -0.6744993 |
| Echdc1 | 1.9608112 |
| Echdc2 | 1.8334116 |
| Echdc2 | -0.3255187 |
| Echdc2 | 0.2908211 |
| Echdc3 | -0.5394317 |
| Echs1 | -0.3718138 |
| Ecm1 | 0.3610308 |
| Ecm1 | -0.4713137 |
| Ecsit | -0.1590531 |
| Eda2r | 1.5178296 |
| Edaradd | 1.1688711 |
| Edc4 | 0.2974961 |
| Edem1 | 1.6303269 |
| Edem2 | -0.5044467 |
| Edem2 | 2.5975703 |
| Edem2 | 1.443971 |
| Edem2 | -0.5136108 |
| Edf1 | -0.6458381 |
| Edg2 | 2.5321014 |
| Edg5 | 1.0556745 |
| Edg7 | -0.0389536 |
| Edg8 | -0.0166459 |
| Edg8 | -0.3662917 |
| Edil3 | 1.539878 |
| Edn1 | 0.4052607 |
| Edn2 | -0.2393942 |
| Ednra | -0.6377967 |
| Ednrb | 3.4130467 |
| Ednrb | 1.6562648 |
| Ednrb | -0.720382 |
| Eed | -0.6393999 |
| Eef1a1 | -0.7822221 |
| Eef1a1 | 0.0474606 |
| Eef1a2 | 1.1521785 |
| Eef1a2 | -0.5072575 |
| Eef1a2 | 0.3568577 |
| Eef1b2 | -0.2568089 |
| Eef1b2 | 1.5378429 |
| Eef1b2 | 0.8594083 |
| Eef1d | 2.3201699 |
| Eef1d | -0.6054811 |
| Eef1d | -0.1625467 |
| Eef1d | 2.7365556 |
| Eef1e1 | -0.4774438 |
| Eef1g | -0.1635471 |
| Eef2 | -0.1940298 |
| Eef2 | 1.492677 |
| Eef2 | -0.1407855 |
| Eef2k | -0.1061591 |
| Eefsec | 0.8557725 |
| Efcab1 | -0.008889 |
| Efcab2 | -0.6580219 |
| Efcab2 | 0.5046784 |
| Efcab3 | -0.4438499 |
| Efcab3 | 0.871673 |
| Efcab3 | 0.0663622 |
| Efcab4a | 0.9504414 |
| Efcab4a | -0.1837662 |
| Efcab5 | -0.3979912 |
| Efemp2 | 1.1225643 |
| Efha1 | 1.8213906 |
| Efha1 | -0.6093671 |
| Efhc2 | 1.0261185 |
| Efhd1 | 0.6038562 |
| Efna1 | 2.3217033 |
| Efna3 | -0.7785317 |
| Efna4 | -0.6963305 |
| Efna4 | -0.7441307 |
| Efna5 | 1.1866045 |
| Efnb1 | 1.0890238 |
| Efnb2 | 0.2474882 |
| Efnb3 | -0.3848175 |
| Efr3a | -0.7212516 |
| Efs | 0.5601359 |
| Eftud1 | 1.7711593 |
| Eftud2 | 0.620091 |
| Eftud2 | -0.4960608 |
| Eftud2 | -0.3979912 |
| EG194588 | 0.3138369 |
| EG208426 | -0.5954473 |
| EG210155 | 2.6818746 |
| EG212225 | 2.8207726 |
| EG214321 | 0.1430541 |
| EG232599 | 2.7401033 |
| EG237009 | -0.433332 |
| EG237009 | -0.6330024 |
| EG237300 | 0.8070825 |
| EG238829 | 0.511107 |
| EG240916 | 0.1437923 |
| EG245297 | -0.5285925 |
| EG245297 | 2.9974859 |
| EG245376 | -0.603931 |
| EG245376 | -0.7611845 |
| EG272350 | -0.3051581 |
| EG277333 | -0.821809 |
| EG317677 | 0.0587637 |
| EG328264 | -0.4209828 |
| EG328280 | -0.598524 |
| EG328644 | -0.6361961 |
| EG328839 | 2.4662977 |
| EG330305 | -0.6629369 |
| EG330503 | -0.1368789 |
| EG330513 | 0.1835835 |
| EG333669 | -0.1761364 |
| EG368203 | -0.3361521 |
| EG380907 | 0.1315422 |
| EG381438 | 2.2063518 |
| EG382109 | 0.8980366 |
| EG382109 | -0.0136223 |
| EG382109 | -0.5893219 |
| EG384639 | 0.4385829 |
| EG384813 | 4.431878 |
| EG384813 | 1.9195578 |
| EG384814 | -0.824697 |
| EG385328 | 1.0371326 |
| EG408196 | 0.0060252 |
| EG432825 | 0.1646072 |
| EG432879 | 0.5696136 |
| EG432939 | 1.1809478 |
| EG433016 | 1.4610022 |
| EG433182 | 0.1667735 |
| EG433224 | 3.1166327 |
| EG433923 | 1.2217958 |
| EG434171 | -0.2106721 |
| EG434197 | -0.4652206 |
| EG434280 | -0.5562814 |
| EG434459 | 2.9299478 |
| EG434459 | 0.5949322 |
| EG434858 | -0.51858 |
| EG435391 | -0.4398928 |
| EG436332 | 0.3571564 |
| EG545047 | 2.6142341 |
| EG545056 | 0.8148885 |
| EG545253 | 1.4049087 |
| EG545370 | 1.7969817 |
| EG545758 | -0.5329142 |
| EG545861 | -0.6937873 |
| EG545963 | 0.3372532 |
| EG546166 | 2.5942952 |
| EG546282 | 0.5766038 |
| EG546325 | 1.1943146 |
| EG546347 | -0.1104229 |
| EG547109 | -0.3057342 |
| EG574403 | 0.2997113 |
| EG622320 | -0.1066319 |
| EG622339 | -0.7361521 |
| EG624866 | 1.7457891 |
| EG625054 | -0.5533307 |
| EG625424 | -0.105214 |
| EG630499 | -0.091609 |
| EG633640 | -0.4877441 |
| EG638695 | -0.527874 |
| EG654453 | -0.7186448 |
| EG665186 | 0.9198051 |
| EG665378 | -0.6946343 |
| EG667977 | 3.1997363 |
| EG668668 | 1.0694711 |
| Egf | -0.7812982 |
| Egfbp2 | -0.785926 |
| Egfl7 | 1.6239707 |
| Egfl7 | -0.5336363 |
| Egflam | 1.5919253 |
| Egflam | 0.9951942 |
| Egfr | -0.6410056 |
| Egfr | -0.3929523 |
| Egfr | 0.7345847 |
| Egfr | -0.6031568 |
| Egln1 | 0.2317186 |
| Egln1 | -0.610146 |
| Egln1 | -0.6645806 |
| Egln2 | 0.2347574 |
| Egln3 | 1.2816767 |
| Egr1 | -0.3736612 |
| Egr2 | -0.730868 |
| Egr3 | -0.0644839 |
| Egr4 | -0.5358057 |
| Ehbp1 | 1.5052839 |
| Ehbp1l1 | -0.6621161 |
| Ehd1 | -0.3397217 |
| Ehd2 | 1.4170898 |
| Ehd3 | -0.6531307 |
| Ehd4 | 0.8254537 |
| Ehmt1 | -0.6563889 |
| Ehmt2 | -0.5023437 |
| Ehmt2 | -0.4011533 |
| Ei24 | -0.2249309 |
| Eid1 | 0.9141491 |
| Eid2 | 1.793682 |
| Eid3 | -0.4235701 |
| Eif1a | -0.0795656 |
| Eif1b | -0.7459123 |
| Eif2a | 1.9488086 |
| Eif2ak1 | -0.6385979 |
| Eif2ak2 | -0.4706349 |
| Eif2b1 | -0.7521729 |
| Eif2b1 | 2.376697 |
| Eif2b2 | 0.1945195 |
| Eif2b4 | 1.536082 |
| Eif2b5 | -0.6744993 |
| Eif2b5 | 0.523083 |
| Eif2b5 | 0.051512 |
| Eif2c2 | 1.4342694 |
| Eif2c3 | -0.703144 |
| Eif2c3 | -0.5079615 |
| Eif2c3 | -0.9249613 |
| Eif2c3 | 1.2566574 |
| Eif2c4 | 1.0534315 |
| Eif2s2 | 0.6184792 |
| Eif2s2 | -0.8314681 |
| Eif2s3x | 0.6624422 |
| Eif2s3y | -0.4815513 |
| Eif3b | 0.2570967 |
| Eif3b | -0.5401584 |
| Eif3d | 0.6562462 |
| Eif3eip | -0.7896436 |
| Eif3eip | 0.101597 |
| Eif3f | -0.5093708 |
| Eif3g | 0.7259218 |
| Eif3i | 0.173961 |
| Eif3i | 0.7887599 |
| Eif3i | -0.7441307 |
| Eif3k | 0.0169633 |
| Eif3s1 | -0.5704163 |
| Eif3s10 | -0.3773663 |
| Eif3s10 | -0.3601913 |
| Eif3s4 | 0.5126438 |
| Eif3s8 | -0.7221221 |
| Eif4a1 | 0.0364397 |
| Eif4a1 | 0.9514357 |
| Eif4a2 | -0.6895627 |
| Eif4a3 | -0.6711822 |
| Eif4b | 1.891 |
| Eif4e | -0.1501248 |
| Eif4e1b | -0.5681712 |
| Eif4e1b | -0.5954473 |
| Eif4e2 | -0.4094215 |
| Eif4e2 | -0.610146 |
| Eif4e3 | 0.6655259 |
| Eif4e3 | 0.7789809 |
| Eif4e3 | 0.2170718 |
| Eif4ebp1 | 2.3957005 |
| Eif4ebp2 | -0.7530704 |
| Eif4el3 | 1.3966344 |
| Eif4enif1 | -0.0541043 |
| Eif4enif1 | -0.7370355 |
| Eif4g1 | -0.3074644 |
| Eif4g2 | -0.4372633 |
| Eif4g2 | -0.0024702 |
| Eif4h | -0.5242892 |
| Eif5 | 0.8866203 |
| Eif5 | -0.527156 |
| Eif5 | -0.368129 |
| Eif5a | -0.2711832 |
| Eif5a | -0.6912504 |
| Eif6 | 0.5281364 |
| Ela1 | -0.0341002 |
| Ela2 | 0.2997113 |
| Ela2a | -0.530751 |
| Ela3b | -0.3179081 |
| Elac2 | -0.342706 |
| Elac2 | 2.4790129 |
| Elac2 | -0.0837182 |
| Elavl1 | -0.6023831 |
| Elavl2 | -0.5438002 |
| Elavl2 | -0.8065445 |
| Elavl3 | 1.4998178 |
| Elavl4 | 3.3726935 |
| Elavl4 | 0.1818084 |
| Elf1 | 1.1934029 |
| Elf2 | -0.2212148 |
| Elf2 | 1.1616357 |
| Elf2 | -0.5659312 |
| Elf2 | -0.0962797 |
| Elf2 | 1.6425032 |
| Elf3 | -0.4801802 |
| Elf3 | 1.3243349 |
| Elf3 | 2.043972 |
| Elf5 | -0.703144 |
| Elfn1 | -0.3361521 |
| Elfn2 | 0.5005424 |
| Elk3 | 1.9948087 |
| Elk3 | 1.9806428 |
| Elk3 | -0.7584725 |
| Ell | 0.093485 |
| Ell3 | 0.1315422 |
| Elmo1 | 1.6712212 |
| Elmo1 | 0.0384897 |
| Elmo1 | 0.4095221 |
| Elmo1 | -0.6670511 |
| Elmo1 | -0.530751 |
| Elmo1 | 1.1010902 |
| Elmo1 | 1.6450875 |
| Elmo1 | 0.6468802 |
| Elmo1 | -0.6820029 |
| Elmo1 | 0.8045948 |
| Elmo1 | -0.7611845 |
| Elmo1 | -0.6786611 |
| Elmo1 | 0.4506301 |
| Elmo2 | -0.6761619 |
| Elmo2 | -0.3973599 |
| Elmo3 | 1.5536453 |
| Elmo3 | -0.6645806 |
| Elmod1 | -0.5423419 |
| Eln | -0.5977539 |
| Elof1 | 0.053935 |
| Elovl1 | -0.7468043 |
| Elovl1 | 0.544139 |
| Elovl1 | -0.5416136 |
| Elovl1 | 0.4511743 |
| Elovl2 | -0.7352695 |
| Elovl4 | 1.3139829 |
| Elovl5 | 0.5741988 |
| Elovl6 | 0.7700981 |
| Elp2 | -0.586273 |
| Elp3 | -0.598524 |
| Elp3 | 1.8476774 |
| Eltd1 | -0.3057342 |
| Emb | -0.7014363 |
| Emcn | 2.0518451 |
| Emd | -0.7221221 |
| Emd | -0.0270822 |
| Emg1 | 4.1658058 |
| Emg1 | 0.2974961 |
| Emid1 | -0.6604764 |
| Emid2 | -0.7134514 |
| Emilin1 | 1.4075464 |
| Emilin2 | -0.5734174 |
| Eml1 | -0.6523178 |
| Eml3 | -0.5416136 |
| Eml3 | -0.6195411 |
| Eml4 | -0.7388046 |
| Eml5 | -0.7639038 |
| Emp1 | -0.6000659 |
| Emp2 | -0.7748548 |
| Emp3 | -0.6031568 |
| Emr1 | -0.2700701 |
| Emr1 | 1.1512279 |
| Emr1 | -0.577936 |
| En2 | 1.2905906 |
| Enah | 1.2076351 |
| Enc1 | 0.651797 |
| Endod1 | -0.037186 |
| Endogl1 | -0.567424 |
| Endogl1 | 0.835166 |
| Eng | 4.7744721 |
| Eno1 | -0.5387054 |
| Eno2 | -0.6720105 |
| Eno3 | 2.0283236 |
| Enoph1 | 0.767716 |
| Enox1 | 0.909325 |
| Enpp1 | 0.9986775 |
| Enpp1 | 2.6513414 |
| Enpp2 | -0.5908497 |
| Enpp4 | -0.5877963 |
| Enpp5 | -0.3992548 |
| Enpp6 | 0.3094635 |
| Ensa | 2.1263885 |
| Ensa | -0.2017967 |
| ENSMUSG00000043795 | -0.0581532 |
| ENSMUSG00000053178 | 0.840727 |
| ENSMUSG00000068790 | 0.2567669 |
| Entpd2 | 1.0302629 |
| Entpd3 | 2.2205198 |
| Entpd4 | 1.0965105 |
| Entpd5 | -0.5839925 |
| Entpd5 | 0.7161386 |
| Entpd5 | 0.4909125 |
| Entpd6 | 0.0746945 |
| Entpd7 | 1.2399995 |
| Ep300 | -0.5129029 |
| Ep400 | 0.1180064 |
| Epas1 | 1.9668199 |
| Epb4.1 | 0.9530907 |
| Epb4.1l1 | -0.3730451 |
| Epb4.1l1 | 0.9316987 |
| Epb4.1l1 | 1.8956863 |
| Epb4.1l2 | 0.3547647 |
| Epb4.1l3 | 1.2483012 |
| Epb4.1l3 | -0.7432411 |
| Epb4.1l3 | 1.3317458 |
| Epb4.1l4a | -0.6670511 |
| Epb4.1l5 | -0.55776 |
| Epb4.1l5 | -0.1325019 |
| Epb4.9 | -0.420337 |
| Epb4.9 | 1.8593908 |
| Epc1 | 1.3004815 |
| Epc1 | 0.4638791 |
| Epc1 | 2.8965474 |
| Epdr1 | -0.8324391 |
| Epgn | -0.650694 |
| Epha1 | 1.641334 |
| Epha1 | -0.257358 |
| Epha3 | 0.8816625 |
| Epha3 | -0.5136108 |
| Epha3 | 1.5154654 |
| Epha4 | -0.7117262 |
| Epha5 | -0.1858106 |
| Epha6 | 0.2905022 |
| Epha7 | 0.0327392 |
| Epha7 | -0.5065541 |
| Epha7 | 2.491462 |
| Ephb1 | -0.6870365 |
| Ephb2 | 0.8837903 |
| Ephb4 | 1.8587848 |
| Ephb6 | 0.2570967 |
| Ephx1 | 0.1304211 |
| Ephx2 | -0.3462988 |
| Epm2a | 0.0127703 |
| Epm2aip1 | 1.8518231 |
| Epm2aip1 | 0.6203211 |
| Epm2aip1 | -0.408145 |
| Epn1 | 1.1016613 |
| Epn2 | 0.9387681 |
| Epn2 | -0.7125884 |
| Epn2 | 0.9997832 |
| Epn2 | -0.603931 |
| Epn2 | 1.1552957 |
| Epor | 0.5278843 |
| Eprs | -0.6539443 |
| Eprs | -0.634598 |
| Eps15 | 0.9044778 |
| Eps15l1 | -0.5666773 |
| Eps8 | 2.1144368 |
| Eps8l1 | -0.6442247 |
| Eps8l2 | 0.5647643 |
| Eps8l2 | -0.5445301 |
| Eps8l3 | 1.7723342 |
| Epsti1 | 0.0281978 |
| Epsti1 | 1.5425544 |
| Eraf | 0.5977589 |
| Eral1 | -0.0003396 |
| Ercc1 | 1.9448582 |
| Ercc2 | 1.319977 |
| Ercc3 | 0.1334078 |
| Ercc3 | 1.5571034 |
| Ercc4 | -0.7757728 |
| Ercc4 | 1.3556561 |
| Ercc5 | -0.3109336 |
| Ercc5 | -0.5481877 |
| Ercc8 | -0.5200043 |
| Ercc8 | 0.2623591 |
| Erdr1 | -0.4274635 |
| Erf | -0.6458381 |
| Erf | 0.3517671 |
| Ergic1 | 1.5645254 |
| Ergic2 | 0.6956425 |
| Ergic2 | 0.5049363 |
| Ergic3 | 0.3894795 |
| Ergic3 | 1.2936625 |
| Erh | -0.5336363 |
| Erich1 | -0.5607237 |
| Erlin1 | 1.2517504 |
| Erlin2 | 2.6884933 |
| Erlin2 | 0.3610308 |
| Ermap | -0.4842989 |
| Ermp1 | -0.5969845 |
| Ern1 | 1.4109127 |
| Ern2 | -0.6596576 |
| Ero1lb | -0.5585001 |
| Erp29 | -0.2159297 |
| Errfi1 | 2.4209698 |
| Es22 | -0.6563889 |
| Esam1 | -0.1132755 |
| Esco2 | 1.5371945 |
| Esd | 0.7494668 |
| Esd | 1.9265726 |
| Esd | 2.8602826 |
| Esd | -0.7575702 |
| Esm1 | -0.4740336 |
| Espl1 | 0.1278004 |
| Espn | 0.5020954 |
| Espn | 1.7604493 |
| Espn | -0.7117262 |
| Espn | -0.4706349 |
| Esr1 | -0.2318688 |
| Esr2 | -0.8490909 |
| Esr2 | -0.3632369 |
| Esr2 | -0.436607 |
| Esrra | -0.5430708 |
| Esrrb | -0.7065681 |
| Etaa1 | 1.913196 |
| Etf1 | 3.0452028 |
| Etfa | -0.5518586 |
| Etfa | -0.5016436 |
| Etfa | -0.4891255 |
| Etfb | 1.0801699 |
| Etfb | 1.4680724 |
| Etfdh | -0.2164569 |
| Ethe1 | 0.3487605 |
| Etnk2 | 0.3693255 |
| Ets1 | 0.8745372 |
| Ets2 | -0.703144 |
| Etsrp71 | 0.8256413 |
| Etv1 | -0.4822375 |
| Etv1 | 1.2174794 |
| Etv4 | -0.7134514 |
| Etv5 | -0.6588394 |
| Etv6 | -0.6845165 |
| Evc | -0.4713137 |
| Evc2 | -0.0781852 |
| Evi1 | 0.792645 |
| Evi1 | -0.1945458 |
| Evi2a | -0.4498148 |
| Evi2a | -0.5741691 |
| Evi5 | 1.7011936 |
| Evi5l | -0.7082845 |
| Evl | -0.4625244 |
| Evx1 | -0.3243441 |
| Exdl1 | -0.5962156 |
| Exdl2 | -0.4100603 |
| Exdl2 | 0.7885652 |
| Exdl2 | 1.8151502 |
| Exoc2 | 0.8521235 |
| Exoc3 | -0.4605069 |
| Exoc3l | 1.2519963 |
| Exoc4 | 1.4751919 |
| Exoc7 | -0.530031 |
| Exoc8 | -0.5756742 |
| Exod1 | -0.6531307 |
| Exosc1 | 0.4851428 |
| Exosc10 | 1.0710905 |
| Exosc2 | 0.4689699 |
| Exosc2 | -0.5939124 |
| Exosc4 | 0.1164912 |
| Exosc5 | -0.3986228 |
| Exosc6 | 1.6155186 |
| Exosc7 | 0.4399591 |
| Exosc8 | -0.1590531 |
| Ext1 | -0.6720105 |
| Ext2 | 0.0384897 |
| Extl1 | 1.3242205 |
| Extl2 | 1.6217578 |
| Extl3 | 1.0149827 |
| Extl3 | 2.6999067 |
| Eya1 | 0.0026356 |
| Eya1 | -0.6211155 |
| Eya2 | -0.5178686 |
| Eya3 | 0.1119318 |
| Eya3 | 2.7850419 |
| Eya3 | -0.6282308 |
| Eya4 | -0.426164 |
| Ezh1 | -0.705711 |
| Ezh2 | 1.368224 |
| Ezh2 | -0.703144 |
| F10 | -0.7335066 |
| F11 | -0.5401584 |
| F11 | -0.4094215 |
| F11r | -0.6637584 |
| F13a1 | -0.6474542 |
| F2r | 0.8670012 |
| F2rl1 | -0.4352957 |
| F2rl3 | 0.7785878 |
| F430201B04Rik | 0.6786364 |
| F5 | 1.231629 |
| F630043A04Rik | -0.6078109 |
| F730014I05Rik | 0.053935 |
| F730047E07Rik | 1.4410193 |
| F730047E07Rik | 0.7541105 |
| F730047E07Rik | -0.7629965 |
| F8 | -0.1786733 |
| F8 | 1.8197816 |
| F8 | -0.5885588 |
| F8a | 1.4319065 |
| Fa2h | -0.0906775 |
| Faah | 0.1548011 |
| Faah | 0.4275045 |
| Fabp3 | -0.643419 |
| Fabp5 | 1.2302481 |
| Fabp9 | 1.7418636 |
| Fadd | 2.1398326 |
| Fadd | 0.5782838 |
| Fadd | 1.1197648 |
| Fads1 | 0.8220698 |
| Fads2 | 0.9138053 |
| Fads3 | -0.5629522 |
| Fads6 | 0.2344202 |
| Faf1 | 0.4177101 |
| Faf1 | 2.8875056 |
| Fah | -0.6720105 |
| Fahd1 | 1.3105084 |
| Fahd2a | -0.5329142 |
| Fahd2a | -0.3638471 |
| Fahd2a | 1.4054368 |
| Fahd2a | 0.6017497 |
| Faim | -0.4151856 |
| Faim | -0.5923799 |
| Faim2 | 0.4106554 |
| Faim2 | 1.9705648 |
| Faim3 | 1.2267244 |
| Fancc | 1.7529214 |
| Fancc | 0.1548011 |
| Fancd2 | 0.9283148 |
| Fancg | -0.5540676 |
| Fanci | -0.6023831 |
| Fancl | 1.7626745 |
| Fancl | -0.6753303 |
| Fank1 | 0.2866667 |
| Fank1 | -0.0456097 |
| Fap | -0.195062 |
| Fars2 | -0.7831468 |
| Farsa | -0.5771815 |
| Farsb | -0.3468989 |
| Farsb | -0.4767608 |
| Fas | -0.7971202 |
| Fas | 0.5937521 |
| Fasl | -0.4788111 |
| Fastk | 0.6423895 |
| Fastk | -0.6266453 |
| Fastkd1 | 1.4525729 |
| Fastkd5 | 1.1867356 |
| Fat1 | 1.3785789 |
| Fat4 | -0.2518805 |
| Fbf1 | -0.0545534 |
| Fblim1 | -0.7379197 |
| Fbln1 | 0.174319 |
| Fbln1 | 0.4196766 |
| Fbln1 | -0.5629522 |
| Fbln2 | 0.2235699 |
| Fbln2 | -0.5408857 |
| Fbn1 | -0.568919 |
| Fbp1 | 1.9626966 |
| Fbp2 | 0.3082104 |
| Fbxl10 | 0.6414889 |
| Fbxl12 | 0.8523063 |
| Fbxl12 | -0.7247379 |
| Fbxl12 | -0.4774438 |
| Fbxl12 | -0.5977539 |
| Fbxl13 | 0.7203133 |
| Fbxl13 | 0.4528051 |
| Fbxl14 | 0.3574549 |
| Fbxl15 | 0.7766198 |
| Fbxl18 | 0.0310902 |
| Fbxl18 | -0.2739711 |
| Fbxl21 | 2.1239059 |
| Fbxl22 | -0.7317467 |
| Fbxl4 | -0.620328 |
| Fbxl5 | -0.3535232 |
| Fbxl6 | -0.4747147 |
| Fbxl6 | -0.650694 |
| Fbxl8 | 0.0719247 |
| Fbxo15 | 0.2824952 |
| Fbxo16 | -0.4379201 |
| Fbxo18 | 0.0454287 |
| Fbxo18 | 0.4557881 |
| Fbxo2 | 0.050703 |
| Fbxo21 | 1.3323137 |
| Fbxo25 | -0.2175123 |
| Fbxo27 | 0.2959108 |
| Fbxo28 | 0.0294384 |
| Fbxo3 | -0.1822356 |
| Fbxo3 | 1.11766 |
| Fbxo30 | 2.4909583 |
| Fbxo31 | 0.4078197 |
| Fbxo31 | -0.7100039 |
| Fbxo32 | -0.1705777 |
| Fbxo32 | -0.2502431 |
| Fbxo32 | -0.6621161 |
| Fbxo33 | 0.0989003 |
| Fbxo34 | -0.3885639 |
| Fbxo34 | -0.6645806 |
| Fbxo34 | 1.2149317 |
| Fbxo36 | 1.0514835 |
| Fbxo38 | 0.374622 |
| Fbxo38 | 0.0735084 |
| Fbxo4 | 0.8713144 |
| Fbxo42 | -0.3385304 |
| Fbxo44 | 2.1084074 |
| Fbxo6 | -0.4706349 |
| Fbxo7 | -0.5704163 |
| Fbxo8 | -0.4431893 |
| Fbxo9 | 0.793614 |
| Fbxo9 | -0.2795702 |
| Fbxw12 | 1.5039441 |
| Fbxw13 | 0.6688196 |
| Fbxw15 | 0.2679204 |
| Fbxw16 | 1.1246589 |
| Fbxw17 | 0.3517671 |
| Fbxw19 | -0.0944088 |
| Fbxw2 | -0.7291127 |
| Fbxw4 | 0.5799611 |
| Fbxw4 | 1.2385054 |
| Fbxw5 | 0.8528544 |
| Fbxw7 | 0.2018609 |
| Fbxw7 | -0.3474993 |
| Fbxw7 | 1.3270776 |
| Fbxw8 | -0.3517122 |
| Fcer1g | -0.0380694 |
| Fcer2a | -0.530751 |
| Fcf1 | -0.6753303 |
| Fcgr2b | 2.0679568 |
| Fcgr2b | 0.092709 |
| Fcgr2b | -0.4856756 |
| Fcgr3 | 0.1084986 |
| Fcgr4 | 0.9174094 |
| Fcgrt | -0.4918939 |
| Fcho1 | -0.4733529 |
| Fcho2 | -0.342706 |
| Fchsd1 | -0.1199631 |
| Fchsd2 | 0.6764631 |
| Fcna | 0.2860261 |
| Fcrla | 1.4567964 |
| Fcrla | -0.0786451 |
| Fcrla | 3.7655813 |
| Fdps | 1.3298129 |
| Fdxr | -0.0635771 |
| Fdxr | 2.1756841 |
| Fem1b | 1.5619972 |
| Fem1c | -0.5221445 |
| Fem1c | -0.7370355 |
| Fen1 | -0.4822375 |
| Fermt2 | 2.242764 |
| Fermt3 | 0.0579606 |
| Fert2 | 2.3183354 |
| Fert2 | 0.1397254 |
| Fes | 2.6695008 |
| Fetub | -0.0920751 |
| Fev | 0.8535847 |
| Fez1 | -0.7343877 |
| Fez1 | 0.7603358 |
| Fez2 | -0.6474542 |
| Fezf2 | 1.8062488 |
| Ffar2 | -0.5599819 |
| Fgd2 | -0.6547585 |
| Fgd4 | -0.3817062 |
| Fgd4 | -0.7548679 |
| Fgd5 | -0.3080417 |
| Fgd6 | 0.7176017 |
| Fgf1 | -0.568919 |
| Fgf1 | 3.2059712 |
| Fgf10 | 1.2141661 |
| Fgf10 | -0.6148326 |
| Fgf12 | 1.6591426 |
| Fgf12 | 0.606891 |
| Fgf13 | 0.5849762 |
| Fgf13 | 2.1522945 |
| Fgf17 | 1.8691047 |
| Fgf21 | 2.7965088 |
| Fgf22 | 1.1661973 |
| Fgf22 | 0.6990521 |
| Fgf5 | -0.0349809 |
| Fgf7 | -0.4326782 |
| Fgf8 | 0.5937521 |
| Fgf9 | 0.5792426 |
| Fgfbp3 | -0.6490728 |
| Fgfr1 | -0.5954473 |
| Fgfr1op | -0.8151023 |
| Fgfr1op2 | -0.5107822 |
| Fgfr1op2 | 0.4695043 |
| Fgfr2 | 0.0992859 |
| Fgfr2 | -0.4222756 |
| Fgfr3 | -0.4126197 |
| Fgfrl1 | -0.6377967 |
| Fgl2 | 0.475364 |
| Fh1 | 0.1793181 |
| Fhad1 | -0.6523178 |
| Fhit | 1.7335853 |
| Fhl1 | 1.912366 |
| Fhl1 | -0.3034319 |
| Fhl2 | 0.5916245 |
| Fhl4 | -0.7584725 |
| Fhl5 | 1.957946 |
| Fhod1 | 2.7613791 |
| Fhod3 | -0.5250052 |
| Fibcd1 | 2.2880791 |
| Fibp | 0.3637044 |
| Figf | -0.7766916 |
| Figla | -0.5719157 |
| Fign | -0.4248662 |
| Fignl1 | -0.6322056 |
| Fip1l1 | -0.2745296 |
| Fis1 | 2.1608673 |
| Fis1 | -0.3942097 |
| Fiz1 | 0.1660519 |
| Fjx1 | 0.7846643 |
| Fkbp10 | 3.1396824 |
| Fkbp11 | -0.0745132 |
| Fkbp14 | -0.461179 |
| Fkbp1a | 1.002936 |
| Fkbp1b | 0.1667735 |
| Fkbp2 | -0.6418094 |
| Fkbp2 | 0.7504781 |
| Fkbp4 | -0.426164 |
| Fkbp5 | -0.0681194 |
| Fkbp6 | 0.4095221 |
| Fkbp7 | 0.1274255 |
| Fkbp8 | -0.1276608 |
| Fkbp9 | -0.4692786 |
| Fkbpl | -0.4235701 |
| Fkbpl | -0.5893219 |
| Fkhl18 | 0.5942244 |
| Fkrp | 1.1482344 |
| Fktn | -0.3155779 |
| Flad1 | 2.2786624 |
| Flcn | 2.2650235 |
| Flcn | -0.5496544 |
| Fli1 | -0.0363034 |
| Flii | 1.1225643 |
| Flna | 2.1653005 |
| Flot1 | 0.7920632 |
| Flot1 | -0.6361961 |
| Flot2 | -0.567424 |
| Flot2 | 2.3762132 |
| Flot2 | -0.2868958 |
| Flrt1 | 1.2977829 |
| Flrt2 | -0.3675162 |
| Flrt3 | -0.3499045 |
| Flt1 | 0.2361051 |
| Flt4 | 0.2844227 |
| Flt4 | -0.3302308 |
| Flvcr2 | 1.6100167 |
| Flywch2 | -0.705711 |
| Fmn2 | 0.0571567 |
| Fmn2 | -0.3644577 |
| Fmnl1 | 1.5012591 |
| Fmnl3 | -0.61405 |
| Fmnl3 | 1.0516334 |
| Fmo1 | -0.5870344 |
| Fmo2 | 1.1911854 |
| Fmo5 | -0.6645806 |
| Fmod | -0.4829242 |
| Fmr1nb | -0.2790089 |
| Fmr1nb | -0.6062571 |
| Fn3k | 1.9736349 |
| Fn3k | 0.1598977 |
| Fnbp1 | 1.0448621 |
| Fnbp1 | 1.1435906 |
| Fnbp1 | 2.1902864 |
| Fnbp1l | 2.5355501 |
| Fnbp4 | -0.7476971 |
| Fndc3 | -0.1161362 |
| Fndc3a | 0.8594083 |
| Fndc3b | 0.8684409 |
| Fndc4 | 0.5637917 |
| Fndc5 | -0.6070337 |
| Fndc5 | 0.178249 |
| Fndc7 | -0.5051487 |
| Fndc7 | -0.5659312 |
| Fndc8 | -0.3361521 |
| Fndc8 | -0.6547585 |
| Fnip1 | -0.5696674 |
| Fnip1 | -0.4981507 |
| Fnta | -0.7005836 |
| Fnta | -0.4946699 |
| Fntb | 1.1513638 |
| Folr1 | -0.4884345 |
| Folr1 | -0.5570204 |
| Folr2 | 1.5709976 |
| Fos | 1.7314477 |
| Fosb | -0.1570622 |
| Foxa1 | 2.2713964 |
| Foxa2 | -0.2270605 |
| Foxa3 | 0.7117365 |
| Foxc1 | -0.7117262 |
| Foxd2 | 2.9787605 |
| Foxg1 | 0.9872327 |
| Foxh1 | 1.1615012 |
| Foxh1 | 0.3166385 |
| Foxj1 | -0.0874238 |
| Foxj2 | -0.8490909 |
| Foxj3 | 2.142683 |
| Foxj3 | -0.4912011 |
| Foxj3 | 1.0495317 |
| Foxj3 | 1.0691764 |
| Foxj3 | 2.6719697 |
| Foxk1 | 0.1874776 |
| Foxk1 | -0.1257309 |
| Foxk1 | 1.6422528 |
| Foxl2 | -0.368129 |
| Foxm1 | -0.3421084 |
| Foxm1 | -0.5916145 |
| Foxn2 | 3.238159 |
| Foxn3 | -0.1466739 |
| Foxo1 | 0.7518923 |
| Foxo6 | -0.6124866 |
| Foxo6 | -0.4870541 |
| Foxp1 | -0.2286608 |
| Foxp2 | 0.4221994 |
| Foxp2 | -0.5764276 |
| Foxp2 | -0.6761619 |
| Foxp3 | 0.4743011 |
| Foxp4 | 1.6128574 |
| Foxq1 | 2.5706344 |
| Foxred1 | -0.042498 |
| Foxred1 | -0.1966122 |
| Foxred1 | -0.0231559 |
| Foxred1 | 1.8843236 |
| Foxred1 | -0.1695704 |
| Foxred2 | 1.1427689 |
| Fpgs | 0.7526995 |
| Fpgs | 0.1821637 |
| Fpgs | -0.0162134 |
| Fpr2 | -0.2007577 |
| Frag1 | 0.3860033 |
| Frag1 | 0.8038281 |
| Frag1 | -0.567424 |
| Frap1 | 2.5987351 |
| Fras1 | 2.2313309 |
| Fras1 | -0.3493027 |
| Frat1 | 0.9115676 |
| Frat1 | -0.5734174 |
| Frat2 | 0.9444548 |
| Freq | 1.8255064 |
| Freq | -0.0327806 |
| Freq | -0.4932809 |
| Frg1 | -0.3237573 |
| Frk | -0.72999 |
| Frmd3 | -0.5540676 |
| Frmd6 | -0.0841807 |
| Frmd8 | 2.1846464 |
| Frmpd4 | -0.7014363 |
| Frrs1 | -0.3196593 |
| Frs2 | 1.6551939 |
| Frs3 | 1.2327574 |
| Fryl | -0.3773663 |
| Fryl | -0.901803 |
| Frzb | -0.809389 |
| Fscn1 | 4.132038 |
| Fscn2 | -0.2965565 |
| Fscn3 | 0.1274255 |
| Fsd1 | 1.7876286 |
| Fsd2 | 1.4399995 |
| Fshb | 0.4590323 |
| Fshr | 1.5778735 |
| Fshr | -0.5250052 |
| Fshr | -0.4665715 |
| Fsip1 | 1.4954814 |
| Fst | -0.5607237 |
| Fstl1 | -0.7675409 |
| Fstl4 | -0.125249 |
| Fstl5 | -0.5401584 |
| Fth1 | 1.3221583 |
| Fth1 | 0.7403189 |
| Ftmt | 1.1201852 |
| Fto | -0.8392624 |
| Ftsj1 | -0.0279568 |
| Ftsj3 | -0.158555 |
| Fubp1 | -0.598524 |
| Fubp3 | 0.6285679 |
| Fuk | 3.6886262 |
| Fuk | 0.5778041 |
| Fundc1 | -0.2426366 |
| Fundc1 | 1.8550058 |
| Fundc2 | 3.8030117 |
| Furin | -0.6662269 |
| Furin | -0.5079615 |
| Fus | 0.922536 |
| Fusip1 | 3.3196123 |
| Fusip1 | -0.426164 |
| Fusip1 | -0.6266453 |
| Fut10 | 0.0750895 |
| Fut10 | 0.0450219 |
| Fut2 | -0.2784479 |
| Fut4 | 0.0169633 |
| Fut8 | -0.5832334 |
| Fut8 | -0.4518109 |
| Fut8 | -0.6997315 |
| Fvt1 | -0.7065681 |
| Fxc1 | -0.2372383 |
| Fxn | -0.7405769 |
| Fxn | -0.4158281 |
| Fxyd1 | 1.9213319 |
| Fxyd2 | 2.6678716 |
| Fxyd2 | 0.4177101 |
| Fxyd2 | -0.9038864 |
| Fxyd3 | -0.673669 |
| Fxyd4 | -0.4222756 |
| Fxyd4 | -0.5445301 |
| Fxyd5 | 1.5103399 |
| Fxyd5 | 0.1206527 |
| Fxyd5 | 0.0954224 |
| Fxyd6 | -0.2739711 |
| Fxyd7 | -0.5408857 |
| Fyb | -0.5445301 |
| Fyco1 | -0.5416136 |
| Fyn | 1.761785 |
| Fyttd1 | -0.7343877 |
| Fzd1 | 0.0339742 |
| Fzd10 | -0.7151796 |
| Fzd10 | -0.5372545 |
| Fzd2 | 0.4140478 |
| Fzd3 | 1.0782729 |
| Fzd4 | -0.5178686 |
| Fzd5 | -0.5143192 |
| Fzd6 | -0.3456991 |
| Fzd7 | -0.6163997 |
| Fzd8 | -0.6054811 |
| Fzr1 | 1.6186858 |
| Fzr1 | 3.1346964 |
| Fzr1 | 0.8154573 |
| G0s2 | 0.1803862 |
| G3bp1 | -0.2857653 |
| G3bp1 | 0.1750347 |
| G3bp2 | 0.3903467 |
| G3bp2 | 0.2965452 |
| G3bp2 | 1.8464508 |
| G430022H21Rik | 0.610846 |
| G430055L02Rik | -0.5741691 |
| G6pc | 0.2350945 |
| G6pc2 | 0.2232289 |
| G6pc2 | -0.4471593 |
| G6pc3 | 0.4901277 |
| G6pd2 | -0.1550752 |
| G6pd2 | -0.1000319 |
| G6pdx | -0.527156 |
| Gaa | -0.1461819 |
| Gab1 | 1.4289193 |
| Gab2 | 1.8316833 |
| Gab2 | 1.3372968 |
| Gab2 | 2.0118501 |
| Gab3 | -0.6482631 |
| Gab3 | -0.3379353 |
| Gab3 | 0.093485 |
| Gabarap | -0.1291107 |
| Gabarapl1 | 1.3909732 |
| Gabarapl2 | 0.9811397 |
| Gabbr1 | -0.6179692 |
| Gabpa | -0.8431824 |
| Gabpa | -0.0170786 |
| Gabpb1 | -0.2612099 |
| Gabpb1 | -0.8131943 |
| Gabpb2 | -0.2617614 |
| Gabra3 | -0.4794954 |
| Gabra4 | -0.7100039 |
| Gabrb1 | -0.7840723 |
| Gabrb2 | -0.7785317 |
| Gabrb3 | -0.6515056 |
| Gabrb3 | -0.6250622 |
| Gabrd | -0.6580219 |
| Gabrd | 0.0996715 |
| Gabrg1 | 1.5114812 |
| Gabrg2 | 2.7196317 |
| Gabrg2 | -0.7396904 |
| Gabrg2 | -0.8112899 |
| Gabrg2 | -0.342706 |
| Gabrp | -0.6482631 |
| Gabrp | -0.6794955 |
| Gad1 | -0.6670511 |
| Gad1 | -0.3992548 |
| Gadd45a | 0.7559218 |
| Gadd45a | 0.8973378 |
| Gadd45b | 0.1345256 |
| Gadd45g | 1.7148038 |
| Gadd45g | 1.5730564 |
| Gak | -0.7711912 |
| Gal | -0.1223624 |
| Gal3st1 | -0.2420955 |
| Gal3st2 | 0.6851282 |
| Gal3st3 | 2.9851292 |
| Galc | -0.8074918 |
| Galc | 0.4838269 |
| Gale | -0.3767479 |
| Gale | -0.8640157 |
| Galk1 | -0.3742778 |
| Galm | 2.7001114 |
| Galns | 1.0946441 |
| Galnt1 | 0.4503579 |
| Galnt1 | -0.0786451 |
| Galnt1 | -0.2999883 |
| Galnt1 | -0.103798 |
| Galnt10 | 1.4132204 |
| Galnt10 | 0.9690048 |
| Galnt10 | 0.4049759 |
| Galnt11 | -0.4788111 |
| Galnt12 | -0.1868343 |
| Galnt13 | -0.7575702 |
| Galnt2 | 0.8449698 |
| Galnt2 | 0.7163477 |
| Galnt2 | -0.5939124 |
| Galnt3 | 1.5300345 |
| Galnt9 | -0.8027642 |
| Galntl2 | 1.0829361 |
| Galntl4 | -0.1700739 |
| Galntl5 | 2.6847589 |
| Galt | 1.1637849 |
| Galt | 0.0806042 |
| Gamt | 2.9651145 |
| Gan | -0.4822375 |
| Ganab | -0.2212148 |
| Ganab | 1.1794956 |
| Ganab | 0.6255976 |
| Ganc | 0.3932319 |
| Gap43 | 0.5937521 |
| Gapdh | -0.7177774 |
| Gapdhs | -0.3595833 |
| Gapvd1 | 2.3293775 |
| Gapvd1 | 1.1540771 |
| Garnl3 | -0.6828401 |
| Garnl4 | -0.5570204 |
| Gars | 3.225056 |
| Gart | -0.5072575 |
| Gart | 0.2731267 |
| Gas2l1 | 1.0273482 |
| Gas2l1 | 3.1017016 |
| Gas2l1 | 2.106091 |
| Gas2l1 | 2.3726991 |
| Gas6 | -0.6242717 |
| Gas7 | 0.7518923 |
| Gas7 | -0.5228589 |
| Gas8 | -0.657205 |
| Gata2 | 0.162074 |
| Gata4 | -0.5394317 |
| Gata6 | -0.7160448 |
| Gatad1 | -0.6811664 |
| Gatad1 | -0.2367001 |
| Gatad2b | 0.9537519 |
| Gats | -0.5585001 |
| Gats | 4.3735712 |
| Gba | -0.0218506 |
| Gba2 | 1.8929553 |
| Gbas | 2.0877947 |
| Gbf1 | -0.7117262 |
| Gbl | -0.4584935 |
| Gbl | -0.2937055 |
| Gbl | 0.4399591 |
| Gbp2 | -0.1156589 |
| Gbp3 | -0.6016101 |
| Gbp3 | 0.8871501 |
| Gbp6 | -0.6402024 |
| Gbp6 | 2.355478 |
| Gbx2 | 1.7929633 |
| Gca | -0.5358057 |
| Gca | 0.3293034 |
| Gcap14 | -0.5372545 |
| Gcap14 | 0.8781061 |
| Gcc1 | -0.5100763 |
| Gcc2 | -0.5614659 |
| Gcdh | 0.709844 |
| Gch1 | -0.8027642 |
| Gch1 | 3.0386602 |
| Gchfr | 1.0975141 |
| Gck | -0.6703546 |
| Gclm | -0.2049203 |
| Gcn5l2 | 2.3797954 |
| Gcnt1 | 3.7056428 |
| Gcnt1 | -0.5794467 |
| Gcnt2 | 1.0391085 |
| Gcnt2 | 0.3078969 |
| Gcs1 | 0.1685752 |
| Gda | 2.1712471 |
| Gdap1 | -0.2852006 |
| Gdap1 | 0.3883221 |
| Gdap1l1 | 1.4482305 |
| Gdap1l1 | -0.798998 |
| Gde1 | 1.6326897 |
| Gde1 | -0.6117058 |
| Gdf10 | -0.3511093 |
| Gdf15 | 1.1281402 |
| Gdf3 | -0.6490728 |
| Gdf9 | 3.0727976 |
| Gdi1 | 1.0412321 |
| Gdi1 | 0.7230176 |
| Gdi1 | 1.4084943 |
| Gdi1 | 1.2179882 |
| Gdi1 | 3.928772 |
| Gdi2 | -0.4692786 |
| Gdnf | -0.31616 |
| Gdpd1 | 2.2564783 |
| Gdpd2 | 1.3024715 |
| Gdpd2 | 1.3420305 |
| Gdpd3 | 0.0591651 |
| Gdpd4 | -0.6211155 |
| Gdpd5 | -0.3391259 |
| Gemin4 | -0.5009441 |
| Gemin4 | 1.0314876 |
| Gemin4 | 0.2201551 |
| Gemin5 | 1.2148041 |
| Gemin6 | -0.7014363 |
| Gemin6 | -0.749485 |
| Gemin7 | 0.50674 |
| Gemin7 | -0.7432411 |
| Gfap | 0.0347967 |
| Gfer | -0.8822219 |
| Gfi1 | -0.2667384 |
| Gfm1 | -0.257358 |
| Gfod1 | -0.5136108 |
| Gfod2 | 0.6479998 |
| Gfpt2 | -0.1047418 |
| Gfra1 | -0.1720907 |
| Gfra4 | 0.3749155 |
| Gga1 | 1.5120513 |
| Gga1 | -0.4733529 |
| Gga1 | -0.3214136 |
| Gga3 | -0.1104229 |
| Ggcx | 0.0950352 |
| Ggcx | -0.5401584 |
| Ggcx | -0.4425291 |
| Ggn | -0.7238652 |
| Ggn | 2.2280203 |
| Ggnbp1 | -0.5445301 |
| Ggnbp2 | 1.8728978 |
| Ggt5 | 1.3268493 |
| Ggt7 | -0.6963305 |
| Ggta1 | -0.0407242 |
| Ggtla1 | -0.1295945 |
| Gh | -0.7803752 |
| Ghitm | -0.5794467 |
| Ghr | -0.6078109 |
| Ghr | -0.6654034 |
| Ghrh | -0.5387054 |
| Ghrhr | 1.5401552 |
| Ghrl | -0.3074644 |
| Ghrl | -0.5214306 |
| Ghsr | 1.0176231 |
| Ghsr | -0.417758 |
| Gif | -0.2739711 |
| Gigyf1 | 0.3874532 |
| Gimap1 | 0.5220693 |
| Gimap4 | -0.4672476 |
| Gimap5 | -0.7221221 |
| Gimap6 | 2.4471257 |
| Gimap8 | -0.2982709 |
| Gimap9 | -0.4638716 |
| Gins3 | 2.1534518 |
| Gins4 | -0.294275 |
| Gipc1 | -0.730868 |
| Gipc1 | 0.6466561 |
| Gipc1 | 0.936249 |
| Gipc2 | -0.0622184 |
| Git1 | 0.1270504 |
| Git2 | -0.6997315 |
| Git2 | 0.8546792 |
| Git2 | 1.0595507 |
| Gja1 | 1.1151285 |
| Gja4 | 0.1270504 |
| Gja5 | -0.2722974 |
| Gja5 | 2.2101621 |
| Gja6 | 0.2623591 |
| Gja7 | 0.2259534 |
| Gjb1 | 0.4587623 |
| Gjb2 | -0.36568 |
| Gjb2 | -0.7091438 |
| Gjb3 | 0.8883851 |
| Gjb6 | 1.2250842 |
| Gjc2 | 1.3517659 |
| Gjc2 | 0.5159655 |
| Gjd2 | 2.0720539 |
| Gjd2 | -0.6539443 |
| Gjd3 | -0.6298188 |
| Gk2 | -0.6904062 |
| Gk5 | -0.5438002 |
| Gkap1 | 0.5564667 |
| Gkap1 | 2.2012329 |
| Glb1 | 1.5851448 |
| Glb1l2 | -0.6580219 |
| Glcci1 | -0.4932809 |
| Gldc | 1.3464063 |
| Gldc | -0.3462988 |
| Gldn | -0.2143495 |
| Gle1 | -0.0188113 |
| Glg1 | -0.6744993 |
| Glipr2 | 2.088814 |
| Glis1 | 1.1005189 |
| Glo1 | -0.7014363 |
| Gloxd1 | 1.5719827 |
| Glp2r | -0.2885939 |
| Glra2 | 2.5674373 |
| Glrb | 0.5618436 |
| Glrp1 | 0.360138 |
| Glrx | 0.4705723 |
| Glrx2 | 1.0635602 |
| Glrx2 | -0.3481001 |
| Glrx2 | -0.4281139 |
| Glrx3 | 0.7388884 |
| Glrx5 | 1.4751919 |
| Gls2 | 2.0471063 |
| Gls2 | 1.2105857 |
| Glt25d1 | 0.2792745 |
| Glt25d2 | -0.5394317 |
| Glt25d2 | -0.0804869 |
| Glt6d1 | -0.6547585 |
| Glt8d1 | 1.1107536 |
| Glt8d1 | -0.4544786 |
| Glt8d1 | 0.6964959 |
| Glt8d2 | 1.4662846 |
| Glt8d3 | -0.0037507 |
| Gltp | 1.3651517 |
| Gltscr1 | -0.6093671 |
| Glud1 | 2.1156906 |
| Glud1 | -0.0305851 |
| Glul | -0.5992946 |
| Glyctk | 0.3631109 |
| Glyctk | -0.6156158 |
| Glyctk | -0.6531307 |
| Gm1006 | 1.0715317 |
| Gm1006 | -0.3992548 |
| Gm1019 | -0.431372 |
| Gm1040 | 2.0587185 |
| Gm1070 | 3.4277208 |
| Gm1082 | -0.1354178 |
| Gm114 | -0.417758 |
| Gm114 | 0.231042 |
| Gm1141 | -0.719513 |
| Gm128 | 0.2821736 |
| Gm129 | -0.1992011 |
| Gm129 | -0.1945458 |
| Gm1322 | -0.2846361 |
| Gm136 | 1.330723 |
| Gm136 | 1.0573162 |
| Gm1381 | -0.2458895 |
| Gm1527 | -0.6156158 |
| Gm1568 | -0.4981507 |
| Gm1574 | 0.8349801 |
| Gm1574 | -0.4268136 |
| Gm1604 | -0.577936 |
| Gm1604 | -0.7620901 |
| Gm166 | -0.7396904 |
| Gm166 | -0.6498831 |
| Gm1661 | -0.0093184 |
| Gm1673 | -0.581717 |
| Gm1673 | -0.0640304 |
| Gm1698 | 2.2265246 |
| Gm1821 | -0.5221445 |
| Gm1943 | -0.7849988 |
| Gm1965 | -0.0892818 |
| Gm1967 | -0.683678 |
| Gm1967 | 1.598922 |
| Gm216 | -0.8314681 |
| Gm239 | 2.530338 |
| Gm249 | 0.9144928 |
| Gm266 | 1.5485275 |
| Gm2a | -0.1909398 |
| Gm347 | -0.4842989 |
| Gm382 | -0.3954686 |
| Gm410 | -0.0823321 |
| Gm428 | -0.705711 |
| Gm443 | -0.1842769 |
| Gm484 | -0.5832334 |
| Gm50 | -0.7512761 |
| Gm525 | -0.2623132 |
| Gm525 | 0.0786382 |
| Gm527 | -0.2281271 |
| Gm527 | 1.3398918 |
| Gm561 | 0.7292307 |
| Gm561 | -0.8122416 |
| Gm597 | -0.2689584 |
| Gm603 | 1.3817188 |
| Gm609 | -0.7405769 |
| Gm614 | -0.5555429 |
| Gm614 | -0.6811664 |
| Gm648 | 1.6030968 |
| Gm648 | 1.521883 |
| Gm71 | -0.3373406 |
| Gm711 | 1.0913334 |
| Gm732 | 1.4928706 |
| Gm732 | -0.6298188 |
| Gm767 | 1.8422144 |
| Gm773 | -0.6008377 |
| Gm773 | -0.6078109 |
| Gm784 | 1.8423514 |
| Gm813 | -0.4190467 |
| Gm813 | -0.3057342 |
| Gm817 | -0.6070337 |
| Gm867 | -0.6337999 |
| Gm884 | -0.5423419 |
| Gm889 | 0.7325289 |
| Gm906 | -0.7602797 |
| Gm93 | 0.4514463 |
| Gm93 | -0.2377769 |
| Gm944 | -0.2420955 |
| Gm949 | 0.8919054 |
| Gm962 | 1.0756404 |
| Gm962 | 2.5592317 |
| Gm97 | 0.1857094 |
| Gm97 | 0.7192712 |
| Gmcl1 | 0.8770368 |
| Gmcl1 | -0.4733529 |
| Gmcl1 | 2.2189658 |
| Gmds | -0.1827456 |
| Gmeb2 | 0.1221617 |
| Gmfg | -0.5749214 |
| Gmfg | 0.0157072 |
| Gmfg | -0.0613136 |
| Gmip | 1.4782418 |
| Gmip | -0.6937873 |
| Gmppa | 1.023346 |
| Gmppa | -0.3255187 |
| Gmppb | 0.900827 |
| Gmpr | 1.2298711 |
| Gmpr2 | -0.3421084 |
| Gna11 | -0.6637584 |
| Gna13 | -0.379224 |
| Gna14 | -0.1700739 |
| Gnai2 | -0.6728394 |
| Gnao1 | -0.0205469 |
| Gnao1 | -0.5445301 |
| Gnaq | 0.9240689 |
| Gnaq | -0.720382 |
| Gna-rs1 | -0.5824749 |
| Gnas | -0.7648118 |
| Gnas | 1.4839235 |
| Gnas | 0.0679546 |
| Gnas | 0.6015154 |
| Gnas | -0.2931363 |
| Gnat1 | -0.6234817 |
| Gnat2 | 3.3743732 |
| Gnaz | 2.7672239 |
| Gnb1 | -0.6645806 |
| Gnb1l | 2.3862834 |
| Gnb2 | 0.1485775 |
| Gnb2l1 | 0.0750895 |
| Gnb3 | 0.3253049 |
| Gnb4 | 0.0619701 |
| Gnb4 | 1.8930204 |
| Gnb5 | 0.2249326 |
| Gnb5 | -0.2350871 |
| Gne | 0.2895447 |
| Gng10 | -0.3632369 |
| Gng11 | 1.060443 |
| Gng13 | 3.572509 |
| Gng2 | -0.2324045 |
| Gng2 | 1.4081785 |
| Gng3 | 1.0357624 |
| Gng4 | -0.6794955 |
| Gng7 | -0.2329404 |
| Gng8 | -0.4017869 |
| Gngt2 | 0.4843534 |
| Gnl2 | -0.0622184 |
| Gnl2 | -0.6418094 |
| Gnl3 | 0.3153943 |
| Gnl3 | -0.1286272 |
| Gnl3 | -0.567424 |
| Gnl3l | 0.939774 |
| Gnl3l | -0.7335066 |
| Gnmt | -0.5474551 |
| Gnmt | -0.0314627 |
| Gnpat | -0.6920953 |
| Gnpda1 | -0.5401584 |
| Gnpda1 | 1.7528466 |
| Gnpda1 | 0.9575457 |
| Gnpda2 | 1.2597122 |
| Gnptab | -0.6980296 |
| Gnptg | 1.3292436 |
| Gnptg | 0.9991515 |
| Gnrhr | 0.9947183 |
| Gns | 2.107671 |
| Golga1 | 0.7706927 |
| Golga2 | -0.7048546 |
| Golga2 | -0.6008377 |
| Golga2 | -0.4184021 |
| Golga2 | -0.5285925 |
| Golga2 | 1.3903303 |
| Golga3 | 0.2557769 |
| Golga4 | -0.6450311 |
| Golga5 | 0.3998369 |
| Golga7 | 0.3900577 |
| Golim4 | -0.61405 |
| Golm1 | 1.2980179 |
| Golm1 | 0.4576818 |
| Golph3l | -0.011468 |
| Golt1a | -0.6070337 |
| Golt1b | 0.7384793 |
| Gopc | -0.515028 |
| Gorasp1 | -0.5416136 |
| Gorasp2 | 1.5277924 |
| Gorasp2 | -0.3589756 |
| Gorasp2 | -0.5178686 |
| Gorasp2 | -0.3255187 |
| Gosr1 | -0.1986828 |
| Gosr2 | 1.7762892 |
| Gosr2 | 1.5963913 |
| Gosr2 | 1.4694607 |
| Gosr2 | 3.7355547 |
| Got1l1 | -0.2101479 |
| Got2 | 1.1274449 |
| Gp1bb | -0.5969845 |
| Gp38 | -0.6498831 |
| Gp5 | 0.2662879 |
| Gp9 | -0.6124866 |
| Gpaa1 | 1.0282695 |
| Gpam | -0.2852006 |
| Gpatch1 | -0.703144 |
| Gpatch2 | -0.0231559 |
| Gpatch3 | 0.0433928 |
| Gpatch4 | -0.7776112 |
| Gpbp1 | 0.6549135 |
| Gpbp1 | -0.6878779 |
| Gpbp1l1 | -0.5221445 |
| Gpc1 | -0.8065445 |
| Gpc3 | -0.4011533 |
| Gpc3 | 0.651351 |
| Gpc4 | -0.2313334 |
| Gpc5 | 0.8422048 |
| Gpc5 | 2.4742681 |
| Gpc5 | -0.7822221 |
| Gpd1 | -0.6887199 |
| Gpd2 | 2.1992646 |
| Gpd2 | 1.3197471 |
| Gper | -0.3091975 |
| Gpha2 | 1.5169791 |
| Gphb5 | 0.2987625 |
| Gphn | 1.6426702 |
| Gphn | -0.2914305 |
| Gphn | -0.4524772 |
| Gpihbp1 | 0.0442077 |
| Gpkow | 0.7261289 |
| Gpld1 | 0.1046702 |
| Gpld1 | -0.2393942 |
| Gpm6a | -0.6963305 |
| Gpm6a | 0.9191212 |
| Gpm6b | 1.0248872 |
| Gpnmb | 0.7007526 |
| Gpnmb | -0.2540679 |
| Gpr1 | -0.5562814 |
| Gpr1 | 1.0610375 |
| Gpr103 | 2.4895179 |
| Gpr107 | -0.6187548 |
| Gpr108 | 0.2692245 |
| Gpr108 | -0.500245 |
| Gpr108 | -0.3296406 |
| Gpr109a | -0.1000319 |
| Gpr113 | 0.3687353 |
| Gpr113 | -0.6662269 |
| Gpr114 | 0.8355377 |
| Gpr12 | 2.4374583 |
| Gpr12 | -0.6711822 |
| Gpr120 | 0.5059674 |
| Gpr123 | -0.0617659 |
| Gpr124 | 0.8477272 |
| Gpr128 | 2.9729093 |
| Gpr135 | 2.0896715 |
| Gpr137 | 2.246256 |
| Gpr137b | -0.2897276 |
| Gpr141 | -0.2350871 |
| Gpr146 | 0.6551357 |
| Gpr146 | 0.5243488 |
| Gpr149 | 0.9228769 |
| Gpr151 | -0.4398928 |
| Gpr153 | -0.5939124 |
| Gpr156 | 2.0889212 |
| Gpr156 | -0.5044467 |
| Gpr158 | 0.2273129 |
| Gpr162 | 1.044258 |
| Gpr162 | -0.6845165 |
| Gpr17 | 1.6459197 |
| Gpr172b | 1.6889926 |
| Gpr172b | -0.500245 |
| Gpr176 | -0.0608615 |
| Gpr177 | 0.5077692 |
| Gpr18 | 3.6267462 |
| Gpr180 | 1.7157357 |
| Gpr182 | 2.0999053 |
| Gpr182 | -0.4988484 |
| Gpr19 | 2.0870967 |
| Gpr20 | -0.2891606 |
| Gpr21 | -0.3005615 |
| Gpr22 | 1.0138935 |
| Gpr23 | -0.6654034 |
| Gpr26 | -0.7282362 |
| Gpr27 | 1.219259 |
| Gpr27 | 2.7274605 |
| Gpr27 | -0.7014363 |
| Gpr34 | 0.2105316 |
| Gpr34 | 1.5173572 |
| Gpr37l1 | -0.0253353 |
| Gpr45 | -0.3804643 |
| Gpr6 | -0.657205 |
| Gpr64 | 0.7730677 |
| Gpr65 | 2.7727358 |
| Gpr68 | -0.5607237 |
| Gpr75 | 1.7478965 |
| Gpr81 | 2.0149125 |
| Gpr83 | 0.3589464 |
| Gpr85 | -0.5430708 |
| Gpr85 | -0.6988802 |
| Gpr89 | 1.593679 |
| Gpr89 | -0.2954151 |
| Gpr97 | -0.5939124 |
| Gprasp1 | -0.703144 |
| Gprasp1 | 1.0128031 |
| Gprc2a-rs5 | 0.6274265 |
| Gprc2a-rs5 | 0.4987276 |
| Gprc5a | -0.6523178 |
| Gprc5b | 3.3342818 |
| Gprc5c | -0.4405512 |
| Gprk6 | -0.105214 |
| Gprk6 | 1.5115762 |
| Gps1 | -0.4747147 |
| Gps1 | -0.3650687 |
| Gps1 | -0.4760783 |
| Gps1 | -0.6070337 |
| Gps2 | 0.711106 |
| Gpsm1 | 1.3142141 |
| Gpsm2 | -0.3149962 |
| Gpsm3 | -0.0093184 |
| Gpsn2 | -0.4829242 |
| Gpsn2 | -0.6054811 |
| Gpsn2 | 1.0076466 |
| Gpt1 | -0.433332 |
| Gpt2 | 1.0023062 |
| Gpx1 | 2.2116822 |
| Gpx2 | 2.1860594 |
| Gpx3 | -0.1595514 |
| Gpx4 | 0.4177101 |
| Gpx4 | -0.3337794 |
| Gpx6 | -0.4631978 |
| Gpx7 | -0.4431893 |
| Gramd1a | -0.3331871 |
| Gramd1b | -0.2568089 |
| Gramd2 | -0.4431893 |
| Gramd2 | -0.2767668 |
| Gramd3 | -0.5946796 |
| Gramd4 | -0.2201555 |
| Grap2 | -0.5824749 |
| Grasp | -0.4788111 |
| Grasp | -0.6385979 |
| Grb10 | 0.0273699 |
| Grb14 | -0.0967479 |
| Grb14 | 1.1221449 |
| Grb2 | 0.5608681 |
| Grb7 | -0.7229933 |
| Grcc10 | 3.1564491 |
| Greb1 | -0.0925414 |
| Greb1 | 1.0709434 |
| Grem1 | 0.5861666 |
| Grem2 | -0.5221445 |
| Grhl1 | 1.2421124 |
| Grhl2 | -0.5832334 |
| Grhpr | -0.6920953 |
| Gria1 | -0.5614659 |
| Gria1 | -0.2661842 |
| Gria1 | -0.5533307 |
| Gria2 | 2.7877801 |
| Gria2 | 0.610846 |
| Gria3 | -0.5711657 |
| Gria3 | 0.1111698 |
| Gria3 | -0.5107822 |
| Grid2 | 2.3870815 |
| Grik1 | -0.3214136 |
| Grik2 | -0.6234817 |
| Grik4 | 2.4072224 |
| Grik5 | -0.6085887 |
| Grin1 | 2.6619969 |
| Grin1 | 1.6943189 |
| Grin2a | 1.7512742 |
| Grin2b | 1.7169769 |
| Grin2c | -0.4068702 |
| Grin3b | -0.7693644 |
| Grina | -0.6450311 |
| Grip1 | 2.5393301 |
| Grip1 | -0.2377769 |
| Grip1 | -0.4068702 |
| Gripap1 | 0.4660257 |
| Gripap1 | 0.0543383 |
| Grit | 0.5339166 |
| Grk4 | 0.557936 |
| Grk4 | -0.6744993 |
| Grk4 | -0.527874 |
| Grk5 | -0.5129029 |
| Grlf1 | -0.5143192 |
| Grm1 | 1.7505246 |
| Grm7 | 1.2163337 |
| Grm8 | -0.0699421 |
| Grn | 1.8024809 |
| Grp | -0.4043253 |
| Grpel1 | 0.606891 |
| Grpr | -0.6450311 |
| Grsf1 | 1.5392309 |
| Grtp1 | -0.3403178 |
| Grwd1 | 0.2682466 |
| Grwd1 | -0.3196593 |
| Gsbs | 0.6129335 |
| Gsc | -0.5916145 |
| Gsdma1 | -0.7739376 |
| Gsdmdc1 | 1.1026597 |
| Gsdmdc1 | 0.5105942 |
| Gsdmdc1 | 1.1449586 |
| Gse1 | -0.6811664 |
| Gsg1 | 1.5875276 |
| Gsg1 | 1.0586575 |
| Gsg1 | -0.5250052 |
| Gsg2 | -0.3891897 |
| Gsk3a | -0.4055969 |
| Gsk3b | -0.3074644 |
| Gsn | 1.3536573 |
| Gspt1 | 0.0611695 |
| Gspt2 | -0.568919 |
| Gss | -0.6971797 |
| Gss | -0.5511233 |
| Gss | 1.2691842 |
| Gsta2 | -0.1929988 |
| Gsta3 | 0.3053853 |
| Gsta3 | 0.3240714 |
| Gsta4 | -0.4733529 |
| Gstcd | 2.0965588 |
| Gstk1 | -0.7396904 |
| Gstm1 | -0.1560682 |
| Gstm1 | 2.7142248 |
| Gstm2 | -0.3841945 |
| Gstm2 | -0.4075074 |
| Gstm2 | -0.4891255 |
| Gstm4 | 1.8458369 |
| Gstm5 | 0.2978129 |
| Gstm6 | -0.515028 |
| Gstm6 | -0.188885 |
| Gsto1 | 3.3014132 |
| Gsto2 | -0.5445301 |
| Gsto2 | -0.6377967 |
| Gstp1 | -0.5518586 |
| Gstp1 | -0.7125884 |
| Gstp2 | -0.5009441 |
| Gstt1 | 0.7832954 |
| Gstt2 | 1.0173128 |
| Gstt3 | -0.1766433 |
| Gstt4 | 0.7706927 |
| Gstz1 | 0.385713 |
| Gtdc1 | -0.7539688 |
| Gtf2a1 | 1.5815157 |
| Gtf2e1 | 1.5150867 |
| Gtf2e2 | -0.7503802 |
| Gtf2e2 | 1.9536636 |
| Gtf2f1 | 0.9596837 |
| Gtf2f2 | -0.3948389 |
| Gtf2h1 | 2.0037155 |
| Gtf2h1 | 0.5069974 |
| Gtf2h2 | 0.7155109 |
| Gtf2h3 | -0.119484 |
| Gtf2h4 | -0.643419 |
| Gtf2h5 | 1.7316769 |
| Gtf2ird1 | -0.4981507 |
| Gtf2ird1 | -0.5614659 |
| Gtf2ird2 | 1.1635165 |
| Gtf2ird2 | 0.2245921 |
| Gtf2ird2 | -0.0398385 |
| Gtf3a | -0.636996 |
| Gtf3a | 0.4196766 |
| Gtf3c1 | -0.4870541 |
| Gtf3c2 | 0.0335627 |
| Gtf3c3 | -0.4713137 |
| Gtf3c4 | 0.7619361 |
| Gtf3c4 | -0.258457 |
| Gtf3c5 | -0.4171143 |
| Gtf3c5 | -0.2491529 |
| Gtf3c5 | 0.0252971 |
| Gtl3 | 0.0770625 |
| Gtpbp1 | 2.0239302 |
| Gtpbp2 | 0.3293034 |
| Gtpbp3 | -0.6904062 |
| Gtpbp4 | -0.6290245 |
| Gtpbp5 | 0.7476438 |
| Gtpbp6 | 0.8731061 |
| Gtpbp8 | 0.3163276 |
| Gtpbp8 | 0.8773934 |
| Gtrgeo22 | 1.3673472 |
| Gtse1 | -0.1590531 |
| Gtse1 | 0.0631698 |
| Gtse1 | -0.5250052 |
| Gtsf1l | 0.0110883 |
| Guca1a | -0.6298188 |
| Guca1b | -0.0925414 |
| Guca2a | 1.8527722 |
| Guca2b | 0.553276 |
| Gucy1a2 | 0.1725276 |
| Gucy1a3 | -0.3034319 |
| Gucy1a3 | -0.1924836 |
| Gucy1a3 | -0.0420543 |
| Gucy1b3 | 0.4029805 |
| Guk1 | 1.2091756 |
| Gulo | 1.5584652 |
| Gulp1 | -0.6426139 |
| Gusb | -0.3179081 |
| Gusb | 0.5010603 |
| Gusb | -0.2937055 |
| Gvin1 | 1.8921084 |
| Gyg | 0.8171618 |
| Gyg | 1.4384678 |
| Gykl1 | 1.4704511 |
| Gyltl1b | -0.5314716 |
| Gyltl1b | 0.965254 |
| Gypa | 0.1761072 |
| Gypc | 1.6138025 |
| Gys1 | 0.3053853 |
| Gys3 | 0.0144496 |
| Gzf1 | -0.2639704 |
| Gzf1 | -0.284072 |
| Gzmd | 2.7950193 |
| Gzmd | 0.7150922 |
| Gzmk | -0.0897468 |
| Gzmm | -0.7108646 |
| Gzmn | 1.1521785 |
| H13 | -0.5372545 |
| H13 | 1.7397435 |
| H13 | -0.4665715 |
| H13 | 0.0388992 |
| H19 | 0.472172 |
| H1f0 | -0.3935808 |
| H1fnt | -0.0314627 |
| H1foo | -0.2132975 |
| H1fx | -0.4781272 |
| H1fx | -0.2064857 |
| H2-Aa | 2.656271 |
| H2-Ab1 | -0.6353967 |
| H2-Ab1 | 1.5862928 |
| H2-Ab1 | 0.823199 |
| H2afj | -0.5100763 |
| H2afx | 3.6703236 |
| H2afy | -0.598524 |
| H2afz | 0.1511448 |
| H2-Bl | 0.1195194 |
| H2-DMa | 1.8550734 |
| H2-DMb1 | -0.5438002 |
| H2-Eb1 | 2.2830943 |
| H2-K1 | -0.6563889 |
| H2-Ke6 | -0.2617614 |
| H2-M10.3 | 1.4251986 |
| H2-M2 | 0.1635224 |
| H2-M3 | -0.2518805 |
| H2-Oa | 1.2725628 |
| H2-Ob | 0.7176017 |
| H2-Q1 | 1.4834349 |
| H2-Q2 | -0.1291107 |
| H2-Q7 | -0.2496978 |
| H2-Q8 | -0.5343589 |
| H2-T10 | -0.5946796 |
| H2-T23 | 0.1214074 |
| H2-T23 | -0.613268 |
| H2-T24 | 4.2761856 |
| H2-Tw3 | -0.0827939 |
| H3f3a | 0.1274255 |
| H3f3a | -0.1161362 |
| H3f3b | 0.853037 |
| H47 | 0.7125764 |
| H47 | -0.6266453 |
| H6pd | 0.3240714 |
| Habp4 | -0.61405 |
| Habp4 | 0.5911511 |
| Hace1 | -0.8382848 |
| Hacl1 | -0.7317467 |
| Hadhb | -0.214876 |
| Hagh | -0.8441647 |
| Haghl | 2.7597266 |
| Haghl | 0.2815301 |
| Hal | -0.1771504 |
| Hamp2 | -0.810339 |
| Hao1 | -0.7352695 |
| Hao3 | -0.2475199 |
| Hap1 | 0.3654829 |
| Hap1 | 1.6232903 |
| Hap1 | 0.789927 |
| Hapln1 | 0.014869 |
| Hapln2 | -0.6258534 |
| Hapln4 | 0.6365214 |
| Harbi1 | -0.3051581 |
| Hars | 1.6044846 |
| Has1 | -0.7423522 |
| Hat1 | 0.7150922 |
| Havcr1 | -0.5794467 |
| Havcr2 | 1.1558368 |
| Hax1 | -0.5893219 |
| Hba-a1 | -0.7666304 |
| Hbb-b1 | -0.304007 |
| Hbegf | -0.3979912 |
| Hbp1 | -0.527156 |
| Hbp1 | 1.3975926 |
| Hbs1l | -0.7396904 |
| Hbs1l | -0.5416136 |
| Hbs1l | 0.1707329 |
| Hbs1l | 0.6823202 |
| Hc | -0.4753963 |
| Hccs | -0.5607237 |
| Hcfc1 | -0.3126727 |
| Hcfc1r1 | -0.5387054 |
| Hcfc2 | 2.994542 |
| Hcls1 | -0.5719157 |
| Hcn2 | 2.5154869 |
| Hcn3 | 1.4342694 |
| Hcn3 | 0.7710889 |
| Hcr | 0.2633427 |
| Hcst | -0.0595064 |
| Hdac10 | -0.3916965 |
| Hdac11 | 0.6626628 |
| Hdac2 | 0.1941685 |
| Hdac3 | -0.4458343 |
| Hdac4 | 0.2924144 |
| Hdac5 | -0.0948762 |
| Hdac6 | 1.8856362 |
| Hdac7 | 1.0032507 |
| Hdac8 | -0.2711832 |
| Hdac9 | -0.6904062 |
| Hdc | -0.620328 |
| Hddc2 | -0.4618515 |
| Hddc2 | -0.4794954 |
| Hdgf | 0.529144 |
| Hdgf | -0.6353967 |
| Hdgf | -0.4307195 |
| Hdgf | 0.1796743 |
| Hdgfl1 | -0.2410141 |
| Hdgfrp2 | -0.785926 |
| Hdhd1a | 2.5234974 |
| Hdhd2 | -0.4884345 |
| Hdhd2 | 1.0345429 |
| Hdhd2 | 0.2167286 |
| Hdlbp | 0.8864437 |
| Hdlbp | 1.7263912 |
| Heatr1 | 0.6637649 |
| Heatr1 | 0.2557769 |
| Heatr3 | -0.4584935 |
| Heatr5a | -0.5756742 |
| Hebp1 | 4.0417566 |
| Hebp2 | -0.567424 |
| Heca | 1.2840615 |
| Hectd2 | -0.3954686 |
| Hectd2 | 2.742798 |
| Hectd3 | -0.5764276 |
| Hecw2 | 0.3943837 |
| Hecw2 | 0.2039487 |
| Hecw2 | -0.6393999 |
| Heg1 | 2.8724342 |
| Hel308 | 1.4613019 |
| Helb | -0.581717 |
| Helb | -0.5726663 |
| Helz | -0.4068702 |
| Hemgn | 1.7785528 |
| Hemk1 | -0.5416136 |
| Hemt1 | -0.7082845 |
| Hepacam | 1.5153708 |
| Heph | 0.620091 |
| Heph | 0.3311436 |
| Heph | -0.2064857 |
| Heph | -0.4068702 |
| Heph | 2.2290473 |
| Herc2 | -0.5228589 |
| Herc3 | -0.4471593 |
| Herc3 | 0.2424821 |
| Herc4 | -0.1247673 |
| Herc4 | 0.6725394 |
| Herpud1 | 0.2245921 |
| Herpud1 | 1.2881026 |
| Herpud2 | -0.6637584 |
| Herpud2 | -0.5908497 |
| Herpud2 | 2.4349888 |
| Herpud2 | 0.0215554 |
| Hes1 | -0.5855123 |
| Hes5 | -0.6290245 |
| Hes5 | 0.8396173 |
| Hes6 | -0.720382 |
| Hes6 | -0.1156589 |
| Hes6 | 1.8652973 |
| Hexa | -0.7739376 |
| Hexb | -0.6670511 |
| Hexdc | -0.4981507 |
| Hexdc | -0.0717681 |
| Hexim1 | -0.3493027 |
| Hexim2 | 2.0096486 |
| Hey1 | -0.1295945 |
| Hey2 | 0.6917928 |
| Hey2 | 1.8206214 |
| Heyl | 0.7009649 |
| Hfe | 1.3773854 |
| Hfe | 0.4506301 |
| Hgfac | -0.5044467 |
| Hgs | 0.4924804 |
| Hgs | 0.4061144 |
| Hgsnat | 1.3973798 |
| Hhat | 1.7439792 |
| Hhat | -0.5474551 |
| Hhat | -0.1630468 |
| Hhatl | -0.5839925 |
| Hhex | 0.0687499 |
| Hhip | -0.4229226 |
| Hibadh | 0.8438647 |
| Hic1 | 0.9238987 |
| Hic2 | -0.6588394 |
| Hif1a | 2.0208377 |
| Hif3a | -0.4788111 |
| Hif3a | 2.6684451 |
| Higd1a | -0.4478225 |
| Higd1b | 0.79051 |
| Higd2a | 1.2903539 |
| Hils1 | -0.3942097 |
| Hint1 | 1.5984861 |
| Hint2 | -0.5079615 |
| Hint3 | -0.2606588 |
| Hint3 | -0.2496978 |
| Hip1 | -0.707426 |
| Hip1r | -0.6124866 |
| Hip2 | -0.0925414 |
| Hipk1 | 1.304691 |
| Hipk2 | 0.0364397 |
| Hipk3 | 3.274245 |
| Hipk4 | -0.1339588 |
| Hira | -0.5900855 |
| Hira | 1.7568787 |
| Hirip3 | 0.3448386 |
| Hisppd1 | -0.4491503 |
| Hist1h1b | -0.5164473 |
| Hist1h1c | -0.2426366 |
| Hist1h2aa | -0.5786911 |
| Hist1h2ac | 0.7704945 |
| Hist1h2ad | 2.2133422 |
| Hist1h2ae | -0.1545791 |
| Hist1h2af | 2.2598779 |
| Hist1h2ag | -0.4788111 |
| Hist1h2ah | 1.2428571 |
| Hist1h2ak | -0.1909398 |
| Hist1h2an | 0.7930327 |
| Hist1h2ao | 0.4235982 |
| Hist1h2bc | -0.6047057 |
| Hist1h2be | -0.5474551 |
| Hist1h2bf | 3.8014922 |
| Hist1h2bg | 1.7768008 |
| Hist1h2bh | 2.102765 |
| Hist1h2bj | -0.5555429 |
| Hist1h2bk | 0.0543383 |
| Hist1h2bm | 0.593516 |
| Hist1h2bn | 1.0419894 |
| Hist1h3a | -0.5824749 |
| Hist1h3d | -0.1204425 |
| Hist1h3e | -0.0818705 |
| Hist1h3f | -0.4339861 |
| Hist1h4c | 1.3876471 |
| Hist1h4d | 1.6793819 |
| Hist1h4f | 1.0657808 |
| Hist1h4h | 1.9756164 |
| Hist1h4i | -0.4836113 |
| Hist1h4j | 1.2908272 |
| Hist1h4k | -0.5969845 |
| Hist1h4m | -0.4679241 |
| Hist2h2aa1 | -0.4884345 |
| Hist2h2ab | 0.3598403 |
| Hist2h2ac | -0.5474551 |
| Hist2h2bb | 0.053128 |
| Hist2h2be | 0.2451551 |
| Hist2h3b | -0.5207172 |
| Hist2h3c1 | -0.5221445 |
| Hist2h3c1 | -0.3450998 |
| Hist2h4 | 1.8955564 |
| Hist3h2a | -0.6621161 |
| Hist3h2bb | 0.0727169 |
| Hist4h4 | -0.3462988 |
| Hk1 | -0.4030553 |
| Hk3 | 1.1540771 |
| Hkdc1 | -0.6195411 |
| Hlcs | 1.7746066 |
| Hlf | -0.6612959 |
| Hltf | -0.5129029 |
| Hlx | -0.598524 |
| Hmg20a | 3.5457865 |
| Hmg20a | -0.5629522 |
| Hmg20b | -0.4458343 |
| Hmga1 | -0.5136108 |
| Hmgb2 | -0.3553374 |
| Hmgb2l1 | 1.5367311 |
| Hmgb2l1 | 1.9851691 |
| Hmgb4 | -0.3693558 |
| Hmgcl | -0.3565487 |
| Hmgcl | 0.5215621 |
| Hmgcl | 3.0941523 |
| Hmgcll1 | -0.4679241 |
| Hmgcs2 | -0.6234817 |
| Hmgn1 | 1.8424883 |
| Hmgn2 | 0.3299172 |
| Hmgn3 | -0.3547323 |
| Hmgn3 | 1.2588578 |
| Hmha1 | -0.4706349 |
| Hmox1 | 0.292096 |
| Hmox2 | -0.5136108 |
| Hmx1 | -0.3080417 |
| Hmx1 | -0.1605489 |
| Hn1 | 2.0475532 |
| Hn1 | 2.400514 |
| Hn1l | -0.7014363 |
| Hnf1b | -0.31616 |
| Hnf1b | -0.5870344 |
| Hnf4a | 3.211164 |
| Hnrnpc | 2.1323116 |
| Hnrpa1 | 0.0013616 |
| Hnrpa1 | -0.6047057 |
| Hnrpab | -0.2507886 |
| Hnrpc | 4.2052072 |
| Hnrpdl | -0.634598 |
| Hnrpdl | -0.515028 |
| Hnrpf | -0.2846361 |
| Hnrph1 | -0.5786911 |
| Hnrph1 | -0.6148326 |
| Hnrph1 | 1.2069925 |
| Hnrph1 | 0.7901214 |
| Hnrph1 | 0.2022092 |
| Hnrph2 | -0.5023437 |
| Hnrph3 | 2.5036171 |
| Hnrpk | 1.4674768 |
| Hnrpl | 0.2163854 |
| Hnrpll | 1.6166325 |
| Hnrpll | -0.4829242 |
| Hnrpm | -0.0649376 |
| Hnrpm | -0.6298188 |
| Hnrpm | 2.6840758 |
| Hnrpul1 | 2.7110187 |
| Hnrpul1 | 0.7255074 |
| Hnrpul1 | -0.4686011 |
| Hnrpul1 | 0.5128997 |
| Hnrpul1 | -0.3179081 |
| Hnt | 0.4410588 |
| Homer1 | 1.5824021 |
| Homer1 | -0.127178 |
| Homer1 | -0.2891606 |
| Homer1 | -0.7361521 |
| Homez | -0.3565487 |
| Homez | 1.6252451 |
| Hook1 | -0.009748 |
| Hook1 | -0.5093708 |
| Hook1 | 3.8121877 |
| Hook1 | 0.1337805 |
| Hook2 | 0.5146892 |
| Hormad2 | -0.4145435 |
| Hoxa13 | 0.4503579 |
| Hoxa2 | -0.6234817 |
| Hoxa2 | 0.753506 |
| Hoxa4 | -0.2902949 |
| Hoxa5 | -0.4815513 |
| Hoxa7 | 1.2777295 |
| Hoxa9 | 1.6510641 |
| Hoxa9 | 2.2268053 |
| Hoxb2 | -0.4222756 |
| Hoxb3 | 2.4677348 |
| Hoxb3 | -0.4471593 |
| Hoxb4 | -0.0016174 |
| Hoxb5 | -0.4268136 |
| Hoxb6 | -0.6402024 |
| Hoxb7 | -0.2623132 |
| Hoxc10 | 1.5949923 |
| Hoxc10 | 1.5130009 |
| Hoxc6 | -0.0772659 |
| Hoxc9 | -0.500245 |
| Hoxd10 | -0.5228589 |
| Hoxd3 | 1.2571468 |
| Hoxd4 | 0.1408362 |
| Hoxd8 | 0.8449698 |
| Hoxd9 | -0.4551467 |
| Hp | 0.2491514 |
| Hp | 0.0438003 |
| Hp | 0.4182724 |
| Hp1bp3 | -0.6385979 |
| Hpca | -0.4412101 |
| Hpcal1 | -0.0986232 |
| Hpcal1 | 1.8773382 |
| Hpcal4 | -0.7091438 |
| Hpgd | -0.5908497 |
| Hpn | 0.0892097 |
| Hpn | -0.5285925 |
| Hprt1 | 1.4285065 |
| Hprt1 | 1.8243222 |
| Hprt1 | -0.2453466 |
| Hps1 | 1.1259135 |
| Hps3 | 0.5823524 |
| Hps3 | -0.6219036 |
| Hps4 | -0.2595573 |
| Hps5 | -0.5387054 |
| Hps6 | -0.6870365 |
| Hpse | -0.471993 |
| Hpx | 1.9750163 |
| Hr | 1.5037525 |
| Hr | -0.5178686 |
| Hras1 | 0.462804 |
| Hrasls | 0.2636704 |
| Hrasls5 | 2.5294032 |
| Hrasls5 | -0.1000319 |
| Hrb | -0.4925872 |
| Hrb | -0.4863646 |
| Hrbl | -0.5659312 |
| Hrbl | 1.0814811 |
| Hrc | 0.523083 |
| Hrg | -0.4618515 |
| Hrh3 | 0.7026622 |
| Hrk | 2.0898858 |
| Hrmt1l2 | 2.7253807 |
| Hrsp12 | 1.1875223 |
| Hrsp12 | -0.6242717 |
| Hrsp12 | -0.650694 |
| Hs1bp3 | 1.5701908 |
| Hs2st1 | -0.0192449 |
| Hs3st1 | -0.2790089 |
| Hs3st3a1 | 1.059253 |
| Hs3st3b1 | 3.6767501 |
| Hs3st6 | 0.7967084 |
| Hs6st1 | 0.2143232 |
| Hs6st2 | -0.527874 |
| Hs6st2 | 0.3848419 |
| Hs6st3 | 2.3366165 |
| Hsbp1 | 1.1641874 |
| Hsd11b1 | -0.4564841 |
| Hsd11b1 | -0.0617659 |
| Hsd11b1 | -0.2818186 |
| Hsd11b1 | 0.0655651 |
| Hsd11b2 | -0.1491376 |
| Hsd17b1 | -0.5741691 |
| Hsd17b10 | -0.3669038 |
| Hsd17b11 | -0.127178 |
| Hsd17b11 | -0.3080417 |
| Hsd17b11 | 3.326251 |
| Hsd17b12 | 0.72985 |
| Hsd17b2 | 0.669696 |
| Hsd17b3 | -0.2377769 |
| Hsd17b4 | -0.2595573 |
| Hsd17b7 | 1.4070194 |
| Hsd3b1 | -0.5474551 |
| Hsd3b2 | -0.5651856 |
| Hsd3b2 | -0.6008377 |
| Hsd3b4 | -0.258457 |
| Hsd3b7 | -0.4445109 |
| Hsd3b7 | -0.3547323 |
| Hsdl1 | -0.2249309 |
| Hsf1 | -0.6258534 |
| Hsf4 | -0.3553374 |
| Hsf5 | -0.6937873 |
| Hsn2 | 2.0145086 |
| Hsp105 | -0.3614084 |
| Hsp90aa1 | -0.1339588 |
| Hsp90ab1 | 0.5986993 |
| Hsp90ab1 | 1.7165892 |
| Hsp90ab1 | 1.3969539 |
| Hsp90b1 | -0.6963305 |
| Hspa12b | 2.6404791 |
| Hspa1a | -0.5741691 |
| Hspa1l | 1.0898905 |
| Hspa2 | -0.613268 |
| Hspa2 | 3.3172363 |
| Hspa4 | -0.5452606 |
| Hspa4l | -0.4960608 |
| Hspa5 | 0.396109 |
| Hspa9 | -0.5379797 |
| Hspa9 | 2.1481071 |
| Hspb1 | -0.5343589 |
| Hspb2 | 1.7242383 |
| Hspb3 | -0.5474551 |
| Hspb6 | -0.6124866 |
| Hspb7 | 1.6678896 |
| Hspb8 | 0.124421 |
| Hspbap1 | -0.5809597 |
| Hspd1 | -0.5570204 |
| Hspd1 | -0.4171143 |
| Hspe1 | 2.5051085 |
| Hspg2 | 0.5778041 |
| Htatip | 1.3412431 |
| Htatip | -0.568919 |
| Htatip2 | -0.6482631 |
| Htatip2 | 0.8635734 |
| Htatsf1 | -0.6937873 |
| Htf9c | -0.643419 |
| Htr1a | -0.6904062 |
| Htr1b | -0.4145435 |
| Htr1d | 0.4098055 |
| Htr1f | -0.6290245 |
| Htr1f | 0.2783063 |
| Htr2b | 1.2839424 |
| Htr3a | 0.7294372 |
| Htr5b | -0.1233237 |
| Htr7 | -0.1771504 |
| Htra1 | 2.6440099 |
| Htra1 | 1.5632621 |
| Htra2 | -0.1919687 |
| Htra3 | 3.6868801 |
| Htra3 | 1.0271946 |
| Htt | 3.1487193 |
| Hus1 | 2.1499255 |
| Hus1 | 1.7746066 |
| Hus1 | -0.3367462 |
| Huwe1 | 2.6536102 |
| Hvcn1 | -0.3601913 |
| Hvcn1 | 0.3271523 |
| Hyal1 | 0.3253049 |
| Hyal2 | -0.4145435 |
| Hyal3 | -0.2846361 |
| Hyal5 | 0.4202378 |
| Hyal6 | -0.603931 |
| Hydin | -0.2954151 |
| Hyi | -0.5525944 |
| Hyou1 | -0.0157811 |
| Iah1 | 1.389687 |
| Iap | -0.8481038 |
| Iars | -0.6250622 |
| Iars | 0.5916245 |
| Iars2 | -0.5518586 |
| Ibtk | 2.0024312 |
| Ica1 | -0.643419 |
| Ica1l | -0.4075074 |
| Icam1 | 1.4751919 |
| Icam2 | -0.2334767 |
| Icam4 | 1.0816267 |
| Icam5 | -0.244804 |
| Ick | 0.1195194 |
| Icmt | -0.4171143 |
| Ict1 | 1.5365457 |
| Id1 | -0.1776578 |
| Id2 | -0.6124866 |
| Id2 | 1.6711401 |
| Id3 | 1.0472749 |
| Id4 | -0.4281139 |
| Ide | 0.1471075 |
| Idh1 | 0.3031826 |
| Idh2 | 2.1382001 |
| Idh3a | -0.5093708 |
| Idh3b | -0.5533307 |
| Idh3g | 2.0437477 |
| Idh3g | 2.7224674 |
| Idi2 | -0.4062334 |
| Ids | -0.634598 |
| Ids | 1.8428989 |
| Ids | 1.5898168 |
| Idua | 0.8876796 |
| Ier3 | -0.173606 |
| Ier3ip1 | -0.6474542 |
| Ier5l | 1.8107799 |
| Ifi202b | -0.5438002 |
| Ifi202b | 0.5751615 |
| Ifi27 | 0.7928389 |
| Ifi30 | -0.7100039 |
| Ifi30 | -0.4692786 |
| Ifi35 | 1.1490517 |
| Ifi47 | 4.4686159 |
| Ifit2 | 1.4345772 |
| Ifit2 | -0.5885588 |
| Ifit3 | -0.7684522 |
| Ifit3 | -0.0595064 |
| Ifitm1 | -0.5136108 |
| Ifitm1 | 0.5433946 |
| Ifitm1 | -0.5489208 |
| Ifitm2 | 0.7846643 |
| Ifitm3 | 1.5935038 |
| Ifitm5 | -0.7868541 |
| Ifitm6 | -0.6878779 |
| Ifna1 | -0.4598353 |
| Ifna6 | -0.6337999 |
| Ifnar2 | 1.4227105 |
| Ifnar2 | 3.2497215 |
| Ifnar2 | 0.0845249 |
| Ifngr1 | -0.4268136 |
| Ifngr2 | 0.0899883 |
| Ifngr2 | 0.0786382 |
| Ifngr2 | -0.6703546 |
| Ifnz | -0.554805 |
| Ifrd1 | 0.4468125 |
| Ifrg15 | -0.1516074 |
| Ift122 | -0.5164473 |
| Ift140 | -0.4774438 |
| Ift140 | 0.6358422 |
| Ift140 | -0.5164473 |
| Ift172 | -0.267293 |
| Ift172 | 1.7418636 |
| Ift172 | -0.6687015 |
| Ift20 | -0.1281439 |
| Ift20 | 2.0123708 |
| Ift20 | -0.0358624 |
| Ift52 | 1.9467119 |
| Ift80 | -0.8650185 |
| Ift81 | 0.7236406 |
| Ift81 | 1.3220436 |
| Ift81 | -0.581717 |
| Ift88 | 1.1842404 |
| Igbp1 | 0.0770625 |
| Igbp1b | -0.6904062 |
| Igf1 | -0.3873136 |
| Igf1 | 0.7079479 |
| Igf1r | 0.7370462 |
| Igf2 | -0.5503886 |
| Igf2bp1 | -0.3523155 |
| Igf2bp2 | -0.6786611 |
| Igf2bp3 | 2.1727283 |
| Igf2bp3 | 4.5593121 |
| Igf2r | -0.0745132 |
| Igf2r | -0.5696674 |
| Igfals | -0.16005 |
| Igfbp2 | -0.126213 |
| Igfbp2 | 0.5833073 |
| Igfbp3 | -0.4571534 |
| Igfbp4 | -0.05231 |
| Igfbp4 | -0.6845165 |
| Igfbp5 | -0.3367462 |
| Igfbp6 | -0.4794954 |
| Igfbp7 | -0.7924408 |
| Igfbpl1 | 0.3293034 |
| Igsf1 | -0.6878779 |
| Igsf1 | -0.5086659 |
| Igsf1 | 2.3639213 |
| Igsf1 | 0.2211807 |
| Igsf11 | -0.4158281 |
| Igsf11 | -0.8037079 |
| Igsf11 | -0.0995621 |
| Igsf21 | -0.6728394 |
| Igsf3 | -0.4235701 |
| Igsf4a | 0.3499642 |
| Igsf5 | -0.0690303 |
| Igsf8 | 0.3475554 |
| Igsf9 | -0.4638716 |
| Igtp | 1.2888141 |
| Ihh | 0.0438003 |
| Ihpk1 | 1.2797648 |
| Ihpk2 | 0.1430541 |
| Ihpk2 | 1.1604247 |
| Iigp2 | 0.3360342 |
| Ik | 0.3740349 |
| Ikbkap | -0.4036901 |
| Ikbkb | -0.643419 |
| Ikbke | 0.4165847 |
| Ikbkg | 2.7423774 |
| Ikbkg | -0.8431824 |
| Il10ra | 0.093485 |
| Il10rb | -0.5121955 |
| Il11 | 0.9323741 |
| Il11ra1 | 2.3448026 |
| Il12a | 1.168604 |
| Il13 | -0.5923799 |
| Il13ra1 | 1.0307223 |
| Il13ra2 | -0.673669 |
| Il15 | -0.1466739 |
| Il15 | 1.6319308 |
| Il15 | 0.1012122 |
| Il15ra | -0.4794954 |
| Il15ra | 0.799023 |
| Il15ra | 1.4723304 |
| Il16 | 0.5210546 |
| Il17d | 1.0062357 |
| Il17d | -0.5954473 |
| Il17ra | 0.6024524 |
| Il17rb | -0.7785317 |
| Il17rc | -0.05231 |
| Il17rc | -0.5570204 |
| Il17rc | -0.5832334 |
| Il17rd | 2.1645547 |
| Il17rd | 2.038238 |
| Il17re | 0.5998736 |
| Il17re | 0.9964622 |
| Il17re | 2.1730736 |
| Il18 | 3.6021823 |
| Il18bp | -0.0644839 |
| Il18r1 | 2.1516902 |
| Il1b | -0.3361521 |
| Il1r1 | 1.7965519 |
| Il1rap | -0.568919 |
| Il1rap | 0.5419039 |
| Il1rap | -0.3675162 |
| Il1rap | -0.7022898 |
| Il1rap | -0.4300675 |
| Il1rl1l | 1.4226067 |
| Il1rl2 | 0.7710889 |
| Il20ra | 0.1710921 |
| Il20rb | -0.3487012 |
| Il20rb | -0.2948449 |
| Il23a | 0.9249195 |
| Il27ra | -0.3998872 |
| Il28ra | -0.6637584 |
| Il28ra | 2.3091983 |
| Il28ra | 2.4493032 |
| Il31ra | -0.3190753 |
| Il33 | -0.6878779 |
| Il3ra | -0.3005615 |
| Il4i1 | -0.1827456 |
| Il6ra | -0.2863304 |
| Il6st | 1.0279625 |
| Il7 | 2.997377 |
| Ildr1 | -0.1315318 |
| Ildr1 | 0.0856981 |
| Ilf2 | -0.1640476 |
| Ilf3 | -0.5121955 |
| Ilf3 | 0.0627701 |
| Ilf3 | 0.5087974 |
| Ilf3 | 2.2563875 |
| Ilk | 1.3568757 |
| Ilkap | -0.5511233 |
| Ilvbl | 0.97598 |
| Ilvbl | -0.5809597 |
| Ilvbl | -0.0358624 |
| Ilvbl | -0.6219036 |
| Immp2l | -0.3767479 |
| Immt | -0.6330024 |
| Imp3 | 0.8621266 |
| Imp4 | -0.3960986 |
| Imp4 | -0.2275937 |
| Impa2 | 3.5816641 |
| Impact | 1.2701507 |
| Impad1 | 0.8952384 |
| Impdh1 | 0.0567546 |
| Impdh2 | 0.334203 |
| Ina | 0.8662805 |
| Inadl | -0.5329142 |
| Inadl | 0.9988355 |
| Incenp | 0.2574264 |
| Indo | 2.0546223 |
| Ing2 | -0.2778872 |
| Ing3 | 0.0034841 |
| Ing3 | 0.6589063 |
| Ing3 | -0.4932809 |
| Ing4 | 1.0516334 |
| Ing4 | -0.6596576 |
| Ing4 | 1.4284033 |
| Ing5 | -0.4794954 |
| Inha | -0.5622088 |
| Inhba | 0.4948276 |
| Inmt | 0.7840778 |
| Inoc1 | -0.5636961 |
| Inpp4a | -0.4551467 |
| Inpp4a | -0.2678478 |
| Inpp4a | 0.0360292 |
| Inpp4b | -0.7343877 |
| Inpp5a | 0.1606236 |
| Inpp5d | 0.0203051 |
| Inpp5e | 0.2734512 |
| Inpp5f | -0.3314123 |
| Inppl1 | -0.6803306 |
| Inppl1 | 0.3012906 |
| Inppl1 | -0.6604764 |
| Ins2 | -0.5072575 |
| Insc | 0.1768217 |
| Insig1 | -0.7238652 |
| Insig1 | 2.2399986 |
| Insig1 | 0.5647643 |
| Insig2 | 1.0589553 |
| Insig2 | -0.4571534 |
| Insl3 | 1.1625765 |
| Insl5 | -0.0851062 |
| Insl6 | -0.6803306 |
| Insm1 | -0.3017087 |
| Ints1 | -0.2700701 |
| Ints12 | -0.1281439 |
| Ints3 | -0.5592407 |
| Ints3 | 2.1830361 |
| Ints4 | 1.2837041 |
| Ints5 | -0.4905087 |
| Ints7 | 1.2183696 |
| Ints9 | 0.0123501 |
| Ints9 | -0.4438499 |
| Ipmk | -0.6629369 |
| Ipmk | 3.0787616 |
| Ipo13 | 0.356559 |
| Ipo4 | -0.4578232 |
| Ipo5 | 3.3956501 |
| Ipp | 1.1148468 |
| Ippk | -0.4960608 |
| Iqcb1 | 2.1852313 |
| Iqcb1 | -0.433332 |
| Iqcd | 0.4566001 |
| Iqce | -0.6980296 |
| Iqcf3 | 0.6506817 |
| Iqcf4 | -0.5711657 |
| Iqgap1 | -0.7450211 |
| Iqgap2 | 1.2616625 |
| Iqgap3 | 2.2514713 |
| Iqsec2 | 0.4930025 |
| Iqsec3 | -0.4842989 |
| Irak1 | -0.703999 |
| Irak1bp1 | -0.5900855 |
| Irak2 | 1.10948 |
| Irak2 | 1.2454591 |
| Irak3 | 0.8412814 |
| Irak4 | 0.6617801 |
| Irak4 | -0.4939752 |
| Irak4 | -0.5540676 |
| Ireb2 | 0.2286705 |
| Irf1 | -0.2750884 |
| Irf1 | -0.6031568 |
| Irf1 | -0.610146 |
| Irf1 | 2.3695382 |
| Irf2 | -0.4544786 |
| Irf2bp1 | 0.6571337 |
| Irf3 | -0.0314627 |
| Irf3 | 1.9127492 |
| Irf3 | 2.8960418 |
| Irf4 | -0.5893219 |
| Irf5 | -0.195062 |
| Irf6 | -0.2190974 |
| Irf6 | 1.7617109 |
| Irf7 | 1.4013103 |
| Irf8 | 0.209495 |
| Irf9 | 0.1533401 |
| Irf9 | 2.5240896 |
| Irgm | 2.1833291 |
| Irgm | -0.603931 |
| Irgq | 0.3100894 |
| Irgq | -0.3385304 |
| Irgq | -0.6234817 |
| Irs2 | 1.1668664 |
| Irs3 | 0.8405422 |
| Irx2 | 0.1172491 |
| Irx3 | -0.4836113 |
| Irx5 | 0.2074187 |
| Isca1 | -0.55776 |
| Iscu | -0.4145435 |
| Isg20 | 0.0273699 |
| Isg20l1 | 0.0969696 |
| Isg20l2 | -0.6988802 |
| Isl1 | -0.2959856 |
| Islr2 | 2.925444 |
| Isy1 | 0.1107887 |
| Isyna1 | -0.5764276 |
| Itfg2 | 0.1012122 |
| Itfg3 | 2.6450914 |
| Itga1 | -0.5771815 |
| Itga11 | 0.3320624 |
| Itga11 | 0.5549954 |
| Itga2b | 1.650733 |
| Itga3 | -0.4464966 |
| Itga5 | -0.4767608 |
| Itga5 | -0.6870365 |
| Itga6 | -0.5164473 |
| Itga7 | 0.1924121 |
| Itgad | 1.3658108 |
| Itgad | -0.5954473 |
| Itgad | -0.3255187 |
| Itgae | 0.366371 |
| Itgae | -0.703144 |
| Itgae | 0.607823 |
| Itgav | 2.3411354 |
| Itgb1 | 1.5587373 |
| Itgb1 | 1.0477267 |
| Itgb1bp1 | -0.6563889 |
| Itgb3bp | -0.2745296 |
| Itgb4 | 0.665086 |
| Itgb4 | -0.1919687 |
| Itgb5 | 0.2511436 |
| Itgb6 | 1.5837301 |
| Itgb6 | 1.1152693 |
| Itgb6 | 2.1688231 |
| Itgb7 | -0.3409143 |
| Itgbl1 | 0.8278905 |
| Itih2 | 0.0735084 |
| Itih3 | 1.8197816 |
| Itih5 | -0.2302635 |
| Itih5 | -0.6728394 |
| Itk | -0.3929523 |
| Itlnb | 0.5842613 |
| Itm2a | 0.987872 |
| Itm2b | -0.3225848 |
| Itm2b | 0.726336 |
| Itpk1 | -0.6258534 |
| Itpka | -0.568919 |
| Itpkc | -0.1339588 |
| Itpr1 | -0.6337999 |
| Itpr2 | -0.6895627 |
| Itpr3 | -0.6769943 |
| Itsn1 | 1.9932772 |
| Itsn2 | -0.6322056 |
| Ivd | -0.2222751 |
| Ivns1abp | -0.6778273 |
| Ivns1abp | -0.6361961 |
| Iws1 | -0.6062571 |
| Iyd | 0.9492801 |
| Izumo1 | 3.3023953 |
| Jag1 | 0.5005424 |
| Jag2 | 1.5820476 |
| Jagn1 | -0.3571549 |
| Jak1 | -0.4068702 |
| Jak2 | 2.5166803 |
| Jak2 | -0.4184021 |
| Jak3 | 1.5663274 |
| Jak3 | -0.4733529 |
| Jakmip1 | 0.2156984 |
| Jam2 | -0.126213 |
| Jam2 | 1.4536808 |
| Jam2 | -0.6274377 |
| Jam2 | 2.543301 |
| Jam3 | -0.2623132 |
| Jam4 | 0.6595702 |
| Jarid1b | 0.4069674 |
| Jarid1c | -0.0389536 |
| Jarid1d | 2.6098941 |
| Jazf1 | -0.5977539 |
| Jdp2 | -0.4767608 |
| Jmjd1a | 0.933555 |
| Jmjd1a | -0.2228057 |
| Jmjd1a | -0.833411 |
| Jmjd2a | -0.5200043 |
| Jmjd2b | 0.4146121 |
| Jmjd2b | -0.4740336 |
| Jmjd2c | -0.554805 |
| Jmjd2d | -0.6703546 |
| Jmjd3 | -0.0622184 |
| Jmjd3 | 2.2376883 |
| Jmjd5 | -0.5923799 |
| Jmjd6 | -0.7151796 |
| Jmy | 1.2383808 |
| Josd1 | 0.0876505 |
| Josd2 | -0.4638716 |
| Josd3 | -0.1786733 |
| Josd3 | 3.3891295 |
| Jph1 | -0.5916145 |
| Jph2 | -0.4471593 |
| Jph3 | 1.8838638 |
| Jph4 | 0.9037834 |
| Jph4 | -0.5719157 |
| Jtb | 0.7121565 |
| Jtv1 | -0.3456991 |
| Jtv1 | -0.4209828 |
| Jun | -0.3835718 |
| Junb | -0.4578232 |
| Jund1 | 1.8326515 |
| Jup | -0.257358 |
| Kal1 | 2.3980707 |
| Kalrn | -0.3904423 |
| Kank1 | -0.2101479 |
| Kank3 | -0.1620469 |
| Kank4 | 0.7908986 |
| Kank4 | 1.5649762 |
| Kank4 | -0.2818186 |
| Kars | 1.1165357 |
| Katna1 | -0.4836113 |
| Katnal1 | 2.0882777 |
| Katnb1 | 0.2448213 |
| Kazald1 | 1.6178308 |
| Kbtbd2 | -0.3261065 |
| Kbtbd2 | -0.6555733 |
| Kbtbd4 | -0.3511093 |
| Kbtbd4 | -0.7566686 |
| Kbtbd7 | 0.1994197 |
| Kbtbd7 | 0.1917086 |
| Kbtbd8 | 0.9244092 |
| Kcmf1 | -0.4564841 |
| Kcna1 | 0.0405356 |
| Kcna2 | -0.6274377 |
| Kcna3 | 0.9318676 |
| Kcna5 | 0.4935243 |
| Kcna6 | -0.3225848 |
| Kcnab1 | -0.6266453 |
| Kcnab1 | -0.5562814 |
| Kcnab1 | 1.7709389 |
| Kcnab2 | -0.3349651 |
| Kcnab3 | 1.6208201 |
| Kcnb1 | -0.7100039 |
| Kcnc1 | 0.8997815 |
| Kcnc1 | 1.1334089 |
| Kcnc4 | -0.6016101 |
| Kcnc4 | 0.5286403 |
| Kcnd2 | -0.6298188 |
| Kcne1 | -0.6946343 |
| Kcne1l | -0.527156 |
| Kcne2 | -0.5877963 |
| Kcne4 | -0.667876 |
| Kcnf1 | -0.3403178 |
| Kcng4 | -0.6266453 |
| Kcnh1 | 3.0244291 |
| Kcnh2 | 2.1238541 |
| Kcnh2 | -0.554805 |
| Kcnh3 | 0.2290097 |
| Kcnh5 | -0.2101479 |
| Kcnip1 | -0.5051487 |
| Kcnip2 | -0.3011349 |
| Kcnip2 | -0.2925673 |
| Kcnip2 | -0.1132755 |
| Kcnip3 | 2.5638953 |
| Kcnip4 | -0.4281139 |
| Kcnj12 | -0.2388548 |
| Kcnj16 | 2.3574516 |
| Kcnj2 | 0.5473588 |
| Kcnj3 | -0.7247379 |
| Kcnj4 | 0.428896 |
| Kcnj6 | 0.0306775 |
| Kcnj6 | 2.1660457 |
| Kcnj8 | 3.1671795 |
| Kcnj9 | 2.6521698 |
| Kcnk1 | 0.4724384 |
| Kcnk10 | 1.3742319 |
| Kcnk12 | -0.6000659 |
| Kcnk13 | -0.5100763 |
| Kcnk2 | -0.6482631 |
| Kcnk2 | 0.9046513 |
| Kcnk3 | -0.3559429 |
| Kcnk4 | 0.3877429 |
| Kcnk5 | -0.1242859 |
| Kcnk5 | 0.4854058 |
| Kcnk6 | 0.5596474 |
| Kcnk6 | 0.0892097 |
| Kcnk9 | 2.0890284 |
| Kcnma1 | -0.0205469 |
| Kcnmb1 | 0.0261268 |
| Kcnmb4 | 1.8182402 |
| Kcnmb4 | 0.3862934 |
| Kcnn1 | 2.4115143 |
| Kcnn2 | -0.673669 |
| Kcnn4 | 0.7978664 |
| Kcnq1 | 0.9101882 |
| Kcnq2 | -0.0781852 |
| Kcnq2 | -0.3290507 |
| Kcnq3 | 1.1113192 |
| Kcnq5 | -0.3337794 |
| Kcnrg | 0.2914588 |
| Kcnrg | -0.6929409 |
| Kcns1 | -0.9718211 |
| Kcns2 | 2.6930094 |
| Kcnt1 | -0.2999883 |
| Kcnu1 | 0.6812381 |
| Kcnv1 | 0.7024502 |
| Kcnv2 | 0.0647673 |
| Kctd10 | -0.4484862 |
| Kctd11 | 2.4271606 |
| Kctd12 | -0.6047057 |
| Kctd12b | 2.5303726 |
| Kctd12b | 0.7205215 |
| Kctd14 | -0.3767479 |
| Kctd14 | -0.8046525 |
| Kctd15 | 0.5113633 |
| Kctd18 | 1.0949314 |
| Kctd19 | 1.0362193 |
| Kctd2 | 0.2170718 |
| Kctd20 | -0.3767479 |
| Kctd20 | -0.5143192 |
| Kctd3 | 0.4976891 |
| Kctd4 | 0.7448016 |
| Kctd5 | 0.817351 |
| Kctd5 | 1.1966552 |
| Kctd6 | 2.0381252 |
| Kctd6 | 1.1190637 |
| Kctd9 | -0.7785317 |
| Kdelc1 | -0.610146 |
| Kdelc1 | 1.8312681 |
| Kdelr2 | -0.673669 |
| Kdelr3 | 1.1257742 |
| Kdr | -0.6753303 |
| Keap1 | 0.0583622 |
| Keap1 | -0.0283944 |
| Keap1 | 0.2776603 |
| Keap1 | -0.719513 |
| Kera | 0.4593022 |
| Khdc1c | 2.8534484 |
| Khdrbs1 | -0.4011533 |
| Khdrbs2 | -0.4815513 |
| Khdrbs3 | 1.4827506 |
| Khk | -0.2700701 |
| Khk | 2.1653999 |
| Khsrp | 0.3006592 |
| Kif11 | -0.7169107 |
| Kif13a | -0.6482631 |
| Kif14 | -0.1238046 |
| Kif15 | 0.0950352 |
| Kif17 | 0.8003708 |
| Kif17 | 0.0043318 |
| Kif18a | -0.4392348 |
| Kif1a | 0.1899478 |
| Kif1b | -0.36568 |
| Kif1b | 2.0965588 |
| Kif1b | 1.0392603 |
| Kif1b | 1.0113994 |
| Kif1b | -0.5037452 |
| Kif1b | 1.7871225 |
| Kif1c | 2.2988028 |
| Kif21a | -0.3462988 |
| Kif21b | -0.7785317 |
| Kif22 | -0.281256 |
| Kif23 | 0.4058299 |
| Kif24 | 1.936599 |
| Kif27 | -0.3595833 |
| Kif2a | -0.6458381 |
| Kif2b | 2.0311663 |
| Kif2c | -0.6728394 |
| Kif3a | 2.2731843 |
| Kif3a | 3.2909852 |
| Kif3b | 1.1065018 |
| Kif3c | 0.6926496 |
| Kif3c | -0.810339 |
| Kif4 | 1.2760502 |
| Kif4 | 0.9742003 |
| Kif4 | 1.0998043 |
| Kif5a | -0.7160448 |
| Kif5a | -0.3445008 |
| Kif5a | 2.7756704 |
| Kif5b | 1.4123819 |
| Kif5c | 0.9631278 |
| Kif9 | -0.785926 |
| Kifap3 | 2.1805914 |
| Kifap3 | 1.2820348 |
| Kifap3 | 0.1757498 |
| Kifc2 | 1.0824998 |
| Kifc2 | -0.1545791 |
| Kifc3 | -0.3225848 |
| Kin | -0.6761619 |
| Kirrel3 | -0.7221221 |
| Kiss1 | -0.5314716 |
| Kiss1r | 0.4890803 |
| Kitl | -0.5839925 |
| Kl | 0.141576 |
| Klb | -0.5143192 |
| Klc1 | -0.6954821 |
| Klc1 | 0.954743 |
| Klc1 | -0.4912011 |
| Klc2 | -0.1960952 |
| Klc2 | -0.0851062 |
| Klc4 | 2.1801018 |
| Klf1 | -0.4359512 |
| Klf10 | -0.0536554 |
| Klf12 | 0.2084574 |
| Klf12 | -0.567424 |
| Klf12 | 0.6670643 |
| Klf13 | 0.5873556 |
| Klf16 | -0.4960608 |
| Klf17 | 0.7437845 |
| Klf17 | -0.0690303 |
| Klf2 | 2.3584371 |
| Klf4 | -0.0613136 |
| Klf4 | 3.0231986 |
| Klf4 | -0.3614084 |
| Klf5 | 2.2110175 |
| Klf6 | -0.6187548 |
| Klf6 | -0.3589756 |
| Klf7 | -0.6000659 |
| Klf9 | 2.1283495 |
| Klhdc1 | 1.4885042 |
| Klhdc1 | 1.7170545 |
| Klhdc1 | -0.7521729 |
| Klhdc2 | -0.1904257 |
| Klhdc3 | -0.5885588 |
| Klhdc4 | 1.8791881 |
| Klhdc4 | 2.208735 |
| Klhdc4 | -0.1170916 |
| Klhdc8b | -0.8630138 |
| Klhdc9 | 0.7785878 |
| Klhl10 | -0.7620901 |
| Klhl11 | -0.2361621 |
| Klhl12 | -0.8863127 |
| Klhl14 | -0.6954821 |
| Klhl15 | 0.0252971 |
| Klhl15 | -0.7379197 |
| Klhl17 | -0.8600141 |
| Klhl2 | 0.9414482 |
| Klhl21 | 2.7597817 |
| Klhl22 | 0.1050537 |
| Klhl22 | -0.2852006 |
| Klhl23 | 1.4576991 |
| Klhl24 | 0.6766806 |
| Klhl24 | 0.0802113 |
| Klhl25 | -0.4912011 |
| Klhl26 | 0.2428166 |
| Klhl26 | -0.2281271 |
| Klhl30 | 2.1086178 |
| Klhl31 | -0.3565487 |
| Klhl32 | 1.4815763 |
| Klhl5 | 0.547606 |
| Klhl5 | -0.3848175 |
| Klhl6 | 3.1944663 |
| Klhl6 | -0.673669 |
| Klhl7 | -0.4484862 |
| Klhl8 | 1.8771398 |
| Klhl9 | 0.5510611 |
| Klhl9 | -0.3132531 |
| Klk1 | 0.1983716 |
| Klk11 | 1.8758161 |
| Klk11 | -0.6282308 |
| Klk15 | -0.2745296 |
| Klk1b1 | -0.6156158 |
| Klk1b11 | 0.3243799 |
| Klk1b16 | 0.1678549 |
| Klk1b21 | 0.8989094 |
| Klk1b21 | 0.3085238 |
| Klk1b22 | 0.233408 |
| Klk1b24 | -0.3571549 |
| Klk1b26 | 1.9947498 |
| Klk1b27 | -0.5121955 |
| Klk1b27 | 0.178249 |
| Klk1b4 | -0.7352695 |
| Klk1b4 | 2.4362433 |
| Klk1b5 | -0.7229933 |
| Klk1b8 | 0.0946479 |
| Klk1b9 | 1.4826528 |
| Klk6 | -0.2275937 |
| Klk8 | -0.471993 |
| Klkb1 | 0.1134539 |
| Klra17 | 0.8614025 |
| Klra4 | -0.7379197 |
| Klrd1 | -0.3779852 |
| Kndc1 | 1.2633658 |
| Kng1 | 0.0417611 |
| Kng1 | -0.8373082 |
| Kng2 | 0.6143228 |
| Kns2 | 0.1000569 |
| Kns2 | -0.4294159 |
| Kntc1 | 1.4110177 |
| Kpna1 | -0.1113729 |
| Kpna2 | -0.7730213 |
| Kpna3 | -0.6946343 |
| Kpna6 | 0.3469523 |
| Kpnb1 | -0.1047418 |
| Kptn | -0.1575596 |
| Kras | 1.3545462 |
| Kras | -0.3296406 |
| Krba1 | -0.4672476 |
| Krba1 | 1.764673 |
| Krba1 | -0.4126197 |
| Krcc1 | 0.1251729 |
| Kremen | 2.8880154 |
| Kremen1 | -0.7512761 |
| Kremen2 | -0.6410056 |
| Krit1 | 0.2955935 |
| Krit1 | 1.792316 |
| Krr1 | -0.5946796 |
| Krr1 | 0.3472539 |
| Krr1 | -0.4190467 |
| Krt10 | 1.2264722 |
| Krt12 | -0.6109256 |
| Krt13 | 2.5978293 |
| Krt14 | -0.0681194 |
| Krt17 | -0.6085887 |
| Krt18 | -0.613268 |
| Krt19 | 0.8680812 |
| Krt2 | -0.5599819 |
| Krt20 | 2.4674402 |
| Krt222 | -0.7414642 |
| Krt23 | 0.0306775 |
| Krt27 | 0.6716654 |
| Krt32 | 2.1034522 |
| Krt4 | 1.5320854 |
| Krt7 | -0.6563889 |
| Krt73 | 0.7167659 |
| Krt75 | -0.1665542 |
| Krt78 | 0.2783063 |
| Krt79 | -0.5121955 |
| Krt8 | -0.5336363 |
| Krt84 | -0.5599819 |
| Krt86 | -0.2106721 |
| Krt86 | -0.515028 |
| Krt9 | -0.720382 |
| Krtap16-4 | -0.107105 |
| Krtap8-2 | -0.7602797 |
| Krtcap2 | -0.0491776 |
| Krtcap3 | 2.6932744 |
| Ksr2 | -0.5214306 |
| Ktelc1 | 0.2471552 |
| Kti12 | 1.1712714 |
| Ktn1 | 1.4852901 |
| Ktn1 | -0.6645806 |
| Ky | -0.2790089 |
| L1cam | 2.727802 |
| L2hgdh | 1.0674062 |
| L3mbtl2 | 1.1152693 |
| L3mbtl3 | 1.7203824 |
| l7Rn6 | 1.611395 |
| Lace1 | 1.6757541 |
| Lad1 | -0.7485906 |
| Lag3 | 2.166145 |
| Lage3 | -0.008889 |
| Lama1 | -0.0568017 |
| Lama2 | 0.3424175 |
| Lamb1-1 | -0.7212516 |
| Lamb2 | 2.2001772 |
| Lamb3 | -0.5916145 |
| Lamc1 | 1.4306715 |
| Lamc2 | -0.3730451 |
| Lamc2 | -0.6621161 |
| Lamc2 | -0.6274377 |
| Lamc3 | 0.1518771 |
| Lamp1 | 0.2879468 |
| Lamp2 | 0.0043318 |
| Lamp2 | -0.7370355 |
| Lamp2 | -0.5474551 |
| Lamp2 | -0.4196916 |
| Lancl1 | -0.4222756 |
| Lancl1 | 0.2773371 |
| Lancl1 | 0.7054142 |
| Laptm4a | -0.5358057 |
| Laptm4b | -0.5704163 |
| Laptm4b | -0.3873136 |
| Laptm5 | -0.6282308 |
| Large | -0.0690303 |
| Larp2 | 1.8261328 |
| Larp5 | -0.258457 |
| Larp6 | -0.3675162 |
| Larp6 | 0.1164912 |
| Lars | 1.3604151 |
| Lars2 | -0.168564 |
| Las1l | -0.4518109 |
| Lasp1 | 1.2517504 |
| Lasp1 | -0.7134514 |
| Lasp1 | -0.4981507 |
| Lass1 | 1.1810797 |
| Lass2 | -0.0595064 |
| Lass2 | -0.6904062 |
| Lass2 | -0.5481877 |
| Lass4 | -0.4995464 |
| Lass4 | 0.0186355 |
| Lass5 | 0.5890179 |
| Lass6 | 0.1363857 |
| Lat2 | -0.5877963 |
| Lat2 | -0.5923799 |
| Lats2 | -0.6811664 |
| Lbh | -0.5164473 |
| Lbh | -0.5438002 |
| Lbp | -0.6385979 |
| Lbr | -0.5228589 |
| Lbx2 | -0.5726663 |
| Lca5 | 1.6493246 |
| Lca5 | -0.4171143 |
| Lca5 | -0.3063106 |
| Lcat | -0.1005019 |
| Lcat | 0.9131173 |
| Lce1c | 0.3955342 |
| Lck | -0.527874 |
| Lcmt1 | -0.5358057 |
| Lcmt1 | 0.8563187 |
| Lcmt1 | -0.5221445 |
| Lcn10 | -0.810339 |
| Lcn12 | 1.7908761 |
| Lcn13 | 1.9076927 |
| Lcn2 | -0.4753963 |
| Lcn3 | -0.5622088 |
| Lcn8 | -0.5178686 |
| Lcor | 0.949612 |
| Lcp1 | -0.2823814 |
| Ldb1 | -0.4451724 |
| Ldb2 | 0.7321172 |
| Ldb2 | -0.4024209 |
| Ldha | -0.6654034 |
| Ldhc | -0.6234817 |
| Ldhc | -0.2196263 |
| Ldhc | 3.0072807 |
| Ldhd | 1.1788349 |
| Ldhd | 0.0186355 |
| Ldoc1 | 2.2328194 |
| Ldoc1l | 2.9378103 |
| Lef1 | 0.0583622 |
| Lef1 | -0.4713137 |
| Lefty1 | 1.1806839 |
| Lefty2 | -0.8461323 |
| Lemd2 | 2.2309118 |
| Leng1 | 1.8877328 |
| Leng8 | 0.8999559 |
| Leng9 | -0.7177774 |
| Leo1 | -0.1496311 |
| Lep | 0.8590453 |
| Lepre1 | -0.6282308 |
| Lepre1 | -0.5939124 |
| Lepre1 | 0.540909 |
| Leprel1 | 0.4748327 |
| Leprel2 | -0.362627 |
| Leprotl1 | 2.3038407 |
| Letm1 | 1.0474255 |
| Letm1 | 2.6904439 |
| Letm1 | 1.5102448 |
| Letmd1 | 2.9101261 |
| Lgals1 | -0.0772659 |
| Lgals2 | -0.8461323 |
| Lgals3 | -0.0953438 |
| Lgals3bp | 0.2537938 |
| Lgals4 | -0.6920953 |
| Lgals4 | 4.5763573 |
| Lgals6 | -0.5518586 |
| Lgals7 | -0.620328 |
| Lgals8 | -0.0028968 |
| Lgals8 | 0.8695194 |
| Lgals9 | 1.1218651 |
| Lgals9 | 0.4026951 |
| Lgi1 | 1.0096811 |
| Lgi1 | 0.7663239 |
| Lgi2 | -0.6490728 |
| Lgi3 | -0.1540832 |
| Lgi4 | 0.4511743 |
| Lgmn | 2.0160658 |
| Lgr5 | 3.2993814 |
| Lgtn | 0.1507784 |
| Lgtn | -0.4307195 |
| Lhb | -0.3409143 |
| Lhcgr | -0.7971202 |
| Lhcgr | 0.2084574 |
| Lhfp | -0.484987 |
| Lhfp | -0.1137517 |
| Lhfp | 0.713206 |
| Lhfpl1 | 1.9566632 |
| Lhfpl2 | -0.8122416 |
| Lhx1 | 3.3182507 |
| Lhx2 | -0.3179081 |
| Lhx3 | -0.5093708 |
| Lhx4 | -0.5372545 |
| Lhx9 | -0.121402 |
| Lhx9 | 0.3338975 |
| Lias | 2.3687261 |
| Lias | 1.8048286 |
| Lias | 2.563527 |
| Lifr | -0.610146 |
| Lig1 | -0.8751026 |
| Lig1 | 0.7165568 |
| Lig1 | -0.3898158 |
| Lig3 | -0.845148 |
| Lig4 | 1.0696184 |
| Lima1 | -0.1009721 |
| Lima1 | 1.3242205 |
| Limd1 | -0.5438002 |
| Limd2 | -0.9196511 |
| Lime1 | 0.2170718 |
| Limk1 | 1.487434 |
| Limk2 | -0.6282308 |
| Limk2 | 2.2313774 |
| Lims2 | 2.7790023 |
| Lims2 | 0.7119465 |
| Lims2 | -0.833411 |
| Lin54 | -0.6402024 |
| Lin7b | 1.4048031 |
| Lin7b | -0.7877831 |
| Lin7b | -0.2959856 |
| Lin7b | -0.6008377 |
| Lin7c | -0.779453 |
| Lincr | 1.2090473 |
| Lincr | -0.7785317 |
| Lingo1 | -0.0786451 |
| Lingo2 | -0.6322056 |
| Lingo2 | -0.5416136 |
| Lingo4 | -0.0791052 |
| Lip1 | -0.6450311 |
| Lipe | -0.7924408 |
| Lipf | 0.3839699 |
| Liph | 1.0090555 |
| Liph | -0.7160448 |
| Litaf | 0.3894795 |
| Lix1 | -0.634598 |
| Lix1 | -0.8510682 |
| Lix1 | -0.0676643 |
| Lix1 | 0.7961289 |
| Llgl1 | 0.853037 |
| Llgl2 | -0.3155779 |
| Lman1 | 0.9355762 |
| Lman2 | 0.7474411 |
| Lman2l | -0.7557679 |
| Lman2l | 1.2348855 |
| Lmbr1 | -0.657205 |
| Lmbr1l | -0.1751235 |
| Lmcd1 | -0.6563889 |
| Lmcd1 | -0.5870344 |
| Lmcd1 | -0.2101479 |
| Lmf1 | 0.3345084 |
| Lmf2 | -0.0028968 |
| Lmna | 2.8519653 |
| Lmna | -0.7352695 |
| Lmna | 0.3271523 |
| Lmna | -0.5555429 |
| Lmna | -0.4981507 |
| Lmnb1 | -0.5893219 |
| Lmnb1 | -0.5749214 |
| Lmnb2 | -0.1868343 |
| Lmo2 | 1.3335617 |
| Lmo3 | 1.4034286 |
| Lmo3 | -0.705711 |
| Lmo4 | 0.7257146 |
| Lmod1 | 0.3160166 |
| Lmtk2 | -0.0777254 |
| Lmtk3 | 0.774648 |
| Lmx1a | -0.3132531 |
| Lmx1b | -0.61405 |
| Lnx1 | -0.2122465 |
| Lnx2 | -0.1650495 |
| Lnx2 | -0.3266947 |
| LOC100038882 | -0.4981507 |
| LOC100038984 | -0.3823277 |
| LOC100039087 | -0.3644577 |
| LOC100039227 | -0.4898169 |
| LOC100039479 | -0.72999 |
| LOC100039532 | 0.3439313 |
| LOC100039571 | -0.8761165 |
| LOC100039728 | -0.7370355 |
| LOC100040573 | -0.5023437 |
| LOC100040592 | 0.3317562 |
| LOC100040601 | -0.4418694 |
| LOC100040799 | -0.1481514 |
| LOC100040899 | 0.3538663 |
| LOC100040919 | -0.0708547 |
| LOC100041103 | -0.6637584 |
| LOC100041290 | 0.3795989 |
| LOC100041463 | 0.2060321 |
| LOC100041483 | 1.7697624 |
| LOC100041500 | -0.2706265 |
| LOC100041504 | 0.81943 |
| LOC100041703 | 1.9366613 |
| LOC100041722 | 2.3349796 |
| LOC100041725 | -0.5839925 |
| LOC100041835 | 2.6823807 |
| LOC100042179 | 0.2747482 |
| LOC100042427 | -0.535082 |
| LOC100042777 | -0.5214306 |
| LOC100043257 | -0.6937873 |
| LOC100043391 | -0.321999 |
| LOC100043671 | 2.3697817 |
| LOC100043822 | 3.3755563 |
| LOC100043986 | 0.0494883 |
| LOC100044087 | 0.4344427 |
| LOC100044089 | -0.5408857 |
| LOC100044101 | -0.16005 |
| LOC100044103 | -0.6980296 |
| LOC100044103 | -0.1412749 |
| LOC100044122 | 0.1088806 |
| LOC100044133 | 0.8493781 |
| LOC100044159 | -0.4385772 |
| LOC100044170 | 3.9978631 |
| LOC100044172 | -0.4274635 |
| LOC100044177 | -0.4392348 |
| LOC100044190 | -0.379224 |
| LOC100044204 | 0.2997113 |
| LOC100044210 | 0.9382648 |
| LOC100044221 | -0.6085887 |
| LOC100044257 | -0.386689 |
| LOC100044275 | -0.6219036 |
| LOC100044289 | 2.070144 |
| LOC100044292 | 0.5301507 |
| LOC100044294 | -0.4005201 |
| LOC100044298 | 0.7240558 |
| LOC100044322 | -0.3979912 |
| LOC100044324 | 0.7617362 |
| LOC100044324 | 0.9025671 |
| LOC100044363 | 0.184647 |
| LOC100044363 | 0.3763814 |
| LOC100044439 | -0.1904257 |
| LOC100044468 | 0.1910047 |
| LOC100044475 | 1.1204654 |
| LOC100044538 | -0.0536554 |
| LOC100044566 | -0.0599579 |
| LOC100044576 | -0.5372545 |
| LOC100044636 | 0.998045 |
| LOC100044692 | 0.1895953 |
| LOC100044708 | 2.7768907 |
| LOC100044736 | 3.7582144 |
| LOC100044756 | -0.4863646 |
| LOC100044776 | 0.3790147 |
| LOC100044779 | -0.7169107 |
| LOC100044812 | -0.6612959 |
| LOC100044829 | 2.5424465 |
| LOC100044862 | 1.0923421 |
| LOC100044873 | 1.4741074 |
| LOC100044934 | 2.6003184 |
| LOC100045005 | 0.1341531 |
| LOC100045019 | -0.8275933 |
| LOC100045040 | -0.3097759 |
| LOC100045098 | 2.6757816 |
| LOC100045228 | 1.2600782 |
| LOC100045241 | 0.31726 |
| LOC100045250 | -0.673669 |
| LOC100045280 | -0.6290245 |
| LOC100045300 | -0.0358624 |
| LOC100045304 | -0.8945446 |
| LOC100045343 | -0.3841945 |
| LOC100045359 | 0.6742851 |
| LOC100045359 | -0.5555429 |
| LOC100045403 | 0.1732445 |
| LOC100045413 | 3.1610697 |
| LOC100045419 | 2.0486697 |
| LOC100045430 | -0.4638716 |
| LOC100045439 | 2.0061045 |
| LOC100045491 | 1.9559906 |
| LOC100045505 | -0.6418094 |
| LOC100045522 | 0.5880684 |
| LOC100045542 | 0.6331204 |
| LOC100045551 | -0.0469462 |
| LOC100045567 | -0.4294159 |
| LOC100045607 | -0.3898158 |
| LOC100045617 | -0.1354178 |
| LOC100045644 | 0.3433261 |
| LOC100045697 | 1.3110883 |
| LOC100045780 | -0.5847521 |
| LOC100045782 | 1.9489318 |
| LOC100045877 | 1.2903539 |
| LOC100045882 | -0.6426139 |
| LOC100045887 | -0.6895627 |
| LOC100045950 | 0.5557313 |
| LOC100045963 | 1.0901793 |
| LOC100045981 | 0.3672582 |
| LOC100046003 | -0.3138338 |
| LOC100046035 | -0.1127995 |
| LOC100046035 | 1.9781926 |
| LOC100046039 | -0.8510682 |
| LOC100046044 | -0.6662269 |
| LOC100046049 | 1.2033865 |
| LOC100046056 | 0.881485 |
| LOC100046068 | 1.2250842 |
| LOC100046080 | 0.2844227 |
| LOC100046081 | 1.2214157 |
| LOC100046120 | -0.6085887 |
| LOC100046128 | 1.3539907 |
| LOC100046129 | 1.1468708 |
| LOC100046163 | -0.4005201 |
| LOC100046163 | -0.4281139 |
| LOC100046207 | -0.7100039 |
| LOC100046207 | 0.7464267 |
| LOC100046207 | 0.4893422 |
| LOC100046232 | -0.461179 |
| LOC100046254 | 1.2813185 |
| LOC100046320 | 0.50674 |
| LOC100046393 | -0.3179081 |
| LOC100046406 | -0.6062571 |
| LOC100046457 | 0.424995 |
| LOC100046518 | -0.3841945 |
| LOC100046568 | 1.4940318 |
| LOC100046586 | 1.8309912 |
| LOC100046608 | -0.4068702 |
| LOC100046608 | 0.4066831 |
| LOC100046608 | -0.4836113 |
| LOC100046608 | 2.7864656 |
| LOC100046616 | -0.1746174 |
| LOC100046641 | 2.1629618 |
| LOC100046650 | 1.6900282 |
| LOC100046690 | 3.3037623 |
| LOC100046690 | 1.7744601 |
| LOC100046741 | 0.4919581 |
| LOC100046744 | -0.5592407 |
| LOC100046744 | -0.5786911 |
| LOC100046746 | 0.6138599 |
| LOC100046775 | -0.7396904 |
| LOC100046781 | -0.7849988 |
| LOC100046781 | -0.1680612 |
| LOC100046792 | -0.5636961 |
| LOC100046796 | 0.5596474 |
| LOC100046802 | 1.5679465 |
| LOC100046841 | -0.6753303 |
| LOC100046855 | -0.7379197 |
| LOC100046883 | 0.5537676 |
| LOC100046891 | 1.193012 |
| LOC100046898 | -0.6539443 |
| LOC100046918 | -0.276207 |
| LOC100046953 | -0.4974536 |
| LOC100046959 | -0.8842652 |
| LOC100046996 | 1.4310833 |
| LOC100047009 | -0.9281609 |
| LOC100047009 | 1.6010985 |
| LOC100047012 | 0.0364397 |
| LOC100047028 | -0.6211155 |
| LOC100047046 | 1.5845261 |
| LOC100047052 | -0.1605489 |
| LOC100047052 | 0.6154791 |
| LOC100047155 | -0.1427445 |
| LOC100047155 | -0.1675586 |
| LOC100047167 | -0.8520583 |
| LOC100047173 | -0.421629 |
| LOC100047184 | 1.2779691 |
| LOC100047200 | -0.3296406 |
| LOC100047214 | 0.8015245 |
| LOC100047214 | 2.5280167 |
| LOC100047238 | 2.0294048 |
| LOC100047261 | 0.0421693 |
| LOC100047323 | -0.7602797 |
| LOC100047323 | 1.9965728 |
| LOC100047339 | 0.3125893 |
| LOC100047353 | -0.5555429 |
| LOC100047368 | 0.0089817 |
| LOC100047369 | 0.9507729 |
| LOC100047369 | -0.7273605 |
| LOC100047391 | 0.3194322 |
| LOC100047393 | -0.4458343 |
| LOC100047427 | -0.7476971 |
| LOC100047480 | 0.9206593 |
| LOC100047490 | 1.8428305 |
| LOC100047579 | 0.1107887 |
| LOC100047583 | -0.2388548 |
| LOC100047606 | -0.7539688 |
| LOC100047615 | 0.0551444 |
| LOC100047619 | 0.0498934 |
| LOC100047634 | 2.1346711 |
| LOC100047651 | -0.4320249 |
| LOC100047653 | 1.91 |
| LOC100047659 | 0.5806791 |
| LOC100047674 | -0.195062 |
| LOC100047707 | -0.6093671 |
| LOC100047717 | 1.785313 |
| LOC100047749 | -0.703999 |
| LOC100047762 | -0.6266453 |
| LOC100047776 | 0.2081113 |
| LOC100047794 | -0.1368789 |
| LOC100047800 | 0.5909143 |
| LOC100047810 | -0.6621161 |
| LOC100047815 | -0.8237334 |
| LOC100047816 | -0.1899118 |
| LOC100047834 | 0.3390789 |
| LOC100047837 | 1.7439037 |
| LOC100047837 | 0.4798687 |
| LOC100047841 | -0.8122416 |
| LOC100047856 | 0.0950352 |
| LOC100047870 | -0.4011533 |
| LOC100047911 | 1.324106 |
| LOC100047934 | 2.655996 |
| LOC100047935 | -0.8431824 |
| LOC100047937 | 2.4934741 |
| LOC100047937 | 0.709844 |
| LOC100047958 | 0.7238482 |
| LOC100047963 | 0.1927636 |
| LOC100047963 | 0.7940013 |
| LOC100047998 | -0.3823277 |
| LOC100048046 | 0.5650074 |
| LOC100048046 | 0.0774567 |
| LOC100048049 | -0.4274635 |
| LOC100048049 | -0.7999382 |
| LOC100048049 | 0.9082883 |
| LOC100048056 | 2.6033809 |
| LOC100048071 | -0.4425291 |
| LOC100048076 | -0.4464966 |
| LOC100048083 | -0.2254629 |
| LOC100048105 | 1.6163756 |
| LOC100048105 | -0.0270822 |
| LOC100048187 | 2.1563644 |
| LOC100048251 | -0.4932809 |
| LOC100048295 | 0.2194707 |
| LOC100048299 | -0.4686011 |
| LOC100048301 | 0.9560629 |
| LOC100048313 | -0.6109256 |
| LOC100048331 | 0.5965821 |
| LOC100048332 | 2.7412831 |
| LOC100048346 | 1.5043271 |
| LOC100048372 | -0.6596576 |
| LOC100048384 | 0.9492801 |
| LOC100048413 | -0.8392624 |
| LOC100048436 | -0.5379797 |
| LOC100048445 | 2.0827369 |
| LOC100048461 | -0.3367462 |
| LOC100048480 | 2.4169504 |
| LOC100048483 | 0.5359194 |
| LOC100048504 | -0.1000319 |
| LOC100048530 | -0.1223624 |
| LOC100048554 | 2.1392717 |
| LOC100048556 | -0.2689584 |
| LOC100048589 | 1.2903539 |
| LOC100048613 | 0.5379182 |
| LOC100048622 | 1.671789 |
| LOC100048645 | 0.9845115 |
| LOC100048684 | 0.8566827 |
| LOC100048703 | 1.2927183 |
| LOC100048710 | -0.4645459 |
| LOC100048721 | -0.6645806 |
| LOC100048721 | -0.6258534 |
| LOC100048726 | 0.7026622 |
| LOC100048733 | -0.4863646 |
| LOC100048816 | -0.6250622 |
| LOC100048832 | 0.7452081 |
| LOC100048832 | 1.5155601 |
| LOC100048858 | 0.9511044 |
| LOC192758 | 1.5714455 |
| LOC194227 | 0.2760435 |
| LOC212390 | -0.9875601 |
| LOC216443 | -0.046055 |
| LOC216963 | -0.3835718 |
| LOC217341 | 4.1364533 |
| LOC218963 | 0.0888201 |
| LOC225922 | -0.1166138 |
| LOC233529 | -0.7822221 |
| LOC234374 | 0.366371 |
| LOC235497 | -0.5870344 |
| LOC236749 | -0.2568089 |
| LOC237408 | -0.3017087 |
| LOC237877 | 0.9189502 |
| LOC238829 | -0.6803306 |
| LOC241593 | -0.7468043 |
| LOC244595 | 0.3909244 |
| LOC245263 | -0.9164784 |
| LOC329575 | 0.8619456 |
| LOC329575 | 1.0002568 |
| LOC333331 | -0.5992946 |
| LOC381066 | -0.2228057 |
| LOC381302 | -0.2734129 |
| LOC381375 | -0.3935808 |
| LOC381483 | 0.6308467 |
| LOC381629 | 0.3769672 |
| LOC381801 | -0.0367446 |
| LOC381936 | -0.3063106 |
| LOC382010 | 1.2233149 |
| LOC382044 | 1.7149592 |
| LOC382106 | -0.2393942 |
| LOC382106 | -0.2212148 |
| LOC382133 | 0.3228364 |
| LOC384806 | -0.3028572 |
| LOC384806 | -0.2959856 |
| LOC386486 | 2.3779053 |
| LOC433479 | -0.4126197 |
| LOC433801 | 2.6426486 |
| LOC433886 | -0.5726663 |
| LOC434172 | -0.5794467 |
| LOC434172 | -0.7117262 |
| LOC434864 | -0.6250622 |
| LOC434960 | 4.0024391 |
| LOC435023 | -0.5058511 |
| LOC435337 | -0.7611845 |
| LOC435565 | -0.0953438 |
| LOC436177 | -0.3403178 |
| LOC544988 | -0.7877831 |
| LOC544988 | 0.665086 |
| LOC545013 | 0.3952467 |
| LOC545013 | -0.5540676 |
| LOC545056 | -0.4774438 |
| LOC545238 | -0.2028368 |
| LOC545703 | 1.2183696 |
| LOC545732 | 2.8212654 |
| LOC545952 | 0.3571564 |
| LOC546015 | 0.472172 |
| LOC546015 | -0.4877441 |
| LOC546723 | -0.7915076 |
| LOC546840 | -0.7933749 |
| LOC547323 | 0.6648659 |
| LOC547343 | -0.4425291 |
| LOC620807 | -0.8590162 |
| LOC621823 | 0.3595424 |
| LOC621824 | 0.2364417 |
| LOC622404 | -0.8771315 |
| LOC622665 | 2.6198864 |
| LOC624855 | -0.720382 |
| LOC625480 | 0.8420202 |
| LOC626152 | -0.6266453 |
| LOC628053 | 1.6707344 |
| LOC631002 | -0.3755121 |
| LOC631784 | -0.6870365 |
| LOC632431 | 2.4239354 |
| LOC639396 | -0.3973599 |
| LOC640963 | 2.03468 |
| LOC641240 | -0.5023437 |
| LOC654426 | 2.1076183 |
| LOC654467 | 1.1060756 |
| LOC654494 | -0.5839925 |
| LOC654842 | 0.0860889 |
| LOC665250 | -0.4760783 |
| LOC666676 | -0.0003396 |
| LOC666904 | 2.6982673 |
| LOC667250 | -0.7776112 |
| LOC667370 | -0.3973599 |
| LOC667609 | 0.5710638 |
| LOC668492 | 0.565736 |
| LOC668837 | 2.7276598 |
| LOC669168 | -0.5533307 |
| LOC669660 | 0.4247158 |
| LOC670044 | -0.2750884 |
| LOC671564 | 0.0364397 |
| LOC672705 | 3.4482188 |
| LOC674706 | 0.7959357 |
| LOC675228 | -0.5726663 |
| LOC675567 | 0.2508118 |
| LOC675567 | 2.7339573 |
| LOC675813 | 3.0762272 |
| LOC676420 | 1.3066727 |
| LOC676420 | -0.4017869 |
| LOC677144 | 0.1240448 |
| LOC677205 | -0.6861958 |
| LOC677317 | -0.3589756 |
| LOC677319 | 1.3416931 |
| LOC677528 | 1.4351925 |
| LOC732482 | 0.256107 |
| Loh11cr2a | 0.9310228 |
| Loh12cr1 | 1.9617239 |
| Loh12cr1 | 0.2461556 |
| Lonp2 | -0.4788111 |
| Lonrf1 | 1.5501753 |
| Lonrf2 | 0.6924354 |
| Lor | -0.4132605 |
| Lox | 1.6322682 |
| Loxl1 | 0.8775716 |
| Lpar2 | -0.8198883 |
| Lpar2 | 1.1023746 |
| Lpar3 | 0.3874532 |
| Lpcat2 | 1.2200207 |
| Lpcat2 | -0.4747147 |
| Lpcat2 | -0.7273605 |
| Lpgat1 | -0.3699697 |
| Lphn1 | 0.0903775 |
| Lphn3 | -0.1615473 |
| Lpin1 | -0.3998872 |
| Lpin1 | -0.2143495 |
| Lpin2 | 0.9644368 |
| Lpin3 | 1.1734002 |
| Lpl | 0.150412 |
| Lpxn | -0.8324391 |
| Lrat | -0.5423419 |
| Lrba | 2.5102757 |
| Lrba | 0.5991692 |
| Lrch1 | 1.3615186 |
| Lrch4 | -0.7485906 |
| Lrch4 | 0.762136 |
| Lrdd | -0.3724292 |
| Lrfn1 | 0.3169493 |
| Lrfn2 | 0.315083 |
| Lrfn3 | 0.620551 |
| Lrfn4 | 1.9266985 |
| Lrg1 | -0.3724292 |
| Lrig1 | 0.0077157 |
| Lrig3 | 1.0239628 |
| Lrmp | -0.7151796 |
| Lrp1 | -0.4438499 |
| Lrp10 | -0.8179712 |
| Lrp11 | -0.1393187 |
| Lrp12 | 1.0537308 |
| Lrp12 | -0.55776 |
| Lrp4 | 0.9688421 |
| Lrp5 | 0.1423153 |
| Lrp8 | -0.420337 |
| Lrp8 | -0.6828401 |
| Lrpap1 | -0.8412205 |
| Lrpprc | -0.5719157 |
| Lrpprc | -0.7048546 |
| Lrpprc | -0.1650495 |
| Lrrc1 | -0.6687015 |
| Lrrc1 | -0.7584725 |
| Lrrc1 | 0.3775526 |
| Lrrc10 | -0.6621161 |
| Lrrc15 | 0.706893 |
| Lrrc16b | 1.067849 |
| Lrrc16b | 0.3003433 |
| Lrrc18 | 1.1983422 |
| Lrrc2 | -0.4158281 |
| Lrrc20 | 2.0301438 |
| Lrrc20 | -0.6474542 |
| Lrrc23 | -0.0123292 |
| Lrrc23 | -0.6242717 |
| Lrrc24 | 0.9160379 |
| Lrrc26 | 1.1744629 |
| Lrrc28 | -0.2852006 |
| Lrrc29 | 1.0121794 |
| Lrrc3 | 0.8904988 |
| Lrrc30 | 1.6339532 |
| Lrrc33 | -0.6078109 |
| Lrrc34 | 1.1809478 |
| Lrrc34 | 2.9921806 |
| Lrrc36 | -0.0411674 |
| Lrrc39 | -0.4699565 |
| Lrrc39 | 0.6473282 |
| Lrrc4 | -0.4504797 |
| Lrrc40 | 3.2884428 |
| Lrrc41 | -0.7693644 |
| Lrrc43 | 1.0900349 |
| Lrrc44 | -0.577936 |
| Lrrc45 | -0.3761298 |
| Lrrc46 | 0.5162206 |
| Lrrc48 | -0.634598 |
| Lrrc48 | 2.1158472 |
| Lrrc48 | 0.794969 |
| Lrrc49 | 2.7676616 |
| Lrrc49 | 2.3479176 |
| Lrrc49 | 2.4467122 |
| Lrrc4c | -0.2722974 |
| Lrrc50 | 0.3424175 |
| Lrrc50 | -0.1986828 |
| Lrrc52 | -0.461179 |
| Lrrc52 | 1.4326262 |
| Lrrc56 | -0.3699697 |
| Lrrc57 | -0.6314094 |
| Lrrc59 | -0.5416136 |
| Lrrc6 | -0.4870541 |
| Lrrc7 | 0.832933 |
| Lrrc8 | -0.5555429 |
| Lrrc8a | 1.8209012 |
| Lrrc8b | -0.5037452 |
| Lrrc8c | 0.5121318 |
| Lrrc8d | 0.7918692 |
| Lrrc9 | 0.7764228 |
| Lrrfip1 | 0.7526995 |
| Lrrfip1 | 2.4317885 |
| Lrriq2 | -0.6385979 |
| Lrriq2 | 1.4820658 |
| Lrrk1 | -0.1422544 |
| Lrrk2 | -0.4119792 |
| Lrrn1 | 0.7908986 |
| Lrrn2 | -0.5051487 |
| Lrrn3 | 1.0481782 |
| Lrrtm1 | -0.4946699 |
| Lrrtm2 | -0.577936 |
| Lrrtm3 | -0.4235701 |
| Lrsam1 | 0.1378714 |
| Lrsam1 | -0.586273 |
| Lrtm1 | -0.0016174 |
| Lrtm1 | -0.0708547 |
| Lsamp | -0.5666773 |
| Lsamp | -0.1388303 |
| Lsg1 | -0.6604764 |
| Lsg1 | 0.366371 |
| Lsm10 | 0.2776603 |
| Lsm11 | 1.4124867 |
| Lsm12 | 3.4917204 |
| Lsm12 | -0.5511233 |
| Lsm12 | 0.0659637 |
| Lsm14a | 0.8601339 |
| Lsm14b | -0.2012771 |
| Lsm14b | 0.0786382 |
| Lsm14b | 0.7382747 |
| Lsm2 | -0.4645459 |
| Lsm2 | -0.088817 |
| Lsm3 | -0.7566686 |
| Lsm4 | 2.8424621 |
| Lsm5 | -0.3935808 |
| Lsm6 | -0.8373082 |
| Lsm6 | 1.5533718 |
| Lsm8 | -0.6156158 |
| Lsr | -0.8431824 |
| Lst1 | -0.6490728 |
| Ltb4dh | -0.0288321 |
| Ltb4r1 | -0.3848175 |
| Ltb4r2 | 1.5705495 |
| Ltbp1 | 0.5108507 |
| Ltbp1 | 1.3996125 |
| Ltbp2 | 0.0152882 |
| Ltbp3 | 2.0317338 |
| Ltbp3 | -0.613268 |
| Ltbp4 | -0.6226923 |
| Ltbp4 | -0.9101628 |
| Ltbp4 | 2.3742759 |
| Ltbr | 0.3766743 |
| Ltc4s | 0.7641324 |
| Ltf | -0.4726727 |
| Ltf | -0.2426366 |
| Ltk | -0.683678 |
| Ltk | -0.1349312 |
| Ltv1 | -0.0465005 |
| Luc7l | -0.9133157 |
| Luc7l | -0.9281609 |
| Luc7l2 | 0.8442332 |
| Luc7l2 | -0.3011349 |
| Lum | 1.5743968 |
| Luzp1 | 2.7003161 |
| Luzp1 | -0.6820029 |
| Luzp1 | -0.643419 |
| Luzp2 | 0.6777676 |
| Lxn | -0.2612099 |
| Ly6a | 2.2043456 |
| Ly6c1 | -0.7238652 |
| Ly6d | 0.5975236 |
| Ly6e | -0.6895627 |
| Ly6f | 1.149324 |
| Ly6g6c | -0.7326263 |
| Ly6g6d | 1.7677002 |
| Ly6g6e | 0.9240689 |
| Ly6k | -0.7812982 |
| Ly86 | 0.6620009 |
| Ly9 | -0.6234817 |
| Ly9 | 0.7115264 |
| Ly96 | -0.6803306 |
| Lyar | 0.1389842 |
| Lyl1 | 1.999682 |
| Lynx1 | 0.4092385 |
| Lynx1 | 1.8242525 |
| Lypd1 | 0.670134 |
| Lypd3 | -0.6547585 |
| Lypd4 | 0.3203618 |
| Lypd5 | -0.4287647 |
| Lypd6 | 1.1433168 |
| Lypla1 | -0.7971202 |
| Lypla1 | 2.4040503 |
| Lypla2 | 0.8919054 |
| Lypla3 | -0.7343877 |
| Lyplal1 | 0.2514752 |
| Lyrm1 | -0.530751 |
| Lyrm2 | -0.3103546 |
| Lyrm2 | 0.9690048 |
| Lyrm5 | 2.1099313 |
| Lysmd1 | -0.6148326 |
| Lysmd1 | -0.2914305 |
| Lysmd2 | 2.4962342 |
| Lysmd4 | -0.4884345 |
| Lyst | -0.4584935 |
| Lyve1 | -0.5629522 |
| Lyz | 0.9105332 |
| Lyz1 | 1.3440524 |
| Lyz1 | -0.417758 |
| Lyz2 | 3.1262801 |
| Lyzl1 | 2.5085091 |
| Lyzl4 | 0.6421644 |
| Lyzl6 | 3.451514 |
| Lyzs | -0.1720907 |
| Lzic | -0.1873466 |
| Lztr1 | -0.063124 |
| Lzts2 | -0.0183778 |
| M6pr | 1.3309504 |
| M6pr | -0.4132605 |
| M6prbp1 | 1.3448377 |
| Mab21l2 | 0.3915018 |
| Macrod1 | -0.3149962 |
| Mad1l1 | -0.204399 |
| Mad2l1 | 0.8896186 |
| Mad2l1bp | -0.6963305 |
| Mad2l1bp | -0.4774438 |
| Mad2l1bp | -0.4100603 |
| Mad2l2 | -0.4043253 |
| Madcam1 | 0.4549754 |
| Madd | 1.7151922 |
| Maea | 1.0371326 |
| Maf | 0.8446016 |
| Maf1 | 0.0494883 |
| Mafg | 0.9680277 |
| Mag | -0.6946343 |
| Magea2 | 0.0615699 |
| Magea5 | -0.5592407 |
| Mageb3 | -0.4870541 |
| Mageb3 | -0.500245 |
| Maged1 | 1.0477267 |
| Maged2 | -0.577936 |
| Magee1 | -0.1873466 |
| Mageh1 | -0.3687422 |
| Magi1 | -0.5072575 |
| Magi1 | 1.7116133 |
| Magi1 | -0.5839925 |
| Magi2 | -0.2259952 |
| Magi3 | 0.851575 |
| Magi3 | -0.7247379 |
| Magix | -0.613268 |
| Magmas | -0.2623132 |
| Magoh | -0.0676643 |
| Magoh | -0.5749214 |
| Magohb | 2.4746338 |
| Mak | 1.7331276 |
| Mak10 | 1.1856858 |
| Mak10 | -0.0227206 |
| Mal | 3.0263565 |
| Mal | 0.9407788 |
| Mall | -0.667876 |
| Malt1 | 0.4937851 |
| Mamdc2 | 0.0766682 |
| Maml1 | -0.1033265 |
| Maml3 | 1.8229273 |
| Man1a | 0.2789519 |
| Man1a2 | -0.6070337 |
| Man1a2 | 0.4252742 |
| Man1b1 | -0.1228429 |
| Man1c1 | -0.3583683 |
| Man1c1 | -0.5329142 |
| Man2a1 | 1.3091153 |
| Man2a2 | 0.6089868 |
| Man2a2 | 2.3617148 |
| Man2a2 | 0.1004421 |
| Man2b1 | 1.2100731 |
| Man2b2 | 1.3130575 |
| Man2c1 | 2.5172764 |
| Manba | -0.5164473 |
| Manba | 2.3833249 |
| Manbal | 0.1771787 |
| Manea | -0.3736612 |
| Mansc1 | 0.029025 |
| Maob | 1.3284462 |
| Map1lc3a | 0.0360292 |
| Map1lc3a | -0.0011913 |
| Map1lc3b | 2.6952451 |
| Map2k1 | 1.1242403 |
| Map2k2 | -0.636996 |
| Map2k2 | -0.0735974 |
| Map2k3 | 2.019173 |
| Map2k4 | -0.7530704 |
| Map2k6 | 1.0963671 |
| Map2k6 | -0.0920751 |
| Map2k6 | -0.3607997 |
| Map2k7 | -0.0358624 |
| Map2k7 | -0.0275194 |
| Map2k7 | -0.0972164 |
| Map3k1 | 0.8999559 |
| Map3k11 | -0.0433861 |
| Map3k12 | 2.2595158 |
| Map3k12 | 1.0829361 |
| Map3k12 | 1.1400249 |
| Map3k14 | 2.3845254 |
| Map3k3 | 0.3743285 |
| Map3k6 | 3.1480068 |
| Map3k7 | 0.5116195 |
| Map3k7ip1 | 0.6338016 |
| Map3k7ip1 | -0.5511233 |
| Map3k7ip1 | 1.6102753 |
| Map3k7ip2 | -0.3284612 |
| Map3k7ip3 | 0.6228481 |
| Map3k8 | 2.1550096 |
| Map3k9 | 0.6967092 |
| Map4k2 | 3.3897495 |
| Map4k4 | -0.6000659 |
| Map4k5 | 0.607823 |
| Map4k5 | 0.6351624 |
| Mapbpip | -0.2127718 |
| Mapk1 | 1.9154909 |
| Mapk1 | -0.3535232 |
| Mapk1 | -0.1127995 |
| Mapk10 | -0.6393999 |
| Mapk10 | 0.5218157 |
| Mapk11 | 0.5766038 |
| Mapk12 | -0.3986228 |
| Mapk12 | -0.5481877 |
| Mapk12 | -0.0681194 |
| Mapk13 | -0.7282362 |
| Mapk13 | -0.0809479 |
| Mapk14 | 0.1649686 |
| Mapk15 | 0.7488595 |
| Mapk15 | -0.4372633 |
| Mapk15 | -0.5228589 |
| Mapk1ip1 | 2.5833226 |
| Mapk1ip1 | -0.471993 |
| Mapk1ip1 | 2.5257249 |
| Mapk3 | -0.6954821 |
| Mapk3 | -0.1132755 |
| Mapk4 | 2.0553985 |
| Mapk6 | -0.5847521 |
| Mapk6 | 0.9030886 |
| Mapk6 | -0.5136108 |
| Mapk8 | -0.6466458 |
| Mapk8ip1 | -0.5969845 |
| Mapk8ip1 | -0.6179692 |
| Mapk8ip2 | -0.4300675 |
| Mapk8ip3 | 2.20597 |
| Mapk8ip3 | 1.8163452 |
| Mapk9 | 0.7844688 |
| Mapk9 | 0.5118757 |
| Mapk9 | 1.6598812 |
| Mapkap1 | 1.7408041 |
| Mapkap1 | -0.2897276 |
| Mapkapk2 | -0.6474542 |
| Mapkapk3 | 0.080997 |
| Mapkbp1 | -0.0860326 |
| Mapre1 | -0.3325952 |
| Mapre2 | -0.6580219 |
| Mapre2 | -0.6946343 |
| Mapre3 | -0.5809597 |
| Mapt | -0.6353967 |
| Mapt | 0.6180183 |
| Mapt | -0.5629522 |
| Mapt | -0.5321926 |
| March1 | -0.5651856 |
| March2 | -0.7065681 |
| March2 | 0.6322116 |
| March4 | -0.6474542 |
| March4 | -0.7352695 |
| March6 | -0.1919687 |
| March7 | -0.6085887 |
| March8 | 2.5424807 |
| March10 | -0.0763475 |
| Marcks | 2.2420273 |
| Marcksl1 | 0.3259211 |
| Marcksl1 | -0.4425291 |
| Marcksl1 | -0.1108978 |
| Mare | -0.5809597 |
| Mark1 | -0.0416107 |
| Mark2 | -0.2612099 |
| Mark2 | 0.8983858 |
| Mark3 | 1.0168472 |
| Mark3 | 0.1474752 |
| Mark4 | -0.3898158 |
| Mars | 0.2940052 |
| Mars2 | 0.3690305 |
| Marveld3 | -0.6171841 |
| Marveld3 | 1.4105976 |
| Mas1 | -0.3302308 |
| Masp1 | -0.6662269 |
| Masp2 | -0.6211155 |
| Mast1 | -0.3439022 |
| Mast1 | -0.0101777 |
| Mast2 | 0.9873926 |
| Mast2 | -0.3693558 |
| Mat1a | -0.2393942 |
| Mat2a | 0.2898639 |
| Mat2a | 1.7567296 |
| Mat2a | 2.2788845 |
| Mat2a | -0.5093708 |
| Mat2b | -0.5786911 |
| Mat2b | -0.7766916 |
| Matk | 1.5332953 |
| Matk | 0.3375577 |
| Matn4 | 0.0360292 |
| Matn4 | -0.3885639 |
| Matn4 | 2.2013289 |
| Matr3 | 1.1708717 |
| Max | -0.5430708 |
| Maz | -0.6290245 |
| Mb | -0.5885588 |
| Mbc2 | 2.9166352 |
| Mbd1 | -0.7221221 |
| Mbd1 | -0.1630468 |
| Mbd3 | 2.1205862 |
| Mbd3l2 | 0.2276525 |
| Mbd4 | 0.8342362 |
| Mbd5 | -0.3343721 |
| Mbd6 | 0.1119318 |
| Mbip | -0.3804643 |
| Mbl2 | 0.5741988 |
| Mbnl1 | -0.6282308 |
| Mbnl2 | -0.158555 |
| Mbnl2 | -0.634598 |
| Mboat1 | 0.1389842 |
| Mboat2 | -0.3571549 |
| Mboat2 | 1.9268874 |
| Mboat2 | 0.2828167 |
| Mboat2 | -0.6912504 |
| Mboat5 | -0.636996 |
| Mboat5 | 2.3943549 |
| Mbp | -0.6171841 |
| Mbp | -0.6523178 |
| Mbp | 0.695429 |
| Mbtd1 | 1.0522331 |
| Mbtps1 | -0.6282308 |
| Mbtps1 | -0.4538111 |
| Mbtps1 | 1.3269635 |
| Mbtps2 | -0.0581532 |
| Mc3r | -0.0393959 |
| Mc4r | 0.4814538 |
| Mc5r | 1.151092 |
| Mcam | -0.6997315 |
| Mcam | 2.0257584 |
| Mcam | -0.2999883 |
| Mcat | 0.0072933 |
| Mcc | -0.0214158 |
| Mccc1 | -0.1127995 |
| Mcee | 1.5971774 |
| Mcf2l | -0.3748948 |
| Mcf2l | 2.1664429 |
| Mcf2l | 1.0351528 |
| Mcfd2 | -0.6298188 |
| Mchr1 | -0.5293115 |
| Mcl1 | 1.6296508 |
| Mcm10 | -0.5459915 |
| Mcm10 | 2.0167571 |
| Mcm2 | -0.6803306 |
| Mcm3 | 2.7920605 |
| Mcm4 | 1.8902168 |
| Mcm5 | -0.6539443 |
| Mcm6 | -0.3992548 |
| Mcm6 | 0.2187859 |
| Mcm6 | -0.4287647 |
| Mcm7 | -0.7221221 |
| Mcoln1 | 0.505452 |
| Mcoln2 | 1.8795841 |
| Mcoln3 | 1.0244251 |
| Mcph1 | -0.4036901 |
| Mcph1 | 0.8784623 |
| Mcph1 | 2.2422115 |
| Mcph1 | 1.4501516 |
| Mcpt4 | -0.568919 |
| Mcrs1 | 0.6459837 |
| Mcrs1 | -0.6703546 |
| Mcrs1 | 3.4131044 |
| Mcts1 | -0.5496544 |
| Mdfic | -0.5200043 |
| Mdga1 | 1.1083465 |
| Mdga2 | -0.6282308 |
| Mdga2 | 0.5930434 |
| Mdh1 | -0.0349809 |
| Mdh1 | 0.0802113 |
| Mdh1b | -0.7748548 |
| Mdh2 | -0.6226923 |
| Mdk | 1.1368601 |
| Mdk | 2.267766 |
| Mdm1 | 0.2273129 |
| Mdm1 | -0.3474993 |
| Mdm1 | -0.5100763 |
| Mdm1 | -0.4464966 |
| Mdm1 | -0.2863304 |
| Mdm1 | -0.0622184 |
| Mdm1 | -0.7441307 |
| Mdm1 | 1.7237763 |
| Mdm1 | 0.4026951 |
| Mdm2 | 1.1073536 |
| Mdm2 | -0.3675162 |
| Mdm4 | 0.4015528 |
| Mdn1 | 0.6992648 |
| Me2 | -0.2518805 |
| Me3 | 1.8950367 |
| Mea1 | -0.4043253 |
| Mecp2 | -0.8560283 |
| Mecr | -0.0726823 |
| Med1 | -0.0995621 |
| Med1 | 0.1934663 |
| Med10 | 1.1540771 |
| Med11 | 0.748657 |
| Med11 | -0.1852991 |
| Med12 | 0.7207298 |
| Med15 | 0.6657459 |
| Med15 | 1.3989751 |
| Med15 | 0.5288922 |
| Med16 | 0.3478568 |
| Med18 | -0.5946796 |
| Med18 | -0.3935808 |
| Med19 | -0.3243441 |
| Med21 | -0.6290245 |
| Med22 | -0.5200043 |
| Med22 | 0.5727531 |
| Med23 | -0.5599819 |
| Med23 | 0.7466296 |
| Med24 | 1.6215021 |
| Med25 | -0.5079615 |
| Med25 | -0.0934747 |
| Med26 | 0.4418828 |
| Med27 | -0.6912504 |
| Med28 | -0.3693558 |
| Med30 | 0.6659657 |
| Med31 | 2.6899418 |
| Med4 | -0.5329142 |
| Med6 | -0.7803752 |
| Med7 | 1.0261185 |
| Med8 | -0.5771815 |
| Med8 | -0.5839925 |
| Med9 | -0.7273605 |
| Mef2b | 1.2318799 |
| Mef2b | -0.5771815 |
| Mef2c | 1.4792237 |
| Mef2c | 0.1761072 |
| Mef2c | 0.25048 |
| Megf10 | 0.0619701 |
| Megf11 | -0.7639038 |
| Megf11 | -0.0319018 |
| Megf11 | -0.5343589 |
| Megf11 | 2.6727512 |
| Megf6 | -0.461179 |
| Megf9 | -0.6563889 |
| Mei1 | -0.5802029 |
| Meis1 | 1.5055707 |
| Meis2 | -0.7379197 |
| Meis3 | 1.9380944 |
| Melk | 0.9570517 |
| Memo1 | -0.7143151 |
| Men1 | 0.5151999 |
| Men1 | -0.5100763 |
| Meox1 | -0.1842769 |
| Meox2 | 1.7116913 |
| Mep1b | -0.5855123 |
| Mepce | -0.5562814 |
| Mertk | -0.657205 |
| Mesdc1 | 0.2266334 |
| Mesp1 | -0.0144852 |
| Mest | 0.0157072 |
| Mest | -0.7370355 |
| Mest | -0.3349651 |
| Metap1 | 1.7319061 |
| Metap2 | -0.5939124 |
| Metap2 | 0.3433261 |
| Metapl1 | -0.5607237 |
| Metrn | -0.5016436 |
| Metrnl | -0.1935142 |
| Metrnl | 0.5208007 |
| Mett11d1 | -0.5749214 |
| Mettl1 | -0.5592407 |
| Mettl1 | 0.7121565 |
| Mettl2 | 0.1278004 |
| Mettl3 | -0.1837662 |
| Mettl4 | -0.6298188 |
| Mettl6 | -0.5016436 |
| Mettl8 | 1.1954856 |
| Mettl9 | -0.1695704 |
| Mettl9 | 0.1077341 |
| Mex3a | 2.5260029 |
| Mex3a | 2.0131802 |
| Mex3c | 1.6337848 |
| Mfap1b | -0.6820029 |
| Mfap2 | 0.8388768 |
| Mfap2 | 0.8447857 |
| Mfap2 | 0.5451308 |
| Mfap3 | 3.7252168 |
| Mfap3 | -0.707426 |
| Mfap3l | -0.5916145 |
| Mfap3l | 0.6127018 |
| Mfap4 | 1.4311862 |
| Mfap5 | 0.7693047 |
| Mfge8 | 1.313636 |
| Mfge8 | 0.020722 |
| Mfi2 | -0.2897276 |
| Mfn1 | -0.6662269 |
| Mfn1 | 1.3422553 |
| Mfn2 | -0.567424 |
| Mfng | -0.535082 |
| Mfrp | -0.5666773 |
| Mfsd1 | -0.5607237 |
| Mfsd10 | 1.5137598 |
| Mfsd10 | 2.7738237 |
| Mfsd11 | 1.9127492 |
| Mfsd2 | 4.7948068 |
| Mfsd3 | -0.4511451 |
| Mfsd4 | 1.8104267 |
| Mfsd7 | -0.276207 |
| Mfsd8 | 1.8030506 |
| Mfsd9 | -0.4049609 |
| Mga | -0.0568017 |
| Mgat1 | -0.1650495 |
| Mgat3 | 1.6437545 |
| Mgat4b | 1.1726024 |
| Mgat4b | -0.0469462 |
| Mgat5 | 1.4000372 |
| MGC107098 | 1.1659296 |
| MGC117608 | -0.5358057 |
| MGC118210 | 0.7217703 |
| MGC18837 | -0.2734129 |
| MGC41689 | -0.55776 |
| MGC67181 | -0.7065681 |
| Mgea6 | 0.5146892 |
| Mgl1 | -0.2706265 |
| Mgl2 | 0.6378786 |
| Mgll | 2.9267762 |
| Mgll | -0.6458381 |
| Mgll | -0.4511451 |
| Mgmt | 0.4916968 |
| Mgp | 2.032698 |
| Mgrn1 | -0.6621161 |
| Mgst1 | -0.6148326 |
| Mgst1 | 0.3702102 |
| Mgst2 | 1.9838014 |
| Mgst3 | -0.1056864 |
| Mgst3 | -0.6803306 |
| Mia1 | -0.1801985 |
| Mia3 | -0.3255187 |
| Mib1 | 0.310715 |
| Mib2 | -0.3331871 |
| Mic2l1 | -0.1610479 |
| Mical1 | 0.9950356 |
| Mical1 | 0.0825665 |
| Mical1 | -0.3190753 |
| Mical2 | 1.3156002 |
| Mical3 | 1.6394938 |
| Micall2 | -0.5107822 |
| Micall2 | 0.0643682 |
| Mid1 | 1.4090205 |
| Mid1 | -0.0640304 |
| Mid1ip1 | 0.0671588 |
| Midn | -0.1575596 |
| Midn | 2.7398222 |
| Mier1 | 0.9729039 |
| Mier1 | 0.5159655 |
| Mif | 0.8756092 |
| Mif4gd | -0.1238046 |
| Mif4gd | -0.5136108 |
| Mill2 | 0.2580855 |
| Mill2 | -0.2117214 |
| Mina | 0.9004787 |
| Mink1 | -0.1630468 |
| Mink1 | -0.3835718 |
| Minpp1 | 0.7685107 |
| Minpp1 | -0.7657207 |
| Miox | -0.3730451 |
| Mipep | -0.6912504 |
| Mir16 | 1.7864715 |
| Mist1 | -0.6466458 |
| Mitf | -0.2106721 |
| Mizf | -0.5908497 |
| Mki67ip | 1.1060756 |
| Mkks | -0.7396904 |
| Mkks | 2.116056 |
| Mkl1 | 1.5155601 |
| Mkl2 | 1.0008878 |
| Mkl2 | -0.471993 |
| Mkl2 | -0.7602797 |
| Mkln1 | 0.7368413 |
| Mknk1 | -0.703999 |
| Mknk2 | 0.4489957 |
| Mkrn1 | 1.2142937 |
| Mkrn2 | -0.2486083 |
| Mkrn3 | 0.6448618 |
| Mkx | -0.1919687 |
| Mlana | -0.1807074 |
| Mlf1 | -0.4686011 |
| Mlf1 | -0.6778273 |
| Mlf2 | 2.2124414 |
| Mlf2 | -0.6306138 |
| Mlh1 | -0.5939124 |
| Mlh3 | 1.0769576 |
| Mlkl | -0.5629522 |
| Mllt1 | 0.1164912 |
| Mllt1 | 0.8429429 |
| Mllt1 | -0.5939124 |
| Mllt10 | -0.3005615 |
| Mllt11 | -0.2914305 |
| Mllt3 | 0.56038 |
| Mllt3 | -0.5178686 |
| Mllt3 | -0.3829496 |
| Mlp | -0.643419 |
| Mlstd2 | 0.8775716 |
| Mlx | 0.3381665 |
| Mlx | -0.7639038 |
| Mlxipl | -0.2954151 |
| Mlycd | -0.4294159 |
| Mmab | -0.1715861 |
| Mmab | 0.0938728 |
| Mmd | -0.8208482 |
| Mmd2 | 0.9424514 |
| Mme | 0.097356 |
| Mme | 0.4885562 |
| Mmel1 | 0.7304688 |
| Mmp10 | 0.0064481 |
| Mmp11 | -0.2222751 |
| Mmp12 | -0.1286272 |
| Mmp13 | -0.2023166 |
| Mmp13 | 0.5902035 |
| Mmp14 | 2.0203787 |
| Mmp15 | 1.0985167 |
| Mmp16 | -0.4571534 |
| Mmp17 | -0.6242717 |
| Mmp1a | 1.5752894 |
| Mmp1a | 0.5100812 |
| Mmp2 | -0.3559429 |
| Mmp23 | -0.6515056 |
| Mmp24 | 2.0228432 |
| Mmp3 | -0.5365298 |
| Mmp3 | -0.6711822 |
| Mmp3 | 0.4120703 |
| Mmp9 | -0.1955784 |
| Mmrn2 | 0.1721689 |
| Mnat1 | -0.1781654 |
| Mnat1 | -0.6870365 |
| Mnd1 | 0.2643254 |
| Mns1 | -0.5214306 |
| Mnt | -0.1776578 |
| Mobk1b | 0.8528544 |
| Mobkl2a | -0.0595064 |
| Mobkl2b | -0.4222756 |
| Mobkl2c | -0.2464327 |
| Mobkl3 | -0.5314716 |
| Mobp | -0.6179692 |
| Mobp | 0.7665229 |
| Mobp | 1.313636 |
| Mocos | -0.426164 |
| Mocs1 | 0.726957 |
| Mocs2 | -0.1496311 |
| Mod1 | -0.824697 |
| Mog | 0.5123878 |
| Mog | -0.3367462 |
| Mon1a | 2.0257584 |
| Mon1a | 1.2433532 |
| Mon1b | 0.0429851 |
| Mon1b | 1.324106 |
| Mon1b | -0.650694 |
| Morc2a | 0.1100259 |
| Morc2b | -0.1407855 |
| Morc3 | -0.7539688 |
| Morf4l1 | 1.9682712 |
| Morf4l1 | 0.1927636 |
| Morf4l2 | -0.6920953 |
| Morn2 | 1.3976991 |
| Morn3 | 0.810136 |
| Morn3 | 0.1732445 |
| Mos | -0.4558152 |
| Mosc2 | -0.4405512 |
| Mospd1 | 0.2815301 |
| Mospd1 | 1.2571468 |
| Mospd1 | -0.5962156 |
| Mospd3 | 2.7291668 |
| Mospd3 | -0.5065541 |
| Mov10 | 1.7011936 |
| Mov10l1 | 1.3118996 |
| Moxd1 | 0.4355484 |
| Moxd1 | -0.0685748 |
| Mpdu1 | 0.8682611 |
| Mpdz | -0.0832559 |
| Mpdz | -0.6547585 |
| Mpg | -0.5962156 |
| Mpg | 0.3589464 |
| Mphosph6 | -0.6761619 |
| Mpnd | 0.8405422 |
| Mpo | 0.8256413 |
| Mpo | 1.3154847 |
| Mpp1 | 1.3891506 |
| Mpp2 | 0.5542589 |
| Mpp3 | -0.8141479 |
| Mpp3 | 2.209972 |
| Mpp5 | 2.5418993 |
| Mpp6 | 2.6421841 |
| Mppe1 | -0.2410141 |
| Mpped1 | -0.4139018 |
| Mpst | 0.3025523 |
| Mpv17 | 0.216042 |
| Mpv17 | -0.535082 |
| Mpv17 | 0.3678493 |
| Mpv17 | -0.6794955 |
| Mpv17l | -0.0292701 |
| Mpv17l | 1.2072496 |
| Mpv17l | 3.0703494 |
| Mpz | 1.260444 |
| Mr1 | 0.1262998 |
| Mrap | -0.3017087 |
| Mras | -0.203878 |
| Mrc1 | -0.7887129 |
| Mrc2 | -0.2383157 |
| Mrc2 | 0.9237285 |
| Mre11a | 0.2860261 |
| Mre11a | -0.6148326 |
| Mre11a | -0.3644577 |
| Mreg | -0.6031568 |
| Mrgpra2 | -0.4788111 |
| Mrgpra2 | 2.6363442 |
| Mrgprb2 | -0.1914541 |
| Mrgpre | 0.1678549 |
| Mrgprf | -0.8112899 |
| Mrgprg | 0.6450863 |
| Mrm1 | -0.7352695 |
| Mro | -0.119484 |
| Mrp63 | -0.683678 |
| Mrpl1 | 1.5223532 |
| Mrpl1 | -0.8008793 |
| Mrpl10 | -0.8112899 |
| Mrpl10 | 2.3971233 |
| Mrpl11 | -0.5809597 |
| Mrpl12 | -0.577936 |
| Mrpl13 | 0.2936872 |
| Mrpl13 | 1.01576 |
| Mrpl14 | -0.51858 |
| Mrpl15 | 0.9293312 |
| Mrpl16 | -0.55776 |
| Mrpl17 | 0.4801331 |
| Mrpl18 | 1.3316322 |
| Mrpl2 | -0.7822221 |
| Mrpl20 | -0.554805 |
| Mrpl22 | 0.366075 |
| Mrpl23 | -0.4511451 |
| Mrpl24 | -0.2931363 |
| Mrpl27 | -0.6531307 |
| Mrpl28 | -0.7247379 |
| Mrpl3 | 0.6810216 |
| Mrpl3 | -0.6531307 |
| Mrpl30 | -0.1300785 |
| Mrpl33 | -0.7521729 |
| Mrpl33 | -0.379224 |
| Mrpl34 | 1.5004907 |
| Mrpl36 | -0.0722251 |
| Mrpl37 | -0.1085256 |
| Mrpl37 | -0.1437254 |
| Mrpl38 | -0.2639704 |
| Mrpl39 | -0.1344449 |
| Mrpl40 | 0.256107 |
| Mrpl43 | 2.9547858 |
| Mrpl43 | -0.7151796 |
| Mrpl45 | -0.0897468 |
| Mrpl46 | 0.7795704 |
| Mrpl47 | 3.6913328 |
| Mrpl47 | 0.9650906 |
| Mrpl48 | 0.0699416 |
| Mrpl48 | 0.5419039 |
| Mrpl48 | 0.3025523 |
| Mrpl49 | 1.5477942 |
| Mrpl49 | -0.785926 |
| Mrpl49 | 2.3846053 |
| Mrpl49 | 1.3362795 |
| Mrpl50 | -0.5681712 |
| Mrpl51 | 1.0474255 |
| Mrpl51 | -0.1605489 |
| Mrpl51 | -0.5992946 |
| Mrpl52 | -0.1766433 |
| Mrpl53 | 0.1927636 |
| Mrpl54 | -0.471993 |
| Mrpl55 | -0.5129029 |
| Mrpl9 | -0.063124 |
| Mrps10 | 0.9141491 |
| Mrps10 | 1.6638934 |
| Mrps11 | -0.4891255 |
| Mrps11 | 1.1997675 |
| Mrps12 | -0.5058511 |
| Mrps14 | 0.3877429 |
| Mrps15 | 0.1757498 |
| Mrps16 | -0.3607997 |
| Mrps17 | -0.0389536 |
| Mrps18a | -0.4491503 |
| Mrps18b | 1.1858171 |
| Mrps18b | 1.3337885 |
| Mrps18c | -0.5607237 |
| Mrps2 | -0.5518586 |
| Mrps21 | -0.2415546 |
| Mrps22 | 1.4001434 |
| Mrps23 | 1.6396613 |
| Mrps24 | 1.5532806 |
| Mrps25 | -0.4652206 |
| Mrps25 | 1.6970902 |
| Mrps25 | 1.8596601 |
| Mrps26 | -0.6442247 |
| Mrps26 | -0.5832334 |
| Mrps27 | 0.080997 |
| Mrps28 | -0.4788111 |
| Mrps30 | 0.3166385 |
| Mrps31 | 1.2543295 |
| Mrps31 | -0.643419 |
| Mrps31 | -0.5931459 |
| Mrps33 | 0.5026125 |
| Mrps33 | -0.6720105 |
| Mrps34 | 0.3648904 |
| Mrps35 | 1.9261947 |
| Mrps5 | 3.1409487 |
| Mrps6 | 0.6725394 |
| Mrps7 | -0.6912504 |
| Mrps7 | 1.4499495 |
| Mrps9 | 0.2320567 |
| Mrrf | -0.01276 |
| Mrrf | 0.0161261 |
| Mrto4 | -0.6482631 |
| Mrvi1 | -0.5489208 |
| Ms4a1 | 3.1988629 |
| Ms4a10 | -0.3445008 |
| Ms4a13 | 1.9854067 |
| Ms4a5 | 2.0455963 |
| Ms4a6d | -0.5518586 |
| Ms4a6d | -0.5171577 |
| Ms4a7 | -0.7229933 |
| Ms4a8a | 1.5728775 |
| Msc | -0.6226923 |
| Msh2 | 0.8526717 |
| Msh3 | 1.2762903 |
| Msh3 | -0.4946699 |
| Msh4 | 1.0035653 |
| Msh5 | -0.281256 |
| Msh5 | -0.6980296 |
| Msh6 | -0.7840723 |
| Msh6 | 1.1168169 |
| Msi2 | 0.2551163 |
| Msi2h | 0.1104074 |
| Msln | -0.2101479 |
| Msn | -0.5977539 |
| Msr2 | -0.7352695 |
| Msrb2 | -0.61405 |
| Msrb2 | 2.7055653 |
| Msrb3 | 2.1341074 |
| Mst1r | -0.6695277 |
| Msto1 | -0.4255149 |
| Msx1 | 2.8735921 |
| Mt1 | 0.083742 |
| Mt3 | 2.7208644 |
| Mta1 | -0.2546155 |
| Mta2 | 1.197564 |
| Mta3 | 1.1138603 |
| Mta3 | 0.7649299 |
| Mtag2 | 1.0865642 |
| Mtap | -0.6912504 |
| Mtap | -0.2249309 |
| Mtap1b | 0.6113103 |
| Mtap1b | -0.4030553 |
| Mtap1b | 0.1393549 |
| Mtap1b | 0.1404661 |
| Mtap1s | -0.6794955 |
| Mtap2 | 1.2650663 |
| Mtap2 | -0.2318688 |
| Mtap2 | -0.1442162 |
| Mtap4 | 1.2621494 |
| Mtap6 | 1.3868945 |
| Mtap6 | -0.6266453 |
| Mtap7 | 2.9870892 |
| Mtap7d1 | -0.5023437 |
| Mtap7d1 | -0.1209221 |
| Mtap7d2 | -0.4898169 |
| Mtap7d3 | 1.3064397 |
| Mtbp | 0.8003708 |
| Mtbp | 2.2913301 |
| Mtbp | -0.0205469 |
| Mtch1 | 1.7845157 |
| Mtch1 | 0.2126015 |
| Mtch2 | -0.6929409 |
| Mtch2 | 0.4885562 |
| Mtch2 | -0.4287647 |
| Mtcp1 | 0.029025 |
| Mtdh | 1.3169844 |
| Mterf | -0.259007 |
| Mterf | -0.6085887 |
| Mterfd1 | -0.408783 |
| Mterfd2 | -0.5659312 |
| Mterfd2 | 1.4669802 |
| Mterfd2 | 2.6053425 |
| Mterfd3 | -0.4359512 |
| Mterfd3 | -0.4339861 |
| Mtf1 | 0.3475554 |
| Mtf1 | 1.9423193 |
| Mtf2 | -0.4294159 |
| Mtfmt | -0.2388548 |
| Mtfmt | 0.0236359 |
| Mtg1 | 0.1217846 |
| Mthfd1 | -0.3916965 |
| Mthfd2 | -0.0327806 |
| Mthfs | -0.5931459 |
| Mthfsd | -0.6728394 |
| Mthfsd | -0.0170786 |
| Mtif3 | -0.5916145 |
| Mtl5 | 0.4427061 |
| Mtl5 | 1.4101773 |
| Mtmr1 | -0.1028552 |
| Mtmr11 | -0.6753303 |
| Mtmr11 | -0.0832559 |
| Mtmr12 | -0.5430708 |
| Mtmr14 | 0.7774075 |
| Mtmr14 | -0.7212516 |
| Mtmr14 | -0.2496978 |
| Mtmr14 | -0.6769943 |
| Mtmr2 | -0.5496544 |
| Mtmr2 | -0.6490728 |
| Mtmr2 | -0.3614084 |
| Mtmr3 | 0.2025573 |
| Mtmr4 | -0.1610479 |
| Mtmr9 | 1.7319061 |
| Mto1 | 0.0152882 |
| Mtpn | -0.6402024 |
| Mtrf1 | 2.7646213 |
| Mtrf1 | 0.5321609 |
| Mtrf1l | -0.581717 |
| Mtrr | -0.5459915 |
| Mtss1 | -0.4591642 |
| Mttp | 1.9701427 |
| Mtus1 | 1.1609631 |
| Mtus1 | -0.4235701 |
| Mtvr2 | 0.833864 |
| Mtvr2 | -0.5659312 |
| Mtx1 | 0.4029805 |
| Mtx1 | 2.9635843 |
| Mtx1 | 3.2506875 |
| Mtx2 | 0.3604357 |
| Muc1 | 4.2361828 |
| Muc1 | -0.673669 |
| Muc1 | -0.4145435 |
| Muc1 | 0.5351688 |
| Muc13 | 0.1149737 |
| Muc5ac | 3.3340323 |
| Mug2 | -0.5037452 |
| Mug2 | -0.6258534 |
| Mug4 | 0.0583622 |
| Mum1 | 0.2384592 |
| Mup1 | 0.8184856 |
| Mup2 | -0.4692786 |
| Mup2 | 1.2821541 |
| Mup3 | 0.3421144 |
| Mupcdh | 0.7488595 |
| Mus81 | -0.5824749 |
| Mus81 | 3.0980227 |
| Mustn1 | 0.050703 |
| Mustn1 | 3.7805162 |
| Muted | 2.3524689 |
| Muted | 0.9849922 |
| Mutyh | 1.3409054 |
| Mvd | -0.6458381 |
| Mvp | -0.1801985 |
| Mx2 | -0.5585001 |
| Mxd1 | 2.4734995 |
| Mxd4 | -0.6163997 |
| Mxi1 | -0.0262084 |
| Mxra7 | 1.4853877 |
| Mxra8 | 1.0465215 |
| Mxra8 | -0.4578232 |
| Myadm | 1.6188567 |
| Mybbp1a | 0.3028675 |
| Mybbp1a | 0.3393829 |
| Mybbp1a | 2.7665671 |
| Mybl2 | -0.5023437 |
| Mybpc2 | -0.3196593 |
| Mybpc3 | -0.7048546 |
| Myc | -0.2529736 |
| Mycbp | 1.1703386 |
| Mycbp2 | -0.8018213 |
| Mycbp2 | 0.7982521 |
| Mycbpap | -0.6054811 |
| Mycl1 | 0.3672582 |
| Myd116 | 0.9081154 |
| Myd88 | -0.7721059 |
| Myef2 | 0.1397254 |
| Myg1 | 1.8010554 |
| Myg1 | 0.464953 |
| Myg1 | 2.9730209 |
| Myh10 | -0.3421084 |
| Myh11 | -0.0541043 |
| Myh8 | 1.6247356 |
| Myh9 | 1.9658512 |
| Myl1 | 0.1221617 |
| Myl2 | -0.3045824 |
| Myl4 | -0.6629369 |
| Myl6 | 2.1273695 |
| Myl6b | -0.5489208 |
| Mylc2b | -0.6171841 |
| Mylc2b | 0.1500453 |
| Mylc2b | -0.6662269 |
| Mylc2pl | -0.3942097 |
| Mylk | 2.3613466 |
| Mylk | 1.0862744 |
| Mynn | 0.544635 |
| Myo10 | -0.6023831 |
| Myo10 | 1.2930725 |
| Myo15 | -0.1089996 |
| Myo18a | 1.2761703 |
| Myo1b | -0.3109336 |
| Myo1c | -0.4011533 |
| Myo1c | -0.6588394 |
| Myo1f | -0.0345405 |
| Myo1g | 0.0615699 |
| Myo3b | -0.2745296 |
| Myo5a | -0.1766433 |
| Myo5b | -0.785926 |
| Myo6 | -0.5832334 |
| Myo7a | 2.3004125 |
| Myo9a | 0.0825665 |
| Myo9b | -0.5749214 |
| Myo9b | 0.7897326 |
| Myoc | 0.5128997 |
| Myocd | -0.554805 |
| Myocd | 2.1492187 |
| Myom1 | -0.7048546 |
| Myom1 | 3.3542287 |
| Myrip | 0.1857094 |
| Mysm1 | -0.2976991 |
| Myst1 | 2.2325405 |
| Myst2 | -0.2334767 |
| Myst4 | -0.6385979 |
| Myst4 | -0.5726663 |
| Myst4 | 0.5008014 |
| Myt1 | 0.9022194 |
| Myt1l | -0.2667384 |
| Myt1l | 0.3917903 |
| Mzf1 | 0.3693255 |
| Mzf1 | -0.568919 |
| N28178 | -0.6418094 |
| N4bp2 | 0.9103607 |
| N6amt1 | -0.7432411 |
| N6amt1 | 0.0735084 |
| N6amt2 | 0.1707329 |
| Naalad2 | 0.1692949 |
| Nab1 | -0.6654034 |
| Nab1 | -0.4692786 |
| Nab1 | -0.5802029 |
| Nab1 | -0.5518586 |
| Nab2 | 1.4779471 |
| Naca | -0.5228589 |
| Naca | -0.436607 |
| Naca | -0.4158281 |
| Nadk | 0.640813 |
| Nadsyn1 | 0.4856687 |
| Nadsyn1 | 0.1430541 |
| Naga | 0.3259211 |
| Nagk | 0.9189502 |
| Nagk | 0.5525383 |
| Nagk | -0.1756298 |
| Nagk | -0.5599819 |
| Naglu | -0.5870344 |
| Nagpa | 2.4530838 |
| Nags | -0.7134514 |
| Nags | 0.4971694 |
| Naif1 | 2.2938272 |
| Naif1 | -0.5452606 |
| Naip5 | 0.2940052 |
| Nalp10 | -0.3986228 |
| Nampt | 0.5873556 |
| Nanos1 | -0.6418094 |
| Nanos2 | -0.3687422 |
| Nans | 2.756026 |
| Nap1l1 | -0.3547323 |
| Nap1l1 | -0.204399 |
| Nap1l3 | -0.5329142 |
| Nap1l4 | -0.5847521 |
| Nap1l5 | -0.4699565 |
| Napa | 3.2444512 |
| Napa | -0.4726727 |
| Napa | 1.3947153 |
| Napb | 0.3619228 |
| Napepld | 0.1382425 |
| Napg | 0.9119122 |
| Napg | 0.0442077 |
| Naprt1 | -0.3474993 |
| Naprt1 | -0.4372633 |
| Napsa | 0.2283313 |
| Napsa | -0.6466458 |
| Napsa | -0.1137517 |
| Narf | -0.5870344 |
| Narfl | 3.0062666 |
| Narg1 | -0.2784479 |
| Narg1 | -0.4030553 |
| Narg1l | 1.6147467 |
| Narg1l | -0.6466458 |
| Narg1l | 1.6721132 |
| Narg2 | -0.4352957 |
| Nars | 0.8980366 |
| Nars2 | -0.4747147 |
| Nasp | -0.2756476 |
| Nasp | -0.1442162 |
| Nasp | -0.6828401 |
| Nasp | -0.61405 |
| Nat1 | -0.3237573 |
| Nat10 | -0.2313334 |
| Nat14 | 0.362814 |
| Nat2 | -0.4145435 |
| Nat5 | -0.6306138 |
| Nat5 | -0.6282308 |
| Nat6 | 2.3586833 |
| Nat6 | -0.6498831 |
| Nav1 | 0.0599673 |
| Nav1 | -0.7620901 |
| Nbl1 | 0.3648904 |
| Nbr1 | 0.9685164 |
| Nbr1 | 0.5258655 |
| Ncald | 0.2222053 |
| Ncam1 | -0.119005 |
| Ncam1 | 0.2580855 |
| Ncam2 | -0.1858106 |
| Ncan | -0.5741691 |
| Ncapd2 | 1.9325378 |
| Ncapd2 | -0.7108646 |
| Ncapd2 | 0.0639689 |
| Ncapd3 | 2.2310981 |
| Ncapg2 | 0.0438003 |
| Ncapg2 | 1.55674 |
| Ncaph | 1.0731479 |
| Ncaph2 | 1.2567798 |
| Ncaph2 | -0.6023831 |
| Ncaph2 | -0.4625244 |
| Ncbp2 | 0.5830686 |
| Ncbp2 | 0.3243799 |
| Ncdn | 1.702846 |
| Ncdn | 0.150412 |
| Ncdn | -0.5741691 |
| Ncdn | -0.4471593 |
| Ncf4 | -0.5870344 |
| Nck1 | -0.1300785 |
| Nckap1 | 1.818801 |
| Nckap1l | 1.1207455 |
| Nckipsd | 1.0179333 |
| Ncl | -0.5518586 |
| Ncl | 0.3992642 |
| Ncoa2 | -0.0046053 |
| Ncoa2 | 2.8355056 |
| Ncoa4 | -0.4294159 |
| Ncoa5 | 0.977111 |
| Ncoa6 | -0.4794954 |
| Ncoa6 | -0.5314716 |
| Ncoa6 | -0.6490728 |
| Ncor1 | -0.6490728 |
| Ncstn | 2.6720599 |
| Ncstn | 0.9601764 |
| Ncstn | 2.5596688 |
| Ndc80 | -0.6314094 |
| Nde1 | 1.764747 |
| Nde1 | 0.7211461 |
| Ndel1 | 1.5729669 |
| Ndfip1 | 0.0060252 |
| Ndfip2 | -0.861013 |
| Ndg2 | -0.4538111 |
| Ndn | 0.4872447 |
| Ndor1 | -0.4645459 |
| Ndor1 | 2.5348613 |
| Ndor1 | -0.3565487 |
| Ndrg1 | -0.2383157 |
| Ndrg2 | -0.2502431 |
| Ndrg3 | -0.4359512 |
| Ndrg4 | -0.6250622 |
| Ndrl | 1.4014164 |
| Ndst1 | -0.2790089 |
| Ndst1 | -0.5459915 |
| Ndst4 | -0.4307195 |
| Ndufa1 | -0.1530921 |
| Ndufa10 | -0.6887199 |
| Ndufa12 | 2.1162647 |
| Ndufa13 | -0.5666773 |
| Ndufa2 | 1.1821344 |
| Ndufa3 | 0.3445363 |
| Ndufa4 | 0.6396854 |
| Ndufa5 | 0.5256129 |
| Ndufa5 | 0.5039042 |
| Ndufa6 | -0.3173251 |
| Ndufa7 | 0.1692949 |
| Ndufa8 | -0.613268 |
| Ndufa8 | -0.6171841 |
| Ndufa9 | 0.8152678 |
| Ndufaf1 | 2.5029417 |
| Ndufb10 | -0.3445008 |
| Ndufb11 | -0.5644406 |
| Ndufb2 | -0.6426139 |
| Ndufb3 | -0.643419 |
| Ndufb4 | 0.8359093 |
| Ndufb5 | -0.703144 |
| Ndufb6 | -0.7221221 |
| Ndufb6 | 1.6367273 |
| Ndufb7 | 0.0711319 |
| Ndufb8 | 1.338313 |
| Ndufb9 | -0.1590531 |
| Ndufc1 | -0.5192919 |
| Ndufc1 | -0.4235701 |
| Ndufc2 | 1.2703922 |
| Ndufs2 | 0.7395017 |
| Ndufs2 | 1.9978059 |
| Ndufs3 | 2.0609809 |
| Ndufs4 | 0.7153016 |
| Ndufs4 | -0.6266453 |
| Ndufs4 | -0.4511451 |
| Ndufs4 | 1.6455869 |
| Ndufs6 | 1.8254368 |
| Ndufs7 | 1.0012032 |
| Ndufs8 | -0.6410056 |
| Ndufv1 | -0.6085887 |
| Ndufv2 | 0.6742851 |
| Necab1 | 2.771129 |
| Necab2 | 0.0298516 |
| Necab3 | 3.0921124 |
| Necap1 | 0.7829039 |
| Necap1 | 2.1583181 |
| Necap1 | 1.6628305 |
| Necap2 | -0.5824749 |
| Nedd1 | 1.940706 |
| Nedd4 | 0.7261289 |
| Nedd4l | 0.3499642 |
| Nedd8 | -0.568919 |
| Nedd9 | -0.4699565 |
| Nedd9 | -0.55776 |
| Nefm | -0.55776 |
| Negr1 | -0.4706349 |
| Negr1 | 1.8645609 |
| Neil1 | -0.6062571 |
| Neil2 | 1.9782524 |
| Nek11 | 1.2938984 |
| Nek3 | 0.0417611 |
| Nek3 | -0.5525944 |
| Nek3 | 2.3606507 |
| Nek4 | 1.1751266 |
| Nek5 | 0.806509 |
| Nek6 | -0.5489208 |
| Nek8 | -0.6242717 |
| Nek9 | 1.7772391 |
| Nek9 | 1.5314333 |
| Nelf | 0.3484594 |
| Nelf | 0.6797212 |
| Nell2 | -0.3553374 |
| Nell2 | -0.5636961 |
| Nell2 | -0.683678 |
| Nell2 | 1.7842257 |
| Nenf | -0.6920953 |
| Neo1 | -0.1960952 |
| Nes | 0.7450048 |
| Net1 | -0.4918939 |
| Net1 | -0.0667546 |
| Neto1 | -0.6997315 |
| Neto1 | -0.6226923 |
| Neto1 | 1.7446583 |
| Neu1 | -0.7229933 |
| Neu2 | -0.7693644 |
| Neu3 | -0.5129029 |
| Neu4 | 2.2968417 |
| Neu4 | -0.826627 |
| Neu4 | -0.1575596 |
| Neurl | -0.634598 |
| Neurod1 | 0.1569885 |
| Neurod2 | 2.0359239 |
| Neurod4 | -0.6887199 |
| Neurog1 | 0.9359126 |
| Neurog2 | 2.9733778 |
| Nf1 | 0.2537938 |
| Nfasc | -0.3860648 |
| Nfasc | 1.7524724 |
| Nfasc | -0.7432411 |
| Nfat5 | 2.9173191 |
| Nfat5 | -0.379224 |
| Nfatc1 | -0.3249312 |
| Nfatc1 | 0.7803557 |
| Nfatc2 | 0.7823164 |
| Nfatc2 | 2.0068027 |
| Nfatc2ip | -0.7441307 |
| Nfatc3 | -0.7657207 |
| Nfatc4 | -0.5666773 |
| Nfe2 | 1.667564 |
| Nfe2l1 | -0.2868958 |
| Nfe2l2 | -0.0622184 |
| Nfe2l3 | -0.0354215 |
| Nfib | -0.1412749 |
| Nfic | 0.3654829 |
| Nfic | 0.133035 |
| Nfic | -0.4094215 |
| Nfix | -0.0986232 |
| Nfix | 0.2146672 |
| Nfkb1 | 0.0034841 |
| Nfkb2 | -0.5072575 |
| Nfkbia | -0.6629369 |
| Nfkbib | 0.8453379 |
| Nfkbid | 1.3354876 |
| Nfkbie | 1.6569233 |
| Nfkbil1 | -0.0722251 |
| Nfkbil2 | 0.8453379 |
| Nfkbiz | 2.1927561 |
| Nfrkb | -0.603931 |
| Nfrkb | 1.943187 |
| Nfs1 | 0.9988355 |
| Nfs1 | 2.307689 |
| Nfs1 | -0.7512761 |
| Nfu1 | -0.1047418 |
| Nfx1 | -0.5855123 |
| Nfya | 0.553276 |
| Nfyb | -0.2486083 |
| Nfyc | 0.4611893 |
| Ngb | 1.2844187 |
| Ngb | 0.9915397 |
| Ngef | 2.0349629 |
| Ngfb | -0.7943099 |
| Ngfb | 0.2300264 |
| Ngfr | -0.4988484 |
| Ngfrap1 | -0.4891255 |
| Ngp | -0.5855123 |
| Nhedc1 | -0.471993 |
| Nhej1 | -0.0451646 |
| Nhlrc1 | 0.5584253 |
| Nhlrc2 | -0.6266453 |
| Nhlrc2 | -0.6645806 |
| Nhp2l1 | 2.1642562 |
| Nhsl1 | -0.6744993 |
| Niban | -0.6703546 |
| Nicn1 | -0.683678 |
| Nicn1 | 3.6121745 |
| Nid1 | -0.7450211 |
| Nid2 | -0.1491376 |
| Nif3l1 | -0.6887199 |
| Nin | 0.830695 |
| Ninj1 | -0.3773663 |
| Ninj2 | -0.8986858 |
| Nip7 | 0.0802113 |
| Nipa1 | -0.2377769 |
| Nipbl | -0.0841807 |
| Nipbl | 1.7700566 |
| Nipsnap1 | -0.3848175 |
| Nipsnap3a | -0.4842989 |
| Nipsnap3a | 1.0407774 |
| Nisch | -0.2891606 |
| Nit1 | 0.2666146 |
| Nit2 | -0.3103546 |
| Nit2 | -0.6523178 |
| Nkain3 | -0.683678 |
| Nkain4 | -0.9217718 |
| Nkd2 | -0.2464327 |
| Nkg7 | -0.3848175 |
| Nkiras1 | -0.809389 |
| Nkiras2 | 1.753445 |
| Nkrf | 1.0469736 |
| Nktr | 2.0175055 |
| Nkx1-2 | -0.4359512 |
| Nkx2-1 | -0.2007577 |
| Nkx2-2 | -0.8256615 |
| Nkx2-2 | 0.5549954 |
| Nkx2-3 | -0.7459123 |
| Nkx2-4 | 0.1266752 |
| Nkx2-6 | 2.8286037 |
| Nkx3-1 | -0.1113729 |
| Nkx6-1 | 2.9058189 |
| Nle1 | 0.9506072 |
| Nlgn1 | 0.7219783 |
| Nlgn2 | 1.5412632 |
| Nlk | -0.5525944 |
| Nln | 0.0228042 |
| Nln | -0.6023831 |
| Nln | -0.7684522 |
| Nlrp14 | 0.7288177 |
| Nlrp4a | -0.2857653 |
| Nlrp4c | 2.4947296 |
| Nlrp5 | 0.1489447 |
| Nlrp5 | 1.8184505 |
| Nlrp5 | -0.7459123 |
| Nlrp9a | -0.7370355 |
| Nlrp9c | -0.9028442 |
| Nlrx1 | -0.9028442 |
| Nlrx1 | 2.9159979 |
| Nmb | -0.2318688 |
| Nmbr | -0.5171577 |
| Nmd3 | -0.6687015 |
| Nme1 | -0.1108978 |
| Nme2 | 1.5598251 |
| Nme2 | -0.821809 |
| Nme2 | -0.6887199 |
| Nme3 | 0.9153515 |
| Nme4 | 3.0393916 |
| Nme4 | 0.124797 |
| Nme4 | -0.9101628 |
| Nme5 | 0.5126438 |
| Nme7 | -0.327872 |
| Nme7 | 1.138375 |
| Nme7 | -0.6621161 |
| Nme7 | 1.8302293 |
| Nme7 | 0.557936 |
| Nmnat1 | 2.1309755 |
| Nmnat2 | 0.101597 |
| Nmnat3 | 1.477259 |
| Nmral1 | -0.0948762 |
| Nmt1 | -0.5314716 |
| Nmu | -0.6410056 |
| Nmur2 | -0.6937873 |
| Nnat | 0.2070723 |
| Nnat | -0.577936 |
| Nnmt | 0.9013494 |
| Nnmt | 0.3228364 |
| Nob1 | -0.5235738 |
| Nob1 | 1.0285765 |
| Nobox | 0.7623358 |
| Noc3l | -0.188885 |
| Noc4l | -0.1151817 |
| Noc4l | -0.8422009 |
| Nod1 | 0.7286111 |
| Nog | -0.0478382 |
| Nol1 | 2.1176725 |
| Nol1 | 0.4481776 |
| Nol1 | 3.5766863 |
| Nol10 | 0.5495818 |
| Nol11 | 0.0161261 |
| Nol12 | -0.7405769 |
| Nol14 | -0.4222756 |
| Nol3 | 0.0938728 |
| Nol4 | 2.6477439 |
| Nol4 | -0.4151856 |
| Nol4 | 1.1013758 |
| Nol5 | 2.1802977 |
| Nol5a | 3.8008837 |
| Nol6 | -0.5555429 |
| Nol7 | -0.3481001 |
| Nol9 | -0.5051487 |
| Nola1 | 3.435028 |
| Nola2 | -0.3967291 |
| Nola3 | -0.5855123 |
| Nola3 | -0.5452606 |
| Nolc1 | 0.7001152 |
| Nomo1 | -0.4733529 |
| Nono | -0.1771504 |
| Nope | -0.2623132 |
| Nos1 | 0.3050709 |
| Nos1ap | -0.5518586 |
| Nos2 | -0.5786911 |
| Nos3 | -0.2372383 |
| Nos3 | -0.5749214 |
| Nos3 | -0.2868958 |
| Nosip | 1.2645808 |
| Nosip | -0.6070337 |
| Nosip | -0.5343589 |
| Nostrin | 0.9238987 |
| Notch1 | -0.3973599 |
| Notch3 | 0.2936872 |
| Notch4 | -0.7256113 |
| Notch4 | -0.3462988 |
| Notum | -0.436607 |
| Nova2 | 0.1775356 |
| Nox4 | 0.6731944 |
| Noxo1 | -0.6515056 |
| Noxo1 | 3.1128332 |
| Npal2 | 0.4363769 |
| Npas1 | 1.3239915 |
| Npas2 | -0.2028368 |
| Npas4 | -0.1056864 |
| Npb | -0.5467231 |
| Npc2 | 1.5143287 |
| Npcd | 1.7532954 |
| Npcd | -0.673669 |
| Npdc1 | -0.581717 |
| Npepl1 | 2.0038905 |
| Npff | -0.530031 |
| Nphp1 | -0.6580219 |
| Nphp1 | -0.946486 |
| Nphp3 | -0.2117214 |
| Nphp4 | -0.2959856 |
| Nphs2 | -0.8500791 |
| Nploc4 | 0.526623 |
| Npm1 | -0.2111966 |
| Npm2 | 1.9436205 |
| Npm3 | -0.4425291 |
| Npm3 | 1.3142141 |
| Npm3-ps1 | 1.5906959 |
| Npr1 | 2.0707992 |
| Npr2 | -0.6410056 |
| Npr3 | -0.327872 |
| Npsr1 | -0.4584935 |
| Nptn | -0.1817259 |
| Nptx1 | -0.4760783 |
| Nptx2 | 0.5787633 |
| Nptxr | 1.4063867 |
| Npy | 0.6586849 |
| Npy1r | -0.4458343 |
| Npy2r | 0.0446149 |
| Npy5r | -0.6258534 |
| Nqo1 | -0.2491529 |
| Nqo2 | 0.9554031 |
| Nqo2 | -0.3942097 |
| Nqo2 | -0.3511093 |
| Nr1d1 | 0.0563523 |
| Nr1d2 | 0.9560629 |
| Nr1h2 | -0.7091438 |
| Nr1h2 | -0.0559018 |
| Nr1h3 | -0.7548679 |
| Nr1h4 | -0.6963305 |
| Nr1h5 | -0.5681712 |
| Nr1i2 | -0.3481001 |
| Nr2c1 | 1.4723304 |
| Nr2c2ap | 1.3327677 |
| Nr2e1 | 4.6692438 |
| Nr2f2 | -0.810339 |
| Nr2f2 | 1.2383808 |
| Nr2f6 | -0.0332203 |
| Nr2f6 | -0.2106721 |
| Nr4a2 | 0.3113401 |
| Nr4a3 | 0.6668447 |
| Nr4a3 | -0.7441307 |
| Nr5a1 | -0.4598353 |
| Nr5a2 | -0.6820029 |
| Nr5a2 | 2.4029122 |
| Nr5a2 | 0.3293034 |
| Nr6a1 | -0.5016436 |
| Nradd | 0.3478568 |
| Nrap | -0.5503886 |
| Nrarp | -0.1315318 |
| Nras | -0.5540676 |
| Nrbf2 | -0.3421084 |
| Nrbp1 | -0.3736612 |
| Nrbp2 | 3.9879799 |
| Nrcam | 1.7486481 |
| Nrd1 | -0.4478225 |
| Nrf1 | -0.7620901 |
| Nrg1 | 1.4218798 |
| Nrg3 | -0.6187548 |
| Nrg4 | -0.4551467 |
| Nrgn | -0.5518586 |
| Nrip2 | -0.5916145 |
| Nrip3 | 1.5211301 |
| Nrl | -0.4438499 |
| Nrm | -0.2012771 |
| Nrn1 | -0.6588394 |
| Nrn1l | -0.554805 |
| Nrp1 | 0.5126438 |
| Nrp1 | 0.4611893 |
| Nrp2 | 0.8482778 |
| Nrp2 | -0.8208482 |
| Nrsn2 | -0.5809597 |
| Nrsn2 | -0.1791814 |
| Nrtn | -0.1137517 |
| Nrxn1 | 1.1868668 |
| Nrxn1 | 2.2845085 |
| Nrxn1 | 1.9966316 |
| Nrxn2 | 0.4611893 |
| Nrxn3 | -0.0841807 |
| Nrxn3 | -0.6531307 |
| Nsbp1 | 0.3280748 |
| Nsd1 | -0.0897468 |
| Nsd1 | -0.5885588 |
| Nsdhl | -0.5832334 |
| Nsdhl | 4.6583723 |
| Nsdhl | -0.6621161 |
| Nsf | -0.4274635 |
| Nsfl1c | -0.4713137 |
| Nsg1 | 1.0610375 |
| Nsg2 | 1.1578635 |
| Nsl1 | 0.2401374 |
| Nsmaf | -0.6330024 |
| Nsmaf | -0.6179692 |
| Nsmce1 | -0.703144 |
| Nsmce2 | -0.2617614 |
| Nsmce2 | 1.7676265 |
| Nsmce2 | -0.3173251 |
| Nsun2 | -0.6156158 |
| Nsun4 | -0.5321926 |
| Nsun5 | -0.5832334 |
| Nsun5 | -0.2954151 |
| Nsun5 | -0.3675162 |
| Nsun5 | -0.426164 |
| Nt5c | -0.6720105 |
| Nt5c1b | -0.5756742 |
| Nt5c2 | 0.9516013 |
| Nt5c2 | -0.4005201 |
| Nt5c2 | -0.4326782 |
| Nt5c3 | 0.8866203 |
| Nt5c3l | -0.3184915 |
| Nt5c3l | -0.7711912 |
| Nt5dc3 | -0.1625467 |
| Nt5dc3 | -0.5214306 |
| Nt5dc3 | 3.1373157 |
| Nt5e | -0.4953651 |
| Nt5e | -0.1868343 |
| Nt5m | -0.2994156 |
| Nt5m | 0.7366364 |
| Ntan1 | -0.6314094 |
| Ntf3 | 0.2978129 |
| Nthl1 | -0.5954473 |
| Ntn2l | -0.6547585 |
| Ntn2l | -0.1620469 |
| Ntn4 | -0.1786733 |
| Ntng1 | -0.6539443 |
| Ntng1 | -0.4974536 |
| Ntng1 | 0.9618172 |
| Ntrk2 | 0.0559498 |
| Ntrk2 | -0.1334729 |
| Ntrk2 | -0.4898169 |
| Ntrk3 | 1.5031777 |
| Ntrk3 | -0.567424 |
| Ntrk3 | 1.3169844 |
| Nts | 0.0523203 |
| Ntsr1 | -0.5644406 |
| Ntsr2 | -0.5734174 |
| Nuak1 | -0.603931 |
| Nuak1 | -0.4753963 |
| Nuak2 | 1.7252384 |
| Nub1 | -0.168564 |
| Nubp1 | -0.3891897 |
| Nubp2 | 0.1092625 |
| Nubp2 | -0.6588394 |
| Nubp2 | 0.0051789 |
| Nucb1 | 0.3556622 |
| Nucb1 | 0.3357293 |
| Nucks1 | 1.2840615 |
| Nudc | -0.3693558 |
| Nudc | -0.5438002 |
| Nudcd2 | -0.1223624 |
| Nudcd3 | 1.7385301 |
| Nudc-ps1 | 1.1613667 |
| Nudt1 | 2.2924261 |
| Nudt10 | -0.3109336 |
| Nudt11 | 0.7897326 |
| Nudt12 | 0.9191212 |
| Nudt13 | 1.1249378 |
| Nudt14 | 0.8526717 |
| Nudt16l1 | -0.6728394 |
| Nudt18 | 1.1644556 |
| Nudt19 | -0.6402024 |
| Nudt19 | 1.8867505 |
| Nudt2 | -0.1868343 |
| Nudt21 | -0.0608615 |
| Nudt22 | 0.0356186 |
| Nudt4 | 0.5488413 |
| Nudt5 | 1.595692 |
| Nudt5 | 1.9443633 |
| Nudt6 | 1.2524879 |
| Nudt7 | -0.6109256 |
| Nudt8 | -0.1344449 |
| Nudt9 | -0.4106995 |
| Nufip1 | -0.636996 |
| Numa1 | 0.8250782 |
| Numb | 0.3194322 |
| Numb | -0.1756298 |
| Numb | -0.0758886 |
| Numb | 1.1978235 |
| Numbl | 0.4793398 |
| Nup107 | 0.980818 |
| Nup133 | -0.0188113 |
| Nup133 | 1.3384259 |
| Nup155 | 0.8331193 |
| Nup155 | 0.1700142 |
| Nup160 | -0.5622088 |
| Nup210 | -0.5264386 |
| Nup37 | 2.0280389 |
| Nup43 | 1.4286097 |
| Nup50 | -0.4558152 |
| Nup50 | -0.4584935 |
| Nup50 | 1.9078851 |
| Nup62 | 0.2381232 |
| Nup85 | 0.1345256 |
| Nup88 | 0.8875031 |
| Nup88 | -0.6179692 |
| Nup88 | -0.2790089 |
| Nup93 | -0.4939752 |
| Nup98 | -0.1863223 |
| Nup98 | -0.0804869 |
| Nupl1 | -0.1496311 |
| Nupl1 | -0.5977539 |
| Nupl1 | -0.7405769 |
| Nupl2 | -0.2852006 |
| Nupr1 | -0.1402963 |
| Nus1 | 1.9132598 |
| Nusap1 | -0.5030442 |
| Nut | 1.4741074 |
| Nutf2 | 0.2574264 |
| Nvl | -0.527874 |
| Nwd1 | -0.7423522 |
| Nxf1 | -0.2410141 |
| Nxf2 | -0.8295288 |
| Nxf3 | -0.3860648 |
| Nxph1 | 1.0635602 |
| Nxph2 | 0.549335 |
| Nxph3 | 1.1660635 |
| Nxt1 | 0.7633343 |
| Nxt1 | 0.513667 |
| Nxt2 | 0.8063177 |
| Nyx | -0.5343589 |
| Oaf | -0.2612099 |
| Oas1b | -0.0967479 |
| Oas1b | 0.0639689 |
| Oas1c | -0.6070337 |
| Oas1d | -0.7005836 |
| Oas1d | 0.8671812 |
| Oas1e | 0.2666146 |
| Oas1g | -0.6290245 |
| Oas1g | 1.1005189 |
| Oas2 | 0.3740349 |
| Oasl1 | 0.987872 |
| Oasl2 | 0.2895447 |
| Oasl2 | -0.5293115 |
| Oat | 1.006863 |
| Oaz2 | -0.6117058 |
| Oaz3 | -0.4767608 |
| Obfc1 | -0.703999 |
| Obfc2a | -0.6258534 |
| Obfc2b | -0.2623132 |
| Obfc2b | 2.2054925 |
| Obox1 | -0.4139018 |
| Obox5 | -0.6163997 |
| Obrgrp | -0.5969845 |
| Obsl1 | -0.321999 |
| Oc90 | -0.6234817 |
| Oca2 | 0.2824952 |
| Ociad1 | -0.5321926 |
| Ociad1 | 0.5329137 |
| Ociad2 | -0.6769943 |
| Ocln | 1.7862544 |
| Ocrl | 0.053935 |
| Odc1 | 1.2798844 |
| Odf1 | -0.2350871 |
| Odf2 | 0.3681447 |
| Odf2 | -0.1620469 |
| Odf2 | 0.3003433 |
| Odf2 | 1.1489155 |
| Odf3 | 1.4967361 |
| Odf3l1 | -0.0336601 |
| Odf4 | -0.554805 |
| Odf4 | -0.2101479 |
| Odz1 | -0.703999 |
| Odz3 | 0.8457059 |
| Odz3 | -0.5157374 |
| Odz4 | -0.0240271 |
| Og9x | 0.1126931 |
| Ogdh | -0.5221445 |
| Ogfod1 | -0.6466458 |
| Ogfod2 | 0.341508 |
| Ogfr | 0.6127018 |
| Ogfrl1 | -0.1705777 |
| Ogg1 | 0.7637334 |
| Ogn | 1.1743301 |
| Ogn | 0.398118 |
| Ogn | -0.2948449 |
| Ogt | -0.3923242 |
| Ogt | 0.2317186 |
| Ogt | 0.2313804 |
| Oit1 | 1.4597023 |
| Oit3 | -0.0140537 |
| Ola1 | 0.6022182 |
| Olfm1 | -0.304007 |
| Olfm1 | -0.6016101 |
| Olfm2 | -0.2377769 |
| Olfm2 | -0.057252 |
| Olfm3 | 0.3562602 |
| Olfm3 | -0.2739711 |
| Olfm4 | 2.2748799 |
| Olfml1 | -0.7212516 |
| Olfml2b | -0.2164569 |
| Olfml3 | 1.9573965 |
| Olfr100 | 0.0421693 |
| Olfr1006 | 2.1749952 |
| Olfr1012 | -0.7370355 |
| Olfr1020 | -0.4294159 |
| Olfr1065 | 1.6747036 |
| Olfr1094 | 1.2495344 |
| Olfr1099 | -0.4055969 |
| Olfr1105 | 3.3677432 |
| Olfr1112 | 1.4445806 |
| Olfr1113 | -0.07543 |
| Olfr1189 | 0.0989003 |
| Olfr1197 | -0.4484862 |
| Olfr1198 | 1.5995319 |
| Olfr1249 | -0.6314094 |
| Olfr1280 | -0.554805 |
| Olfr133 | -0.2535206 |
| Olfr1330 | -0.6637584 |
| Olfr134 | 1.1767175 |
| Olfr1340 | -0.6117058 |
| Olfr1384 | 2.9555579 |
| Olfr1386 | -0.0713113 |
| Olfr1390 | 1.2544522 |
| Olfr1394 | -0.6361961 |
| Olfr1412 | 1.7426197 |
| Olfr1437 | 1.1872602 |
| Olfr144 | 1.717132 |
| Olfr1443 | 1.8679707 |
| Olfr1443 | 0.1206527 |
| Olfr1459 | -0.3535232 |
| Olfr1500 | 0.3654829 |
| Olfr1508 | -0.4326782 |
| Olfr166 | -0.3120927 |
| Olfr168 | 1.9532343 |
| Olfr206 | 2.4690228 |
| Olfr213 | -0.046055 |
| Olfr215 | 0.1825188 |
| Olfr232 | 0.2249326 |
| Olfr257 | 0.2901831 |
| Olfr267 | -0.4692786 |
| Olfr272 | -0.6547585 |
| Olfr303 | 2.4663346 |
| Olfr310 | 3.8022767 |
| Olfr352 | -0.7396904 |
| Olfr371 | 2.2864056 |
| Olfr378 | 1.7564315 |
| Olfr419 | 2.1339536 |
| Olfr437 | -0.6695277 |
| Olfr464 | -0.6330024 |
| Olfr47 | 1.2015786 |
| Olfr49 | -0.8520583 |
| Olfr503 | -0.7317467 |
| Olfr51 | 2.8484625 |
| Olfr536 | 2.1830361 |
| Olfr538 | -0.657205 |
| Olfr54 | -0.8131943 |
| Olfr550 | 1.4824572 |
| Olfr574 | -0.2117214 |
| Olfr591 | 3.3958115 |
| Olfr593 | 0.1046702 |
| Olfr608 | -0.636996 |
| Olfr638 | -0.471993 |
| Olfr65 | 2.6635143 |
| Olfr655 | 0.6242238 |
| Olfr658 | -0.4017869 |
| Olfr665 | -0.8324391 |
| Olfr675 | -0.7125884 |
| Olfr691 | 0.5172402 |
| Olfr692 | 0.3865835 |
| Olfr702 | -0.4538111 |
| Olfr724 | 0.6145542 |
| Olfr725 | -0.2773268 |
| Olfr748 | 0.2377872 |
| Olfr786 | -0.2557116 |
| Olfr802 | -0.6728394 |
| Olfr824 | -0.3517122 |
| Olfr91 | 1.8958161 |
| Olfr910 | 2.2598326 |
| Olfr920 | -0.7117262 |
| Olfr924 | 2.2168429 |
| Olfr952 | 2.1452719 |
| Olfr965 | 0.3203618 |
| Olfr982 | -0.7693644 |
| Olfr983 | 0.7738582 |
| Olfr984 | -0.7335066 |
| Olig1 | -0.7896436 |
| Olig3 | -0.5379797 |
| Oma1 | -0.5171577 |
| Omd | 0.9554031 |
| Omg | -0.0599579 |
| Omt2b | 0.1345256 |
| Onecut2 | -0.6937873 |
| Onecut3 | -0.8550343 |
| Onecut3 | 0.6595702 |
| Oog4 | 0.1489447 |
| Oosp1 | -0.4524772 |
| Opa1 | -0.4287647 |
| Opa1 | 1.7442811 |
| Ophn1 | 0.1255487 |
| Oplah | 2.7688916 |
| Opn1mw | 0.0425773 |
| Opn3 | -0.2367001 |
| Oprl1 | 0.2307036 |
| Oprl1 | -0.7125884 |
| Oprl1 | 0.5503217 |
| Oprs1 | -0.5157374 |
| Optc | 0.1983716 |
| Optn | 0.3225274 |
| Optn | 0.3249967 |
| Orai1 | -0.6769943 |
| Orai3 | -0.5430708 |
| Oraov1 | 0.5837844 |
| Orc2l | 2.829067 |
| Orc3l | -0.6274377 |
| Orc5l | -0.5570204 |
| Orc5l | -0.7441307 |
| Orc6l | 0.0252971 |
| ORF19 | 1.7421661 |
| ORF28 | -0.4075074 |
| ORF34 | 0.1456354 |
| ORF61 | 0.7182281 |
| ORF63 | -0.4294159 |
| ORF9 | -0.3553374 |
| ORF9 | -0.8037079 |
| ORF9 | -0.7849988 |
| Orm1 | 0.7337629 |
| Ormdl2 | 0.427226 |
| Ormdl3 | -0.4918939 |
| Os9 | -0.4652206 |
| Osbp2 | -0.272855 |
| Osbpl10 | 0.1562599 |
| Osbpl11 | 1.0801699 |
| Osbpl2 | 0.1485775 |
| Osbpl3 | 0.1389842 |
| Osbpl3 | -0.0663 |
| Osbpl6 | -0.6393999 |
| Osbpl8 | -0.6148326 |
| Osbpl9 | -0.5044467 |
| Oscar | -0.4062334 |
| Osgep | 0.5185133 |
| Osgepl1 | 1.1405743 |
| Osgin1 | 1.0230375 |
| Osm | -0.7693644 |
| Osmr | 1.6971693 |
| Osr1 | 1.6629941 |
| Osr2 | -0.4119792 |
| Ostf1 | -0.0279568 |
| Ostm1 | -0.6920953 |
| Otof | 0.1130736 |
| Otop1 | 0.0013616 |
| Otop2 | -0.1209221 |
| Ott | -0.6703546 |
| Ott | 1.6609472 |
| OTTMUSG00000000421 | 1.2892882 |
| OTTMUSG00000000971 | -0.6778273 |
| OTTMUSG00000000990 | 1.8982156 |
| OTTMUSG00000001305 | -0.6980296 |
| OTTMUSG00000002038 | 1.2337594 |
| OTTMUSG00000004461 | -0.1645485 |
| OTTMUSG00000004551 | -0.6629369 |
| OTTMUSG00000005148 | 2.2943958 |
| OTTMUSG00000005491 | -0.0626711 |
| OTTMUSG00000008540 | 2.4953747 |
| OTTMUSG00000010009 | 0.0667606 |
| OTTMUSG00000010328 | 2.1028707 |
| OTTMUSG00000015762 | -0.5719157 |
| OTTMUSG00000015859 | 0.8190523 |
| OTTMUSG00000016293 | -0.1257309 |
| OTTMUSG00000016790 | -0.4856756 |
| OTTMUSG00000016790 | 0.0699416 |
| OTTMUSG00000017677 | 0.0368501 |
| OTTMUSG00000020946 | 1.037741 |
| Otub1 | -0.5525944 |
| Otub2 | 0.0739039 |
| Otub2 | 0.0833503 |
| Otub2 | -0.527874 |
| Otud5 | 0.4336127 |
| Otud6b | 0.3604357 |
| Otud7b | 0.5859287 |
| Otud7b | 2.3390458 |
| Otx1 | -0.3798439 |
| Otx2 | -0.3644577 |
| Ovca2 | -0.824697 |
| Ovgp1 | 1.3979118 |
| Ovgp1 | 2.2706355 |
| Ovol1 | 0.8919054 |
| Ovol2 | 1.5305942 |
| Ovol2 | -0.4733529 |
| Oxct1 | -0.3325952 |
| Oxct2a | -0.5030442 |
| Oxct2b | 2.6580104 |
| Oxgr1 | 0.3308371 |
| Oxnad1 | -0.2661842 |
| Oxr1 | 0.3749155 |
| Oxr1 | -0.7014363 |
| Oxt | 0.4617278 |
| P140 | 0.4617278 |
| P2rx2 | -0.158555 |
| P2rx3 | -0.6377967 |
| P2rx4 | 0.231042 |
| P2rx4 | -0.6282308 |
| P2rx5 | 0.3427204 |
| P2rx7 | -0.5666773 |
| P2rx7 | 1.9192406 |
| P2ry1 | 0.3147716 |
| P2ry12 | -0.4255149 |
| P2ry13 | 2.7791645 |
| P2ry14 | -0.3823277 |
| P2ry14 | -0.5696674 |
| P2ry5 | -0.6047057 |
| P2ry6 | 1.1779091 |
| P42pop | 0.2901831 |
| P4ha1 | -0.5365298 |
| P4ha2 | -0.1731006 |
| P4hb | 1.3437157 |
| Pa2g4 | -0.3391259 |
| Pabpc1 | -0.3954686 |
| Pabpc1 | -0.6466458 |
| Pabpc1 | -0.2259952 |
| Pabpc2 | -0.0559018 |
| Pabpc4 | -0.3225848 |
| Pabpn1 | -0.620328 |
| Pabpnl1 | -0.8620129 |
| Pacrg | 2.7456532 |
| Pacs1 | -0.2976991 |
| Pacs2 | 0.5220693 |
| Pacsin1 | -0.2307983 |
| Pacsin1 | 1.154348 |
| Pacsin1 | -0.8112899 |
| Pacsin2 | 1.0631155 |
| Pacsin2 | -0.5257216 |
| Pacsin3 | 0.5508147 |
| Pacsin3 | 0.3903467 |
| Padi1 | 1.6304114 |
| Padi2 | -0.01276 |
| Padi6 | -0.2502431 |
| Paf1 | 0.2136349 |
| Paf1 | 0.553276 |
| Pafah1b1 | -0.535082 |
| Pafah1b1 | 0.3203618 |
| Pafah1b3 | 0.6907208 |
| Pafah2 | 0.1352701 |
| Pafah2 | -0.7877831 |
| Paip1 | -0.2885939 |
| Paip2 | 2.1462854 |
| Paip2b | -0.4918939 |
| Paip2b | 0.8610402 |
| Pak1 | -0.7943099 |
| Pak3 | 1.0535812 |
| Pak4 | 0.3326744 |
| Pak6 | 1.7117692 |
| Pak7 | -0.4863646 |
| Palld | 1.682756 |
| Palm | 1.4233331 |
| Palm | -0.4062334 |
| Palm2 | -0.5250052 |
| Palmd | -0.5555429 |
| Palmd | 3.9114329 |
| Palmd | 3.1842047 |
| Pam | 3.0231774 |
| Pank1 | -0.4398928 |
| Pank1 | -0.6008377 |
| Pank2 | 2.8980625 |
| Pank3 | 1.8311296 |
| Pank4 | 2.0112712 |
| Panx1 | -0.6234817 |
| Panx3 | -0.6450311 |
| Paox | -0.4815513 |
| Paox | 1.8954265 |
| Paox | 0.3634077 |
| Paox | 2.1336459 |
| Papd1 | -0.5726663 |
| Papd1 | -0.2988431 |
| Papd4 | 0.2692245 |
| Papln | -0.2291947 |
| Papola | 1.191316 |
| Papolb | -0.5962156 |
| Papolg | -0.4036901 |
| Papss1 | -0.5711657 |
| Papss1 | -0.657205 |
| Papss2 | -0.4320249 |
| Paqr3 | 1.456696 |
| Paqr6 | 0.4748327 |
| Paqr7 | -0.0672093 |
| Paqr9 | -0.4740336 |
| Paqr9 | 0.0802113 |
| Paqr9 | 1.7266216 |
| Pard3 | 0.3586483 |
| Pard3 | -0.6482631 |
| Pard6a | -0.6523178 |
| Pard6a | 0.6502352 |
| Pard6b | -0.4151856 |
| Pard6g | -0.3433039 |
| Park7 | -0.2054418 |
| Parl | 0.6161722 |
| Parp1 | 0.973066 |
| Parp1 | -0.0341002 |
| Parp1 | 3.7560307 |
| Parp12 | -0.7915076 |
| Parp14 | -0.416471 |
| Parp16 | -0.703144 |
| Parp2 | -0.2835081 |
| Parp2 | -0.7432411 |
| Parp3 | 0.0742993 |
| Parp6 | -0.7575702 |
| Parp8 | 0.8141296 |
| Pars2 | -0.6828401 |
| Pars2 | -0.4119792 |
| Parva | -0.5923799 |
| Parvg | -0.5877963 |
| Pask | -0.3910692 |
| Patl1 | -0.4960608 |
| Patz1 | -0.6794955 |
| Pawr | -0.1590531 |
| Pax8 | -0.4024209 |
| Paxip1 | -0.5969845 |
| Pbk | 1.9260056 |
| Pbld | 0.1685752 |
| Pbp2 | 0.5331645 |
| Pbrm1 | 0.3141486 |
| Pbrm1 | -0.3829496 |
| Pbx1 | -0.7169107 |
| Pbx1 | -0.4068702 |
| Pbx2 | 0.6010466 |
| Pbx2 | 1.0331691 |
| Pbx2 | -0.5962156 |
| Pbx3 | -0.3391259 |
| Pbx3 | 0.7395017 |
| Pbx4 | 1.6747036 |
| Pcaf | 0.097356 |
| Pcaf | -0.8027642 |
| Pcbd1 | 2.661602 |
| Pcbd2 | -0.3761298 |
| Pcbp1 | -0.6258534 |
| Pcbp2 | -0.2874615 |
| Pcbp3 | -0.7915076 |
| Pcca | 1.1151285 |
| Pcca | -0.408145 |
| Pccb | -0.6629369 |
| Pccb | 0.2940052 |
| Pcdh1 | 0.6026865 |
| Pcdh10 | -0.6124866 |
| Pcdh10 | 2.7908584 |
| Pcdh10 | 0.8067002 |
| Pcdh15 | 1.2142937 |
| Pcdh17 | -0.6156158 |
| Pcdh20 | 1.9880173 |
| Pcdh21 | -0.8373082 |
| Pcdh7 | 3.1488505 |
| Pcdh7 | 2.3887953 |
| Pcdh8 | -0.6466458 |
| Pcdh9 | -0.1019132 |
| Pcdha10 | 0.7492644 |
| Pcdha11 | -0.4753963 |
| Pcdha2 | -0.1832557 |
| Pcdha6 | 5.1716669 |
| Pcdhac1 | 1.5670473 |
| Pcdhac2 | -0.7849988 |
| Pcdhb1 | -0.636996 |
| Pcdhb10 | 3.4090008 |
| Pcdhb12 | -0.4106995 |
| Pcdhb14 | 0.6331204 |
| Pcdhb19 | 0.95127 |
| Pcdhb20 | -0.9028442 |
| Pcdhb22 | -0.6963305 |
| Pcdhb3 | -0.1590531 |
| Pcdhb7 | -0.1099482 |
| Pcdhb8 | -0.1393187 |
| Pcdhga10 | -0.4132605 |
| Pcdhga12 | -0.6234817 |
| Pcdhga4 | -0.0855693 |
| Pcdhga7 | -0.730868 |
| Pcdhga8 | 2.617477 |
| Pcdhgb2 | -0.7022898 |
| Pcdhgb6 | 2.3360712 |
| Pcdhgc3 | 3.0698834 |
| Pcdhgc4 | -0.3237573 |
| Pcdhgc5 | 2.3420952 |
| Pcf11 | 1.6094131 |
| Pcgf1 | -0.3397217 |
| Pcgf2 | 1.6150041 |
| Pcgf3 | 1.7772391 |
| Pcgf5 | -0.6442247 |
| Pcgf6 | -0.6187548 |
| Pck1 | 0.5498285 |
| Pclo | 0.1408362 |
| Pclo | 3.0655369 |
| Pcmt1 | -0.0676643 |
| Pcmt1 | -0.3138338 |
| Pcmt1 | -0.3748948 |
| Pcmtd1 | -0.3632369 |
| Pcmtd2 | 0.3157055 |
| Pcna | -0.1491376 |
| Pcnt | 2.5176269 |
| Pcnx | -0.0270822 |
| Pcnxl2 | -0.5372545 |
| Pcnxl2 | 0.2643254 |
| Pcolce | 2.3324983 |
| Pcolce2 | 0.7617362 |
| Pcolce2 | 0.8273287 |
| Pcsk1n | 3.3877404 |
| Pcsk2 | 1.1657957 |
| Pcsk2 | -0.5336363 |
| Pcsk2 | 1.1040844 |
| Pcsk2 | 1.640498 |
| Pcsk4 | -0.0527583 |
| Pcsk4 | -0.6330024 |
| Pcsk4 | 0.2136349 |
| Pcsk4 | 0.0954224 |
| Pcsk4 | 2.1583682 |
| Pcsk9 | 0.7829039 |
| Pctk1 | -0.6377967 |
| Pcx | -0.6219036 |
| Pcyox1 | 2.6566985 |
| Pcyox1l | -0.4877441 |
| Pcyt1a | 0.0277839 |
| Pcyt1b | -0.7186448 |
| Pcyt1b | 0.8497446 |
| Pcyt2 | 0.2256132 |
| Pcyt2 | -0.9640428 |
| Pcyt2 | 1.1252167 |
| Pdcd10 | -0.5214306 |
| Pdcd2 | -0.5467231 |
| Pdcd2l | 2.6348478 |
| Pdcd4 | 0.9437875 |
| Pdcd5 | 1.5971774 |
| Pdcd5 | -0.5423419 |
| Pdcd6ip | 1.4398975 |
| Pdcl | -0.4412101 |
| Pdcl3 | 1.0986599 |
| Pddc1 | -0.1056864 |
| Pde10a | -0.4981507 |
| Pde1a | 0.1104074 |
| Pde1a | 0.7870066 |
| Pde1a | 0.8484613 |
| Pde1b | 1.5660573 |
| Pde1c | -0.4139018 |
| Pde2a | 1.0872882 |
| Pde3a | -0.554805 |
| Pde4a | 0.1850012 |
| Pde4a | -0.527156 |
| Pde4a | 0.1274255 |
| Pde4a | -0.7022898 |
| Pde4b | 1.5216948 |
| Pde4d | -0.3445008 |
| Pde4dip | 0.3749155 |
| Pde4dip | -0.2948449 |
| Pde4dip | 0.1555307 |
| Pde4dip | 1.7679214 |
| Pde4dip | -0.0772659 |
| Pde5a | -0.5533307 |
| Pde6a | -0.3421084 |
| Pde6d | -0.5741691 |
| Pde6d | -0.3711987 |
| Pde6d | -0.7134514 |
| Pde6d | 2.7321458 |
| Pde6h | -0.1842769 |
| Pde7a | 0.5640349 |
| Pdgfa | -0.5525944 |
| Pdgfb | 2.520217 |
| Pdgfc | -0.0500716 |
| Pdgfra | -0.5079615 |
| Pdgfrb | -0.1670563 |
| Pdgfrl | 0.2792745 |
| Pdha1 | 0.2721526 |
| Pdhb | -0.5438002 |
| Pdhb | -0.0170786 |
| Pdia3 | -0.0478382 |
| Pdia4 | -0.6211155 |
| Pdia5 | -0.3829496 |
| Pdia5 | -0.4405512 |
| Pdia6 | 0.6771156 |
| Pdik1l | 0.6542465 |
| Pdk1 | 2.5567401 |
| Pdk2 | -0.6629369 |
| Pdk3 | -0.4898169 |
| Pdk4 | 0.7223941 |
| Pdlim1 | 1.3578724 |
| Pdlim2 | 0.0265413 |
| Pdlim2 | -0.4584935 |
| Pdlim2 | 1.3300405 |
| Pdlim2 | -0.7256113 |
| Pdlim3 | 1.3446134 |
| Pdlim3 | 0.8053609 |
| Pdlim4 | -0.3080417 |
| Pdlim5 | 2.4120982 |
| Pdlim5 | -0.4884345 |
| Pdlim7 | -0.6895627 |
| Pdpk1 | -0.634598 |
| Pdpk1 | 3.9125408 |
| Pdrg1 | -0.3823277 |
| Pdss1 | -0.6016101 |
| Pdxdc1 | 0.9158664 |
| Pdxdc1 | 1.0814811 |
| Pdxk | -0.1986828 |
| Pdxp | -0.6442247 |
| Pdyn | 0.4476317 |
| Pdyn | 1.1387877 |
| Pdzd11 | 0.3249967 |
| Pdzd11 | -0.3266947 |
| Pdzd11 | 0.3929438 |
| Pdzd8 | 2.1850364 |
| Pdzk1 | 0.4772213 |
| Pdzk1ip1 | 0.2132905 |
| Pdzrn3 | -0.5802029 |
| Pdzrn3 | 1.1371357 |
| Pea15 | 1.6092406 |
| Pear1 | 0.428896 |
| Pear1 | 0.3728596 |
| Pebp1 | -0.5401584 |
| Pecam1 | -0.5496544 |
| Pecam1 | -0.730868 |
| Peci | 0.871135 |
| Pef1 | 1.8223688 |
| Peg10 | -0.6402024 |
| Peg3 | 0.2184433 |
| Peli1 | -0.809389 |
| Peli1 | 0.0298516 |
| Peli2 | 1.2926002 |
| Peli3 | -0.2897276 |
| Pelo | -0.4190467 |
| Pelp1 | -0.6187548 |
| Pemt | 0.881485 |
| Peo1 | 3.7019787 |
| Pepd | -0.6929409 |
| Per1 | -0.1204425 |
| Per1 | -0.6016101 |
| Per2 | 1.0791489 |
| Per2 | -0.8520583 |
| Per2 | 1.1065018 |
| Per3 | 0.1537056 |
| Perld1 | 0.7030861 |
| Pes1 | -0.7503802 |
| Pet112l | -0.2823814 |
| Pet112l | 0.2488189 |
| Pet112l | -0.0855693 |
| Pet112l | 0.864296 |
| Pex11a | 0.2249326 |
| Pex11a | -0.6298188 |
| Pex11a | 1.0872882 |
| Pex11b | -0.6402024 |
| Pex11c | -0.3349651 |
| Pex14 | 0.0607689 |
| Pex16 | 0.6500119 |
| Pex19 | -0.0358624 |
| Pex19 | -0.5016436 |
| Pex2 | -0.61405 |
| Pex26 | 0.3699154 |
| Pex3 | 0.2434853 |
| Pex3 | 3.013408 |
| Pex5 | -0.1094738 |
| Pex5 | -0.2361621 |
| Pex5 | 2.6558127 |
| Pex5l | -0.2546155 |
| Pex6 | 0.5715467 |
| Pex6 | 1.3583151 |
| Pex6 | -0.4171143 |
| Pex7 | -0.51858 |
| Pfdn1 | -0.5023437 |
| Pfdn2 | 0.836095 |
| Pfdn4 | -0.2196263 |
| Pfdn5 | -0.705711 |
| Pfdn5 | 1.2761703 |
| Pfkfb2 | 1.1542125 |
| Pfkfb2 | -0.6912504 |
| Pfkfb4 | 0.4221994 |
| Pfkl | 1.188963 |
| Pfkl | -0.0319018 |
| Pfkm | 0.0663622 |
| Pfkm | 0.1888899 |
| Pfkm | 0.4435287 |
| Pfkp | -0.5666773 |
| Pfkp | 1.0599969 |
| Pfkp | 1.0366761 |
| Pfn1 | 3.3766931 |
| Pfn1 | -0.6547585 |
| Pfn2 | 1.1364466 |
| Pfn2 | 1.6054376 |
| Pfn2 | -0.3462988 |
| Pfn3 | -0.5802029 |
| Pfn4 | 0.3816411 |
| Pftk1 | -0.6322056 |
| Pga5 | 1.3761904 |
| Pgam1 | -0.1461819 |
| Pgam2 | 1.6412504 |
| Pgam5 | 0.8731061 |
| Pgbd5 | -0.1407855 |
| Pgcp | -0.5954473 |
| Pgd | -0.3730451 |
| Pgk1 | 0.6688196 |
| Pgk2 | -0.7169107 |
| Pgls | 2.0384071 |
| Pglyrp1 | 0.0486777 |
| Pglyrp4 | -0.3910692 |
| Pgm2 | -0.3385304 |
| Pgm2l1 | -0.5711657 |
| Pgm3 | -0.3017087 |
| Pgm5 | 0.0009366 |
| Pgm5 | -0.3493027 |
| Pgpep1 | -0.636996 |
| Pgpep1 | 0.544635 |
| Pgr | 0.2763671 |
| Pgrmc1 | -0.636996 |
| Pgs1 | 0.5471115 |
| Phactr1 | -0.683678 |
| Phactr1 | -0.5257216 |
| Phactr1 | -0.567424 |
| Phactr4 | -0.31616 |
| Phb2 | 0.6628833 |
| Phc1 | -0.7503802 |
| Phc1 | -0.3355584 |
| Phc2 | 1.3618494 |
| Phca | 0.0723209 |
| Phca | 3.2171887 |
| Phex | 1.675431 |
| Phf1 | 1.0496819 |
| Phf11 | 2.136974 |
| Phf11 | -0.5704163 |
| Phf12 | 0.9274671 |
| Phf12 | 0.8042115 |
| Phf13 | 0.0470545 |
| Phf13 | -0.4359512 |
| Phf14 | 0.5261181 |
| Phf16 | -0.5136108 |
| Phf17 | -0.5570204 |
| Phf19 | 1.7056724 |
| Phf2 | 0.0352077 |
| Phf20l1 | 2.736217 |
| Phf21a | 0.3915018 |
| Phf5a | 2.0408842 |
| Phf6 | -0.3314123 |
| Phf7 | 0.3535667 |
| Phgdh | 1.6501533 |
| Phka1 | -0.4346407 |
| Phka1 | 0.1367574 |
| Phka2 | -0.6861958 |
| Phka2 | -0.6377967 |
| Phka2 | -0.5749214 |
| Phkb | 1.6997751 |
| Phkg1 | 0.8982112 |
| Phkg2 | -0.0007654 |
| Phlda1 | 1.4704511 |
| Phlda2 | 1.3460704 |
| Phlda3 | 0.7378654 |
| Phlda3 | -0.6290245 |
| Phldb1 | -0.1185263 |
| Phldb1 | 0.1969726 |
| Phlpp | -0.5100763 |
| Phospho1 | 1.7855303 |
| Phospho2 | 0.1903002 |
| Phox2a | 0.1456354 |
| Phox2b | 0.6491181 |
| Phpt1 | 0.1304211 |
| Phtf1 | -0.6937873 |
| Phtf2 | -0.7459123 |
| Phyhip | -0.2524269 |
| Pi16 | -0.6711822 |
| Pi4k2b | -0.1741115 |
| Pi4k2b | -0.4300675 |
| Pi4ka | 1.6978016 |
| Pi4kb | -0.730868 |
| Pi4kb | 1.0725605 |
| Pias1 | -0.7785317 |
| Pias2 | 0.2933692 |
| Pias3 | 0.4061144 |
| Pias3 | 3.3973653 |
| Pias4 | 0.605258 |
| Pibf1 | -0.936743 |
| Pibf1 | -0.4822375 |
| Picalm | 0.8695194 |
| Pick1 | 0.5057097 |
| Pick1 | 1.7085691 |
| Pif1 | 0.1548011 |
| Piga | 0.5220693 |
| Piga | 3.3454926 |
| Pigb | 0.4798687 |
| Pigc | -0.4891255 |
| Pigf | -0.657205 |
| Pigh | -0.0209813 |
| Pigk | -0.6531307 |
| Pigk | 0.7801594 |
| Pigl | 0.0228042 |
| Pign | -0.643419 |
| Pigo | 3.3578909 |
| Pigo | -0.6085887 |
| Pigo | -0.3034319 |
| Pigp | -0.7414642 |
| Pigp | 1.3611877 |
| Pigq | -0.3517122 |
| Pigt | 1.5074811 |
| Pigx | -0.4359512 |
| Pigy | -0.3296406 |
| Pigyl | -0.6226923 |
| Pigz | -0.3687422 |
| Pih1d1 | 0.918608 |
| Pik3ap1 | 1.032558 |
| Pik3c2a | -0.0599579 |
| Pik3c3 | -0.3620175 |
| Pik3ca | 0.5256129 |
| Pik3cb | -0.6008377 |
| Pik3cd | 1.8604673 |
| Pik3cg | -0.1233237 |
| Pik3ip1 | 0.6138599 |
| Pik3r1 | -0.5726663 |
| Pik3r1 | 0.0631698 |
| Pik3r1 | 0.0110883 |
| Pik3r2 | 0.256437 |
| Pik3r2 | -0.3367462 |
| Pik3r3 | 0.0930971 |
| Pik3r4 | 1.8980861 |
| Pik3r6 | 2.6025759 |
| Pik3r6 | -0.6604764 |
| Pim1 | -0.5129029 |
| Pim2 | 0.0310902 |
| Pim2 | 1.2921277 |
| Pim3 | 0.9486159 |
| Pip4k2a | -0.6195411 |
| Pip4k2b | -0.5474551 |
| Pip4k2b | 0.7961289 |
| Pip4k2c | -0.4431893 |
| Pip5k1a | -0.8441647 |
| Pip5k1a | 0.050703 |
| Pip5k1b | -0.3898158 |
| Pip5kl1 | -0.1233237 |
| Pipox | -0.7503802 |
| Pir | 2.3240414 |
| Pira11 | 0.9340607 |
| Pira4 | -0.2852006 |
| Pisd | -0.2372383 |
| Pit1 | -0.3891897 |
| Pitpna | 2.805347 |
| Pitpnb | 2.2575674 |
| Pitpnc1 | 2.1009124 |
| Pitpnm1 | -0.7091438 |
| Pitpnm2 | 2.4816313 |
| Pitpnm3 | 0.9115676 |
| Pitx1 | 0.6973487 |
| Pitx2 | -0.7388046 |
| Pitx2 | -0.5570204 |
| Pitx2 | 0.0343856 |
| Pitx2 | -0.496757 |
| Piwil1 | 0.3442338 |
| Piwil2 | -0.6290245 |
| Piwil4 | -0.7100039 |
| Pja1 | -0.500245 |
| Pja2 | 1.4548879 |
| Pja2 | 2.9848647 |
| Pkd1 | 0.1307949 |
| Pkd1l3 | -0.5242892 |
| Pkd2 | -0.3397217 |
| Pkd2l1 | -0.3835718 |
| Pkd2l2 | -0.8700479 |
| Pkdrej | 0.173961 |
| Pkhd1l1 | 2.1596185 |
| Pkhd1l1 | 1.3856031 |
| Pkia | 0.1000569 |
| Pkib | 1.6586498 |
| Pkib | -0.6861958 |
| Pkig | -0.0131911 |
| Pkig | -0.2623132 |
| Pkig | -0.1123237 |
| Pkm2 | 0.3442338 |
| Pkmyt1 | 0.154436 |
| Pkn1 | -0.673669 |
| Pkn1 | 0.6909353 |
| Pkn2 | 1.6491588 |
| Pkn2 | -0.5764276 |
| Pkn2 | 1.5235749 |
| Pkn3 | -0.6466458 |
| Pknox1 | 2.2935209 |
| Pknox1 | 0.0190532 |
| Pknox2 | -0.730868 |
| Pknox2 | 1.3039906 |
| Pknox2 | 0.6260552 |
| Pknox2 | -0.2868958 |
| Pknox2 | 2.0524567 |
| Pkp1 | 1.9038996 |
| Pkp2 | -0.6242717 |
| Pkp2 | -0.6078109 |
| Pkp2 | 1.5202824 |
| Pkp3 | -0.7100039 |
| Pla1a | -0.1746174 |
| Pla1a | -0.6621161 |
| Pla1a | -0.4822375 |
| Pla2g10 | -0.2551634 |
| Pla2g12a | -0.7014363 |
| Pla2g1b | 0.5208007 |
| Pla2g2c | -0.5786911 |
| Pla2g2d | -0.4605069 |
| Pla2g2f | -0.5114886 |
| Pla2g4c | -0.0253353 |
| Pla2g4d | -0.31616 |
| Pla2g4e | -0.4158281 |
| Pla2g4f | 1.3627311 |
| Pla2g5 | 2.1518413 |
| Pla2g6 | -0.0781852 |
| Pla2g7 | 0.1054371 |
| Plaa | -0.0496245 |
| Plac1 | -0.3948389 |
| Plac1l | -0.6920953 |
| Plac8 | 0.6751569 |
| Plag1 | 1.5704598 |
| Plag1 | 3.7945716 |
| Plagl2 | -0.4558152 |
| Plau | -0.1427445 |
| Plb1 | 0.9266186 |
| Plcb2 | 1.7983414 |
| Plcb3 | 0.7066818 |
| Plcb4 | 0.6214705 |
| Plcd1 | -0.4346407 |
| Plcd3 | -0.6523178 |
| Plcd4 | -0.703144 |
| Plcd4 | -0.1142282 |
| Plcd4 | -0.5207172 |
| Plce1 | 1.9257536 |
| Plcg2 | 0.4063988 |
| Plch2 | 0.3028675 |
| Plcl2 | 2.2742109 |
| Plcxd1 | -0.284072 |
| Plcxd1 | -0.2399339 |
| Plcz1 | 1.8376163 |
| Pld1 | 0.6885735 |
| Pld2 | -0.7125884 |
| Pld3 | -0.0367446 |
| Pld3 | -0.0658457 |
| Pld4 | -0.3462988 |
| Pld5 | -0.5900855 |
| Pldn | 0.5642781 |
| Plec1 | 0.5126438 |
| Plec1 | -0.1295945 |
| Plec1 | 0.8451539 |
| Plec1 | 1.2050623 |
| Plek | 1.6629123 |
| Plek2 | -0.4392348 |
| Plekha1 | 0.7049913 |
| Plekha1 | 0.8934856 |
| Plekha2 | 1.5782295 |
| Plekha3 | -0.5636961 |
| Plekha4 | 2.5816795 |
| Plekha4 | -0.327872 |
| Plekha5 | -0.3184915 |
| Plekha6 | -0.0563517 |
| Plekha7 | -0.4808655 |
| Plekha8 | -0.6988802 |
| Plekhb1 | 0.3305305 |
| Plekhb2 | -0.1113729 |
| Plekhf1 | -0.5401584 |
| Plekhf1 | -0.3565487 |
| Plekhf2 | -0.4733529 |
| Plekhf2 | -0.5923799 |
| Plekhg2 | 1.4323178 |
| Plekhg2 | -0.1560682 |
| Plekhg2 | 0.4867196 |
| Plekhg3 | 1.8062488 |
| Plekhg3 | 1.305624 |
| Plekhg4 | 0.0671588 |
| Plekhg5 | -0.5321926 |
| Plekhg6 | -0.3355584 |
| Plekhj1 | 2.7242392 |
| Plekhm1 | -0.01276 |
| Plekhm3 | -0.496757 |
| Plekho2 | 1.7942567 |
| Plin | -0.6393999 |
| Plk1 | 2.3078616 |
| Plk2 | -0.2281271 |
| Plk3 | 0.505452 |
| Plk4 | -0.2458895 |
| Pllp | -0.500245 |
| Plod1 | 3.6858903 |
| Plod2 | -0.2169845 |
| Plod3 | 0.6484473 |
| Plp1 | -0.5711657 |
| Plp2 | 3.2658028 |
| Plrg1 | -0.0635771 |
| Pls3 | 0.0409443 |
| Plscr1 | 1.7272357 |
| Plscr1 | 1.181739 |
| Plscr3 | -0.9196511 |
| Plscr4 | 1.1221449 |
| Pltp | -0.4740336 |
| Plunc | -0.4024209 |
| Plvap | -0.5072575 |
| Plxdc1 | -0.6093671 |
| Plxdc1 | 1.8216003 |
| Plxna2 | -0.3948389 |
| Plxnb1 | -0.6054811 |
| Plxnb3 | -0.6853558 |
| Plxnc1 | 0.0619701 |
| Plxnd1 | 0.6964959 |
| Pmaip1 | 0.1786055 |
| Pmch | 0.3752088 |
| Pmepa1 | -0.2818186 |
| Pmf1 | 2.6496826 |
| Pmfbp1 | 0.136014 |
| Pml | 1.1121669 |
| Pml | 0.4827729 |
| Pmm1 | -0.6306138 |
| Pmm1 | 0.0458354 |
| Pmm2 | 0.345745 |
| Pmm2 | 0.0458354 |
| Pmm2 | 0.8568646 |
| Pmm2 | 0.3865835 |
| Pmp2 | 0.4833 |
| Pmp22 | 0.0482721 |
| Pmp22 | -0.5228589 |
| Pmpca | 2.0386325 |
| Pmpcb | 0.2613745 |
| Pms2 | -0.6687015 |
| Pmvk | 0.0438003 |
| Pnck | 3.0153994 |
| Pnkd | -0.6861958 |
| Pnkd | 0.0186355 |
| Pnkp | 0.0152882 |
| Pnkp | 0.2081113 |
| Pnliprp1 | -0.5171577 |
| Pnma1 | 0.5235895 |
| Pnma2 | 0.7984449 |
| Pno1 | 1.8969842 |
| Pnpla2 | 2.2072104 |
| Pnpla6 | 0.0535316 |
| Pnpla7 | -0.613268 |
| Pnpla8 | -0.3144148 |
| Pnpla8 | -0.3391259 |
| Pnpo | 1.8241131 |
| Pnpo | -0.1481514 |
| Pnrc2 | 0.9004787 |
| Podn | -0.5555429 |
| Podxl | -0.2579073 |
| Podxl2 | -0.1393187 |
| Pof1b | 0.8420202 |
| Pofut1 | 0.0579606 |
| Pofut1 | 1.2418641 |
| Pofut2 | -0.0389536 |
| Pogz | -0.6878779 |
| Pogz | 1.1483707 |
| Pola2 | -0.2557116 |
| Polb | 2.8224058 |
| Pold1 | 0.2786291 |
| Pold2 | -0.5343589 |
| Pold3 | 1.752697 |
| Pold4 | -0.6085887 |
| Poldip2 | -0.5794467 |
| Poldip3 | -0.5430708 |
| Poldip3 | 1.7864715 |
| Pole | 0.1786055 |
| Pole3 | -0.4918939 |
| Pole3 | -0.5408857 |
| Pole4 | 0.0248821 |
| Polg | 1.8095783 |
| Polg2 | 2.9904278 |
| Polh | -0.6322056 |
| Poli | -0.5511233 |
| Polk | -0.3086194 |
| Poll | 3.9757564 |
| Poll | 0.5530302 |
| Polm | 1.7941849 |
| Polm | 0.1153533 |
| Poln | 2.0734159 |
| Polq | -0.2469761 |
| Polr1e | 0.6509049 |
| Polr2d | 1.1029448 |
| Polr2d | -0.6555733 |
| Polr2d | 1.2561678 |
| Polr2d | -0.5847521 |
| Polr2e | -0.810339 |
| Polr2e | -0.6124866 |
| Polr2f | 1.384849 |
| Polr2g | -0.6031568 |
| Polr2h | 0.5844997 |
| Polr2j | -0.5969845 |
| Polr3a | -0.4625244 |
| Polr3a | 0.0364397 |
| Polr3b | -0.2595573 |
| Polr3d | 0.2521382 |
| Polr3e | 3.3450607 |
| Polr3g | -0.6393999 |
| Polr3h | -0.2908625 |
| Polr3h | -0.2486083 |
| Polr3h | -0.6621161 |
| Polr3k | -0.7539688 |
| Polr3k | 3.1075757 |
| Polrmt | 3.186139 |
| Pols | 0.080997 |
| Pom121 | -0.7186448 |
| Pomc | -0.6912504 |
| Pomgnt1 | -0.3468989 |
| Pomgnt1 | -0.1437254 |
| Pomp | 1.3686622 |
| Pomt1 | 0.1371288 |
| Pomt1 | -0.1359046 |
| Pomt2 | -0.6870365 |
| Pon1 | 3.4925604 |
| Pon2 | 1.9033842 |
| Pon3 | -0.0041779 |
| Pon3 | 0.2330703 |
| Pop4 | 1.6691098 |
| Pop5 | 0.0739039 |
| Pop5 | 0.4700384 |
| Pop7 | 1.1135783 |
| Popdc2 | 0.3598403 |
| Popdc2 | 1.836997 |
| Popdc3 | -0.6474542 |
| Por | 0.9196342 |
| Por | -0.6109256 |
| Porcn | -0.4829242 |
| Porcn | -0.4075074 |
| Pou2af1 | -0.5358057 |
| Pou2f1 | -0.5585001 |
| Pou2f1 | 0.0992859 |
| Pou2f2 | -0.3361521 |
| Pou3f1 | -0.5719157 |
| Pou3f3 | -0.3742778 |
| Pou5f1 | 1.9111518 |
| Pou5f1 | 0.0352077 |
| Pou6f1 | 1.069029 |
| Ppa1 | 1.5847913 |
| Ppa2 | -0.7108646 |
| Ppa2 | 2.3592985 |
| Ppa2 | -0.7521729 |
| Ppap2a | -0.4498148 |
| Ppap2a | -0.6744993 |
| Ppap2a | -0.7730213 |
| Ppap2b | -0.6377967 |
| Ppap2b | 1.8728978 |
| Ppap2c | -0.4438499 |
| Ppapdc1 | 1.6974855 |
| Ppapdc1a | 2.3468803 |
| Ppapdc2 | -0.5200043 |
| Ppapdc3 | 0.5540132 |
| Ppapdc3 | 0.5713053 |
| Pparbp | -0.5885588 |
| Pparg | -0.7048546 |
| Ppargc1a | 2.9650021 |
| Ppargc1b | 1.6043112 |
| Ppat | -0.9324428 |
| Ppcs | -0.4953651 |
| Ppef2 | 0.9160379 |
| Ppfia3 | 2.8017575 |
| Ppfia3 | -0.2568089 |
| Ppfibp1 | 1.2696676 |
| Ppfibp2 | 1.6542868 |
| Ppfibp2 | 2.7397941 |
| Ppia | 0.8859136 |
| Ppic | -0.6803306 |
| Ppic | -0.5044467 |
| Ppid | -0.1104229 |
| Ppie | -0.2180404 |
| Ppie | -0.5416136 |
| Ppif | -0.7933749 |
| Ppil2 | -0.2524269 |
| Ppil3 | -0.3718138 |
| Ppl | 1.0940691 |
| Ppm1a | -0.7512761 |
| Ppm1a | 1.6669124 |
| Ppm1a | -0.6171841 |
| Ppm1b | -0.8295288 |
| Ppm1b | -0.4346407 |
| Ppm1b | 0.3169493 |
| Ppm1e | -0.7221221 |
| Ppm1e | -0.620328 |
| Ppm1f | 2.5963069 |
| Ppm1f | -0.6187548 |
| Ppm1f | -0.5969845 |
| Ppm1g | -0.7229933 |
| Ppm1h | 1.1036572 |
| Ppm1j | -0.6385979 |
| Ppm1k | 0.9704688 |
| Ppm1l | -0.1368789 |
| Ppm1l | -0.5962156 |
| Ppm1m | -0.1180478 |
| Ppm1m | 1.027809 |
| Ppm2c | 1.3936476 |
| Ppme1 | -0.7022898 |
| Ppnr | -0.3935808 |
| Ppox | 0.7514884 |
| Ppox | -0.5916145 |
| Ppp1ca | -0.4248662 |
| Ppp1ca | -0.7432411 |
| Ppp1ca | 0.3556622 |
| Ppp1cb | 0.0778507 |
| Ppp1cb | 0.0758792 |
| Ppp1cc | -0.6547585 |
| Ppp1r10 | 0.9279758 |
| Ppp1r10 | 1.1332706 |
| Ppp1r11 | 1.2891697 |
| Ppp1r12c | -0.3403178 |
| Ppp1r13b | 2.523811 |
| Ppp1r13b | -0.6547585 |
| Ppp1r13b | 3.0253191 |
| Ppp1r14a | 2.0352456 |
| Ppp1r14a | -0.705711 |
| Ppp1r14c | 0.6251399 |
| Ppp1r14d | 1.4450883 |
| Ppp1r15b | 0.5031294 |
| Ppp1r16a | -0.8151023 |
| Ppp1r1a | -0.0708547 |
| Ppp1r1b | 2.357123 |
| Ppp1r1b | 0.713206 |
| Ppp1r2 | -0.6290245 |
| Ppp1r3c | -0.5518586 |
| Ppp1r3f | -0.9596249 |
| Ppp1r8 | -0.7125884 |
| Ppp1r9a | -0.4988484 |
| Ppp1r9b | 2.7804075 |
| Ppp2ca | 0.1764645 |
| Ppp2cb | -0.6016101 |
| Ppp2cz | -0.5100763 |
| Ppp2r1a | -0.2667384 |
| Ppp2r1b | -0.6242717 |
| Ppp2r2b | -0.833411 |
| Ppp2r2b | 2.7188283 |
| Ppp2r2b | 1.4822615 |
| Ppp2r2c | 1.0394121 |
| Ppp2r2c | 1.3409054 |
| Ppp2r2d | -1.0244848 |
| Ppp2r3c | 0.0880405 |
| Ppp2r4 | -0.5496544 |
| Ppp2r5b | -0.8863127 |
| Ppp2r5c | 0.0778507 |
| Ppp2r5c | 0.8316281 |
| Ppp2r5c | 0.1198973 |
| Ppp2r5d | 0.2283313 |
| Ppp2r5d | -0.9530341 |
| Ppp2r5d | 0.1482102 |
| Ppp2r5d | 0.6432892 |
| Ppp2r5e | -0.5809597 |
| Ppp2r5e | -0.1812165 |
| Ppp3ca | -0.1914541 |
| Ppp3ca | -0.9080663 |
| Ppp3ca | -0.379224 |
| Ppp3cb | 0.2143232 |
| Ppp3cc | 0.046242 |
| Ppp3r1 | -0.6377967 |
| Ppp4c | 0.7106855 |
| Ppp4r1 | -0.5171577 |
| Ppp4r1 | 2.7175357 |
| Ppp4r1 | -0.8935119 |
| Ppp4r1l | -0.4471593 |
| Ppp5c | -0.4618515 |
| Ppp5c | -0.7766916 |
| Ppp6c | 0.356559 |
| Pps | 0.6117743 |
| Ppt1 | 0.8966385 |
| Ppt2 | -0.7575702 |
| Pptc7 | -0.0649376 |
| Pqbp1 | 0.3854227 |
| Pqlc1 | -0.2410141 |
| Pqlc2 | -0.4405512 |
| Pqlc3 | 1.5826678 |
| Pqlc3 | 0.3345084 |
| Pqlc3 | 1.581161 |
| Pqlc3 | -0.7503802 |
| Pramef12 | 2.3422203 |
| Prap1 | 1.4776522 |
| Prc1 | -0.8720668 |
| Prcp | -0.5607237 |
| Prdm10 | 0.9792079 |
| Prdm4 | -0.4856756 |
| Prdm8 | -0.4740336 |
| Prdm9 | -0.2122465 |
| Prdx1 | 1.9652454 |
| Prdx2 | -0.3103546 |
| Prdx2 | 1.4527744 |
| Prdx2 | 0.01403 |
| Prdx3 | 0.4500856 |
| Prdx4 | -0.7877831 |
| Prdx5 | 0.9629641 |
| Prdx5 | -0.3583683 |
| Prdx6 | -0.0777254 |
| Prdx6-rs1 | -0.4898169 |
| Prdx6-rs1 | 0.9441212 |
| Preb | 2.1200145 |
| Preb | 1.954644 |
| Prei3 | 0.4606505 |
| Prei4 | 0.3943837 |
| Prei4 | -0.0214158 |
| Prelp | 2.0986318 |
| Prep | -0.1476587 |
| Prep | -0.3923242 |
| Prf1 | 0.092709 |
| Prickle1 | -0.1986828 |
| Prickle2 | 0.3277674 |
| Prickle3 | 0.0742993 |
| Prickle4 | -0.2513344 |
| Prim2 | 0.1736028 |
| Prim2 | -0.433332 |
| Prima1 | 2.1650519 |
| Prkaa2 | 1.2424848 |
| Prkaa2 | 1.6538742 |
| Prkab1 | -0.634598 |
| Prkab2 | -0.0786451 |
| Prkaca | -0.6946343 |
| Prkaca | -0.4392348 |
| Prkacb | 0.6681617 |
| Prkag1 | -0.3565487 |
| Prkag2 | -0.8208482 |
| Prkar1a | 2.7646487 |
| Prkar1b | 1.2527337 |
| Prkar2b | -0.5207172 |
| Prkca | 0.0302646 |
| Prkcb1 | -0.9112126 |
| Prkcbp1 | 1.7501496 |
| Prkcbp1 | -0.9898287 |
| Prkcc | -0.36568 |
| Prkcd | 0.7364314 |
| Prkcdbp | 2.2616412 |
| Prkcdbp | -0.8893918 |
| Prkce | -0.613268 |
| Prkch | 0.1664128 |
| Prkcq | -0.7905751 |
| Prkcsh | -0.214876 |
| Prkcz | 0.4984681 |
| Prkcz | -0.2007577 |
| Prkcz | -0.527874 |
| Prkd1 | 1.2850138 |
| Prkd1 | -0.5343589 |
| Prkd2 | -0.7648118 |
| Prkd3 | -0.6929409 |
| Prkd3 | 0.0899883 |
| Prkd3 | -0.6482631 |
| Prkg1 | 1.8981508 |
| Prkg2 | 0.3430233 |
| Prkra | 0.8586822 |
| Prkrip1 | -0.6920953 |
| Prkrip1 | 0.9932892 |
| Prkrir | 1.7589631 |
| Prkx | 1.6099305 |
| Prkx | 1.5648861 |
| Prl | 0.0244668 |
| Prlr | -0.7370355 |
| Prlr | 0.1761072 |
| Prm1 | 2.234677 |
| Prm3 | 3.0987517 |
| Prmt2 | -0.6963305 |
| Prmt2 | -0.2689584 |
| Prmt3 | -0.5946796 |
| Prmt5 | 1.4551895 |
| Prmt5 | -0.707426 |
| Prmt5 | 0.1667735 |
| Prmt7 | 0.9150081 |
| Prmt7 | -0.5562814 |
| Prmt7 | -0.7575702 |
| Prmt7 | -0.1675586 |
| Prmt8 | 0.1895953 |
| Prnp | 0.3900577 |
| Prnpip1 | -0.6629369 |
| Prnpip1 | -0.6466458 |
| Prnpip1 | -0.9740546 |
| Proc | 1.155431 |
| Proc | 2.2627247 |
| Procr | 0.1489447 |
| Prodh | 0.707526 |
| Prok2 | 0.1627985 |
| Prok2 | -0.9028442 |
| Prokr1 | 0.9074235 |
| Prokr2 | 0.4877695 |
| Prom1 | -0.883243 |
| Prom2 | -0.2684029 |
| Prom2 | 1.9889057 |
| Pros1 | 0.6958559 |
| Prosapip1 | 1.5080535 |
| Prosapip1 | 0.7553184 |
| Prosapip1 | -0.8710568 |
| Prosc | 1.4490398 |
| Prosc | -0.0170786 |
| Prosc | 0.3091503 |
| Prox1 | -0.7468043 |
| Prox1 | 1.6326054 |
| Prpf18 | -0.515028 |
| Prpf19 | 0.1393549 |
| Prpf19 | -0.7868541 |
| Prpf19 | 3.5119116 |
| Prpf3 | -0.496757 |
| Prpf3 | -0.4822375 |
| Prpf3 | -0.5644406 |
| Prpf3 | -0.4398928 |
| Prpf3 | -0.3929523 |
| Prpf31 | 0.1689351 |
| Prpf38a | -0.7831468 |
| Prpf38a | -0.8600141 |
| Prpf38a | -0.6242717 |
| Prpf38b | 2.975383 |
| Prpf39 | 0.8301347 |
| Prpf40a | -0.3034319 |
| Prpf40a | -0.1295945 |
| Prpf40b | 0.5103377 |
| Prpf40b | -0.2399339 |
| Prpf40b | -0.4665715 |
| Prpf6 | 1.5380281 |
| Prpf8 | -0.3184915 |
| Prpf8 | 0.5508147 |
| Prph2 | 0.4269474 |
| Prps1 | -0.2925673 |
| Prps1l1 | -0.5416136 |
| Prps2 | -0.1904257 |
| Prpsap2 | -0.5877963 |
| Prpsap2 | 0.9529253 |
| Prr11 | -0.8650185 |
| Prr13 | -0.527156 |
| Prr14 | -0.785926 |
| Prr15 | 2.7718646 |
| Prr16 | -0.1878591 |
| Prr18 | 0.5816356 |
| Prr19 | 1.0807529 |
| Prr3 | 1.0859845 |
| Prr7 | 0.031915 |
| Prrc1 | 2.3882775 |
| Prrg2 | -0.0640304 |
| Prrt1 | -0.5257216 |
| Prrt1 | -0.7739376 |
| Prrt1 | -0.3589756 |
| Prrt3 | 1.1264707 |
| Prrx1 | 0.1393549 |
| Prrx1 | 0.3332861 |
| Prrx1 | 0.1750347 |
| Prss21 | -0.6670511 |
| Prss23 | 0.489866 |
| Prss27 | 0.8156469 |
| Prss28 | -0.046055 |
| Prss29 | 2.8138472 |
| Prss29 | -0.3860648 |
| Prss34 | 0.0169633 |
| Prss35 | 1.3565432 |
| Prss36 | 0.4457191 |
| Prss7 | -0.4268136 |
| Prss8 | 0.9522635 |
| Prss8 | 2.1109284 |
| Prtn3 | 0.393808 |
| Prune | 1.6524702 |
| Prune | 1.6546992 |
| Prune | 1.3306092 |
| Prune | 2.2086873 |
| Psap | -0.3553374 |
| Psat1 | -0.0080308 |
| Psca | 1.340455 |
| Pscd1 | -0.7620901 |
| Pscd2 | -0.5533307 |
| Pscd3 | 1.3744497 |
| Pscdbp | -0.4425291 |
| Psd2 | -0.6870365 |
| Psd3 | 1.9534796 |
| Psd4 | 0.2307036 |
| Psen1 | -0.9101628 |
| Psen2 | 0.780552 |
| Psenen | -0.2891606 |
| Psg19 | 1.2345103 |
| Psg23 | -0.6980296 |
| Psg27 | -0.36568 |
| Psip1 | -0.7441307 |
| Psip1 | 2.2393984 |
| Pskh1 | 2.6380562 |
| Psma2 | 0.4872447 |
| Psma4 | -0.6997315 |
| Psma5 | 2.1251996 |
| Psma7 | 2.2887389 |
| Psma7 | -0.284072 |
| Psmb1 | -0.6769943 |
| Psmb10 | 1.6367273 |
| Psmb10 | -0.3149962 |
| Psmb2 | -0.7022898 |
| Psmb2 | 0.4511743 |
| Psmb3 | 0.8131802 |
| Psmb4 | -0.0231559 |
| Psmb5 | -0.0420543 |
| Psmb6 | -0.8295288 |
| Psmb7 | -0.3063106 |
| Psmb7 | -0.5756742 |
| Psmb7 | -0.5445301 |
| Psmb8 | -0.5771815 |
| Psmb9 | 1.3635019 |
| Psmc1 | 1.8281484 |
| Psmc2 | -0.7575702 |
| Psmc3 | -0.9519398 |
| Psmc3ip | -0.5016436 |
| Psmc4 | 0.9982032 |
| Psmc5 | -0.6588394 |
| Psmc5 | 1.0188632 |
| Psmd1 | 1.4060702 |
| Psmd10 | -0.9122636 |
| Psmd10 | -0.2994156 |
| Psmd10 | 0.3684401 |
| Psmd11 | -0.9281609 |
| Psmd11 | 0.7573284 |
| Psmd12 | 1.9678481 |
| Psmd12 | 1.8053259 |
| Psmd13 | 1.0239628 |
| Psmd14 | 2.0264431 |
| Psmd2 | 1.5483442 |
| Psmd2 | 0.1721689 |
| Psmd4 | 0.4943065 |
| Psmd5 | -0.613268 |
| Psmd6 | -0.6555733 |
| Psmd6 | -0.3138338 |
| Psmd7 | -0.2175123 |
| Psmd7 | 0.9228769 |
| Psmd8 | -0.9313706 |
| Psmd9 | 1.1565128 |
| Psmd9 | 0.799023 |
| Psme1 | -0.2437197 |
| Psme1 | 0.6372003 |
| Psme2 | -0.8710568 |
| Psme3 | -0.2324045 |
| Psme4 | -0.8771315 |
| Psme4 | -0.8751026 |
| Psmf1 | -0.4781272 |
| Psmf1 | -0.6563889 |
| Psmg2 | 0.7872016 |
| Psmg2 | 1.7117692 |
| Psmg3 | 2.6318482 |
| Psors1c2 | 1.5470605 |
| Psp | 0.600343 |
| Pspc1 | 1.1737988 |
| Pstpip1 | 0.2421474 |
| Pstpip2 | 1.6215874 |
| Ptbp1 | -0.7757728 |
| Ptbp1 | -0.1924836 |
| Ptcd3 | 2.1525462 |
| Ptch1 | 0.9249195 |
| Ptch2 | -0.8074918 |
| Ptdss2 | 0.7223941 |
| Pten | 3.1531329 |
| Pter | -0.5644406 |
| Ptgds | -0.5235738 |
| Ptgds2 | 3.1040358 |
| Ptgds2 | 0.8042115 |
| Ptger2 | -0.2745296 |
| Ptger2 | -0.5489208 |
| Ptger3 | -0.1295945 |
| Ptges | -0.3904423 |
| Ptges | 0.0727169 |
| Ptges2 | 0.124797 |
| Ptges3 | 0.5606241 |
| Ptgfr | 1.4204244 |
| Ptgir | -0.6811664 |
| Ptgis | -0.2801318 |
| Ptgs1 | -1.0268381 |
| Ptgs1 | -0.4863646 |
| Pth2 | 0.0954224 |
| Pth2r | -0.9175349 |
| Pthlh | -0.9080663 |
| Pthr1 | -0.9989545 |
| Pthr1 | -0.6490728 |
| Pthr1 | 1.4027936 |
| Ptk2 | 0.4233186 |
| Ptk2b | 3.534868 |
| Ptk7 | 1.5812497 |
| Ptms | 0.2679204 |
| Ptn | -0.2201555 |
| Ptp4a2 | 0.6140914 |
| Ptp4a2 | 2.0473856 |
| Ptp4a2 | -0.2281271 |
| Ptp4a2 | -0.4248662 |
| Ptp4a3 | -0.2002386 |
| Ptp4a3 | -0.1075783 |
| Ptpdc1 | 0.7039333 |
| Ptpdc1 | 0.4673651 |
| Ptpdc1 | 1.0993753 |
| Ptpla | -0.705711 |
| Ptpla | 0.2081113 |
| Ptplad1 | -0.2233366 |
| Ptplad2 | -0.4491503 |
| Ptplb | -0.7143151 |
| Ptpn1 | -0.8680332 |
| Ptpn11 | 0.4806616 |
| Ptpn11 | -0.015349 |
| Ptpn11 | 0.5642781 |
| Ptpn12 | -0.5459915 |
| Ptpn12 | -0.1945458 |
| Ptpn12 | 0.3871633 |
| Ptpn12 | -0.3786044 |
| Ptpn12 | -0.568919 |
| Ptpn14 | 1.3415806 |
| Ptpn18 | 2.0204361 |
| Ptpn20 | 2.0427941 |
| Ptpn21 | -0.3916965 |
| Ptpn22 | -0.5481877 |
| Ptpn5 | 2.1030293 |
| Ptpn6 | -0.9475744 |
| Ptpn6 | -0.127178 |
| Ptpra | 0.8913782 |
| Ptprb | -0.8314681 |
| Ptprd | 1.3142141 |
| Ptprd | 0.0347967 |
| Ptpre | 0.3969705 |
| Ptpre | 0.6571337 |
| Ptprf | -0.6845165 |
| Ptprf | -0.6171841 |
| Ptprg | 1.3622903 |
| Ptprj | -0.5969845 |
| Ptprk | 1.3387643 |
| Ptprk | -0.3225848 |
| Ptprn | 1.363612 |
| Ptprn2 | -0.4255149 |
| Ptpro | 1.0021487 |
| Ptpro | -0.6490728 |
| Ptprq | -0.7868541 |
| Ptprr | 1.8147281 |
| Ptprr | 1.8413924 |
| Ptprs | 0.8222581 |
| Ptprs | -0.8720668 |
| Ptprt | -0.5622088 |
| Ptpru | 0.9039571 |
| Ptprv | -0.7014363 |
| Ptrf | -0.7575702 |
| Ptrh1 | 3.1835169 |
| Ptrh1 | -0.707426 |
| Ptrh2 | 2.1016009 |
| Pts | 0.3616256 |
| Pts | -0.8422009 |
| Pttg1 | -0.8997238 |
| Pttg1ip | 1.8806392 |
| Ptx3 | -0.8324391 |
| Ptx3 | -0.2002386 |
| Puf60 | 1.5426465 |
| Puf60 | -0.9154231 |
| Pum1 | -0.1170916 |
| Pum2 | -0.2002386 |
| Pum2 | 0.356559 |
| Punc | 1.0969408 |
| Purb | -0.5946796 |
| Purg | 0.6154791 |
| Pus1 | -0.8074918 |
| Pus1 | -0.817014 |
| Pus10 | -0.5358057 |
| Pus10 | 0.1126931 |
| Pus10 | -0.3415112 |
| Pus3 | -0.705711 |
| Pus7l | -0.6124866 |
| Pvalb | -0.7739376 |
| Pvr | -0.61405 |
| Pvr | -0.8945446 |
| Pvrl1 | 1.357651 |
| Pvrl2 | -0.4645459 |
| Pvrl2 | 0.0115091 |
| Pvrl2 | 0.8544969 |
| Pvrl3 | 0.4100889 |
| Pvrl3 | 2.4881837 |
| Pvrl3 | -0.0118985 |
| Pwp1 | 2.8033955 |
| Pwp2 | -0.2169845 |
| Pwwp2b | -0.8761165 |
| Pxdn | 2.0128334 |
| Pxmp2 | 0.802677 |
| Pxmp4 | -0.1383421 |
| Pxn | -0.824697 |
| Pxt1 | 0.6315294 |
| Pycr2 | 2.0911704 |
| Pycrl | 2.4953747 |
| Pygb | -0.6523178 |
| Pygl | -0.1909398 |
| Pygl | 0.360138 |
| Pygm | -0.4774438 |
| Pygo1 | -0.4504797 |
| Pygo2 | 1.1201852 |
| Pyroxd1 | -0.7177774 |
| Pyy | -0.4760783 |
| Qars | 0.3427204 |
| Qdpr | 0.3769672 |
| Qpct | 0.8484613 |
| Qprt | -0.0310238 |
| Qrich2 | 0.6486709 |
| Qrich2 | 1.8255064 |
| Qrsl1 | 1.4009922 |
| Qsox1 | -0.4740336 |
| Qtrt1 | 0.6001083 |
| R3hdm1 | 3.7128027 |
| R3hdm1 | -0.4753963 |
| R3hdm2 | -0.2054418 |
| R74862 | -0.5599819 |
| Rab1 | 0.287627 |
| Rab10 | 0.8954135 |
| Rab10 | -0.7952458 |
| Rab11a | 0.4058299 |
| Rab11b | 1.0782729 |
| Rab11fip2 | 1.2888141 |
| Rab11fip3 | -0.527156 |
| Rab11fip5 | 1.529381 |
| Rab11fip5 | 3.2813525 |
| Rab11fip5 | 1.2240736 |
| Rab12 | 0.491174 |
| Rab13 | 2.3983863 |
| Rab13 | -0.1151817 |
| Rab14 | -0.6621161 |
| Rab15 | 0.1976723 |
| Rab17 | 1.2093039 |
| Rab17 | -0.7468043 |
| Rab18 | -0.4679241 |
| Rab19 | -0.610146 |
| Rab19 | 1.0363716 |
| Rab1b | -0.6963305 |
| Rab1b | -0.6845165 |
| Rab1b | -0.119484 |
| Rab21 | 2.1184017 |
| Rab22a | 2.8186427 |
| Rab22a | -0.3992548 |
| Rab23 | 1.1980829 |
| Rab24 | -0.5877963 |
| Rab24 | -0.6604764 |
| Rab24 | 0.0502983 |
| Rab25 | 1.0279625 |
| Rab27a | 2.9541723 |
| Rab27b | 2.3967678 |
| Rab28 | -0.0257717 |
| Rab28 | 1.2428571 |
| Rab2a | 1.3820431 |
| Rab31 | 0.9146646 |
| Rab32 | -0.431372 |
| Rab33a | 1.7749726 |
| Rab33b | -0.6670511 |
| Rab34 | -0.6937873 |
| Rab34 | -0.5802029 |
| Rab34 | 0.0310902 |
| Rab35 | -0.152102 |
| Rab36 | -0.5839925 |
| Rab38 | -0.7414642 |
| Rab38 | -0.6250622 |
| Rab39 | -0.667876 |
| Rab39b | -0.6887199 |
| Rab3a | -0.5847521 |
| Rab3b | -0.4672476 |
| Rab3d | -0.436607 |
| Rab3gap1 | -0.7143151 |
| Rab3gap2 | -0.586273 |
| Rab3il1 | -0.2388548 |
| Rab3il1 | -0.2623132 |
| Rab3ip | 0.1315422 |
| Rab40b | -0.6274377 |
| Rab40c | 0.1685752 |
| Rab43 | -0.4075074 |
| Rab4a | -0.6353967 |
| Rab4b | 0.2344202 |
| Rab5a | 0.8597712 |
| Rab5a | -0.5250052 |
| Rab5b | -0.6769943 |
| Rab5c | -0.6769943 |
| Rab6 | -0.2442617 |
| Rab6 | 0.1289244 |
| Rab6 | -0.4524772 |
| Rab6b | -0.7639038 |
| Rab6ip1 | 1.6144035 |
| Rab7 | 0.3523673 |
| Rab7 | 3.2461713 |
| Rab7 | -0.7503802 |
| Rab7l1 | -0.6687015 |
| Rab8a | -0.5908497 |
| Rab8a | -0.7117262 |
| Rab8b | -0.2324045 |
| Rab8b | 0.4603809 |
| Rab9 | -0.61405 |
| Rab9 | -0.0786451 |
| Rab9b | 0.1278004 |
| Rabac1 | -0.5467231 |
| Rabac1 | -0.603931 |
| Rabep1 | -0.5438002 |
| Rabep1 | -0.243178 |
| Rabep2 | 0.0595663 |
| Rabgap1 | 1.7651166 |
| Rabgap1 | -0.3109336 |
| Rabgap1l | 0.7339684 |
| Rabgap1l | 0.1682151 |
| Rabgef1 | 0.7779978 |
| Rabggta | 0.7047798 |
| Rabggtb | -0.4836113 |
| Rabif | 2.2982367 |
| Rabif | -0.3385304 |
| Rabl2a | 2.5792098 |
| Rabl2a | 0.1518771 |
| Rabl3 | -0.4544786 |
| Rabl4 | -0.3638471 |
| Rabl4 | 0.7695031 |
| Rabl5 | 0.5490882 |
| Rac1 | 2.3791122 |
| Rac2 | -0.5992946 |
| Rac3 | 0.1363857 |
| Rac3 | 0.0727169 |
| Rad1 | -0.2700701 |
| Rad17 | 0.9088068 |
| Rad18 | 0.0856981 |
| Rad23a | -0.4024209 |
| Rad23a | -0.4524772 |
| Rad23a | -0.304007 |
| Rad23b | 1.0238086 |
| Rad50 | 2.033378 |
| Rad51 | -0.5250052 |
| Rad51 | -0.3773663 |
| Rad51ap1 | 1.038197 |
| Rad51c | 1.0259646 |
| Rad51l3 | 0.3003433 |
| Rad51l3 | 1.5027942 |
| Rad51l3 | -0.5749214 |
| Rad51l3 | 1.9013845 |
| Rad52 | -0.3620175 |
| Rad54l | 0.707737 |
| Rad9 | 0.0880405 |
| Rad9 | 0.6979878 |
| Rad9 | -0.2639704 |
| Rad9b | -0.6179692 |
| Rae1 | -0.3948389 |
| Raet1b | -0.6786611 |
| Raet1b | -0.0559018 |
| Raf1 | -0.1992011 |
| Rag1 | 0.8579556 |
| Rag1ap1 | -0.703999 |
| Rage | -0.0305851 |
| Rage | -0.2259952 |
| Rai1 | -0.087888 |
| Rai12 | 0.3363391 |
| Rai12 | 0.3752088 |
| Rai12 | -0.4753963 |
| Rai12 | 2.794034 |
| Rai14 | 0.300975 |
| Rala | 2.8401486 |
| Ralb | -0.535082 |
| Ralbp1 | -0.7151796 |
| Ralgds | -0.5401584 |
| Ralgps1 | 2.1628123 |
| Ralgps1 | 0.6099168 |
| Ralgps1 | -0.5200043 |
| Ralgps2 | 0.3025523 |
| Ralgps2 | -0.6588394 |
| Raly | 0.211222 |
| Raly | -0.8500791 |
| Ralyl | -0.5855123 |
| Ramp1 | -0.6047057 |
| Ramp2 | -0.3373406 |
| Ramp3 | 2.0361499 |
| Ramp3 | 1.2179882 |
| Ran | -0.5607237 |
| Ranbp1 | 0.6602337 |
| Ranbp10 | -0.6062571 |
| Ranbp17 | -0.3266947 |
| Ranbp2 | -0.6353967 |
| Ranbp3l | 0.1482102 |
| Ranbp6 | 3.6272361 |
| Ranbp9 | 1.1442748 |
| Rangap1 | -0.5681712 |
| Rangrf | 1.6866783 |
| Rap1b | -0.3385304 |
| Rap1gap | 2.5500429 |
| Rap2a | 2.1148549 |
| Rap2b | -0.6085887 |
| Rap2c | 0.0595663 |
| Rap2c | -0.6711822 |
| Rap2c | 0.1903002 |
| Rap2ip | 1.088301 |
| Rapgef1 | -0.6887199 |
| Rapgef1 | -0.7100039 |
| Rapgef1 | 0.2215224 |
| Rapgef1 | -0.4372633 |
| Rapgef3 | -0.7256113 |
| Rapgef4 | -0.3462988 |
| Rapgef5 | 2.8946924 |
| Rapgef6 | -0.7352695 |
| Rapgefl1 | 0.9753332 |
| Rapgefl1 | -0.1359046 |
| Rapsn | -0.7282362 |
| Rapsn | -0.01276 |
| Rara | 0.5359194 |
| Rarb | 0.3332861 |
| Rarb | -0.4740336 |
| Rarg | -0.4184021 |
| Rarg | 0.3517671 |
| Rarg | -0.4856756 |
| Rarres2 | 1.4570974 |
| Rars | -0.6887199 |
| Rars | -0.243178 |
| Rars2 | -0.0358624 |
| Rars2 | 1.5489855 |
| Rars2 | -0.2054418 |
| Rasa1 | -0.4740336 |
| Rasa2 | -0.4524772 |
| Rasal1 | -0.5293115 |
| Rasd1 | -0.4281139 |
| Rasef | -0.6117058 |
| Rasgef1b | -0.4300675 |
| Rasgef1b | -0.1746174 |
| Rasgef1c | 0.0215554 |
| Rasgef1c | -0.107105 |
| Rasgrf1 | 1.17632 |
| Rasgrf1 | 0.8940118 |
| Rasgrf1 | -0.657205 |
| Rasgrf1 | -0.4398928 |
| Rasgrp1 | 0.6595702 |
| Rasgrp1 | -0.3261065 |
| Rasgrp1 | -0.3208285 |
| Rasgrp3 | -0.2324045 |
| Rasip1 | 1.2073781 |
| Rasl10a | 0.4867196 |
| Rasl10b | 1.5431072 |
| Rasl10b | 0.2454887 |
| Rasl11a | 1.8147984 |
| Rasl11b | 1.5931532 |
| Rasl11b | 2.0218696 |
| Rasl12 | 2.5169959 |
| Rasl2-9 | -0.5570204 |
| Rassf1 | 1.6730044 |
| Rassf1 | -0.5445301 |
| Rassf10 | 0.3784301 |
| Rassf2 | -0.4960608 |
| Rassf2 | 0.0173816 |
| Rassf3 | -0.1161362 |
| Rassf4 | 0.620091 |
| Rassf4 | 0.2232289 |
| Rassf4 | -0.3742778 |
| Rassf4 | -0.6093671 |
| Rassf5 | -0.5058511 |
| Rassf7 | -0.5314716 |
| Rassf7 | -0.8580192 |
| Raver1 | 0.5169854 |
| Raver2 | 0.2249326 |
| Rb1 | 0.651797 |
| Rb1 | 0.7452081 |
| Rbak | -0.7831468 |
| Rbak | -0.5200043 |
| Rbbp5 | -0.4544786 |
| Rbbp5 | -0.3190753 |
| Rbbp6 | -0.7291127 |
| Rbbp7 | -0.5250052 |
| Rbbp7 | -0.1266954 |
| Rbbp9 | -0.2919988 |
| Rbck1 | -0.1945458 |
| Rbed1 | -0.7521729 |
| Rbed1 | -0.2535206 |
| Rbj | 0.1386134 |
| Rbks | -0.1447073 |
| Rbl1 | 2.8813672 |
| Rbl2 | 0.6310743 |
| Rbm10 | 1.7044957 |
| Rbm10 | 1.1442748 |
| Rbm10 | 2.5280167 |
| Rbm11 | -0.7961825 |
| Rbm12 | 2.0492833 |
| Rbm12 | 2.0049398 |
| Rbm12 | 2.1200665 |
| Rbm12 | -0.1705777 |
| Rbm13 | -0.4912011 |
| Rbm13 | 1.0201019 |
| Rbm14 | -0.2835081 |
| Rbm14 | 0.526623 |
| Rbm15 | 0.0265413 |
| Rbm16 | 0.5557313 |
| Rbm18 | -0.4385772 |
| Rbm18 | 0.2408078 |
| Rbm19 | -0.4658958 |
| Rbm24 | 1.394822 |
| Rbm26 | -0.4625244 |
| Rbm28 | 1.6609472 |
| Rbm28 | 1.1536705 |
| Rbm3 | -0.3237573 |
| Rbm35a | -0.4808655 |
| Rbm38 | 0.2428166 |
| Rbm38 | 0.4029805 |
| Rbm38 | -0.3179081 |
| Rbm4 | 0.9461213 |
| Rbm41 | 0.4932634 |
| Rbm42 | -0.3891897 |
| Rbm42 | 0.2468221 |
| Rbm43 | -0.5285925 |
| Rbm45 | -0.5459915 |
| Rbm47 | 0.5918611 |
| Rbm47 | -0.3841945 |
| Rbm47 | 0.2623591 |
| Rbm47 | 1.8821541 |
| Rbm4b | -0.7711912 |
| Rbm5 | -0.5900855 |
| Rbm5 | 2.4152839 |
| Rbm6 | 0.2167286 |
| Rbm6 | 2.809711 |
| Rbm6 | 1.7439037 |
| Rbm8a | -0.6466458 |
| Rbm9 | -0.276207 |
| Rbm9 | 1.4289193 |
| Rbms1 | 0.4487231 |
| Rbms1 | -0.6861958 |
| Rbms2 | -0.6929409 |
| Rbms2 | -0.6124866 |
| Rbms3 | -0.6711822 |
| Rbmx | 1.3322001 |
| Rbmy1a1 | 2.315984 |
| Rbp1 | 0.794582 |
| Rbp7 | 3.0683013 |
| Rbpms | 1.7310655 |
| Rbpms | 1.2723219 |
| Rbpms | -0.3632369 |
| Rbpms2 | 1.9436205 |
| Rbx1 | -0.119484 |
| Rbx1 | -0.5157374 |
| Rc3h1 | 1.9444871 |
| Rcan1 | -0.5629522 |
| Rcan2 | 0.4185534 |
| Rcan2 | 1.6099305 |
| Rcan2 | -0.5489208 |
| Rcan3 | -0.0473921 |
| Rcbtb2 | -0.7326263 |
| Rcbtb2 | -0.4981507 |
| Rcc1 | 0.6677229 |
| Rccd1 | 1.0348479 |
| Rce1 | -0.1842769 |
| Rchy1 | -0.152597 |
| Rcl1 | 2.0550659 |
| Rcl1 | -0.7450211 |
| Rcl1 | -0.4781272 |
| Rcn3 | 0.0169633 |
| Rcor1 | 1.1660635 |
| Rcor2 | 0.046242 |
| Rcsd1 | -0.1960952 |
| Rdbp | -0.0297082 |
| Rdbp | 0.578044 |
| Rdh11 | -0.577936 |
| Rdh12 | -0.6786611 |
| Rdh14 | -0.7648118 |
| Rdh14 | -0.4036901 |
| Rdh5 | 0.9330491 |
| Rdh7 | 0.9567222 |
| Rdh9 | 1.4283001 |
| Rdm1 | -0.7117262 |
| Rec8 | 1.8000564 |
| Reck | 0.7738582 |
| Recql | 1.1124494 |
| Recql4 | 0.0687499 |
| Recql5 | -0.5666773 |
| Reep1 | 1.4369339 |
| Reep2 | -0.5540676 |
| Reep3 | 3.1720079 |
| Reep4 | 0.1983716 |
| Reep5 | 1.038045 |
| Reep6 | 0.8704173 |
| Reep6 | 0.8431273 |
| Refbp2 | -0.5969845 |
| Reg1 | 0.3505655 |
| Rela | -0.6117058 |
| Relb | 0.1386134 |
| Rell1 | 1.7548647 |
| Rell2 | 2.1026592 |
| Rell2 | -0.6393999 |
| Reln | -0.2350871 |
| Relt | 0.3185018 |
| Relt | 0.7205215 |
| Rem1 | -0.3742778 |
| Rem2 | 2.1244236 |
| Renbp | 0.0269557 |
| Repin1 | -0.6753303 |
| Repin1 | -0.259007 |
| Reps1 | -0.6621161 |
| Reps1 | 0.2222053 |
| Rer1 | 0.502871 |
| Rer1 | 0.8460737 |
| Rerg | 0.2018609 |
| Resp18 | 2.4052263 |
| Rest | -0.417758 |
| Ret | 1.9894384 |
| Retnla | -0.61405 |
| Retnlg | -0.7177774 |
| Retsat | -0.603931 |
| Retsat | -0.6393999 |
| Rev1 | 1.7072392 |
| Rev3l | 1.0734414 |
| Rex3 | 1.8658995 |
| Rexo1 | -0.4801802 |
| Rexo1 | -0.5009441 |
| Rexo2 | 0.4769562 |
| Rfc1 | 3.2327761 |
| Rfc2 | 0.2692245 |
| Rfc3 | 0.5554861 |
| Rfc4 | -0.7143151 |
| Rfc4 | -0.527156 |
| Rfc5 | 1.1880464 |
| Rfesd | 0.6879284 |
| Rffl | 1.2961368 |
| Rffl | -0.6450311 |
| Rffl | 2.5791768 |
| Rffl | -0.5496544 |
| Rffl | -0.5336363 |
| Rfng | 3.2690684 |
| Rfpl4 | 0.8357235 |
| Rfpl4 | -0.2750884 |
| Rft1 | -0.484987 |
| Rftn1 | 0.1732445 |
| Rftn2 | 1.3446134 |
| Rftn2 | 0.730675 |
| Rfwd2 | -0.7177774 |
| Rfwd3 | 0.45199 |
| Rfwd3 | 1.3429296 |
| Rfx1 | 1.8430358 |
| Rfx1 | 1.595692 |
| Rfx2 | 0.9486159 |
| Rfx3 | -0.6163997 |
| Rfx4 | -0.6418094 |
| Rfx4 | -0.3421084 |
| Rfx4 | 0.4283396 |
| Rfxank | 2.1739609 |
| Rfxap | 3.0530643 |
| Rfxdc1 | 1.154348 |
| Rg9mtd1 | -0.3028572 |
| Rg9mtd2 | 1.1659296 |
| Rgag4 | 0.8030608 |
| Rgl1 | 0.6306191 |
| Rgl2 | 0.2428166 |
| Rgl2 | -0.7169107 |
| Rgl3 | 0.3920788 |
| Rgma | 1.3808536 |
| Rgmb | 1.2570245 |
| Rgmb | 2.6162701 |
| Rgnef | 0.8347942 |
| Rgs1 | -0.9143688 |
| Rgs1 | 1.6309181 |
| Rgs1 | -0.8720668 |
| Rgs10 | -0.535082 |
| Rgs12 | 3.5084279 |
| Rgs14 | 3.5725951 |
| Rgs16 | -0.5900855 |
| Rgs17 | 1.6098443 |
| Rgs2 | 1.6291435 |
| Rgs3 | 0.5020954 |
| Rgs3 | 2.3927299 |
| Rgs3 | -0.0136223 |
| Rgs3 | 1.6435044 |
| Rgs3 | -0.830498 |
| Rgs3 | 0.4579521 |
| Rgs3 | -0.1852991 |
| Rgs4 | 3.6834228 |
| Rgs5 | 0.5478532 |
| Rgs6 | -0.4618515 |
| Rgs7 | 1.3398918 |
| Rgs7bp | -0.7831468 |
| Rgs8 | 0.8349801 |
| Rgs9 | -0.9270932 |
| Rgs9bp | -0.9399802 |
| Rhag | 3.5511075 |
| Rhbdd1 | 0.9003044 |
| Rhbdd3 | -0.703144 |
| Rhbdf1 | -0.5877963 |
| Rhbdf2 | 0.1580804 |
| Rhbdl1 | -0.4043253 |
| Rhbdl2 | 1.2004147 |
| Rhbdl3 | -0.07543 |
| Rhbdl7 | 1.010931 |
| Rhcg | 1.9562964 |
| Rheb | 0.5157104 |
| Rhebl1 | -0.6794955 |
| Rhob | -0.2617614 |
| Rhobtb2 | 0.2968623 |
| Rhobtb2 | 1.0585085 |
| Rhobtb2 | -0.7273605 |
| Rhobtb2 | 2.15043 |
| Rhobtb3 | 0.5725119 |
| Rhoc | -0.4679241 |
| Rhoc | 0.2984461 |
| Rhod | 2.9335039 |
| Rhof | 0.0060252 |
| Rhog | 1.2434772 |
| Rhoj | -0.7212516 |
| Rhot1 | 0.9673758 |
| Rhot2 | 2.3229793 |
| Rhot2 | 0.3110276 |
| Rhot2 | 0.2108769 |
| Rhou | -0.4618515 |
| Rhov | -0.1801985 |
| Rhox11 | -0.7238652 |
| Rhox12 | 1.4545863 |
| Rhox3a | -0.6778273 |
| Rhox3f | 0.1573526 |
| Rhox4d | -0.4055969 |
| Rhox5 | -0.7943099 |
| Rhox8 | 0.1460037 |
| Rhpn1 | -0.1766433 |
| Rhpn1 | -0.0319018 |
| Rhpn1 | 0.4269474 |
| Ribc1 | -0.4733529 |
| Ric3 | -0.2101479 |
| Ric3 | -0.3804643 |
| Ric3 | 1.3518773 |
| Ric8b | -0.8008793 |
| Ric8b | 1.2608097 |
| Rif1 | 0.4305632 |
| Rilp | -0.7361521 |
| Rilpl1 | -0.5336363 |
| Rilpl1 | -0.5809597 |
| Rilpl2 | 1.9152998 |
| Rims1 | -0.3798439 |
| Rims2 | -0.1680612 |
| Rims3 | 2.8218876 |
| Rin1 | -0.6531307 |
| Rin2 | -0.7256113 |
| Rin2 | -0.8363325 |
| Rin2 | 1.2372587 |
| Rin3 | -0.2415546 |
| Riok1 | 0.5108507 |
| Riok1 | 2.3536241 |
| Riok3 | -0.6330024 |
| Riok3 | 0.8396173 |
| Ripk1 | 0.053128 |
| Ripk2 | -0.1565651 |
| Ripk4 | 3.5198035 |
| Ripk5 | 1.4215681 |
| Rit1 | -0.730868 |
| Rit2 | -0.4788111 |
| Rlbp1 | -0.6085887 |
| Rlbp1l1 | 0.7352006 |
| Rlbp1l1 | -0.3711987 |
| Rln1 | -0.6820029 |
| Rltpr | -0.1156589 |
| Rmnd1 | -0.6912504 |
| Rmnd5b | -0.3595833 |
| Rmnd5b | 1.2333838 |
| Rnase1 | 1.2806017 |
| Rnase4 | 0.4029805 |
| Rnase4 | 1.1645897 |
| Rnase6 | -0.7530704 |
| Rnaseh1 | 0.5625746 |
| Rnaseh1 | -0.6728394 |
| Rnaseh2c | 0.3523673 |
| Rnasek | -0.7151796 |
| Rnasen | 1.2426089 |
| Rnaset2 | -0.824697 |
| Rnaset2b | -0.1204425 |
| Rnd2 | -0.4405512 |
| Rnd3 | 1.5818704 |
| Rnf10 | -0.0482845 |
| Rnf103 | -0.0332203 |
| Rnf11 | -0.6250622 |
| Rnf11 | -0.2334767 |
| Rnf11 | -0.6786611 |
| Rnf111 | 0.031915 |
| Rnf113a1 | 2.2189187 |
| Rnf113a2 | 0.5854526 |
| Rnf12 | -0.1339588 |
| Rnf121 | -0.5764276 |
| Rnf123 | -0.8055981 |
| Rnf125 | -0.7566686 |
| Rnf125 | -0.5847521 |
| Rnf128 | -0.7840723 |
| Rnf133 | 0.3012906 |
| Rnf135 | 1.1864733 |
| Rnf135 | -0.5786911 |
| Rnf135 | -0.2639704 |
| Rnf138 | 2.2249331 |
| Rnf138 | 0.2218639 |
| Rnf138 | 1.5753786 |
| Rnf138 | 0.8427584 |
| Rnf139 | 0.1609864 |
| Rnf139 | -0.6778273 |
| Rnf14 | -0.4471593 |
| Rnf14 | -0.3742778 |
| Rnf14 | -0.4836113 |
| Rnf141 | 3.5662724 |
| Rnf141 | 2.6478363 |
| Rnf144a | -0.7291127 |
| Rnf145 | -0.1466739 |
| Rnf150 | 0.3231453 |
| Rnf166 | -0.0518619 |
| Rnf167 | -0.2540679 |
| Rnf167 | -0.3823277 |
| Rnf167 | -0.0363034 |
| Rnf167 | -0.4774438 |
| Rnf167 | -0.4652206 |
| Rnf168 | -0.7539688 |
| Rnf17 | 0.7113162 |
| Rnf170 | -0.6148326 |
| Rnf181 | 2.5595343 |
| Rnf182 | -0.7014363 |
| Rnf182 | 0.9804962 |
| Rnf183 | -0.636996 |
| Rnf185 | -0.8018213 |
| Rnf185 | 1.0747614 |
| Rnf185 | 1.3654813 |
| Rnf185 | -0.6811664 |
| Rnf186 | -0.5321926 |
| Rnf19a | -0.830498 |
| Rnf2 | 1.0090555 |
| Rnf20 | 0.9117399 |
| Rnf207 | 1.1920991 |
| Rnf208 | 3.9547145 |
| Rnf214 | 0.7472383 |
| Rnf215 | -0.5832334 |
| Rnf216 | 2.8066371 |
| Rnf216 | 0.0005113 |
| Rnf219 | 0.1707329 |
| Rnf24 | -0.379224 |
| Rnf24 | 0.9494461 |
| Rnf25 | -0.5164473 |
| Rnf25 | -0.1655508 |
| Rnf25 | -0.1595514 |
| Rnf26 | -0.80844 |
| Rnf31 | 1.0201019 |
| Rnf32 | 0.9067312 |
| Rnf34 | 0.1642458 |
| Rnf34 | -0.272855 |
| Rnf34 | 1.3205515 |
| Rnf38 | 0.4969095 |
| Rnf38 | 0.5139227 |
| Rnf38 | 3.8820574 |
| Rnf4 | -0.8600141 |
| Rnf4 | -0.8018213 |
| Rnf40 | -0.6156158 |
| Rnf41 | -0.5100763 |
| Rnf41 | -0.6219036 |
| Rnf41 | -0.5771815 |
| Rnf44 | -0.8314681 |
| Rnf5 | -0.810339 |
| Rnf6 | -0.5908497 |
| Rnf6 | -0.8189293 |
| Rnf8 | -0.0416107 |
| Rnf8 | -0.7721059 |
| Rngtt | -0.1080519 |
| Rnmt | 2.5093221 |
| Rnmtl1 | 2.1683773 |
| Rnpc2 | -0.2678478 |
| Rnpep | -0.7186448 |
| Rnpepl1 | 0.8554082 |
| Rnps1 | -0.5016436 |
| Rnps1 | 0.7352006 |
| Rnu6 | -0.4100603 |
| Rnuxa | -0.304007 |
| Rnuxa | -0.6988802 |
| Robo1 | -0.8751026 |
| Robo2 | -0.6418094 |
| Robo4 | -0.259007 |
| Rock1 | -0.386689 |
| Rock2 | 1.0980872 |
| Rock2 | -0.244804 |
| Rod1 | -0.3885639 |
| Rogdi | -0.55776 |
| Rom1 | -0.6426139 |
| Ropn1 | -0.0869599 |
| Ror1 | -0.5992946 |
| Ror2 | 1.298605 |
| Rora | 1.166465 |
| Rorb | -0.730868 |
| Rorb | -0.5285925 |
| Rorc | -0.5923799 |
| Rorc | 3.052219 |
| Ros1 | -0.4431893 |
| Rp23-297j14.5 | 2.9575082 |
| Rp23-438h3.2 | 0.8141296 |
| rp9 | -0.3214136 |
| rp9 | 1.2689424 |
| Rpa1 | -0.6515056 |
| Rpa2 | -0.7666304 |
| Rpa2 | -0.6637584 |
| Rpa3 | 0.799023 |
| Rpain | 1.5945547 |
| Rpap1 | -0.6629369 |
| Rpap1 | 1.1238216 |
| Rpap2 | -0.6563889 |
| Rpap2 | 0.4609199 |
| Rpap3 | 1.087433 |
| Rpe | 0.9249195 |
| Rpgr | -0.0855693 |
| Rpgrip1 | 1.7955484 |
| Rpgrip1l | 2.8623762 |
| Rph3a | 0.5737171 |
| Rph3al | 2.4378376 |
| Rpia | 0.1176279 |
| Rpl12 | 0.1793181 |
| Rpl13a | -0.4551467 |
| Rpl18 | 0.256437 |
| Rpl19 | -0.5659312 |
| Rpl21 | -0.7611845 |
| Rpl21 | 1.4902531 |
| Rpl22 | 0.8532196 |
| Rpl22l1 | -0.6728394 |
| Rpl23 | 1.127584 |
| Rpl23 | 0.578044 |
| Rpl23 | 0.5118757 |
| Rpl24 | 0.206379 |
| Rpl27 | -0.4564841 |
| Rpl27a | -0.4438499 |
| Rpl28 | -0.5164473 |
| Rpl29 | -0.4877441 |
| Rpl29 | 0.2705269 |
| Rpl3 | 0.5635484 |
| Rpl30 | 0.9182657 |
| Rpl31 | -0.703999 |
| Rpl31 | -0.4618515 |
| Rpl34 | 2.7740683 |
| Rpl35 | -0.6458381 |
| Rpl36a | 0.4713725 |
| Rpl36al | -0.6008377 |
| Rpl37 | -0.61405 |
| Rpl38 | 0.1566242 |
| Rpl38 | -0.6078109 |
| Rpl39 | 0.7265431 |
| Rpl39l | -0.8412205 |
| Rpl3l | 2.1104562 |
| Rpl4 | -0.8570233 |
| Rpl41 | 0.5031294 |
| Rpl7a | -0.4645459 |
| Rpl7l1 | 0.639911 |
| Rpl7l1 | 0.6145542 |
| Rpl9 | -0.3249312 |
| Rplp1 | -0.6912504 |
| Rpn2 | 0.7223941 |
| Rpo1-1 | 0.1081164 |
| Rpo1-3 | 0.41433 |
| Rpo1-3 | 0.8666409 |
| Rpo1-4 | 3.1775056 |
| Rpo1-4 | 0.1266752 |
| Rpo1-4 | -0.6895627 |
| Rpo2tc1 | 2.3544897 |
| Rpp21 | 0.4859315 |
| Rpp25 | -0.0336601 |
| Rpp30 | 3.306603 |
| Rpp38 | 2.4660764 |
| Rpp40 | 1.5625395 |
| Rprm | -0.5489208 |
| Rprml | -0.3620175 |
| Rps10 | -0.1127995 |
| Rps11 | 1.9144716 |
| Rps12 | 0.1969726 |
| Rps13 | -0.1368789 |
| Rps14 | 0.6319842 |
| Rps15 | -0.3415112 |
| Rps15a | -0.7702774 |
| Rps16 | -0.7100039 |
| Rps19 | -0.2823814 |
| Rps19 | 0.9678648 |
| Rps19bp1 | -0.6878779 |
| Rps2 | 2.1617655 |
| Rps21 | 0.4636104 |
| Rps24 | 4.5581592 |
| Rps24 | 0.7842733 |
| Rps25 | -0.8471176 |
| Rps26 | -1.0386878 |
| Rps27a | -0.4352957 |
| Rps27a | 0.4772213 |
| Rps27l | -0.1909398 |
| Rps3 | 0.8250782 |
| Rps3 | 1.6181728 |
| Rps3a | -0.6778273 |
| Rps4x | -0.5065541 |
| Rps4y2 | 1.8175387 |
| Rps5 | -0.4995464 |
| Rps6 | -0.577936 |
| Rps6ka1 | 2.4393537 |
| Rps6ka3 | 3.8782098 |
| Rps6ka4 | -0.2897276 |
| Rps6ka5 | -0.6070337 |
| Rps6kb1 | 2.1684268 |
| Rps6kl1 | 0.2330703 |
| Rps7 | 1.257636 |
| Rps8 | -0.730868 |
| Rps9 | -0.3272832 |
| Rps9 | -0.1781654 |
| Rpsa | -0.4024209 |
| Rpusd1 | 1.122005 |
| Rpusd4 | -0.2458895 |
| Rqcd1 | -0.785926 |
| Rrad | 3.4522356 |
| Rragb | 0.7060482 |
| Rragc | -0.8324391 |
| Rragd | -0.4158281 |
| Rras | -0.4995464 |
| Rras2 | -0.6670511 |
| Rrbp1 | 0.1363857 |
| Rrbp1 | 1.155972 |
| Rreb1 | -0.1266954 |
| Rreb1 | -0.3086194 |
| Rrh | -0.6515056 |
| Rrm1 | -0.3261065 |
| Rrm2 | 1.8705704 |
| Rrm2 | -0.673669 |
| Rrm2b | -0.5178686 |
| Rrn3 | 1.2325068 |
| Rrp1 | -0.530751 |
| Rrp12 | 0.5008014 |
| Rrp15 | 0.1789619 |
| Rrp1b | -0.6498831 |
| Rrp1b | -0.1650495 |
| Rrp1b | 1.497411 |
| Rrp9 | -0.2383157 |
| Rs1 | -0.4591642 |
| Rsad1 | -0.5496544 |
| Rsad2 | 0.9988355 |
| Rsf1 | 2.0434673 |
| Rshl1 | -0.4491503 |
| Rshl2a | -0.1451986 |
| Rsl1d1 | 1.5784074 |
| Rsl1d1 | -0.0608615 |
| Rsn | 0.2357684 |
| Rsph1 | -0.311513 |
| Rspo1 | -0.385441 |
| Rspo2 | -0.0380694 |
| Rspo3 | 1.7591862 |
| Rspo4 | -0.5962156 |
| Rspry1 | -0.3973599 |
| Rsrc1 | 0.8355377 |
| Rsrc1 | 0.2256132 |
| Rsrc2 | 0.0762738 |
| Rsu1 | -0.6179692 |
| Rtbdn | -0.461179 |
| Rtel1 | 0.0173816 |
| Rtel1 | -0.6008377 |
| Rtkn | 0.9486159 |
| Rtn1 | 0.1692949 |
| Rtn1 | -0.3120927 |
| Rtn1 | 0.7016018 |
| Rtn1 | 2.4174537 |
| Rtn2 | -0.749485 |
| Rtn3 | -0.0608615 |
| Rtn4 | -0.8412205 |
| Rtn4 | -0.8945446 |
| Rtn4 | -0.0911431 |
| Rtn4ip1 | 1.5661474 |
| Rtn4r | 0.0490831 |
| Rtn4rl1 | -0.5445301 |
| Rtn4rl2 | 1.0444091 |
| Rtp1 | -0.554805 |
| Rtp4 | -0.6171841 |
| Rttn | -0.5726663 |
| Rufy1 | 0.5411578 |
| Rufy2 | 1.0049799 |
| Rufy3 | 3.0797053 |
| Rufy3 | 0.7510844 |
| Rufy3 | 1.2040314 |
| Rufy4 | -0.5503886 |
| Rundc1 | -0.5314716 |
| Rundc3b | -0.3923242 |
| Runx1 | -0.2480639 |
| Runx1t1 | 0.6301636 |
| Runx1t1 | -0.6337999 |
| Runx2 | 2.7564962 |
| Rusc1 | -0.5659312 |
| Rusc2 | -0.2426366 |
| Rusc2 | -0.5044467 |
| Ruvbl1 | 0.1624363 |
| Ruvbl1 | -0.5235738 |
| Ruvbl1 | -0.3748948 |
| Ruvbl2 | 1.3294713 |
| Rwdd1 | -0.610146 |
| Rwdd1 | 2.3151275 |
| Rwdd3 | -0.0473921 |
| Rwdd3 | -0.2222751 |
| Rwdd4a | -0.6353967 |
| Rxfp1 | 0.4919581 |
| Rxfp3 | -0.0726823 |
| Rxra | 0.1198973 |
| Rxrb | 0.0659637 |
| Rxrb | -0.2902949 |
| Rxrg | -0.2096239 |
| Rya3 | -0.3711987 |
| Ryk | 0.9562277 |
| Ryk | -0.5916145 |
| Ryr1 | 0.4835635 |
| Ryr1 | 3.7253332 |
| Ryr1 | 0.6734126 |
| Ryr3 | -0.5496544 |
| S100a1 | -0.1085256 |
| S100a10 | -0.7748548 |
| S100a11 | 0.1941685 |
| S100a13 | 1.0345429 |
| S100a16 | -0.3779852 |
| S100a4 | -0.6761619 |
| S100a6 | 0.526623 |
| S100a8 | -0.2874615 |
| S100a9 | -0.2628653 |
| S100b | -0.6498831 |
| S100pbp | 0.4708391 |
| S100pbp | 0.6401366 |
| S3-12 | 1.9271392 |
| Saa3 | -0.5696674 |
| Sac3d1 | -0.1471662 |
| Sacm1l | -0.0301466 |
| Sacs | -0.6629369 |
| Sae1 | 0.2317186 |
| Sae1 | 0.3412047 |
| Sae1 | -0.1914541 |
| Sag | 0.5608681 |
| Sall1 | 0.0380801 |
| Sall1 | -0.4385772 |
| Sall2 | -0.643419 |
| Sall4 | -0.2954151 |
| Sall4 | 2.4816313 |
| Samd10 | -0.515028 |
| Samd11 | -0.0376276 |
| Samd12 | -0.4094215 |
| Samd14 | 1.4666822 |
| Samd4 | -0.4126197 |
| Samd4 | 2.0156046 |
| Samd4b | 0.4391336 |
| Samd5 | -0.436607 |
| Samd7 | -0.7414642 |
| Samd8 | 0.4352721 |
| Samd9l | -0.6662269 |
| Samhd1 | 0.0478664 |
| Samm50 | 1.1019466 |
| Samsn1 | -0.4531439 |
| Sap130 | -0.6490728 |
| Sap30 | 2.2069243 |
| Sap30bp | 2.9947822 |
| Sap30l | -0.5824749 |
| Saps1 | 1.0314876 |
| Saps2 | 0.4160215 |
| Saps3 | 0.5278843 |
| Saps3 | 2.6669654 |
| Sar1a | -0.16005 |
| Sar1b | 0.4429803 |
| Sar1b | -0.5336363 |
| Sarm1 | -0.4036901 |
| Sars | -0.3589756 |
| Sars | 2.1771588 |
| Sars2 | -0.8520583 |
| Sart1 | -0.381085 |
| Sart3 | -0.436607 |
| Sas | 2.0930943 |
| Sass6 | -0.6353967 |
| Sat2 | -0.6385979 |
| Satb1 | 0.918608 |
| Sav1 | 2.4415858 |
| Sbds | -0.2361621 |
| Sbf1 | -0.1620469 |
| Sbk | -0.5023437 |
| Sbno1 | 0.2734512 |
| Sbno2 | 0.2750722 |
| Sc4mol | -0.3487012 |
| Sc5d | -0.2313334 |
| Scaf1 | -0.613268 |
| Scamp1 | 0.2441535 |
| Scamp2 | -0.5009441 |
| Scamp3 | -0.7100039 |
| Scamp4 | -0.4733529 |
| Scamp5 | 1.972432 |
| Scamp5 | -0.6531307 |
| Scand1 | 1.021957 |
| Scap | 0.8154573 |
| Scaper | 0.0478664 |
| Scara3 | -0.4458343 |
| Scara5 | 0.5637917 |
| Scarb1 | 1.9682712 |
| Scarb2 | -0.5802029 |
| Scarf1 | -0.3214136 |
| Scarf2 | 0.7856409 |
| Sccpdh | -0.6258534 |
| Scd1 | 0.0119297 |
| Scd2 | 1.3461823 |
| Scd4 | -0.3355584 |
| Scel | -0.5636961 |
| Scg2 | 0.326229 |
| Scg3 | -0.1610479 |
| Scg5 | 0.6421644 |
| Scgn | 1.8402268 |
| Scgn | -0.6828401 |
| Scgn | 2.360405 |
| Schip1 | 0.6401366 |
| Schip1 | 0.6935056 |
| Scin | 0.2901831 |
| scl0003251.1_21 | 2.0866132 |
| Sclt1 | -0.6234817 |
| Scly | -0.7521729 |
| Scmh1 | -0.5023437 |
| Scmh1 | 0.2646528 |
| Scml2 | 0.8807746 |
| Scml4 | -0.3992548 |
| Scn10a | 2.0237015 |
| Scn11a | -0.7169107 |
| Scn1a | 0.6657459 |
| Scn2b | 1.3658108 |
| Scn3b | -0.4196916 |
| Scn3b | 1.101804 |
| Scn3b | -0.2711832 |
| Scn3b | -0.4171143 |
| Scn4b | -0.809389 |
| Scn5a | -0.2372383 |
| Scn7a | -0.1878591 |
| Scn8a | 1.9368484 |
| Scnm1 | 0.4579521 |
| Scnm1 | 1.2828698 |
| Scnn1a | -0.8160577 |
| Scnn1b | 0.7347901 |
| Scnn1g | -0.381085 |
| Scoc | -0.6266453 |
| Scoc | 1.1699386 |
| Scotin | -0.1605489 |
| Scotin | -0.7352695 |
| Scotin | 0.0005113 |
| Scp2 | -0.2185688 |
| Scp2 | 2.0425696 |
| Scp2 | -0.0740552 |
| Scrg1 | -0.5540676 |
| Scrib | 1.8904779 |
| Scrib | 1.193924 |
| Scrn1 | -0.2064857 |
| Scrn1 | -0.105214 |
| Scrn1 | -0.3848175 |
| Scrn2 | -0.7602797 |
| Sct | -0.6687015 |
| Sctr | 3.9534496 |
| Scube1 | 1.4468127 |
| Scube2 | 0.6622215 |
| Scube3 | 0.8512092 |
| Scube3 | -0.6458381 |
| Scx | -0.6695277 |
| Scye1 | -0.1883719 |
| Scyl1 | 1.8467235 |
| Scyl1bp1 | 1.4804987 |
| Scyl3 | 4.6111202 |
| Scyl3 | -0.5143192 |
| Sdad1 | -0.6266453 |
| Sdc1 | 1.0403225 |
| Sdc1 | -0.3237573 |
| Sdc2 | 0.1718101 |
| Sdc3 | -0.1725955 |
| Sdc4 | 5.0464114 |
| Sdcbp | 3.1411187 |
| Sdcbp | 3.3402051 |
| Sdcbp2 | 0.2828167 |
| Sdcbp2 | -0.2291947 |
| Sdccag1 | 0.5720295 |
| Sdccag1 | -0.4856756 |
| Sdccag3 | -0.61405 |
| Sdccag3 | 0.2428166 |
| Sdf2 | -0.603931 |
| Sdf2 | 0.0119297 |
| Sdf4 | -0.7221221 |
| Sdhb | 0.1255487 |
| Sdhb | 2.4834456 |
| Sdhb | -0.5100763 |
| Sdhc | -0.4564841 |
| Sdhd | -0.1940298 |
| Sdk1 | 0.7335573 |
| Sdpr | -0.1645485 |
| Sds | -0.6490728 |
| Sdsl | -0.4518109 |
| Sec1 | -0.7082845 |
| Sec11a | -0.6047057 |
| Sec11c | 2.9545359 |
| Sec13 | 1.5288206 |
| Sec14l1 | 0.6917928 |
| Sec14l1 | 0.3320624 |
| Sec14l1 | -0.3841945 |
| Sec14l2 | 3.1751166 |
| Sec14l3 | 0.2643254 |
| Sec14l3 | -0.8560283 |
| Sec14l4 | -0.1565651 |
| Sec15l1 | 0.9141491 |
| Sec16a | 0.1081164 |
| Sec16b | 0.4191152 |
| Sec22a | -0.4478225 |
| Sec22b | -0.7896436 |
| Sec22c | 1.8822199 |
| Sec22c | -0.4584935 |
| Sec23a | -0.3028572 |
| Sec23ip | -0.0283944 |
| Sec24b | 0.4349957 |
| Sec24c | 0.5540132 |
| Sec24d | 2.9064387 |
| Sec61a1 | -0.5977539 |
| Sec61a2 | -0.5562814 |
| Sec61a2 | -0.7177774 |
| Sec61b | 2.3367003 |
| Sec61b | 0.0567546 |
| Sec61g | 0.1664128 |
| Sec63 | 0.7317053 |
| Sec63 | 1.4810867 |
| Secisbp2 | 0.2740999 |
| Sectm1b | -0.705711 |
| Seh1l | -0.8151023 |
| Seh1l | 0.1750347 |
| Sel1l | -0.0777254 |
| Sel1l | -0.3415112 |
| Sel1l | 0.2518068 |
| Sel1l2 | 1.6873173 |
| Sel1l2 | 0.7374559 |
| Selenbp2 | 0.610846 |
| Selk | 1.1547542 |
| Sell | 1.1577285 |
| Selp | -0.1344449 |
| Selplg | -0.577936 |
| Sema3a | -0.2734129 |
| Sema3b | -0.5659312 |
| Sema3b | -0.0763475 |
| Sema3b | 2.0204935 |
| Sema3e | -0.6466458 |
| Sema3f | 0.1039028 |
| Sema3f | 0.6958559 |
| Sema3g | -0.7441307 |
| Sema4a | 2.3875202 |
| Sema4b | -0.6695277 |
| Sema4d | 1.11766 |
| Sema4f | 0.184647 |
| Sema4g | 1.7430731 |
| Sema5a | -0.7548679 |
| Sema5a | -0.6242717 |
| Sema5b | 2.1370763 |
| Sema6b | -0.8074918 |
| Sema6b | 1.1156916 |
| Sema6b | -0.4425291 |
| Sema6d | 0.5916245 |
| Sema6d | -0.6474542 |
| Sema7a | -0.7831468 |
| Sema7a | -0.8422009 |
| Senp1 | -0.6887199 |
| Senp2 | 0.5039042 |
| Senp3 | 0.4330589 |
| Senp5 | -0.7877831 |
| Senp5 | 0.2692245 |
| Senp5 | -0.2201555 |
| Senp6 | 0.0806042 |
| Senp6 | -0.7822221 |
| Senp7 | 0.529144 |
| Senp7 | 0.5319099 |
| Senp7 | 2.0941082 |
| Senp8 | -0.7593757 |
| Sephs1 | -0.2329404 |
| Sephs2 | -0.3005615 |
| Sepm | 0.0571567 |
| Sepn1 | 1.6541218 |
| Sepp1 | 1.3304955 |
| Sepp1 | -0.2818186 |
| Sepsecs | -0.6803306 |
| Sept2 | -0.2437197 |
| Sept3 | 3.2044826 |
| Sept3 | 0.0228042 |
| Sept5 | -0.4538111 |
| Sept6 | 0.6405876 |
| Sept6 | -0.4385772 |
| Sept7 | 0.188537 |
| Sept8 | -0.6853558 |
| Sept9 | -0.015349 |
| Sept10 | 1.009212 |
| Sept10 | -0.7238652 |
| Sept12 | -0.2313334 |
| Sept15 | 0.8635734 |
| Sepw1 | -0.4815513 |
| Sepx1 | -0.0496245 |
| Sepx1 | -0.6426139 |
| Serac1 | -0.1223624 |
| Serbp1 | 2.1655986 |
| Serbp1 | -0.8046525 |
| Serbp1 | -0.7082845 |
| Serf1 | -0.7388046 |
| Serf2 | -0.8441647 |
| Sergef | -0.7999382 |
| Serhl | 0.1202751 |
| Serhl | 0.4049759 |
| Serinc1 | -0.705711 |
| Serinc2 | 2.2593347 |
| Serinc3 | 0.2882666 |
| Serpina1a | 3.022774 |
| Serpina1a | -0.4255149 |
| Serpina1b | 0.8141296 |
| Serpina1b | -0.6306138 |
| Serpina1d | 1.2460776 |
| Serpina1e | -0.0058885 |
| Serpina3b | -0.6353967 |
| Serpina3c | 0.9313608 |
| Serpina3g | -0.2475199 |
| Serpina3h | 1.0007301 |
| Serpina3k | -0.7896436 |
| Serpina3n | -0.8027642 |
| Serpina5 | 0.1255487 |
| Serpina5 | 3.0346806 |
| Serpina7 | -0.4974536 |
| Serpina9 | -0.2965565 |
| Serpinb11 | 0.2441535 |
| Serpinb12 | 1.5541921 |
| Serpinb1a | 0.9070774 |
| Serpinb3a | 0.0438003 |
| Serpinb6a | -0.2132975 |
| Serpinb6b | 0.4473587 |
| Serpinb6c | 0.6071241 |
| Serpind1 | -0.3493027 |
| Serpine2 | 0.4825092 |
| Serpinf1 | -0.5870344 |
| Serping1 | 0.092709 |
| Serpinh1 | -0.7108646 |
| Serpini1 | 1.0285765 |
| Sertad1 | 0.1270504 |
| Sertad2 | 0.8477272 |
| Sertad2 | -0.4692786 |
| Sertad3 | -0.5511233 |
| Sertad4 | 1.4452913 |
| Sesn1 | 0.0619701 |
| Sesn1 | 0.4679003 |
| Sesn2 | -0.7125884 |
| Sesn3 | -0.1873466 |
| Setd1b | -0.6662269 |
| Setd2 | -0.6393999 |
| Setd3 | 1.001676 |
| Setd4 | -0.6498831 |
| Setd4 | -0.6580219 |
| Setd5 | 3.5311037 |
| Setd6 | -0.8198883 |
| Setd6 | 0.3363391 |
| Setd7 | -0.5343589 |
| Setd7 | -0.168564 |
| Setd8 | 1.182793 |
| Setd8 | -0.8008793 |
| Setd8 | -0.6482631 |
| Setdb1 | 0.5594031 |
| Setdb2 | -0.0487309 |
| Setmar | -0.527156 |
| Setx | -0.408145 |
| Sez6 | -0.8761165 |
| Sez6l | -0.6179692 |
| Sez6l2 | -0.598524 |
| Sez6l2 | -0.4747147 |
| Sf1 | 2.6525378 |
| Sf1 | -0.6179692 |
| Sf3a1 | -0.125249 |
| Sf3a2 | 1.6440879 |
| Sf3a3 | 2.6570648 |
| Sf3b1 | -0.6314094 |
| Sf3b2 | -0.2623132 |
| Sf3b3 | 1.9489318 |
| Sf3b4 | 0.2357684 |
| Sf3b4 | -0.362627 |
| Sf3b5 | -0.2291947 |
| Sf4 | 1.1900094 |
| Sfi1 | -0.0398385 |
| Sfi1 | -0.2846361 |
| Sfmbt1 | 1.020566 |
| Sfmbt1 | 1.3249069 |
| Sfmbt1 | -0.1741115 |
| Sfmbt2 | -0.4055969 |
| Sfrp1 | -0.304007 |
| Sfrp2 | 0.8398023 |
| Sfrp4 | 1.5884086 |
| Sfrp5 | -0.0496245 |
| Sfrs1 | 2.656271 |
| Sfrs1 | -0.6377967 |
| Sfrs11 | 1.2981353 |
| Sfrs12 | 0.4644161 |
| Sfrs14 | 0.2866667 |
| Sfrs16 | 0.1721689 |
| Sfrs2 | -0.0559018 |
| Sfrs2 | 1.038349 |
| Sfrs2 | -0.72999 |
| Sfrs3 | 0.335119 |
| Sfrs3 | -0.6803306 |
| Sfrs4 | 1.3257072 |
| Sfrs5 | -0.7924408 |
| Sfrs5 | -0.3620175 |
| Sfrs6 | 4.6854282 |
| Sfrs7 | -0.0433861 |
| Sfrs8 | -0.2101479 |
| Sfrs9 | -0.6845165 |
| Sft2d2 | 0.7793739 |
| Sft2d2 | -0.6250622 |
| Sft2d2 | -0.703144 |
| Sftpc | 0.1157327 |
| Sftpd | -0.4320249 |
| Sfxn1 | 1.1858171 |
| Sfxn1 | -0.125249 |
| Sfxn3 | -0.6490728 |
| Sfxn3 | -0.5072575 |
| Sfxn4 | -0.6163997 |
| Sfxn4 | -0.515028 |
| Sfxn5 | 0.7555196 |
| Sgca | 0.8297611 |
| Sgca | 0.6228481 |
| Sgcb | 2.3663265 |
| Sgcb | -0.7134514 |
| Sgcz | -0.6482631 |
| Sgip1 | 1.4519681 |
| Sgip1 | 0.4806616 |
| Sgip1 | -0.3005615 |
| Sgip1 | -0.4645459 |
| Sgip1 | -0.1373664 |
| Sgk1 | -0.4815513 |
| Sgk2 | 1.6886737 |
| Sgk2 | -0.833411 |
| Sgk3 | 0.9139772 |
| Sgk3 | 0.4361008 |
| Sgk3 | -0.7414642 |
| Sgk3 | -0.6612959 |
| Sgk3 | 0.8366519 |
| Sgms2 | -0.657205 |
| Sgol1 | -0.244804 |
| Sgpl1 | 1.2982528 |
| Sgpl1 | -0.4005201 |
| Sgpp1 | 2.6347542 |
| Sgpp2 | -0.496757 |
| Sgsm1 | -0.7022898 |
| Sgsm2 | -0.4242179 |
| Sgsm3 | 1.0288833 |
| Sgta | 0.263015 |
| Sgtb | 0.0252971 |
| Sgtb | 1.6513124 |
| Sh2b1 | 0.0715284 |
| Sh2b1 | 0.9657441 |
| Sh2b3 | -0.7291127 |
| Sh2d2a | -0.5525944 |
| Sh3bgrl | 2.054955 |
| Sh3bgrl3 | -0.5343589 |
| Sh3bp2 | 0.4777514 |
| Sh3bp5 | -0.5607237 |
| Sh3bp5l | -0.6442247 |
| Sh3gl1 | 2.2081635 |
| Sh3gl2 | -0.8055981 |
| Sh3gl2 | 0.5123878 |
| Sh3gl2 | -0.6078109 |
| Sh3gl3 | 0.4838269 |
| Sh3glb1 | -0.4591642 |
| Sh3glb2 | -0.1276608 |
| Sh3kbp1 | 1.1293905 |
| Sh3kbp1 | -0.1580572 |
| Sh3kbp1 | -0.8343839 |
| Sh3kbp1 | 1.5698321 |
| Sh3md4 | 0.3314499 |
| Sh3md4 | -0.4753963 |
| Sh3pxd2a | 1.2278583 |
| Sh3pxd2b | -0.4679241 |
| Sh3rf1 | -0.6234817 |
| Sh3rf2 | -0.257358 |
| Sh3tc1 | -0.4005201 |
| Sh3tc2 | -0.3391259 |
| Sh3yl1 | 0.4676327 |
| Shank2 | -0.8590162 |
| Sharpin | -0.5164473 |
| Shbg | -0.6662269 |
| Shc1 | 1.0330164 |
| Shc1 | 1.7142598 |
| Shc3 | -0.2852006 |
| Shcbp1 | 1.7383024 |
| Shd | -0.5257216 |
| She | -0.6687015 |
| Shf | -0.5756742 |
| Shf | 1.4467113 |
| Shf | -0.4458343 |
| Shfm1 | -0.4392348 |
| Shh | 0.6529112 |
| Shisa2 | 0.1463718 |
| Shisa3 | 0.7510844 |
| Shisa4 | -0.3022828 |
| Shkbp1 | 1.7679214 |
| Shkbp1 | -0.7575702 |
| Shmt1 | -0.4760783 |
| Shmt2 | -0.0703983 |
| Shoc2 | 2.3331298 |
| Shox2 | 0.1771787 |
| Shprh | 1.4403056 |
| Shq1 | -0.4836113 |
| Shroom3 | 0.1482102 |
| Shroom3 | -0.6954821 |
| Si | -0.6054811 |
| Si | 0.7339684 |
| Siah1a | -0.6878779 |
| Siah1b | -0.4767608 |
| Siah1b | -0.5562814 |
| Siah1b | -0.8670273 |
| Siah1b | 1.8318217 |
| Siah2 | -0.1832557 |
| Siat7f | -0.1924836 |
| Siat9 | 1.520565 |
| Sidt1 | 0.5339166 |
| Sidt2 | -0.1417645 |
| Sidt2 | -0.2902949 |
| Sidt2 | 0.6549135 |
| Sigirr | -0.2806938 |
| Siglec1 | 0.3631109 |
| Siglech | -0.7022898 |
| Siglech | -0.2291947 |
| Sil1 | 0.1685752 |
| Sin3a | -0.7593757 |
| Sin3b | -0.4638716 |
| Sip1 | 1.1527213 |
| Sip1 | -0.1127995 |
| Sipa1 | -0.3103546 |
| Sipa1l1 | 0.3047565 |
| Sirpa | -0.5314716 |
| Sirpa | 0.540909 |
| Sirpb1 | -0.620328 |
| Sirt1 | -0.515028 |
| Sirt2 | -0.4884345 |
| Sirt3 | 1.2871532 |
| Sirt5 | -0.6211155 |
| Sirt5 | 2.0247305 |
| Sirt6 | -0.2404738 |
| Sirt7 | -0.3045824 |
| Siva1 | 0.5453786 |
| Six1 | 0.7914811 |
| Six2 | 0.003908 |
| Six3 | 1.8653642 |
| Six5 | -0.5459915 |
| Six6os1 | 2.1655986 |
| Skap2 | -0.4385772 |
| Skil | 0.031915 |
| Skiv2l | -0.057252 |
| Skiv2l | -0.6008377 |
| Skiv2l2 | -0.6070337 |
| Skp1a | 1.3720514 |
| Skp2 | 0.5547499 |
| Skp2 | 1.4355 |
| Skp2 | 4.7410512 |
| Sla | 0.4391336 |
| Slain1 | -0.6878779 |
| Slain2 | -0.091609 |
| Slamf7 | 1.5591 |
| Slamf8 | -0.0354215 |
| Slamf9 | -0.2265277 |
| Slc10a3 | 0.2156984 |
| Slc10a4 | 1.1813435 |
| Slc10a6 | -0.6274377 |
| Slc10a6 | -0.0354215 |
| Slc10a7 | 1.4053312 |
| Slc10a7 | -0.7757728 |
| Slc10a7 | -0.3669038 |
| Slc11a1 | -0.6410056 |
| Slc11a1 | 1.6931289 |
| Slc11a1 | 0.7970946 |
| Slc11a1 | 2.7231537 |
| Slc11a2 | 0.1793181 |
| Slc12a2 | -0.4075074 |
| Slc12a2 | 2.396254 |
| Slc12a3 | -0.3848175 |
| Slc12a5 | -0.7450211 |
| Slc12a6 | -0.7849988 |
| Slc12a7 | -0.2372383 |
| Slc12a8 | -0.6124866 |
| Slc12a9 | 0.2656342 |
| Slc12a9 | -0.6753303 |
| Slc13a2 | -0.8122416 |
| Slc13a3 | -0.3091975 |
| Slc13a3 | -0.4248662 |
| Slc13a4 | 0.3589464 |
| Slc14a1 | 2.522347 |
| Slc14a1 | 0.5503217 |
| Slc14a1 | 0.0077157 |
| Slc14a2 | -0.6078109 |
| Slc14a2 | -0.7702774 |
| Slc15a2 | -0.7648118 |
| Slc15a3 | -0.5143192 |
| Slc15a4 | -0.2410141 |
| Slc15a4 | -0.1315318 |
| Slc16a10 | -0.5030442 |
| Slc16a11 | 2.0913844 |
| Slc16a4 | 1.1165357 |
| Slc16a6 | -0.4631978 |
| Slc16a6 | -0.7702774 |
| Slc16a6 | 2.4016155 |
| Slc16a8 | -0.0367446 |
| Slc16a9 | -0.6450311 |
| Slc17a2 | 0.1448986 |
| Slc17a5 | -0.613268 |
| Slc17a6 | -0.1175696 |
| Slc17a7 | 0.5904405 |
| Slc17a8 | -0.5452606 |
| Slc18a2 | 1.7532954 |
| Slc19a1 | -0.4645459 |
| Slc19a2 | -0.749485 |
| Slc1a1 | 1.9716494 |
| Slc1a1 | 1.8945167 |
| Slc1a2 | -0.7675409 |
| Slc1a2 | -0.1359046 |
| Slc1a2 | 1.5109107 |
| Slc1a2 | 0.0655651 |
| Slc1a3 | 1.7526221 |
| Slc1a4 | -0.7657207 |
| Slc1a6 | -0.5423419 |
| Slc1a7 | 1.8424883 |
| Slc20a1 | -0.3184915 |
| Slc22a1 | 0.0806042 |
| Slc22a12 | -0.3173251 |
| Slc22a13 | -0.3063106 |
| Slc22a14 | -0.8324391 |
| Slc22a17 | -0.8141479 |
| Slc22a17 | 1.0715317 |
| Slc22a18 | 2.9682576 |
| Slc22a21 | -0.1570622 |
| Slc22a3 | 2.7833469 |
| Slc22a4 | -0.6219036 |
| Slc22a5 | -0.673669 |
| Slc22a5 | -0.3761298 |
| Slc22a5 | -0.5885588 |
| Slc22a6 | 1.0008878 |
| Slc22a7 | -0.3823277 |
| Slc22a8 | 0.1058203 |
| Slc23a2 | 0.029025 |
| Slc23a3 | 1.7607463 |
| Slc24a1 | 0.124421 |
| Slc24a3 | -0.2469761 |
| Slc24a4 | -0.6282308 |
| Slc24a5 | -0.1094738 |
| Slc24a6 | 1.2589799 |
| Slc25a1 | 1.0632637 |
| Slc25a1 | -0.7022898 |
| Slc25a10 | 2.6572479 |
| Slc25a11 | -0.4932809 |
| Slc25a11 | -0.2404738 |
| Slc25a11 | 0.4840902 |
| Slc25a12 | 1.92506 |
| Slc25a14 | -0.6016101 |
| Slc25a15 | 0.7685107 |
| Slc25a15 | 0.9264488 |
| Slc25a16 | -0.4242179 |
| Slc25a17 | -0.7943099 |
| Slc25a18 | -0.7666304 |
| Slc25a19 | 0.4673651 |
| Slc25a19 | 1.7347285 |
| Slc25a20 | 2.4564036 |
| Slc25a20 | 0.1262998 |
| Slc25a23 | -0.8046525 |
| Slc25a24 | -0.4359512 |
| Slc25a24 | 0.4924804 |
| Slc25a25 | -0.4558152 |
| Slc25a26 | -0.7264855 |
| Slc25a27 | 0.5705806 |
| Slc25a28 | -0.9207109 |
| Slc25a29 | 1.4397954 |
| Slc25a3 | -0.1334729 |
| Slc25a3 | -0.2818186 |
| Slc25a30 | -0.703144 |
| Slc25a32 | 0.1278004 |
| Slc25a33 | -0.6523178 |
| Slc25a34 | 0.4341661 |
| Slc25a35 | -0.1981647 |
| Slc25a36 | 1.4934514 |
| Slc25a37 | -0.7335066 |
| Slc25a38 | -0.0694861 |
| Slc25a38 | 3.9457839 |
| Slc25a38 | 1.9057015 |
| Slc25a39 | -0.2874615 |
| Slc25a41 | -0.2874615 |
| Slc25a42 | 1.0366761 |
| Slc25a44 | 1.4379568 |
| Slc25a45 | 1.6215874 |
| Slc25a46 | 0.7064707 |
| Slc25a5 | -0.720382 |
| Slc26a4 | -0.1997197 |
| Slc26a6 | -0.4196916 |
| Slc26a7 | -0.657205 |
| Slc26a8 | -0.8373082 |
| Slc27a1 | -0.1180478 |
| Slc27a2 | 2.5290914 |
| Slc27a3 | -0.5877963 |
| Slc27a4 | 0.0735084 |
| Slc27a6 | -0.719513 |
| Slc29a1 | -0.8018213 |
| Slc29a2 | -0.5086659 |
| Slc29a3 | -0.6078109 |
| Slc29a4 | 2.3830847 |
| Slc2a1 | 0.837765 |
| Slc2a10 | -0.6912504 |
| Slc2a10 | -0.4524772 |
| Slc2a13 | -0.1180478 |
| Slc2a3 | 1.1284182 |
| Slc2a4 | -0.5143192 |
| Slc2a5 | -0.707426 |
| Slc2a6 | -0.7343877 |
| Slc2a8 | -0.1817259 |
| Slc2a8 | -0.0763475 |
| Slc2a8 | 1.6692723 |
| Slc2a9 | 0.0081379 |
| Slc30a1 | 0.9703062 |
| Slc30a10 | -0.3835718 |
| Slc30a3 | 0.5942244 |
| Slc30a5 | 1.4960607 |
| Slc30a5 | -0.6769943 |
| Slc30a6 | -0.0058885 |
| Slc30a7 | 3.0559658 |
| Slc30a7 | 1.4055424 |
| Slc30a9 | -0.3296406 |
| Slc31a1 | -0.169067 |
| Slc31a2 | 0.4932634 |
| Slc32a1 | 0.4432545 |
| Slc34a2 | 0.0131904 |
| Slc35a1 | 0.0938728 |
| Slc35a2 | 0.5243488 |
| Slc35a3 | -0.3650687 |
| Slc35a4 | 0.7119465 |
| Slc35a5 | 1.5185849 |
| Slc35a5 | 1.6075137 |
| Slc35b1 | 0.1195194 |
| Slc35b2 | 0.4830365 |
| Slc35b3 | 0.6668447 |
| Slc35b3 | -0.7785317 |
| Slc35b3 | -0.6498831 |
| Slc35b4 | -0.3761298 |
| Slc35c1 | -0.4248662 |
| Slc35c1 | 1.9986858 |
| Slc35c2 | -0.8027642 |
| Slc35c2 | -0.3325952 |
| Slc35c2 | -0.3255187 |
| Slc35e1 | -0.2700701 |
| Slc35e3 | -0.5467231 |
| Slc35e4 | -0.1228429 |
| Slc35f1 | 0.4780163 |
| Slc35f2 | 2.0749935 |
| Slc35f3 | -0.0663 |
| Slc35f4 | -0.5877963 |
| Slc35f4 | -0.5893219 |
| Slc36a1 | -0.3589756 |
| Slc36a3 | 0.7647306 |
| Slc37a1 | -0.4498148 |
| Slc37a3 | -0.4184021 |
| Slc38a1 | 2.145424 |
| Slc38a10 | -0.7748548 |
| Slc38a11 | -0.5749214 |
| Slc38a2 | 0.1927636 |
| Slc38a2 | -0.5962156 |
| Slc38a2 | -0.7108646 |
| Slc38a3 | -0.007602 |
| Slc38a4 | 0.1217846 |
| Slc38a5 | -0.2313334 |
| Slc38a8 | 0.5645213 |
| Slc38a9 | 0.8603152 |
| Slc38a9 | -0.7620901 |
| Slc39a10 | -0.6786611 |
| Slc39a11 | 0.9544127 |
| Slc39a12 | 0.3854227 |
| Slc39a13 | -0.5916145 |
| Slc39a13 | -0.304007 |
| Slc39a14 | -0.1349312 |
| Slc39a3 | 0.1598977 |
| Slc39a3 | -0.6482631 |
| Slc39a4 | -0.7169107 |
| Slc39a5 | -0.2491529 |
| Slc39a5 | 0.3163276 |
| Slc39a5 | 0.1363857 |
| Slc39a6 | -0.568919 |
| Slc39a7 | -0.6393999 |
| Slc39a7 | -0.4405512 |
| Slc39a7 | -0.581717 |
| Slc39a8 | -0.2388548 |
| Slc3a2 | 0.7129962 |
| Slc40a1 | -0.3589756 |
| Slc41a2 | -0.2356245 |
| Slc41a3 | 1.274849 |
| Slc41a3 | -0.3385304 |
| Slc41a3 | -0.7273605 |
| Slc43a2 | 1.2072496 |
| Slc43a2 | -0.5585001 |
| Slc44a1 | -0.1061591 |
| Slc44a1 | 3.3708306 |
| Slc44a2 | -0.5214306 |
| Slc44a2 | 0.9659074 |
| Slc44a2 | -0.3132531 |
| Slc44a3 | 0.726957 |
| Slc44a4 | 2.7389784 |
| Slc45a1 | -0.7620901 |
| Slc45a2 | 0.1718101 |
| Slc45a3 | -0.5939124 |
| Slc45a4 | -0.1199631 |
| Slc46a1 | 0.9355762 |
| Slc46a3 | -0.5171577 |
| Slc47a1 | -0.2222751 |
| Slc4a1 | -0.7108646 |
| Slc4a1ap | -0.015349 |
| Slc4a2 | 1.8066744 |
| Slc4a3 | -0.4445109 |
| Slc4a3 | -0.7757728 |
| Slc4a3 | -0.2222751 |
| Slc4a3 | 1.4814784 |
| Slc4a8 | -0.3954686 |
| Slc4a8 | -0.4151856 |
| Slc5a4a | -0.5100763 |
| Slc5a4b | -0.5065541 |
| Slc5a5 | 0.9380969 |
| Slc5a6 | -0.6148326 |
| Slc5a7 | 1.7414853 |
| Slc5a8 | 1.9598977 |
| Slc5a9 | 0.960833 |
| Slc6a1 | 0.1315422 |
| Slc6a11 | -0.4326782 |
| Slc6a12 | -0.3798439 |
| Slc6a13 | -0.730868 |
| Slc6a15 | -0.4119792 |
| Slc6a15 | 0.9806571 |
| Slc6a17 | -0.7877831 |
| Slc6a2 | 1.4160455 |
| Slc6a4 | 0.019888 |
| Slc6a6 | -0.4307195 |
| Slc6a7 | -0.5336363 |
| Slc6a8 | -0.6258534 |
| Slc6a9 | -0.2091002 |
| Slc7a1 | 0.1609864 |
| Slc7a10 | -0.4953651 |
| Slc7a11 | -0.7887129 |
| Slc7a14 | 3.1580659 |
| Slc7a14 | 0.792645 |
| Slc7a14 | 1.0270409 |
| Slc7a2 | 2.939034 |
| Slc7a3 | -0.2513344 |
| Slc7a4 | -0.2185688 |
| Slc7a6 | -0.152102 |
| Slc7a6os | -0.719513 |
| Slc7a7 | 0.250148 |
| Slc7a7 | 0.3883221 |
| Slc7a8 | 0.4405091 |
| Slc7a8 | 2.1525462 |
| Slc8a1 | 0.0774567 |
| Slc8a2 | -0.5666773 |
| Slc8a2 | 1.1329939 |
| Slc9a1 | 2.1321575 |
| Slc9a10 | -0.7639038 |
| Slc9a3r1 | -0.4352957 |
| Slc9a3r2 | 0.6967092 |
| Slc9a4 | -0.8510682 |
| Slc9a6 | -0.9101628 |
| Slc9a6 | 1.3025885 |
| Slc9a8 | -0.3080417 |
| Slc9a8 | 1.7502996 |
| Slc9a8 | -0.5629522 |
| Slc9a8 | 2.7085857 |
| Slc9a9 | 0.257756 |
| Slco1a4 | -0.5164473 |
| Slco1c1 | 2.3981496 |
| Slco2a1 | -0.1266954 |
| Slco2b1 | -0.8275933 |
| Slco3a1 | -0.5809597 |
| Slco3a1 | -0.3314123 |
| Slco3a1 | -0.7766916 |
| Slco4a1 | 1.0081165 |
| Slfnl1 | -0.923897 |
| Slit1 | 0.046242 |
| Slit2 | -0.7999382 |
| Slit3 | 0.300975 |
| Slitrk1 | 0.9369214 |
| Slitrk3 | -0.8893918 |
| Slitrk4 | 0.7388884 |
| Slitrk5 | -0.0491776 |
| Slitrk5 | -0.719513 |
| Slitrk6 | 0.3607333 |
| Slk | 0.0056021 |
| Slk | 1.9380944 |
| Slmap | 1.6500705 |
| Slmap | -0.5540676 |
| Slmap | -0.3320036 |
| Slmo1 | 0.1289244 |
| Slmo2 | -0.0345405 |
| Sln | -0.2201555 |
| Slpi | 1.3708501 |
| Sltm | 0.4774864 |
| Sltm | -0.5408857 |
| Slu7 | -0.4017869 |
| Slu7 | 2.207115 |
| Sly | 3.3826556 |
| Smad1 | 0.0068708 |
| Smad1 | -0.2925673 |
| Smad2 | 1.6692723 |
| Smad3 | 2.5800996 |
| Smad5 | 0.2702015 |
| Smad5 | 1.0474255 |
| Smad6 | -0.7831468 |
| Smap2 | -0.5336363 |
| Smap2 | -0.5314716 |
| Smap2 | 2.2227288 |
| Smarca1 | -0.5607237 |
| Smarca2 | 1.4978928 |
| Smarca2 | -0.9475744 |
| Smarca4 | -0.257358 |
| Smarcad1 | -0.0581532 |
| Smarcal1 | -0.1746174 |
| Smarcb1 | -0.2601079 |
| Smarcc1 | 0.3469523 |
| Smarcc1 | -0.0279568 |
| Smarcc2 | 1.074175 |
| Smarcd1 | 2.1232843 |
| Smarcd1 | 0.9427856 |
| Smarcd1 | 3.7593811 |
| Smarcd2 | 1.2957837 |
| Smarcd3 | 1.1448219 |
| Smarce1 | -0.577936 |
| Smarce1 | 1.0371326 |
| Smc1a | 0.1558954 |
| Smc1a | -0.5644406 |
| Smc1a | -0.7530704 |
| Smc1a | -0.7238652 |
| Smc1b | -0.3705841 |
| Smc3 | 4.1914666 |
| Smc5l1 | -0.6337999 |
| Smc6 | 0.1821637 |
| Smchd1 | 0.5396639 |
| Smcp | -0.4631978 |
| Smcr7 | 2.2501929 |
| Smcr7l | 1.3289019 |
| Smek1 | -0.0080308 |
| Smek2 | -0.6171841 |
| Smek2 | 3.2463925 |
| Smg5 | 0.5854526 |
| Smg6 | -0.515028 |
| Smn1 | 0.3332861 |
| Smo | 1.6006636 |
| Smo | -0.3904423 |
| Smo | -0.3493027 |
| Smoc1 | 2.1563143 |
| Smox | -0.2313334 |
| Smox | 0.0470545 |
| Smox | 1.3532126 |
| Smox | 1.8828778 |
| Smox | 1.7015873 |
| Smpd1 | 3.8043724 |
| Smpd1 | 2.4105015 |
| Smpd1 | 1.0535812 |
| Smpd2 | 0.6747211 |
| Smpd3 | 2.1155339 |
| Smpd4 | 0.2091493 |
| Smpdl3a | 0.3722715 |
| Smpdl3b | -0.2356245 |
| Smpx | 1.0992322 |
| Smtn | 2.0905818 |
| Smtn | -0.0667546 |
| Smtn | 2.030087 |
| Smtnl1 | 2.6452767 |
| Smtnl2 | -0.4326782 |
| Smu1 | -0.4379201 |
| Smu1 | 0.866821 |
| Smug1 | 0.1434232 |
| Smurf1 | 1.0073333 |
| Smyd1 | 0.8594083 |
| Smyd2 | -0.603931 |
| Smyd2 | -0.6482631 |
| Smyd3 | 2.5683375 |
| Smyd4 | 0.3511665 |
| Smyd5 | -0.4747147 |
| Smyd5 | -0.2706265 |
| Snai1 | -0.3730451 |
| Snai3 | 1.7286161 |
| Snap23 | 0.4861943 |
| Snap29 | 0.5471115 |
| Snap91 | -0.5681712 |
| Snap91 | 0.3129014 |
| Snapc1 | 0.0707353 |
| Snapc3 | -0.6523178 |
| Snapc3 | 1.244964 |
| Snapc3 | 0.1456354 |
| Snapc3 | 1.1761875 |
| Snapc4 | 1.3752116 |
| Snapin | -0.7666304 |
| Snca | 0.7060482 |
| Snca | 1.2108418 |
| Snca | -0.3891897 |
| Sncaip | -0.0024702 |
| Sncg | -0.4652206 |
| Sncg | 4.7886406 |
| Snd1 | 0.2824952 |
| Snf1lk | 1.4543851 |
| Snf1lk2 | 0.2245921 |
| Snf8 | 0.2962281 |
| Snip1 | 0.5351688 |
| Snip1 | -0.5592407 |
| Snn | 2.5524834 |
| Snora65 | -0.6322056 |
| Snph | -0.7335066 |
| Snrk | -0.0667546 |
| Snrk | 0.1430541 |
| Snrk | 3.1997185 |
| Snrp70 | 0.3129014 |
| Snrpa | 0.5401621 |
| Snrpb | -0.6258534 |
| Snrpb | -0.8353577 |
| Snrpd1 | -0.6274377 |
| Snrpd1 | -0.4974536 |
| Snrpd2 | -0.4392348 |
| Snrpd3 | 0.280564 |
| Snrpg | 0.1569885 |
| Snta1 | -0.4106995 |
| Sntb2 | -0.4988484 |
| Sntb2 | -0.1580572 |
| Sntg2 | -0.6637584 |
| Snupn | 2.2905842 |
| Snurf | 2.6247827 |
| Snurf | 1.2827505 |
| Snx1 | -0.7476971 |
| Snx10 | 0.050703 |
| Snx11 | -0.3711987 |
| Snx12 | -0.2233366 |
| Snx13 | 0.2708523 |
| Snx14 | 1.2639735 |
| Snx14 | -0.1705777 |
| Snx14 | -0.7125884 |
| Snx15 | -0.657205 |
| Snx16 | 0.1850012 |
| Snx17 | -0.3736612 |
| Snx17 | 0.2776603 |
| Snx18 | 0.3481582 |
| Snx19 | 1.3914016 |
| Snx19 | -0.5489208 |
| Snx2 | 1.7236993 |
| Snx2 | -0.5401584 |
| Snx20 | -0.4652206 |
| Snx21 | -0.3403178 |
| Snx22 | 3.6744159 |
| Snx24 | -0.4699565 |
| Snx25 | 0.4369288 |
| Snx25 | -0.6000659 |
| Snx26 | 1.9468353 |
| Snx29 | -0.5314716 |
| Snx3 | -0.0157811 |
| Snx30 | -0.6988802 |
| Snx33 | -0.2070081 |
| Snx4 | 0.7454113 |
| Snx6 | -0.6314094 |
| Snx7 | 0.1172491 |
| Snx8 | -0.5093708 |
| Snx9 | -0.4094215 |
| Soat1 | 1.6979596 |
| Socs2 | 2.4799592 |
| Socs3 | -0.3403178 |
| Socs4 | -0.417758 |
| Socs5 | -0.4385772 |
| Socs6 | 0.1054371 |
| Socs7 | 0.051512 |
| Socs7 | 0.0746945 |
| Sod1 | 0.1660519 |
| Sod2 | -0.6490728 |
| Sod3 | 0.388901 |
| Sohlh1 | 1.7706449 |
| Sohlh1 | -0.1185263 |
| Son | 0.5471115 |
| Sorbs1 | -0.5908497 |
| Sorbs2 | 3.1638431 |
| Sorbs3 | -0.420337 |
| Sorcs1 | 1.217861 |
| Sorcs1 | -0.6555733 |
| Sorcs2 | -0.3450998 |
| Sorcs3 | 2.7155216 |
| Sorcs3 | -0.5533307 |
| Sord | 1.1987312 |
| Sorl1 | 0.6686003 |
| Sort1 | -0.4740336 |
| Sort1 | -0.6687015 |
| Sort1 | -0.6266453 |
| Sort1 | 0.8871501 |
| Sostdc1 | 0.9719306 |
| Sox12 | 1.2701507 |
| Sox13 | -0.6612959 |
| Sox17 | 2.0335479 |
| Sox17 | -0.7379197 |
| Sox18 | 0.2518068 |
| Sox2 | 0.7325289 |
| Sox2 | 0.9915397 |
| Sox21 | 0.9105332 |
| Sox21 | 0.2317186 |
| Sox30 | 0.5892551 |
| Sox5 | -0.2196263 |
| Sox5 | -0.4281139 |
| Sox5 | -0.5264386 |
| Sox5 | 0.4352721 |
| Sox7 | -0.385441 |
| Sox7 | 0.201164 |
| Sox8 | -0.0925414 |
| Sox9 | 1.633869 |
| Sp100 | 2.6283059 |
| Sp140 | 1.8202716 |
| Sp2 | 2.8639186 |
| Sp4 | 1.6106199 |
| Sp5 | -0.3208285 |
| Sp6 | 0.9693303 |
| Sp7 | 2.774503 |
| Sp8 | -0.613268 |
| Sp9 | 0.4703054 |
| Spaca3 | 2.1213133 |
| Spaca4 | 1.8133903 |
| Spag1 | 3.0524665 |
| Spag1 | 1.1239612 |
| Spag16 | 0.0005113 |
| Spag16 | 0.7870066 |
| Spag4 | 1.6360555 |
| Spag4 | -0.5969845 |
| Spag4l | 0.6716654 |
| Spag4l | 0.2067257 |
| Spag5 | 0.355064 |
| Spag5 | -0.4106995 |
| Spag6 | 0.0535316 |
| Spag6 | -0.7212516 |
| Spag7 | 0.887856 |
| Spag8 | -0.0136223 |
| Spag9 | 0.6394597 |
| Spag9 | -0.7134514 |
| Spag9 | -0.0131911 |
| Spag9 | -0.4870541 |
| Spam1 | -0.0786451 |
| Spam1 | 0.2194707 |
| Sparc | -0.1334729 |
| Sparc | -0.6596576 |
| Sparcl1 | -0.6845165 |
| Spast | -0.4281139 |
| Spast | -0.6728394 |
| Spast | 0.4814538 |
| Spata16 | -0.4598353 |
| Spata16 | 0.7838823 |
| Spata17 | -0.3761298 |
| Spata17 | 0.5727531 |
| Spata19 | 0.4188343 |
| Spata2 | 0.7472383 |
| Spata20 | -0.244804 |
| Spata20 | -0.6604764 |
| Spata21 | -0.1680612 |
| Spata22 | 1.0351528 |
| Spata2L | 3.2876103 |
| Spata3 | 0.6956425 |
| Spata3 | -0.6687015 |
| Spata3 | 0.7378654 |
| Spata3 | -0.276207 |
| Spata4 | -0.7238652 |
| Spata6 | 0.5334153 |
| Spata7 | -0.0033237 |
| Spata7 | 0.8442332 |
| Spatc1 | -0.1056864 |
| Spatc1 | -0.461179 |
| Spats1 | -0.5474551 |
| Spc24 | -0.2790089 |
| Spc24 | 0.1008272 |
| Spc25 | -0.304007 |
| Spc25 | -0.6637584 |
| Spcs1 | -0.1320168 |
| Spcs1 | -0.0814091 |
| Spcs3 | 0.3345084 |
| Spdef | 0.1888899 |
| Specc1l | 1.0201019 |
| Speer1-ps1 | -0.1725955 |
| Speer1-ps1 | 0.0081379 |
| Speer2 | 0.6131652 |
| Speer2 | -0.1373664 |
| Speer3 | 1.2284878 |
| Speer4b | -0.3126727 |
| Speer4d | -0.6234817 |
| Speer4f | 1.54154 |
| Speer6-ps1 | -0.0319018 |
| Spef1 | 0.5366694 |
| Spen | -0.3450998 |
| Spert | -0.6539443 |
| Spesp1 | -0.643419 |
| Spg20 | -0.3687422 |
| Spg21 | 0.8756092 |
| Spg7 | 2.4430963 |
| Spg7 | 1.864427 |
| Sphk1 | 1.1246589 |
| Sphk2 | 0.6600126 |
| Sphk2 | -0.4538111 |
| Spic | 1.7019021 |
| Spin | -0.5562814 |
| Spin2 | -0.603931 |
| Spink10 | -0.6450311 |
| Spink2 | -0.6588394 |
| Spink4 | -0.6290245 |
| Spink8 | 1.3742319 |
| Spinlw1 | -0.6282308 |
| Spinlw1 | -0.4767608 |
| Spinlw1 | 1.9397119 |
| Spint1 | 2.7451219 |
| Spint2 | 0.7553184 |
| Spire1 | -0.0545534 |
| Spire1 | 0.1019817 |
| Spire1 | 0.4066831 |
| Spire2 | 1.5520944 |
| Spna1 | -0.214876 |
| Spnb1 | 0.9593551 |
| Spnb2 | 0.8563187 |
| Spnb2 | 1.3117838 |
| Spnb2 | -0.2790089 |
| Spnb2 | 1.4939351 |
| Spnb3 | -0.3687422 |
| Spnb4 | -0.817014 |
| Spns1 | -0.4974536 |
| Spo11 | 1.5971774 |
| Spo11 | 2.9335039 |
| Spock1 | 0.7815326 |
| Spock2 | -0.6555733 |
| Spock3 | 1.2484246 |
| Spon1 | 0.6127018 |
| Spon2 | -0.5235738 |
| Spop | 2.0276402 |
| Spp1 | -0.6912504 |
| Spp2 | -0.4829242 |
| Sppl3 | -0.515028 |
| Spr | 1.1211655 |
| Spred1 | 1.9985685 |
| Spred2 | 1.158673 |
| Spred2 | 2.5041144 |
| Sprn | 0.7589335 |
| Sprn | 0.4297299 |
| Sprr2a | 1.3228462 |
| Sprr2a | 1.4773573 |
| Sprr2f | -0.6612959 |
| Spry3 | -0.5214306 |
| Spry4 | 2.5225214 |
| Spryd4 | 1.0483287 |
| Spsb1 | -0.6794955 |
| Spsb2 | -0.2106721 |
| Spsb3 | -0.4960608 |
| Spsb3 | -0.267293 |
| Spsb4 | -0.3080417 |
| Sptlc1 | -0.5379797 |
| Sptlc2 | -0.6109256 |
| Spz1 | -0.4385772 |
| Sqle | 0.4355484 |
| Sqle | 1.7235453 |
| Sqrdl | -0.5734174 |
| Sqstm1 | -0.5992946 |
| Sra1 | -0.4781272 |
| Sra1 | -0.4339861 |
| Src | 1.3897942 |
| Srd5a1 | -0.6322056 |
| Srd5a2 | 0.8086104 |
| Srd5a3 | 2.3327089 |
| Srd5a3 | 0.5157104 |
| Srd5a3 | -0.7108646 |
| Srebf1 | -0.2971276 |
| Srebf2 | 1.6813916 |
| Srf | 1.1121669 |
| Srf | 2.3168824 |
| Srfbp1 | -0.5030442 |
| Srgn | -0.3120927 |
| Srgn | 0.2418127 |
| Sri | -0.2070081 |
| Sri | -0.567424 |
| Sri | -0.105214 |
| Srm | -0.169067 |
| Srm | 0.684265 |
| Srm | 2.1742073 |
| Srp14 | 3.4311261 |
| Srp19 | -0.2846361 |
| Srp19 | -0.7169107 |
| Srp54 | 1.3976991 |
| Srp68 | 2.7483888 |
| Srp72 | -0.7048546 |
| Srp9 | 0.2567669 |
| Srp9 | -0.5969845 |
| Srpk1 | 0.428896 |
| Srpk2 | 0.8626694 |
| Srpk3 | 0.2987625 |
| Srpr | -0.1720907 |
| Srprb | 1.1400249 |
| Srprb | -0.6912504 |
| Srpx | -0.5459915 |
| Srpx | -0.0332203 |
| Srpx2 | 0.4517182 |
| Srpx2 | 1.3388771 |
| Srpx2 | -0.5416136 |
| Srr | 1.7553872 |
| Srrd | -0.2717401 |
| Srrm1 | 1.3139829 |
| Srrm2 | -0.6654034 |
| Srrm2 | 0.366075 |
| Srrp | 1.9556847 |
| Srxn1 | -0.6929409 |
| Srxn1 | 0.346047 |
| Srxn1 | -0.1945458 |
| Ss18 | 2.31825 |
| Ss18 | 0.360138 |
| Ss18l1 | -0.152597 |
| Ss18l1 | 1.9481307 |
| Ss18l1 | 1.0906122 |
| Ssbp1 | 0.8416509 |
| Ssbp2 | -0.5525944 |
| Ssbp2 | 0.8256413 |
| Ssbp3 | 0.2721526 |
| Ssbp3 | 1.9264466 |
| Ssbp4 | -0.6870365 |
| Ssfa2 | -0.4794954 |
| Ssh2 | 2.6556293 |
| Ssh3 | 0.5281364 |
| Ssh3 | 1.4693616 |
| Ssna1 | 0.4372047 |
| Sspn | 1.0573162 |
| Ssr2 | -0.6234817 |
| Ssr3 | -0.3736612 |
| Ssr4 | -0.5719157 |
| Ssrp1 | -0.1204425 |
| Sssca1 | -0.0118985 |
| Sssca1 | -0.01276 |
| Sst | -0.3517122 |
| Sstr1 | -0.5900855 |
| Sstr5 | 2.4592298 |
| Ssty2 | -0.6753303 |
| Ssu72 | -0.5570204 |
| Ssx2ip | -0.6904062 |
| Ssxb2 | -0.7521729 |
| Ssxb5 | 0.1828738 |
| St13 | -0.6778273 |
| St14 | -0.5107822 |
| St14 | 2.9695345 |
| St18 | -0.5511233 |
| St3gal1 | -0.5228589 |
| St3gal2 | 0.1292988 |
| St3gal2 | -0.1286272 |
| St3gal3 | 0.9151798 |
| St3gal3 | 0.9527599 |
| St3gal4 | 0.5647643 |
| St3gal5 | 0.9238987 |
| St3gal6 | -0.833411 |
| St5 | -0.5200043 |
| St5 | 0.2682466 |
| St6gal1 | 0.1035189 |
| St6galnac2 | -0.0851062 |
| St6galnac3 | 0.8095642 |
| St6galnac4 | -0.2982709 |
| St6galnac4 | 1.9175899 |
| St6galnac4 | 0.4481776 |
| St6galnac5 | 0.3915018 |
| St6galnac6 | 0.0782445 |
| St6galnac6 | -0.6954821 |
| St7 | -0.1447073 |
| St7l | 0.2167286 |
| St7l | 0.4171476 |
| St8sia1 | -0.530751 |
| St8sia1 | -0.7125884 |
| St8sia2 | -0.577936 |
| St8sia3 | -0.6786611 |
| St8sia4 | 0.0022111 |
| St8sia4 | -0.2313334 |
| St8sia5 | -0.3767479 |
| Stab1 | 0.2060321 |
| Stab2 | 0.5684034 |
| Stac | -0.515028 |
| Stac2 | -0.7108646 |
| Stac3 | 0.203601 |
| Stag1 | -0.5329142 |
| Stag2 | -0.5749214 |
| Stag2 | -0.1108978 |
| Stag3 | 0.5057097 |
| Stam | -0.5946796 |
| Stam | 2.2221653 |
| Stam | 1.0170024 |
| Stam2 | 0.1584441 |
| Stam2 | -0.3051581 |
| Stambp | 1.0719727 |
| Stambpl1 | 0.2792745 |
| Stap1 | 2.792274 |
| Stap2 | 0.5806791 |
| Star | 1.1847662 |
| Stard10 | -0.6093671 |
| Stard10 | 2.7033523 |
| Stard13 | -0.7151796 |
| Stard3 | -0.6539443 |
| Stard3nl | -0.5946796 |
| Stard4 | 0.5339166 |
| Stard5 | 1.4308774 |
| Stard5 | 0.2698759 |
| Stard6 | 2.9910197 |
| Stard7 | -0.5379797 |
| Stard7 | 0.4462659 |
| Stard8 | 2.4382169 |
| Stard8 | -0.1822356 |
| Stat1 | -0.2367001 |
| Stat1 | -0.0041779 |
| Stat2 | 1.3237626 |
| Stat3 | -0.3196593 |
| Stat3 | 0.0711319 |
| Stat3 | -0.4863646 |
| Stat4 | -0.4988484 |
| Stat4 | 1.9733944 |
| Stat5a | -0.4139018 |
| Stat5a | 0.3219091 |
| Stat6 | -0.5585001 |
| Statip1 | 0.2187859 |
| Stau1 | 2.8309438 |
| Stau2 | 1.8544648 |
| Stbd1 | -0.5401584 |
| Stc2 | 2.9233838 |
| Steap1 | -0.3045824 |
| Steap2 | -0.3535232 |
| Steap3 | -0.4564841 |
| Stfa1 | -0.5809597 |
| Stfa2 | 0.5825912 |
| Stim1 | -0.7450211 |
| Stip1 | -0.5107822 |
| Stk11 | 0.2317186 |
| Stk11ip | 0.1292988 |
| Stk17b | -0.2085768 |
| Stk19 | 0.2959108 |
| Stk19 | 1.8422829 |
| Stk19 | 0.4555173 |
| Stk22b | 1.6278739 |
| Stk22s1 | -0.8314681 |
| Stk24 | -0.3960986 |
| Stk25 | 0.7409313 |
| Stk25 | 2.6980915 |
| Stk3 | -0.0658457 |
| Stk31 | -0.4339861 |
| Stk32a | 1.8460416 |
| Stk32b | 0.871135 |
| Stk32c | -0.5401584 |
| Stk35 | 2.7220097 |
| Stk35 | 0.3110276 |
| Stk36 | -0.4665715 |
| Stk38l | -0.3017087 |
| Stk39 | -0.4352957 |
| Stk4 | 0.3119649 |
| Stk40 | 0.1100259 |
| Stmn1 | -0.6418094 |
| Stmn2 | -0.5321926 |
| Stmn3 | 0.2837806 |
| Stmn4 | -0.5489208 |
| Stmn4 | 0.2122568 |
| Stoml1 | 0.998045 |
| Stoml1 | 0.9069043 |
| Stoml1 | -0.7143151 |
| Stoml2 | 0.6455351 |
| Stoml3 | -0.008889 |
| Stra13 | 1.4481293 |
| Stra13 | 0.4319505 |
| Stra13 | 0.7941949 |
| Stra6 | -0.7273605 |
| Strap | 0.4266687 |
| Strbp | -0.586273 |
| Strn4 | 0.4835635 |
| Strn4 | -0.1776578 |
| Stt3a | -0.6963305 |
| Stt3b | 0.3197422 |
| Stx12 | -0.5459915 |
| Stx17 | 1.7127039 |
| Stx18 | -0.6887199 |
| Stx1a | -0.4294159 |
| Stx2 | 0.0294384 |
| Stx3 | 1.2235678 |
| Stx3 | 0.6047909 |
| Stx4a | 0.0013616 |
| Stx5a | -0.1132755 |
| Stx5a | 0.081782 |
| Stx6 | -0.634598 |
| Stx7 | -0.6179692 |
| Stx8 | -0.6124866 |
| Stx8 | 0.0127703 |
| Stx8 | -0.5824749 |
| Stx8 | 2.0070353 |
| Stxbp1 | -0.3755121 |
| Stxbp1 | -0.5870344 |
| Stxbp1 | 0.2511436 |
| Stxbp2 | 1.9339142 |
| Stxbp4 | -0.6670511 |
| Stxbp4 | 2.4184982 |
| Stxbp5l | -0.5423419 |
| Stxbp5l | -0.6861958 |
| Styx | -0.0162134 |
| Styxl1 | -0.657205 |
| Styxl1 | -0.1085256 |
| Styxl1 | 1.9861194 |
| Suclg1 | 0.4872447 |
| Suclg1 | 0.0327392 |
| Suclg2 | 2.9536948 |
| Suclg2 | -0.6778273 |
| Suds3 | -0.1014425 |
| Sufu | -0.6853558 |
| Suhw2 | 0.0615699 |
| Suhw4 | 0.3484594 |
| Sulf1 | 1.6574169 |
| Sult1a1 | -0.3724292 |
| Sult1c2 | 2.5339307 |
| Sult1d1 | 0.7508824 |
| Sult1d1 | -0.3929523 |
| Sult2b1 | -0.3028572 |
| Sult2b1 | 0.4880318 |
| Sult4a1 | -0.4905087 |
| Sult5a1 | 0.7553184 |
| Sult5a1 | 2.0535678 |
| Sumf2 | 0.5371691 |
| Sumo1 | 0.2408078 |
| Sumo1 | 0.4888183 |
| Sumo1 | 1.40237 |
| Sumo2 | 0.9717682 |
| Sumo3 | 0.3369486 |
| Sumo3 | 2.322299 |
| Sunc1 | 2.9602685 |
| Suox | -0.2546155 |
| Supt3h | -0.4905087 |
| Supt4h1 | -0.1715861 |
| Supt4h2 | 0.8019088 |
| Supt5h | 1.1063597 |
| Supt5h | -0.0869599 |
| Supt6h | -0.4558152 |
| Supv3l1 | 1.5580115 |
| Surf1 | -0.3986228 |
| Surf1 | -0.2897276 |
| Surf2 | 2.1233361 |
| Surf4 | -0.3748948 |
| Susd2 | -0.5511233 |
| Susd3 | 1.8906737 |
| Susd4 | 1.2148041 |
| Suv39h1 | -0.8018213 |
| Suv420h1 | 0.0675568 |
| Suv420h1 | 0.4006952 |
| Suv420h1 | 2.299064 |
| Suv420h2 | 2.3424705 |
| Suz12 | 1.5998802 |
| Sv2a | -0.8710568 |
| Sv2a | 4.9086796 |
| Sv2b | -0.923897 |
| Sval2 | 0.7079479 |
| Svep1 | -0.1033265 |
| Svil | 0.5288922 |
| Svil | 1.5370092 |
| Svop | 0.0068708 |
| Svopl | 0.0635694 |
| Svs4 | 0.959848 |
| Svs5 | -0.6820029 |
| Svs6 | 2.6655742 |
| Swap70 | 0.2296876 |
| Swap70 | -0.5562814 |
| Swap70 | -0.0416107 |
| Syap1 | 2.2945707 |
| Syap1 | -0.1511129 |
| Sybl1 | 0.0364397 |
| Syce2 | 1.655029 |
| Sycp1 | -0.1295945 |
| Syf2 | -0.6720105 |
| Syf2 | 1.1826613 |
| Syn1 | 0.371683 |
| Syn1 | -0.5459915 |
| Syn2 | -0.7134514 |
| Sync | -0.6085887 |
| Syncrip | -0.6971797 |
| Syne1 | 0.5033877 |
| Syne1 | -0.5939124 |
| Syngr1 | -0.4274635 |
| Syngr1 | 0.5151999 |
| Syngr1 | 1.7316005 |
| Syngr1 | 2.5274963 |
| Syngr2 | 0.1707329 |
| Syngr2 | 0.6080559 |
| Syngr2 | -0.1746174 |
| Syngr3 | 1.5503583 |
| Syngr4 | 2.5073414 |
| Syngr4 | -0.7702774 |
| Syngr4 | -0.4808655 |
| Synj2 | 0.6177877 |
| Synj2bp | 2.8986391 |
| Synj2bp | 1.2057061 |
| Synpo2l | -0.5839925 |
| Syp | -0.4713137 |
| Sypl | 0.41433 |
| Sypl | 1.9654878 |
| Sypl2 | 0.8595898 |
| Sys1 | -0.51858 |
| Syt1 | -0.6878779 |
| Syt11 | 2.6098621 |
| Syt11 | -0.5719157 |
| Syt12 | 0.0895991 |
| Syt13 | 0.0786382 |
| Syt15 | -0.4658958 |
| Syt16 | -0.7503802 |
| Syt3 | 0.1168702 |
| Syt4 | 2.0907424 |
| Syt5 | 1.76652 |
| Syt9 | 0.7575291 |
| Sytl2 | -0.657205 |
| Sytl3 | 0.0119297 |
| Sytl3 | -0.5726663 |
| Sytl3 | 1.3505402 |
| Sytl4 | -0.008889 |
| Sytl5 | 1.2526108 |
| Syvn1 | -0.4699565 |
| Taar2 | -0.3942097 |
| Taar3 | 2.4248963 |
| Taar4 | 1.5489855 |
| Tac1 | 0.0695445 |
| Tacc1 | -0.5651856 |
| Tacc1 | -0.368129 |
| Tacr3 | 0.9211715 |
| Tacr3 | -0.3421084 |
| Tacstd2 | -0.6171841 |
| Tada1l | 1.3533238 |
| Tada2l | -0.5157374 |
| Tada2l | -0.5072575 |
| Tada3l | -0.4171143 |
| Tada3l | 2.1938197 |
| Taf10 | 0.8164046 |
| Taf11 | 0.2721526 |
| Taf12 | -0.471993 |
| Taf12 | -0.5489208 |
| Taf12 | 1.8962056 |
| Taf13 | 0.4503579 |
| Taf1c | 0.8320011 |
| Taf3 | 0.2371147 |
| Taf5 | -0.4794954 |
| Taf5 | -0.6887199 |
| Taf5 | -0.6820029 |
| Taf5l | -0.2891606 |
| Taf6 | 0.0923208 |
| Taf6 | 1.9154272 |
| Taf6l | -0.5977539 |
| Taf7l | 0.7228098 |
| Taf9 | -0.6695277 |
| Taf9 | -0.3998872 |
| Taf9 | 0.0864795 |
| Taf9b | -0.4584935 |
| Tagap | 3.0345127 |
| Tagap | 2.2061132 |
| Tagln | 1.7196867 |
| Tagln3 | 0.5258655 |
| Tal1 | 1.5306875 |
| Tal2 | 1.4246808 |
| Taldo1 | 3.4625445 |
| Tank | -0.1329873 |
| Tank | -0.4808655 |
| Tank | -0.4706349 |
| Tap1 | 1.7863991 |
| Tap2 | -0.9007629 |
| Tapbp | 0.8947129 |
| Tapbp | -0.3325952 |
| Tapbpl | 1.6135448 |
| Tapbpl | -0.4605069 |
| Tapt1 | -0.381085 |
| Tarbp2 | -0.3237573 |
| Tarbp2 | -0.5969845 |
| Tardbp | -0.6828401 |
| Tardbp | 2.4877145 |
| Tardbp | 2.0235871 |
| Tars | -0.3761298 |
| Tarsl2 | -0.6250622 |
| Tarsl2 | -0.7134514 |
| Tas1r1 | 0.9544127 |
| Tasp1 | -0.5207172 |
| Tatdn2 | 1.013582 |
| Tax1bp1 | 0.3842607 |
| Tax1bp3 | 2.3932058 |
| Tax1bp3 | 0.8666409 |
| Taz | -0.3559429 |
| Taz | -0.4584935 |
| Tbc1d1 | 0.8141296 |
| Tbc1d10a | -0.6920953 |
| Tbc1d10a | -0.3505067 |
| Tbc1d10c | 2.1321575 |
| Tbc1d12 | -0.3373406 |
| Tbc1d13 | 2.7359631 |
| Tbc1d14 | -0.6410056 |
| Tbc1d15 | -0.5599819 |
| Tbc1d16 | -0.650694 |
| Tbc1d17 | 0.151511 |
| Tbc1d19 | -0.6703546 |
| Tbc1d2 | -0.6000659 |
| Tbc1d20 | -0.6845165 |
| Tbc1d22a | -0.4504797 |
| Tbc1d22b | -0.0586041 |
| Tbc1d23 | 2.6148708 |
| Tbc1d24 | 2.2901012 |
| Tbc1d25 | -0.3823277 |
| Tbc1d2b | -0.6226923 |
| Tbc1d5 | -0.4584935 |
| Tbc1d5 | -0.5016436 |
| Tbc1d7 | -0.2201555 |
| Tbc1d7 | -0.703999 |
| Tbc1d8 | -0.6054811 |
| Tbc1d9 | 0.2046436 |
| Tbc1d9 | -0.500245 |
| Tbc1d9b | -0.527874 |
| Tbca | 2.7428821 |
| Tbcb | 0.8795302 |
| Tbcc | -0.2650767 |
| Tbcd | 0.1871242 |
| Tbcel | -0.3214136 |
| Tbk1 | 1.6123415 |
| Tbkbp1 | -0.5065541 |
| Tbl1x | 0.2398019 |
| Tbl1xr1 | -0.3841945 |
| Tbl2 | 2.8266194 |
| Tbl3 | -0.5962156 |
| Tbp | -0.4946699 |
| Tbpl1 | -0.5474551 |
| Tbr1 | -0.421629 |
| Tbrg1 | -0.3403178 |
| Tbrg1 | -0.4229226 |
| Tbrg1 | -0.5214306 |
| Tbrg4 | 0.5727531 |
| Tbrg4 | -0.4981507 |
| Tbx1 | -0.7414642 |
| Tbx19 | -0.7548679 |
| Tbx2 | 0.2724774 |
| Tbx22 | -0.4856756 |
| Tbx3 | 2.2254483 |
| Tbx3 | -0.0183778 |
| Tbx5 | 0.6922213 |
| Tbx6 | -0.7657207 |
| Tbxa2r | -0.5870344 |
| Tbxa2r | -0.5931459 |
| Tcam1 | 1.158673 |
| Tcam1 | -0.6219036 |
| Tcam1 | -0.6687015 |
| Tcap | 1.535154 |
| Tcea2 | 1.3947153 |
| Tcea3 | 1.04622 |
| Tceal1 | 1.9080776 |
| Tceal1 | 0.4066831 |
| Tceal3 | -0.7702774 |
| Tceal5 | 0.0013616 |
| Tceal6 | -0.643419 |
| Tceal6 | -0.6695277 |
| Tceal8 | -0.0595064 |
| Tceb1 | 0.634709 |
| Tceb2 | -0.1660524 |
| Tceb2 | 2.0188282 |
| Tceb3 | 0.5026125 |
| Tcerg1 | -0.5592407 |
| Tcf12 | -0.5228589 |
| Tcf12 | 2.3649004 |
| Tcf15 | 0.5090543 |
| Tcf19 | -0.1175696 |
| Tcf20 | -0.3571549 |
| Tcf20 | -0.1807074 |
| Tcf20 | -0.6156158 |
| Tcf20 | 0.9519325 |
| Tcf20 | -0.7134514 |
| Tcf21 | -0.3891897 |
| Tcf23 | -0.7273605 |
| Tcf25 | -0.5452606 |
| Tcf25 | 1.461202 |
| Tcf25 | -0.7459123 |
| Tcf3 | 0.5424011 |
| Tcf3 | 1.469956 |
| Tcf4 | -0.7238652 |
| Tcf7 | -0.6761619 |
| Tcf7 | -0.6695277 |
| Tcf7l2 | 2.9945201 |
| Tcf7l2 | 1.8622813 |
| Tcfap2b | -0.5072575 |
| Tcfap2b | -0.3097759 |
| Tcfap2c | -0.4960608 |
| Tcfap2c | 0.8945376 |
| Tcfcp2 | -0.779453 |
| Tcfe3 | 1.0957931 |
| Tcfl5 | 0.8957637 |
| Tchp | 1.991271 |
| Tcirg1 | -0.496757 |
| Tcirg1 | 0.8299479 |
| Tcirg1 | -0.4558152 |
| Tcirg1 | -0.5893219 |
| Tcl1 | 3.719444 |
| Tcl1 | -0.4274635 |
| Tcl1b2 | 0.7996008 |
| Tcl1b2 | -0.3973599 |
| Tcl1b3 | 0.651797 |
| Tcl1b4 | 0.0535316 |
| Tcn2 | 0.8532196 |
| Tcof1 | -0.6954821 |
| Tcp1 | 0.5351688 |
| Tcp1 | 0.5220693 |
| Tcp10a | 0.7522959 |
| Tcp10a | 2.2985851 |
| Tcp11 | 0.4112216 |
| Tcstv1 | -0.7924408 |
| Tcte3 | -0.5452606 |
| Tctex1d1 | 1.0957931 |
| Tctex1d2 | 0.4029805 |
| Tctex1d4 | -0.5394317 |
| Tctn3 | -0.643419 |
| Tctn3 | -0.5065541 |
| Tctn3 | 0.247821 |
| Tdg | -0.6670511 |
| Tdp1 | -0.6604764 |
| Tdpoz1 | 1.9775941 |
| Tdpoz2 | 0.7789809 |
| Tdrd1 | -0.7291127 |
| Tdrd12 | 0.7559218 |
| Tdrd3 | 0.7750427 |
| Tdrd3 | 1.1538061 |
| Tdrd3 | 0.6362951 |
| Tdrd6 | -0.5030442 |
| Tdrd7 | -0.5802029 |
| Tdrkh | -0.5136108 |
| Tead2 | -0.1695704 |
| Tec | -0.2175123 |
| Tecta | 1.0602944 |
| Tef | 2.8627744 |
| Tef | 0.2757198 |
| Tegt | -0.4538111 |
| Tek | -0.4326782 |
| Tekt1 | -0.4981507 |
| Tekt1 | 0.5991692 |
| Tekt3 | -0.8373082 |
| Tenc1 | 3.4812084 |
| Tenc1 | -0.3361521 |
| Terf2 | -0.3873136 |
| Terf2 | -0.3138338 |
| Terf2ip | 1.3718331 |
| Terf2ip | 0.36222 |
| Terf2ip | -0.2164569 |
| Terf2ip | -0.0253353 |
| Tes | -0.4788111 |
| Tes | -0.379224 |
| Tes | -0.705711 |
| Tesc | 0.3006592 |
| Tesk1 | -0.6306138 |
| Tesp1 | -0.2064857 |
| Tesp2 | 1.0342378 |
| Tessp2 | 0.8141296 |
| Tessp3 | -0.3179081 |
| Tex10 | 1.0869987 |
| Tex10 | 1.2878654 |
| Tex101 | -0.1329873 |
| Tex11 | 1.8109918 |
| Tex13 | -0.6282308 |
| Tex14 | 0.4112216 |
| Tex16 | 3.2710957 |
| Tex19 | -0.7952458 |
| Tex2 | -0.6290245 |
| Tex2 | -0.7169107 |
| Tex22 | 0.4029805 |
| Tex24 | -0.7238652 |
| Tex261 | -0.5408857 |
| Tex261 | 1.2211622 |
| Tex264 | 1.6563472 |
| Tex264 | -0.7476971 |
| Tex9 | -0.6744993 |
| Tfam | -0.4300675 |
| Tfam | 0.3813496 |
| Tfb1m | 0.0829585 |
| Tfb2m | 1.5552848 |
| Tfdp1 | 0.3707996 |
| Tfdp2 | -0.7238652 |
| Tfdp2 | 1.5248889 |
| Tfg | 0.3308371 |
| Tfip11 | 1.2865594 |
| Tfpi | -0.3761298 |
| Tfpt | 0.9824255 |
| Tfpt | -0.1451986 |
| Tfpt | 1.9145991 |
| Tfrc | 1.7340427 |
| Tfrc | -0.8997238 |
| Tg | -0.7022898 |
| Tgfb1 | -0.6361961 |
| Tgfb1i1 | -0.0726823 |
| Tgfb1i1 | -0.8160577 |
| Tgfb2 | 0.5840229 |
| Tgfb3 | -0.1402963 |
| Tgfbi | -0.610146 |
| Tgfbi | -0.4248662 |
| Tgfbr1 | 0.3144602 |
| Tgfbr1 | -0.5257216 |
| Tgfbr1 | -0.6744993 |
| Tgfbr2 | 2.0700893 |
| Tgfbr2 | -0.7812982 |
| Tgfbr3 | 2.6023181 |
| Tgfbr3 | 2.329462 |
| Tgfbrap1 | -0.6070337 |
| Tgif1 | -0.6226923 |
| Tgif1 | -0.6629369 |
| Tgif1 | 1.1306393 |
| Tgif2 | 0.6758102 |
| Tgif2lx | 1.9218382 |
| Tgm2 | -0.7291127 |
| Tgm4 | 0.7437845 |
| Tgoln1 | -0.3361521 |
| Tgs1 | -0.6282308 |
| Tgs1 | 0.2201551 |
| Th1l | 0.7988303 |
| Tha1 | -0.4445109 |
| Thada | 2.8801204 |
| Thada | -0.1863223 |
| Thap1 | -0.2070081 |
| Thap1 | 0.6071241 |
| Thap1 | -0.7361521 |
| Thap11 | 1.0872882 |
| Thap3 | -0.6498831 |
| Thap4 | -0.6711822 |
| Thap7 | -0.4248662 |
| Thbd | 0.3063279 |
| Thbs1 | -0.1695704 |
| Thbs2 | 0.7123665 |
| Thbs3 | 0.0144496 |
| Thbs3 | 0.5652503 |
| Theg | 0.0429851 |
| Theg | 0.4235982 |
| Theg | 0.0064481 |
| Them2 | 0.7627353 |
| Them4 | -0.4918939 |
| Them5 | -0.4932809 |
| Thg1l | 0.5059674 |
| Thnsl1 | 0.2799195 |
| Thnsl2 | -0.2778872 |
| Thnsl2 | 0.6414889 |
| Thoc1 | 1.253225 |
| Thoc3 | -0.6861958 |
| Thoc4 | -0.3296406 |
| Thoc4 | 0.0591651 |
| Thoc5 | 0.3598403 |
| Thoc6 | 2.2630406 |
| Thop1 | 0.6939333 |
| Thra | 0.1341531 |
| Thra | 0.9063849 |
| Thrap2 | 1.1338236 |
| Thrap3 | -0.4884345 |
| Thrsp | -0.3748948 |
| Thsd1 | 0.749062 |
| Thsd4 | 2.8660543 |
| Thsd4 | -0.7264855 |
| Thsd7b | -0.3614084 |
| Thsd7b | 0.398118 |
| Thsd7b | -0.7151796 |
| Thtpa | -0.5916145 |
| Thumpd1 | 2.9800242 |
| Thumpd3 | 0.6269696 |
| Thy1 | -0.3126727 |
| Thyn1 | 0.5809183 |
| Tia1 | -0.7822221 |
| Tial1 | -0.7999382 |
| Tial1 | 0.1938175 |
| Tiam1 | -0.5900855 |
| Tiam2 | -0.496757 |
| Ticam1 | 1.4707481 |
| Tie1 | -0.3051581 |
| Tigd2 | 0.81943 |
| Tigd3 | 1.2726833 |
| Timeless | -0.8122416 |
| Timm10 | -0.0536554 |
| Timm13 | 2.1702584 |
| Timm17a | -0.61405 |
| Timm17b | 0.3915018 |
| Timm22 | -0.1471662 |
| Timm44 | 1.9130684 |
| Timm44 | 0.5164756 |
| Timm44 | -0.6418094 |
| Timm50 | -0.7160448 |
| Timm8a1 | -0.5711657 |
| Timm8a2 | 1.1800239 |
| Timm8a2 | -0.2175123 |
| Timm8b | -0.6410056 |
| Timm8b | -0.6670511 |
| Timm9 | 2.6336303 |
| Timm9 | 0.8211278 |
| Timm9 | 0.8876796 |
| Timp1 | 1.2974304 |
| Timp1 | -0.5786911 |
| Timp2 | -0.5129029 |
| Timp3 | 0.4241572 |
| Timp3 | 0.4533481 |
| Timp4 | -0.3730451 |
| Tinagl | -0.5452606 |
| Tipin | 0.2812081 |
| Tipin | -0.496757 |
| Tirap | 0.1558954 |
| Tirap | 3.090823 |
| Tirap | -0.5681712 |
| Tjap1 | -0.610146 |
| Tjap1 | 1.5705495 |
| Tjap1 | 0.0876505 |
| Tjp1 | 3.7676226 |
| Tjp2 | -0.7766916 |
| Tjp3 | 0.3050709 |
| Tjp3 | 0.490651 |
| Tk1 | -0.6250622 |
| Tk2 | 4.0187208 |
| Tkt | 1.8612067 |
| Tkt | 2.0483349 |
| Tkt | -0.0613136 |
| Tktl1 | -0.6242717 |
| Tktl1 | -0.7476971 |
| Tktl2 | 1.8041178 |
| Tlcd1 | 1.1839774 |
| Tle1 | -0.7014363 |
| Tle1 | -0.5931459 |
| Tle2 | 1.0561225 |
| Tle3 | -0.6008377 |
| Tle3 | -0.7831468 |
| Tle3 | -0.8131943 |
| Tle4 | 0.0595663 |
| Tle4 | 2.074341 |
| Tle4 | 0.0294384 |
| Tle6 | 1.3132889 |
| Tlk1 | -0.5023437 |
| Tlk2 | 1.6350469 |
| Tln1 | -0.5214306 |
| Tln2 | -0.294275 |
| Tlr13 | 1.9718903 |
| Tlr2 | 1.4577993 |
| Tlr3 | -0.6861958 |
| Tlr4 | 0.5169854 |
| Tlr6 | 0.1062033 |
| Tlr7 | 3.1718796 |
| Tm2d2 | 0.0880405 |
| Tm2d3 | 2.2080206 |
| Tm2d3 | -0.3607997 |
| Tm4sf4 | -0.2706265 |
| Tm4sf5 | 1.0669632 |
| Tm6sf1 | -0.3184915 |
| Tm6sf2 | 0.530905 |
| Tm9sf2 | 2.6020281 |
| Tm9sf2 | -0.3916965 |
| Tm9sf2 | 2.9307159 |
| Tm9sf3 | -0.5221445 |
| Tm9sf4 | 0.9650906 |
| Tm9sf4 | 2.248684 |
| Tmbim1 | -0.7405769 |
| Tmbim4 | -0.6458381 |
| Tmc1 | -0.4578232 |
| Tmc4 | 0.0989003 |
| Tmc5 | -0.2122465 |
| Tmc6 | 0.9733902 |
| Tmc6 | -0.496757 |
| Tmc6 | 2.5840119 |
| Tmc7 | -0.1056864 |
| Tmcc2 | -0.4605069 |
| Tmcc3 | 0.8047864 |
| Tmco3 | -0.3638471 |
| Tmco3 | 1.3593104 |
| Tmco4 | -0.6171841 |
| Tmco4 | -0.703144 |
| Tmco5 | -0.5387054 |
| Tmco6 | 1.1941844 |
| Tmco6 | -0.4912011 |
| Tmco7 | -0.6895627 |
| Tmed1 | 1.8876673 |
| Tmed1 | -0.5372545 |
| Tmed10 | -0.379224 |
| Tmed2 | -0.4498148 |
| Tmed3 | -0.5214306 |
| Tmed3 | 3.0046896 |
| Tmed4 | 1.6198816 |
| Tmed4 | -0.4017869 |
| Tmed4 | 1.3688812 |
| Tmed4 | -0.4822375 |
| Tmed8 | 2.3568764 |
| Tmed8 | -0.7512761 |
| Tmed9 | 0.1764645 |
| Tmed9 | -0.2143495 |
| Tmeff2 | 0.0687499 |
| Tmem10 | -0.2480639 |
| Tmem100 | 2.7267487 |
| Tmem101 | -0.798998 |
| Tmem102 | -0.4672476 |
| Tmem102 | -0.6250622 |
| Tmem106a | -0.2734129 |
| Tmem106c | 1.7043387 |
| Tmem107 | 2.2959252 |
| Tmem107 | -0.705711 |
| Tmem107 | 1.1739317 |
| Tmem108 | 0.0127703 |
| Tmem109 | 2.2538869 |
| Tmem11 | 1.0659287 |
| Tmem110 | -0.730868 |
| Tmem111 | 1.3088829 |
| Tmem111 | -0.2307983 |
| Tmem111 | 2.9182382 |
| Tmem115 | -0.4726727 |
| Tmem116 | -0.7530704 |
| Tmem116 | -0.7639038 |
| Tmem117 | -0.3296406 |
| Tmem118 | -0.8074918 |
| Tmem119 | 1.3421429 |
| Tmem120a | 1.7676265 |
| Tmem120b | 1.6094131 |
| Tmem121 | 1.2671274 |
| Tmem125 | 0.1533401 |
| Tmem126a | 2.6575225 |
| Tmem126b | 0.4216393 |
| Tmem127 | -0.6853558 |
| Tmem128 | 0.1832287 |
| Tmem130 | -0.720382 |
| Tmem131 | 0.7037216 |
| Tmem132a | -0.2557116 |
| Tmem132a | -0.577936 |
| Tmem132c | 0.8906747 |
| Tmem132d | 0.8250782 |
| Tmem132e | -0.785926 |
| Tmem134 | 0.019888 |
| Tmem134 | 2.3066527 |
| Tmem135 | -0.3798439 |
| Tmem136 | 1.6299044 |
| Tmem138 | 1.6530485 |
| Tmem139 | 0.1793181 |
| Tmem140 | -0.6887199 |
| Tmem141 | -0.8690401 |
| Tmem143 | -0.4268136 |
| Tmem143 | 1.8948417 |
| Tmem143 | -0.0791052 |
| Tmem143 | -0.749485 |
| Tmem144 | 1.9394632 |
| Tmem146 | 2.1628621 |
| Tmem147 | 0.545874 |
| Tmem14c | -0.0953438 |
| Tmem14c | -0.2801318 |
| Tmem14c | -0.1986828 |
| Tmem150 | 1.6825154 |
| Tmem150 | 0.8606778 |
| Tmem151a | 1.0953623 |
| Tmem154 | -0.2458895 |
| Tmem158 | -0.5651856 |
| Tmem159 | 2.6421221 |
| Tmem159 | -0.4287647 |
| Tmem160 | -0.7125884 |
| Tmem160 | -0.3742778 |
| Tmem161a | -0.7566686 |
| Tmem162 | 0.280564 |
| Tmem164 | 2.3255265 |
| Tmem166 | -0.152102 |
| Tmem167 | 0.7399104 |
| Tmem168 | 0.3952467 |
| Tmem168 | -0.6490728 |
| Tmem168 | -0.5178686 |
| Tmem169 | 1.4192794 |
| Tmem16a | 2.0019638 |
| Tmem16b | 1.4239554 |
| Tmem16d | -0.5599819 |
| Tmem16e | -0.1108978 |
| Tmem16g | 0.0599673 |
| Tmem16j | 1.3953554 |
| Tmem16k | -0.5877963 |
| Tmem17 | 2.6261683 |
| Tmem17 | -0.2085768 |
| Tmem170 | -0.6023831 |
| Tmem176a | -0.6402024 |
| Tmem176a | -0.6093671 |
| Tmem176b | -0.6148326 |
| Tmem177 | 0.8375795 |
| Tmem178 | 1.8024809 |
| Tmem179b | -0.4320249 |
| Tmem180 | 2.5447348 |
| Tmem183a | 0.8641154 |
| Tmem183a | -0.4877441 |
| Tmem184a | -0.3998872 |
| Tmem184a | -0.0545534 |
| Tmem184b | 0.6054915 |
| Tmem185b | -0.1665542 |
| Tmem186 | -0.667876 |
| Tmem19 | 1.2403727 |
| Tmem192 | -0.0301466 |
| Tmem198 | 1.0422922 |
| Tmem199 | 1.5460506 |
| Tmem2 | 2.6169372 |
| Tmem2 | 0.9798523 |
| Tmem2 | 0.787981 |
| Tmem201 | -0.4431893 |
| Tmem204 | -0.598524 |
| Tmem205 | -0.4307195 |
| Tmem208 | -0.4774438 |
| Tmem209 | -0.5969845 |
| Tmem209 | -0.0235914 |
| Tmem23 | -0.169067 |
| Tmem25 | 0.173961 |
| Tmem25 | -0.1147048 |
| Tmem25 | -0.5946796 |
| Tmem27 | -0.6337999 |
| Tmem32 | 1.3432666 |
| Tmem33 | 0.3589464 |
| Tmem33 | 1.1644556 |
| Tmem34 | 0.6024524 |
| Tmem35 | 0.5637917 |
| Tmem38a | -0.5518586 |
| Tmem38b | 1.5280729 |
| Tmem38b | -0.5100763 |
| Tmem39a | 0.1555307 |
| Tmem40 | 0.7419513 |
| Tmem41a | -0.5207172 |
| Tmem41b | -0.4618515 |
| Tmem42 | -0.657205 |
| Tmem43 | -0.720382 |
| Tmem43 | 2.0940016 |
| Tmem44 | -0.5666773 |
| Tmem45a | 1.243973 |
| Tmem45b | -0.6621161 |
| Tmem47 | -0.5030442 |
| Tmem47 | -0.1094738 |
| Tmem48 | -0.1575596 |
| Tmem49 | 0.1104074 |
| Tmem49 | -0.5030442 |
| Tmem49 | -0.5794467 |
| Tmem49 | 1.6042245 |
| Tmem5 | -0.1228429 |
| Tmem50a | -0.55776 |
| Tmem50b | -0.6156158 |
| Tmem50b | -0.4925872 |
| Tmem51 | 0.2850643 |
| Tmem53 | 0.033151 |
| Tmem53 | 0.2377872 |
| Tmem53 | 0.7997934 |
| Tmem54 | -0.6555733 |
| Tmem55a | -0.0301466 |
| Tmem55b | -0.7160448 |
| Tmem56 | 1.053132 |
| Tmem57 | 0.2084574 |
| Tmem59 | 1.5587373 |
| Tmem59l | -0.5257216 |
| Tmem60 | -0.7396904 |
| Tmem62 | -0.1412749 |
| Tmem63a | 0.4538909 |
| Tmem63b | -0.2965565 |
| Tmem63b | -0.1645485 |
| Tmem63c | -0.6474542 |
| Tmem65 | 3.1353234 |
| Tmem66 | -0.1670563 |
| Tmem66 | -0.6912504 |
| Tmem66 | -0.258457 |
| Tmem68 | 0.2201551 |
| Tmem69 | -0.2767668 |
| Tmem70 | 0.6967092 |
| Tmem71 | 2.5561331 |
| Tmem74 | -0.1486444 |
| Tmem74 | -0.7379197 |
| Tmem74 | -0.7134514 |
| Tmem77 | 0.302237 |
| Tmem77 | 1.040929 |
| Tmem77 | -0.4491503 |
| Tmem79 | -0.6093671 |
| Tmem8 | 0.5942244 |
| Tmem8 | -0.4638716 |
| Tmem8 | 1.2788075 |
| Tmem81 | 1.0505831 |
| Tmem82 | -0.4281139 |
| Tmem85 | -0.7711912 |
| Tmem85 | 2.7974651 |
| Tmem86a | 1.2903539 |
| Tmem86b | -0.3391259 |
| Tmem87a | 0.4840902 |
| Tmem88 | -0.598524 |
| Tmem9 | 0.2600602 |
| Tmem90a | 0.3595424 |
| Tmem91 | 0.178249 |
| Tmem93 | 1.7736543 |
| Tmem97 | -0.5696674 |
| Tmem98 | 1.1796277 |
| Tmem98 | 0.838321 |
| Tmem9b | 3.3760949 |
| Tmem9b | 2.8485635 |
| Tmem9b | -0.3296406 |
| Tmie | 2.4379135 |
| Tmod1 | -0.6187548 |
| Tmod2 | -0.3711987 |
| Tmod2 | -0.5562814 |
| Tmod3 | -0.6904062 |
| Tmod4 | 3.3899413 |
| Tmod4 | 1.3349215 |
| Tmod4 | -0.7343877 |
| Tmpo | -0.4339861 |
| Tmpo | -0.6654034 |
| Tmpo | 2.9710333 |
| Tmprss11a | -0.3632369 |
| Tmprss11c | -0.5459915 |
| Tmprss13 | -0.4781272 |
| Tmprss13 | -0.4113392 |
| Tmprss2 | 0.4224793 |
| Tmprss4 | 0.020722 |
| Tmprss4 | -0.311513 |
| Tmprss4 | -0.6047057 |
| Tmprss7 | -0.0681194 |
| Tmprss9 | 0.8816625 |
| Tmsb10 | -0.581717 |
| Tmsb10 | -0.0527583 |
| Tmsb10 | -0.259007 |
| Tmsb4x | 0.544139 |
| Tmtc2 | 0.2394664 |
| Tmtc4 | -0.5977539 |
| Tmub1 | 0.0034841 |
| Tmub1 | 0.225273 |
| Tnc | -0.6322056 |
| Tnc | 1.1776444 |
| Tnc | 2.5042565 |
| Tnc | -0.1655508 |
| Tnfaip1 | 1.0640047 |
| Tnfaip2 | 0.4492683 |
| Tnfaip2 | -0.3086194 |
| Tnfaip2 | 1.2087907 |
| Tnfaip3 | -0.7980586 |
| Tnfaip8 | 0.2056852 |
| Tnfaip8l2 | 2.5863061 |
| Tnfaip8l2 | 0.902741 |
| Tnfaip8l3 | -0.4898169 |
| Tnfrsf10b | 1.4037459 |
| Tnfrsf10b | -0.4891255 |
| Tnfrsf10b | 2.4762052 |
| Tnfrsf11a | 1.6024891 |
| Tnfrsf11b | 0.4280613 |
| Tnfrsf12a | -0.7022898 |
| Tnfrsf13c | -0.6778273 |
| Tnfrsf19 | 0.3713886 |
| Tnfrsf19 | 0.7007526 |
| Tnfrsf1a | -0.7273605 |
| Tnfrsf1a | -0.6258534 |
| Tnfrsf1a | -0.3391259 |
| Tnfrsf1b | 1.69154 |
| Tnfrsf21 | 1.2220492 |
| Tnfrsf21 | -0.4113392 |
| Tnfrsf22 | -0.0536554 |
| Tnfrsf22 | -0.5214306 |
| Tnfrsf25 | 1.9462796 |
| Tnfrsf4 | -0.5734174 |
| Tnfsf13b | -0.6971797 |
| Tnfsf13b | 2.1653502 |
| Tnip1 | 1.8783957 |
| Tnip1 | -0.5832334 |
| Tnip1 | -0.643419 |
| Tnip1 | 3.1085904 |
| Tnip2 | 3.7490689 |
| Tnks | -0.673669 |
| Tnks1bp1 | -0.4405512 |
| Tnnc1 | 0.2266334 |
| Tnnc2 | 0.486457 |
| Tnni2 | 1.6901079 |
| Tnni3 | 0.5596474 |
| Tnnt1 | 0.0323272 |
| Tnp1 | 0.8519407 |
| Tnp2 | -0.4753963 |
| Tnpo1 | 1.4929674 |
| Tnpo2 | -0.530751 |
| Tnr | -0.1047418 |
| Tnrc15 | -0.6531307 |
| Tnrc18 | -0.4326782 |
| Tnrc18 | -0.6195411 |
| Tnrc18 | 0.0068708 |
| Tnrc6a | 2.2043456 |
| Tnrc6a | 0.4617278 |
| Tnrc6b | -0.2154027 |
| Tnrc6b | -0.7620901 |
| Tnrc6c | 1.3425925 |
| Tns4 | 3.1646552 |
| Tnxb | 1.2328827 |
| Tnxb | -0.6023831 |
| Tnxb | 3.6806506 |
| Tob1 | -0.7666304 |
| Tob2 | 1.6252451 |
| Toe1 | -0.4030553 |
| Toe1 | -0.5644406 |
| Tollip | 2.7429661 |
| Tollip | 1.8138129 |
| Tom1 | 1.7947592 |
| Tom1l2 | 1.4215681 |
| Tom1l2 | -0.0345405 |
| Tom1l2 | -0.386689 |
| Tomm22 | 3.0918943 |
| Tomm34 | 0.3034975 |
| Tomm34 | 2.465892 |
| Tomm7 | 3.0452859 |
| Tomm70a | 0.0587637 |
| Top1 | -0.6070337 |
| Top1mt | 0.0417611 |
| Top2a | -0.830498 |
| Top3a | -0.7684522 |
| Top3b | -0.257358 |
| Topors | 3.578034 |
| Tor1a | -0.6629369 |
| Tor1a | -0.6695277 |
| Tor1aip1 | 0.3675538 |
| Tor1aip2 | 0.3000274 |
| Tor1aip2 | -0.5877963 |
| Tor1b | 2.6222586 |
| Tor3a | 0.952429 |
| Tox3 | 1.500971 |
| Tpbg | 1.4922895 |
| Tpcn1 | 2.5380602 |
| Tpcn1 | -0.6853558 |
| Tpcn2 | 1.351209 |
| Tpd52 | -0.8018213 |
| Tpd52l1 | -0.61405 |
| Tpd52l1 | -0.7849988 |
| Tpd52l2 | -0.5599819 |
| Tph2 | 0.9289925 |
| Tpi1 | 1.6756733 |
| Tpi1 | 1.5674071 |
| Tpi1 | 0.366371 |
| Tpk1 | 0.0310902 |
| Tpk1 | 0.256107 |
| Tpk1 | 0.2936872 |
| Tpm1 | 1.6704097 |
| Tpm2 | 1.1619046 |
| Tpm2 | 1.9188599 |
| Tpm3 | -0.6426139 |
| Tpm3 | 0.4109386 |
| Tpm4 | 0.136014 |
| Tpo | 2.7254092 |
| Tpp1 | -0.5954473 |
| Tpp2 | 0.1400958 |
| Tppp | 0.610846 |
| Tppp | -0.603931 |
| Tppp3 | -0.5430708 |
| Tpr | -0.6466458 |
| Tpra40 | 0.0123501 |
| Tprgl | -0.7282362 |
| Tprgl | 1.8333425 |
| Tprkb | -0.5809597 |
| Tprkb | 1.7596321 |
| Tpsab1 | 3.4599599 |
| Tpst1 | -0.7134514 |
| Tpst2 | -0.7557679 |
| Tpte | 0.4750984 |
| Tpte | 0.5002833 |
| Tpx2 | -0.4544786 |
| Tra2a | -0.1929988 |
| Trabd | 0.5324119 |
| Trabd | -0.610146 |
| Traf1 | -0.7335066 |
| Traf2 | -0.6498831 |
| Traf3 | -0.3699697 |
| Traf3 | -0.5786911 |
| Traf3ip1 | -0.5136108 |
| Traf3ip2 | 0.3019217 |
| Traf4 | -0.3601913 |
| Traf4 | 1.8151502 |
| Traf4 | 0.1367574 |
| Traf4 | 2.1578175 |
| Traf5 | 1.59709 |
| Traf6 | -0.3662917 |
| Traf6 | -0.6912504 |
| Traf6 | -0.272855 |
| Traf7 | 1.3717239 |
| Trafd1 | -0.707426 |
| Trafd1 | -0.5511233 |
| Trafd1 | 0.5436428 |
| Traip | 1.7864715 |
| Trak1 | 0.4352721 |
| Trak2 | 0.0017865 |
| Trak2 | 1.3770596 |
| Trak2 | 0.2808861 |
| Tram1 | -0.7785317 |
| Tram1 | -0.379224 |
| Tram1 | -0.7370355 |
| Tram2 | -0.6778273 |
| Tram2 | -0.6109256 |
| Trap1 | -0.3499045 |
| Trappc1 | 0.1980221 |
| Trappc1 | 0.5187677 |
| Trappc2 | 0.6187097 |
| Trappc2l | -0.3541276 |
| Trappc3 | 1.8913261 |
| Trappc3 | 0.2491514 |
| Trappc3 | -0.2835081 |
| Trappc3 | -0.6853558 |
| Trappc4 | -0.5051487 |
| Trappc4 | 0.1796743 |
| Trappc5 | -0.4822375 |
| Trappc6a | 1.3433789 |
| Trat1 | 2.4980582 |
| Trcg1 | -0.1368789 |
| Trdmt1 | -0.6498831 |
| Trdmt1 | 3.4890492 |
| Trem2 | -0.6023831 |
| Treml1 | 0.5235895 |
| Treml2 | 0.8917297 |
| Treml4 | -0.2307983 |
| Trerf1 | 3.2055992 |
| Trerf1 | 0.9382648 |
| Trex1 | -0.2711832 |
| Trex1 | -0.7396904 |
| Trex2 | 0.8040198 |
| Trf | 0.5281364 |
| Trfp | -0.3373406 |
| Trfr2 | -0.5734174 |
| Trfr2 | 0.0884304 |
| Trh | 1.5250765 |
| Trhde | -0.4551467 |
| Trhr | -0.2524269 |
| Triap1 | -0.5992946 |
| Trib2 | 1.1161137 |
| Trib3 | -0.1935142 |
| Trib3 | -0.4564841 |
| Trim11 | 1.0937814 |
| Trim16 | 1.9215851 |
| Trim17 | -0.4478225 |
| Trim2 | -0.6047057 |
| Trim2 | -0.7352695 |
| Trim21 | 0.051512 |
| Trim21 | 1.3360533 |
| Trim23 | 1.0517834 |
| Trim23 | -0.4781272 |
| Trim23 | -0.5192919 |
| Trim25 | -0.6402024 |
| Trim26 | -0.6078109 |
| Trim28 | -0.7108646 |
| Trim3 | 2.4337327 |
| Trim3 | 0.9659074 |
| Trim30 | -0.381085 |
| Trim32 | -0.126213 |
| Trim32 | 1.0018336 |
| Trim33 | 2.6777569 |
| Trim33 | 0.7537076 |
| Trim33 | -0.2557116 |
| Trim35 | 0.1088806 |
| Trim36 | -0.4625244 |
| Trim36 | -0.5741691 |
| Trim36 | 0.8359093 |
| Trim37 | 0.8076558 |
| Trim38 | -0.1132755 |
| Trim38 | -0.5939124 |
| Trim39 | 3.0159341 |
| Trim40 | 3.0666151 |
| Trim41 | 1.8457005 |
| Trim42 | 0.0115091 |
| Trim44 | 0.3737412 |
| Trim45 | 0.0265413 |
| Trim45 | 2.1155862 |
| Trim46 | 1.632774 |
| Trim47 | 0.3845513 |
| Trim54 | -0.4511451 |
| Trim56 | -0.6031568 |
| Trim59 | -0.5121955 |
| Trim61 | 0.3713886 |
| Trim62 | 0.2547858 |
| Trim67 | -0.5250052 |
| Trim68 | 0.7415434 |
| Trim69 | 0.6864216 |
| Trim72 | -0.5622088 |
| Trim8 | 0.3499642 |
| Trim9 | -0.5525944 |
| Trim9 | -0.5481877 |
| Trim9 | 0.9991515 |
| Trim9 | -0.6016101 |
| Triml1 | -0.3724292 |
| Triobp | 1.0718258 |
| Triobp | -0.6645806 |
| Trip10 | -0.1516074 |
| Trip12 | -0.2442617 |
| Trip12 | -0.586273 |
| Trip12 | 1.4048031 |
| Trip6 | -0.4912011 |
| Trip6 | 0.0623702 |
| Trit1 | -0.4953651 |
| Trmt1 | 1.7742404 |
| Trmt12 | 1.8857673 |
| Trmu | -0.634598 |
| Tro | -0.6156158 |
| Tro | 0.2815301 |
| Tro | -0.1945458 |
| Trove2 | -0.2908625 |
| Trp53 | -0.7291127 |
| Trp53bp1 | -0.7221221 |
| Trp53bp1 | -0.5037452 |
| Trp53bp2 | -0.4158281 |
| Trp53i11 | -0.657205 |
| Trp53i11 | 1.250643 |
| Trp53inp1 | 0.0802113 |
| Trp53inp1 | 1.4911263 |
| Trp53inp2 | 0.4638791 |
| Trp53rk | 0.6862061 |
| Trp63 | -0.6687015 |
| Trpc1 | 0.0228042 |
| Trpc1 | -0.2988431 |
| Trpc2 | 0.3778452 |
| Trpc3 | 0.2387951 |
| Trpc4 | 0.8097548 |
| Trpc4ap | -0.3891897 |
| Trpc4ap | -0.7620901 |
| Trpc5 | -0.55776 |
| Trpc6 | 0.8920811 |
| Trpc7 | 0.5591587 |
| Trpd52l3 | -0.4884345 |
| Trpm1 | -0.4017869 |
| Trpm2 | -0.5533307 |
| Trpm3 | 2.4410566 |
| Trpm3 | -0.2897276 |
| Trpm3 | -0.2437197 |
| Trpm3 | -0.2518805 |
| Trpm3 | -0.7160448 |
| Trpm4 | -0.6778273 |
| Trpm6 | -0.7933749 |
| Trpm6 | 0.4454455 |
| Trpm6 | 0.8067002 |
| Trpm8 | 0.4137656 |
| Trps1 | -0.5555429 |
| Trpt1 | 0.3384707 |
| Trpt1 | 0.6180183 |
| Trpt1 | -0.6954821 |
| Trpv2 | 0.4086712 |
| Trpv4 | 0.287627 |
| Trpv6 | 2.1319007 |
| Trrap | 1.6006636 |
| Trub1 | 1.1239612 |
| Trub1 | 4.9430296 |
| Trub1 | -0.3798439 |
| Trub2 | 1.8143762 |
| Tsc1 | -0.2486083 |
| Tsc2 | -0.0800261 |
| Tsc2 | -0.6410056 |
| Tsc22d1 | 1.2136554 |
| Tsc22d1 | 0.6308467 |
| Tsc22d2 | -0.6195411 |
| Tsc22d3 | 1.0753475 |
| Tsc22d3 | -0.7082845 |
| Tsc22d4 | -0.1645485 |
| Tsen2 | 1.8183103 |
| Tsen34 | -0.4605069 |
| Tsen54 | -0.577936 |
| Tsfm | -0.6563889 |
| Tsg101 | 2.4213169 |
| Tsga10 | 2.6390509 |
| Tsga10 | 2.4234738 |
| Tsga14 | -0.6621161 |
| Tsga8 | -0.6187548 |
| Tshb | 1.7052019 |
| Tshr | 0.9678648 |
| Tshz3 | -0.5562814 |
| Tsku | -0.36568 |
| Tsn | -0.2617614 |
| Tsnaxip1 | -0.3284612 |
| Tsnaxip1 | -0.5931459 |
| Tspan1 | -0.1242859 |
| Tspan11 | -0.7575702 |
| Tspan12 | -0.3705841 |
| Tspan13 | -0.6580219 |
| Tspan14 | -0.51858 |
| Tspan17 | 1.1495962 |
| Tspan17 | 0.5164756 |
| Tspan18 | -0.61405 |
| Tspan18 | 1.6763193 |
| Tspan2 | -0.3379353 |
| Tspan3 | 0.4448982 |
| Tspan31 | -0.5794467 |
| Tspan33 | -0.436607 |
| Tspan6 | 0.9772724 |
| Tspan6 | 1.0267335 |
| Tspan7 | -0.1481514 |
| Tspan8 | -0.0786451 |
| Tspo | -0.1407855 |
| Tspo | -0.5992946 |
| Tspo | -0.1771504 |
| Tspyl1 | 4.2435762 |
| Tspyl3 | 1.6667494 |
| Tsr1 | -0.4418694 |
| Tsr2 | -0.0235914 |
| Tsr2 | 1.0353052 |
| Tsr2 | 0.8720315 |
| Tssc1 | -0.3755121 |
| Tssc1 | -0.6426139 |
| Tssc1 | -0.7352695 |
| Tssc1 | 2.0259296 |
| Tssc1 | 0.3427204 |
| Tssc4 | 1.776143 |
| Tssc4 | 0.620551 |
| Tssk4 | -0.3935808 |
| Tssk5 | 0.9704688 |
| Tssk6 | -0.1476587 |
| Tst | 1.0731479 |
| Tsta3 | 0.1955715 |
| Tsx | -0.6588394 |
| Ttbk2 | -0.6306138 |
| Ttc1 | -0.3261065 |
| Ttc12 | -0.1971294 |
| Ttc13 | 0.9970957 |
| Ttc14 | -0.4591642 |
| Ttc16 | 0.0687499 |
| Ttc17 | -0.1132755 |
| Ttc19 | 3.2988809 |
| Ttc19 | -0.5916145 |
| Ttc21a | 0.7198966 |
| Ttc22 | 0.3360342 |
| Ttc23 | 0.2753961 |
| Ttc24 | 0.5314075 |
| Ttc24 | 1.950409 |
| Ttc26 | 2.7962695 |
| Ttc27 | 1.8824173 |
| Ttc29 | -0.5228589 |
| Ttc29 | -0.3632369 |
| Ttc3 | 1.9600805 |
| Ttc3 | -0.6337999 |
| Ttc3 | -0.1817259 |
| Ttc3 | -0.6937873 |
| Ttc3 | 0.1084986 |
| Ttc3 | 2.3309392 |
| Ttc30a1 | -0.2420955 |
| Ttc30a2 | -0.119005 |
| Ttc33 | 0.3929438 |
| Ttc33 | -0.1731006 |
| Ttc33 | 1.7439792 |
| Ttc35 | 0.9853126 |
| Ttc4 | 3.0994211 |
| Ttc5 | -0.5839925 |
| Ttc5 | 3.1052693 |
| Ttc5 | 0.1566242 |
| Ttc7 | -0.4438499 |
| Ttc8 | 0.1300471 |
| Ttc8 | 0.8154573 |
| Ttf2 | -0.4551467 |
| Ttk | 0.1910047 |
| Ttl | 0.1104074 |
| Ttll1 | -0.7915076 |
| Ttll1 | -0.431372 |
| Ttll1 | 0.4817178 |
| Ttll10 | -0.2464327 |
| Ttll11 | 0.4922193 |
| Ttll13 | -0.568919 |
| Ttll4 | 3.0994014 |
| Ttll5 | -0.7317467 |
| Ttll5 | -0.7924408 |
| Ttll6 | 1.90628 |
| Ttll8 | -0.5636961 |
| Ttll9 | 2.8221468 |
| Ttn | 2.5312028 |
| Ttn | -0.1858106 |
| Ttr | 0.1371288 |
| Ttyh1 | -0.4498148 |
| Ttyh1 | 2.015374 |
| Ttyh2 | 0.2307036 |
| Ttyh2 | 0.3929438 |
| Ttyh3 | -0.4431893 |
| Tub | -0.5408857 |
| Tub | -0.4638716 |
| Tuba1a | -0.7748548 |
| Tuba1b | -0.6937873 |
| Tuba3a | -0.385441 |
| Tuba3a | 1.2776096 |
| Tuba3a | -0.6330024 |
| Tuba3a | -0.3898158 |
| Tuba3b | 0.6827527 |
| Tuba4a | -0.1863223 |
| Tuba6 | -0.6654034 |
| Tuba8 | 0.178249 |
| Tubb2b | 0.0169633 |
| Tubb2b | -0.7831468 |
| Tubb2b | 0.4888183 |
| Tubb2b | 1.7877732 |
| Tubb2b | 2.3634722 |
| Tubb2c | -0.6870365 |
| Tubb4 | -0.6929409 |
| Tubb5 | -0.3935808 |
| Tubb6 | 2.2893544 |
| Tubd1 | 2.4424169 |
| Tubd1 | -0.1113729 |
| Tube1 | -0.730868 |
| Tubg1 | -0.4788111 |
| Tubgcp2 | 0.2721526 |
| Tubgcp4 | 0.096583 |
| Tufm | -0.5192919 |
| Tuft1 | -0.7405769 |
| Tug1 | -0.7143151 |
| Tulp1 | -0.4578232 |
| Tulp2 | 1.7786257 |
| Tulp3 | 2.1287103 |
| Tulp4 | 1.6350469 |
| Tusc2 | -0.2324045 |
| Tusc2 | 0.5000242 |
| Tusc4 | 0.2841017 |
| Tut1 | -0.7282362 |
| Twf2 | 1.3116679 |
| Twist1 | -0.7335066 |
| Twist2 | 2.888064 |
| Twistnb | 1.3566541 |
| Twistnb | -0.809389 |
| Twsg1 | -0.2243992 |
| Txk | -0.431372 |
| Txlna | -0.5379797 |
| Txn1 | 1.059253 |
| Txn2 | 0.6801549 |
| Txndc1 | -0.5086659 |
| Txndc11 | 1.1764525 |
| Txndc11 | -0.2117214 |
| Txndc11 | 2.2276466 |
| Txndc12 | 2.1834267 |
| Txndc13 | 1.2117379 |
| Txndc13 | 3.3683765 |
| Txndc15 | 2.130924 |
| Txndc2 | -0.7980586 |
| Txndc5 | -0.719513 |
| Txndc8 | -0.3779852 |
| Txndc9 | 0.1322888 |
| Txnip | 1.0712376 |
| Txnl2 | 0.3807664 |
| Txnl4 | -0.6988802 |
| Txnl4a | 2.8943789 |
| Txnl4a | -0.5293115 |
| Txnl4a | -0.707426 |
| Txnl4b | 0.2084574 |
| Txnrd2 | -0.5756742 |
| Txnrd3 | -0.6887199 |
| Tyk2 | 1.9566632 |
| Tyk2 | 1.3339019 |
| Tyk2 | -0.7291127 |
| Tyki | -0.3367462 |
| Tyms | 0.3978312 |
| Tyms | -0.5908497 |
| Tyms | -0.7684522 |
| Tyms-ps | 0.3743285 |
| Tyro3 | -0.7343877 |
| Tyrobp | 1.4700551 |
| Tyw1 | 0.3104022 |
| Tyw1 | 0.8843215 |
| U2af1l4 | 1.2058348 |
| U2af2 | 0.7037216 |
| U2af2 | -0.613268 |
| Uaca | 0.1649686 |
| Uap1 | 0.0954224 |
| Uap1l1 | -0.4418694 |
| Uba1 | 0.4221994 |
| Uba1 | -0.6466458 |
| Uba2 | -0.567424 |
| Uba5 | 0.4160215 |
| Ubac1 | -0.3403178 |
| Ubac1 | 1.4649915 |
| Ubac1 | -0.5962156 |
| Ubac2 | -0.8189293 |
| Ubap1 | 2.4152451 |
| Ubap2 | -0.7143151 |
| Ubap2l | 0.1807419 |
| Ubap2l | 1.1401623 |
| Ubap2l | -0.5969845 |
| Ubb | 0.7102648 |
| Ubd | 0.6624422 |
| Ube1c | -0.7335066 |
| Ube1l | -0.3266947 |
| Ube1x | 3.4710486 |
| Ube2a | 1.5961291 |
| Ube2b | 1.4986632 |
| Ube2d1 | -0.2028368 |
| Ube2d2 | -0.5607237 |
| Ube2d3 | -0.7503802 |
| Ube2e1 | -0.6250622 |
| Ube2e2 | -0.0275194 |
| Ube2e3 | 3.4825015 |
| Ube2f | -0.8791646 |
| Ube2f | -0.3167424 |
| Ube2f | 0.2039487 |
| Ube2g1 | 1.3063233 |
| Ube2g1 | -0.5401584 |
| Ube2g1 | -0.5481877 |
| Ube2g2 | -0.4326782 |
| Ube2i | 1.1046537 |
| Ube2j1 | -0.5100763 |
| Ube2j2 | 2.5738546 |
| Ube2j2 | -0.6980296 |
| Ube2j2 | 0.8346082 |
| Ube2k | -0.1630468 |
| Ube2k | 0.7510844 |
| Ube2l3 | 2.360364 |
| Ube2l3 | 0.1255487 |
| Ube2l6 | 1.5269503 |
| Ube2m | -0.3499045 |
| Ube2n | -0.3379353 |
| Ube2n | -0.779453 |
| Ube2o | 0.3072696 |
| Ube2q1 | -0.0336601 |
| Ube2q1 | -0.7108646 |
| Ube2q2 | -0.4598353 |
| Ube2q2 | 2.1476014 |
| Ube2q2 | -0.6670511 |
| Ube2r2 | 2.7980491 |
| Ube2s | 0.8169726 |
| Ube2t | 0.4772213 |
| Ube2u | 2.8302758 |
| Ube2u | -0.8660224 |
| Ube3a | -0.4863646 |
| Ube3a | -0.6179692 |
| Ube3b | -0.5726663 |
| Ube3b | -0.5877963 |
| Ube3c | 0.0161261 |
| Ube4a | -0.2965565 |
| Ube4b | 0.7384793 |
| Ubfd1 | -0.1442162 |
| Ubiad1 | 0.0631698 |
| Ubie | -0.7343877 |
| Ubl3 | 2.2269455 |
| Ubl4 | -0.5756742 |
| Ubl4b | -0.6988802 |
| Ubl5 | -0.6070337 |
| Ubl7 | -0.6109256 |
| Ublcp1 | 0.6437388 |
| Ubox5 | -0.5107822 |
| Ubox5 | 1.0070198 |
| Ubp1 | -0.3367462 |
| Ubp1 | -0.667876 |
| Ubqln1 | 0.1289244 |
| Ubqln1 | 0.0543383 |
| Ubqln1 | 1.0601457 |
| Ubqln3 | -0.7539688 |
| Ubqln4 | -0.6904062 |
| Ubqlnl | -0.1873466 |
| Ubr1 | 1.5762704 |
| Ubr5 | -0.3718138 |
| Ubtd1 | -0.7256113 |
| Ubtd2 | 0.2323947 |
| Ubtd2 | -0.650694 |
| Ubtd2 | 0.2844227 |
| Ubtf | -0.3767479 |
| Ubxd1 | -0.3718138 |
| Ubxd1 | -0.7229933 |
| Ubxd1 | 0.0727169 |
| Ubxd3 | -0.31616 |
| Ubxd4 | -0.4268136 |
| Ubxd5 | 1.7496993 |
| Ubxd8 | -0.5614659 |
| Uchl1 | 0.2662879 |
| Uchl3 | -0.5321926 |
| Uchl5 | -0.0809479 |
| Uchl5 | 0.0051789 |
| Uchl5 | -0.6845165 |
| Uck1 | 0.1202751 |
| Uck1 | 1.4239554 |
| Uck1 | -0.5771815 |
| Uck1 | -0.0763475 |
| Uck2 | 0.5741988 |
| Uck2 | -0.4652206 |
| Uck2 | 0.1789619 |
| Uevld | 2.4108522 |
| Ufc1 | 1.9895567 |
| Ufsp1 | 1.7120809 |
| Ufsp2 | -0.4692786 |
| Ugcg | -0.276207 |
| Ugp2 | 0.8501109 |
| Ugt1a10 | -0.6720105 |
| Ugt1a6a | 2.1050891 |
| Ugt1a6a | 0.5637917 |
| Ugt1a6a | -0.7868541 |
| Ugt3a2 | -0.6211155 |
| Ugt8a | -0.0341002 |
| Uhmk1 | 0.0551444 |
| Uhrf1 | 1.4041689 |
| Uhrf1bp1 | -0.6588394 |
| Uhrf1bp1l | -0.8189293 |
| Uhrf2 | -0.011468 |
| Uhrf2 | 0.5773241 |
| Uhrf2 | -0.5824749 |
| Uhrf2 | 1.4920958 |
| Uimc1 | 1.9958675 |
| Ulk1 | -0.7169107 |
| Ulk1 | -0.7212516 |
| Ulk2 | 2.1537033 |
| Ulk2 | -0.3231709 |
| Umodl1 | -0.0011913 |
| Umps | -0.3986228 |
| Umps | 0.4120703 |
| Umps | -0.6442247 |
| Unc119 | 0.5567118 |
| Unc119 | -0.5171577 |
| Unc119b | -0.1127995 |
| Unc119b | -0.6629369 |
| Unc119b | 0.1678549 |
| Unc13b | 1.9392145 |
| Unc13c | -0.3848175 |
| Unc13d | -0.5100763 |
| Unc45a | -0.0667546 |
| Unc45a | 2.539913 |
| Unc45b | -0.6078109 |
| Unc5a | -0.3559429 |
| Unc5a | 1.4718362 |
| Unc5b | 2.0465473 |
| Unc5c | 0.2718277 |
| Unc84a | -0.5257216 |
| Unc84a | 1.9295282 |
| Unc84b | -0.6226923 |
| Unc93a | -0.0438304 |
| Unc93b1 | -0.5489208 |
| Unc93b1 | -0.5051487 |
| Ung | -0.1660524 |
| Ung | 2.0572812 |
| Unk | 0.0711319 |
| Unkl | 1.0625222 |
| Uox | 1.0486296 |
| Upb1 | -0.4418694 |
| Upb1 | -0.2175123 |
| Upb1 | 0.5842613 |
| Upf1 | -0.530031 |
| Upf1 | -0.4222756 |
| Upf1 | 0.6057248 |
| Upf2 | -0.6703546 |
| Upf3a | 1.0616317 |
| Upk1a | -0.7459123 |
| Upk1a | -0.4918939 |
| Upk1b | -0.673669 |
| Upk2 | -0.3910692 |
| Upk3a | 0.9614892 |
| Upk3b | -0.5293115 |
| Upk3b | 0.7148828 |
| Upk3b | -0.5481877 |
| Upp1 | 1.6813916 |
| Upp1 | -0.267293 |
| Upp1 | -0.6219036 |
| Upp2 | 1.5927148 |
| Upp2 | 3.2881328 |
| Upp2 | 1.4881152 |
| Upp2 | 0.0119297 |
| Uqcc | 2.4951239 |
| Uqcr | 0.7783912 |
| Uqcrc1 | -0.7405769 |
| Uqcrc2 | -0.5696674 |
| Uqcrh | -0.5954473 |
| Urm1 | -0.3290507 |
| Urod | 2.5283982 |
| Uros | -0.342706 |
| Uros | -0.6563889 |
| Use1 | 0.9560629 |
| Use1 | -0.6988802 |
| Use1 | -0.4190467 |
| Usf1 | 0.2882666 |
| Usf1 | -0.7485906 |
| Usf2 | -0.4068702 |
| Usf2 | -0.7238652 |
| Ush1c | -0.4484862 |
| Ush1g | 2.1415133 |
| Ush2a | -0.6654034 |
| Ush3a | -0.4891255 |
| Ushbp1 | 2.3372034 |
| Usmg5 | -0.5467231 |
| Uso1 | 1.8703041 |
| Usp1 | 1.9736349 |
| Usp1 | -0.4891255 |
| Usp1 | -0.568919 |
| Usp10 | 1.155025 |
| Usp10 | -0.2243992 |
| Usp10 | 0.7809443 |
| Usp11 | 1.2475605 |
| Usp11 | 1.334695 |
| Usp12 | -0.5503886 |
| Usp12 | 0.6857751 |
| Usp13 | -0.0786451 |
| Usp14 | 0.4191152 |
| Usp15 | -0.6761619 |
| Usp16 | -0.2318688 |
| Usp18 | -0.4679241 |
| Usp18 | 3.1911472 |
| Usp19 | 1.6255847 |
| Usp2 | 0.4681678 |
| Usp2 | 0.709844 |
| Usp2 | 0.9997832 |
| Usp20 | 0.0567546 |
| Usp21 | 1.1699386 |
| Usp22 | -0.3804643 |
| Usp25 | 0.1548011 |
| Usp28 | 2.6065946 |
| Usp29 | -0.1305627 |
| Usp3 | -0.3355584 |
| Usp3 | 0.5039042 |
| Usp33 | 1.5549207 |
| Usp37 | -0.8570233 |
| Usp38 | -0.6385979 |
| Usp38 | 0.3348138 |
| Usp39 | -0.0681194 |
| Usp39 | -0.1540832 |
| Usp39 | -0.7933749 |
| Usp39 | 1.3289019 |
| Usp4 | -0.6361961 |
| Usp4 | -0.6195411 |
| Usp43 | 2.319701 |
| Usp45 | 0.6857751 |
| Usp46 | -0.6290245 |
| Usp47 | -0.1981647 |
| Usp48 | -0.4829242 |
| Usp49 | 0.3986913 |
| Usp5 | 0.9386003 |
| Usp50 | 0.6799381 |
| Usp52 | 0.0323272 |
| Usp52 | 0.2181006 |
| Usp52 | 0.3868735 |
| Usp53 | -0.0262084 |
| Usp6nl | 2.6331304 |
| Usp6nl | -0.6612959 |
| Usp7 | 1.2718398 |
| Usp8 | -0.1971294 |
| Usp8 | 0.8748947 |
| Usp9x | 1.028423 |
| Usp9y | 1.2197669 |
| Uspl1 | -0.6997315 |
| Utp11l | 0.6271981 |
| Utp15 | -0.6385979 |
| Utp6 | 0.9851524 |
| Utx | -0.72999 |
| Utx | -0.3523155 |
| Uty | 1.2794059 |
| Uvrag | -0.5923799 |
| Uxt | 0.8933101 |
| Uxt | 0.7056256 |
| V1ra2 | 1.5250765 |
| V1rd2 | 2.6009639 |
| V1rd20 | 1.3500941 |
| V1rd21 | -0.4222756 |
| V2r8 | 1.2163337 |
| Vac14 | -0.2356245 |
| Vamp1 | -0.3565487 |
| Vamp1 | 0.0535316 |
| Vamp2 | 0.0376702 |
| Vamp3 | -0.1417645 |
| Vamp3 | 0.2414779 |
| Vamp4 | -0.5931459 |
| Vamp5 | -0.6062571 |
| Vamp8 | -0.2249309 |
| Vamp8 | -0.5518586 |
| Vangl2 | -0.158555 |
| Vapa | -0.4498148 |
| Vapb | -0.5704163 |
| Vars | -0.0218506 |
| Vars | 3.6605216 |
| Vars | 2.2148575 |
| Vars2 | -0.7082845 |
| Vash1 | 0.1255487 |
| Vash1 | -0.5939124 |
| Vasn | 0.178249 |
| Vasp | 0.7760287 |
| Vat1 | 0.0433928 |
| Vav1 | 0.4582222 |
| Vav2 | 1.8911305 |
| Vav3 | 0.9201469 |
| Vax1 | 0.6265125 |
| Vbp1 | 0.7319113 |
| Vcam1 | 4.1246881 |
| Vcl | -0.6523178 |
| Vcp | -0.5143192 |
| Vcpip1 | 2.4320174 |
| Vdac1 | -0.6828401 |
| Vdac2 | -0.667876 |
| Vdac3 | -0.7776112 |
| Vdr | -0.4438499 |
| Vdr | 0.5645213 |
| Vdr | 0.1962723 |
| Vegfa | 0.9198051 |
| Vegfa | -0.6466458 |
| Vegfb | 1.823904 |
| Vegfc | 0.0707353 |
| Vezf1 | -0.2925673 |
| Vezt | 0.2927328 |
| Vgf | 2.3888749 |
| Vgll2 | -0.6458381 |
| Vgll4 | -0.5870344 |
| Vhlh | 0.1278004 |
| Vil1 | -0.7530704 |
| Vill | -0.421629 |
| Vim | -0.4024209 |
| Vip | 2.0406592 |
| Vit | -0.6306138 |
| Vkorc1 | -0.6803306 |
| Vkorc1 | 0.0663622 |
| Vkorc1 | -0.5962156 |
| Vkorc1l1 | 0.817351 |
| Vkorc1l1 | 0.3883221 |
| Vkorc1l1 | 0.5854526 |
| Vldlr | -0.5143192 |
| Vldlr | 0.3830972 |
| Vldlr | 0.4547044 |
| Vldlr | -0.6410056 |
| Vldlr | -0.7620901 |
| Vmn2r81 | -0.6179692 |
| Vmo1 | 0.1142141 |
| Vnn3 | 0.5157104 |
| Vnn3 | -0.6490728 |
| Vpreb1 | -0.6330024 |
| Vpreb3 | 0.5725119 |
| Vps11 | -0.3916965 |
| Vps13a | -0.7666304 |
| Vps16 | 1.8724328 |
| Vps16 | -0.5839925 |
| Vps16 | -0.6596576 |
| Vps16 | 0.398118 |
| Vps18 | 1.0769576 |
| Vps24 | 2.6927737 |
| Vps25 | -0.5681712 |
| Vps25 | -0.8065445 |
| Vps26 | -0.5336363 |
| Vps26a | -0.6971797 |
| Vps26b | -0.1660524 |
| Vps28 | 1.2816767 |
| Vps29 | 1.7193774 |
| Vps29 | -0.6988802 |
| Vps29 | -0.7352695 |
| Vps33a | 1.5290075 |
| Vps33a | 0.2042962 |
| Vps33b | -0.2475199 |
| Vps33b | -0.817014 |
| Vps35 | 0.1400958 |
| Vps36 | -0.051414 |
| Vps37b | -0.7915076 |
| Vps37c | -0.4551467 |
| Vps37d | 1.5527333 |
| Vps39 | -0.2994156 |
| Vps39 | -0.5592407 |
| Vps41 | 1.7609689 |
| Vps41 | 2.6626041 |
| Vps45 | -0.6109256 |
| Vps4a | -0.2784479 |
| Vps4b | -0.581717 |
| Vps53 | 0.8975125 |
| Vps53 | 0.6258264 |
| Vps53 | 0.9300082 |
| Vps54 | -0.2988431 |
| Vps72 | 0.7092123 |
| Vrk1 | 2.2546149 |
| Vrk1 | -0.6904062 |
| Vrk2 | 3.1234782 |
| Vrk3 | -0.7575702 |
| Vsig2 | 0.0144496 |
| Vsig4 | -0.7748548 |
| Vsnl1 | 3.1957372 |
| Vstm2a | 0.6278832 |
| Vstm2a | -0.7915076 |
| Vstm2a | 0.2022092 |
| Vstm2b | 0.9215128 |
| Vsx2 | 0.4909125 |
| Vti1a | -0.5178686 |
| Vti1a | 2.6667538 |
| Vti1a | -0.4591642 |
| Vti1b | 0.7482518 |
| Vti1b | -0.5445301 |
| Vtn | 0.7555196 |
| Vwa2 | 2.9211314 |
| Vwa3a | -0.7639038 |
| Vwf | -0.2383157 |
| Wars | -0.6274377 |
| Wars | -0.7125884 |
| Wars | -0.5058511 |
| Wars | -0.0041779 |
| Wars2 | 1.9405818 |
| Wars2 | -0.5711657 |
| Was | -0.6861958 |
| Wasf1 | -0.4747147 |
| Wasf2 | 0.0853072 |
| Wasf3 | -0.4538111 |
| Wasl | 1.3692096 |
| Wbp1 | -0.281256 |
| Wbp1 | 0.083742 |
| Wbp11 | -0.0478382 |
| Wbp2 | 2.5320669 |
| Wbp2nl | 0.2042962 |
| Wbp4 | -0.8189293 |
| Wbp5 | -0.1276608 |
| Wbp7 | -0.6187548 |
| Wbscr16 | -0.6187548 |
| Wbscr18 | -0.4068702 |
| Wbscr22 | -0.1756298 |
| Wbscr22 | -0.1580572 |
| Wbscr22 | -0.1142282 |
| Wbscr27 | 1.5101496 |
| Wbscr28 | 0.6285679 |
| Wbscr28 | -0.1066319 |
| Wdfy1 | 0.106969 |
| Wdfy3 | 0.3836791 |
| Wdhd1 | 0.1660519 |
| Wdr1 | -0.6523178 |
| Wdr1 | 1.9786711 |
| Wdr13 | -0.703144 |
| Wdr13 | -0.6377967 |
| Wdr16 | 0.155166 |
| Wdr17 | -0.1204425 |
| Wdr18 | -0.0874238 |
| Wdr20a | 1.0540301 |
| Wdr20b | -0.5711657 |
| Wdr21 | 0.3375577 |
| Wdr22 | -0.5592407 |
| Wdr23 | 1.8379602 |
| Wdr24 | -0.5065541 |
| Wdr27 | 0.8233871 |
| Wdr3 | -0.36568 |
| Wdr31 | -0.5885588 |
| Wdr31 | 0.0186355 |
| Wdr31 | -0.4686011 |
| Wdr32 | 0.0356186 |
| Wdr33 | -0.8740896 |
| Wdr34 | 0.2917774 |
| Wdr37 | -0.7459123 |
| Wdr37 | -0.3214136 |
| Wdr37 | -0.6687015 |
| Wdr37 | 0.3836791 |
| Wdr38 | 0.0397177 |
| Wdr41 | 0.0315027 |
| Wdr45 | 1.1182217 |
| Wdr45l | -0.5430708 |
| Wdr45l | 1.0959366 |
| Wdr46 | -0.5037452 |
| Wdr46 | -0.5946796 |
| Wdr47 | -0.2233366 |
| Wdr5 | -0.7803752 |
| Wdr51b | -0.5228589 |
| Wdr53 | -0.6266453 |
| Wdr54 | -0.6621161 |
| Wdr55 | 0.0409443 |
| Wdr59 | -0.3804643 |
| Wdr5b | -0.7014363 |
| Wdr6 | -0.7117262 |
| Wdr60 | -0.0559018 |
| Wdr61 | -0.8112899 |
| Wdr62 | -0.703144 |
| Wdr63 | 0.5688877 |
| Wdr66 | 1.243973 |
| Wdr67 | 1.4135347 |
| Wdr68 | 2.1546581 |
| Wdr69 | 1.17685 |
| Wdr70 | 0.2354315 |
| Wdr70 | -0.4652206 |
| Wdr74 | -0.1014425 |
| Wdr75 | -0.7952458 |
| Wdr75 | 0.8664607 |
| Wdr76 | -0.6179692 |
| Wdr77 | -0.4726727 |
| Wdr78 | -0.703144 |
| Wdr8 | 1.753445 |
| Wdr8 | -0.0456097 |
| Wdr9 | 0.8394322 |
| Wdr93 | 0.193115 |
| Wdsof1 | -0.6219036 |
| Wfdc1 | -0.61405 |
| Wfdc10 | 2.2463018 |
| Wfdc10 | 0.748657 |
| Wfdc12 | 0.4798687 |
| Wfdc15b | -0.385441 |
| Wfdc15b | -0.4255149 |
| Wfdc2 | -0.5285925 |
| Wfdc3 | -0.5121955 |
| Wfdc6a | -0.568919 |
| Wfdc6b | 1.7053587 |
| Wfdc6b | -0.6853558 |
| Wfikkn2 | 0.0356186 |
| Wfs1 | 0.4333358 |
| Whdc1 | 0.6597914 |
| Whrn | -0.6219036 |
| Whrn | 2.1075657 |
| Whsc2 | -0.4307195 |
| Wif1 | 0.3845513 |
| Wipf1 | 2.2105899 |
| Wipf2 | 0.4369288 |
| Wipi1 | 1.0163814 |
| Wipi1 | 0.0778507 |
| Wipi1 | -0.4106995 |
| Wipi2 | 0.1292988 |
| Wipi2 | -0.5741691 |
| Wipi2 | -0.4538111 |
| Wisp1 | 0.2354315 |
| Wisp1 | 1.4042746 |
| Wisp2 | -0.5681712 |
| Wiz | 4.7267144 |
| Wiz | 0.206379 |
| Wnk1 | 0.4416082 |
| Wnk1 | 0.3508661 |
| Wnk2 | 0.6120063 |
| Wnk4 | 1.78582 |
| Wnt10a | -0.1610479 |
| Wnt10b | -0.5394317 |
| Wnt16 | -0.0640304 |
| Wnt16 | -0.530751 |
| Wnt16 | 1.1418093 |
| Wnt2 | 0.1104074 |
| Wnt3a | -0.258457 |
| Wnt4 | -0.6062571 |
| Wnt4 | 0.837765 |
| Wnt5a | 2.4585611 |
| Wnt5a | 1.4844118 |
| Wnt5b | 0.6868523 |
| Wnt6 | 1.9509009 |
| Wnt7a | -0.107105 |
| Wnt7b | 0.1983716 |
| Wnt7b | -0.1822356 |
| Wrb | 0.2508118 |
| Wrn | -0.6016101 |
| Wsb1 | 0.1689351 |
| Wsb2 | -0.1047418 |
| Wscd1 | 1.354324 |
| Wscd2 | 0.1533401 |
| Wtap | 3.1626792 |
| Wtip | -0.3017087 |
| Wwc1 | -0.386689 |
| Wwc1 | -0.5533307 |
| Wwc2 | 4.8699131 |
| Wwc2 | -0.7169107 |
| Wwox | -0.3804643 |
| Wwox | -0.8441647 |
| Wwp1 | 3.305673 |
| Wwp2 | -0.5802029 |
| Wwp2 | -0.3450998 |
| Wwp2 | 0.1270504 |
| Wwtr1 | 1.1208855 |
| X99384 | 3.3494171 |
| Xab1 | -0.2790089 |
| Xab1 | -0.5794467 |
| Xbp1 | -0.5931459 |
| Xdh | -0.6282308 |
| Xist | -0.7739376 |
| Xist | -0.0270822 |
| Xk | 1.1703386 |
| Xkr4 | -0.4939752 |
| Xkr5 | -0.7748548 |
| Xkr6 | 0.8955886 |
| Xkr8 | 0.720938 |
| Xkrx | -0.1325019 |
| Xlr4a | 2.1390677 |
| Xpc | -0.6946343 |
| Xpnpep1 | 1.6643019 |
| Xpnpep1 | -0.3644577 |
| Xpnpep1 | 0.4617278 |
| Xpnpep2 | 2.0001505 |
| Xpnpep2 | 0.7140447 |
| Xpnpep3 | -0.7143151 |
| Xpo4 | -0.6887199 |
| Xpo4 | -0.703999 |
| Xpo5 | -0.7915076 |
| Xpo6 | -0.3361521 |
| Xpo7 | 0.4089549 |
| Xpo7 | -0.5365298 |
| Xpr1 | 0.2705269 |
| Xrcc1 | -0.5877963 |
| Xrcc2 | 1.3439402 |
| Xrcc2 | -0.6062571 |
| Xrcc3 | 1.3991876 |
| Xrcc6 | 0.124797 |
| Xrcc6 | -0.6703546 |
| Xrcc6 | 1.6425867 |
| Xrn1 | 2.7748018 |
| Xrn2 | 1.9227236 |
| Xylb | -0.5445301 |
| Xylt2 | 0.1764645 |
| Yaf2 | -0.6330024 |
| Yap1 | 0.8436804 |
| Ybx2 | 1.3361664 |
| Ybx2 | 0.0327392 |
| Yeats2 | 2.9332951 |
| Yeats4 | 0.9062116 |
| Yeats4 | -0.6555733 |
| Yif1a | -0.4932809 |
| Yif1a | -0.6450311 |
| Yif1a | 1.5514551 |
| Yif1b | 2.1593686 |
| Yipf1 | 2.527531 |
| Yipf2 | 1.4525729 |
| Yipf3 | 0.0068708 |
| Yipf3 | -0.821809 |
| Yipf3 | 0.1062033 |
| Yipf4 | -0.0253353 |
| Yipf6 | -0.4190467 |
| Yipf6 | -0.2185688 |
| Yipf6 | -0.5423419 |
| Yipf6 | -0.3986228 |
| Yipf7 | -0.4747147 |
| Ykt6 | 0.1674946 |
| Ykt6 | 2.8092912 |
| Ylpm1 | 2.2001772 |
| Yme1l1 | -0.2706265 |
| Yme1l1 | -0.2667384 |
| Yme1l1 | -0.8620129 |
| Yod1 | 1.4442758 |
| Ypel1 | 0.1311686 |
| Ypel3 | -0.8065445 |
| Ypel3 | 0.6775503 |
| Ypel4 | -0.3973599 |
| Ypel4 | 0.4435287 |
| Ypel5 | 0.0884304 |
| Yrdc | -0.4119792 |
| Ythdf1 | 1.435705 |
| Ythdf2 | -0.6466458 |
| Ythdf2 | -0.4287647 |
| Ythdf3 | -0.5452606 |
| Ythdf3 | -0.5496544 |
| Ywhab | -0.0855693 |
| Ywhab | -0.3817062 |
| Ywhae | -0.496757 |
| Ywhag | 1.3950354 |
| Ywhah | -0.527874 |
| Ywhaq | -0.4094215 |
| Ywhaz | -0.8237334 |
| Yy2 | 0.0323272 |
| Zadh1 | -0.6997315 |
| Zadh2 | 0.1463718 |
| Zan | -0.7803752 |
| Zap70 | 1.4944186 |
| Zap70 | 1.6542868 |
| Zbbx | -0.1339588 |
| Zbed4 | 0.2554466 |
| Zbp1 | 1.6457533 |
| Zbtb17 | 0.2454887 |
| Zbtb2 | 0.8521235 |
| Zbtb22 | -0.4094215 |
| Zbtb24 | -0.6828401 |
| Zbtb3 | -0.7721059 |
| Zbtb32 | -0.3848175 |
| Zbtb33 | -0.7282362 |
| Zbtb38 | 0.0442077 |
| Zbtb39 | -0.5503886 |
| Zbtb40 | 0.5925707 |
| Zbtb41 | 0.3775526 |
| Zbtb43 | 0.093485 |
| Zbtb45 | -0.1242859 |
| Zbtb46 | 0.7039333 |
| Zbtb5 | -0.643419 |
| Zbtb7a | 1.3228462 |
| Zbtb7c | -0.8122416 |
| Zbtb8 | 1.5584652 |
| Zbtb9 | 0.7354059 |
| Zbtb9 | -0.6426139 |
| Zc3h10 | -0.0491776 |
| Zc3h11a | -0.6878779 |
| Zc3h12a | 1.6403307 |
| Zc3h12b | -0.7082845 |
| Zc3h13 | -0.3929523 |
| Zc3h14 | 0.3816411 |
| Zc3h14 | 0.230365 |
| Zc3h15 | 0.4950881 |
| Zc3h18 | -0.4242179 |
| Zc3h3 | 1.2077635 |
| Zc3h3 | -0.6878779 |
| Zc3h3 | 0.0615699 |
| Zc3h6 | 2.4948372 |
| Zc3h7a | -0.0196787 |
| Zc3h7a | -0.6687015 |
| Zc3h8 | 2.9629986 |
| Zc3hc1 | 0.1081164 |
| Zc3hc1 | -0.5496544 |
| Zc3hc1 | -0.0196787 |
| Zc3hc1 | 0.986913 |
| Zcchc12 | -0.7022898 |
| Zcchc14 | 2.7620119 |
| Zcchc16 | -0.3693558 |
| Zcchc17 | -0.5372545 |
| Zcchc17 | 3.2165755 |
| Zcchc18 | -0.8208482 |
| Zcchc18 | 1.5254515 |
| Zcchc3 | -0.3296406 |
| Zcchc3 | 1.7886401 |
| Zcchc3 | -0.6377967 |
| Zcchc5 | -0.1731006 |
| Zcchc6 | -0.421629 |
| Zcchc8 | -0.3761298 |
| Zcchc8 | 0.8661002 |
| Zcchc8 | 1.5390459 |
| Zcchc9 | 0.1008272 |
| Zcrb1 | -0.5037452 |
| Zcwpw1 | -0.7441307 |
| Zdhhc1 | -0.0157811 |
| Zdhhc12 | 0.4089549 |
| Zdhhc12 | 1.1296682 |
| Zdhhc13 | 1.0070198 |
| Zdhhc13 | -0.6523178 |
| Zdhhc13 | -0.471993 |
| Zdhhc14 | -0.636996 |
| Zdhhc15 | -0.5358057 |
| Zdhhc16 | 1.0526827 |
| Zdhhc16 | -0.6580219 |
| Zdhhc17 | -0.3823277 |
| Zdhhc19 | 0.0571567 |
| Zdhhc19 | -0.6728394 |
| Zdhhc21 | 3.1597914 |
| Zdhhc24 | -0.5855123 |
| Zdhhc3 | 0.9020454 |
| Zdhhc3 | 2.4745607 |
| Zdhhc4 | 0.9982032 |
| Zdhhc6 | 1.4540833 |
| Zdhhc7 | 1.5586466 |
| Zdhhc9 | -0.6000659 |
| Zeb1 | 0.1602607 |
| Zeb2 | -0.4706349 |
| Zer1 | 0.6493416 |
| Zf | -0.1766433 |
| Zfa | -0.6604764 |
| Zfand2a | -0.7693644 |
| Zfand2b | -0.7432411 |
| Zfand2b | 0.7464267 |
| Zfand2b | 1.3953554 |
| Zfand2b | 0.475895 |
| Zfand3 | -0.6008377 |
| Zfand6 | 1.2082772 |
| Zfand6 | -0.1997197 |
| Zfat | 2.8849281 |
| Zfh4 | 1.9215218 |
| Zfhx2 | 0.3000274 |
| Zfhx2 | -0.5023437 |
| Zfhx3 | -0.0735974 |
| Zfhx3 | 2.3881579 |
| Zfml | 1.2066068 |
| Zfp1 | 0.0923208 |
| Zfp101 | 2.4337327 |
| Zfp105 | -0.4645459 |
| Zfp106 | 1.032558 |
| Zfp106 | -0.2410141 |
| Zfp109 | -0.6070337 |
| Zfp11 | 0.2936872 |
| Zfp119 | -0.3650687 |
| Zfp12 | -0.2634177 |
| Zfp128 | -0.7620901 |
| Zfp13 | 1.9736349 |
| Zfp131 | -0.2080536 |
| Zfp142 | -0.3022828 |
| Zfp143 | 0.9795301 |
| Zfp148 | -0.7952458 |
| Zfp160 | -0.1132755 |
| Zfp161 | -0.4484862 |
| Zfp161 | -0.3126727 |
| Zfp169 | 0.4809258 |
| Zfp179 | -0.527874 |
| Zfp182 | -0.6645806 |
| Zfp184 | -0.1014425 |
| Zfp185 | -0.2767668 |
| Zfp187 | -0.2891606 |
| Zfp187 | -0.2117214 |
| Zfp189 | 2.1736159 |
| Zfp191 | 0.8976872 |
| Zfp2 | -0.2212148 |
| Zfp2 | 2.4797046 |
| Zfp202 | 0.6446373 |
| Zfp207 | 1.540617 |
| Zfp212 | -0.3718138 |
| Zfp212 | 1.2975479 |
| Zfp212 | 1.4887959 |
| Zfp212 | -0.2222751 |
| Zfp213 | 0.5797217 |
| Zfp217 | 1.9864755 |
| Zfp219 | -0.6654034 |
| Zfp235 | 1.052383 |
| Zfp236 | -0.7256113 |
| Zfp236 | -0.0731397 |
| Zfp238 | -0.3373406 |
| Zfp238 | -0.6124866 |
| Zfp238 | 0.4940458 |
| Zfp238 | -0.8955783 |
| Zfp238 | -0.535082 |
| Zfp239 | 0.3191222 |
| Zfp251 | -1.0081636 |
| Zfp259 | 0.3535667 |
| Zfp26 | -0.4884345 |
| Zfp263 | 1.1020893 |
| Zfp27 | -1.0233102 |
| Zfp27 | -0.5065541 |
| Zfp275 | 2.0275262 |
| Zfp276 | -0.6539443 |
| Zfp277 | -0.6163997 |
| Zfp277 | 1.3247925 |
| Zfp280c | 1.7576981 |
| Zfp281 | 2.6427414 |
| Zfp282 | -0.4891255 |
| Zfp286 | -1.0543035 |
| Zfp288 | -0.8945446 |
| Zfp292 | 0.8756092 |
| Zfp292 | -0.4352957 |
| Zfp295 | -0.4538111 |
| Zfp296 | -0.6266453 |
| Zfp296 | 1.833895 |
| Zfp3 | 3.4634091 |
| Zfp30 | 3.1405897 |
| Zfp313 | -0.0016174 |
| Zfp313 | -0.5072575 |
| Zfp316 | 2.0214684 |
| Zfp316 | -0.887338 |
| Zfp318 | -0.4113392 |
| Zfp322a | 0.0619701 |
| Zfp324 | -0.119005 |
| Zfp326 | 0.9474526 |
| Zfp326 | 0.385713 |
| Zfp326 | 2.0160658 |
| Zfp329 | -0.7961825 |
| Zfp329 | 0.1352701 |
| Zfp330 | -0.1242859 |
| Zfp330 | 1.2668851 |
| Zfp334 | -0.7849988 |
| Zfp335 | -0.9080663 |
| Zfp339 | -0.7657207 |
| Zfp346 | 0.079425 |
| Zfp346 | -0.6377967 |
| Zfp35 | -0.0550026 |
| Zfp354a | 0.8405422 |
| Zfp36 | 0.8188634 |
| Zfp365 | -0.4438499 |
| Zfp367 | 1.1557015 |
| Zfp367 | -0.1089996 |
| Zfp36l1 | -0.7915076 |
| Zfp36l1 | -0.0841807 |
| Zfp36l1 | -0.613268 |
| Zfp36l2 | -0.703144 |
| Zfp37 | 1.2655517 |
| Zfp371 | -0.1530921 |
| Zfp382 | 0.4325048 |
| Zfp385a | -0.8392624 |
| Zfp385a | -0.2334767 |
| Zfp385c | -0.0976851 |
| Zfp386 | 0.3384707 |
| Zfp386 | 0.371683 |
| Zfp39 | 0.0294384 |
| Zfp393 | 1.4805967 |
| Zfp395 | 0.1232919 |
| Zfp397 | 0.0352077 |
| Zfp398 | 1.861677 |
| Zfp398 | -0.6124866 |
| Zfp398 | -0.229729 |
| Zfp41 | 1.4395913 |
| Zfp41 | -0.1127995 |
| Zfp410 | -0.8690401 |
| Zfp410 | -0.610146 |
| Zfp410 | 0.1198973 |
| Zfp414 | -0.5157374 |
| Zfp418 | -0.1700739 |
| Zfp42 | -0.5416136 |
| Zfp423 | 0.0352077 |
| Zfp423 | -0.9038864 |
| Zfp428 | -0.6596576 |
| Zfp428 | -0.6458381 |
| Zfp446 | 0.8532196 |
| Zfp446 | 0.0627701 |
| Zfp449 | -0.6047057 |
| Zfp451 | 2.6569732 |
| Zfp457 | 3.1970958 |
| Zfp46 | 1.3239915 |
| Zfp467 | 0.7294372 |
| Zfp472 | -0.2259952 |
| Zfp473 | -0.9228339 |
| Zfp473 | 0.2968623 |
| Zfp474 | 0.2276525 |
| Zfp474 | 0.3909244 |
| Zfp512 | 2.2460266 |
| Zfp513 | 0.6040899 |
| Zfp516 | 0.2259534 |
| Zfp52 | 1.5903443 |
| Zfp521 | -0.7256113 |
| Zfp523 | -0.3028572 |
| Zfp523 | 2.1233361 |
| Zfp524 | -0.4504797 |
| Zfp526 | 0.8059351 |
| Zfp532 | -0.1310472 |
| Zfp532 | 1.2982528 |
| Zfp536 | 0.1707329 |
| Zfp551 | 1.375538 |
| Zfp553 | -0.3126727 |
| Zfp553 | -0.2064857 |
| Zfp560 | 0.5894923 |
| Zfp566 | 1.7091161 |
| Zfp57 | -0.0144852 |
| Zfp574 | -0.901803 |
| Zfp579 | -0.7335066 |
| Zfp592 | -0.4638716 |
| Zfp593 | 1.38431 |
| Zfp595 | 0.2404726 |
| Zfp598 | 1.9444871 |
| Zfp60 | 1.2141661 |
| Zfp606 | 0.4940458 |
| Zfp607 | -0.7361521 |
| Zfp608 | 1.3941816 |
| Zfp609 | -0.7396904 |
| Zfp61 | 0.2534629 |
| Zfp612 | -0.4043253 |
| Zfp617 | 0.703298 |
| Zfp619 | -0.8122416 |
| Zfp62 | 0.2056852 |
| Zfp622 | 0.4038362 |
| Zfp623 | -0.527156 |
| Zfp629 | 2.1425305 |
| Zfp637 | 0.3631109 |
| Zfp639 | -0.408783 |
| Zfp64 | 0.6475521 |
| Zfp64 | -0.3266947 |
| Zfp64 | -0.051414 |
| Zfp641 | 0.6351624 |
| Zfp644 | -0.7657207 |
| Zfp646 | 0.388901 |
| Zfp653 | 3.6351463 |
| Zfp654 | 1.6817128 |
| Zfp654 | 1.1988608 |
| Zfp655 | -0.4126197 |
| Zfp661 | -0.0672093 |
| Zfp667 | -0.4274635 |
| Zfp668 | 3.0192 |
| Zfp67 | -0.6588394 |
| Zfp672 | 0.7840778 |
| Zfp68 | 2.5283982 |
| Zfp68 | 0.6836172 |
| Zfp68 | 2.0547332 |
| Zfp687 | -0.5719157 |
| Zfp688 | -0.9785363 |
| Zfp689 | -0.9059742 |
| Zfp69 | -1.0035485 |
| Zfp691 | 1.0030933 |
| Zfp692 | -0.7414642 |
| Zfp692 | 0.136014 |
| Zfp704 | 0.7409313 |
| Zfp704 | 0.201164 |
| Zfp706 | 1.7657077 |
| Zfp709 | 1.9591053 |
| Zfp709 | 1.5437518 |
| Zfp710 | -0.634598 |
| Zfp711 | -1.172319 |
| Zfp715 | -0.4801802 |
| Zfp71-rs1 | -0.4794954 |
| Zfp74 | 1.2755699 |
| Zfp75 | 1.48187 |
| Zfp75 | -0.1909398 |
| Zfp75 | 3.4543418 |
| Zfp750 | -0.7343877 |
| Zfp758 | 0.7395017 |
| Zfp760 | -0.2243992 |
| Zfp764 | -0.8237334 |
| Zfp768 | 0.0277839 |
| Zfp770 | -0.7212516 |
| Zfp771 | -0.8986858 |
| Zfp771 | 1.024271 |
| Zfp78 | -0.7999382 |
| Zfp78 | 0.7883705 |
| Zfp780b | -0.4498148 |
| Zfp786 | 2.3511883 |
| Zfp787 | 1.4018403 |
| Zfp787 | -0.1147048 |
| Zfp790 | -0.5719157 |
| Zfp804a | -0.1142282 |
| Zfp809 | -0.9898287 |
| Zfp810 | 0.3194322 |
| Zfp811 | 1.0911892 |
| Zfp817 | 1.0851145 |
| Zfp819 | 0.4416082 |
| Zfp82 | 1.9283344 |
| Zfp825 | -0.6466458 |
| Zfp828 | 1.1459152 |
| Zfp85-rs1 | 0.1793181 |
| Zfp87 | 1.5790298 |
| Zfp87 | 0.0397177 |
| Zfp90 | -0.4905087 |
| Zfp91 | -0.024463 |
| Zfp91-cntf | -0.8863127 |
| Zfp92 | -0.1945458 |
| Zfp93 | -0.7317467 |
| Zfp93 | 1.4526737 |
| Zfp94 | 2.1199626 |
| Zfp97 | 2.8856096 |
| Zfpl1 | 3.1710734 |
| Zfpm2 | 0.2350945 |
| Zfr | -0.80844 |
| Zfx | -0.6195411 |
| Zfx | -0.730868 |
| Zfy1 | 0.393808 |
| Zfyve1 | -0.5044467 |
| Zfyve16 | -0.1914541 |
| Zfyve19 | 0.7569267 |
| Zfyve20 | -0.6031568 |
| Zfyve21 | 0.7631347 |
| Zfyve26 | 0.8773934 |
| Zfyve27 | 0.0232201 |
| Zgpat | -0.8324391 |
| Zgpat | 2.3344334 |
| Zhx1 | 0.0302646 |
| Zhx1 | -0.5401584 |
| Zhx1 | -0.4891255 |
| Zhx2 | -0.1966122 |
| Zhx3 | -0.826627 |
| Zic1 | -0.6929409 |
| Zic2 | 2.2006092 |
| Zic3 | -0.1560682 |
| Zic4 | -0.7971202 |
| Zkscan1 | -0.4801802 |
| Zkscan14 | 0.2792745 |
| Zkscan17 | -0.809389 |
| Zkscan3 | 1.3529902 |
| Zkscan6 | -0.1170916 |
| Zkscan6 | -0.6744993 |
| Zmat1 | -0.7326263 |
| Zmat2 | -0.272855 |
| Zmat3 | 1.088301 |
| Zmat4 | -0.342706 |
| Zmat5 | 1.1247984 |
| Zmiz1 | -0.6588394 |
| Zmiz1 | -0.7134514 |
| Zmiz2 | 0.5671919 |
| Zmiz2 | -0.6786611 |
| Zmpste24 | 0.1404661 |
| Zmpste24 | 1.1204654 |
| Zmpste24 | -0.7065681 |
| Zmym3 | -0.4672476 |
| Zmym3 | 1.0463708 |
| Zmym5 | 0.1725276 |
| Zmym6 | 3.1142694 |
| Zmynd10 | -0.530751 |
| Zmynd11 | 1.8936062 |
| Zmynd11 | -0.4504797 |
| Zmynd11 | -0.5824749 |
| Zmynd12 | 0.852489 |
| Zmynd15 | -0.7831468 |
| Znhit2 | 0.6356156 |
| Znhit3 | -0.5143192 |
| Znhit4 | 0.1157327 |
| Znrd1 | 0.133035 |
| Znrd1 | -0.1009721 |
| Znrd1 | -0.1486444 |
| Znrf1 | 1.5674071 |
| Znrf2 | -0.5496544 |
| Znrf3 | -0.5855123 |
| Znrf4 | -0.3517122 |
| Zp1 | -0.2914305 |
| Zp3 | -0.7169107 |
| Zp3r | 0.097356 |
| Zpbp | -0.1329873 |
| Zpbp2 | -0.6274377 |
| Zpbp2 | 3.1364813 |
| Zranb1 | 1.8358263 |
| Zranb2 | -0.7370355 |
| Zranb3 | 1.891 |
| Zrsr2 | -0.673669 |
| Zscan10 | 0.5026125 |
| Zscan12 | -0.707426 |
| Zscan2 | 0.0186355 |
| Zscan2 | 0.4266687 |
| Zscan2 | 0.4227591 |
| Zscan20 | 1.7588143 |
| Zscan20 | -0.431372 |
| Zscan21 | 0.4462659 |
| Zscan21 | -0.5946796 |
| Zswim1 | 0.1732445 |
| Zswim2 | 0.1345256 |
| Zswim3 | 0.9649272 |
| Zswim4 | -0.2784479 |
| Zswim5 | 1.8703707 |
| Zw10 | -0.2999883 |
| Zw10 | 0.3484594 |
| Zw10 | -0.8500791 |
| Zwilch | 0.8156469 |
| Zxda | 1.2162063 |
| Zxdc | 2.627772 |
| Zxdc | 0.7778011 |
| Zxdc | 0.9426185 |
| Zyg11b | -0.3730451 |
| Zyx | -0.6980296 |
| Zzz3 | 1.8600638 |
| Zzz3 | -0.1247673 |
| Zzz3 | 0.6943609 |
| Zzz3 | 0.0360292 |

| **Gene Symbol** | **WT5** |
| --- | --- |
| 0610006I08Rik | -0.4191118 |
| 0610006I08Rik | -0.0085324 |
| 0610007C21Rik | -0.6674313 |
| 0610007C21Rik | 0.11288 |
| 0610007P08Rik | 1.1405018 |
| 0610007P14Rik | -0.5576429 |
| 0610007P22Rik | -0.6199123 |
| 0610009B22Rik | 0.0775496 |
| 0610009D07Rik | -0.3161065 |
| 0610009O20Rik | 0.1481883 |
| 0610010E21Rik | 1.6337577 |
| 0610010K06Rik | -0.1161253 |
| 0610010K14Rik | -0.5182775 |
| 0610012A05Rik | -0.321153 |
| 0610012D14Rik | -0.395303 |
| 0610012G03Rik | -0.7067715 |
| 0610012H03Rik | 1.1053808 |
| 0610025P10Rik | -0.5428496 |
| 0610031J06Rik | -0.1367569 |
| 0610031J06Rik | 1.4564469 |
| 0610031J06Rik | 2.883691 |
| 0610037D15Rik | 0.0627723 |
| 0610037L13Rik | -0.4804711 |
| 0610037L13Rik | 0.740028 |
| 0610038F07Rik | -0.0103576 |
| 0610040J01Rik | 1.7026525 |
| 0710008K08Rik | 0.210196 |
| 0910001A06Rik | 1.3034417 |
| 0910001A06Rik | -0.7067715 |
| 0910001L09Rik | 1.1363329 |
| 1110001A07Rik | 0.262948 |
| 1110001J03Rik | 1.0405366 |
| 1110002B05Rik | -0.813281 |
| 1110002N22Rik | 0.2254454 |
| 1110003E01Rik | 1.7631526 |
| 1110006G06Rik | -0.1815911 |
| 1110007A13Rik | -0.6769318 |
| 1110007A13Rik | -0.4174313 |
| 1110007C09Rik | 1.5429847 |
| 1110007L15Rik | 2.9027921 |
| 1110007L15Rik | 0.9092662 |
| 1110007L15Rik | -0.3715399 |
| 1110007L15Rik | -0.1608339 |
| 1110007M04Rik | -0.5358548 |
| 1110008F13Rik | 1.0156618 |
| 1110008J03Rik | 1.9253924 |
| 1110008J03Rik | -0.6020908 |
| 1110008J03Rik | -0.2467158 |
| 1110008P14Rik | -0.2457802 |
| 1110012D08Rik | 0.1714249 |
| 1110012J17Rik | -0.6458612 |
| 1110012L19Rik | -0.414637 |
| 1110012L19Rik | 0.1747417 |
| 1110012M11Rik | 2.1279596 |
| 1110013L07Rik | -0.4349405 |
| 1110017D15Rik | -0.6939755 |
| 1110017D15Rik | 0.2305696 |
| 1110017D15Rik | 0.0501665 |
| 1110018G07Rik | 0.4473092 |
| 1110018J18Rik | -0.2868632 |
| 1110018J18Rik | 0.6413527 |
| 1110019N10Rik | 0.0999466 |
| 1110019N10Rik | 0.3959501 |
| 1110020C03Rik | 1.6624474 |
| 1110020P15Rik | 2.2409454 |
| 1110020P15Rik | -0.7159083 |
| 1110031B06Rik | -0.4224816 |
| 1110031I02Rik | 1.1921709 |
| 1110032A13Rik | -0.6630781 |
| 1110032A13Rik | -0.4973598 |
| 1110032E23Rik | 0.7388617 |
| 1110034A24Rik | 0.4197652 |
| 1110036O03Rik | 0.1753436 |
| 1110038D17Rik | 0.730321 |
| 1110038F14Rik | 1.0159113 |
| 1110038F14Rik | 0.2319881 |
| 1110039B18Rik | -0.660909 |
| 1110039B18Rik | -0.6479965 |
| 1110039B18Rik | 0.089143 |
| 1110048D14Rik | 2.2052276 |
| 1110049B09Rik | 1.3909548 |
| 1110049B09Rik | 0.8542978 |
| 1110049F12Rik | 0.3095456 |
| 1110049F12Rik | -0.6027701 |
| 1110057K04Rik | -0.6401906 |
| 1110057K04Rik | 0.9667727 |
| 1110057K04Rik | 2.4191589 |
| 1110058L19Rik | -0.525771 |
| 1110059E24Rik | -0.7807991 |
| 1110059G10Rik | 1.0349287 |
| 1110067D22Rik | -0.7391405 |
| 1110067I12Rik | -0.2927389 |
| 1190002A17Rik | 0.096359 |
| 1190002H23Rik | 1.7186188 |
| 1190002N15Rik | -0.621297 |
| 1190003J15Rik | -0.6082222 |
| 1190003M12Rik | 1.0114105 |
| 1190005F20Rik | -0.6296481 |
| 1190005I06Rik | 1.2431551 |
| 1190005P17Rik | 0.1711228 |
| 1190005P17Rik | 1.1004847 |
| 1190007F08Rik | -0.6359596 |
| 1190017O12Rik | 3.1237188 |
| 1190020J12Rik | -0.660909 |
| 1200002N14Rik | -0.5238921 |
| 1200003C05Rik | 0.4445903 |
| 1200003C05Rik | 0.8186272 |
| 1200003C05Rik | 0.0979914 |
| 1200003I07Rik | -0.3662207 |
| 1200009F10Rik | -0.3865933 |
| 1200009I06Rik | 2.4411645 |
| 1200009I06Rik | 2.1014024 |
| 1200009O22Rik | 0.0755479 |
| 1200011O22Rik | 2.8458087 |
| 1200013B08Rik | 0.2604748 |
| 1200013P24Rik | 0.0481065 |
| 1200013P24Rik | -0.5201454 |
| 1200014J11Rik | 2.9329591 |
| 1200015F23Rik | 0.0705253 |
| 1200015F23Rik | 0.6551015 |
| 1200015N20Rik | 1.7644633 |
| 1200016B10Rik | -0.5157927 |
| 1200016B10Rik | -0.2947055 |
| 1200016D23Rik | 2.1230043 |
| 1300001I01Rik | 1.160113 |
| 1300007L22Rik | 3.6186061 |
| 1300010M03Rik | 0.6040749 |
| 1300013J15Rik | -0.1206261 |
| 1300013J15Rik | -0.7446436 |
| 1300014I06Rik | 0.3737278 |
| 1300017J02Rik | -0.3080872 |
| 1300018I05Rik | -0.6408976 |
| 1300018J18Rik | -0.7407096 |
| 1500001L15Rik | -0.7758985 |
| 1500001M20Rik | -0.5022406 |
| 1500001M20Rik | 1.8429267 |
| 1500001M20Rik | -0.6401906 |
| 1500002O20Rik | -0.5270257 |
| 1500003O03Rik | 1.5429847 |
| 1500005A01Rik | 0.8377571 |
| 1500010J02Rik | -0.766979 |
| 1500010J02Rik | 0.1426011 |
| 1500011H22Rik | -0.3525279 |
| 1500012F01Rik | -0.5700388 |
| 1500015O10Rik | 0.1488071 |
| 1500019C06Rik | 0.2410169 |
| 1500019G21Rik | 2.370672 |
| 1500031L02Rik | 0.2043777 |
| 1500032D16Rik | -0.3838877 |
| 1500032D16Rik | -0.0140186 |
| 1500032D16Rik | 0.9322777 |
| 1500032L24Rik | -0.1392629 |
| 1500041J02Rik | 2.3142582 |
| 1500041N16Rik | -0.3262263 |
| 1520401A03Rik | -0.0366601 |
| 1600002K03Rik | 0.7991062 |
| 1600012H06Rik | -0.4383668 |
| 1600012H06Rik | 1.4180206 |
| 1600014C10Rik | -0.5010181 |
| 1600016N20Rik | -0.5207688 |
| 1600021P15Rik | 2.0269901 |
| 1600027N09Rik | 0.028312 |
| 1600029D21Rik | 2.710063 |
| 1700001C02Rik | 0.4125186 |
| 1700001C19Rik | -0.2680132 |
| 1700001E04Rik | -0.5550537 |
| 1700001J03Rik | 1.6376678 |
| 1700001O22Rik | 1.7094935 |
| 1700001O22Rik | 0.1132011 |
| 1700001P01Rik | -0.7973184 |
| 1700003F12Rik | 1.091654 |
| 1700003M02Rik | 1.0459908 |
| 1700007G11Rik | 1.6548764 |
| 1700007I06Rik | -0.1330101 |
| 1700007K09Rik | 1.0013321 |
| 1700008F21Rik | 1.8189175 |
| 1700008I05Rik | 0.538722 |
| 1700008P20Rik | -0.0056192 |
| 1700009P17Rik | 0.2496829 |
| 1700010A17Rik | -0.7120907 |
| 1700010A17Rik | -0.0571614 |
| 1700010D01Rik | -0.3130914 |
| 1700010M22Rik | 1.0401718 |
| 1700010M22Rik | 0.2151139 |
| 1700011A15Rik | -0.3457545 |
| 1700011F03Rik | -0.4906893 |
| 1700011F14Rik | 3.4159705 |
| 1700011H14Rik | -0.2849127 |
| 1700011K15Rik | -0.3410932 |
| 1700012A03Rik | 0.1671868 |
| 1700012B09Rik | 0.4343248 |
| 1700012G19Rik | -0.1442948 |
| 1700012H05Rik | -0.5089926 |
| 1700012H17Rik | -0.6240726 |
| 1700012L04Rik | 0.4306481 |
| 1700013B16Rik | 0.911495 |
| 1700013G24Rik | -0.3752811 |
| 1700013N18Rik | -0.6573046 |
| 1700013N18Rik | 0.9748797 |
| 1700015E13Rik | -0.7143795 |
| 1700016C15Rik | -0.5466867 |
| 1700016D06Rik | -0.2208591 |
| 1700016G05Rik | 0.1589597 |
| 1700016K19Rik | 0.3776299 |
| 1700016K19Rik | 0.1067584 |
| 1700017N19Rik | 3.4498406 |
| 1700018B24Rik | -0.6732666 |
| 1700018C11Rik | 2.0371021 |
| 1700018F24Rik | -0.3920275 |
| 1700019D03Rik | -0.4412315 |
| 1700019E19Rik | -0.6289493 |
| 1700019G17Rik | 0.2841182 |
| 1700019H03Rik | -0.5634946 |
| 1700019L03Rik | -0.5825977 |
| 1700019M22Rik | 2.4036574 |
| 1700019N19Rik | -0.2853999 |
| 1700020C11Rik | -0.6082222 |
| 1700020D05Rik | -0.6387782 |
| 1700020L24Rik | 0.4315686 |
| 1700020N01Rik | 2.2908564 |
| 1700020O03Rik | 2.8810243 |
| 1700021C14Rik | 0.0874954 |
| 1700021F05Rik | 2.5785528 |
| 1700021K02Rik | 1.2408913 |
| 1700021K02Rik | 1.3743907 |
| 1700021K19Rik | -0.6739985 |
| 1700022C21Rik | 0.8576896 |
| 1700023B02Rik | 1.7513323 |
| 1700023B02Rik | 2.5518677 |
| 1700023E05Rik | -0.5595895 |
| 1700023E05Rik | -0.6537138 |
| 1700023I07Rik | -0.2699295 |
| 1700023M03Rik | -0.4574344 |
| 1700024G13Rik | 0.1057883 |
| 1700025D03Rik | -0.3226721 |
| 1700025E21Rik | 1.2085189 |
| 1700025F22Rik | -0.1881714 |
| 1700025G04Rik | -0.3252095 |
| 1700025K23Rik | -0.6850452 |
| 1700026D08Rik | -0.6537138 |
| 1700026L06Rik | 0.96651 |
| 1700027A23Rik | 0.0205608 |
| 1700027D21Rik | -0.4574344 |
| 1700027J05Rik | 0.2480118 |
| 1700027N10Rik | -0.7605439 |
| 1700028K03Rik | -0.706014 |
| 1700029F12Rik | 1.3286712 |
| 1700029F12Rik | -0.5661069 |
| 1700029G01Rik | 3.1437513 |
| 1700029H14Rik | 0.1164062 |
| 1700029H14Rik | -0.196572 |
| 1700029I01Rik | -0.7022357 |
| 1700029I08Rik | 2.5838063 |
| 1700029I15Rik | -0.4441048 |
| 1700029J07Rik | -0.5700388 |
| 1700029P11Rik | -0.604811 |
| 1700029P11Rik | 1.6633974 |
| 1700029P11Rik | -0.5028524 |
| 1700030J22Rik | 2.2057658 |
| 1700030K09Rik | -0.6784018 |
| 1700034E13Rik | 0.2727772 |
| 1700034H14Rik | 0.2859954 |
| 1700034H14Rik | -0.1169421 |
| 1700034H14Rik | 2.1021624 |
| 1700034H14Rik | -0.433232 |
| 1700034I23Rik | 2.2978527 |
| 1700034I23Rik | 1.7646911 |
| 1700034O15Rik | 1.5656235 |
| 1700036D21Rik | -0.3546218 |
| 1700037C18Rik | 0.6625347 |
| 1700037C18Rik | 1.2051488 |
| 1700037H04Rik | 3.4729707 |
| 1700039E15Rik | -0.5779515 |
| 1700040I03Rik | 1.5914995 |
| 1700042B14Rik | 1.1495448 |
| 1700045I19Rik | -0.5441269 |
| 1700047I17Rik1 | 0.8954996 |
| 1700047I17Rik1 | -0.0668039 |
| 1700049G17Rik | 0.6605461 |
| 1700049K14Rik | -0.7718339 |
| 1700049L16Rik | 0.9903778 |
| 1700052N19Rik | 0.0927578 |
| 1700052N19Rik | 0.4904398 |
| 1700054O13Rik | -0.754152 |
| 1700055M20Rik | 1.7286043 |
| 1700057G04Rik | 1.86926 |
| 1700057K13Rik | -0.5550537 |
| 1700058C13Rik | -0.766979 |
| 1700060H10Rik | 2.4242516 |
| 1700061G19Rik | -0.5806037 |
| 1700063I17Rik | 3.0645944 |
| 1700065I17Rik | -0.0332856 |
| 1700065O13Rik | -0.754152 |
| 1700065O13Rik | 0.4343248 |
| 1700067C01Rik | -0.804007 |
| 1700067K01Rik | -0.2217706 |
| 1700067P10Rik | 0.1503523 |
| 1700071K01Rik | -0.6472842 |
| 1700072E05Rik | -0.7621486 |
| 1700072E05Rik | 1.2121818 |
| 1700072E05Rik | 2.2289423 |
| 1700073E17Rik | 0.5331547 |
| 1700080E11Rik | -0.6718044 |
| 1700081D17Rik | -0.0389164 |
| 1700081D17Rik | 0.6929271 |
| 1700084C01Rik | -0.0239741 |
| 1700084C01Rik | 1.8059196 |
| 1700088E04Rik | -0.4627015 |
| 1700093K21Rik | 2.3272332 |
| 1700094C09Rik | 1.878957 |
| 1700095K08Rik | 1.6417563 |
| 1700102P08Rik | 1.273917 |
| 1700108M19Rik | -0.3641013 |
| 1700109H08Rik | 0.7356888 |
| 1700112C13Rik | -0.2010233 |
| 1700113I22Rik | -0.6565854 |
| 1700113I22Rik | 1.1252742 |
| 1700113O17Rik | 0.6040749 |
| 1700120B06Rik | -0.1472423 |
| 1700123D08Rik | -0.6909894 |
| 1700123K08Rik | -0.3457545 |
| 1700123L14Rik | 1.6700216 |
| 1700123O20Rik | -0.2145029 |
| 1700123O20Rik | -0.750972 |
| 1700127D06Rik | -0.1334257 |
| 1700129C05Rik | -0.203704 |
| 1700129I15Rik | -0.196128 |
| 1810007M14Rik | 1.4179387 |
| 1810007P19Rik | -0.5726691 |
| 1810007P19Rik | 3.4565619 |
| 1810008A18Rik | -0.0563942 |
| 1810008A18Rik | -0.6939755 |
| 1810009A15Rik | -0.2897965 |
| 1810009A15Rik | 0.4569889 |
| 1810009N02Rik | 1.5377999 |
| 1810009O10Rik | -0.4230444 |
| 1810009O10Rik | 0.0668252 |
| 1810010M01Rik | 3.1484939 |
| 1810011O10Rik | -0.3333745 |
| 1810014F10Rik | -0.6465724 |
| 1810015A11Rik | 2.0069695 |
| 1810015C04Rik | -0.571353 |
| 1810019J16Rik | -0.632448 |
| 1810020D17Rik | -0.4985777 |
| 1810020G14Rik | 0.2288646 |
| 1810020G14Rik | 2.5163912 |
| 1810021J13Rik | 0.3000895 |
| 1810021J13Rik | 0.0934136 |
| 1810022C23Rik | 0.2496829 |
| 1810026J23Rik | 0.8779354 |
| 1810027O10Rik | 1.4886848 |
| 1810030J14Rik | -0.3619866 |
| 1810030N24Rik | -0.7621486 |
| 1810030N24Rik | -0.5213927 |
| 1810030N24Rik | 0.1555875 |
| 1810031K17Rik | 2.2203964 |
| 1810035L17Rik | 2.4100794 |
| 1810035L17Rik | 0.9315967 |
| 1810037C20Rik | -0.5674157 |
| 1810043G02Rik | 0.9096845 |
| 1810044A24Rik | -0.847785 |
| 1810046J19Rik | 1.3605816 |
| 1810048J11Rik | 0.3887604 |
| 1810049H13Rik | 1.0152874 |
| 1810049H19Rik | -0.2490589 |
| 1810055E12Rik | -0.8399122 |
| 1810055E12Rik | 1.779941 |
| 1810055E12Rik | -0.6769318 |
| 1810055G02Rik | -0.7321113 |
| 1810063B05Rik | -0.3272442 |
| 1810064F22Rik | 0.8258079 |
| 1810065E05Rik | 0.0030674 |
| 1810073G14Rik | -0.6992237 |
| 1810074P20Rik | -0.5582913 |
| 1810074P20Rik | 0.028312 |
| 2010001J22Rik | -0.421357 |
| 2010001J22Rik | 0.0698536 |
| 2010001M09Rik | -0.0879701 |
| 2010003J03Rik | 0.6101991 |
| 2010003O18Rik | 1.2169428 |
| 2010004A03Rik | -0.3514827 |
| 2010005H15Rik | -0.1430343 |
| 2010005J08Rik | -0.3731415 |
| 2010005J08Rik | 0.4384443 |
| 2010005J08Rik | -0.2290941 |
| 2010007H12Rik | 0.2692394 |
| 2010007H12Rik | 0.0360007 |
| 2010011I20Rik | -0.8166759 |
| 2010012O05Rik | 0.6249642 |
| 2010100O12Rik | -0.771023 |
| 2010100O12Rik | -0.1019439 |
| 2010106G01Rik | -0.4029897 |
| 2010106G01Rik | 1.1282727 |
| 2010107E04Rik | 1.7352037 |
| 2010107G12Rik | 0.5362516 |
| 2010109I03Rik | -0.7824382 |
| 2010110K16Rik | -0.6969709 |
| 2010111I01Rik | -0.7462218 |
| 2010200O16Rik | -0.6843047 |
| 2010301N04Rik | -0.1772293 |
| 2010305A19Rik | 1.3453341 |
| 2010305C02Rik | -0.6508508 |
| 2010305C02Rik | 0.7607816 |
| 2010308M01Rik | 0.7852318 |
| 2010311D03Rik | -0.5028524 |
| 2010316F05Rik | 1.3200027 |
| 2010321M09Rik | 1.1659932 |
| 2010321M09Rik | -0.4185513 |
| 2200001I15Rik | -0.7189735 |
| 2210009G21Rik | -0.4096279 |
| 2210009G21Rik | -0.73601 |
| 2210009G21Rik | 1.2169428 |
| 2210010B09Rik | -0.1970162 |
| 2210010L05Rik | 0.3029888 |
| 2210012G02Rik | 0.256891 |
| 2210012G02Rik | 1.5592199 |
| 2210012G02Rik | -0.7501787 |
| 2210012G02Rik | 0.4663497 |
| 2210016L21Rik | 0.4856701 |
| 2210016L21Rik | 4.2560179 |
| 2210016L21Rik | -0.6275534 |
| 2210021J22Rik | 3.4042751 |
| 2210023G05Rik | -0.7136159 |
| 2210023G05Rik | 1.6052563 |
| 2210407C18Rik | -0.7052572 |
| 2210412D01Rik | 0.6191233 |
| 2210417D09Rik | 0.8833963 |
| 2210418O10Rik | 1.2831064 |
| 2300002D11Rik | 0.5517904 |
| 2300002D11Rik | -0.4400846 |
| 2300002M23Rik | -0.8023305 |
| 2300005B03Rik | 0.6823729 |
| 2310001A20Rik | -0.5358548 |
| 2310002B06Rik | 0.5446658 |
| 2310003C23Rik | 1.0077693 |
| 2310003F16Rik | 0.1253236 |
| 2310003H01Rik | 1.4433842 |
| 2310003L22Rik | 1.3003911 |
| 2310003L22Rik | 2.3450388 |
| 2310004I24Rik | -0.4326632 |
| 2310005E10Rik | 0.3056169 |
| 2310005E10Rik | -0.1737542 |
| 2310005G13Rik | 0.9512838 |
| 2310005N01Rik | 1.3632728 |
| 2310005N03Rik | -0.6850452 |
| 2310005P05Rik | -0.1342574 |
| 2310006J04Rik | 0.5436436 |
| 2310007A19Rik | -0.6828256 |
| 2310007B03Rik | -0.3447167 |
| 2310007F21Rik | -0.4961435 |
| 2310007F21Rik | -0.4101832 |
| 2310008H04Rik | -0.7282284 |
| 2310008H09Rik | -0.524518 |
| 2310008H09Rik | 2.9885082 |
| 2310008M10Rik | 2.2953412 |
| 2310009E04Rik | 0.6346823 |
| 2310010B21Rik | 0.2432608 |
| 2310010M24Rik | 2.4192163 |
| 2310011J03Rik | 1.2855546 |
| 2310014L17Rik | -0.6366635 |
| 2310016C08Rik | -0.607539 |
| 2310016C08Rik | -0.4504566 |
| 2310016C16Rik | -0.7266797 |
| 2310016C16Rik | 2.5612085 |
| 2310016C16Rik | 0.3492223 |
| 2310016E02Rik | 1.4140011 |
| 2310016M24Rik | 1.754445 |
| 2310021P13Rik | -0.310586 |
| 2310022A10Rik | -0.0781126 |
| 2310022B05Rik | 2.1573519 |
| 2310031L18Rik | -0.0749795 |
| 2310031L18Rik | 0.3027256 |
| 2310033E01Rik | -0.222683 |
| 2310033K02Rik | 1.4686349 |
| 2310033P09Rik | -0.5232667 |
| 2310035C23Rik | -0.4338012 |
| 2310035C23Rik | -0.1063783 |
| 2310035K24Rik | -0.695472 |
| 2310036D22Rik | 2.0406481 |
| 2310036O22Rik | -0.297663 |
| 2310037I24Rik | 0.6242126 |
| 2310037I24Rik | 0.3077142 |
| 2310038H17Rik | 0.9471375 |
| 2310039E09Rik | 0.9324138 |
| 2310039H08Rik | 2.6397185 |
| 2310040C09Rik | -0.1120519 |
| 2310042D19Rik | 0.8777913 |
| 2310044G17Rik | 0.0825355 |
| 2310044H10Rik | 1.702531 |
| 2310045A20Rik | -0.6761976 |
| 2310046K01Rik | 0.5254723 |
| 2310046O06Rik | -0.6444404 |
| 2310047C04Rik | -0.5077614 |
| 2310047D13Rik | 1.2453155 |
| 2310047D13Rik | 1.1603274 |
| 2310047M10Rik | 1.8637843 |
| 2310047M10Rik | 1.2472752 |
| 2310047O13Rik | -0.6472842 |
| 2310056P07Rik | 0.1186433 |
| 2310057J16Rik | 0.8639987 |
| 2310058J06Rik | -0.712853 |
| 2310061C15Rik | -0.0476153 |
| 2310061F22Rik | -0.675464 |
| 2310061F22Rik | -0.5139333 |
| 2310065K24Rik | 0.593068 |
| 2310065K24Rik | -0.4236076 |
| 2310066E14Rik | -0.5232667 |
| 2310067B10Rik | 0.675984 |
| 2310076L09Rik | 0.8344445 |
| 2310076L09Rik | -0.3493958 |
| 2310079F23Rik | -0.4586024 |
| 2310079N02Rik | -0.6123318 |
| 2400001E08Rik | 2.2693196 |
| 2400003C14Rik | 2.5964458 |
| 2400003C14Rik | -0.4504566 |
| 2400003C14Rik | 0.1469495 |
| 2400006N03Rik | 0.0701895 |
| 2400010D15Rik | 1.9296171 |
| 2410001C21Rik | 0.5366638 |
| 2410001C21Rik | 1.6097535 |
| 2410001C21Rik | 0.1674901 |
| 2410002F23Rik | -0.2761842 |
| 2410002F23Rik | 3.4867888 |
| 2410002F23Rik | -0.6565854 |
| 2410002I01Rik | 2.0977943 |
| 2410002O22Rik | 0.0148839 |
| 2410003P15Rik | -0.0899539 |
| 2410004A20Rik | 0.9876837 |
| 2410004B18Rik | 1.3434773 |
| 2410004L22Rik | 0.2894717 |
| 2410005O16Rik | 0.6616313 |
| 2410008K03Rik | 0.8587195 |
| 2410014A08Rik | 1.3332445 |
| 2410014A08Rik | 0.291871 |
| 2410014A08Rik | 0.8178601 |
| 2410014A08Rik | 1.2818799 |
| 2410015M20Rik | 0.349473 |
| 2410015N17Rik | 0.5534114 |
| 2410016F19Rik | 0.813553 |
| 2410016F19Rik | 1.3731903 |
| 2410016O06Rik | -0.3588233 |
| 2410017P07Rik | 0.8575424 |
| 2410018C17Rik | -0.8115881 |
| 2410018G20Rik | 0.7512843 |
| 2410018M08Rik | 0.2634967 |
| 2410019A14Rik | -0.5602392 |
| 2410019G02Rik | 2.2655196 |
| 2410022L05Rik | -0.2771501 |
| 2410025L10Rik | 0.8918137 |
| 2410042D21Rik | -0.597349 |
| 2410042D21Rik | -0.7120907 |
| 2410066E13Rik | -0.3191312 |
| 2410081M15Rik | 1.424059 |
| 2410091C18Rik | -0.5518269 |
| 2410116G06Rik | 2.57178 |
| 2410127E18Rik | 0.3810312 |
| 2410127L17Rik | 1.0663191 |
| 2410127L17Rik | -0.771023 |
| 2410129H14Rik | 1.1960002 |
| 2410137M14Rik | 0.0262042 |
| 2410146L05Rik | -0.7045009 |
| 2410166I05Rik | -0.3893065 |
| 2500002L14Rik | 0.4430007 |
| 2500003M10Rik | -0.0610067 |
| 2500003M10Rik | -0.5865982 |
| 2500003M10Rik | -0.8065274 |
| 2510002A14Rik | 0.1189625 |
| 2510003E04Rik | -0.7948222 |
| 2510006D16Rik | -0.5201454 |
| 2510009E07Rik | -0.6338511 |
| 2510010F15Rik | -0.3328621 |
| 2510012J08Rik | 1.1708836 |
| 2510039O18Rik | 0.1256405 |
| 2510040D07Rik | 0.2851913 |
| 2510048L02Rik | 0.2008695 |
| 2510049I19Rik | -0.4129644 |
| 2600001B17Rik | -0.4258634 |
| 2600009E05Rik | -0.7305562 |
| 2600009E05Rik | -0.4320947 |
| 2600010E01Rik | -0.6178389 |
| 2600011E07Rik | -0.2245104 |
| 2600011E07Rik | 1.9563056 |
| 2610002J02Rik | -0.6082222 |
| 2610002M06Rik | -0.8451535 |
| 2610003J06Rik | 0.4042703 |
| 2610016F04Rik | 1.1673779 |
| 2610019A05Rik | 1.5348348 |
| 2610019P18Rik | -0.5004074 |
| 2610020C11Rik | 2.307704 |
| 2610020H08Rik | -0.1952407 |
| 2610020H08Rik | 3.7868672 |
| 2610020H08Rik | -0.6089059 |
| 2610020O08Rik | 2.2856578 |
| 2610021K21Rik | 2.1404862 |
| 2610021K21Rik | 0.8677934 |
| 2610024G14Rik | -0.4697697 |
| 2610024G14Rik | 2.02361 |
| 2610024G14Rik | -0.3646307 |
| 2610027C15Rik | 1.8132527 |
| 2610027C15Rik | -0.3431621 |
| 2610027L16Rik | 0.564282 |
| 2610028A01Rik | 1.0402934 |
| 2610028H24Rik | 0.181341 |
| 2610029G23Rik | -0.9001403 |
| 2610029G23Rik | 1.1405018 |
| 2610034E13Rik | -0.3272442 |
| 2610034E13Rik | 1.0025977 |
| 2610034M16Rik | 1.1493279 |
| 2610036D13Rik | 0.9835638 |
| 2610039C10Rik | -0.7259063 |
| 2610042L04Rik | 0.5734456 |
| 2610044O15Rik | -0.3625149 |
| 2610101N10Rik | -0.8364339 |
| 2610101N10Rik | 0.0698536 |
| 2610103J23Rik | -0.7890231 |
| 2610110G12Rik | -0.9198274 |
| 2610200G18Rik | 1.1662064 |
| 2610204K14Rik | 2.9854218 |
| 2610204L23Rik | -0.5700388 |
| 2610204M08Rik | 0.9592582 |
| 2610205E22Rik | 1.2169428 |
| 2610207I05Rik | -0.7653661 |
| 2610208M17Rik | -0.771023 |
| 2610209A20Rik | -0.4627015 |
| 2610209A20Rik | -0.2575418 |
| 2610209M04Rik | -0.1518926 |
| 2610301B20Rik | 0.7189835 |
| 2610301F02Rik | 2.8877562 |
| 2610304G08Rik | -0.5667611 |
| 2610305D13Rik | -0.8835827 |
| 2610318N02Rik | -0.6089059 |
| 2610507B11Rik | 0.2345361 |
| 2610507B11Rik | -0.8141286 |
| 2610510B01Rik | 0.4949705 |
| 2610510J17Rik | 0.2754897 |
| 2610524A10Rik | -0.61577 |
| 2610524H06Rik | 1.1134105 |
| 2610528E23Rik | 0.4880579 |
| 2610528J11Rik | 0.6549195 |
| 2610528O22Rik | 2.990576 |
| 2700007P21Rik | -0.0967298 |
| 2700007P21Rik | -0.4967515 |
| 2700038C09Rik | 2.4435444 |
| 2700038C09Rik | 2.7052493 |
| 2700050L05Rik | -0.5667611 |
| 2700055A20Rik | 1.0644242 |
| 2700060E02Rik | -0.1019439 |
| 2700062C07Rik | -0.8781268 |
| 2700062C07Rik | 1.4741309 |
| 2700078K21Rik | -0.5133143 |
| 2700081O15Rik | 0.267329 |
| 2700087H15Rik | 0.6417219 |
| 2700094K13Rik | 0.281161 |
| 2700097O09Rik | 1.4933864 |
| 2810002N01Rik | 1.5039932 |
| 2810002O09Rik | -0.6969709 |
| 2810003C17Rik | -0.3359405 |
| 2810004N20Rik | -0.5906156 |
| 2810004N23Rik | 0.3446984 |
| 2810008M24Rik | 0.5156235 |
| 2810021B07Rik | 1.2558507 |
| 2810021J22Rik | 0.6866086 |
| 2810021J22Rik | -0.9293469 |
| 2810022L02Rik | -0.4269933 |
| 2810022L02Rik | -0.5371227 |
| 2810022L02Rik | -0.541574 |
| 2810030E01Rik | 1.5829289 |
| 2810046L04Rik | -0.5479692 |
| 2810046M22Rik | -0.5647998 |
| 2810403A07Rik | 0.3499743 |
| 2810405J04Rik | -0.6014119 |
| 2810405K02Rik | 1.8222468 |
| 2810408A11Rik | 0.0940689 |
| 2810408M09Rik | -0.675464 |
| 2810410C14Rik | -0.4135216 |
| 2810410P22Rik | 1.9250555 |
| 2810410P22Rik | 0.1917468 |
| 2810417H13Rik | -0.9418658 |
| 2810422O20Rik | 1.5411876 |
| 2810428I15Rik | 0.7252611 |
| 2810432D09Rik | -0.0656416 |
| 2810432L12Rik | 0.4736408 |
| 2810439F02Rik | 0.6892464 |
| 2810439F02Rik | -0.5886048 |
| 2810441K11Rik | -0.4236076 |
| 2810452K22Rik | -0.8826712 |
| 2810453I06Rik | -0.8260743 |
| 2810453I06Rik | 1.2398067 |
| 2810470D21Rik | -0.5238921 |
| 2810485I05Rik | 0.2429806 |
| 2900006K08Rik | 1.5895179 |
| 2900010M23Rik | 0.6111524 |
| 2900011O08Rik | 2.155879 |
| 2900011O08Rik | 2.2988944 |
| 2900024C23Rik | -0.524518 |
| 2900024O10Rik | -0.6857862 |
| 2900026A02Rik | 0.7576808 |
| 2900046G09Rik | -0.632448 |
| 2900062L11Rik | -0.7998212 |
| 2900064A13Rik | 1.1070828 |
| 2900073G15Rik | 0.3014085 |
| 2900092C05Rik | -1.0039392 |
| 3000004C01Rik | -0.9094645 |
| 3000004C01Rik | 0.7283002 |
| 3000004C01Rik | -0.0254578 |
| 3010003L21Rik | 0.4317986 |
| 3010026O09Rik | 1.7881424 |
| 3110001A13Rik | -0.5307996 |
| 3110001D03Rik | -0.1235014 |
| 3110004L20Rik | -0.8260743 |
| 3110007F17Rik | -0.8574963 |
| 3110009E18Rik | -0.7067715 |
| 3110023B02Rik | 0.8209248 |
| 3110035E14Rik | 0.0269073 |
| 3110035E14Rik | -0.6401906 |
| 3110037I16Rik | 0.1732355 |
| 3110040N11Rik | 0.3358401 |
| 3110040N11Rik | 0.3844204 |
| 3110043J09Rik | -0.960463 |
| 3110043J09Rik | 1.937441 |
| 3110043J09Rik | -0.4709528 |
| 3110048E14Rik | -0.5602392 |
| 3110049J23Rik | -0.5460461 |
| 3110050N22Rik | -0.3598766 |
| 3110050N22Rik | 2.2109888 |
| 3110056O03Rik | -0.2742553 |
| 3110057O12Rik | 1.20002 |
| 3110057O12Rik | 0.876926 |
| 3110070M22Rik | 0.2678753 |
| 3110082I17Rik | 0.5298401 |
| 3200001I04Rik | 1.3835153 |
| 3200002M19Rik | -0.743068 |
| 3200002M19Rik | 0.3335489 |
| 3300001G02Rik | -0.7407096 |
| 3300001P08Rik | 0.8749041 |
| 3300001P08Rik | -0.4258634 |
| 3300001P08Rik | -0.3349133 |
| 3300002I08Rik | -0.3426444 |
| 3321401G04Rik | -0.5746465 |
| 3322402L07Rik | -0.3855101 |
| 3632451O06Rik | 1.0800624 |
| 3830402I07Rik | -0.9207751 |
| 3830406C13Rik | 1.3626658 |
| 3830406C13Rik | 1.5358483 |
| 3830406C13Rik | 2.4672681 |
| 3830408P04Rik | 1.9613232 |
| 3930401K13Rik | 0.4379874 |
| 4121402D02Rik | 2.2242392 |
| 4121402D02Rik | -0.5792767 |
| 4430402I18Rik | 0.3557193 |
| 4432405B04Rik | 0.0556381 |
| 4432416J03Rik | 0.1626249 |
| 4631426J05Rik | 2.577241 |
| 4631427C17Rik | -0.0939339 |
| 4632404H12Rik | 0.9625613 |
| 4632404H12Rik | 3.020579 |
| 4632404H12Rik | -0.6688868 |
| 4632404H22Rik | -0.6226838 |
| 4632411B12Rik | -0.7718339 |
| 4632411B12Rik | 1.8864539 |
| 4632412N22Rik | 3.3415194 |
| 4632415K11Rik | 1.3000207 |
| 4632417K18Rik | 2.044096 |
| 4632417N05Rik | -0.0426888 |
| 4632419K20Rik | 1.1832105 |
| 4632434I11Rik | 1.7120838 |
| 4732415M23Rik | 0.7737281 |
| 4732418C07Rik | 0.4141597 |
| 4732418C07Rik | 0.8987484 |
| 4732418C07Rik | 0.5946205 |
| 4732429D16Rik | -0.0518038 |
| 4732456N10Rik | -0.6331493 |
| 4732471D19Rik | -0.2213148 |
| 4732473B16Rik | -0.8303772 |
| 4732479N06Rik | -0.822646 |
| 4732479N06Rik | 0.1064352 |
| 4732496O08Rik | 0.7094029 |
| 4733401H18Rik | -0.122679 |
| 4831426I19Rik | 1.0599086 |
| 4833418A01Rik | 0.1344725 |
| 4833420G17Rik | 0.6520024 |
| 4833421E05Rik | -0.6275534 |
| 4833424O15Rik | 1.4088908 |
| 4833424O15Rik | -0.5792767 |
| 4833426J09Rik | -0.4586024 |
| 4833426J09Rik | 0.3043038 |
| 4833427G06Rik | 0.7759758 |
| 4833436C18Rik | -0.4372234 |
| 4833439L19Rik | -0.3694087 |
| 4833439L19Rik | 1.0239844 |
| 4833439L19Rik | 1.6684486 |
| 4833442J19Rik | 0.1243721 |
| 4833446K15Rik | -0.8691022 |
| 4921501E09Rik | -0.015487 |
| 4921504E06Rik | 0.7308255 |
| 4921504I05Rik | 0.8107739 |
| 4921505C17Rik | -0.4441048 |
| 4921505C17Rik | -0.1169421 |
| 4921507P07Rik | 1.3553513 |
| 4921511H03Rik | -0.3031091 |
| 4921511H03Rik | 0.1528195 |
| 4921511K06Rik | 0.5542209 |
| 4921511K06Rik | -0.2420469 |
| 4921515J06Rik | 3.3793235 |
| 4921517D21Rik | -0.4627015 |
| 4921517D22Rik | 1.2036131 |
| 4921520G13Rik | 1.2728692 |
| 4921521F21Rik | 1.6909154 |
| 4921521F21Rik | 2.4300266 |
| 4921523A10Rik | 0.9099632 |
| 4921523A10Rik | -0.5120776 |
| 4921523A10Rik | 1.3395751 |
| 4921525H12Rik | -0.5403001 |
| 4921528G01Rik | -0.607539 |
| 4921528I01Rik | -0.7462218 |
| 4921530G04Rik | -0.2771501 |
| 4921530L21Rik | -0.3876777 |
| 4921530L21Rik | 1.6893764 |
| 4921536K21Rik | -0.695472 |
| 4921537P18Rik | 0.3029888 |
| 4922502D21Rik | 1.1712017 |
| 4922503N01Rik | -0.4429544 |
| 4922503N01Rik | 0.3727498 |
| 4922505E12Rik | 0.7410265 |
| 4922505G16Rik | 2.7759341 |
| 4922505G16Rik | -0.6430217 |
| 4930401F20Rik | -0.6820869 |
| 4930402H24Rik | 2.6572936 |
| 4930403C10Rik | 0.4448172 |
| 4930403L05Rik | 0.1463295 |
| 4930403O06Rik | 0.3170954 |
| 4930404H21Rik | 1.5003956 |
| 4930404N11Rik | -0.4185513 |
| 4930408O21Rik | -0.7105679 |
| 4930408O21Rik | -0.5422116 |
| 4930412F15Rik | 0.5430298 |
| 4930415L06Rik | -0.6178389 |
| 4930418G15Rik | 0.1552803 |
| 4930427A07Rik | -0.0415555 |
| 4930428D18Rik | 0.1641479 |
| 4930428E23Rik | 1.841877 |
| 4930430D24Rik | -0.6999758 |
| 4930430E16Rik | -0.4937156 |
| 4930430E16Rik | -0.6580244 |
| 4930431L04Rik | -0.5213927 |
| 4930432H15Rik | 0.1237373 |
| 4930432K21Rik | -0.1272109 |
| 4930432O21Rik | -0.2661008 |
| 4930432O21Rik | 0.4567649 |
| 4930438O05Rik | -0.3374834 |
| 4930438O05Rik | -0.4580182 |
| 4930449I24Rik | 1.0125377 |
| 4930451C15Rik | -0.3135932 |
| 4930451I11Rik | -0.8700008 |
| 4930452B06Rik | 1.2588533 |
| 4930453N24Rik | 1.4508479 |
| 4930455C21Rik | -0.8566094 |
| 4930455F23Rik | -0.4906893 |
| 4930467M19Rik | -0.5188998 |
| 4930470P17Rik | 0.1531275 |
| 4930473A06Rik | 1.9520572 |
| 4930473A06Rik | -0.5621911 |
| 4930473A06Rik | -0.6761976 |
| 4930474N05Rik | -0.3758167 |
| 4930478A21Rik | 1.2388196 |
| 4930481F22Rik | 0.3564661 |
| 4930481F22Rik | 0.3759247 |
| 4930481M05 | -0.9113403 |
| 4930486L24Rik | 0.4712166 |
| 4930502E18Rik | -0.122679 |
| 4930503B20Rik | 3.3858808 |
| 4930503L19Rik | -0.353051 |
| 4930504E06Rik | -0.5832633 |
| 4930504E06Rik | 4.0867337 |
| 4930504O13Rik | -0.2045993 |
| 4930506M07Rik | -0.706014 |
| 4930507C10Rik | -0.6828256 |
| 4930511I11Rik | 1.7564571 |
| 4930511J11Rik | -0.448142 |
| 4930515G01Rik | -0.6192206 |
| 4930518C23Rik | -0.2728112 |
| 4930519F16Rik | 1.4088082 |
| 4930519G04Rik | -0.3374834 |
| 4930519N13Rik | -0.5700388 |
| 4930519N16Rik | -0.0525673 |
| 4930522H14Rik | -0.5403001 |
| 4930524B15Rik | 2.0712813 |
| 4930524B15Rik | 0.8663357 |
| 4930524E20Rik | -0.5687263 |
| 4930526D03Rik | -0.756544 |
| 4930535B03Rik | 2.4915075 |
| 4930535E21Rik | -0.2689709 |
| 4930538K18Rik | 1.1015115 |
| 4930539E08Rik | -0.4906893 |
| 4930540L03Rik | -0.7212788 |
| 4930544G11Rik | -0.4668182 |
| 4930544G21Rik | 1.5138914 |
| 4930547C10Rik | 2.3697057 |
| 4930547N16Rik | -0.5339559 |
| 4930549C01Rik | -0.6007336 |
| 4930550C14Rik | -0.4918987 |
| 4930550L24Rik | -0.5759671 |
| 4930555G01Rik | -0.5912868 |
| 4930555I21Rik | 0.6558293 |
| 4930562D19Rik | -0.0939339 |
| 4930562D19Rik | 0.0307651 |
| 4930563D23Rik | -0.3646307 |
| 4930563P21Rik | 1.7014366 |
| 4930570C03Rik | -0.6718044 |
| 4930572J05Rik | 2.2041862 |
| 4930572J05Rik | 0.9967619 |
| 4930572J05Rik | -0.4768906 |
| 4930573I19Rik | 0.529425 |
| 4930578I06Rik | 2.5919321 |
| 4930579C15Rik | 0.8362528 |
| 4930579E17Rik | 0.0391283 |
| 4930579E17Rik | -0.5441269 |
| 4930579J09Rik | -0.1824658 |
| 4930583H14Rik | 0.7790178 |
| 4930590J08Rik | -0.0695213 |
| 4930595M18Rik | 1.461466 |
| 4930596D02Rik | -0.5933032 |
| 4930599N23Rik | 1.0334604 |
| 4931406B18Rik | -0.7939916 |
| 4931406C07Rik | -0.466229 |
| 4931406H21Rik | -0.4168718 |
| 4931406I20Rik | 1.6661789 |
| 4931406I20Rik | 0.4698917 |
| 4931406P16Rik | 0.8455416 |
| 4931407G18Rik | -0.2072901 |
| 4931408A02Rik | -0.1754902 |
| 4931408C20Rik | 2.7126402 |
| 4931408C20Rik | -0.851305 |
| 4931409K22Rik | 1.1933109 |
| 4931417E11Rik | -0.8065274 |
| 4931417G12Rik | -0.6587447 |
| 4931428L18Rik | 1.4585675 |
| 4931432M23Rik | -0.4864685 |
| 4931433A01Rik | 0.0080983 |
| 4931440B09Rik | 0.5346012 |
| 4931440F15Rik | 0.2294332 |
| 4931440F15Rik | -0.5806037 |
| 4931440L10Rik | 1.053335 |
| 4932409I22Rik | -0.3181219 |
| 4932411E22Rik | -0.2704092 |
| 4932411G14Rik | 0.6541911 |
| 4932412H11Rik | 0.8230642 |
| 4932413O14Rik | 2.0663127 |
| 4932414J04Rik | 0.6100083 |
| 4932414N04Rik | 0.9854972 |
| 4932418E24Rik | 1.925248 |
| 4932431H17Rik | 0.6080983 |
| 4932431H17Rik | -0.807369 |
| 4932441K18Rik | 3.3993655 |
| 4932441K18Rik | 0.210196 |
| 4932442K08Rik | 0.7973829 |
| 4932702K14Rik | 0.4172001 |
| 4933400A11Rik | -0.6451506 |
| 4933400C05Rik | 0.8523754 |
| 4933400E14Rik | 1.8102719 |
| 4933402J07Rik | -0.6515658 |
| 4933402N03Rik | 0.7614331 |
| 4933402P03Rik | -0.6007336 |
| 4933403G14Rik | -0.8082113 |
| 4933404M02Rik | -0.6902444 |
| 4933405O20Rik | 0.1335301 |
| 4933406M09Rik | 1.2383257 |
| 4933407N01Rik | 1.7259991 |
| 4933407N01Rik | -0.5492533 |
| 4933407N01Rik | 0.6157154 |
| 4933407P14Rik | -0.1059743 |
| 4933407P14Rik | 0.7284688 |
| 4933407P14Rik | 0.7396949 |
| 4933409N07Rik | 1.9352053 |
| 4933411G06Rik | -0.2350861 |
| 4933411K20Rik | 0.1106292 |
| 4933412E14Rik | -0.7931617 |
| 4933413G11Rik | -0.360931 |
| 4933415F23Rik | -0.5589402 |
| 4933416C03Rik | -0.7791628 |
| 4933417A18Rik | -0.5759671 |
| 4933417E01Rik | -0.5537617 |
| 4933421E11Rik | -0.2323159 |
| 4933421E11Rik | 1.4167919 |
| 4933421E11Rik | 0.1653646 |
| 4933422H20Rik | -0.3181219 |
| 4933424B01Rik | 0.3800606 |
| 4933424B01Rik | -0.0621633 |
| 4933424B01Rik | -0.6544309 |
| 4933425O20Rik | 1.3487729 |
| 4933426M11Rik | -0.3525279 |
| 4933427D14Rik | 0.1515867 |
| 4933428G09Rik | 3.5158244 |
| 4933428G20Rik | -0.7052572 |
| 4933429E10Rik | 1.7197534 |
| 4933429E10Rik | -0.1886117 |
| 4933430I17Rik | -0.4925039 |
| 4933434E20Rik | -0.7734577 |
| 4933434E20Rik | -0.0195366 |
| 4933434E20Rik | -0.3656905 |
| 4933434I06Rik | 0.9174595 |
| 4933436I01Rik | 0.7150602 |
| 4933437F05Rik | 1.9106522 |
| 4933439C20Rik | 0.4625719 |
| 4933440H19Rik | -0.8854083 |
| 5031414D18Rik | -0.5759671 |
| 5031414D18Rik | -0.7677864 |
| 5033411D12Rik | -0.5333238 |
| 5033414D02Rik | -0.4691787 |
| 5033414K04Rik | -0.2504675 |
| 5133400G04Rik | -0.7321113 |
| 5133400G04Rik | -0.6551485 |
| 5133400G04Rik | 3.1525614 |
| 5133401N09Rik | 0.4999082 |
| 5230400G24Rik | -0.2304735 |
| 5230400G24Rik | 1.8116279 |
| 5330401P04Rik | 0.4301875 |
| 5330417C22Rik | 0.5481329 |
| 5330431N19Rik | -0.6696153 |
| 5330431N19Rik | 0.3225256 |
| 5330431N19Rik | -0.8065274 |
| 5330437I02Rik | 1.4931594 |
| 5330439C02Rik | 0.175945 |
| 5330439J01Rik | -0.3226721 |
| 5330439J01Rik | 0.9609772 |
| 5430407P10Rik | -0.3828076 |
| 5430432M24Rik | -0.5582913 |
| 5430432M24Rik | -0.8141286 |
| 5430432M24Rik | 0.2093255 |
| 5430435G22Rik | 2.5642198 |
| 5430437P03Rik | 1.1362229 |
| 5530601I19Rik | -0.485266 |
| 5630401D24Rik | -0.5939763 |
| 5630401D24Rik | -0.6623545 |
| 5630401D24Rik | 2.7610312 |
| 5730403M16Rik | -0.5479692 |
| 5730409E04Rik | 1.6227893 |
| 5730410E15Rik | 0.9010019 |
| 5730410E15Rik | 0.3966661 |
| 5730410I19Rik | -0.278117 |
| 5730410I19Rik | 0.5726522 |
| 5730410I19Rik | -0.3176176 |
| 5730419I09Rik | -0.1116456 |
| 5730437N04Rik | -0.7166737 |
| 5730446C15Rik | -0.1582707 |
| 5730449L18Rik | 1.3438312 |
| 5730449L18Rik | -0.606174 |
| 5730449L18Rik | -0.4556852 |
| 5730453I16Rik | 0.8019194 |
| 5730453I16Rik | -0.541574 |
| 5730469M10Rik | 1.6440202 |
| 5730472N09Rik | 0.2867987 |
| 5730494M16Rik | -0.7159083 |
| 5730494N06Rik | -0.2824801 |
| 5730502D15Rik | 2.6905094 |
| 5730507A09Rik | 1.8073907 |
| 5730507A09Rik | -0.6508508 |
| 5730528L13Rik | 1.1016256 |
| 5730589K01Rik | 0.6352399 |
| 5730593F17Rik | 0.3276685 |
| 5730596K20Rik | -0.5939763 |
| 5730596K20Rik | 0.1466396 |
| 5730596K20Rik | -0.3763527 |
| 5730596K20Rik | -0.3125898 |
| 5830403L16Rik | 1.1764873 |
| 5830404H04Rik | 1.0514141 |
| 5830405N20Rik | -0.4733235 |
| 5830415F09Rik | -0.5959985 |
| 5830415L20Rik | 0.022328 |
| 5830417C01Rik | 0.4470829 |
| 5830426I05Rik | 0.502047 |
| 5830427D03Rik | -0.5383924 |
| 5830434P21Rik | 0.1503523 |
| 5830435K17Rik | -0.3593498 |
| 5830457O10Rik | -0.2300135 |
| 5830457O10Rik | -0.1591243 |
| 5830457O10Rik | -0.7105679 |
| 5830467P10Rik | -0.6487092 |
| 5830468K18Rik | 4.0528329 |
| 5830472M02Rik | 0.8124732 |
| 5830472M02Rik | 0.6752714 |
| 5830472M02Rik | -0.0085324 |
| 5930416I19Rik | -0.5195224 |
| 5930431H10 | -0.2378643 |
| 5930434B04Rik | -0.7259063 |
| 5930434B04Rik | -0.4539392 |
| 5930434B04Rik | -0.179844 |
| 6030405A18Rik | -0.4510362 |
| 6030408B16Rik | -0.3176176 |
| 6030408B16Rik | 0.8409085 |
| 6030419C18Rik | -0.7454324 |
| 6030429G01Rik | 0.8842557 |
| 6030443O07Rik | 0.4061619 |
| 6030446N20Rik | -0.749386 |
| 6030458C11Rik | 1.183943 |
| 6230409E13Rik | 2.2260319 |
| 6230409E13Rik | 0.7727632 |
| 6230416J20Rik | -0.5906156 |
| 6230427J02Rik | 1.0626443 |
| 6330403K07Rik | -0.0224926 |
| 6330406I15Rik | 0.8764931 |
| 6330406I15Rik | 0.7890358 |
| 6330407J23Rik | 0.3019355 |
| 6330408A02Rik | -0.6130184 |
| 6330409N04Rik | -0.7120907 |
| 6330416G13Rik | -0.7677864 |
| 6330439K17Rik | 2.7249854 |
| 6330442E10Rik | 0.6200678 |
| 6330442E10Rik | 0.505885 |
| 6330500D04Rik | 1.8014925 |
| 6330503C03Rik | -0.4406579 |
| 6330503K22Rik | -0.3452354 |
| 6330503K22Rik | -0.3871353 |
| 6330503K22Rik | -0.6999758 |
| 6330505N24Rik | 3.0548413 |
| 6330512M04Rik | 0.271146 |
| 6330534C20Rik | -0.1894929 |
| 6330548G22Rik | -0.1903749 |
| 6330569M22Rik | -0.6014119 |
| 6330577E15Rik | -0.7758985 |
| 6330578E17Rik | 0.5048204 |
| 6332401O19Rik | -0.5602392 |
| 6430411K18Rik | 2.4450533 |
| 6430502M16Rik | 1.508769 |
| 6430510M02Rik | 0.7198342 |
| 6430517E21Rik | -0.7605439 |
| 6430526N21Rik | -0.4264282 |
| 6430527G18Rik | 0.4301875 |
| 6430527G18Rik | 0.378846 |
| 6430531B16Rik | -0.7948222 |
| 6430537H07Rik | -0.34368 |
| 6430537H07Rik | 0.9653271 |
| 6430548M08Rik | 1.9609061 |
| 6430550H21Rik | -0.607539 |
| 6430571L13Rik | 0.1221483 |
| 6430573F11Rik | 1.2932349 |
| 6430598A04Rik | -0.001628 |
| 6430604K15Rik | -0.4858671 |
| 6430706D22Rik | -0.6345534 |
| 6530404N21Rik | 1.6209379 |
| 6530406M24Rik | -0.5170343 |
| 6530418L21Rik | -0.2757016 |
| 6530418L21Rik | 1.715267 |
| 6530418L21Rik | 4.2143742 |
| 6530421E24Rik | -0.5892746 |
| 6620401K05Rik | -0.1733207 |
| 6720456B07Rik | -0.7259063 |
| 6720456B07Rik | -0.6776665 |
| 6720456H20Rik | -0.2570686 |
| 6720456H20Rik | 1.2086208 |
| 6720458F09Rik | 1.1095737 |
| 6720463M24Rik | 0.6017682 |
| 6720467C03Rik | 1.4573114 |
| 6720467C03Rik | 0.4627945 |
| 6820408C15Rik | 0.4290351 |
| 7420416P09Rik | -0.606174 |
| 7530404M11Rik | -0.4991872 |
| 8030411F24Rik | 0.3988109 |
| 8030451F13Rik | -0.2599116 |
| 8030451F13Rik | 2.4132021 |
| 8030451F13Rik | 1.7945649 |
| 8430408G22Rik | 0.2800833 |
| 8430410A17Rik | -0.4068564 |
| 8430410K20Rik | 1.3719026 |
| 8430415E04Rik | -0.7849022 |
| 8430415E04Rik | -0.4846654 |
| 8430419L09Rik | -0.7344486 |
| 8430427H17Rik | 2.0583507 |
| 8430432M10Rik | 0.1077276 |
| 9030025P20Rik | -0.5046903 |
| 9030025P20Rik | -0.5083768 |
| 9030025P20Rik | -0.3985897 |
| 9030205A07Rik | -0.5157927 |
| 9030221M09Rik | 1.3123527 |
| 9030224M15Rik | 1.6979627 |
| 9030227G01Rik | -0.6537138 |
| 9030409G11Rik | -0.4931096 |
| 9030418K01Rik | 0.7356888 |
| 9030425E11Rik | 0.3992869 |
| 9030607L17Rik | 2.0354742 |
| 9030607L17Rik | 0.0644631 |
| 9030611O19Rik | -0.227258 |
| 9030612M13Rik | -0.6565854 |
| 9030617O03Rik | 0.4533986 |
| 9030624J02Rik | 1.0413871 |
| 9030625A04Rik | -0.8312401 |
| 9130005N14Rik | -0.0808625 |
| 9130011E15Rik | 2.0583088 |
| 9130011J15Rik | -0.1877313 |
| 9130014G24Rik | 0.8093812 |
| 9130017N09Rik | 0.0134593 |
| 9130206N08Rik | 1.335032 |
| 9130211I03Rik | 0.7479874 |
| 9130213B05Rik | -0.6254634 |
| 9130213B05Rik | 1.5107024 |
| 9130218O11Rik | 1.0258377 |
| 9130221H12Rik | 0.3499743 |
| 9130227C08Rik | -0.5886048 |
| 9130401M01Rik | 0.4324882 |
| 9130404D08Rik | -0.2565955 |
| 9130404D08Rik | 0.4607889 |
| 9130404D14Rik | -0.4768906 |
| 9130422G05Rik | -0.2267995 |
| 9230104L09Rik | -0.1859729 |
| 9330129D05Rik | 1.3110717 |
| 9330134C04Rik | 0.1341585 |
| 9330176C04Rik | -0.181154 |
| 9330186A19Rik | 0.9102419 |
| 9430015G10Rik | 1.1650335 |
| 9430015G10Rik | 4.5667859 |
| 9430023L20Rik | 1.3058384 |
| 9430025C20Rik | -0.4745111 |
| 9430028L06Rik | 1.223388 |
| 9430029K10Rik | 0.5797687 |
| 9430031J16Rik | 0.5544231 |
| 9430034D17Rik | 0.3436902 |
| 9430057O19Rik | 0.9239364 |
| 9430069J07Rik | 2.607249 |
| 9430077A04Rik | 0.1491164 |
| 9430079M16Rik | 0.4153302 |
| 9530015I07Rik | 0.7203443 |
| 9530027K23Rik | 2.43014 |
| 9530058B02Rik | 0.8891111 |
| 9530058B02Rik | 1.5063095 |
| 9530068E07Rik | -0.4315265 |
| 9530077C05Rik | 0.2878688 |
| 9530096D07Rik | 1.1459596 |
| 9530096D07Rik | -0.409073 |
| 9630015D15Rik | 0.1887853 |
| 9630020C08Rik | 0.0570011 |
| 9630028B13Rik | -0.2761842 |
| 9630031F12Rik | -0.2613363 |
| 9630050M13Rik | -0.6947235 |
| 9630058J23Rik | -0.0567777 |
| 9830163H01Rik | 0.5495569 |
| 9830169C18Rik | -0.2425128 |
| 9930013L23Rik | -0.5667611 |
| 9930021D14Rik | -0.6437308 |
| 9930023K05Rik | -0.7865484 |
| 9930023K05Rik | 0.479565 |
| 9930104L06Rik | -0.5479692 |
| a | -0.2981569 |
| A030003K21Rik | 1.4000067 |
| A030007L17Rik | 0.1488071 |
| A030007L17Rik | -0.6380728 |
| A030007L17Rik | 1.2177509 |
| A030007L17Rik | 2.0679303 |
| A030009H04Rik | 1.8171408 |
| A030010B05Rik | -0.6331493 |
| A030012M09Rik | 1.279233 |
| A130010J15Rik | 0.8826796 |
| A130038H09Rik | 1.3184594 |
| A130042E20Rik | -0.5040773 |
| A130090K04Rik | -0.7653661 |
| A130092J06Rik | 0.0742111 |
| A230050P20Rik | -0.5759671 |
| A230051G13Rik | -0.6082222 |
| A230054D04Rik | 1.9081569 |
| A230067G21Rik | -0.4679977 |
| A230069A22Rik | 1.5776108 |
| A230074B11Rik | 0.2966517 |
| A230074B11Rik | 1.3510585 |
| A230074B11Rik | -0.1510454 |
| A230106M15Rik | -0.239721 |
| A230106N23Rik | 1.4393102 |
| A230107C01Rik | 1.0071402 |
| A2bp1 | 2.0174256 |
| A2bp1 | 1.1843613 |
| A330021E22Rik | -0.4597718 |
| A330021E22Rik | 1.8017664 |
| A330049M08Rik | 2.4061035 |
| A330080J22Rik | 1.2787595 |
| A3galt2 | 0.1592656 |
| A430005L14Rik | -0.829515 |
| A430005L14Rik | 1.160113 |
| A430005L14Rik | 2.1796194 |
| A430005L14Rik | -0.5195224 |
| A430033K04Rik | -0.5188998 |
| A430079E08 | 0.3439424 |
| A430083B19Rik | -0.1557144 |
| A430083B19Rik | 1.4171197 |
| A430107D22Rik | 0.0467307 |
| A430107P09Rik | 1.4711159 |
| A430110N23Rik | 0.4000004 |
| A4galt | -0.5120776 |
| A4gnt | -0.135923 |
| A530050D06Rik | -0.7022357 |
| A530082C11Rik | 0.7570267 |
| A530082C11Rik | 0.7353541 |
| A530088H08Rik | -0.5518269 |
| A530088H08Rik | 1.1032205 |
| A530098C11Rik | 2.2436039 |
| A630042L21Rik | -0.3216591 |
| A630054L15Rik | 0.0758818 |
| A630077B13Rik | -0.4429544 |
| A630095E13Rik | 1.5912264 |
| A730008H23Rik | -0.6054923 |
| A730008L03Rik | 0.3431857 |
| A730008L03Rik | -0.6594656 |
| A730017C20Rik | -0.6479965 |
| A730024F05Rik | -0.7478026 |
| A730037C10Rik | 0.2507954 |
| A730042J05Rik | 0.8737468 |
| A730098D12Rik | 1.7733625 |
| A830006F12Rik | 0.1246894 |
| A830007P12Rik | -0.6282511 |
| A830053O21Rik | -0.5912868 |
| A830059I20Rik | -0.7313334 |
| A830073O21Rik | 1.5842366 |
| A830080D01Rik | 2.2650815 |
| A830093I24Rik | -0.4961435 |
| A930001N09Rik | -0.0879701 |
| A930005I04Rik | -0.3221655 |
| A930008G19Rik | -0.3715399 |
| A930010I20Rik | 1.5110738 |
| A930016P21Rik | 0.2934672 |
| A930018P22Rik | -0.2346238 |
| A930024E05Rik | 0.7304892 |
| A930025D01Rik | 2.3229912 |
| A930025D01Rik | 2.6134012 |
| A930025D01Rik | -0.6352563 |
| A930034L06Rik | -0.6116456 |
| A930038C07Rik | 0.7000726 |
| A930041I02Rik | -0.6551485 |
| AA388235 | -0.2805384 |
| AA407270 | 0.3181321 |
| AA407659 | -0.0009041 |
| AA407659 | 0.8297571 |
| AA408296 | 1.0862235 |
| AA408296 | -0.5634946 |
| AA408296 | -0.7367916 |
| AA409316 | -0.4464097 |
| AA415398 | -0.1908162 |
| AA467197 | 0.4080498 |
| AA536717 | -0.6027701 |
| AA536717 | -0.5133143 |
| AA536749 | -0.6296481 |
| AA536749 | -0.1177595 |
| AA536749 | -0.4435294 |
| AA536749 | -0.8863225 |
| AA673488 | 0.0001807 |
| AA691260 | 0.5973313 |
| AA792892 | 0.0023465 |
| AA881470 | -0.5865982 |
| AA881470 | 1.2302884 |
| AA960436 | 1.4361026 |
| AA960436 | -0.3292833 |
| Aaas | -0.0633214 |
| Aaas | -0.342127 |
| Aacs | 1.7516209 |
| Aadacl1 | -0.5195224 |
| Aak1 | -0.3390287 |
| Aak1 | 1.1719435 |
| Aamp | 0.210196 |
| Aard | -0.5706957 |
| Aars | 0.1941094 |
| Aars2 | 1.2993721 |
| Aarsd1 | 1.1290485 |
| Aasdh | -0.5276536 |
| Aatf | -0.7075296 |
| Aatf | 1.3811359 |
| Aatk | -0.2401857 |
| AB112350 | 2.6491441 |
| AB182283 | 0.187301 |
| AB182283 | 1.2585632 |
| Abat | 3.0159225 |
| Abat | -0.5251443 |
| Abat | -0.597349 |
| Abca14 | 1.4251971 |
| Abca14 | -0.5557003 |
| Abca15 | -0.7758985 |
| Abca15 | -0.1916995 |
| Abca15 | -0.2829661 |
| Abca15 | 2.1823437 |
| Abca17 | 0.2046694 |
| Abca2 | 0.7507905 |
| Abca3 | -0.4452565 |
| Abca4 | -0.6408976 |
| Abca4 | 0.5975246 |
| Abca5 | 0.8637062 |
| Abca6 | -0.5089926 |
| Abca6 | -0.1463992 |
| Abca7 | -0.6725352 |
| Abca7 | 0.4464038 |
| Abca8a | -0.7399247 |
| Abca8b | -0.5845958 |
| Abca9 | -0.7407096 |
| Abca9 | -0.6947235 |
| Abca9 | 1.1192488 |
| Abcb11 | -0.8346995 |
| Abcb1b | 1.7253467 |
| Abcb4 | 0.1687025 |
| Abcb6 | 0.4122839 |
| Abcb8 | -0.7251335 |
| Abcb8 | -0.3374834 |
| Abcb8 | -0.5786138 |
| Abcb9 | -0.7182063 |
| Abcb9 | -0.4864685 |
| Abcc1 | -0.6451506 |
| Abcc10 | -0.735229 |
| Abcc10 | 1.2558507 |
| Abcc12 | -0.163404 |
| Abcc3 | 0.660003 |
| Abcc5 | -0.4129644 |
| Abcc6 | 2.0043153 |
| Abcc8 | -0.5390279 |
| Abcc9 | -0.1247363 |
| Abcc9 | -0.6345534 |
| Abcd1 | -0.3833475 |
| Abcd2 | -0.5933032 |
| Abcd2 | -0.7956536 |
| Abcd3 | 0.1202381 |
| Abcd4 | -0.2086383 |
| Abcd4 | 1.0141635 |
| Abce1 | -0.3333745 |
| Abcf1 | -0.8927459 |
| Abcf1 | 0.4181337 |
| Abcf1 | 2.8630294 |
| Abcf2 | 1.9261619 |
| Abcf2 | -0.6537138 |
| Abcf2 | -0.1301061 |
| Abcf2 | -0.0983311 |
| Abcf3 | -0.7791628 |
| Abcg1 | 0.5946205 |
| Abcg4 | 2.3364122 |
| Abcg5 | -0.6999758 |
| Abhd1 | 2.1768134 |
| Abhd1 | -0.239721 |
| Abhd10 | -0.1947973 |
| Abhd11 | 0.6895976 |
| Abhd12 | -0.3191312 |
| Abhd14a | -0.0078032 |
| Abhd14b | 0.3116343 |
| Abhd2 | 3.7304109 |
| Abhd2 | 2.0286325 |
| Abhd2 | 1.9342524 |
| Abhd3 | 0.4806581 |
| Abhd4 | 0.383937 |
| Abhd5 | 0.186112 |
| Abhd6 | -0.7290037 |
| Abhd7 | 0.2681483 |
| Abhd7 | 0.3452022 |
| Abhd8 | 1.0937257 |
| Abi1 | -0.4360813 |
| Abi1 | -0.5608894 |
| Abi2 | 0.1976424 |
| Abi2 | -0.0879701 |
| Abi2 | -0.6857862 |
| Abi3 | -0.5845958 |
| Abi3bp | 2.3700077 |
| Abi3bp | -0.3156033 |
| Abl1 | 1.4060768 |
| Ablim1 | -0.5993783 |
| Ablim2 | 0.4239478 |
| Ablim2 | -0.4040929 |
| Ablim3 | -0.7407096 |
| Ablim3 | 0.8311203 |
| Abp1 | -0.0757618 |
| Abr | -0.6185295 |
| Abr | 0.4281121 |
| Abtb1 | 1.7026525 |
| Acaa1a | 2.2133049 |
| Acaa1b | 0.481095 |
| Acaa2 | 1.5549033 |
| Acaa2 | -0.5753066 |
| Acaa2 | 1.0596704 |
| Acaa2 | 1.3551765 |
| Acad10 | 0.7455072 |
| Acad10 | 1.3708711 |
| Acad8 | -0.4721374 |
| Acad8 | 0.0113185 |
| Acad9 | -0.1903749 |
| Acad9 | 2.2922663 |
| Acadl | -0.1392629 |
| Acadl | 1.9535065 |
| Acadm | -0.5089926 |
| Acads | -0.7549487 |
| Acads | 0.6257153 |
| Acadsb | -0.4085183 |
| Acadvl | -0.595324 |
| Acan | 4.5565865 |
| Acat2 | -0.5792767 |
| Acbd3 | -0.6089059 |
| Acbd4 | -0.0840149 |
| Acbd4 | -0.6401906 |
| Acbd5 | -0.7182063 |
| Acbd6 | 2.2974943 |
| Accn1 | 1.5484272 |
| Accn1 | -0.7197413 |
| Accn2 | 1.3022412 |
| Accn3 | -0.8381714 |
| Accn3 | 1.0947598 |
| Accs | -0.5739869 |
| Acd | 1.5161099 |
| Ace | 0.7040578 |
| Ace | 1.3540392 |
| Ache | -0.2627632 |
| Acin1 | -0.5505393 |
| Acly | 4.3674791 |
| Acn9 | -0.7029901 |
| Aco1 | -0.1116456 |
| Aco2 | -0.6487092 |
| Acot1 | -0.6185295 |
| Acot1 | -0.2267995 |
| Acot10 | -0.852187 |
| Acot11 | -0.0005424 |
| Acot11 | -0.4441048 |
| Acot2 | -0.3462738 |
| Acot2 | -0.6437308 |
| Acot3 | 1.1849884 |
| Acot5 | -0.5621911 |
| Acot7 | 0.1265907 |
| Acot7 | -0.3257177 |
| Acot7 | 0.5783891 |
| Acot7 | -0.6932281 |
| Acot8 | -0.6338511 |
| Acot8 | -0.5959985 |
| Acot8 | 0.2549557 |
| Acot9 | -0.2786008 |
| Acox2 | -0.1724542 |
| Acox3 | 1.2551714 |
| Acox3 | -0.1413563 |
| Acoxl | -0.6296481 |
| Acp2 | -0.1621181 |
| Acp6 | -0.5595895 |
| Acpl2 | -0.815826 |
| Acpp | 2.24653 |
| Acr | 1.0551323 |
| Acr | 1.0273178 |
| Acrbp | -0.2902863 |
| Acrbp | -0.5383924 |
| Acrv1 | -0.5680708 |
| Acsbg1 | -0.771023 |
| Acsbg1 | -0.5892746 |
| Acsbg2 | 0.547522 |
| Acsf3 | -0.4858671 |
| Acsl1 | -0.7159083 |
| Acsl3 | 3.0037767 |
| Acsl3 | 0.5746343 |
| Acsl4 | -0.5859303 |
| Acsl4 | 0.2055442 |
| Acsl5 | -0.0664163 |
| Acsl6 | 2.3209585 |
| Acsl6 | 1.2594334 |
| Acsl6 | -0.1149015 |
| Acsm3 | -0.5739869 |
| Acss1 | 3.647329 |
| Acss2 | -0.7067715 |
| Acss2 | -0.5812679 |
| Acta1 | -0.2771501 |
| Acta2 | -0.3135932 |
| Acta2 | 0.4663497 |
| Acta2 | 0.352976 |
| Acta2 | -0.5621911 |
| Actb | 0.5378992 |
| Actb | 0.0387813 |
| Actb | 0.5862459 |
| Actb | 1.0891159 |
| Actb | 0.2645932 |
| Actg2 | 0.1795457 |
| Actl6a | -0.7446436 |
| Actl6b | -0.5441269 |
| Actl6b | 2.0048467 |
| Actl6b | -0.4521963 |
| Actl6b | 1.4907351 |
| Actl7a | -0.7865484 |
| Actl7b | -0.0034401 |
| Actn1 | 0.0711965 |
| Actn2 | 0.7254302 |
| Actn3 | -0.5220169 |
| Actn3 | 1.0987708 |
| Actn4 | 0.6580092 |
| Actn4 | 0.217995 |
| Actn4 | 1.638253 |
| Actr10 | -0.4185513 |
| Actr10 | 0.13792 |
| Actr1a | -0.2406507 |
| Actr1b | -0.5733278 |
| Actr3 | 0.9504827 |
| Actr3b | -0.6872699 |
| Actr5 | 0.5596671 |
| Actr5 | -0.5872666 |
| Actr6 | 0.737026 |
| Actrt1 | 2.0566328 |
| Actrt2 | 0.2942643 |
| Acvr1 | -0.5819325 |
| Acvr1c | 0.5215031 |
| Acvr2a | 1.7373153 |
| Acvr2b | -0.3344001 |
| Acvrl1 | -0.1480861 |
| Acy1 | -0.4985777 |
| Acy1l2 | -0.7022357 |
| Acy3 | 1.9813158 |
| Ada | -0.5505393 |
| Adal | 3.0984103 |
| Adal | 0.4162656 |
| Adam11 | -0.2819943 |
| Adam11 | -0.5441269 |
| Adam12 | 1.2879965 |
| Adam15 | -0.5739869 |
| Adam17 | 0.1831329 |
| Adam1b | -0.7037452 |
| Adam1b | -0.466229 |
| Adam21 | -0.798152 |
| Adam22 | 2.5086126 |
| Adam22 | -0.8235019 |
| Adam22 | -0.7029901 |
| Adam23 | 0.0177266 |
| Adam24 | 1.1001422 |
| Adam26a | 0.5726522 |
| Adam28 | 1.2427618 |
| Adam29 | 0.8248942 |
| Adam29 | 1.9295692 |
| Adam3 | -0.0679676 |
| Adam30 | -0.6594656 |
| Adam32 | -0.5188998 |
| Adam33 | -0.5301696 |
| Adam39 | -0.4715449 |
| Adam4 | 0.6309563 |
| Adam4 | -0.0518038 |
| Adam4 | -0.6769318 |
| Adam5 | -0.2300135 |
| Adam6 | -0.4967515 |
| Adam8 | -0.5933032 |
| Adam8 | -0.5845958 |
| Adam9 | 2.0240007 |
| Adamdec1 | 1.5014463 |
| Adamts1 | 0.3703003 |
| Adamts13 | -0.6529972 |
| Adamts15 | -0.6054923 |
| Adamts16 | 0.2689668 |
| Adamts17 | 0.8896807 |
| Adamts18 | -0.3726073 |
| Adamts19 | 1.2191633 |
| Adamts2 | 0.1457091 |
| Adamts2 | -0.8098982 |
| Adamts2 | 1.5358483 |
| Adamts20 | -0.5102254 |
| Adamts20 | 0.5718582 |
| Adamts4 | -0.2457802 |
| Adamts7 | 0.3899624 |
| Adamtsl2 | -0.5102254 |
| Adamtsl4 | 0.2488477 |
| Adamtsl5 | -0.2937217 |
| Adar | 0.9925532 |
| Adar | -0.2641921 |
| Adar | 0.7421902 |
| Adarb1 | -0.4912938 |
| Adarb1 | 0.7179616 |
| Adarb1 | -0.7143795 |
| Adat1 | 0.0300648 |
| Adc | -0.5886048 |
| Adc | -0.4355107 |
| Adck1 | 0.0102463 |
| Adck2 | 3.2120432 |
| Adck4 | -0.3349133 |
| Adck5 | 1.3439197 |
| Adcy1 | -0.2135983 |
| Adcy10 | 1.3266015 |
| Adcy2 | 1.4107073 |
| Adcy4 | 1.5726808 |
| Adcy4 | 0.8835396 |
| Adcy6 | 0.8058131 |
| Adcy7 | 2.0168137 |
| Adcy8 | -0.2713693 |
| Adcy9 | 1.3197305 |
| Adcyap1 | 2.486361 |
| Adcyap1r1 | 1.5012212 |
| Add1 | -0.7321113 |
| Add1 | -0.7923326 |
| Add1 | -0.2290941 |
| Add1 | 2.1516365 |
| Add3 | -0.4355107 |
| Add3 | 3.3530553 |
| Add3 | 0.2345361 |
| Adfp | -0.8355663 |
| Adh1 | -0.3359405 |
| Adh1 | 1.8522703 |
| Adh4 | 0.7007669 |
| Adh5 | 0.3105905 |
| Adh7 | 0.526514 |
| Adhfe1 | 0.270057 |
| Adi1 | 1.1086687 |
| Adipoq | -0.203704 |
| Adipor1 | -0.4377949 |
| Adipor2 | -0.1794077 |
| Adk | -0.3801128 |
| Adk | 0.1632344 |
| Adm | -0.7446436 |
| Adm2 | -0.7174397 |
| Adnp | 0.4676795 |
| Ado | -0.0879701 |
| Adora1 | -0.2956903 |
| Adora1 | 1.4083948 |
| Adora1 | -0.1746218 |
| Adora2b | 0.3376184 |
| Adora3 | -0.3036058 |
| Adpgk | 1.9682947 |
| Adprh | 0.2998255 |
| Adprhl1 | -0.0560108 |
| Adprhl1 | 0.0394752 |
| Adprhl1 | 1.1987849 |
| Adprhl2 | 0.9240737 |
| Adra1a | 1.2675176 |
| Adra1a | 0.0775496 |
| Adra1d | -0.3980411 |
| Adra2a | -0.7391405 |
| Adra2c | 0.5154129 |
| Adra2c | -0.5563474 |
| Adrb1 | 0.1723306 |
| Adrb2 | -0.6580244 |
| Adrbk1 | 0.3178731 |
| Adrbk2 | -0.4810691 |
| Adrm1 | 1.9088915 |
| Adsl | -0.3473133 |
| Adss | 0.716256 |
| Adssl1 | 0.564282 |
| Adssl1 | 1.269528 |
| Adssl1 | 0.1422897 |
| Aebp1 | -0.2204037 |
| Aebp2 | -0.179844 |
| Aes | 0.3302297 |
| Afap1 | -0.6984722 |
| Afap1 | 0.2822374 |
| Afap1 | 0.3150187 |
| Afap1l1 | 0.5781918 |
| Aff1 | 1.1618273 |
| Aff4 | -0.2126946 |
| Afg3l1 | -0.7923326 |
| Afg3l1 | -0.4846654 |
| Afg3l1 | 1.5631657 |
| Afg3l1 | -0.6394841 |
| Afg3l2 | 2.8079852 |
| Afmid | -0.348354 |
| Aftph | 0.7746921 |
| Agbl1 | 1.6702731 |
| Ager | 0.4192993 |
| Ager | 0.7920365 |
| Aggf1 | -0.6776665 |
| Aggf1 | -0.6843047 |
| Aggf1 | -0.2641921 |
| Agpat1 | -0.395303 |
| Agpat2 | 1.265791 |
| Agpat3 | 0.3056169 |
| Agpat4 | -0.6718044 |
| Agpat5 | -0.1055705 |
| Agpat6 | -0.5238921 |
| Agpat6 | -0.5550537 |
| Agpat7 | 1.2203723 |
| Agpat9 | -0.665978 |
| Agpat9 | 2.0973121 |
| Agps | -0.6289493 |
| Agps | -0.6857862 |
| Agrn | -0.5065318 |
| Agrp | -0.6776665 |
| Agt | 2.0381715 |
| Agtpbp1 | 1.3256101 |
| Agtr1a | -0.6984722 |
| Agtrap | -0.2689709 |
| Agtrl1 | 1.7143672 |
| Agxt2l1 | 0.0363487 |
| Agxt2l1 | 0.3887604 |
| Agxt2l2 | 0.7530928 |
| Agxt2l2 | -0.5700388 |
| Agxt2l2 | 1.3510585 |
| Agxt2l2 | -0.4632885 |
| Ahctf1 | -0.2849127 |
| Ahcy | 0.2043777 |
| Ahcyl1 | -0.0529493 |
| Ahdc1 | -0.6924813 |
| Ahi1 | -0.1837795 |
| Ahnak | -0.7282284 |
| Ahnak | -0.1724542 |
| Ahnak2 | -0.4035411 |
| Ahrr | -0.4412315 |
| Ahsa1 | -0.6430217 |
| Ahsa2 | 1.7794926 |
| AI118078 | 0.1410433 |
| AI132487 | -0.2708891 |
| AI314180 | -0.3390287 |
| AI314976 | -0.6268563 |
| AI316807 | 0.8042575 |
| AI317395 | 0.5177275 |
| AI413582 | 1.746412 |
| AI413782 | -0.4973598 |
| AI427515 | -0.421357 |
| AI428936 | 1.2664628 |
| AI449175 | 2.2892472 |
| AI449175 | -0.6909894 |
| AI450540 | -0.7923326 |
| AI451557 | 0.340153 |
| AI451617 | 0.428343 |
| AI467606 | 1.2035106 |
| AI480653 | -0.149776 |
| AI504432 | -0.6345534 |
| AI553587 | -0.3774255 |
| AI593442 | -0.3931181 |
| AI593442 | 0.2005766 |
| AI593442 | -0.2912666 |
| AI595366 | -0.5151725 |
| AI595406 | 1.9452484 |
| AI597468 | -0.7067715 |
| AI597479 | -0.4343707 |
| AI606181 | 1.2084169 |
| AI646023 | 1.934014 |
| AI661453 | 1.472586 |
| AI662250 | -0.2834524 |
| AI747699 | 1.6512927 |
| AI836003 | 0.0711965 |
| AI837181 | 0.5835034 |
| AI842396 | -0.4751054 |
| AI846148 | 1.3885096 |
| AI847670 | 0.0600608 |
| AI851790 | -0.7075296 |
| AI894139 | 1.1137483 |
| AI987692 | 3.0207776 |
| AI987692 | 0.0815404 |
| AI987944 | -0.4320947 |
| Aicda | 1.8853005 |
| Aif1 | -0.2599116 |
| Aif1 | -0.3985897 |
| Aif1 | 1.2550743 |
| Aifm1 | -0.6102748 |
| Aifm1 | 4.2049315 |
| Aifm2 | -0.6394841 |
| Aifm2 | -0.2249677 |
| Aifm2 | 1.2011507 |
| Aifm3 | 1.0576428 |
| Aig1 | 0.1762456 |
| Aig1-pending | -0.1455569 |
| Aim1 | 0.0661509 |
| Aim2 | -0.3196362 |
| Aip | -0.4991872 |
| Ak1 | 0.0861753 |
| AK122209 | -0.6430217 |
| AK122525 | 0.1999905 |
| AK129128 | 0.2496829 |
| AK129302 | -0.604811 |
| AK129302 | -0.4051973 |
| AK129302 | -0.5498961 |
| AK129341 | -0.0329114 |
| AK162044 | -0.5301696 |
| Ak2 | -0.6465724 |
| Ak2 | 1.3659564 |
| Ak2 | 0.7002463 |
| Ak3 | 2.2214909 |
| Ak3 | 4.3653667 |
| Ak3l1 | -0.5693823 |
| Ak5 | 1.0820429 |
| Akap1 | 3.1634247 |
| Akap1 | 0.8545932 |
| Akap1 | 0.5126701 |
| Akap10 | -0.6969709 |
| Akap10 | 0.1762456 |
| Akap12 | -0.4224816 |
| Akap14 | 0.1450883 |
| Akap2 | 0.4913044 |
| Akap2 | -0.5492533 |
| Akap3 | 1.7709334 |
| Akap4 | 0.2067093 |
| Akap4 | -0.4096279 |
| Akap4 | 0.2838498 |
| Akap4 | -0.5333238 |
| Akap6 | -0.3426444 |
| Akap7 | 1.6409141 |
| Akap8 | 1.1260525 |
| Akap8l | 2.9510921 |
| Akap9 | -0.4894815 |
| Akap9 | -0.5059176 |
| Akap9 | 1.8717062 |
| Akna | -0.5220169 |
| Akp2 | -0.5201454 |
| Akp5 | 1.2573048 |
| Akr1a4 | -0.5544074 |
| Akr1a4 | -0.675464 |
| Akr1b3 | -0.2996398 |
| Akr1b8 | -0.2713693 |
| Akr1c12 | 0.5572504 |
| Akr1c14 | 0.1753436 |
| Akr1c14 | -0.5114598 |
| Akr1c18 | 1.1657801 |
| Akr1c19 | 1.5542647 |
| Akr1e1 | -0.4107388 |
| Akr7a5 | -0.5806037 |
| Akr7a5 | 0.1416667 |
| Akr7a5 | 0.8785117 |
| Akt1s1 | 1.7349101 |
| Akt3 | 1.0018385 |
| Akt3 | 2.610377 |
| Aktip | -0.5819325 |
| Alad | -0.1565657 |
| Alas1 | -0.5364885 |
| Alas2 | -0.2504675 |
| Alcam | -0.6095901 |
| Aldh16a1 | 0.3539744 |
| Aldh18a1 | -0.829515 |
| Aldh1a1 | 0.0927578 |
| Aldh1a2 | -0.2641921 |
| Aldh1a2 | 1.6186186 |
| Aldh1a7 | -0.0317896 |
| Aldh1b1 | 2.3849611 |
| Aldh1l1 | -0.2622873 |
| Aldh1l2 | -0.6359596 |
| Aldh1l2 | -0.7589419 |
| Aldh2 | 1.0812279 |
| Aldh3a1 | -0.2689709 |
| Aldh3a2 | 0.7923518 |
| Aldh3b1 | 0.2574433 |
| Aldh3b1 | 0.3754369 |
| Aldh3b1 | 3.1537184 |
| Aldh4a1 | 0.9551464 |
| Aldh5a1 | 0.9299604 |
| Aldh5a1 | 2.9541112 |
| Aldh6a1 | 0.4558684 |
| Aldh8a1 | 2.0291074 |
| Aldh8a1 | -0.6551485 |
| Aldoa | -0.494322 |
| Aldoc | -0.1565657 |
| Alg1 | 1.5194281 |
| Alg10b | -0.571353 |
| Alg11 | 2.0473198 |
| Alg11 | 2.3424932 |
| Alg12 | 0.2449403 |
| Alg12 | -0.0317896 |
| Alg14 | 0.1855169 |
| Alg2 | -0.4446805 |
| Alg3 | 0.0106039 |
| Alg3 | -0.3075882 |
| Alg3 | 0.6417219 |
| Alg5 | 1.4718899 |
| Alg5 | -0.5852628 |
| Alg5 | -0.5498961 |
| Alg8 | -0.7422812 |
| Alg8 | 0.1522033 |
| Alg8 | -0.6494226 |
| Alg8 | -0.1908162 |
| Alg9 | 0.1917468 |
| Alg9 | 1.4954266 |
| Alg9 | 1.0259612 |
| Alk | -0.5479692 |
| Alkbh2 | -0.3577712 |
| Alkbh3 | -0.5428496 |
| Alkbh4 | -0.5906156 |
| Alkbh5 | -0.4650517 |
| Alkbh7 | -0.2873515 |
| Alkbh8 | 1.5829289 |
| Allc | 1.4040854 |
| Allc | 1.167165 |
| Alox12 | -0.0757618 |
| Alox12b | -0.2902863 |
| Alox12e | 0.8184738 |
| Alox15 | 1.3114378 |
| Alox5ap | 1.5003205 |
| Alox8 | -0.1625465 |
| Aloxe3 | 0.1240547 |
| Alpk3 | 0.136981 |
| Als2 | 1.1516029 |
| Als2 | -0.7136159 |
| Als2 | -0.753356 |
| Als2cl | -0.5946499 |
| Als2cr13 | 0.1952885 |
| Als2cr2 | -0.5608894 |
| Als2cr2 | -0.3630434 |
| Als2cr4 | 1.4844183 |
| Als2cr4 | 0.3302297 |
| Als2cr4 | -0.4798734 |
| Alx3 | 0.9447217 |
| Amac1 | 0.3111125 |
| Ambp | 0.7700239 |
| Ambra1 | 1.338597 |
| Ambra1 | -0.5226416 |
| Amd2 | 0.9273638 |
| Amdhd2 | 0.028312 |
| Amdhd2 | -0.149776 |
| Amfr | -0.785725 |
| Amh | 0.1237373 |
| Amhr2 | 2.1440899 |
| Amhr2 | 2.2303766 |
| Amhr2 | 1.0159113 |
| Amigo1 | 1.6020213 |
| Amigo1 | 0.286531 |
| Amigo2 | 0.5071609 |
| Amigo3 | 1.2015615 |
| Ammecr1l | -0.5959985 |
| Amn | -0.3364545 |
| Amotl2 | 1.4759816 |
| Ampd1 | 0.0037877 |
| Ampd1 | 1.715207 |
| Ampd2 | 2.6269506 |
| Amph | -0.6769318 |
| Amph | 0.4324882 |
| Amy1 | -0.7029901 |
| Amz2 | -0.5766281 |
| Anapc10 | 2.3096364 |
| Anapc11 | -0.4751054 |
| Anapc13 | -0.52954 |
| Anapc2 | 0.8913875 |
| Anapc4 | 0.5534114 |
| Anapc5 | 0.1717269 |
| Anapc5 | 2.3693128 |
| Anapc5 | -0.2966762 |
| Anapc7 | 0.5831109 |
| Ang | -0.2425128 |
| Angel1 | -0.5661069 |
| Angel2 | -0.1540139 |
| Angpt1 | -0.4574344 |
| Angpt2 | -0.6977213 |
| Angpt2 | 0.3183911 |
| Angptl1 | 0.031115 |
| Angptl2 | 1.4945204 |
| Angptl3 | 1.069744 |
| Angptl4 | -0.3980411 |
| Angptl7 | -0.7605439 |
| Ank | 1.7451925 |
| Ank1 | -0.3252095 |
| Ank2 | 0.547522 |
| Ank2 | 0.4893579 |
| Ank2 | 1.0752688 |
| Ank3 | -0.6178389 |
| Ank3 | -0.6014119 |
| Ank3 | 2.6273428 |
| Ankar | -0.4674078 |
| Ankar | -0.2476524 |
| Ankfy1 | 1.7600682 |
| Ankle2 | 0.2154024 |
| Ankle2 | 3.7191635 |
| Ankle2 | 0.6960708 |
| Ankmy1 | 1.0052501 |
| Ankmy1 | -0.4858671 |
| Ankmy1 | -0.61577 |
| Ankmy2 | 1.8042823 |
| Ankmy2 | 1.7727417 |
| Ankrd10 | 1.3732761 |
| Ankrd12 | -0.3525279 |
| Ankrd12 | -0.7791628 |
| Ankrd13a | -0.4372234 |
| Ankrd13b | 0.1528195 |
| Ankrd13d | -0.1051669 |
| Ankrd17 | 1.1018536 |
| Ankrd17 | -0.7067715 |
| Ankrd17 | -0.6296481 |
| Ankrd22 | 0.3853866 |
| Ankrd23 | 0.3863517 |
| Ankrd24 | 0.7395283 |
| Ankrd25 | 1.4697211 |
| Ankrd27 | 2.4397057 |
| Ankrd34a | -0.1489307 |
| Ankrd35 | -0.5232667 |
| Ankrd36 | -0.5089926 |
| Ankrd37 | -0.0923399 |
| Ankrd39 | 1.3645722 |
| Ankrd40 | -0.5680708 |
| Ankrd40 | -0.6289493 |
| Ankrd40 | 0.9708348 |
| Ankrd46 | 0.8751932 |
| Ankrd47 | -0.0034401 |
| Ankrd49 | -0.0125525 |
| Ankrd5 | 2.0156325 |
| Ankrd50 | -0.6007336 |
| Ankrd53 | 0.8569533 |
| Ankrd54 | 1.4627947 |
| Ankrd54 | 0.3108515 |
| Ankrd54 | 0.0321641 |
| Ankrd56 | -0.7029901 |
| Ankrd58 | 0.0874954 |
| Ankrd6 | -0.6068563 |
| Ankrd6 | 1.8797649 |
| Ankrd6 | -0.5133143 |
| Ankrd7 | 1.8218178 |
| Ankrd9 | -0.0140186 |
| Anks1 | 2.44951 |
| Anks3 | 4.6469484 |
| Anks3 | 1.3306467 |
| Anks6 | 2.2850307 |
| Anks6 | 0.9789075 |
| Ankzf1 | -0.1247363 |
| Ankzf1 | 0.4856701 |
| Anln | -0.5792767 |
| Anln | -0.5207688 |
| Anp32a | -0.6909894 |
| Anp32a | -0.0785049 |
| Anp32a | -0.2117917 |
| Anp32e | -0.1112393 |
| Anpep | 1.2810299 |
| Antxr1 | 2.6005351 |
| Antxr1 | 0.1208753 |
| Antxrl | 2.6500456 |
| Anxa1 | -0.1193965 |
| Anxa11 | 4.2443753 |
| Anxa11 | 1.2312843 |
| Anxa2 | 0.4524989 |
| Anxa3 | 2.7366795 |
| Anxa3 | 3.2828367 |
| Anxa4 | 1.4852578 |
| Anxa5 | -0.3318382 |
| Anxa6 | -0.7336689 |
| Anxa8 | -0.4751054 |
| Anxa8 | 0.2857274 |
| Anxa9 | -0.4828656 |
| Aoah | 1.0781946 |
| Aoc2 | 2.1266355 |
| Aoc2 | 0.7746921 |
| Aoc3 | -0.5505393 |
| Aof1 | -0.0445806 |
| Aof1 | -0.7251335 |
| Aof2 | -0.0848046 |
| Aox3 | -0.8031684 |
| Aox3l1 | -0.4046449 |
| Ap1b1 | -0.3678134 |
| Ap1g1 | -0.7282284 |
| Ap1g1 | -0.7824382 |
| Ap1g2 | 1.4066568 |
| Ap1gbp1 | 0.2480118 |
| Ap1gbp1 | 1.105835 |
| Ap1m1 | -0.6843047 |
| Ap1m2 | 0.0758818 |
| Ap1m2 | -0.5647998 |
| Ap1m2 | 0.5444614 |
| Ap1m2 | 1.1430163 |
| Ap1s1 | -0.6472842 |
| Ap1s1 | 0.3276685 |
| Ap1s1 | 1.5255183 |
| Ap1s3 | 0.5385164 |
| Ap2a1 | -0.813281 |
| Ap2a1 | -0.2824801 |
| Ap2a2 | -0.621297 |
| Ap2b1 | 2.7975235 |
| Ap2b1 | -0.4191118 |
| Ap2b1 | -0.0552445 |
| Ap2b1 | -0.5276536 |
| Ap2m1 | 1.8682389 |
| Ap2s1 | -0.6219902 |
| Ap2s1 | 1.7011932 |
| Ap2s1 | 1.9262581 |
| Ap3b1 | -0.7143795 |
| Ap3b1 | 2.7885645 |
| Ap3b2 | 2.3188897 |
| Ap3d1 | 1.5740026 |
| Ap3m1 | 0.0450082 |
| Ap3m2 | 3.0552982 |
| Ap3s2 | -0.2570686 |
| Ap3s2 | 0.1083732 |
| Ap4b1 | -0.4191118 |
| Ap4m1 | 1.7922445 |
| Ap4m1 | -0.5276536 |
| Ap4m1 | -0.4786792 |
| Ap4s1 | 0.986784 |
| Apaf1 | 1.2210768 |
| Apaf1 | 0.8658979 |
| Apba1 | -0.7702128 |
| Apba2 | -0.1222681 |
| Apba2 | 0.2254454 |
| Apba3 | -0.5912868 |
| Apbb1 | -0.4757001 |
| Apbb1ip | 2.051798 |
| Apbb1ip | -0.0832258 |
| Apbb1ip | -0.6437308 |
| Apbb2 | 0.1054647 |
| Apbb3 | -0.2627632 |
| Apc | -0.5926306 |
| Apc2 | 0.5341881 |
| Apcdd1 | -0.4721374 |
| Apeh | -0.7166737 |
| Apex1 | -0.5108424 |
| Aph1a | 0.454522 |
| Aph1b | 2.6459145 |
| Apip | 1.7724029 |
| Apip | -0.4085183 |
| Apitd1 | -0.6137056 |
| Aplp1 | -0.1930259 |
| Aplp2 | 1.4401103 |
| Apoa1 | 1.1942427 |
| Apoa1bp | -0.3742107 |
| Apoa2 | -0.6865278 |
| Apoa4 | -0.3985897 |
| Apob48r | -0.5892746 |
| Apobec1 | 0.2929355 |
| Apobec3 | -0.4112947 |
| Apoc1 | -0.3272442 |
| Apoc1 | -0.2786008 |
| Apoc2 | 1.6330391 |
| Apoc3 | 0.3150187 |
| Apoe | 0.1632344 |
| Apof | -0.2467158 |
| Apoh | -0.5377574 |
| Apol7c | 1.0124125 |
| Apol9b | -0.1855338 |
| App | -0.0660289 |
| Appbp1 | -0.145136 |
| Appbp1 | 0.4698917 |
| Appbp2 | -0.080076 |
| Appl1 | -0.5872666 |
| Appl2 | -0.5544074 |
| Aprin | 1.0017119 |
| Aprt | -0.3090859 |
| Aptx | -0.6835649 |
| Aqp1 | -0.9010686 |
| Aqp1 | -0.7029901 |
| Aqp1 | -0.756544 |
| Aqp11 | 1.1999171 |
| Aqp11 | -0.6718044 |
| Aqp3 | -0.756544 |
| Aqp4 | -0.8252161 |
| Aqp5 | 1.6007377 |
| Aqp7 | 1.4292507 |
| Aqp7 | 0.6772296 |
| Aqp8 | 0.6101991 |
| Aqp8 | 0.0148839 |
| Aqp9 | 1.4979143 |
| Aqr | -0.2439118 |
| Aqr | 0.3985729 |
| Ar | 0.1973485 |
| Araf | 0.1122374 |
| Araf | 2.1741845 |
| Arbp | 1.8735368 |
| Arc | 1.0328479 |
| Arcn1 | -0.1668416 |
| Ard1 | 0.8294539 |
| Ard1 | -0.0606215 |
| Areg | -0.7037452 |
| Arf2 | -0.6000557 |
| Arf3 | 0.9064727 |
| Arf5 | -0.8090544 |
| Arf6 | -0.4697697 |
| Arfgap1 | -0.5912868 |
| Arfgap1 | 0.2148254 |
| Arfgap1 | -0.8031684 |
| Arfgap2 | 0.033561 |
| Arfgap2 | 2.0024974 |
| Arfgap2 | -0.3985897 |
| Arfgap2 | 0.286531 |
| Arfgap3 | -0.2723303 |
| Arfip2 | -0.5839293 |
| Arfip2 | -0.3651605 |
| Arfip2 | -0.6587447 |
| Arfrp1 | 0.6830802 |
| Arfrp1 | -0.7151436 |
| Arg1 | 0.4764978 |
| Arg2 | 0.3786029 |
| Arglu1 | 2.3127201 |
| Arhgap1 | -0.7816183 |
| Arhgap1 | 2.887844 |
| Arhgap1 | 2.3175504 |
| Arhgap10 | 0.67438 |
| Arhgap12 | -0.3577712 |
| Arhgap15 | -0.607539 |
| Arhgap15 | 0.19823 |
| Arhgap17 | 0.621954 |
| Arhgap18 | 0.2645932 |
| Arhgap19 | -0.3784995 |
| Arhgap20 | 0.4941092 |
| Arhgap22 | 0.7233991 |
| Arhgap24 | -0.5102254 |
| Arhgap24 | -0.4343707 |
| Arhgap25 | -0.5595895 |
| Arhgap26 | -0.1859729 |
| Arhgap27 | 0.1565083 |
| Arhgap28 | 1.607474 |
| Arhgap29 | -0.0537138 |
| Arhgap30 | 1.7907498 |
| Arhgap4 | 1.2933282 |
| Arhgap6 | 0.5026878 |
| Arhgap8 | 2.6665481 |
| Arhgap9 | 0.538105 |
| Arhgdib | 3.2927466 |
| Arhgdig | -0.6199123 |
| Arhgef1 | -0.4168718 |
| Arhgef1 | 1.4677806 |
| Arhgef10 | 2.2083451 |
| Arhgef10l | -0.5188998 |
| Arhgef10l | -0.6739985 |
| Arhgef15 | -0.4157537 |
| Arhgef18 | -0.0250867 |
| Arhgef19 | 0.5389276 |
| Arhgef2 | 1.9970227 |
| Arhgef3 | -0.674731 |
| Arhgef4 | -0.5164133 |
| Arhgef6 | 0.1472594 |
| Arid2 | 3.4118107 |
| Arid3a | -0.2028096 |
| Arid3b | 1.4020066 |
| Arid4b | -0.5615401 |
| Arid5a | -0.6034499 |
| Arid5b | -0.6219902 |
| Arih1 | -0.6865278 |
| Arih2 | -0.7174397 |
| Arl1 | 0.3940381 |
| Arl11 | -0.665978 |
| Arl16 | 2.8760952 |
| Arl2 | -0.5524714 |
| Arl2bp | 0.0120327 |
| Arl2bp | 0.6494427 |
| Arl2bp | -0.3817288 |
| Arl3 | -0.2126946 |
| Arl3 | -0.4864685 |
| Arl4a | -0.4810691 |
| Arl4a | 0.1294356 |
| Arl4c | -0.4140792 |
| Arl5a | -0.7718339 |
| Arl5b | 1.4961811 |
| Arl6 | 0.0651386 |
| Arl6ip1 | 0.9575361 |
| Arl6ip2 | 0.4614579 |
| Arl6ip2 | 2.7870436 |
| Arl6ip2 | -0.7336689 |
| Arl6ip2 | 1.1296023 |
| Arl6ip4 | 2.1855001 |
| Arl6ip4 | -0.4644636 |
| Arl6ip5 | 1.2229865 |
| Arl6ip5 | 0.5777972 |
| Arl8a | 1.6840015 |
| Arl8b | -0.607539 |
| Armc10 | 1.1592546 |
| Armc3 | 0.8582783 |
| Armc6 | 1.740474 |
| Armc6 | 0.785073 |
| Armc8 | 0.8572479 |
| Armc8 | -0.3181219 |
| Armc8 | 1.1883261 |
| Armc9 | -0.2387922 |
| Armc9 | -0.6123318 |
| Armcx1 | -0.6226838 |
| Armcx2 | 0.1580412 |
| Armcx3 | -0.1140864 |
| Armcx5 | 3.2321363 |
| Armcx6 | 1.3267817 |
| Armcx6 | 1.3231722 |
| Armetl1 | 1.3051937 |
| Arnt | 2.2349105 |
| Arnt | -0.1698597 |
| Arnt2 | -0.2406507 |
| Arntl | 0.141355 |
| Arpc1a | 0.5724538 |
| Arpc1a | -0.2873515 |
| Arpc1a | -0.353051 |
| Arpc1b | -0.1321795 |
| Arpc1b | 3.7613766 |
| Arpc1b | -0.6199123 |
| Arpc3 | -0.3742107 |
| Arpc4 | -0.606174 |
| Arpc5 | -0.6199123 |
| Arpc5 | -0.5615401 |
| Arpc5l | 1.1459596 |
| Arpp19 | 1.2273943 |
| Arpp21 | 0.2607499 |
| Arpp21 | 0.0614175 |
| Arrb1 | 1.2590467 |
| Arrb1 | -0.4236076 |
| Arrb1 | -0.1255605 |
| Arrb1 | -0.2453128 |
| Arrb2 | -0.5720108 |
| Arrdc1 | -0.2839389 |
| Arrdc2 | -0.0931366 |
| Arrdc3 | 0.2741344 |
| Arrdc4 | -0.6776665 |
| Ars2 | 2.647971 |
| Ars2 | 2.6863333 |
| Ars2 | -0.524518 |
| Arsa | -0.571353 |
| Arsb | 0.8344445 |
| Arsb | 0.0765493 |
| Arsg | 0.1985236 |
| Arsi | 0.114805 |
| Arsj | -0.631047 |
| Arsj | 1.8129279 |
| Art1 | -0.6338511 |
| Art2b | -0.3206471 |
| Art3 | 0.0446633 |
| Art3 | -0.7274537 |
| Art4 | 0.019499 |
| Art5 | 0.0808764 |
| Artn | 0.0907878 |
| Artn | 0.9149673 |
| Arv1 | -0.61096 |
| As3mt | -0.6977213 |
| As3mt | 1.802697 |
| Asah1 | 0.2477329 |
| Asah1 | 0.7321695 |
| Asah2 | 0.0460421 |
| Asah3 | -0.3267351 |
| Asah3l | 0.5086473 |
| Asb1 | -0.4029897 |
| Asb1 | -0.0895568 |
| Asb1 | -0.5263982 |
| Asb12 | -0.660187 |
| Asb13 | -0.1380091 |
| Asb15 | -0.7742706 |
| Asb17 | 0.9078705 |
| Asb2 | 0.3599435 |
| Asb3 | -0.5879355 |
| Asb3 | -0.2776334 |
| Asb4 | -0.5582913 |
| Asb4 | 1.6638405 |
| Asb6 | 0.4813133 |
| Asb6 | -0.4810691 |
| Asb7 | -0.6776665 |
| Asb7 | -0.632448 |
| Asb8 | -0.4562679 |
| Asb9 | -0.355146 |
| Ascc1 | 0.6170421 |
| Ascc1 | -0.815826 |
| Ascc2 | 2.9879866 |
| Ascc2 | -0.7328898 |
| Ascc2 | 0.2532938 |
| Ascc2 | -0.2863752 |
| Ascc3l1 | 1.7796047 |
| Ascl1 | 1.54241 |
| Ascl2 | -0.6494226 |
| Asf1a | -0.5270257 |
| Asf1b | -0.8863225 |
| Asgr1 | -0.7220485 |
| Ash1l | -0.6380728 |
| Ash1l | 0.6853752 |
| Ash1l | 1.4850289 |
| Ash2l | -0.4423798 |
| Ash2l | -0.4774864 |
| Asl | 0.0265559 |
| Asl | 1.52771 |
| Asna1 | -0.7478026 |
| Asns | 0.3103294 |
| Asns | 0.0216215 |
| Asns | 0.523803 |
| Asnsd1 | 0.4037968 |
| Asnsd1 | -0.6000557 |
| Asph | 0.0778828 |
| Asph | -0.5706957 |
| Asph | -0.2704092 |
| Asph | -0.8407837 |
| Asph | -0.8486638 |
| Asphd1 | 0.0758818 |
| Asphd2 | -0.2518782 |
| Aspm | -0.2095382 |
| Asprv1 | 2.0310908 |
| Aspscr1 | -0.2113406 |
| Aspscr1 | 1.1163341 |
| Aspscr1 | 0.0894722 |
| Asrgl1 | -0.9057239 |
| Ass1 | -0.2113406 |
| Aste1 | 0.6253398 |
| Astn1 | -0.785725 |
| Astn2 | 1.4580967 |
| Asz1 | 0.6725947 |
| Atad1 | 1.6471806 |
| Atad2 | 2.3140661 |
| Atad3a | -0.0088971 |
| Atad4 | 2.2626174 |
| Atad4 | 0.2145367 |
| Atcay | 2.097272 |
| Ate1 | 1.01977 |
| Ate1 | 0.9390608 |
| Atf2 | -0.421357 |
| Atf2 | -0.0460967 |
| Atf2 | 0.9721415 |
| Atf3 | -0.424171 |
| Atf4 | -0.5933032 |
| Atf4 | 2.4607264 |
| Atf5 | -0.1447153 |
| Atf7 | -0.4124075 |
| Atf7ip | 0.4569889 |
| Atg10 | -0.448142 |
| Atg10 | -0.7438555 |
| Atg12 | 0.7717973 |
| Atg16l1 | 1.1580732 |
| Atg2a | -0.2350861 |
| Atg2a | 1.5252257 |
| Atg2b | -0.3303044 |
| Atg2b | -0.564147 |
| Atg3 | -0.6939755 |
| Atg3 | -0.52954 |
| Atg4b | -0.4650517 |
| Atg4c | -0.4967515 |
| Atg4c | 1.7995732 |
| Atg4d | -0.7485939 |
| Atg5 | -0.2561228 |
| Atg5 | -0.2932302 |
| Atg7 | -0.2556502 |
| Atg9b | 0.0244443 |
| Athl1 | -0.5466867 |
| Atic | 2.9330596 |
| Atic | 0.0091729 |
| Atic | -0.8338334 |
| Atm | -0.494322 |
| Atm | 1.3749903 |
| Atmin | -0.5786138 |
| Atmin | 0.6398744 |
| Atmin | -0.5016291 |
| Atn1 | 0.9651956 |
| Atoh8 | 0.7700239 |
| Atox1 | -0.0136519 |
| Atp10a | 0.8480728 |
| Atp10d | -0.5126957 |
| Atp11c | -0.2028096 |
| Atp11c | -0.6451506 |
| Atp12a | -0.203704 |
| Atp13a1 | -0.5133143 |
| Atp13a1 | 0.4824044 |
| Atp13a2 | -0.7075296 |
| Atp13a4 | -0.4715449 |
| Atp13a5 | -0.7220485 |
| Atp1a1 | -0.61577 |
| Atp1a2 | 1.1765927 |
| Atp1b1 | -0.6732666 |
| Atp1b2 | 0.5787835 |
| Atp1b3 | -0.4372234 |
| Atp2a1 | 1.2048419 |
| Atp2a2 | 2.1997203 |
| Atp2a2 | 2.4722468 |
| Atp2a3 | 1.3875804 |
| Atp2a3 | 1.5917725 |
| Atp2a3 | 3.7294441 |
| Atp2b1 | -0.8166759 |
| Atp2b2 | -0.675464 |
| Atp2b2 | -0.8936671 |
| Atp2b2 | -0.53143 |
| Atp2b3 | 1.1304877 |
| Atp2b3 | 2.2781058 |
| Atp2b4 | -0.4140792 |
| Atp2b4 | 0.3865929 |
| Atp2c1 | 0.737026 |
| Atp2c1 | 2.5687925 |
| Atp5a1 | 0.3621723 |
| Atp5b | 2.4753353 |
| Atp5c1 | 0.7365248 |
| Atp5d | 0.4262636 |
| Atp5e | 0.3472144 |
| Atp5f1 | -0.8014933 |
| Atp5g2 | 0.5354267 |
| Atp5g3 | 0.6281522 |
| Atp5h | -0.7645607 |
| Atp5j | 0.9213234 |
| Atp5j | 0.0019859 |
| Atp5j2 | -0.7313334 |
| Atp5l | -0.5505393 |
| Atp5o | -0.5383924 |
| Atp5s | 1.0628818 |
| Atp5sl | 0.40356 |
| Atp6ap1 | 0.9746192 |
| Atp6ap2 | -0.8200828 |
| Atp6ap2 | 2.5677128 |
| Atp6ap2 | -0.0979305 |
| Atp6ap2 | -0.0571614 |
| Atp6v0a1 | -0.2641921 |
| Atp6v0a1 | 0.7507905 |
| Atp6v0a2 | -0.7082882 |
| Atp6v0b | 1.3779827 |
| Atp6v0d1 | -0.7501787 |
| Atp6v0d1 | 0.6641586 |
| Atp6v0d1 | -0.785725 |
| Atp6v0d1 | -0.379037 |
| Atp6v0e | -0.548611 |
| Atp6v0e2 | 1.5937493 |
| Atp6v1a | 0.022681 |
| Atp6v1b1 | 0.4082855 |
| Atp6v1b2 | 1.2863066 |
| Atp6v1c1 | 0.2280109 |
| Atp6v1c2 | -0.7446436 |
| Atp6v1d | -0.7783457 |
| Atp6v1e1 | 0.4370731 |
| Atp6v1e2 | 1.2677092 |
| Atp6v1g1 | 1.687835 |
| Atp6v1g2 | 0.9343177 |
| Atp6v1g2 | 1.6606079 |
| Atp6v1h | 1.0010788 |
| Atp7a | -0.2032567 |
| Atp8a1 | 0.7056127 |
| Atp8a2 | 0.2084543 |
| Atp8b1 | 2.6852702 |
| Atp8b3 | 3.23518 |
| Atp8b3 | -0.6880127 |
| Atp8b3 | 0.4904398 |
| Atp8b3 | 0.3199436 |
| Atp9a | 0.7203443 |
| Atp9a | 3.4824091 |
| Atp9b | 3.692801 |
| Atp9b | 0.9776101 |
| Atpaf1 | -0.6437308 |
| Atpaf2 | -0.7136159 |
| Atpaf2 | -0.0491363 |
| Atpbd1b | 2.0586018 |
| Atpbd1c | 0.2741344 |
| Atpbd3 | 0.1525115 |
| Atpif1 | 1.6237797 |
| Atrx | 0.332784 |
| Atxn1 | 2.5044765 |
| Atxn10 | 1.7573181 |
| Atxn2 | 0.9215988 |
| Atxn2l | -0.5176557 |
| Atxn3 | -0.7948222 |
| Atxn3 | 0.855774 |
| Atxn3 | 0.6512718 |
| Atxn7l1 | 2.3195267 |
| Atxn7l2 | -0.5746465 |
| AU014645 | -0.8243586 |
| AU017455 | 1.8020402 |
| AU017455 | -0.4504566 |
| AU018778 | 1.8195628 |
| AU018829 | -0.3865933 |
| AU019823 | 3.2938261 |
| AU021034 | 0.3383795 |
| AU021092 | 1.124384 |
| AU021838 | -0.528282 |
| AU022252 | 1.265791 |
| AU022751 | -0.7375739 |
| AU022870 | 1.6610522 |
| AU023871 | 0.4136911 |
| AU040320 | 2.4259906 |
| AU040320 | -0.7159083 |
| AU040320 | -0.6380728 |
| AU040829 | 0.2557856 |
| AU042651 | -0.6494226 |
| Aup1 | 0.6790063 |
| Aup1 | 0.1141637 |
| Aurka | -0.2883287 |
| Aurkaip1 | -0.2190387 |
| Aurkc | -0.4768906 |
| Auts2 | 1.166952 |
| Auts2 | 0.8382079 |
| AV028368 | 0.8458397 |
| AV249152 | 1.9399084 |
| AV249152 | 0.9476735 |
| Aven | 0.3240714 |
| Avil | 1.6908538 |
| Avp | 0.1741395 |
| Avpi1 | 0.9188412 |
| Avpi1 | 1.028057 |
| Avpr1a | -0.4727303 |
| AW049604 | -0.7799806 |
| AW061290 | 0.2482905 |
| AW111922 | -0.5595895 |
| AW121567 | 0.9998111 |
| AW125753 | -0.5498961 |
| AW146242 | 0.9432423 |
| AW146299 | -0.4751054 |
| AW146299 | 2.0739618 |
| AW540478 | -0.7939916 |
| AW544981 | -0.6681587 |
| AW548124 | -0.4751054 |
| AW549877 | -0.5004074 |
| AW551984 | 1.2810299 |
| AW551984 | 0.3492223 |
| AW551984 | -0.129692 |
| AW555464 | 1.5099593 |
| Axin2 | -0.7282284 |
| Axl | -0.5164133 |
| Axud1 | -0.1177595 |
| Azgp1 | -0.2594372 |
| Azgp1 | 1.8397742 |
| Azi1 | 3.597322 |
| Azi2 | 1.1284944 |
| Azin1 | 0.3105905 |
| B020018G12Rik | -0.4751054 |
| B230118H07Rik | 0.2334045 |
| B230120H23Rik | 0.7890358 |
| B230206H07Rik | 2.9782132 |
| B230209C24Rik | 0.502047 |
| B230217C12Rik | 0.0494803 |
| B230219D22Rik | -0.0773284 |
| B230312A22Rik | -0.5926306 |
| B230312I18Rik | -0.7143795 |
| B230317C12Rik | -0.6275534 |
| B230317C12Rik | -0.4040929 |
| B230339H12Rik | 2.6783634 |
| B230339M05Rik | 1.392049 |
| B230339M05Rik | 1.887656 |
| B230340J04Rik | -0.3920275 |
| B230342M21Rik | 1.7694616 |
| B230373P09Rik | 0.8922397 |
| B230378H13Rik | -0.0781126 |
| B230380D07Rik | 0.4111096 |
| B230396O12Rik | 0.2081636 |
| B230399E16Rik | 0.1457091 |
| B2m | 0.0943964 |
| B2m | 1.0826247 |
| B3galnt1 | -0.5693823 |
| B3galnt2 | -0.5647998 |
| B3galt1 | 2.4645638 |
| B3galt4 | -0.6206044 |
| B3galt4 | -0.6373678 |
| B3galt5 | -0.6969709 |
| B3galt6 | -0.7136159 |
| B3gat1 | 1.3114378 |
| B3gat1 | -0.5145527 |
| B3gat1 | 0.5319131 |
| B3gat2 | -0.3060928 |
| B3gat3 | 1.4625604 |
| B3gat3 | 0.7435184 |
| B3gnt1 | -0.3540979 |
| B3gnt3 | -0.2374007 |
| B3gnt5 | 0.1265907 |
| B3gnt7 | -0.5919585 |
| B3gnt8 | 0.2090352 |
| B3gnt8 | 1.8662958 |
| B430119L13Rik | 0.2538481 |
| B4galnt3 | -0.2467158 |
| B4galnt4 | -0.5993783 |
| B4galt1 | -0.660187 |
| B4galt3 | -0.6969709 |
| B4galt3 | -0.632448 |
| B4galt4 | 0.0970123 |
| B4galt7 | 1.9168864 |
| B630005N14Rik | 1.4346557 |
| B830045N13Rik | 2.1878788 |
| B930006L02Rik | 0.5191976 |
| B930011P16Rik | -0.2937217 |
| B930037P14Rik | -0.6820869 |
| B930041F14Rik | 1.0569262 |
| B930041G04 | 0.6793612 |
| B930076A02 | -0.6387782 |
| B9d1 | 1.5914312 |
| B9d2 | 3.1046292 |
| Baalc | 0.4720988 |
| Baalc | -0.5786138 |
| Bace1 | 2.6418337 |
| Bace2 | -0.4372234 |
| Bace2 | 0.1671868 |
| Bace2 | 2.0934868 |
| Bach1 | -0.2518782 |
| Bach2 | -0.2072901 |
| Bad | 2.4013805 |
| Bag1 | -0.2364742 |
| Bag2 | 0.9035308 |
| Bag3 | 0.6551015 |
| Bag4 | -0.7621486 |
| Bag5 | 1.6830097 |
| Bahcc1 | 1.9810889 |
| Bahd1 | 0.5409809 |
| Bai1 | 2.6725951 |
| Bai2 | 0.5438481 |
| Bai2 | 2.9416289 |
| Bai3 | -0.3667513 |
| Baiap2 | 0.4910883 |
| Baiap2 | 1.1214849 |
| Banf1 | -0.5065318 |
| Banf1 | 0.4736408 |
| Banf1 | -0.6703445 |
| Banf1 | -0.433232 |
| Banf2 | 1.4632632 |
| Bap1 | 1.4584106 |
| Bard1 | -0.1689964 |
| Barx2 | 0.8749041 |
| Basp1 | 0.8329349 |
| Bat1a | 2.723464 |
| Bat1a | -0.6268563 |
| Bat2 | -0.1120519 |
| Bat3 | -0.2863752 |
| Bat4 | 3.1573665 |
| Bat4 | -0.6939755 |
| Bat5 | -0.4810691 |
| Batf | 0.6962451 |
| Bax | -0.7597426 |
| Baz1a | -0.6401906 |
| Baz1b | 2.0510812 |
| Baz2a | -0.4372234 |
| BB014433 | -0.6380728 |
| BB128963 | 0.5050334 |
| BB146404 | -0.2622873 |
| Bbc3 | -0.4101832 |
| Bbc3 | 0.6939762 |
| Bbox1 | -0.6857862 |
| Bbs1 | 0.8073661 |
| Bbs12 | 1.381221 |
| Bbs2 | 2.6664374 |
| Bbs4 | -0.5371227 |
| Bbs5 | -0.3016207 |
| Bbs7 | -0.3191312 |
| Bbs9 | -0.4632885 |
| Bbs9 | -0.6185295 |
| Bbx | 1.2662709 |
| BC002163 | 1.4045006 |
| BC002199 | 1.9691684 |
| BC002216 | 0.6632568 |
| BC003236 | -0.1974606 |
| BC003266 | -0.4792761 |
| BC003885 | -0.2090882 |
| BC003885 | 0.9042321 |
| BC003993 | 1.4718899 |
| BC003993 | -0.4157537 |
| BC004004 | -0.807369 |
| BC004044 | -0.7849022 |
| BC004044 | -0.0325373 |
| BC004728 | 0.3678446 |
| BC004728 | -0.6479965 |
| BC005537 | 2.2870754 |
| BC005682 | -0.6041302 |
| BC005764 | -0.6652522 |
| BC006662 | 1.4607617 |
| BC006779 | -0.3806511 |
| BC006779 | 0.6050344 |
| BC008155 | 2.8860689 |
| BC008163 | -0.1096162 |
| BC008163 | 0.5578551 |
| BC010462 | 0.4756196 |
| BC011248 | -0.5352214 |
| BC011248 | -0.6791377 |
| BC011487 | -0.6902444 |
| BC013491 | -0.1153092 |
| BC013491 | -0.2603863 |
| BC013529 | -0.5595895 |
| BC013712 | 0.4681223 |
| BC016201 | -0.2704092 |
| BC016423 | 1.8468528 |
| BC016495 | 0.1917468 |
| BC017158 | 1.7011932 |
| BC017612 | 0.9333662 |
| BC017643 | 0.5852673 |
| BC017647 | 1.8210667 |
| BC018242 | 2.5245437 |
| BC018242 | -0.7734577 |
| BC018465 | -0.3021166 |
| BC020002 | 0.0688452 |
| BC020535 | 0.1786468 |
| BC021381 | 0.0617564 |
| BC021395 | -0.6739985 |
| BC021614 | -0.6784018 |
| BC021790 | 1.535776 |
| BC021891 | -0.3075882 |
| BC022224 | -0.6820869 |
| BC022687 | 1.8401951 |
| BC023055 | -0.4446805 |
| BC023151 | 1.9659919 |
| BC023744 | 0.67634 |
| BC023814 | 1.3169136 |
| BC023829 | 1.3853806 |
| BC024561 | 1.2804628 |
| BC024814 | -0.4762952 |
| BC024868 | -0.4900852 |
| BC024997 | -0.0521855 |
| BC025076 | -0.7251335 |
| BC025546 | -0.8303772 |
| BC025575 | -0.5933032 |
| BC025920 | -0.753356 |
| BC026439 | -0.2217706 |
| BC026585 | 3.8528064 |
| BC026590 | -0.5812679 |
| BC026590 | -0.6703445 |
| BC027072 | 0.677763 |
| BC027231 | 0.2735918 |
| BC027231 | -0.4002373 |
| BC027231 | -0.0306692 |
| BC027344 | -0.4551028 |
| BC028528 | 1.9708214 |
| BC028528 | 1.2487424 |
| BC029169 | 2.0596056 |
| BC029169 | 4.2029138 |
| BC029214 | 2.749674 |
| BC030183 | -0.8460299 |
| BC030307 | -0.3779623 |
| BC030476 | -0.753356 |
| BC030499 | 0.0742111 |
| BC030500 | 0.0570011 |
| BC030867 | -0.4834651 |
| BC031181 | -0.5634946 |
| BC031353 | 0.5622783 |
| BC031575 | -0.4973598 |
| BC031748 | -0.761346 |
| BC031781 | 0.5325341 |
| BC031853 | 0.0460421 |
| BC032265 | 1.5353417 |
| BC034076 | 0.2084543 |
| BC037034 | 0.3426809 |
| BC037594 | -0.6338511 |
| BC038156 | -0.7305562 |
| BC038286 | -0.5720108 |
| BC038613 | -0.5201454 |
| BC038822 | 0.2058356 |
| BC038822 | -0.2561228 |
| BC038822 | -0.5786138 |
| BC038925 | -0.6732666 |
| BC039093 | -0.2737737 |
| BC040758 | -0.1921414 |
| BC043098 | -0.4638759 |
| BC043118 | 0.510555 |
| BC043301 | -0.1846562 |
| BC046331 | -0.771023 |
| BC046404 | 1.4476789 |
| BC048355 | -0.0911462 |
| BC048502 | 0.0681724 |
| BC048546 | -0.3001347 |
| BC048546 | 1.5388824 |
| BC048562 | 0.8603357 |
| BC048644 | -0.5993783 |
| BC048651 | -0.5403001 |
| BC048671 | -0.3828076 |
| BC049635 | -0.258963 |
| BC049635 | 1.0742134 |
| BC049730 | -0.7939916 |
| BC049762 | 1.649817 |
| BC049806 | 0.3006173 |
| BC049806 | -0.4768906 |
| BC049806 | -0.5320609 |
| BC050196 | 3.1789803 |
| BC050210 | -0.0269439 |
| BC050777 | 1.4819716 |
| BC050811 | 1.3682013 |
| BC050811 | -0.2217706 |
| BC051019 | -0.4051973 |
| BC051070 | 2.4742798 |
| BC051142 | 1.2244914 |
| BC051227 | 1.5994524 |
| BC051628 | 2.1059134 |
| BC051665 | 1.6846827 |
| BC052484 | -0.6761976 |
| BC053749 | 1.2060691 |
| BC053749 | 1.911629 |
| BC054059 | -0.6254634 |
| BC055107 | 0.020207 |
| BC055111 | -0.6558667 |
| BC055324 | 0.8938002 |
| BC055368 | -0.8056865 |
| BC056474 | -0.3931181 |
| BC056923 | 0.4092278 |
| BC057022 | 2.4086883 |
| BC057371 | 2.3633095 |
| BC057371 | -0.6041302 |
| BC057371 | 1.1554903 |
| BC057552 | 0.16232 |
| BC057627 | -0.2858874 |
| BC057893 | -0.3635722 |
| BC060267 | 0.8529673 |
| BC060615 | 2.5044765 |
| BC060632 | 0.5505728 |
| BC061039 | 0.8285437 |
| BC061237 | 0.0818722 |
| BC062115 | -0.3811898 |
| BC062650 | -0.6020908 |
| BC063749 | 1.0461117 |
| BC064033 | -0.2786008 |
| BC065085 | 1.1208146 |
| BC065397 | 0.2706017 |
| BC066028 | -0.5589402 |
| BC066107 | 0.341165 |
| BC067047 | 0.5620776 |
| BC067068 | -0.5188998 |
| BC068157 | 2.9046227 |
| BC068281 | 0.2463377 |
| BC072620 | -0.6880127 |
| BC085271 | 1.1096868 |
| BC087945 | -0.1455569 |
| BC088983 | 2.6085438 |
| BC089491 | 0.7157437 |
| BC099439 | -0.3683449 |
| BC107230 | -0.4400846 |
| Bcam | 1.4605269 |
| Bcan | 1.5502107 |
| Bcan | 0.1031964 |
| Bcap29 | -0.5674157 |
| Bcap29 | 1.5186918 |
| Bcap31 | 0.1394829 |
| Bcap31 | -0.6178389 |
| Bcar1 | -0.5182775 |
| Bcar3 | -0.4230444 |
| Bcas1 | -0.2907763 |
| Bcas2 | -0.4326632 |
| Bcas3 | 0.1167261 |
| Bcat1 | 0.6565565 |
| Bcat2 | -0.7645607 |
| Bccip | -0.0074389 |
| Bcdin3d | -0.2561228 |
| Bcdo2 | -0.6352563 |
| Bckdha | -0.1711561 |
| Bckdhb | -0.5746465 |
| Bckdk | 0.0116757 |
| Bckdk | -0.7501787 |
| Bcl10 | -0.2222267 |
| Bcl11a | 0.7947134 |
| Bcl11b | 2.8652974 |
| Bcl2 | 0.5795717 |
| Bcl2 | -0.0110887 |
| Bcl2l1 | 0.7191537 |
| Bcl2l1 | 1.1630042 |
| Bcl2l1 | -0.7478026 |
| Bcl2l10 | 0.710434 |
| Bcl2l11 | -0.6275534 |
| Bcl2l11 | -0.0213831 |
| Bcl2l11 | 0.6691933 |
| Bcl2l12 | 2.1183769 |
| Bcl2l13 | -0.3699411 |
| Bcl2l14 | -0.0468557 |
| Bcl2l14 | 1.0070143 |
| Bcl2l2 | -0.7832589 |
| Bcl2l2 | -0.3715399 |
| Bcl3 | -0.4286908 |
| Bcl6 | -0.6494226 |
| Bcl6b | 0.3705455 |
| Bcl7a | 1.0312536 |
| Bcl7b | -0.6451506 |
| Bcl9l | -0.5403001 |
| Bclaf1 | -0.7525607 |
| Bclaf1 | -0.232777 |
| Bclp2 | -0.1715886 |
| Bcmo1 | -0.6769318 |
| Bcor | -0.6813487 |
| Bcor | -0.7098074 |
| Bcorl1 | 0.4004758 |
| Bcs1l | 1.3689772 |
| Bdh1 | 1.7266511 |
| Bdh2 | 0.3345679 |
| Bdkrb1 | -0.1974606 |
| Bdnf | -0.6573046 |
| Bdnf | -0.122679 |
| Becn1 | -0.6977213 |
| Becn1 | 0.3887604 |
| Bet1 | -0.2476524 |
| Bet1l | 1.4001735 |
| Bex2 | 2.7259637 |
| Bex2 | -0.4286908 |
| Bex2 | 0.7640347 |
| Bex4 | -0.5505393 |
| Bex4 | 2.2107033 |
| Bex6 | -0.8530698 |
| Bfar | -0.3338871 |
| Bfar | 1.0233658 |
| Bglap2 | 1.3624055 |
| Bgn | 1.1778568 |
| Bhlhb2 | 0.5166761 |
| Bhlhb5 | -0.7090475 |
| Bhlhb8 | 0.3861105 |
| Bhlhb8 | 0.1577348 |
| Bhlhb9 | 1.9430782 |
| Bhmt | -0.6909894 |
| Bhmt2 | -0.6828256 |
| Bicc1 | -0.2387922 |
| Bicd1 | -0.5987014 |
| Bicd2 | 2.008382 |
| Bicd2 | 0.5162552 |
| Bid | 0.3554702 |
| Bid | -0.239721 |
| Bik | 1.7945649 |
| Bin1 | 2.0159826 |
| Bin1 | -0.231855 |
| Bin1 | 0.5285942 |
| Bin3 | 0.6941509 |
| Birc2 | -0.6806111 |
| Birc2 | 0.3214936 |
| Birc2 | 0.9328221 |
| Birc4 | 0.5793747 |
| Birc5 | -0.0749795 |
| Birc5 | 1.5740026 |
| Birc5 | 1.026948 |
| Blcap | 1.2693367 |
| Blm | 0.9514173 |
| Blm | 4.2209729 |
| Blmh | 0.4190663 |
| Blnk | -0.7067715 |
| Bloc1s1 | -0.545406 |
| Bloc1s3 | -0.8192299 |
| Blvra | -0.2429789 |
| Blvrb | 1.5544776 |
| Bmf | 1.1958969 |
| Bmi1 | 1.7367879 |
| Bmp1 | -0.5766281 |
| Bmp1 | 1.4008405 |
| Bmp1 | 0.307976 |
| Bmp1 | 0.259649 |
| Bmp15 | 0.5835034 |
| Bmp2k | 1.4609965 |
| Bmp2k | 0.9196693 |
| Bmp3 | 0.6496257 |
| Bmp4 | 0.8737468 |
| Bmp6 | -0.5364885 |
| Bmp7 | 1.9077649 |
| Bmp8a | 1.9343954 |
| Bmper | -0.2090882 |
| Bmpr1a | 1.3738764 |
| Bmpr1a | 1.3121698 |
| Bmpr1b | 3.4464915 |
| Bmpr1b | 1.4120262 |
| Bmpr2 | -0.7235897 |
| Bms1 | 1.1159972 |
| Bnc1 | 1.7014366 |
| Bnc2 | -0.5693823 |
| Bnc2 | -0.813281 |
| Bnip1 | 0.0631107 |
| Bnip2 | -0.7197413 |
| Bnip3l | 1.4168739 |
| Bnipl | 2.8140369 |
| Bnipl | 0.1852193 |
| Bok | -0.2986509 |
| Bok | -0.3176176 |
| Bola1 | 1.4762897 |
| Bola2 | 2.0025861 |
| Bola3 | -0.4900852 |
| Boll | 1.6172908 |
| Bop1 | 0.6899486 |
| Bpi | 0.0668252 |
| Bpil1 | 2.3540183 |
| Bpil3 | -0.5352214 |
| Bpnt1 | -0.103957 |
| Bpnt1 | -0.4527769 |
| Bpnt1 | -0.4991872 |
| Bpnt1 | -0.4051973 |
| Brap | -0.7750842 |
| Brca2 | 0.5961705 |
| Brca2 | 0.2939987 |
| Brcc3 | 0.0422457 |
| Brcc3 | -0.7973184 |
| Brcc3 | 2.2261021 |
| Brd2 | 1.6274679 |
| Brd2 | -0.5647998 |
| Brd2 | -0.2010233 |
| Brd4 | 1.4961811 |
| Brd4 | -0.7783457 |
| Brd8 | -0.3577712 |
| Brd8 | -0.3525279 |
| Brd9 | 2.3525737 |
| Brd9 | 0.030415 |
| Brdt | -0.494322 |
| Brdt | -0.6451506 |
| Bre | 0.1674901 |
| Bre | 0.5913184 |
| Bre | 0.2480118 |
| Brf1 | 3.1091242 |
| Brf2 | 1.6074068 |
| Bri3 | 0.5797687 |
| Brip1 | -0.556995 |
| Brms1 | 2.0355599 |
| Brp16 | 0.7014607 |
| Brp16 | 0.0758818 |
| Brp17 | 2.2168218 |
| Brp44 | 0.4852354 |
| Brpf1 | -0.2708891 |
| Brpf1 | -0.5557003 |
| Brs3 | -0.4562679 |
| Brsk1 | -0.1591243 |
| Brsk2 | -0.8700008 |
| Brsk2 | -0.763756 |
| Brunol4 | 0.7200043 |
| Brunol4 | -0.1342574 |
| Brwd1 | -0.4888781 |
| Brwd1 | -0.2332384 |
| Brwd2 | -0.2323159 |
| Bscl2 | -0.5959985 |
| Bsdc1 | -0.7082882 |
| Bsg | 0.8701211 |
| Bsg | 1.1891587 |
| Bspry | -0.813281 |
| Bst2 | -0.6924813 |
| Btbd1 | 2.0503217 |
| Btbd10 | -0.5582913 |
| Btbd11 | -0.5383924 |
| Btbd12 | -0.0460967 |
| Btbd14a | -0.5557003 |
| Btbd14b | -0.7045009 |
| Btbd16 | 0.0494803 |
| Btbd3 | 0.600998 |
| Btbd3 | 0.4994798 |
| Btbd6 | 2.401439 |
| Btbd9 | -0.0195366 |
| Btc | -0.4562679 |
| Btd | 0.1837295 |
| Btf3 | -0.6508508 |
| Btg1 | 1.5886283 |
| Btg1 | 0.9113559 |
| Btg3 | -0.390394 |
| Btg3 | -0.1149015 |
| Btg4 | 1.5325868 |
| Btk | 0.6523676 |
| Btla | 0.3574609 |
| Btrc | 0.5346012 |
| Btrc | 1.2370403 |
| Btrc | 1.3205468 |
| Bub1b | -0.1003365 |
| Bub3 | 1.3051016 |
| Bud13 | 0.9149673 |
| Bud31 | -0.0404236 |
| Bves | 1.40367 |
| Bxdc1 | -0.524518 |
| Bxdc1 | -0.1574178 |
| Bxdc2 | 0.1048172 |
| Bxdc2 | 0.3703003 |
| Bxdc5 | 2.5617028 |
| Bxdc5 | 2.6215353 |
| Bxdc5 | -0.5621911 |
| Bzrap1 | 1.3383301 |
| Bzrpl1 | -0.664527 |
| Bzw1 | -0.5852628 |
| Bzw2 | 3.1919908 |
| Bzw2 | 2.9076716 |
| C030006K11Rik | 3.7935277 |
| C030011O14Rik | -0.5700388 |
| C030014K22Rik | -0.6917351 |
| C030018G13Rik | 2.5660663 |
| C030030A07Rik | -0.5301696 |
| C030039L03Rik | 0.565882 |
| C030045D06Rik | -0.0118203 |
| C030045D06Rik | -0.7336689 |
| C030046I01Rik | -0.7321113 |
| C030048B08Rik | -0.6487092 |
| C030048B08Rik | -0.4475642 |
| C130022K22Rik | 1.3563123 |
| C130032J12Rik | -0.2795691 |
| C130034I18Rik | -0.5364885 |
| C130038G02Rik | -0.6082222 |
| C130060K24Rik | 2.144396 |
| C130073D16Rik | 0.9861409 |
| C130074G19Rik | -0.6969709 |
| C130090K23Rik | -0.6275534 |
| C130090K23Rik | 2.3885521 |
| C1galt1 | 0.4108746 |
| C1galt1c1 | -0.6843047 |
| C1qa | 0.6578277 |
| C1qb | 0.5889806 |
| C1qbp | 1.4844946 |
| C1qc | -0.5926306 |
| C1qdc2 | 1.3060225 |
| C1ql2 | -0.5693823 |
| C1ql3 | 0.8892535 |
| C1qtnf1 | 0.3933202 |
| C1qtnf2 | 1.4901281 |
| C1qtnf3 | 1.7145472 |
| C1qtnf3 | -0.6401906 |
| C1qtnf3 | 1.766568 |
| C1qtnf4 | 1.4789813 |
| C1qtnf6 | -0.5733278 |
| C1qtnf7 | -0.4315265 |
| C1qtnf9 | 0.6183671 |
| C1rl | 0.4361579 |
| C1s | 3.2696969 |
| C1s | -0.6444404 |
| C2 | 0.022681 |
| C230021P08Rik | 0.5154129 |
| C230029F24Rik | -0.4961435 |
| C230052I12Rik | 1.0150378 |
| C230052I12Rik | -0.6962212 |
| C230055K05Rik | 3.3298314 |
| C230071H18Rik | -0.3546218 |
| C230078M08Rik | 0.7183023 |
| C230078M14Rik | -0.5946499 |
| C230090D14 | 0.6370964 |
| C230093N12Rik | 0.9667727 |
| C230096C10Rik | -0.5371227 |
| C2cd3 | -0.0903512 |
| C3 | -0.5276536 |
| C330001K17Rik | 0.0812084 |
| C330005M16Rik | -0.6387782 |
| C330016O10Rik | -0.7605439 |
| C330019G07Rik | -0.7734577 |
| C330023M02Rik | 0.1227842 |
| C330027C09Rik | -0.3795747 |
| C430003P19Rik | 1.173531 |
| C430004E15Rik | 0.335077 |
| C4b | -0.7758985 |
| C530028I08Rik | 2.6259578 |
| C530028I08Rik | 0.0782159 |
| C530028O21Rik | 1.5968767 |
| C530043G21Rik | -0.5505393 |
| C530044N13Rik | 3.5589434 |
| C6 | -0.7767136 |
| C630004H02Rik | -0.6962212 |
| C630004H02Rik | 0.615336 |
| C630007B19Rik | 0.1481883 |
| C630028N24Rik | 0.2277262 |
| C630035N08Rik | 0.1738383 |
| C630035N08Rik | -0.8899877 |
| C730025P13Rik | -0.0121864 |
| C730027P07Rik | 0.3810312 |
| C78339 | 0.4211614 |
| C79127 | 3.4593936 |
| C79267 | 0.1552803 |
| C79407 | -0.8691022 |
| C79407 | 2.7368852 |
| C80008 | 1.7344988 |
| C80913 | -0.2350861 |
| C85492 | 0.4607889 |
| C85492 | -0.3151004 |
| C85492 | -0.4709528 |
| C85627 | -0.3736759 |
| C87436 | 1.9001378 |
| C87436 | 1.8508148 |
| C87499 | -0.8539535 |
| C8b | 1.8389844 |
| C8g | 0.7165973 |
| C9 | 2.8868074 |
| C9 | 1.5333126 |
| C920005C14Rik | 2.3980136 |
| Cab39l | -0.6143932 |
| Cabc1 | 1.1170075 |
| Cables2 | -0.6835649 |
| Cabp2 | -0.3556704 |
| Cabp7 | 0.5868326 |
| Cabyr | 2.0771686 |
| Cabyr | 2.071818 |
| Cachd1 | 2.2281717 |
| Cacna1a | -0.6835649 |
| Cacna1b | -0.6020908 |
| Cacna1b | 0.0772163 |
| Cacna1b | 0.92709 |
| Cacna1d | -0.3525279 |
| Cacna1g | -0.4876726 |
| Cacna1h | 2.1616068 |
| Cacna2d1 | 0.6029222 |
| Cacna2d3 | -0.5251443 |
| Cacnb1 | -0.5479692 |
| Cacnb1 | -0.5270257 |
| Cacnb2 | 1.7174231 |
| Cacnb2 | -0.2059437 |
| Cacnb3 | -0.5799399 |
| Cacnb3 | -0.2369373 |
| Cacnb3 | 1.405911 |
| Cacnb3 | -0.7470118 |
| Cacnb4 | 1.8089139 |
| Cacnb4 | 1.7987495 |
| Cacng2 | 0.3251005 |
| Cacng3 | -0.5077614 |
| Cacng3 | -0.34161 |
| Cacng4 | 1.2503049 |
| Cacng5 | -0.3742107 |
| Cacng5 | -0.5126957 |
| Cacng5 | -0.1859729 |
| Cacng7 | 0.8209248 |
| Cacng8 | -0.5164133 |
| Cacybp | -0.118987 |
| Cad | 0.0109613 |
| Cadm2 | -0.2304735 |
| Cadm3 | 1.4475202 |
| Cadps | -0.4002373 |
| Cadps2 | 2.4445506 |
| Cadps2 | 0.8648757 |
| Cage1 | -0.40741 |
| Calb2 | 0.4943246 |
| Calb2 | -0.5460461 |
| Calca | 1.1480255 |
| Calca | 1.1032205 |
| Calcoco1 | -0.6696153 |
| Cald1 | 1.8638869 |
| Cald1 | -0.7290037 |
| Calm2 | 2.2706947 |
| Calm3 | -0.6902444 |
| Calml4 | -0.6688868 |
| Caln1 | -0.0859905 |
| Calr | 0.1341585 |
| Calr3 | -0.6247678 |
| Calr3 | 1.4597436 |
| Calr3 | -0.34161 |
| Calr4 | 1.9056551 |
| Calu | 0.0474189 |
| Calu | 0.1723306 |
| Calu | 1.7629244 |
| Caly | -0.749386 |
| Camk1 | 2.9302587 |
| Camk1d | 1.2305873 |
| Camk1g | 1.3069425 |
| Camk1g | 1.7044128 |
| Camk2a | 0.8545932 |
| Camk2a | 0.3663681 |
| Camk2a | 0.4767172 |
| Camk2b | -0.2800537 |
| Camk2d | -0.4355107 |
| Camk2d | -0.4366522 |
| Camk2d | -0.1943541 |
| Camk2g | 0.6023454 |
| Camk2g | 1.2304877 |
| Camk2n1 | 0.7408602 |
| Camk4 | 0.0641252 |
| Camkk1 | 0.9970163 |
| Camkk2 | 1.5869838 |
| Camkk2 | 2.0873747 |
| Camkv | -0.4858671 |
| Caml | 0.8207718 |
| Camp | 1.4961057 |
| Camsap1l1 | 1.0633566 |
| Camta2 | -0.4264282 |
| Cant1 | -0.7197413 |
| Cant1 | -0.4709528 |
| Cap1 | -0.1177595 |
| Capg | -0.4202337 |
| Capg | 0.2735918 |
| Capn1 | -0.1604062 |
| Capn10 | -0.3641013 |
| Capn11 | 0.2593735 |
| Capn2 | -0.1350899 |
| Capn2 | -0.6020908 |
| Capn2 | 0.1086958 |
| Capn3 | 0.419998 |
| Capn5 | -0.6034499 |
| Capn6 | -0.53143 |
| Capn7 | -0.3985897 |
| Capn8 | 1.8915523 |
| Capns1 | -0.4816676 |
| Caprin1 | -0.6565854 |
| Caprin1 | -0.3535743 |
| Capsl | -0.8557233 |
| Capza1 | 1.0355399 |
| Capza3 | 0.181341 |
| Capzb | -0.4586024 |
| Capzb | -0.5345885 |
| Capzb | 1.1013975 |
| Car11 | -0.6014119 |
| Car12 | -0.5345885 |
| Car13 | 0.8190872 |
| Car14 | 2.1207326 |
| Car15 | 0.2838498 |
| Car2 | -0.4876726 |
| Car3 | 1.6907308 |
| Car4 | -0.1544387 |
| Car5b | -0.4326632 |
| Car5b | -0.8090544 |
| Car5b | -0.1785357 |
| Car7 | -0.5320609 |
| Car9 | 2.4615225 |
| Card10 | -0.0738073 |
| Card11 | -0.395303 |
| Carhsp1 | 2.9653805 |
| Carm1 | -0.0769365 |
| Cart | 2.0084702 |
| Cartpt | 2.4015266 |
| Casc1 | 0.3469631 |
| Casc1 | 2.1765545 |
| Casc1 | 4.0600825 |
| Casc3 | -0.7067715 |
| Casc4 | -0.4007872 |
| Casc4 | -0.6784018 |
| Casc4 | -0.4973598 |
| Cask | -0.6718044 |
| Caskin1 | -0.7305562 |
| Caskin1 | 2.4038324 |
| Caskin1 | -0.1112393 |
| Caskin2 | -0.8200828 |
| Casp1 | 0.9740981 |
| Casp2 | 0.5829147 |
| Casp4 | -0.7159083 |
| Casp4 | -0.7816183 |
| Casp4 | 0.4202308 |
| Casp6 | 0.1765461 |
| Casp6 | -0.278117 |
| Casp7 | 0.0325135 |
| Casp8 | 1.3802847 |
| Casp8 | -0.4668182 |
| Casp9 | 1.1765927 |
| Casq2 | -0.3630434 |
| Cass4 | 1.0837871 |
| Cast | -0.7685945 |
| Cat | -0.6508508 |
| Catsper1 | 3.1105019 |
| Catsper2 | -0.7045009 |
| Catsper3 | -0.660909 |
| Catsper4 | 1.8861532 |
| Cav1 | -0.6206044 |
| Cav2 | -0.7849022 |
| Cbara1 | 1.345069 |
| Cbfa2t3h | 0.6025377 |
| Cbfa2t3h | -0.34161 |
| Cbfb | -0.6508508 |
| Cbfb | -0.2570686 |
| Cbfb | 0.54854 |
| Cbl | 0.3675986 |
| Cblb | -0.8141286 |
| Cblc | -0.5301696 |
| Cbln1 | -0.7105679 |
| Cbln2 | 0.5372817 |
| Cbln4 | 2.2130201 |
| Cbr1 | 0.5308772 |
| Cbr2 | 0.2790045 |
| Cbr3 | 1.4924025 |
| Cbr3 | -0.0891599 |
| Cbs | -0.750972 |
| Cbs | 0.8014511 |
| Cbwd1 | 2.3359428 |
| Cbx2 | 1.5819641 |
| Cbx5 | 1.992414 |
| Cbx7 | -0.1947973 |
| Cbx8 | -0.7807991 |
| Cby1 | 1.0238607 |
| Cc2d1a | 1.4348166 |
| Cc2d1b | -0.6102748 |
| Cc2d2a | 1.5631657 |
| Ccar1 | -0.4281246 |
| Ccbe1 | -0.3452354 |
| Ccbl1 | 1.9356339 |
| Ccbl1 | 1.2120802 |
| Ccbl2 | 0.3497237 |
| Ccbp2 | -0.0832258 |
| Ccdc100 | 0.6124855 |
| Ccdc101 | -0.2429789 |
| Ccdc108 | 0.8031671 |
| Ccdc109a | -0.6537138 |
| Ccdc109a | 0.2792743 |
| Ccdc109b | 0.2379229 |
| Ccdc11 | -0.3277535 |
| Ccdc110 | 0.2971814 |
| Ccdc111 | -0.3546218 |
| Ccdc113 | -0.4870704 |
| Ccdc114 | 1.0702155 |
| Ccdc115 | 0.4190663 |
| Ccdc116 | -0.5114598 |
| Ccdc117 | -0.5096088 |
| Ccdc12 | -0.7677864 |
| Ccdc120 | 1.74304 |
| Ccdc123 | -0.3672822 |
| Ccdc124 | -0.6437308 |
| Ccdc125 | 1.5351969 |
| Ccdc126 | -0.3374834 |
| Ccdc127 | 0.0515373 |
| Ccdc127 | 3.0362794 |
| Ccdc13 | -0.6338511 |
| Ccdc130 | 1.1681226 |
| Ccdc131 | 0.856069 |
| Ccdc132 | 1.1280509 |
| Ccdc134 | -0.5466867 |
| Ccdc137 | 3.7812076 |
| Ccdc137 | -0.5852628 |
| Ccdc18 | -0.5102254 |
| Ccdc21 | 1.752371 |
| Ccdc23 | 0.0412077 |
| Ccdc25 | 0.744016 |
| Ccdc27 | -0.6275534 |
| Ccdc27 | 1.3369944 |
| Ccdc27 | -0.766979 |
| Ccdc28a | -0.7266797 |
| Ccdc28b | -0.0262006 |
| Ccdc28b | -0.0979305 |
| Ccdc3 | 1.2272944 |
| Ccdc32 | -0.0243448 |
| Ccdc38 | 0.2308534 |
| Ccdc39 | 0.1227842 |
| Ccdc40 | -0.6178389 |
| Ccdc41 | 2.2345973 |
| Ccdc42 | -0.6178389 |
| Ccdc43 | 0.5003363 |
| Ccdc45 | -0.2185842 |
| Ccdc46 | -0.1651213 |
| Ccdc46 | -0.2627632 |
| Ccdc47 | 0.6372819 |
| Ccdc49 | -0.1198062 |
| Ccdc5 | -0.6303473 |
| Ccdc50 | -0.595324 |
| Ccdc51 | 3.6901902 |
| Ccdc53 | 2.4248221 |
| Ccdc53 | -0.2819943 |
| Ccdc53 | 0.24242 |
| Ccdc54 | 1.2822575 |
| Ccdc56 | 0.1831329 |
| Ccdc57 | 1.6117604 |
| Ccdc58 | -0.8583841 |
| Ccdc59 | -0.3969449 |
| Ccdc59 | -0.9057239 |
| Ccdc64 | -0.6999758 |
| Ccdc65 | 2.4291753 |
| Ccdc65 | 0.4576606 |
| Ccdc66 | 0.7249228 |
| Ccdc67 | -0.5993783 |
| Ccdc68 | 0.2524617 |
| Ccdc70 | -0.5157927 |
| Ccdc71 | -0.6164591 |
| Ccdc73 | 0.4352418 |
| Ccdc77 | -0.3257177 |
| Ccdc79 | 2.4513701 |
| Ccdc80 | -0.4900852 |
| Ccdc82 | 1.3830908 |
| Ccdc84 | 1.9220184 |
| Ccdc85a | 0.4479877 |
| Ccdc85b | 0.8816752 |
| Ccdc86 | 1.8036266 |
| Ccdc87 | -0.6932281 |
| Ccdc88a | -0.6909894 |
| Ccdc88b | 1.4449771 |
| Ccdc88c | -0.930304 |
| Ccdc90b | -0.0891599 |
| Ccdc91 | 1.3631861 |
| Ccdc92 | -0.5518269 |
| Ccdc93 | -0.5466867 |
| Ccdc94 | 2.6336423 |
| Ccdc95 | 1.1574281 |
| Ccdc96 | 2.8372194 |
| Ccdc97 | 0.531706 |
| Ccdc98 | 1.0783114 |
| Ccdc99 | 0.6499918 |
| Cchcr1 | -0.7775293 |
| Ccin | 0.596364 |
| Cck | 0.8747595 |
| Cckbr | -0.7037452 |
| Ccl11 | -0.5852628 |
| Ccl17 | -0.632448 |
| Ccl19 | -0.495536 |
| Ccl21b | 0.1789466 |
| Ccl21c | 1.5321511 |
| Ccl25 | -0.6178389 |
| Ccl25 | 0.1999905 |
| Ccl27 | 0.2200066 |
| Ccl27 | 0.2760313 |
| Ccl28 | 1.0400502 |
| Ccl3 | 0.6523676 |
| Ccl4 | 1.3359245 |
| Ccl4 | -0.1031513 |
| Ccl5 | 2.2018838 |
| Ccl6 | -0.9001403 |
| Ccl7 | -0.5403001 |
| Ccl9 | -0.247184 |
| Ccm2 | -0.1426145 |
| Ccna1 | 1.1057214 |
| Ccnb1 | -0.8700008 |
| Ccnb1 | 0.0109613 |
| Ccnb3 | 0.4292657 |
| Ccnc | -0.8175265 |
| Ccnd1 | -0.8434032 |
| Ccnd1 | 0.8227589 |
| Ccnd1 | -0.7090475 |
| Ccnd2 | -0.6130184 |
| Ccnd3 | -0.5301696 |
| Ccnd3 | 1.5479272 |
| Ccndbp1 | 0.5507758 |
| Ccne1 | -0.7470118 |
| Ccne2 | 0.8441988 |
| Ccnf | 0.2305696 |
| Ccng1 | -0.4697697 |
| Ccng1 | -0.5176557 |
| Ccng1 | -0.7375739 |
| Ccng2 | 1.2261942 |
| Ccnk | -0.5145527 |
| Ccnl1 | -0.5145527 |
| Ccno | 0.7828466 |
| Ccr4 | 0.9236617 |
| Ccr5 | 0.5448701 |
| Ccr5 | -0.1591243 |
| Ccr5 | 1.805265 |
| Ccr6 | 1.6687005 |
| Ccr6 | 1.1015115 |
| Ccrk | -0.8772205 |
| Ccrk | 1.9372034 |
| Ccrk | -0.5839293 |
| Ccrk | 1.8588446 |
| Ccrl2 | -0.8486638 |
| Ccrn4l | -0.7589419 |
| Ccrn4l | 1.0901551 |
| Ccs | 1.5238345 |
| Cct2 | 0.7097468 |
| Cct3 | 2.015326 |
| Cct3 | -0.8918256 |
| Cct4 | -0.1116456 |
| Cct5 | -0.5819325 |
| Cct6a | -0.4650517 |
| Cct6b | -0.6784018 |
| Cct7 | 0.2670558 |
| Cct8 | -0.6487092 |
| Cd109 | 0.8397092 |
| Cd109 | 3.0285069 |
| Cd14 | -0.6034499 |
| Cd151 | -0.804007 |
| Cd151 | 1.614363 |
| Cd151 | -0.8115881 |
| Cd160 | -0.2467158 |
| Cd163 | -0.2942135 |
| Cd164 | 0.209906 |
| Cd177 | -0.2728112 |
| Cd180 | 2.3139059 |
| Cd1d1 | 0.7169386 |
| Cd2 | 1.0528551 |
| Cd200 | 0.4531738 |
| Cd200 | -0.6254634 |
| Cd226 | -0.4822664 |
| Cd247 | -0.2476524 |
| Cd248 | 1.3087799 |
| Cd248 | 0.1357276 |
| Cd27 | -0.631047 |
| Cd274 | -0.8381714 |
| Cd276 | -0.7898494 |
| Cd2bp2 | 1.5687743 |
| Cd300lg | -0.9274354 |
| Cd320 | -0.3400604 |
| Cd34 | 0.0681724 |
| Cd3d | -0.5077614 |
| Cd3eap | 1.09223 |
| Cd3g | -0.4967515 |
| Cd4 | 0.4042703 |
| Cd40 | -0.1833414 |
| Cd40 | -0.1763593 |
| Cd44 | 2.1263626 |
| Cd44 | -0.7998212 |
| Cd44 | -0.7685945 |
| Cd44 | -0.9122796 |
| Cd47 | -0.0344091 |
| Cd52 | -0.0158544 |
| Cd53 | -0.7840802 |
| Cd55 | 2.0266873 |
| Cd59a | -0.8260743 |
| Cd59b | 1.2359513 |
| Cd59b | -0.7014818 |
| Cd6 | 0.8928074 |
| Cd6 | 0.5648823 |
| Cd6 | -0.6020908 |
| Cd63 | -0.3556704 |
| Cd63 | 1.5590081 |
| Cd63 | -0.8918256 |
| Cd68 | 0.3404061 |
| Cd74 | -0.5251443 |
| Cd74 | -0.2917571 |
| Cd79b | -0.7266797 |
| Cd79b | 0.9049329 |
| Cd81 | 0.1656686 |
| Cd82 | -0.0359092 |
| Cd83 | -0.7462218 |
| Cd84 | -0.4709528 |
| Cd86 | -0.7120907 |
| Cd86 | -0.5333238 |
| Cd86 | -0.2495282 |
| Cd8b1 | 1.9542071 |
| Cd9 | -0.0081677 |
| Cd93 | 2.6083085 |
| Cd96 | 1.2998354 |
| Cd97 | -0.8217908 |
| Cd97 | -0.6630781 |
| Cd97 | -0.8964359 |
| Cd99l2 | -0.3540979 |
| Cdan1 | -0.7274537 |
| Cdc123 | 1.4863253 |
| Cdc14b | -0.4674078 |
| Cdc14b | -0.3145977 |
| Cdc14b | 1.0964809 |
| Cdc16 | -0.8183778 |
| Cdc20 | -0.8844951 |
| Cdc20 | 1.6529583 |
| Cdc20 | 0.3983347 |
| Cdc20 | -0.0107231 |
| Cdc23 | -0.7849022 |
| Cdc25a | -0.2286347 |
| Cdc25b | 0.2604748 |
| Cdc25c | -0.0907486 |
| Cdc25c | -0.8992128 |
| Cdc27 | 1.2465898 |
| Cdc2a | 1.5406838 |
| Cdc2l1 | -0.8243586 |
| Cdc2l1 | -0.3452354 |
| Cdc2l5 | 3.7356276 |
| Cdc2l6 | 2.8378117 |
| Cdc34 | 0.0091729 |
| Cdc37 | 1.7870863 |
| Cdc37l1 | 1.0989995 |
| Cdc37l1 | 1.9536467 |
| Cdc37l1 | -0.485266 |
| Cdc37l1 | -0.3216591 |
| Cdc42 | 2.38612 |
| Cdc42bpb | 1.7221375 |
| Cdc42ep1 | 0.1460194 |
| Cdc42ep2 | 0.2593735 |
| Cdc42ep3 | -0.5621911 |
| Cdc42ep4 | 2.4920392 |
| Cdc42ep5 | -0.5289108 |
| Cdc42se1 | 2.3027586 |
| Cdc42se1 | -0.6813487 |
| Cdc45l | -0.1574178 |
| Cdc5l | 0.8724431 |
| Cdc5l | 2.3074138 |
| Cdc5l | -0.5383924 |
| Cdc5l | 1.3980025 |
| Cdc6 | 0.5878095 |
| Cdc7 | 0.037392 |
| Cdc73 | 1.2816911 |
| Cdca2 | 0.5969445 |
| Cdca3 | -0.7220485 |
| Cdca3 | -0.0359092 |
| Cdca4 | -0.52954 |
| Cdca4 | 1.5469264 |
| Cdca4 | 1.2627138 |
| Cdca5 | 2.0716942 |
| Cdca7 | -0.7328898 |
| Cdca8 | -0.7478026 |
| Cdcp1 | -0.7274537 |
| Cdgap | -0.5010181 |
| Cdh1 | 0.256891 |
| Cdh11 | -0.6345534 |
| Cdh11 | -0.8808509 |
| Cdh12 | -0.5615401 |
| Cdh13 | 1.9267387 |
| Cdh13 | 0.3582064 |
| Cdh16 | -0.1759246 |
| Cdh22 | -0.1894929 |
| Cdh23 | -0.6303473 |
| Cdh23 | 0.3522264 |
| Cdh23 | -0.6178389 |
| Cdh24 | 0.6479769 |
| Cdh26 | 0.6174209 |
| Cdh3 | 0.4230199 |
| Cdh5 | -0.6247678 |
| Cdh6 | -0.688756 |
| Cdh7 | 0.0088149 |
| Cdh8 | 0.625152 |
| Cdh8 | -0.0879701 |
| Cdh8 | -0.2467158 |
| Cdipt | 0.1265907 |
| Cdipt | -0.6007336 |
| Cdk10 | -0.6408976 |
| Cdk10 | 0.3749489 |
| Cdk10 | -0.621297 |
| Cdk2 | 1.3876649 |
| Cdk2 | 0.4368444 |
| Cdk2 | 1.4032544 |
| Cdk2 | -0.4320947 |
| Cdk2ap2 | -0.6430217 |
| Cdk3 | 1.3346748 |
| Cdk5 | -0.6880127 |
| Cdk5r1 | 2.0959046 |
| Cdk5rap1 | -0.4709528 |
| Cdk5rap2 | 3.4909899 |
| Cdk5rap3 | 0.8273287 |
| Cdk6 | -0.1342574 |
| Cdk8 | -0.7383569 |
| Cdk9 | -0.3963973 |
| Cdkal1 | -0.0769365 |
| Cdkl1 | -0.5812679 |
| Cdkl2 | -0.556995 |
| Cdkl2 | 0.1765461 |
| Cdkl2 | -0.4418055 |
| Cdkl2 | 0.8037904 |
| Cdkl3 | 0.3340586 |
| Cdkl4 | 0.307976 |
| Cdkn1a | -0.0753706 |
| Cdkn1a | 1.4236522 |
| Cdkn1a | 0.2125135 |
| Cdkn1b | 1.7908606 |
| Cdkn1b | 0.1278562 |
| Cdkn1c | 0.7418579 |
| Cdkn2a | 1.0461117 |
| Cdkn2a | -0.4219191 |
| Cdkn2aipnl | 0.9181506 |
| Cdkn2b | -0.0931366 |
| Cdkn2c | 0.0805442 |
| Cdkn2c | 3.1145541 |
| Cdkn2d | -0.1685651 |
| Cdo1 | 0.5975246 |
| Cdr2 | -0.6909894 |
| Cdrt4 | -0.6551485 |
| Cds1 | 1.3141797 |
| Cds2 | -0.6894999 |
| Cdsn | 0.7064754 |
| Cdt1 | 1.8142802 |
| Cdv3 | 0.4676795 |
| Cdv3 | 2.0270766 |
| Cdyl | -0.660187 |
| Cdyl | 0.8779354 |
| Cdyl | 1.1965165 |
| Cdyl2 | 0.5187778 |
| Ceacam1 | -0.4822664 |
| Ceacam13 | -0.155289 |
| Ceacam2 | 0.0432826 |
| Cebpa | 0.241859 |
| Cebpb | -0.4870704 |
| Cecr5 | 0.946467 |
| Cecr6 | 0.6396895 |
| Celsr1 | -0.4469868 |
| Celsr2 | -0.0617776 |
| Celsr3 | 0.8819623 |
| Cend1 | -0.2001313 |
| Cenpa | 1.0776102 |
| Cenpb | -0.2790848 |
| Cenph | 0.7040578 |
| Cenpi | -0.448142 |
| Cenpj | -0.6123318 |
| Cenpk | -0.4864685 |
| Cenpl | -0.771023 |
| Cenpl | -0.3914827 |
| Cenpm | -0.2140505 |
| Cenpm | -0.6947235 |
| Cenpm | 1.6228554 |
| Cenpn | 0.1834313 |
| Cenpp | -0.6594656 |
| Cenpq | 1.4937645 |
| Cenpt | -0.6451506 |
| Centa1 | -0.1664112 |
| Centa1 | 1.5572409 |
| Centa2 | -0.7865484 |
| Centb5 | 1.5009211 |
| Centb5 | 0.5774024 |
| Centd2 | 2.1360974 |
| Centd2 | 0.1795457 |
| Centd3 | 0.0778828 |
| Centg1 | 1.8760734 |
| Centg1 | 0.2552325 |
| Centg2 | -0.0734169 |
| Centg2 | 0.4520488 |
| Centg3 | -0.5004074 |
| Centg3 | -0.4309587 |
| Cep135 | 0.4486658 |
| Cep164 | -0.4774864 |
| Cep170 | 1.7694616 |
| Cep55 | -0.7677864 |
| Cep57 | -0.6116456 |
| Cep57 | -0.409073 |
| Cep63 | 0.1999905 |
| Cep68 | -0.1400997 |
| Cep70 | 0.727457 |
| Cep72 | -0.19835 |
| Cep78 | 1.9119706 |
| Cep97 | 2.1863425 |
| Cept1 | -0.2313943 |
| Cercam | -0.0983311 |
| Ces3 | 0.341165 |
| Cetn2 | -0.2406507 |
| Cetn3 | 2.626004 |
| Cetn4 | -0.460357 |
| Cetn4 | 0.2446606 |
| Cfd | 1.8686985 |
| Cfdp1 | 1.8262586 |
| Cfh | 0.5811463 |
| Cfhr1 | -0.7718339 |
| Cfi | 1.4053305 |
| Cfl1 | 0.0318145 |
| Cfl2 | -0.7422812 |
| Cfl2 | 1.0559699 |
| Cfp | 1.7370223 |
| Cftr | 0.1284883 |
| Cga | 0.0934136 |
| Cggbp1 | 0.4211614 |
| Cgnl1 | 2.2494129 |
| Cgrrf1 | 2.5507186 |
| Ch25h | -0.0125525 |
| Chac1 | 1.865835 |
| Chad | 0.3624196 |
| Chaf1a | -0.4715449 |
| Chaf1b | 2.250372 |
| Chat | 2.1758145 |
| Chchd1 | 0.6790063 |
| Chchd2 | 0.0251486 |
| Chchd4 | -0.6850452 |
| Chchd5 | 0.2336875 |
| Chchd6 | 0.7849141 |
| Chchd7 | -0.5647998 |
| Chchd8 | 0.6419065 |
| Chd1 | -0.4906893 |
| Chd1 | -0.0114544 |
| Chd1l | -0.3181219 |
| Chd1l | 0.0718673 |
| Chd4 | -0.4349405 |
| Chd4 | 0.893233 |
| Chd4 | -0.7067715 |
| Chd5 | -0.6667044 |
| Chd8 | 1.0291648 |
| Chdh | 0.4068703 |
| Chdh | -0.6909894 |
| Chek1 | 1.3961617 |
| Chek1 | -0.7052572 |
| Chek2 | 0.1463295 |
| Chek2 | 0.5489469 |
| Cherp | -0.3860516 |
| Chfr | -0.5939763 |
| Chfr | 1.4344948 |
| Chfr | 0.3173547 |
| Chga | 2.7328043 |
| Chga | -0.5628426 |
| Chga | 0.2032097 |
| Chgb | -0.665978 |
| Chi3l1 | 4.1535392 |
| Chi3l3 | 1.3771287 |
| Chi3l3 | 2.0307031 |
| Chia | 1.8638869 |
| Chia | 0.8540023 |
| Chic1 | 4.2422822 |
| Chic2 | -0.1802805 |
| Chic2 | 0.187895 |
| Chit1 | 0.3258716 |
| Chka | 1.8532568 |
| Chka | -0.6522812 |
| Chka | -0.3504387 |
| Chkb | 0.4605657 |
| Chl1 | 4.1487791 |
| Chl1 | -0.2632393 |
| Chl1 | 1.1173441 |
| Chm | -0.7220485 |
| Chmp1b | 0.0034276 |
| Chmp2a | 2.1500787 |
| Chmp2a | 1.3382411 |
| Chmp2b | -0.5133143 |
| Chmp4b | 0.0405151 |
| Chmp5 | 0.7815721 |
| Chmp7 | 1.7087693 |
| Chmp7 | -0.7677864 |
| Chmp7 | -0.1952407 |
| Chn2 | 0.7174502 |
| Chodl | 2.6658394 |
| Chordc1 | 1.3372617 |
| Chpf | 1.145633 |
| Chpt1 | -0.753356 |
| Chpt1 | 0.6309563 |
| Chpt1 | -0.5865982 |
| Chrac1 | -0.5845958 |
| Chrd | 1.8284979 |
| Chrm1 | 0.8395592 |
| Chrm2 | -0.3651605 |
| Chrm3 | 1.402173 |
| Chrna1 | 0.2113555 |
| Chrna4 | -0.5766281 |
| Chrna4 | 1.1315933 |
| Chrna4 | -0.2637156 |
| Chrna5 | -0.4068564 |
| Chrna6 | -0.8065274 |
| Chrna6 | -0.2675348 |
| Chrna7 | -0.1384268 |
| Chrna9 | -0.460357 |
| Chrnb1 | 0.3299739 |
| Chrnb1 | 0.7866601 |
| Chrnb2 | 0.1332158 |
| Chrnb2 | -0.0745886 |
| Chrnb3 | 0.191451 |
| Chrnb3 | -0.4366522 |
| Chrnb3 | 0.9845955 |
| Chrne | 0.1846236 |
| Chst1 | 0.7976964 |
| Chst10 | 1.7013758 |
| Chst12 | 0.985626 |
| Chst14 | -0.6345534 |
| Chst2 | 0.5187778 |
| Chst3 | 0.526514 |
| Chst3 | -0.3593498 |
| Chst7 | 1.8324306 |
| Chst8 | 1.8061377 |
| Chtf18 | 1.0303941 |
| Chuk | -0.2902863 |
| Churc1 | 3.2248236 |
| Ciao1 | 2.6279885 |
| Ciapin1 | 2.2712642 |
| Cib1 | 0.5680776 |
| Cib2 | 2.2898715 |
| Cib2 | -0.4191118 |
| Cic | 0.932686 |
| Cidea | -0.1763593 |
| Cideb | 0.1515867 |
| Cidec | 1.6774115 |
| Ciita | -0.203704 |
| Cirbp | 1.6056599 |
| Cirh1a | -0.3374834 |
| Cisd1 | -0.6909894 |
| Cish | -0.2485898 |
| Cit | 2.1521679 |
| Cited1 | 3.3505603 |
| Cited2 | 0.8347461 |
| Cited4 | -0.5383924 |
| Ciz1 | 1.3067586 |
| Ciz1 | -0.6551485 |
| Ckap2l | -0.2637156 |
| Ckap5 | 0.2830439 |
| Ckb | 1.4866301 |
| Cklf | 1.5226615 |
| Cklf | -0.6423131 |
| Ckmt1 | 0.9683472 |
| Ckmt2 | -0.2761842 |
| Ckmt2 | -0.2415813 |
| Ckt2 | -0.0239741 |
| Ckt2 | 1.3393973 |
| Clca5 | 1.7779217 |
| Clcc1 | -0.6317473 |
| Clcf1 | 0.9680849 |
| Clcf1 | -0.3080872 |
| Clcf1 | 0.8825361 |
| Clcn1 | 0.2665091 |
| Clcn2 | -0.6494226 |
| Clcn3 | -0.6688868 |
| Clcn3 | 0.2703294 |
| Clcn3 | 3.467848 |
| Clcn4-2 | -0.5511829 |
| Clcn7 | -0.4269933 |
| Clcnka | 0.1668833 |
| Cldn1 | -0.5034647 |
| Cldn10 | 1.1036757 |
| Cldn10 | 1.460292 |
| Cldn10 | 0.7286373 |
| Cldn11 | -0.5799399 |
| Cldn12 | -0.0923399 |
| Cldn15 | -0.0963298 |
| Cldn15 | -0.5213927 |
| Cldn17 | -0.7422812 |
| Cldn2 | -0.5307996 |
| Cldn23 | 0.5654823 |
| Cldn3 | -0.7266797 |
| Cldn4 | 1.5355589 |
| Cldn5 | 0.5722553 |
| Cldn6 | 0.0752139 |
| Cldn9 | 0.3006173 |
| Cldnd1 | -0.5071464 |
| Cldnd2 | -0.001266 |
| Clec11a | 0.8835396 |
| Clec16a | -0.813281 |
| Clec1a | -0.3431621 |
| Clec2d | 3.3235106 |
| Clec2e | 0.419998 |
| Clec3b | 0.4551954 |
| Clec4a1 | 1.7443786 |
| Clec4a3 | -0.1582707 |
| Clec4b1 | 0.7299845 |
| Clec4b1 | 1.1271632 |
| Clec4d | -0.525771 |
| Clec4g | -0.5621911 |
| Clec4n | 0.3756808 |
| Clec7a | 0.9112167 |
| Clecsf12 | 1.459195 |
| Clgn | -0.5720108 |
| Clic1 | 1.7020447 |
| Clic3 | -0.0210135 |
| Clic4 | 0.5821291 |
| Clic6 | 2.1354412 |
| Clip1 | 0.2507954 |
| Clip1 | -0.1485083 |
| Clip4 | -0.822646 |
| Clk1 | 0.8221479 |
| Clk2 | 2.4003278 |
| Clk2 | -0.6226838 |
| Clk2 | -0.6054923 |
| Clk3 | 1.3154565 |
| Clk4 | 1.1288269 |
| Clk4 | -0.751766 |
| Clk4 | -0.8090544 |
| Clm3 | 0.5014059 |
| Clm3 | 1.1760656 |
| Clmn | -0.5602392 |
| Clmn | -0.6408976 |
| Cln3 | -0.3292833 |
| Cln5 | 0.2156909 |
| Cln6 | 2.0684689 |
| Cln6 | 1.5721933 |
| Cln8 | -0.753356 |
| Clns1a | 1.1053808 |
| Clns1a | -0.3893065 |
| Clns1a | 1.5107024 |
| Clock | -0.5946499 |
| Clock | -0.0362845 |
| Clp1 | -0.5852628 |
| Clpb | 1.5678651 |
| Clpp | 0.8364033 |
| Clptm1 | 2.0801601 |
| Clptm1l | 0.5022607 |
| Clpx | 1.3165496 |
| Clpx | 0.0498235 |
| Clrn1 | 1.7450181 |
| Clspn | -0.6924813 |
| Clspn | -0.4168718 |
| Clspn | 1.4340925 |
| Clstn1 | -0.6894999 |
| Clstn3 | 1.5823088 |
| Clstn3 | 0.3371106 |
| Clta | 1.074448 |
| Cltb | -0.4757001 |
| Cluap1 | 1.5961977 |
| Cma1 | 2.5690623 |
| Cma1 | 0.7383614 |
| Cmas | 0.7834832 |
| Cmbl | -0.5608894 |
| Cml1 | -0.4858671 |
| Cml3 | -0.8090544 |
| Cml4 | 1.6359754 |
| Cmpk | -0.7849022 |
| Cmtm2a | 2.6474742 |
| Cmtm2b | -0.6206044 |
| Cmtm4 | -0.6444404 |
| Cmtm4 | -0.9198274 |
| Cmtm5 | -0.4338012 |
| Cmtm6 | -0.4586024 |
| Cmtm7 | 4.5343255 |
| Cmtm7 | 0.9033905 |
| Cmtm7 | 2.9913956 |
| Cmtm8 | -0.8286537 |
| Cmtm8 | -0.6718044 |
| Cmya1 | -0.3779623 |
| CN716893 | 3.0992268 |
| Cnbp2 | 1.7372567 |
| Cnbp2 | -0.2956903 |
| Cndp1 | 1.1583955 |
| Cnga4 | -0.5505393 |
| Cnih | -0.2145029 |
| Cnih3 | 0.7313297 |
| Cnih4 | 1.3308261 |
| Cnksr2 | 1.3884251 |
| Cnksr2 | -0.2829661 |
| Cnksr3 | 0.32895 |
| Cnn1 | 1.4203099 |
| Cnn2 | -0.4810691 |
| Cnn2 | -0.1956842 |
| Cnnm1 | 1.4256844 |
| Cnnm1 | -0.0548616 |
| Cnnm2 | -0.5959985 |
| Cnnm3 | -0.448142 |
| Cno | -0.3313267 |
| Cnot10 | 1.1925856 |
| Cnot2 | -0.6487092 |
| Cnot2 | 1.0504522 |
| Cnot2 | 0.701634 |
| Cnot2 | 1.1926893 |
| Cnot3 | 1.3636195 |
| Cnot3 | -0.5919585 |
| Cnot4 | -0.675464 |
| Cnot4 | 1.5546195 |
| Cnot4 | -0.4118509 |
| Cnot6l | -0.0362845 |
| Cnot6l | -0.5232667 |
| Cnot7 | 0.6330074 |
| Cnp | 0.4945399 |
| Cnp | 0.8020755 |
| Cnpy2 | 0.9695263 |
| Cnpy3 | -0.7159083 |
| Cnr1 | -0.3110865 |
| Cnr1 | -0.5004074 |
| Cntd1 | -0.6247678 |
| Cntd1 | 3.2668323 |
| Cntfr | 0.085845 |
| Cntln | -0.5077614 |
| Cntn1 | 0.5604713 |
| Cntn4 | 1.2286927 |
| Cntn6 | 0.2870664 |
| Cntnap1 | -0.5537617 |
| Cntnap2 | 1.6864147 |
| Cntnap4 | 2.2471829 |
| Cntnap4 | 0.8717181 |
| Coasy | -0.3582971 |
| Cobl | -0.5654531 |
| Cobll1 | -0.9905852 |
| Cobll1 | 2.8392354 |
| Coch | -0.0005424 |
| Cog1 | 2.1740733 |
| Cog2 | -0.711329 |
| Cog3 | -0.5409368 |
| Cog4 | -0.0903512 |
| Cog6 | 0.8871148 |
| Cog6 | -0.7501787 |
| Cog8 | -0.5524714 |
| Coil | 3.6226325 |
| Coil | 0.0106039 |
| Col11a1 | 2.7130198 |
| Col12a1 | 1.0005719 |
| Col14a1 | 0.4856701 |
| Col15a1 | -0.0943328 |
| Col16a1 | 0.9236617 |
| Col17a1 | 0.2692394 |
| Col17a1 | -0.6020908 |
| Col17a1 | 2.4419492 |
| Col18a1 | 1.6321237 |
| Col18a1 | -0.2383282 |
| Col1a1 | -0.664527 |
| Col1a2 | 0.3671066 |
| Col24a1 | 0.0412077 |
| Col24a1 | -0.8637281 |
| Col25a1 | 1.3777266 |
| Col25a1 | 1.5112965 |
| Col25a1 | 2.0243911 |
| Col27a1 | -0.7573426 |
| Col28a1 | -0.1055705 |
| Col2a1 | 0.4237159 |
| Col2a1 | 0.2665091 |
| Col3a1 | 0.8458397 |
| Col3a1 | -0.2728112 |
| Col4a1 | 0.1604886 |
| Col4a2 | 0.7068203 |
| Col4a3 | -0.7143795 |
| Col4a3bp | 1.4239776 |
| Col4a3bp | -0.6338511 |
| Col4a3bp | -0.0610067 |
| Col4a4 | 2.3087351 |
| Col4a4 | 0.6331936 |
| Col4a5 | -0.5687263 |
| Col4a6 | 1.353689 |
| Col4a6 | 1.6249008 |
| Col5a1 | -0.6638023 |
| Col5a3 | 1.1728963 |
| Col6a1 | -0.3267351 |
| Col6a1 | 0.873602 |
| Col6a1 | -0.2603863 |
| Col6a2 | -0.6387782 |
| Col7a1 | -0.1472423 |
| Col8a2 | -0.556995 |
| Col9a2 | 0.1491164 |
| Colec12 | 0.6827266 |
| Commd1 | 0.5208749 |
| Commd1 | -0.3860516 |
| Commd10 | -0.3400604 |
| Commd2 | -0.5557003 |
| Commd2 | -0.4757001 |
| Commd4 | 0.2336875 |
| Commd5 | -0.6674313 |
| Commd6 | -0.5926306 |
| Commd8 | -0.5753066 |
| Commd9 | -0.3577712 |
| Comp | -0.4556852 |
| Comtd1 | -0.4207952 |
| Copa | -0.5133143 |
| Copb2 | -0.3614587 |
| Cope | -0.0449594 |
| Cope | -0.7235897 |
| Cope | -0.6791377 |
| Cope | 1.3558756 |
| Copg | 0.7885611 |
| Copg | -0.5120776 |
| Copg | -0.3206471 |
| Copg | -0.631047 |
| Cops2 | -0.7151436 |
| Cops3 | -0.749386 |
| Cops4 | -0.4389391 |
| Cops4 | 0.4917365 |
| Cops5 | -0.7391405 |
| Cops5 | 0.3253576 |
| Cops6 | -0.2805384 |
| Cops7a | -0.5654531 |
| Cops7a | -0.5766281 |
| Cops7a | -0.4510362 |
| Cops7b | -0.7029901 |
| Cops7b | 4.1420946 |
| Cops8 | 2.2232535 |
| Copz1 | -0.3520052 |
| Copz2 | -0.7022357 |
| Copz2 | -0.5195224 |
| Copz2 | 2.3299458 |
| Copz2 | 0.9116342 |
| Coq10b | -0.7383569 |
| Coq10b | 1.6323199 |
| Coq2 | 0.020207 |
| Coq3 | -0.7136159 |
| Coq3 | -0.7037452 |
| Coq4 | 0.3240714 |
| Coq4 | -0.5059176 |
| Coq5 | 1.5499256 |
| Coq5 | -0.6872699 |
| Coq5 | 2.6537999 |
| Coq6 | 0.8795194 |
| Coq6 | 0.9940858 |
| Coq7 | -0.6387782 |
| Coq9 | -0.1728874 |
| Corin | 0.1568151 |
| Coro1a | 1.5893811 |
| Coro1a | -0.1140864 |
| Coro1a | 0.0134593 |
| Coro1a | 2.7655325 |
| Coro1b | -0.5065318 |
| Coro1b | -0.6587447 |
| Coro1c | 1.706291 |
| Coro2a | 1.5714966 |
| Coro2a | -0.6850452 |
| Coro2b | 0.695024 |
| Coro2b | 2.7258805 |
| Coro6 | -0.7832589 |
| Coro7 | 0.4941092 |
| Cort | -0.1375915 |
| Cort | -0.7143795 |
| Cotl1 | 0.2947954 |
| Cova1 | -0.7367916 |
| Cox10 | -0.2072901 |
| Cox11 | 2.2561401 |
| Cox15 | -0.5933032 |
| Cox17 | -0.5939763 |
| Cox18 | -0.1112393 |
| Cox18 | -0.2167681 |
| Cox19 | 1.3552639 |
| Cox4i1 | 1.4122733 |
| Cox4nb | -0.851305 |
| Cox4nb | -0.1088057 |
| Cox5a | 0.5969445 |
| Cox5b | 2.2355366 |
| Cox6a1 | -0.5195224 |
| Cox6a1 | 0.12754 |
| Cox6a2 | -0.4446805 |
| Cox6b1 | -0.2332384 |
| Cox6b1 | 1.4737449 |
| Cox6b2 | -0.3426444 |
| Cox6b2 | -0.6969709 |
| Cox6c | 1.0861076 |
| Cox7a1 | 0.8090715 |
| Cox7a2 | -0.2907763 |
| Cox7a2l | 0.4813133 |
| Cox7b | -0.4040929 |
| Cox7b2 | -0.5396638 |
| Cox8b | -0.3252095 |
| Cox8c | 1.1003705 |
| Cp | -0.4627015 |
| Cpa1 | -0.2580153 |
| Cpa2 | -0.4281246 |
| Cpa2 | -0.8006569 |
| Cpa3 | 0.1747417 |
| Cpa3 | -0.7998212 |
| Cpa3 | -0.6089059 |
| Cpa4 | 0.9872983 |
| Cpa5 | -0.3619866 |
| Cpa6 | -0.5333238 |
| Cpb2 | -0.4355107 |
| Cpeb1 | 1.5080244 |
| Cpeb2 | -0.1334257 |
| Cpeb3 | -0.6394841 |
| Cpeb4 | 0.4160319 |
| Cphx | -0.6041302 |
| Cplx1 | 0.0145279 |
| Cplx2 | 0.2387677 |
| Cpn1 | -0.771023 |
| Cpne2 | -0.4721374 |
| Cpne4 | 2.4091812 |
| Cpne5 | -0.5232667 |
| Cpne6 | 0.7147183 |
| Cpne7 | 2.5851815 |
| Cpne8 | 3.5008073 |
| Cpne8 | -0.3493958 |
| Cpne9 | -0.5819325 |
| Cpsf1 | 0.9544816 |
| Cpsf1 | -0.6143932 |
| Cpsf2 | 0.2351014 |
| Cpsf3l | 0.5304625 |
| Cpsf4 | 2.1441664 |
| Cpsf4l | 1.4315133 |
| Cpsf4l | -0.4816676 |
| Cpsf6 | -0.0445806 |
| Cpsf6 | -0.5812679 |
| Cpt1b | 2.4652201 |
| Cpt1b | 0.1565083 |
| Cpt1c | -0.129692 |
| Cpt2 | -0.4151952 |
| Cpxcr1 | -0.2966762 |
| Cpxm1 | 0.7602927 |
| Cpxm2 | 0.1801445 |
| Cpz | -0.6522812 |
| Crabp2 | -0.6465724 |
| Crat | 0.9645377 |
| Crat | -0.2476524 |
| Crat | 0.222873 |
| Crb1 | -0.7259063 |
| Crbn | -0.4372234 |
| Crbn | 1.0585975 |
| Crbn | 3.5747918 |
| Crcp | 0.2165556 |
| Creb1 | -0.6710741 |
| Creb1 | -0.6551485 |
| Creb3 | 1.7353212 |
| Creb3l1 | 0.4687862 |
| Creb3l2 | -0.2542339 |
| Creb3l2 | 3.129462 |
| Creb3l3 | -0.6000557 |
| Creb3l4 | 1.3741336 |
| Creb5 | 1.126608 |
| Crebl1 | -0.2771501 |
| Crebl2 | 1.2003285 |
| Creg2 | 2.4042404 |
| Creld1 | -0.5524714 |
| Creld2 | 0.3668605 |
| Creld2 | 1.3079535 |
| Crem | -0.7906764 |
| Crem | -0.674731 |
| Crem | 0.9370309 |
| Crem | 0.96651 |
| Crhbp | -0.3021166 |
| Crhr1 | 0.1884886 |
| Crim1 | 0.281161 |
| Crim2 | -0.5053038 |
| Crip2 | -0.5492533 |
| Crip3 | 1.0231182 |
| Crip3 | 0.7493075 |
| Cript | -0.621297 |
| Cript | -0.813281 |
| Crisp2 | -0.4151952 |
| Crispld2 | -0.0828315 |
| Crk | 0.1911552 |
| Crk | 2.6604404 |
| Crk | -0.6880127 |
| Crkl | 1.242565 |
| Crkrs | 1.9848487 |
| Crkrs | -0.4556852 |
| Crlf3 | 1.4716578 |
| Crlf3 | -0.5460461 |
| Crls1 | 0.0109613 |
| Crls1 | 0.4488917 |
| Crls1 | -0.7865484 |
| Crls1 | -0.7898494 |
| Crmp1 | 1.3752472 |
| Crmp1 | 0.7517779 |
| Crnkl1 | -0.2981569 |
| Crocc | 0.7410265 |
| Crry | 2.2913156 |
| Crry | 0.8167852 |
| Crtac1 | 1.4703413 |
| Crtam | 1.6814579 |
| Crtap | 0.0566605 |
| Crtap | 0.5588622 |
| Crtc1 | -0.3252095 |
| Crtc3 | 0.2214409 |
| Crxos1 | -0.448142 |
| Cry2 | -0.0088971 |
| Cryab | 0.9457961 |
| Cryba2 | -0.4644636 |
| Cryba4 | 0.1199194 |
| Crybb1 | -0.8338334 |
| Crybb2 | 0.730321 |
| Crybb3 | 0.3304854 |
| Crygn | -0.6638023 |
| Crygn | 0.3227834 |
| Cryl1 | 0.0722025 |
| Crym | 1.5019712 |
| Cryz | 0.6960708 |
| Cryz | 2.700817 |
| Cryz | 0.1354139 |
| Cryzl1 | -0.7022357 |
| Cryzl1 | -0.1243245 |
| Cs | -0.7189735 |
| Csad | -0.6380728 |
| Csda | -0.632448 |
| Csda | 1.3219057 |
| Csda | -0.289307 |
| Csdc2 | -0.6587447 |
| Csde1 | 2.0703723 |
| Csde1 | 0.0614175 |
| Csde1 | 0.5279706 |
| Cse1l | -0.7549487 |
| Csf1 | 2.7348469 |
| Csf1r | 0.709231 |
| Csf1r | -0.4858671 |
| Csf2ra | -0.0563942 |
| Csgalnact1 | -0.7151436 |
| Csgalnact2 | -0.421357 |
| Csk | 3.3849945 |
| Csl | 0.239893 |
| Csl | -0.2429789 |
| Csmd1 | 0.402612 |
| Csnk1d | -0.1198062 |
| Csnk1d | 1.2201709 |
| Csnk1d | 0.2265865 |
| Csnk1d | 1.2839545 |
| Csnk1d | 0.1054647 |
| Csnk1e | 1.398671 |
| Csnk1e | 0.4092278 |
| Csnk1g1 | 3.9798721 |
| Csnk1g1 | -0.664527 |
| Csnk1g1 | -0.4013373 |
| Csnk1g2 | -0.8338334 |
| Csnk1g2 | 0.5473182 |
| Csnk1g2 | 2.6112454 |
| Csnk1g3 | -0.5326921 |
| Csnk2a1 | 0.199404 |
| Csnk2a2 | 2.2065905 |
| Csnk2a2 | 0.9164911 |
| Csnrp2 | -0.3115874 |
| Csnrp3 | 1.8741462 |
| Cspg4 | -0.6835649 |
| Cspg5 | 1.5432001 |
| Cspp1 | 0.7325053 |
| Csprs | 1.7340286 |
| Csrp1 | 1.4510851 |
| Csrp2 | -0.1363399 |
| Csrp2bp | 0.8398592 |
| Cst11 | 0.6067591 |
| Cst3 | -0.02806 |
| Cst8 | 3.6205236 |
| Cst9 | 0.2384862 |
| Cstad | 2.485291 |
| Cstb | -0.5114598 |
| Cstf1 | 4.1986567 |
| Cstf2 | 0.4087568 |
| Cstf3 | -0.5053038 |
| Cstf3 | -0.5661069 |
| Cstl1 | 0.1015729 |
| Ctage5 | 0.5981044 |
| Ctcf | -0.762952 |
| Ctdp1 | 1.8726728 |
| Ctdp1 | 0.6088628 |
| Ctdp1 | 0.2379229 |
| Ctdsp1 | 1.3445387 |
| Ctdsp1 | 1.9808165 |
| Ctdsp1 | -0.7783457 |
| Ctdsp2 | -0.5959985 |
| Ctdspl | -0.5661069 |
| Ctdspl2 | -0.5845958 |
| Ctdspl2 | 0.4771559 |
| Ctf1 | -0.5004074 |
| Ctgf | -0.3156033 |
| Ctla4 | -0.5753066 |
| Ctnna1 | -0.4487201 |
| Ctnna3 | 0.51119 |
| Ctnnal1 | -0.6515658 |
| Ctnnb1 | -0.7589419 |
| Ctnnb1 | -0.4580182 |
| Ctnnb1 | 0.7994192 |
| Ctnnbip1 | 0.5572504 |
| Ctnnbl1 | 1.1983729 |
| Ctnnbl1 | -0.2956903 |
| Ctnnd2 | -0.4632885 |
| Ctnnd2 | -0.4292572 |
| Ctnnd2 | -0.4458329 |
| Ctns | 1.260882 |
| Ctns | -0.0269439 |
| Ctps | -0.61096 |
| Ctps | 0.0408614 |
| Ctps | 1.3550016 |
| Ctps2 | -0.5339559 |
| Ctps2 | 1.6770995 |
| Ctps2 | 1.1099129 |
| Ctr9 | -0.5959985 |
| Ctrb1 | -0.4834651 |
| Ctrl | -0.1363399 |
| Ctsa | -0.0753706 |
| Ctsa | -0.3504387 |
| Ctsa | -0.3720735 |
| Ctsa | -0.7075296 |
| Ctsb | 1.0279339 |
| Ctsc | 1.4819716 |
| Ctsc | 2.4718126 |
| Ctsc | -0.4991872 |
| Ctse | -0.5779515 |
| Ctsf | -0.5071464 |
| Ctsh | 0.6695519 |
| Ctsk | -0.7873726 |
| Ctsl | 0.5760194 |
| Ctso | -0.5358548 |
| Ctsr | -0.258489 |
| Ctss | -0.7422812 |
| Ctsw | 0.7002463 |
| Ctsz | 0.596364 |
| Ctsz | -0.5946499 |
| Cttn | -0.660187 |
| Cttn | 1.9117266 |
| Cttnbp2nl | -0.2561228 |
| Ctxn1 | 0.9757907 |
| Cuedc1 | 0.676518 |
| Cuedc2 | 0.6514545 |
| Cugbp1 | 2.3884928 |
| Cugbp1 | -0.5621911 |
| Cugbp1 | 1.7552503 |
| Cugbp1 | -0.6164591 |
| Cugbp2 | 1.7006454 |
| Cugbp2 | 0.2401741 |
| Cul1 | 2.8992765 |
| Cul2 | -0.5615401 |
| Cul2 | -0.4400846 |
| Cul3 | -0.0695213 |
| Cul4a | 0.9170446 |
| Cul4a | 1.0752688 |
| Cul4a | -0.4264282 |
| Cul4b | 0.1366678 |
| Cul5 | 0.9214611 |
| Cul7 | 0.1875981 |
| Cuta | 0.9490123 |
| Cuta | -0.4556852 |
| Cuta | -0.6373678 |
| Cutc | -0.2364742 |
| Cutc | 0.2008695 |
| Cux1 | -0.7243613 |
| Cux1 | -0.5276536 |
| Cux1 | 0.0924298 |
| Cux1 | 2.239873 |
| Cux1 | 1.9386755 |
| Cux2 | 3.6466948 |
| Cux2 | 0.4815317 |
| Cuzd1 | -0.0449594 |
| Cwf19l2 | 0.4585554 |
| Cx3cl1 | -0.6352563 |
| Cx3cr1 | 2.6022417 |
| Cxadr | -0.3287731 |
| Cxadr | 0.390923 |
| Cxadr | 1.0685644 |
| Cxcl1 | 0.6837869 |
| Cxcl12 | -0.750972 |
| Cxcl12 | -0.7082882 |
| Cxcl12 | 2.3860903 |
| Cxcl13 | -0.4533579 |
| Cxcl14 | -0.5654531 |
| Cxcl14 | -0.6082222 |
| Cxcl15 | 1.0286726 |
| Cxcl16 | -0.3520052 |
| Cxcl17 | -0.2947055 |
| Cxcl4 | -0.2907763 |
| Cxcl9 | -0.5906156 |
| Cxcr3 | -0.7881975 |
| Cxcr4 | 0.1741395 |
| Cxcr5 | -0.3016207 |
| Cxcr7 | -0.2126946 |
| Cxx1a | -0.0434451 |
| Cxx1b | -0.8200828 |
| Cxx1c | -0.0389164 |
| Cxxc1 | -0.6451506 |
| Cxxc1 | -0.0521855 |
| Cxxc4 | 0.0198531 |
| Cxxc4 | 3.2055065 |
| Cyb5 | 1.3328866 |
| Cyb561 | -0.1019439 |
| Cyb561d1 | 1.6651684 |
| Cyb561d1 | 2.1696875 |
| Cyb561d2 | 1.048646 |
| Cyb5b | 1.2967733 |
| Cyb5d1 | 2.393604 |
| Cyb5d2 | 1.5164054 |
| Cyb5d2 | -0.7758985 |
| Cyb5r1 | -0.5766281 |
| Cyb5r2 | 0.7133494 |
| Cyb5r3 | -0.5753066 |
| Cyb5r3 | 2.1217908 |
| Cyb5r3 | -0.3688766 |
| Cyb5r4 | 0.0415538 |
| Cyba | 1.0259612 |
| Cybasc3 | -0.4562679 |
| Cyc1 | -0.675464 |
| Cycs | -0.6247678 |
| Cycs | -0.7726454 |
| Cyfip1 | 0.8905345 |
| Cyfip2 | 2.0130023 |
| Cyfip2 | 0.0871656 |
| Cygb | -0.5040773 |
| Cyhr1 | 1.1064024 |
| Cyhr1 | 3.190227 |
| Cyhr1 | -0.3936639 |
| Cyhr1 | -0.545406 |
| Cyld | -0.6573046 |
| Cyln2 | 4.2307023 |
| Cyp11a1 | -0.2346238 |
| Cyp11b2 | -0.0067106 |
| Cyp17a1 | -0.7313334 |
| Cyp19a1 | -0.0487558 |
| Cyp1b1 | -0.4264282 |
| Cyp20a1 | -0.5511829 |
| Cyp24a1 | -0.6969709 |
| Cyp26a1 | 1.2821631 |
| Cyp26b1 | -0.8909062 |
| Cyp27a1 | 0.2499612 |
| Cyp27a1 | -0.3090859 |
| Cyp27a1 | -0.3822681 |
| Cyp2a12 | 0.7021539 |
| Cyp2a5 | 1.6859201 |
| Cyp2a5 | -0.4615285 |
| Cyp2b10 | 0.1525115 |
| Cyp2c55 | 3.9142687 |
| Cyp2d10 | 0.3452022 |
| Cyp2d22 | -0.2323159 |
| Cyp2d26 | 0.0470749 |
| Cyp2d26 | -0.0560108 |
| Cyp2d26 | 1.3503558 |
| Cyp2e1 | 2.4721383 |
| Cyp2f2 | -0.5621911 |
| Cyp2j11 | 2.2535504 |
| Cyp2j9 | -0.3572456 |
| Cyp2s1 | -0.7589419 |
| Cyp2u1 | -0.7159083 |
| Cyp39a1 | 0.3824852 |
| Cyp3a13 | 0.3396466 |
| Cyp3a13 | -0.6068563 |
| Cyp46a1 | -0.7873726 |
| Cyp4b1 | 0.8763488 |
| Cyp4f13 | 0.8407587 |
| Cyp4f14 | 1.6200771 |
| Cyp4f15 | 1.2481558 |
| Cyp4f16 | -0.1185777 |
| Cyp4f16 | -0.6902444 |
| Cyp4f16 | 2.1958136 |
| Cyp4f18 | 2.6657951 |
| Cyp4f39 | 0.3309967 |
| Cyp4v3 | 2.7336507 |
| Cyp51 | -0.0434451 |
| Cyp7b1 | -0.8434032 |
| Cypt10 | 0.241859 |
| Cypt2 | -0.6835649 |
| Cypt3 | 1.14987 |
| Cypt6 | -0.7849022 |
| Cypt8 | -0.1992403 |
| Cypt9 | 1.0217556 |
| Cyr61 | -0.2135983 |
| Cyr61 | -0.4650517 |
| Cysltr1 | -0.3520052 |
| Cytl1 | 0.9379787 |
| D030011O10Rik | -0.1561399 |
| D030013I16Rik | 2.0095722 |
| D030018L15Rik | 2.2453262 |
| D030028O16Rik | -0.4539392 |
| D030051N19Rik | -0.5434881 |
| D030056L22Rik | -0.5628426 |
| D030070L09Rik | -0.6969709 |
| D030070L09Rik | -0.169428 |
| D030070L09Rik | -0.7462218 |
| D030070L09Rik | 0.2963867 |
| D030074E01Rik | 2.3770491 |
| D0H4S114 | -0.0983311 |
| D0H8S2298E | 0.0251486 |
| D0HXS9928E | 0.8204657 |
| D0HXS9928E | -0.5428496 |
| D10627 | -0.7478026 |
| D10Bwg1364e | -0.6798741 |
| D10Bwg1379e | -0.6947235 |
| D10Ertd322e | 1.6074068 |
| D10Ertd610e | 0.3335489 |
| D10Ertd610e | -0.6373678 |
| D10Ertd610e | 0.7040578 |
| D10Ertd641e | -0.3942099 |
| D10Jhu81e | 3.3812944 |
| D10Wsu102e | 0.7261063 |
| D10Wsu52e | -0.5980249 |
| D11Bwg0517e | 2.2122366 |
| D11Wsu47e | 0.2348188 |
| D11Wsu47e | 1.0223752 |
| D12Ertd551e | -0.6027701 |
| D12Ertd551e | -0.5912868 |
| D12Ertd553e | -0.276667 |
| D12Ertd647e | 0.358703 |
| D12Ertd647e | -0.2888178 |
| D130040H23Rik | 1.04587 |
| D130043K22Rik | -0.7022357 |
| D130059P03Rik | 1.10504 |
| D13Ertd608e | -0.6219902 |
| D14Ertd171e | 1.1398447 |
| D14Ertd231e | -0.6007336 |
| D14Ertd449e | -0.7906764 |
| D14Ertd500e | 0.8286954 |
| D14Ertd668e | -0.7282284 |
| D14Ertd668e | -0.7422812 |
| D15Ertd621e | -0.6761976 |
| D15Ertd682e | -0.5674157 |
| D15Mgi27 | -0.0540962 |
| D15Wsu169e | 1.1299344 |
| D15Wsu75e | 0.6320756 |
| D16Ertd472e | -0.5226416 |
| D16H22S680E | 1.841877 |
| D17H6S56E-5 | 1.3007614 |
| D17Wsu104e | 1.5073537 |
| D17Wsu92e | -0.1301061 |
| D19Bwg1357e | -0.1899338 |
| D19Ertd721e | -0.621297 |
| D19Ertd737e | -0.3206471 |
| D19Wsu162e | 0.067499 |
| D1Bwg0212e | 1.1533326 |
| D1Bwg0212e | 1.8770862 |
| D1Pas1 | -0.4051973 |
| D230007K08Rik | 0.5602703 |
| D230014K01Rik | -0.664527 |
| D2Ertd750e | 1.9867005 |
| D2Ertd750e | 0.8311203 |
| D330014H01Rik | -0.2045993 |
| D330017J20Rik | -0.6501364 |
| D330028D13Rik | -0.4452565 |
| D330045A20Rik | -0.0202748 |
| D330045A20Rik | 1.5976908 |
| D330045A20Rik | 1.5306245 |
| D3Bwg0562e | -0.4498774 |
| D3Ertd300e | -0.6494226 |
| D3Ertd751e | 0.2087448 |
| D3Ucla1 | 0.4880579 |
| D430015B01Rik | -0.5792767 |
| D430028G21Rik | -0.6551485 |
| D430039N05Rik | -0.5126957 |
| D430041B17Rik | -0.5779515 |
| D430042O09Rik | -0.6095901 |
| D4Bwg0951e | -0.7313334 |
| D4Bwg1540e | 0.8479241 |
| D4Ertd22e | -0.525771 |
| D4Ertd22e | -0.4586024 |
| D4Wsu114e | -0.3390287 |
| D4Wsu132e | 3.2462701 |
| D4Wsu132e | 0.1935193 |
| D530004J12Rik | -0.7266797 |
| D530033C11Rik | 1.6616866 |
| D5Ertd135e | 0.7839604 |
| D5Ertd579e | -0.3262263 |
| D5Wsu178e | -0.5647998 |
| D630003M21Rik | -0.3752811 |
| D630003M21Rik | 1.2718204 |
| D630014A15Rik | -0.5434881 |
| D630023F18Rik | 0.1488071 |
| D630042P16Rik | -0.5108424 |
| D630048P19Rik | -0.4498774 |
| D6Ertd474e | -0.4858671 |
| D6Mm5e | 1.7497721 |
| D6Wsu116e | 0.1438455 |
| D6Wsu163e | -0.5661069 |
| D6Wsu163e | -0.595324 |
| D6Wsu163e | 0.0725375 |
| D6Wsu176e | 1.8375612 |
| D6Wsu176e | 0.2662357 |
| D730039F16Rik | 0.0247965 |
| D730039F16Rik | -0.0445806 |
| D7Bwg0611e | -0.6331493 |
| D830014E11Rik | -0.5170343 |
| D830030K20Rik | 1.3377961 |
| D830030K20Rik | 0.0566605 |
| D830050J10Rik | 0.7341821 |
| D8Ertd738e | 2.5534641 |
| D8Ertd82e | 1.9514487 |
| D930001I22Rik | -0.4219191 |
| D930005D10Rik | 0.3604392 |
| D930014E17Rik | 1.2679008 |
| D930015E06Rik | -0.3817288 |
| D930020B18Rik | 2.5669759 |
| D930028F11Rik | -0.7205098 |
| D930049A15Rik | 1.3406409 |
| D9Ertd280e | 0.7553896 |
| D9Ertd392e | 1.1548435 |
| D9Ertd402e | -0.3328621 |
| D9Wsu20e | 0.8708473 |
| Daam1 | -0.1132721 |
| Daam2 | 1.0451444 |
| Dab1 | -0.6199123 |
| Dab1 | 0.2008695 |
| Dab1 | 0.7200043 |
| Dab2 | -0.5792767 |
| Dab2 | -0.0491363 |
| Dab2ip | 1.1936216 |
| Dact1 | 1.6996708 |
| Dact2 | -0.2185842 |
| Dad1 | 1.6502022 |
| Daf2 | 1.6695814 |
| Dag1 | 0.5009782 |
| Dag1 | -0.706014 |
| Dagla | 0.1237373 |
| Daglb | 0.7468308 |
| Dak | -0.5409368 |
| Dalrd3 | -0.7090475 |
| Dap | -0.6820869 |
| Dap3 | -0.5333238 |
| Dap3 | 0.1768465 |
| Dap3 | -0.103554 |
| Dap3 | -0.5865982 |
| Dapk1 | -0.6082222 |
| Dapk1 | 2.2406342 |
| Dapk2 | 0.2819684 |
| Dapk2 | -0.5779515 |
| Dapl1 | 2.0893219 |
| Dapp1 | -0.3931181 |
| Dapp1 | -0.5966735 |
| Darc | -0.6835649 |
| Dars | 0.815709 |
| Dars | -0.5371227 |
| Daxx | -0.5040773 |
| Dazap1 | 2.1228869 |
| Dazap1 | -0.433232 |
| Dazap2 | 0.2727772 |
| Dazl | -0.6828256 |
| Dbc1 | 1.4004237 |
| Dbf4 | -0.212243 |
| Dbi | -0.3452354 |
| Dbi | -0.6761976 |
| Dbil5 | 0.8911032 |
| Dbn1 | 0.8323304 |
| Dbndd1 | -0.4475642 |
| Dbndd2 | 0.2632224 |
| Dbndd2 | 0.222873 |
| Dbndd2 | -0.524518 |
| Dbnl | -0.1288643 |
| Dbp | -0.6501364 |
| Dbpht2 | -0.0785049 |
| Dbr1 | 0.8427046 |
| Dbt | 0.2454995 |
| Dcakd | -0.6902444 |
| Dcbld1 | 1.305378 |
| Dcbld2 | 0.4311085 |
| Dci | 0.3144988 |
| Dck | 0.8175531 |
| Dclk1 | 0.1512783 |
| Dclk2 | 1.1341313 |
| Dclk3 | -0.2713693 |
| Dclre1b | 1.6711526 |
| Dclre1b | -0.695472 |
| Dclre1c | -0.6917351 |
| Dclre1c | 0.6255276 |
| Dclre1c | 0.0566605 |
| Dcn | -0.2467158 |
| Dcn | -0.7973184 |
| Dcp1a | 1.0278107 |
| Dcp1a | 0.9522176 |
| Dcp1a | 1.4870109 |
| Dcp1a | -0.6465724 |
| Dcp1b | 0.4590025 |
| Dcp1b | 0.7588244 |
| Dcpp1 | -0.1930259 |
| Dcpp2 | -0.7391405 |
| Dcpp2 | 0.8980431 |
| Dcpp3 | -0.1116456 |
| Dcps | -0.595324 |
| Dcps | -0.7274537 |
| Dct | 0.5654823 |
| Dctd | 2.2264532 |
| Dctn1 | -0.2824801 |
| Dctn2 | 0.259649 |
| Dctn2 | 1.761783 |
| Dctn2 | 1.3275019 |
| Dctn3 | -0.6776665 |
| Dctn3 | -0.7212788 |
| Dctn4 | -0.201916 |
| Dctn4 | -0.2185842 |
| Dctn5 | 0.0293641 |
| Dctn6 | 1.4519545 |
| Dcun1d2 | 1.2120802 |
| Dcun1d3 | -0.6178389 |
| Dcun1d4 | -0.0228628 |
| Dcun1d4 | 2.5557791 |
| Dcun1d5 | 1.9368232 |
| Dcx | 1.0682102 |
| Dcx | 0.6594597 |
| Dcxr | 1.8537237 |
| Dcxr | 0.338633 |
| Ddah1 | -0.6387782 |
| Ddah2 | -0.6494226 |
| Ddb1 | 1.213501 |
| Ddb2 | -0.8303772 |
| Ddb2 | 0.1313276 |
| Ddb2 | -0.5188998 |
| Ddc | -0.6331493 |
| Ddc8 | 1.114311 |
| Ddef2 | -0.5720108 |
| Ddef2 | -0.0389164 |
| Ddefl1 | -0.3045998 |
| Ddhd1 | 0.6383938 |
| Ddhd1 | -0.5120776 |
| Ddi2 | -0.5004074 |
| Ddit3 | 0.1272237 |
| Ddit3 | 0.9128854 |
| Ddit4 | 0.9188412 |
| Ddit4l | -0.6739985 |
| Ddo | 1.7812848 |
| Ddo | 0.2499612 |
| Ddo | 2.7356916 |
| Ddost | -0.2014695 |
| Ddr1 | -0.3855101 |
| Ddr2 | 1.2814078 |
| Ddt | 1.4711159 |
| Ddx1 | 0.1093407 |
| Ddx1 | -0.5792767 |
| Ddx17 | 0.844945 |
| Ddx17 | -0.7151436 |
| Ddx17 | 0.329718 |
| Ddx19a | -0.5157927 |
| Ddx19b | -0.3488747 |
| Ddx19b | -0.0009041 |
| Ddx20 | -0.6247678 |
| Ddx21 | -0.3855101 |
| Ddx24 | -0.306591 |
| Ddx24 | 2.0029855 |
| Ddx24 | 0.7947134 |
| Ddx25 | -0.5693823 |
| Ddx26b | -0.6359596 |
| Ddx27 | -0.571353 |
| Ddx28 | -0.2374007 |
| Ddx3x | 0.3579579 |
| Ddx3y | -0.7898494 |
| Ddx4 | -0.606174 |
| Ddx4 | -0.0213831 |
| Ddx41 | 0.5493536 |
| Ddx46 | 0.9531505 |
| Ddx47 | -0.3196362 |
| Ddx47 | 0.1173656 |
| Ddx47 | -0.6894999 |
| Ddx49 | 2.3404705 |
| Ddx5 | -0.4900852 |
| Ddx50 | -0.4810691 |
| Ddx50 | 3.2971144 |
| Ddx51 | 1.1289377 |
| Ddx52 | 1.2408913 |
| Ddx54 | 1.768101 |
| Ddx58 | -0.6667044 |
| Ddx6 | 0.9544816 |
| Ddx6 | 0.8724431 |
| Ddx6 | 0.4994798 |
| Ddx6 | -0.5065318 |
| Deadc1 | -0.8277931 |
| Deb1 | 0.8812444 |
| Decr1 | 1.841982 |
| Decr2 | 2.0873341 |
| Decr2 | 0.6063761 |
| Dedd | 1.0884225 |
| Dedd | -0.5919585 |
| Dedd2 | -0.40575 |
| Def6 | 0.8093812 |
| Def8 | 1.4879243 |
| Defb11 | 0.8510422 |
| Defb14 | 0.222873 |
| Defb15 | 0.2843866 |
| Defb19 | -0.2680132 |
| Defb20 | 0.2781945 |
| Defb21 | 0.0818722 |
| Defb25 | -0.5987014 |
| Defb26 | 0.33914 |
| Defb30 | -0.414637 |
| Defb36 | 0.6023454 |
| Defb36 | -0.3441982 |
| Defb4 | -0.414637 |
| Defcr20 | 2.5179186 |
| Defcr6 | -0.675464 |
| Defcr-rs7 | 1.5525599 |
| Degs1 | 1.8634763 |
| Degs2 | -0.3768889 |
| Dek | -0.2608612 |
| Dek | -0.3452354 |
| Dennd1a | -0.660187 |
| Dennd1c | 1.1149859 |
| Dennd2a | -0.5602392 |
| Dennd3 | -0.3216591 |
| Denr | -0.6902444 |
| Denr | 1.5705202 |
| Depdc1b | 0.1920424 |
| Depdc5 | 2.0558777 |
| Depdc5 | -0.6331493 |
| Depdc6 | -0.2883287 |
| Depdc6 | 0.9595229 |
| Dera | 0.2819684 |
| Derl2 | 1.4054964 |
| Derl3 | 0.6734878 |
| Des | 0.3066661 |
| Det1 | 0.3921223 |
| Dexi | 0.1391705 |
| Dexi | -0.5700388 |
| Dffa | 1.9814065 |
| Dfna5h | 0.0898013 |
| Dgat1 | 0.1338443 |
| Dgat2 | -0.3593498 |
| Dgcr2 | 0.6352399 |
| Dgcr2 | -0.6573046 |
| Dgcr6 | 0.6065676 |
| Dgcr6 | 2.4591046 |
| Dgcr8 | 1.3733619 |
| Dgka | -0.3838877 |
| Dgka | 0.1497345 |
| Dgkb | -0.4118509 |
| Dgke | -0.6054923 |
| Dgkg | -0.6565854 |
| Dgkh | -0.545406 |
| Dgki | 0.6272156 |
| Dgkk | 1.9276511 |
| Dgkq | 0.0861753 |
| Dgkz | 2.4237949 |
| Dgkz | 0.8876856 |
| Dgkz | 2.1864157 |
| Dguok | 2.2388341 |
| Dhcr24 | 1.3515852 |
| Dhcr24 | -0.2378643 |
| Dhcr24 | 0.1668833 |
| Dhcr7 | 0.4136911 |
| Dhdds | -0.6494226 |
| Dhdds | 1.4133432 |
| Dhdh | 3.6872074 |
| Dhdh | 0.5793747 |
| Dhdh | 2.3106654 |
| Dhh | 0.2610249 |
| Dhodh | -0.6630781 |
| Dhps | 1.5592199 |
| Dhrs1 | 0.5048204 |
| Dhrs4 | 1.0980844 |
| Dhrs7 | -0.2448455 |
| Dhrs7 | 1.1459596 |
| Dhrs7 | -0.2341618 |
| Dhrs7b | -0.4107388 |
| Dhrs7b | -0.1136792 |
| Dhrs7b | 0.070861 |
| Dhrsx | -0.4900852 |
| Dhrsx | -0.2829661 |
| Dhtkd1 | 1.74496 |
| Dhx15 | 1.2069885 |
| Dhx15 | 1.3550016 |
| Dhx16 | -0.4185513 |
| Dhx29 | -0.1934684 |
| Dhx30 | 1.1781726 |
| Dhx32 | -0.0887631 |
| Dhx33 | 0.0080983 |
| Dhx36 | 1.3734477 |
| Dhx37 | 0.1005974 |
| Dhx38 | -0.7621486 |
| Dhx57 | -0.4475642 |
| Dhx58 | -0.6247678 |
| Dhx8 | -0.3196362 |
| Dhx9 | 1.7658859 |
| Diablo | -0.5176557 |
| Diap1 | -0.414637 |
| Diap3 | 2.8011453 |
| Dicer1 | -0.4931096 |
| Dido1 | -0.4292572 |
| Dido1 | 1.4917965 |
| Dido1 | 0.3916428 |
| Dio1 | -0.384969 |
| Dio1 | 2.0766349 |
| Dio3 | -0.6359596 |
| Dip2b | -0.2873515 |
| Dip2b | 1.0480432 |
| Diras1 | 1.007266 |
| Diras2 | 0.8664815 |
| Dirc2 | 0.5101314 |
| Dis3 | 0.0828669 |
| Dis3l | 0.1447778 |
| Dis3l2 | 0.7116356 |
| Disp1 | 1.04587 |
| Dixdc1 | 1.7868638 |
| Dkk3 | 0.2819684 |
| Dkk3 | -0.771023 |
| Dkk3 | 0.189971 |
| Dkkl1 | -0.606174 |
| Dkkl1 | 1.9077649 |
| Dlat | 1.964007 |
| Dlc1 | -0.3473133 |
| Dld | -0.6116456 |
| Dleu7 | -0.4810691 |
| Dlg1 | 0.9139963 |
| Dlg2 | 1.6295691 |
| Dlg3 | 1.6340189 |
| Dlg4 | -0.3328621 |
| Dlg7 | 0.0269073 |
| Dlgap1 | 0.0394752 |
| Dlgap2 | 1.2764837 |
| Dlgap2 | -0.688756 |
| Dlgap2 | -0.2277167 |
| Dlgap3 | 0.8600421 |
| Dlgap4 | -0.7462218 |
| Dlgap4 | 0.3322737 |
| Dlgap4 | 0.6166633 |
| Dlgap4 | -0.3344001 |
| Dlk1 | -0.1612618 |
| Dlk2 | 0.3952336 |
| Dll1 | 0.1234197 |
| Dlst | -0.1198062 |
| Dlx1 | -0.5120776 |
| Dlx1as | 0.3014085 |
| Dlx2 | 0.2843866 |
| Dlx3 | 0.052222 |
| Dmap1 | 2.3914801 |
| Dmbt1 | 0.4565409 |
| Dmkn | 0.0356525 |
| Dmkn | 2.3608122 |
| Dmkn | -0.5680708 |
| Dmn | 1.0926904 |
| Dmn | -0.6813487 |
| Dmpk | 0.33914 |
| Dmrt2 | 2.1117104 |
| Dmrt3 | -0.528282 |
| Dmrta1 | 0.9871697 |
| Dmrta2 | 0.8050356 |
| Dmrtc1a | 1.0522549 |
| Dmrtc1a | 1.1670585 |
| Dmrtc1a | 0.3732389 |
| Dmrtc1b | -0.4286908 |
| Dmrtc2 | -0.7422812 |
| Dmtf1 | -0.6580244 |
| Dmtf1 | -0.7014818 |
| Dmtf1 | 2.1072268 |
| Dmwd | 0.0812084 |
| Dmwd | -0.7816183 |
| Dmwd | 1.1664195 |
| Dnahc11 | -0.3374834 |
| Dnahc2 | -0.5511829 |
| Dnahc5 | 2.3257159 |
| Dnahc8 | 0.3431857 |
| Dnaic1 | 4.0897055 |
| Dnaic1 | 0.8773588 |
| Dnaja1 | -0.2523489 |
| Dnaja2 | 1.9320093 |
| Dnaja2 | -0.5263982 |
| Dnaja3 | 0.4411807 |
| Dnajb1 | -0.528282 |
| Dnajb10 | -0.4230444 |
| Dnajb12 | 0.6090538 |
| Dnajb13 | -0.2457802 |
| Dnajb3 | 1.1436711 |
| Dnajb4 | -0.7478026 |
| Dnajb4 | 3.2485353 |
| Dnajb5 | 1.3668204 |
| Dnajb6 | -0.6813487 |
| Dnajb6 | 0.4007134 |
| Dnajb7 | 1.3340493 |
| Dnajb8 | 4.367192 |
| Dnajb9 | -0.1363399 |
| Dnajc10 | 1.7684414 |
| Dnajc10 | 3.6675618 |
| Dnajc12 | 1.1859283 |
| Dnajc14 | -0.5028524 |
| Dnajc15 | -0.2618117 |
| Dnajc16 | 0.240736 |
| Dnajc17 | -0.5390279 |
| Dnajc18 | 0.971227 |
| Dnajc19 | -0.5004074 |
| Dnajc19 | -0.4925039 |
| Dnajc2 | 1.9548138 |
| Dnajc2 | -0.1720213 |
| Dnajc3a | -0.0228628 |
| Dnajc4 | -0.1569917 |
| Dnajc5 | -0.548611 |
| Dnajc5b | 0.7420241 |
| Dnajc5g | 1.3837699 |
| Dnajc6 | 1.7247532 |
| Dnajc6 | -0.6338511 |
| Dnajc7 | -0.7597426 |
| Dnajc8 | -0.2637156 |
| Dnalc1 | 1.5549742 |
| Dnali1 | 1.638253 |
| Dnase1l1 | 0.1064352 |
| Dnase1l2 | -0.7243613 |
| Dnase1l2 | 0.3790891 |
| Dnase2a | 3.7795528 |
| Dnd1 | 0.0911164 |
| Dner | -0.379037 |
| Dnm | 0.6326348 |
| Dnm1 | 1.135783 |
| Dnm1l | -0.6850452 |
| Dnm1l | 0.9409515 |
| Dnmbp | 0.9313242 |
| Dnmt1 | 1.8481579 |
| Dnmt3b | 1.8904549 |
| Dnmt3l | -0.6373678 |
| Dnmt3l | -0.712853 |
| Dnttip1 | 1.0729217 |
| Doc2a | -0.5845958 |
| Doc2b | 1.2403984 |
| Doc2b | -0.7840802 |
| Dock1 | -0.73601 |
| Dock3 | -0.6219902 |
| Dock4 | -0.4925039 |
| Dock6 | 0.8733124 |
| Dock8 | 1.108895 |
| Dock9 | -0.6769318 |
| Dok1 | -0.3499171 |
| Dok1 | -0.5157927 |
| Dok2 | -0.6423131 |
| Dok3 | -0.4151952 |
| Dok4 | -0.595324 |
| Dok5 | 2.4786564 |
| Dok5 | -0.7989862 |
| Dok7 | 0.9684782 |
| Dolk | 0.573842 |
| Dolpp1 | -0.3683449 |
| Dolpp1 | -0.4562679 |
| Dolpp1 | 0.3926016 |
| Dom3z | -0.1767942 |
| Dom3z | -0.5746465 |
| Donson | -0.5396638 |
| Donson | -0.660187 |
| Dos | -0.4096279 |
| Dos | -0.4230444 |
| Dot1l | 0.1958775 |
| Dpagt1 | 0.0286628 |
| Dpep1 | 0.7516134 |
| Dpep2 | 1.5687044 |
| Dpep2 | 3.0301119 |
| Dpep3 | 1.9644228 |
| Dpf1 | -0.2737737 |
| Dpf1 | 0.6965937 |
| Dpf3 | 0.3969047 |
| Dph2 | -0.0753706 |
| Dph2 | 2.3958995 |
| Dph2 | -0.4355107 |
| Dph3 | 2.5933434 |
| Dph3 | 0.0943964 |
| Dph3 | 0.3189089 |
| Dph4 | 0.8596014 |
| Dpm1 | -0.541574 |
| Dpm1 | 1.6514851 |
| Dpm2 | -0.1996857 |
| Dpm3 | -0.7151436 |
| Dpp10 | -0.6027701 |
| Dpp3 | 0.7738888 |
| Dpp3 | -0.621297 |
| Dpp4 | -0.7399247 |
| Dpp6 | 0.6702688 |
| Dpp7 | -0.7159083 |
| Dpp8 | -0.4366522 |
| Dpp9 | -0.5102254 |
| Dpp9 | -0.1371741 |
| Dppa3 | 1.0857599 |
| Dppa4 | 1.0522549 |
| Dppa5 | 1.5377277 |
| Dppa5 | 3.0186211 |
| Dpt | 1.9492456 |
| Dpy30 | -0.5403001 |
| Dpyd | -0.7501787 |
| Dpysl2 | -0.3667513 |
| Dpysl2 | 0.0788817 |
| Dpysl2 | 1.1819535 |
| Dpysl3 | 0.7903004 |
| Dpysl4 | 0.3320184 |
| Dpysl5 | 1.2151223 |
| Drbp1 | -0.541574 |
| Drctnnb1a | -0.3252095 |
| Drd1a | -0.4679977 |
| Drd2 | -0.525771 |
| Drd4 | 2.0505327 |
| Drd5 | 0.955678 |
| Drg1 | 1.7765168 |
| Drg1 | -0.6703445 |
| Drg2 | -0.1255605 |
| Drp2 | 2.9797932 |
| Dscam | -0.7290037 |
| Dscam | 1.2821631 |
| Dscc1 | 1.0063846 |
| Dscr1 | -0.2181299 |
| Dscr1l2 | -0.2927389 |
| Dscr2 | 3.091938 |
| Dscr3 | -0.6458612 |
| Dscr3 | -0.276667 |
| Dscr6 | 1.2035106 |
| Dsg2 | -0.1746218 |
| Dsg2 | 0.2342533 |
| Dsn1 | -0.6268563 |
| Dsp | -0.2637156 |
| Dst | 0.0563198 |
| Dst | 0.9059131 |
| Dst | 1.0786619 |
| Dstn | 0.4740809 |
| Dtd1 | 0.6851988 |
| Dtl | 1.7233868 |
| Dtna | -0.6894999 |
| Dtna | -0.4040929 |
| Dtna | -0.6171488 |
| Dtna | 2.5965173 |
| Dtnb | 1.5032448 |
| Dtnbp1 | -0.0217528 |
| Dtwd1 | 1.5914995 |
| Dtx1 | -0.3667513 |
| Dtx2 | 1.7031992 |
| Dtx2 | -0.6616315 |
| Dtx2 | -0.4533579 |
| Dtx4 | 0.7586611 |
| Dtx4 | -0.6171488 |
| Dtymk | -0.0005424 |
| Dus1l | 2.0110689 |
| Dus1l | -0.5213927 |
| Dus2l | -0.3525279 |
| Dus3l | -0.2786008 |
| Dus3l | 1.7862517 |
| Dus3l | 0.7980099 |
| Dus4l | 0.7018074 |
| Dusp1 | 3.3164418 |
| Dusp11 | 2.102682 |
| Dusp11 | 0.0387813 |
| Dusp13 | 0.3980965 |
| Dusp13 | 1.7735318 |
| Dusp13 | -0.3855101 |
| Dusp14 | -0.7290037 |
| Dusp15 | 0.2950608 |
| Dusp15 | 2.2672702 |
| Dusp16 | 0.7514489 |
| Dusp16 | 1.2365454 |
| Dusp16 | 0.60042 |
| Dusp18 | -0.041178 |
| Dusp19 | 1.5203846 |
| Dusp2 | 2.4967057 |
| Dusp2 | 0.0353043 |
| Dusp21 | -0.2718497 |
| Dusp22 | 0.9340459 |
| Dusp23 | 0.8637062 |
| Dusp26 | 0.9189793 |
| Dusp28 | 1.0164101 |
| Dusp3 | 1.3945691 |
| Dusp3 | -0.5946499 |
| Dusp3 | -0.4709528 |
| Dusp4 | -0.1442948 |
| Dusp4 | -0.3216591 |
| Dusp4 | -0.4040929 |
| Dusp6 | 0.3286938 |
| Dusp6 | 1.2042277 |
| Dusp6 | -0.5661069 |
| Dusp7 | 0.2873339 |
| Dusp8 | 1.9349672 |
| Dut | 0.3214936 |
| Duxbl | 1.1654602 |
| Duxbl | -0.2457802 |
| Dvl1 | -0.1007381 |
| Dvl1 | -0.4912938 |
| Dvl2 | -0.4925039 |
| DXBwg1396e | -0.2181299 |
| Dydc2 | -0.3145977 |
| Dym | -0.6515658 |
| Dync1h1 | 0.9389256 |
| Dync1i1 | -0.4446805 |
| Dync1i1 | 0.0297145 |
| Dync1i2 | -0.695472 |
| Dync1li1 | 0.3675986 |
| Dync1li2 | 2.5126529 |
| Dync2li1 | 0.0095309 |
| Dynll1 | 2.4216228 |
| Dynll2 | 0.5048204 |
| Dynlrb1 | 0.0109613 |
| Dynlrb2 | 0.2265865 |
| Dynlt3 | 1.8971193 |
| Dyrk1a | -0.5422116 |
| Dyrk1b | 1.0820429 |
| Dyrk1b | -0.1476641 |
| Dyrk2 | 2.0257781 |
| Dyrk2 | -0.0273158 |
| Dyrk3 | -0.4627015 |
| Dyrk4 | 0.3066661 |
| Dysf | 0.7365248 |
| Dysfip1 | -0.5966735 |
| Dzip1 | 1.9484006 |
| E030010A14Rik | 0.0798796 |
| E030034P13Rik | -0.4967515 |
| E130009J12Rik | -0.5409368 |
| E130012A19Rik | -0.6303473 |
| E130014J05Rik | 1.8726219 |
| E130016E03Rik | 0.797226 |
| E130203B14Rik | -0.5926306 |
| E130203B14Rik | -0.7525607 |
| E130303B06Rik | -0.7159083 |
| E130306D19Rik | 1.1801699 |
| E130308A19Rik | -0.4510362 |
| E130309D02Rik | -0.5615401 |
| E130309D02Rik | -0.4834651 |
| E130309D14Rik | 1.8207447 |
| E130309F12Rik | -0.3036058 |
| E130309F12Rik | 1.6890067 |
| E130309F12Rik | -0.766979 |
| E230008N13Rik | 2.0914672 |
| E2f1 | -0.6784018 |
| E2f2 | -0.4258634 |
| E2f2 | -0.5628426 |
| E2f3 | -0.5919585 |
| E2f4 | -0.6529972 |
| E2f5 | 3.3855681 |
| E2f6 | -0.1463992 |
| E2f6 | 2.4168612 |
| E2f6 | -0.7375739 |
| E330016A19Rik | 0.1093407 |
| E330017A01Rik | 1.0670287 |
| E330018D03Rik | -0.3338871 |
| E330021D16Rik | 0.3995248 |
| E330034G19Rik | -0.0637077 |
| E330034G19Rik | -0.1820284 |
| E330036I19Rik | -0.5933032 |
| E430002D04Rik | -0.5966735 |
| E430002G05Rik | -0.2888178 |
| E430018J23Rik | -0.6116456 |
| E430025E21Rik | 1.0281802 |
| E430028B21Rik | -0.4412315 |
| E4f1 | 0.9684782 |
| Eaf1 | 0.0762156 |
| Ear11 | -0.0765448 |
| Ear2 | 1.0469572 |
| Ear4 | 0.0077398 |
| Ears2 | -0.8200828 |
| Ebag9 | 0.8573952 |
| Ebf2 | -0.6472842 |
| Ebf3 | 0.8412081 |
| Ebf4 | 0.6029222 |
| Ebi2 | 1.2977022 |
| Ebi3 | -0.6380728 |
| Ebna1bp2 | 2.5410217 |
| Ebna1bp2 | 1.6365616 |
| Ebp | -0.7414951 |
| Ebpl | 2.2854268 |
| Ebpl | 2.0942934 |
| Ecd | 1.6055253 |
| Ecd | 1.0341948 |
| Ece1 | -0.4668182 |
| Ece1 | 2.0099686 |
| Ece2 | -0.6865278 |
| Ecel1 | 2.7160726 |
| Ech1 | -0.7775293 |
| Echdc1 | 1.6178885 |
| Echdc2 | 1.7471662 |
| Echdc2 | -0.5441269 |
| Echdc2 | 0.240736 |
| Echdc3 | 1.2639651 |
| Echs1 | -0.3303044 |
| Ecm1 | 2.0380005 |
| Ecm1 | -0.5232667 |
| Ecsit | -0.0788975 |
| Eda2r | 1.1814293 |
| Edaradd | 1.0234895 |
| Edc4 | 0.269512 |
| Edem1 | 1.5944981 |
| Edem2 | -0.5933032 |
| Edem2 | 2.5287986 |
| Edem2 | 1.659655 |
| Edem2 | -0.6296481 |
| Edf1 | -0.762952 |
| Edg2 | 2.5954923 |
| Edg5 | 1.2600131 |
| Edg7 | 0.3663681 |
| Edg8 | 0.0815404 |
| Edg8 | -0.2966762 |
| Edil3 | 1.4369055 |
| Edn1 | 0.1253236 |
| Edn2 | -0.2149555 |
| Ednra | -0.4961435 |
| Ednrb | 2.6137991 |
| Ednrb | 1.6743498 |
| Ednrb | -0.7742706 |
| Eed | -0.7029901 |
| Eef1a1 | -0.7266797 |
| Eef1a1 | 0.4436823 |
| Eef1a2 | 1.2360503 |
| Eef1a2 | -0.4751054 |
| Eef1a2 | 0.6204454 |
| Eef1b2 | 0.022681 |
| Eef1b2 | 1.3293002 |
| Eef1b2 | 0.8695397 |
| Eef1d | 2.2033954 |
| Eef1d | -0.6247678 |
| Eef1d | 0.0293641 |
| Eef1d | 2.5065468 |
| Eef1e1 | -0.5479692 |
| Eef1g | -0.3045998 |
| Eef2 | -0.1599787 |
| Eef2 | 1.4360223 |
| Eef2 | -0.1540139 |
| Eef2k | 0.085184 |
| Eefsec | 0.5321201 |
| Efcab1 | 0.0874954 |
| Efcab2 | -0.7022357 |
| Efcab2 | 0.7641971 |
| Efcab3 | -0.4574344 |
| Efcab3 | 0.833086 |
| Efcab3 | 0.2049611 |
| Efcab4a | 1.0043668 |
| Efcab4a | -0.1019439 |
| Efcab5 | -0.3196362 |
| Efemp2 | 0.9596552 |
| Efha1 | 2.0257348 |
| Efha1 | -0.7151436 |
| Efhc2 | 1.2413838 |
| Efhd1 | 0.5678782 |
| Efna1 | 2.3524814 |
| Efna3 | -0.4864685 |
| Efna4 | -0.6962212 |
| Efna4 | -0.8399122 |
| Efna5 | 1.1932073 |
| Efnb1 | 1.0648982 |
| Efnb2 | 0.3335489 |
| Efnb3 | -0.4556852 |
| Efr3a | -0.4492986 |
| Efs | 0.625152 |
| Eftud1 | 1.5571702 |
| Eftud2 | 0.6391344 |
| Eftud2 | -0.5511829 |
| Eftud2 | -0.5939763 |
| EG194588 | 0.4106395 |
| EG208426 | -0.3942099 |
| EG210155 | 2.7180884 |
| EG212225 | 2.7259429 |
| EG214321 | 0.4979791 |
| EG232599 | 2.8462123 |
| EG237009 | -0.4286908 |
| EG237009 | -0.571353 |
| EG237300 | 0.8501523 |
| EG238829 | 0.4652401 |
| EG240916 | 0.3248433 |
| EG245297 | 0.7242459 |
| EG245297 | 2.9364031 |
| EG245376 | -0.6226838 |
| EG245376 | -0.8700008 |
| EG272350 | -0.4527769 |
| EG277333 | -0.7143795 |
| EG317677 | -0.0288047 |
| EG328264 | -0.3303044 |
| EG328280 | -0.6880127 |
| EG328644 | -0.7581419 |
| EG328839 | 2.3603547 |
| EG330305 | -0.8166759 |
| EG330503 | -0.2063923 |
| EG330513 | 0.1488071 |
| EG333669 | -0.1728874 |
| EG368203 | -0.3704737 |
| EG380907 | -0.0726365 |
| EG381438 | 2.0208712 |
| EG382109 | 0.9725331 |
| EG382109 | 0.028312 |
| EG382109 | -0.5220169 |
| EG384639 | 0.6212 |
| EG384813 | 4.0737749 |
| EG384813 | 1.6825134 |
| EG384814 | -0.8312401 |
| EG385328 | 0.9073116 |
| EG408196 | -0.3909382 |
| EG432825 | -0.0415555 |
| EG432879 | 0.6474266 |
| EG432939 | 0.89025 |
| EG433016 | 1.2837661 |
| EG433182 | 0.136981 |
| EG433224 | 2.7818779 |
| EG433923 | 1.2753438 |
| EG434171 | 0.1035208 |
| EG434197 | -0.4230444 |
| EG434280 | -0.6594656 |
| EG434459 | 2.7017776 |
| EG434459 | 0.3160576 |
| EG434858 | -0.6703445 |
| EG435391 | -0.196128 |
| EG436332 | 0.3003534 |
| EG545047 | 2.363127 |
| EG545056 | 0.7632225 |
| EG545253 | 1.2676134 |
| EG545370 | 2.0129145 |
| EG545758 | -0.7321113 |
| EG545861 | -0.8166759 |
| EG545963 | 0.7445133 |
| EG546166 | 2.2822535 |
| EG546282 | 0.5304625 |
| EG546325 | 1.0885381 |
| EG546347 | -0.1015418 |
| EG547109 | -0.4107388 |
| EG574403 | 0.073542 |
| EG622320 | -0.0702992 |
| EG622339 | -0.5164133 |
| EG624866 | 1.5072046 |
| EG625054 | -0.6219902 |
| EG625424 | -0.0919418 |
| EG630499 | 0.0059455 |
| EG633640 | -0.4979686 |
| EG638695 | -0.6544309 |
| EG654453 | -0.6387782 |
| EG665186 | 0.8886837 |
| EG665378 | -0.9341424 |
| EG667977 | 3.0507047 |
| EG668668 | 0.8919557 |
| Egf | -0.8373023 |
| Egfbp2 | 0.1224663 |
| Egfl7 | 1.7630385 |
| Egfl7 | -0.4219191 |
| Egflam | 1.4253595 |
| Egflam | 1.0779609 |
| Egfr | -0.7022357 |
| Egfr | -0.460357 |
| Egfr | 0.5783891 |
| Egfr | -0.6924813 |
| Egln1 | 0.3152785 |
| Egln1 | -0.545406 |
| Egln1 | -0.6776665 |
| Egln2 | 0.2781945 |
| Egln3 | 1.2292914 |
| Egr1 | -0.165981 |
| Egr2 | -0.5207688 |
| Egr3 | 0.2870664 |
| Egr4 | -0.1079959 |
| Ehbp1 | 1.5243473 |
| Ehbp1l1 | -0.4650517 |
| Ehd1 | -0.4389391 |
| Ehd2 | 1.7633807 |
| Ehd3 | -0.5182775 |
| Ehd4 | 0.9476735 |
| Ehmt1 | -0.6791377 |
| Ehmt2 | -0.5753066 |
| Ehmt2 | -0.3844282 |
| Ei24 | -0.1646917 |
| Eid1 | 1.5175124 |
| Eid2 | 2.0725194 |
| Eid3 | -0.4925039 |
| Eif1a | -0.0202748 |
| Eif1b | -0.8303772 |
| Eif2a | 2.3731436 |
| Eif2ak1 | -0.6494226 |
| Eif2ak2 | -0.1027486 |
| Eif2b1 | -0.8682044 |
| Eif2b1 | 2.3323994 |
| Eif2b2 | 0.5037547 |
| Eif2b4 | 1.4095518 |
| Eif2b5 | -0.706014 |
| Eif2b5 | 0.5823256 |
| Eif2b5 | -0.0325373 |
| Eif2c2 | 1.3287611 |
| Eif2c3 | -0.6806111 |
| Eif2c3 | -0.6587447 |
| Eif2c3 | -0.903859 |
| Eif2c3 | 1.1591473 |
| Eif2c4 | 1.1630042 |
| Eif2s2 | 1.2746782 |
| Eif2s2 | -0.8504238 |
| Eif2s3x | 0.6100083 |
| Eif2s3y | -0.6014119 |
| Eif3b | 0.3464603 |
| Eif3b | -0.7029901 |
| Eif3d | 1.5930681 |
| Eif3eip | -0.7313334 |
| Eif3eip | 0.2223004 |
| Eif3f | -0.5733278 |
| Eif3g | 0.6457739 |
| Eif3i | 0.3103294 |
| Eif3i | 0.9852397 |
| Eif3i | -0.7462218 |
| Eif3k | -0.0295501 |
| Eif3s1 | -0.5126957 |
| Eif3s10 | -0.2883287 |
| Eif3s10 | -0.3546218 |
| Eif3s4 | 0.637838 |
| Eif3s8 | -0.5912868 |
| Eif4a1 | -0.0254578 |
| Eif4a1 | 0.8826796 |
| Eif4a2 | -0.7791628 |
| Eif4a3 | -0.4458329 |
| Eif4b | 1.9096256 |
| Eif4e | -0.2532909 |
| Eif4e1b | -0.5053038 |
| Eif4e1b | -0.660187 |
| Eif4e2 | -0.4068564 |
| Eif4e2 | -0.6515658 |
| Eif4e3 | 1.09223 |
| Eif4e3 | 0.7346846 |
| Eif4e3 | 0.1385454 |
| Eif4ebp1 | 2.0312631 |
| Eif4ebp2 | -0.8286537 |
| Eif4el3 | 1.3691495 |
| Eif4enif1 | 0.0624338 |
| Eif4enif1 | -0.712853 |
| Eif4g1 | -0.3196362 |
| Eif4g2 | -0.6437308 |
| Eif4g2 | -0.0114544 |
| Eif4h | 0.0066636 |
| Eif5 | 1.0379806 |
| Eif5 | -0.5933032 |
| Eif5 | -0.2453128 |
| Eif5a | -0.3806511 |
| Eif5a | 0.5536138 |
| Eif6 | 0.5450743 |
| Ela1 | 0.8597483 |
| Ela2 | 1.0049978 |
| Ela2a | -0.6472842 |
| Ela3b | -0.4002373 |
| Elac2 | -0.2323159 |
| Elac2 | 2.5078025 |
| Elac2 | -0.0518038 |
| Elavl1 | -0.763756 |
| Elavl2 | -0.6373678 |
| Elavl2 | -0.7661722 |
| Elavl3 | 1.5284395 |
| Elavl4 | 3.3200664 |
| Elavl4 | -0.0633214 |
| Elf1 | 1.2909934 |
| Elf2 | -0.1388448 |
| Elf2 | 1.2535196 |
| Elf2 | -0.6710741 |
| Elf2 | -0.0518038 |
| Elf2 | 1.8356074 |
| Elf3 | -0.6303473 |
| Elf3 | 1.3415282 |
| Elf3 | 1.5091412 |
| Elf5 | -0.9179349 |
| Elfn1 | -0.5022406 |
| Elfn2 | 0.5501666 |
| Elk3 | 1.9015201 |
| Elk3 | 1.8250835 |
| Elk3 | -0.7407096 |
| Ell | -0.2032567 |
| Ell3 | 0.1911552 |
| Elmo1 | 1.8016021 |
| Elmo1 | -0.0184305 |
| Elmo1 | 0.8945086 |
| Elmo1 | -0.5403001 |
| Elmo1 | -0.5145527 |
| Elmo1 | 1.3001133 |
| Elmo1 | 1.5030202 |
| Elmo1 | 0.5576536 |
| Elmo1 | -0.6095901 |
| Elmo1 | 0.7777382 |
| Elmo1 | -0.6054923 |
| Elmo1 | -0.7136159 |
| Elmo1 | 0.4565409 |
| Elmo2 | -0.5726691 |
| Elmo2 | -0.3504387 |
| Elmo3 | 1.2473731 |
| Elmo3 | -0.6969709 |
| Elmod1 | -0.3292833 |
| Eln | -0.807369 |
| Elof1 | 0.1816399 |
| Elovl1 | -0.61577 |
| Elovl1 | 0.6272156 |
| Elovl1 | 0.6044588 |
| Elovl1 | 0.1074047 |
| Elovl2 | -0.7470118 |
| Elovl4 | 1.3392195 |
| Elovl5 | 0.4598961 |
| Elovl6 | 0.6641586 |
| Elp2 | -0.5120776 |
| Elp3 | -0.4757001 |
| Elp3 | 1.7327932 |
| Eltd1 | -0.1104274 |
| Emb | -0.5595895 |
| Emcn | 1.8853005 |
| Emd | -0.7865484 |
| Emd | -0.1259728 |
| Emg1 | 3.9058297 |
| Emg1 | 0.3431857 |
| Emid1 | -0.7478026 |
| Emid2 | -0.7220485 |
| Emilin1 | 1.552631 |
| Emilin2 | -0.414637 |
| Eml1 | -0.6192206 |
| Eml3 | -0.6835649 |
| Eml3 | -0.6041302 |
| Eml4 | -0.8881533 |
| Eml5 | -0.6616315 |
| Emp1 | -0.5544074 |
| Emp2 | -0.5766281 |
| Emp3 | -0.756544 |
| Emr1 | -0.2254253 |
| Emr1 | 1.4247095 |
| Emr1 | -0.5251443 |
| En2 | 1.1248292 |
| Enah | 1.0083981 |
| Enc1 | 0.5992629 |
| Endod1 | 0.3509759 |
| Endogl1 | -0.3567202 |
| Endogl1 | 0.8704116 |
| Eng | 4.3724728 |
| Eno1 | -0.3125898 |
| Eno2 | -0.6472842 |
| Eno3 | 1.8739431 |
| Enoph1 | 0.6141968 |
| Enox1 | 0.6969422 |
| Enpp1 | 1.2383257 |
| Enpp1 | 2.6232512 |
| Enpp2 | -0.5289108 |
| Enpp4 | -0.5126957 |
| Enpp5 | -0.2613363 |
| Enpp6 | 0.0728725 |
| Ensa | 1.9550004 |
| Ensa | -0.2117917 |
| ENSMUSG00000043795 | 0.067499 |
| ENSMUSG00000053178 | 0.9277742 |
| ENSMUSG00000068790 | 0.0019859 |
| Entpd2 | 1.0436917 |
| Entpd3 | 2.1608927 |
| Entpd4 | 1.0066365 |
| Entpd5 | 0.5026878 |
| Entpd5 | 0.671164 |
| Entpd5 | 0.2530165 |
| Entpd6 | 0.8766375 |
| Entpd7 | 1.122601 |
| Ep300 | -0.4864685 |
| Ep400 | 0.2662357 |
| Epas1 | 0.8756267 |
| Epb4.1 | 0.7682473 |
| Epb4.1l1 | -0.1899338 |
| Epb4.1l1 | 0.8604825 |
| Epb4.1l1 | 1.9658075 |
| Epb4.1l2 | 0.1941094 |
| Epb4.1l3 | 1.1048128 |
| Epb4.1l3 | -0.712853 |
| Epb4.1l3 | 1.1928965 |
| Epb4.1l4a | -0.7501787 |
| Epb4.1l5 | -0.6820869 |
| Epb4.1l5 | -0.0959301 |
| Epb4.9 | -0.5326921 |
| Epb4.9 | 1.5463541 |
| Epc1 | 1.0652536 |
| Epc1 | 0.2816993 |
| Epc1 | 2.8204086 |
| Epdr1 | -0.8557233 |
| Epgn | -0.5345885 |
| Epha1 | 1.785305 |
| Epha1 | -0.4129644 |
| Epha3 | 1.9771319 |
| Epha3 | -0.495536 |
| Epha3 | 1.6404603 |
| Epha4 | -0.8691022 |
| Epha5 | 0.767115 |
| Epha6 | 0.3464603 |
| Epha7 | -0.0140186 |
| Epha7 | -0.6143932 |
| Epha7 | 2.2268042 |
| Ephb1 | -0.5576429 |
| Ephb2 | 1.019273 |
| Ephb4 | 1.8221931 |
| Ephb6 | 0.4237159 |
| Ephx1 | 0.4281121 |
| Ephx2 | -0.4174313 |
| Epm2a | 0.0339099 |
| Epm2aip1 | 1.8089683 |
| Epm2aip1 | 0.2838498 |
| Epm2aip1 | -0.4668182 |
| Epn1 | 0.9018456 |
| Epn2 | 1.0936107 |
| Epn2 | -0.6732666 |
| Epn2 | 1.036761 |
| Epn2 | -0.6984722 |
| Epn2 | 1.2110641 |
| Epor | 0.5077982 |
| Eprs | -0.6178389 |
| Eprs | -0.7082882 |
| Eps15 | 1.1366626 |
| Eps15l1 | -0.5628426 |
| Eps8 | 1.9405716 |
| Eps8l1 | -0.6932281 |
| Eps8l2 | 0.6829034 |
| Eps8l2 | -0.4870704 |
| Eps8l3 | 1.9993856 |
| Epsti1 | 0.0332119 |
| Epsti1 | 1.7572033 |
| Eraf | 0.543439 |
| Eral1 | 0.3304854 |
| Ercc1 | 1.7682712 |
| Ercc2 | 1.4555029 |
| Ercc3 | 0.3256147 |
| Ercc3 | 1.4691006 |
| Ercc4 | -0.751766 |
| Ercc4 | 1.8189175 |
| Ercc5 | -0.3257177 |
| Ercc5 | -0.6034499 |
| Ercc8 | -0.5628426 |
| Ercc8 | 0.3790891 |
| Erdr1 | -0.5145527 |
| Erf | -0.674731 |
| Erf | 0.2626735 |
| Ergic1 | 1.6572369 |
| Ergic2 | 0.7435184 |
| Ergic2 | 0.1705183 |
| Ergic3 | 0.3256147 |
| Ergic3 | 1.3727613 |
| Erh | -0.6430217 |
| Erich1 | -0.4816676 |
| Erlin1 | 1.1377611 |
| Erlin2 | 3.0332986 |
| Erlin2 | 0.5177275 |
| Ermap | -0.5563474 |
| Ermp1 | -0.7228188 |
| Ern1 | 1.2558507 |
| Ern2 | -0.6820869 |
| Ero1lb | -0.6472842 |
| Erp29 | 0.1693082 |
| Errfi1 | 2.41089 |
| Es22 | -0.6857862 |
| Esam1 | -0.2420469 |
| Esco2 | 1.3050095 |
| Esd | 0.7474919 |
| Esd | 1.7000364 |
| Esd | 2.626512 |
| Esd | -0.766979 |
| Esm1 | -0.4124075 |
| Espl1 | -0.0773284 |
| Espn | 0.5487435 |
| Espn | 1.5330949 |
| Espn | -0.7037452 |
| Espn | -0.6423131 |
| Esr1 | -0.2086383 |
| Esr2 | -0.706014 |
| Esr2 | -0.3478335 |
| Esr2 | -0.4372234 |
| Esrra | 2.6562647 |
| Esrrb | -0.2986509 |
| Etaa1 | 1.5314972 |
| Etf1 | 2.9913641 |
| Etfa | -0.4574344 |
| Etfa | -0.33235 |
| Etfa | -0.5371227 |
| Etfb | 1.2401026 |
| Etfb | 1.4535332 |
| Etfdh | -0.3216591 |
| Ethe1 | 0.2119347 |
| Etnk2 | 0.3404061 |
| Ets1 | 1.1263859 |
| Ets2 | -0.7775293 |
| Etsrp71 | 1.4043345 |
| Etv1 | 0.0788817 |
| Etv1 | 1.6877733 |
| Etv4 | -0.7274537 |
| Etv5 | -0.7597426 |
| Etv6 | -0.564147 |
| Evc | -0.5615401 |
| Evc2 | 0.1855169 |
| Evi1 | 0.8022315 |
| Evi1 | 0.0901303 |
| Evi2a | -0.3683449 |
| Evi2a | -0.6102748 |
| Evi5 | 2.1341661 |
| Evi5l | -0.6798741 |
| Evl | -0.5912868 |
| Evx1 | -0.2897965 |
| Exdl1 | -0.6630781 |
| Exdl2 | -0.3467934 |
| Exdl2 | 0.6352399 |
| Exdl2 | 1.5479986 |
| Exoc2 | 0.876926 |
| Exoc3 | -0.4418055 |
| Exoc3l | 1.8084246 |
| Exoc4 | 1.5444917 |
| Exoc7 | -0.5434881 |
| Exoc8 | -0.7136159 |
| Exod1 | -0.0468557 |
| Exosc1 | 0.4049801 |
| Exosc10 | 1.0715107 |
| Exosc2 | 0.5158341 |
| Exosc2 | -0.695472 |
| Exosc4 | 0.2787346 |
| Exosc5 | -0.466229 |
| Exosc6 | 1.5193545 |
| Exosc7 | 0.3497237 |
| Exosc8 | -0.155289 |
| Ext1 | -0.6206044 |
| Ext2 | 0.0059455 |
| Extl1 | 1.4108722 |
| Extl2 | 1.5446351 |
| Extl3 | 1.1085555 |
| Extl3 | 2.359439 |
| Eya1 | -0.1934684 |
| Eya1 | -0.6451506 |
| Eya2 | -0.0434451 |
| Eya3 | 0.0418998 |
| Eya3 | 2.0586436 |
| Eya3 | -0.7166737 |
| Eya4 | -0.3726073 |
| Ezh1 | -0.5693823 |
| Ezh2 | 1.3975009 |
| Ezh2 | -0.7007285 |
| F10 | -0.6638023 |
| F11 | -0.5307996 |
| F11 | -0.5933032 |
| F11r | -0.7939916 |
| F13a1 | -0.6296481 |
| F2r | 1.0223752 |
| F2rl1 | -0.2045993 |
| F2rl3 | 0.96651 |
| F430201B04Rik | 0.6830802 |
| F5 | 1.0640684 |
| F630043A04Rik | -0.4366522 |
| F730014I05Rik | -0.0176937 |
| F730047E07Rik | 1.4702638 |
| F730047E07Rik | 0.7817315 |
| F730047E07Rik | -0.5151725 |
| F8 | -0.231855 |
| F8 | 1.5434155 |
| F8 | -0.6820869 |
| F8a | 1.2036131 |
| Fa2h | -0.1979052 |
| Faah | 0.2365132 |
| Faah | 0.3666143 |
| Fabp3 | -0.7336689 |
| Fabp5 | 1.4334483 |
| Fabp9 | 1.8371391 |
| Fadd | 2.1218691 |
| Fadd | 0.6645191 |
| Fadd | 1.1024234 |
| Fads1 | 0.5750303 |
| Fads2 | 0.7980099 |
| Fads3 | -0.556995 |
| Fads6 | 0.24522 |
| Faf1 | 0.8787997 |
| Faf1 | 2.6387161 |
| Fah | -0.753356 |
| Fahd1 | 1.185824 |
| Fahd2a | -0.6587447 |
| Fahd2a | -0.4168718 |
| Fahd2a | 1.4619351 |
| Fahd2a | 0.6025377 |
| Faim | -0.4157537 |
| Faim | -0.6089059 |
| Faim2 | 1.3137232 |
| Faim2 | 1.9018654 |
| Faim3 | 1.3337811 |
| Fancc | 1.6846827 |
| Fancc | 0.0992953 |
| Fancd2 | 0.7512843 |
| Fancg | -0.5852628 |
| Fanci | -0.5839293 |
| Fancl | 1.5815504 |
| Fancl | -0.6806111 |
| Fank1 | 0.3906829 |
| Fank1 | 0.1253236 |
| Fap | -0.2295537 |
| Fars2 | -0.7045009 |
| Farsa | -0.5879355 |
| Farsb | -0.3520052 |
| Farsb | -0.5071464 |
| Fas | -0.7282284 |
| Fas | 0.6461414 |
| Fasl | -0.460357 |
| Fastk | 0.6623541 |
| Fastk | -0.6423131 |
| Fastkd1 | 1.4189206 |
| Fastkd5 | 0.7680856 |
| Fat1 | 1.3001133 |
| Fat4 | -0.2556502 |
| Fbf1 | 0.0342587 |
| Fblim1 | -0.7105679 |
| Fbln1 | 0.3474656 |
| Fbln1 | 0.2894717 |
| Fbln1 | -0.5602392 |
| Fbln2 | 0.16232 |
| Fbln2 | -0.0792901 |
| Fbn1 | -0.5674157 |
| Fbp1 | 1.9016681 |
| Fbp2 | 0.1650606 |
| Fbxl10 | 0.6892464 |
| Fbxl12 | 0.8132446 |
| Fbxl12 | -0.7375739 |
| Fbxl12 | -0.5201454 |
| Fbxl12 | -0.5220169 |
| Fbxl13 | 0.5922908 |
| Fbxl13 | 0.2125135 |
| Fbxl14 | 0.4306481 |
| Fbxl15 | 0.7614331 |
| Fbxl18 | 0.0855145 |
| Fbxl18 | -0.0840149 |
| Fbxl21 | 1.7911376 |
| Fbxl22 | -0.6020908 |
| Fbxl4 | -0.7313334 |
| Fbxl5 | -0.3974929 |
| Fbxl6 | -0.5422116 |
| Fbxl6 | -0.6289493 |
| Fbxl8 | 0.5750303 |
| Fbxo15 | 0.3557193 |
| Fbxo16 | -0.34161 |
| Fbxo18 | 0.1141637 |
| Fbxo18 | 0.3824852 |
| Fbxo2 | -0.031416 |
| Fbxo21 | 1.2054557 |
| Fbxo25 | -0.3272442 |
| Fbxo27 | 0.8058131 |
| Fbxo28 | 0.0841916 |
| Fbxo3 | -0.2392565 |
| Fbxo3 | 0.9718803 |
| Fbxo30 | 2.2549143 |
| Fbxo31 | 0.5852673 |
| Fbxo31 | -0.7328898 |
| Fbxo32 | -0.1672722 |
| Fbxo32 | -0.2786008 |
| Fbxo32 | -0.6296481 |
| Fbxo33 | 0.1224663 |
| Fbxo34 | -0.407964 |
| Fbxo34 | -0.7407096 |
| Fbxo34 | 1.0121621 |
| Fbxo36 | 0.6549195 |
| Fbxo38 | 0.3574609 |
| Fbxo38 | 0.2348188 |
| Fbxo4 | 0.3562172 |
| Fbxo42 | -0.4997971 |
| Fbxo44 | 2.1635209 |
| Fbxo6 | -0.4627015 |
| Fbxo7 | -0.7383569 |
| Fbxo8 | -0.3865933 |
| Fbxo9 | 0.6958964 |
| Fbxo9 | -0.3206471 |
| Fbxw12 | 2.044096 |
| Fbxw13 | 1.0345619 |
| Fbxw15 | 0.405453 |
| Fbxw16 | 1.3536015 |
| Fbxw17 | 0.370055 |
| Fbxw19 | -0.1288643 |
| Fbxw2 | -0.6674313 |
| Fbxw4 | 0.6733092 |
| Fbxw4 | 1.5602784 |
| Fbxw5 | 0.8918137 |
| Fbxw7 | 0.1577348 |
| Fbxw7 | -0.1430343 |
| Fbxw7 | 1.293608 |
| Fbxw8 | -0.2888178 |
| Fcer1g | -0.0983311 |
| Fcer2a | -0.4236076 |
| Fcf1 | -0.6739985 |
| Fcgr2b | 2.7029078 |
| Fcgr2b | -0.0967298 |
| Fcgr2b | -0.6880127 |
| Fcgr3 | 0.0023465 |
| Fcgr4 | 0.7471614 |
| Fcgrt | -0.353051 |
| Fcho1 | -0.5133143 |
| Fcho2 | -0.5114598 |
| Fchsd1 | -0.1616898 |
| Fchsd2 | 0.9604486 |
| Fcna | 0.256891 |
| Fcrla | 1.6680707 |
| Fcrla | 0.0566605 |
| Fcrla | 3.5038518 |
| Fdps | 1.57282 |
| Fdxr | 0.0180813 |
| Fdxr | 1.989539 |
| Fem1b | 1.8980605 |
| Fem1c | -0.4372234 |
| Fem1c | -0.6992237 |
| Fen1 | -0.606174 |
| Fermt2 | 2.2142298 |
| Fermt3 | 0.302199 |
| Fert2 | 2.5619746 |
| Fert2 | -0.0514223 |
| Fes | 2.2813923 |
| Fetub | -0.1259728 |
| Fev | 0.7233991 |
| Fez1 | -0.3405766 |
| Fez1 | 0.9040919 |
| Fez2 | -0.6843047 |
| Fezf2 | 1.8093487 |
| Ffar2 | 0.0247965 |
| Fgd2 | -0.7621486 |
| Fgd4 | -0.3865933 |
| Fgd4 | -0.5680708 |
| Fgd5 | -0.4230444 |
| Fgd6 | 0.6193123 |
| Fgf1 | -0.6068563 |
| Fgf1 | 3.2601858 |
| Fgf10 | 1.04587 |
| Fgf10 | -0.2907763 |
| Fgf12 | 1.824442 |
| Fgf12 | 0.5428251 |
| Fgf13 | 0.7695397 |
| Fgf13 | 2.0813459 |
| Fgf17 | 1.7434476 |
| Fgf21 | 2.5473133 |
| Fgf22 | 1.1011694 |
| Fgf22 | 0.5880048 |
| Fgf5 | 0.1404194 |
| Fgf7 | -0.4140792 |
| Fgf8 | 0.5750303 |
| Fgf9 | 0.4169666 |
| Fgfbp3 | -0.7694033 |
| Fgfr1 | -0.5022406 |
| Fgfr1op | -0.7067715 |
| Fgfr1op2 | -0.6430217 |
| Fgfr1op2 | 0.5950082 |
| Fgfr2 | 0.063449 |
| Fgfr2 | -0.3656905 |
| Fgfr3 | -0.3991386 |
| Fgfrl1 | -0.6416051 |
| Fgl2 | 0.4734206 |
| Fh1 | 0.1401073 |
| Fhad1 | -0.6226838 |
| Fhit | 1.6639038 |
| Fhl1 | 2.0055106 |
| Fhl1 | -0.2742553 |
| Fhl2 | 0.8312716 |
| Fhl4 | -0.7525607 |
| Fhl5 | 2.1320372 |
| Fhod1 | 2.6622879 |
| Fhod3 | -0.5674157 |
| Fibcd1 | 2.2416021 |
| Fibp | 0.1604886 |
| Figf | -0.8557233 |
| Figla | -0.5466867 |
| Fign | -0.4586024 |
| Fignl1 | -0.7197413 |
| Fip1l1 | -0.3196362 |
| Fis1 | 2.1117896 |
| Fis1 | -0.2300135 |
| Fiz1 | 0.0762156 |
| Fjx1 | 0.7366919 |
| Fkbp10 | 3.0305051 |
| Fkbp11 | 0.2703294 |
| Fkbp14 | -0.3774255 |
| Fkbp1a | 0.9063328 |
| Fkbp1b | 0.2257308 |
| Fkbp2 | -0.7305562 |
| Fkbp2 | 1.0712754 |
| Fkbp4 | -0.3855101 |
| Fkbp5 | -0.0919418 |
| Fkbp6 | 1.0468365 |
| Fkbp7 | 0.2297174 |
| Fkbp8 | -0.150199 |
| Fkbp9 | -0.7205098 |
| Fkbpl | -0.1578442 |
| Fkbpl | -0.6494226 |
| Fkhl18 | 0.5499634 |
| Fkrp | 0.9609772 |
| Fktn | -0.196572 |
| Flad1 | 1.9029989 |
| Flcn | 2.3608427 |
| Flcn | -0.5263982 |
| Fli1 | -0.0114544 |
| Flii | 0.7762965 |
| Flna | 1.8754146 |
| Flot1 | 0.7435184 |
| Flot1 | -0.7075296 |
| Flot2 | -0.5441269 |
| Flot2 | 2.3948407 |
| Flot2 | -0.2077393 |
| Flrt1 | 1.3096056 |
| Flrt2 | -0.3694087 |
| Flrt3 | -0.409073 |
| Flt1 | 0.2681483 |
| Flt4 | 0.5107667 |
| Flt4 | -0.297663 |
| Flvcr2 | 1.8344438 |
| Flywch2 | -0.6630781 |
| Fmn2 | -0.0718568 |
| Fmn2 | -0.289307 |
| Fmnl1 | 1.2426634 |
| Fmnl3 | -0.5589402 |
| Fmnl3 | 0.9692644 |
| Fmo1 | -0.6082222 |
| Fmo2 | 1.0447815 |
| Fmo5 | -0.7399247 |
| Fmod | -0.4372234 |
| Fmr1nb | -0.3231791 |
| Fmr1nb | -0.6359596 |
| Fn3k | 1.9871967 |
| Fn3k | -0.0836203 |
| Fnbp1 | 1.1159972 |
| Fnbp1 | 1.0546533 |
| Fnbp1 | 2.0399655 |
| Fnbp1l | 2.6712081 |
| Fnbp4 | -0.6806111 |
| Fndc3 | -0.0781126 |
| Fndc3a | 0.8229115 |
| Fndc3b | 0.7826874 |
| Fndc4 | 0.6981608 |
| Fndc5 | -0.6667044 |
| Fndc5 | 0.3061417 |
| Fndc7 | -0.5511829 |
| Fndc7 | -0.3509606 |
| Fndc8 | 0.7345171 |
| Fndc8 | -0.7328898 |
| Fnip1 | -0.771023 |
| Fnip1 | -0.5919585 |
| Fnta | -0.6924813 |
| Fnta | -0.6041302 |
| Fntb | 1.1650335 |
| Folr1 | -0.1455569 |
| Folr1 | -0.40575 |
| Folr2 | 1.5604195 |
| Fos | 1.6789698 |
| Fosb | -0.1535893 |
| Foxa1 | 2.2884251 |
| Foxa2 | -0.3855101 |
| Foxa3 | 0.4683437 |
| Foxc1 | -0.7525607 |
| Foxd2 | 2.796616 |
| Foxg1 | 1.135893 |
| Foxh1 | 1.0295337 |
| Foxh1 | 0.2168436 |
| Foxj1 | -0.258963 |
| Foxj2 | -0.8346995 |
| Foxj3 | 1.9757636 |
| Foxj3 | -0.5498961 |
| Foxj3 | 1.1933109 |
| Foxj3 | 1.0818101 |
| Foxj3 | 2.5843132 |
| Foxk1 | -0.0310425 |
| Foxk1 | -0.0679676 |
| Foxk1 | 1.7301994 |
| Foxl2 | -0.3364545 |
| Foxm1 | -0.3318382 |
| Foxm1 | -0.4870704 |
| Foxn2 | 3.0613496 |
| Foxn3 | -0.2295537 |
| Foxo1 | 0.4917365 |
| Foxo6 | -0.6020908 |
| Foxo6 | -0.5176557 |
| Foxp1 | -0.2575418 |
| Foxp2 | 1.2325775 |
| Foxp2 | -0.5779515 |
| Foxp2 | -0.3920275 |
| Foxp3 | 0.3997626 |
| Foxp4 | 1.5788567 |
| Foxq1 | 2.2638671 |
| Foxred1 | -0.231855 |
| Foxred1 | -0.3641013 |
| Foxred1 | 0.0768828 |
| Foxred1 | 1.7698581 |
| Foxred1 | -0.1668416 |
| Foxred2 | 1.384194 |
| Fpgs | 0.7468308 |
| Fpgs | 0.1306974 |
| Fpgs | 0.1426011 |
| Fpr2 | 0.5891756 |
| Frag1 | 0.3609347 |
| Frag1 | 0.7071651 |
| Frag1 | -0.4632885 |
| Frap1 | 2.5370745 |
| Fras1 | 2.0310047 |
| Fras1 | -0.3191312 |
| Frat1 | 1.0004451 |
| Frat1 | -0.675464 |
| Frat2 | 1.0297796 |
| Freq | 1.7664544 |
| Freq | -0.3115874 |
| Freq | -0.5511829 |
| Frg1 | -0.2495282 |
| Frk | -0.7259063 |
| Frmd3 | -0.5270257 |
| Frmd6 | -0.2131463 |
| Frmd8 | 2.4518137 |
| Frmpd4 | -0.8082113 |
| Frrs1 | -0.2095382 |
| Frs2 | 1.4349775 |
| Frs3 | 1.1277181 |
| Fryl | -0.4372234 |
| Fryl | -0.9283906 |
| Frzb | -0.7799806 |
| Fscn1 | 3.7615594 |
| Fscn2 | -0.3865933 |
| Fscn3 | -0.0258291 |
| Fsd1 | 1.5944301 |
| Fsd2 | 1.4846473 |
| Fshb | 0.6040749 |
| Fshr | 1.9341094 |
| Fshr | -0.5661069 |
| Fshr | -0.6703445 |
| Fsip1 | 1.2795169 |
| Fst | -0.545406 |
| Fstl1 | -0.8574963 |
| Fstl4 | -0.030296 |
| Fstl5 | -0.6522812 |
| Fth1 | 0.9300968 |
| Fth1 | 0.6750932 |
| Ftmt | 1.1203675 |
| Fto | -0.8390414 |
| Ftsj1 | 1.2701016 |
| Ftsj3 | -0.1489307 |
| Fubp1 | -0.4418055 |
| Fubp3 | 0.6545554 |
| Fuk | 3.2989084 |
| Fuk | 0.2524617 |
| Fundc1 | -0.1472423 |
| Fundc1 | 1.715207 |
| Fundc2 | 3.5762615 |
| Furin | -0.7881975 |
| Furin | -0.4002373 |
| Fus | 0.8531152 |
| Fusip1 | 3.3396397 |
| Fusip1 | -0.6020908 |
| Fusip1 | -0.5589402 |
| Fut10 | -0.1485083 |
| Fut10 | -0.0804691 |
| Fut2 | -0.39585 |
| Fut4 | 0.0668252 |
| Fut8 | -0.5226416 |
| Fut8 | -0.1193965 |
| Fut8 | -0.8235019 |
| Fvt1 | -0.2077393 |
| Fxc1 | -0.2951978 |
| Fxn | -0.7501787 |
| Fxn | -0.4129644 |
| Fxyd1 | 2.0061298 |
| Fxyd2 | 2.7949917 |
| Fxyd2 | 0.1595715 |
| Fxyd2 | -0.798152 |
| Fxyd3 | -0.7098074 |
| Fxyd4 | -0.384969 |
| Fxyd4 | -0.5799399 |
| Fxyd5 | 1.571148 |
| Fxyd5 | 0.1469495 |
| Fxyd5 | 0.043628 |
| Fxyd6 | -0.2752192 |
| Fxyd7 | -0.6199123 |
| Fyb | -0.7159083 |
| Fyco1 | -0.6718044 |
| Fyn | 2.1548958 |
| Fyttd1 | -0.7923326 |
| Fzd1 | -0.0295501 |
| Fzd10 | -0.3036058 |
| Fzd10 | -0.6718044 |
| Fzd2 | 0.2632224 |
| Fzd3 | 1.0785451 |
| Fzd4 | -0.6674313 |
| Fzd5 | -0.485266 |
| Fzd6 | 0.2297174 |
| Fzd7 | -0.5289108 |
| Fzd8 | -0.5046903 |
| Fzr1 | 1.5318605 |
| Fzr1 | 2.8242638 |
| Fzr1 | 0.6844931 |
| G0s2 | 0.3032519 |
| G3bp1 | -0.2532909 |
| G3bp1 | 0.0930858 |
| G3bp2 | 0.3271555 |
| G3bp2 | -0.248121 |
| G3bp2 | 1.7632666 |
| G430022H21Rik | 0.8983253 |
| G430055L02Rik | -0.6932281 |
| G6pc | 0.1472594 |
| G6pc2 | 0.2854594 |
| G6pc2 | -0.4281246 |
| G6pc3 | 0.5944266 |
| G6pd2 | -0.1472423 |
| G6pd2 | -0.2258832 |
| G6pdx | -0.597349 |
| Gaa | 1.3157299 |
| Gab1 | 1.3071264 |
| Gab2 | 1.6057944 |
| Gab2 | 1.2544915 |
| Gab2 | 1.7812848 |
| Gab3 | -0.5602392 |
| Gab3 | -0.3161065 |
| Gab3 | 0.3178731 |
| Gabarap | -0.1877313 |
| Gabarapl1 | 1.1603274 |
| Gabarapl2 | 0.60273 |
| Gabbr1 | -0.7174397 |
| Gabpa | -0.6850452 |
| Gabpa | -0.0213831 |
| Gabpb1 | -0.3758167 |
| Gabpb1 | -0.7767136 |
| Gabpb2 | -0.4236076 |
| Gabra3 | -0.5213927 |
| Gabra4 | -0.763756 |
| Gabrb1 | -0.7726454 |
| Gabrb2 | -0.7228188 |
| Gabrb3 | -0.6835649 |
| Gabrb3 | -0.6924813 |
| Gabrd | -0.6739985 |
| Gabrd | 0.0240919 |
| Gabrg1 | 1.4226751 |
| Gabrg2 | 1.6668099 |
| Gabrg2 | -0.7832589 |
| Gabrg2 | -0.7174397 |
| Gabrg2 | -0.379037 |
| Gabrp | -0.751766 |
| Gabrp | -0.6616315 |
| Gad1 | -0.7336689 |
| Gad1 | -0.3736759 |
| Gadd45a | 0.8918137 |
| Gadd45a | 0.9445873 |
| Gadd45b | 0.2779244 |
| Gadd45g | 1.7028955 |
| Gadd45g | 1.5543357 |
| Gak | -0.7597426 |
| Gal | 0.0983175 |
| Gal3st1 | -0.2694501 |
| Gal3st2 | 0.5688748 |
| Gal3st3 | 2.4899904 |
| Galc | -0.771023 |
| Galc | 0.5872235 |
| Gale | 0.1580412 |
| Gale | -0.7685945 |
| Galk1 | -0.0963298 |
| Galm | 2.601839 |
| Galns | 1.2900579 |
| Galnt1 | 0.3436902 |
| Galnt1 | 0.0398219 |
| Galnt1 | -0.2504675 |
| Galnt1 | -0.0078032 |
| Galnt10 | 1.4159719 |
| Galnt10 | 0.9080101 |
| Galnt10 | 0.573049 |
| Galnt11 | -0.4452565 |
| Galnt12 | -0.1872914 |
| Galnt13 | -0.7525607 |
| Galnt2 | 0.8519312 |
| Galnt2 | 1.1962068 |
| Galnt2 | -0.4504566 |
| Galnt3 | 1.5927955 |
| Galnt9 | -0.753356 |
| Galntl2 | 1.1576432 |
| Galntl4 | -0.2323159 |
| Galntl5 | 2.6311872 |
| Galt | 1.0424794 |
| Galt | 0.3815161 |
| Gamt | 2.9418281 |
| Gan | -0.7067715 |
| Ganab | -0.258489 |
| Ganab | 1.6212688 |
| Ganab | 0.8709925 |
| Ganc | 0.9965073 |
| Gap43 | 0.6790063 |
| Gapdh | -0.7581419 |
| Gapdhs | 0.0450082 |
| Gapvd1 | 2.2355018 |
| Gapvd1 | 1.1968261 |
| Garnl3 | -0.8355663 |
| Garnl4 | -0.1750559 |
| Gars | 2.7975428 |
| Gart | -0.5120776 |
| Gart | -0.0419331 |
| Gas2l1 | 1.221278 |
| Gas2l1 | 2.9963924 |
| Gas2l1 | 1.9660841 |
| Gas2l1 | 2.4332262 |
| Gas6 | -0.6820869 |
| Gas7 | 0.6955475 |
| Gas7 | -0.6710741 |
| Gas8 | -0.5647998 |
| Gata2 | 0.0265559 |
| Gata4 | -0.4973598 |
| Gata6 | -0.6791377 |
| Gatad1 | -0.8098982 |
| Gatad1 | -0.3410932 |
| Gatad2b | 0.709231 |
| Gats | -0.660187 |
| Gats | 4.0408627 |
| Gba | -0.0191678 |
| Gba2 | 1.7482094 |
| Gbas | 2.2161833 |
| Gbf1 | -0.5865982 |
| Gbl | -0.5654531 |
| Gbl | -0.4135216 |
| Gbl | 0.5622783 |
| Gbp2 | -0.2411159 |
| Gbp3 | -0.6394841 |
| Gbp3 | 1.1480255 |
| Gbp6 | -0.7228188 |
| Gbp6 | 2.2319834 |
| Gbx2 | 1.901076 |
| Gca | -0.5563474 |
| Gca | 0.2026252 |
| Gcap14 | -0.6674313 |
| Gcap14 | 0.6874887 |
| Gcc1 | -0.4615285 |
| Gcc2 | -0.4446805 |
| Gcdh | 1.1757491 |
| Gch1 | -0.7581419 |
| Gch1 | 3.0440338 |
| Gchfr | 1.2499145 |
| Gck | -0.7422812 |
| Gclm | -0.3257177 |
| Gcn5l2 | 2.3289058 |
| Gcnt1 | 3.5549268 |
| Gcnt1 | -0.6992237 |
| Gcnt2 | 1.413261 |
| Gcnt2 | 0.1644523 |
| Gcs1 | 0.0286628 |
| Gda | 1.9438336 |
| Gdap1 | -0.179844 |
| Gdap1 | 0.4258009 |
| Gdap1l1 | 1.2499145 |
| Gdap1l1 | -0.7407096 |
| Gde1 | 1.6133628 |
| Gde1 | -0.6835649 |
| Gdf10 | 0.5967511 |
| Gdf15 | 1.0899242 |
| Gdf3 | -0.5819325 |
| Gdf9 | 2.8155154 |
| Gdi1 | 1.1314828 |
| Gdi1 | 0.4977645 |
| Gdi1 | 1.5420507 |
| Gdi1 | 1.1941392 |
| Gdi1 | 3.5395857 |
| Gdi2 | -0.5845958 |
| Gdnf | -0.1561399 |
| Gdpd1 | 2.1511429 |
| Gdpd2 | 1.2981664 |
| Gdpd2 | 1.4022563 |
| Gdpd3 | 0.0792144 |
| Gdpd4 | -0.6880127 |
| Gdpd5 | -0.2537623 |
| Gemin4 | -0.4406579 |
| Gemin4 | 0.9922975 |
| Gemin4 | 0.2300016 |
| Gemin5 | 1.2068864 |
| Gemin6 | -0.7807991 |
| Gemin6 | -0.761346 |
| Gemin7 | 0.4773751 |
| Gemin7 | -0.7313334 |
| Gfap | 0.105141 |
| Gfer | -0.754152 |
| Gfi1 | -0.3272442 |
| Gfm1 | -0.3795747 |
| Gfod1 | -0.5912868 |
| Gfod2 | 0.465018 |
| Gfpt2 | -0.0773284 |
| Gfra1 | -0.1750559 |
| Gfra4 | 0.138858 |
| Gga1 | 1.6938019 |
| Gga1 | -0.3006298 |
| Gga1 | -0.4580182 |
| Gga3 | -0.1447153 |
| Ggcx | 0.1288042 |
| Ggcx | -0.5065318 |
| Ggcx | -0.5479692 |
| Ggn | -0.851305 |
| Ggn | 2.1142405 |
| Ggnbp1 | -0.5615401 |
| Ggnbp2 | 1.6619403 |
| Ggt5 | 1.1507369 |
| Ggt7 | -0.7344486 |
| Ggta1 | 0.3144988 |
| Ggtla1 | -0.2014695 |
| Gh | -0.5460461 |
| Ghitm | -0.7597426 |
| Ghr | -0.6616315 |
| Ghr | -0.6444404 |
| Ghrh | -0.6725352 |
| Ghrhr | 1.4155617 |
| Ghrl | -0.3614587 |
| Ghrl | -0.4768906 |
| Ghsr | 0.8842557 |
| Ghsr | -0.4906893 |
| Gif | -0.1664112 |
| Gigyf1 | 0.2356663 |
| Gimap1 | 0.499694 |
| Gimap4 | -0.355146 |
| Gimap5 | -0.8124342 |
| Gimap6 | 2.2663953 |
| Gimap8 | -0.3385133 |
| Gimap9 | -0.4539392 |
| Gins3 | 2.0499417 |
| Gins4 | -0.3130914 |
| Gipc1 | -0.706014 |
| Gipc1 | 0.5479293 |
| Gipc1 | 0.9680849 |
| Gipc2 | -0.0525673 |
| Git1 | 0.0768828 |
| Git2 | -0.7478026 |
| Git2 | 0.704922 |
| Git2 | 0.9844666 |
| Gja1 | 0.9008612 |
| Gja4 | 0.3131978 |
| Gja5 | -0.456851 |
| Gja5 | 2.2156154 |
| Gja6 | 0.1683996 |
| Gja7 | 0.0037877 |
| Gjb1 | 0.3147588 |
| Gjb2 | -0.4224816 |
| Gjb2 | -0.6681587 |
| Gjb3 | 0.8195469 |
| Gjb6 | 1.4496607 |
| Gjc2 | 1.4874677 |
| Gjc2 | 0.5269304 |
| Gjd2 | 2.0349595 |
| Gjd2 | -0.7082882 |
| Gjd3 | -0.761346 |
| Gk2 | 0.2648672 |
| Gk5 | -0.4876726 |
| Gkap1 | 0.7174502 |
| Gkap1 | 1.9629437 |
| Glb1 | 1.5157405 |
| Glb1l2 | -0.4973598 |
| Glcci1 | -0.4230444 |
| Gldc | 1.0745653 |
| Gldc | -0.3257177 |
| Gldn | -0.1925835 |
| Gle1 | 0.1009227 |
| Glg1 | 0.1647565 |
| Glipr2 | 2.0182991 |
| Glis1 | 1.3061146 |
| Glo1 | -0.6206044 |
| Gloxd1 | 1.4636535 |
| Glp2r | -0.1574178 |
| Glra2 | 2.4475349 |
| Glrb | 0.7405274 |
| Glrp1 | 0.3766558 |
| Glrx | 0.5005503 |
| Glrx2 | 1.0264547 |
| Glrx2 | -0.4096279 |
| Glrx2 | -0.4757001 |
| Glrx3 | 0.513937 |
| Glrx5 | 1.1109296 |
| Gls2 | 2.1106014 |
| Gls2 | 1.1830011 |
| Glt25d1 | 0.2878688 |
| Glt25d2 | -0.4586024 |
| Glt25d2 | 0.4696707 |
| Glt6d1 | -0.5661069 |
| Glt8d1 | 1.4605269 |
| Glt8d1 | -0.2608612 |
| Glt8d1 | 0.7193238 |
| Glt8d2 | 1.4857155 |
| Glt8d3 | 0.1332158 |
| Gltp | 1.3031648 |
| Gltscr1 | -0.6623545 |
| Glud1 | 1.8767319 |
| Glud1 | 0.0191447 |
| Glul | -0.6587447 |
| Glyctk | 0.6756278 |
| Glyctk | -0.5460461 |
| Glyctk | -0.6909894 |
| Gm1006 | 0.8998758 |
| Gm1006 | -0.4372234 |
| Gm1019 | -0.34368 |
| Gm1040 | 1.719634 |
| Gm1070 | 3.3639844 |
| Gm1082 | -0.1608339 |
| Gm114 | -0.5892746 |
| Gm114 | 0.1253236 |
| Gm1141 | -0.5320609 |
| Gm128 | 0.725092 |
| Gm129 | -0.2849127 |
| Gm129 | -0.3252095 |
| Gm1322 | -0.3651605 |
| Gm136 | 1.2958434 |
| Gm136 | 1.1159972 |
| Gm1381 | -0.2819943 |
| Gm1527 | -0.6185295 |
| Gm1568 | -0.5345885 |
| Gm1574 | 0.8896807 |
| Gm1574 | -0.548611 |
| Gm1604 | -0.460357 |
| Gm1604 | -0.751766 |
| Gm166 | -0.7282284 |
| Gm166 | -0.6501364 |
| Gm1661 | 0.1018978 |
| Gm1673 | -0.7166737 |
| Gm1673 | -0.1140864 |
| Gm1698 | 2.0849759 |
| Gm1821 | -0.4051973 |
| Gm1943 | -0.751766 |
| Gm1965 | -0.0400465 |
| Gm1967 | -0.7998212 |
| Gm1967 | 1.389016 |
| Gm216 | -0.9351044 |
| Gm239 | 2.2523213 |
| Gm249 | 0.978259 |
| Gm266 | 1.5437026 |
| Gm2a | -0.1476641 |
| Gm347 | -0.5226416 |
| Gm382 | 0.0924298 |
| Gm410 | 0.096359 |
| Gm428 | -0.6401906 |
| Gm443 | -0.348354 |
| Gm484 | -0.6872699 |
| Gm50 | -0.2337 |
| Gm525 | -0.2790848 |
| Gm525 | 0.2319881 |
| Gm527 | -0.1463992 |
| Gm527 | 1.4133432 |
| Gm561 | 0.600998 |
| Gm561 | -0.8407837 |
| Gm597 | -0.4492986 |
| Gm603 | 1.598843 |
| Gm609 | -0.7274537 |
| Gm614 | -0.6199123 |
| Gm614 | -0.763756 |
| Gm648 | 1.6092846 |
| Gm648 | 1.175327 |
| Gm71 | -0.4168718 |
| Gm711 | 1.1425794 |
| Gm732 | 1.3540392 |
| Gm732 | -0.5466867 |
| Gm767 | 1.8719607 |
| Gm773 | -0.6909894 |
| Gm773 | -0.7438555 |
| Gm784 | 1.8940913 |
| Gm813 | -0.5766281 |
| Gm813 | -0.3619866 |
| Gm817 | -0.621297 |
| Gm867 | -0.6537138 |
| Gm884 | -0.5195224 |
| Gm889 | 0.8854004 |
| Gm906 | -0.8772205 |
| Gm93 | 0.3824852 |
| Gm93 | -0.2462479 |
| Gm944 | 0.3312522 |
| Gm949 | 0.6666793 |
| Gm962 | 1.0564482 |
| Gm962 | 2.4625922 |
| Gm97 | 0.5054593 |
| Gm97 | 0.6472431 |
| Gmcl1 | 0.8285437 |
| Gmcl1 | -0.5040773 |
| Gmcl1 | 2.045582 |
| Gmds | -0.1859729 |
| Gmeb2 | 0.4299572 |
| Gmfg | -0.7865484 |
| Gmfg | 0.3045665 |
| Gmfg | 0.0924298 |
| Gmip | 1.4626385 |
| Gmip | -0.3656905 |
| Gmppa | 1.0742134 |
| Gmppa | 0.4945399 |
| Gmppb | 1.2959364 |
| Gmpr | 1.2574016 |
| Gmpr2 | 0.1941094 |
| Gna11 | -0.7367916 |
| Gna13 | -0.6199123 |
| Gna14 | -0.2495282 |
| Gnai2 | -0.7791628 |
| Gnao1 | -0.1943541 |
| Gnao1 | -0.5176557 |
| Gnaq | 0.8839693 |
| Gnaq | -0.7305562 |
| Gna-rs1 | -0.528282 |
| Gnas | -0.6924813 |
| Gnas | 1.0913083 |
| Gnas | 0.7767773 |
| Gnas | 0.856806 |
| Gnas | -0.2594372 |
| Gnat1 | -0.7067715 |
| Gnat2 | 3.1601958 |
| Gnaz | 2.669466 |
| Gnb1 | -0.7120907 |
| Gnb1l | 2.266429 |
| Gnb2 | -0.0121864 |
| Gnb2l1 | 0.2623989 |
| Gnb3 | 0.5676788 |
| Gnb4 | -0.0919418 |
| Gnb4 | 1.8442896 |
| Gnb5 | 0.2412977 |
| Gnb5 | 0.0276099 |
| Gne | 0.2052527 |
| Gng10 | -0.2689709 |
| Gng11 | 1.1384196 |
| Gng13 | 3.2045618 |
| Gng2 | -0.2457802 |
| Gng2 | 0.9750099 |
| Gng3 | 1.0654905 |
| Gng4 | -0.7022357 |
| Gng7 | -0.4040929 |
| Gng8 | -0.5126957 |
| Gngt2 | 0.7529285 |
| Gnl2 | 1.3458639 |
| Gnl2 | -0.7734577 |
| Gnl3 | 0.2749478 |
| Gnl3 | -0.1255605 |
| Gnl3 | -0.4638759 |
| Gnl3l | 1.7137068 |
| Gnl3l | -0.7931617 |
| Gnmt | -0.6725352 |
| Gnmt | 0.6499918 |
| Gnpat | -0.6143932 |
| Gnpda1 | -0.5726691 |
| Gnpda1 | 1.6656738 |
| Gnpda1 | 1.7899185 |
| Gnpda2 | 1.433851 |
| Gnptab | -0.6451506 |
| Gnptg | 1.1921709 |
| Gnptg | 1.2356541 |
| Gnrhr | 0.9059131 |
| Gns | 2.1681589 |
| Golga1 | 0.7931397 |
| Golga2 | -0.6843047 |
| Golga2 | -0.7865484 |
| Golga2 | -0.4068564 |
| Golga2 | -0.5926306 |
| Golga2 | 1.1934145 |
| Golga3 | 0.32895 |
| Golga4 | -0.6681587 |
| Golga5 | 0.541186 |
| Golga7 | 0.6092448 |
| Golim4 | -0.5693823 |
| Golm1 | 1.3264213 |
| Golm1 | 0.6339382 |
| Golph3l | 0.0080983 |
| Golt1a | -0.6261596 |
| Golt1b | 0.7361904 |
| Gopc | -0.6544309 |
| Gorasp1 | -0.5232667 |
| Gorasp2 | 1.7281903 |
| Gorasp2 | -0.4949288 |
| Gorasp2 | -0.5390279 |
| Gorasp2 | -0.0753706 |
| Gosr1 | -0.3318382 |
| Gosr2 | 1.7589233 |
| Gosr2 | 1.8154687 |
| Gosr2 | 1.6016161 |
| Gosr2 | 3.4509122 |
| Got1l1 | -0.1681339 |
| Got2 | 1.6725958 |
| Gp1bb | -0.7143795 |
| Gp38 | -0.846907 |
| Gp5 | 0.1041692 |
| Gp9 | -0.6089059 |
| Gpaa1 | 1.0535748 |
| Gpam | -0.1728874 |
| Gpatch1 | -0.7702128 |
| Gpatch2 | 0.0627723 |
| Gpatch3 | 0.0106039 |
| Gpatch4 | -0.7235897 |
| Gpbp1 | 0.6523676 |
| Gpbp1 | -0.8031684 |
| Gpbp1l1 | -0.4918987 |
| Gpc1 | -0.852187 |
| Gpc3 | -0.079683 |
| Gpc3 | 0.7061304 |
| Gpc4 | -0.1144939 |
| Gpc5 | 0.7184726 |
| Gpc5 | 2.3163372 |
| Gpc5 | -0.395303 |
| Gpd1 | -0.7923326 |
| Gpd2 | 1.9944302 |
| Gpd2 | 1.3877494 |
| Gper | -0.545406 |
| Gpha2 | 1.2166397 |
| Gphb5 | 0.3240714 |
| Gphn | 1.6918375 |
| Gphn | -0.2023627 |
| Gphn | -0.5145527 |
| Gpihbp1 | 0.1890818 |
| Gpkow | 0.5118246 |
| Gpld1 | 1.0287957 |
| Gpld1 | -0.265146 |
| Gpm6a | -0.8082113 |
| Gpm6a | 1.6559617 |
| Gpm6b | 0.9853685 |
| Gpnmb | 0.611343 |
| Gpnmb | 0.9800737 |
| Gpr1 | -0.5151725 |
| Gpr1 | 1.2553655 |
| Gpr103 | 2.3555833 |
| Gpr107 | -0.6282511 |
| Gpr108 | 0.2356663 |
| Gpr108 | -0.4281246 |
| Gpr108 | -0.3282632 |
| Gpr109a | -0.0668039 |
| Gpr113 | 0.3937989 |
| Gpr113 | -0.761346 |
| Gpr114 | 1.0656089 |
| Gpr12 | 2.4460857 |
| Gpr12 | -0.5946499 |
| Gpr120 | 0.4780326 |
| Gpr123 | 0.0446633 |
| Gpr124 | 0.586637 |
| Gpr128 | 2.7603692 |
| Gpr135 | 2.4991095 |
| Gpr137 | 2.1844736 |
| Gpr137b | 0.2958565 |
| Gpr141 | -0.1903749 |
| Gpr146 | 0.6505406 |
| Gpr146 | 0.5242206 |
| Gpr149 | 0.7430205 |
| Gpr151 | -0.7045009 |
| Gpr153 | -0.5706957 |
| Gpr156 | 1.892499 |
| Gpr156 | -0.6178389 |
| Gpr158 | 0.0583622 |
| Gpr162 | 0.8722981 |
| Gpr162 | -0.7266797 |
| Gpr17 | 1.546998 |
| Gpr172b | 1.5531285 |
| Gpr172b | -0.5059176 |
| Gpr176 | -0.1548638 |
| Gpr177 | 0.4703335 |
| Gpr18 | 3.4003925 |
| Gpr180 | 1.7321454 |
| Gpr182 | 2.2156864 |
| Gpr182 | -0.5667611 |
| Gpr19 | 1.8254042 |
| Gpr20 | -0.3582971 |
| Gpr21 | -0.3236863 |
| Gpr22 | 1.1527924 |
| Gpr23 | -0.6969709 |
| Gpr26 | -0.7775293 |
| Gpr27 | 1.4508479 |
| Gpr27 | 2.5550827 |
| Gpr27 | -0.7367916 |
| Gpr34 | 0.1357276 |
| Gpr34 | 1.426415 |
| Gpr37l1 | 0.209906 |
| Gpr45 | -0.495536 |
| Gpr6 | -0.7375739 |
| Gpr64 | 0.9224245 |
| Gpr65 | 3.6378984 |
| Gpr68 | -0.5892746 |
| Gpr75 | 1.8704329 |
| Gpr81 | 1.9038852 |
| Gpr83 | 0.3820008 |
| Gpr85 | -0.494322 |
| Gpr85 | -0.7501787 |
| Gpr89 | 1.4501358 |
| Gpr89 | -0.348354 |
| Gpr97 | -0.6487092 |
| Gprasp1 | -0.7143795 |
| Gprasp1 | 0.7871357 |
| Gprc2a-rs5 | 0.5436436 |
| Gprc2a-rs5 | 0.4101692 |
| Gprc5a | -0.6791377 |
| Gprc5b | 3.2253416 |
| Gprc5c | -0.4834651 |
| Gprk6 | 0.0610785 |
| Gprk6 | 1.7773038 |
| Gps1 | -0.6068563 |
| Gps1 | -0.3176176 |
| Gps1 | -0.4925039 |
| Gps1 | -0.5276536 |
| Gps2 | 0.6498088 |
| Gpsm1 | 1.6086144 |
| Gpsm2 | -0.2439118 |
| Gpsm3 | -0.0415555 |
| Gpsn2 | -0.4961435 |
| Gpsn2 | -0.6784018 |
| Gpsn2 | 1.2676134 |
| Gpt1 | -0.5621911 |
| Gpt2 | 0.9968891 |
| Gpx1 | 2.0564651 |
| Gpx2 | 2.2029278 |
| Gpx3 | 0.0738767 |
| Gpx4 | 0.5120361 |
| Gpx4 | 0.1735369 |
| Gpx6 | -0.4650517 |
| Gpx7 | -0.5326921 |
| Gramd1a | -0.4586024 |
| Gramd1b | -0.3931181 |
| Gramd2 | -0.0107231 |
| Gramd2 | -0.3619866 |
| Gramd3 | -0.5518269 |
| Gramd4 | 0.0349558 |
| Grap2 | -0.5879355 |
| Grasp | -0.7266797 |
| Grasp | -0.6178389 |
| Grb10 | 0.0134593 |
| Grb14 | 0.1484978 |
| Grb14 | 1.1170075 |
| Grb2 | 0.5204558 |
| Grb7 | -0.7075296 |
| Grcc10 | 3.011598 |
| Greb1 | -0.085595 |
| Greb1 | 1.1939322 |
| Grem1 | 0.3281813 |
| Grem2 | -0.4521963 |
| Grhl1 | 1.3998398 |
| Grhl2 | 0.4350126 |
| Grhpr | -0.61577 |
| Gria1 | -0.6784018 |
| Gria1 | -0.3196362 |
| Gria1 | -0.6587447 |
| Gria2 | 2.8676138 |
| Gria2 | 0.6936266 |
| Gria3 | -0.5912868 |
| Gria3 | 0.2396118 |
| Gria3 | -0.735229 |
| Grid2 | 2.208524 |
| Grik1 | -0.3241937 |
| Grik2 | -0.6843047 |
| Grik4 | 2.4337066 |
| Grik5 | -0.495536 |
| Grin1 | 2.4806489 |
| Grin1 | 1.595858 |
| Grin2a | 1.7727982 |
| Grin2b | 2.216999 |
| Grin2c | -0.3833475 |
| Grin3b | -0.7052572 |
| Grina | -0.5466867 |
| Grip1 | 2.5459829 |
| Grip1 | 0.7038849 |
| Grip1 | -0.6089059 |
| Gripap1 | 0.4292657 |
| Gripap1 | -0.1413563 |
| Grit | 0.3949946 |
| Grk4 | 0.4947552 |
| Grk4 | -0.6116456 |
| Grk4 | -0.6000557 |
| Grk5 | -0.5733278 |
| Grlf1 | -0.4745111 |
| Grm1 | 1.9488701 |
| Grm7 | 1.4521125 |
| Grm8 | -0.1992403 |
| Grn | 1.7643494 |
| Grp | -0.5046903 |
| Grpel1 | 0.2477329 |
| Grpr | -0.4762952 |
| Grsf1 | 1.4511642 |
| Grtp1 | -0.4024386 |
| Grwd1 | 0.0992953 |
| Grwd1 | -0.4377949 |
| Gsbs | 0.5269304 |
| Gsc | -0.595324 |
| Gsdma1 | -0.5307996 |
| Gsdmdc1 | 1.2336703 |
| Gsdmdc1 | 0.2460584 |
| Gsdmdc1 | 1.2505976 |
| Gse1 | -0.7205098 |
| Gsg1 | 1.5661143 |
| Gsg1 | 1.0028506 |
| Gsg1 | -0.4912938 |
| Gsg2 | -0.4562679 |
| Gsk3a | 0.5503697 |
| Gsk3b | -0.39585 |
| Gsn | 1.485868 |
| Gspt1 | 0.1555875 |
| Gspt2 | -0.2532909 |
| Gss | -0.1655511 |
| Gss | -0.5886048 |
| Gss | 1.2486447 |
| Gsta2 | 0.1025473 |
| Gsta3 | 0.3343133 |
| Gsta3 | 0.181042 |
| Gsta4 | -0.3504387 |
| Gstcd | 2.1184949 |
| Gstk1 | -0.5108424 |
| Gstm1 | -0.3338871 |
| Gstm1 | 2.7211042 |
| Gstm2 | 0.0508522 |
| Gstm2 | -0.2163147 |
| Gstm2 | -0.395303 |
| Gstm4 | 1.8648614 |
| Gstm5 | 0.16232 |
| Gstm6 | -0.5674157 |
| Gstm6 | -0.1514689 |
| Gsto1 | 3.1526146 |
| Gsto2 | -0.5492533 |
| Gsto2 | -0.7159083 |
| Gstp1 | -0.5022406 |
| Gstp1 | -0.7446436 |
| Gstp2 | -0.4135216 |
| Gstt1 | 0.5936505 |
| Gstt2 | 0.949146 |
| Gstt3 | -0.3045998 |
| Gstt4 | 0.850449 |
| Gstz1 | 0.384662 |
| Gtdc1 | -0.9122796 |
| Gtf2a1 | 1.3961617 |
| Gtf2e1 | 1.3766159 |
| Gtf2e2 | -0.6020908 |
| Gtf2e2 | 1.8135232 |
| Gtf2f1 | 0.9812385 |
| Gtf2f2 | -0.0579292 |
| Gtf2h1 | 1.958074 |
| Gtf2h1 | 0.3562172 |
| Gtf2h2 | 0.9105205 |
| Gtf2h3 | 0.2412977 |
| Gtf2h4 | -0.6969709 |
| Gtf2h5 | 1.5298967 |
| Gtf2ird1 | -0.4864685 |
| Gtf2ird1 | -0.6718044 |
| Gtf2ird2 | 1.2932349 |
| Gtf2ird2 | 0.2308534 |
| Gtf2ird2 | 0.0030674 |
| Gtf3a | -0.5232667 |
| Gtf3a | 0.5842878 |
| Gtf3c1 | -0.597349 |
| Gtf3c2 | 0.0487937 |
| Gtf3c3 | -0.456851 |
| Gtf3c4 | 0.6426443 |
| Gtf3c4 | -0.1409372 |
| Gtf3c5 | -0.2665785 |
| Gtf3c5 | -0.0935351 |
| Gtf3c5 | -0.0483755 |
| Gtl3 | 0.1070816 |
| Gtpbp1 | 1.8512829 |
| Gtpbp2 | 0.2339704 |
| Gtpbp3 | -0.7228188 |
| Gtpbp4 | -0.7478026 |
| Gtpbp5 | 0.7031929 |
| Gtpbp6 | 0.5990699 |
| Gtpbp8 | 0.1173656 |
| Gtpbp8 | 0.602153 |
| Gtrgeo22 | 1.2528386 |
| Gtse1 | -0.310586 |
| Gtse1 | 0.022681 |
| Gtse1 | -0.3747457 |
| Gtsf1l | -0.0552445 |
| Guca1a | -0.6501364 |
| Guca1b | -0.129692 |
| Guca2a | 2.0500683 |
| Guca2b | 0.481095 |
| Gucy1a2 | 1.8501903 |
| Gucy1a3 | -0.3630434 |
| Gucy1a3 | -0.0158544 |
| Gucy1a3 | -0.34161 |
| Gucy1b3 | 0.9667727 |
| Guk1 | 1.2436465 |
| Gulo | 1.7303175 |
| Gulp1 | -0.5628426 |
| Gusb | -0.2951978 |
| Gusb | 0.2825063 |
| Gusb | -0.3303044 |
| Gvin1 | 1.7057461 |
| Gyg | 0.783165 |
| Gyg | 1.3617979 |
| Gykl1 | 1.4498983 |
| Gyltl1b | -0.4834651 |
| Gyltl1b | 1.060742 |
| Gypa | 0.3549719 |
| Gypc | 1.3300186 |
| Gys1 | 0.2746768 |
| Gys3 | 0.0539316 |
| Gzf1 | -0.0423109 |
| Gzf1 | -0.433232 |
| Gzmd | 2.6113627 |
| Gzmd | 0.7068203 |
| Gzmk | -0.2023627 |
| Gzmm | -0.8425292 |
| Gzmn | 1.1032205 |
| H13 | -0.541574 |
| H13 | 1.8513869 |
| H13 | -0.545406 |
| H13 | 0.4694497 |
| H19 | 1.3348534 |
| H1f0 | -0.4129644 |
| H1fnt | -0.203704 |
| H1foo | -0.2661008 |
| H1fx | -0.5403001 |
| H1fx | 0.1762456 |
| H2-Aa | 2.2790697 |
| H2-Ab1 | -0.6275534 |
| H2-Ab1 | 1.5200904 |
| H2-Ab1 | 0.8544456 |
| H2afj | -0.3656905 |
| H2afx | 3.3057791 |
| H2afy | -0.6947235 |
| H2afz | 0.2148254 |
| H2-Bl | 0.0808764 |
| H2-DMa | 1.8054287 |
| H2-DMb1 | -0.4709528 |
| H2-Eb1 | 2.2203964 |
| H2-K1 | -0.6688868 |
| H2-Ke6 | -0.2514077 |
| H2-M10.3 | 1.8244955 |
| H2-M2 | 0.2798137 |
| H2-M3 | -0.33235 |
| H2-Oa | 1.3813061 |
| H2-Ob | 0.6899486 |
| H2-Q1 | 1.6208718 |
| H2-Q2 | -0.0832258 |
| H2-Q7 | -0.3026127 |
| H2-Q8 | -0.5176557 |
| H2-T10 | 0.2263014 |
| H2-T23 | 0.1555875 |
| H2-T23 | -0.6416051 |
| H2-T24 | 3.9882332 |
| H2-Tw3 | -0.3811898 |
| H3f3a | -0.1489307 |
| H3f3a | -0.145136 |
| H3f3b | 0.8987484 |
| H47 | 0.9314604 |
| H47 | -0.7182063 |
| H6pd | 0.2429806 |
| Habp4 | -0.6798741 |
| Habp4 | 0.4345541 |
| Hace1 | -0.8141286 |
| Hacl1 | -0.8200828 |
| Hadhb | -0.2286347 |
| Hagh | -0.8235019 |
| Haghl | 2.7597068 |
| Haghl | 2.2093463 |
| Hal | -0.0030774 |
| Hamp2 | -0.6894999 |
| Hao1 | -0.9179349 |
| Hao3 | -0.2281756 |
| Hap1 | 0.3786029 |
| Hap1 | 1.6249668 |
| Hap1 | 0.7294795 |
| Hapln1 | -0.0005424 |
| Hapln2 | -0.7014818 |
| Hapln4 | 0.7030198 |
| Harbi1 | -0.3145977 |
| Hars | 1.8262586 |
| Has1 | -0.6089059 |
| Hat1 | 0.803323 |
| Havcr1 | -0.4709528 |
| Havcr2 | 1.3296595 |
| Hax1 | -0.6261596 |
| Hba-a1 | -0.8056865 |
| Hbb-b1 | -0.2341618 |
| Hbegf | -0.4264282 |
| Hbp1 | -0.5133143 |
| Hbp1 | 1.4363436 |
| Hbs1l | -0.6352563 |
| Hbs1l | -0.4395117 |
| Hbs1l | 0.2482905 |
| Hbs1l | 0.6426443 |
| Hc | -0.548611 |
| Hccs | -0.7621486 |
| Hcfc1 | -0.3085864 |
| Hcfc1r1 | -0.6359596 |
| Hcfc2 | 2.933361 |
| Hcls1 | -0.5479692 |
| Hcn2 | 2.446922 |
| Hcn3 | 1.3617979 |
| Hcn3 | 0.7239073 |
| Hcr | 0.2460584 |
| Hcst | -0.0078032 |
| Hdac10 | -0.406303 |
| Hdac11 | 0.581343 |
| Hdac2 | 0.0989695 |
| Hdac3 | -0.5511829 |
| Hdac4 | 0.0177266 |
| Hdac5 | -0.0269439 |
| Hdac6 | 2.0105409 |
| Hdac7 | 1.0688004 |
| Hdac8 | -0.2932302 |
| Hdac9 | -0.7685945 |
| Hdc | -0.621297 |
| Hddc2 | -0.4632885 |
| Hddc2 | -0.5170343 |
| Hdgf | 0.528802 |
| Hdgf | -0.6999758 |
| Hdgf | -0.4207952 |
| Hdgf | 0.0542731 |
| Hdgfl1 | 0.6481602 |
| Hdgfrp2 | -0.7589419 |
| Hdhd1a | 2.3099902 |
| Hdhd2 | -0.3344001 |
| Hdhd2 | 0.9135799 |
| Hdhd2 | 0.3890009 |
| Hdlbp | 0.8356504 |
| Hdlbp | 1.7179016 |
| Heatr1 | 0.6132465 |
| Heatr1 | -0.0468557 |
| Heatr3 | -0.2708891 |
| Heatr5a | -0.4906893 |
| Hebp1 | 3.7814211 |
| Hebp2 | -0.6143932 |
| Heca | 1.1221547 |
| Hectd2 | -0.5492533 |
| Hectd2 | 2.6057873 |
| Hectd3 | -0.5441269 |
| Hecw2 | 0.2859954 |
| Hecw2 | 0.0957052 |
| Hecw2 | -0.6479965 |
| Heg1 | 2.6612199 |
| Hel308 | 1.5702411 |
| Helb | -0.632448 |
| Helb | -0.607539 |
| Helz | -0.4185513 |
| Hemgn | 1.5474984 |
| Hemk1 | -0.6843047 |
| Hemt1 | -0.763756 |
| Hepacam | 1.536427 |
| Heph | 0.7430205 |
| Heph | 0.7620842 |
| Heph | 0.9400066 |
| Heph | -0.5582913 |
| Heph | 2.2845354 |
| Herc2 | -0.53143 |
| Herc3 | -0.3736759 |
| Herc3 | 0.4118144 |
| Herc4 | -0.3779623 |
| Herc4 | 0.5503697 |
| Herpud1 | 0.9542155 |
| Herpud1 | 1.1797498 |
| Herpud2 | -0.6828256 |
| Herpud2 | -0.6394841 |
| Herpud2 | 2.2390766 |
| Herpud2 | -0.041178 |
| Hes1 | -0.494322 |
| Hes5 | -0.7344486 |
| Hes5 | 0.6693727 |
| Hes6 | -0.763756 |
| Hes6 | -0.1434543 |
| Hes6 | 1.7520826 |
| Hexa | -0.1595514 |
| Hexb | -0.6529972 |
| Hexdc | -0.4400846 |
| Hexdc | 0.030415 |
| Hexim1 | 0.6951986 |
| Hexim2 | 2.1192414 |
| Hey1 | -0.485266 |
| Hey2 | 0.6543732 |
| Hey2 | 1.8072273 |
| Heyl | 0.6955475 |
| Hfe | 1.1118325 |
| Hfe | 0.4141597 |
| Hgfac | -0.4786792 |
| Hgs | 0.531706 |
| Hgs | 0.721024 |
| Hgsnat | 1.6297659 |
| Hhat | 1.8459643 |
| Hhat | -0.6444404 |
| Hhat | -0.2415813 |
| Hhatl | -0.6401906 |
| Hhex | -0.0895568 |
| Hhip | -0.4129644 |
| Hibadh | 0.7617587 |
| Hic1 | 0.5092836 |
| Hic2 | -0.8243586 |
| Hif1a | 2.0355171 |
| Hif3a | -0.6345534 |
| Hif3a | 2.7386318 |
| Higd1a | -0.548611 |
| Higd1b | 0.5515875 |
| Higd2a | 1.1341313 |
| Hils1 | -0.163833 |
| Hint1 | 1.5551869 |
| Hint2 | -0.5557003 |
| Hint3 | -0.1059743 |
| Hint3 | -0.2542339 |
| Hip1 | -0.6732666 |
| Hip1r | -0.528282 |
| Hip2 | -0.2005772 |
| Hipk1 | 1.1836291 |
| Hipk2 | -0.0556276 |
| Hipk3 | 3.0804832 |
| Hipk4 | -0.0202748 |
| Hira | -0.7174397 |
| Hira | 2.6551451 |
| Hirip3 | 0.6718796 |
| Hisppd1 | -0.2883287 |
| Hist1h1b | -0.5892746 |
| Hist1h1c | -0.1434543 |
| Hist1h2aa | -0.6095901 |
| Hist1h2ac | 1.0024712 |
| Hist1h2ad | 2.3669221 |
| Hist1h2ae | -0.0749795 |
| Hist1h2af | 1.9052621 |
| Hist1h2ag | -0.4834651 |
| Hist1h2ah | 1.2253933 |
| Hist1h2ak | -0.1417755 |
| Hist1h2an | 0.6013832 |
| Hist1h2ao | 0.3990489 |
| Hist1h2bc | -0.7313334 |
| Hist1h2be | -0.1263854 |
| Hist1h2bf | 3.4423156 |
| Hist1h2bg | 1.3076779 |
| Hist1h2bh | 2.0400509 |
| Hist1h2bj | -0.604811 |
| Hist1h2bk | -0.0927381 |
| Hist1h2bm | 0.5770075 |
| Hist1h2bn | 1.4219417 |
| Hist1h3a | -0.5892746 |
| Hist1h3d | -0.2757016 |
| Hist1h3e | 0.0258525 |
| Hist1h3f | -0.4377949 |
| Hist1h4c | 1.2955642 |
| Hist1h4d | 1.6039771 |
| Hist1h4f | 0.8800948 |
| Hist1h4h | 1.1879096 |
| Hist1h4i | -0.3593498 |
| Hist1h4j | 1.5832043 |
| Hist1h4k | -0.7344486 |
| Hist1h4m | -0.4406579 |
| Hist2h2aa1 | -0.5746465 |
| Hist2h2ab | 0.1908593 |
| Hist2h2ac | -0.6331493 |
| Hist2h2bb | 0.037392 |
| Hist2h2be | 0.7355215 |
| Hist2h3b | -0.5460461 |
| Hist2h3c1 | -0.5825977 |
| Hist2h3c1 | -0.4435294 |
| Hist2h4 | 1.5590787 |
| Hist3h2a | -0.6776665 |
| Hist3h2bb | 0.2014551 |
| Hist4h4 | 0.2002836 |
| Hk1 | -0.3462738 |
| Hk3 | 0.7978532 |
| Hkdc1 | -0.8082113 |
| Hlcs | 1.8949858 |
| Hlf | -0.7653661 |
| Hltf | -0.3252095 |
| Hlx | -0.604811 |
| Hmg20a | 3.384431 |
| Hmg20a | -0.7037452 |
| Hmg20b | -0.5518269 |
| Hmga1 | -0.6317473 |
| Hmgb2 | -0.085595 |
| Hmgb2l1 | 1.5289498 |
| Hmgb2l1 | 1.8736384 |
| Hmgb4 | -0.5059176 |
| Hmgcl | -0.3588233 |
| Hmgcl | 0.5517904 |
| Hmgcl | 2.9302419 |
| Hmgcll1 | -0.5912868 |
| Hmgcs2 | -0.6761976 |
| Hmgn1 | 1.9923691 |
| Hmgn2 | 0.4884914 |
| Hmgn3 | -0.1565657 |
| Hmgn3 | 1.1325873 |
| Hmha1 | -0.5120776 |
| Hmox1 | 0.4253379 |
| Hmox2 | -0.4858671 |
| Hmx1 | -0.2718497 |
| Hmx1 | -0.2041515 |
| Hn1 | 1.9586318 |
| Hn1 | 2.4647552 |
| Hn1l | -0.6261596 |
| Hnf1b | 0.0380869 |
| Hnf1b | -0.4985777 |
| Hnf4a | 3.0377513 |
| Hnrnpc | 2.0992793 |
| Hnrpa1 | 0.1798452 |
| Hnrpa1 | 0.3507256 |
| Hnrpab | -0.0521855 |
| Hnrpc | 3.8960458 |
| Hnrpdl | -0.621297 |
| Hnrpdl | -0.429824 |
| Hnrpf | 0.3256147 |
| Hnrph1 | -0.3369688 |
| Hnrph1 | -0.5647998 |
| Hnrph1 | 1.0376149 |
| Hnrph1 | 0.6798934 |
| Hnrph1 | 0.2781945 |
| Hnrph2 | -0.4888781 |
| Hnrph3 | 2.5958977 |
| Hnrpk | 1.6960681 |
| Hnrpl | 0.3954725 |
| Hnrpll | 1.3811359 |
| Hnrpll | -0.5170343 |
| Hnrpm | 0.1083732 |
| Hnrpm | -0.4258634 |
| Hnrpm | 3.0869764 |
| Hnrpul1 | 1.9484006 |
| Hnrpul1 | 0.5145698 |
| Hnrpul1 | -0.5151725 |
| Hnrpul1 | 0.4594494 |
| Hnrpul1 | -0.4691787 |
| Hnt | 0.3698097 |
| Homer1 | 1.5724023 |
| Homer1 | -0.1181685 |
| Homer1 | -0.2675348 |
| Homer1 | -0.53143 |
| Homez | -0.3001347 |
| Homez | 1.9244296 |
| Hook1 | 0.1497345 |
| Hook1 | -0.4258634 |
| Hook1 | 3.3763911 |
| Hook1 | 0.6981608 |
| Hook2 | 0.9878122 |
| Hormad2 | -0.4446805 |
| Hoxa13 | 0.8612161 |
| Hoxa2 | -0.7120907 |
| Hoxa2 | 0.4718783 |
| Hoxa4 | -0.3405766 |
| Hoxa5 | -0.2467158 |
| Hoxa7 | 1.2947263 |
| Hoxa9 | 1.4758275 |
| Hoxa9 | 2.1219083 |
| Hoxb2 | 1.3079535 |
| Hoxb3 | 2.5070179 |
| Hoxb3 | -0.3931181 |
| Hoxb4 | -0.3774255 |
| Hoxb5 | -0.5687263 |
| Hoxb6 | -0.604811 |
| Hoxb7 | -0.3731415 |
| Hoxc10 | 1.79787 |
| Hoxc10 | 1.6408493 |
| Hoxc6 | 0.1543583 |
| Hoxc9 | -0.5628426 |
| Hoxd10 | -0.6352563 |
| Hoxd3 | 1.1712017 |
| Hoxd4 | 0.3519764 |
| Hoxd8 | 0.570666 |
| Hoxd9 | -0.5151725 |
| Hp | 0.5748323 |
| Hp | 0.2200066 |
| Hp | 0.459226 |
| Hp1bp3 | -0.7182063 |
| Hpca | -0.4798734 |
| Hpcal1 | -0.0419331 |
| Hpcal1 | 1.7859177 |
| Hpcal4 | -0.5859303 |
| Hpgd | -0.688756 |
| Hpn | 0.002707 |
| Hpn | -0.4562679 |
| Hprt1 | 1.4494231 |
| Hprt1 | 1.7326165 |
| Hprt1 | -0.0132853 |
| Hps1 | 0.9692644 |
| Hps3 | 0.7667912 |
| Hps3 | -0.606174 |
| Hps4 | -0.2684919 |
| Hps5 | -0.4650517 |
| Hps6 | -0.695472 |
| Hpse | -0.4219191 |
| Hpx | 1.9555133 |
| Hr | 1.9715094 |
| Hr | -0.5096088 |
| Hras1 | 0.2787346 |
| Hrasls | 0.0059455 |
| Hrasls5 | 2.4065687 |
| Hrasls5 | -0.0749795 |
| Hrb | -0.5301696 |
| Hrb | -0.5195224 |
| Hrbl | -0.571353 |
| Hrbl | 1.2818799 |
| Hrc | 0.3095456 |
| Hrg | -0.3980411 |
| Hrh3 | 0.7550618 |
| Hrk | 1.9628512 |
| Hrmt1l2 | 2.6415156 |
| Hrsp12 | 1.1813244 |
| Hrsp12 | -0.6630781 |
| Hrsp12 | -0.6303473 |
| Hs1bp3 | 1.6877116 |
| Hs2st1 | 0.7943989 |
| Hs3st1 | -0.3504387 |
| Hs3st3a1 | 1.1268301 |
| Hs3st3b1 | 3.6999835 |
| Hs3st6 | 0.9300968 |
| Hs6st1 | 0.3725051 |
| Hs6st2 | -0.545406 |
| Hs6st2 | 0.2849232 |
| Hs6st3 | 2.2309358 |
| Hsbp1 | 1.1842567 |
| Hsd11b1 | -0.3395444 |
| Hsd11b1 | -0.2752192 |
| Hsd11b1 | -0.390394 |
| Hsd11b1 | 0.0792144 |
| Hsd11b2 | -0.181154 |
| Hsd17b1 | -0.6551485 |
| Hsd17b10 | -0.40741 |
| Hsd17b11 | -0.4516161 |
| Hsd17b11 | -0.2095382 |
| Hsd17b11 | 3.2117306 |
| Hsd17b12 | 0.7499669 |
| Hsd17b2 | 0.4784707 |
| Hsd17b3 | -0.3125898 |
| Hsd17b4 | -0.3909382 |
| Hsd17b7 | 1.3885096 |
| Hsd3b1 | -0.4412315 |
| Hsd3b2 | -0.7438555 |
| Hsd3b2 | -0.5544074 |
| Hsd3b4 | 0.0443183 |
| Hsd3b7 | -0.4556852 |
| Hsd3b7 | -0.3625149 |
| Hsdl1 | -0.0110887 |
| Hsf1 | -0.6007336 |
| Hsf4 | -0.495536 |
| Hsf5 | -0.5980249 |
| Hsn2 | 1.9814519 |
| Hsp105 | -0.424171 |
| Hsp90aa1 | -0.1161253 |
| Hsp90ab1 | 0.5170968 |
| Hsp90ab1 | 1.7371981 |
| Hsp90ab1 | 1.5863663 |
| Hsp90b1 | -0.6616315 |
| Hspa12b | 2.7593252 |
| Hspa1a | -0.3344001 |
| Hspa1l | 1.6349324 |
| Hspa2 | -0.4591869 |
| Hspa2 | 2.9558297 |
| Hspa4 | -0.5726691 |
| Hspa4l | -0.4632885 |
| Hspa5 | 0.3966661 |
| Hspa9 | -0.5326921 |
| Hspa9 | 2.1126995 |
| Hspb1 | -0.4918987 |
| Hspb2 | 1.6787207 |
| Hspb3 | -0.5016291 |
| Hspb6 | -0.6408976 |
| Hspb7 | 1.3314537 |
| Hspb8 | 0.3761685 |
| Hspbap1 | -0.5022406 |
| Hspd1 | -0.5220169 |
| Hspd1 | -0.4888781 |
| Hspe1 | 2.4705086 |
| Hspg2 | 0.5073734 |
| Htatip | 1.3143622 |
| Htatip | -0.6580244 |
| Htatip2 | -0.6924813 |
| Htatip2 | 1.0492485 |
| Htatsf1 | -0.6014119 |
| Htf9c | -0.6761976 |
| Htr1a | -0.7266797 |
| Htr1b | -0.4949288 |
| Htr1d | 0.1491164 |
| Htr1f | -0.5207688 |
| Htr1f | 0.1656686 |
| Htr2b | 1.1441075 |
| Htr3a | 0.6525501 |
| Htr5b | -0.1405184 |
| Htr7 | -0.28784 |
| Htra1 | 2.7228381 |
| Htra1 | 1.403005 |
| Htra2 | -0.1582707 |
| Htra3 | 3.7423312 |
| Htra3 | 1.1166709 |
| Htt | 3.0668098 |
| Hus1 | 1.8615234 |
| Hus1 | 1.8734352 |
| Hus1 | -0.1591243 |
| Huwe1 | 2.7647139 |
| Hvcn1 | -0.2514077 |
| Hvcn1 | 0.3027256 |
| Hyal1 | 0.4040336 |
| Hyal2 | -0.5498961 |
| Hyal3 | -0.2443785 |
| Hyal5 | 0.2905388 |
| Hyal6 | -0.4562679 |
| Hydin | -0.3630434 |
| Hyi | -0.6192206 |
| Hyou1 | -0.1214468 |
| Iah1 | 1.4224307 |
| Iap | -0.7052572 |
| Iars | -0.712853 |
| Iars | 0.3751929 |
| Iars2 | -0.6465724 |
| Ibtk | 2.1998286 |
| Ica1 | -0.751766 |
| Ica1l | -0.2694501 |
| Icam1 | 1.4420281 |
| Icam2 | -0.4539392 |
| Icam4 | 1.8225147 |
| Icam5 | -0.1442948 |
| Ick | -0.0005424 |
| Icmt | 0.030415 |
| Ict1 | 1.7862517 |
| Id1 | -0.1872914 |
| Id2 | -0.7197413 |
| Id2 | 1.7044735 |
| Id3 | 1.0046192 |
| Id4 | -0.3201416 |
| Ide | 0.1180047 |
| Idh1 | 0.4000004 |
| Idh2 | 2.0580576 |
| Idh3a | -0.5608894 |
| Idh3b | -0.556995 |
| Idh3g | 2.1624329 |
| Idh3g | 2.604725 |
| Idi2 | -0.5182775 |
| Ids | -0.73601 |
| Ids | 2.0329412 |
| Ids | 1.3805402 |
| Idua | 1.3716448 |
| Ier3 | -0.2834524 |
| Ier3ip1 | -0.6137056 |
| Ier5l | 1.857089 |
| Ifi202b | 1.0411441 |
| Ifi202b | 0.5746343 |
| Ifi27 | 0.7709916 |
| Ifi30 | -0.6623545 |
| Ifi30 | -0.6240726 |
| Ifi35 | 1.3682875 |
| Ifi47 | 4.1242983 |
| Ifit2 | 1.3050095 |
| Ifit2 | -0.4858671 |
| Ifit3 | -0.6551485 |
| Ifit3 | 0.0808764 |
| Ifitm1 | -0.466229 |
| Ifitm1 | 0.8250465 |
| Ifitm1 | -0.52954 |
| Ifitm2 | 0.9287313 |
| Ifitm3 | 1.5016713 |
| Ifitm5 | -0.7259063 |
| Ifitm6 | -0.6820869 |
| Ifna1 | -0.5213927 |
| Ifna6 | -0.7454324 |
| Ifnar2 | 1.3054701 |
| Ifnar2 | 2.9085655 |
| Ifnar2 | 0.0233867 |
| Ifngr1 | -0.3747457 |
| Ifngr2 | 0.052222 |
| Ifngr2 | -0.1075912 |
| Ifngr2 | -0.7212788 |
| Ifnz | -0.2295537 |
| Ifrd1 | 0.3424284 |
| Ifrg15 | -0.198795 |
| Ift122 | -0.6150814 |
| Ift140 | -0.5693823 |
| Ift140 | 0.5109784 |
| Ift140 | -0.3996878 |
| Ift172 | -0.3070895 |
| Ift172 | 1.6327776 |
| Ift172 | -0.5892746 |
| Ift20 | -0.0816496 |
| Ift20 | 2.1636334 |
| Ift20 | 0.0163063 |
| Ift52 | 1.618685 |
| Ift80 | -0.8303772 |
| Ift81 | 0.4986225 |
| Ift81 | 1.2501097 |
| Ift81 | -0.5706957 |
| Ift88 | 1.1336904 |
| Igbp1 | 0.2061269 |
| Igbp1b | -0.7832589 |
| Igf1 | -0.4002373 |
| Igf1 | 0.9300968 |
| Igf1r | 0.7685706 |
| Igf2 | -0.5352214 |
| Igf2bp1 | -0.5772896 |
| Igf2bp2 | -0.6573046 |
| Igf2bp3 | 2.2329601 |
| Igf2bp3 | 4.3608163 |
| Igf2r | -0.1092109 |
| Igf2r | -0.6472842 |
| Igfals | -0.2637156 |
| Igfbp2 | -0.0306692 |
| Igfbp2 | 0.7682473 |
| Igfbp3 | -0.3779623 |
| Igfbp4 | -0.1007381 |
| Igfbp4 | -0.3457545 |
| Igfbp5 | 0.2449403 |
| Igfbp6 | -0.5447662 |
| Igfbp7 | -0.7865484 |
| Igfbpl1 | 0.3222677 |
| Igsf1 | -0.7328898 |
| Igsf1 | -0.5213927 |
| Igsf1 | 2.3309846 |
| Igsf1 | 0.2365132 |
| Igsf11 | -0.3001347 |
| Igsf11 | -0.753356 |
| Igsf11 | -0.3257177 |
| Igsf21 | -0.1447153 |
| Igsf3 | -0.4762952 |
| Igsf4a | 0.1005974 |
| Igsf5 | -0.1886117 |
| Igsf8 | 0.2966517 |
| Igsf9 | -0.524518 |
| Igtp | 1.2788542 |
| Ihh | 0.0818722 |
| Ihpk1 | 1.5049652 |
| Ihpk2 | 0.0328628 |
| Ihpk2 | 1.0929206 |
| Iigp2 | 0.4096986 |
| Ik | 0.5170968 |
| Ikbkap | -0.4498774 |
| Ikbkb | -0.8056865 |
| Ikbke | 0.3918826 |
| Ikbkg | 2.4635237 |
| Ikbkg | -0.7791628 |
| Il10ra | 0.0989695 |
| Il10rb | -0.4627015 |
| Il11 | 0.9785185 |
| Il11ra1 | 2.3250196 |
| Il12a | 0.9662473 |
| Il13 | -0.5667611 |
| Il13ra1 | 0.7667912 |
| Il13ra2 | -0.7414951 |
| Il15 | 0.1196005 |
| Il15 | 1.6310765 |
| Il15 | 0.1141637 |
| Il15ra | -0.5157927 |
| Il15ra | 1.0019651 |
| Il15ra | 1.5430565 |
| Il16 | 0.3995248 |
| Il17d | 1.6801525 |
| Il17d | -0.5812679 |
| Il17ra | 0.4061619 |
| Il17rb | -0.8269334 |
| Il17rc | 0.0460421 |
| Il17rc | -0.6638023 |
| Il17rc | -0.5819325 |
| Il17rd | 1.786029 |
| Il17rd | 2.0354313 |
| Il17re | 0.4729802 |
| Il17re | 1.6735985 |
| Il17re | 2.2203964 |
| Il18 | 3.5835085 |
| Il18bp | 0.6046507 |
| Il18r1 | 2.05243 |
| Il1b | -0.421357 |
| Il1r1 | 1.761783 |
| Il1rap | -0.4360813 |
| Il1rap | 0.5967511 |
| Il1rap | -0.3784995 |
| Il1rap | -0.8217908 |
| Il1rap | -0.3604037 |
| Il1rl1l | 1.6600363 |
| Il1rl2 | 0.5990699 |
| Il20ra | 0.2610249 |
| Il20rb | -0.34161 |
| Il20rb | -0.3499171 |
| Il23a | 1.0028506 |
| Il27ra | -0.2932302 |
| Il28ra | -0.4967515 |
| Il28ra | 2.2871083 |
| Il28ra | 2.4149325 |
| Il31ra | -0.212243 |
| Il33 | -0.7045009 |
| Il3ra | 0.020207 |
| Il4i1 | 0.8629745 |
| Il6ra | -0.3045998 |
| Il6st | 1.1693979 |
| Il7 | 2.714558 |
| Ildr1 | 0.0233867 |
| Ildr1 | 0.2305696 |
| Ilf2 | 0.0460421 |
| Ilf3 | -0.5182775 |
| Ilf3 | -0.0224926 |
| Ilf3 | 0.3754369 |
| Ilf3 | 2.1128577 |
| Ilk | 1.4050816 |
| Ilkap | -0.6095901 |
| Ilvbl | 1.3456873 |
| Ilvbl | -0.7212788 |
| Ilvbl | -0.2937217 |
| Ilvbl | -0.7205098 |
| Immp2l | -0.1535893 |
| Immt | -0.6999758 |
| Imp3 | 0.8327838 |
| Imp4 | -0.4804711 |
| Imp4 | -0.3441982 |
| Impa2 | 3.3620415 |
| Impact | 1.6406548 |
| Impad1 | 0.8386586 |
| Impdh1 | -0.1096162 |
| Impdh2 | 0.3050919 |
| Ina | 0.6541911 |
| Inadl | -0.6902444 |
| Inadl | 1.2684754 |
| Incenp | 0.1693082 |
| Indo | 1.7947305 |
| Ing2 | -0.4029897 |
| Ing3 | -0.0510409 |
| Ing3 | 0.4169666 |
| Ing3 | 0.2697846 |
| Ing4 | 1.2974236 |
| Ing4 | -0.7407096 |
| Ing4 | 1.2956573 |
| Ing5 | -0.2732923 |
| Inha | -0.4846654 |
| Inhba | 0.4683437 |
| Inmt | 0.8776471 |
| Inoc1 | -0.6437308 |
| Inpp4a | -0.6041302 |
| Inpp4a | 0.2787346 |
| Inpp4a | 0.0450082 |
| Inpp4b | -0.712853 |
| Inpp5a | 0.4934627 |
| Inpp5d | 0.3082378 |
| Inpp5e | 0.3100682 |
| Inpp5f | -0.3614587 |
| Inppl1 | -0.7597426 |
| Inppl1 | 0.1469495 |
| Inppl1 | -0.5899448 |
| Ins2 | -0.1561399 |
| Insc | 0.1837295 |
| Insig1 | -0.6130184 |
| Insig1 | 1.9909776 |
| Insig1 | 0.6155257 |
| Insig2 | 1.0545335 |
| Insig2 | -0.3882203 |
| Insl3 | 1.0682102 |
| Insl5 | -0.0702992 |
| Insl6 | -0.6894999 |
| Insm1 | -0.1578442 |
| Ints1 | -0.2313943 |
| Ints12 | -0.1198062 |
| Ints3 | -0.3828076 |
| Ints3 | 2.1203404 |
| Ints4 | 1.1619344 |
| Ints5 | -0.5028524 |
| Ints7 | 1.1199202 |
| Ints9 | -0.2126946 |
| Ints9 | -0.4124075 |
| Ipmk | -0.755746 |
| Ipmk | 2.9235335 |
| Ipo13 | 0.5179377 |
| Ipo4 | -0.1384268 |
| Ipo5 | 3.3061077 |
| Ipp | 0.9213234 |
| Ippk | -0.4912938 |
| Iqcb1 | 2.8154018 |
| Iqcb1 | -0.4609426 |
| Iqcd | 0.423484 |
| Iqce | -0.8023305 |
| Iqcf3 | 0.5644822 |
| Iqcf4 | -0.5046903 |
| Iqgap1 | -0.7182063 |
| Iqgap2 | 1.3569234 |
| Iqgap3 | 2.3192082 |
| Iqsec2 | 0.4981936 |
| Iqsec3 | -0.3688766 |
| Irak1 | -0.5879355 |
| Irak1bp1 | -0.7243613 |
| Irak2 | 1.0120369 |
| Irak2 | 1.2742025 |
| Irak3 | 0.9417606 |
| Irak4 | 0.8079866 |
| Irak4 | -0.4040929 |
| Irak4 | -0.5845958 |
| Ireb2 | 0.5075858 |
| Irf1 | -0.3641013 |
| Irf1 | -0.7321113 |
| Irf1 | -0.6054923 |
| Irf1 | 1.4815888 |
| Irf2 | -0.4264282 |
| Irf2bp1 | 0.4910883 |
| Irf3 | -0.0074389 |
| Irf3 | 1.8154147 |
| Irf3 | 2.7461364 |
| Irf4 | -0.6515658 |
| Irf5 | -0.28784 |
| Irf6 | -0.150199 |
| Irf6 | 1.6436325 |
| Irf7 | 1.2210768 |
| Irf8 | 0.3058793 |
| Irf9 | 0.0610785 |
| Irf9 | 2.2873389 |
| Irgm | 2.0177751 |
| Irgm | -0.564147 |
| Irgq | 0.0792144 |
| Irgq | 0.7445133 |
| Irgq | -0.5832633 |
| Irs2 | 0.9366244 |
| Irs3 | 0.7191537 |
| Irx2 | 0.1122374 |
| Irx3 | -0.2873515 |
| Irx5 | 0.291871 |
| Isca1 | -0.5912868 |
| Iscu | -0.5114598 |
| Isg20 | -0.1247363 |
| Isg20l1 | 0.0191447 |
| Isg20l2 | -0.6681587 |
| Isl1 | -0.4487201 |
| Islr2 | 2.6279654 |
| Isy1 | 1.510851 |
| Isyna1 | -0.4174313 |
| Itfg2 | 0.2585467 |
| Itfg3 | 2.4551623 |
| Itga1 | -0.5845958 |
| Itga11 | 0.3614299 |
| Itga11 | 0.5254723 |
| Itga2b | 1.692943 |
| Itga3 | -0.4469868 |
| Itga5 | -0.5089926 |
| Itga5 | -0.7661722 |
| Itga6 | -0.4967515 |
| Itga7 | 0.7056127 |
| Itgad | 1.2664628 |
| Itgad | -0.4389391 |
| Itgad | -0.3509606 |
| Itgae | 0.541391 |
| Itgae | -0.6401906 |
| Itgae | 0.6040749 |
| Itgav | 2.3026614 |
| Itgb1 | 1.6061305 |
| Itgb1 | 1.0910778 |
| Itgb1bp1 | -0.6416051 |
| Itgb3bp | 0.3474656 |
| Itgb4 | 0.6634372 |
| Itgb4 | -0.0262006 |
| Itgb5 | 0.240736 |
| Itgb6 | 2.3517736 |
| Itgb6 | 0.9378434 |
| Itgb6 | 2.0622358 |
| Itgb7 | -0.2383282 |
| Itgbl1 | 0.8279364 |
| Itih2 | 0.1466396 |
| Itih3 | 1.6869089 |
| Itih5 | -0.0457175 |
| Itih5 | -0.6501364 |
| Itk | -0.3535743 |
| Itlnb | 0.600998 |
| Itm2a | 0.7602927 |
| Itm2b | -0.3467934 |
| Itm2b | 0.660003 |
| Itpk1 | -0.6472842 |
| Itpka | -0.4691787 |
| Itpkc | 0.2237313 |
| Itpr1 | -0.5865982 |
| Itpr2 | -0.7228188 |
| Itpr3 | -0.7290037 |
| Itsn1 | 1.8514909 |
| Itsn2 | -0.6345534 |
| Ivd | -0.3963973 |
| Ivns1abp | -0.660187 |
| Ivns1abp | -0.7212788 |
| Iws1 | -0.6667044 |
| Iyd | 1.3575341 |
| Izumo1 | 3.0180698 |
| Jag1 | 0.4389009 |
| Jag2 | 2.3007473 |
| Jagn1 | -0.4269933 |
| Jak1 | -0.4912938 |
| Jak2 | 2.5086388 |
| Jak2 | -0.5428496 |
| Jak3 | 1.7340286 |
| Jak3 | -0.4196726 |
| Jakmip1 | 0.2223004 |
| Jam2 | -0.4174313 |
| Jam2 | 1.1247179 |
| Jam2 | -0.5772896 |
| Jam2 | 2.45378 |
| Jam3 | -0.2374007 |
| Jam4 | 0.4213939 |
| Jarid1b | 0.5922908 |
| Jarid1c | -0.1846562 |
| Jarid1d | 2.6956986 |
| Jazf1 | -0.5946499 |
| Jdp2 | -0.4967515 |
| Jmjd1a | 0.4281121 |
| Jmjd1a | -0.2509375 |
| Jmjd1a | -0.8124342 |
| Jmjd2a | -0.5371227 |
| Jmjd2b | 1.0747999 |
| Jmjd2b | -0.3221655 |
| Jmjd2c | -0.6667044 |
| Jmjd2d | -0.6638023 |
| Jmjd3 | -0.1842178 |
| Jmjd3 | 2.1955236 |
| Jmjd5 | -0.4906893 |
| Jmjd6 | -0.6761976 |
| Jmy | 1.8464348 |
| Josd1 | -0.0019902 |
| Josd2 | -0.5987014 |
| Josd3 | -0.0745886 |
| Josd3 | 3.1295301 |
| Jph1 | -0.7014818 |
| Jph2 | 0.0573416 |
| Jph3 | 1.8487837 |
| Jph4 | 0.9332302 |
| Jph4 | -0.5589402 |
| Jtb | 0.6260906 |
| Jtv1 | -0.4527769 |
| Jtv1 | -0.4906893 |
| Jun | 0.4301875 |
| Junb | -0.4870704 |
| Jund1 | 1.8885065 |
| Jup | -0.3287731 |
| Kal1 | 2.3461543 |
| Kalrn | -0.3561952 |
| Kank1 | -0.2204037 |
| Kank3 | -0.1565657 |
| Kank4 | 0.6632568 |
| Kank4 | 1.4472027 |
| Kank4 | -0.2723303 |
| Kars | 1.0919996 |
| Katna1 | -0.5753066 |
| Katnal1 | 1.9289463 |
| Katnb1 | 0.1303821 |
| Kazald1 | 1.692329 |
| Kbtbd2 | -0.258489 |
| Kbtbd2 | -0.6769318 |
| Kbtbd4 | -0.2981569 |
| Kbtbd4 | -0.7182063 |
| Kbtbd7 | 0.1419783 |
| Kbtbd7 | -0.0288047 |
| Kbtbd8 | 0.8503007 |
| Kcmf1 | -0.4360813 |
| Kcna1 | 0.0871656 |
| Kcna2 | -0.5371227 |
| Kcna3 | 0.879951 |
| Kcna5 | 0.4157981 |
| Kcna6 | -0.4727303 |
| Kcnab1 | -0.7029901 |
| Kcnab1 | -0.5301696 |
| Kcnab1 | 1.7229111 |
| Kcnab2 | -0.3909382 |
| Kcnab3 | 1.4525863 |
| Kcnb1 | -0.7573426 |
| Kcnc1 | 0.6908256 |
| Kcnc1 | 0.9881975 |
| Kcnc4 | -0.5518269 |
| Kcnc4 | 0.5781918 |
| Kcnd2 | -0.7758985 |
| Kcne1 | -0.8090544 |
| Kcne1l | -0.524518 |
| Kcne2 | -0.4751054 |
| Kcne4 | -0.6977213 |
| Kcnf1 | -0.421357 |
| Kcng4 | -0.5071464 |
| Kcnh1 | 2.861045 |
| Kcnh2 | 1.8942404 |
| Kcnh2 | -0.706014 |
| Kcnh3 | 0.3651359 |
| Kcnh5 | -0.1724542 |
| Kcnip1 | -0.3742107 |
| Kcnip2 | -0.3369688 |
| Kcnip2 | -0.4101832 |
| Kcnip2 | -0.2800537 |
| Kcnip3 | 2.4064815 |
| Kcnip4 | -0.5345885 |
| Kcnj12 | -0.1011399 |
| Kcnj16 | 2.4778744 |
| Kcnj2 | 0.6025377 |
| Kcnj3 | -0.8107428 |
| Kcnj4 | 0.4936782 |
| Kcnj6 | 0.0768828 |
| Kcnj6 | 1.9914716 |
| Kcnj8 | 2.8228364 |
| Kcnj9 | 2.4241945 |
| Kcnk1 | 0.4356999 |
| Kcnk10 | 1.3593638 |
| Kcnk12 | -0.597349 |
| Kcnk13 | -0.5164133 |
| Kcnk2 | -0.7189735 |
| Kcnk2 | 0.8998758 |
| Kcnk3 | -0.0844097 |
| Kcnk4 | 0.4011884 |
| Kcnk5 | 0.1086958 |
| Kcnk5 | 0.4186001 |
| Kcnk6 | 0.4687862 |
| Kcnk6 | 0.1012478 |
| Kcnk9 | 2.2894772 |
| Kcnma1 | 0.030415 |
| Kcnmb1 | 0.0491371 |
| Kcnmb4 | 1.901076 |
| Kcnmb4 | 0.1741395 |
| Kcnn1 | 2.31771 |
| Kcnn2 | -0.6580244 |
| Kcnn4 | 0.7895102 |
| Kcnq1 | 1.0049978 |
| Kcnq2 | 0.0173717 |
| Kcnq2 | -0.448142 |
| Kcnq3 | 1.0243553 |
| Kcnq5 | -0.3947563 |
| Kcnrg | 0.270057 |
| Kcnrg | -0.6894999 |
| Kcns1 | -0.9844842 |
| Kcns2 | 2.4539736 |
| Kcnt1 | -0.1267981 |
| Kcnu1 | 1.5923182 |
| Kcnv1 | 0.6417219 |
| Kcnv2 | -0.0096271 |
| Kctd10 | -0.3619866 |
| Kctd11 | 2.1752962 |
| Kctd12 | -0.674731 |
| Kctd12b | 2.5633075 |
| Kctd12b | 0.9710963 |
| Kctd14 | -0.7336689 |
| Kctd14 | -0.8399122 |
| Kctd15 | 0.587028 |
| Kctd18 | 1.2309856 |
| Kctd19 | 1.1097998 |
| Kctd2 | -0.0247157 |
| Kctd20 | -0.276667 |
| Kctd20 | -0.4973598 |
| Kctd3 | 0.4042703 |
| Kctd4 | 0.2177073 |
| Kctd5 | 0.6545554 |
| Kctd5 | 1.0589553 |
| Kctd6 | 1.9166436 |
| Kctd6 | 0.9939581 |
| Kctd9 | -0.8048464 |
| Kdelc1 | -0.4774864 |
| Kdelc1 | 1.6255597 |
| Kdelr2 | -0.5919585 |
| Kdelr3 | 1.163966 |
| Kdr | -0.7090475 |
| Keap1 | 0.0718673 |
| Keap1 | -0.1903749 |
| Keap1 | 0.4551954 |
| Keap1 | -0.6544309 |
| Kera | 0.2894717 |
| Khdc1c | 2.7312953 |
| Khdrbs1 | -0.4281246 |
| Khdrbs2 | -0.4733235 |
| Khdrbs3 | 1.1857196 |
| Khk | -0.3100857 |
| Khk | 2.1693522 |
| Khsrp | 0.2443808 |
| Kif11 | -0.7923326 |
| Kif13a | -0.7266797 |
| Kif14 | -0.2737737 |
| Kif15 | 0.1589597 |
| Kif17 | 0.8582783 |
| Kif17 | 0.2557856 |
| Kif18a | -0.5667611 |
| Kif1a | 0.5135149 |
| Kif1b | -0.34161 |
| Kif1b | 2.0724369 |
| Kif1b | 0.9243483 |
| Kif1b | 1.1594693 |
| Kif1b | -0.5479692 |
| Kif1b | 1.7550778 |
| Kif1c | 2.099199 |
| Kif21a | -0.414637 |
| Kif21b | -0.7305562 |
| Kif22 | -0.1388448 |
| Kif23 | 0.3207189 |
| Kif24 | 2.1485184 |
| Kif27 | -0.1493533 |
| Kif2a | 0.3544733 |
| Kif2b | 1.9604424 |
| Kif2c | -0.4780826 |
| Kif3a | 2.1745181 |
| Kif3a | 3.2436366 |
| Kif3b | 0.9420302 |
| Kif3c | 0.4969057 |
| Kif3c | -0.8973607 |
| Kif4 | 1.1100259 |
| Kif4 | 0.8341427 |
| Kif4 | 1.1340211 |
| Kif5a | -0.7791628 |
| Kif5a | -0.384969 |
| Kif5a | 2.6774884 |
| Kif5b | 1.2096396 |
| Kif5c | 0.9191173 |
| Kif9 | -0.7549487 |
| Kifap3 | 1.9967548 |
| Kifap3 | 1.2271944 |
| Kifap3 | 0.2773839 |
| Kifc2 | 0.9865268 |
| Kifc2 | -0.163404 |
| Kifc3 | 0.9402767 |
| Kin | -0.8141286 |
| Kirrel3 | -0.6345534 |
| Kiss1 | -0.6359596 |
| Kiss1r | 0.4923842 |
| Kitl | -0.6565854 |
| Kl | 0.3861105 |
| Klb | -0.5654531 |
| Klc1 | -0.212243 |
| Klc1 | 0.8976196 |
| Klc1 | -0.5289108 |
| Klc2 | -0.1210364 |
| Klc2 | 0.0508522 |
| Klc4 | 1.9001872 |
| Klf1 | -0.5120776 |
| Klf10 | -0.0971298 |
| Klf12 | 0.172934 |
| Klf12 | -0.4985777 |
| Klf12 | 1.1309301 |
| Klf13 | 0.6461414 |
| Klf16 | -0.5164133 |
| Klf17 | 0.5250552 |
| Klf17 | -0.2599116 |
| Klf2 | 2.2611291 |
| Klf4 | -0.2090882 |
| Klf4 | 2.4255918 |
| Klf4 | -0.4638759 |
| Klf5 | 1.4944448 |
| Klf6 | -0.6537138 |
| Klf6 | -0.2304735 |
| Klf7 | -0.5576429 |
| Klf9 | 1.9765848 |
| Klhdc1 | 1.805865 |
| Klhdc1 | 1.6197459 |
| Klhdc1 | -0.8048464 |
| Klhdc2 | -0.3925727 |
| Klhdc3 | -0.7045009 |
| Klhdc4 | 1.8400899 |
| Klhdc4 | 2.0343158 |
| Klhdc4 | -0.1527406 |
| Klhdc8b | -0.6150814 |
| Klhdc9 | 0.5175173 |
| Klhl10 | -0.8709003 |
| Klhl11 | -0.1305205 |
| Klhl12 | -0.5825977 |
| Klhl14 | -0.6932281 |
| Klhl15 | -0.0370358 |
| Klhl15 | -0.395303 |
| Klhl17 | -0.7799806 |
| Klhl2 | 1.1957936 |
| Klhl21 | 2.7188642 |
| Klhl22 | 0.0914449 |
| Klhl22 | -0.2406507 |
| Klhl23 | 1.0271946 |
| Klhl24 | 0.8167852 |
| Klhl24 | 0.2643192 |
| Klhl25 | -0.4475642 |
| Klhl26 | 0.0566605 |
| Klhl26 | -0.0633214 |
| Klhl30 | 2.1884634 |
| Klhl31 | -0.4124075 |
| Klhl32 | 1.3272319 |
| Klhl5 | 0.5354267 |
| Klhl5 | -0.3763527 |
| Klhl6 | 2.9798889 |
| Klhl6 | -0.6007336 |
| Klhl7 | -0.52954 |
| Klhl8 | 1.6021563 |
| Klhl9 | 0.7107775 |
| Klhl9 | -0.0702992 |
| Klk1 | 0.2188577 |
| Klk11 | 1.9917409 |
| Klk11 | -0.6430217 |
| Klk15 | -0.4179911 |
| Klk1b1 | -0.7525607 |
| Klk1b11 | 0.3189089 |
| Klk1b16 | 0.0405151 |
| Klk1b21 | 0.8467336 |
| Klk1b21 | 0.2257308 |
| Klk1b22 | 0.0979914 |
| Klk1b24 | -0.5176557 |
| Klk1b26 | 1.9783617 |
| Klk1b27 | -0.4870704 |
| Klk1b27 | 0.3199436 |
| Klk1b4 | -0.8065274 |
| Klk1b4 | 2.4473121 |
| Klk1b5 | -0.785725 |
| Klk1b8 | 0.0123895 |
| Klk1b9 | 1.2565295 |
| Klk6 | 0.0230339 |
| Klk8 | -0.6630781 |
| Klkb1 | 0.0106039 |
| Klra17 | 0.6995516 |
| Klra4 | -0.7653661 |
| Klrd1 | 0.5787835 |
| Kndc1 | 1.2590467 |
| Kng1 | -0.0722465 |
| Kng1 | -0.755746 |
| Kng2 | 0.5969445 |
| Kns2 | 0.1641479 |
| Kns2 | -0.4527769 |
| Kntc1 | 1.1977545 |
| Kpna1 | 0.0048672 |
| Kpna2 | -0.6977213 |
| Kpna3 | -0.6123318 |
| Kpna6 | 0.390923 |
| Kpnb1 | -0.0879701 |
| Kptn | -0.0610067 |
| Kras | 1.4007571 |
| Kras | -0.3374834 |
| Krba1 | -0.5518269 |
| Krba1 | 1.7487306 |
| Krba1 | -0.4792761 |
| Krcc1 | 0.1109511 |
| Kremen | 2.8482278 |
| Kremen1 | -0.6739985 |
| Kremen2 | -0.5980249 |
| Krit1 | 0.1680965 |
| Krit1 | 1.779941 |
| Krr1 | -0.5213927 |
| Krr1 | 0.0219748 |
| Krr1 | -0.5059176 |
| Krt10 | 2.096146 |
| Krt12 | -0.6806111 |
| Krt13 | 2.852574 |
| Krt14 | -0.0586977 |
| Krt17 | -0.7422812 |
| Krt18 | -0.5550537 |
| Krt19 | 1.0384679 |
| Krt2 | -0.6338511 |
| Krt20 | 2.2629553 |
| Krt222 | -0.7653661 |
| Krt23 | 0.2557856 |
| Krt27 | 0.4085212 |
| Krt32 | 2.2160768 |
| Krt4 | 1.4003403 |
| Krt7 | -0.6776665 |
| Krt73 | 0.4108746 |
| Krt75 | 0.0265559 |
| Krt78 | 3.1470629 |
| Krt79 | -0.3694087 |
| Krt8 | -0.6458612 |
| Krt84 | -0.5739869 |
| Krt86 | -0.2235962 |
| Krt86 | -0.4906893 |
| Krt9 | -0.7454324 |
| Krtap16-4 | -0.0644808 |
| Krtap8-2 | -0.6529972 |
| Krtcap2 | -0.0679676 |
| Krtcap3 | 2.903915 |
| Ksr2 | -0.6416051 |
| Ktelc1 | 0.3574609 |
| Kti12 | 1.3581444 |
| Ktn1 | 1.2802737 |
| Ktn1 | -0.7235897 |
| Ky | -0.265146 |
| L1cam | 2.7365149 |
| L2hgdh | 0.8422559 |
| L3mbtl2 | 1.1264969 |
| L3mbtl3 | 0.7709916 |
| l7Rn6 | 1.3283116 |
| Lace1 | 1.6327123 |
| Lad1 | -0.7422812 |
| Lag3 | 2.1524334 |
| Lage3 | -0.145978 |
| Lama1 | -0.2172218 |
| Lama2 | 0.3441945 |
| Lamb1-1 | -0.7120907 |
| Lamb2 | 2.2494472 |
| Lamb3 | -0.4580182 |
| Lamc1 | 1.6820789 |
| Lamc2 | -0.4013373 |
| Lamc2 | 0.096359 |
| Lamc2 | -0.6508508 |
| Lamc3 | 0.3183911 |
| Lamp1 | 0.2555091 |
| Lamp2 | -0.0129189 |
| Lamp2 | -0.623378 |
| Lamp2 | -0.4539392 |
| Lamp2 | -0.4286908 |
| Lancl1 | -0.6529972 |
| Lancl1 | 0.4745207 |
| Lancl1 | 0.6623541 |
| Laptm4a | -0.6289493 |
| Laptm4b | -0.495536 |
| Laptm4b | -0.0103576 |
| Laptm5 | -0.6352563 |
| Large | 0.0607394 |
| Larp2 | 1.674162 |
| Larp5 | -0.33235 |
| Larp6 | -0.4326632 |
| Larp6 | 0.3522264 |
| Lars | 1.4520335 |
| Lars2 | 0.0973387 |
| Las1l | -0.5550537 |
| Lasp1 | 1.3903652 |
| Lasp1 | -0.6289493 |
| Lasp1 | -0.4668182 |
| Lass1 | 1.0455073 |
| Lass2 | -0.1400997 |
| Lass2 | -0.6268563 |
| Lass2 | -0.5511829 |
| Lass4 | -0.5133143 |
| Lass4 | -0.009262 |
| Lass5 | 0.3990489 |
| Lass6 | 0.4464038 |
| Lat2 | -0.5409368 |
| Lat2 | -0.5170343 |
| Lats2 | -0.6178389 |
| Lbh | -0.5886048 |
| Lbh | -0.5779515 |
| Lbp | -0.8557233 |
| Lbr | -0.468588 |
| Lbx2 | -0.6494226 |
| Lca5 | 1.9067842 |
| Lca5 | -0.163404 |
| Lca5 | -0.3011251 |
| Lcat | 0.0566605 |
| Lcat | 1.1220431 |
| Lce1c | 0.1979362 |
| Lck | -0.5383924 |
| Lcmt1 | -0.2665785 |
| Lcmt1 | 0.8276326 |
| Lcmt1 | -0.5071464 |
| Lcn10 | -0.7824382 |
| Lcn12 | 1.8212277 |
| Lcn13 | 1.7198727 |
| Lcn2 | -0.5550537 |
| Lcn3 | -0.6458612 |
| Lcn8 | -0.6289493 |
| Lcor | 0.9747495 |
| Lcp1 | -0.3272442 |
| Ldb1 | -0.2570686 |
| Ldb2 | 0.787611 |
| Ldb2 | -0.3699411 |
| Ldha | -0.632448 |
| Ldhc | -0.2374007 |
| Ldhc | -0.265146 |
| Ldhc | 2.6004402 |
| Ldhd | 1.249524 |
| Ldhd | -0.2014695 |
| Ldoc1 | 2.3473303 |
| Ldoc1l | 2.6935058 |
| Lef1 | 0.121512 |
| Lef1 | 0.1426011 |
| Lefty1 | 1.2827292 |
| Lefty2 | -0.7597426 |
| Lemd2 | 1.9669598 |
| Leng1 | 1.6385129 |
| Leng8 | 0.8789437 |
| Leng9 | -0.7228188 |
| Leo1 | -0.155289 |
| Lep | 0.801295 |
| Lepre1 | -0.7251335 |
| Lepre1 | -0.5251443 |
| Lepre1 | 0.4756196 |
| Leprel1 | 0.3363485 |
| Leprel2 | -0.407964 |
| Leprotl1 | 2.3433632 |
| Letm1 | 1.0468365 |
| Letm1 | 2.6146877 |
| Letm1 | 0.8756267 |
| Letmd1 | 3.5593781 |
| Lgals1 | -0.2135983 |
| Lgals2 | -0.807369 |
| Lgals3 | -0.1850949 |
| Lgals3bp | 0.7242459 |
| Lgals4 | -0.4516161 |
| Lgals4 | 4.5250935 |
| Lgals6 | -0.1321795 |
| Lgals7 | -0.623378 |
| Lgals8 | 0.2549557 |
| Lgals8 | 0.6702688 |
| Lgals9 | 0.9144126 |
| Lgals9 | 0.3798178 |
| Lgi1 | 0.9007205 |
| Lgi1 | 1.1892628 |
| Lgi2 | -0.6289493 |
| Lgi3 | -0.4168718 |
| Lgi4 | 0.2838498 |
| Lgmn | 2.1773679 |
| Lgr5 | 3.1495358 |
| Lgtn | 0.0871656 |
| Lgtn | -0.3333745 |
| Lhb | -0.1794077 |
| Lhcgr | -0.118987 |
| Lhcgr | 0.2538481 |
| Lhfp | -0.414637 |
| Lhfp | -0.0586977 |
| Lhfp | 0.7682473 |
| Lhfpl1 | 1.728486 |
| Lhfpl2 | -0.7832589 |
| Lhx1 | 3.1520289 |
| Lhx2 | -0.424171 |
| Lhx3 | -0.5832633 |
| Lhx4 | -0.5473277 |
| Lhx9 | -0.0734169 |
| Lhx9 | 0.3519764 |
| Lias | 2.3292211 |
| Lias | 1.5987075 |
| Lias | 2.3004225 |
| Lifr | -0.5544074 |
| Lig1 | -0.8252161 |
| Lig1 | 0.8919557 |
| Lig1 | 1.061099 |
| Lig3 | -0.8373023 |
| Lig4 | 1.0502116 |
| Lima1 | -0.0664163 |
| Lima1 | 1.1923783 |
| Limd1 | -0.2708891 |
| Limd2 | -0.8592727 |
| Lime1 | 1.5309882 |
| Limk1 | 1.5350521 |
| Limk2 | -0.61577 |
| Limk2 | 2.5061802 |
| Lims2 | 2.4767137 |
| Lims2 | 0.8053467 |
| Lims2 | -0.8899877 |
| Lin54 | -0.5825977 |
| Lin7b | 1.365524 |
| Lin7b | -0.7290037 |
| Lin7b | -0.1877313 |
| Lin7b | -0.8460299 |
| Lin7c | -0.5706957 |
| Lincr | 1.309147 |
| Lincr | -0.6681587 |
| Lingo1 | -0.1051669 |
| Lingo2 | -0.4107388 |
| Lingo2 | -0.456851 |
| Lingo4 | -0.2786008 |
| Lip1 | -0.5531163 |
| Lipe | -0.8434032 |
| Lipf | 0.2557856 |
| Liph | 0.8410583 |
| Liph | -0.5879355 |
| Litaf | 0.3892414 |
| Lix1 | -0.2790848 |
| Lix1 | -0.7605439 |
| Lix1 | -0.0844097 |
| Lix1 | 1.0080209 |
| Llgl1 | 0.9861409 |
| Llgl2 | -0.1421949 |
| Lman1 | 0.8635599 |
| Lman2 | 0.6855515 |
| Lman2l | -0.750972 |
| Lman2l | 1.4387496 |
| Lmbr1 | -0.665978 |
| Lmbr1l | -0.2883287 |
| Lmcd1 | -0.6850452 |
| Lmcd1 | -0.7259063 |
| Lmcd1 | 0.8464357 |
| Lmf1 | 0.4725396 |
| Lmf2 | 0.0678358 |
| Lmna | 2.7728691 |
| Lmna | -0.7336689 |
| Lmna | 0.1167261 |
| Lmna | -0.4780826 |
| Lmna | -0.5674157 |
| Lmnb1 | -0.4768906 |
| Lmnb1 | -0.6423131 |
| Lmnb2 | -0.3593498 |
| Lmo2 | 1.2966803 |
| Lmo3 | 1.3231722 |
| Lmo3 | -0.6681587 |
| Lmo4 | 0.5998416 |
| Lmod1 | 0.3322737 |
| Lmtk2 | -0.079683 |
| Lmtk3 | 0.8872575 |
| Lmx1a | -0.1707238 |
| Lmx1b | -0.8646217 |
| Lnx1 | -0.2401857 |
| Lnx2 | -0.0347839 |
| Lnx2 | -0.2819943 |
| LOC100038882 | -0.5615401 |
| LOC100038984 | -0.4051973 |
| LOC100039087 | -0.4219191 |
| LOC100039227 | -0.4343707 |
| LOC100039479 | -0.5832633 |
| LOC100039532 | 0.2738632 |
| LOC100039571 | -0.8192299 |
| LOC100039728 | -0.6366635 |
| LOC100040573 | -0.495536 |
| LOC100040592 | 0.6029222 |
| LOC100040601 | -0.5544074 |
| LOC100040799 | -0.0675795 |
| LOC100040899 | 0.32895 |
| LOC100040919 | -0.2675348 |
| LOC100041103 | -0.6761976 |
| LOC100041290 | 0.2862632 |
| LOC100041463 | 0.1780471 |
| LOC100041483 | 1.4508479 |
| LOC100041500 | 0.2113555 |
| LOC100041504 | 0.7080264 |
| LOC100041703 | 1.5754614 |
| LOC100041722 | 2.1617946 |
| LOC100041725 | -0.6638023 |
| LOC100041835 | 2.6373474 |
| LOC100042179 | 0.1828345 |
| LOC100042427 | -0.5010181 |
| LOC100042777 | 1.3451574 |
| LOC100043257 | -0.6494226 |
| LOC100043391 | -0.321153 |
| LOC100043671 | 2.3872182 |
| LOC100043822 | 3.1394437 |
| LOC100043986 | 0.0711965 |
| LOC100044087 | 0.4639069 |
| LOC100044089 | -0.2996398 |
| LOC100044101 | 0.0474189 |
| LOC100044103 | -0.6696153 |
| LOC100044103 | -0.2608612 |
| LOC100044122 | -0.0741979 |
| LOC100044133 | 1.0179048 |
| LOC100044159 | -0.3806511 |
| LOC100044170 | 3.5901121 |
| LOC100044172 | -0.4632885 |
| LOC100044177 | -0.3641013 |
| LOC100044190 | -0.3985897 |
| LOC100044204 | 0.3539744 |
| LOC100044210 | 0.6843166 |
| LOC100044221 | -0.7243613 |
| LOC100044257 | -0.4816676 |
| LOC100044275 | -0.6769318 |
| LOC100044289 | 2.1079821 |
| LOC100044292 | 0.4775944 |
| LOC100044294 | -0.4349405 |
| LOC100044298 | 0.7045764 |
| LOC100044322 | -0.5182775 |
| LOC100044324 | 1.1382002 |
| LOC100044324 | 0.9552793 |
| LOC100044363 | 0.2942643 |
| LOC100044363 | 0.3609347 |
| LOC100044439 | -0.0656416 |
| LOC100044468 | 0.2926695 |
| LOC100044475 | 0.9406816 |
| LOC100044538 | -0.0781126 |
| LOC100044566 | -0.085595 |
| LOC100044576 | -0.604811 |
| LOC100044636 | 1.1571054 |
| LOC100044692 | 0.1048172 |
| LOC100044708 | 2.6301988 |
| LOC100044736 | 3.5091518 |
| LOC100044756 | -0.4395117 |
| LOC100044776 | 0.6103898 |
| LOC100044779 | -0.6917351 |
| LOC100044812 | -0.5766281 |
| LOC100044829 | 2.6147345 |
| LOC100044862 | 1.1571054 |
| LOC100044873 | 0.3930807 |
| LOC100044934 | 2.6655292 |
| LOC100045005 | 0.0446633 |
| LOC100045019 | -0.9584883 |
| LOC100045040 | -0.4236076 |
| LOC100045098 | 2.5624191 |
| LOC100045228 | 1.3457756 |
| LOC100045241 | 0.6904749 |
| LOC100045250 | -0.6688868 |
| LOC100045280 | -0.6718044 |
| LOC100045300 | -0.080076 |
| LOC100045304 | -0.8691022 |
| LOC100045343 | -0.4822664 |
| LOC100045359 | 0.6520024 |
| LOC100045359 | -0.5634946 |
| LOC100045403 | 0.0106039 |
| LOC100045413 | 3.1179822 |
| LOC100045419 | 1.9822682 |
| LOC100045430 | -0.5479692 |
| LOC100045439 | 2.2209614 |
| LOC100045491 | 1.7436222 |
| LOC100045505 | 0.6750932 |
| LOC100045522 | 0.5775998 |
| LOC100045542 | 0.8535589 |
| LOC100045551 | -0.0552445 |
| LOC100045567 | -0.3844282 |
| LOC100045607 | -0.4309587 |
| LOC100045617 | -0.1527406 |
| LOC100045644 | 0.1714249 |
| LOC100045697 | 1.3515852 |
| LOC100045780 | -0.5667611 |
| LOC100045782 | 1.8093487 |
| LOC100045877 | 1.1595766 |
| LOC100045882 | -0.5799399 |
| LOC100045887 | -0.6917351 |
| LOC100045950 | 0.5177275 |
| LOC100045963 | 1.1450885 |
| LOC100045981 | 0.2873339 |
| LOC100046003 | -0.2185842 |
| LOC100046035 | -0.0706883 |
| LOC100046035 | 1.8568306 |
| LOC100046039 | -0.771023 |
| LOC100046044 | -0.6813487 |
| LOC100046049 | 1.0539344 |
| LOC100046056 | 0.7458383 |
| LOC100046068 | 1.0171577 |
| LOC100046080 | 0.2162674 |
| LOC100046081 | 1.1083291 |
| LOC100046120 | -0.4230444 |
| LOC100046128 | 1.2528386 |
| LOC100046129 | 1.273917 |
| LOC100046163 | -0.3882203 |
| LOC100046163 | -0.4275588 |
| LOC100046207 | -0.7807991 |
| LOC100046207 | 0.3532257 |
| LOC100046207 | 0.3464603 |
| LOC100046232 | -0.6394841 |
| LOC100046254 | 1.45684 |
| LOC100046320 | 0.7661433 |
| LOC100046393 | -0.2805384 |
| LOC100046406 | -0.4286908 |
| LOC100046457 | 0.258271 |
| LOC100046518 | -0.4627015 |
| LOC100046568 | 1.3719026 |
| LOC100046586 | 1.9846226 |
| LOC100046608 | -0.2675348 |
| LOC100046608 | 0.3542239 |
| LOC100046608 | -0.5133143 |
| LOC100046608 | 2.4531435 |
| LOC100046616 | -0.2961831 |
| LOC100046641 | 1.6947825 |
| LOC100046650 | 1.5754614 |
| LOC100046690 | 3.2472467 |
| LOC100046690 | 2.1637083 |
| LOC100046741 | 0.5852673 |
| LOC100046744 | -0.4786792 |
| LOC100046744 | -0.6894999 |
| LOC100046746 | 1.1921709 |
| LOC100046775 | -0.7989862 |
| LOC100046781 | -0.6652522 |
| LOC100046781 | -0.1850949 |
| LOC100046792 | -0.606174 |
| LOC100046796 | 0.3727498 |
| LOC100046802 | 1.4494231 |
| LOC100046841 | -0.7182063 |
| LOC100046855 | -0.8346995 |
| LOC100046883 | 0.4709959 |
| LOC100046891 | 1.126608 |
| LOC100046898 | -0.6718044 |
| LOC100046918 | 0.2415784 |
| LOC100046953 | -0.1664112 |
| LOC100046959 | -0.9293469 |
| LOC100046996 | 1.3693218 |
| LOC100047009 | -0.876315 |
| LOC100047009 | 1.4597436 |
| LOC100047012 | -0.2661008 |
| LOC100047028 | -0.6508508 |
| LOC100047046 | 1.7482094 |
| LOC100047052 | 0.0795471 |
| LOC100047052 | 0.6034987 |
| LOC100047155 | -0.212243 |
| LOC100047155 | -0.0675795 |
| LOC100047167 | 0.2776542 |
| LOC100047173 | -0.3333745 |
| LOC100047184 | 1.3274119 |
| LOC100047200 | -0.3100857 |
| LOC100047214 | 0.8130904 |
| LOC100047214 | 2.5417284 |
| LOC100047238 | 2.1071472 |
| LOC100047261 | 0.2752188 |
| LOC100047323 | -0.8504238 |
| LOC100047323 | 1.7347339 |
| LOC100047339 | 0.1744407 |
| LOC100047353 | -0.4029897 |
| LOC100047368 | 0.1189625 |
| LOC100047369 | 0.9119124 |
| LOC100047369 | -0.5046903 |
| LOC100047391 | 0.4080498 |
| LOC100047393 | -0.5276536 |
| LOC100047427 | -0.7344486 |
| LOC100047480 | 0.9362177 |
| LOC100047490 | 1.8958795 |
| LOC100047579 | 0.1935193 |
| LOC100047583 | -0.3635722 |
| LOC100047606 | -0.807369 |
| LOC100047615 | 0.191451 |
| LOC100047619 | -0.1438744 |
| LOC100047634 | 2.7117326 |
| LOC100047651 | -0.2922479 |
| LOC100047653 | 2.0096603 |
| LOC100047659 | 0.6816652 |
| LOC100047674 | -0.2800537 |
| LOC100047707 | 1.1324769 |
| LOC100047717 | 1.7819001 |
| LOC100047749 | -0.7964857 |
| LOC100047762 | -0.631047 |
| LOC100047776 | 0.1322721 |
| LOC100047794 | 0.244101 |
| LOC100047800 | 0.5722553 |
| LOC100047810 | -0.6573046 |
| LOC100047815 | -0.6268563 |
| LOC100047816 | -0.0544788 |
| LOC100047834 | 0.2684212 |
| LOC100047837 | 1.857089 |
| LOC100047837 | 0.2494046 |
| LOC100047841 | -0.8664114 |
| LOC100047856 | 0.3302297 |
| LOC100047870 | -0.28784 |
| LOC100047911 | 1.4931594 |
| LOC100047934 | 2.7208741 |
| LOC100047935 | -0.706014 |
| LOC100047937 | 2.4819123 |
| LOC100047937 | 0.5041811 |
| LOC100047958 | 0.6008054 |
| LOC100047963 | 0.1747417 |
| LOC100047963 | 0.7225515 |
| LOC100047998 | -0.155289 |
| LOC100048046 | 0.6668591 |
| LOC100048046 | -0.1239129 |
| LOC100048049 | -0.5004074 |
| LOC100048049 | -0.7266797 |
| LOC100048049 | 0.8846851 |
| LOC100048056 | 2.4935795 |
| LOC100048071 | -0.5544074 |
| LOC100048076 | -0.4586024 |
| LOC100048083 | -0.3457545 |
| LOC100048105 | 1.4985917 |
| LOC100048105 | 0.0098887 |
| LOC100048187 | 1.9283229 |
| LOC100048251 | -0.5409368 |
| LOC100048295 | 0.2843866 |
| LOC100048299 | -0.4609426 |
| LOC100048301 | 1.1638592 |
| LOC100048313 | -0.5661069 |
| LOC100048331 | 0.6363543 |
| LOC100048332 | 2.8012799 |
| LOC100048346 | 1.4005904 |
| LOC100048372 | -0.761346 |
| LOC100048384 | 1.0997995 |
| LOC100048413 | -0.8200828 |
| LOC100048436 | -0.495536 |
| LOC100048445 | 1.8901554 |
| LOC100048461 | -0.4521963 |
| LOC100048480 | 2.352789 |
| LOC100048483 | -0.0426888 |
| LOC100048504 | -0.0613921 |
| LOC100048530 | -0.1092109 |
| LOC100048554 | 1.9515891 |
| LOC100048556 | -0.2737737 |
| LOC100048589 | 1.4075676 |
| LOC100048613 | 0.4395854 |
| LOC100048622 | 1.6716548 |
| LOC100048645 | 1.0861076 |
| LOC100048684 | 0.6281522 |
| LOC100048703 | 1.3524624 |
| LOC100048710 | -0.5263982 |
| LOC100048721 | -0.6565854 |
| LOC100048721 | -0.6401906 |
| LOC100048726 | 0.684846 |
| LOC100048733 | -0.2158614 |
| LOC100048816 | -0.5759671 |
| LOC100048832 | 0.9615056 |
| LOC100048832 | 1.3546518 |
| LOC100048858 | 0.7406938 |
| LOC192758 | 1.2427618 |
| LOC194227 | 0.1481883 |
| LOC212390 | -0.8451535 |
| LOC216443 | 0.1074047 |
| LOC216963 | -0.5498961 |
| LOC217341 | 3.9116944 |
| LOC218963 | -0.0023524 |
| LOC225922 | -0.0506597 |
| LOC233529 | -0.750972 |
| LOC234374 | 0.2733203 |
| LOC235497 | -0.6857862 |
| LOC236749 | -0.297663 |
| LOC237408 | -0.0284323 |
| LOC237877 | 0.8365538 |
| LOC238829 | -0.7726454 |
| LOC241593 | -0.6924813 |
| LOC244595 | 0.2768431 |
| LOC245263 | -0.926481 |
| LOC329575 | 0.8190872 |
| LOC329575 | 0.9510169 |
| LOC333331 | -0.7243613 |
| LOC381066 | -0.1956842 |
| LOC381302 | -0.2337 |
| LOC381375 | -0.4870704 |
| LOC381483 | 1.6749754 |
| LOC381629 | 1.1778568 |
| LOC381801 | -0.0344091 |
| LOC381936 | -0.2443785 |
| LOC382010 | 1.4441013 |
| LOC382044 | 1.5974874 |
| LOC382106 | -0.3374834 |
| LOC382106 | -0.278117 |
| LOC382133 | 0.3827273 |
| LOC384806 | -0.4627015 |
| LOC384806 | -0.2694501 |
| LOC386486 | 2.1748146 |
| LOC433479 | -0.3201416 |
| LOC433801 | 2.7921815 |
| LOC433886 | -0.674731 |
| LOC434172 | -0.761346 |
| LOC434172 | -0.7605439 |
| LOC434864 | -0.5733278 |
| LOC434960 | 3.7872164 |
| LOC435023 | -0.6247678 |
| LOC435337 | -0.8269334 |
| LOC435565 | -0.1970162 |
| LOC436177 | -0.494322 |
| LOC544988 | -0.6857862 |
| LOC544988 | 0.6661397 |
| LOC545013 | 0.8118556 |
| LOC545013 | -0.4609426 |
| LOC545056 | -0.4135216 |
| LOC545238 | -0.0262006 |
| LOC545703 | 1.1278291 |
| LOC545732 | 2.6246633 |
| LOC545952 | 0.22516 |
| LOC546015 | 0.5060978 |
| LOC546015 | -0.4498774 |
| LOC546723 | -0.7923326 |
| LOC546840 | -0.5441269 |
| LOC547323 | 0.3890009 |
| LOC547343 | 1.2514751 |
| LOC620807 | -0.6947235 |
| LOC621823 | 0.5088594 |
| LOC621824 | 0.2415784 |
| LOC622404 | -0.8808509 |
| LOC622665 | 2.6975006 |
| LOC624855 | -0.6458612 |
| LOC625480 | 0.7575173 |
| LOC626152 | -0.6917351 |
| LOC628053 | 1.5760859 |
| LOC631002 | -0.3110865 |
| LOC631784 | -0.7197413 |
| LOC632431 | 2.4681678 |
| LOC639396 | -0.33235 |
| LOC640963 | 1.8688517 |
| LOC641240 | -0.6739985 |
| LOC654426 | 2.2026039 |
| LOC654467 | 1.0330929 |
| LOC654494 | -0.5371227 |
| LOC654842 | -0.0875739 |
| LOC665250 | -0.5779515 |
| LOC666676 | 0.0269073 |
| LOC666904 | 2.7501609 |
| LOC667250 | -0.749386 |
| LOC667370 | -0.3385133 |
| LOC667609 | 0.4800024 |
| LOC668492 | 0.3338038 |
| LOC668837 | 2.8017412 |
| LOC669168 | -0.4967515 |
| LOC669660 | 0.3424284 |
| LOC670044 | -0.4247348 |
| LOC671564 | -0.0832258 |
| LOC672705 | 3.3634617 |
| LOC674706 | 0.9264053 |
| LOC675228 | 0.000542 |
| LOC675567 | 0.4701126 |
| LOC675567 | 2.5285428 |
| LOC675813 | 2.8812188 |
| LOC676420 | 1.1351227 |
| LOC676420 | -0.3467934 |
| LOC677144 | -0.018799 |
| LOC677205 | -0.6850452 |
| LOC677317 | -0.379037 |
| LOC677319 | 1.1213732 |
| LOC677528 | 1.2088247 |
| LOC732482 | 0.0353043 |
| Loh11cr2a | 0.9670353 |
| Loh12cr1 | 1.8309976 |
| Loh12cr1 | 0.267329 |
| Lonp2 | -0.2699295 |
| Lonrf1 | 1.3607555 |
| Lonrf2 | 0.4115795 |
| Lor | -0.5933032 |
| Lox | 1.4896725 |
| Loxl1 | 0.8878282 |
| Lpar2 | -0.8863225 |
| Lpar2 | 1.1948634 |
| Lpar3 | 0.4218588 |
| Lpcat2 | 1.4320781 |
| Lpcat2 | -0.6565854 |
| Lpcat2 | -0.7824382 |
| Lpgat1 | 0.5708648 |
| Lphn1 | 0.0240919 |
| Lphn3 | -0.0987318 |
| Lpin1 | -0.5661069 |
| Lpin1 | 0.2325549 |
| Lpin2 | 1.185824 |
| Lpin3 | 1.3104305 |
| Lpl | 0.2654148 |
| Lpxn | -0.9379963 |
| Lrat | -0.5364885 |
| Lrba | 2.3226422 |
| Lrba | 0.923112 |
| Lrch1 | 1.1075362 |
| Lrch4 | -0.7151436 |
| Lrch4 | 0.980721 |
| Lrdd | -0.4937156 |
| Lrfn1 | 0.5680776 |
| Lrfn2 | 0.2886706 |
| Lrfn3 | 0.4085212 |
| Lrfn4 | 2.7390423 |
| Lrg1 | -0.3774255 |
| Lrig1 | -0.1468207 |
| Lrig3 | 1.8095117 |
| Lrmp | -0.6616315 |
| Lrp1 | -0.4035411 |
| Lrp10 | -0.711329 |
| Lrp11 | -0.3514827 |
| Lrp12 | 1.9168378 |
| Lrp12 | -0.6451506 |
| Lrp4 | 1.6347367 |
| Lrp5 | 0.4630171 |
| Lrp8 | -0.456851 |
| Lrp8 | -0.6296481 |
| Lrpap1 | -0.7581419 |
| Lrpprc | -0.6289493 |
| Lrpprc | -0.6939755 |
| Lrpprc | -0.1646917 |
| Lrrc1 | -0.6472842 |
| Lrrc1 | -0.7573426 |
| Lrrc1 | 0.5955895 |
| Lrrc10 | -0.7367916 |
| Lrrc15 | 0.8759156 |
| Lrrc16b | 1.0492485 |
| Lrrc16b | 0.2471751 |
| Lrrc18 | 1.4173655 |
| Lrrc2 | -0.3196362 |
| Lrrc20 | 1.9836725 |
| Lrrc20 | -0.755746 |
| Lrrc23 | -0.1612618 |
| Lrrc23 | -0.6580244 |
| Lrrc24 | 0.6846696 |
| Lrrc26 | 1.0842516 |
| Lrrc28 | -0.2350861 |
| Lrrc29 | 0.9071719 |
| Lrrc3 | 0.9336381 |
| Lrrc30 | 1.6841873 |
| Lrrc33 | -0.5460461 |
| Lrrc34 | 1.1170075 |
| Lrrc34 | 3.060544 |
| Lrrc36 | -0.0808625 |
| Lrrc39 | -0.3431621 |
| Lrrc39 | 0.8839693 |
| Lrrc4 | -0.4870704 |
| Lrrc40 | 2.7824075 |
| Lrrc41 | -0.6479965 |
| Lrrc43 | 1.0339501 |
| Lrrc44 | -0.3774255 |
| Lrrc45 | -0.460357 |
| Lrrc46 | 0.6234603 |
| Lrrc48 | -0.0824374 |
| Lrrc48 | 1.8136855 |
| Lrrc48 | 0.8458397 |
| Lrrc49 | 2.7370703 |
| Lrrc49 | 2.3390676 |
| Lrrc49 | 2.4265886 |
| Lrrc4c | -0.1468207 |
| Lrrc50 | 0.6913514 |
| Lrrc50 | -0.2485898 |
| Lrrc52 | -0.5307996 |
| Lrrc52 | 0.9033905 |
| Lrrc56 | -0.3996878 |
| Lrrc57 | -0.6776665 |
| Lrrc59 | -0.484065 |
| Lrrc6 | 1.0302712 |
| Lrrc7 | 0.735856 |
| Lrrc8 | -0.5576429 |
| Lrrc8a | 1.8080982 |
| Lrrc8b | -0.3125898 |
| Lrrc8c | 0.4636845 |
| Lrrc8d | 0.6356115 |
| Lrrc9 | 1.1325873 |
| Lrrfip1 | 0.7026736 |
| Lrrfip1 | 2.3392236 |
| Lrriq2 | -0.541574 |
| Lrriq2 | 1.4465673 |
| Lrrk1 | 0.2067093 |
| Lrrk2 | -0.4469868 |
| Lrrn1 | 0.7542419 |
| Lrrn2 | -0.5016291 |
| Lrrn3 | 1.0027242 |
| Lrrtm1 | -0.5276536 |
| Lrrtm2 | -0.5799399 |
| Lrrtm3 | -0.4487201 |
| Lrsam1 | 0.0966857 |
| Lrsam1 | -0.5364885 |
| Lrtm1 | 0.1534353 |
| Lrtm1 | 0.0184359 |
| Lsamp | -0.5188998 |
| Lsamp | -0.1161253 |
| Lsg1 | -0.7775293 |
| Lsg1 | 0.6603651 |
| Lsm10 | 0.3019355 |
| Lsm11 | 1.4799024 |
| Lsm12 | 3.3849319 |
| Lsm12 | -0.524518 |
| Lsm12 | 0.181042 |
| Lsm14a | 0.8981842 |
| Lsm14b | -0.3758167 |
| Lsm14b | 0.3366026 |
| Lsm14b | 0.5971379 |
| Lsm2 | -0.2277167 |
| Lsm2 | 0.1494255 |
| Lsm3 | -0.761346 |
| Lsm4 | 2.6909195 |
| Lsm5 | -0.4219191 |
| Lsm6 | -0.8566094 |
| Lsm6 | 1.5417631 |
| Lsm8 | -0.6345534 |
| Lsr | -0.8927459 |
| Lst1 | -0.6430217 |
| Ltb4dh | -0.0671916 |
| Ltb4r1 | -0.3931181 |
| Ltb4r2 | 1.5122611 |
| Ltbp1 | 0.632262 |
| Ltbp1 | 1.6239776 |
| Ltbp2 | 0.2563385 |
| Ltbp3 | 2.140755 |
| Ltbp3 | -0.7022357 |
| Ltbp4 | -0.6150814 |
| Ltbp4 | -0.960463 |
| Ltbp4 | 2.2401845 |
| Ltbr | 1.7205289 |
| Ltc4s | 0.8789437 |
| Ltf | -0.4281246 |
| Ltf | -0.3593498 |
| Ltk | -0.8927459 |
| Ltk | -0.0544788 |
| Ltv1 | 0.0023465 |
| Luc7l | -0.9236238 |
| Luc7l | -0.9447782 |
| Luc7l2 | 0.9117733 |
| Luc7l2 | -0.4355107 |
| Lum | 1.3693218 |
| Luzp1 | 2.5714866 |
| Luzp1 | -0.7605439 |
| Luzp1 | -0.6573046 |
| Luzp2 | 0.7124929 |
| Lxn | -0.2258832 |
| Ly6a | 2.1074256 |
| Ly6c1 | -0.3441982 |
| Ly6d | 0.8218423 |
| Ly6e | -0.4668182 |
| Ly6f | 1.3575341 |
| Ly6g6c | -0.6681587 |
| Ly6g6d | 1.6842492 |
| Ly6g6e | 0.8785117 |
| Ly6k | -0.914161 |
| Ly86 | 0.7501317 |
| Ly9 | -0.6345534 |
| Ly9 | 0.4902235 |
| Ly96 | -0.8149769 |
| Lyar | 0.0772163 |
| Lyl1 | 2.0344017 |
| Lynx1 | 0.2684212 |
| Lynx1 | 1.6623206 |
| Lypd1 | 0.6195012 |
| Lypd3 | -0.4315265 |
| Lypd4 | 0.3800606 |
| Lypd5 | 0.0088149 |
| Lypd6 | 0.9835638 |
| Lypla1 | -0.8048464 |
| Lypla1 | 2.4712151 |
| Lypla2 | 1.036639 |
| Lypla3 | -0.6857862 |
| Lyplal1 | 0.4037968 |
| Lyrm1 | -0.6681587 |
| Lyrm2 | -0.2374007 |
| Lyrm2 | 1.0968247 |
| Lyrm5 | 1.7652602 |
| Lysmd1 | -0.6638023 |
| Lysmd1 | -0.3036058 |
| Lysmd2 | 1.8804208 |
| Lysmd4 | -0.5028524 |
| Lyst | -0.3050972 |
| Lyve1 | -0.5071464 |
| Lyz | 0.6657798 |
| Lyz1 | 1.3691495 |
| Lyz1 | -0.3710067 |
| Lyz2 | 3.1204626 |
| Lyzl1 | 2.1926549 |
| Lyzl4 | 0.8118556 |
| Lyzl6 | 3.0701958 |
| Lyzs | -0.3860516 |
| Lzic | -0.1837795 |
| Lztr1 | -0.0923399 |
| Lzts2 | 0.0346073 |
| M6pr | 1.3215435 |
| M6pr | -0.4739171 |
| M6prbp1 | 1.2370403 |
| Mab21l2 | 0.2760313 |
| Macrod1 | -0.3667513 |
| Mad1l1 | -0.1396812 |
| Mad2l1 | 1.7480356 |
| Mad2l1bp | -0.7197413 |
| Mad2l1bp | -0.5301696 |
| Mad2l1bp | -0.5010181 |
| Mad2l2 | -0.6366635 |
| Madcam1 | 0.6610889 |
| Madd | 1.8088596 |
| Maea | 0.9248973 |
| Maf | 0.7121501 |
| Maf1 | -0.0761532 |
| Mafg | 0.8610694 |
| Mag | -0.735229 |
| Magea2 | 2.8063228 |
| Magea5 | -0.6095901 |
| Mageb3 | -0.3441982 |
| Mageb3 | -0.5582913 |
| Maged1 | 1.1709897 |
| Maged2 | -0.6828256 |
| Magee1 | -0.0792901 |
| Mageh1 | -0.4551028 |
| Magi1 | -0.6558667 |
| Magi1 | 1.8349729 |
| Magi1 | -0.621297 |
| Magi2 | -0.247184 |
| Magi3 | 0.8695397 |
| Magi3 | -0.8566094 |
| Magix | -0.6947235 |
| Magmas | 0.8020755 |
| Magoh | -0.1604062 |
| Magoh | -0.5582913 |
| Magohb | 2.2850307 |
| Mak | 1.377812 |
| Mak10 | 1.0117864 |
| Mak10 | 0.0745455 |
| Mal | 2.9771583 |
| Mal | 0.9456619 |
| Mall | -0.7022357 |
| Malt1 | 0.5922908 |
| Mamdc2 | -0.0621633 |
| Maml1 | -0.0438234 |
| Maml3 | 1.7530052 |
| Man1a | 0.4104044 |
| Man1a2 | -0.61096 |
| Man1a2 | 0.3904428 |
| Man1b1 | 0.3207189 |
| Man1c1 | -0.2742553 |
| Man1c1 | -0.6268563 |
| Man2a1 | 2.5641458 |
| Man2a2 | 0.600998 |
| Man2a2 | 2.1795457 |
| Man2a2 | 0.3113734 |
| Man2b1 | 0.9592582 |
| Man2b2 | 0.9510169 |
| Man2c1 | 1.4975378 |
| Manba | -0.4679977 |
| Manba | 2.5678355 |
| Manbal | 0.2735918 |
| Manea | -0.4002373 |
| Mansc1 | 1.2811244 |
| Maob | 1.1651402 |
| Map1lc3a | 0.0943964 |
| Map1lc3a | 0.9871697 |
| Map1lc3b | 2.0873341 |
| Map2k1 | 1.0880757 |
| Map2k2 | -0.606174 |
| Map2k2 | 0.1173656 |
| Map2k3 | 1.8465915 |
| Map2k4 | -0.6977213 |
| Map2k6 | 1.1207029 |
| Map2k6 | -0.1642622 |
| Map2k6 | -0.3075882 |
| Map2k7 | 0.071532 |
| Map2k7 | 0.0293641 |
| Map2k7 | 0.0159509 |
| Map3k1 | 0.7335117 |
| Map3k11 | -0.0781126 |
| Map3k12 | 2.5679337 |
| Map3k12 | 0.9099632 |
| Map3k12 | 1.0880757 |
| Map3k14 | 2.1948344 |
| Map3k3 | 0.841807 |
| Map3k6 | 3.0038078 |
| Map3k7 | 0.5152022 |
| Map3k7ip1 | 0.5924851 |
| Map3k7ip1 | -0.5993783 |
| Map3k7ip1 | 1.3531635 |
| Map3k7ip2 | -0.2235962 |
| Map3k7ip3 | 0.6816652 |
| Map3k8 | 1.9241888 |
| Map3k9 | 0.7540778 |
| Map4k2 | 3.1968816 |
| Map4k4 | -0.7052572 |
| Map4k5 | 0.586637 |
| Map4k5 | 0.5223401 |
| Mapbpip | -0.1720213 |
| Mapk1 | 1.802697 |
| Mapk1 | -0.3349133 |
| Mapk1 | -0.0792901 |
| Mapk10 | -0.6529972 |
| Mapk10 | 0.5570487 |
| Mapk11 | 0.8144776 |
| Mapk12 | -0.2485898 |
| Mapk12 | -0.5912868 |
| Mapk12 | -0.1092109 |
| Mapk13 | -0.7159083 |
| Mapk13 | 0.6002273 |
| Mapk14 | -0.0434451 |
| Mapk15 | 0.4590025 |
| Mapk15 | -0.545406 |
| Mapk15 | -0.5608894 |
| Mapk1ip1 | 2.4266739 |
| Mapk1ip1 | -0.3942099 |
| Mapk1ip1 | 2.3787565 |
| Mapk3 | -0.6565854 |
| Mapk3 | -0.0575452 |
| Mapk4 | 1.9391973 |
| Mapk6 | -0.6275534 |
| Mapk6 | 0.8609227 |
| Mapk6 | -0.5531163 |
| Mapk8 | -0.6710741 |
| Mapk8ip1 | -0.6102748 |
| Mapk8ip1 | -0.7228188 |
| Mapk8ip2 | -0.3969449 |
| Mapk8ip3 | 2.1471081 |
| Mapk8ip3 | 1.3969989 |
| Mapk9 | 0.8952166 |
| Mapk9 | 0.6247764 |
| Mapk9 | 1.5527021 |
| Mapkap1 | 1.7830736 |
| Mapkap1 | -0.2873515 |
| Mapkapk2 | -0.7274537 |
| Mapkapk3 | 0.1583474 |
| Mapkbp1 | -0.02806 |
| Mapre1 | -0.5537617 |
| Mapre2 | -0.6041302 |
| Mapre2 | -0.6850452 |
| Mapre3 | -0.751766 |
| Mapt | -0.7391405 |
| Mapt | 0.6006127 |
| Mapt | -0.6544309 |
| Mapt | -0.621297 |
| March1 | -0.695472 |
| March2 | -0.7105679 |
| March2 | 0.5538162 |
| March4 | -0.5022406 |
| March4 | -0.7948222 |
| March6 | -0.2059437 |
| March7 | -0.6123318 |
| March8 | 2.5778971 |
| March10 | -0.0085324 |
| Marcks | 2.1422131 |
| Marcksl1 | 0.0258525 |
| Marcksl1 | 0.1629297 |
| Marcksl1 | -0.4275588 |
| Mare | -0.7702128 |
| Mark1 | 0.8563638 |
| Mark2 | -0.3672822 |
| Mark2 | 0.833388 |
| Mark3 | 0.7466654 |
| Mark3 | 0.0356525 |
| Mark4 | -0.3811898 |
| Mars | 0.3720156 |
| Mars2 | 0.2947954 |
| Marveld3 | -0.6992237 |
| Marveld3 | 1.1854063 |
| Mas1 | -0.4303912 |
| Masp1 | -0.6865278 |
| Masp2 | -0.7014818 |
| Mast1 | -0.2570686 |
| Mast1 | 0.0255006 |
| Mast2 | 0.8572479 |
| Mast2 | 1.0666739 |
| Mat1a | -0.5004074 |
| Mat2a | 0.1771468 |
| Mat2a | 1.7646341 |
| Mat2a | 2.1945441 |
| Mat2a | -0.5511829 |
| Mat2b | -0.6102748 |
| Mat2b | -0.7931617 |
| Matk | 1.4762897 |
| Matk | 0.9476735 |
| Matn4 | -0.1438744 |
| Matn4 | -0.4792761 |
| Matn4 | 2.122613 |
| Matr3 | 1.1940357 |
| Max | -0.524518 |
| Maz | -0.7297797 |
| Mb | -0.1903749 |
| Mbc2 | 2.9495791 |
| Mbd1 | -0.6947235 |
| Mbd1 | -0.2126946 |
| Mbd3 | 2.0635689 |
| Mbd3l2 | 0.1164062 |
| Mbd4 | 0.7560448 |
| Mbd5 | 0.0576819 |
| Mbd6 | 0.7435184 |
| Mbip | -0.2897965 |
| Mbl2 | 0.6955475 |
| Mbnl1 | 0.7563722 |
| Mbnl2 | -0.2839389 |
| Mbnl2 | -0.6296481 |
| Mboat1 | 0.0943964 |
| Mboat2 | -0.3297937 |
| Mboat2 | 1.918487 |
| Mboat2 | 0.4181337 |
| Mboat2 | -0.755746 |
| Mboat5 | -0.6784018 |
| Mboat5 | 2.4914277 |
| Mbp | -0.122679 |
| Mbp | -0.6835649 |
| Mbp | 0.9008612 |
| Mbtd1 | 1.3917124 |
| Mbtps1 | -0.8090544 |
| Mbtps1 | -0.3504387 |
| Mbtps1 | 1.5298239 |
| Mbtps2 | -0.0288047 |
| Mc3r | -0.1434543 |
| Mc4r | 0.6140068 |
| Mc5r | 1.1132979 |
| Mcam | -0.8065274 |
| Mcam | 2.2097393 |
| Mcam | -0.321153 |
| Mcat | 0.0116757 |
| Mcc | -0.0085324 |
| Mccc1 | 0.2274414 |
| Mcee | 1.1874928 |
| Mcf2l | -0.2675348 |
| Mcf2l | 2.3721497 |
| Mcf2l | 1.1020815 |
| Mcfd2 | -0.2113406 |
| Mchr1 | -0.2622873 |
| Mcl1 | 1.7598965 |
| Mcm10 | -0.6688868 |
| Mcm10 | 1.8795125 |
| Mcm2 | -0.6317473 |
| Mcm3 | 2.6152018 |
| Mcm4 | 1.8314224 |
| Mcm5 | -0.706014 |
| Mcm6 | 0.0240919 |
| Mcm6 | 0.229149 |
| Mcm6 | -0.5739869 |
| Mcm7 | -0.8107428 |
| Mcoln1 | 0.676518 |
| Mcoln2 | 1.9277471 |
| Mcoln3 | 1.0154122 |
| Mcph1 | -0.3060928 |
| Mcph1 | 0.6162842 |
| Mcph1 | 2.0560456 |
| Mcph1 | 1.8278586 |
| Mcpt4 | -0.1970162 |
| Mcrs1 | 0.6986826 |
| Mcrs1 | -0.5906156 |
| Mcrs1 | 3.1775553 |
| Mcts1 | -0.485266 |
| Mdfic | -0.5460461 |
| Mdga1 | 0.7903004 |
| Mdga2 | -0.5680708 |
| Mdga2 | 1.6274679 |
| Mdh1 | -0.2374007 |
| Mdh1 | 0.1973485 |
| Mdh1b | -0.5065318 |
| Mdh2 | -0.6437308 |
| Mdk | 1.0355399 |
| Mdk | 2.1450461 |
| Mdm1 | 0.3469631 |
| Mdm1 | -0.495536 |
| Mdm1 | -0.5053038 |
| Mdm1 | -0.4292572 |
| Mdm1 | -0.1711561 |
| Mdm1 | 0.0377395 |
| Mdm1 | -0.7956536 |
| Mdm1 | 1.7480356 |
| Mdm1 | 0.4255694 |
| Mdm2 | 1.2268945 |
| Mdm2 | -0.4804711 |
| Mdm4 | 0.4155642 |
| Mdn1 | 0.6479769 |
| Me2 | -0.3140953 |
| Me3 | 2.0517559 |
| Mea1 | -0.4757001 |
| Mecp2 | -0.7931617 |
| Mecr | 0.067499 |
| Med1 | -0.1251483 |
| Med1 | 0.0898013 |
| Med10 | 1.182687 |
| Med11 | 0.6191233 |
| Med11 | -0.1371741 |
| Med12 | 0.7271195 |
| Med15 | 0.7378608 |
| Med15 | 1.3670795 |
| Med15 | 0.4134567 |
| Med16 | 0.3256147 |
| Med18 | -0.674731 |
| Med18 | -0.5176557 |
| Med19 | -0.3535743 |
| Med21 | -0.5133143 |
| Med22 | -0.4858671 |
| Med22 | 0.5552318 |
| Med23 | -0.7478026 |
| Med23 | 0.8753377 |
| Med24 | 1.4874677 |
| Med25 | -0.3561952 |
| Med25 | -0.2108897 |
| Med26 | 0.270057 |
| Med27 | -0.7734577 |
| Med28 | -0.4372234 |
| Med30 | 0.9029694 |
| Med31 | 2.4439358 |
| Med4 | -0.5899448 |
| Med6 | -0.7581419 |
| Med7 | 1.2090285 |
| Med8 | -0.6791377 |
| Med8 | -0.4101832 |
| Med9 | -0.6416051 |
| Mef2b | 1.0570457 |
| Mef2b | -0.6296481 |
| Mef2c | 1.0844838 |
| Mef2c | 0.5473182 |
| Mef2c | 0.6228957 |
| Megf10 | 0.7368589 |
| Megf11 | -0.6373678 |
| Megf11 | -0.103957 |
| Megf11 | -0.6261596 |
| Megf11 | 2.997661 |
| Megf6 | -0.4418055 |
| Megf9 | -0.6268563 |
| Mei1 | 0.0066636 |
| Meis1 | 1.4221863 |
| Meis2 | -0.5301696 |
| Meis3 | 1.6919604 |
| Melk | 0.8248942 |
| Memo1 | -0.7948222 |
| Men1 | 0.4290351 |
| Men1 | -0.6776665 |
| Meox1 | -0.1367569 |
| Meox2 | 1.4123556 |
| Mep1b | 0.0321641 |
| Mepce | -0.6116456 |
| Mertk | -0.4768906 |
| Mesdc1 | 0.2002836 |
| Mesp1 | 0.1429124 |
| Mest | 0.0180813 |
| Mest | -0.3026127 |
| Mest | -0.2902863 |
| Metap1 | 1.4769826 |
| Metap2 | -0.548611 |
| Metap2 | 0.3877976 |
| Metapl1 | -0.4774864 |
| Metrn | -0.6282511 |
| Metrnl | -0.0567777 |
| Metrnl | 0.5362516 |
| Mett11d1 | 0.3492223 |
| Mettl1 | -0.6387782 |
| Mettl1 | 0.6616313 |
| Mettl2 | -0.4973598 |
| Mettl3 | -0.3556704 |
| Mettl4 | -0.5772896 |
| Mettl6 | -0.5852628 |
| Mettl8 | 1.2730598 |
| Mettl9 | -0.1330101 |
| Mettl9 | 0.1460194 |
| Mex3a | 2.6163459 |
| Mex3a | 1.8827883 |
| Mex3c | 1.4728179 |
| Mfap1b | -0.7407096 |
| Mfap2 | 0.8913875 |
| Mfap2 | 0.9730551 |
| Mfap2 | 0.6499918 |
| Mfap3 | 3.4249966 |
| Mfap3 | -0.7313334 |
| Mfap3l | -0.7151436 |
| Mfap3l | 0.5499634 |
| Mfap4 | 1.2984448 |
| Mfap5 | 1.0202668 |
| Mfge8 | 1.3282217 |
| Mfge8 | -0.02806 |
| Mfi2 | -0.129692 |
| Mfn1 | -0.6902444 |
| Mfn1 | 1.253714 |
| Mfn2 | -0.5865982 |
| Mfng | -0.6000557 |
| Mfrp | -0.595324 |
| Mfsd1 | -0.73601 |
| Mfsd10 | 1.4801325 |
| Mfsd10 | 2.5261608 |
| Mfsd11 | 2.0275091 |
| Mfsd2 | 4.5014841 |
| Mfsd3 | -0.5352214 |
| Mfsd4 | 1.9151858 |
| Mfsd7 | -0.3947563 |
| Mfsd8 | 1.7326754 |
| Mfsd9 | -0.3768889 |
| Mga | -0.2304735 |
| Mgat1 | -0.0594667 |
| Mgat3 | 1.7039276 |
| Mgat4b | 1.2453155 |
| Mgat4b | 0.0785488 |
| Mgat5 | 1.25895 |
| MGC107098 | 1.1755381 |
| MGC117608 | -0.5022406 |
| MGC118210 | 0.6145767 |
| MGC18837 | -0.1724542 |
| MGC41689 | -0.4068564 |
| MGC67181 | -0.6587447 |
| Mgea6 | 0.5298401 |
| Mgl1 | -0.3156033 |
| Mgl2 | 0.5546254 |
| Mgll | 2.7782806 |
| Mgll | -0.6408976 |
| Mgll | -0.5726691 |
| Mgmt | 0.3803033 |
| Mgp | 2.1249192 |
| Mgrn1 | -0.6902444 |
| Mgst1 | -0.4906893 |
| Mgst1 | 0.3798178 |
| Mgst2 | 1.6581921 |
| Mgst3 | 0.2670558 |
| Mgst3 | -0.9104019 |
| Mia1 | -0.1746218 |
| Mia3 | -0.2420469 |
| Mib1 | 0.3029888 |
| Mib2 | -0.4046449 |
| Mic2l1 | -0.0613921 |
| Mical1 | 0.8892535 |
| Mical1 | 0.0549559 |
| Mical1 | -0.3925727 |
| Mical2 | 1.3113463 |
| Mical3 | 1.7891418 |
| Micall2 | 0.8250465 |
| Micall2 | -0.0221226 |
| Mid1 | 1.5135211 |
| Mid1 | -0.2185842 |
| Mid1ip1 | 0.1840276 |
| Midn | -0.1655511 |
| Midn | 2.6647088 |
| Mier1 | 0.8763488 |
| Mier1 | 0.2005766 |
| Mif | 0.9899934 |
| Mif4gd | -0.2023627 |
| Mif4gd | -0.2277167 |
| Mill2 | 0.458779 |
| Mill2 | -0.2081887 |
| Mina | 1.0541741 |
| Mink1 | -0.1375915 |
| Mink1 | -0.5492533 |
| Minpp1 | 0.8618026 |
| Minpp1 | -0.7677864 |
| Miox | -0.1350899 |
| Mipep | -0.5016291 |
| Mir16 | 1.5246402 |
| Mist1 | -0.6444404 |
| Mitf | -0.0078032 |
| Mizf | -0.6331493 |
| Mki67ip | 1.5450651 |
| Mkks | -0.750972 |
| Mkks | 2.0316506 |
| Mkl1 | 0.6788288 |
| Mkl2 | 0.5740401 |
| Mkl2 | -0.1540139 |
| Mkl2 | -0.3011251 |
| Mkln1 | 0.745176 |
| Mknk1 | -0.5919585 |
| Mknk2 | 0.5893706 |
| Mkrn1 | 1.2022801 |
| Mkrn2 | -0.2504675 |
| Mkrn3 | 0.5343946 |
| Mkx | -0.2675348 |
| Mlana | 0.1031964 |
| Mlf1 | -0.4320947 |
| Mlf1 | -0.7235897 |
| Mlf2 | 2.1870744 |
| Mlf2 | -0.660909 |
| Mlh1 | -0.6872699 |
| Mlh3 | 0.8623887 |
| Mlkl | -0.6616315 |
| Mllt1 | 0.3168361 |
| Mllt1 | 1.0094033 |
| Mllt1 | -0.632448 |
| Mllt10 | -0.3140953 |
| Mllt11 | -0.1438744 |
| Mllt3 | 0.4639069 |
| Mllt3 | -0.4435294 |
| Mllt3 | -0.3704737 |
| Mlp | -0.6638023 |
| Mlstd2 | 1.052495 |
| Mlx | 0.9206345 |
| Mlx | -0.7948222 |
| Mlxipl | -0.3100857 |
| Mlycd | -0.4179911 |
| Mmab | -0.5071464 |
| Mmab | 0.1512783 |
| Mmd | -0.7243613 |
| Mmd2 | 1.0643056 |
| Mme | 0.059042 |
| Mme | 0.4336365 |
| Mmel1 | 0.7031929 |
| Mmp10 | 0.0401686 |
| Mmp11 | -0.196572 |
| Mmp12 | 0.979426 |
| Mmp13 | 0.246896 |
| Mmp13 | 0.7552257 |
| Mmp14 | 2.0294095 |
| Mmp15 | 1.3300186 |
| Mmp16 | 0.6096266 |
| Mmp17 | -0.7197413 |
| Mmp1a | 1.3007614 |
| Mmp1a | 0.3191677 |
| Mmp2 | -0.1426145 |
| Mmp23 | -0.6275534 |
| Mmp24 | 1.9148453 |
| Mmp3 | -0.4900852 |
| Mmp3 | 0.4569889 |
| Mmp3 | 0.9646693 |
| Mmp9 | -0.3349133 |
| Mmrn2 | 0.556847 |
| Mnat1 | 0.1481883 |
| Mnat1 | -0.7832589 |
| Mnd1 | 0.4900072 |
| Mns1 | -0.61096 |
| Mnt | -0.1785357 |
| Mobk1b | 0.9838218 |
| Mobkl2a | -0.2485898 |
| Mobkl2b | -0.2713693 |
| Mobkl2c | -0.3070895 |
| Mobkl3 | -0.5602392 |
| Mobp | 0.0023465 |
| Mobp | 0.7390284 |
| Mobp | 1.7091918 |
| Mocos | -0.4469868 |
| Mocs1 | 0.8011388 |
| Mocs2 | 0.1325868 |
| Mod1 | -0.8023305 |
| Mog | 0.7174502 |
| Mog | -0.2131463 |
| Mon1a | 1.4454544 |
| Mon1a | 1.5251525 |
| Mon1b | -0.1802805 |
| Mon1b | 1.0906165 |
| Mon1b | -0.7375739 |
| Morc2a | 0.268694 |
| Morc2b | -0.0221226 |
| Morc3 | -0.8390414 |
| Morf4l1 | 1.8191327 |
| Morf4l1 | 0.0855145 |
| Morf4l2 | -0.6857862 |
| Morn2 | 1.2431551 |
| Morn3 | 0.9404117 |
| Morn3 | 0.1617099 |
| Mos | -0.4931096 |
| Mosc2 | -0.4492986 |
| Mospd1 | 1.14683 |
| Mospd1 | 1.3033494 |
| Mospd1 | -0.6437308 |
| Mospd3 | 2.354663 |
| Mospd3 | -0.5213927 |
| Mov10 | 1.5648517 |
| Mov10l1 | 1.3912916 |
| Moxd1 | 0.3131978 |
| Moxd1 | -0.1746218 |
| Mpdu1 | 0.8285437 |
| Mpdz | -0.0085324 |
| Mpdz | -0.7865484 |
| Mpg | -0.3241937 |
| Mpg | 0.5035414 |
| Mphosph6 | -0.7305562 |
| Mpnd | 0.7511198 |
| Mpo | 0.739195 |
| Mpo | 1.0435706 |
| Mpp1 | 1.1600057 |
| Mpp2 | 1.1025373 |
| Mpp3 | -0.8183778 |
| Mpp3 | 2.396869 |
| Mpp5 | 2.4618244 |
| Mpp6 | 2.555804 |
| Mppe1 | 0.1540507 |
| Mpped1 | -0.2627632 |
| Mpst | 0.4211614 |
| Mpv17 | 0.0155953 |
| Mpv17 | -0.4315265 |
| Mpv17 | 0.541391 |
| Mpv17 | -0.8286537 |
| Mpv17l | -0.0306692 |
| Mpv17l | 1.2924883 |
| Mpv17l | 2.9118759 |
| Mpz | 1.3284914 |
| Mr1 | 0.1875981 |
| Mrap | -0.232777 |
| Mras | -0.1750559 |
| Mrc1 | -0.7597426 |
| Mrc2 | -0.0923399 |
| Mrc2 | 1.0677378 |
| Mre11a | 0.187895 |
| Mre11a | -0.597349 |
| Mre11a | -0.3045998 |
| Mreg | -0.7478026 |
| Mrgpra2 | -0.4979686 |
| Mrgpra2 | 2.3716373 |
| Mrgprb2 | -0.0859905 |
| Mrgpre | 0.095051 |
| Mrgprf | -0.6843047 |
| Mrgprg | 0.6556474 |
| Mrm1 | -0.7022357 |
| Mro | -0.2286347 |
| Mrp63 | -0.632448 |
| Mrpl1 | 1.817033 |
| Mrpl1 | -0.6710741 |
| Mrpl10 | -0.6909894 |
| Mrpl10 | 2.3978082 |
| Mrpl11 | -0.6696153 |
| Mrpl12 | -0.5746465 |
| Mrpl13 | 0.2248744 |
| Mrpl13 | 1.0255908 |
| Mrpl14 | -0.5550537 |
| Mrpl15 | 0.6997253 |
| Mrpl16 | -0.3535743 |
| Mrpl17 | 0.4698917 |
| Mrpl18 | 1.3845332 |
| Mrpl2 | -0.5779515 |
| Mrpl20 | -0.6020908 |
| Mrpl22 | 0.2897386 |
| Mrpl23 | -0.6296481 |
| Mrpl24 | -0.0840149 |
| Mrpl27 | -0.7915041 |
| Mrpl28 | -0.8955121 |
| Mrpl3 | 0.7822095 |
| Mrpl3 | -0.7220485 |
| Mrpl30 | -0.222683 |
| Mrpl33 | -0.7767136 |
| Mrpl33 | -0.4035411 |
| Mrpl34 | 1.4301402 |
| Mrpl36 | -0.0959301 |
| Mrpl37 | -0.2158614 |
| Mrpl37 | -0.127624 |
| Mrpl38 | -0.2718497 |
| Mrpl39 | -0.2443785 |
| Mrpl40 | 0.1332158 |
| Mrpl43 | 2.807737 |
| Mrpl43 | -0.8260743 |
| Mrpl45 | -0.1514689 |
| Mrpl46 | 0.8970547 |
| Mrpl47 | 3.2719308 |
| Mrpl47 | 1.2242909 |
| Mrpl48 | -0.039293 |
| Mrpl48 | 0.5704671 |
| Mrpl48 | 0.267329 |
| Mrpl49 | 1.1248292 |
| Mrpl49 | -0.4858671 |
| Mrpl49 | 2.4111504 |
| Mrpl49 | 1.1118325 |
| Mrpl50 | -0.4858671 |
| Mrpl51 | 0.9103812 |
| Mrpl51 | -0.0074389 |
| Mrpl51 | -0.5993783 |
| Mrpl52 | -0.2117917 |
| Mrpl53 | 0.0765493 |
| Mrpl54 | -0.424171 |
| Mrpl55 | -0.4679977 |
| Mrpl9 | 0.2090352 |
| Mrps10 | 1.0255908 |
| Mrps10 | 1.5367162 |
| Mrps11 | -0.4487201 |
| Mrps11 | 1.0944152 |
| Mrps12 | -0.5352214 |
| Mrps14 | 0.4839298 |
| Mrps15 | 0.1641479 |
| Mrps16 | -0.3535743 |
| Mrps17 | 0.2322715 |
| Mrps18a | -0.4551028 |
| Mrps18b | 1.0174068 |
| Mrps18b | 1.5006208 |
| Mrps18c | -0.6394841 |
| Mrps2 | -0.5720108 |
| Mrps21 | 0.0191447 |
| Mrps22 | 1.3902809 |
| Mrps23 | 1.5519909 |
| Mrps24 | 1.4865539 |
| Mrps25 | -0.5959985 |
| Mrps25 | 1.7083466 |
| Mrps25 | 1.7313203 |
| Mrps26 | -0.7525607 |
| Mrps26 | -0.6962212 |
| Mrps27 | 0.180743 |
| Mrps28 | -0.73601 |
| Mrps30 | 0.362914 |
| Mrps31 | 1.4106248 |
| Mrps31 | -0.6806111 |
| Mrps31 | -0.6674313 |
| Mrps33 | 1.0428433 |
| Mrps33 | -0.8399122 |
| Mrps34 | 0.4641292 |
| Mrps35 | 1.7953923 |
| Mrps5 | 3.0117214 |
| Mrps6 | 0.6124855 |
| Mrps7 | -0.7454324 |
| Mrps7 | 1.6487247 |
| Mrps9 | 0.1756443 |
| Mrrf | 0.0034276 |
| Mrrf | -0.1047634 |
| Mrto4 | -0.6317473 |
| Mrvi1 | -0.5779515 |
| Ms4a1 | 3.274909 |
| Ms4a10 | -0.4498774 |
| Ms4a13 | 1.6696443 |
| Ms4a5 | 1.8961772 |
| Ms4a6d | -0.5010181 |
| Ms4a6d | -0.4168718 |
| Ms4a7 | -0.8200828 |
| Ms4a8a | 1.6036401 |
| Msc | -0.6068563 |
| Msh2 | 0.9400066 |
| Msh3 | 1.0546533 |
| Msh3 | -0.5879355 |
| Msh4 | 1.0250968 |
| Msh5 | -0.0498977 |
| Msh5 | -0.5759671 |
| Msh6 | -0.4366522 |
| Msh6 | 0.8474779 |
| Msi2 | 0.541801 |
| Msi2h | -0.0423109 |
| Msln | -0.1612618 |
| Msn | -0.4292572 |
| Msr2 | -0.73601 |
| Msrb2 | -0.5946499 |
| Msrb2 | 2.7268994 |
| Msrb3 | 2.2115951 |
| Mst1r | -0.6095901 |
| Msto1 | -0.2805384 |
| Msx1 | 2.9657366 |
| Mt1 | -0.145136 |
| Mt3 | 2.7894798 |
| Mta1 | 0.3214936 |
| Mta2 | 1.3989216 |
| Mta3 | 1.2465898 |
| Mta3 | 0.8030113 |
| Mtag2 | 1.3448923 |
| Mtap | -0.7807991 |
| Mtap | -0.2646689 |
| Mtap1b | 0.5401601 |
| Mtap1b | -0.3811898 |
| Mtap1b | -0.525771 |
| Mtap1b | -0.0202748 |
| Mtap1s | -0.751766 |
| Mtap2 | 1.2428601 |
| Mtap2 | 0.1112728 |
| Mtap2 | -0.2518782 |
| Mtap4 | 1.2545887 |
| Mtap6 | 1.5330223 |
| Mtap6 | -0.2406507 |
| Mtap7 | 2.9125948 |
| Mtap7d1 | -0.1698597 |
| Mtap7d1 | 0.1460194 |
| Mtap7d2 | -0.6352563 |
| Mtap7d3 | 1.2734409 |
| Mtbp | 0.93391 |
| Mtbp | 2.0639019 |
| Mtbp | -0.3006298 |
| Mtch1 | 1.7028348 |
| Mtch1 | 0.253571 |
| Mtch2 | -0.7098074 |
| Mtch2 | 0.2947954 |
| Mtch2 | -0.4644636 |
| Mtcp1 | 0.0815404 |
| Mtdh | 1.0164101 |
| Mterf | -0.2637156 |
| Mterf | -0.674731 |
| Mterfd1 | -0.276667 |
| Mterfd2 | -0.5403001 |
| Mterfd2 | 1.5098849 |
| Mterfd2 | 2.7761908 |
| Mterfd3 | -0.4412315 |
| Mterfd3 | -0.1733207 |
| Mtf1 | 0.3304854 |
| Mtf1 | 1.9047705 |
| Mtf2 | -0.4207952 |
| Mtfmt | -0.2689709 |
| Mtfmt | 1.1362229 |
| Mtg1 | 0.1002721 |
| Mthfd1 | -0.3080872 |
| Mthfd2 | 0.2910719 |
| Mthfs | -0.6544309 |
| Mthfsd | -0.8107428 |
| Mthfsd | -0.0063466 |
| Mtif3 | -0.6394841 |
| Mtl5 | 0.9776101 |
| Mtl5 | 1.3289408 |
| Mtmr1 | -0.2485898 |
| Mtmr11 | 0.2260161 |
| Mtmr11 | -0.2776334 |
| Mtmr12 | -0.5479692 |
| Mtmr14 | 0.6437501 |
| Mtmr14 | -0.7758985 |
| Mtmr14 | -0.1112393 |
| Mtmr14 | -0.7007285 |
| Mtmr2 | -0.4135216 |
| Mtmr2 | -0.6623545 |
| Mtmr2 | -0.3882203 |
| Mtmr3 | 0.3163172 |
| Mtmr4 | -0.203704 |
| Mtmr9 | 1.709795 |
| Mto1 | 0.0016251 |
| Mtpn | -0.5557003 |
| Mtrf1 | 3.1129741 |
| Mtrf1 | 0.5260975 |
| Mtrf1l | -0.6089059 |
| Mtrr | -0.5326921 |
| Mtss1 | -0.5859303 |
| Mttp | 2.2087743 |
| Mtus1 | 1.1330287 |
| Mtus1 | -0.3974929 |
| Mtvr2 | 0.7753342 |
| Mtvr2 | -0.4638759 |
| Mtx1 | 0.5934563 |
| Mtx1 | 2.3783973 |
| Mtx1 | 3.1839122 |
| Mtx2 | 0.3194264 |
| Muc1 | 3.9411245 |
| Muc1 | -0.7791628 |
| Muc1 | -0.5232667 |
| Muc1 | 0.5530064 |
| Muc13 | 0.3439424 |
| Muc5ac | 3.2003814 |
| Mug2 | -0.4487201 |
| Mug2 | -0.6027701 |
| Mug4 | -0.297663 |
| Mum1 | 0.1397952 |
| Mup1 | 1.5775416 |
| Mup2 | -0.4609426 |
| Mup2 | 1.4012571 |
| Mup3 | 0.1789466 |
| Mupcdh | 0.9235243 |
| Mus81 | -0.5151725 |
| Mus81 | 2.8986169 |
| Mustn1 | 0.8282401 |
| Mustn1 | 3.0321973 |
| Muted | 2.3215625 |
| Muted | 0.8141695 |
| Mutyh | 1.4005904 |
| Mvd | -0.674731 |
| Mvp | -0.4040929 |
| Mx2 | -0.5582913 |
| Mxd1 | 2.1553498 |
| Mxd4 | -0.7832589 |
| Mxi1 | -0.018062 |
| Mxra7 | 1.8264187 |
| Mxra8 | 1.0270713 |
| Mxra8 | -0.4406579 |
| Myadm | 1.4854867 |
| Mybbp1a | 0.5807529 |
| Mybbp1a | 0.2819684 |
| Mybbp1a | 2.5941319 |
| Mybl2 | -0.3090859 |
| Mybpc2 | -0.3936639 |
| Mybpc3 | -0.6331493 |
| Myc | -0.1079959 |
| Mycbp | 0.8906767 |
| Mycbp2 | -0.735229 |
| Mycbp2 | 0.6738448 |
| Mycbpap | -0.6537138 |
| Mycl1 | 0.573842 |
| Myd116 | 0.9804622 |
| Myd88 | -0.7702128 |
| Myef2 | 0.1543583 |
| Myg1 | 1.8322184 |
| Myg1 | 0.4104044 |
| Myg1 | 2.7454436 |
| Myh10 | -0.3257177 |
| Myh11 | 0.0265559 |
| Myh8 | 1.563728 |
| Myh9 | 1.7844688 |
| Myl1 | 0.2432608 |
| Myl2 | -0.456851 |
| Myl4 | 0.0318145 |
| Myl6 | 1.1553825 |
| Myl6b | 0.1744407 |
| Mylc2b | -0.6082222 |
| Mylc2b | 0.0012642 |
| Mylc2b | -0.5812679 |
| Mylc2pl | -0.3898501 |
| Mylk | 2.1246068 |
| Mylk | 1.1403923 |
| Mynn | 0.4213939 |
| Myo10 | -0.8235019 |
| Myo10 | 1.3452457 |
| Myo15 | 0.3168361 |
| Myo18a | 1.2174479 |
| Myo1b | -0.2378643 |
| Myo1c | -0.4925039 |
| Myo1c | -0.6394841 |
| Myo1f | -0.0381637 |
| Myo1g | -0.0594667 |
| Myo3b | -0.3572456 |
| Myo5a | -0.0859905 |
| Myo5b | -0.7045009 |
| Myo6 | -0.7098074 |
| Myo7a | 2.0767991 |
| Myo9a | 0.085184 |
| Myo9b | -0.6192206 |
| Myo9b | 0.8197001 |
| Myoc | 0.4643515 |
| Myocd | -0.6143932 |
| Myocd | 2.0433308 |
| Myom1 | -0.7399247 |
| Myom1 | 3.9889595 |
| Myrip | 0.1999905 |
| Mysm1 | -0.4002373 |
| Myst1 | 2.1256997 |
| Myst2 | -0.2341618 |
| Myst4 | -0.6947235 |
| Myst4 | -0.6143932 |
| Myst4 | 0.4441364 |
| Myt1 | 0.8419566 |
| Myt1l | -0.1272109 |
| Myt1l | 0.4797837 |
| Mzf1 | 0.2934672 |
| Mzf1 | -0.5602392 |
| N28178 | -0.5919585 |
| N4bp2 | 1.1554903 |
| N6amt1 | -0.7029901 |
| N6amt1 | 0.0216215 |
| N6amt2 | 0.0549559 |
| Naalad2 | 0.0600608 |
| Nab1 | -0.3075882 |
| Nab1 | -0.1629752 |
| Nab1 | -0.6116456 |
| Nab1 | -0.6380728 |
| Nab2 | 1.5241276 |
| Naca | -0.4739171 |
| Naca | -0.1908162 |
| Naca | -0.3216591 |
| Nadk | 0.7007669 |
| Nadsyn1 | 0.467458 |
| Nadsyn1 | 0.3183911 |
| Naga | 0.1702159 |
| Nagk | 1.2295906 |
| Nagk | 0.5783891 |
| Nagk | -0.1974606 |
| Nagk | -0.4774864 |
| Naglu | -0.5912868 |
| Nagpa | 2.9038978 |
| Nags | -0.7075296 |
| Nags | 0.3217517 |
| Naif1 | 2.4758491 |
| Naif1 | -0.5059176 |
| Naip5 | 0.3472144 |
| Nalp10 | -0.2337 |
| Nampt | 0.7081986 |
| Nanos1 | -0.0445806 |
| Nanos2 | 0.4248748 |
| Nans | 2.5682528 |
| Nap1l1 | -0.2387922 |
| Nap1l1 | -0.1136792 |
| Nap1l3 | -0.5680708 |
| Nap1l4 | -0.5739869 |
| Nap1l5 | -0.4464097 |
| Napa | 3.1143602 |
| Napa | -0.4140792 |
| Napa | 1.5274181 |
| Napb | 0.3973816 |
| Napepld | 0.2093255 |
| Napg | 0.8606293 |
| Napg | 0.2884034 |
| Naprt1 | 0.4129878 |
| Naprt1 | -0.4051973 |
| Napsa | -0.0001808 |
| Napsa | -0.6894999 |
| Napsa | 0.2023328 |
| Narf | -0.5759671 |
| Narfl | 2.8988253 |
| Narg1 | -0.2001313 |
| Narg1 | -0.3236863 |
| Narg1l | 1.8065192 |
| Narg1l | -0.4656402 |
| Narg1l | 1.9498086 |
| Narg2 | -0.3822681 |
| Nars | 0.8545932 |
| Nars2 | -0.5886048 |
| Nasp | -0.3090859 |
| Nasp | -0.2369373 |
| Nasp | -0.6102748 |
| Nasp | -0.756544 |
| Nat1 | -0.4383668 |
| Nat10 | -0.1859729 |
| Nat14 | 0.6993779 |
| Nat2 | -0.5628426 |
| Nat5 | -0.6338511 |
| Nat5 | -0.5806037 |
| Nat6 | 2.420277 |
| Nat6 | -0.7105679 |
| Nav1 | 0.3634082 |
| Nav1 | -0.7597426 |
| Nbl1 | 0.3142387 |
| Nbr1 | 0.7555534 |
| Nbr1 | 0.6157154 |
| Ncald | 0.071532 |
| Ncam1 | -0.0023524 |
| Ncam1 | 0.2070003 |
| Ncam2 | 0.2692394 |
| Ncan | -0.6082222 |
| Ncapd2 | 2.0388553 |
| Ncapd2 | -0.7775293 |
| Ncapd2 | 0.2197195 |
| Ncapd3 | 2.1350163 |
| Ncapg2 | -0.1434543 |
| Ncapg2 | 1.6402009 |
| Ncaph | 1.2141093 |
| Ncaph2 | 1.3765304 |
| Ncaph2 | -0.6703445 |
| Ncaph2 | -0.5232667 |
| Ncbp2 | 0.7496373 |
| Ncbp2 | 0.3643959 |
| Ncdn | 1.7958332 |
| Ncdn | 0.1819387 |
| Ncdn | -0.632448 |
| Ncdn | -0.2041515 |
| Ncf4 | -0.5263982 |
| Nck1 | -0.2332384 |
| Nckap1 | 1.9821322 |
| Nckap1l | 1.1604346 |
| Nckipsd | 0.9159372 |
| Ncl | -0.5746465 |
| Ncl | 0.0841916 |
| Ncoa2 | 0.1955831 |
| Ncoa2 | 2.2356756 |
| Ncoa4 | -0.4046449 |
| Ncoa5 | 1.0379806 |
| Ncoa6 | -0.5550537 |
| Ncoa6 | -0.3001347 |
| Ncoa6 | -0.6247678 |
| Ncor1 | -0.8329682 |
| Ncstn | 2.440772 |
| Ncstn | 0.9351324 |
| Ncstn | 2.4598746 |
| Ndc80 | -0.6865278 |
| Nde1 | 1.6637139 |
| Nde1 | 0.7368589 |
| Ndel1 | 1.5561791 |
| Ndfip1 | 0.6208228 |
| Ndfip2 | -0.7573426 |
| Ndg2 | -0.6282511 |
| Ndn | 0.3678446 |
| Ndor1 | -0.4621148 |
| Ndor1 | 2.7376048 |
| Ndor1 | -0.2028096 |
| Ndrg1 | -0.2570686 |
| Ndrg2 | -0.2383282 |
| Ndrg3 | 0.1846236 |
| Ndrg4 | -0.6501364 |
| Ndrl | 0.9482092 |
| Ndst1 | 1.0614558 |
| Ndst1 | -0.6137056 |
| Ndst4 | -0.3980411 |
| Ndufa1 | -0.0374116 |
| Ndufa10 | -0.6992237 |
| Ndufa12 | 1.9996972 |
| Ndufa13 | -0.6894999 |
| Ndufa2 | 1.1586104 |
| Ndufa3 | 0.3053544 |
| Ndufa4 | 0.7807746 |
| Ndufa5 | 0.6901241 |
| Ndufa5 | 0.2924034 |
| Ndufa6 | -0.3085864 |
| Ndufa7 | 0.2593735 |
| Ndufa8 | -0.2742553 |
| Ndufa8 | -0.7098074 |
| Ndufa9 | 0.7383614 |
| Ndufaf1 | 2.3209903 |
| Ndufb10 | -0.3768889 |
| Ndufb11 | -0.623378 |
| Ndufb2 | -0.6558667 |
| Ndufb3 | -0.6776665 |
| Ndufb4 | 0.5158341 |
| Ndufb5 | -0.7645607 |
| Ndufb6 | -0.8023305 |
| Ndufb6 | 1.6629542 |
| Ndufb7 | -0.2095382 |
| Ndufb8 | 1.1609705 |
| Ndufb9 | -0.2752192 |
| Ndufc1 | -0.7067715 |
| Ndufc1 | -0.3546218 |
| Ndufc2 | 1.1911333 |
| Ndufs2 | 0.5564434 |
| Ndufs2 | 2.0255616 |
| Ndufs3 | 2.1417145 |
| Ndufs4 | 1.0233658 |
| Ndufs4 | -0.5544074 |
| Ndufs4 | -0.409073 |
| Ndufs4 | 1.7769104 |
| Ndufs6 | 1.8537237 |
| Ndufs7 | 0.871573 |
| Ndufs8 | -0.6784018 |
| Ndufv1 | -0.6430217 |
| Ndufv2 | 0.8556265 |
| Necab1 | 2.8238508 |
| Necab2 | 0.2916047 |
| Necab3 | 3.2560634 |
| Necap1 | 0.9824019 |
| Necap1 | 1.8510229 |
| Necap1 | 1.4186752 |
| Necap2 | -0.4372234 |
| Nedd1 | 2.1562569 |
| Nedd4 | 0.9103812 |
| Nedd4l | 0.358703 |
| Nedd8 | -0.4997971 |
| Nedd9 | -0.4762952 |
| Nedd9 | -0.6020908 |
| Nefm | -0.4834651 |
| Negr1 | -0.0613921 |
| Negr1 | 2.0907392 |
| Neil1 | -0.631047 |
| Neil2 | 1.9873771 |
| Nek11 | 1.5561082 |
| Nek3 | 0.0155953 |
| Nek3 | -0.4864685 |
| Nek3 | 2.2766748 |
| Nek4 | 1.0511737 |
| Nek5 | 0.615336 |
| Nek6 | -0.7151436 |
| Nek8 | -0.73601 |
| Nek9 | 1.7502346 |
| Nek9 | 1.4352991 |
| Nelf | 0.2945299 |
| Nelf | 0.7123215 |
| Nell2 | -0.0567777 |
| Nell2 | -0.4085183 |
| Nell2 | -0.3625149 |
| Nell2 | 1.7244564 |
| Nenf | -0.7336689 |
| Neo1 | -0.1489307 |
| Nes | 0.9018456 |
| Net1 | -0.4864685 |
| Net1 | 0.0429371 |
| Neto1 | -0.2090882 |
| Neto1 | -0.7274537 |
| Neto1 | 1.7082258 |
| Neu1 | -0.5139333 |
| Neu2 | -0.8854083 |
| Neu3 | -0.4644636 |
| Neu4 | 1.9948777 |
| Neu4 | -0.5865982 |
| Neu4 | 0.4762783 |
| Neurl | -0.755746 |
| Neurod1 | 0.2961217 |
| Neurod2 | 2.2721681 |
| Neurod4 | -0.3577712 |
| Neurog1 | 1.0719813 |
| Neurog2 | 3.1018687 |
| Nf1 | 0.0681724 |
| Nfasc | -0.4013373 |
| Nfasc | 1.8567272 |
| Nfasc | -0.6544309 |
| Nfat5 | 3.0020489 |
| Nfat5 | -0.3656905 |
| Nfatc1 | -0.40575 |
| Nfatc1 | 0.8986074 |
| Nfatc2 | 0.5901502 |
| Nfatc2 | 1.7831853 |
| Nfatc2ip | -0.6999758 |
| Nfatc3 | -0.8808509 |
| Nfatc4 | -0.5886048 |
| Nfe2 | 1.5034694 |
| Nfe2l1 | -0.201916 |
| Nfe2l2 | -0.0879701 |
| Nfe2l3 | -0.1305205 |
| Nfib | -0.149776 |
| Nfic | 0.4197652 |
| Nfic | 0.1574283 |
| Nfic | -0.4979686 |
| Nfix | 0.12754 |
| Nfix | 0.2474541 |
| Nfkb1 | -0.0560108 |
| Nfkb2 | -0.6718044 |
| Nfkbia | -0.6813487 |
| Nfkbib | 0.9468694 |
| Nfkbid | 1.1950702 |
| Nfkbie | 1.6466009 |
| Nfkbil1 | 0.0159509 |
| Nfkbil2 | 0.5932622 |
| Nfkbiz | 1.8472706 |
| Nfrkb | -0.5872666 |
| Nfrkb | 2.007102 |
| Nfs1 | 0.8693943 |
| Nfs1 | 2.2177787 |
| Nfs1 | -0.8329682 |
| Nfu1 | -0.2172218 |
| Nfx1 | -0.2622873 |
| Nfya | 0.3567149 |
| Nfyb | -0.1824658 |
| Nfyc | 0.5833072 |
| Ngb | 1.2252931 |
| Ngb | 0.9229745 |
| Ngef | 1.9489171 |
| Ngfb | -0.8023305 |
| Ngfb | 0.1870039 |
| Ngfr | -0.2337 |
| Ngfrap1 | -0.4168718 |
| Ngp | -0.4469868 |
| Nhedc1 | -0.5053038 |
| Nhej1 | -0.1067824 |
| Nhlrc1 | 0.5385164 |
| Nhlrc2 | -0.6739985 |
| Nhlrc2 | -0.8434032 |
| Nhp2l1 | 2.3444806 |
| Nhsl1 | -0.3828076 |
| Niban | -0.5505393 |
| Nicn1 | -0.6164591 |
| Nicn1 | 3.3237775 |
| Nid1 | -0.846907 |
| Nid2 | -0.0859905 |
| Nif3l1 | -0.564147 |
| Nin | 0.8988894 |
| Ninj1 | -0.4846654 |
| Ninj2 | -0.9447782 |
| Nip7 | -0.0765448 |
| Nipa1 | -0.181154 |
| Nipbl | -0.2050472 |
| Nipbl | 2.0182118 |
| Nipsnap1 | -0.1582707 |
| Nipsnap3a | -0.6282511 |
| Nipsnap3a | 0.8152474 |
| Nisch | -0.1405184 |
| Nit1 | 0.2471751 |
| Nit2 | -0.2163147 |
| Nit2 | -0.7573426 |
| Nkain3 | -0.6977213 |
| Nkain4 | -0.8557233 |
| Nkd2 | -0.3344001 |
| Nkg7 | -0.1970162 |
| Nkiras1 | -0.8592727 |
| Nkiras2 | 1.5644305 |
| Nkrf | 1.2730598 |
| Nktr | 3.0233559 |
| Nkx1-2 | 0.3856279 |
| Nkx2-1 | -0.1442948 |
| Nkx2-2 | -0.8664114 |
| Nkx2-2 | 0.5909291 |
| Nkx2-3 | -0.7321113 |
| Nkx2-4 | -0.0247157 |
| Nkx2-6 | 2.8683668 |
| Nkx3-1 | 0.4477616 |
| Nkx6-1 | 2.7064391 |
| Nle1 | 0.9751401 |
| Nlgn1 | 0.7555534 |
| Nlgn2 | 1.4873155 |
| Nlk | -0.5589402 |
| Nln | 0.0758818 |
| Nln | -0.5792767 |
| Nln | -0.9001403 |
| Nlrp14 | 0.6479769 |
| Nlrp4a | -0.1754902 |
| Nlrp4c | 2.4397618 |
| Nlrp5 | 0.2645932 |
| Nlrp5 | 1.7738702 |
| Nlrp5 | -0.7328898 |
| Nlrp9a | -0.6894999 |
| Nlrp9c | -0.8460299 |
| Nlrx1 | -0.9496521 |
| Nlrx1 | 2.4306504 |
| Nmb | 0.445044 |
| Nmbr | -0.4129644 |
| Nmd3 | 1.8777945 |
| Nme1 | 0.2640451 |
| Nme2 | 1.2385233 |
| Nme2 | -0.6806111 |
| Nme2 | -0.606174 |
| Nme3 | 0.9829185 |
| Nme4 | 2.8447439 |
| Nme4 | 0.0917733 |
| Nme4 | -0.8637281 |
| Nme5 | 0.7833241 |
| Nme7 | -0.4786792 |
| Nme7 | 1.0987708 |
| Nme7 | -0.6917351 |
| Nme7 | 1.7527746 |
| Nme7 | 0.6964194 |
| Nmnat1 | 2.1769613 |
| Nmnat2 | 0.1970545 |
| Nmnat3 | 1.6045159 |
| Nmral1 | 0.2162674 |
| Nmt1 | -0.484065 |
| Nmu | -0.3604037 |
| Nmur2 | -0.6014119 |
| Nnat | 0.4099339 |
| Nnat | -0.1711561 |
| Nnmt | 1.2373371 |
| Nnmt | -0.1829035 |
| Nob1 | -0.4876726 |
| Nob1 | 1.0898088 |
| Nobox | 0.6938014 |
| Noc3l | -0.0648676 |
| Noc4l | -0.1829035 |
| Noc4l | -0.7605439 |
| Nod1 | 0.7056127 |
| Nog | 0.7136918 |
| Nol1 | 2.2627864 |
| Nol1 | 0.0360007 |
| Nol1 | 3.4399746 |
| Nol10 | 0.5337749 |
| Nol11 | -0.0453384 |
| Nol12 | -0.846907 |
| Nol14 | -0.3969449 |
| Nol3 | 0.0728725 |
| Nol4 | 2.4456952 |
| Nol4 | -0.61096 |
| Nol4 | 0.7658192 |
| Nol5 | 2.3307958 |
| Nol5a | 3.6994133 |
| Nol6 | -0.3385133 |
| Nol7 | -0.348354 |
| Nol9 | -0.6558667 |
| Nola1 | 3.1742569 |
| Nola2 | -0.3016207 |
| Nola3 | -0.5301696 |
| Nola3 | -0.6776665 |
| Nolc1 | 0.670985 |
| Nomo1 | -0.4804711 |
| Nono | 0.0009032 |
| Nope | -0.2467158 |
| Nos1 | 0.2979754 |
| Nos1ap | -0.6544309 |
| Nos2 | -0.5799399 |
| Nos3 | -0.1015418 |
| Nos3 | -0.6317473 |
| Nos3 | -0.248121 |
| Nosip | 1.3443619 |
| Nosip | -0.6437308 |
| Nosip | -0.5726691 |
| Nostrin | 0.6179887 |
| Notch1 | -0.3860516 |
| Notch3 | 0.6330074 |
| Notch4 | -0.756544 |
| Notch4 | -0.2971695 |
| Notum | -0.210439 |
| Nova2 | 0.1262741 |
| Nox4 | 0.924211 |
| Noxo1 | -0.6784018 |
| Noxo1 | 3.159945 |
| Npal2 | 0.483712 |
| Npas1 | 1.1365527 |
| Npas2 | -0.1321795 |
| Npas4 | -0.0533315 |
| Npb | -0.5428496 |
| Npc2 | 1.51906 |
| Npcd | 1.6411085 |
| Npcd | -0.766979 |
| Npdc1 | -0.5706957 |
| Npepl1 | 2.0070137 |
| Npff | -0.665978 |
| Nphp1 | -0.4040929 |
| Nphp1 | -0.9104019 |
| Nphp3 | -0.1776646 |
| Nphp4 | -0.0769365 |
| Nphs2 | -0.7758985 |
| Nploc4 | 0.5640818 |
| Npm1 | -0.1489307 |
| Npm2 | 2.3675885 |
| Npm3 | -0.6387782 |
| Npm3 | 1.3761029 |
| Npm3-ps1 | 1.7381938 |
| Npr1 | 1.9082549 |
| Npr2 | -0.6623545 |
| Npr3 | -0.1112393 |
| Npsr1 | -0.4292572 |
| Nptn | -0.3338871 |
| Nptx1 | -0.5114598 |
| Nptx2 | 0.7553896 |
| Nptxr | 1.4060768 |
| Npy | 0.6786512 |
| Npy1r | -0.1430343 |
| Npy2r | 0.172934 |
| Npy5r | -0.7159083 |
| Nqo1 | -0.2661008 |
| Nqo2 | 1.0305169 |
| Nqo2 | -0.4185513 |
| Nqo2 | -0.0947319 |
| Nr1d1 | 0.352976 |
| Nr1d2 | 1.3519362 |
| Nr1h2 | -0.7750842 |
| Nr1h2 | 0.019499 |
| Nr1h3 | -0.7391405 |
| Nr1h4 | -0.7653661 |
| Nr1h5 | -0.7906764 |
| Nr1i2 | -0.2608612 |
| Nr2c1 | 1.3074022 |
| Nr2c2ap | 1.2366444 |
| Nr2e1 | 4.412281 |
| Nr2f2 | -0.7166737 |
| Nr2f2 | 1.1120581 |
| Nr2f6 | 0.1288042 |
| Nr2f6 | 0.4581081 |
| Nr4a2 | 0.3189089 |
| Nr4a3 | 0.7687321 |
| Nr4a3 | -0.4931096 |
| Nr5a1 | -0.5251443 |
| Nr5a2 | -0.7485939 |
| Nr5a2 | 2.2592655 |
| Nr5a2 | 0.1926334 |
| Nr6a1 | -0.4551028 |
| Nradd | 0.8000449 |
| Nrap | -0.4882752 |
| Nrarp | -0.1850949 |
| Nras | -0.571353 |
| Nrbf2 | -0.2961831 |
| Nrbp1 | -0.4281246 |
| Nrbp2 | 3.6422509 |
| Nrcam | 1.7120237 |
| Nrd1 | -0.4876726 |
| Nrf1 | -0.7014818 |
| Nrg1 | 0.2524617 |
| Nrg3 | -0.7174397 |
| Nrg4 | -0.4733235 |
| Nrgn | -0.3833475 |
| Nrip2 | -0.2732923 |
| Nrip3 | 1.3135406 |
| Nrl | -0.5447662 |
| Nrm | -0.0987318 |
| Nrn1 | -0.6932281 |
| Nrn1l | -0.5511829 |
| Nrp1 | 0.3524763 |
| Nrp1 | 0.4141597 |
| Nrp2 | 0.8593076 |
| Nrp2 | -0.7783457 |
| Nrsn2 | -0.6014119 |
| Nrsn2 | -0.0883666 |
| Nrtn | -0.2077393 |
| Nrxn1 | 1.1575356 |
| Nrxn1 | 2.2126285 |
| Nrxn1 | 2.0325112 |
| Nrxn2 | 0.2154024 |
| Nrxn3 | -0.2145029 |
| Nrxn3 | -0.5706957 |
| Nsbp1 | 0.2889377 |
| Nsd1 | -0.2240532 |
| Nsd1 | -0.6688868 |
| Nsdhl | -0.2723303 |
| Nsdhl | 4.3828998 |
| Nsdhl | -0.743068 |
| Nsf | -0.0613921 |
| Nsfl1c | -0.4925039 |
| Nsg1 | 1.0246026 |
| Nsg2 | 1.0187757 |
| Nsl1 | 0.2507954 |
| Nsmaf | -0.6865278 |
| Nsmaf | -0.5576429 |
| Nsmce1 | -0.6939755 |
| Nsmce2 | -0.348354 |
| Nsmce2 | 1.8317409 |
| Nsmce2 | -0.3871353 |
| Nsun2 | -0.6082222 |
| Nsun4 | -0.5345885 |
| Nsun5 | -0.4360813 |
| Nsun5 | 0.7005934 |
| Nsun5 | -0.2149555 |
| Nsun5 | -0.4446805 |
| Nt5c | -0.766979 |
| Nt5c1b | -0.6359596 |
| Nt5c2 | 1.0815773 |
| Nt5c2 | -0.4733235 |
| Nt5c2 | -0.5010181 |
| Nt5c3 | 0.8050356 |
| Nt5c3l | -0.5589402 |
| Nt5c3l | -0.3452354 |
| Nt5dc3 | -0.321153 |
| Nt5dc3 | -0.6472842 |
| Nt5dc3 | 2.6221617 |
| Nt5e | -0.621297 |
| Nt5e | -0.407964 |
| Nt5m | -0.4309587 |
| Nt5m | 0.7887193 |
| Ntan1 | -0.7438555 |
| Ntf3 | 0.302199 |
| Nthl1 | -0.5251443 |
| Ntn2l | -0.5628426 |
| Ntn2l | -0.1019439 |
| Ntn4 | -0.0979305 |
| Ntng1 | -0.7470118 |
| Ntng1 | -0.3947563 |
| Ntng1 | 0.8631209 |
| Ntrk2 | 0.1022226 |
| Ntrk2 | -0.3364545 |
| Ntrk2 | -0.4691787 |
| Ntrk3 | 1.356487 |
| Ntrk3 | -0.6082222 |
| Ntrk3 | 1.4260904 |
| Nts | -0.0506597 |
| Ntsr1 | -0.607539 |
| Ntsr2 | 0.4466302 |
| Nuak1 | -0.556995 |
| Nuak1 | -0.5053038 |
| Nuak2 | 1.7049585 |
| Nub1 | -0.232777 |
| Nubp1 | 0.0325135 |
| Nubp2 | 0.103845 |
| Nubp2 | -0.6880127 |
| Nubp2 | 0.7807746 |
| Nucb1 | 0.6204454 |
| Nucb1 | 0.3651359 |
| Nucks1 | 1.2570141 |
| Nudc | -0.4441048 |
| Nudc | -0.3525279 |
| Nudcd2 | 0.0901303 |
| Nudcd3 | 1.6061305 |
| Nudc-ps1 | 1.0337053 |
| Nudt1 | 2.3605072 |
| Nudt10 | -0.0434451 |
| Nudt11 | 0.6610889 |
| Nudt12 | 0.9611093 |
| Nudt13 | 1.1530085 |
| Nudt14 | 0.8124732 |
| Nudt16l1 | -0.7702128 |
| Nudt18 | 1.0595512 |
| Nudt19 | -0.6020908 |
| Nudt19 | 1.8250301 |
| Nudt2 | 0.0209145 |
| Nudt21 | -0.3855101 |
| Nudt22 | 0.0966857 |
| Nudt4 | 0.6058013 |
| Nudt5 | 1.3695802 |
| Nudt5 | 1.3372617 |
| Nudt6 | 1.2669424 |
| Nudt7 | -0.664527 |
| Nudt8 | -0.2309338 |
| Nudt9 | -0.4452565 |
| Nufip1 | -0.597349 |
| Numa1 | 0.8079866 |
| Numb | 0.1908593 |
| Numb | -0.0891599 |
| Numb | -0.2090882 |
| Numb | 1.1684416 |
| Numbl | 0.2725056 |
| Nup107 | 0.253571 |
| Nup133 | 0.020207 |
| Nup133 | 1.3570979 |
| Nup155 | 1.019273 |
| Nup155 | 0.299033 |
| Nup160 | -0.6423131 |
| Nup210 | -0.6226838 |
| Nup37 | 1.8828386 |
| Nup43 | 1.5104052 |
| Nup50 | -0.3667513 |
| Nup50 | -0.4721374 |
| Nup50 | 1.6172908 |
| Nup62 | 0.1693082 |
| Nup85 | -0.3467934 |
| Nup88 | 0.8702664 |
| Nup88 | -0.6494226 |
| Nup88 | -0.2942135 |
| Nup93 | 0.3266422 |
| Nup98 | -0.1405184 |
| Nup98 | -0.0583134 |
| Nupl1 | -0.3736759 |
| Nupl1 | -0.3656905 |
| Nupl1 | -0.5733278 |
| Nupl2 | -0.39585 |
| Nupr1 | -0.2580153 |
| Nus1 | 1.799683 |
| Nusap1 | -0.4679977 |
| Nut | 1.6648524 |
| Nutf2 | 0.2643192 |
| Nvl | -0.6137056 |
| Nwd1 | 0.0992953 |
| Nxf1 | 1.4362633 |
| Nxf2 | -0.916046 |
| Nxf3 | -0.4745111 |
| Nxph1 | 1.0794791 |
| Nxph2 | 1.1814293 |
| Nxph3 | 1.3686324 |
| Nxt1 | 1.0332154 |
| Nxt1 | 0.6545554 |
| Nxt2 | 0.4518236 |
| Nyx | -0.5839293 |
| Oaf | -0.2603863 |
| Oas1b | 0.0614175 |
| Oas1b | -0.004529 |
| Oas1c | -0.4780826 |
| Oas1d | -0.429824 |
| Oas1d | 0.733847 |
| Oas1e | 1.0327254 |
| Oas1g | -0.4406579 |
| Oas1g | 1.1986819 |
| Oas2 | 0.335077 |
| Oasl1 | 1.137212 |
| Oasl2 | 0.1246894 |
| Oasl2 | -0.664527 |
| Oat | 1.5622511 |
| Oaz2 | -0.7022357 |
| Oaz3 | 0.210486 |
| Obfc1 | -0.766979 |
| Obfc2a | -0.7205098 |
| Obfc2b | -0.0927381 |
| Obfc2b | 2.3413735 |
| Obox1 | -0.4355107 |
| Obox5 | -0.5301696 |
| Obrgrp | -0.6247678 |
| Obsl1 | 0.0418998 |
| Oc90 | -0.5700388 |
| Oca2 | 0.4767172 |
| Ociad1 | -0.5786138 |
| Ociad1 | 0.5143589 |
| Ociad2 | -0.5201454 |
| Ocln | 1.6980238 |
| Ocrl | -0.0434451 |
| Odc1 | 1.6322545 |
| Odf1 | -0.247184 |
| Odf2 | 0.263771 |
| Odf2 | -0.1974606 |
| Odf2 | 0.1335301 |
| Odf2 | 1.097741 |
| Odf3 | 1.5504958 |
| Odf3l1 | -0.2439118 |
| Odf4 | -0.5226416 |
| Odf4 | -0.2425128 |
| Odz1 | -0.7485939 |
| Odz3 | 0.6008054 |
| Odz3 | -0.433232 |
| Odz4 | 0.085184 |
| Og9x | 0.1211937 |
| Ogdh | -0.5028524 |
| Ogfod1 | -0.4303912 |
| Ogfod2 | -0.0151197 |
| Ogfr | 0.7177911 |
| Ogfrl1 | -0.2613363 |
| Ogg1 | 0.7471614 |
| Ogn | 1.4761357 |
| Ogn | 0.4590025 |
| Ogn | -0.4040929 |
| Ogt | -0.3985897 |
| Ogt | 0.3227834 |
| Ogt | 0.8410583 |
| Oit1 | 1.2653108 |
| Oit3 | 0.0752139 |
| Ola1 | 0.6149564 |
| Olfm1 | -0.2208591 |
| Olfm1 | -0.6681587 |
| Olfm2 | -0.1063783 |
| Olfm2 | 0.1132011 |
| Olfm3 | 0.3507256 |
| Olfm3 | -0.3379982 |
| Olfm4 | 2.0488853 |
| Olfml1 | -0.7791628 |
| Olfml2b | -0.3651605 |
| Olfml3 | 1.7698015 |
| Olfr100 | 0.1272237 |
| Olfr1006 | 2.1703578 |
| Olfr1012 | -0.7075296 |
| Olfr1020 | -0.4487201 |
| Olfr1065 | 1.7286634 |
| Olfr1094 | 1.0956781 |
| Olfr1099 | -0.5176557 |
| Olfr1105 | 3.2537442 |
| Olfr1112 | 1.4211261 |
| Olfr1113 | -0.227258 |
| Olfr1189 | 0.3574609 |
| Olfr1197 | -0.4315265 |
| Olfr1198 | 1.6974742 |
| Olfr1249 | -0.6992237 |
| Olfr1280 | -0.5706957 |
| Olfr133 | -0.3026127 |
| Olfr1330 | -0.6240726 |
| Olfr134 | 1.0890004 |
| Olfr1340 | -0.6408976 |
| Olfr1384 | 2.9656072 |
| Olfr1386 | -0.0096271 |
| Olfr1390 | 1.0352955 |
| Olfr1394 | -0.6219902 |
| Olfr1412 | 1.826312 |
| Olfr1437 | 1.2095378 |
| Olfr144 | 1.8568306 |
| Olfr1443 | 1.7224351 |
| Olfr1443 | -0.0844097 |
| Olfr1459 | -0.2415813 |
| Olfr1500 | 0.5708648 |
| Olfr1508 | -0.5759671 |
| Olfr166 | -0.2912666 |
| Olfr168 | 2.0744147 |
| Olfr206 | 2.0489276 |
| Olfr213 | -0.1198062 |
| Olfr215 | 0.0914449 |
| Olfr232 | 0.3512261 |
| Olfr257 | -0.1881714 |
| Olfr267 | -0.3991386 |
| Olfr272 | -0.6667044 |
| Olfr303 | 2.2428796 |
| Olfr310 | 3.3877758 |
| Olfr352 | -0.7313334 |
| Olfr371 | 2.0210018 |
| Olfr378 | 1.9790897 |
| Olfr419 | 2.2134117 |
| Olfr437 | -0.6185295 |
| Olfr464 | -0.6932281 |
| Olfr47 | 1.0704511 |
| Olfr49 | -0.903859 |
| Olfr503 | -0.9057239 |
| Olfr51 | 2.717417 |
| Olfr536 | 2.0388981 |
| Olfr538 | -0.7939916 |
| Olfr54 | -0.851305 |
| Olfr550 | 1.3972499 |
| Olfr574 | -0.3991386 |
| Olfr591 | 3.5105888 |
| Olfr593 | 0.1160861 |
| Olfr608 | -0.7581419 |
| Olfr638 | -0.4562679 |
| Olfr65 | 2.2128065 |
| Olfr655 | 0.8952166 |
| Olfr658 | -0.5270257 |
| Olfr665 | -0.8107428 |
| Olfr675 | 0.1012478 |
| Olfr691 | 0.4470829 |
| Olfr692 | 0.4966909 |
| Olfr702 | -0.6199123 |
| Olfr724 | 0.7057853 |
| Olfr725 | -0.2332384 |
| Olfr748 | 0.6747367 |
| Olfr786 | -0.0625492 |
| Olfr802 | -0.785725 |
| Olfr824 | -0.3514827 |
| Olfr91 | 1.7085882 |
| Olfr910 | 2.293805 |
| Olfr920 | -0.73601 |
| Olfr924 | 2.1083794 |
| Olfr952 | 2.2646431 |
| Olfr965 | 0.3302297 |
| Olfr982 | -0.1899338 |
| Olfr983 | 0.7687321 |
| Olfr984 | -0.7014818 |
| Olig1 | -0.8124342 |
| Olig3 | -0.4458329 |
| Oma1 | -0.4487201 |
| Omd | 0.7522712 |
| Omg | 0.0216215 |
| Omt2b | 0.1884886 |
| Onecut2 | -0.7549487 |
| Onecut3 | -0.8637281 |
| Onecut3 | 1.1457419 |
| Oog4 | -0.0085324 |
| Oosp1 | -0.4937156 |
| Opa1 | -0.3277535 |
| Opa1 | 1.6928202 |
| Ophn1 | 0.16232 |
| Oplah | 2.7496943 |
| Opn1mw | -0.1084007 |
| Opn3 | -0.1375915 |
| Oprl1 | 0.3176139 |
| Oprl1 | -0.7446436 |
| Oprl1 | 0.7852318 |
| Oprs1 | -0.6296481 |
| Optc | 0.3768994 |
| Optn | 0.2599243 |
| Optn | 0.3768994 |
| Orai1 | -0.5647998 |
| Orai3 | -0.61096 |
| Oraov1 | 0.5033281 |
| Orc2l | 2.6200489 |
| Orc3l | -0.6416051 |
| Orc5l | -0.548611 |
| Orc5l | -0.6487092 |
| Orc6l | 0.5260975 |
| ORF19 | 1.8778957 |
| ORF28 | -0.4464097 |
| ORF34 | 0.0265559 |
| ORF61 | 0.8258079 |
| ORF63 | -0.2771501 |
| ORF9 | -0.4230444 |
| ORF9 | -0.7645607 |
| ORF9 | -0.6652522 |
| Orm1 | 0.8708473 |
| Ormdl2 | 0.5728506 |
| Ormdl3 | -0.407964 |
| Os9 | -0.5779515 |
| Osbp2 | -0.1239129 |
| Osbpl10 | 0.2862632 |
| Osbpl11 | 1.1805899 |
| Osbpl2 | 0.0508522 |
| Osbpl3 | 0.2188577 |
| Osbpl3 | -0.0919418 |
| Osbpl6 | -0.5926306 |
| Osbpl8 | -0.6932281 |
| Osbpl9 | -0.6007336 |
| Oscar | -0.3593498 |
| Osgep | 0.3489716 |
| Osgepl1 | 1.1457419 |
| Osgin1 | 1.0654905 |
| Osm | -0.8243586 |
| Osmr | 1.6965574 |
| Osr1 | 1.5702411 |
| Osr2 | -0.4703611 |
| Ostf1 | 0.0732073 |
| Ostm1 | -0.6932281 |
| Otof | 0.2977108 |
| Otop1 | -0.0396697 |
| Otop2 | 0.0251486 |
| Ott | -0.7685945 |
| Ott | 1.6550042 |
| OTTMUSG00000000421 | 1.2848019 |
| OTTMUSG00000000971 | -0.688756 |
| OTTMUSG00000000990 | 1.6387728 |
| OTTMUSG00000001305 | -0.8416561 |
| OTTMUSG00000002038 | 1.1887425 |
| OTTMUSG00000004461 | -0.0652545 |
| OTTMUSG00000004551 | -0.7067715 |
| OTTMUSG00000005148 | 2.1657305 |
| OTTMUSG00000005491 | 0.0066636 |
| OTTMUSG00000008540 | 2.1863791 |
| OTTMUSG00000010009 | -0.1455569 |
| OTTMUSG00000010328 | 2.1536465 |
| OTTMUSG00000015762 | -0.595324 |
| OTTMUSG00000015859 | 0.9378434 |
| OTTMUSG00000016293 | -0.258489 |
| OTTMUSG00000016790 | -0.4135216 |
| OTTMUSG00000016790 | 0.002707 |
| OTTMUSG00000017677 | 0.1568151 |
| OTTMUSG00000020946 | 1.0210115 |
| Otub1 | -0.5071464 |
| Otub2 | -0.0351588 |
| Otub2 | -0.2883287 |
| Otub2 | -0.6366635 |
| Otud5 | 0.3904428 |
| Otud6b | 0.4511478 |
| Otud7b | 0.5789806 |
| Otud7b | 2.4757409 |
| Otx1 | -0.4504566 |
| Otx2 | -0.4492986 |
| Ovca2 | -0.7807991 |
| Ovgp1 | 1.2600131 |
| Ovgp1 | 2.2721012 |
| Ovol1 | 0.9684782 |
| Ovol2 | 1.4631852 |
| Ovol2 | -0.0560108 |
| Oxct1 | -0.4487201 |
| Oxct2a | -0.5906156 |
| Oxct2b | 2.5858563 |
| Oxgr1 | 0.4459507 |
| Oxnad1 | -0.1829035 |
| Oxr1 | 0.4676795 |
| Oxr1 | -0.004529 |
| Oxt | 0.2557856 |
| P140 | 0.568277 |
| P2rx2 | -0.2140505 |
| P2rx3 | -0.2145029 |
| P2rx4 | 0.2538481 |
| P2rx4 | -0.5505393 |
| P2rx5 | 0.6971164 |
| P2rx7 | -0.6366635 |
| P2rx7 | 1.969536 |
| P2ry1 | 3.2554184 |
| P2ry12 | -0.429824 |
| P2ry13 | 2.5525166 |
| P2ry14 | -0.2561228 |
| P2ry14 | -0.5634946 |
| P2ry5 | -0.6623545 |
| P2ry6 | 1.2496216 |
| P42pop | 0.2708739 |
| P4ha1 | -0.4949288 |
| P4ha2 | -0.0963298 |
| P4hb | 1.5942259 |
| Pa2g4 | -0.1846562 |
| Pabpc1 | -0.421357 |
| Pabpc1 | -0.4349405 |
| Pabpc1 | -0.1222681 |
| Pabpc2 | -0.0254578 |
| Pabpc4 | -0.2346238 |
| Pabpn1 | -0.762952 |
| Pabpnl1 | -0.6776665 |
| Pacrg | 2.6023127 |
| Pacs1 | -0.466229 |
| Pacs2 | 0.5290097 |
| Pacsin1 | -0.0919418 |
| Pacsin1 | 0.8044132 |
| Pacsin1 | -0.7573426 |
| Pacsin2 | 1.1691855 |
| Pacsin2 | -0.5352214 |
| Pacsin3 | 0.6374673 |
| Pacsin3 | 0.450246 |
| Padi1 | 1.866091 |
| Padi2 | -0.1239129 |
| Padi6 | -0.2108897 |
| Paf1 | 0.0016251 |
| Paf1 | 0.5217124 |
| Pafah1b1 | -0.5232667 |
| Pafah1b1 | 0.2524617 |
| Pafah1b3 | 0.7620842 |
| Pafah2 | 0.0861753 |
| Pafah2 | -0.7677864 |
| Paip1 | -0.4096279 |
| Paip2 | 2.1215557 |
| Paip2b | -0.556995 |
| Paip2b | 0.9896089 |
| Pak1 | -0.7313334 |
| Pak3 | 1.036761 |
| Pak4 | 0.7826874 |
| Pak6 | 1.6535981 |
| Pak7 | -0.1642622 |
| Palld | 1.5084713 |
| Palm | 1.5655533 |
| Palm | -0.2883287 |
| Palm2 | -0.4864685 |
| Palmd | -0.5550537 |
| Palmd | 3.6629726 |
| Palmd | 3.004383 |
| Pam | 2.8052704 |
| Pank1 | -0.4219191 |
| Pank1 | -0.5872666 |
| Pank2 | 2.4088913 |
| Pank3 | 1.8176257 |
| Pank4 | 1.8254576 |
| Panx1 | -0.7367916 |
| Panx3 | -0.6580244 |
| Paox | -0.5886048 |
| Paox | 1.6390326 |
| Paox | 0.3307411 |
| Paox | 2.0453698 |
| Papd1 | -0.804007 |
| Papd1 | -0.2661008 |
| Papd4 | -0.1149015 |
| Papln | 0.0251486 |
| Papola | 1.1769089 |
| Papolb | -0.61577 |
| Papolg | -0.4774864 |
| Papss1 | -0.1894929 |
| Papss1 | -0.6451506 |
| Papss2 | 0.6396895 |
| Paqr3 | 1.3999232 |
| Paqr6 | 0.0106039 |
| Paqr7 | -0.0329114 |
| Paqr9 | -0.4349405 |
| Paqr9 | -0.0476153 |
| Paqr9 | 1.5902017 |
| Pard3 | 0.6071419 |
| Pard3 | -0.6164591 |
| Pard6a | -0.7956536 |
| Pard6a | 0.7325053 |
| Pard6b | -0.0479953 |
| Pard6g | -0.4013373 |
| Park7 | -0.2249677 |
| Parl | 0.6682962 |
| Parp1 | 1.2118771 |
| Parp1 | 0.0838606 |
| Parp1 | 3.8543582 |
| Parp12 | -0.7694033 |
| Parp14 | -0.390394 |
| Parp16 | -0.665978 |
| Parp2 | -0.4377949 |
| Parp2 | -0.7067715 |
| Parp3 | 0.0037877 |
| Parp6 | -0.804007 |
| Parp8 | 0.867502 |
| Pars2 | -0.6947235 |
| Pars2 | -0.5674157 |
| Parva | -0.6178389 |
| Parvg | -0.7205098 |
| Pask | -0.4858671 |
| Patl1 | -0.2499977 |
| Patz1 | -0.6345534 |
| Pawr | -0.1059743 |
| Pax8 | -0.2824801 |
| Paxip1 | -0.6894999 |
| Pbk | 1.9655307 |
| Pbld | 0.3439424 |
| Pbp2 | 0.6837869 |
| Pbrm1 | 0.3747048 |
| Pbrm1 | -0.5906156 |
| Pbx1 | -0.5759671 |
| Pbx1 | -0.4834651 |
| Pbx2 | 0.5889806 |
| Pbx2 | 0.856069 |
| Pbx2 | -0.524518 |
| Pbx3 | 0.3781165 |
| Pbx3 | 0.5582581 |
| Pbx4 | 1.6562806 |
| Pcaf | 0.0563198 |
| Pcaf | -0.8381714 |
| Pcbd1 | 2.3554607 |
| Pcbd2 | -0.4876726 |
| Pcbp1 | -0.6623545 |
| Pcbp2 | -0.4124075 |
| Pcbp3 | -0.6444404 |
| Pcca | 0.9317329 |
| Pcca | -0.4949288 |
| Pccb | -0.4691787 |
| Pccb | 0.4294962 |
| Pcdh1 | 0.5750303 |
| Pcdh10 | -0.6872699 |
| Pcdh10 | 2.9075168 |
| Pcdh10 | 0.5517904 |
| Pcdh15 | 1.3139971 |
| Pcdh17 | -0.5726691 |
| Pcdh20 | 1.8099462 |
| Pcdh21 | -0.8973607 |
| Pcdh7 | 3.0829054 |
| Pcdh7 | 2.2547098 |
| Pcdh8 | -0.8124342 |
| Pcdh9 | 1.2353568 |
| Pcdha10 | 0.6786512 |
| Pcdha11 | -0.0625492 |
| Pcdha2 | -0.118987 |
| Pcdha6 | 4.7020002 |
| Pcdhac1 | 1.2394119 |
| Pcdhac2 | -0.2222267 |
| Pcdhb1 | -0.2406507 |
| Pcdhb10 | 3.3295992 |
| Pcdhb12 | -0.4269933 |
| Pcdhb14 | 0.5990699 |
| Pcdhb19 | 0.6547375 |
| Pcdhb20 | -0.7832589 |
| Pcdhb22 | -0.3567202 |
| Pcdhb3 | -0.1824658 |
| Pcdhb7 | -0.0679676 |
| Pcdhb8 | -0.2005772 |
| Pcdhga10 | -0.4018878 |
| Pcdhga12 | -0.4937156 |
| Pcdhga4 | 0.0976651 |
| Pcdhga7 | -0.6522812 |
| Pcdhga8 | 2.5289009 |
| Pcdhgb2 | -0.5557003 |
| Pcdhgb6 | 1.9362525 |
| Pcdhgc3 | 2.9410313 |
| Pcdhgc4 | -0.4709528 |
| Pcdhgc5 | 2.3844853 |
| Pcf11 | 1.4948225 |
| Pcgf1 | -0.4400846 |
| Pcgf2 | 1.4939157 |
| Pcgf3 | 1.6184196 |
| Pcgf5 | -0.4258634 |
| Pcgf6 | -0.5859303 |
| Pck1 | 0.4430007 |
| Pclo | 0.2401741 |
| Pclo | 3.0554308 |
| Pcmt1 | -0.0529493 |
| Pcmt1 | -0.3161065 |
| Pcmt1 | -0.342127 |
| Pcmtd1 | -0.276667 |
| Pcmtd2 | 0.4172001 |
| Pcna | -0.1153092 |
| Pcnt | 2.2112741 |
| Pcnx | -0.276667 |
| Pcnxl2 | -0.5040773 |
| Pcnxl2 | 0.5208749 |
| Pcolce | 2.3784272 |
| Pcolce2 | 0.8878282 |
| Pcolce2 | 0.7469961 |
| Pcsk1n | 3.3828117 |
| Pcsk2 | 1.106062 |
| Pcsk2 | -0.5946499 |
| Pcsk2 | 1.0010788 |
| Pcsk2 | 1.618884 |
| Pcsk4 | 0.2963867 |
| Pcsk4 | -0.7136159 |
| Pcsk4 | -0.0140186 |
| Pcsk4 | -0.0594667 |
| Pcsk4 | 2.0093079 |
| Pcsk9 | 0.7716362 |
| Pctk1 | -0.6806111 |
| Pcx | -0.33235 |
| Pcyox1 | 1.4442606 |
| Pcyox1l | -0.4007872 |
| Pcyt1a | -0.2068411 |
| Pcyt1b | -0.6401906 |
| Pcyt1b | 0.5331547 |
| Pcyt2 | 0.2156909 |
| Pcyt2 | -0.8425292 |
| Pcyt2 | 1.1441075 |
| Pdcd10 | -0.5919585 |
| Pdcd2 | -0.5016291 |
| Pdcd2l | 2.4372318 |
| Pdcd4 | 1.3056543 |
| Pdcd5 | 1.4970858 |
| Pdcd5 | -0.6472842 |
| Pdcd6ip | 1.3769578 |
| Pdcl | -0.424171 |
| Pdcl3 | 1.1278291 |
| Pddc1 | -0.118987 |
| Pde10a | -0.6437308 |
| Pde1a | -0.0832258 |
| Pde1a | 0.8731676 |
| Pde1a | 0.8581312 |
| Pde1b | 1.3727613 |
| Pde1c | -0.181154 |
| Pde2a | 0.6305829 |
| Pde3a | -0.6345534 |
| Pde4a | 0.6330074 |
| Pde4a | -0.5980249 |
| Pde4a | -0.414637 |
| Pde4a | -0.7022357 |
| Pde4b | 1.6805256 |
| Pde4d | -0.2996398 |
| Pde4dip | 0.2950608 |
| Pde4dip | -0.2490589 |
| Pde4dip | 0.2610249 |
| Pde4dip | 2.0224807 |
| Pde4dip | -0.1689964 |
| Pde5a | -0.5720108 |
| Pde6a | -0.2981569 |
| Pde6d | -0.3893065 |
| Pde6d | -0.5980249 |
| Pde6d | -0.7189735 |
| Pde6d | 2.7982953 |
| Pde6h | -0.2932302 |
| Pde7a | 0.4897908 |
| Pdgfa | -0.7105679 |
| Pdgfb | 2.3090893 |
| Pdgfc | -0.1518926 |
| Pdgfra | -0.5700388 |
| Pdgfrb | -0.3646307 |
| Pdgfrl | 0.6472431 |
| Pdha1 | 0.4932471 |
| Pdhb | -0.7589419 |
| Pdhb | 0.0173717 |
| Pdia3 | -0.1405184 |
| Pdia4 | -0.5498961 |
| Pdia5 | -0.3478335 |
| Pdia5 | -0.3176176 |
| Pdia6 | 0.4751802 |
| Pdik1l | 0.652185 |
| Pdk1 | 2.5665581 |
| Pdk2 | -0.6820869 |
| Pdk3 | -0.5473277 |
| Pdk4 | 0.4402694 |
| Pdlim1 | 1.2300891 |
| Pdlim2 | 0.3048292 |
| Pdlim2 | -0.6116456 |
| Pdlim2 | 1.4642776 |
| Pdlim2 | -0.7906764 |
| Pdlim3 | 1.3080454 |
| Pdlim3 | 1.0600277 |
| Pdlim4 | -0.3410932 |
| Pdlim5 | 2.3741967 |
| Pdlim5 | -0.4828656 |
| Pdlim7 | -0.8065274 |
| Pdpk1 | -0.6014119 |
| Pdpk1 | 3.6384586 |
| Pdrg1 | -0.310586 |
| Pdss1 | -0.6444404 |
| Pdxdc1 | 0.9352682 |
| Pdxdc1 | 1.4020898 |
| Pdxk | -0.2158614 |
| Pdxp | -0.7462218 |
| Pdyn | 0.3663681 |
| Pdyn | 1.2930483 |
| Pdzd11 | 0.1366678 |
| Pdzd11 | -0.4423798 |
| Pdzd11 | 0.3897221 |
| Pdzd8 | 2.2564803 |
| Pdzk1 | 0.343438 |
| Pdzk1ip1 | 0.5003363 |
| Pdzrn3 | -0.631047 |
| Pdzrn3 | 0.9829185 |
| Pea15 | 1.3446271 |
| Pear1 | 0.4101692 |
| Pear1 | 0.3183911 |
| Pebp1 | -0.5993783 |
| Pecam1 | -0.5422116 |
| Pecam1 | -0.8166759 |
| Peci | 0.9494135 |
| Pef1 | 1.7218398 |
| Peg10 | -0.7120907 |
| Peg3 | 0.0805442 |
| Peli1 | -0.6977213 |
| Peli1 | 1.4959548 |
| Peli2 | 1.3950723 |
| Peli3 | -0.1930259 |
| Pelo | -0.4510362 |
| Pelp1 | -0.524518 |
| Pemt | 0.9275006 |
| Peo1 | 3.6226569 |
| Pepd | -0.3715399 |
| Per1 | -0.0254578 |
| Per1 | -0.5447662 |
| Per2 | 1.2117755 |
| Per2 | -0.7621486 |
| Per2 | 1.4245469 |
| Per3 | 0.1938144 |
| Perld1 | 0.6715219 |
| Pes1 | -0.8637281 |
| Pet112l | -0.0041659 |
| Pet112l | 0.2285801 |
| Pet112l | 0.0037877 |
| Pet112l | 1.6985731 |
| Pex11a | 0.4632396 |
| Pex11a | -0.8082113 |
| Pex11a | 0.9684782 |
| Pex11b | -0.6247678 |
| Pex11c | -0.2795691 |
| Pex14 | -0.1251483 |
| Pex16 | 0.7620842 |
| Pex19 | 0.0576819 |
| Pex19 | 0.5596671 |
| Pex2 | -0.7251335 |
| Pex26 | 0.4037968 |
| Pex3 | 0.4533986 |
| Pex3 | 3.1423534 |
| Pex5 | -0.0999351 |
| Pex5 | -0.2439118 |
| Pex5 | 2.7892656 |
| Pex5l | -0.4492986 |
| Pex6 | 0.5450743 |
| Pex6 | 1.2925817 |
| Pex6 | -0.2627632 |
| Pex7 | -0.484065 |
| Pfdn1 | -0.6254634 |
| Pfdn2 | 0.7703466 |
| Pfdn4 | -0.129692 |
| Pfdn5 | -0.1100218 |
| Pfdn5 | 1.3836002 |
| Pfkfb2 | 1.3009465 |
| Pfkfb2 | -0.706014 |
| Pfkfb4 | 0.5116131 |
| Pfkl | 1.2174479 |
| Pfkl | 0.0255006 |
| Pfkm | -0.0389164 |
| Pfkm | 1.7159862 |
| Pfkm | 0.2530165 |
| Pfkp | -0.5333238 |
| Pfkp | 0.9713577 |
| Pfkp | 1.1479168 |
| Pfn1 | 3.2092383 |
| Pfn1 | -0.5188998 |
| Pfn2 | 1.0363948 |
| Pfn2 | 1.3594508 |
| Pfn2 | -0.1079959 |
| Pfn3 | 0.2017478 |
| Pfn4 | 0.291871 |
| Pftk1 | -0.5993783 |
| Pga5 | 1.4592734 |
| Pgam1 | -0.1754902 |
| Pgam2 | 1.6053908 |
| Pgam5 | 1.1648201 |
| Pgbd5 | -0.0210135 |
| Pgcp | -0.5628426 |
| Pgd | -0.4967515 |
| Pgk1 | 0.1999905 |
| Pgk2 | -0.6472842 |
| Pgls | 1.9805895 |
| Pglyrp1 | 0.1580412 |
| Pglyrp4 | -0.2135983 |
| Pgm2 | -0.2374007 |
| Pgm2l1 | -0.5845958 |
| Pgm3 | -0.3763527 |
| Pgm5 | -0.0560108 |
| Pgm5 | -0.3171137 |
| Pgpep1 | -0.9784221 |
| Pgpep1 | 0.5352204 |
| Pgr | 0.3404061 |
| Pgrmc1 | -0.6487092 |
| Pgs1 | 0.6065676 |
| Phactr1 | -0.7136159 |
| Phactr1 | -0.5825977 |
| Phactr1 | -0.5859303 |
| Phactr4 | 0.3952336 |
| Phb2 | 0.6598219 |
| Phc1 | -0.7549487 |
| Phc1 | -0.2694501 |
| Phc2 | 1.3527254 |
| Phca | 0.0237394 |
| Phca | 2.9374706 |
| Phex | 1.6451178 |
| Phf1 | 0.9518175 |
| Phf11 | 1.7843015 |
| Phf11 | -0.6240726 |
| Phf12 | 1.026948 |
| Phf12 | 0.9490123 |
| Phf13 | 0.4297267 |
| Phf13 | -0.5563474 |
| Phf14 | 0.637838 |
| Phf16 | -0.5582913 |
| Phf17 | -0.5919585 |
| Phf19 | 1.6775363 |
| Phf2 | -0.0571614 |
| Phf20l1 | 2.7904719 |
| Phf21a | 0.5109784 |
| Phf5a | 2.108578 |
| Phf6 | -0.5518269 |
| Phf7 | 0.6170421 |
| Phgdh | 1.5304063 |
| Phka1 | -0.4745111 |
| Phka1 | 0.6040749 |
| Phka2 | -0.5839293 |
| Phka2 | -0.6289493 |
| Phka2 | -0.5301696 |
| Phkb | 1.4889128 |
| Phkg1 | 1.2427618 |
| Phkg2 | 0.1284883 |
| Phlda1 | 1.1453063 |
| Phlda2 | 1.5033945 |
| Phlda3 | 0.7748526 |
| Phlda3 | -0.7367916 |
| Phldb1 | 0.055979 |
| Phldb1 | 0.0583622 |
| Phlpp | -0.4510362 |
| Phospho1 | 1.7963291 |
| Phospho2 | 0.1044932 |
| Phox2a | 0.0429371 |
| Phox2b | 0.839109 |
| Phpt1 | 0.6463251 |
| Phtf1 | -0.8530698 |
| Phtf2 | -0.8252161 |
| Phyhip | -0.3145977 |
| Pi16 | -0.6857862 |
| Pi4k2b | 0.4636845 |
| Pi4k2b | -0.4551028 |
| Pi4ka | 1.6251645 |
| Pi4kb | -0.7791628 |
| Pi4kb | 1.0985421 |
| Pias1 | -0.9001403 |
| Pias2 | 0.4237159 |
| Pias3 | 0.7177911 |
| Pias3 | 2.7244438 |
| Pias4 | 0.4132223 |
| Pibf1 | -0.9179349 |
| Pibf1 | -0.485266 |
| Picalm | 0.9972707 |
| Pick1 | 0.4179004 |
| Pick1 | 1.6090836 |
| Pif1 | 0.270057 |
| Piga | 0.8198533 |
| Piga | 3.2297213 |
| Pigb | 0.7506259 |
| Pigc | -0.4395117 |
| Pigf | -0.6416051 |
| Pigh | -0.0525673 |
| Pigk | -0.4112947 |
| Pigk | 0.7799765 |
| Pigl | 0.2790045 |
| Pign | -0.6872699 |
| Pigo | 3.429209 |
| Pigo | -0.6007336 |
| Pigo | -0.4527769 |
| Pigp | -0.5772896 |
| Pigp | 1.4345753 |
| Pigq | -0.4418055 |
| Pigt | 1.3807956 |
| Pigx | -0.4810691 |
| Pigy | -0.5145527 |
| Pigyl | -0.7375739 |
| Pigz | -0.4157537 |
| Pih1d1 | 0.9731855 |
| Pik3ap1 | 1.0910778 |
| Pik3c2a | -0.155289 |
| Pik3c3 | -0.1820284 |
| Pik3ca | 0.5126701 |
| Pik3cb | -0.7014818 |
| Pik3cd | 1.7920232 |
| Pik3cg | -0.0140186 |
| Pik3ip1 | 0.6941509 |
| Pik3r1 | -0.6296481 |
| Pik3r1 | 0.031115 |
| Pik3r1 | 0.0878252 |
| Pik3r2 | 0.4134567 |
| Pik3r2 | -0.2675348 |
| Pik3r3 | 0.1475691 |
| Pik3r4 | 1.7632666 |
| Pik3r6 | 2.5372267 |
| Pik3r6 | -0.6696153 |
| Pim1 | -0.4638759 |
| Pim2 | 0.1335301 |
| Pim2 | 1.0844838 |
| Pim3 | 0.7097468 |
| Pip4k2a | -0.3535743 |
| Pip4k2b | -0.4703611 |
| Pip4k2b | 0.8160166 |
| Pip4k2c | -0.2383282 |
| Pip5k1a | -0.8141286 |
| Pip5k1a | 0.1855169 |
| Pip5k1b | -0.2290941 |
| Pip5kl1 | 0.1313276 |
| Pipox | -0.846907 |
| Pir | 2.1536086 |
| Pira11 | 0.8888262 |
| Pira4 | -0.1979052 |
| Pisd | -0.1591243 |
| Pit1 | -0.429824 |
| Pitpna | 2.5636282 |
| Pitpnb | 2.0876589 |
| Pitpnc1 | 2.4258767 |
| Pitpnm1 | -0.7235897 |
| Pitpnm2 | 2.3176781 |
| Pitpnm3 | 0.8537067 |
| Pitx1 | 0.5932622 |
| Pitx2 | -0.7282284 |
| Pitx2 | -0.2081887 |
| Pitx2 | 0.0470749 |
| Pitx2 | -0.0887631 |
| Piwil1 | 0.271962 |
| Piwil2 | -0.6939755 |
| Piwil4 | -0.8031684 |
| Pja1 | -0.6185295 |
| Pja2 | 1.1627903 |
| Pja2 | 2.8448541 |
| Pkd1 | 0.1935193 |
| Pkd1l3 | -0.6171488 |
| Pkd2 | -0.2776334 |
| Pkd2l1 | -0.4018878 |
| Pkd2l2 | -0.8425292 |
| Pkdrej | 0.16232 |
| Pkhd1l1 | 1.9985837 |
| Pkhd1l1 | 1.3824963 |
| Pkia | 0.24522 |
| Pkib | 1.5807224 |
| Pkib | -0.7082882 |
| Pkig | 0.067499 |
| Pkig | -0.4157537 |
| Pkig | 0.115766 |
| Pkm2 | 0.3695643 |
| Pkmyt1 | 0.5668806 |
| Pkn1 | -0.6014119 |
| Pkn1 | 1.2744879 |
| Pkn2 | 2.2906595 |
| Pkn2 | -0.4562679 |
| Pkn2 | 1.5394592 |
| Pkn3 | -0.6130184 |
| Pknox1 | 2.2537551 |
| Pknox1 | 0.4791275 |
| Pknox2 | -0.7105679 |
| Pknox2 | 1.1688667 |
| Pknox2 | 0.5306698 |
| Pknox2 | -0.3151004 |
| Pknox2 | 1.9363476 |
| Pkp1 | 1.8321123 |
| Pkp2 | -0.5825977 |
| Pkp2 | -0.5345885 |
| Pkp2 | 1.3608424 |
| Pkp3 | -0.2063923 |
| Pla1a | -0.2742553 |
| Pla1a | -0.8056865 |
| Pla1a | -0.4035411 |
| Pla2g10 | -0.3779623 |
| Pla2g12a | -0.7775293 |
| Pla2g1b | 1.2341666 |
| Pla2g2c | -0.5139333 |
| Pla2g2d | 0.4749604 |
| Pla2g2f | -0.1380091 |
| Pla2g4c | -0.0640942 |
| Pla2g4d | -0.2108897 |
| Pla2g4e | -0.2858874 |
| Pla2g4f | 1.3484207 |
| Pla2g5 | 1.9182932 |
| Pla2g6 | -0.1317645 |
| Pla2g7 | 0.059042 |
| Plaa | 0.3983347 |
| Plac1 | -0.6652522 |
| Plac1l | -0.6501364 |
| Plac8 | 0.7759758 |
| Plag1 | 1.74902 |
| Plag1 | 3.6112183 |
| Plagl2 | -0.5667611 |
| Plau | -0.0351588 |
| Plb1 | 0.9288679 |
| Plcb2 | 1.7903619 |
| Plcb3 | 1.0361506 |
| Plcb4 | 0.5821291 |
| Plcd1 | -0.4798734 |
| Plcd3 | -0.7159083 |
| Plcd4 | -0.421357 |
| Plcd4 | -0.041178 |
| Plcd4 | -0.5207688 |
| Plce1 | 2.0334569 |
| Plcg2 | 0.442546 |
| Plch2 | 0.5988769 |
| Plcl2 | 2.1378322 |
| Plcxd1 | -0.3196362 |
| Plcxd1 | -0.2425128 |
| Plcz1 | 1.7403572 |
| Pld1 | 0.5446658 |
| Pld2 | -0.7205098 |
| Pld3 | 0.5114016 |
| Pld3 | -0.1301061 |
| Pld4 | 0.1902672 |
| Pld5 | -0.4644636 |
| Pldn | 0.5304625 |
| Plec1 | 0.4743008 |
| Plec1 | -0.201916 |
| Plec1 | 0.6754496 |
| Plec1 | 1.1294916 |
| Plek | 1.7046554 |
| Plek2 | -0.4834651 |
| Plekha1 | 1.547141 |
| Plekha1 | 0.9989227 |
| Plekha2 | 1.5142615 |
| Plekha3 | -0.5537617 |
| Plekha4 | 2.4794378 |
| Plekha4 | -0.3257177 |
| Plekha5 | -0.3641013 |
| Plekha6 | 1.4694109 |
| Plekha7 | -0.5022406 |
| Plekha8 | -0.762952 |
| Plekhb1 | 0.3406591 |
| Plekhb2 | -0.0785049 |
| Plekhf1 | -0.6537138 |
| Plekhf1 | -0.3447167 |
| Plekhf2 | -0.0598515 |
| Plekhf2 | -0.4168718 |
| Plekhg2 | 1.6779728 |
| Plekhg2 | -0.1284507 |
| Plekhg2 | 0.6407986 |
| Plekhg3 | 2.0529353 |
| Plekhg3 | 0.988711 |
| Plekhg4 | 0.0546146 |
| Plekhg5 | -0.494322 |
| Plekhg6 | -0.5307996 |
| Plekhj1 | 2.6814417 |
| Plekhm1 | -0.0243448 |
| Plekhm3 | -0.6199123 |
| Plekho2 | 1.6314039 |
| Plin | -0.8090544 |
| Plk1 | 1.7137068 |
| Plk2 | -0.3236863 |
| Plk3 | 0.627403 |
| Plk4 | -0.122679 |
| Pllp | -0.6401906 |
| Plod1 | 3.6213873 |
| Plod2 | -0.1485083 |
| Plod3 | 0.8431531 |
| Plp1 | -0.4985777 |
| Plp2 | 2.9640683 |
| Plrg1 | -0.122679 |
| Pls3 | 0.0911164 |
| Plscr1 | 1.4540851 |
| Plscr1 | 1.33226 |
| Plscr3 | -0.8399122 |
| Plscr4 | 0.9110775 |
| Pltp | -0.5966735 |
| Plunc | -0.2542339 |
| Plvap | -0.4650517 |
| Plxdc1 | -0.4745111 |
| Plxdc1 | 1.45684 |
| Plxna2 | -0.4338012 |
| Plxnb1 | -0.5524714 |
| Plxnb3 | -0.7037452 |
| Plxnc1 | 0.0314648 |
| Plxnd1 | 0.7433525 |
| Pmaip1 | 0.0610785 |
| Pmch | 0.2159792 |
| Pmepa1 | -0.1947973 |
| Pmf1 | 2.6116911 |
| Pmfbp1 | 0.0924298 |
| Pml | 0.9341818 |
| Pml | 0.2945299 |
| Pmm1 | -0.4446805 |
| Pmm1 | -0.0598515 |
| Pmm2 | 0.262948 |
| Pmm2 | 0.0884843 |
| Pmm2 | 1.0669104 |
| Pmm2 | 0.5135149 |
| Pmp2 | 0.2825063 |
| Pmp22 | -0.1263854 |
| Pmp22 | -0.4632885 |
| Pmpca | 2.2160059 |
| Pmpcb | 0.5532089 |
| Pms2 | -0.5602392 |
| Pmvk | -0.2054954 |
| Pnck | 2.7340221 |
| Pnkd | -0.4882752 |
| Pnkd | 0.0705253 |
| Pnkp | -0.1868517 |
| Pnkp | -0.1447153 |
| Pnliprp1 | -0.3359405 |
| Pnma1 | 0.0237394 |
| Pnma2 | 0.7742102 |
| Pno1 | 1.7329109 |
| Pnpla2 | 2.0365455 |
| Pnpla6 | -0.0030774 |
| Pnpla7 | -0.5819325 |
| Pnpla8 | -0.3354267 |
| Pnpla8 | -0.4101832 |
| Pnpo | 1.7645772 |
| Pnpo | 0.3266422 |
| Pnrc2 | 1.0340725 |
| Podn | -0.6565854 |
| Podxl | 1.0317445 |
| Podxl2 | -0.1807171 |
| Pof1b | 0.7281316 |
| Pofut1 | 0.3129374 |
| Pofut1 | 1.5351969 |
| Pofut2 | -0.0377876 |
| Pogz | -0.8530698 |
| Pogz | 1.0545335 |
| Pola2 | -0.1518926 |
| Polb | 2.6687374 |
| Pold1 | 0.8009826 |
| Pold2 | -0.5232667 |
| Pold3 | 1.768101 |
| Pold4 | -0.6123318 |
| Poldip2 | -0.6594656 |
| Poldip3 | -0.6652522 |
| Poldip3 | 1.4591166 |
| Pole | 0.0501665 |
| Pole3 | -0.468588 |
| Pole3 | -0.4135216 |
| Pole4 | -0.0765448 |
| Polg | 1.8080438 |
| Polg2 | 2.6880449 |
| Polh | -0.7228188 |
| Poli | -0.5608894 |
| Polk | -0.3050972 |
| Poll | 4.0255032 |
| Poll | 0.9597875 |
| Polm | 1.7352624 |
| Polm | 0.2513512 |
| Poln | 1.9411397 |
| Polq | -0.4085183 |
| Polr1e | 0.4122839 |
| Polr2d | 1.2779067 |
| Polr2d | -0.7367916 |
| Polr2d | 1.0959075 |
| Polr2d | -0.5004074 |
| Polr2e | -0.785725 |
| Polr2e | -0.5980249 |
| Polr2f | 1.5559666 |
| Polr2g | -0.6268563 |
| Polr2h | 0.7012873 |
| Polr2j | -0.7282284 |
| Polr3a | -0.3844282 |
| Polr3a | 0.1460194 |
| Polr3b | -0.3704737 |
| Polr3d | 0.3449503 |
| Polr3e | 3.3528072 |
| Polr3g | -0.4024386 |
| Polr3h | -0.2277167 |
| Polr3h | -0.3318382 |
| Polr3h | -0.5687263 |
| Polr3k | -0.6977213 |
| Polr3k | 2.8511335 |
| Polrmt | 3.5031976 |
| Pols | -0.0804691 |
| Pom121 | -0.7677864 |
| Pomc | -0.6813487 |
| Pomgnt1 | -0.4281246 |
| Pomgnt1 | 0.3312522 |
| Pomp | 1.4603703 |
| Pomt1 | 0.2961217 |
| Pomt1 | -0.039293 |
| Pomt2 | -0.753356 |
| Pon1 | 3.2404013 |
| Pon2 | 1.4514804 |
| Pon3 | 0.099621 |
| Pon3 | 0.1382328 |
| Pop4 | 1.7126251 |
| Pop5 | 0.265141 |
| Pop5 | -0.1342574 |
| Pop7 | 0.9597875 |
| Popdc2 | 0.3739721 |
| Popdc2 | 1.7178418 |
| Popdc3 | -0.7259063 |
| Por | 0.7767773 |
| Por | -0.7414951 |
| Porcn | -0.3525279 |
| Porcn | -0.1604062 |
| Pou2af1 | -0.5377574 |
| Pou2f1 | -0.4906893 |
| Pou2f1 | 0.2156909 |
| Pou2f2 | -0.4937156 |
| Pou3f1 | -0.5182775 |
| Pou3f3 | -0.2844256 |
| Pou5f1 | 1.7502346 |
| Pou5f1 | -0.0254578 |
| Pou6f1 | 1.1291593 |
| Ppa1 | 1.4457725 |
| Ppa2 | -0.7939916 |
| Ppa2 | 2.4106295 |
| Ppa2 | -0.8619434 |
| Ppap2a | -0.3604037 |
| Ppap2a | -0.7525607 |
| Ppap2a | -0.7783457 |
| Ppap2b | -0.7007285 |
| Ppap2b | 1.7252281 |
| Ppap2c | -0.5126957 |
| Ppapdc1 | 1.8479492 |
| Ppapdc1a | 2.1711764 |
| Ppapdc2 | -0.4320947 |
| Ppapdc3 | 0.4524989 |
| Ppapdc3 | 1.1492194 |
| Pparbp | -0.6416051 |
| Pparg | -0.755746 |
| Ppargc1a | 2.8785807 |
| Ppargc1b | 1.5675152 |
| Ppat | -0.6254634 |
| Ppcs | -0.3303044 |
| Ppef2 | 0.8086067 |
| Ppfia3 | 2.618723 |
| Ppfia3 | -0.2805384 |
| Ppfibp1 | 1.2820687 |
| Ppfibp2 | 1.3692357 |
| Ppfibp2 | 2.6138927 |
| Ppia | 1.1699288 |
| Ppic | -0.7037452 |
| Ppic | -0.5946499 |
| Ppid | -0.2387922 |
| Ppie | -0.3604037 |
| Ppie | -0.4383668 |
| Ppif | -0.8664114 |
| Ppil2 | 0.4895744 |
| Ppil3 | -0.2922479 |
| Ppl | 0.9591258 |
| Ppm1a | -0.8425292 |
| Ppm1a | 1.4943693 |
| Ppm1a | -0.761346 |
| Ppm1b | -0.7470118 |
| Ppm1b | -0.2646689 |
| Ppm1b | 0.1976424 |
| Ppm1e | -0.7282284 |
| Ppm1e | -0.7906764 |
| Ppm1f | 2.5395813 |
| Ppm1f | -0.4258634 |
| Ppm1f | 0.2387677 |
| Ppm1g | -0.7694033 |
| Ppm1h | 1.1096868 |
| Ppm1j | -0.7205098 |
| Ppm1k | 0.9265423 |
| Ppm1l | -0.0695213 |
| Ppm1l | -0.8023305 |
| Ppm1m | -0.196128 |
| Ppm1m | 0.9523509 |
| Ppm2c | 1.4914176 |
| Ppme1 | -0.8329682 |
| Ppnr | -0.3556704 |
| Ppox | 0.6691933 |
| Ppox | -0.6472842 |
| Ppp1ca | -0.5114598 |
| Ppp1ca | -0.7906764 |
| Ppp1ca | 0.3027256 |
| Ppp1cb | -0.0788975 |
| Ppp1cb | -0.0184305 |
| Ppp1cc | -0.7391405 |
| Ppp1r10 | 0.9224245 |
| Ppp1r10 | 1.0321124 |
| Ppp1r11 | 1.3583187 |
| Ppp1r12c | -0.3262263 |
| Ppp1r13b | 2.4326607 |
| Ppp1r13b | -0.6718044 |
| Ppp1r13b | 2.8492705 |
| Ppp1r14a | 1.8836935 |
| Ppp1r14a | -0.5899448 |
| Ppp1r14c | 0.6699104 |
| Ppp1r14d | 1.3407297 |
| Ppp1r15b | 0.3544733 |
| Ppp1r16a | -0.8321037 |
| Ppp1r1a | -0.0741979 |
| Ppp1r1b | 2.4186137 |
| Ppp1r1b | 1.0494893 |
| Ppp1r2 | -0.8115881 |
| Ppp1r3c | -0.1908162 |
| Ppp1r3f | -1.0269726 |
| Ppp1r8 | -0.6352563 |
| Ppp1r9a | -0.4870704 |
| Ppp1r9b | 2.6376441 |
| Ppp2ca | 0.4160319 |
| Ppp2cb | -0.7120907 |
| Ppp2cz | -0.4846654 |
| Ppp2r1a | -0.4533579 |
| Ppp2r1b | -0.7189735 |
| Ppp2r2b | -0.632448 |
| Ppp2r2b | 2.7742941 |
| Ppp2r2b | 1.3646588 |
| Ppp2r2c | 1.3323495 |
| Ppp2r2c | 1.2157295 |
| Ppp2r2d | -1.0322858 |
| Ppp2r3c | 0.0884843 |
| Ppp2r4 | -0.4429544 |
| Ppp2r5b | -0.9188807 |
| Ppp2r5c | 0.1435346 |
| Ppp2r5c | 0.7540778 |
| Ppp2r5c | 0.3124163 |
| Ppp2r5d | 0.2513512 |
| Ppp2r5d | -0.2420469 |
| Ppp2r5d | -0.0468557 |
| Ppp2r5d | 0.6344963 |
| Ppp2r5e | -0.6275534 |
| Ppp2r5e | -0.227258 |
| Ppp3ca | -0.1746218 |
| Ppp3ca | -0.9198274 |
| Ppp3ca | -0.2350861 |
| Ppp3cb | 0.0113185 |
| Ppp3cc | -0.0034401 |
| Ppp3r1 | -0.541574 |
| Ppp4c | 0.450697 |
| Ppp4r1 | 0.1644523 |
| Ppp4r1 | 2.496203 |
| Ppp4r1 | -0.3947563 |
| Ppp4r1l | -0.149776 |
| Ppp5c | -0.4810691 |
| Ppp5c | -0.904791 |
| Ppp6c | 0.244101 |
| Pps | 0.6677576 |
| Ppt1 | 0.8084517 |
| Ppt2 | -0.8610524 |
| Pptc7 | 0.882823 |
| Pqbp1 | 0.3906829 |
| Pqlc1 | -0.2204037 |
| Pqlc2 | -0.5987014 |
| Pqlc3 | 0.7591508 |
| Pqlc3 | 1.1424702 |
| Pqlc3 | 1.5624623 |
| Pqlc3 | -0.6522812 |
| Pramef12 | 2.3292841 |
| Prap1 | 1.4620133 |
| Prc1 | -0.9104019 |
| Prcp | -0.6522812 |
| Prdm10 | 0.9070321 |
| Prdm4 | -0.5661069 |
| Prdm8 | -0.2392565 |
| Prdm9 | -0.1413563 |
| Prdx1 | 1.9430782 |
| Prdx2 | -0.5016291 |
| Prdx2 | 1.3065746 |
| Prdx2 | -0.0963298 |
| Prdx3 | 0.5946205 |
| Prdx4 | -0.665978 |
| Prdx5 | 0.8079866 |
| Prdx5 | -0.2561228 |
| Prdx6 | -0.0613921 |
| Prdx6-rs1 | -0.5071464 |
| Prdx6-rs1 | 1.0480432 |
| Preb | 1.9662686 |
| Preb | 2.1181411 |
| Prei3 | 0.3006173 |
| Prei4 | 0.2825063 |
| Prei4 | -0.0675795 |
| Prelp | 1.9164979 |
| Prep | 0.022328 |
| Prep | -0.5016291 |
| Prf1 | 0.3309967 |
| Prickle1 | -0.2383282 |
| Prickle2 | 0.6073333 |
| Prickle3 | 0.1973485 |
| Prickle4 | -0.155289 |
| Prim2 | 0.2263014 |
| Prim2 | -0.3525279 |
| Prima1 | 2.0850166 |
| Prkaa2 | 1.2521571 |
| Prkaa2 | 1.0826247 |
| Prkab1 | -0.5674157 |
| Prkab2 | -0.1802805 |
| Prkaca | -0.2443785 |
| Prkaca | -0.6240726 |
| Prkacb | 0.4479877 |
| Prkag1 | -0.5595895 |
| Prkag2 | -0.8425292 |
| Prkar1a | 2.5911178 |
| Prkar1b | 0.2897386 |
| Prkar2b | -0.3865933 |
| Prkca | -0.1409372 |
| Prkcb1 | -0.8927459 |
| Prkcbp1 | 1.6245053 |
| Prkcbp1 | -0.9774154 |
| Prkcc | -0.4360813 |
| Prkcd | 0.5955895 |
| Prkcdbp | 2.3330275 |
| Prkcdbp | -0.525771 |
| Prkce | -0.4721374 |
| Prkch | 0.080212 |
| Prkcq | -0.8601621 |
| Prkcsh | -0.406303 |
| Prkcz | 0.402612 |
| Prkcz | -0.0851997 |
| Prkcz | -0.4834651 |
| Prkd1 | 1.5217807 |
| Prkd1 | -0.4627015 |
| Prkd2 | -0.8592727 |
| Prkd3 | -0.7282284 |
| Prkd3 | 0.0934136 |
| Prkd3 | -0.5786138 |
| Prkg1 | 1.7049585 |
| Prkg2 | 0.2746768 |
| Prkra | 0.9337741 |
| Prkrip1 | -0.6206044 |
| Prkrip1 | 2.6513286 |
| Prkrir | 1.4724313 |
| Prkx | 1.6970465 |
| Prkx | 1.4774442 |
| Prl | 0.1447778 |
| Prlr | -0.804007 |
| Prlr | 0.4554198 |
| Prm1 | 1.969536 |
| Prm3 | 2.9669981 |
| Prmt2 | -0.4157537 |
| Prmt2 | -0.0792901 |
| Prmt3 | -0.4739171 |
| Prmt5 | 1.8264187 |
| Prmt5 | -0.753356 |
| Prmt5 | 0.099621 |
| Prmt7 | 0.3424284 |
| Prmt7 | 0.1668833 |
| Prmt7 | -0.914161 |
| Prmt7 | -0.0228628 |
| Prmt8 | 0.9649325 |
| Prnp | 0.1744407 |
| Prnpip1 | -0.6835649 |
| Prnpip1 | -0.5028524 |
| Prnpip1 | -0.8149769 |
| Proc | 1.0126628 |
| Proc | 2.4147885 |
| Procr | 0.9366244 |
| Prodh | 0.566681 |
| Prok2 | 0.7164267 |
| Prok2 | -0.9283906 |
| Prokr1 | 0.9074514 |
| Prokr2 | 0.6955475 |
| Prom1 | -0.7422812 |
| Prom2 | -0.0239741 |
| Prom2 | 2.2059452 |
| Pros1 | 0.6619928 |
| Prosapip1 | 1.3874114 |
| Prosapip1 | 0.1955831 |
| Prosapip1 | -0.8909062 |
| Prosc | 1.7281903 |
| Prosc | -0.1850949 |
| Prosc | 0.4891413 |
| Prox1 | -0.5872666 |
| Prox1 | 1.7849149 |
| Prpf18 | -0.5390279 |
| Prpf19 | 0.1306974 |
| Prpf19 | -0.753356 |
| Prpf19 | 3.3393224 |
| Prpf3 | -0.33235 |
| Prpf3 | 0.3966661 |
| Prpf3 | 1.0680921 |
| Prpf3 | -0.0971298 |
| Prpf3 | -0.4452565 |
| Prpf31 | 0.0401686 |
| Prpf38a | -0.73601 |
| Prpf38a | -0.9584883 |
| Prpf38a | -0.5987014 |
| Prpf38b | 2.8499284 |
| Prpf39 | 0.8198533 |
| Prpf40a | -0.6014119 |
| Prpf40a | -0.0971298 |
| Prpf40b | 0.4960461 |
| Prpf40b | -0.3201416 |
| Prpf40b | -0.2453128 |
| Prpf6 | 1.173531 |
| Prpf8 | -0.3493958 |
| Prpf8 | 0.3942774 |
| Prph2 | 0.5248466 |
| Prps1 | -0.39585 |
| Prps1l1 | -0.5832633 |
| Prps2 | -0.1899338 |
| Prpsap2 | -0.712853 |
| Prpsap2 | 0.7861842 |
| Prr11 | -0.9654179 |
| Prr13 | -0.5621911 |
| Prr14 | -0.9875298 |
| Prr15 | 2.7597068 |
| Prr16 | -0.0540962 |
| Prr18 | 0.7812532 |
| Prr19 | 1.036883 |
| Prr3 | 1.2149198 |
| Prr7 | -0.0001808 |
| Prrc1 | 2.3199406 |
| Prrg2 | 0.2382046 |
| Prrt1 | -0.4562679 |
| Prrt1 | -0.7989862 |
| Prrt1 | -0.409073 |
| Prrt3 | 0.270057 |
| Prrx1 | 0.1665798 |
| Prrx1 | 0.2876014 |
| Prrx1 | -0.1247363 |
| Prss21 | -0.7274537 |
| Prss23 | 0.8126275 |
| Prss27 | 0.7580077 |
| Prss28 | 0.2285801 |
| Prss29 | 2.6575618 |
| Prss29 | -0.2570686 |
| Prss34 | -0.2485898 |
| Prss35 | 1.596673 |
| Prss36 | 0.5339815 |
| Prss7 | -0.34368 |
| Prss8 | 1.1100259 |
| Prss8 | 2.1961398 |
| Prtn3 | 0.6266533 |
| Prune | 1.465213 |
| Prune | 1.8009993 |
| Prune | 1.803408 |
| Prune | 1.9818148 |
| Psap | -0.2902863 |
| Psat1 | -0.0377876 |
| Psca | 1.3351213 |
| Pscd1 | -0.7807991 |
| Pscd2 | -0.1523165 |
| Pscd3 | 1.6408493 |
| Pscdbp | -0.4406579 |
| Psd2 | -0.7939916 |
| Psd3 | 1.9550004 |
| Psd4 | 0.4973352 |
| Psen1 | -0.8709003 |
| Psen2 | 0.9354039 |
| Psenen | -0.1088057 |
| Psg19 | 1.0804122 |
| Psg23 | -0.7931617 |
| Psg27 | -0.1715886 |
| Psip1 | -0.8090544 |
| Psip1 | 2.4719212 |
| Pskh1 | 2.4169187 |
| Psma2 | 0.7005934 |
| Psma4 | -0.8835827 |
| Psma5 | 2.0898485 |
| Psma7 | 2.3791454 |
| Psma7 | 0.2886706 |
| Psmb1 | -0.355146 |
| Psmb10 | 1.5581603 |
| Psmb10 | -0.3241937 |
| Psmb2 | -0.7022357 |
| Psmb2 | 0.5346012 |
| Psmb3 | 0.7945562 |
| Psmb4 | -0.0434451 |
| Psmb5 | 0.0474189 |
| Psmb6 | -0.9226733 |
| Psmb7 | -0.1850949 |
| Psmb7 | -0.6718044 |
| Psmb7 | -0.5059176 |
| Psmb8 | -0.4967515 |
| Psmb9 | 1.5461394 |
| Psmc1 | 1.5152231 |
| Psmc2 | -0.7694033 |
| Psmc3 | -0.4051973 |
| Psmc3ip | -0.4309587 |
| Psmc4 | 1.4790581 |
| Psmc5 | -0.3871353 |
| Psmc5 | 0.8585725 |
| Psmd1 | 1.3219962 |
| Psmd10 | -0.7750842 |
| Psmd10 | -0.1059743 |
| Psmd10 | 0.513726 |
| Psmd11 | -0.8566094 |
| Psmd11 | 0.6485268 |
| Psmd12 | 1.7848034 |
| Psmd12 | 1.7951717 |
| Psmd13 | 0.9554122 |
| Psmd14 | 1.9238997 |
| Psmd2 | 1.2935147 |
| Psmd2 | 0.2862632 |
| Psmd4 | 0.4271883 |
| Psmd5 | -0.7159083 |
| Psmd6 | -0.6703445 |
| Psmd6 | -0.0975301 |
| Psmd7 | 0.1176852 |
| Psmd7 | 0.8219951 |
| Psmd8 | -1.07144 |
| Psmd9 | 0.971619 |
| Psmd9 | 0.7732458 |
| Psme1 | -0.1067824 |
| Psme1 | 0.6561929 |
| Psme2 | -0.7375739 |
| Psme3 | -0.1535893 |
| Psme4 | -0.9814483 |
| Psme4 | -0.9399291 |
| Psmf1 | -0.5307996 |
| Psmf1 | -0.5441269 |
| Psmg2 | 1.5388103 |
| Psmg2 | 1.7358494 |
| Psmg3 | 2.4024613 |
| Psors1c2 | 1.4496607 |
| Psp | 0.7014607 |
| Pspc1 | 1.6251645 |
| Pstpip1 | 0.3577095 |
| Pstpip2 | 1.5460678 |
| Ptbp1 | -0.1321795 |
| Ptbp1 | -0.1400997 |
| Ptcd3 | 2.1206934 |
| Ptch1 | 1.7356147 |
| Ptch2 | -0.8425292 |
| Ptdss2 | 0.7111208 |
| Pten | 2.9183264 |
| Pter | -0.6515658 |
| Ptgds | -0.2618117 |
| Ptgds2 | 2.9836764 |
| Ptgds2 | 0.7865015 |
| Ptger2 | -0.4650517 |
| Ptger2 | -0.6171488 |
| Ptger3 | 0.0041477 |
| Ptges | -0.4343707 |
| Ptges | 0.2208675 |
| Ptges2 | 0.2771135 |
| Ptges3 | 0.8117011 |
| Ptgfr | 1.1995055 |
| Ptgir | -0.7597426 |
| Ptgis | -0.2154084 |
| Ptgs1 | -1.0571151 |
| Ptgs1 | -0.4961435 |
| Pth2 | -0.1063783 |
| Pth2r | -0.8504238 |
| Pthlh | -0.9824592 |
| Pthr1 | -1.0950441 |
| Pthr1 | -0.5460461 |
| Pthr1 | 1.590475 |
| Ptk2 | 1.0306397 |
| Ptk2b | 3.3662744 |
| Ptk7 | 1.0383461 |
| Ptms | 0.1662761 |
| Ptn | 0.000542 |
| Ptp4a2 | 0.7033659 |
| Ptp4a2 | 2.2593673 |
| Ptp4a2 | -0.466229 |
| Ptp4a2 | 1.3534263 |
| Ptp4a3 | -0.0741979 |
| Ptp4a3 | -0.0169576 |
| Ptpdc1 | 0.6627153 |
| Ptpdc1 | 0.5696713 |
| Ptpdc1 | 0.9852397 |
| Ptpla | -0.5647998 |
| Ptpla | 0.222873 |
| Ptplad1 | -0.2532909 |
| Ptplad2 | 0.0573416 |
| Ptplb | -0.623378 |
| Ptpn1 | -0.852187 |
| Ptpn11 | 0.3626669 |
| Ptpn11 | -0.0213831 |
| Ptpn11 | 0.6369109 |
| Ptpn12 | -0.4973598 |
| Ptpn12 | -0.3001347 |
| Ptpn12 | -0.0023524 |
| Ptpn12 | -0.5466867 |
| Ptpn12 | -0.6178389 |
| Ptpn14 | 1.2149198 |
| Ptpn18 | 1.9834914 |
| Ptpn20 | 1.8647589 |
| Ptpn21 | -0.5582913 |
| Ptpn22 | -0.19835 |
| Ptpn5 | 1.9721968 |
| Ptpn6 | -0.9664119 |
| Ptpn6 | 0.0658136 |
| Ptpra | 0.6300225 |
| Ptprb | -0.7898494 |
| Ptprd | 1.25895 |
| Ptprd | -0.0525673 |
| Ptpre | 0.6305829 |
| Ptpre | 0.5758216 |
| Ptprf | -0.664527 |
| Ptprf | -0.6494226 |
| Ptprg | 1.2047395 |
| Ptprj | -0.6501364 |
| Ptprk | 1.4782131 |
| Ptprk | -0.2277167 |
| Ptprn | 1.6204083 |
| Ptprn2 | -0.3257177 |
| Ptpro | 0.9730551 |
| Ptpro | -0.5608894 |
| Ptprq | 0.1099852 |
| Ptprr | 1.8761241 |
| Ptprr | 1.7671929 |
| Ptprs | 0.83369 |
| Ptprs | 0.1472594 |
| Ptprt | -0.6806111 |
| Ptpru | 0.7599666 |
| Ptprv | -0.6143932 |
| Ptrf | -0.7989862 |
| Ptrh1 | 2.8966878 |
| Ptrh1 | -0.7573426 |
| Ptrh2 | 1.8455459 |
| Pts | 0.2615748 |
| Pts | -0.8486638 |
| Pttg1 | -0.660187 |
| Pttg1ip | 1.9670058 |
| Ptx3 | -0.9255277 |
| Ptx3 | -0.0518038 |
| Puf60 | 1.760011 |
| Puf60 | -0.7702128 |
| Pum1 | -0.2081887 |
| Pum2 | -0.1523165 |
| Pum2 | 0.2908054 |
| Punc | 1.0541741 |
| Purb | -0.6192206 |
| Purg | 0.2942643 |
| Pus1 | -0.756544 |
| Pus1 | -0.8700008 |
| Pus10 | -0.1595514 |
| Pus10 | 0.1552803 |
| Pus10 | -0.2800537 |
| Pus3 | -0.0449594 |
| Pus7l | -0.4733235 |
| Pvalb | -0.7767136 |
| Pvr | -0.5083768 |
| Pvr | -0.7939916 |
| Pvrl1 | 1.5372943 |
| Pvrl2 | -0.494322 |
| Pvrl2 | 0.7378608 |
| Pvrl2 | 1.0187757 |
| Pvrl3 | 0.5864415 |
| Pvrl3 | 2.4717854 |
| Pvrl3 | -0.1625465 |
| Pwp1 | 2.6999409 |
| Pwp2 | -0.0575452 |
| Pwwp2b | -0.6240726 |
| Pxdn | 1.940098 |
| Pxmp2 | 0.8279364 |
| Pxmp4 | -0.1235014 |
| Pxn | -0.4870704 |
| Pxt1 | 0.6775852 |
| Pycr2 | 1.8617806 |
| Pycrl | 2.4344409 |
| Pygb | -0.6082222 |
| Pygl | -0.1485083 |
| Pygl | 0.338633 |
| Pygm | -0.5966735 |
| Pygo1 | -0.3161065 |
| Pygo2 | 0.9779995 |
| Pyroxd1 | -0.6465724 |
| Pyy | -0.4510362 |
| Qars | 0.5260975 |
| Qdpr | 0.4317986 |
| Qpct | 1.0259612 |
| Qprt | 0.1080505 |
| Qrich2 | 0.5346012 |
| Qrich2 | 1.6564082 |
| Qrsl1 | 1.3822414 |
| Qsox1 | -0.4627015 |
| Qtrt1 | 0.575426 |
| R3hdm1 | 3.5175511 |
| R3hdm1 | -0.3582971 |
| R3hdm2 | -0.1925835 |
| R74862 | -0.6359596 |
| Rab1 | 0.3276685 |
| Rab10 | 0.9021267 |
| Rab10 | -0.9312622 |
| Rab11a | 0.299033 |
| Rab11b | 0.9532837 |
| Rab11fip2 | 1.2398067 |
| Rab11fip3 | -0.6268563 |
| Rab11fip5 | 1.4294934 |
| Rab11fip5 | 2.9496449 |
| Rab11fip5 | 1.2272944 |
| Rab12 | 0.9905059 |
| Rab13 | 2.5510685 |
| Rab13 | -0.0745886 |
| Rab14 | -0.6171488 |
| Rab15 | 0.2510733 |
| Rab17 | 1.1447616 |
| Rab17 | -0.6537138 |
| Rab18 | -0.4697697 |
| Rab19 | -0.6206044 |
| Rab19 | 1.0978555 |
| Rab1b | -0.6992237 |
| Rab1b | -0.7235897 |
| Rab1b | -0.1763593 |
| Rab21 | 1.9827668 |
| Rab22a | 2.7003683 |
| Rab22a | -0.3985897 |
| Rab23 | 1.4011738 |
| Rab24 | -0.5772896 |
| Rab24 | -0.2213148 |
| Rab24 | 0.0573416 |
| Rab25 | 1.2490356 |
| Rab27a | 3.1213568 |
| Rab27b | 2.2384875 |
| Rab28 | 0.1450883 |
| Rab28 | 1.2782859 |
| Rab2a | 1.4495816 |
| Rab31 | 0.8250465 |
| Rab32 | -0.3236863 |
| Rab33a | 1.853983 |
| Rab33b | -0.6674313 |
| Rab34 | -0.6373678 |
| Rab34 | -0.4580182 |
| Rab34 | 0.0691815 |
| Rab35 | -0.0637077 |
| Rab36 | -0.5263982 |
| Rab38 | -0.73601 |
| Rab38 | -0.6696153 |
| Rab39 | -0.5608894 |
| Rab39b | -0.6137056 |
| Rab3a | -0.6247678 |
| Rab3b | -0.5164133 |
| Rab3d | -0.1899338 |
| Rab3gap1 | -0.7136159 |
| Rab3gap2 | -0.5792767 |
| Rab3il1 | -0.2378643 |
| Rab3il1 | -0.3026127 |
| Rab3ip | 0.4278813 |
| Rab40b | -0.6317473 |
| Rab40c | 0.0775496 |
| Rab43 | -0.3262263 |
| Rab4a | -0.5966735 |
| Rab4b | 0.2825063 |
| Rab5a | 1.0449025 |
| Rab5a | -0.5602392 |
| Rab5b | -0.6268563 |
| Rab5c | -0.7399247 |
| Rab6 | -0.0254578 |
| Rab6 | -0.0078032 |
| Rab6 | -0.4085183 |
| Rab6b | -0.7742706 |
| Rab6ip1 | 1.4427462 |
| Rab7 | 0.3019355 |
| Rab7 | 3.076687 |
| Rab7 | -0.7549487 |
| Rab7l1 | -0.5832633 |
| Rab8a | -0.6769318 |
| Rab8a | -0.8209364 |
| Rab8b | -0.0563942 |
| Rab8b | 0.4775944 |
| Rab9 | -0.6226838 |
| Rab9 | -0.0005424 |
| Rab9b | 0.3574609 |
| Rabac1 | -0.674731 |
| Rabac1 | -0.495536 |
| Rabep1 | -0.5779515 |
| Rabep1 | -0.3016207 |
| Rabep2 | 0.0973387 |
| Rabgap1 | 1.7772476 |
| Rabgap1 | -0.0991328 |
| Rabgap1l | 0.9861409 |
| Rabgap1l | 0.3858693 |
| Rabgef1 | 0.8443481 |
| Rabggta | 0.5905397 |
| Rabggtb | -0.595324 |
| Rabif | 2.3001301 |
| Rabif | -0.1947973 |
| Rabl2a | 2.3834733 |
| Rabl2a | -0.0048923 |
| Rabl3 | -0.3016207 |
| Rabl4 | -0.1728874 |
| Rabl4 | 0.7455072 |
| Rabl5 | 0.5598682 |
| Rac1 | 2.2650815 |
| Rac2 | -0.4834651 |
| Rac3 | 0.2168436 |
| Rac3 | -0.0121864 |
| Rad1 | -0.2561228 |
| Rad17 | 0.9688714 |
| Rad18 | 0.8501523 |
| Rad23a | -0.3577712 |
| Rad23a | -0.6034499 |
| Rad23a | -0.3499171 |
| Rad23b | 0.7196641 |
| Rad50 | 2.0297978 |
| Rad51 | -0.6465724 |
| Rad51 | -0.4539392 |
| Rad51ap1 | 0.9207723 |
| Rad51c | 1.0582396 |
| Rad51l3 | 0.3507256 |
| Rad51l3 | 1.4714256 |
| Rad51l3 | -0.5879355 |
| Rad51l3 | 1.6813958 |
| Rad52 | -0.5390279 |
| Rad54l | 0.4301875 |
| Rad9 | 0.8149396 |
| Rad9 | 0.8144776 |
| Rad9 | -0.5946499 |
| Rad9b | -0.6068563 |
| Rae1 | -0.239721 |
| Raet1b | -0.7007285 |
| Raet1b | 0.2670558 |
| Raf1 | -0.348354 |
| Rag1 | 0.8247418 |
| Rag1ap1 | -0.8183778 |
| Rage | -0.227258 |
| Rage | -0.2981569 |
| Rai1 | -0.0498977 |
| Rai12 | 0.5799656 |
| Rai12 | 0.4288044 |
| Rai12 | -0.3683449 |
| Rai12 | 2.6502257 |
| Rai14 | 0.2507954 |
| Rala | 2.6557275 |
| Ralb | -0.6116456 |
| Ralbp1 | -0.7151436 |
| Ralgds | 0.1335301 |
| Ralgps1 | 2.093285 |
| Ralgps1 | 0.777418 |
| Ralgps1 | -0.5182775 |
| Ralgps2 | 0.2052527 |
| Ralgps2 | -0.6387782 |
| Raly | 0.9031098 |
| Raly | -0.6999758 |
| Ralyl | -0.4366522 |
| Ramp1 | -0.6102748 |
| Ramp2 | -0.3226721 |
| Ramp3 | 2.1433626 |
| Ramp3 | 0.9547476 |
| Ran | -0.6095901 |
| Ranbp1 | 0.8152474 |
| Ranbp10 | -0.6501364 |
| Ranbp17 | -0.2986509 |
| Ranbp2 | -0.6725352 |
| Ranbp3l | 0.1855169 |
| Ranbp6 | 3.0169354 |
| Ranbp9 | 1.6196133 |
| Rangap1 | -0.4207952 |
| Rangrf | 1.6317311 |
| Rap1b | -0.5170343 |
| Rap1gap | 2.5169869 |
| Rap2a | 1.8758708 |
| Rap2b | -0.5289108 |
| Rap2c | 0.0831983 |
| Rap2c | -0.6962212 |
| Rap2c | 0.0339099 |
| Rap2ip | 1.4042515 |
| Rapgef1 | -0.5102254 |
| Rapgef1 | -0.6932281 |
| Rapgef1 | 0.4368444 |
| Rapgef1 | -0.3556704 |
| Rapgef3 | -0.4858671 |
| Rapgef4 | -0.227258 |
| Rapgef5 | 2.5655004 |
| Rapgef6 | -0.7470118 |
| Rapgefl1 | 0.8476266 |
| Rapgefl1 | -0.0423109 |
| Rapsn | -0.7775293 |
| Rapsn | -0.1108333 |
| Rara | 0.737193 |
| Rarb | 0.2348188 |
| Rarb | 0.8634136 |
| Rarg | -0.2476524 |
| Rarg | 0.2446606 |
| Rarg | -0.5126957 |
| Rarres2 | 1.2139066 |
| Rars | -0.813281 |
| Rars | -0.4768906 |
| Rars2 | 0.4705544 |
| Rars2 | 1.4351383 |
| Rars2 | -0.3100857 |
| Rasa1 | -0.5563474 |
| Rasa2 | 1.3672522 |
| Rasal1 | -0.4900852 |
| Rasd1 | -0.3166099 |
| Rasef | -0.6102748 |
| Rasgef1b | -0.1750559 |
| Rasgef1b | 0.3396466 |
| Rasgef1c | 0.0888137 |
| Rasgef1c | -0.2815088 |
| Rasgrf1 | 0.704922 |
| Rasgrf1 | 0.7413592 |
| Rasgrf1 | -0.5825977 |
| Rasgrf1 | -0.3364545 |
| Rasgrp1 | 0.6745584 |
| Rasgrp1 | -0.53143 |
| Rasgrp1 | -0.4668182 |
| Rasgrp3 | 0.0088149 |
| Rasip1 | 0.9738375 |
| Rasl10a | 0.922562 |
| Rasl10b | 1.5271991 |
| Rasl10b | 1.1078761 |
| Rasl11a | 1.6998536 |
| Rasl11b | 1.8820837 |
| Rasl11b | 1.6781598 |
| Rasl12 | 2.4779553 |
| Rasl2-9 | -0.4418055 |
| Rassf1 | 1.6519337 |
| Rassf1 | -0.6423131 |
| Rassf10 | 0.3841787 |
| Rassf2 | -0.5498961 |
| Rassf2 | 0.0346073 |
| Rassf3 | 0.6115335 |
| Rassf4 | 0.4246431 |
| Rassf4 | 0.0904591 |
| Rassf4 | -0.1956842 |
| Rassf4 | -0.5628426 |
| Rassf5 | -0.5819325 |
| Rassf7 | -0.623378 |
| Rassf7 | -0.7948222 |
| Raver1 | 0.4037968 |
| Raver2 | 0.0456976 |
| Rb1 | 0.5403653 |
| Rb1 | 0.6050344 |
| Rbak | -0.7501787 |
| Rbak | -0.4751054 |
| Rbbp5 | -0.3667513 |
| Rbbp5 | -0.2699295 |
| Rbbp6 | -0.5872666 |
| Rbbp7 | -0.4679977 |
| Rbbp7 | 0.1208753 |
| Rbbp9 | -0.2313943 |
| Rbck1 | 0.0091729 |
| Rbed1 | -0.7090475 |
| Rbed1 | -0.2742553 |
| Rbj | 0.0576819 |
| Rbks | -0.3252095 |
| Rbl1 | 2.821802 |
| Rbl2 | 0.6011906 |
| Rbm10 | 1.9485884 |
| Rbm10 | 1.0461117 |
| Rbm10 | 2.3709134 |
| Rbm11 | -0.8583841 |
| Rbm12 | 2.1266355 |
| Rbm12 | 2.1073461 |
| Rbm12 | 1.7170042 |
| Rbm12 | -0.2337 |
| Rbm13 | -0.5524714 |
| Rbm13 | 0.883253 |
| Rbm14 | 0.2781945 |
| Rbm14 | 0.5086473 |
| Rbm15 | 0.3207189 |
| Rbm16 | 0.6130563 |
| Rbm18 | -0.4101832 |
| Rbm18 | 0.2234453 |
| Rbm19 | -0.3567202 |
| Rbm24 | 2.0502794 |
| Rbm26 | -0.4715449 |
| Rbm28 | 1.6324507 |
| Rbm28 | 1.2636765 |
| Rbm3 | -0.0824374 |
| Rbm35a | -0.4551028 |
| Rbm38 | 0.2825063 |
| Rbm38 | 0.496476 |
| Rbm38 | -0.4219191 |
| Rbm4 | 0.9551464 |
| Rbm41 | 0.6339382 |
| Rbm42 | -0.4429544 |
| Rbm42 | 0.1256405 |
| Rbm43 | -0.5899448 |
| Rbm45 | -0.6289493 |
| Rbm47 | 0.6407986 |
| Rbm47 | -0.5608894 |
| Rbm47 | 0.3807886 |
| Rbm47 | 1.7966595 |
| Rbm4b | -0.9486753 |
| Rbm5 | -0.5608894 |
| Rbm5 | 1.6498812 |
| Rbm6 | 0.3368567 |
| Rbm6 | 2.4975781 |
| Rbm6 | 1.8926484 |
| Rbm8a | -0.7014818 |
| Rbm9 | -0.1367569 |
| Rbm9 | 0.8494102 |
| Rbms1 | 0.4160319 |
| Rbms1 | -0.623378 |
| Rbms2 | -0.541574 |
| Rbms2 | -0.753356 |
| Rbms3 | -0.8023305 |
| Rbmx | 1.3126269 |
| Rbmy1a1 | 2.0097484 |
| Rbp1 | 0.6472431 |
| Rbp7 | 2.819164 |
| Rbpms | 1.9394818 |
| Rbpms | 1.1715197 |
| Rbpms | -0.4894815 |
| Rbpms2 | 2.0458365 |
| Rbx1 | 0.0947238 |
| Rbx1 | -0.5170343 |
| Rc3h1 | 1.7887533 |
| Rcan1 | -0.2090882 |
| Rcan2 | 0.3858693 |
| Rcan2 | 1.6676926 |
| Rcan2 | -0.3431621 |
| Rcan3 | -0.0911462 |
| Rcbtb2 | -0.8090544 |
| Rcbtb2 | -0.5518269 |
| Rcc1 | 0.3624196 |
| Rccd1 | 0.9883259 |
| Rce1 | -0.3303044 |
| Rchy1 | -0.0325373 |
| Rcl1 | 2.6155756 |
| Rcl1 | -0.6696153 |
| Rcl1 | -0.2158614 |
| Rcn3 | -0.0959301 |
| Rcor1 | 1.0468365 |
| Rcor2 | 0.1795457 |
| Rcsd1 | 0.0631107 |
| Rdbp | 0.0216215 |
| Rdbp | 0.5177275 |
| Rdh11 | -0.5628426 |
| Rdh12 | -0.6123318 |
| Rdh14 | -0.6580244 |
| Rdh14 | -0.3838877 |
| Rdh5 | 0.6888951 |
| Rdh7 | 0.8766375 |
| Rdh9 | 1.7198727 |
| Rdm1 | 0.085845 |
| Rec8 | 1.7661134 |
| Reck | 0.6851988 |
| Recql | 0.9367599 |
| Recql4 | 0.0450082 |
| Recql5 | -0.6345534 |
| Reep1 | 1.4721993 |
| Reep2 | -0.5505393 |
| Reep3 | 3.0782562 |
| Reep4 | 0.0607394 |
| Reep5 | 0.7976964 |
| Reep6 | 0.8836829 |
| Reep6 | 1.5656235 |
| Refbp2 | -0.6594656 |
| Reg1 | 0.0418998 |
| Rela | -0.6573046 |
| Relb | 0.0658136 |
| Rell1 | 1.7764606 |
| Rell2 | 2.0930832 |
| Rell2 | -0.7873726 |
| Reln | 0.1106292 |
| Relt | 0.4687862 |
| Relt | 0.9300968 |
| Rem1 | -0.3742107 |
| Rem2 | 2.0377867 |
| Renbp | 0.0600608 |
| Repin1 | -0.6857862 |
| Repin1 | -0.1243245 |
| Reps1 | -0.6331493 |
| Reps1 | 0.3890009 |
| Rer1 | 0.2217275 |
| Rer1 | 0.9987958 |
| Rerg | 0.2017478 |
| Resp18 | 2.4154799 |
| Rest | -0.5307996 |
| Ret | 1.9717386 |
| Retnla | -0.6739985 |
| Retnlg | -0.852187 |
| Retsat | -0.5028524 |
| Retsat | -0.5326921 |
| Rev1 | 1.7622397 |
| Rev3l | 1.0639498 |
| Rex3 | 2.1893397 |
| Rexo1 | -0.6674313 |
| Rexo1 | -0.1890522 |
| Rexo2 | 0.5712623 |
| Rfc1 | 2.8927479 |
| Rfc2 | 0.2806223 |
| Rfc3 | 0.5007643 |
| Rfc4 | -0.7220485 |
| Rfc4 | -0.6638023 |
| Rfc5 | 0.8973372 |
| Rfesd | 0.9257202 |
| Rffl | 1.331364 |
| Rffl | -0.6739985 |
| Rffl | 2.3729329 |
| Rffl | -0.3593498 |
| Rffl | -0.5582913 |
| Rfng | 3.2191314 |
| Rfpl4 | 0.8912453 |
| Rfpl4 | -0.2406507 |
| Rft1 | -0.395303 |
| Rftn1 | 0.1475691 |
| Rftn2 | 1.5605605 |
| Rftn2 | 0.8864008 |
| Rfwd2 | -0.4309587 |
| Rfwd3 | 0.5428251 |
| Rfwd3 | 1.3859734 |
| Rfx1 | 1.9035406 |
| Rfx1 | 1.3388639 |
| Rfx2 | 1.0310081 |
| Rfx3 | -0.7989862 |
| Rfx4 | -0.8192299 |
| Rfx4 | -0.3726073 |
| Rfx4 | 0.5744363 |
| Rfxank | 2.164982 |
| Rfxap | 2.9222132 |
| Rfxdc1 | 1.0328479 |
| Rg9mtd1 | -0.3844282 |
| Rg9mtd2 | 1.4118614 |
| Rgag4 | 1.0554913 |
| Rgl1 | 0.9128854 |
| Rgl2 | 0.1911552 |
| Rgl2 | -0.7501787 |
| Rgl3 | 0.3207189 |
| Rgma | 1.2272944 |
| Rgmb | 1.5717753 |
| Rgmb | 2.5123407 |
| Rgnef | 0.8757712 |
| Rgs1 | -0.9804385 |
| Rgs1 | 2.3193993 |
| Rgs1 | -0.9236238 |
| Rgs10 | -0.5806037 |
| Rgs12 | 3.3631842 |
| Rgs14 | 3.3973698 |
| Rgs16 | -0.7022357 |
| Rgs17 | 1.4638096 |
| Rgs2 | 1.6456339 |
| Rgs3 | 0.4533986 |
| Rgs3 | 2.2523555 |
| Rgs3 | 0.2916047 |
| Rgs3 | 1.6227893 |
| Rgs3 | -0.8183778 |
| Rgs3 | 0.0463865 |
| Rgs3 | -0.1480861 |
| Rgs4 | 3.4762283 |
| Rgs5 | 0.4565409 |
| Rgs6 | 0.8733124 |
| Rgs7 | 1.5743501 |
| Rgs7bp | -0.9179349 |
| Rgs8 | 0.8163241 |
| Rgs9 | -0.9506299 |
| Rgs9bp | -0.9255277 |
| Rhag | 3.3607156 |
| Rhbdd1 | 0.8329349 |
| Rhbdd3 | -0.6776665 |
| Rhbdf1 | -0.5339559 |
| Rhbdf2 | 0.0532481 |
| Rhbdl1 | -0.3231791 |
| Rhbdl2 | 1.0490075 |
| Rhbdl3 | -0.1092109 |
| Rhbdl7 | 1.1079894 |
| Rhcg | 1.6226572 |
| Rheb | 0.2881362 |
| Rhebl1 | -0.7783457 |
| Rhob | -0.3196362 |
| Rhobtb2 | 0.5168865 |
| Rhobtb2 | 1.1071962 |
| Rhobtb2 | -0.2932302 |
| Rhobtb2 | 2.1410238 |
| Rhobtb3 | 0.6443025 |
| Rhoc | 0.1614047 |
| Rhoc | 0.5339815 |
| Rhod | 2.8436226 |
| Rhof | -0.1157172 |
| Rhog | 1.3838547 |
| Rhoj | -0.8090544 |
| Rhot1 | 0.9691334 |
| Rhot2 | 2.2536528 |
| Rhot2 | 0.3043038 |
| Rhot2 | 0.0911164 |
| Rhou | 0.1964662 |
| Rhov | -0.2045993 |
| Rhox11 | -0.847785 |
| Rhox12 | 1.2195665 |
| Rhox3a | -0.6703445 |
| Rhox3f | 0.2300016 |
| Rhox4d | -0.5201454 |
| Rhox5 | -0.8303772 |
| Rhox8 | -0.3942099 |
| Rhpn1 | -0.1833414 |
| Rhpn1 | -0.0426888 |
| Rhpn1 | 0.3105905 |
| Ribc1 | -0.6850452 |
| Ric3 | -0.1930259 |
| Ric3 | -0.2131463 |
| Ric3 | 1.239708 |
| Ric8b | -0.9001403 |
| Ric8b | 1.2206743 |
| Rif1 | 0.2585467 |
| Rilp | -0.6776665 |
| Rilpl1 | -0.360931 |
| Rilpl1 | -0.735229 |
| Rilpl2 | 1.973433 |
| Rims1 | -0.4281246 |
| Rims2 | -0.1388448 |
| Rims3 | 2.7997984 |
| Rin1 | -0.6769318 |
| Rin2 | -0.6739985 |
| Rin2 | -0.7399247 |
| Rin2 | 1.1073096 |
| Rin3 | -0.2937217 |
| Riok1 | 0.3935596 |
| Riok1 | 2.2097036 |
| Riok3 | -0.6681587 |
| Riok3 | 0.8041018 |
| Ripk1 | 0.1109511 |
| Ripk2 | -0.0556276 |
| Ripk4 | 3.5736281 |
| Ripk5 | 1.3433002 |
| Rit1 | -0.607539 |
| Rit2 | -0.7470118 |
| Rlbp1 | -0.6652522 |
| Rlbp1l1 | 1.1747991 |
| Rlbp1l1 | -0.2392565 |
| Rln1 | -0.7391405 |
| Rltpr | -0.4366522 |
| Rmnd1 | -0.7212788 |
| Rmnd5b | -0.2699295 |
| Rmnd5b | 1.0433282 |
| Rnase1 | 1.1612919 |
| Rnase4 | 0.423252 |
| Rnase4 | 1.2363474 |
| Rnase6 | -0.754152 |
| Rnaseh1 | 0.5018334 |
| Rnaseh1 | -0.5879355 |
| Rnaseh2c | 0.4129878 |
| Rnasek | -0.7159083 |
| Rnasen | 1.2532278 |
| Rnaset2 | -0.9516086 |
| Rnaset2b | 0.1272237 |
| Rnd2 | -0.342127 |
| Rnd3 | 1.4159719 |
| Rnf10 | -0.0583134 |
| Rnf103 | -0.0495169 |
| Rnf11 | -0.7605439 |
| Rnf11 | -0.2570686 |
| Rnf11 | -0.6289493 |
| Rnf111 | 0.6075246 |
| Rnf113a1 | 2.009396 |
| Rnf113a2 | 0.7962846 |
| Rnf12 | 0.9171829 |
| Rnf121 | -0.6261596 |
| Rnf123 | -0.9057239 |
| Rnf125 | -0.8407837 |
| Rnf125 | -0.5396638 |
| Rnf128 | -0.8574963 |
| Rnf133 | 0.0116757 |
| Rnf135 | 1.1520356 |
| Rnf135 | -0.1677029 |
| Rnf135 | -0.2599116 |
| Rnf138 | 2.3172632 |
| Rnf138 | -0.0625492 |
| Rnf138 | 1.5174386 |
| Rnf138 | 1.1743766 |
| Rnf139 | 0.2963867 |
| Rnf139 | -0.8338334 |
| Rnf14 | -0.4516161 |
| Rnf14 | -0.4715449 |
| Rnf14 | -0.5428496 |
| Rnf141 | 3.2653554 |
| Rnf141 | 2.6031648 |
| Rnf144a | -0.7422812 |
| Rnf145 | -0.3828076 |
| Rnf150 | 0.3281813 |
| Rnf166 | 0.0401686 |
| Rnf167 | -0.201916 |
| Rnf167 | -0.4281246 |
| Rnf167 | -0.0935351 |
| Rnf167 | -0.5906156 |
| Rnf167 | -0.5145527 |
| Rnf168 | -0.7998212 |
| Rnf17 | 0.8541501 |
| Rnf170 | -0.7422812 |
| Rnf181 | 2.631371 |
| Rnf182 | -0.6623545 |
| Rnf182 | 1.1388583 |
| Rnf183 | -0.4157537 |
| Rnf185 | -0.7621486 |
| Rnf185 | 1.0166594 |
| Rnf185 | 1.365524 |
| Rnf185 | -0.7391405 |
| Rnf186 | -0.2439118 |
| Rnf19a | -0.8808509 |
| Rnf2 | 0.9695263 |
| Rnf20 | 0.7228906 |
| Rnf207 | 1.3645722 |
| Rnf208 | 3.7591726 |
| Rnf214 | 0.6534621 |
| Rnf215 | -0.5687263 |
| Rnf216 | 2.6078142 |
| Rnf216 | -0.0254578 |
| Rnf219 | -0.0502786 |
| Rnf24 | -0.4107388 |
| Rnf24 | 0.7833241 |
| Rnf25 | -0.5120776 |
| Rnf25 | -0.1023462 |
| Rnf25 | -0.2575418 |
| Rnf26 | -0.8808509 |
| Rnf31 | 0.9113559 |
| Rnf32 | 1.1090082 |
| Rnf34 | 0.2263014 |
| Rnf34 | -0.2542339 |
| Rnf34 | 1.2748684 |
| Rnf38 | 0.4276503 |
| Rnf38 | 0.9024077 |
| Rnf38 | 3.6700726 |
| Rnf4 | -0.8031684 |
| Rnf4 | -0.735229 |
| Rnf40 | -0.5906156 |
| Rnf41 | -0.5531163 |
| Rnf41 | -0.6465724 |
| Rnf41 | -0.5812679 |
| Rnf44 | -0.3914827 |
| Rnf5 | -0.7873726 |
| Rnf6 | -0.5232667 |
| Rnf6 | -0.750972 |
| Rnf8 | -0.1518926 |
| Rnf8 | -0.7391405 |
| Rngtt | -0.34161 |
| Rnmt | 2.6448054 |
| Rnmtl1 | 1.9014708 |
| Rnpc2 | -0.1759246 |
| Rnpep | -0.7840802 |
| Rnpepl1 | 1.0786619 |
| Rnps1 | 0.1189625 |
| Rnps1 | 0.8308175 |
| Rnu6 | -0.4786792 |
| Rnuxa | -0.3181219 |
| Rnuxa | -0.7775293 |
| Robo1 | -0.7367916 |
| Robo2 | -0.6769318 |
| Robo4 | -0.2966762 |
| Rock1 | -0.276667 |
| Rock2 | 1.0742134 |
| Rock2 | -0.1527406 |
| Rod1 | -0.1785357 |
| Rogdi | -0.5466867 |
| Rom1 | -0.7166737 |
| Ropn1 | -0.1604062 |
| Ror1 | -0.5466867 |
| Ror2 | 1.3951562 |
| Rora | 1.2752487 |
| Rorb | -0.8098982 |
| Rorb | -0.4786792 |
| Rorc | -0.5926306 |
| Rorc | 2.8330257 |
| Ros1 | -0.4931096 |
| Rp23-297j14.5 | 2.7569316 |
| Rp23-438h3.2 | 1.0862235 |
| rp9 | -0.2167681 |
| rp9 | 1.3332445 |
| Rpa1 | -0.6185295 |
| Rpa2 | -0.7274537 |
| Rpa2 | -0.7775293 |
| Rpa3 | 0.7406938 |
| Rpain | 1.5250793 |
| Rpap1 | -0.7251335 |
| Rpap1 | 1.1991968 |
| Rpap2 | -0.6206044 |
| Rpap2 | 0.4729802 |
| Rpap3 | 0.9908901 |
| Rpe | 0.7794973 |
| Rpgr | 0.0722025 |
| Rpgrip1 | 1.5919089 |
| Rpgrip1l | 2.9565162 |
| Rph3a | 0.6955475 |
| Rph3al | 2.1160549 |
| Rpia | -0.0355339 |
| Rpl12 | 0.2894717 |
| Rpl13a | -0.5083768 |
| Rpl18 | 0.2325549 |
| Rpl19 | -0.4475642 |
| Rpl21 | -0.7948222 |
| Rpl21 | 1.4652909 |
| Rpl22 | 0.7751737 |
| Rpl22l1 | -0.5126957 |
| Rpl23 | 1.0500912 |
| Rpl23 | 0.7124929 |
| Rpl23 | 0.3268989 |
| Rpl24 | 0.3322737 |
| Rpl27 | -0.5621911 |
| Rpl27a | -0.4469868 |
| Rpl28 | -0.5114598 |
| Rpl29 | -0.5739869 |
| Rpl29 | 0.1941094 |
| Rpl3 | 0.4882747 |
| Rpl30 | 1.0156618 |
| Rpl31 | -0.7367916 |
| Rpl31 | -0.4870704 |
| Rpl34 | 2.7756577 |
| Rpl35 | -0.1877313 |
| Rpl36a | 0.6443025 |
| Rpl36al | -0.5390279 |
| Rpl37 | -0.6458612 |
| Rpl38 | 0.1385454 |
| Rpl38 | -0.5238921 |
| Rpl39 | 0.922837 |
| Rpl39l | -0.8390414 |
| Rpl3l | 1.8198315 |
| Rpl4 | -0.8149769 |
| Rpl41 | 0.5200366 |
| Rpl7a | -0.3996878 |
| Rpl7l1 | 0.5391331 |
| Rpl7l1 | 0.7294795 |
| Rpl9 | -0.0430668 |
| Rplp1 | -0.7105679 |
| Rpn2 | 0.6857277 |
| Rpo1-1 | 0.1870039 |
| Rpo1-3 | 0.426726 |
| Rpo1-3 | 0.8268727 |
| Rpo1-4 | 3.1226895 |
| Rpo1-4 | 0.7724413 |
| Rpo1-4 | -0.7305562 |
| Rpo2tc1 | 2.2671693 |
| Rpp21 | 0.658372 |
| Rpp25 | -0.1604062 |
| Rpp30 | 2.1897775 |
| Rpp38 | 2.4343562 |
| Rpp40 | 2.2472515 |
| Rprm | 0.4404973 |
| Rprml | -0.129692 |
| Rps10 | 0.0501665 |
| Rps11 | 1.809077 |
| Rps12 | 0.1674901 |
| Rps13 | 0.12754 |
| Rps14 | 0.8070557 |
| Rps15 | -0.3662207 |
| Rps15a | -0.8682044 |
| Rps16 | -0.7159083 |
| Rps19 | -0.0514223 |
| Rps19 | 1.1736367 |
| Rps19bp1 | -0.8056865 |
| Rps2 | 2.1735911 |
| Rps21 | 0.268694 |
| Rps24 | 4.3129577 |
| Rps24 | 0.857984 |
| Rps25 | -0.7605439 |
| Rps26 | -0.6932281 |
| Rps27a | -0.4870704 |
| Rps27a | 0.5187778 |
| Rps27l | -0.2131463 |
| Rps3 | 0.7413592 |
| Rps3 | 1.5590787 |
| Rps3a | -0.7573426 |
| Rps4x | -0.4360813 |
| Rps4y2 | 1.7605258 |
| Rps5 | -0.5151725 |
| Rps6 | -0.1781001 |
| Rps6ka1 | 2.2250832 |
| Rps6ka3 | 3.6419481 |
| Rps6ka4 | -0.3055949 |
| Rps6ka5 | -0.5276536 |
| Rps6kb1 | 2.1586718 |
| Rps6kl1 | 0.3479679 |
| Rps7 | 1.230388 |
| Rps8 | -0.4429544 |
| Rps9 | -0.3795747 |
| Rps9 | -0.0487558 |
| Rpsa | -0.1629752 |
| Rpusd1 | 0.9487447 |
| Rpusd4 | -0.1850949 |
| Rqcd1 | -0.8574963 |
| Rrad | 3.2864754 |
| Rragb | 0.8089166 |
| Rragc | -0.7849022 |
| Rragd | -0.5383924 |
| Rras | -0.545406 |
| Rras2 | -0.7597426 |
| Rrbp1 | 0.1837295 |
| Rrbp1 | 1.3663021 |
| Rreb1 | -0.1894929 |
| Rreb1 | -0.1405184 |
| Rrh | -0.660909 |
| Rrm1 | -0.4389391 |
| Rrm2 | 1.9382484 |
| Rrm2 | -0.8610524 |
| Rrm2b | -0.409073 |
| Rrn3 | 1.2620394 |
| Rrp1 | -0.5182775 |
| Rrp12 | 0.4033231 |
| Rrp15 | 0.111916 |
| Rrp1b | -0.5872666 |
| Rrp1b | -0.3036058 |
| Rrp1b | 1.3900281 |
| Rrp9 | -0.3031091 |
| Rs1 | -0.3747457 |
| Rsad1 | -0.6551485 |
| Rsad2 | 0.9564747 |
| Rsf1 | 2.0274226 |
| Rshl1 | -0.5926306 |
| Rshl2a | -0.150199 |
| Rsl1d1 | 1.345069 |
| Rsl1d1 | 0.0597213 |
| Rsn | 0.4949705 |
| Rsph1 | -0.3075882 |
| Rspo1 | -0.2937217 |
| Rspo2 | 0.340153 |
| Rspo3 | 1.6191493 |
| Rspo4 | -0.6317473 |
| Rspry1 | -0.4264282 |
| Rsrc1 | 0.8465847 |
| Rsrc1 | 0.3202021 |
| Rsrc2 | 0.3661218 |
| Rsu1 | -0.6359596 |
| Rtbdn | -0.485266 |
| Rtel1 | 0.0120327 |
| Rtel1 | -0.3763527 |
| Rtkn | 0.8455416 |
| Rtn1 | 0.3599435 |
| Rtn1 | -0.2853999 |
| Rtn1 | 0.8178601 |
| Rtn1 | 2.1362903 |
| Rtn2 | -0.7328898 |
| Rtn3 | -0.0347839 |
| Rtn4 | -0.9179349 |
| Rtn4 | -0.9341424 |
| Rtn4 | -0.018799 |
| Rtn4ip1 | 1.5078009 |
| Rtn4r | 0.0973387 |
| Rtn4rl1 | -0.6275534 |
| Rtn4rl2 | 1.080179 |
| Rtp1 | -0.5270257 |
| Rtp4 | -0.7438555 |
| Rttn | -0.6303473 |
| Rufy1 | 0.6895976 |
| Rufy2 | 0.9482092 |
| Rufy3 | 2.5674427 |
| Rufy3 | 0.6962451 |
| Rufy3 | 1.1638592 |
| Rufy4 | -0.5892746 |
| Rundc1 | -0.6289493 |
| Rundc3b | -0.3710067 |
| Runx1 | -0.3282632 |
| Runx1t1 | 0.8065899 |
| Runx1t1 | -0.6373678 |
| Runx2 | 2.5633569 |
| Rusc1 | -0.631047 |
| Rusc2 | 0.0219748 |
| Rusc2 | -0.6529972 |
| Ruvbl1 | 0.0943964 |
| Ruvbl1 | -0.5912868 |
| Ruvbl1 | -0.321153 |
| Ruvbl2 | 1.4583321 |
| Rwdd1 | -0.6594656 |
| Rwdd1 | 2.1961398 |
| Rwdd3 | 0.0539316 |
| Rwdd3 | 0.9688714 |
| Rwdd4a | -0.5102254 |
| Rxfp1 | 0.8042575 |
| Rxfp3 | -0.196128 |
| Rxra | 0.0762156 |
| Rxrb | 0.0576819 |
| Rxrb | -0.5010181 |
| Rxrg | -0.3540979 |
| Rya3 | -0.456851 |
| Ryk | 1.2153247 |
| Ryk | -0.6387782 |
| Ryr1 | 0.4999082 |
| Ryr1 | 3.6649821 |
| Ryr1 | 1.0106583 |
| Ryr3 | -0.6226838 |
| S100a1 | -0.169428 |
| S100a10 | -0.813281 |
| S100a11 | 0.3011448 |
| S100a13 | 0.6044588 |
| S100a16 | -0.456851 |
| S100a4 | -0.6616315 |
| S100a6 | 0.6071419 |
| S100a8 | -0.1974606 |
| S100a9 | -0.3656905 |
| S100b | -0.6437308 |
| S100pbp | 0.5650823 |
| S100pbp | 0.6476101 |
| S3-12 | 1.8139559 |
| Saa3 | -0.6178389 |
| Sac3d1 | -0.2172218 |
| Sacm1l | -0.0491363 |
| Sacs | -0.5966735 |
| Sae1 | 0.3614299 |
| Sae1 | 0.3542239 |
| Sae1 | -0.4258634 |
| Sag | 0.3499743 |
| Sall1 | -0.4556852 |
| Sall1 | -0.5661069 |
| Sall2 | -0.674731 |
| Sall4 | -0.3201416 |
| Sall4 | 2.3161135 |
| Samd10 | -0.6458612 |
| Samd11 | 0.0597213 |
| Samd12 | -0.4185513 |
| Samd14 | 1.4007571 |
| Samd4 | -0.3540979 |
| Samd4 | 1.8858523 |
| Samd4b | 0.5215031 |
| Samd5 | -0.6020908 |
| Samd7 | -0.9283906 |
| Samd8 | 0.4619036 |
| Samd9l | -0.6014119 |
| Samhd1 | -0.03854 |
| Samm50 | 1.4365042 |
| Samsn1 | -0.0769365 |
| Sap130 | -0.6437308 |
| Sap30 | 1.9912471 |
| Sap30bp | 3.1475179 |
| Sap30l | -0.6776665 |
| Saps1 | 1.1831058 |
| Saps2 | 0.4040336 |
| Saps3 | 0.1491164 |
| Saps3 | 2.3587363 |
| Sar1a | -0.3344001 |
| Sar1b | 0.4464038 |
| Sar1b | -0.6054923 |
| Sarm1 | -0.6123318 |
| Sars | -0.2622873 |
| Sars | 2.0651496 |
| Sars2 | -0.7166737 |
| Sart1 | -0.3333745 |
| Sart3 | -0.5053038 |
| Sas | 2.1805039 |
| Sass6 | -0.5926306 |
| Sat2 | -0.7014818 |
| Satb1 | 1.7245157 |
| Sav1 | 2.4518137 |
| Sbds | -0.0699102 |
| Sbf1 | -0.1104274 |
| Sbk | -0.390394 |
| Sbno1 | 0.2070003 |
| Sbno2 | 0.3621723 |
| Sc4mol | -0.3267351 |
| Sc5d | -0.3914827 |
| Scaf1 | -0.6850452 |
| Scamp1 | 0.6067591 |
| Scamp2 | -0.6034499 |
| Scamp3 | -0.7549487 |
| Scamp4 | -0.4157537 |
| Scamp5 | 2.0105409 |
| Scamp5 | -0.0063466 |
| Scand1 | 1.0286726 |
| Scap | 1.0112852 |
| Scaper | 0.0691815 |
| Scara3 | -0.4562679 |
| Scara5 | 1.0814608 |
| Scarb1 | 2.3823708 |
| Scarb2 | 0.1083732 |
| Scarf1 | -0.5667611 |
| Scarf2 | 0.8995941 |
| Sccpdh | -0.6054923 |
| Scd1 | -0.0232331 |
| Scd2 | 1.2602063 |
| Scd4 | -0.3036058 |
| Scel | -0.7274537 |
| Scg2 | 0.2365132 |
| Scg3 | -0.2632393 |
| Scg5 | 0.8866865 |
| Scgn | 1.6505231 |
| Scgn | -0.6924813 |
| Scgn | 2.1347073 |
| Schip1 | 1.9065879 |
| Schip1 | 0.6527325 |
| Scin | 0.1515867 |
| scl0003251.1_21 | 1.9425113 |
| Sclt1 | -0.7462218 |
| Scly | -0.8700008 |
| Scmh1 | -0.4258634 |
| Scmh1 | 0.5424156 |
| Scml2 | 1.0569262 |
| Scml4 | -0.0430668 |
| Scn10a | 2.1719944 |
| Scn11a | -0.6551485 |
| Scn1a | 0.9247601 |
| Scn2b | 1.3275019 |
| Scn3b | -0.4469868 |
| Scn3b | 1.0301483 |
| Scn3b | -0.1625465 |
| Scn3b | 0.9708348 |
| Scn4b | -0.7989862 |
| Scn5a | -0.1468207 |
| Scn7a | -0.3252095 |
| Scn8a | 1.6785961 |
| Scnm1 | 0.5973313 |
| Scnm1 | 1.2373371 |
| Scnn1a | -0.7758985 |
| Scnn1b | 1.3056543 |
| Scnn1g | -0.247184 |
| Scoc | -0.3672822 |
| Scoc | 1.5076518 |
| Scotin | -0.130935 |
| Scotin | -0.7259063 |
| Scotin | -0.0217528 |
| Scp2 | -0.2868632 |
| Scp2 | 1.9757636 |
| Scp2 | -0.0438234 |
| Scrg1 | -0.5201454 |
| Scrib | 1.8326957 |
| Scrib | 0.7615959 |
| Scrn1 | -0.2054954 |
| Scrn1 | -0.0749795 |
| Scrn1 | -0.40741 |
| Scrn2 | -0.7915041 |
| Sct | -0.7143795 |
| Sctr | 3.6571696 |
| Scube1 | 1.3005763 |
| Scube2 | 0.6915266 |
| Scube3 | 0.8070557 |
| Scube3 | -0.61577 |
| Scx | -0.7344486 |
| Scye1 | -0.1672722 |
| Scyl1 | 1.8000123 |
| Scyl1bp1 | 1.545495 |
| Scyl3 | 4.31422 |
| Scyl3 | -0.5145527 |
| Sdad1 | -0.5083768 |
| Sdc1 | 0.5521959 |
| Sdc1 | -0.433232 |
| Sdc2 | 0.1849215 |
| Sdc3 | -0.3499171 |
| Sdc4 | 4.5449282 |
| Sdcbp | 2.8205593 |
| Sdcbp | 3.2761024 |
| Sdcbp2 | 0.2665091 |
| Sdcbp2 | 0.1227842 |
| Sdccag1 | 0.8093812 |
| Sdccag1 | 0.1577348 |
| Sdccag3 | -0.631047 |
| Sdccag3 | 0.340153 |
| Sdf2 | -0.6373678 |
| Sdf2 | -0.4236076 |
| Sdf4 | -0.6813487 |
| Sdhb | 0.663798 |
| Sdhb | 2.3076395 |
| Sdhb | -0.6171488 |
| Sdhc | -0.53143 |
| Sdhd | -0.1850949 |
| Sdk1 | 0.8016073 |
| Sdpr | -0.1668416 |
| Sds | -0.6331493 |
| Sdsl | -0.6296481 |
| Sec1 | -0.6458612 |
| Sec11a | -0.5544074 |
| Sec11c | 2.2199724 |
| Sec13 | 1.4917207 |
| Sec14l1 | 0.7865015 |
| Sec14l1 | 0.3186501 |
| Sec14l1 | -0.4219191 |
| Sec14l2 | 2.9063292 |
| Sec14l3 | 0.3690732 |
| Sec14l3 | -0.8381714 |
| Sec14l4 | -0.2528198 |
| Sec15l1 | 0.8098457 |
| Sec16a | 0.1693082 |
| Sec16b | 0.0415538 |
| Sec22a | -0.4151952 |
| Sec22b | -0.914161 |
| Sec22c | 1.7832412 |
| Sec22c | -0.4745111 |
| Sec23a | 0.1586536 |
| Sec23ip | -0.1894929 |
| Sec24b | 0.5018334 |
| Sec24c | 0.64871 |
| Sec24d | 2.4963353 |
| Sec61a1 | -0.3488747 |
| Sec61a2 | -0.429824 |
| Sec61a2 | -0.6345534 |
| Sec61b | 2.1372928 |
| Sec61b | 0.333039 |
| Sec61g | 0.1288042 |
| Sec63 | 0.5260975 |
| Sec63 | 1.5034694 |
| Secisbp2 | 1.4927053 |
| Sectm1b | -0.8014933 |
| Seh1l | -0.8364339 |
| Seh1l | 0.6788288 |
| Sel1l | 0.0155953 |
| Sel1l | -0.3540979 |
| Sel1l | 0.5483365 |
| Sel1l2 | 1.5653429 |
| Sel1l2 | 0.8036346 |
| Selenbp2 | 0.5033281 |
| Selk | 1.1174562 |
| Sell | 1.0743307 |
| Selp | -0.1646917 |
| Selplg | -0.6430217 |
| Sema3a | -0.3426444 |
| Sema3b | 0.5946205 |
| Sema3b | 0.2714181 |
| Sema3b | 2.0315645 |
| Sema3e | -0.6820869 |
| Sema3f | 0.19823 |
| Sema3f | 0.423484 |
| Sema3g | -0.8149769 |
| Sema4a | 1.416218 |
| Sema4b | -0.6123318 |
| Sema4d | 1.0989995 |
| Sema4f | 0.1028719 |
| Sema4g | 1.7018623 |
| Sema5a | -0.7881975 |
| Sema5a | -0.5201454 |
| Sema5b | 2.0644428 |
| Sema6b | -0.8303772 |
| Sema6b | 1.1111554 |
| Sema6b | -0.3055949 |
| Sema6d | 0.5391331 |
| Sema6d | -0.6587447 |
| Sema7a | -0.8243586 |
| Sema7a | -0.8183778 |
| Senp1 | -0.7573426 |
| Senp2 | 0.5007643 |
| Senp3 | 0.4845828 |
| Senp5 | -0.7653661 |
| Senp5 | 0.22516 |
| Senp5 | -0.0875739 |
| Senp6 | 0.0970123 |
| Senp6 | -0.6784018 |
| Senp7 | 0.4819681 |
| Senp7 | 0.6823729 |
| Senp7 | 2.3143543 |
| Senp8 | -0.7391405 |
| Sephs1 | -0.1259728 |
| Sephs2 | -0.0979305 |
| Sepm | 0.037392 |
| Sepn1 | 1.5055629 |
| Sepp1 | 1.2115723 |
| Sepp1 | -0.2718497 |
| Sepsecs | -0.706014 |
| Sept2 | -0.2462479 |
| Sept3 | 2.7828976 |
| Sept3 | 0.0868356 |
| Sept5 | -0.4621148 |
| Sept6 | 0.6986826 |
| Sept6 | -0.3742107 |
| Sept7 | 0.0986436 |
| Sept8 | -0.7881975 |
| Sept9 | -0.0851997 |
| Sept10 | 1.0362727 |
| Sept10 | -0.5799399 |
| Sept12 | -0.4452565 |
| Sept15 | -0.1544387 |
| Sepw1 | -0.528282 |
| Sepx1 | -0.0602364 |
| Sepx1 | -0.7174397 |
| Serac1 | -0.1664112 |
| Serbp1 | 2.1362131 |
| Serbp1 | -0.8574963 |
| Serbp1 | -0.8141286 |
| Serf1 | -0.6725352 |
| Serf2 | -0.6820869 |
| Sergef | -0.6828256 |
| Serhl | -0.0232331 |
| Serhl | 0.3959501 |
| Serinc1 | -0.6872699 |
| Serinc2 | 2.1691658 |
| Serinc3 | 0.5292174 |
| Serpina1a | 2.7248396 |
| Serpina1a | -0.4068564 |
| Serpina1b | 0.5955895 |
| Serpina1b | -0.6999758 |
| Serpina1d | 1.2342659 |
| Serpina1e | -0.0147525 |
| Serpina3b | -0.631047 |
| Serpina3c | 0.5462989 |
| Serpina3g | -0.0426888 |
| Serpina3h | 0.8912453 |
| Serpina3k | -0.5333238 |
| Serpina3n | -0.8346995 |
| Serpina5 | -0.0472354 |
| Serpina5 | 2.8710512 |
| Serpina7 | -0.5602392 |
| Serpina9 | -0.2937217 |
| Serpinb11 | -0.3672822 |
| Serpinb12 | 1.3674248 |
| Serpinb1a | 0.7445133 |
| Serpinb3a | 0.0321641 |
| Serpinb6a | -0.1355063 |
| Serpinb6b | 0.6115335 |
| Serpinb6c | 0.4895744 |
| Serpind1 | -0.348354 |
| Serpine2 | 0.5946205 |
| Serpinf1 | -0.4556852 |
| Serping1 | -0.0453384 |
| Serpinh1 | 0.3961888 |
| Serpini1 | 1.1360029 |
| Sertad1 | 0.1132011 |
| Sertad2 | 0.8933748 |
| Sertad2 | -0.6494226 |
| Sertad3 | -0.6843047 |
| Sertad4 | 1.5886283 |
| Sesn1 | 0.2574433 |
| Sesn1 | 0.5584595 |
| Sesn2 | -0.771023 |
| Sesn3 | -0.1707238 |
| Setd1b | -0.7694033 |
| Setd2 | -0.6515658 |
| Setd3 | 0.8497071 |
| Setd4 | -0.4864685 |
| Setd4 | -0.756544 |
| Setd5 | 3.3470714 |
| Setd6 | -0.6880127 |
| Setd6 | 0.1849215 |
| Setd7 | 0.0187904 |
| Setd7 | -0.4973598 |
| Setd8 | 1.2062735 |
| Setd8 | -0.945751 |
| Setd8 | -0.6437308 |
| Setdb1 | 0.5469107 |
| Setdb2 | -0.212243 |
| Setmar | -0.4858671 |
| Setx | -0.4586024 |
| Sez6 | -0.8390414 |
| Sez6l | -0.7694033 |
| Sez6l2 | -0.5892746 |
| Sez6l2 | -0.0038029 |
| Sf1 | 2.6220458 |
| Sf1 | -0.5059176 |
| Sf3a1 | -0.0575452 |
| Sf3a2 | 1.6972298 |
| Sf3a3 | 2.3079941 |
| Sf3b1 | -0.5544074 |
| Sf3b2 | -0.4804711 |
| Sf3b3 | 1.9523379 |
| Sf3b4 | 0.5920964 |
| Sf3b4 | 0.0297145 |
| Sf3b5 | -0.2518782 |
| Sf4 | 0.8779354 |
| Sfi1 | 0.3675986 |
| Sfi1 | -0.4349405 |
| Sfmbt1 | 1.2097414 |
| Sfmbt1 | 1.3858887 |
| Sfmbt1 | -0.2364742 |
| Sfmbt2 | -0.7274537 |
| Sfrp1 | -0.2632393 |
| Sfrp2 | 0.7640347 |
| Sfrp4 | 1.7632666 |
| Sfrp5 | -0.0419331 |
| Sfrs1 | 2.6124645 |
| Sfrs1 | -0.61577 |
| Sfrs11 | 1.2675176 |
| Sfrs12 | 0.384662 |
| Sfrs14 | 0.2151139 |
| Sfrs16 | -0.0871778 |
| Sfrs2 | 0.180743 |
| Sfrs2 | 1.5640793 |
| Sfrs2 | -0.753356 |
| Sfrs3 | 0.5692731 |
| Sfrs3 | -0.6558667 |
| Sfrs4 | 1.4048326 |
| Sfrs5 | -0.7136159 |
| Sfrs5 | -0.3201416 |
| Sfrs6 | 4.4207591 |
| Sfrs7 | -0.2355486 |
| Sfrs8 | -0.1051669 |
| Sfrs9 | -0.7865484 |
| Sft2d2 | 0.8034788 |
| Sft2d2 | -0.6123318 |
| Sft2d2 | -0.6999758 |
| Sftpc | 0.1404194 |
| Sftpd | -0.4286908 |
| Sfxn1 | 2.3223882 |
| Sfxn1 | -0.0438234 |
| Sfxn3 | -0.8619434 |
| Sfxn3 | -0.4366522 |
| Sfxn4 | -0.6522812 |
| Sfxn4 | 0.2752188 |
| Sfxn5 | 0.6561929 |
| Sgca | 0.9160757 |
| Sgca | 0.8233694 |
| Sgcb | 2.1224173 |
| Sgcb | 0.2846549 |
| Sgcz | -0.7775293 |
| Sgip1 | 1.2014588 |
| Sgip1 | 0.5217124 |
| Sgip1 | -0.2699295 |
| Sgip1 | -0.524518 |
| Sgip1 | -0.0792901 |
| Sgk1 | -0.6034499 |
| Sgk2 | 1.6467942 |
| Sgk2 | -0.750972 |
| Sgk3 | 1.1086687 |
| Sgk3 | 0.6307696 |
| Sgk3 | -0.6725352 |
| Sgk3 | -0.7758985 |
| Sgk3 | 1.0078951 |
| Sgms2 | -0.6465724 |
| Sgol1 | -0.2081887 |
| Sgpl1 | 1.5785799 |
| Sgpl1 | 0.0566605 |
| Sgpp1 | 2.3854367 |
| Sgpp2 | -0.5434881 |
| Sgsm1 | -0.7282284 |
| Sgsm2 | -0.6761976 |
| Sgsm3 | 1.1708836 |
| Sgta | 0.1577348 |
| Sgtb | 0.0808764 |
| Sgtb | 1.6583831 |
| Sh2b1 | 0.5426204 |
| Sh2b1 | 0.9637476 |
| Sh2b3 | -0.6977213 |
| Sh2d2a | -0.5333238 |
| Sh3bgrl | 1.7490779 |
| Sh3bgrl3 | -0.6894999 |
| Sh3bp2 | 0.4841475 |
| Sh3bp5 | -0.3011251 |
| Sh3bp5l | -0.6939755 |
| Sh3gl1 | 2.2636308 |
| Sh3gl2 | -0.8592727 |
| Sh3gl2 | 0.4647959 |
| Sh3gl2 | -0.597349 |
| Sh3gl3 | 0.1491164 |
| Sh3glb1 | -0.5396638 |
| Sh3glb2 | -0.0625492 |
| Sh3kbp1 | 1.1004847 |
| Sh3kbp1 | -0.1720213 |
| Sh3kbp1 | -0.8601621 |
| Sh3kbp1 | 1.5042176 |
| Sh3md4 | 0.3189089 |
| Sh3md4 | -0.4828656 |
| Sh3pxd2a | 0.9945961 |
| Sh3pxd2b | -0.5524714 |
| Sh3rf1 | -0.7266797 |
| Sh3rf2 | -0.2981569 |
| Sh3tc1 | 0.0511948 |
| Sh3tc2 | -0.0265722 |
| Sh3yl1 | 0.3398998 |
| Shank2 | -0.771023 |
| Sharpin | -0.34161 |
| Shbg | -0.8890701 |
| Shc1 | 1.2219819 |
| Shc1 | 1.5666749 |
| Shc3 | -0.3226721 |
| Shcbp1 | 2.4647826 |
| Shd | -0.5806037 |
| She | -0.7923326 |
| Shf | -0.5441269 |
| Shf | 1.2986303 |
| Shf | -0.3588233 |
| Shfm1 | -0.5301696 |
| Shh | 0.6906503 |
| Shisa2 | 0.0233867 |
| Shisa3 | 0.7193238 |
| Shisa4 | -0.3036058 |
| Shkbp1 | 1.6991221 |
| Shkbp1 | -0.8286537 |
| Shmt1 | -0.3509606 |
| Shmt2 | -0.0434451 |
| Shoc2 | 2.1945804 |
| Shox2 | 0.1503523 |
| Shprh | 1.221278 |
| Shq1 | -0.6150814 |
| Shroom3 | 0.0297145 |
| Shroom3 | -0.7881975 |
| Si | -0.6501364 |
| Si | 0.5831109 |
| Siah1a | -0.3006298 |
| Siah1b | -0.5145527 |
| Siah1b | -0.4894815 |
| Siah1b | -0.807369 |
| Siah1b | 1.7242188 |
| Siah2 | -0.1363399 |
| Siat7f | -0.1664112 |
| Siat9 | 1.1738482 |
| Sidt1 | 0.4725396 |
| Sidt2 | -0.3779623 |
| Sidt2 | -0.3635722 |
| Sidt2 | 0.5011921 |
| Sigirr | -0.2231395 |
| Siglec1 | 0.2618496 |
| Siglech | -0.5201454 |
| Siglech | -0.227258 |
| Sil1 | 0.1568151 |
| Sin3a | -0.6857862 |
| Sin3b | -0.3359405 |
| Sip1 | 1.1291593 |
| Sip1 | -0.0284323 |
| Sipa1 | -0.2185842 |
| Sipa1l1 | 0.4216264 |
| Sirpa | -0.5307996 |
| Sirpa | 0.3183911 |
| Sirpb1 | -0.7037452 |
| Sirt1 | -0.4018878 |
| Sirt2 | -0.5004074 |
| Sirt3 | 1.2938877 |
| Sirt5 | -0.4510362 |
| Sirt5 | 2.0266873 |
| Sirt6 | -0.3313267 |
| Sirt7 | -0.4556852 |
| Siva1 | 0.5335682 |
| Six1 | 0.7809341 |
| Six2 | 0.0755479 |
| Six3 | 2.0101888 |
| Six5 | -0.4816676 |
| Six6os1 | 2.0334999 |
| Skap2 | -0.4918987 |
| Skil | -0.1346735 |
| Skiv2l | 0.1109511 |
| Skiv2l | -0.3478335 |
| Skiv2l2 | -0.2323159 |
| Skp1a | 1.4085602 |
| Skp2 | 0.6733092 |
| Skp2 | 1.7985846 |
| Skp2 | 4.5860692 |
| Sla | 0.566681 |
| Slain1 | -0.631047 |
| Slain2 | -0.0907486 |
| Slamf7 | 1.40367 |
| Slamf8 | 0.12754 |
| Slamf9 | -0.1877313 |
| Slc10a3 | 2.2088816 |
| Slc10a4 | 1.2990013 |
| Slc10a6 | -0.7297797 |
| Slc10a6 | -0.0034401 |
| Slc10a7 | 1.2162353 |
| Slc10a7 | -0.8014933 |
| Slc10a7 | -0.5071464 |
| Slc11a1 | -0.6041302 |
| Slc11a1 | 1.6554511 |
| Slc11a1 | 0.9262683 |
| Slc11a1 | 2.3131369 |
| Slc11a2 | 0.3651359 |
| Slc12a2 | -0.0291773 |
| Slc12a2 | 2.5236442 |
| Slc12a3 | -0.2757016 |
| Slc12a5 | -0.7478026 |
| Slc12a6 | -0.7832589 |
| Slc12a7 | -0.2547058 |
| Slc12a8 | -0.6820869 |
| Slc12a9 | 0.1057883 |
| Slc12a9 | -0.3221655 |
| Slc13a2 | -0.8610524 |
| Slc13a3 | -0.1251483 |
| Slc13a3 | 0.0728725 |
| Slc13a4 | 0.3168361 |
| Slc14a1 | 2.3620617 |
| Slc14a1 | 0.526514 |
| Slc14a1 | 0.1297512 |
| Slc14a2 | -0.6984722 |
| Slc14a2 | -0.7964857 |
| Slc15a2 | -0.7581419 |
| Slc15a3 | -0.6178389 |
| Slc15a4 | -0.4286908 |
| Slc15a4 | 0.5783891 |
| Slc16a10 | -0.5083768 |
| Slc16a11 | 2.0392398 |
| Slc16a4 | 1.0081467 |
| Slc16a6 | -0.4303912 |
| Slc16a6 | -0.7685945 |
| Slc16a6 | 2.4575076 |
| Slc16a8 | -0.0660289 |
| Slc16a9 | -0.7758985 |
| Slc17a2 | 1.5814124 |
| Slc17a5 | -0.5759671 |
| Slc17a6 | -0.2897965 |
| Slc17a7 | 1.611493 |
| Slc17a8 | -0.7259063 |
| Slc18a2 | 1.8129279 |
| Slc19a1 | -0.5108424 |
| Slc19a2 | -0.749386 |
| Slc1a1 | 1.846487 |
| Slc1a1 | 1.6390976 |
| Slc1a2 | -0.7501787 |
| Slc1a2 | -0.2010233 |
| Slc1a2 | 1.5095131 |
| Slc1a2 | 0.0838606 |
| Slc1a3 | 1.7379596 |
| Slc1a4 | -0.6479965 |
| Slc1a6 | -0.4464097 |
| Slc1a7 | 1.2119787 |
| Slc20a1 | 0.1855169 |
| Slc22a1 | 0.3069283 |
| Slc22a12 | -0.3646307 |
| Slc22a13 | -0.2425128 |
| Slc22a14 | -0.8286537 |
| Slc22a17 | -0.750972 |
| Slc22a17 | 0.8708473 |
| Slc22a18 | 2.5597983 |
| Slc22a21 | -0.1438744 |
| Slc22a3 | 2.675363 |
| Slc22a4 | -0.5046903 |
| Slc22a5 | -0.632448 |
| Slc22a5 | -0.460357 |
| Slc22a5 | -0.621297 |
| Slc22a6 | 1.0647797 |
| Slc22a7 | 0.0230339 |
| Slc22a8 | 0.1176852 |
| Slc23a2 | -0.0683558 |
| Slc23a3 | 1.8399847 |
| Slc24a1 | -0.029923 |
| Slc24a3 | -0.1903749 |
| Slc24a4 | -0.7367916 |
| Slc24a5 | -0.0979305 |
| Slc24a6 | 1.1635386 |
| Slc25a1 | 0.7627348 |
| Slc25a1 | -0.7881975 |
| Slc25a10 | 2.5585349 |
| Slc25a11 | -0.564147 |
| Slc25a11 | -0.3001347 |
| Slc25a11 | 0.4306481 |
| Slc25a12 | 1.7683279 |
| Slc25a14 | -0.4423798 |
| Slc25a15 | 0.9329581 |
| Slc25a15 | 0.9894806 |
| Slc25a16 | -0.231855 |
| Slc25a17 | -0.7865484 |
| Slc25a18 | -0.7151436 |
| Slc25a19 | 0.2162674 |
| Slc25a19 | 1.9182932 |
| Slc25a20 | 2.236336 |
| Slc25a20 | -0.0136519 |
| Slc25a23 | -0.8451535 |
| Slc25a24 | -0.4446805 |
| Slc25a24 | 0.4443634 |
| Slc25a25 | -0.3318382 |
| Slc25a26 | -0.6939755 |
| Slc25a27 | 0.7094029 |
| Slc25a28 | -0.6451506 |
| Slc25a29 | 1.3325286 |
| Slc25a3 | 0.5940386 |
| Slc25a3 | -0.0457175 |
| Slc25a30 | -0.7014818 |
| Slc25a32 | 0.210196 |
| Slc25a33 | 0.5185678 |
| Slc25a34 | 0.343438 |
| Slc25a35 | -0.3625149 |
| Slc25a36 | 1.2485469 |
| Slc25a37 | -0.7462218 |
| Slc25a38 | 0.0098887 |
| Slc25a38 | 3.7578251 |
| Slc25a38 | 1.7770228 |
| Slc25a39 | -0.3267351 |
| Slc25a41 | -0.5157927 |
| Slc25a42 | 0.7940842 |
| Slc25a44 | 1.6755381 |
| Slc25a45 | 1.4622478 |
| Slc25a46 | 0.5052464 |
| Slc25a5 | -0.7898494 |
| Slc26a4 | -0.3473133 |
| Slc26a6 | -0.5040773 |
| Slc26a7 | -0.6638023 |
| Slc26a8 | -0.8727018 |
| Slc27a1 | -0.1672722 |
| Slc27a2 | 2.2810277 |
| Slc27a3 | -0.5409368 |
| Slc27a4 | -0.196128 |
| Slc27a6 | 0.0617564 |
| Slc29a1 | -0.8619434 |
| Slc29a2 | -0.5524714 |
| Slc29a3 | -0.6580244 |
| Slc29a4 | 2.2553231 |
| Slc2a1 | 0.8981842 |
| Slc2a10 | -0.4912938 |
| Slc2a10 | -0.6041302 |
| Slc2a13 | -0.1846562 |
| Slc2a3 | 1.4010071 |
| Slc2a4 | -0.5301696 |
| Slc2a5 | -0.8006569 |
| Slc2a6 | -0.8065274 |
| Slc2a8 | -0.0140186 |
| Slc2a8 | 0.0701895 |
| Slc2a8 | 1.9860234 |
| Slc2a9 | 0.2727772 |
| Slc30a1 | 0.8422559 |
| Slc30a10 | -0.4068564 |
| Slc30a3 | 0.3338038 |
| Slc30a5 | 1.2216803 |
| Slc30a5 | -0.6178389 |
| Slc30a6 | -0.4650517 |
| Slc30a7 | 2.8289257 |
| Slc30a7 | 1.4061597 |
| Slc30a9 | 0.4723192 |
| Slc31a1 | -0.1144939 |
| Slc31a2 | 0.6400593 |
| Slc32a1 | 0.5221309 |
| Slc34a2 | 0.0366966 |
| Slc35a1 | 0.2294332 |
| Slc35a2 | 0.5692731 |
| Slc35a3 | -0.4383668 |
| Slc35a4 | 0.67438 |
| Slc35a5 | 1.4985917 |
| Slc35a5 | 1.4149049 |
| Slc35b1 | -0.0136519 |
| Slc35b2 | 0.4420911 |
| Slc35b3 | 0.5787835 |
| Slc35b3 | -0.9066576 |
| Slc35b3 | -0.665978 |
| Slc35b4 | -0.4338012 |
| Slc35c1 | -0.3974929 |
| Slc35c1 | 2.1704695 |
| Slc35c2 | -0.7750842 |
| Slc35c2 | 0.1580412 |
| Slc35c2 | -0.353051 |
| Slc35e1 | -0.4650517 |
| Slc35e3 | -0.6494226 |
| Slc35e4 | 0.2177073 |
| Slc35f1 | 0.5063105 |
| Slc35f2 | 1.9670058 |
| Slc35f3 | -0.1230901 |
| Slc35f4 | -0.4441048 |
| Slc35f4 | -0.5102254 |
| Slc36a1 | 0.307976 |
| Slc36a3 | 0.6648795 |
| Slc37a1 | 0.3040409 |
| Slc37a3 | -0.3303044 |
| Slc38a1 | 2.0189973 |
| Slc38a10 | -0.706014 |
| Slc38a11 | -0.3811898 |
| Slc38a2 | 0.5343946 |
| Slc38a2 | -0.4107388 |
| Slc38a2 | -0.7399247 |
| Slc38a3 | 0.12754 |
| Slc38a4 | 0.2471751 |
| Slc38a5 | -0.2295537 |
| Slc38a8 | 0.4089923 |
| Slc38a9 | 0.9373018 |
| Slc38a9 | -0.6522812 |
| Slc39a10 | -0.7414951 |
| Slc39a11 | 0.721024 |
| Slc39a12 | 0.4101692 |
| Slc39a13 | -0.5693823 |
| Slc39a13 | -0.1346735 |
| Slc39a14 | -0.0288047 |
| Slc39a3 | 0.2574433 |
| Slc39a3 | -0.7525607 |
| Slc39a4 | -0.8023305 |
| Slc39a5 | -0.1346735 |
| Slc39a5 | 0.3183911 |
| Slc39a5 | 0.6565565 |
| Slc39a6 | -0.4007872 |
| Slc39a7 | -0.8048464 |
| Slc39a7 | -0.3561952 |
| Slc39a7 | -0.5576429 |
| Slc39a8 | -0.4931096 |
| Slc3a2 | 0.7730849 |
| Slc40a1 | -0.3985897 |
| Slc41a2 | -0.0250867 |
| Slc41a3 | 1.5338203 |
| Slc41a3 | -0.2956903 |
| Slc41a3 | -0.4366522 |
| Slc43a2 | 1.2323786 |
| Slc43a2 | -0.6123318 |
| Slc44a1 | -0.0048923 |
| Slc44a1 | 3.3278288 |
| Slc44a2 | -0.4733235 |
| Slc44a2 | 0.8541501 |
| Slc44a2 | -0.2249677 |
| Slc44a3 | 0.5580566 |
| Slc44a4 | 2.6098368 |
| Slc45a1 | -0.8056865 |
| Slc45a2 | 0.5473182 |
| Slc45a3 | -0.6268563 |
| Slc45a4 | -0.1899338 |
| Slc46a1 | 0.92709 |
| Slc46a3 | -0.5096088 |
| Slc47a1 | -0.2637156 |
| Slc4a1 | -0.8610524 |
| Slc4a1ap | 0.0387813 |
| Slc4a2 | 1.7776409 |
| Slc4a3 | -0.4185513 |
| Slc4a3 | -0.2185842 |
| Slc4a3 | -0.1746218 |
| Slc4a3 | 1.3622319 |
| Slc4a8 | -0.3125898 |
| Slc4a8 | -0.3145977 |
| Slc5a4a | -0.5852628 |
| Slc5a4b | 0.5325341 |
| Slc5a5 | 1.253714 |
| Slc5a6 | -0.6616315 |
| Slc5a7 | 1.6754756 |
| Slc5a8 | 1.7995732 |
| Slc5a9 | 0.7223819 |
| Slc6a1 | 0.0525642 |
| Slc6a11 | -0.5531163 |
| Slc6a12 | -0.1759246 |
| Slc6a13 | -0.6638023 |
| Slc6a15 | -0.4452565 |
| Slc6a15 | 1.018527 |
| Slc6a17 | -0.7998212 |
| Slc6a2 | 1.3547392 |
| Slc6a4 | 0.0943964 |
| Slc6a6 | -0.5289108 |
| Slc6a7 | -0.4949288 |
| Slc6a8 | -0.1165336 |
| Slc6a9 | -0.2258832 |
| Slc7a1 | 0.0401686 |
| Slc7a10 | -0.5364885 |
| Slc7a11 | -0.660909 |
| Slc7a14 | 2.9235504 |
| Slc7a14 | 0.1911552 |
| Slc7a14 | 0.9876837 |
| Slc7a2 | 2.9090465 |
| Slc7a3 | -0.3752811 |
| Slc7a4 | -0.3328621 |
| Slc7a6 | -0.2185842 |
| Slc7a6os | -0.815826 |
| Slc7a7 | 0.1849215 |
| Slc7a7 | 0.6477935 |
| Slc7a8 | 0.2449403 |
| Slc7a8 | 2.071818 |
| Slc8a1 | 0.1224663 |
| Slc8a2 | -0.6909894 |
| Slc8a2 | 1.074448 |
| Slc9a1 | 2.0203922 |
| Slc9a10 | -0.8601621 |
| Slc9a3r1 | -0.3313267 |
| Slc9a3r2 | 0.6623541 |
| Slc9a4 | -0.9438064 |
| Slc9a6 | -1.0636999 |
| Slc9a6 | 1.5425537 |
| Slc9a8 | -0.5182775 |
| Slc9a8 | 1.7284269 |
| Slc9a8 | -0.6594656 |
| Slc9a8 | 2.463195 |
| Slc9a9 | 0.2549557 |
| Slco1a4 | -0.5746465 |
| Slco1c1 | 2.060149 |
| Slco2a1 | -0.4464097 |
| Slco2b1 | -0.8321037 |
| Slco3a1 | -0.5071464 |
| Slco3a1 | -0.4973598 |
| Slco3a1 | -0.7414951 |
| Slco4a1 | 1.1616132 |
| Slfnl1 | -0.9634328 |
| Slit1 | 0.2585467 |
| Slit2 | -0.8936671 |
| Slit3 | 0.2892048 |
| Slitrk1 | 0.7992627 |
| Slitrk3 | -0.9447782 |
| Slitrk4 | 0.7868186 |
| Slitrk5 | 0.1762456 |
| Slitrk5 | -0.6123318 |
| Slitrk6 | 0.3807886 |
| Slk | -0.3410932 |
| Slk | 1.9608597 |
| Slmap | 1.6661158 |
| Slmap | -0.4521963 |
| Slmap | -0.4632885 |
| Slmo1 | 1.5168483 |
| Slmo2 | -0.0464761 |
| Sln | -0.3070895 |
| Slpi | 1.4070709 |
| Sltm | 0.4439093 |
| Sltm | -0.7742706 |
| Slu7 | -0.1071867 |
| Slu7 | 2.251159 |
| Sly | 3.2074182 |
| Smad1 | -0.2824801 |
| Smad1 | -0.0804691 |
| Smad2 | 1.9699953 |
| Smad3 | 2.5050273 |
| Smad5 | 0.4014258 |
| Smad5 | 1.1024234 |
| Smad6 | -0.1430343 |
| Smap2 | -0.3752811 |
| Smap2 | -0.6458612 |
| Smap2 | 2.2133405 |
| Smarca1 | -0.5524714 |
| Smarca2 | 1.7864743 |
| Smarca2 | -0.9824592 |
| Smarca4 | 1.1191369 |
| Smarcad1 | -0.1124585 |
| Smarcal1 | -0.2922479 |
| Smarcb1 | -0.4780826 |
| Smarcc1 | 0.4696707 |
| Smarcc1 | -0.1173507 |
| Smarcc2 | 1.0000648 |
| Smarcd1 | 1.9180508 |
| Smarcd1 | 0.9163526 |
| Smarcd1 | 3.6021123 |
| Smarcd2 | 1.2310852 |
| Smarcd3 | 0.8835396 |
| Smarce1 | -0.674731 |
| Smarce1 | 0.8150935 |
| Smc1a | 0.7676004 |
| Smc1a | -0.5251443 |
| Smc1a | -0.749386 |
| Smc1a | -0.7485939 |
| Smc1b | -0.456851 |
| Smc3 | 3.6072756 |
| Smc5l1 | -0.6732666 |
| Smc6 | 0.2107759 |
| Smchd1 | 0.5073734 |
| Smcp | -0.5466867 |
| Smcr7 | 1.95905 |
| Smcr7l | 1.3070345 |
| Smek1 | -0.0048923 |
| Smek2 | -0.7029901 |
| Smek2 | 3.2880932 |
| Smg5 | 0.6409834 |
| Smg6 | -0.5839293 |
| Smn1 | 0.3168361 |
| Smo | 1.563236 |
| Smo | -0.7220485 |
| Smo | -0.466229 |
| Smoc1 | 2.0008094 |
| Smox | -0.2108897 |
| Smox | 0.1064352 |
| Smox | 1.1941392 |
| Smox | 1.9006317 |
| Smox | 1.7122643 |
| Smpd1 | 3.2301999 |
| Smpd1 | 2.3652845 |
| Smpd1 | 0.6361686 |
| Smpd2 | 0.968216 |
| Smpd3 | 2.3120464 |
| Smpd4 | 0.3222677 |
| Smpdl3a | 0.2670558 |
| Smpdl3b | -0.1181685 |
| Smpx | 1.5338928 |
| Smtn | 1.9961295 |
| Smtn | 0.073542 |
| Smtn | 1.8916021 |
| Smtnl1 | 2.4554661 |
| Smtnl2 | -0.1737542 |
| Smu1 | -0.5460461 |
| Smu1 | 0.9622975 |
| Smug1 | 0.2827752 |
| Smurf1 | 0.5746343 |
| Smyd1 | 0.9189793 |
| Smyd2 | -0.4429544 |
| Smyd2 | -0.7454324 |
| Smyd3 | 2.4801646 |
| Smyd4 | 0.3286938 |
| Smyd5 | -0.4135216 |
| Smyd5 | -0.1007381 |
| Snai1 | -0.3452354 |
| Snai3 | 1.6764128 |
| Snap23 | 0.4220911 |
| Snap29 | 0.5624788 |
| Snap91 | -0.5602392 |
| Snap91 | 0.4164993 |
| Snapc1 | -0.0741979 |
| Snapc3 | -0.8364339 |
| Snapc3 | 2.0323391 |
| Snapc3 | -0.0099923 |
| Snapc3 | 1.2868702 |
| Snapc4 | 1.5164792 |
| Snapin | -0.7775293 |
| Snca | 1.1954836 |
| Snca | 1.2538113 |
| Snca | -0.3462738 |
| Sncaip | 0.0937413 |
| Sncg | -0.3742107 |
| Sncg | 4.4069411 |
| Snd1 | 0.2779244 |
| Snf1lk | 1.2862126 |
| Snf1lk2 | 0.382243 |
| Snf8 | 0.2819684 |
| Snip1 | 0.4990513 |
| Snip1 | -0.6137056 |
| Snn | 2.475768 |
| Snora65 | -0.5263982 |
| Snph | -0.7750842 |
| Snrk | -0.231855 |
| Snrk | -0.0679676 |
| Snrk | 3.029234 |
| Snrp70 | 0.4315686 |
| Snrpa | 0.4132223 |
| Snrpb | -0.6054923 |
| Snrpb | -0.7328898 |
| Snrpd1 | -0.6623545 |
| Snrpd1 | -0.4609426 |
| Snrpd2 | -0.3535743 |
| Snrpd3 | 0.1659724 |
| Snrpg | -0.0633214 |
| Snta1 | -0.4894815 |
| Sntb2 | -0.5706957 |
| Sntb2 | 0.0016251 |
| Sntg2 | -0.6992237 |
| Snupn | 2.2939685 |
| Snurf | 2.4263039 |
| Snurf | 1.0932657 |
| Snx1 | -0.5720108 |
| Snx10 | -0.4638759 |
| Snx11 | -0.4949288 |
| Snx12 | -0.306591 |
| Snx13 | 0.3547226 |
| Snx14 | 1.0866869 |
| Snx14 | -0.4179911 |
| Snx14 | -0.7589419 |
| Snx15 | -0.3779623 |
| Snx16 | 0.1170459 |
| Snx17 | -0.4338012 |
| Snx17 | 0.1846236 |
| Snx18 | 0.4793463 |
| Snx19 | 1.351673 |
| Snx19 | -0.7791628 |
| Snx2 | 1.4955021 |
| Snx2 | -0.6034499 |
| Snx20 | -0.6254634 |
| Snx21 | -0.3855101 |
| Snx22 | 3.3637711 |
| Snx24 | -0.4349405 |
| Snx25 | 0.6065676 |
| Snx25 | -0.6007336 |
| Snx26 | 1.8756173 |
| Snx29 | -0.5364885 |
| Snx3 | 0.2075822 |
| Snx30 | -0.5077614 |
| Snx33 | -0.1728874 |
| Snx4 | 0.7423563 |
| Snx6 | -0.660909 |
| Snx7 | 0.2148254 |
| Snx8 | -0.6082222 |
| Snx9 | 2.6111281 |
| Soat1 | 1.8900057 |
| Socs2 | 2.2345973 |
| Socs3 | -0.3656905 |
| Socs4 | -0.2956903 |
| Socs5 | -0.3478335 |
| Socs6 | 0.3621723 |
| Socs7 | 0.0073812 |
| Socs7 | -0.0129189 |
| Sod1 | 0.1512783 |
| Sod2 | -0.7446436 |
| Sod3 | 0.3582064 |
| Sohlh1 | 1.6841873 |
| Sohlh1 | -0.0967298 |
| Son | 0.3155383 |
| Sorbs1 | -0.7375739 |
| Sorbs2 | 3.1046852 |
| Sorbs3 | 0.1798452 |
| Sorcs1 | 1.4005904 |
| Sorcs1 | -0.6725352 |
| Sorcs2 | -0.2613363 |
| Sorcs3 | 2.7497754 |
| Sorcs3 | -0.5276536 |
| Sord | 1.2749635 |
| Sorl1 | 0.8540023 |
| Sort1 | -0.4967515 |
| Sort1 | -0.5812679 |
| Sort1 | -0.7422812 |
| Sort1 | 1.2250928 |
| Sostdc1 | 0.9133022 |
| Sox12 | 1.3604947 |
| Sox13 | -0.854838 |
| Sox17 | 1.8589478 |
| Sox17 | -0.6835649 |
| Sox18 | 0.4420911 |
| Sox2 | 0.8482215 |
| Sox2 | 1.052495 |
| Sox21 | 0.7524356 |
| Sox21 | 0.4281121 |
| Sox30 | 0.7465 |
| Sox5 | -0.0381637 |
| Sox5 | -0.1850949 |
| Sox5 | -0.5674157 |
| Sox5 | 0.1644523 |
| Sox7 | -0.4650517 |
| Sox7 | 0.2482905 |
| Sox8 | 0.0023465 |
| Sox9 | 1.7727417 |
| Sp100 | 2.2752083 |
| Sp140 | 2.0700416 |
| Sp2 | 2.7212505 |
| Sp4 | 1.566745 |
| Sp5 | -0.4412315 |
| Sp6 | 0.8610694 |
| Sp7 | 2.5800568 |
| Sp8 | -0.6366635 |
| Sp9 | 0.6270282 |
| Spaca3 | 2.2080589 |
| Spaca4 | 1.3355676 |
| Spag1 | 2.8909994 |
| Spag1 | 1.087613 |
| Spag16 | -0.0907486 |
| Spag16 | 0.7687321 |
| Spag4 | 1.7523133 |
| Spag4 | -0.7767136 |
| Spag4l | 1.0042405 |
| Spag4l | -0.1055705 |
| Spag5 | 0.4000004 |
| Spag5 | -0.6430217 |
| Spag6 | -0.2509375 |
| Spag6 | -0.7259063 |
| Spag7 | 0.797226 |
| Spag8 | 0.0077398 |
| Spag9 | 0.6695519 |
| Spag9 | -0.7881975 |
| Spag9 | -0.122679 |
| Spag9 | -0.5034647 |
| Spam1 | -0.1128652 |
| Spam1 | 0.2507954 |
| Sparc | -0.0629352 |
| Sparc | -0.8252161 |
| Sparcl1 | -0.4315265 |
| Spast | -0.5040773 |
| Spast | -0.7915041 |
| Spast | 0.1253236 |
| Spata16 | -0.4786792 |
| Spata16 | 0.8276326 |
| Spata17 | -0.4360813 |
| Spata17 | 0.4718783 |
| Spata19 | 0.5364577 |
| Spata2 | 0.7501317 |
| Spata20 | -0.041178 |
| Spata20 | -0.6739985 |
| Spata21 | -0.1181685 |
| Spata22 | 1.2746782 |
| Spata2L | 3.2065508 |
| Spata3 | 1.4062425 |
| Spata3 | -0.7243613 |
| Spata3 | 0.2282956 |
| Spata3 | 0.675984 |
| Spata4 | -0.8434032 |
| Spata6 | 0.5992629 |
| Spata7 | -0.0377876 |
| Spata7 | 0.7124929 |
| Spatc1 | -0.1259728 |
| Spatc1 | -0.4780826 |
| Spats1 | -0.762952 |
| Spc24 | 0.1656686 |
| Spc24 | 0.0240919 |
| Spc25 | 0.0134593 |
| Spc25 | -0.5687263 |
| Spcs1 | -0.2323159 |
| Spcs1 | -0.0224926 |
| Spcs3 | 0.5507758 |
| Spdef | 0.244101 |
| Specc1l | 1.1981668 |
| Speer1-ps1 | -0.1850949 |
| Speer1-ps1 | -0.0254578 |
| Speer2 | 0.4356999 |
| Speer2 | 0.2656885 |
| Speer3 | 1.0361506 |
| Speer4b | -0.179844 |
| Speer4d | -0.5576429 |
| Speer4f | 1.5792716 |
| Speer6-ps1 | -0.1400997 |
| Spef1 | 0.9522176 |
| Spen | -0.258489 |
| Spert | -0.6254634 |
| Spesp1 | -0.712853 |
| Spg20 | -0.306591 |
| Spg21 | 0.9373018 |
| Spg7 | 2.5406681 |
| Spg7 | 1.761326 |
| Sphk1 | 1.3414395 |
| Sphk2 | 0.6697312 |
| Sphk2 | -0.5799399 |
| Spic | 1.9181962 |
| Spin | -0.2300135 |
| Spin2 | -0.0041659 |
| Spink10 | -0.7783457 |
| Spink2 | -0.695472 |
| Spink4 | -0.6261596 |
| Spink8 | 1.4818185 |
| Spinlw1 | -0.8881533 |
| Spinlw1 | -0.3026127 |
| Spinlw1 | 1.7547902 |
| Spint1 | 2.4189007 |
| Spint2 | 0.7820502 |
| Spire1 | 0.0247965 |
| Spire1 | 0.2654148 |
| Spire1 | 0.3754369 |
| Spire2 | 1.3666477 |
| Spna1 | -0.2523489 |
| Spnb1 | 1.0067624 |
| Spnb2 | 1.1573205 |
| Spnb2 | 1.3269618 |
| Spnb2 | -0.1463992 |
| Spnb2 | 1.4762897 |
| Spnb3 | -0.5164133 |
| Spnb4 | -0.8927459 |
| Spns1 | -0.3758167 |
| Spo11 | 1.4555816 |
| Spo11 | 2.8891776 |
| Spock1 | 0.6645191 |
| Spock2 | -0.6487092 |
| Spock3 | 1.3412621 |
| Spon1 | 0.7223819 |
| Spon2 | -0.4627015 |
| Spop | 1.950465 |
| Spp1 | -0.7266797 |
| Spp2 | -0.5083768 |
| Sppl3 | -0.5207688 |
| Spr | 0.9644061 |
| Spred1 | 2.0021422 |
| Spred2 | 1.0589553 |
| Spred2 | 2.5633569 |
| Sprn | 0.744679 |
| Sprn | 0.450697 |
| Sprr2a | 1.563728 |
| Sprr2a | 1.8929471 |
| Sprr2f | -0.751766 |
| Spry3 | 0.2648672 |
| Spry4 | 2.4133464 |
| Spryd4 | 0.9778697 |
| Spsb1 | -0.7220485 |
| Spsb2 | -0.1463992 |
| Spsb3 | -0.6219902 |
| Spsb3 | -0.2286347 |
| Spsb4 | -0.414637 |
| Sptlc1 | -0.4118509 |
| Sptlc2 | -0.7438555 |
| Spz1 | -0.2135983 |
| Sqle | 0.416733 |
| Sqle | 1.5815504 |
| Sqrdl | -0.621297 |
| Sqstm1 | -0.0777204 |
| Sra1 | -0.6594656 |
| Sra1 | -0.40741 |
| Src | 1.4094692 |
| Srd5a1 | -0.5746465 |
| Srd5a2 | 0.4155642 |
| Srd5a3 | 2.1230043 |
| Srd5a3 | 0.6011906 |
| Srd5a3 | -0.7873726 |
| Srebf1 | -0.1317645 |
| Srebf2 | 1.7743776 |
| Srf | 1.0105329 |
| Srf | 2.0658144 |
| Srfbp1 | -0.4096279 |
| Srgn | -0.2054954 |
| Srgn | 0.2790045 |
| Sri | 0.0752139 |
| Sri | -0.5492533 |
| Sri | -0.222683 |
| Srm | -0.2565955 |
| Srm | 1.1922746 |
| Srm | 2.0184737 |
| Srp14 | 3.2421476 |
| Srp19 | -0.3947563 |
| Srp19 | -0.8056865 |
| Srp54 | 1.1655668 |
| Srp68 | 2.3871885 |
| Srp72 | -0.7228188 |
| Srp9 | 0.5114016 |
| Srp9 | -0.4780826 |
| Srpk1 | 0.4806581 |
| Srpk2 | 0.8285437 |
| Srpk3 | 0.241859 |
| Srpr | 0.0432826 |
| Srprb | 1.0352955 |
| Srprb | -0.6616315 |
| Srpx | -0.6150814 |
| Srpx | 0.4049801 |
| Srpx2 | 0.3066661 |
| Srpx2 | 1.3877494 |
| Srpx2 | -0.6725352 |
| Srr | 1.923996 |
| Srrd | -0.5220169 |
| Srrm1 | 1.160113 |
| Srrm2 | -0.6674313 |
| Srrm2 | 0.4099339 |
| Srrp | 2.0029855 |
| Srxn1 | -0.222683 |
| Srxn1 | 1.455188 |
| Srxn1 | -0.3267351 |
| Ss18 | 2.1918908 |
| Ss18 | 0.3449503 |
| Ss18l1 | -0.2149555 |
| Ss18l1 | 1.8424545 |
| Ss18l1 | 1.0259612 |
| Ssbp1 | 0.9347251 |
| Ssbp2 | -0.2947055 |
| Ssbp2 | 0.7874526 |
| Ssbp3 | 0.3890009 |
| Ssbp3 | 1.8923496 |
| Ssbp4 | -0.8407837 |
| Ssfa2 | -0.571353 |
| Ssh2 | 2.3868028 |
| Ssh3 | 0.6720584 |
| Ssh3 | 1.2368424 |
| Ssna1 | 0.3653825 |
| Sspn | 0.9691334 |
| Ssr2 | -0.3369688 |
| Ssr3 | -0.4492986 |
| Ssr4 | -0.4858671 |
| Ssrp1 | -0.1586974 |
| Sssca1 | -0.0560108 |
| Sssca1 | -0.127624 |
| Sst | -0.33235 |
| Sstr1 | -0.6331493 |
| Sstr5 | 2.4265032 |
| Ssty2 | -0.753356 |
| Ssu72 | 0.1103073 |
| Ssx2ip | -0.8790339 |
| Ssxb2 | -0.7742706 |
| Ssxb5 | 0.3053544 |
| St13 | -0.3774255 |
| St14 | -0.4326632 |
| St14 | 2.735671 |
| St18 | -0.429824 |
| St3gal1 | -0.4389391 |
| St3gal2 | 0.3803033 |
| St3gal2 | -0.2231395 |
| St3gal3 | 0.9182888 |
| St3gal3 | 0.9273638 |
| St3gal4 | 0.4667932 |
| St3gal5 | 0.992681 |
| St3gal6 | -0.4157537 |
| St5 | -0.7589419 |
| St5 | -0.016222 |
| St6gal1 | 0.481095 |
| St6galnac2 | -0.1737542 |
| St6galnac3 | 0.7857081 |
| St6galnac4 | -0.1380091 |
| St6galnac4 | 1.9648846 |
| St6galnac4 | 0.3829694 |
| St6galnac5 | 0.5319131 |
| St6galnac6 | -0.0206441 |
| St6galnac6 | -0.785725 |
| St7 | -0.414637 |
| St7l | 0.3061417 |
| St7l | 0.3906829 |
| St8sia1 | -0.4774864 |
| St8sia1 | -0.665978 |
| St8sia2 | -0.0777204 |
| St8sia3 | -0.5016291 |
| St8sia4 | 0.2610249 |
| St8sia4 | -0.1051669 |
| St8sia5 | -0.2355486 |
| Stab1 | 0.0113185 |
| Stab2 | 0.6212 |
| Stac | -0.5065318 |
| Stac2 | -0.5409368 |
| Stac3 | 0.3304854 |
| Stag1 | -0.6494226 |
| Stag2 | -0.5700388 |
| Stag2 | -0.1569917 |
| Stag3 | 0.089143 |
| Stam | -0.4556852 |
| Stam | 2.0778251 |
| Stam | 1.272488 |
| Stam2 | 0.4529489 |
| Stam2 | -0.2713693 |
| Stambp | 1.3699246 |
| Stambpl1 | 0.2746768 |
| Stap1 | 2.6601953 |
| Stap2 | 0.911495 |
| Star | 1.2441377 |
| Stard10 | -0.665978 |
| Stard10 | 2.6083085 |
| Stard13 | -0.6164591 |
| Stard3 | -0.6479965 |
| Stard3nl | -0.6703445 |
| Stard4 | 0.490656 |
| Stard5 | 1.4997195 |
| Stard5 | 0.1543583 |
| Stard6 | 2.7126191 |
| Stard7 | -0.5946499 |
| Stard7 | 0.4049801 |
| Stard8 | 2.2503377 |
| Stard8 | -0.2332384 |
| Stat1 | -0.1239129 |
| Stat1 | 0.0401686 |
| Stat2 | 1.182687 |
| Stat3 | -0.278117 |
| Stat3 | -0.0295501 |
| Stat3 | -0.3635722 |
| Stat4 | -0.5428496 |
| Stat4 | 1.779997 |
| Stat5a | -0.2086383 |
| Stat5a | 0.433407 |
| Stat6 | -0.6387782 |
| Statip1 | 0.8072109 |
| Stau1 | 2.7350118 |
| Stau2 | 1.8137395 |
| Stbd1 | -0.5912868 |
| Stc2 | 2.5134589 |
| Steap1 | 0.4497948 |
| Steap2 | -0.3855101 |
| Steap3 | -0.5865982 |
| Stfa1 | -0.7029901 |
| Stfa2 | 0.5067358 |
| Stim1 | -0.7581419 |
| Stip1 | -0.409073 |
| Stk11 | 0.0725375 |
| Stk11ip | -0.1157172 |
| Stk17b | 1.3641393 |
| Stk19 | 0.3729944 |
| Stk19 | 1.7608688 |
| Stk19 | 0.4030861 |
| Stk22b | 1.5261032 |
| Stk22s1 | -0.7328898 |
| Stk24 | -0.3876777 |
| Stk25 | 0.9275006 |
| Stk25 | 2.5240555 |
| Stk3 | -0.0344091 |
| Stk31 | -0.2309338 |
| Stk32a | 1.7302584 |
| Stk32b | 0.7812532 |
| Stk32c | -0.7220485 |
| Stk35 | 2.6543158 |
| Stk35 | 0.2916047 |
| Stk36 | -0.528282 |
| Stk38l | -0.2523489 |
| Stk39 | -0.448142 |
| Stk4 | 0.1518951 |
| Stk40 | 0.1202381 |
| Stmn1 | -0.7336689 |
| Stmn2 | -0.4580182 |
| Stmn3 | 0.4556441 |
| Stmn4 | -0.5270257 |
| Stmn4 | 0.3747048 |
| Stoml1 | 1.2542972 |
| Stoml1 | 0.7818908 |
| Stoml1 | -0.6820869 |
| Stoml2 | 0.6946749 |
| Stoml3 | 0.0487937 |
| Stra13 | 1.7912483 |
| Stra13 | 0.4643515 |
| Stra13 | 0.9253088 |
| Stra6 | -0.7964857 |
| Strap | 1.6699588 |
| Strbp | -0.6027701 |
| Strn4 | 0.4734206 |
| Strn4 | 0.0894722 |
| Stt3a | -0.6857862 |
| Stt3b | 0.0752139 |
| Stx12 | -0.4487201 |
| Stx17 | 2.0116406 |
| Stx18 | -0.6784018 |
| Stx1a | -0.4429544 |
| Stx2 | 0.0698536 |
| Stx3 | 0.9381141 |
| Stx3 | 0.5202462 |
| Stx4a | -0.1181685 |
| Stx5a | -0.0714671 |
| Stx5a | 0.2367953 |
| Stx6 | -0.5126957 |
| Stx7 | -0.6696153 |
| Stx8 | -0.5108424 |
| Stx8 | 0.4375304 |
| Stx8 | 0.0009032 |
| Stx8 | 1.6848065 |
| Stxbp1 | -0.1565657 |
| Stxbp1 | -0.4412315 |
| Stxbp1 | 0.12912 |
| Stxbp2 | 2.3040216 |
| Stxbp4 | -0.6623545 |
| Stxbp4 | 2.2149051 |
| Stxbp5l | -0.6130184 |
| Stxbp5l | -0.6894999 |
| Styx | -0.3806511 |
| Styxl1 | -0.3282632 |
| Styxl1 | -0.0851997 |
| Styxl1 | 1.7926316 |
| Suclg1 | 0.5944266 |
| Suclg1 | -0.0621633 |
| Suclg2 | 2.8872644 |
| Suclg2 | -0.6992237 |
| Suds3 | -0.0741979 |
| Sufu | -0.6696153 |
| Suhw2 | 0.1822374 |
| Suhw4 | 0.671164 |
| Sulf1 | 1.8246559 |
| Sult1a1 | 0.9456619 |
| Sult1c2 | 2.3248613 |
| Sult1d1 | 0.4926 |
| Sult1d1 | -0.390394 |
| Sult2b1 | -0.297663 |
| Sult2b1 | 0.7415254 |
| Sult4a1 | -0.4275588 |
| Sult5a1 | 0.5793747 |
| Sult5a1 | 1.8257247 |
| Sumf2 | 0.4533986 |
| Sumo1 | 0.2822374 |
| Sumo1 | 0.362914 |
| Sumo1 | 1.5439179 |
| Sumo2 | 1.4405901 |
| Sumo3 | 0.1926334 |
| Sumo3 | 2.2588924 |
| Sunc1 | 3.1263622 |
| Suox | -0.3619866 |
| Supt3h | -0.541574 |
| Supt4h1 | 0.2217275 |
| Supt4h2 | 0.9082894 |
| Supt5h | 0.7516134 |
| Supt5h | 0.0391283 |
| Supt6h | -0.4107388 |
| Supv3l1 | 1.5198697 |
| Surf1 | -0.407964 |
| Surf1 | -0.3478335 |
| Surf2 | 1.9171776 |
| Surf4 | -0.3731415 |
| Susd2 | -0.4492986 |
| Susd3 | 1.8143343 |
| Susd4 | 1.0417513 |
| Suv39h1 | -0.631047 |
| Suv420h1 | 0.1656686 |
| Suv420h1 | 0.3431857 |
| Suv420h1 | 2.2546758 |
| Suv420h2 | 2.2019559 |
| Suz12 | 1.4906593 |
| Sv2a | -0.9066576 |
| Sv2a | 4.4480114 |
| Sv2b | -0.8808509 |
| Sval2 | 0.5314988 |
| Svep1 | 0.0792144 |
| Svil | 0.5982976 |
| Svil | 1.5127802 |
| Svop | 0.0116757 |
| Svopl | -0.1544387 |
| Svs4 | 0.8183204 |
| Svs5 | -0.7621486 |
| Svs6 | 2.6249177 |
| Swap70 | 0.2835812 |
| Swap70 | -0.5102254 |
| Swap70 | -0.1689964 |
| Syap1 | 2.2924629 |
| Syap1 | -0.1247363 |
| Sybl1 | -0.0899539 |
| Syce2 | 1.4219417 |
| Sycp1 | 0.4209289 |
| Syf2 | -0.632448 |
| Syf2 | 1.067974 |
| Syn1 | 0.4448172 |
| Syn1 | -0.1063783 |
| Syn2 | 0.1269072 |
| Sync | -0.7143795 |
| Syncrip | -0.7290037 |
| Syne1 | 0.468565 |
| Syne1 | -0.6178389 |
| Syngr1 | -0.4882752 |
| Syngr1 | 0.6285265 |
| Syngr1 | 1.6394871 |
| Syngr1 | 2.4297146 |
| Syngr2 | 0.1457091 |
| Syngr2 | 0.7687321 |
| Syngr2 | 0.0418998 |
| Syngr3 | 1.3004837 |
| Syngr4 | 2.2277862 |
| Syngr4 | -0.8031684 |
| Syngr4 | -0.2849127 |
| Synj2 | 0.5821291 |
| Synj2bp | 2.8793428 |
| Synj2bp | 1.0352955 |
| Synpo2l | -0.761346 |
| Syp | 0.0432826 |
| Sypl | 2.7572537 |
| Sypl | 1.7915805 |
| Sypl2 | 1.6068697 |
| Sys1 | -0.7399247 |
| Syt1 | -0.1763593 |
| Syt11 | 2.0706616 |
| Syt11 | -0.5819325 |
| Syt12 | 0.1540507 |
| Syt13 | 0.1996973 |
| Syt15 | -0.3588233 |
| Syt16 | -0.6116456 |
| Syt3 | 0.1565083 |
| Syt4 | 1.8544495 |
| Syt5 | 1.6050544 |
| Syt9 | 0.8051912 |
| Sytl2 | -0.7321113 |
| Sytl3 | -0.0067106 |
| Sytl3 | -0.6917351 |
| Sytl3 | 1.3631861 |
| Sytl4 | 0.1629297 |
| Sytl5 | 1.0317445 |
| Syvn1 | -0.5912868 |
| Taar2 | -0.4118509 |
| Taar3 | 2.3253362 |
| Taar4 | 1.5666048 |
| Tac1 | -0.1463992 |
| Tacc1 | -0.5460461 |
| Tacc1 | -0.2868632 |
| Tacr3 | 0.9747495 |
| Tacr3 | -0.433232 |
| Tacstd2 | -0.4219191 |
| Tada1l | 1.2044325 |
| Tada2l | -0.6068563 |
| Tada2l | -0.5582913 |
| Tada3l | -0.4315265 |
| Tada3l | 1.6032357 |
| Taf10 | 0.7294795 |
| Taf11 | 0.2900054 |
| Taf12 | -0.6359596 |
| Taf12 | -0.5226416 |
| Taf12 | 1.5785108 |
| Taf13 | 0.1450883 |
| Taf1c | 0.6581906 |
| Taf3 | 0.3040409 |
| Taf5 | -0.3828076 |
| Taf5 | -0.8416561 |
| Taf5 | -0.5201454 |
| Taf5l | -0.2263412 |
| Taf6 | 0.0681724 |
| Taf6 | 1.9494333 |
| Taf6l | -0.5120776 |
| Taf7l | 0.7165973 |
| Taf9 | -0.6544309 |
| Taf9 | -0.4372234 |
| Taf9 | 0.3658754 |
| Taf9b | -0.5133143 |
| Tagap | 2.8674345 |
| Tagap | 2.2113098 |
| Tagln | 1.562603 |
| Tagln3 | 0.4951857 |
| Tal1 | 1.5447067 |
| Tal2 | 1.9840798 |
| Taldo1 | 3.2564574 |
| Tank | -0.135923 |
| Tank | -0.5089926 |
| Tank | -0.4157537 |
| Tap1 | 1.7900293 |
| Tap2 | -0.9506299 |
| Tapbp | 0.7729241 |
| Tapbp | -0.4498774 |
| Tapbpl | 1.3270518 |
| Tapbpl | -0.4739171 |
| Tapt1 | -0.4412315 |
| Tarbp2 | -0.2504675 |
| Tarbp2 | -0.6444404 |
| Tardbp | -0.5550537 |
| Tardbp | 2.3159857 |
| Tardbp | 1.681023 |
| Tars | -0.6226838 |
| Tarsl2 | -0.749386 |
| Tarsl2 | -0.749386 |
| Tas1r1 | 0.9871697 |
| Tasp1 | -0.5531163 |
| Tatdn2 | 0.923112 |
| Tax1bp1 | 0.0775496 |
| Tax1bp3 | 2.2755751 |
| Tax1bp3 | 1.0015853 |
| Taz | -0.1737542 |
| Taz | -0.406303 |
| Tbc1d1 | 0.8441988 |
| Tbc1d10a | -0.7143795 |
| Tbc1d10a | -0.2942135 |
| Tbc1d10c | 2.1907983 |
| Tbc1d12 | 0.4104044 |
| Tbc1d13 | 3.154967 |
| Tbc1d14 | -0.660187 |
| Tbc1d15 | -0.6123318 |
| Tbc1d16 | -0.6465724 |
| Tbc1d17 | 0.3624196 |
| Tbc1d19 | -0.688756 |
| Tbc1d2 | -0.3893065 |
| Tbc1d20 | -0.5164133 |
| Tbc1d22a | -0.6843047 |
| Tbc1d22b | -0.0295501 |
| Tbc1d23 | 2.5407439 |
| Tbc1d24 | 2.2387301 |
| Tbc1d25 | -0.3504387 |
| Tbc1d2b | -0.7166737 |
| Tbc1d5 | -0.5139333 |
| Tbc1d5 | -0.5226416 |
| Tbc1d7 | -0.1859729 |
| Tbc1d7 | -0.7391405 |
| Tbc1d8 | -0.5959985 |
| Tbc1d9 | 0.1558945 |
| Tbc1d9 | -0.5083768 |
| Tbc1d9b | 0.4023749 |
| Tbca | 3.2348748 |
| Tbcb | 0.8455416 |
| Tbcc | -0.2332384 |
| Tbcd | 0.9455276 |
| Tbcel | -0.2594372 |
| Tbk1 | 1.6100883 |
| Tbkbp1 | -0.5608894 |
| Tbl1x | 0.2969165 |
| Tbl1xr1 | -0.3942099 |
| Tbl2 | 2.8233061 |
| Tbl3 | -0.7653661 |
| Tbp | -0.3166099 |
| Tbpl1 | -0.6178389 |
| Tbr1 | -0.4247348 |
| Tbrg1 | -0.0415555 |
| Tbrg1 | -0.2996398 |
| Tbrg1 | -0.4918987 |
| Tbrg4 | 0.716256 |
| Tbrg4 | -0.5176557 |
| Tbx1 | -0.6791377 |
| Tbx19 | -0.6638023 |
| Tbx2 | 0.1789466 |
| Tbx22 | -0.5746465 |
| Tbx3 | 2.0828159 |
| Tbx3 | 0.0691815 |
| Tbx5 | 0.7928246 |
| Tbx6 | -0.6917351 |
| Tbxa2r | -0.2570686 |
| Tbxa2r | -0.5320609 |
| Tcam1 | 1.1600057 |
| Tcam1 | -0.7344486 |
| Tcam1 | -0.6164591 |
| Tcap | 1.5836174 |
| Tcea2 | 1.3217246 |
| Tcea3 | 0.9679538 |
| Tceal1 | 1.5075773 |
| Tceal1 | 0.5760194 |
| Tceal3 | -0.7873726 |
| Tceal5 | 0.3019355 |
| Tceal6 | -0.7344486 |
| Tceal6 | -0.6359596 |
| Tceal8 | 0.2502393 |
| Tceb1 | 0.6339382 |
| Tceb2 | -0.2108897 |
| Tceb2 | 2.0719831 |
| Tceb3 | 0.4002381 |
| Tcerg1 | -0.52954 |
| Tcf12 | -0.3936639 |
| Tcf12 | 2.3925131 |
| Tcf15 | 0.7198342 |
| Tcf19 | 0.0269073 |
| Tcf20 | -0.409073 |
| Tcf20 | -0.2023627 |
| Tcf20 | -0.5865982 |
| Tcf20 | 0.980721 |
| Tcf20 | -0.9122796 |
| Tcf21 | -0.5746465 |
| Tcf23 | -0.771023 |
| Tcf25 | -0.6718044 |
| Tcf25 | 1.5450651 |
| Tcf25 | -0.7446436 |
| Tcf3 | 0.5957832 |
| Tcf3 | 1.585199 |
| Tcf4 | -0.6522812 |
| Tcf7 | -0.7014818 |
| Tcf7 | -0.8056865 |
| Tcf7l2 | 2.7918903 |
| Tcf7l2 | 1.8988029 |
| Tcfap2b | -0.6171488 |
| Tcfap2b | -0.1455569 |
| Tcfap2c | -0.4366522 |
| Tcfap2c | 1.0600277 |
| Tcfcp2 | -0.798152 |
| Tcfe3 | 1.00978 |
| Tcfl5 | 0.7965985 |
| Tchp | 2.272302 |
| Tcirg1 | -0.4458329 |
| Tcirg1 | 0.881388 |
| Tcirg1 | -0.4644636 |
| Tcirg1 | -0.6130184 |
| Tcl1 | 3.7135896 |
| Tcl1 | -0.379037 |
| Tcl1b2 | 0.9272269 |
| Tcl1b2 | -0.5872666 |
| Tcl1b3 | 0.4513731 |
| Tcl1b4 | 0.1083732 |
| Tcn2 | 1.0625255 |
| Tcof1 | -0.8628354 |
| Tcp1 | 0.5764147 |
| Tcp1 | 0.1988172 |
| Tcp10a | 0.7047492 |
| Tcp10a | 2.2823529 |
| Tcp11 | 0.4030861 |
| Tcstv1 | -0.7865484 |
| Tcte3 | -0.4751054 |
| Tctex1d1 | 1.2477645 |
| Tctex1d2 | 0.3240714 |
| Tctex1d4 | -0.5473277 |
| Tctn3 | -0.798152 |
| Tctn3 | -0.541574 |
| Tctn3 | 0.1262741 |
| Tdg | -0.1243245 |
| Tdp1 | -0.6268563 |
| Tdpoz1 | 1.7109401 |
| Tdpoz2 | 0.8995941 |
| Tdrd1 | -0.8557233 |
| Tdrd12 | 0.6170421 |
| Tdrd3 | 0.7159145 |
| Tdrd3 | 1.3315433 |
| Tdrd3 | 0.6289007 |
| Tdrd6 | -0.4168718 |
| Tdrd7 | -0.6718044 |
| Tdrkh | -0.4586024 |
| Tead2 | -0.0059828 |
| Tec | -0.2771501 |
| Tecta | 1.1692917 |
| Tef | 2.6850531 |
| Tef | 0.3155383 |
| Tegt | -0.2176757 |
| Tek | -0.5899448 |
| Tekt1 | -0.3969449 |
| Tekt1 | 0.8922397 |
| Tekt3 | -0.8628354 |
| Tenc1 | 3.3640164 |
| Tenc1 | -0.2439118 |
| Terf2 | -0.3090859 |
| Terf2 | 0.7443476 |
| Terf2ip | 1.1044718 |
| Terf2ip | 0.1941094 |
| Terf2ip | 0.2544021 |
| Terf2ip | -0.0202748 |
| Tes | -0.4961435 |
| Tes | -0.2453128 |
| Tes | -0.7098074 |
| Tesc | 0.2463377 |
| Tesk1 | -0.8023305 |
| Tesp1 | -0.232777 |
| Tesp2 | 1.1575356 |
| Tessp2 | 0.7917211 |
| Tessp3 | -0.342127 |
| Tex10 | 1.2305873 |
| Tex10 | 1.6420153 |
| Tex101 | -0.1763593 |
| Tex11 | 1.4456135 |
| Tex13 | -0.6558667 |
| Tex14 | 0.1054647 |
| Tex16 | 3.1906619 |
| Tex19 | -0.6638023 |
| Tex2 | -0.815826 |
| Tex2 | -0.735229 |
| Tex22 | 0.4190663 |
| Tex24 | -0.5993783 |
| Tex261 | -0.494322 |
| Tex261 | 1.3667341 |
| Tex264 | 1.9234658 |
| Tex264 | -0.8235019 |
| Tex9 | -0.6366635 |
| Tfam | -0.348354 |
| Tfam | 0.4313386 |
| Tfb1m | -0.1833414 |
| Tfb2m | 1.6339536 |
| Tfdp1 | 0.3567149 |
| Tfdp2 | -0.7305562 |
| Tfdp2 | 1.5599257 |
| Tfg | 0.9220117 |
| Tfip11 | 1.0988852 |
| Tfpi | -0.3828076 |
| Tfpt | 0.8201595 |
| Tfpt | -0.0030774 |
| Tfpt | 1.7758418 |
| Tfrc | 1.496709 |
| Tfrc | -0.9113403 |
| Tg | -0.6472842 |
| Tgfb1 | -0.6917351 |
| Tgfb1i1 | -0.0487558 |
| Tgfb1i1 | -0.7478026 |
| Tgfb2 | 0.4386726 |
| Tgfb3 | -0.1668416 |
| Tgfbi | -0.6000557 |
| Tgfbi | -0.5772896 |
| Tgfbr1 | 0.5462989 |
| Tgfbr1 | -0.4906893 |
| Tgfbr1 | -0.6261596 |
| Tgfbr2 | 2.1334312 |
| Tgfbr2 | -0.3176176 |
| Tgfbr3 | 2.0836315 |
| Tgfbr3 | 2.1005617 |
| Tgfbrap1 | -0.6199123 |
| Tgif1 | -0.7082882 |
| Tgif1 | -0.6416051 |
| Tgif1 | 1.1287161 |
| Tgif2 | 0.6775852 |
| Tgif2lx | 1.9711884 |
| Tgm2 | -0.7151436 |
| Tgm4 | 0.6955475 |
| Tgoln1 | -0.3410932 |
| Tgs1 | -0.5089926 |
| Tgs1 | 0.1671868 |
| Th1l | 1.0351732 |
| Tha1 | -0.4275588 |
| Thada | 2.6158791 |
| Thada | -0.2443785 |
| Thap1 | -0.1903749 |
| Thap1 | 0.5862459 |
| Thap1 | -0.8992128 |
| Thap11 | 0.8447958 |
| Thap3 | -0.6977213 |
| Thap4 | -0.3678134 |
| Thap7 | -0.4703611 |
| Thbd | 0.3398998 |
| Thbs1 | -0.6226838 |
| Thbs2 | 0.6046507 |
| Thbs3 | -0.0085324 |
| Thbs3 | 0.523803 |
| Theg | 0.1173656 |
| Theg | 0.3715258 |
| Theg | 0.1568151 |
| Them2 | 0.7823688 |
| Them4 | -0.34161 |
| Them5 | -0.3795747 |
| Thg1l | 0.2081636 |
| Thnsl1 | 0.3325289 |
| Thnsl2 | -0.3833475 |
| Thnsl2 | 0.5600692 |
| Thoc1 | 1.2122833 |
| Thoc3 | -0.807369 |
| Thoc4 | -0.3206471 |
| Thoc4 | 0.1531275 |
| Thoc5 | 0.3256147 |
| Thoc6 | 2.359439 |
| Thop1 | 0.8998758 |
| Thra | 0.243821 |
| Thra | 1.0023447 |
| Thrap2 | 1.1961035 |
| Thrap3 | -0.4492986 |
| Thrsp | -0.4423798 |
| Thsd1 | 0.8854004 |
| Thsd4 | 2.7986809 |
| Thsd4 | -0.6444404 |
| Thsd7b | -0.2378643 |
| Thsd7b | 0.3737278 |
| Thsd7b | -0.7266797 |
| Thtpa | -0.5447662 |
| Thumpd1 | 2.6077907 |
| Thumpd3 | 0.5758216 |
| Thy1 | -0.1677029 |
| Thyn1 | 3.766784 |
| Tia1 | -0.6850452 |
| Tial1 | -0.750972 |
| Tial1 | 0.12912 |
| Tiam1 | -0.3593498 |
| Tiam2 | -0.4627015 |
| Ticam1 | 1.5878749 |
| Tie1 | 0.1771468 |
| Tigd2 | 1.2213786 |
| Tigd3 | 1.3668204 |
| Timeless | -0.8303772 |
| Timm10 | -0.1161253 |
| Timm13 | 2.1388331 |
| Timm17a | -0.6515658 |
| Timm17b | 0.7565359 |
| Timm22 | -0.2849127 |
| Timm44 | 1.9162065 |
| Timm44 | 0.6413527 |
| Timm44 | -0.7166737 |
| Timm50 | -0.468588 |
| Timm8a1 | -0.7718339 |
| Timm8a2 | 1.4395503 |
| Timm8a2 | -0.3855101 |
| Timm8b | -0.5939763 |
| Timm8b | -0.6681587 |
| Timm9 | 2.210025 |
| Timm9 | 0.7118071 |
| Timm9 | 0.6836103 |
| Timp1 | 1.1856151 |
| Timp1 | -0.7840802 |
| Timp2 | -0.5906156 |
| Timp3 | 1.6765377 |
| Timp3 | 0.5724538 |
| Timp4 | -0.3567202 |
| Tinagl | -0.5065318 |
| Tipin | 0.4285737 |
| Tipin | -0.5441269 |
| Tirap | 0.000542 |
| Tirap | 2.5156654 |
| Tirap | -0.5799399 |
| Tjap1 | -0.6681587 |
| Tjap1 | 1.8348142 |
| Tjap1 | -0.0971298 |
| Tjp1 | 3.5584651 |
| Tjp2 | -0.7767136 |
| Tjp3 | 0.2585467 |
| Tjp3 | 0.5016197 |
| Tk1 | -0.6739985 |
| Tk2 | 3.4125711 |
| Tkt | 1.9219701 |
| Tkt | 2.1557278 |
| Tkt | 0.3092842 |
| Tktl1 | -0.7758985 |
| Tktl1 | -0.7228188 |
| Tktl2 | 1.770311 |
| Tlcd1 | 1.1634318 |
| Tle1 | -0.7414951 |
| Tle1 | -0.7228188 |
| Tle2 | 1.0506928 |
| Tle3 | -0.6798741 |
| Tle3 | -0.6902444 |
| Tle3 | -0.7220485 |
| Tle4 | 0.240736 |
| Tle4 | 1.9322959 |
| Tle4 | 0.1834313 |
| Tle6 | 0.3547226 |
| Tlk1 | -0.5120776 |
| Tlk2 | 1.4854867 |
| Tln1 | -0.5608894 |
| Tln2 | 0.4513731 |
| Tlr13 | 3.316296 |
| Tlr2 | 1.684435 |
| Tlr3 | -0.7989862 |
| Tlr4 | 0.5075858 |
| Tlr6 | 0.1002721 |
| Tlr7 | 2.9132276 |
| Tm2d2 | 0.5285942 |
| Tm2d3 | 2.1968642 |
| Tm2d3 | -0.3667513 |
| Tm4sf4 | -0.2032567 |
| Tm4sf5 | 0.865606 |
| Tm6sf1 | -0.278117 |
| Tm6sf2 | 0.4839298 |
| Tm9sf2 | 2.5425859 |
| Tm9sf2 | -0.4810691 |
| Tm9sf2 | 2.9647327 |
| Tm9sf3 | -0.5220169 |
| Tm9sf4 | 0.8270247 |
| Tm9sf4 | 2.033371 |
| Tmbim1 | -0.8637281 |
| Tmbim4 | -0.7470118 |
| Tmc1 | -0.6638023 |
| Tmc4 | -0.0236035 |
| Tmc5 | -0.3625149 |
| Tmc6 | 1.2621357 |
| Tmc6 | -0.6894999 |
| Tmc6 | 2.606872 |
| Tmc7 | 0.0610785 |
| Tmcc2 | 1.1578582 |
| Tmcc3 | 0.778858 |
| Tmco3 | -0.4906893 |
| Tmco3 | 1.0210115 |
| Tmco4 | -0.7259063 |
| Tmco4 | -0.7677864 |
| Tmco5 | -0.61577 |
| Tmco6 | 1.2818799 |
| Tmco6 | 0.1416667 |
| Tmco7 | -0.7485939 |
| Tmed1 | 1.8385629 |
| Tmed1 | -0.4292572 |
| Tmed10 | -0.3817288 |
| Tmed2 | -0.5479692 |
| Tmed3 | -0.5634946 |
| Tmed3 | 3.1122942 |
| Tmed4 | 1.5006959 |
| Tmed4 | -0.0955305 |
| Tmed4 | 1.4822778 |
| Tmed4 | -0.6137056 |
| Tmed8 | 2.3123672 |
| Tmed8 | -0.5799399 |
| Tmed9 | 0.0163063 |
| Tmed9 | -0.1375915 |
| Tmeff2 | 0.1391705 |
| Tmem10 | -0.2172218 |
| Tmem100 | 2.2958636 |
| Tmem101 | -0.8781268 |
| Tmem102 | -0.597349 |
| Tmem102 | -0.7407096 |
| Tmem106a | -0.5096088 |
| Tmem106c | 1.5770566 |
| Tmem107 | 2.1171182 |
| Tmem107 | -0.7581419 |
| Tmem107 | 1.2691454 |
| Tmem108 | 0.7128356 |
| Tmem109 | 2.290561 |
| Tmem11 | 1.2782859 |
| Tmem110 | -0.6917351 |
| Tmem111 | 1.3854653 |
| Tmem111 | 0.0276099 |
| Tmem111 | 2.3058646 |
| Tmem115 | -0.4949288 |
| Tmem116 | -0.706014 |
| Tmem116 | -0.8175265 |
| Tmem117 | -0.424171 |
| Tmem118 | -0.8115881 |
| Tmem119 | 1.1233814 |
| Tmem120a | 1.7357908 |
| Tmem120b | 1.6004673 |
| Tmem121 | 1.0938406 |
| Tmem125 | 0.0627723 |
| Tmem126a | 2.2251184 |
| Tmem126b | 0.5787835 |
| Tmem127 | -0.660909 |
| Tmem128 | 0.1180047 |
| Tmem130 | -0.8955121 |
| Tmem131 | 0.7223819 |
| Tmem132a | -0.1144939 |
| Tmem132a | -0.6813487 |
| Tmem132c | 0.8842557 |
| Tmem132d | 1.0096544 |
| Tmem132e | -0.8098982 |
| Tmem134 | -0.0059828 |
| Tmem134 | 2.0297547 |
| Tmem135 | -0.5263982 |
| Tmem136 | 1.5780263 |
| Tmem138 | 1.5921136 |
| Tmem139 | 0.2607499 |
| Tmem140 | -0.61577 |
| Tmem141 | -0.7189735 |
| Tmem143 | -0.4516161 |
| Tmem143 | 1.9135308 |
| Tmem143 | -0.2613363 |
| Tmem143 | -0.7136159 |
| Tmem144 | 1.797485 |
| Tmem146 | 2.2224431 |
| Tmem147 | 0.4301875 |
| Tmem14c | 0.2336875 |
| Tmem14c | -0.1886117 |
| Tmem14c | -0.3699411 |
| Tmem150 | 1.9087936 |
| Tmem150 | 1.0216316 |
| Tmem151a | 1.1650335 |
| Tmem154 | -0.2332384 |
| Tmem158 | -0.6095901 |
| Tmem159 | 2.4364996 |
| Tmem159 | -0.4487201 |
| Tmem160 | -0.6501364 |
| Tmem160 | -0.3379982 |
| Tmem161a | -0.8700008 |
| Tmem162 | 0.239893 |
| Tmem164 | 2.3747679 |
| Tmem166 | -0.3262263 |
| Tmem167 | 0.9302333 |
| Tmem168 | 0.4009509 |
| Tmem168 | -0.6725352 |
| Tmem168 | -0.5326921 |
| Tmem169 | 1.2810299 |
| Tmem16a | 1.8427693 |
| Tmem16b | 1.3036263 |
| Tmem16d | -0.6261596 |
| Tmem16e | -0.0879701 |
| Tmem16g | 0.0219748 |
| Tmem16j | 1.3388639 |
| Tmem16k | -0.7243613 |
| Tmem17 | 2.4141542 |
| Tmem17 | -0.1434543 |
| Tmem170 | -0.6616315 |
| Tmem176a | -0.7136159 |
| Tmem176a | -0.4828656 |
| Tmem176b | -0.7865484 |
| Tmem177 | 0.833237 |
| Tmem178 | 1.6934952 |
| Tmem179b | -0.4219191 |
| Tmem180 | 2.7519643 |
| Tmem183a | 0.8936584 |
| Tmem183a | -0.4894815 |
| Tmem184a | -0.3405766 |
| Tmem184a | 0.0412077 |
| Tmem184b | 0.5750303 |
| Tmem185b | -0.1438744 |
| Tmem186 | -0.6219902 |
| Tmem19 | 1.6546848 |
| Tmem192 | -0.1075912 |
| Tmem198 | 1.0732742 |
| Tmem199 | 1.5856112 |
| Tmem2 | 2.5459327 |
| Tmem2 | 0.8720081 |
| Tmem2 | 0.6538267 |
| Tmem201 | -0.4230444 |
| Tmem204 | -0.6761976 |
| Tmem205 | -0.3016207 |
| Tmem208 | -0.5859303 |
| Tmem209 | -0.6150814 |
| Tmem209 | -0.0812559 |
| Tmem23 | 0.0874954 |
| Tmem25 | 0.3574609 |
| Tmem25 | -0.0691327 |
| Tmem25 | -0.6508508 |
| Tmem27 | -0.5479692 |
| Tmem32 | 1.5509232 |
| Tmem33 | 0.0023465 |
| Tmem33 | 1.292955 |
| Tmem34 | 0.532741 |
| Tmem35 | 0.2122241 |
| Tmem38a | -0.5779515 |
| Tmem38b | 1.4864015 |
| Tmem38b | -0.4991872 |
| Tmem39a | 0.335077 |
| Tmem40 | 0.8763488 |
| Tmem41a | -0.5959985 |
| Tmem41b | -0.5120776 |
| Tmem42 | -0.5726691 |
| Tmem43 | -0.8416561 |
| Tmem43 | 2.0335858 |
| Tmem44 | -0.6296481 |
| Tmem45a | 1.2892151 |
| Tmem45b | -0.7336689 |
| Tmem47 | -0.1612618 |
| Tmem47 | 0.4000004 |
| Tmem48 | -0.0396697 |
| Tmem49 | 0.1475691 |
| Tmem49 | -0.7151436 |
| Tmem49 | -0.5263982 |
| Tmem49 | 1.2325775 |
| Tmem5 | -0.0773284 |
| Tmem50a | -0.6020908 |
| Tmem50b | -0.6813487 |
| Tmem50b | -0.5270257 |
| Tmem51 | 0.3381258 |
| Tmem53 | 0.2035018 |
| Tmem53 | 0.0624338 |
| Tmem53 | 0.8516349 |
| Tmem54 | -0.8175265 |
| Tmem55a | -0.145978 |
| Tmem55b | -0.6806111 |
| Tmem56 | 1.2062735 |
| Tmem57 | -0.0621633 |
| Tmem59 | 1.4634974 |
| Tmem59l | -0.5946499 |
| Tmem60 | -0.3431621 |
| Tmem62 | -0.2947055 |
| Tmem63a | 0.4815317 |
| Tmem63b | -0.1724542 |
| Tmem63b | -0.2996398 |
| Tmem63c | -0.4846654 |
| Tmem65 | 2.8711048 |
| Tmem66 | -0.1548638 |
| Tmem66 | -0.5251443 |
| Tmem66 | -0.2805384 |
| Tmem68 | 0.3406591 |
| Tmem69 | -0.406303 |
| Tmem70 | 0.7411929 |
| Tmem71 | 2.296451 |
| Tmem74 | 0.0505094 |
| Tmem74 | -0.5010181 |
| Tmem74 | -0.807369 |
| Tmem77 | 0.2555091 |
| Tmem77 | 1.3577085 |
| Tmem77 | -0.2805384 |
| Tmem79 | -0.6178389 |
| Tmem8 | 0.4843652 |
| Tmem8 | -0.4309587 |
| Tmem8 | 1.2199695 |
| Tmem81 | 1.4659139 |
| Tmem82 | -0.4727303 |
| Tmem85 | -0.8183778 |
| Tmem85 | 2.5866029 |
| Tmem86a | 0.5598682 |
| Tmem86b | 2.693807 |
| Tmem87a | 0.486322 |
| Tmem88 | -0.5906156 |
| Tmem9 | 0.1028719 |
| Tmem90a | 0.5240118 |
| Tmem91 | -0.0525673 |
| Tmem93 | 1.8717571 |
| Tmem97 | 0.2257308 |
| Tmem98 | 1.8916021 |
| Tmem98 | 0.7650085 |
| Tmem9b | 2.8185978 |
| Tmem9b | 2.6870053 |
| Tmem9b | -0.5270257 |
| Tmie | 2.304507 |
| Tmod1 | -0.2467158 |
| Tmod2 | -0.3075882 |
| Tmod2 | -0.4412315 |
| Tmod3 | -0.5766281 |
| Tmod4 | 3.2860936 |
| Tmod4 | 1.2244914 |
| Tmod4 | -0.6102748 |
| Tmpo | -0.4912938 |
| Tmpo | -0.5628426 |
| Tmpo | 2.8152313 |
| Tmprss11a | -0.4894815 |
| Tmprss11c | -0.5441269 |
| Tmprss13 | -0.4792761 |
| Tmprss13 | -0.4733235 |
| Tmprss2 | 0.3363485 |
| Tmprss4 | -0.3509606 |
| Tmprss4 | -0.2095382 |
| Tmprss4 | 0.3421759 |
| Tmprss7 | -0.2406507 |
| Tmprss9 | 0.7578443 |
| Tmsb10 | -0.6962212 |
| Tmsb10 | 0.033561 |
| Tmsb10 | -0.0396697 |
| Tmsb4x | 0.4204636 |
| Tmtc2 | 0.0106039 |
| Tmtc4 | -0.5926306 |
| Tmub1 | -0.0617776 |
| Tmub1 | 0.2626735 |
| Tnc | -0.6401906 |
| Tnc | 1.3435658 |
| Tnc | 2.8616768 |
| Tnc | -0.2632393 |
| Tnfaip1 | 1.1134105 |
| Tnfaip2 | 0.3202021 |
| Tnfaip2 | -0.4768906 |
| Tnfaip2 | 1.2429584 |
| Tnfaip3 | -0.6739985 |
| Tnfaip8 | 0.467458 |
| Tnfaip8l2 | 2.2760085 |
| Tnfaip8l2 | 0.9781293 |
| Tnfaip8l3 | -0.3509606 |
| Tnfrsf10b | 1.3302878 |
| Tnfrsf10b | -0.4780826 |
| Tnfrsf10b | 2.5008755 |
| Tnfrsf11a | 1.798035 |
| Tnfrsf11b | 0.4848004 |
| Tnfrsf12a | -0.8048464 |
| Tnfrsf13c | -0.5403001 |
| Tnfrsf19 | 0.4690074 |
| Tnfrsf19 | 0.6650596 |
| Tnfrsf1a | -0.7906764 |
| Tnfrsf1a | -0.6000557 |
| Tnfrsf1a | -0.1807171 |
| Tnfrsf1b | 1.5991139 |
| Tnfrsf21 | 1.6770371 |
| Tnfrsf21 | -0.3604037 |
| Tnfrsf22 | -0.039293 |
| Tnfrsf22 | -0.5557003 |
| Tnfrsf25 | 1.8510749 |
| Tnfrsf4 | -0.6565854 |
| Tnfsf13b | -0.761346 |
| Tnfsf13b | 2.1670762 |
| Tnip1 | 1.8368752 |
| Tnip1 | -0.6352563 |
| Tnip1 | -0.4822664 |
| Tnip1 | 2.9418281 |
| Tnip2 | 3.7173828 |
| Tnks | -0.5595895 |
| Tnks1bp1 | -0.3909382 |
| Tnnc1 | 0.2214409 |
| Tnnc2 | 0.1629297 |
| Tnni2 | 1.4895207 |
| Tnni3 | 0.4919524 |
| Tnnt1 | -0.0640942 |
| Tnp1 | 0.944184 |
| Tnp2 | -0.2665785 |
| Tnpo1 | 1.1627903 |
| Tnpo2 | -0.5207688 |
| Tnr | -0.0291773 |
| Tnrc15 | -0.6739985 |
| Tnrc18 | -0.5473277 |
| Tnrc18 | -0.6843047 |
| Tnrc18 | 0.1515867 |
| Tnrc6a | 1.8121698 |
| Tnrc6a | 0.2376411 |
| Tnrc6b | -0.1447153 |
| Tnrc6b | -0.8200828 |
| Tnrc6c | 0.3404061 |
| Tns4 | 3.0298244 |
| Tnxb | 1.1854063 |
| Tnxb | -0.595324 |
| Tnxb | 3.8478917 |
| Tob1 | -0.7906764 |
| Tob2 | 1.6553873 |
| Toe1 | 0.3214936 |
| Toe1 | -0.571353 |
| Tollip | 2.6171156 |
| Tollip | 1.6061977 |
| Tom1 | 1.6397467 |
| Tom1l2 | 1.654557 |
| Tom1l2 | 0.1970545 |
| Tom1l2 | -0.3936639 |
| Tomm22 | 2.9274665 |
| Tomm34 | 0.3142387 |
| Tomm34 | 2.6423789 |
| Tomm7 | 2.7820741 |
| Tomm70a | 0.2090352 |
| Top1 | -0.712853 |
| Top1mt | -0.1472423 |
| Top2a | -0.9236238 |
| Top3a | -0.5403001 |
| Top3b | -0.2181299 |
| Topors | 3.2516832 |
| Tor1a | -0.6843047 |
| Tor1a | -0.6394841 |
| Tor1aip1 | 0.3286938 |
| Tor1aip2 | 0.3725051 |
| Tor1aip2 | -0.5939763 |
| Tor1b | 2.7142842 |
| Tor3a | 0.6369109 |
| Tox3 | 1.6578738 |
| Tpbg | 1.4452158 |
| Tpcn1 | 2.472084 |
| Tpcn1 | -0.7022357 |
| Tpcn2 | 1.0073919 |
| Tpd52 | -0.7367916 |
| Tpd52l1 | -0.4269933 |
| Tpd52l1 | -0.7807991 |
| Tpd52l2 | -0.6089059 |
| Tph2 | 0.7964416 |
| Tpi1 | 1.6661158 |
| Tpi1 | 1.4003403 |
| Tpi1 | 0.4491175 |
| Tpk1 | -0.0460967 |
| Tpk1 | 0.020207 |
| Tpk1 | 0.3844204 |
| Tpm1 | 1.6507797 |
| Tpm2 | 0.8995941 |
| Tpm2 | 1.9782251 |
| Tpm3 | -0.8243586 |
| Tpm3 | 3.2858274 |
| Tpm4 | 0.0098887 |
| Tpo | 2.4431249 |
| Tpp1 | 0.7609445 |
| Tpp2 | 0.2382046 |
| Tppp | 0.3639022 |
| Tppp | -0.5700388 |
| Tppp3 | -0.5447662 |
| Tpr | -0.6296481 |
| Tpra40 | 2.6386021 |
| Tprgl | -0.8014933 |
| Tprgl | 1.7511013 |
| Tprkb | -0.766979 |
| Tprkb | 1.8769344 |
| Tpsab1 | 3.3180544 |
| Tpst1 | -0.6296481 |
| Tpst2 | -0.8700008 |
| Tpte | 0.5907345 |
| Tpte | 0.2924034 |
| Tpx2 | 0.7068203 |
| Tra2a | -0.3026127 |
| Trabd | 0.3966661 |
| Trabd | -0.597349 |
| Traf1 | -0.8124342 |
| Traf2 | -0.5139333 |
| Traf3 | 0.160183 |
| Traf3 | -0.6192206 |
| Traf3ip1 | -0.5133143 |
| Traf3ip2 | 0.3557193 |
| Traf4 | -0.4107388 |
| Traf4 | 1.6935566 |
| Traf4 | 0.0318145 |
| Traf4 | 2.0791776 |
| Traf5 | 1.4936889 |
| Traf6 | -0.2337 |
| Traf6 | -0.751766 |
| Traf6 | 0.2449403 |
| Traf7 | 1.1544121 |
| Trafd1 | -0.8056865 |
| Trafd1 | -0.604811 |
| Trafd1 | 1.8957306 |
| Traip | 1.3393973 |
| Trak1 | 0.5848756 |
| Trak2 | -0.103554 |
| Trak2 | 1.2705793 |
| Trak2 | 0.1864094 |
| Tram1 | -0.8610524 |
| Tram1 | -0.3699411 |
| Tram1 | -0.631047 |
| Tram2 | -0.7251335 |
| Tram2 | -0.6501364 |
| Trap1 | -0.1079959 |
| Trappc1 | 0.2382046 |
| Trappc1 | 0.54854 |
| Trappc2 | 0.4085212 |
| Trappc2l | -0.0498977 |
| Trappc3 | 2.0533983 |
| Trappc3 | 0.3675986 |
| Trappc3 | -0.4292572 |
| Trappc3 | -0.5792767 |
| Trappc4 | -0.6573046 |
| Trappc4 | 0.1711228 |
| Trappc5 | 0.1401073 |
| Trappc6a | 1.3188228 |
| Trat1 | 2.3938986 |
| Trcg1 | 0.022681 |
| Trdmt1 | -0.5819325 |
| Trdmt1 | 3.2238237 |
| Trem2 | -0.5576429 |
| Treml1 | 0.4443634 |
| Treml2 | 1.4110372 |
| Treml4 | -0.0009041 |
| Trerf1 | 3.1954068 |
| Trerf1 | 1.0343172 |
| Trex1 | -0.163833 |
| Trex1 | -0.52954 |
| Trex2 | 0.9038114 |
| Trf | 0.5660818 |
| Trfp | -0.2509375 |
| Trfr2 | -0.6171488 |
| Trfr2 | 0.1435346 |
| Trh | 1.3733619 |
| Trhde | -0.3556704 |
| Trhr | -0.3457545 |
| Triap1 | -0.4230444 |
| Trib2 | 1.1065158 |
| Trib3 | -0.2912666 |
| Trib3 | -0.5201454 |
| Trim11 | 1.2861186 |
| Trim16 | 1.9498555 |
| Trim17 | -0.1019439 |
| Trim2 | -0.7082882 |
| Trim2 | -0.6688868 |
| Trim21 | -0.0063466 |
| Trim21 | 1.4025891 |
| Trim23 | 0.9164911 |
| Trim23 | -0.5959985 |
| Trim23 | -0.6178389 |
| Trim25 | -0.6992237 |
| Trim26 | -0.7182063 |
| Trim28 | -0.7931617 |
| Trim3 | 2.3405328 |
| Trim3 | 0.640429 |
| Trim30 | -0.4621148 |
| Trim32 | 2.2435004 |
| Trim32 | 0.9634841 |
| Trim33 | 2.5671724 |
| Trim33 | 0.8676477 |
| Trim33 | -0.0434451 |
| Trim35 | 0.2382046 |
| Trim36 | -0.4679977 |
| Trim36 | -0.6917351 |
| Trim36 | 0.7059579 |
| Trim37 | 1.106062 |
| Trim38 | -0.1177595 |
| Trim38 | -0.6479965 |
| Trim39 | 2.9613733 |
| Trim40 | 2.863948 |
| Trim41 | 1.4621696 |
| Trim42 | 0.0016251 |
| Trim44 | 0.4824044 |
| Trim45 | -0.1992403 |
| Trim45 | 1.8924492 |
| Trim46 | 1.5102566 |
| Trim47 | 0.1583474 |
| Trim54 | -0.4644636 |
| Trim56 | 0.0429371 |
| Trim59 | -0.6014119 |
| Trim61 | 0.1478788 |
| Trim62 | 0.2955914 |
| Trim67 | -0.5726691 |
| Trim68 | 0.7177911 |
| Trim69 | 0.9285946 |
| Trim72 | -0.6472842 |
| Trim8 | 0.5308772 |
| Trim9 | -0.4674078 |
| Trim9 | -0.7783457 |
| Trim9 | 1.0985421 |
| Trim9 | -0.6437308 |
| Triml1 | -0.4556852 |
| Triobp | 1.1434529 |
| Triobp | -0.7243613 |
| Trip10 | 0.1920424 |
| Trip12 | -0.429824 |
| Trip12 | -0.6537138 |
| Trip12 | 1.3612768 |
| Trip6 | -0.6352563 |
| Trip6 | 0.6758059 |
| Trit1 | -0.5358548 |
| Trmt1 | 1.8089139 |
| Trmt12 | 1.7792123 |
| Trmu | -0.7890231 |
| Tro | -0.5766281 |
| Tro | 0.2058356 |
| Tro | -0.0544788 |
| Trove2 | 0.3507256 |
| Trp53 | -0.6150814 |
| Trp53bp1 | -0.8425292 |
| Trp53bp1 | -0.3646307 |
| Trp53bp2 | -0.494322 |
| Trp53i11 | -0.6558667 |
| Trp53i11 | 1.4650572 |
| Trp53inp1 | -0.1518926 |
| Trp53inp1 | 1.5039932 |
| Trp53inp2 | 0.3648893 |
| Trp53rk | 0.6130563 |
| Trp63 | -0.7189735 |
| Trpc1 | 0.8591606 |
| Trpc1 | -0.5188998 |
| Trpc2 | 0.2792743 |
| Trpc3 | 0.3467117 |
| Trpc4 | 0.8031671 |
| Trpc4ap | -0.4085183 |
| Trpc4ap | -0.1100218 |
| Trpc5 | -0.5812679 |
| Trpc6 | 1.039807 |
| Trpc7 | 0.8510422 |
| Trpd52l3 | -0.4556852 |
| Trpm1 | -0.4900852 |
| Trpm2 | -0.5139333 |
| Trpm3 | 2.3238158 |
| Trpm3 | -0.2863752 |
| Trpm3 | -0.3026127 |
| Trpm3 | 0.0131029 |
| Trpm3 | -0.5993783 |
| Trpm4 | -0.597349 |
| Trpm6 | -0.7282284 |
| Trpm6 | 0.3472144 |
| Trpm6 | 0.9976522 |
| Trpm8 | 0.7727632 |
| Trps1 | -0.4286908 |
| Trpt1 | 0.9435114 |
| Trpt1 | 0.6514545 |
| Trpt1 | -0.606174 |
| Trpv2 | 0.4625719 |
| Trpv4 | 0.2494046 |
| Trpv6 | 1.8736892 |
| Trrap | 1.5382331 |
| Trub1 | 1.060861 |
| Trub1 | 4.5842409 |
| Trub1 | -0.4931096 |
| Trub2 | 1.8516468 |
| Tsc1 | -0.4046449 |
| Tsc2 | -0.0741979 |
| Tsc2 | -0.6688868 |
| Tsc22d1 | 1.3783241 |
| Tsc22d1 | 0.4436823 |
| Tsc22d2 | -0.7313334 |
| Tsc22d3 | 1.2073969 |
| Tsc22d3 | -0.8736038 |
| Tsc22d4 | -0.0510409 |
| Tsen2 | 1.6084803 |
| Tsen34 | -0.5409368 |
| Tsen54 | -0.8574963 |
| Tsfm | -0.7029901 |
| Tsg101 | 2.307188 |
| Tsga10 | 2.4960707 |
| Tsga10 | 2.0699175 |
| Tsga14 | -0.8416561 |
| Tsga8 | -0.7661722 |
| Tshb | 1.7838552 |
| Tshr | 0.8255034 |
| Tshz3 | -0.2323159 |
| Tsku | -0.2996398 |
| Tsn | -0.2551779 |
| Tsnaxip1 | -0.0859905 |
| Tsnaxip1 | -0.6254634 |
| Tspan1 | -0.0734169 |
| Tspan11 | -0.7336689 |
| Tspan12 | -0.3985897 |
| Tspan13 | -0.7159083 |
| Tspan14 | -0.5926306 |
| Tspan17 | 1.141049 |
| Tspan17 | 0.4701126 |
| Tspan18 | -0.7501787 |
| Tspan18 | 1.842087 |
| Tspan2 | -0.163833 |
| Tspan3 | 0.4734206 |
| Tspan31 | -0.5806037 |
| Tspan33 | 0.0398219 |
| Tspan6 | 0.9799442 |
| Tspan6 | 1.7620685 |
| Tspan7 | -0.1523165 |
| Tspan8 | -0.0136519 |
| Tspo | -0.1214468 |
| Tspo | -0.6565854 |
| Tspo | -0.2971695 |
| Tspyl1 | 3.9205643 |
| Tspyl3 | 1.5350521 |
| Tsr1 | -0.3390287 |
| Tsr2 | -0.1112393 |
| Tsr2 | 1.0654905 |
| Tsr2 | 0.6585534 |
| Tssc1 | -0.2761842 |
| Tssc1 | -0.4320947 |
| Tssc1 | -0.7621486 |
| Tssc1 | 1.9357291 |
| Tssc1 | 0.1999905 |
| Tssc4 | 1.8398268 |
| Tssc4 | 0.4614579 |
| Tssk4 | -0.3855101 |
| Tssk5 | 1.1710957 |
| Tssk6 | -0.4846654 |
| Tst | 1.2721066 |
| Tsta3 | 0.1022226 |
| Tsx | -0.632448 |
| Ttbk2 | -0.5912868 |
| Ttc1 | -0.5518269 |
| Ttc12 | -0.2800537 |
| Ttc13 | 1.0829735 |
| Ttc14 | -0.4609426 |
| Ttc16 | 0.1093407 |
| Ttc17 | -0.0895568 |
| Ttc19 | 3.1883318 |
| Ttc19 | -0.4762952 |
| Ttc21a | 1.1663129 |
| Ttc22 | 0.0658136 |
| Ttc23 | 0.2285801 |
| Ttc24 | 0.6869607 |
| Ttc24 | 1.845912 |
| Ttc26 | 2.3557673 |
| Ttc27 | 1.8332258 |
| Ttc29 | 0.5955895 |
| Ttc29 | -0.4191118 |
| Ttc3 | 1.9274591 |
| Ttc3 | -0.6828256 |
| Ttc3 | -0.1829035 |
| Ttc3 | -0.7422812 |
| Ttc3 | 0.2342533 |
| Ttc3 | 2.2505431 |
| Ttc30a1 | -0.2912666 |
| Ttc30a2 | 0.0346073 |
| Ttc33 | 0.2084543 |
| Ttc33 | -0.1582707 |
| Ttc33 | 1.705625 |
| Ttc35 | 1.1375415 |
| Ttc4 | 2.4114975 |
| Ttc5 | -0.5004074 |
| Ttc5 | 3.254474 |
| Ttc5 | 0.0671622 |
| Ttc7 | -0.5492533 |
| Ttc8 | 1.5544776 |
| Ttc8 | 1.3157299 |
| Ttf2 | -0.5263982 |
| Ttk | -0.0250867 |
| Ttl | 0.454971 |
| Ttll1 | -0.7290037 |
| Ttll1 | -0.4118509 |
| Ttll1 | 0.5552318 |
| Ttll10 | -0.5251443 |
| Ttll11 | 0.4192993 |
| Ttll13 | -0.6587447 |
| Ttll4 | 2.9644897 |
| Ttll5 | -0.8235019 |
| Ttll5 | -0.6652522 |
| Ttll6 | 1.9676505 |
| Ttll8 | -0.6289493 |
| Ttll9 | 2.7008383 |
| Ttn | 2.5941558 |
| Ttn | 0.0593817 |
| Ttr | 0.2588224 |
| Ttyh1 | -0.5654531 |
| Ttyh1 | 2.1039998 |
| Ttyh2 | 0.0353043 |
| Ttyh2 | 0.1780471 |
| Ttyh3 | -0.4498774 |
| Tub | -0.5576429 |
| Tub | -0.2364742 |
| Tuba1a | -0.7383569 |
| Tuba1b | -0.7212788 |
| Tuba3a | -0.2873515 |
| Tuba3a | 1.2035106 |
| Tuba3a | -0.7174397 |
| Tuba3a | -0.5812679 |
| Tuba3b | 0.6507234 |
| Tuba4a | -0.0506597 |
| Tuba6 | -0.5498961 |
| Tuba8 | 0.4771559 |
| Tubb2b | -0.0344091 |
| Tubb2b | -0.847785 |
| Tubb2b | 0.9547476 |
| Tubb2b | 1.723684 |
| Tubb2b | 2.3235939 |
| Tubb2c | -0.8124342 |
| Tubb4 | -0.766979 |
| Tubb5 | -0.4275588 |
| Tubb6 | 2.1172363 |
| Tubd1 | 2.4595997 |
| Tubd1 | -0.2254253 |
| Tube1 | -0.4309587 |
| Tubg1 | -0.5939763 |
| Tubgcp2 | 0.0917733 |
| Tubgcp4 | -0.1400997 |
| Tufm | -0.5270257 |
| Tuft1 | -0.8664114 |
| Tug1 | -0.6185295 |
| Tulp1 | 0.0772163 |
| Tulp2 | 1.6804635 |
| Tulp3 | 1.9253443 |
| Tulp4 | 1.6198121 |
| Tusc2 | -0.2888178 |
| Tusc2 | 0.5994559 |
| Tusc4 | 0.4363868 |
| Tut1 | -0.6465724 |
| Twf2 | 1.3398416 |
| Twist1 | -0.8854083 |
| Twist2 | 3.2410444 |
| Twistnb | 1.4711933 |
| Twistnb | -0.8442779 |
| Twsg1 | 0.0498235 |
| Txk | 0.1041692 |
| Txlna | -0.4007872 |
| Txn1 | 0.8852574 |
| Txn2 | 0.5256807 |
| Txndc1 | -0.4168718 |
| Txndc11 | 1.1268301 |
| Txndc11 | -0.0273158 |
| Txndc11 | 2.1995759 |
| Txndc12 | 2.1279985 |
| Txndc13 | 1.1300451 |
| Txndc13 | 3.0624471 |
| Txndc15 | 2.0904154 |
| Txndc2 | -0.6917351 |
| Txndc5 | -0.7266797 |
| Txndc8 | -0.5654531 |
| Txndc9 | 0.0924298 |
| Txnip | 1.0201426 |
| Txnl2 | 0.3479679 |
| Txnl4 | -0.73601 |
| Txnl4a | 2.7847381 |
| Txnl4a | -0.4888781 |
| Txnl4a | -0.6444404 |
| Txnl4b | 0.3155383 |
| Txnrd2 | -0.5089926 |
| Txnrd3 | -0.7407096 |
| Tyk2 | 2.0052894 |
| Tyk2 | 1.2094359 |
| Tyk2 | -0.6054923 |
| Tyki | -0.3774255 |
| Tyms | 0.222873 |
| Tyms | -0.6366635 |
| Tyms | -0.8023305 |
| Tyms-ps | 0.3591994 |
| Tyro3 | -0.7220485 |
| Tyrobp | 1.4540063 |
| Tyw1 | 0.2090352 |
| Tyw1 | 0.6428287 |
| U2af1l4 | 1.4176112 |
| U2af2 | 0.6157154 |
| U2af2 | -0.6338511 |
| Uaca | 0.3472144 |
| Uap1 | 0.12912 |
| Uap1l1 | -0.4574344 |
| Uba1 | 0.2681483 |
| Uba1 | -0.6909894 |
| Uba2 | -0.5333238 |
| Uba5 | 0.3902027 |
| Ubac1 | -0.3969449 |
| Ubac1 | 2.0314354 |
| Ubac1 | -0.798152 |
| Ubac2 | -0.8209364 |
| Ubap1 | 2.4051142 |
| Ubap2 | -0.6102748 |
| Ubap2l | 0.1316425 |
| Ubap2l | 1.1157726 |
| Ubap2l | -0.7581419 |
| Ubb | 1.2165386 |
| Ubd | 0.5734456 |
| Ube1c | -0.7589419 |
| Ube1l | -0.248121 |
| Ube1x | 2.8659444 |
| Ube2a | 1.8798154 |
| Ube2b | 1.6185523 |
| Ube2d1 | -0.2126946 |
| Ube2d2 | -0.5441269 |
| Ube2d3 | -0.6969709 |
| Ube2e1 | -0.7136159 |
| Ube2e2 | 0.1768465 |
| Ube2e3 | 3.174491 |
| Ube2f | -0.6962212 |
| Ube2f | -0.2149555 |
| Ube2f | 0.524638 |
| Ube2g1 | 1.2779067 |
| Ube2g1 | -0.6219902 |
| Ube2g1 | -0.2844256 |
| Ube2g2 | -0.4985777 |
| Ube2i | 1.238128 |
| Ube2j1 | 0.3619249 |
| Ube2j2 | 2.2739065 |
| Ube2j2 | -0.8006569 |
| Ube2j2 | 0.7283002 |
| Ube2k | 0.3322737 |
| Ube2k | 1.0193973 |
| Ube2l3 | 2.4231381 |
| Ube2l3 | -0.1472423 |
| Ube2l6 | 1.5267609 |
| Ube2m | -0.4668182 |
| Ube2n | -0.5046903 |
| Ube2n | -0.8286537 |
| Ube2o | 0.293733 |
| Ube2q1 | -0.0483755 |
| Ube2q1 | -0.306591 |
| Ube2q2 | -0.5358548 |
| Ube2q2 | 1.9639146 |
| Ube2q2 | -0.6529972 |
| Ube2r2 | 2.457039 |
| Ube2s | 0.9796852 |
| Ube2t | 0.3204606 |
| Ube2u | 2.6548538 |
| Ube2u | -0.8700008 |
| Ube3a | -0.4096279 |
| Ube3a | -0.4973598 |
| Ube3b | -0.5946499 |
| Ube3b | -0.5498961 |
| Ube3c | 0.2513512 |
| Ube4a | -0.3641013 |
| Ube4b | 1.2748684 |
| Ubfd1 | 0.3875568 |
| Ubiad1 | 0.3256147 |
| Ubie | -0.829515 |
| Ubl3 | 2.2477666 |
| Ubl4 | -0.5720108 |
| Ubl4b | -0.7182063 |
| Ubl5 | -0.5602392 |
| Ubl7 | -0.6366635 |
| Ublcp1 | 0.4315686 |
| Ubox5 | -0.3747457 |
| Ubox5 | 0.9295508 |
| Ubp1 | -0.3026127 |
| Ubp1 | -0.6116456 |
| Ubqln1 | 0.085845 |
| Ubqln1 | -0.0644808 |
| Ubqln1 | 1.0731567 |
| Ubqln3 | -0.6791377 |
| Ubqln4 | -0.7438555 |
| Ubqlnl | -0.3876777 |
| Ubr1 | 1.6956398 |
| Ubr5 | -0.3598766 |
| Ubtd1 | -0.761346 |
| Ubtd2 | 0.3230411 |
| Ubtd2 | -0.6501364 |
| Ubtd2 | 0.0432826 |
| Ubtf | -0.1582707 |
| Ubxd1 | -0.3561952 |
| Ubxd1 | -0.807369 |
| Ubxd1 | 0.9893524 |
| Ubxd3 | -0.3768889 |
| Ubxd4 | -0.3991386 |
| Ubxd5 | 1.8033533 |
| Ubxd8 | -0.5396638 |
| Uchl1 | 0.2593735 |
| Uchl3 | -0.5700388 |
| Uchl5 | -0.0979305 |
| Uchl5 | 0.510555 |
| Uchl5 | -0.7266797 |
| Uck1 | 0.0237394 |
| Uck1 | 1.2193649 |
| Uck1 | -0.3349133 |
| Uck1 | -0.0351588 |
| Uck2 | 0.4153302 |
| Uck2 | -0.4024386 |
| Uck2 | 0.646876 |
| Uevld | 2.2464613 |
| Ufc1 | 1.3074022 |
| Ufsp1 | 1.6573006 |
| Ufsp2 | -0.595324 |
| Ugcg | -0.1728874 |
| Ugp2 | 1.0017119 |
| Ugt1a10 | -0.7726454 |
| Ugt1a6a | 2.0371449 |
| Ugt1a6a | 0.4764978 |
| Ugt1a6a | -0.829515 |
| Ugt3a2 | -0.4179911 |
| Ugt8a | -0.0506597 |
| Uhmk1 | 0.180743 |
| Uhrf1 | 1.3867349 |
| Uhrf1bp1 | -0.5563474 |
| Uhrf1bp1l | -0.7645607 |
| Uhrf2 | -0.1202161 |
| Uhrf2 | 0.7890358 |
| Uhrf2 | -0.5531163 |
| Uhrf2 | 1.5060856 |
| Uimc1 | 1.966499 |
| Ulk1 | -0.6999758 |
| Ulk1 | -0.706014 |
| Ulk2 | 1.8998413 |
| Ulk2 | -0.2608612 |
| Umodl1 | 0.256891 |
| Umps | -0.4906893 |
| Umps | 0.6663196 |
| Umps | -0.5524714 |
| Unc119 | 0.8868293 |
| Unc119 | -0.5059176 |
| Unc119b | -0.0598515 |
| Unc119b | -0.5654531 |
| Unc119b | 0.341165 |
| Unc13b | 1.6719686 |
| Unc13c | -0.3297937 |
| Unc13d | -0.6014119 |
| Unc45a | 0.0664881 |
| Unc45a | 2.2026039 |
| Unc45b | -0.6199123 |
| Unc5a | -0.5034647 |
| Unc5a | 1.2920214 |
| Unc5b | 1.9887739 |
| Unc5c | 0.5720568 |
| Unc84a | -0.548611 |
| Unc84a | 1.7893638 |
| Unc84b | -0.6828256 |
| Unc93a | 0.1240547 |
| Unc93b1 | -0.4185513 |
| Unc93b1 | -0.5083768 |
| Ung | -0.1544387 |
| Ung | 1.9462375 |
| Unk | 0.0960321 |
| Unkl | 0.9617696 |
| Uox | 0.8753377 |
| Upb1 | -0.5687263 |
| Upb1 | -0.1330101 |
| Upb1 | 0.4753999 |
| Upf1 | -0.5505393 |
| Upf1 | -0.4757001 |
| Upf1 | 0.5785863 |
| Upf2 | -0.460357 |
| Upf3a | 0.8821058 |
| Upk1a | -0.3582971 |
| Upk1a | -0.6192206 |
| Upk1b | -0.7645607 |
| Upk2 | -0.3678134 |
| Upk3a | 0.8572479 |
| Upk3b | -0.5016291 |
| Upk3b | 0.8219951 |
| Upk3b | -0.0525673 |
| Upp1 | 2.1647948 |
| Upp1 | -0.2523489 |
| Upp1 | -0.7052572 |
| Upp2 | 1.4371463 |
| Upp2 | 3.4034262 |
| Upp2 | 1.3907864 |
| Upp2 | 0.115766 |
| Uqcc | 2.3114685 |
| Uqcr | 0.6317026 |
| Uqcrc1 | -0.9217237 |
| Uqcrc2 | -0.6537138 |
| Uqcrh | -0.6261596 |
| Urm1 | -0.3135932 |
| Urod | 2.3978082 |
| Uros | -0.3206471 |
| Uros | -0.5473277 |
| Use1 | 0.7845963 |
| Use1 | -0.9331814 |
| Use1 | -0.53143 |
| Usf1 | 0.4181337 |
| Usf1 | -0.7783457 |
| Usf2 | -0.4615285 |
| Usf2 | -0.852187 |
| Ush1c | -0.3974929 |
| Ush1g | 1.9365854 |
| Ush2a | -0.7228188 |
| Ush3a | -0.4638759 |
| Ushbp1 | 2.2551528 |
| Usmg5 | -0.4870704 |
| Uso1 | 1.9900787 |
| Usp1 | 1.8123323 |
| Usp1 | -0.4107388 |
| Usp1 | -0.6226838 |
| Usp10 | 1.740182 |
| Usp10 | -0.2222267 |
| Usp10 | 0.6056096 |
| Usp11 | 1.0242317 |
| Usp11 | 1.1984759 |
| Usp12 | -0.145136 |
| Usp12 | 0.719494 |
| Usp13 | -0.155289 |
| Usp14 | 0.2488477 |
| Usp15 | -0.7939916 |
| Usp16 | -0.1939112 |
| Usp18 | -0.606174 |
| Usp18 | 3.0795356 |
| Usp19 | 1.5326594 |
| Usp2 | -0.0099923 |
| Usp2 | 0.4612349 |
| Usp2 | 0.884542 |
| Usp20 | 0.0009032 |
| Usp21 | 1.225994 |
| Usp22 | -0.4129644 |
| Usp25 | 0.2884034 |
| Usp28 | 2.4895109 |
| Usp29 | -0.1837795 |
| Usp3 | -0.3758167 |
| Usp3 | 0.0976651 |
| Usp33 | 1.4172836 |
| Usp37 | -0.8601621 |
| Usp38 | -0.7143795 |
| Usp38 | 0.4554198 |
| Usp39 | -0.0621633 |
| Usp39 | -0.2032567 |
| Usp39 | -0.6969709 |
| Usp39 | 1.0152874 |
| Usp4 | -0.7605439 |
| Usp4 | -0.7075296 |
| Usp43 | 2.1324246 |
| Usp45 | 0.5331547 |
| Usp46 | -0.8772205 |
| Usp47 | -0.3287731 |
| Usp48 | -0.4591869 |
| Usp49 | 0.1580412 |
| Usp5 | 1.0198943 |
| Usp50 | 1.7491358 |
| Usp52 | -0.0005424 |
| Usp52 | 0.3050919 |
| Usp52 | 0.4188332 |
| Usp53 | 0.0474189 |
| Usp6nl | 2.7180045 |
| Usp6nl | -0.545406 |
| Usp7 | 1.425928 |
| Usp8 | -0.1384268 |
| Usp8 | 0.670985 |
| Usp9x | 1.3632728 |
| Usp9y | 0.9636159 |
| Uspl1 | -0.632448 |
| Utp11l | 0.7959706 |
| Utp15 | -0.7391405 |
| Utp6 | 0.9178742 |
| Utx | -0.7414951 |
| Utx | -0.4101832 |
| Uty | 1.7554228 |
| Uvrag | -0.5839293 |
| Uxt | 1.0106583 |
| Uxt | 0.6354258 |
| V1ra2 | 1.408064 |
| V1rd2 | 2.6632437 |
| V1rd20 | 1.6463431 |
| V1rd21 | -0.1051669 |
| V2r8 | 1.1573205 |
| Vac14 | -0.0947319 |
| Vamp1 | -0.1574178 |
| Vamp1 | 0.7075097 |
| Vamp2 | -0.222683 |
| Vamp3 | -0.0502786 |
| Vamp3 | 0.1804438 |
| Vamp4 | -0.7446436 |
| Vamp5 | -0.7136159 |
| Vamp8 | 0.2049611 |
| Vamp8 | -0.6000557 |
| Vangl2 | 0.0758818 |
| Vapa | -0.4556852 |
| Vapb | -0.4580182 |
| Vars | -0.1438744 |
| Vars | 3.4201323 |
| Vars | 2.1325795 |
| Vars2 | -0.660187 |
| Vash1 | 0.1103073 |
| Vash1 | -0.5939763 |
| Vasn | 0.3014085 |
| Vasp | 0.7360232 |
| Vat1 | 0.1205567 |
| Vav1 | 0.92709 |
| Vav2 | 1.8416144 |
| Vav3 | 1.0077693 |
| Vax1 | -0.2537623 |
| Vbp1 | 0.5493536 |
| Vcam1 | 4.1617643 |
| Vcl | -0.5959985 |
| Vcp | -0.4745111 |
| Vcpip1 | 2.5570214 |
| Vdac1 | -0.7923326 |
| Vdac2 | -0.7414951 |
| Vdac3 | -1.0070475 |
| Vdr | -0.421357 |
| Vdr | 0.5202462 |
| Vdr | 0.8531152 |
| Vegfa | 0.9984147 |
| Vegfa | -0.3582971 |
| Vegfb | 1.8844471 |
| Vegfc | 0.6244005 |
| Vezf1 | -0.395303 |
| Vezt | 0.3163172 |
| Vgf | 2.0817953 |
| Vgll2 | -0.5544074 |
| Vgll4 | -0.0765448 |
| Vhlh | 0.541596 |
| Vil1 | -0.7840802 |
| Vill | -0.407964 |
| Vim | -0.5345885 |
| Vip | 1.8408787 |
| Vit | -0.3855101 |
| Vkorc1 | -0.6739985 |
| Vkorc1 | 0.031115 |
| Vkorc1 | 0.4125186 |
| Vkorc1l1 | 0.1653646 |
| Vkorc1l1 | 0.9973979 |
| Vkorc1l1 | 0.3098069 |
| Vldlr | -0.2337 |
| Vldlr | 0.301672 |
| Vldlr | 0.3230411 |
| Vldlr | -0.7166737 |
| Vldlr | -0.6240726 |
| Vmn2r81 | -0.6102748 |
| Vmo1 | 0.0339099 |
| Vnn3 | 0.8582783 |
| Vnn3 | -0.6917351 |
| Vpreb1 | -0.5926306 |
| Vpreb3 | 0.3914029 |
| Vps11 | -0.1750559 |
| Vps13a | -0.7573426 |
| Vps16 | 1.810109 |
| Vps16 | -0.5358548 |
| Vps16 | -0.6192206 |
| Vps16 | 0.454971 |
| Vps18 | 1.0965955 |
| Vps24 | 2.5550081 |
| Vps25 | -0.7645607 |
| Vps25 | -0.8192299 |
| Vps26 | -0.5059176 |
| Vps26a | -0.6000557 |
| Vps26b | -0.1405184 |
| Vps28 | 1.3033494 |
| Vps29 | 1.698329 |
| Vps29 | -0.851305 |
| Vps29 | -0.5466867 |
| Vps33a | 1.6705244 |
| Vps33a | 0.2913384 |
| Vps33b | -0.2355486 |
| Vps33b | -0.6947235 |
| Vps35 | 0.3902027 |
| Vps36 | -0.1886117 |
| Vps37b | -0.7931617 |
| Vps37c | -0.5621911 |
| Vps37d | 1.6449242 |
| Vps39 | -0.3349133 |
| Vps39 | -0.4991872 |
| Vps41 | 2.1143984 |
| Vps41 | 2.7590841 |
| Vps45 | -0.5537617 |
| Vps4a | -0.2922479 |
| Vps4b | -0.807369 |
| Vps53 | 0.7939269 |
| Vps53 | 0.5756239 |
| Vps53 | 0.8341427 |
| Vps54 | -0.1136792 |
| Vps72 | 0.590345 |
| Vrk1 | 2.1595005 |
| Vrk1 | -0.7220485 |
| Vrk2 | 3.0166286 |
| Vrk3 | -0.0103576 |
| Vsig2 | -0.0788975 |
| Vsig4 | 0.191451 |
| Vsnl1 | 3.1292575 |
| Vstm2a | 0.7237379 |
| Vstm2a | -0.8557233 |
| Vstm2a | 0.1768465 |
| Vstm2b | 0.713863 |
| Vsx2 | 0.3868339 |
| Vti1a | -0.4798734 |
| Vti1a | 1.9556532 |
| Vti1a | -0.3619866 |
| Vti1b | 1.3011316 |
| Vti1b | 0.0620952 |
| Vtn | 0.2390491 |
| Vwa2 | 2.7470526 |
| Vwa3a | -0.7807991 |
| Vwf | -0.3504387 |
| Wars | -0.8023305 |
| Wars | -0.7525607 |
| Wars | -0.4810691 |
| Wars | -0.0947319 |
| Wars2 | 1.989674 |
| Wars2 | -0.5852628 |
| Was | -0.7661722 |
| Wasf1 | -0.4632885 |
| Wasf2 | 0.0491371 |
| Wasf3 | -0.3969449 |
| Wasl | 1.6553234 |
| Wbp1 | -0.2332384 |
| Wbp1 | 0.1677934 |
| Wbp11 | 0.2113555 |
| Wbp2 | 2.687482 |
| Wbp2nl | 0.265141 |
| Wbp4 | -0.7501787 |
| Wbp5 | -0.0621633 |
| Wbp7 | -0.7243613 |
| Wbscr16 | -0.766979 |
| Wbscr18 | -0.4834651 |
| Wbscr22 | -0.1112393 |
| Wbscr22 | -0.2245104 |
| Wbscr22 | -0.3206471 |
| Wbscr27 | 1.4346557 |
| Wbscr28 | 0.5686756 |
| Wbscr28 | 0.0055862 |
| Wdfy1 | 0.0661509 |
| Wdfy3 | 0.3191677 |
| Wdhd1 | -0.0059828 |
| Wdr1 | -0.7098074 |
| Wdr1 | 1.8358188 |
| Wdr13 | -0.6515658 |
| Wdr13 | -0.4627015 |
| Wdr16 | 0.1265907 |
| Wdr17 | -0.1664112 |
| Wdr18 | 0.0498235 |
| Wdr20a | 1.0396853 |
| Wdr20b | -0.6681587 |
| Wdr21 | 0.3355858 |
| Wdr22 | -0.5428496 |
| Wdr23 | 2.1081808 |
| Wdr24 | -0.4644636 |
| Wdr27 | 1.0152874 |
| Wdr3 | -0.3710067 |
| Wdr31 | -0.5441269 |
| Wdr31 | 0.0001807 |
| Wdr31 | -0.4792761 |
| Wdr32 | 0.5503697 |
| Wdr33 | -0.7694033 |
| Wdr34 | 0.3199436 |
| Wdr37 | -0.8031684 |
| Wdr37 | -0.4656402 |
| Wdr37 | -0.1711561 |
| Wdr37 | 0.6536444 |
| Wdr38 | 0.2342533 |
| Wdr41 | 0.0332119 |
| Wdr45 | 1.207907 |
| Wdr45l | -0.5946499 |
| Wdr45l | 0.9649325 |
| Wdr46 | -0.5188998 |
| Wdr46 | -0.3942099 |
| Wdr47 | -0.004529 |
| Wdr5 | -0.8183778 |
| Wdr51b | 0.6650596 |
| Wdr53 | -0.3390287 |
| Wdr54 | -0.7228188 |
| Wdr55 | 0.0989695 |
| Wdr59 | -0.466229 |
| Wdr5b | -0.429824 |
| Wdr6 | -0.8183778 |
| Wdr60 | -0.1707238 |
| Wdr61 | -0.7653661 |
| Wdr62 | -0.8048464 |
| Wdr63 | 0.5221309 |
| Wdr66 | 2.241982 |
| Wdr67 | 1.5034694 |
| Wdr68 | 2.4962559 |
| Wdr69 | 1.4606835 |
| Wdr70 | 0.3517264 |
| Wdr70 | -0.5993783 |
| Wdr74 | -0.0691327 |
| Wdr75 | -0.8338334 |
| Wdr75 | 1.0518947 |
| Wdr76 | -0.4441048 |
| Wdr77 | -0.2718497 |
| Wdr78 | -0.7799806 |
| Wdr8 | 1.5558249 |
| Wdr8 | -0.0844097 |
| Wdr9 | 0.9761809 |
| Wdr93 | 0.031115 |
| Wdsof1 | -0.4644636 |
| Wfdc1 | 0.3181321 |
| Wfdc10 | 2.128932 |
| Wfdc10 | 0.6904749 |
| Wfdc12 | 0.6580092 |
| Wfdc15b | -0.4709528 |
| Wfdc15b | -0.2637156 |
| Wfdc2 | -0.7143795 |
| Wfdc3 | -0.5263982 |
| Wfdc6a | -0.5919585 |
| Wfdc6b | 2.1883538 |
| Wfdc6b | -0.8235019 |
| Wfikkn2 | -0.0919418 |
| Wfs1 | 0.4379874 |
| Whdc1 | 1.3130839 |
| Whrn | -0.6703445 |
| Whrn | 1.669204 |
| Whsc2 | -0.4991872 |
| Wif1 | 0.3978583 |
| Wipf1 | 2.147642 |
| Wipf2 | 2.1248802 |
| Wipi1 | 1.4329649 |
| Wipi1 | 0.8666274 |
| Wipi1 | -0.3822681 |
| Wipi2 | 0.0637872 |
| Wipi2 | -0.7407096 |
| Wipi2 | -0.4002373 |
| Wisp1 | 0.1376071 |
| Wisp1 | 1.5036939 |
| Wisp2 | -0.1794077 |
| Wiz | 4.4562778 |
| Wiz | 0.2841182 |
| Wnk1 | 0.2331213 |
| Wnk1 | 0.4871903 |
| Wnk2 | 0.6318891 |
| Wnk4 | 1.821496 |
| Wnt10a | -0.029923 |
| Wnt10b | -0.6565854 |
| Wnt16 | -0.004529 |
| Wnt16 | -0.5164133 |
| Wnt16 | 1.2994648 |
| Wnt2 | 0.0610785 |
| Wnt3a | -0.1970162 |
| Wnt4 | -0.5320609 |
| Wnt4 | 0.8224534 |
| Wnt5a | 2.2646431 |
| Wnt5a | 1.3223582 |
| Wnt5b | 1.3102473 |
| Wnt6 | 1.8580189 |
| Wnt7a | -0.1934684 |
| Wnt7b | 0.0805442 |
| Wnt7b | -0.1557144 |
| Wrb | 0.3552211 |
| Wrn | -0.5819325 |
| Wsb1 | 0.3098069 |
| Wsb2 | 0.2211542 |
| Wscd1 | 1.202793 |
| Wscd2 | 0.5187778 |
| Wtap | 2.831779 |
| Wtip | -0.2059437 |
| Wwc1 | -0.2108897 |
| Wwc1 | -0.5276536 |
| Wwc2 | 4.6243489 |
| Wwc2 | -0.7816183 |
| Wwox | -0.3667513 |
| Wwox | -0.9188807 |
| Wwp1 | 3.1080373 |
| Wwp2 | -0.3026127 |
| Wwp2 | -0.395303 |
| Wwp2 | 0.315798 |
| Wwtr1 | 1.2103521 |
| X99384 | 3.0818312 |
| Xab1 | -0.1214468 |
| Xab1 | -0.4185513 |
| Xbp1 | -0.664527 |
| Xdh | -0.3520052 |
| Xist | -0.8065274 |
| Xist | 0.0134593 |
| Xk | 0.9333662 |
| Xkr4 | -0.5518269 |
| Xkr5 | -0.8373023 |
| Xkr6 | 0.6920521 |
| Xkr8 | 0.5656822 |
| Xkrx | 0.0914449 |
| Xlr4a | 2.0587273 |
| Xpc | -0.4768906 |
| Xpnpep1 | 2.0495615 |
| Xpnpep1 | -0.3656905 |
| Xpnpep1 | 0.5022607 |
| Xpnpep2 | 2.0398375 |
| Xpnpep2 | 0.8865437 |
| Xpnpep3 | -0.5772896 |
| Xpo4 | -0.2387922 |
| Xpo4 | -0.7266797 |
| Xpo5 | -0.5473277 |
| Xpo6 | -0.2199485 |
| Xpo7 | 0.4350126 |
| Xpo7 | -0.5479692 |
| Xpr1 | 0.1466396 |
| Xrcc1 | -0.278117 |
| Xrcc2 | 1.1717316 |
| Xrcc2 | -0.606174 |
| Xrcc3 | 1.4908868 |
| Xrcc6 | 1.105835 |
| Xrcc6 | -0.6828256 |
| Xrcc6 | 1.5036191 |
| Xrn1 | 2.5320666 |
| Xrn2 | 1.8115737 |
| Xylb | -0.6820869 |
| Xylt2 | 0.1902672 |
| Yaf2 | -0.6932281 |
| Yap1 | 0.9290045 |
| Ybx2 | 1.1967229 |
| Ybx2 | 0.0470749 |
| Yeats2 | 2.9491839 |
| Yeats4 | 1.0730392 |
| Yeats4 | -0.6192206 |
| Yif1a | -0.2966762 |
| Yif1a | -0.7750842 |
| Yif1a | 1.5706598 |
| Yif1b | 1.993221 |
[truncated: 4,795,438 more chars]
