# Supplementary material for: Plurigon: three dimensional visualization and classification of high-dimensionality data
Source: Front Physiol. 2013 Jul 22;4:190. doi: 10.3389/fphys.2013.00190 (PMC3717481; doi:10.3389/fphys.2013.00190)
Supplement: Table S9 — GIT2 heterozygous (HET) murine transcriptomic data from multiple isolated tissues. Datasets are arranged alphanumerically with the following nomenclature: HET1 to HET19. [file 46440_Maudsley_Data_Sheet_4.ZIP › Table-S9.doc]

**Table S9. GIT2 heterozygous (HET) murine transcriptomic data from multiple isolated tissues.** Datasets are arranged alphanumerically with the following nomenclature: HET1 to HET19.

| **Gene Symbol** | **HET1** |
| --- | --- |
| 0610006I08Rik | -0.6077935 |
| 0610006I08Rik | -0.2834533 |
| 0610007C21Rik | -0.4313749 |
| 0610007C21Rik | -0.0476741 |
| 0610007P08Rik | 3.50282347 |
| 0610007P14Rik | -0.4092386 |
| 0610007P22Rik | -0.6193265 |
| 0610009B22Rik | 0.31706763 |
| 0610009D07Rik | -0.3201182 |
| 0610009O20Rik | -0.1928418 |
| 0610010E21Rik | 1.40059513 |
| 0610010K06Rik | -0.4368207 |
| 0610010K14Rik | 0.4191522 |
| 0610012A05Rik | -0.45683 |
| 0610012D14Rik | -0.4443604 |
| 0610012G03Rik | -0.3445547 |
| 0610012H03Rik | 0.43576932 |
| 0610025P10Rik | -0.7225978 |
| 0610031J06Rik | -0.5217848 |
| 0610031J06Rik | 0.95245617 |
| 0610031J06Rik | 1.78390621 |
| 0610037D15Rik | -0.3830869 |
| 0610037L13Rik | -0.0812185 |
| 0610037L13Rik | 0.16044059 |
| 0610038F07Rik | 0.07132019 |
| 0610040J01Rik | 1.05710801 |
| 0710008K08Rik | 0.41280974 |
| 0910001A06Rik | 1.76864399 |
| 0910001A06Rik | -0.5669372 |
| 0910001L09Rik | 0.51670218 |
| 1110001A07Rik | 2.5702365 |
| 1110001J03Rik | 1.24455634 |
| 1110002B05Rik | -0.7479109 |
| 1110002N22Rik | 1.27911608 |
| 1110003E01Rik | 1.52188393 |
| 1110006G06Rik | -0.1331483 |
| 1110007A13Rik | -0.6487931 |
| 1110007A13Rik | 0.07330699 |
| 1110007C09Rik | 1.97420848 |
| 1110007L15Rik | 1.67516918 |
| 1110007L15Rik | 2.3429521 |
| 1110007L15Rik | -0.6792126 |
| 1110007L15Rik | -0.2266255 |
| 1110007M04Rik | 0.18613792 |
| 1110008F13Rik | 0.42953827 |
| 1110008J03Rik | 1.7263259 |
| 1110008J03Rik | -0.2299072 |
| 1110008J03Rik | -0.2448181 |
| 1110008P14Rik | -0.6020797 |
| 1110012D08Rik | -0.6625772 |
| 1110012J17Rik | -0.6783297 |
| 1110012L19Rik | 0.15608862 |
| 1110012L19Rik | -0.1592123 |
| 1110012M11Rik | 3.05192848 |
| 1110013L07Rik | -0.5202916 |
| 1110017D15Rik | -0.6827526 |
| 1110017D15Rik | 0.72339789 |
| 1110017D15Rik | -0.6961474 |
| 1110018G07Rik | -0.6135424 |
| 1110018J18Rik | -0.2582736 |
| 1110018J18Rik | 0.18367015 |
| 1110019N10Rik | 0.59076268 |
| 1110019N10Rik | 0.37642186 |
| 1110020C03Rik | 2.30400196 |
| 1110020P15Rik | -0.4052692 |
| 1110020P15Rik | -0.7161448 |
| 1110031B06Rik | -0.3464132 |
| 1110031I02Rik | 0.96191881 |
| 1110032A13Rik | -0.3557616 |
| 1110032A13Rik | -0.717984 |
| 1110032E23Rik | 0.15899217 |
| 1110034A24Rik | 0.98554356 |
| 1110036O03Rik | 0.67189311 |
| 1110038D17Rik | -0.7300272 |
| 1110038F14Rik | 0.42435957 |
| 1110038F14Rik | -0.1164888 |
| 1110039B18Rik | -0.6599773 |
| 1110039B18Rik | -0.5708652 |
| 1110039B18Rik | -0.1068251 |
| 1110048D14Rik | 3.7153432 |
| 1110049B09Rik | 0.18367015 |
| 1110049B09Rik | 0.16188679 |
| 1110049F12Rik | 0.0377112 |
| 1110049F12Rik | -0.8087522 |
| 1110057K04Rik | -0.5915593 |
| 1110057K04Rik | -0.0929868 |
| 1110057K04Rik | 2.2364008 |
| 1110058L19Rik | 0.67692162 |
| 1110059E24Rik | -0.0972597 |
| 1110059G10Rik | -0.1960075 |
| 1110067D22Rik | -0.7143092 |
| 1110067I12Rik | -0.0476741 |
| 1190002A17Rik | 0.61428264 |
| 1190002H23Rik | 1.46504816 |
| 1190002N15Rik | -0.7661412 |
| 1190003J15Rik | -0.5779772 |
| 1190003M12Rik | 0.26284529 |
| 1190005F20Rik | -0.6783297 |
| 1190005I06Rik | 1.47045737 |
| 1190005P17Rik | 0.03275118 |
| 1190005P17Rik | -0.0854381 |
| 1190007F08Rik | 0.63984086 |
| 1190017O12Rik | 3.29352811 |
| 1190020J12Rik | -0.0906213 |
| 1200002N14Rik | -0.6854163 |
| 1200003C05Rik | -0.273071 |
| 1200003C05Rik | 0.05162423 |
| 1200003C05Rik | -0.5795649 |
| 1200003I07Rik | -0.7393973 |
| 1200009F10Rik | -0.3219291 |
| 1200009I06Rik | 3.72429581 |
| 1200009I06Rik | -0.5996414 |
| 1200009O22Rik | -0.0222891 |
| 1200011O22Rik | 2.67786418 |
| 1200013B08Rik | -0.0010013 |
| 1200013P24Rik | -0.6318422 |
| 1200013P24Rik | 1.43323324 |
| 1200014J11Rik | 2.6399216 |
| 1200015F23Rik | -0.4765618 |
| 1200015F23Rik | 0.66366401 |
| 1200015N20Rik | 0.60485486 |
| 1200016B10Rik | -0.5188007 |
| 1200016B10Rik | -0.6730495 |
| 1200016D23Rik | 3.35659824 |
| 1300001I01Rik | 2.13157091 |
| 1300007L22Rik | 2.89544168 |
| 1300010M03Rik | -0.149605 |
| 1300013J15Rik | -0.0355642 |
| 1300013J15Rik | -0.6160169 |
| 1300014I06Rik | -0.096784 |
| 1300017J02Rik | 0.27829414 |
| 1300018I05Rik | -0.4059296 |
| 1300018J18Rik | -0.5779772 |
| 1500001L15Rik | 0.36455561 |
| 1500001M20Rik | -0.2271717 |
| 1500001M20Rik | 1.89777628 |
| 1500001M20Rik | -0.4471172 |
| 1500002O20Rik | -0.4239381 |
| 1500003O03Rik | 1.5119587 |
| 1500005A01Rik | 0.27445565 |
| 1500010J02Rik | 1.55672274 |
| 1500010J02Rik | 0.24779822 |
| 1500011H22Rik | -0.3171078 |
| 1500012F01Rik | -0.4909137 |
| 1500015O10Rik | 0.02442591 |
| 1500019C06Rik | 0.86511335 |
| 1500019G21Rik | 1.78061496 |
| 1500031L02Rik | 2.26877142 |
| 1500032D16Rik | -0.6053405 |
| 1500032D16Rik | 0.14842389 |
| 1500032D16Rik | 0.51620653 |
| 1500032L24Rik | -0.4457378 |
| 1500041J02Rik | 2.31340566 |
| 1500041N16Rik | -0.717984 |
| 1520401A03Rik | -0.6836397 |
| 1600002K03Rik | -0.131173 |
| 1600012H06Rik | -0.6730495 |
| 1600012H06Rik | 0.98704756 |
| 1600014C10Rik | -0.5740194 |
| 1600016N20Rik | -0.5843444 |
| 1600021P15Rik | 0.67838323 |
| 1600027N09Rik | -0.1648196 |
| 1600029D21Rik | 2.22235831 |
| 1700001C02Rik | -0.4306964 |
| 1700001C19Rik | -0.277095 |
| 1700001E04Rik | -0.515826 |
| 1700001J03Rik | 1.62892631 |
| 1700001O22Rik | 1.71862382 |
| 1700001O22Rik | -0.5947838 |
| 1700001P01Rik | 0.33436855 |
| 1700003F12Rik | 1.01415439 |
| 1700003M02Rik | 0.61249414 |
| 1700007G11Rik | 2.30570527 |
| 1700007I06Rik | 0.00285687 |
| 1700007K09Rik | 0.25076121 |
| 1700008F21Rik | -0.4293409 |
| 1700008I05Rik | 0.16152545 |
| 1700008P20Rik | 0.98207522 |
| 1700009P17Rik | 0.29944256 |
| 1700010A17Rik | -0.5843444 |
| 1700010A17Rik | 0.67021095 |
| 1700010D01Rik | 0.18824801 |
| 1700010M22Rik | -0.3863164 |
| 1700010M22Rik | -0.2098501 |
| 1700011A15Rik | -0.6765663 |
| 1700011F03Rik | 0.34215576 |
| 1700011F14Rik | 2.74060967 |
| 1700011H14Rik | 0.44169108 |
| 1700011K15Rik | -0.3830869 |
| 1700012A03Rik | 0.2481279 |
| 1700012B09Rik | 0.81972713 |
| 1700012G19Rik | -0.3476543 |
| 1700012H05Rik | -0.658248 |
| 1700012H17Rik | 0.90759308 |
| 1700012L04Rik | -0.3576425 |
| 1700013B16Rik | 0.53728726 |
| 1700013G24Rik | -0.4996312 |
| 1700013N18Rik | -0.107787 |
| 1700013N18Rik | 0.41253301 |
| 1700015E13Rik | -0.6310026 |
| 1700016C15Rik | -0.4815601 |
| 1700016D06Rik | -0.3457933 |
| 1700016G05Rik | 1.17150131 |
| 1700016K19Rik | 0.2435018 |
| 1700016K19Rik | -0.5622452 |
| 1700017N19Rik | 2.8581882 |
| 1700018B24Rik | -0.760346 |
| 1700018C11Rik | 1.40878944 |
| 1700018F24Rik | -0.1699463 |
| 1700019D03Rik | -0.5180561 |
| 1700019E19Rik | -0.6094325 |
| 1700019G17Rik | -0.1997144 |
| 1700019H03Rik | -0.4873052 |
| 1700019L03Rik | 0.13958704 |
| 1700019M22Rik | 2.53021557 |
| 1700019N19Rik | -0.4631274 |
| 1700020C11Rik | -0.4085759 |
| 1700020D05Rik | -0.5498463 |
| 1700020L24Rik | -0.0240483 |
| 1700020N01Rik | 0.59053365 |
| 1700020O03Rik | 2.56769171 |
| 1700021C14Rik | -0.1267436 |
| 1700021F05Rik | 2.24614671 |
| 1700021K02Rik | -0.3908563 |
| 1700021K02Rik | 2.34053097 |
| 1700021K19Rik | 0.26608479 |
| 1700022C21Rik | -0.2470474 |
| 1700023B02Rik | 1.42788726 |
| 1700023B02Rik | 3.41299467 |
| 1700023E05Rik | -0.4901909 |
| 1700023E05Rik | -0.4645325 |
| 1700023I07Rik | 0.28655753 |
| 1700023M03Rik | -0.2354022 |
| 1700024G13Rik | -0.5323045 |
| 1700025D03Rik | -0.7290953 |
| 1700025E21Rik | 0.78684323 |
| 1700025F22Rik | 0.27413506 |
| 1700025G04Rik | -0.3960719 |
| 1700025K23Rik | -0.5843444 |
| 1700026D08Rik | -0.3947653 |
| 1700026L06Rik | -0.3570151 |
| 1700027A23Rik | 0.8021493 |
| 1700027D21Rik | -0.7651729 |
| 1700027J05Rik | -0.6251465 |
| 1700027N10Rik | -0.7290953 |
| 1700028K03Rik | -0.6020797 |
| 1700029F12Rik | 0.4821314 |
| 1700029F12Rik | -0.6880877 |
| 1700029G01Rik | 2.40942512 |
| 1700029H14Rik | 0.2435018 |
| 1700029H14Rik | -0.7986691 |
| 1700029I01Rik | 0.73741414 |
| 1700029I08Rik | 2.5291951 |
| 1700029I15Rik | -0.6556599 |
| 1700029J07Rik | 0.15572505 |
| 1700029P11Rik | -0.0035822 |
| 1700029P11Rik | 1.60927708 |
| 1700029P11Rik | -0.5867432 |
| 1700030J22Rik | 1.76975641 |
| 1700030K09Rik | 0.01686965 |
| 1700034E13Rik | 0.76388321 |
| 1700034H14Rik | -0.4132249 |
| 1700034H14Rik | -0.2282649 |
| 1700034H14Rik | 1.44835532 |
| 1700034H14Rik | 1.77529888 |
| 1700034I23Rik | 2.30703616 |
| 1700034I23Rik | 1.2588482 |
| 1700034O15Rik | 0.67378195 |
| 1700036D21Rik | 2.30492805 |
| 1700037C18Rik | 0.79166474 |
| 1700037C18Rik | 1.74899403 |
| 1700037H04Rik | 3.8186797 |
| 1700039E15Rik | -0.7143092 |
| 1700040I03Rik | -0.3941127 |
| 1700042B14Rik | 1.0237421 |
| 1700045I19Rik | -0.5875442 |
| 1700047I17Rik1 | 0.17015953 |
| 1700047I17Rik1 | 0.18119589 |
| 1700049G17Rik | -0.3377711 |
| 1700049K14Rik | -0.6168432 |
| 1700049L16Rik | 1.85246754 |
| 1700052N19Rik | 0.54284597 |
| 1700052N19Rik | 0.63810034 |
| 1700054O13Rik | -0.0427216 |
| 1700055M20Rik | 1.99172585 |
| 1700057G04Rik | 1.24306935 |
| 1700057K13Rik | 0.56804824 |
| 1700058C13Rik | -0.7719723 |
| 1700060H10Rik | 2.6986307 |
| 1700061G19Rik | -0.8026894 |
| 1700063I17Rik | 2.63633601 |
| 1700065I17Rik | -0.2354022 |
| 1700065O13Rik | -0.7143092 |
| 1700065O13Rik | 0.18895033 |
| 1700067C01Rik | -0.2794021 |
| 1700067K01Rik | -0.4259605 |
| 1700067P10Rik | -0.001431 |
| 1700071K01Rik | -0.6880877 |
| 1700072E05Rik | -0.056746 |
| 1700072E05Rik | 1.93344957 |
| 1700072E05Rik | 2.67556985 |
| 1700073E17Rik | 0.32651635 |
| 1700080E11Rik | -0.6774476 |
| 1700081D17Rik | -0.3093256 |
| 1700081D17Rik | 0.79480396 |
| 1700084C01Rik | 0.69983514 |
| 1700084C01Rik | 2.04107954 |
| 1700088E04Rik | 0.35841931 |
| 1700093K21Rik | 2.20047991 |
| 1700094C09Rik | 0.64914137 |
| 1700095K08Rik | 1.87384441 |
| 1700102P08Rik | 0.05405826 |
| 1700108M19Rik | 0.30442337 |
| 1700109H08Rik | -0.5708652 |
| 1700112C13Rik | -0.3677378 |
| 1700113I22Rik | -0.1881131 |
| 1700113I22Rik | 0.76102118 |
| 1700113O17Rik | 0.02275198 |
| 1700120B06Rik | -0.6625772 |
| 1700123D08Rik | -0.2968887 |
| 1700123K08Rik | -0.1366151 |
| 1700123L14Rik | 1.8166984 |
| 1700123O20Rik | -0.4981726 |
| 1700123O20Rik | -0.787701 |
| 1700127D06Rik | -0.3986905 |
| 1700129C05Rik | -0.3928088 |
| 1700129I15Rik | -0.5653706 |
| 1810007M14Rik | 0.72100818 |
| 1810007P19Rik | -0.5376088 |
| 1810007P19Rik | 3.7543095 |
| 1810008A18Rik | 0.25535184 |
| 1810008A18Rik | 0.09450124 |
| 1810009A15Rik | -0.0481257 |
| 1810009A15Rik | 0.0088272 |
| 1810009N02Rik | 1.7708022 |
| 1810009O10Rik | -0.3501414 |
| 1810009O10Rik | 1.42345621 |
| 1810010M01Rik | 2.05740532 |
| 1810011O10Rik | 0.44890811 |
| 1810014F10Rik | -0.4375036 |
| 1810015A11Rik | 1.11197182 |
| 1810015C04Rik | -0.6818663 |
| 1810019J16Rik | -0.3464132 |
| 1810020D17Rik | -0.5875442 |
| 1810020G14Rik | -0.4603233 |
| 1810020G14Rik | 3.59927623 |
| 1810021J13Rik | -0.717984 |
| 1810021J13Rik | -0.0816864 |
| 1810022C23Rik | -0.1981239 |
| 1810026J23Rik | 2.10047903 |
| 1810027O10Rik | 1.64302443 |
| 1810030J14Rik | -0.1546492 |
| 1810030N24Rik | -0.6739275 |
| 1810030N24Rik | -0.6591122 |
| 1810030N24Rik | -0.7198268 |
| 1810031K17Rik | 2.69672838 |
| 1810035L17Rik | 2.85619144 |
| 1810035L17Rik | 0.28307034 |
| 1810037C20Rik | -0.5188007 |
| 1810043G02Rik | 0.4552771 |
| 1810044A24Rik | 0.24581769 |
| 1810046J19Rik | 1.48650086 |
| 1810048J11Rik | 0.17123324 |
| 1810049H13Rik | 1.73505176 |
| 1810049H19Rik | 0.94084764 |
| 1810055E12Rik | 0.05162423 |
| 1810055E12Rik | 1.09269486 |
| 1810055E12Rik | -0.572441 |
| 1810055G02Rik | 0.67817456 |
| 1810063B05Rik | -0.3645714 |
| 1810064F22Rik | 0.90742954 |
| 1810065E05Rik | -0.1430876 |
| 1810073G14Rik | -0.8480852 |
| 1810074P20Rik | 1.82752346 |
| 1810074P20Rik | -0.5931702 |
| 2010001J22Rik | -0.3908563 |
| 2010001J22Rik | -0.3159064 |
| 2010001M09Rik | 0.5464536 |
| 2010003J03Rik | 0.21252229 |
| 2010003O18Rik | 1.16816027 |
| 2010004A03Rik | -0.6845276 |
| 2010005H15Rik | -0.3111158 |
| 2010005J08Rik | -0.4815601 |
| 2010005J08Rik | -0.6284884 |
| 2010005J08Rik | -0.1839296 |
| 2010007H12Rik | 0.06092065 |
| 2010007H12Rik | -0.058571 |
| 2010011I20Rik | 0.57412817 |
| 2010012O05Rik | 0.4363092 |
| 2010100O12Rik | -0.5544768 |
| 2010100O12Rik | 0.3689143 |
| 2010106G01Rik | 2.97892676 |
| 2010106G01Rik | 0.80013504 |
| 2010107E04Rik | 0.83786348 |
| 2010107G12Rik | -0.5537035 |
| 2010109I03Rik | -0.6522203 |
| 2010110K16Rik | 0.27027953 |
| 2010111I01Rik | -0.4471172 |
| 2010200O16Rik | -0.3347038 |
| 2010301N04Rik | 0.7340771 |
| 2010305A19Rik | 1.72365108 |
| 2010305C02Rik | -0.4631274 |
| 2010305C02Rik | 1.62603544 |
| 2010308M01Rik | 0.90218127 |
| 2010311D03Rik | -0.6402795 |
| 2010316F05Rik | 1.00387769 |
| 2010321M09Rik | -0.0165942 |
| 2010321M09Rik | -0.3464132 |
| 2200001I15Rik | 1.25274969 |
| 2210009G21Rik | -0.2857762 |
| 2210009G21Rik | -0.6428255 |
| 2210009G21Rik | 0.59851678 |
| 2210010B09Rik | 0.03026132 |
| 2210010L05Rik | 3.68905995 |
| 2210012G02Rik | -0.4974442 |
| 2210012G02Rik | -0.1740679 |
| 2210012G02Rik | -0.6176703 |
| 2210012G02Rik | 0.23116502 |
| 2210016L21Rik | 0.15499748 |
| 2210016L21Rik | 4.59777736 |
| 2210016L21Rik | -0.5128607 |
| 2210021J22Rik | 2.91901971 |
| 2210023G05Rik | -0.3908563 |
| 2210023G05Rik | 0.77185089 |
| 2210407C18Rik | -0.5947838 |
| 2210412D01Rik | 0.47023285 |
| 2210417D09Rik | 0.87903806 |
| 2210418O10Rik | 1.60523519 |
| 2300002D11Rik | 0.61361235 |
| 2300002D11Rik | 0.17266296 |
| 2300002M23Rik | -0.7115625 |
| 2300005B03Rik | -0.7161448 |
| 2310001A20Rik | -0.6428255 |
| 2310002B06Rik | 0.06932919 |
| 2310003C23Rik | 0.76635662 |
| 2310003F16Rik | -0.0774835 |
| 2310003H01Rik | 0.55624446 |
| 2310003L22Rik | 1.10616389 |
| 2310003L22Rik | -0.5740194 |
| 2310004I24Rik | -0.6045242 |
| 2310005E10Rik | -0.0057384 |
| 2310005E10Rik | -0.5513873 |
| 2310005G13Rik | 0.80342885 |
| 2310005N01Rik | 0.41253301 |
| 2310005N03Rik | 2.21195285 |
| 2310005P05Rik | 0.48828011 |
| 2310006J04Rik | -0.2222677 |
| 2310007A19Rik | -0.5180561 |
| 2310007B03Rik | -0.3779426 |
| 2310007F21Rik | -0.3576425 |
| 2310007F21Rik | -0.1511154 |
| 2310008H04Rik | -0.3563881 |
| 2310008H09Rik | -0.7431716 |
| 2310008H09Rik | -0.6402795 |
| 2310008M10Rik | 1.96580393 |
| 2310009E04Rik | 0.01855407 |
| 2310010B21Rik | -0.3105187 |
| 2310010M24Rik | 2.1660985 |
| 2310011J03Rik | 0.01137435 |
| 2310014L17Rik | -0.6496487 |
| 2310016C08Rik | -0.7088237 |
| 2310016C08Rik | -0.7207495 |
| 2310016C16Rik | 1.12074741 |
| 2310016C16Rik | 2.27540441 |
| 2310016C16Rik | 1.62389963 |
| 2310016E02Rik | 1.56983586 |
| 2310016M24Rik | 1.57628434 |
| 2310021P13Rik | 0.6229532 |
| 2310022A10Rik | -0.6462309 |
| 2310022B05Rik | 1.7019039 |
| 2310031L18Rik | 0.7827441 |
| 2310031L18Rik | 0.89574511 |
| 2310033E01Rik | -0.6756859 |
| 2310033K02Rik | -0.538369 |
| 2310033P09Rik | -0.3557616 |
| 2310035C23Rik | -0.4327334 |
| 2310035C23Rik | -0.3207215 |
| 2310035K24Rik | -0.6094325 |
| 2310036D22Rik | 1.65617678 |
| 2310036O22Rik | -0.4815601 |
| 2310037I24Rik | 1.22795242 |
| 2310037I24Rik | 1.43023573 |
| 2310038H17Rik | -0.2476055 |
| 2310039E09Rik | 0.3546005 |
| 2310039H08Rik | 2.56951012 |
| 2310040C09Rik | 0.52729604 |
| 2310042D19Rik | 0.05567745 |
| 2310044G17Rik | -0.4246118 |
| 2310044H10Rik | 1.42477828 |
| 2310045A20Rik | -0.5915593 |
| 2310046K01Rik | 0.13699391 |
| 2310046O06Rik | -0.4125593 |
| 2310047C04Rik | -0.4981726 |
| 2310047D13Rik | -0.3261681 |
| 2310047D13Rik | 0.93721021 |
| 2310047M10Rik | 1.47824589 |
| 2310047M10Rik | 0.03109201 |
| 2310047O13Rik | -0.3408485 |
| 2310056P07Rik | 0.97036986 |
| 2310057J16Rik | 0.89009794 |
| 2310058J06Rik | -0.6086127 |
| 2310061C15Rik | -0.6462309 |
| 2310061F22Rik | -0.6979479 |
| 2310061F22Rik | -0.6453784 |
| 2310065K24Rik | 3.39547932 |
| 2310065K24Rik | -0.0798161 |
| 2310066E14Rik | -0.6997518 |
| 2310067B10Rik | -0.3207215 |
| 2310076L09Rik | 0.96438514 |
| 2310076L09Rik | -0.8502016 |
| 2310079F23Rik | -0.5444728 |
| 2310079N02Rik | 1.34966688 |
| 2400001E08Rik | 1.78151959 |
| 2400003C14Rik | 1.96213317 |
| 2400003C14Rik | -0.1756181 |
| 2400003C14Rik | -0.6625772 |
| 2400006N03Rik | 0.28687391 |
| 2400010D15Rik | 1.81364493 |
| 2410001C21Rik | -0.4006593 |
| 2410001C21Rik | 0.97995778 |
| 2410001C21Rik | 1.84639875 |
| 2410002F23Rik | -0.4815601 |
| 2410002F23Rik | 3.63982534 |
| 2410002F23Rik | -0.5099047 |
| 2410002I01Rik | 2.5939795 |
| 2410002O22Rik | -0.1272347 |
| 2410003P15Rik | 0.04141413 |
| 2410004A20Rik | 1.49818188 |
| 2410004B18Rik | 1.24089183 |
| 2410004L22Rik | -0.4624256 |
| 2410005O16Rik | 1.02547487 |
| 2410008K03Rik | 0.27541674 |
| 2410014A08Rik | 1.23398214 |
| 2410014A08Rik | -0.6343655 |
| 2410014A08Rik | 1.04291253 |
| 2410014A08Rik | 0.69554274 |
| 2410015M20Rik | 0.75757521 |
| 2410015N17Rik | 1.98514299 |
| 2410016F19Rik | 1.15580727 |
| 2410016F19Rik | 1.29722895 |
| 2410016O06Rik | -0.2029034 |
| 2410017P07Rik | 1.45046967 |
| 2410018C17Rik | -0.7024642 |
| 2410018G20Rik | 0.15062019 |
| 2410018M08Rik | -0.0001426 |
| 2410019A14Rik | -0.6686719 |
| 2410019G02Rik | 2.01024071 |
| 2410022L05Rik | 0.51620653 |
| 2410025L10Rik | 0.77693756 |
| 2410042D21Rik | -0.6792126 |
| 2410042D21Rik | -0.0581144 |
| 2410066E13Rik | -0.572441 |
| 2410081M15Rik | 0.61651349 |
| 2410091C18Rik | -0.6854163 |
| 2410116G06Rik | 2.36724262 |
| 2410127E18Rik | -0.5003613 |
| 2410127L17Rik | 1.43500851 |
| 2410127L17Rik | -0.6547988 |
| 2410129H14Rik | 0.83327359 |
| 2410137M14Rik | 2.12791336 |
| 2410146L05Rik | -0.4858656 |
| 2410166I05Rik | -0.2398215 |
| 2500002L14Rik | 0.57389505 |
| 2500003M10Rik | -0.1592123 |
| 2500003M10Rik | -0.7051845 |
| 2500003M10Rik | -0.7356381 |
| 2510002A14Rik | 0.26511411 |
| 2510003E04Rik | -0.7574618 |
| 2510006D16Rik | -0.6201557 |
| 2510009E07Rik | -0.7613094 |
| 2510010F15Rik | -0.2387147 |
| 2510012J08Rik | 1.01648767 |
| 2510039O18Rik | 0.08081879 |
| 2510040D07Rik | -0.6284884 |
| 2510048L02Rik | 0.10951659 |
| 2510049I19Rik | -0.950733 |
| 2600001B17Rik | -0.6310026 |
| 2600009E05Rik | -0.0297886 |
| 2600009E05Rik | -0.7450645 |
| 2600010E01Rik | -0.7700246 |
| 2600011E07Rik | 0.06051836 |
| 2600011E07Rik | 1.76464149 |
| 2610002J02Rik | -0.6201557 |
| 2610002M06Rik | -0.5552507 |
| 2610003J06Rik | -0.2431496 |
| 2610016F04Rik | -0.7469612 |
| 2610019A05Rik | 1.34093858 |
| 2610019P18Rik | -0.6907666 |
| 2610020C11Rik | 2.87689854 |
| 2610020H08Rik | -0.6037087 |
| 2610020H08Rik | 4.07396905 |
| 2610020H08Rik | -0.4464272 |
| 2610020O08Rik | 2.2159091 |
| 2610021K21Rik | 2.21195285 |
| 2610021K21Rik | 0.80306344 |
| 2610024G14Rik | -0.4293409 |
| 2610024G14Rik | 1.24592687 |
| 2610024G14Rik | -0.7916746 |
| 2610027C15Rik | 1.90210419 |
| 2610027C15Rik | -0.8344433 |
| 2610027L16Rik | 1.04644626 |
| 2610028A01Rik | 1.04177892 |
| 2610028H24Rik | -0.2163084 |
| 2610029G23Rik | -0.8251135 |
| 2610029G23Rik | 1.31362003 |
| 2610034E13Rik | -0.4395554 |
| 2610034E13Rik | 1.53883786 |
| 2610034M16Rik | 2.26807859 |
| 2610036D13Rik | 0.70065052 |
| 2610039C10Rik | 0.45791817 |
| 2610042L04Rik | -0.6168432 |
| 2610044O15Rik | -0.3093256 |
| 2610101N10Rik | 0.81088866 |
| 2610101N10Rik | -0.770998 |
| 2610103J23Rik | -0.4533496 |
| 2610110G12Rik | 2.08699285 |
| 2610200G18Rik | -0.4687606 |
| 2610204K14Rik | 2.69848451 |
| 2610204L23Rik | -0.8608557 |
| 2610204M08Rik | -0.8344433 |
| 2610205E22Rik | 3.27068911 |
| 2610207I05Rik | -0.2109234 |
| 2610208M17Rik | -0.1844515 |
| 2610209A20Rik | -0.4457378 |
| 2610209A20Rik | 2.00383757 |
| 2610209M04Rik | -0.541416 |
| 2610301B20Rik | 0.45580591 |
| 2610301F02Rik | 3.42454178 |
| 2610304G08Rik | -0.4286638 |
| 2610305D13Rik | -0.7956651 |
| 2610318N02Rik | -0.7290953 |
| 2610507B11Rik | -0.2622347 |
| 2610507B11Rik | -0.717984 |
| 2610510B01Rik | -0.1010742 |
| 2610510J17Rik | -0.1341375 |
| 2610524A10Rik | -0.2409296 |
| 2610524H06Rik | 1.52951304 |
| 2610528E23Rik | -0.5300404 |
| 2610528J11Rik | 1.1455782 |
| 2610528O22Rik | 2.63135069 |
| 2700007P21Rik | -0.1546492 |
| 2700007P21Rik | -0.3658367 |
| 2700038C09Rik | 2.53637321 |
| 2700038C09Rik | 1.25873563 |
| 2700050L05Rik | -0.7272343 |
| 2700055A20Rik | 0.79921804 |
| 2700060E02Rik | 1.39690857 |
| 2700062C07Rik | -0.5490768 |
| 2700062C07Rik | 1.63807271 |
| 2700078K21Rik | -0.4851467 |
| 2700081O15Rik | 1.92804865 |
| 2700087H15Rik | 0.68005085 |
| 2700094K13Rik | -0.5121208 |
| 2700097O09Rik | 1.48976505 |
| 2810002N01Rik | 0.38129844 |
| 2810002O09Rik | -0.5506165 |
| 2810003C17Rik | -0.2168486 |
| 2810004N20Rik | -0.9013596 |
| 2810004N23Rik | 0.19000282 |
| 2810008M24Rik | 0.5435686 |
| 2810021B07Rik | 1.3365957 |
| 2810021J22Rik | 0.99572364 |
| 2810021J22Rik | -0.7347006 |
| 2810022L02Rik | -0.7431716 |
| 2810022L02Rik | -0.658248 |
| 2810022L02Rik | -0.6836397 |
| 2810030E01Rik | 1.97326079 |
| 2810046L04Rik | -0.5483079 |
| 2810046M22Rik | -0.5851433 |
| 2810403A07Rik | 0.71941166 |
| 2810405J04Rik | -0.7671106 |
| 2810405K02Rik | -0.2881048 |
| 2810408A11Rik | -0.2228113 |
| 2810408M09Rik | 1.86383822 |
| 2810410C14Rik | -0.7613094 |
| 2810410P22Rik | 1.36396934 |
| 2810410P22Rik | -0.9929262 |
| 2810417H13Rik | -0.7837441 |
| 2810422O20Rik | 2.13946189 |
| 2810428I15Rik | -0.0826229 |
| 2810432D09Rik | 0.12504773 |
| 2810432L12Rik | 0.83927126 |
| 2810439F02Rik | 0.81342242 |
| 2810439F02Rik | -0.5452386 |
| 2810441K11Rik | -0.4273112 |
| 2810452K22Rik | -0.8168973 |
| 2810453I06Rik | -0.8417643 |
| 2810453I06Rik | 0.67000046 |
| 2810470D21Rik | -0.6599773 |
| 2810485I05Rik | -0.1875891 |
| 2900006K08Rik | 1.29949569 |
| 2900010M23Rik | 0.73072819 |
| 2900011O08Rik | 2.57588867 |
| 2900011O08Rik | 2.2027527 |
| 2900024C23Rik | -0.6470842 |
| 2900024O10Rik | -0.6479383 |
| 2900026A02Rik | -0.1917889 |
| 2900046G09Rik | -0.4172281 |
| 2900062L11Rik | -0.2337504 |
| 2900064A13Rik | 0.671683 |
| 2900073G15Rik | 0.02442591 |
| 2900092C05Rik | -0.9425168 |
| 3000004C01Rik | -0.8480852 |
| 3000004C01Rik | -0.7996725 |
| 3000004C01Rik | -0.5552507 |
| 3010003L21Rik | -0.7450645 |
| 3010026O09Rik | 1.54774 |
| 3110001A13Rik | -0.6045242 |
| 3110001D03Rik | -0.8705487 |
| 3110004L20Rik | -0.8138346 |
| 3110007F17Rik | -0.8597849 |
| 3110009E18Rik | 1.99306786 |
| 3110023B02Rik | -0.2851949 |
| 3110035E14Rik | 0.56922051 |
| 3110035E14Rik | 1.39056894 |
| 3110037I16Rik | 1.09510945 |
| 3110040N11Rik | 0.24746842 |
| 3110040N11Rik | 0.36484682 |
| 3110043J09Rik | -0.7006551 |
| 3110043J09Rik | 1.74785676 |
| 3110043J09Rik | -0.7263052 |
| 3110048E14Rik | -0.5277817 |
| 3110049J23Rik | -0.6326825 |
| 3110050N22Rik | -0.5240292 |
| 3110050N22Rik | 1.21495051 |
| 3110056O03Rik | -0.4794147 |
| 3110057O12Rik | 1.77432317 |
| 3110057O12Rik | -0.1390993 |
| 3110070M22Rik | -0.041824 |
| 3110082I17Rik | 0.39662321 |
| 3200001I04Rik | 0.66324005 |
| 3200002M19Rik | -0.3016072 |
| 3200002M19Rik | 0.39690466 |
| 3300001G02Rik | -0.6748063 |
| 3300001P08Rik | 0.50372957 |
| 3300001P08Rik | -0.1521237 |
| 3300001P08Rik | 0.24084806 |
| 3300002I08Rik | -0.03601 |
| 3321401G04Rik | 0.11901727 |
| 3322402L07Rik | -0.5899512 |
| 3632451O06Rik | 1.19540549 |
| 3830402I07Rik | 0.36455561 |
| 3830406C13Rik | 0.86168559 |
| 3830406C13Rik | -0.8608557 |
| 3830406C13Rik | 2.73265383 |
| 3830408P04Rik | 2.36415347 |
| 3930401K13Rik | -0.6110743 |
| 4121402D02Rik | 2.33792487 |
| 4121402D02Rik | 0.01560435 |
| 4430402I18Rik | 0.98162188 |
| 4432405B04Rik | 0.37498274 |
| 4432416J03Rik | 0.08239261 |
| 4631426J05Rik | 1.3365957 |
| 4631427C17Rik | -0.0640671 |
| 4632404H12Rik | 1.26714173 |
| 4632404H12Rik | 3.37270587 |
| 4632404H12Rik | -0.8117984 |
| 4632404H22Rik | -0.6243128 |
| 4632411B12Rik | -0.8189446 |
| 4632411B12Rik | 0.21798384 |
| 4632412N22Rik | 2.67439559 |
| 4632415K11Rik | 1.2956065 |
| 4632417K18Rik | 1.68545384 |
| 4632417N05Rik | 0.46239105 |
| 4632419K20Rik | 1.39056894 |
| 4632434I11Rik | 1.4596986 |
| 4732415M23Rik | 0.74599661 |
| 4732418C07Rik | -0.1756181 |
| 4732418C07Rik | 0.31122922 |
| 4732418C07Rik | -0.5859429 |
| 4732429D16Rik | 1.58714633 |
| 4732456N10Rik | -0.5099047 |
| 4732471D19Rik | -0.2222677 |
| 4732473B16Rik | -0.7328285 |
| 4732479N06Rik | -0.8097665 |
| 4732479N06Rik | 0.05121795 |
| 4732496O08Rik | 1.2619949 |
| 4733401H18Rik | 0.3977485 |
| 4831426I19Rik | 0.55434203 |
| 4833418A01Rik | 0.11408889 |
| 4833420G17Rik | 0.18261055 |
| 4833421E05Rik | -0.5513873 |
| 4833424O15Rik | 1.33669934 |
| 4833424O15Rik | -0.6069752 |
| 4833426J09Rik | -0.3734644 |
| 4833426J09Rik | -0.3760208 |
| 4833427G06Rik | 1.09510945 |
| 4833436C18Rik | -0.218471 |
| 4833439L19Rik | -0.3177091 |
| 4833439L19Rik | 1.01488416 |
| 4833439L19Rik | 1.47984929 |
| 4833442J19Rik | 0.05365302 |
| 4833446K15Rik | -0.8640757 |
| 4921501E09Rik | -0.1390993 |
| 4921504E06Rik | -0.7906796 |
| 4921504I05Rik | 2.33042338 |
| 4921505C17Rik | -0.5506165 |
| 4921505C17Rik | -0.5444728 |
| 4921507P07Rik | 1.80976709 |
| 4921511H03Rik | 0.87464518 |
| 4921511H03Rik | 0.11332838 |
| 4921511K06Rik | 0.39606005 |
| 4921511K06Rik | 0.05890749 |
| 4921515J06Rik | -0.0048754 |
| 4921517D21Rik | -0.6110743 |
| 4921517D22Rik | 1.91530593 |
| 4921520G13Rik | 1.90748623 |
| 4921521F21Rik | 2.06134694 |
| 4921521F21Rik | 0.37354142 |
| 4921523A10Rik | 0.57552567 |
| 4921523A10Rik | -0.7079126 |
| 4921523A10Rik | -0.4219201 |
| 4921525H12Rik | -0.2968887 |
| 4921528G01Rik | -0.658248 |
| 4921528I01Rik | -0.6284884 |
| 4921530G04Rik | -0.4199064 |
| 4921530L21Rik | -0.6496487 |
| 4921530L21Rik | 1.2637883 |
| 4921536K21Rik | -0.3588985 |
| 4921537P18Rik | 0.59533165 |
| 4922502D21Rik | -0.0324499 |
| 4922503N01Rik | -0.8107819 |
| 4922503N01Rik | 0.85634774 |
| 4922505E12Rik | -0.3129094 |
| 4922505G16Rik | 4.01439076 |
| 4922505G16Rik | -0.5859429 |
| 4930401F20Rik | -0.5851433 |
| 4930402H24Rik | 2.39190115 |
| 4930403C10Rik | 0.26414243 |
| 4930403L05Rik | 0.27925132 |
| 4930403O06Rik | 0.29568956 |
| 4930404H21Rik | 1.21093172 |
| 4930404N11Rik | -0.502555 |
| 4930408O21Rik | -0.7622738 |
| 4930408O21Rik | -0.5498463 |
| 4930412F15Rik | 0.36455561 |
| 4930415L06Rik | -0.5779772 |
| 4930418G15Rik | 0.12166035 |
| 4930427A07Rik | -0.2093139 |
| 4930428D18Rik | 0.21354874 |
| 4930428E23Rik | 1.8191837 |
| 4930430D24Rik | -0.538369 |
| 4930430E16Rik | -0.7555438 |
| 4930430E16Rik | -0.4484986 |
| 4930431L04Rik | -0.4687606 |
| 4930432H15Rik | -0.3633078 |
| 4930432K21Rik | -0.5136011 |
| 4930432O21Rik | 0.34334809 |
| 4930432O21Rik | 0.3062844 |
| 4930438O05Rik | -0.1130952 |
| 4930438O05Rik | -0.6094325 |
| 4930449I24Rik | 0.49210275 |
| 4930451C15Rik | -0.2582736 |
| 4930451I11Rik | 0.01179821 |
| 4930452B06Rik | 0.25535184 |
| 4930453N24Rik | 1.04319572 |
| 4930455C21Rik | -0.7070024 |
| 4930455F23Rik | -0.0499339 |
| 4930467M19Rik | 3.94518499 |
| 4930470P17Rik | -0.5622452 |
| 4930473A06Rik | 1.77432317 |
| 4930473A06Rik | -0.7015592 |
| 4930473A06Rik | -0.6863059 |
| 4930474N05Rik | 1.1259478 |
| 4930478A21Rik | 2.45791943 |
| 4930481F22Rik | 0.34602532 |
| 4930481F22Rik | 0.47361077 |
| 4930481M05 | -0.5947838 |
| 4930486L24Rik | 1.1342091 |
| 4930502E18Rik | 1.01561337 |
| 4930503B20Rik | 2.58277557 |
| 4930503L19Rik | -0.6037087 |
| 4930504E06Rik | -0.1725202 |
| 4930504E06Rik | 3.55829843 |
| 4930504O13Rik | -0.2933653 |
| 4930506M07Rik | 0.42435957 |
| 4930507C10Rik | -0.541416 |
| 4930511I11Rik | 1.75625339 |
| 4930511J11Rik | -0.4801293 |
| 4930515G01Rik | 1.40561659 |
| 4930518C23Rik | -0.2093139 |
| 4930519F16Rik | 3.81293604 |
| 4930519G04Rik | -0.3213251 |
| 4930519N13Rik | 0.7731723 |
| 4930519N16Rik | -0.2168486 |
| 4930522H14Rik | -0.5645883 |
| 4930524B15Rik | 2.46634659 |
| 4930524B15Rik | 0.58961696 |
| 4930524E20Rik | -0.3645714 |
| 4930526D03Rik | -0.6765663 |
| 4930535B03Rik | 1.89463178 |
| 4930535E21Rik | -0.4858656 |
| 4930538K18Rik | 0.23851988 |
| 4930539E08Rik | -0.5915593 |
| 4930540L03Rik | -0.4561329 |
| 4930544G11Rik | 0.1502545 |
| 4930544G21Rik | 2.01175886 |
| 4930547C10Rik | 2.46049631 |
| 4930547N16Rik | -0.5345741 |
| 4930549C01Rik | -0.3856696 |
| 4930550C14Rik | -0.3741028 |
| 4930550L24Rik | 1.62359411 |
| 4930555G01Rik | 0.84645325 |
| 4930555I21Rik | 0.6831697 |
| 4930562D19Rik | -0.3715516 |
| 4930562D19Rik | 0.02358931 |
| 4930563D23Rik | -0.7079126 |
| 4930563P21Rik | 0.95073961 |
| 4930570C03Rik | -0.5891482 |
| 4930572J05Rik | 2.42887071 |
| 4930572J05Rik | 0.41557259 |
| 4930572J05Rik | 0.25338717 |
| 4930573I19Rik | -0.1612474 |
| 4930578I06Rik | 2.47735362 |
| 4930579C15Rik | 2.2857017 |
| 4930579E17Rik | 0.47205324 |
| 4930579E17Rik | -0.6209857 |
| 4930579J09Rik | -0.2548917 |
| 4930583H14Rik | 0.30783245 |
| 4930590J08Rik | 0.04305521 |
| 4930595M18Rik | 0.72934575 |
| 4930596D02Rik | -0.6259808 |
| 4930599N23Rik | 0.65257868 |
| 4931406B18Rik | -0.638586 |
| 4931406C07Rik | -0.6053405 |
| 4931406H21Rik | -0.4026322 |
| 4931406I20Rik | 1.39359779 |
| 4931406I20Rik | 0.27701638 |
| 4931406P16Rik | 2.33168379 |
| 4931407G18Rik | -0.4967164 |
| 4931408A02Rik | -0.7300272 |
| 4931408C20Rik | 2.29382918 |
| 4931408C20Rik | 0.07924234 |
| 4931409K22Rik | 1.51804605 |
| 4931417E11Rik | -0.569292 |
| 4931417G12Rik | -0.6818663 |
| 4931428L18Rik | 1.59725606 |
| 4931432M23Rik | -0.0887332 |
| 4931433A01Rik | -0.5475396 |
| 4931440B09Rik | 2.25000128 |
| 4931440F15Rik | -0.6651844 |
| 4931440F15Rik | -0.4673491 |
| 4931440L10Rik | 0.73976259 |
| 4932409I22Rik | 2.45106588 |
| 4932411E22Rik | -0.5947838 |
| 4932411G14Rik | -0.076552 |
| 4932412H11Rik | 2.36321431 |
| 4932413O14Rik | 2.26934839 |
| 4932414J04Rik | 0.42381278 |
| 4932414N04Rik | 0.52607031 |
| 4932418E24Rik | 0.85063205 |
| 4932431H17Rik | 0.72478909 |
| 4932431H17Rik | -0.6774476 |
| 4932441K18Rik | 3.05411835 |
| 4932441K18Rik | 0.3604692 |
| 4932442K08Rik | 0.66026703 |
| 4932702K14Rik | 0.52582497 |
| 4933400A11Rik | -0.3207215 |
| 4933400C05Rik | -0.149605 |
| 4933400E14Rik | 1.38231239 |
| 4933402J07Rik | 0.10989846 |
| 4933402N03Rik | -0.2811362 |
| 4933402P03Rik | 0.6777571 |
| 4933403G14Rik | -0.7584222 |
| 4933404M02Rik | -0.6643145 |
| 4933405O20Rik | -0.1034661 |
| 4933406M09Rik | 0.89325788 |
| 4933407N01Rik | 1.10125205 |
| 4933407N01Rik | -0.6069752 |
| 4933407N01Rik | 0.56475808 |
| 4933407P14Rik | -0.1607382 |
| 4933407P14Rik | 2.55880099 |
| 4933407P14Rik | 1.07479595 |
| 4933409N07Rik | 1.64713105 |
| 4933411G06Rik | -0.3696428 |
| 4933411K20Rik | -0.061315 |
| 4933412E14Rik | -0.5136011 |
| 4933413G11Rik | -0.0027211 |
| 4933415F23Rik | 3.5007693 |
| 4933416C03Rik | -0.706093 |
| 4933417A18Rik | -0.6377404 |
| 4933417E01Rik | -0.5188007 |
| 4933421E11Rik | -0.6234799 |
| 4933421E11Rik | 0.92957344 |
| 4933421E11Rik | 1.33192026 |
| 4933422H20Rik | -0.6783297 |
| 4933424B01Rik | -0.3105187 |
| 4933424B01Rik | -0.3690074 |
| 4933424B01Rik | -0.6677988 |
| 4933425O20Rik | 1.51273252 |
| 4933426M11Rik | -0.4293409 |
| 4933427D14Rik | 0.23518389 |
| 4933428G09Rik | 3.77668798 |
| 4933428G20Rik | -0.5669372 |
| 4933429E10Rik | 2.2608505 |
| 4933429E10Rik | -0.515826 |
| 4933430I17Rik | 0.489301 |
| 4933434E20Rik | -0.528534 |
| 4933434E20Rik | -0.7015592 |
| 4933434E20Rik | -0.4286638 |
| 4933434I06Rik | 0.19768529 |
| 4933436I01Rik | 0.33557079 |
| 4933437F05Rik | 1.39301232 |
| 4933439C20Rik | 0.57506007 |
| 4933440H19Rik | 1.01619633 |
| 5031414D18Rik | -0.6259808 |
| 5031414D18Rik | -0.4354563 |
| 5033411D12Rik | -0.5202916 |
| 5033414D02Rik | -0.4873052 |
| 5033414K04Rik | -0.1371114 |
| 5133400G04Rik | 1.50088381 |
| 5133400G04Rik | -0.6916613 |
| 5133400G04Rik | 2.21019372 |
| 5133401N09Rik | -0.546005 |
| 5230400G24Rik | -0.6168432 |
| 5230400G24Rik | 1.54931199 |
| 5330401P04Rik | 1.18827353 |
| 5330417C22Rik | 0.92909408 |
| 5330431N19Rik | -0.6209857 |
| 5330431N19Rik | 0.08944239 |
| 5330431N19Rik | -0.6268159 |
| 5330437I02Rik | 2.37476672 |
| 5330439C02Rik | 0.37382986 |
| 5330439J01Rik | -0.5972095 |
| 5330439J01Rik | 0.04714544 |
| 5430407P10Rik | -0.3464132 |
| 5430432M24Rik | -0.5330604 |
| 5430432M24Rik | -0.5202916 |
| 5430432M24Rik | 0.96960471 |
| 5430435G22Rik | 3.39177819 |
| 5430437P03Rik | 1.14900026 |
| 5530601I19Rik | -0.5452386 |
| 5630401D24Rik | -0.1912629 |
| 5630401D24Rik | -0.4865851 |
| 5630401D24Rik | 1.92450471 |
| 5730403M16Rik | -0.2759435 |
| 5730409E04Rik | 1.3445424 |
| 5730410E15Rik | 0.43008174 |
| 5730410E15Rik | 0.67294293 |
| 5730410I19Rik | -0.7749015 |
| 5730410I19Rik | 0.69390237 |
| 5730410I19Rik | -0.4471172 |
| 5730419I09Rik | -0.5437077 |
| 5730437N04Rik | -0.5843444 |
| 5730446C15Rik | -0.5307945 |
| 5730449L18Rik | 1.96074627 |
| 5730449L18Rik | -0.6086127 |
| 5730449L18Rik | -0.4974442 |
| 5730453I16Rik | 0.72716923 |
| 5730453I16Rik | -0.170975 |
| 5730469M10Rik | 1.55170628 |
| 5730472N09Rik | 0.42572515 |
| 5730494M16Rik | -0.6352081 |
| 5730494N06Rik | -0.149102 |
| 5730502D15Rik | 2.39244109 |
| 5730507A09Rik | 2.35324327 |
| 5730507A09Rik | -0.7235233 |
| 5730528L13Rik | 1.052213 |
| 5730589K01Rik | 0.74502526 |
| 5730593F17Rik | 0.28529095 |
| 5730596K20Rik | -0.6251465 |
| 5730596K20Rik | 0.13364935 |
| 5730596K20Rik | -0.3334797 |
| 5730596K20Rik | -0.3279907 |
| 5830403L16Rik | 1.35191283 |
| 5830404H04Rik | 1.00106882 |
| 5830405N20Rik | -0.5859429 |
| 5830415F09Rik | -0.6608431 |
| 5830415L20Rik | -0.6748063 |
| 5830417C01Rik | -0.3396163 |
| 5830426I05Rik | -0.3390009 |
| 5830427D03Rik | -0.2846141 |
| 5830434P21Rik | -0.2326507 |
| 5830435K17Rik | -0.8261456 |
| 5830457O10Rik | -0.45683 |
| 5830457O10Rik | -0.7906796 |
| 5830457O10Rik | -0.3099219 |
| 5830467P10Rik | -0.4032908 |
| 5830468K18Rik | 3.52028832 |
| 5830472M02Rik | -0.237057 |
| 5830472M02Rik | 0.69656651 |
| 5830472M02Rik | 0.37382986 |
| 5930416I19Rik | -0.7106487 |
| 5930431H10 | -0.6988494 |
| 5930434B04Rik | -0.7719723 |
| 5930434B04Rik | 0.09798765 |
| 5930434B04Rik | -0.1450882 |
| 6030405A18Rik | -0.3741028 |
| 6030408B16Rik | -0.3934605 |
| 6030408B16Rik | 0.09837424 |
| 6030419C18Rik | -0.7225978 |
| 6030429G01Rik | 0.59053365 |
| 6030443O07Rik | 0.69533785 |
| 6030446N20Rik | 0.2230754 |
| 6030458C11Rik | 0.52607031 |
| 6230409E13Rik | 2.35552669 |
| 6230409E13Rik | 0.46239105 |
| 6230416J20Rik | -0.6669265 |
| 6230427J02Rik | 0.76673657 |
| 6330403K07Rik | 0.57785023 |
| 6330406I15Rik | -0.0868489 |
| 6330406I15Rik | 0.64569145 |
| 6330407J23Rik | -0.0196564 |
| 6330408A02Rik | -0.6748063 |
| 6330409N04Rik | -0.6020797 |
| 6330416G13Rik | -0.3420823 |
| 6330439K17Rik | 1.67111079 |
| 6330442E10Rik | 0.22002377 |
| 6330442E10Rik | -0.7966653 |
| 6330500D04Rik | 2.97832972 |
| 6330503C03Rik | -0.7347006 |
| 6330503K22Rik | -0.389557 |
| 6330503K22Rik | -0.6428255 |
| 6330503K22Rik | -0.7593836 |
| 6330505N24Rik | 3.23739887 |
| 6330512M04Rik | -0.3420823 |
| 6330534C20Rik | -0.3147064 |
| 6330548G22Rik | -0.6193265 |
| 6330569M22Rik | -0.6428255 |
| 6330577E15Rik | -0.73096 |
| 6330578E17Rik | 0.57389505 |
| 6332401O19Rik | -0.7097358 |
| 6430411K18Rik | 2.33038735 |
| 6430502M16Rik | 0.71981104 |
| 6430510M02Rik | 1.56252809 |
| 6430517E21Rik | -0.6988494 |
| 6430526N21Rik | -0.5756005 |
| 6430527G18Rik | 1.12400102 |
| 6430527G18Rik | 0.37958018 |
| 6430531B16Rik | -0.6783297 |
| 6430537H07Rik | -0.5803598 |
| 6430537H07Rik | 0.94273986 |
| 6430548M08Rik | 4.41855174 |
| 6430550H21Rik | 1.74086931 |
| 6430571L13Rik | 0.0256794 |
| 6430573F11Rik | 0.4717934 |
| 6430598A04Rik | 0.34155903 |
| 6430604K15Rik | 2.22033502 |
| 6430706D22Rik | -0.4279873 |
| 6530404N21Rik | -0.0010013 |
| 6530406M24Rik | -0.5787707 |
| 6530418L21Rik | 3.01229432 |
| 6530418L21Rik | 1.82116723 |
| 6530418L21Rik | 3.2243228 |
| 6530421E24Rik | -0.6143665 |
| 6620401K05Rik | -0.4000026 |
| 6720456B07Rik | -0.6591122 |
| 6720456B07Rik | -0.5262789 |
| 6720456H20Rik | -0.8271788 |
| 6720456H20Rik | 0.21593947 |
| 6720458F09Rik | 0.19420103 |
| 6720463M24Rik | -0.0284608 |
| 6720467C03Rik | 2.03275972 |
| 6720467C03Rik | 0.08670708 |
| 6820408C15Rik | 0.14952268 |
| 7420416P09Rik | -0.5708652 |
| 7530404M11Rik | -0.4192361 |
| 8030411F24Rik | -0.0705201 |
| 8030451F13Rik | -0.3225335 |
| 8030451F13Rik | 1.83034835 |
| 8030451F13Rik | 1.43398113 |
| 8430408G22Rik | 0.29725513 |
| 8430410A17Rik | -0.2013075 |
| 8430410K20Rik | 0.03523446 |
| 8430415E04Rik | -0.5398912 |
| 8430415E04Rik | -0.4687606 |
| 8430419L09Rik | -0.7488617 |
| 8430427H17Rik | 1.51513589 |
| 8430432M10Rik | 0.06533449 |
| 9030025P20Rik | 0.59738047 |
| 9030025P20Rik | -0.3824423 |
| 9030025P20Rik | -0.1581964 |
| 9030205A07Rik | -0.5756005 |
| 9030221M09Rik | 0.73761007 |
| 9030224M15Rik | 0.87988049 |
| 9030227G01Rik | -0.4901909 |
| 9030409G11Rik | -0.0540153 |
| 9030418K01Rik | 0.58386734 |
| 9030425E11Rik | 0.008402 |
| 9030607L17Rik | 3.14928738 |
| 9030607L17Rik | -0.2365051 |
| 9030611O19Rik | 0.11256726 |
| 9030612M13Rik | -0.5165688 |
| 9030617O03Rik | 0.67210317 |
| 9030624J02Rik | 0.08905212 |
| 9030625A04Rik | 0.92380491 |
| 9130005N14Rik | 0.07410054 |
| 9130011E15Rik | 0.69615714 |
| 9130011J15Rik | -0.2136121 |
| 9130014G24Rik | 0.38415526 |
| 9130017N09Rik | -0.0631488 |
| 9130206N08Rik | 1.44863138 |
| 9130211I03Rik | 3.840724 |
| 9130213B05Rik | -0.6209857 |
| 9130213B05Rik | 0.96299862 |
| 9130218O11Rik | 0.51322712 |
| 9130221H12Rik | 3.20807593 |
| 9130227C08Rik | -0.7729477 |
| 9130401M01Rik | -0.0035822 |
| 9130404D08Rik | -0.5421792 |
| 9130404D08Rik | 1.50687006 |
| 9130404D14Rik | -0.5669372 |
| 9130422G05Rik | -0.4491901 |
| 9230104L09Rik | -0.5300404 |
| 9330129D05Rik | 0.89275965 |
| 9330134C04Rik | -0.2713517 |
| 9330176C04Rik | -0.6394323 |
| 9330186A19Rik | 0.59624279 |
| 9430015G10Rik | 0.689995 |
| 9430015G10Rik | 3.78038323 |
| 9430023L20Rik | 0.9010293 |
| 9430025C20Rik | -0.239268 |
| 9430028L06Rik | -0.0053068 |
| 9430029K10Rik | 0.43387727 |
| 9430031J16Rik | 0.50523557 |
| 9430034D17Rik | 0.29317922 |
| 9430057O19Rik | 0.72100818 |
| 9430069J07Rik | 2.39065134 |
| 9430077A04Rik | -0.0200946 |
| 9430079M16Rik | -0.3021987 |
| 9530015I07Rik | 0.84295669 |
| 9530027K23Rik | 3.93612743 |
| 9530058B02Rik | 0.82259527 |
| 9530058B02Rik | 0.22679178 |
| 9530068E07Rik | -0.5315492 |
| 9530077C05Rik | 0.63504667 |
| 9530096D07Rik | 1.2250397 |
| 9530096D07Rik | -0.8036971 |
| 9630015D15Rik | 0.42572515 |
| 9630020C08Rik | 0.76977063 |
| 9630028B13Rik | -0.5121208 |
| 9630031F12Rik | -0.4638297 |
| 9630050M13Rik | -0.5708652 |
| 9630058J23Rik | 0.06613479 |
| 9830163H01Rik | -0.4851467 |
| 9830169C18Rik | -0.5915593 |
| 9930013L23Rik | -0.4638297 |
| 9930021D14Rik | -0.7170639 |
| 9930023K05Rik | -0.5827485 |
| 9930023K05Rik | 0.98554356 |
| 9930104L06Rik | -0.0719086 |
| a | 1.71050997 |
| A030003K21Rik | -0.3715516 |
| A030007L17Rik | 0.3821564 |
| A030007L17Rik | -0.7097358 |
| A030007L17Rik | 0.27027953 |
| A030007L17Rik | 1.49249123 |
| A030009H04Rik | 1.84862651 |
| A030010B05Rik | 0.65021688 |
| A030012M09Rik | 3.05053837 |
| A130010J15Rik | 0.52312202 |
| A130038H09Rik | 1.70709097 |
| A130042E20Rik | -0.7680809 |
| A130090K04Rik | 0.62228906 |
| A130092J06Rik | 0.16296997 |
| A230050P20Rik | -0.4589245 |
| A230051G13Rik | -0.502555 |
| A230054D04Rik | 1.81433126 |
| A230067G21Rik | -0.6651844 |
| A230069A22Rik | 0.9796549 |
| A230074B11Rik | -0.1792454 |
| A230074B11Rik | -0.177171 |
| A230074B11Rik | -0.2834533 |
| A230106M15Rik | -0.1986538 |
| A230106N23Rik | 2.12424152 |
| A230107C01Rik | 1.21730651 |
| A2bp1 | -0.7798038 |
| A2bp1 | 0.98779866 |
| A330021E22Rik | -0.6871964 |
| A330021E22Rik | 1.95855506 |
| A330049M08Rik | 1.41013227 |
| A330080J22Rik | 0.70937155 |
| A3galt2 | 2.10488601 |
| A430005L14Rik | -0.7739241 |
| A430005L14Rik | 1.5758826 |
| A430005L14Rik | 1.87863314 |
| A430005L14Rik | -0.4715899 |
| A430033K04Rik | -0.5262789 |
| A430079E08 | 0.1329045 |
| A430083B19Rik | -0.1425882 |
| A430083B19Rik | 1.16219119 |
| A430107D22Rik | 0.12767395 |
| A430107P09Rik | 1.18157604 |
| A430110N23Rik | -0.1561678 |
| A4galt | 0.01264535 |
| A4gnt | 0.76216704 |
| A530050D06Rik | -0.5537035 |
| A530082C11Rik | 0.88374602 |
| A530082C11Rik | 0.65514845 |
| A530088H08Rik | -0.6845276 |
| A530088H08Rik | 1.46278511 |
| A530098C11Rik | 1.83120642 |
| A630042L21Rik | -0.7365765 |
| A630054L15Rik | -0.2398215 |
| A630077B13Rik | -0.5521587 |
| A630095E13Rik | 1.14570516 |
| A730008H23Rik | 0.16080235 |
| A730008L03Rik | 1.8726141 |
| A730008L03Rik | -0.3292077 |
| A730017C20Rik | -0.0293458 |
| A730024F05Rik | -0.6513623 |
| A730037C10Rik | -0.0535609 |
| A730042J05Rik | 0.47049312 |
| A730098D12Rik | 1.03808525 |
| A830006F12Rik | -0.0253699 |
| A830007P12Rik | -0.4645325 |
| A830053O21Rik | -0.5732299 |
| A830059I20Rik | -0.6004535 |
| A830073O21Rik | -0.5069579 |
| A830080D01Rik | 2.40680395 |
| A830093I24Rik | -0.5255284 |
| A930001N09Rik | -0.3967259 |
| A930005I04Rik | -0.3702786 |
| A930008G19Rik | -0.0445195 |
| A930010I20Rik | 0.74948518 |
| A930016P21Rik | 1.25952338 |
| A930018P22Rik | 1.40078876 |
| A930024E05Rik | 1.31605954 |
| A930025D01Rik | -0.3876113 |
| A930025D01Rik | 2.32304322 |
| A930025D01Rik | -0.6086127 |
| A930034L06Rik | -0.3960719 |
| A930038C07Rik | 0.33587111 |
| A930041I02Rik | -0.6310026 |
| AA388235 | -0.3377711 |
| AA407270 | 0.98524248 |
| AA407659 | 0.68627825 |
| AA407659 | 0.78423676 |
| AA408296 | 0.2517468 |
| AA408296 | -0.7051845 |
| AA408296 | -0.5121208 |
| AA409316 | -0.7272343 |
| AA415398 | -0.6110743 |
| AA467197 | 1.0933662 |
| AA536717 | -0.6037087 |
| AA536717 | -0.5955917 |
| AA536749 | -0.4158918 |
| AA536749 | -0.3111158 |
| AA536749 | -0.528534 |
| AA536749 | 0.55148116 |
| AA673488 | -0.2645057 |
| AA691260 | 0.8021493 |
| AA792892 | -0.6377404 |
| AA881470 | -0.5716528 |
| AA881470 | 1.18705934 |
| AA960436 | 2.41098109 |
| AA960436 | -0.3595271 |
| Aaas | 1.47708618 |
| Aaas | -0.3993463 |
| Aacs | 0.52312202 |
| Aadacl1 | -0.5891482 |
| Aak1 | -0.237057 |
| Aak1 | 0.58202002 |
| Aamp | -0.3021987 |
| Aard | -0.1849737 |
| Aars | -0.6028938 |
| Aars2 | -0.4952623 |
| Aarsd1 | 0.14915656 |
| Aasdh | -0.6419761 |
| Aatf | -0.6522203 |
| Aatf | 1.02026695 |
| Aatk | -0.4192361 |
| AB112350 | 1.89680547 |
| AB182283 | -0.2376093 |
| AB182283 | 1.24329827 |
| Abat | 3.14647633 |
| Abat | -0.4645325 |
| Abat | -0.528534 |
| Abca14 | -0.3231383 |
| Abca14 | 1.25986079 |
| Abca15 | -0.832362 |
| Abca15 | -0.6004535 |
| Abca15 | -0.3316464 |
| Abca15 | 1.56740624 |
| Abca17 | -0.1546492 |
| Abca2 | 0.15899217 |
| Abca3 | -0.8608557 |
| Abca4 | -0.7375158 |
| Abca4 | -0.1287096 |
| Abca5 | 0.69267022 |
| Abca6 | -0.4631274 |
| Abca6 | -0.0152849 |
| Abca7 | -0.618498 |
| Abca7 | 0.85254114 |
| Abca8a | 0.64849547 |
| Abca8b | -0.6573845 |
| Abca9 | -0.3934605 |
| Abca9 | -0.6522203 |
| Abca9 | 1.3403194 |
| Abcb11 | -0.7946659 |
| Abcb1b | 1.20212515 |
| Abcb4 | 1.14074077 |
| Abcb6 | -0.8968958 |
| Abcb8 | 0.81685022 |
| Abcb8 | -0.7622738 |
| Abcb8 | -0.5622452 |
| Abcb9 | -0.7365765 |
| Abcb9 | 0.1672903 |
| Abcc1 | -0.5121208 |
| Abcc10 | -0.7479109 |
| Abcc10 | 1.32993918 |
| Abcc12 | -0.3785841 |
| Abcc3 | 0.5119829 |
| Abcc5 | -0.7498134 |
| Abcc6 | 2.35391131 |
| Abcc8 | -0.4554363 |
| Abcc9 | -0.2093139 |
| Abcc9 | 0.0189747 |
| Abcd1 | -0.5875442 |
| Abcd2 | -0.505488 |
| Abcd2 | -0.3614155 |
| Abcd3 | 0.62758907 |
| Abcd4 | -0.3532596 |
| Abcd4 | 0.44088575 |
| Abce1 | -0.4219201 |
| Abcf1 | -0.7700246 |
| Abcf1 | 0.20219563 |
| Abcf1 | 3.06032586 |
| Abcf2 | 1.94909048 |
| Abcf2 | -0.7517196 |
| Abcf2 | 0.14106563 |
| Abcf2 | -0.3279907 |
| Abcf3 | -0.6069752 |
| Abcg1 | 1.22270301 |
| Abcg4 | 2.55284578 |
| Abcg5 | -0.6608431 |
| Abhd1 | 2.06057914 |
| Abhd1 | 4.59464613 |
| Abhd10 | -0.4945362 |
| Abhd11 | 0.81810995 |
| Abhd12 | -0.4603233 |
| Abhd14a | 0.79830015 |
| Abhd14b | 0.01981541 |
| Abhd2 | 3.55220187 |
| Abhd2 | 1.58579542 |
| Abhd2 | 1.17998601 |
| Abhd3 | 0.30937795 |
| Abhd4 | 0.72478909 |
| Abhd5 | -0.4880258 |
| Abhd6 | -0.3588985 |
| Abhd7 | 0.35929838 |
| Abhd7 | 0.20874899 |
| Abhd8 | -0.2840335 |
| Abi1 | -0.4645325 |
| Abi1 | -0.6677988 |
| Abi2 | 0.04305521 |
| Abi2 | -0.017031 |
| Abi2 | -0.7642055 |
| Abi3 | 0.92925389 |
| Abi3bp | 3.58019954 |
| Abi3bp | 0.12542335 |
| Abl1 | 1.52485816 |
| Ablim1 | 0.65043183 |
| Ablim2 | 0.30721353 |
| Ablim2 | -0.6004535 |
| Ablim3 | -0.6836397 |
| Ablim3 | 0.87278046 |
| Abp1 | 0.1797791 |
| Abr | -0.6453784 |
| Abr | -0.7976667 |
| Abtb1 | 0.44276377 |
| Acaa1a | 1.96186662 |
| Acaa1b | -0.6077935 |
| Acaa2 | 0.95370261 |
| Acaa2 | -0.5653706 |
| Acaa2 | 1.25569094 |
| Acaa2 | 0.74851743 |
| Acad10 | 0.55814304 |
| Acad10 | 0.13884688 |
| Acad8 | -0.6880877 |
| Acad8 | 0.4473091 |
| Acad9 | -0.1425882 |
| Acad9 | 0.73995803 |
| Acadl | 1.07232859 |
| Acadl | 1.5652142 |
| Acadm | -0.5915593 |
| Acads | -0.6845276 |
| Acads | 0.23451527 |
| Acadsb | 2.9636285 |
| Acadvl | -0.528534 |
| Acan | 3.98146521 |
| Acat2 | -0.6783297 |
| Acbd3 | -0.5180561 |
| Acbd4 | 1.46820731 |
| Acbd4 | 0.55861709 |
| Acbd5 | -0.7536298 |
| Acbd6 | 1.84216957 |
| Accn1 | 1.79993865 |
| Accn1 | -0.6318422 |
| Accn2 | 0.93578303 |
| Accn3 | 0.84854497 |
| Accn3 | 0.67524844 |
| Accs | -0.7051845 |
| Acd | -0.0826229 |
| Ace | 1.12309114 |
| Ace | 2.02765273 |
| Ache | -0.4952623 |
| Acin1 | -0.7170639 |
| Acly | 4.66131035 |
| Acn9 | -0.671296 |
| Aco1 | -0.4680546 |
| Aco2 | -0.4989016 |
| Acot1 | -0.7956651 |
| Acot1 | -0.5867432 |
| Acot10 | -0.8417643 |
| Acot11 | -0.3129094 |
| Acot11 | -0.3507642 |
| Acot2 | -0.5568006 |
| Acot2 | -0.2476055 |
| Acot3 | 0.66599233 |
| Acot5 | -0.5756005 |
| Acot7 | -0.5614655 |
| Acot7 | -0.3123111 |
| Acot7 | -0.6045242 |
| Acot7 | -0.6453784 |
| Acot8 | -0.0135419 |
| Acot8 | -0.5537035 |
| Acot8 | -0.6556599 |
| Acot9 | 2.68097254 |
| Acox2 | -0.3383858 |
| Acox3 | 0.6510764 |
| Acox3 | -0.5345741 |
| Acoxl | -0.6845276 |
| Acp2 | -0.2299072 |
| Acp6 | -0.6077935 |
| Acpl2 | -0.6259808 |
| Acpp | 2.14509566 |
| Acr | 1.21317963 |
| Acr | -0.6907666 |
| Acrbp | 0.11370871 |
| Acrbp | -0.5980194 |
| Acrv1 | -0.5003613 |
| Acsbg1 | -0.7253769 |
| Acsbg1 | -0.1597207 |
| Acsbg2 | -0.5173122 |
| Acsf3 | -0.7441176 |
| Acsl1 | -0.5047539 |
| Acsl3 | 1.98779204 |
| Acsl3 | 0.28529095 |
| Acsl4 | -0.3734644 |
| Acsl4 | 0.67273306 |
| Acsl5 | -0.3863164 |
| Acsl6 | 2.20411374 |
| Acsl6 | 0.99765735 |
| Acsl6 | -0.3850232 |
| Acsm3 | 1.21187887 |
| Acss1 | 4.26433537 |
| Acss2 | -0.248164 |
| Acss2 | -0.6045242 |
| Acta1 | -0.5262789 |
| Acta2 | -0.7124771 |
| Acta2 | 0.82616819 |
| Acta2 | 0.65941586 |
| Acta2 | -0.4484986 |
| Actb | 0.83592433 |
| Actb | -0.2381618 |
| Actb | 0.53898252 |
| Actb | 0.91411137 |
| Actb | 0.45368889 |
| Actg2 | -0.658248 |
| Actl6a | 0.02358931 |
| Actl6b | -0.4989016 |
| Actl6b | 1.66213487 |
| Actl6b | -0.4019741 |
| Actl6b | 0.95867191 |
| Actl7a | -0.8097665 |
| Actl7b | 0.15608862 |
| Actn1 | -0.5591301 |
| Actn2 | 2.04742971 |
| Actn3 | -0.4652359 |
| Actn3 | -0.6326825 |
| Actn4 | 0.50899006 |
| Actn4 | 1.17001787 |
| Actn4 | 1.2509351 |
| Actr10 | -0.3225335 |
| Actr10 | -0.5748096 |
| Actr1a | -0.2650743 |
| Actr1b | -0.3934605 |
| Actr3 | 0.30349147 |
| Actr3b | -0.5980194 |
| Actr5 | 0.52778589 |
| Actr5 | -0.5429431 |
| Actr6 | 0.3858652 |
| Actrt1 | 2.9649695 |
| Actrt2 | 0.29034706 |
| Acvr1 | 0.24680848 |
| Acvr1c | 0.08435619 |
| Acvr2a | 1.45541521 |
| Acvr2b | -0.4409258 |
| Acvrl1 | 0.52065803 |
| Acy1 | -0.3383858 |
| Acy1l2 | -0.7006551 |
| Acy3 | 2.27597733 |
| Ada | -0.5544768 |
| Adal | 2.43842876 |
| Adal | 1.70176331 |
| Adam11 | -0.5795649 |
| Adam11 | 0.17087548 |
| Adam12 | 0.8468022 |
| Adam15 | -0.501092 |
| Adam17 | 0.52287591 |
| Adam1b | -0.7450645 |
| Adam1b | -0.7244496 |
| Adam21 | -0.8179204 |
| Adam22 | 1.92528109 |
| Adam22 | -0.3658367 |
| Adam22 | 0.4510359 |
| Adam23 | 0.33976655 |
| Adam24 | 1.53221077 |
| Adam26a | 0.33496986 |
| Adam28 | 1.59418724 |
| Adam29 | 0.94037399 |
| Adam29 | 2.04382025 |
| Adam3 | -0.1949511 |
| Adam30 | -0.4429851 |
| Adam32 | -0.5429431 |
| Adam33 | -0.653079 |
| Adam39 | 0.12542335 |
| Adam4 | 0.20046344 |
| Adam4 | 0.94021606 |
| Adam4 | -0.6419761 |
| Adam5 | -0.4772742 |
| Adam6 | -0.6352081 |
| Adam8 | -0.4945362 |
| Adam8 | -0.5490768 |
| Adam9 | 2.50947353 |
| Adamdec1 | 0.58617141 |
| Adamts1 | 0.21457408 |
| Adamts13 | -0.4395554 |
| Adamts15 | -0.4138909 |
| Adamts16 | -0.2753683 |
| Adamts17 | 0.36542897 |
| Adamts18 | -0.3353165 |
| Adamts19 | 0.98494129 |
| Adamts2 | 0.60304833 |
| Adamts2 | -0.3433176 |
| Adamts2 | 1.28515604 |
| Adamts20 | -0.289855 |
| Adamts20 | 0.53388754 |
| Adamts4 | 1.10444103 |
| Adamts7 | 0.08592411 |
| Adamtsl2 | -0.5498463 |
| Adamtsl4 | 0.61025372 |
| Adamtsl5 | -0.5165688 |
| Adar | -0.0590278 |
| Adar | -0.7281644 |
| Adar | 1.35547493 |
| Adarb1 | -0.5867432 |
| Adarb1 | 0.79940151 |
| Adarb1 | -0.7622738 |
| Adat1 | 0.10568929 |
| Adc | 0.1193953 |
| Adc | -0.6643145 |
| Adck1 | -0.0746919 |
| Adck2 | 2.45046341 |
| Adck4 | -0.5700783 |
| Adck5 | 0.44757579 |
| Adcy1 | 0.45448333 |
| Adcy10 | 1.10589904 |
| Adcy2 | 0.9038245 |
| Adcy4 | 1.3398031 |
| Adcy4 | 0.57785023 |
| Adcy6 | 1.18547856 |
| Adcy7 | 1.32544024 |
| Adcy8 | -0.1571815 |
| Adcy9 | 1.90562017 |
| Adcyap1 | 2.79277486 |
| Adcyap1r1 | 1.58141149 |
| Add1 | -0.6617098 |
| Add1 | 0.00413942 |
| Add1 | 0.19803301 |
| Add1 | 1.91988869 |
| Add3 | 2.0853577 |
| Add3 | 3.83577596 |
| Add3 | 0.3047338 |
| Adfp | -0.6608431 |
| Adh1 | -0.1541436 |
| Adh1 | 0.91216062 |
| Adh4 | 0.67880041 |
| Adh5 | 0.10376978 |
| Adh7 | 0.14915656 |
| Adhfe1 | 0.48084535 |
| Adi1 | 0.65856393 |
| Adipoq | -0.6268159 |
| Adipor1 | -0.7857205 |
| Adipor2 | -0.1287096 |
| Adk | 0.55671946 |
| Adk | -0.5606864 |
| Adm | -0.5591301 |
| Adm2 | -0.2260797 |
| Adnp | 0.30659421 |
| Ado | -0.1366151 |
| Adora1 | -0.7565023 |
| Adora1 | 1.02345299 |
| Adora1 | 1.78615827 |
| Adora2b | 2.59509531 |
| Adora3 | -0.4844283 |
| Adpgk | 1.73308188 |
| Adprh | -0.1058642 |
| Adprhl1 | -0.4603233 |
| Adprhl1 | -0.4422981 |
| Adprhl1 | 2.1985748 |
| Adprhl2 | 1.3642713 |
| Adra1a | 0.34543104 |
| Adra1a | 0.21389065 |
| Adra1d | -0.6352081 |
| Adra2a | -0.706093 |
| Adra2c | 0.47852601 |
| Adra2c | -0.4505746 |
| Adrb1 | 1.052213 |
| Adrb2 | -0.4659398 |
| Adrbk1 | -0.3690074 |
| Adrbk2 | -0.2794021 |
| Adrm1 | 1.2137703 |
| Adsl | -0.7097358 |
| Adss | 1.35730163 |
| Adssl1 | 0.23651971 |
| Adssl1 | 1.80265804 |
| Adssl1 | -0.3601562 |
| Aebp1 | -0.6419761 |
| Aebp2 | -0.5398912 |
| Aes | -0.4687606 |
| Afap1 | 0.66704876 |
| Afap1 | 0.36076167 |
| Afap1 | 0.01560435 |
| Afap1l1 | 0.44222757 |
| Aff1 | 0.57808237 |
| Aff4 | -0.5368492 |
| Afg3l1 | -0.2684931 |
| Afg3l1 | -0.6818663 |
| Afg3l1 | 2.4841108 |
| Afg3l1 | -0.6143665 |
| Afg3l2 | 2.62491698 |
| Afmid | -0.6565218 |
| Aftph | 1.14125115 |
| Agbl1 | 1.2816466 |
| Ager | 0.54956907 |
| Ager | 0.02149457 |
| Aggf1 | -0.4464272 |
| Aggf1 | -0.3620459 |
| Aggf1 | -0.7460124 |
| Agpat1 | -0.6053405 |
| Agpat2 | 1.82856843 |
| Agpat3 | -0.6721723 |
| Agpat4 | -0.5996414 |
| Agpat5 | -0.3147064 |
| Agpat6 | -0.4938106 |
| Agpat6 | 0.7121936 |
| Agpat7 | 0.65429264 |
| Agpat9 | -0.5955917 |
| Agpat9 | 0.78591317 |
| Agps | -0.5452386 |
| Agps | 0.20323341 |
| Agrn | -0.4052692 |
| Agrp | -0.5300404 |
| Agt | 1.64891742 |
| Agtpbp1 | 0.00584677 |
| Agtr1a | -0.3390009 |
| Agtrap | -0.3734644 |
| Agtrl1 | 1.98373753 |
| Agxt2l1 | 0.04510255 |
| Agxt2l1 | 0.41474458 |
| Agxt2l2 | 1.16094285 |
| Agxt2l2 | -0.5732299 |
| Agxt2l2 | 2.35120107 |
| Agxt2l2 | -0.1704605 |
| Ahctf1 | -0.3420823 |
| Ahcy | -0.1536382 |
| Ahcyl1 | -0.3941127 |
| Ahdc1 | 1.15794006 |
| Ahi1 | -0.2811362 |
| Ahnak | -0.7189049 |
| Ahnak | -0.253767 |
| Ahnak2 | -0.5964003 |
| Ahrr | -0.4199064 |
| Ahsa1 | -0.5069579 |
| Ahsa2 | 1.03022359 |
| AI118078 | -0.2980661 |
| AI132487 | 0.1129479 |
| AI314180 | -0.6505051 |
| AI314976 | -0.5217848 |
| AI316807 | -0.3396163 |
| AI317395 | -0.2881048 |
| AI413582 | 1.39515726 |
| AI413782 | -0.0812185 |
| AI427515 | -0.3773016 |
| AI428936 | 1.25839781 |
| AI449175 | 0.51023824 |
| AI449175 | -0.5795649 |
| AI450540 | 1.51152852 |
| AI451557 | -0.4205771 |
| AI451617 | 0.50121419 |
| AI467606 | 1.08433019 |
| AI480653 | -0.2684931 |
| AI504432 | -0.6061575 |
| AI553587 | -0.2628019 |
| AI593442 | -0.7807873 |
| AI593442 | -0.4286638 |
| AI593442 | -0.6845276 |
| AI595366 | -0.0066023 |
| AI595406 | 0.89806059 |
| AI597468 | -0.658248 |
| AI597479 | -0.638586 |
| AI606181 | 0.28687391 |
| AI646023 | 1.66250142 |
| AI661453 | 0.98809893 |
| AI662250 | 2.02616003 |
| AI747699 | 0.58548078 |
| AI836003 | -0.4909137 |
| AI837181 | -0.5591301 |
| AI842396 | -0.533817 |
| AI846148 | 0.20668444 |
| AI847670 | -0.6765663 |
| AI851790 | -0.0337833 |
| AI894139 | 0.55861709 |
| AI987692 | 2.97142724 |
| AI987692 | -0.3033827 |
| AI987944 | -0.3576425 |
| Aicda | 1.93042226 |
| Aif1 | -0.2442616 |
| Aif1 | -0.6020797 |
| Aif1 | 1.10231624 |
| Aifm1 | -0.515826 |
| Aifm1 | 4.58779966 |
| Aifm2 | -0.5923644 |
| Aifm2 | -0.3798684 |
| Aifm2 | 0.76102118 |
| Aifm3 | 3.01838423 |
| Aig1 | -0.0677491 |
| Aig1-pending | 0.53388754 |
| Aim1 | -0.0728355 |
| Aim2 | -0.4266356 |
| Aip | -0.5106428 |
| Ak1 | -0.4059296 |
| AK122209 | -0.6094325 |
| AK122525 | 1.02301917 |
| AK129128 | -0.3292077 |
| AK129302 | -0.1020302 |
| AK129302 | 0.02776486 |
| AK129302 | 0.44490548 |
| AK129341 | -0.3304263 |
| AK162044 | -0.5300404 |
| Ak2 | -0.7469612 |
| Ak2 | 0.92010085 |
| Ak2 | 0.5555315 |
| Ak3 | 1.49424587 |
| Ak3 | 4.24532978 |
| Ak3l1 | 0.53145162 |
| Ak5 | 0.22577968 |
| Akap1 | 1.21235208 |
| Akap1 | -0.4858656 |
| Akap1 | 0.12842296 |
| Akap10 | -0.7739241 |
| Akap10 | -0.2222677 |
| Akap12 | -0.4152243 |
| Akap14 | -0.8673066 |
| Akap2 | 0.82563312 |
| Akap2 | -0.5732299 |
| Akap3 | 1.87571608 |
| Akap4 | 0.03812336 |
| Akap4 | -0.438187 |
| Akap4 | -0.541416 |
| Akap4 | -0.0113678 |
| Akap6 | -0.4327334 |
| Akap7 | 3.00225601 |
| Akap8 | 2.00439831 |
| Akap8l | 2.99405141 |
| Akap9 | -0.6487931 |
| Akap9 | -0.5591301 |
| Akap9 | 2.17293972 |
| Akna | -0.7328285 |
| Akp2 | -0.6243128 |
| Akp5 | 1.1031136 |
| Akr1a4 | 1.24467063 |
| Akr1a4 | -0.546772 |
| Akr1b3 | -0.6411274 |
| Akr1b8 | 0.73898043 |
| Akr1c12 | -0.0027211 |
| Akr1c14 | 0.18754517 |
| Akr1c14 | -0.1536382 |
| Akr1c18 | 0.99676536 |
| Akr1c19 | 1.08717172 |
| Akr1e1 | -0.3051617 |
| Akr7a5 | -0.3980352 |
| Akr7a5 | 1.01093665 |
| Akr7a5 | 0.78143607 |
| Akt1s1 | -0.5498463 |
| Akt3 | 0.62051573 |
| Akt3 | 3.57529015 |
| Aktip | -0.5740194 |
| Alad | -0.3347038 |
| Alas1 | -0.6168432 |
| Alas2 | -0.3445547 |
| Alcam | -0.2420389 |
| Aldh16a1 | 0.95975546 |
| Aldh18a1 | -0.5345741 |
| Aldh1a1 | -0.1576888 |
| Aldh1a2 | 0.24317049 |
| Aldh1a2 | -0.546005 |
| Aldh1a7 | -0.0788823 |
| Aldh1b1 | 1.45860682 |
| Aldh1l1 | -0.6037087 |
| Aldh1l2 | -0.8375739 |
| Aldh1l2 | -0.1938958 |
| Aldh2 | 1.12737289 |
| Aldh3a1 | -0.541416 |
| Aldh3a2 | -0.06269 |
| Aldh3b1 | -0.0280186 |
| Aldh3b1 | -0.5544768 |
| Aldh3b1 | 0.54139903 |
| Aldh4a1 | 0.771473 |
| Aldh5a1 | 0.3878562 |
| Aldh5a1 | 2.46774899 |
| Aldh6a1 | 0.52803071 |
| Aldh8a1 | 0.87040183 |
| Aldh8a1 | 2.84261702 |
| Aldoa | -0.328599 |
| Aldoc | 3.25286747 |
| Alg1 | 1.17912872 |
| Alg10b | 1.98008392 |
| Alg11 | 2.15847198 |
| Alg11 | 1.70330863 |
| Alg12 | 0.1487903 |
| Alg12 | 0.03233666 |
| Alg14 | -0.2776712 |
| Alg2 | -0.1044246 |
| Alg3 | 0.05162423 |
| Alg3 | -0.3876113 |
| Alg3 | -0.0802833 |
| Alg5 | 1.25648125 |
| Alg5 | -0.5173122 |
| Alg5 | 0.56263689 |
| Alg8 | -0.7115625 |
| Alg8 | -0.4652359 |
| Alg8 | -0.5835461 |
| Alg8 | -0.0485774 |
| Alg9 | 0.79535685 |
| Alg9 | 1.31499966 |
| Alg9 | 0.96315278 |
| Alk | -0.5084301 |
| Alkbh2 | -0.541416 |
| Alkbh3 | -0.5811554 |
| Alkbh4 | -0.546005 |
| Alkbh5 | -0.4006593 |
| Alkbh7 | -0.6979479 |
| Alkbh8 | 1.56081474 |
| Allc | -0.3863164 |
| Allc | 1.21601144 |
| Alox12 | -0.5537035 |
| Alox12b | 0.91702993 |
| Alox12e | 1.59757025 |
| Alox15 | 0.94525692 |
| Alox5ap | 1.58770202 |
| Alox8 | -0.6952485 |
| Aloxe3 | -0.3507642 |
| Alpk3 | 0.17230573 |
| Als2 | 2.75290177 |
| Als2 | -0.528534 |
| Als2 | -0.6889798 |
| Als2cl | -0.6836397 |
| Als2cr13 | 1.08080017 |
| Als2cr2 | -0.4617243 |
| Als2cr2 | -0.2639374 |
| Als2cr4 | 0.71319943 |
| Als2cr4 | 0.4725727 |
| Als2cr4 | -0.2986553 |
| Alx3 | 1.43873499 |
| Amac1 | -0.5498463 |
| Ambp | 1.34649299 |
| Ambra1 | -0.2667821 |
| Ambra1 | -0.7079126 |
| Amd2 | 0.67817456 |
| Amdhd2 | 0.30659421 |
| Amdhd2 | -0.8417643 |
| Amfr | -0.4152243 |
| Amh | -0.0503865 |
| Amhr2 | 2.86315139 |
| Amhr2 | 2.26406554 |
| Amhr2 | 0.24283906 |
| Amigo1 | 1.42033252 |
| Amigo1 | 0.18296388 |
| Amigo2 | -0.5988301 |
| Amigo3 | 1.09751785 |
| Ammecr1l | -0.3582703 |
| Amn | -0.6818663 |
| Amotl2 | 2.48908614 |
| Ampd1 | 1.96197325 |
| Ampd1 | 1.55631256 |
| Ampd2 | 1.80316311 |
| Amph | -0.6880877 |
| Amph | 0.97722833 |
| Amy1 | -0.6871964 |
| Amz2 | -0.4471172 |
| Anapc10 | 2.13383859 |
| Anapc11 | -0.4450489 |
| Anapc13 | -0.6565218 |
| Anapc2 | 0.52090472 |
| Anapc4 | -0.2013075 |
| Anapc5 | 0.82670296 |
| Anapc5 | -0.7088237 |
| Anapc5 | 0.173377 |
| Anapc7 | 0.43116774 |
| Ang | 0.28908558 |
| Angel1 | -0.6004535 |
| Angel2 | -0.4582258 |
| Angpt1 | 2.01155658 |
| Angpt2 | -0.2696355 |
| Angpt2 | 0.15899217 |
| Angptl1 | 0.23183602 |
| Angptl2 | 1.23236255 |
| Angptl3 | 0.6229532 |
| Angptl4 | -0.5277817 |
| Angptl7 | -0.6961474 |
| Ank | 1.51307623 |
| Ank1 | -0.3231383 |
| Ank2 | 0.64461075 |
| Ank2 | -0.014413 |
| Ank2 | -0.1602293 |
| Ank3 | -0.8067269 |
| Ank3 | -0.6160169 |
| Ank3 | 1.64713105 |
| Ankar | -0.5708652 |
| Ankar | -0.1198946 |
| Ankfy1 | 0.59987854 |
| Ankle2 | -0.7622738 |
| Ankle2 | 3.08706254 |
| Ankle2 | 0.20046344 |
| Ankmy1 | 0.17194837 |
| Ankmy1 | -0.7143092 |
| Ankmy1 | -0.6513623 |
| Ankmy2 | 1.70337882 |
| Ankmy2 | 1.19985064 |
| Ankrd10 | -0.6487931 |
| Ankrd12 | -0.3677378 |
| Ankrd12 | -0.5210379 |
| Ankrd13a | 0.53922445 |
| Ankrd13b | -0.0816864 |
| Ankrd13d | -0.6259808 |
| Ankrd17 | 0.50923983 |
| Ankrd17 | -0.3843773 |
| Ankrd17 | -0.6143665 |
| Ankrd22 | -0.6201557 |
| Ankrd23 | 1.01677892 |
| Ankrd24 | 0.55410396 |
| Ankrd25 | 0.99929046 |
| Ankrd27 | 2.73886048 |
| Ankrd34a | 0.06051836 |
| Ankrd35 | -0.7024642 |
| Ankrd36 | -0.6168432 |
| Ankrd37 | 0.24018345 |
| Ankrd39 | 0.82937226 |
| Ankrd40 | -0.3195154 |
| Ankrd40 | -0.7106487 |
| Ankrd40 | -0.2310036 |
| Ankrd46 | 2.17676616 |
| Ankrd47 | 0.11180552 |
| Ankrd49 | -0.1121278 |
| Ankrd5 | 3.16056763 |
| Ankrd50 | -0.5483079 |
| Ankrd53 | 0.64677091 |
| Ankrd54 | 0.23484964 |
| Ankrd54 | 0.03894713 |
| Ankrd54 | 0.19070382 |
| Ankrd56 | -0.3837319 |
| Ankrd58 | -0.6721723 |
| Ankrd6 | -0.3402322 |
| Ankrd6 | 1.55302466 |
| Ankrd6 | -0.1870654 |
| Ankrd7 | 1.89245305 |
| Ankrd9 | 0.68067545 |
| Anks1 | 2.17442963 |
| Anks3 | 4.49759787 |
| Anks3 | 1.10775143 |
| Anks6 | 2.03641177 |
| Anks6 | 1.58364586 |
| Ankzf1 | -0.7441176 |
| Ankzf1 | 0.5253341 |
| Anln | -0.5907549 |
| Anln | -0.3690074 |
| Anp32a | -0.5091671 |
| Anp32a | -0.12087 |
| Anp32a | 0.41832735 |
| Anp32e | -0.3709149 |
| Anpep | 1.88553956 |
| Antxr1 | 2.01180942 |
| Antxr1 | 0.75334625 |
| Antxrl | 2.68587525 |
| Anxa1 | -0.6556599 |
| Anxa11 | 3.30592552 |
| Anxa11 | -0.4989016 |
| Anxa2 | -0.3960719 |
| Anxa3 | 0.24680848 |
| Anxa3 | 3.44799958 |
| Anxa4 | -0.1218464 |
| Anxa5 | -0.5630256 |
| Anxa6 | -0.6952485 |
| Anxa8 | -0.7337641 |
| Anxa8 | 0.97067575 |
| Anxa9 | -0.5947838 |
| Aoah | 0.56922051 |
| Aoc2 | 1.10417569 |
| Aoc2 | 1.55795221 |
| Aoc3 | -0.4673491 |
| Aof1 | -0.7097358 |
| Aof1 | -0.7565023 |
| Aof2 | -0.8117984 |
| Aox3 | -0.6118963 |
| Aox3l1 | -0.031562 |
| Ap1b1 | -0.1823656 |
| Ap1g1 | -0.2822941 |
| Ap1g1 | -0.6565218 |
| Ap1g2 | 1.15956776 |
| Ap1gbp1 | -0.3805112 |
| Ap1gbp1 | 1.22585617 |
| Ap1m1 | -0.0821545 |
| Ap1m2 | 0.32378293 |
| Ap1m2 | -0.5685064 |
| Ap1m2 | 1.15593286 |
| Ap1m2 | 0.46919104 |
| Ap1s1 | -0.5330604 |
| Ap1s1 | 0.94305486 |
| Ap1s1 | 1.74302483 |
| Ap1s3 | 0.60033202 |
| Ap2a1 | -0.7189049 |
| Ap2a1 | -0.6836397 |
| Ap2a2 | -0.6037087 |
| Ap2b1 | 1.10894033 |
| Ap2b1 | -0.0821545 |
| Ap2b1 | -0.3908563 |
| Ap2b1 | -0.4158918 |
| Ap2m1 | 2.09280959 |
| Ap2s1 | -0.7384561 |
| Ap2s1 | 1.37597268 |
| Ap2s1 | -0.6436757 |
| Ap3b1 | -0.5899512 |
| Ap3b1 | 2.06765751 |
| Ap3b2 | 2.38563545 |
| Ap3d1 | 1.18401701 |
| Ap3m1 | 0.92653335 |
| Ap3m2 | 3.24179955 |
| Ap3s2 | -0.538369 |
| Ap3s2 | 0.06613479 |
| Ap4b1 | 0.9282946 |
| Ap4m1 | 0.69102483 |
| Ap4m1 | -0.7263052 |
| Ap4m1 | -0.770998 |
| Ap4s1 | 1.03908113 |
| Apaf1 | -0.1571815 |
| Apaf1 | 0.46814809 |
| Apba1 | -0.4680546 |
| Apba2 | -0.2381618 |
| Apba2 | 0.16369141 |
| Apba3 | -0.4815601 |
| Apbb1 | -0.7088237 |
| Apbb1ip | 0.55338937 |
| Apbb1ip | 0.21080903 |
| Apbb1ip | -0.5315492 |
| Apbb2 | 0.01306864 |
| Apbb3 | -0.2431496 |
| Apc | -0.7097358 |
| Apc2 | -0.7170639 |
| Apcdd1 | 0.33105446 |
| Apeh | -0.6756859 |
| Apex1 | -0.5537035 |
| Aph1a | 0.46344041 |
| Aph1b | 2.75023017 |
| Apip | 1.8913618 |
| Apip | 0.41584843 |
| Apitd1 | -0.5292869 |
| Aplp1 | -0.3817982 |
| Aplp2 | 1.27137233 |
| Apoa1 | 1.58976316 |
| Apoa1bp | -0.2492819 |
| Apoa2 | -0.1991839 |
| Apoa4 | 1.77165104 |
| Apob48r | -0.5923644 |
| Apobec1 | 0.50398073 |
| Apobec3 | -0.2233552 |
| Apoc1 | -0.5763921 |
| Apoc1 | -0.1792454 |
| Apoc2 | 1.45138748 |
| Apoc3 | -0.3734644 |
| Apoe | 2.31274494 |
| Apof | -0.5552507 |
| Apoh | -0.4478077 |
| Apol7c | 0.67294293 |
| Apol9b | 0.07489342 |
| App | -0.3986905 |
| Appbp1 | -0.3830869 |
| Appbp1 | 0.34006554 |
| Appbp2 | 0.59236434 |
| Appl1 | -0.3171078 |
| Appl2 | 1.60015828 |
| Aprin | 0.42134829 |
| Aprt | -0.6218164 |
| Aptx | -0.7263052 |
| Aqp1 | -0.7613094 |
| Aqp1 | -0.7253769 |
| Aqp1 | -0.6020797 |
| Aqp11 | 0.97586063 |
| Aqp11 | -0.6012662 |
| Aqp3 | -0.6677988 |
| Aqp4 | -0.7517196 |
| Aqp5 | 1.10337924 |
| Aqp7 | 2.12576573 |
| Aqp7 | 1.17335233 |
| Aqp8 | -0.6677988 |
| Aqp8 | 1.281207 |
| Aqp9 | 0.56287281 |
| Aqr | -0.6234799 |
| Aqr | -0.2945383 |
| Ar | -0.1714898 |
| Araf | 0.39746731 |
| Araf | 1.92256096 |
| Arbp | -0.1356233 |
| Arc | 1.08554904 |
| Arcn1 | 0.23217135 |
| Ard1 | 0.00028652 |
| Ard1 | -0.4673491 |
| Areg | -0.6704205 |
| Arf2 | -0.4808445 |
| Arf3 | -0.0972597 |
| Arf5 | -0.4715899 |
| Arf6 | -0.6836397 |
| Arfgap1 | 0.99795449 |
| Arfgap1 | 0.04959106 |
| Arfgap1 | -0.7033701 |
| Arfgap2 | -0.6470842 |
| Arfgap2 | 1.81862508 |
| Arfgap2 | -0.4185663 |
| Arfgap2 | -0.3237435 |
| Arfgap3 | -0.6419761 |
| Arfip2 | -0.3595271 |
| Arfip2 | -0.0594848 |
| Arfip2 | -0.7593836 |
| Arfrp1 | 0.06252808 |
| Arfrp1 | -0.3322571 |
| Arg1 | 1.663527 |
| Arg2 | 0.37986678 |
| Arglu1 | 2.60265331 |
| Arhgap1 | -0.6765663 |
| Arhgap1 | 3.57874757 |
| Arhgap1 | 2.44995577 |
| Arhgap10 | 0.02859775 |
| Arhgap12 | -0.14159 |
| Arhgap15 | -0.6127189 |
| Arhgap15 | -0.2684931 |
| Arhgap17 | 0.05081149 |
| Arhgap18 | -0.0844988 |
| Arhgap19 | 0.44490548 |
| Arhgap20 | 0.12917136 |
| Arhgap22 | -0.3715516 |
| Arhgap24 | 0.69267022 |
| Arhgap24 | -0.2238995 |
| Arhgap25 | -0.3869636 |
| Arhgap26 | 0.85807294 |
| Arhgap27 | -0.2736448 |
| Arhgap28 | 0.63722888 |
| Arhgap29 | 0.06533449 |
| Arhgap30 | 0.80689319 |
| Arhgap4 | 0.8934239 |
| Arhgap6 | -0.1597207 |
| Arhgap8 | 2.57874359 |
| Arhgap9 | -0.4865851 |
| Arhgdib | 1.2447849 |
| Arhgdig | 1.19021297 |
| Arhgef1 | 0.68731216 |
| Arhgef1 | 0.81522808 |
| Arhgef10 | 1.83420351 |
| Arhgef10l | -0.5622452 |
| Arhgef10l | 1.68559689 |
| Arhgef15 | -0.4125593 |
| Arhgef18 | 1.31616546 |
| Arhgef19 | 0.05040486 |
| Arhgef2 | 2.91699358 |
| Arhgef3 | -0.706093 |
| Arhgef4 | -0.4772742 |
| Arhgef6 | 1.05052883 |
| Arid2 | 3.69359758 |
| Arid3a | 0.0377112 |
| Arid3b | 1.88420865 |
| Arid4b | -0.7051845 |
| Arid5a | -0.6028938 |
| Arid5b | -0.4945362 |
| Arih1 | 0.13662288 |
| Arih2 | -0.5421792 |
| Arl1 | 3.68528599 |
| Arl11 | -0.5835461 |
| Arl16 | 2.79336986 |
| Arl2 | -0.5700783 |
| Arl2bp | -0.6496487 |
| Arl2bp | 1.20355886 |
| Arl2bp | -0.0289032 |
| Arl3 | -0.2190125 |
| Arl3 | -0.3273827 |
| Arl4a | -0.5217848 |
| Arl4a | 0.60800796 |
| Arl4c | -0.7235233 |
| Arl5a | -0.7097358 |
| Arl5b | 0.74034879 |
| Arl6 | -0.0939347 |
| Arl6ip1 | 0.68731216 |
| Arl6ip2 | 0.42872247 |
| Arl6ip2 | 1.99785252 |
| Arl6ip2 | -0.7300272 |
| Arl6ip2 | 0.54741331 |
| Arl6ip4 | -0.3377711 |
| Arl6ip4 | -0.4772742 |
| Arl6ip5 | 1.01707009 |
| Arl6ip5 | -0.1381048 |
| Arl8a | 0.69697571 |
| Arl8b | -0.541416 |
| Armc10 | 2.18129521 |
| Armc3 | 2.08090505 |
| Armc6 | 2.4463309 |
| Armc6 | -0.7996725 |
| Armc8 | 0.62824948 |
| Armc8 | -0.653079 |
| Armc8 | 0.95743204 |
| Armc9 | -0.1198946 |
| Armc9 | -0.6218164 |
| Armcx1 | -0.5506165 |
| Armcx2 | -0.1839296 |
| Armcx3 | 0.02693123 |
| Armcx5 | 3.25056672 |
| Armcx6 | 1.00476296 |
| Armcx6 | -0.0040131 |
| Armetl1 | 0.86989134 |
| Arnt | 2.34045967 |
| Arnt | -0.6721723 |
| Arnt2 | -0.3677378 |
| Arntl | 0.36076167 |
| Arpc1a | 0.30504412 |
| Arpc1a | -0.6739275 |
| Arpc1a | -0.2515218 |
| Arpc1b | -0.4212484 |
| Arpc1b | 2.9548187 |
| Arpc1b | -0.5255284 |
| Arpc3 | 0.14842389 |
| Arpc4 | -0.6496487 |
| Arpc5 | -0.3470336 |
| Arpc5 | -0.5376088 |
| Arpc5l | -0.2742189 |
| Arpp19 | 0.5877809 |
| Arpp21 | -0.2487228 |
| Arpp21 | -0.7124771 |
| Arrb1 | 0.01264535 |
| Arrb1 | -0.502555 |
| Arrb1 | -0.1029873 |
| Arrb1 | -0.3773016 |
| Arrb2 | -0.4744278 |
| Arrdc1 | -0.1287096 |
| Arrdc2 | -0.1029873 |
| Arrdc3 | 0.30999545 |
| Arrdc4 | 1.27856506 |
| Ars2 | 1.76009346 |
| Ars2 | 2.18015442 |
| Ars2 | -0.4666442 |
| Arsa | -0.4659398 |
| Arsb | -0.8107819 |
| Arsb | -0.2554545 |
| Arsg | -0.0844988 |
| Arsi | 1.89720533 |
| Arsj | -0.5232805 |
| Arsj | 2.27693141 |
| Art1 | -0.8673066 |
| Art2b | 1.99887831 |
| Art3 | -0.4858656 |
| Art3 | -0.5498463 |
| Art4 | 0.07251278 |
| Art5 | 0.10338541 |
| Artn | 0.24449502 |
| Artn | 0.59555952 |
| Arv1 | -0.6310026 |
| As3mt | -0.4409258 |
| As3mt | 1.19901127 |
| Asah1 | 0.07092233 |
| Asah1 | 1.65151649 |
| Asah2 | -0.2616678 |
| Asah3 | -0.0617731 |
| Asah3l | 0.82883901 |
| Asb1 | -0.3033827 |
| Asb1 | -0.5421792 |
| Asb1 | -0.4138909 |
| Asb12 | -0.6143665 |
| Asb13 | -0.2504012 |
| Asb15 | -0.671296 |
| Asb17 | 0.57342864 |
| Asb2 | 0.08278566 |
| Asb3 | -0.5300404 |
| Asb3 | -0.546005 |
| Asb4 | 1.48800203 |
| Asb4 | 0.48572303 |
| Asb6 | -0.6193265 |
| Asb6 | -0.7936677 |
| Asb7 | -0.6739275 |
| Asb7 | -0.7115625 |
| Asb8 | -0.3882594 |
| Asb9 | -0.5875442 |
| Ascc1 | 0.94447106 |
| Ascc1 | -0.4894686 |
| Ascc2 | 0.43143905 |
| Ascc2 | -0.501092 |
| Ascc2 | 1.92494843 |
| Ascc2 | -0.3715516 |
| Ascc3l1 | 2.58799693 |
| Ascl1 | 1.79230782 |
| Ascl2 | -0.6110743 |
| Asf1a | -0.7207495 |
| Asf1b | -0.6907666 |
| Asgr1 | -0.3967259 |
| Ash1l | -0.4865851 |
| Ash1l | 1.69640531 |
| Ash1l | 1.00903007 |
| Ash2l | -0.6792126 |
| Ash2l | -0.6394323 |
| Asl | -0.7613094 |
| Asl | 1.104043 |
| Asna1 | -0.8365292 |
| Asns | 1.53917217 |
| Asns | 0.26640813 |
| Asns | 1.34196965 |
| Asnsd1 | -0.8240825 |
| Asnsd1 | -0.5437077 |
| Asph | -0.5859429 |
| Asph | -0.7006551 |
| Asph | -0.4327334 |
| Asph | 2.76976847 |
| Asph | -0.5091671 |
| Asphd1 | 1.1919067 |
| Asphd2 | -0.4327334 |
| Aspm | -0.6863059 |
| Asprv1 | 2.65820711 |
| Aspscr1 | 2.27906506 |
| Aspscr1 | 0.50548634 |
| Aspscr1 | 0.51123558 |
| Asrgl1 | -0.8313231 |
| Ass1 | -0.4185663 |
| Aste1 | 0.04510255 |
| Astn1 | 0.09605235 |
| Astn2 | 4.1498615 |
| Asz1 | -0.0882618 |
| Atad1 | 1.71419415 |
| Atad2 | -0.6730495 |
| Atad3a | 0.00755103 |
| Atad4 | 2.51922385 |
| Atad4 | 0.57901036 |
| Atcay | 1.90465742 |
| Ate1 | 1.22141536 |
| Ate1 | 0.4510359 |
| Atf2 | -0.3601562 |
| Atf2 | -0.0812185 |
| Atf2 | 0.06613479 |
| Atf3 | -0.7896857 |
| Atf4 | -0.4192361 |
| Atf4 | 2.40090457 |
| Atf5 | -0.5677215 |
| Atf7 | -0.6608431 |
| Atf7ip | 0.11901727 |
| Atg10 | -0.4205771 |
| Atg10 | -0.5614655 |
| Atg12 | 0.12391996 |
| Atg16l1 | 0.68875772 |
| Atg2a | 0.42599803 |
| Atg2a | 1.50859811 |
| Atg2b | -0.276519 |
| Atg2b | -0.5018232 |
| Atg3 | -0.5827485 |
| Atg3 | -0.6143665 |
| Atg4b | -0.4013165 |
| Atg4c | -0.2304553 |
| Atg4c | 1.96866673 |
| Atg4d | -0.9309025 |
| Atg5 | -0.2633695 |
| Atg5 | 0.84067693 |
| Atg7 | 0.07370385 |
| Atg9b | 0.05810102 |
| Athl1 | -0.4865851 |
| Atic | 3.10464805 |
| Atic | -0.2736448 |
| Atic | -0.6436757 |
| Atm | -0.5988301 |
| Atm | 1.43799087 |
| Atmin | 0.37382986 |
| Atmin | -0.3734644 |
| Atmin | 0.09333623 |
| Atn1 | 0.38501062 |
| Atoh8 | 0.79738136 |
| Atox1 | -0.5232805 |
| Atp10a | 0.78572705 |
| Atp10d | -0.7593836 |
| Atp11c | -0.4631274 |
| Atp11c | -0.8057159 |
| Atp12a | -0.2321014 |
| Atp13a1 | -0.6634455 |
| Atp13a1 | -0.0873196 |
| Atp13a2 | -0.6479383 |
| Atp13a4 | -0.5700783 |
| Atp13a5 | -0.2299072 |
| Atp1a1 | -0.6695458 |
| Atp1a2 | 1.88507685 |
| Atp1b1 | -0.5217848 |
| Atp1b2 | -0.2459321 |
| Atp1b3 | -0.5232805 |
| Atp2a1 | 1.42364519 |
| Atp2a2 | 0.54525259 |
| Atp2a2 | 2.26425887 |
| Atp2a3 | 1.31138711 |
| Atp2a3 | 1.3992386 |
| Atp2a3 | 3.92365505 |
| Atp2b1 | -0.7469612 |
| Atp2b2 | -0.7837441 |
| Atp2b2 | -0.0227286 |
| Atp2b2 | -0.2974772 |
| Atp2b3 | -0.7235233 |
| Atp2b3 | 1.86708718 |
| Atp2b4 | 1.39544937 |
| Atp2b4 | 0.27797486 |
| Atp2c1 | 0.37180892 |
| Atp2c1 | 3.03176457 |
| Atp5a1 | 0.24018345 |
| Atp5b | 2.32086152 |
| Atp5c1 | 0.36426431 |
| Atp5d | 1.12957105 |
| Atp5e | 0.56145638 |
| Atp5f1 | -0.7431716 |
| Atp5g2 | 1.06765029 |
| Atp5g3 | 0.2712449 |
| Atp5h | -0.6565218 |
| Atp5j | -0.6505051 |
| Atp5j | -0.3377711 |
| Atp5j2 | 0.12767395 |
| Atp5l | 1.88721498 |
| Atp5o | -0.7170639 |
| Atp5s | 1.07012993 |
| Atp5sl | 0.12354373 |
| Atp6ap1 | 0.29097717 |
| Atp6ap2 | -0.7356381 |
| Atp6ap2 | 2.5630036 |
| Atp6ap2 | 0.0377112 |
| Atp6ap2 | -0.4659398 |
| Atp6v0a1 | -0.7393973 |
| Atp6v0a1 | 0.84155442 |
| Atp6v0a2 | -0.7272343 |
| Atp6v0b | 1.80461366 |
| Atp6v0d1 | -0.5018232 |
| Atp6v0d1 | 0.1152285 |
| Atp6v0d1 | -0.7375158 |
| Atp6v0d1 | -0.4794147 |
| Atp6v0e | -0.4617243 |
| Atp6v0e2 | -0.45683 |
| Atp6v1a | 0.54429068 |
| Atp6v1b1 | -0.4974442 |
| Atp6v1b2 | 0.85202086 |
| Atp6v1c1 | -0.750766 |
| Atp6v1c2 | -0.8087522 |
| Atp6v1d | -0.3545098 |
| Atp6v1e1 | -0.5972095 |
| Atp6v1e2 | 1.83016438 |
| Atp6v1g1 | 0.58640151 |
| Atp6v1g2 | 0.65021688 |
| Atp6v1g2 | 0.31982059 |
| Atp6v1h | 2.35345428 |
| Atp7a | -0.6934532 |
| Atp8a1 | 0.75795872 |
| Atp8a2 | -0.3353165 |
| Atp8b1 | 2.01216329 |
| Atp8b3 | -0.7412826 |
| Atp8b3 | -0.5323045 |
| Atp8b3 | 0.22948541 |
| Atp8b3 | 0.26187126 |
| Atp9a | 0.54813244 |
| Atp9a | 3.37812875 |
| Atp9b | 2.74764347 |
| Atp9b | 1.064057 |
| Atpaf1 | -0.5173122 |
| Atpaf2 | -0.760346 |
| Atpaf2 | -0.0427216 |
| Atpbd1b | 0.53655978 |
| Atpbd1c | 0.44276377 |
| Atpbd3 | -0.5429431 |
| Atpif1 | 1.22748699 |
| Atrx | 0.14254189 |
| Atxn1 | 2.36526535 |
| Atxn10 | 1.09510945 |
| Atxn2 | 1.31701241 |
| Atxn2l | -0.6086127 |
| Atxn3 | -0.7272343 |
| Atxn3 | 0.38043971 |
| Atxn3 | 0.36076167 |
| Atxn7l1 | 2.79021428 |
| Atxn7l2 | -0.4498821 |
| AU014645 | -0.6916613 |
| AU017455 | 2.20687103 |
| AU017455 | -0.4638297 |
| AU018778 | 1.40561659 |
| AU018829 | -0.3747417 |
| AU019823 | 2.83189384 |
| AU021034 | -0.0677491 |
| AU021092 | 1.69520263 |
| AU021838 | -0.5575765 |
| AU022252 | 1.40715628 |
| AU022751 | -0.8565796 |
| AU022870 | 1.39379286 |
| AU023871 | -0.0324499 |
| AU040320 | 2.76239801 |
| AU040320 | -0.5591301 |
| AU040320 | -0.6845276 |
| AU040829 | 0.768634 |
| AU042651 | -0.6513623 |
| Aup1 | 1.00520528 |
| Aup1 | 0.10989846 |
| Aurka | -0.1541436 |
| Aurkaip1 | -0.3359295 |
| Aurkc | -0.2599692 |
| Auts2 | -0.2588385 |
| Auts2 | 0.30876006 |
| AV028368 | 0.64222883 |
| AV249152 | 0.40055588 |
| AV249152 | 1.95501699 |
| Aven | 0.94462829 |
| Avil | 1.29083111 |
| Avp | -0.4145574 |
| Avpi1 | 0.44997261 |
| Avpi1 | 0.60958056 |
| Avpr1a | -0.5391298 |
| AW049604 | -0.6651844 |
| AW061290 | 0.52705102 |
| AW111922 | -0.5099047 |
| AW121567 | 0.65771122 |
| AW125753 | -0.3177091 |
| AW146242 | 0.82688115 |
| AW146299 | 0.50799033 |
| AW146299 | 1.53212659 |
| AW540478 | -0.6547988 |
| AW544981 | -0.5475396 |
| AW548124 | -0.5398912 |
| AW549877 | -0.4652359 |
| AW551984 | 0.72359676 |
| AW551984 | -0.2217243 |
| AW551984 | -0.4099018 |
| AW555464 | 0.89624176 |
| Axin2 | -0.324955 |
| Axl | 1.03893893 |
| Axud1 | 0.09372472 |
| Azgp1 | -0.059942 |
| Azgp1 | 2.00958208 |
| Azi1 | 3.49680391 |
| Azi2 | 1.40754081 |
| Azin1 | -0.324349 |
| B020018G12Rik | -0.3476543 |
| B230118H07Rik | 0.819368 |
| B230120H23Rik | 0.65300747 |
| B230206H07Rik | 2.60588548 |
| B230209C24Rik | 0.47594224 |
| B230217C12Rik | -0.4039498 |
| B230219D22Rik | -0.7170639 |
| B230312A22Rik | -0.4112296 |
| B230312I18Rik | -0.572441 |
| B230317C12Rik | -0.7225978 |
| B230317C12Rik | -0.2163084 |
| B230339H12Rik | 1.22515638 |
| B230339M05Rik | 1.45860682 |
| B230339M05Rik | 2.34412481 |
| B230340J04Rik | -0.5529308 |
| B230342M21Rik | 1.89971491 |
| B230373P09Rik | -0.6961474 |
| B230378H13Rik | 0.4910849 |
| B230380D07Rik | 0.33707143 |
| B230396O12Rik | -0.0622315 |
| B230399E16Rik | -0.131173 |
| B2m | 1.60749147 |
| B2m | 1.5767662 |
| B3galnt1 | -0.4471172 |
| B3galnt2 | -0.5606864 |
| B3galt1 | 1.23974373 |
| B3galt4 | -0.3334797 |
| B3galt4 | -0.6310026 |
| B3galt5 | -0.7498134 |
| B3galt6 | -0.3237435 |
| B3gat1 | 1.152662 |
| B3gat1 | 1.53313634 |
| B3gat1 | 0.26511411 |
| B3gat2 | -0.3671037 |
| B3gat3 | 0.99154517 |
| B3gat3 | 1.67495221 |
| B3gnt1 | 0.21115193 |
| B3gnt3 | -0.1126114 |
| B3gnt5 | -0.1356233 |
| B3gnt7 | -0.8036971 |
| B3gnt8 | 0.05567745 |
| B3gnt8 | 0.66196705 |
| B430119L13Rik | 0.29160685 |
| B4galnt3 | -0.1218464 |
| B4galnt4 | 0.25926891 |
| B4galt1 | -0.7441176 |
| B4galt3 | -0.5537035 |
| B4galt3 | -0.7244496 |
| B4galt4 | 0.22070277 |
| B4galt7 | 2.4213435 |
| B630005N14Rik | 1.33773506 |
| B830045N13Rik | 2.18268763 |
| B930006L02Rik | -0.3111158 |
| B930011P16Rik | -0.2986553 |
| B930037P14Rik | -0.546772 |
| B930041F14Rik | -0.2437055 |
| B930041G04 | 0.93085055 |
| B930076A02 | -0.6748063 |
| B9d1 | 1.87431268 |
| B9d2 | 1.80976709 |
| Baalc | 0.67545775 |
| Baalc | -0.4779872 |
| Bace1 | 1.47323999 |
| Bace2 | -0.7758799 |
| Bace2 | -0.7290953 |
| Bace2 | 2.05648965 |
| Bach1 | -0.0553797 |
| Bach2 | -0.2811362 |
| Bad | 2.05058879 |
| Bag1 | -0.4172281 |
| Bag2 | 0.24217586 |
| Bag3 | 0.84120352 |
| Bag4 | -0.7469612 |
| Bag5 | 2.01584568 |
| Bahcc1 | 1.53523651 |
| Bahd1 | 0.25567889 |
| Bai1 | 0.60553142 |
| Bai2 | 0.48367235 |
| Bai2 | 3.46840681 |
| Bai3 | -0.3340915 |
| Baiap2 | -0.208778 |
| Baiap2 | 1.22082948 |
| Banf1 | -0.3004253 |
| Banf1 | -0.3785841 |
| Banf1 | -0.5899512 |
| Banf1 | -0.4491901 |
| Banf2 | 0.23585203 |
| Bap1 | 0.88692734 |
| Bard1 | 1.99821167 |
| Barx2 | 0.75546311 |
| Basp1 | 1.23375094 |
| Bat1a | -0.0590278 |
| Bat1a | -0.6037087 |
| Bat2 | -0.4259605 |
| Bat3 | -0.7161448 |
| Bat4 | 3.4951441 |
| Bat4 | -0.4610236 |
| Bat5 | -0.6377404 |
| Batf | 0.88659297 |
| Bax | -0.7347006 |
| Baz1a | -0.4837104 |
| Baz1b | 2.75970762 |
| Baz2a | -0.3201182 |
| BB014433 | -0.4779872 |
| BB128963 | 0.4966695 |
| BB146404 | -0.5225324 |
| Bbc3 | 0.25992018 |
| Bbc3 | 0.86013901 |
| Bbox1 | -0.2881048 |
| Bbs1 | 0.06172471 |
| Bbs12 | 0.71360146 |
| Bbs2 | 2.6293334 |
| Bbs4 | -0.3811545 |
| Bbs5 | -0.4744278 |
| Bbs7 | -0.2453749 |
| Bbs9 | -0.4873052 |
| Bbs9 | 0.13476553 |
| Bbx | -0.1740679 |
| BC002163 | 0.49006594 |
| BC002199 | -0.3359295 |
| BC002216 | 0.25763878 |
| BC003236 | 0.07449706 |
| BC003266 | -0.4246118 |
| BC003885 | 0.39999507 |
| BC003885 | 0.64720234 |
| BC003993 | 1.37358424 |
| BC003993 | -0.3760208 |
| BC004004 | -0.6608431 |
| BC004044 | -0.4744278 |
| BC004044 | 0.04755348 |
| BC004728 | 0.22611717 |
| BC004728 | -0.4533496 |
| BC005537 | 1.72468076 |
| BC005682 | -0.4751386 |
| BC005764 | -0.7545863 |
| BC006662 | 1.69435274 |
| BC006779 | -0.6988494 |
| BC006779 | 1.76048982 |
| BC008155 | 1.93816374 |
| BC008163 | -0.2487228 |
| BC008163 | 0.936259 |
| BC010462 | 0.29223612 |
| BC011248 | -0.546005 |
| BC011248 | -0.7033701 |
| BC011487 | -0.6748063 |
| BC013491 | -0.4765618 |
| BC013491 | 0.21115193 |
| BC013529 | 0.50623823 |
| BC013712 | -0.2968887 |
| BC016201 | -0.1782076 |
| BC016423 | -0.7996725 |
| BC016495 | 0.69615714 |
| BC017158 | 1.73437295 |
| BC017612 | 1.44642066 |
| BC017643 | 0.15281138 |
| BC017647 | 1.14532422 |
| BC018242 | 2.91743778 |
| BC018242 | -0.8158753 |
| BC018465 | 0.843482 |
| BC020002 | -0.2690641 |
| BC020535 | 0.19210427 |
| BC021381 | -0.1891618 |
| BC021395 | -0.5980194 |
| BC021614 | -0.6809809 |
| BC021790 | 2.85056958 |
| BC021891 | -0.3267752 |
| BC022224 | -0.2679224 |
| BC022687 | 2.62145877 |
| BC023055 | -0.5947838 |
| BC023151 | 1.25749639 |
| BC023744 | 2.44057882 |
| BC023814 | 1.05836262 |
| BC023829 | 2.41425016 |
| BC024561 | 0.25240329 |
| BC024814 | -0.7272343 |
| BC024868 | -0.5202916 |
| BC024997 | 0.15208155 |
| BC025076 | 2.20929242 |
| BC025546 | -0.3869636 |
| BC025575 | 0.5435686 |
| BC025920 | 0.35988397 |
| BC026439 | -0.4450489 |
| BC026585 | 3.7490755 |
| BC026590 | -0.5851433 |
| BC026590 | -0.5947838 |
| BC027072 | -0.0240483 |
| BC027231 | -0.0925132 |
| BC027231 | -0.6462309 |
| BC027231 | 0.51297841 |
| BC027344 | -0.653079 |
| BC028528 | 1.14950617 |
| BC028528 | 1.23455989 |
| BC029169 | 0.91622013 |
| BC029169 | 4.44152143 |
| BC029214 | 1.9176001 |
| BC030183 | 1.05431398 |
| BC030307 | -0.0311183 |
| BC030476 | -0.6547988 |
| BC030499 | -0.2315523 |
| BC030500 | 0.96945161 |
| BC030867 | -0.5106428 |
| BC031181 | -0.8705487 |
| BC031353 | 0.46134052 |
| BC031575 | -0.6069752 |
| BC031748 | -0.7079126 |
| BC031781 | 0.22239812 |
| BC031853 | -0.0355642 |
| BC032265 | 1.67748043 |
| BC034076 | -0.2594037 |
| BC037034 | 0.50372957 |
| BC037594 | -0.7133927 |
| BC038156 | -0.4300184 |
| BC038286 | -0.5521587 |
| BC038613 | -0.4751386 |
| BC038822 | 0.3548948 |
| BC038822 | -0.2103866 |
| BC038822 | -0.4779872 |
| BC038925 | -0.7422266 |
| BC039093 | -0.1331483 |
| BC040758 | -0.1233129 |
| BC043098 | -0.5262789 |
| BC043118 | 0.47826795 |
| BC043301 | -0.3507642 |
| BC046331 | 0.90987962 |
| BC046404 | 1.02864334 |
| BC048355 | -0.6160169 |
| BC048502 | -0.0540153 |
| BC048546 | 0.42654355 |
| BC048546 | 1.62938195 |
| BC048562 | 2.1475061 |
| BC048644 | -0.5307945 |
| BC048651 | -0.501092 |
| BC048671 | -0.1184335 |
| BC049635 | 0.19733745 |
| BC049635 | 0.90119395 |
| BC049730 | -0.7680809 |
| BC049762 | 3.77430699 |
| BC049806 | 0.07726805 |
| BC049806 | -0.3051617 |
| BC049806 | -0.5255284 |
| BC050196 | 3.21742827 |
| BC050210 | -0.7739241 |
| BC050777 | 0.47775162 |
| BC050811 | -0.1839296 |
| BC050811 | 0.14695683 |
| BC051019 | -0.3414652 |
| BC051070 | 1.87220364 |
| BC051142 | 1.31202563 |
| BC051227 | 1.4256272 |
| BC051628 | 2.66960843 |
| BC051665 | 1.4207117 |
| BC052484 | -0.4138909 |
| BC053749 | 1.07342599 |
| BC053749 | 2.2258685 |
| BC054059 | -0.6479383 |
| BC055107 | 0.60304833 |
| BC055111 | -0.5376088 |
| BC055324 | 0.42545219 |
| BC055368 | 0.44329965 |
| BC056474 | -0.4279873 |
| BC056923 | 0.00456655 |
| BC057022 | 1.36447255 |
| BC057371 | 2.11418283 |
| BC057371 | -0.4582258 |
| BC057371 | 1.57233989 |
| BC057552 | -0.6836397 |
| BC057627 | 0.20081013 |
| BC057893 | -0.7133927 |
| BC060267 | 0.83451153 |
| BC060615 | 3.04569883 |
| BC060632 | 0.78888611 |
| BC061039 | 1.24100656 |
| BC061237 | 0.01939515 |
| BC062115 | 1.19275243 |
| BC062650 | -0.6118963 |
| BC063749 | 0.4823884 |
| BC064033 | -0.2332004 |
| BC065085 | 0.5612201 |
| BC065397 | -0.2315523 |
| BC066028 | -0.7281644 |
| BC066107 | 1.68273181 |
| BC067047 | 0.65792447 |
| BC067068 | 0.1152285 |
| BC068157 | 1.6176167 |
| BC068281 | -0.0104995 |
| BC072620 | -0.6880877 |
| BC085271 | 2.36575138 |
| BC087945 | -0.1954791 |
| BC088983 | 2.08024709 |
| BC089491 | -0.6226478 |
| BC099439 | 0.65172053 |
| BC107230 | -0.3588985 |
| Bcam | 1.68994941 |
| Bcan | 1.36497549 |
| Bcan | -0.2061032 |
| Bcap29 | -0.7106487 |
| Bcap29 | -0.7161448 |
| Bcap31 | -0.4666442 |
| Bcap31 | -0.533817 |
| Bcar1 | -0.5099047 |
| Bcar3 | -0.6479383 |
| Bcas1 | 0.68834493 |
| Bcas2 | -0.6898728 |
| Bcas3 | 2.86609232 |
| Bcat1 | 0.749872 |
| Bcat2 | -0.7024642 |
| Bccip | -0.3520111 |
| Bcdin3d | -0.137608 |
| Bcdo2 | -0.6445267 |
| Bckdha | -0.7384561 |
| Bckdhb | -0.2282649 |
| Bckdk | 0.26705447 |
| Bckdk | -0.6695458 |
| Bcl10 | 2.30792237 |
| Bcl11a | 0.40335489 |
| Bcl11b | 2.75363743 |
| Bcl2 | 0.29192154 |
| Bcl2 | -0.6251465 |
| Bcl2l1 | 0.85617505 |
| Bcl2l1 | 0.7206093 |
| Bcl2l1 | -0.6792126 |
| Bcl2l10 | -0.7847318 |
| Bcl2l11 | -0.4673491 |
| Bcl2l11 | -0.7006551 |
| Bcl2l11 | 0.31920952 |
| Bcl2l12 | 3.29753268 |
| Bcl2l13 | -0.2023711 |
| Bcl2l14 | 0.50749006 |
| Bcl2l14 | 0.49235704 |
| Bcl2l2 | -0.7033701 |
| Bcl2l2 | 0.77562143 |
| Bcl3 | -0.546772 |
| Bcl6 | -0.3626766 |
| Bcl6b | 0.714405 |
| Bcl7a | 0.53898252 |
| Bcl7b | -0.0122368 |
| Bcl9l | -0.5606864 |
| Bclaf1 | -0.4125593 |
| Bclaf1 | -0.7097358 |
| Bclp2 | 0.14695683 |
| Bcmo1 | -0.5429431 |
| Bcor | -0.281715 |
| Bcor | 0.46579719 |
| Bcorl1 | 0.0151822 |
| Bcs1l | 1.2144786 |
| Bdh1 | 1.64451982 |
| Bdh2 | -0.1501082 |
| Bdkrb1 | -0.5232805 |
| Bdnf | 0.22510434 |
| Bdnf | -0.6343655 |
| Becn1 | -0.4409258 |
| Becn1 | -0.4996312 |
| Bet1 | -0.0948835 |
| Bet1l | 1.63437951 |
| Bex2 | 3.42765634 |
| Bex2 | -0.4429851 |
| Bex2 | 1.11892046 |
| Bex4 | -0.1356233 |
| Bex4 | 2.21444274 |
| Bex6 | -0.750766 |
| Bfar | -0.6243128 |
| Bfar | 0.88659297 |
| Bglap2 | 1.30711922 |
| Bgn | 1.29960349 |
| Bhlhb2 | 3.54935572 |
| Bhlhb5 | -0.4512676 |
| Bhlhb8 | 0.104154 |
| Bhlhb8 | 1.11722082 |
| Bhlhb9 | 1.37696608 |
| Bhmt | -0.2348513 |
| Bhmt2 | -0.5069579 |
| Bicc1 | -0.0481257 |
| Bicd1 | -0.4395554 |
| Bicd2 | 2.21383109 |
| Bicd2 | 0.92316177 |
| Bid | -0.6916613 |
| Bid | -0.2437055 |
| Bik | 1.28985134 |
| Bin1 | 1.50097084 |
| Bin1 | -0.2788248 |
| Bin1 | 0.48828011 |
| Bin3 | 0.39239131 |
| Birc2 | -0.5899512 |
| Birc2 | -0.1607382 |
| Birc2 | -0.2420389 |
| Birc4 | 1.38939386 |
| Birc5 | 0.1329045 |
| Birc5 | 2.44684049 |
| Birc5 | 0.67441073 |
| Blcap | 1.35038208 |
| Blm | -0.308134 |
| Blm | 3.50810427 |
| Blmh | -0.0280186 |
| Blnk | -0.6809809 |
| Bloc1s1 | -0.3830869 |
| Bloc1s3 | -0.7393973 |
| Blvra | 0.03399364 |
| Blvrb | 0.84732537 |
| Bmf | 1.85533805 |
| Bmi1 | 1.3691896 |
| Bmp1 | -0.6135424 |
| Bmp1 | 0.15681535 |
| Bmp1 | 0.25043245 |
| Bmp1 | 1.60578031 |
| Bmp15 | 1.67567525 |
| Bmp2k | 1.32470581 |
| Bmp2k | -0.0377951 |
| Bmp3 | 0.74152009 |
| Bmp4 | 1.35669313 |
| Bmp6 | -0.5047539 |
| Bmp7 | 2.31860176 |
| Bmp8a | 0.9002056 |
| Bmper | -0.7460124 |
| Bmpr1a | 1.88235382 |
| Bmpr1a | 0.98554356 |
| Bmpr1b | 1.4019497 |
| Bmpr1b | 1.81333279 |
| Bmpr2 | -0.6243128 |
| Bms1 | -0.5552507 |
| Bnc1 | 1.67125603 |
| Bnc2 | 0.12954535 |
| Bnc2 | 0.22002377 |
| Bnip1 | -0.4199064 |
| Bnip2 | 1.24238226 |
| Bnip3l | 1.65299845 |
| Bnipl | 2.17268407 |
| Bnipl | -0.5732299 |
| Bok | 0.31338443 |
| Bok | -0.6513623 |
| Bola1 | 1.22468957 |
| Bola2 | 2.35197622 |
| Bola3 | -0.7365765 |
| Boll | 1.37118971 |
| Bop1 | 0.00114409 |
| Bpi | -0.2560176 |
| Bpil1 | 1.85468099 |
| Bpil3 | -0.5996414 |
| Bpnt1 | -0.5552507 |
| Bpnt1 | -0.6069752 |
| Bpnt1 | -0.5867432 |
| Bpnt1 | -0.5529308 |
| Brap | -0.7431716 |
| Brca2 | 0.25829117 |
| Brca2 | -0.6660551 |
| Brcc3 | -0.3837319 |
| Brcc3 | -0.8814371 |
| Brcc3 | 1.91480158 |
| Brd2 | 2.34635958 |
| Brd2 | -0.6748063 |
| Brd2 | 0.72080876 |
| Brd4 | 2.06220997 |
| Brd4 | 0.93879343 |
| Brd8 | -0.6800963 |
| Brd8 | -0.5899512 |
| Brd9 | 2.10616749 |
| Brd9 | -0.2875221 |
| Brdt | -0.5811554 |
| Brdt | -0.7460124 |
| Bre | 0.18437589 |
| Bre | -0.2206387 |
| Bre | -0.572441 |
| Brf1 | 2.00383757 |
| Brf2 | 1.68781134 |
| Bri3 | 2.82200914 |
| Brip1 | -0.6127189 |
| Brms1 | 1.50218841 |
| Brp16 | 0.71641086 |
| Brp16 | 0.07884781 |
| Brp17 | 2.28524811 |
| Brp44 | -0.4019741 |
| Brpf1 | -0.0161575 |
| Brpf1 | -0.501092 |
| Brs3 | -0.3741028 |
| Brsk1 | -0.4624256 |
| Brsk2 | -0.6268159 |
| Brsk2 | -0.7574618 |
| Brunol4 | -0.9103513 |
| Brunol4 | -0.7042769 |
| Brwd1 | -0.6547988 |
| Brwd1 | -0.3934605 |
| Brwd2 | 0.98494129 |
| Bscl2 | -0.0249292 |
| Bsdc1 | -0.6925568 |
| Bsg | 0.7525753 |
| Bsg | 1.19817116 |
| Bspry | 0.1600787 |
| Bst2 | -0.7700246 |
| Btbd1 | 1.97167916 |
| Btbd10 | -0.7690523 |
| Btbd11 | 0.57970575 |
| Btbd12 | -0.1292018 |
| Btbd14a | -0.7244496 |
| Btbd14b | -0.6284884 |
| Btbd16 | -0.0882618 |
| Btbd3 | 0.17942456 |
| Btbd3 | -0.1818448 |
| Btbd6 | 2.82068329 |
| Btbd9 | 1.10629629 |
| Btc | -0.7124771 |
| Btd | 2.46441095 |
| Btf3 | -0.6695458 |
| Btg1 | 1.15555603 |
| Btg1 | 0.15317608 |
| Btg3 | -0.6479383 |
| Btg3 | -0.4118942 |
| Btg4 | 0.75795872 |
| Btk | -0.3614155 |
| Btla | -0.6243128 |
| Btrc | -0.2453749 |
| Btrc | -0.4032908 |
| Btrc | 1.87372731 |
| Bub1b | -0.1179469 |
| Bub3 | 1.23870924 |
| Bud13 | 0.90792006 |
| Bud31 | -0.0377951 |
| Bves | 0.96623065 |
| Bxdc1 | 1.08392356 |
| Bxdc1 | -0.3652038 |
| Bxdc2 | 0.10223135 |
| Bxdc2 | 2.27295603 |
| Bxdc5 | 1.57901162 |
| Bxdc5 | 2.29048886 |
| Bxdc5 | -0.558353 |
| Bzrap1 | 0.65621712 |
| Bzrpl1 | -0.6037087 |
| Bzw1 | -0.4617243 |
| Bzw2 | 2.95929855 |
| Bzw2 | 0.96546213 |
| C030006K11Rik | 0.94068979 |
| C030011O14Rik | -0.4894686 |
| C030014K22Rik | -0.0840295 |
| C030018G13Rik | 2.00026135 |
| C030030A07Rik | -0.4279873 |
| C030039L03Rik | 0.61962782 |
| C030045D06Rik | 0.31122922 |
| C030045D06Rik | -0.5988301 |
| C030046I01Rik | -0.5851433 |
| C030048B08Rik | -0.5843444 |
| C030048B08Rik | -0.5599079 |
| C130022K22Rik | 1.76293032 |
| C130032J12Rik | 2.0150902 |
| C130034I18Rik | -0.4708818 |
| C130038G02Rik | -0.4429851 |
| C130060K24Rik | 4.0024815 |
| C130073D16Rik | 1.89026927 |
| C130074G19Rik | -0.8240825 |
| C130090K23Rik | -0.7328285 |
| C130090K23Rik | 2.88853823 |
| C1galt1 | 0.26543778 |
| C1galt1c1 | -0.7244496 |
| C1qa | -0.2543292 |
| C1qb | 0.5216444 |
| C1qbp | 1.37138949 |
| C1qc | -0.770998 |
| C1qdc2 | 0.71340046 |
| C1ql2 | -0.3837319 |
| C1ql3 | 0.29694222 |
| C1qtnf1 | 0.35283273 |
| C1qtnf2 | 1.07205404 |
| C1qtnf3 | 2.52137366 |
| C1qtnf3 | -0.4680546 |
| C1qtnf3 | 1.19696969 |
| C1qtnf4 | 0.93689325 |
| C1qtnf6 | -0.2211813 |
| C1qtnf7 | -0.4561329 |
| C1qtnf9 | 0.45580591 |
| C1rl | 0.70166876 |
| C1s | 4.19606737 |
| C1s | 0.20254168 |
| C2 | -0.3219291 |
| C230021P08Rik | 1.17075989 |
| C230029F24Rik | -0.6037087 |
| C230052I12Rik | -0.5756005 |
| C230052I12Rik | 0.7849822 |
| C230055K05Rik | 3.39921223 |
| C230071H18Rik | -0.2828735 |
| C230078M08Rik | -0.4765618 |
| C230078M14Rik | -0.7006551 |
| C230090D14 | -0.0868489 |
| C230093N12Rik | 0.80031833 |
| C230096C10Rik | -0.2476055 |
| C2cd3 | 0.63264038 |
| C3 | -0.6634455 |
| C330001K17Rik | 1.11735167 |
| C330005M16Rik | -0.653079 |
| C330016O10Rik | -0.7393973 |
| C330019G07Rik | -0.4631274 |
| C330023M02Rik | -0.6854163 |
| C330027C09Rik | -0.5165688 |
| C430003P19Rik | -0.6783297 |
| C430004E15Rik | 0.0240077 |
| C4b | 0.24283906 |
| C530028I08Rik | 2.5679157 |
| C530028I08Rik | -0.3129094 |
| C530028O21Rik | 0.29192154 |
| C530043G21Rik | -0.6462309 |
| C530044N13Rik | 3.71900695 |
| C6 | 0.26737747 |
| C630004H02Rik | -0.4252859 |
| C630004H02Rik | -0.2498414 |
| C630007B19Rik | -0.170975 |
| C630028N24Rik | 0.9471403 |
| C630035N08Rik | -0.4533496 |
| C630035N08Rik | -0.7593836 |
| C730025P13Rik | -0.7235233 |
| C730027P07Rik | -0.1287096 |
| C78339 | 0.05203034 |
| C79127 | 3.36930809 |
| C79267 | 0.55386582 |
| C79407 | -0.7347006 |
| C79407 | 3.08519047 |
| C80008 | 1.2787855 |
| C80913 | 0.28908558 |
| C85492 | 1.01925095 |
| C85492 | -0.6037087 |
| C85492 | -0.4575277 |
| C85627 | -0.1673794 |
| C87436 | 3.14550714 |
| C87436 | 1.84976845 |
| C87499 | -0.8814371 |
| C8b | 1.95560759 |
| C8g | 1.90233143 |
| C9 | 2.50121525 |
| C9 | 1.09658198 |
| C920005C14Rik | 3.98912878 |
| Cab39l | 0.28719018 |
| Cabc1 | 0.69983514 |
| Cables2 | -0.6453784 |
| Cabp2 | -0.3105187 |
| Cabp7 | 0.71601003 |
| Cabyr | 2.32496619 |
| Cabyr | 1.84513199 |
| Cachd1 | 1.27635776 |
| Cacna1a | -0.5225324 |
| Cacna1b | -0.4429851 |
| Cacna1b | 0.1193953 |
| Cacna1b | 0.42572515 |
| Cacna1d | -0.4471172 |
| Cacna1g | -0.638586 |
| Cacna1h | 2.27521336 |
| Cacna2d1 | 0.26381831 |
| Cacna2d3 | -0.4974442 |
| Cacnb1 | -0.5591301 |
| Cacnb1 | -0.4327334 |
| Cacnb2 | 2.77173173 |
| Cacnb2 | 0.32712269 |
| Cacnb3 | -0.3570151 |
| Cacnb3 | -0.4478077 |
| Cacnb3 | 2.22683452 |
| Cacnb3 | 0.97082865 |
| Cacnb4 | 1.3201817 |
| Cacnb4 | 2.53593885 |
| Cacng2 | 1.26513111 |
| Cacng3 | -0.5437077 |
| Cacng3 | -0.3237435 |
| Cacng4 | 0.93260375 |
| Cacng5 | -0.6151914 |
| Cacng5 | -0.0849683 |
| Cacng5 | -0.5645883 |
| Cacng7 | 1.92550279 |
| Cacng8 | -0.3255614 |
| Cacybp | -0.1986538 |
| Cad | -0.3377711 |
| Cadm2 | -0.5429431 |
| Cadm3 | -0.3741028 |
| Cadps | -0.5195458 |
| Cadps2 | 2.25987995 |
| Cadps2 | 0.29819322 |
| Cage1 | -0.4246118 |
| Calb2 | 1.12750233 |
| Calb2 | -0.653079 |
| Calca | 1.01064358 |
| Calca | 1.38349634 |
| Calcoco1 | -0.6226478 |
| Cald1 | 1.84802493 |
| Cald1 | -0.5923644 |
| Calm2 | 2.35028417 |
| Calm3 | -0.4779872 |
| Calml4 | -0.6591122 |
| Caln1 | -0.0526529 |
| Calr | -0.3993463 |
| Calr3 | -0.6505051 |
| Calr3 | -0.2650743 |
| Calr3 | -0.3921575 |
| Calr4 | 1.61723224 |
| Calu | -0.328599 |
| Calu | 0.07251278 |
| Calu | 1.10894033 |
| Caly | -0.6234799 |
| Camk1 | 2.17655399 |
| Camk1d | 0.99318893 |
| Camk1g | 0.33105446 |
| Camk1g | 1.47895885 |
| Camk2a | -0.7207495 |
| Camk2a | 0.76426416 |
| Camk2a | 0.46396465 |
| Camk2b | -0.501092 |
| Camk2d | -0.1928418 |
| Camk2d | 1.08581968 |
| Camk2d | -0.5113815 |
| Camk2g | 0.9202622 |
| Camk2g | 1.10337924 |
| Camk2n1 | 0.83680626 |
| Camk4 | 0.38358459 |
| Camkk1 | 1.08663114 |
| Camkk2 | 2.16399339 |
| Camkk2 | 1.40020775 |
| Camkv | 0.08553237 |
| Caml | 0.15317608 |
| Camp | 2.04884045 |
| Camsap1l1 | 2.39321672 |
| Camta2 | -0.2200963 |
| Cant1 | -0.313508 |
| Cant1 | -0.5732299 |
| Cap1 | -0.4145574 |
| Capg | -0.5630256 |
| Capg | -0.6522203 |
| Capn1 | 0.05971327 |
| Capn10 | -0.6539385 |
| Capn11 | -0.03601 |
| Capn2 | 2.25828609 |
| Capn2 | -0.6487931 |
| Capn2 | 0.22002377 |
| Capn3 | 0.32226093 |
| Capn5 | 0.15426934 |
| Capn6 | -0.4596236 |
| Capn7 | -0.4715899 |
| Capn8 | 1.1697704 |
| Capns1 | -0.4808445 |
| Caprin1 | -0.7926706 |
| Caprin1 | -0.546772 |
| Capsl | -0.5740194 |
| Capza1 | 1.66491707 |
| Capza3 | 0.12579883 |
| Capzb | -0.5732299 |
| Capzb | 1.56610787 |
| Capzb | 1.52689222 |
| Car11 | -0.4065905 |
| Car12 | 0.32924184 |
| Car13 | 1.2384792 |
| Car14 | 0.31768009 |
| Car15 | 0.03067676 |
| Car2 | -0.4395554 |
| Car3 | 0.40363434 |
| Car4 | 0.23150058 |
| Car5b | -0.4032908 |
| Car5b | -0.3607856 |
| Car5b | -0.0732992 |
| Car7 | -0.6513623 |
| Car9 | 2.1857185 |
| Card10 | -0.0705201 |
| Card11 | -0.4652359 |
| Carhsp1 | 2.77456782 |
| Carm1 | 0.4306249 |
| Cart | 2.69797264 |
| Cartpt | 0.91150947 |
| Casc1 | 1.68000187 |
| Casc1 | 2.51280414 |
| Casc1 | 4.42485364 |
| Casc3 | -0.2939516 |
| Casc4 | 0.10913456 |
| Casc4 | -0.4959891 |
| Casc4 | -0.2863578 |
| Cask | -0.7768594 |
| Caskin1 | -0.4361382 |
| Caskin1 | 1.72926628 |
| Caskin1 | -0.4019741 |
| Caskin2 | -0.8067269 |
| Casp1 | -0.2130737 |
| Casp2 | 1.75459334 |
| Casp4 | -0.7651729 |
| Casp4 | -0.5032874 |
| Casp4 | 0.36513794 |
| Casp6 | -0.5429431 |
| Casp6 | -0.5121208 |
| Casp7 | 0.33527037 |
| Casp8 | 1.36678385 |
| Casp8 | -0.6934532 |
| Casp9 | 0.64935657 |
| Casq2 | -0.3255614 |
| Cass4 | 0.36134635 |
| Cast | -0.7768594 |
| Cat | 0.13958704 |
| Catsper1 | 3.79112337 |
| Catsper2 | -0.5568006 |
| Catsper3 | -0.6765663 |
| Catsper4 | 1.34813246 |
| Cav1 | -0.6677988 |
| Cav2 | 0.94132105 |
| Cbara1 | 2.03221563 |
| Cbfa2t3h | -0.3292077 |
| Cbfa2t3h | -0.1155179 |
| Cbfb | -0.8057159 |
| Cbfb | -0.3298168 |
| Cbfb | 0.36630151 |
| Cbl | 0.472313 |
| Cblb | -0.7106487 |
| Cblc | -0.0275767 |
| Cbln1 | 0.78031346 |
| Cbln2 | 0.58984622 |
| Cbln4 | 2.3640144 |
| Cbr1 | 0.07845312 |
| Cbr2 | -0.5851433 |
| Cbr3 | 0.87278046 |
| Cbr3 | -0.4457378 |
| Cbs | -0.7281644 |
| Cbs | 1.32805852 |
| Cbwd1 | 1.16343788 |
| Cbx2 | 1.33066954 |
| Cbx5 | 0.60868225 |
| Cbx7 | 0.43089636 |
| Cbx8 | 0.34810236 |
| Cby1 | -0.3177091 |
| Cc2d1a | -0.1975944 |
| Cc2d1b | -0.7189049 |
| Cc2d2a | 1.62869841 |
| Ccar1 | -0.5560253 |
| Ccbe1 | -0.6318422 |
| Ccbl1 | 1.45550655 |
| Ccbl1 | 1.21187887 |
| Ccbl2 | -0.1073059 |
| Ccbp2 | 0.29223612 |
| Ccdc100 | 0.15935449 |
| Ccdc101 | -0.4526551 |
| Ccdc108 | 0.60800796 |
| Ccdc109a | 0.23418078 |
| Ccdc109a | 1.51152852 |
| Ccdc109b | 1.80813613 |
| Ccdc11 | -0.7198268 |
| Ccdc110 | 0.24581769 |
| Ccdc111 | -0.5232805 |
| Ccdc113 | -0.6765663 |
| Ccdc114 | 0.39239131 |
| Ccdc115 | 0.056486 |
| Ccdc116 | -0.5851433 |
| Ccdc117 | -0.2916085 |
| Ccdc12 | -0.7690523 |
| Ccdc120 | 2.2872121 |
| Ccdc123 | -0.3728264 |
| Ccdc124 | 0.4610777 |
| Ccdc125 | 0.210466 |
| Ccdc126 | -0.5032874 |
| Ccdc127 | 0.34483638 |
| Ccdc127 | 2.6202679 |
| Ccdc13 | -0.5307945 |
| Ccdc130 | 0.16441229 |
| Ccdc131 | 0.46134052 |
| Ccdc132 | 1.22012594 |
| Ccdc134 | -0.3563881 |
| Ccdc137 | 2.80318791 |
| Ccdc137 | -0.5811554 |
| Ccdc18 | -0.4512676 |
| Ccdc21 | 2.51428723 |
| Ccdc23 | -0.4505746 |
| Ccdc25 | 0.23351145 |
| Ccdc27 | -0.6259808 |
| Ccdc27 | 0.47981524 |
| Ccdc27 | -0.7431716 |
| Ccdc28a | -0.0113678 |
| Ccdc28b | -0.2840335 |
| Ccdc28b | -0.7565023 |
| Ccdc3 | 1.09350041 |
| Ccdc32 | 0.17480346 |
| Ccdc38 | -0.6952485 |
| Ccdc39 | 0.08357125 |
| Ccdc40 | -0.4673491 |
| Ccdc41 | 2.87391037 |
| Ccdc42 | -0.6352081 |
| Ccdc43 | 0.39097643 |
| Ccdc45 | -0.4561329 |
| Ccdc46 | 0.51868219 |
| Ccdc46 | -0.5444728 |
| Ccdc47 | 1.89828984 |
| Ccdc49 | -0.5552507 |
| Ccdc5 | -0.5614655 |
| Ccdc50 | -0.4974442 |
| Ccdc51 | 2.77972287 |
| Ccdc53 | 2.37528223 |
| Ccdc53 | -0.4361382 |
| Ccdc53 | 0.40335489 |
| Ccdc54 | 0.70451397 |
| Ccdc56 | 0.22341386 |
| Ccdc57 | 0.10261619 |
| Ccdc58 | -0.5915593 |
| Ccdc59 | -0.7584222 |
| Ccdc59 | -0.4901909 |
| Ccdc64 | -0.5247785 |
| Ccdc65 | 1.90403393 |
| Ccdc65 | 0.79443518 |
| Ccdc66 | 0.68441436 |
| Ccdc67 | -0.8470288 |
| Ccdc68 | -0.0109336 |
| Ccdc70 | -0.5827485 |
| Ccdc71 | -0.5716528 |
| Ccdc73 | -0.4457378 |
| Ccdc77 | 1.44798712 |
| Ccdc79 | 2.43075123 |
| Ccdc80 | -0.7006551 |
| Ccdc82 | 0.80671118 |
| Ccdc84 | 2.0568271 |
| Ccdc85a | -0.6293257 |
| Ccdc85b | 0.53970812 |
| Ccdc86 | 1.00210464 |
| Ccdc87 | -0.7033701 |
| Ccdc88a | -0.9103513 |
| Ccdc88b | 1.0629486 |
| Ccdc88c | -0.4450489 |
| Ccdc90b | 0.65365027 |
| Ccdc91 | 0.9779873 |
| Ccdc92 | -0.8694668 |
| Ccdc93 | -0.4989016 |
| Ccdc94 | 2.93389791 |
| Ccdc95 | 1.14074077 |
| Ccdc96 | 2.97874588 |
| Ccdc97 | 1.34176353 |
| Ccdc98 | 0.73132004 |
| Ccdc99 | 0.35753942 |
| Cchcr1 | -0.7253769 |
| Ccin | 0.05567745 |
| Cck | 0.72280103 |
| Cckbr | -0.717984 |
| Ccl11 | -0.5353319 |
| Ccl17 | -0.6301638 |
| Ccl19 | -0.8428149 |
| Ccl21b | -0.5521587 |
| Ccl21c | 1.9686138 |
| Ccl25 | -0.541416 |
| Ccl25 | -0.1678923 |
| Ccl27 | 0.02693123 |
| Ccl27 | 0.7633115 |
| Ccl28 | 1.06017189 |
| Ccl3 | -0.4533496 |
| Ccl4 | 1.28449902 |
| Ccl4 | -0.0271349 |
| Ccl5 | 2.54816869 |
| Ccl6 | -0.9390175 |
| Ccl7 | -0.5421792 |
| Ccl9 | -0.5653706 |
| Ccm2 | -0.4617243 |
| Ccna1 | 1.8010147 |
| Ccnb1 | 2.4335327 |
| Ccnb1 | -0.7024642 |
| Ccnb3 | 0.38955943 |
| Ccnc | -0.3292077 |
| Ccnd1 | -0.4498821 |
| Ccnd1 | -0.0320058 |
| Ccnd1 | 1.28438947 |
| Ccnd2 | -0.3741028 |
| Ccnd3 | -0.5529308 |
| Ccnd3 | -0.8880312 |
| Ccndbp1 | 1.83132894 |
| Ccne1 | -0.183408 |
| Ccne2 | 1.41805422 |
| Ccnf | -0.5150838 |
| Ccng1 | -0.6818663 |
| Ccng1 | -0.324955 |
| Ccng1 | -0.6470842 |
| Ccng2 | 1.09818576 |
| Ccnk | 0.19559629 |
| Ccnl1 | -0.5756005 |
| Ccno | 0.1329045 |
| Ccr4 | 1.39661691 |
| Ccr5 | -0.5875442 |
| Ccr5 | -0.6226478 |
| Ccr5 | 1.00608931 |
| Ccr6 | 1.3195487 |
| Ccr6 | 1.95345817 |
| Ccrk | 1.26390027 |
| Ccrk | 2.28581507 |
| Ccrk | -0.131173 |
| Ccrk | 2.18651669 |
| Ccrl2 | -0.822024 |
| Ccrn4l | 0.0601159 |
| Ccrn4l | 0.55243575 |
| Ccs | 2.09034768 |
| Cct2 | -0.5552507 |
| Cct3 | 1.48597046 |
| Cct3 | -0.7133927 |
| Cct4 | -0.2605351 |
| Cct5 | -0.163287 |
| Cct6a | -0.4772742 |
| Cct6b | -0.787701 |
| Cct7 | -0.0266933 |
| Cct8 | -0.3721888 |
| Cd109 | 0.39040989 |
| Cd109 | 2.91455646 |
| Cd14 | 0.1787151 |
| Cd151 | 0.04999805 |
| Cd151 | -0.2753683 |
| Cd151 | -0.8694668 |
| Cd160 | -0.0920399 |
| Cd163 | -0.8683861 |
| Cd164 | -0.6573845 |
| Cd177 | 0.95136418 |
| Cd180 | 1.95211228 |
| Cd1d1 | -0.3439359 |
| Cd2 | 0.94210955 |
| Cd200 | -0.7526742 |
| Cd200 | -0.538369 |
| Cd226 | -0.8138346 |
| Cd247 | -0.1954791 |
| Cd248 | 1.08243106 |
| Cd248 | -0.5113815 |
| Cd27 | -0.6293257 |
| Cd274 | -0.7235233 |
| Cd276 | -0.7574618 |
| Cd2bp2 | 1.17814801 |
| Cd300lg | -0.2939516 |
| Cd320 | -0.3973803 |
| Cd34 | -0.6934532 |
| Cd3d | -0.7469612 |
| Cd3eap | 0.90906363 |
| Cd3g | -0.5700783 |
| Cd4 | 0.54139903 |
| Cd40 | -0.0066023 |
| Cd40 | 0.11028018 |
| Cd44 | 2.32706608 |
| Cd44 | -0.73096 |
| Cd44 | -0.5202916 |
| Cd44 | -0.8261456 |
| Cd47 | -0.0218498 |
| Cd52 | 0.19245405 |
| Cd53 | -0.5988301 |
| Cd55 | 2.46883831 |
| Cd59a | -0.7798038 |
| Cd59b | 1.02229568 |
| Cd59b | -0.4715899 |
| Cd6 | 0.32499876 |
| Cd6 | -0.2098501 |
| Cd6 | 0.07211542 |
| Cd63 | -0.5779772 |
| Cd63 | 0.04796135 |
| Cd63 | -0.8138346 |
| Cd68 | -0.0695955 |
| Cd74 | -0.3195154 |
| Cd74 | -0.4844283 |
| Cd79b | -0.7956651 |
| Cd79b | 0.25763878 |
| Cd81 | -0.658248 |
| Cd82 | -0.804706 |
| Cd83 | -0.9661847 |
| Cd84 | -0.7986691 |
| Cd86 | -0.2071722 |
| Cd86 | -0.7403395 |
| Cd86 | -0.4894686 |
| Cd8b1 | 2.28077611 |
| Cd9 | 0.43549926 |
| Cd93 | 0.10568929 |
| Cd96 | 0.53315743 |
| Cd97 | 1.94285441 |
| Cd97 | -0.638586 |
| Cd97 | -0.6695458 |
| Cd99l2 | -0.5498463 |
| Cdan1 | -0.7375158 |
| Cdc123 | 1.81283315 |
| Cdc14b | -0.7033701 |
| Cdc14b | -0.6310026 |
| Cdc14b | 0.68813846 |
| Cdc16 | -0.4266356 |
| Cdc20 | -0.8587152 |
| Cdc20 | 1.65121981 |
| Cdc20 | 0.95401396 |
| Cdc20 | 0.06813256 |
| Cdc23 | -0.7024642 |
| Cdc25a | -0.3304263 |
| Cdc25b | -0.5614655 |
| Cdc25c | -0.2359535 |
| Cdc25c | -0.770998 |
| Cdc27 | 1.30957562 |
| Cdc2a | 0.82008613 |
| Cdc2l1 | -0.8006771 |
| Cdc2l1 | -0.7778398 |
| Cdc2l5 | 3.7617837 |
| Cdc2l6 | 2.70116117 |
| Cdc34 | -0.3408485 |
| Cdc37 | 0.77900205 |
| Cdc37l1 | 1.50401182 |
| Cdc37l1 | 1.96229307 |
| Cdc37l1 | -0.5076937 |
| Cdc37l1 | 0.05689001 |
| Cdc42 | 1.99697972 |
| Cdc42bpb | 1.30733307 |
| Cdc42ep1 | 0.33105446 |
| Cdc42ep2 | -0.3396163 |
| Cdc42ep3 | -0.610253 |
| Cdc42ep4 | 2.92761582 |
| Cdc42ep5 | -0.8716319 |
| Cdc42se1 | 0.4341478 |
| Cdc42se1 | -0.5756005 |
| Cdc45l | 0.12015093 |
| Cdc5l | 0.50121419 |
| Cdc5l | 1.78712179 |
| Cdc5l | -0.3734644 |
| Cdc5l | 0.94557108 |
| Cdc6 | 0.74424746 |
| Cdc7 | 0.55076458 |
| Cdc73 | 0.85306113 |
| Cdca2 | -0.4687606 |
| Cdca3 | -0.1326541 |
| Cdca3 | -0.1116445 |
| Cdca4 | -0.5195458 |
| Cdca4 | 1.12568847 |
| Cdca4 | 0.95774216 |
| Cdca5 | 3.90802207 |
| Cdca7 | -0.0915668 |
| Cdca8 | 0.11028018 |
| Cdcp1 | -0.389557 |
| Cdgap | -0.7006551 |
| Cdh1 | -0.7916746 |
| Cdh11 | -0.7088237 |
| Cdh11 | -0.1740679 |
| Cdh12 | 1.85903287 |
| Cdh13 | 1.35699743 |
| Cdh13 | -0.0066023 |
| Cdh16 | -0.2249889 |
| Cdh22 | -0.7545863 |
| Cdh23 | -0.6151914 |
| Cdh23 | 0.43171027 |
| Cdh23 | -0.5638066 |
| Cdh24 | 0.96484685 |
| Cdh26 | 0.50899006 |
| Cdh3 | 0.97036986 |
| Cdh5 | -0.1435874 |
| Cdh6 | -0.4822763 |
| Cdh7 | -0.6268159 |
| Cdh8 | 0.31061253 |
| Cdh8 | -0.7253769 |
| Cdh8 | -0.528534 |
| Cdipt | -0.1262527 |
| Cdipt | -0.4079136 |
| Cdk10 | -0.7281644 |
| Cdk10 | 0.34126052 |
| Cdk10 | -0.5899512 |
| Cdk2 | 0.87295014 |
| Cdk2 | 1.24146535 |
| Cdk2 | 0.8880967 |
| Cdk2 | -0.3501414 |
| Cdk2ap2 | -0.5076937 |
| Cdk3 | 0.38614989 |
| Cdk5 | -0.7584222 |
| Cdk5r1 | -0.0737632 |
| Cdk5rap1 | -0.6800963 |
| Cdk5rap2 | 2.32489369 |
| Cdk5rap3 | 1.74262104 |
| Cdk6 | -0.6045242 |
| Cdk8 | -0.6268159 |
| Cdk9 | -0.760346 |
| Cdkal1 | 0.44115427 |
| Cdkl1 | -0.5980194 |
| Cdkl2 | -0.4072518 |
| Cdkl2 | -0.3057554 |
| Cdkl2 | -0.6343655 |
| Cdkl2 | 0.96376916 |
| Cdkl3 | 1.52714616 |
| Cdkl4 | 1.19612775 |
| Cdkn1a | 0.50573703 |
| Cdkn1a | 1.54914664 |
| Cdkn1a | -0.4673491 |
| Cdkn1b | -0.0915668 |
| Cdkn1b | -0.4787007 |
| Cdkn1c | 0.10261619 |
| Cdkn2a | 1.37567446 |
| Cdkn2a | 0.05608181 |
| Cdkn2aipnl | 0.26931317 |
| Cdkn2b | -0.5819516 |
| Cdkn2c | -0.2034359 |
| Cdkn2c | -0.0686718 |
| Cdkn2d | 1.18984963 |
| Cdo1 | 0.9883991 |
| Cdr2 | 0.34750939 |
| Cdrt4 | -0.505488 |
| Cds1 | 1.24387033 |
| Cds2 | -0.4212484 |
| Cdsn | 0.69677114 |
| Cdt1 | 1.37696608 |
| Cdv3 | -0.4715899 |
| Cdv3 | 3.01463055 |
| Cdyl | -0.0152849 |
| Cdyl | 0.54188159 |
| Cdyl | -0.1991839 |
| Cdyl2 | -0.546005 |
| Ceacam1 | 0.51496625 |
| Ceacam13 | -0.1410914 |
| Ceacam2 | -0.7015592 |
| Cebpa | 0.58801061 |
| Cebpb | -0.6004535 |
| Cecr5 | 0.95120808 |
| Cecr6 | 0.11712479 |
| Celsr1 | -0.3538845 |
| Celsr2 | 0.16260905 |
| Celsr3 | 1.63218688 |
| Cend1 | -0.1425882 |
| Cenpa | 1.72413173 |
| Cenpb | -0.3010161 |
| Cenph | 0.64396173 |
| Cenpi | -0.6565218 |
| Cenpj | -0.4801293 |
| Cenpk | 0.47955753 |
| Cenpl | 1.55096387 |
| Cenpl | -0.5180561 |
| Cenpm | -0.2200963 |
| Cenpm | -0.6660551 |
| Cenpm | -0.2277181 |
| Cenpn | 1.77873831 |
| Cenpp | -0.6836397 |
| Cenpq | 1.16605055 |
| Cenpt | -0.6925568 |
| Centa1 | 0.14364757 |
| Centa1 | 1.25422147 |
| Centa2 | -0.5121208 |
| Centb5 | 2.31725061 |
| Centb5 | 0.09450124 |
| Centd2 | 1.74825831 |
| Centd2 | 0.04877656 |
| Centd3 | 0.3139993 |
| Centg1 | 2.07212573 |
| Centg1 | 0.08827109 |
| Centg2 | -0.6522203 |
| Centg2 | 0.50372957 |
| Centg3 | 0.2988181 |
| Centg3 | -0.3837319 |
| Cep135 | 2.14592906 |
| Cep164 | -0.5591301 |
| Cep170 | 2.25137409 |
| Cep55 | -0.546005 |
| Cep57 | -0.5867432 |
| Cep57 | -0.1886373 |
| Cep63 | 0.57947401 |
| Cep68 | -0.3721888 |
| Cep70 | 2.37737572 |
| Cep72 | -0.1097138 |
| Cep78 | 1.89003911 |
| Cep97 | 0.3921085 |
| Cept1 | -0.1981239 |
| Cercam | -0.2904391 |
| Ces3 | 0.12542335 |
| Cetn2 | -0.2498414 |
| Cetn3 | 2.4432675 |
| Cetn4 | -0.3595271 |
| Cetn4 | 0.68793195 |
| Cfd | 1.20248378 |
| Cfdp1 | 1.22888259 |
| Cfh | 1.0233084 |
| Cfhr1 | -0.6176703 |
| Cfi | 0.02651414 |
| Cfl1 | 0.1797791 |
| Cfl2 | -0.8597849 |
| Cfl2 | 1.25274969 |
| Cfp | 1.12061703 |
| Cftr | 0.29975463 |
| Cga | 0.80288069 |
| Cggbp1 | 0.23685336 |
| Cgnl1 | 2.17349339 |
| Cgrrf1 | 2.32111631 |
| Ch25h | 0.48007287 |
| Chac1 | 1.77171631 |
| Chad | 1.25873563 |
| Chaf1a | 0.56593446 |
| Chaf1b | 1.06585536 |
| Chat | 1.5196684 |
| Chchd1 | 0.83150226 |
| Chchd2 | -0.3576425 |
| Chchd4 | -0.2645057 |
| Chchd5 | 1.00505787 |
| Chchd6 | 2.16536869 |
| Chchd7 | -0.3328682 |
| Chchd8 | 0.26705447 |
| Chd1 | 0.98914912 |
| Chd1 | -0.5136011 |
| Chd1l | -0.4306964 |
| Chd1l | -0.0409272 |
| Chd4 | -0.5560253 |
| Chd4 | 0.51620653 |
| Chd4 | 2.35836444 |
| Chd5 | -0.6522203 |
| Chd8 | 0.4887907 |
| Chdh | 0.01939515 |
| Chdh | 2.7290988 |
| Chek1 | -0.5732299 |
| Chek1 | -0.5452386 |
| Chek2 | -0.3457933 |
| Chek2 | 0.14622245 |
| Cherp | -0.3734644 |
| Chfr | -0.7886928 |
| Chfr | -0.7328285 |
| Chfr | -0.5180561 |
| Chga | 1.35415344 |
| Chga | 0.82545469 |
| Chga | 0.08278566 |
| Chgb | -0.717984 |
| Chi3l1 | 3.45793249 |
| Chi3l3 | 2.02665786 |
| Chi3l3 | 1.5060914 |
| Chia | 2.29812783 |
| Chia | 0.74152009 |
| Chic1 | 4.46656643 |
| Chic2 | -0.1566745 |
| Chic2 | 0.15062019 |
| Chit1 | 1.14112358 |
| Chka | 1.48658923 |
| Chka | -0.6792126 |
| Chka | 2.50798283 |
| Chkb | -0.5076937 |
| Chl1 | -0.7441176 |
| Chl1 | -0.5429431 |
| Chl1 | 0.69983514 |
| Chm | -0.6318422 |
| Chmp1b | -0.6127189 |
| Chmp2a | 2.15929362 |
| Chmp2a | 1.26881398 |
| Chmp2b | -0.6961474 |
| Chmp4b | 0.02275198 |
| Chmp5 | 0.3543061 |
| Chmp7 | 2.3151653 |
| Chmp7 | -0.7079126 |
| Chmp7 | -0.505488 |
| Chn2 | 0.37699689 |
| Chodl | 1.43482179 |
| Chordc1 | 1.79147742 |
| Chpf | 1.87290718 |
| Chpt1 | -0.7916746 |
| Chpt1 | -0.2639374 |
| Chpt1 | -0.3863164 |
| Chrac1 | 1.55458785 |
| Chrd | 1.83755626 |
| Chrm1 | -0.0873196 |
| Chrm2 | 0.43089636 |
| Chrm3 | 0.97388155 |
| Chrna1 | -0.1933686 |
| Chrna4 | -0.5040204 |
| Chrna4 | 0.87379804 |
| Chrna4 | -0.5315492 |
| Chrna5 | -0.3267752 |
| Chrna6 | -0.7536298 |
| Chrna6 | 0.386719 |
| Chrna7 | -0.6061575 |
| Chrna9 | -0.2206387 |
| Chrnb1 | 0.3604692 |
| Chrnb1 | 1.27768276 |
| Chrnb2 | -0.6845276 |
| Chrnb2 | -0.6168432 |
| Chrnb3 | -0.5498463 |
| Chrnb3 | -0.618498 |
| Chrnb3 | 0.61762693 |
| Chrne | -0.2673521 |
| Chst1 | 0.4105936 |
| Chst10 | 1.37994 |
| Chst12 | 0.83962287 |
| Chst14 | -0.6704205 |
| Chst2 | 1.54317466 |
| Chst3 | 0.41942699 |
| Chst3 | -0.2359535 |
| Chst7 | 1.84826561 |
| Chst8 | 1.72687365 |
| Chtf18 | -0.6748063 |
| Chuk | -0.4300184 |
| Churc1 | 1.52375454 |
| Ciao1 | 2.97633683 |
| Ciapin1 | 1.93701478 |
| Cib1 | 0.21320672 |
| Cib2 | 2.6772662 |
| Cib2 | -0.4132249 |
| Cic | 1.11708995 |
| Cidea | -0.0709827 |
| Cideb | -0.3954184 |
| Cidec | 2.29382918 |
| Ciita | 1.02979288 |
| Cirbp | 3.24854554 |
| Cirh1a | -0.4052692 |
| Cisd1 | -0.6251465 |
| Cish | -0.6094325 |
| Cit | 2.74033002 |
| Cited1 | 3.48483813 |
| Cited2 | -0.2163084 |
| Cited4 | -0.3563881 |
| Ciz1 | 1.00062454 |
| Ciz1 | -0.6961474 |
| Ckap2l | -0.2980661 |
| Ckap5 | -0.2093139 |
| Ckb | 1.52077681 |
| Cklf | -0.5819516 |
| Cklf | -0.1361191 |
| Ckmt1 | 0.39352168 |
| Ckmt2 | -0.2114605 |
| Ckmt2 | -0.6077935 |
| Ckt2 | -0.4464272 |
| Ckt2 | 0.76806517 |
| Clca5 | 2.0798709 |
| Clcc1 | 0.01729104 |
| Clcf1 | 0.94068979 |
| Clcf1 | 0.04755348 |
| Clcf1 | 0.56098376 |
| Clcn1 | -0.3817982 |
| Clcn2 | -0.4039498 |
| Clcn3 | 1.84549409 |
| Clcn3 | -0.3921575 |
| Clcn3 | 2.86827166 |
| Clcn4-2 | -0.5875442 |
| Clcn7 | -0.5763921 |
| Clcnka | -0.423265 |
| Cldn1 | -0.7024642 |
| Cldn10 | 1.34700563 |
| Cldn10 | 0.79443518 |
| Cldn10 | 0.44329965 |
| Cldn11 | 2.02810008 |
| Cldn12 | -0.5210379 |
| Cldn15 | 0.06573473 |
| Cldn15 | -0.6419761 |
| Cldn17 | -0.5939767 |
| Cldn2 | -0.4996312 |
| Cldn23 | 0.55028656 |
| Cldn3 | -0.5121208 |
| Cldn4 | 1.52968188 |
| Cldn5 | -0.6284884 |
| Cldn6 | -0.2509614 |
| Cldn9 | 0.45130154 |
| Cldnd1 | -0.5756005 |
| Cldnd2 | -0.1720048 |
| Clec11a | 0.36746365 |
| Clec16a | -0.8251135 |
| Clec1a | -0.6462309 |
| Clec2d | 1.30957562 |
| Clec2e | -0.4099018 |
| Clec3b | 0.34543104 |
| Clec4a1 | 1.96213317 |
| Clec4a3 | 1.87472222 |
| Clec4b1 | 0.24713851 |
| Clec4b1 | 1.84368217 |
| Clec4d | -0.5360902 |
| Clec4g | 0.19210427 |
| Clec4n | 0.668104 |
| Clec7a | 1.41290777 |
| Clecsf12 | -0.5529308 |
| Clgn | 0.25992018 |
| Clic1 | 1.63656703 |
| Clic3 | -0.7966653 |
| Clic4 | 0.11446891 |
| Clic6 | 2.30132969 |
| Clip1 | -0.1087499 |
| Clip1 | 3.11048477 |
| Clip4 | -0.8179204 |
| Clk1 | 0.80871142 |
| Clk2 | 1.53237913 |
| Clk2 | -0.5771843 |
| Clk2 | -0.6522203 |
| Clk3 | 0.90069991 |
| Clk4 | 1.13073271 |
| Clk4 | -0.7739241 |
| Clk4 | -0.7986691 |
| Clm3 | 0.689995 |
| Clm3 | 1.18851618 |
| Clmn | -0.5128607 |
| Clmn | -0.7070024 |
| Cln3 | -0.5069579 |
| Cln5 | 0.35253777 |
| Cln6 | 1.0387967 |
| Cln6 | -0.4266356 |
| Cln8 | -0.8673066 |
| Clns1a | -0.3671037 |
| Clns1a | -0.1818448 |
| Clns1a | 1.54525256 |
| Clock | -0.2611013 |
| Clock | -0.1020302 |
| Clp1 | -0.4589245 |
| Clpb | 1.81277068 |
| Clpp | 0.08278566 |
| Clptm1 | 1.63029258 |
| Clptm1l | -0.7798038 |
| Clpx | 1.78281041 |
| Clpx | -0.12087 |
| Clrn1 | 0.76178524 |
| Clspn | -0.6326825 |
| Clspn | 0.57249513 |
| Clspn | 1.20032994 |
| Clstn1 | 2.7336872 |
| Clstn3 | -0.6854163 |
| Clstn3 | 0.46055185 |
| Clta | -0.2007762 |
| Cltb | 0.31522783 |
| Cluap1 | 0.88357828 |
| Cma1 | 1.81775546 |
| Cma1 | 0.31706763 |
| Cmas | 0.3028697 |
| Cmbl | -0.5188007 |
| Cml1 | -0.4751386 |
| Cml3 | -0.5091671 |
| Cml4 | 1.74651705 |
| Cmpk | -0.6591122 |
| Cmtm2a | 2.88752225 |
| Cmtm2b | 0.66556943 |
| Cmtm4 | -0.874889 |
| Cmtm4 | -0.8057159 |
| Cmtm5 | -0.4779872 |
| Cmtm6 | -0.4722986 |
| Cmtm7 | 4.42161236 |
| Cmtm7 | 0.36251462 |
| Cmtm7 | 3.41161405 |
| Cmtm8 | -0.6352081 |
| Cmtm8 | 1.47503087 |
| Cmya1 | -0.6301638 |
| CN716893 | 1.5266382 |
| Cnbp2 | 1.88692633 |
| Cnbp2 | -0.5391298 |
| Cndp1 | 1.27370215 |
| Cnga4 | -0.5032874 |
| Cnih | -0.2211813 |
| Cnih3 | -0.0240483 |
| Cnih4 | 1.59984496 |
| Cnksr2 | 1.49512196 |
| Cnksr2 | -0.3377711 |
| Cnksr3 | -0.0944089 |
| Cnn1 | 1.56862183 |
| Cnn2 | -0.7555438 |
| Cnn2 | 0.42326567 |
| Cnnm1 | 2.08498355 |
| Cnnm1 | -0.248164 |
| Cnnm2 | -0.3396163 |
| Cnnm3 | -0.6925568 |
| Cno | -0.6539385 |
| Cnot10 | 0.1769391 |
| Cnot2 | 0.10338541 |
| Cnot2 | 0.63088648 |
| Cnot2 | 0.93053144 |
| Cnot2 | -0.2498414 |
| Cnot3 | 1.15480192 |
| Cnot3 | 1.19323536 |
| Cnot4 | -0.6704205 |
| Cnot4 | 1.60484563 |
| Cnot4 | -0.3773016 |
| Cnot6l | -0.4246118 |
| Cnot6l | -0.505488 |
| Cnot7 | -0.6809809 |
| Cnp | -0.0096321 |
| Cnp | -0.0779495 |
| Cnpy2 | 2.42974659 |
| Cnpy3 | -0.6988494 |
| Cnr1 | -0.2071722 |
| Cnr1 | -0.7526742 |
| Cntd1 | -0.1658427 |
| Cntd1 | 3.83242382 |
| Cntfr | -0.2980661 |
| Cntln | -0.5630256 |
| Cntn1 | 1.4456826 |
| Cntn4 | 0.71239485 |
| Cntn6 | 0.94352717 |
| Cntnap1 | -0.5996414 |
| Cntnap2 | 1.03409116 |
| Cntnap4 | 2.78396019 |
| Cntnap4 | 1.30743998 |
| Coasy | 0.4560702 |
| Cobl | 1.42600426 |
| Cobll1 | -0.8344433 |
| Cobll1 | -0.3237435 |
| Coch | -0.2532052 |
| Cog1 | 2.24484518 |
| Cog2 | -0.4375036 |
| Cog3 | -0.6143665 |
| Cog4 | -0.0035822 |
| Cog6 | 1.76048982 |
| Cog6 | -0.5795649 |
| Cog8 | -0.2103866 |
| Coil | 3.10019098 |
| Coil | -0.1501082 |
| Col11a1 | 2.05426908 |
| Col12a1 | 1.32722146 |
| Col14a1 | 3.03516106 |
| Col15a1 | -0.5225324 |
| Col16a1 | 2.25622171 |
| Col17a1 | -0.3177091 |
| Col17a1 | -0.4471172 |
| Col17a1 | 3.24305701 |
| Col18a1 | 0.49818684 |
| Col18a1 | -0.4457378 |
| Col1a1 | -0.7347006 |
| Col1a2 | 0.46893041 |
| Col24a1 | 0.58016906 |
| Col24a1 | -0.7817719 |
| Col25a1 | 1.6064807 |
| Col25a1 | 1.95098024 |
| Col25a1 | 1.82942812 |
| Col27a1 | -0.7412826 |
| Col28a1 | -0.2520826 |
| Col2a1 | -0.787701 |
| Col2a1 | -0.4589245 |
| Col3a1 | 0.98403716 |
| Col3a1 | -0.1010742 |
| Col4a1 | -0.0467717 |
| Col4a2 | 0.16260905 |
| Col4a3 | -0.5195458 |
| Col4a3bp | 2.00724784 |
| Col4a3bp | -0.7189049 |
| Col4a3bp | 0.07092233 |
| Col4a4 | 1.94018469 |
| Col4a4 | 0.58984622 |
| Col4a5 | -0.1029873 |
| Col4a6 | 1.32523046 |
| Col4a6 | -0.6061575 |
| Col5a1 | -0.6037087 |
| Col5a3 | 0.64070991 |
| Col6a1 | 2.72907522 |
| Col6a1 | 1.01152251 |
| Col6a1 | 1.43304618 |
| Col6a2 | -0.5099047 |
| Col7a1 | -0.6094325 |
| Col8a2 | -0.3785841 |
| Col9a2 | -0.1169746 |
| Colec12 | 0.30906906 |
| Commd1 | 0.59806242 |
| Commd1 | -0.671296 |
| Commd10 | -0.1776891 |
| Commd2 | -0.2881048 |
| Commd2 | -0.2886878 |
| Commd4 | -0.1663547 |
| Commd5 | -0.7403395 |
| Commd6 | 1.01648767 |
| Commd8 | -0.3902064 |
| Commd9 | -0.5575765 |
| Comp | -0.5708652 |
| Comtd1 | 0.04469344 |
| Copa | -0.5875442 |
| Copb2 | -0.4526551 |
| Cope | -0.5915593 |
| Cope | -0.6077935 |
| Cope | -0.7024642 |
| Cope | 0.82509774 |
| Copg | -0.122335 |
| Copg | -0.7739241 |
| Copg | -0.5240292 |
| Copg | 0.51917654 |
| Cops2 | -0.5732299 |
| Cops3 | -0.8902395 |
| Cops4 | -0.5630256 |
| Cops4 | 1.93168941 |
| Cops5 | 1.7009897 |
| Cops5 | 0.59327836 |
| Cops6 | 1.08188774 |
| Cops7a | -0.0148488 |
| Cops7a | -0.5630256 |
| Cops7a | -0.2238995 |
| Cops7b | -0.7857205 |
| Cops7b | 3.57272479 |
| Cops8 | 1.44355745 |
| Copz1 | 0.16296997 |
| Copz2 | -0.618498 |
| Copz2 | -0.3696428 |
| Copz2 | 3.90086764 |
| Copz2 | -0.0400312 |
| Coq10b | 0.6804673 |
| Coq10b | 0.58938765 |
| Coq2 | 0.01771224 |
| Coq3 | 2.24397649 |
| Coq3 | -0.7189049 |
| Coq4 | -0.2599692 |
| Coq4 | 2.04781909 |
| Coq5 | 1.00254822 |
| Coq5 | -0.5645883 |
| Coq5 | 2.16575513 |
| Coq6 | 0.30814175 |
| Coq6 | 0.19245405 |
| Coq7 | -0.4880258 |
| Coq9 | -0.6453784 |
| Corin | 0.25926891 |
| Coro1a | 2.07160395 |
| Coro1a | -0.5300404 |
| Coro1a | -0.3520111 |
| Coro1a | 2.84198962 |
| Coro1b | -0.4617243 |
| Coro1b | -0.7006551 |
| Coro1c | 0.71661121 |
| Coro2a | 1.30604921 |
| Coro2a | -0.3570151 |
| Coro2b | -0.1039453 |
| Coro2b | 1.81607605 |
| Coro6 | 2.20423738 |
| Coro7 | 2.12033007 |
| Cort | -0.658248 |
| Cort | -0.5113815 |
| Cotl1 | -0.7356381 |
| Cova1 | -0.3967259 |
| Cox10 | -0.5307945 |
| Cox11 | 1.94676357 |
| Cox15 | -0.6343655 |
| Cox17 | -0.6721723 |
| Cox18 | -0.6845276 |
| Cox18 | -0.5232805 |
| Cox19 | 2.19116631 |
| Cox4i1 | 1.04503436 |
| Cox4nb | -0.8230527 |
| Cox4nb | 0.65771122 |
| Cox5a | -0.4118942 |
| Cox5b | 1.94269117 |
| Cox6a1 | -0.2588385 |
| Cox6a1 | -0.7272343 |
| Cox6a2 | 3.42080647 |
| Cox6b1 | -0.3261681 |
| Cox6b1 | 1.7826169 |
| Cox6b2 | 0.22881272 |
| Cox6b2 | -0.6625772 |
| Cox6c | 0.52900939 |
| Cox7a1 | 0.30193626 |
| Cox7a2 | -0.533817 |
| Cox7a2l | 0.58317502 |
| Cox7b | -0.6310026 |
| Cox7b2 | -0.6077935 |
| Cox8b | -0.5560253 |
| Cox8c | 1.03065411 |
| Cp | -0.7337641 |
| Cpa1 | -0.2822941 |
| Cpa2 | -0.1233129 |
| Cpa2 | 2.47253258 |
| Cpa3 | -0.0196564 |
| Cpa3 | -0.0640671 |
| Cpa3 | -0.6739275 |
| Cpa4 | 1.60156694 |
| Cpa5 | -0.546005 |
| Cpa6 | -0.6310026 |
| Cpb2 | 0.1152285 |
| Cpeb1 | 2.60154644 |
| Cpeb2 | 0.2271289 |
| Cpeb3 | -0.2359535 |
| Cpeb4 | 1.52638411 |
| Cphx | 2.69242176 |
| Cplx1 | -0.3658367 |
| Cplx2 | 0.80324617 |
| Cpn1 | 0.38700343 |
| Cpne2 | -0.6326825 |
| Cpne4 | 2.01815871 |
| Cpne5 | -0.6335236 |
| Cpne6 | 0.50146603 |
| Cpne7 | 2.24717089 |
| Cpne8 | 2.19216861 |
| Cpne8 | -0.3582703 |
| Cpne9 | -0.4340939 |
| Cpsf1 | 1.25535204 |
| Cpsf1 | -0.569292 |
| Cpsf2 | 0.12015093 |
| Cpsf3l | 1.12995843 |
| Cpsf4 | 1.44059271 |
| Cpsf4l | 0.27605692 |
| Cpsf4l | -0.6505051 |
| Cpsf6 | -0.0100657 |
| Cpsf6 | -0.7161448 |
| Cpt1b | 1.94111177 |
| Cpt1b | -0.4313749 |
| Cpt1c | -0.1928418 |
| Cpt2 | -0.149102 |
| Cpxcr1 | -0.6539385 |
| Cpxm1 | 0.07647717 |
| Cpxm2 | -0.5645883 |
| Cpz | -0.6310026 |
| Crabp2 | -0.4631274 |
| Crat | 1.71848571 |
| Crat | -0.149102 |
| Crat | -0.7798038 |
| Crb1 | 1.32291981 |
| Crbn | -0.3414652 |
| Crbn | 0.07489342 |
| Crbn | 4.23015107 |
| Crcp | -0.3195154 |
| Creb1 | -0.7403395 |
| Creb1 | -0.7033701 |
| Creb3 | 2.19316986 |
| Creb3l1 | -0.5835461 |
| Creb3l2 | 0.08081879 |
| Creb3l2 | 3.4237418 |
| Creb3l3 | -0.6916613 |
| Creb3l4 | 1.64519197 |
| Creb5 | 1.27922624 |
| Crebl1 | -0.0760867 |
| Crebl2 | 0.92893424 |
| Creg2 | 2.5334156 |
| Creld1 | -0.6625772 |
| Creld2 | 0.26737747 |
| Creld2 | 0.43981092 |
| Crem | 1.06044993 |
| Crem | 2.27936948 |
| Crem | 1.07136731 |
| Crem | 0.95510288 |
| Crhbp | -0.3195154 |
| Crhr1 | -0.7097358 |
| Crim1 | 0.77806421 |
| Crim2 | -0.2892712 |
| Crip2 | -0.7152265 |
| Crip3 | 1.42392859 |
| Crip3 | 1.0870366 |
| Cript | -0.706093 |
| Cript | -0.2684931 |
| Crisp2 | -0.6094325 |
| Crispld2 | -0.2921937 |
| Crk | -0.2343007 |
| Crk | 3.05313271 |
| Crk | -0.6898728 |
| Crkl | 1.27381295 |
| Crkrs | 0.35871243 |
| Crkrs | 4.61320903 |
| Crlf3 | 1.03836989 |
| Crlf3 | -0.4945362 |
| Crls1 | -0.6943504 |
| Crls1 | 0.68088356 |
| Crls1 | -0.7488617 |
| Crls1 | -0.7798038 |
| Crmp1 | 1.39056894 |
| Crmp1 | 0.26187126 |
| Crnkl1 | -0.6360514 |
| Crocc | 0.2712449 |
| Crry | 3.06165191 |
| Crry | 0.58869938 |
| Crtac1 | 1.42506134 |
| Crtam | 1.25817254 |
| Crtap | -0.2277181 |
| Crtap | 0.4823884 |
| Crtc1 | -0.7393973 |
| Crtc3 | 0.00157258 |
| Crxos1 | 0.50874023 |
| Cry2 | 1.60391003 |
| Cryab | -0.2018392 |
| Cryba2 | -0.2332004 |
| Cryba4 | -0.4212484 |
| Crybb1 | -0.4092386 |
| Crybb2 | 0.6113746 |
| Crybb3 | -0.618498 |
| Crygn | -0.6445267 |
| Crygn | -0.7143092 |
| Cryl1 | -0.5763921 |
| Crym | 0.8955795 |
| Cryz | 0.63592016 |
| Cryz | 1.56708181 |
| Cryz | 0.33466926 |
| Cryzl1 | -0.528534 |
| Cryzl1 | -0.1648196 |
| Cs | 2.30411314 |
| Csad | -0.6660551 |
| Csda | -0.5353319 |
| Csda | 0.95743204 |
| Csda | -0.0770176 |
| Csdc2 | 1.00711962 |
| Csde1 | 2.02735438 |
| Csde1 | -0.076552 |
| Csde1 | 0.75122462 |
| Cse1l | -0.5376088 |
| Csf1 | 1.67451812 |
| Csf1r | -0.3715516 |
| Csf1r | -0.0135419 |
| Csf2ra | -0.4887469 |
| Csgalnact1 | -0.7253769 |
| Csgalnact2 | -0.0581144 |
| Csk | 2.20068675 |
| Csl | -0.2707793 |
| Csl | 0.48187433 |
| Csmd1 | 0.30255865 |
| Csnk1d | -0.3021987 |
| Csnk1d | 1.37288646 |
| Csnk1d | 1.31796432 |
| Csnk1d | 1.82524509 |
| Csnk1d | -0.0458702 |
| Csnk1e | 0.91036887 |
| Csnk1e | -0.1587042 |
| Csnk1g1 | 0.60259616 |
| Csnk1g1 | -0.4533496 |
| Csnk1g1 | -0.5076937 |
| Csnk1g2 | -0.7161448 |
| Csnk1g2 | 1.35221869 |
| Csnk1g2 | 1.0691664 |
| Csnk1g3 | -0.4865851 |
| Csnk2a1 | -0.1082683 |
| Csnk2a2 | 2.26615138 |
| Csnk2a2 | 0.2988181 |
| Csnrp2 | -0.4638297 |
| Csnrp3 | 0.87430642 |
| Cspg4 | -0.076552 |
| Cspg5 | 1.44114931 |
| Cspp1 | 0.77599766 |
| Csprs | 0.63132526 |
| Csrp1 | 0.98674695 |
| Csrp2 | -0.6880877 |
| Csrp2bp | 0.91540963 |
| Cst11 | 0.00028652 |
| Cst3 | 1.1824311 |
| Cst8 | 2.35102482 |
| Cst9 | 0.65086159 |
| Cstad | 3.09923655 |
| Cstb | -0.6077935 |
| Cstf1 | 3.99263255 |
| Cstf2 | 0.35577717 |
| Cstf3 | -0.3908563 |
| Cstf3 | 0.06573473 |
| Cstl1 | 0.09837424 |
| Ctage5 | 0.10837003 |
| Ctcf | 1.11787489 |
| Ctdp1 | -0.6934532 |
| Ctdp1 | -0.4464272 |
| Ctdp1 | 0.34126052 |
| Ctdsp1 | 1.5413423 |
| Ctdsp1 | 1.91804709 |
| Ctdsp1 | -0.7788213 |
| Ctdsp2 | -0.7042769 |
| Ctdspl | -0.533817 |
| Ctdspl2 | -0.5483079 |
| Ctdspl2 | -0.0440697 |
| Ctf1 | -0.4039498 |
| Ctgf | -0.3993463 |
| Ctla4 | -0.5217848 |
| Ctnna1 | -0.1063445 |
| Ctnna3 | 0.03482103 |
| Ctnnal1 | -0.5202916 |
| Ctnnb1 | -0.6143665 |
| Ctnnb1 | 0.06132276 |
| Ctnnb1 | 0.73917603 |
| Ctnnbip1 | -0.1475948 |
| Ctnnbl1 | 0.65365027 |
| Ctnnbl1 | -0.4340939 |
| Ctnnd2 | -0.4165597 |
| Ctnnd2 | 0.11446891 |
| Ctnnd2 | -0.5121208 |
| Ctns | -0.4938106 |
| Ctns | -0.1184335 |
| Ctps | -0.6934532 |
| Ctps | -0.2039688 |
| Ctps | 0.5583801 |
| Ctps2 | -0.5677215 |
| Ctps2 | 0.79885099 |
| Ctps2 | 0.40670276 |
| Ctr9 | -0.4624256 |
| Ctrb1 | -0.4219201 |
| Ctrl | 1.58213026 |
| Ctsa | -0.0636079 |
| Ctsa | -0.3027905 |
| Ctsa | -0.3702786 |
| Ctsa | -0.2409296 |
| Ctsb | 1.01005717 |
| Ctsc | 1.83968362 |
| Ctsc | 2.81553331 |
| Ctsc | -0.5843444 |
| Ctse | -0.6284884 |
| Ctsf | 1.2185998 |
| Ctsh | -0.2163084 |
| Ctsk | 0.05931047 |
| Ctsl | -0.7244496 |
| Ctso | 0.40475129 |
| Ctsr | -0.1272347 |
| Ctss | -0.6377404 |
| Ctsw | -0.2376093 |
| Ctsz | -0.1965362 |
| Ctsz | -0.0793491 |
| Cttn | -0.6907666 |
| Cttn | 1.41729355 |
| Cttnbp2nl | -0.4132249 |
| Ctxn1 | -0.0581144 |
| Cuedc1 | 0.49032078 |
| Cuedc2 | 0.21730287 |
| Cugbp1 | 1.57949208 |
| Cugbp1 | -0.4638297 |
| Cugbp1 | 1.84856637 |
| Cugbp1 | -0.5779772 |
| Cugbp2 | -0.3811545 |
| Cugbp2 | -0.1435874 |
| Cul1 | 1.53296812 |
| Cul2 | 1.16256537 |
| Cul2 | -0.6453784 |
| Cul3 | -0.4185663 |
| Cul4a | 0.65514845 |
| Cul4a | -0.0549247 |
| Cul4a | -0.5232805 |
| Cul4b | 1.35089261 |
| Cul5 | -0.0227286 |
| Cul7 | 0.19872806 |
| Cuta | 0.60259616 |
| Cuta | -0.6335236 |
| Cuta | -0.7632392 |
| Cutc | -0.2277181 |
| Cutc | 0.15754152 |
| Cux1 | 0.59144944 |
| Cux1 | -0.313508 |
| Cux1 | -0.4199064 |
| Cux1 | 1.94040291 |
| Cux1 | 1.59174077 |
| Cux2 | 3.43035776 |
| Cux2 | 0.35165236 |
| Cuzd1 | -0.2233552 |
| Cwf19l2 | 0.25240329 |
| Cx3cl1 | -0.186019 |
| Cx3cr1 | 2.64403596 |
| Cxadr | 0.5808636 |
| Cxadr | -0.541416 |
| Cxadr | -0.2152289 |
| Cxcl1 | 1.29528168 |
| Cxcl12 | -0.6453784 |
| Cxcl12 | -0.6335236 |
| Cxcl12 | 1.89846096 |
| Cxcl13 | -0.1485994 |
| Cxcl14 | 0.07449706 |
| Cxcl14 | -0.7133927 |
| Cxcl15 | 1.03337614 |
| Cxcl16 | -0.2696355 |
| Cxcl17 | 1.42298359 |
| Cxcl4 | -0.5040204 |
| Cxcl9 | -0.572441 |
| Cxcr3 | -0.7788213 |
| Cxcr4 | 0.45315889 |
| Cxcr5 | -0.6783297 |
| Cxcr7 | -0.4313749 |
| Cxx1a | -0.4368207 |
| Cxx1b | -0.4930855 |
| Cxx1c | -0.4603233 |
| Cxxc1 | -0.4737175 |
| Cxxc1 | -0.5330604 |
| Cxxc4 | -0.2249889 |
| Cxxc4 | 3.0189044 |
| Cyb5 | 0.75392405 |
| Cyb561 | -0.2190125 |
| Cyb561d1 | 1.24695345 |
| Cyb561d1 | 1.78840496 |
| Cyb561d2 | 1.14862064 |
| Cyb5b | 0.69492793 |
| Cyb5d1 | 2.06970412 |
| Cyb5d2 | 1.51213071 |
| Cyb5d2 | -0.6961474 |
| Cyb5r1 | 0.7633115 |
| Cyb5r2 | -0.2453749 |
| Cyb5r3 | -0.5972095 |
| Cyb5r3 | 0.64978682 |
| Cyb5r3 | 3.06269486 |
| Cyb5r4 | 0.29568956 |
| Cyba | 0.03688634 |
| Cybasc3 | -0.388908 |
| Cyc1 | -0.6997518 |
| Cycs | 2.58059603 |
| Cycs | -0.6818663 |
| Cyfip1 | 0.81396448 |
| Cyfip2 | 1.2565941 |
| Cyfip2 | 0.68813846 |
| Cygb | -0.6053405 |
| Cyhr1 | 0.46684276 |
| Cyhr1 | 3.127441 |
| Cyhr1 | -0.5947838 |
| Cyhr1 | -0.6634455 |
| Cyld | -0.6037087 |
| Cyln2 | 3.80371628 |
| Cyp11a1 | -0.3954184 |
| Cyp11b2 | -0.0022909 |
| Cyp17a1 | -0.7024642 |
| Cyp19a1 | -0.438871 |
| Cyp1b1 | -0.5444728 |
| Cyp20a1 | -0.5323045 |
| Cyp24a1 | -0.6539385 |
| Cyp26a1 | 1.23444437 |
| Cyp26b1 | -0.8067269 |
| Cyp27a1 | -0.3915067 |
| Cyp27a1 | -0.3117133 |
| Cyp27a1 | -0.3850232 |
| Cyp2a12 | -0.6704205 |
| Cyp2a5 | 2.13068014 |
| Cyp2a5 | -0.5740194 |
| Cyp2b10 | -0.1155179 |
| Cyp2c55 | 3.43839359 |
| Cyp2d10 | 1.01313176 |
| Cyp2d22 | -0.5106428 |
| Cyp2d26 | 0.11370871 |
| Cyp2d26 | -0.3414652 |
| Cyp2d26 | 1.27059444 |
| Cyp2e1 | 2.2249821 |
| Cyp2f2 | -0.5996414 |
| Cyp2j11 | 1.4850858 |
| Cyp2j9 | -0.2337504 |
| Cyp2s1 | -0.5106428 |
| Cyp2u1 | -0.5225324 |
| Cyp39a1 | -0.1865421 |
| Cyp3a13 | 0.39887246 |
| Cyp3a13 | -0.7729477 |
| Cyp46a1 | -0.3734644 |
| Cyp4b1 | 0.74871106 |
| Cyp4f13 | 0.80013504 |
| Cyp4f14 | 1.08568437 |
| Cyp4f15 | 1.06433389 |
| Cyp4f16 | -0.6827526 |
| Cyp4f16 | -0.7337641 |
| Cyp4f16 | 1.50184069 |
| Cyp4f18 | 2.40310867 |
| Cyp4f39 | -0.4589245 |
| Cyp4v3 | 1.18620848 |
| Cyp51 | 0.24713851 |
| Cyp7b1 | -0.706093 |
| Cypt10 | -0.6226478 |
| Cypt2 | -0.1740679 |
| Cypt3 | 1.01765214 |
| Cypt6 | 1.97961314 |
| Cypt8 | 0.29819322 |
| Cypt9 | 0.26479033 |
| Cyr61 | -0.2939516 |
| Cyr61 | -0.6436757 |
| Cysltr1 | -0.4974442 |
| Cytl1 | 0.4712735 |
| D030011O10Rik | 1.35618574 |
| D030013I16Rik | 2.04854875 |
| D030018L15Rik | 2.79879561 |
| D030028O16Rik | -0.4498821 |
| D030051N19Rik | 0.14585504 |
| D030056L22Rik | -0.6436757 |
| D030070L09Rik | -0.0572019 |
| D030070L09Rik | -0.4610236 |
| D030070L09Rik | 0.0701261 |
| D030070L09Rik | 0.18296388 |
| D030074E01Rik | 2.13241637 |
| D0H4S114 | 0.13810613 |
| D0H8S2298E | -0.0821545 |
| D0HXS9928E | -0.3476543 |
| D0HXS9928E | -0.6218164 |
| D10627 | -0.6118963 |
| D10Bwg1364e | -0.5661536 |
| D10Bwg1379e | 0.22239812 |
| D10Ertd322e | 0.73623771 |
| D10Ertd610e | 0.87684418 |
| D10Ertd610e | -0.6774476 |
| D10Ertd610e | 1.06419546 |
| D10Ertd641e | -0.4205771 |
| D10Jhu81e | 3.38678542 |
| D10Wsu102e | 1.17248898 |
| D10Wsu52e | -0.4212484 |
| D11Bwg0517e | 1.84597668 |
| D11Wsu47e | -0.0174681 |
| D11Wsu47e | 0.67859184 |
| D12Ertd551e | 3.35151208 |
| D12Ertd551e | -0.3986905 |
| D12Ertd553e | -0.5521587 |
| D12Ertd647e | -0.1238022 |
| D12Ertd647e | -0.6118963 |
| D130040H23Rik | 0.3010019 |
| D130043K22Rik | -0.6135424 |
| D130059P03Rik | 1.83034835 |
| D13Ertd608e | -0.6411274 |
| D14Ertd171e | 0.78162304 |
| D14Ertd231e | -0.546005 |
| D14Ertd449e | -0.6028938 |
| D14Ertd500e | -0.6961474 |
| D14Ertd668e | -0.7088237 |
| D14Ertd668e | 1.82079564 |
| D15Ertd621e | -0.5475396 |
| D15Ertd682e | -0.5630256 |
| D15Mgi27 | -0.706093 |
| D15Wsu169e | 0.43198142 |
| D15Wsu75e | 1.00180881 |
| D16Ertd472e | -0.5907549 |
| D16H22S680E | 0.58686153 |
| D17H6S56E-5 | 0.59487575 |
| D17Wsu104e | 0.45633442 |
| D17Wsu92e | -0.7422266 |
| D19Bwg1357e | -0.8168973 |
| D19Ertd721e | -0.6445267 |
| D19Ertd737e | -0.6209857 |
| D19Wsu162e | -0.0859081 |
| D1Bwg0212e | 1.40850144 |
| D1Bwg0212e | 1.89360038 |
| D1Pas1 | -0.2707793 |
| D230007K08Rik | 0.008402 |
| D230014K01Rik | -0.5240292 |
| D2Ertd750e | 0.92476879 |
| D2Ertd750e | 0.01137435 |
| D330014H01Rik | -0.501092 |
| D330017J20Rik | -0.4505746 |
| D330028D13Rik | 1.00888325 |
| D330045A20Rik | 0.16224799 |
| D330045A20Rik | 0.83944708 |
| D330045A20Rik | 1.20272279 |
| D3Bwg0562e | -0.5779772 |
| D3Ertd300e | -0.610253 |
| D3Ertd751e | 1.80050848 |
| D3Ucla1 | 1.95951768 |
| D430015B01Rik | -0.6077935 |
| D430028G21Rik | -0.5376088 |
| D430039N05Rik | -0.6565218 |
| D430041B17Rik | -0.5069579 |
| D430042O09Rik | -0.5437077 |
| D4Bwg0951e | 1.18096481 |
| D4Bwg1540e | 0.19280371 |
| D4Ertd22e | -0.3683724 |
| D4Ertd22e | -0.6360514 |
| D4Wsu114e | -0.3383858 |
| D4Wsu132e | 3.45053374 |
| D4Wsu132e | -0.3237435 |
| D530004J12Rik | -0.7079126 |
| D530033C11Rik | 1.54000743 |
| D5Ertd135e | 3.59329915 |
| D5Ertd579e | 3.74524694 |
| D5Wsu178e | -0.208778 |
| D630003M21Rik | -0.6617098 |
| D630003M21Rik | 0.50799033 |
| D630014A15Rik | -0.5763921 |
| D630023F18Rik | 0.1600787 |
| D630042P16Rik | -0.6660551 |
| D630048P19Rik | -0.1576888 |
| D6Ertd474e | -0.6218164 |
| D6Mm5e | 2.09308789 |
| D6Wsu116e | 0.2230754 |
| D6Wsu163e | -0.5277817 |
| D6Wsu163e | -0.6952485 |
| D6Wsu163e | -0.2195542 |
| D6Wsu176e | 2.38035348 |
| D6Wsu176e | -0.5255284 |
| D730039F16Rik | 0.11863908 |
| D730039F16Rik | 0.8975649 |
| D7Bwg0611e | -0.569292 |
| D830014E11Rik | -0.5345741 |
| D830030K20Rik | 0.1006904 |
| D830030K20Rik | -0.4279873 |
| D830050J10Rik | 0.74619076 |
| D8Ertd738e | 1.76798901 |
| D8Ertd82e | 0.44356748 |
| D930001I22Rik | -0.6453784 |
| D930005D10Rik | -0.2359535 |
| D930014E17Rik | 1.49240341 |
| D930015E06Rik | -0.4519611 |
| D930020B18Rik | 2.76678189 |
| D930028F11Rik | -0.4443604 |
| D930049A15Rik | 0.99199376 |
| D9Ertd280e | 0.19000282 |
| D9Ertd392e | 2.05054027 |
| D9Ertd402e | -0.5323045 |
| D9Wsu20e | 1.23916916 |
| Daam1 | -0.569292 |
| Daam2 | 0.74152009 |
| Dab1 | -0.7143092 |
| Dab1 | 1.39320752 |
| Dab1 | 1.15693699 |
| Dab2 | -0.6436757 |
| Dab2 | 0.00328459 |
| Dab2ip | 0.52852018 |
| Dact1 | 1.62816642 |
| Dact2 | 0.46605869 |
| Dad1 | 1.4818053 |
| Daf2 | 0.81088866 |
| Dag1 | 1.21176053 |
| Dag1 | -0.6565218 |
| Dagla | -0.2611013 |
| Daglb | 1.10483889 |
| Dak | -0.5391298 |
| Dalrd3 | 1.78545106 |
| Dap | -0.6677988 |
| Dap3 | 1.1774118 |
| Dap3 | -0.0882618 |
| Dap3 | 0.2435018 |
| Dap3 | 0.23116502 |
| Dapk1 | -0.6565218 |
| Dapk1 | 1.08690147 |
| Dapk2 | -0.3501414 |
| Dapk2 | -0.3652038 |
| Dapl1 | 3.31088231 |
| Dapp1 | -0.8057159 |
| Dapp1 | -0.5315492 |
| Darc | -0.6496487 |
| Dars | -0.6470842 |
| Dars | -0.610253 |
| Daxx | -0.7300272 |
| Dazap1 | 1.90346677 |
| Dazap1 | -0.3830869 |
| Dazap2 | 0.58132634 |
| Dazl | 0.11484878 |
| Dbc1 | 1.83773879 |
| Dbf4 | -0.3526351 |
| Dbi | 2.56021335 |
| Dbi | -0.6943504 |
| Dbil5 | -0.5843444 |
| Dbn1 | -0.0816864 |
| Dbndd1 | -0.2554545 |
| Dbndd2 | -0.6453784 |
| Dbndd2 | 0.14327916 |
| Dbndd2 | -0.6979479 |
| Dbnl | 0.62383798 |
| Dbp | -0.6599773 |
| Dbpht2 | 1.66637809 |
| Dbr1 | 0.95712182 |
| Dbt | -0.1450882 |
| Dcakd | -0.4694672 |
| Dcbld1 | 1.47377762 |
| Dcbld2 | 0.83398118 |
| Dci | -0.0214107 |
| Dck | 0.70836162 |
| Dclk1 | -0.2222677 |
| Dclk2 | 0.90906363 |
| Dclk3 | 0.0256794 |
| Dclre1b | 1.01137608 |
| Dclre1b | -0.6318422 |
| Dclre1c | -0.6135424 |
| Dclre1c | -0.389557 |
| Dclre1c | -0.3340915 |
| Dcn | -0.5923644 |
| Dcn | 0.70573072 |
| Dcp1a | 1.33150354 |
| Dcp1a | 1.09013939 |
| Dcp1a | 1.00373006 |
| Dcp1a | -0.3690074 |
| Dcp1b | 0.40027551 |
| Dcp1b | 1.23270984 |
| Dcpp1 | -0.5915593 |
| Dcpp2 | -0.7956651 |
| Dcpp2 | -0.1272347 |
| Dcpp3 | -0.7124771 |
| Dcps | -0.6151914 |
| Dcps | -0.6143665 |
| Dct | 0.83398118 |
| Dctd | 2.74502645 |
| Dctn1 | 0.84697662 |
| Dctn2 | 0.19907539 |
| Dctn2 | 1.23951394 |
| Dctn2 | 0.4725727 |
| Dctn3 | -0.5988301 |
| Dctn3 | -0.6547988 |
| Dctn4 | 0.20702884 |
| Dctn4 | -0.1366151 |
| Dctn5 | 0.87751977 |
| Dctn6 | -0.1316664 |
| Dcun1d2 | 0.00028652 |
| Dcun1d3 | -0.6377404 |
| Dcun1d4 | -0.0910939 |
| Dcun1d4 | 1.82696977 |
| Dcun1d5 | 1.79632172 |
| Dcx | 0.57226161 |
| Dcx | 0.66873658 |
| Dcxr | 1.20475194 |
| Dcxr | 0.35165236 |
| Ddah1 | -0.6377404 |
| Ddah2 | -0.0284608 |
| Ddb1 | 0.20427005 |
| Ddb2 | -0.7807873 |
| Ddb2 | 0.16872601 |
| Ddb2 | -0.4694672 |
| Ddc | -0.5630256 |
| Ddc8 | 1.3940854 |
| Ddef2 | -0.5964003 |
| Ddef2 | 0.00413942 |
| Ddefl1 | 0.05608181 |
| Ddhd1 | 0.70552804 |
| Ddhd1 | -0.6916613 |
| Ddi2 | 0.03647365 |
| Ddit3 | -0.2560176 |
| Ddit3 | 0.58640151 |
| Ddit4 | 2.00719703 |
| Ddit4l | -0.4758499 |
| Ddo | 1.67509686 |
| Ddo | 0.36134635 |
| Ddo | 2.87364744 |
| Ddost | 3.18637866 |
| Ddr1 | -0.3451738 |
| Ddr2 | 1.25670693 |
| Ddt | 0.96346102 |
| Ddx1 | -0.2673521 |
| Ddx1 | 1.5981983 |
| Ddx17 | 0.69102483 |
| Ddx17 | -0.3255614 |
| Ddx17 | 0.35106163 |
| Ddx19a | -0.3470336 |
| Ddx19b | -0.5708652 |
| Ddx19b | -0.0719086 |
| Ddx20 | -0.170975 |
| Ddx21 | -0.4212484 |
| Ddx24 | -0.4901909 |
| Ddx24 | 2.9386293 |
| Ddx24 | 0.09411306 |
| Ddx25 | 0.50473384 |
| Ddx26b | -0.6226478 |
| Ddx27 | -0.3798684 |
| Ddx28 | 0.11977319 |
| Ddx3x | 0.05405826 |
| Ddx3y | -0.6634455 |
| Ddx4 | -0.5552507 |
| Ddx4 | 0.68171553 |
| Ddx41 | 0.58063214 |
| Ddx46 | 0.69554274 |
| Ddx47 | -0.4085759 |
| Ddx47 | -0.1566745 |
| Ddx47 | 1.30412013 |
| Ddx49 | 3.10539148 |
| Ddx5 | -0.6756859 |
| Ddx50 | -0.5827485 |
| Ddx50 | 3.19282673 |
| Ddx51 | 0.98147072 |
| Ddx52 | -0.4858656 |
| Ddx54 | 2.14820616 |
| Ddx58 | -0.658248 |
| Ddx6 | 0.71017872 |
| Ddx6 | -0.4851467 |
| Ddx6 | -0.4279873 |
| Ddx6 | -0.6428255 |
| Deadc1 | -0.7749015 |
| Deb1 | 0.13253185 |
| Decr1 | 1.19287318 |
| Decr2 | 1.23386655 |
| Decr2 | 0.02651414 |
| Dedd | 0.63897101 |
| Dedd | -0.6617098 |
| Dedd2 | -0.5173122 |
| Def6 | 0.72498767 |
| Def8 | 0.5292539 |
| Defb11 | 0.71380241 |
| Defb14 | -0.2921937 |
| Defb15 | -0.0152849 |
| Defb19 | 0.79885099 |
| Defb20 | 0.97311925 |
| Defb21 | -0.502555 |
| Defb25 | -0.4138909 |
| Defb26 | 0.88139498 |
| Defb30 | -0.3843773 |
| Defb36 | 0.40279576 |
| Defb36 | -0.5421792 |
| Defb4 | -0.7042769 |
| Defcr20 | 2.56586977 |
| Defcr6 | -0.618498 |
| Defcr-rs7 | 1.02835574 |
| Degs1 | 0.82331094 |
| Degs2 | -0.7115625 |
| Dek | 0.31584149 |
| Dek | 0.37670941 |
| Dennd1a | -0.6669265 |
| Dennd1c | -0.0807508 |
| Dennd2a | 0.87667521 |
| Dennd3 | -0.3213251 |
| Denr | -0.7574618 |
| Denr | 1.76792349 |
| Depdc1b | -0.1720048 |
| Depdc5 | 1.60531308 |
| Depdc5 | -0.6004535 |
| Depdc6 | -0.4547402 |
| Depdc6 | 0.81703029 |
| Dera | -0.5575765 |
| Derl2 | 1.1243907 |
| Derl3 | 1.51864408 |
| Des | -0.1782076 |
| Det1 | 1.95464096 |
| Dexi | -0.0010013 |
| Dexi | 2.57973938 |
| Dffa | 1.82936674 |
| Dfna5h | 0.30968675 |
| Dgat1 | 0.68627825 |
| Dgat2 | -0.5939767 |
| Dgcr2 | 0.05284203 |
| Dgcr2 | 2.04479715 |
| Dgcr6 | 0.25043245 |
| Dgcr6 | 2.41819751 |
| Dgcr8 | 2.58881872 |
| Dgka | -0.8512616 |
| Dgka | 0.01939515 |
| Dgkb | -0.546772 |
| Dgke | -0.7479109 |
| Dgkg | -0.4464272 |
| Dgkh | -0.7906796 |
| Dgki | 0.27605692 |
| Dgkk | 1.67992992 |
| Dgkq | 0.16549258 |
| Dgkz | 2.34929572 |
| Dgkz | 0.69779358 |
| Dgkz | 2.60910659 |
| Dguok | 3.2367803 |
| Dhcr24 | -0.3010161 |
| Dhcr24 | 0.4821314 |
| Dhcr24 | 2.4700817 |
| Dhcr7 | -0.1001191 |
| Dhdds | -0.4844283 |
| Dhdds | 1.01721564 |
| Dhdh | 2.98006552 |
| Dhdh | 0.68937656 |
| Dhdh | 2.4619388 |
| Dhh | 0.62626686 |
| Dhodh | -0.4989016 |
| Dhps | 0.68129963 |
| Dhrs1 | 0.47205324 |
| Dhrs4 | 0.31184549 |
| Dhrs7 | -0.6176703 |
| Dhrs7 | 1.66747236 |
| Dhrs7 | -0.2846141 |
| Dhrs7b | -0.2442616 |
| Dhrs7b | -0.6077935 |
| Dhrs7b | 0.44837541 |
| Dhrsx | -0.5939767 |
| Dhrsx | 0.14217304 |
| Dhtkd1 | 2.98670784 |
| Dhx15 | 0.3604692 |
| Dhx15 | 0.80050159 |
| Dhx16 | -0.492361 |
| Dhx29 | -0.4152243 |
| Dhx30 | 0.57761803 |
| Dhx32 | 0.41612419 |
| Dhx33 | -0.0535609 |
| Dhx36 | -0.1844515 |
| Dhx37 | -0.0654462 |
| Dhx38 | -0.4185663 |
| Dhx57 | -0.5677215 |
| Dhx58 | -0.4844283 |
| Dhx8 | -0.6020797 |
| Dhx9 | 1.29625582 |
| Diablo | -0.6028938 |
| Diap1 | -0.7431716 |
| Diap3 | 2.8050392 |
| Dicer1 | -0.1145481 |
| Dido1 | -0.2788248 |
| Dido1 | 1.55557378 |
| Dido1 | 0.83327359 |
| Dio1 | -0.4989016 |
| Dio1 | 1.88865692 |
| Dio3 | -0.4974442 |
| Dip2b | 0.23484964 |
| Dip2b | 0.73250263 |
| Diras1 | 1.35517014 |
| Diras2 | -0.2233552 |
| Dirc2 | -0.4808445 |
| Dis3 | 0.26122135 |
| Dis3l | 0.7883294 |
| Dis3l2 | 0.35988397 |
| Disp1 | 1.23027612 |
| Dixdc1 | 1.72337631 |
| Dkk3 | 0.07805826 |
| Dkk3 | -0.4416117 |
| Dkk3 | -0.1622667 |
| Dkkl1 | -0.6310026 |
| Dkkl1 | 0.69020106 |
| Dlat | 1.83719108 |
| Dlc1 | -0.2799798 |
| Dld | -0.6020797 |
| Dleu7 | -0.6916613 |
| Dlg1 | 0.84993687 |
| Dlg2 | 1.32323523 |
| Dlg3 | 2.49412615 |
| Dlg4 | -0.6077935 |
| Dlg7 | -0.6020797 |
| Dlgap1 | -0.4402403 |
| Dlgap2 | -0.5748096 |
| Dlgap2 | -0.7263052 |
| Dlgap2 | -0.1386019 |
| Dlgap3 | 0.2696354 |
| Dlgap4 | -0.6268159 |
| Dlgap4 | 0.14988866 |
| Dlgap4 | 0.67671264 |
| Dlgap4 | 0.60598218 |
| Dlk1 | -0.7124771 |
| Dlk2 | 0.07805826 |
| Dll1 | 0.25600582 |
| Dlst | 0.32073646 |
| Dlx1 | -0.6845276 |
| Dlx1as | 0.52262974 |
| Dlx2 | 0.08042493 |
| Dlx3 | 3.0693885 |
| Dmap1 | 1.5333886 |
| Dmbt1 | 0.47309188 |
| Dmkn | -0.492361 |
| Dmkn | 1.31074816 |
| Dmkn | -0.6479383 |
| Dmn | 1.60133231 |
| Dmn | -0.5867432 |
| Dmpk | 0.89690356 |
| Dmrt2 | 0.24118019 |
| Dmrt3 | -0.1849737 |
| Dmrta1 | 0.39097643 |
| Dmrta2 | 0.33256231 |
| Dmrtc1a | 1.46251318 |
| Dmrtc1a | 1.00844265 |
| Dmrtc1a | 0.61873906 |
| Dmrtc1b | -0.0802833 |
| Dmrtc2 | -0.8006771 |
| Dmtf1 | -0.6765663 |
| Dmtf1 | -0.7671106 |
| Dmtf1 | 2.16395038 |
| Dmwd | 0.28148096 |
| Dmwd | 2.15439647 |
| Dmwd | 0.22914913 |
| Dnahc11 | 0.11332838 |
| Dnahc2 | -0.5345741 |
| Dnahc5 | 1.34320545 |
| Dnahc8 | -0.0887332 |
| Dnaic1 | 0.26122135 |
| Dnaic1 | 1.22702133 |
| Dnaja1 | -0.6479383 |
| Dnaja2 | 0.22510434 |
| Dnaja2 | -0.7412826 |
| Dnaja3 | -0.6617098 |
| Dnajb1 | -0.501092 |
| Dnajb10 | -0.6301638 |
| Dnajb12 | 0.20357908 |
| Dnajb13 | 0.75103151 |
| Dnajb3 | 1.28054721 |
| Dnajb4 | -0.5143422 |
| Dnajb4 | 3.06738211 |
| Dnajb5 | 1.5799723 |
| Dnajb6 | -0.142089 |
| Dnajb6 | 0.27957016 |
| Dnajb7 | 1.2185998 |
| Dnajb8 | 4.22021646 |
| Dnajb9 | 0.73976259 |
| Dnajc10 | 1.27159447 |
| Dnajc10 | 0.83468825 |
| Dnajc12 | 0.57993744 |
| Dnajc14 | -0.3027905 |
| Dnajc15 | -0.6086127 |
| Dnajc16 | 0.15681535 |
| Dnajc17 | -0.6135424 |
| Dnajc18 | 1.04827857 |
| Dnajc19 | -0.5452386 |
| Dnajc19 | -0.4059296 |
| Dnajc2 | 1.64451982 |
| Dnajc2 | -0.2055691 |
| Dnajc3a | -0.6193265 |
| Dnajc4 | 0.22510434 |
| Dnajc5 | -0.3850232 |
| Dnajc5b | -0.149605 |
| Dnajc5g | -0.4039498 |
| Dnajc6 | 0.64417812 |
| Dnajc6 | -0.4340939 |
| Dnajc7 | -0.4085759 |
| Dnajc8 | 0.04877656 |
| Dnalc1 | 0.91977806 |
| Dnali1 | 1.80038188 |
| Dnase1l1 | -0.4046093 |
| Dnase1l2 | -0.5360902 |
| Dnase1l2 | -0.2904391 |
| Dnase2a | 3.57068289 |
| Dnd1 | 0.18895033 |
| Dner | -0.505488 |
| Dnm | 0.19245405 |
| Dnm1 | 0.55243575 |
| Dnm1l | -0.6952485 |
| Dnm1l | 0.70126159 |
| Dnmbp | -0.4252859 |
| Dnmt1 | -0.1111615 |
| Dnmt3b | 0.15463348 |
| Dnmt3l | -0.6748063 |
| Dnmt3l | -0.0377951 |
| Dnttip1 | -0.3863164 |
| Doc2a | -0.3402322 |
| Doc2b | 1.06668421 |
| Doc2b | -0.7088237 |
| Dock1 | -0.6643145 |
| Dock3 | -0.3087296 |
| Dock4 | -0.7690523 |
| Dock6 | 0.28084446 |
| Dock8 | 0.17515973 |
| Dock9 | -0.1886373 |
| Dok1 | -0.1761355 |
| Dok1 | -0.3359295 |
| Dok2 | -0.6774476 |
| Dok3 | 1.15278801 |
| Dok4 | -0.5875442 |
| Dok5 | 1.69619319 |
| Dok5 | -0.1082683 |
| Dok7 | 0.48315898 |
| Dolk | 0.17551587 |
| Dolpp1 | 0.15681535 |
| Dolpp1 | 0.99929046 |
| Dolpp1 | 2.00378658 |
| Dom3z | -0.3426997 |
| Dom3z | -0.6573845 |
| Donson | -0.7469612 |
| Donson | 2.63521596 |
| Dos | -0.0126717 |
| Dos | -0.4105655 |
| Dot1l | -0.1164888 |
| Dpagt1 | 0.87227126 |
| Dpep1 | 0.65386445 |
| Dpep2 | 0.62714854 |
| Dpep2 | 1.90397723 |
| Dpep3 | 1.95399599 |
| Dpf1 | 0.2435018 |
| Dpf1 | 1.15366963 |
| Dpf3 | 0.70451397 |
| Dph2 | -0.6573845 |
| Dph2 | -0.1410914 |
| Dph2 | -0.4638297 |
| Dph3 | 2.11685458 |
| Dph3 | -0.4484986 |
| Dph3 | -0.6045242 |
| Dph4 | -0.0742275 |
| Dpm1 | -0.6565218 |
| Dpm1 | 1.31436316 |
| Dpm2 | 0.06092065 |
| Dpm3 | -0.0840295 |
| Dpp10 | -0.7700246 |
| Dpp3 | -0.6110743 |
| Dpp3 | -0.5560253 |
| Dpp4 | -0.3526351 |
| Dpp6 | 3.45479676 |
| Dpp7 | 0.69431273 |
| Dpp8 | -0.7216732 |
| Dpp9 | -0.5247785 |
| Dpp9 | -0.4065905 |
| Dppa3 | 0.56358023 |
| Dppa4 | 0.85063205 |
| Dppa5 | 1.04531691 |
| Dppa5 | 2.2420388 |
| Dpt | 1.3298348 |
| Dpy30 | -0.5716528 |
| Dpyd | -0.7729477 |
| Dpysl2 | -0.3677378 |
| Dpysl2 | 1.04404477 |
| Dpysl2 | 1.29538996 |
| Dpysl3 | -0.277095 |
| Dpysl4 | 0.93910976 |
| Dpysl5 | 0.95307959 |
| Drbp1 | -0.3582703 |
| Drctnnb1a | -0.533817 |
| Drd1a | -0.6599773 |
| Drd2 | -0.1034661 |
| Drd4 | -0.6193265 |
| Drd5 | 0.72359676 |
| Drg1 | 1.7546598 |
| Drg1 | -0.6061575 |
| Drg2 | -0.4938106 |
| Drp2 | 3.75294415 |
| Dscam | -0.5947838 |
| Dscam | 1.43733929 |
| Dscc1 | 0.68813846 |
| Dscr1 | 0.51073704 |
| Dscr1l2 | -0.5955917 |
| Dscr2 | 3.32867071 |
| Dscr3 | -0.52703 |
| Dscr3 | 1.14849407 |
| Dscr6 | -0.001431 |
| Dsg2 | 0.16800843 |
| Dsg2 | -0.1531331 |
| Dsn1 | -0.5939767 |
| Dsp | -0.5677215 |
| Dst | -0.6061575 |
| Dst | 0.98071456 |
| Dst | 0.87718203 |
| Dstn | 0.49590991 |
| Dtd1 | -0.0373485 |
| Dtl | 0.08944239 |
| Dtna | -0.6135424 |
| Dtna | 1.08229526 |
| Dtna | -0.5406533 |
| Dtna | 0.91816249 |
| Dtnb | 2.19246075 |
| Dtnbp1 | -0.4909137 |
| Dtwd1 | 1.53103147 |
| Dtx1 | -0.6069752 |
| Dtx2 | 0.68855134 |
| Dtx2 | -0.5599079 |
| Dtx2 | -0.4006593 |
| Dtx4 | 0.51248079 |
| Dtx4 | -0.3785841 |
| Dtymk | 0.03192196 |
| Dus1l | 1.79492199 |
| Dus1l | -0.5475396 |
| Dus2l | -0.4680546 |
| Dus3l | -0.3563881 |
| Dus3l | 1.72481796 |
| Dus3l | 0.86989134 |
| Dus4l | 0.30783245 |
| Dusp1 | 2.58440005 |
| Dusp11 | 1.36868891 |
| Dusp11 | 0.30690392 |
| Dusp13 | 0.88893106 |
| Dusp13 | 0.31461376 |
| Dusp13 | 0.00285687 |
| Dusp14 | -0.4313749 |
| Dusp15 | -0.0925132 |
| Dusp15 | 3.13001681 |
| Dusp16 | 1.76575866 |
| Dusp16 | 0.96500071 |
| Dusp16 | 0.62846951 |
| Dusp18 | 0.16800843 |
| Dusp19 | 0.05162423 |
| Dusp2 | 2.4474771 |
| Dusp2 | 0.11028018 |
| Dusp21 | -0.2951253 |
| Dusp22 | 0.86185727 |
| Dusp23 | -0.2945383 |
| Dusp26 | 0.81810995 |
| Dusp28 | 1.39875366 |
| Dusp3 | 1.42948486 |
| Dusp3 | -0.2840335 |
| Dusp3 | -0.5188007 |
| Dusp4 | -0.73096 |
| Dusp4 | -0.3773016 |
| Dusp4 | -0.501092 |
| Dusp6 | -0.0342282 |
| Dusp6 | 0.45686263 |
| Dusp6 | -0.3902064 |
| Dusp7 | -0.2776712 |
| Dusp8 | 0.87091204 |
| Dut | 0.39295666 |
| Duxbl | 3.28905128 |
| Duxbl | -0.5452386 |
| Dvl1 | -0.3063495 |
| Dvl1 | -0.569292 |
| Dvl2 | -0.1622667 |
| DXBwg1396e | -0.6916613 |
| Dydc2 | -0.4422981 |
| Dym | -0.6445267 |
| Dync1h1 | 0.61159861 |
| Dync1i1 | -0.6651844 |
| Dync1i1 | -0.1020302 |
| Dync1i2 | 0.44088575 |
| Dync1li1 | -0.558353 |
| Dync1li2 | 2.38281073 |
| Dync2li1 | -0.1082683 |
| Dynll1 | 0.09022245 |
| Dynll2 | 0.35871243 |
| Dynlrb1 | -0.5537035 |
| Dynlrb2 | -0.1015521 |
| Dynlt3 | 1.4097488 |
| Dyrk1a | -0.4219201 |
| Dyrk1b | -0.0920399 |
| Dyrk1b | 0.06813256 |
| Dyrk2 | 4.28460019 |
| Dyrk2 | 0.01939515 |
| Dyrk3 | -0.7272343 |
| Dyrk4 | 0.21183736 |
| Dysf | 0.59851678 |
| Dysfip1 | -0.6293257 |
| Dzip1 | 2.11372924 |
| E030010A14Rik | -0.5827485 |
| E030034P13Rik | -0.3954184 |
| E130009J12Rik | -0.4340939 |
| E130012A19Rik | -0.3954184 |
| E130014J05Rik | 2.51238846 |
| E130016E03Rik | 0.42817821 |
| E130203B14Rik | -0.6028938 |
| E130203B14Rik | 0.46291587 |
| E130303B06Rik | -0.8313231 |
| E130306D19Rik | 0.6613299 |
| E130308A19Rik | -0.5429431 |
| E130309D02Rik | -0.5277817 |
| E130309D02Rik | -0.3457933 |
| E130309D14Rik | 1.08676631 |
| E130309F12Rik | -0.6127189 |
| E130309F12Rik | 1.85216802 |
| E130309F12Rik | -0.5779772 |
| E230008N13Rik | 2.76198811 |
| E2f1 | 1.09081255 |
| E2f2 | 0.78479589 |
| E2f2 | -0.5988301 |
| E2f3 | -0.5795649 |
| E2f4 | -0.183408 |
| E2f5 | 3.15215024 |
| E2f6 | -0.4730078 |
| E2f6 | 0.85080576 |
| E2f6 | -0.7207495 |
| E330016A19Rik | -0.0183428 |
| E330017A01Rik | 1.34073223 |
| E330018D03Rik | -0.5708652 |
| E330021D16Rik | 0.38899203 |
| E330034G19Rik | -0.6326825 |
| E330034G19Rik | -0.5748096 |
| E330036I19Rik | 1.87407857 |
| E430002D04Rik | -0.6721723 |
| E430002G05Rik | -0.6226478 |
| E430018J23Rik | -0.569292 |
| E430025E21Rik | 1.00018006 |
| E430028B21Rik | -0.7422266 |
| E4f1 | 1.05654987 |
| Eaf1 | -0.2034359 |
| Ear11 | 0.48955605 |
| Ear2 | -0.177171 |
| Ear4 | -0.2348513 |
| Ears2 | 2.48771521 |
| Ebag9 | 0.4191522 |
| Ebf2 | 0.11446891 |
| Ebf3 | -0.3075388 |
| Ebf4 | 1.62389963 |
| Ebi2 | 1.03580499 |
| Ebi3 | -0.6505051 |
| Ebna1bp2 | 2.37349393 |
| Ebna1bp2 | 0.83291959 |
| Ebp | -0.2921937 |
| Ebpl | 0.03109201 |
| Ebpl | 0.9482372 |
| Ecd | 0.38814029 |
| Ecd | 0.12128322 |
| Ece1 | 1.0559914 |
| Ece1 | 1.87869139 |
| Ece2 | -0.5568006 |
| Ecel1 | 1.72131296 |
| Ech1 | -0.7574618 |
| Echdc1 | 1.00991051 |
| Echdc2 | 1.37069008 |
| Echdc2 | -0.7768594 |
| Echdc2 | 0.19594478 |
| Echdc3 | 0.26673135 |
| Echs1 | 1.47395675 |
| Ecm1 | 0.32378293 |
| Ecm1 | -0.4059296 |
| Ecsit | 0.26931317 |
| Eda2r | 1.40571289 |
| Edaradd | 0.5243516 |
| Edc4 | 0.26252073 |
| Edem1 | 2.07855306 |
| Edem2 | -0.5143422 |
| Edem2 | 1.05151163 |
| Edem2 | 1.17630641 |
| Edem2 | -0.5568006 |
| Edf1 | -0.6677988 |
| Edg2 | -0.2639374 |
| Edg5 | 1.31245107 |
| Edg7 | 1.5204359 |
| Edg8 | 0.35548314 |
| Edg8 | -0.149102 |
| Edil3 | 1.37776005 |
| Edn1 | 0.32590961 |
| Edn2 | -0.2282649 |
| Ednra | -0.5591301 |
| Ednrb | 2.14588521 |
| Ednrb | 1.14456186 |
| Ednrb | -0.7847318 |
| Eed | 0.78124906 |
| Eef1a1 | -0.7412826 |
| Eef1a1 | 1.34598007 |
| Eef1a2 | 0.71179097 |
| Eef1a2 | -0.4894686 |
| Eef1a2 | 1.25274969 |
| Eef1b2 | -0.5763921 |
| Eef1b2 | 0.80580057 |
| Eef1b2 | 0.93863523 |
| Eef1d | 2.79281895 |
| Eef1d | 1.04898236 |
| Eef1d | 0.49464257 |
| Eef1d | 1.37298618 |
| Eef1e1 | 1.34392558 |
| Eef1g | -0.770998 |
| Eef2 | -0.0458702 |
| Eef2 | 0.52115135 |
| Eef2 | 0.27445565 |
| Eef2k | -0.0048754 |
| Eefsec | 0.10453805 |
| Efcab1 | -0.1891618 |
| Efcab2 | -0.4610236 |
| Efcab2 | 2.63644013 |
| Efcab3 | -0.7170639 |
| Efcab3 | 0.55814304 |
| Efcab3 | 0.22409042 |
| Efcab4a | 0.78851501 |
| Efcab4a | -0.638586 |
| Efcab5 | 0.11408889 |
| Efemp2 | 0.90612021 |
| Efha1 | 1.87863314 |
| Efha1 | -0.6730495 |
| Efhc2 | 0.83944708 |
| Efhd1 | 0.88424904 |
| Efna1 | 1.32418087 |
| Efna3 | -0.8449195 |
| Efna4 | -0.7545863 |
| Efna4 | -0.0391361 |
| Efna5 | 1.2634523 |
| Efnb1 | 0.61562178 |
| Efnb2 | -0.0458702 |
| Efnb3 | -0.3915067 |
| Efr3a | -0.770998 |
| Efs | -0.1169746 |
| Eftud1 | 0.70532531 |
| Eftud2 | 0.75584748 |
| Eftud2 | -0.6783297 |
| Eftud2 | -0.2851949 |
| EG194588 | 0.22611717 |
| EG208426 | -0.6889798 |
| EG210155 | 0.27861331 |
| EG212225 | 1.82339371 |
| EG214321 | 0.35401161 |
| EG232599 | 0.76559626 |
| EG237009 | -0.3633078 |
| EG237009 | -0.5552507 |
| EG237300 | 0.29412138 |
| EG238829 | 1.44049991 |
| EG240916 | 2.07236281 |
| EG245297 | -0.4887469 |
| EG245297 | 2.81359478 |
| EG245376 | -0.7375158 |
| EG245376 | -0.7051845 |
| EG272350 | -0.4306964 |
| EG277333 | 1.00136488 |
| EG317677 | -0.1189203 |
| EG328264 | -0.6730495 |
| EG328280 | -0.0779495 |
| EG328644 | -0.6765663 |
| EG328839 | 1.02950563 |
| EG330305 | -0.6907666 |
| EG330503 | -0.5360902 |
| EG330513 | -0.4865851 |
| EG333669 | -0.0490294 |
| EG368203 | -0.3069439 |
| EG380907 | -0.3645714 |
| EG381438 | 1.67314217 |
| EG382109 | 1.71197147 |
| EG382109 | -0.6436757 |
| EG382109 | -0.5915593 |
| EG384639 | -0.142089 |
| EG384813 | 3.489054 |
| EG384813 | 1.91266912 |
| EG384814 | -0.8587152 |
| EG385328 | 0.55695688 |
| EG408196 | 0.04714544 |
| EG432825 | 0.62846951 |
| EG432879 | 1.24455634 |
| EG432939 | 2.06560644 |
| EG433016 | 1.88952103 |
| EG433182 | -0.2696355 |
| EG433224 | 2.17523745 |
| EG433923 | 2.23524521 |
| EG434171 | 0.93387675 |
| EG434197 | 0.96161006 |
| EG434280 | -0.5032874 |
| EG434459 | 2.45036825 |
| EG434459 | -0.3488971 |
| EG434858 | 0.5243516 |
| EG435391 | -0.4252859 |
| EG436332 | -0.5099047 |
| EG545047 | 1.99012326 |
| EG545056 | 0.11180552 |
| EG545253 | 1.6063251 |
| EG545370 | 1.21671806 |
| EG545758 | 1.82813829 |
| EG545861 | -0.7347006 |
| EG545963 | 0.00200087 |
| EG546166 | 2.62151167 |
| EG546282 | 1.21294326 |
| EG546325 | 1.47359844 |
| EG546347 | -0.4464272 |
| EG547109 | -0.7906796 |
| EG574403 | 1.63889981 |
| EG622320 | -0.2071722 |
| EG622339 | -0.6053405 |
| EG624866 | 1.52188393 |
| EG625054 | -0.5614655 |
| EG625424 | 0.1658524 |
| EG630499 | 0.54717347 |
| EG633640 | 0.79590942 |
| EG638695 | 2.04689404 |
| EG654453 | -0.5537035 |
| EG665186 | 0.42107406 |
| EG665378 | -0.3551355 |
| EG667977 | 2.91232657 |
| EG668668 | -0.1326541 |
| Egf | 1.09242619 |
| Egfbp2 | -0.7488617 |
| Egfl7 | 1.83536308 |
| Egfl7 | 0.06453352 |
| Egflam | 1.46024402 |
| Egflam | 1.51804605 |
| Egfr | -0.6394323 |
| Egfr | -0.3470336 |
| Egfr | 0.50623823 |
| Egfr | -0.5376088 |
| Egln1 | 0.09798765 |
| Egln1 | -0.5899512 |
| Egln1 | -0.8694668 |
| Egln2 | 0.17159088 |
| Egln3 | 1.14583211 |
| Egr1 | 0.01560435 |
| Egr2 | -0.1808042 |
| Egr3 | 2.15513484 |
| Egr4 | -0.7244496 |
| Ehbp1 | 1.45806046 |
| Ehbp1l1 | -0.5330604 |
| Ehd1 | -0.6037087 |
| Ehd2 | 0.52582497 |
| Ehd3 | 0.34036443 |
| Ehd4 | 1.19672921 |
| Ehmt1 | -0.6453784 |
| Ehmt2 | -0.4464272 |
| Ehmt2 | -0.5591301 |
| Ei24 | -0.3633078 |
| Eid1 | 0.9085737 |
| Eid2 | 2.77728335 |
| Eid3 | -0.7006551 |
| Eif1a | -0.2582736 |
| Eif1b | 0.38301358 |
| Eif2a | 1.89549041 |
| Eif2ak1 | -0.6756859 |
| Eif2ak2 | -0.5638066 |
| Eif2b1 | 1.56570176 |
| Eif2b1 | 2.33592076 |
| Eif2b2 | 0.81378383 |
| Eif2b4 | 1.5613862 |
| Eif2b5 | -0.4737175 |
| Eif2b5 | 1.01517591 |
| Eif2b5 | -0.3824423 |
| Eif2c2 | 0.42953827 |
| Eif2c3 | -0.6774476 |
| Eif2c3 | -0.5018232 |
| Eif2c3 | -0.5136011 |
| Eif2c3 | 0.96438514 |
| Eif2c4 | 1.20582443 |
| Eif2s2 | 0.25207511 |
| Eif2s2 | -0.7450645 |
| Eif2s3x | 0.49692256 |
| Eif2s3y | -0.6765663 |
| Eif3b | 0.0548682 |
| Eif3b | -0.4744278 |
| Eif3d | 0.36630151 |
| Eif3eip | -0.0183428 |
| Eif3eip | 0.24680848 |
| Eif3f | -0.5787707 |
| Eif3g | 0.80543608 |
| Eif3i | 0.16333076 |
| Eif3i | 1.25229637 |
| Eif3i | 1.95162729 |
| Eif3k | -0.0351187 |
| Eif3s1 | -0.4858656 |
| Eif3s10 | -0.3316464 |
| Eif3s10 | -0.438871 |
| Eif3s4 | 0.94682667 |
| Eif3s8 | 2.85857882 |
| Eif4a1 | 0.08160603 |
| Eif4a1 | 0.68462164 |
| Eif4a2 | -0.5076937 |
| Eif4a3 | -0.3433176 |
| Eif4b | 1.69901762 |
| Eif4e | -0.2921937 |
| Eif4e1b | -0.5779772 |
| Eif4e1b | 0.45554154 |
| Eif4e2 | -0.8087522 |
| Eif4e2 | -0.653079 |
| Eif4e3 | 0.06212648 |
| Eif4e3 | 0.49641637 |
| Eif4e3 | 0.86271523 |
| Eif4ebp1 | 0.00584677 |
| Eif4ebp2 | 2.47191271 |
| Eif4el3 | 1.37098989 |
| Eif4enif1 | 0.00285687 |
| Eif4enif1 | -0.2077072 |
| Eif4g1 | 0.21423243 |
| Eif4g2 | -0.1238022 |
| Eif4g2 | 0.04592024 |
| Eif4h | -0.5716528 |
| Eif5 | -0.2662125 |
| Eif5 | -0.4582258 |
| Eif5 | 0.79110967 |
| Eif5a | -0.4505746 |
| Eif5a | -0.7460124 |
| Eif6 | 0.19664137 |
| Ela1 | 3.18171028 |
| Ela2 | -0.2492819 |
| Ela2a | 1.39135151 |
| Ela3b | -0.6209857 |
| Elac2 | 3.24816524 |
| Elac2 | 2.79204713 |
| Elac2 | 0.82152074 |
| Elavl1 | -0.4730078 |
| Elavl2 | -0.4000026 |
| Elavl2 | -0.7837441 |
| Elavl3 | 2.01996495 |
| Elavl4 | 3.77307924 |
| Elavl4 | -0.1954791 |
| Elf1 | 1.88409283 |
| Elf2 | 0.01939515 |
| Elf2 | 0.88290703 |
| Elf2 | -0.8261456 |
| Elf2 | -0.0719086 |
| Elf2 | 2.67599423 |
| Elf3 | -0.4152243 |
| Elf3 | 1.1281493 |
| Elf3 | 1.27492024 |
| Elf5 | -0.8491428 |
| Elfn1 | -0.3513875 |
| Elfn2 | -0.4533496 |
| Elk3 | 1.87044248 |
| Elk3 | 1.80044518 |
| Elk3 | 0.50973916 |
| Ell | 1.18790944 |
| Ell3 | 0.15971666 |
| Elmo1 | 0.85634774 |
| Elmo1 | -0.423265 |
| Elmo1 | 0.78516847 |
| Elmo1 | -0.2437055 |
| Elmo1 | -0.2359535 |
| Elmo1 | 1.20236425 |
| Elmo1 | 2.48838569 |
| Elmo1 | -0.0152849 |
| Elmo1 | -0.438187 |
| Elmo1 | 0.60935606 |
| Elmo1 | -0.7536298 |
| Elmo1 | -0.501092 |
| Elmo1 | -0.2656432 |
| Elmo2 | -0.3908563 |
| Elmo2 | -0.3231383 |
| Elmo3 | 1.07698374 |
| Elmo3 | -0.638586 |
| Elmod1 | -0.8533852 |
| Eln | -0.6419761 |
| Elof1 | -0.4687606 |
| Elovl1 | -0.8261456 |
| Elovl1 | 0.32621303 |
| Elovl1 | -0.2565811 |
| Elovl1 | -0.1839296 |
| Elovl2 | -0.6487931 |
| Elovl4 | 0.14438397 |
| Elovl5 | 1.06530238 |
| Elovl6 | 0.6831697 |
| Elp2 | 0.87328939 |
| Elp3 | -0.6462309 |
| Elp3 | 1.37517721 |
| Eltd1 | 1.16306404 |
| Emb | -0.6004535 |
| Emcn | 1.55030347 |
| Emd | -0.3785841 |
| Emd | -0.4205771 |
| Emg1 | 2.20847231 |
| Emg1 | -0.0070345 |
| Emid1 | -0.6335236 |
| Emid2 | -0.0391361 |
| Emilin1 | 1.18547856 |
| Emilin2 | -0.056746 |
| Eml1 | -0.5345741 |
| Eml3 | -0.3607856 |
| Eml3 | -0.1306798 |
| Eml4 | -0.4808445 |
| Eml5 | 1.34196965 |
| Emp1 | -0.5136011 |
| Emp2 | -0.5661536 |
| Emp3 | -0.7356381 |
| Emr1 | -0.3328682 |
| Emr1 | 2.18978639 |
| Emr1 | 1.2361757 |
| En2 | 3.67279175 |
| Enah | 0.79443518 |
| Enc1 | 0.57179438 |
| Endod1 | 1.4115689 |
| Endogl1 | -0.5240292 |
| Endogl1 | 0.46344041 |
| Eng | 4.21155266 |
| Eno1 | -0.2206387 |
| Eno2 | -0.5292869 |
| Eno3 | 1.74463823 |
| Enoph1 | 0.49286542 |
| Enox1 | 0.21389065 |
| Enpp1 | 0.05810102 |
| Enpp1 | 1.97825179 |
| Enpp2 | 2.30954492 |
| Enpp4 | -0.1135792 |
| Enpp5 | -0.3785841 |
| Enpp6 | 0.32530248 |
| Ensa | 2.59338023 |
| Ensa | -0.1891618 |
| ENSMUSG00000043795 | -0.5490768 |
| ENSMUSG00000053178 | -0.804706 |
| ENSMUSG00000068790 | -0.4967164 |
| Entpd2 | 1.08879161 |
| Entpd3 | 2.01468703 |
| Entpd4 | 0.75334625 |
| Entpd5 | -0.122335 |
| Entpd5 | 1.27370215 |
| Entpd5 | 0.24317049 |
| Entpd6 | 1.06003284 |
| Entpd7 | 1.90957409 |
| Ep300 | -0.2828735 |
| Ep400 | -0.3231383 |
| Epas1 | -0.9058447 |
| Epb4.1 | 0.95898162 |
| Epb4.1l1 | 0.82866119 |
| Epb4.1l1 | 0.72677296 |
| Epb4.1l1 | 1.85384408 |
| Epb4.1l2 | 0.73289649 |
| Epb4.1l3 | 0.44007974 |
| Epb4.1l3 | 0.21080903 |
| Epb4.1l3 | 0.93132902 |
| Epb4.1l4a | -0.6730495 |
| Epb4.1l5 | -0.2409296 |
| Epb4.1l5 | 0.10989846 |
| Epb4.9 | -0.6151914 |
| Epb4.9 | 0.66345205 |
| Epc1 | 1.07575373 |
| Epc1 | 0.53825635 |
| Epc1 | 1.51118424 |
| Epdr1 | -0.7956651 |
| Epgn | 1.01881518 |
| Epha1 | 1.34217573 |
| Epha1 | -0.6284884 |
| Epha3 | 0.57645618 |
| Epha3 | -0.4631274 |
| Epha3 | 1.24786503 |
| Epha4 | -0.6774476 |
| Epha5 | 0.48418544 |
| Epha6 | 0.11484878 |
| Epha7 | -0.1571815 |
| Epha7 | -0.3747417 |
| Epha7 | 2.81093897 |
| Ephb1 | -0.2782478 |
| Ephb2 | 1.94225574 |
| Ephb4 | 0.86082669 |
| Ephb6 | 0.86528441 |
| Ephx1 | -0.3402322 |
| Ephx2 | -0.4687606 |
| Epm2a | 0.13773554 |
| Epm2aip1 | 1.43789782 |
| Epm2aip1 | 0.72954336 |
| Epm2aip1 | -0.423265 |
| Epn1 | -0.5421792 |
| Epn2 | 0.79738136 |
| Epn2 | -0.6259808 |
| Epn2 | 0.99393514 |
| Epn2 | -0.5292869 |
| Epn2 | 0.8155888 |
| Epor | 0.75795872 |
| Eprs | -0.4751386 |
| Eprs | -0.6739275 |
| Eps15 | 0.29568956 |
| Eps15l1 | -0.6020797 |
| Eps8 | 1.65676692 |
| Eps8l1 | -0.7106487 |
| Eps8l2 | -0.1184335 |
| Eps8l2 | -0.5136011 |
| Eps8l3 | 1.85897339 |
| Epsti1 | -0.5614655 |
| Epsti1 | 1.63603947 |
| Eraf | 0.59053365 |
| Eral1 | 0.16152545 |
| Ercc1 | 1.67690315 |
| Ercc2 | 0.08592411 |
| Ercc3 | -0.1366151 |
| Ercc3 | 1.75671768 |
| Ercc4 | -0.7584222 |
| Ercc4 | 3.7097292 |
| Ercc5 | -0.0436202 |
| Ercc5 | -0.4858656 |
| Ercc8 | -0.4464272 |
| Ercc8 | 0.52508857 |
| Erdr1 | -0.6284884 |
| Erf | -0.6880877 |
| Erf | 0.07290997 |
| Ergic1 | 1.24649733 |
| Ergic2 | 0.89093053 |
| Ergic2 | 0.28052605 |
| Ergic3 | 0.15390506 |
| Ergic3 | 2.0714616 |
| Erh | -0.3464132 |
| Erich1 | -0.7778398 |
| Erlin1 | 0.76616659 |
| Erlin2 | -0.0788823 |
| Erlin2 | 2.10922684 |
| Ermap | -0.5763921 |
| Ermp1 | -0.6020797 |
| Ern1 | 1.2907223 |
| Ern2 | 1.72000382 |
| Ero1lb | -0.6193265 |
| Erp29 | -0.3153062 |
| Errfi1 | 1.98685793 |
| Es22 | -0.5763921 |
| Esam1 | 1.60974234 |
| Esco2 | 0.59099166 |
| Esd | 0.08748941 |
| Esd | 1.75711546 |
| Esd | 1.98133819 |
| Esd | -0.7070024 |
| Esm1 | -0.5165688 |
| Espl1 | -0.2304553 |
| Espn | 1.06350296 |
| Espn | 1.78718599 |
| Espn | 0.03026132 |
| Espn | -0.5907549 |
| Esr1 | 0.10376978 |
| Esr2 | 0.71961137 |
| Esr2 | -0.3792261 |
| Esr2 | -0.3683724 |
| Esrra | -0.7207495 |
| Esrrb | 0.45262859 |
| Etaa1 | 0.93736865 |
| Etf1 | 2.46815374 |
| Etfa | -0.3876113 |
| Etfa | 1.53254745 |
| Etfa | -0.3557616 |
| Etfb | 0.58938765 |
| Etfb | 1.47036747 |
| Etfdh | 0.40530926 |
| Ethe1 | 0.30442337 |
| Etnk2 | -0.0337833 |
| Ets1 | 1.41443558 |
| Ets2 | -0.3488971 |
| Etsrp71 | -0.324955 |
| Etv1 | -0.6201557 |
| Etv1 | 2.95238472 |
| Etv4 | -0.3267752 |
| Etv5 | -0.5568006 |
| Etv6 | -0.7690523 |
| Evc | -0.4340939 |
| Evc2 | -0.1306798 |
| Evi1 | 1.00210464 |
| Evi1 | 0.30037847 |
| Evi2a | -0.5376088 |
| Evi2a | -0.5003613 |
| Evi5 | 0.53558893 |
| Evi5l | -0.7469612 |
| Evl | -0.4429851 |
| Evx1 | -0.2927793 |
| Exdl1 | -0.6143665 |
| Exdl2 | -0.7244496 |
| Exdl2 | 1.30111145 |
| Exdl2 | 1.73260578 |
| Exoc2 | 0.0601159 |
| Exoc3 | -0.2881048 |
| Exoc3l | -0.2822941 |
| Exoc4 | 1.39778301 |
| Exoc7 | -0.5003613 |
| Exoc8 | -0.6617098 |
| Exod1 | -0.3396163 |
| Exosc1 | -0.0152849 |
| Exosc10 | 1.20725251 |
| Exosc2 | -0.1587042 |
| Exosc2 | -0.6625772 |
| Exosc4 | -0.2299072 |
| Exosc5 | -0.328599 |
| Exosc6 | 1.72762627 |
| Exosc7 | -0.3004253 |
| Exosc8 | -0.2498414 |
| Ext1 | -0.7347006 |
| Ext2 | 0.02317074 |
| Extl1 | 1.89766212 |
| Extl2 | 0.34602532 |
| Extl3 | 1.44872338 |
| Extl3 | 2.75451028 |
| Eya1 | -0.376661 |
| Eya1 | 0.0160263 |
| Eya2 | -0.3837319 |
| Eya3 | -0.3420823 |
| Eya3 | 1.47368803 |
| Eya3 | -0.6979479 |
| Eya4 | -0.4887469 |
| Ezh1 | 2.81376725 |
| Ezh2 | 1.16766429 |
| Ezh2 | -0.6343655 |
| F10 | -0.4575277 |
| F11 | -0.6176703 |
| F11 | -0.5498463 |
| F11r | -0.7798038 |
| F13a1 | -0.6012662 |
| F2r | 0.84837084 |
| F2rl1 | -0.0253699 |
| F2rl3 | -0.5907549 |
| F430201B04Rik | 0.42217051 |
| F5 | 1.5562305 |
| F630043A04Rik | -0.7115625 |
| F730014I05Rik | 0.06693441 |
| F730047E07Rik | 0.52656079 |
| F730047E07Rik | 0.02023548 |
| F730047E07Rik | -0.2459321 |
| F8 | -0.2271717 |
| F8 | 1.74080187 |
| F8 | -0.6168432 |
| F8a | 1.57467636 |
| Fa2h | -0.1965362 |
| Faah | -0.1029873 |
| Faah | 0.2162805 |
| Fabp3 | -0.7006551 |
| Fabp5 | 1.2816466 |
| Fabp9 | 2.02246788 |
| Fadd | 2.18785948 |
| Fadd | 1.84886704 |
| Fadd | 1.73647564 |
| Fads1 | 0.50623823 |
| Fads2 | 0.87616811 |
| Fads3 | -0.5955917 |
| Fads6 | 0.18965212 |
| Faf1 | 0.93228523 |
| Faf1 | -0.5345741 |
| Fah | -0.7886928 |
| Fahd1 | 0.75218959 |
| Fahd2a | 0.13588038 |
| Fahd2a | -0.4118942 |
| Fahd2a | 0.98162188 |
| Fahd2a | 0.89076407 |
| Faim | -0.3728264 |
| Faim | -0.5614655 |
| Faim2 | -0.1756181 |
| Faim2 | 1.67509686 |
| Faim3 | 2.47101317 |
| Fancc | 1.16518047 |
| Fancc | -0.822024 |
| Fancd2 | 0.53242675 |
| Fancg | -0.717984 |
| Fanci | 0.12166035 |
| Fancl | 0.4327944 |
| Fancl | -0.5795649 |
| Fank1 | 0.27477612 |
| Fank1 | -0.0422727 |
| Fap | -0.3626766 |
| Fars2 | -0.0174681 |
| Farsa | -0.6069752 |
| Farsb | -0.3779426 |
| Farsb | -0.3402322 |
| Fas | 0.07647717 |
| Fas | -0.1316664 |
| Fasl | -0.4000026 |
| Fastk | 0.78684323 |
| Fastk | -0.5819516 |
| Fastkd1 | 0.26866838 |
| Fastkd5 | 0.79590942 |
| Fat1 | 0.936259 |
| Fat4 | 0.01813325 |
| Fbf1 | -0.0793491 |
| Fblim1 | -0.7469612 |
| Fbln1 | 0.09139132 |
| Fbln1 | 0.26252073 |
| Fbln1 | -0.6284884 |
| Fbln2 | 0.27349357 |
| Fbln2 | -0.5560253 |
| Fbn1 | -0.6293257 |
| Fbp1 | 2.03443945 |
| Fbp2 | -0.0826229 |
| Fbxl10 | 0.01052607 |
| Fbxl12 | 0.69656651 |
| Fbxl12 | 0.81522808 |
| Fbxl12 | 0.73643389 |
| Fbxl12 | 1.67978601 |
| Fbxl13 | 0.20806131 |
| Fbxl13 | -0.1865421 |
| Fbxl14 | 0.09876066 |
| Fbxl15 | 0.8362772 |
| Fbxl18 | -0.1053841 |
| Fbxl18 | 1.06378002 |
| Fbxl21 | 0.80998209 |
| Fbxl22 | 0.43873485 |
| Fbxl4 | -0.7300272 |
| Fbxl5 | -0.5599079 |
| Fbxl6 | -0.4901909 |
| Fbxl6 | -0.5452386 |
| Fbxl8 | 0.0819994 |
| Fbxo15 | 0.3047338 |
| Fbxo16 | -0.6028938 |
| Fbxo18 | -0.2851949 |
| Fbxo18 | 0.14327916 |
| Fbxo2 | 0.66429958 |
| Fbxo21 | 0.62846951 |
| Fbxo25 | -0.45683 |
| Fbxo27 | -0.2002451 |
| Fbxo28 | 0.21080903 |
| Fbxo3 | -0.4981726 |
| Fbxo3 | 1.05780523 |
| Fbxo30 | 1.96532594 |
| Fbxo31 | 1.17937374 |
| Fbxo31 | 0.26122135 |
| Fbxo32 | 1.31849274 |
| Fbxo32 | -0.3715516 |
| Fbxo32 | -0.6243128 |
| Fbxo33 | 0.17123324 |
| Fbxo34 | -0.7460124 |
| Fbxo34 | -0.6411274 |
| Fbxo34 | 0.42681619 |
| Fbxo36 | 0.24482586 |
| Fbxo38 | 0.41446842 |
| Fbxo38 | -0.4478077 |
| Fbxo4 | -0.3153062 |
| Fbxo42 | -0.1001191 |
| Fbxo44 | 2.20970221 |
| Fbxo6 | -0.2799798 |
| Fbxo7 | -0.7328285 |
| Fbxo8 | -0.5843444 |
| Fbxo9 | 0.42736124 |
| Fbxo9 | 0.08944239 |
| Fbxw12 | 1.62573062 |
| Fbxw13 | -0.7996725 |
| Fbxw15 | 0.27220927 |
| Fbxw16 | 0.3858652 |
| Fbxw17 | 0.67859184 |
| Fbxw19 | -0.5803598 |
| Fbxw2 | 2.74053976 |
| Fbxw4 | 1.12165955 |
| Fbxw4 | -0.2425941 |
| Fbxw5 | -0.3817982 |
| Fbxw7 | 0.13029286 |
| Fbxw7 | -0.1331483 |
| Fbxw7 | 0.87464518 |
| Fbxw8 | -0.6268159 |
| Fcer1g | 0.77185089 |
| Fcer2a | -0.1965362 |
| Fcf1 | -0.5763921 |
| Fcgr2b | 2.83842641 |
| Fcgr2b | 0.07924234 |
| Fcgr2b | -0.5996414 |
| Fcgr3 | -0.8036971 |
| Fcgr4 | 2.53106028 |
| Fcgrt | -0.4409258 |
| Fcho1 | -0.7070024 |
| Fcho2 | -0.1049042 |
| Fchsd1 | -0.1912629 |
| Fchsd2 | 0.75565531 |
| Fcna | 0.5590909 |
| Fcrla | 2.10963704 |
| Fcrla | 0.28813836 |
| Fcrla | 2.9945852 |
| Fdps | 1.03509127 |
| Fdxr | -0.2582736 |
| Fdxr | 1.91216336 |
| Fem1b | 2.90070416 |
| Fem1c | -0.2332004 |
| Fem1c | 1.44355745 |
| Fen1 | -0.6704205 |
| Fermt2 | 1.64167655 |
| Fermt3 | 0.51073704 |
| Fert2 | 1.50001306 |
| Fert2 | -0.5232805 |
| Fes | 2.11631179 |
| Fetub | -0.4273112 |
| Fev | 0.34215576 |
| Fez1 | 0.36134635 |
| Fez1 | 1.29170117 |
| Fez2 | -0.5606864 |
| Fezf2 | 2.67796381 |
| Ffar2 | -0.45683 |
| Fgd2 | -0.6608431 |
| Fgd4 | 2.89354234 |
| Fgd4 | -0.6522203 |
| Fgd5 | -0.5661536 |
| Fgd6 | 0.63395366 |
| Fgf1 | -0.4701742 |
| Fgf1 | 3.1879731 |
| Fgf10 | -0.0944089 |
| Fgf10 | -0.6836397 |
| Fgf12 | 1.22562297 |
| Fgf12 | 0.69963118 |
| Fgf13 | 0.29034706 |
| Fgf13 | 1.64167655 |
| Fgf17 | 1.47054726 |
| Fgf21 | 2.21088958 |
| Fgf22 | 1.02561912 |
| Fgf22 | 0.71641086 |
| Fgf5 | 0.63504667 |
| Fgf7 | -0.6539385 |
| Fgf8 | 0.69492793 |
| Fgf9 | 0.81161329 |
| Fgfbp3 | 0.01813325 |
| Fgfr1 | -0.3960719 |
| Fgfr1op | -0.4652359 |
| Fgfr1op2 | -0.6634455 |
| Fgfr1op2 | 0.53437396 |
| Fgfr2 | 0.03606076 |
| Fgfr2 | -0.4293409 |
| Fgfr3 | -0.5300404 |
| Fgfrl1 | -0.5452386 |
| Fgl2 | 0.98914912 |
| Fh1 | 0.04223503 |
| Fhad1 | 2.21427967 |
| Fhit | 2.92100301 |
| Fhl1 | 2.21031655 |
| Fhl1 | 1.03637557 |
| Fhl2 | 1.28252519 |
| Fhl4 | -0.8565796 |
| Fhl5 | 1.58213026 |
| Fhod1 | 2.69521352 |
| Fhod3 | -0.2759435 |
| Fibcd1 | 1.81183309 |
| Fibp | 0.33346587 |
| Figf | -0.9251487 |
| Figla | -0.5498463 |
| Fign | -0.6135424 |
| Fignl1 | -0.6836397 |
| Fip1l1 | -0.2348513 |
| Fis1 | 1.62305923 |
| Fis1 | -0.7318938 |
| Fiz1 | -0.289855 |
| Fjx1 | 1.02026695 |
| Fkbp10 | 2.17323789 |
| Fkbp11 | 0.2963161 |
| Fkbp14 | -0.5947838 |
| Fkbp1a | 1.23859423 |
| Fkbp1b | 0.50473384 |
| Fkbp2 | -0.3986905 |
| Fkbp2 | -0.1870654 |
| Fkbp4 | -0.308134 |
| Fkbp5 | 0.22780279 |
| Fkbp6 | 1.41491251 |
| Fkbp7 | 2.01951372 |
| Fkbp8 | 0.65771122 |
| Fkbp9 | -0.3093256 |
| Fkbpl | 0.35988397 |
| Fkbpl | -0.5323045 |
| Fkhl18 | 0.0294299 |
| Fkrp | 1.39564406 |
| Fktn | -0.0603994 |
| Flad1 | 0.00413942 |
| Flcn | 2.05981071 |
| Flcn | -0.4478077 |
| Fli1 | -0.2799798 |
| Flii | 1.49398288 |
| Flna | 2.73741202 |
| Flot1 | 0.34155903 |
| Flot1 | 0.05850434 |
| Flot2 | -0.4540447 |
| Flot2 | 1.91115101 |
| Flot2 | 0.92797462 |
| Flrt1 | 1.43566171 |
| Flrt2 | -0.3027905 |
| Flrt3 | -0.5803598 |
| Flt1 | 0.23518389 |
| Flt4 | 1.23247833 |
| Flt4 | -0.4952623 |
| Flvcr2 | 1.36577964 |
| Flywch2 | 0.41529667 |
| Fmn2 | -0.2337504 |
| Fmn2 | -0.2951253 |
| Fmnl1 | 0.06972773 |
| Fmnl3 | 1.47538864 |
| Fmnl3 | 0.71138816 |
| Fmo1 | -0.5677215 |
| Fmo2 | 1.15228386 |
| Fmo5 | -0.059942 |
| Fmod | -0.4981726 |
| Fmr1nb | 0.69102483 |
| Fmr1nb | -0.6871964 |
| Fn3k | 1.41491251 |
| Fn3k | -0.1174606 |
| Fnbp1 | 0.94021606 |
| Fnbp1 | 1.67509686 |
| Fnbp1 | 1.14672027 |
| Fnbp1l | 2.58203123 |
| Fnbp4 | -0.7290953 |
| Fndc3 | -0.0863784 |
| Fndc3a | 1.61807786 |
| Fndc3b | 0.31737391 |
| Fndc4 | 3.36509452 |
| Fndc5 | 1.87138218 |
| Fndc5 | -0.4708818 |
| Fndc7 | -0.4561329 |
| Fndc7 | -0.3696428 |
| Fndc8 | -0.2851949 |
| Fndc8 | -0.5165688 |
| Fnip1 | -0.6301638 |
| Fnip1 | -0.7574618 |
| Fnta | -0.4596236 |
| Fnta | -0.2146897 |
| Fntb | -0.2018392 |
| Folr1 | 0.75680772 |
| Folr1 | 0.66345205 |
| Folr2 | 1.90130844 |
| Fos | 1.32344545 |
| Fosb | -0.5490768 |
| Foxa1 | 2.22090198 |
| Foxa2 | -0.533817 |
| Foxa3 | -0.1116445 |
| Foxc1 | -0.6343655 |
| Foxd2 | 2.06718091 |
| Foxg1 | 0.41170232 |
| Foxh1 | 0.79517259 |
| Foxh1 | 0.61873906 |
| Foxj1 | 0.00669929 |
| Foxj2 | -0.8428149 |
| Foxj3 | 1.50357799 |
| Foxj3 | -0.6176703 |
| Foxj3 | 2.4759346 |
| Foxj3 | 0.70187227 |
| Foxj3 | 0.67000046 |
| Foxk1 | -0.4638297 |
| Foxk1 | 0.10261619 |
| Foxk1 | 1.33742446 |
| Foxl2 | -0.5915593 |
| Foxm1 | -0.324349 |
| Foxm1 | -0.3664699 |
| Foxn2 | 3.08950998 |
| Foxn3 | -0.5748096 |
| Foxo1 | 1.9051106 |
| Foxo6 | -0.6127189 |
| Foxo6 | -0.5939767 |
| Foxp1 | -0.4327334 |
| Foxp2 | 0.56027438 |
| Foxp2 | -0.6160169 |
| Foxp2 | 0.31369191 |
| Foxp3 | 0.42599803 |
| Foxp4 | 1.62892631 |
| Foxq1 | 1.49748335 |
| Foxred1 | -0.7412826 |
| Foxred1 | -0.0714456 |
| Foxred1 | -0.0508394 |
| Foxred1 | 0.88508683 |
| Foxred1 | -0.0436202 |
| Foxred2 | 0.54621352 |
| Fpgs | 0.52484298 |
| Fpgs | 0.43549926 |
| Fpgs | -0.3464132 |
| Fpr2 | -0.546772 |
| Frag1 | 0.27861331 |
| Frag1 | 1.53431301 |
| Frag1 | -0.6818663 |
| Frap1 | 3.40069201 |
| Fras1 | 2.62325546 |
| Fras1 | -0.423265 |
| Frat1 | 0.76654661 |
| Frat1 | -0.4484986 |
| Frat2 | 0.62890942 |
| Freq | 1.7050616 |
| Freq | 0.40223629 |
| Freq | -0.5669372 |
| Frg1 | -0.7916746 |
| Frk | -0.3201182 |
| Frmd3 | -0.6209857 |
| Frmd6 | -0.4730078 |
| Frmd8 | 2.15422265 |
| Frmpd4 | -0.6377404 |
| Frrs1 | -0.1891618 |
| Frs2 | 1.37477922 |
| Frs3 | 1.32302496 |
| Fryl | -0.2724975 |
| Fryl | -0.1587042 |
| Frzb | 0.44195936 |
| Fscn1 | 3.83244572 |
| Fscn2 | -0.3153062 |
| Fscn3 | -0.6293257 |
| Fsd1 | 0.4821314 |
| Fsd2 | 1.064057 |
| Fshb | 0.35076612 |
| Fshr | 3.26980002 |
| Fshr | -0.4554363 |
| Fshr | -0.3087296 |
| Fsip1 | 0.75006535 |
| Fst | -0.4794147 |
| Fstl1 | -0.7788213 |
| Fstl4 | 0.01222188 |
| Fstl5 | -0.5980194 |
| Fth1 | -0.7375158 |
| Fth1 | 0.9038245 |
| Ftmt | 0.84732537 |
| Fto | -0.2381618 |
| Ftsj1 | -0.3671037 |
| Ftsj3 | -0.7788213 |
| Fubp1 | -0.6479383 |
| Fubp3 | 0.57319535 |
| Fuk | 3.20643002 |
| Fuk | 1.49020529 |
| Fundc1 | -0.3390009 |
| Fundc1 | 2.08264175 |
| Fundc2 | 1.26959334 |
| Furin | -0.7079126 |
| Furin | -0.7198268 |
| Fus | 1.83755626 |
| Fusip1 | 2.48172062 |
| Fusip1 | -0.5899512 |
| Fusip1 | -0.4967164 |
| Fut10 | 1.43229755 |
| Fut10 | 0.53607448 |
| Fut2 | -0.3304263 |
| Fut4 | 0.33527037 |
| Fut8 | 0.21491562 |
| Fut8 | -0.1960075 |
| Fut8 | 0.31461376 |
| Fvt1 | -0.6871964 |
| Fxc1 | -0.2886878 |
| Fxn | -0.4327334 |
| Fxn | -0.4347748 |
| Fxyd1 | 2.39054993 |
| Fxyd2 | 3.69010512 |
| Fxyd2 | 0.15536133 |
| Fxyd2 | -0.7042769 |
| Fxyd3 | -0.6889798 |
| Fxyd4 | -0.4730078 |
| Fxyd4 | -0.6028938 |
| Fxyd5 | 1.90726024 |
| Fxyd5 | 0.09450124 |
| Fxyd5 | -0.1326541 |
| Fxyd6 | 0.62073758 |
| Fxyd7 | -0.5490768 |
| Fyb | -0.7015592 |
| Fyco1 | -0.6168432 |
| Fyn | 2.77849271 |
| Fyttd1 | -0.0346734 |
| Fzd1 | -0.1198946 |
| Fzd10 | -0.6756859 |
| Fzd10 | -0.4873052 |
| Fzd2 | -0.618498 |
| Fzd3 | 1.07012993 |
| Fzd4 | -0.653079 |
| Fzd5 | -0.6625772 |
| Fzd6 | -0.2753683 |
| Fzd7 | -0.5099047 |
| Fzd8 | 0.88976469 |
| Fzr1 | 1.58332699 |
| Fzr1 | 0.76958129 |
| Fzr1 | 0.48802472 |
| G0s2 | -0.8168973 |
| G3bp1 | -0.2206387 |
| G3bp1 | -0.8057159 |
| G3bp2 | -0.0018608 |
| G3bp2 | 0.01981541 |
| G3bp2 | 1.58061221 |
| G430022H21Rik | 0.99079706 |
| G430055L02Rik | -0.8209964 |
| G6pc | -0.0844988 |
| G6pc2 | 0.40781607 |
| G6pc2 | -0.6251465 |
| G6pc3 | 0.84295669 |
| G6pd2 | 0.64396173 |
| G6pd2 | -0.5300404 |
| G6pdx | -0.6505051 |
| Gaa | -0.3805112 |
| Gab1 | 1.86283173 |
| Gab2 | 1.19721011 |
| Gab2 | 1.70443091 |
| Gab2 | 1.50001306 |
| Gab3 | -0.6625772 |
| Gab3 | -0.4645325 |
| Gab3 | -0.5661536 |
| Gabarap | -0.0078996 |
| Gabarapl1 | 0.96500071 |
| Gabarapl2 | 0.63766471 |
| Gabbr1 | -0.4132249 |
| Gabpa | -0.5811554 |
| Gabpa | -0.6218164 |
| Gabpb1 | -0.1526283 |
| Gabpb1 | -0.0760867 |
| Gabpb2 | -0.4125593 |
| Gabra3 | 0.28307034 |
| Gabra4 | 2.19187639 |
| Gabrb1 | -0.1571815 |
| Gabrb2 | -0.6487931 |
| Gabrb3 | -0.6943504 |
| Gabrb3 | -0.2702072 |
| Gabrd | -0.6135424 |
| Gabrd | -0.0531068 |
| Gabrg1 | 1.89003911 |
| Gabrg2 | -0.1341375 |
| Gabrg2 | 0.0160263 |
| Gabrg2 | -0.5429431 |
| Gabrg2 | -0.438187 |
| Gabrp | -0.5398912 |
| Gabrp | -0.5899512 |
| Gad1 | -0.6209857 |
| Gad1 | -0.4758499 |
| Gadd45a | 1.62214157 |
| Gadd45a | 0.89524819 |
| Gadd45b | -0.2190125 |
| Gadd45g | 1.43817696 |
| Gadd45g | 1.09108168 |
| Gak | 0.35724594 |
| Gal | 0.61227034 |
| Gal3st1 | -0.2141507 |
| Gal3st2 | 0.13103978 |
| Gal3st3 | 0.87142198 |
| Galc | -0.3027905 |
| Galc | 0.20874899 |
| Gale | -0.5195458 |
| Gale | -0.7798038 |
| Galk1 | -0.4340939 |
| Galm | 2.67950667 |
| Galns | 1.1215293 |
| Galnt1 | 0.64569145 |
| Galnt1 | -0.0631488 |
| Galnt1 | 0.91751548 |
| Galnt1 | -0.2939516 |
| Galnt10 | 0.68441436 |
| Galnt10 | 0.50923983 |
| Galnt10 | 0.55529372 |
| Galnt11 | -0.3677378 |
| Galnt12 | -0.5143422 |
| Galnt13 | -0.6318422 |
| Galnt2 | 1.43182935 |
| Galnt2 | 1.53933929 |
| Galnt2 | -0.6110743 |
| Galnt3 | 1.13664777 |
| Galnt9 | -0.4603233 |
| Galntl2 | 0.63875342 |
| Galntl4 | -0.3876113 |
| Galntl5 | 2.98785514 |
| Galt | 1.04729239 |
| Galt | -0.2152289 |
| Gamt | 2.95685764 |
| Gan | -0.6513623 |
| Ganab | -0.4638297 |
| Ganab | 1.06474908 |
| Ganab | 1.0712299 |
| Ganc | 1.55868912 |
| Gap43 | -0.3292077 |
| Gapdh | 0.82670296 |
| Gapdhs | -0.2599692 |
| Gapvd1 | 2.48908614 |
| Gapvd1 | 0.72914809 |
| Garnl3 | -0.0508394 |
| Garnl4 | -0.4808445 |
| Gars | 1.93091831 |
| Gart | -0.658248 |
| Gart | -0.7807873 |
| Gas2l1 | 0.31982059 |
| Gas2l1 | 3.20394151 |
| Gas2l1 | 1.80291061 |
| Gas2l1 | 2.24709215 |
| Gas6 | -0.5661536 |
| Gas7 | 0.70268589 |
| Gas7 | -0.4822763 |
| Gas8 | -0.3292077 |
| Gata2 | -0.2200963 |
| Gata4 | -0.5677215 |
| Gata6 | -0.5875442 |
| Gatad1 | -0.6739275 |
| Gatad1 | -0.2348513 |
| Gatad2b | 1.45724032 |
| Gats | -0.538369 |
| Gats | 3.95618728 |
| Gba | -0.0640671 |
| Gba2 | 1.39359779 |
| Gbas | 1.93789031 |
| Gbf1 | -0.7300272 |
| Gbl | -0.4694672 |
| Gbl | -0.3396163 |
| Gbl | -0.1960075 |
| Gbp2 | -0.3159064 |
| Gbp3 | -0.8502016 |
| Gbp3 | -0.3563881 |
| Gbp6 | 1.65506928 |
| Gbp6 | 2.60154644 |
| Gbx2 | 2.04708886 |
| Gca | -0.2662125 |
| Gca | -0.4185663 |
| Gcap14 | 0.66069232 |
| Gcap14 | 0.91492299 |
| Gcc1 | -0.6880877 |
| Gcc2 | -0.6871964 |
| Gcdh | 1.53237913 |
| Gch1 | -0.7729477 |
| Gch1 | 2.85886654 |
| Gchfr | 0.97813903 |
| Gck | -0.7384561 |
| Gclm | 0.16441229 |
| Gcn5l2 | 1.34884883 |
| Gcnt1 | 3.76077586 |
| Gcnt1 | -0.6880877 |
| Gcnt2 | 2.15989857 |
| Gcnt2 | -0.2633695 |
| Gcs1 | -0.5406533 |
| Gda | 0.6363566 |
| Gdap1 | -0.8282132 |
| Gdap1 | 0.65129116 |
| Gdap1l1 | 1.70134142 |
| Gdap1l1 | -0.822024 |
| Gde1 | 1.72371976 |
| Gde1 | 1.152662 |
| Gdf10 | -0.6368955 |
| Gdf15 | 0.83662994 |
| Gdf3 | 0.47516573 |
| Gdf9 | 2.36904283 |
| Gdi1 | 0.61361235 |
| Gdi1 | 1.13408058 |
| Gdi1 | 1.24341271 |
| Gdi1 | 0.5752929 |
| Gdi1 | 4.10578868 |
| Gdi2 | -0.4498821 |
| Gdnf | -0.5195458 |
| Gdpd1 | 1.10907234 |
| Gdpd2 | 1.40310921 |
| Gdpd2 | 0.32256553 |
| Gdpd3 | 0.11826073 |
| Gdpd4 | -0.3798684 |
| Gdpd5 | -0.4092386 |
| Gemin4 | -0.4026322 |
| Gemin4 | 0.59099166 |
| Gemin4 | 0.133277 |
| Gemin5 | 1.17038895 |
| Gemin6 | -0.7337641 |
| Gemin6 | -0.5599079 |
| Gemin7 | 0.21832413 |
| Gemin7 | -0.7318938 |
| Gfap | -0.1203822 |
| Gfer | -0.6943504 |
| Gfi1 | -0.5032874 |
| Gfm1 | -0.1257621 |
| Gfod1 | -0.4909137 |
| Gfod2 | 0.79388175 |
| Gfpt2 | -0.1501082 |
| Gfra1 | -0.376661 |
| Gfra4 | -0.4464272 |
| Gga1 | 1.53808523 |
| Gga1 | 0.41446842 |
| Gga1 | -0.4730078 |
| Gga3 | -0.1511154 |
| Ggcx | -0.2974772 |
| Ggcx | 0.1373648 |
| Ggcx | -0.8651514 |
| Ggn | -0.5247785 |
| Ggn | 2.25750761 |
| Ggnbp1 | -0.6053405 |
| Ggnbp2 | 1.25444769 |
| Ggt5 | 1.01122963 |
| Ggt7 | -0.6487931 |
| Ggta1 | 0.26802315 |
| Ggtla1 | 0.04264521 |
| Gh | -0.7124771 |
| Ghitm | -0.7189049 |
| Ghr | 0.20288761 |
| Ghr | 0.75795872 |
| Ghrh | -0.5406533 |
| Ghrhr | 1.17495361 |
| Ghrl | -0.6061575 |
| Ghrl | -0.6625772 |
| Ghsr | 0.89209493 |
| Ghsr | -0.2509614 |
| Gif | -0.6259808 |
| Gigyf1 | 3.71166513 |
| Gimap1 | 0.58409801 |
| Gimap4 | -0.4801293 |
| Gimap5 | -0.7460124 |
| Gimap6 | 2.81409056 |
| Gimap8 | -0.4300184 |
| Gimap9 | -0.3159064 |
| Gins3 | 1.69009177 |
| Gins4 | -0.4772742 |
| Gipc1 | -0.1917889 |
| Gipc1 | -0.1287096 |
| Gipc1 | 0.94729708 |
| Gipc2 | -0.4158918 |
| Git1 | -0.2886878 |
| Git2 | -0.6686719 |
| Git2 | 0.19420103 |
| Git2 | 0.64525932 |
| Gja1 | 0.11826073 |
| Gja4 | 0.84453175 |
| Gja5 | -0.515826 |
| Gja5 | 3.13637211 |
| Gja6 | 0.39915324 |
| Gja7 | -0.7033701 |
| Gjb1 | 0.24515659 |
| Gjb2 | -0.6479383 |
| Gjb2 | -0.5811554 |
| Gjb3 | 1.50166678 |
| Gjb6 | 1.8376171 |
| Gjc2 | 1.21081326 |
| Gjc2 | 0.210466 |
| Gjd2 | 0.94980199 |
| Gjd2 | 0.36280646 |
| Gjd3 | -0.7198268 |
| Gk2 | -0.5575765 |
| Gk5 | -0.492361 |
| Gkap1 | 1.5627726 |
| Gkap1 | 1.68866715 |
| Glb1 | 1.45860682 |
| Glb1l2 | -0.7097358 |
| Glcci1 | -0.4246118 |
| Gldc | 0.67273306 |
| Gldc | -0.7253769 |
| Gldn | -0.1381048 |
| Gle1 | -0.253767 |
| Glg1 | 0.55576921 |
| Glipr2 | 1.75206445 |
| Glis1 | 1.84483013 |
| Glo1 | -0.6704205 |
| Gloxd1 | 2.17532244 |
| Glp2r | 0.08788033 |
| Glra2 | 2.19662429 |
| Glrb | 0.87176179 |
| Glrp1 | -0.8814371 |
| Glrx | -0.6505051 |
| Glrx2 | 1.03151455 |
| Glrx2 | -0.5835461 |
| Glrx2 | -0.6988494 |
| Glrx3 | 0.06733396 |
| Glrx5 | -0.7926706 |
| Gls2 | 1.63942577 |
| Gls2 | 1.64197624 |
| Glt25d1 | 0.37871987 |
| Glt25d2 | -0.5614655 |
| Glt25d2 | 0.49032078 |
| Glt6d1 | 1.10761924 |
| Glt8d1 | -0.2986553 |
| Glt8d1 | -0.502555 |
| Glt8d1 | 0.26284529 |
| Glt8d2 | 1.48366861 |
| Glt8d3 | -0.4865851 |
| Gltp | 1.02590756 |
| Gltscr1 | -0.6970472 |
| Glud1 | 3.49459018 |
| Glud1 | 0.04346504 |
| Glul | -0.5964003 |
| Glyctk | 0.55529372 |
| Glyctk | -0.5939767 |
| Glyctk | -0.7263052 |
| Gm1006 | -0.3470336 |
| Gm1006 | -0.6617098 |
| Gm1019 | -0.4730078 |
| Gm1040 | 0.72080876 |
| Gm1070 | 3.9715626 |
| Gm1082 | -0.2509614 |
| Gm114 | -0.349519 |
| Gm114 | 0.55766876 |
| Gm1141 | -0.6997518 |
| Gm128 | 1.9292638 |
| Gm129 | -0.2315523 |
| Gm129 | -0.1130952 |
| Gm1322 | -0.7593836 |
| Gm136 | 1.10417569 |
| Gm136 | 0.91281132 |
| Gm1381 | -0.7642055 |
| Gm1527 | -0.5128607 |
| Gm1568 | 0.86271523 |
| Gm1574 | 0.69143645 |
| Gm1574 | -0.4981726 |
| Gm1604 | -0.6168432 |
| Gm1604 | -0.822024 |
| Gm166 | -0.770998 |
| Gm166 | -0.6127189 |
| Gm1661 | 0.01306864 |
| Gm1673 | -0.4540447 |
| Gm1673 | -0.2271717 |
| Gm1698 | 3.04714658 |
| Gm1821 | -0.6226478 |
| Gm1943 | -0.7966653 |
| Gm1965 | -0.3671037 |
| Gm1967 | -0.4099018 |
| Gm1967 | 1.03323307 |
| Gm216 | -0.7337641 |
| Gm239 | 2.44448129 |
| Gm249 | 1.26434804 |
| Gm266 | 2.01765636 |
| Gm2a | 0.04141413 |
| Gm347 | -0.1531331 |
| Gm382 | -0.3928088 |
| Gm410 | 0.28624105 |
| Gm428 | -0.1787263 |
| Gm443 | 0.19315323 |
| Gm484 | -0.5748096 |
| Gm50 | -0.5475396 |
| Gm525 | -0.6201557 |
| Gm525 | 0.22982157 |
| Gm527 | -0.4006593 |
| Gm527 | 0.71380241 |
| Gm561 | -0.5867432 |
| Gm561 | -0.81997 |
| Gm597 | -0.5398912 |
| Gm603 | 2.16579806 |
| Gm609 | -0.7837441 |
| Gm614 | -0.208778 |
| Gm614 | -0.7189049 |
| Gm648 | 1.11722082 |
| Gm648 | 1.24123598 |
| Gm71 | -0.4582258 |
| Gm711 | 0.9202622 |
| Gm732 | 1.32774471 |
| Gm732 | -0.5330604 |
| Gm767 | 1.05010732 |
| Gm773 | 1.62428138 |
| Gm773 | -0.8396668 |
| Gm784 | 1.72262027 |
| Gm813 | -0.6547988 |
| Gm813 | -0.4006593 |
| Gm817 | -0.7133927 |
| Gm867 | -0.6419761 |
| Gm884 | -0.3760208 |
| Gm889 | 0.50146603 |
| Gm906 | -0.8292487 |
| Gm93 | -0.1823656 |
| Gm93 | -0.4989016 |
| Gm944 | -0.4582258 |
| Gm949 | 0.37209789 |
| Gm962 | 1.57989228 |
| Gm962 | 2.62206686 |
| Gm97 | -0.3129094 |
| Gm97 | 0.98479066 |
| Gmcl1 | 0.53922445 |
| Gmcl1 | -0.7051845 |
| Gmcl1 | 1.93602885 |
| Gmds | -0.1776891 |
| Gmeb2 | 1.70028586 |
| Gmfg | -0.6110743 |
| Gmfg | -0.6234799 |
| Gmfg | 1.24547025 |
| Gmip | 1.10894033 |
| Gmip | -0.7152265 |
| Gmppa | 1.38968777 |
| Gmppa | -0.5513873 |
| Gmppb | 1.38762848 |
| Gmpr | 2.51487981 |
| Gmpr2 | -0.5630256 |
| Gna11 | -0.5763921 |
| Gna13 | -0.0631488 |
| Gna14 | -0.4610236 |
| Gnai2 | 1.86099357 |
| Gnao1 | -0.3551355 |
| Gnao1 | -0.7051845 |
| Gnaq | 0.39943393 |
| Gnaq | -0.7244496 |
| Gna-rs1 | -0.618498 |
| Gnas | -0.4751386 |
| Gnas | -0.1516194 |
| Gnas | -0.0723719 |
| Gnas | 0.03399364 |
| Gnas | -0.308134 |
| Gnat1 | -0.7593836 |
| Gnat2 | 2.82303431 |
| Gnaz | 2.25485596 |
| Gnb1 | -0.5143422 |
| Gnb1l | 2.08357917 |
| Gnb2 | -0.5232805 |
| Gnb2l1 | -0.4409258 |
| Gnb3 | 0.01264535 |
| Gnb4 | 0.17551587 |
| Gnb4 | 1.69250869 |
| Gnb5 | 0.78684323 |
| Gnb5 | 0.94855037 |
| Gne | -0.5106428 |
| Gng10 | 1.17075989 |
| Gng11 | 1.70555185 |
| Gng13 | 2.31695821 |
| Gng2 | -0.2650743 |
| Gng2 | 0.72954336 |
| Gng3 | 0.97220368 |
| Gng4 | -0.7042769 |
| Gng7 | -0.7593836 |
| Gng8 | -0.4013165 |
| Gngt2 | 0.21832413 |
| Gnl2 | -0.6160169 |
| Gnl2 | 0.20565046 |
| Gnl3 | -0.0906213 |
| Gnl3 | -0.2799798 |
| Gnl3 | -0.528534 |
| Gnl3l | 1.46369099 |
| Gnl3l | -0.7015592 |
| Gnmt | -0.5345741 |
| Gnmt | -0.1145481 |
| Gnpat | -0.5084301 |
| Gnpda1 | -0.3986905 |
| Gnpda1 | -0.7584222 |
| Gnpda1 | -0.3316464 |
| Gnpda2 | 1.2707056 |
| Gnptab | -0.5891482 |
| Gnptg | 1.31170643 |
| Gnptg | 0.6831697 |
| Gnrhr | -0.4779872 |
| Gns | 1.47467297 |
| Golga1 | 0.59510373 |
| Golga2 | -0.638586 |
| Golga2 | -0.5980194 |
| Golga2 | -0.4959891 |
| Golga2 | 0.95276793 |
| Golga2 | 1.40030461 |
| Golga3 | 0.80816632 |
| Golga4 | -0.6739275 |
| Golga5 | 0.36397292 |
| Golga7 | 0.6802591 |
| Golim4 | -0.5947838 |
| Golm1 | -0.3359295 |
| Golm1 | 0.1006904 |
| Golph3l | -0.4026322 |
| Golt1a | -0.1839296 |
| Golt1b | 0.62029383 |
| Gopc | -0.5859429 |
| Gorasp1 | -0.5188007 |
| Gorasp2 | 1.01503005 |
| Gorasp2 | -0.0958332 |
| Gorasp2 | -0.4354563 |
| Gorasp2 | 0.28750635 |
| Gosr1 | -0.4491901 |
| Gosr2 | 1.88923308 |
| Gosr2 | 2.04762442 |
| Gosr2 | 1.00343474 |
| Gosr2 | 2.66001118 |
| Got1l1 | -0.4967164 |
| Got2 | 0.40027551 |
| Gp1bb | -0.6925568 |
| Gp38 | 1.00623657 |
| Gp5 | 0.07251278 |
| Gp9 | -0.4989016 |
| Gpaa1 | 1.50868442 |
| Gpam | -0.2442616 |
| Gpatch1 | 1.56991674 |
| Gpatch2 | 0.03026132 |
| Gpatch3 | -0.4219201 |
| Gpatch4 | -0.7498134 |
| Gpbp1 | 0.45633442 |
| Gpbp1 | -0.750766 |
| Gpbp1l1 | 2.99590059 |
| Gpc1 | -0.8344433 |
| Gpc3 | 0.26154636 |
| Gpc3 | 1.4462362 |
| Gpc4 | 1.44401985 |
| Gpc5 | 0.64244561 |
| Gpc5 | 2.83482857 |
| Gpc5 | 0.79351261 |
| Gpd1 | -0.6925568 |
| Gpd2 | 2.21048031 |
| Gpd2 | 1.39232881 |
| Gper | -0.832362 |
| Gpha2 | 0.210466 |
| Gphb5 | 0.12467196 |
| Gphn | 0.82098302 |
| Gphn | 0.63570186 |
| Gphn | -0.528534 |
| Gpihbp1 | 0.0092522 |
| Gpkow | -0.8087522 |
| Gpld1 | -0.0925132 |
| Gpld1 | 0.12316736 |
| Gpm6a | 0.65472064 |
| Gpm6a | -0.7225978 |
| Gpm6b | 0.72100818 |
| Gpnmb | 0.16044059 |
| Gpnmb | -0.7300272 |
| Gpr1 | -0.2409296 |
| Gpr1 | 2.77443296 |
| Gpr103 | 1.8036679 |
| Gpr107 | -0.6428255 |
| Gpr108 | 0.43360667 |
| Gpr108 | -0.5819516 |
| Gpr108 | -0.2702072 |
| Gpr109a | -0.4436725 |
| Gpr113 | -0.1445876 |
| Gpr113 | 1.50687006 |
| Gpr114 | -0.6756859 |
| Gpr12 | 2.37428533 |
| Gpr12 | -0.6643145 |
| Gpr120 | 0.05162423 |
| Gpr123 | -0.4751386 |
| Gpr124 | 0.03067676 |
| Gpr128 | 2.7722726 |
| Gpr135 | 0.31061253 |
| Gpr137 | 1.34341126 |
| Gpr137b | 0.98388639 |
| Gpr141 | -0.3010161 |
| Gpr146 | -0.3532596 |
| Gpr146 | 0.37151986 |
| Gpr149 | 0.56405155 |
| Gpr151 | -0.4219201 |
| Gpr153 | -0.5345741 |
| Gpr156 | 1.82252848 |
| Gpr156 | -0.7347006 |
| Gpr158 | -0.4730078 |
| Gpr162 | -0.1949511 |
| Gpr162 | -0.5843444 |
| Gpr17 | 2.28879541 |
| Gpr172b | 0.90906363 |
| Gpr172b | 0.14548749 |
| Gpr176 | -0.0040131 |
| Gpr177 | -0.0986883 |
| Gpr18 | 3.0704065 |
| Gpr180 | 1.12607745 |
| Gpr182 | 2.33315812 |
| Gpr182 | -0.4808445 |
| Gpr19 | 1.38250982 |
| Gpr20 | -0.4013165 |
| Gpr21 | -0.5099047 |
| Gpr22 | 0.51297841 |
| Gpr23 | 0.94761056 |
| Gpr26 | -0.653079 |
| Gpr27 | 1.34884883 |
| Gpr27 | 2.50481472 |
| Gpr27 | -0.6352081 |
| Gpr34 | 0.36163856 |
| Gpr34 | 2.39815893 |
| Gpr37l1 | -0.5779772 |
| Gpr45 | -0.5173122 |
| Gpr6 | -0.2520826 |
| Gpr64 | 0.31951511 |
| Gpr65 | 1.09148524 |
| Gpr68 | -0.6479383 |
| Gpr75 | 1.69569804 |
| Gpr81 | 2.40390888 |
| Gpr83 | 0.3878562 |
| Gpr85 | -0.5255284 |
| Gpr85 | -0.7290953 |
| Gpr89 | 0.98809893 |
| Gpr89 | -0.5827485 |
| Gpr97 | -0.5988301 |
| Gprasp1 | -0.7300272 |
| Gprasp1 | 1.25263638 |
| Gprc2a-rs5 | 1.10748703 |
| Gprc2a-rs5 | 0.35929838 |
| Gprc5a | -0.7115625 |
| Gprc5b | 2.59468724 |
| Gprc5c | -0.6343655 |
| Gprk6 | -0.4300184 |
| Gprk6 | 0.75603961 |
| Gps1 | -0.3582703 |
| Gps1 | -0.5560253 |
| Gps1 | -0.4744278 |
| Gps1 | -0.4596236 |
| Gps2 | 0.65984154 |
| Gpsm1 | 1.10111894 |
| Gpsm2 | -0.6402795 |
| Gpsm3 | -0.273071 |
| Gpsn2 | -0.4395554 |
| Gpsn2 | 0.05162423 |
| Gpsn2 | 0.45130154 |
| Gpt1 | -0.5923644 |
| Gpt2 | 0.80707517 |
| Gpx1 | 1.87606661 |
| Gpx2 | 1.07903018 |
| Gpx3 | -0.218471 |
| Gpx4 | 0.7927739 |
| Gpx4 | 1.86241697 |
| Gpx6 | -0.5292869 |
| Gpx7 | -0.6487931 |
| Gramd1a | -0.3709149 |
| Gramd1b | -0.3779426 |
| Gramd2 | -0.4701742 |
| Gramd2 | -0.1844515 |
| Gramd3 | -0.5330604 |
| Gramd4 | -0.3033827 |
| Grap2 | -0.3928088 |
| Grasp | -0.7272343 |
| Grasp | 0.01391463 |
| Grb10 | -0.0148488 |
| Grb14 | -0.501092 |
| Grb14 | 0.83451153 |
| Grb2 | -0.502555 |
| Grb7 | 0.35548314 |
| Grcc10 | 1.62763413 |
| Greb1 | 0.02609687 |
| Greb1 | 1.11341822 |
| Grem1 | -0.1818448 |
| Grem2 | -0.4185663 |
| Grhl1 | 0.9319666 |
| Grhl2 | -0.1627767 |
| Grhpr | -0.3934605 |
| Gria1 | -0.6004535 |
| Gria1 | -0.6143665 |
| Gria1 | -0.6565218 |
| Gria2 | 2.05639321 |
| Gria2 | -0.5437077 |
| Gria3 | -0.7632392 |
| Gria3 | 0.25829117 |
| Gria3 | -0.2811362 |
| Grid2 | 2.13192699 |
| Grik1 | -0.1792454 |
| Grik2 | 1.33804556 |
| Grik4 | 2.51951863 |
| Grik5 | -0.5360902 |
| Grin1 | 1.89811868 |
| Grin1 | 1.49398288 |
| Grin2a | 2.07482489 |
| Grin2b | 1.53111575 |
| Grin2c | 0.4931195 |
| Grin3b | -0.6539385 |
| Grina | -0.6276518 |
| Grip1 | 3.05776794 |
| Grip1 | 0.05689001 |
| Grip1 | -0.5827485 |
| Gripap1 | -0.4554363 |
| Gripap1 | -0.7300272 |
| Grit | -0.5685064 |
| Grk4 | 1.18693784 |
| Grk4 | -0.6176703 |
| Grk4 | 1.59702036 |
| Grk5 | -0.5315492 |
| Grlf1 | -0.5391298 |
| Grm1 | 2.49160952 |
| Grm7 | -0.4666442 |
| Grm8 | -0.0682104 |
| Grn | 1.10191731 |
| Grp | -0.5779772 |
| Grpel1 | 0.37238677 |
| Grpr | -0.6952485 |
| Grsf1 | 1.10191731 |
| Grtp1 | -0.3709149 |
| Grwd1 | -0.2945383 |
| Grwd1 | -0.2939516 |
| Gsbs | 0.02442591 |
| Gsc | -0.7441176 |
| Gsdma1 | -0.4065905 |
| Gsdmdc1 | 0.96222745 |
| Gsdmdc1 | 0.0898325 |
| Gsdmdc1 | -0.1933686 |
| Gse1 | -0.7365765 |
| Gsg1 | -0.935531 |
| Gsg1 | 0.77109496 |
| Gsg1 | -0.4575277 |
| Gsg2 | -0.2381618 |
| Gsk3a | 0.75122462 |
| Gsk3b | -0.3033827 |
| Gsn | 1.54475428 |
| Gspt1 | -0.5429431 |
| Gspt2 | -0.4416117 |
| Gss | -0.6845276 |
| Gss | -0.5980194 |
| Gss | 1.32355055 |
| Gsta2 | -0.4989016 |
| Gsta3 | 1.61492214 |
| Gsta3 | 0.04632881 |
| Gsta4 | -0.2776712 |
| Gstcd | 0.60191749 |
| Gstk1 | -0.7642055 |
| Gstm1 | 0.27669667 |
| Gstm1 | -0.1203822 |
| Gstm2 | -0.314107 |
| Gstm2 | 0.30224751 |
| Gstm2 | 3.19230789 |
| Gstm4 | 1.63619024 |
| Gstm5 | 0.31798618 |
| Gstm6 | -0.5330604 |
| Gstm6 | -0.5700783 |
| Gsto1 | 3.66640347 |
| Gsto2 | -0.4252859 |
| Gsto2 | -0.1648196 |
| Gstp1 | -0.5188007 |
| Gstp1 | -0.6565218 |
| Gstp2 | -0.4409258 |
| Gstt1 | 0.59783516 |
| Gstt2 | 0.81197539 |
| Gstt3 | -0.3934605 |
| Gstt4 | 0.44276377 |
| Gstz1 | 0.14548749 |
| Gtdc1 | -0.917152 |
| Gtf2a1 | 1.05822331 |
| Gtf2e1 | 0.29255059 |
| Gtf2e2 | -0.3728264 |
| Gtf2e2 | 1.83493604 |
| Gtf2f1 | 0.58409801 |
| Gtf2f2 | -0.2904391 |
| Gtf2h1 | 1.09992011 |
| Gtf2h1 | 1.05543259 |
| Gtf2h2 | 0.90464504 |
| Gtf2h3 | -0.4930855 |
| Gtf2h4 | 0.0240077 |
| Gtf2h5 | 1.58205042 |
| Gtf2ird1 | -0.5748096 |
| Gtf2ird1 | 1.31616546 |
| Gtf2ird2 | 1.19757062 |
| Gtf2ird2 | 0.27893237 |
| Gtf2ird2 | 0.10798753 |
| Gtf3a | -0.0849683 |
| Gtf3a | 0.40475129 |
| Gtf3c1 | 0.44169108 |
| Gtf3c2 | 0.50498474 |
| Gtf3c3 | 0.31522783 |
| Gtf3c4 | 1.26535472 |
| Gtf3c4 | -0.4192361 |
| Gtf3c5 | -0.5498463 |
| Gtf3c5 | -0.1933686 |
| Gtf3c5 | 0.23985097 |
| Gtl3 | -0.2425941 |
| Gtpbp1 | 1.55055117 |
| Gtpbp2 | -0.0364559 |
| Gtpbp3 | -0.5483079 |
| Gtpbp4 | -0.5835461 |
| Gtpbp5 | 0.41777704 |
| Gtpbp6 | -0.804706 |
| Gtpbp8 | 1.95501699 |
| Gtpbp8 | 0.03026132 |
| Gtrgeo22 | 1.80518067 |
| Gtse1 | -0.4498821 |
| Gtse1 | -0.1684053 |
| Gtse1 | -0.5923644 |
| Gtsf1l | 0.39380406 |
| Guca1a | -0.5599079 |
| Guca1b | -0.4554363 |
| Guca2a | 2.91153029 |
| Guca2b | 0.22442851 |
| Gucy1a2 | 1.63142962 |
| Gucy1a3 | -0.6326825 |
| Gucy1a3 | 0.29412138 |
| Gucy1a3 | -0.1169746 |
| Gucy1b3 | 1.85378427 |
| Guk1 | 1.87519005 |
| Gulo | 2.2609281 |
| Gulp1 | -0.3921575 |
| Gusb | -0.4694672 |
| Gusb | 0.1193953 |
| Gusb | -0.6625772 |
| Gvin1 | 1.3382525 |
| Gyg | 1.24123598 |
| Gyg | 0.68524321 |
| Gykl1 | 1.25501301 |
| Gyltl1b | -0.6127189 |
| Gyltl1b | 0.88742867 |
| Gypa | 0.43387727 |
| Gypc | -0.6880877 |
| Gys1 | -0.1643084 |
| Gys3 | 1.404653 |
| Gzf1 | -0.7768594 |
| Gzf1 | -0.324349 |
| Gzmd | 2.96020304 |
| Gzmd | 0.80852975 |
| Gzmk | -0.3195154 |
| Gzmm | -0.5677215 |
| Gzmn | 1.10138514 |
| H13 | -0.5136011 |
| H13 | 2.4663154 |
| H13 | -0.4794147 |
| H13 | -0.6800963 |
| H19 | 1.81164546 |
| H1f0 | -0.6774476 |
| H1fnt | 0.02901392 |
| H1foo | 0.13402156 |
| H1fx | -0.4708818 |
| H1fx | -0.5391298 |
| H2-Aa | 0.56546409 |
| H2-Ab1 | 0.90300325 |
| H2-Ab1 | 1.21953925 |
| H2-Ab1 | 0.43495891 |
| H2afj | -0.2179299 |
| H2afx | 2.60386668 |
| H2afy | -0.5915593 |
| H2afz | 0.50096229 |
| H2-Bl | 0.07410054 |
| H2-DMa | 1.11970387 |
| H2-DMb1 | -0.7700246 |
| H2-Eb1 | -0.2582736 |
| H2-K1 | -0.6411274 |
| H2-Ke6 | -0.2759435 |
| H2-M10.3 | 0.11674584 |
| H2-M2 | 0.93705174 |
| H2-M3 | 0.16764943 |
| H2-Oa | 1.91648169 |
| H2-Ob | 0.90628398 |
| H2-Q1 | 1.09965349 |
| H2-Q2 | -0.2255341 |
| H2-Q7 | 0.20254168 |
| H2-Q8 | -0.5891482 |
| H2-T10 | -0.6193265 |
| H2-T23 | 0.33436855 |
| H2-T23 | -0.6539385 |
| H2-T24 | 3.55002618 |
| H2-Tw3 | -0.1622667 |
| H3f3a | 0.40837223 |
| H3f3a | -0.299245 |
| H3f3b | 0.81990665 |
| H47 | 2.60558966 |
| H47 | -0.6496487 |
| H6pd | -0.1430876 |
| Habp4 | -0.6301638 |
| Habp4 | 2.48683066 |
| Hace1 | -0.7749015 |
| Hacl1 | -0.7170639 |
| Hadhb | -0.5883458 |
| Hagh | -0.3201182 |
| Haghl | 2.94697403 |
| Haghl | 4.01021215 |
| Hal | 2.70125836 |
| Hamp2 | -0.6660551 |
| Hao1 | -0.8334021 |
| Hao3 | -0.4293409 |
| Hap1 | 0.38728777 |
| Hap1 | 1.00873641 |
| Hap1 | 1.08609024 |
| Hapln1 | -0.06269 |
| Hapln2 | -0.388908 |
| Hapln4 | 0.95261206 |
| Harbi1 | -0.3010161 |
| Hars | 1.05417406 |
| Has1 | -0.7393973 |
| Hat1 | -0.4498821 |
| Havcr1 | -0.515826 |
| Havcr2 | 1.59324098 |
| Hax1 | -0.5803598 |
| Hba-a1 | -0.8491428 |
| Hbb-b1 | -0.7450645 |
| Hbegf | -0.4610236 |
| Hbp1 | -0.5084301 |
| Hbp1 | 1.21730651 |
| Hbs1l | -0.5630256 |
| Hbs1l | -0.7926706 |
| Hbs1l | -0.0485774 |
| Hbs1l | 0.42025089 |
| Hc | -0.3153062 |
| Hccs | -0.4079136 |
| Hcfc1 | -0.3677378 |
| Hcfc1r1 | 1.65166479 |
| Hcfc2 | 2.99021704 |
| Hcls1 | -0.5225324 |
| Hcn2 | 2.10355691 |
| Hcn3 | 1.39027531 |
| Hcn3 | 0.96883894 |
| Hcr | -0.7097358 |
| Hcst | -0.1597207 |
| Hdac10 | -0.5867432 |
| Hdac11 | -0.0663667 |
| Hdac2 | -0.5173122 |
| Hdac3 | -0.6487931 |
| Hdac4 | -0.3677378 |
| Hdac5 | -0.1179469 |
| Hdac6 | -0.0320058 |
| Hdac7 | 2.66953305 |
| Hdac8 | -0.0977357 |
| Hdac9 | -0.7613094 |
| Hdc | -0.6889798 |
| Hddc2 | -0.4252859 |
| Hddc2 | -0.5003613 |
| Hdgf | -0.2125357 |
| Hdgf | -0.6176703 |
| Hdgf | -0.5143422 |
| Hdgf | -0.041824 |
| Hdgfl1 | -0.1949511 |
| Hdgfrp2 | -0.6201557 |
| Hdhd1a | 3.03832462 |
| Hdhd2 | -0.3129094 |
| Hdhd2 | 1.08771199 |
| Hdhd2 | 0.05850434 |
| Hdlbp | -0.2348513 |
| Hdlbp | 1.80069834 |
| Heatr1 | 2.34568616 |
| Heatr1 | -0.5307945 |
| Heatr3 | -0.1954791 |
| Heatr5a | -0.6774476 |
| Hebp1 | 3.18103939 |
| Hebp2 | -0.6800963 |
| Heca | 1.4141493 |
| Hectd2 | -0.299835 |
| Hectd2 | 2.22582822 |
| Hectd3 | 0.20461534 |
| Hecw2 | 0.20565046 |
| Hecw2 | 0.00456655 |
| Hecw2 | -0.5795649 |
| Heg1 | 2.55888579 |
| Hel308 | 0.87971207 |
| Helb | -0.3445547 |
| Helb | 0.0092522 |
| Helz | -0.4673491 |
| Hemgn | 1.02662828 |
| Hemk1 | -0.6360514 |
| Hemt1 | 0.45923593 |
| Hepacam | 1.49896711 |
| Heph | -0.1740679 |
| Heph | 0.38301358 |
| Heph | -0.2881048 |
| Heph | -0.3563881 |
| Heph | 1.78235882 |
| Herc2 | -0.52703 |
| Herc3 | -0.3902064 |
| Herc3 | 0.76901303 |
| Herc4 | -0.492361 |
| Herc4 | 0.92861447 |
| Herpud1 | 0.39634167 |
| Herpud1 | 0.84715101 |
| Herpud2 | -0.2326507 |
| Herpud2 | -0.750766 |
| Herpud2 | 0.9549474 |
| Herpud2 | -0.1668669 |
| Hes1 | -0.4996312 |
| Hes5 | -0.6599773 |
| Hes5 | -0.5032874 |
| Hes6 | -0.7198268 |
| Hes6 | 0.66175472 |
| Hes6 | 1.04813775 |
| Hexa | -0.6259808 |
| Hexb | 0.55195858 |
| Hexdc | 0.95370261 |
| Hexdc | 2.41273261 |
| Hexim1 | 3.38761744 |
| Hexim2 | 1.9758384 |
| Hey1 | 3.47059714 |
| Hey2 | 1.17211873 |
| Hey2 | 1.79409389 |
| Heyl | 0.71861238 |
| Hfe | -0.1101961 |
| Hfe | 0.0007154 |
| Hgfac | -0.5560253 |
| Hgs | 0.44837541 |
| Hgs | 0.43954202 |
| Hgsnat | 1.22608932 |
| Hhat | 2.57157604 |
| Hhat | -0.6845276 |
| Hhat | -0.2724975 |
| Hhatl | -0.5202916 |
| Hhex | -0.1870654 |
| Hhip | -0.2045019 |
| Hibadh | 0.8817312 |
| Hic1 | -0.2376093 |
| Hic2 | -0.5891482 |
| Hif1a | 2.423435 |
| Hif3a | -0.6651844 |
| Hif3a | 2.85050739 |
| Higd1a | 0.06853161 |
| Higd1b | -0.0139774 |
| Higd2a | 1.08080017 |
| Hils1 | -0.5330604 |
| Hint1 | 2.44979708 |
| Hint2 | -0.4722986 |
| Hint3 | 0.62780926 |
| Hint3 | -0.3261681 |
| Hip1 | 0.26834582 |
| Hip1r | -0.4327334 |
| Hip2 | 0.38757203 |
| Hipk1 | 1.91165732 |
| Hipk2 | -0.7015592 |
| Hipk3 | 2.53140959 |
| Hipk4 | 0.09178062 |
| Hira | -0.4858656 |
| Hira | 2.04704016 |
| Hirip3 | 0.6877254 |
| Hisppd1 | -0.4722986 |
| Hist1h1b | -0.4645325 |
| Hist1h1c | -0.5771843 |
| Hist1h2aa | -0.7024642 |
| Hist1h2ac | -0.0672881 |
| Hist1h2ad | 2.03058148 |
| Hist1h2ae | -0.4909137 |
| Hist1h2af | 2.19250248 |
| Hist1h2ag | -0.6907666 |
| Hist1h2ah | 1.01997678 |
| Hist1h2ak | -0.5685064 |
| Hist1h2an | -0.0723719 |
| Hist1h2ao | -0.276519 |
| Hist1h2bc | -0.7244496 |
| Hist1h2be | -0.6193265 |
| Hist1h2bf | 1.90011355 |
| Hist1h2bg | 1.18108709 |
| Hist1h2bh | 1.57266251 |
| Hist1h2bj | -0.4603233 |
| Hist1h2bk | -0.2974772 |
| Hist1h2bm | 1.10523659 |
| Hist1h2bn | 1.09148524 |
| Hist1h3a | 2.39590957 |
| Hist1h3d | -0.5552507 |
| Hist1h3e | -0.3482755 |
| Hist1h3f | -0.4708818 |
| Hist1h4c | 1.36878907 |
| Hist1h4d | 0.24449502 |
| Hist1h4f | 0.49894459 |
| Hist1h4h | 1.84210901 |
| Hist1h4i | 2.78451631 |
| Hist1h4j | -0.3322571 |
| Hist1h4k | -0.5740194 |
| Hist1h4m | 1.05347415 |
| Hist2h2aa1 | 0.36862435 |
| Hist2h2ab | -0.0104995 |
| Hist2h2ac | -0.5136011 |
| Hist2h2bb | -0.0732992 |
| Hist2h2be | 0.33165789 |
| Hist2h3b | -0.4464272 |
| Hist2h3c1 | -0.5210379 |
| Hist2h3c1 | -0.4561329 |
| Hist2h4 | 0.69983514 |
| Hist3h2a | -0.6251465 |
| Hist3h2bb | -0.4199064 |
| Hist4h4 | 1.52409426 |
| Hk1 | -0.3201182 |
| Hk3 | -0.760346 |
| Hkdc1 | -0.6854163 |
| Hlcs | 0.73956711 |
| Hlf | 0.4834157 |
| Hltf | 2.63101041 |
| Hlx | 0.31676124 |
| Hmg20a | 2.50780379 |
| Hmg20a | -0.653079 |
| Hmg20b | -0.3279907 |
| Hmga1 | -0.558353 |
| Hmgb2 | 0.73072819 |
| Hmgb2l1 | 1.76995259 |
| Hmgb2l1 | 0.77693756 |
| Hmgb4 | 0.03357967 |
| Hmgcl | -0.4794147 |
| Hmgcl | 0.54019155 |
| Hmgcl | 2.61391766 |
| Hmgcll1 | -0.5225324 |
| Hmgcs2 | -0.6836397 |
| Hmgn1 | 1.54009092 |
| Hmgn2 | -0.6352081 |
| Hmgn3 | 0.13847658 |
| Hmgn3 | 1.21953925 |
| Hmha1 | -0.4259605 |
| Hmox1 | 0.69205353 |
| Hmox2 | -0.3614155 |
| Hmx1 | -0.4368207 |
| Hmx1 | -0.0035822 |
| Hn1 | 1.3691896 |
| Hn1 | 1.22340463 |
| Hn1l | -0.1766531 |
| Hnf1b | 1.01079013 |
| Hnf1b | -0.6695458 |
| Hnf4a | 1.41596091 |
| Hnrnpc | 1.730017 |
| Hnrpa1 | 0.73721817 |
| Hnrpa1 | -0.2332004 |
| Hnrpab | 1.94887427 |
| Hnrpc | 3.87407767 |
| Hnrpdl | -0.4996312 |
| Hnrpdl | -0.4540447 |
| Hnrpf | -0.6445267 |
| Hnrph1 | -0.4666442 |
| Hnrph1 | -0.6077935 |
| Hnrph1 | 0.92845455 |
| Hnrph1 | 0.31920952 |
| Hnrph1 | -0.3908563 |
| Hnrph2 | -0.5165688 |
| Hnrph3 | 0.30690392 |
| Hnrpk | 1.96654697 |
| Hnrpl | 0.07687269 |
| Hnrpll | 0.41805223 |
| Hnrpll | -0.2702072 |
| Hnrpm | -0.7778398 |
| Hnrpm | -0.5851433 |
| Hnrpm | 2.89346311 |
| Hnrpul1 | 0.84383205 |
| Hnrpul1 | 0.41253301 |
| Hnrpul1 | -0.3620459 |
| Hnrpul1 | 0.89275965 |
| Hnrpul1 | -0.1917889 |
| Hnt | -0.0377951 |
| Homer1 | 1.21187887 |
| Homer1 | -0.6293257 |
| Homer1 | -0.3371569 |
| Homer1 | -0.6168432 |
| Homez | -0.237057 |
| Homez | 1.44982668 |
| Hook1 | -0.0562904 |
| Hook1 | -0.6695458 |
| Hook1 | 3.50424218 |
| Hook1 | 0.42817821 |
| Hook2 | 0.07647717 |
| Hormad2 | -0.4880258 |
| Hoxa13 | -0.6243128 |
| Hoxa2 | -0.6160169 |
| Hoxa2 | -0.06269 |
| Hoxa4 | -0.2498414 |
| Hoxa5 | 0.24911626 |
| Hoxa7 | 1.5799723 |
| Hoxa9 | 1.53271574 |
| Hoxa9 | 1.90946135 |
| Hoxb2 | -0.4125593 |
| Hoxb3 | 2.77091984 |
| Hoxb3 | -0.4596236 |
| Hoxb4 | -0.7088237 |
| Hoxb5 | -0.5180561 |
| Hoxb6 | -0.4118942 |
| Hoxb7 | -0.4673491 |
| Hoxc10 | 1.11931224 |
| Hoxc10 | 1.72241397 |
| Hoxc6 | 2.29390411 |
| Hoxc9 | -0.4039498 |
| Hoxd10 | -0.5972095 |
| Hoxd3 | 2.77436553 |
| Hoxd4 | 0.20392463 |
| Hoxd8 | 0.66894735 |
| Hoxd9 | 0.51719758 |
| Hp | 1.91793536 |
| Hp | 0.15317608 |
| Hp | 0.66111742 |
| Hp1bp3 | 2.07439922 |
| Hpca | -0.4575277 |
| Hpcal1 | -0.3779426 |
| Hpcal1 | 1.58754328 |
| Hpcal4 | -0.3741028 |
| Hpgd | -0.5779772 |
| Hpn | -0.2018392 |
| Hpn | 0.09488926 |
| Hprt1 | 1.92511478 |
| Hprt1 | 2.23200999 |
| Hprt1 | -0.5779772 |
| Hps1 | 1.7564524 |
| Hps3 | -0.3986905 |
| Hps3 | -0.2639374 |
| Hps4 | -0.4105655 |
| Hps5 | -0.0490294 |
| Hps6 | -0.7281644 |
| Hpse | -0.538369 |
| Hpx | 2.12661642 |
| Hr | -0.2707793 |
| Hr | -0.3334797 |
| Hras1 | 1.88871455 |
| Hrasls | -0.4751386 |
| Hrasls5 | 3.28687656 |
| Hrasls5 | -0.3396163 |
| Hrb | -0.5756005 |
| Hrb | 0.08905212 |
| Hrbl | -0.7015592 |
| Hrbl | 1.25184283 |
| Hrc | 1.03280374 |
| Hrg | -0.5568006 |
| Hrh3 | 0.61740435 |
| Hrk | 1.78532242 |
| Hrmt1l2 | 2.30440955 |
| Hrsp12 | 0.20737313 |
| Hrsp12 | -0.6377404 |
| Hrsp12 | 0.14364757 |
| Hs1bp3 | 2.0591859 |
| Hs2st1 | 2.14509566 |
| Hs3st1 | -0.5568006 |
| Hs3st3a1 | 0.46344041 |
| Hs3st3b1 | 2.58571959 |
| Hs3st6 | 1.23548355 |
| Hs6st1 | -0.1776891 |
| Hs6st2 | -0.502555 |
| Hs6st2 | 0.81576911 |
| Hs6st3 | 2.34621785 |
| Hsbp1 | 0.71601003 |
| Hsd11b1 | -0.5677215 |
| Hsd11b1 | -0.438871 |
| Hsd11b1 | -0.5475396 |
| Hsd11b1 | 0.1797791 |
| Hsd11b2 | -0.122335 |
| Hsd17b1 | -0.6809809 |
| Hsd17b10 | -0.5972095 |
| Hsd17b11 | -0.5669372 |
| Hsd17b11 | -0.6565218 |
| Hsd17b11 | 2.33211554 |
| Hsd17b12 | 0.33647146 |
| Hsd17b2 | -0.5931702 |
| Hsd17b3 | -0.2724975 |
| Hsd17b4 | -0.7152265 |
| Hsd17b7 | 1.89937309 |
| Hsd3b1 | -0.658248 |
| Hsd3b2 | -0.5622452 |
| Hsd3b2 | -0.4645325 |
| Hsd3b4 | 0.29223612 |
| Hsd3b7 | -0.5819516 |
| Hsd3b7 | -0.3402322 |
| Hsdl1 | -0.5121208 |
| Hsf1 | -0.2061032 |
| Hsf4 | -0.6176703 |
| Hsf5 | -0.5779772 |
| Hsn2 | 1.45787827 |
| Hsp105 | 0.06453352 |
| Hsp90aa1 | -0.3721888 |
| Hsp90ab1 | 0.59236434 |
| Hsp90ab1 | 1.43845601 |
| Hsp90ab1 | 1.55244809 |
| Hsp90b1 | -0.6889798 |
| Hspa12b | 1.90562017 |
| Hspa1a | 0.97036986 |
| Hspa1l | 1.73274183 |
| Hspa2 | -0.7661412 |
| Hspa2 | 1.15580727 |
| Hspa4 | -0.6045242 |
| Hspa4l | -0.5923644 |
| Hspa5 | 0.92251819 |
| Hspa9 | -0.4533496 |
| Hspa9 | 1.11144531 |
| Hspb1 | -0.5345741 |
| Hspb2 | 1.34680061 |
| Hspb3 | -0.4873052 |
| Hspb6 | -0.5165688 |
| Hspb7 | -0.0723719 |
| Hspb8 | -0.3165069 |
| Hspbap1 | -0.4844283 |
| Hspd1 | 0.35283273 |
| Hspd1 | -0.3377711 |
| Hspe1 | 2.68627032 |
| Hspg2 | -0.0686718 |
| Htatip | 0.62472194 |
| Htatip | 1.27624726 |
| Htatip2 | -0.1247817 |
| Htatip2 | -0.4673491 |
| Htatsf1 | -0.0386889 |
| Htf9c | 1.65047772 |
| Htr1a | -0.6677988 |
| Htr1b | -0.5763921 |
| Htr1d | -0.4026322 |
| Htr1f | -0.5376088 |
| Htr1f | -0.2050354 |
| Htr2b | 1.62634016 |
| Htr3a | 0.17515973 |
| Htr5b | 0.30597448 |
| Htr7 | -0.6301638 |
| Htra1 | 2.85707712 |
| Htra1 | 1.44881537 |
| Htra2 | -0.0944089 |
| Htra3 | 1.94345269 |
| Htra3 | 0.65835082 |
| Htt | 2.35042529 |
| Hus1 | 1.36728556 |
| Hus1 | 1.47744317 |
| Hus1 | 0.2011567 |
| Huwe1 | 2.2249821 |
| Hvcn1 | 0.32073646 |
| Hvcn1 | 0.39352168 |
| Hyal1 | 0.72716923 |
| Hyal2 | -0.5091671 |
| Hyal3 | -0.4779872 |
| Hyal5 | 0.28687391 |
| Hyal6 | -0.6470842 |
| Hydin | -0.2945383 |
| Hyi | -0.4138909 |
| Hyou1 | -0.3201182 |
| Iah1 | 2.23068874 |
| Iap | -0.1450882 |
| Iars | -0.7536298 |
| Iars | 0.72498767 |
| Iars2 | 0.47049312 |
| Ibtk | 2.50923517 |
| Ica1 | -0.6513623 |
| Ica1l | -0.4172281 |
| Icam1 | 0.48649089 |
| Icam2 | -0.6617098 |
| Icam4 | 2.01191054 |
| Icam5 | -0.4478077 |
| Ick | 0.02191389 |
| Icmt | -0.6765663 |
| Ict1 | 2.17757198 |
| Id1 | -0.4192361 |
| Id2 | -0.7347006 |
| Id2 | 1.2572709 |
| Id3 | 1.56756841 |
| Id4 | -0.3219291 |
| Ide | -0.1010742 |
| Idh1 | 1.53322043 |
| Idh2 | 2.14478844 |
| Idh3a | -0.4737175 |
| Idh3b | -0.1475948 |
| Idh3g | 1.63324603 |
| Idh3g | 2.21387188 |
| Idi2 | -0.4938106 |
| Ids | -0.0431708 |
| Ids | 1.87594978 |
| Ids | 1.12957105 |
| Idua | 1.33545496 |
| Ier3 | -0.6135424 |
| Ier3ip1 | -0.6462309 |
| Ier5l | 1.71176282 |
| Ifi202b | -0.6961474 |
| Ifi202b | 0.82990522 |
| Ifi27 | 0.46291587 |
| Ifi30 | -0.52703 |
| Ifi30 | -0.5972095 |
| Ifi35 | 0.82954995 |
| Ifi47 | 3.84543737 |
| Ifit2 | 1.51522161 |
| Ifit2 | -0.0463209 |
| Ifit3 | -0.5195458 |
| Ifit3 | 1.81208321 |
| Ifitm1 | -0.52703 |
| Ifitm1 | 0.26316975 |
| Ifitm1 | -0.3941127 |
| Ifitm2 | 0.82919455 |
| Ifitm3 | 1.51736211 |
| Ifitm5 | -0.1034661 |
| Ifitm6 | -0.6547988 |
| Ifna1 | -0.1751011 |
| Ifna6 | -0.7079126 |
| Ifnar2 | 1.06516409 |
| Ifnar2 | 2.6095888 |
| Ifnar2 | 3.12833639 |
| Ifngr1 | 0.2284762 |
| Ifngr2 | -0.2071722 |
| Ifngr2 | -0.4436725 |
| Ifngr2 | -0.1198946 |
| Ifnz | -0.3414652 |
| Ifrd1 | 0.14401584 |
| Ifrg15 | -0.061315 |
| Ift122 | -0.1160032 |
| Ift140 | -0.3639393 |
| Ift140 | 0.28148096 |
| Ift140 | -0.45683 |
| Ift172 | -0.3069439 |
| Ift172 | 1.66892942 |
| Ift172 | -0.7070024 |
| Ift20 | -0.3588985 |
| Ift20 | 1.64899178 |
| Ift20 | -0.5803598 |
| Ift52 | 1.5606514 |
| Ift80 | 2.21676342 |
| Ift81 | 0.20565046 |
| Ift81 | 1.44300227 |
| Ift81 | -0.3973803 |
| Ift88 | 1.12165955 |
| Igbp1 | 0.75122462 |
| Igbp1b | -0.3721888 |
| Igf1 | -0.2851949 |
| Igf1 | 0.39182561 |
| Igf1r | 1.04757426 |
| Igf2 | -0.4013165 |
| Igf2bp1 | -0.6094325 |
| Igf2bp2 | -0.5669372 |
| Igf2bp3 | 1.58348644 |
| Igf2bp3 | 3.55450988 |
| Igf2r | -0.4212484 |
| Igf2r | -0.2114605 |
| Igfals | 0.04059252 |
| Igfbp2 | -0.5883458 |
| Igfbp2 | 0.49565658 |
| Igfbp3 | -0.6462309 |
| Igfbp4 | 0.63701088 |
| Igfbp4 | -0.4837104 |
| Igfbp5 | 1.75976304 |
| Igfbp6 | -0.6318422 |
| Igfbp7 | -0.7807873 |
| Igfbpl1 | 0.64978682 |
| Igsf1 | -0.706093 |
| Igsf1 | -0.6318422 |
| Igsf1 | 2.02506387 |
| Igsf1 | 0.01306864 |
| Igsf11 | 0.2481279 |
| Igsf11 | -0.4293409 |
| Igsf11 | -0.6505051 |
| Igsf21 | -0.8459735 |
| Igsf3 | -0.5575765 |
| Igsf4a | 0.33976655 |
| Igsf5 | -0.546005 |
| Igsf8 | -0.3310361 |
| Igsf9 | -0.4212484 |
| Igtp | 1.47216383 |
| Ihh | 0.84102803 |
| Ihpk1 | 1.38221366 |
| Ihpk2 | 0.36659218 |
| Ihpk2 | 0.99378594 |
| Iigp2 | 0.53461708 |
| Ik | 0.91783904 |
| Ikbkap | -0.3741028 |
| Ikbkb | -0.8006771 |
| Ikbke | 0.73013596 |
| Ikbkg | 1.92788283 |
| Ikbkg | -0.8640757 |
| Il10ra | 0.10683911 |
| Il10rb | -0.5475396 |
| Il11 | 1.1431626 |
| Il11ra1 | 0.9106949 |
| Il12a | 0.67021095 |
| Il13 | -0.5740194 |
| Il13ra1 | -0.4603233 |
| Il13ra2 | -0.6739275 |
| Il15 | 0.05890749 |
| Il15 | 1.06613172 |
| Il15 | -0.3261681 |
| Il15ra | -0.3063495 |
| Il15ra | 0.3977485 |
| Il15ra | 1.18401701 |
| Il16 | 0.572962 |
| Il17d | 1.00520528 |
| Il17d | -0.5756005 |
| Il17ra | -0.3639393 |
| Il17rb | -0.7536298 |
| Il17rc | -0.6997518 |
| Il17rc | -0.6880877 |
| Il17rc | -0.5300404 |
| Il17rd | 2.02988735 |
| Il17rd | 1.93339462 |
| Il17re | 0.67378195 |
| Il17re | 0.36397292 |
| Il17re | 1.91871718 |
| Il18 | 2.47098213 |
| Il18bp | -0.5843444 |
| Il18r1 | 2.20719972 |
| Il1b | -0.6470842 |
| Il1r1 | 1.42863941 |
| Il1rap | -0.6809809 |
| Il1rap | 1.21353408 |
| Il1rap | -0.4981726 |
| Il1rap | 1.53992394 |
| Il1rap | 0.07330699 |
| Il1rl1l | 1.02964926 |
| Il1rl2 | 0.75103151 |
| Il20ra | -0.546772 |
| Il20rb | -0.5653706 |
| Il20rb | -0.5128607 |
| Il23a | 0.75603961 |
| Il27ra | -0.6643145 |
| Il28ra | -0.5638066 |
| Il28ra | -0.0174681 |
| Il28ra | 1.604222 |
| Il31ra | 0.58040063 |
| Il33 | -0.3532596 |
| Il3ra | -0.4596236 |
| Il4i1 | 1.58960477 |
| Il6ra | -0.1813244 |
| Il6st | 0.85807294 |
| Il7 | 2.27395145 |
| Ildr1 | 0.79738136 |
| Ildr1 | 0.22948541 |
| Ilf2 | 0.54669362 |
| Ilf3 | -0.4422981 |
| Ilf3 | 0.10875237 |
| Ilf3 | 0.42790597 |
| Ilf3 | 1.09537735 |
| Ilk | 1.29766113 |
| Ilkap | -0.6318422 |
| Ilvbl | 0.8584176 |
| Ilvbl | -0.3117133 |
| Ilvbl | -0.1891618 |
| Ilvbl | -0.7337641 |
| Immp2l | 0.00797661 |
| Immt | 3.1831962 |
| Imp3 | 0.36630151 |
| Imp4 | -0.2163084 |
| Imp4 | -0.2605351 |
| Impa2 | 3.19443905 |
| Impact | 1.12879582 |
| Impad1 | 0.69861073 |
| Impdh1 | 0.13884688 |
| Impdh2 | 0.23618593 |
| Ina | -0.0691336 |
| Inadl | -0.3470336 |
| Inadl | 0.38358459 |
| Incenp | -0.0549247 |
| Indo | 2.1197442 |
| Ing2 | -0.1938958 |
| Ing3 | -0.3980352 |
| Ing3 | 0.69225914 |
| Ing3 | 0.03523446 |
| Ing4 | 2.24172206 |
| Ing4 | -0.7106487 |
| Ing4 | -0.6625772 |
| Ing5 | 0.0800309 |
| Inha | -0.5300404 |
| Inhba | -0.0422727 |
| Inmt | 1.66645108 |
| Inoc1 | -0.4505746 |
| Inpp4a | -0.5740194 |
| Inpp4a | -0.1068251 |
| Inpp4a | 0.27669667 |
| Inpp4b | 1.04347882 |
| Inpp5a | -0.5062227 |
| Inpp5d | -0.5106428 |
| Inpp5e | 0.38444047 |
| Inpp5f | -0.6318422 |
| Inppl1 | 3.73889837 |
| Inppl1 | 0.17302004 |
| Inppl1 | -0.7584222 |
| Ins2 | -0.5247785 |
| Insc | -0.0572019 |
| Insig1 | -0.804706 |
| Insig1 | 2.13023443 |
| Insig1 | 0.31369191 |
| Insig2 | 0.5664046 |
| Insig2 | -0.2851949 |
| Insl3 | 1.46269448 |
| Insl5 | -0.0253699 |
| Insl6 | -0.2560176 |
| Insm1 | 0.45315889 |
| Ints1 | -0.1720048 |
| Ints12 | -0.6151914 |
| Ints3 | 0.19070382 |
| Ints3 | 1.57467636 |
| Ints4 | 1.09175418 |
| Ints5 | -0.4178969 |
| Ints7 | 1.12853726 |
| Ints9 | -0.4085759 |
| Ints9 | -0.3882594 |
| Ipmk | -0.8057159 |
| Ipmk | 2.34359194 |
| Ipo13 | -0.14159 |
| Ipo4 | -0.7827575 |
| Ipo5 | 2.42327177 |
| Ipp | -0.6402795 |
| Ippk | -0.5498463 |
| Iqcb1 | 0.13178611 |
| Iqcb1 | 0.514718 |
| Iqcd | 0.03935875 |
| Iqce | -0.148097 |
| Iqcf3 | 0.34513376 |
| Iqcf4 | -0.5391298 |
| Iqgap1 | 0.53096368 |
| Iqgap2 | 0.8840814 |
| Iqgap3 | 2.14241502 |
| Iqsec2 | 0.57272859 |
| Iqsec3 | -0.4422981 |
| Irak1 | -0.6854163 |
| Irak1bp1 | -0.2927793 |
| Irak2 | 1.00254822 |
| Irak2 | 0.27445565 |
| Irak3 | 0.77562143 |
| Irak4 | 0.33496986 |
| Irak4 | -0.2332004 |
| Irak4 | -0.6077935 |
| Ireb2 | -0.5040204 |
| Irf1 | -0.6556599 |
| Irf1 | -0.6961474 |
| Irf1 | -0.5537035 |
| Irf1 | 0.71560903 |
| Irf2 | -0.5685064 |
| Irf2bp1 | -0.6854163 |
| Irf3 | 0.88776274 |
| Irf3 | 1.18328538 |
| Irf3 | 2.34781111 |
| Irf4 | -0.5498463 |
| Irf5 | 1.63950088 |
| Irf6 | -0.2271717 |
| Irf6 | 2.06436408 |
| Irf7 | 0.96607699 |
| Irf8 | 0.20254168 |
| Irf9 | -0.0531068 |
| Irf9 | 2.51232906 |
| Irgm | 2.84591446 |
| Irgm | -0.7097358 |
| Irgq | 1.02979288 |
| Irgq | -0.4822763 |
| Irgq | -0.1938958 |
| Irs2 | 0.13439362 |
| Irs3 | -0.041824 |
| Irx2 | -0.8158753 |
| Irx3 | -0.6952485 |
| Irx5 | 0.73289649 |
| Isca1 | -0.6004535 |
| Iscu | -0.4457378 |
| Isg20 | -0.0183428 |
| Isg20l1 | -0.2851949 |
| Isg20l2 | -0.658248 |
| Isl1 | -0.3954184 |
| Islr2 | 2.35478958 |
| Isy1 | 0.29944256 |
| Isyna1 | 2.17769915 |
| Itfg2 | 0.20565046 |
| Itfg3 | 2.06665638 |
| Itga1 | -0.4851467 |
| Itga11 | 0.22577968 |
| Itga11 | 0.86151387 |
| Itga2b | 1.24158001 |
| Itga3 | -0.6086127 |
| Itga5 | -0.5143422 |
| Itga5 | -0.2846141 |
| Itga6 | 1.88980888 |
| Itga7 | -0.2916085 |
| Itgad | 1.3318161 |
| Itgad | -0.6997518 |
| Itgad | -0.4118942 |
| Itgae | 1.14786095 |
| Itgae | -0.6069752 |
| Itgae | 0.72637652 |
| Itgav | 1.77328129 |
| Itgb1 | 2.09623622 |
| Itgb1 | 0.40809419 |
| Itgb1bp1 | 0.47023285 |
| Itgb3bp | -0.4610236 |
| Itgb4 | 1.19009187 |
| Itgb4 | -0.3488971 |
| Itgb5 | -0.3488971 |
| Itgb6 | 4.34068268 |
| Itgb6 | 0.85582957 |
| Itgb6 | 1.74450389 |
| Itgb7 | -0.4652359 |
| Itgbl1 | -0.0849683 |
| Itih2 | -0.3004253 |
| Itih3 | 1.63392628 |
| Itih5 | -0.0209718 |
| Itih5 | -0.6135424 |
| Itk | -0.6591122 |
| Itlnb | 0.64157816 |
| Itm2a | 1.00092075 |
| Itm2b | 0.03150707 |
| Itm2b | 0.32347873 |
| Itpk1 | -0.6377404 |
| Itpka | -0.4659398 |
| Itpkc | -0.4765618 |
| Itpr1 | -0.7375158 |
| Itpr2 | -0.6487931 |
| Itpr3 | -0.4212484 |
| Itsn1 | 1.97961314 |
| Itsn2 | -0.4981726 |
| Ivd | -0.1366151 |
| Ivns1abp | -0.1440874 |
| Ivns1abp | 0.50923983 |
| Iws1 | -0.4858656 |
| Iyd | 1.21495051 |
| Izumo1 | 1.49652202 |
| Jag1 | -0.5483079 |
| Jag2 | 1.11105023 |
| Jagn1 | -0.3171078 |
| Jak1 | -0.4645325 |
| Jak2 | 0.99631905 |
| Jak2 | -0.4065905 |
| Jak3 | 2.06718091 |
| Jak3 | 0.00755103 |
| Jakmip1 | 0.00797661 |
| Jam2 | -0.4072518 |
| Jam2 | 0.61495245 |
| Jam2 | 2.53906177 |
| Jam2 | 3.10521754 |
| Jam3 | -0.1316664 |
| Jam4 | 0.31030404 |
| Jarid1b | 0.72677296 |
| Jarid1c | -0.3099219 |
| Jarid1d | 3.33257843 |
| Jazf1 | -0.6094325 |
| Jdp2 | -0.4132249 |
| Jmjd1a | -0.1425882 |
| Jmjd1a | -0.4680546 |
| Jmjd1a | -0.6617098 |
| Jmjd2a | -0.3457933 |
| Jmjd2b | 0.1672903 |
| Jmjd2b | -0.515826 |
| Jmjd2c | -0.5513873 |
| Jmjd2d | -0.6925568 |
| Jmjd3 | 0.27060143 |
| Jmjd3 | -0.1607382 |
| Jmjd5 | -0.6335236 |
| Jmjd6 | -0.6086127 |
| Jmy | 2.7172065 |
| Josd1 | -0.1891618 |
| Josd2 | -0.5398912 |
| Josd3 | -0.2957127 |
| Josd3 | 3.05100755 |
| Jph1 | -0.7431716 |
| Jph2 | -0.3696428 |
| Jph3 | 2.38110499 |
| Jph4 | 0.48418544 |
| Jph4 | -0.4512676 |
| Jtb | 0.74288476 |
| Jtv1 | -0.6943504 |
| Jtv1 | -0.6326825 |
| Jun | 2.1871044 |
| Junb | -0.163287 |
| Jund1 | 1.98675409 |
| Jup | -0.3570151 |
| Kal1 | 2.78815635 |
| Kalrn | -0.3195154 |
| Kank1 | -0.3316464 |
| Kank3 | -0.1673794 |
| Kank4 | 0.22036333 |
| Kank4 | 1.50574512 |
| Kank4 | -0.1189203 |
| Kars | 0.66683757 |
| Katna1 | -0.7024642 |
| Katnal1 | 1.85712768 |
| Katnb1 | -0.5740194 |
| Kazald1 | 1.16056803 |
| Kbtbd2 | -0.0608571 |
| Kbtbd2 | -0.5437077 |
| Kbtbd4 | -0.3671037 |
| Kbtbd4 | -0.8344433 |
| Kbtbd7 | 0.20323341 |
| Kbtbd7 | -0.5931702 |
| Kbtbd8 | 0.64914137 |
| Kcmf1 | -0.1808042 |
| Kcna1 | -0.3267752 |
| Kcna2 | 3.11742575 |
| Kcna3 | 0.72120756 |
| Kcna5 | -0.1944233 |
| Kcna6 | 1.92909819 |
| Kcnab1 | -0.7300272 |
| Kcnab1 | -0.5875442 |
| Kcnab1 | 1.31531775 |
| Kcnab2 | -0.6118963 |
| Kcnab3 | 1.60187969 |
| Kcnb1 | -0.7088237 |
| Kcnc1 | -0.5040204 |
| Kcnc1 | 0.25763878 |
| Kcnc4 | -0.6651844 |
| Kcnc4 | 0.03688634 |
| Kcnd2 | -0.0342282 |
| Kcne1 | -0.6539385 |
| Kcne1l | -0.6470842 |
| Kcne2 | -0.277095 |
| Kcne4 | -0.7847318 |
| Kcnf1 | -0.3063495 |
| Kcng4 | -0.6739275 |
| Kcnh1 | 2.62040029 |
| Kcnh2 | 1.88669534 |
| Kcnh2 | -0.6069752 |
| Kcnh3 | 0.21968409 |
| Kcnh5 | -0.1668669 |
| Kcnip1 | -0.5136011 |
| Kcnip2 | -0.6513623 |
| Kcnip2 | 0.98584456 |
| Kcnip2 | -0.0892049 |
| Kcnip3 | 2.90388283 |
| Kcnip4 | -0.4610236 |
| Kcnj12 | -0.3639393 |
| Kcnj16 | 2.34586343 |
| Kcnj2 | 1.37388312 |
| Kcnj3 | -0.73096 |
| Kcnj4 | 0.2162805 |
| Kcnj6 | 0.24416406 |
| Kcnj6 | 1.85730645 |
| Kcnj8 | 1.54699446 |
| Kcnj9 | 2.10854278 |
| Kcnk1 | 0.31982059 |
| Kcnk10 | 0.61249414 |
| Kcnk12 | -0.6135424 |
| Kcnk13 | -0.1933686 |
| Kcnk2 | -0.6800963 |
| Kcnk2 | 1.14379888 |
| Kcnk3 | -0.4032908 |
| Kcnk4 | 1.57796977 |
| Kcnk5 | 0.65600348 |
| Kcnk5 | 0.2331766 |
| Kcnk6 | 0.10683911 |
| Kcnk6 | 0.06493409 |
| Kcnk9 | 1.66827403 |
| Kcnma1 | -0.2381618 |
| Kcnmb1 | -0.1430876 |
| Kcnmb4 | 1.70841818 |
| Kcnmb4 | 0.40223629 |
| Kcnn1 | 1.86590704 |
| Kcnn2 | -0.4945362 |
| Kcnn4 | 0.58525046 |
| Kcnq1 | 0.57970575 |
| Kcnq2 | -0.1001191 |
| Kcnq2 | 1.60966481 |
| Kcnq3 | 0.46134052 |
| Kcnq5 | -0.4822763 |
| Kcnrg | -0.3402322 |
| Kcnrg | 0.00242897 |
| Kcns1 | 0.04551148 |
| Kcns2 | 2.58972185 |
| Kcnt1 | -0.2398215 |
| Kcnu1 | -0.0640671 |
| Kcnv1 | 0.667471 |
| Kcnv2 | 0.1502545 |
| Kctd10 | -0.3709149 |
| Kctd11 | 0.44517286 |
| Kctd12 | -0.5165688 |
| Kctd12b | 1.47628247 |
| Kctd12b | 0.33677149 |
| Kctd14 | -0.6669265 |
| Kctd14 | -0.8365292 |
| Kctd15 | -0.3507642 |
| Kctd18 | 1.06861537 |
| Kctd19 | 0.50723983 |
| Kctd2 | 0.59965171 |
| Kctd20 | -0.3850232 |
| Kctd20 | -0.7143092 |
| Kctd3 | 0.18824801 |
| Kctd4 | -0.5262789 |
| Kctd5 | 0.07171789 |
| Kctd5 | 1.1297002 |
| Kctd6 | 1.36084342 |
| Kctd6 | 0.79517259 |
| Kctd9 | -0.4765618 |
| Kdelc1 | -0.5795649 |
| Kdelc1 | 1.82869129 |
| Kdelr2 | -0.6997518 |
| Kdelr3 | 0.82563312 |
| Kdr | -0.6934532 |
| Keap1 | -0.2238995 |
| Keap1 | -0.0742275 |
| Keap1 | 0.20668444 |
| Keap1 | -0.6487931 |
| Kera | -0.0018608 |
| Khdc1c | 3.26352607 |
| Khdrbs1 | -0.3824423 |
| Khdrbs2 | -0.3908563 |
| Khdrbs3 | 0.54621352 |
| Khk | -0.3709149 |
| Khk | 0.7321086 |
| Khsrp | 0.68233902 |
| Kif11 | -0.4952623 |
| Kif13a | 2.38899363 |
| Kif14 | -0.4072518 |
| Kif15 | -0.1802843 |
| Kif17 | 0.72359676 |
| Kif17 | -0.3219291 |
| Kif18a | -0.5591301 |
| Kif1a | 1.03409116 |
| Kif1b | -0.6160169 |
| Kif1b | 2.84023066 |
| Kif1b | 0.77392656 |
| Kif1b | 0.57436123 |
| Kif1b | -0.5345741 |
| Kif1b | 1.39904465 |
| Kif1c | 2.06278488 |
| Kif21a | -0.4306964 |
| Kif21b | -0.7024642 |
| Kif22 | -0.3696428 |
| Kif23 | 0.53461708 |
| Kif24 | 3.65151536 |
| Kif27 | -0.1287096 |
| Kif2a | -0.3316464 |
| Kif2b | 1.89994273 |
| Kif2c | -0.3856696 |
| Kif3a | -0.3129094 |
| Kif3a | 3.85154469 |
| Kif3b | 0.63482817 |
| Kif3c | -0.1058642 |
| Kif3c | -0.8148544 |
| Kif4 | 0.17373381 |
| Kif4 | 0.40586691 |
| Kif4 | 0.86322963 |
| Kif5a | -0.658248 |
| Kif5a | -0.3177091 |
| Kif5a | 2.6115152 |
| Kif5b | 1.69130101 |
| Kif5c | 0.73937159 |
| Kif9 | -0.7632392 |
| Kifap3 | 1.65322054 |
| Kifap3 | 0.6877254 |
| Kifap3 | 0.4039137 |
| Kifc2 | -0.73096 |
| Kifc2 | 0.30566447 |
| Kifc3 | 0.48418544 |
| Kin | -0.6748063 |
| Kirrel3 | -0.6335236 |
| Kiss1 | -0.5622452 |
| Kiss1r | 0.2011567 |
| Kitl | -0.7690523 |
| Kl | -0.2986553 |
| Klb | -0.5513873 |
| Klc1 | 1.98008392 |
| Klc1 | 0.66493472 |
| Klc1 | -0.6251465 |
| Klc2 | 0.37065214 |
| Klc2 | 0.19315323 |
| Klc4 | 2.57283014 |
| Klf1 | 1.09564518 |
| Klf10 | -0.1949511 |
| Klf12 | -0.4443604 |
| Klf12 | 0.14548749 |
| Klf12 | 1.52765385 |
| Klf13 | -0.7758799 |
| Klf16 | -0.4787007 |
| Klf17 | 0.01391463 |
| Klf17 | 1.86235771 |
| Klf2 | 0.63810034 |
| Klf4 | 0.00114409 |
| Klf4 | 0.86562644 |
| Klf4 | -0.0581144 |
| Klf5 | -0.5756005 |
| Klf6 | 0.2237522 |
| Klf6 | -0.5391298 |
| Klf7 | -0.6226478 |
| Klf9 | 1.22106387 |
| Klhdc1 | 1.52799216 |
| Klhdc1 | 0.44329965 |
| Klhdc1 | -0.8026894 |
| Klhdc2 | -0.73096 |
| Klhdc3 | -0.6907666 |
| Klhdc4 | 2.24721026 |
| Klhdc4 | 1.51204471 |
| Klhdc4 | 0.18048776 |
| Klhdc8b | -0.7460124 |
| Klhdc9 | 0.06453352 |
| Klhl10 | -0.6880877 |
| Klhl11 | -0.4279873 |
| Klhl12 | -0.706093 |
| Klhl14 | -0.4815601 |
| Klhl15 | 0.06693441 |
| Klhl15 | 0.82080371 |
| Klhl17 | 0.85427332 |
| Klhl2 | -0.7272343 |
| Klhl21 | 3.08211607 |
| Klhl22 | -0.3928088 |
| Klhl22 | 0.86955086 |
| Klhl23 | 1.17335233 |
| Klhl24 | 0.84906717 |
| Klhl24 | 0.86425758 |
| Klhl25 | -0.4901909 |
| Klhl26 | -0.7651729 |
| Klhl26 | 0.41114813 |
| Klhl30 | 1.95474841 |
| Klhl31 | -0.5560253 |
| Klhl32 | 1.1131554 |
| Klhl5 | 0.84102803 |
| Klhl5 | -0.6318422 |
| Klhl6 | 3.6705313 |
| Klhl6 | -0.7565023 |
| Klhl7 | -0.8189446 |
| Klhl8 | 0.02693123 |
| Klhl9 | 1.0441862 |
| Klhl9 | -0.6234799 |
| Klk1 | -0.7593836 |
| Klk11 | 1.16605055 |
| Klk11 | -0.7375158 |
| Klk15 | -0.1802843 |
| Klk1b1 | -0.546772 |
| Klk1b11 | -0.0631488 |
| Klk1b16 | 0.09294757 |
| Klk1b21 | 1.36728556 |
| Klk1b21 | -0.299245 |
| Klk1b22 | -0.2071722 |
| Klk1b24 | -0.4694672 |
| Klk1b26 | 1.40127266 |
| Klk1b27 | -0.5763921 |
| Klk1b27 | -0.6428255 |
| Klk1b4 | -0.8107819 |
| Klk1b4 | 1.8666743 |
| Klk1b5 | -0.7651729 |
| Klk1b8 | -0.1247817 |
| Klk1b9 | 0.67000046 |
| Klk6 | -0.438187 |
| Klk8 | -0.5653706 |
| Klkb1 | -0.1870654 |
| Klra17 | -0.4105655 |
| Klra4 | -0.840715 |
| Klrd1 | -0.3520111 |
| Kndc1 | 1.09711688 |
| Kng1 | -0.0691336 |
| Kng1 | -0.4808445 |
| Kng2 | 1.05975468 |
| Kns2 | 0.92653335 |
| Kns2 | -0.5398912 |
| Kntc1 | 1.25997323 |
| Kpna1 | 0.46658147 |
| Kpna2 | -0.6871964 |
| Kpna3 | -0.7916746 |
| Kpna6 | 0.47542464 |
| Kpnb1 | 0.1429106 |
| Kptn | -0.2980661 |
| Kras | 1.72878824 |
| Kras | -0.6774476 |
| Krba1 | -0.6880877 |
| Krba1 | 1.97945617 |
| Krba1 | -0.6496487 |
| Krcc1 | -0.5099047 |
| Kremen | 3.7457914 |
| Kremen1 | -0.840715 |
| Kremen2 | -0.6565218 |
| Krit1 | -0.6800963 |
| Krit1 | -0.3111158 |
| Krr1 | 0.23685336 |
| Krr1 | 0.04428415 |
| Krr1 | -0.4457378 |
| Krt10 | 3.19935968 |
| Krt12 | -0.3057554 |
| Krt13 | 0.96853246 |
| Krt14 | -0.1228238 |
| Krt17 | 1.83010304 |
| Krt18 | -0.4744278 |
| Krt19 | 0.45659856 |
| Krt2 | -0.6827526 |
| Krt20 | 1.2698159 |
| Krt222 | -0.7079126 |
| Krt23 | -0.0929868 |
| Krt27 | -0.0562904 |
| Krt32 | 2.0537373 |
| Krt4 | 2.04528521 |
| Krt7 | -0.3087296 |
| Krt73 | 2.36042607 |
| Krt75 | 0.20427005 |
| Krt78 | -0.7300272 |
| Krt79 | -0.546772 |
| Krt8 | -0.7936677 |
| Krt84 | -0.4916371 |
| Krt86 | -0.5300404 |
| Krt86 | 1.28076719 |
| Krt9 | -0.6360514 |
| Krtap16-4 | -0.0293458 |
| Krtap8-2 | -0.6293257 |
| Krtcap2 | -0.0467717 |
| Krtcap3 | 1.47368803 |
| Ksr2 | -0.5544768 |
| Ktelc1 | 0.53801417 |
| Kti12 | 1.35017779 |
| Ktn1 | 1.04912305 |
| Ktn1 | -0.81997 |
| Ky | -0.4422981 |
| L1cam | 2.62085029 |
| L2hgdh | 0.57970575 |
| L3mbtl2 | 1.26992716 |
| L3mbtl3 | -0.7106487 |
| l7Rn6 | 1.3483372 |
| Lace1 | 1.43500851 |
| Lad1 | -0.7143092 |
| Lag3 | 2.75195836 |
| Lage3 | 0.46840893 |
| Lama1 | -0.3960719 |
| Lama2 | 0.5808636 |
| Lamb1-1 | -0.6970472 |
| Lamb2 | 2.20514353 |
| Lamb3 | -0.6487931 |
| Lamc1 | 1.78872547 |
| Lamc2 | -0.4354563 |
| Lamc2 | 1.6176167 |
| Lamc2 | 0.32499876 |
| Lamc3 | 0.28719018 |
| Lamp1 | 0.61495245 |
| Lamp2 | -0.1097138 |
| Lamp2 | -0.7393973 |
| Lamp2 | -0.183408 |
| Lamp2 | -0.3231383 |
| Lancl1 | -0.5247785 |
| Lancl1 | -0.186019 |
| Lancl1 | 0.23818682 |
| Laptm4a | -0.7272343 |
| Laptm4b | -0.7384561 |
| Laptm4b | -0.438187 |
| Laptm5 | 0.47412937 |
| Large | -0.6845276 |
| Larp2 | 1.75366243 |
| Larp5 | 0.72815918 |
| Larp6 | -0.1097138 |
| Larp6 | 0.53704483 |
| Lars | 0.63439102 |
| Lars2 | -0.6318422 |
| Las1l | -0.6513623 |
| Lasp1 | 0.74112982 |
| Lasp1 | -0.2673521 |
| Lasp1 | -0.6836397 |
| Lass1 | 0.72855487 |
| Lass2 | -0.1928418 |
| Lass2 | -0.5113815 |
| Lass2 | -0.6436757 |
| Lass4 | -0.3371569 |
| Lass4 | -0.5406533 |
| Lass5 | -0.1063445 |
| Lass6 | -0.1326541 |
| Lat2 | -0.6368955 |
| Lat2 | -0.4680546 |
| Lats2 | -0.6394323 |
| Lbh | -0.2045019 |
| Lbh | -0.4526551 |
| Lbp | -0.6854163 |
| Lbr | -0.6970472 |
| Lbx2 | -0.6209857 |
| Lca5 | 1.31010878 |
| Lca5 | -0.3683724 |
| Lca5 | 0.55505589 |
| Lcat | -0.1802843 |
| Lcat | 0.35283273 |
| Lce1c | 0.14254189 |
| Lck | -0.658248 |
| Lcmt1 | -0.0266933 |
| Lcmt1 | 1.32638366 |
| Lcmt1 | -0.653079 |
| Lcn10 | -0.515826 |
| Lcn12 | 1.52833035 |
| Lcn13 | 1.07164206 |
| Lcn2 | -0.2974772 |
| Lcn3 | -0.658248 |
| Lcn8 | -0.0535609 |
| Lcor | 0.67629453 |
| Lcp1 | -0.5225324 |
| Ldb1 | -0.4737175 |
| Ldb2 | 0.94147881 |
| Ldb2 | 0.4867467 |
| Ldha | -0.6284884 |
| Ldhc | -0.6617098 |
| Ldhc | -0.3159064 |
| Ldhc | -0.6326825 |
| Ldhd | 0.73427373 |
| Ldhd | -0.4974442 |
| Ldoc1 | 2.37407894 |
| Ldoc1l | 3.14342911 |
| Lef1 | -0.5521587 |
| Lef1 | -0.0196564 |
| Lefty1 | 0.65129116 |
| Lefty2 | -0.5669372 |
| Lemd2 | 2.86439833 |
| Leng1 | 2.97190113 |
| Leng8 | 2.21289244 |
| Leng9 | -0.7365765 |
| Leo1 | -0.1262527 |
| Lep | 1.630065 |
| Lepre1 | -0.3292077 |
| Lepre1 | -0.5638066 |
| Lepre1 | 2.03572193 |
| Leprel1 | -0.3334797 |
| Leprel2 | -0.0896768 |
| Leprotl1 | 1.69392751 |
| Letm1 | 1.07821214 |
| Letm1 | 3.02868177 |
| Letm1 | 1.70695115 |
| Letmd1 | 0.82062437 |
| Lgals1 | -0.1179469 |
| Lgals2 | 2.68364991 |
| Lgals3 | -0.7015592 |
| Lgals3bp | 0.65450667 |
| Lgals4 | -0.538369 |
| Lgals4 | 2.3599722 |
| Lgals6 | -0.7412826 |
| Lgals7 | -0.5988301 |
| Lgals8 | -0.3824423 |
| Lgals8 | 0.51620653 |
| Lgals9 | 3.75148134 |
| Lgals9 | 0.1571785 |
| Lgi1 | 1.61059472 |
| Lgi1 | 1.32207816 |
| Lgi2 | -0.6377404 |
| Lgi3 | -0.6617098 |
| Lgi4 | -0.7106487 |
| Lgmn | 1.95254317 |
| Lgr5 | 2.23205 |
| Lgtn | 0.23985097 |
| Lgtn | -0.4652359 |
| Lhb | -0.4967164 |
| Lhcgr | 0.97859404 |
| Lhcgr | -0.3741028 |
| Lhfp | 2.52007847 |
| Lhfp | -0.4945362 |
| Lhfp | 0.34839871 |
| Lhfpl1 | 1.41729355 |
| Lhfpl2 | -0.8417643 |
| Lhx1 | 2.41852571 |
| Lhx2 | -0.6916613 |
| Lhx3 | -0.4596236 |
| Lhx4 | -0.7574618 |
| Lhx9 | -0.6608431 |
| Lhx9 | 0.22746591 |
| Lias | 1.95076446 |
| Lias | 2.0245652 |
| Lias | 2.87855008 |
| Lifr | -0.211998 |
| Lig1 | -0.9297489 |
| Lig1 | 0.20840521 |
| Lig1 | 0.94729708 |
| Lig3 | -0.6176703 |
| Lig4 | -0.0558349 |
| Lima1 | -0.4645325 |
| Lima1 | 0.86733472 |
| Limd1 | -0.3921575 |
| Limd2 | -0.7788213 |
| Lime1 | 0.20427005 |
| Limk1 | -0.5771843 |
| Limk2 | -0.7555438 |
| Limk2 | 1.99826297 |
| Lims2 | 1.7139166 |
| Lims2 | -0.096784 |
| Lims2 | -0.4687606 |
| Lin54 | -0.7498134 |
| Lin7b | 1.48791379 |
| Lin7b | 1.24775113 |
| Lin7b | -0.1410914 |
| Lin7b | -0.4715899 |
| Lin7c | -0.6827526 |
| Lincr | 1.83530209 |
| Lincr | -0.7024642 |
| Lingo1 | -0.7328285 |
| Lingo2 | -0.9047215 |
| Lingo2 | 1.24649733 |
| Lingo4 | -0.6599773 |
| Lip1 | -0.1521237 |
| Lipe | -0.4533496 |
| Lipf | -0.3741028 |
| Liph | 1.33669934 |
| Liph | -0.4533496 |
| Litaf | 1.92011167 |
| Lix1 | -0.1975944 |
| Lix1 | 0.33496986 |
| Lix1 | -0.2492819 |
| Lix1 | 1.38063257 |
| Llgl1 | 0.63373491 |
| Llgl2 | 0.10989846 |
| Lman1 | 0.81828978 |
| Lman2 | 0.26576134 |
| Lman2l | -0.6428255 |
| Lman2l | 1.51118424 |
| Lmbr1 | -0.7661412 |
| Lmbr1l | -0.6110743 |
| Lmcd1 | -0.717984 |
| Lmcd1 | -0.6160169 |
| Lmcd1 | -0.5630256 |
| Lmf1 | -0.1371114 |
| Lmf2 | -0.6268159 |
| Lmna | 3.390658 |
| Lmna | 0.42817821 |
| Lmna | -0.2431496 |
| Lmna | -0.6110743 |
| Lmna | -0.6310026 |
| Lmnb1 | -0.6625772 |
| Lmnb1 | -0.8026894 |
| Lmnb2 | -0.7517196 |
| Lmo2 | 0.9319666 |
| Lmo3 | 0.01010164 |
| Lmo3 | -0.4185663 |
| Lmo4 | -0.7207495 |
| Lmod1 | 1.75837399 |
| Lmtk2 | 0.39323921 |
| Lmtk3 | 0.67294293 |
| Lmx1a | -0.5685064 |
| Lmx1b | -0.4715899 |
| Lnx1 | -0.3021987 |
| Lnx2 | 0.47361077 |
| Lnx2 | -0.4205771 |
| LOC100038882 | -0.5653706 |
| LOC100038984 | -0.2476055 |
| LOC100039087 | -0.1970651 |
| LOC100039227 | 2.67299454 |
| LOC100039479 | -0.2594037 |
| LOC100039532 | -0.2851949 |
| LOC100039571 | -0.6704205 |
| LOC100039728 | -0.7106487 |
| LOC100040573 | -0.4246118 |
| LOC100040592 | 2.02998654 |
| LOC100040601 | -0.5121208 |
| LOC100040799 | -0.1536382 |
| LOC100040899 | 0.36659218 |
| LOC100040919 | -0.1917889 |
| LOC100041103 | -0.5875442 |
| LOC100041290 | 0.27477612 |
| LOC100041463 | 0.14695683 |
| LOC100041483 | 1.38477728 |
| LOC100041500 | -0.5988301 |
| LOC100041504 | 0.64396173 |
| LOC100041703 | 0.03192196 |
| LOC100041722 | 1.94584204 |
| LOC100041725 | -0.6209857 |
| LOC100041835 | 2.6339902 |
| LOC100042179 | 0.31737391 |
| LOC100042427 | -0.4638297 |
| LOC100042777 | -0.6730495 |
| LOC100043257 | -0.6377404 |
| LOC100043391 | -0.5406533 |
| LOC100043671 | 3.20093229 |
| LOC100043822 | 3.16596472 |
| LOC100043986 | -0.0540153 |
| LOC100044087 | 0.05608181 |
| LOC100044089 | 3.36700015 |
| LOC100044101 | -0.0048754 |
| LOC100044103 | -0.5859429 |
| LOC100044103 | -0.7365765 |
| LOC100044122 | -0.5452386 |
| LOC100044133 | 1.13279434 |
| LOC100044159 | -0.1465914 |
| LOC100044170 | 2.78424941 |
| LOC100044172 | -0.237057 |
| LOC100044177 | -0.5669372 |
| LOC100044190 | -0.2387147 |
| LOC100044204 | -0.0231683 |
| LOC100044210 | 0.02776486 |
| LOC100044221 | -0.6419761 |
| LOC100044257 | 1.83181885 |
| LOC100044275 | -0.5980194 |
| LOC100044289 | 3.07955179 |
| LOC100044292 | -0.0649862 |
| LOC100044294 | 0.25338717 |
| LOC100044298 | 0.91199787 |
| LOC100044322 | 0.42790597 |
| LOC100044324 | 0.98824903 |
| LOC100044324 | -0.5955917 |
| LOC100044363 | -0.12087 |
| LOC100044363 | -0.3532596 |
| LOC100044439 | 1.4235507 |
| LOC100044468 | 0.33316478 |
| LOC100044475 | 1.4852628 |
| LOC100044538 | 1.3193376 |
| LOC100044566 | -0.1213581 |
| LOC100044576 | -0.5756005 |
| LOC100044636 | 1.10085266 |
| LOC100044692 | -0.0369021 |
| LOC100044708 | 2.26684563 |
| LOC100044736 | 0.23250655 |
| LOC100044756 | -0.2045019 |
| LOC100044776 | 0.73309336 |
| LOC100044779 | 1.73158474 |
| LOC100044812 | -0.6310026 |
| LOC100044829 | 0.14032662 |
| LOC100044862 | 0.93387675 |
| LOC100044873 | 0.57249513 |
| LOC100044934 | 1.47485194 |
| LOC100045005 | -0.0183428 |
| LOC100045019 | -0.8292487 |
| LOC100045040 | -0.2588385 |
| LOC100045098 | 2.70057782 |
| LOC100045228 | 1.86584799 |
| LOC100045241 | 0.29725513 |
| LOC100045250 | -0.6704205 |
| LOC100045280 | 1.10722255 |
| LOC100045300 | -0.2476055 |
| LOC100045304 | -0.3464132 |
| LOC100045343 | -0.3457933 |
| LOC100045359 | 0.38386997 |
| LOC100045359 | -0.638586 |
| LOC100045403 | -0.0849683 |
| LOC100045413 | 2.7055248 |
| LOC100045419 | 1.59339875 |
| LOC100045430 | -0.4125593 |
| LOC100045439 | 1.94116628 |
| LOC100045491 | 0.08631568 |
| LOC100045505 | -0.1960075 |
| LOC100045522 | 0.04796135 |
| LOC100045542 | 0.73682611 |
| LOC100045551 | -0.6143665 |
| LOC100045567 | -0.4125593 |
| LOC100045607 | 1.64750349 |
| LOC100045617 | -0.5763921 |
| LOC100045644 | 0.25404252 |
| LOC100045697 | 0.38358459 |
| LOC100045780 | -0.076552 |
| LOC100045782 | 1.77184682 |
| LOC100045877 | 0.42627083 |
| LOC100045882 | -0.5955917 |
| LOC100045887 | -0.6863059 |
| LOC100045950 | 0.1797791 |
| LOC100045963 | 1.11630436 |
| LOC100045981 | 0.25207511 |
| LOC100046003 | 0.71761233 |
| LOC100046035 | -0.0100657 |
| LOC100046035 | 2.16450936 |
| LOC100046039 | 0.08514047 |
| LOC100046044 | -0.6669265 |
| LOC100046049 | 0.70775514 |
| LOC100046056 | 0.9057926 |
| LOC100046068 | 1.02849955 |
| LOC100046080 | -0.6970472 |
| LOC100046081 | 0.1571785 |
| LOC100046120 | -0.3588985 |
| LOC100046128 | 0.94855037 |
| LOC100046129 | 0.92989288 |
| LOC100046163 | -0.0830915 |
| LOC100046163 | -0.6234799 |
| LOC100046207 | -0.7272343 |
| LOC100046207 | -0.7375158 |
| LOC100046207 | 0.21662142 |
| LOC100046232 | -0.5552507 |
| LOC100046254 | 1.23386655 |
| LOC100046320 | 1.16729214 |
| LOC100046393 | -0.3830869 |
| LOC100046406 | -0.3457933 |
| LOC100046457 | 0.30162491 |
| LOC100046518 | -0.6094325 |
| LOC100046568 | 1.21306146 |
| LOC100046586 | 3.28854386 |
| LOC100046608 | 1.43957142 |
| LOC100046608 | 0.24515659 |
| LOC100046608 | 0.16369141 |
| LOC100046608 | 1.71655001 |
| LOC100046616 | -0.2376093 |
| LOC100046641 | 0.51843491 |
| LOC100046650 | 0.94917639 |
| LOC100046690 | 3.7867194 |
| LOC100046690 | 0.28687391 |
| LOC100046741 | 0.49210275 |
| LOC100046744 | -0.5475396 |
| LOC100046744 | -0.671296 |
| LOC100046746 | 1.42090124 |
| LOC100046775 | 1.47027757 |
| LOC100046781 | -0.6845276 |
| LOC100046781 | -0.2736448 |
| LOC100046792 | -0.4293409 |
| LOC100046796 | 0.04592024 |
| LOC100046802 | 1.2887615 |
| LOC100046841 | -0.6143665 |
| LOC100046855 | -0.717984 |
| LOC100046883 | 0.45739055 |
| LOC100046891 | 0.75546311 |
| LOC100046898 | -0.5136011 |
| LOC100046918 | -0.706093 |
| LOC100046953 | -0.4505746 |
| LOC100046959 | -0.9047215 |
| LOC100046996 | 1.04262926 |
| LOC100047009 | -0.807739 |
| LOC100047009 | 0.39943393 |
| LOC100047012 | -0.6997518 |
| LOC100047028 | -0.5398912 |
| LOC100047046 | 1.62221808 |
| LOC100047052 | 1.15303998 |
| LOC100047052 | 1.28340296 |
| LOC100047155 | -0.4751386 |
| LOC100047155 | -0.502555 |
| LOC100047167 | -0.2238995 |
| LOC100047173 | -0.1044246 |
| LOC100047184 | 1.68523923 |
| LOC100047200 | -0.0183428 |
| LOC100047214 | 0.79958495 |
| LOC100047214 | 0.24779822 |
| LOC100047238 | 2.49009022 |
| LOC100047261 | 2.27030922 |
| LOC100047323 | -0.8502016 |
| LOC100047323 | 1.95060259 |
| LOC100047339 | 0.04510255 |
| LOC100047353 | 3.60154638 |
| LOC100047368 | 1.25161598 |
| LOC100047369 | 1.26356431 |
| LOC100047369 | -0.5003613 |
| LOC100047391 | 0.25731242 |
| LOC100047393 | -0.1627767 |
| LOC100047427 | -0.7161448 |
| LOC100047480 | 1.60865638 |
| LOC100047490 | 2.06541541 |
| LOC100047579 | 0.69922313 |
| LOC100047583 | -0.6487931 |
| LOC100047606 | -0.4801293 |
| LOC100047615 | -0.1130952 |
| LOC100047619 | -0.3760208 |
| LOC100047634 | 1.95743075 |
| LOC100047651 | -0.5406533 |
| LOC100047653 | 2.01971429 |
| LOC100047659 | -0.4722986 |
| LOC100047674 | 0.15608862 |
| LOC100047707 | -0.0531068 |
| LOC100047717 | 1.65993257 |
| LOC100047749 | 1.34567218 |
| LOC100047762 | -0.6889798 |
| LOC100047776 | -0.7966653 |
| LOC100047794 | -0.0944089 |
| LOC100047800 | 0.74696696 |
| LOC100047810 | -0.5247785 |
| LOC100047815 | -0.9035995 |
| LOC100047816 | -0.7244496 |
| LOC100047834 | -0.3189129 |
| LOC100047837 | 0.99378594 |
| LOC100047837 | 0.10223135 |
| LOC100047841 | -0.5923644 |
| LOC100047856 | 0.49413515 |
| LOC100047870 | -0.4638297 |
| LOC100047911 | -0.2282649 |
| LOC100047934 | 2.03960801 |
| LOC100047935 | -0.8770666 |
| LOC100047937 | 2.27735091 |
| LOC100047937 | 0.24317049 |
| LOC100047958 | 0.39690466 |
| LOC100047963 | -0.5859429 |
| LOC100047963 | 0.35253777 |
| LOC100047998 | -0.6268159 |
| LOC100048046 | -0.059942 |
| LOC100048046 | -0.1997144 |
| LOC100048049 | -0.5262789 |
| LOC100048049 | -0.9058447 |
| LOC100048049 | 0.85202086 |
| LOC100048056 | 2.38946756 |
| LOC100048071 | -0.4409258 |
| LOC100048076 | -0.5653706 |
| LOC100048083 | -0.041824 |
| LOC100048105 | 2.34625329 |
| LOC100048105 | 0.58594126 |
| LOC100048187 | 1.97083454 |
| LOC100048251 | -0.4930855 |
| LOC100048295 | 0.17515973 |
| LOC100048299 | -0.3033827 |
| LOC100048301 | 1.14392609 |
| LOC100048313 | -0.6293257 |
| LOC100048331 | 0.31184549 |
| LOC100048332 | 2.00978478 |
| LOC100048346 | 1.00991051 |
| LOC100048372 | 2.7842939 |
| LOC100048384 | -0.0087654 |
| LOC100048413 | -0.2851949 |
| LOC100048436 | -0.3279907 |
| LOC100048445 | 1.00800185 |
| LOC100048461 | -0.5069579 |
| LOC100048480 | 2.3044466 |
| LOC100048483 | -0.8271788 |
| LOC100048504 | 0.04428415 |
| LOC100048530 | -0.2013075 |
| LOC100048554 | 2.88304686 |
| LOC100048556 | -0.5398912 |
| LOC100048589 | 1.55819791 |
| LOC100048613 | 0.42654355 |
| LOC100048622 | 2.67392047 |
| LOC100048645 | 0.38955943 |
| LOC100048684 | 0.64633927 |
| LOC100048703 | 2.2539183 |
| LOC100048710 | 2.69999411 |
| LOC100048721 | -0.5360902 |
| LOC100048721 | -0.5827485 |
| LOC100048726 | 0.54861156 |
| LOC100048733 | -0.7460124 |
| LOC100048816 | -0.5677215 |
| LOC100048832 | 0.6229532 |
| LOC100048832 | -0.4192361 |
| LOC100048858 | -0.0078996 |
| LOC192758 | 0.74735481 |
| LOC194227 | -0.5843444 |
| LOC212390 | -0.9758212 |
| LOC216443 | -0.4165597 |
| LOC216963 | -0.6827526 |
| LOC217341 | 2.74193688 |
| LOC218963 | -0.5859429 |
| LOC225922 | -0.7986691 |
| LOC233529 | -0.8480852 |
| LOC234374 | -0.5398912 |
| LOC235497 | -0.5803598 |
| LOC236749 | -0.2420389 |
| LOC237408 | 0.89192867 |
| LOC237877 | 0.82080371 |
| LOC238829 | -0.5599079 |
| LOC241593 | -0.7403395 |
| LOC244595 | 1.24146535 |
| LOC245263 | -0.5599079 |
| LOC329575 | 0.10951659 |
| LOC329575 | 0.749872 |
| LOC333331 | 2.89346311 |
| LOC381066 | -0.7106487 |
| LOC381302 | 0.92685382 |
| LOC381375 | -0.3328682 |
| LOC381483 | 0.32833422 |
| LOC381629 | 2.23008756 |
| LOC381801 | 0.74366369 |
| LOC381936 | 0.30535435 |
| LOC382010 | 1.14481605 |
| LOC382044 | 1.54425574 |
| LOC382106 | -0.3980352 |
| LOC382106 | -0.6343655 |
| LOC382133 | -0.2799798 |
| LOC384806 | -0.6028938 |
| LOC384806 | -0.6739275 |
| LOC386486 | 1.50210149 |
| LOC433479 | -0.7216732 |
| LOC433801 | 3.1602992 |
| LOC433886 | -0.7006551 |
| LOC434172 | 0.53510313 |
| LOC434172 | -0.6765663 |
| LOC434864 | -0.6094325 |
| LOC434960 | 3.19676629 |
| LOC435023 | -0.6479383 |
| LOC435337 | -0.8619278 |
| LOC435565 | -0.6061575 |
| LOC436177 | -0.8902395 |
| LOC544988 | -0.3576425 |
| LOC544988 | 0.0281814 |
| LOC545013 | 0.9057926 |
| LOC545013 | -0.8608557 |
| LOC545056 | -0.4099018 |
| LOC545238 | -0.2916085 |
| LOC545703 | 0.95634582 |
| LOC545732 | 2.66691594 |
| LOC545952 | 0.13215906 |
| LOC546015 | -0.1617569 |
| LOC546015 | -0.2520826 |
| LOC546723 | 1.63029258 |
| LOC546840 | 1.60578031 |
| LOC547323 | 0.37354142 |
| LOC547343 | -0.5040204 |
| LOC620807 | -0.6394323 |
| LOC621823 | -0.0302317 |
| LOC621824 | 0.61784946 |
| LOC622404 | -0.7768594 |
| LOC622665 | 2.25458257 |
| LOC624855 | -0.6310026 |
| LOC625480 | 0.54621352 |
| LOC626152 | -0.3876113 |
| LOC628053 | 1.46793695 |
| LOC631002 | -0.0454198 |
| LOC631784 | -0.7015592 |
| LOC632431 | 1.94138427 |
| LOC639396 | -0.2639374 |
| LOC640963 | -0.4533496 |
| LOC641240 | -0.4652359 |
| LOC654426 | 1.32889484 |
| LOC654467 | 0.92364416 |
| LOC654494 | -0.5811554 |
| LOC654842 | -0.276519 |
| LOC665250 | -0.4225923 |
| LOC666676 | -0.6053405 |
| LOC666904 | 2.53385113 |
| LOC667250 | 0.93483037 |
| LOC667370 | 1.12892507 |
| LOC667609 | 0.70977522 |
| LOC668492 | 0.32621303 |
| LOC668837 | 2.47110626 |
| LOC669168 | -0.0521992 |
| LOC669660 | 0.37065214 |
| LOC670044 | -0.2029034 |
| LOC671564 | 1.14163375 |
| LOC672705 | 3.04712976 |
| LOC674706 | 0.61762693 |
| LOC675228 | 0.58409801 |
| LOC675567 | -0.5716528 |
| LOC675567 | 2.68181624 |
| LOC675813 | 2.51030729 |
| LOC676420 | 0.71961137 |
| LOC676420 | -0.5121208 |
| LOC677144 | -0.1870654 |
| LOC677205 | 1.48038315 |
| LOC677317 | -0.3863164 |
| LOC677319 | 1.15076977 |
| LOC677528 | 1.17100709 |
| LOC732482 | 0.65514845 |
| Loh11cr2a | 0.40195643 |
| Loh12cr1 | 1.75989522 |
| Loh12cr1 | 0.02859775 |
| Lonp2 | -0.4300184 |
| Lonrf1 | 1.8760082 |
| Lonrf2 | -0.4624256 |
| Lor | -0.3414652 |
| Lox | 0.67566701 |
| Loxl1 | -0.5498463 |
| Lpar2 | 0.26284529 |
| Lpar2 | 1.23351969 |
| Lpar3 | -0.0118022 |
| Lpcat2 | -0.5330604 |
| Lpcat2 | 3.00772718 |
| Lpcat2 | -0.5859429 |
| Lpgat1 | 1.82339371 |
| Lphn1 | -0.7006551 |
| Lphn3 | -0.2487228 |
| Lpin1 | -0.4246118 |
| Lpin1 | -0.7024642 |
| Lpin2 | 1.98706559 |
| Lpin3 | 1.49450878 |
| Lpl | -0.7300272 |
| Lpxn | -0.7768594 |
| Lrat | -0.7517196 |
| Lrba | 2.45640773 |
| Lrba | 0.87785737 |
| Lrch1 | 0.4306249 |
| Lrch4 | 2.34362748 |
| Lrch4 | 1.81632504 |
| Lrdd | -0.6547988 |
| Lrfn1 | -0.717984 |
| Lrfn2 | -0.1823656 |
| Lrfn3 | -0.3165069 |
| Lrfn4 | 2.183699 |
| Lrg1 | -0.9297489 |
| Lrig1 | 0.14475195 |
| Lrig3 | 2.07212573 |
| Lrmp | -0.7450645 |
| Lrp1 | -0.4865851 |
| Lrp10 | -0.4484986 |
| Lrp11 | -0.5875442 |
| Lrp12 | 1.84071517 |
| Lrp12 | -0.8128159 |
| Lrp4 | -0.5121208 |
| Lrp5 | 0.91686803 |
| Lrp8 | -0.2304553 |
| Lrp8 | -0.5972095 |
| Lrpap1 | -0.8087522 |
| Lrpprc | -0.501092 |
| Lrpprc | -0.5787707 |
| Lrpprc | -0.2173891 |
| Lrrc1 | -0.3464132 |
| Lrrc1 | -0.7847318 |
| Lrrc1 | 1.12478022 |
| Lrrc10 | -0.7051845 |
| Lrrc15 | 1.0586412 |
| Lrrc16b | 1.39952945 |
| Lrrc16b | -0.3792261 |
| Lrrc18 | 2.49986189 |
| Lrrc2 | 1.66294109 |
| Lrrc20 | 1.82543003 |
| Lrrc20 | -0.0728355 |
| Lrrc23 | -0.7042769 |
| Lrrc23 | 0.21832413 |
| Lrrc24 | 0.92235723 |
| Lrrc26 | 1.0125469 |
| Lrrc28 | -0.2228113 |
| Lrrc29 | 0.70288919 |
| Lrrc3 | 0.85306113 |
| Lrrc30 | 1.74772288 |
| Lrrc33 | -0.7088237 |
| Lrrc34 | 1.28974241 |
| Lrrc34 | 1.12257082 |
| Lrrc36 | 0.48033043 |
| Lrrc39 | -0.7403395 |
| Lrrc39 | 0.64417812 |
| Lrrc4 | -0.2071722 |
| Lrrc40 | -0.5292869 |
| Lrrc41 | -0.7088237 |
| Lrrc43 | 1.03108443 |
| Lrrc44 | -0.7384561 |
| Lrrc45 | -0.546005 |
| Lrrc46 | -0.0422727 |
| Lrrc48 | -0.7170639 |
| Lrrc48 | 2.15073952 |
| Lrrc48 | 0.51571062 |
| Lrrc49 | 1.64906613 |
| Lrrc49 | 3.09473772 |
| Lrrc49 | 2.97275718 |
| Lrrc4c | 2.30983963 |
| Lrrc50 | 3.47859566 |
| Lrrc50 | -0.4938106 |
| Lrrc52 | -0.6368955 |
| Lrrc52 | 1.19624807 |
| Lrrc56 | 0.4191522 |
| Lrrc57 | -0.2055691 |
| Lrrc59 | -0.3805112 |
| Lrrc6 | -0.4680546 |
| Lrrc7 | 1.33212855 |
| Lrrc8 | -0.5180561 |
| Lrrc8a | 2.04601684 |
| Lrrc8b | 0.04796135 |
| Lrrc8c | 0.47981524 |
| Lrrc8d | -0.3677378 |
| Lrrc9 | 0.133277 |
| Lrrfip1 | 0.66026703 |
| Lrrfip1 | 1.64690751 |
| Lrriq2 | -0.4065905 |
| Lrriq2 | 1.96803131 |
| Lrrk1 | 0.80434176 |
| Lrrk2 | -0.2910236 |
| Lrrn1 | 1.31096119 |
| Lrrn2 | -0.4737175 |
| Lrrn3 | 0.97464323 |
| Lrrtm1 | -0.5262789 |
| Lrrtm2 | -0.5398912 |
| Lrrtm3 | 1.28395115 |
| Lrsam1 | -0.2013075 |
| Lrsam1 | -0.5368492 |
| Lrtm1 | 0.54525259 |
| Lrtm1 | 0.45897253 |
| Lsamp | -0.2939516 |
| Lsamp | -0.2662125 |
| Lsg1 | -0.6643145 |
| Lsg1 | 0.07449706 |
| Lsm10 | 1.02619591 |
| Lsm11 | 1.16306404 |
| Lsm12 | 3.61312906 |
| Lsm12 | -0.5891482 |
| Lsm12 | 0.37036272 |
| Lsm14a | 1.43957142 |
| Lsm14b | -0.6069752 |
| Lsm14b | -0.2690641 |
| Lsm14b | 0.30999545 |
| Lsm2 | 2.02486444 |
| Lsm2 | 1.25150253 |
| Lsm3 | -0.5018232 |
| Lsm4 | 2.29097751 |
| Lsm5 | -0.3532596 |
| Lsm6 | 1.46242252 |
| Lsm6 | 1.20032994 |
| Lsm8 | -0.3231383 |
| Lsr | -0.7124771 |
| Lst1 | -0.6453784 |
| Ltb4dh | -0.2179299 |
| Ltb4r1 | -0.7926706 |
| Ltb4r2 | 1.37746239 |
| Ltbp1 | 0.19420103 |
| Ltbp1 | 0.69820224 |
| Ltbp2 | -0.4758499 |
| Ltbp3 | 2.39483336 |
| Ltbp3 | -0.4989016 |
| Ltbp4 | -0.5429431 |
| Ltbp4 | -0.8480852 |
| Ltbp4 | 2.16549752 |
| Ltbr | 2.23695816 |
| Ltc4s | -0.5353319 |
| Ltf | 0.24416406 |
| Ltf | -0.3520111 |
| Ltk | 0.20046344 |
| Ltk | -0.0174681 |
| Ltv1 | -0.1678923 |
| Luc7l | -0.2520826 |
| Luc7l | -0.1782076 |
| Luc7l2 | 0.16188679 |
| Luc7l2 | -0.2255341 |
| Lum | 1.62824243 |
| Luzp1 | 1.99692835 |
| Luzp1 | -0.7070024 |
| Luzp1 | -0.6234799 |
| Luzp2 | 0.32012598 |
| Lxn | -0.3664699 |
| Ly6a | 1.84886704 |
| Ly6c1 | -0.7555438 |
| Ly6d | 0.99034791 |
| Ly6e | -0.5003613 |
| Ly6f | 0.26122135 |
| Ly6g6c | 0.06051836 |
| Ly6g6d | 0.11598748 |
| Ly6g6e | 0.13773554 |
| Ly6k | -0.6617098 |
| Ly86 | 0.2271289 |
| Ly9 | -0.6259808 |
| Ly9 | 2.01745535 |
| Ly96 | 0.71017872 |
| Lyar | -0.5851433 |
| Lyl1 | 2.22792012 |
| Lynx1 | 0.38614989 |
| Lynx1 | 1.49669688 |
| Lypd1 | 0.008402 |
| Lypd3 | -0.7124771 |
| Lypd4 | 0.12354373 |
| Lypd5 | -0.3785841 |
| Lypd6 | 1.05137129 |
| Lypla1 | -0.7996725 |
| Lypla1 | 0.19559629 |
| Lypla2 | 0.37986678 |
| Lypla3 | -0.7498134 |
| Lyplal1 | -0.528534 |
| Lyrm1 | -0.5859429 |
| Lyrm2 | -0.4758499 |
| Lyrm2 | 0.92492934 |
| Lyrm5 | -0.4512676 |
| Lysmd1 | -0.671296 |
| Lysmd1 | -0.1242918 |
| Lysmd2 | 0.42107406 |
| Lysmd4 | 0.54067473 |
| Lyst | -0.7088237 |
| Lyve1 | -0.1896867 |
| Lyz | 0.36775396 |
| Lyz1 | 1.08825196 |
| Lyz1 | -0.5113815 |
| Lyz2 | 3.71444986 |
| Lyzl1 | 2.80037133 |
| Lyzl4 | 1.87296578 |
| Lyzl6 | 3.91216268 |
| Lyzs | -0.1252718 |
| Lzic | -0.5076937 |
| Lztr1 | -0.6651844 |
| Lzts2 | -0.4409258 |
| M6pr | 0.66683757 |
| M6pr | -0.4730078 |
| M6prbp1 | 0.61204648 |
| Mab21l2 | 0.61517561 |
| Macrod1 | -0.5490768 |
| Mad1l1 | -0.5740194 |
| Mad2l1 | 1.16691983 |
| Mad2l1bp | -0.7986691 |
| Mad2l1bp | -0.6845276 |
| Mad2l1bp | 2.80725595 |
| Mad2l2 | -0.7565023 |
| Madcam1 | 0.53096368 |
| Madd | 0.29537614 |
| Maea | 0.84958909 |
| Maf | 0.05890749 |
| Maf1 | -0.4730078 |
| Mafg | 1.4291092 |
| Mag | -0.6069752 |
| Magea2 | 1.9971338 |
| Magea5 | -0.4967164 |
| Mageb3 | -0.5262789 |
| Mageb3 | -0.4138909 |
| Maged1 | 1.04177892 |
| Maged2 | 0.10951659 |
| Magee1 | 0.10261619 |
| Mageh1 | -0.5661536 |
| Magi1 | -0.4887469 |
| Magi1 | 0.9883991 |
| Magi1 | -0.569292 |
| Magi2 | -0.5210379 |
| Magi3 | -0.1556613 |
| Magi3 | -0.8148544 |
| Magix | -0.5669372 |
| Magmas | 3.1046164 |
| Magoh | 1.0069725 |
| Magoh | -0.5150838 |
| Magohb | 1.53708078 |
| Mak | -0.4851467 |
| Mak10 | 0.77052762 |
| Mak10 | -0.2082425 |
| Mal | 2.11662846 |
| Mal | 0.5877809 |
| Mall | -0.6226478 |
| Malt1 | 0.60823278 |
| Mamdc2 | 0.24944548 |
| Maml1 | -0.653079 |
| Maml3 | 1.02648418 |
| Man1a | 0.84854497 |
| Man1a2 | -0.7170639 |
| Man1a2 | 1.54334106 |
| Man1b1 | 2.78458302 |
| Man1c1 | 0.31030404 |
| Man1c1 | -0.4981726 |
| Man2a1 | 3.59296022 |
| Man2a2 | 1.6434733 |
| Man2a2 | 1.85533805 |
| Man2a2 | 0.4610777 |
| Man2b1 | 0.09216977 |
| Man2b2 | 1.10669337 |
| Man2c1 | -0.7263052 |
| Manba | -0.6234799 |
| Manba | 2.15439647 |
| Manbal | -0.1997144 |
| Manea | -0.3856696 |
| Mansc1 | 0.7673062 |
| Maob | 2.1136385 |
| Map1lc3a | -0.2892712 |
| Map1lc3a | 0.20081013 |
| Map1lc3b | 1.57201717 |
| Map2k1 | 1.57572186 |
| Map2k2 | 0.19664137 |
| Map2k2 | -0.5376088 |
| Map2k3 | 0.43873485 |
| Map2k4 | -0.7300272 |
| Map2k6 | 0.71821249 |
| Map2k6 | 0.06653469 |
| Map2k6 | 0.07132019 |
| Map2k7 | 0.6249428 |
| Map2k7 | -0.0342282 |
| Map2k7 | -0.6625772 |
| Map3k1 | 0.50096229 |
| Map3k11 | 0.31676124 |
| Map3k12 | 3.43252673 |
| Map3k12 | 0.63044749 |
| Map3k12 | 0.90135858 |
| Map3k14 | 2.85340538 |
| Map3k3 | 0.32165144 |
| Map3k6 | 2.76093977 |
| Map3k7 | 0.53704483 |
| Map3k7ip1 | 0.46919104 |
| Map3k7ip1 | -0.4478077 |
| Map3k7ip1 | -0.7365765 |
| Map3k7ip2 | -0.5827485 |
| Map3k7ip3 | -0.4212484 |
| Map3k8 | 2.15135 |
| Map3k9 | 0.1914043 |
| Map4k2 | 3.01855765 |
| Map4k4 | -0.7431716 |
| Map4k5 | 0.69595238 |
| Map4k5 | 0.8990512 |
| Mapbpip | -0.2776712 |
| Mapk1 | 1.63874949 |
| Mapk1 | -0.3928088 |
| Mapk1 | -0.6020797 |
| Mapk10 | 0.96745898 |
| Mapk10 | 0.19070382 |
| Mapk11 | 0.50648873 |
| Mapk12 | 0.02065536 |
| Mapk12 | -0.5391298 |
| Mapk12 | 0.27381437 |
| Mapk13 | -0.706093 |
| Mapk13 | -0.6643145 |
| Mapk14 | 0.133277 |
| Mapk15 | 0.31951511 |
| Mapk15 | -0.3075388 |
| Mapk15 | -0.6209857 |
| Mapk1ip1 | 1.7641811 |
| Mapk1ip1 | -0.5180561 |
| Mapk1ip1 | 2.13928529 |
| Mapk3 | -0.7318938 |
| Mapk3 | 1.22713777 |
| Mapk4 | 0.99587252 |
| Mapk6 | 1.0586412 |
| Mapk6 | 0.54741331 |
| Mapk6 | -0.4561329 |
| Mapk8 | -0.5980194 |
| Mapk8ip1 | -0.4808445 |
| Mapk8ip1 | 2.96243285 |
| Mapk8ip2 | -0.299245 |
| Mapk8ip3 | 2.43382331 |
| Mapk8ip3 | 0.22409042 |
| Mapk9 | 0.61851674 |
| Mapk9 | -0.1213581 |
| Mapk9 | 0.94384191 |
| Mapkap1 | 0.71841246 |
| Mapkap1 | -0.4909137 |
| Mapkapk2 | 0.13921703 |
| Mapkapk3 | 0.04714544 |
| Mapkbp1 | 0.18190348 |
| Mapre1 | -0.6845276 |
| Mapre2 | -0.5653706 |
| Mapre2 | -0.6880877 |
| Mapre3 | -0.7365765 |
| Mapt | -0.4730078 |
| Mapt | 0.34750939 |
| Mapt | -0.6827526 |
| Mapt | -0.6871964 |
| March2 | -0.3551355 |
| March3 | -0.7479109 |
| March3 | 0.71841246 |
| March5 | -0.389557 |
| March5 | -0.0996419 |
| March7 | -0.3967259 |
| March8 | -0.7079126 |
| March9 | 3.02526177 |
| March11 | -0.4498821 |
| Marcks | 0.60169116 |
| Marcksl1 | -0.0311183 |
| Marcksl1 | -0.0306749 |
| Marcksl1 | -0.239268 |
| Mare | 0.00797661 |
| Mark1 | 2.31156919 |
| Mark2 | -0.1501082 |
| Mark2 | 0.84767399 |
| Mark3 | 0.09022245 |
| Mark3 | -0.4519611 |
| Mark4 | 0.27027953 |
| Mars | 0.43089636 |
| Mars2 | -0.2939516 |
| Marveld3 | -0.558353 |
| Marveld3 | 2.45340865 |
| Mas1 | -0.4402403 |
| Masp1 | -0.6368955 |
| Masp2 | -0.5748096 |
| Mast1 | -0.3069439 |
| Mast1 | -0.0562904 |
| Mast2 | 0.17551587 |
| Mast2 | 0.45554154 |
| Mat1a | -0.1068251 |
| Mat2a | 0.4327944 |
| Mat2a | 1.31891527 |
| Mat2a | 0.82830545 |
| Mat2a | -0.4436725 |
| Mat2b | -0.5923644 |
| Mat2b | -0.7690523 |
| Matk | 0.26640813 |
| Matk | 0.56546409 |
| Matn4 | -0.4967164 |
| Matn4 | -0.6836397 |
| Matn4 | 2.02226789 |
| Matr3 | 0.61450596 |
| Max | -0.4787007 |
| Maz | -0.6445267 |
| Mb | 0.4505044 |
| Mbc2 | -0.5398912 |
| Mbd1 | -0.5819516 |
| Mbd1 | -0.4436725 |
| Mbd3 | 1.28865244 |
| Mbd3l2 | -0.0892049 |
| Mbd4 | 0.43711845 |
| Mbd5 | -0.4199064 |
| Mbd6 | 2.53527244 |
| Mbip | -0.3677378 |
| Mbl2 | 0.30162491 |
| Mbnl1 | -0.6209857 |
| Mbnl2 | 0.19315323 |
| Mbnl2 | -0.5947838 |
| Mboat1 | 0.0151822 |
| Mboat2 | -0.2656432 |
| Mboat2 | 1.55154135 |
| Mboat2 | 1.86212061 |
| Mboat2 | -0.6943504 |
| Mboat5 | -0.7375158 |
| Mboat5 | 1.6003932 |
| Mbp | -0.6368955 |
| Mbp | -0.653079 |
| Mbp | -0.2146897 |
| Mbtd1 | 0.86631013 |
| Mbtps1 | -0.7033701 |
| Mbtps1 | -0.6988494 |
| Mbtps1 | 1.45239603 |
| Mbtps2 | 0.54932978 |
| Mc3r | -0.0271349 |
| Mc4r | 0.38386997 |
| Mc5r | 0.17658349 |
| Mcam | -0.7739241 |
| Mcam | 1.99027847 |
| Mcam | 0.28940111 |
| Mcat | 0.65086159 |
| Mcc | -0.5292869 |
| Mccc1 | -0.3811545 |
| Mcee | -0.5032874 |
| Mcf2l | -0.1029873 |
| Mcf2l | 3.76654843 |
| Mcf2l | -0.2034359 |
| Mcfd2 | -0.7042769 |
| Mchr1 | -0.2013075 |
| Mcl1 | -0.7375158 |
| Mcm10 | -0.2822941 |
| Mcm10 | -0.7798038 |
| Mcm2 | 2.54122438 |
| Mcm3 | 3.19984604 |
| Mcm4 | 1.60156694 |
| Mcm5 | -0.6863059 |
| Mcm6 | -0.822024 |
| Mcm6 | 0.20150314 |
| Mcm6 | 0.76995994 |
| Mcm7 | 0.42627083 |
| Mcoln1 | 0.39239131 |
| Mcoln2 | 1.79154132 |
| Mcoln3 | 0.27188792 |
| Mcph1 | -0.3785841 |
| Mcph1 | 0.95556917 |
| Mcph1 | 1.51015064 |
| Mcph1 | 0.58938765 |
| Mcpt4 | 0.92845455 |
| Mcrs1 | -0.6143665 |
| Mcrs1 | -0.5685064 |
| Mcrs1 | 3.24984828 |
| Mcts1 | -0.5708652 |
| Mdfic | -0.4758499 |
| Mdga1 | 1.52892188 |
| Mdga2 | -0.5483079 |
| Mdga2 | 1.21495051 |
| Mdh1 | -0.147093 |
| Mdh1 | 0.54043317 |
| Mdh1b | -0.7024642 |
| Mdh2 | -0.6127189 |
| Mdk | 1.02460888 |
| Mdk | 3.80831124 |
| Mdm1 | 0.4360393 |
| Mdm1 | -0.8597849 |
| Mdm1 | 2.54590737 |
| Mdm1 | -0.6453784 |
| Mdm1 | -0.5669372 |
| Mdm1 | -0.5345741 |
| Mdm1 | -0.807739 |
| Mdm1 | 1.4081173 |
| Mdm1 | -0.3353165 |
| Mdm2 | -0.1282177 |
| Mdm2 | -0.3850232 |
| Mdm4 | 0.08788033 |
| Mdn1 | 0.43765756 |
| Me2 | -0.3683724 |
| Me3 | 2.22029451 |
| Mea1 | -0.538369 |
| Mecp2 | -0.2968887 |
| Mecr | 0.48521078 |
| Med1 | -0.0422727 |
| Med1 | -0.6617098 |
| Med10 | 1.42600426 |
| Med11 | 0.84505619 |
| Med11 | 0.19210427 |
| Med12 | 0.99453168 |
| Med15 | 0.84558033 |
| Med15 | 1.75485915 |
| Med15 | -0.0714456 |
| Med16 | 1.0801198 |
| Med18 | -0.4293409 |
| Med18 | 1.38595814 |
| Med19 | -0.5406533 |
| Med21 | -0.6979479 |
| Med22 | -0.2822941 |
| Med22 | 0.1406962 |
| Med23 | -0.505488 |
| Med23 | 2.17493991 |
| Med24 | 1.6880967 |
| Med25 | -0.4758499 |
| Med25 | -0.3322571 |
| Med26 | -0.0549247 |
| Med27 | -0.1020302 |
| Med28 | -0.4610236 |
| Med30 | 1.26322823 |
| Med31 | 2.15217787 |
| Med4 | -0.14609 |
| Med6 | 0.48649089 |
| Med7 | 1.43957142 |
| Med8 | -0.5552507 |
| Med8 | 2.0891376 |
| Med9 | -0.7006551 |
| Mef2b | 0.98764849 |
| Mef2b | -0.6326825 |
| Mef2c | -0.6677988 |
| Mef2c | 1.70155239 |
| Mef2c | -0.4887469 |
| Megf10 | -0.5521587 |
| Megf11 | -0.8026894 |
| Megf11 | -0.4894686 |
| Megf11 | -0.7632392 |
| Megf11 | 2.703394 |
| Megf6 | -0.3817982 |
| Megf9 | -0.6651844 |
| Mei1 | 2.47883206 |
| Meis1 | 1.21436059 |
| Meis2 | -0.7867102 |
| Meis3 | 1.36668348 |
| Melk | 1.92228295 |
| Memo1 | -0.1371114 |
| Men1 | 2.32009672 |
| Men1 | -0.6565218 |
| Meox1 | -0.6651844 |
| Meox2 | 1.46748616 |
| Mep1b | -0.3507642 |
| Mepce | -0.3171078 |
| Mertk | -0.760346 |
| Mesdc1 | -0.2002451 |
| Mesp1 | -0.7622738 |
| Mest | -0.3507642 |
| Mest | -0.8148544 |
| Mest | -0.3626766 |
| Metap1 | 2.13840179 |
| Metap2 | -0.7115625 |
| Metap2 | 0.6300083 |
| Metapl1 | -0.4638297 |
| Metrn | -0.5955917 |
| Metrnl | -0.1321601 |
| Metrnl | -0.4967164 |
| Mett11d1 | -0.1386019 |
| Mettl1 | -0.7632392 |
| Mettl1 | 0.56992318 |
| Mettl2 | -0.6934532 |
| Mettl3 | -0.6259808 |
| Mettl4 | -0.5795649 |
| Mettl6 | -0.6608431 |
| Mettl8 | 0.80141734 |
| Mettl9 | -0.4519611 |
| Mettl9 | -0.0944089 |
| Mex3a | 3.17035761 |
| Mex3a | 0.19489892 |
| Mex3c | 0.22442851 |
| Mfap1b | -0.5180561 |
| Mfap2 | 1.99543751 |
| Mfap2 | 0.57389505 |
| Mfap2 | -0.2543292 |
| Mfap3 | 3.26459467 |
| Mfap3 | 2.22582822 |
| Mfap3l | -0.5330604 |
| Mfap3l | -0.1551551 |
| Mfap4 | 0.9067751 |
| Mfap5 | 3.11725402 |
| Mfge8 | 1.73552662 |
| Mfge8 | 0.16044059 |
| Mfi2 | 0.02609687 |
| Mfn1 | -0.7133927 |
| Mfn1 | 0.79110967 |
| Mfn2 | -0.5040204 |
| Mfng | -0.558353 |
| Mfrp | -0.5188007 |
| Mfsd1 | -0.6037087 |
| Mfsd10 | -0.4887469 |
| Mfsd10 | 2.60781947 |
| Mfsd11 | 1.38939386 |
| Mfsd2 | 4.71778834 |
| Mfsd3 | -0.5173122 |
| Mfsd4 | 0.60755817 |
| Mfsd7 | -0.505488 |
| Mfsd8 | 1.00888325 |
| Mfsd9 | 0.51223188 |
| Mga | -0.0139774 |
| Mgat1 | 0.37094147 |
| Mgat3 | 1.29712087 |
| Mgat4b | 0.50096229 |
| Mgat4b | -0.4491901 |
| Mgat5 | -0.6704205 |
| MGC107098 | 0.97555643 |
| MGC117608 | -0.5537035 |
| MGC118210 | 0.98418791 |
| MGC18837 | 0.6269282 |
| MGC41689 | -0.6293257 |
| MGC67181 | -0.7867102 |
| Mgea6 | 0.41474458 |
| Mgl1 | -0.8016827 |
| Mgl2 | 0.36222269 |
| Mgll | 2.20682994 |
| Mgll | -0.6556599 |
| Mgll | -0.6127189 |
| Mgmt | 0.37151986 |
| Mgp | 0.72974094 |
| Mgrn1 | 3.16035886 |
| Mgst1 | -0.6925568 |
| Mgst1 | -0.2548917 |
| Mgst2 | 0.87988049 |
| Mgst3 | 1.12296109 |
| Mgst3 | -0.770998 |
| Mia1 | -0.2271717 |
| Mia3 | -0.1902118 |
| Mib1 | 2.25544153 |
| Mib2 | 0.74230015 |
| Mic2l1 | 0.14842389 |
| Mical1 | 1.22818505 |
| Mical1 | -0.0553797 |
| Mical1 | -0.638586 |
| Mical2 | 0.60055868 |
| Mical3 | 1.63105076 |
| Micall2 | -0.6436757 |
| Micall2 | 0.24680848 |
| Mid1 | 1.66381982 |
| Mid1 | 0.14952268 |
| Mid1ip1 | -0.0654462 |
| Midn | -0.388908 |
| Midn | 1.25286298 |
| Mier1 | 0.8805539 |
| Mier1 | -0.2639374 |
| Mif | 0.03399364 |
| Mif4gd | -0.2702072 |
| Mif4gd | -0.502555 |
| Mill2 | 1.84192732 |
| Mill2 | -0.3639393 |
| Mina | 0.40558813 |
| Mink1 | -0.3779426 |
| Mink1 | -0.7272343 |
| Minpp1 | 1.11289251 |
| Minpp1 | -0.73096 |
| Miox | -0.1886373 |
| Mipep | 3.62875906 |
| Mir16 | -0.7847318 |
| Mist1 | -0.5899512 |
| Mitf | -0.6077935 |
| Mizf | -0.2639374 |
| Mki67ip | -0.5622452 |
| Mkks | -0.0581144 |
| Mkks | 1.72967584 |
| Mkl1 | 2.01029135 |
| Mkl2 | 0.68834493 |
| Mkl2 | -0.1296942 |
| Mkl2 | -0.4225923 |
| Mkln1 | 1.23502183 |
| Mknk1 | -0.6377404 |
| Mknk2 | 0.84208051 |
| Mkrn1 | 1.26769948 |
| Mkrn2 | -0.4880258 |
| Mkrn3 | 0.68275445 |
| Mkx | 0.69779358 |
| Mlana | 0.29725513 |
| Mlf1 | -0.438187 |
| Mlf1 | -0.2974772 |
| Mlf2 | 2.35566703 |
| Mlf2 | -0.6907666 |
| Mlh1 | -0.6276518 |
| Mlh3 | 1.34546687 |
| Mlkl | -0.2910236 |
| Mllt1 | -0.2805578 |
| Mllt1 | 0.31092093 |
| Mllt1 | -0.515826 |
| Mllt10 | -0.2249889 |
| Mllt11 | 0.00755103 |
| Mllt3 | -0.0816864 |
| Mllt3 | -0.6045242 |
| Mllt3 | -0.5771843 |
| Mlp | -0.7244496 |
| Mlstd2 | 0.97980635 |
| Mlx | 0.45554154 |
| Mlx | -0.7498134 |
| Mlxipl | -0.5748096 |
| Mlycd | -0.2939516 |
| Mmab | -0.2869398 |
| Mmab | 0.28465703 |
| Mmd | 0.45474799 |
| Mmd2 | 1.35527175 |
| Mme | -0.2260797 |
| Mme | 1.99466545 |
| Mmel1 | 0.88324272 |
| Mmp10 | -0.4320539 |
| Mmp11 | -0.1058642 |
| Mmp12 | 3.19384915 |
| Mmp13 | 0.95354689 |
| Mmp13 | 1.26423611 |
| Mmp14 | 1.9475218 |
| Mmp15 | -0.4779872 |
| Mmp16 | -0.6907666 |
| Mmp17 | -0.058571 |
| Mmp1a | 0.05769752 |
| Mmp1a | 0.14952268 |
| Mmp2 | 0.29192154 |
| Mmp23 | -0.5996414 |
| Mmp24 | 1.68480986 |
| Mmp3 | 2.15942329 |
| Mmp3 | -0.4039498 |
| Mmp3 | 0.87278046 |
| Mmp9 | -0.6792126 |
| Mmrn2 | -0.0302317 |
| Mnat1 | 0.81703029 |
| Mnat1 | -0.7798038 |
| Mnd1 | 0.48700244 |
| Mns1 | -0.4617243 |
| Mnt | 0.03067676 |
| Mobk1b | 1.45028601 |
| Mobkl2a | -0.4715899 |
| Mobkl2b | -0.7106487 |
| Mobkl2c | -0.6651844 |
| Mobkl3 | -0.7719723 |
| Mobp | 0.33014859 |
| Mobp | -0.5875442 |
| Mobp | 2.6512379 |
| Mocos | -0.4751386 |
| Mocs1 | 0.61784946 |
| Mocs2 | -0.3153062 |
| Mod1 | -0.7051845 |
| Mog | 1.86448891 |
| Mog | -0.2690641 |
| Mon1a | 0.68067545 |
| Mon1a | 1.56375 |
| Mon1b | 0.38101229 |
| Mon1b | -0.7867102 |
| Mon1b | 0.22881272 |
| Morc2a | -0.0240483 |
| Morc2b | 0.64136118 |
| Morc3 | -0.8117984 |
| Morf4l1 | -0.7412826 |
| Morf4l1 | 0.64569145 |
| Morf4l2 | -0.5106428 |
| Morn2 | 1.8476036 |
| Morn3 | 0.81685022 |
| Morn3 | -0.1044246 |
| Mos | -0.4152243 |
| Mosc2 | -0.5980194 |
| Mospd1 | -0.4751386 |
| Mospd1 | 1.44798712 |
| Mospd1 | -0.5972095 |
| Mospd3 | -0.5622452 |
| Mospd3 | -0.3747417 |
| Mov10 | 1.49730863 |
| Mov10l1 | 1.74932825 |
| Moxd1 | 0.79129473 |
| Moxd1 | -0.6077935 |
| Mpdu1 | 0.36949394 |
| Mpdz | -0.3671037 |
| Mpdz | -0.7244496 |
| Mpg | -0.3105187 |
| Mpg | 2.11454555 |
| Mphosph6 | -0.4589245 |
| Mpnd | -0.1073059 |
| Mpo | 0.65386445 |
| Mpo | 0.94242476 |
| Mpp1 | -0.6151914 |
| Mpp2 | 0.41970171 |
| Mpp3 | 0.57132693 |
| Mpp3 | 2.32554594 |
| Mpp5 | 2.15643643 |
| Mpp6 | 2.37366603 |
| Mppe1 | 0.31030404 |
| Mpped1 | -0.2673521 |
| Mpst | 1.17495361 |
| Mpv17 | 0.20874899 |
| Mpv17 | -0.7300272 |
| Mpv17 | 0.96899215 |
| Mpv17 | -0.6445267 |
| Mpv17l | 0.18578578 |
| Mpv17l | 0.36920416 |
| Mpv17l | 3.36492654 |
| Mpz | 1.0833811 |
| Mr1 | 0.77298364 |
| Mrap | 2.24385797 |
| Mras | -0.4165597 |
| Mrc1 | -0.8148544 |
| Mrc2 | -0.0636079 |
| Mrc2 | 1.02893086 |
| Mre11a | 0.41502067 |
| Mre11a | -0.4822763 |
| Mre11a | -0.4974442 |
| Mreg | 0.61405926 |
| Mrgpra2 | -0.4894686 |
| Mrgpra2 | 2.35563194 |
| Mrgprb2 | -0.2939516 |
| Mrgpre | -0.610253 |
| Mrgprf | -0.8016827 |
| Mrgprg | 0.1790699 |
| Mrm1 | -0.7584222 |
| Mro | -0.7300272 |
| Mrp63 | -0.1242918 |
| Mrpl1 | 0.79351261 |
| Mrpl1 | -0.7079126 |
| Mrpl10 | -0.2071722 |
| Mrpl10 | 2.59721442 |
| Mrpl11 | -0.5716528 |
| Mrpl12 | -0.6979479 |
| Mrpl13 | 0.16549258 |
| Mrpl13 | 0.95120808 |
| Mrpl14 | -0.4829931 |
| Mrpl15 | 1.08527832 |
| Mrpl16 | -0.5202916 |
| Mrpl17 | 1.29495674 |
| Mrpl18 | 0.33587111 |
| Mrpl2 | -0.1782076 |
| Mrpl20 | -0.6599773 |
| Mrpl22 | -0.2747934 |
| Mrpl23 | 0.27381437 |
| Mrpl24 | -0.3219291 |
| Mrpl27 | -0.6428255 |
| Mrpl28 | -0.8026894 |
| Mrpl3 | 0.42926641 |
| Mrpl3 | -0.6470842 |
| Mrpl30 | 0.28813836 |
| Mrpl33 | -0.4575277 |
| Mrpl33 | -0.5165688 |
| Mrpl34 | 1.5196684 |
| Mrpl36 | -0.6854163 |
| Mrpl37 | 0.43981092 |
| Mrpl37 | 0.14585504 |
| Mrpl38 | 0.82456206 |
| Mrpl39 | -0.2310036 |
| Mrpl40 | -0.4273112 |
| Mrpl43 | 1.59686318 |
| Mrpl43 | 1.07780285 |
| Mrpl45 | -0.5875442 |
| Mrpl46 | 0.09333623 |
| Mrpl47 | -0.6836397 |
| Mrpl47 | 0.62162445 |
| Mrpl48 | -0.2077072 |
| Mrpl48 | 0.40530926 |
| Mrpl48 | -0.1896867 |
| Mrpl49 | 1.87507312 |
| Mrpl49 | -0.6176703 |
| Mrpl49 | 2.2440555 |
| Mrpl49 | 1.09912004 |
| Mrpl50 | -0.6988494 |
| Mrpl51 | 0.19872806 |
| Mrpl51 | -0.4402403 |
| Mrpl51 | -0.5498463 |
| Mrpl52 | -0.438871 |
| Mrpl53 | 0.21696221 |
| Mrpl54 | 0.04632881 |
| Mrpl55 | -0.3973803 |
| Mrpl9 | -0.3607856 |
| Mrps10 | 0.67000046 |
| Mrps10 | 2.37394132 |
| Mrps11 | -0.3620459 |
| Mrps11 | 0.9516763 |
| Mrps12 | -0.6695458 |
| Mrps14 | 0.50096229 |
| Mrps15 | -0.6792126 |
| Mrps16 | -0.4079136 |
| Mrps17 | -0.5939767 |
| Mrps18a | -0.5210379 |
| Mrps18b | 0.39521469 |
| Mrps18b | 1.02157164 |
| Mrps18c | -0.5143422 |
| Mrps2 | -0.5883458 |
| Mrps21 | -0.2442616 |
| Mrps22 | 1.12087777 |
| Mrps23 | 2.40170665 |
| Mrps24 | 1.31499966 |
| Mrps25 | -0.5292869 |
| Mrps25 | 0.3289394 |
| Mrps25 | 1.35068843 |
| Mrps26 | 0.16224799 |
| Mrps26 | -0.3129094 |
| Mrps27 | 2.57001306 |
| Mrps28 | -0.6127189 |
| Mrps30 | 0.74327431 |
| Mrps31 | 1.96117322 |
| Mrps31 | -0.5708652 |
| Mrps31 | -0.6988494 |
| Mrps33 | 0.27637685 |
| Mrps33 | -0.7545863 |
| Mrps34 | 0.65300747 |
| Mrps35 | 1.58316751 |
| Mrps5 | 3.2063306 |
| Mrps6 | -0.7384561 |
| Mrps7 | -0.6625772 |
| Mrps7 | 0.94352717 |
| Mrps9 | -0.0709827 |
| Mrrf | 0.36076167 |
| Mrrf | -0.0982119 |
| Mrto4 | -0.7143092 |
| Mrvi1 | -0.4666442 |
| Ms4a1 | 3.78553078 |
| Ms4a10 | -0.5568006 |
| Ms4a13 | 1.58730513 |
| Ms4a5 | 0.9779873 |
| Ms4a6d | -0.5084301 |
| Ms4a6d | 0.55434203 |
| Ms4a7 | -0.7097358 |
| Ms4a8a | 1.81819037 |
| Msc | -0.4596236 |
| Msh2 | 0.25240329 |
| Msh3 | 1.3323368 |
| Msh3 | -0.5819516 |
| Msh4 | 1.09040871 |
| Msh5 | 0.84540565 |
| Msh5 | -0.7300272 |
| Msh6 | 0.46344041 |
| Msh6 | 0.89690356 |
| Msi2 | -0.1627767 |
| Msi2h | 0.50272422 |
| Msln | -0.2492819 |
| Msn | -0.5421792 |
| Msr2 | -0.6318422 |
| Msrb2 | -0.5955917 |
| Msrb2 | 3.77595958 |
| Msrb3 | 1.41309888 |
| Mst1r | -0.6695458 |
| Msto1 | -0.7216732 |
| Msx1 | 2.86790539 |
| Mt1 | -0.2548917 |
| Mt3 | 0.99810302 |
| Mta1 | 0.49464257 |
| Mta2 | 0.80324617 |
| Mta3 | 1.18693784 |
| Mta3 | 0.68067545 |
| Mtag2 | 2.03606692 |
| Mtap | -0.6479383 |
| Mtap | -0.2077072 |
| Mtap1b | -0.0109336 |
| Mtap1b | -0.3563881 |
| Mtap1b | 1.73294588 |
| Mtap1b | 1.46658393 |
| Mtap1s | -0.7042769 |
| Mtap2 | 1.09751785 |
| Mtap2 | 0.22510434 |
| Mtap2 | -0.3414652 |
| Mtap4 | 0.89441941 |
| Mtap6 | 1.51410663 |
| Mtap6 | -0.6522203 |
| Mtap7 | 2.96680339 |
| Mtap7d1 | -0.7403395 |
| Mtap7d1 | -0.7441176 |
| Mtap7d2 | -0.6368955 |
| Mtap7d3 | 1.91698514 |
| Mtbp | 0.32469495 |
| Mtbp | 2.00348056 |
| Mtbp | 0.13995691 |
| Mtch1 | 2.67933261 |
| Mtch1 | 0.20806131 |
| Mtch2 | -0.7916746 |
| Mtch2 | -0.145589 |
| Mtch2 | -0.3057554 |
| Mtcp1 | 0.35283273 |
| Mtdh | 1.21906964 |
| Mterf | 3.98139667 |
| Mterf | -0.6513623 |
| Mterfd1 | -0.183408 |
| Mterfd2 | -0.3863164 |
| Mterfd2 | 2.23272989 |
| Mterfd2 | 3.59215006 |
| Mterfd3 | -0.3063495 |
| Mterfd3 | -0.3033827 |
| Mtf1 | 0.26316975 |
| Mtf1 | 1.88014644 |
| Mtf2 | -0.6513623 |
| Mtfmt | 0.24680848 |
| Mtfmt | -0.3310361 |
| Mtg1 | -0.0275767 |
| Mthfd1 | 0.55719423 |
| Mthfd2 | -0.0324499 |
| Mthfs | -0.2622347 |
| Mthfsd | -0.6094325 |
| Mthfsd | -0.0944089 |
| Mtif3 | -0.6827526 |
| Mtl5 | 0.64914137 |
| Mtl5 | 1.79943185 |
| Mtmr1 | -0.3947653 |
| Mtmr11 | -0.7097358 |
| Mtmr11 | -0.8502016 |
| Mtmr12 | -0.0925132 |
| Mtmr14 | 0.31153741 |
| Mtmr14 | 1.7903904 |
| Mtmr14 | -0.4505746 |
| Mtmr14 | -0.5568006 |
| Mtmr2 | -0.6118963 |
| Mtmr2 | -0.6916613 |
| Mtmr2 | 0.10184635 |
| Mtmr3 | -0.0018608 |
| Mtmr4 | 0.0812125 |
| Mtmr9 | 0.69983514 |
| Mto1 | 0.28971653 |
| Mtpn | -0.4851467 |
| Mtrf1 | -0.6677988 |
| Mtrf1 | 0.26543778 |
| Mtrf1l | -0.7051845 |
| Mtrr | -0.4989016 |
| Mtss1 | -0.658248 |
| Mttp | 2.19058113 |
| Mtus1 | -0.2594037 |
| Mtus1 | -0.5091671 |
| Mtvr2 | 0.56922051 |
| Mtvr2 | -0.5165688 |
| Mtx1 | 0.41336296 |
| Mtx1 | -0.1607382 |
| Mtx1 | 3.57475268 |
| Mtx2 | 0.04018144 |
| Muc1 | 3.45132222 |
| Muc1 | -0.142089 |
| Muc1 | -0.4013165 |
| Muc1 | 0.01222188 |
| Muc13 | 0.14915656 |
| Muc5ac | 3.64174058 |
| Mug2 | 0.32469495 |
| Mug2 | 0.73976259 |
| Mug4 | -0.7584222 |
| Mum1 | -0.2782478 |
| Mup1 | 2.41635747 |
| Mup2 | -0.4118942 |
| Mup2 | 1.74053206 |
| Mup3 | 0.40195643 |
| Mupcdh | 0.55124236 |
| Mus81 | -0.5732299 |
| Mus81 | 1.97945617 |
| Mustn1 | -0.6094325 |
| Mustn1 | 3.32722299 |
| Muted | 2.1871044 |
| Muted | 0.60146477 |
| Mutyh | 0.53486014 |
| Mvd | -0.7916746 |
| Mvp | -0.2504012 |
| Mx2 | -0.6453784 |
| Mxd1 | 2.64279578 |
| Mxd4 | -0.6625772 |
| Mxi1 | -0.3488971 |
| Mxra7 | 1.58881242 |
| Mxra8 | 1.05165195 |
| Mxra8 | -0.3177091 |
| Myadm | 1.5058317 |
| Mybbp1a | -0.0236082 |
| Mybbp1a | 0.4510359 |
| Mybbp1a | 1.83560702 |
| Mybl2 | -0.0788823 |
| Mybpc2 | -0.2916085 |
| Mybpc3 | -0.6419761 |
| Myc | -0.6160169 |
| Mycbp | 0.63918855 |
| Mycbp2 | -0.7207495 |
| Mycbp2 | -0.1435874 |
| Mycbpap | -0.5099047 |
| Mycl1 | -0.3010161 |
| Myd116 | 0.8515003 |
| Myd88 | -0.6827526 |
| Myef2 | -0.2963005 |
| Myg1 | 2.30370541 |
| Myg1 | -0.1174606 |
| Myg1 | 2.43698192 |
| Myh10 | -0.2588385 |
| Myh11 | 2.03847827 |
| Myh8 | 1.91485763 |
| Myh9 | 1.65240594 |
| Myl1 | 0.90152317 |
| Myl2 | -0.492361 |
| Myl4 | -0.2244441 |
| Myl6 | -0.3390009 |
| Myl6b | -0.3645714 |
| Mylc2b | -0.4505746 |
| Mylc2b | -0.7015592 |
| Mylc2b | -0.5552507 |
| Mylc2pl | -0.1155179 |
| Mylk | 1.5737908 |
| Mylk | 1.11578026 |
| Mynn | -0.4457378 |
| Myo10 | -0.7460124 |
| Myo10 | 1.25783453 |
| Myo15 | 0.13476553 |
| Myo18a | 1.92250536 |
| Myo1b | -0.7632392 |
| Myo1c | -0.5475396 |
| Myo1c | 0.59419149 |
| Myo1f | 0.31276916 |
| Myo1g | -0.3507642 |
| Myo3b | -0.5622452 |
| Myo5a | 0.1658524 |
| Myo5b | -0.7996725 |
| Myo6 | -0.5669372 |
| Myo7a | 1.79211625 |
| Myo9a | 0.08748941 |
| Myo9b | -0.6061575 |
| Myo9b | 0.5664046 |
| Myoc | -0.515826 |
| Myocd | -0.2974772 |
| Myocd | 0.86271523 |
| Myom1 | -0.4730078 |
| Myom1 | 4.73934456 |
| Myrip | -0.2293594 |
| Mysm1 | 1.75685029 |
| Myst1 | 2.24949087 |
| Myst2 | -0.4092386 |
| Myst4 | -0.6301638 |
| Myst4 | -0.6436757 |
| Myst4 | 0.13141302 |
| Myt1 | 0.76293017 |
| Myt1l | -0.0122368 |
| Myt1l | 0.06813256 |
| Mzf1 | -0.1881131 |
| Mzf1 | -0.6800963 |
| N28178 | -0.4484986 |
| N4bp2 | 0.75680772 |
| N6amt1 | -0.7235233 |
| N6amt1 | -0.3219291 |
| N6amt2 | -0.2565811 |
| Naalad2 | 0.28971653 |
| Nab1 | -0.3677378 |
| Nab1 | -0.5429431 |
| Nab1 | -0.5859429 |
| Nab1 | -0.5599079 |
| Nab2 | 2.20851333 |
| Naca | -0.12087 |
| Naca | -0.5606864 |
| Naca | -0.4751386 |
| Nadk | 0.87768859 |
| Nadsyn1 | 0.33617133 |
| Nadsyn1 | -0.1063445 |
| Naga | -0.6462309 |
| Nagk | 1.58029231 |
| Nagk | 0.51991758 |
| Nagk | -0.4533496 |
| Nagk | -0.6695458 |
| Naglu | -0.3347038 |
| Nagpa | 2.77330839 |
| Nags | -0.6556599 |
| Nags | 0.66514633 |
| Naif1 | 1.91519387 |
| Naif1 | -0.6037087 |
| Naip5 | -0.5099047 |
| Nalp10 | -0.6377404 |
| Nampt | -0.2157685 |
| Nanos1 | -0.5964003 |
| Nanos2 | -0.6880877 |
| Nans | 2.27624457 |
| Nap1l1 | -0.5867432 |
| Nap1l1 | -0.5076937 |
| Nap1l3 | -0.0756215 |
| Nap1l4 | -0.0113678 |
| Nap1l5 | -0.4687606 |
| Napa | 3.39449145 |
| Napa | 0.0256794 |
| Napa | 1.95716286 |
| Napb | -0.1870654 |
| Napepld | -0.1445876 |
| Napg | 0.91036887 |
| Napg | -0.0445195 |
| Naprt1 | 0.23015761 |
| Naprt1 | -0.5740194 |
| Napsa | -0.0222891 |
| Napsa | -0.2409296 |
| Napsa | 0.96822588 |
| Narf | -0.5032874 |
| Narfl | 2.16596975 |
| Narg1 | -0.1435874 |
| Narg1 | -0.6730495 |
| Narg1l | 1.38576143 |
| Narg1l | -0.4645325 |
| Narg1l | 1.99646593 |
| Narg2 | -0.4065905 |
| Nars | 0.25796503 |
| Nars2 | -0.2228113 |
| Nasp | -0.5843444 |
| Nasp | -0.1511154 |
| Nasp | -0.6634455 |
| Nasp | -0.4019741 |
| Nat1 | -0.7216732 |
| Nat10 | -0.2425941 |
| Nat14 | 1.24192391 |
| Nat2 | -0.6943504 |
| Nat5 | -0.7024642 |
| Nat5 | -0.4901909 |
| Nat6 | 1.64705654 |
| Nat6 | 2.98159976 |
| Nav1 | 1.25308953 |
| Nav1 | -0.6979479 |
| Nbl1 | 0.4097612 |
| Nbr1 | 1.03466278 |
| Nbr1 | 0.56804824 |
| Ncald | -0.2869398 |
| Ncam1 | -0.1233129 |
| Ncam1 | 0.36338987 |
| Ncam2 | 0.2843399 |
| Ncan | 1.07342599 |
| Ncapd2 | 1.91799123 |
| Ncapd2 | 0.26608479 |
| Ncapd2 | 0.41419218 |
| Ncapd3 | 1.57981226 |
| Ncapg2 | -0.4952623 |
| Ncapg2 | 2.75004564 |
| Ncaph | 0.59327836 |
| Ncaph2 | 1.23606038 |
| Ncaph2 | -0.5360902 |
| Ncaph2 | -0.7749015 |
| Ncbp2 | 0.54765308 |
| Ncbp2 | 0.06333077 |
| Ncdn | 1.17912872 |
| Ncdn | -0.541416 |
| Ncdn | -0.7469612 |
| Ncdn | -0.6961474 |
| Ncf4 | -0.2365051 |
| Nck1 | 0.18225708 |
| Nckap1 | 0.45501258 |
| Nckap1l | 0.6400582 |
| Nckipsd | 0.83150226 |
| Ncl | -0.5552507 |
| Ncl | -0.4694672 |
| Ncoa2 | 1.19371804 |
| Ncoa2 | 3.18425822 |
| Ncoa4 | 0.02651414 |
| Ncoa5 | 0.76445458 |
| Ncoa6 | -0.5084301 |
| Ncoa6 | -0.7143092 |
| Ncoa6 | -0.6934532 |
| Ncor1 | -0.6012662 |
| Ncstn | 2.51161599 |
| Ncstn | 0.89275965 |
| Ncstn | 2.10061706 |
| Ndc80 | -0.6686719 |
| Nde1 | 1.68823934 |
| Nde1 | 0.91281132 |
| Ndel1 | 3.36835212 |
| Ndfip1 | 1.58476106 |
| Ndfip2 | -0.6800963 |
| Ndg2 | -0.6774476 |
| Ndn | -0.2588385 |
| Ndor1 | -0.7170639 |
| Ndor1 | 2.26707693 |
| Ndor1 | -0.3824423 |
| Ndrg1 | -0.4582258 |
| Ndrg2 | -0.0187804 |
| Ndrg3 | -0.186019 |
| Ndrg4 | -0.4694672 |
| Ndrl | 1.9636777 |
| Ndst1 | -0.2114605 |
| Ndst1 | -0.5032874 |
| Ndst4 | -0.3734644 |
| Ndufa1 | -0.3805112 |
| Ndufa10 | 1.53833618 |
| Ndufa12 | 1.29798514 |
| Ndufa13 | -0.7088237 |
| Ndufa2 | 1.89130433 |
| Ndufa3 | 0.12467196 |
| Ndufa4 | -0.2560176 |
| Ndufa5 | 2.43566175 |
| Ndufa5 | 0.48649089 |
| Ndufa6 | -0.1745843 |
| Ndufa7 | 0.30442337 |
| Ndufa8 | -0.6591122 |
| Ndufa8 | -0.3189129 |
| Ndufa9 | 0.87040183 |
| Ndufaf1 | 1.82370253 |
| Ndufb10 | -0.2963005 |
| Ndufb11 | -0.7189049 |
| Ndufb2 | 0.13215906 |
| Ndufb3 | -0.7133927 |
| Ndufb4 | 0.36688276 |
| Ndufb5 | -0.7403395 |
| Ndufb6 | 0.66366401 |
| Ndufb6 | 1.90431739 |
| Ndufb7 | 0.98162188 |
| Ndufb8 | 0.92957344 |
| Ndufb9 | -0.4039498 |
| Ndufc1 | -0.2195542 |
| Ndufc1 | -0.3520111 |
| Ndufc2 | 1.00358241 |
| Ndufs2 | 0.42926641 |
| Ndufs2 | 1.07109248 |
| Ndufs3 | 2.60437852 |
| Ndufs4 | 1.4968717 |
| Ndufs4 | -0.7015592 |
| Ndufs4 | -0.7916746 |
| Ndufs4 | 0.97418629 |
| Ndufs6 | 1.93075299 |
| Ndufs7 | 0.99691408 |
| Ndufs8 | -0.7253769 |
| Ndufv1 | -0.5475396 |
| Ndufv2 | 0.90267454 |
| Necab1 | 3.07312671 |
| Necab2 | 1.05431398 |
| Necab3 | 2.38583931 |
| Necap1 | 0.36193067 |
| Necap1 | 0.27349357 |
| Necap1 | 1.69810061 |
| Necap2 | -0.5240292 |
| Nedd1 | 2.48248737 |
| Nedd4 | 0.76121226 |
| Nedd4l | 0.08827109 |
| Nedd8 | -0.388908 |
| Nedd9 | -0.4006593 |
| Nedd9 | -0.7469612 |
| Nefm | -0.6168432 |
| Negr1 | -0.4327334 |
| Negr1 | 1.42741686 |
| Neil1 | -0.7403395 |
| Neil2 | 1.42929705 |
| Nek11 | 0.7849822 |
| Nek3 | -0.4457378 |
| Nek3 | -0.5700783 |
| Nek3 | 2.63922115 |
| Nek4 | 0.6877254 |
| Nek5 | 1.5196684 |
| Nek6 | -0.5552507 |
| Nek8 | -0.5143422 |
| Nek9 | 0.85790056 |
| Nek9 | 1.47332962 |
| Nelf | -0.5323045 |
| Nelf | 0.03233666 |
| Nell2 | -0.2933653 |
| Nell2 | -0.6020797 |
| Nell2 | -0.5323045 |
| Nell2 | 1.02677235 |
| Nenf | -0.7642055 |
| Neo1 | -0.1828867 |
| Nes | 1.40706012 |
| Net1 | -0.7225978 |
| Net1 | -0.5708652 |
| Neto1 | 0.34364594 |
| Neto1 | -0.750766 |
| Neto1 | 2.06689484 |
| Neu1 | -0.4561329 |
| Neu2 | -0.4347748 |
| Neu3 | -0.4822763 |
| Neu4 | 1.48349131 |
| Neu4 | -0.6127189 |
| Neu4 | 1.38772665 |
| Neurl | -0.7729477 |
| Neurod1 | 0.53704483 |
| Neurod2 | 1.69449444 |
| Neurod4 | -0.6462309 |
| Neurog1 | 1.03609033 |
| Neurog2 | 2.23918432 |
| Nf1 | -0.5003613 |
| Nfasc | -0.5787707 |
| Nfasc | 2.77085216 |
| Nfasc | -0.6069752 |
| Nfat5 | 1.04574058 |
| Nfat5 | -0.0972597 |
| Nfatc1 | -0.3607856 |
| Nfatc1 | 0.49464257 |
| Nfatc2 | 0.33286359 |
| Nfatc2 | 1.40185301 |
| Nfatc2ip | -0.5763921 |
| Nfatc3 | -0.7642055 |
| Nfatc4 | -0.4758499 |
| Nfe2 | 1.51941242 |
| Nfe2l1 | -0.4478077 |
| Nfe2l2 | -0.423265 |
| Nfe2l3 | -0.4347748 |
| Nfib | 1.282086 |
| Nfic | 0.23951838 |
| Nfic | 0.74346902 |
| Nfic | -0.4989016 |
| Nfix | -0.3470336 |
| Nfix | 0.34066322 |
| Nfkb1 | -0.2403754 |
| Nfkb2 | -0.5980194 |
| Nfkbia | -0.4429851 |
| Nfkbib | 0.63831809 |
| Nfkbid | 0.94886343 |
| Nfkbie | 2.01936326 |
| Nfkbil1 | -0.5003613 |
| Nfkbil2 | 1.89003911 |
| Nfkbiz | 0.82777159 |
| Nfrkb | -0.4603233 |
| Nfrkb | 1.1036448 |
| Nfs1 | 0.92573168 |
| Nfs1 | 2.25219682 |
| Nfs1 | 0.40865019 |
| Nfu1 | 0.03109201 |
| Nfx1 | -0.7161448 |
| Nfya | 1.2772413 |
| Nfyb | -0.1627767 |
| Nfyc | 1.28010709 |
| Ngb | 0.85306113 |
| Ngb | 0.37584647 |
| Ngef | 1.36407 |
| Ngfb | -0.4099018 |
| Ngfb | -0.3213251 |
| Ngfr | -0.6686719 |
| Ngfrap1 | -0.6617098 |
| Ngp | -0.6226478 |
| Nhedc1 | -0.0877906 |
| Nhej1 | -0.2354022 |
| Nhlrc1 | 0.69184788 |
| Nhlrc2 | -0.4837104 |
| Nhlrc2 | -0.7479109 |
| Nhp2l1 | 1.45395258 |
| Nhsl1 | -0.6591122 |
| Niban | -0.706093 |
| Nicn1 | -0.4185663 |
| Nicn1 | 3.0737483 |
| Nid1 | -0.1155179 |
| Nid2 | 0.54885103 |
| Nif3l1 | -0.81997 |
| Nin | 0.02859775 |
| Ninj1 | -0.3219291 |
| Ninj2 | -0.8770666 |
| Nip7 | -0.1714898 |
| Nipa1 | 1.21518638 |
| Nipbl | -0.4687606 |
| Nipbl | 3.36929614 |
| Nipsnap1 | -0.2431496 |
| Nipsnap3a | -0.7778398 |
| Nipsnap3a | 0.1053057 |
| Nisch | 0.59760784 |
| Nit1 | 0.35283273 |
| Nit2 | -0.2459321 |
| Nit2 | -0.6943504 |
| Nkain3 | -0.1198946 |
| Nkain4 | -0.7431716 |
| Nkd2 | -0.5513873 |
| Nkg7 | 0.11142441 |
| Nkiras1 | -0.7356381 |
| Nkiras2 | 1.90533711 |
| Nkrf | 0.88424904 |
| Nktr | -0.0645266 |
| Nkx1-2 | 0.83874359 |
| Nkx2-1 | -0.6513623 |
| Nkx2-2 | -0.7545863 |
| Nkx2-2 | -0.3824423 |
| Nkx2-3 | -1.0204501 |
| Nkx2-4 | 0.92074608 |
| Nkx2-6 | 2.85172977 |
| Nkx3-1 | -0.273071 |
| Nkx6-1 | 1.98986452 |
| Nle1 | 0.64892612 |
| Nlgn1 | 1.10231624 |
| Nlgn2 | 0.71500721 |
| Nlk | -0.5360902 |
| Nln | -0.2448181 |
| Nln | -0.4409258 |
| Nln | -0.7318938 |
| Nlrp14 | 0.45077019 |
| Nlrp4a | -0.2986553 |
| Nlrp4c | 2.37713572 |
| Nlrp5 | -0.3439359 |
| Nlrp5 | 1.73233361 |
| Nlrp5 | -0.3261681 |
| Nlrp9a | -0.3614155 |
| Nlrp9c | 0.73956711 |
| Nlrx1 | -0.2822941 |
| Nlrx1 | 0.25207511 |
| Nmb | 0.36630151 |
| Nmbr | -0.4375036 |
| Nmd3 | -0.658248 |
| Nme1 | 0.43441825 |
| Nme2 | 1.94002099 |
| Nme2 | -0.7024642 |
| Nme2 | -0.1130952 |
| Nme3 | 1.25489997 |
| Nme4 | 2.4142172 |
| Nme4 | 0.24383299 |
| Nme4 | -0.7680809 |
| Nme5 | 0.56757892 |
| Nme7 | -0.2039688 |
| Nme7 | 0.62604632 |
| Nme7 | -0.6721723 |
| Nme7 | 2.28183919 |
| Nme7 | 0.91913217 |
| Nmnat1 | 0.77185089 |
| Nmnat2 | -0.5091671 |
| Nmnat3 | 1.21530429 |
| Nmral1 | -0.0275767 |
| Nmt1 | -0.3045683 |
| Nmu | -0.6863059 |
| Nmur2 | -0.0152849 |
| Nnat | -0.2875221 |
| Nnat | -0.8386198 |
| Nnmt | -0.2007762 |
| Nnmt | -0.7216732 |
| Nob1 | -0.45683 |
| Nob1 | 1.05417406 |
| Nobox | 0.64287904 |
| Noc3l | -0.3482755 |
| Noc4l | -0.2548917 |
| Noc4l | -0.8107819 |
| Nod1 | 0.6875188 |
| Nog | -0.7412826 |
| Nol1 | 1.70890668 |
| Nol1 | -0.5180561 |
| Nol1 | 2.50070421 |
| Nol10 | 0.22881272 |
| Nol11 | -0.4039498 |
| Nol12 | 1.83627753 |
| Nol14 | 3.22095876 |
| Nol3 | -0.5376088 |
| Nol4 | 1.52909082 |
| Nol4 | 0.46919104 |
| Nol4 | 0.75161073 |
| Nol5 | 1.73667888 |
| Nol5a | 3.19253852 |
| Nol6 | 0.00627313 |
| Nol7 | -0.7536298 |
| Nol9 | -0.5795649 |
| Nola1 | 3.23432954 |
| Nola2 | -0.2904391 |
| Nola3 | -0.7244496 |
| Nola3 | 0.42953827 |
| Nolc1 | 1.14659344 |
| Nomo1 | -0.4945362 |
| Nono | 3.99382628 |
| Nope | -0.5506165 |
| Nos1 | 1.4242119 |
| Nos1ap | -0.4880258 |
| Nos2 | 1.73471242 |
| Nos3 | -0.1787263 |
| Nos3 | -0.5661536 |
| Nos3 | -0.7006551 |
| Nosip | 0.29975463 |
| Nosip | -0.4737175 |
| Nosip | -0.1351278 |
| Nostrin | 0.60462924 |
| Notch1 | -0.7749015 |
| Notch3 | 0.71560903 |
| Notch4 | -0.4019741 |
| Notch4 | 1.02446447 |
| Notum | -0.5980194 |
| Nova2 | 1.00106882 |
| Nox4 | 0.63329725 |
| Noxo1 | -0.4491901 |
| Noxo1 | 2.64036236 |
| Npal2 | 0.58478965 |
| Npas1 | 0.67252314 |
| Npas2 | -0.3470336 |
| Npas4 | 0.0377112 |
| Npb | -0.5143422 |
| Npc2 | 1.47708618 |
| Npcd | 1.27657873 |
| Npcd | -0.7189049 |
| Npdc1 | 0.12429603 |
| Npepl1 | 2.70644343 |
| Npff | -0.5047539 |
| Nphp1 | -0.4981726 |
| Nphp1 | -0.8980098 |
| Nphp3 | -0.1802843 |
| Nphp4 | -0.807739 |
| Nphs2 | -0.6160169 |
| Nploc4 | 0.80050159 |
| Npm1 | -0.6077935 |
| Npm2 | -0.3856696 |
| Npm3 | -0.4708818 |
| Npm3 | 0.72697112 |
| Npm3-ps1 | 0.9521443 |
| Npr1 | 2.19466972 |
| Npr2 | -0.4582258 |
| Npr3 | -0.4178969 |
| Npsr1 | -0.4478077 |
| Nptn | -0.4443604 |
| Nptx1 | -0.5360902 |
| Nptx2 | 0.35312759 |
| Nptxr | 2.30843894 |
| Npy | 0.12128322 |
| Npy1r | -0.5859429 |
| Npy2r | -0.1571815 |
| Npy5r | -0.0040131 |
| Nqo1 | -0.3501414 |
| Nqo2 | 1.2625557 |
| Nqo2 | -0.7996725 |
| Nqo2 | -0.6721723 |
| Nr1d1 | 0.43765756 |
| Nr1d2 | -0.5803598 |
| Nr1h2 | -0.8057159 |
| Nr1h2 | -0.0645266 |
| Nr1h3 | -0.6176703 |
| Nr1h4 | -0.8271788 |
| Nr1h5 | 0.43981092 |
| Nr1i2 | -0.3439359 |
| Nr2c1 | 2.34565071 |
| Nr2c2ap | 1.42440073 |
| Nr2e1 | 3.6658982 |
| Nr2f2 | -0.8006771 |
| Nr2f2 | -0.3304263 |
| Nr2f6 | 0.27413506 |
| Nr2f6 | 0.63285939 |
| Nr4a2 | 0.77449186 |
| Nr4a3 | 0.96822588 |
| Nr4a3 | -0.2266255 |
| Nr5a1 | -0.4844283 |
| Nr5a2 | 1.1824311 |
| Nr5a2 | 2.45634469 |
| Nr5a2 | 0.30504412 |
| Nr6a1 | -0.2109234 |
| Nradd | 0.35017482 |
| Nrap | -0.4185663 |
| Nrarp | 0.2884542 |
| Nras | -0.3607856 |
| Nrbf2 | -0.7318938 |
| Nrbp1 | -0.2141507 |
| Nrbp2 | 3.42550986 |
| Nrcam | 1.30722615 |
| Nrd1 | -0.502555 |
| Nrf1 | -0.6428255 |
| Nrg1 | -0.3105187 |
| Nrg3 | -0.8006771 |
| Nrg4 | 0.22577968 |
| Nrgn | 1.74778982 |
| Nrip2 | -0.4085759 |
| Nrip3 | 0.99393514 |
| Nrl | -0.3328682 |
| Nrm | 0.13364935 |
| Nrn1 | -0.5907549 |
| Nrn1l | -0.6496487 |
| Nrp1 | -0.4981726 |
| Nrp1 | 1.14621285 |
| Nrp2 | 0.45395378 |
| Nrp2 | -0.3863164 |
| Nrsn2 | -0.253767 |
| Nrsn2 | -0.0868489 |
| Nrtn | -0.0087654 |
| Nrxn1 | 0.4931195 |
| Nrxn1 | 3.03102904 |
| Nrxn1 | 1.30936228 |
| Nrxn2 | 1.01298558 |
| Nrxn3 | -0.389557 |
| Nrxn3 | -0.3279907 |
| Nsbp1 | 0.33617133 |
| Nsd1 | -0.4837104 |
| Nsd1 | -0.5062227 |
| Nsdhl | -0.7328285 |
| Nsdhl | 4.77047988 |
| Nsdhl | -0.6792126 |
| Nsf | -0.6411274 |
| Nsfl1c | -0.4512676 |
| Nsg1 | 0.74112982 |
| Nsg2 | 0.08278566 |
| Nsl1 | -0.0139774 |
| Nsmaf | -0.6643145 |
| Nsmaf | -0.5677215 |
| Nsmce1 | -0.5891482 |
| Nsmce2 | -0.4259605 |
| Nsmce2 | 2.43343581 |
| Nsmce2 | -0.137608 |
| Nsun2 | -0.4967164 |
| Nsun4 | -0.5188007 |
| Nsun5 | 2.41678495 |
| Nsun5 | 0.54091623 |
| Nsun5 | -0.5300404 |
| Nsun5 | -0.4909137 |
| Nt5c | -0.7651729 |
| Nt5c1b | -0.558353 |
| Nt5c2 | 1.08892647 |
| Nt5c2 | -0.3709149 |
| Nt5c2 | -0.6293257 |
| Nt5c3 | 0.59738047 |
| Nt5c3l | -0.4722986 |
| Nt5c3l | -0.8158753 |
| Nt5dc3 | 0.03482103 |
| Nt5dc3 | -0.6792126 |
| Nt5dc3 | 1.59324098 |
| Nt5e | -0.5867432 |
| Nt5e | -0.7680809 |
| Nt5m | -0.4059296 |
| Nt5m | 0.61338881 |
| Ntan1 | -0.4829931 |
| Ntf3 | -0.058571 |
| Nthl1 | 0.17836017 |
| Ntn2l | -0.4631274 |
| Ntn2l | 0.03109201 |
| Ntn4 | -0.2315523 |
| Ntng1 | -0.5165688 |
| Ntng1 | -0.3353165 |
| Ntng1 | 0.79406626 |
| Ntrk2 | 0.008402 |
| Ntrk2 | -0.0844988 |
| Ntrk2 | -0.3316464 |
| Ntrk3 | 1.36938981 |
| Ntrk3 | 1.34330836 |
| Ntrk3 | 0.61002939 |
| Nts | 0.65257868 |
| Ntsr1 | -0.3614155 |
| Ntsr2 | 2.9048226 |
| Nuak1 | -0.6259808 |
| Nuak1 | -0.5891482 |
| Nuak2 | 1.52018014 |
| Nub1 | -0.0018608 |
| Nubp1 | 0.03606076 |
| Nubp2 | 0.06653469 |
| Nubp2 | 1.10550163 |
| Nubp2 | 1.44300227 |
| Nucb1 | 0.2843399 |
| Nucb1 | 0.31492085 |
| Nucks1 | 0.05810102 |
| Nudc | -0.6916613 |
| Nudc | -0.3322571 |
| Nudcd2 | -0.3908563 |
| Nudcd3 | 1.13240813 |
| Nudc-ps1 | 1.04686942 |
| Nudt1 | 0.70370193 |
| Nudt10 | 0.59327836 |
| Nudt11 | 1.13330905 |
| Nudt12 | 0.70410804 |
| Nudt13 | 0.95650107 |
| Nudt14 | 0.80050159 |
| Nudt16l1 | -0.5180561 |
| Nudt18 | 1.19395929 |
| Nudt19 | -0.6284884 |
| Nudt19 | 0.67273306 |
| Nudt2 | -0.1592123 |
| Nudt21 | -0.4680546 |
| Nudt22 | 0.46317818 |
| Nudt4 | 1.32249908 |
| Nudt5 | 1.44281714 |
| Nudt5 | -0.0972597 |
| Nudt6 | 0.42899448 |
| Nudt7 | -0.5653706 |
| Nudt8 | -0.1306798 |
| Nudt9 | -0.6176703 |
| Nufip1 | -0.7318938 |
| Numa1 | -0.1213581 |
| Numb | 0.47153348 |
| Numb | -0.0485774 |
| Numb | -0.6310026 |
| Numb | 1.07520655 |
| Numbl | 0.01475987 |
| Nup107 | -0.2504012 |
| Nup133 | -0.4354563 |
| Nup133 | 1.5716136 |
| Nup155 | 0.76312086 |
| Nup155 | 0.11977319 |
| Nup160 | -0.5136011 |
| Nup210 | 0.06212648 |
| Nup37 | 2.21158493 |
| Nup43 | 2.05233388 |
| Nup50 | -0.6160169 |
| Nup50 | -0.1272347 |
| Nup50 | 1.2619949 |
| Nup62 | 1.33918316 |
| Nup85 | -0.2633695 |
| Nup88 | 2.03783913 |
| Nup88 | -0.4794147 |
| Nup88 | -0.2228113 |
| Nup93 | -0.4631274 |
| Nup98 | 1.13971919 |
| Nup98 | -0.131173 |
| Nupl1 | -0.0422727 |
| Nupl1 | -0.6428255 |
| Nupl1 | -0.5677215 |
| Nupl2 | -0.4443604 |
| Nupr1 | 0.14217304 |
| Nus1 | 2.20604881 |
| Nusap1 | 0.01264535 |
| Nut | 0.74541392 |
| Nutf2 | -0.4787007 |
| Nvl | -0.6110743 |
| Nwd1 | 0.61070223 |
| Nxf1 | -0.73096 |
| Nxf2 | -0.7778398 |
| Nxf3 | -0.4589245 |
| Nxph1 | 1.74383188 |
| Nxph2 | -0.375381 |
| Nxph3 | 1.82752346 |
| Nxt1 | 0.54909043 |
| Nxt1 | 1.02648418 |
| Nxt2 | 0.0491839 |
| Nyx | -0.4313749 |
| Oaf | -0.4172281 |
| Oas1b | 0.65792447 |
| Oas1b | -0.3779426 |
| Oas1c | -0.4219201 |
| Oas1d | 0.43549926 |
| Oas1d | 0.16405192 |
| Oas1e | 0.44624159 |
| Oas1g | -0.6251465 |
| Oas1g | 1.32028716 |
| Oas2 | -0.0139774 |
| Oasl1 | 0.8758299 |
| Oasl2 | 0.04673721 |
| Oasl2 | -0.5202916 |
| Oat | 0.1658524 |
| Oaz2 | -0.2464896 |
| Oaz3 | -0.5883458 |
| Obfc1 | 0.25535184 |
| Obfc2a | -0.5452386 |
| Obfc2b | -0.4306964 |
| Obfc2b | 1.49293019 |
| Obox1 | -0.4554363 |
| Obox5 | -0.6765663 |
| Obrgrp | 0.59555952 |
| Obsl1 | -0.2173891 |
| Oc90 | -0.6301638 |
| Oca2 | -0.0364559 |
| Ociad1 | -0.2656432 |
| Ociad1 | 0.12090594 |
| Ociad2 | -0.7290953 |
| Ocln | 1.15165328 |
| Ocrl | 0.11180552 |
| Odc1 | 2.44773163 |
| Odf1 | -0.4694672 |
| Odf2 | -0.7170639 |
| Odf2 | -0.2690641 |
| Odf2 | -0.5521587 |
| Odf2 | -0.0963085 |
| Odf3 | -0.541416 |
| Odf3l1 | -0.5128607 |
| Odf4 | 2.97908952 |
| Odf4 | -0.3334797 |
| Odz1 | -0.7216732 |
| Odz3 | -0.1745843 |
| Odz3 | -0.1405929 |
| Odz4 | -0.0896768 |
| Og9x | -0.1933686 |
| Ogdh | -0.6251465 |
| Ogfod1 | -0.7867102 |
| Ogfod2 | 0.73623771 |
| Ogfr | 1.07081756 |
| Ogfrl1 | -0.7070024 |
| Ogg1 | 0.09139132 |
| Ogn | 2.51229936 |
| Ogn | 0.42790597 |
| Ogn | -0.6730495 |
| Ogt | -0.2650743 |
| Ogt | 0.18613792 |
| Ogt | 1.11289251 |
| Oit1 | 1.24547025 |
| Oit3 | -0.6818663 |
| Ola1 | 0.73937159 |
| Olfm1 | 1.07246583 |
| Olfm1 | -0.6276518 |
| Olfm2 | -0.5787707 |
| Olfm2 | 0.75527086 |
| Olfm3 | 0.27285164 |
| Olfm3 | -0.1390993 |
| Olfm4 | 2.08503033 |
| Olfml1 | -0.4959891 |
| Olfml2b | -0.6479383 |
| Olfml3 | 1.28318359 |
| Olfr100 | -0.1944233 |
| Olfr1006 | 2.04122657 |
| Olfr1012 | -0.4837104 |
| Olfr1020 | -0.4744278 |
| Olfr1065 | 1.22176671 |
| Olfr1094 | 0.69861073 |
| Olfr1099 | -0.5277817 |
| Olfr1105 | 3.33972944 |
| Olfr1112 | 1.7352553 |
| Olfr1113 | -0.0821545 |
| Olfr1189 | 0.05810102 |
| Olfr1197 | -0.3390009 |
| Olfr1198 | 1.55582011 |
| Olfr1249 | -0.3045683 |
| Olfr1280 | -0.4340939 |
| Olfr133 | -0.1092317 |
| Olfr1330 | -0.5591301 |
| Olfr134 | 0.04551148 |
| Olfr1340 | -0.4909137 |
| Olfr1384 | 3.33855782 |
| Olfr1386 | -0.6765663 |
| Olfr1390 | -0.1068251 |
| Olfr1394 | -0.5964003 |
| Olfr1412 | 2.15621963 |
| Olfr1437 | 0.73388044 |
| Olfr144 | 1.69037644 |
| Olfr1443 | 1.38900184 |
| Olfr1443 | -0.7749015 |
| Olfr1459 | -0.3347038 |
| Olfr1500 | 3.73378849 |
| Olfr1508 | -0.6135424 |
| Olfr166 | -0.6284884 |
| Olfr168 | 1.56700069 |
| Olfr206 | 0.88391372 |
| Olfr213 | -0.2195542 |
| Olfr215 | 0.13588038 |
| Olfr232 | 0.22273682 |
| Olfr257 | -0.3482755 |
| Olfr267 | -0.4708818 |
| Olfr272 | -0.2315523 |
| Olfr303 | 2.30218415 |
| Olfr310 | 3.80922455 |
| Olfr352 | -0.6625772 |
| Olfr371 | 1.15366963 |
| Olfr378 | 1.22129821 |
| Olfr419 | 1.27126124 |
| Olfr437 | -0.6997518 |
| Olfr464 | -0.6368955 |
| Olfr47 | 0.93085055 |
| Olfr49 | -0.7986691 |
| Olfr503 | -0.6748063 |
| Olfr51 | 2.56995719 |
| Olfr536 | 1.71516492 |
| Olfr538 | -0.7632392 |
| Olfr54 | -0.7356381 |
| Olfr550 | 0.33135622 |
| Olfr574 | -0.7337641 |
| Olfr591 | 3.37132439 |
| Olfr593 | -0.281715 |
| Olfr608 | 1.76595567 |
| Olfr638 | -0.3219291 |
| Olfr65 | 1.89388699 |
| Olfr655 | 1.10748703 |
| Olfr658 | -0.3532596 |
| Olfr665 | -0.2238995 |
| Olfr675 | 1.37408233 |
| Olfr691 | 0.54693358 |
| Olfr692 | 0.8311476 |
| Olfr702 | 0.28719018 |
| Olfr724 | -0.0131067 |
| Olfr725 | -0.3316464 |
| Olfr748 | 0.13253185 |
| Olfr786 | 0.41225619 |
| Olfr802 | 1.4260985 |
| Olfr824 | 0.12316736 |
| Olfr91 | 1.36858874 |
| Olfr910 | 2.55588481 |
| Olfr920 | -0.6756859 |
| Olfr924 | 1.93366936 |
| Olfr952 | 1.28712437 |
| Olfr965 | 0.06373185 |
| Olfr982 | -0.1516194 |
| Olfr983 | 0.35871243 |
| Olfr984 | -0.5669372 |
| Olig1 | -0.3947653 |
| Olig3 | -0.7097358 |
| Oma1 | -0.3798684 |
| Omd | 0.4712735 |
| Omg | -0.1802843 |
| Omt2b | -0.569292 |
| Onecut2 | -0.5748096 |
| Onecut3 | -0.8428149 |
| Onecut3 | 1.7338975 |
| Oog4 | -0.0355642 |
| Oosp1 | -0.4252859 |
| Opa1 | 0.00669929 |
| Opa1 | 1.06447231 |
| Ophn1 | 1.42364519 |
| Oplah | 1.7023958 |
| Opn1mw | -0.1198946 |
| Opn3 | -0.5437077 |
| Oprl1 | 2.76230694 |
| Oprl1 | -0.7700246 |
| Oprl1 | 1.07534337 |
| Oprs1 | -0.5429431 |
| Optc | -0.186019 |
| Optn | -0.5835461 |
| Optn | 0.33286359 |
| Orai1 | -0.5622452 |
| Orai3 | -0.5429431 |
| Oraov1 | 0.12166035 |
| Orc2l | 1.37716464 |
| Orc3l | -0.6608431 |
| Orc5l | -0.5630256 |
| Orc5l | -0.7216732 |
| Orc6l | 1.66871101 |
| ORF19 | 2.01100009 |
| ORF28 | -0.6539385 |
| ORF34 | 0.04592024 |
| ORF61 | 0.44249571 |
| ORF63 | 0.26349409 |
| ORF9 | -0.2332004 |
| ORF9 | -0.1912629 |
| ORF9 | -0.6854163 |
| Orm1 | 1.36033827 |
| Ormdl2 | 0.10837003 |
| Ormdl3 | 0.61002939 |
| Os9 | -0.5136011 |
| Osbp2 | -0.1005965 |
| Osbpl10 | 2.69244628 |
| Osbpl11 | 0.89392179 |
| Osbpl2 | -0.2476055 |
| Osbpl3 | 0.21354874 |
| Osbpl3 | 0.12879723 |
| Osbpl6 | 2.39358744 |
| Osbpl8 | -0.1766531 |
| Osbpl9 | -0.5988301 |
| Oscar | -0.3837319 |
| Osgep | 0.46317818 |
| Osgepl1 | 1.20224471 |
| Osgin1 | 0.98869918 |
| Osm | -0.8230527 |
| Osmr | 1.7137778 |
| Osr1 | 1.98139041 |
| Osr2 | -0.3601562 |
| Ostf1 | 2.96629027 |
| Ostm1 | -0.7253769 |
| Otof | 0.68855134 |
| Otop1 | 0.01264535 |
| Otop2 | -0.3967259 |
| Ott | 0.76502559 |
| Ott | 2.16712786 |
| OTTMUSG00000000421 | 1.67227212 |
| OTTMUSG00000000971 | -0.0840295 |
| OTTMUSG00000000990 | 1.06156127 |
| OTTMUSG00000001305 | -0.3869636 |
| OTTMUSG00000002038 | 1.08906132 |
| OTTMUSG00000004461 | -0.5835461 |
| OTTMUSG00000004551 | -0.658248 |
| OTTMUSG00000005148 | 2.51591592 |
| OTTMUSG00000005491 | -0.5165688 |
| OTTMUSG00000008540 | 1.64317408 |
| OTTMUSG00000010009 | 2.60964237 |
| OTTMUSG00000010328 | 1.8010147 |
| OTTMUSG00000015762 | -0.5568006 |
| OTTMUSG00000015859 | 1.1697704 |
| OTTMUSG00000016293 | -0.0821545 |
| OTTMUSG00000016790 | -0.6479383 |
| OTTMUSG00000016790 | 1.07520655 |
| OTTMUSG00000017677 | -0.4822763 |
| OTTMUSG00000020946 | 0.4717934 |
| Otub1 | -0.5188007 |
| Otub2 | -0.0449695 |
| Otub2 | -0.7651729 |
| Otub2 | -0.6243128 |
| Otud5 | -0.3159064 |
| Otud6b | 0.67399159 |
| Otud7b | 0.21696221 |
| Otud7b | 1.63301917 |
| Otx1 | -0.546772 |
| Otx2 | -0.4638297 |
| Ovca2 | -0.7916746 |
| Ovgp1 | 1.57193647 |
| Ovgp1 | 0.42790597 |
| Ovol1 | -0.336543 |
| Ovol2 | 1.11025957 |
| Ovol2 | 0.15354064 |
| Oxct1 | -0.4375036 |
| Oxct2a | -0.7422266 |
| Oxct2b | 1.98300794 |
| Oxgr1 | 1.89982883 |
| Oxnad1 | -0.7328285 |
| Oxr1 | 0.04346504 |
| Oxr1 | -0.8097665 |
| Oxt | -0.6086127 |
| P140 | -0.056746 |
| P2rx2 | -0.3371569 |
| P2rx3 | 2.7220908 |
| P2rx4 | 0.34780592 |
| P2rx4 | -0.610253 |
| P2rx5 | 0.3028697 |
| P2rx7 | -0.7015592 |
| P2rx7 | 2.81188987 |
| P2ry1 | 1.24900326 |
| P2ry12 | -0.7170639 |
| P2ry13 | 2.80211904 |
| P2ry14 | -0.5645883 |
| P2ry14 | -0.6522203 |
| P2ry5 | -0.7450645 |
| P2ry6 | 0.36833431 |
| P42pop | 0.01729104 |
| P4ha1 | 0.34424135 |
| P4ha2 | -0.2448181 |
| P4hb | 1.22865013 |
| Pa2g4 | -0.4582258 |
| Pabpc1 | 1.41147319 |
| Pabpc1 | -0.6988494 |
| Pabpc1 | 0.13141302 |
| Pabpc2 | 0.10453805 |
| Pabpc4 | -0.3004253 |
| Pabpn1 | -0.1823656 |
| Pabpnl1 | 0.36688276 |
| Pacrg | 1.94160222 |
| Pacs1 | -0.4313749 |
| Pacs2 | 1.77458346 |
| Pacsin1 | 0.1502545 |
| Pacsin1 | 2.33394827 |
| Pacsin1 | -0.3671037 |
| Pacsin2 | 1.80725675 |
| Pacsin2 | -0.7263052 |
| Pacsin3 | 0.4560702 |
| Pacsin3 | 0.72716923 |
| Padi1 | 1.86714615 |
| Padi2 | -0.3225335 |
| Padi6 | 0.20771728 |
| Paf1 | -0.3811545 |
| Paf1 | 0.6229532 |
| Pafah1b1 | -0.1917889 |
| Pafah1b1 | 0.15899217 |
| Pafah1b3 | -0.3010161 |
| Pafah2 | 0.0601159 |
| Pafah2 | -0.8117984 |
| Paip1 | 0.09876066 |
| Paip2 | 1.90164956 |
| Paip2b | -0.8179204 |
| Paip2b | 0.87768859 |
| Pak1 | -0.7115625 |
| Pak3 | 1.59047557 |
| Pak4 | 0.32256553 |
| Pak6 | 2.86490889 |
| Pak7 | -0.6952485 |
| Palld | 0.7527681 |
| Palm | 1.7930737 |
| Palm | -0.6127189 |
| Palm2 | -0.5513873 |
| Palmd | -0.5756005 |
| Palmd | 1.92655518 |
| Palmd | 1.62831844 |
| Pam | 2.95968629 |
| Pank1 | 0.06172471 |
| Pank1 | -0.5980194 |
| Pank2 | 2.21737318 |
| Pank3 | 1.58587494 |
| Pank4 | 0.80816632 |
| Panx1 | 1.28592199 |
| Panx3 | -0.7235233 |
| Paox | -0.8271788 |
| Paox | 1.1824311 |
| Paox | 1.4011759 |
| Paox | 3.39533992 |
| Papd1 | -0.7207495 |
| Papd1 | -0.3614155 |
| Papd4 | -0.3123111 |
| Papln | 1.10986399 |
| Papola | 1.18169824 |
| Papolb | -0.6651844 |
| Papolg | -0.4105655 |
| Papss1 | -0.5740194 |
| Papss1 | -0.7207495 |
| Papss2 | 0.49919704 |
| Paqr3 | 0.84383205 |
| Paqr6 | -0.1761355 |
| Paqr7 | -0.5763921 |
| Paqr9 | -0.6505051 |
| Paqr9 | -0.5513873 |
| Paqr9 | 1.76792349 |
| Pard3 | 0.26995752 |
| Pard3 | -0.5232805 |
| Pard6a | -0.6889798 |
| Pard6a | 1.07766638 |
| Pard6b | -0.3093256 |
| Pard6g | -0.3470336 |
| Park7 | -0.5069579 |
| Parl | 1.22445607 |
| Parp1 | 0.75776698 |
| Parp1 | -0.336543 |
| Parp1 | 2.35829448 |
| Parp12 | 1.29278761 |
| Parp14 | -0.1179469 |
| Parp16 | -0.5421792 |
| Parp2 | -0.6020797 |
| Parp2 | -0.7526742 |
| Parp3 | 0.5583801 |
| Parp6 | -0.7700246 |
| Parp8 | 0.22813956 |
| Pars2 | -0.277095 |
| Pars2 | -0.6226478 |
| Parva | -0.5113815 |
| Parvg | -0.6428255 |
| Pask | 0.28592446 |
| Patl1 | -0.6251465 |
| Patz1 | -0.4787007 |
| Pawr | -0.5599079 |
| Pax8 | -0.5851433 |
| Paxip1 | -0.8148544 |
| Pbk | 2.27061648 |
| Pbld | 0.28307034 |
| Pbp2 | 0.42681619 |
| Pbrm1 | 0.22070277 |
| Pbrm1 | -0.6259808 |
| Pbx1 | -0.7926706 |
| Pbx1 | 0.1006904 |
| Pbx2 | 0.72319898 |
| Pbx2 | 0.75296086 |
| Pbx2 | -0.2332004 |
| Pbx3 | -0.3837319 |
| Pbx3 | 0.9057926 |
| Pbx4 | 1.54575058 |
| Pcaf | -0.1612474 |
| Pcaf | 0.48751372 |
| Pcbd1 | 2.33981769 |
| Pcbd2 | -0.6310026 |
| Pcbp1 | -0.7431716 |
| Pcbp2 | -0.6419761 |
| Pcbp3 | -0.6643145 |
| Pcca | 0.74638487 |
| Pcca | -0.2599692 |
| Pccb | -0.1828867 |
| Pccb | 0.0356477 |
| Pcdh1 | 0.63220221 |
| Pcdh10 | -0.832362 |
| Pcdh10 | 2.46703247 |
| Pcdh10 | 3.45201159 |
| Pcdh15 | 1.1105232 |
| Pcdh17 | -0.4851467 |
| Pcdh20 | 1.73593346 |
| Pcdh21 | -0.807739 |
| Pcdh7 | 4.0135782 |
| Pcdh7 | 1.52332973 |
| Pcdh8 | -0.6151914 |
| Pcdh9 | 0.51496625 |
| Pcdha10 | 0.06051836 |
| Pcdha11 | 0.94933283 |
| Pcdha2 | 0.33376686 |
| Pcdha6 | 4.57469527 |
| Pcdhac1 | 1.96175998 |
| Pcdhac2 | 2.03936253 |
| Pcdhb1 | -0.5188007 |
| Pcdhb10 | 2.41487626 |
| Pcdhb12 | 1.21199719 |
| Pcdhb14 | 0.73092551 |
| Pcdhb19 | 0.75603961 |
| Pcdhb20 | -0.7235233 |
| Pcdhb22 | -0.873802 |
| Pcdhb3 | -0.3779426 |
| Pcdhb7 | -0.4246118 |
| Pcdhb8 | -0.1720048 |
| Pcdhga10 | 0.02651414 |
| Pcdhga12 | -0.6368955 |
| Pcdhga4 | -0.0499339 |
| Pcdhga7 | -0.5923644 |
| Pcdhga8 | 2.3098028 |
| Pcdhgb2 | -0.0135419 |
| Pcdhgb6 | 1.06696033 |
| Pcdhgc3 | 2.03975526 |
| Pcdhgc4 | -0.3760208 |
| Pcdhgc5 | 2.63074858 |
| Pcf11 | 1.57427394 |
| Pcgf1 | -0.8608557 |
| Pcgf2 | 0.22070277 |
| Pcgf3 | 1.29928004 |
| Pcgf5 | -0.7867102 |
| Pcgf6 | -0.6135424 |
| Pck1 | 0.55243575 |
| Pclo | 0.20323341 |
| Pclo | 3.20950666 |
| Pcmt1 | 0.2162805 |
| Pcmt1 | -0.3010161 |
| Pcmt1 | -0.3798684 |
| Pcmtd1 | -0.2945383 |
| Pcmtd2 | 0.31338443 |
| Pcna | -0.3664699 |
| Pcnt | 0.57366188 |
| Pcnx | -0.45683 |
| Pcnxl2 | -0.6127189 |
| Pcnxl2 | 0.30318064 |
| Pcolce | 2.17430202 |
| Pcolce2 | 0.36222269 |
| Pcolce2 | 0.50096229 |
| Pcsk1n | 3.02039394 |
| Pcsk2 | 1.26546651 |
| Pcsk2 | -0.7170639 |
| Pcsk2 | 1.23559895 |
| Pcsk2 | 1.59229375 |
| Pcsk4 | -0.2013075 |
| Pcsk4 | -0.572441 |
| Pcsk4 | 0.2271289 |
| Pcsk4 | -0.3482755 |
| Pcsk4 | 2.07591183 |
| Pcsk9 | 1.26401223 |
| Pctk1 | -0.438187 |
| Pcx | -0.3532596 |
| Pcyox1 | -0.3715516 |
| Pcyox1l | -0.618498 |
| Pcyt1a | -0.7198268 |
| Pcyt1b | -0.5076937 |
| Pcyt1b | 0.870742 |
| Pcyt2 | 0.26931317 |
| Pcyt2 | -0.177171 |
| Pcyt2 | 0.12354373 |
| Pdcd10 | -0.349519 |
| Pdcd2 | -0.4715899 |
| Pdcd2l | 1.34721061 |
| Pdcd4 | 1.5883367 |
| Pdcd5 | 0.31246137 |
| Pdcd5 | -0.2957127 |
| Pdcd6ip | 1.5553274 |
| Pdcl | -0.4652359 |
| Pdcl3 | 0.98192413 |
| Pddc1 | 0.12052851 |
| Pde10a | -0.3696428 |
| Pde1a | -0.7431716 |
| Pde1a | 0.14143491 |
| Pde1a | 1.11996486 |
| Pde1b | 1.97014773 |
| Pde1c | 0.008402 |
| Pde2a | 0.11901727 |
| Pde3a | -0.7079126 |
| Pde4a | 2.48560922 |
| Pde4a | -0.2776712 |
| Pde4a | -0.7837441 |
| Pde4a | -0.5032874 |
| Pde4b | 0.79811646 |
| Pde4d | -0.533817 |
| Pde4dip | 1.08554904 |
| Pde4dip | -0.5875442 |
| Pde4dip | -0.3482755 |
| Pde4dip | 0.39380406 |
| Pde4dip | -0.3237435 |
| Pde5a | 0.6291293 |
| Pde6a | -0.6739275 |
| Pde6d | -0.4429851 |
| Pde6d | -0.5859429 |
| Pde6d | -0.4887469 |
| Pde6d | 2.7882228 |
| Pde6h | -0.2662125 |
| Pde7a | -0.163287 |
| Pdgfa | -0.8107819 |
| Pdgfb | 2.69350026 |
| Pdgfc | -0.299835 |
| Pdgfra | 3.17873137 |
| Pdgfrb | -0.6160169 |
| Pdgfrl | 1.34073223 |
| Pdha1 | 1.25036732 |
| Pdhb | -0.3183108 |
| Pdhb | -0.4989016 |
| Pdia3 | -0.7488617 |
| Pdia4 | 0.1983806 |
| Pdia5 | -0.5353319 |
| Pdia5 | -0.2565811 |
| Pdia6 | -0.2282649 |
| Pdik1l | 0.25502468 |
| Pdk1 | 1.82493678 |
| Pdk2 | -0.6436757 |
| Pdk3 | -0.3633078 |
| Pdk4 | 0.07290997 |
| Pdlim1 | 0.63831809 |
| Pdlim2 | 0.51818758 |
| Pdlim2 | -0.5947838 |
| Pdlim2 | 2.13206048 |
| Pdlim2 | -0.7198268 |
| Pdlim3 | 1.22911499 |
| Pdlim3 | -0.2459321 |
| Pdlim4 | -0.6453784 |
| Pdlim5 | 2.54539138 |
| Pdlim5 | -0.4561329 |
| Pdlim7 | -0.7143092 |
| Pdpk1 | -0.3850232 |
| Pdpk1 | 2.70475052 |
| Pdrg1 | -0.8814371 |
| Pdss1 | -0.2588385 |
| Pdxdc1 | 1.11813639 |
| Pdxdc1 | 2.0723154 |
| Pdxk | -0.0253699 |
| Pdxp | -0.8087522 |
| Pdyn | -0.4246118 |
| Pdyn | 0.97525212 |
| Pdzd11 | -0.4673491 |
| Pdzd11 | 0.00328459 |
| Pdzd11 | 0.16800843 |
| Pdzd8 | 1.24604099 |
| Pdzk1 | -0.0760867 |
| Pdzk1ip1 | -0.1658427 |
| Pdzrn3 | -0.3702786 |
| Pdzrn3 | 1.24809278 |
| Pea15 | -0.6151914 |
| Pear1 | 0.57808237 |
| Pear1 | 0.50121419 |
| Pebp1 | -0.5091671 |
| Pecam1 | -0.289855 |
| Pecam1 | -0.8251135 |
| Peci | 0.39239131 |
| Pef1 | 0.76464495 |
| Peg10 | -0.3551355 |
| Peg3 | 0.21730287 |
| Peli1 | -0.7106487 |
| Peli1 | 0.16549258 |
| Peli2 | -0.3728264 |
| Peli3 | 0.04387468 |
| Pelo | 0.00285687 |
| Pelp1 | -0.6686719 |
| Pemt | 1.46378153 |
| Peo1 | 2.90219673 |
| Pepd | 1.27746206 |
| Per1 | -0.6411274 |
| Per1 | -0.6934532 |
| Per2 | 0.46632012 |
| Per2 | -0.7170639 |
| Per2 | 0.91524744 |
| Per3 | 0.44329965 |
| Perld1 | 0.81918838 |
| Pes1 | -0.4138909 |
| Pet112l | -0.3954184 |
| Pet112l | 0.35312759 |
| Pet112l | 0.85582957 |
| Pet112l | 0.38043971 |
| Pex11a | 0.54188159 |
| Pex11a | -0.7642055 |
| Pex11a | -0.618498 |
| Pex11b | -0.6625772 |
| Pex11c | -0.2431496 |
| Pex14 | -0.3551355 |
| Pex16 | 1.64250048 |
| Pex19 | -0.1730358 |
| Pex19 | -0.618498 |
| Pex2 | 0.83256546 |
| Pex26 | 1.02634006 |
| Pex3 | 1.07725685 |
| Pex3 | 2.33445075 |
| Pex5 | -0.0700577 |
| Pex5 | -0.0494815 |
| Pex5 | -0.8158753 |
| Pex5l | -0.3069439 |
| Pex6 | 0.23183602 |
| Pex6 | 1.00136488 |
| Pex6 | 0.00157258 |
| Pex7 | -0.4624256 |
| Pfdn1 | -0.5851433 |
| Pfdn2 | 1.05263357 |
| Pfdn4 | -0.0253699 |
| Pfdn5 | -0.6045242 |
| Pfdn5 | 1.60180151 |
| Pfkfb2 | 2.53068171 |
| Pfkfb2 | -0.5763921 |
| Pfkfb4 | 0.30906906 |
| Pfkl | 1.1956463 |
| Pfkl | -0.1381048 |
| Pfkm | -0.1440874 |
| Pfkm | 3.449273 |
| Pfkm | -0.0732992 |
| Pfkp | -0.8057159 |
| Pfkp | 0.29317922 |
| Pfkp | 1.10178429 |
| Pfn1 | 3.46722362 |
| Pfn1 | -0.7488617 |
| Pfn2 | 0.61807194 |
| Pfn2 | 1.85802122 |
| Pfn2 | -0.0485774 |
| Pfn3 | 1.2184823 |
| Pfn4 | -0.3099219 |
| Pftk1 | -0.4491901 |
| Pga5 | 1.29830903 |
| Pgam1 | -0.3414652 |
| Pgam2 | 0.88508683 |
| Pgam5 | 1.45166265 |
| Pgbd5 | -0.3316464 |
| Pgcp | -0.5121208 |
| Pgd | 0.64092705 |
| Pgk1 | -0.0929868 |
| Pgk2 | -0.5939767 |
| Pgls | 1.68409381 |
| Pglyrp1 | -0.0463209 |
| Pglyrp4 | -0.3340915 |
| Pgm2 | -0.3476543 |
| Pgm2l1 | -0.1912629 |
| Pgm3 | -0.6086127 |
| Pgm5 | 0.40279576 |
| Pgm5 | -0.1663547 |
| Pgpep1 | -0.7347006 |
| Pgpep1 | 0.06972773 |
| Pgr | 0.10300088 |
| Pgrmc1 | -0.4252859 |
| Pgs1 | -0.2332004 |
| Phactr1 | -0.5827485 |
| Phactr1 | -0.4491901 |
| Phactr1 | -0.4519611 |
| Phactr4 | -0.6360514 |
| Phb2 | 0.51719758 |
| Phc1 | -0.658248 |
| Phc1 | -0.4032908 |
| Phc2 | 1.76812004 |
| Phca | 0.21866431 |
| Phca | 2.8652355 |
| Phex | 0.84505619 |
| Phf1 | 1.35770708 |
| Phf11 | 2.03665802 |
| Phf11 | -0.5277817 |
| Phf12 | 0.96269023 |
| Phf12 | 0.58432861 |
| Phf13 | -0.0306749 |
| Phf13 | -0.6151914 |
| Phf14 | 0.46997251 |
| Phf16 | -0.5136011 |
| Phf17 | -0.3902064 |
| Phf19 | 1.43622123 |
| Phf2 | 0.85132671 |
| Phf20l1 | 2.26850205 |
| Phf21a | 0.1672903 |
| Phf5a | 1.84301693 |
| Phf6 | 1.24604099 |
| Phf7 | 2.24602847 |
| Phgdh | 2.16218525 |
| Phka1 | 1.89021174 |
| Phka1 | -0.208778 |
| Phka2 | -0.1001191 |
| Phka2 | -0.4246118 |
| Phka2 | -0.4815601 |
| Phkb | -0.2828735 |
| Phkg1 | 1.10165126 |
| Phkg2 | 0.2230754 |
| Phlda1 | 1.17199528 |
| Phlda2 | 1.22270301 |
| Phlda3 | 0.76711636 |
| Phlda3 | 0.39915324 |
| Phldb1 | -0.4554363 |
| Phldb1 | -0.1587042 |
| Phlpp | -0.5113815 |
| Phospho1 | 1.44290971 |
| Phospho2 | -0.2526438 |
| Phox2a | -0.0784158 |
| Phox2b | 2.19237729 |
| Phpt1 | 0.91719181 |
| Phtf1 | -0.7124771 |
| Phtf2 | -0.4694672 |
| Phyhip | -0.6556599 |
| Pi16 | -0.7384561 |
| Pi4k2b | 0.3858652 |
| Pi4k2b | 2.78709243 |
| Pi4ka | 1.66477084 |
| Pi4kb | -0.2414841 |
| Pi4kb | 1.76365466 |
| Pias1 | 0.47749335 |
| Pias2 | 0.36338987 |
| Pias3 | 0.73545261 |
| Pias3 | 1.796576 |
| Pias4 | 0.46475046 |
| Pibf1 | -0.5498463 |
| Pibf1 | -0.6669265 |
| Picalm | -0.1521237 |
| Pick1 | 1.68909477 |
| Pick1 | 0.85858988 |
| Pif1 | -0.1015521 |
| Piga | 1.22992793 |
| Piga | 2.61870425 |
| Pigb | 1.69831231 |
| Pigc | -0.5599079 |
| Pigf | -0.6871964 |
| Pigh | -0.0906213 |
| Pigk | -0.4300184 |
| Pigk | 0.19420103 |
| Pigl | 0.14511979 |
| Pign | -0.4105655 |
| Pigo | 2.37871175 |
| Pigo | -0.6411274 |
| Pigo | 0.53120769 |
| Pigp | -0.5843444 |
| Pigp | 3.01859233 |
| Pigq | -0.6988494 |
| Pigt | 1.3564902 |
| Pigx | -0.4989016 |
| Pigy | -0.7798038 |
| Pigyl | 1.33576621 |
| Pigz | -0.0953582 |
| Pih1d1 | 0.57085925 |
| Pik3ap1 | 1.03266058 |
| Pik3c2a | -0.6118963 |
| Pik3c3 | -0.6765663 |
| Pik3ca | 0.48392893 |
| Pik3cb | -0.2788248 |
| Pik3cd | 1.69591028 |
| Pik3cg | -0.5202916 |
| Pik3ip1 | -0.3545098 |
| Pik3r1 | -0.7365765 |
| Pik3r1 | 0.19594478 |
| Pik3r1 | 0.05567745 |
| Pik3r2 | 0.66641505 |
| Pik3r2 | 0.82883901 |
| Pik3r3 | -0.3231383 |
| Pik3r4 | 1.86714615 |
| Pik3r6 | 2.17498242 |
| Pik3r6 | -0.7216732 |
| Pim1 | -0.107787 |
| Pim2 | 0.06853161 |
| Pim2 | 1.53699703 |
| Pim3 | -0.0915668 |
| Pip4k2a | 1.91143233 |
| Pip4k2b | 2.38848559 |
| Pip4k2b | 0.87142198 |
| Pip4k2c | -0.2045019 |
| Pip5k1a | -0.7966653 |
| Pip5k1a | -0.3824423 |
| Pip5k1b | -0.3696428 |
| Pip5kl1 | -0.3921575 |
| Pipox | -0.5225324 |
| Pir | 1.87863314 |
| Pira11 | 1.45495838 |
| Pira4 | 1.96362448 |
| Pisd | -0.5931702 |
| Pit1 | -0.3702786 |
| Pitpna | 1.05305396 |
| Pitpnb | 1.12296109 |
| Pitpnc1 | 3.5218333 |
| Pitpnm1 | -0.2881048 |
| Pitpnm2 | 2.21472805 |
| Pitpnm3 | 0.89109696 |
| Pitx1 | 0.31492085 |
| Pitx2 | -0.6880877 |
| Pitx2 | -0.3470336 |
| Pitx2 | -0.5591301 |
| Pitx2 | 3.8068721 |
| Piwil1 | -0.7143092 |
| Piwil2 | -0.4851467 |
| Piwil4 | 1.18547856 |
| Pja1 | -0.4416117 |
| Pja2 | 0.82670296 |
| Pja2 | 2.32674054 |
| Pkd1 | -0.0677491 |
| Pkd1l3 | 2.56049557 |
| Pkd2 | -0.5353319 |
| Pkd2l1 | -0.7015592 |
| Pkd2l2 | -0.8128159 |
| Pkdrej | -0.0122368 |
| Pkhd1l1 | 1.48764903 |
| Pkhd1l1 | 1.44826328 |
| Pkia | 0.34661923 |
| Pkib | 2.29105267 |
| Pkib | -0.671296 |
| Pkig | -0.3652038 |
| Pkig | -0.3902064 |
| Pkig | -0.5368492 |
| Pkm2 | 0.46344041 |
| Pkmyt1 | -0.2565811 |
| Pkn1 | -0.5568006 |
| Pkn1 | 1.1331804 |
| Pkn2 | 1.57861104 |
| Pkn2 | 0.19524767 |
| Pkn2 | 1.47207409 |
| Pkn3 | 0.16296997 |
| Pknox1 | 2.85303328 |
| Pknox1 | 2.54099393 |
| Pknox2 | 0.10683911 |
| Pknox2 | 1.25580389 |
| Pknox2 | 1.02950563 |
| Pknox2 | -0.5429431 |
| Pknox2 | 1.36999016 |
| Pkp1 | 0.92412631 |
| Pkp2 | -0.6599773 |
| Pkp2 | -0.1725202 |
| Pkp2 | 1.21329779 |
| Pkp3 | -0.8792494 |
| Pla1a | -0.3652038 |
| Pla1a | -0.6907666 |
| Pla1a | -0.6028938 |
| Pla2g10 | -0.5173122 |
| Pla2g12a | -0.7768594 |
| Pla2g1b | -0.2163084 |
| Pla2g2c | -0.5875442 |
| Pla2g2d | 0.66005431 |
| Pla2g2f | -0.5315492 |
| Pla2g4c | -0.3557616 |
| Pla2g4d | -0.4851467 |
| Pla2g4e | -0.7042769 |
| Pla2g4f | 2.21574628 |
| Pla2g5 | 2.16102103 |
| Pla2g6 | 0.63504667 |
| Pla2g7 | 0.28465703 |
| Plaa | 0.80598276 |
| Plac1 | -0.7318938 |
| Plac1l | -0.3645714 |
| Plac8 | 0.30037847 |
| Plag1 | 0.69840651 |
| Plag1 | 3.23372274 |
| Plagl2 | -0.6045242 |
| Plau | 0.12166035 |
| Plb1 | 0.63066701 |
| Plcb2 | 1.78332624 |
| Plcb3 | 1.30604921 |
| Plcb4 | 0.18965212 |
| Plcd1 | -0.7290953 |
| Plcd3 | -0.4901909 |
| Plcd4 | -0.7916746 |
| Plcd4 | -0.4624256 |
| Plcd4 | -0.6547988 |
| Plce1 | 2.23926373 |
| Plcg2 | 0.73427373 |
| Plch2 | 0.26479033 |
| Plcl2 | 0.33256231 |
| Plcxd1 | 0.19315323 |
| Plcxd1 | -0.3677378 |
| Plcz1 | 3.02346947 |
| Pld1 | 1.15492765 |
| Pld2 | -0.6792126 |
| Pld3 | 0.30752304 |
| Pld3 | 0.77825185 |
| Pld4 | 0.76901303 |
| Pld5 | -0.5645883 |
| Pldn | -0.0849683 |
| Plec1 | 0.54260497 |
| Plec1 | -0.0240483 |
| Plec1 | 0.16260905 |
| Plec1 | 0.20427005 |
| Plek | 2.62037382 |
| Plek2 | -0.5875442 |
| Plekha1 | 1.54608244 |
| Plekha1 | 0.25273137 |
| Plekha2 | 1.08865672 |
| Plekha3 | -0.5939767 |
| Plekha4 | 2.76487639 |
| Plekha4 | -0.6453784 |
| Plekha5 | -0.4680546 |
| Plekha6 | -0.5875442 |
| Plekha7 | -0.5537035 |
| Plekha8 | -0.7106487 |
| Plekhb1 | 0.54115766 |
| Plekhb2 | -0.2515218 |
| Plekhf1 | -0.0031516 |
| Plekhf1 | -0.2776712 |
| Plekhf2 | -0.5980194 |
| Plekhf2 | 2.2077336 |
| Plekhg2 | 1.03065411 |
| Plekhg2 | -0.0868489 |
| Plekhg2 | 1.32805852 |
| Plekhg3 | 1.94914453 |
| Plekhg3 | 0.53340086 |
| Plekhg4 | -0.1991839 |
| Plekhg5 | -0.5972095 |
| Plekhg6 | -0.5498463 |
| Plekhj1 | 2.33867531 |
| Plekhm1 | 0.2162805 |
| Plekhm3 | -0.515826 |
| Plekho2 | 1.34936019 |
| Plin | -0.7479109 |
| Plk1 | 0.23384617 |
| Plk2 | -0.2179299 |
| Plk3 | 1.05165195 |
| Plk4 | 0.14401584 |
| Pllp | -0.5851433 |
| Plod1 | 2.56043914 |
| Plod2 | 0.01771224 |
| Plod3 | 0.12879723 |
| Plp1 | -0.5964003 |
| Plp2 | 1.09618061 |
| Plrg1 | -0.2980661 |
| Pls3 | -0.7337641 |
| Plscr1 | 1.7524642 |
| Plscr1 | 0.62029383 |
| Plscr3 | -0.7115625 |
| Plscr4 | 0.45580591 |
| Pltp | -0.5653706 |
| Plunc | 0.73505981 |
| Plvap | -0.4334134 |
| Plxdc1 | 0.7853547 |
| Plxdc1 | 1.64190133 |
| Plxna2 | -0.6411274 |
| Plxnb1 | -0.5483079 |
| Plxnb3 | -0.5732299 |
| Plxnc1 | 0.58063214 |
| Plxnd1 | 0.29819322 |
| Pmaip1 | -0.4354563 |
| Pmch | -0.1272347 |
| Pmepa1 | 0.12128322 |
| Pmf1 | 2.27184451 |
| Pmfbp1 | -0.5779772 |
| Pml | 1.32648842 |
| Pml | -0.5490768 |
| Pmm1 | -0.5599079 |
| Pmm1 | 0.39971454 |
| Pmm2 | -0.2702072 |
| Pmm2 | -0.0222891 |
| Pmm2 | 0.85669304 |
| Pmm2 | 1.17199528 |
| Pmp2 | 0.08553237 |
| Pmp22 | -0.5575765 |
| Pmp22 | -0.7517196 |
| Pmpca | 1.83901554 |
| Pmpcb | 0.23918566 |
| Pms2 | -0.7749015 |
| Pmvk | -0.502555 |
| Pnck | 2.11413748 |
| Pnkd | -0.7488617 |
| Pnkd | 2.0983139 |
| Pnkp | 0.36630151 |
| Pnkp | 3.39429375 |
| Pnliprp1 | -0.5150838 |
| Pnma1 | -0.5988301 |
| Pnma2 | 0.57319535 |
| Pno1 | 1.62122302 |
| Pnpla2 | 0.84313183 |
| Pnpla6 | 0.91118373 |
| Pnpla7 | -0.4873052 |
| Pnpla8 | 0.76140329 |
| Pnpla8 | -0.1986538 |
| Pnpo | 1.66250142 |
| Pnpo | 0.1193953 |
| Pnrc2 | 0.921552 |
| Podn | -0.4880258 |
| Podxl | -0.4286638 |
| Podxl2 | -0.2152289 |
| Pof1b | 0.53413078 |
| Pofut1 | 0.14217304 |
| Pofut1 | -0.7393973 |
| Pofut2 | -0.6326825 |
| Pogz | -0.8016827 |
| Pogz | 0.6819234 |
| Pola2 | -0.1247817 |
| Polb | 1.20998363 |
| Pold1 | -0.7798038 |
| Pold2 | -0.6863059 |
| Pold3 | 1.28427991 |
| Pold4 | -0.5851433 |
| Poldip2 | -0.6118963 |
| Poldip3 | -0.6411274 |
| Poldip3 | 0.25567889 |
| Pole | -0.0788823 |
| Pole3 | -0.2857762 |
| Pole3 | -0.8240825 |
| Pole4 | -0.313508 |
| Polg | 1.65971205 |
| Polg2 | 2.27268786 |
| Polh | -0.840715 |
| Poli | -0.5368492 |
| Polk | -0.6045242 |
| Poll | 2.68925264 |
| Poll | 1.79581297 |
| Polm | 3.1147056 |
| Polm | -0.3027905 |
| Poln | 2.18428847 |
| Polq | -0.7024642 |
| Polr1e | 0.1769391 |
| Polr2d | 1.32585965 |
| Polr2d | -0.5247785 |
| Polr2d | -0.6961474 |
| Polr2d | -0.7526742 |
| Polr2e | -0.6979479 |
| Polr2e | -0.2526438 |
| Polr2f | 1.20570533 |
| Polr2g | -0.7097358 |
| Polr2h | 0.68710547 |
| Polr2j | -0.7161448 |
| Polr3a | -0.3856696 |
| Polr3a | -0.6748063 |
| Polr3b | -0.4865851 |
| Polr3d | 2.05504205 |
| Polr3e | 3.27033092 |
| Polr3g | 0.19070382 |
| Polr3h | -0.5150838 |
| Polr3h | 0.04673721 |
| Polr3h | -0.7847318 |
| Polr3k | -0.7886928 |
| Polr3k | 1.54075853 |
| Polrmt | 0.72240292 |
| Pols | -0.3696428 |
| Pom121 | -0.7356381 |
| Pomc | -0.7115625 |
| Pomgnt1 | -0.1607382 |
| Pomgnt1 | 2.68122076 |
| Pomp | 1.93014657 |
| Pomt1 | 2.60181653 |
| Pomt1 | -0.5240292 |
| Pomt2 | -0.7133927 |
| Pon1 | 0.78814376 |
| Pon2 | 1.17298242 |
| Pon3 | 0.29850571 |
| Pon3 | -0.2315523 |
| Pop4 | 0.97995778 |
| Pop5 | 0.26284529 |
| Pop5 | -0.4808445 |
| Pop7 | 0.69287569 |
| Popdc2 | -0.7837441 |
| Popdc2 | 1.47180483 |
| Popdc3 | -0.7460124 |
| Por | 0.3548948 |
| Por | -0.3588985 |
| Porcn | 0.27541674 |
| Porcn | -0.3111158 |
| Pou2af1 | 0.81468675 |
| Pou2f1 | -0.3347038 |
| Pou2f1 | -0.558353 |
| Pou2f2 | -0.336543 |
| Pou3f1 | -0.5091671 |
| Pou3f3 | 0.17051757 |
| Pou5f1 | 1.45239603 |
| Pou5f1 | -0.0431708 |
| Pou6f1 | 0.66620371 |
| Ppa1 | 1.40936517 |
| Ppa2 | 1.21081326 |
| Ppa2 | 2.02944085 |
| Ppa2 | -0.7536298 |
| Ppap2a | -0.6704205 |
| Ppap2a | 2.10881646 |
| Ppap2a | -0.8781574 |
| Ppap2b | -0.7517196 |
| Ppap2b | 1.34526151 |
| Ppap2c | -0.4873052 |
| Ppapdc1 | 1.44485159 |
| Ppapdc1a | 1.52638411 |
| Ppapdc2 | -0.7441176 |
| Ppapdc3 | 0.88726159 |
| Ppapdc3 | 2.7849832 |
| Pparbp | -0.6234799 |
| Pparg | -0.6943504 |
| Ppargc1a | 3.86528565 |
| Ppargc1b | 1.62321208 |
| Ppat | 0.00456655 |
| Ppcs | -0.2398215 |
| Ppef2 | -0.0148488 |
| Ppfia3 | 2.64923718 |
| Ppfia3 | -0.208778 |
| Ppfibp1 | -0.7632392 |
| Ppfibp2 | 1.60857877 |
| Ppfibp2 | 1.5803723 |
| Ppia | 0.38927577 |
| Ppic | -0.1782076 |
| Ppic | -0.6402795 |
| Ppid | 0.22442851 |
| Ppie | -0.0351187 |
| Ppie | -0.4829931 |
| Ppif | -0.8619278 |
| Ppil2 | -0.2916085 |
| Ppil3 | -0.3798684 |
| Ppl | 1.02388662 |
| Ppm1a | -0.7469612 |
| Ppm1a | -0.03601 |
| Ppm1a | -0.3353165 |
| Ppm1b | 0.15862972 |
| Ppm1b | -0.4498821 |
| Ppm1b | 0.22138127 |
| Ppm1e | -0.7778398 |
| Ppm1e | -0.7613094 |
| Ppm1f | 2.07680879 |
| Ppm1f | -0.6988494 |
| Ppm1f | 4.25953061 |
| Ppm1g | 0.07410054 |
| Ppm1h | 0.75603961 |
| Ppm1j | -0.6487931 |
| Ppm1k | 0.90612021 |
| Ppm1l | -0.4178969 |
| Ppm1l | -0.8587152 |
| Ppm1m | -0.4225923 |
| Ppm1m | 0.48367235 |
| Ppm2c | 0.45474799 |
| Ppme1 | -0.8026894 |
| Ppnr | 1.48861946 |
| Ppox | 0.51794017 |
| Ppox | -0.289855 |
| Ppp1ca | -0.658248 |
| Ppp1ca | 0.44061716 |
| Ppp1ca | 0.09372472 |
| Ppp1cb | 0.16116397 |
| Ppp1cb | 0.10913456 |
| Ppp1cc | -0.5899512 |
| Ppp1r10 | 1.15404721 |
| Ppp1r10 | 0.67545775 |
| Ppp1r11 | 0.99854849 |
| Ppp1r12c | -0.4079136 |
| Ppp1r13b | 1.88235382 |
| Ppp1r13b | -0.0078996 |
| Ppp1r13b | 2.433242 |
| Ppp1r14a | 1.2606476 |
| Ppp1r14a | -0.7680809 |
| Ppp1r14c | 1.81308301 |
| Ppp1r14d | 2.32641489 |
| Ppp1r15b | -0.0812185 |
| Ppp1r16a | -0.6765663 |
| Ppp1r1a | -0.717984 |
| Ppp1r1b | 0.91216062 |
| Ppp1r1b | 0.74657894 |
| Ppp1r2 | -0.947203 |
| Ppp1r3c | -0.388908 |
| Ppp1r3f | -0.3439359 |
| Ppp1r8 | -0.6110743 |
| Ppp1r9a | -0.4873052 |
| Ppp1r9b | 0.18084189 |
| Ppp2ca | -0.3347038 |
| Ppp2cb | -0.5972095 |
| Ppp2cz | -0.4687606 |
| Ppp2r1a | -0.4092386 |
| Ppp2r1b | -0.3588985 |
| Ppp2r2b | 0.48495455 |
| Ppp2r2b | 3.55147392 |
| Ppp2r2b | 0.97662071 |
| Ppp2r2c | -0.5964003 |
| Ppp2r2c | 1.0572475 |
| Ppp2r2d | 0.39577835 |
| Ppp2r3c | 0.25108986 |
| Ppp2r4 | -0.7651729 |
| Ppp2r5b | 1.42515567 |
| Ppp2r5c | -0.2206387 |
| Ppp2r5c | 0.5517199 |
| Ppp2r5c | -0.4395554 |
| Ppp2r5d | 0.4552771 |
| Ppp2r5d | -0.8036971 |
| Ppp2r5d | -0.2679224 |
| Ppp2r5d | 0.93721021 |
| Ppp2r5e | -0.4491901 |
| Ppp2r5e | 0.3047338 |
| Ppp3ca | -0.3513875 |
| Ppp3ca | -0.9343718 |
| Ppp3ca | -0.5437077 |
| Ppp3cb | -1.0766895 |
| Ppp3cc | -0.6479383 |
| Ppp3r1 | -0.4844283 |
| Ppp4c | -0.7526742 |
| Ppp4r1 | -0.9309025 |
| Ppp4r1 | 1.01283937 |
| Ppp4r1 | -0.5677215 |
| Ppp4r1l | 0.10683911 |
| Ppp5c | -0.7680809 |
| Ppp5c | -0.7817719 |
| Ppp6c | 0.41970171 |
| Pps | 0.35724594 |
| Ppt1 | -0.0691336 |
| Ppt2 | -0.7079126 |
| Pptc7 | -0.4450489 |
| Pqbp1 | 0.31061253 |
| Pqlc1 | -0.1813244 |
| Pqlc2 | -0.9843348 |
| Pqlc3 | 0.95960074 |
| Pqlc3 | 0.44597452 |
| Pqlc3 | 1.45046967 |
| Pqlc3 | -0.6377404 |
| Pramef12 | 0.61940571 |
| Prap1 | 1.10748703 |
| Prc1 | -0.9332139 |
| Prcp | -0.3213251 |
| Prdm10 | 1.07260306 |
| Prdm4 | -0.4000026 |
| Prdm8 | -0.3709149 |
| Prdm9 | -0.5740194 |
| Prdx1 | 1.31775287 |
| Prdx2 | -0.2747934 |
| Prdx2 | 2.34646587 |
| Prdx2 | -0.4327334 |
| Prdx3 | 0.24944548 |
| Prdx4 | -0.6721723 |
| Prdx5 | 0.27156646 |
| Prdx5 | -0.8087522 |
| Prdx6 | -0.6004535 |
| Prdx6-rs1 | -0.2945383 |
| Prdx6-rs1 | 1.37855334 |
| Preb | 0.2950626 |
| Preb | 2.05629677 |
| Prei3 | 0.26608479 |
| Prei4 | 1.01444637 |
| Prei4 | 0.01813325 |
| Prelp | 1.67791316 |
| Prep | -0.5300404 |
| Prep | -0.4701742 |
| Prf1 | -0.1296942 |
| Prickle1 | 0.03233666 |
| Prickle2 | -0.3557616 |
| Prickle3 | -0.4443604 |
| Prickle4 | 0.23951838 |
| Prim2 | -0.026252 |
| Prim2 | -0.2622347 |
| Prima1 | 1.92882211 |
| Prkaa2 | 0.97113439 |
| Prkaa2 | -0.3039753 |
| Prkab1 | 0.36833431 |
| Prkab2 | -0.0784158 |
| Prkaca | 1.92139277 |
| Prkaca | -0.7827575 |
| Prkacb | 0.9640772 |
| Prkag1 | -0.6608431 |
| Prkag2 | -0.8240825 |
| Prkar1a | 0.18084189 |
| Prkar1b | -0.7024642 |
| Prkar2b | 0.27765548 |
| Prkca | 0.58016906 |
| Prkcb1 | -0.9343718 |
| Prkcbp1 | 1.27735169 |
| Prkcbp1 | -0.81997 |
| Prkcc | -0.1597207 |
| Prkcd | 0.91783904 |
| Prkcdbp | 3.360211 |
| Prkcdbp | -0.886929 |
| Prkce | -0.4505746 |
| Prkch | -0.2238995 |
| Prkcq | -0.8673066 |
| Prkcsh | -0.2776712 |
| Prkcz | -0.0445195 |
| Prkcz | -0.1287096 |
| Prkcz | -0.5740194 |
| Prkd1 | -0.5568006 |
| Prkd1 | -0.6069752 |
| Prkd2 | 0.32590961 |
| Prkd3 | -0.4279873 |
| Prkd3 | 3.67729642 |
| Prkd3 | 0.82223723 |
| Prkg1 | -0.5972095 |
| Prkg2 | -0.1425882 |
| Prkra | 0.26931317 |
| Prkrip1 | -0.7574618 |
| Prkrip1 | -0.2679224 |
| Prkrir | 1.53858705 |
| Prkx | 0.92106853 |
| Prkx | 1.09711688 |
| Prl | -0.2805578 |
| Prlr | -0.5996414 |
| Prlr | -0.0521992 |
| Prm1 | 2.1056185 |
| Prm3 | 2.57280228 |
| Prmt2 | -0.6012662 |
| Prmt2 | -0.1870654 |
| Prmt3 | -0.1475948 |
| Prmt5 | -0.4909137 |
| Prmt5 | -0.376661 |
| Prmt5 | -0.3869636 |
| Prmt7 | 0.28275268 |
| Prmt7 | 0.30814175 |
| Prmt7 | -0.5568006 |
| Prmt7 | 0.46866971 |
| Prmt8 | -0.1526283 |
| Prnp | 0.00542023 |
| Prnpip1 | -0.4554363 |
| Prnpip1 | -0.2157685 |
| Prnpip1 | -0.8694668 |
| Proc | 1.05654987 |
| Proc | 2.5304778 |
| Procr | 2.19932074 |
| Prodh | 0.80379412 |
| Prok2 | -0.4930855 |
| Prok2 | -0.7926706 |
| Prokr1 | 0.47075332 |
| Prokr2 | -0.0005718 |
| Prom1 | -0.7886928 |
| Prom2 | 0.37267557 |
| Prom2 | 2.13800394 |
| Pros1 | -0.4205771 |
| Prosapip1 | 0.33827021 |
| Prosapip1 | -0.927446 |
| Prosapip1 | -0.5779772 |
| Prosc | 0.34245398 |
| Prosc | -0.1699463 |
| Prosc | -0.0691336 |
| Prox1 | -0.5150838 |
| Prox1 | 2.12719803 |
| Prpf18 | -0.5003613 |
| Prpf19 | 0.01052607 |
| Prpf19 | -0.7318938 |
| Prpf19 | 3.31740472 |
| Prpf3 | -0.671296 |
| Prpf3 | 0.51297841 |
| Prpf3 | 2.32253461 |
| Prpf3 | 0.3000666 |
| Prpf3 | -0.6765663 |
| Prpf31 | 0.69020106 |
| Prpf38a | -0.7632392 |
| Prpf38a | -0.7281644 |
| Prpf38a | -0.1296942 |
| Prpf38b | 3.48632638 |
| Prpf39 | 0.83345053 |
| Prpf40a | -0.5084301 |
| Prpf40a | 1.68352057 |
| Prpf40b | -0.3057554 |
| Prpf40b | -0.7042769 |
| Prpf40b | -0.2168486 |
| Prpf6 | 1.13664777 |
| Prpf8 | -0.5835461 |
| Prpf8 | -0.1912629 |
| Prph2 | 0.01010164 |
| Prps1 | -0.5521587 |
| Prps1l1 | -0.8459735 |
| Prps2 | -0.1169746 |
| Prpsap2 | -0.6952485 |
| Prpsap2 | 1.266472 |
| Prr11 | -0.8630011 |
| Prr13 | -0.6462309 |
| Prr14 | -0.1361191 |
| Prr15 | 2.32217114 |
| Prr16 | -0.5803598 |
| Prr18 | 0.35783281 |
| Prr19 | 1.25851043 |
| Prr3 | 1.14023012 |
| Prr7 | 0.2798889 |
| Prrc1 | 2.52659517 |
| Prrg2 | -0.0454198 |
| Prrt1 | -0.8292487 |
| Prrt1 | -0.2599692 |
| Prrt1 | -0.4464272 |
| Prrt3 | -0.5315492 |
| Prrx1 | 0.12692435 |
| Prrx1 | -0.2071722 |
| Prrx1 | -0.2968887 |
| Prss21 | -0.6880877 |
| Prss23 | 0.5877809 |
| Prss27 | -0.1087499 |
| Prss28 | -0.1087499 |
| Prss29 | 1.990175 |
| Prss29 | 0.30906906 |
| Prss34 | -0.4858656 |
| Prss35 | 1.65240594 |
| Prss36 | -0.2442616 |
| Prss7 | -0.5859429 |
| Prss8 | 0.27605692 |
| Prss8 | 1.37348459 |
| Prtn3 | 0.9784424 |
| Prune | 0.80561834 |
| Prune | 0.10722208 |
| Prune | 1.65344258 |
| Prune | 2.39859475 |
| Psap | -0.6086127 |
| Psat1 | 1.08676631 |
| Psca | 0.64612338 |
| Pscd1 | -0.8491428 |
| Pscd2 | 2.33624314 |
| Pscd3 | 0.31584149 |
| Pscdbp | -0.5136011 |
| Psd2 | -0.0467717 |
| Psd3 | 1.20200558 |
| Psd4 | 0.23585203 |
| Psen1 | -0.7565023 |
| Psen2 | 0.85392714 |
| Psenen | -0.1272347 |
| Psg19 | 1.78158417 |
| Psg23 | -0.7097358 |
| Psg27 | 0.26834582 |
| Psip1 | -0.8138346 |
| Psip1 | 3.00799026 |
| Pskh1 | 2.61319756 |
| Psma2 | 0.63504667 |
| Psma4 | 0.30659421 |
| Psma5 | 0.58824025 |
| Psma7 | 3.01273062 |
| Psma7 | -0.2249889 |
| Psmb1 | -0.7565023 |
| Psmb10 | 1.47341924 |
| Psmb10 | -0.5421792 |
| Psmb2 | -0.9697868 |
| Psmb2 | 0.01981541 |
| Psmb3 | -0.1720048 |
| Psmb4 | 0.42025089 |
| Psmb5 | -0.2560176 |
| Psmb6 | -0.1521237 |
| Psmb7 | -0.4708818 |
| Psmb7 | -0.8006771 |
| Psmb7 | -0.52703 |
| Psmb8 | -0.1126114 |
| Psmb9 | 1.63747073 |
| Psmc1 | 1.00062454 |
| Psmc2 | -0.6792126 |
| Psmc3 | -0.7393973 |
| Psmc3ip | -0.6176703 |
| Psmc4 | 1.43295263 |
| Psmc5 | -1.0474961 |
| Psmc5 | 0.98584456 |
| Psmd1 | 0.28876994 |
| Psmd10 | -0.7479109 |
| Psmd10 | -0.314107 |
| Psmd10 | -0.289855 |
| Psmd11 | -0.0695955 |
| Psmd11 | 1.83243087 |
| Psmd12 | 1.98722131 |
| Psmd12 | 1.55934367 |
| Psmd13 | 0.77956431 |
| Psmd14 | 2.13281659 |
| Psmd2 | 1.2346754 |
| Psmd2 | 0.26057099 |
| Psmd4 | -0.1073059 |
| Psmd5 | -0.6695458 |
| Psmd6 | 1.31245107 |
| Psmd6 | 0.47800982 |
| Psmd7 | -0.5232805 |
| Psmd7 | 1.6928636 |
| Psmd8 | 0.17051757 |
| Psmd9 | 1.55187118 |
| Psmd9 | 0.2435018 |
| Psme1 | -0.1787263 |
| Psme1 | 0.3139993 |
| Psme2 | 1.09256053 |
| Psme3 | 0.29412138 |
| Psme4 | -0.8673066 |
| Psme4 | -0.7015592 |
| Psmf1 | 0.59897092 |
| Psmf1 | -0.6226478 |
| Psmg2 | 2.30477994 |
| Psmg2 | 2.06388582 |
| Psmg3 | 2.26437485 |
| Psors1c2 | 2.01236544 |
| Psp | 0.76235788 |
| Pspc1 | -0.1140635 |
| Pstpip1 | 0.61002939 |
| Pstpip2 | 0.82812753 |
| Ptbp1 | -0.7244496 |
| Ptbp1 | 0.07528961 |
| Ptcd3 | 2.72522244 |
| Ptch1 | 1.52196904 |
| Ptch2 | -0.8502016 |
| Ptdss2 | 0.73329019 |
| Pten | 3.39564193 |
| Pter | -0.5653706 |
| Ptgds | 1.5196684 |
| Ptgds2 | 2.23846934 |
| Ptgds2 | 0.74541392 |
| Ptger2 | -0.5544768 |
| Ptger2 | -0.6276518 |
| Ptger3 | -0.2921937 |
| Ptges | -0.4354563 |
| Ptges | 0.00584677 |
| Ptges2 | -0.3347038 |
| Ptges3 | 1.06156127 |
| Ptgfr | 0.06613479 |
| Ptgir | -0.7024642 |
| Ptgis | -0.5811554 |
| Ptgs1 | -0.9831138 |
| Ptgs1 | -0.9205708 |
| Pth2 | -0.4959891 |
| Pth2r | -0.6462309 |
| Pthlh | 0.16188679 |
| Pthr1 | -0.9578331 |
| Pthr1 | -0.8230527 |
| Pthr1 | 0.48776925 |
| Ptk2 | 0.82474065 |
| Ptk2b | 2.49829558 |
| Ptk7 | -0.7642055 |
| Ptms | 0.37642186 |
| Ptn | -0.7051845 |
| Ptp4a2 | -0.0972597 |
| Ptp4a2 | 1.91547398 |
| Ptp4a2 | -0.760346 |
| Ptp4a2 | -0.6402795 |
| Ptp4a3 | -0.6012662 |
| Ptp4a3 | 0.03399364 |
| Ptpdc1 | 1.06599355 |
| Ptpdc1 | 0.62405905 |
| Ptpdc1 | 0.10837003 |
| Ptpla | -0.6505051 |
| Ptpla | 0.13253185 |
| Ptplad1 | -0.3207215 |
| Ptplad2 | 0.28211703 |
| Ptplb | -0.4046093 |
| Ptpn1 | -0.7574618 |
| Ptpn11 | -0.2834533 |
| Ptpn11 | 0.05608181 |
| Ptpn11 | -0.2719244 |
| Ptpn12 | -0.6226478 |
| Ptpn12 | -0.5165688 |
| Ptpn12 | -0.4498821 |
| Ptpn12 | -0.653079 |
| Ptpn12 | 0.1406962 |
| Ptpn14 | 1.06156127 |
| Ptpn18 | 2.46093556 |
| Ptpn20 | 1.73870884 |
| Ptpn21 | -0.3902064 |
| Ptpn22 | -0.8480852 |
| Ptpn5 | 2.00673963 |
| Ptpn6 | -0.9818945 |
| Ptpn6 | -0.0682104 |
| Ptpra | -0.2277181 |
| Ptprb | 0.12617415 |
| Ptprd | 1.07397421 |
| Ptprd | -0.4916371 |
| Ptpre | -0.1356233 |
| Ptpre | -0.1063445 |
| Ptprf | 0.59076268 |
| Ptprf | -0.8640757 |
| Ptprg | 0.37728427 |
| Ptprj | -0.4952623 |
| Ptprk | 1.06114467 |
| Ptprk | 0.10030477 |
| Ptprn | 1.41328995 |
| Ptprn2 | -0.3183108 |
| Ptpro | 0.89475101 |
| Ptpro | -0.8240825 |
| Ptprq | 1.00770787 |
| Ptprr | 1.68423706 |
| Ptprr | 1.38978572 |
| Ptprs | 0.3657199 |
| Ptprs | -0.8438666 |
| Ptprt | -0.2565811 |
| Ptpru | 0.80050159 |
| Ptprv | -0.6547988 |
| Ptrf | -0.6151914 |
| Ptrh1 | 3.1915291 |
| Ptrh1 | -0.5513873 |
| Ptrh2 | 1.36878907 |
| Pts | -0.4212484 |
| Pts | -0.5521587 |
| Pttg1 | 0.49032078 |
| Pttg1ip | 1.96335835 |
| Ptx3 | 1.05024784 |
| Ptx3 | -0.2520826 |
| Puf60 | 1.56415695 |
| Puf60 | -0.8282132 |
| Pum1 | -0.0179053 |
| Pum2 | -0.5537035 |
| Pum2 | -0.0018608 |
| Punc | 2.00689212 |
| Purb | -0.7517196 |
| Purg | 1.26781099 |
| Pus1 | 0.44490548 |
| Pus1 | -0.8282132 |
| Pus10 | -0.148097 |
| Pus10 | 0.64374529 |
| Pus10 | 0.55980117 |
| Pus3 | -0.822024 |
| Pus7l | -0.1247817 |
| Pvalb | -0.4306964 |
| Pvr | 0.72240292 |
| Pvr | -0.7412826 |
| Pvrl1 | 1.76042377 |
| Pvrl2 | -0.0187804 |
| Pvrl2 | 2.43752876 |
| Pvrl2 | 0.89789539 |
| Pvrl3 | -0.4808445 |
| Pvrl3 | 1.44642066 |
| Pvrl3 | -0.5195458 |
| Pwp1 | 2.46359805 |
| Pwp2 | -0.0668272 |
| Pwwp2b | -0.5891482 |
| Pxdn | 1.45065331 |
| Pxmp2 | 0.82634648 |
| Pxmp4 | -0.2875221 |
| Pxn | -0.6721723 |
| Pxt1 | -0.1169746 |
| Pycr2 | 1.82351725 |
| Pycrl | 2.3509543 |
| Pygb | -0.5121208 |
| Pygl | -0.1704605 |
| Pygl | 0.09255875 |
| Pygm | -0.3915067 |
| Pygo1 | -0.5947838 |
| Pygo2 | 0.47387011 |
| Pyroxd1 | -0.7450645 |
| Pyy | -0.9460293 |
| Qars | 0.06092065 |
| Qdpr | 0.31829216 |
| Qpct | 0.6521497 |
| Qprt | -0.2398215 |
| Qrich2 | 1.43463504 |
| Qrich2 | 1.55803412 |
| Qrsl1 | 1.25817254 |
| Qsox1 | -0.6617098 |
| Qtrt1 | 0.65429264 |
| R3hdm1 | 3.76567108 |
| R3hdm1 | -0.5255284 |
| R3hdm2 | 0.04428415 |
| R74862 | -0.1044246 |
| Rab1 | 0.14106563 |
| Rab10 | 1.24077708 |
| Rab10 | -1.0435844 |
| Rab11a | 0.04141413 |
| Rab11b | 0.79480396 |
| Rab11fip2 | 1.01532175 |
| Rab11fip3 | -0.5972095 |
| Rab11fip5 | 0.90808352 |
| Rab11fip5 | 1.43901388 |
| Rab11fip5 | 0.74015343 |
| Rab12 | 0.66111742 |
| Rab13 | 1.07465904 |
| Rab13 | -0.1218464 |
| Rab14 | -0.4138909 |
| Rab15 | 0.2798889 |
| Rab17 | 3.25105441 |
| Rab17 | -0.7441176 |
| Rab18 | 0.41225619 |
| Rab19 | -0.6445267 |
| Rab19 | 1.02950563 |
| Rab1b | 0.87311978 |
| Rab1b | -0.6318422 |
| Rab1b | 0.11863908 |
| Rab21 | 0.91719181 |
| Rab22a | 1.69760647 |
| Rab22a | -0.4422981 |
| Rab23 | 1.26847977 |
| Rab24 | -0.6176703 |
| Rab24 | -0.0906213 |
| Rab24 | -0.0723719 |
| Rab25 | 1.41643707 |
| Rab27a | 2.48667806 |
| Rab27b | 1.59576225 |
| Rab28 | -0.4715899 |
| Rab28 | 1.06875316 |
| Rab2a | 1.98212125 |
| Rab31 | 2.33796062 |
| Rab32 | -0.2476055 |
| Rab33a | -0.6394323 |
| Rab33b | -0.3195154 |
| Rab34 | -0.0714456 |
| Rab34 | 2.82427158 |
| Rab34 | 0.17087548 |
| Rab35 | 0.02442591 |
| Rab36 | -0.5891482 |
| Rab38 | -0.0948835 |
| Rab38 | -0.5323045 |
| Rab39 | -0.7422266 |
| Rab39b | -0.3830869 |
| Rab3a | -0.4533496 |
| Rab3b | -0.0553797 |
| Rab3d | -0.6809809 |
| Rab3gap1 | -0.8016827 |
| Rab3gap2 | -0.5040204 |
| Rab3il1 | -0.4547402 |
| Rab3il1 | -0.1351278 |
| Rab3ip | -0.4758499 |
| Rab40b | -0.5859429 |
| Rab40c | -0.1390993 |
| Rab43 | -0.7133927 |
| Rab4a | 0.37785877 |
| Rab4b | 0.87345897 |
| Rab5a | 2.03473556 |
| Rab5a | -0.5150838 |
| Rab5b | -0.6739275 |
| Rab5c | 0.00328459 |
| Rab6 | -0.6069752 |
| Rab6 | 0.13884688 |
| Rab6 | -0.3671037 |
| Rab6b | -0.5076937 |
| Rab6ip1 | 0.63022792 |
| Rab7 | 0.33737127 |
| Rab7 | 2.18087285 |
| Rab7 | -0.6251465 |
| Rab7l1 | -0.5150838 |
| Rab8a | -0.2719244 |
| Rab8a | -0.5907549 |
| Rab8b | -0.1228238 |
| Rab8b | 0.01855407 |
| Rab9 | -0.5568006 |
| Rab9 | 0.26576134 |
| Rab9b | 1.57371025 |
| Rabac1 | 0.5668745 |
| Rabac1 | 2.15613289 |
| Rabep1 | -0.5740194 |
| Rabep1 | -0.4092386 |
| Rabep2 | -0.4484986 |
| Rabgap1 | 0.52582497 |
| Rabgap1 | -0.2255341 |
| Rabgap1l | 0.34661923 |
| Rabgap1l | -0.2359535 |
| Rabgef1 | -0.1336428 |
| Rabggta | 0.58640151 |
| Rabggtb | -0.5307945 |
| Rabif | 0.69840651 |
| Rabif | -0.5513873 |
| Rabl2a | 2.32627012 |
| Rabl2a | -0.6863059 |
| Rabl3 | -0.3359295 |
| Rabl4 | -0.5779772 |
| Rabl4 | 0.43954202 |
| Rabl5 | 0.63307835 |
| Rac1 | 1.1386965 |
| Rac2 | -0.4554363 |
| Rac3 | 1.37736315 |
| Rac3 | 0.1776499 |
| Rad1 | -0.6651844 |
| Rad17 | 1.12672539 |
| Rad18 | -0.1761355 |
| Rad23a | -0.4138909 |
| Rad23a | -0.2249889 |
| Rad23a | -0.541416 |
| Rad23b | 0.23618593 |
| Rad50 | 1.91429696 |
| Rad51 | -0.3563881 |
| Rad51 | -0.5353319 |
| Rad51ap1 | 0.77731326 |
| Rad51c | 0.68399966 |
| Rad51l3 | 0.40920586 |
| Rad51l3 | 1.99831426 |
| Rad51l3 | -0.6012662 |
| Rad51l3 | 2.08381338 |
| Rad52 | -0.5947838 |
| Rad54l | -0.0346734 |
| Rad9 | -0.1928418 |
| Rad9 | -0.5606864 |
| Rad9 | -0.5996414 |
| Rad9b | -0.515826 |
| Rae1 | -0.4989016 |
| Raet1b | -0.5955917 |
| Raet1b | 0.2454872 |
| Raf1 | -0.1766531 |
| Rag1 | 0.43468862 |
| Rag1ap1 | -0.7272343 |
| Rage | -0.6934532 |
| Rage | -0.5521587 |
| Rai1 | -0.3004253 |
| Rai12 | -0.1678923 |
| Rai12 | 0.41253301 |
| Rai12 | 0.01179821 |
| Rai12 | 2.68730657 |
| Rai14 | -0.4052692 |
| Rala | 2.18382535 |
| Ralb | -0.0581144 |
| Ralbp1 | -0.5292869 |
| Ralgds | 1.67596431 |
| Ralgps1 | 1.91715289 |
| Ralgps1 | 0.66175472 |
| Ralgps1 | -0.2168486 |
| Ralgps2 | 0.06413277 |
| Ralgps2 | -0.0719086 |
| Raly | 1.69477778 |
| Raly | -0.5360902 |
| Ralyl | 1.9291534 |
| Ramp1 | -0.228812 |
| Ramp2 | -0.5779772 |
| Ramp3 | 1.80461366 |
| Ramp3 | 0.20150314 |
| Ran | -0.6591122 |
| Ranbp1 | 0.23985097 |
| Ranbp10 | 0.61539872 |
| Ranbp17 | -0.3445547 |
| Ranbp2 | -0.7024642 |
| Ranbp3l | 0.41114813 |
| Ranbp6 | 1.83865093 |
| Ranbp9 | 2.19175112 |
| Rangap1 | -0.6660551 |
| Rangrf | 1.09925343 |
| Rap1b | -0.5630256 |
| Rap1gap | 3.09657495 |
| Rap2a | -0.6660551 |
| Rap2b | -0.5544768 |
| Rap2c | 0.12654933 |
| Rap2c | -0.804706 |
| Rap2c | -0.0328942 |
| Rap2ip | 1.59607693 |
| Rapgef1 | 0.28813836 |
| Rapgef1 | -0.4981726 |
| Rapgef1 | -0.1371114 |
| Rapgef1 | -0.313508 |
| Rapgef3 | -0.6193265 |
| Rapgef4 | 0.68627825 |
| Rapgef5 | 2.41283166 |
| Rapgef6 | -0.7403395 |
| Rapgefl1 | 0.85755571 |
| Rapgefl1 | -0.5173122 |
| Rapsn | -0.6765663 |
| Rapsn | -0.1541436 |
| Rara | 0.70552804 |
| Rarb | -0.7817719 |
| Rarb | 0.33135622 |
| Rarg | -0.5851433 |
| Rarg | -0.2713517 |
| Rarg | -0.4916371 |
| Rarres2 | 2.08493677 |
| Rars | -0.6783297 |
| Rars | -0.6730495 |
| Rars2 | -0.5606864 |
| Rars2 | 1.63799749 |
| Rars2 | -0.1576888 |
| Rasa1 | -0.5483079 |
| Rasa2 | -0.6118963 |
| Rasal1 | -0.2109234 |
| Rasd1 | 1.45587182 |
| Rasef | -0.6411274 |
| Rasgef1b | -0.253767 |
| Rasgef1b | -0.5429431 |
| Rasgef1c | -0.3426997 |
| Rasgef1c | -0.6352081 |
| Rasgrf1 | -0.6997518 |
| Rasgrf1 | 0.27317266 |
| Rasgrf1 | -0.7574618 |
| Rasgrf1 | -0.0244886 |
| Rasgrp1 | -0.3869636 |
| Rasgrp1 | -0.7375158 |
| Rasgrp1 | -0.5475396 |
| Rasgrp3 | 0.30814175 |
| Rasip1 | 0.52262974 |
| Rasl10a | -0.3273827 |
| Rasl10b | 1.27713091 |
| Rasl10b | -0.6547988 |
| Rasl11a | 0.437927 |
| Rasl11b | 0.78311748 |
| Rasl11b | 1.68258832 |
| Rasl12 | 1.02041201 |
| Rasl2-9 | -0.4959891 |
| Rassf1 | 1.81264572 |
| Rassf1 | -0.5732299 |
| Rassf10 | -0.2851949 |
| Rassf2 | -0.52703 |
| Rassf2 | -0.2611013 |
| Rassf3 | 2.15959615 |
| Rassf4 | 0.10913456 |
| Rassf4 | 0.52041128 |
| Rassf4 | -0.2238995 |
| Rassf4 | -0.5277817 |
| Rassf5 | 0.25108986 |
| Rassf7 | -0.5867432 |
| Rassf7 | 2.3211527 |
| Raver1 | -0.0118022 |
| Raver2 | 0.32347873 |
| Rb1 | 0.57202802 |
| Rb1 | 0.69799793 |
| Rbak | -0.289855 |
| Rbak | -0.4165597 |
| Rbbp5 | -0.1501082 |
| Rbbp5 | -0.1981239 |
| Rbbp6 | -0.4561329 |
| Rbbp7 | -0.4013165 |
| Rbbp7 | 1.04390331 |
| Rbbp9 | -0.7033701 |
| Rbck1 | -0.4158918 |
| Rbed1 | -0.8189446 |
| Rbed1 | -0.2588385 |
| Rbj | -0.1902118 |
| Rbks | -0.1949511 |
| Rbl1 | 0.75661575 |
| Rbl2 | 0.39267403 |
| Rbm10 | 1.71744922 |
| Rbm10 | -0.6470842 |
| Rbm10 | 2.28316635 |
| Rbm11 | -0.7365765 |
| Rbm12 | 3.18682827 |
| Rbm12 | 2.25544153 |
| Rbm12 | 1.82672358 |
| Rbm12 | 0.06653469 |
| Rbm13 | -0.4751386 |
| Rbm13 | 1.39817139 |
| Rbm14 | -0.3696428 |
| Rbm14 | 0.51397285 |
| Rbm15 | 1.15831593 |
| Rbm16 | 0.92781458 |
| Rbm18 | -0.6268159 |
| Rbm18 | -0.1356233 |
| Rbm19 | 0.29850571 |
| Rbm24 | 1.34196965 |
| Rbm26 | -0.4457378 |
| Rbm28 | 1.76595567 |
| Rbm28 | 0.68254676 |
| Rbm3 | -0.4266356 |
| Rbm35a | -0.492361 |
| Rbm38 | 0.03729886 |
| Rbm38 | -0.3683724 |
| Rbm38 | -0.2515218 |
| Rbm4 | 0.84120352 |
| Rbm41 | 0.28876994 |
| Rbm42 | 0.03482103 |
| Rbm42 | 0.13773554 |
| Rbm43 | -0.4673491 |
| Rbm45 | -0.5851433 |
| Rbm47 | 0.36746365 |
| Rbm47 | -0.4751386 |
| Rbm47 | 0.94870691 |
| Rbm47 | 2.77602755 |
| Rbm4b | -0.8480852 |
| Rbm5 | -0.1991839 |
| Rbm5 | 1.89766212 |
| Rbm6 | 0.18930129 |
| Rbm6 | 2.3622742 |
| Rbm6 | 2.36272696 |
| Rbm8a | -0.5498463 |
| Rbm9 | -0.4152243 |
| Rbm9 | -0.2753683 |
| Rbms1 | 0.47955753 |
| Rbms1 | -0.6077935 |
| Rbms2 | 1.86170554 |
| Rbms2 | -0.7290953 |
| Rbms3 | 0.89259351 |
| Rbmx | 0.95183233 |
| Rbmy1a1 | 1.23698257 |
| Rbp1 | -0.3051617 |
| Rbp7 | 2.81486604 |
| Rbpms | 2.32067948 |
| Rbpms | 1.18705934 |
| Rbpms | -0.5360902 |
| Rbpms2 | 2.18672663 |
| Rbx1 | -0.0427216 |
| Rbx1 | -0.5040204 |
| Rc3h1 | 1.43892093 |
| Rcan1 | 0.04510255 |
| Rcan2 | -0.5262789 |
| Rcan2 | 1.43248476 |
| Rcan2 | -0.7403395 |
| Rcan3 | -0.5661536 |
| Rcbtb2 | -0.8354857 |
| Rcbtb2 | -0.5867432 |
| Rcc1 | 0.33617133 |
| Rccd1 | 0.89872112 |
| Rce1 | -0.0179053 |
| Rchy1 | 0.03440743 |
| Rcl1 | 1.69115883 |
| Rcl1 | -0.6445267 |
| Rcl1 | 0.64136118 |
| Rcn3 | -0.1813244 |
| Rcor1 | 0.18154975 |
| Rcor2 | 0.14401584 |
| Rcsd1 | 2.09382962 |
| Rdbp | -0.336543 |
| Rdbp | -0.0311183 |
| Rdh11 | -0.4974442 |
| Rdh12 | 0.99587252 |
| Rdh14 | -0.6310026 |
| Rdh14 | -0.2736448 |
| Rdh5 | 0.02901392 |
| Rdh7 | 0.41474458 |
| Rdh9 | 2.06331158 |
| Rdm1 | -0.6470842 |
| Rec8 | 1.72158833 |
| Reck | 0.54573318 |
| Recql | 1.18352932 |
| Recql4 | -0.717984 |
| Recql5 | 1.13189293 |
| Reep1 | 1.9845186 |
| Reep2 | -0.3741028 |
| Reep3 | 3.16461464 |
| Reep4 | -0.4603233 |
| Reep5 | -0.3279907 |
| Reep6 | 1.4139584 |
| Reep6 | 1.94676357 |
| Refbp2 | -0.5606864 |
| Reg1 | -0.3051617 |
| Rela | -0.6077935 |
| Relb | 0.57668867 |
| Rell1 | 1.38595814 |
| Rell2 | 2.22449826 |
| Rell2 | 1.29668844 |
| Reln | 2.13791551 |
| Relt | 0.09100186 |
| Relt | 0.81703029 |
| Rem1 | -0.0205331 |
| Rem2 | 2.38895977 |
| Renbp | 1.20820337 |
| Repin1 | -0.7290953 |
| Repin1 | 0.0800309 |
| Reps1 | -0.4829931 |
| Reps1 | 0.00499349 |
| Rer1 | 0.41557259 |
| Rer1 | 0.51347576 |
| Rerg | 0.31061253 |
| Resp18 | 1.38526948 |
| Rest | -0.3010161 |
| Ret | 1.9476842 |
| Retnla | -0.7403395 |
| Retnlg | -0.6889798 |
| Retsat | -0.6326825 |
| Retsat | -0.4938106 |
| Rev1 | 1.55942546 |
| Rev3l | 0.63744682 |
| Rex3 | 2.52989497 |
| Rexo1 | -0.2892712 |
| Rexo1 | -0.6988494 |
| Rexo2 | 3.33970479 |
| Rfc1 | 2.78979421 |
| Rfc2 | 1.94007556 |
| Rfc3 | -0.7170639 |
| Rfc4 | -0.099165 |
| Rfc4 | -0.6522203 |
| Rfc5 | 0.5624009 |
| Rfesd | 1.35405171 |
| Rffl | 1.18047553 |
| Rffl | -0.6863059 |
| Rffl | 2.53958125 |
| Rffl | -0.6730495 |
| Rffl | -0.4952623 |
| Rfng | 3.44417998 |
| Rfpl4 | 2.45650229 |
| Rfpl4 | -0.52703 |
| Rft1 | 0.2595946 |
| Rftn1 | 0.07171789 |
| Rftn2 | 1.13189293 |
| Rftn2 | 2.13924113 |
| Rfwd2 | -0.8662284 |
| Rfwd3 | 0.66196705 |
| Rfwd3 | 1.59686318 |
| Rfx1 | 1.19504415 |
| Rfx1 | 1.12478022 |
| Rfx2 | 1.33025226 |
| Rfx3 | -0.7235233 |
| Rfx4 | -0.3902064 |
| Rfx4 | 0.41639986 |
| Rfx4 | -0.0280186 |
| Rfxank | 2.6082487 |
| Rfxap | 2.76592036 |
| Rfxdc1 | 1.12244069 |
| Rg9mtd1 | -0.7143092 |
| Rg9mtd2 | 1.19781088 |
| Rgag4 | -0.1725202 |
| Rgl1 | 1.74598063 |
| Rgl2 | -0.3709149 |
| Rgl2 | -0.5661536 |
| Rgl3 | 1.367787 |
| Rgma | 0.92396562 |
| Rgmb | 2.77728335 |
| Rgmb | 2.41819751 |
| Rgnef | 2.07293155 |
| Rgs1 | -0.5121208 |
| Rgs1 | -0.3856696 |
| Rgs1 | -0.7946659 |
| Rgs10 | -0.5638066 |
| Rgs12 | 2.89314616 |
| Rgs14 | 2.4633165 |
| Rgs16 | 0.72835705 |
| Rgs17 | 1.27999703 |
| Rgs2 | 1.62374688 |
| Rgs3 | 0.41003875 |
| Rgs3 | 1.00121686 |
| Rgs3 | -0.277095 |
| Rgs3 | 1.15931753 |
| Rgs3 | -0.8006771 |
| Rgs3 | -0.8470288 |
| Rgs3 | -0.706093 |
| Rgs4 | 3.64207133 |
| Rgs5 | -0.1516194 |
| Rgs6 | 0.03399364 |
| Rgs7 | 1.07807573 |
| Rgs7bp | 1.3335853 |
| Rgs8 | 0.04714544 |
| Rgs9 | 2.35232835 |
| Rgs9bp | -0.8189446 |
| Rhag | 3.25633502 |
| Rhbdd1 | 1.71738008 |
| Rhbdd3 | -0.6845276 |
| Rhbdf1 | 2.64095836 |
| Rhbdf2 | -0.3576425 |
| Rhbdl1 | -0.4006593 |
| Rhbdl2 | 2.29259202 |
| Rhbdl3 | -0.0253699 |
| Rhbdl7 | 0.52607031 |
| Rhcg | 1.71530352 |
| Rheb | 0.64417812 |
| Rhebl1 | -0.6360514 |
| Rhob | -0.2963005 |
| Rhobtb2 | 1.07957515 |
| Rhobtb2 | 1.09229182 |
| Rhobtb2 | -0.8396668 |
| Rhobtb2 | 1.48349131 |
| Rhobtb3 | 0.77900205 |
| Rhoc | -0.6889798 |
| Rhoc | 0.50448287 |
| Rhod | 3.62836919 |
| Rhof | 0.39380406 |
| Rhog | -0.1087499 |
| Rhoj | -0.7365765 |
| Rhot1 | 1.26177048 |
| Rhot2 | 1.82308479 |
| Rhot2 | 0.01475987 |
| Rhot2 | 0.12203733 |
| Rhou | -0.5398912 |
| Rhov | -0.1506117 |
| Rhox11 | -0.7281644 |
| Rhox12 | 0.68503607 |
| Rhox3a | -0.5406533 |
| Rhox3f | 1.83267557 |
| Rhox4d | -0.6209857 |
| Rhox5 | -0.8209964 |
| Rhox8 | -0.8544488 |
| Rhpn1 | 0.4510359 |
| Rhpn1 | -0.0617731 |
| Rhpn1 | 0.33856967 |
| Ribc1 | 0.03067676 |
| Ric3 | 1.57371025 |
| Ric3 | -0.4575277 |
| Ric3 | 2.05851256 |
| Ric8b | -0.7006551 |
| Ric8b | 0.20392463 |
| Rif1 | 0.41502067 |
| Rilp | -0.7632392 |
| Rilpl1 | 0.16872601 |
| Rilpl1 | -0.4659398 |
| Rilpl2 | 0.38386997 |
| Rims1 | 0.28529095 |
| Rims2 | -0.4561329 |
| Rims3 | 2.26437485 |
| Rin1 | -0.4334134 |
| Rin2 | -0.5128607 |
| Rin2 | -0.6677988 |
| Rin2 | 0.91378653 |
| Rin3 | -0.7584222 |
| Riok1 | 0.1790699 |
| Riok1 | 1.6698753 |
| Riok3 | -0.4659398 |
| Riok3 | -0.0485774 |
| Ripk1 | -0.2039688 |
| Ripk2 | 0.14695683 |
| Ripk4 | 2.44534277 |
| Ripk5 | 1.14912676 |
| Rit1 | -0.2326507 |
| Rit2 | -0.4617243 |
| Rlbp1 | -0.4715899 |
| Rlbp1l1 | 1.57957214 |
| Rlbp1l1 | -0.7152265 |
| Rln1 | 1.15417304 |
| Rltpr | 1.04827857 |
| Rmnd1 | 1.51684884 |
| Rmnd5b | -0.5875442 |
| Rmnd5b | 0.4821314 |
| Rnase1 | 1.27558398 |
| Rnase4 | -0.658248 |
| Rnase4 | 1.3939879 |
| Rnase6 | -0.6871964 |
| Rnaseh1 | 0.21593947 |
| Rnaseh1 | -0.4478077 |
| Rnaseh2c | 0.34750939 |
| Rnasek | -0.3747417 |
| Rnasen | 1.01196167 |
| Rnaset2 | -0.0622315 |
| Rnaset2b | -0.6201557 |
| Rnd2 | 0.1329045 |
| Rnd3 | 1.53917217 |
| Rnf10 | -0.1252718 |
| Rnf103 | -0.7337641 |
| Rnf11 | -0.7235233 |
| Rnf11 | -0.3734644 |
| Rnf11 | -0.5891482 |
| Rnf111 | -0.5606864 |
| Rnf113a1 | 1.65388651 |
| Rnf113a2 | 3.66417125 |
| Rnf12 | -0.5622452 |
| Rnf121 | -0.6462309 |
| Rnf123 | -0.8006771 |
| Rnf125 | -0.0695955 |
| Rnf125 | -0.558353 |
| Rnf128 | -0.3934605 |
| Rnf133 | 2.96011078 |
| Rnf135 | 2.33027923 |
| Rnf135 | 0.59783516 |
| Rnf135 | -0.5867432 |
| Rnf138 | 1.82122915 |
| Rnf138 | -0.0320058 |
| Rnf138 | -0.6470842 |
| Rnf138 | 1.53069425 |
| Rnf139 | 0.11636673 |
| Rnf139 | -0.3570151 |
| Rnf14 | -0.4118942 |
| Rnf14 | -0.6127189 |
| Rnf14 | -0.1551551 |
| Rnf141 | 4.13297804 |
| Rnf141 | 1.92005593 |
| Rnf144a | -0.3954184 |
| Rnf145 | -0.7642055 |
| Rnf150 | -0.6643145 |
| Rnf166 | 0.02065536 |
| Rnf167 | -0.52703 |
| Rnf167 | -0.4165597 |
| Rnf167 | -0.3231383 |
| Rnf167 | -0.6889798 |
| Rnf167 | 0.16152545 |
| Rnf168 | -0.1395969 |
| Rnf17 | 0.7673062 |
| Rnf170 | -0.6792126 |
| Rnf181 | 2.85139844 |
| Rnf182 | 0.49768133 |
| Rnf182 | 1.3363884 |
| Rnf183 | -0.6686719 |
| Rnf185 | 0.37814589 |
| Rnf185 | -0.1981239 |
| Rnf185 | 1.74725413 |
| Rnf185 | 0.06853161 |
| Rnf186 | 2.51937125 |
| Rnf19a | -0.7479109 |
| Rnf2 | 0.95463635 |
| Rnf20 | 1.07287745 |
| Rnf207 | 1.37148936 |
| Rnf208 | 3.39785758 |
| Rnf214 | 0.40419298 |
| Rnf215 | -0.3093256 |
| Rnf216 | 2.28035814 |
| Rnf216 | -0.5106428 |
| Rnf219 | 0.01306864 |
| Rnf24 | -0.6276518 |
| Rnf24 | 0.6269282 |
| Rnf25 | -0.4436725 |
| Rnf25 | -0.6854163 |
| Rnf25 | 0.20150314 |
| Rnf26 | -0.8148544 |
| Rnf31 | 1.58643138 |
| Rnf32 | 1.13099066 |
| Rnf34 | -0.2093139 |
| Rnf34 | -0.0925132 |
| Rnf34 | 3.8787732 |
| Rnf38 | 1.49275463 |
| Rnf38 | 2.39812539 |
| Rnf38 | 3.47083305 |
| Rnf4 | -0.6360514 |
| Rnf4 | -0.2238995 |
| Rnf40 | -0.8251135 |
| Rnf41 | -0.2662125 |
| Rnf41 | -0.3715516 |
| Rnf41 | -0.5811554 |
| Rnf44 | -0.8619278 |
| Rnf5 | -0.4443604 |
| Rnf6 | -0.2679224 |
| Rnf6 | -0.658248 |
| Rnf8 | -0.4099018 |
| Rnf8 | -0.7896857 |
| Rngtt | 0.13439362 |
| Rnmt | 1.59497508 |
| Rnmtl1 | 0.54549292 |
| Rnpc2 | 0.0151822 |
| Rnpep | -0.7198268 |
| Rnpepl1 | 0.52778589 |
| Rnps1 | -0.3396163 |
| Rnps1 | 0.4191522 |
| Rnu6 | -0.6943504 |
| Rnuxa | -0.0476741 |
| Rnuxa | -0.6539385 |
| Robo1 | -0.9137455 |
| Robo2 | -0.6889798 |
| Robo4 | -0.2431496 |
| Rock1 | -0.3482755 |
| Rock2 | 0.82545469 |
| Rock2 | -0.1301869 |
| Rod1 | 0.48700244 |
| Rogdi | 0.08239261 |
| Rom1 | 0.70957341 |
| Ropn1 | 0.42353927 |
| Ror1 | -0.3830869 |
| Ror2 | 1.4889721 |
| Rora | 1.3535429 |
| Rorb | -0.750766 |
| Rorb | -0.5521587 |
| Rorc | -0.5931702 |
| Rorc | 2.82674124 |
| Ros1 | -0.0218498 |
| Rp23-297j14.5 | 2.02346718 |
| Rp23-438h3.2 | 0.71078363 |
| rp9 | -0.2633695 |
| rp9 | 0.94336976 |
| Rpa1 | -0.2846141 |
| Rpa2 | -0.502555 |
| Rpa2 | -0.5851433 |
| Rpa3 | 0.81882904 |
| Rpain | 0.96807255 |
| Rpap1 | -0.6259808 |
| Rpap1 | 0.90562875 |
| Rpap2 | -0.7749015 |
| Rpap2 | 0.48776925 |
| Rpap3 | 1.26580179 |
| Rpe | -0.3967259 |
| Rpgr | -0.4708818 |
| Rpgrip1 | 1.38369352 |
| Rpgrip1l | 3.50775383 |
| Rph3a | -0.2013075 |
| Rph3al | 1.16989415 |
| Rpia | 0.20461534 |
| Rpl12 | -0.3545098 |
| Rpl13a | -0.3856696 |
| Rpl18 | 0.78106202 |
| Rpl19 | 0.99854849 |
| Rpl21 | -0.5771843 |
| Rpl21 | 1.06128356 |
| Rpl22 | 0.5302313 |
| Rpl22l1 | -0.6118963 |
| Rpl23 | 0.93546559 |
| Rpl23 | 0.33946748 |
| Rpl23 | 0.10300088 |
| Rpl24 | 0.49464257 |
| Rpl27 | -0.5883458 |
| Rpl27a | -0.6318422 |
| Rpl28 | -0.4519611 |
| Rpl29 | -0.5740194 |
| Rpl29 | -0.2077072 |
| Rpl3 | -0.7517196 |
| Rpl30 | 0.99214324 |
| Rpl31 | -0.5988301 |
| Rpl31 | -0.5173122 |
| Rpl34 | 2.62307066 |
| Rpl35 | -0.4751386 |
| Rpl36a | -0.3396163 |
| Rpl36al | -0.3165069 |
| Rpl37 | -0.6394323 |
| Rpl38 | 0.10184635 |
| Rpl38 | 0.15171642 |
| Rpl39 | 0.62405905 |
| Rpl39l | 1.88519255 |
| Rpl3l | 1.71057962 |
| Rpl4 | -0.6284884 |
| Rpl41 | 0.75661575 |
| Rpl7a | -0.5106428 |
| Rpl7l1 | 0.76901303 |
| Rpl7l1 | 1.49503439 |
| Rpl9 | -0.5195458 |
| Rplp1 | -0.7632392 |
| Rpn2 | 0.43576932 |
| Rpo1-1 | 0.03109201 |
| Rpo1-3 | 0.4552771 |
| Rpo1-3 | 1.98368543 |
| Rpo1-4 | 2.70820566 |
| Rpo1-4 | 0.17729456 |
| Rpo1-4 | -0.4484986 |
| Rpo2tc1 | 2.04893767 |
| Rpp21 | 0.1406962 |
| Rpp25 | -0.2963005 |
| Rpp30 | -0.3882594 |
| Rpp38 | 1.94849577 |
| Rpp40 | -0.1797647 |
| Rprm | 3.08188914 |
| Rprml | -0.7189049 |
| Rps10 | -0.9697868 |
| Rps11 | 1.09027406 |
| Rps12 | 0.33527037 |
| Rps13 | 0.16944305 |
| Rps14 | 1.55401224 |
| Rps15 | -0.6243128 |
| Rps15a | -0.8334021 |
| Rps16 | -0.638586 |
| Rps19 | -0.5360902 |
| Rps19 | -0.0929868 |
| Rps19bp1 | -0.8128159 |
| Rps2 | 1.166299 |
| Rps21 | 0.29850571 |
| Rps24 | 3.76884758 |
| Rps24 | 0.35577717 |
| Rps25 | 0.10223135 |
| Rps26 | -0.5645883 |
| Rps27a | 0.3657199 |
| Rps27a | 0.70836162 |
| Rps27l | 0.28971653 |
| Rps3 | 0.2454872 |
| Rps3 | 2.17097796 |
| Rps3a | -0.6318422 |
| Rps4x | -0.6368955 |
| Rps4y2 | 0.01264535 |
| Rps5 | -0.1756181 |
| Rps6 | 0.65043183 |
| Rps6ka1 | 1.62030357 |
| Rps6ka3 | 3.32460894 |
| Rps6ka4 | -0.7422266 |
| Rps6ka5 | -0.14159 |
| Rps6kb1 | 2.06984674 |
| Rps6kl1 | -0.1725202 |
| Rps7 | 1.40059513 |
| Rps8 | 0.81342242 |
| Rps9 | 0.02317074 |
| Rps9 | -0.4145574 |
| Rpsa | -0.8705487 |
| Rpusd1 | 0.89458522 |
| Rpusd4 | 0.37382986 |
| Rqcd1 | -0.7384561 |
| Rrad | 2.35419244 |
| Rragb | 3.08596557 |
| Rragc | -0.807739 |
| Rragd | -0.6251465 |
| Rras | -0.4737175 |
| Rras2 | -0.4225923 |
| Rrbp1 | -0.2157685 |
| Rrbp1 | 0.54956907 |
| Rreb1 | -0.4199064 |
| Rreb1 | -0.5787707 |
| Rrh | 0.74307956 |
| Rrm1 | -0.515826 |
| Rrm2 | 2.04264664 |
| Rrm2 | -0.5732299 |
| Rrm2b | 0.53583174 |
| Rrn3 | 1.68215774 |
| Rrp1 | -0.4916371 |
| Rrp12 | -0.142089 |
| Rrp15 | 1.27226056 |
| Rrp1b | -0.1823656 |
| Rrp1b | 0.03647365 |
| Rrp1b | 1.0323742 |
| Rrp9 | -0.3677378 |
| Rs1 | -0.4967164 |
| Rsad1 | -0.6943504 |
| Rsad2 | 1.16915145 |
| Rsf1 | 2.45902018 |
| Rshl1 | -0.505488 |
| Rshl2a | 0.07884781 |
| Rsl1d1 | 0.06613479 |
| Rsl1d1 | 0.08081879 |
| Rsn | 0.23484964 |
| Rsph1 | 0.12316736 |
| Rspo1 | -0.122335 |
| Rspo2 | -0.7070024 |
| Rspo3 | 1.61260635 |
| Rspo4 | -0.2875221 |
| Rspry1 | -0.5195458 |
| Rsrc1 | 1.57764897 |
| Rsrc1 | -0.3402322 |
| Rsrc2 | 3.66850876 |
| Rsu1 | -0.7253769 |
| Rtbdn | -0.2233552 |
| Rtel1 | -0.4334134 |
| Rtel1 | -0.6863059 |
| Rtkn | 0.70836162 |
| Rtn1 | 0.25926891 |
| Rtn1 | -0.3947653 |
| Rtn1 | 0.104154 |
| Rtn1 | 2.33092775 |
| Rtn2 | 0.53364423 |
| Rtn3 | -0.0431708 |
| Rtn4 | -0.7431716 |
| Rtn4 | -0.8282132 |
| Rtn4 | -0.2759435 |
| Rtn4ip1 | 0.00413942 |
| Rtn4r | 0.53071962 |
| Rtn4rl1 | -0.5106428 |
| Rtn4rl2 | 3.15563417 |
| Rtp1 | -0.5996414 |
| Rtp4 | -0.8097665 |
| Rttn | -0.6012662 |
| Rufy1 | 0.60598218 |
| Rufy2 | 1.44346495 |
| Rufy3 | 2.33595658 |
| Rufy3 | 0.87396753 |
| Rufy3 | 0.38244221 |
| Rufy4 | -0.6377404 |
| Rundc1 | -0.5669372 |
| Rundc3b | -0.4715899 |
| Runx1 | -0.4340939 |
| Runx1t1 | 0.37440648 |
| Runx1t1 | -0.0963085 |
| Runx2 | 2.6486978 |
| Rusc1 | 0.76312086 |
| Rusc2 | -0.6677988 |
| Rusc2 | -0.515826 |
| Ruvbl1 | 0.42845038 |
| Ruvbl1 | -0.5811554 |
| Ruvbl1 | 1.66257471 |
| Ruvbl2 | 1.37527668 |
| Rwdd1 | -0.6505051 |
| Rwdd1 | 2.32405962 |
| Rwdd3 | -0.0849683 |
| Rwdd3 | 1.48535128 |
| Rwdd4a | -0.5748096 |
| Rxfp1 | 1.17704348 |
| Rxfp3 | 0.04387468 |
| Rxra | -0.0087654 |
| Rxrb | -0.1797647 |
| Rxrb | -0.0413755 |
| Rxrg | -0.8117984 |
| Rya3 | 0.38643449 |
| Ryk | -0.2359535 |
| Ryk | -0.4916371 |
| Ryr1 | -0.1184335 |
| Ryr1 | 1.24238226 |
| Ryr1 | 1.00003185 |
| Ryr3 | -0.7393973 |
| S100a1 | -0.0526529 |
| S100a10 | -0.8138346 |
| S100a11 | -0.0044441 |
| S100a13 | 0.18190348 |
| S100a16 | -0.4192361 |
| S100a4 | -0.7778398 |
| S100a6 | 0.51620653 |
| S100a8 | -0.3334797 |
| S100a9 | 0.10223135 |
| S100b | 1.62198854 |
| S100pbp | 0.41336296 |
| S100pbp | -0.5475396 |
| S3-12 | 2.14219496 |
| Saa3 | -0.5947838 |
| Sac3d1 | -0.1440874 |
| Sacm1l | -0.0549247 |
| Sacs | -0.6176703 |
| Sae1 | 0.33346587 |
| Sae1 | 0.39380406 |
| Sae1 | -0.5330604 |
| Sag | 0.05243627 |
| Sall1 | -0.770998 |
| Sall1 | -0.4959891 |
| Sall2 | -0.7244496 |
| Sall4 | 0.51496625 |
| Sall4 | 1.99831426 |
| Samd10 | -0.5437077 |
| Samd11 | 0.23618593 |
| Samd12 | -0.6086127 |
| Samd14 | 0.75083836 |
| Samd4 | -0.4938106 |
| Samd4 | 3.32414541 |
| Samd4b | 0.53994987 |
| Samd5 | -0.5368492 |
| Samd7 | -0.73096 |
| Samd8 | 0.21286456 |
| Samd9l | -0.6686719 |
| Samhd1 | 0.23418078 |
| Samm50 | 1.08243106 |
| Samsn1 | -0.4959891 |
| Sap130 | -0.5552507 |
| Sap30 | 1.42080647 |
| Sap30bp | 2.79710753 |
| Sap30l | -0.6765663 |
| Saps1 | -0.1044246 |
| Saps2 | 1.82499845 |
| Saps3 | -0.3532596 |
| Saps3 | 1.41061139 |
| Sar1a | -0.7079126 |
| Sar1b | 0.60123833 |
| Sar1b | -0.4540447 |
| Sarm1 | -0.6445267 |
| Sars | 0.32772865 |
| Sars | 2.46484838 |
| Sars2 | -0.7300272 |
| Sart1 | 1.34987128 |
| Sart3 | -0.5202916 |
| Sas | 0.90300325 |
| Sass6 | -0.5964003 |
| Sat2 | -0.6453784 |
| Satb1 | 1.42128019 |
| Sav1 | 3.29138968 |
| Sbds | 0.93657618 |
| Sbf1 | -0.6854163 |
| Sbk | 2.83497612 |
| Sbno1 | -0.1561678 |
| Sbno2 | 0.15826712 |
| Sc4mol | -0.388908 |
| Sc5d | -0.7431716 |
| Scaf1 | -0.6251465 |
| Scamp1 | 2.03355055 |
| Scamp2 | -0.5529308 |
| Scamp3 | 1.58801941 |
| Scamp4 | -0.3557616 |
| Scamp5 | 2.30507615 |
| Scamp5 | 3.89661555 |
| Scand1 | 0.60733319 |
| Scap | 0.32469495 |
| Scaper | 0.23785363 |
| Scara3 | -0.4286638 |
| Scara5 | 1.99980056 |
| Scarb1 | 1.66191487 |
| Scarb2 | -0.5843444 |
| Scarf1 | -0.2945383 |
| Scarf2 | 0.35548314 |
| Sccpdh | -0.6251465 |
| Scd1 | -0.12087 |
| Scd2 | 0.33346587 |
| Scd4 | -0.5685064 |
| Scel | -0.6143665 |
| Scg2 | 0.02149457 |
| Scg3 | -0.257145 |
| Scg5 | -0.7290953 |
| Scgn | 0.70410804 |
| Scgn | -0.7827575 |
| Scgn | 1.52315975 |
| Schip1 | 1.3396998 |
| Schip1 | 0.88122682 |
| Scin | -0.2354022 |
| scl0003251.1_21 | 1.66674299 |
| Sclt1 | -0.4938106 |
| Scly | -0.6394323 |
| Scmh1 | -0.5716528 |
| Scmh1 | -0.1277261 |
| Scml2 | 1.32060347 |
| Scml4 | -0.3626766 |
| Scn10a | 1.41672265 |
| Scn11a | -0.6756859 |
| Scn1a | -0.0617731 |
| Scn2b | 1.21105017 |
| Scn3b | -0.4708818 |
| Scn3b | 1.11025957 |
| Scn3b | -0.5099047 |
| Scn3b | -0.8168973 |
| Scn4b | -0.8189446 |
| Scn5a | 0.7273673 |
| Scn7a | -0.6259808 |
| Scn8a | -0.1044246 |
| Scnm1 | 0.43981092 |
| Scnm1 | 0.86545544 |
| Scnn1a | -0.7263052 |
| Scnn1b | 0.58686153 |
| Scnn1g | -0.3934605 |
| Scoc | 2.62370409 |
| Scoc | 1.36306289 |
| Scotin | -0.4659398 |
| Scotin | -0.6863059 |
| Scotin | -0.349519 |
| Scp2 | 0.37382986 |
| Scp2 | 1.36608101 |
| Scp2 | -0.8523228 |
| Scrg1 | -0.5113815 |
| Scrib | 2.00312341 |
| Scrib | 1.21742415 |
| Scrn1 | -0.1571815 |
| Scrn1 | -0.3792261 |
| Scrn1 | -0.2173891 |
| Scrn2 | -0.6045242 |
| Sct | -0.5113815 |
| Sctr | 0.81793009 |
| Scube1 | 1.41491251 |
| Scube2 | 0.98884919 |
| Scube3 | 0.2454872 |
| Scube3 | -0.6591122 |
| Scx | 0.10989846 |
| Scye1 | -0.3304263 |
| Scyl1 | 1.29582299 |
| Scyl1bp1 | 0.88876425 |
| Scyl3 | 3.53360127 |
| Scyl3 | -0.6251465 |
| Sdad1 | -0.4026322 |
| Sdc1 | 1.4456826 |
| Sdc1 | 0.16333076 |
| Sdc2 | 0.21491562 |
| Sdc3 | -0.5262789 |
| Sdc4 | 4.56993412 |
| Sdcbp | 2.10653331 |
| Sdcbp | 3.35953542 |
| Sdcbp2 | -0.0631488 |
| Sdcbp2 | 0.2537149 |
| Sdccag1 | 0.19803301 |
| Sdccag1 | -0.8354857 |
| Sdccag3 | -0.4901909 |
| Sdccag3 | 0.60755817 |
| Sdf2 | -0.7051845 |
| Sdf2 | -0.7661412 |
| Sdf4 | -0.3334797 |
| Sdhb | -0.375381 |
| Sdhb | 2.76380853 |
| Sdhb | -0.5292869 |
| Sdhc | -0.4429851 |
| Sdhd | 0.84488141 |
| Sdk1 | 0.68834493 |
| Sdpr | -0.4125593 |
| Sds | -0.4334134 |
| Sdsl | -0.7133927 |
| Sec1 | -0.7300272 |
| Sec11a | -0.5827485 |
| Sec11c | 1.21329779 |
| Sec13 | 2.7477591 |
| Sec14l1 | 0.70775514 |
| Sec14l1 | 0.99646784 |
| Sec14l1 | -0.0214107 |
| Sec14l2 | 2.83930799 |
| Sec14l3 | 0.06373185 |
| Sec14l3 | -0.7479109 |
| Sec14l4 | 0.19733745 |
| Sec15l1 | -0.1361191 |
| Sec16a | 0.05040486 |
| Sec16b | -0.2403754 |
| Sec22a | -0.3960719 |
| Sec22b | -0.4554363 |
| Sec22c | 1.58714633 |
| Sec22c | -0.6739275 |
| Sec23a | 2.66411462 |
| Sec23ip | 0.19594478 |
| Sec24b | 0.01264535 |
| Sec24c | 0.24779822 |
| Sec24d | 2.87318208 |
| Sec61a1 | -0.6827526 |
| Sec61a2 | -0.3457933 |
| Sec61a2 | 1.22071226 |
| Sec61b | 0.45554154 |
| Sec61b | 2.79537246 |
| Sec61g | -0.1766531 |
| Sec63 | 0.61204648 |
| Sec63 | -0.501092 |
| Secisbp2 | -0.6086127 |
| Sectm1b | -0.653079 |
| Seh1l | -0.1058642 |
| Seh1l | 1.28219581 |
| Sel1l | -0.4666442 |
| Sel1l | -0.3824423 |
| Sel1l | 0.37411821 |
| Sel1l2 | 1.28919759 |
| Sel1l2 | 1.51830239 |
| Selenbp2 | 0.58340585 |
| Selk | 2.19591779 |
| Sell | 3.40456541 |
| Selp | -0.4680546 |
| Selplg | -0.6970472 |
| Sema3a | -0.5835461 |
| Sema3b | 0.01349173 |
| Sema3b | 0.32560609 |
| Sema3b | 2.22445793 |
| Sema3e | -0.5716528 |
| Sema3f | 1.19468267 |
| Sema3f | 0.43576932 |
| Sema3g | -0.5262789 |
| Sema4a | -0.2526438 |
| Sema4b | -0.6360514 |
| Sema4d | 1.21789459 |
| Sema4f | -0.6748063 |
| Sema4g | 1.78846907 |
| Sema5a | -0.7161448 |
| Sema5a | -0.4498821 |
| Sema5b | 2.892948 |
| Sema6b | -0.9148796 |
| Sema6b | 0.9105319 |
| Sema6b | -0.2742189 |
| Sema6d | 0.34275211 |
| Sema6d | -0.6479383 |
| Sema7a | 0.79774898 |
| Sema7a | -0.5972095 |
| Senp1 | -0.6513623 |
| Senp2 | -0.4561329 |
| Senp3 | 0.04264521 |
| Senp5 | -0.1761355 |
| Senp5 | -0.4652359 |
| Senp5 | 0.47904191 |
| Senp6 | -0.2650743 |
| Senp6 | 0.8715919 |
| Senp7 | 0.81179436 |
| Senp7 | -0.5827485 |
| Senp7 | 1.39525464 |
| Senp8 | 2.32038815 |
| Sephs1 | -0.7545863 |
| Sephs2 | 0.46840893 |
| Sepm | -0.7488617 |
| Sepn1 | 0.47205324 |
| Sepp1 | -0.0603994 |
| Sepp1 | 0.27188792 |
| Sept3 | -0.3021987 |
| Sept4 | 3.22839655 |
| Sept4 | -0.3526351 |
| Sept6 | -0.1521237 |
| Sept7 | 1.28930658 |
| Sept7 | -0.4794147 |
| Sept8 | 0.24911626 |
| Sept9 | -0.5988301 |
| Sept10 | 0.34572823 |
| Sept11 | 0.76939191 |
| Sept11 | -0.7729477 |
| Sept13 | -0.1063445 |
| Sept16 | 1.23236255 |
| Sepsecs | -0.6863059 |
| Sepw1 | -0.5128607 |
| Sepx1 | -0.7006551 |
| Sepx1 | 0.54284597 |
| Serac1 | -0.0091987 |
| Serbp1 | 0.21149471 |
| Serbp1 | 1.32879034 |
| Serbp1 | -0.1106787 |
| Serf1 | -0.7574618 |
| Serf2 | 1.30218708 |
| Sergef | -0.1730358 |
| Serhl | -0.546005 |
| Serhl | 0.72498767 |
| Serinc1 | 1.4066754 |
| Serinc2 | 1.58260913 |
| Serinc3 | -0.4046093 |
| Serpina1a | 1.96591011 |
| Serpina1a | 0.23250655 |
| Serpina1b | -0.2304553 |
| Serpina1b | -0.2548917 |
| Serpina1d | 0.78907161 |
| Serpina1e | -0.1745843 |
| Serpina3b | -0.5506165 |
| Serpina3c | 1.10761924 |
| Serpina3g | 0.23985097 |
| Serpina3h | 1.79154132 |
| Serpina3k | 1.15303998 |
| Serpina3n | -0.8344433 |
| Serpina5 | -0.2951253 |
| Serpina5 | 2.85367403 |
| Serpina7 | -0.7328285 |
| Serpina9 | 0.11484878 |
| Serpinb11 | 0.90316755 |
| Serpinb12 | 1.18815218 |
| Serpinb1a | 1.04729239 |
| Serpinb3a | -0.163287 |
| Serpinb6a | -0.5599079 |
| Serpinb6b | 0.13921703 |
| Serpinb6c | -0.4519611 |
| Serpind1 | -0.5217848 |
| Serpine2 | 0.20427005 |
| Serpinf1 | -0.45683 |
| Serping1 | -0.6599773 |
| Serpinh1 | -0.8138346 |
| Serpini1 | 0.8155888 |
| Sertad1 | -0.0774835 |
| Sertad2 | 0.49413515 |
| Sertad2 | -0.1865421 |
| Sertad3 | -0.7070024 |
| Sertad4 | 3.83175195 |
| Sesn1 | 0.09991898 |
| Sesn1 | 0.13141302 |
| Sesn2 | -0.6565218 |
| Sesn3 | -0.1658427 |
| Setd1b | -0.5150838 |
| Setd2 | -0.6012662 |
| Setd3 | 1.04503436 |
| Setd4 | 1.13047469 |
| Setd4 | 1.05529284 |
| Setd5 | 2.52995327 |
| Setd6 | -0.8148544 |
| Setd6 | 0.35135704 |
| Setd7 | 1.49407055 |
| Setd7 | -0.538369 |
| Setd8 | 1.12633668 |
| Setd8 | -0.4145574 |
| Setd8 | -0.5360902 |
| Setdb1 | 0.71821249 |
| Setdb2 | -0.324349 |
| Setmar | -0.6952485 |
| Setx | -0.4225923 |
| Sez6 | -0.4293409 |
| Sez6l | 0.67441073 |
| Sez6l2 | -0.4631274 |
| Sez6l2 | -0.4989016 |
| Sf1 | 3.11481518 |
| Sf1 | -0.3470336 |
| Sf3a1 | -0.1267436 |
| Sf3a2 | 1.5083391 |
| Sf3a3 | 1.06086684 |
| Sf3b1 | -0.7584222 |
| Sf3b2 | -0.502555 |
| Sf3b3 | 1.36938981 |
| Sf3b4 | 0.31890383 |
| Sf3b4 | -0.6669265 |
| Sf3b5 | -0.2157685 |
| Sf4 | 0.43252348 |
| Sfi1 | 0.88004889 |
| Sfi1 | -0.6061575 |
| Sfmbt1 | 0.68813846 |
| Sfmbt1 | 0.46736511 |
| Sfmbt1 | 1.1351083 |
| Sfmbt2 | -0.5915593 |
| Sfrp1 | -0.7798038 |
| Sfrp2 | 0.11256726 |
| Sfrp4 | 1.86336471 |
| Sfrp5 | -0.5638066 |
| Sfrs1 | 2.95702427 |
| Sfrs1 | -0.5955917 |
| Sfrs11 | 1.15013819 |
| Sfrs12 | 0.16260905 |
| Sfrs14 | 0.7085637 |
| Sfrs16 | 0.12316736 |
| Sfrs2 | 0.69492793 |
| Sfrs2 | 2.37939615 |
| Sfrs2 | -0.7729477 |
| Sfrs3 | 0.45501258 |
| Sfrs3 | -0.7033701 |
| Sfrs4 | 2.12087055 |
| Sfrs5 | -0.5315492 |
| Sfrs5 | -0.5851433 |
| Sfrs6 | 3.81430641 |
| Sfrs7 | -0.6800963 |
| Sfrs8 | 2.14929896 |
| Sfrs9 | -0.6651844 |
| Sft2d2 | 0.51272963 |
| Sft2d2 | -0.5195458 |
| Sft2d2 | -0.7015592 |
| Sftpc | 0.10913456 |
| Sftpd | -0.7384561 |
| Sfxn1 | 1.91508181 |
| Sfxn1 | 0.04387468 |
| Sfxn3 | -0.8230527 |
| Sfxn3 | -0.6765663 |
| Sfxn4 | 1.65084884 |
| Sfxn4 | -0.5661536 |
| Sfxn5 | 0.72716923 |
| Sgca | -0.4680546 |
| Sgca | 0.7273673 |
| Sgcb | 1.73008521 |
| Sgcb | -0.6774476 |
| Sgcz | -0.7565023 |
| Sgip1 | 1.41510322 |
| Sgip1 | -0.0901489 |
| Sgip1 | 0.8039767 |
| Sgip1 | -0.6970472 |
| Sgip1 | -0.3195154 |
| Sgk1 | -0.7498134 |
| Sgk2 | 1.63972618 |
| Sgk2 | -0.7441176 |
| Sgk3 | 1.68738314 |
| Sgk3 | 0.62472194 |
| Sgk3 | -0.6343655 |
| Sgk3 | -0.7479109 |
| Sgk3 | 0.89358989 |
| Sgms2 | -0.0490294 |
| Sgol1 | -0.6419761 |
| Sgpl1 | 1.91513784 |
| Sgpl1 | -0.7584222 |
| Sgpp1 | 1.54989048 |
| Sgpp2 | -0.4596236 |
| Sgsm1 | -0.6069752 |
| Sgsm2 | 0.23985097 |
| Sgsm3 | 0.95851702 |
| Sgta | -0.1597207 |
| Sgtb | -0.1410914 |
| Sgtb | 0.93689325 |
| Sh2b1 | 1.95737718 |
| Sh2b1 | 0.58846985 |
| Sh2b3 | -0.7216732 |
| Sh2d2a | -0.4722986 |
| Sh3bgrl | -0.5923644 |
| Sh3bgrl3 | 1.58754328 |
| Sh3bp2 | -0.06269 |
| Sh3bp5 | 1.66645108 |
| Sh3bp5l | -0.7642055 |
| Sh3gl1 | 2.34195592 |
| Sh3gl2 | -0.7088237 |
| Sh3gl2 | -0.3414652 |
| Sh3gl2 | -0.610253 |
| Sh3gl3 | 0.59692556 |
| Sh3glb1 | -0.5136011 |
| Sh3glb2 | -0.376661 |
| Sh3kbp1 | -0.0174681 |
| Sh3kbp1 | 0.1571785 |
| Sh3kbp1 | -0.717984 |
| Sh3kbp1 | 1.72117524 |
| Sh3md4 | 0.94100547 |
| Sh3md4 | -0.750766 |
| Sh3pxd2a | 0.3010019 |
| Sh3pxd2b | -0.3087296 |
| Sh3rf1 | -0.5972095 |
| Sh3rf2 | -0.4052692 |
| Sh3tc1 | -0.7124771 |
| Sh3tc2 | -0.4026322 |
| Sh3yl1 | -0.610253 |
| Shank2 | -0.7700246 |
| Sharpin | 0.10453805 |
| Shbg | -0.4967164 |
| Shc1 | 1.17285908 |
| Shc1 | 1.25036732 |
| Shc3 | -0.1390993 |
| Shcbp1 | -0.1792454 |
| Shd | -0.6453784 |
| She | -0.2266255 |
| Shf | -0.4554363 |
| Shf | 0.88642574 |
| Shf | -0.1643084 |
| Shfm1 | -0.4059296 |
| Shh | 0.84697662 |
| Shisa2 | -0.1116445 |
| Shisa3 | 0.34036443 |
| Shisa4 | -0.1228238 |
| Shkbp1 | 2.3685931 |
| Shkbp1 | -0.7207495 |
| Shmt1 | -0.1923152 |
| Shmt2 | -0.4192361 |
| Shoc2 | 1.82850699 |
| Shox2 | -0.2255341 |
| Shprh | 0.87937512 |
| Shq1 | -0.4092386 |
| Shroom3 | -0.4361382 |
| Shroom3 | -0.1566745 |
| Si | -0.3954184 |
| Si | 0.42107406 |
| Siah1a | 2.95604259 |
| Siah1b | 0.69840651 |
| Siah1b | -0.3921575 |
| Siah1b | -0.8814371 |
| Siah1b | 1.11970387 |
| Siah2 | -0.1351278 |
| Siat7f | -0.0958332 |
| Siat9 | 0.99199376 |
| Sidt1 | 0.62780926 |
| Sidt2 | -0.2927793 |
| Sidt2 | -0.145589 |
| Sidt2 | -0.0218498 |
| Sigirr | -0.2776712 |
| Siglec1 | -0.1218464 |
| Siglech | 0.28560776 |
| Siglech | -0.1179469 |
| Sil1 | -0.2565811 |
| Sin3a | -0.7946659 |
| Sin3b | -0.1039453 |
| Sip1 | 0.97342424 |
| Sip1 | 0.17015953 |
| Sipa1 | -0.3207215 |
| Sipa1l1 | 0.18930129 |
| Sirpa | -0.5614655 |
| Sirpa | -0.1881131 |
| Sirpb1 | -0.5032874 |
| Sirt1 | -0.4273112 |
| Sirt2 | -0.4779872 |
| Sirt3 | 0.44570737 |
| Sirt5 | -0.5980194 |
| Sirt5 | 1.41185596 |
| Sirt6 | -0.6176703 |
| Sirt7 | -0.2794021 |
| Siva1 | 0.04264521 |
| Six1 | 0.3139993 |
| Six2 | 0.13588038 |
| Six3 | 2.1871044 |
| Six5 | -0.5498463 |
| Six6os1 | 2.18150631 |
| Skap2 | -0.6360514 |
| Skil | -0.558353 |
| Skiv2l | 0.3010019 |
| Skiv2l | 0.33946748 |
| Skiv2l2 | -0.2611013 |
| Skp1a | 2.25466069 |
| Skp2 | 1.42213227 |
| Skp2 | 0.24317049 |
| Skp2 | 4.28324492 |
| Sla | 0.62670781 |
| Slain1 | -0.4205771 |
| Slain2 | -0.2696355 |
| Slamf7 | 1.41252543 |
| Slamf8 | 1.97924682 |
| Slamf9 | 0.0256794 |
| Slc10a3 | 4.05489859 |
| Slc10a4 | 0.93736865 |
| Slc10a6 | -0.6565218 |
| Slc10a6 | -0.228812 |
| Slc10a7 | 1.22270301 |
| Slc10a7 | -0.4436725 |
| Slc10a7 | -0.6952485 |
| Slc11a1 | -0.52703 |
| Slc11a1 | 1.77067154 |
| Slc11a1 | 1.49038133 |
| Slc11a1 | 1.39447531 |
| Slc11a2 | 1.55261286 |
| Slc12a2 | 0.40223629 |
| Slc12a2 | 2.62562816 |
| Slc12a3 | -0.6573845 |
| Slc12a5 | -0.832362 |
| Slc12a6 | -0.6470842 |
| Slc12a7 | -0.2066375 |
| Slc12a8 | -0.6809809 |
| Slc12a9 | 0.25633264 |
| Slc12a9 | -0.5779772 |
| Slc13a2 | -0.7758799 |
| Slc13a3 | -0.5763921 |
| Slc13a3 | -0.0535609 |
| Slc13a4 | -0.4192361 |
| Slc14a1 | 2.75471689 |
| Slc14a1 | 0.35135704 |
| Slc14a1 | -0.7584222 |
| Slc14a2 | -0.6952485 |
| Slc14a2 | -0.7936677 |
| Slc15a2 | -0.8814371 |
| Slc15a3 | -0.4659398 |
| Slc15a4 | -0.3476543 |
| Slc15a4 | -0.7837441 |
| Slc16a10 | -0.7651729 |
| Slc16a11 | 1.73951959 |
| Slc16a4 | 1.89440268 |
| Slc16a6 | -0.2326507 |
| Slc16a6 | -0.6377404 |
| Slc16a6 | 1.49538463 |
| Slc16a8 | 0.5769211 |
| Slc16a9 | -0.6863059 |
| Slc17a2 | 0.30442337 |
| Slc17a5 | -0.6259808 |
| Slc17a6 | -0.4436725 |
| Slc17a7 | 0.09022245 |
| Slc17a8 | -0.5947838 |
| Slc18a2 | 1.06086684 |
| Slc19a1 | -0.0231683 |
| Slc19a2 | -0.7517196 |
| Slc1a1 | 1.56627026 |
| Slc1a1 | 0.01855407 |
| Slc1a2 | 1.6995815 |
| Slc1a2 | -0.2719244 |
| Slc1a2 | 1.49205206 |
| Slc1a2 | 0.47594224 |
| Slc1a3 | 0.95727695 |
| Slc1a4 | -0.5391298 |
| Slc1a6 | -0.1326541 |
| Slc1a7 | 0.56003781 |
| Slc20a1 | -0.6809809 |
| Slc22a1 | -0.3105187 |
| Slc22a12 | -0.3928088 |
| Slc22a13 | -0.2066375 |
| Slc22a14 | 0.66493472 |
| Slc22a17 | -0.5955917 |
| Slc22a17 | 0.77712543 |
| Slc22a18 | -0.505488 |
| Slc22a21 | 0.02233303 |
| Slc22a3 | 2.46021383 |
| Slc22a4 | -0.6453784 |
| Slc22a5 | 0.48572303 |
| Slc22a5 | -0.2293594 |
| Slc22a5 | -0.7441176 |
| Slc22a6 | 0.20011662 |
| Slc22a7 | 0.38814029 |
| Slc22a8 | 0.04182467 |
| Slc23a2 | 0.75834207 |
| Slc23a3 | 2.29472791 |
| Slc24a1 | 1.09013939 |
| Slc24a3 | -0.4645325 |
| Slc24a4 | -0.6827526 |
| Slc24a5 | 0.2284762 |
| Slc24a6 | 1.49835643 |
| Slc25a1 | 0.0151822 |
| Slc25a1 | -0.541416 |
| Slc25a10 | 2.93461862 |
| Slc25a11 | -0.6077935 |
| Slc25a11 | -0.3457933 |
| Slc25a11 | 0.69164219 |
| Slc25a12 | 1.50314397 |
| Slc25a14 | -0.5240292 |
| Slc25a15 | 0.73995803 |
| Slc25a15 | 0.72776333 |
| Slc25a16 | 0.16657162 |
| Slc25a17 | -0.4631274 |
| Slc25a18 | 0.85893435 |
| Slc25a19 | -0.3760208 |
| Slc25a19 | 3.29427865 |
| Slc25a20 | 1.19275243 |
| Slc25a20 | -0.5292869 |
| Slc25a23 | -0.7356381 |
| Slc25a24 | -0.6193265 |
| Slc25a24 | -0.1627767 |
| Slc25a25 | 0.0256794 |
| Slc25a26 | -0.4952623 |
| Slc25a27 | 1.0066782 |
| Slc25a28 | -0.8107819 |
| Slc25a29 | 1.62084003 |
| Slc25a3 | 0.10453805 |
| Slc25a3 | -0.1923152 |
| Slc25a30 | -0.7143092 |
| Slc25a32 | -0.7051845 |
| Slc25a33 | -0.7857205 |
| Slc25a34 | 0.0160263 |
| Slc25a35 | -0.4225923 |
| Slc25a36 | 0.7633115 |
| Slc25a37 | -0.5406533 |
| Slc25a38 | -0.6898728 |
| Slc25a38 | 3.42514976 |
| Slc25a38 | 1.61700148 |
| Slc25a39 | -0.2387147 |
| Slc25a41 | -0.2195542 |
| Slc25a42 | 0.23284163 |
| Slc25a44 | 1.25467386 |
| Slc25a45 | 1.85974633 |
| Slc25a46 | 0.47412937 |
| Slc25a5 | -0.4158918 |
| Slc26a4 | -0.3960719 |
| Slc26a6 | -0.4737175 |
| Slc26a7 | -0.2442616 |
| Slc26a8 | -0.7847318 |
| Slc27a1 | -0.5763921 |
| Slc27a2 | 0.61784946 |
| Slc27a3 | -0.5307945 |
| Slc27a4 | -0.0503865 |
| Slc27a6 | -0.3099219 |
| Slc29a1 | -0.8097665 |
| Slc29a2 | -0.5606864 |
| Slc29a3 | -0.7450645 |
| Slc29a4 | 1.29744506 |
| Slc2a1 | 1.08852182 |
| Slc2a10 | -0.1684053 |
| Slc2a10 | -0.5763921 |
| Slc2a13 | -0.1405929 |
| Slc2a3 | 1.24945817 |
| Slc2a4 | -0.6496487 |
| Slc2a5 | -0.6310026 |
| Slc2a6 | -0.7290953 |
| Slc2a8 | -0.0572019 |
| Slc2a8 | -0.3027905 |
| Slc2a8 | 1.1344661 |
| Slc2a9 | -0.4118942 |
| Slc30a1 | 0.53534606 |
| Slc30a10 | 0.30783245 |
| Slc30a3 | 0.08553237 |
| Slc30a5 | 0.99453168 |
| Slc30a5 | -0.4000026 |
| Slc30a6 | -0.7318938 |
| Slc30a7 | 2.91246246 |
| Slc30a7 | 1.59387193 |
| Slc30a9 | -0.4192361 |
| Slc31a1 | -0.0100657 |
| Slc31a2 | 1.20320064 |
| Slc32a1 | 0.13476553 |
| Slc34a2 | -0.4801293 |
| Slc35a1 | 0.03192196 |
| Slc35a2 | 0.74735481 |
| Slc35a3 | -0.5121208 |
| Slc35a4 | -0.3201182 |
| Slc35a5 | 1.82826121 |
| Slc35a5 | 2.10995597 |
| Slc35b1 | 0.16405192 |
| Slc35b2 | 0.70977522 |
| Slc35b3 | 2.30374249 |
| Slc35b3 | 1.74517542 |
| Slc35b3 | -0.4582258 |
| Slc35b4 | -0.5345741 |
| Slc35c1 | -0.218471 |
| Slc35c1 | 1.65159064 |
| Slc35c2 | -0.6748063 |
| Slc35c2 | -0.5113815 |
| Slc35c2 | -0.3279907 |
| Slc35e1 | -0.6110743 |
| Slc35e3 | -0.7729477 |
| Slc35e4 | -0.2125357 |
| Slc35f1 | -0.3520111 |
| Slc35f2 | 1.32795393 |
| Slc35f3 | -0.4185663 |
| Slc35f4 | -0.1813244 |
| Slc35f4 | -0.6774476 |
| Slc36a1 | 0.49032078 |
| Slc36a3 | 1.08039201 |
| Slc37a1 | -0.4099018 |
| Slc37a3 | -0.3792261 |
| Slc38a1 | 0.60643273 |
| Slc38a10 | 2.18878154 |
| Slc38a11 | -0.1997144 |
| Slc38a2 | 0.28307034 |
| Slc38a2 | -0.5032874 |
| Slc38a2 | -0.3683724 |
| Slc38a3 | -0.5653706 |
| Slc38a4 | 1.08879161 |
| Slc38a5 | -0.1430876 |
| Slc38a8 | 0.94588513 |
| Slc38a9 | 1.02561912 |
| Slc38a9 | -0.7936677 |
| Slc39a10 | -0.7328285 |
| Slc39a11 | 0.03026132 |
| Slc39a12 | 0.4821314 |
| Slc39a13 | 0.2049605 |
| Slc39a13 | -0.3129094 |
| Slc39a14 | 0.4467755 |
| Slc39a3 | 0.02023548 |
| Slc39a3 | -0.5513873 |
| Slc39a4 | -0.6979479 |
| Slc39a5 | -0.0048754 |
| Slc39a5 | -0.336543 |
| Slc39a5 | 1.91614591 |
| Slc39a6 | -0.8230527 |
| Slc39a7 | -0.7469612 |
| Slc39a7 | -0.2707793 |
| Slc39a7 | -0.4212484 |
| Slc39a8 | -0.4105655 |
| Slc3a2 | 0.61896133 |
| Slc40a1 | -0.2130737 |
| Slc41a2 | 0.28243491 |
| Slc41a3 | 3.26977347 |
| Slc41a3 | -0.546005 |
| Slc41a3 | -0.6686719 |
| Slc43a2 | 1.41443558 |
| Slc43a2 | -0.4099018 |
| Slc44a1 | -0.3408485 |
| Slc44a1 | 3.46579106 |
| Slc44a2 | -0.6343655 |
| Slc44a2 | 1.17199528 |
| Slc44a2 | -0.3396163 |
| Slc44a3 | -0.8128159 |
| Slc44a4 | 2.69092451 |
| Slc45a1 | -0.7189049 |
| Slc45a2 | -0.4450489 |
| Slc45a3 | 0.05850434 |
| Slc45a4 | -0.0677491 |
| Slc46a1 | 0.90546487 |
| Slc46a3 | -0.4361382 |
| Slc47a1 | -0.3588985 |
| Slc4a1 | -0.7555438 |
| Slc4a1ap | -0.4519611 |
| Slc4a2 | 1.89754794 |
| Slc4a3 | -0.3664699 |
| Slc4a3 | -0.0053068 |
| Slc4a3 | -0.2018392 |
| Slc4a3 | 0.77543326 |
| Slc4a8 | -0.502555 |
| Slc4a8 | 0.33496986 |
| Slc5a4a | -0.5803598 |
| Slc5a4b | -0.492361 |
| Slc5a5 | 0.38814029 |
| Slc5a6 | -0.4505746 |
| Slc5a7 | 1.24318382 |
| Slc5a8 | 1.71876191 |
| Slc5a9 | 0.62868949 |
| Slc6a1 | -0.5247785 |
| Slc6a11 | 0.05405826 |
| Slc6a12 | -0.505488 |
| Slc6a13 | -0.8365292 |
| Slc6a15 | 1.43789782 |
| Slc6a15 | 0.36630151 |
| Slc6a17 | -0.6522203 |
| Slc6a2 | 0.83574785 |
| Slc6a4 | -0.1981239 |
| Slc6a6 | -0.1395969 |
| Slc6a7 | -0.6704205 |
| Slc6a8 | -0.4212484 |
| Slc6a9 | -0.1849737 |
| Slc7a1 | 0.52949834 |
| Slc7a10 | -0.3201182 |
| Slc7a11 | -0.7318938 |
| Slc7a14 | 2.36935406 |
| Slc7a14 | 0.56804824 |
| Slc7a14 | 0.87295014 |
| Slc7a2 | 2.07680879 |
| Slc7a3 | -0.4219201 |
| Slc7a4 | -0.6961474 |
| Slc7a6 | 0.32104155 |
| Slc7a6os | -0.6845276 |
| Slc7a7 | 0.86767601 |
| Slc7a7 | 2.05431741 |
| Slc7a8 | 0.26024564 |
| Slc7a8 | 2.64214921 |
| Slc8a1 | 0.07805826 |
| Slc8a2 | -0.6756859 |
| Slc8a2 | 0.68565736 |
| Slc9a1 | 1.91888463 |
| Slc9a10 | -0.4701742 |
| Slc9a3r1 | -0.2599692 |
| Slc9a3r2 | 0.82438343 |
| Slc9a4 | -0.8630011 |
| Slc9a6 | -0.9855573 |
| Slc9a6 | 2.08005901 |
| Slc9a8 | -0.9240021 |
| Slc9a8 | 1.19202757 |
| Slc9a8 | -0.6168432 |
| Slc9a8 | 3.2366978 |
| Slc9a9 | -0.0100657 |
| Slco1a4 | -0.2321014 |
| Slco1c1 | 1.00003185 |
| Slco2a1 | -0.7431716 |
| Slco2b1 | -0.6651844 |
| Slco3a1 | -0.4491901 |
| Slco3a1 | 0.11826073 |
| Slco3a1 | -0.5923644 |
| Slco4a1 | 1.15177943 |
| Slfnl1 | -0.9297489 |
| Slit1 | 0.43089636 |
| Slit2 | -0.6419761 |
| Slit3 | 0.44837541 |
| Slitrk1 | 0.56945479 |
| Slitrk3 | -0.1073059 |
| Slitrk4 | 1.92283888 |
| Slitrk5 | 0.38072604 |
| Slitrk5 | -0.7613094 |
| Slitrk6 | 0.35224273 |
| Slk | -0.4596236 |
| Slk | 1.49108516 |
| Slmap | 1.25014012 |
| Slmap | -0.1881131 |
| Slmap | -0.6301638 |
| Slmo1 | -0.6669265 |
| Slmo2 | -0.4512676 |
| Sln | -0.7976667 |
| Slpi | 0.31706763 |
| Sltm | 0.17159088 |
| Sltm | 2.77683504 |
| Slu7 | 1.06156127 |
| Slu7 | 1.34956466 |
| Sly | 3.17178789 |
| Smad1 | -0.6411274 |
| Smad1 | -0.8036971 |
| Smad2 | 1.2030812 |
| Smad3 | 1.72433765 |
| Smad5 | 0.44061716 |
| Smad5 | 1.6404768 |
| Smad6 | -0.7817719 |
| Smap2 | -0.4225923 |
| Smap2 | -0.5307945 |
| Smap2 | 1.51778963 |
| Smarca1 | -0.6301638 |
| Smarca2 | -0.7225978 |
| Smarca2 | -0.807739 |
| Smarca4 | 2.23675914 |
| Smarcad1 | 0.67252314 |
| Smarcal1 | 0.14401584 |
| Smarcb1 | -0.2082425 |
| Smarcc1 | 0.39718602 |
| Smarcc1 | -0.5150838 |
| Smarcc2 | 0.94384191 |
| Smarcd1 | 2.00623114 |
| Smarcd1 | 0.60304833 |
| Smarcd1 | 3.70605412 |
| Smarcd2 | 1.25489997 |
| Smarcd3 | -0.4395554 |
| Smarce1 | 0.41695098 |
| Smarce1 | 0.96715205 |
| Smc1a | 0.88709448 |
| Smc1a | -0.4894686 |
| Smc1a | -0.0118022 |
| Smc1a | -0.7651729 |
| Smc1b | -0.6352081 |
| Smc3 | 2.22655287 |
| Smc5l1 | 0.4931195 |
| Smc6 | 0.88189927 |
| Smchd1 | 0.1776499 |
| Smcp | -0.5353319 |
| Smcr7 | 2.18584458 |
| Smcr7l | 1.26277993 |
| Smek1 | 0.40837223 |
| Smek2 | -0.2476055 |
| Smek2 | 2.59889545 |
| Smg5 | -0.1203822 |
| Smg6 | -0.2321014 |
| Smn1 | 0.31614818 |
| Smo | 0.5605109 |
| Smo | -0.7300272 |
| Smo | -0.6118963 |
| Smoc1 | 1.59835525 |
| Smox | -0.3016072 |
| Smox | 0.37584647 |
| Smox | 2.06288067 |
| Smox | 1.74315938 |
| Smox | 2.18848825 |
| Smpd1 | 3.46294098 |
| Smpd1 | 2.06808624 |
| Smpd1 | 0.48264533 |
| Smpd2 | 1.62862243 |
| Smpd3 | -0.0022909 |
| Smpd4 | 0.49032078 |
| Smpdl3a | 0.93371772 |
| Smpdl3b | -0.3626766 |
| Smpx | 1.62015025 |
| Smtn | 1.65941795 |
| Smtn | -0.4744278 |
| Smtn | 1.24638326 |
| Smtnl1 | 2.43588727 |
| Smtnl2 | 0.36978362 |
| Smu1 | -0.4279873 |
| Smu1 | 0.70309244 |
| Smug1 | 1.21282506 |
| Smurf1 | 0.14842389 |
| Smyd1 | 0.39295666 |
| Smyd2 | -0.6276518 |
| Smyd2 | 1.71113661 |
| Smyd3 | 1.93881966 |
| Smyd4 | 0.17587188 |
| Smyd5 | -0.4072518 |
| Smyd5 | 0.44034849 |
| Snai1 | 0.24746842 |
| Snai3 | 1.51890026 |
| Snap23 | 0.0189747 |
| Snap29 | -0.1663547 |
| Snap91 | -0.5247785 |
| Snap91 | -0.5803598 |
| Snapc1 | -0.5661536 |
| Snapc3 | -0.6368955 |
| Snapc3 | 0.85254114 |
| Snapc3 | 0.03482103 |
| Snapc3 | 0.00413942 |
| Snapc4 | 1.64839675 |
| Snapin | 0.33195946 |
| Snca | 0.99691408 |
| Snca | 0.71721201 |
| Snca | -0.0695955 |
| Sncaip | -0.4582258 |
| Sncg | 0.46840893 |
| Sncg | 4.19265879 |
| Snd1 | -0.1371114 |
| Snf1lk | 0.7121936 |
| Snf1lk2 | 0.28687391 |
| Snf8 | 0.28402267 |
| Snip1 | -0.73096 |
| Snip1 | -0.4526551 |
| Snn | 2.05923398 |
| Snora65 | -0.2846141 |
| Snph | -0.8107819 |
| Snrk | -0.6004535 |
| Snrk | -0.0934606 |
| Snrk | -0.0302317 |
| Snrp70 | 0.11104316 |
| Snrpa | -0.1938958 |
| Snrpb | -0.4779872 |
| Snrpb | -0.6651844 |
| Snrpd1 | -0.6343655 |
| Snrpd1 | -0.7106487 |
| Snrpd2 | -0.8006771 |
| Snrpd3 | -0.1597207 |
| Snrpg | 0.10376978 |
| Snta1 | -0.501092 |
| Sntb2 | -0.7788213 |
| Sntb2 | 1.08148005 |
| Sntg2 | -0.2098501 |
| Snupn | 2.63077476 |
| Snurf | 2.67519525 |
| Snurf | 1.56635145 |
| Snx1 | -0.8523228 |
| Snx10 | -0.8067269 |
| Snx11 | -0.3147064 |
| Snx12 | -0.0187804 |
| Snx13 | 0.96099227 |
| Snx14 | 0.9116723 |
| Snx14 | -0.7498134 |
| Snx14 | -0.5128607 |
| Snx15 | -0.7024642 |
| Snx16 | -0.2130737 |
| Snx17 | -0.3967259 |
| Snx17 | 0.12279083 |
| Snx18 | -0.1058642 |
| Snx19 | 1.43313971 |
| Snx19 | -0.5685064 |
| Snx2 | 1.47458347 |
| Snx2 | -0.6889798 |
| Snx20 | -0.4554363 |
| Snx21 | -0.5964003 |
| Snx22 | 2.83731276 |
| Snx24 | -0.6721723 |
| Snx25 | 1.09684947 |
| Snx25 | -0.5398912 |
| Snx26 | 1.87155826 |
| Snx29 | -0.6284884 |
| Snx3 | -0.2109234 |
| Snx30 | -0.7916746 |
| Snx33 | 1.27624726 |
| Snx4 | 1.06100577 |
| Snx6 | -0.3177091 |
| Snx7 | -0.076552 |
| Snx8 | -0.5787707 |
| Snx9 | -0.4540447 |
| Soat1 | 1.11643533 |
| Socs2 | 1.73810031 |
| Socs3 | -0.6774476 |
| Socs4 | -0.1287096 |
| Socs5 | -0.5173122 |
| Socs6 | -0.1991839 |
| Socs7 | -0.4687606 |
| Socs7 | 0.00157258 |
| Sod1 | -0.1865421 |
| Sod2 | 0.92122971 |
| Sod3 | 0.78180998 |
| Sohlh1 | 1.02950563 |
| Sohlh1 | -0.6352081 |
| Son | -0.0306749 |
| Sorbs1 | -0.1787263 |
| Sorbs2 | 3.01400347 |
| Sorbs3 | 0.76939191 |
| Sorcs1 | 1.3281631 |
| Sorcs1 | -0.6748063 |
| Sorcs2 | 1.8476036 |
| Sorcs3 | 2.67943208 |
| Sorcs3 | -0.4959891 |
| Sord | 1.02026695 |
| Sorl1 | 0.35753942 |
| Sort1 | -0.4039498 |
| Sort1 | -0.6809809 |
| Sort1 | -0.4540447 |
| Sort1 | 1.08039201 |
| Sostdc1 | 1.36074241 |
| Sox12 | 0.72954336 |
| Sox13 | -0.7613094 |
| Sox17 | 1.42939096 |
| Sox17 | -0.7966653 |
| Sox18 | 0.40614561 |
| Sox2 | 1.12763176 |
| Sox2 | 0.3878562 |
| Sox21 | 0.67462022 |
| Sox21 | 0.00797661 |
| Sox30 | 1.47288141 |
| Sox5 | -0.4491901 |
| Sox5 | -0.6765663 |
| Sox5 | -0.2409296 |
| Sox5 | 0.23116502 |
| Sox7 | -0.5398912 |
| Sox7 | 0.17480346 |
| Sox8 | -0.4072518 |
| Sox9 | 0.74657894 |
| Sp100 | 1.97204845 |
| Sp140 | 0.14327916 |
| Sp2 | 2.54119557 |
| Sp4 | -0.4046093 |
| Sp5 | -0.6970472 |
| Sp6 | 0.60530595 |
| Sp7 | 2.49358095 |
| Sp8 | -0.6809809 |
| Sp9 | 0.17836017 |
| Spaca3 | 1.42704038 |
| Spaca4 | 1.1085442 |
| Spag1 | 3.73119059 |
| Spag1 | 0.79921804 |
| Spag16 | 0.07330699 |
| Spag16 | 0.671683 |
| Spag4 | 1.42014287 |
| Spag4 | -0.7133927 |
| Spag4l | 0.45448333 |
| Spag4l | -0.6335236 |
| Spag5 | -0.1116445 |
| Spag5 | -0.1981239 |
| Spag6 | -0.6836397 |
| Spag6 | -0.6836397 |
| Spag7 | 0.33014859 |
| Spag8 | 1.59031731 |
| Spag9 | 0.93673473 |
| Spag9 | 1.26925941 |
| Spag9 | -0.0481257 |
| Spag9 | -0.3741028 |
| Spam1 | -0.3377711 |
| Spam1 | -0.1005965 |
| Sparc | -0.1802843 |
| Sparc | -0.717984 |
| Sparcl1 | -0.1954791 |
| Spast | -0.5128607 |
| Spast | -0.6970472 |
| Spast | -0.52703 |
| Spata16 | -0.4673491 |
| Spata16 | 2.36140289 |
| Spata17 | -0.6880877 |
| Spata17 | -0.14609 |
| Spata19 | 0.22205929 |
| Spata2 | 0.8352182 |
| Spata20 | -0.4225923 |
| Spata20 | -0.653079 |
| Spata21 | -0.4873052 |
| Spata22 | 0.80943773 |
| Spata2L | 2.02899414 |
| Spata3 | 1.97825179 |
| Spata3 | -0.4178969 |
| Spata3 | 0.00797661 |
| Spata3 | 3.63953862 |
| Spata4 | -0.6686719 |
| Spata6 | 0.38301358 |
| Spata7 | -0.281715 |
| Spata7 | 0.78591317 |
| Spatc1 | -0.5964003 |
| Spatc1 | -0.3177091 |
| Spats1 | -0.6591122 |
| Spc24 | -0.6151914 |
| Spc24 | 0.66957936 |
| Spc25 | -0.7006551 |
| Spc25 | -0.4065905 |
| Spcs1 | -0.6176703 |
| Spcs1 | -0.0830915 |
| Spcs3 | 0.0160263 |
| Spdef | 0.15317608 |
| Specc1l | 0.44195936 |
| Speer1-ps1 | 0.49261126 |
| Speer1-ps1 | -0.1287096 |
| Speer2 | 0.25108986 |
| Speer2 | 3.30721502 |
| Speer3 | 0.28940111 |
| Speer4b | 0.09837424 |
| Speer4d | -0.7170639 |
| Speer4f | 1.34114489 |
| Speer6-ps1 | 0.1854335 |
| Spef1 | -0.1556613 |
| Spen | 0.81288005 |
| Spert | -0.3261681 |
| Spesp1 | -0.7460124 |
| Spg20 | -0.1130952 |
| Spg21 | 0.32012598 |
| Spg7 | 2.71691981 |
| Spg7 | 0.38272794 |
| Sphk1 | -0.2986553 |
| Sphk2 | 0.40753787 |
| Sphk2 | -0.4118942 |
| Spic | 1.19057617 |
| Spin | -0.8781574 |
| Spin2 | 0.32134655 |
| Spink10 | -0.7079126 |
| Spink2 | 2.31955006 |
| Spink4 | 0.07568563 |
| Spink8 | 0.99154517 |
| Spinlw1 | -0.3734644 |
| Spinlw1 | -0.6643145 |
| Spinlw1 | 1.51727659 |
| Spint1 | -0.8662284 |
| Spint2 | 0.5991979 |
| Spire1 | -0.376661 |
| Spire1 | -0.7290953 |
| Spire1 | -0.2548917 |
| Spire2 | 1.24215311 |
| Spna1 | -0.3805112 |
| Spnb1 | -0.6268159 |
| Spnb2 | 1.30722615 |
| Spnb2 | 0.33226094 |
| Spnb2 | -0.7584222 |
| Spnb2 | 1.28897957 |
| Spnb3 | -0.4052692 |
| Spnb4 | -0.4887469 |
| Spns1 | -0.3869636 |
| Spo11 | 0.72815918 |
| Spo11 | 1.30068085 |
| Spock1 | 1.02547487 |
| Spock2 | 0.46658147 |
| Spock3 | 1.29647216 |
| Spon1 | 0.53607448 |
| Spon2 | -0.5421792 |
| Spop | 2.21802314 |
| Spp1 | -0.376661 |
| Spp2 | -0.1516194 |
| Sppl3 | -0.6069752 |
| Spr | 1.85730645 |
| Spred1 | 1.83090006 |
| Spred2 | 1.04827857 |
| Spred2 | 2.68010323 |
| Sprn | 0.08553237 |
| Sprn | 0.49843949 |
| Sprr2a | 0.46239105 |
| Sprr2a | 0.57668867 |
| Sprr2f | -0.5661536 |
| Spry3 | 0.46762617 |
| Spry4 | 1.9373432 |
| Spryd4 | 1.17630641 |
| Spsb1 | -0.7015592 |
| Spsb2 | -0.1252718 |
| Spsb3 | -0.1179469 |
| Spsb3 | -0.2315523 |
| Spsb4 | 0.73545261 |
| Sptlc1 | -0.7263052 |
| Sptlc2 | -0.6845276 |
| Spz1 | 0.37180892 |
| Sqle | -0.1346325 |
| Sqle | 1.86932538 |
| Sqrdl | -0.5819516 |
| Sqstm1 | -0.7441176 |
| Sra1 | -0.4512676 |
| Sra1 | 0.78367729 |
| Src | 1.98431038 |
| Srd5a1 | -0.8449195 |
| Srd5a2 | -0.2007762 |
| Srd5a3 | 0.67880041 |
| Srd5a3 | 1.0379429 |
| Srd5a3 | -0.8705487 |
| Srebf1 | -0.6086127 |
| Srebf2 | 1.6448933 |
| Srf | 0.65984154 |
| Srf | 2.3238782 |
| Srfbp1 | -0.2163084 |
| Srgn | 0.02651414 |
| Srgn | 0.75315357 |
| Sri | -0.5475396 |
| Sri | -0.6792126 |
| Sri | -0.2442616 |
| Srm | -0.6209857 |
| Srm | 0.64157816 |
| Srm | 1.38743213 |
| Srp14 | 3.59893945 |
| Srp19 | -0.1242918 |
| Srp19 | -0.7986691 |
| Srp54 | 0.34958316 |
| Srp68 | 2.13321663 |
| Srp72 | 0.72280103 |
| Srp9 | -0.7015592 |
| Srp9 | -0.2554545 |
| Srpk1 | 0.7894425 |
| Srpk2 | 2.11431887 |
| Srpk3 | 0.22205929 |
| Srpr | 1.46829742 |
| Srprb | 0.74424746 |
| Srprb | -0.5988301 |
| Srpx | -0.4680546 |
| Srpx | 0.85945082 |
| Srpx2 | 0.88609119 |
| Srpx2 | 1.6732871 |
| Srpx2 | -0.4758499 |
| Srr | 0.55267425 |
| Srrd | -0.6411274 |
| Srrm1 | 0.14988866 |
| Srrm2 | -0.7347006 |
| Srrm2 | 0.834158 |
| Srrp | 1.54325786 |
| Srxn1 | -0.4765618 |
| Srxn1 | 3.28357231 |
| Srxn1 | 0.15536133 |
| Ss18 | 2.23890634 |
| Ss18 | -0.0788823 |
| Ss18l1 | -0.5091671 |
| Ss18l1 | 2.47101317 |
| Ss18l1 | 1.19985064 |
| Ssbp1 | 2.07420997 |
| Ssbp2 | -0.5513873 |
| Ssbp2 | 0.20323341 |
| Ssbp3 | -0.3709149 |
| Ssbp3 | 0.72140689 |
| Ssbp4 | 1.6176167 |
| Ssfa2 | -0.6037087 |
| Ssh2 | 2.56443778 |
| Ssh3 | 0.19210427 |
| Ssh3 | 0.78199688 |
| Ssna1 | -0.149102 |
| Sspn | 2.28259779 |
| Ssr2 | -0.7545863 |
| Ssr3 | 0.06932919 |
| Ssr4 | 0.1740905 |
| Ssrp1 | 0.77562143 |
| Sssca1 | 0.91459843 |
| Sssca1 | 0.12090594 |
| Sst | 0.40948357 |
| Sstr1 | 0.17194837 |
| Sstr5 | 2.13579062 |
| Ssty2 | -0.7412826 |
| Ssu72 | -0.6053405 |
| Ssx2ip | 1.17912872 |
| Ssxb2 | -0.6513623 |
| Ssxb5 | -0.0751566 |
| St13 | 1.48597046 |
| St14 | -0.0400312 |
| St14 | 2.95587579 |
| St18 | -0.6168432 |
| St3gal1 | -0.4582258 |
| St3gal2 | 0.38927577 |
| St3gal2 | -0.281715 |
| St3gal3 | 0.80178339 |
| St3gal3 | -0.7613094 |
| St3gal4 | 1.13395203 |
| St3gal5 | 0.7317144 |
| St3gal6 | -0.717984 |
| St5 | 0.36659218 |
| St5 | -0.7143092 |
| St6gal1 | 1.22387208 |
| St6galnac2 | -0.3779426 |
| St6galnac3 | 0.52803071 |
| St6galnac4 | -0.2271717 |
| St6galnac4 | 2.02061635 |
| St6galnac4 | -0.0695955 |
| St6galnac5 | -0.3973803 |
| St6galnac6 | 0.16549258 |
| St6galnac6 | -0.7986691 |
| St7 | 0.37814589 |
| St7l | 1.15076977 |
| St7l | 0.65450667 |
| St8sia1 | -0.7946659 |
| St8sia1 | 0.9450998 |
| St8sia2 | -0.3340915 |
| St8sia3 | -0.8067269 |
| St8sia4 | 1.14836748 |
| St8sia4 | -0.3934605 |
| St8sia5 | -0.2098501 |
| Stab1 | -0.0445195 |
| Stab2 | -0.1116445 |
| Stac | -0.5398912 |
| Stac2 | -0.6573845 |
| Stac3 | -0.2299072 |
| Stag1 | -0.3741028 |
| Stag2 | 1.22760337 |
| Stag2 | -0.4722986 |
| Stag3 | 3.43341969 |
| Stam | -0.6402795 |
| Stam | 2.11978928 |
| Stam | 0.14511979 |
| Stam2 | -0.1135792 |
| Stam2 | -0.4293409 |
| Stambp | -0.3457933 |
| Stambpl1 | 0.58294414 |
| Stap1 | 2.91878837 |
| Stap2 | -0.5047539 |
| Star | 1.58794007 |
| Stard10 | -0.5452386 |
| Stard10 | 3.35848489 |
| Stard13 | -0.2690641 |
| Stard3 | -0.658248 |
| Stard3nl | -0.4829931 |
| Stard4 | 0.39999507 |
| Stard5 | 0.77468021 |
| Stard5 | -0.2794021 |
| Stard6 | 2.59261697 |
| Stard7 | -0.658248 |
| Stard7 | 0.12917136 |
| Stard8 | 2.69215194 |
| Stard8 | -0.6730495 |
| Stat1 | -0.2980661 |
| Stat1 | 0.33406775 |
| Stat2 | 0.61092641 |
| Stat3 | -0.1073059 |
| Stat3 | -0.7393973 |
| Stat3 | -0.0027211 |
| Stat4 | -0.4996312 |
| Stat4 | 2.04362475 |
| Stat5a | 0.591907 |
| Stat5a | 0.27285164 |
| Stat6 | -0.5225324 |
| Statip1 | -0.5506165 |
| Stau1 | 2.93622873 |
| Stau2 | 0.63373491 |
| Stbd1 | -0.281715 |
| Stc2 | 1.57796977 |
| Steap1 | 1.88594425 |
| Steap2 | -0.5444728 |
| Steap3 | -0.5376088 |
| Stfa1 | -0.6453784 |
| Stfa2 | -0.7189049 |
| Stim1 | -0.7469612 |
| Stip1 | -0.2599692 |
| Stk11 | 0.18472856 |
| Stk11ip | -0.4026322 |
| Stk17b | 1.1344661 |
| Stk19 | 0.91686803 |
| Stk19 | 1.96935463 |
| Stk19 | -0.2713517 |
| Stk22b | 1.36487492 |
| Stk22s1 | -0.7133927 |
| Stk24 | -0.4938106 |
| Stk25 | 0.96975779 |
| Stk25 | 2.92876114 |
| Stk3 | -0.0636079 |
| Stk31 | -0.4652359 |
| Stk32a | 2.23116941 |
| Stk32b | 0.47568347 |
| Stk32c | 0.37670941 |
| Stk35 | 2.16811328 |
| Stk35 | 0.71540847 |
| Stk36 | -0.4787007 |
| Stk38l | 0.79147975 |
| Stk39 | -0.7412826 |
| Stk4 | -0.0179053 |
| Stk40 | 0.00712525 |
| Stmn1 | -0.6677988 |
| Stmn2 | -0.5173122 |
| Stmn3 | 0.29317922 |
| Stmn4 | -0.4582258 |
| Stmn4 | -0.3039753 |
| Stoml1 | 1.24272587 |
| Stoml1 | -0.3721888 |
| Stoml1 | 0.09372472 |
| Stoml2 | 0.4515671 |
| Stoml3 | -0.3117133 |
| Stra13 | 0.34899112 |
| Stra13 | 0.36717325 |
| Stra13 | 0.62184604 |
| Stra6 | -0.7700246 |
| Strap | 0.92090732 |
| Strbp | -0.5173122 |
| Strn4 | 0.23885283 |
| Strn4 | -0.7106487 |
| Stt3a | 0.87954361 |
| Stt3b | -0.6513623 |
| Stx12 | -0.6436757 |
| Stx17 | 2.82614571 |
| Stx18 | 0.8307928 |
| Stx1a | -0.4072518 |
| Stx2 | 0.44810895 |
| Stx3 | -0.1581964 |
| Stx3 | -0.6209857 |
| Stx4a | -0.026252 |
| Stx5a | -0.1782076 |
| Stx5a | 0.06573473 |
| Stx6 | -0.6226478 |
| Stx7 | -0.5811554 |
| Stx8 | -0.6505051 |
| Stx8 | 0.02776486 |
| Stx8 | -0.4858656 |
| Stx8 | 1.0399339 |
| Stxbp1 | 2.5334156 |
| Stxbp1 | -0.281715 |
| Stxbp1 | 0.41114813 |
| Stxbp2 | 1.21128701 |
| Stxbp4 | -0.5915593 |
| Stxbp4 | 2.05102536 |
| Stxbp5l | -0.5003613 |
| Stxbp5l | -0.6660551 |
| Styx | -0.6854163 |
| Styxl1 | -0.7916746 |
| Styxl1 | 0.19976967 |
| Styxl1 | 1.72557223 |
| Suclg1 | 0.41695098 |
| Suclg1 | 0.06413277 |
| Suclg2 | 2.68599873 |
| Suclg2 | -0.7642055 |
| Suds3 | -0.2702072 |
| Sufu | -0.787701 |
| Suhw2 | 0.26511411 |
| Suhw4 | 1.73280985 |
| Sulf1 | 1.13240813 |
| Sult1a1 | -0.170975 |
| Sult1c2 | 2.4332743 |
| Sult1d1 | 0.36542897 |
| Sult1d1 | -0.6792126 |
| Sult2b1 | -0.4974442 |
| Sult2b1 | 0.22881272 |
| Sult4a1 | -0.0499339 |
| Sult5a1 | 0.72399437 |
| Sult5a1 | 2.0773277 |
| Sumf2 | 0.83698254 |
| Sumo1 | 0.09721401 |
| Sumo1 | -0.0906213 |
| Sumo1 | 0.77918951 |
| Sumo2 | 0.22510434 |
| Sumo3 | -0.2713517 |
| Sumo3 | 0.79682965 |
| Sunc1 | 1.59276748 |
| Suox | -0.2892712 |
| Supt3h | -0.4340939 |
| Supt4h1 | 1.22141536 |
| Supt4h2 | 0.80798455 |
| Supt5h | 0.08474841 |
| Supt5h | -0.0391361 |
| Supt6h | -0.6243128 |
| Supv3l1 | 1.97046478 |
| Surf1 | -0.4313749 |
| Surf1 | 0.05689001 |
| Surf2 | 2.13937359 |
| Surf4 | -0.8016827 |
| Susd2 | -0.3967259 |
| Susd3 | 1.67683096 |
| Susd4 | 1.70049707 |
| Suv39h1 | -0.7536298 |
| Suv420h1 | 0.11636673 |
| Suv420h1 | 0.86955086 |
| Suv420h1 | 3.0504378 |
| Suv420h2 | 2.24681649 |
| Suz12 | 1.37865246 |
| Sv2a | -0.8006771 |
| Sv2a | 4.49767706 |
| Sv2b | 0.15499748 |
| Sval2 | 0.70349881 |
| Svep1 | 0.16116397 |
| Svil | 0.21320672 |
| Svil | 1.86034046 |
| Svop | -0.7488617 |
| Svopl | 0.04264521 |
| Svs4 | 0.52361404 |
| Svs5 | -0.5859429 |
| Svs6 | 2.39711885 |
| Swap70 | -0.4205771 |
| Swap70 | -0.3551355 |
| Swap70 | 1.01837921 |
| Syap1 | 2.11558758 |
| Syap1 | -0.658248 |
| Sybl1 | -0.211998 |
| Syce2 | -0.6721723 |
| Sycp1 | -0.7632392 |
| Syf2 | -0.7758799 |
| Syf2 | 0.84976299 |
| Syn1 | -0.2233552 |
| Syn1 | 0.01179821 |
| Syn2 | -0.4239381 |
| Sync | -0.1169746 |
| Syncrip | -0.6160169 |
| Syne1 | 1.41852932 |
| Syne1 | -0.5915593 |
| Syngr1 | -0.6889798 |
| Syngr1 | 2.66676473 |
| Syngr1 | 1.49450878 |
| Syngr1 | 2.65471619 |
| Syngr2 | -0.7526742 |
| Syngr2 | 0.21900436 |
| Syngr2 | -0.1813244 |
| Syngr3 | 0.78962788 |
| Syngr4 | 2.96226718 |
| Syngr4 | -0.6118963 |
| Syngr4 | -0.6110743 |
| Synj2 | -0.3439359 |
| Synj2bp | 2.24417399 |
| Synj2bp | 0.89076407 |
| Synpo2l | -0.7916746 |
| Syp | -0.5803598 |
| Sypl | -0.324349 |
| Sypl | 1.19323536 |
| Sypl2 | 1.85414306 |
| Sys1 | -0.5307945 |
| Syt1 | 0.80415925 |
| Syt11 | -0.6880877 |
| Syt11 | -0.5360902 |
| Syt12 | -0.1981239 |
| Syt13 | 0.29662922 |
| Syt15 | 0.26414243 |
| Syt16 | -0.8026894 |
| Syt3 | 0.71901211 |
| Syt4 | 1.35252444 |
| Syt5 | 0.96623065 |
| Syt9 | 0.40251606 |
| Sytl2 | -0.5376088 |
| Sytl3 | 0.06733396 |
| Sytl3 | -0.0982119 |
| Sytl3 | 0.98734807 |
| Sytl4 | 0.48367235 |
| Sytl5 | 0.68171553 |
| Syvn1 | -0.502555 |
| Taar2 | -0.0400312 |
| Taar3 | 1.42798131 |
| Taar4 | 0.76958129 |
| Tac1 | -0.0342282 |
| Tacc1 | -0.1551551 |
| Tacc1 | 0.40307537 |
| Tacr3 | 0.96837918 |
| Tacr3 | -0.3747417 |
| Tacstd2 | -0.7837441 |
| Tada1l | 1.24558442 |
| Tada2l | -0.163287 |
| Tada2l | -0.4666442 |
| Tada3l | 0.30069023 |
| Tada3l | 2.44664943 |
| Taf10 | -0.2957127 |
| Taf11 | 1.24832049 |
| Taf12 | -0.6037087 |
| Taf12 | -0.7729477 |
| Taf12 | 2.67963099 |
| Taf13 | -0.2504012 |
| Taf1c | 0.50799033 |
| Taf3 | -0.1135792 |
| Taf5 | -0.4582258 |
| Taf5 | -0.1475948 |
| Taf5 | -0.5677215 |
| Taf5l | -0.2974772 |
| Taf6 | 0.37411821 |
| Taf6 | 0.11218646 |
| Taf6l | -0.610253 |
| Taf7l | 0.671683 |
| Taf9 | -0.1917889 |
| Taf9 | -0.6961474 |
| Taf9 | -0.0631488 |
| Taf9b | -0.6505051 |
| Tagap | 2.66721828 |
| Tagap | 2.01488864 |
| Tagln | 2.6392471 |
| Tagln3 | 0.32317443 |
| Tal1 | -0.4199064 |
| Tal2 | 0.99840002 |
| Taldo1 | 3.43355356 |
| Tank | -0.2459321 |
| Tank | -0.4354563 |
| Tank | 0.51843491 |
| Tap1 | 0.85358083 |
| Tap2 | -0.6268159 |
| Tapbp | 1.72323889 |
| Tapbp | -0.2846141 |
| Tapbpl | 1.96490087 |
| Tapbpl | -0.5106428 |
| Tapt1 | -0.1965362 |
| Tarbp2 | -0.5931702 |
| Tarbp2 | -0.6004535 |
| Tardbp | -0.5907549 |
| Tardbp | 2.57896497 |
| Tardbp | 0.96592331 |
| Tars | -0.6352081 |
| Tarsl2 | 0.91232334 |
| Tarsl2 | 1.2076092 |
| Tas1r1 | 0.8975649 |
| Tasp1 | -0.5529308 |
| Tatdn2 | 1.19745046 |
| Tax1bp1 | 0.35900545 |
| Tax1bp3 | 1.29050464 |
| Tax1bp3 | 0.90922689 |
| Taz | -0.4701742 |
| Taz | -0.4505746 |
| Tbc1d1 | 0.37209789 |
| Tbc1d10a | -0.5795649 |
| Tbc1d10a | 0.39465069 |
| Tbc1d10c | 2.12433125 |
| Tbc1d12 | 3.66614216 |
| Tbc1d13 | -0.2696355 |
| Tbc1d14 | 0.29286496 |
| Tbc1d15 | -0.6143665 |
| Tbc1d16 | -0.6845276 |
| Tbc1d17 | 0.55315106 |
| Tbc1d19 | -0.6276518 |
| Tbc1d2 | 1.78242335 |
| Tbc1d20 | -0.6335236 |
| Tbc1d22a | -0.4320539 |
| Tbc1d22b | -0.572441 |
| Tbc1d23 | 2.16093474 |
| Tbc1d24 | -0.7976667 |
| Tbc1d25 | 0.46448859 |
| Tbc1d2b | -0.6677988 |
| Tbc1d5 | -0.4478077 |
| Tbc1d5 | -0.6907666 |
| Tbc1d7 | -0.3075388 |
| Tbc1d7 | -0.3908563 |
| Tbc1d8 | -0.3709149 |
| Tbc1d9 | -0.3607856 |
| Tbc1d9 | -0.4547402 |
| Tbc1d9b | -0.1351278 |
| Tbca | 4.57540141 |
| Tbcb | 0.61249414 |
| Tbcc | -0.4112296 |
| Tbcd | 0.39662321 |
| Tbcel | -0.2980661 |
| Tbk1 | 1.91793536 |
| Tbkbp1 | -0.2381618 |
| Tbl1x | 1.91435304 |
| Tbl1xr1 | -0.7079126 |
| Tbl2 | 2.73131344 |
| Tbl3 | -0.770998 |
| Tbp | -0.0714456 |
| Tbpl1 | -0.5708652 |
| Tbr1 | -0.5277817 |
| Tbrg1 | -0.4909137 |
| Tbrg1 | -0.3664699 |
| Tbrg1 | -0.5202916 |
| Tbrg4 | 1.79714786 |
| Tbrg4 | -0.4526551 |
| Tbx1 | 0.84278152 |
| Tbx19 | -0.7412826 |
| Tbx2 | 1.30872195 |
| Tbx22 | -0.6343655 |
| Tbx3 | 2.25466069 |
| Tbx3 | 0.91297392 |
| Tbx5 | 0.48700244 |
| Tbx6 | -0.4395554 |
| Tbxa2r | 1.25116212 |
| Tbxa2r | -0.7263052 |
| Tcam1 | 1.19793099 |
| Tcam1 | -0.6880877 |
| Tcam1 | -0.6436757 |
| Tcap | 0.85634774 |
| Tcea2 | 0.63766471 |
| Tcea3 | 0.2454872 |
| Tceal1 | 0.45023854 |
| Tceal1 | 0.10645599 |
| Tceal3 | -0.7469612 |
| Tceal5 | -0.4145574 |
| Tceal6 | 0.55148116 |
| Tceal6 | -0.3876113 |
| Tceal8 | -0.2548917 |
| Tceb1 | 0.30442337 |
| Tceb2 | -0.2260797 |
| Tceb2 | 1.38526948 |
| Tceb3 | 0.10837003 |
| Tcerg1 | -0.3063495 |
| Tcf12 | 1.22841762 |
| Tcf12 | 1.89817574 |
| Tcf15 | 1.52120278 |
| Tcf19 | -0.4829931 |
| Tcf20 | -0.0409272 |
| Tcf20 | 0.71199231 |
| Tcf20 | 0.72160618 |
| Tcf20 | -0.2834533 |
| Tcf20 | -0.7916746 |
| Tcf21 | -0.5763921 |
| Tcf23 | -0.8814371 |
| Tcf25 | -0.7006551 |
| Tcf25 | 0.20909265 |
| Tcf25 | -0.3728264 |
| Tcf3 | 0.54621352 |
| Tcf3 | 0.77430346 |
| Tcf4 | -0.5883458 |
| Tcf7 | -0.7263052 |
| Tcf7 | -0.0770176 |
| Tcf7l2 | 3.05084001 |
| Tcf7l2 | 1.69406928 |
| Tcfap2b | -0.6625772 |
| Tcfap2b | 0.76044773 |
| Tcfap2c | -0.5779772 |
| Tcfap2c | 1.11025957 |
| Tcfcp2 | -0.7170639 |
| Tcfe3 | 1.00062454 |
| Tcfl5 | 1.05696851 |
| Tchp | 2.16566927 |
| Tcirg1 | -0.3051617 |
| Tcirg1 | 2.55310178 |
| Tcirg1 | -0.5819516 |
| Tcirg1 | -0.5513873 |
| Tcl1 | 3.04357368 |
| Tcl1 | 0.30937795 |
| Tcl1b2 | 0.42353927 |
| Tcl1b2 | -0.5202916 |
| Tcl1b3 | 0.14988866 |
| Tcl1b4 | 0.42681619 |
| Tcn2 | -0.0737632 |
| Tcof1 | -0.5150838 |
| Tcp1 | 0.46971209 |
| Tcp1 | -0.5069579 |
| Tcp10a | 0.71801248 |
| Tcp10a | 1.50687006 |
| Tcp11 | -0.0481257 |
| Tcstv1 | -0.8026894 |
| Tcte3 | -0.5875442 |
| Tctex1d1 | 0.76616659 |
| Tctex1d2 | -0.1351278 |
| Tctex1d4 | -0.5062227 |
| Tctn3 | -0.3501414 |
| Tctn3 | -0.4673491 |
| Tctn3 | 1.23039215 |
| Tdg | -0.2875221 |
| Tdp1 | -0.1689187 |
| Tdpoz1 | 1.52748465 |
| Tdpoz2 | 1.20725251 |
| Tdrd1 | -0.7916746 |
| Tdrd12 | 0.67189311 |
| Tdrd3 | 0.49616318 |
| Tdrd3 | -0.1277261 |
| Tdrd3 | 0.37757156 |
| Tdrd6 | -0.6310026 |
| Tdrd7 | 2.12616879 |
| Tdrkh | 0.49006594 |
| Tead2 | -0.2628019 |
| Tec | 0.6875188 |
| Tecta | 2.23687856 |
| Tef | 2.20374275 |
| Tef | -0.7768594 |
| Tegt | 0.60598218 |
| Tek | 1.19829122 |
| Tekt1 | -0.9602117 |
| Tekt1 | -0.2034359 |
| Tekt3 | -0.7916746 |
| Tenc1 | 3.32017727 |
| Tenc1 | -0.3785841 |
| Terf2 | -0.4829931 |
| Terf2 | -0.4039498 |
| Terf2ip | 1.3042274 |
| Terf2ip | 0.53291393 |
| Terf2ip | 0.93371772 |
| Terf2ip | -0.1073059 |
| Tes | -0.6301638 |
| Tes | -0.6997518 |
| Tes | -0.6730495 |
| Tesc | 0.57412817 |
| Tesk1 | -0.750766 |
| Tesp1 | 0.98569407 |
| Tesp2 | 0.57877845 |
| Tessp2 | 1.67241718 |
| Tessp3 | -0.2173891 |
| Tex10 | -0.3986905 |
| Tex10 | 0.9324445 |
| Tex101 | -0.1228238 |
| Tex11 | 2.37163324 |
| Tex13 | -0.6496487 |
| Tex14 | -0.2431496 |
| Tex16 | -0.2560176 |
| Tex19 | 0.58455916 |
| Tex2 | -0.7161448 |
| Tex2 | -0.7070024 |
| Tex22 | 1.46640338 |
| Tex24 | -0.7337641 |
| Tex261 | -0.5923644 |
| Tex261 | 0.81197539 |
| Tex264 | 1.51727659 |
| Tex264 | -0.8792494 |
| Tex9 | 0.18048776 |
| Tfam | 0.69287569 |
| Tfam | 0.47205324 |
| Tfb1m | -0.760346 |
| Tfb2m | 2.60870455 |
| Tfdp1 | 2.12907461 |
| Tfdp2 | 0.65536228 |
| Tfdp2 | 2.68515877 |
| Tfg | 3.42492463 |
| Tfip11 | 0.68503607 |
| Tfpi | -0.0214107 |
| Tfpt | 1.38556468 |
| Tfpt | -0.3177091 |
| Tfpt | 1.72220763 |
| Tfrc | 0.91783904 |
| Tfrc | -0.6979479 |
| Tg | -0.2157685 |
| Tgfb1 | -0.6453784 |
| Tgfb1i1 | 1.34803008 |
| Tgfb1i1 | -0.6961474 |
| Tgfb2 | -0.1694324 |
| Tgfb3 | -0.3785841 |
| Tgfbi | -0.5843444 |
| Tgfbi | 0.64741799 |
| Tgfbr1 | -0.0873196 |
| Tgfbr1 | -0.3099219 |
| Tgfbr1 | -0.7170639 |
| Tgfbr2 | 2.50541329 |
| Tgfbr2 | -0.7170639 |
| Tgfbr3 | 1.26300411 |
| Tgfbr3 | 1.43724617 |
| Tgfbrap1 | -0.5827485 |
| Tgif1 | -0.7642055 |
| Tgif1 | -0.6898728 |
| Tgif1 | 0.20530554 |
| Tgif2 | 0.55004745 |
| Tgif2lx | 2.25164841 |
| Tgm2 | -0.6591122 |
| Tgm4 | 0.34572823 |
| Tgoln1 | -0.3153062 |
| Tgs1 | -0.558353 |
| Tgs1 | -0.1087499 |
| Th1l | 1.09081255 |
| Tha1 | -0.6402795 |
| Thada | 2.69119468 |
| Thada | 0.32226093 |
| Thap1 | -0.3973803 |
| Thap1 | -0.2622347 |
| Thap1 | -0.787701 |
| Thap11 | 0.70471687 |
| Thap3 | -0.5740194 |
| Thap4 | -0.653079 |
| Thap7 | -0.7088237 |
| Thbd | 0.50573703 |
| Thbs1 | -0.8016827 |
| Thbs2 | 0.68171553 |
| Thbs3 | 0.18859924 |
| Thbs3 | -0.3340915 |
| Theg | -0.170975 |
| Theg | 0.17194837 |
| Theg | 0.37527074 |
| Them2 | 0.71340046 |
| Them4 | -0.7300272 |
| Them5 | -0.4687606 |
| Thg1l | 0.39182561 |
| Thnsl1 | 0.78814376 |
| Thnsl2 | -0.6020797 |
| Thnsl2 | 0.67545775 |
| Thoc1 | 1.52985068 |
| Thoc3 | -0.5811554 |
| Thoc4 | 0.17942456 |
| Thoc4 | 0.28655753 |
| Thoc5 | -0.273071 |
| Thoc6 | 2.0567789 |
| Thop1 | 0.4515671 |
| Thra | 0.40948357 |
| Thra | 0.89839091 |
| Thrap2 | 1.64593824 |
| Thrap3 | 0.73132004 |
| Thrsp | -0.0544699 |
| Thsd1 | 0.30535435 |
| Thsd4 | 2.72678436 |
| Thsd4 | -0.6565218 |
| Thsd7b | 0.95027092 |
| Thsd7b | 0.19976967 |
| Thsd7b | -0.5867432 |
| Thtpa | -0.6135424 |
| Thumpd1 | 2.8156624 |
| Thumpd3 | -0.7517196 |
| Thy1 | 0.07410054 |
| Thyn1 | -0.5552507 |
| Tia1 | -0.3545098 |
| Tial1 | -0.7788213 |
| Tial1 | 0.31246137 |
| Tiam1 | -0.0793491 |
| Tiam2 | -0.3177091 |
| Ticam1 | 0.42435957 |
| Tie1 | 2.16106418 |
| Tigd2 | 1.20415559 |
| Tigd3 | 1.86803023 |
| Timeless | -0.6961474 |
| Timm10 | -0.0010013 |
| Timm13 | 2.66408935 |
| Timm17a | -0.4794147 |
| Timm17b | 0.31859805 |
| Timm22 | -0.3677378 |
| Timm44 | 1.77653333 |
| Timm44 | 3.61129314 |
| Timm44 | -0.6020797 |
| Timm50 | 3.52089849 |
| Timm8a1 | -0.8107819 |
| Timm8a2 | 1.50885704 |
| Timm8a2 | -0.6591122 |
| Timm8b | -0.5106428 |
| Timm8b | -0.3785841 |
| Timm9 | 1.26210709 |
| Timm9 | 1.11970387 |
| Timm9 | -0.5835461 |
| Timp1 | 0.06653469 |
| Timp1 | 0.15062019 |
| Timp2 | -0.4225923 |
| Timp3 | 0.8371588 |
| Timp3 | -0.375381 |
| Timp4 | -0.4582258 |
| Tinagl | -0.6907666 |
| Tipin | -0.2271717 |
| Tipin | -0.4464272 |
| Tirap | -0.3426997 |
| Tirap | 2.54504722 |
| Tirap | -0.5330604 |
| Tjap1 | -0.5795649 |
| Tjap1 | 1.14392609 |
| Tjap1 | -0.4512676 |
| Tjp1 | 4.1883131 |
| Tjp2 | -0.6053405 |
| Tjp3 | 0.51173386 |
| Tjp3 | 0.63875342 |
| Tk1 | -0.6513623 |
| Tk2 | 3.65187806 |
| Tkt | 1.94018469 |
| Tkt | 2.11626654 |
| Tkt | 3.16569783 |
| Tktl1 | -0.3633078 |
| Tktl1 | -0.4561329 |
| Tktl2 | 1.11970387 |
| Tlcd1 | 0.80743903 |
| Tle1 | -0.8365292 |
| Tle1 | -0.7207495 |
| Tle2 | 1.13754464 |
| Tle3 | -0.2963005 |
| Tle3 | -0.7015592 |
| Tle3 | 0.21286456 |
| Tle4 | 0.21968409 |
| Tle4 | 1.71972798 |
| Tle4 | 1.58571589 |
| Tle6 | 2.6961667 |
| Tlk1 | -0.3671037 |
| Tlk2 | 0.83839164 |
| Tln1 | -0.4624256 |
| Tln2 | -0.1844515 |
| Tlr13 | 3.43895942 |
| Tlr2 | 0.38955943 |
| Tlr3 | -0.7235233 |
| Tlr4 | 0.47929976 |
| Tlr6 | 0.16333076 |
| Tlr7 | 2.58318884 |
| Tm2d2 | -0.1673794 |
| Tm2d3 | 1.55844355 |
| Tm2d3 | -0.6677988 |
| Tm4sf4 | -0.7896857 |
| Tm4sf5 | 1.68172696 |
| Tm6sf1 | 0.22645453 |
| Tm6sf2 | 0.39125958 |
| Tm9sf2 | 1.59804133 |
| Tm9sf2 | -0.4945362 |
| Tm9sf2 | 2.34501226 |
| Tm9sf3 | -0.4533496 |
| Tm9sf4 | 0.91605808 |
| Tm9sf4 | 2.36241353 |
| Tmbim1 | -0.4758499 |
| Tmbim4 | -0.7235233 |
| Tmc1 | -0.569292 |
| Tmc4 | 0.21934428 |
| Tmc5 | -0.1371114 |
| Tmc6 | 1.43622123 |
| Tmc6 | 0.02358931 |
| Tmc6 | 2.92956204 |
| Tmc7 | 1.20044973 |
| Tmcc2 | 3.054519 |
| Tmcc3 | 1.41357649 |
| Tmco3 | -0.4930855 |
| Tmco3 | 0.01222188 |
| Tmco4 | -0.6428255 |
| Tmco4 | -0.7281644 |
| Tmco5 | -0.4512676 |
| Tmco6 | 1.17113067 |
| Tmco6 | -0.3614155 |
| Tmco7 | -0.7216732 |
| Tmed1 | 0.63613841 |
| Tmed1 | -0.6943504 |
| Tmed10 | -0.6979479 |
| Tmed2 | -0.2034359 |
| Tmed3 | -0.5638066 |
| Tmed3 | 2.17829238 |
| Tmed4 | 1.39535201 |
| Tmed4 | -0.7161448 |
| Tmed4 | 2.17888525 |
| Tmed4 | -0.6004535 |
| Tmed8 | 1.29419811 |
| Tmed8 | 1.7154421 |
| Tmed9 | -0.4894686 |
| Tmed9 | -0.0355642 |
| Tmeff2 | -0.4178969 |
| Tmem10 | -0.142089 |
| Tmem100 | 1.42317266 |
| Tmem101 | -0.807739 |
| Tmem102 | -0.5076937 |
| Tmem102 | -0.5843444 |
| Tmem106a | -0.7070024 |
| Tmem106c | 1.45568921 |
| Tmem107 | 2.47534785 |
| Tmem107 | -0.5955917 |
| Tmem107 | -0.1082683 |
| Tmem108 | 0.93148845 |
| Tmem109 | 3.6872132 |
| Tmem11 | 1.19793099 |
| Tmem110 | -0.6880877 |
| Tmem111 | 0.99869693 |
| Tmem111 | -0.3267752 |
| Tmem111 | 2.17110603 |
| Tmem115 | -0.1917889 |
| Tmem116 | -0.4000026 |
| Tmem116 | -0.8449195 |
| Tmem117 | -0.3371569 |
| Tmem118 | -0.7384561 |
| Tmem119 | 1.22340463 |
| Tmem120a | 1.20092873 |
| Tmem120b | 1.38171985 |
| Tmem121 | 0.81125104 |
| Tmem125 | -0.4722986 |
| Tmem126a | 1.48773729 |
| Tmem126b | 0.13884688 |
| Tmem127 | -0.5195458 |
| Tmem128 | 0.40837223 |
| Tmem130 | 0.08631568 |
| Tmem131 | 0.82598987 |
| Tmem132a | 0.65193514 |
| Tmem132a | 0.00157258 |
| Tmem132c | 0.58248219 |
| Tmem132d | 1.33607735 |
| Tmem132e | -0.7936677 |
| Tmem134 | -0.2968887 |
| Tmem134 | 1.20522876 |
| Tmem135 | -0.8148544 |
| Tmem136 | 1.42477828 |
| Tmem138 | 1.58013232 |
| Tmem139 | -0.1597207 |
| Tmem140 | -0.4701742 |
| Tmem141 | -0.0490294 |
| Tmem143 | -0.2055691 |
| Tmem143 | 0.84871907 |
| Tmem143 | -0.7143092 |
| Tmem143 | -0.3147064 |
| Tmem144 | 0.60327434 |
| Tmem146 | 2.46175077 |
| Tmem147 | -0.3129094 |
| Tmem14c | 0.58340585 |
| Tmem14c | 0.11370871 |
| Tmem14c | 1.65669317 |
| Tmem150 | 1.7594325 |
| Tmem150 | 0.35135704 |
| Tmem151a | 0.09566481 |
| Tmem154 | -0.3237435 |
| Tmem158 | -0.5669372 |
| Tmem159 | 1.46937802 |
| Tmem159 | -0.4787007 |
| Tmem160 | -0.4851467 |
| Tmem160 | -0.3513875 |
| Tmem161a | -0.7788213 |
| Tmem162 | -0.4402403 |
| Tmem164 | 1.46432458 |
| Tmem166 | -0.2018392 |
| Tmem167 | -0.0686718 |
| Tmem168 | -0.3626766 |
| Tmem168 | -0.6686719 |
| Tmem168 | -0.4375036 |
| Tmem169 | 2.4241855 |
| Tmem16a | 1.40725242 |
| Tmem16b | 1.4681172 |
| Tmem16d | -0.0654462 |
| Tmem16e | 0.14032662 |
| Tmem16g | -0.2359535 |
| Tmem16j | 1.76227135 |
| Tmem16k | -0.3588985 |
| Tmem17 | 1.2948484 |
| Tmem17 | -0.3123111 |
| Tmem170 | 1.84386352 |
| Tmem176a | -0.6800963 |
| Tmem176a | 2.37775274 |
| Tmem176b | -0.5277817 |
| Tmem177 | 0.65086159 |
| Tmem178 | 1.82887555 |
| Tmem179b | -0.5195458 |
| Tmem180 | 0.64049273 |
| Tmem183a | 0.99408431 |
| Tmem183a | 1.10005338 |
| Tmem184a | -0.6704205 |
| Tmem184a | 0.69840651 |
| Tmem184b | 0.43143905 |
| Tmem185b | 0.19210427 |
| Tmem186 | -0.7680809 |
| Tmem19 | 0.26995752 |
| Tmem192 | -0.3850232 |
| Tmem198 | 1.16181686 |
| Tmem199 | 1.5940296 |
| Tmem2 | 2.28505905 |
| Tmem2 | 0.65792447 |
| Tmem2 | 0.83274254 |
| Tmem201 | -0.541416 |
| Tmem204 | -0.6151914 |
| Tmem205 | -0.3171078 |
| Tmem208 | -0.4212484 |
| Tmem209 | -0.5859429 |
| Tmem209 | -0.4000026 |
| Tmem23 | 0.40698121 |
| Tmem25 | -0.0668272 |
| Tmem25 | -0.0986883 |
| Tmem25 | -0.4118942 |
| Tmem27 | 0.24018345 |
| Tmem32 | 0.50573703 |
| Tmem33 | -0.5740194 |
| Tmem33 | 0.60440355 |
| Tmem34 | 0.72240292 |
| Tmem35 | 0.85738324 |
| Tmem38a | -0.4694672 |
| Tmem38b | 0.85184737 |
| Tmem38b | -0.0205331 |
| Tmem39a | 1.15806537 |
| Tmem40 | 0.71761233 |
| Tmem41a | 0.15062019 |
| Tmem41b | 1.62106984 |
| Tmem42 | -0.4306964 |
| Tmem43 | -0.7300272 |
| Tmem43 | 2.68478796 |
| Tmem44 | -0.4286638 |
| Tmem45a | 1.29809311 |
| Tmem45b | -0.6496487 |
| Tmem47 | 1.6995815 |
| Tmem47 | 3.42199299 |
| Tmem48 | -0.1233129 |
| Tmem49 | -0.2875221 |
| Tmem49 | -0.7124771 |
| Tmem49 | -0.3773016 |
| Tmem49 | 0.6300083 |
| Tmem5 | -0.5483079 |
| Tmem50a | -0.2222677 |
| Tmem50b | -0.6970472 |
| Tmem50b | -0.6284884 |
| Tmem51 | 0.56098376 |
| Tmem53 | 0.31920952 |
| Tmem53 | 0.00797661 |
| Tmem53 | 0.20150314 |
| Tmem54 | 0.44837541 |
| Tmem55a | -0.2387147 |
| Tmem55b | -0.8354857 |
| Tmem56 | 2.03547543 |
| Tmem57 | -0.3779426 |
| Tmem59 | 1.12283102 |
| Tmem59l | -0.4540447 |
| Tmem60 | 0.67252314 |
| Tmem62 | -0.5787707 |
| Tmem63a | 0.00499349 |
| Tmem63b | -0.2974772 |
| Tmem63b | -0.0227286 |
| Tmem63c | -0.4498821 |
| Tmem65 | 3.25907839 |
| Tmem66 | 0.04223503 |
| Tmem66 | -0.5062227 |
| Tmem66 | -0.3856696 |
| Tmem68 | 1.30679834 |
| Tmem69 | -0.0906213 |
| Tmem70 | 0.51322712 |
| Tmem71 | 2.54642309 |
| Tmem74 | -0.2577091 |
| Tmem74 | -0.6053405 |
| Tmem74 | 1.82856843 |
| Tmem77 | -0.0719086 |
| Tmem77 | 2.61226329 |
| Tmem77 | -0.8502016 |
| Tmem79 | -0.3980352 |
| Tmem8 | 0.10453805 |
| Tmem8 | -0.6470842 |
| Tmem8 | 0.89142973 |
| Tmem81 | -0.2939516 |
| Tmem82 | 0.03067676 |
| Tmem85 | -0.0728355 |
| Tmem85 | 2.01675146 |
| Tmem86a | 0.6777571 |
| Tmem86b | -0.7526742 |
| Tmem87a | -0.4617243 |
| Tmem88 | -0.5819516 |
| Tmem9 | -0.6343655 |
| Tmem90a | -0.0222891 |
| Tmem91 | -0.5575765 |
| Tmem93 | 0.93974209 |
| Tmem97 | 1.18157604 |
| Tmem98 | 3.86541959 |
| Tmem98 | 0.96346102 |
| Tmem9b | 2.53402528 |
| Tmem9b | 1.9310836 |
| Tmem9b | -0.2921937 |
| Tmie | 2.6885141 |
| Tmod1 | -0.546772 |
| Tmod2 | -0.423265 |
| Tmod2 | -0.3513875 |
| Tmod3 | -0.6686719 |
| Tmod4 | 3.04916298 |
| Tmod4 | 0.79147975 |
| Tmod4 | 2.47990862 |
| Tmpo | -0.4652359 |
| Tmpo | 2.31134857 |
| Tmpo | 2.73645294 |
| Tmprss11a | -0.6783297 |
| Tmprss11c | -0.5391298 |
| Tmprss13 | -0.4808445 |
| Tmprss13 | -0.3824423 |
| Tmprss2 | 0.00456655 |
| Tmprss4 | -0.5614655 |
| Tmprss4 | -0.4293409 |
| Tmprss4 | 2.48279389 |
| Tmprss7 | -0.541416 |
| Tmprss9 | 0.58132634 |
| Tmsb10 | -0.4219201 |
| Tmsb10 | 0.21354874 |
| Tmsb10 | 1.07943893 |
| Tmsb4x | 0.20392463 |
| Tmtc2 | -0.3526351 |
| Tmtc4 | -0.7070024 |
| Tmub1 | 0.11408889 |
| Tmub1 | 0.61940571 |
| Tnc | 0.06292951 |
| Tnc | 1.32648842 |
| Tnc | 2.16136614 |
| Tnc | -0.2082425 |
| Tnfaip1 | 1.37646952 |
| Tnfaip2 | -0.149605 |
| Tnfaip2 | -0.336543 |
| Tnfaip2 | 1.11722082 |
| Tnfaip3 | -0.4652359 |
| Tnfaip8 | 1.17961869 |
| Tnfaip8l2 | 2.47682945 |
| Tnfaip8l2 | 1.40281947 |
| Tnfaip8l3 | -0.6360514 |
| Tnfrsf10b | 1.00653101 |
| Tnfrsf10b | -0.610253 |
| Tnfrsf10b | 2.15230852 |
| Tnfrsf11a | 1.67921015 |
| Tnfrsf11b | 0.45262859 |
| Tnfrsf12a | -0.7788213 |
| Tnfrsf13c | -0.7976667 |
| Tnfrsf19 | 0.53655978 |
| Tnfrsf19 | 1.96628167 |
| Tnfrsf1a | -0.1745843 |
| Tnfrsf1a | -0.4974442 |
| Tnfrsf1a | -0.4989016 |
| Tnfrsf1b | 1.30978892 |
| Tnfrsf21 | 0.85755571 |
| Tnfrsf21 | -0.6479383 |
| Tnfrsf22 | 0.01771224 |
| Tnfrsf22 | -0.5406533 |
| Tnfrsf25 | 1.64175148 |
| Tnfrsf4 | -0.6651844 |
| Tnfsf13b | -0.1366151 |
| Tnfsf13b | 0.88575652 |
| Tnip1 | 0.04428415 |
| Tnip1 | -0.671296 |
| Tnip1 | -0.3189129 |
| Tnip1 | 2.85352938 |
| Tnip2 | 3.11479953 |
| Tnks | 0.34066322 |
| Tnks1bp1 | -0.3902064 |
| Tnnc1 | -0.1296942 |
| Tnnc2 | 0.10989846 |
| Tnni2 | 1.3584162 |
| Tnni3 | 1.24957186 |
| Tnnt1 | 1.57548069 |
| Tnp1 | 0.77392656 |
| Tnp2 | 3.59347798 |
| Tnpo1 | 0.63264038 |
| Tnpo2 | -0.5444728 |
| Tnr | 0.45501258 |
| Tnrc15 | -0.5915593 |
| Tnrc18 | -0.7263052 |
| Tnrc18 | -0.5883458 |
| Tnrc18 | -0.8261456 |
| Tnrc6a | 1.9552318 |
| Tnrc6a | 0.41142527 |
| Tnrc6b | -0.8470288 |
| Tnrc6b | -0.804706 |
| Tnrc6c | -0.3658367 |
| Tns4 | 2.65520111 |
| Tnxb | 0.75719154 |
| Tnxb | -0.6143665 |
| Tnxb | 3.97961288 |
| Tob1 | -0.4395554 |
| Tob2 | 0.76006523 |
| Toe1 | -0.4484986 |
| Toe1 | -0.4737175 |
| Tollip | 3.1696638 |
| Tollip | 1.0387967 |
| Tom1 | 1.61538461 |
| Tom1l2 | 1.59662738 |
| Tom1l2 | -0.4610236 |
| Tom1l2 | -0.4680546 |
| Tomm22 | 2.68449121 |
| Tomm34 | -0.349519 |
| Tomm34 | 1.47931513 |
| Tomm7 | 0.74444197 |
| Tomm70a | -0.4844283 |
| Top1 | -0.6251465 |
| Top1mt | -0.5748096 |
| Top2a | -0.7393973 |
| Top3a | 1.21824726 |
| Top3b | -0.3147064 |
| Topors | 2.55389777 |
| Tor1a | -0.4967164 |
| Tor1a | -0.3588985 |
| Tor1aip1 | -0.1663547 |
| Tor1aip2 | -0.1179469 |
| Tor1aip2 | -0.3075388 |
| Tor1b | 2.16089159 |
| Tor3a | 0.30876006 |
| Tox3 | 0.90497306 |
| Tpbg | 1.88663759 |
| Tpcn1 | 1.80398326 |
| Tpcn1 | -0.5232805 |
| Tpcn2 | 1.05891969 |
| Tpd52 | -0.7207495 |
| Tpd52l1 | -0.4787007 |
| Tpd52l1 | -0.7088237 |
| Tpd52l2 | -0.5575765 |
| Tph2 | -0.1341375 |
| Tpi1 | 1.49818188 |
| Tpi1 | 1.93289987 |
| Tpi1 | 0.00755103 |
| Tpk1 | -0.4402403 |
| Tpk1 | -0.0915668 |
| Tpk1 | -0.5763921 |
| Tpm1 | 1.56910763 |
| Tpm2 | -0.3967259 |
| Tpm2 | 1.47886976 |
| Tpm3 | -0.7996725 |
| Tpm3 | 1.4508369 |
| Tpm4 | 0.17942456 |
| Tpo | 1.41462638 |
| Tpp1 | 0.27637685 |
| Tpp2 | 1.11105023 |
| Tppp | 0.27285164 |
| Tppp | -0.6625772 |
| Tppp3 | -0.6505051 |
| Tpr | -0.6934532 |
| Tpra40 | -0.618498 |
| Tprgl | -0.5552507 |
| Tprgl | 1.88831106 |
| Tprkb | -0.4730078 |
| Tprkb | 2.95787546 |
| Tpsab1 | 2.30184989 |
| Tpst1 | 1.66725361 |
| Tpst2 | -0.653079 |
| Tpte | -0.6774476 |
| Tpte | -0.1179469 |
| Tpx2 | -0.4327334 |
| Tra2a | -0.3709149 |
| Trabd | 0.26381831 |
| Trabd | -0.8683861 |
| Traf1 | -0.6402795 |
| Traf2 | -0.7024642 |
| Traf3 | 1.10391028 |
| Traf3 | -0.1839296 |
| Traf3ip1 | -0.4880258 |
| Traf3ip2 | 1.44133477 |
| Traf4 | 0.07924234 |
| Traf4 | 2.35633334 |
| Traf4 | 0.10683911 |
| Traf4 | 3.1310944 |
| Traf5 | 0.94886343 |
| Traf6 | -0.5091671 |
| Traf6 | -0.7015592 |
| Traf6 | -0.5756005 |
| Traf7 | 1.99569472 |
| Trafd1 | -0.7115625 |
| Trafd1 | -0.5771843 |
| Trafd1 | -0.9137455 |
| Traip | 1.12074741 |
| Trak1 | -0.2414841 |
| Trak2 | -0.6721723 |
| Trak2 | 0.29317922 |
| Trak2 | 0.93578303 |
| Tram1 | -0.7460124 |
| Tram1 | -0.299245 |
| Tram1 | -0.6284884 |
| Tram2 | -0.4099018 |
| Tram2 | -0.3576425 |
| Trap1 | -0.6539385 |
| Trappc1 | 0.45183258 |
| Trappc1 | 0.95323539 |
| Trappc2 | 1.31584766 |
| Trappc2l | 0.12052851 |
| Trappc3 | 2.38481953 |
| Trappc3 | -0.7946659 |
| Trappc3 | -0.324349 |
| Trappc3 | 0.95774216 |
| Trappc4 | -0.5521587 |
| Trappc4 | -0.4185663 |
| Trappc5 | -0.6377404 |
| Trappc6a | 0.35841931 |
| Trat1 | 2.39742093 |
| Trcg1 | -0.1965362 |
| Trdmt1 | -0.4158918 |
| Trdmt1 | 1.65203544 |
| Trem2 | -0.3639393 |
| Treml1 | 0.48725812 |
| Treml2 | 1.43873499 |
| Treml4 | -0.5353319 |
| Trerf1 | 3.90833191 |
| Trerf1 | 0.94948924 |
| Trex1 | -0.6226478 |
| Trex1 | 0.10338541 |
| Trex2 | -0.1440874 |
| Trf | 0.37958018 |
| Trfp | -0.541416 |
| Trfr2 | -0.6774476 |
| Trfr2 | 0.33135622 |
| Trh | 1.18291936 |
| Trhde | -0.1735517 |
| Trhr | -0.505488 |
| Triap1 | -0.2299072 |
| Trib2 | 0.8955795 |
| Trib3 | -0.177171 |
| Trib3 | -0.6748063 |
| Trim11 | 3.3978344 |
| Trim16 | 1.71724179 |
| Trim17 | -0.4259605 |
| Trim2 | -0.8459735 |
| Trim2 | -0.7161448 |
| Trim21 | 0.59738047 |
| Trim21 | 1.67791316 |
| Trim23 | 0.34275211 |
| Trim23 | -0.5544768 |
| Trim23 | -0.4052692 |
| Trim25 | -0.7235233 |
| Trim26 | -0.7281644 |
| Trim28 | -0.6377404 |
| Trim3 | 2.25516831 |
| Trim3 | -0.2622347 |
| Trim30 | -0.7152265 |
| Trim32 | -0.7365765 |
| Trim32 | 0.79793274 |
| Trim33 | 1.37845422 |
| Trim33 | 0.55624446 |
| Trim33 | -0.0562904 |
| Trim35 | -0.0788823 |
| Trim36 | -0.4436725 |
| Trim36 | -0.6201557 |
| Trim36 | 0.33316478 |
| Trim37 | 0.44517286 |
| Trim38 | 1.24203852 |
| Trim38 | -0.7042769 |
| Trim39 | 2.17913922 |
| Trim40 | 1.24569858 |
| Trim41 | 1.14290796 |
| Trim42 | -0.438871 |
| Trim44 | 0.49057556 |
| Trim45 | -0.5875442 |
| Trim45 | 1.4081173 |
| Trim46 | 1.48747248 |
| Trim47 | 0.3047338 |
| Trim54 | -0.5606864 |
| Trim56 | -0.6961474 |
| Trim59 | 0.19105413 |
| Trim61 | 0.34543104 |
| Trim62 | 0.38129844 |
| Trim67 | -0.5996414 |
| Trim68 | 1.85012878 |
| Trim69 | -0.2387147 |
| Trim72 | -0.5069579 |
| Trim8 | 0.08081879 |
| Trim9 | -0.4192361 |
| Trim9 | -0.6651844 |
| Trim9 | 0.79609354 |
| Trim9 | -0.6218164 |
| Triml1 | -0.2013075 |
| Triobp | 0.18190348 |
| Triobp | -0.7384561 |
| Trip10 | 0.17123324 |
| Trip12 | -0.349519 |
| Trip12 | 3.19871559 |
| Trip12 | 1.05641028 |
| Trip6 | -0.5771843 |
| Trip6 | 0.24018345 |
| Trit1 | -0.4758499 |
| Trmt1 | 1.84368217 |
| Trmt12 | 1.47126608 |
| Trmu | -0.6730495 |
| Tro | -0.6360514 |
| Tro | 0.42244442 |
| Tro | 0.05405826 |
| Trove2 | 0.18190348 |
| Trp53 | -0.0476741 |
| Trp53bp1 | -0.7244496 |
| Trp53bp1 | -0.5217848 |
| Trp53bp2 | -0.6617098 |
| Trp53i11 | -0.0863784 |
| Trp53i11 | -0.2365051 |
| Trp53inp1 | -0.4952623 |
| Trp53inp1 | 1.23097211 |
| Trp53inp2 | 0.49235704 |
| Trp53rk | -0.0840295 |
| Trp63 | -0.6453784 |
| Trpc1 | -0.1849737 |
| Trpc1 | -0.6284884 |
| Trpc2 | 0.71158959 |
| Trpc3 | -0.4443604 |
| Trpc4 | -0.0018608 |
| Trpc4ap | -0.5591301 |
| Trpc4ap | -0.7651729 |
| Trpc5 | -0.4844283 |
| Trpc6 | 0.21423243 |
| Trpc7 | 1.2867966 |
| Trpd52l3 | -0.6748063 |
| Trpm1 | -0.2326507 |
| Trpm2 | -0.4981726 |
| Trpm3 | 0.08435619 |
| Trpm3 | -0.3207215 |
| Trpm3 | -0.6168432 |
| Trpm3 | -0.5859429 |
| Trpm3 | 2.04455302 |
| Trpm4 | -0.7613094 |
| Trpm6 | -0.5173122 |
| Trpm6 | -0.2029034 |
| Trpm6 | 0.90135858 |
| Trpm8 | 0.19872806 |
| Trps1 | -0.7207495 |
| Trpt1 | 2.26522491 |
| Trpt1 | 0.42381278 |
| Trpt1 | -0.5622452 |
| Trpv2 | 0.94745383 |
| Trpv4 | -0.3986905 |
| Trpv6 | 0.48597905 |
| Trrap | 1.58332699 |
| Trub1 | 3.31024665 |
| Trub1 | 4.39045181 |
| Trub1 | -0.4858656 |
| Trub2 | 1.93865572 |
| Tsc1 | -0.3255614 |
| Tsc2 | -0.5173122 |
| Tsc2 | 0.34424135 |
| Tsc22d1 | 0.80561834 |
| Tsc22d1 | 0.34810236 |
| Tsc22d2 | -0.7106487 |
| Tsc22d3 | 0.56804824 |
| Tsc22d3 | -0.6818663 |
| Tsc22d4 | -0.4822763 |
| Tsen2 | 1.44022146 |
| Tsen34 | -0.3316464 |
| Tsen54 | -0.6871964 |
| Tsfm | -0.299245 |
| Tsg101 | -0.6988494 |
| Tsga10 | 2.72429827 |
| Tsga10 | -0.8705487 |
| Tsga14 | -0.7161448 |
| Tsga8 | -0.7384561 |
| Tshb | 1.69477778 |
| Tshr | 0.56875178 |
| Tshz3 | -0.5787707 |
| Tsku | -0.4464272 |
| Tsn | -0.6318422 |
| Tsnaxip1 | 1.36527712 |
| Tsnaxip1 | -0.6505051 |
| Tspan1 | -0.0608571 |
| Tspan11 | -0.6997518 |
| Tspan12 | -0.5475396 |
| Tspan13 | -0.6634455 |
| Tspan14 | -0.0302317 |
| Tspan17 | 1.27115014 |
| Tspan17 | 0.07766324 |
| Tspan18 | 0.60913151 |
| Tspan18 | 1.14035781 |
| Tspan2 | 0.19559629 |
| Tspan3 | -0.1169746 |
| Tspan31 | 0.6804673 |
| Tspan33 | -0.4801293 |
| Tspan6 | 0.43198142 |
| Tspan6 | 1.87132347 |
| Tspan7 | -0.0284608 |
| Tspan8 | -0.5996414 |
| Tspo | -0.2163084 |
| Tspo | 2.56869926 |
| Tspo | -0.6970472 |
| Tspyl1 | 3.09015299 |
| Tspyl3 | 0.31614818 |
| Tsr1 | 1.19985064 |
| Tsr2 | -0.1425882 |
| Tsr2 | 1.35027994 |
| Tsr2 | 0.45923593 |
| Tssc1 | -0.2742189 |
| Tssc1 | -0.7235233 |
| Tssc1 | 0.00028652 |
| Tssc1 | 1.48189411 |
| Tssc1 | 0.21934428 |
| Tssc4 | 1.35577963 |
| Tssc4 | -0.4945362 |
| Tssk4 | -0.4079136 |
| Tssk5 | 1.12009533 |
| Tssk6 | -0.5421792 |
| Tst | -0.239268 |
| Tsta3 | -0.3016072 |
| Tsx | -0.2163084 |
| Ttbk2 | -0.6268159 |
| Ttc1 | -0.5883458 |
| Ttc12 | -0.7479109 |
| Ttc13 | 0.87498382 |
| Ttc14 | -0.7936677 |
| Ttc16 | -0.3520111 |
| Ttc17 | 0.61695902 |
| Ttc19 | 3.95212711 |
| Ttc19 | 0.84365704 |
| Ttc21a | 0.98614545 |
| Ttc22 | 0.57552567 |
| Ttc23 | -0.1381048 |
| Ttc24 | 0.71299835 |
| Ttc24 | 1.22235201 |
| Ttc26 | 0.5921357 |
| Ttc27 | 2.46250264 |
| Ttc29 | 1.87413711 |
| Ttc29 | -0.5398912 |
| Ttc3 | 2.16429441 |
| Ttc3 | -0.5972095 |
| Ttc3 | -0.1684053 |
| Ttc3 | -0.6792126 |
| Ttc3 | 0.23183602 |
| Ttc3 | 1.70667146 |
| Ttc30a1 | 0.1690846 |
| Ttc30a2 | -0.0010013 |
| Ttc33 | 0.66324005 |
| Ttc33 | 0.00669929 |
| Ttc33 | 1.7755589 |
| Ttc35 | 1.84561476 |
| Ttc4 | 3.10128738 |
| Ttc5 | -0.5568006 |
| Ttc5 | 0.74483087 |
| Ttc5 | 0.2284762 |
| Ttc7 | -0.0779495 |
| Ttc8 | -0.5708652 |
| Ttc8 | 1.233057 |
| Ttf2 | -0.0122368 |
| Ttk | -0.6669265 |
| Ttl | -0.0714456 |
| Ttll1 | 0.79351261 |
| Ttll1 | 0.43035336 |
| Ttll1 | 0.42926641 |
| Ttll10 | -0.1135792 |
| Ttll11 | -0.2543292 |
| Ttll13 | -0.3322571 |
| Ttll4 | 3.45851928 |
| Ttll5 | -0.8428149 |
| Ttll5 | 0.98177302 |
| Ttll6 | 2.74128515 |
| Ttll8 | 2.78640467 |
| Ttll9 | 1.0874419 |
| Ttn | 1.56122296 |
| Ttn | -0.4099018 |
| Ttr | -0.2029034 |
| Ttyh1 | 0.41970171 |
| Ttyh1 | 2.77042335 |
| Ttyh2 | -0.4589245 |
| Ttyh2 | -0.1875891 |
| Ttyh3 | -0.5779772 |
| Tub | -0.4375036 |
| Tub | -0.5748096 |
| Tuba1a | 0.87718203 |
| Tuba1b | -0.7070024 |
| Tuba3a | -0.7749015 |
| Tuba3a | 1.48011626 |
| Tuba3a | -0.7632392 |
| Tuba3a | -0.1292018 |
| Tuba3b | 3.53225633 |
| Tuba4a | 0.85219432 |
| Tuba6 | -0.3980352 |
| Tuba8 | 3.34486727 |
| Tubb2b | -0.5406533 |
| Tubb2b | 0.09450124 |
| Tubb2b | -0.3588985 |
| Tubb2b | 1.62983737 |
| Tubb2b | 2.65075094 |
| Tubb2c | -0.7347006 |
| Tubb4 | -0.706093 |
| Tubb5 | -0.6643145 |
| Tubb6 | 1.82573818 |
| Tubd1 | 2.10826901 |
| Tubd1 | -0.4829931 |
| Tube1 | -0.8117984 |
| Tubg1 | -0.5787707 |
| Tubgcp2 | 1.15768939 |
| Tubgcp4 | 1.48304791 |
| Tufm | -0.7133927 |
| Tuft1 | -0.4851467 |
| Tug1 | 0.75064517 |
| Tulp1 | -0.4178969 |
| Tulp2 | 1.99569472 |
| Tulp3 | 2.91187996 |
| Tulp4 | 1.17986359 |
| Tusc2 | -0.6783297 |
| Tusc2 | 0.90168774 |
| Tusc4 | 0.30876006 |
| Tut1 | -0.7613094 |
| Twf2 | 0.59533165 |
| Twist1 | -0.73096 |
| Twist2 | 0.02984571 |
| Twistnb | 0.93037184 |
| Twistnb | 1.14176125 |
| Twsg1 | -0.7225978 |
| Txk | -0.6061575 |
| Txlna | -0.6845276 |
| Txn1 | 1.57088675 |
| Txn2 | 0.51073704 |
| Txndc1 | -0.610253 |
| Txndc11 | 1.07040504 |
| Txndc11 | -0.3532596 |
| Txndc11 | 3.69235297 |
| Txndc12 | 1.64586364 |
| Txndc13 | -0.12087 |
| Txndc13 | 2.8658476 |
| Txndc15 | 1.77601376 |
| Txndc2 | -0.7225978 |
| Txndc5 | -0.6377404 |
| Txndc8 | -0.5136011 |
| Txndc9 | 0.05446331 |
| Txnip | 1.53657816 |
| Txnl2 | 0.43549926 |
| Txnl4 | -0.7403395 |
| Txnl4a | 2.34280985 |
| Txnl4a | -0.6293257 |
| Txnl4a | 1.09040871 |
| Txnl4b | 0.02317074 |
| Txnrd2 | -0.8067269 |
| Txnrd3 | -0.8006771 |
| Tyk2 | 1.20320064 |
| Tyk2 | 1.81076935 |
| Tyk2 | 1.41548451 |
| Tyki | 0.06132276 |
| Tyms | -0.0324499 |
| Tyms | 0.28529095 |
| Tyms | -0.8597849 |
| Tyms-ps | 0.554818 |
| Tyro3 | -0.6643145 |
| Tyrobp | 1.55868912 |
| Tyw1 | -0.0788823 |
| Tyw1 | 0.55719423 |
| U2af1l4 | 2.96443701 |
| U2af2 | 0.24283906 |
| U2af2 | -0.4680546 |
| Uaca | -0.0617731 |
| Uap1 | 0.24713851 |
| Uap1l1 | -0.5084301 |
| Uba1 | 0.10607272 |
| Uba1 | -0.6686719 |
| Uba2 | -0.6916613 |
| Uba5 | -0.0342282 |
| Ubac1 | -0.7412826 |
| Ubac1 | 1.51221671 |
| Ubac1 | -0.7097358 |
| Ubac2 | 0.34632232 |
| Ubap1 | 1.83591186 |
| Ubap2 | -0.6539385 |
| Ubap2l | -0.2260797 |
| Ubap2l | 0.56992318 |
| Ubap2l | -0.7817719 |
| Ubb | 3.52743286 |
| Ubd | 0.06693441 |
| Ube1c | -0.7798038 |
| Ube1l | -0.2611013 |
| Ube1x | 0.07251278 |
| Ube2a | 1.91664954 |
| Ube2b | 1.48020523 |
| Ube2d1 | -0.6453784 |
| Ube2d2 | -0.5939767 |
| Ube2d3 | -0.6573845 |
| Ube2e1 | -0.3279907 |
| Ube2e2 | -0.3402322 |
| Ube2e3 | 3.18466525 |
| Ube2f | -0.6997518 |
| Ube2f | -0.2409296 |
| Ube2f | 0.46919104 |
| Ube2g1 | 1.10444103 |
| Ube2g1 | -0.5091671 |
| Ube2g1 | -0.52703 |
| Ube2g2 | -0.5018232 |
| Ube2i | 0.2011567 |
| Ube2j1 | -0.3396163 |
| Ube2j2 | 2.52219695 |
| Ube2j2 | 0.2878224 |
| Ube2j2 | 0.62648736 |
| Ube2k | 1.78955831 |
| Ube2k | 0.66704876 |
| Ube2l3 | 1.91064443 |
| Ube2l3 | 0.43089636 |
| Ube2l6 | 0.7883294 |
| Ube2m | -0.0540153 |
| Ube2n | -0.5315492 |
| Ube2n | -0.7778398 |
| Ube2o | -0.3189129 |
| Ube2q1 | -0.6352081 |
| Ube2q1 | -0.6774476 |
| Ube2q2 | -0.3334797 |
| Ube2q2 | 1.79428506 |
| Ube2q2 | -0.4858656 |
| Ube2r2 | 1.90216101 |
| Ube2s | 1.66878382 |
| Ube2t | 0.24878692 |
| Ube2u | 1.51907101 |
| Ube2u | -0.7235233 |
| Ube3a | 0.12767395 |
| Ube3a | -0.2453749 |
| Ube3b | -0.4666442 |
| Ube3b | -0.3921575 |
| Ube3c | -0.5708652 |
| Ube4a | -0.3798684 |
| Ube4b | 0.61271789 |
| Ubfd1 | 1.0063838 |
| Ubiad1 | 1.04036 |
| Ubie | -0.7403395 |
| Ubl3 | 1.66513637 |
| Ubl4 | -0.5255284 |
| Ubl4b | -0.5614655 |
| Ubl5 | -0.5923644 |
| Ubl7 | -0.6599773 |
| Ublcp1 | -0.7006551 |
| Ubox5 | -0.3328682 |
| Ubox5 | -0.7642055 |
| Ubp1 | -0.5368492 |
| Ubp1 | -0.6343655 |
| Ubqln1 | -0.5069579 |
| Ubqln1 | -0.2599692 |
| Ubqln1 | 0.92733432 |
| Ubqln3 | -0.6143665 |
| Ubqln4 | -0.4694672 |
| Ubqlnl | -0.6556599 |
| Ubr1 | 1.78210068 |
| Ubr5 | -0.2103866 |
| Ubtd1 | -0.7847318 |
| Ubtd2 | 1.07698374 |
| Ubtd2 | -0.5240292 |
| Ubtd2 | 0.02484393 |
| Ubtf | -0.1740679 |
| Ubxd1 | -0.1637976 |
| Ubxd1 | -0.7161448 |
| Ubxd1 | -0.0449695 |
| Ubxd3 | 0.76692648 |
| Ubxd4 | -0.5069579 |
| Ubxd5 | 1.23525272 |
| Ubxd8 | -0.6293257 |
| Uchl1 | -0.3476543 |
| Uchl3 | -0.4744278 |
| Uchl5 | -0.3093256 |
| Uchl5 | 0.19629314 |
| Uchl5 | 0.2843399 |
| Uck1 | 0.20046344 |
| Uck1 | 1.22748699 |
| Uck1 | 0.84837084 |
| Uck1 | 0.15244654 |
| Uck2 | -0.1561678 |
| Uck2 | -0.3039753 |
| Uck2 | 0.16477252 |
| Uevld | 0.36513794 |
| Ufc1 | -0.4624256 |
| Ufsp1 | 1.47788921 |
| Ufsp2 | 0.54308691 |
| Ugcg | -0.0083324 |
| Ugp2 | 0.84260632 |
| Ugt1a10 | -0.5150838 |
| Ugt1a6a | 2.11739706 |
| Ugt1a6a | 0.64849547 |
| Ugt1a6a | 0.14769065 |
| Ugt3a2 | 0.03688634 |
| Ugt8a | 0.768634 |
| Uhmk1 | -0.3027905 |
| Uhrf1 | 0.95058341 |
| Uhrf1bp1 | -0.5700783 |
| Uhrf1bp1l | -0.7337641 |
| Uhrf2 | -0.4981726 |
| Uhrf2 | 0.12128322 |
| Uhrf2 | -0.4596236 |
| Uhrf2 | 1.02403111 |
| Uimc1 | 1.92005593 |
| Ulk1 | -0.6069752 |
| Ulk1 | 0.42381278 |
| Ulk2 | 1.43659407 |
| Ulk2 | -0.4974442 |
| Umodl1 | -0.5899512 |
| Umps | -0.5544768 |
| Umps | -0.6818663 |
| Umps | -0.6462309 |
| Unc119 | 0.82313208 |
| Unc119 | -0.3582703 |
| Unc119b | -0.6209857 |
| Unc119b | -0.1155179 |
| Unc119b | 0.11484878 |
| Unc13b | 1.65210955 |
| Unc13c | -0.5653706 |
| Unc13d | -0.5195458 |
| Unc45a | -0.2554545 |
| Unc45a | 2.14496401 |
| Unc45b | -0.4118942 |
| Unc5a | -0.4006593 |
| Unc5a | 1.67089288 |
| Unc5b | 1.84615759 |
| Unc5c | 1.39768589 |
| Unc84a | -0.8386198 |
| Unc84a | 1.4852628 |
| Unc84b | -0.5740194 |
| Unc93a | -0.3016072 |
| Unc93b1 | -0.5069579 |
| Unc93b1 | -0.4132249 |
| Ung | -0.2753683 |
| Ung | 1.67870597 |
| Unk | 0.04755348 |
| Unkl | -0.3093256 |
| Uox | 0.14180405 |
| Upb1 | -0.1189203 |
| Upb1 | -0.5143422 |
| Upb1 | 0.03440743 |
| Upf1 | -0.6827526 |
| Upf1 | -0.5843444 |
| Upf1 | -0.0472228 |
| Upf2 | 0.88156311 |
| Upf3a | 0.82402607 |
| Upk1a | -0.5803598 |
| Upk1a | -0.7729477 |
| Upk1b | -0.5421792 |
| Upk2 | -0.5803598 |
| Upk3a | 0.64157816 |
| Upk3b | -0.2577091 |
| Upk3b | 1.82838411 |
| Upk3b | 1.34628786 |
| Upp1 | 2.62055914 |
| Upp1 | -0.2846141 |
| Upp1 | -0.0835604 |
| Upp2 | 1.23062418 |
| Upp2 | 2.88913536 |
| Upp2 | 1.18145383 |
| Upp2 | 0.3792935 |
| Uqcc | 2.33301439 |
| Uqcr | 0.92845455 |
| Uqcrc1 | -0.5653706 |
| Uqcrc2 | 0.09061223 |
| Uqcrh | 0.62428007 |
| Urm1 | -0.6854163 |
| Urod | 1.48136111 |
| Uros | -0.4450489 |
| Uros | -0.313508 |
| Use1 | 0.5877809 |
| Use1 | -0.8026894 |
| Use1 | -0.5084301 |
| Usf1 | 1.11682816 |
| Usf1 | -0.4347748 |
| Usf2 | 0.8527145 |
| Usf2 | -0.4313749 |
| Ush1c | -0.5630256 |
| Ush1g | 0.53777193 |
| Ush2a | -0.7263052 |
| Ush3a | -0.376661 |
| Ushbp1 | 1.69350209 |
| Usmg5 | -0.5835461 |
| Uso1 | 1.06488744 |
| Usp1 | 1.80549552 |
| Usp1 | -0.8240825 |
| Usp1 | -0.505488 |
| Usp10 | -0.3087296 |
| Usp10 | 0.75987393 |
| Usp10 | -0.336543 |
| Usp11 | 0.50723983 |
| Usp11 | 1.06957946 |
| Usp12 | -0.6997518 |
| Usp12 | -0.2249889 |
| Usp13 | 0.40363434 |
| Usp14 | -0.2354022 |
| Usp15 | -0.7124771 |
| Usp16 | 0.87751977 |
| Usp18 | -0.5047539 |
| Usp18 | 3.13316912 |
| Usp19 | 1.05375418 |
| Usp2 | -0.7788213 |
| Usp2 | 0.86682256 |
| Usp2 | 1.37467969 |
| Usp20 | -0.147093 |
| Usp21 | 1.00077266 |
| Usp22 | -0.4125593 |
| Usp25 | 0.45474799 |
| Usp28 | 1.81868717 |
| Usp29 | 0.2284762 |
| Usp3 | -0.3099219 |
| Usp3 | -0.7886928 |
| Usp33 | 1.26970463 |
| Usp37 | -0.5899512 |
| Usp38 | -0.3595271 |
| Usp38 | 1.14252588 |
| Usp39 | -0.3027905 |
| Usp39 | -0.3129094 |
| Usp39 | -0.8006771 |
| Usp39 | -0.5819516 |
| Usp4 | 0.51843491 |
| Usp4 | -0.6343655 |
| Usp43 | 2.14649885 |
| Usp45 | 0.55885403 |
| Usp46 | 1.29398125 |
| Usp47 | 1.33327333 |
| Usp48 | -0.3470336 |
| Usp49 | 0.36775396 |
| Usp5 | 0.69677114 |
| Usp50 | 1.35740301 |
| Usp52 | 0.28497404 |
| Usp52 | -0.0131067 |
| Usp52 | -0.1725202 |
| Usp53 | 2.22792012 |
| Usp6nl | 2.69085082 |
| Usp6nl | -0.5795649 |
| Usp7 | 0.23484964 |
| Usp8 | 0.5808636 |
| Usp8 | 0.4552771 |
| Usp9x | 2.86190281 |
| Usp9y | 0.43981092 |
| Uspl1 | -0.6061575 |
| Utp11l | 1.11983438 |
| Utp15 | -0.6168432 |
| Utp6 | 0.1672903 |
| Utx | 0.0151822 |
| Utx | -0.0413755 |
| Uty | 1.54691159 |
| Uvrag | -0.4013165 |
| Uxt | 1.21565793 |
| Uxt | 2.50995005 |
| V1ra2 | 0.99169473 |
| V1rd2 | 0.11712479 |
| V1rd20 | 1.80593614 |
| V1rd21 | -0.6077935 |
| V2r8 | 1.39515726 |
| Vac14 | 3.61367268 |
| Vamp1 | -0.6970472 |
| Vamp1 | -0.5173122 |
| Vamp2 | -0.3255614 |
| Vamp3 | 0.26511411 |
| Vamp3 | -0.1965362 |
| Vamp4 | -0.7479109 |
| Vamp5 | -0.438871 |
| Vamp8 | 0.31737391 |
| Vamp8 | -0.5972095 |
| Vangl2 | -0.3709149 |
| Vapa | -0.538369 |
| Vapb | 2.9937488 |
| Vars | -0.7403395 |
| Vars | 1.63377515 |
| Vars | 1.07671054 |
| Vars2 | 0.88993133 |
| Vash1 | -0.5552507 |
| Vash1 | -0.6487931 |
| Vasn | -0.4779872 |
| Vasp | 0.16800843 |
| Vat1 | 0.04264521 |
| Vav1 | 0.38072604 |
| Vav2 | 2.07643123 |
| Vav3 | 0.83856763 |
| Vax1 | -0.4429851 |
| Vbp1 | -0.1776891 |
| Vcam1 | 0.66514633 |
| Vcl | -0.6268159 |
| Vcp | -0.6573845 |
| Vcpip1 | 2.2398194 |
| Vdac1 | -0.7272343 |
| Vdac2 | -0.3231383 |
| Vdac3 | 0.57808237 |
| Vdr | -0.2200963 |
| Vdr | 0.41253301 |
| Vdr | 0.54573318 |
| Vegfa | 0.76768576 |
| Vegfa | -0.3973803 |
| Vegfb | 2.68454068 |
| Vegfc | -0.2742189 |
| Vezf1 | -0.7526742 |
| Vezt | 0.31614818 |
| Vgf | 2.00220441 |
| Vgll2 | -0.1607382 |
| Vgll4 | 1.8070682 |
| Vhlh | 1.64921482 |
| Vil1 | 1.01430039 |
| Vill | -0.3947653 |
| Vim | -0.8240825 |
| Vip | 2.00434735 |
| Vit | -0.3255614 |
| Vkorc1 | -0.6445267 |
| Vkorc1 | 0.33045064 |
| Vkorc1 | -0.0590278 |
| Vkorc1l1 | 2.00714622 |
| Vkorc1l1 | 0.3887082 |
| Vkorc1l1 | 0.50673916 |
| Vldlr | -0.6952485 |
| Vldlr | -0.2238995 |
| Vldlr | 0.04632881 |
| Vldlr | -0.6160169 |
| Vldlr | 0.76293017 |
| Vmn2r81 | 0.26866838 |
| Vmo1 | -0.2951253 |
| Vnn3 | -0.0022909 |
| Vnn3 | 0.65172053 |
| Vpreb1 | -0.5606864 |
| Vpreb3 | 0.27893237 |
| Vps11 | 0.2331766 |
| Vps13a | -0.7281644 |
| Vps16 | 1.09778507 |
| Vps16 | -0.4252859 |
| Vps16 | -0.6284884 |
| Vps16 | -0.7244496 |
| Vps18 | 0.99527683 |
| Vps24 | 0.83874359 |
| Vps25 | -0.0859081 |
| Vps25 | -0.7097358 |
| Vps26 | -0.6028938 |
| Vps26a | -0.6127189 |
| Vps26b | -0.5202916 |
| Vps28 | 1.15417304 |
| Vps29 | 0.4626535 |
| Vps29 | -0.8924531 |
| Vps29 | -0.618498 |
| Vps33a | 2.896765 |
| Vps33a | -0.1296942 |
| Vps33b | 0.75025866 |
| Vps33b | 2.64905742 |
| Vps35 | 0.55932772 |
| Vps36 | 0.76083007 |
| Vps37b | -0.7642055 |
| Vps37c | -0.5708652 |
| Vps37d | 1.43388768 |
| Vps39 | -0.3383858 |
| Vps39 | -0.6916613 |
| Vps41 | 1.51864408 |
| Vps41 | -0.0236082 |
| Vps45 | -0.5669372 |
| Vps4a | -0.6135424 |
| Vps4b | 0.56475808 |
| Vps53 | -0.7906796 |
| Vps53 | 0.69595238 |
| Vps53 | 0.92412631 |
| Vps54 | -0.1121278 |
| Vps72 | 0.48955605 |
| Vrk1 | 1.24135067 |
| Vrk1 | -0.5740194 |
| Vrk2 | 2.84643515 |
| Vrk3 | -0.6020797 |
| Vsig2 | -0.273071 |
| Vsig4 | -0.770998 |
| Vsnl1 | 3.52950021 |
| Vstm2a | 1.32732613 |
| Vstm2a | -0.5552507 |
| Vstm2a | 0.36775396 |
| Vstm2b | 0.14915656 |
| Vsx2 | -0.7189049 |
| Vti1a | -0.5225324 |
| Vti1a | 2.39849419 |
| Vti1a | 0.0294299 |
| Vti1b | 1.62839445 |
| Vti1b | -0.6539385 |
| Vtn | 0.06292951 |
| Vwa2 | 2.51751227 |
| Vwa3a | -0.6547988 |
| Vwf | -0.6377404 |
| Wars | -0.8957832 |
| Wars | -0.6783297 |
| Wars | -0.4865851 |
| Wars | -0.1720048 |
| Wars2 | 2.39919785 |
| Wars2 | -0.6863059 |
| Was | -0.8026894 |
| Wasf1 | -0.4901909 |
| Wasf2 | -0.0910939 |
| Wasf3 | 0.23518389 |
| Wasl | 1.88008828 |
| Wbp1 | -0.4085759 |
| Wbp1 | -0.3347038 |
| Wbp11 | 0.7014652 |
| Wbp2 | 2.67041217 |
| Wbp2nl | 0.40251606 |
| Wbp4 | 0.38043971 |
| Wbp5 | 0.06413277 |
| Wbp7 | -0.5851433 |
| Wbscr16 | 1.13124854 |
| Wbscr18 | -0.4967164 |
| Wbscr22 | -0.2881048 |
| Wbscr22 | 0.35312759 |
| Wbscr22 | -0.6898728 |
| Wbscr27 | 1.15693699 |
| Wbscr28 | -0.3420823 |
| Wbscr28 | 0.09139132 |
| Wdfy1 | 0.22813956 |
| Wdfy3 | 0.02651414 |
| Wdhd1 | -0.0148488 |
| Wdr1 | -0.7161448 |
| Wdr1 | 1.5368295 |
| Wdr13 | -0.6368955 |
| Wdr13 | -0.6907666 |
| Wdr16 | -0.5136011 |
| Wdr17 | 1.22946348 |
| Wdr18 | -0.6547988 |
| Wdr20a | 0.69922313 |
| Wdr20b | 0.4097612 |
| Wdr21 | -0.3696428 |
| Wdr22 | -0.6625772 |
| Wdr23 | 1.84676036 |
| Wdr24 | -0.1386019 |
| Wdr27 | 1.24444204 |
| Wdr3 | 0.33376686 |
| Wdr31 | -0.6436757 |
| Wdr31 | -0.1986538 |
| Wdr31 | 0.32408704 |
| Wdr32 | 0.64633927 |
| Wdr33 | 1.59811982 |
| Wdr34 | -0.5716528 |
| Wdr37 | 0.69779358 |
| Wdr37 | -0.4199064 |
| Wdr37 | -0.6739275 |
| Wdr37 | 0.30597448 |
| Wdr38 | 0.08278566 |
| Wdr41 | 0.29160685 |
| Wdr45 | 0.46239105 |
| Wdr45l | -0.3633078 |
| Wdr45l | 1.67487988 |
| Wdr46 | -0.2206387 |
| Wdr46 | -0.4687606 |
| Wdr47 | -0.1174606 |
| Wdr5 | -0.8386198 |
| Wdr51b | -0.6193265 |
| Wdr53 | -0.6677988 |
| Wdr54 | -0.2916085 |
| Wdr55 | -0.3702786 |
| Wdr59 | -0.5835461 |
| Wdr5b | 0.71901211 |
| Wdr6 | -0.0709827 |
| Wdr60 | -0.2921937 |
| Wdr61 | -0.7422266 |
| Wdr62 | 0.19976967 |
| Wdr63 | 0.1938519 |
| Wdr66 | -0.2387147 |
| Wdr67 | 1.1220502 |
| Wdr68 | 0.78460955 |
| Wdr69 | 1.67248971 |
| Wdr70 | -0.5591301 |
| Wdr70 | -0.5136011 |
| Wdr74 | 0.38015328 |
| Wdr75 | -0.8179204 |
| Wdr75 | 1.72468076 |
| Wdr76 | -0.4471172 |
| Wdr77 | -0.2577091 |
| Wdr78 | 0.56922051 |
| Wdr8 | 1.37974203 |
| Wdr8 | -0.6151914 |
| Wdr9 | 0.49388134 |
| Wdr93 | -0.3601562 |
| Wdsof1 | -0.6251465 |
| Wfdc1 | -0.7460124 |
| Wfdc10 | 1.6605938 |
| Wfdc10 | 0.68875772 |
| Wfdc12 | 0.81125104 |
| Wfdc15b | -0.6143665 |
| Wfdc15b | -0.610253 |
| Wfdc2 | -0.5018232 |
| Wfdc3 | -0.4787007 |
| Wfdc6a | -0.6377404 |
| Wfdc6b | 0.38643449 |
| Wfdc6b | -0.8683861 |
| Wfikkn2 | -0.1571815 |
| Wfs1 | -0.2409296 |
| Whdc1 | 0.57645618 |
| Whrn | -0.7758799 |
| Whrn | -0.2453749 |
| Whsc2 | -0.4225923 |
| Wif1 | -0.3513875 |
| Wipf1 | 1.62022691 |
| Wipf2 | -0.147093 |
| Wipi1 | 1.82401124 |
| Wipi1 | 0.31246137 |
| Wipi1 | -0.5575765 |
| Wipi2 | 0.01306864 |
| Wipi2 | -0.7498134 |
| Wipi2 | 0.581095 |
| Wisp1 | 0.70471687 |
| Wisp1 | 1.41900418 |
| Wisp2 | 1.53708078 |
| Wiz | 4.84109815 |
| Wiz | 0.12504773 |
| Wnk1 | -0.7976667 |
| Wnk1 | 0.29255059 |
| Wnk2 | -0.1526283 |
| Wnk4 | 0.62626686 |
| Wnt10a | -0.3882594 |
| Wnt10b | 0.90562875 |
| Wnt16 | -0.1997144 |
| Wnt16 | -0.5099047 |
| Wnt16 | 1.45523251 |
| Wnt2 | -0.2195542 |
| Wnt3a | 0.01306864 |
| Wnt4 | -0.5883458 |
| Wnt4 | 0.26834582 |
| Wnt5a | -0.2904391 |
| Wnt5a | 0.80561834 |
| Wnt5b | 2.1362337 |
| Wnt6 | 1.01721564 |
| Wnt7a | 1.25059448 |
| Wnt7b | 1.06737437 |
| Wnt7b | 0.11028018 |
| Wrb | 0.15281138 |
| Wrn | -0.4687606 |
| Wsb1 | 0.53801417 |
| Wsb2 | -0.2759435 |
| Wscd1 | 1.5091159 |
| Wscd2 | 2.20530819 |
| Wtap | 2.98086002 |
| Wtip | -0.1390993 |
| Wwc1 | -0.4361382 |
| Wwc1 | -0.5437077 |
| Wwc2 | 4.01386719 |
| Wwc2 | -0.8026894 |
| Wwox | -0.4772742 |
| Wwox | -0.7207495 |
| Wwp1 | 2.43456557 |
| Wwp2 | 1.31053508 |
| Wwp2 | -0.1053841 |
| Wwp2 | 0.13402156 |
| Wwtr1 | 1.13047469 |
| X99384 | 0.72954336 |
| Xab1 | -0.4981726 |
| Xab1 | 0.49083026 |
| Xbp1 | -0.6522203 |
| Xdh | 0.40363434 |
| Xist | 0.42134829 |
| Xist | 0.13253185 |
| Xk | -0.4192361 |
| Xkr4 | 1.70897645 |
| Xkr5 | -0.6970472 |
| Xkr6 | 0.64309568 |
| Xkr8 | 0.45501258 |
| Xkrx | 0.31092093 |
| Xlr4a | 2.07060702 |
| Xpc | 2.85720064 |
| Xpnpep1 | 3.10228732 |
| Xpnpep1 | 0.63679284 |
| Xpnpep1 | 0.2481279 |
| Xpnpep2 | 1.07315176 |
| Xpnpep2 | 1.81308301 |
| Xpnpep3 | -0.6643145 |
| Xpo4 | -0.8282132 |
| Xpo4 | -0.6730495 |
| Xpo5 | -0.6436757 |
| Xpo6 | -0.533817 |
| Xpo7 | 1.82091952 |
| Xpo7 | -0.7244496 |
| Xpr1 | 0.21491562 |
| Xrcc1 | 1.56326142 |
| Xrcc2 | 1.2392841 |
| Xrcc2 | -0.0087654 |
| Xrcc3 | 0.749872 |
| Xrcc6 | 2.64240789 |
| Xrcc6 | -0.2504012 |
| Xrcc6 | 2.10603028 |
| Xrn1 | 2.04205928 |
| Xrn2 | 1.5159071 |
| Xylb | -0.5391298 |
| Xylt2 | -0.7517196 |
| Yaf2 | 0.53340086 |
| Yap1 | 0.5316955 |
| Ybx2 | 1.12750233 |
| Ybx2 | -0.7393973 |
| Yeats2 | 3.04468746 |
| Yeats4 | 0.78460955 |
| Yeats4 | 0.00413942 |
| Yif1a | -0.4751386 |
| Yif1a | -0.7642055 |
| Yif1a | 1.52672288 |
| Yif1b | 1.30132667 |
| Yipf1 | 2.3875365 |
| Yipf2 | 1.763523 |
| Yipf3 | 0.33557079 |
| Yipf3 | 0.1502545 |
| Yipf3 | 3.33919931 |
| Yipf4 | 1.58316751 |
| Yipf6 | -0.6845276 |
| Yipf6 | 0.1193953 |
| Yipf6 | -0.6721723 |
| Yipf6 | -0.6226478 |
| Yipf7 | -0.2453749 |
| Ykt6 | 0.29725513 |
| Ykt6 | 2.48386593 |
| Ylpm1 | 1.66827403 |
| Yme1l1 | -0.569292 |
| Yme1l1 | 0.0548682 |
| Yme1l1 | -0.6925568 |
| Yod1 | 1.06142243 |
| Ypel1 | -0.7837441 |
| Ypel3 | -0.8057159 |
| Ypel3 | -0.4880258 |
| Ypel4 | 2.7821115 |
| Ypel4 | -0.5040204 |
| Ypel5 | 0.23484964 |
| Yrdc | -0.2321014 |
| Ythdf1 | 1.438363 |
| Ythdf2 | -1.0090928 |
| Ythdf2 | -0.3021987 |
| Ythdf3 | 0.38899203 |
| Ythdf3 | -0.3626766 |
| Ywhab | -0.2921937 |
| Ywhab | -0.8438666 |
| Ywhae | -0.5803598 |
| Ywhag | 0.99825153 |
| Ywhah | 1.28087716 |
| Ywhaq | -0.4575277 |
| Ywhaz | -0.4722986 |
| Yy2 | 0.16944305 |
| Zadh1 | -0.7235233 |
| Zadh2 | 1.24272587 |
| Zan | -0.6591122 |
| Zap70 | 0.51645439 |
| Zap70 | 2.40220761 |
| Zbbx | -0.2173891 |
| Zbed4 | 0.19594478 |
| Zbp1 | 1.10058631 |
| Zbtb17 | 0.44704234 |
| Zbtb2 | 1.48064997 |
| Zbtb22 | -0.6925568 |
| Zbtb24 | -0.3652038 |
| Zbtb3 | -0.9366917 |
| Zbtb32 | 0.91557178 |
| Zbtb33 | -0.9137455 |
| Zbtb38 | 0.21389065 |
| Zbtb39 | -0.6259808 |
| Zbtb40 | -0.0192183 |
| Zbtb41 | 0.86305819 |
| Zbtb43 | 0.06893048 |
| Zbtb45 | -0.4645325 |
| Zbtb46 | 0.33135622 |
| Zbtb5 | -0.6094325 |
| Zbtb7a | 0.61047801 |
| Zbtb7c | -0.8179204 |
| Zbtb8 | 1.372687 |
| Zbtb9 | 0.55338937 |
| Zbtb9 | -0.5506165 |
| Zc3h10 | -0.6487931 |
| Zc3h11a | -0.4575277 |
| Zc3h12a | 1.93745265 |
| Zc3h12b | -0.7088237 |
| Zc3h13 | -0.5763921 |
| Zc3h14 | -0.336543 |
| Zc3h14 | 2.70462949 |
| Zc3h15 | -0.0139774 |
| Zc3h18 | -0.7700246 |
| Zc3h3 | 2.19346168 |
| Zc3h3 | -0.6651844 |
| Zc3h3 | 0.05203034 |
| Zc3h6 | 2.08661935 |
| Zc3h7a | -0.7207495 |
| Zc3h7a | 0.63810034 |
| Zc3h8 | 3.23188619 |
| Zc3hc1 | 0.02609687 |
| Zc3hc1 | -0.4851467 |
| Zc3hc1 | -0.5923644 |
| Zc3hc1 | -0.1828867 |
| Zcchc12 | -0.6660551 |
| Zcchc14 | 2.85948279 |
| Zcchc16 | -0.3501414 |
| Zcchc17 | -0.6193265 |
| Zcchc17 | 2.67584447 |
| Zcchc18 | -0.7375158 |
| Zcchc18 | 1.02012188 |
| Zcchc3 | 2.26213008 |
| Zcchc3 | 3.02417646 |
| Zcchc3 | -0.7498134 |
| Zcchc5 | -0.2719244 |
| Zcchc6 | -0.4996312 |
| Zcchc8 | -0.6061575 |
| Zcchc8 | 0.61204648 |
| Zcchc8 | 0.6829621 |
| Zcchc9 | -0.7328285 |
| Zcrb1 | -0.1907372 |
| Zcwpw1 | 0.93276297 |
| Zdhhc1 | 0.07489342 |
| Zdhhc12 | 0.25305932 |
| Zdhhc12 | 0.05162423 |
| Zdhhc13 | 1.19323536 |
| Zdhhc13 | -0.7966653 |
| Zdhhc13 | -0.572441 |
| Zdhhc14 | -0.5787707 |
| Zdhhc15 | -0.6845276 |
| Zdhhc16 | -0.8781574 |
| Zdhhc16 | -0.7170639 |
| Zdhhc17 | -0.5437077 |
| Zdhhc19 | 1.00594203 |
| Zdhhc19 | -0.7375158 |
| Zdhhc21 | 2.65200628 |
| Zdhhc24 | 1.22981184 |
| Zdhhc3 | 0.40948357 |
| Zdhhc3 | -0.0165942 |
| Zdhhc4 | -0.026252 |
| Zdhhc6 | 1.35517014 |
| Zdhhc7 | 1.09818576 |
| Zdhhc9 | -0.4354563 |
| Zeb1 | -0.1808042 |
| Zeb2 | -0.6591122 |
| Zer1 | 0.59419149 |
| Zf | 0.70552804 |
| Zfa | -0.6110743 |
| Zfand2a | -0.8067269 |
| Zfand2b | 0.1850811 |
| Zfand2b | 2.02660809 |
| Zfand2b | 2.39735381 |
| Zfand2b | 0.06373185 |
| Zfand3 | -0.7758799 |
| Zfand6 | 1.59623424 |
| Zfand6 | -0.6889798 |
| Zfat | 3.10271555 |
| Zfh4 | 2.0853577 |
| Zfhx2 | 1.43276551 |
| Zfhx2 | -0.2910236 |
| Zfhx3 | 0.15935449 |
| Zfhx3 | 2.13747322 |
| Zfml | 1.48153881 |
| Zfp1 | -0.1954791 |
| Zfp101 | 2.3365654 |
| Zfp105 | -0.6686719 |
| Zfp106 | 0.45023854 |
| Zfp106 | 0.91102081 |
| Zfp109 | 0.210466 |
| Zfp11 | 0.32621303 |
| Zfp119 | -0.5779772 |
| Zfp12 | -0.7042769 |
| Zfp128 | -0.7526742 |
| Zfp13 | -0.3027905 |
| Zfp131 | 0.18084189 |
| Zfp142 | 0.19698947 |
| Zfp143 | 1.40214305 |
| Zfp148 | -0.7015592 |
| Zfp160 | -0.0608571 |
| Zfp161 | -0.4967164 |
| Zfp161 | -0.211998 |
| Zfp169 | -0.0022909 |
| Zfp179 | 0.61873906 |
| Zfp182 | -0.6505051 |
| Zfp184 | -0.3033827 |
| Zfp185 | 0.09411306 |
| Zfp187 | -0.1997144 |
| Zfp187 | -0.2266255 |
| Zfp189 | -0.3099219 |
| Zfp191 | 1.26580179 |
| Zfp2 | -0.2673521 |
| Zfp2 | -0.2337504 |
| Zfp202 | -0.211998 |
| Zfp207 | 2.40350886 |
| Zfp212 | -0.3721888 |
| Zfp212 | -0.4266356 |
| Zfp212 | 1.68923726 |
| Zfp212 | -0.4533496 |
| Zfp213 | -0.3353165 |
| Zfp217 | 0.60845754 |
| Zfp219 | -0.5345741 |
| Zfp235 | -0.1865421 |
| Zfp236 | -0.5255284 |
| Zfp236 | 0.62361687 |
| Zfp238 | -0.2337504 |
| Zfp238 | 0.68441436 |
| Zfp238 | -0.1092317 |
| Zfp238 | -0.237057 |
| Zfp238 | -0.5300404 |
| Zfp239 | -0.546005 |
| Zfp251 | -0.9002417 |
| Zfp259 | -0.5240292 |
| Zfp26 | -0.5763921 |
| Zfp263 | 0.63264038 |
| Zfp27 | -0.7946659 |
| Zfp27 | -0.4873052 |
| Zfp275 | 1.21707117 |
| Zfp276 | -0.6209857 |
| Zfp277 | -0.3147064 |
| Zfp277 | 0.58915828 |
| Zfp280c | 1.3237607 |
| Zfp281 | 2.22118533 |
| Zfp282 | -0.3439359 |
| Zfp286 | -0.7170639 |
| Zfp288 | -0.3219291 |
| Zfp292 | 1.166299 |
| Zfp292 | -0.6730495 |
| Zfp295 | 0.1053057 |
| Zfp296 | -0.7584222 |
| Zfp296 | 2.22308563 |
| Zfp3 | 3.21358856 |
| Zfp30 | 3.32269069 |
| Zfp313 | -0.0668272 |
| Zfp313 | -0.5018232 |
| Zfp316 | -0.2628019 |
| Zfp316 | -0.760346 |
| Zfp318 | -0.5638066 |
| Zfp322a | -0.2577091 |
| Zfp324 | 2.98679753 |
| Zfp326 | 2.4569119 |
| Zfp326 | 0.46893041 |
| Zfp326 | 0.54067473 |
| Zfp329 | -0.4666442 |
| Zfp329 | 2.91038324 |
| Zfp330 | -0.4617243 |
| Zfp330 | 2.07098693 |
| Zfp334 | 2.27402798 |
| Zfp335 | 0.39915324 |
| Zfp339 | -0.3570151 |
| Zfp346 | 1.23455989 |
| Zfp346 | -0.4865851 |
| Zfp35 | 0.81504767 |
| Zfp354a | -0.6143665 |
| Zfp36 | 0.64266235 |
| Zfp365 | -0.8891347 |
| Zfp367 | 0.46344041 |
| Zfp367 | 0.03606076 |
| Zfp36l1 | -0.9590216 |
| Zfp36l1 | -0.2387147 |
| Zfp36l1 | -0.8138346 |
| Zfp36l2 | 0.64633927 |
| Zfp37 | 1.20415559 |
| Zfp371 | -0.026252 |
| Zfp382 | 0.78795808 |
| Zfp385a | -0.9413489 |
| Zfp385a | -0.147093 |
| Zfp385c | -0.7412826 |
| Zfp386 | 0.21696221 |
| Zfp386 | -0.671296 |
| Zfp39 | 0.04223503 |
| Zfp393 | 1.43257835 |
| Zfp395 | 0.04836904 |
| Zfp397 | 1.44605171 |
| Zfp398 | 0.19664137 |
| Zfp398 | -0.7235233 |
| Zfp398 | -0.5939767 |
| Zfp41 | 1.57933196 |
| Zfp41 | -0.2582736 |
| Zfp410 | -0.9320575 |
| Zfp410 | -0.750766 |
| Zfp410 | -0.6419761 |
| Zfp414 | -0.6020797 |
| Zfp418 | -0.1126114 |
| Zfp42 | -0.0109336 |
| Zfp423 | 0.09527712 |
| Zfp423 | 0.57226161 |
| Zfp428 | 0.33165789 |
| Zfp428 | -0.4930855 |
| Zfp446 | 0.59076268 |
| Zfp446 | -0.1465914 |
| Zfp449 | -0.873802 |
| Zfp451 | 1.56122296 |
| Zfp457 | 1.11486239 |
| Zfp46 | 1.22176671 |
| Zfp467 | 2.66928172 |
| Zfp472 | -0.3219291 |
| Zfp473 | -0.4996312 |
| Zfp473 | 2.21117597 |
| Zfp474 | -0.4865851 |
| Zfp474 | -0.1198946 |
| Zfp512 | 1.80926556 |
| Zfp513 | 0.49843949 |
| Zfp516 | 0.10376978 |
| Zfp52 | 1.52018014 |
| Zfp521 | -0.4313749 |
| Zfp523 | -0.007467 |
| Zfp523 | 0.64157816 |
| Zfp524 | 0.08631568 |
| Zfp526 | 1.24615509 |
| Zfp532 | -0.2365051 |
| Zfp532 | 2.10066307 |
| Zfp536 | 0.938477 |
| Zfp551 | 1.04021799 |
| Zfp553 | -0.0253699 |
| Zfp553 | -0.0244886 |
| Zfp560 | 0.67336254 |
| Zfp566 | 1.63769652 |
| Zfp57 | -0.5003613 |
| Zfp574 | 0.29380744 |
| Zfp579 | 1.22270301 |
| Zfp592 | -0.8148544 |
| Zfp593 | 1.43510185 |
| Zfp595 | 0.1053057 |
| Zfp598 | 1.92566904 |
| Zfp60 | 0.25404252 |
| Zfp606 | 0.64201199 |
| Zfp607 | 1.69463612 |
| Zfp608 | 1.20451345 |
| Zfp609 | -0.2939516 |
| Zfp61 | 0.61673628 |
| Zfp612 | -0.4327334 |
| Zfp617 | 1.39184029 |
| Zfp619 | 1.49695911 |
| Zfp62 | -0.1735517 |
| Zfp622 | 0.13215906 |
| Zfp623 | -0.4765618 |
| Zfp629 | 1.8854239 |
| Zfp637 | 0.27156646 |
| Zfp639 | -0.4851467 |
| Zfp64 | -0.0929868 |
| Zfp64 | -0.3377711 |
| Zfp64 | -0.2459321 |
| Zfp641 | 0.71360146 |
| Zfp644 | 0.13364935 |
| Zfp646 | 0.69942718 |
| Zfp653 | 3.72199996 |
| Zfp654 | 2.43923159 |
| Zfp654 | 0.56145638 |
| Zfp655 | -0.6669265 |
| Zfp661 | -0.7584222 |
| Zfp667 | -0.4765618 |
| Zfp668 | 2.53358984 |
| Zfp67 | -0.6251465 |
| Zfp672 | 1.82924397 |
| Zfp68 | 2.54418628 |
| Zfp68 | -0.3915067 |
| Zfp68 | 1.89325633 |
| Zfp687 | -0.3153062 |
| Zfp688 | -0.610253 |
| Zfp689 | -1.0065875 |
| Zfp69 | -0.7807873 |
| Zfp691 | 0.77843946 |
| Zfp692 | -0.760346 |
| Zfp692 | 1.96031911 |
| Zfp704 | 2.73837012 |
| Zfp704 | 1.17581471 |
| Zfp706 | 1.55302466 |
| Zfp709 | 1.15530473 |
| Zfp709 | 0.26802315 |
| Zfp710 | -0.4252859 |
| Zfp711 | -0.0631488 |
| Zfp715 | -0.5614655 |
| Zfp71-rs1 | -0.3359295 |
| Zfp74 | 1.18815218 |
| Zfp75 | 1.88420865 |
| Zfp75 | 1.70855778 |
| Zfp75 | 2.82788871 |
| Zfp750 | -0.7584222 |
| Zfp758 | 1.46649366 |
| Zfp760 | -0.3464132 |
| Zfp764 | -0.6310026 |
| Zfp768 | -0.2217243 |
| Zfp770 | 2.58511503 |
| Zfp771 | -0.9013596 |
| Zfp771 | 0.83592433 |
| Zfp78 | -0.7817719 |
| Zfp78 | 0.92637307 |
| Zfp780b | -0.6436757 |
| Zfp786 | 1.60461182 |
| Zfp787 | -0.6151914 |
| Zfp787 | -0.6411274 |
| Zfp790 | -0.3316464 |
| Zfp804a | 0.14438397 |
| Zfp809 | -0.9047215 |
| Zfp810 | 0.52115135 |
| Zfp811 | 1.48543976 |
| Zfp817 | 0.7069459 |
| Zfp819 | 0.12128322 |
| Zfp82 | 1.18560025 |
| Zfp825 | 1.49310572 |
| Zfp828 | 1.08973526 |
| Zfp85-rs1 | -0.0948835 |
| Zfp87 | 2.16631304 |
| Zfp87 | -0.6360514 |
| Zfp90 | -0.6151914 |
| Zfp91 | -0.2904391 |
| Zfp91-cntf | 1.152662 |
| Zfp92 | -0.2588385 |
| Zfp93 | 0.7075529 |
| Zfp93 | 1.61105932 |
| Zfp94 | 2.45886301 |
| Zfp97 | 1.99805777 |
| Zfpl1 | 2.91465329 |
| Zfpm2 | -0.349519 |
| Zfr | -0.4996312 |
| Zfx | -0.5032874 |
| Zfx | 0.44624159 |
| Zfy1 | 1.66922056 |
| Zfyve1 | -0.5490768 |
| Zfyve16 | -0.7088237 |
| Zfyve19 | 0.79147975 |
| Zfyve20 | -0.4105655 |
| Zfyve21 | 0.78311748 |
| Zfyve26 | 0.86305819 |
| Zfyve27 | -0.0986883 |
| Zgpat | 0.29003185 |
| Zgpat | 1.42298359 |
| Zhx1 | -0.760346 |
| Zhx1 | -0.6765663 |
| Zhx1 | -0.4981726 |
| Zhx2 | -0.6343655 |
| Zhx3 | -0.7936677 |
| Zic1 | -0.3614155 |
| Zic2 | 3.72355847 |
| Zic3 | -0.2163084 |
| Zic4 | -0.9217132 |
| Zkscan1 | -0.6037087 |
| Zkscan14 | 0.34661923 |
| Zkscan17 | -0.770998 |
| Zkscan3 | 0.8468022 |
| Zkscan6 | -0.7758799 |
| Zkscan6 | 1.27359134 |
| Zmat1 | -0.8781574 |
| Zmat2 | -0.5851433 |
| Zmat3 | 0.91751548 |
| Zmat4 | 1.12801994 |
| Zmat5 | 1.48233805 |
| Zmiz1 | -0.7365765 |
| Zmiz1 | -0.7412826 |
| Zmiz2 | 0.54405005 |
| Zmiz2 | -0.6836397 |
| Zmpste24 | 0.386719 |
| Zmpste24 | 0.84715101 |
| Zmpste24 | 0.18154975 |
| Zmym3 | 0.10683911 |
| Zmym3 | 0.08239261 |
| Zmym5 | 0.18154975 |
| Zmym6 | 3.69120003 |
| Zmynd10 | -0.5575765 |
| Zmynd11 | 0.76559626 |
| Zmynd11 | 2.99705448 |
| Zmynd11 | -0.5638066 |
| Zmynd12 | 0.12467196 |
| Zmynd15 | -0.7051845 |
| Znhit2 | -0.0594848 |
| Znhit3 | -0.706093 |
| Znhit4 | -0.1607382 |
| Znrd1 | 0.06853161 |
| Znrd1 | -0.4901909 |
| Znrd1 | 0.10951659 |
| Znrf1 | 1.41510322 |
| Znrf2 | -0.538369 |
| Znrf3 | 0.46684276 |
| Znrf4 | -0.0854381 |
| Zp1 | 4.13223521 |
| Zp3 | -0.4844283 |
| Zp3r | -0.3255614 |
| Zpbp | -0.3601562 |
| Zpbp2 | -0.4540447 |
| Zpbp2 | 3.31679872 |
| Zranb1 | 2.08978937 |
| Zranb2 | -0.7225978 |
| Zranb3 | 1.28919759 |
| Zrsr2 | -0.0540153 |
| Zscan10 | -0.3538845 |
| Zscan12 | -0.0422727 |
| Zscan2 | -0.5136011 |
| Zscan2 | -0.3677378 |
| Zscan2 | 0.17551587 |
| Zscan20 | 1.69215365 |
| Zscan20 | 1.00535268 |
| Zscan21 | 0.11180552 |
| Zscan21 | -0.276519 |
| Zswim1 | 0.25108986 |
| Zswim2 | -0.0297886 |
| Zswim3 | -0.7926706 |
| Zswim4 | -0.4225923 |
| Zswim5 | 2.36241353 |
| Zw10 | 0.05365302 |
| Zw10 | 0.6417951 |
| Zw10 | -0.7671106 |
| Zwilch | 1.21812972 |
| Zxda | 0.15426934 |
| Zxdc | 1.9261676 |
| Zxdc | 0.68233902 |
| Zxdc | -0.1849737 |
| Zyg11b | 0.73289649 |
| Zyx | -0.2459321 |
| Zzz3 | 1.60904437 |
| Zzz3 | -0.7857205 |
| Zzz3 | 1.69186952 |
| Zzz3 | 0.31338443 |

| **Gene Symbol** | **HET2** |
| --- | --- |
| 0610006I08Rik | -0.6430801 |
| 0610006I08Rik | -0.3072643 |
| 0610007C21Rik | -0.4591344 |
| 0610007C21Rik | -0.0848725 |
| 0610007P08Rik | 3.4678854 |
| 0610007P14Rik | -0.5018338 |
| 0610007P22Rik | -0.4631822 |
| 0610009B22Rik | 0.23003033 |
| 0610009D07Rik | -0.4195934 |
| 0610009O20Rik | -0.102619 |
| 0610010E21Rik | 1.43578212 |
| 0610010K06Rik | -0.4208848 |
| 0610010K14Rik | 0.31306174 |
| 0610012A05Rik | -0.2757668 |
| 0610012D14Rik | -0.6414418 |
| 0610012G03Rik | -0.4183038 |
| 0610012H03Rik | 0.3109998 |
| 0610025P10Rik | -0.7863604 |
| 0610031J06Rik | -0.417016 |
| 0610031J06Rik | 1.02758504 |
| 0610031J06Rik | 1.74850745 |
| 0610037D15Rik | -0.4267183 |
| 0610037L13Rik | -0.0238116 |
| 0610037L13Rik | 0.43661966 |
| 0610038F07Rik | 0.13425998 |
| 0610040J01Rik | 1.10851811 |
| 0710008K08Rik | 0.4620443 |
| 0910001A06Rik | 1.78899553 |
| 0910001A06Rik | -0.5912541 |
| 0910001L09Rik | 0.51446846 |
| 1110001A07Rik | 2.55492435 |
| 1110001J03Rik | 1.19140438 |
| 1110002B05Rik | -0.7361155 |
| 1110002N22Rik | 1.21910943 |
| 1110003E01Rik | 1.48045039 |
| 1110006G06Rik | -0.2587773 |
| 1110007A13Rik | -0.7005673 |
| 1110007A13Rik | 0.24439223 |
| 1110007C09Rik | 1.9556317 |
| 1110007L15Rik | 1.77448623 |
| 1110007L15Rik | 2.35495412 |
| 1110007L15Rik | -0.5842999 |
| 1110007L15Rik | -0.1007832 |
| 1110007M04Rik | 0.05934039 |
| 1110008F13Rik | 0.52037745 |
| 1110008J03Rik | 1.77926628 |
| 1110008J03Rik | -0.0736716 |
| 1110008J03Rik | -0.2463691 |
| 1110008P14Rik | -0.6398063 |
| 1110012D08Rik | -0.7479665 |
| 1110012J17Rik | -0.744304 |
| 1110012L19Rik | 0.14277435 |
| 1110012L19Rik | -0.1717284 |
| 1110012M11Rik | 3.08897022 |
| 1110013L07Rik | -0.5789266 |
| 1110017D15Rik | -0.7146245 |
| 1110017D15Rik | 0.72271472 |
| 1110017D15Rik | -0.7825443 |
| 1110018G07Rik | -0.5232181 |
| 1110018J18Rik | -0.2571494 |
| 1110018J18Rik | 0.12422734 |
| 1110019N10Rik | 0.46904078 |
| 1110019N10Rik | 0.27369784 |
| 1110020C03Rik | 2.29150024 |
| 1110020P15Rik | -0.4080507 |
| 1110020P15Rik | -0.7014397 |
| 1110031B06Rik | -0.3321929 |
| 1110031I02Rik | 0.7765191 |
| 1110032A13Rik | -0.4286709 |
| 1110032A13Rik | -0.6832886 |
| 1110032E23Rik | 0.33083976 |
| 1110034A24Rik | 0.95669178 |
| 1110036O03Rik | 0.58302019 |
| 1110038D17Rik | -0.698825 |
| 1110038F14Rik | 0.28589444 |
| 1110038F14Rik | -0.0322938 |
| 1110039B18Rik | -0.6962175 |
| 1110039B18Rik | -0.6045331 |
| 1110039B18Rik | -0.0989511 |
| 1110048D14Rik | 3.79210995 |
| 1110049B09Rik | 0.01261993 |
| 1110049B09Rik | 0.17811786 |
| 1110049F12Rik | -0.009151 |
| 1110049F12Rik | -0.627631 |
| 1110057K04Rik | -0.5224973 |
| 1110057K04Rik | -0.1526314 |
| 1110057K04Rik | 2.18985607 |
| 1110058L19Rik | 0.59744873 |
| 1110059E24Rik | -0.2992897 |
| 1110059G10Rik | -0.1358196 |
| 1110067D22Rik | -0.8936298 |
| 1110067I12Rik | -0.0687855 |
| 1190002A17Rik | 0.55846545 |
| 1190002H23Rik | 1.61076777 |
| 1190002N15Rik | -0.8455574 |
| 1190003J15Rik | -0.472696 |
| 1190003M12Rik | 0.19863215 |
| 1190005F20Rik | -0.69535 |
| 1190005I06Rik | 1.38131322 |
| 1190005P17Rik | 0.1195324 |
| 1190005P17Rik | -0.1937536 |
| 1190007F08Rik | 0.64502467 |
| 1190017O12Rik | 3.27969112 |
| 1190020J12Rik | -0.0839715 |
| 1200002N14Rik | -0.6284377 |
| 1200003C05Rik | -0.2034277 |
| 1200003C05Rik | 0.23548195 |
| 1200003C05Rik | -0.4906261 |
| 1200003I07Rik | -0.813519 |
| 1200009F10Rik | -0.3153077 |
| 1200009I06Rik | 3.63989696 |
| 1200009I06Rik | -0.568272 |
| 1200009O22Rik | -0.1599302 |
| 1200011O22Rik | 2.74334523 |
| 1200013B08Rik | 0.0852383 |
| 1200013P24Rik | -0.6722266 |
| 1200013P24Rik | 1.36658986 |
| 1200014J11Rik | 2.63533824 |
| 1200015F23Rik | -0.4138042 |
| 1200015F23Rik | 0.61698364 |
| 1200015N20Rik | 0.4722714 |
| 1200016B10Rik | -0.4941141 |
| 1200016B10Rik | -0.6892998 |
| 1200016D23Rik | 3.26324164 |
| 1300001I01Rik | 1.8331805 |
| 1300007L22Rik | 2.84529856 |
| 1300010M03Rik | -0.1334425 |
| 1300013J15Rik | -0.0808247 |
| 1300013J15Rik | -0.6414418 |
| 1300014I06Rik | -0.2658643 |
| 1300017J02Rik | 0.22163045 |
| 1300018I05Rik | -0.3953907 |
| 1300018J18Rik | -0.6398063 |
| 1500001L15Rik | 0.43558813 |
| 1500001M20Rik | -0.2593205 |
| 1500001M20Rik | 1.92445419 |
| 1500001M20Rik | -0.4189484 |
| 1500002O20Rik | -0.4042346 |
| 1500003O03Rik | 1.5278915 |
| 1500005A01Rik | 0.13675137 |
| 1500010J02Rik | 1.6373326 |
| 1500010J02Rik | 0.3443102 |
| 1500011H22Rik | -0.227273 |
| 1500012F01Rik | -0.350587 |
| 1500015O10Rik | -0.036136 |
| 1500019C06Rik | 0.75697857 |
| 1500019G21Rik | 1.68309231 |
| 1500031L02Rik | 2.21371835 |
| 1500032D16Rik | -0.4740631 |
| 1500032D16Rik | 0.06741526 |
| 1500032D16Rik | 0.6264947 |
| 1500032L24Rik | -0.4864577 |
| 1500041J02Rik | 2.25899233 |
| 1500041N16Rik | -0.7023129 |
| 1520401A03Rik | -0.5720633 |
| 1600002K03Rik | 0.03151019 |
| 1600012H06Rik | -0.645543 |
| 1600012H06Rik | 1.04372472 |
| 1600014C10Rik | -0.7711889 |
| 1600016N20Rik | -0.602176 |
| 1600021P15Rik | 0.59135476 |
| 1600027N09Rik | -0.2013827 |
| 1600029D21Rik | 2.24618422 |
| 1700001C02Rik | -0.5297301 |
| 1700001C19Rik | -0.2309556 |
| 1700001E04Rik | -0.6092653 |
| 1700001J03Rik | 1.44404838 |
| 1700001O22Rik | 1.91244894 |
| 1700001O22Rik | -0.5951401 |
| 1700001P01Rik | 0.38058673 |
| 1700003F12Rik | 1.12825296 |
| 1700003M02Rik | 0.49747994 |
| 1700007G11Rik | 2.35419525 |
| 1700007I06Rik | 0.0852383 |
| 1700007K09Rik | 0.30597319 |
| 1700008F21Rik | -0.3257521 |
| 1700008I05Rik | 0.18015578 |
| 1700008P20Rik | 0.94465832 |
| 1700009P17Rik | 0.24058395 |
| 1700010A17Rik | -0.6180038 |
| 1700010A17Rik | 0.70876445 |
| 1700010D01Rik | 0.20493081 |
| 1700010M22Rik | -0.2658643 |
| 1700010M22Rik | -0.164337 |
| 1700011A15Rik | -0.7352101 |
| 1700011F03Rik | 0.36902014 |
| 1700011F14Rik | 2.76116202 |
| 1700011H14Rik | 0.51066697 |
| 1700011K15Rik | -0.423473 |
| 1700012A03Rik | 0.28559146 |
| 1700012B09Rik | 0.99925042 |
| 1700012G19Rik | -0.3922524 |
| 1700012H05Rik | -0.4969139 |
| 1700012H17Rik | 0.54134522 |
| 1700012L04Rik | -0.3369052 |
| 1700013B16Rik | 0.44585255 |
| 1700013G24Rik | -0.6447213 |
| 1700013N18Rik | -0.0844219 |
| 1700013N18Rik | 0.44890998 |
| 1700015E13Rik | -0.5974795 |
| 1700016C15Rik | -0.5153202 |
| 1700016D06Rik | -0.2576917 |
| 1700016G05Rik | 1.23348268 |
| 1700016K19Rik | 0.23580164 |
| 1700016K19Rik | -0.5261067 |
| 1700017N19Rik | 2.96414373 |
| 1700018B24Rik | -0.652971 |
| 1700018C11Rik | 1.34542316 |
| 1700018F24Rik | -0.3153077 |
| 1700019D03Rik | -0.551028 |
| 1700019E19Rik | -0.610848 |
| 1700019G17Rik | -0.213203 |
| 1700019H03Rik | -0.5935838 |
| 1700019L03Rik | 0.18725364 |
| 1700019M22Rik | 2.53002634 |
| 1700019N19Rik | -0.3991707 |
| 1700020C11Rik | -0.349988 |
| 1700020D05Rik | -0.4774896 |
| 1700020L24Rik | -0.0314421 |
| 1700020N01Rik | 0.43558813 |
| 1700020O03Rik | 2.49325193 |
| 1700021C14Rik | -0.2065036 |
| 1700021F05Rik | 2.22462838 |
| 1700021K02Rik | -0.2501281 |
| 1700021K02Rik | 2.28948587 |
| 1700021K19Rik | 0.27246937 |
| 1700022C21Rik | -0.2383648 |
| 1700023B02Rik | 1.32833537 |
| 1700023B02Rik | 3.45882687 |
| 1700023E05Rik | -0.5253837 |
| 1700023E05Rik | -0.3960196 |
| 1700023I07Rik | 0.27339087 |
| 1700023M03Rik | -0.30213 |
| 1700024G13Rik | -0.4768033 |
| 1700025D03Rik | -0.7711889 |
| 1700025E21Rik | 0.76716177 |
| 1700025F22Rik | 0.28316407 |
| 1700025G04Rik | -0.4892346 |
| 1700025K23Rik | -0.4983169 |
| 1700026D08Rik | -0.2658643 |
| 1700026L06Rik | -0.4118824 |
| 1700027A23Rik | 0.86770582 |
| 1700027D21Rik | -0.7562601 |
| 1700027J05Rik | -0.6140216 |
| 1700027N10Rik | -0.7181721 |
| 1700028K03Rik | -0.7244131 |
| 1700029F12Rik | 0.68734148 |
| 1700029F12Rik | -0.6596236 |
| 1700029G01Rik | 2.35118672 |
| 1700029H14Rik | 0.12566719 |
| 1700029H14Rik | -0.8324088 |
| 1700029I01Rik | 0.67204725 |
| 1700029I08Rik | 2.54251794 |
| 1700029I15Rik | -0.6300534 |
| 1700029J07Rik | 0.34743497 |
| 1700029P11Rik | -0.128234 |
| 1700029P11Rik | 1.58131073 |
| 1700029P11Rik | -0.6471886 |
| 1700030J22Rik | 1.86850433 |
| 1700030K09Rik | 0.02672338 |
| 1700034E13Rik | 0.6146439 |
| 1700034H14Rik | -0.2998571 |
| 1700034H14Rik | -0.3547912 |
| 1700034H14Rik | 1.5994225 |
| 1700034H14Rik | 1.55401116 |
| 1700034I23Rik | 2.43732185 |
| 1700034I23Rik | 1.18576326 |
| 1700034O15Rik | 0.60091321 |
| 1700036D21Rik | 2.34083093 |
| 1700037C18Rik | 0.77077186 |
| 1700037C18Rik | 1.68003799 |
| 1700037H04Rik | 3.76442704 |
| 1700039E15Rik | -0.6389897 |
| 1700040I03Rik | -0.3779505 |
| 1700042B14Rik | 1.18726321 |
| 1700045I19Rik | -0.6164089 |
| 1700047I17Rik1 | 0.01586242 |
| 1700047I17Rik1 | 0.08636446 |
| 1700049G17Rik | -0.3170403 |
| 1700049K14Rik | -0.56299 |
| 1700049L16Rik | 1.85743999 |
| 1700052N19Rik | 0.53742057 |
| 1700052N19Rik | 0.55279354 |
| 1700054O13Rik | 0.11080226 |
| 1700055M20Rik | 1.90221805 |
| 1700057G04Rik | 1.2542066 |
| 1700057K13Rik | 0.5116188 |
| 1700058C13Rik | -0.7988715 |
| 1700060H10Rik | 2.76533521 |
| 1700061G19Rik | -0.705814 |
| 1700063I17Rik | 2.64662513 |
| 1700065I17Rik | -0.0839715 |
| 1700065O13Rik | -0.6260195 |
| 1700065O13Rik | 0.20493081 |
| 1700067C01Rik | -0.2576917 |
| 1700067K01Rik | -0.4864577 |
| 1700067P10Rik | -0.0200197 |
| 1700071K01Rik | -0.6747677 |
| 1700072E05Rik | -0.1463516 |
| 1700072E05Rik | 1.943511 |
| 1700072E05Rik | 2.71702874 |
| 1700073E17Rik | 0.28407508 |
| 1700080E11Rik | -0.6996958 |
| 1700081D17Rik | -0.2209935 |
| 1700081D17Rik | 0.85363572 |
| 1700084C01Rik | 0.73869964 |
| 1700084C01Rik | 2.11926889 |
| 1700088E04Rik | 0.27920605 |
| 1700093K21Rik | 2.18926541 |
| 1700094C09Rik | 0.64130661 |
| 1700095K08Rik | 1.82361211 |
| 1700102P08Rik | -0.1511784 |
| 1700108M19Rik | 0.35224366 |
| 1700109H08Rik | -0.703187 |
| 1700112C13Rik | -0.3269199 |
| 1700113I22Rik | -0.2697028 |
| 1700113I22Rik | 0.70471242 |
| 1700113O17Rik | 0.2625829 |
| 1700120B06Rik | -0.6053201 |
| 1700123D08Rik | -0.3095554 |
| 1700123K08Rik | -0.2034277 |
| 1700123L14Rik | 1.88785604 |
| 1700123O20Rik | -0.5465815 |
| 1700123O20Rik | -0.795004 |
| 1700127D06Rik | -0.3434236 |
| 1700129C05Rik | -0.4625064 |
| 1700129I15Rik | -0.4093262 |
| 1810007M14Rik | 0.8396849 |
| 1810007P19Rik | -0.6612942 |
| 1810007P19Rik | 3.70059247 |
| 1810008A18Rik | 0.3227214 |
| 1810008A18Rik | 0.1519114 |
| 1810009A15Rik | -0.030166 |
| 1810009A15Rik | -0.0133163 |
| 1810009N02Rik | 1.88146822 |
| 1810009O10Rik | -0.2469052 |
| 1810009O10Rik | 1.41216673 |
| 1810010M01Rik | 2.03382555 |
| 1810011O10Rik | 0.46679763 |
| 1810014F10Rik | -0.5819933 |
| 1810015A11Rik | 1.12616277 |
| 1810015C04Rik | -0.7844504 |
| 1810019J16Rik | -0.3222577 |
| 1810020D17Rik | -0.5667598 |
| 1810020G14Rik | -0.4260683 |
| 1810020G14Rik | 3.57789792 |
| 1810021J13Rik | -0.7825443 |
| 1810021J13Rik | -0.0808247 |
| 1810022C23Rik | -0.1747014 |
| 1810026J23Rik | 2.04997068 |
| 1810027O10Rik | 1.72619482 |
| 1810030J14Rik | -0.1239941 |
| 1810030N24Rik | -0.6236076 |
| 1810030N24Rik | -0.6884387 |
| 1810030N24Rik | -0.657956 |
| 1810031K17Rik | 2.75435126 |
| 1810035L17Rik | 2.93887776 |
| 1810035L17Rik | 0.25039039 |
| 1810037C20Rik | -0.5495435 |
| 1810043G02Rik | 0.5602732 |
| 1810044A24Rik | 0.21217126 |
| 1810046J19Rik | 1.51904682 |
| 1810048J11Rik | 0.10970523 |
| 1810049H13Rik | 1.67040252 |
| 1810049H19Rik | 0.99032322 |
| 1810055E12Rik | 0.08298188 |
| 1810055E12Rik | 0.92699798 |
| 1810055E12Rik | -0.6463654 |
| 1810055G02Rik | 0.61592085 |
| 1810063B05Rik | -0.3469984 |
| 1810064F22Rik | 0.87948478 |
| 1810065E05Rik | -0.189206 |
| 1810073G14Rik | -0.6832886 |
| 1810074P20Rik | 1.72794018 |
| 1810074P20Rik | -0.6807241 |
| 2010001J22Rik | -0.2813139 |
| 2010001J22Rik | -0.2404926 |
| 2010001M09Rik | 0.56207746 |
| 2010003J03Rik | 0.20691106 |
| 2010003O18Rik | 1.12628585 |
| 2010004A03Rik | -0.6471886 |
| 2010005H15Rik | -0.341048 |
| 2010005J08Rik | -0.4672477 |
| 2010005J08Rik | -0.6398063 |
| 2010005J08Rik | -0.0799276 |
| 2010007H12Rik | -0.0246564 |
| 2010007H12Rik | -0.1230544 |
| 2010011I20Rik | 0.57438817 |
| 2010012O05Rik | 0.39981948 |
| 2010100O12Rik | -0.5282791 |
| 2010100O12Rik | 0.38821889 |
| 2010106G01Rik | 2.95709574 |
| 2010106G01Rik | 0.80282317 |
| 2010107E04Rik | 0.87868273 |
| 2010107G12Rik | -0.6076852 |
| 2010109I03Rik | -0.6638057 |
| 2010110K16Rik | 0.35308969 |
| 2010111I01Rik | -0.4312808 |
| 2010200O16Rik | -0.3222577 |
| 2010301N04Rik | 0.8101057 |
| 2010305A19Rik | 1.73347783 |
| 2010305C02Rik | -0.4843805 |
| 2010305C02Rik | 1.60060768 |
| 2010308M01Rik | 0.90432476 |
| 2010311D03Rik | -0.535557 |
| 2010316F05Rik | 1.00319081 |
| 2010321M09Rik | -0.0468927 |
| 2010321M09Rik | -0.3170403 |
| 2200001I15Rik | 1.32892984 |
| 2210009G21Rik | -0.4293227 |
| 2210009G21Rik | -0.7040618 |
| 2210009G21Rik | 0.67124585 |
| 2210010B09Rik | 0.07731686 |
| 2210010L05Rik | 3.71489058 |
| 2210012G02Rik | -0.5562425 |
| 2210012G02Rik | -0.0331462 |
| 2210012G02Rik | -0.6538 |
| 2210012G02Rik | 0.22422309 |
| 2210016L21Rik | 0.24944587 |
| 2210016L21Rik | 4.4873167 |
| 2210016L21Rik | -0.5517711 |
| 2210021J22Rik | 3.03650787 |
| 2210023G05Rik | -0.3626496 |
| 2210023G05Rik | 0.79565878 |
| 2210407C18Rik | -0.5554958 |
| 2210412D01Rik | 0.41074779 |
| 2210417D09Rik | 0.98332031 |
| 2210418O10Rik | 1.68714925 |
| 2300002D11Rik | 0.77616094 |
| 2300002D11Rik | 0.02911986 |
| 2300002M23Rik | -0.6398063 |
| 2300005B03Rik | -0.7590411 |
| 2310001A20Rik | -0.5443661 |
| 2310002B06Rik | 0.05314086 |
| 2310003C23Rik | 0.7511093 |
| 2310003F16Rik | -0.187695 |
| 2310003H01Rik | 0.35646615 |
| 2310003L22Rik | 1.11651196 |
| 2310003L22Rik | -0.7721299 |
| 2310004I24Rik | -0.6414418 |
| 2310005E10Rik | -0.0137339 |
| 2310005E10Rik | -0.6204013 |
| 2310005G13Rik | 0.78134096 |
| 2310005N01Rik | 0.40596575 |
| 2310005N03Rik | 2.21827334 |
| 2310005P05Rik | 0.54318642 |
| 2310006J04Rik | -0.2168305 |
| 2310007A19Rik | -0.4365225 |
| 2310007B03Rik | -0.4733793 |
| 2310007F21Rik | -0.3269199 |
| 2310007F21Rik | -0.1599302 |
| 2310008H04Rik | -0.5458424 |
| 2310008H09Rik | -0.813519 |
| 2310008H09Rik | -0.4477599 |
| 2310008M10Rik | 2.05857426 |
| 2310009E04Rik | 0.15330919 |
| 2310010B21Rik | -0.2818704 |
| 2310010M24Rik | 2.23466224 |
| 2310011J03Rik | -0.025925 |
| 2310014L17Rik | -0.5174675 |
| 2310016C08Rik | -0.6841449 |
| 2310016C08Rik | -0.813519 |
| 2310016C16Rik | 1.25022781 |
| 2310016C16Rik | 2.20326146 |
| 2310016C16Rik | 1.76317443 |
| 2310016E02Rik | 1.46448486 |
| 2310016M24Rik | 1.73116374 |
| 2310021P13Rik | 0.53347934 |
| 2310022A10Rik | -0.5766332 |
| 2310022B05Rik | 1.86371472 |
| 2310031L18Rik | 0.81613136 |
| 2310031L18Rik | 0.84502412 |
| 2310033E01Rik | -0.6284377 |
| 2310033K02Rik | -0.5406855 |
| 2310033P09Rik | -0.3754854 |
| 2310035C23Rik | -0.3985396 |
| 2310035C23Rik | -0.2970237 |
| 2310035K24Rik | -0.6196014 |
| 2310036D22Rik | 1.61405707 |
| 2310036O22Rik | -0.5096179 |
| 2310037I24Rik | 1.13254162 |
| 2310037I24Rik | 1.59369771 |
| 2310038H17Rik | -0.1348681 |
| 2310039E09Rik | 0.52155477 |
| 2310039H08Rik | 2.64742097 |
| 2310040C09Rik | 0.4327455 |
| 2310042D19Rik | 0.12746389 |
| 2310044G17Rik | -0.5480613 |
| 2310044H10Rik | 1.46234106 |
| 2310045A20Rik | -0.5951401 |
| 2310046K01Rik | 0.21837948 |
| 2310046O06Rik | -0.3327806 |
| 2310047C04Rik | -0.5131777 |
| 2310047D13Rik | -0.2528223 |
| 2310047D13Rik | 1.09487122 |
| 2310047M10Rik | 1.65180937 |
| 2310047M10Rik | -0.0560252 |
| 2310047O13Rik | -0.2346529 |
| 2310056P07Rik | 0.90260576 |
| 2310057J16Rik | 0.91333658 |
| 2310058J06Rik | -0.6116404 |
| 2310061C15Rik | -0.6471886 |
| 2310061F22Rik | -0.7452183 |
| 2310061F22Rik | -0.5998248 |
| 2310065K24Rik | 3.41377907 |
| 2310065K24Rik | -0.1687648 |
| 2310066E14Rik | -0.7352101 |
| 2310067B10Rik | -0.4517586 |
| 2310076L09Rik | 0.93941026 |
| 2310076L09Rik | -0.8174641 |
| 2310079F23Rik | -0.5827615 |
| 2310079N02Rik | 1.32892984 |
| 2400001E08Rik | 1.89241534 |
| 2400003C14Rik | 1.95790584 |
| 2400003C14Rik | -0.4584614 |
| 2400003C14Rik | -0.5974795 |
| 2400006N03Rik | 0.43790749 |
| 2400010D15Rik | 1.76696029 |
| 2410001C21Rik | -0.3730268 |
| 2410001C21Rik | 0.89585548 |
| 2410001C21Rik | 1.63512375 |
| 2410002F23Rik | -0.5406855 |
| 2410002F23Rik | 3.55626278 |
| 2410002F23Rik | -0.5697867 |
| 2410002I01Rik | 2.74701391 |
| 2410002O22Rik | -0.0736716 |
| 2410003P15Rik | 0.08973482 |
| 2410004A20Rik | 1.48372845 |
| 2410004B18Rik | 1.20235846 |
| 2410004L22Rik | -0.4299749 |
| 2410005O16Rik | 1.02209664 |
| 2410008K03Rik | 0.38604464 |
| 2410014A08Rik | 1.17612939 |
| 2410014A08Rik | -0.4983169 |
| 2410014A08Rik | 1.04157086 |
| 2410014A08Rik | 0.59375357 |
| 2410015M20Rik | 0.68319513 |
| 2410015N17Rik | 1.88190639 |
| 2410016F19Rik | 1.48146025 |
| 2410016F19Rik | 1.32595368 |
| 2410016O06Rik | -0.3181972 |
| 2410017P07Rik | 1.43807587 |
| 2410018C17Rik | -0.7618305 |
| 2410018G20Rik | 0.20162103 |
| 2410018M08Rik | -5.23E-05 |
| 2410019A14Rik | -0.5959193 |
| 2410019G02Rik | 2.16152042 |
| 2410022L05Rik | 0.50852181 |
| 2410025L10Rik | 0.64027118 |
| 2410042D21Rik | -0.6284377 |
| 2410042D21Rik | 0.02110756 |
| 2410066E13Rik | -0.5304565 |
| 2410081M15Rik | 0.6392346 |
| 2410091C18Rik | -0.6260195 |
| 2410116G06Rik | 2.25895578 |
| 2410127E18Rik | -0.5174675 |
| 2410127L17Rik | 1.47749871 |
| 2410127L17Rik | -0.6053201 |
| 2410129H14Rik | 0.96579885 |
| 2410137M14Rik | 2.12797461 |
| 2410146L05Rik | -0.5644961 |
| 2410166I05Rik | -0.3339572 |
| 2500002L14Rik | 0.54548281 |
| 2500003M10Rik | -0.1367722 |
| 2500003M10Rik | -0.6970859 |
| 2500003M10Rik | -0.7507229 |
| 2510002A14Rik | 0.22098116 |
| 2510003E04Rik | -0.7581132 |
| 2510006D16Rik | -0.417016 |
| 2510009E07Rik | -0.739746 |
| 2510010F15Rik | -0.219951 |
| 2510012J08Rik | 1.1916339 |
| 2510039O18Rik | 0.065499 |
| 2510040D07Rik | -0.6324821 |
| 2510048L02Rik | -0.0989511 |
| 2510049I19Rik | -0.9895538 |
| 2600001B17Rik | -0.5743455 |
| 2600009E05Rik | -0.1988327 |
| 2600009E05Rik | -0.8254048 |
| 2600010E01Rik | -0.7146245 |
| 2600011E07Rik | 0.11481372 |
| 2600011E07Rik | 1.78839041 |
| 2610002J02Rik | -0.6324821 |
| 2610002M06Rik | -0.4286709 |
| 2610003J06Rik | -0.4345534 |
| 2610016F04Rik | -0.8661548 |
| 2610019A05Rik | 1.33001873 |
| 2610019P18Rik | -0.8787351 |
| 2610020C11Rik | 2.91638421 |
| 2610020H08Rik | -0.5690291 |
| 2610020H08Rik | 3.93481209 |
| 2610020H08Rik | -0.4099646 |
| 2610020O08Rik | 2.1981253 |
| 2610021K21Rik | 1.96519613 |
| 2610021K21Rik | 0.88921469 |
| 2610024G14Rik | -0.5935838 |
| 2610024G14Rik | 1.27384999 |
| 2610024G14Rik | -0.9197302 |
| 2610027C15Rik | 2.01801157 |
| 2610027C15Rik | -0.8047029 |
| 2610027L16Rik | 1.0092099 |
| 2610028A01Rik | 1.11227376 |
| 2610028H24Rik | -0.186689 |
| 2610029G23Rik | -0.8588939 |
| 2610029G23Rik | 1.34152254 |
| 2610034E13Rik | -0.5525149 |
| 2610034E13Rik | 1.58778749 |
| 2610034M16Rik | 2.24873777 |
| 2610036D13Rik | 0.71068782 |
| 2610039C10Rik | 0.29072878 |
| 2610042L04Rik | -0.6430801 |
| 2610044O15Rik | -0.4524267 |
| 2610101N10Rik | 0.7511093 |
| 2610101N10Rik | -0.9319413 |
| 2610103J23Rik | -0.3644725 |
| 2610110G12Rik | 2.15127916 |
| 2610200G18Rik | -0.3835208 |
| 2610204K14Rik | 2.66196355 |
| 2610204L23Rik | -0.8344196 |
| 2610204M08Rik | -0.8968527 |
| 2610205E22Rik | 3.24332041 |
| 2610207I05Rik | -0.2863347 |
| 2610208M17Rik | -0.1202413 |
| 2610209A20Rik | -0.3669086 |
| 2610209A20Rik | 2.03126287 |
| 2610209M04Rik | -0.6140216 |
| 2610301B20Rik | 0.47301534 |
| 2610301F02Rik | 3.4219706 |
| 2610304G08Rik | -0.4906261 |
| 2610305D13Rik | -0.820434 |
| 2610318N02Rik | -0.7424781 |
| 2610507B11Rik | -0.1202413 |
| 2610507B11Rik | -0.7892327 |
| 2610510B01Rik | -0.1667943 |
| 2610510J17Rik | -0.0464601 |
| 2610524A10Rik | -0.5146055 |
| 2610524H06Rik | 1.51199162 |
| 2610528E23Rik | -0.4850724 |
| 2610528J11Rik | 1.14263375 |
| 2610528O22Rik | 2.82106069 |
| 2700007P21Rik | -0.1502111 |
| 2700007P21Rik | -0.4878451 |
| 2700038C09Rik | 2.47475622 |
| 2700038C09Rik | 1.26051628 |
| 2700050L05Rik | -0.8872173 |
| 2700055A20Rik | 0.66480978 |
| 2700060E02Rik | 1.42735284 |
| 2700062C07Rik | -0.5290043 |
| 2700062C07Rik | 1.72392744 |
| 2700078K21Rik | -0.4754322 |
| 2700081O15Rik | 1.88436727 |
| 2700087H15Rik | 0.76426361 |
| 2700094K13Rik | -0.417016 |
| 2700097O09Rik | 1.45033083 |
| 2810002N01Rik | 0.52131942 |
| 2810002O09Rik | -0.6268249 |
| 2810003C17Rik | -0.2752139 |
| 2810004N20Rik | -0.8455574 |
| 2810004N23Rik | 0.25227656 |
| 2810008M24Rik | 0.70141926 |
| 2810021B07Rik | 1.31143352 |
| 2810021J22Rik | 0.95905052 |
| 2810021J22Rik | -0.7825443 |
| 2810022L02Rik | -0.5592354 |
| 2810022L02Rik | -0.5713038 |
| 2810022L02Rik | -0.717284 |
| 2810030E01Rik | 1.98207047 |
| 2810046L04Rik | -0.4658906 |
| 2810046M22Rik | -0.723519 |
| 2810403A07Rik | 0.70818666 |
| 2810405J04Rik | -0.666324 |
| 2810405K02Rik | -0.3210957 |
| 2810408A11Rik | -0.2183894 |
| 2810408M09Rik | 1.78012165 |
| 2810410C14Rik | -0.8829666 |
| 2810410P22Rik | 1.53028921 |
| 2810410P22Rik | -0.755335 |
| 2810417H13Rik | -0.8630361 |
| 2810422O20Rik | 2.07202522 |
| 2810428I15Rik | -0.1487619 |
| 2810432D09Rik | 0.07920903 |
| 2810432L12Rik | 0.99089257 |
| 2810439F02Rik | 0.72385209 |
| 2810439F02Rik | -0.5866122 |
| 2810441K11Rik | -0.6646444 |
| 2810452K22Rik | -0.8808484 |
| 2810453I06Rik | -0.9386699 |
| 2810453I06Rik | 0.65077904 |
| 2810470D21Rik | -0.5966991 |
| 2810485I05Rik | -0.2080453 |
| 2900006K08Rik | 1.37718395 |
| 2900010M23Rik | 0.87304898 |
| 2900011O08Rik | 2.54386569 |
| 2900011O08Rik | 2.16257487 |
| 2900024C23Rik | -0.6068962 |
| 2900024O10Rik | -0.6944833 |
| 2900026A02Rik | 0.02872087 |
| 2900046G09Rik | -0.5196198 |
| 2900062L11Rik | -0.2868942 |
| 2900064A13Rik | 0.60930437 |
| 2900073G15Rik | -0.0560252 |
| 2900092C05Rik | -0.9033321 |
| 3000004C01Rik | -0.8334137 |
| 3000004C01Rik | -0.717284 |
| 3000004C01Rik | -0.5728234 |
| 3010003L21Rik | -0.7646281 |
| 3010026O09Rik | 1.56998183 |
| 3110001A13Rik | -0.6398063 |
| 3110001D03Rik | -0.8599277 |
| 3110004L20Rik | -0.8568297 |
| 3110007F17Rik | -0.8979295 |
| 3110009E18Rik | 2.12797461 |
| 3110023B02Rik | -0.2085598 |
| 3110035E14Rik | 0.59113638 |
| 3110035E14Rik | 1.38896025 |
| 3110037I16Rik | 1.14914158 |
| 3110040N11Rik | 0.02351848 |
| 3110040N11Rik | 0.35083191 |
| 3110043J09Rik | -0.8344196 |
| 3110043J09Rik | 1.78444757 |
| 3110043J09Rik | -0.6357302 |
| 3110048E14Rik | -0.5025388 |
| 3110049J23Rik | -0.7075694 |
| 3110050N22Rik | -0.5525149 |
| 3110050N22Rik | 1.06657007 |
| 3110056O03Rik | -0.4740631 |
| 3110057O12Rik | 1.70809753 |
| 3110057O12Rik | -0.0516652 |
| 3110070M22Rik | -0.1183706 |
| 3110082I17Rik | 0.35026661 |
| 3200001I04Rik | 0.63674209 |
| 3200002M19Rik | -0.1305979 |
| 3200002M19Rik | 0.3238855 |
| 3300001G02Rik | -0.6406237 |
| 3300001P08Rik | 0.54088435 |
| 3300001P08Rik | -0.128234 |
| 3300001P08Rik | 0.22065635 |
| 3300002I08Rik | 0.14171429 |
| 3321401G04Rik | 0.14171429 |
| 3322402L07Rik | -0.6092653 |
| 3632451O06Rik | 1.15261788 |
| 3830402I07Rik | 0.52202528 |
| 3830406C13Rik | 0.73944516 |
| 3830406C13Rik | -0.8234133 |
| 3830406C13Rik | 2.77295197 |
| 3830408P04Rik | 2.31812267 |
| 3930401K13Rik | -0.532639 |
| 4121402D02Rik | 2.34580673 |
| 4121402D02Rik | -0.0529711 |
| 4430402I18Rik | 0.84385877 |
| 4432405B04Rik | 0.41339392 |
| 4432416J03Rik | 0.10750729 |
| 4631426J05Rik | 1.20145052 |
| 4631427C17Rik | -0.1492447 |
| 4632404H12Rik | 1.36220362 |
| 4632404H12Rik | 3.34247057 |
| 4632404H12Rik | -0.7730719 |
| 4632404H22Rik | -0.6496624 |
| 4632411B12Rik | -0.7969357 |
| 4632411B12Rik | 0.21740198 |
| 4632412N22Rik | 2.75448023 |
| 4632415K11Rik | 1.22289002 |
| 4632417K18Rik | 1.5945922 |
| 4632417N05Rik | 0.45802263 |
| 4632419K20Rik | 1.35201701 |
| 4632434I11Rik | 1.53004972 |
| 4732415M23Rik | 0.93064797 |
| 4732418C07Rik | -0.0404238 |
| 4732418C07Rik | 0.38604464 |
| 4732418C07Rik | -0.5181844 |
| 4732429D16Rik | 1.55385552 |
| 4732456N10Rik | -0.6996958 |
| 4732471D19Rik | -0.2708024 |
| 4732473B16Rik | -0.617206 |
| 4732479N06Rik | -0.8324088 |
| 4732479N06Rik | -0.1628658 |
| 4732496O08Rik | 1.18576326 |
| 4733401H18Rik | 0.15051151 |
| 4831426I19Rik | 0.54617063 |
| 4833418A01Rik | -0.0200197 |
| 4833420G17Rik | 0.2659932 |
| 4833421E05Rik | -0.5253837 |
| 4833424O15Rik | 1.30860341 |
| 4833424O15Rik | -0.645543 |
| 4833426J09Rik | -0.3422351 |
| 4833426J09Rik | -0.2474416 |
| 4833427G06Rik | 1.02415857 |
| 4833436C18Rik | -0.2680558 |
| 4833439L19Rik | -0.3292598 |
| 4833439L19Rik | 1.0266269 |
| 4833439L19Rik | 1.5157286 |
| 4833442J19Rik | 0.0465084 |
| 4833446K15Rik | -0.7361155 |
| 4921501E09Rik | -0.1377257 |
| 4921504E06Rik | -0.7119726 |
| 4921504I05Rik | 2.335256 |
| 4921505C17Rik | -0.5562425 |
| 4921505C17Rik | -0.6571233 |
| 4921507P07Rik | 1.81087695 |
| 4921511H03Rik | 0.93519046 |
| 4921511H03Rik | 0.02512231 |
| 4921511K06Rik | 0.40329843 |
| 4921511K06Rik | 0.08335833 |
| 4921515J06Rik | 0.09903354 |
| 4921517D21Rik | -0.6236076 |
| 4921517D22Rik | 1.94027605 |
| 4921520G13Rik | 1.95679473 |
| 4921521F21Rik | 2.05911785 |
| 4921521F21Rik | 0.35477945 |
| 4921523A10Rik | 0.5685888 |
| 4921523A10Rik | -0.5622379 |
| 4921523A10Rik | -0.4955129 |
| 4921525H12Rik | -0.3004248 |
| 4921528G01Rik | -0.6713811 |
| 4921528I01Rik | -0.5904789 |
| 4921530G04Rik | -0.3374959 |
| 4921530L21Rik | -0.5525149 |
| 4921530L21Rik | 1.48305696 |
| 4921536K21Rik | -0.4941141 |
| 4921537P18Rik | 0.74056234 |
| 4922502D21Rik | 0.0089586 |
| 4922503N01Rik | -0.851689 |
| 4922503N01Rik | 0.7779504 |
| 4922505E12Rik | -0.3072643 |
| 4922505G16Rik | 3.88646167 |
| 4922505G16Rik | -0.5728234 |
| 4930401F20Rik | -0.6747677 |
| 4930402H24Rik | 2.28587031 |
| 4930403C10Rik | 0.30121739 |
| 4930403L05Rik | 0.29042737 |
| 4930403O06Rik | 0.41708595 |
| 4930404H21Rik | 1.20947782 |
| 4930404N11Rik | -0.5751074 |
| 4930408O21Rik | -0.7664979 |
| 4930408O21Rik | -0.5232181 |
| 4930412F15Rik | 0.3177577 |
| 4930415L06Rik | -0.7863604 |
| 4930418G15Rik | 0.03349747 |
| 4930427A07Rik | -0.1067627 |
| 4930428D18Rik | 0.15957348 |
| 4930428E23Rik | 1.87232947 |
| 4930430D24Rik | -0.622805 |
| 4930430E16Rik | -0.703187 |
| 4930430E16Rik | -0.4788637 |
| 4930431L04Rik | -0.3991707 |
| 4930432H15Rik | -0.2840999 |
| 4930432K21Rik | -0.5637428 |
| 4930432O21Rik | 0.52928805 |
| 4930432O21Rik | 0.25509869 |
| 4930438O05Rik | -0.1584659 |
| 4930438O05Rik | -0.7749587 |
| 4930449I24Rik | 0.57461051 |
| 4930451C15Rik | -0.3044084 |
| 4930451I11Rik | 0.10420063 |
| 4930452B06Rik | 0.23099469 |
| 4930453N24Rik | 0.89617053 |
| 4930455C21Rik | -0.6504884 |
| 4930455F23Rik | -0.1372488 |
| 4930467M19Rik | 3.87255318 |
| 4930470P17Rik | -0.6124335 |
| 4930473A06Rik | 1.89549651 |
| 4930473A06Rik | -0.5812257 |
| 4930473A06Rik | -0.5720633 |
| 4930474N05Rik | 1.2349079 |
| 4930478A21Rik | 2.48751846 |
| 4930481F22Rik | 0.12025624 |
| 4930481F22Rik | 0.48899011 |
| 4930481M05 | -0.4099646 |
| 4930486L24Rik | 1.08373086 |
| 4930502E18Rik | 1.0079533 |
| 4930503B20Rik | 2.57748786 |
| 4930503L19Rik | -0.5599851 |
| 4930504E06Rik | -0.2533621 |
| 4930504E06Rik | 3.5206814 |
| 4930504O13Rik | -0.2987227 |
| 4930506M07Rik | 0.35506077 |
| 4930507C10Rik | -0.7844504 |
| 4930511I11Rik | 1.77116256 |
| 4930511J11Rik | -0.535557 |
| 4930515G01Rik | 1.34921063 |
| 4930518C23Rik | -0.0318678 |
| 4930519F16Rik | 3.68864195 |
| 4930519G04Rik | -0.4299749 |
| 4930519N13Rik | 0.80647154 |
| 4930519N16Rik | -0.164828 |
| 4930522H14Rik | -0.5399512 |
| 4930524B15Rik | 2.40687803 |
| 4930524B15Rik | 0.57816067 |
| 4930524E20Rik | -0.3620427 |
| 4930526D03Rik | -0.6646444 |
| 4930535B03Rik | 1.95365629 |
| 4930535E21Rik | -0.5562425 |
| 4930538K18Rik | 0.26165065 |
| 4930539E08Rik | -0.5866122 |
| 4930540L03Rik | -0.5362879 |
| 4930544G11Rik | 0.20987357 |
| 4930544G21Rik | 2.04382236 |
| 4930547C10Rik | 2.50120878 |
| 4930547N16Rik | -0.5319109 |
| 4930549C01Rik | -0.2469052 |
| 4930550C14Rik | -0.3748701 |
| 4930550L24Rik | 1.66198439 |
| 4930555G01Rik | 0.92593071 |
| 4930555I21Rik | 0.78365373 |
| 4930562D19Rik | -0.3578058 |
| 4930562D19Rik | -0.0327199 |
| 4930563D23Rik | -0.703187 |
| 4930563P21Rik | 1.01851178 |
| 4930570C03Rik | -0.6053201 |
| 4930572J05Rik | 2.36954881 |
| 4930572J05Rik | 0.37178712 |
| 4930572J05Rik | 0.22292767 |
| 4930573I19Rik | -0.2891357 |
| 4930578I06Rik | 2.61753806 |
| 4930579C15Rik | 2.17325472 |
| 4930579E17Rik | 0.57793918 |
| 4930579E17Rik | -0.6936174 |
| 4930579J09Rik | -0.2774275 |
| 4930583H14Rik | 0.26846561 |
| 4930590J08Rik | 0.10346423 |
| 4930595M18Rik | 0.88108682 |
| 4930596D02Rik | -0.6807241 |
| 4930599N23Rik | 0.75276377 |
| 4931406B18Rik | -0.6100563 |
| 4931406C07Rik | -0.5174675 |
| 4931406H21Rik | -0.4611561 |
| 4931406I20Rik | 1.38691476 |
| 4931406I20Rik | 0.22648576 |
| 4931406P16Rik | 2.40287643 |
| 4931407G18Rik | -0.3779505 |
| 4931408A02Rik | -0.851689 |
| 4931408C20Rik | 2.33683783 |
| 4931408C20Rik | 0.03111223 |
| 4931409K22Rik | 1.55587691 |
| 4931417E11Rik | -0.7361155 |
| 4931417G12Rik | -0.719951 |
| 4931428L18Rik | 1.54909577 |
| 4931432M23Rik | 0.03508425 |
| 4931433A01Rik | -0.5525149 |
| 4931440B09Rik | 2.22618201 |
| 4931440F15Rik | -0.6398063 |
| 4931440F15Rik | -0.4920198 |
| 4931440L10Rik | 0.72062592 |
| 4932409I22Rik | 2.4096503 |
| 4932411E22Rik | -0.633293 |
| 4932411G14Rik | -0.1732137 |
| 4932412H11Rik | 2.41380882 |
| 4932413O14Rik | 2.23462472 |
| 4932414J04Rik | 0.29253522 |
| 4932414N04Rik | 0.44432006 |
| 4932418E24Rik | 0.79548335 |
| 4932431H17Rik | 0.70741572 |
| 4932431H17Rik | -0.5124647 |
| 4932441K18Rik | 3.13548694 |
| 4932441K18Rik | 0.27031563 |
| 4932442K08Rik | 0.62733545 |
| 4932702K14Rik | 0.4620443 |
| 4933400A11Rik | -0.3608302 |
| 4933400C05Rik | -0.2236048 |
| 4933400E14Rik | 1.479608 |
| 4933402J07Rik | -0.0116479 |
| 4933402N03Rik | -0.3363148 |
| 4933402P03Rik | 0.66258708 |
| 4933403G14Rik | -0.719951 |
| 4933404M02Rik | -0.6349171 |
| 4933405O20Rik | -0.0095667 |
| 4933406M09Rik | 0.81148643 |
| 4933407N01Rik | 1.22775992 |
| 4933407N01Rik | -0.5217771 |
| 4933407N01Rik | 0.49747994 |
| 4933407P14Rik | -0.1912244 |
| 4933407P14Rik | 2.58204958 |
| 4933407P14Rik | 1.15929431 |
| 4933409N07Rik | 1.67232388 |
| 4933411G06Rik | -0.2189096 |
| 4933411K20Rik | -0.049927 |
| 4933412E14Rik | -0.350587 |
| 4933413G11Rik | -0.0271952 |
| 4933415F23Rik | 3.54873325 |
| 4933416C03Rik | -0.6927523 |
| 4933417A18Rik | -0.6604585 |
| 4933417E01Rik | -0.5046569 |
| 4933421E11Rik | -0.5690291 |
| 4933421E11Rik | 0.84285875 |
| 4933421E11Rik | 1.30077604 |
| 4933422H20Rik | -0.7759035 |
| 4933424B01Rik | -0.3245859 |
| 4933424B01Rik | -0.3730268 |
| 4933424B01Rik | -0.6554601 |
| 4933425O20Rik | 1.49407512 |
| 4933426M11Rik | -0.3903744 |
| 4933427D14Rik | 0.16095984 |
| 4933428G09Rik | 3.82673006 |
| 4933428G20Rik | -0.6140216 |
| 4933429E10Rik | 2.26946077 |
| 4933429E10Rik | -0.3541894 |
| 4933430I17Rik | 0.66480978 |
| 4933434E20Rik | -0.5858408 |
| 4933434E20Rik | -0.7005673 |
| 4933434E20Rik | -0.4591344 |
| 4933434I06Rik | 0.12494754 |
| 4933436I01Rik | 0.33974632 |
| 4933437F05Rik | 1.42395969 |
| 4933439C20Rik | 0.51066697 |
| 4933440H19Rik | 0.96944605 |
| 5031414D18Rik | -0.6244109 |
| 5031414D18Rik | -0.2790911 |
| 5033411D12Rik | -0.6268249 |
| 5033414D02Rik | -0.4215312 |
| 5033414K04Rik | -0.1254052 |
| 5133400G04Rik | 1.56990533 |
| 5133400G04Rik | -0.5103288 |
| 5133400G04Rik | 2.25899233 |
| 5133401N09Rik | -0.461831 |
| 5230400G24Rik | -0.617206 |
| 5230400G24Rik | 1.55719622 |
| 5330401P04Rik | 1.01823545 |
| 5330417C22Rik | 0.89664291 |
| 5330431N19Rik | -0.6554601 |
| 5330431N19Rik | 0.13532853 |
| 5330431N19Rik | -0.6084749 |
| 5330437I02Rik | 2.40218693 |
| 5330439C02Rik | 0.53068724 |
| 5330439J01Rik | -0.5858408 |
| 5330439J01Rik | 0.03310035 |
| 5430407P10Rik | -0.219951 |
| 5430432M24Rik | -0.4665689 |
| 5430432M24Rik | -0.6696923 |
| 5430432M24Rik | 0.89522505 |
| 5430435G22Rik | 3.32223601 |
| 5430437P03Rik | 1.22820139 |
| 5530601I19Rik | -0.5268303 |
| 5630401D24Rik | -0.1973061 |
| 5630401D24Rik | -0.6447213 |
| 5630401D24Rik | 1.85699017 |
| 5730403M16Rik | -0.1628658 |
| 5730409E04Rik | 1.28460998 |
| 5730410E15Rik | 0.45448939 |
| 5730410E15Rik | 0.63653408 |
| 5730410I19Rik | -0.7433906 |
| 5730410I19Rik | 0.69616496 |
| 5730410I19Rik | -0.472696 |
| 5730419I09Rik | -0.3164624 |
| 5730437N04Rik | -0.6705363 |
| 5730446C15Rik | -0.4625064 |
| 5730449L18Rik | 1.83289199 |
| 5730449L18Rik | -0.5275544 |
| 5730449L18Rik | -0.4106034 |
| 5730453I16Rik | 0.74000392 |
| 5730453I16Rik | -0.1072243 |
| 5730469M10Rik | 1.54651066 |
| 5730472N09Rik | 0.45246437 |
| 5730494M16Rik | -0.6850021 |
| 5730494N06Rik | -0.1067627 |
| 5730502D15Rik | 2.41739382 |
| 5730507A09Rik | 2.25441193 |
| 5730507A09Rik | -0.7163966 |
| 5730528L13Rik | 1.21004053 |
| 5730589K01Rik | 0.7779504 |
| 5730593F17Rik | 0.29523834 |
| 5730596K20Rik | -0.6164089 |
| 5730596K20Rik | 0.07199829 |
| 5730596K20Rik | -0.3298457 |
| 5730596K20Rik | -0.350587 |
| 5830403L16Rik | 1.20552933 |
| 5830404H04Rik | 1.04962234 |
| 5830405N20Rik | -0.5827615 |
| 5830415F09Rik | -0.6284377 |
| 5830415L20Rik | -0.58507 |
| 5830417C01Rik | -0.30213 |
| 5830426I05Rik | -0.3724132 |
| 5830427D03Rik | -0.423473 |
| 5830434P21Rik | -0.3547912 |
| 5830435K17Rik | -0.820434 |
| 5830457O10Rik | -0.5362879 |
| 5830457O10Rik | -0.7711889 |
| 5830457O10Rik | -0.5189018 |
| 5830467P10Rik | -0.3205153 |
| 5830468K18Rik | 3.53786231 |
| 5830472M02Rik | -0.2388963 |
| 5830472M02Rik | 0.59244589 |
| 5830472M02Rik | 0.39309252 |
| 5930416I19Rik | -0.6832886 |
| 5930431H10 | -0.7516435 |
| 5930434B04Rik | -0.85477 |
| 5930434B04Rik | 0.22260353 |
| 5930434B04Rik | -0.182676 |
| 6030405A18Rik | -0.4163728 |
| 6030408B16Rik | -0.3141544 |
| 6030408B16Rik | 0.09346544 |
| 6030419C18Rik | -0.6722266 |
| 6030429G01Rik | 0.6392346 |
| 6030443O07Rik | 0.7181513 |
| 6030446N20Rik | 0.22551671 |
| 6030458C11Rik | 0.55642753 |
| 6230409E13Rik | 2.21272017 |
| 6230409E13Rik | 0.44380868 |
| 6230416J20Rik | -0.7636946 |
| 6230427J02Rik | 0.92103613 |
| 6330403K07Rik | 0.58081446 |
| 6330406I15Rik | 0.17437007 |
| 6330406I15Rik | 0.67803589 |
| 6330407J23Rik | 0.05818111 |
| 6330408A02Rik | -0.4990192 |
| 6330409N04Rik | -0.5735841 |
| 6330416G13Rik | -0.376101 |
| 6330439K17Rik | 1.77497762 |
| 6330442E10Rik | 0.32009685 |
| 6330442E10Rik | -0.871376 |
| 6330500D04Rik | 2.95579753 |
| 6330503C03Rik | -0.752565 |
| 6330503K22Rik | -0.3773336 |
| 6330503K22Rik | -0.7023129 |
| 6330503K22Rik | -0.6722266 |
| 6330505N24Rik | 3.26972266 |
| 6330512M04Rik | -0.310129 |
| 6330534C20Rik | -0.3015612 |
| 6330548G22Rik | -0.5392174 |
| 6330569M22Rik | -0.5827615 |
| 6330577E15Rik | -0.8066549 |
| 6330578E17Rik | 0.60307199 |
| 6332401O19Rik | -0.726204 |
| 6430411K18Rik | 2.38857508 |
| 6430502M16Rik | 0.67922902 |
| 6430510M02Rik | 1.53697019 |
| 6430517E21Rik | -0.6521427 |
| 6430526N21Rik | -0.5660047 |
| 6430527G18Rik | 1.16438862 |
| 6430527G18Rik | 0.37068131 |
| 6430531B16Rik | -0.7279982 |
| 6430537H07Rik | -0.5873843 |
| 6430537H07Rik | 0.76208406 |
| 6430548M08Rik | 4.4264398 |
| 6430550H21Rik | 1.7034681 |
| 6430571L13Rik | -0.0074901 |
| 6430573F11Rik | 0.60004829 |
| 6430598A04Rik | 0.30863771 |
| 6430604K15Rik | 2.24235935 |
| 6430706D22Rik | -0.3693511 |
| 6530404N21Rik | 0.27123927 |
| 6530406M24Rik | -0.6739199 |
| 6530418L21Rik | 2.80871161 |
| 6530418L21Rik | 1.65837312 |
| 6530418L21Rik | 3.35656639 |
| 6530421E24Rik | -0.5667598 |
| 6620401K05Rik | -0.5554958 |
| 6720456B07Rik | -0.6646444 |
| 6720456B07Rik | -0.6910244 |
| 6720456H20Rik | -0.7721299 |
| 6720456H20Rik | 0.26475453 |
| 6720458F09Rik | 0.23067335 |
| 6720463M24Rik | -0.0037637 |
| 6720467C03Rik | 2.05771292 |
| 6720467C03Rik | 0.25947177 |
| 6820408C15Rik | 0.06703232 |
| 7420416P09Rik | -0.5554958 |
| 7530404M11Rik | -0.5428922 |
| 8030411F24Rik | -0.0604056 |
| 8030451F13Rik | -0.3773336 |
| 8030451F13Rik | 1.66571982 |
| 8030451F13Rik | 1.37322525 |
| 8430408G22Rik | 0.22487013 |
| 8430410A17Rik | -0.2116527 |
| 8430410K20Rik | 0.07352098 |
| 8430415E04Rik | -0.5781615 |
| 8430415E04Rik | -0.4850724 |
| 8430419L09Rik | -0.7014397 |
| 8430427H17Rik | 1.39118661 |
| 8430432M10Rik | 0.05236301 |
| 9030025P20Rik | 0.54318642 |
| 9030025P20Rik | -0.264224 |
| 9030025P20Rik | -0.2746613 |
| 9030205A07Rik | -0.6341047 |
| 9030221M09Rik | 0.8348197 |
| 9030224M15Rik | 0.90619643 |
| 9030227G01Rik | -0.5384841 |
| 9030409G11Rik | -0.0714475 |
| 9030418K01Rik | 0.69284144 |
| 9030425E11Rik | -0.0701155 |
| 9030607L17Rik | 3.19644394 |
| 9030607L17Rik | -0.260408 |
| 9030611O19Rik | 0.22260353 |
| 9030612M13Rik | -0.6013916 |
| 9030617O03Rik | 0.68517188 |
| 9030624J02Rik | 0.15365831 |
| 9030625A04Rik | 0.82942159 |
| 9130005N14Rik | 0.04376363 |
| 9130011E15Rik | 0.67962639 |
| 9130011J15Rik | -0.1756945 |
| 9130014G24Rik | 0.33083976 |
| 9130017N09Rik | -0.0112313 |
| 9130206N08Rik | 1.39875347 |
| 9130211I03Rik | 3.81159017 |
| 9130213B05Rik | -0.6875784 |
| 9130213B05Rik | 1.04157086 |
| 9130218O11Rik | 0.41365812 |
| 9130221H12Rik | 3.22797237 |
| 9130227C08Rik | -0.7343056 |
| 9130401M01Rik | -0.0767943 |
| 9130404D08Rik | -0.6268249 |
| 9130404D08Rik | 1.46465615 |
| 9130404D14Rik | -0.41573 |
| 9130422G05Rik | -0.3428292 |
| 9230104L09Rik | -0.6471886 |
| 9330129D05Rik | 0.94779304 |
| 9330134C04Rik | -0.2779817 |
| 9330176C04Rik | -0.7815927 |
| 9330186A19Rik | 0.623757 |
| 9430015G10Rik | 0.63653408 |
| 9430015G10Rik | 3.81615454 |
| 9430023L20Rik | 0.98375057 |
| 9430025C20Rik | -0.455104 |
| 9430028L06Rik | 0.09160199 |
| 9430029K10Rik | 0.63861209 |
| 9430031J16Rik | 0.44941858 |
| 9430034D17Rik | 0.29643722 |
| 9430057O19Rik | 0.71796067 |
| 9430069J07Rik | 2.37246644 |
| 9430077A04Rik | 0.04061682 |
| 9430079M16Rik | -0.0812736 |
| 9530015I07Rik | 0.90057012 |
| 9530027K23Rik | 3.99387578 |
| 9530058B02Rik | 0.89206653 |
| 9530058B02Rik | 0.30567666 |
| 9530068E07Rik | -0.5465815 |
| 9530077C05Rik | 0.44636282 |
| 9530096D07Rik | 1.26805314 |
| 9530096D07Rik | -0.7618305 |
| 9630015D15Rik | 0.445342 |
| 9630020C08Rik | 0.77759278 |
| 9630028B13Rik | -0.3541894 |
| 9630031F12Rik | -0.4997221 |
| 9630050M13Rik | -0.4878451 |
| 9630058J23Rik | -0.0468927 |
| 9830163H01Rik | -0.4055049 |
| 9830169C18Rik | -0.5502854 |
| 9930013L23Rik | -0.5089076 |
| 9930021D14Rik | -0.5920301 |
| 9930023K05Rik | -0.5607354 |
| 9930023K05Rik | 0.95017435 |
| 9930104L06Rik | -0.1492447 |
| a | 1.58500649 |
| A030003K21Rik | -0.4286709 |
| A030007L17Rik | 0.3177577 |
| A030007L17Rik | -0.6671649 |
| A030007L17Rik | 0.34060375 |
| A030007L17Rik | 1.62911205 |
| A030009H04Rik | 1.84828872 |
| A030010B05Rik | 0.62227953 |
| A030012M09Rik | 2.99316398 |
| A130010J15Rik | 0.35280777 |
| A130038H09Rik | 1.70697535 |
| A130042E20Rik | -0.7217333 |
| A130090K04Rik | 0.72081601 |
| A130092J06Rik | 0.29012586 |
| A230050P20Rik | -0.4871512 |
| A230051G13Rik | -0.5131777 |
| A230054D04Rik | 1.92946317 |
| A230067G21Rik | -0.5897043 |
| A230069A22Rik | 1.0758397 |
| A230074B11Rik | -0.0661311 |
| A230074B11Rik | -0.1618862 |
| A230074B11Rik | -0.391 |
| A230106M15Rik | -0.1516625 |
| A230106N23Rik | 2.049559 |
| A230107C01Rik | 1.26720696 |
| A2bp1 | -0.6790184 |
| A2bp1 | 1.1329083 |
| A330021E22Rik | -0.7093282 |
| A330021E22Rik | 1.83069636 |
| A330049M08Rik | 1.23818859 |
| A330080J22Rik | 0.79249597 |
| A3galt2 | 2.09731634 |
| A430005L14Rik | -0.8047029 |
| A430005L14Rik | 1.65152903 |
| A430005L14Rik | 2.04896403 |
| A430005L14Rik | -0.5982606 |
| A430033K04Rik | -0.5110402 |
| A430079E08 | 0.26165065 |
| A430083B19Rik | -0.2388963 |
| A430083B19Rik | 1.30250982 |
| A430107D22Rik | 0.09160199 |
| A430107P09Rik | 1.23172557 |
| A430110N23Rik | -0.2829845 |
| A4galt | -0.0378488 |
| A4gnt | 0.81148643 |
| A530050D06Rik | -0.5414205 |
| A530082C11Rik | 0.95551017 |
| A530082C11Rik | 0.66380012 |
| A530088H08Rik | -0.5592354 |
| A530088H08Rik | 1.52049988 |
| A530098C11Rik | 1.74165952 |
| A630042L21Rik | -0.5943616 |
| A630054L15Rik | -0.3095554 |
| A630077B13Rik | -0.5174675 |
| A630095E13Rik | 1.22996514 |
| A730008H23Rik | 0.0223138 |
| A730008L03Rik | 1.78371784 |
| A730008L03Rik | -0.2735571 |
| A730017C20Rik | -0.0408537 |
| A730024F05Rik | -0.5584863 |
| A730037C10Rik | -0.021282 |
| A730042J05Rik | 0.48459364 |
| A730098D12Rik | 1.06064691 |
| A830006F12Rik | -0.067015 |
| A830007P12Rik | -0.5904789 |
| A830053O21Rik | -0.5889303 |
| A830059I20Rik | -0.6100563 |
| A830073O21Rik | -0.4983169 |
| A830080D01Rik | 2.41581935 |
| A830093I24Rik | -0.4906261 |
| A930001N09Rik | -0.4067769 |
| A930005I04Rik | -0.3872529 |
| A930008G19Rik | -0.0826216 |
| A930010I20Rik | 0.73645948 |
| A930016P21Rik | 1.32972188 |
| A930018P22Rik | 1.27353473 |
| A930024E05Rik | 1.28034173 |
| A930025D01Rik | -0.3286742 |
| A930025D01Rik | 2.27759412 |
| A930025D01Rik | -0.6875784 |
| A930034L06Rik | -0.4048696 |
| A930038C07Rik | 0.43067268 |
| A930041I02Rik | -0.6268249 |
| AA388235 | -0.3798035 |
| AA407270 | 0.91549887 |
| AA407659 | 0.72593368 |
| AA407659 | 0.78098464 |
| AA408296 | 0.22227928 |
| AA408296 | -0.7796925 |
| AA408296 | -0.7324991 |
| AA409316 | -0.698825 |
| AA415398 | -0.6713811 |
| AA467197 | 1.09804802 |
| AA536717 | -0.6471886 |
| AA536717 | -0.5751074 |
| AA536749 | -0.4591344 |
| AA536749 | -0.3292598 |
| AA536749 | -0.5311834 |
| AA536749 | 0.61187116 |
| AA673488 | -0.4208848 |
| AA691260 | 0.64399336 |
| AA792892 | -0.627631 |
| AA881470 | -0.6892998 |
| AA881470 | 1.17273975 |
| AA960436 | 2.48840503 |
| AA960436 | -0.3985396 |
| Aaas | 1.43904461 |
| Aaas | -0.4692871 |
| Aacs | 0.57572138 |
| Aadacl1 | -0.6406237 |
| Aak1 | -0.0590893 |
| Aak1 | 0.63090023 |
| Aamp | -0.311852 |
| Aard | -0.0893908 |
| Aars | -0.703187 |
| Aars2 | -0.545104 |
| Aarsd1 | 0.25666286 |
| Aasdh | -0.5392174 |
| Aatf | -0.719951 |
| Aatf | 1.01352529 |
| Aatk | -0.4437783 |
| AB112350 | 2.01786958 |
| AB182283 | -0.1917297 |
| AB182283 | 1.19060059 |
| Abat | 3.01637087 |
| Abat | -0.4378375 |
| Abat | -0.4802398 |
| Abca14 | -0.3210957 |
| Abca14 | 1.44579775 |
| Abca15 | -0.8017826 |
| Abca15 | -0.6562913 |
| Abca15 | -0.2840999 |
| Abca15 | 1.77804296 |
| Abca17 | -0.1922353 |
| Abca2 | 0.14242113 |
| Abca3 | -0.8008112 |
| Abca4 | -0.8445393 |
| Abca4 | -0.2320105 |
| Abca5 | 0.86949027 |
| Abca6 | -0.4997221 |
| Abca6 | 0.14665095 |
| Abca7 | -0.6430801 |
| Abca7 | 0.84369218 |
| Abca8a | 0.82484252 |
| Abca8b | -0.5414205 |
| Abca9 | -0.3572021 |
| Abca9 | -0.5943616 |
| Abca9 | 1.25173529 |
| Abcb11 | -0.7768493 |
| Abcb1b | 1.19186337 |
| Abcb4 | 1.18368242 |
| Abcb6 | -0.8115526 |
| Abcb8 | 0.80264908 |
| Abcb8 | -0.7306962 |
| Abcb8 | -0.6341047 |
| Abcb9 | -0.7244131 |
| Abcb9 | 0.15295994 |
| Abcc1 | -0.4927174 |
| Abcc10 | -0.5789266 |
| Abcc10 | 1.39220525 |
| Abcc12 | -0.3891246 |
| Abcc3 | 0.62817545 |
| Abcc5 | -0.7740148 |
| Abcc6 | 2.34313846 |
| Abcc8 | -0.5743455 |
| Abcc9 | -0.2230819 |
| Abcc9 | 0.00487354 |
| Abcd1 | -0.5532592 |
| Abcd2 | -0.5046569 |
| Abcd2 | -0.3310185 |
| Abcd3 | 0.74742218 |
| Abcd4 | -0.2506663 |
| Abcd4 | 0.49554621 |
| Abce1 | -0.5189018 |
| Abcf1 | -0.8037285 |
| Abcf1 | 0.19596731 |
| Abcf1 | 3.08212721 |
| Abcf2 | 1.90601443 |
| Abcf2 | -0.5131777 |
| Abcf2 | 0.01989976 |
| Abcf2 | -0.3422351 |
| Abcf3 | -0.7516435 |
| Abcg1 | 1.16757321 |
| Abcg4 | 2.34410659 |
| Abcg5 | -0.4774896 |
| Abhd1 | 2.01331452 |
| Abhd1 | 4.46221423 |
| Abhd10 | -0.4598078 |
| Abhd11 | 0.81269289 |
| Abhd12 | -0.4774896 |
| Abhd14a | 0.90930756 |
| Abhd14b | 0.25603751 |
| Abhd2 | 3.58219195 |
| Abhd2 | 1.6263797 |
| Abhd2 | 1.23960665 |
| Abhd3 | 0.27829027 |
| Abhd4 | 0.82518248 |
| Abhd5 | -0.5138913 |
| Abhd6 | -0.4228253 |
| Abhd7 | 0.38140745 |
| Abhd7 | 0.19529991 |
| Abhd8 | -0.2168305 |
| Abi1 | -0.4802398 |
| Abi1 | -0.58507 |
| Abi2 | 0.23676007 |
| Abi2 | -0.0857745 |
| Abi2 | -0.7777961 |
| Abi3 | 0.93760411 |
| Abi3bp | 3.46299004 |
| Abi3bp | 0.15714236 |
| Abl1 | 1.34230397 |
| Ablim1 | 0.76008166 |
| Ablim2 | 0.3041926 |
| Ablim2 | -0.4484252 |
| Ablim3 | -0.7646281 |
| Ablim3 | 0.91441835 |
| Abp1 | 0.30240861 |
| Abr | -0.6292452 |
| Abr | -0.7014397 |
| Abtb1 | 0.53973118 |
| Acaa1a | 1.92701422 |
| Acaa1b | -0.6236076 |
| Acaa2 | 0.95787189 |
| Acaa2 | -0.6076852 |
| Acaa2 | 1.24807002 |
| Acaa2 | 0.89475196 |
| Acad10 | 0.5187267 |
| Acad10 | -0.0255019 |
| Acad8 | -0.5131777 |
| Acad8 | 0.35224366 |
| Acad9 | -0.1672866 |
| Acad9 | 0.63424291 |
| Acadl | 1.04251379 |
| Acadl | 1.54108218 |
| Acadm | -0.5660047 |
| Acads | -0.6521427 |
| Acads | 0.26846561 |
| Acadsb | 2.87513784 |
| Acadvl | -0.4228253 |
| Acan | 3.91413695 |
| Acat2 | -0.6521427 |
| Acbd3 | -0.636544 |
| Acbd4 | 1.53942375 |
| Acbd4 | 0.50397687 |
| Acbd5 | -0.6790184 |
| Acbd6 | 1.79135173 |
| Accn1 | 1.81582733 |
| Accn1 | -0.535557 |
| Accn2 | 1.03846594 |
| Accn3 | 0.93292198 |
| Accn3 | 0.78826215 |
| Accs | -0.6764657 |
| Acd | -0.1137101 |
| Ace | 1.12690102 |
| Ace | 2.00018995 |
| Ache | -0.5319109 |
| Acin1 | -0.6422606 |
| Acly | 4.60436284 |
| Acn9 | -0.7590411 |
| Aco1 | -0.4208848 |
| Aco2 | -0.5089076 |
| Acot1 | -0.6341047 |
| Acot1 | -0.602961 |
| Acot10 | -0.8850895 |
| Acot11 | -0.3363148 |
| Acot11 | -0.2593205 |
| Acot2 | -0.5392174 |
| Acot2 | -0.1856842 |
| Acot3 | 0.7804499 |
| Acot5 | -0.4941141 |
| Acot7 | -0.4983169 |
| Acot7 | -0.1536013 |
| Acot7 | -0.6927523 |
| Acot7 | -0.6970859 |
| Acot8 | -0.0310165 |
| Acot8 | -0.4920198 |
| Acot8 | -0.6790184 |
| Acot9 | 2.75071138 |
| Acox2 | -0.3675186 |
| Acox3 | 0.62438949 |
| Acox3 | -0.4672477 |
| Acoxl | -0.8435224 |
| Acp2 | -0.3158849 |
| Acp6 | -0.58507 |
| Acpl2 | -0.6252148 |
| Acpp | 2.18709645 |
| Acr | 1.14986188 |
| Acr | -0.7226257 |
| Acrbp | 0.05197384 |
| Acrbp | -0.6439004 |
| Acrv1 | -0.4699679 |
| Acsbg1 | -0.6705363 |
| Acsbg1 | -0.1099984 |
| Acsbg2 | -0.5781615 |
| Acsf3 | -0.698825 |
| Acsl1 | -0.5131777 |
| Acsl3 | 1.91319057 |
| Acsl3 | 0.24629055 |
| Acsl4 | -0.4843805 |
| Acsl4 | 0.51470554 |
| Acsl5 | -0.2034277 |
| Acsl6 | 2.22667408 |
| Acsl6 | 0.85676254 |
| Acsl6 | -0.3626496 |
| Acsm3 | 1.17834353 |
| Acss1 | 4.22295822 |
| Acss2 | -0.38228 |
| Acss2 | -0.7190611 |
| Acta1 | -0.5039503 |
| Acta2 | -0.739746 |
| Acta2 | 0.86819283 |
| Acta2 | 0.65731229 |
| Acta2 | -0.5174675 |
| Actb | 0.86770582 |
| Actb | -0.1957819 |
| Actb | 0.64811178 |
| Actb | 0.8818868 |
| Actb | 0.52648336 |
| Actg2 | -0.4352093 |
| Actl6a | -0.043006 |
| Actl6b | -0.5203384 |
| Actl6b | 1.66661686 |
| Actl6b | -0.4809287 |
| Actl6b | 0.96287079 |
| Actl7a | -0.8334137 |
| Actl7b | 0.19796665 |
| Actn1 | -0.7023129 |
| Actn2 | 2.05694158 |
| Actn3 | -0.5025388 |
| Actn3 | -0.602176 |
| Actn4 | 0.50828315 |
| Actn4 | 1.23216516 |
| Actn4 | 1.23720559 |
| Actr10 | -0.2458333 |
| Actr10 | -0.423473 |
| Actr1a | -0.2320105 |
| Actr1b | -0.2121692 |
| Actr3 | 0.43893646 |
| Actr3b | -0.6244109 |
| Actr5 | 0.63048155 |
| Actr5 | -0.5181844 |
| Actr6 | 0.33257026 |
| Actrt1 | 2.93101319 |
| Actrt2 | 0.3941721 |
| Acvr1 | 0.18725364 |
| Acvr1c | 0.03864467 |
| Acvr2a | 1.4290448 |
| Acvr2b | -0.5138913 |
| Acvrl1 | 0.44890998 |
| Acy1 | -0.3469984 |
| Acy1l2 | -0.7352101 |
| Acy3 | 2.29217074 |
| Ada | -0.5103288 |
| Adal | 2.31502976 |
| Adal | 1.63448149 |
| Adam11 | -0.4061407 |
| Adam11 | 0.180495 |
| Adam12 | 0.83951756 |
| Adam15 | -0.4976151 |
| Adam17 | 0.57372085 |
| Adam1b | -0.7711889 |
| Adam1b | -0.5592354 |
| Adam21 | -0.8435224 |
| Adam22 | 1.98054451 |
| Adam22 | -0.3304319 |
| Adam22 | 0.42807517 |
| Adam23 | 0.35308969 |
| Adam24 | 1.46867248 |
| Adam26a | 0.34913499 |
| Adam28 | 1.65934676 |
| Adam29 | 0.96550646 |
| Adam29 | 2.07340877 |
| Adam3 | -0.1405922 |
| Adam30 | -0.69535 |
| Adam32 | -0.5458424 |
| Adam33 | -0.5928066 |
| Adam39 | 0.14946021 |
| Adam4 | 0.16786096 |
| Adam4 | 0.95477091 |
| Adam4 | -0.627631 |
| Adam5 | -0.368129 |
| Adam6 | -0.5935838 |
| Adam8 | -0.4754322 |
| Adam8 | -0.5253837 |
| Adam9 | 2.49796062 |
| Adamdec1 | 0.83817772 |
| Adamts1 | 0.16579598 |
| Adamts13 | -0.461831 |
| Adamts15 | -0.5990424 |
| Adamts16 | -0.2774275 |
| Adamts17 | 0.3098195 |
| Adamts18 | -0.2840999 |
| Adamts19 | 0.93110321 |
| Adamts2 | 0.41603257 |
| Adamts2 | -0.2790911 |
| Adamts2 | 1.28709943 |
| Adamts20 | -0.3380869 |
| Adamts20 | 0.6227019 |
| Adamts4 | 1.17121625 |
| Adamts7 | 0.1346163 |
| Adamtsl2 | -0.6447213 |
| Adamtsl4 | 0.59026235 |
| Adamtsl5 | -0.4850724 |
| Adar | -0.2044518 |
| Adar | -0.7562601 |
| Adar | 1.30159234 |
| Adarb1 | -0.457117 |
| Adarb1 | 0.78578353 |
| Adarb1 | -0.6713811 |
| Adat1 | 0.03706394 |
| Adc | 0.20393911 |
| Adc | -0.6389897 |
| Adck1 | -0.2908204 |
| Adck2 | 2.41470636 |
| Adck4 | -0.6068962 |
| Adck5 | 0.34573185 |
| Adcy1 | 0.30002464 |
| Adcy10 | 1.05747245 |
| Adcy2 | 0.96944605 |
| Adcy4 | 1.42957846 |
| Adcy4 | 0.40569936 |
| Adcy6 | 1.18206077 |
| Adcy7 | 1.24341381 |
| Adcy8 | 0.06319428 |
| Adcy9 | 1.93123076 |
| Adcyap1 | 2.84424499 |
| Adcyap1r1 | 1.36649472 |
| Add1 | -0.6373585 |
| Add1 | 0.05236301 |
| Add1 | 0.00977347 |
| Add1 | 1.93739108 |
| Add3 | 2.14518738 |
| Add3 | 3.84492689 |
| Add3 | 0.37702189 |
| Adfp | -0.617206 |
| Adh1 | -0.1081481 |
| Adh1 | 0.95669178 |
| Adh4 | 0.61549539 |
| Adh5 | 0.08072 |
| Adh7 | 0.30091934 |
| Adhfe1 | 0.29703608 |
| Adi1 | 0.64502467 |
| Adipoq | -0.6116404 |
| Adipor1 | -0.7110903 |
| Adipor2 | -0.5584863 |
| Adk | 0.52578087 |
| Adk | -0.5751074 |
| Adm | -0.5011294 |
| Adm2 | -0.2992897 |
| Adnp | 0.2625829 |
| Ado | -0.2018935 |
| Adora1 | -0.7479665 |
| Adora1 | 0.93790538 |
| Adora1 | 1.72152449 |
| Adora2b | 2.63161895 |
| Adora3 | -0.3742553 |
| Adpgk | 1.75956046 |
| Adprh | -0.1570038 |
| Adprhl1 | -0.3327806 |
| Adprhl1 | -0.376101 |
| Adprhl1 | 2.11236513 |
| Adprhl2 | 1.51426808 |
| Adra1a | 0.2730838 |
| Adra1a | 0.27798481 |
| Adra1d | -0.5873843 |
| Adra2a | -0.6587894 |
| Adra2c | 0.52413966 |
| Adra2c | -0.4470952 |
| Adrb1 | 1.15691535 |
| Adrb2 | -0.4424549 |
| Adrbk1 | -0.3565988 |
| Adrbk2 | -0.2998571 |
| Adrm1 | 1.08218376 |
| Adsl | -0.7271007 |
| Adss | 1.33090869 |
| Adssl1 | 0.14875869 |
| Adssl1 | 1.96108034 |
| Adssl1 | -0.3392702 |
| Aebp1 | -0.6538 |
| Aebp2 | -0.4899301 |
| Aes | -0.422178 |
| Afap1 | 0.83111185 |
| Afap1 | 0.42338134 |
| Afap1 | -0.0095667 |
| Afap1l1 | 0.46829366 |
| Aff1 | 0.66359805 |
| Aff4 | -0.6212018 |
| Afg3l1 | -0.2593205 |
| Afg3l1 | -0.7066913 |
| Afg3l1 | 2.56234788 |
| Afg3l1 | -0.7433906 |
| Afg3l2 | 2.61234638 |
| Afmid | -0.6061078 |
| Aftph | 1.06381061 |
| Agbl1 | 1.3076919 |
| Ager | 0.68319513 |
| Ager | -0.1263472 |
| Aggf1 | -0.4899301 |
| Aggf1 | -0.5592354 |
| Aggf1 | -0.6204013 |
| Agpat1 | -0.5348266 |
| Agpat2 | 1.90606779 |
| Agpat3 | -0.6471886 |
| Agpat4 | -0.5174675 |
| Agpat5 | -0.3147309 |
| Agpat6 | -0.4144457 |
| Agpat6 | 0.60091321 |
| Agpat7 | 0.78914579 |
| Agpat9 | -0.6236076 |
| Agpat9 | 0.83901534 |
| Agps | -0.5562425 |
| Agps | 0.20789961 |
| Agrn | -0.5025388 |
| Agrp | -0.505364 |
| Agt | 1.58643561 |
| Agtpbp1 | -0.1377257 |
| Agtr1a | -0.4023324 |
| Agtrap | -0.274109 |
| Agtrl1 | 1.96594705 |
| Agxt2l1 | 0.0788309 |
| Agxt2l1 | 0.34203105 |
| Agxt2l2 | 1.24882581 |
| Agxt2l2 | -0.542156 |
| Agxt2l2 | 2.44271099 |
| Agxt2l2 | -0.0054181 |
| Ahctf1 | -0.2813139 |
| Ahcy | -0.1179035 |
| Ahcyl1 | -0.4795515 |
| Ahdc1 | 1.20360545 |
| Ahi1 | -0.3985396 |
| Ahnak | -0.6596236 |
| Ahnak | -0.2325384 |
| Ahnak2 | -0.385385 |
| Ahrr | -0.4658906 |
| Ahsa1 | -0.4899301 |
| Ahsa2 | 0.98875571 |
| AI118078 | -0.3216765 |
| AI132487 | 0.03230561 |
| AI314180 | -0.7023129 |
| AI314976 | -0.5406855 |
| AI316807 | -0.3669086 |
| AI317395 | -0.2818704 |
| AI413582 | 1.23873425 |
| AI413782 | -0.102619 |
| AI427515 | -0.4345534 |
| AI428936 | 1.32007271 |
| AI449175 | 0.40142677 |
| AI449175 | -0.4802398 |
| AI450540 | 1.45657125 |
| AI451557 | -0.4484252 |
| AI451617 | 0.54937377 |
| AI467606 | 1.08089255 |
| AI480653 | -0.1983236 |
| AI504432 | -0.6013916 |
| AI553587 | -0.1897102 |
| AI593442 | -0.7740148 |
| AI593442 | -0.3620427 |
| AI593442 | -0.5569898 |
| AI595366 | -0.0617237 |
| AI595406 | 0.98418064 |
| AI597468 | -0.7470495 |
| AI597479 | -0.6156124 |
| AI606181 | 0.24850039 |
| AI646023 | 1.63996215 |
| AI661453 | 1.06565116 |
| AI662250 | 1.92696204 |
| AI747699 | 0.62396788 |
| AI836003 | -0.4424549 |
| AI837181 | -0.5428922 |
| AI842396 | -0.5333677 |
| AI846148 | 0.18354245 |
| AI847670 | -0.6596236 |
| AI851790 | -0.1118524 |
| AI894139 | 0.56612433 |
| AI987692 | 2.90907386 |
| AI987692 | -0.2925081 |
| AI987944 | -0.2675074 |
| Aicda | 1.8672258 |
| Aif1 | -0.1766886 |
| Aif1 | -0.5428922 |
| Aif1 | 1.08373086 |
| Aifm1 | -0.5246613 |
| Aifm1 | 4.51097999 |
| Aifm2 | -0.6316718 |
| Aifm2 | -0.457117 |
| Aifm2 | 0.81784585 |
| Aifm3 | 2.99509241 |
| Aig1 | 0.05547051 |
| Aig1-pending | 0.53045419 |
| Aim1 | 0.09866337 |
| Aim2 | -0.5275544 |
| Aip | -0.6546297 |
| Ak1 | -0.4823079 |
| AK122209 | -0.6164089 |
| AK122525 | 1.1125236 |
| AK129128 | -0.2730055 |
| AK129302 | -0.4371798 |
| AK129302 | -0.0365639 |
| AK129302 | 0.29163244 |
| AK129341 | -0.3147309 |
| AK162044 | -0.6504884 |
| Ak2 | -0.6140216 |
| Ak2 | 0.98231559 |
| Ak2 | 0.64852263 |
| Ak3 | 1.42225847 |
| Ak3 | 4.29828478 |
| Ak3l1 | 0.3041926 |
| Ak5 | 0.15714236 |
| Akap1 | 1.17542909 |
| Akap1 | -0.2598641 |
| Akap1 | 0.22745379 |
| Akap10 | -0.6447213 |
| Akap10 | -0.1401138 |
| Akap12 | -0.4358657 |
| Akap14 | -0.831405 |
| Akap2 | 0.91023888 |
| Akap2 | -0.5060716 |
| Akap3 | 1.7264536 |
| Akap4 | 0.18117307 |
| Akap4 | -0.3897493 |
| Akap4 | -0.5827615 |
| Akap4 | -0.0387063 |
| Akap6 | -0.4378375 |
| Akap7 | 2.95336935 |
| Akap8 | 1.94186925 |
| Akap8l | 2.9436456 |
| Akap9 | -0.627631 |
| Akap9 | -0.5959193 |
| Akap9 | 2.1493078 |
| Akna | -0.6739199 |
| Akp2 | -0.6970859 |
| Akp5 | 1.05055717 |
| Akr1a4 | 1.19940441 |
| Akr1a4 | -0.5819933 |
| Akr1b3 | -0.627631 |
| Akr1b8 | 0.79372723 |
| Akr1c12 | -0.0099826 |
| Akr1c14 | 0.21870509 |
| Akr1c14 | -0.1216468 |
| Akr1c18 | 1.05109093 |
| Akr1c19 | 1.1380268 |
| Akr1e1 | -0.2953278 |
| Akr7a5 | -0.3922524 |
| Akr7a5 | 0.88603572 |
| Akr7a5 | 0.89853003 |
| Akt1s1 | -0.6538 |
| Akt3 | 0.63902714 |
| Akt3 | 3.47229726 |
| Aktip | -0.4836891 |
| Alad | -0.1791787 |
| Alas1 | -0.5705449 |
| Alas2 | -0.4733793 |
| Alcam | -0.3669086 |
| Aldh16a1 | 0.95787189 |
| Aldh18a1 | -0.4490909 |
| Aldh1a1 | -0.1492447 |
| Aldh1a2 | 0.26753923 |
| Aldh1a2 | -0.5827615 |
| Aldh1a7 | -0.1978147 |
| Aldh1b1 | 1.47851176 |
| Aldh1l1 | -0.4086882 |
| Aldh1l2 | -0.8304023 |
| Aldh1l2 | -0.088938 |
| Aldh2 | 1.18760901 |
| Aldh3a1 | -0.4720133 |
| Aldh3a2 | -0.1902146 |
| Aldh3b1 | 0.05934039 |
| Aldh3b1 | -0.380422 |
| Aldh3b1 | 0.34031803 |
| Aldh4a1 | 0.8195572 |
| Aldh5a1 | 0.33372215 |
| Aldh5a1 | 2.41331325 |
| Aldh6a1 | 0.56342837 |
| Aldh8a1 | 0.98102221 |
| Aldh8a1 | 2.79083774 |
| Aldoa | -0.4871512 |
| Aldoc | 3.21651606 |
| Alg1 | 1.11874795 |
| Alg10b | 2.14782654 |
| Alg11 | 2.19589865 |
| Alg11 | 1.73834436 |
| Alg12 | 0.18151192 |
| Alg12 | -0.0525356 |
| Alg14 | -0.3363148 |
| Alg2 | 0.0089586 |
| Alg3 | 0.05895412 |
| Alg3 | -0.4378375 |
| Alg3 | -0.0187591 |
| Alg5 | 1.27069254 |
| Alg5 | -0.5304565 |
| Alg5 | 0.47351097 |
| Alg8 | -0.755335 |
| Alg8 | -0.5743455 |
| Alg8 | -0.636544 |
| Alg8 | -0.0327199 |
| Alg9 | 0.67863264 |
| Alg9 | 1.40086491 |
| Alg9 | 0.85478892 |
| Alk | -0.5117522 |
| Alkbh2 | -0.5660047 |
| Alkbh3 | -0.6236076 |
| Alkbh4 | -0.6292452 |
| Alkbh5 | -0.5873843 |
| Alkbh7 | -0.6646444 |
| Alkbh8 | 1.57615755 |
| Allc | -0.2367722 |
| Allc | 1.3183713 |
| Alox12 | -0.5599851 |
| Alox12b | 0.85264612 |
| Alox12e | 1.53084779 |
| Alox15 | 1.01046481 |
| Alox5ap | 1.53004972 |
| Alox8 | -0.6308622 |
| Aloxe3 | -0.4504238 |
| Alpk3 | 0.22260353 |
| Als2 | 2.73786972 |
| Als2 | -0.602176 |
| Als2 | -0.6389897 |
| Als2cl | -0.729796 |
| Als2cr13 | 1.20133696 |
| Als2cr2 | -0.4524267 |
| Als2cr2 | -0.4195934 |
| Als2cr4 | 0.78258701 |
| Als2cr4 | 0.46629842 |
| Als2cr4 | -0.391626 |
| Alx3 | 1.45041779 |
| Amac1 | -0.5311834 |
| Ambp | 1.39893727 |
| Ambra1 | -0.2724542 |
| Ambra1 | -0.7119726 |
| Amd2 | 0.63048155 |
| Amdhd2 | 0.1346163 |
| Amdhd2 | -0.8475968 |
| Amfr | -0.4093262 |
| Amh | -0.1502111 |
| Amhr2 | 2.84786893 |
| Amhr2 | 2.39788168 |
| Amhr2 | 0.2967367 |
| Amigo1 | 1.24839401 |
| Amigo1 | 0.19596731 |
| Amigo2 | -0.5275544 |
| Amigo3 | 1.06853613 |
| Ammecr1l | -0.2691534 |
| Amn | -0.7279982 |
| Amotl2 | 2.35896913 |
| Ampd1 | 1.83554296 |
| Ampd1 | 1.53601869 |
| Ampd2 | 1.87005845 |
| Amph | -0.737929 |
| Amph | 1.04412802 |
| Amy1 | -0.7711889 |
| Amz2 | -0.5835304 |
| Anapc10 | 2.10730411 |
| Anapc11 | -0.4706492 |
| Anapc13 | -0.602176 |
| Anapc2 | 0.48140548 |
| Anapc4 | -0.0158244 |
| Anapc5 | 0.8138978 |
| Anapc5 | -0.7721299 |
| Anapc5 | 0.12134094 |
| Anapc7 | 0.40409943 |
| Ang | 0.32533858 |
| Angel1 | -0.4118824 |
| Angel2 | -0.3511865 |
| Angpt1 | 1.97332398 |
| Angpt2 | -0.3216765 |
| Angpt2 | 0.06588257 |
| Angptl1 | 0.06626598 |
| Angptl2 | 1.26837026 |
| Angptl3 | 0.64109961 |
| Angptl4 | -0.5804587 |
| Angptl7 | -0.6414418 |
| Ank | 1.67287212 |
| Ank1 | -0.3816603 |
| Ank2 | 0.61783301 |
| Ank2 | 0.04963543 |
| Ank2 | -0.3095554 |
| Ank3 | -0.6884387 |
| Ank3 | -0.5607354 |
| Ank3 | 1.54752989 |
| Ankar | -0.5735841 |
| Ankar | -0.0674573 |
| Ankfy1 | 0.53464025 |
| Ankle2 | -0.8829666 |
| Ankle2 | 3.07317393 |
| Ankle2 | 0.2849852 |
| Ankmy1 | 0.13425998 |
| Ankmy1 | -0.6124335 |
| Ankmy1 | -0.6480124 |
| Ankmy2 | 1.69232994 |
| Ankmy2 | 1.27573935 |
| Ankrd10 | -0.6996958 |
| Ankrd12 | -0.4878451 |
| Ankrd12 | -0.6006079 |
| Ankrd13a | 0.46329753 |
| Ankrd13b | 0.08373463 |
| Ankrd13d | -0.6604585 |
| Ankrd17 | 0.63299081 |
| Ankrd17 | -0.3773336 |
| Ankrd17 | -0.4004341 |
| Ankrd22 | -0.5812257 |
| Ankrd23 | 0.93473721 |
| Ankrd24 | 0.65649813 |
| Ankrd25 | 1.04654414 |
| Ankrd27 | 2.79257253 |
| Ankrd34a | -0.030166 |
| Ankrd35 | -0.6790184 |
| Ankrd36 | -0.7137397 |
| Ankrd37 | 0.16026692 |
| Ankrd39 | 0.99401782 |
| Ankrd40 | -0.4537644 |
| Ankrd40 | -0.6739199 |
| Ankrd40 | 0.0465084 |
| Ankrd46 | 2.30756468 |
| Ankrd47 | 0.16854827 |
| Ankrd49 | -0.1806759 |
| Ankrd5 | 3.1351011 |
| Ankrd50 | -0.5103288 |
| Ankrd53 | 0.6458489 |
| Ankrd54 | 0.3570277 |
| Ankrd54 | -0.0246564 |
| Ankrd54 | 0.11735751 |
| Ankrd56 | -0.3481931 |
| Ankrd58 | -0.752565 |
| Ankrd6 | -0.3493893 |
| Ankrd6 | 1.52452433 |
| Ankrd6 | -0.2394281 |
| Ankrd7 | 1.90007242 |
| Ankrd9 | 0.62523214 |
| Anks1 | 2.11433066 |
| Anks3 | 4.41680287 |
| Anks3 | 1.07141221 |
| Anks6 | 2.05757685 |
| Anks6 | 1.52869142 |
| Ankzf1 | -0.726204 |
| Ankzf1 | 0.54318642 |
| Anln | -0.4843805 |
| Anln | -0.4138042 |
| Anp32a | -0.5160354 |
| Anp32a | -0.1816754 |
| Anp32a | 0.45246437 |
| Anp32e | -0.2658643 |
| Anpep | 1.65369943 |
| Antxr1 | 2.09431212 |
| Antxr1 | 0.8825263 |
| Antxrl | 2.69325911 |
| Anxa1 | -0.6164089 |
| Anxa11 | 3.38064784 |
| Anxa11 | -0.5465815 |
| Anxa2 | -0.2614967 |
| Anxa3 | 0.16545137 |
| Anxa3 | 3.35031146 |
| Anxa4 | -0.030166 |
| Anxa5 | -0.5110402 |
| Anxa6 | -0.6546297 |
| Anxa8 | -0.7102088 |
| Anxa8 | 0.82314085 |
| Anxa9 | -0.5904789 |
| Aoah | 0.61229827 |
| Aoc2 | 1.0297714 |
| Aoc2 | 1.68322783 |
| Aoc3 | -0.5096179 |
| Aof1 | -0.869284 |
| Aof1 | -0.6596236 |
| Aof2 | -0.831405 |
| Aox3 | -0.4658906 |
| Aox3l1 | -0.1526314 |
| Ap1b1 | -0.2987227 |
| Ap1g1 | -0.3084091 |
| Ap1g1 | -0.7562601 |
| Ap1g2 | 1.11077331 |
| Ap1gbp1 | -0.376101 |
| Ap1gbp1 | 1.19552724 |
| Ap1m1 | -0.1063014 |
| Ap1m2 | 0.26320388 |
| Ap1m2 | -0.5436289 |
| Ap1m2 | 1.23786104 |
| Ap1m2 | 0.27645601 |
| Ap1s1 | -0.4557745 |
| Ap1s1 | 0.96609115 |
| Ap1s1 | 1.82855353 |
| Ap1s3 | 0.47820639 |
| Ap2a1 | -0.7163966 |
| Ap2a1 | -0.5920301 |
| Ap2a2 | -0.7075694 |
| Ap2b1 | 0.7770561 |
| Ap2b1 | -0.0875809 |
| Ap2b1 | -0.2394281 |
| Ap2b1 | -0.2106206 |
| Ap2m1 | 2.04589162 |
| Ap2s1 | -0.6680066 |
| Ap2s1 | 1.17799427 |
| Ap2s1 | -0.6841449 |
| Ap3b1 | -0.7005673 |
| Ap3b1 | 2.21628566 |
| Ap3b2 | 2.32879998 |
| Ap3d1 | 1.36057739 |
| Ap3m1 | 1.01171804 |
| Ap3m2 | 3.2243684 |
| Ap3s2 | -0.5943616 |
| Ap3s2 | -0.1072243 |
| Ap4b1 | 0.92318067 |
| Ap4m1 | 0.71068782 |
| Ap4m1 | -0.58507 |
| Ap4m1 | -0.821426 |
| Ap4s1 | 1.15213916 |
| Apaf1 | -0.2137204 |
| Apaf1 | 0.40356551 |
| Apba1 | -0.4299749 |
| Apba2 | -0.1978147 |
| Apba2 | -0.0037637 |
| Apba3 | -0.6398063 |
| Apbb1 | -0.7873168 |
| Apbb1ip | 0.57216191 |
| Apbb1ip | 0.31129465 |
| Apbb1ip | -0.6884387 |
| Apbb2 | 0.06932759 |
| Apbb3 | -0.3257521 |
| Apc | -0.831405 |
| Apc2 | -0.7674342 |
| Apcdd1 | 0.39767213 |
| Apeh | -0.6037467 |
| Apex1 | -0.5311834 |
| Aph1a | 0.26537407 |
| Aph1b | 2.86325901 |
| Apip | 1.94545683 |
| Apip | 0.62122275 |
| Apitd1 | -0.4299749 |
| Aplp1 | -0.457789 |
| Aplp2 | 1.34415725 |
| Apoa1 | 1.59466671 |
| Apoa1bp | -0.3158849 |
| Apoa2 | -0.3089821 |
| Apoa4 | 1.73367041 |
| Apob48r | -0.703187 |
| Apobec1 | 0.39471142 |
| Apobec3 | -0.2785362 |
| Apoc1 | -0.5982606 |
| Apoc1 | -0.0634842 |
| Apoc2 | 1.44553556 |
| Apoc3 | -0.4195934 |
| Apoe | 2.37149491 |
| Apof | -0.6463654 |
| Apoh | -0.4645354 |
| Apol7c | 0.74594324 |
| Apol9b | 0.12818161 |
| App | -0.4241211 |
| Appbp1 | -0.4497571 |
| Appbp1 | 0.40569936 |
| Appbp2 | 0.63090023 |
| Appl1 | -0.3835208 |
| Appl2 | 1.5945177 |
| Aprin | 0.54157557 |
| Aprt | -0.6867188 |
| Aptx | -0.6680066 |
| Aqp1 | -0.8274006 |
| Aqp1 | -0.6996958 |
| Aqp1 | -0.6504884 |
| Aqp11 | 1.06709476 |
| Aqp11 | -0.4699679 |
| Aqp3 | -0.5261067 |
| Aqp4 | -0.7023129 |
| Aqp5 | 1.21183892 |
| Aqp7 | 2.07980883 |
| Aqp7 | 1.1078907 |
| Aqp8 | -0.7119726 |
| Aqp8 | 1.3207724 |
| Aqp9 | 0.49989149 |
| Aqr | -0.705814 |
| Aqr | -0.3280891 |
| Ar | -0.0534068 |
| Araf | 0.3221388 |
| Araf | 1.84282309 |
| Arbp | -0.1003249 |
| Arc | 1.19780988 |
| Arcn1 | 0.045725 |
| Ard1 | 0.15505349 |
| Ard1 | -0.5562425 |
| Areg | -0.7075694 |
| Arf2 | -0.4477599 |
| Arf3 | 0.01586242 |
| Arf5 | -0.4477599 |
| Arf6 | -0.7740148 |
| Arfgap1 | 1.01712931 |
| Arfgap1 | 0.12566719 |
| Arfgap1 | -0.7361155 |
| Arfgap2 | -0.5622379 |
| Arfgap2 | 1.79892368 |
| Arfgap2 | -0.4444407 |
| Arfgap2 | -0.1717284 |
| Arfgap3 | -0.5675156 |
| Arfip2 | -0.4345534 |
| Arfip2 | -0.1179035 |
| Arfip2 | -0.7137397 |
| Arfrp1 | 0.03587663 |
| Arfrp1 | -0.3386784 |
| Arg1 | 1.775346 |
| Arg2 | 0.34003222 |
| Arglu1 | 2.72011113 |
| Arhgap1 | -0.8154895 |
| Arhgap1 | 3.48428899 |
| Arhgap1 | 2.47536516 |
| Arhgap10 | 0.03468781 |
| Arhgap12 | -0.2054771 |
| Arhgap15 | -0.6807241 |
| Arhgap15 | -0.2614967 |
| Arhgap17 | -0.0989511 |
| Arhgap18 | -0.2262234 |
| Arhgap19 | 0.3570277 |
| Arhgap20 | 0.15714236 |
| Arhgap22 | -0.3141544 |
| Arhgap24 | 0.68556673 |
| Arhgap24 | -0.3687399 |
| Arhgap25 | -0.4665689 |
| Arhgap26 | 0.97583071 |
| Arhgap27 | -0.2399602 |
| Arhgap28 | 0.69382018 |
| Arhgap29 | 0.10309581 |
| Arhgap30 | 0.98718556 |
| Arhgap4 | 0.87723729 |
| Arhgap6 | -0.0494929 |
| Arhgap8 | 2.65994404 |
| Arhgap9 | -0.5297301 |
| Arhgdib | 1.17145079 |
| Arhgdig | 1.05109093 |
| Arhgef1 | 0.68022213 |
| Arhgef1 | 0.7398177 |
| Arhgef10 | 1.83709547 |
| Arhgef10l | -0.5990424 |
| Arhgef10l | 1.57554942 |
| Arhgef15 | -0.5117522 |
| Arhgef18 | 1.37048202 |
| Arhgef19 | -0.0246564 |
| Arhgef2 | 2.82011962 |
| Arhgef3 | -0.6944833 |
| Arhgef4 | -0.4892346 |
| Arhgef6 | 0.99515168 |
| Arid2 | 3.58059731 |
| Arid3a | -0.1334425 |
| Arid3b | 1.90253947 |
| Arid4b | -0.6867188 |
| Arid5a | -0.4564455 |
| Arid5b | -0.5370194 |
| Arih1 | 0.13568444 |
| Arih2 | -0.6910244 |
| Arl1 | 3.65388684 |
| Arl11 | -0.6092653 |
| Arl16 | 2.6477102 |
| Arl2 | -0.451091 |
| Arl2bp | -0.551028 |
| Arl2bp | 1.31032271 |
| Arl2bp | -0.0630438 |
| Arl3 | -0.0750086 |
| Arl3 | -0.1781819 |
| Arl4a | -0.5812257 |
| Arl4a | 0.44380868 |
| Arl4c | -0.705814 |
| Arl5a | -0.6587894 |
| Arl5b | 0.89742967 |
| Arl6 | -0.1978147 |
| Arl6ip1 | 0.59375357 |
| Arl6ip2 | 0.2730838 |
| Arl6ip2 | 1.95790584 |
| Arl6ip2 | -0.7128557 |
| Arl6ip2 | 0.6146439 |
| Arl6ip4 | -0.380422 |
| Arl6ip4 | -0.4247697 |
| Arl6ip5 | 1.06275723 |
| Arl6ip5 | -0.0095667 |
| Arl8a | 0.7198652 |
| Arl8b | -0.6148167 |
| Armc10 | 2.31901427 |
| Armc3 | 1.91941816 |
| Armc6 | 2.36188724 |
| Armc6 | -0.7163966 |
| Armc8 | 0.59222777 |
| Armc8 | -0.5789266 |
| Armc8 | 0.76535147 |
| Armc9 | -0.2614967 |
| Armc9 | -0.5599851 |
| Armcx1 | -0.6132272 |
| Armcx2 | -0.1922353 |
| Armcx3 | -0.0204403 |
| Armcx5 | 3.26410091 |
| Armcx6 | 1.1095211 |
| Armcx6 | -0.0395647 |
| Armetl1 | 0.91147919 |
| Arnt | 2.49583614 |
| Arnt | -0.5340969 |
| Arnt2 | -0.2431591 |
| Arntl | 0.37866889 |
| Arpc1a | 0.22680855 |
| Arpc1a | -0.6970859 |
| Arpc1a | -0.602961 |
| Arpc1b | -0.3724132 |
| Arpc1b | 2.92137593 |
| Arpc1b | -0.57587 |
| Arpc3 | 0.06280961 |
| Arpc4 | -0.6268249 |
| Arpc5 | -0.4125226 |
| Arpc5 | -0.6180038 |
| Arpc5l | -0.2576917 |
| Arpp19 | 0.53115317 |
| Arpp21 | -0.0468927 |
| Arpp21 | -0.726204 |
| Arrb1 | -0.0934761 |
| Arrb1 | -0.481618 |
| Arrb1 | -0.0772413 |
| Arrb1 | -0.4067769 |
| Arrb2 | -0.6587894 |
| Arrdc1 | 0.07807419 |
| Arrdc2 | 0.04846402 |
| Arrdc3 | 0.30181319 |
| Arrdc4 | 1.31425507 |
| Ars2 | 1.77454767 |
| Ars2 | 2.26620198 |
| Ars2 | -0.4920198 |
| Arsa | -0.4557745 |
| Arsb | -0.7618305 |
| Arsb | -0.2431591 |
| Arsg | -0.0438683 |
| Arsi | 1.75806092 |
| Arsj | -0.5174675 |
| Arsj | 2.24072063 |
| Art1 | -0.7361155 |
| Art2b | 1.87183143 |
| Art3 | -0.5167512 |
| Art3 | -0.6084749 |
| Art4 | 0.02191189 |
| Art5 | 0.02110756 |
| Artn | 0.23771751 |
| Artn | 0.43403868 |
| Arv1 | -0.6430801 |
| As3mt | -0.4557745 |
| As3mt | 1.36382703 |
| Asah1 | 0.03190798 |
| Asah1 | 1.57204495 |
| Asah2 | -0.2919452 |
| Asah3 | 0.12170223 |
| Asah3l | 0.85215092 |
| Asb1 | -0.2919452 |
| Asb1 | -0.6722266 |
| Asb1 | -0.3222577 |
| Asb12 | -0.7452183 |
| Asb13 | -0.1881984 |
| Asb15 | -0.6790184 |
| Asb17 | 0.61783301 |
| Asb2 | 0.1038325 |
| Asb3 | -0.5473211 |
| Asb3 | -0.6084749 |
| Asb4 | 1.61032832 |
| Asb4 | 0.51541642 |
| Asb6 | -0.6867188 |
| Asb6 | -0.6944833 |
| Asb7 | -0.717284 |
| Asb7 | -0.7516435 |
| Asb8 | -0.3493893 |
| Asb9 | -0.4280196 |
| Ascc1 | 0.93895905 |
| Ascc1 | -0.461831 |
| Ascc2 | 0.5229656 |
| Ascc2 | -0.4920198 |
| Ascc2 | 1.95365629 |
| Ascc2 | -0.4150876 |
| Ascc3l1 | 2.70262165 |
| Ascl1 | 1.83939073 |
| Ascl2 | -0.719951 |
| Asf1a | -0.7759035 |
| Asf1b | -0.7433906 |
| Asgr1 | -0.2746613 |
| Ash1l | -0.4530953 |
| Ash1l | 1.60658462 |
| Ash1l | 0.90338752 |
| Ash2l | -0.5644961 |
| Ash2l | -0.6538 |
| Asl | -0.6053201 |
| Asl | 1.03467204 |
| Asna1 | -0.7806421 |
| Asns | 1.4740291 |
| Asns | 0.09606802 |
| Asns | 1.25517186 |
| Asnsd1 | -0.7155101 |
| Asnsd1 | -0.4260683 |
| Asph | -0.4665689 |
| Asph | -0.6790184 |
| Asph | -0.5773971 |
| Asph | 2.81053496 |
| Asph | -0.4537644 |
| Asphd1 | 1.17227124 |
| Asphd2 | -0.3693511 |
| Aspm | -0.7014397 |
| Asprv1 | 2.76955327 |
| Aspscr1 | 2.20717239 |
| Aspscr1 | 0.32939523 |
| Aspscr1 | 0.48361383 |
| Asrgl1 | -0.7749587 |
| Ass1 | -0.5117522 |
| Aste1 | 0.09234782 |
| Astn1 | -0.0029377 |
| Astn2 | 4.03284384 |
| Asz1 | 0.06779804 |
| Atad1 | 1.7699903 |
| Atad2 | -0.652971 |
| Atad3a | -0.1482793 |
| Atad4 | 2.4325693 |
| Atad4 | 0.45827449 |
| Atcay | 1.90835939 |
| Ate1 | 1.33465698 |
| Ate1 | 0.41866378 |
| Atf2 | -0.376101 |
| Atf2 | -0.0037637 |
| Atf2 | 0.16992138 |
| Atf3 | -0.7084484 |
| Atf4 | -0.3602246 |
| Atf4 | 2.41665322 |
| Atf5 | -0.5473211 |
| Atf7 | -0.6116404 |
| Atf7ip | 0.28891884 |
| Atg10 | -0.3333687 |
| Atg10 | -0.4720133 |
| Atg12 | 0.08109736 |
| Atg16l1 | 0.58235902 |
| Atg2a | 0.35139687 |
| Atg2a | 1.50217192 |
| Atg2b | -0.2752139 |
| Atg2b | -0.5637428 |
| Atg3 | -0.5819933 |
| Atg3 | -0.7271007 |
| Atg4b | -0.4202389 |
| Atg4c | -0.2383648 |
| Atg4c | 1.87823036 |
| Atg4d | -0.8264022 |
| Atg5 | -0.147315 |
| Atg5 | 0.80699158 |
| Atg7 | 0.01018064 |
| Atg9b | -0.0772413 |
| Athl1 | -0.4404732 |
| Atic | 3.16486551 |
| Atic | -0.1324933 |
| Atic | -0.7674342 |
| Atm | -0.6722266 |
| Atm | 1.37849979 |
| Atmin | 0.37482136 |
| Atmin | -0.3469984 |
| Atmin | -0.0759011 |
| Atn1 | 0.18927172 |
| Atoh8 | 0.77077186 |
| Atox1 | -0.6781667 |
| Atp10a | 0.79758634 |
| Atp10d | -0.7969357 |
| Atp11c | -0.4451036 |
| Atp11c | -0.6252148 |
| Atp12a | -0.4306276 |
| Atp13a1 | -0.5928066 |
| Atp13a1 | -0.0271952 |
| Atp13a2 | -0.6292452 |
| Atp13a4 | -0.5912541 |
| Atp13a5 | -0.1851821 |
| Atp1a1 | -0.602176 |
| Atp1a2 | 1.78231758 |
| Atp1b1 | -0.5189018 |
| Atp1b2 | -0.1086103 |
| Atp1b3 | -0.4892346 |
| Atp2a1 | 1.38187489 |
| Atp2a2 | 0.50205657 |
| Atp2a2 | 2.28675777 |
| Atp2a3 | 1.21318544 |
| Atp2a3 | 1.40352037 |
| Atp2a3 | 3.78905147 |
| Atp2b1 | -0.738837 |
| Atp2b2 | -0.7988715 |
| Atp2b2 | -0.0608448 |
| Atp2b2 | -0.4029661 |
| Atp2b3 | -0.8008112 |
| Atp2b3 | 1.86533257 |
| Atp2b4 | 1.45872834 |
| Atp2b4 | 0.27431147 |
| Atp2c1 | 0.41918914 |
| Atp2c1 | 3.03974303 |
| Atp5a1 | 0.32852743 |
| Atp5b | 2.34454025 |
| Atp5c1 | 0.40035555 |
| Atp5d | 1.17834353 |
| Atp5e | 0.36374015 |
| Atp5f1 | -0.8086109 |
| Atp5g2 | 1.18530125 |
| Atp5g3 | 0.16407166 |
| Atp5h | -0.7005673 |
| Atp5j | -0.5705449 |
| Atp5j | -0.2452979 |
| Atp5j2 | 0.02632337 |
| Atp5l | 1.847039 |
| Atp5o | -0.6562913 |
| Atp5s | 1.1995182 |
| Atp5sl | 0.09420977 |
| Atp6ap1 | 0.31951261 |
| Atp6ap2 | -0.6480124 |
| Atp6ap2 | 2.63702172 |
| Atp6ap2 | 0.0465084 |
| Atp6ap2 | -0.4424549 |
| Atp6v0a1 | -0.7834969 |
| Atp6v0a1 | 0.72442026 |
| Atp6v0a2 | -0.7683715 |
| Atp6v0b | 1.89689858 |
| Atp6v0d1 | -0.3947622 |
| Atp6v0d1 | 0.25415893 |
| Atp6v0d1 | -0.6858601 |
| Atp6v0d1 | -0.4138042 |
| Atp6v0e | -0.5362879 |
| Atp6v0e2 | -0.5196198 |
| Atp6v1a | 0.5056539 |
| Atp6v1b1 | -0.5004255 |
| Atp6v1b2 | 0.73458856 |
| Atp6v1c1 | -0.8154895 |
| Atp6v1c2 | -0.7488844 |
| Atp6v1d | -0.3363148 |
| Atp6v1e1 | -0.4768033 |
| Atp6v1e2 | 1.85558311 |
| Atp6v1g1 | 0.68457929 |
| Atp6v1g2 | 0.78631521 |
| Atp6v1g2 | 0.4094219 |
| Atp6v1h | 2.36247632 |
| Atp7a | -0.6936174 |
| Atp8a1 | 0.65405138 |
| Atp8a2 | -0.2691534 |
| Atp8b1 | 2.12477224 |
| Atp8b3 | -0.6841449 |
| Atp8b3 | -0.5167512 |
| Atp8b3 | 0.42911504 |
| Atp8b3 | 0.14665095 |
| Atp9a | 0.54639979 |
| Atp9a | 3.46467823 |
| Atp9b | 2.79689544 |
| Atp9b | 0.99783909 |
| Atpaf1 | -0.4665689 |
| Atpaf2 | -0.8703294 |
| Atpaf2 | -0.0037637 |
| Atpbd1b | 0.51730944 |
| Atpbd1c | 0.47721984 |
| Atpbd3 | -0.4857648 |
| Atpif1 | 1.27772953 |
| Atrx | 0.15086168 |
| Atxn1 | 2.25771208 |
| Atxn10 | 0.99188811 |
| Atxn2 | 1.15476909 |
| Atxn2l | -0.4720133 |
| Atxn3 | -0.8076324 |
| Atxn3 | 0.44073445 |
| Atxn3 | 0.27614995 |
| Atxn7l1 | 2.72126998 |
| Atxn7l2 | -0.4638586 |
| AU014645 | -0.7507229 |
| AU017455 | 2.03074949 |
| AU017455 | -0.4990192 |
| AU018778 | 1.42476444 |
| AU018829 | -0.2818704 |
| AU019823 | 2.84882129 |
| AU021034 | -0.182676 |
| AU021092 | 1.70446206 |
| AU021838 | -0.5517711 |
| AU022252 | 1.44780548 |
| AU022751 | -0.7806421 |
| AU022870 | 1.17997164 |
| AU023871 | -0.0652479 |
| AU040320 | 2.73758525 |
| AU040320 | -0.5089076 |
| AU040320 | -0.5399512 |
| AU040829 | 0.64006395 |
| AU042651 | -0.7190611 |
| Aup1 | 0.96066869 |
| Aup1 | 0.10162067 |
| Aurka | -0.2863347 |
| Aurkaip1 | -0.2528223 |
| Aurkc | -0.368129 |
| Auts2 | -0.2941989 |
| Auts2 | 0.35618524 |
| AV028368 | 0.50804443 |
| AV249152 | 0.28164374 |
| AV249152 | 1.9661472 |
| Aven | 1.0149131 |
| Avil | 1.11027263 |
| Avp | -0.3351353 |
| Avpi1 | 0.52037745 |
| Avpi1 | 0.62058811 |
| Avpr1a | -0.5735841 |
| AW049604 | -0.5982606 |
| AW061290 | 0.59157309 |
| AW111922 | -0.5819933 |
| AW121567 | 0.77293123 |
| AW125753 | -0.2528223 |
| AW146242 | 0.77257168 |
| AW146299 | 0.6311095 |
| AW146299 | 1.54029282 |
| AW540478 | -0.5644961 |
| AW544981 | -0.4843805 |
| AW548124 | -0.6037467 |
| AW549877 | -0.5959193 |
| AW551984 | 0.70200125 |
| AW551984 | -0.1482793 |
| AW551984 | -0.3687399 |
| AW555464 | 0.73608559 |
| Axin2 | -0.2970237 |
| Axl | 0.94495731 |
| Axud1 | 0.08673955 |
| Azgp1 | -0.310703 |
| Azgp1 | 2.0233441 |
| Azi1 | 3.47568678 |
| Azi2 | 1.32037264 |
| Azin1 | -0.3565988 |
| B020018G12Rik | -0.3327806 |
| B230118H07Rik | 0.97987104 |
| B230120H23Rik | 0.75331461 |
| B230206H07Rik | 2.67239513 |
| B230209C24Rik | 0.33314638 |
| B230217C12Rik | -0.3614363 |
| B230219D22Rik | -0.6381738 |
| B230312A22Rik | -0.5377515 |
| B230312I18Rik | -0.622003 |
| B230317C12Rik | -0.6204013 |
| B230317C12Rik | -0.0661311 |
| B230339H12Rik | 1.0204438 |
| B230339M05Rik | 1.34824095 |
| B230339M05Rik | 2.41445885 |
| B230340J04Rik | -0.7075694 |
| B230342M21Rik | 1.91883937 |
| B230373P09Rik | -0.7370218 |
| B230378H13Rik | 0.31335593 |
| B230380D07Rik | 0.4471277 |
| B230396O12Rik | -0.1072243 |
| B230399E16Rik | -0.1463516 |
| B2m | 1.61849746 |
| B2m | 1.63448149 |
| B3galnt1 | -0.4665689 |
| B3galnt2 | -0.5751074 |
| B3galt1 | 1.1504617 |
| B3galt4 | -0.4080507 |
| B3galt4 | -0.627631 |
| B3galt5 | -0.7049375 |
| B3galt6 | -0.2713527 |
| B3gat1 | 1.0089308 |
| B3gat1 | 1.28782427 |
| B3gat1 | 0.19929717 |
| B3gat2 | -0.4371798 |
| B3gat3 | 0.9388086 |
| B3gat3 | 1.71198172 |
| B3gnt1 | 0.14559529 |
| B3gnt3 | -0.0425751 |
| B3gnt5 | -0.0875809 |
| B3gnt7 | -0.7119726 |
| B3gnt8 | 0.12134094 |
| B3gnt8 | 0.71337389 |
| B430119L13Rik | 0.33141695 |
| B4galnt3 | -0.2691534 |
| B4galnt4 | 0.24026589 |
| B4galt1 | -0.617206 |
| B4galt3 | -0.5675156 |
| B4galt3 | -0.6571233 |
| B4galt4 | 0.13994485 |
| B4galt7 | 2.43777465 |
| B630005N14Rik | 1.3808449 |
| B830045N13Rik | 2.27097765 |
| B930006L02Rik | -0.2183894 |
| B930011P16Rik | -0.2018935 |
| B930037P14Rik | -0.5167512 |
| B930041F14Rik | -0.1781819 |
| B930041G04 | 0.9466001 |
| B930076A02 | -0.5835304 |
| B9d1 | 1.90580096 |
| B9d2 | 1.84942337 |
| Baalc | 0.65241665 |
| Baalc | -0.3736409 |
| Bace1 | 1.46294182 |
| Bace2 | -0.6480124 |
| Bace2 | -0.8066549 |
| Bace2 | 2.18543675 |
| Bach1 | -0.1058403 |
| Bach2 | -0.3130025 |
| Bad | 2.09496603 |
| Bag1 | -0.4125226 |
| Bag2 | 0.18893568 |
| Bag3 | 0.89443643 |
| Bag4 | -0.7730719 |
| Bag5 | 1.86828211 |
| Bahcc1 | 1.55905561 |
| Bahd1 | 0.08034249 |
| Bai1 | 0.74093443 |
| Bai2 | 0.5548394 |
| Bai2 | 3.2140371 |
| Bai3 | -0.2885749 |
| Baiap2 | -0.189206 |
| Baiap2 | 1.14577282 |
| Banf1 | -0.4795515 |
| Banf1 | -0.4699679 |
| Banf1 | -0.5217771 |
| Banf1 | -0.4754322 |
| Banf2 | 0.18421831 |
| Bap1 | 1.11514289 |
| Bard1 | 2.16018036 |
| Barx2 | 0.82654109 |
| Basp1 | 1.22244604 |
| Bat1a | -0.0116479 |
| Bat1a | -0.5697867 |
| Bat2 | -0.391626 |
| Bat3 | -0.7415665 |
| Bat4 | 3.49848434 |
| Bat4 | -0.3339572 |
| Bat5 | -0.6996958 |
| Batf | 0.7868466 |
| Bax | -0.7110903 |
| Baz1a | -0.5004255 |
| Baz1b | 2.83846833 |
| Baz2a | -0.3669086 |
| BB014433 | -0.5377515 |
| BB128963 | 0.33716929 |
| BB146404 | -0.5928066 |
| Bbc3 | 0.22648576 |
| Bbc3 | 0.8628217 |
| Bbox1 | -0.2680558 |
| Bbs1 | 0.07617972 |
| Bbs12 | 0.84152371 |
| Bbs2 | 2.60000059 |
| Bbs4 | -0.3339572 |
| Bbs5 | -0.3718 |
| Bbs7 | -0.2680558 |
| Bbs9 | -0.4093262 |
| Bbs9 | 0.07047311 |
| Bbx | -0.2874541 |
| BC002163 | 0.50157587 |
| BC002199 | -0.2593205 |
| BC002216 | 0.33716929 |
| BC003236 | 0.08072 |
| BC003266 | -0.3032685 |
| BC003885 | 0.44124753 |
| BC003885 | 0.49965062 |
| BC003993 | 1.3747353 |
| BC003993 | -0.5131777 |
| BC004004 | -0.819443 |
| BC004044 | -0.455104 |
| BC004044 | 0.04022272 |
| BC004728 | 0.15749005 |
| BC004728 | -0.6790184 |
| BC005537 | 1.61303498 |
| BC005682 | -0.4692871 |
| BC005764 | -0.6781667 |
| BC006662 | 1.73540186 |
| BC006779 | -0.7110903 |
| BC006779 | 1.65334971 |
| BC008155 | 1.9862418 |
| BC008163 | -0.3130025 |
| BC008163 | 0.89664291 |
| BC010462 | 0.41101274 |
| BC011248 | -0.5210575 |
| BC011248 | -0.6781667 |
| BC011487 | -0.5966991 |
| BC013491 | -0.4955129 |
| BC013491 | 0.01383718 |
| BC013529 | 0.36929721 |
| BC013712 | -0.3428292 |
| BC016201 | -0.2111365 |
| BC016423 | -0.7693097 |
| BC016495 | 0.74852985 |
| BC017158 | 1.70267217 |
| BC017612 | 1.45440916 |
| BC017643 | 0.20493081 |
| BC017647 | 1.14057556 |
| BC018242 | 2.82656787 |
| BC018242 | -0.7618305 |
| BC018465 | 0.83009806 |
| BC020002 | -0.1444278 |
| BC020535 | 0.35534202 |
| BC021381 | -0.1492447 |
| BC021395 | -0.5835304 |
| BC021614 | -0.6621306 |
| BC021790 | 2.81775276 |
| BC021891 | -0.1141751 |
| BC022224 | -0.3941341 |
| BC022687 | 2.57018429 |
| BC023055 | -0.6496624 |
| BC023151 | 1.17074698 |
| BC023744 | 2.50455806 |
| BC023814 | 0.97857427 |
| BC023829 | 2.30763407 |
| BC024561 | 0.23099469 |
| BC024814 | -0.7244131 |
| BC024868 | -0.4768033 |
| BC024997 | 0.12422734 |
| BC025076 | 2.23904169 |
| BC025546 | -0.3038383 |
| BC025575 | 0.64934377 |
| BC025920 | 0.37454593 |
| BC026439 | -0.2941989 |
| BC026585 | 3.73573993 |
| BC026590 | -0.6308622 |
| BC026590 | -0.5675156 |
| BC027072 | -0.0902971 |
| BC027231 | -0.0175003 |
| BC027231 | -0.744304 |
| BC027231 | 0.59397134 |
| BC027344 | -0.6373585 |
| BC028528 | 1.25270311 |
| BC028528 | 1.2722726 |
| BC029169 | 0.85396535 |
| BC029169 | 4.42112065 |
| BC029214 | 2.05097624 |
| BC030183 | 1.09614324 |
| BC030307 | -0.049927 |
| BC030476 | -0.7479665 |
| BC030499 | -0.2730055 |
| BC030500 | 0.99727396 |
| BC030867 | -0.5032443 |
| BC031181 | -0.820434 |
| BC031353 | 0.5854405 |
| BC031575 | -0.5181844 |
| BC031748 | -0.6596236 |
| BC031781 | 0.16957829 |
| BC031853 | -0.0305912 |
| BC032265 | 1.67300913 |
| BC034076 | -0.2840999 |
| BC037034 | 0.70297043 |
| BC037594 | -0.6962175 |
| BC038156 | -0.5153202 |
| BC038286 | -0.5912541 |
| BC038613 | -0.4241211 |
| BC038822 | 0.41471418 |
| BC038822 | -0.2549834 |
| BC038822 | -0.4325884 |
| BC038925 | -0.7433906 |
| BC039093 | -0.2059902 |
| BC040758 | 0.09755196 |
| BC043098 | -0.5540042 |
| BC043118 | 0.55393068 |
| BC043301 | -0.311852 |
| BC046331 | 0.99642563 |
| BC046404 | 1.07258624 |
| BC048355 | -0.6546297 |
| BC048502 | 0.05469461 |
| BC048546 | 0.32301256 |
| BC048546 | 1.57744852 |
| BC048562 | 2.16326367 |
| BC048644 | -0.6092653 |
| BC048651 | -0.568272 |
| BC048671 | -0.3044084 |
| BC049635 | 0.09420977 |
| BC049635 | 0.97525209 |
| BC049730 | -0.7102088 |
| BC049762 | 3.82116957 |
| BC049806 | 0.03864467 |
| BC049806 | -0.2902585 |
| BC049806 | -0.532639 |
| BC050196 | 3.11808092 |
| BC050210 | -0.802755 |
| BC050777 | 0.47746658 |
| BC050811 | -0.122585 |
| BC050811 | 0.04807322 |
| BC051019 | -0.2987227 |
| BC051070 | 1.89306485 |
| BC051142 | 1.46148215 |
| BC051227 | 1.35288625 |
| BC051628 | 2.66435754 |
| BC051665 | 1.3668752 |
| BC052484 | -0.5189018 |
| BC053749 | 1.10272993 |
| BC053749 | 2.24023591 |
| BC054059 | -0.6084749 |
| BC055107 | 0.60134536 |
| BC055111 | -0.4036001 |
| BC055324 | 0.48483843 |
| BC055368 | 0.40703055 |
| BC056474 | -0.5096179 |
| BC056923 | -0.1604188 |
| BC057022 | 1.30596774 |
| BC057371 | 2.08135779 |
| BC057371 | -0.4774896 |
| BC057371 | 1.63290965 |
| BC057552 | -0.819443 |
| BC057627 | 0.25070502 |
| BC057893 | -0.8154895 |
| BC060267 | 0.68061907 |
| BC060615 | 3.02353441 |
| BC060632 | 0.90119695 |
| BC061039 | 1.16674862 |
| BC061237 | -0.0352808 |
| BC062115 | 1.15917551 |
| BC062650 | -0.6260195 |
| BC063749 | 0.55142713 |
| BC064033 | -0.2533621 |
| BC065085 | 0.51423132 |
| BC065397 | -0.2647705 |
| BC066028 | -0.752565 |
| BC066107 | 1.54917399 |
| BC067047 | 0.77131217 |
| BC067068 | 0.02632337 |
| BC068157 | 1.696347 |
| BC068281 | -0.1348681 |
| BC072620 | -0.4417939 |
| BC085271 | 2.4125384 |
| BC087945 | -0.210105 |
| BC088983 | 2.13325971 |
| BC089491 | -0.6807241 |
| BC099439 | 0.93760411 |
| BC107230 | -0.2908204 |
| Bcam | 1.65173929 |
| Bcan | 1.46414218 |
| Bcan | -0.2735571 |
| Bcap29 | -0.7306962 |
| Bcap29 | -0.7646281 |
| Bcap31 | -0.4384958 |
| Bcap31 | -0.3785677 |
| Bcar1 | -0.4899301 |
| Bcar3 | -0.737929 |
| Bcas1 | 0.82416222 |
| Bcas2 | -0.7415665 |
| Bcas3 | 2.85273709 |
| Bcat1 | 0.8825263 |
| Bcat2 | -0.6521427 |
| Bccip | -0.3608302 |
| Bcdin3d | -0.2620415 |
| Bcdo2 | -0.4906261 |
| Bckdha | -0.6654838 |
| Bckdhb | -0.0808247 |
| Bckdk | 0.25822441 |
| Bckdk | -0.6892998 |
| Bcl10 | 2.51431196 |
| Bcl11a | 0.4655491 |
| Bcl11b | 2.66563498 |
| Bcl2 | 0.34913499 |
| Bcl2 | -0.6439004 |
| Bcl2l1 | 0.81114144 |
| Bcl2l1 | 0.67044377 |
| Bcl2l1 | -0.698825 |
| Bcl2l10 | -0.7146245 |
| Bcl2l11 | -0.5495435 |
| Bcl2l11 | -0.7190611 |
| Bcl2l11 | 0.30330103 |
| Bcl2l12 | 3.33700694 |
| Bcl2l13 | -0.2919452 |
| Bcl2l14 | 0.38331967 |
| Bcl2l14 | 0.55142713 |
| Bcl2l2 | -0.6696923 |
| Bcl2l2 | 0.85905982 |
| Bcl3 | -0.4836891 |
| Bcl6 | -0.3240033 |
| Bcl6b | 0.87417845 |
| Bcl7a | 0.54433532 |
| Bcl7b | 0.01545773 |
| Bcl9l | -0.622003 |
| Bclaf1 | -0.423473 |
| Bclaf1 | -0.6513152 |
| Bclp2 | -0.1434674 |
| Bcmo1 | -0.5253837 |
| Bcor | -0.2691534 |
| Bcor | 0.42023895 |
| Bcorl1 | -0.0200197 |
| Bcs1l | 1.10989694 |
| Bdh1 | 1.55276526 |
| Bdh2 | -0.1049188 |
| Bdkrb1 | -0.4431164 |
| Bdnf | 0.28316407 |
| Bdnf | -0.4398136 |
| Becn1 | -0.4885396 |
| Becn1 | -0.5081978 |
| Bet1 | -0.0975793 |
| Bet1l | 1.682889 |
| Bex2 | 3.39881613 |
| Bex2 | -0.4530953 |
| Bex2 | 1.01975425 |
| Bex4 | -0.1801766 |
| Bex4 | 2.28149165 |
| Bex6 | -0.744304 |
| Bfar | -0.7352101 |
| Bfar | 0.90510508 |
| Bglap2 | 1.36153434 |
| Bgn | 1.45596637 |
| Bhlhb2 | 3.58631651 |
| Bhlhb5 | -0.4754322 |
| Bhlhb8 | 0.07845262 |
| Bhlhb8 | 1.21228797 |
| Bhlhb9 | 1.32376511 |
| Bhmt | -0.2256991 |
| Bhmt2 | -0.5253837 |
| Bicc1 | -0.0058321 |
| Bicd1 | -0.5697867 |
| Bicd2 | 2.23919105 |
| Bicd2 | 0.92654073 |
| Bid | -0.6341047 |
| Bid | -0.1453892 |
| Bik | 1.22996514 |
| Bin1 | 1.36592372 |
| Bin1 | -0.2533621 |
| Bin1 | 0.35534202 |
| Bin3 | 0.52952139 |
| Birc2 | -0.542156 |
| Birc2 | -0.1841789 |
| Birc2 | -0.2774275 |
| Birc4 | 1.31807073 |
| Birc5 | 0.20822889 |
| Birc5 | 2.34753702 |
| Birc5 | 0.65180288 |
| Blcap | 1.33760555 |
| Blm | -0.3275043 |
| Blm | 3.470543 |
| Blmh | -0.0263482 |
| Blnk | -0.7930763 |
| Bloc1s1 | -0.4061407 |
| Bloc1s3 | -0.7969357 |
| Blvra | 0.12134094 |
| Blvrb | 0.84933985 |
| Bmf | 1.79532565 |
| Bmi1 | 1.38486476 |
| Bmp1 | -0.6513152 |
| Bmp1 | 0.15400731 |
| Bmp1 | 0.05391805 |
| Bmp1 | 1.57013481 |
| Bmp15 | 1.71073264 |
| Bmp2k | 1.25087417 |
| Bmp2k | 0.05547051 |
| Bmp3 | 0.75147722 |
| Bmp4 | 1.35250002 |
| Bmp6 | -0.5297301 |
| Bmp7 | 2.38898857 |
| Bmp8a | 0.90697519 |
| Bmper | -0.6629678 |
| Bmpr1a | 1.9032355 |
| Bmpr1a | 0.92699798 |
| Bmpr1b | 1.39451621 |
| Bmpr1b | 1.84942337 |
| Bmpr2 | -0.6962175 |
| Bms1 | -0.8304023 |
| Bnc1 | 1.76205435 |
| Bnc2 | -0.0191791 |
| Bnc2 | 0.17879766 |
| Bnip1 | -0.4962131 |
| Bnip2 | 1.16438862 |
| Bnip3l | 1.61106063 |
| Bnipl | 2.10837866 |
| Bnipl | -0.5362879 |
| Bok | 0.34317132 |
| Bok | -0.6773158 |
| Bola1 | 1.22875292 |
| Bola2 | 2.26264053 |
| Bola3 | -0.6884387 |
| Boll | 1.48732941 |
| Bop1 | -0.0595279 |
| Bpi | -0.0799276 |
| Bpil1 | 1.87453978 |
| Bpil3 | -0.5622379 |
| Bpnt1 | -0.6212018 |
| Bpnt1 | -0.7093282 |
| Bpnt1 | -0.4424549 |
| Bpnt1 | -0.6316718 |
| Brap | -0.821426 |
| Brca2 | 0.33716929 |
| Brca2 | -0.831405 |
| Brcc3 | -0.4138042 |
| Brcc3 | -0.7998409 |
| Brcc3 | 2.01398018 |
| Brd2 | 2.37656809 |
| Brd2 | -0.7066913 |
| Brd2 | 0.89048325 |
| Brd4 | 2.11624946 |
| Brd4 | 0.98174098 |
| Brd8 | -0.6713811 |
| Brd8 | -0.7424781 |
| Brd9 | 2.0301424 |
| Brd9 | -0.2746613 |
| Brdt | -0.4871512 |
| Brdt | -0.7461334 |
| Bre | 0.21085899 |
| Bre | -0.1291789 |
| Bre | -0.5089076 |
| Brf1 | 1.77975516 |
| Brf2 | 1.74768582 |
| Bri3 | 2.87040444 |
| Brip1 | -0.645543 |
| Brms1 | 1.51605285 |
| Brp16 | 0.73102345 |
| Brp16 | 0.1314045 |
| Brp17 | 2.34500705 |
| Brp44 | -0.4325884 |
| Brpf1 | 0.01707546 |
| Brpf1 | -0.4099646 |
| Brs3 | -0.4312808 |
| Brsk1 | -0.457117 |
| Brsk2 | -0.5018338 |
| Brsk2 | -0.7882743 |
| Brunol4 | -0.7424781 |
| Brunol4 | -0.717284 |
| Brwd1 | -0.7075694 |
| Brwd1 | -0.451091 |
| Brwd2 | 1.03589319 |
| Bscl2 | -0.1761914 |
| Bsdc1 | -0.6901617 |
| Bsg | 0.73851316 |
| Bsg | 1.24373942 |
| Bspry | 0.28377151 |
| Bst2 | -0.6546297 |
| Btbd1 | 2.09505319 |
| Btbd10 | -0.5046569 |
| Btbd11 | 0.60026459 |
| Btbd12 | -0.0058321 |
| Btbd14a | -0.6867188 |
| Btbd14b | -0.7163966 |
| Btbd16 | -0.0994088 |
| Btbd3 | 0.24723827 |
| Btbd3 | -0.189206 |
| Btbd6 | 2.75682002 |
| Btbd9 | 1.08373086 |
| Btc | -0.5607354 |
| Btd | 2.41325129 |
| Btf3 | -0.5584863 |
| Btg1 | 1.11887201 |
| Btg1 | 0.25947177 |
| Btg3 | -0.6546297 |
| Btg3 | -0.4934155 |
| Btg4 | 0.79811135 |
| Btk | -0.457789 |
| Btla | -0.691888 |
| Btrc | -0.2539022 |
| Btrc | -0.5333677 |
| Btrc | 1.86343549 |
| Bub1b | -0.1254052 |
| Bub3 | 1.25141247 |
| Bud13 | 0.97987104 |
| Bud31 | -0.088033 |
| Bves | 1.09512576 |
| Bxdc1 | 0.92897683 |
| Bxdc1 | -0.5607354 |
| Bxdc2 | 0.23356148 |
| Bxdc2 | 2.1708875 |
| Bxdc5 | 1.54313141 |
| Bxdc5 | 2.25935779 |
| Bxdc5 | -0.5562425 |
| Bzrap1 | 0.56140127 |
| Bzrpl1 | -0.7049375 |
| Bzw1 | -0.4557745 |
| Bzw2 | 2.95430669 |
| Bzw2 | 1.02744823 |
| C030006K11Rik | 0.89554032 |
| C030011O14Rik | -0.535557 |
| C030014K22Rik | 0.02552283 |
| C030018G13Rik | 1.81382677 |
| C030030A07Rik | -0.4788637 |
| C030039L03Rik | 0.46154254 |
| C030045D06Rik | 0.04219155 |
| C030045D06Rik | -0.6389897 |
| C030046I01Rik | -0.6480124 |
| C030048B08Rik | -0.602176 |
| C030048B08Rik | -0.4969139 |
| C130022K22Rik | 1.94433079 |
| C130032J12Rik | 1.91069847 |
| C130034I18Rik | -0.4645354 |
| C130038G02Rik | -0.38228 |
| C130060K24Rik | 4.02361327 |
| C130073D16Rik | 1.94063604 |
| C130074G19Rik | -0.7988715 |
| C130090K23Rik | -0.7608998 |
| C130090K23Rik | 2.79279947 |
| C1galt1 | 0.31805041 |
| C1galt1c1 | -0.8164763 |
| C1qa | -0.2309556 |
| C1qb | 0.53254957 |
| C1qbp | 1.35722032 |
| C1qc | -0.6850021 |
| C1qdc2 | 0.78702366 |
| C1ql2 | -0.3866298 |
| C1ql3 | 0.35393495 |
| C1qtnf1 | 0.25164826 |
| C1qtnf2 | 1.0873308 |
| C1qtnf3 | 2.53751423 |
| C1qtnf3 | -0.4358657 |
| C1qtnf3 | 1.2908212 |
| C1qtnf4 | 1.01712931 |
| C1qtnf6 | -0.3446137 |
| C1qtnf7 | -0.5728234 |
| C1qtnf9 | 0.43996431 |
| C1rl | 0.79495686 |
| C1s | 4.0806314 |
| C1s | 0.3227214 |
| C2 | -0.2560658 |
| C230021P08Rik | 0.99032322 |
| C230029F24Rik | -0.7479665 |
| C230052I12Rik | -0.4809287 |
| C230052I12Rik | 0.75294742 |
| C230055K05Rik | 3.38820833 |
| C230071H18Rik | -0.2885749 |
| C230078M08Rik | -0.6300534 |
| C230078M14Rik | -0.6638057 |
| C230090D14 | -0.082172 |
| C230093N12Rik | 0.82314085 |
| C230096C10Rik | -0.3055497 |
| C2cd3 | 0.76099238 |
| C3 | -0.6596236 |
| C330001K17Rik | 0.93110321 |
| C330005M16Rik | -0.5842999 |
| C330016O10Rik | -0.7873168 |
| C330019G07Rik | -0.3316055 |
| C330023M02Rik | -0.7882743 |
| C330027C09Rik | -0.4537644 |
| C430003P19Rik | -0.7244131 |
| C430004E15Rik | 0.13959056 |
| C4b | 0.25196246 |
| C530028I08Rik | 2.59578081 |
| C530028I08Rik | -0.2730055 |
| C530028O21Rik | 0.41918914 |
| C530043G21Rik | -0.5146055 |
| C530044N13Rik | 3.81413406 |
| C6 | 0.25728779 |
| C630004H02Rik | -0.3066924 |
| C630004H02Rik | -0.3038383 |
| C630007B19Rik | -0.30213 |
| C630028N24Rik | 0.88380398 |
| C630035N08Rik | -0.5060716 |
| C630035N08Rik | -0.6538 |
| C730025P13Rik | -0.7352101 |
| C730027P07Rik | -0.0004639 |
| C78339 | 0.2690827 |
| C79127 | 3.40053837 |
| C79267 | 0.55233829 |
| C79407 | -0.8095904 |
| C79407 | 3.10718773 |
| C80008 | 1.34064265 |
| C80913 | 0.30478651 |
| C85492 | 1.09346999 |
| C85492 | -0.6596236 |
| C85492 | -0.5384841 |
| C85627 | -0.1155715 |
| C87436 | 3.17524619 |
| C87436 | 1.84652727 |
| C87499 | -0.705814 |
| C8b | 2.01083788 |
| C8g | 1.9307632 |
| C9 | 2.549157 |
| C9 | 1.1203595 |
| C920005C14Rik | 3.97447053 |
| Cab39l | 0.26413458 |
| Cabc1 | 0.62249074 |
| Cables2 | -0.5253837 |
| Cabp2 | -0.2807577 |
| Cabp7 | 0.64646659 |
| Cabyr | 2.31681802 |
| Cabyr | 1.89046408 |
| Cachd1 | 1.20654625 |
| Cacna1a | -0.6156124 |
| Cacna1b | -0.4280196 |
| Cacna1b | 0.05469461 |
| Cacna1b | 0.55620082 |
| Cacna1d | -0.4948133 |
| Cacna1g | -0.6430801 |
| Cacna1h | 2.39006912 |
| Cacna2d1 | 0.21511715 |
| Cacna2d3 | -0.5781615 |
| Cacnb1 | -0.705814 |
| Cacnb1 | -0.3559959 |
| Cacnb2 | 2.8085088 |
| Cacnb2 | 0.22938686 |
| Cacnb3 | -0.4948133 |
| Cacnb3 | -0.4955129 |
| Cacnb3 | 2.32349365 |
| Cacnb3 | 1.15118098 |
| Cacnb4 | 1.38037635 |
| Cacnb4 | 2.42007356 |
| Cacng2 | 1.24156656 |
| Cacng3 | -0.6422606 |
| Cacng3 | -0.1382029 |
| Cacng4 | 1.08321544 |
| Cacng5 | -0.6414418 |
| Cacng5 | -0.2194302 |
| Cacng5 | -0.5081978 |
| Cacng7 | 1.81805819 |
| Cacng8 | -0.3891246 |
| Cacybp | -0.1324933 |
| Cad | -0.3386784 |
| Cadm2 | -0.636544 |
| Cadm3 | -0.4144457 |
| Cadps | -0.4761175 |
| Cadps2 | 2.3087437 |
| Cadps2 | 0.42023895 |
| Cage1 | -0.4598078 |
| Calb2 | 1.21453002 |
| Calb2 | -0.5025388 |
| Calca | 1.1175064 |
| Calca | 1.25087417 |
| Calcoco1 | -0.698825 |
| Cald1 | 1.92382617 |
| Cald1 | -0.6654838 |
| Calm2 | 2.3621491 |
| Calm3 | -0.5951401 |
| Calml4 | -0.6341047 |
| Caln1 | 0.04415623 |
| Calr | -0.3316055 |
| Calr3 | -0.5812257 |
| Calr3 | 0.02792239 |
| Calr3 | -0.4809287 |
| Calr4 | 1.58943714 |
| Calu | -0.2902585 |
| Calu | 0.06280961 |
| Calu | 1.23216516 |
| Caly | -0.7288967 |
| Camk1 | 2.15798337 |
| Camk1d | 0.95240132 |
| Camk1g | 0.24121974 |
| Camk1g | 1.36620926 |
| Camk2a | -0.7479665 |
| Camk2a | 0.68081749 |
| Camk2a | 0.52882118 |
| Camk2b | -0.4099646 |
| Camk2d | -0.1434674 |
| Camk2d | 1.02936185 |
| Camk2d | -0.4247697 |
| Camk2g | 1.08011697 |
| Camk2g | 1.18252438 |
| Camk2n1 | 0.93125491 |
| Camk4 | 0.38086038 |
| Camkk1 | 1.24384793 |
| Camkk2 | 2.19217571 |
| Camkk2 | 1.48849883 |
| Camkv | 0.26009483 |
| Caml | 0.09420977 |
| Camp | 1.8883456 |
| Camsap1l1 | 2.29022851 |
| Camta2 | -0.1067627 |
| Cant1 | -0.3960196 |
| Cant1 | -0.5959193 |
| Cap1 | -0.2528223 |
| Capg | -0.5827615 |
| Capg | -0.6730728 |
| Capn1 | -0.0839715 |
| Capn10 | -0.6612942 |
| Capn11 | 0.02832172 |
| Capn2 | 2.32489119 |
| Capn2 | -0.5974795 |
| Capn2 | 0.28133938 |
| Capn3 | 0.384138 |
| Capn5 | 0.24058395 |
| Capn6 | -0.5644961 |
| Capn7 | -0.5110402 |
| Capn8 | 1.17332505 |
| Capns1 | -0.5569898 |
| Caprin1 | -0.6061078 |
| Caprin1 | -0.5275544 |
| Capsl | -0.6471886 |
| Capza1 | 1.61987623 |
| Capza3 | 0.18286609 |
| Capzb | -0.4843805 |
| Capzb | 1.46148215 |
| Capzb | 1.41956601 |
| Car11 | -0.3754854 |
| Car12 | 0.28437855 |
| Car13 | 1.069583 |
| Car14 | 0.21642345 |
| Car15 | -0.0862257 |
| Car2 | -0.445767 |
| Car3 | 0.35730835 |
| Car4 | 0.08598922 |
| Car5b | -0.3872529 |
| Car5b | -0.2925081 |
| Car5b | -0.2686044 |
| Car7 | -0.4768033 |
| Car9 | 2.37414804 |
| Card10 | -0.0451633 |
| Card11 | -0.366299 |
| Carhsp1 | 2.85779165 |
| Carm1 | 0.39740336 |
| Cart | 2.61304695 |
| Cartpt | 0.87675499 |
| Casc1 | 1.77841012 |
| Casc1 | 2.54596432 |
| Casc1 | 4.43322683 |
| Casc3 | -0.2620415 |
| Casc4 | 0.16476177 |
| Casc4 | -0.5414205 |
| Casc4 | -0.4398136 |
| Cask | -0.8008112 |
| Caskin1 | -0.3897493 |
| Caskin1 | 1.71865884 |
| Caskin1 | -0.6053201 |
| Caskin2 | -0.6638057 |
| Casp1 | -0.3032685 |
| Casp2 | 1.87343528 |
| Casp4 | -0.622805 |
| Casp4 | -0.4010665 |
| Casp4 | 0.27400471 |
| Casp6 | -0.5592354 |
| Casp6 | -0.4631822 |
| Casp7 | 0.10603911 |
| Casp8 | 1.30474876 |
| Casp8 | -0.6389897 |
| Casp9 | 0.57771764 |
| Casq2 | -0.4836891 |
| Cass4 | 0.38058673 |
| Cast | -0.69535 |
| Cat | 0.10198967 |
| Catsper1 | 3.72424915 |
| Catsper2 | -0.6696923 |
| Catsper3 | -0.5904789 |
| Catsper4 | 1.39488543 |
| Cav1 | -0.6962175 |
| Cav2 | 0.8529761 |
| Cbara1 | 2.0638608 |
| Cbfa2t3h | -0.3590144 |
| Cbfa2t3h | -0.1405922 |
| Cbfb | -0.6292452 |
| Cbfb | -0.2090746 |
| Cbfb | 0.30508332 |
| Cbl | 0.63736584 |
| Cblb | -0.6705363 |
| Cblc | -0.2013827 |
| Cbln1 | 0.63090023 |
| Cbln2 | 0.43507193 |
| Cbln4 | 2.39630546 |
| Cbr1 | 0.14171429 |
| Cbr2 | -0.6944833 |
| Cbr3 | 0.79302385 |
| Cbr3 | -0.505364 |
| Cbs | -0.6996958 |
| Cbs | 1.29740125 |
| Cbwd1 | 1.21564902 |
| Cbx2 | 1.17822712 |
| Cbx5 | 0.83330463 |
| Cbx7 | 0.35252576 |
| Cbx8 | 0.2749247 |
| Cby1 | -0.3972788 |
| Cc2d1a | -0.1109249 |
| Cc2d1b | -0.6252148 |
| Cc2d2a | 1.68843028 |
| Ccar1 | -0.4686068 |
| Ccbe1 | -0.6944833 |
| Ccbl1 | 1.63018841 |
| Ccbl1 | 1.12640892 |
| Ccbl2 | 0.01788327 |
| Ccbp2 | 0.24850039 |
| Ccdc100 | 0.24597443 |
| Ccdc101 | -0.3210957 |
| Ccdc108 | 0.46704714 |
| Ccdc109a | 0.28528838 |
| Ccdc109a | 1.4694396 |
| Ccdc109b | 1.72178456 |
| Ccdc11 | -0.6807241 |
| Ccdc110 | 0.16786096 |
| Ccdc111 | -0.5392174 |
| Ccdc113 | -0.6747677 |
| Ccdc114 | 0.5001323 |
| Ccdc115 | 0.07466137 |
| Ccdc116 | -0.4286709 |
| Ccdc117 | -0.2485152 |
| Ccdc12 | -0.739746 |
| Ccdc120 | 2.15936726 |
| Ccdc123 | -0.471331 |
| Ccdc124 | 0.4986865 |
| Ccdc125 | 0.25227656 |
| Ccdc126 | -0.4686068 |
| Ccdc127 | 0.28831474 |
| Ccdc127 | 2.78827095 |
| Ccdc13 | -0.5436289 |
| Ccdc130 | 0.10787397 |
| Ccdc131 | 0.50732792 |
| Ccdc132 | 1.15476909 |
| Ccdc134 | -0.3675186 |
| Ccdc137 | 2.9215197 |
| Ccdc137 | -0.5370194 |
| Ccdc18 | -0.5773971 |
| Ccdc21 | 2.54470033 |
| Ccdc23 | -0.5261067 |
| Ccdc25 | 0.22842083 |
| Ccdc27 | -0.7516435 |
| Ccdc27 | 0.5199061 |
| Ccdc27 | -0.7271007 |
| Ccdc28a | -0.1053794 |
| Ccdc28b | -0.2891357 |
| Ccdc28b | -0.7815927 |
| Ccdc3 | 1.07440958 |
| Ccdc32 | 0.29253522 |
| Ccdc38 | -0.6324821 |
| Ccdc39 | 0.16303554 |
| Ccdc40 | -0.445767 |
| Ccdc41 | 2.73939999 |
| Ccdc42 | -0.617206 |
| Ccdc43 | 0.31247308 |
| Ccdc45 | -0.4144457 |
| Ccdc46 | 0.45297104 |
| Ccdc46 | -0.4892346 |
| Ccdc47 | 1.96349178 |
| Ccdc49 | -0.5124647 |
| Ccdc5 | -0.5982606 |
| Ccdc50 | -0.58507 |
| Ccdc51 | 2.75112131 |
| Ccdc53 | 2.30238012 |
| Ccdc53 | -0.3903744 |
| Ccdc53 | 0.53765189 |
| Ccdc54 | 0.7693295 |
| Ccdc56 | 0.33141695 |
| Ccdc57 | 0.12925714 |
| Ccdc58 | -0.4391545 |
| Ccdc59 | -0.6422606 |
| Ccdc59 | -0.3089821 |
| Ccdc64 | -0.5370194 |
| Ccdc65 | 1.98536001 |
| Ccdc65 | 0.87787998 |
| Ccdc66 | 0.69244966 |
| Ccdc67 | -0.7969357 |
| Ccdc68 | -0.0412838 |
| Ccdc70 | -0.682433 |
| Ccdc71 | -0.3773336 |
| Ccdc73 | -0.3055497 |
| Ccdc77 | 1.50635792 |
| Ccdc79 | 2.38774756 |
| Ccdc80 | -0.6841449 |
| Ccdc82 | 0.92775957 |
| Ccdc84 | 2.04064056 |
| Ccdc85a | -0.7488844 |
| Ccdc85b | 0.45221094 |
| Ccdc86 | 1.0079533 |
| Ccdc87 | -0.6180038 |
| Ccdc88a | -0.8455574 |
| Ccdc88b | 1.02949838 |
| Ccdc88c | -0.4530953 |
| Ccdc90b | 0.5948419 |
| Ccdc91 | 1.11961605 |
| Ccdc92 | -0.7969357 |
| Ccdc93 | -0.467927 |
| Ccdc94 | 2.9430312 |
| Ccdc95 | 1.14251281 |
| Ccdc96 | 3.11482921 |
| Ccdc97 | 1.30981736 |
| Ccdc98 | 0.74167818 |
| Ccdc99 | 0.22130586 |
| Cchcr1 | -0.8394658 |
| Ccin | 0.03508425 |
| Cck | 0.75844013 |
| Cckbr | -0.545104 |
| Ccl11 | -0.4927174 |
| Ccl17 | -0.6381738 |
| Ccl19 | -0.8364348 |
| Ccl21b | -0.5819933 |
| Ccl21c | 1.87735035 |
| Ccl25 | -0.602961 |
| Ccl25 | -0.1155715 |
| Ccl27 | 0.21772793 |
| Ccl27 | 0.80247496 |
| Ccl28 | 1.05295672 |
| Ccl3 | -0.4955129 |
| Ccl4 | 1.25944984 |
| Ccl4 | 0.06165464 |
| Ccl5 | 2.54437733 |
| Ccl6 | -0.9557067 |
| Ccl7 | -0.5697867 |
| Ccl9 | -0.4802398 |
| Ccm2 | -0.3181972 |
| Ccna1 | 1.83098555 |
| Ccnb1 | 2.48278541 |
| Ccnb1 | -0.7646281 |
| Ccnb3 | 0.36707835 |
| Ccnc | -0.3614363 |
| Ccnd1 | -0.6006079 |
| Ccnd1 | -0.0830713 |
| Ccnd1 | 1.35923601 |
| Ccnd2 | -0.4490909 |
| Ccnd3 | -0.4564455 |
| Ccnd3 | -0.85477 |
| Ccndbp1 | 1.5262896 |
| Ccne1 | -0.2309556 |
| Ccne2 | 1.38858868 |
| Ccnf | -0.4878451 |
| Ccng1 | -0.648837 |
| Ccng1 | -0.4280196 |
| Ccng1 | -0.6430801 |
| Ccng2 | 1.02539355 |
| Ccnk | 0.28982425 |
| Ccnl1 | -0.5904789 |
| Ccno | 0.0585677 |
| Ccr4 | 1.26530022 |
| Ccr5 | -0.6037467 |
| Ccr5 | -0.5217771 |
| Ccr5 | 0.82789773 |
| Ccr6 | 1.18287194 |
| Ccr6 | 1.91878674 |
| Ccrk | 1.29586321 |
| Ccrk | 2.26616571 |
| Ccrk | -0.2697028 |
| Ccrk | 2.08060576 |
| Ccrl2 | -0.719951 |
| Ccrn4l | 0.05275201 |
| Ccrn4l | 0.62670496 |
| Ccs | 1.9486739 |
| Cct2 | -0.5827615 |
| Cct3 | 1.43197619 |
| Cct3 | -0.7352101 |
| Cct4 | -0.1988327 |
| Cct5 | -0.568272 |
| Cct6a | -0.4477599 |
| Cct6b | -0.8425066 |
| Cct7 | -0.0736716 |
| Cct8 | -0.3656897 |
| Cd109 | 0.42520953 |
| Cd109 | 2.91363368 |
| Cd14 | 0.17266155 |
| Cd151 | -0.1221158 |
| Cd151 | -0.5297301 |
| Cd151 | -0.755335 |
| Cd160 | -0.0246564 |
| Cd163 | -0.7854049 |
| Cd164 | -0.7023129 |
| Cd177 | 1.01269161 |
| Cd180 | 2.04363821 |
| Cd1d1 | -0.4061407 |
| Cd2 | 0.98604197 |
| Cd200 | -0.7979031 |
| Cd200 | -0.5392174 |
| Cd226 | -0.7882743 |
| Cd247 | -0.2852166 |
| Cd248 | 1.13168547 |
| Cd248 | -0.4260683 |
| Cd27 | -0.5392174 |
| Cd274 | -0.8506643 |
| Cd276 | -0.6722266 |
| Cd2bp2 | 1.2053032 |
| Cd300lg | -0.2341238 |
| Cd320 | -0.3928792 |
| Cd34 | -0.4990192 |
| Cd3d | -0.5713038 |
| Cd3eap | 0.99585964 |
| Cd3g | -0.5146055 |
| Cd4 | 0.68200706 |
| Cd40 | 0.15086168 |
| Cd40 | -0.0062463 |
| Cd44 | 2.4095881 |
| Cd44 | -0.7415665 |
| Cd44 | -0.4497571 |
| Cd44 | -0.8947029 |
| Cd47 | 0.04101075 |
| Cd52 | 0.19929717 |
| Cd53 | -0.5705449 |
| Cd55 | 2.55835182 |
| Cd59a | -0.657956 |
| Cd59b | 1.01947829 |
| Cd59b | -0.4464308 |
| Cd6 | 0.22130586 |
| Cd6 | -0.2631321 |
| Cd6 | 0.10493646 |
| Cd63 | -0.6284377 |
| Cd63 | 0.16026692 |
| Cd63 | -0.752565 |
| Cd68 | -0.0305912 |
| Cd74 | -0.3626496 |
| Cd74 | -0.5131777 |
| Cd79b | -0.698825 |
| Cd79b | 0.04297792 |
| Cd81 | -0.7190611 |
| Cd82 | -0.6884387 |
| Cd83 | -0.8264022 |
| Cd84 | -0.7730719 |
| Cd86 | -0.293635 |
| Cd86 | -0.6696923 |
| Cd86 | -0.5928066 |
| Cd8b1 | 2.20968068 |
| Cd9 | 0.445342 |
| Cd93 | 0.07123601 |
| Cd96 | 0.56095021 |
| Cd97 | 2.02174283 |
| Cd97 | -0.5897043 |
| Cd97 | -0.7740148 |
| Cd99l2 | -0.274109 |
| Cdan1 | -0.648837 |
| Cdc123 | 1.75899842 |
| Cdc14b | -0.568272 |
| Cdc14b | -0.6471886 |
| Cdc14b | 0.78967556 |
| Cdc16 | -0.3493893 |
| Cdc20 | -0.8766266 |
| Cdc20 | 1.69192728 |
| Cdc20 | 1.17449455 |
| Cdc20 | 0.0597265 |
| Cdc23 | -0.7664979 |
| Cdc25a | -0.233595 |
| Cdc25b | -0.622805 |
| Cdc25c | -0.2506663 |
| Cdc25c | -0.7969357 |
| Cdc27 | 1.22399905 |
| Cdc2a | 0.66258708 |
| Cdc2l1 | -0.7618305 |
| Cdc2l1 | -0.7711889 |
| Cdc2l5 | 3.73813954 |
| Cdc2l6 | 2.64205401 |
| Cdc34 | -0.3572021 |
| Cdc37 | 0.74000392 |
| Cdc37l1 | 1.47029121 |
| Cdc37l1 | 1.91382579 |
| Cdc37l1 | -0.4557745 |
| Cdc37l1 | 0.1346163 |
| Cdc42 | 1.99409203 |
| Cdc42bpb | 1.31586357 |
| Cdc42ep1 | 0.36066961 |
| Cdc42ep2 | -0.6116404 |
| Cdc42ep3 | -0.4927174 |
| Cdc42ep4 | 2.8386451 |
| Cdc42ep5 | -0.8703294 |
| Cdc42se1 | 0.23994772 |
| Cdc42se1 | -0.4557745 |
| Cdc45l | 0.08109736 |
| Cdc5l | 0.62817545 |
| Cdc5l | 1.80077723 |
| Cdc5l | -0.5203384 |
| Cdc5l | 0.87514549 |
| Cdc6 | 0.6448185 |
| Cdc7 | 0.445342 |
| Cdc73 | 0.97756442 |
| Cdca2 | -0.5333677 |
| Cdca3 | -0.0179197 |
| Cdca3 | -0.1063014 |
| Cdca4 | -0.5340969 |
| Cdca4 | 1.22996514 |
| Cdca4 | 0.95136273 |
| Cdca5 | 3.98487058 |
| Cdca7 | -0.0710033 |
| Cdca8 | 0.11262779 |
| Cdcp1 | -0.349988 |
| Cdgap | -0.7352101 |
| Cdh1 | -0.6901617 |
| Cdh11 | -0.6936174 |
| Cdh11 | -0.1751978 |
| Cdh12 | 1.74850745 |
| Cdh13 | 1.31877191 |
| Cdh13 | 0.00814302 |
| Cdh16 | -0.2452979 |
| Cdh22 | -0.7618305 |
| Cdh23 | -0.6944833 |
| Cdh23 | 0.27523116 |
| Cdh23 | -0.7461334 |
| Cdh24 | 0.97929492 |
| Cdh26 | 0.42833525 |
| Cdh3 | 0.90244933 |
| Cdh5 | -0.1722232 |
| Cdh6 | -0.5210575 |
| Cdh7 | -0.7674342 |
| Cdh8 | 0.43403868 |
| Cdh8 | -0.7208417 |
| Cdh8 | -0.4733793 |
| Cdipt | -0.1132453 |
| Cdipt | -0.2807577 |
| Cdk10 | -0.6832886 |
| Cdk10 | 0.31188405 |
| Cdk10 | -0.7084484 |
| Cdk2 | 0.84917423 |
| Cdk2 | 1.33799799 |
| Cdk2 | 0.98989598 |
| Cdk2 | -0.3112773 |
| Cdk2ap2 | -0.5089076 |
| Cdk3 | 0.52155477 |
| Cdk5 | -0.6629678 |
| Cdk5r1 | -0.0817227 |
| Cdk5rap1 | -0.691888 |
| Cdk5rap2 | 2.3143066 |
| Cdk5rap3 | 1.83766981 |
| Cdk6 | -0.7334019 |
| Cdk8 | -0.6422606 |
| Cdk9 | -0.7452183 |
| Cdkal1 | 0.31863557 |
| Cdkl1 | -0.5554958 |
| Cdkl2 | -0.226748 |
| Cdkl2 | -0.3205153 |
| Cdkl2 | -0.5319109 |
| Cdkl2 | 0.93941026 |
| Cdkl3 | 1.57547337 |
| Cdkl4 | 1.229855 |
| Cdkn1a | 0.65323437 |
| Cdkn1a | 1.62204909 |
| Cdkn1a | -0.467927 |
| Cdkn1b | 0.01667129 |
| Cdkn1b | -0.5465815 |
| Cdkn1c | -0.0648067 |
| Cdkn2a | 1.44719489 |
| Cdkn2a | 0.07123601 |
| Cdkn2aipnl | 0.36150803 |
| Cdkn2b | -0.3327806 |
| Cdkn2c | -0.0934761 |
| Cdkn2c | -0.1511784 |
| Cdkn2d | 1.20643332 |
| Cdo1 | 1.05972214 |
| Cdr2 | 0.38250063 |
| Cdrt4 | -0.5599851 |
| Cds1 | 1.31001954 |
| Cds2 | -0.3878764 |
| Cdsn | 0.69811445 |
| Cdt1 | 1.44053977 |
| Cdv3 | -0.3193556 |
| Cdv3 | 2.90932878 |
| Cdyl | -0.0365639 |
| Cdyl | 0.63528505 |
| Cdyl | -0.2415583 |
| Cdyl2 | -0.6414418 |
| Ceacam1 | 0.4091565 |
| Ceacam13 | -0.186689 |
| Ceacam2 | -0.7882743 |
| Cebpa | 0.64213413 |
| Cebpb | -0.5562425 |
| Cecr5 | 1.03670641 |
| Cecr6 | 0.17573465 |
| Celsr1 | -0.3935064 |
| Celsr2 | 0.0585677 |
| Celsr3 | 1.58191512 |
| Cend1 | 0.01869039 |
| Cenpa | 1.65677135 |
| Cenpb | -0.2998571 |
| Cenph | 0.65507164 |
| Cenpi | -0.6188023 |
| Cenpj | -0.4913227 |
| Cenpk | 0.44355288 |
| Cenpl | 1.482385 |
| Cenpl | -0.5599851 |
| Cenpm | -0.2008721 |
| Cenpm | -0.6587894 |
| Cenpm | -0.2846581 |
| Cenpn | 1.78122026 |
| Cenpp | -0.6713811 |
| Cenpq | 1.16792639 |
| Cenpt | -0.6349171 |
| Centa1 | 0.17539369 |
| Centa1 | 1.32645036 |
| Centa2 | -0.6260195 |
| Centb5 | 2.33875262 |
| Centb5 | -0.0008758 |
| Centd2 | 1.72243444 |
| Centd2 | 0.24217262 |
| Centd3 | 0.28952254 |
| Centg1 | 2.13150133 |
| Centg1 | 0.20228392 |
| Centg2 | -0.5333677 |
| Centg2 | 0.59614622 |
| Centg3 | 0.33458513 |
| Centg3 | -0.3245859 |
| Cep135 | 2.01810621 |
| Cep164 | -0.4850724 |
| Cep170 | 2.06183105 |
| Cep55 | -0.4983169 |
| Cep57 | -0.6300534 |
| Cep57 | -0.1687648 |
| Cep63 | 0.63131872 |
| Cep68 | -0.3559959 |
| Cep70 | 2.33252356 |
| Cep72 | -0.1618862 |
| Cep78 | 1.72379771 |
| Cep97 | 0.20526114 |
| Cept1 | -0.2008721 |
| Cercam | -0.3841418 |
| Ces3 | 0.21577053 |
| Cetn2 | -0.2708024 |
| Cetn3 | 2.56994858 |
| Cetn4 | -0.4384958 |
| Cetn4 | 0.55574724 |
| Cfd | 1.18414523 |
| Cfdp1 | 1.27615869 |
| Cfh | 1.05042368 |
| Cfhr1 | -0.6006079 |
| Cfi | 0.04454867 |
| Cfl1 | 0.36624492 |
| Cfl2 | -0.8224191 |
| Cfl2 | 1.29647873 |
| Cfp | 1.06025069 |
| Cftr | 0.36262476 |
| Cga | 0.48410386 |
| Cggbp1 | 0.11226297 |
| Cgnl1 | 2.25283105 |
| Cgrrf1 | 2.33461577 |
| Ch25h | 0.41445028 |
| Chac1 | 1.88709398 |
| Chad | 1.23161564 |
| Chaf1a | 0.47894562 |
| Chaf1b | 1.01768255 |
| Chat | 1.44018818 |
| Chchd1 | 0.88412313 |
| Chchd2 | -0.274109 |
| Chchd4 | -0.4892346 |
| Chchd5 | 0.91194389 |
| Chchd6 | 2.30468019 |
| Chchd7 | -0.381041 |
| Chchd8 | 0.04415623 |
| Chd1 | 0.96696751 |
| Chd1 | -0.6084749 |
| Chd1l | -0.5081978 |
| Chd1l | 0.10603911 |
| Chd4 | -0.4202389 |
| Chd4 | 0.65118871 |
| Chd4 | 2.35531684 |
| Chd5 | -0.7892327 |
| Chd8 | 0.56004743 |
| Chdh | 0.11626816 |
| Chdh | 2.66186862 |
| Chek1 | -0.6124335 |
| Chek1 | -0.451091 |
| Chek2 | -0.4029661 |
| Chek2 | 0.19730068 |
| Cherp | -0.3428292 |
| Chfr | -0.6773158 |
| Chfr | -0.6688491 |
| Chfr | -0.6006079 |
| Chga | 1.52460464 |
| Chga | 0.84485773 |
| Chga | 0.13211919 |
| Chgb | -0.697955 |
| Chi3l1 | 3.40750249 |
| Chi3l3 | 1.88687614 |
| Chi3l3 | 1.46139622 |
| Chia | 2.34243677 |
| Chia | 0.76281114 |
| Chic1 | 4.39398987 |
| Chic2 | -0.1821756 |
| Chic2 | 0.12961538 |
| Chit1 | 1.15429147 |
| Chka | 1.43100008 |
| Chka | -0.6571233 |
| Chka | 2.40466051 |
| Chkb | -0.56299 |
| Chl1 | -0.6730728 |
| Chl1 | -0.6061078 |
| Chl1 | 0.72536644 |
| Chm | -0.726204 |
| Chmp1b | -0.6260195 |
| Chmp2a | 2.22311016 |
| Chmp2a | 1.18818506 |
| Chmp2b | -0.755335 |
| Chmp4b | -0.1254052 |
| Chmp5 | 0.31365003 |
| Chmp7 | 2.37653586 |
| Chmp7 | -0.8425066 |
| Chmp7 | -0.5974795 |
| Chn2 | 0.35026661 |
| Chodl | 1.55253147 |
| Chordc1 | 1.81087695 |
| Chpf | 1.84709584 |
| Chpt1 | -0.69535 |
| Chpt1 | -0.2752139 |
| Chpt1 | -0.5124647 |
| Chrac1 | 1.30921058 |
| Chrd | 1.77202539 |
| Chrm1 | -0.1334425 |
| Chrm2 | 0.39713451 |
| Chrm3 | 0.93140659 |
| Chrna1 | -0.3481931 |
| Chrna4 | -0.5990424 |
| Chrna4 | 0.95254959 |
| Chrna4 | -0.4273687 |
| Chrna5 | -0.4067769 |
| Chrna6 | -0.7998409 |
| Chrna6 | 0.44814656 |
| Chrna7 | -0.75997 |
| Chrna9 | -0.1035382 |
| Chrnb1 | 0.26692112 |
| Chrnb1 | 1.44045189 |
| Chrnb2 | -0.7406558 |
| Chrnb2 | -0.6389897 |
| Chrnb3 | -0.5089076 |
| Chrnb3 | -0.6053201 |
| Chrnb3 | 0.57816067 |
| Chrne | -0.0284673 |
| Chst1 | 0.4254704 |
| Chst10 | 1.48615853 |
| Chst12 | 0.90479303 |
| Chst14 | -0.4983169 |
| Chst2 | 1.56584185 |
| Chst3 | 0.37123438 |
| Chst3 | -0.1978147 |
| Chst7 | 1.88031705 |
| Chst8 | 1.69514369 |
| Chtf18 | -0.7507229 |
| Chuk | -0.3193556 |
| Churc1 | 1.65131872 |
| Ciao1 | 3.09234307 |
| Ciapin1 | 1.97068894 |
| Cib1 | 0.08561384 |
| Cib2 | 2.7502365 |
| Cib2 | -0.3147309 |
| Cic | 1.24287088 |
| Cidea | -0.0705593 |
| Cideb | -0.2730055 |
| Cidec | 2.31729889 |
| Ciita | 1.07479983 |
| Cirbp | 3.19497058 |
| Cirh1a | -0.4444407 |
| Cisd1 | -0.6841449 |
| Cish | -0.5781615 |
| Cit | 2.65249354 |
| Cited1 | 3.53137937 |
| Cited2 | -0.3158849 |
| Cited4 | -0.4692871 |
| Ciz1 | 0.82348143 |
| Ciz1 | -0.7279982 |
| Ckap2l | -0.4093262 |
| Ckap5 | -0.296458 |
| Ckb | 1.6187153 |
| Cklf | -0.6212018 |
| Cklf | -0.1967978 |
| Ckmt1 | 0.29972621 |
| Ckmt2 | -0.1258761 |
| Ckmt2 | -0.6308622 |
| Ckt2 | -0.5781615 |
| Ckt2 | 0.81492934 |
| Clca5 | 2.00115422 |
| Clcc1 | -0.0083202 |
| Clcf1 | 0.84185764 |
| Clcf1 | 0.03349747 |
| Clcf1 | 0.47820639 |
| Clcn1 | -0.4431164 |
| Clcn2 | -0.5690291 |
| Clcn3 | 2.04749787 |
| Clcn3 | -0.4404732 |
| Clcn3 | 2.97770981 |
| Clcn4-2 | -0.6962175 |
| Clcn7 | -0.6680066 |
| Clcnka | -0.3316055 |
| Cldn1 | -0.6061078 |
| Cldn10 | 1.24308809 |
| Cldn10 | 0.81784585 |
| Cldn10 | 0.57149299 |
| Cldn11 | 2.07976453 |
| Cldn12 | -0.636544 |
| Cldn15 | -0.0835213 |
| Cldn15 | -0.6204013 |
| Cldn17 | -0.6504884 |
| Cldn2 | -0.3675186 |
| Cldn23 | 0.45271774 |
| Cldn3 | -0.454434 |
| Cldn4 | 1.55097135 |
| Cldn5 | -0.3590144 |
| Cldn6 | -0.2947632 |
| Cldn9 | 0.56095021 |
| Cldnd1 | -0.6252148 |
| Cldnd2 | -0.1653192 |
| Clec11a | 0.25133395 |
| Clec16a | -0.7901921 |
| Clec1a | -0.6439004 |
| Clec2d | 1.40150658 |
| Clec2e | -0.4378375 |
| Clec3b | 0.38331967 |
| Clec4a1 | 2.0189576 |
| Clec4a3 | 1.87575322 |
| Clec4b1 | 0.38441061 |
| Clec4b1 | 1.74844428 |
| Clec4d | -0.5866122 |
| Clec4g | 0.22745379 |
| Clec4n | 0.67324806 |
| Clec7a | 1.33110634 |
| Clecsf12 | -0.6140216 |
| Clgn | 0.20195253 |
| Clic1 | 1.66930282 |
| Clic3 | -0.622003 |
| Clic4 | 0.06434735 |
| Clic6 | 2.23765895 |
| Clip1 | -0.0339995 |
| Clip1 | 3.04168824 |
| Clip4 | -0.813519 |
| Clk1 | 0.84834569 |
| Clk2 | 1.67833682 |
| Clk2 | -0.633293 |
| Clk2 | -0.6447213 |
| Clk3 | 0.90025655 |
| Clk4 | 1.179623 |
| Clk4 | -0.6646444 |
| Clk4 | -0.6841449 |
| Clm3 | 0.55005873 |
| Clm3 | 1.25495745 |
| Clmn | -0.4969139 |
| Clmn | -0.6521427 |
| Cln3 | -0.6414418 |
| Cln5 | 0.26102863 |
| Cln6 | 0.97264384 |
| Cln6 | -0.3620427 |
| Cln8 | -0.890418 |
| Clns1a | -0.4652127 |
| Clns1a | -0.0516652 |
| Clns1a | 1.56214639 |
| Clock | -0.182676 |
| Clock | -0.0037637 |
| Clp1 | -0.3187762 |
| Clpb | 1.74489953 |
| Clpp | -0.1123165 |
| Clptm1 | 1.48657687 |
| Clptm1l | -0.6188023 |
| Clpx | 1.66737522 |
| Clpx | 0.19529991 |
| Clrn1 | 0.75331461 |
| Clspn | -0.739746 |
| Clspn | 0.50325722 |
| Clspn | 1.13656724 |
| Clstn1 | 2.65527055 |
| Clstn3 | -0.739746 |
| Clstn3 | 0.54410565 |
| Clta | -0.102619 |
| Cltb | 0.27185452 |
| Cluap1 | 0.98102221 |
| Cma1 | 1.87547758 |
| Cma1 | 0.37344338 |
| Cmas | 0.23931106 |
| Cmbl | -0.5246613 |
| Cml1 | -0.6236076 |
| Cml3 | -0.5117522 |
| Cml4 | 1.87255074 |
| Cmpk | -0.682433 |
| Cmtm2a | 2.98137414 |
| Cmtm2b | 0.68141246 |
| Cmtm4 | -0.7093282 |
| Cmtm4 | -0.8145037 |
| Cmtm5 | -0.5232181 |
| Cmtm6 | -0.5399512 |
| Cmtm7 | 4.40027984 |
| Cmtm7 | 0.40782834 |
| Cmtm7 | 3.48587041 |
| Cmtm8 | -0.7921139 |
| Cmtm8 | 1.43055606 |
| Cmya1 | -0.7217333 |
| CN716893 | 1.49249716 |
| Cnbp2 | 1.94653042 |
| Cnbp2 | -0.4781764 |
| Cndp1 | 1.21609625 |
| Cnga4 | -0.5835304 |
| Cnih | -0.2549834 |
| Cnih3 | 0.11226297 |
| Cnih4 | 1.4786805 |
| Cnksr2 | 1.5942196 |
| Cnksr2 | -0.3529871 |
| Cnksr3 | -0.187695 |
| Cnn1 | 1.55727377 |
| Cnn2 | -0.7674342 |
| Cnn2 | 0.51042886 |
| Cnnm1 | 2.07363173 |
| Cnnm1 | -0.3112773 |
| Cnnm2 | -0.4183038 |
| Cnnm3 | -0.6180038 |
| Cno | -0.5304565 |
| Cnot10 | -0.0050042 |
| Cnot2 | -0.0943864 |
| Cnot2 | 0.74982047 |
| Cnot2 | 0.80073196 |
| Cnot2 | -0.2947632 |
| Cnot3 | 1.17039487 |
| Cnot3 | 1.32565554 |
| Cnot4 | -0.7049375 |
| Cnot4 | 1.74604038 |
| Cnot4 | -0.340455 |
| Cnot6l | -0.3626496 |
| Cnot6l | -0.4384958 |
| Cnot7 | -0.7352101 |
| Cnp | 0.03508425 |
| Cnp | 0.00446405 |
| Cnpy2 | 2.37724458 |
| Cnpy3 | -0.6730728 |
| Cnr1 | -0.1667943 |
| Cnr1 | -0.7608998 |
| Cntd1 | -0.1104615 |
| Cntd1 | 3.88578425 |
| Cntfr | -0.3327806 |
| Cntln | -0.5096179 |
| Cntn1 | 1.42037457 |
| Cntn4 | 0.70025419 |
| Cntn6 | 0.89900122 |
| Cntnap1 | -0.6284377 |
| Cntnap2 | 0.94136299 |
| Cntnap4 | 2.82559252 |
| Cntnap4 | 1.32505897 |
| Coasy | 0.19529991 |
| Cobl | 1.47046144 |
| Cobll1 | -0.8404783 |
| Cobll1 | -0.2469052 |
| Coch | -0.2080453 |
| Cog1 | 2.19292149 |
| Cog2 | -0.4857648 |
| Cog3 | -0.5858408 |
| Cog4 | -0.029741 |
| Cog6 | 1.56668667 |
| Cog6 | -0.5502854 |
| Cog8 | -0.2049643 |
| Coil | 3.10346907 |
| Coil | -0.1791787 |
| Col11a1 | 1.8864403 |
| Col12a1 | 1.27762489 |
| Col14a1 | 3.06830412 |
| Col15a1 | -0.4699679 |
| Col16a1 | 2.24023591 |
| Col17a1 | -0.1742052 |
| Col17a1 | -0.5637428 |
| Col17a1 | 3.30738197 |
| Col18a1 | 0.57060036 |
| Col18a1 | -0.3754854 |
| Col1a1 | -0.7181721 |
| Col1a2 | 0.49940968 |
| Col24a1 | 0.61974125 |
| Col24a1 | -0.7470495 |
| Col25a1 | 1.60871523 |
| Col25a1 | 1.80578135 |
| Col25a1 | 1.7784713 |
| Col27a1 | -0.7759035 |
| Col28a1 | -0.1287063 |
| Col2a1 | -0.7479665 |
| Col2a1 | -0.4202389 |
| Col3a1 | 0.95860871 |
| Col3a1 | -0.2549834 |
| Col4a1 | -0.0250791 |
| Col4a2 | 0.26568369 |
| Col4a3 | -0.5766332 |
| Col4a3bp | 1.95881394 |
| Col4a3bp | -0.6850021 |
| Col4a3bp | 0.13639586 |
| Col4a4 | 1.99185584 |
| Col4a4 | 0.7401901 |
| Col4a5 | -0.1786802 |
| Col4a6 | 1.49341104 |
| Col4a6 | -0.5340969 |
| Col5a1 | -0.5812257 |
| Col5a3 | 0.57171602 |
| Col6a1 | 2.56263832 |
| Col6a1 | 1.0216837 |
| Col6a1 | 1.33337636 |
| Col6a2 | -0.4983169 |
| Col7a1 | -0.532639 |
| Col8a2 | -0.4029661 |
| Col9a2 | -0.0037637 |
| Colec12 | 0.36624492 |
| Commd1 | 0.70857189 |
| Commd1 | -0.5720633 |
| Commd10 | -0.3357249 |
| Commd2 | -0.3638645 |
| Commd2 | -0.3960196 |
| Commd4 | 0.01058763 |
| Commd5 | -0.7608998 |
| Commd6 | 1.00837235 |
| Commd8 | -0.4464308 |
| Commd9 | -0.5217771 |
| Comp | -0.5290043 |
| Comtd1 | 0.09122886 |
| Copa | -0.6546297 |
| Copb2 | -0.4365225 |
| Cope | -0.6053201 |
| Cope | -0.5990424 |
| Cope | -0.6148167 |
| Cope | 0.77239185 |
| Copg | -0.0790313 |
| Copg | -0.7516435 |
| Copg | -0.5920301 |
| Copg | 0.445342 |
| Cops2 | -0.4604817 |
| Cops3 | -0.7854049 |
| Cops4 | -0.5873843 |
| Cops4 | 1.89479468 |
| Cops5 | 1.91578165 |
| Cops5 | 0.3941721 |
| Cops6 | 1.02826883 |
| Cops7a | 0.07617972 |
| Cops7a | -0.4843805 |
| Cops7a | -0.1628658 |
| Cops7b | -0.6116404 |
| Cops7b | 3.57121363 |
| Cops8 | 1.60636393 |
| Copz1 | 0.14100691 |
| Copz2 | -0.5011294 |
| Copz2 | -0.4955129 |
| Copz2 | 3.88158096 |
| Copz2 | 0.17197726 |
| Coq10b | 0.68734148 |
| Coq10b | 0.81938621 |
| Coq2 | -0.0288917 |
| Coq3 | 2.27094157 |
| Coq3 | -0.717284 |
| Coq4 | -0.3135783 |
| Coq4 | 2.16225055 |
| Coq5 | 1.04641008 |
| Coq5 | -0.6140216 |
| Coq5 | 2.17365535 |
| Coq6 | 0.45145021 |
| Coq6 | 0.26506435 |
| Coq7 | -0.5348266 |
| Coq9 | -0.6621306 |
| Corin | 0.18826323 |
| Coro1a | 2.07527999 |
| Coro1a | -0.4016992 |
| Coro1a | -0.3816603 |
| Coro1a | 2.90212786 |
| Coro1b | -0.4604817 |
| Coro1b | -0.7534874 |
| Coro1c | 0.59157309 |
| Coro2a | 1.34376739 |
| Coro2a | -0.2614967 |
| Coro2b | -0.0464601 |
| Coro2b | 1.69607973 |
| Coro6 | 2.25301501 |
| Coro7 | 2.12041457 |
| Cort | -0.5311834 |
| Cort | -0.4280196 |
| Cotl1 | -0.7066913 |
| Cova1 | -0.381041 |
| Cox10 | -0.5781615 |
| Cox11 | 1.85428673 |
| Cox15 | -0.6268249 |
| Cox17 | -0.6480124 |
| Cox18 | -0.7787438 |
| Cox18 | -0.5039503 |
| Cox19 | 2.09147318 |
| Cox4i1 | 1.01975425 |
| Cox4nb | -0.7777961 |
| Cox4nb | 0.70837929 |
| Cox5a | -0.3742553 |
| Cox5b | 2.06304943 |
| Cox6a1 | -0.3903744 |
| Cox6a1 | -0.7137397 |
| Cox6a2 | 3.30154092 |
| Cox6b1 | -0.3493893 |
| Cox6b1 | 1.61544241 |
| Cox6b2 | 0.18489368 |
| Cox6b2 | -0.7084484 |
| Cox6c | 0.56095021 |
| Cox7a1 | 0.32155584 |
| Cox7a2 | -0.5584863 |
| Cox7a2l | 0.36485421 |
| Cox7b | -0.6798709 |
| Cox7b2 | -0.6252148 |
| Cox8b | -0.5735841 |
| Cox8c | 1.06275723 |
| Cp | -0.6252148 |
| Cpa1 | -0.2746613 |
| Cpa2 | -0.067015 |
| Cpa2 | 2.43819706 |
| Cpa3 | 0.04807322 |
| Cpa3 | 0.02030253 |
| Cpa3 | -0.6092653 |
| Cpa4 | 1.31344978 |
| Cpa5 | -0.5773971 |
| Cpa6 | -0.8066549 |
| Cpb2 | -0.1007832 |
| Cpeb1 | 2.60918732 |
| Cpeb2 | 0.15783761 |
| Cpeb3 | -0.2506663 |
| Cpeb4 | 1.33268607 |
| Cphx | 2.54572241 |
| Cplx1 | -0.2768736 |
| Cplx2 | 0.93971095 |
| Cpn1 | 0.36206656 |
| Cpne2 | -0.5517711 |
| Cpne4 | 2.05780363 |
| Cpne5 | -0.7693097 |
| Cpne6 | 0.52037745 |
| Cpne7 | 2.22273021 |
| Cpne8 | 2.21552004 |
| Cpne8 | -0.2512048 |
| Cpne9 | -0.3711871 |
| Cpsf1 | 1.18691728 |
| Cpsf1 | -0.4658906 |
| Cpsf2 | 0.09940357 |
| Cpsf3l | 1.06578248 |
| Cpsf4 | 1.42494318 |
| Cpsf4l | 0.19128545 |
| Cpsf4l | -0.7208417 |
| Cpsf6 | -0.007905 |
| Cpsf6 | -0.7352101 |
| Cpt1b | 1.88649479 |
| Cpt1b | -0.4036001 |
| Cpt1c | -0.2724542 |
| Cpt2 | -0.0608448 |
| Cpxcr1 | -0.6006079 |
| Cpxm1 | 0.14277435 |
| Cpxm2 | -0.5138913 |
| Cpz | -0.697955 |
| Crabp2 | -0.5275544 |
| Crat | 1.74204128 |
| Crat | -0.2075311 |
| Crat | -0.6944833 |
| Crb1 | 1.30627223 |
| Crbn | -0.3374959 |
| Crbn | 0.10235853 |
| Crbn | 4.20699691 |
| Crcp | -0.3452093 |
| Creb1 | -0.6292452 |
| Creb1 | -0.6513152 |
| Creb3 | 2.06070151 |
| Creb3l1 | -0.6284377 |
| Creb3l2 | 0.02911986 |
| Creb3l2 | 3.44670591 |
| Creb3l3 | -0.6654838 |
| Creb3l4 | 1.8182927 |
| Creb5 | 1.49291267 |
| Crebl1 | -0.0582128 |
| Crebl2 | 1.0727166 |
| Creg2 | 2.52767229 |
| Creld1 | -0.652971 |
| Creld2 | 0.24597443 |
| Creld2 | 0.43584612 |
| Crem | 0.93519046 |
| Crem | 2.42433908 |
| Crem | 1.00094119 |
| Crem | 0.9439104 |
| Crhbp | -0.2178695 |
| Crhr1 | -0.7370218 |
| Crim1 | 0.71299064 |
| Crim2 | -0.3321929 |
| Crip2 | -0.7461334 |
| Crip3 | 1.36172561 |
| Crip3 | 1.22177968 |
| Cript | -0.6892998 |
| Cript | -0.3089821 |
| Crisp2 | -0.5392174 |
| Crispld2 | -0.4189484 |
| Crk | -0.2953278 |
| Crk | 3.14230554 |
| Crk | -0.7155101 |
| Crkl | 1.25527905 |
| Crkrs | 0.30538004 |
| Crkrs | 4.49590651 |
| Crlf3 | 1.10738847 |
| Crlf3 | -0.4927174 |
| Crls1 | -0.6546297 |
| Crls1 | 0.67604394 |
| Crls1 | -0.7424781 |
| Crls1 | -0.6513152 |
| Crmp1 | 1.40260555 |
| Crmp1 | 0.33400989 |
| Crnkl1 | -0.6773158 |
| Crocc | 0.20757021 |
| Crry | 3.09637446 |
| Crry | 0.66339595 |
| Crtac1 | 1.51410566 |
| Crtam | 1.28128034 |
| Crtap | -0.080376 |
| Crtap | 0.2749247 |
| Crtc1 | -0.7749587 |
| Crtc3 | 0.01424258 |
| Crxos1 | 0.46604871 |
| Cry2 | 1.70399834 |
| Cryab | -0.2998571 |
| Cryba2 | -0.2469052 |
| Cryba4 | -0.5637428 |
| Crybb1 | -0.3422351 |
| Crybb2 | 0.71241546 |
| Crybb3 | -0.56299 |
| Crygn | -0.6546297 |
| Crygn | -0.7674342 |
| Cryl1 | -0.542156 |
| Crym | 0.82992899 |
| Cryz | 0.60737463 |
| Cryz | 1.53204362 |
| Cryz | 0.28377151 |
| Cryzl1 | -0.5743455 |
| Cryzl1 | -0.0916582 |
| Cs | 2.38723796 |
| Csad | -0.6680066 |
| Csda | -0.5502854 |
| Csda | 0.87353321 |
| Csda | -0.1932472 |
| Csdc2 | 0.93307338 |
| Csde1 | 1.78292665 |
| Csde1 | -0.0630438 |
| Csde1 | 0.86884178 |
| Cse1l | -0.633293 |
| Csf1 | 1.69854903 |
| Csf1r | -0.3321929 |
| Csf1r | 0.13497248 |
| Csf2ra | -0.5547497 |
| Csgalnact1 | -0.6389897 |
| Csgalnact2 | -0.1254052 |
| Csk | 2.09068549 |
| Csl | -0.3280891 |
| Csl | 0.3227214 |
| Csmd1 | 0.30002464 |
| Csnk1d | -0.3038383 |
| Csnk1d | 1.20167759 |
| Csnk1d | 1.39100128 |
| Csnk1d | 1.81676729 |
| Csnk1d | -0.1521468 |
| Csnk1e | 0.92164936 |
| Csnk1e | -0.0263482 |
| Csnk1g1 | 0.53208433 |
| Csnk1g1 | -0.5018338 |
| Csnk1g1 | -0.622003 |
| Csnk1g2 | -0.6996958 |
| Csnk1g2 | 1.37010301 |
| Csnk1g2 | 1.11874795 |
| Csnk1g3 | -0.4016992 |
| Csnk2a1 | -0.0808247 |
| Csnk2a2 | 2.22656055 |
| Csnk2a2 | 0.33516 |
| Csnrp2 | -0.6308622 |
| Csnrp3 | 0.92973681 |
| Cspg4 | -0.2528223 |
| Cspg5 | 1.41677562 |
| Cspp1 | 0.88140689 |
| Csprs | 0.81027841 |
| Csrp1 | 0.85741948 |
| Csrp2 | -0.6430801 |
| Csrp2bp | 0.93277055 |
| Cst11 | -0.0503612 |
| Cst3 | 1.13899858 |
| Cst8 | 2.46454827 |
| Cst9 | 0.58653856 |
| Cstad | 3.00506963 |
| Cstb | -0.4941141 |
| Cstf1 | 3.95907248 |
| Cstf2 | 0.51375686 |
| Cstf3 | -0.4202389 |
| Cstf3 | 0.11626816 |
| Cstl1 | 0.04297792 |
| Ctage5 | 0.18759029 |
| Ctcf | 1.15142062 |
| Ctdp1 | -0.6756163 |
| Ctdp1 | -0.5181844 |
| Ctdp1 | 0.44201661 |
| Ctdsp1 | 1.57866191 |
| Ctdsp1 | 1.95542929 |
| Ctdsp1 | -0.7128557 |
| Ctdsp2 | -0.6680066 |
| Ctdspl | -0.5362879 |
| Ctdspl2 | -0.4809287 |
| Ctdspl2 | 0.05080537 |
| Ctf1 | -0.3596193 |
| Ctgf | -0.5074885 |
| Ctla4 | -0.6244109 |
| Ctnna1 | -0.2746613 |
| Ctnna3 | -0.1207096 |
| Ctnnal1 | -0.5224973 |
| Ctnnb1 | -0.7787438 |
| Ctnnb1 | 0.13390352 |
| Ctnnb1 | 0.76860748 |
| Ctnnbip1 | -0.0962096 |
| Ctnnbl1 | 0.68734148 |
| Ctnnbl1 | -0.4809287 |
| Ctnnd2 | -0.5935838 |
| Ctnnd2 | 0.1195324 |
| Ctnnd2 | -0.4829982 |
| Ctns | -0.5096179 |
| Ctns | -0.0626035 |
| Ctps | -0.6357302 |
| Ctps | -0.3529871 |
| Ctps | 0.36457582 |
| Ctps2 | -0.5577378 |
| Ctps2 | 0.82518248 |
| Ctps2 | 0.37206337 |
| Ctr9 | -0.5547497 |
| Ctrb1 | -0.5951401 |
| Ctrl | 1.62709951 |
| Ctsa | -0.1846804 |
| Ctsa | -0.2790911 |
| Ctsa | -0.3614363 |
| Ctsa | -0.1927411 |
| Ctsb | 0.92425109 |
| Ctsc | 1.7035344 |
| Ctsc | 2.79378909 |
| Ctsc | -0.5842999 |
| Ctse | -0.6713811 |
| Ctsf | 1.31475802 |
| Ctsh | -0.2404926 |
| Ctsk | 0.0239197 |
| Ctsl | -0.6596236 |
| Ctso | 0.4542365 |
| Ctsr | -0.1127808 |
| Ctss | -0.6471886 |
| Ctsw | -0.226748 |
| Ctsz | -0.0683426 |
| Ctsz | -0.1058403 |
| Cttn | -0.6308622 |
| Cttn | 1.17601271 |
| Cttnbp2nl | -0.4557745 |
| Ctxn1 | -0.0455953 |
| Cuedc1 | 0.37866889 |
| Cuedc2 | 0.21184337 |
| Cugbp1 | 1.48732941 |
| Cugbp1 | -0.5443661 |
| Cugbp1 | 1.85940538 |
| Cugbp1 | -0.5728234 |
| Cugbp2 | -0.433898 |
| Cugbp2 | -0.1663023 |
| Cul1 | 1.39608439 |
| Cul2 | 1.18714791 |
| Cul2 | -0.5562425 |
| Cul3 | -0.5340969 |
| Cul4a | 0.61379162 |
| Cul4a | -0.0547151 |
| Cul4a | -0.4584614 |
| Cul4b | 1.28544053 |
| Cul5 | -0.1244642 |
| Cul7 | 0.06280961 |
| Cuta | 0.49554621 |
| Cuta | -0.6132272 |
| Cuta | -0.6496624 |
| Cutc | -0.1856842 |
| Cutc | 0.26165065 |
| Cux1 | 0.60630083 |
| Cux1 | -0.3222577 |
| Cux1 | -0.3321929 |
| Cux1 | 2.12591118 |
| Cux1 | 1.51751058 |
| Cux2 | 3.47732246 |
| Cux2 | 0.35421653 |
| Cuzd1 | -0.3380869 |
| Cwf19l2 | 0.3098195 |
| Cx3cl1 | -0.2283237 |
| Cx3cr1 | 2.61596863 |
| Cxadr | 0.62249074 |
| Cxadr | -0.627631 |
| Cxadr | -0.1570038 |
| Cxcl1 | 1.2845061 |
| Cxcl12 | -0.8808484 |
| Cxcl12 | -0.7324991 |
| Cxcl12 | 1.67465166 |
| Cxcl13 | -0.2147559 |
| Cxcl14 | 0.12242439 |
| Cxcl14 | -0.7279982 |
| Cxcl15 | 0.91240835 |
| Cxcl16 | -0.1942603 |
| Cxcl17 | 1.31606443 |
| Cxcl4 | -0.4823079 |
| Cxcl9 | -0.636544 |
| Cxcr3 | -0.7279982 |
| Cxcr4 | 0.53138605 |
| Cxcr5 | -0.7049375 |
| Cxcr7 | -0.3078366 |
| Cxx1a | -0.5812257 |
| Cxx1b | -0.4504238 |
| Cxx1c | -0.4598078 |
| Cxxc1 | -0.5167512 |
| Cxxc1 | -0.5554958 |
| Cxxc4 | -0.3055497 |
| Cxxc4 | 3.04126159 |
| Cyb5 | 0.65303001 |
| Cyb561 | -0.2178695 |
| Cyb561d1 | 1.25055105 |
| Cyb561d1 | 1.89781419 |
| Cyb561d2 | 1.15727258 |
| Cyb5b | 0.63131872 |
| Cyb5d1 | 1.98403577 |
| Cyb5d2 | 1.46491303 |
| Cyb5d2 | -0.636544 |
| Cyb5r1 | 0.65139348 |
| Cyb5r2 | -0.2790911 |
| Cyb5r3 | -0.6381738 |
| Cyb5r3 | 0.71929425 |
| Cyb5r3 | 3.01706862 |
| Cyb5r4 | 0.22938686 |
| Cyba | 0.20393911 |
| Cybasc3 | -0.4768033 |
| Cyc1 | -0.6781667 |
| Cycs | 2.60392358 |
| Cycs | -0.5804587 |
| Cyfip1 | 0.8135537 |
| Cyfip2 | 1.18483901 |
| Cyfip2 | 0.79443007 |
| Cygb | -0.6612942 |
| Cyhr1 | 0.367356 |
| Cyhr1 | 3.12414568 |
| Cyhr1 | -0.5812257 |
| Cyhr1 | -0.6373585 |
| Cyld | -0.5998248 |
| Cyln2 | 3.76042114 |
| Cyp11a1 | -0.461831 |
| Cyp11b2 | -0.210105 |
| Cyp17a1 | -0.5812257 |
| Cyp19a1 | -0.4352093 |
| Cyp1b1 | -0.3847632 |
| Cyp20a1 | -0.5458424 |
| Cyp24a1 | -0.6480124 |
| Cyp26a1 | 1.31797051 |
| Cyp26b1 | -0.6596236 |
| Cyp27a1 | -0.3798035 |
| Cyp27a1 | -0.1160374 |
| Cyp27a1 | -0.4365225 |
| Cyp2a12 | -0.6807241 |
| Cyp2a5 | 2.06350027 |
| Cyp2a5 | -0.4969139 |
| Cyp2b10 | 0.06319428 |
| Cyp2c55 | 3.46034107 |
| Cyp2d10 | 0.94256263 |
| Cyp2d22 | -0.4892346 |
| Cyp2d26 | 0.00282429 |
| Cyp2d26 | -0.323421 |
| Cyp2d26 | 1.2927795 |
| Cyp2e1 | 2.2567599 |
| Cyp2f2 | -0.682433 |
| Cyp2j11 | 1.44106691 |
| Cyp2j9 | -0.3061209 |
| Cyp2s1 | -0.5436289 |
| Cyp2u1 | -0.5675156 |
| Cyp39a1 | -0.2325384 |
| Cyp3a13 | 0.4887464 |
| Cyp3a13 | -0.7271007 |
| Cyp46a1 | -0.3847632 |
| Cyp4b1 | 0.65547943 |
| Cyp4f13 | 0.81630295 |
| Cyp4f14 | 1.23117579 |
| Cyp4f15 | 0.93956062 |
| Cyp4f16 | -0.4444407 |
| Cyp4f16 | -0.7226257 |
| Cyp4f16 | 1.48666052 |
| Cyp4f18 | 2.38704678 |
| Cyp4f39 | -0.5781615 |
| Cyp4v3 | 1.13119588 |
| Cyp51 | 0.1346163 |
| Cyp7b1 | -0.6730728 |
| Cypt10 | -0.6538 |
| Cypt2 | -0.2555244 |
| Cypt3 | 1.02470766 |
| Cypt6 | 1.98580101 |
| Cypt8 | 0.08373463 |
| Cypt9 | 0.28771025 |
| Cyr61 | -0.2404926 |
| Cyr61 | -0.6124335 |
| Cysltr1 | -0.5224973 |
| Cytl1 | 0.62249074 |
| D030011O10Rik | 1.28460998 |
| D030013I16Rik | 2.08122512 |
| D030018L15Rik | 2.77751332 |
| D030028O16Rik | -0.2852166 |
| D030051N19Rik | 0.15887952 |
| D030056L22Rik | -0.6006079 |
| D030070L09Rik | -0.0412838 |
| D030070L09Rik | -0.2309556 |
| D030070L09Rik | -0.1358196 |
| D030070L09Rik | 0.32910605 |
| D030074E01Rik | 2.14183645 |
| D0H4S114 | 0.35618524 |
| D0H8S2298E | 0.065499 |
| D0HXS9928E | -0.4016992 |
| D0HXS9928E | -0.7693097 |
| D10627 | -0.5392174 |
| D10Bwg1364e | -0.6196014 |
| D10Bwg1379e | 0.10346423 |
| D10Ertd322e | 0.74797618 |
| D10Ertd610e | 0.8396849 |
| D10Ertd610e | -0.6521427 |
| D10Ertd610e | 0.91039401 |
| D10Ertd641e | -0.2919452 |
| D10Jhu81e | 3.45152433 |
| D10Wsu102e | 1.18055242 |
| D10Wsu52e | -0.4384958 |
| D11Bwg0517e | 1.93868012 |
| D11Wsu47e | 0.01505285 |
| D11Wsu47e | 0.68457929 |
| D12Ertd551e | 3.35546097 |
| D12Ertd551e | -0.4431164 |
| D12Ertd553e | -0.532639 |
| D12Ertd647e | -0.1540866 |
| D12Ertd647e | -0.645543 |
| D130040H23Rik | 0.26537407 |
| D130043K22Rik | -0.6790184 |
| D130059P03Rik | 1.84333685 |
| D13Ertd608e | -0.7806421 |
| D14Ertd171e | 0.8049097 |
| D14Ertd231e | -0.5181844 |
| D14Ertd449e | -0.6357302 |
| D14Ertd500e | -0.6013916 |
| D14Ertd668e | -0.7306962 |
| D14Ertd668e | 2.03345324 |
| D15Ertd621e | -0.4733793 |
| D15Ertd682e | -0.5690291 |
| D15Mgi27 | -0.7825443 |
| D15Wsu169e | 0.36011024 |
| D15Wsu75e | 1.11551647 |
| D16Ertd472e | -0.6308622 |
| D16H22S680E | 0.71719777 |
| D17H6S56E-5 | 0.6509839 |
| D17Wsu104e | 0.62079971 |
| D17Wsu92e | -0.6996958 |
| D19Bwg1357e | -0.705814 |
| D19Ertd721e | -0.4878451 |
| D19Ertd737e | -0.4645354 |
| D19Wsu162e | -0.0547151 |
| D1Bwg0212e | 1.34930754 |
| D1Bwg0212e | 1.91530628 |
| D1Pas1 | -0.3553933 |
| D230007K08Rik | 0.12925714 |
| D230014K01Rik | -0.5920301 |
| D2Ertd750e | 0.84602183 |
| D2Ertd750e | 0.12566719 |
| D330014H01Rik | -0.4906261 |
| D330017J20Rik | -0.5990424 |
| D330028D13Rik | 0.94226287 |
| D330045A20Rik | 0.18421831 |
| D330045A20Rik | 0.87240295 |
| D330045A20Rik | 0.5732757 |
| D3Bwg0562e | -0.5796923 |
| D3Ertd300e | -0.6389897 |
| D3Ertd751e | 1.72865032 |
| D3Ucla1 | 1.8831103 |
| D430015B01Rik | -0.5866122 |
| D430028G21Rik | -0.5131777 |
| D430039N05Rik | -0.5943616 |
| D430041B17Rik | -0.5018338 |
| D430042O09Rik | -0.5532592 |
| D4Bwg0951e | 1.13461759 |
| D4Bwg1540e | 0.20261519 |
| D4Ertd22e | -0.451091 |
| D4Ertd22e | -0.5720633 |
| D4Wsu114e | -0.3009928 |
| D4Wsu132e | 3.48122657 |
| D4Wsu132e | -0.2490525 |
| D530004J12Rik | -0.657956 |
| D530033C11Rik | 1.54823486 |
| D5Ertd135e | 3.55788136 |
| D5Ertd579e | 3.79348724 |
| D5Wsu178e | -0.1263472 |
| D630003M21Rik | -0.648837 |
| D630003M21Rik | 0.61804523 |
| D630014A15Rik | -0.5735841 |
| D630023F18Rik | 0.08185163 |
| D630042P16Rik | -0.6268249 |
| D630048P19Rik | -0.3078366 |
| D6Ertd474e | -0.5480613 |
| D6Mm5e | 2.0066316 |
| D6Wsu116e | 0.16889174 |
| D6Wsu163e | -0.5304565 |
| D6Wsu163e | -0.6140216 |
| D6Wsu163e | -0.2593205 |
| D6Wsu176e | 2.1511971 |
| D6Wsu176e | -0.3928792 |
| D730039F16Rik | 0.12350658 |
| D730039F16Rik | 0.7018073 |
| D7Bwg0611e | -0.6962175 |
| D830014E11Rik | -0.738837 |
| D830030K20Rik | 0.25916009 |
| D830030K20Rik | -0.535557 |
| D830050J10Rik | 0.78702366 |
| D8Ertd738e | 1.75724766 |
| D8Ertd82e | 0.4986865 |
| D930001I22Rik | -0.6513152 |
| D930005D10Rik | -0.2367722 |
| D930014E17Rik | 1.45760727 |
| D930015E06Rik | -0.4564455 |
| D930020B18Rik | 2.8999236 |
| D930028F11Rik | -0.5540042 |
| D930049A15Rik | 1.05255722 |
| D9Ertd280e | 0.2238994 |
| D9Ertd392e | 2.0382355 |
| D9Ertd402e | -0.5428922 |
| D9Wsu20e | 1.19461262 |
| Daam1 | -0.6284377 |
| Daam2 | 0.76462637 |
| Dab1 | -0.5990424 |
| Dab1 | 1.29781097 |
| Dab1 | 1.13070604 |
| Dab2 | -0.57587 |
| Dab2 | 0.02632337 |
| Dab2ip | 0.55869161 |
| Dact1 | 1.7431221 |
| Dact2 | 0.65036919 |
| Dad1 | 1.3715235 |
| Daf2 | 0.6528256 |
| Dag1 | 1.21453002 |
| Dag1 | -0.6092653 |
| Dagla | -0.2824273 |
| Daglb | 1.12295691 |
| Dak | -0.6124335 |
| Dalrd3 | 1.70366698 |
| Dap | -0.6841449 |
| Dap3 | 1.25055105 |
| Dap3 | 0.05624577 |
| Dap3 | 0.24597443 |
| Dap3 | 0.50109493 |
| Dapk1 | -0.6773158 |
| Dapk1 | 0.94435922 |
| Dapk2 | -0.4417939 |
| Dapk2 | -0.2582343 |
| Dapl1 | 3.2326188 |
| Dapp1 | -0.6546297 |
| Dapp1 | -0.542156 |
| Darc | -0.6156124 |
| Dars | -0.6164089 |
| Dars | -0.5046569 |
| Daxx | -0.698825 |
| Dazap1 | 1.98678026 |
| Dazap1 | -0.4417939 |
| Dazap2 | 0.49409328 |
| Dazl | 0.01828692 |
| Dbc1 | 1.73193574 |
| Dbf4 | -0.3644725 |
| Dbi | 2.68013135 |
| Dbi | -0.6236076 |
| Dbil5 | -0.5525149 |
| Dbn1 | -0.1434674 |
| Dbndd1 | -0.4067769 |
| Dbndd2 | -0.6068962 |
| Dbndd2 | 0.30240861 |
| Dbndd2 | -0.7721299 |
| Dbnl | 0.57238477 |
| Dbp | -0.6604585 |
| Dbpht2 | 1.72262931 |
| Dbr1 | 0.83750708 |
| Dbt | -0.1086103 |
| Dcakd | -0.3991707 |
| Dcbld1 | 1.32187084 |
| Dcbld2 | 0.86396362 |
| Dci | -0.1058403 |
| Dck | 0.75037304 |
| Dclk1 | -0.1781819 |
| Dclk2 | 0.89695769 |
| Dclk3 | 0.10051278 |
| Dclre1b | 0.96652943 |
| Dclre1b | -0.505364 |
| Dclre1c | -0.6341047 |
| Dclre1c | -0.4260683 |
| Dclre1c | -0.3547912 |
| Dcn | -0.4733793 |
| Dcn | 0.65507164 |
| Dcp1a | 1.33888038 |
| Dcp1a | 1.14613434 |
| Dcp1a | 1.03575759 |
| Dcp1a | -0.4378375 |
| Dcp1b | 0.39201169 |
| Dcp1b | 1.25248813 |
| Dcpp1 | -0.6349171 |
| Dcpp2 | -0.6688491 |
| Dcpp2 | -0.1742052 |
| Dcpp3 | -0.7119726 |
| Dcps | -0.5428922 |
| Dcps | -0.729796 |
| Dct | 0.73571156 |
| Dctd | 2.73040107 |
| Dctn1 | 0.78400904 |
| Dctn2 | 0.1038325 |
| Dctn2 | 1.39035235 |
| Dctn2 | 0.39011722 |
| Dctn3 | -0.636544 |
| Dctn3 | -0.7571862 |
| Dctn4 | 0.1438332 |
| Dctn4 | -0.1687648 |
| Dctn5 | 0.83951756 |
| Dctn6 | -0.2697028 |
| Dcun1d2 | -0.147797 |
| Dcun1d3 | -0.7498032 |
| Dcun1d4 | -0.0808247 |
| Dcun1d4 | 1.83531274 |
| Dcun1d5 | 1.70187556 |
| Dcx | 0.5001323 |
| Dcx | 0.64646659 |
| Dcxr | 1.13522729 |
| Dcxr | 0.2828602 |
| Ddah1 | -0.6910244 |
| Ddah2 | -0.102619 |
| Ddb1 | 0.21249904 |
| Ddb2 | -0.8947029 |
| Ddb2 | 0.19396367 |
| Ddb2 | -0.4864577 |
| Ddc | -0.5675156 |
| Ddc8 | 1.28440222 |
| Ddef2 | -0.5414205 |
| Ddef2 | 0.01018064 |
| Ddefl1 | 0.10014319 |
| Ddhd1 | 0.66883752 |
| Ddhd1 | -0.5607354 |
| Ddi2 | 0.07390126 |
| Ddit3 | -0.1786802 |
| Ddit3 | 0.80003385 |
| Ddit4 | 2.01824816 |
| Ddit4l | -0.4584614 |
| Ddo | 1.7034018 |
| Ddo | 0.44432006 |
| Ddo | 2.78965872 |
| Ddost | 3.00771121 |
| Ddr1 | -0.2774275 |
| Ddr2 | 1.1659233 |
| Ddt | 0.73064738 |
| Ddx1 | -0.368129 |
| Ddx1 | 1.66011105 |
| Ddx17 | 0.55869161 |
| Ddx17 | -0.2675074 |
| Ddx17 | 0.25947177 |
| Ddx19a | -0.3369052 |
| Ddx19b | -0.5153202 |
| Ddx19b | -0.0464601 |
| Ddx20 | -0.2085598 |
| Ddx21 | -0.472696 |
| Ddx24 | -0.532639 |
| Ddx24 | 2.99620443 |
| Ddx24 | 0.34601592 |
| Ddx25 | 0.64995916 |
| Ddx26b | -0.6156124 |
| Ddx27 | -0.3464017 |
| Ddx28 | 0.21903058 |
| Ddx3x | 0.06203979 |
| Ddx3y | -0.7279982 |
| Ddx4 | -0.5584863 |
| Ddx4 | 0.68615869 |
| Ddx41 | 0.68852278 |
| Ddx46 | 0.71318228 |
| Ddx47 | -0.4997221 |
| Ddx47 | -0.1429876 |
| Ddx47 | 1.31827112 |
| Ddx49 | 3.11888117 |
| Ddx5 | -0.6970859 |
| Ddx50 | -0.5990424 |
| Ddx50 | 3.19336062 |
| Ddx51 | 0.79495686 |
| Ddx52 | -0.451091 |
| Ddx54 | 2.21991381 |
| Ddx58 | -0.5089076 |
| Ddx6 | 0.7381401 |
| Ddx6 | -0.4273687 |
| Ddx6 | -0.3565988 |
| Ddx6 | -0.6654838 |
| Deadc1 | -0.8008112 |
| Deb1 | 0.20691106 |
| Decr1 | 1.0198922 |
| Decr2 | 1.32057254 |
| Decr2 | 0.05158451 |
| Dedd | 0.55005873 |
| Dedd | -0.5735841 |
| Dedd2 | -0.5547497 |
| Def6 | 0.90088359 |
| Def8 | 0.58829279 |
| Defb11 | 0.63299081 |
| Defb14 | -0.3009928 |
| Defb15 | 0.02472161 |
| Defb19 | 0.82705006 |
| Defb20 | 0.88523929 |
| Defb21 | -0.3251688 |
| Defb25 | -0.4280196 |
| Defb26 | 0.81045108 |
| Defb30 | -0.3998022 |
| Defb36 | 0.60264063 |
| Defb36 | -0.4584614 |
| Defb4 | -0.6554601 |
| Defcr20 | 2.52627347 |
| Defcr6 | -0.5217771 |
| Defcr-rs7 | 1.0949985 |
| Degs1 | 0.86510414 |
| Degs2 | -0.6414418 |
| Dek | 0.23195807 |
| Dek | 0.36846576 |
| Dennd1a | -0.7777961 |
| Dennd1c | -0.0817227 |
| Dennd2a | 0.67883148 |
| Dennd3 | -0.3339572 |
| Denr | -0.6747677 |
| Denr | 1.73917425 |
| Depdc1b | -0.1917297 |
| Depdc5 | 1.45691672 |
| Depdc5 | -0.6381738 |
| Depdc6 | -0.5652501 |
| Depdc6 | 0.79179167 |
| Dera | -0.6180038 |
| Derl2 | 0.99684989 |
| Derl3 | 1.55982927 |
| Des | -0.2653172 |
| Det1 | 1.93294317 |
| Dexi | 0.04022272 |
| Dexi | 2.60473144 |
| Dffa | 1.74749611 |
| Dfna5h | 0.31453177 |
| Dgat1 | 0.61846952 |
| Dgat2 | -0.5399512 |
| Dgcr2 | 0.08373463 |
| Dgcr2 | 2.05325759 |
| Dgcr6 | 0.07655892 |
| Dgcr6 | 2.42127233 |
| Dgcr8 | 2.55201843 |
| Dgka | -0.723519 |
| Dgka | -0.0723365 |
| Dgkb | -0.6730728 |
| Dgke | -0.7863604 |
| Dgkg | -0.4720133 |
| Dgkh | -0.7406558 |
| Dgki | 0.55142713 |
| Dgkk | 1.71937608 |
| Dgkq | 0.2036083 |
| Dgkz | 2.17805096 |
| Dgkz | 0.59505941 |
| Dgkz | 2.66563498 |
| Dguok | 3.32215414 |
| Dhcr24 | -0.1301247 |
| Dhcr24 | 0.22551671 |
| Dhcr24 | 2.42681577 |
| Dhcr7 | -0.1202413 |
| Dhdds | -0.5377515 |
| Dhdds | 0.99501002 |
| Dhdh | 3.01718215 |
| Dhdh | 0.58478104 |
| Dhdh | 2.49433192 |
| Dhh | 0.71145605 |
| Dhodh | -0.3878764 |
| Dhps | 0.73795352 |
| Dhrs1 | 0.44814656 |
| Dhrs4 | 0.23356148 |
| Dhrs7 | -0.6807241 |
| Dhrs7 | 1.75624576 |
| Dhrs7 | -0.4444407 |
| Dhrs7b | -0.2024046 |
| Dhrs7b | -0.6116404 |
| Dhrs7b | 0.38550028 |
| Dhrsx | -0.5584863 |
| Dhrsx | -0.0162431 |
| Dhtkd1 | 3.07428827 |
| Dhx15 | 0.45827449 |
| Dhx15 | 0.75697857 |
| Dhx16 | -0.666324 |
| Dhx29 | -0.4517586 |
| Dhx30 | 0.69342881 |
| Dhx32 | 0.50517505 |
| Dhx33 | -0.1329678 |
| Dhx36 | -0.1747014 |
| Dhx37 | 0.07314054 |
| Dhx38 | -0.5124647 |
| Dhx57 | -0.5473211 |
| Dhx58 | -0.5569898 |
| Dhx8 | -0.6596236 |
| Dhx9 | 1.2465566 |
| Diablo | -0.6316718 |
| Diap1 | -0.7721299 |
| Diap3 | 2.75786994 |
| Dicer1 | -0.2116527 |
| Dido1 | -0.2054771 |
| Dido1 | 1.50570254 |
| Dido1 | 0.81492934 |
| Dio1 | -0.5428922 |
| Dio1 | 1.84817518 |
| Dio3 | -0.4189484 |
| Dip2b | 0.26692112 |
| Dip2b | 0.73252621 |
| Diras1 | 1.3684905 |
| Diras2 | -0.2293756 |
| Dirc2 | -0.5584863 |
| Dis3 | 0.21020216 |
| Dis3l | 0.77812916 |
| Dis3l2 | 0.4542365 |
| Disp1 | 1.31405381 |
| Dixdc1 | 1.61464062 |
| Dkk3 | 0.1346163 |
| Dkk3 | -0.461831 |
| Dkk3 | -0.2614967 |
| Dkkl1 | -0.6316718 |
| Dkkl1 | 0.81835959 |
| Dlat | 1.85445592 |
| Dlc1 | -0.3369052 |
| Dld | -0.6587894 |
| Dleu7 | -0.6439004 |
| Dlg1 | 0.8996291 |
| Dlg2 | 1.33229139 |
| Dlg3 | 2.52090562 |
| Dlg4 | -0.5554958 |
| Dlg7 | -0.5660047 |
| Dlgap1 | -0.5743455 |
| Dlgap2 | -0.5217771 |
| Dlgap2 | -0.6713811 |
| Dlgap2 | -0.0781359 |
| Dlgap3 | 0.065499 |
| Dlgap4 | -0.7693097 |
| Dlgap4 | -0.1090728 |
| Dlgap4 | 0.66843553 |
| Dlgap4 | 0.6227019 |
| Dlk1 | -0.505364 |
| Dlk2 | 0.21446331 |
| Dll1 | 0.30804625 |
| Dlst | 0.51446846 |
| Dlx1 | -0.6798709 |
| Dlx1as | 0.45347743 |
| Dlx2 | 0.03943403 |
| Dlx3 | 3.03580862 |
| Dmap1 | 1.56260912 |
| Dmbt1 | 0.45474221 |
| Dmkn | -0.4747474 |
| Dmkn | 1.25581481 |
| Dmkn | -0.691888 |
| Dmn | 1.44203254 |
| Dmn | -0.6236076 |
| Dmpk | 0.98747124 |
| Dmrt2 | 0.14206778 |
| Dmrt3 | -0.3263358 |
| Dmrta1 | 0.51849064 |
| Dmrta2 | 0.46479918 |
| Dmrtc1a | 1.59362313 |
| Dmrtc1a | 0.7714922 |
| Dmrtc1a | 0.85889592 |
| Dmrtc1b | -0.1207096 |
| Dmrtc2 | -0.6629678 |
| Dmtf1 | -0.7844504 |
| Dmtf1 | -0.7049375 |
| Dmtf1 | 2.09861951 |
| Dmwd | 0.21020216 |
| Dmwd | 2.24729532 |
| Dmwd | 0.32184737 |
| Dnahc11 | 0.20987357 |
| Dnahc2 | -0.5224973 |
| Dnahc5 | 1.33150152 |
| Dnahc8 | 0.09197498 |
| Dnaic1 | 0.03270306 |
| Dnaic1 | 1.38337102 |
| Dnaja1 | -0.6722266 |
| Dnaja2 | 0.21903058 |
| Dnaja2 | -0.6439004 |
| Dnaja3 | -0.6944833 |
| Dnajb1 | -0.5246613 |
| Dnajb10 | -0.5196198 |
| Dnajb12 | 0.07123601 |
| Dnajb13 | 0.77759278 |
| Dnajb3 | 1.28398657 |
| Dnajb4 | -0.5261067 |
| Dnajb4 | 3.15742922 |
| Dnajb5 | 1.6834988 |
| Dnajb6 | -0.2236048 |
| Dnajb6 | 0.32881678 |
| Dnajb7 | 1.21251242 |
| Dnajb8 | 4.15856664 |
| Dnajb9 | 0.54708694 |
| Dnajc10 | 1.219555 |
| Dnajc10 | 0.88762656 |
| Dnajc12 | 0.26723023 |
| Dnajc14 | -0.5319109 |
| Dnajc15 | -0.7075694 |
| Dnajc16 | 0.17231947 |
| Dnajc17 | -0.5414205 |
| Dnajc18 | 1.11014742 |
| Dnajc19 | -0.5517711 |
| Dnajc19 | -0.4829982 |
| Dnajc2 | 1.73277136 |
| Dnajc2 | -0.3298457 |
| Dnajc3a | -0.6013916 |
| Dnajc4 | 0.24786955 |
| Dnajc5 | -0.568272 |
| Dnajc5b | -0.1123165 |
| Dnajc5g | -0.2479782 |
| Dnajc6 | 0.59440672 |
| Dnajc6 | -0.472696 |
| Dnajc7 | -0.2157926 |
| Dnajc8 | 0.02991731 |
| Dnalc1 | 0.86608062 |
| Dnali1 | 1.76726993 |
| Dnase1l1 | -0.3650809 |
| Dnase1l2 | -0.5804587 |
| Dnase1l2 | -0.2620415 |
| Dnase2a | 3.55537957 |
| Dnd1 | 0.18354245 |
| Dner | -0.4955129 |
| Dnm | 0.20658131 |
| Dnm1 | 0.49336596 |
| Dnm1l | -0.7306962 |
| Dnm1l | 0.67164664 |
| Dnmbp | -0.5943616 |
| Dnmt1 | 0.01058763 |
| Dnmt3b | 0.15156163 |
| Dnmt3l | -0.6252148 |
| Dnmt3l | -0.0808247 |
| Dnttip1 | -0.423473 |
| Doc2a | -0.350587 |
| Doc2b | 1.14214988 |
| Doc2b | -0.7288967 |
| Dock1 | -0.5959193 |
| Dock3 | -0.3523865 |
| Dock4 | -0.6538 |
| Dock6 | 0.40062347 |
| Dock8 | 0.21282671 |
| Dock9 | -0.2885749 |
| Dok1 | -0.2367722 |
| Dok1 | -0.3644725 |
| Dok2 | -0.7137397 |
| Dok3 | 1.25806165 |
| Dok4 | -0.6357302 |
| Dok5 | 1.65962476 |
| Dok5 | -0.1871919 |
| Dok7 | 0.56297828 |
| Dolk | 0.16338105 |
| Dolpp1 | 0.27645601 |
| Dolpp1 | 1.39063052 |
| Dolpp1 | 2.03939255 |
| Dom3z | -0.3493893 |
| Dom3z | -0.7479665 |
| Donson | -0.7711889 |
| Donson | 2.59896012 |
| Dos | -0.1174366 |
| Dos | -0.3972788 |
| Dot1l | -0.1751978 |
| Dpagt1 | 0.86640589 |
| Dpep1 | 0.62628439 |
| Dpep2 | 0.64316749 |
| Dpep2 | 1.92125736 |
| Dpep3 | 1.82145293 |
| Dpf1 | 0.21118723 |
| Dpf1 | 1.24308809 |
| Dpf3 | 0.74705266 |
| Dph2 | -0.5974795 |
| Dph2 | -0.1967978 |
| Dph2 | -0.4672477 |
| Dph3 | 2.18095666 |
| Dph3 | -0.2680558 |
| Dph3 | -0.4692871 |
| Dph4 | 0.09940357 |
| Dpm1 | -0.6596236 |
| Dpm1 | 1.34444953 |
| Dpm2 | 0.04376363 |
| Dpm3 | -0.0141516 |
| Dpp10 | -0.7730719 |
| Dpp3 | -0.5789266 |
| Dpp3 | -0.6324821 |
| Dpp4 | -0.3998022 |
| Dpp6 | 3.35591232 |
| Dpp7 | 0.68180891 |
| Dpp8 | -0.6439004 |
| Dpp9 | -0.4843805 |
| Dpp9 | -0.3072643 |
| Dppa3 | 0.51825452 |
| Dppa4 | 0.87996568 |
| Dppa5 | 0.9580193 |
| Dppa5 | 2.25032559 |
| Dpt | 1.2929854 |
| Dpy30 | -0.5547497 |
| Dpyd | -0.737929 |
| Dpysl2 | -0.3316055 |
| Dpysl2 | 1.12258629 |
| Dpysl2 | 1.20868946 |
| Dpysl3 | -0.2571494 |
| Dpysl4 | 1.11177388 |
| Dpysl5 | 1.05282358 |
| Drbp1 | -0.4384958 |
| Drctnnb1a | -0.5697867 |
| Drd1a | -0.5912541 |
| Drd2 | -0.2625867 |
| Drd4 | -0.6850021 |
| Drd5 | 0.77400904 |
| Drg1 | 1.62450557 |
| Drg1 | -0.5189018 |
| Drg2 | -0.3584099 |
| Drp2 | 3.79665082 |
| Dscam | -0.6944833 |
| Dscam | 1.56129744 |
| Dscc1 | 0.65036919 |
| Dscr1 | 0.48434879 |
| Dscr1l2 | -0.5889303 |
| Dscr2 | 3.4658421 |
| Dscr3 | -0.568272 |
| Dscr3 | 1.20235846 |
| Dscr6 | 0.02110756 |
| Dsg2 | 0.16372642 |
| Dsg2 | -0.1123165 |
| Dsn1 | -0.5990424 |
| Dsp | -0.545104 |
| Dst | -0.6204013 |
| Dst | 0.97394888 |
| Dst | 0.92593071 |
| Dstn | 0.54111481 |
| Dtd1 | -0.030166 |
| Dtl | 0.07047311 |
| Dtna | -0.5966991 |
| Dtna | 1.0266269 |
| Dtna | -0.617206 |
| Dtna | 1.08771568 |
| Dtnb | 2.21724181 |
| Dtnbp1 | -0.451091 |
| Dtwd1 | 1.44972189 |
| Dtx1 | -0.461831 |
| Dtx2 | 0.6384045 |
| Dtx2 | -0.5117522 |
| Dtx2 | -0.3304319 |
| Dtx4 | 0.40169438 |
| Dtx4 | -0.2796463 |
| Dtymk | 0.04768226 |
| Dus1l | 1.79008375 |
| Dus1l | -0.4788637 |
| Dus2l | -0.5951401 |
| Dus3l | -0.4747474 |
| Dus3l | 1.81034499 |
| Dus3l | 0.80421471 |
| Dus4l | 0.32910605 |
| Dusp1 | 2.63423868 |
| Dusp11 | 1.37577205 |
| Dusp11 | 0.19362932 |
| Dusp13 | 0.91426389 |
| Dusp13 | 0.3089333 |
| Dusp13 | -0.0280431 |
| Dusp14 | -0.4504238 |
| Dusp15 | 0.03190798 |
| Dusp15 | 3.09937918 |
| Dusp16 | 1.74038585 |
| Dusp16 | 0.99812153 |
| Dusp16 | 0.54731587 |
| Dusp18 | 0.12458751 |
| Dusp19 | -0.0033506 |
| Dusp2 | 2.47776804 |
| Dusp2 | 0.20029382 |
| Dusp21 | -0.2447627 |
| Dusp22 | 0.85824001 |
| Dusp23 | -0.381041 |
| Dusp26 | 0.82024086 |
| Dusp28 | 1.42797655 |
| Dusp3 | 1.53172488 |
| Dusp3 | -0.2168305 |
| Dusp3 | -0.4484252 |
| Dusp4 | -0.5781615 |
| Dusp4 | -0.3669086 |
| Dusp4 | -0.422178 |
| Dusp6 | -0.082172 |
| Dusp6 | 0.46754595 |
| Dusp6 | -0.4118824 |
| Dusp7 | -0.2824273 |
| Dusp8 | 0.72932994 |
| Dut | 0.47400634 |
| Duxbl | 3.19723324 |
| Duxbl | -0.4215312 |
| Dvl1 | -0.4464308 |
| Dvl1 | -0.58507 |
| Dvl2 | -0.1922353 |
| DXBwg1396e | -0.6910244 |
| Dydc2 | -0.3903744 |
| Dym | -0.7452183 |
| Dync1h1 | 0.43713501 |
| Dync1i1 | -0.5488021 |
| Dync1i1 | -0.0957534 |
| Dync1i2 | 0.56724535 |
| Dync1li1 | -0.5812257 |
| Dync1li2 | 2.32236728 |
| Dync2li1 | -0.0745627 |
| Dynll1 | 0.26102863 |
| Dynll2 | 0.46654806 |
| Dynlrb1 | -0.3638645 |
| Dynlrb2 | 0.02351848 |
| Dynlt3 | 1.53020938 |
| Dyrk1a | -0.433243 |
| Dyrk1b | 0.01424258 |
| Dyrk1b | 0.03587663 |
| Dyrk2 | 4.29244869 |
| Dyrk2 | 0.03031579 |
| Dyrk3 | -0.682433 |
| Dyrk4 | 0.3041926 |
| Dysf | 0.75642989 |
| Dysfip1 | -0.5167512 |
| Dzip1 | 2.10415934 |
| E030010A14Rik | -0.5713038 |
| E030034P13Rik | -0.3596193 |
| E130009J12Rik | -0.4042346 |
| E130012A19Rik | -0.2807577 |
| E130014J05Rik | 2.5647742 |
| E130016E03Rik | 0.20493081 |
| E130203B14Rik | -0.6140216 |
| E130203B14Rik | 0.49263807 |
| E130303B06Rik | -0.752565 |
| E130306D19Rik | 0.78471924 |
| E130308A19Rik | -0.6316718 |
| E130309D02Rik | -0.5039503 |
| E130309D02Rik | -0.3446137 |
| E130309D14Rik | 1.06302069 |
| E130309F12Rik | -0.7137397 |
| E130309F12Rik | 1.94417714 |
| E130309F12Rik | -0.4260683 |
| E230008N13Rik | 2.90513195 |
| E2f1 | 1.18066854 |
| E2f2 | 0.88667238 |
| E2f2 | -0.6571233 |
| E2f3 | -0.5982606 |
| E2f4 | -0.2774275 |
| E2f5 | 3.12272733 |
| E2f6 | -0.4836891 |
| E2f6 | 0.85380055 |
| E2f6 | -0.7137397 |
| E330016A19Rik | -0.147315 |
| E330017A01Rik | 0.96886346 |
| E330018D03Rik | -0.622003 |
| E330021D16Rik | 0.37729659 |
| E330034G19Rik | -0.5644961 |
| E330034G19Rik | -0.5781615 |
| E330036I19Rik | 1.94943824 |
| E430002D04Rik | -0.6260195 |
| E430002G05Rik | -0.6381738 |
| E430018J23Rik | -0.6414418 |
| E430025E21Rik | 0.94361106 |
| E430028B21Rik | -0.9076768 |
| E4f1 | 0.87272602 |
| Eaf1 | -0.1377257 |
| Ear11 | 0.32068072 |
| Ear2 | -0.1618862 |
| Ear4 | -0.2571494 |
| Ears2 | 2.50010849 |
| Ebag9 | 0.41918914 |
| Ebf2 | 0.22970865 |
| Ebf3 | -0.2490525 |
| Ebf4 | 1.61405707 |
| Ebi2 | 0.91318194 |
| Ebi3 | -0.6430801 |
| Ebna1bp2 | 2.50427707 |
| Ebna1bp2 | 0.87546761 |
| Ebp | -0.3742553 |
| Ebpl | -0.1339174 |
| Ebpl | 0.97133698 |
| Ecd | 0.25447229 |
| Ecd | 0.01707546 |
| Ece1 | 0.9681347 |
| Ece1 | 1.83789945 |
| Ece2 | -0.6638057 |
| Ecel1 | 1.65753776 |
| Ech1 | -0.6739199 |
| Echdc1 | 0.95017435 |
| Echdc2 | 1.34620133 |
| Echdc2 | -0.6341047 |
| Echdc2 | 0.16957829 |
| Echdc3 | 0.20888711 |
| Echs1 | 1.48707863 |
| Ecm1 | 0.26846561 |
| Ecm1 | -0.4055049 |
| Ecsit | 0.25572467 |
| Eda2r | 1.35385112 |
| Edaradd | 0.60069705 |
| Edc4 | 0.34544769 |
| Edem1 | 2.09744674 |
| Edem2 | -0.5897043 |
| Edem2 | 1.04197509 |
| Edem2 | 1.15976937 |
| Edem2 | -0.6439004 |
| Edf1 | -0.6188023 |
| Edg2 | -0.2463691 |
| Edg5 | 1.19197809 |
| Edg7 | 1.32246944 |
| Edg8 | 0.26568369 |
| Edg8 | -0.2479782 |
| Edil3 | 1.38477147 |
| Edn1 | 0.53254957 |
| Edn2 | -0.2958927 |
| Ednra | -0.6140216 |
| Ednrb | 2.27184334 |
| Ednrb | 1.05733994 |
| Ednrb | -0.7075694 |
| Eed | 0.76281114 |
| Eef1a1 | -0.6910244 |
| Eef1a1 | 1.20360545 |
| Eef1a2 | 0.71164802 |
| Eef1a2 | -0.4857648 |
| Eef1a2 | 1.12554711 |
| Eef1b2 | -0.535557 |
| Eef1b2 | 1.02648994 |
| Eef1b2 | 0.9466001 |
| Eef1d | 2.74060058 |
| Eef1d | 1.02826883 |
| Eef1d | 0.55665419 |
| Eef1d | 1.40242248 |
| Eef1e1 | 1.02648994 |
| Eef1g | -0.818453 |
| Eef2 | 0.11116766 |
| Eef2 | 0.50133543 |
| Eef2 | 0.16614046 |
| Eef2k | -0.0477586 |
| Eefsec | 0.10677349 |
| Efcab1 | -0.0678998 |
| Efcab2 | -0.296458 |
| Efcab2 | 2.54582993 |
| Efcab3 | -0.6798709 |
| Efcab3 | 0.69577457 |
| Efcab3 | 0.22487013 |
| Efcab4a | 0.99642563 |
| Efcab4a | -0.5480613 |
| Efcab5 | 0.02672338 |
| Efemp2 | 0.74464724 |
| Efha1 | 1.90516026 |
| Efha1 | -0.6756163 |
| Efhc2 | 0.90385626 |
| Efhd1 | 0.75642989 |
| Efna1 | 1.39294538 |
| Efna3 | -0.7979031 |
| Efna4 | -0.8037285 |
| Efna4 | -0.164337 |
| Efna5 | 1.20065534 |
| Efnb1 | 0.76408217 |
| Efnb2 | -0.1574909 |
| Efnb3 | -0.2913826 |
| Efr3a | -0.7217333 |
| Efs | -0.2346529 |
| Eftud1 | 0.76770417 |
| Eftud2 | 0.77544419 |
| Eftud2 | -0.5517711 |
| Eftud2 | -0.2357119 |
| EG194588 | 0.08185163 |
| EG208426 | -0.698825 |
| EG210155 | 0.28952254 |
| EG212225 | 1.78814826 |
| EG214321 | 0.45499496 |
| EG232599 | 0.62523214 |
| EG237009 | -0.2544426 |
| EG237009 | -0.5517711 |
| EG237300 | 0.35843012 |
| EG238829 | 1.38103226 |
| EG240916 | 2.08862558 |
| EG245297 | -0.4016992 |
| EG245297 | 2.84082248 |
| EG245376 | -0.6756163 |
| EG245376 | -0.6132272 |
| EG272350 | -0.4306276 |
| EG277333 | 0.90088359 |
| EG317677 | -0.1021597 |
| EG328264 | -0.588157 |
| EG328280 | -0.0395647 |
| EG328644 | -0.7128557 |
| EG328839 | 1.00865162 |
| EG330305 | -0.6341047 |
| EG330503 | -0.7470495 |
| EG330513 | -0.5592354 |
| EG333669 | -0.0438683 |
| EG368203 | -0.3493893 |
| EG380907 | -0.391626 |
| EG381438 | 1.67294062 |
| EG382109 | 1.70505792 |
| EG382109 | -0.5473211 |
| EG382109 | -0.4740631 |
| EG384639 | 0.10640637 |
| EG384813 | 3.49335797 |
| EG384813 | 1.94079028 |
| EG384814 | -0.8244085 |
| EG385328 | 0.59766563 |
| EG408196 | 0.20195253 |
| EG432825 | 0.46179345 |
| EG432879 | 1.11314791 |
| EG432939 | 1.9862418 |
| EG433016 | 2.02089398 |
| EG433182 | -0.2719033 |
| EG433224 | 2.31935696 |
| EG433923 | 2.38292703 |
| EG434171 | 0.94211295 |
| EG434197 | 0.84018675 |
| EG434280 | -0.5951401 |
| EG434459 | 2.54222117 |
| EG434459 | -0.2785362 |
| EG434858 | 0.76281114 |
| EG435391 | -0.6414418 |
| EG436332 | -0.397909 |
| EG545047 | 2.1992951 |
| EG545056 | 0.08260528 |
| EG545253 | 1.63076196 |
| EG545370 | 1.22244604 |
| EG545758 | 1.81805819 |
| EG545861 | -0.7146245 |
| EG545963 | -0.025925 |
| EG546166 | 2.5677195 |
| EG546282 | 1.08784394 |
| EG546325 | 1.44937374 |
| EG546347 | -0.3791854 |
| EG547109 | -0.9109487 |
| EG574403 | 1.62428908 |
| EG622320 | -0.1146403 |
| EG622339 | -0.7959694 |
| EG624866 | 1.46739255 |
| EG625054 | -0.5311834 |
| EG625424 | 0.11080226 |
| EG630499 | 0.45726663 |
| EG633640 | 0.8478482 |
| EG638695 | 2.09072927 |
| EG654453 | -0.5781615 |
| EG665186 | 0.37371914 |
| EG665378 | -0.4267183 |
| EG667977 | 2.86766706 |
| EG668668 | -0.1638464 |
| Egf | 1.0613069 |
| Egfbp2 | -0.7424781 |
| Egfl7 | 1.72113424 |
| Egfl7 | 0.11262779 |
| Egflam | 1.27741557 |
| Egflam | 1.52331882 |
| Egfr | -0.6308622 |
| Egfr | -0.455104 |
| Egfr | 0.43842212 |
| Egfr | -0.56299 |
| Egln1 | 0.22325169 |
| Egln1 | -0.6053201 |
| Egln1 | -0.7315972 |
| Egln2 | 0.30270618 |
| Egln3 | 1.23567443 |
| Egr1 | 0.03548052 |
| Egr2 | -0.0826216 |
| Egr3 | 2.21682122 |
| Egr4 | -0.719951 |
| Ehbp1 | 1.59511359 |
| Ehbp1l1 | -0.6406237 |
| Ehd1 | -0.602176 |
| Ehd2 | 0.39713451 |
| Ehd3 | 0.32126422 |
| Ehd4 | 1.14553173 |
| Ehmt1 | -0.5789266 |
| Ehmt2 | -0.4208848 |
| Ehmt2 | -0.6124335 |
| Ei24 | -0.1420287 |
| Eid1 | 0.99274057 |
| Eid2 | 2.72216042 |
| Eid3 | -0.7315972 |
| Eif1a | -0.3565988 |
| Eif1b | 0.51351954 |
| Eif2a | 1.72890842 |
| Eif2ak1 | -0.622003 |
| Eif2ak2 | -0.5599851 |
| Eif2b1 | 1.65068752 |
| Eif2b1 | 2.31057863 |
| Eif2b2 | 0.75018889 |
| Eif2b4 | 1.57608155 |
| Eif2b5 | -0.5239394 |
| Eif2b5 | 0.97307906 |
| Eif2b5 | -0.5117522 |
| Eif2c2 | 0.58982503 |
| Eif2c3 | -0.5502854 |
| Eif2c3 | -0.5096179 |
| Eif2c3 | -0.5011294 |
| Eif2c3 | 0.72081601 |
| Eif2c4 | 1.22177968 |
| Eif2s2 | 0.11408565 |
| Eif2s2 | -0.820434 |
| Eif2s3x | 0.43326299 |
| Eif2s3y | -0.7324991 |
| Eif3b | -0.0162431 |
| Eif3b | -0.4829982 |
| Eif3d | 0.34856866 |
| Eif3eip | -0.0374203 |
| Eif3eip | 0.14840773 |
| Eif3f | -0.5858408 |
| Eif3g | 0.86380057 |
| Eif3i | 0.05119502 |
| Eif3i | 1.24221895 |
| Eif3i | 1.79448411 |
| Eif3k | -0.0348535 |
| Eif3s1 | -0.5297301 |
| Eif3s10 | -0.2636779 |
| Eif3s10 | -0.3972788 |
| Eif3s4 | 0.91271786 |
| Eif3s8 | 2.82423727 |
| Eif4a1 | 0.0597265 |
| Eif4a1 | 0.65364297 |
| Eif4a2 | -0.6268249 |
| Eif4a3 | -0.4144457 |
| Eif4b | 1.70207478 |
| Eif4e | -0.164337 |
| Eif4e1b | -0.5697867 |
| Eif4e1b | 0.43996431 |
| Eif4e2 | -0.7119726 |
| Eif4e2 | -0.542156 |
| Eif4e3 | -0.1396357 |
| Eif4e3 | 0.44073445 |
| Eif4e3 | 0.76426361 |
| Eif4ebp1 | -0.0643656 |
| Eif4ebp2 | 2.34203557 |
| Eif4el3 | 1.36953421 |
| Eif4enif1 | -0.0481919 |
| Eif4enif1 | -0.0568996 |
| Eif4g1 | 0.24818502 |
| Eif4g2 | -0.1174366 |
| Eif4g2 | -0.0128989 |
| Eif4h | -0.5196198 |
| Eif5 | -0.1687648 |
| Eif5 | -0.4768033 |
| Eif5 | 0.8306051 |
| Eif5a | -0.5011294 |
| Eif5a | -0.5912541 |
| Eif6 | 0.18624296 |
| Ela1 | 3.25741763 |
| Ela2 | -0.1183706 |
| Ela2a | 1.46354219 |
| Ela3b | -0.5261067 |
| Elac2 | 3.07040524 |
| Elac2 | 2.69900195 |
| Elac2 | 0.86461552 |
| Elavl1 | -0.4411333 |
| Elavl2 | -0.4391545 |
| Elavl2 | -0.6638057 |
| Elavl3 | 1.92826555 |
| Elavl4 | 3.70362822 |
| Elavl4 | -0.2857755 |
| Elf1 | 1.89484869 |
| Elf2 | 0.10088222 |
| Elf2 | 0.89333122 |
| Elf2 | -0.8364348 |
| Elf2 | 0.06049823 |
| Elf2 | 2.72920935 |
| Elf3 | -0.3785677 |
| Elf3 | 1.20902741 |
| Elf3 | 1.38327758 |
| Elf5 | -0.7882743 |
| Elfn1 | -0.3339572 |
| Elfn2 | -0.4241211 |
| Elk3 | 1.89484869 |
| Elk3 | 1.7483811 |
| Elk3 | 0.46129156 |
| Ell | 1.25033557 |
| Ell3 | 0.09160199 |
| Elmo1 | 0.9359454 |
| Elmo1 | -0.3416413 |
| Elmo1 | 0.59896602 |
| Elmo1 | -0.2458333 |
| Elmo1 | -0.3440185 |
| Elmo1 | 1.16462489 |
| Elmo1 | 2.44153947 |
| Elmo1 | 0.12314599 |
| Elmo1 | -0.5232181 |
| Elmo1 | 0.73869964 |
| Elmo1 | -0.7590411 |
| Elmo1 | -0.3960196 |
| Elmo1 | -0.368129 |
| Elmo2 | -0.2620415 |
| Elmo2 | -0.2341238 |
| Elmo3 | 1.0763592 |
| Elmo3 | -0.6638057 |
| Elmod1 | -0.8008112 |
| Eln | -0.6308622 |
| Elof1 | -0.4665689 |
| Elovl1 | -0.7759035 |
| Elovl1 | 0.30002464 |
| Elovl1 | -0.4306276 |
| Elovl1 | -0.2544426 |
| Elovl2 | -0.7093282 |
| Elovl4 | 0.02110756 |
| Elovl5 | 0.99755657 |
| Elovl6 | 0.54272646 |
| Elp2 | 0.92425109 |
| Elp3 | -0.5584863 |
| Elp3 | 1.47529997 |
| Eltd1 | 1.07791598 |
| Emb | -0.682433 |
| Emcn | 1.49291267 |
| Emd | -0.2309556 |
| Emd | -0.2970237 |
| Emg1 | 2.12079615 |
| Emg1 | -0.0327199 |
| Emid1 | -0.6696923 |
| Emid2 | -0.0776885 |
| Emilin1 | 1.26530022 |
| Emilin2 | 0.14910952 |
| Eml1 | -0.7226257 |
| Eml3 | -0.3416413 |
| Eml3 | 0.03349747 |
| Eml4 | -0.5540042 |
| Eml5 | 1.41126035 |
| Emp1 | -0.5660047 |
| Emp2 | -0.6850021 |
| Emp3 | -0.7479665 |
| Emr1 | -0.2070172 |
| Emr1 | 2.01730141 |
| Emr1 | 1.29452819 |
| En2 | 3.61316598 |
| Enah | 0.78808533 |
| Enc1 | 0.53649472 |
| Endod1 | 1.43224223 |
| Endogl1 | -0.396649 |
| Endogl1 | 0.35758892 |
| Eng | 4.16703252 |
| Eno1 | -0.2807577 |
| Eno2 | -0.4029661 |
| Eno3 | 1.81140861 |
| Enoph1 | 0.57771764 |
| Enox1 | 0.14453843 |
| Enpp1 | 0.03349747 |
| Enpp1 | 1.87696509 |
| Enpp2 | 2.33407629 |
| Enpp4 | -0.0438683 |
| Enpp5 | -0.2095897 |
| Enpp6 | 0.31011472 |
| Ensa | 2.4963748 |
| Ensa | -0.3553933 |
| ENSMUSG00000043795 | -0.568272 |
| ENSMUSG00000053178 | -0.8947029 |
| ENSMUSG00000068790 | -0.5046569 |
| Entpd2 | 1.00333123 |
| Entpd3 | 1.89915903 |
| Entpd4 | 0.79548335 |
| Entpd5 | -0.0280431 |
| Entpd5 | 1.18772425 |
| Entpd5 | 0.30745442 |
| Entpd6 | 0.92394538 |
| Entpd7 | 1.88256326 |
| Ep300 | -0.3446137 |
| Ep400 | -0.3711871 |
| Epas1 | -0.8284001 |
| Epb4.1 | 1.02195901 |
| Epb4.1l1 | 0.89254099 |
| Epb4.1l1 | 0.82484252 |
| Epb4.1l1 | 2.01250557 |
| Epb4.1l2 | 0.7204358 |
| Epb4.1l3 | 0.32765882 |
| Epb4.1l3 | 0.17402862 |
| Epb4.1l3 | 1.01310854 |
| Epb4.1l4a | -0.6116404 |
| Epb4.1l5 | -0.2998571 |
| Epb4.1l5 | -0.0538427 |
| Epb4.9 | -0.610848 |
| Epb4.9 | 0.5466289 |
| Epc1 | 1.15417203 |
| Epc1 | 0.46679763 |
| Epc1 | 1.40370323 |
| Epdr1 | -0.6756163 |
| Epgn | 1.03086266 |
| Epha1 | 1.17915794 |
| Epha1 | -0.6300534 |
| Epha3 | 0.55936976 |
| Epha3 | -0.5443661 |
| Epha3 | 1.34074046 |
| Epha4 | -0.6739199 |
| Epha5 | 0.61783301 |
| Epha6 | 0.12242439 |
| Epha7 | -0.1197733 |
| Epha7 | -0.2458333 |
| Epha7 | 2.7960119 |
| Ephb1 | -0.3078366 |
| Ephb2 | 2.04745201 |
| Ephb4 | 0.72119606 |
| Ephb6 | 0.71260723 |
| Ephx1 | -0.4118824 |
| Ephx2 | -0.2746613 |
| Epm2a | 0.06126933 |
| Epm2aip1 | 1.46422786 |
| Epm2aip1 | 0.75221261 |
| Epm2aip1 | -0.2998571 |
| Epn1 | -0.5789266 |
| Epn2 | 0.62058811 |
| Epn2 | -0.5904789 |
| Epn2 | 1.09817487 |
| Epn2 | -0.6100563 |
| Epn2 | 0.83364153 |
| Epor | 0.72460957 |
| Eprs | -0.703187 |
| Eprs | -0.7014397 |
| Eps15 | 0.37949131 |
| Eps15l1 | -0.3779505 |
| Eps8 | 1.63669186 |
| Eps8l1 | -0.6571233 |
| Eps8l2 | -0.0310165 |
| Eps8l2 | -0.5004255 |
| Eps8l3 | 1.94330594 |
| Epsti1 | -0.6447213 |
| Epsti1 | 1.84116569 |
| Eraf | 0.73515023 |
| Eral1 | 0.13425998 |
| Ercc1 | 1.6264517 |
| Ercc2 | 0.08373463 |
| Ercc3 | -0.0648067 |
| Ercc3 | 1.73776938 |
| Ercc4 | -0.75997 |
| Ercc4 | 3.52579187 |
| Ercc5 | -0.0621635 |
| Ercc5 | -0.5268303 |
| Ercc8 | -0.4537644 |
| Ercc8 | 0.60737463 |
| Erdr1 | -0.4477599 |
| Erf | -0.7102088 |
| Erf | -0.0626035 |
| Ergic1 | 1.29729879 |
| Ergic2 | 0.85610513 |
| Ergic2 | 0.31188405 |
| Ergic3 | 0.21380901 |
| Ergic3 | 2.07381006 |
| Erh | -0.3392702 |
| Erich1 | -0.7806421 |
| Erlin1 | 0.74538803 |
| Erlin2 | 0.02150981 |
| Erlin2 | 2.20376576 |
| Ermap | -0.5812257 |
| Ermp1 | -0.5414205 |
| Ern1 | 1.43463313 |
| Ern2 | 1.59183144 |
| Ero1lb | -0.4941141 |
| Erp29 | -0.233595 |
| Errfi1 | 2.00004522 |
| Es22 | -0.5690291 |
| Esam1 | 1.5549445 |
| Esco2 | 0.52695139 |
| Esd | -0.0233896 |
| Esd | 1.86377055 |
| Esd | 2.04905559 |
| Esd | -0.697955 |
| Esm1 | -0.3205153 |
| Espl1 | -0.1932472 |
| Espn | 1.15524647 |
| Espn | 1.90312845 |
| Espn | 0.02951867 |
| Espn | -0.6196014 |
| Esr1 | 0.11989439 |
| Esr2 | 0.79020504 |
| Esr2 | -0.3210957 |
| Esr2 | -0.3699627 |
| Esrra | -0.717284 |
| Esrrb | 0.45094272 |
| Etaa1 | 1.01408066 |
| Etf1 | 2.38273497 |
| Etfa | -0.4189484 |
| Etfa | 1.51928916 |
| Etfa | -0.3535881 |
| Etfb | 0.59918257 |
| Etfb | 1.50987277 |
| Etfdh | 0.49651358 |
| Ethe1 | 0.11735751 |
| Etnk2 | -0.2204721 |
| Ets1 | 1.36620926 |
| Ets2 | -0.3493893 |
| Etsrp71 | -0.2609522 |
| Etv1 | -0.6841449 |
| Etv1 | 2.93129775 |
| Etv4 | -0.366299 |
| Etv5 | -0.7244131 |
| Etv6 | -0.6705363 |
| Evc | -0.3009928 |
| Evc2 | -0.0233896 |
| Evi1 | 0.91472719 |
| Evi1 | 0.26846561 |
| Evi2a | -0.6654838 |
| Evi2a | -0.5232181 |
| Evi5 | 0.50061374 |
| Evi5l | -0.652971 |
| Evl | -0.4267183 |
| Evx1 | -0.2757668 |
| Exdl1 | -0.5153202 |
| Exdl2 | -0.6554601 |
| Exdl2 | 1.28388263 |
| Exdl2 | 1.66233089 |
| Exoc2 | 0.06664923 |
| Exoc3 | -0.3608302 |
| Exoc3l | -0.3785677 |
| Exoc4 | 1.33711477 |
| Exoc7 | -0.5812257 |
| Exoc8 | -0.6604585 |
| Exod1 | -0.4048696 |
| Exosc1 | 0.12134094 |
| Exosc10 | 1.12246272 |
| Exosc2 | -0.0705593 |
| Exosc2 | -0.6773158 |
| Exosc4 | -0.1604188 |
| Exosc5 | -0.380422 |
| Exosc6 | 1.75524277 |
| Exosc7 | -0.2868942 |
| Exosc8 | -0.0989511 |
| Ext1 | -0.7806421 |
| Ext2 | 0.0635788 |
| Extl1 | 1.98638868 |
| Extl2 | 0.2625829 |
| Extl3 | 1.49680944 |
| Extl3 | 2.95828851 |
| Eya1 | -0.3222577 |
| Eya1 | 0.09085557 |
| Eya2 | -0.3998022 |
| Eya3 | -0.4809287 |
| Eya3 | 1.32664896 |
| Eya3 | -0.6944833 |
| Eya4 | -0.4788637 |
| Ezh1 | 2.83200319 |
| Ezh2 | 1.10939578 |
| Ezh2 | -0.6496624 |
| F10 | -0.3885002 |
| F11 | -0.5990424 |
| F11 | -0.5428922 |
| F11r | -0.7163966 |
| F13a1 | -0.5773971 |
| F2r | 0.83650022 |
| F2rl1 | 0.07009143 |
| F2rl3 | -0.5804587 |
| F430201B04Rik | 0.35562318 |
| F5 | 1.58996142 |
| F630043A04Rik | -0.6252148 |
| F730014I05Rik | 0.05663316 |
| F730047E07Rik | 0.39065889 |
| F730047E07Rik | 0.00035922 |
| F730047E07Rik | -0.3928792 |
| F8 | 0.02991731 |
| F8 | 1.78213479 |
| F8 | -0.6406237 |
| F8a | 1.4895834 |
| Fa2h | -0.1329678 |
| Faah | -0.1560304 |
| Faah | 0.27216199 |
| Fabp3 | -0.7901921 |
| Fabp5 | 1.30169433 |
| Fabp9 | 2.12969765 |
| Fadd | 2.15279591 |
| Fadd | 1.89679081 |
| Fadd | 1.79934254 |
| Fads1 | 0.51943452 |
| Fads2 | 0.91734827 |
| Fads3 | -0.5781615 |
| Fads6 | 0.07731686 |
| Faf1 | 0.95432707 |
| Faf1 | -0.5584863 |
| Fah | -0.7217333 |
| Fahd1 | 0.80456227 |
| Fahd2a | 0.12997348 |
| Fahd2a | -0.4215312 |
| Fahd2a | 0.87723729 |
| Fahd2a | 0.7669809 |
| Faim | -0.4036001 |
| Faim | -0.5959193 |
| Faim2 | -0.0745627 |
| Faim2 | 1.77448623 |
| Faim3 | 2.46531043 |
| Fancc | 1.28606297 |
| Fancc | -0.6858601 |
| Fancd2 | 0.52202528 |
| Fancg | -0.7343056 |
| Fanci | 0.13710674 |
| Fancl | 0.45474221 |
| Fancl | -0.5637428 |
| Fank1 | 0.19128545 |
| Fank1 | -0.0335728 |
| Fap | -0.2913826 |
| Fars2 | -0.0434371 |
| Farsa | -0.4720133 |
| Farsb | -0.3632568 |
| Farsb | -0.2713527 |
| Fas | 0.09085557 |
| Fas | 0.08486261 |
| Fasl | -0.3620427 |
| Fastk | 0.79038147 |
| Fastk | -0.6447213 |
| Fastkd1 | 0.37095789 |
| Fastkd5 | 0.75147722 |
| Fat1 | 1.01310854 |
| Fat4 | 0.08561384 |
| Fbf1 | -0.0408537 |
| Fblim1 | -0.7562601 |
| Fbln1 | 0.12638629 |
| Fbln1 | 0.29343713 |
| Fbln1 | -0.6164089 |
| Fbln2 | 0.15365831 |
| Fbln2 | -0.451091 |
| Fbn1 | -0.723519 |
| Fbp1 | 2.14287197 |
| Fbp2 | 0.00610094 |
| Fbxl10 | 0.23420208 |
| Fbxl12 | 0.74483249 |
| Fbxl12 | 0.78507413 |
| Fbxl12 | 0.66763102 |
| Fbxl12 | 1.68559618 |
| Fbxl13 | 0.1730035 |
| Fbxl13 | -0.082172 |
| Fbxl14 | 0.06626598 |
| Fbxl15 | 0.69675023 |
| Fbxl18 | -0.102619 |
| Fbxl18 | 0.95919774 |
| Fbxl21 | 0.71279895 |
| Fbxl22 | 0.33516 |
| Fbxl4 | -0.691888 |
| Fbxl5 | -0.3953907 |
| Fbxl6 | -0.4969139 |
| Fbxl6 | -0.5067798 |
| Fbxl8 | -0.009151 |
| Fbxo15 | 0.36374015 |
| Fbxo16 | -0.6414418 |
| Fbxo18 | -0.2059902 |
| Fbxo18 | 0.17334533 |
| Fbxo2 | 0.81131395 |
| Fbxo21 | 0.77347029 |
| Fbxo25 | -0.3608302 |
| Fbxo27 | -0.1574909 |
| Fbxo28 | 0.08448677 |
| Fbxo3 | -0.545104 |
| Fbxo3 | 0.8847611 |
| Fbxo30 | 1.96609717 |
| Fbxo31 | 1.00962839 |
| Fbxo31 | 0.16717314 |
| Fbxo32 | 1.2918524 |
| Fbxo32 | -0.3705747 |
| Fbxo32 | -0.5377515 |
| Fbxo33 | 0.18455605 |
| Fbxo34 | -0.7102088 |
| Fbxo34 | -0.648837 |
| Fbxo34 | 0.5116188 |
| Fbxo36 | 0.00936613 |
| Fbxo38 | 0.37371914 |
| Fbxo38 | -0.4625064 |
| Fbxo4 | -0.3164624 |
| Fbxo42 | -0.1123165 |
| Fbxo44 | 2.13091447 |
| Fbxo6 | -0.3176186 |
| Fbxo7 | -0.739746 |
| Fbxo8 | -0.5637428 |
| Fbxo9 | 0.34317132 |
| Fbxo9 | -0.0718919 |
| Fbxw12 | 1.56314869 |
| Fbxw13 | -0.6680066 |
| Fbxw15 | 0.31188405 |
| Fbxw16 | 0.3863167 |
| Fbxw17 | 0.66561672 |
| Fbxw19 | -0.3972788 |
| Fbxw2 | 2.68553886 |
| Fbxw4 | 1.01088274 |
| Fbxw4 | -0.1947672 |
| Fbxw5 | -0.5074885 |
| Fbxw7 | 0.17947696 |
| Fbxw7 | -0.1063014 |
| Fbxw7 | 0.92882476 |
| Fbxw8 | -0.5599851 |
| Fcer1g | 0.81510115 |
| Fcer2a | -0.067015 |
| Fcf1 | -0.8154895 |
| Fcgr2b | 2.76329353 |
| Fcgr2b | 0.07314054 |
| Fcgr2b | -0.532639 |
| Fcgr3 | -0.755335 |
| Fcgr4 | 2.57457329 |
| Fcgrt | -0.4920198 |
| Fcho1 | -0.7834969 |
| Fcho2 | -0.007905 |
| Fchsd1 | 0.0223138 |
| Fchsd2 | 0.60587096 |
| Fcna | 0.63881964 |
| Fcrla | 2.22219803 |
| Fcrla | 0.17641619 |
| Fcrla | 2.98619851 |
| Fdps | 1.0927048 |
| Fdxr | -0.340455 |
| Fdxr | 1.9742668 |
| Fem1b | 2.8262495 |
| Fem1c | -0.3286742 |
| Fem1c | 1.3715235 |
| Fen1 | -0.6252148 |
| Fermt2 | 1.57926802 |
| Fermt3 | 0.39955134 |
| Fert2 | 1.55120554 |
| Fert2 | -0.5018338 |
| Fes | 2.20794489 |
| Fetub | -0.5089076 |
| Fev | 0.38222746 |
| Fez1 | 0.45094272 |
| Fez1 | 1.08089255 |
| Fez2 | -0.5897043 |
| Fezf2 | 2.57334749 |
| Ffar2 | -0.5751074 |
| Fgd2 | -0.8414919 |
| Fgd4 | 2.88395343 |
| Fgd4 | -0.7315972 |
| Fgd5 | -0.5943616 |
| Fgd6 | 0.58522073 |
| Fgf1 | -0.6164089 |
| Fgf1 | 3.19955713 |
| Fgf10 | -0.1761914 |
| Fgf10 | -0.7636946 |
| Fgf12 | 1.32704604 |
| Fgf12 | 0.66015627 |
| Fgf13 | 0.39659659 |
| Fgf13 | 1.67554016 |
| Fgf17 | 1.48924982 |
| Fgf21 | 2.13363607 |
| Fgf22 | 0.91147919 |
| Fgf22 | 0.80924171 |
| Fgf5 | 0.6569053 |
| Fgf7 | -0.5319109 |
| Fgf8 | 0.6384045 |
| Fgf9 | 0.73664636 |
| Fgfbp3 | 0.10235853 |
| Fgfr1 | -0.4273687 |
| Fgfr1op | -0.5261067 |
| Fgfr1op2 | -0.6884387 |
| Fgfr1op2 | 0.61293857 |
| Fgfr2 | -0.0070753 |
| Fgfr2 | -0.3280891 |
| Fgfr3 | -0.5253837 |
| Fgfrl1 | -0.6084749 |
| Fgl2 | 1.05707488 |
| Fh1 | 0.03071409 |
| Fhad1 | 2.15045826 |
| Fhit | 2.87990371 |
| Fhl1 | 2.15275495 |
| Fhl1 | 1.03629989 |
| Fhl2 | 1.3253573 |
| Fhl4 | -0.7627621 |
| Fhl5 | 1.5744081 |
| Fhod1 | 2.7567557 |
| Fhod3 | -0.3517863 |
| Fibcd1 | 1.83996367 |
| Fibp | 0.36874299 |
| Figf | -0.7901921 |
| Figla | -0.4692871 |
| Fign | -0.7190611 |
| Fignl1 | -0.5990424 |
| Fip1l1 | -0.2415583 |
| Fis1 | 1.66045825 |
| Fis1 | -0.622805 |
| Fiz1 | -0.1732137 |
| Fjx1 | 0.97770875 |
| Fkbp10 | 2.07790229 |
| Fkbp11 | 0.33573453 |
| Fkbp14 | -0.5912541 |
| Fkbp1a | 1.32833537 |
| Fkbp1b | 0.56140127 |
| Fkbp2 | -0.3891246 |
| Fkbp2 | -0.186689 |
| Fkbp4 | -0.5959193 |
| Fkbp5 | 0.12386703 |
| Fkbp6 | 1.37350857 |
| Fkbp7 | 2.17625526 |
| Fkbp8 | 0.70703002 |
| Fkbp9 | -0.4306276 |
| Fkbpl | 0.48091403 |
| Fkbpl | -0.3004248 |
| Fkhl18 | -0.0179197 |
| Fkrp | 1.38271676 |
| Fktn | -0.2469052 |
| Flad1 | -0.0975793 |
| Flcn | 1.95926766 |
| Flcn | -0.3773336 |
| Fli1 | -0.380422 |
| Flii | 1.60356404 |
| Flna | 2.86652412 |
| Flot1 | 0.40196191 |
| Flot1 | 0.02271553 |
| Flot2 | -0.4299749 |
| Flot2 | 1.93309868 |
| Flot2 | 0.84235833 |
| Flrt1 | 1.50693101 |
| Flrt2 | -0.3493893 |
| Flrt3 | -0.6045331 |
| Flt1 | 0.19696752 |
| Flt4 | 1.17484511 |
| Flt4 | -0.5333677 |
| Flvcr2 | 1.3461041 |
| Flywch2 | 0.56881252 |
| Fmn2 | -0.1174366 |
| Fmn2 | -0.2293756 |
| Fmnl1 | 0.08034249 |
| Fmnl3 | 1.49000021 |
| Fmnl3 | 0.73458856 |
| Fmo1 | -0.5428922 |
| Fmo2 | 1.18379814 |
| Fmo5 | 0.03111223 |
| Fmod | -0.5032443 |
| Fmr1nb | 0.72100605 |
| Fmr1nb | -0.6850021 |
| Fn3k | 1.50439038 |
| Fn3k | -0.1179035 |
| Fnbp1 | 0.867381 |
| Fnbp1 | 1.74178679 |
| Fnbp1 | 1.08938169 |
| Fnbp1l | 2.53199262 |
| Fnbp4 | -0.6037467 |
| Fndc3 | 0.07693797 |
| Fndc3a | 1.58244364 |
| Fndc3b | 0.40303128 |
| Fndc4 | 3.2689057 |
| Fndc5 | 1.98746512 |
| Fndc5 | -0.5224973 |
| Fndc7 | -0.4686068 |
| Fndc7 | -0.4067769 |
| Fndc8 | -0.3517863 |
| Fndc8 | -0.3620427 |
| Fnip1 | -0.6100563 |
| Fnip1 | -0.6832886 |
| Fnta | -0.5124647 |
| Fnta | -0.2152741 |
| Fntb | -0.3590144 |
| Folr1 | 0.89490968 |
| Folr1 | 0.73252621 |
| Folr2 | 1.87199747 |
| Fos | 1.48078713 |
| Fosb | -0.5319109 |
| Foxa1 | 2.16107394 |
| Foxa2 | -0.5275544 |
| Foxa3 | -0.0608448 |
| Foxc1 | -0.5660047 |
| Foxd2 | 2.29386247 |
| Foxg1 | 0.38794739 |
| Foxh1 | 0.8135537 |
| Foxh1 | 0.68536933 |
| Foxj1 | -0.0542788 |
| Foxj2 | -0.703187 |
| Foxj3 | 1.48036618 |
| Foxj3 | -0.5720633 |
| Foxj3 | 2.37343696 |
| Foxj3 | 0.68950605 |
| Foxj3 | 0.89317323 |
| Foxk1 | -0.381041 |
| Foxk1 | -0.1072243 |
| Foxk1 | 1.43108886 |
| Foxl2 | -0.4990192 |
| Foxm1 | -0.3632568 |
| Foxm1 | -0.4345534 |
| Foxn2 | 2.96996244 |
| Foxn3 | -0.5488021 |
| Foxo1 | 1.63112026 |
| Foxo6 | -0.6471886 |
| Foxo6 | -0.6292452 |
| Foxp1 | -0.3240033 |
| Foxp2 | 0.35730835 |
| Foxp2 | -0.602176 |
| Foxp2 | 0.31541268 |
| Foxp3 | 0.34402561 |
| Foxp4 | 1.55440017 |
| Foxq1 | 1.40269707 |
| Foxred1 | -0.5990424 |
| Foxred1 | -0.127762 |
| Foxred1 | -0.0718919 |
| Foxred1 | 0.78454174 |
| Foxred1 | -0.1401138 |
| Foxred2 | 0.66561672 |
| Fpgs | 0.61251175 |
| Fpgs | 0.45448939 |
| Fpgs | -0.2891357 |
| Fpr2 | -0.4850724 |
| Frag1 | 0.19362932 |
| Frag1 | 1.55859111 |
| Frag1 | -0.617206 |
| Frap1 | 3.22669054 |
| Fras1 | 2.63240319 |
| Fras1 | -0.3748701 |
| Frat1 | 0.75624693 |
| Frat1 | -0.4099646 |
| Frat2 | 0.70297043 |
| Freq | 1.66730631 |
| Freq | 0.30715836 |
| Freq | -0.6357302 |
| Frg1 | -0.8527149 |
| Frk | -0.4774896 |
| Frmd3 | -0.666324 |
| Frmd6 | -0.5074885 |
| Frmd8 | 2.21256651 |
| Frmpd4 | -0.5889303 |
| Frrs1 | -0.2236048 |
| Frs2 | 1.54705961 |
| Frs3 | 1.26147503 |
| Fryl | -0.1444278 |
| Fryl | -0.0608448 |
| Frzb | 0.57860349 |
| Fscn1 | 3.87840958 |
| Fscn2 | -0.2373028 |
| Fscn3 | -0.6621306 |
| Fsd1 | 0.56342837 |
| Fsd2 | 1.05495165 |
| Fshb | 0.34913499 |
| Fshr | 3.19676509 |
| Fshr | -0.4955129 |
| Fshr | -0.2813139 |
| Fsip1 | 0.7770561 |
| Fst | -0.4774896 |
| Fstl1 | -0.8364348 |
| Fstl4 | 0.02150981 |
| Fstl5 | -0.3565988 |
| Fth1 | -0.7646281 |
| Fth1 | 0.84202457 |
| Ftmt | 0.72706714 |
| Fto | -0.1761914 |
| Ftsj1 | -0.4658906 |
| Ftsj3 | -0.7940396 |
| Fubp1 | -0.5675156 |
| Fubp3 | 0.62544269 |
| Fuk | 3.17180336 |
| Fuk | 1.41506124 |
| Fundc1 | -0.1937536 |
| Fundc1 | 2.10717508 |
| Fundc2 | 1.30951402 |
| Furin | -0.6850021 |
| Furin | -0.738837 |
| Fus | 1.91181278 |
| Fusip1 | 2.54041154 |
| Fusip1 | -0.7370218 |
| Fusip1 | -0.6164089 |
| Fut10 | 1.30474876 |
| Fut10 | 0.59744873 |
| Fut2 | -0.4195934 |
| Fut4 | 0.30151534 |
| Fut8 | 0.26351422 |
| Fut8 | -0.298156 |
| Fut8 | 0.26846561 |
| Fvt1 | -0.617206 |
| Fxc1 | -0.1658106 |
| Fxn | -0.3251688 |
| Fxn | -0.4761175 |
| Fxyd1 | 2.32318659 |
| Fxyd2 | 3.6035436 |
| Fxyd2 | 0.25603751 |
| Fxyd2 | -0.7507229 |
| Fxyd3 | -0.633293 |
| Fxyd4 | -0.2399602 |
| Fxyd4 | -0.6053201 |
| Fxyd5 | 1.90718765 |
| Fxyd5 | -0.0652479 |
| Fxyd5 | -0.1988327 |
| Fxyd6 | 0.74982047 |
| Fxyd7 | -0.5827615 |
| Fyb | -0.7084484 |
| Fyco1 | -0.6654838 |
| Fyn | 2.85256298 |
| Fyttd1 | 0.00773496 |
| Fzd1 | -0.0732264 |
| Fzd10 | -0.8787351 |
| Fzd10 | -0.5297301 |
| Fzd2 | -0.6439004 |
| Fzd3 | 1.16497918 |
| Fzd4 | -0.7768493 |
| Fzd5 | -0.7674342 |
| Fzd6 | -0.3693511 |
| Fzd7 | -0.4934155 |
| Fzd8 | 0.95610117 |
| Fzr1 | 1.68214315 |
| Fzr1 | 0.64749518 |
| Fzr1 | 0.61187116 |
| G0s2 | -0.7452183 |
| G3bp1 | -0.2362419 |
| G3bp1 | -0.6962175 |
| G3bp2 | 0.01099445 |
| G3bp2 | -0.0975793 |
| G3bp2 | 1.64900221 |
| G430022H21Rik | 0.96081566 |
| G430055L02Rik | -0.7693097 |
| G6pc | -0.1831767 |
| G6pc2 | 0.44508662 |
| G6pc2 | -0.7110903 |
| G6pc3 | 0.76317447 |
| G6pd2 | 0.62417871 |
| G6pd2 | -0.4352093 |
| G6pdx | -0.5974795 |
| Gaa | -0.4524267 |
| Gab1 | 2.00043111 |
| Gab2 | 1.2543139 |
| Gab2 | 1.57972234 |
| Gab2 | 1.46799007 |
| Gab3 | -0.7693097 |
| Gab3 | -0.3044084 |
| Gab3 | -0.6596236 |
| Gabarap | 0.00405438 |
| Gabarapl1 | 1.02936185 |
| Gabarapl2 | 0.68200706 |
| Gabbr1 | -0.4706492 |
| Gabpa | -0.5039503 |
| Gabpa | -0.6260195 |
| Gabpb1 | -0.0962096 |
| Gabpb1 | -0.1377257 |
| Gabpb2 | -0.2560658 |
| Gabra3 | 0.10933927 |
| Gabra4 | 2.19350984 |
| Gabrb1 | -0.155058 |
| Gabrb2 | -0.6496624 |
| Gabrb3 | -0.6292452 |
| Gabrb3 | -0.1806759 |
| Gabrd | -0.7128557 |
| Gabrd | -0.147797 |
| Gabrg1 | 2.04446662 |
| Gabrg2 | -0.0108149 |
| Gabrg2 | 0.13746198 |
| Gabrg2 | -0.5304565 |
| Gabrg2 | -0.2802018 |
| Gabrp | -0.3049789 |
| Gabrp | -0.4010665 |
| Gad1 | -0.7646281 |
| Gad1 | -0.4781764 |
| Gadd45a | 1.45033083 |
| Gadd45a | 0.84118965 |
| Gadd45b | -0.0399942 |
| Gadd45g | 1.43675324 |
| Gadd45g | 1.06157076 |
| Gak | 0.3783946 |
| Gal | 0.59200959 |
| Gal3st1 | -0.2587773 |
| Gal3st2 | 0.25384546 |
| Gal3st3 | 0.85544726 |
| Galc | -0.3205153 |
| Galc | 0.04611678 |
| Gale | -0.6944833 |
| Gale | -0.7730719 |
| Galk1 | -0.3748701 |
| Galm | 2.66818462 |
| Galns | 1.03521498 |
| Galnt1 | 0.52061303 |
| Galnt1 | -0.1141751 |
| Galnt1 | 0.92409825 |
| Galnt1 | -0.3170403 |
| Galnt10 | 0.6466724 |
| Galnt10 | 0.40782834 |
| Galnt10 | 0.64234089 |
| Galnt11 | -0.3903744 |
| Galnt12 | -0.4411333 |
| Galnt13 | -0.717284 |
| Galnt2 | 1.36535236 |
| Galnt2 | 1.66045825 |
| Galnt2 | -0.6722266 |
| Galnt3 | 1.22842204 |
| Galnt9 | -0.4391545 |
| Galntl2 | 0.58697743 |
| Galntl4 | -0.4306276 |
| Galntl5 | 2.92345842 |
| Galt | 1.08192566 |
| Galt | -0.1836776 |
| Gamt | 2.87999766 |
| Gan | -0.4530953 |
| Ganab | -0.4997221 |
| Ganab | 1.03426464 |
| Ganab | 0.96711349 |
| Ganc | 1.60053365 |
| Gap43 | -0.1796775 |
| Gapdh | 0.83851286 |
| Gapdhs | -0.2533621 |
| Gapvd1 | 2.46334514 |
| Gapvd1 | 0.84867719 |
| Garnl3 | -0.1324933 |
| Garnl4 | -0.5384841 |
| Gars | 1.93599693 |
| Gart | -0.6680066 |
| Gart | -0.7825443 |
| Gas2l1 | 0.29823266 |
| Gas2l1 | 3.13814058 |
| Gas2l1 | 1.7167653 |
| Gas2l1 | 2.16265593 |
| Gas6 | -0.6236076 |
| Gas7 | 0.75551471 |
| Gas7 | -0.6061078 |
| Gas8 | -0.4055049 |
| Gata2 | -0.1697516 |
| Gata4 | -0.5928066 |
| Gata6 | -0.505364 |
| Gatad1 | -0.5982606 |
| Gatad1 | -0.0794793 |
| Gatad2b | 1.30799584 |
| Gats | -0.5858408 |
| Gats | 3.95728592 |
| Gba | -0.0058321 |
| Gba2 | 1.52356005 |
| Gbas | 1.96624725 |
| Gbf1 | -0.7730719 |
| Gbl | -0.4086882 |
| Gbl | -0.3767171 |
| Gbl | -0.2539022 |
| Gbp2 | -0.298156 |
| Gbp3 | -0.7155101 |
| Gbp3 | -0.380422 |
| Gbp6 | 1.76819822 |
| Gbp6 | 2.64343496 |
| Gbx2 | 2.09015999 |
| Gca | -0.3398625 |
| Gca | -0.588157 |
| Gcap14 | 0.62565319 |
| Gcap14 | 1.04520262 |
| Gcc1 | -0.703187 |
| Gcc2 | -0.4795515 |
| Gcdh | 1.42931167 |
| Gch1 | -0.7005673 |
| Gch1 | 2.78926539 |
| Gchfr | 1.04520262 |
| Gck | -0.6901617 |
| Gclm | 0.27737359 |
| Gcn5l2 | 1.31726873 |
| Gcnt1 | 3.77560903 |
| Gcnt1 | -0.7040618 |
| Gcnt2 | 2.07683648 |
| Gcnt2 | -0.4365225 |
| Gcs1 | -0.5210575 |
| Gda | 0.5175458 |
| Gdap1 | -0.8294007 |
| Gdap1 | 0.76317447 |
| Gdap1l1 | 1.67745098 |
| Gdap1l1 | -0.7253081 |
| Gde1 | 1.5830473 |
| Gde1 | 1.29483644 |
| Gdf10 | -0.7155101 |
| Gdf15 | 0.8095874 |
| Gdf3 | 0.60199322 |
| Gdf9 | 2.37824232 |
| Gdi1 | 0.58982503 |
| Gdi1 | 1.10738847 |
| Gdi1 | 1.23117579 |
| Gdi1 | 0.60694526 |
| Gdi1 | 3.90048059 |
| Gdi2 | -0.461831 |
| Gdnf | -0.4371798 |
| Gdpd1 | 1.18530125 |
| Gdpd2 | 1.3076919 |
| Gdpd2 | 0.3944418 |
| Gdpd3 | 0.05663316 |
| Gdpd4 | -0.385385 |
| Gdpd5 | -0.3742553 |
| Gemin4 | -0.4920198 |
| Gemin4 | 0.4254704 |
| Gemin4 | 0.06319428 |
| Gemin5 | 1.18887585 |
| Gemin6 | -0.802755 |
| Gemin6 | -0.56299 |
| Gemin7 | 0.31892801 |
| Gemin7 | -0.7911525 |
| Gfap | -0.0599666 |
| Gfer | -0.7146245 |
| Gfi1 | -0.5488021 |
| Gfm1 | -0.0656894 |
| Gfod1 | -0.4761175 |
| Gfod2 | 0.90713086 |
| Gfpt2 | -0.1947672 |
| Gfra1 | -0.341048 |
| Gfra4 | -0.3553933 |
| Gga1 | 1.55905561 |
| Gga1 | 0.17947696 |
| Gga1 | -0.4829982 |
| Gga3 | -0.2713527 |
| Ggcx | -0.2485152 |
| Ggcx | 0.09866337 |
| Ggcx | -0.8017826 |
| Ggn | -0.4878451 |
| Ggn | 2.25694309 |
| Ggnbp1 | -0.6713811 |
| Ggnbp2 | 1.33317919 |
| Ggt5 | 0.99444318 |
| Ggt7 | -0.6944833 |
| Ggta1 | 0.29493838 |
| Ggtla1 | 0.00241389 |
| Gh | -0.5138913 |
| Ghitm | -0.7740148 |
| Ghr | 0.06856313 |
| Ghr | 0.77131217 |
| Ghrh | -0.5253837 |
| Ghrhr | 1.35462229 |
| Ghrl | -0.6571233 |
| Ghrl | -0.6447213 |
| Ghsr | 0.85577625 |
| Ghsr | -0.3181972 |
| Gif | -0.4941141 |
| Gigyf1 | 3.68991252 |
| Gimap1 | 0.48630584 |
| Gimap4 | -0.2880143 |
| Gimap5 | -0.7749587 |
| Gimap6 | 2.86207234 |
| Gimap8 | -0.4055049 |
| Gimap9 | -0.5773971 |
| Gins3 | 1.83329588 |
| Gins4 | -0.5174675 |
| Gipc1 | -0.1811756 |
| Gipc1 | -0.0154059 |
| Gipc1 | 1.04801759 |
| Gipc2 | -0.3578058 |
| Git1 | -0.3084091 |
| Git2 | -0.6061078 |
| Git2 | 0.30300365 |
| Git2 | 0.65609079 |
| Gja1 | 0.10346423 |
| Gja4 | 0.8302671 |
| Gja5 | -0.551028 |
| Gja5 | 3.04195677 |
| Gja6 | 0.23131593 |
| Gja7 | -0.7315972 |
| Gjb1 | 0.40756249 |
| Gjb2 | -0.6654838 |
| Gjb2 | -0.6562913 |
| Gjb3 | 1.51003593 |
| Gjb6 | 1.8266382 |
| Gjc2 | 1.23611216 |
| Gjc2 | 0.25666286 |
| Gjd2 | 0.95963927 |
| Gjd2 | 0.34060375 |
| Gjd3 | -0.6739199 |
| Gk2 | -0.6867188 |
| Gk5 | -0.6045331 |
| Gkap1 | 1.46311339 |
| Gkap1 | 1.68843028 |
| Glb1 | 1.42467506 |
| Glb1l2 | -0.7534874 |
| Glcci1 | -0.3199353 |
| Gldc | 0.79618487 |
| Gldc | -0.69535 |
| Gldn | -0.1291789 |
| Gle1 | -0.2479782 |
| Glg1 | 0.46629842 |
| Glipr2 | 1.71624227 |
| Glis1 | 1.8672258 |
| Glo1 | -0.6373585 |
| Gloxd1 | 2.1371415 |
| Glp2r | 0.07047311 |
| Glra2 | 2.29779796 |
| Glrb | 0.746683 |
| Glrp1 | -0.7618305 |
| Glrx | -0.6084749 |
| Glrx2 | 0.9681347 |
| Glrx2 | -0.652971 |
| Glrx2 | -0.8047029 |
| Glrx3 | 0.18893568 |
| Glrx5 | -0.6463654 |
| Gls2 | 1.6336959 |
| Gls2 | 1.65306983 |
| Glt25d1 | 0.29163244 |
| Glt25d2 | -0.5319109 |
| Glt25d2 | 0.48165111 |
| Glt6d1 | 1.0128306 |
| Glt8d1 | -0.2383648 |
| Glt8d1 | -0.4906261 |
| Glt8d1 | 0.33917426 |
| Glt8d2 | 1.57881348 |
| Glt8d3 | -0.3860072 |
| Gltp | 1.07609949 |
| Gltscr1 | -0.7093282 |
| Glud1 | 3.37915257 |
| Glud1 | 0.01909369 |
| Glul | -0.6308622 |
| Glyctk | 0.58434114 |
| Glyctk | -0.5998248 |
| Glyctk | -0.8056784 |
| Gm1006 | -0.334546 |
| Gm1006 | -0.6116404 |
| Gm1019 | -0.5743455 |
| Gm1040 | 0.79618487 |
| Gm1070 | 3.95058253 |
| Gm1082 | -0.2891357 |
| Gm114 | -0.2404926 |
| Gm114 | 0.63236413 |
| Gm1141 | -0.6447213 |
| Gm128 | 1.9495401 |
| Gm129 | -0.0621635 |
| Gm129 | -0.1254052 |
| Gm1322 | -0.8294007 |
| Gm136 | 1.18182887 |
| Gm136 | 0.86835511 |
| Gm1381 | -0.6722266 |
| Gm1527 | -0.4706492 |
| Gm1568 | 0.87771935 |
| Gm1574 | 0.65812574 |
| Gm1574 | -0.4802398 |
| Gm1604 | -0.6013916 |
| Gm1604 | -0.819443 |
| Gm166 | -0.6546297 |
| Gm166 | -0.5773971 |
| Gm1661 | 0.06741526 |
| Gm1673 | -0.340455 |
| Gm1673 | -0.1917297 |
| Gm1698 | 3.10243348 |
| Gm1821 | -0.6562913 |
| Gm1943 | -0.7128557 |
| Gm1965 | -0.289697 |
| Gm1967 | -0.3626496 |
| Gm1967 | 1.05029017 |
| Gm216 | -0.8724238 |
| Gm239 | 2.49364997 |
| Gm249 | 1.16190418 |
| Gm266 | 2.0053852 |
| Gm2a | 0.02951867 |
| Gm347 | -0.1444278 |
| Gm382 | -0.3398625 |
| Gm410 | 0.02792239 |
| Gm428 | -0.1429876 |
| Gm443 | 0.05314086 |
| Gm484 | -0.5488021 |
| Gm50 | -0.5789266 |
| Gm525 | -0.6621306 |
| Gm525 | 0.20921605 |
| Gm527 | -0.3363148 |
| Gm527 | 0.72233528 |
| Gm561 | -0.4431164 |
| Gm561 | -0.810571 |
| Gm597 | -0.5117522 |
| Gm603 | 2.12591118 |
| Gm609 | -0.7343056 |
| Gm614 | -0.1072243 |
| Gm614 | -0.7424781 |
| Gm648 | 1.30505365 |
| Gm648 | 1.21397001 |
| Gm71 | -0.4564455 |
| Gm711 | 0.97727569 |
| Gm732 | 1.35095352 |
| Gm732 | -0.3928792 |
| Gm767 | 1.25076648 |
| Gm773 | 1.65096811 |
| Gm773 | -0.7137397 |
| Gm784 | 1.51199162 |
| Gm813 | -0.7005673 |
| Gm813 | -0.5607354 |
| Gm817 | -0.5904789 |
| Gm867 | -0.5488021 |
| Gm884 | -0.3779505 |
| Gm889 | 0.63445143 |
| Gm906 | -0.7796925 |
| Gm93 | -0.3004248 |
| Gm93 | -0.396649 |
| Gm944 | -0.4829982 |
| Gm949 | 0.29823266 |
| Gm962 | 1.65621357 |
| Gm962 | 2.53805647 |
| Gm97 | -0.2283237 |
| Gm97 | 1.05149106 |
| Gmcl1 | 0.59331788 |
| Gmcl1 | -0.9011673 |
| Gmcl1 | 1.7624901 |
| Gmds | -0.3669086 |
| Gmeb2 | 1.74699003 |
| Gmfg | -0.6471886 |
| Gmfg | -0.6496624 |
| Gmfg | 1.22787031 |
| Gmip | 1.15834345 |
| Gmip | -0.7516435 |
| Gmppa | 1.40031456 |
| Gmppa | -0.5766332 |
| Gmppb | 1.32157139 |
| Gmpr | 2.47927031 |
| Gmpr2 | -0.5414205 |
| Gna11 | -0.5920301 |
| Gna13 | -0.041714 |
| Gna14 | -0.451091 |
| Gnai2 | 1.72890842 |
| Gnao1 | -0.4983169 |
| Gnao1 | -0.5735841 |
| Gnaq | 0.48312354 |
| Gnaq | -0.697955 |
| Gna-rs1 | -0.7226257 |
| Gnas | -0.4312808 |
| Gnas | -0.2431591 |
| Gnas | -0.147797 |
| Gnas | 0.03982846 |
| Gnas | -0.1922353 |
| Gnat1 | -0.4878451 |
| Gnat2 | 2.7747203 |
| Gnaz | 2.28796337 |
| Gnb1 | -0.4720133 |
| Gnb1l | 1.91223694 |
| Gnb2 | -0.4477599 |
| Gnb2l1 | -0.3872529 |
| Gnb3 | -0.0314421 |
| Gnb4 | 0.09940357 |
| Gnb4 | 1.60090373 |
| Gnb5 | 0.75752692 |
| Gnb5 | 1.03344929 |
| Gne | -0.457117 |
| Gng10 | 1.37341414 |
| Gng11 | 1.66461458 |
| Gng13 | 2.42111873 |
| Gng2 | -0.1781819 |
| Gng2 | 0.72668948 |
| Gng3 | 1.07036739 |
| Gng4 | -0.5990424 |
| Gng7 | -0.8435224 |
| Gng8 | -0.2785362 |
| Gngt2 | 0.26382445 |
| Gnl2 | -0.705814 |
| Gnl2 | 0.21053063 |
| Gnl3 | -0.0391354 |
| Gnl3 | -0.2719033 |
| Gnl3 | -0.5751074 |
| Gnl3l | 1.444311 |
| Gnl3l | -0.7066913 |
| Gnmt | -0.4864577 |
| Gnmt | -0.1415496 |
| Gnpat | -0.5011294 |
| Gnpda1 | -0.2571494 |
| Gnpda1 | -0.6244109 |
| Gnpda1 | -0.3675186 |
| Gnpda2 | 1.16816176 |
| Gnptab | -0.5637428 |
| Gnptg | 1.33780179 |
| Gnptg | 0.66561672 |
| Gnrhr | -0.4048696 |
| Gns | 1.44124255 |
| Golga1 | 0.59157309 |
| Golga2 | -0.6621306 |
| Golga2 | -0.6688491 |
| Golga2 | -0.5004255 |
| Golga2 | 1.01255259 |
| Golga2 | 1.37029254 |
| Golga3 | 0.72517728 |
| Golga4 | -0.6944833 |
| Golga5 | 0.51849064 |
| Golga7 | 0.65995341 |
| Golim4 | -0.5547497 |
| Golm1 | -0.2669594 |
| Golm1 | -0.0149876 |
| Golph3l | -0.2902585 |
| Golt1a | -0.376101 |
| Golt1b | 0.7204358 |
| Gopc | -0.5297301 |
| Gorasp1 | -0.4645354 |
| Gorasp2 | 1.19666926 |
| Gorasp2 | -0.1296516 |
| Gorasp2 | -0.5297301 |
| Gorasp2 | 0.30686221 |
| Gosr1 | -0.5488021 |
| Gosr2 | 1.93852553 |
| Gosr2 | 1.99321764 |
| Gosr2 | 1.04841904 |
| Gosr2 | 2.72338339 |
| Got1l1 | -0.648837 |
| Got2 | 0.40623206 |
| Gp1bb | -0.737929 |
| Gp38 | 1.07141221 |
| Gp5 | 0.03230561 |
| Gp9 | -0.4131632 |
| Gpaa1 | 1.50930148 |
| Gpam | -0.2675074 |
| Gpatch1 | 1.58251912 |
| Gpatch2 | 0.00650971 |
| Gpatch3 | -0.4131632 |
| Gpatch4 | -0.7636946 |
| Gpbp1 | 0.47845286 |
| Gpbp1 | -0.7315972 |
| Gpbp1l1 | 3.02084039 |
| Gpc1 | -0.8537419 |
| Gpc3 | 0.15922656 |
| Gpc3 | 1.36773071 |
| Gpc4 | 1.46027836 |
| Gpc5 | 0.6182574 |
| Gpc5 | 2.95638628 |
| Gpc5 | 0.89095852 |
| Gpd1 | -0.7084484 |
| Gpd2 | 2.29706132 |
| Gpd2 | 1.26889856 |
| Gper | -0.7674342 |
| Gpha2 | 0.10272724 |
| Gphb5 | 0.2865001 |
| Gphn | 0.63131872 |
| Gphn | 0.52812045 |
| Gphn | -0.6006079 |
| Gpihbp1 | 0.19763373 |
| Gpkow | -0.7119726 |
| Gpld1 | -0.1747014 |
| Gpld1 | 0.22033142 |
| Gpm6a | 0.54777358 |
| Gpm6a | -0.703187 |
| Gpm6b | 0.69830917 |
| Gpnmb | 0.05275201 |
| Gpnmb | -0.6349171 |
| Gpr1 | -0.3669086 |
| Gpr1 | 2.70009617 |
| Gpr103 | 1.78984203 |
| Gpr107 | -0.705814 |
| Gpr108 | 0.6466724 |
| Gpr108 | -0.5517711 |
| Gpr108 | -0.3816603 |
| Gpr109a | -0.4189484 |
| Gpr113 | -0.1555441 |
| Gpr113 | 1.47411388 |
| Gpr114 | -0.6439004 |
| Gpr12 | 2.27439962 |
| Gpr12 | -0.7777961 |
| Gpr120 | -0.1296516 |
| Gpr123 | -0.610848 |
| Gpr124 | -0.0608448 |
| Gpr128 | 2.82196082 |
| Gpr135 | 0.35224366 |
| Gpr137 | 1.44071552 |
| Gpr137b | 0.85099445 |
| Gpr141 | -0.3446137 |
| Gpr146 | -0.3357249 |
| Gpr146 | 0.38441061 |
| Gpr149 | 0.62943403 |
| Gpr151 | -0.4504238 |
| Gpr153 | -0.5607354 |
| Gpr156 | 1.8681154 |
| Gpr156 | -0.657956 |
| Gpr158 | -0.4042346 |
| Gpr162 | -0.0288917 |
| Gpr162 | -0.4740631 |
| Gpr17 | 2.20205778 |
| Gpr172b | 0.92623577 |
| Gpr172b | -0.0365639 |
| Gpr176 | 0.14348038 |
| Gpr177 | -0.1348681 |
| Gpr18 | 3.16039694 |
| Gpr180 | 1.23205528 |
| Gpr182 | 2.30248479 |
| Gpr182 | -0.5392174 |
| Gpr19 | 1.42984518 |
| Gpr20 | -0.4431164 |
| Gpr21 | -0.3458053 |
| Gpr22 | 0.6458489 |
| Gpr23 | 0.9141094 |
| Gpr26 | -0.7137397 |
| Gpr27 | 1.27919326 |
| Gpr27 | 2.48680287 |
| Gpr27 | -0.652971 |
| Gpr34 | 0.50661085 |
| Gpr34 | 2.56408914 |
| Gpr37l1 | -0.5443661 |
| Gpr45 | -0.4652127 |
| Gpr6 | -0.1796775 |
| Gpr64 | 0.30538004 |
| Gpr65 | 1.30931174 |
| Gpr68 | -0.6204013 |
| Gpr75 | 1.68795853 |
| Gpr81 | 2.42363462 |
| Gpr83 | 0.37784576 |
| Gpr85 | -0.5392174 |
| Gpr85 | -0.7674342 |
| Gpr89 | 0.96638336 |
| Gpr89 | -0.461831 |
| Gpr97 | -0.617206 |
| Gprasp1 | -0.7014397 |
| Gprasp1 | 1.32007271 |
| Gprc2a-rs5 | 1.08037557 |
| Gprc2a-rs5 | 0.24058395 |
| Gprc5a | -0.6260195 |
| Gprc5b | 2.56798202 |
| Gprc5c | -0.348791 |
| Gprk6 | -0.4497571 |
| Gprk6 | 0.80386702 |
| Gps1 | -0.2277982 |
| Gps1 | -0.5060716 |
| Gps1 | -0.6324821 |
| Gps1 | -0.471331 |
| Gps2 | 0.83431495 |
| Gpsm1 | 1.17997164 |
| Gpsm2 | -0.5181844 |
| Gpsm3 | -0.2620415 |
| Gpsn2 | -0.422178 |
| Gpsn2 | 0.08072 |
| Gpsn2 | 0.3857725 |
| Gpt1 | -0.6308622 |
| Gpt2 | 0.9467493 |
| Gpx1 | 1.78535894 |
| Gpx2 | 0.82109473 |
| Gpx3 | -0.274109 |
| Gpx4 | 0.77472689 |
| Gpx4 | 1.71983222 |
| Gpx6 | -0.4319344 |
| Gpx7 | -0.6068962 |
| Gramd1a | -0.3245859 |
| Gramd1b | -0.5067798 |
| Gramd2 | -0.4768033 |
| Gramd2 | -0.2299019 |
| Gramd3 | -0.505364 |
| Gramd4 | -0.3245859 |
| Grap2 | -0.3903744 |
| Grasp | -0.7075694 |
| Grasp | 0.0342912 |
| Grb10 | -0.036136 |
| Grb14 | -0.7208417 |
| Grb14 | 0.98675687 |
| Grb2 | -0.4645354 |
| Grb7 | 0.43584612 |
| Grcc10 | 1.5715105 |
| Greb1 | 0.03666833 |
| Greb1 | 1.10159325 |
| Grem1 | -0.1584659 |
| Grem2 | -0.4195934 |
| Grhl1 | 1.18657122 |
| Grhl2 | -0.1396357 |
| Grhpr | -0.3748701 |
| Gria1 | -0.5282791 |
| Gria1 | -0.5458424 |
| Gria1 | -0.617206 |
| Gria2 | 2.07554701 |
| Gria2 | -0.4254188 |
| Gria3 | -0.5889303 |
| Gria3 | 0.30270618 |
| Gria3 | -0.2941989 |
| Grid2 | 2.13057895 |
| Grik1 | -0.3124271 |
| Grik2 | 1.31706812 |
| Grik4 | 2.7058425 |
| Grik5 | -0.5297301 |
| Grin1 | 1.87619408 |
| Grin1 | 1.39746591 |
| Grin2a | 2.01193412 |
| Grin2b | 1.5121544 |
| Grin2c | 0.62333511 |
| Grin3b | -0.7498032 |
| Grina | -0.6858601 |
| Grip1 | 3.07314338 |
| Grip1 | 0.12566719 |
| Grip1 | -0.6406237 |
| Gripap1 | -0.4652127 |
| Gripap1 | -0.6562913 |
| Grit | -0.6513152 |
| Grk4 | 1.20688498 |
| Grk4 | -0.7084484 |
| Grk4 | 1.80411629 |
| Grk5 | -0.4934155 |
| Grlf1 | -0.5304565 |
| Grm1 | 2.52426758 |
| Grm7 | -0.391 |
| Grm8 | 0.00282429 |
| Grn | 1.14227087 |
| Grp | -0.6061078 |
| Grpel1 | 0.41021766 |
| Grpr | -0.7352101 |
| Grsf1 | 1.10726286 |
| Grtp1 | -0.481618 |
| Grwd1 | -0.293635 |
| Grwd1 | -0.5124647 |
| Gsbs | -0.007905 |
| Gsc | -0.6790184 |
| Gsdma1 | -0.433243 |
| Gsdmdc1 | 0.99444318 |
| Gsdmdc1 | -0.0012878 |
| Gsdmdc1 | -0.1287063 |
| Gse1 | -0.7415665 |
| Gsg1 | -0.535557 |
| Gsg1 | 0.73776689 |
| Gsg1 | -0.3669086 |
| Gsg2 | -0.2044518 |
| Gsk3a | 0.75368165 |
| Gsk3b | -0.1420287 |
| Gsn | 1.53562194 |
| Gspt1 | -0.5705449 |
| Gspt2 | -0.5039503 |
| Gss | -0.744304 |
| Gss | -0.6324821 |
| Gss | 1.43992441 |
| Gsta2 | -0.4312808 |
| Gsta3 | 1.57067004 |
| Gsta3 | 0.02150981 |
| Gsta4 | -0.1653192 |
| Gstcd | 0.73963145 |
| Gstk1 | -0.648837 |
| Gstm1 | 0.32794845 |
| Gstm1 | -0.0564623 |
| Gstm2 | -0.3748701 |
| Gstm2 | 0.3363087 |
| Gstm2 | 3.22065917 |
| Gstm4 | 1.73955703 |
| Gstm5 | 0.35196148 |
| Gstm6 | -0.5873843 |
| Gstm6 | -0.627631 |
| Gsto1 | 3.61953187 |
| Gsto2 | -0.467927 |
| Gsto2 | -0.1372488 |
| Gstp1 | -0.6061078 |
| Gstp1 | -0.705814 |
| Gstp2 | -0.4029661 |
| Gstt1 | 0.45550025 |
| Gstt2 | 0.72932994 |
| Gstt3 | -0.3730268 |
| Gstt4 | 0.50732792 |
| Gstz1 | 0.29763456 |
| Gtdc1 | -0.7969357 |
| Gtf2a1 | 1.1125236 |
| Gtf2e1 | 0.23067335 |
| Gtf2e2 | -0.5224973 |
| Gtf2e2 | 1.76242786 |
| Gtf2f1 | 0.50732792 |
| Gtf2f2 | -0.2399602 |
| Gtf2h1 | 1.22974485 |
| Gtf2h1 | 1.12677802 |
| Gtf2h2 | 0.98389395 |
| Gtf2h3 | -0.4740631 |
| Gtf2h4 | 0.03310035 |
| Gtf2h5 | 1.67088323 |
| Gtf2ird1 | -0.6053201 |
| Gtf2ird1 | 1.37322525 |
| Gtf2ird2 | 1.14793985 |
| Gtf2ird2 | 0.40756249 |
| Gtf2ird2 | 0.08748927 |
| Gtf3a | 0.03943403 |
| Gtf3a | 0.33917426 |
| Gtf3c1 | 0.43093204 |
| Gtf3c2 | 0.5548394 |
| Gtf3c3 | 0.31892801 |
| Gtf3c4 | 1.37793608 |
| Gtf3c4 | -0.341048 |
| Gtf3c5 | -0.4990192 |
| Gtf3c5 | -0.2501281 |
| Gtf3c5 | 0.16957829 |
| Gtl3 | -0.3009928 |
| Gtpbp1 | 1.44071552 |
| Gtpbp2 | 0.08711448 |
| Gtpbp3 | -0.5842999 |
| Gtpbp4 | -0.6260195 |
| Gtpbp5 | 0.60479543 |
| Gtpbp6 | -0.648837 |
| Gtpbp8 | 1.87221882 |
| Gtpbp8 | -0.0255019 |
| Gtrgeo22 | 1.69794899 |
| Gtse1 | -0.3529871 |
| Gtse1 | -0.2669594 |
| Gtse1 | -0.5592354 |
| Gtsf1l | 0.33026222 |
| Guca1a | -0.5789266 |
| Guca1b | -0.4470952 |
| Guca2a | 2.85971367 |
| Guca2b | 0.46179345 |
| Gucy1a2 | 1.82285405 |
| Gucy1a3 | -0.7479665 |
| Gucy1a3 | 0.27216199 |
| Gucy1a3 | -0.186689 |
| Gucy1b3 | 1.68336333 |
| Guk1 | 1.82727708 |
| Gulo | 2.29365117 |
| Gulp1 | -0.3928792 |
| Gusb | -0.4598078 |
| Gusb | 0.20493081 |
| Gusb | -0.5728234 |
| Gvin1 | 1.3431823 |
| Gyg | 1.24395643 |
| Gyg | 0.88730861 |
| Gykl1 | 1.24839401 |
| Gyltl1b | -0.6439004 |
| Gyltl1b | 0.76517025 |
| Gypa | 0.41286529 |
| Gypc | -0.6430801 |
| Gys1 | -0.0634842 |
| Gys3 | 1.40251402 |
| Gzf1 | -0.7979031 |
| Gzf1 | -0.5399512 |
| Gzmd | 2.92166345 |
| Gzmd | 0.78560623 |
| Gzmk | -0.2506663 |
| Gzmm | -0.6773158 |
| Gzmn | 1.10449536 |
| H13 | -0.4010665 |
| H13 | 2.52278099 |
| H13 | -0.6244109 |
| H13 | -0.5990424 |
| H19 | 1.7034018 |
| H1f0 | -0.6061078 |
| H1fnt | 0.02030253 |
| H1foo | 0.16130612 |
| H1fx | -0.6196014 |
| H1fx | -0.4378375 |
| H2-Aa | 0.41127761 |
| H2-Ab1 | 1.04533686 |
| H2-Ab1 | 1.20609442 |
| H2-Ab1 | 0.47003601 |
| H2afj | -0.0826216 |
| H2afx | 2.70228077 |
| H2afy | -0.6300534 |
| H2afz | 0.58235902 |
| H2-Bl | 0.09755196 |
| H2-DMa | 1.13070604 |
| H2-DMb1 | -0.8224191 |
| H2-Eb1 | -0.0599666 |
| H2-K1 | -0.5990424 |
| H2-Ke6 | -0.2378336 |
| H2-M10.3 | 0.19329484 |
| H2-M2 | 0.99896832 |
| H2-M3 | 0.2619615 |
| H2-Oa | 1.92518634 |
| H2-Ob | 0.86656848 |
| H2-Q1 | 1.15822453 |
| H2-Q2 | -0.2442279 |
| H2-Q7 | 0.14348038 |
| H2-Q8 | -0.341048 |
| H2-T10 | -0.6148167 |
| H2-T23 | 0.39390232 |
| H2-T23 | -0.7084484 |
| H2-T24 | 3.54235339 |
| H2-Tw3 | -0.1497278 |
| H3f3a | 0.4254704 |
| H3f3a | -0.2768736 |
| H3f3b | 0.74390586 |
| H47 | 2.45579763 |
| H47 | -0.6747677 |
| H6pd | -0.1141751 |
| Habp4 | -0.6463654 |
| Habp4 | 2.5080913 |
| Hace1 | -0.818453 |
| Hacl1 | -0.7815927 |
| Hadhb | -0.6596236 |
| Hagh | -0.4055049 |
| Haghl | 3.03804756 |
| Haghl | 4.09715376 |
| Hal | 2.56939836 |
| Hamp2 | -0.6901617 |
| Hao1 | -0.8334137 |
| Hao3 | -0.56299 |
| Hap1 | 0.36902014 |
| Hap1 | 1.03222494 |
| Hap1 | 1.12060718 |
| Hapln1 | -0.004177 |
| Hapln2 | -0.3176186 |
| Hapln4 | 0.96243078 |
| Harbi1 | -0.3773336 |
| Hars | 1.15691535 |
| Has1 | -0.6406237 |
| Hat1 | -0.5282791 |
| Havcr1 | -0.5812257 |
| Havcr2 | 1.47292628 |
| Hax1 | -0.5362879 |
| Hba-a1 | -0.810571 |
| Hbb-b1 | -0.7777961 |
| Hbegf | -0.5203384 |
| Hbp1 | -0.5039503 |
| Hbp1 | 1.13620199 |
| Hbs1l | -0.6730728 |
| Hbs1l | -0.7740148 |
| Hbs1l | 0.03071409 |
| Hbs1l | 0.46279644 |
| Hc | -0.2415583 |
| Hccs | -0.4411333 |
| Hcfc1 | -0.4740631 |
| Hcfc1r1 | 1.73019784 |
| Hcfc2 | 2.89363125 |
| Hcls1 | -0.5096179 |
| Hcn2 | 2.11236513 |
| Hcn3 | 1.29452819 |
| Hcn3 | 1.02539355 |
| Hcr | -0.6389897 |
| Hcst | -0.2157926 |
| Hdac10 | -0.4976151 |
| Hdac11 | -0.0975793 |
| Hdac2 | -0.5392174 |
| Hdac3 | -0.6815782 |
| Hdac4 | -0.2388963 |
| Hdac5 | 0.05624577 |
| Hdac6 | 0.09903354 |
| Hdac7 | 2.60369625 |
| Hdac8 | -0.2299019 |
| Hdac9 | -0.6858601 |
| Hdc | -0.6053201 |
| Hddc2 | -0.3517863 |
| Hddc2 | -0.4962131 |
| Hdgf | -0.2539022 |
| Hdgf | -0.6638057 |
| Hdgf | -0.5637428 |
| Hdgf | 0.03071409 |
| Hdgfl1 | -0.2085598 |
| Hdgfrp2 | -0.4871512 |
| Hdhd1a | 3.02208351 |
| Hdhd2 | -0.1415496 |
| Hdhd2 | 1.07505991 |
| Hdhd2 | -0.0008758 |
| Hdlbp | -0.3004248 |
| Hdlbp | 1.75367345 |
| Heatr1 | 2.39542162 |
| Heatr1 | -0.4913227 |
| Heatr3 | -0.1776839 |
| Heatr5a | -0.75997 |
| Hebp1 | 3.21506096 |
| Hebp2 | -0.6398063 |
| Heca | 1.26008985 |
| Hectd2 | -0.3304319 |
| Hectd2 | 2.23765895 |
| Hectd3 | 0.15051151 |
| Hecw2 | 0.33802909 |
| Hecw2 | 0.09010855 |
| Hecw2 | -0.7288967 |
| Heg1 | 2.53223812 |
| Hel308 | 0.97191803 |
| Helb | -0.2404926 |
| Helb | -0.1072243 |
| Helz | -0.4490909 |
| Hemgn | 1.10436937 |
| Hemk1 | -0.56299 |
| Hemt1 | 0.43196873 |
| Hepacam | 1.4006815 |
| Heph | -0.1353437 |
| Heph | 0.28831474 |
| Heph | -0.2152741 |
| Heph | -0.3687399 |
| Heph | 1.75499186 |
| Herc2 | -0.4850724 |
| Herc3 | -0.3398625 |
| Herc3 | 0.80820386 |
| Herc4 | -0.6132272 |
| Herc4 | 0.82143606 |
| Herpud1 | 0.4141863 |
| Herpud1 | 0.86656848 |
| Herpud2 | -0.0683426 |
| Herpud2 | -0.7005673 |
| Herpud2 | 0.90229288 |
| Herpud2 | -0.0604056 |
| Hes1 | -0.4941141 |
| Hes5 | -0.5990424 |
| Hes5 | -0.5081978 |
| Hes6 | -0.698825 |
| Hes6 | 0.62628439 |
| Hes6 | 1.00697477 |
| Hexa | -0.6646444 |
| Hexb | 0.5229656 |
| Hexdc | 1.07258624 |
| Hexdc | 2.3736956 |
| Hexim1 | 3.44479001 |
| Hexim2 | 1.95091419 |
| Hey1 | 3.43774843 |
| Hey2 | 1.24904164 |
| Hey2 | 1.76503825 |
| Heyl | 0.79302385 |
| Hfe | -0.1244642 |
| Hfe | -0.0012878 |
| Hgfac | -0.5392174 |
| Hgs | 0.57948851 |
| Hgs | 0.40995248 |
| Hgsnat | 1.13412954 |
| Hhat | 2.61736381 |
| Hhat | -0.6013916 |
| Hhat | -0.2571494 |
| Hhatl | -0.4927174 |
| Hhex | -0.1291789 |
| Hhip | -0.2802018 |
| Hibadh | 1.00065961 |
| Hic1 | -0.1682718 |
| Hic2 | -0.5547497 |
| Hif1a | 2.36398004 |
| Hif3a | -0.7306962 |
| Hif3a | 2.7438671 |
| Higd1a | -0.0683426 |
| Higd1b | -0.1239941 |
| Higd2a | 1.20394524 |
| Hils1 | -0.5540042 |
| Hint1 | 2.47582885 |
| Hint2 | -0.4934155 |
| Hint3 | 0.64934377 |
| Hint3 | -0.2874541 |
| Hip1 | 0.30508332 |
| Hip1r | -0.3632568 |
| Hip2 | 0.37178712 |
| Hipk1 | 1.82646389 |
| Hipk2 | -0.6587894 |
| Hipk3 | 2.49535392 |
| Hipk4 | 0.13994485 |
| Hira | -0.4247697 |
| Hira | 1.96749706 |
| Hirip3 | 0.63570158 |
| Hisppd1 | -0.4437783 |
| Hist1h1b | -0.5032443 |
| Hist1h1c | -0.4927174 |
| Hist1h2aa | -0.5660047 |
| Hist1h2ac | -0.1663023 |
| Hist1h2ad | 1.96999165 |
| Hist1h2ae | -0.5046569 |
| Hist1h2af | 2.18985607 |
| Hist1h2ag | -0.739746 |
| Hist1h2ah | 0.99769784 |
| Hist1h2ak | -0.6013916 |
| Hist1h2an | -0.1618862 |
| Hist1h2ao | -0.2796463 |
| Hist1h2bc | -0.6910244 |
| Hist1h2be | -0.6414418 |
| Hist1h2bf | 1.83887479 |
| Hist1h2bg | 1.27363983 |
| Hist1h2bh | 1.74654698 |
| Hist1h2bj | -0.3985396 |
| Hist1h2bk | -0.3135783 |
| Hist1h2bm | 1.07258624 |
| Hist1h2bn | 1.01754427 |
| Hist1h3a | 2.23653631 |
| Hist1h3d | -0.6196014 |
| Hist1h3e | -0.2941989 |
| Hist1h3f | -0.5004255 |
| Hist1h4c | 1.33071099 |
| Hist1h4d | 0.26102863 |
| Hist1h4f | 0.49239532 |
| Hist1h4h | 1.89214457 |
| Hist1h4i | 2.81676814 |
| Hist1h4j | -0.2852166 |
| Hist1h4k | -0.4829982 |
| Hist1h4m | 1.11949209 |
| Hist2h2aa1 | 0.35786941 |
| Hist2h2ab | 0.02150981 |
| Hist2h2ac | -0.3147309 |
| Hist2h2bb | -0.0246564 |
| Hist2h2be | 0.27706783 |
| Hist2h3b | -0.5253837 |
| Hist2h3c1 | -0.5502854 |
| Hist2h3c1 | -0.3985396 |
| Hist2h4 | 0.64564291 |
| Hist3h2a | -0.6850021 |
| Hist3h2bb | -0.3644725 |
| Hist4h4 | 1.54431161 |
| Hk1 | -0.4913227 |
| Hk3 | -0.5998248 |
| Hkdc1 | -0.7137397 |
| Hlcs | 0.79355143 |
| Hlf | 0.31276746 |
| Hltf | 2.54418887 |
| Hlx | 0.28133938 |
| Hmg20a | 2.54663596 |
| Hmg20a | -0.6260195 |
| Hmg20b | -0.2075311 |
| Hmga1 | -0.5990424 |
| Hmgb2 | 0.68477687 |
| Hmgb2l1 | 1.8154746 |
| Hmgb2l1 | 0.87932442 |
| Hmgb4 | 0.09977345 |
| Hmgcl | -0.4411333 |
| Hmgcl | 0.64811178 |
| Hmgcl | 2.57295594 |
| Hmgcll1 | -0.5796923 |
| Hmgcs2 | -0.6798709 |
| Hmgn1 | 1.55928778 |
| Hmgn2 | -0.6236076 |
| Hmgn3 | 0.06511528 |
| Hmgn3 | 1.30688093 |
| Hmha1 | -0.5167512 |
| Hmox1 | 0.57216191 |
| Hmox2 | -0.4352093 |
| Hmx1 | -0.451091 |
| Hmx1 | 0.00241389 |
| Hn1 | 1.280759 |
| Hn1 | 1.00206668 |
| Hn1l | -0.1570038 |
| Hnf1b | 0.92608325 |
| Hnf1b | -0.6646444 |
| Hnf4a | 1.39912102 |
| Hnrnpc | 1.7374498 |
| Hnrpa1 | 0.83380993 |
| Hnrpa1 | -0.3147309 |
| Hnrpab | 1.97689177 |
| Hnrpc | 3.83972265 |
| Hnrpdl | -0.3730268 |
| Hnrpdl | -0.5011294 |
| Hnrpf | -0.5268303 |
| Hnrph1 | -0.3935064 |
| Hnrph1 | -0.6156124 |
| Hnrph1 | 0.98461051 |
| Hnrph1 | 0.44763727 |
| Hnrph1 | -0.2474416 |
| Hnrph2 | -0.5584863 |
| Hnrph3 | 0.37371914 |
| Hnrpk | 1.81840994 |
| Hnrpl | 0.21935596 |
| Hnrpll | 0.33516 |
| Hnrpll | -0.2724542 |
| Hnrpm | -0.7581132 |
| Hnrpm | -0.7014397 |
| Hnrpm | 2.89743756 |
| Hnrpul1 | 0.89569791 |
| Hnrpul1 | 0.57104678 |
| Hnrpul1 | -0.348791 |
| Hnrpul1 | 0.82211835 |
| Hnrpul1 | -0.2399602 |
| Hnt | 0.08335833 |
| Homer1 | 1.08089255 |
| Homer1 | -0.5224973 |
| Homer1 | -0.1781819 |
| Homer1 | -0.6688491 |
| Homez | -0.1044584 |
| Homez | 1.4314439 |
| Hook1 | -0.0555883 |
| Hook1 | -0.5766332 |
| Hook1 | 3.51071979 |
| Hook1 | 0.5770527 |
| Hook2 | 0.03864467 |
| Hormad2 | -0.4437783 |
| Hoxa13 | -0.5569898 |
| Hoxa2 | -0.7334019 |
| Hoxa2 | -0.0045905 |
| Hoxa4 | -0.2220371 |
| Hoxa5 | 0.22000638 |
| Hoxa7 | 1.42886684 |
| Hoxa9 | 1.51734872 |
| Hoxa9 | 1.83738268 |
| Hoxb2 | -0.481618 |
| Hoxb3 | 2.80668148 |
| Hoxb3 | -0.4591344 |
| Hoxb4 | -0.8619988 |
| Hoxb5 | -0.4352093 |
| Hoxb6 | -0.5224973 |
| Hoxb7 | -0.2953278 |
| Hoxc10 | 1.11127373 |
| Hoxc10 | 1.71434381 |
| Hoxc6 | 2.27328436 |
| Hoxc9 | -0.4202389 |
| Hoxd10 | -0.4260683 |
| Hoxd3 | 2.7845948 |
| Hoxd4 | 0.24565821 |
| Hoxd8 | 0.66843553 |
| Hoxd9 | 0.55324856 |
| Hp | 1.96489559 |
| Hp | 0.14206778 |
| Hp | 0.66984175 |
| Hp1bp3 | 2.00134695 |
| Hpca | -0.5275544 |
| Hpcal1 | -0.5117522 |
| Hpcal1 | 1.75134599 |
| Hpcal4 | -0.5081978 |
| Hpgd | -0.739746 |
| Hpn | -0.1254052 |
| Hpn | 0.07845262 |
| Hprt1 | 1.95906604 |
| Hprt1 | 2.27407598 |
| Hprt1 | -0.4878451 |
| Hps1 | 1.64244275 |
| Hps3 | -0.264224 |
| Hps3 | -0.3773336 |
| Hps4 | -0.5554958 |
| Hps5 | -0.0551516 |
| Hps6 | -0.8324088 |
| Hpse | -0.5488021 |
| Hpx | 2.17325472 |
| Hr | -0.2697028 |
| Hr | -0.3210957 |
| Hras1 | 1.78917699 |
| Hrasls | -0.5160354 |
| Hrasls5 | 3.32217753 |
| Hrasls5 | -0.2913826 |
| Hrb | -0.636544 |
| Hrb | 0.02911986 |
| Hrbl | -0.6962175 |
| Hrbl | 1.387287 |
| Hrc | 1.00403301 |
| Hrg | -0.5384841 |
| Hrh3 | 0.62628439 |
| Hrk | 1.7288439 |
| Hrmt1l2 | 2.29463683 |
| Hrsp12 | 0.30389551 |
| Hrsp12 | -0.7796925 |
| Hrsp12 | 0.24280733 |
| Hs1bp3 | 2.08510887 |
| Hs2st1 | 2.08369847 |
| Hs3st1 | -0.6092653 |
| Hs3st3a1 | 0.5759434 |
| Hs3st3b1 | 2.60903662 |
| Hs3st6 | 1.3081984 |
| Hs6st1 | -0.3275043 |
| Hs6st2 | -0.5275544 |
| Hs6st2 | 0.88842096 |
| Hs6st3 | 2.33188145 |
| Hsbp1 | 0.62964363 |
| Hsd11b1 | -0.6422606 |
| Hsd11b1 | -0.4645354 |
| Hsd11b1 | -0.3897493 |
| Hsd11b1 | 0.2910301 |
| Hsd11b2 | -0.1104615 |
| Hsd17b1 | -0.645543 |
| Hsd17b10 | -0.6936174 |
| Hsd17b11 | -0.602176 |
| Hsd17b11 | -0.6244109 |
| Hsd17b11 | 2.2439952 |
| Hsd17b12 | 0.34231625 |
| Hsd17b2 | -0.5974795 |
| Hsd17b3 | -0.340455 |
| Hsd17b4 | -0.85477 |
| Hsd17b7 | 1.82326231 |
| Hsd3b1 | -0.5443661 |
| Hsd3b2 | -0.6236076 |
| Hsd3b2 | -0.4781764 |
| Hsd3b4 | 0.24629055 |
| Hsd3b7 | -0.5943616 |
| Hsd3b7 | -0.2566074 |
| Hsdl1 | -0.5789266 |
| Hsf1 | -0.009151 |
| Hsf4 | -0.6910244 |
| Hsf5 | -0.5959193 |
| Hsn2 | 1.36706538 |
| Hsp105 | 0.06779804 |
| Hsp90aa1 | -0.4183038 |
| Hsp90ab1 | 0.67723962 |
| Hsp90ab1 | 1.44027609 |
| Hsp90ab1 | 1.59317553 |
| Hsp90b1 | -0.7075694 |
| Hspa12b | 2.04960475 |
| Hspa1a | 0.97002829 |
| Hspa1l | 1.57668934 |
| Hspa2 | -0.6406237 |
| Hspa2 | 1.16320638 |
| Hspa4 | -0.6892998 |
| Hspa4l | -0.5110402 |
| Hspa5 | 0.90572887 |
| Hspa9 | -0.5362879 |
| Hspa9 | 1.13996934 |
| Hspb1 | -0.4927174 |
| Hspb2 | 1.31817093 |
| Hspb3 | -0.4023324 |
| Hspb6 | -0.5540042 |
| Hspb7 | -0.1301247 |
| Hspb8 | -0.2958927 |
| Hspbap1 | -0.5819933 |
| Hspd1 | 0.29763456 |
| Hspd1 | -0.4754322 |
| Hspe1 | 2.51306 |
| Hspg2 | -0.0242339 |
| Htatip | 0.59505941 |
| Htatip | 1.34015346 |
| Htatip2 | -0.0050042 |
| Htatip2 | -0.5117522 |
| Htatsf1 | -0.0701155 |
| Htf9c | 1.78808771 |
| Htr1a | -0.7674342 |
| Htr1b | -0.5873843 |
| Htr1d | -0.3748701 |
| Htr1f | -0.4976151 |
| Htr1f | -0.2183894 |
| Htr2b | 1.69948165 |
| Htr3a | 0.03706394 |
| Htr5b | 0.35534202 |
| Htr7 | -0.6430801 |
| Htra1 | 2.79744979 |
| Htra1 | 1.44492348 |
| Htra2 | -0.1305979 |
| Htra3 | 1.99404347 |
| Htra3 | 0.67264785 |
| Htt | 2.28224068 |
| Hus1 | 1.40251402 |
| Hus1 | 1.51386198 |
| Hus1 | 0.27767925 |
| Huwe1 | 2.33029119 |
| Hvcn1 | 0.35674697 |
| Hvcn1 | 0.44941858 |
| Hyal1 | 0.63403435 |
| Hyal2 | -0.5858408 |
| Hyal3 | -0.5690291 |
| Hyal5 | 0.41207179 |
| Hyal6 | -0.5935838 |
| Hydin | -0.2189096 |
| Hyi | -0.3428292 |
| Hyou1 | -0.4299749 |
| Iah1 | 2.24907033 |
| Iap | -0.0564623 |
| Iars | -0.7655626 |
| Iars | 0.72290438 |
| Iars2 | 0.61698364 |
| Ibtk | 2.54779005 |
| Ica1 | -0.6696923 |
| Ica1l | -0.4464308 |
| Icam1 | 0.60285634 |
| Icam2 | -0.6389897 |
| Icam4 | 1.90915746 |
| Icam5 | -0.3687399 |
| Ick | 0.19162065 |
| Icmt | -0.6901617 |
| Ict1 | 2.21869327 |
| Id1 | -0.445767 |
| Id2 | -0.7834969 |
| Id2 | 1.39847772 |
| Id3 | 1.69661419 |
| Id4 | -0.3153077 |
| Ide | -0.0661311 |
| Idh1 | 1.41442883 |
| Idh2 | 1.91101698 |
| Idh3a | -0.4036001 |
| Idh3b | -0.2947632 |
| Idh3g | 1.57028776 |
| Idh3g | 2.22727935 |
| Idi2 | -0.5652501 |
| Ids | 0.00487354 |
| Ids | 1.80933935 |
| Ids | 1.01588334 |
| Idua | 1.20643332 |
| Ier3 | -0.5124647 |
| Ier3ip1 | -0.602176 |
| Ier5l | 1.67280361 |
| Ifi202b | -0.6730728 |
| Ifi202b | 0.75807496 |
| Ifi27 | 0.44610772 |
| Ifi30 | -0.4524267 |
| Ifi30 | -0.4524267 |
| Ifi35 | 0.84202457 |
| Ifi47 | 3.81590653 |
| Ifit2 | 1.63590813 |
| Ifit2 | -0.1756945 |
| Ifit3 | -0.6204013 |
| Ifit3 | 1.67383075 |
| Ifitm1 | -0.6414418 |
| Ifitm1 | 0.33285837 |
| Ifitm1 | -0.5599851 |
| Ifitm2 | 0.89648547 |
| Ifitm3 | 1.47241685 |
| Ifitm5 | -0.0732264 |
| Ifitm6 | -0.6587894 |
| Ifna1 | -0.0674573 |
| Ifna6 | -0.545104 |
| Ifnar2 | 1.070106 |
| Ifnar2 | 2.70466433 |
| Ifnar2 | 3.03257571 |
| Ifngr1 | 0.17368704 |
| Ifngr2 | -0.1401138 |
| Ifngr2 | -0.3596193 |
| Ifngr2 | -0.1021597 |
| Ifnz | -0.423473 |
| Ifrd1 | 0.14277435 |
| Ifrg15 | -0.0066607 |
| Ift122 | -0.2049643 |
| Ift140 | -0.4004341 |
| Ift140 | 0.37427041 |
| Ift140 | -0.4761175 |
| Ift172 | -0.3240033 |
| Ift172 | 1.71683066 |
| Ift172 | -0.7190611 |
| Ift20 | -0.3669086 |
| Ift20 | 1.66038882 |
| Ift20 | -0.4112427 |
| Ift52 | 1.57028776 |
| Ift80 | 2.28480424 |
| Ift81 | 0.35730835 |
| Ift81 | 1.40998994 |
| Ift81 | -0.4437783 |
| Ift88 | 1.35645128 |
| Igbp1 | 0.67684123 |
| Igbp1b | -0.4254188 |
| Igf1 | -0.3228392 |
| Igf1 | 0.40409943 |
| Igf1r | 0.93140659 |
| Igf2 | -0.4437783 |
| Igf2bp1 | -0.627631 |
| Igf2bp2 | -0.5720633 |
| Igf2bp3 | 1.67273509 |
| Igf2bp3 | 3.60102267 |
| Igf2r | -0.2636779 |
| Igf2r | -0.0971225 |
| Igfals | 0.02832172 |
| Igfbp2 | -0.3928792 |
| Igfbp2 | 0.55005873 |
| Igfbp3 | -0.744304 |
| Igfbp4 | 0.62481091 |
| Igfbp4 | -0.4892346 |
| Igfbp5 | 1.64900221 |
| Igfbp6 | -0.7618305 |
| Igfbp7 | -0.818453 |
| Igfbpl1 | 0.73421393 |
| Igsf1 | -0.617206 |
| Igsf1 | -0.738837 |
| Igsf1 | 2.02625702 |
| Igsf1 | 0.05236301 |
| Igsf11 | 0.25853641 |
| Igsf11 | -0.2609522 |
| Igsf11 | -0.5268303 |
| Igsf21 | -0.7627621 |
| Igsf3 | -0.5160354 |
| Igsf4a | 0.41445028 |
| Igsf5 | -0.5966991 |
| Igsf8 | -0.2768736 |
| Igsf9 | -0.4788637 |
| Igtp | 1.52797153 |
| Ihh | 0.75935245 |
| Ihpk1 | 1.45397614 |
| Ihpk2 | 0.4262526 |
| Ihpk2 | 0.88667238 |
| Iigp2 | 0.4141863 |
| Ik | 0.97046472 |
| Ikbkap | -0.5282791 |
| Ikbkb | -0.7969357 |
| Ikbke | 0.7414923 |
| Ikbkg | 1.99825842 |
| Ikbkg | -0.691888 |
| Il10ra | 0.11808304 |
| Il10rb | -0.5532592 |
| Il11 | 1.26476989 |
| Il11ra1 | 1.00234784 |
| Il12a | 0.72423091 |
| Il13 | -0.5103288 |
| Il13ra1 | -0.5311834 |
| Il13ra2 | -0.5304565 |
| Il15 | -0.036992 |
| Il15 | 0.97409377 |
| Il15 | -0.3339572 |
| Il15ra | -0.4280196 |
| Il15ra | 0.40782834 |
| Il15ra | 1.14456673 |
| Il16 | 0.62079971 |
| Il17d | 0.88028614 |
| Il17d | -0.6910244 |
| Il17ra | -0.467927 |
| Il17rb | -0.6713811 |
| Il17rc | -0.7190611 |
| Il17rc | -0.5131777 |
| Il17rc | -0.6875784 |
| Il17rd | 2.06056587 |
| Il17rd | 1.87845023 |
| Il17re | 0.6940158 |
| Il17re | 0.39038809 |
| Il17re | 1.91032673 |
| Il18 | 2.3456735 |
| Il18bp | -0.6148167 |
| Il18r1 | 2.21168247 |
| Il1b | -0.6180038 |
| Il1r1 | 1.41749652 |
| Il1rap | -0.7343056 |
| Il1rap | 1.23807942 |
| Il1rap | -0.6006079 |
| Il1rap | 1.53792072 |
| Il1rap | 0.07504118 |
| Il1rl1l | 1.04157086 |
| Il1rl2 | 0.82109473 |
| Il20ra | -0.3816603 |
| Il20rb | -0.5667598 |
| Il20rb | -0.5160354 |
| Il23a | 0.63590978 |
| Il27ra | -0.7488844 |
| Il28ra | -0.5232181 |
| Il28ra | -0.0137339 |
| Il28ra | 1.65418882 |
| Il31ra | 0.57683094 |
| Il33 | -0.3935064 |
| Il3ra | -0.5311834 |
| Il4i1 | 1.70902067 |
| Il6ra | -0.1003249 |
| Il6st | 0.67284796 |
| Il7 | 2.21306581 |
| Ildr1 | 0.69088089 |
| Ildr1 | 0.24375859 |
| Ilf2 | 0.55279354 |
| Ilf3 | -0.4497571 |
| Ilf3 | 0.10567171 |
| Ilf3 | 0.57104678 |
| Ilf3 | 1.08476083 |
| Ilk | 1.24752979 |
| Ilkap | -0.5473211 |
| Ilvbl | 0.73552449 |
| Ilvbl | -0.3578058 |
| Ilvbl | -0.212686 |
| Ilvbl | -0.6463654 |
| Immp2l | -0.1183706 |
| Immt | 3.06428812 |
| Imp3 | 0.40089131 |
| Imp4 | -0.0812736 |
| Imp4 | -0.1268186 |
| Impa2 | 3.29362721 |
| Impact | 0.95993351 |
| Impad1 | 0.82975989 |
| Impdh1 | 0.16889174 |
| Impdh2 | 0.38740414 |
| Ina | -0.030166 |
| Inadl | -0.3903744 |
| Inadl | 0.58279986 |
| Incenp | -0.1109249 |
| Indo | 2.0233441 |
| Ing2 | -0.2320105 |
| Ing3 | -0.3998022 |
| Ing3 | 0.94286231 |
| Ing3 | 0.27246937 |
| Ing4 | 2.12350512 |
| Ing4 | -0.7979031 |
| Ing4 | -0.5660047 |
| Ing5 | -0.0621635 |
| Inha | -0.4260683 |
| Inhba | 0.01424258 |
| Inmt | 1.5945922 |
| Inoc1 | -0.5532592 |
| Inpp4a | -0.5039503 |
| Inpp4a | -0.2152741 |
| Inpp4a | 0.38031299 |
| Inpp4b | 1.00697477 |
| Inpp5a | -0.4899301 |
| Inpp5d | -0.5998248 |
| Inpp5e | 0.26846561 |
| Inpp5f | -0.58507 |
| Inppl1 | 3.77722635 |
| Inppl1 | 0.23548195 |
| Inppl1 | -0.7306962 |
| Ins2 | -0.5577378 |
| Insc | -0.127762 |
| Insig1 | -0.820434 |
| Insig1 | 2.21666824 |
| Insig1 | 0.26382445 |
| Insig2 | 0.74520289 |
| Insig2 | -0.3638645 |
| Insl3 | 1.5904854 |
| Insl5 | -0.0750086 |
| Insl6 | -0.2625867 |
| Insm1 | 0.53371164 |
| Ints1 | -0.2785362 |
| Ints12 | -0.6571233 |
| Ints3 | 0.19863215 |
| Ints3 | 1.53823735 |
| Ints4 | 1.25998321 |
| Ints5 | -0.293635 |
| Ints7 | 1.13534919 |
| Ints9 | -0.4955129 |
| Ints9 | -0.4768033 |
| Ipmk | -0.6892998 |
| Ipmk | 2.16156099 |
| Ipo13 | 0.0342912 |
| Ipo4 | -0.7306962 |
| Ipo5 | 2.45898918 |
| Ipp | -0.588157 |
| Ippk | -0.5160354 |
| Iqcb1 | 0.09977345 |
| Iqcb1 | 0.5967977 |
| Iqcd | 0.1195324 |
| Iqce | -0.0759011 |
| Iqcf3 | 0.41074779 |
| Iqcf4 | -0.5607354 |
| Iqgap1 | 0.51660001 |
| Iqgap2 | 0.84718448 |
| Iqgap3 | 2.33895395 |
| Iqsec2 | 0.5390386 |
| Iqsec3 | -0.3644725 |
| Irak1 | -0.6875784 |
| Irak1bp1 | -0.310703 |
| Irak2 | 1.08810041 |
| Irak2 | 0.25164826 |
| Irak3 | 0.8138978 |
| Irak4 | 0.2849852 |
| Irak4 | -0.2320105 |
| Irak4 | -0.5935838 |
| Ireb2 | -0.4899301 |
| Irf1 | -0.6100563 |
| Irf1 | -0.6680066 |
| Irf1 | -0.6116404 |
| Irf1 | 0.62438949 |
| Irf2 | -0.4836891 |
| Irf2bp1 | -0.6349171 |
| Irf3 | 0.79126309 |
| Irf3 | 1.29164625 |
| Irf3 | 2.39986393 |
| Irf4 | -0.5167512 |
| Irf5 | 1.52468495 |
| Irf6 | -0.4010665 |
| Irf6 | 1.79742619 |
| Irf7 | 0.93639807 |
| Irf8 | 0.16130612 |
| Irf9 | -0.0025249 |
| Irf9 | 2.62079305 |
| Irgm | 2.82096062 |
| Irgm | -0.6504884 |
| Irgq | 1.05388822 |
| Irgq | -0.6815782 |
| Irgq | -0.147797 |
| Irs2 | 0.08861272 |
| Irs3 | -0.1049188 |
| Irx2 | -0.7244131 |
| Irx3 | -0.6196014 |
| Irx5 | 0.81183129 |
| Isca1 | -0.4774896 |
| Iscu | -0.391 |
| Isg20 | -0.0276191 |
| Isg20l1 | -0.1012418 |
| Isg20l2 | -0.6680066 |
| Isl1 | -0.472696 |
| Islr2 | 2.38736538 |
| Isy1 | 0.14171429 |
| Isyna1 | 2.10885106 |
| Itfg2 | 0.17163493 |
| Itfg3 | 2.1576167 |
| Itga1 | -0.5465815 |
| Itga11 | 0.29912908 |
| Itga11 | 0.93730275 |
| Itga2b | 1.3048504 |
| Itga3 | -0.5004255 |
| Itga5 | -0.4417939 |
| Itga5 | -0.1922353 |
| Itga6 | 1.79514539 |
| Itga7 | -0.3754854 |
| Itgad | 1.36554285 |
| Itgad | -0.7190611 |
| Itgad | -0.481618 |
| Itgae | 1.21754826 |
| Itgae | -0.6061078 |
| Itgae | 0.7219557 |
| Itgav | 1.8219202 |
| Itgb1 | 1.98270965 |
| Itgb1 | 0.4254704 |
| Itgb1bp1 | 0.44124753 |
| Itgb3bp | -0.3935064 |
| Itgb4 | 1.04237914 |
| Itgb4 | -0.455104 |
| Itgb5 | -0.2824273 |
| Itgb6 | 4.26287846 |
| Itgb6 | 1.03616434 |
| Itgb6 | 1.66647891 |
| Itgb7 | -0.4564455 |
| Itgbl1 | -0.1211781 |
| Itih2 | -0.2325384 |
| Itih3 | 1.38467817 |
| Itih5 | -0.1049188 |
| Itih5 | -0.7516435 |
| Itk | -0.7271007 |
| Itlnb | 0.52155477 |
| Itm2a | 0.99699127 |
| Itm2b | -0.1348681 |
| Itm2b | 0.31511913 |
| Itpk1 | -0.6841449 |
| Itpka | -0.5046569 |
| Itpkc | -0.4913227 |
| Itpr1 | -0.5889303 |
| Itpr2 | -0.6546297 |
| Itpr3 | -0.4652127 |
| Itsn1 | 2.04878088 |
| Itsn2 | -0.5046569 |
| Ivd | -0.1536013 |
| Ivns1abp | -0.2075311 |
| Ivns1abp | 0.47375869 |
| Iws1 | -0.3998022 |
| Iyd | 1.0806341 |
| Izumo1 | 1.4843156 |
| Jag1 | -0.5067798 |
| Jag2 | 0.71164802 |
| Jagn1 | -0.274109 |
| Jak1 | -0.5547497 |
| Jak2 | 1.03507927 |
| Jak2 | -0.2941989 |
| Jak3 | 2.04593755 |
| Jak3 | 0.05352954 |
| Jakmip1 | -0.082172 |
| Jam2 | -0.4138042 |
| Jam2 | 0.52601509 |
| Jam2 | 2.56677381 |
| Jam2 | 3.09150474 |
| Jam3 | -0.0781359 |
| Jam4 | 0.45246437 |
| Jarid1b | 0.87996568 |
| Jarid1c | -0.2802018 |
| Jarid1d | 3.26524538 |
| Jazf1 | -0.5399512 |
| Jdp2 | -0.4254188 |
| Jmjd1a | -0.2691534 |
| Jmjd1a | -0.4254188 |
| Jmjd1a | -0.645543 |
| Jmjd2a | -0.3998022 |
| Jmjd2b | 0.09606802 |
| Jmjd2b | -0.5074885 |
| Jmjd2c | -0.5319109 |
| Jmjd2d | -0.7102088 |
| Jmjd3 | 0.25447229 |
| Jmjd3 | -0.1658106 |
| Jmjd5 | -0.617206 |
| Jmjd6 | -0.5392174 |
| Jmy | 2.83636419 |
| Josd1 | -0.2653172 |
| Josd2 | -0.6292452 |
| Josd3 | -0.4706492 |
| Josd3 | 2.99462729 |
| Jph1 | -0.6850021 |
| Jph2 | -0.4086882 |
| Jph3 | 2.37447107 |
| Jph4 | 0.49603002 |
| Jph4 | -0.4692871 |
| Jtb | 0.81887304 |
| Jtv1 | -0.7581132 |
| Jtv1 | -0.5644961 |
| Jun | 2.10074411 |
| Junb | 0.04415623 |
| Jund1 | 2.03107622 |
| Jup | -0.1927411 |
| Kal1 | 2.96290686 |
| Kalrn | -0.213203 |
| Kank1 | -0.2713527 |
| Kank3 | -0.0391354 |
| Kank4 | 0.19195573 |
| Kank4 | 1.4935771 |
| Kank4 | -0.1160374 |
| Kars | 0.6186816 |
| Katna1 | -0.7128557 |
| Katnal1 | 1.87492604 |
| Katnb1 | -0.6156124 |
| Kazald1 | 1.04855281 |
| Kbtbd2 | -0.2262234 |
| Kbtbd2 | -0.5319109 |
| Kbtbd4 | -0.2970237 |
| Kbtbd4 | -0.7343056 |
| Kbtbd7 | 0.15226105 |
| Kbtbd7 | -0.6084749 |
| Kbtbd8 | 0.60264063 |
| Kcmf1 | -0.2708024 |
| Kcna1 | -0.3269199 |
| Kcna2 | 3.11864844 |
| Kcna3 | 0.78062818 |
| Kcna5 | -0.1811756 |
| Kcna6 | 1.92382617 |
| Kcnab1 | -0.6061078 |
| Kcnab1 | -0.6910244 |
| Kcnab1 | 1.3343616 |
| Kcnab2 | -0.5928066 |
| Kcnab3 | 1.62081843 |
| Kcnb1 | -0.6850021 |
| Kcnc1 | -0.4042346 |
| Kcnc1 | 0.17231947 |
| Kcnc4 | -0.5319109 |
| Kcnc4 | 0.11844559 |
| Kcnd2 | -0.0830713 |
| Kcne1 | -0.6832886 |
| Kcne1l | -0.4417939 |
| Kcne2 | -0.3529871 |
| Kcne4 | -0.8304023 |
| Kcnf1 | -0.2593205 |
| Kcng4 | -0.5982606 |
| Kcnh1 | 2.58445106 |
| Kcnh2 | 1.91154759 |
| Kcnh2 | -0.5897043 |
| Kcnh3 | 0.20228392 |
| Kcnh5 | -0.2587773 |
| Kcnip1 | -0.5622379 |
| Kcnip2 | -0.5644961 |
| Kcnip2 | 0.85676254 |
| Kcnip2 | -0.1771861 |
| Kcnip3 | 2.88666216 |
| Kcnip4 | -0.4451036 |
| Kcnj12 | -0.455104 |
| Kcnj16 | 2.3389875 |
| Kcnj2 | 1.4626844 |
| Kcnj3 | -0.85477 |
| Kcnj4 | 0.26568369 |
| Kcnj6 | 0.27093149 |
| Kcnj6 | 1.86337964 |
| Kcnj8 | 1.76782701 |
| Kcnj9 | 2.17521617 |
| Kcnk1 | 0.29313659 |
| Kcnk10 | 0.53440819 |
| Kcnk12 | -0.6638057 |
| Kcnk13 | -0.341048 |
| Kcnk2 | -0.6901617 |
| Kcnk2 | 1.09232197 |
| Kcnk3 | -0.3687399 |
| Kcnk4 | 1.55229762 |
| Kcnk5 | 0.68339299 |
| Kcnk5 | 0.32852743 |
| Kcnk6 | -0.0124817 |
| Kcnk6 | 0.1519114 |
| Kcnk9 | 1.74882327 |
| Kcnma1 | -0.1761914 |
| Kcnmb1 | -0.1482793 |
| Kcnmb4 | 1.59272772 |
| Kcnmb4 | 0.39686559 |
| Kcnn1 | 1.92356437 |
| Kcnn2 | -0.6349171 |
| Kcnn4 | 0.44610772 |
| Kcnq1 | 0.58279986 |
| Kcnq2 | -0.0839715 |
| Kcnq2 | 1.65047702 |
| Kcnq3 | 0.65200751 |
| Kcnq5 | -0.3327806 |
| Kcnrg | -0.454434 |
| Kcnrg | -0.069672 |
| Kcns1 | 0.0878639 |
| Kcns2 | 2.64025814 |
| Kcnt1 | -0.3084091 |
| Kcnu1 | -0.0494929 |
| Kcnv1 | 0.62943403 |
| Kcnv2 | 0.15051151 |
| Kctd10 | -0.5562425 |
| Kctd11 | 0.29403792 |
| Kctd12 | -0.617206 |
| Kctd12b | 1.40233093 |
| Kctd12b | 0.40035555 |
| Kctd14 | -0.6858601 |
| Kctd14 | -0.755335 |
| Kctd15 | -0.3650809 |
| Kctd18 | 1.13608021 |
| Kctd19 | 0.58037268 |
| Kctd2 | 0.61804523 |
| Kctd20 | -0.471331 |
| Kctd20 | -0.698825 |
| Kctd3 | 0.17879766 |
| Kctd4 | -0.6100563 |
| Kctd5 | 0.10125152 |
| Kctd5 | 1.11489375 |
| Kctd6 | 1.42583637 |
| Kctd6 | 0.81045108 |
| Kctd9 | -0.3257521 |
| Kdelc1 | -0.6910244 |
| Kdelc1 | 2.01839009 |
| Kdelr2 | -0.7361155 |
| Kdelr3 | 0.88204672 |
| Kdr | -0.7370218 |
| Keap1 | -0.1410708 |
| Keap1 | -0.1937536 |
| Keap1 | 0.20393911 |
| Keap1 | -0.5982606 |
| Kera | -0.0714475 |
| Khdc1c | 3.40210715 |
| Khdrbs1 | -0.3847632 |
| Khdrbs2 | -0.3638645 |
| Khdrbs3 | 0.4655491 |
| Khk | -0.3009928 |
| Khk | 0.76553266 |
| Khsrp | 0.78134096 |
| Kif11 | -0.717284 |
| Kif13a | 2.52871319 |
| Kif14 | -0.2998571 |
| Kif15 | -0.1737093 |
| Kif17 | 0.64954894 |
| Kif17 | -0.3559959 |
| Kif18a | -0.4747474 |
| Kif1a | 1.09868209 |
| Kif1b | -0.7253081 |
| Kif1b | 2.86593304 |
| Kif1b | 0.84102257 |
| Kif1b | 0.55165501 |
| Kif1b | -0.5414205 |
| Kif1b | 1.38486476 |
| Kif1c | 2.03308077 |
| Kif21a | -0.4795515 |
| Kif21b | -0.7190611 |
| Kif22 | -0.3724132 |
| Kif23 | 0.34203105 |
| Kif24 | 3.67874982 |
| Kif27 | -0.0772413 |
| Kif2a | -0.3469984 |
| Kif2b | 1.81981563 |
| Kif2c | -0.4878451 |
| Kif3a | -0.3112773 |
| Kif3a | 3.8396622 |
| Kif3b | 0.64399336 |
| Kif3c | 0.00610094 |
| Kif3c | -0.7040618 |
| Kif4 | 0.08335833 |
| Kif4 | 0.44304107 |
| Kif4 | 0.81733184 |
| Kif5a | -0.7155101 |
| Kif5a | -0.2829845 |
| Kif5a | 2.49453073 |
| Kif5b | 1.61025506 |
| Kif5c | 0.56365333 |
| Kif9 | -0.7334019 |
| Kifap3 | 1.7808542 |
| Kifap3 | 0.7720321 |
| Kifap3 | 0.39065889 |
| Kifc2 | -0.7571862 |
| Kifc2 | 0.33170541 |
| Kifc3 | 0.48728283 |
| Kin | -0.6204013 |
| Kirrel3 | -0.6324821 |
| Kiss1 | -0.4010665 |
| Kiss1r | 0.14946021 |
| Kitl | -0.737929 |
| Kl | -0.4228253 |
| Klb | -0.627631 |
| Klc1 | 1.80459232 |
| Klc1 | 0.62943403 |
| Klc1 | -0.7882743 |
| Klc2 | 0.12206338 |
| Klc2 | 0.33688251 |
| Klc4 | 2.44367114 |
| Klf1 | 1.03751891 |
| Klf10 | -0.1151058 |
| Klf12 | -0.381041 |
| Klf12 | 0.24121974 |
| Klf12 | 1.40388605 |
| Klf13 | -0.7534874 |
| Klf16 | -0.4247697 |
| Klf17 | -0.1410708 |
| Klf17 | 1.88474949 |
| Klf2 | 0.5063717 |
| Klf4 | 0.03230561 |
| Klf4 | 0.73308912 |
| Klf4 | -0.0898439 |
| Klf5 | -0.5525149 |
| Klf6 | 0.17368704 |
| Klf6 | -0.4733793 |
| Klf7 | -0.6084749 |
| Klf9 | 1.29862986 |
| Klhdc1 | 1.54336757 |
| Klhdc1 | 0.4141863 |
| Klhdc1 | -0.8334137 |
| Klhdc2 | -0.6148167 |
| Klhdc3 | -0.802755 |
| Klhdc4 | 2.22440081 |
| Klhdc4 | 1.58432877 |
| Klhdc4 | 0.31070487 |
| Klhdc8b | -0.7361155 |
| Klhdc9 | 0.31011472 |
| Klhl10 | -0.5607354 |
| Klhl11 | -0.5196198 |
| Klhl12 | -0.8154895 |
| Klhl14 | -0.2796463 |
| Klhl15 | 0.05002557 |
| Klhl15 | 0.80543061 |
| Klhl17 | 0.79495686 |
| Klhl2 | -0.5912541 |
| Klhl21 | 3.09241788 |
| Klhl22 | -0.2517437 |
| Klhl22 | 0.80716486 |
| Klhl23 | 1.19266608 |
| Klhl24 | 0.84219146 |
| Klhl24 | 0.73496304 |
| Klhl25 | -0.455104 |
| Klhl26 | -0.648837 |
| Klhl26 | 0.25321822 |
| Klhl30 | 1.98379033 |
| Klhl31 | -0.6747677 |
| Klhl32 | 1.17799427 |
| Klhl5 | 0.68852278 |
| Klhl5 | -0.4823079 |
| Klhl6 | 3.63283095 |
| Klhl6 | -0.6045331 |
| Klhl7 | -0.6284377 |
| Klhl8 | 0.19496603 |
| Klhl9 | 1.00459406 |
| Klhl9 | -0.5974795 |
| Klk1 | -0.7562601 |
| Klk11 | 1.19917678 |
| Klk11 | -0.6521427 |
| Klk15 | -0.1207096 |
| Klk1b1 | -0.682433 |
| Klk1b11 | 0.15330919 |
| Klk1b16 | 0.11299247 |
| Klk1b21 | 1.3926679 |
| Klk1b21 | -0.1682718 |
| Klk1b22 | -0.1727183 |
| Klk1b24 | -0.3953907 |
| Klk1b26 | 1.40616829 |
| Klk1b27 | -0.6944833 |
| Klk1b27 | -0.5458424 |
| Klk1b4 | -0.744304 |
| Klk1b4 | 1.85417391 |
| Klk1b5 | -0.7571862 |
| Klk1b8 | -0.1821756 |
| Klk1b9 | 0.639442 |
| Klk6 | -0.5011294 |
| Klk8 | -0.5480613 |
| Klkb1 | -0.2469052 |
| Klra17 | -0.4564455 |
| Klra4 | -0.6764657 |
| Klrd1 | -0.3095554 |
| Kndc1 | 1.12923497 |
| Kng1 | 0.01909369 |
| Kng1 | -0.451091 |
| Kng2 | 1.18217669 |
| Kns2 | 1.07674864 |
| Kns2 | -0.4384958 |
| Kntc1 | 1.35230685 |
| Kpna1 | 0.38984626 |
| Kpna2 | -0.9109487 |
| Kpna3 | -0.6807241 |
| Kpna6 | 0.38794739 |
| Kpnb1 | -0.041714 |
| Kptn | -0.2230819 |
| Kras | 1.8449904 |
| Kras | -0.6284377 |
| Krba1 | -0.6148167 |
| Krba1 | 1.99312043 |
| Krba1 | -0.6936174 |
| Krcc1 | -0.5959193 |
| Kremen | 3.67012213 |
| Kremen1 | -0.7244131 |
| Kremen2 | -0.6124335 |
| Krit1 | -0.5959193 |
| Krit1 | -0.3158849 |
| Krr1 | 0.21446331 |
| Krr1 | 0.16545137 |
| Krr1 | -0.4010665 |
| Krt10 | 3.100611 |
| Krt12 | -0.3816603 |
| Krt13 | 0.99953243 |
| Krt14 | -0.1497278 |
| Krt17 | 1.87624917 |
| Krt18 | -0.532639 |
| Krt19 | 0.47968426 |
| Krt2 | -0.723519 |
| Krt20 | 1.34678453 |
| Krt222 | -0.7571862 |
| Krt23 | -0.1367722 |
| Krt27 | -0.1291789 |
| Krt32 | 1.9183129 |
| Krt4 | 2.04211753 |
| Krt7 | -0.3422351 |
| Krt73 | 2.28352334 |
| Krt75 | 0.08748927 |
| Krt78 | -0.8324088 |
| Krt79 | -0.5943616 |
| Krt8 | -0.7646281 |
| Krt84 | -0.4437783 |
| Krt86 | -0.4899301 |
| Krt86 | 1.50389784 |
| Krt9 | -0.6124335 |
| Krtap16-4 | 0.19596731 |
| Krtap8-2 | -0.6381738 |
| Krtcap2 | 0.08222853 |
| Krtcap3 | 1.47182216 |
| Ksr2 | -0.5473211 |
| Ktelc1 | 0.6264947 |
| Kti12 | 1.3883099 |
| Ktn1 | 0.96008059 |
| Ktn1 | -0.7674342 |
| Ky | -0.4125226 |
| L1cam | 2.53368249 |
| L2hgdh | 0.88523929 |
| L3mbtl2 | 1.29483644 |
| L3mbtl3 | -0.6850021 |
| l7Rn6 | 1.47952372 |
| Lace1 | 1.23348268 |
| Lad1 | -0.8264022 |
| Lag3 | 2.91183784 |
| Lage3 | 0.4620443 |
| Lama1 | -0.4774896 |
| Lama2 | 0.47721984 |
| Lamb1-1 | -0.7516435 |
| Lamb2 | 2.19987944 |
| Lamb3 | -0.5690291 |
| Lamc1 | 1.83000193 |
| Lamc2 | -0.4699679 |
| Lamc2 | 1.79183432 |
| Lamc2 | 0.29193347 |
| Lamc3 | 0.12746389 |
| Lamp1 | 0.53672627 |
| Lamp2 | 0.05624577 |
| Lamp2 | -0.744304 |
| Lamp2 | -0.1179035 |
| Lamp2 | -0.2891357 |
| Lancl1 | -0.5751074 |
| Lancl1 | -0.147797 |
| Lancl1 | 0.23803644 |
| Laptm4a | -0.6236076 |
| Laptm4b | -0.7040618 |
| Laptm4b | -0.4484252 |
| Laptm5 | 0.50109493 |
| Large | -0.6910244 |
| Larp2 | 1.71309789 |
| Larp5 | 0.86265846 |
| Larp6 | -0.0276191 |
| Larp6 | 0.43481372 |
| Lars | 0.63881964 |
| Lars2 | -0.645543 |
| Las1l | -0.5889303 |
| Lasp1 | 0.85709107 |
| Lasp1 | -0.3240033 |
| Lasp1 | -0.7516435 |
| Lass1 | 0.90759772 |
| Lass2 | -0.1044584 |
| Lass2 | -0.6587894 |
| Lass2 | -0.5124647 |
| Lass4 | -0.4892346 |
| Lass4 | -0.5046569 |
| Lass5 | -0.0718919 |
| Lass6 | -0.2495901 |
| Lat2 | -0.5174675 |
| Lat2 | -0.5146055 |
| Lats2 | -0.6538 |
| Lbh | -0.2070172 |
| Lbh | -0.3724132 |
| Lbp | -0.8154895 |
| Lbr | -0.717284 |
| Lbx2 | -0.6406237 |
| Lca5 | 1.2234447 |
| Lca5 | -0.4885396 |
| Lca5 | 0.61719606 |
| Lcat | -0.298156 |
| Lcat | 0.06011244 |
| Lce1c | 0.10824051 |
| Lck | -0.5011294 |
| Lcmt1 | 0.04729114 |
| Lcmt1 | 1.29493917 |
| Lcmt1 | -0.6832886 |
| Lcn10 | -0.4036001 |
| Lcn12 | 1.5232384 |
| Lcn13 | 0.97467311 |
| Lcn2 | -0.2992897 |
| Lcn3 | -0.666324 |
| Lcn8 | -0.0934761 |
| Lcor | 0.68180891 |
| Lcp1 | -0.4955129 |
| Ldb1 | -0.5644961 |
| Ldb2 | 0.86461552 |
| Ldb2 | 0.45373052 |
| Ldha | -0.417016 |
| Ldhc | -0.6308622 |
| Ldhc | -0.1811756 |
| Ldhc | -0.6092653 |
| Ldhd | 0.81972816 |
| Ldhd | -0.471331 |
| Ldoc1 | 2.29516443 |
| Ldoc1l | 3.07442556 |
| Lef1 | -0.5743455 |
| Lef1 | 0.05740746 |
| Lefty1 | 0.50589323 |
| Lefty2 | -0.5842999 |
| Lemd2 | 2.7576986 |
| Leng1 | 3.03893529 |
| Leng8 | 2.15932659 |
| Leng9 | -0.744304 |
| Leo1 | -0.0412838 |
| Lep | 1.60533337 |
| Lepre1 | -0.3947622 |
| Lepre1 | -0.6260195 |
| Lepre1 | 2.04492654 |
| Leprel1 | -0.3298457 |
| Leprel2 | -0.1737093 |
| Leprotl1 | 1.64272583 |
| Letm1 | 1.03548632 |
| Letm1 | 3.07639119 |
| Letm1 | 1.72638891 |
| Letmd1 | 0.87030026 |
| Lgals1 | 0.00405438 |
| Lgals2 | 2.81513822 |
| Lgals3 | -0.5819933 |
| Lgals3bp | 0.64995916 |
| Lgals4 | -0.6654838 |
| Lgals4 | 2.53010836 |
| Lgals6 | -0.6798709 |
| Lgals7 | -0.5406855 |
| Lgals8 | -0.5232181 |
| Lgals8 | 0.56567554 |
| Lgals9 | 3.72661022 |
| Lgals9 | 0.48923375 |
| Lgi1 | 1.71826739 |
| Lgi1 | 1.11849977 |
| Lgi2 | -0.7049375 |
| Lgi3 | -0.6092653 |
| Lgi4 | -0.6875784 |
| Lgmn | 1.92758795 |
| Lgr5 | 2.31537392 |
| Lgtn | 0.26939108 |
| Lgtn | -0.3705747 |
| Lhb | -0.5858408 |
| Lhcgr | 1.11713361 |
| Lhcgr | -0.3922524 |
| Lhfp | 2.51714352 |
| Lhfp | -0.3935064 |
| Lhfp | 0.25415893 |
| Lhfpl1 | 1.34853196 |
| Lhfpl2 | -0.7825443 |
| Lhx1 | 2.51567329 |
| Lhx2 | -0.7163966 |
| Lhx3 | -0.4892346 |
| Lhx4 | -0.8234133 |
| Lhx9 | -0.7146245 |
| Lhx9 | 0.28164374 |
| Lias | 2.07599185 |
| Lias | 1.87183143 |
| Lias | 2.84763555 |
| Lifr | -0.0998667 |
| Lig1 | -0.7892327 |
| Lig1 | 0.10970523 |
| Lig1 | 0.9614033 |
| Lig3 | -0.6773158 |
| Lig4 | -0.0516652 |
| Lima1 | -0.4878451 |
| Lima1 | 1.01157887 |
| Limd1 | -0.5333677 |
| Limd2 | -0.7315972 |
| Lime1 | 0.08335833 |
| Limk1 | -0.4299749 |
| Limk2 | -0.6927523 |
| Limk2 | 1.96619723 |
| Lims2 | 1.84322271 |
| Lims2 | -0.0741171 |
| Lims2 | -0.3650809 |
| Lin54 | -0.6292452 |
| Lin7b | 1.46148215 |
| Lin7b | 1.29955026 |
| Lin7b | -0.2925081 |
| Lin7b | -0.4740631 |
| Lin7c | -0.5866122 |
| Lincr | 1.67095189 |
| Lincr | -0.7882743 |
| Lingo1 | -0.7137397 |
| Lingo2 | -0.8703294 |
| Lingo2 | 1.20767487 |
| Lingo4 | -0.5584863 |
| Lip1 | -0.0335728 |
| Lipe | -0.3386784 |
| Lipf | -0.4906261 |
| Liph | 1.25044331 |
| Liph | -0.5004255 |
| Litaf | 1.93770061 |
| Lix1 | -0.3124271 |
| Lix1 | 0.25259055 |
| Lix1 | -0.4686068 |
| Lix1 | 1.3748296 |
| Llgl1 | 0.70780127 |
| Llgl2 | 0.07996482 |
| Lman1 | 0.75221261 |
| Lman2 | 0.25290444 |
| Lman2l | -0.7352101 |
| Lman2l | 1.47139715 |
| Lmbr1 | -0.7253081 |
| Lmbr1l | -0.6308622 |
| Lmcd1 | -0.6398063 |
| Lmcd1 | -0.7208417 |
| Lmcd1 | -0.6204013 |
| Lmf1 | -0.1560304 |
| Lmf2 | -0.645543 |
| Lmna | 3.36269007 |
| Lmna | 0.48214218 |
| Lmna | -0.2885749 |
| Lmna | -0.6268249 |
| Lmna | -0.6562913 |
| Lmnb1 | -0.4997221 |
| Lmnb1 | -0.7969357 |
| Lmnb2 | -0.7452183 |
| Lmo2 | 1.04143608 |
| Lmo3 | 0.11226297 |
| Lmo3 | -0.3953907 |
| Lmo4 | -0.6841449 |
| Lmod1 | 1.67635956 |
| Lmtk2 | 0.2730838 |
| Lmtk3 | 0.67384788 |
| Lmx1a | -0.5210575 |
| Lmx1b | -0.381041 |
| Lnx1 | -0.2992897 |
| Lnx2 | 0.45726663 |
| Lnx2 | -0.5297301 |
| LOC100038882 | -0.5443661 |
| LOC100038984 | -0.0898439 |
| LOC100039087 | -0.11697 |
| LOC100039227 | 2.64689049 |
| LOC100039479 | -0.4444407 |
| LOC100039532 | -0.2571494 |
| LOC100039571 | -0.5644961 |
| LOC100039728 | -0.645543 |
| LOC100040573 | -0.3222577 |
| LOC100040592 | 1.83485213 |
| LOC100040601 | -0.6587894 |
| LOC100040799 | -0.2215151 |
| LOC100040899 | 0.31040984 |
| LOC100040919 | -0.2582343 |
| LOC100041103 | -0.6612942 |
| LOC100041290 | 0.25541173 |
| LOC100041463 | 0.20658131 |
| LOC100041483 | 1.49208147 |
| LOC100041500 | -0.5110402 |
| LOC100041504 | 0.7018073 |
| LOC100041703 | -0.0745627 |
| LOC100041722 | 1.90756063 |
| LOC100041725 | -0.5866122 |
| LOC100041835 | 2.65856351 |
| LOC100042179 | 0.33831552 |
| LOC100042427 | -0.4665689 |
| LOC100042777 | -0.7825443 |
| LOC100043257 | -0.56299 |
| LOC100043391 | -0.5362879 |
| LOC100043671 | 3.11175668 |
| LOC100043822 | 3.22606841 |
| LOC100043986 | -0.0630438 |
| LOC100044087 | 0.02351848 |
| LOC100044089 | 3.42577792 |
| LOC100044101 | 0.0239197 |
| LOC100044103 | -0.6807241 |
| LOC100044103 | -0.7424781 |
| LOC100044122 | -0.4934155 |
| LOC100044133 | 1.08205472 |
| LOC100044159 | -0.0857745 |
| LOC100044170 | 2.72651134 |
| LOC100044172 | -0.2189096 |
| LOC100044177 | -0.6398063 |
| LOC100044190 | -0.3754854 |
| LOC100044204 | -0.0907506 |
| LOC100044210 | -0.0425751 |
| LOC100044221 | -0.6422606 |
| LOC100044257 | 1.88009762 |
| LOC100044275 | -0.4768033 |
| LOC100044289 | 3.07751689 |
| LOC100044292 | -0.0217031 |
| LOC100044294 | 0.11481372 |
| LOC100044298 | 0.75037304 |
| LOC100044322 | 0.5118566 |
| LOC100044324 | 1.15118098 |
| LOC100044324 | -0.6164089 |
| LOC100044363 | -0.1497278 |
| LOC100044363 | -0.2090746 |
| LOC100044439 | 1.47943943 |
| LOC100044468 | 0.37261562 |
| LOC100044475 | 1.40971748 |
| LOC100044538 | 1.14468741 |
| LOC100044566 | -0.2241279 |
| LOC100044576 | -0.5146055 |
| LOC100044636 | 1.04426241 |
| LOC100044692 | 0.01302586 |
| LOC100044708 | 2.2883885 |
| LOC100044736 | 0.44073445 |
| LOC100044756 | -0.3135783 |
| LOC100044776 | 0.61804523 |
| LOC100044779 | 1.81811682 |
| LOC100044812 | -0.5146055 |
| LOC100044829 | 0.00282429 |
| LOC100044862 | 1.16533334 |
| LOC100044873 | 0.62733545 |
| LOC100044934 | 1.58478064 |
| LOC100045005 | -0.0732264 |
| LOC100045019 | -0.8037285 |
| LOC100045040 | -0.2157926 |
| LOC100045098 | 2.84864644 |
| LOC100045228 | 1.85117937 |
| LOC100045241 | 0.24407546 |
| LOC100045250 | -0.617206 |
| LOC100045280 | 1.05588116 |
| LOC100045300 | -0.2367722 |
| LOC100045304 | -0.5060716 |
| LOC100045343 | -0.3928792 |
| LOC100045359 | 0.4275548 |
| LOC100045359 | -0.568272 |
| LOC100045403 | -0.0025249 |
| LOC100045413 | 2.72533681 |
| LOC100045419 | 1.53847475 |
| LOC100045430 | -0.5297301 |
| LOC100045439 | 1.84168037 |
| LOC100045491 | 0.09160199 |
| LOC100045505 | -0.1917297 |
| LOC100045522 | 0.12818161 |
| LOC100045542 | 0.67644267 |
| LOC100045551 | -0.4470952 |
| LOC100045567 | -0.3363148 |
| LOC100045607 | 1.64130958 |
| LOC100045617 | -0.5290043 |
| LOC100045644 | 0.14348038 |
| LOC100045697 | 0.45524764 |
| LOC100045780 | -0.0826216 |
| LOC100045782 | 1.78626942 |
| LOC100045877 | 0.36846576 |
| LOC100045882 | -0.7163966 |
| LOC100045887 | -0.6116404 |
| LOC100045950 | 0.15505349 |
| LOC100045963 | 1.127024 |
| LOC100045981 | 0.24818502 |
| LOC100046003 | 0.88984919 |
| LOC100046035 | -0.3363148 |
| LOC100046035 | 2.20454109 |
| LOC100046039 | 0.15783761 |
| LOC100046044 | -0.795004 |
| LOC100046049 | 0.7989857 |
| LOC100046056 | 0.87433969 |
| LOC100046068 | 1.11302308 |
| LOC100046080 | -0.6132272 |
| LOC100046081 | 0.06511528 |
| LOC100046120 | -0.4948133 |
| LOC100046128 | 0.87191813 |
| LOC100046129 | 0.8877855 |
| LOC100046163 | 0.03824974 |
| LOC100046163 | -0.5728234 |
| LOC100046207 | -0.8164763 |
| LOC100046207 | -0.69535 |
| LOC100046207 | 0.27890089 |
| LOC100046232 | -0.5766332 |
| LOC100046254 | 1.34639577 |
| LOC100046320 | 1.22477459 |
| LOC100046393 | -0.4241211 |
| LOC100046406 | -0.3941341 |
| LOC100046457 | 0.38168087 |
| LOC100046518 | -0.6480124 |
| LOC100046568 | 1.35616273 |
| LOC100046586 | 3.31604022 |
| LOC100046608 | 1.52299709 |
| LOC100046608 | 0.20888711 |
| LOC100046608 | 0.25635023 |
| LOC100046608 | 1.75467812 |
| LOC100046616 | -0.2625867 |
| LOC100046641 | 0.61357843 |
| LOC100046650 | 1.08385967 |
| LOC100046690 | 3.80210022 |
| LOC100046690 | 0.09940357 |
| LOC100046741 | 0.4471277 |
| LOC100046744 | -0.5812257 |
| LOC100046744 | -0.6832886 |
| LOC100046746 | 1.31777006 |
| LOC100046775 | 1.48314092 |
| LOC100046781 | -0.6629678 |
| LOC100046781 | -0.2495901 |
| LOC100046792 | -0.4962131 |
| LOC100046796 | 0.16854827 |
| LOC100046802 | 1.41857683 |
| LOC100046841 | -0.6562913 |
| LOC100046855 | -0.6604585 |
| LOC100046883 | 0.48140548 |
| LOC100046891 | 0.92928089 |
| LOC100046898 | -0.5735841 |
| LOC100046918 | -0.7969357 |
| LOC100046953 | -0.4990192 |
| LOC100046959 | -0.8861528 |
| LOC100046996 | 0.9096181 |
| LOC100047009 | -0.8609627 |
| LOC100047009 | 0.37702189 |
| LOC100047012 | -0.7217333 |
| LOC100047028 | -0.5290043 |
| LOC100047046 | 1.57349404 |
| LOC100047052 | 1.17729538 |
| LOC100047052 | 1.33927228 |
| LOC100047155 | -0.4598078 |
| LOC100047155 | -0.588157 |
| LOC100047167 | -0.2283237 |
| LOC100047173 | -0.3985396 |
| LOC100047184 | 1.65892961 |
| LOC100047200 | -0.0925667 |
| LOC100047214 | 0.93654891 |
| LOC100047214 | 0.18691687 |
| LOC100047238 | 2.35396417 |
| LOC100047261 | 2.20543192 |
| LOC100047323 | -0.8990075 |
| LOC100047323 | 2.0484603 |
| LOC100047339 | 0.08486261 |
| LOC100047353 | 3.50045747 |
| LOC100047368 | 1.26094252 |
| LOC100047369 | 1.20598143 |
| LOC100047369 | -0.4080507 |
| LOC100047391 | 0.4471277 |
| LOC100047393 | -0.4611561 |
| LOC100047427 | -0.5340969 |
| LOC100047480 | 1.56967579 |
| LOC100047490 | 2.1167179 |
| LOC100047579 | 0.66197997 |
| LOC100047583 | -0.705814 |
| LOC100047606 | -0.7075694 |
| LOC100047615 | 0.02632337 |
| LOC100047619 | -0.3767171 |
| LOC100047634 | 1.83853067 |
| LOC100047651 | -0.8125353 |
| LOC100047653 | 1.89236119 |
| LOC100047659 | -0.472696 |
| LOC100047674 | 0.26692112 |
| LOC100047707 | -0.0939311 |
| LOC100047717 | 1.63612194 |
| LOC100047749 | 1.35414038 |
| LOC100047762 | -0.6562913 |
| LOC100047776 | -0.8990075 |
| LOC100047794 | -0.1235241 |
| LOC100047800 | 0.74000392 |
| LOC100047810 | -0.4927174 |
| LOC100047815 | -0.869284 |
| LOC100047816 | -0.682433 |
| LOC100047834 | -0.2351822 |
| LOC100047837 | 0.9924565 |
| LOC100047837 | -0.0839715 |
| LOC100047841 | -0.535557 |
| LOC100047856 | 0.60177732 |
| LOC100047870 | -0.4878451 |
| LOC100047911 | -0.2840999 |
| LOC100047934 | 1.99049205 |
| LOC100047935 | -0.9660802 |
| LOC100047937 | 2.36863923 |
| LOC100047937 | 0.32620935 |
| LOC100047958 | 0.33573453 |
| LOC100047963 | -0.5239394 |
| LOC100047963 | 0.30211095 |
| LOC100047998 | -0.622805 |
| LOC100048046 | -0.1618862 |
| LOC100048046 | -0.1137101 |
| LOC100048049 | -0.5540042 |
| LOC100048049 | -0.7882743 |
| LOC100048049 | 0.96594501 |
| LOC100048056 | 2.43550844 |
| LOC100048071 | -0.58507 |
| LOC100048076 | -0.5517711 |
| LOC100048083 | -0.0595279 |
| LOC100048105 | 2.3445736 |
| LOC100048105 | 0.60328759 |
| LOC100048187 | 1.99907981 |
| LOC100048251 | -0.4247697 |
| LOC100048295 | 0.16786096 |
| LOC100048299 | -0.210105 |
| LOC100048301 | 1.09627035 |
| LOC100048313 | -0.6621306 |
| LOC100048331 | 0.36818844 |
| LOC100048332 | 2.10160991 |
| LOC100048346 | 0.95240132 |
| LOC100048372 | 2.79673112 |
| LOC100048384 | -0.082172 |
| LOC100048413 | -0.3170403 |
| LOC100048436 | -0.4286709 |
| LOC100048445 | 1.15739163 |
| LOC100048461 | -0.4692871 |
| LOC100048480 | 2.26978603 |
| LOC100048483 | -0.7479665 |
| LOC100048504 | 0.10787397 |
| LOC100048530 | -0.1463516 |
| LOC100048554 | 2.82855524 |
| LOC100048556 | -0.551028 |
| LOC100048589 | 1.43745888 |
| LOC100048613 | 0.66035908 |
| LOC100048622 | 2.68096889 |
| LOC100048645 | 0.45398355 |
| LOC100048684 | 0.63674209 |
| LOC100048703 | 2.24533148 |
| LOC100048710 | 2.78812584 |
| LOC100048721 | -0.6292452 |
| LOC100048721 | -0.4941141 |
| LOC100048726 | 0.55393068 |
| LOC100048733 | -0.7253081 |
| LOC100048816 | -0.542156 |
| LOC100048832 | 0.61058864 |
| LOC100048832 | -0.3292598 |
| LOC100048858 | 0.16751711 |
| LOC192758 | 0.78843895 |
| LOC194227 | -0.6604585 |
| LOC212390 | -0.7988715 |
| LOC216443 | -0.2620415 |
| LOC216963 | -0.698825 |
| LOC217341 | 2.81445321 |
| LOC218963 | -0.5473211 |
| LOC225922 | -0.7479665 |
| LOC233529 | -0.8017826 |
| LOC234374 | -0.4913227 |
| LOC235497 | -0.4720133 |
| LOC236749 | -0.2653172 |
| LOC237408 | 0.87094775 |
| LOC237877 | 0.72404152 |
| LOC238829 | -0.5297301 |
| LOC241593 | -0.7488844 |
| LOC244595 | 1.09715961 |
| LOC245263 | -0.6504884 |
| LOC329575 | 0.2235756 |
| LOC329575 | 0.65792245 |
| LOC333331 | 2.86896071 |
| LOC381066 | -0.6638057 |
| LOC381302 | 0.95329062 |
| LOC381375 | -0.2708024 |
| LOC381483 | 0.4141863 |
| LOC381629 | 2.28934435 |
| LOC381801 | 0.69655518 |
| LOC381936 | 0.40968723 |
| LOC382010 | 1.19666926 |
| LOC382044 | 1.70446206 |
| LOC382106 | -0.2555244 |
| LOC382106 | -0.5525149 |
| LOC382133 | -0.2669594 |
| LOC384806 | -0.6571233 |
| LOC384806 | -0.645543 |
| LOC386486 | 1.58108398 |
| LOC433479 | -0.7636946 |
| LOC433801 | 3.13786986 |
| LOC433886 | -0.6292452 |
| LOC434172 | 0.54088435 |
| LOC434172 | -0.6308622 |
| LOC434864 | -0.7014397 |
| LOC434960 | 3.21445728 |
| LOC435023 | -0.4055049 |
| LOC435337 | -0.8893499 |
| LOC435565 | -0.6414418 |
| LOC436177 | -0.9330594 |
| LOC544988 | -0.3791854 |
| LOC544988 | 0.01707546 |
| LOC545013 | 0.60199322 |
| LOC545013 | -0.7815927 |
| LOC545056 | -0.3816603 |
| LOC545238 | -0.2194302 |
| LOC545703 | 1.10864355 |
| LOC545732 | 2.7871302 |
| LOC545952 | 0.01989976 |
| LOC546015 | -0.2506663 |
| LOC546015 | -0.1771861 |
| LOC546723 | 1.66045825 |
| LOC546840 | 1.56891026 |
| LOC547323 | 0.57660914 |
| LOC547343 | -0.5297301 |
| LOC620807 | -0.6944833 |
| LOC621823 | -0.0785835 |
| LOC621824 | 0.49166668 |
| LOC622404 | -0.8404783 |
| LOC622665 | 2.33566012 |
| LOC624855 | -0.5532592 |
| LOC625480 | 0.4267737 |
| LOC626152 | -0.4983169 |
| LOC628053 | 1.50979118 |
| LOC631002 | 0.0089586 |
| LOC631784 | -0.8661548 |
| LOC632431 | 1.85423032 |
| LOC639396 | -0.3339572 |
| LOC640963 | -0.5414205 |
| LOC641240 | -0.6124335 |
| LOC654426 | 1.16839708 |
| LOC654467 | 1.03711275 |
| LOC654494 | -0.4920198 |
| LOC654842 | -0.3578058 |
| LOC665250 | -0.5340969 |
| LOC666676 | -0.666324 |
| LOC666904 | 2.41964286 |
| LOC667250 | 0.87482325 |
| LOC667370 | 1.05002309 |
| LOC667609 | 0.74446195 |
| LOC668492 | 0.32009685 |
| LOC668837 | 2.61207102 |
| LOC669168 | -0.1086103 |
| LOC669660 | 0.32562893 |
| LOC670044 | -0.433898 |
| LOC671564 | 1.16733769 |
| LOC672705 | 3.03579272 |
| LOC674706 | 0.69460242 |
| LOC675228 | 0.69928219 |
| LOC675567 | -0.283542 |
| LOC675567 | 2.48774733 |
| LOC675813 | 2.57621385 |
| LOC676420 | 0.45877801 |
| LOC676420 | -0.350587 |
| LOC677144 | -0.0434371 |
| LOC677205 | 1.49904068 |
| LOC677317 | -0.3903744 |
| LOC677319 | 1.16816176 |
| LOC677528 | 1.2344696 |
| LOC732482 | 0.66601993 |
| Loh11cr2a | 0.35449803 |
| Loh12cr1 | 1.89030129 |
| Loh12cr1 | -0.0723365 |
| Lonp2 | -0.3369052 |
| Lonrf1 | 1.73968459 |
| Lonrf2 | -0.6292452 |
| Lor | -0.2533621 |
| Lox | 0.75880517 |
| Loxl1 | -0.5540042 |
| Lpar2 | 0.10198967 |
| Lpar2 | 1.24914954 |
| Lpar3 | 0.00936613 |
| Lpcat2 | -0.4183038 |
| Lpcat2 | 3.01210816 |
| Lpcat2 | -0.723519 |
| Lpgat1 | 1.87894475 |
| Lphn1 | -0.738837 |
| Lphn3 | -0.2757668 |
| Lpin1 | -0.4686068 |
| Lpin1 | -0.7102088 |
| Lpin2 | 1.92987938 |
| Lpin3 | 1.41623457 |
| Lpl | -0.5998248 |
| Lpxn | -0.869284 |
| Lrat | -0.7424781 |
| Lrba | 2.46563269 |
| Lrba | 0.9788626 |
| Lrch1 | 0.43015376 |
| Lrch4 | 2.33421119 |
| Lrch4 | 1.84783447 |
| Lrdd | -0.7315972 |
| Lrfn1 | -0.697955 |
| Lrfn2 | -0.2947632 |
| Lrfn3 | -0.3816603 |
| Lrfn4 | 2.23926572 |
| Lrg1 | -0.7562601 |
| Lrig1 | 0.18252773 |
| Lrig3 | 1.9077737 |
| Lrmp | -0.7084484 |
| Lrp1 | -0.422178 |
| Lrp10 | -0.4444407 |
| Lrp11 | -0.481618 |
| Lrp12 | 1.58342438 |
| Lrp12 | -0.8661548 |
| Lrp4 | -0.5622379 |
| Lrp5 | 0.99217235 |
| Lrp8 | -0.1806759 |
| Lrp8 | -0.6006079 |
| Lrpap1 | -0.7655626 |
| Lrpprc | -0.5096179 |
| Lrpprc | -0.5488021 |
| Lrpprc | -0.2351822 |
| Lrrc1 | -0.2631321 |
| Lrrc1 | -0.7825443 |
| Lrrc1 | 1.05998645 |
| Lrrc10 | -0.8364348 |
| Lrrc15 | 0.93216459 |
| Lrrc16b | 1.30698234 |
| Lrrc16b | -0.298156 |
| Lrrc18 | 2.45015622 |
| Lrrc2 | 1.80756204 |
| Lrrc20 | 1.76850745 |
| Lrrc20 | -0.167779 |
| Lrrc23 | -0.5943616 |
| Lrrc23 | 0.34060375 |
| Lrrc24 | 0.65812574 |
| Lrrc26 | 0.8879444 |
| Lrrc28 | -0.0998667 |
| Lrrc29 | 0.7414923 |
| Lrrc3 | 0.84735046 |
| Lrrc30 | 1.57478869 |
| Lrrc33 | -0.5735841 |
| Lrrc34 | 1.28398657 |
| Lrrc34 | 1.13095099 |
| Lrrc36 | 0.5229656 |
| Lrrc39 | -0.6092653 |
| Lrrc39 | 0.69577457 |
| Lrrc4 | -0.2044518 |
| Lrrc40 | -0.4781764 |
| Lrrc41 | -0.6596236 |
| Lrrc43 | 1.02580485 |
| Lrrc44 | -0.6357302 |
| Lrrc45 | -0.5667598 |
| Lrrc46 | 0.0342912 |
| Lrrc48 | -0.755335 |
| Lrrc48 | 2.19917818 |
| Lrrc48 | 0.52202528 |
| Lrrc49 | 1.79316016 |
| Lrrc49 | 3.14239063 |
| Lrrc49 | 2.96570465 |
| Lrrc4c | 2.1703651 |
| Lrrc50 | 3.50027387 |
| Lrrc50 | -0.4962131 |
| Lrrc52 | -0.481618 |
| Lrrc52 | 1.32654967 |
| Lrrc56 | 0.44789195 |
| Lrrc57 | -0.3066924 |
| Lrrc59 | -0.4029661 |
| Lrrc6 | -0.5495435 |
| Lrrc7 | 1.28055039 |
| Lrrc8 | -0.542156 |
| Lrrc8a | 2.06033977 |
| Lrrc8b | -0.1183706 |
| Lrrc8c | 0.55074318 |
| Lrrc8d | -0.3147309 |
| Lrrc9 | 0.23548195 |
| Lrrfip1 | 0.58235902 |
| Lrrfip1 | 1.73501737 |
| Lrriq2 | -0.4444407 |
| Lrriq2 | 1.87354579 |
| Lrrk1 | 0.68240325 |
| Lrrk2 | -0.4106034 |
| Lrrn1 | 1.25205801 |
| Lrrn2 | -0.4788637 |
| Lrrn3 | 0.82891391 |
| Lrrtm1 | -0.4598078 |
| Lrrtm2 | -0.5146055 |
| Lrrtm3 | 1.22996514 |
| Lrsam1 | -0.3292598 |
| Lrsam1 | -0.5081978 |
| Lrtm1 | 0.61357843 |
| Lrtm1 | 0.40436628 |
| Lsamp | -0.3847632 |
| Lsamp | -0.2474416 |
| Lsg1 | -0.7288967 |
| Lsg1 | -0.1320191 |
| Lsm10 | 1.1095211 |
| Lsm11 | 0.98647098 |
| Lsm12 | 3.65666145 |
| Lsm12 | -0.5473211 |
| Lsm12 | 0.39928311 |
| Lsm14a | 1.49166559 |
| Lsm14b | -0.5873843 |
| Lsm14b | -0.2533621 |
| Lsm14b | 0.35337153 |
| Lsm2 | 1.99869336 |
| Lsm2 | 1.30759057 |
| Lsm3 | -0.5827615 |
| Lsm4 | 2.20322265 |
| Lsm5 | -0.3816603 |
| Lsm6 | 1.56052501 |
| Lsm6 | 1.16710211 |
| Lsm8 | -0.3972788 |
| Lsr | -0.818453 |
| Lst1 | -0.5138913 |
| Ltb4dh | -0.3228392 |
| Ltb4r1 | -0.6596236 |
| Ltb4r2 | 1.25087417 |
| Ltbp1 | 0.40623206 |
| Ltbp1 | 0.62691517 |
| Ltbp2 | -0.1766886 |
| Ltbp3 | 2.33272623 |
| Ltbp3 | -0.5210575 |
| Ltbp4 | -0.5039503 |
| Ltbp4 | -0.7343056 |
| Ltbp4 | 2.07144441 |
| Ltbr | 2.21348809 |
| Ltc4s | -0.5592354 |
| Ltf | 0.29343713 |
| Ltf | -0.617206 |
| Ltk | 0.25290444 |
| Ltk | -0.2367722 |
| Ltv1 | -0.1536013 |
| Luc7l | -0.2277982 |
| Luc7l | -0.1263472 |
| Luc7l2 | 0.17879766 |
| Luc7l2 | -0.0503612 |
| Lum | 1.71696136 |
| Luzp1 | 2.1786886 |
| Luzp1 | -0.6756163 |
| Luzp1 | -0.5261067 |
| Luzp2 | 0.51066697 |
| Lxn | -0.5690291 |
| Ly6a | 1.84487645 |
| Ly6c1 | -0.682433 |
| Ly6d | 1.02539355 |
| Ly6e | -0.4048696 |
| Ly6f | 0.34003222 |
| Ly6g6c | 0.23739847 |
| Ly6g6d | 0.06165464 |
| Ly6g6e | 0.3909296 |
| Ly6k | -0.691888 |
| Ly86 | 0.36011024 |
| Ly9 | -0.6164089 |
| Ly9 | 2.04717684 |
| Ly96 | 0.69694524 |
| Lyar | -0.6596236 |
| Lyl1 | 2.13747466 |
| Lynx1 | 0.30567666 |
| Lynx1 | 1.5270909 |
| Lypd1 | 0.06779804 |
| Lypd3 | -0.7787438 |
| Lypd4 | 0.19262552 |
| Lypd5 | -0.3216765 |
| Lypd6 | 1.02402125 |
| Lypla1 | -0.7873168 |
| Lypla1 | 0.31306174 |
| Lypla2 | 0.35111443 |
| Lypla3 | -0.7324991 |
| Lyplal1 | -0.4299749 |
| Lyrm1 | -0.633293 |
| Lyrm2 | -0.4004341 |
| Lyrm2 | 0.9597864 |
| Lyrm5 | -0.5117522 |
| Lysmd1 | -0.6084749 |
| Lysmd1 | -0.2614967 |
| Lysmd2 | 0.31511913 |
| Lysmd4 | 0.52882118 |
| Lyst | -0.6463654 |
| Lyve1 | -0.1594418 |
| Lyz | 0.25384546 |
| Lyz1 | 1.10121405 |
| Lyz1 | -0.5697867 |
| Lyz2 | 3.66294942 |
| Lyzl1 | 2.97635527 |
| Lyzl4 | 1.75273058 |
| Lyzl6 | 3.93838405 |
| Lyzs | -0.1334425 |
| Lzic | -0.5174675 |
| Lztr1 | -0.7049375 |
| Lzts2 | -0.4352093 |
| M6pr | 0.58982503 |
| M6pr | -0.5562425 |
| M6prbp1 | 0.80595122 |
| Mab21l2 | 0.5087604 |
| Macrod1 | -0.6188023 |
| Mad1l1 | -0.4836891 |
| Mad2l1 | 1.14854091 |
| Mad2l1bp | -0.869284 |
| Mad2l1bp | -0.7049375 |
| Mad2l1bp | 2.80138238 |
| Mad2l2 | -0.6722266 |
| Madcam1 | 0.56970687 |
| Madd | 0.34601592 |
| Maea | 0.94286231 |
| Maf | 0.12889877 |
| Maf1 | -0.4150876 |
| Mafg | 1.46319917 |
| Mag | -0.5819933 |
| Magea2 | 1.85727133 |
| Magea5 | -0.5502854 |
| Mageb3 | -0.5032443 |
| Mageb3 | -0.3960196 |
| Maged1 | 1.0933425 |
| Maged2 | 0.27123927 |
| Magee1 | 0.04297792 |
| Mageh1 | -0.551028 |
| Magi1 | -0.3891246 |
| Magi1 | 0.98732841 |
| Magi1 | -0.610848 |
| Magi2 | -0.5362879 |
| Magi3 | -0.2013827 |
| Magi3 | -0.726204 |
| Magix | -0.5495435 |
| Magmas | 3.24658574 |
| Magoh | 0.9681347 |
| Magoh | -0.366299 |
| Magohb | 1.48916641 |
| Mak | -0.4444407 |
| Mak10 | 0.75441531 |
| Mak10 | -0.2719033 |
| Mal | 2.04960475 |
| Mal | 0.40489974 |
| Mall | -0.737929 |
| Malt1 | 0.58081446 |
| Mamdc2 | 0.38277373 |
| Maml1 | -0.729796 |
| Maml3 | 1.04399361 |
| Man1a | 0.88220661 |
| Man1a2 | -0.7075694 |
| Man1a2 | 1.5126426 |
| Man1b1 | 2.78052795 |
| Man1c1 | 0.45474221 |
| Man1c1 | -0.4864577 |
| Man2a1 | 3.52014232 |
| Man2a2 | 1.73866364 |
| Man2a2 | 2.00018995 |
| Man2a2 | 0.36818844 |
| Man2b1 | 0.02752289 |
| Man2b2 | 1.0860467 |
| Man2c1 | -0.6284377 |
| Manba | -0.729796 |
| Manba | 2.09322124 |
| Manbal | -0.0017 |
| Manea | -0.3872529 |
| Mansc1 | 0.83414664 |
| Maob | 1.96609717 |
| Map1lc3a | -0.3416413 |
| Map1lc3a | 0.18927172 |
| Map1lc3b | 1.6344101 |
| Map2k1 | 1.60915544 |
| Map2k2 | 0.17709723 |
| Map2k2 | -0.5966991 |
| Map2k3 | 0.48655019 |
| Map2k4 | -0.7618305 |
| Map2k6 | 0.77973644 |
| Map2k6 | 0.20789961 |
| Map2k6 | -0.0939311 |
| Map2k7 | 0.53371164 |
| Map2k7 | -0.0103986 |
| Map2k7 | -0.6629678 |
| Map3k1 | 0.54249639 |
| Map3k11 | 0.30389551 |
| Map3k12 | 3.47617283 |
| Map3k12 | 0.72725592 |
| Map3k12 | 1.06025069 |
| Map3k14 | 2.71640208 |
| Map3k3 | 0.4557528 |
| Map3k6 | 2.70667992 |
| Map3k7 | 0.66803336 |
| Map3k7ip1 | 0.57416578 |
| Map3k7ip1 | -0.4976151 |
| Map3k7ip1 | -0.7244131 |
| Map3k7ip2 | -0.6148167 |
| Map3k7ip3 | -0.4106034 |
| Map3k8 | 2.12717505 |
| Map3k9 | 0.33429755 |
| Map4k2 | 3.03451998 |
| Map4k4 | -0.739746 |
| Map4k5 | 0.71433133 |
| Map4k5 | 0.92547293 |
| Mapbpip | -0.2697028 |
| Mapk1 | 1.65781629 |
| Mapk1 | -0.4484252 |
| Mapk1 | -0.5866122 |
| Mapk10 | 0.76135641 |
| Mapk10 | 0.11808304 |
| Mapk11 | -0.041714 |
| Mapk12 | -0.0162431 |
| Mapk12 | -0.5488021 |
| Mapk12 | 0.29493838 |
| Mapk13 | -0.7882743 |
| Mapk13 | -0.8344196 |
| Mapk14 | 0.01302586 |
| Mapk15 | 0.31423795 |
| Mapk15 | -0.2691534 |
| Mapk15 | -0.5928066 |
| Mapk1ip1 | 1.80554366 |
| Mapk1ip1 | -0.6781667 |
| Mapk1ip1 | 2.16411385 |
| Mapk3 | -0.6571233 |
| Mapk3 | 1.10449536 |
| Mapk4 | 1.05588116 |
| Mapk6 | 1.02936185 |
| Mapk6 | 0.58169738 |
| Mapk6 | -0.5089076 |
| Mapk8 | -0.6730728 |
| Mapk8ip1 | -0.4358657 |
| Mapk8ip1 | 2.95170066 |
| Mapk8ip2 | -0.1761914 |
| Mapk8ip3 | 2.45836946 |
| Mapk8ip3 | 0.28437855 |
| Mapk9 | 0.60801832 |
| Mapk9 | -0.1221158 |
| Mapk9 | 0.97915083 |
| Mapkap1 | 0.84967101 |
| Mapkap1 | -0.5773971 |
| Mapkapk2 | 0.14840773 |
| Mapkapk3 | 0.28316407 |
| Mapkbp1 | 0.19362932 |
| Mapre1 | -0.7711889 |
| Mapre2 | -0.7049375 |
| Mapre2 | -0.6629678 |
| Mapre3 | -0.7636946 |
| Mapt | -0.5067798 |
| Mapt | 0.37178712 |
| Mapt | -0.6944833 |
| Mapt | -0.6414418 |
| March2 | -0.4150876 |
| March3 | -0.7452183 |
| March3 | 0.69362451 |
| March5 | -0.4384958 |
| March5 | -0.2241279 |
| March7 | -0.3608302 |
| March8 | -0.6188023 |
| March9 | 3.0548964 |
| March11 | -0.3742553 |
| Marcks | 0.66823447 |
| Marcksl1 | -0.0460276 |
| Marcksl1 | -0.102619 |
| Marcksl1 | -0.1756945 |
| Mare | -0.0033506 |
| Mark1 | 2.39822809 |
| Mark2 | -0.0844219 |
| Mark2 | 0.91580735 |
| Mark3 | 0.14946021 |
| Mark3 | -0.5735841 |
| Mark4 | 0.08335833 |
| Mars | 0.45474221 |
| Mars2 | -0.4417939 |
| Marveld3 | -0.6654838 |
| Marveld3 | 2.41541759 |
| Mas1 | -0.5060716 |
| Masp1 | -0.633293 |
| Masp2 | -0.4864577 |
| Mast1 | -0.2571494 |
| Mast1 | -0.087129 |
| Mast2 | 0.15540196 |
| Mast2 | 0.52413966 |
| Mat1a | -0.102619 |
| Mat2a | 0.4642989 |
| Mat2a | 1.33011765 |
| Mat2a | 0.96110953 |
| Mat2a | -0.5167512 |
| Mat2b | -0.5081978 |
| Mat2b | -0.75997 |
| Matk | 0.3871324 |
| Matk | 0.54548281 |
| Matn4 | -0.3866298 |
| Matn4 | -0.7516435 |
| Matn4 | 1.91371995 |
| Matr3 | 0.6452308 |
| Max | -0.4490909 |
| Maz | -0.737929 |
| Mb | 0.38031299 |
| Mbc2 | -0.57587 |
| Mbd1 | -0.6212018 |
| Mbd1 | -0.5196198 |
| Mbd3 | 1.3939621 |
| Mbd3l2 | -0.1339174 |
| Mbd4 | 0.4557528 |
| Mbd5 | -0.5146055 |
| Mbd6 | 2.52311155 |
| Mbip | -0.3044084 |
| Mbl2 | 0.11226297 |
| Mbnl1 | -0.7315972 |
| Mbnl2 | 0.28831474 |
| Mbnl2 | -0.5690291 |
| Mboat1 | -0.067015 |
| Mboat2 | -0.3049789 |
| Mboat2 | 1.55463349 |
| Mboat2 | 1.96764692 |
| Mboat2 | -0.666324 |
| Mboat5 | -0.755335 |
| Mboat5 | 1.54776494 |
| Mbp | -0.5599851 |
| Mbp | -0.6236076 |
| Mbp | -0.3061209 |
| Mbtd1 | 0.84252516 |
| Mbtps1 | -0.7084484 |
| Mbtps1 | -0.6406237 |
| Mbtps1 | 1.51516089 |
| Mbtps2 | 0.59113638 |
| Mc3r | -0.0229677 |
| Mc4r | 0.31892801 |
| Mc5r | 0.16165226 |
| Mcam | -0.7730719 |
| Mcam | 1.99946609 |
| Mcam | 0.22227928 |
| Mcat | 0.49093747 |
| Mcc | -0.5935838 |
| Mccc1 | -0.3644725 |
| Mcee | -0.4631822 |
| Mcf2l | -0.0745627 |
| Mcf2l | 3.78813623 |
| Mcf2l | -0.0568996 |
| Mcfd2 | -0.6496624 |
| Mchr1 | -0.380422 |
| Mcl1 | -0.6892998 |
| Mcm10 | -0.1831767 |
| Mcm10 | -0.8284001 |
| Mcm2 | 2.55145753 |
| Mcm3 | 3.22591281 |
| Mcm4 | 1.6650292 |
| Mcm5 | -0.6798709 |
| Mcm6 | -0.8154895 |
| Mcm6 | 0.01018064 |
| Mcm6 | 0.73139936 |
| Mcm7 | 0.5087604 |
| Mcoln1 | 0.26537407 |
| Mcoln2 | 1.71571894 |
| Mcoln3 | 0.30389551 |
| Mcph1 | -0.467927 |
| Mcph1 | 0.83901534 |
| Mcph1 | 1.53307877 |
| Mcph1 | 0.65670174 |
| Mcpt4 | 0.62880495 |
| Mcrs1 | -0.6944833 |
| Mcrs1 | -0.4836891 |
| Mcrs1 | 3.202527 |
| Mcts1 | -0.6646444 |
| Mdfic | -0.4969139 |
| Mdga1 | 1.49274649 |
| Mdga2 | -0.4692871 |
| Mdga2 | 1.16048152 |
| Mdh1 | -0.182676 |
| Mdh1 | 0.3783946 |
| Mdh1b | -0.7507229 |
| Mdh2 | -0.7702488 |
| Mdk | 0.97554144 |
| Mdk | 3.73807213 |
| Mdm1 | 0.38686058 |
| Mdm1 | -0.7825443 |
| Mdm1 | 2.37204557 |
| Mdm1 | -0.6148167 |
| Mdm1 | -0.4927174 |
| Mdm1 | -0.5067798 |
| Mdm1 | -0.8936298 |
| Mdm1 | 1.38477147 |
| Mdm1 | -0.4293227 |
| Mdm2 | -0.1086103 |
| Mdm2 | -0.3578058 |
| Mdm4 | -0.0293162 |
| Mdn1 | 0.58982503 |
| Me2 | -0.260408 |
| Me3 | 2.25767548 |
| Mea1 | -0.5697867 |
| Mecp2 | -0.1628658 |
| Mecr | 0.39282243 |
| Med1 | 0.05041555 |
| Med1 | -0.6300534 |
| Med10 | 1.37746607 |
| Med11 | 0.96755129 |
| Med11 | 0.22454666 |
| Med12 | 0.8204117 |
| Med15 | 0.91857918 |
| Med15 | 1.92361673 |
| Med15 | -0.0687855 |
| Med16 | 1.14456673 |
| Med18 | -0.472696 |
| Med18 | 1.40944495 |
| Med19 | -0.4843805 |
| Med21 | -0.5796923 |
| Med22 | -0.2724542 |
| Med22 | 0.1195324 |
| Med23 | -0.4260683 |
| Med23 | 2.19083967 |
| Med24 | 1.80762134 |
| Med25 | -0.3620427 |
| Med25 | -0.3866298 |
| Med26 | -0.058651 |
| Med27 | -0.1263472 |
| Med28 | -0.505364 |
| Med30 | 1.17973923 |
| Med31 | 2.14720866 |
| Med4 | -0.1851821 |
| Med6 | 0.22842083 |
| Med7 | 1.46960999 |
| Med8 | -0.5781615 |
| Med8 | 2.08581328 |
| Med9 | -0.7844504 |
| Mef2b | 0.93352745 |
| Mef2b | -0.6671649 |
| Mef2c | -0.7479665 |
| Mef2c | 1.54234378 |
| Mef2c | -0.6463654 |
| Megf10 | -0.6970859 |
| Megf11 | -0.719951 |
| Megf11 | -0.4391545 |
| Megf11 | -0.633293 |
| Megf11 | 2.72995987 |
| Megf6 | -0.4099646 |
| Megf9 | -0.5290043 |
| Mei1 | 2.5868721 |
| Meis1 | 1.21105255 |
| Meis2 | -0.698825 |
| Meis3 | 1.4186668 |
| Melk | 1.91144149 |
| Memo1 | -0.2713527 |
| Men1 | 2.50744734 |
| Men1 | -0.6554601 |
| Meox1 | -0.6587894 |
| Meox2 | 1.44018818 |
| Mep1b | -0.5789266 |
| Mepce | -0.1831767 |
| Mertk | -0.7768493 |
| Mesdc1 | -0.1320191 |
| Mesp1 | -0.6815782 |
| Mest | -0.349988 |
| Mest | -0.9131363 |
| Mest | -0.3428292 |
| Metap1 | 2.19699311 |
| Metap2 | -0.7796925 |
| Metap2 | 0.89333122 |
| Metapl1 | -0.6324821 |
| Metrn | -0.6447213 |
| Metrnl | -0.0790313 |
| Metrnl | -0.532639 |
| Mett11d1 | -0.0939311 |
| Mettl1 | -0.729796 |
| Mettl1 | 0.64872798 |
| Mettl2 | -0.8145037 |
| Mettl3 | -0.4490909 |
| Mettl4 | -0.5743455 |
| Mettl6 | -0.7128557 |
| Mettl8 | 0.62880495 |
| Mettl9 | -0.4118824 |
| Mettl9 | -0.0776885 |
| Mex3a | 3.26033418 |
| Mex3a | 0.05702039 |
| Mex3c | 0.2238994 |
| Mfap1b | -0.4672477 |
| Mfap2 | 2.06875905 |
| Mfap2 | 0.62565319 |
| Mfap2 | -0.4144457 |
| Mfap3 | 3.29502509 |
| Mfap3 | 2.29277024 |
| Mfap3l | -0.4530953 |
| Mfap3l | -0.2807577 |
| Mfap4 | 0.86608062 |
| Mfap5 | 3.07123212 |
| Mfge8 | 1.64187634 |
| Mfge8 | 0.00691831 |
| Mfi2 | -0.1732137 |
| Mfn1 | -0.6389897 |
| Mfn1 | 0.91302727 |
| Mfn2 | -0.5644961 |
| Mfng | -0.6884387 |
| Mfrp | -0.5781615 |
| Mfsd1 | -0.6196014 |
| Mfsd10 | -0.6156124 |
| Mfsd10 | 2.58414154 |
| Mfsd11 | 1.4254792 |
| Mfsd2 | 4.7235089 |
| Mfsd3 | -0.6236076 |
| Mfsd4 | 0.8348197 |
| Mfsd7 | -0.7759035 |
| Mfsd8 | 1.09295993 |
| Mfsd9 | 0.69048829 |
| Mga | 0.06664923 |
| Mgat1 | 0.25478554 |
| Mgat3 | 1.2920585 |
| Mgat4b | 0.54845971 |
| Mgat4b | -0.4470952 |
| Mgat5 | -0.7146245 |
| MGC107098 | 1.03440046 |
| MGC117608 | -0.6612942 |
| MGC118210 | 1.01130048 |
| MGC18837 | 0.68852278 |
| MGC41689 | -0.5652501 |
| MGC67181 | -0.7066913 |
| Mgea6 | 0.50109493 |
| Mgl1 | -0.7796925 |
| Mgl2 | 0.17505261 |
| Mgll | 2.06232762 |
| Mgll | -0.5812257 |
| Mgll | -0.4645354 |
| Mgmt | 0.35983043 |
| Mgp | 0.70374505 |
| Mgrn1 | 3.25411457 |
| Mgst1 | -0.6349171 |
| Mgst1 | -0.3440185 |
| Mgst2 | 0.99628417 |
| Mgst3 | 1.02539355 |
| Mgst3 | -0.8990075 |
| Mia1 | -0.260408 |
| Mia3 | -0.1806759 |
| Mib1 | 2.2337989 |
| Mib2 | 0.59592896 |
| Mic2l1 | 0.09420977 |
| Mical1 | 1.22077923 |
| Mical1 | -0.0280431 |
| Mical1 | -0.5644961 |
| Mical2 | 0.5267174 |
| Mical3 | 1.60016341 |
| Micall2 | -0.6406237 |
| Micall2 | 0.16751711 |
| Mid1 | 1.62118056 |
| Mid1 | 0.19128545 |
| Mid1ip1 | -0.128234 |
| Midn | -0.3773336 |
| Midn | 1.22266806 |
| Mier1 | 0.91132424 |
| Mier1 | -0.2178695 |
| Mif | 0.01788327 |
| Mif4gd | -0.3481931 |
| Mif4gd | -0.4598078 |
| Mill2 | 1.78705778 |
| Mill2 | -0.3055497 |
| Mina | 0.44687281 |
| Mink1 | -0.3620427 |
| Mink1 | -0.602961 |
| Minpp1 | 1.12911227 |
| Minpp1 | -0.719951 |
| Miox | -0.2209935 |
| Mipep | 3.57250245 |
| Mir16 | -0.7759035 |
| Mist1 | -0.4976151 |
| Mitf | -0.5196198 |
| Mizf | -0.1633559 |
| Mki67ip | -0.3941341 |
| Mkks | 0.05508264 |
| Mkks | 1.79538573 |
| Mkl1 | 1.89365984 |
| Mkl2 | 0.77293123 |
| Mkl2 | -0.1296516 |
| Mkl2 | -0.4228253 |
| Mkln1 | 1.18217669 |
| Mknk1 | -0.7969357 |
| Mknk2 | 0.80142955 |
| Mkrn1 | 1.35192038 |
| Mkrn2 | -0.622805 |
| Mkrn3 | 0.57505502 |
| Mkx | 0.86314811 |
| Mlana | 0.23292045 |
| Mlf1 | -0.4464308 |
| Mlf1 | -0.3847632 |
| Mlf2 | 2.22625773 |
| Mlf2 | -0.5384841 |
| Mlh1 | -0.6730728 |
| Mlh3 | 1.28741015 |
| Mlkl | -0.4625064 |
| Mllt1 | -0.3572021 |
| Mllt1 | 0.34941802 |
| Mllt1 | -0.5406855 |
| Mllt10 | -0.0980364 |
| Mllt11 | -0.0195993 |
| Mllt3 | -0.2152741 |
| Mllt3 | -0.6053201 |
| Mllt3 | -0.6260195 |
| Mlp | -0.6252148 |
| Mlstd2 | 0.96959165 |
| Mlx | 0.26289344 |
| Mlx | -0.813519 |
| Mlxipl | -0.5443661 |
| Mlycd | -0.2404926 |
| Mmab | -0.2669594 |
| Mmab | 0.09048214 |
| Mmd | 0.48728283 |
| Mmd2 | 1.43383684 |
| Mme | -0.2970237 |
| Mme | 2.01212464 |
| Mmel1 | 0.72631166 |
| Mmp10 | -0.2880143 |
| Mmp11 | -0.1521468 |
| Mmp12 | 3.11588033 |
| Mmp13 | 0.90244933 |
| Mmp13 | 1.29975467 |
| Mmp14 | 1.8853498 |
| Mmp15 | -0.4260683 |
| Mmp16 | -0.7998409 |
| Mmp17 | -0.5966991 |
| Mmp1a | 0.08486261 |
| Mmp1a | 0.13781708 |
| Mmp2 | 0.39578913 |
| Mmp23 | -0.6596236 |
| Mmp24 | 1.85648387 |
| Mmp3 | 2.06192136 |
| Mmp3 | -0.4658906 |
| Mmp3 | 0.92425109 |
| Mmp9 | -0.7014397 |
| Mmrn2 | -0.0621635 |
| Mnat1 | 0.83985221 |
| Mnat1 | -0.7571862 |
| Mnd1 | 0.42128759 |
| Mns1 | -0.5124647 |
| Mnt | 0.11989439 |
| Mobk1b | 1.31274459 |
| Mobkl2a | -0.471331 |
| Mobkl2b | -0.7618305 |
| Mobkl2c | -0.6546297 |
| Mobkl3 | -0.7498032 |
| Mobp | 0.23259976 |
| Mobp | -0.5920301 |
| Mobp | 2.70175784 |
| Mocos | -0.5951401 |
| Mocs1 | 0.65200751 |
| Mocs2 | -0.2730055 |
| Mod1 | -0.6867188 |
| Mog | 1.95896521 |
| Mog | -0.2941989 |
| Mon1a | 0.4655491 |
| Mon1a | 1.54313141 |
| Mon1b | 0.29883037 |
| Mon1b | -0.5766332 |
| Mon1b | 0.27339087 |
| Morc2a | -0.164828 |
| Morc2b | 0.49142367 |
| Morc3 | -0.9660802 |
| Morf4l1 | -0.6037467 |
| Morf4l1 | 0.52998791 |
| Morf4l2 | -0.3210957 |
| Morn2 | 1.84065073 |
| Morn3 | 0.95742953 |
| Morn3 | -0.1786802 |
| Mos | -0.4080507 |
| Mosc2 | -0.729796 |
| Mospd1 | -0.3960196 |
| Mospd1 | 1.46414218 |
| Mospd1 | -0.5812257 |
| Mospd3 | -0.636544 |
| Mospd3 | -0.4464308 |
| Mov10 | 1.4843156 |
| Mov10l1 | 1.74070443 |
| Moxd1 | 0.75404855 |
| Moxd1 | -0.6268249 |
| Mpdu1 | 0.5054145 |
| Mpdz | -0.5982606 |
| Mpdz | -0.821426 |
| Mpg | -0.535557 |
| Mpg | 2.10734712 |
| Mphosph6 | -0.5935838 |
| Mpnd | -0.0421445 |
| Mpo | 0.74390586 |
| Mpo | 1.10159325 |
| Mpp1 | -0.5622379 |
| Mpp2 | 0.50613249 |
| Mpp3 | 0.55233829 |
| Mpp3 | 2.33781251 |
| Mpp5 | 2.14757944 |
| Mpp6 | 2.43514534 |
| Mppe1 | 0.23803644 |
| Mpped1 | -0.2874541 |
| Mpst | 1.0989356 |
| Mpv17 | 0.32649942 |
| Mpv17 | -0.7343056 |
| Mpv17 | 1.00122269 |
| Mpv17 | -0.6076852 |
| Mpv17l | 0.16751711 |
| Mpv17l | 0.48361383 |
| Mpv17l | 3.18375061 |
| Mpz | 0.98446724 |
| Mr1 | 0.84851145 |
| Mrap | 2.28227633 |
| Mras | -0.4254188 |
| Mrc1 | -0.755335 |
| Mrc2 | -0.1035382 |
| Mrc2 | 1.12542393 |
| Mre11a | 0.27798481 |
| Mre11a | -0.5167512 |
| Mre11a | -0.5319109 |
| Mreg | 0.58302019 |
| Mrgpra2 | -0.5362879 |
| Mrgpra2 | 2.4034715 |
| Mrgprb2 | -0.2039396 |
| Mrgpre | -0.5943616 |
| Mrgprf | -0.8154895 |
| Mrgprg | 0.18286609 |
| Mrm1 | -0.6696923 |
| Mro | -0.7146245 |
| Mrp63 | -0.1035382 |
| Mrpl1 | 0.95254959 |
| Mrpl1 | -0.8145037 |
| Mrpl10 | -0.1358196 |
| Mrpl10 | 2.52789152 |
| Mrpl11 | -0.4990192 |
| Mrpl12 | -0.7181721 |
| Mrpl13 | 0.11408565 |
| Mrpl13 | 0.93367875 |
| Mrpl14 | -0.4934155 |
| Mrpl15 | 1.09295993 |
| Mrpl16 | -0.4969139 |
| Mrpl17 | 1.3017963 |
| Mrpl18 | 0.38113396 |
| Mrpl2 | -0.0812736 |
| Mrpl20 | -0.5675156 |
| Mrpl22 | -0.2189096 |
| Mrpl23 | 0.3838653 |
| Mrpl24 | -0.4241211 |
| Mrpl27 | -0.7787438 |
| Mrpl28 | -0.719951 |
| Mrpl3 | 0.49409328 |
| Mrpl3 | -0.6212018 |
| Mrpl30 | 0.22519348 |
| Mrpl33 | -0.380422 |
| Mrpl33 | -0.4843805 |
| Mrpl34 | 1.56337983 |
| Mrpl36 | -0.6316718 |
| Mrpl37 | 0.43970745 |
| Mrpl37 | -0.0547151 |
| Mrpl38 | 0.86949027 |
| Mrpl39 | -0.1221158 |
| Mrpl40 | -0.3529871 |
| Mrpl43 | 1.65271987 |
| Mrpl43 | 0.94181304 |
| Mrpl45 | -0.5096179 |
| Mrpl46 | 0.02191189 |
| Mrpl47 | -0.705814 |
| Mrpl47 | 0.58960629 |
| Mrpl48 | -0.1468332 |
| Mrpl48 | 0.41365812 |
| Mrpl48 | -0.1751978 |
| Mrpl49 | 1.99034581 |
| Mrpl49 | -0.532639 |
| Mrpl49 | 2.35699669 |
| Mrpl49 | 1.21419405 |
| Mrpl50 | -0.610848 |
| Mrpl51 | 0.27000755 |
| Mrpl51 | -0.274109 |
| Mrpl51 | -0.5858408 |
| Mrpl52 | -0.3947622 |
| Mrpl53 | 0.19796665 |
| Mrpl54 | 0.09643923 |
| Mrpl55 | -0.2609522 |
| Mrpl9 | -0.4260683 |
| Mrps10 | 0.57638728 |
| Mrps10 | 2.39561108 |
| Mrps11 | -0.4163728 |
| Mrps11 | 0.63361707 |
| Mrps12 | -0.5675156 |
| Mrps14 | 0.49603002 |
| Mrps15 | -0.6944833 |
| Mrps16 | -0.4273687 |
| Mrps17 | -0.4885396 |
| Mrps18a | -0.4118824 |
| Mrps18b | 0.60887588 |
| Mrps18b | 1.07115112 |
| Mrps18c | -0.5897043 |
| Mrps2 | -0.5282791 |
| Mrps21 | -0.1821756 |
| Mrps22 | 1.15500781 |
| Mrps23 | 2.38847964 |
| Mrps24 | 1.28388263 |
| Mrps25 | -0.4948133 |
| Mrps25 | 0.30775038 |
| Mrps25 | 1.32615238 |
| Mrps26 | 0.26009483 |
| Mrps26 | -0.2730055 |
| Mrps27 | 2.44941139 |
| Mrps28 | -0.5660047 |
| Mrps30 | 0.78329829 |
| Mrps31 | 1.88878055 |
| Mrps31 | -0.4286709 |
| Mrps31 | -0.5011294 |
| Mrps33 | 0.32562893 |
| Mrps33 | -0.7693097 |
| Mrps34 | 0.55529343 |
| Mrps35 | 1.69018042 |
| Mrps5 | 3.09120516 |
| Mrps6 | -0.5781615 |
| Mrps7 | -0.6037467 |
| Mrps7 | 0.99075027 |
| Mrps9 | -0.1339174 |
| Mrrf | 0.41286529 |
| Mrrf | -0.1584659 |
| Mrto4 | -0.697955 |
| Mrvi1 | -0.4477599 |
| Ms4a1 | 3.81021857 |
| Ms4a10 | -0.5637428 |
| Ms4a13 | 1.74591369 |
| Ms4a5 | 0.88699055 |
| Ms4a6d | -0.5660047 |
| Ms4a6d | 0.56702125 |
| Ms4a7 | -0.7155101 |
| Ms4a8a | 1.78917699 |
| Msc | -0.3369052 |
| Msh2 | 0.26939108 |
| Msh3 | 1.33120515 |
| Msh3 | -0.3866298 |
| Msh4 | 1.06905971 |
| Msh5 | 0.89695769 |
| Msh5 | -0.627631 |
| Msh6 | 0.39120024 |
| Msh6 | 0.78009324 |
| Msi2 | -0.1545722 |
| Msi2h | 0.60887588 |
| Msln | -0.2522829 |
| Msn | -0.5781615 |
| Msr2 | -0.7163966 |
| Msrb2 | -0.602176 |
| Msrb2 | 3.62315617 |
| Msrb3 | 1.46576878 |
| Mst1r | -0.648837 |
| Msto1 | -0.5912541 |
| Msx1 | 2.91854997 |
| Mt1 | -0.2024046 |
| Mt3 | 0.77669814 |
| Mta1 | 0.44610772 |
| Mta2 | 0.7381401 |
| Mta3 | 1.17985544 |
| Mta3 | 0.73233849 |
| Mtag2 | 2.04137934 |
| Mtap | -0.6688491 |
| Mtap | -0.2147559 |
| Mtap1b | 0.01099445 |
| Mtap1b | -0.3541894 |
| Mtap1b | 1.58040341 |
| Mtap1b | 1.45432258 |
| Mtap1s | -0.6244109 |
| Mtap2 | 1.09359746 |
| Mtap2 | 0.26475453 |
| Mtap2 | -0.2987227 |
| Mtap4 | 0.83094297 |
| Mtap6 | 1.52524689 |
| Mtap6 | -0.682433 |
| Mtap7 | 2.93744794 |
| Mtap7d1 | -0.636544 |
| Mtap7d1 | -0.7315972 |
| Mtap7d2 | -0.6406237 |
| Mtap7d3 | 1.92549994 |
| Mtbp | 0.30300365 |
| Mtbp | 1.96524621 |
| Mtbp | 0.17675677 |
| Mtch1 | 2.71267822 |
| Mtch1 | 0.15435617 |
| Mtch2 | -0.8066549 |
| Mtch2 | -0.1386802 |
| Mtch2 | -0.2970237 |
| Mtcp1 | 0.22648576 |
| Mtdh | 1.24026041 |
| Mterf | 3.969025 |
| Mterf | -0.6422606 |
| Mterfd1 | -0.2320105 |
| Mterfd2 | -0.3711871 |
| Mterfd2 | 2.27029175 |
| Mterfd2 | 3.61792317 |
| Mterfd3 | -0.3298457 |
| Mterfd3 | -0.1320191 |
| Mtf1 | 0.28225217 |
| Mtf1 | 1.86678068 |
| Mtf2 | -0.6471886 |
| Mtfmt | 0.317172 |
| Mtfmt | -0.6140216 |
| Mtg1 | -0.0229677 |
| Mthfd1 | 0.39578913 |
| Mthfd2 | -0.1638464 |
| Mthfs | -0.3181972 |
| Mthfsd | -0.8174641 |
| Mthfsd | -0.0723365 |
| Mtif3 | -0.6045331 |
| Mtl5 | 0.56252798 |
| Mtl5 | 1.72541805 |
| Mtmr1 | -0.3602246 |
| Mtmr11 | -0.6713811 |
| Mtmr11 | -0.7516435 |
| Mtmr12 | 0.04807322 |
| Mtmr14 | 0.47943811 |
| Mtmr14 | 1.78717901 |
| Mtmr14 | -0.4138042 |
| Mtmr14 | -0.7911525 |
| Mtmr2 | -0.5443661 |
| Mtmr2 | -0.691888 |
| Mtmr2 | 0.05740746 |
| Mtmr3 | 0.03349747 |
| Mtmr4 | 0.02070513 |
| Mtmr9 | 0.70083691 |
| Mto1 | 0.22033142 |
| Mtpn | -0.3602246 |
| Mtrf1 | -0.7023129 |
| Mtrf1 | 0.20426979 |
| Mtrf1l | -0.6513152 |
| Mtrr | -0.602176 |
| Mtss1 | -0.6132272 |
| Mttp | 2.16009908 |
| Mtus1 | -0.3066924 |
| Mtus1 | -0.5146055 |
| Mtvr2 | 0.63048155 |
| Mtvr2 | -0.5858408 |
| Mtx1 | 0.23771751 |
| Mtx1 | -0.0503612 |
| Mtx1 | 3.4403901 |
| Mtx2 | -0.0004639 |
| Muc1 | 3.43622814 |
| Muc1 | -0.2168305 |
| Muc1 | -0.4802398 |
| Muc1 | -0.0408537 |
| Muc13 | 0.19128545 |
| Muc5ac | 3.69038559 |
| Mug2 | 0.2409019 |
| Mug2 | 0.75917006 |
| Mug4 | -0.7608998 |
| Mum1 | -0.0455953 |
| Mup1 | 2.37888545 |
| Mup2 | -0.4672477 |
| Mup2 | 1.78061008 |
| Mup3 | 0.37729659 |
| Mupcdh | 0.44201661 |
| Mus81 | -0.5554958 |
| Mus81 | 1.9803967 |
| Mustn1 | -0.6463654 |
| Mustn1 | 3.21986334 |
| Muted | 2.20019095 |
| Muted | 0.84452487 |
| Mutyh | 0.56836503 |
| Mvd | -0.6730728 |
| Mvp | -0.2560658 |
| Mx2 | -0.6756163 |
| Mxd1 | 2.64631143 |
| Mxd4 | -0.8234133 |
| Mxi1 | -0.2752139 |
| Mxra7 | 1.57501698 |
| Mxra8 | 0.86884178 |
| Mxra8 | -0.4286709 |
| Myadm | 1.64237197 |
| Mybbp1a | -0.0966659 |
| Mybbp1a | 0.52531224 |
| Mybbp1a | 1.92987938 |
| Mybl2 | -0.0029377 |
| Mybpc2 | -0.1988327 |
| Mybpc3 | -0.5920301 |
| Myc | -0.622003 |
| Mycbp | 0.5770527 |
| Mycbp2 | -0.7863604 |
| Mycbp2 | -0.2194302 |
| Mycbpap | -0.6513152 |
| Mycl1 | -0.3718 |
| Myd116 | 0.9571345 |
| Myd88 | -0.7181721 |
| Myef2 | -0.2490525 |
| Myg1 | 2.27497472 |
| Myg1 | -0.1216468 |
| Myg1 | 2.51211297 |
| Myh10 | -0.2941989 |
| Myh11 | 1.94765381 |
| Myh8 | 1.86220592 |
| Myh9 | 1.62045616 |
| Myl1 | 1.09359746 |
| Myl2 | -0.5110402 |
| Myl4 | -0.2891357 |
| Myl6 | -0.3860072 |
| Myl6b | -0.5614864 |
| Mylc2b | -0.5117522 |
| Mylc2b | -0.7040618 |
| Mylc2b | -0.6504884 |
| Mylc2pl | -0.2479782 |
| Mylk | 1.64230118 |
| Mylk | 1.0622301 |
| Mynn | -0.5232181 |
| Myo10 | -0.7433906 |
| Myo10 | 1.20258531 |
| Myo15 | 0.09829305 |
| Myo18a | 2.00806769 |
| Myo1b | -0.7721299 |
| Myo1c | -0.588157 |
| Myo1c | 0.53254957 |
| Myo1f | 0.26506435 |
| Myo1g | -0.3286742 |
| Myo3b | -0.3960196 |
| Myo5a | 0.14665095 |
| Myo5b | -0.7315972 |
| Myo6 | -0.6430801 |
| Myo7a | 1.90387752 |
| Myo9a | 0.02872087 |
| Myo9b | -0.6068962 |
| Myo9b | 0.4882588 |
| Myoc | -0.5495435 |
| Myocd | -0.289697 |
| Myocd | 0.99003841 |
| Myom1 | -0.4189484 |
| Myom1 | 4.73966382 |
| Myrip | -0.1415496 |
| Mysm1 | 1.81688472 |
| Myst1 | 2.19738368 |
| Myst2 | -0.4131632 |
| Myst4 | -0.7093282 |
| Myst4 | -0.5046569 |
| Myst4 | 0.09755196 |
| Myt1 | 0.67124585 |
| Myt1l | -0.1589537 |
| Myt1l | -0.036992 |
| Mzf1 | -0.1202413 |
| Mzf1 | -0.6722266 |
| N28178 | -0.264224 |
| N4bp2 | 0.72763336 |
| N6amt1 | -0.729796 |
| N6amt1 | -0.4451036 |
| N6amt2 | -0.2111365 |
| Naalad2 | 0.23452221 |
| Nab1 | -0.396649 |
| Nab1 | -0.5928066 |
| Nab1 | -0.6156124 |
| Nab1 | -0.5311834 |
| Nab2 | 2.2053932 |
| Naca | -0.122585 |
| Naca | -0.645543 |
| Naca | -0.4934155 |
| Nadk | 0.89095852 |
| Nadsyn1 | 0.20724069 |
| Nadsyn1 | -0.0621635 |
| Naga | -0.6996958 |
| Nagk | 1.65886006 |
| Nagk | 0.41655941 |
| Nagk | -0.4976151 |
| Nagk | -0.7288967 |
| Naglu | -0.3112773 |
| Nagpa | 2.89881951 |
| Nags | -0.6970859 |
| Nags | 0.73027116 |
| Naif1 | 1.95152424 |
| Naif1 | -0.6910244 |
| Naip5 | -0.5502854 |
| Nalp10 | -0.6646444 |
| Nampt | -0.1410708 |
| Nanos1 | -0.6100563 |
| Nanos2 | -0.5920301 |
| Nans | 2.3029731 |
| Nap1l1 | -0.5644961 |
| Nap1l1 | -0.4055049 |
| Nap1l3 | -0.0898439 |
| Nap1l4 | -0.1449084 |
| Nap1l5 | -0.5607354 |
| Napa | 3.40398407 |
| Napa | 0.04297792 |
| Napa | 1.87927429 |
| Napb | -0.2225594 |
| Napepld | -0.2194302 |
| Napg | 0.87627243 |
| Napg | 0.02191189 |
| Naprt1 | 0.37123438 |
| Naprt1 | -0.4906261 |
| Napsa | -0.0776885 |
| Napsa | -0.311852 |
| Napsa | 0.80647154 |
| Narf | -0.4424549 |
| Narfl | 2.12189762 |
| Narg1 | -0.1555441 |
| Narg1 | -0.5607354 |
| Narg1l | 1.29113068 |
| Narg1l | -0.5196198 |
| Narg1l | 2.02602245 |
| Narg2 | -0.3493893 |
| Nars | 0.20625144 |
| Nars2 | -0.0839715 |
| Nasp | -0.5362879 |
| Nasp | -0.182676 |
| Nasp | -0.737929 |
| Nasp | -0.5720633 |
| Nat1 | -0.737929 |
| Nat10 | -0.2346529 |
| Nat14 | 1.13351912 |
| Nat2 | -0.6430801 |
| Nat5 | -0.627631 |
| Nat5 | -0.5290043 |
| Nat6 | 1.72074382 |
| Nat6 | 3.00296414 |
| Nav1 | 1.22033424 |
| Nav1 | -0.7181721 |
| Nbl1 | 0.3990148 |
| Nbr1 | 0.97351407 |
| Nbr1 | 0.55936976 |
| Ncald | -0.2576917 |
| Ncam1 | -0.0045905 |
| Ncam1 | 0.46704714 |
| Ncam2 | 0.29072878 |
| Ncan | 1.194727 |
| Ncapd2 | 1.94402345 |
| Ncapd2 | 0.09903354 |
| Ncapd2 | 0.33055103 |
| Ncapd3 | 1.53514562 |
| Ncapg2 | -0.4658906 |
| Ncapg2 | 2.75996624 |
| Ncaph | 0.76860748 |
| Ncaph2 | 1.17973923 |
| Ncaph2 | -0.588157 |
| Ncaph2 | -0.602961 |
| Ncbp2 | 0.65588705 |
| Ncbp2 | 0.17505261 |
| Ncdn | 1.14166575 |
| Ncdn | -0.5232181 |
| Ncdn | -0.7470495 |
| Ncdn | -0.6832886 |
| Ncf4 | -0.4504238 |
| Nck1 | 0.11735751 |
| Nckap1 | 0.17709723 |
| Nckap1l | 0.72005544 |
| Nckipsd | 0.96257748 |
| Ncl | -0.6140216 |
| Ncl | -0.396649 |
| Ncoa2 | 1.13534919 |
| Ncoa2 | 3.27658893 |
| Ncoa4 | 0.13068926 |
| Ncoa5 | 0.72157595 |
| Ncoa6 | -0.4892346 |
| Ncoa6 | -0.6496624 |
| Ncoa6 | -0.6076852 |
| Ncor1 | -0.7155101 |
| Ncstn | 2.577228 |
| Ncstn | 0.78080642 |
| Ncstn | 2.02625702 |
| Ndc80 | -0.729796 |
| Nde1 | 1.51296793 |
| Nde1 | 1.06394219 |
| Ndel1 | 3.37314741 |
| Ndfip1 | 1.58183959 |
| Ndfip2 | -0.6798709 |
| Ndg2 | -0.4809287 |
| Ndn | -0.2018935 |
| Ndor1 | -0.6430801 |
| Ndor1 | 2.16156099 |
| Ndor1 | -0.5480613 |
| Ndrg1 | -0.5261067 |
| Ndrg2 | -0.2908204 |
| Ndrg3 | -0.1263472 |
| Ndrg4 | -0.5622379 |
| Ndrl | 1.96384293 |
| Ndst1 | -0.3130025 |
| Ndst1 | -0.4638586 |
| Ndst4 | -0.3440185 |
| Ndufa1 | -0.4899301 |
| Ndufa10 | 1.43657675 |
| Ndufa12 | 1.1516602 |
| Ndufa13 | -0.666324 |
| Ndufa2 | 1.82570814 |
| Ndufa3 | 0.17641619 |
| Ndufa4 | -0.3199353 |
| Ndufa5 | 2.60614351 |
| Ndufa5 | 0.62122275 |
| Ndufa6 | -0.1439475 |
| Ndufa7 | 0.45195743 |
| Ndufa8 | -0.6841449 |
| Ndufa8 | -0.3903744 |
| Ndufa9 | 0.94943084 |
| Ndufaf1 | 1.92424489 |
| Ndufb10 | -0.3124271 |
| Ndufb11 | -0.7361155 |
| Ndufb2 | 0.06434735 |
| Ndufb3 | -0.7217333 |
| Ndufb4 | 0.36122864 |
| Ndufb5 | -0.7244131 |
| Ndufb6 | 0.61208474 |
| Ndufb6 | 1.81653239 |
| Ndufb7 | 0.92486221 |
| Ndufb8 | 1.0327693 |
| Ndufb9 | -0.4645354 |
| Ndufc1 | -0.0812736 |
| Ndufc1 | -0.2262234 |
| Ndufc2 | 0.90401245 |
| Ndufs2 | 0.41550544 |
| Ndufs2 | 1.05069064 |
| Ndufs3 | 2.61661665 |
| Ndufs4 | 1.59986712 |
| Ndufs4 | -0.6671649 |
| Ndufs4 | -0.7075694 |
| Ndufs4 | 1.0052949 |
| Ndufs6 | 1.94918353 |
| Ndufs7 | 0.98518335 |
| Ndufs8 | -0.6471886 |
| Ndufv1 | -0.4969139 |
| Ndufv2 | 1.06117494 |
| Necab1 | 3.1386818 |
| Necab2 | 0.98804233 |
| Necab3 | 2.38026669 |
| Necap1 | 0.25039039 |
| Necap1 | 0.27000755 |
| Necap1 | 1.72438136 |
| Necap2 | -0.6236076 |
| Nedd1 | 2.45839898 |
| Nedd4 | 0.81613136 |
| Nedd4l | 0.11663142 |
| Nedd8 | -0.551028 |
| Nedd9 | -0.3263358 |
| Nedd9 | -0.6076852 |
| Nefm | -0.5920301 |
| Negr1 | -0.423473 |
| Negr1 | 1.24914954 |
| Neil1 | -0.6546297 |
| Neil2 | 1.42082346 |
| Nek11 | 0.85215092 |
| Nek3 | -0.4138042 |
| Nek3 | -0.5495435 |
| Nek3 | 2.58743782 |
| Nek4 | 0.70722289 |
| Nek5 | 1.55073711 |
| Nek6 | -0.5835304 |
| Nek8 | -0.588157 |
| Nek9 | 1.03358523 |
| Nek9 | 1.55680838 |
| Nelf | -0.5540042 |
| Nelf | 0.02872087 |
| Nell2 | -0.4398136 |
| Nell2 | -0.535557 |
| Nell2 | -0.6300534 |
| Nell2 | 1.21710172 |
| Nenf | -0.682433 |
| Neo1 | -0.2625867 |
| Nes | 1.53997689 |
| Net1 | -0.7361155 |
| Net1 | -0.5124647 |
| Neto1 | 0.50349716 |
| Neto1 | -0.7452183 |
| Neto1 | 2.0567146 |
| Neu1 | -0.3547912 |
| Neu2 | -0.3860072 |
| Neu3 | -0.4345534 |
| Neu4 | 1.40835401 |
| Neu4 | -0.6850021 |
| Neu4 | 1.34425469 |
| Neurl | -0.752565 |
| Neurod1 | 0.63131872 |
| Neurod2 | 1.79664654 |
| Neurod4 | -0.6841449 |
| Neurog1 | 1.01046481 |
| Neurog2 | 2.20275688 |
| Nf1 | -0.5667598 |
| Nfasc | -0.6596236 |
| Nfasc | 2.78874759 |
| Nfasc | -0.6858601 |
| Nfat5 | 1.05707488 |
| Nfat5 | 0.0465084 |
| Nfatc1 | -0.4176597 |
| Nfatc1 | 0.56230275 |
| Nfatc2 | 0.14524314 |
| Nfatc2 | 1.40962665 |
| Nfatc2ip | -0.4598078 |
| Nfatc3 | -0.7306962 |
| Nfatc4 | -0.5089076 |
| Nfe2 | 1.45284935 |
| Nfe2l1 | -0.2992897 |
| Nfe2l2 | -0.3170403 |
| Nfe2l3 | -0.2802018 |
| Nfib | 1.17904164 |
| Nfic | 0.11844559 |
| Nfic | 0.6522121 |
| Nfic | -0.5842999 |
| Nfix | -0.3245859 |
| Nfix | 0.13211919 |
| Nfkb1 | -0.2598641 |
| Nfkb2 | -0.5103288 |
| Nfkbia | -0.5495435 |
| Nfkbib | 0.68950605 |
| Nfkbid | 0.96900914 |
| Nfkbie | 2.07095267 |
| Nfkbil1 | -0.3061209 |
| Nfkbil2 | 2.03675237 |
| Nfkbiz | 0.7989857 |
| Nfrkb | -0.3590144 |
| Nfrkb | 1.15022182 |
| Nfs1 | 0.64728955 |
| Nfs1 | 2.11009536 |
| Nfs1 | 0.4267737 |
| Nfu1 | -0.0612842 |
| Nfx1 | -0.7049375 |
| Nfya | 1.30556159 |
| Nfyb | -0.1221158 |
| Nfyc | 1.25076648 |
| Ngb | 0.84152371 |
| Ngb | 0.4542365 |
| Ngef | 1.43153263 |
| Ngfb | -0.310703 |
| Ngfb | -0.3736409 |
| Ngfr | -0.7334019 |
| Ngfrap1 | -0.5652501 |
| Ngp | -0.4990192 |
| Nhedc1 | -0.0045905 |
| Nhej1 | -0.1846804 |
| Nhlrc1 | 0.6986985 |
| Nhlrc2 | -0.4829982 |
| Nhlrc2 | -0.8304023 |
| Nhp2l1 | 1.41938624 |
| Nhsl1 | -0.5160354 |
| Niban | -0.6962175 |
| Nicn1 | -0.4530953 |
| Nicn1 | 3.10820335 |
| Nid1 | 0.113357 |
| Nid2 | 0.30922879 |
| Nif3l1 | -0.7534874 |
| Nin | 0.07996482 |
| Ninj1 | -0.3841418 |
| Ninj2 | -0.9131363 |
| Nip7 | -0.2283237 |
| Nipa1 | 1.16450676 |
| Nipbl | -0.5167512 |
| Nipbl | 3.29640886 |
| Nipsnap1 | -0.1821756 |
| Nipsnap3a | -0.8557993 |
| Nipsnap3a | 0.27400471 |
| Nisch | 0.74816077 |
| Nit1 | 0.23003033 |
| Nit2 | -0.3286742 |
| Nit2 | -0.5866122 |
| Nkain3 | -0.0772413 |
| Nkain4 | -0.8979295 |
| Nkd2 | -0.6140216 |
| Nkg7 | 0.22260353 |
| Nkiras1 | -0.7979031 |
| Nkiras2 | 1.88720289 |
| Nkrf | 0.87562863 |
| Nktr | 0.01626694 |
| Nkx1-2 | 0.76752341 |
| Nkx2-1 | -0.5261067 |
| Nkx2-2 | -0.8066549 |
| Nkx2-2 | -0.2121692 |
| Nkx2-3 | -0.8968527 |
| Nkx2-4 | 1.10121405 |
| Nkx2-6 | 2.87660976 |
| Nkx3-1 | -0.2757668 |
| Nkx6-1 | 2.09199795 |
| Nle1 | 0.60371865 |
| Nlgn1 | 0.8135537 |
| Nlgn2 | 0.60823279 |
| Nlk | -0.3650809 |
| Nln | -0.381041 |
| Nln | -0.5046569 |
| Nln | -0.8076324 |
| Nlrp14 | 0.35646615 |
| Nlrp4a | -0.2735571 |
| Nlrp4c | 2.31863716 |
| Nlrp5 | -0.2479782 |
| Nlrp5 | 1.76124457 |
| Nlrp5 | -0.4836891 |
| Nlrp9a | -0.4176597 |
| Nlrp9c | 0.74982047 |
| Nlrx1 | -0.2277982 |
| Nlrx1 | 0.17505261 |
| Nmb | 0.45145021 |
| Nmbr | -0.4325884 |
| Nmd3 | -0.9087662 |
| Nme1 | 0.53603145 |
| Nme2 | 1.83046494 |
| Nme2 | -0.7608998 |
| Nme2 | -0.2383648 |
| Nme3 | 1.23665904 |
| Nme4 | 2.43035124 |
| Nme4 | 0.29912908 |
| Nme4 | -0.8914874 |
| Nme5 | 0.62460023 |
| Nme7 | -0.2941989 |
| Nme7 | 0.75807496 |
| Nme7 | -0.7693097 |
| Nme7 | 2.35274179 |
| Nme7 | 1.0204438 |
| Nmnat1 | 0.61677118 |
| Nmnat2 | -0.2713527 |
| Nmnat3 | 1.26211366 |
| Nmral1 | 0.08373463 |
| Nmt1 | -0.214238 |
| Nmu | -0.6867188 |
| Nmur2 | 0.05158451 |
| Nnat | -0.4163728 |
| Nnat | -0.851689 |
| Nnmt | 0.05624577 |
| Nnmt | -0.7226257 |
| Nob1 | -0.4598078 |
| Nob1 | 1.10914511 |
| Nobox | 0.51280721 |
| Noc3l | -0.4150876 |
| Noc4l | -0.341048 |
| Noc4l | -0.7488844 |
| Nod1 | 0.66197997 |
| Nog | -0.6970859 |
| Nol1 | 1.74642036 |
| Nol1 | -0.5804587 |
| Nol1 | 2.656465 |
| Nol10 | 0.21935596 |
| Nol11 | -0.4215312 |
| Nol12 | 1.92644013 |
| Nol14 | 3.34452834 |
| Nol3 | -0.5138913 |
| Nol4 | 1.66696165 |
| Nol4 | 0.57104678 |
| Nol4 | 0.66177751 |
| Nol5 | 1.79556595 |
| Nol5a | 3.32826312 |
| Nol6 | -0.017081 |
| Nol7 | -0.7208417 |
| Nol9 | -0.5951401 |
| Nola1 | 3.14987692 |
| Nola2 | -0.2947632 |
| Nola3 | -0.6260195 |
| Nola3 | 0.49433559 |
| Nolc1 | 1.17566258 |
| Nomo1 | -0.4293227 |
| Nono | 4.07719571 |
| Nope | -0.455104 |
| Nos1 | 1.24232764 |
| Nos1ap | -0.481618 |
| Nos2 | 1.79376219 |
| Nos3 | -0.0477586 |
| Nos3 | -0.6841449 |
| Nos3 | -0.6562913 |
| Nosip | 0.20228392 |
| Nosip | -0.4638586 |
| Nosip | -0.1076861 |
| Nostrin | 0.59831605 |
| Notch1 | -0.6892998 |
| Notch3 | 0.73102345 |
| Notch4 | -0.4345534 |
| Notch4 | 0.94465832 |
| Notum | -0.6815782 |
| Nova2 | 1.1298482 |
| Nox4 | 0.80543061 |
| Noxo1 | -0.5384841 |
| Noxo1 | 2.52476258 |
| Npal2 | 0.60651569 |
| Npas1 | 0.71624327 |
| Npas2 | -0.3199353 |
| Npas4 | -0.0179197 |
| Npb | -0.4391545 |
| Npc2 | 1.48506997 |
| Npcd | 1.30759057 |
| Npcd | -0.7911525 |
| Npdc1 | 0.17743756 |
| Npepl1 | 2.72144813 |
| Npff | -0.5766332 |
| Nphp1 | -0.5074885 |
| Nphp1 | -0.7253081 |
| Nphp3 | -0.3199353 |
| Nphp4 | -0.8425066 |
| Nphs2 | -0.6756163 |
| Nploc4 | 0.79073422 |
| Npm1 | -0.4969139 |
| Npm2 | -0.3304319 |
| Npm3 | -0.4686068 |
| Npm3 | 0.72970654 |
| Npm3-ps1 | 0.97684243 |
| Npr1 | 2.1703651 |
| Npr2 | -0.4740631 |
| Npr3 | -0.3434236 |
| Npsr1 | -0.4631822 |
| Nptn | -0.4997221 |
| Nptx1 | -0.5117522 |
| Nptx2 | 0.36206656 |
| Nptxr | 2.15361475 |
| Npy | 0.04807322 |
| Npy1r | -0.5340969 |
| Npy2r | -0.0652479 |
| Npy5r | -0.1053794 |
| Nqo1 | -0.5547497 |
| Nqo2 | 1.21486586 |
| Nqo2 | -0.5928066 |
| Nqo2 | -0.4941141 |
| Nr1d1 | 0.41365812 |
| Nr1d2 | -0.4885396 |
| Nr1h2 | -0.7693097 |
| Nr1h2 | 0.03230561 |
| Nr1h3 | -0.5232181 |
| Nr1h4 | -0.7693097 |
| Nr1h5 | 0.44585255 |
| Nr1i2 | -0.3434236 |
| Nr2c1 | 2.25815122 |
| Nr2c2ap | 1.4726716 |
| Nr2e1 | 3.67758382 |
| Nr2f2 | -0.7279982 |
| Nr2f2 | -0.3835208 |
| Nr2f6 | 0.21511715 |
| Nr2f6 | 0.8488429 |
| Nr4a2 | 0.64934377 |
| Nr4a3 | 0.81887304 |
| Nr4a3 | -0.2304286 |
| Nr5a1 | -0.535557 |
| Nr5a2 | 1.01602186 |
| Nr5a2 | 2.50078574 |
| Nr5a2 | 0.28528838 |
| Nr6a1 | -0.2293756 |
| Nradd | 0.39981948 |
| Nrap | -0.3614363 |
| Nrarp | 0.35758892 |
| Nras | -0.4652127 |
| Nrbf2 | -0.636544 |
| Nrbp1 | -0.186689 |
| Nrbp2 | 3.48978786 |
| Nrcam | 1.2615815 |
| Nrd1 | -0.5928066 |
| Nrf1 | -0.7730719 |
| Nrg1 | -0.2609522 |
| Nrg3 | -0.7863604 |
| Nrg4 | 0.16923508 |
| Nrgn | 1.56837402 |
| Nrip2 | -0.4029661 |
| Nrip3 | 0.95506669 |
| Nrl | -0.3553933 |
| Nrm | 0.045725 |
| Nrn1 | -0.7190611 |
| Nrn1l | -0.6045331 |
| Nrp1 | -0.3878764 |
| Nrp1 | 1.19025589 |
| Nrp2 | 0.19829946 |
| Nrp2 | -0.4990192 |
| Nrsn2 | -0.3316055 |
| Nrsn2 | -0.080376 |
| Nrtn | -0.0538427 |
| Nrxn1 | 0.43970745 |
| Nrxn1 | 3.18558035 |
| Nrxn1 | 1.18460781 |
| Nrxn2 | 1.01712931 |
| Nrxn3 | -0.3644725 |
| Nrxn3 | -0.2299019 |
| Nsbp1 | 0.35167922 |
| Nsd1 | -0.4470952 |
| Nsd1 | -0.5110402 |
| Nsdhl | -0.6781667 |
| Nsdhl | 4.66371846 |
| Nsdhl | -0.6252148 |
| Nsf | -0.6300534 |
| Nsfl1c | -0.5089076 |
| Nsg1 | 0.71279895 |
| Nsg2 | 0.12242439 |
| Nsl1 | -0.0665729 |
| Nsmaf | -0.6910244 |
| Nsmaf | -0.6357302 |
| Nsmce1 | -0.5835304 |
| Nsmce2 | -0.3903744 |
| Nsmce2 | 2.26754309 |
| Nsmce2 | -0.3446137 |
| Nsun2 | -0.4672477 |
| Nsun4 | -0.5081978 |
| Nsun5 | 2.39542162 |
| Nsun5 | 0.54019262 |
| Nsun5 | -0.6252148 |
| Nsun5 | -0.6196014 |
| Nt5c | -0.6875784 |
| Nt5c1b | -0.5866122 |
| Nt5c2 | 0.91163412 |
| Nt5c2 | -0.4604817 |
| Nt5c2 | -0.5982606 |
| Nt5c3 | 0.69186168 |
| Nt5c3l | -0.4319344 |
| Nt5c3l | -0.8047029 |
| Nt5dc3 | 0.06741526 |
| Nt5dc3 | -0.6927523 |
| Nt5dc3 | 1.63669186 |
| Nt5e | -0.648837 |
| Nt5e | -0.6300534 |
| Nt5m | -0.5735841 |
| Nt5m | 0.52741919 |
| Ntan1 | -0.3742553 |
| Ntf3 | -0.0568996 |
| Nthl1 | 0.2006258 |
| Ntn2l | -0.5540042 |
| Ntn2l | -0.0037637 |
| Ntn4 | -0.2163114 |
| Ntng1 | -0.5804587 |
| Ntng1 | -0.4036001 |
| Ntng1 | 0.81904413 |
| Ntrk2 | 0.04846402 |
| Ntrk2 | -0.1468332 |
| Ntrk2 | -0.4398136 |
| Ntrk3 | 1.41243847 |
| Ntrk3 | 1.3622992 |
| Ntrk3 | 0.52507784 |
| Nts | 0.51209434 |
| Ntsr1 | -0.3112773 |
| Ntsr2 | 2.86392824 |
| Nuak1 | -0.6140216 |
| Nuak1 | -0.5046569 |
| Nuak2 | 1.40744391 |
| Nub1 | -0.0608448 |
| Nubp1 | -0.1141751 |
| Nubp2 | 0.04337086 |
| Nubp2 | 0.99316652 |
| Nubp2 | 1.38747306 |
| Nucb1 | 0.31834304 |
| Nucb1 | 0.33802909 |
| Nucks1 | -0.0137339 |
| Nudc | -0.6381738 |
| Nudc | -0.3699627 |
| Nudcd2 | -0.2458333 |
| Nudcd3 | 1.20699786 |
| Nudc-ps1 | 0.95136273 |
| Nudt1 | 0.72404152 |
| Nudt10 | 0.688326 |
| Nudt11 | 1.21944363 |
| Nudt12 | 0.67284796 |
| Nudt13 | 1.04076188 |
| Nudt14 | 0.78009324 |
| Nudt16l1 | -0.568272 |
| Nudt18 | 1.26847595 |
| Nudt19 | -0.5920301 |
| Nudt19 | 0.71662519 |
| Nudt2 | -0.227273 |
| Nudt21 | -0.4524267 |
| Nudt22 | 0.57549931 |
| Nudt4 | 1.42868885 |
| Nudt5 | 1.33347493 |
| Nudt5 | -0.1272901 |
| Nudt6 | 0.50469596 |
| Nudt7 | -0.4913227 |
| Nudt8 | -0.2506663 |
| Nudt9 | -0.5690291 |
| Nufip1 | -0.6927523 |
| Numa1 | -0.007905 |
| Numb | 0.4577707 |
| Numb | -0.0648067 |
| Numb | -0.5959193 |
| Numb | 1.07427946 |
| Numbl | -0.0674573 |
| Nup107 | -0.2367722 |
| Nup133 | -0.3935064 |
| Nup133 | 1.59704764 |
| Nup155 | 0.92516762 |
| Nup155 | 0.08072 |
| Nup160 | -0.4080507 |
| Nup210 | 0.16820468 |
| Nup37 | 2.17285392 |
| Nup43 | 2.08524099 |
| Nup50 | -0.6148167 |
| Nup50 | -0.2631321 |
| Nup50 | 1.44579775 |
| Nup62 | 1.35673975 |
| Nup85 | -0.3130025 |
| Nup88 | 2.18071818 |
| Nup88 | -0.602961 |
| Nup88 | -0.1367722 |
| Nup93 | -0.4990192 |
| Nup98 | 1.03562197 |
| Nup98 | -0.1663023 |
| Nupl1 | -0.2178695 |
| Nupl1 | -0.7146245 |
| Nupl1 | -0.6180038 |
| Nupl2 | -0.5428922 |
| Nupr1 | 0.20954487 |
| Nus1 | 2.1353487 |
| Nusap1 | -0.0434371 |
| Nut | 0.67783689 |
| Nutf2 | -0.3369052 |
| Nvl | -0.57587 |
| Nwd1 | 0.36541075 |
| Nxf1 | -0.6927523 |
| Nxf2 | -0.7749587 |
| Nxf3 | -0.2653172 |
| Nxph1 | 1.72477025 |
| Nxph2 | -0.1747014 |
| Nxph3 | 1.80607836 |
| Nxt1 | 0.37178712 |
| Nxt1 | 0.96272414 |
| Nxt2 | -0.0652479 |
| Nyx | -0.4176597 |
| Oaf | -0.3835208 |
| Oas1b | 0.73645948 |
| Oas1b | -0.532639 |
| Oas1c | -0.3699627 |
| Oas1d | 0.30745442 |
| Oas1d | 0.08598922 |
| Oas1e | 0.49892762 |
| Oas1g | -0.5458424 |
| Oas1g | 1.25838218 |
| Oas2 | 0.12494754 |
| Oasl1 | 0.66278936 |
| Oasl2 | 0.065499 |
| Oasl2 | -0.535557 |
| Oat | 0.25101954 |
| Oaz2 | -0.1053794 |
| Oaz3 | -0.726204 |
| Obfc1 | 0.2409019 |
| Obfc2a | -0.535557 |
| Obfc2b | -0.4036001 |
| Obfc2b | 1.32406385 |
| Obox1 | -0.5246613 |
| Obox5 | -0.622805 |
| Obrgrp | 0.59462433 |
| Obsl1 | -0.2256991 |
| Oc90 | -0.6562913 |
| Oca2 | -0.067015 |
| Ociad1 | -0.3228392 |
| Ociad1 | 0.28377151 |
| Ociad2 | -0.7226257 |
| Ocln | 1.12579342 |
| Ocrl | 0.05702039 |
| Odc1 | 2.4802801 |
| Odf1 | -0.5614864 |
| Odf2 | -0.6439004 |
| Odf2 | -0.2533621 |
| Odf2 | -0.5174675 |
| Odf2 | -0.0442998 |
| Odf3 | -0.5735841 |
| Odf3l1 | -0.3565988 |
| Odf4 | 2.88770619 |
| Odf4 | -0.1952744 |
| Odz1 | -0.744304 |
| Odz3 | -0.1329678 |
| Odz3 | -0.0781359 |
| Odz4 | -0.0902971 |
| Og9x | -0.1682718 |
| Ogdh | -0.6571233 |
| Ogfod1 | -0.7507229 |
| Ogfod2 | 0.68339299 |
| Ogfr | 1.23993358 |
| Ogfrl1 | -0.7066913 |
| Ogg1 | 0.06779804 |
| Ogn | 2.42800592 |
| Ogn | 0.55710733 |
| Ogn | -0.6244109 |
| Ogt | -0.4061407 |
| Ogt | 0.27431147 |
| Ogt | 1.06748808 |
| Oit1 | 1.126655 |
| Oit3 | -0.545104 |
| Ola1 | 0.73383914 |
| Olfm1 | 1.14866107 |
| Olfm1 | -0.5873843 |
| Olfm2 | -0.6236076 |
| Olfm2 | 0.56297828 |
| Olfm3 | 0.29853156 |
| Olfm3 | 0.01180754 |
| Olfm4 | 2.09230392 |
| Olfml1 | -0.4795515 |
| Olfml2b | -0.5103288 |
| Olfml3 | 1.38346445 |
| Olfr100 | -0.2256991 |
| Olfr1006 | 1.98123396 |
| Olfr1012 | -0.4504238 |
| Olfr1020 | -0.5607354 |
| Olfr1065 | 1.18206077 |
| Olfr1094 | 0.70857189 |
| Olfr1099 | -0.5614864 |
| Olfr1105 | 3.35772679 |
| Olfr1112 | 1.64003311 |
| Olfr1113 | -0.1339174 |
| Olfr1189 | 0.01464781 |
| Olfr1197 | -0.3572021 |
| Olfr1198 | 1.55572157 |
| Olfr1249 | -0.3351353 |
| Olfr1280 | -0.4990192 |
| Olfr133 | -0.0547151 |
| Olfr1330 | -0.5796923 |
| Olfr134 | 0.17334533 |
| Olfr1340 | -0.3903744 |
| Olfr1384 | 3.34810718 |
| Olfr1386 | -0.755335 |
| Olfr1390 | -0.1463516 |
| Olfr1394 | -0.6996958 |
| Olfr1412 | 2.1486909 |
| Olfr1437 | 0.69928219 |
| Olfr144 | 1.67362541 |
| Olfr1443 | 1.37228022 |
| Olfr1443 | -0.6045331 |
| Olfr1459 | -0.3847632 |
| Olfr1500 | 3.70012455 |
| Olfr1508 | -0.5311834 |
| Olfr166 | -0.6612942 |
| Olfr168 | 1.48716224 |
| Olfr206 | 1.06104296 |
| Olfr213 | -0.2293756 |
| Olfr215 | 0.02030253 |
| Olfr232 | 0.13817204 |
| Olfr257 | -0.2024046 |
| Olfr267 | -0.4444407 |
| Olfr272 | -0.1263472 |
| Olfr303 | 2.28540849 |
| Olfr310 | 3.8369311 |
| Olfr352 | -0.6832886 |
| Olfr371 | 1.21486586 |
| Olfr378 | 1.12456122 |
| Olfr419 | 1.19529867 |
| Olfr437 | -0.6875784 |
| Olfr464 | -0.5502854 |
| Olfr47 | 1.07075934 |
| Olfr49 | -0.7930763 |
| Olfr503 | -0.7208417 |
| Olfr51 | 2.59374035 |
| Olfr536 | 1.81764764 |
| Olfr538 | -0.6867188 |
| Olfr54 | -0.7406558 |
| Olfr550 | 0.37866889 |
| Olfr574 | -0.6164089 |
| Olfr591 | 3.29351866 |
| Olfr593 | -0.212686 |
| Olfr608 | 1.81488642 |
| Olfr638 | -0.4625064 |
| Olfr65 | 2.01625887 |
| Olfr655 | 1.20519011 |
| Olfr658 | -0.4638586 |
| Olfr665 | -0.2774275 |
| Olfr675 | 1.23040556 |
| Olfr691 | 0.5177821 |
| Olfr692 | 0.82280014 |
| Olfr702 | 0.40115908 |
| Olfr724 | -0.1118524 |
| Olfr725 | -0.3724132 |
| Olfr748 | -0.0175003 |
| Olfr786 | 0.57616537 |
| Olfr802 | 1.26498206 |
| Olfr824 | 0.14629919 |
| Olfr91 | 1.3657333 |
| Olfr910 | 2.52349705 |
| Olfr920 | -0.7146245 |
| Olfr924 | 1.92272612 |
| Olfr952 | 1.42824373 |
| Olfr965 | -0.0948418 |
| Olfr982 | -0.1099984 |
| Olfr983 | 0.32939523 |
| Olfr984 | -0.5804587 |
| Olig1 | -0.3547912 |
| Olig3 | -0.7075694 |
| Oma1 | -0.422178 |
| Omd | 0.3041926 |
| Omg | -0.1957819 |
| Omt2b | -0.739746 |
| Onecut2 | -0.6204013 |
| Onecut3 | -0.813519 |
| Onecut3 | 1.70730554 |
| Oog4 | -0.2947632 |
| Oosp1 | -0.3590144 |
| Opa1 | -0.0395647 |
| Opa1 | 1.09219432 |
| Ophn1 | 1.35770064 |
| Oplah | 1.7303911 |
| Opn1mw | -0.0387063 |
| Opn3 | -0.5290043 |
| Oprl1 | 2.7267992 |
| Oprl1 | -0.7488844 |
| Oprl1 | 1.1504617 |
| Oprs1 | -0.5637428 |
| Optc | -0.0293162 |
| Optn | -0.5399512 |
| Optn | 0.25509869 |
| Orai1 | -0.5210575 |
| Orai3 | -0.6496624 |
| Oraov1 | 0.21870509 |
| Orc2l | 1.33169905 |
| Orc3l | -0.7306962 |
| Orc5l | -0.5827615 |
| Orc5l | -0.7306962 |
| Orc6l | 1.81311966 |
| ORF19 | 2.06169558 |
| ORF28 | -0.7244131 |
| ORF34 | -0.0074901 |
| ORF61 | 0.44124753 |
| ORF63 | 0.22777625 |
| ORF9 | -0.1727183 |
| ORF9 | -0.1962897 |
| ORF9 | -0.7406558 |
| Orm1 | 1.22974485 |
| Ormdl2 | 0.0788309 |
| Ormdl3 | 0.61400476 |
| Os9 | -0.6447213 |
| Osbp2 | -0.0767943 |
| Osbpl10 | 2.71669308 |
| Osbpl11 | 0.84867719 |
| Osbpl2 | -0.2383648 |
| Osbpl3 | 0.10530416 |
| Osbpl3 | 0.28891884 |
| Osbpl6 | 2.44460034 |
| Osbpl8 | -0.186689 |
| Osbpl9 | -0.5705449 |
| Oscar | -0.3158849 |
| Osgep | 0.60479543 |
| Osgepl1 | 1.35394755 |
| Osgin1 | 0.99798032 |
| Osm | -0.7882743 |
| Osmr | 1.79442397 |
| Osr1 | 2.06187621 |
| Osr2 | -0.2958927 |
| Ostf1 | 2.9117289 |
| Ostm1 | -0.703187 |
| Otof | 0.81148643 |
| Otop1 | 0.11772035 |
| Otop2 | -0.4074136 |
| Ott | 0.82806717 |
| Ott | 2.14345136 |
| OTTMUSG00000000421 | 1.71303227 |
| OTTMUSG00000000971 | -0.1405922 |
| OTTMUSG00000000990 | 1.19071546 |
| OTTMUSG00000001305 | -0.2479782 |
| OTTMUSG00000002038 | 1.07791598 |
| OTTMUSG00000004461 | -0.5804587 |
| OTTMUSG00000004551 | -0.7433906 |
| OTTMUSG00000005148 | 2.56211018 |
| OTTMUSG00000005491 | -0.422178 |
| OTTMUSG00000008540 | 1.52677046 |
| OTTMUSG00000010009 | 2.65294897 |
| OTTMUSG00000010328 | 1.78018272 |
| OTTMUSG00000015762 | -0.622805 |
| OTTMUSG00000015859 | 1.17051225 |
| OTTMUSG00000016293 | -0.1692581 |
| OTTMUSG00000016790 | -0.697955 |
| OTTMUSG00000016790 | 1.0546859 |
| OTTMUSG00000017677 | -0.5835304 |
| OTTMUSG00000020946 | 0.49433559 |
| Otub1 | -0.4631822 |
| Otub2 | -0.2003618 |
| Otub2 | -0.7424781 |
| Otub2 | -0.6638057 |
| Otud5 | -0.2330665 |
| Otud6b | 0.47672618 |
| Otud7b | 0.24249003 |
| Otud7b | 1.5624549 |
| Otx1 | -0.4131632 |
| Otx2 | -0.4176597 |
| Ovca2 | -0.7979031 |
| Ovgp1 | 1.60952213 |
| Ovgp1 | 0.33141695 |
| Ovol1 | -0.4969139 |
| Ovol2 | 1.08501814 |
| Ovol2 | 0.2619615 |
| Oxct1 | -0.4699679 |
| Oxct2a | -0.7155101 |
| Oxct2b | 2.06426621 |
| Oxgr1 | 1.91239594 |
| Oxnad1 | -0.752565 |
| Oxr1 | 0.01788327 |
| Oxr1 | -0.8872173 |
| Oxt | -0.6781667 |
| P140 | -0.0652479 |
| P2rx2 | -0.3584099 |
| P2rx3 | 2.64681813 |
| P2rx4 | 0.33400989 |
| P2rx4 | -0.648837 |
| P2rx5 | 0.33458513 |
| P2rx7 | -0.7740148 |
| P2rx7 | 2.86147844 |
| P2ry1 | 1.25442119 |
| P2ry12 | -0.58507 |
| P2ry13 | 2.76601478 |
| P2ry14 | -0.6381738 |
| P2ry14 | -0.5667598 |
| P2ry5 | -0.6781667 |
| P2ry6 | 0.41312965 |
| P42pop | 0.17505261 |
| P4ha1 | 0.29223439 |
| P4ha2 | -0.2085598 |
| P4hb | 1.23589332 |
| Pa2g4 | -0.3798035 |
| Pabpc1 | 1.48473477 |
| Pabpc1 | -0.7146245 |
| Pabpc1 | 0.13959056 |
| Pabpc2 | 0.08486261 |
| Pabpc4 | -0.3112773 |
| Pabpn1 | -0.1268186 |
| Pabpnl1 | 0.41021766 |
| Pacrg | 1.95477114 |
| Pacs1 | -0.3187762 |
| Pacs2 | 1.74553351 |
| Pacsin1 | 0.19262552 |
| Pacsin1 | 2.35998658 |
| Pacsin1 | -0.2829845 |
| Pacsin2 | 1.91562322 |
| Pacsin2 | -0.6901617 |
| Pacsin3 | 0.39498096 |
| Pacsin3 | 0.7779504 |
| Padi1 | 1.75033772 |
| Padi2 | -0.2925081 |
| Padi6 | 0.14171429 |
| Paf1 | -0.4990192 |
| Paf1 | 0.5665729 |
| Pafah1b1 | -0.2039396 |
| Pafah1b1 | 0.09234782 |
| Pafah1b3 | -0.293635 |
| Pafah2 | -0.0412838 |
| Pafah2 | -0.7119726 |
| Paip1 | 0.07390126 |
| Paip2 | 1.86119869 |
| Paip2b | -0.8274006 |
| Paip2b | 0.92973681 |
| Pak1 | -0.6807241 |
| Pak3 | 1.52957054 |
| Pak4 | 0.4194517 |
| Pak6 | 2.917576 |
| Pak7 | -0.7636946 |
| Palld | 0.88937336 |
| Palm | 1.72483505 |
| Palm | -0.5959193 |
| Palm2 | -0.5268303 |
| Palmd | -0.5751074 |
| Palmd | 1.84755045 |
| Palmd | 1.64583404 |
| Pam | 2.9998135 |
| Pank1 | -0.0344264 |
| Pank1 | -0.6739199 |
| Pank2 | 2.21206694 |
| Pank3 | 1.48849883 |
| Pank4 | 0.74316389 |
| Panx1 | 1.24319668 |
| Panx3 | -0.6037467 |
| Paox | -0.691888 |
| Paox | 1.08976573 |
| Paox | 1.39746591 |
| Paox | 3.42355625 |
| Papd1 | -0.6739199 |
| Papd1 | -0.3112773 |
| Papd4 | -0.3015612 |
| Papln | 1.0092099 |
| Papola | 1.21374591 |
| Papolb | -0.622003 |
| Papolg | -0.3947622 |
| Papss1 | -0.5959193 |
| Papss1 | -0.7544107 |
| Papss2 | 0.45195743 |
| Paqr3 | 1.03331333 |
| Paqr6 | -0.0626035 |
| Paqr7 | -0.3626496 |
| Paqr9 | -0.648837 |
| Paqr9 | -0.5167512 |
| Paqr9 | 1.60695231 |
| Pard3 | 0.12314599 |
| Pard3 | -0.542156 |
| Pard6a | -0.7137397 |
| Pard6a | 1.03222494 |
| Pard6b | -0.2152741 |
| Pard6g | -0.27632 |
| Park7 | -0.5275544 |
| Parl | 1.0622301 |
| Parp1 | 0.81458562 |
| Parp1 | -0.3216765 |
| Parp1 | 2.32103436 |
| Parp12 | 1.35846864 |
| Parp14 | -0.1235241 |
| Parp16 | -0.6604585 |
| Parp2 | -0.4823079 |
| Parp2 | -0.8724238 |
| Parp3 | 0.60823279 |
| Parp6 | -0.6781667 |
| Parp8 | 0.19262552 |
| Pars2 | -0.1113885 |
| Pars2 | -0.633293 |
| Parva | -0.4920198 |
| Parvg | -0.622003 |
| Pask | 0.36429735 |
| Patl1 | -0.6212018 |
| Patz1 | -0.4106034 |
| Pawr | -0.6116404 |
| Pax8 | -0.6236076 |
| Paxip1 | -0.7415665 |
| Pbk | 2.23586208 |
| Pbld | 0.19829946 |
| Pbp2 | 0.44967278 |
| Pbrm1 | 0.33199378 |
| Pbrm1 | -0.6430801 |
| Pbx1 | -0.7424781 |
| Pbx1 | 0.09977345 |
| Pbx2 | 0.63798918 |
| Pbx2 | 0.72876476 |
| Pbx2 | -0.1296516 |
| Pbx3 | -0.4016992 |
| Pbx3 | 1.00557509 |
| Pbx4 | 1.49133275 |
| Pcaf | -0.0477586 |
| Pcaf | 0.45953279 |
| Pcbd1 | 2.18420984 |
| Pcbd2 | -0.5392174 |
| Pcbp1 | -0.7921139 |
| Pcbp2 | -0.7066913 |
| Pcbp3 | -0.5577378 |
| Pcca | 0.85478892 |
| Pcca | -0.4325884 |
| Pccb | -0.1613968 |
| Pccb | 0.08636446 |
| Pcdh1 | 0.6509839 |
| Pcdh10 | -0.9109487 |
| Pcdh10 | 2.39684168 |
| Pcdh10 | 3.37693825 |
| Pcdh15 | 1.32226995 |
| Pcdh17 | -0.4857648 |
| Pcdh20 | 1.7158498 |
| Pcdh21 | -0.9044164 |
| Pcdh7 | 4.03352074 |
| Pcdh7 | 1.57196862 |
| Pcdh8 | -0.4969139 |
| Pcdh9 | 0.51399412 |
| Pcdha10 | 0.02911986 |
| Pcdha11 | 0.89900122 |
| Pcdha2 | 0.36318262 |
| Pcdha6 | 4.60662714 |
| Pcdhac1 | 2.06043021 |
| Pcdhac2 | 2.08440394 |
| Pcdhb1 | -0.5290043 |
| Pcdhb10 | 2.32475494 |
| Pcdhb12 | 1.24558238 |
| Pcdhb14 | 0.70895696 |
| Pcdhb19 | 0.75679571 |
| Pcdhb20 | -0.7740148 |
| Pcdhb22 | -0.9297092 |
| Pcdhb3 | -0.4809287 |
| Pcdhb7 | -0.3773336 |
| Pcdhb8 | -0.1771861 |
| Pcdhga10 | -0.2664117 |
| Pcdhga12 | -0.657956 |
| Pcdhga4 | 0.03903943 |
| Pcdhga7 | -0.648837 |
| Pcdhga8 | 2.19311764 |
| Pcdhgb2 | 0.02191189 |
| Pcdhgb6 | 1.24752979 |
| Pcdhgc3 | 2.06647041 |
| Pcdhgc4 | -0.4099646 |
| Pcdhgc5 | 2.61409681 |
| Pcf11 | 1.51726778 |
| Pcgf1 | -0.8947029 |
| Pcgf2 | 0.08298188 |
| Pcgf3 | 1.57904078 |
| Pcgf5 | -0.7979031 |
| Pcgf6 | -0.568272 |
| Pck1 | 0.4342971 |
| Pclo | 0.38495561 |
| Pclo | 3.15342949 |
| Pcmt1 | 0.20162103 |
| Pcmt1 | -0.2533621 |
| Pcmt1 | -0.4138042 |
| Pcmtd1 | -0.310703 |
| Pcmtd2 | 0.18286609 |
| Pcna | -0.4016992 |
| Pcnt | 0.72138602 |
| Pcnx | -0.4591344 |
| Pcnxl2 | -0.5333677 |
| Pcnxl2 | 0.27154695 |
| Pcolce | 2.22455253 |
| Pcolce2 | 0.16130612 |
| Pcolce2 | 0.46504922 |
| Pcsk1n | 3.00882467 |
| Pcsk2 | 1.36392243 |
| Pcsk2 | -0.752565 |
| Pcsk2 | 1.29668382 |
| Pcsk2 | 1.52009648 |
| Pcsk4 | -0.1449084 |
| Pcsk4 | -0.7208417 |
| Pcsk4 | 0.20658131 |
| Pcsk4 | -0.2880143 |
| Pcsk4 | 2.09827217 |
| Pcsk9 | 1.25827535 |
| Pctk1 | -0.3872529 |
| Pcx | -0.2111365 |
| Pcyox1 | -0.3693511 |
| Pcyox1l | -0.5599851 |
| Pcyt1a | -0.7084484 |
| Pcyt1b | -0.6739199 |
| Pcyt1b | 0.86510414 |
| Pcyt2 | 0.25196246 |
| Pcyt2 | -0.1811756 |
| Pcyt2 | 0.14594731 |
| Pdcd10 | -0.2528223 |
| Pdcd2 | -0.4795515 |
| Pdcd2l | 1.30677951 |
| Pdcd4 | 1.52869142 |
| Pdcd5 | 0.36429735 |
| Pdcd5 | -0.2658643 |
| Pdcd6ip | 1.60186527 |
| Pdcl | -0.4720133 |
| Pdcl3 | 1.0178208 |
| Pddc1 | 0.08411077 |
| Pde10a | -0.588157 |
| Pde1a | -0.6252148 |
| Pde1a | 0.09829305 |
| Pde1a | 1.13376334 |
| Pde1b | 1.96879508 |
| Pde1c | 0.01828692 |
| Pde2a | 0.01949681 |
| Pde3a | -0.7882743 |
| Pde4a | 2.47033676 |
| Pde4a | -0.1952744 |
| Pde4a | -0.738837 |
| Pde4a | -0.4850724 |
| Pde4b | 0.7422356 |
| Pde4d | -0.4490909 |
| Pde4dip | 1.14974187 |
| Pde4dip | -0.6406237 |
| Pde4dip | -0.5804587 |
| Pde4dip | 0.28861684 |
| Pde4dip | -0.4080507 |
| Pde5a | 0.78578353 |
| Pde6a | -0.5532592 |
| Pde6d | -0.396649 |
| Pde6d | -0.6629678 |
| Pde6d | -0.5138913 |
| Pde6d | 2.63250117 |
| Pde6h | -0.2436934 |
| Pde7a | -0.2724542 |
| Pdgfa | -0.6688491 |
| Pdgfb | 2.66681624 |
| Pdgfc | -0.2908204 |
| Pdgfra | 3.1369431 |
| Pdgfrb | -0.6604585 |
| Pdgfrl | 1.35923601 |
| Pdha1 | 1.4531095 |
| Pdhb | -0.0451633 |
| Pdhb | -0.4843805 |
| Pdia3 | -0.6316718 |
| Pdia4 | 0.16579598 |
| Pdia5 | -0.5319109 |
| Pdia5 | -0.2442279 |
| Pdia6 | -0.2204721 |
| Pdik1l | 0.30091934 |
| Pdk1 | 1.99965918 |
| Pdk2 | -0.6324821 |
| Pdk3 | -0.4564455 |
| Pdk4 | 0.07920903 |
| Pdlim1 | 0.49627183 |
| Pdlim2 | 0.4620443 |
| Pdlim2 | -0.7315972 |
| Pdlim2 | 2.2099504 |
| Pdlim2 | -0.7217333 |
| Pdlim3 | 1.1770623 |
| Pdlim3 | -0.1137101 |
| Pdlim4 | -0.5443661 |
| Pdlim5 | 2.56480054 |
| Pdlim5 | -0.5096179 |
| Pdlim7 | -0.7627621 |
| Pdpk1 | -0.4273687 |
| Pdpk1 | 2.63833711 |
| Pdrg1 | -0.8661548 |
| Pdss1 | -0.3380869 |
| Pdxdc1 | 1.16745546 |
| Pdxdc1 | 2.01250557 |
| Pdxk | -0.049059 |
| Pdxp | -0.7844504 |
| Pdyn | -0.4665689 |
| Pdyn | 1.02209664 |
| Pdzd11 | -0.4497571 |
| Pdzd11 | -0.0683426 |
| Pdzd11 | 0.1730035 |
| Pdzd8 | 1.14239185 |
| Pdzk1 | -0.0687855 |
| Pdzk1ip1 | -0.3135783 |
| Pdzrn3 | -0.4969139 |
| Pdzrn3 | 1.29288246 |
| Pea15 | -0.723519 |
| Pear1 | 0.66561672 |
| Pear1 | 0.45676229 |
| Pebp1 | -0.6148167 |
| Pecam1 | -0.2293756 |
| Pecam1 | -0.7507229 |
| Peci | 0.32009685 |
| Pef1 | 0.82262974 |
| Peg10 | -0.5912541 |
| Peg3 | 0.24565821 |
| Peli1 | -0.7181721 |
| Peli1 | -0.0357083 |
| Peli2 | -0.3333687 |
| Peli3 | 0.12710483 |
| Pelo | 0.0647314 |
| Pelp1 | -0.7005673 |
| Pemt | 1.53633596 |
| Peo1 | 2.85783013 |
| Pepd | 1.25613612 |
| Per1 | -0.6596236 |
| Per1 | -0.6439004 |
| Per2 | 0.41997661 |
| Per2 | -0.6132272 |
| Per2 | 0.87562863 |
| Per3 | 0.36957419 |
| Perld1 | 0.67444732 |
| Pes1 | -0.3693511 |
| Pet112l | -0.5117522 |
| Pet112l | 0.3443102 |
| Pet112l | 0.78896913 |
| Pet112l | 0.34658379 |
| Pex11a | 0.43403868 |
| Pex11a | -0.75997 |
| Pex11a | -0.6236076 |
| Pex11b | -0.7137397 |
| Pex11c | -0.3327806 |
| Pex14 | -0.4036001 |
| Pex16 | 1.5489393 |
| Pex19 | -0.2189096 |
| Pex19 | -0.6381738 |
| Pex2 | 0.73533738 |
| Pex26 | 0.84585562 |
| Pex3 | 1.02922529 |
| Pex3 | 2.3697436 |
| Pex5 | -0.102619 |
| Pex5 | -0.0912043 |
| Pex5 | -0.7911525 |
| Pex5l | -0.1791787 |
| Pex6 | 0.24312453 |
| Pex6 | 0.86997635 |
| Pex6 | -0.0037637 |
| Pex7 | -0.4733793 |
| Pfdn1 | -0.5713038 |
| Pfdn2 | 1.06394219 |
| Pfdn4 | 0.04846402 |
| Pfdn5 | -0.6300534 |
| Pfdn5 | 1.58206616 |
| Pfkfb2 | 2.53218357 |
| Pfkfb2 | -0.7049375 |
| Pfkfb4 | 0.34345617 |
| Pfkl | 1.13754053 |
| Pfkl | -0.2049643 |
| Pfkm | -0.2724542 |
| Pfkm | 3.50557391 |
| Pfkm | 0.0239197 |
| Pfkp | -0.8354267 |
| Pfkp | 0.09569667 |
| Pfkp | 1.07947016 |
| Pfn1 | 3.44373305 |
| Pfn1 | -0.6901617 |
| Pfn2 | 0.51090502 |
| Pfn2 | 1.8266382 |
| Pfn2 | -0.0604056 |
| Pfn3 | 1.24666478 |
| Pfn4 | -0.3736409 |
| Pftk1 | -0.5203384 |
| Pga5 | 1.22089044 |
| Pgam1 | -0.3263358 |
| Pgam2 | 0.98546965 |
| Pgam5 | 1.388124 |
| Pgbd5 | -0.1216468 |
| Pgcp | -0.6284377 |
| Pgd | 0.66722851 |
| Pgk1 | -0.1420287 |
| Pgk2 | -0.4899301 |
| Pgls | 1.68478485 |
| Pglyrp1 | -0.1628658 |
| Pglyrp4 | -0.3541894 |
| Pgm2 | -0.4055049 |
| Pgm2l1 | -0.1396357 |
| Pgm3 | -0.6546297 |
| Pgm5 | 0.54410565 |
| Pgm5 | -0.210105 |
| Pgpep1 | -0.8776803 |
| Pgpep1 | -0.0994088 |
| Pgr | 0.0647314 |
| Pgrmc1 | -0.5275544 |
| Pgs1 | -0.2003618 |
| Phactr1 | -0.5866122 |
| Phactr1 | -0.5436289 |
| Phactr1 | -0.6100563 |
| Phactr4 | -0.7226257 |
| Phb2 | 0.60715997 |
| Phc1 | -0.75997 |
| Phc1 | -0.5304565 |
| Phc2 | 1.70756961 |
| Phca | 0.31541268 |
| Phca | 2.90905565 |
| Phex | 0.84402534 |
| Phf1 | 1.43410234 |
| Phf11 | 2.12927769 |
| Phf11 | -0.56299 |
| Phf12 | 1.02813211 |
| Phf12 | 0.38984626 |
| Phf13 | 0.08335833 |
| Phf13 | -0.57587 |
| Phf14 | 0.52131942 |
| Phf16 | -0.467927 |
| Phf17 | -0.3434236 |
| Phf19 | 1.31797051 |
| Phf2 | 0.68989907 |
| Phf20l1 | 2.32550408 |
| Phf21a | 0.24723827 |
| Phf5a | 1.87066829 |
| Phf6 | 1.32674825 |
| Phf7 | 2.23642398 |
| Phgdh | 2.17844954 |
| Phka1 | 2.01226751 |
| Phka1 | -0.0158244 |
| Phka2 | -0.0276191 |
| Phka2 | -0.4836891 |
| Phka2 | -0.4899301 |
| Phkb | -0.2388963 |
| Phkg1 | 1.09206666 |
| Phkg2 | 0.14875869 |
| Phlda1 | 1.31213971 |
| Phlda2 | 1.23007527 |
| Phlda3 | 0.78578353 |
| Phlda3 | 0.56724535 |
| Phldb1 | -0.4490909 |
| Phldb1 | -0.233595 |
| Phlpp | -0.542156 |
| Phospho1 | 1.44876418 |
| Phospho2 | -0.1584659 |
| Phox2a | 0.16407166 |
| Phox2b | 2.19593776 |
| Phpt1 | 1.05442008 |
| Phtf1 | -0.7863604 |
| Phtf2 | -0.5614864 |
| Phyhip | -0.6308622 |
| Pi16 | -0.7979031 |
| Pi4k2b | 0.31247308 |
| Pi4k2b | 2.83565519 |
| Pi4ka | 1.685461 |
| Pi4kb | -0.1492447 |
| Pi4kb | 1.79664654 |
| Pias1 | 0.50301721 |
| Pias2 | 0.38903293 |
| Pias3 | 0.59571165 |
| Pias3 | 1.90398449 |
| Pias4 | 0.46579894 |
| Pibf1 | -0.5592354 |
| Pibf1 | -0.8164763 |
| Picalm | -0.1053794 |
| Pick1 | 1.64406928 |
| Pick1 | 0.91980846 |
| Pif1 | -0.0477586 |
| Piga | 1.31495913 |
| Piga | 2.66827892 |
| Pigb | 1.67280361 |
| Pigc | -0.7023129 |
| Pigf | -0.7424781 |
| Pigh | -0.058651 |
| Pigk | -0.5370194 |
| Pigk | 0.22292767 |
| Pigl | 0.01343161 |
| Pign | -0.5217771 |
| Pigo | 2.56081499 |
| Pigo | -0.6471886 |
| Pigo | 0.50205657 |
| Pigp | -0.752565 |
| Pigp | 3.08507254 |
| Pigq | -0.6747677 |
| Pigt | 1.35645128 |
| Pigx | -0.3847632 |
| Pigy | -0.7664979 |
| Pigyl | 1.36296793 |
| Pigz | -0.1239941 |
| Pih1d1 | 0.66096724 |
| Pik3ap1 | 0.97061016 |
| Pik3c2a | -0.5480613 |
| Pik3c3 | -0.6068962 |
| Pik3ca | 0.47177511 |
| Pik3cb | -0.376101 |
| Pik3cd | 1.68647432 |
| Pik3cg | -0.5480613 |
| Pik3ip1 | -0.457117 |
| Pik3r1 | -0.755335 |
| Pik3r1 | 0.03230561 |
| Pik3r1 | -0.0250791 |
| Pik3r2 | 0.72176584 |
| Pik3r2 | 0.83532417 |
| Pik3r3 | -0.3991707 |
| Pik3r4 | 1.80868806 |
| Pik3r6 | 2.11693075 |
| Pik3r6 | -0.6629678 |
| Pim1 | -0.1197733 |
| Pim2 | 0.02351848 |
| Pim2 | 1.55112748 |
| Pim3 | 0.09346544 |
| Pip4k2a | 2.07135502 |
| Pip4k2b | 2.23225792 |
| Pip4k2b | 0.85149026 |
| Pip4k2c | -0.2730055 |
| Pip5k1a | -0.8745228 |
| Pip5k1a | -0.4517586 |
| Pip5k1b | -0.4477599 |
| Pip5kl1 | -0.4754322 |
| Pipox | -0.4920198 |
| Pir | 1.82302904 |
| Pira11 | 1.54163434 |
| Pira4 | 1.96113064 |
| Pisd | -0.7479665 |
| Pit1 | -0.302699 |
| Pitpna | 0.99060794 |
| Pitpnb | 1.14878122 |
| Pitpnc1 | 3.46532055 |
| Pitpnm1 | -0.1584659 |
| Pitpnm2 | 2.18843785 |
| Pitpnm3 | 0.86867958 |
| Pitx1 | 0.32736911 |
| Pitx2 | -0.8324088 |
| Pitx2 | -0.4391545 |
| Pitx2 | -0.5781615 |
| Pitx2 | 3.82387198 |
| Piwil1 | -0.4365225 |
| Piwil2 | -0.5607354 |
| Piwil4 | 1.24417339 |
| Pja1 | -0.4829982 |
| Pja2 | 0.91888665 |
| Pja2 | 2.29505893 |
| Pkd1 | -0.1044584 |
| Pkd1l3 | 2.62512368 |
| Pkd2 | -0.5428922 |
| Pkd2l1 | -0.871376 |
| Pkd2l2 | -0.8671967 |
| Pkdrej | 0.06894544 |
| Pkhd1l1 | 1.5367324 |
| Pkhd1l1 | 1.35866054 |
| Pkia | 0.30508332 |
| Pkib | 2.25569671 |
| Pkib | -0.7721299 |
| Pkig | -0.4215312 |
| Pkig | -0.472696 |
| Pkig | -0.5766332 |
| Pkm2 | 0.54111481 |
| Pkmyt1 | -0.227273 |
| Pkn1 | -0.5160354 |
| Pkn1 | 1.21374591 |
| Pkn2 | 1.56875708 |
| Pkn2 | 0.23292045 |
| Pkn2 | 1.59310091 |
| Pkn3 | 0.17879766 |
| Pknox1 | 2.91981101 |
| Pknox1 | 2.60883565 |
| Pknox2 | 0.13959056 |
| Pknox2 | 1.26307077 |
| Pknox2 | 0.98989598 |
| Pknox2 | -0.4036001 |
| Pknox2 | 1.42046436 |
| Pkp1 | 0.94615235 |
| Pkp2 | -0.5990424 |
| Pkp2 | -0.156517 |
| Pkp2 | 1.23029547 |
| Pkp3 | -0.8264022 |
| Pla1a | -0.5804587 |
| Pla1a | -0.6901617 |
| Pla1a | -0.6781667 |
| Pla2g10 | -0.6430801 |
| Pla2g12a | -0.7155101 |
| Pla2g1b | -0.1439475 |
| Pla2g2c | -0.645543 |
| Pla2g2d | 0.6227019 |
| Pla2g2f | -0.4948133 |
| Pla2g4c | -0.334546 |
| Pla2g4d | -0.472696 |
| Pla2g4e | -0.7146245 |
| Pla2g4f | 2.01145765 |
| Pla2g5 | 2.05516963 |
| Pla2g6 | 0.66702719 |
| Pla2g7 | 0.24913082 |
| Plaa | 0.84768232 |
| Plac1 | -0.737929 |
| Plac1l | -0.4080507 |
| Plac8 | 0.29643722 |
| Plag1 | 0.5836809 |
| Plag1 | 3.24232711 |
| Plagl2 | -0.7352101 |
| Plau | 0.00814302 |
| Plb1 | 0.7204358 |
| Plcb2 | 1.83893213 |
| Plcb3 | 1.24558238 |
| Plcb4 | 0.17231947 |
| Plcd1 | -0.6596236 |
| Plcd3 | -0.7163966 |
| Plcd4 | -0.7324991 |
| Plcd4 | -0.5103288 |
| Plcd4 | -0.5866122 |
| Plce1 | 2.36388204 |
| Plcg2 | 0.72574464 |
| Plch2 | 0.19396367 |
| Plcl2 | 0.38930412 |
| Plcxd1 | 0.18556856 |
| Plcxd1 | -0.3263358 |
| Plcz1 | 3.02226096 |
| Pld1 | 1.1992906 |
| Pld2 | -0.698825 |
| Pld3 | 0.35449803 |
| Pld3 | 0.61208474 |
| Pld4 | 0.69303727 |
| Pld5 | -0.6439004 |
| Pldn | -0.1747014 |
| Plec1 | 0.61995304 |
| Plec1 | -0.1707395 |
| Plec1 | 0.15400731 |
| Plec1 | 0.2006258 |
| Plek | 2.5462599 |
| Plek2 | -0.4934155 |
| Plekha1 | 1.62190439 |
| Plekha1 | 0.39336253 |
| Plekha2 | 0.9788626 |
| Plekha3 | -0.5362879 |
| Plekha4 | 2.74653714 |
| Plekha4 | -0.6381738 |
| Plekha5 | -0.4112427 |
| Plekha6 | -0.5998248 |
| Plekha7 | -0.5443661 |
| Plekha8 | -0.7488844 |
| Plekhb1 | 0.65751572 |
| Plekhb2 | -0.1151058 |
| Plekhf1 | -0.0916582 |
| Plekhf1 | -0.167779 |
| Plekhf2 | -0.568272 |
| Plekhf2 | 2.16192611 |
| Plekhg2 | 1.12898956 |
| Plekhg2 | -0.0776885 |
| Plekhg2 | 1.35327232 |
| Plekhg3 | 1.98462455 |
| Plekhg3 | 0.49457784 |
| Plekhg4 | -0.1030785 |
| Plekhg5 | -0.5720633 |
| Plekhg6 | -0.6196014 |
| Plekhj1 | 2.43077703 |
| Plekhm1 | 0.10677349 |
| Plekhm3 | -0.472696 |
| Plekho2 | 1.23927959 |
| Plin | -0.7702488 |
| Plk1 | 0.25666286 |
| Plk2 | -0.1937536 |
| Plk3 | 1.07036739 |
| Plk4 | 0.17094989 |
| Pllp | -0.719951 |
| Plod1 | 2.59169541 |
| Plod2 | -0.1506946 |
| Plod3 | 0.01383718 |
| Plp1 | -0.6730728 |
| Plp2 | 1.13180782 |
| Plrg1 | -0.3216765 |
| Pls3 | -0.7873168 |
| Plscr1 | 1.76055882 |
| Plscr1 | 0.70837929 |
| Plscr3 | -0.8755741 |
| Plscr4 | 0.51446846 |
| Pltp | -0.4686068 |
| Plunc | 0.67704045 |
| Plvap | -0.5167512 |
| Plxdc1 | 0.95210469 |
| Plxdc1 | 1.75354779 |
| Plxna2 | -0.6538 |
| Plxnb1 | -0.5735841 |
| Plxnb3 | -0.648837 |
| Plxnc1 | 0.67644267 |
| Plxnd1 | 0.35308969 |
| Pmaip1 | -0.5174675 |
| Pmch | 0.04179812 |
| Pmepa1 | -0.155058 |
| Pmf1 | 2.26336846 |
| Pmfbp1 | -0.4312808 |
| Pml | 1.26794741 |
| Pml | -0.4254188 |
| Pmm1 | -0.5399512 |
| Pmm1 | 0.30478651 |
| Pmm2 | -0.3158849 |
| Pmm2 | -0.1353437 |
| Pmm2 | 0.65425553 |
| Pmm2 | 1.18275611 |
| Pmp2 | 0.15156163 |
| Pmp22 | -0.4906261 |
| Pmp22 | -0.7581132 |
| Pmpca | 1.94994745 |
| Pmpcb | 0.19061469 |
| Pms2 | -0.802755 |
| Pmvk | -0.4754322 |
| Pnck | 2.16500368 |
| Pnkd | -0.6357302 |
| Pnkd | 2.21544344 |
| Pnkp | 0.34828536 |
| Pnkp | 3.52122016 |
| Pnliprp1 | -0.4345534 |
| Pnma1 | -0.5261067 |
| Pnma2 | 0.59788249 |
| Pno1 | 1.64872103 |
| Pnpla2 | 0.90494907 |
| Pnpla6 | 1.01768255 |
| Pnpla7 | -0.6196014 |
| Pnpla8 | 0.83918278 |
| Pnpla8 | -0.2173498 |
| Pnpo | 1.59078469 |
| Pnpo | 0.01343161 |
| Pnrc2 | 1.01408066 |
| Podn | -0.5592354 |
| Podxl | -0.4843805 |
| Podxl2 | -0.1786802 |
| Pof1b | 0.60608592 |
| Pofut1 | 0.26753923 |
| Pofut1 | -0.6892998 |
| Pofut2 | -0.6053201 |
| Pogz | -0.7702488 |
| Pogz | 0.61357843 |
| Pola2 | -0.1339174 |
| Polb | 1.28377868 |
| Pold1 | -0.6430801 |
| Pold2 | -0.7882743 |
| Pold3 | 1.25377728 |
| Pold4 | -0.5443661 |
| Poldip2 | -0.6621306 |
| Poldip3 | -0.6324821 |
| Poldip3 | 0.40569936 |
| Pole | -0.0516652 |
| Pole3 | -0.3044084 |
| Pole3 | -0.8066549 |
| Pole4 | -0.1801766 |
| Polg | 1.69139014 |
| Polg2 | 2.3736956 |
| Polh | -0.682433 |
| Poli | -0.4941141 |
| Polk | -0.691888 |
| Poll | 2.52704171 |
| Poll | 1.92617906 |
| Polm | 3.09740191 |
| Polm | -0.4864577 |
| Poln | 2.28327407 |
| Polq | -0.6132272 |
| Polr1e | 0.07693797 |
| Polr2d | 1.38234269 |
| Polr2d | -0.3626496 |
| Polr2d | -0.5637428 |
| Polr2d | -0.8125353 |
| Polr2e | -0.698825 |
| Polr2e | -0.2913826 |
| Polr2f | 1.25613612 |
| Polr2g | -0.7683715 |
| Polr2h | 0.57461051 |
| Polr2j | -0.7208417 |
| Polr3a | -0.423473 |
| Polr3a | -0.691888 |
| Polr3b | -0.4652127 |
| Polr3d | 2.12818491 |
| Polr3e | 3.22562748 |
| Polr3g | 0.23771751 |
| Polr3h | -0.4850724 |
| Polr3h | 0.05663316 |
| Polr3h | -0.7226257 |
| Polr3k | -0.810571 |
| Polr3k | 1.67533519 |
| Polrmt | 0.88012592 |
| Pols | -0.2262234 |
| Pom121 | -0.7787438 |
| Pomc | -0.7571862 |
| Pomgnt1 | -0.1386802 |
| Pomgnt1 | 2.69834024 |
| Pomp | 1.95750195 |
| Pomt1 | 2.59197686 |
| Pomt1 | -0.5766332 |
| Pomt2 | -0.7825443 |
| Pon1 | 1.09078906 |
| Pon2 | 1.11014742 |
| Pon3 | 0.32359461 |
| Pon3 | -0.2757668 |
| Pop4 | 1.06381061 |
| Pop5 | 0.07996482 |
| Pop5 | -0.4906261 |
| Pop7 | 0.73064738 |
| Popdc2 | -0.6373585 |
| Popdc2 | 1.49000021 |
| Popdc3 | -0.7461334 |
| Por | 0.13852687 |
| Por | -0.417016 |
| Porcn | 0.43196873 |
| Porcn | -0.3584099 |
| Pou2af1 | 0.88571723 |
| Pou2f1 | -0.4920198 |
| Pou2f1 | -0.3736409 |
| Pou2f2 | -0.2485152 |
| Pou3f1 | -0.5261067 |
| Pou3f3 | 0.17026434 |
| Pou5f1 | 1.48716224 |
| Pou5f1 | -0.0727813 |
| Pou6f1 | 0.60608592 |
| Ppa1 | 1.42055415 |
| Ppa2 | 1.15524647 |
| Ppa2 | 2.04639674 |
| Ppa2 | -0.813519 |
| Ppap2a | -0.7163966 |
| Ppap2a | 2.19158652 |
| Ppap2a | -0.8651141 |
| Ppap2b | -0.7470495 |
| Ppap2b | 1.52259476 |
| Ppap2c | -0.6705363 |
| Ppapdc1 | 1.36382703 |
| Ppapdc1a | 1.53204362 |
| Ppapdc2 | -0.627631 |
| Ppapdc3 | 0.94853785 |
| Ppapdc3 | 2.64619075 |
| Pparbp | -0.6730728 |
| Pparg | -0.7825443 |
| Ppargc1a | 3.86536032 |
| Ppargc1b | 1.49390914 |
| Ppat | 0.14981078 |
| Ppcs | -0.3181972 |
| Ppef2 | -0.1897102 |
| Ppfia3 | 2.5670366 |
| Ppfia3 | -0.2757668 |
| Ppfibp1 | -0.7921139 |
| Ppfibp2 | 1.56168342 |
| Ppfibp2 | 1.62702756 |
| Ppia | 0.43481372 |
| Ppic | -0.2357119 |
| Ppic | -0.7343056 |
| Ppid | 0.31129465 |
| Ppie | -0.1197733 |
| Ppie | -0.376101 |
| Ppif | -0.7825443 |
| Ppil2 | -0.3991707 |
| Ppil3 | -0.2746613 |
| Ppl | 1.10713725 |
| Ppm1a | -0.7534874 |
| Ppm1a | -0.0229677 |
| Ppm1a | -0.2790911 |
| Ppm1b | 0.24249003 |
| Ppm1b | -0.5377515 |
| Ppm1b | 0.37040466 |
| Ppm1e | -0.7646281 |
| Ppm1e | -0.8557993 |
| Ppm1f | 2.09883653 |
| Ppm1f | -0.8017826 |
| Ppm1f | 4.12647552 |
| Ppm1g | 0.09940357 |
| Ppm1h | 0.77239185 |
| Ppm1j | -0.5268303 |
| Ppm1k | 0.87980541 |
| Ppm1l | -0.4537644 |
| Ppm1l | -0.8284001 |
| Ppm1m | -0.3829002 |
| Ppm1m | 0.45373052 |
| Ppm2c | 0.44355288 |
| Ppme1 | -0.7892327 |
| Ppnr | 1.34756157 |
| Ppox | 0.54180586 |
| Ppox | -0.3205153 |
| Ppp1ca | -0.657956 |
| Ppp1ca | 0.31247308 |
| Ppp1ca | 0.19796665 |
| Ppp1cb | 0.20460036 |
| Ppp1cb | 0.13532853 |
| Ppp1cc | -0.6521427 |
| Ppp1r10 | 1.25923641 |
| Ppp1r10 | 0.70297043 |
| Ppp1r11 | 0.94420964 |
| Ppp1r12c | -0.3602246 |
| Ppp1r13b | 1.91799687 |
| Ppp1r13b | -0.0217031 |
| Ppp1r13b | 2.48894799 |
| Ppp1r14a | 1.14613434 |
| Ppp1r14a | -0.8037285 |
| Ppp1r14c | 1.9174172 |
| Ppp1r14d | 2.45621197 |
| Ppp1r15b | -0.0162431 |
| Ppp1r16a | -0.7873168 |
| Ppp1r1a | -0.8264022 |
| Ppp1r1b | 0.96316401 |
| Ppp1r1b | 0.77293123 |
| Ppp1r2 | -0.96724 |
| Ppp1r3c | -0.350587 |
| Ppp1r3f | -0.3705747 |
| Ppp1r8 | -0.505364 |
| Ppp1r9a | -0.4378375 |
| Ppp1r9b | 0.16476177 |
| Ppp2ca | -0.3669086 |
| Ppp2cb | -0.622003 |
| Ppp2cz | -0.3632568 |
| Ppp2r1a | -0.3578058 |
| Ppp2r1b | -0.3434236 |
| Ppp2r2b | 0.47276743 |
| Ppp2r2b | 3.54921939 |
| Ppp2r2b | 0.98317684 |
| Ppp2r2c | -0.4306276 |
| Ppp2r2c | 1.07284694 |
| Ppp2r2d | 0.36262476 |
| Ppp2r3c | 0.28012093 |
| Ppp2r4 | -0.7777961 |
| Ppp2r5b | 1.36000275 |
| Ppp2r5c | -0.2970237 |
| Ppp2r5c | 0.58916866 |
| Ppp2r5c | -0.5138913 |
| Ppp2r5d | 0.37233954 |
| Ppp2r5d | -0.8425066 |
| Ppp2r5d | -0.2065036 |
| Ppp2r5d | 0.96169699 |
| Ppp2r5e | -0.5025388 |
| Ppp2r5e | 0.47721984 |
| Ppp3ca | -0.4163728 |
| Ppp3ca | -0.9022491 |
| Ppp3ca | -0.5517711 |
| Ppp3cb | -1.0003127 |
| Ppp3cc | -0.7128557 |
| Ppp3r1 | -0.4163728 |
| Ppp4c | -0.6447213 |
| Ppp4r1 | -0.8486181 |
| Ppp4r1 | 0.90104028 |
| Ppp4r1 | -0.5060716 |
| Ppp4r1l | 0.15016121 |
| Ppp5c | -0.5540042 |
| Ppp5c | -0.7749587 |
| Ppp6c | 0.30863771 |
| Pps | 0.3698511 |
| Ppt1 | 0.0032345 |
| Ppt2 | -0.5414205 |
| Pptc7 | -0.3860072 |
| Pqbp1 | 0.18523118 |
| Pqlc1 | -0.0425751 |
| Pqlc2 | -0.9208337 |
| Pqlc3 | 0.99600117 |
| Pqlc3 | 0.59766563 |
| Pqlc3 | 1.57782787 |
| Pqlc3 | -0.5414205 |
| Pramef12 | 0.79020504 |
| Prap1 | 1.11414593 |
| Prc1 | -0.810571 |
| Prcp | -0.2469052 |
| Prdm10 | 1.0798583 |
| Prdm4 | -0.4692871 |
| Prdm8 | -0.5607354 |
| Prdm9 | -0.4638586 |
| Prdx1 | 1.20439811 |
| Prdx2 | -0.3572021 |
| Prdx2 | 2.32161561 |
| Prdx2 | -0.2796463 |
| Prdx3 | 0.21020216 |
| Prdx4 | -0.5239394 |
| Prdx5 | 0.47400634 |
| Prdx5 | -1.0015159 |
| Prdx6 | -0.6406237 |
| Prdx6-rs1 | -0.2587773 |
| Prdx6-rs1 | 1.05015664 |
| Preb | 0.29942769 |
| Preb | 2.08638521 |
| Prei3 | 0.23516214 |
| Prei4 | 1.05561568 |
| Prei4 | -0.049059 |
| Prelp | 1.65040685 |
| Prep | -0.5577378 |
| Prep | -0.433243 |
| Prf1 | -0.2018935 |
| Prickle1 | 0.03151019 |
| Prickle2 | -0.2447627 |
| Prickle3 | -0.4004341 |
| Prickle4 | 0.324467 |
| Prim2 | -0.017081 |
| Prim2 | -0.289697 |
| Prima1 | 1.89214457 |
| Prkaa2 | 0.96900914 |
| Prkaa2 | -0.3434236 |
| Prkab1 | 0.33257026 |
| Prkab2 | 0.01343161 |
| Prkaca | 1.87773545 |
| Prkaca | -0.9545612 |
| Prkacb | 0.97307906 |
| Prkag1 | -0.657956 |
| Prkag2 | -0.9274824 |
| Prkar1a | 0.22551671 |
| Prkar1b | -0.810571 |
| Prkar2b | 0.19229068 |
| Prkca | 0.57771764 |
| Prkcb1 | -1.0408588 |
| Prkcbp1 | 1.20846409 |
| Prkcbp1 | -0.958002 |
| Prkcc | -0.1035382 |
| Prkcd | 0.96257748 |
| Prkcdbp | 3.3571862 |
| Prkcdbp | -0.7730719 |
| Prkce | -0.3596193 |
| Prkch | -0.2658643 |
| Prkcq | -0.7740148 |
| Prkcsh | -0.2039396 |
| Prkcz | 0.08711448 |
| Prkcz | -0.0408537 |
| Prkcz | -0.5599851 |
| Prkd1 | -0.5720633 |
| Prkd1 | -0.6480124 |
| Prkd2 | 0.18388044 |
| Prkd3 | -0.3578058 |
| Prkd3 | 3.78408135 |
| Prkd3 | 0.71624327 |
| Prkg1 | -0.5743455 |
| Prkg2 | -0.2075311 |
| Prkra | 0.33831552 |
| Prkrip1 | -0.7854049 |
| Prkrip1 | -0.2790911 |
| Prkrir | 1.63932321 |
| Prkx | 0.88156689 |
| Prkx | 1.04600777 |
| Prl | -0.1907194 |
| Prlr | -0.5525149 |
| Prlr | -0.2070172 |
| Prm1 | 2.23825714 |
| Prm3 | 2.61497079 |
| Prmt2 | -0.5502854 |
| Prmt2 | -0.0512304 |
| Prmt3 | -0.3841418 |
| Prmt5 | -0.4055049 |
| Prmt5 | -0.296458 |
| Prmt5 | -0.3626496 |
| Prmt7 | 0.43403868 |
| Prmt7 | 0.26537407 |
| Prmt7 | -0.6381738 |
| Prmt7 | 0.55005873 |
| Prmt8 | -0.0534068 |
| Prnp | -0.0857745 |
| Prnpip1 | -0.4384958 |
| Prnpip1 | -0.3321929 |
| Prnpip1 | -0.7110903 |
| Proc | 1.11986393 |
| Proc | 2.50143432 |
| Procr | 2.22174164 |
| Prodh | 0.78080642 |
| Prok2 | -0.7721299 |
| Prok2 | -0.7110903 |
| Prokr1 | 0.36290373 |
| Prokr2 | 0.08486261 |
| Prom1 | -0.6513152 |
| Prom2 | 0.35562318 |
| Prom2 | 2.24499759 |
| Pros1 | -0.3669086 |
| Prosapip1 | 0.43739257 |
| Prosapip1 | -0.7969357 |
| Prosapip1 | -0.6996958 |
| Prosc | 1.13936273 |
| Prosc | -0.1090728 |
| Prosc | -0.1511784 |
| Prox1 | -0.467927 |
| Prox1 | 2.09927041 |
| Prpf18 | -0.5436289 |
| Prpf19 | 0.00528285 |
| Prpf19 | -0.698825 |
| Prpf19 | 3.30394137 |
| Prpf3 | -0.6076852 |
| Prpf3 | 0.49190962 |
| Prpf3 | 2.33566012 |
| Prpf3 | 0.30478651 |
| Prpf3 | -0.717284 |
| Prpf31 | 0.78896913 |
| Prpf38a | -0.8568297 |
| Prpf38a | -0.9055019 |
| Prpf38a | -0.0455953 |
| Prpf38b | 3.54202077 |
| Prpf39 | 0.87771935 |
| Prpf40a | -0.4665689 |
| Prpf40a | 1.6684772 |
| Prpf40b | -0.1756945 |
| Prpf40b | -0.6381738 |
| Prpf40b | -0.1526314 |
| Prpf6 | 1.0079533 |
| Prpf8 | -0.4417939 |
| Prpf8 | -0.2236048 |
| Prph2 | 0.16234416 |
| Prps1 | -0.4754322 |
| Prps1l1 | -0.8066549 |
| Prps2 | -0.1907194 |
| Prpsap2 | -0.627631 |
| Prpsap2 | 1.19369711 |
| Prr11 | -0.7693097 |
| Prr13 | -0.5562425 |
| Prr14 | -0.2495901 |
| Prr15 | 2.35992098 |
| Prr16 | -0.4074136 |
| Prr18 | 0.27000755 |
| Prr19 | 1.37756009 |
| Prr3 | 1.15417203 |
| Prr7 | 0.16407166 |
| Prrc1 | 2.56800827 |
| Prrg2 | -0.0293162 |
| Prrt1 | -0.8425066 |
| Prrt1 | -0.396649 |
| Prrt1 | -0.3286742 |
| Prrt3 | -0.7334019 |
| Prrx1 | 0.12818161 |
| Prrx1 | -0.2790911 |
| Prrx1 | -0.3374959 |
| Prss21 | -0.8145037 |
| Prss23 | 0.64358052 |
| Prss27 | -0.0348535 |
| Prss28 | -0.0175003 |
| Prss29 | 2.01160062 |
| Prss29 | 0.10750729 |
| Prss34 | -0.4497571 |
| Prss35 | 1.54123998 |
| Prss36 | -0.396649 |
| Prss7 | -0.6140216 |
| Prss8 | 0.20888711 |
| Prss8 | 1.3223697 |
| Prtn3 | 0.99131936 |
| Prune | 0.69714021 |
| Prune | 0.1115329 |
| Prune | 1.66902769 |
| Prune | 2.46656955 |
| Psap | -0.6504884 |
| Psat1 | 0.99995528 |
| Psca | 0.48115979 |
| Pscd1 | -0.8244085 |
| Pscd2 | 2.45798561 |
| Pscd3 | 0.5734983 |
| Pscdbp | -0.4444407 |
| Psd2 | 0.05041555 |
| Psd3 | 1.26699529 |
| Psd4 | 0.22970865 |
| Psen1 | -0.6621306 |
| Psen2 | 0.83212455 |
| Psenen | -0.2230819 |
| Psg19 | 1.76757946 |
| Psg23 | -0.8066549 |
| Psg27 | 0.25415893 |
| Psip1 | -0.7796925 |
| Psip1 | 2.94532875 |
| Pskh1 | 2.58772055 |
| Psma2 | 0.7414923 |
| Psma4 | 0.43300428 |
| Psma5 | 0.48532783 |
| Psma7 | 3.00432996 |
| Psma7 | -0.3511865 |
| Psmb1 | -0.7618305 |
| Psmb10 | 1.61762561 |
| Psmb10 | -0.4358657 |
| Psmb2 | -0.8254048 |
| Psmb2 | -0.0547151 |
| Psmb3 | -0.1663023 |
| Psmb4 | 0.28528838 |
| Psmb5 | -0.283542 |
| Psmb6 | -0.2049643 |
| Psmb7 | -0.5495435 |
| Psmb7 | -0.8008112 |
| Psmb7 | -0.481618 |
| Psmb8 | -0.2587773 |
| Psmb9 | 1.55525541 |
| Psmc1 | 0.96360367 |
| Psmc2 | -0.8344196 |
| Psmc3 | -0.9011673 |
| Psmc3ip | -0.5362879 |
| Psmc4 | 1.3400556 |
| Psmc5 | -0.802755 |
| Psmc5 | 1.0727166 |
| Psmd1 | 0.23195807 |
| Psmd10 | -0.8364348 |
| Psmd10 | -0.4189484 |
| Psmd10 | -0.4871512 |
| Psmd11 | 0.12206338 |
| Psmd11 | 1.68451425 |
| Psmd12 | 1.97530777 |
| Psmd12 | 1.69172589 |
| Psmd13 | 0.76281114 |
| Psmd14 | 2.0076371 |
| Psmd2 | 1.27185151 |
| Psmd2 | 0.48263299 |
| Psmd4 | -0.2246514 |
| Psmd5 | -0.610848 |
| Psmd6 | 1.01588334 |
| Psmd6 | 0.75935245 |
| Psmd7 | -0.6140216 |
| Psmd7 | 1.6390391 |
| Psmd8 | 0.15226105 |
| Psmd9 | 1.4468458 |
| Psmd9 | 0.04963543 |
| Psme1 | -0.1502111 |
| Psme1 | 0.45802263 |
| Psme2 | 1.06459986 |
| Psme3 | 0.3311284 |
| Psme4 | -0.917527 |
| Psme4 | -0.6996958 |
| Psmf1 | 0.65731229 |
| Psmf1 | -0.5644961 |
| Psmg2 | 2.27227588 |
| Psmg2 | 2.12011768 |
| Psmg3 | 2.20964214 |
| Psors1c2 | 2.06133422 |
| Psp | 0.88076663 |
| Pspc1 | -0.0391354 |
| Pstpip1 | 0.38740414 |
| Pstpip2 | 0.75386512 |
| Ptbp1 | -0.698825 |
| Ptbp1 | 0.21118723 |
| Ptcd3 | 2.73077593 |
| Ptch1 | 1.61689845 |
| Ptch2 | -0.7655626 |
| Ptdss2 | 0.68081749 |
| Pten | 3.36025664 |
| Pter | -0.6764657 |
| Ptgds | 1.67314611 |
| Ptgds2 | 2.27623152 |
| Ptgds2 | 0.7680656 |
| Ptger2 | -0.7424781 |
| Ptger2 | -0.6996958 |
| Ptger3 | -0.2992897 |
| Ptges | -0.4138042 |
| Ptges | -0.1003249 |
| Ptges2 | -0.3632568 |
| Ptges3 | 0.85495355 |
| Ptgfr | -0.1058403 |
| Ptgir | -0.7424781 |
| Ptgis | -0.6815782 |
| Ptgs1 | -0.9488546 |
| Ptgs1 | -0.9341788 |
| Pth2 | -0.4125226 |
| Pth2r | -0.5275544 |
| Pthlh | 0.19929717 |
| Pthr1 | -0.8766266 |
| Pthr1 | -0.8829666 |
| Pthr1 | 0.52413966 |
| Ptk2 | 0.81475749 |
| Ptk2b | 2.63436091 |
| Ptk7 | -0.7892327 |
| Ptms | 0.50421663 |
| Ptn | -0.729796 |
| Ptp4a2 | -0.1570038 |
| Ptp4a2 | 1.8335266 |
| Ptp4a2 | -0.8364348 |
| Ptp4a2 | -0.8224191 |
| Ptp4a3 | -0.3584099 |
| Ptp4a3 | -0.1174366 |
| Ptpdc1 | 1.11289823 |
| Ptpdc1 | 0.47894562 |
| Ptpdc1 | 0.00200332 |
| Ptpla | -0.657956 |
| Ptpla | 0.12566719 |
| Ptplad1 | -0.3263358 |
| Ptplad2 | 0.18556856 |
| Ptplb | -0.3481931 |
| Ptpn1 | -0.6204013 |
| Ptpn11 | -0.4843805 |
| Ptpn11 | 0.04061682 |
| Ptpn11 | -0.2947632 |
| Ptpn12 | -0.6006079 |
| Ptpn12 | -0.3935064 |
| Ptpn12 | -0.4631822 |
| Ptpn12 | -0.5473211 |
| Ptpn12 | 0.24249003 |
| Ptpn14 | 1.03657092 |
| Ptpn18 | 2.36368602 |
| Ptpn20 | 1.76813636 |
| Ptpn21 | -0.3392702 |
| Ptpn22 | -0.9352995 |
| Ptpn5 | 2.12633266 |
| Ptpn6 | -1.0148537 |
| Ptpn6 | -0.3363148 |
| Ptpra | -0.2970237 |
| Ptprb | 0.16957829 |
| Ptprd | 0.96871775 |
| Ptprd | -0.4611561 |
| Ptpre | -0.1952744 |
| Ptpre | -0.2609522 |
| Ptprf | 0.67644267 |
| Ptprf | -0.7581132 |
| Ptprg | 0.39201169 |
| Ptprj | -0.5517711 |
| Ptprk | 1.0546859 |
| Ptprk | 0.14805664 |
| Ptprn | 1.478343 |
| Ptprn2 | -0.3614363 |
| Ptpro | 0.96900914 |
| Ptpro | -0.7627621 |
| Ptprq | 0.98145354 |
| Ptprr | 1.6932688 |
| Ptprr | 1.44308483 |
| Ptprs | 0.37399482 |
| Ptprs | -0.6927523 |
| Ptprt | -0.2147559 |
| Ptpru | 0.76444501 |
| Ptprv | -0.57587 |
| Ptrf | -0.5675156 |
| Ptrh1 | 3.19360229 |
| Ptrh1 | -0.6284377 |
| Ptrh2 | 1.3578927 |
| Pts | -0.5966991 |
| Pts | -0.691888 |
| Pttg1 | 0.45018096 |
| Pttg1ip | 2.0184374 |
| Ptx3 | 1.1380268 |
| Ptx3 | -0.2735571 |
| Puf60 | 1.58070595 |
| Puf60 | -0.8095904 |
| Pum1 | -0.0534068 |
| Pum2 | -0.4371798 |
| Pum2 | 0.18421831 |
| Punc | 2.01050398 |
| Purb | -0.7844504 |
| Purg | 1.15976937 |
| Pus1 | 0.21413622 |
| Pus1 | -0.7683715 |
| Pus10 | -0.0710033 |
| Pus10 | 0.62754552 |
| Pus10 | 0.5229656 |
| Pus3 | -0.7768493 |
| Pus7l | 0.03508425 |
| Pvalb | -0.4948133 |
| Pvr | 0.81527293 |
| Pvr | -0.8086109 |
| Pvrl1 | 1.73411962 |
| Pvrl2 | 0.13104695 |
| Pvrl2 | 2.51467332 |
| Pvrl2 | 0.99981435 |
| Pvrl3 | -0.6116404 |
| Pvrl3 | 1.42512189 |
| Pvrl3 | -0.5951401 |
| Pwp1 | 2.45887117 |
| Pwp2 | -0.0116479 |
| Pwwp2b | -0.467927 |
| Pxdn | 1.50282979 |
| Pxmp2 | 0.85873199 |
| Pxmp4 | -0.2080453 |
| Pxn | -0.5160354 |
| Pxt1 | -0.0344264 |
| Pycr2 | 1.8559773 |
| Pycrl | 2.38497321 |
| Pygb | -0.5458424 |
| Pygl | -0.302699 |
| Pygl | 0.11226297 |
| Pygm | -0.4431164 |
| Pygo1 | -0.6815782 |
| Pygo2 | 0.68635592 |
| Pyroxd1 | -0.7721299 |
| Pyy | -0.9557067 |
| Qars | 0.13781708 |
| Qdpr | 0.41813813 |
| Qpct | 0.71375698 |
| Qprt | -0.0468927 |
| Qrich2 | 1.32346627 |
| Qrich2 | 1.4498089 |
| Qrsl1 | 1.25955654 |
| Qsox1 | -0.645543 |
| Qtrt1 | 0.58697743 |
| R3hdm1 | 3.78886708 |
| R3hdm1 | -0.2970237 |
| R3hdm2 | 0.12638629 |
| R74862 | -0.1638464 |
| Rab1 | 0.16476177 |
| Rab10 | 1.15345505 |
| Rab10 | -0.8354267 |
| Rab11a | 0.10088222 |
| Rab11b | 0.67544553 |
| Rab11fip2 | 1.13119588 |
| Rab11fip3 | -0.6037467 |
| Rab11fip5 | 0.98317684 |
| Rab11fip5 | 1.5405297 |
| Rab11fip5 | 0.70238905 |
| Rab12 | 0.4921525 |
| Rab13 | 1.04868657 |
| Rab13 | -0.0943864 |
| Rab14 | -0.5348266 |
| Rab15 | 0.34658379 |
| Rab17 | 3.23994142 |
| Rab17 | -0.7507229 |
| Rab18 | 0.4511965 |
| Rab19 | -0.5067798 |
| Rab19 | 1.00655509 |
| Rab1b | 0.94495731 |
| Rab1b | -0.7023129 |
| Rab1b | 0.09085557 |
| Rab21 | 0.87643331 |
| Rab22a | 1.72600069 |
| Rab22a | -0.4598078 |
| Rab23 | 1.32097221 |
| Rab24 | -0.682433 |
| Rab24 | -0.1183706 |
| Rab24 | 0.00732673 |
| Rab25 | 1.39746591 |
| Rab27a | 2.47837502 |
| Rab27b | 1.41902659 |
| Rab28 | -0.4048696 |
| Rab28 | 0.96696751 |
| Rab2a | 1.93759745 |
| Rab31 | 2.3386855 |
| Rab32 | -0.3216765 |
| Rab33a | -0.6447213 |
| Rab33b | -0.3095554 |
| Rab34 | -0.0741171 |
| Rab34 | 2.85279513 |
| Rab34 | 0.140299 |
| Rab35 | 0.13033144 |
| Rab36 | -0.6867188 |
| Rab38 | -0.1310714 |
| Rab38 | -0.6654838 |
| Rab39 | -0.7066913 |
| Rab39b | -0.4920198 |
| Rab3a | -0.3602246 |
| Rab3b | -0.0930213 |
| Rab3d | -0.6381738 |
| Rab3gap1 | -0.6798709 |
| Rab3gap2 | -0.5858408 |
| Rab3il1 | -0.57587 |
| Rab3il1 | -0.2075311 |
| Rab3ip | -0.5866122 |
| Rab40b | -0.6373585 |
| Rab40c | 0.00077052 |
| Rab43 | -0.7084484 |
| Rab4a | 0.34828536 |
| Rab4b | 0.83650022 |
| Rab5a | 2.01412276 |
| Rab5a | -0.6084749 |
| Rab5b | -0.610848 |
| Rab5c | 0.06511528 |
| Rab6 | -0.5920301 |
| Rab6 | 0.12889877 |
| Rab6 | -0.4517586 |
| Rab6b | -0.4208848 |
| Rab6ip1 | 0.46829366 |
| Rab7 | 0.39874642 |
| Rab7 | 2.25404452 |
| Rab7 | -0.7146245 |
| Rab7l1 | -0.5348266 |
| Rab8a | -0.1372488 |
| Rab8a | -0.5781615 |
| Rab8b | -0.1942603 |
| Rab8b | 0.16441678 |
| Rab9 | -0.5203384 |
| Rab9 | 0.3098195 |
| Rab9b | 1.34044701 |
| Rabac1 | 0.6146439 |
| Rabac1 | 2.08638521 |
| Rabep1 | -0.5275544 |
| Rabep1 | -0.4042346 |
| Rabep2 | -0.4934155 |
| Rabgap1 | 0.38794739 |
| Rabgap1 | -0.3216765 |
| Rabgap1l | 0.45018096 |
| Rabgap1l | -0.1962897 |
| Rabgef1 | -0.1633559 |
| Rabggta | 0.7496362 |
| Rabggtb | -0.6045331 |
| Rabif | 0.79513239 |
| Rabif | -0.4665689 |
| Rabl2a | 2.28288223 |
| Rabl2a | -0.5377515 |
| Rabl3 | -0.4665689 |
| Rabl4 | -0.4665689 |
| Rabl4 | 0.44559731 |
| Rabl5 | 0.63403435 |
| Rac1 | 1.18991107 |
| Rac2 | -0.5060716 |
| Rac3 | 1.51936992 |
| Rac3 | 0.18556856 |
| Rad1 | -0.6156124 |
| Rad17 | 0.99359226 |
| Rad18 | -0.4189484 |
| Rad23a | -0.5060716 |
| Rad23a | -0.2560658 |
| Rad23a | -0.5966991 |
| Rad23b | 0.23931106 |
| Rad50 | 1.94959103 |
| Rad51 | -0.4080507 |
| Rad51 | -0.5644961 |
| Rad51ap1 | 0.76535147 |
| Rad51c | 0.78914579 |
| Rad51l3 | 0.45676229 |
| Rad51l3 | 2.08739624 |
| Rad51l3 | -0.5904789 |
| Rad51l3 | 1.92847394 |
| Rad52 | -0.3866298 |
| Rad54l | 0.05041555 |
| Rad9 | -0.2533621 |
| Rad9 | -0.5637428 |
| Rad9 | -0.6300534 |
| Rad9b | -0.4391545 |
| Rae1 | -0.5153202 |
| Raet1b | -0.6439004 |
| Raet1b | 0.24121974 |
| Raf1 | -0.2351822 |
| Rag1 | 0.39038809 |
| Rag1ap1 | -0.6962175 |
| Rage | -0.5873843 |
| Rage | -0.6901617 |
| Rai1 | -0.3565988 |
| Rai12 | -0.0687855 |
| Rai12 | 0.42885518 |
| Rai12 | -0.0438683 |
| Rai12 | 2.88001644 |
| Rai14 | -0.3199353 |
| Rala | 2.25213168 |
| Ralb | 0.06703232 |
| Ralbp1 | -0.57587 |
| Ralgds | 1.56637955 |
| Ralgps1 | 1.98477169 |
| Ralgps1 | 0.59026235 |
| Ralgps1 | -0.0551516 |
| Ralgps2 | 0.14100691 |
| Ralgps2 | -0.0280431 |
| Raly | 1.61965867 |
| Raly | -0.6398063 |
| Ralyl | 1.88801925 |
| Ramp1 | -0.1796775 |
| Ramp2 | -0.6100563 |
| Ramp3 | 1.85083975 |
| Ramp3 | 0.17913737 |
| Ran | -0.691888 |
| Ranbp1 | 0.08748927 |
| Ranbp10 | 0.55529343 |
| Ranbp17 | -0.3773336 |
| Ranbp2 | -0.69535 |
| Ranbp3l | 0.51209434 |
| Ranbp6 | 2.02747587 |
| Ranbp9 | 2.19632877 |
| Rangap1 | -0.6739199 |
| Rangrf | 1.12690102 |
| Rap1b | -0.6562913 |
| Rap1gap | 3.1638266 |
| Rap2a | -0.6045331 |
| Rap2b | -0.7646281 |
| Rap2c | 0.16889174 |
| Rap2c | -0.8264022 |
| Rap2c | 0.01909369 |
| Rap2ip | 1.56860387 |
| Rapgef1 | 0.10235853 |
| Rapgef1 | -0.5773971 |
| Rapgef1 | -0.1482793 |
| Rapgef1 | -0.4484252 |
| Rapgef3 | -0.5458424 |
| Rapgef4 | 0.64192732 |
| Rapgef5 | 2.42320556 |
| Rapgef6 | -0.6646444 |
| Rapgefl1 | 1.04868657 |
| Rapgefl1 | -0.4099646 |
| Rapsn | -0.7863604 |
| Rapsn | -0.1836776 |
| Rara | 0.80055749 |
| Rarb | -0.7306962 |
| Rarb | 0.29853156 |
| Rarg | -0.6841449 |
| Rarg | -0.2576917 |
| Rarg | -0.5781615 |
| Rarres2 | 2.12801667 |
| Rars | -0.7544107 |
| Rars | -0.6398063 |
| Rars2 | -0.6406237 |
| Rars2 | 1.66820182 |
| Rars2 | -0.2436934 |
| Rasa1 | -0.4733793 |
| Rasa2 | -0.5827615 |
| Rasal1 | -0.2152741 |
| Rasd1 | 1.50897488 |
| Rasef | -0.6538 |
| Rasgef1b | -0.2325384 |
| Rasgef1b | -0.622003 |
| Rasgef1c | -0.3626496 |
| Rasgef1c | -0.6373585 |
| Rasgrf1 | -0.7768493 |
| Rasgrf1 | 0.38441061 |
| Rasgrf1 | -0.7288967 |
| Rasgrf1 | 0.03982846 |
| Rasgrp1 | -0.3422351 |
| Rasgrp1 | -0.7271007 |
| Rasgrp1 | -0.3835208 |
| Rasgrp3 | 0.32678941 |
| Rasip1 | 0.57015372 |
| Rasl10a | -0.2474416 |
| Rasl10b | 1.29699136 |
| Rasl10b | -0.7479665 |
| Rasl11a | 0.44304107 |
| Rasl11b | 0.75202881 |
| Rasl11b | 1.81494526 |
| Rasl12 | 0.96066869 |
| Rasl2-9 | -0.4913227 |
| Rassf1 | 1.74006716 |
| Rassf1 | -0.6180038 |
| Rassf10 | -0.298156 |
| Rassf2 | -0.5690291 |
| Rassf2 | -0.1947672 |
| Rassf3 | 2.12570037 |
| Rassf4 | 0.01464781 |
| Rassf4 | 0.54456493 |
| Rassf4 | -0.3416413 |
| Rassf4 | -0.5253837 |
| Rassf5 | 0.24280733 |
| Rassf7 | -0.5735841 |
| Rassf7 | 2.24932891 |
| Raver1 | 0.045725 |
| Raver2 | 0.12710483 |
| Rb1 | 0.40676446 |
| Rb1 | 0.80125521 |
| Rbak | -0.2790911 |
| Rbak | -0.4584614 |
| Rbbp5 | -0.2163114 |
| Rbbp5 | -0.1618862 |
| Rbbp6 | -0.5835304 |
| Rbbp7 | -0.4106034 |
| Rbbp7 | 0.8204117 |
| Rbbp9 | -0.7306962 |
| Rbck1 | -0.2785362 |
| Rbed1 | -0.7352101 |
| Rbed1 | -0.2840999 |
| Rbj | -0.2802018 |
| Rbks | -0.2675074 |
| Rbl1 | 0.85049838 |
| Rbl2 | 0.50157587 |
| Rbm10 | 1.75561902 |
| Rbm10 | -0.6053201 |
| Rbm10 | 2.28530189 |
| Rbm11 | -0.7370218 |
| Rbm12 | 3.19080581 |
| Rbm12 | 2.27702064 |
| Rbm12 | 1.88179687 |
| Rbm12 | 0.05469461 |
| Rbm13 | -0.5032443 |
| Rbm13 | 1.40771704 |
| Rbm14 | -0.3650809 |
| Rbm14 | 0.42963455 |
| Rbm15 | 1.16154871 |
| Rbm16 | 1.09461661 |
| Rbm18 | -0.6996958 |
| Rbm18 | -0.0481919 |
| Rbm19 | 0.17913737 |
| Rbm24 | 1.20959039 |
| Rbm26 | -0.4477599 |
| Rbm28 | 1.81323755 |
| Rbm28 | 0.69146949 |
| Rbm3 | -0.1861865 |
| Rbm35a | -0.5131777 |
| Rbm38 | 0.08072 |
| Rbm38 | -0.2863347 |
| Rbm38 | -0.2868942 |
| Rbm4 | 0.7714922 |
| Rbm41 | 0.34060375 |
| Rbm42 | 0.11772035 |
| Rbm42 | 0.03903943 |
| Rbm43 | -0.4398136 |
| Rbm45 | -0.6076852 |
| Rbm47 | 0.44278506 |
| Rbm47 | -0.4665689 |
| Rbm47 | 1.01629885 |
| Rbm47 | 2.69213386 |
| Rbm4b | -0.7343056 |
| Rbm5 | -0.1463516 |
| Rbm5 | 1.84487645 |
| Rbm6 | 0.17879766 |
| Rbm6 | 2.43417637 |
| Rbm6 | 2.29502376 |
| Rbm8a | -0.4823079 |
| Rbm9 | -0.3193556 |
| Rbm9 | -0.2829845 |
| Rbms1 | 0.34117493 |
| Rbms1 | -0.6504884 |
| Rbms2 | 1.9493873 |
| Rbms2 | -0.7806421 |
| Rbms3 | 0.77920099 |
| Rbmx | 1.13473956 |
| Rbmy1a1 | 1.33041437 |
| Rbp1 | -0.2913826 |
| Rbp7 | 2.83870401 |
| Rbpms | 2.43369151 |
| Rbpms | 1.20360545 |
| Rbpms | -0.5297301 |
| Rbpms2 | 2.288601 |
| Rbx1 | 0.05158451 |
| Rbx1 | -0.433898 |
| Rc3h1 | 1.43303987 |
| Rcan1 | 0.21315426 |
| Rcan2 | -0.4228253 |
| Rcan2 | 1.35346529 |
| Rcan2 | -0.6884387 |
| Rcan3 | -0.6045331 |
| Rcbtb2 | -0.8324088 |
| Rcbtb2 | -0.697955 |
| Rcc1 | 0.18927172 |
| Rccd1 | 0.90557296 |
| Rce1 | -0.0921124 |
| Rchy1 | 0.24375859 |
| Rcl1 | 1.6326951 |
| Rcl1 | -0.739746 |
| Rcl1 | 0.5770527 |
| Rcn3 | -0.227273 |
| Rcor1 | 0.30538004 |
| Rcor2 | 0.23035189 |
| Rcsd1 | 1.98383943 |
| Rdbp | -0.2095897 |
| Rdbp | -0.0772413 |
| Rdh11 | -0.5697867 |
| Rdh12 | 1.00023707 |
| Rdh14 | -0.6463654 |
| Rdh14 | -0.296458 |
| Rdh5 | 0.0342912 |
| Rdh7 | 0.35899049 |
| Rdh9 | 1.97317502 |
| Rdm1 | -0.6504884 |
| Rec8 | 1.73328521 |
| Reck | 0.52390496 |
| Recql | 1.28627035 |
| Recql4 | -0.6300534 |
| Recql5 | 1.21553718 |
| Reep1 | 1.93133463 |
| Reep2 | -0.2941989 |
| Reep3 | 3.15720591 |
| Reep4 | -0.323421 |
| Reep5 | -0.2582343 |
| Reep6 | 1.38047008 |
| Reep6 | 1.87166535 |
| Refbp2 | -0.3872529 |
| Reg1 | -0.227273 |
| Rela | -0.7740148 |
| Relb | 0.48606143 |
| Rell1 | 1.44972189 |
| Rell2 | 2.30115808 |
| Rell2 | 1.33150152 |
| Reln | 2.1439477 |
| Relt | 0.01788327 |
| Relt | 0.70722289 |
| Rem1 | -0.0767943 |
| Rem2 | 2.43117223 |
| Renbp | 1.21888657 |
| Repin1 | -0.7406558 |
| Repin1 | 0.11699454 |
| Reps1 | -0.41573 |
| Reps1 | -0.0687855 |
| Rer1 | 0.38903293 |
| Rer1 | 0.51423132 |
| Rerg | 0.49675526 |
| Resp18 | 1.41884671 |
| Rest | -0.5443661 |
| Ret | 1.90734752 |
| Retnla | -0.8154895 |
| Retnlg | -0.5525149 |
| Retsat | -0.4878451 |
| Retsat | -0.4055049 |
| Rev1 | 1.57074648 |
| Rev3l | 0.59418905 |
| Rex3 | 2.52635581 |
| Rexo1 | -0.0826216 |
| Rexo1 | -0.6790184 |
| Rexo2 | 3.25646433 |
| Rfc1 | 2.79808587 |
| Rfc2 | 1.95142259 |
| Rfc3 | -0.7049375 |
| Rfc4 | -0.1137101 |
| Rfc4 | -0.7226257 |
| Rfc5 | 0.64770076 |
| Rfesd | 1.24026041 |
| Rffl | 1.29965247 |
| Rffl | -0.8154895 |
| Rffl | 2.42949908 |
| Rffl | -0.8703294 |
| Rffl | -0.3669086 |
| Rfng | 3.34556693 |
| Rfpl4 | 2.49968496 |
| Rfpl4 | -0.5282791 |
| Rft1 | 0.06319428 |
| Rftn1 | 0.21772793 |
| Rftn2 | 1.02635296 |
| Rftn2 | 2.1621289 |
| Rfwd2 | -0.7608998 |
| Rfwd3 | 0.62312409 |
| Rfwd3 | 1.63240897 |
| Rfx1 | 1.12886684 |
| Rfx1 | 1.13559292 |
| Rfx2 | 1.2722726 |
| Rfx3 | -0.7806421 |
| Rfx4 | -0.5103288 |
| Rfx4 | 0.43661966 |
| Rfx4 | -0.0674573 |
| Rfxank | 2.60715083 |
| Rfxap | 2.8742876 |
| Rfxdc1 | 1.1314407 |
| Rg9mtd1 | -0.6807241 |
| Rg9mtd2 | 1.15786766 |
| Rgag4 | -0.2178695 |
| Rgl1 | 1.84282309 |
| Rgl2 | -0.2220371 |
| Rgl2 | -0.6260195 |
| Rgl3 | 1.42225847 |
| Rgma | 0.98059069 |
| Rgmb | 2.74595169 |
| Rgmb | 2.42335881 |
| Rgnef | 1.94048177 |
| Rgs1 | -0.5046569 |
| Rgs1 | -0.4365225 |
| Rgs1 | -0.8527149 |
| Rgs10 | -0.7084484 |
| Rgs12 | 2.8615551 |
| Rgs14 | 2.52457011 |
| Rgs16 | 0.58390103 |
| Rgs17 | 1.36744563 |
| Rgs2 | 1.55120554 |
| Rgs3 | 0.2910301 |
| Rgs3 | 1.10373916 |
| Rgs3 | -0.289697 |
| Rgs3 | 1.21643153 |
| Rgs3 | -0.6798709 |
| Rgs3 | -0.820434 |
| Rgs3 | -0.5319109 |
| Rgs4 | 3.69867252 |
| Rgs5 | -0.041714 |
| Rgs6 | 0.05430641 |
| Rgs7 | 1.02840553 |
| Rgs7bp | 1.37350857 |
| Rgs8 | 0.00282429 |
| Rgs9 | 2.26169337 |
| Rgs9bp | -0.8755741 |
| Rhag | 3.2618951 |
| Rhbdd1 | 1.73968459 |
| Rhbdd3 | -0.8125353 |
| Rhbdf1 | 2.5807559 |
| Rhbdf2 | -0.3158849 |
| Rhbdl1 | -0.4976151 |
| Rhbdl2 | 2.24551693 |
| Rhbdl3 | 0.10198967 |
| Rhbdl7 | 0.59026235 |
| Rhcg | 1.76677446 |
| Rheb | 0.78967556 |
| Rhebl1 | -0.682433 |
| Rhob | -0.2941989 |
| Rhobtb2 | 1.10449536 |
| Rhobtb2 | 1.16533334 |
| Rhobtb2 | -0.8154895 |
| Rhobtb2 | 1.52227278 |
| Rhobtb3 | 0.81510115 |
| Rhoc | -0.8599277 |
| Rhoc | 0.55302108 |
| Rhod | 3.60388959 |
| Rhof | 0.43713501 |
| Rhog | -0.0408537 |
| Rhoj | -0.7452183 |
| Rhot1 | 1.34435212 |
| Rhot2 | 1.69881559 |
| Rhot2 | 0.05119502 |
| Rhot2 | 0.21609704 |
| Rhou | -0.4464308 |
| Rhov | -0.3199353 |
| Rhox11 | -0.7208417 |
| Rhox12 | 0.84219146 |
| Rhox3a | -0.5751074 |
| Rhox3f | 1.78006058 |
| Rhox4d | -0.666324 |
| Rhox5 | -0.8840274 |
| Rhox8 | -0.8294007 |
| Rhpn1 | 0.46704714 |
| Rhpn1 | 0.1137214 |
| Rhpn1 | 0.48581696 |
| Ribc1 | -0.0564623 |
| Ric3 | 1.47199211 |
| Ric3 | -0.3626496 |
| Ric3 | 2.05744076 |
| Ric8b | -0.820434 |
| Ric8b | 0.12278526 |
| Rif1 | 0.4064983 |
| Rilp | -0.7190611 |
| Rilpl1 | 0.11989439 |
| Rilpl1 | -0.6037467 |
| Rilpl2 | 0.45676229 |
| Rims1 | 0.08936094 |
| Rims2 | -0.4490909 |
| Rims3 | 2.34943001 |
| Rin1 | -0.5261067 |
| Rin2 | -0.3718 |
| Rin2 | -0.691888 |
| Rin2 | 0.89837292 |
| Rin3 | -0.810571 |
| Riok1 | 0.14735406 |
| Riok1 | 1.63097696 |
| Riok3 | -0.4871512 |
| Riok3 | 0.16130612 |
| Ripk1 | -0.1697516 |
| Ripk2 | 0.15957348 |
| Ripk4 | 2.63986939 |
| Ripk5 | 1.01130048 |
| Rit1 | -0.3147309 |
| Rit2 | -0.5103288 |
| Rlbp1 | -0.5377515 |
| Rlbp1l1 | 1.55175174 |
| Rlbp1l1 | -0.648837 |
| Rln1 | 1.27153557 |
| Rltpr | 1.08899749 |
| Rmnd1 | 1.50987277 |
| Rmnd5b | -0.5789266 |
| Rmnd5b | 0.43558813 |
| Rnase1 | 1.37114492 |
| Rnase4 | -0.7217333 |
| Rnase4 | 1.3815941 |
| Rnase6 | -0.7370218 |
| Rnaseh1 | 0.32417629 |
| Rnaseh1 | -0.4280196 |
| Rnaseh2c | 0.36011024 |
| Rnasek | -0.5032443 |
| Rnasen | 0.97104632 |
| Rnaset2 | -0.2116527 |
| Rnaset2b | -0.5495435 |
| Rnd2 | 0.22422309 |
| Rnd3 | 1.48246902 |
| Rnf10 | -0.1109249 |
| Rnf103 | -0.7534874 |
| Rnf11 | -0.6910244 |
| Rnf11 | -0.2512048 |
| Rnf11 | -0.6349171 |
| Rnf111 | -0.5414205 |
| Rnf113a1 | 1.81004933 |
| Rnf113a2 | 3.69524425 |
| Rnf12 | -0.5873843 |
| Rnf121 | -0.7777961 |
| Rnf123 | -0.7911525 |
| Rnf125 | -0.1881984 |
| Rnf125 | -0.3767171 |
| Rnf128 | -0.4384958 |
| Rnf133 | 2.8209406 |
| Rnf135 | 2.32011045 |
| Rnf135 | 0.45877801 |
| Rnf135 | -0.5959193 |
| Rnf138 | 1.82535913 |
| Rnf138 | -0.0512304 |
| Rnf138 | -0.3656897 |
| Rnf138 | 1.50324072 |
| Rnf139 | 0.21282671 |
| Rnf139 | -0.3559959 |
| Rnf14 | -0.6061078 |
| Rnf14 | -0.622003 |
| Rnf14 | -0.1463516 |
| Rnf141 | 4.15029291 |
| Rnf141 | 2.0091672 |
| Rnf144a | -0.2362419 |
| Rnf145 | -0.7226257 |
| Rnf150 | -0.6341047 |
| Rnf166 | 0.39820943 |
| Rnf167 | -0.5842999 |
| Rnf167 | -0.4871512 |
| Rnf167 | -0.213203 |
| Rnf167 | -0.56299 |
| Rnf167 | 0.13068926 |
| Rnf168 | -0.1771861 |
| Rnf17 | 0.70200125 |
| Rnf170 | -0.6349171 |
| Rnf181 | 2.91880231 |
| Rnf182 | 0.56612433 |
| Rnf182 | 1.35914012 |
| Rnf183 | -0.6447213 |
| Rnf185 | 0.28316407 |
| Rnf185 | -0.2299019 |
| Rnf185 | 1.74134127 |
| Rnf185 | 0.15226105 |
| Rnf186 | 2.43332769 |
| Rnf19a | -0.7361155 |
| Rnf2 | 0.85577625 |
| Rnf20 | 1.14745872 |
| Rnf207 | 1.43002295 |
| Rnf208 | 3.37600026 |
| Rnf214 | 0.4141863 |
| Rnf215 | -0.4029661 |
| Rnf216 | 2.31561476 |
| Rnf216 | -0.5819933 |
| Rnf219 | 0.14946021 |
| Rnf24 | -0.7271007 |
| Rnf24 | 0.64089257 |
| Rnf25 | -0.3147309 |
| Rnf25 | -0.6604585 |
| Rnf25 | 0.22130586 |
| Rnf26 | -0.8354267 |
| Rnf31 | 1.5841781 |
| Rnf32 | 1.19495571 |
| Rnf34 | -0.1851821 |
| Rnf34 | 0.03824974 |
| Rnf34 | 3.83241333 |
| Rnf38 | 1.5270108 |
| Rnf38 | 2.27623152 |
| Rnf38 | 3.47861906 |
| Rnf4 | -0.8568297 |
| Rnf4 | -0.1272901 |
| Rnf40 | -0.810571 |
| Rnf41 | -0.4273687 |
| Rnf41 | -0.5032443 |
| Rnf41 | -0.4934155 |
| Rnf44 | -0.8234133 |
| Rnf5 | -0.4247697 |
| Rnf6 | -0.2852166 |
| Rnf6 | -0.6927523 |
| Rnf8 | -0.2277982 |
| Rnf8 | -0.8578612 |
| Rngtt | 0.1314045 |
| Rnmt | 1.60385916 |
| Rnmtl1 | 0.54387593 |
| Rnpc2 | 0.07504118 |
| Rnpep | -0.7711889 |
| Rnpepl1 | 0.39228201 |
| Rnps1 | -0.3181972 |
| Rnps1 | 0.54983046 |
| Rnu6 | -0.652971 |
| Rnuxa | 0.0971812 |
| Rnuxa | -0.6389897 |
| Robo1 | -0.9164274 |
| Robo2 | -0.5713038 |
| Robo4 | -0.1391578 |
| Rock1 | -0.4293227 |
| Rock2 | 0.76117441 |
| Rock2 | -0.1449084 |
| Rod1 | 0.51825452 |
| Rogdi | -0.1113885 |
| Rom1 | 0.75789231 |
| Ropn1 | 0.41813813 |
| Ror1 | -0.5081978 |
| Ror2 | 1.46431354 |
| Rora | 1.39654513 |
| Rorb | -0.8086109 |
| Rorb | -0.588157 |
| Rorc | -0.5046569 |
| Rorc | 2.86738146 |
| Ros1 | -0.1570038 |
| Rp23-297j14.5 | 1.94366476 |
| Rp23-438h3.2 | 0.63131872 |
| rp9 | -0.2075311 |
| rp9 | 0.82891391 |
| Rpa1 | -0.3049789 |
| Rpa2 | -0.5569898 |
| Rpa2 | -0.6604585 |
| Rpa3 | 0.68694738 |
| Rpain | 1.00459406 |
| Rpap1 | -0.5502854 |
| Rpap1 | 0.90057012 |
| Rpap2 | -0.7854049 |
| Rpap2 | 0.50756682 |
| Rpap3 | 1.31334907 |
| Rpe | -0.366299 |
| Rpgr | -0.4138042 |
| Rpgrip1 | 1.47665365 |
| Rpgrip1l | 3.51363944 |
| Rph3a | -0.1109249 |
| Rph3al | 1.16214107 |
| Rpia | 0.15156163 |
| Rpl12 | -0.2555244 |
| Rpl13a | -0.4906261 |
| Rpl18 | 0.69967111 |
| Rpl19 | 1.08617519 |
| Rpl21 | -0.6901617 |
| Rpl21 | 1.01227449 |
| Rpl22 | 0.49554621 |
| Rpl22l1 | -0.5304565 |
| Rpl23 | 0.77866523 |
| Rpl23 | 0.41866378 |
| Rpl23 | -0.0108149 |
| Rpl24 | 0.56612433 |
| Rpl27 | -0.5465815 |
| Rpl27a | -0.7406558 |
| Rpl28 | -0.4761175 |
| Rpl29 | -0.5866122 |
| Rpl29 | -0.3015612 |
| Rpl3 | -0.6398063 |
| Rpl30 | 1.31977269 |
| Rpl31 | -0.6398063 |
| Rpl31 | -0.5268303 |
| Rpl34 | 2.55014746 |
| Rpl35 | -0.6944833 |
| Rpl36a | -0.3791854 |
| Rpl36al | -0.2757668 |
| Rpl37 | -0.5827615 |
| Rpl38 | 0.06049823 |
| Rpl38 | 0.13710674 |
| Rpl39 | 0.55165501 |
| Rpl39l | 1.95065988 |
| Rpl3l | 1.78310929 |
| Rpl4 | -0.69535 |
| Rpl41 | 0.72782202 |
| Rpl7a | -0.5789266 |
| Rpl7l1 | 0.71413992 |
| Rpl7l1 | 1.42512189 |
| Rpl9 | -0.636544 |
| Rplp1 | -0.8234133 |
| Rpn2 | 0.44201661 |
| Rpo1-1 | 0.08673955 |
| Rpo1-3 | 0.52788676 |
| Rpo1-3 | 1.82267902 |
| Rpo1-4 | 2.74958855 |
| Rpo1-4 | 0.1747114 |
| Rpo1-4 | -0.4048696 |
| Rpo2tc1 | 2.06083713 |
| Rpp21 | 0.10530416 |
| Rpp25 | -0.1742052 |
| Rpp30 | -0.4795515 |
| Rpp38 | 2.06444634 |
| Rpp40 | -0.2383648 |
| Rprm | 3.03994882 |
| Rprml | -0.7618305 |
| Rps10 | -0.9252609 |
| Rps11 | 1.07998764 |
| Rps12 | 0.41074779 |
| Rps13 | -0.0468927 |
| Rps14 | 1.50840305 |
| Rps15 | -0.6688491 |
| Rps15a | -0.7854049 |
| Rps16 | -0.7636946 |
| Rps19 | -0.5167512 |
| Rps19 | -0.0599666 |
| Rps19bp1 | -0.7759035 |
| Rps2 | 1.219555 |
| Rps21 | 0.35562318 |
| Rps24 | 3.738162 |
| Rps24 | 0.39874642 |
| Rps25 | 0.31040984 |
| Rps26 | -0.56299 |
| Rps27a | 0.32330363 |
| Rps27a | 0.71413992 |
| Rps27l | 0.3623457 |
| Rps3 | 0.37674711 |
| Rps3 | 2.05083918 |
| Rps3a | -0.5210575 |
| Rps4x | -0.5982606 |
| Rps4y2 | 0.07617972 |
| Rps5 | -0.1035382 |
| Rps6 | 0.79302385 |
| Rps6ka1 | 1.66433807 |
| Rps6ka3 | 3.41673251 |
| Rps6ka4 | -0.6188023 |
| Rps6ka5 | -0.0687855 |
| Rps6kb1 | 2.11531187 |
| Rps6kl1 | -0.2868942 |
| Rps7 | 1.35114697 |
| Rps8 | 0.88044633 |
| Rps9 | -0.0008758 |
| Rps9 | -0.2807577 |
| Rpsa | -0.892558 |
| Rpusd1 | 0.82840596 |
| Rpusd4 | 0.29643722 |
| Rqcd1 | -0.7759035 |
| Rrad | 2.36313041 |
| Rragb | 3.11307472 |
| Rragc | -0.8724238 |
| Rragd | -0.6875784 |
| Rras | -0.4591344 |
| Rras2 | -0.380422 |
| Rrbp1 | -0.1449084 |
| Rrbp1 | 0.17709723 |
| Rreb1 | -0.3124271 |
| Rreb1 | -0.5131777 |
| Rrh | 0.72725592 |
| Rrm1 | -0.4781764 |
| Rrm2 | 2.08726443 |
| Rrm2 | -0.5897043 |
| Rrm2b | 0.7429783 |
| Rrn3 | 1.59845844 |
| Rrp1 | -0.5599851 |
| Rrp12 | -0.1511784 |
| Rrp15 | 1.17355906 |
| Rrp1b | -0.2593205 |
| Rrp1b | 0.17607548 |
| Rrp1b | 1.09283237 |
| Rrp9 | -0.4208848 |
| Rs1 | -0.4934155 |
| Rsad1 | -0.6430801 |
| Rsad2 | 1.13461759 |
| Rsf1 | 2.53561392 |
| Rshl1 | -0.4990192 |
| Rshl2a | 0.17845782 |
| Rsl1d1 | 0.18691687 |
| Rsl1d1 | 0.06779804 |
| Rsn | 0.0647314 |
| Rsph1 | 0.19996172 |
| Rspo1 | -0.1806759 |
| Rspo2 | -0.5812257 |
| Rspo3 | 1.63376734 |
| Rspo4 | -0.214238 |
| Rspry1 | -0.4023324 |
| Rsrc1 | 1.51702492 |
| Rsrc1 | -0.3416413 |
| Rsrc2 | 3.61505638 |
| Rsu1 | -0.69535 |
| Rtbdn | -0.2095897 |
| Rtel1 | -0.5912541 |
| Rtel1 | -0.622003 |
| Rtkn | 0.67604394 |
| Rtn1 | 0.41761219 |
| Rtn1 | -0.4451036 |
| Rtn1 | 0.08861272 |
| Rtn1 | 2.33279378 |
| Rtn2 | 0.46654806 |
| Rtn3 | -0.004177 |
| Rtn4 | -1.0039268 |
| Rtn4 | -0.8425066 |
| Rtn4 | -0.3257521 |
| Rtn4ip1 | -0.0486254 |
| Rtn4r | 0.57749604 |
| Rtn4rl1 | -0.5414205 |
| Rtn4rl2 | 3.01354208 |
| Rtp1 | -0.5275544 |
| Rtp4 | -0.8017826 |
| Rttn | -0.4658906 |
| Rufy1 | 0.54731587 |
| Rufy2 | 1.44675851 |
| Rufy3 | 2.11236513 |
| Rufy3 | 0.83868038 |
| Rufy3 | 0.40383251 |
| Rufy4 | -0.5167512 |
| Rundc1 | -0.6100563 |
| Rundc3b | -0.5046569 |
| Runx1 | -0.4564455 |
| Runx1t1 | 0.45499496 |
| Runx1t1 | -0.214238 |
| Runx2 | 2.51583985 |
| Rusc1 | 0.72290438 |
| Rusc2 | -0.6892998 |
| Rusc2 | -0.5622379 |
| Ruvbl1 | 0.48189668 |
| Ruvbl1 | -0.5974795 |
| Ruvbl1 | 1.74989626 |
| Ruvbl2 | 1.24134899 |
| Rwdd1 | -0.6196014 |
| Rwdd1 | 2.45981485 |
| Rwdd3 | -0.0284673 |
| Rwdd3 | 1.42762019 |
| Rwdd4a | -0.4990192 |
| Rxfp1 | 1.25602903 |
| Rxfp3 | 0.11844559 |
| Rxra | -0.0521003 |
| Rxrb | -0.0617237 |
| Rxrb | -0.1751978 |
| Rxrg | -0.7534874 |
| Rya3 | 0.3783946 |
| Ryk | -0.1747014 |
| Ryk | -0.5174675 |
| Ryr1 | -0.0665729 |
| Ryr1 | 0.8818868 |
| Ryr1 | 0.86086088 |
| Ryr3 | -0.7102088 |
| S100a1 | 0.00035922 |
| S100a10 | -0.6901617 |
| S100a11 | 0.14489085 |
| S100a13 | 0.26630261 |
| S100a16 | -0.6292452 |
| S100a4 | -0.7516435 |
| S100a6 | 0.49796275 |
| S100a8 | -0.4042346 |
| S100a9 | 0.20228392 |
| S100b | 1.55463349 |
| S100pbp | 0.36429735 |
| S100pbp | -0.5189018 |
| S3-12 | 2.09051035 |
| Saa3 | -0.5766332 |
| Sac3d1 | -0.1377257 |
| Sacm1l | -0.1742052 |
| Sacs | -0.5990424 |
| Sae1 | 0.28073035 |
| Sae1 | 0.43533006 |
| Sae1 | -0.6571233 |
| Sag | 0.18590582 |
| Sall1 | -0.8703294 |
| Sall1 | -0.4470952 |
| Sall2 | -0.7226257 |
| Sall4 | 0.49288077 |
| Sall4 | 2.14204364 |
| Samd10 | -0.6406237 |
| Samd11 | 0.20987357 |
| Samd12 | -0.633293 |
| Samd14 | 0.56297828 |
| Samd4 | -0.4150876 |
| Samd4 | 3.33251804 |
| Samd4b | 0.55165501 |
| Samd5 | -0.6196014 |
| Samd7 | -0.7452183 |
| Samd8 | 0.25791231 |
| Samd9l | -0.7562601 |
| Samhd1 | 0.29942769 |
| Samm50 | 1.14613434 |
| Samsn1 | -0.5340969 |
| Sap130 | -0.5897043 |
| Sap30 | 1.46867248 |
| Sap30bp | 2.82035998 |
| Sap30l | -0.7507229 |
| Saps1 | -0.0395647 |
| Saps2 | 1.74318563 |
| Saps3 | -0.4829982 |
| Saps3 | 1.56160624 |
| Sar1a | -0.6892998 |
| Sar1b | 0.66015627 |
| Sar1b | -0.4537644 |
| Sarm1 | -0.6513152 |
| Sars | 0.33974632 |
| Sars | 2.44732268 |
| Sars2 | -0.633293 |
| Sart1 | 1.43117763 |
| Sart3 | -0.467927 |
| Sas | 0.68477687 |
| Sass6 | -0.5842999 |
| Sat2 | -0.7452183 |
| Satb1 | 1.26519418 |
| Sav1 | 3.35020932 |
| Sbds | 1.02703765 |
| Sbf1 | -0.4299749 |
| Sbk | 2.74068784 |
| Sbno1 | -0.1104615 |
| Sbno2 | 0.24565821 |
| Sc4mol | -0.3572021 |
| Sc5d | -0.5812257 |
| Scaf1 | -0.7014397 |
| Scamp1 | 1.95735045 |
| Scamp2 | -0.6781667 |
| Scamp3 | 1.61142656 |
| Scamp4 | -0.3009928 |
| Scamp5 | 2.26801383 |
| Scamp5 | 3.94169556 |
| Scand1 | 0.56095021 |
| Scap | 0.32736911 |
| Scaper | 0.21020216 |
| Scara3 | -0.5181844 |
| Scara5 | 2.05771292 |
| Scarb1 | 1.49208147 |
| Scarb2 | -0.6116404 |
| Scarf1 | -0.2506663 |
| Scarf2 | 0.39498096 |
| Sccpdh | -0.6722266 |
| Scd1 | -0.1732137 |
| Scd2 | 0.27706783 |
| Scd4 | -0.4788637 |
| Scel | -0.7181721 |
| Scg2 | 0.01140108 |
| Scg3 | -0.3130025 |
| Scg5 | -0.7534874 |
| Scgn | 0.7194846 |
| Scgn | -0.6841449 |
| Scgn | 1.58334898 |
| Schip1 | 1.4148806 |
| Schip1 | 0.81114144 |
| Scin | -0.3493893 |
| scl0003251.1_21 | 1.61478645 |
| Sclt1 | -0.4754322 |
| Scly | -0.622003 |
| Scmh1 | -0.6841449 |
| Scmh1 | -0.2902585 |
| Scml2 | 1.29339706 |
| Scml4 | -0.3357249 |
| Scn10a | 1.37896928 |
| Scn11a | -0.7911525 |
| Scn1a | 0.13104695 |
| Scn2b | 1.27668259 |
| Scn3b | -0.4652127 |
| Scn3b | 1.23194539 |
| Scn3b | -0.5246613 |
| Scn3b | -0.7181721 |
| Scn4b | -0.8174641 |
| Scn5a | 0.7398177 |
| Scn7a | -0.5614864 |
| Scn8a | 0.02311709 |
| Scnm1 | 0.29972621 |
| Scnm1 | 0.82688044 |
| Scnn1a | -0.5473211 |
| Scnn1b | 0.66662442 |
| Scnn1g | -0.4345534 |
| Scoc | 2.62334437 |
| Scoc | 1.20733643 |
| Scotin | -0.3860072 |
| Scotin | -0.6252148 |
| Scotin | -0.2631321 |
| Scp2 | 0.24058395 |
| Scp2 | 1.34998565 |
| Scp2 | -0.8086109 |
| Scrg1 | -0.5246613 |
| Scrib | 2.00255064 |
| Scrib | 1.27992427 |
| Scrn1 | -0.0750086 |
| Scrn1 | -0.2431591 |
| Scrn1 | -0.3754854 |
| Scrn2 | -0.7562601 |
| Sct | -0.4273687 |
| Sctr | 0.78169714 |
| Scube1 | 1.44299718 |
| Scube2 | 0.86037003 |
| Scube3 | 0.40276405 |
| Scube3 | -0.6892998 |
| Scx | 0.22777625 |
| Scye1 | -0.41573 |
| Scyl1 | 1.23468878 |
| Scyl1bp1 | 0.90197988 |
| Scyl3 | 3.47778786 |
| Scyl3 | -0.6671649 |
| Sdad1 | -0.3422351 |
| Sdc1 | 1.48398013 |
| Sdc1 | 0.07580036 |
| Sdc2 | 0.17879766 |
| Sdc3 | -0.433243 |
| Sdc4 | 4.48910664 |
| Sdcbp | 1.96589701 |
| Sdcbp | 3.39387938 |
| Sdcbp2 | -0.0447314 |
| Sdcbp2 | 0.32678941 |
| Sdccag1 | 0.35674697 |
| Sdccag1 | -0.7128557 |
| Sdccag3 | -0.5858408 |
| Sdccag3 | 0.53138605 |
| Sdf2 | -0.6962175 |
| Sdf2 | -0.7424781 |
| Sdf4 | -0.4927174 |
| Sdhb | -0.0179197 |
| Sdhb | 2.85273709 |
| Sdhb | -0.5282791 |
| Sdhc | -0.5297301 |
| Sdhd | 0.82501251 |
| Sdk1 | 0.63465991 |
| Sdpr | -0.4537644 |
| Sds | -0.5067798 |
| Sdsl | -0.6781667 |
| Sec1 | -0.7244131 |
| Sec11a | -0.6901617 |
| Sec11c | 1.16320638 |
| Sec13 | 2.64188428 |
| Sec14l1 | 0.68911286 |
| Sec14l1 | 1.12147356 |
| Sec14l1 | 0.09420977 |
| Sec14l2 | 2.85765696 |
| Sec14l3 | 0.16476177 |
| Sec14l3 | -0.5858408 |
| Sec14l4 | 0.01586242 |
| Sec15l1 | -0.0083202 |
| Sec16a | -0.0534068 |
| Sec16b | -0.2891357 |
| Sec22a | -0.433898 |
| Sec22b | -0.454434 |
| Sec22c | 1.55424459 |
| Sec22c | -0.6504884 |
| Sec23a | 2.49914821 |
| Sec23ip | 0.18185065 |
| Sec24b | -0.0516652 |
| Sec24c | 0.30240861 |
| Sec24d | 2.94588913 |
| Sec61a1 | -0.7988715 |
| Sec61a2 | -0.4371798 |
| Sec61a2 | 1.16863234 |
| Sec61b | 0.44763727 |
| Sec61b | 2.80944138 |
| Sec61g | -0.0521003 |
| Sec63 | 0.5708236 |
| Sec63 | -0.4885396 |
| Secisbp2 | -0.610848 |
| Sectm1b | -0.6521427 |
| Seh1l | -0.1526314 |
| Seh1l | 1.47436817 |
| Sel1l | -0.5599851 |
| Sel1l | -0.385385 |
| Sel1l | 0.3109998 |
| Sel1l2 | 1.19209279 |
| Sel1l2 | 1.47088688 |
| Selenbp2 | 0.4189265 |
| Selk | 2.25782189 |
| Sell | 3.40021569 |
| Selp | -0.481618 |
| Selplg | -0.7244131 |
| Sema3a | -0.610848 |
| Sema3b | 0.05158451 |
| Sema3b | 0.31453177 |
| Sema3b | 2.29569173 |
| Sema3e | -0.6260195 |
| Sema3f | 1.23172557 |
| Sema3f | 0.41997661 |
| Sema3g | -0.5998248 |
| Sema4a | 0.11116766 |
| Sema4b | -0.6324821 |
| Sema4d | 1.15858126 |
| Sema4f | -0.6521427 |
| Sema4g | 1.94279309 |
| Sema5a | -0.6587894 |
| Sema5a | -0.4920198 |
| Sema5b | 2.8031589 |
| Sema6b | -0.7854049 |
| Sema6b | 0.97900673 |
| Sema6b | -0.2246514 |
| Sema6d | 0.47053323 |
| Sema6d | -0.56299 |
| Sema7a | 0.87175647 |
| Sema7a | -0.7110903 |
| Senp1 | -0.6858601 |
| Senp2 | -0.3535881 |
| Senp3 | 0.12925714 |
| Senp5 | -0.2539022 |
| Senp5 | -0.5377515 |
| Senp5 | 0.46579894 |
| Senp6 | -0.2293756 |
| Senp6 | 0.75478192 |
| Senp7 | 0.92973681 |
| Senp7 | -0.5982606 |
| Senp7 | 1.35491132 |
| Senp8 | 2.40337757 |
| Sephs1 | -0.9065887 |
| Sephs2 | 0.41971419 |
| Sepm | -0.682433 |
| Sepn1 | 0.41445028 |
| Sepp1 | 0.12278526 |
| Sepp1 | 0.24913082 |
| Sept3 | -0.2283237 |
| Sept4 | 3.30055986 |
| Sept4 | -0.341048 |
| Sept6 | -0.0318678 |
| Sept7 | 1.31375185 |
| Sept7 | -0.5912541 |
| Sept8 | 0.34941802 |
| Sept9 | -0.7470495 |
| Sept10 | 0.31040984 |
| Sept11 | 0.73252621 |
| Sept11 | -0.6180038 |
| Sept13 | -0.0776885 |
| Sept16 | 1.29893676 |
| Sepsecs | -0.7415665 |
| Sepw1 | -0.4584614 |
| Sepx1 | -0.6116404 |
| Sepx1 | 0.52249556 |
| Serac1 | 0.02552283 |
| Serbp1 | 0.0788309 |
| Serbp1 | 1.24504071 |
| Serbp1 | -0.0481919 |
| Serf1 | -0.7424781 |
| Serf2 | 1.28460998 |
| Sergef | -0.2163114 |
| Serhl | -0.451091 |
| Serhl | 0.81200368 |
| Serinc1 | 1.34103382 |
| Serinc2 | 1.58470534 |
| Serinc3 | -0.3422351 |
| Serpina1a | 1.93759745 |
| Serpina1a | 0.25447229 |
| Serpina1b | -0.2598641 |
| Serpina1b | -0.210105 |
| Serpina1d | 0.70645116 |
| Serpina1e | -0.2593205 |
| Serpina3b | -0.5819933 |
| Serpina3c | 1.09219432 |
| Serpina3g | 0.25133395 |
| Serpina3h | 1.90734752 |
| Serpina3k | 1.10801622 |
| Serpina3n | -0.6406237 |
| Serpina5 | -0.3164624 |
| Serpina5 | 2.91595045 |
| Serpina7 | -0.6521427 |
| Serpina9 | 0.01828692 |
| Serpinb11 | 0.89569791 |
| Serpinb12 | 1.23654969 |
| Serpinb1a | 1.05428715 |
| Serpinb3a | -0.2571494 |
| Serpinb6a | -0.5812257 |
| Serpinb6b | 0.16026692 |
| Serpinb6c | -0.3269199 |
| Serpind1 | -0.6521427 |
| Serpine2 | 0.37178712 |
| Serpinf1 | -0.4955129 |
| Serping1 | -0.7863604 |
| Serpinh1 | -0.7226257 |
| Serpini1 | 0.72819923 |
| Sertad1 | -0.1692581 |
| Sertad2 | 0.52061303 |
| Sertad2 | -0.4004341 |
| Sertad3 | -0.6092653 |
| Sertad4 | 3.83011567 |
| Sesn1 | 0.09866337 |
| Sesn1 | 0.12854026 |
| Sesn2 | -0.6629678 |
| Sesn3 | -0.0723365 |
| Setd1b | -0.3903744 |
| Setd2 | -0.4829982 |
| Setd3 | 1.07141221 |
| Setd4 | 1.18599418 |
| Setd4 | 1.06025069 |
| Setd5 | 2.53095538 |
| Setd6 | -0.7406558 |
| Setd6 | 0.48581696 |
| Setd7 | 1.59287701 |
| Setd7 | -0.6204013 |
| Setd8 | 1.1662771 |
| Setd8 | -0.391 |
| Setd8 | -0.645543 |
| Setdb1 | 0.57771764 |
| Setdb2 | -0.2426252 |
| Setmar | -0.729796 |
| Setx | -0.2774275 |
| Sez6 | -0.4692871 |
| Sez6l | 0.80577771 |
| Sez6l2 | -0.5297301 |
| Sez6l2 | -0.4477599 |
| Sf1 | 3.04657288 |
| Sf1 | -0.2824273 |
| Sf3a1 | -0.1420287 |
| Sf3a2 | 1.43745888 |
| Sf3a3 | 0.8847611 |
| Sf3b1 | -0.729796 |
| Sf3b2 | -0.5210575 |
| Sf3b3 | 1.23315347 |
| Sf3b4 | 0.19229068 |
| Sf3b4 | -0.5644961 |
| Sf3b5 | -0.2394281 |
| Sf4 | 0.47598519 |
| Sfi1 | 0.84219146 |
| Sfi1 | -0.5858408 |
| Sfmbt1 | 0.53068724 |
| Sfmbt1 | 0.47326319 |
| Sfmbt1 | 1.19860748 |
| Sfmbt2 | -0.6076852 |
| Sfrp1 | -0.6381738 |
| Sfrp2 | 0.045725 |
| Sfrp4 | 1.75781077 |
| Sfrp5 | -0.4948133 |
| Sfrs1 | 3.00762929 |
| Sfrs1 | -0.7279982 |
| Sfrs11 | 1.05574843 |
| Sfrs12 | 0.19229068 |
| Sfrs14 | 0.6054409 |
| Sfrs16 | 0.14665095 |
| Sfrs2 | 0.98804233 |
| Sfrs2 | 2.07670317 |
| Sfrs2 | -0.8578612 |
| Sfrs3 | 0.50804443 |
| Sfrs3 | -0.6100563 |
| Sfrs4 | 1.93589357 |
| Sfrs5 | -0.5743455 |
| Sfrs5 | -0.6100563 |
| Sfrs6 | 3.82661427 |
| Sfrs7 | -0.5789266 |
| Sfrs8 | 2.02141281 |
| Sfrs9 | -0.5458424 |
| Sft2d2 | 0.45398355 |
| Sft2d2 | -0.5577378 |
| Sft2d2 | -0.6480124 |
| Sftpc | 0.05158451 |
| Sftpd | -0.6463654 |
| Sfxn1 | 2.05243695 |
| Sfxn1 | -0.088938 |
| Sfxn3 | -0.7796925 |
| Sfxn3 | -0.7901921 |
| Sfxn4 | 1.59622988 |
| Sfxn4 | -0.472696 |
| Sfxn5 | 0.71203182 |
| Sgca | -0.5103288 |
| Sgca | 0.87627243 |
| Sgcb | 1.75310784 |
| Sgcb | -0.6538 |
| Sgcz | -0.6798709 |
| Sgip1 | 1.46867248 |
| Sgip1 | -0.1197733 |
| Sgip1 | 0.93775476 |
| Sgip1 | -0.69535 |
| Sgip1 | -0.3187762 |
| Sgk1 | -0.7181721 |
| Sgk2 | 1.596899 |
| Sgk2 | -0.6696923 |
| Sgk3 | 1.59503913 |
| Sgk3 | 0.56545107 |
| Sgk3 | -0.737929 |
| Sgk3 | -0.8017826 |
| Sgk3 | 0.77920099 |
| Sgms2 | 0.03548052 |
| Sgol1 | -0.4955129 |
| Sgpl1 | 1.86091871 |
| Sgpl1 | -0.6713811 |
| Sgpp1 | 1.6454108 |
| Sgpp2 | -0.5096179 |
| Sgsm1 | -0.5067798 |
| Sgsm2 | 0.33487261 |
| Sgsm3 | 1.06275723 |
| Sgta | -0.020861 |
| Sgtb | -0.2288495 |
| Sgtb | 1.01519041 |
| Sh2b1 | 1.94509869 |
| Sh2b1 | 0.8204117 |
| Sh2b3 | -0.6006079 |
| Sh2d2a | -0.542156 |
| Sh3bgrl | -0.6471886 |
| Sh3bgrl3 | 1.76379612 |
| Sh3bp2 | -0.0037637 |
| Sh3bp5 | 1.57044069 |
| Sh3bp5l | -0.738837 |
| Sh3gl1 | 2.23176878 |
| Sh3gl2 | -0.7315972 |
| Sh3gl2 | -0.4345534 |
| Sh3gl2 | -0.5622379 |
| Sh3gl3 | 0.45474221 |
| Sh3glb1 | -0.6756163 |
| Sh3glb2 | -0.3141544 |
| Sh3kbp1 | -0.0701155 |
| Sh3kbp1 | 0.18657997 |
| Sh3kbp1 | -0.6638057 |
| Sh3kbp1 | 1.65012609 |
| Sh3md4 | 1.01505177 |
| Sh3md4 | -0.7119726 |
| Sh3pxd2a | 0.37233954 |
| Sh3pxd2b | -0.3257521 |
| Sh3rf1 | -0.56299 |
| Sh3rf2 | -0.226748 |
| Sh3tc1 | -0.7361155 |
| Sh3tc2 | -0.5217771 |
| Sh3yl1 | -0.602176 |
| Shank2 | -0.7498032 |
| Sharpin | 0.08673955 |
| Shbg | -0.4850724 |
| Shc1 | 1.24579896 |
| Shc1 | 1.21161431 |
| Shc3 | -0.0975793 |
| Shcbp1 | -0.1391578 |
| Shd | -0.5046569 |
| She | -0.1249346 |
| Shf | -0.4273687 |
| Shf | 0.88268611 |
| Shf | -0.4451036 |
| Shfm1 | -0.3351353 |
| Shh | 0.92302765 |
| Shisa2 | -0.0952975 |
| Shisa3 | 0.45474221 |
| Shisa4 | -0.0626035 |
| Shkbp1 | 2.42910317 |
| Shkbp1 | -0.719951 |
| Shmt1 | -0.2178695 |
| Shmt2 | -0.4530953 |
| Shoc2 | 1.77270277 |
| Shox2 | -0.2664117 |
| Shprh | 0.7779504 |
| Shq1 | -0.4016992 |
| Shroom3 | -0.4464308 |
| Shroom3 | -0.2225594 |
| Si | -0.391 |
| Si | 0.59135476 |
| Siah1a | 2.67295823 |
| Siah1b | 0.56207746 |
| Siah1b | -0.4371798 |
| Siah1b | -0.8755741 |
| Siah1b | 1.05348912 |
| Siah2 | 0.32910605 |
| Siat7f | -0.0661311 |
| Siat9 | 1.02867886 |
| Sidt1 | 0.62185696 |
| Sidt2 | -0.4118824 |
| Sidt2 | -0.2746613 |
| Sidt2 | -0.0438683 |
| Sigirr | -0.1998519 |
| Siglec1 | -0.122585 |
| Siglech | 0.28225217 |
| Siglech | -0.0344264 |
| Sil1 | -0.2846581 |
| Sin3a | -0.8404783 |
| Sin3b | 0.01909369 |
| Sip1 | 0.96022765 |
| Sip1 | 0.10051278 |
| Sipa1 | -0.3055497 |
| Sipa1l1 | 0.23835526 |
| Sirpa | -0.4604817 |
| Sirpa | -0.2330665 |
| Sirpb1 | -0.3565988 |
| Sirt1 | -0.4537644 |
| Sirt2 | -0.4464308 |
| Sirt3 | 0.48752692 |
| Sirt5 | -0.6596236 |
| Sirt5 | 1.40178144 |
| Sirt6 | -0.6471886 |
| Sirt7 | -0.3860072 |
| Siva1 | 0.10530416 |
| Six1 | 0.20987357 |
| Six2 | 0.15295994 |
| Six3 | 2.21781502 |
| Six5 | -0.5189018 |
| Six6os1 | 2.20221318 |
| Skap2 | -0.6705363 |
| Skil | -0.5138913 |
| Skiv2l | 0.26102863 |
| Skiv2l | 0.42259674 |
| Skiv2l2 | -0.2246514 |
| Skp1a | 2.1118517 |
| Skp2 | 1.38365128 |
| Skp2 | 0.24058395 |
| Skp2 | 4.27796549 |
| Sla | 0.72725592 |
| Slain1 | -0.2757668 |
| Slain2 | -0.4202389 |
| Slamf7 | 1.31666677 |
| Slamf8 | 1.80673147 |
| Slamf9 | 0.05352954 |
| Slc10a3 | 4.02480377 |
| Slc10a4 | 1.01864992 |
| Slc10a6 | -0.4591344 |
| Slc10a6 | -0.1291789 |
| Slc10a7 | 1.21206347 |
| Slc10a7 | -0.5146055 |
| Slc10a7 | -0.6807241 |
| Slc11a1 | -0.4404732 |
| Slc11a1 | 1.74274078 |
| Slc11a1 | 1.51191021 |
| Slc11a1 | 1.3883099 |
| Slc11a2 | 1.49961827 |
| Slc12a2 | 0.4178752 |
| Slc12a2 | 2.53016303 |
| Slc12a3 | -0.5246613 |
| Slc12a5 | -0.6006079 |
| Slc12a6 | -0.6244109 |
| Slc12a7 | -0.1095355 |
| Slc12a8 | -0.7066913 |
| Slc12a9 | 0.19329484 |
| Slc12a9 | -0.6612942 |
| Slc13a2 | -0.8125353 |
| Slc13a3 | -0.6604585 |
| Slc13a3 | 0.03270306 |
| Slc13a4 | -0.542156 |
| Slc14a1 | 2.62845077 |
| Slc14a1 | 0.2588483 |
| Slc14a1 | -0.7721299 |
| Slc14a2 | -0.8619988 |
| Slc14a2 | -0.8578612 |
| Slc15a2 | -0.6962175 |
| Slc15a3 | -0.2220371 |
| Slc15a4 | -0.2925081 |
| Slc15a4 | -0.8537419 |
| Slc16a10 | -0.6638057 |
| Slc16a11 | 1.84042177 |
| Slc16a4 | 1.99243971 |
| Slc16a6 | -0.213203 |
| Slc16a6 | -0.6696923 |
| Slc16a6 | 1.52909113 |
| Slc16a8 | 0.65139348 |
| Slc16a9 | -0.627631 |
| Slc17a2 | 0.07085464 |
| Slc17a5 | -0.6349171 |
| Slc17a6 | -0.4871512 |
| Slc17a7 | 0.08335833 |
| Slc17a8 | -0.5827615 |
| Slc18a2 | 0.90572887 |
| Slc19a1 | 0.01221382 |
| Slc19a2 | -0.7768493 |
| Slc1a1 | 1.83548541 |
| Slc1a1 | -0.0037637 |
| Slc1a2 | 1.75354779 |
| Slc1a2 | -0.1526314 |
| Slc1a2 | 1.35616273 |
| Slc1a2 | 0.55597406 |
| Slc1a3 | 1.02621597 |
| Slc1a4 | -0.5436289 |
| Slc1a6 | -0.212686 |
| Slc1a7 | 0.65057414 |
| Slc20a1 | -0.6349171 |
| Slc22a1 | -0.3141544 |
| Slc22a12 | -0.5614864 |
| Slc22a13 | -0.2410253 |
| Slc22a14 | 0.63861209 |
| Slc22a17 | -0.7181721 |
| Slc22a17 | 0.82501251 |
| Slc22a18 | -0.4774896 |
| Slc22a21 | -0.0442998 |
| Slc22a3 | 2.4569514 |
| Slc22a4 | -0.5858408 |
| Slc22a5 | 0.43893646 |
| Slc22a5 | -0.3928792 |
| Slc22a5 | -0.7787438 |
| Slc22a6 | 0.17709723 |
| Slc22a7 | 0.36902014 |
| Slc22a8 | 0.09160199 |
| Slc23a2 | 0.76607601 |
| Slc23a3 | 2.25719949 |
| Slc24a1 | 1.20970294 |
| Slc24a3 | -0.417016 |
| Slc24a4 | -0.622003 |
| Slc24a5 | 0.36902014 |
| Slc24a6 | 1.42806562 |
| Slc25a1 | -0.0551516 |
| Slc25a1 | -0.551028 |
| Slc25a10 | 2.95349091 |
| Slc25a11 | -0.610848 |
| Slc25a11 | -0.2686044 |
| Slc25a11 | 0.59157309 |
| Slc25a12 | 1.45475543 |
| Slc25a14 | -0.472696 |
| Slc25a15 | 0.78737769 |
| Slc25a15 | 0.65200751 |
| Slc25a16 | 0.06280961 |
| Slc25a17 | -0.4809287 |
| Slc25a18 | 0.91441835 |
| Slc25a19 | -0.3269199 |
| Slc25a19 | 3.22653504 |
| Slc25a20 | 1.2349079 |
| Slc25a20 | -0.58507 |
| Slc25a23 | -0.7825443 |
| Slc25a24 | -0.6480124 |
| Slc25a24 | -0.0830713 |
| Slc25a25 | 0.1346163 |
| Slc25a26 | -0.368129 |
| Slc25a27 | 0.84452487 |
| Slc25a28 | -0.6892998 |
| Slc25a29 | 1.71937608 |
| Slc25a3 | -0.0087355 |
| Slc25a3 | -0.189206 |
| Slc25a30 | -0.5713038 |
| Slc25a32 | -0.719951 |
| Slc25a33 | -0.8496406 |
| Slc25a34 | -0.0732264 |
| Slc25a35 | -0.2930714 |
| Slc25a36 | 0.80924171 |
| Slc25a37 | -0.4913227 |
| Slc25a38 | -0.7740148 |
| Slc25a38 | 3.3943017 |
| Slc25a38 | 1.79700646 |
| Slc25a39 | -0.2152741 |
| Slc25a41 | -0.2987227 |
| Slc25a42 | 0.36038996 |
| Slc25a44 | 1.12110236 |
| Slc25a45 | 1.90793347 |
| Slc25a46 | 0.45018096 |
| Slc25a5 | -0.2351822 |
| Slc26a4 | -0.385385 |
| Slc26a6 | -0.3791854 |
| Slc26a7 | -0.3339572 |
| Slc26a8 | -0.8264022 |
| Slc27a1 | -0.6430801 |
| Slc27a2 | 0.36929721 |
| Slc27a3 | -0.4962131 |
| Slc27a4 | 0.05624577 |
| Slc27a6 | -0.3897493 |
| Slc29a1 | -0.8274006 |
| Slc29a2 | -0.5897043 |
| Slc29a3 | -0.739746 |
| Slc29a4 | 1.41261959 |
| Slc2a1 | 1.01477441 |
| Slc2a10 | -0.1917297 |
| Slc2a10 | -0.6422606 |
| Slc2a13 | -0.1722232 |
| Slc2a3 | 1.2692154 |
| Slc2a4 | -0.6124335 |
| Slc2a5 | -0.7253081 |
| Slc2a6 | -0.851689 |
| Slc2a8 | -0.1482793 |
| Slc2a8 | -0.2116527 |
| Slc2a8 | 1.00641515 |
| Slc2a9 | -0.3656897 |
| Slc30a1 | 0.52202528 |
| Slc30a10 | 0.40249675 |
| Slc30a3 | 0.02991731 |
| Slc30a5 | 1.13217479 |
| Slc30a5 | -0.5781615 |
| Slc30a6 | -0.5982606 |
| Slc30a7 | 2.9819975 |
| Slc30a7 | 1.43719433 |
| Slc30a9 | -0.6300534 |
| Slc31a1 | 0.12818161 |
| Slc31a2 | 1.16980773 |
| Slc32a1 | 0.44278506 |
| Slc34a2 | -0.5584863 |
| Slc35a1 | 0.00364453 |
| Slc35a2 | 0.68319513 |
| Slc35a3 | -0.4086882 |
| Slc35a4 | -0.2378336 |
| Slc35a5 | 1.9307632 |
| Slc35a5 | 2.16738585 |
| Slc35b1 | 0.30597319 |
| Slc35b2 | 0.66460793 |
| Slc35b3 | 2.41343717 |
| Slc35b3 | 1.57608155 |
| Slc35b3 | -0.4625064 |
| Slc35b4 | -0.6381738 |
| Slc35c1 | -0.3718 |
| Slc35c1 | 1.51726778 |
| Slc35c2 | -0.7181721 |
| Slc35c2 | -0.4564455 |
| Slc35c2 | -0.3475956 |
| Slc35e1 | -0.5644961 |
| Slc35e3 | -0.6596236 |
| Slc35e4 | -0.1449084 |
| Slc35f1 | -0.2752139 |
| Slc35f2 | 1.33829221 |
| Slc35f3 | -0.3210957 |
| Slc35f4 | -0.2512048 |
| Slc35f4 | -0.717284 |
| Slc36a1 | 0.56589996 |
| Slc36a3 | 1.06091096 |
| Slc37a1 | -0.457789 |
| Slc37a3 | -0.3228392 |
| Slc38a1 | 0.51730944 |
| Slc38a10 | 2.16863508 |
| Slc38a11 | -0.2614967 |
| Slc38a2 | 0.37894311 |
| Slc38a2 | -0.5032443 |
| Slc38a2 | -0.4864577 |
| Slc38a3 | -0.5196198 |
| Slc38a4 | 1.09766738 |
| Slc38a5 | -0.214238 |
| Slc38a8 | 0.90977333 |
| Slc38a9 | 1.05826707 |
| Slc38a9 | -0.7181721 |
| Slc39a10 | -0.6756163 |
| Slc39a11 | 0.10272724 |
| Slc39a12 | 0.55688079 |
| Slc39a13 | 0.10714046 |
| Slc39a13 | -0.3263358 |
| Slc39a14 | 0.45347743 |
| Slc39a3 | 0.0342912 |
| Slc39a3 | -0.4118824 |
| Slc39a4 | -0.8224191 |
| Slc39a5 | -0.0391354 |
| Slc39a5 | -0.2495901 |
| Slc39a5 | 1.94647933 |
| Slc39a6 | -0.6646444 |
| Slc39a7 | -0.6164089 |
| Slc39a7 | -0.2824273 |
| Slc39a7 | -0.5011294 |
| Slc39a8 | -0.2614967 |
| Slc3a2 | 0.62985318 |
| Slc40a1 | -0.0335728 |
| Slc41a2 | 0.18859951 |
| Slc41a3 | 3.2797768 |
| Slc41a3 | -0.5819933 |
| Slc41a3 | -0.6013916 |
| Slc43a2 | 1.41812685 |
| Slc43a2 | -0.4099646 |
| Slc44a1 | -0.3791854 |
| Slc44a1 | 3.45221647 |
| Slc44a2 | -0.5502854 |
| Slc44a2 | 1.17133353 |
| Slc44a2 | -0.2669594 |
| Slc44a3 | -0.7315972 |
| Slc44a4 | 2.68535372 |
| Slc45a1 | -0.802755 |
| Slc45a2 | -0.4920198 |
| Slc45a3 | -0.0103986 |
| Slc45a4 | -0.0447314 |
| Slc46a1 | 0.87868273 |
| Slc46a3 | -0.4254188 |
| Slc47a1 | -0.2625867 |
| Slc4a1 | -0.6496624 |
| Slc4a1ap | -0.4948133 |
| Slc4a2 | 1.84561684 |
| Slc4a3 | -0.293635 |
| Slc4a3 | -0.0763476 |
| Slc4a3 | -0.2664117 |
| Slc4a3 | 0.86069729 |
| Slc4a8 | -0.5370194 |
| Slc4a8 | 0.22713123 |
| Slc5a4a | -0.5502854 |
| Slc5a4b | -0.5110402 |
| Slc5a5 | 0.24439223 |
| Slc5a6 | -0.5735841 |
| Slc5a7 | 1.14661615 |
| Slc5a8 | 1.75071595 |
| Slc5a9 | 0.64151356 |
| Slc6a1 | -0.3885002 |
| Slc6a11 | 0.04494095 |
| Slc6a12 | -0.5067798 |
| Slc6a13 | -0.8244085 |
| Slc6a15 | 1.39562344 |
| Slc6a15 | 0.55846545 |
| Slc6a17 | -0.6061078 |
| Slc6a2 | 0.95210469 |
| Slc6a4 | -0.2065036 |
| Slc6a6 | -0.1007832 |
| Slc6a7 | -0.7155101 |
| Slc6a8 | -0.4247697 |
| Slc6a9 | -0.0799276 |
| Slc7a1 | 0.52998791 |
| Slc7a10 | -0.2241279 |
| Slc7a11 | -0.7288967 |
| Slc7a14 | 2.32737432 |
| Slc7a14 | 0.51919864 |
| Slc7a14 | 0.93488832 |
| Slc7a2 | 1.985311 |
| Slc7a3 | -0.4228253 |
| Slc7a4 | -0.6260195 |
| Slc7a6 | 0.34374093 |
| Slc7a6os | -0.69535 |
| Slc7a7 | 0.97351407 |
| Slc7a7 | 1.95542929 |
| Slc7a8 | 0.28952254 |
| Slc7a8 | 2.6958718 |
| Slc8a1 | 0.06396316 |
| Slc8a2 | -0.6037467 |
| Slc8a2 | 0.76153838 |
| Slc9a1 | 1.88589521 |
| Slc9a10 | -0.4871512 |
| Slc9a3r1 | -0.1197733 |
| Slc9a3r2 | 0.84718448 |
| Slc9a4 | -0.8486181 |
| Slc9a6 | -1.0027205 |
| Slc9a6 | 2.0825951 |
| Slc9a8 | -0.8766266 |
| Slc9a8 | 1.21899801 |
| Slc9a8 | -0.7343056 |
| Slc9a8 | 3.36624467 |
| Slc9a9 | 0.05663316 |
| Slco1a4 | -0.2852166 |
| Slco1c1 | 0.86265846 |
| Slco2a1 | -0.8224191 |
| Slco2b1 | -0.7507229 |
| Slco3a1 | -0.4010665 |
| Slco3a1 | 0.10603911 |
| Slco3a1 | -0.7271007 |
| Slco4a1 | 1.37671355 |
| Slfnl1 | -0.9614555 |
| Slit1 | 0.53254957 |
| Slit2 | -0.7415665 |
| Slit3 | 0.76752341 |
| Slitrk1 | 0.64089257 |
| Slitrk3 | -0.3572021 |
| Slitrk4 | 1.79892368 |
| Slitrk5 | 0.47993034 |
| Slitrk5 | -0.7940396 |
| Slitrk6 | 0.33516 |
| Slk | -0.4524267 |
| Slk | 1.65488751 |
| Slmap | 1.21754826 |
| Slmap | -0.1239941 |
| Slmap | -0.6815782 |
| Slmo1 | -0.7181721 |
| Slmo2 | -0.391626 |
| Sln | -0.7702488 |
| Slpi | 0.35421653 |
| Sltm | 0.10272724 |
| Sltm | 2.69525366 |
| Slu7 | 1.19780988 |
| Slu7 | 1.41171365 |
| Sly | 3.1523918 |
| Smad1 | -0.7208417 |
| Smad1 | -0.9319413 |
| Smad2 | 1.18956613 |
| Smad3 | 1.62125296 |
| Smad5 | 0.3098195 |
| Smad5 | 1.56776079 |
| Smad6 | -0.7740148 |
| Smap2 | -0.5480613 |
| Smap2 | -0.4962131 |
| Smap2 | 1.49473872 |
| Smarca1 | -0.6398063 |
| Smarca2 | -0.7226257 |
| Smarca2 | -0.7406558 |
| Smarca4 | 2.21903671 |
| Smarcad1 | 0.75789231 |
| Smarcal1 | 0.19929717 |
| Smarcb1 | -0.1160374 |
| Smarcc1 | 0.25478554 |
| Smarcc1 | -0.5569898 |
| Smarcc2 | 1.05002309 |
| Smarcd1 | 2.1762952 |
| Smarcd1 | 0.69264557 |
| Smarcd1 | 3.74257006 |
| Smarcd2 | 1.35635511 |
| Smarcd3 | -0.5311834 |
| Smarce1 | 0.41180714 |
| Smarce1 | 0.90992854 |
| Smc1a | 1.00948892 |
| Smc1a | -0.4969139 |
| Smc1a | 0.11590477 |
| Smc1a | -0.6850021 |
| Smc1b | -0.610848 |
| Smc3 | 2.09046657 |
| Smc5l1 | 0.48679447 |
| Smc6 | 0.8725645 |
| Smchd1 | 0.23899257 |
| Smcp | -0.4983169 |
| Smcr7 | 2.23184405 |
| Smcr7l | 1.18148092 |
| Smek1 | 0.53603145 |
| Smek2 | -0.2930714 |
| Smek2 | 2.59634115 |
| Smg5 | -0.128234 |
| Smg6 | -0.1957819 |
| Smn1 | 0.18286609 |
| Smo | 0.62101125 |
| Smo | -0.7834969 |
| Smo | -0.6688491 |
| Smoc1 | 1.52170904 |
| Smox | -0.3216765 |
| Smox | 0.27062361 |
| Smox | 1.94320339 |
| Smox | 1.77405605 |
| Smox | 2.30422759 |
| Smpd1 | 3.57124945 |
| Smpd1 | 1.98980941 |
| Smpd1 | 0.56230275 |
| Smpd2 | 1.75247899 |
| Smpd3 | 0.27277664 |
| Smpd4 | 0.58500091 |
| Smpdl3a | 0.98575585 |
| Smpdl3b | -0.323421 |
| Smpx | 1.54131886 |
| Smtn | 1.7146059 |
| Smtn | -0.4451036 |
| Smtn | 1.24968883 |
| Smtnl1 | 2.44105842 |
| Smtnl2 | 0.41048276 |
| Smu1 | -0.5167512 |
| Smu1 | 0.68180891 |
| Smug1 | 1.14613434 |
| Smurf1 | 0.29343713 |
| Smyd1 | 0.42181147 |
| Smyd2 | -0.6406237 |
| Smyd2 | 1.56083406 |
| Smyd3 | 2.07945442 |
| Smyd4 | 0.17573465 |
| Smyd5 | -0.3590144 |
| Smyd5 | 0.40276405 |
| Snai1 | 0.01545773 |
| Snai3 | 1.50282979 |
| Snap23 | 0.10235853 |
| Snap29 | -0.1609077 |
| Snap91 | -0.3656897 |
| Snap91 | -0.5181844 |
| Snapc1 | -0.535557 |
| Snapc3 | -0.4990192 |
| Snapc3 | 0.82772826 |
| Snapc3 | 0.04729114 |
| Snapc3 | -0.0305912 |
| Snapc4 | 1.56276331 |
| Snapin | 0.30032297 |
| Snca | 0.92882476 |
| Snca | 0.947644 |
| Snca | -0.1927411 |
| Sncaip | -0.5858408 |
| Sncg | 0.5001323 |
| Sncg | 4.22746382 |
| Snd1 | -0.3440185 |
| Snf1lk | 0.96022765 |
| Snf1lk2 | 0.03031579 |
| Snf8 | 0.30508332 |
| Snip1 | -0.666324 |
| Snip1 | -0.5842999 |
| Snn | 2.08893267 |
| Snora65 | -0.2757668 |
| Snph | -0.6260195 |
| Snrk | -0.6546297 |
| Snrk | -0.1589537 |
| Snrk | -0.0322938 |
| Snrp70 | 0.12961538 |
| Snrpa | -0.1942603 |
| Snrpb | -0.5096179 |
| Snrpb | -0.7226257 |
| Snrpd1 | -0.5982606 |
| Snrpd1 | -0.6316718 |
| Snrpd2 | -0.831405 |
| Snrpd3 | -0.1076861 |
| Snrpg | -0.0322938 |
| Snta1 | -0.5660047 |
| Sntb2 | -0.6781667 |
| Sntb2 | 1.14661615 |
| Sntg2 | -0.2658643 |
| Snupn | 2.55505743 |
| Snurf | 2.72173757 |
| Snurf | 1.58236816 |
| Snx1 | -0.7306962 |
| Snx10 | -0.6308622 |
| Snx11 | -0.3669086 |
| Snx12 | -0.0412838 |
| Snx13 | 1.04788374 |
| Snx14 | 0.90135359 |
| Snx14 | -0.7361155 |
| Snx14 | -0.6341047 |
| Snx15 | -0.6521427 |
| Snx16 | -0.1811756 |
| Snx17 | -0.4358657 |
| Snx17 | 0.12925714 |
| Snx18 | -0.1053794 |
| Snx19 | 1.3491137 |
| Snx19 | -0.7415665 |
| Snx2 | 1.40689743 |
| Snx2 | -0.7146245 |
| Snx20 | -0.4692871 |
| Snx21 | -0.5819933 |
| Snx22 | 2.81511808 |
| Snx24 | -0.7163966 |
| Snx25 | 1.0379249 |
| Snx25 | -0.4885396 |
| Snx26 | 1.86047057 |
| Snx29 | -0.617206 |
| Snx3 | -0.2724542 |
| Snx30 | -0.7911525 |
| Snx33 | 1.30118428 |
| Snx4 | 1.1096464 |
| Snx6 | -0.4484252 |
| Snx7 | -0.0639248 |
| Snx8 | -0.56299 |
| Snx9 | -0.4720133 |
| Soat1 | 0.98889832 |
| Socs2 | 1.77054577 |
| Socs3 | -0.8086109 |
| Socs4 | -0.3596193 |
| Socs5 | -0.5181844 |
| Socs6 | -0.167779 |
| Socs7 | -0.3304319 |
| Socs7 | -0.1396357 |
| Sod1 | -0.2582343 |
| Sod2 | 0.97220843 |
| Sod3 | 0.67324806 |
| Sohlh1 | 0.97972704 |
| Sohlh1 | -0.6212018 |
| Son | 0.03111223 |
| Sorbs1 | -0.2152741 |
| Sorbs2 | 3.03639666 |
| Sorbs3 | 0.67544553 |
| Sorcs1 | 1.47504593 |
| Sorcs1 | -0.6562913 |
| Sorcs2 | 1.87993301 |
| Sorcs3 | 2.65953961 |
| Sorcs3 | -0.6910244 |
| Sord | 1.14444604 |
| Sorl1 | 0.2238994 |
| Sort1 | -0.4378375 |
| Sort1 | -0.7128557 |
| Sort1 | -0.5584863 |
| Sort1 | 1.09423456 |
| Sostdc1 | 1.39728182 |
| Sox12 | 0.73139936 |
| Sox13 | -0.8334137 |
| Sox17 | 1.478343 |
| Sox17 | -0.837444 |
| Sox18 | 0.32823798 |
| Sox2 | 1.16320638 |
| Sox2 | 0.50708895 |
| Sox21 | 0.56522654 |
| Sox21 | 0.1195324 |
| Sox30 | 1.45232884 |
| Sox5 | -0.4345534 |
| Sox5 | -0.7128557 |
| Sox5 | -0.3779505 |
| Sox5 | 0.23163705 |
| Sox7 | -0.6422606 |
| Sox7 | 0.21446331 |
| Sox8 | -0.4150876 |
| Sox9 | 0.6629916 |
| Sp100 | 1.97406839 |
| Sp140 | 0.25353189 |
| Sp2 | 2.67980543 |
| Sp4 | -0.4761175 |
| Sp5 | -0.717284 |
| Sp6 | 0.63611793 |
| Sp7 | 2.57298205 |
| Sp8 | -0.7834969 |
| Sp9 | 0.00364453 |
| Spaca3 | 1.41686576 |
| Spaca4 | 1.05813468 |
| Spag1 | 3.70612724 |
| Spag1 | 0.71681609 |
| Spag16 | -0.0994088 |
| Spag16 | 0.62291302 |
| Spag4 | 1.3491137 |
| Spag4 | -0.7163966 |
| Spag4l | 0.56185212 |
| Spag4l | -0.666324 |
| Spag5 | -0.2320105 |
| Spag5 | 0.03468781 |
| Spag6 | -0.7627621 |
| Spag6 | -0.6212018 |
| Spag7 | 0.27859563 |
| Spag8 | 1.57280791 |
| Spag9 | 0.82160668 |
| Spag9 | 1.19105998 |
| Spag9 | 0.01424258 |
| Spag9 | -0.4099646 |
| Spam1 | -0.3602246 |
| Spam1 | -0.0348535 |
| Sparc | -0.0573372 |
| Sparc | -0.818453 |
| Sparcl1 | -0.2080453 |
| Spast | -0.4809287 |
| Spast | -0.6884387 |
| Spast | -0.5897043 |
| Spata16 | -0.4131632 |
| Spata16 | 2.24492338 |
| Spata17 | -0.5406855 |
| Spata17 | -0.1254052 |
| Spata19 | 0.19128545 |
| Spata2 | 0.77991486 |
| Spata20 | -0.4016992 |
| Spata20 | -0.698825 |
| Spata21 | -0.5599851 |
| Spata22 | 0.82365167 |
| Spata2L | 2.05280177 |
| Spata3 | 1.98202128 |
| Spata3 | -0.4325884 |
| Spata3 | 0.02191189 |
| Spata3 | 3.56323534 |
| Spata4 | -0.7217333 |
| Spata6 | 0.46754595 |
| Spata7 | -0.3044084 |
| Spata7 | 0.90572887 |
| Spatc1 | -0.633293 |
| Spatc1 | -0.4802398 |
| Spats1 | -0.6996958 |
| Spc24 | -0.5637428 |
| Spc24 | 0.60458017 |
| Spc25 | -0.7796925 |
| Spc25 | -0.3885002 |
| Spcs1 | -0.4740631 |
| Spcs1 | -0.1756945 |
| Spcs3 | 0.00569199 |
| Spdef | 0.17026434 |
| Specc1l | 0.32243014 |
| Speer1-ps1 | 0.54341631 |
| Speer1-ps1 | -0.088033 |
| Speer2 | 0.30626962 |
| Speer2 | 3.37917457 |
| Speer3 | 0.32910605 |
| Speer4b | 0.03627256 |
| Speer4d | -0.7190611 |
| Speer4f | 1.33750741 |
| Speer6-ps1 | 0.14770541 |
| Spef1 | -0.1193055 |
| Spen | 0.88108682 |
| Spert | -0.4048696 |
| Spesp1 | -0.7534874 |
| Spg20 | -0.2807577 |
| Spg21 | 0.14489085 |
| Spg7 | 2.71790088 |
| Spg7 | 0.44483117 |
| Sphk1 | -0.3724132 |
| Sphk2 | 0.44585255 |
| Sphk2 | -0.3644725 |
| Spic | 1.08257078 |
| Spin | -0.7759035 |
| Spin2 | 0.22551671 |
| Spink10 | -0.7834969 |
| Spink2 | 2.1989833 |
| Spink4 | 0.00569199 |
| Spink8 | 1.01795904 |
| Spinlw1 | -0.4093262 |
| Spinlw1 | -0.6546297 |
| Spinlw1 | 1.387287 |
| Spint1 | -0.8364348 |
| Spint2 | 0.66702719 |
| Spire1 | -0.4424549 |
| Spire1 | -0.7424781 |
| Spire1 | -0.2902585 |
| Spire2 | 1.16261469 |
| Spna1 | -0.617206 |
| Spnb1 | -0.6773158 |
| Spnb2 | 1.28896217 |
| Spnb2 | 0.38359253 |
| Spnb2 | -0.7873168 |
| Spnb2 | 1.31234138 |
| Spnb3 | -0.366299 |
| Spnb4 | -0.4464308 |
| Spns1 | -0.5637428 |
| Spo11 | 0.6144309 |
| Spo11 | 1.17320802 |
| Spock1 | 1.06064691 |
| Spock2 | 0.61762074 |
| Spock3 | 1.25259562 |
| Spon1 | 0.48752692 |
| Spon2 | -0.6901617 |
| Spop | 2.15394208 |
| Spp1 | -0.5577378 |
| Spp2 | -0.3705747 |
| Sppl3 | -0.737929 |
| Spr | 1.73834436 |
| Spred1 | 1.80011994 |
| Spred2 | 1.01864992 |
| Spred2 | 2.6028622 |
| Sprn | 0.07009143 |
| Sprn | 0.40222937 |
| Sprr2a | 0.31335593 |
| Sprr2a | 0.58037268 |
| Sprr2f | -0.7119726 |
| Spry3 | 0.60479543 |
| Spry4 | 1.91662616 |
| Spryd4 | 1.31465746 |
| Spsb1 | -0.7075694 |
| Spsb2 | 0.04376363 |
| Spsb3 | -0.2746613 |
| Spsb3 | -0.1801766 |
| Spsb4 | 0.74056234 |
| Sptlc1 | -0.6936174 |
| Sptlc2 | -0.645543 |
| Spz1 | 0.418401 |
| Sqle | -0.1362958 |
| Sqle | 1.89073533 |
| Sqrdl | -0.6308622 |
| Sqstm1 | -0.6538 |
| Sra1 | -0.4431164 |
| Sra1 | 0.89285717 |
| Src | 1.85146228 |
| Srd5a1 | -0.7488844 |
| Srd5a2 | -0.2070172 |
| Srd5a3 | 0.65812574 |
| Srd5a3 | 0.9605217 |
| Srd5a3 | -0.7959694 |
| Srebf1 | -0.5569898 |
| Srebf2 | 1.61740752 |
| Srf | 0.70934187 |
| Srf | 2.45698096 |
| Srfbp1 | -0.2163114 |
| Srgn | -0.0305912 |
| Srgn | 0.80421471 |
| Sri | -0.5297301 |
| Sri | -0.7190611 |
| Sri | -0.30213 |
| Srm | -0.5495435 |
| Srm | 0.72479885 |
| Srm | 1.37718395 |
| Srp14 | 3.57035342 |
| Srp19 | -0.0648067 |
| Srp19 | -0.719951 |
| Srp54 | 0.52131942 |
| Srp68 | 2.06952069 |
| Srp72 | 0.78027159 |
| Srp9 | -0.8537419 |
| Srp9 | -0.1367722 |
| Srpk1 | 0.81664604 |
| Srpk2 | 2.16338517 |
| Srpk3 | 0.13994485 |
| Srpr | 1.42476444 |
| Srprb | 0.91534458 |
| Srprb | -0.698825 |
| Srpx | -0.5406855 |
| Srpx | 0.8874676 |
| Srpx2 | 0.99003841 |
| Srpx2 | 1.71290102 |
| Srpx2 | -0.4927174 |
| Srr | 0.49892762 |
| Srrd | -0.6180038 |
| Srrm1 | 0.15365831 |
| Srrm2 | -0.7655626 |
| Srrm2 | 0.91596156 |
| Srrp | 1.46816072 |
| Srxn1 | -0.2779817 |
| Srxn1 | 3.345373 |
| Srxn1 | 0.17026434 |
| Ss18 | 2.2082923 |
| Ss18 | -0.2215151 |
| Ss18l1 | -0.4280196 |
| Ss18l1 | 2.47403077 |
| Ss18l1 | 1.0204438 |
| Ssbp1 | 2.07479027 |
| Ssbp2 | -0.4074136 |
| Ssbp2 | 0.24976082 |
| Ssbp3 | -0.396649 |
| Ssbp3 | 0.83901534 |
| Ssbp4 | 1.65264987 |
| Ssfa2 | -0.6513152 |
| Ssh2 | 2.64169026 |
| Ssh3 | 0.30121739 |
| Ssh3 | 0.84485773 |
| Ssna1 | -0.088033 |
| Sspn | 2.31527069 |
| Ssr2 | -0.7137397 |
| Ssr3 | -0.0723365 |
| Ssr4 | 0.2418551 |
| Ssrp1 | 0.89238287 |
| Sssca1 | 0.82738922 |
| Sssca1 | -0.0029377 |
| Sst | 0.48850264 |
| Sstr1 | 0.16854827 |
| Sstr5 | 2.18654354 |
| Ssty2 | -0.7498032 |
| Ssu72 | -0.627631 |
| Ssx2ip | 1.25998321 |
| Ssxb2 | -0.6300534 |
| Ssxb5 | -0.2152741 |
| St13 | 1.47724527 |
| St14 | 0.07580036 |
| St14 | 3.06558251 |
| St18 | -0.6414418 |
| St3gal1 | -0.4195934 |
| St3gal2 | 0.23388183 |
| St3gal2 | -0.3141544 |
| St3gal3 | 0.66843553 |
| St3gal3 | -0.810571 |
| St3gal4 | 1.11064817 |
| St3gal5 | 0.62586363 |
| St3gal6 | -0.8354267 |
| St5 | 0.38495561 |
| St5 | -0.7433906 |
| St6gal1 | 1.12874409 |
| St6galnac2 | -0.3972788 |
| St6galnac3 | 0.59397134 |
| St6galnac4 | -0.1861865 |
| St6galnac4 | 1.98177532 |
| St6galnac4 | 0.02592319 |
| St6galnac5 | -0.3292598 |
| St6galnac6 | 0.0597265 |
| St6galnac6 | -0.755335 |
| St7 | 0.54502399 |
| St7l | 0.98288985 |
| St7l | 0.57860349 |
| St8sia1 | -0.7636946 |
| St8sia1 | 0.89522505 |
| St8sia2 | -0.2598641 |
| St8sia3 | -0.8086109 |
| St8sia4 | 1.17332505 |
| St8sia4 | -0.505364 |
| St8sia5 | -0.1536013 |
| Stab1 | -0.2593205 |
| Stab2 | -0.0447314 |
| Stac | -0.472696 |
| Stac2 | -0.6068962 |
| Stac3 | -0.3263358 |
| Stag1 | -0.5443661 |
| Stag2 | 1.02470766 |
| Stag2 | -0.4067769 |
| Stag3 | 3.41887557 |
| Stam | -0.6781667 |
| Stam | 2.0567146 |
| Stam | 0.13247633 |
| Stam2 | -0.0267716 |
| Stam2 | -0.4352093 |
| Stambp | -0.4099646 |
| Stambpl1 | 0.58982503 |
| Stap1 | 3.04405497 |
| Stap2 | -0.5414205 |
| Star | 1.51936992 |
| Stard10 | -0.5370194 |
| Stard10 | 3.43299318 |
| Stard13 | -0.2947632 |
| Stard3 | -0.717284 |
| Stard3nl | -0.5547497 |
| Stard4 | 0.29643722 |
| Stard5 | 0.5667971 |
| Stard5 | -0.2987227 |
| Stard6 | 2.65321253 |
| Stard7 | -0.551028 |
| Stard7 | 0.079587 |
| Stard8 | 2.65665597 |
| Stard8 | -0.6546297 |
| Stat1 | -0.2415583 |
| Stat1 | 0.31892801 |
| Stat2 | 0.60458017 |
| Stat3 | -0.0217031 |
| Stat3 | -0.6538 |
| Stat3 | -0.1188379 |
| Stat4 | -0.4665689 |
| Stat4 | 2.06871423 |
| Stat5a | 0.48508316 |
| Stat5a | 0.16130612 |
| Stat6 | -0.4451036 |
| Statip1 | -0.627631 |
| Stau1 | 2.94167808 |
| Stau2 | 0.67384788 |
| Stbd1 | -0.2587773 |
| Stc2 | 1.59377229 |
| Steap1 | 1.812058 |
| Steap2 | -0.58507 |
| Steap3 | -0.5203384 |
| Stfa1 | -0.5904789 |
| Stfa2 | -0.6807241 |
| Stim1 | -0.633293 |
| Stip1 | -0.2277982 |
| Stk11 | 0.31276746 |
| Stk11ip | -0.4843805 |
| Stk17b | 1.0759696 |
| Stk19 | 0.98432395 |
| Stk19 | 2.01957201 |
| Stk19 | -0.3124271 |
| Stk22b | 1.4427342 |
| Stk22s1 | -0.7271007 |
| Stk24 | -0.5384841 |
| Stk25 | 0.97031927 |
| Stk25 | 2.95210072 |
| Stk3 | -0.1570038 |
| Stk31 | -0.6324821 |
| Stk32a | 2.27637504 |
| Stk32b | 0.53138605 |
| Stk32c | 0.46404866 |
| Stk35 | 2.02906735 |
| Stk35 | 0.72725592 |
| Stk36 | -0.4955129 |
| Stk38l | 0.73888608 |
| Stk39 | -0.645543 |
| Stk4 | 0.24502543 |
| Stk40 | 0.00118163 |
| Stmn1 | -0.666324 |
| Stmn2 | -0.4809287 |
| Stmn3 | 0.16061345 |
| Stmn4 | -0.451091 |
| Stmn4 | -0.2730055 |
| Stoml1 | 1.19849358 |
| Stoml1 | -0.4598078 |
| Stoml1 | 0.09383768 |
| Stoml2 | 0.3941721 |
| Stoml3 | -0.2512048 |
| Stra13 | 0.24565821 |
| Stra13 | 0.33573453 |
| Stra13 | 0.61974125 |
| Stra6 | -0.7863604 |
| Strap | 0.98789959 |
| Strbp | -0.5858408 |
| Strn4 | 0.34686761 |
| Strn4 | -0.6798709 |
| Stt3a | 0.84102257 |
| Stt3b | -0.726204 |
| Stx12 | -0.7873168 |
| Stx17 | 2.68981018 |
| Stx18 | 0.87175647 |
| Stx1a | -0.4444407 |
| Stx2 | 0.29703608 |
| Stx3 | -0.2137204 |
| Stx3 | -0.6300534 |
| Stx4a | -0.0318678 |
| Stx5a | -0.210105 |
| Stx5a | 0.12206338 |
| Stx6 | -0.6996958 |
| Stx7 | -0.5046569 |
| Stx8 | -0.6850021 |
| Stx8 | 0.11226297 |
| Stx8 | -0.4080507 |
| Stx8 | 0.9717728 |
| Stxbp1 | 2.65139002 |
| Stxbp1 | -0.2593205 |
| Stxbp1 | 0.51660001 |
| Stxbp2 | 1.26657181 |
| Stxbp4 | -0.657956 |
| Stxbp4 | 1.91773343 |
| Stxbp5l | -0.5146055 |
| Stxbp5l | -0.5920301 |
| Styx | -0.6521427 |
| Styxl1 | -0.7787438 |
| Styxl1 | 0.05702039 |
| Styxl1 | 1.75033772 |
| Suclg1 | 0.4194517 |
| Suclg1 | 0.16889174 |
| Suclg2 | 2.57036757 |
| Suclg2 | -0.6867188 |
| Suds3 | -0.349988 |
| Sufu | -0.6373585 |
| Suhw2 | 0.0788309 |
| Suhw4 | 1.5489393 |
| Sulf1 | 1.13899858 |
| Sult1a1 | -0.087129 |
| Sult1c2 | 2.3324222 |
| Sult1d1 | 0.36178734 |
| Sult1d1 | -0.6092653 |
| Sult2b1 | -0.4365225 |
| Sult2b1 | 0.03706394 |
| Sult4a1 | -0.1742052 |
| Sult5a1 | 0.6569053 |
| Sult5a1 | 2.12083854 |
| Sumf2 | 0.88284589 |
| Sumo1 | 0.0635788 |
| Sumo1 | -0.0267716 |
| Sumo1 | 0.86167839 |
| Sumo2 | 0.20162103 |
| Sumo3 | -0.0794793 |
| Sumo3 | 0.85561177 |
| Sunc1 | 1.65411892 |
| Suox | -0.4557745 |
| Supt3h | -0.5117522 |
| Supt4h1 | 1.16190418 |
| Supt4h2 | 0.85000204 |
| Supt5h | 0.0089586 |
| Supt5h | -0.0391354 |
| Supt6h | -0.5532592 |
| Supv3l1 | 2.12032975 |
| Surf1 | -0.4163728 |
| Surf1 | 0.16268991 |
| Surf2 | 2.18610889 |
| Surf4 | -0.7488844 |
| Susd2 | -0.4391545 |
| Susd3 | 1.6344101 |
| Susd4 | 1.73981213 |
| Suv39h1 | -0.6341047 |
| Suv420h1 | 0.23612123 |
| Suv420h1 | 0.89427862 |
| Suv420h1 | 3.07845901 |
| Suv420h2 | 2.30801566 |
| Suz12 | 1.43578212 |
| Sv2a | -0.8154895 |
| Sv2a | 4.49564052 |
| Sv2b | 0.32243014 |
| Sval2 | 0.76208406 |
| Svep1 | 0.27951111 |
| Svil | 0.31629275 |
| Svil | 1.83611826 |
| Svop | -0.8037285 |
| Svopl | 0.2418551 |
| Svs4 | 0.64296091 |
| Svs5 | -0.7084484 |
| Svs6 | 2.29502376 |
| Swap70 | -0.3767171 |
| Swap70 | -0.3392702 |
| Swap70 | 0.87288751 |
| Syap1 | 2.10394356 |
| Syap1 | -0.6430801 |
| Sybl1 | -0.1497278 |
| Syce2 | -0.5796923 |
| Sycp1 | -0.6798709 |
| Syf2 | -0.7940396 |
| Syf2 | 0.93910948 |
| Syn1 | -0.4055049 |
| Syn1 | -0.1358196 |
| Syn2 | -0.4241211 |
| Sync | -0.2230819 |
| Syncrip | -0.7093282 |
| Syne1 | 1.3498888 |
| Syne1 | -0.568272 |
| Syngr1 | -0.6196014 |
| Syngr1 | 2.67197259 |
| Syngr1 | 1.55284318 |
| Syngr1 | 2.69532236 |
| Syngr2 | -0.6638057 |
| Syngr2 | 0.21935596 |
| Syngr2 | -0.2479782 |
| Syngr3 | 0.88667238 |
| Syngr4 | 2.90686771 |
| Syngr4 | -0.7244131 |
| Syngr4 | -0.610848 |
| Synj2 | -0.3953907 |
| Synj2bp | 2.30860507 |
| Synj2bp | 0.82857531 |
| Synpo2l | -0.8037285 |
| Syp | -0.4990192 |
| Sypl | -0.4365225 |
| Sypl | 1.26328333 |
| Sypl2 | 1.80017971 |
| Sys1 | -0.4809287 |
| Syt1 | 0.74982047 |
| Syt11 | -0.6970859 |
| Syt11 | -0.2970237 |
| Syt12 | -0.349988 |
| Syt13 | 0.45145021 |
| Syt15 | 0.24660657 |
| Syt16 | -0.7507229 |
| Syt3 | 0.66561672 |
| Syt4 | 1.4733506 |
| Syt5 | 1.00122269 |
| Syt9 | 0.34743497 |
| Sytl2 | -0.6850021 |
| Sytl3 | 0.05585822 |
| Sytl3 | 0.07047311 |
| Sytl3 | 0.84252516 |
| Sytl4 | 0.56185212 |
| Sytl5 | 0.45094272 |
| Syvn1 | -0.5160354 |
| Taar2 | -0.0875809 |
| Taar3 | 1.55975193 |
| Taar4 | 0.55936976 |
| Tac1 | 0.0585677 |
| Tacc1 | -0.1258761 |
| Tacc1 | 0.34288638 |
| Tacr3 | 0.80751132 |
| Tacr3 | -0.5488021 |
| Tacstd2 | -0.6910244 |
| Tada1l | 1.26328333 |
| Tada2l | -0.0116479 |
| Tada2l | -0.4537644 |
| Tada3l | 0.40516636 |
| Tada3l | 2.43475182 |
| Taf10 | -0.3049789 |
| Taf11 | 1.48490238 |
| Taf12 | -0.6373585 |
| Taf12 | -0.6148167 |
| Taf12 | 2.61999899 |
| Taf13 | -0.2587773 |
| Taf1c | 0.60887588 |
| Taf3 | 0.05119502 |
| Taf5 | -0.5096179 |
| Taf5 | -0.1324933 |
| Taf5 | -0.5239394 |
| Taf5l | -0.3263358 |
| Taf6 | 0.2588483 |
| Taf6 | 0.16268991 |
| Taf6l | -0.3847632 |
| Taf7l | 0.70741572 |
| Taf9 | -0.3009928 |
| Taf9 | -0.5697867 |
| Taf9 | -0.1053794 |
| Taf9b | -0.6867188 |
| Tagap | 2.78209422 |
| Tagap | 2.06408605 |
| Tagln | 2.58127359 |
| Tagln3 | 0.26133969 |
| Tal1 | -0.5873843 |
| Tal2 | 0.88635411 |
| Taldo1 | 3.34101597 |
| Tank | -0.1952744 |
| Tank | -0.5203384 |
| Tank | 0.49409328 |
| Tap1 | 0.7637192 |
| Tap2 | -0.5436289 |
| Tapbp | 1.78012165 |
| Tapbp | -0.3205153 |
| Tapbpl | 2.00997904 |
| Tapbpl | -0.4591344 |
| Tapt1 | -0.2571494 |
| Tarbp2 | -0.4829982 |
| Tarbp2 | -0.5495435 |
| Tardbp | -0.5667598 |
| Tardbp | 2.55734348 |
| Tardbp | 0.94645087 |
| Tars | -0.738837 |
| Tarsl2 | 0.99174595 |
| Tarsl2 | 1.26868728 |
| Tas1r1 | 0.86233188 |
| Tasp1 | -0.6013916 |
| Tatdn2 | 1.02484488 |
| Tax1bp1 | 0.2364407 |
| Tax1bp3 | 1.18090072 |
| Tax1bp3 | 0.72650059 |
| Taz | -0.5117522 |
| Taz | -0.3718 |
| Tbc1d1 | 0.5087604 |
| Tbc1d10a | -0.4490909 |
| Tbc1d10a | 0.31365003 |
| Tbc1d10c | 2.18764902 |
| Tbc1d12 | 3.61682357 |
| Tbc1d13 | -0.0790313 |
| Tbc1d14 | 0.35421653 |
| Tbc1d15 | -0.5562425 |
| Tbc1d16 | -0.7516435 |
| Tbc1d17 | 0.60220908 |
| Tbc1d19 | -0.6832886 |
| Tbc1d2 | 1.75367345 |
| Tbc1d20 | -0.7361155 |
| Tbc1d22a | -0.698825 |
| Tbc1d22b | -0.5697867 |
| Tbc1d23 | 2.15724989 |
| Tbc1d24 | -0.6756163 |
| Tbc1d25 | 0.39282243 |
| Tbc1d2b | -0.739746 |
| Tbc1d5 | -0.4795515 |
| Tbc1d5 | -0.6204013 |
| Tbc1d7 | -0.3263358 |
| Tbc1d7 | -0.3572021 |
| Tbc1d8 | -0.4761175 |
| Tbc1d9 | -0.4189484 |
| Tbc1d9 | -0.4299749 |
| Tbc1d9b | -0.0767943 |
| Tbca | 4.55374913 |
| Tbcb | 0.68556673 |
| Tbcc | -0.3061209 |
| Tbcd | 0.33199378 |
| Tbcel | -0.3269199 |
| Tbk1 | 1.89824475 |
| Tbkbp1 | -0.3644725 |
| Tbl1x | 1.93831937 |
| Tbl1xr1 | -0.6604585 |
| Tbl2 | 2.60526122 |
| Tbl3 | -0.6798709 |
| Tbp | -0.0817227 |
| Tbpl1 | -0.5920301 |
| Tbr1 | -0.5728234 |
| Tbrg1 | -0.4781764 |
| Tbrg1 | -0.4189484 |
| Tbrg1 | -0.5812257 |
| Tbrg4 | 1.8177063 |
| Tbrg4 | -0.4299749 |
| Tbx1 | 0.75459863 |
| Tbx19 | -0.729796 |
| Tbx2 | 1.25838218 |
| Tbx22 | -0.6970859 |
| Tbx3 | 2.3631631 |
| Tbx3 | 0.93307338 |
| Tbx5 | 0.52179006 |
| Tbx6 | -0.4280196 |
| Tbxa2r | 1.4309113 |
| Tbxa2r | -0.7959694 |
| Tcam1 | 1.07674864 |
| Tcam1 | -0.7137397 |
| Tcam1 | -0.6629678 |
| Tcap | 0.8847611 |
| Tcea2 | 0.50613249 |
| Tcea3 | 0.17777777 |
| Tceal1 | 0.45726663 |
| Tceal1 | 0.06664923 |
| Tceal3 | -0.7655626 |
| Tceal5 | -0.3748701 |
| Tceal6 | 0.72951826 |
| Tceal6 | -0.4411333 |
| Tceal8 | -0.2785362 |
| Tceb1 | 0.3388881 |
| Tceb2 | -0.1579783 |
| Tceb2 | 1.29924356 |
| Tceb3 | 0.06894544 |
| Tcerg1 | -0.2609522 |
| Tcf12 | 1.18899093 |
| Tcf12 | 2.02367344 |
| Tcf15 | 1.29236757 |
| Tcf19 | -0.3428292 |
| Tcf20 | -0.0494929 |
| Tcf20 | 0.76734261 |
| Tcf20 | 0.74816077 |
| Tcf20 | -0.4138042 |
| Tcf20 | -0.8599277 |
| Tcf21 | -0.648837 |
| Tcf23 | -0.7777961 |
| Tcf25 | -0.7023129 |
| Tcf25 | 0.16441678 |
| Tcf25 | -0.4934155 |
| Tcf3 | 0.45726663 |
| Tcf3 | 0.84801406 |
| Tcf4 | -0.6236076 |
| Tcf7 | -0.6739199 |
| Tcf7 | -0.233595 |
| Tcf7l2 | 2.9390894 |
| Tcf7l2 | 1.6043754 |
| Tcfap2b | -0.5873843 |
| Tcfap2b | 0.72782202 |
| Tcfap2c | -0.5377515 |
| Tcfap2c | 1.08553256 |
| Tcfcp2 | -0.7315972 |
| Tcfe3 | 0.97611988 |
| Tcfl5 | 1.08617519 |
| Tchp | 2.10256136 |
| Tcirg1 | -0.27632 |
| Tcirg1 | 2.44537896 |
| Tcirg1 | -0.7507229 |
| Tcirg1 | -0.6406237 |
| Tcl1 | 2.99164837 |
| Tcl1 | 0.36150803 |
| Tcl1b2 | 0.52928805 |
| Tcl1b2 | -0.4497571 |
| Tcl1b3 | 0.05352954 |
| Tcl1b4 | 0.46928969 |
| Tcn2 | -0.0066607 |
| Tcof1 | -0.6196014 |
| Tcp1 | 0.57060036 |
| Tcp1 | -0.4878451 |
| Tcp10a | 0.83717158 |
| Tcp10a | 1.42225847 |
| Tcp11 | 0.12458751 |
| Tcstv1 | -0.7721299 |
| Tcte3 | -0.6764657 |
| Tctex1d1 | 0.87996568 |
| Tctex1d2 | -0.1579783 |
| Tctex1d4 | -0.4665689 |
| Tctn3 | -0.5131777 |
| Tctn3 | -0.5773971 |
| Tctn3 | 1.29009869 |
| Tdg | -0.2189096 |
| Tdp1 | -0.2415583 |
| Tdpoz1 | 1.58906248 |
| Tdpoz2 | 1.30240792 |
| Tdrd1 | -0.7146245 |
| Tdrd12 | 0.55733382 |
| Tdrd3 | 0.54868831 |
| Tdrd3 | -0.2054771 |
| Tdrd3 | 0.35083191 |
| Tdrd6 | -0.4761175 |
| Tdrd7 | 2.17860892 |
| Tdrkh | 0.49263807 |
| Tead2 | -0.2458333 |
| Tec | 0.75055716 |
| Tecta | 2.20310623 |
| Tef | 2.28913202 |
| Tef | -0.7911525 |
| Tegt | 0.54157557 |
| Tek | 1.1504617 |
| Tekt1 | -0.820434 |
| Tekt1 | -0.1434674 |
| Tekt3 | -0.8829666 |
| Tenc1 | 3.46548106 |
| Tenc1 | -0.3072643 |
| Terf2 | -0.4955129 |
| Terf2 | -0.3135783 |
| Terf2ip | 1.33946817 |
| Terf2ip | 0.43403868 |
| Terf2ip | 1.04130128 |
| Terf2ip | -0.0577749 |
| Tes | -0.729796 |
| Tes | -0.802755 |
| Tes | -0.7759035 |
| Tesc | 0.76734261 |
| Tesk1 | -0.7040618 |
| Tesp1 | 0.97972704 |
| Tesp2 | 0.5948419 |
| Tessp2 | 1.68492012 |
| Tessp3 | -0.2080453 |
| Tex10 | -0.4955129 |
| Tex10 | 0.86314811 |
| Tex101 | -0.1301247 |
| Tex11 | 2.27404001 |
| Tex13 | -0.6688491 |
| Tex14 | -0.2975897 |
| Tex16 | -0.2539022 |
| Tex19 | 0.64109961 |
| Tex2 | -0.7190611 |
| Tex2 | -0.6268249 |
| Tex22 | 1.65271987 |
| Tex24 | -0.6188023 |
| Tex261 | -0.5275544 |
| Tex261 | 0.84319221 |
| Tex264 | 1.53989789 |
| Tex264 | -0.8609627 |
| Tex9 | 0.18320433 |
| Tfam | 0.57616537 |
| Tfam | 0.42442646 |
| Tfb1m | -0.7343056 |
| Tfb2m | 2.59448053 |
| Tfdp1 | 2.00216563 |
| Tfdp2 | 0.770952 |
| Tfdp2 | 2.80452471 |
| Tfg | 3.3928607 |
| Tfip11 | 0.59658059 |
| Tfpi | 0.03627256 |
| Tfpt | 1.47436817 |
| Tfpt | -0.3124271 |
| Tfpt | 1.81170384 |
| Tfrc | 0.90119695 |
| Tfrc | -0.7636946 |
| Tg | -0.1296516 |
| Tgfb1 | -0.7664979 |
| Tgfb1i1 | 1.28813475 |
| Tgfb1i1 | -0.8745228 |
| Tgfb2 | -0.0714475 |
| Tgfb3 | -0.3078366 |
| Tgfbi | -0.5873843 |
| Tgfbi | 0.61037472 |
| Tgfbr1 | -0.0652479 |
| Tgfbr1 | -0.5333677 |
| Tgfbr1 | -0.7306962 |
| Tgfbr2 | 2.4486063 |
| Tgfbr2 | -0.8056784 |
| Tgfbr3 | 1.29576059 |
| Tgfbr3 | 1.44290953 |
| Tgfbrap1 | -0.4747474 |
| Tgif1 | -0.7119726 |
| Tgif1 | -0.7825443 |
| Tgif1 | 0.39011722 |
| Tgif2 | 0.41576904 |
| Tgif2lx | 2.11287828 |
| Tgm2 | -0.6688491 |
| Tgm4 | 0.43481372 |
| Tgoln1 | -0.3199353 |
| Tgs1 | -0.4761175 |
| Tgs1 | -0.1521468 |
| Th1l | 1.13193016 |
| Tha1 | -0.7040618 |
| Thada | 2.78773186 |
| Thada | 0.30091934 |
| Thap1 | -0.4286709 |
| Thap1 | -0.2080453 |
| Thap1 | -0.7479665 |
| Thap11 | 0.74982047 |
| Thap3 | -0.6688491 |
| Thap4 | -0.7618305 |
| Thap7 | -0.7749587 |
| Thbd | 0.49675526 |
| Thbs1 | -0.7759035 |
| Thbs2 | 0.65139348 |
| Thbs3 | 0.07352098 |
| Thbs3 | -0.381041 |
| Theg | -0.1692581 |
| Theg | 0.12638629 |
| Theg | 0.36485421 |
| Them2 | 0.74279268 |
| Them4 | -0.6996958 |
| Them5 | -0.4878451 |
| Thg1l | 0.3857725 |
| Thnsl1 | 0.78080642 |
| Thnsl2 | -0.6638057 |
| Thnsl2 | 0.68891621 |
| Thoc1 | 1.62616364 |
| Thoc3 | -0.5928066 |
| Thoc4 | 0.18725364 |
| Thoc4 | 0.05663316 |
| Thoc5 | -0.1998519 |
| Thoc6 | 2.0088327 |
| Thop1 | 0.4471277 |
| Thra | 0.50205657 |
| Thra | 1.04882031 |
| Thrap2 | 1.5357013 |
| Thrap3 | 0.91811778 |
| Thrsp | 0.05585822 |
| Thsd1 | 0.38658868 |
| Thsd4 | 2.65006888 |
| Thsd4 | -0.6875784 |
| Thsd7b | 0.97742007 |
| Thsd7b | 0.11226297 |
| Thsd7b | -0.5743455 |
| Thtpa | -0.5333677 |
| Thumpd1 | 2.8263888 |
| Thumpd3 | -0.7040618 |
| Thy1 | -0.0029377 |
| Thyn1 | -0.6910244 |
| Tia1 | -0.5146055 |
| Tial1 | -0.7959694 |
| Tial1 | 0.23259976 |
| Tiam1 | -0.0754547 |
| Tiam2 | -0.3176186 |
| Ticam1 | 0.48263299 |
| Tie1 | 2.12641694 |
| Tigd2 | 1.37322525 |
| Tigd3 | 1.88964985 |
| Timeless | -0.9033321 |
| Timm10 | 0.03151019 |
| Timm13 | 2.72617908 |
| Timm17a | -0.3304319 |
| Timm17b | 0.31863557 |
| Timm22 | -0.4497571 |
| Timm44 | 1.79646653 |
| Timm44 | 3.48545813 |
| Timm44 | -0.5951401 |
| Timm50 | 3.40224671 |
| Timm8a1 | -0.4358657 |
| Timm8a2 | 1.58312273 |
| Timm8a2 | -0.5990424 |
| Timm8b | -0.5577378 |
| Timm8b | -0.4010665 |
| Timm9 | 1.24211025 |
| Timm9 | 1.06328407 |
| Timm9 | -0.4131632 |
| Timp1 | -0.0395647 |
| Timp1 | 0.09048214 |
| Timp2 | -0.4906261 |
| Timp3 | 0.90119695 |
| Timp3 | -0.4023324 |
| Timp4 | -0.5290043 |
| Tinagl | -0.5751074 |
| Tipin | -0.1287063 |
| Tipin | -0.5532592 |
| Tirap | -0.3374959 |
| Tirap | 2.39339825 |
| Tirap | -0.5261067 |
| Tjap1 | -0.5743455 |
| Tjap1 | 1.07921131 |
| Tjap1 | -0.2420916 |
| Tjp1 | 4.16171587 |
| Tjp2 | -0.5766332 |
| Tjp3 | 0.52624925 |
| Tjp3 | 0.51589003 |
| Tk1 | -0.6850021 |
| Tk2 | 3.62992558 |
| Tkt | 1.99307183 |
| Tkt | 2.02892703 |
| Tkt | 3.10711409 |
| Tktl1 | -0.4280196 |
| Tktl1 | -0.3257521 |
| Tktl2 | 1.12628585 |
| Tlcd1 | 0.84402534 |
| Tle1 | -0.7470495 |
| Tle1 | -0.6398063 |
| Tle2 | 1.15620048 |
| Tle3 | -0.3620427 |
| Tle3 | -0.691888 |
| Tle3 | 0.08373463 |
| Tle4 | 0.05469461 |
| Tle4 | 1.7108642 |
| Tle4 | 1.58032776 |
| Tle6 | 2.58150646 |
| Tlk1 | -0.4625064 |
| Tlk2 | 0.89931521 |
| Tln1 | -0.5480613 |
| Tln2 | -0.1506946 |
| Tlr13 | 3.39290407 |
| Tlr2 | 0.32910605 |
| Tlr3 | -0.7415665 |
| Tlr4 | 0.43196873 |
| Tlr6 | 0.25070502 |
| Tlr7 | 2.57717602 |
| Tm2d2 | -0.3158849 |
| Tm2d3 | 1.65976372 |
| Tm2d3 | -0.461831 |
| Tm4sf4 | -0.6422606 |
| Tm4sf5 | 1.71722272 |
| Tm6sf1 | 0.14524314 |
| Tm6sf2 | 0.27553752 |
| Tm9sf2 | 1.45752098 |
| Tm9sf2 | -0.5966991 |
| Tm9sf2 | 2.21552004 |
| Tm9sf3 | -0.4280196 |
| Tm9sf4 | 0.88730861 |
| Tm9sf4 | 2.38628169 |
| Tmbim1 | -0.6398063 |
| Tmbim4 | -0.6562913 |
| Tmc1 | -0.6068962 |
| Tmc4 | 0.07466137 |
| Tmc5 | -0.3084091 |
| Tmc6 | 1.34950133 |
| Tmc6 | -0.0451633 |
| Tmc6 | 2.93420952 |
| Tmc7 | 1.19289529 |
| Tmcc2 | 2.9418715 |
| Tmcc3 | 1.56530384 |
| Tmco3 | -0.3559959 |
| Tmco3 | -0.1396357 |
| Tmco4 | -0.5804587 |
| Tmco4 | -0.7470495 |
| Tmco5 | -0.5004255 |
| Tmco6 | 1.22111283 |
| Tmco6 | -0.3638645 |
| Tmco7 | -0.6773158 |
| Tmed1 | 0.66116988 |
| Tmed1 | -0.57587 |
| Tmed10 | -0.6496624 |
| Tmed2 | -0.1516625 |
| Tmed3 | -0.648837 |
| Tmed3 | 2.3330977 |
| Tmed4 | 1.41893665 |
| Tmed4 | -0.6562913 |
| Tmed4 | 2.20659259 |
| Tmed4 | -0.5781615 |
| Tmed8 | 1.19186337 |
| Tmed8 | 2.02381454 |
| Tmed9 | -0.41573 |
| Tmed9 | -0.2256991 |
| Tmeff2 | -0.5904789 |
| Tmem10 | -0.1049188 |
| Tmem100 | 1.51256125 |
| Tmem101 | -0.705814 |
| Tmem102 | -0.5146055 |
| Tmem102 | -0.535557 |
| Tmem106a | -0.7244131 |
| Tmem106c | 1.35365822 |
| Tmem107 | 2.49046091 |
| Tmem107 | -0.4604817 |
| Tmem107 | -0.0661311 |
| Tmem108 | 1.14335909 |
| Tmem109 | 3.70264852 |
| Tmem11 | 1.16367945 |
| Tmem110 | -0.6308622 |
| Tmem111 | 0.89412079 |
| Tmem111 | -0.2458333 |
| Tmem111 | 2.07825729 |
| Tmem115 | 0.01180754 |
| Tmem116 | -0.2880143 |
| Tmem116 | -0.7498032 |
| Tmem117 | -0.2746613 |
| Tmem118 | -0.723519 |
| Tmem119 | 1.22809104 |
| Tmem120a | 1.18275611 |
| Tmem120b | 1.44413593 |
| Tmem121 | 0.81544468 |
| Tmem125 | -0.5253837 |
| Tmem126a | 1.46474179 |
| Tmem126b | 0.0776956 |
| Tmem127 | -0.6124335 |
| Tmem128 | 0.33257026 |
| Tmem130 | 0.27369784 |
| Tmem131 | 0.76734261 |
| Tmem132a | 0.5116188 |
| Tmem132a | -0.0250791 |
| Tmem132c | 0.34003222 |
| Tmem132d | 1.19037081 |
| Tmem132e | -0.7590411 |
| Tmem134 | -0.3517863 |
| Tmem134 | 1.24860994 |
| Tmem135 | -0.7306962 |
| Tmem136 | 1.42592565 |
| Tmem138 | 1.68849765 |
| Tmem139 | -0.0862257 |
| Tmem140 | -0.4625064 |
| Tmem141 | -0.0701155 |
| Tmem143 | -0.1796775 |
| Tmem143 | 0.80108082 |
| Tmem143 | -0.5904789 |
| Tmem143 | -0.2533621 |
| Tmem144 | 0.56095021 |
| Tmem146 | 2.29720168 |
| Tmem147 | -0.3298457 |
| Tmem14c | 0.49965062 |
| Tmem14c | 0.01989976 |
| Tmem14c | 1.39737387 |
| Tmem150 | 1.60871523 |
| Tmem150 | 0.45297104 |
| Tmem151a | 0.07009143 |
| Tmem154 | -0.4215312 |
| Tmem158 | -0.5562425 |
| Tmem159 | 1.5065217 |
| Tmem159 | -0.4378375 |
| Tmem160 | -0.5458424 |
| Tmem160 | -0.3210957 |
| Tmem161a | -0.7921139 |
| Tmem162 | -0.3547912 |
| Tmem164 | 1.47893355 |
| Tmem166 | -0.1353437 |
| Tmem167 | -0.0781359 |
| Tmem168 | -0.3724132 |
| Tmem168 | -0.6236076 |
| Tmem168 | -0.4293227 |
| Tmem169 | 2.42525714 |
| Tmem16a | 1.35153376 |
| Tmem16b | 1.52965042 |
| Tmem16d | -0.0246564 |
| Tmem16e | 0.09085557 |
| Tmem16g | -0.016662 |
| Tmem16j | 1.67328308 |
| Tmem16k | -0.3985396 |
| Tmem17 | 1.26051628 |
| Tmem17 | -0.3960196 |
| Tmem170 | 1.81217602 |
| Tmem176a | -0.6944833 |
| Tmem176a | 2.39434725 |
| Tmem176b | -0.5196198 |
| Tmem177 | 0.69850386 |
| Tmem178 | 1.70578569 |
| Tmem179b | -0.6798709 |
| Tmem180 | 0.59113638 |
| Tmem183a | 0.9383571 |
| Tmem183a | 0.88412313 |
| Tmem184a | -0.7271007 |
| Tmem184a | 0.61315191 |
| Tmem184b | 0.35421653 |
| Tmem185b | 0.16579598 |
| Tmem186 | -0.7693097 |
| Tmem19 | 0.42573121 |
| Tmem192 | -0.4118824 |
| Tmem198 | 1.05588116 |
| Tmem199 | 1.67246097 |
| Tmem2 | 2.18804352 |
| Tmem2 | 0.48459364 |
| Tmem2 | 0.7204358 |
| Tmem201 | -0.5912541 |
| Tmem204 | -0.6180038 |
| Tmem205 | -0.30213 |
| Tmem208 | -0.4934155 |
| Tmem209 | -0.6116404 |
| Tmem209 | -0.3829002 |
| Tmem23 | 0.20987357 |
| Tmem25 | -0.0516652 |
| Tmem25 | -0.1160374 |
| Tmem25 | -0.5333677 |
| Tmem27 | 0.15679454 |
| Tmem32 | 0.60414951 |
| Tmem33 | -0.5189018 |
| Tmem33 | 0.58785454 |
| Tmem34 | 0.66923934 |
| Tmem35 | 0.77454748 |
| Tmem38a | -0.4948133 |
| Tmem38b | 0.91271786 |
| Tmem38b | -0.0179197 |
| Tmem39a | 1.05866414 |
| Tmem40 | 0.71203182 |
| Tmem41a | 0.29883037 |
| Tmem41b | 1.53116683 |
| Tmem42 | -0.4138042 |
| Tmem43 | -0.6781667 |
| Tmem43 | 2.80213833 |
| Tmem44 | -0.3257521 |
| Tmem45a | 1.25098186 |
| Tmem45b | -0.6730728 |
| Tmem47 | 1.53799989 |
| Tmem47 | 3.54546131 |
| Tmem48 | 0.01586242 |
| Tmem49 | -0.1095355 |
| Tmem49 | -0.7049375 |
| Tmem49 | -0.5174675 |
| Tmem49 | 0.5772744 |
| Tmem5 | -0.6513152 |
| Tmem50a | -0.1771861 |
| Tmem50b | -0.7208417 |
| Tmem50b | -0.5812257 |
| Tmem51 | 0.52952139 |
| Tmem53 | 0.19429791 |
| Tmem53 | -0.0512304 |
| Tmem53 | 0.17129247 |
| Tmem54 | 0.48923375 |
| Tmem55a | -0.2824273 |
| Tmem55b | -0.6798709 |
| Tmem56 | 2.0701027 |
| Tmem57 | -0.3434236 |
| Tmem59 | 1.13826984 |
| Tmem59l | -0.4490909 |
| Tmem60 | 0.99003841 |
| Tmem62 | -0.5637428 |
| Tmem63a | 0.06049823 |
| Tmem63b | -0.2614967 |
| Tmem63b | 0.00610094 |
| Tmem63c | -0.3953907 |
| Tmem65 | 3.32306603 |
| Tmem66 | -0.0750086 |
| Tmem66 | -0.622805 |
| Tmem66 | -0.3779505 |
| Tmem68 | 1.3724693 |
| Tmem69 | -0.0391354 |
| Tmem70 | 0.54318642 |
| Tmem71 | 2.42940773 |
| Tmem74 | -0.1049188 |
| Tmem74 | -0.5920301 |
| Tmem74 | 1.88529525 |
| Tmem77 | -0.0921124 |
| Tmem77 | 2.75310367 |
| Tmem77 | -0.8274006 |
| Tmem79 | -0.6696923 |
| Tmem8 | 0.09643923 |
| Tmem8 | -0.568272 |
| Tmem8 | 0.8049097 |
| Tmem81 | -0.3240033 |
| Tmem82 | 0.02351848 |
| Tmem85 | 0.03903943 |
| Tmem85 | 1.9517275 |
| Tmem86a | 0.70451902 |
| Tmem86b | -0.8017826 |
| Tmem87a | -0.5333677 |
| Tmem88 | -0.5959193 |
| Tmem9 | -0.6901617 |
| Tmem90a | 0.21805376 |
| Tmem91 | -0.4948133 |
| Tmem93 | 0.80889589 |
| Tmem97 | 1.21564902 |
| Tmem98 | 3.75407437 |
| Tmem98 | 0.87530656 |
| Tmem9b | 2.59766427 |
| Tmem9b | 1.98442833 |
| Tmem9b | -0.3947622 |
| Tmie | 2.7502365 |
| Tmod1 | -0.6300534 |
| Tmod2 | -0.3638645 |
| Tmod2 | -0.2490525 |
| Tmod3 | -0.6316718 |
| Tmod4 | 2.97159882 |
| Tmod4 | 0.8195572 |
| Tmod4 | 2.63208467 |
| Tmpo | -0.4080507 |
| Tmpo | 2.37840314 |
| Tmpo | 2.68189862 |
| Tmprss11a | -0.7279982 |
| Tmprss11c | -0.4537644 |
| Tmprss13 | -0.4983169 |
| Tmprss13 | -0.4299749 |
| Tmprss2 | 0.07580036 |
| Tmprss4 | -0.4611561 |
| Tmprss4 | -0.4850724 |
| Tmprss4 | 2.28063487 |
| Tmprss7 | -0.4864577 |
| Tmprss9 | 0.52155477 |
| Tmsb10 | -0.4365225 |
| Tmsb10 | 0.0788309 |
| Tmsb10 | 1.03602878 |
| Tmsb4x | 0.12638629 |
| Tmtc2 | -0.4631822 |
| Tmtc4 | -0.6389897 |
| Tmub1 | 0.23292045 |
| Tmub1 | 0.55914377 |
| Tnc | 0.02911986 |
| Tnc | 1.36877527 |
| Tnc | 2.1613175 |
| Tnc | -0.1540866 |
| Tnfaip1 | 1.26593623 |
| Tnfaip2 | -0.1560304 |
| Tnfaip2 | -0.4761175 |
| Tnfaip2 | 0.99896832 |
| Tnfaip3 | -0.5607354 |
| Tnfaip8 | 1.25848901 |
| Tnfaip8l2 | 2.49396259 |
| Tnfaip8l2 | 1.3736974 |
| Tnfaip8l3 | -0.5181844 |
| Tnfrsf10b | 1.07895239 |
| Tnfrsf10b | -0.6781667 |
| Tnfrsf10b | 2.1965633 |
| Tnfrsf11a | 1.68660934 |
| Tnfrsf11b | 0.44073445 |
| Tnfrsf12a | -0.7066913 |
| Tnfrsf13c | -0.717284 |
| Tnfrsf19 | 0.60307199 |
| Tnfrsf19 | 1.89862133 |
| Tnfrsf1a | -0.2054771 |
| Tnfrsf1a | -0.4093262 |
| Tnfrsf1a | -0.7049375 |
| Tnfrsf1b | 1.40835401 |
| Tnfrsf21 | 0.8204117 |
| Tnfrsf21 | -0.6140216 |
| Tnfrsf22 | 0.14136067 |
| Tnfrsf22 | -0.5827615 |
| Tnfrsf25 | 1.74274078 |
| Tnfrsf4 | -0.657956 |
| Tnfsf13b | 0.06049823 |
| Tnfsf13b | 0.98346375 |
| Tnip1 | 0.11262779 |
| Tnip1 | -0.7155101 |
| Tnip1 | -0.2691534 |
| Tnip1 | 2.80444323 |
| Tnip2 | 3.06045606 |
| Tnks | 0.16854827 |
| Tnks1bp1 | -0.4437783 |
| Tnnc1 | 0.01058763 |
| Tnnc2 | 0.17879766 |
| Tnni2 | 1.43002295 |
| Tnni3 | 1.10272993 |
| Tnnt1 | 1.48808135 |
| Tnp1 | 0.80020843 |
| Tnp2 | 3.56002302 |
| Tnpo1 | 0.74760688 |
| Tnpo2 | -0.5384841 |
| Tnr | 0.41682272 |
| Tnrc15 | -0.682433 |
| Tnrc18 | -0.5443661 |
| Tnrc18 | -0.5577378 |
| Tnrc18 | -0.6680066 |
| Tnrc6a | 1.99088191 |
| Tnrc6a | 0.34516345 |
| Tnrc6b | -0.821426 |
| Tnrc6b | -0.7181721 |
| Tnrc6c | -0.2008721 |
| Tns4 | 2.62403673 |
| Tnxb | 0.81441371 |
| Tnxb | -0.7244131 |
| Tnxb | 4.01998834 |
| Tob1 | -0.3596193 |
| Tob2 | 0.74705266 |
| Toe1 | -0.4036001 |
| Toe1 | -0.5282791 |
| Tollip | 3.04859801 |
| Tollip | 1.1065089 |
| Tom1 | 1.60326882 |
| Tom1l2 | 1.68647432 |
| Tom1l2 | -0.4273687 |
| Tom1l2 | -0.5998248 |
| Tomm22 | 2.56680009 |
| Tomm34 | -0.289697 |
| Tomm34 | 1.31052478 |
| Tomm7 | 0.68517188 |
| Tomm70a | -0.4273687 |
| Top1 | -0.652971 |
| Top1mt | -0.4990192 |
| Top2a | -0.7636946 |
| Top3a | 1.21127729 |
| Top3b | -0.0975793 |
| Topors | 2.48837644 |
| Tor1a | -0.4665689 |
| Tor1a | -0.3935064 |
| Tor1aip1 | -0.0984936 |
| Tor1aip2 | -0.004177 |
| Tor1aip2 | -0.4036001 |
| Tor1b | 2.16528663 |
| Tor3a | 0.27246937 |
| Tox3 | 0.87369456 |
| Tpbg | 1.87729532 |
| Tpcn1 | 1.82808957 |
| Tpcn1 | -0.5275544 |
| Tpcn2 | 1.05680974 |
| Tpd52 | -0.7075694 |
| Tpd52l1 | -0.5920301 |
| Tpd52l1 | -0.7590411 |
| Tpd52l2 | -0.5189018 |
| Tph2 | -0.0754547 |
| Tpi1 | 1.48707863 |
| Tpi1 | 1.84459153 |
| Tpi1 | 0.12350658 |
| Tpk1 | -0.4652127 |
| Tpk1 | 0.00200332 |
| Tpk1 | -0.5796923 |
| Tpm1 | 1.65656223 |
| Tpm2 | -0.27632 |
| Tpm2 | 1.44343533 |
| Tpm3 | -0.6867188 |
| Tpm3 | 1.27206208 |
| Tpm4 | 0.11663142 |
| Tpo | 1.39792596 |
| Tpp1 | 0.35308969 |
| Tpp2 | 1.10663461 |
| Tppp | 0.22260353 |
| Tppp | -0.682433 |
| Tppp3 | -0.5297301 |
| Tpr | -0.6790184 |
| Tpra40 | -0.4857648 |
| Tprgl | -0.4955129 |
| Tprgl | 1.93335782 |
| Tprkb | -0.5210575 |
| Tprkb | 2.85256298 |
| Tpsab1 | 2.2455911 |
| Tpst1 | 1.73559405 |
| Tpst2 | -0.6884387 |
| Tpte | -0.461831 |
| Tpte | -0.0221244 |
| Tpx2 | -0.5246613 |
| Tra2a | -0.3547912 |
| Trabd | 0.18388044 |
| Trabd | -0.7306962 |
| Traf1 | -0.6850021 |
| Traf2 | -0.7854049 |
| Traf3 | 1.1495018 |
| Traf3 | -0.2246514 |
| Traf3ip1 | -0.4699679 |
| Traf3ip2 | 1.51321184 |
| Traf4 | 0.23163705 |
| Traf4 | 2.37763093 |
| Traf4 | 0.12566719 |
| Traf4 | 3.12233618 |
| Traf5 | 0.84169069 |
| Traf6 | -0.5804587 |
| Traf6 | -0.8819069 |
| Traf6 | -0.5495435 |
| Traf7 | 1.86382638 |
| Trafd1 | -0.7334019 |
| Trafd1 | -0.5089076 |
| Trafd1 | -0.9375451 |
| Traip | 1.15034177 |
| Trak1 | -0.1594418 |
| Trak2 | -0.6612942 |
| Trak2 | 0.21577053 |
| Trak2 | 0.83195584 |
| Tram1 | -0.7988715 |
| Tram1 | -0.1952744 |
| Tram1 | -0.5735841 |
| Tram2 | -0.4638586 |
| Tram2 | -0.4913227 |
| Trap1 | -0.5751074 |
| Trappc1 | 0.47771325 |
| Trappc1 | 0.89931521 |
| Trappc2 | 1.41035309 |
| Trappc2l | 0.09606802 |
| Trappc3 | 2.38956078 |
| Trappc3 | -0.9364216 |
| Trappc3 | -0.323421 |
| Trappc3 | 0.95106577 |
| Trappc4 | -0.4464308 |
| Trappc4 | -0.4557745 |
| Trappc5 | -0.4899301 |
| Trappc6a | 0.48777095 |
| Trat1 | 2.30251968 |
| Trcg1 | -0.2571494 |
| Trdmt1 | -0.5789266 |
| Trdmt1 | 1.58395205 |
| Trem2 | -0.5406855 |
| Treml1 | 0.52928805 |
| Treml2 | 1.41370556 |
| Treml4 | -0.5196198 |
| Trerf1 | 3.9411294 |
| Trerf1 | 1.0327693 |
| Trex1 | -0.5502854 |
| Trex1 | 0.2849852 |
| Trex2 | -0.1123165 |
| Trf | 0.28589444 |
| Trfp | -0.5488021 |
| Trfr2 | -0.6730728 |
| Trfr2 | 0.41074779 |
| Trh | 1.1640341 |
| Trhde | -0.0551516 |
| Trhr | -0.5297301 |
| Triap1 | -0.2824273 |
| Trib2 | 0.91811778 |
| Trib3 | -0.1751978 |
| Trib3 | -0.717284 |
| Trim11 | 3.2648599 |
| Trim16 | 1.71375385 |
| Trim17 | -0.381041 |
| Trim2 | -0.69535 |
| Trim2 | -0.7334019 |
| Trim21 | 0.56634864 |
| Trim21 | 1.6965474 |
| Trim23 | 0.41392225 |
| Trim23 | -0.6037467 |
| Trim23 | -0.4969139 |
| Trim25 | -0.6268249 |
| Trim26 | -0.813519 |
| Trim28 | -0.8496406 |
| Trim3 | 2.3713653 |
| Trim3 | -0.3440185 |
| Trim30 | -0.8174641 |
| Trim32 | -0.6773158 |
| Trim32 | 0.77973644 |
| Trim33 | 1.35085677 |
| Trim33 | 0.52882118 |
| Trim33 | -0.0975793 |
| Trim35 | -0.1021597 |
| Trim36 | -0.3872529 |
| Trim36 | -0.6236076 |
| Trim36 | 0.19128545 |
| Trim37 | 0.50085437 |
| Trim38 | 1.25570768 |
| Trim38 | -0.5974795 |
| Trim39 | 2.20007414 |
| Trim40 | 1.25827535 |
| Trim41 | 1.22100164 |
| Trim42 | -0.293635 |
| Trim44 | 0.5732757 |
| Trim45 | -0.6841449 |
| Trim45 | 1.21284899 |
| Trim46 | 1.67150093 |
| Trim47 | 0.47697304 |
| Trim54 | -0.4280196 |
| Trim56 | -0.7343056 |
| Trim59 | 0.23035189 |
| Trim61 | 0.39282243 |
| Trim62 | 0.49142367 |
| Trim67 | -0.5951401 |
| Trim68 | 1.93169809 |
| Trim69 | -0.1947672 |
| Trim72 | -0.5697867 |
| Trim8 | 0.01464781 |
| Trim9 | -0.4699679 |
| Trim9 | -0.5713038 |
| Trim9 | 0.80073196 |
| Trim9 | -0.691888 |
| Triml1 | -0.127762 |
| Triobp | 0.03943403 |
| Triobp | -0.6037467 |
| Trip10 | 0.13283333 |
| Trip12 | -0.4029661 |
| Trip12 | 3.05966606 |
| Trip12 | 1.07193419 |
| Trip6 | -0.6463654 |
| Trip6 | 0.30211095 |
| Trit1 | -0.4557745 |
| Trmt1 | 1.92256886 |
| Trmt12 | 1.4680754 |
| Trmu | -0.7049375 |
| Tro | -0.5951401 |
| Tro | 0.43326299 |
| Tro | 0.22227928 |
| Trove2 | 0.32184737 |
| Trp53 | 0.08185163 |
| Trp53bp1 | -0.7683715 |
| Trp53bp1 | -0.433898 |
| Trp53bp2 | -0.6252148 |
| Trp53i11 | -0.0612842 |
| Trp53i11 | -0.298156 |
| Trp53inp1 | -0.4557745 |
| Trp53inp1 | 1.2666777 |
| Trp53inp2 | 0.64068549 |
| Trp53rk | -0.0512304 |
| Trp63 | -0.7119726 |
| Trpc1 | -0.1633559 |
| Trpc1 | -0.4864577 |
| Trpc2 | 0.69107713 |
| Trpc3 | -0.5117522 |
| Trpc4 | 0.07428139 |
| Trpc4ap | -0.6722266 |
| Trpc4ap | -0.7226257 |
| Trpc5 | -0.5370194 |
| Trpc6 | 0.14348038 |
| Trpc7 | 1.24145778 |
| Trpd52l3 | -0.7730719 |
| Trpm1 | -0.2802018 |
| Trpm2 | -0.6471886 |
| Trpm3 | 0.3044896 |
| Trpm3 | -0.4163728 |
| Trpm3 | -0.5667598 |
| Trpm3 | -0.6688491 |
| Trpm3 | 2.11005248 |
| Trpm4 | -0.7834969 |
| Trpm6 | -0.457117 |
| Trpm6 | -0.1155715 |
| Trpm6 | 0.83884788 |
| Trpm8 | 0.19529991 |
| Trps1 | -0.7571862 |
| Trpt1 | 2.28572824 |
| Trpt1 | 0.39363246 |
| Trpt1 | -0.5217771 |
| Trpv2 | 1.19197809 |
| Trpv4 | -0.1821756 |
| Trpv6 | 0.43533006 |
| Trrap | 1.49282959 |
| Trub1 | 3.35350637 |
| Trub1 | 4.35314638 |
| Trub1 | -0.4451036 |
| Trub2 | 1.81940586 |
| Tsc1 | -0.4189484 |
| Tsc2 | -0.457789 |
| Tsc2 | 0.19962951 |
| Tsc22d1 | 0.82789773 |
| Tsc22d1 | 0.47919189 |
| Tsc22d2 | -0.7452183 |
| Tsc22d3 | 0.76026388 |
| Tsc22d3 | -0.8394658 |
| Tsc22d4 | -0.4280196 |
| Tsen2 | 1.47580783 |
| Tsen34 | -0.2304286 |
| Tsen54 | -0.6260195 |
| Tsfm | -0.3614363 |
| Tsg101 | -0.8056784 |
| Tsga10 | 2.70708704 |
| Tsga10 | -0.755335 |
| Tsga14 | -0.7208417 |
| Tsga8 | -0.7711889 |
| Tshb | 1.72858579 |
| Tshr | 0.56455263 |
| Tshz3 | -0.5268303 |
| Tsku | -0.4016992 |
| Tsn | -0.7693097 |
| Tsnaxip1 | 1.29995903 |
| Tsnaxip1 | -0.682433 |
| Tspan1 | 0.09495352 |
| Tspan11 | -0.7315972 |
| Tspan12 | -0.5268303 |
| Tspan13 | -0.8578612 |
| Tspan14 | -0.0087355 |
| Tspan17 | 1.31294613 |
| Tspan17 | -0.0012878 |
| Tspan18 | 0.40115908 |
| Tspan18 | 1.28699584 |
| Tspan2 | 0.17777777 |
| Tspan3 | -0.0582128 |
| Tspan31 | 0.44073445 |
| Tspan33 | -0.5495435 |
| Tspan6 | 0.39551982 |
| Tspan6 | 1.95911645 |
| Tspan7 | -0.0103986 |
| Tspan8 | -0.6944833 |
| Tspo | -0.0617237 |
| Tspo | 2.42898131 |
| Tspo | -0.5592354 |
| Tspyl1 | 3.10095206 |
| Tspyl3 | 0.3041926 |
| Tsr1 | 1.20936524 |
| Tsr2 | -0.1962897 |
| Tsr2 | 1.3169678 |
| Tsr2 | 0.46154254 |
| Tssc1 | -0.2680558 |
| Tssc1 | -0.8125353 |
| Tssc1 | -0.041714 |
| Tssc1 | 1.37934471 |
| Tssc1 | 0.34088938 |
| Tssc4 | 1.39118661 |
| Tssc4 | -0.58507 |
| Tssk4 | -0.4299749 |
| Tssk5 | 1.04721418 |
| Tssk6 | -0.5592354 |
| Tst | -0.3187762 |
| Tsta3 | -0.2919452 |
| Tsx | -0.3038383 |
| Ttbk2 | -0.7343056 |
| Ttc1 | -0.6406237 |
| Ttc12 | -0.6480124 |
| Ttc13 | 0.81578808 |
| Ttc14 | -0.5842999 |
| Ttc16 | -0.3228392 |
| Ttc17 | 0.61506974 |
| Ttc19 | 4.00602077 |
| Ttc19 | 0.71528779 |
| Ttc21a | 1.04359025 |
| Ttc22 | 0.63674209 |
| Ttc23 | -0.0648067 |
| Ttc24 | 0.66460793 |
| Ttc24 | 1.16568736 |
| Ttc26 | 0.53996193 |
| Ttc27 | 2.44361116 |
| Ttc29 | 1.78456914 |
| Ttc29 | -0.6604585 |
| Ttc3 | 2.05033646 |
| Ttc3 | -0.6268249 |
| Ttc3 | -0.0763476 |
| Ttc3 | -0.7507229 |
| Ttc3 | 0.2418551 |
| Ttc3 | 1.6964806 |
| Ttc30a1 | 0.15261056 |
| Ttc30a2 | -0.1216468 |
| Ttc33 | 0.53672627 |
| Ttc33 | 0.12961538 |
| Ttc33 | 1.77951075 |
| Ttc35 | 1.79796556 |
| Ttc4 | 3.01238534 |
| Ttc5 | -0.6646444 |
| Ttc5 | 0.65914156 |
| Ttc5 | 0.30151534 |
| Ttc7 | -0.2106206 |
| Ttc8 | -0.6357302 |
| Ttc8 | 1.16415229 |
| Ttf2 | 0.01261993 |
| Ttk | -0.739746 |
| Ttl | -0.030166 |
| Ttll1 | 0.74501771 |
| Ttll1 | 0.43015376 |
| Ttll1 | 0.51849064 |
| Ttll10 | 0.02951867 |
| Ttll11 | -0.1531162 |
| Ttll13 | -0.4802398 |
| Ttll4 | 3.52112567 |
| Ttll5 | -0.8455574 |
| Ttll5 | 0.94779304 |
| Ttll6 | 2.72538116 |
| Ttll8 | 2.78006801 |
| Ttll9 | 1.00122269 |
| Ttn | 1.42957846 |
| Ttn | -0.3286742 |
| Ttr | -0.1692581 |
| Ttyh1 | 0.26661192 |
| Ttyh1 | 2.63365171 |
| Ttyh2 | -0.2880143 |
| Ttyh2 | -0.0857745 |
| Ttyh3 | -0.6781667 |
| Tub | -0.2746613 |
| Tub | -0.6284377 |
| Tuba1a | 0.69830917 |
| Tuba1b | -0.717284 |
| Tuba3a | -0.6773158 |
| Tuba3a | 1.48574 |
| Tuba3a | -0.8164763 |
| Tuba3a | -0.2802018 |
| Tuba3b | 3.44634762 |
| Tuba4a | 1.03629989 |
| Tuba6 | -0.5517711 |
| Tuba8 | 3.41752485 |
| Tubb2b | -0.5261067 |
| Tubb2b | 0.13675137 |
| Tubb2b | -0.3885002 |
| Tubb2b | 1.68964226 |
| Tubb2b | 2.74171248 |
| Tubb2c | -0.7102088 |
| Tubb4 | -0.7315972 |
| Tubb5 | -0.6076852 |
| Tubb6 | 1.91852352 |
| Tubd1 | 2.08836229 |
| Tubd1 | -0.5181844 |
| Tube1 | -0.8095904 |
| Tubg1 | -0.5540042 |
| Tubgcp2 | 1.22388821 |
| Tubgcp4 | 1.55812638 |
| Tufm | -0.6875784 |
| Tuft1 | -0.5370194 |
| Tug1 | 0.53440819 |
| Tulp1 | -0.4517586 |
| Tulp2 | 2.11786671 |
| Tulp3 | 2.7931707 |
| Tulp4 | 1.249581 |
| Tusc2 | -0.602961 |
| Tusc2 | 0.90930756 |
| Tusc4 | 0.36401879 |
| Tut1 | -0.6858601 |
| Twf2 | 0.63985668 |
| Twist1 | -0.7815927 |
| Twist2 | -0.1604188 |
| Twistnb | 0.92440391 |
| Twistnb | 1.20790043 |
| Twsg1 | -0.7279982 |
| Txk | -0.6439004 |
| Txlna | -0.6850021 |
| Txn1 | 1.71381942 |
| Txn2 | 0.47993034 |
| Txndc1 | -0.5117522 |
| Txndc11 | 1.1313183 |
| Txndc11 | -0.3553933 |
| Txndc11 | 3.64401493 |
| Txndc12 | 1.71132452 |
| Txndc13 | 0.05895412 |
| Txndc13 | 2.87898258 |
| Txndc15 | 1.75987257 |
| Txndc2 | -0.7806421 |
| Txndc5 | -0.7146245 |
| Txndc8 | -0.6538 |
| Txndc9 | 0.05002557 |
| Txnip | 1.5462753 |
| Txnl2 | 0.45322427 |
| Txnl4 | -0.7066913 |
| Txnl4a | 2.19099695 |
| Txnl4a | -0.7137397 |
| Txnl4a | 0.93745344 |
| Txnl4b | 0.01949681 |
| Txnrd2 | -0.7498032 |
| Txnrd3 | -0.7749587 |
| Tyk2 | 1.06722588 |
| Tyk2 | 1.71388498 |
| Tyk2 | 1.65788592 |
| Tyki | 0.15121172 |
| Tyms | 0.05779436 |
| Tyms | 0.19296024 |
| Tyms | -0.8766266 |
| Tyms-ps | 0.40862546 |
| Tyro3 | -0.7664979 |
| Tyrobp | 1.39479314 |
| Tyw1 | -0.1727183 |
| Tyw1 | 0.59200959 |
| U2af1l4 | 2.85982887 |
| U2af2 | 0.25697538 |
| U2af2 | -0.4969139 |
| Uaca | -0.0590893 |
| Uap1 | 0.31922035 |
| Uap1l1 | -0.4906261 |
| Uba1 | -0.0158244 |
| Uba1 | -0.682433 |
| Uba2 | -0.7343056 |
| Uba5 | 0.11444975 |
| Ubac1 | -0.7217333 |
| Ubac1 | 1.58123515 |
| Ubac1 | -0.6747677 |
| Ubac2 | 0.41997661 |
| Ubap1 | 1.92298815 |
| Ubap2 | -0.7618305 |
| Ubap2l | -0.127762 |
| Ubap2l | 0.62922438 |
| Ubap2l | -0.8506643 |
| Ubb | 3.48153228 |
| Ubd | 0.18489368 |
| Ube1c | -0.6807241 |
| Ube1l | -0.3124271 |
| Ube1x | 0.04885465 |
| Ube2a | 1.8676707 |
| Ube2b | 1.3107268 |
| Ube2d1 | -0.7217333 |
| Ube2d2 | -0.6705363 |
| Ube2d3 | -0.6604585 |
| Ube2e1 | -0.3369052 |
| Ube2e2 | -0.4672477 |
| Ube2e3 | 3.19673833 |
| Ube2f | -0.7516435 |
| Ube2f | -0.2669594 |
| Ube2f | 0.57616537 |
| Ube2g1 | 1.08024628 |
| Ube2g1 | -0.666324 |
| Ube2g1 | -0.5203384 |
| Ube2g2 | -0.5131777 |
| Ube2i | 0.11735751 |
| Ube2j1 | -0.4352093 |
| Ube2j2 | 2.4355992 |
| Ube2j2 | 0.3006212 |
| Ube2j2 | 0.60069705 |
| Ube2k | 1.81217602 |
| Ube2k | 0.88028614 |
| Ube2l3 | 1.78917699 |
| Ube2l3 | 0.30181319 |
| Ube2l6 | 0.88124687 |
| Ube2m | 0.02150981 |
| Ube2n | -0.6244109 |
| Ube2n | -0.738837 |
| Ube2o | -0.2436934 |
| Ube2q1 | -0.6381738 |
| Ube2q1 | -0.7415665 |
| Ube2q2 | -0.3872529 |
| Ube2q2 | 1.69239703 |
| Ube2q2 | -0.5547497 |
| Ube2r2 | 1.99156377 |
| Ube2s | 1.56160624 |
| Ube2t | 0.31129465 |
| Ube2u | 1.57494089 |
| Ube2u | -0.8671967 |
| Ube3a | 0.19763373 |
| Ube3a | -0.3773336 |
| Ube3b | -0.5812257 |
| Ube3b | -0.2220371 |
| Ube3c | -0.666324 |
| Ube4a | -0.380422 |
| Ube4b | 0.55688079 |
| Ubfd1 | 1.19128959 |
| Ubiad1 | 1.01920225 |
| Ubie | -0.6815782 |
| Ubl3 | 1.7313568 |
| Ubl4 | -0.5370194 |
| Ubl4b | -0.7005673 |
| Ubl5 | -0.5525149 |
| Ubl7 | -0.5904789 |
| Ublcp1 | -0.5866122 |
| Ubox5 | -0.3158849 |
| Ubox5 | -0.7825443 |
| Ubp1 | -0.5607354 |
| Ubp1 | -0.6398063 |
| Ubqln1 | -0.3785677 |
| Ubqln1 | -0.1897102 |
| Ubqln1 | 1.0015041 |
| Ubqln3 | -0.6045331 |
| Ubqln4 | -0.6092653 |
| Ubqlnl | -0.6148167 |
| Ubr1 | 1.92795288 |
| Ubr5 | -0.1796775 |
| Ubtd1 | -0.802755 |
| Ubtd2 | 1.11202385 |
| Ubtd2 | -0.6807241 |
| Ubtd2 | 0.10198967 |
| Ubtf | -0.1258761 |
| Ubxd1 | -0.2813139 |
| Ubxd1 | -0.7315972 |
| Ubxd1 | -0.0839715 |
| Ubxd3 | 0.82620162 |
| Ubxd4 | -0.4378375 |
| Ubxd5 | 1.02744823 |
| Ubxd8 | -0.719951 |
| Uchl1 | -0.2209935 |
| Uchl3 | -0.457789 |
| Uchl5 | -0.3434236 |
| Uchl5 | 0.12566719 |
| Uchl5 | 0.40756249 |
| Uck1 | 0.17368704 |
| Uck1 | 1.17215408 |
| Uck1 | 0.96243078 |
| Uck1 | 0.11262779 |
| Uck2 | -0.0516652 |
| Uck2 | -0.423473 |
| Uck2 | 0.12782282 |
| Uevld | 0.38686058 |
| Ufc1 | -0.4010665 |
| Ufsp1 | 1.46747794 |
| Ufsp2 | 0.64995916 |
| Ugcg | -0.1727183 |
| Ugp2 | 0.76335608 |
| Ugt1a10 | -0.5004255 |
| Ugt1a6a | 2.14262355 |
| Ugt1a6a | 0.66823447 |
| Ugt1a6a | 0.30330103 |
| Ugt3a2 | 0.01058763 |
| Ugt8a | 0.81613136 |
| Uhmk1 | -0.3245859 |
| Uhrf1 | 0.91534458 |
| Uhrf1bp1 | -0.6260195 |
| Uhrf1bp1l | -0.7844504 |
| Uhrf2 | -0.4774896 |
| Uhrf2 | 0.04611678 |
| Uhrf2 | -0.5081978 |
| Uhrf2 | 1.00879122 |
| Uimc1 | 1.78711839 |
| Ulk1 | -0.5858408 |
| Ulk1 | 0.56117577 |
| Ulk2 | 1.48339276 |
| Ulk2 | -0.5046569 |
| Umodl1 | -0.5217771 |
| Umps | -0.5067798 |
| Umps | -0.719951 |
| Umps | -0.5004255 |
| Unc119 | 0.71624327 |
| Unc119 | -0.3124271 |
| Unc119b | -0.6756163 |
| Unc119b | -0.0124817 |
| Unc119b | 0.07655892 |
| Unc13b | 1.76782701 |
| Unc13c | -0.5842999 |
| Unc13d | -0.5146055 |
| Unc45a | -0.2394281 |
| Unc45a | 2.23788332 |
| Unc45b | -0.3428292 |
| Unc5a | -0.5131777 |
| Unc5a | 1.66703059 |
| Unc5b | 1.86471923 |
| Unc5c | 1.41225732 |
| Unc84a | -0.7424781 |
| Unc84a | 1.40808107 |
| Unc84b | -0.58507 |
| Unc93a | -0.2095897 |
| Unc93b1 | -0.6037467 |
| Unc93b1 | -0.3452093 |
| Ung | -0.2018935 |
| Ung | 1.62587549 |
| Unk | -0.0033506 |
| Unkl | -0.2863347 |
| Uox | 0.04179812 |
| Upb1 | -0.1638464 |
| Upb1 | -0.617206 |
| Upb1 | 0.10420063 |
| Upf1 | -0.69535 |
| Upf1 | -0.5443661 |
| Upf1 | -0.1947672 |
| Upf2 | 0.70297043 |
| Upf3a | 0.94031203 |
| Upk1a | -0.7711889 |
| Upk1a | -0.6430801 |
| Upk1b | -0.5897043 |
| Upk2 | -0.5025388 |
| Upk3a | 0.73290152 |
| Upk3b | -0.381041 |
| Upk3b | 1.78790604 |
| Upk3b | 1.40962665 |
| Upp1 | 2.52459761 |
| Upp1 | -0.2614967 |
| Upp1 | -0.1531162 |
| Upp2 | 1.30789454 |
| Upp2 | 2.81845542 |
| Upp2 | 1.22177968 |
| Upp2 | 0.43222773 |
| Uqcc | 2.26154757 |
| Uqcr | 0.94256263 |
| Uqcrc1 | -0.7217333 |
| Uqcrc2 | 0.00241389 |
| Uqcrh | 0.58982503 |
| Urm1 | -0.7146245 |
| Urod | 1.44597251 |
| Uros | -0.4692871 |
| Uros | -0.2857755 |
| Use1 | 0.53347934 |
| Use1 | -0.6713811 |
| Use1 | -0.6927523 |
| Usf1 | 1.12097859 |
| Usf1 | -0.4906261 |
| Usf2 | 0.83532417 |
| Usf2 | -0.4611561 |
| Ush1c | -0.467927 |
| Ush1g | 0.58169738 |
| Ush2a | -0.6671649 |
| Ush3a | -0.3565988 |
| Ushbp1 | 1.78426519 |
| Usmg5 | -0.5804587 |
| Uso1 | 1.02264693 |
| Usp1 | 1.7061163 |
| Usp1 | -0.7683715 |
| Usp1 | -0.4864577 |
| Usp10 | -0.0577749 |
| Usp10 | 0.78098464 |
| Usp10 | -0.4411333 |
| Usp11 | 0.55756026 |
| Usp11 | 1.21385797 |
| Usp12 | -0.7969357 |
| Usp12 | -0.274109 |
| Usp13 | 0.56769338 |
| Usp14 | -0.3181972 |
| Usp15 | -0.7571862 |
| Usp16 | 1.06459986 |
| Usp18 | -0.5146055 |
| Usp18 | 3.11292836 |
| Usp19 | 1.16320638 |
| Usp2 | -0.8703294 |
| Usp2 | 0.89695769 |
| Usp2 | 1.35952361 |
| Usp20 | -0.3130025 |
| Usp21 | 0.96871775 |
| Usp22 | -0.38228 |
| Usp25 | 0.5667971 |
| Usp28 | 1.72295401 |
| Usp29 | 0.25978335 |
| Usp3 | -0.3205153 |
| Usp3 | -0.682433 |
| Usp33 | 1.24200154 |
| Usp37 | -0.6970859 |
| Usp38 | -0.3292598 |
| Usp38 | 1.22753912 |
| Usp39 | -0.3181972 |
| Usp39 | -0.2085598 |
| Usp39 | -0.7618305 |
| Usp39 | -0.5495435 |
| Usp4 | 0.70818666 |
| Usp4 | -0.5766332 |
| Usp43 | 2.16358764 |
| Usp45 | 0.58894977 |
| Usp46 | 1.31686747 |
| Usp47 | 1.37435802 |
| Usp48 | -0.340455 |
| Usp49 | 0.40222937 |
| Usp5 | 0.6146439 |
| Usp50 | 1.31526071 |
| Usp52 | 0.34715133 |
| Usp52 | -0.0582128 |
| Usp52 | -0.0617237 |
| Usp53 | 2.22935698 |
| Usp6nl | 2.70853314 |
| Usp6nl | -0.5246613 |
| Usp7 | 0.32504815 |
| Usp8 | 0.53464025 |
| Usp8 | 0.64358052 |
| Usp9x | 2.97562639 |
| Usp9y | 0.39228201 |
| Uspl1 | -0.6680066 |
| Utp11l | 0.96988276 |
| Utp15 | -0.5622379 |
| Utp6 | 0.19463203 |
| Utx | 0.01221382 |
| Utx | 0.16854827 |
| Uty | 1.26636 |
| Uvrag | -0.4611561 |
| Uxt | 1.07947016 |
| Uxt | 2.39029143 |
| V1ra2 | 1.01227449 |
| V1rd2 | 0.16545137 |
| V1rd20 | 1.81188093 |
| V1rd21 | -0.4823079 |
| V2r8 | 1.26423924 |
| Vac14 | 3.48019036 |
| Vamp1 | -0.6554601 |
| Vamp1 | -0.6850021 |
| Vamp2 | -0.4484252 |
| Vamp3 | 0.43996431 |
| Vamp3 | -0.0893908 |
| Vamp4 | -0.7768493 |
| Vamp5 | -0.4699679 |
| Vamp8 | 0.44406441 |
| Vamp8 | -0.457117 |
| Vangl2 | -0.4976151 |
| Vapa | -0.5160354 |
| Vapb | 2.87828642 |
| Vars | -0.6100563 |
| Vars | 1.71539171 |
| Vars | 1.28169719 |
| Vars2 | 0.8204117 |
| Vash1 | -0.368129 |
| Vash1 | -0.6554601 |
| Vasn | -0.4864577 |
| Vasp | 0.2849852 |
| Vat1 | 0.19262552 |
| Vav1 | 0.79811135 |
| Vav2 | 2.1504172 |
| Vav3 | 0.76734261 |
| Vax1 | -0.1707395 |
| Vbp1 | -0.1109249 |
| Vcam1 | 0.63778145 |
| Vcl | -0.6807241 |
| Vcp | -0.6747677 |
| Vcpip1 | 2.18622745 |
| Vdac1 | -0.821426 |
| Vdac2 | -0.2669594 |
| Vdac3 | 0.62101125 |
| Vdr | -0.1816754 |
| Vdr | 0.49069427 |
| Vdr | 0.71872295 |
| Vegfa | 0.87964511 |
| Vegfa | -0.5502854 |
| Vegfb | 2.712341 |
| Vegfc | -0.2447627 |
| Vezf1 | -0.726204 |
| Vezt | 0.16061345 |
| Vgf | 2.1771735 |
| Vgll2 | -0.2320105 |
| Vgll4 | 1.8506699 |
| Vhlh | 1.64463437 |
| Vil1 | 1.03358523 |
| Vill | -0.4604817 |
| Vim | -0.8578612 |
| Vip | 1.97610011 |
| Vit | -0.3446137 |
| Vkorc1 | -0.6621306 |
| Vkorc1 | 0.18960764 |
| Vkorc1 | -0.0233896 |
| Vkorc1l1 | 1.85406109 |
| Vkorc1l1 | 0.26846561 |
| Vkorc1l1 | 0.51541642 |
| Vldlr | -0.6504884 |
| Vldlr | -0.219951 |
| Vldlr | 0.06703232 |
| Vldlr | -0.5705449 |
| Vldlr | 0.94420964 |
| Vmn2r81 | 0.29613765 |
| Vmo1 | -0.2436934 |
| Vnn3 | -0.0250791 |
| Vnn3 | 0.74316389 |
| Vpreb1 | -0.6204013 |
| Vpreb3 | 0.30508332 |
| Vps11 | 0.07085464 |
| Vps13a | -0.7137397 |
| Vps16 | 1.09155583 |
| Vps16 | -0.5751074 |
| Vps16 | -0.5465815 |
| Vps16 | -0.7664979 |
| Vps18 | 0.86151494 |
| Vps24 | 0.70548559 |
| Vps25 | 0.02351848 |
| Vps25 | -0.7815927 |
| Vps26 | -0.5697867 |
| Vps26a | -0.657956 |
| Vps26b | -0.4631822 |
| Vps28 | 1.13632375 |
| Vps29 | 0.44278506 |
| Vps29 | -0.7882743 |
| Vps29 | -0.7137397 |
| Vps33a | 2.835734 |
| Vps33a | 0.01302586 |
| Vps33b | 0.53765189 |
| Vps33b | 2.61676613 |
| Vps35 | 0.3944418 |
| Vps36 | 0.69908767 |
| Vps37b | -0.8568297 |
| Vps37c | -0.4706492 |
| Vps37d | 1.3913719 |
| Vps39 | -0.4398136 |
| Vps39 | -0.5766332 |
| Vps41 | 1.58229266 |
| Vps41 | 0.00077052 |
| Vps45 | -0.6212018 |
| Vps4a | -0.4892346 |
| Vps4b | 0.90946284 |
| Vps53 | -0.7815927 |
| Vps53 | 0.52507784 |
| Vps53 | 0.85314105 |
| Vps54 | 0.03706394 |
| Vps72 | 0.26475453 |
| Vrk1 | 1.18818506 |
| Vrk1 | -0.5210575 |
| Vrk2 | 2.91241869 |
| Vrk3 | -0.5951401 |
| Vsig2 | -0.2325384 |
| Vsig4 | -0.8066549 |
| Vsnl1 | 3.5432121 |
| Vstm2a | 1.20564238 |
| Vstm2a | -0.6156124 |
| Vstm2a | 0.5234354 |
| Vstm2b | 0.28528838 |
| Vsx2 | -0.7343056 |
| Vti1a | -0.2625867 |
| Vti1a | 2.43705006 |
| Vti1a | 0.02911986 |
| Vti1b | 1.59018602 |
| Vti1b | -0.622003 |
| Vtn | -0.0331462 |
| Vwa2 | 2.44388104 |
| Vwa3a | -0.6764657 |
| Vwf | -0.7208417 |
| Wars | -0.8145037 |
| Wars | -0.6513152 |
| Wars | -0.433243 |
| Wars | -0.0648067 |
| Wars2 | 2.44316118 |
| Wars2 | -0.5540042 |
| Was | -0.755335 |
| Wasf1 | -0.3626496 |
| Wasf2 | -0.1468332 |
| Wasf3 | 0.03627256 |
| Wasl | 1.97580306 |
| Wbp1 | -0.3547912 |
| Wbp1 | -0.3193556 |
| Wbp11 | 0.71356546 |
| Wbp2 | 2.68414936 |
| Wbp2nl | 0.4189265 |
| Wbp4 | 0.40062347 |
| Wbp5 | -0.0004639 |
| Wbp7 | -0.5959193 |
| Wbscr16 | 1.10676029 |
| Wbscr18 | -0.5480613 |
| Wbscr22 | -0.1049188 |
| Wbscr22 | 0.46354797 |
| Wbscr22 | -0.6571233 |
| Wbscr27 | 1.25130483 |
| Wbscr28 | -0.2442279 |
| Wbscr28 | 0.05275201 |
| Wdfy1 | 0.36763356 |
| Wdfy3 | 0.13354693 |
| Wdhd1 | -0.0066607 |
| Wdr1 | -0.4564455 |
| Wdr1 | 1.6311919 |
| Wdr13 | -0.6858601 |
| Wdr13 | -0.8008112 |
| Wdr16 | -0.5196198 |
| Wdr17 | 1.19894911 |
| Wdr18 | -0.6764657 |
| Wdr20a | 0.49409328 |
| Wdr20b | 0.51494256 |
| Wdr21 | -0.2325384 |
| Wdr22 | -0.5697867 |
| Wdr23 | 1.80673147 |
| Wdr24 | -0.213203 |
| Wdr27 | 1.24178407 |
| Wdr3 | 0.62670496 |
| Wdr31 | -0.4857648 |
| Wdr31 | -0.0246564 |
| Wdr31 | 0.32533858 |
| Wdr32 | 0.69323306 |
| Wdr33 | 1.66875249 |
| Wdr34 | -0.5990424 |
| Wdr37 | 0.83549227 |
| Wdr37 | -0.433243 |
| Wdr37 | -0.6629678 |
| Wdr37 | 0.31511913 |
| Wdr38 | 0.12278526 |
| Wdr41 | 0.17197726 |
| Wdr45 | 0.55506644 |
| Wdr45l | -0.4260683 |
| Wdr45l | 1.79490498 |
| Wdr46 | -0.186689 |
| Wdr46 | -0.5982606 |
| Wdr47 | -0.0447314 |
| Wdr5 | -0.7777961 |
| Wdr51b | -0.5074885 |
| Wdr53 | -0.5866122 |
| Wdr54 | -0.1570038 |
| Wdr55 | -0.2582343 |
| Wdr59 | -0.6587894 |
| Wdr5b | 0.73832665 |
| Wdr6 | 0.00487354 |
| Wdr60 | -0.3590144 |
| Wdr61 | -0.7664979 |
| Wdr62 | 0.13390352 |
| Wdr63 | 0.18421831 |
| Wdr66 | -0.2410253 |
| Wdr67 | 1.16945527 |
| Wdr68 | 0.90899691 |
| Wdr69 | 1.68261785 |
| Wdr70 | -0.5131777 |
| Wdr70 | -0.455104 |
| Wdr74 | 0.45928127 |
| Wdr75 | -0.7415665 |
| Wdr75 | 1.69908207 |
| Wdr76 | -0.3656897 |
| Wdr77 | -0.2686044 |
| Wdr78 | 0.52882118 |
| Wdr8 | 1.38121957 |
| Wdr8 | -0.6156124 |
| Wdr9 | 0.58037268 |
| Wdr93 | -0.2631321 |
| Wdsof1 | -0.6867188 |
| Wfdc1 | -0.818453 |
| Wfdc10 | 1.59003629 |
| Wfdc10 | 0.78613802 |
| Wfdc12 | 0.69382018 |
| Wfdc15b | -0.6061078 |
| Wfdc15b | -0.6471886 |
| Wfdc2 | -0.3675186 |
| Wfdc3 | -0.4241211 |
| Wfdc6a | -0.610848 |
| Wfdc6b | 0.36568889 |
| Wfdc6b | -0.7825443 |
| Wfikkn2 | -0.2436934 |
| Wfs1 | -0.3565988 |
| Whdc1 | 0.52788676 |
| Whrn | -0.705814 |
| Whrn | -0.298156 |
| Whsc2 | -0.3141544 |
| Wif1 | -0.3084091 |
| Wipf1 | 1.54139774 |
| Wipf2 | -0.2631321 |
| Wipi1 | 1.70862515 |
| Wipi1 | 0.32243014 |
| Wipi1 | -0.4927174 |
| Wipi2 | -0.0577749 |
| Wipi2 | -0.7049375 |
| Wipi2 | 0.63319961 |
| Wisp1 | 0.71011122 |
| Wisp1 | 1.36115168 |
| Wisp2 | 1.55276526 |
| Wiz | 4.73467113 |
| Wiz | 0.08109736 |
| Wnk1 | -0.7146245 |
| Wnk1 | 0.25415893 |
| Wnk2 | -0.1672866 |
| Wnk4 | 0.59505941 |
| Wnt10a | -0.341048 |
| Wnt10b | 1.05906103 |
| Wnt16 | -0.0952975 |
| Wnt16 | -0.4631822 |
| Wnt16 | 1.41162301 |
| Wnt2 | -0.1957819 |
| Wnt3a | -0.1310714 |
| Wnt4 | -0.5547497 |
| Wnt4 | 0.28891884 |
| Wnt5a | -0.348791 |
| Wnt5a | 0.88364437 |
| Wnt5b | 2.17501621 |
| Wnt6 | 0.90041335 |
| Wnt7a | 1.20076898 |
| Wnt7b | 1.32466105 |
| Wnt7b | 0.08147457 |
| Wrb | 0.04611678 |
| Wrn | -0.5436289 |
| Wsb1 | 0.52507784 |
| Wsb2 | -0.1983236 |
| Wscd1 | 1.5082396 |
| Wscd2 | 2.10519441 |
| Wtap | 2.95809846 |
| Wtip | -0.1555441 |
| Wwc1 | -0.4584614 |
| Wwc1 | -0.588157 |
| Wwc2 | 3.94189424 |
| Wwc2 | -0.6730728 |
| Wwox | -0.457789 |
| Wwox | -0.5146055 |
| Wwp1 | 2.23331057 |
| Wwp2 | 1.1718025 |
| Wwp2 | -0.1594418 |
| Wwp2 | 0.10125152 |
| Wwtr1 | 1.09487122 |
| X99384 | 0.72328358 |
| Xab1 | -0.6244109 |
| Xab1 | 0.28012093 |
| Xbp1 | -0.698825 |
| Xdh | 0.48115979 |
| Xist | 0.38849032 |
| Xist | 0.06741526 |
| Xk | -0.4345534 |
| Xkr4 | 1.70479314 |
| Xkr5 | -0.5392174 |
| Xkr6 | 0.56275316 |
| Xkr8 | 0.4262526 |
| Xkrx | 0.32009685 |
| Xlr4a | 2.07162316 |
| Xpc | 2.70396124 |
| Xpnpep1 | 3.1216404 |
| Xpnpep1 | 0.73795352 |
| Xpnpep1 | 0.23676007 |
| Xpnpep2 | 1.00711462 |
| Xpnpep2 | 1.85191477 |
| Xpnpep3 | -0.7406558 |
| Xpo4 | -0.723519 |
| Xpo4 | -0.7005673 |
| Xpo5 | -0.532639 |
| Xpo6 | -0.4241211 |
| Xpo7 | 1.9839376 |
| Xpo7 | -0.7940396 |
| Xpr1 | 0.22422309 |
| Xrcc1 | 1.49780176 |
| Xrcc2 | 1.26519418 |
| Xrcc2 | -0.2885749 |
| Xrcc3 | 0.74852985 |
| Xrcc6 | 2.65524665 |
| Xrcc6 | -0.2039396 |
| Xrcc6 | 1.99729115 |
| Xrn1 | 2.03042264 |
| Xrn2 | 1.37812402 |
| Xylb | -0.697955 |
| Xylt2 | -0.6722266 |
| Yaf2 | 0.56252798 |
| Yap1 | 0.50661085 |
| Ybx2 | 1.02594191 |
| Ybx2 | -0.682433 |
| Yeats2 | 3.06465813 |
| Yeats4 | 0.82857531 |
| Yeats4 | 0.24502543 |
| Yif1a | -0.457789 |
| Yif1a | -0.7324991 |
| Yif1a | 1.52877138 |
| Yif1b | 1.36763569 |
| Yipf1 | 2.25043627 |
| Yipf2 | 1.86572267 |
| Yipf3 | 0.31834304 |
| Yipf3 | 0.14065303 |
| Yipf3 | 3.39063524 |
| Yipf4 | 1.69219574 |
| Yipf6 | -0.6596236 |
| Yipf6 | -0.0652479 |
| Yipf6 | -0.6562913 |
| Yipf6 | -0.5167512 |
| Yipf7 | -0.2868942 |
| Ykt6 | 0.26009483 |
| Ykt6 | 2.52184378 |
| Ylpm1 | 1.58515702 |
| Yme1l1 | -0.4927174 |
| Yme1l1 | 0.05352954 |
| Yme1l1 | -0.703187 |
| Yod1 | 1.0758397 |
| Ypel1 | -0.697955 |
| Ypel3 | -0.7988715 |
| Ypel3 | -0.7005673 |
| Ypel4 | 2.89732692 |
| Ypel4 | -0.5261067 |
| Ypel5 | 0.22648576 |
| Yrdc | -0.2680558 |
| Ythdf1 | 1.49986571 |
| Ythdf2 | -0.869284 |
| Ythdf2 | -0.2625867 |
| Ythdf3 | 0.3270793 |
| Ythdf3 | -0.3547912 |
| Ywhab | -0.3897493 |
| Ywhab | -0.6798709 |
| Ywhae | -0.5406855 |
| Ywhag | 0.96345714 |
| Ywhah | 1.32037264 |
| Ywhaq | -0.4920198 |
| Ywhaz | -0.4829982 |
| Yy2 | 0.19796665 |
| Zadh1 | -0.5835304 |
| Zadh2 | 1.21754826 |
| Zan | -0.744304 |
| Zap70 | 0.54868831 |
| Zap70 | 2.38882955 |
| Zbbx | -0.2241279 |
| Zbed4 | 0.05508264 |
| Zbp1 | 1.24828602 |
| Zbtb17 | 0.47647925 |
| Zbtb2 | 1.52251428 |
| Zbtb22 | -0.6439004 |
| Zbtb24 | -0.5348266 |
| Zbtb3 | -0.8115526 |
| Zbtb32 | 0.86461552 |
| Zbtb33 | -0.7075694 |
| Zbtb38 | 0.37206337 |
| Zbtb39 | -0.744304 |
| Zbtb40 | 0.03349747 |
| Zbtb41 | 0.90635223 |
| Zbtb43 | 0.08636446 |
| Zbtb45 | -0.4740631 |
| Zbtb46 | 0.50277714 |
| Zbtb5 | -0.5720633 |
| Zbtb7a | 0.6986985 |
| Zbtb7c | -0.8254048 |
| Zbtb8 | 1.53490737 |
| Zbtb9 | 0.55959571 |
| Zbtb9 | -0.535557 |
| Zc3h10 | -0.4451036 |
| Zc3h11a | -0.6349171 |
| Zc3h12a | 1.81817545 |
| Zc3h12b | -0.6850021 |
| Zc3h13 | -0.5443661 |
| Zc3h14 | -0.4080507 |
| Zc3h14 | 2.72106951 |
| Zc3h15 | -0.2288495 |
| Zc3h18 | -0.7075694 |
| Zc3h3 | 2.24451509 |
| Zc3h3 | -0.703187 |
| Zc3h3 | 0.11080226 |
| Zc3h6 | 2.22489382 |
| Zc3h7a | -0.7181721 |
| Zc3h7a | 0.70991894 |
| Zc3h8 | 3.17961711 |
| Zc3hc1 | -0.0438683 |
| Zc3hc1 | -0.3872529 |
| Zc3hc1 | -0.4672477 |
| Zc3hc1 | -0.3084091 |
| Zcchc12 | -0.69535 |
| Zcchc14 | 2.88632633 |
| Zcchc16 | -0.4183038 |
| Zcchc17 | -0.6646444 |
| Zcchc17 | 2.67335688 |
| Zcchc18 | -0.810571 |
| Zcchc18 | 0.93004063 |
| Zcchc3 | 2.17541608 |
| Zcchc3 | 3.09330023 |
| Zcchc3 | -0.7040618 |
| Zcchc5 | -0.4464308 |
| Zcchc6 | -0.58507 |
| Zcchc8 | -0.4706492 |
| Zcchc8 | 0.77257168 |
| Zcchc8 | 0.73271388 |
| Zcchc9 | -0.7863604 |
| Zcrb1 | -0.2357119 |
| Zcwpw1 | 0.92943289 |
| Zdhhc1 | 0.08260528 |
| Zdhhc12 | 0.27216199 |
| Zdhhc12 | 0.02592319 |
| Zdhhc13 | 1.21273681 |
| Zdhhc13 | -0.7516435 |
| Zdhhc13 | -0.6006079 |
| Zdhhc14 | -0.6447213 |
| Zdhhc15 | -0.7796925 |
| Zdhhc16 | -0.7102088 |
| Zdhhc16 | -0.752565 |
| Zdhhc17 | -0.6730728 |
| Zdhhc19 | 0.94166304 |
| Zdhhc19 | -0.6308622 |
| Zdhhc21 | 2.65784864 |
| Zdhhc24 | 1.45354292 |
| Zdhhc3 | 0.48091403 |
| Zdhhc3 | 0.26506435 |
| Zdhhc4 | 0.00650971 |
| Zdhhc6 | 1.34959822 |
| Zdhhc7 | 1.04318672 |
| Zdhhc9 | -0.5174675 |
| Zeb1 | -0.1821756 |
| Zeb2 | -0.5377515 |
| Zer1 | 0.59896602 |
| Zf | 0.688326 |
| Zfa | -0.6596236 |
| Zfand2a | -0.7608998 |
| Zfand2b | 0.16407166 |
| Zfand2b | 1.97004147 |
| Zfand2b | 2.34437348 |
| Zfand2b | 0.11408565 |
| Zfand3 | -0.8037285 |
| Zfand6 | 1.59727054 |
| Zfand6 | -0.5966991 |
| Zfat | 3.20289924 |
| Zfh4 | 2.0324752 |
| Zfhx2 | 1.47614625 |
| Zfhx2 | -0.3374959 |
| Zfhx3 | 0.14700257 |
| Zfhx3 | 2.14303755 |
| Zfml | 1.50274758 |
| Zfp1 | -0.293635 |
| Zfp101 | 2.28934435 |
| Zfp105 | -0.6268249 |
| Zfp106 | 0.48557243 |
| Zfp106 | 0.86786818 |
| Zfp109 | 0.39011722 |
| Zfp11 | 0.27369784 |
| Zfp119 | -0.5239394 |
| Zfp12 | -0.7271007 |
| Zfp128 | -0.6944833 |
| Zfp13 | -0.3559959 |
| Zfp131 | 0.08373463 |
| Zfp142 | 0.13888157 |
| Zfp143 | 1.38589032 |
| Zfp148 | -0.6316718 |
| Zfp160 | 0.02150981 |
| Zfp161 | -0.4699679 |
| Zfp161 | -0.1249346 |
| Zfp169 | -0.1687648 |
| Zfp179 | 0.39282243 |
| Zfp182 | -0.6439004 |
| Zfp184 | -0.1449084 |
| Zfp185 | 0.01099445 |
| Zfp187 | -0.1722232 |
| Zfp187 | -0.3730268 |
| Zfp189 | -0.2039396 |
| Zfp191 | 1.30352819 |
| Zfp2 | -0.417016 |
| Zfp2 | -0.2442279 |
| Zfp202 | -0.2116527 |
| Zfp207 | 2.45916617 |
| Zfp212 | -0.4638586 |
| Zfp212 | -0.3972788 |
| Zfp212 | 1.59645297 |
| Zfp212 | -0.4241211 |
| Zfp213 | -0.3245859 |
| Zfp217 | 0.58763534 |
| Zfp219 | -0.4437783 |
| Zfp235 | -0.1521468 |
| Zfp236 | -0.705814 |
| Zfp236 | 0.41813813 |
| Zfp238 | -0.2852166 |
| Zfp238 | 0.64047836 |
| Zfp238 | -0.0595279 |
| Zfp238 | -0.128234 |
| Zfp238 | -0.568272 |
| Zfp239 | -0.6100563 |
| Zfp251 | -1.0039268 |
| Zfp259 | -0.5488021 |
| Zfp26 | -0.3779505 |
| Zfp263 | 0.63674209 |
| Zfp27 | -0.8755741 |
| Zfp27 | -0.4144457 |
| Zfp275 | 1.29216154 |
| Zfp276 | -0.5348266 |
| Zfp277 | -0.3711871 |
| Zfp277 | 0.59440672 |
| Zfp280c | 1.27353473 |
| Zfp281 | 2.19417619 |
| Zfp282 | -0.3416413 |
| Zfp286 | -0.8294007 |
| Zfp288 | -0.2431591 |
| Zfp292 | 1.29360282 |
| Zfp292 | -0.5796923 |
| Zfp295 | 0.11917027 |
| Zfp296 | -0.8445393 |
| Zfp296 | 2.34657245 |
| Zfp3 | 3.20730156 |
| Zfp30 | 3.31759303 |
| Zfp313 | 0.02191189 |
| Zfp313 | -0.4899301 |
| Zfp316 | 0.1314045 |
| Zfp316 | -0.6076852 |
| Zfp318 | -0.3693511 |
| Zfp322a | -0.3004248 |
| Zfp324 | 2.86090334 |
| Zfp326 | 2.46422563 |
| Zfp326 | 0.44763727 |
| Zfp326 | 0.68950605 |
| Zfp329 | -0.5239394 |
| Zfp329 | 2.98356249 |
| Zfp330 | -0.391626 |
| Zfp330 | 2.12439228 |
| Zfp334 | 2.29881428 |
| Zfp335 | 0.53857659 |
| Zfp339 | -0.227273 |
| Zfp346 | 1.13960542 |
| Zfp346 | -0.5319109 |
| Zfp35 | 0.79828628 |
| Zfp354a | -0.622003 |
| Zfp36 | 0.58741609 |
| Zfp365 | -0.8324088 |
| Zfp367 | 0.50397687 |
| Zfp367 | -0.017081 |
| Zfp36l1 | -0.8364348 |
| Zfp36l1 | -0.2598641 |
| Zfp36l1 | -0.7702488 |
| Zfp36l2 | 0.5685888 |
| Zfp37 | 1.18876075 |
| Zfp371 | -0.2070172 |
| Zfp382 | 0.81252064 |
| Zfp385a | -0.9044164 |
| Zfp385a | -0.0745627 |
| Zfp385c | -0.8344196 |
| Zfp386 | 0.12818161 |
| Zfp386 | -0.4934155 |
| Zfp39 | 0.13781708 |
| Zfp393 | 1.38607667 |
| Zfp395 | -0.0339995 |
| Zfp397 | 1.55026845 |
| Zfp398 | 0.30686221 |
| Zfp398 | -0.5827615 |
| Zfp398 | -0.6764657 |
| Zfp41 | 1.52925096 |
| Zfp41 | -0.1113885 |
| Zfp410 | -0.9000868 |
| Zfp410 | -0.5990424 |
| Zfp410 | -0.6406237 |
| Zfp414 | -0.6164089 |
| Zfp418 | -0.2106206 |
| Zfp42 | -0.1367722 |
| Zfp423 | 0.05547051 |
| Zfp423 | 0.48017636 |
| Zfp428 | 0.32417629 |
| Zfp428 | -0.4740631 |
| Zfp446 | 0.52037745 |
| Zfp446 | -0.1039982 |
| Zfp449 | -0.7433906 |
| Zfp451 | 1.44623458 |
| Zfp457 | 1.09461661 |
| Zfp46 | 1.13376334 |
| Zfp467 | 2.61354703 |
| Zfp472 | -0.2163114 |
| Zfp473 | -0.5920301 |
| Zfp473 | 2.10609915 |
| Zfp474 | -0.6901617 |
| Zfp474 | -0.2168305 |
| Zfp512 | 1.65306983 |
| Zfp513 | 0.54548281 |
| Zfp516 | 0.02191189 |
| Zfp52 | 1.60260425 |
| Zfp521 | -0.5690291 |
| Zfp523 | -0.0095667 |
| Zfp523 | 0.46928969 |
| Zfp524 | 0.01869039 |
| Zfp526 | 1.38616982 |
| Zfp532 | -0.1506946 |
| Zfp532 | 1.90382404 |
| Zfp536 | 1.03589319 |
| Zfp551 | 1.10726286 |
| Zfp553 | -0.1449084 |
| Zfp553 | -0.1653192 |
| Zfp560 | 0.85577625 |
| Zfp566 | 1.69179303 |
| Zfp57 | -0.5697867 |
| Zfp574 | 0.15365831 |
| Zfp579 | 1.19232215 |
| Zfp592 | -0.8599277 |
| Zfp593 | 1.33534581 |
| Zfp595 | 0.16026692 |
| Zfp598 | 1.95477114 |
| Zfp60 | 0.28528838 |
| Zfp606 | 0.81544468 |
| Zfp607 | 1.64808807 |
| Zfp608 | 1.04412802 |
| Zfp609 | -0.2251751 |
| Zfp61 | 0.60587096 |
| Zfp612 | -0.6148167 |
| Zfp617 | 1.50938312 |
| Zfp619 | 1.37737204 |
| Zfp62 | -0.0128989 |
| Zfp622 | -0.0590893 |
| Zfp623 | -0.3523865 |
| Zfp629 | 1.83450652 |
| Zfp637 | 0.3227214 |
| Zfp639 | -0.5773971 |
| Zfp64 | -0.0934761 |
| Zfp64 | -0.3434236 |
| Zfp64 | -0.2863347 |
| Zfp641 | 0.70335782 |
| Zfp644 | 0.15400731 |
| Zfp646 | 0.72024564 |
| Zfp653 | 3.67444595 |
| Zfp654 | 2.43538742 |
| Zfp654 | 0.52390496 |
| Zfp655 | -0.633293 |
| Zfp661 | -0.7137397 |
| Zfp667 | -0.5174675 |
| Zfp668 | 2.46651103 |
| Zfp67 | -0.7155101 |
| Zfp672 | 1.77810416 |
| Zfp68 | 2.57358234 |
| Zfp68 | -0.2664117 |
| Zfp68 | 1.8608067 |
| Zfp687 | -0.4260683 |
| Zfp688 | -0.5480613 |
| Zfp689 | -0.819443 |
| Zfp69 | -0.7969357 |
| Zfp691 | 0.81252064 |
| Zfp692 | -0.8164763 |
| Zfp692 | 1.99869336 |
| Zfp704 | 2.63658284 |
| Zfp704 | 1.09601612 |
| Zfp706 | 1.46508425 |
| Zfp709 | 1.05442008 |
| Zfp709 | -0.020861 |
| Zfp710 | -0.3004248 |
| Zfp711 | 0.13390352 |
| Zfp715 | -0.691888 |
| Zfp71-rs1 | -0.3590144 |
| Zfp74 | 1.10864355 |
| Zfp75 | 1.94607046 |
| Zfp75 | 1.76055882 |
| Zfp75 | 2.75626243 |
| Zfp750 | -0.813519 |
| Zfp758 | 1.46909874 |
| Zfp760 | -0.4093262 |
| Zfp764 | -0.7334019 |
| Zfp768 | -0.0930213 |
| Zfp770 | 2.52344199 |
| Zfp771 | -0.8224191 |
| Zfp771 | 0.8109689 |
| Zfp78 | -0.7279982 |
| Zfp78 | 0.87948478 |
| Zfp780b | -0.4404732 |
| Zfp786 | 1.5775244 |
| Zfp787 | -0.5998248 |
| Zfp787 | -0.7674342 |
| Zfp790 | -0.3475956 |
| Zfp804a | 0.16234416 |
| Zfp809 | -0.8819069 |
| Zfp810 | 0.55642753 |
| Zfp811 | 1.36924968 |
| Zfp817 | 0.80941457 |
| Zfp819 | 0.08673955 |
| Zfp82 | 1.24124019 |
| Zfp825 | 1.53148576 |
| Zfp828 | 1.1137718 |
| Zfp85-rs1 | -0.0817227 |
| Zfp87 | 2.20558676 |
| Zfp87 | -0.5268303 |
| Zfp90 | -0.5959193 |
| Zfp91 | -0.2941989 |
| Zfp91-cntf | 1.14673656 |
| Zfp92 | -0.2049643 |
| Zfp93 | 0.7414923 |
| Zfp93 | 1.63341007 |
| Zfp94 | 2.53995182 |
| Zfp97 | 2.13070479 |
| Zfpl1 | 2.83289283 |
| Zfpm2 | -0.3724132 |
| Zfr | -0.5096179 |
| Zfx | -0.5443661 |
| Zfx | 0.46754595 |
| Zfy1 | 1.67396762 |
| Zfyve1 | -0.3885002 |
| Zfyve16 | -0.8174641 |
| Zfyve19 | 0.89032478 |
| Zfyve20 | -0.4138042 |
| Zfyve21 | 0.85347085 |
| Zfyve26 | 0.91132424 |
| Zfyve27 | -0.2024046 |
| Zgpat | 0.38331967 |
| Zgpat | 1.45076554 |
| Zhx1 | -0.7796925 |
| Zhx1 | -0.5920301 |
| Zhx1 | -0.5232181 |
| Zhx2 | -0.4955129 |
| Zhx3 | -0.8651141 |
| Zic1 | -0.4112427 |
| Zic2 | 3.72728479 |
| Zic3 | -0.3210957 |
| Zic4 | -0.7110903 |
| Zkscan1 | -0.717284 |
| Zkscan14 | 0.48385888 |
| Zkscan17 | -0.8496406 |
| Zkscan3 | 0.76643807 |
| Zkscan6 | -0.7415665 |
| Zkscan6 | 1.23622156 |
| Zmat1 | -0.7674342 |
| Zmat2 | -0.6164089 |
| Zmat3 | 1.00094119 |
| Zmat4 | 1.10121405 |
| Zmat5 | 1.31052478 |
| Zmiz1 | -0.7049375 |
| Zmiz1 | -0.5858408 |
| Zmiz2 | 0.57394334 |
| Zmiz2 | -0.6756163 |
| Zmpste24 | 0.14665095 |
| Zmpste24 | 0.94645087 |
| Zmpste24 | 0.12638629 |
| Zmym3 | -0.1099984 |
| Zmym3 | -0.0099826 |
| Zmym5 | 0.22809859 |
| Zmym6 | 3.76626007 |
| Zmynd10 | -0.5644961 |
| Zmynd11 | 0.83868038 |
| Zmynd11 | 2.87932106 |
| Zmynd11 | -0.5889303 |
| Zmynd12 | 0.13959056 |
| Zmynd15 | -0.7217333 |
| Znhit2 | -0.1090728 |
| Znhit3 | -0.6815782 |
| Znhit4 | -0.1239941 |
| Znrd1 | -0.0799276 |
| Znrd1 | -0.6781667 |
| Znrd1 | 0.21609704 |
| Znrf1 | 1.45803861 |
| Znrf2 | -0.4384958 |
| Znrf3 | 0.52741919 |
| Znrf4 | -0.1697516 |
| Zp1 | 4.03834844 |
| Zp3 | -0.5370194 |
| Zp3r | -0.2309556 |
| Zpbp | -0.3327806 |
| Zpbp2 | -0.4638586 |
| Zpbp2 | 3.58483482 |
| Zranb1 | 2.15553619 |
| Zranb2 | -0.610848 |
| Zranb3 | 1.15405257 |
| Zrsr2 | -0.1555441 |
| Zscan10 | -0.3321929 |
| Zscan12 | 0.02150981 |
| Zscan2 | -0.5189018 |
| Zscan2 | -0.3351353 |
| Zscan2 | 0.05508264 |
| Zscan20 | 1.80619715 |
| Zscan20 | 0.9771313 |
| Zscan21 | 0.23803644 |
| Zscan21 | -0.293635 |
| Zswim1 | 0.23003033 |
| Zswim2 | 0.07655892 |
| Zswim3 | -0.813519 |
| Zswim4 | -0.4016992 |
| Zswim5 | 2.32137632 |
| Zw10 | 0.08598922 |
| Zw10 | 0.52928805 |
| Zw10 | -0.7023129 |
| Zwilch | 1.14673656 |
| Zxda | 0.06703232 |
| Zxdc | 2.01635369 |
| Zxdc | 0.75129328 |
| Zxdc | -0.2054771 |
| Zyg11b | 0.64913855 |
| Zyx | 0.08748927 |
| Zzz3 | 1.60908209 |
| Zzz3 | -0.617206 |
| Zzz3 | 1.60966876 |
| Zzz3 | 0.09940357 |

| **Gene Symbol** | **HET3** |
| --- | --- |
| 0610006I08Rik | -0.5006208 |
| 0610006I08Rik | -0.2380807 |
| 0610007C21Rik | -0.3347031 |
| 0610007C21Rik | -0.2605218 |
| 0610007P08Rik | 3.4203359 |
| 0610007P14Rik | -0.5355125 |
| 0610007P22Rik | -0.5390059 |
| 0610009B22Rik | 0.30200085 |
| 0610009D07Rik | -0.2528094 |
| 0610009O20Rik | -0.1710684 |
| 0610010E21Rik | 1.390034 |
| 0610010K06Rik | -0.3038719 |
| 0610010K14Rik | 0.40363331 |
| 0610012A05Rik | -0.4586377 |
| 0610012D14Rik | -0.6333067 |
| 0610012G03Rik | -0.2589741 |
| 0610012H03Rik | 0.49169906 |
| 0610025P10Rik | -0.8165634 |
| 0610031J06Rik | -0.4793885 |
| 0610031J06Rik | 0.89871674 |
| 0610031J06Rik | 1.76910651 |
| 0610037D15Rik | -0.2719522 |
| 0610037L13Rik | 0.05034919 |
| 0610037L13Rik | 0.25649488 |
| 0610038F07Rik | 0.05511546 |
| 0610040J01Rik | 1.02118071 |
| 0710008K08Rik | 0.29019865 |
| 0910001A06Rik | 1.724444 |
| 0910001A06Rik | -0.5908496 |
| 0910001L09Rik | 0.70794465 |
| 1110001A07Rik | 2.68939539 |
| 1110001J03Rik | 1.14863396 |
| 1110002B05Rik | -0.781226 |
| 1110002N22Rik | 1.04379778 |
| 1110003E01Rik | 1.45816007 |
| 1110006G06Rik | -0.2176537 |
| 1110007A13Rik | -0.6697192 |
| 1110007A13Rik | 0.19928307 |
| 1110007C09Rik | 2.04277651 |
| 1110007L15Rik | 1.44264213 |
| 1110007L15Rik | 2.3689542 |
| 1110007L15Rik | -0.724632 |
| 1110007L15Rik | -0.1866523 |
| 1110007M04Rik | 0.10041698 |
| 1110008F13Rik | 0.55291525 |
| 1110008J03Rik | 1.7525553 |
| 1110008J03Rik | -0.0274915 |
| 1110008J03Rik | -0.3158606 |
| 1110008P14Rik | -0.6489605 |
| 1110012D08Rik | -0.7410711 |
| 1110012J17Rik | -0.7428188 |
| 1110012L19Rik | 0.05108408 |
| 1110012L19Rik | -0.2211091 |
| 1110012M11Rik | 2.97591307 |
| 1110013L07Rik | -0.6810951 |
| 1110017D15Rik | -0.781226 |
| 1110017D15Rik | 0.71169917 |
| 1110017D15Rik | -0.7000968 |
| 1110018G07Rik | -0.590109 |
| 1110018J18Rik | -0.2088279 |
| 1110018J18Rik | 0.00976205 |
| 1110019N10Rik | 0.71169917 |
| 1110019N10Rik | 0.42413909 |
| 1110020C03Rik | 2.32422521 |
| 1110020P15Rik | -0.4057003 |
| 1110020P15Rik | -0.7358478 |
| 1110031B06Rik | -0.3924836 |
| 1110031I02Rik | 0.86795461 |
| 1110032A13Rik | -0.4722022 |
| 1110032A13Rik | -0.8127788 |
| 1110032E23Rik | 0.08462059 |
| 1110034A24Rik | 1.11710838 |
| 1110036O03Rik | 0.62020398 |
| 1110038D17Rik | -0.7367163 |
| 1110038F14Rik | 0.35725518 |
| 1110038F14Rik | 0.08462059 |
| 1110039B18Rik | -0.6286624 |
| 1110039B18Rik | -0.6379748 |
| 1110039B18Rik | 0.0370192 |
| 1110048D14Rik | 3.66915243 |
| 1110049B09Rik | 0.15151395 |
| 1110049B09Rik | 0.04444871 |
| 1110049F12Rik | 0.07644032 |
| 1110049F12Rik | -0.7721492 |
| 1110057K04Rik | -0.5566762 |
| 1110057K04Rik | 0.01703415 |
| 1110057K04Rik | 2.1754888 |
| 1110058L19Rik | 0.89842582 |
| 1110059E24Rik | -0.4683061 |
| 1110059G10Rik | -0.2436396 |
| 1110067D22Rik | -0.6664947 |
| 1110067I12Rik | -0.0387673 |
| 1190002A17Rik | 0.75303673 |
| 1190002H23Rik | 1.45468151 |
| 1190002N15Rik | -0.8435037 |
| 1190003J15Rik | -0.4972706 |
| 1190003M12Rik | 0.1045837 |
| 1190005F20Rik | -0.7533759 |
| 1190005I06Rik | 1.4847563 |
| 1190005P17Rik | 0.13490843 |
| 1190005P17Rik | -0.1809547 |
| 1190007F08Rik | 0.59339212 |
| 1190017O12Rik | 3.22110388 |
| 1190020J12Rik | 0.06746497 |
| 1200002N14Rik | -0.7821387 |
| 1200003C05Rik | -0.1254033 |
| 1200003C05Rik | 0.03065633 |
| 1200003C05Rik | -0.5175584 |
| 1200003I07Rik | -0.8854624 |
| 1200009F10Rik | -0.2335575 |
| 1200009I06Rik | 3.64948137 |
| 1200009I06Rik | -0.4853105 |
| 1200009O22Rik | -0.0359353 |
| 1200011O22Rik | 2.70232862 |
| 1200013B08Rik | 0.02952886 |
| 1200013P24Rik | -0.6371951 |
| 1200013P24Rik | 1.41162426 |
| 1200014J11Rik | 2.58415451 |
| 1200015F23Rik | -0.4892798 |
| 1200015F23Rik | 0.55736133 |
| 1200015N20Rik | 0.79535939 |
| 1200016B10Rik | -0.5251114 |
| 1200016B10Rik | -0.6640838 |
| 1200016D23Rik | 3.34239189 |
| 1300001I01Rik | 1.82409843 |
| 1300007L22Rik | 2.79412557 |
| 1300010M03Rik | 0.0261381 |
| 1300013J15Rik | 0.06818625 |
| 1300013J15Rik | -0.7203546 |
| 1300014I06Rik | -0.0183321 |
| 1300017J02Rik | 0.2739339 |
| 1300018I05Rik | -0.4117716 |
| 1300018J18Rik | -0.5237335 |
| 1500001L15Rik | 0.51610486 |
| 1500001M20Rik | -0.1701324 |
| 1500001M20Rik | 1.89329963 |
| 1500001M20Rik | -0.5168748 |
| 1500002O20Rik | -0.5355125 |
| 1500003O03Rik | 1.53985889 |
| 1500005A01Rik | 0.14158685 |
| 1500010J02Rik | 1.4413571 |
| 1500010J02Rik | 0.32733333 |
| 1500011H22Rik | -0.2310542 |
| 1500012F01Rik | -0.2315543 |
| 1500015O10Rik | -0.1081939 |
| 1500019C06Rik | 0.96477415 |
| 1500019G21Rik | 1.707117 |
| 1500031L02Rik | 2.27922529 |
| 1500032D16Rik | -0.4308528 |
| 1500032D16Rik | 0.15019655 |
| 1500032D16Rik | 0.71223427 |
| 1500032L24Rik | -0.4191107 |
| 1500041J02Rik | 2.34675845 |
| 1500041N16Rik | -0.8184617 |
| 1520401A03Rik | -0.563841 |
| 1600002K03Rik | 0.14953713 |
| 1600012H06Rik | -0.7127053 |
| 1600012H06Rik | 0.9794049 |
| 1600014C10Rik | -0.5975416 |
| 1600016N20Rik | -0.6737658 |
| 1600021P15Rik | 0.53836832 |
| 1600027N09Rik | -0.0403896 |
| 1600029D21Rik | 2.19152355 |
| 1700001C02Rik | -0.5624036 |
| 1700001C19Rik | -0.1631428 |
| 1700001E04Rik | -0.5432158 |
| 1700001J03Rik | 1.5087365 |
| 1700001O22Rik | 1.73482482 |
| 1700001O22Rik | -0.8251366 |
| 1700001P01Rik | 0.2260637 |
| 1700003F12Rik | 1.04788427 |
| 1700003M02Rik | 0.58112733 |
| 1700007G11Rik | 2.31204906 |
| 1700007I06Rik | 0.04481861 |
| 1700007K09Rik | 0.49509653 |
| 1700008F21Rik | -0.3087574 |
| 1700008I05Rik | 0.14391282 |
| 1700008P20Rik | 1.009659 |
| 1700009P17Rik | 0.40037952 |
| 1700010A17Rik | -0.6125903 |
| 1700010A17Rik | 0.72691607 |
| 1700010D01Rik | 0.03103184 |
| 1700010M22Rik | -0.4859708 |
| 1700010M22Rik | -0.106447 |
| 1700011A15Rik | -0.6876597 |
| 1700011F03Rik | 0.42803972 |
| 1700011F14Rik | 2.58597354 |
| 1700011H14Rik | 0.50005679 |
| 1700011K15Rik | -0.2703851 |
| 1700012A03Rik | 0.2782422 |
| 1700012B09Rik | 0.91885457 |
| 1700012G19Rik | -0.3936773 |
| 1700012H05Rik | -0.6118319 |
| 1700012H17Rik | 0.62158324 |
| 1700012L04Rik | -0.2872411 |
| 1700013B16Rik | 0.54095229 |
| 1700013G24Rik | -0.5509845 |
| 1700013N18Rik | 0.21138521 |
| 1700013N18Rik | 0.51566286 |
| 1700015E13Rik | -0.607295 |
| 1700016C15Rik | -0.5134647 |
| 1700016D06Rik | -0.2260682 |
| 1700016G05Rik | 1.25999427 |
| 1700016K19Rik | 0.21015131 |
| 1700016K19Rik | -0.5975416 |
| 1700017N19Rik | 2.895844 |
| 1700018B24Rik | -0.8175121 |
| 1700018C11Rik | 1.3553829 |
| 1700018F24Rik | -0.2777214 |
| 1700019D03Rik | -0.433968 |
| 1700019E19Rik | -0.4859708 |
| 1700019G17Rik | -0.2400982 |
| 1700019H03Rik | -0.4879548 |
| 1700019L03Rik | 0.21844804 |
| 1700019M22Rik | 2.59640318 |
| 1700019N19Rik | -0.5320323 |
| 1700020C11Rik | -0.4228025 |
| 1700020D05Rik | -0.4992793 |
| 1700020L24Rik | 0.06746497 |
| 1700020N01Rik | 0.60489333 |
| 1700020O03Rik | 2.54215548 |
| 1700021C14Rik | -0.1923856 |
| 1700021F05Rik | 2.33382049 |
| 1700021K02Rik | -0.3677699 |
| 1700021K02Rik | 2.23077863 |
| 1700021K19Rik | 0.42438337 |
| 1700022C21Rik | -0.3527989 |
| 1700023B02Rik | 1.28369243 |
| 1700023B02Rik | 3.47290446 |
| 1700023E05Rik | -0.5938178 |
| 1700023E05Rik | -0.4567163 |
| 1700023I07Rik | 0.07858136 |
| 1700023M03Rik | -0.4142114 |
| 1700024G13Rik | -0.5073583 |
| 1700025D03Rik | -0.7419445 |
| 1700025E21Rik | 0.76943121 |
| 1700025F22Rik | 0.23060285 |
| 1700025G04Rik | -0.4579968 |
| 1700025K23Rik | -0.3966683 |
| 1700026D08Rik | -0.3608298 |
| 1700026L06Rik | -0.4271285 |
| 1700027A23Rik | 0.81293787 |
| 1700027D21Rik | -0.7766763 |
| 1700027J05Rik | -0.7254899 |
| 1700027N10Rik | -0.7160971 |
| 1700028K03Rik | -0.6302079 |
| 1700029F12Rik | 0.3624969 |
| 1700029F12Rik | -0.6942718 |
| 1700029G01Rik | 2.36204631 |
| 1700029H14Rik | 0.0916749 |
| 1700029H14Rik | -0.7587005 |
| 1700029I01Rik | 0.84279428 |
| 1700029I08Rik | 2.45018375 |
| 1700029I15Rik | -0.6186797 |
| 1700029J07Rik | 0.14391282 |
| 1700029P11Rik | -0.1402223 |
| 1700029P11Rik | 1.67069608 |
| 1700029P11Rik | -0.5754227 |
| 1700030J22Rik | 1.99787611 |
| 1700030K09Rik | -0.0061301 |
| 1700034E13Rik | 0.74274677 |
| 1700034H14Rik | -0.4945993 |
| 1700034H14Rik | -0.2310542 |
| 1700034H14Rik | 1.76030135 |
| 1700034H14Rik | 1.63015334 |
| 1700034I23Rik | 2.42068188 |
| 1700034I23Rik | 1.32823244 |
| 1700034O15Rik | 0.61248126 |
| 1700036D21Rik | 2.29684196 |
| 1700037C18Rik | 0.70794465 |
| 1700037C18Rik | 1.69446689 |
| 1700037H04Rik | 3.70824151 |
| 1700039E15Rik | -0.7349802 |
| 1700040I03Rik | -0.4050954 |
| 1700042B14Rik | 1.06973789 |
| 1700045I19Rik | -0.5908496 |
| 1700047I17Rik1 | 0.15414309 |
| 1700047I17Rik1 | 0.30923142 |
| 1700049G17Rik | -0.3625599 |
| 1700049K14Rik | -0.7254899 |
| 1700049L16Rik | 1.81136817 |
| 1700052N19Rik | 0.53469516 |
| 1700052N19Rik | 0.67008725 |
| 1700054O13Rik | 0.11697118 |
| 1700055M20Rik | 1.66913095 |
| 1700057G04Rik | 1.26488074 |
| 1700057K13Rik | 0.65975945 |
| 1700058C13Rik | -0.8299344 |
| 1700060H10Rik | 2.76055546 |
| 1700061G19Rik | -0.7375856 |
| 1700063I17Rik | 2.5197056 |
| 1700065I17Rik | -0.0934505 |
| 1700065O13Rik | -0.7110141 |
| 1700065O13Rik | 0.1429167 |
| 1700067C01Rik | -0.2335575 |
| 1700067K01Rik | -0.4459035 |
| 1700067P10Rik | -0.0002838 |
| 1700071K01Rik | -0.6802779 |
| 1700072E05Rik | -0.1130151 |
| 1700072E05Rik | 1.89359384 |
| 1700072E05Rik | 2.81744327 |
| 1700073E17Rik | 0.38597471 |
| 1700080E11Rik | -0.8289728 |
| 1700081D17Rik | -0.2275611 |
| 1700081D17Rik | 0.6804844 |
| 1700084C01Rik | 0.57679552 |
| 1700084C01Rik | 1.98550027 |
| 1700088E04Rik | 0.2969614 |
| 1700093K21Rik | 2.11733299 |
| 1700094C09Rik | 0.72146003 |
| 1700095K08Rik | 1.72579959 |
| 1700102P08Rik | 0.00706822 |
| 1700108M19Rik | 0.50342343 |
| 1700109H08Rik | -0.6012806 |
| 1700112C13Rik | -0.2656997 |
| 1700113I22Rik | -0.3853542 |
| 1700113I22Rik | 0.77793458 |
| 1700113O17Rik | 0.31282538 |
| 1700120B06Rik | -0.5710624 |
| 1700123D08Rik | -0.3175076 |
| 1700123K08Rik | -0.0999256 |
| 1700123L14Rik | 1.74075393 |
| 1700123O20Rik | -0.5299505 |
| 1700123O20Rik | -0.7721492 |
| 1700127D06Rik | -0.355659 |
| 1700129C05Rik | -0.3942747 |
| 1700129I15Rik | -0.4191107 |
| 1810007M14Rik | 0.92622287 |
| 1810007P19Rik | -0.6672998 |
| 1810007P19Rik | 3.60351308 |
| 1810008A18Rik | 0.28081749 |
| 1810008A18Rik | 0.10907632 |
| 1810009A15Rik | -0.0006724 |
| 1810009A15Rik | 0.03589955 |
| 1810009N02Rik | 1.80900509 |
| 1810009O10Rik | -0.3476732 |
| 1810009O10Rik | 1.43296014 |
| 1810010M01Rik | 2.04064986 |
| 1810011O10Rik | 0.47428857 |
| 1810014F10Rik | -0.4945993 |
| 1810015A11Rik | 1.09625284 |
| 1810015C04Rik | -0.7569222 |
| 1810019J16Rik | -0.3285644 |
| 1810020D17Rik | -0.5531148 |
| 1810020G14Rik | -0.4709017 |
| 1810020G14Rik | 3.48116486 |
| 1810021J13Rik | -0.7280684 |
| 1810021J13Rik | -0.079788 |
| 1810022C23Rik | -0.261555 |
| 1810026J23Rik | 1.99599311 |
| 1810027O10Rik | 1.63556431 |
| 1810030J14Rik | -0.079788 |
| 1810030N24Rik | -0.672145 |
| 1810030N24Rik | -0.7000968 |
| 1810030N24Rik | -0.7428188 |
| 1810031K17Rik | 2.57449238 |
| 1810035L17Rik | 2.85074808 |
| 1810035L17Rik | 0.32624638 |
| 1810037C20Rik | -0.5141457 |
| 1810043G02Rik | 0.64183384 |
| 1810044A24Rik | 0.28138879 |
| 1810046J19Rik | 1.37443027 |
| 1810048J11Rik | 0.13020436 |
| 1810049H13Rik | 1.57940557 |
| 1810049H19Rik | 0.97459855 |
| 1810055E12Rik | -0.0990596 |
| 1810055E12Rik | 1.06877225 |
| 1810055E12Rik | -0.6133492 |
| 1810055G02Rik | 0.48074157 |
| 1810063B05Rik | -0.3224667 |
| 1810064F22Rik | 0.87574265 |
| 1810065E05Rik | -0.0456818 |
| 1810073G14Rik | -0.7297915 |
| 1810074P20Rik | 1.51898262 |
| 1810074P20Rik | -0.5893691 |
| 2010001J22Rik | -0.403283 |
| 2010001J22Rik | -0.30009 |
| 2010001M09Rik | 0.64029192 |
| 2010003J03Rik | 0.2482415 |
| 2010003O18Rik | 1.1594162 |
| 2010004A03Rik | -0.6348601 |
| 2010005H15Rik | -0.3420114 |
| 2010005J08Rik | -0.477423 |
| 2010005J08Rik | -0.6640838 |
| 2010005J08Rik | -0.1200735 |
| 2010007H12Rik | -0.0709317 |
| 2010007H12Rik | -0.0073039 |
| 2010011I20Rik | 0.60709631 |
| 2010012O05Rik | 0.41973092 |
| 2010100O12Rik | -0.5264914 |
| 2010100O12Rik | 0.26757812 |
| 2010106G01Rik | 2.87074737 |
| 2010106G01Rik | 0.7840546 |
| 2010107E04Rik | 0.62551255 |
| 2010107G12Rik | -0.668912 |
| 2010109I03Rik | -0.7358478 |
| 2010110K16Rik | 0.41899415 |
| 2010111I01Rik | -0.3579535 |
| 2010200O16Rik | -0.4579968 |
| 2010301N04Rik | 0.92622287 |
| 2010305A19Rik | 1.68914906 |
| 2010305C02Rik | -0.5182424 |
| 2010305C02Rik | 1.62877922 |
| 2010308M01Rik | 0.84449334 |
| 2010311D03Rik | -0.5107457 |
| 2010316F05Rik | 1.15260956 |
| 2010321M09Rik | 0.07321933 |
| 2010321M09Rik | -0.30009 |
| 2200001I15Rik | 1.30744374 |
| 2210009G21Rik | -0.4846506 |
| 2210009G21Rik | -0.7940868 |
| 2210009G21Rik | 0.7304189 |
| 2210010B09Rik | 0.01283104 |
| 2210010L05Rik | 3.72388028 |
| 2210012G02Rik | -0.5915907 |
| 2210012G02Rik | -0.0448656 |
| 2210012G02Rik | -0.631756 |
| 2210012G02Rik | 0.17266016 |
| 2210016L21Rik | 0.32896133 |
| 2210016L21Rik | 4.39549297 |
| 2210016L21Rik | -0.4728532 |
| 2210021J22Rik | 2.84764273 |
| 2210023G05Rik | -0.2688206 |
| 2210023G05Rik | 0.86930729 |
| 2210407C18Rik | -0.6271195 |
| 2210412D01Rik | 0.26030164 |
| 2210417D09Rik | 0.97285671 |
| 2210418O10Rik | 1.62134946 |
| 2300002D11Rik | 0.56639836 |
| 2300002D11Rik | 0.06710412 |
| 2300002M23Rik | -0.6225063 |
| 2300005B03Rik | -0.7393267 |
| 2310001A20Rik | -0.7332473 |
| 2310002B06Rik | 0.10907632 |
| 2310003C23Rik | 0.64337317 |
| 2310003F16Rik | -0.0776716 |
| 2310003H01Rik | 0.56577076 |
| 2310003L22Rik | 1.09929484 |
| 2310003L22Rik | -0.668912 |
| 2310004I24Rik | -0.6163914 |
| 2310005E10Rik | -0.0274915 |
| 2310005E10Rik | -0.5107457 |
| 2310005G13Rik | 0.94147804 |
| 2310005N01Rik | 0.51389272 |
| 2310005N03Rik | 2.26479863 |
| 2310005P05Rik | 0.66484403 |
| 2310006J04Rik | -0.1800085 |
| 2310007A19Rik | -0.5674446 |
| 2310007B03Rik | -0.3625599 |
| 2310007F21Rik | -0.27457 |
| 2310007F21Rik | -0.3252333 |
| 2310008H04Rik | -0.4154338 |
| 2310008H09Rik | -0.7640563 |
| 2310008H09Rik | -0.5502755 |
| 2310008M10Rik | 1.94335103 |
| 2310009E04Rik | 0.05912916 |
| 2310010B21Rik | -0.2543467 |
| 2310010M24Rik | 2.11560376 |
| 2310011J03Rik | 0.09832649 |
| 2310014L17Rik | -0.5369083 |
| 2310016C08Rik | -0.7042804 |
| 2310016C08Rik | -0.6705271 |
| 2310016C16Rik | 1.17953746 |
| 2310016C16Rik | 2.35250653 |
| 2310016C16Rik | 1.54905001 |
| 2310016E02Rik | 1.35423443 |
| 2310016M24Rik | 1.7556958 |
| 2310021P13Rik | 0.4663911 |
| 2310022A10Rik | -0.5674446 |
| 2310022B05Rik | 1.7557528 |
| 2310031L18Rik | 0.84078224 |
| 2310031L18Rik | 0.92268782 |
| 2310033E01Rik | -0.4728532 |
| 2310033K02Rik | -0.495934 |
| 2310033P09Rik | -0.4670111 |
| 2310035C23Rik | -0.3996691 |
| 2310035C23Rik | -0.2186397 |
| 2310035K24Rik | -0.724632 |
| 2310036D22Rik | 1.60341609 |
| 2310036O22Rik | -0.4541606 |
| 2310037I24Rik | 1.22839165 |
| 2310037I24Rik | 1.73348254 |
| 2310038H17Rik | 0.03103184 |
| 2310039E09Rik | 0.57140374 |
| 2310039H08Rik | 2.60893881 |
| 2310040C09Rik | 0.32543032 |
| 2310042D19Rik | 0.17747142 |
| 2310044G17Rik | -0.5390059 |
| 2310044H10Rik | 1.33403469 |
| 2310045A20Rik | -0.7017679 |
| 2310046K01Rik | 0.21875389 |
| 2310046O06Rik | -0.4440086 |
| 2310047C04Rik | -0.4966021 |
| 2310047D13Rik | -0.3358237 |
| 2310047D13Rik | 1.08148495 |
| 2310047M10Rik | 1.56048113 |
| 2310047M10Rik | 0.0024317 |
| 2310047O13Rik | -0.1857002 |
| 2310056P07Rik | 0.77327585 |
| 2310057J16Rik | 0.84818944 |
| 2310058J06Rik | -0.5046573 |
| 2310061C15Rik | -0.6333067 |
| 2310061F22Rik | -0.6141088 |
| 2310061F22Rik | -0.6255792 |
| 2310065K24Rik | 3.37627326 |
| 2310065K24Rik | -0.1691974 |
| 2310066E14Rik | -0.6529161 |
| 2310067B10Rik | -0.3972676 |
| 2310076L09Rik | 0.87574265 |
| 2310076L09Rik | -0.7254899 |
| 2310079F23Rik | -0.4459035 |
| 2310079N02Rik | 1.4177476 |
| 2400001E08Rik | 1.84159723 |
| 2400003C14Rik | 1.87572172 |
| 2400003C14Rik | -0.4320976 |
| 2400003C14Rik | -0.7940868 |
| 2400006N03Rik | 0.36197408 |
| 2400010D15Rik | 1.69890525 |
| 2410001C21Rik | -0.2406032 |
| 2410001C21Rik | 1.0739506 |
| 2410001C21Rik | 1.67742681 |
| 2410002F23Rik | -0.583471 |
| 2410002F23Rik | 3.50606334 |
| 2410002F23Rik | -0.4277481 |
| 2410002I01Rik | 2.54210722 |
| 2410002O22Rik | -0.0159577 |
| 2410003P15Rik | 0.13457322 |
| 2410004A20Rik | 1.5225933 |
| 2410004B18Rik | 1.36303199 |
| 2410004L22Rik | -0.4271285 |
| 2410005O16Rik | 1.05722674 |
| 2410008K03Rik | 0.36301942 |
| 2410014A08Rik | 1.13761113 |
| 2410014A08Rik | -0.5446234 |
| 2410014A08Rik | 0.98696369 |
| 2410014A08Rik | 0.64510183 |
| 2410015M20Rik | 0.65217442 |
| 2410015N17Rik | 1.88219796 |
| 2410016F19Rik | 1.4119563 |
| 2410016F19Rik | 1.30399482 |
| 2410016O06Rik | 0.13188716 |
| 2410017P07Rik | 1.19880674 |
| 2410018C17Rik | -0.8552968 |
| 2410018G20Rik | 0.25178778 |
| 2410018M08Rik | 0.0089932 |
| 2410019A14Rik | -0.6225063 |
| 2410019G02Rik | 2.09199261 |
| 2410022L05Rik | 0.64644409 |
| 2410025L10Rik | 0.71134226 |
| 2410042D21Rik | -0.7051194 |
| 2410042D21Rik | -0.0934505 |
| 2410066E13Rik | -0.6656904 |
| 2410081M15Rik | 0.73495263 |
| 2410091C18Rik | -0.6012806 |
| 2410116G06Rik | 2.42814701 |
| 2410127E18Rik | -0.5842062 |
| 2410127L17Rik | 1.4346619 |
| 2410127L17Rik | -0.6255792 |
| 2410129H14Rik | 0.76641105 |
| 2410137M14Rik | 2.12729863 |
| 2410146L05Rik | -0.7237749 |
| 2410166I05Rik | -0.4014743 |
| 2500002L14Rik | 0.6106898 |
| 2500003M10Rik | -0.2471947 |
| 2500003M10Rik | -0.6656904 |
| 2500003M10Rik | -0.840579 |
| 2510002A14Rik | 0.19928307 |
| 2510003E04Rik | -0.7803143 |
| 2510006D16Rik | -0.6851925 |
| 2510009E07Rik | -0.8957427 |
| 2510010F15Rik | -0.3352632 |
| 2510012J08Rik | 1.15843128 |
| 2510039O18Rik | 0.17040622 |
| 2510040D07Rik | -0.5967956 |
| 2510048L02Rik | 0.08320318 |
| 2510049I19Rik | -0.9599754 |
| 2600001B17Rik | -0.5609684 |
| 2600009E05Rik | 0.09588152 |
| 2600009E05Rik | -0.6959323 |
| 2600010E01Rik | -0.7195015 |
| 2600011E07Rik | 0.22241609 |
| 2600011E07Rik | 1.76882561 |
| 2610002J02Rik | -0.6434512 |
| 2610002M06Rik | -0.4945993 |
| 2610003J06Rik | -0.3136691 |
| 2610016F04Rik | -0.7848821 |
| 2610019A05Rik | 1.40034662 |
| 2610019P18Rik | -0.848399 |
| 2610020C11Rik | 2.88439495 |
| 2610020H08Rik | -0.6648867 |
| 2610020H08Rik | 3.91496172 |
| 2610020H08Rik | -0.5271822 |
| 2610020O08Rik | 2.24113152 |
| 2610021K21Rik | 2.22554086 |
| 2610021K21Rik | 0.80909627 |
| 2610024G14Rik | -0.4117716 |
| 2610024G14Rik | 1.43027953 |
| 2610024G14Rik | -0.982429 |
| 2610027C15Rik | 2.00402354 |
| 2610027C15Rik | -0.759591 |
| 2610027L16Rik | 1.0252426 |
| 2610028A01Rik | 1.09929484 |
| 2610028H24Rik | -0.1981551 |
| 2610029G23Rik | -0.716947 |
| 2610029G23Rik | 1.51572818 |
| 2610034E13Rik | -0.5383062 |
| 2610034E13Rik | 1.63217805 |
| 2610034M16Rik | 2.31724759 |
| 2610036D13Rik | 0.66409254 |
| 2610039C10Rik | 0.45960073 |
| 2610042L04Rik | -0.4722022 |
| 2610044O15Rik | -0.4370939 |
| 2610101N10Rik | 0.75966368 |
| 2610101N10Rik | -0.9294382 |
| 2610103J23Rik | -0.5251114 |
| 2610110G12Rik | 2.06157476 |
| 2610200G18Rik | -0.4142114 |
| 2610204K14Rik | 2.58631852 |
| 2610204L23Rik | -0.7042804 |
| 2610204M08Rik | -0.9009265 |
| 2610205E22Rik | 3.34007444 |
| 2610207I05Rik | -0.0452736 |
| 2610208M17Rik | 0.0231135 |
| 2610209A20Rik | -0.3625599 |
| 2610209A20Rik | 2.05629589 |
| 2610209M04Rik | -0.7393267 |
| 2610301B20Rik | 0.47127699 |
| 2610301F02Rik | 3.29341494 |
| 2610304G08Rik | -0.4683061 |
| 2610305D13Rik | -0.7703446 |
| 2610318N02Rik | -0.6893083 |
| 2610507B11Rik | -0.3109371 |
| 2610507B11Rik | -0.8289728 |
| 2610510B01Rik | -0.1654667 |
| 2610510J17Rik | -0.0080872 |
| 2610524A10Rik | -0.553826 |
| 2610524H06Rik | 1.4974677 |
| 2610528E23Rik | -0.4813582 |
| 2610528J11Rik | 1.20006309 |
| 2610528O22Rik | 2.51777456 |
| 2700007P21Rik | -0.3912915 |
| 2700007P21Rik | -0.3033307 |
| 2700038C09Rik | 2.60383236 |
| 2700038C09Rik | 1.25871951 |
| 2700050L05Rik | -0.8328252 |
| 2700055A20Rik | 0.64009899 |
| 2700060E02Rik | 1.37287248 |
| 2700062C07Rik | -0.5411084 |
| 2700062C07Rik | 1.68914906 |
| 2700078K21Rik | -0.4069114 |
| 2700081O15Rik | 2.15766309 |
| 2700087H15Rik | 0.6178347 |
| 2700094K13Rik | -0.5739669 |
| 2700097O09Rik | 1.34150509 |
| 2810002N01Rik | 0.67437182 |
| 2810002O09Rik | -0.6379748 |
| 2810003C17Rik | -0.3324661 |
| 2810004N20Rik | -0.8156158 |
| 2810004N23Rik | 0.33247877 |
| 2810008M24Rik | 0.57513989 |
| 2810021B07Rik | 1.31642424 |
| 2810021J22Rik | 1.14896592 |
| 2810021J22Rik | -0.7419445 |
| 2810022L02Rik | -0.6616792 |
| 2810022L02Rik | -0.6179163 |
| 2810022L02Rik | -0.7516079 |
| 2810030E01Rik | 2.01469389 |
| 2810046L04Rik | -0.4715517 |
| 2810046M22Rik | -0.6217397 |
| 2810403A07Rik | 0.82041459 |
| 2810405J04Rik | -0.7017679 |
| 2810405K02Rik | -0.313122 |
| 2810408A11Rik | -0.2803558 |
| 2810408M09Rik | 1.7854188 |
| 2810410C14Rik | -0.848399 |
| 2810410P22Rik | 1.40495801 |
| 2810410P22Rik | -0.951146 |
| 2810417H13Rik | -0.8137235 |
| 2810422O20Rik | 2.026726 |
| 2810428I15Rik | 0.04444871 |
| 2810432D09Rik | 0.239913 |
| 2810432L12Rik | 0.96179256 |
| 2810439F02Rik | 0.52926653 |
| 2810439F02Rik | -0.433968 |
| 2810441K11Rik | -0.5446234 |
| 2810452K22Rik | -0.7959389 |
| 2810453I06Rik | -0.9294382 |
| 2810453I06Rik | 0.7209303 |
| 2810470D21Rik | -0.6217397 |
| 2810485I05Rik | -0.2230895 |
| 2900006K08Rik | 1.49504562 |
| 2900010M23Rik | 0.77244143 |
| 2900011O08Rik | 2.45685683 |
| 2900011O08Rik | 2.09593106 |
| 2900024C23Rik | -0.6632816 |
| 2900024O10Rik | -0.5967956 |
| 2900026A02Rik | 0.02007888 |
| 2900046G09Rik | -0.5559628 |
| 2900062L11Rik | -0.2411086 |
| 2900064A13Rik | 0.8602514 |
| 2900073G15Rik | -0.0227013 |
| 2900092C05Rik | -0.8052561 |
| 3000004C01Rik | -0.7551474 |
| 3000004C01Rik | -0.8732766 |
| 3000004C01Rik | -0.6917867 |
| 3010003L21Rik | -0.7393267 |
| 3010026O09Rik | 1.54854975 |
| 3110001A13Rik | -0.632531 |
| 3110001D03Rik | -0.766746 |
| 3110004L20Rik | -0.95776 |
| 3110007F17Rik | -0.7894729 |
| 3110009E18Rik | 2.11518056 |
| 3110023B02Rik | -0.246686 |
| 3110035E14Rik | 0.71614884 |
| 3110035E14Rik | 1.39376672 |
| 3110037I16Rik | 1.11044003 |
| 3110040N11Rik | 0.196154 |
| 3110040N11Rik | 0.33139792 |
| 3110043J09Rik | -0.8376636 |
| 3110043J09Rik | 1.77960648 |
| 3110043J09Rik | -0.8203638 |
| 3110048E14Rik | -0.3924836 |
| 3110049J23Rik | -0.7332473 |
| 3110050N22Rik | -0.6232736 |
| 3110050N22Rik | 1.06913449 |
| 3110056O03Rik | -0.3510871 |
| 3110057O12Rik | 1.73569916 |
| 3110057O12Rik | -0.089159 |
| 3110070M22Rik | -0.0424214 |
| 3110082I17Rik | 0.40936182 |
| 3200001I04Rik | 0.70255418 |
| 3200002M19Rik | -0.2370736 |
| 3200002M19Rik | 0.29724209 |
| 3300001G02Rik | -0.581269 |
| 3300001P08Rik | 0.58461908 |
| 3300001P08Rik | -0.1502366 |
| 3300001P08Rik | 0.42046709 |
| 3300002I08Rik | 0.07858136 |
| 3321401G04Rik | 0.04333812 |
| 3322402L07Rik | -0.5362101 |
| 3632451O06Rik | 1.20880959 |
| 3830402I07Rik | 0.45299634 |
| 3830406C13Rik | 0.74291922 |
| 3830406C13Rik | -0.9071861 |
| 3830406C13Rik | 2.68601991 |
| 3830408P04Rik | 2.4267793 |
| 3930401K13Rik | -0.5559628 |
| 4121402D02Rik | 2.4267793 |
| 4121402D02Rik | -0.0250933 |
| 4430402I18Rik | 0.93072789 |
| 4432405B04Rik | 0.44705901 |
| 4432416J03Rik | 0.0314072 |
| 4631426J05Rik | 1.41972478 |
| 4631427C17Rik | -0.1534461 |
| 4632404H12Rik | 1.40871375 |
| 4632404H12Rik | 3.28964896 |
| 4632404H12Rik | -0.6786455 |
| 4632404H22Rik | -0.6967637 |
| 4632411B12Rik | -0.7977948 |
| 4632411B12Rik | 0.43143918 |
| 4632412N22Rik | 2.62241091 |
| 4632415K11Rik | 1.27746474 |
| 4632417K18Rik | 1.69665836 |
| 4632417N05Rik | 0.44896315 |
| 4632419K20Rik | 1.37209259 |
| 4632434I11Rik | 1.55417972 |
| 4732415M23Rik | 0.71951617 |
| 4732418C07Rik | -0.2015375 |
| 4732418C07Rik | 0.2488335 |
| 4732418C07Rik | -0.590109 |
| 4732429D16Rik | 1.64042636 |
| 4732456N10Rik | -0.461206 |
| 4732471D19Rik | -0.0772489 |
| 4732473B16Rik | -0.6753895 |
| 4732479N06Rik | -0.8184617 |
| 4732479N06Rik | 0.12242335 |
| 4732496O08Rik | 1.15678738 |
| 4733401H18Rik | 0.20953373 |
| 4831426I19Rik | 0.48028215 |
| 4833418A01Rik | -0.0428283 |
| 4833420G17Rik | 0.21354055 |
| 4833421E05Rik | -0.4327206 |
| 4833424O15Rik | 1.34873715 |
| 4833424O15Rik | -0.6271195 |
| 4833426J09Rik | -0.4383472 |
| 4833426J09Rik | -0.3835805 |
| 4833427G06Rik | 1.10162797 |
| 4833436C18Rik | -0.1552852 |
| 4833439L19Rik | -0.2280593 |
| 4833439L19Rik | 0.99576947 |
| 4833439L19Rik | 1.51089921 |
| 4833442J19Rik | -0.0227013 |
| 4833446K15Rik | -0.7839667 |
| 4921501E09Rik | -0.2260682 |
| 4921504E06Rik | -0.7000968 |
| 4921504I05Rik | 2.31514698 |
| 4921505C17Rik | -0.4637815 |
| 4921505C17Rik | -0.6309816 |
| 4921507P07Rik | 1.64641965 |
| 4921511H03Rik | 0.90999102 |
| 4921511H03Rik | 0.00397982 |
| 4921511K06Rik | 0.3653671 |
| 4921511K06Rik | -0.0407956 |
| 4921515J06Rik | -0.1081939 |
| 4921517D21Rik | -0.5667227 |
| 4921517D22Rik | 1.88199937 |
| 4921520G13Rik | 2.10208042 |
| 4921521F21Rik | 2.01855114 |
| 4921521F21Rik | 0.3690071 |
| 4921523A10Rik | 0.58809755 |
| 4921523A10Rik | -0.6340831 |
| 4921523A10Rik | -0.5271822 |
| 4921525H12Rik | -0.2872411 |
| 4921528G01Rik | -0.6705271 |
| 4921528I01Rik | -0.5886297 |
| 4921530G04Rik | -0.3347031 |
| 4921530L21Rik | -0.6893083 |
| 4921530L21Rik | 1.36992274 |
| 4921536K21Rik | -0.4780777 |
| 4921537P18Rik | 0.53750537 |
| 4922502D21Rik | 0.03515235 |
| 4922503N01Rik | -0.8396062 |
| 4922503N01Rik | 0.94022803 |
| 4922505E12Rik | -0.2161768 |
| 4922505G16Rik | 3.85591755 |
| 4922505G16Rik | -0.606541 |
| 4930401F20Rik | -0.6753895 |
| 4930402H24Rik | 2.26345791 |
| 4930403C10Rik | 0.19207014 |
| 4930403L05Rik | 0.21076847 |
| 4930403O06Rik | 0.43022654 |
| 4930404H21Rik | 1.20048149 |
| 4930404N11Rik | -0.5566762 |
| 4930408O21Rik | -0.8146692 |
| 4930408O21Rik | -0.409946 |
| 4930412F15Rik | 0.42657893 |
| 4930415L06Rik | -0.7220632 |
| 4930418G15Rik | 0.02576057 |
| 4930427A07Rik | -0.0195216 |
| 4930428D18Rik | 0.16588161 |
| 4930428E23Rik | 2.00271903 |
| 4930430D24Rik | -0.6481714 |
| 4930430E16Rik | -0.7913157 |
| 4930430E16Rik | -0.3044134 |
| 4930431L04Rik | -0.3591029 |
| 4930432H15Rik | -0.4586377 |
| 4930432K21Rik | -0.4992793 |
| 4930432O21Rik | 0.72936946 |
| 4930432O21Rik | 0.28253032 |
| 4930438O05Rik | -0.01754 |
| 4930438O05Rik | -0.8175121 |
| 4930449I24Rik | 0.57223532 |
| 4930451C15Rik | -0.4289887 |
| 4930451I11Rik | -0.0065212 |
| 4930452B06Rik | 0.26438359 |
| 4930453N24Rik | 1.03469259 |
| 4930455C21Rik | -0.6418832 |
| 4930455F23Rik | -0.0571854 |
| 4930467M19Rik | 3.88043823 |
| 4930470P17Rik | -0.6005316 |
| 4930473A06Rik | 1.71656548 |
| 4930473A06Rik | -0.5776107 |
| 4930473A06Rik | -0.632531 |
| 4930474N05Rik | 1.02460912 |
| 4930478A21Rik | 2.40598653 |
| 4930481F22Rik | 0.35383189 |
| 4930481F22Rik | 0.52621236 |
| 4930481M05 | -0.491271 |
| 4930486L24Rik | 1.2706134 |
| 4930502E18Rik | 1.05182957 |
| 4930503B20Rik | 2.54164858 |
| 4930503L19Rik | -0.7498434 |
| 4930504E06Rik | -0.1271868 |
| 4930504E06Rik | 3.37337889 |
| 4930504O13Rik | -0.3375071 |
| 4930506M07Rik | 0.2986443 |
| 4930507C10Rik | -0.6762024 |
| 4930511I11Rik | 1.78514286 |
| 4930511J11Rik | -0.477423 |
| 4930515G01Rik | 1.38491926 |
| 4930518C23Rik | -0.149779 |
| 4930519F16Rik | 3.81617825 |
| 4930519G04Rik | -0.4014743 |
| 4930519N13Rik | 0.64471797 |
| 4930519N16Rik | -0.1343558 |
| 4930522H14Rik | -0.5732399 |
| 4930524B15Rik | 2.35789412 |
| 4930524B15Rik | 0.51389272 |
| 4930524E20Rik | -0.0768264 |
| 4930526D03Rik | -0.7101696 |
| 4930535B03Rik | 2.03981453 |
| 4930535E21Rik | -0.5148272 |
| 4930538K18Rik | 0.48532315 |
| 4930539E08Rik | -0.631756 |
| 4930540L03Rik | -0.4741565 |
| 4930544G11Rik | 0.11286063 |
| 4930544G21Rik | 2.1383958 |
| 4930547C10Rik | 2.27564797 |
| 4930547N16Rik | -0.5237335 |
| 4930549C01Rik | -0.293643 |
| 4930550C14Rik | -0.5616857 |
| 4930550L24Rik | 1.68201019 |
| 4930555G01Rik | 1.11710838 |
| 4930555I21Rik | 0.72110691 |
| 4930562D19Rik | -0.3942747 |
| 4930562D19Rik | -0.0522376 |
| 4930563D23Rik | -0.6411001 |
| 4930563P21Rik | 1.05796026 |
| 4930570C03Rik | -0.5667227 |
| 4930572J05Rik | 2.32480683 |
| 4930572J05Rik | 0.46569098 |
| 4930572J05Rik | 0.402133 |
| 4930573I19Rik | -0.2840568 |
| 4930578I06Rik | 2.55675843 |
| 4930579C15Rik | 2.21445679 |
| 4930579E17Rik | 0.48165972 |
| 4930579E17Rik | -0.5725135 |
| 4930579J09Rik | -0.3420114 |
| 4930583H14Rik | 0.28310055 |
| 4930590J08Rik | 0.10873152 |
| 4930595M18Rik | 0.7449861 |
| 4930596D02Rik | -0.5746945 |
| 4930599N23Rik | 0.63351528 |
| 4931406B18Rik | -0.5945613 |
| 4931406C07Rik | -0.5790722 |
| 4931406H21Rik | -0.424654 |
| 4931406I20Rik | 1.43910395 |
| 4931406I20Rik | 0.23120637 |
| 4931406P16Rik | 2.40920131 |
| 4931407G18Rik | -0.4926009 |
| 4931408A02Rik | -0.8165634 |
| 4931408C20Rik | 2.25027365 |
| 4931408C20Rik | 0.18765166 |
| 4931409K22Rik | 1.52801931 |
| 4931417E11Rik | -0.7401985 |
| 4931417G12Rik | -0.6648867 |
| 4931428L18Rik | 1.53450285 |
| 4931432M23Rik | 0.06602071 |
| 4931433A01Rik | -0.4452714 |
| 4931440B09Rik | 2.21872787 |
| 4931440F15Rik | -0.5616857 |
| 4931440F15Rik | -0.5397062 |
| 4931440L10Rik | 0.66428047 |
| 4932409I22Rik | 2.3446647 |
| 4932411E22Rik | -0.6356378 |
| 4932411G14Rik | 0.03103184 |
| 4932412H11Rik | 2.38602775 |
| 4932413O14Rik | 2.31117976 |
| 4932414J04Rik | 0.41035443 |
| 4932414N04Rik | 0.62296042 |
| 4932418E24Rik | 1.01479768 |
| 4932431H17Rik | 0.72709153 |
| 4932431H17Rik | -0.6876597 |
| 4932441K18Rik | 2.96747775 |
| 4932441K18Rik | 0.21107689 |
| 4932442K08Rik | 0.6749292 |
| 4932702K14Rik | 0.59825237 |
| 4933400A11Rik | -0.2543467 |
| 4933400C05Rik | -0.0501838 |
| 4933400E14Rik | 1.30846659 |
| 4933402J07Rik | 0.14158685 |
| 4933402N03Rik | -0.4308528 |
| 4933402P03Rik | 0.81149916 |
| 4933403G14Rik | -0.8280123 |
| 4933404M02Rik | -0.6232736 |
| 4933405O20Rik | -0.0925906 |
| 4933406M09Rik | 0.8886262 |
| 4933407N01Rik | 1.13368218 |
| 4933407N01Rik | -0.5990353 |
| 4933407N01Rik | 0.5480208 |
| 4933407P14Rik | -0.1325583 |
| 4933407P14Rik | 2.60617542 |
| 4933407P14Rik | 0.90393752 |
| 4933409N07Rik | 1.58526153 |
| 4933411G06Rik | -0.0943112 |
| 4933411K20Rik | -0.0692544 |
| 4933412E14Rik | -0.440859 |
| 4933413G11Rik | 0.13624802 |
| 4933415F23Rik | 3.50307121 |
| 4933416C03Rik | -0.6992624 |
| 4933417A18Rik | -0.6884836 |
| 4933417E01Rik | -0.5033099 |
| 4933421E11Rik | -0.626349 |
| 4933421E11Rik | 1.04924239 |
| 4933421E11Rik | 1.27370768 |
| 4933422H20Rik | -0.5545377 |
| 4933424B01Rik | -0.2925729 |
| 4933424B01Rik | -0.3369456 |
| 4933424B01Rik | -0.6125903 |
| 4933425O20Rik | 1.39613418 |
| 4933426M11Rik | -0.3895063 |
| 4933427D14Rik | 0.02083848 |
| 4933428G09Rik | 3.72378742 |
| 4933428G20Rik | -0.7306542 |
| 4933429E10Rik | 2.23267586 |
| 4933429E10Rik | -0.4478024 |
| 4933430I17Rik | 0.62158324 |
| 4933434E20Rik | -0.6876597 |
| 4933434E20Rik | -0.7220632 |
| 4933434E20Rik | -0.5039833 |
| 4933434I06Rik | 0.1445763 |
| 4933436I01Rik | 0.33652063 |
| 4933437F05Rik | 1.4298727 |
| 4933439C20Rik | 0.45983572 |
| 4933440H19Rik | 0.780917 |
| 5031414D18Rik | -0.5516941 |
| 5031414D18Rik | -0.430231 |
| 5033411D12Rik | -0.4728532 |
| 5033414D02Rik | -0.5616857 |
| 5033414K04Rik | -0.0444578 |
| 5133400G04Rik | 1.47852671 |
| 5133400G04Rik | -0.5746945 |
| 5133400G04Rik | 2.27432307 |
| 5133401N09Rik | -0.447169 |
| 5230400G24Rik | -0.6309816 |
| 5230400G24Rik | 1.49261712 |
| 5330401P04Rik | 1.12871886 |
| 5330417C22Rik | 0.8830212 |
| 5330431N19Rik | -0.7177977 |
| 5330431N19Rik | 0.38801017 |
| 5330431N19Rik | -0.4966021 |
| 5330437I02Rik | 2.12665282 |
| 5330439C02Rik | 0.51853205 |
| 5330439J01Rik | -0.5938178 |
| 5330439J01Rik | -0.0139837 |
| 5430407P10Rik | -0.4129907 |
| 5430432M24Rik | -0.4352171 |
| 5430432M24Rik | -0.6762024 |
| 5430432M24Rik | 0.98140012 |
| 5430435G22Rik | 3.42279081 |
| 5430437P03Rik | 1.17331043 |
| 5530601I19Rik | -0.5915907 |
| 5630401D24Rik | -0.2756193 |
| 5630401D24Rik | -0.6552978 |
| 5630401D24Rik | 1.86879641 |
| 5730403M16Rik | -0.3274527 |
| 5730409E04Rik | 1.31771315 |
| 5730410E15Rik | 0.71454945 |
| 5730410E15Rik | 0.6561644 |
| 5730410I19Rik | -0.7658485 |
| 5730410I19Rik | 0.58420897 |
| 5730410I19Rik | -0.4063056 |
| 5730419I09Rik | -0.27457 |
| 5730437N04Rik | -0.7839667 |
| 5730446C15Rik | -0.4478024 |
| 5730449L18Rik | 1.78811864 |
| 5730449L18Rik | -0.5990353 |
| 5730449L18Rik | -0.4002704 |
| 5730453I16Rik | 0.69604341 |
| 5730453I16Rik | -0.1231788 |
| 5730469M10Rik | 1.59441783 |
| 5730472N09Rik | 0.44156243 |
| 5730494M16Rik | -0.7186492 |
| 5730494N06Rik | -0.0925906 |
| 5730502D15Rik | 2.29718896 |
| 5730507A09Rik | 2.30328733 |
| 5730507A09Rik | -0.7931622 |
| 5730528L13Rik | 1.03919414 |
| 5730589K01Rik | 0.80427147 |
| 5730593F17Rik | 0.42120267 |
| 5730596K20Rik | -0.6860142 |
| 5730596K20Rik | 0.07214314 |
| 5730596K20Rik | -0.1645364 |
| 5730596K20Rik | -0.291504 |
| 5830403L16Rik | 1.29148891 |
| 5830404H04Rik | 1.16214655 |
| 5830405N20Rik | -0.5960502 |
| 5830415F09Rik | -0.6917867 |
| 5830415L20Rik | -0.632531 |
| 5830417C01Rik | -0.313122 |
| 5830426I05Rik | -0.4148224 |
| 5830427D03Rik | -0.3358237 |
| 5830434P21Rik | -0.2594897 |
| 5830435K17Rik | -0.7748628 |
| 5830457O10Rik | -0.5306439 |
| 5830457O10Rik | -0.7507252 |
| 5830457O10Rik | -0.5327273 |
| 5830467P10Rik | -0.2275611 |
| 5830468K18Rik | 3.43870685 |
| 5830472M02Rik | -0.2171612 |
| 5830472M02Rik | 0.48760536 |
| 5830472M02Rik | 0.40787088 |
| 5930416I19Rik | -0.6141088 |
| 5930431H10 | -0.6992624 |
| 5930434B04Rik | -0.7950124 |
| 5930434B04Rik | 0.15315805 |
| 5930434B04Rik | -0.1819018 |
| 6030405A18Rik | -0.3602538 |
| 6030408B16Rik | -0.3341433 |
| 6030408B16Rik | 0.13825373 |
| 6030419C18Rik | -0.6713357 |
| 6030429G01Rik | 0.73129252 |
| 6030443O07Rik | 0.56283625 |
| 6030446N20Rik | 0.20612959 |
| 6030458C11Rik | 0.59582547 |
| 6230409E13Rik | 2.26231185 |
| 6230409E13Rik | 0.33058645 |
| 6230416J20Rik | -0.7349802 |
| 6230427J02Rik | 0.76152417 |
| 6330403K07Rik | 0.53685763 |
| 6330406I15Rik | 0.04036995 |
| 6330406I15Rik | 0.70002772 |
| 6330407J23Rik | -0.0734533 |
| 6330408A02Rik | -0.5251114 |
| 6330409N04Rik | -0.518927 |
| 6330416G13Rik | -0.3363845 |
| 6330439K17Rik | 1.65096889 |
| 6330442E10Rik | 0.36406358 |
| 6330442E10Rik | -0.8885344 |
| 6330500D04Rik | 3.05561334 |
| 6330503C03Rik | -0.656889 |
| 6330503K22Rik | -0.4979397 |
| 6330503K22Rik | -0.6951017 |
| 6330503K22Rik | -0.677016 |
| 6330505N24Rik | 3.15322532 |
| 6330512M04Rik | -0.4741565 |
| 6330534C20Rik | -0.3631373 |
| 6330548G22Rik | -0.4547988 |
| 6330569M22Rik | -0.5033099 |
| 6330577E15Rik | -0.7712464 |
| 6330578E17Rik | 0.58543873 |
| 6332401O19Rik | -0.7551474 |
| 6430411K18Rik | 2.46749639 |
| 6430502M16Rik | 0.7665791 |
| 6430510M02Rik | 1.59169419 |
| 6430517E21Rik | -0.7067997 |
| 6430526N21Rik | -0.5271822 |
| 6430527G18Rik | 1.02321391 |
| 6430527G18Rik | 0.46545749 |
| 6430531B16Rik | -0.5975416 |
| 6430537H07Rik | -0.5516941 |
| 6430537H07Rik | 0.76792238 |
| 6430548M08Rik | 4.31591987 |
| 6430550H21Rik | 1.7917974 |
| 6430571L13Rik | 0.00436644 |
| 6430573F11Rik | 0.77793458 |
| 6430598A04Rik | 0.40338343 |
| 6430604K15Rik | 2.30974983 |
| 6430706D22Rik | -0.4879548 |
| 6530404N21Rik | 0.07572552 |
| 6530406M24Rik | -0.6465953 |
| 6530418L21Rik | 2.85742331 |
| 6530418L21Rik | 1.77877313 |
| 6530418L21Rik | 3.01236826 |
| 6530421E24Rik | -0.5930748 |
| 6620401K05Rik | -0.3375071 |
| 6720456B07Rik | -0.7026046 |
| 6720456B07Rik | -0.5467389 |
| 6720456H20Rik | -0.7578109 |
| 6720456H20Rik | 0.29161168 |
| 6720458F09Rik | 0.02500505 |
| 6720463M24Rik | 0.01893829 |
| 6720467C03Rik | 1.9390693 |
| 6720467C03Rik | 0.27795561 |
| 6820408C15Rik | 0.09343001 |
| 7420416P09Rik | -0.6088048 |
| 7530404M11Rik | -0.3782799 |
| 8030411F24Rik | 0.06348775 |
| 8030451F13Rik | -0.3972676 |
| 8030451F13Rik | 1.71448085 |
| 8030451F13Rik | 1.45531496 |
| 8430408G22Rik | 0.42389475 |
| 8430410A17Rik | -0.3341433 |
| 8430410K20Rik | 0.1544712 |
| 8430415E04Rik | -0.6356378 |
| 8430415E04Rik | -0.4741565 |
| 8430419L09Rik | -0.6942718 |
| 8430427H17Rik | 1.24098392 |
| 8430432M10Rik | 0.09448146 |
| 9030025P20Rik | 0.72339977 |
| 9030025P20Rik | -0.459921 |
| 9030025P20Rik | 0.03253236 |
| 9030205A07Rik | -0.5446234 |
| 9030221M09Rik | 0.70471418 |
| 9030224M15Rik | 0.77210745 |
| 9030227G01Rik | -0.6967637 |
| 9030409G11Rik | 0.11731289 |
| 9030418K01Rik | 0.69495377 |
| 9030425E11Rik | -0.0956038 |
| 9030607L17Rik | 3.04595273 |
| 9030607L17Rik | -0.1429426 |
| 9030611O19Rik | 0.20519898 |
| 9030612M13Rik | -0.5087117 |
| 9030617O03Rik | 0.84124695 |
| 9030624J02Rik | 0.09272837 |
| 9030625A04Rik | 0.72936946 |
| 9130005N14Rik | -0.0853138 |
| 9130011E15Rik | 0.73842489 |
| 9130011J15Rik | -0.2107818 |
| 9130014G24Rik | 0.44012309 |
| 9130017N09Rik | -0.210293 |
| 9130206N08Rik | 1.25537708 |
| 9130211I03Rik | 3.6977243 |
| 9130213B05Rik | -0.6209738 |
| 9130213B05Rik | 0.98907428 |
| 9130218O11Rik | 0.38060998 |
| 9130221H12Rik | 3.08475827 |
| 9130227C08Rik | -0.7730528 |
| 9130401M01Rik | 0.12615296 |
| 9130404D08Rik | -0.6481714 |
| 9130404D08Rik | 1.72779985 |
| 9130404D14Rik | -0.606541 |
| 9130422G05Rik | -0.2667388 |
| 9230104L09Rik | -0.6868366 |
| 9330129D05Rik | 0.90437125 |
| 9330134C04Rik | -0.2814117 |
| 9330176C04Rik | -0.6163914 |
| 9330186A19Rik | 0.73199082 |
| 9430015G10Rik | 0.6684052 |
| 9430015G10Rik | 3.73751984 |
| 9430023L20Rik | 0.96680149 |
| 9430025C20Rik | -0.2667388 |
| 9430028L06Rik | 0.12004202 |
| 9430029K10Rik | 0.48120076 |
| 9430031J16Rik | 0.72357591 |
| 9430034D17Rik | 0.32679001 |
| 9430057O19Rik | 0.57119573 |
| 9430069J07Rik | 2.33424462 |
| 9430077A04Rik | 0.0024317 |
| 9430079M16Rik | -0.0219053 |
| 9530015I07Rik | 0.8207314 |
| 9530027K23Rik | 3.9135667 |
| 9530058B02Rik | 0.95141713 |
| 9530058B02Rik | 0.46872097 |
| 9530068E07Rik | -0.6737658 |
| 9530077C05Rik | 0.52861292 |
| 9530096D07Rik | 1.33213488 |
| 9530096D07Rik | -0.7775845 |
| 9630015D15Rik | 0.37495584 |
| 9630020C08Rik | 0.78635965 |
| 9630028B13Rik | -0.38299 |
| 9630031F12Rik | -0.5376069 |
| 9630050M13Rik | -0.583471 |
| 9630058J23Rik | 0.10700554 |
| 9830163H01Rik | -0.4735046 |
| 9830169C18Rik | -0.6819131 |
| 9930013L23Rik | -0.4986092 |
| 9930021D14Rik | -0.6893083 |
| 9930023K05Rik | -0.6103171 |
| 9930023K05Rik | 0.96342009 |
| 9930104L06Rik | -0.1047034 |
| a | 1.72544615 |
| A030003K21Rik | -0.3403197 |
| A030007L17Rik | 0.40762216 |
| A030007L17Rik | -0.6005316 |
| A030007L17Rik | 0.2008436 |
| A030007L17Rik | 1.55567049 |
| A030009H04Rik | 1.69233124 |
| A030010B05Rik | 0.38750173 |
| A030012M09Rik | 2.8852412 |
| A130010J15Rik | 0.27652131 |
| A130038H09Rik | 1.68847448 |
| A130042E20Rik | -0.7676443 |
| A130090K04Rik | 0.77327585 |
| A130092J06Rik | 0.22848738 |
| A230050P20Rik | -0.5271822 |
| A230051G13Rik | -0.5334228 |
| A230054D04Rik | 1.83487832 |
| A230067G21Rik | -0.590109 |
| A230069A22Rik | 1.06792649 |
| A230074B11Rik | -0.3614061 |
| A230074B11Rik | -0.1819018 |
| A230074B11Rik | -0.2625894 |
| A230106M15Rik | -0.1899924 |
| A230106N23Rik | 2.06694504 |
| A230107C01Rik | 1.33204431 |
| A2bp1 | -0.7560344 |
| A2bp1 | 1.11424005 |
| A330021E22Rik | -0.6802779 |
| A330021E22Rik | 1.79196177 |
| A330049M08Rik | 1.38115019 |
| A330080J22Rik | 0.81469322 |
| A3galt2 | 2.262148 |
| A430005L14Rik | -0.7472028 |
| A430005L14Rik | 1.71585128 |
| A430005L14Rik | 2.02362645 |
| A430005L14Rik | -0.5320323 |
| A430033K04Rik | -0.5864153 |
| A430079E08 | 0.17233851 |
| A430083B19Rik | -0.2522975 |
| A430083B19Rik | 1.3441903 |
| A430107D22Rik | 0.21569082 |
| A430107P09Rik | 1.09331827 |
| A430110N23Rik | -0.2625894 |
| A4galt | 0.03813748 |
| A4gnt | 0.83425137 |
| A530050D06Rik | -0.358528 |
| A530082C11Rik | 0.70435453 |
| A530082C11Rik | 0.67362811 |
| A530088H08Rik | -0.6489605 |
| A530088H08Rik | 1.49897825 |
| A530098C11Rik | 1.74963354 |
| A630042L21Rik | -0.590109 |
| A630054L15Rik | -0.210293 |
| A630077B13Rik | -0.4087309 |
| A630095E13Rik | 1.17921641 |
| A730008H23Rik | 0.20209011 |
| A730008L03Rik | 1.86874603 |
| A730008L03Rik | -0.3545139 |
| A730017C20Rik | -0.1640717 |
| A730024F05Rik | -0.5960502 |
| A730037C10Rik | -0.1196308 |
| A730042J05Rik | 0.39484661 |
| A730098D12Rik | 0.98973284 |
| A830006F12Rik | -0.055946 |
| A830007P12Rik | -0.5285653 |
| A830053O21Rik | -0.4728532 |
| A830059I20Rik | -0.716947 |
| A830073O21Rik | -0.3895063 |
| A830080D01Rik | 2.39610403 |
| A830093I24Rik | -0.5060068 |
| A930001N09Rik | -0.3573793 |
| A930005I04Rik | -0.3735939 |
| A930008G19Rik | -0.1012262 |
| A930010I20Rik | 0.72410411 |
| A930016P21Rik | 1.51164379 |
| A930018P22Rik | 1.31881648 |
| A930024E05Rik | 1.34998136 |
| A930025D01Rik | -0.3125753 |
| A930025D01Rik | 2.17368568 |
| A930025D01Rik | -0.6967637 |
| A930034L06Rik | -0.4592791 |
| A930038C07Rik | 0.45038866 |
| A930041I02Rik | -0.6225063 |
| AA388235 | -0.3386311 |
| AA407270 | 0.8952196 |
| AA407659 | 0.75627124 |
| AA407659 | 0.94008903 |
| AA408296 | 0.2947127 |
| AA408296 | -0.7110141 |
| AA408296 | -0.6371951 |
| AA409316 | -0.7000968 |
| AA415398 | -0.716947 |
| AA467197 | 1.16388971 |
| AA536717 | -0.8156158 |
| AA536717 | -0.6118319 |
| AA536749 | -0.3759339 |
| AA536749 | -0.2461775 |
| AA536749 | -0.4892798 |
| AA536749 | 0.66503181 |
| AA673488 | -0.2350627 |
| AA691260 | 0.7436087 |
| AA792892 | -0.6403178 |
| AA881470 | -0.6057877 |
| AA881470 | 1.28120644 |
| AA960436 | 2.41695402 |
| AA960436 | -0.4296096 |
| Aaas | 1.41865434 |
| Aaas | -0.461206 |
| Aacs | 0.57762221 |
| Aadacl1 | -0.5334228 |
| Aak1 | -0.0246942 |
| Aak1 | 0.78289989 |
| Aamp | -0.3324661 |
| Aard | -0.2073651 |
| Aars | -0.659281 |
| Aars2 | -0.5609684 |
| Aarsd1 | 0.23090466 |
| Aasdh | -0.6624801 |
| Aatf | -0.7533759 |
| Aatf | 0.95045556 |
| Aatk | -0.4234192 |
| AB112350 | 2.00254496 |
| AB182283 | -0.0692544 |
| AB182283 | 1.12327346 |
| Abat | 3.03191799 |
| Abat | -0.3865386 |
| Abat | -0.4932665 |
| Abca14 | -0.2385846 |
| Abca14 | 1.30137617 |
| Abca15 | -0.8080699 |
| Abca15 | -0.5121042 |
| Abca15 | -0.3871314 |
| Abca15 | 1.67019553 |
| Abca17 | -0.1795358 |
| Abca2 | 0.03888224 |
| Abca3 | -0.7876338 |
| Abca4 | -0.7959389 |
| Abca4 | -0.0481347 |
| Abca5 | 0.68453689 |
| Abca6 | -0.4452714 |
| Abca6 | -0.0112273 |
| Abca7 | -0.7220632 |
| Abca7 | 0.83347077 |
| Abca8a | 0.81165912 |
| Abca8b | -0.6110742 |
| Abca9 | -0.2729984 |
| Abca9 | -0.6576856 |
| Abca9 | 1.38269396 |
| Abcb11 | -0.6537093 |
| Abcb1b | 1.17234036 |
| Abcb4 | 1.22747829 |
| Abcb6 | -0.7640563 |
| Abcb8 | 0.82547039 |
| Abcb8 | -0.8043201 |
| Abcb8 | -0.6125903 |
| Abcb9 | -0.8337908 |
| Abcb9 | -0.0069124 |
| Abcc1 | -0.5202977 |
| Abcc10 | -0.647383 |
| Abcc10 | 1.37417082 |
| Abcc12 | -0.225571 |
| Abcc3 | 0.55947095 |
| Abcc5 | -0.6851925 |
| Abcc6 | 2.39782918 |
| Abcc8 | -0.5341188 |
| Abcc9 | -0.1876053 |
| Abcc9 | -0.0968982 |
| Abcd1 | -0.5327273 |
| Abcd2 | -0.6379748 |
| Abcd2 | -0.4221862 |
| Abcd3 | 0.83938668 |
| Abcd4 | -0.2925729 |
| Abcd4 | 0.28736601 |
| Abce1 | -0.4446398 |
| Abcf1 | -0.7160971 |
| Abcf1 | 0.2816743 |
| Abcf1 | 2.89794009 |
| Abcf2 | 1.8985322 |
| Abcf2 | -0.5667227 |
| Abcf2 | -0.019125 |
| Abcf2 | -0.2558866 |
| Abcf3 | -0.5327273 |
| Abcg1 | 1.23223807 |
| Abcg4 | 2.3188507 |
| Abcg5 | -0.5754227 |
| Abhd1 | 1.94691931 |
| Abhd1 | 4.35235652 |
| Abhd10 | -0.4741565 |
| Abhd11 | 0.90523808 |
| Abhd12 | -0.4919357 |
| Abhd14a | 0.89185558 |
| Abhd14b | 0.17040622 |
| Abhd2 | 3.42551486 |
| Abhd2 | 1.61399199 |
| Abhd2 | 1.20037691 |
| Abhd3 | 0.12885588 |
| Abhd4 | 0.84093717 |
| Abhd5 | -0.587891 |
| Abhd6 | -0.358528 |
| Abhd7 | 0.51189722 |
| Abhd7 | 0.28138879 |
| Abhd8 | -0.1585125 |
| Abi1 | -0.4209547 |
| Abi1 | -0.6088048 |
| Abi2 | 0.05767167 |
| Abi2 | 0.00706822 |
| Abi2 | -0.6608791 |
| Abi3 | 1.00617401 |
| Abi3bp | 3.45582764 |
| Abi3bp | 0.28338554 |
| Abl1 | 1.52692204 |
| Ablim1 | 0.56618921 |
| Ablim2 | 0.27795561 |
| Ablim2 | -0.5798039 |
| Ablim3 | -0.7067997 |
| Ablim3 | 1.11124742 |
| Abp1 | 0.44252073 |
| Abr | -0.607295 |
| Abr | -0.6802779 |
| Abtb1 | 0.58952603 |
| Acaa1a | 1.92515575 |
| Acaa1b | -0.6333067 |
| Acaa2 | 0.97151456 |
| Acaa2 | -0.5175584 |
| Acaa2 | 1.17072129 |
| Acaa2 | 0.76809015 |
| Acad10 | 0.68066899 |
| Acad10 | 0.02576057 |
| Acad8 | -0.5271822 |
| Acad8 | 0.34721242 |
| Acad9 | -0.3109371 |
| Acad9 | 0.56073439 |
| Acadl | 1.12372848 |
| Acadl | 1.59808194 |
| Acadm | -0.6209738 |
| Acads | -0.583471 |
| Acads | 0.44705901 |
| Acadsb | 2.95888742 |
| Acadvl | -0.5244222 |
| Acan | 3.87057648 |
| Acat2 | -0.607295 |
| Acbd3 | -0.6271195 |
| Acbd4 | 1.62530198 |
| Acbd4 | 0.6420264 |
| Acbd5 | -0.7622676 |
| Acbd6 | 1.82420422 |
| Accn1 | 1.78866864 |
| Accn1 | -0.553826 |
| Accn2 | 0.84772825 |
| Accn3 | 1.04528589 |
| Accn3 | 0.62079535 |
| Accs | -0.766746 |
| Acd | -0.1267406 |
| Ace | 1.23827863 |
| Ace | 1.67891583 |
| Ache | -0.5602516 |
| Acin1 | -0.6827319 |
| Acly | 4.46599443 |
| Acn9 | -0.8328252 |
| Aco1 | -0.545328 |
| Aco2 | -0.6027805 |
| Acot1 | -0.7757691 |
| Acot1 | -0.6545032 |
| Acot10 | -0.8864852 |
| Acot11 | -0.491271 |
| Acot11 | -0.3197083 |
| Acot2 | -0.5982882 |
| Acot2 | -0.291504 |
| Acot3 | 0.74687665 |
| Acot5 | -0.5864153 |
| Acot7 | -0.4172704 |
| Acot7 | -0.1986375 |
| Acot7 | -0.6340831 |
| Acot7 | -0.7272081 |
| Acot8 | -0.0518265 |
| Acot8 | -0.5073583 |
| Acot8 | -0.7959389 |
| Acot9 | 2.54893533 |
| Acox2 | -0.2260682 |
| Acox3 | 0.67400004 |
| Acox3 | -0.5093892 |
| Acoxl | -0.759591 |
| Acp2 | -0.1866523 |
| Acp6 | -0.5230453 |
| Acpl2 | -0.6497503 |
| Acpp | 2.2020127 |
| Acr | 1.24188391 |
| Acr | -0.7186492 |
| Acrbp | 0.01855778 |
| Acrbp | -0.5967956 |
| Acrv1 | -0.4820158 |
| Acsbg1 | -0.716947 |
| Acsbg1 | -0.2688206 |
| Acsbg2 | -0.4605633 |
| Acsf3 | -0.6802779 |
| Acsl1 | -0.5258011 |
| Acsl3 | 1.87271715 |
| Acsl3 | 0.34214939 |
| Acsl4 | -0.4926009 |
| Acsl4 | 0.56073439 |
| Acsl5 | -0.1866523 |
| Acsl6 | 2.163449 |
| Acsl6 | 0.96626131 |
| Acsl6 | -0.4228025 |
| Acsm3 | 1.02359463 |
| Acss1 | 4.15963389 |
| Acss2 | -0.4402304 |
| Acss2 | -0.6333067 |
| Acta1 | -0.6209738 |
| Acta2 | -0.7480822 |
| Acta2 | 0.79389275 |
| Acta2 | 0.48257695 |
| Acta2 | -0.4547988 |
| Actb | 0.77876401 |
| Actb | -0.1622149 |
| Actb | 0.73181629 |
| Actb | 1.03707117 |
| Actb | 0.2509025 |
| Actg2 | -0.5039833 |
| Actl6a | -0.0938807 |
| Actl6b | -0.6125903 |
| Actl6b | 1.79420528 |
| Actl6b | -0.4050954 |
| Actl6b | 1.09613563 |
| Actl7a | -0.7026046 |
| Actl7b | 0.30867724 |
| Actn1 | -0.672145 |
| Actn2 | 2.09608825 |
| Actn3 | -0.5624036 |
| Actn3 | -0.5754227 |
| Actn4 | 0.4541792 |
| Actn4 | 1.39672509 |
| Actn4 | 1.2604841 |
| Actr10 | -0.3114829 |
| Actr10 | -0.518927 |
| Actr1a | -0.2579438 |
| Actr1b | -0.2073651 |
| Actr3 | 0.3155804 |
| Actr3b | -0.5481519 |
| Actr5 | 0.66126894 |
| Actr5 | -0.5645606 |
| Actr6 | 0.33274879 |
| Actrt1 | 2.91070712 |
| Actrt2 | 0.49735453 |
| Acvr1 | 0.25502651 |
| Acvr1c | 0.239913 |
| Acvr2a | 1.48995353 |
| Acvr2b | -0.5278735 |
| Acvrl1 | 0.4451509 |
| Acy1 | -0.491271 |
| Acy1l2 | -0.6217397 |
| Acy3 | 2.27796999 |
| Ada | -0.5127842 |
| Adal | 2.23908373 |
| Adal | 1.76092501 |
| Adam11 | -0.3871314 |
| Adam11 | 0.28338554 |
| Adam12 | 0.84526459 |
| Adam15 | -0.5093892 |
| Adam17 | 0.63156984 |
| Adam1b | -0.7212085 |
| Adam1b | -0.6513318 |
| Adam21 | -0.7712464 |
| Adam22 | 1.79157818 |
| Adam22 | -0.3637151 |
| Adam22 | 0.48578005 |
| Adam23 | 0.49712898 |
| Adam24 | 1.40520887 |
| Adam26a | 0.38163426 |
| Adam28 | 1.50896046 |
| Adam29 | 0.92988488 |
| Adam29 | 2.07264688 |
| Adam3 | -0.2250742 |
| Adam30 | -0.5703377 |
| Adam32 | -0.6975958 |
| Adam33 | -0.626349 |
| Adam39 | 0.0514513 |
| Adam4 | 0.14920724 |
| Adam4 | 0.88435181 |
| Adam4 | -0.5754227 |
| Adam5 | -0.4800446 |
| Adam6 | -0.7110141 |
| Adam8 | -0.4586377 |
| Adam8 | -0.5581047 |
| Adam9 | 2.48317581 |
| Adamdec1 | 0.90263512 |
| Adamts1 | 0.06094776 |
| Adamts13 | -0.4063056 |
| Adamts15 | -0.4702521 |
| Adamts16 | -0.2766697 |
| Adamts17 | 0.39333181 |
| Adamts18 | -0.2507637 |
| Adamts19 | 1.01377225 |
| Adamts2 | 0.33085702 |
| Adamts2 | -0.3666096 |
| Adamts2 | 1.39832707 |
| Adamts20 | -0.3093018 |
| Adamts20 | 0.54417202 |
| Adamts4 | 1.09789209 |
| Adamts7 | 0.42487173 |
| Adamtsl2 | -0.6843715 |
| Adamtsl4 | 0.63118025 |
| Adamtsl5 | -0.56889 |
| Adar | -0.0347243 |
| Adar | -0.7569222 |
| Adar | 1.29479655 |
| Adarb1 | -0.6434512 |
| Adarb1 | 0.66578252 |
| Adarb1 | -0.7160971 |
| Adat1 | 0.0543838 |
| Adc | 0.11045425 |
| Adc | -0.6057877 |
| Adck1 | -0.2034759 |
| Adck2 | 2.35173428 |
| Adck4 | -0.5990353 |
| Adck5 | 0.43820044 |
| Adcy1 | 0.39610703 |
| Adcy10 | 0.97218588 |
| Adcy2 | 1.00384328 |
| Adcy4 | 1.43320344 |
| Adcy4 | 0.26234489 |
| Adcy6 | 1.22218313 |
| Adcy7 | 1.34704587 |
| Adcy8 | 0.02952886 |
| Adcy9 | 1.89388796 |
| Adcyap1 | 2.78907303 |
| Adcyap1r1 | 1.38157928 |
| Add1 | -0.7212085 |
| Add1 | 0.01091413 |
| Add1 | 0.109421 |
| Add1 | 1.78613586 |
| Add3 | 2.13229769 |
| Add3 | 3.72253297 |
| Add3 | 0.3627582 |
| Adfp | -0.6225063 |
| Adh1 | -0.104268 |
| Adh1 | 0.92268782 |
| Adh4 | 0.71685878 |
| Adh5 | 0.12378133 |
| Adh7 | 0.25119769 |
| Adhfe1 | 0.29976452 |
| Adi1 | 0.68490441 |
| Adipoq | -0.6035314 |
| Adipor1 | -0.7254899 |
| Adipor2 | -0.2456694 |
| Adk | 0.58564353 |
| Adk | -0.6020302 |
| Adm | -0.5244222 |
| Adm2 | -0.1828499 |
| Adnp | 0.15643741 |
| Ado | -0.1706003 |
| Adora1 | -0.7739574 |
| Adora1 | 0.93100871 |
| Adora1 | 1.74618419 |
| Adora2b | 2.67423202 |
| Adora3 | -0.4075175 |
| Adpgk | 1.71406336 |
| Adprh | -0.1529869 |
| Adprhl1 | -0.3579535 |
| Adprhl1 | -0.4057003 |
| Adprhl1 | 2.01550955 |
| Adprhl2 | 1.54223732 |
| Adra1a | 0.34721242 |
| Adra1a | 0.46709068 |
| Adra1d | -0.5404071 |
| Adra2a | -0.6745773 |
| Adra2c | 0.51742958 |
| Adra2c | -0.4906068 |
| Adrb1 | 0.7994211 |
| Adrb2 | -0.3235723 |
| Adrbk1 | -0.3895063 |
| Adrbk2 | -0.2300548 |
| Adrm1 | 1.22768134 |
| Adsl | -0.7186492 |
| Adss | 1.35361542 |
| Adssl1 | 0.32787632 |
| Adssl1 | 1.85258438 |
| Adssl1 | -0.2646618 |
| Aebp1 | -0.6624801 |
| Aebp2 | -0.5930748 |
| Aes | -0.4859708 |
| Afap1 | 0.81835267 |
| Afap1 | 0.40637751 |
| Afap1 | 0.02764666 |
| Afap1l1 | 0.43940257 |
| Aff1 | 0.70830288 |
| Aff4 | -0.4945993 |
| Afg3l1 | -0.3065828 |
| Afg3l1 | -0.7059591 |
| Afg3l1 | 2.51270481 |
| Afg3l1 | -0.7913157 |
| Afg3l2 | 2.56557429 |
| Afmid | -0.6640838 |
| Aftph | 1.27698393 |
| Agbl1 | 1.39037397 |
| Ager | 0.64625246 |
| Ager | -0.1205165 |
| Aggf1 | -0.446536 |
| Aggf1 | -0.6005316 |
| Aggf1 | -0.8533207 |
| Agpat1 | -0.5696135 |
| Agpat2 | 1.83414616 |
| Agpat3 | -0.7685435 |
| Agpat4 | -0.6411001 |
| Agpat5 | -0.2819401 |
| Agpat6 | -0.5237335 |
| Agpat6 | 0.79324016 |
| Agpat7 | 0.70147226 |
| Agpat9 | -0.656093 |
| Agpat9 | 0.75881675 |
| Agps | -0.5696135 |
| Agps | 0.0692671 |
| Agrn | -0.4136009 |
| Agrp | -0.4370939 |
| Agt | 1.59366964 |
| Agtpbp1 | -0.1020943 |
| Agtr1a | -0.4320976 |
| Agtrap | -0.3235723 |
| Agtrl1 | 1.90890842 |
| Agxt2l1 | 0.25854664 |
| Agxt2l1 | 0.34960095 |
| Agxt2l2 | 1.08895776 |
| Agxt2l2 | -0.4503404 |
| Agxt2l2 | 2.41098553 |
| Agxt2l2 | -0.0542959 |
| Ahctf1 | -0.2973982 |
| Ahcy | -0.0128014 |
| Ahcyl1 | -0.5134647 |
| Ahdc1 | 1.08267519 |
| Ahi1 | -0.3906961 |
| Ahnak | -0.7685435 |
| Ahnak | -0.1608247 |
| Ahnak2 | -0.4879548 |
| Ahrr | -0.4807012 |
| Ahsa1 | -0.6278906 |
| Ahsa2 | 0.8824292 |
| AI118078 | -0.4050954 |
| AI132487 | 0.0867426 |
| AI314180 | -0.6624801 |
| AI314976 | -0.4670111 |
| AI316807 | -0.2750945 |
| AI317395 | -0.183799 |
| AI413582 | 1.40504164 |
| AI413782 | -0.0335149 |
| AI427515 | -0.3499477 |
| AI428936 | 1.30651288 |
| AI449175 | 0.65521598 |
| AI449175 | -0.4459035 |
| AI450540 | 1.86242719 |
| AI451557 | -0.3403197 |
| AI451617 | 0.43168152 |
| AI467606 | 1.12883197 |
| AI480653 | -0.3602538 |
| AI504432 | -0.631756 |
| AI553587 | -0.0810602 |
| AI593442 | -0.563841 |
| AI593442 | -0.3539419 |
| AI593442 | -0.5696135 |
| AI595366 | -0.0913022 |
| AI595406 | 0.89419707 |
| AI597468 | -0.7042804 |
| AI597479 | -0.7017679 |
| AI606181 | 0.33947335 |
| AI646023 | 1.85083939 |
| AI661453 | 1.03944358 |
| AI662250 | 1.95671814 |
| AI747699 | 0.7191623 |
| AI836003 | -0.5087117 |
| AI837181 | -0.6884836 |
| AI842396 | -0.3307919 |
| AI846148 | 0.29132925 |
| AI847670 | -0.6403178 |
| AI851790 | -0.0254925 |
| AI894139 | 0.5964328 |
| AI987692 | 2.92058978 |
| AI987692 | -0.2522975 |
| AI987944 | -0.3274527 |
| Aicda | 1.84123391 |
| Aif1 | -0.197191 |
| Aif1 | -0.5790722 |
| Aif1 | 1.06356443 |
| Aifm1 | -0.6020302 |
| Aifm1 | 4.28993507 |
| Aifm2 | -0.6411001 |
| Aifm2 | -0.3637151 |
| Aifm2 | 0.97258843 |
| Aifm3 | 2.98167784 |
| Aig1 | -0.1330073 |
| Aig1-pending | 0.32896133 |
| Aim1 | 0.13524351 |
| Aim2 | -0.5175584 |
| Aip | -0.6057877 |
| Ak1 | -0.5244222 |
| AK122209 | -0.6489605 |
| AK122525 | 1.0069496 |
| AK129128 | -0.3017089 |
| AK129302 | -0.1178619 |
| AK129302 | 0.2290923 |
| AK129302 | 0.374698 |
| AK129341 | -0.3319077 |
| AK162044 | -0.5871528 |
| Ak2 | -0.6656904 |
| Ak2 | 1.01697291 |
| Ak2 | 0.6178347 |
| Ak3 | 1.42325658 |
| Ak3 | 4.20352512 |
| Ak3l1 | 0.46778973 |
| Ak5 | 0.34694664 |
| Akap1 | 1.16149201 |
| Akap1 | -0.4618492 |
| Akap1 | 0.24289617 |
| Akap10 | -0.7315178 |
| Akap10 | -0.2740459 |
| Akap12 | -0.4478024 |
| Akap14 | -0.7533759 |
| Akap2 | 0.94908012 |
| Akap2 | -0.5725135 |
| Akap3 | 1.82953055 |
| Akap4 | 0.27249327 |
| Akap4 | -0.3274527 |
| Akap4 | -0.5278735 |
| Akap4 | -0.019125 |
| Akap6 | -0.4327206 |
| Akap7 | 3.02047301 |
| Akap8 | 1.99234749 |
| Akap8l | 3.04475537 |
| Akap9 | -0.7051194 |
| Akap9 | -0.5710624 |
| Akap9 | 2.09289996 |
| Akna | -0.6584829 |
| Akp2 | -0.7454467 |
| Akp5 | 1.15678738 |
| Akr1a4 | 1.21139665 |
| Akr1a4 | -0.4892798 |
| Akr1b3 | -0.631756 |
| Akr1b8 | 0.90898488 |
| Akr1c12 | 0.1452393 |
| Akr1c14 | 0.27565965 |
| Akr1c14 | -0.0501838 |
| Akr1c18 | 1.09108168 |
| Akr1c19 | 1.18444617 |
| Akr1e1 | -0.2240813 |
| Akr7a5 | -0.2798283 |
| Akr7a5 | 1.06405013 |
| Akr7a5 | 0.84757446 |
| Akt1s1 | -0.590109 |
| Akt3 | 0.6608918 |
| Akt3 | 3.44992363 |
| Aktip | -0.6255792 |
| Alad | -0.3358237 |
| Alas1 | -0.6133492 |
| Alas2 | -0.5039833 |
| Alcam | -0.2703851 |
| Aldh16a1 | 1.05404135 |
| Aldh18a1 | -0.4586377 |
| Aldh1a1 | -0.0772489 |
| Aldh1a2 | 0.31337705 |
| Aldh1a2 | -0.5798039 |
| Aldh1a7 | -0.2543467 |
| Aldh1b1 | 1.53195862 |
| Aldh1l1 | -0.451612 |
| Aldh1l2 | -0.743694 |
| Aldh1l2 | -0.0159577 |
| Aldh2 | 1.15744529 |
| Aldh3a1 | -0.3771061 |
| Aldh3a2 | -0.1007925 |
| Aldh3b1 | -0.088731 |
| Aldh3b1 | -0.3448379 |
| Aldh3b1 | 0.39937603 |
| Aldh4a1 | 0.7772705 |
| Aldh5a1 | 0.54844745 |
| Aldh5a1 | 2.2839734 |
| Aldh6a1 | 0.69749426 |
| Aldh8a1 | 0.89565747 |
| Aldh8a1 | 2.93745936 |
| Aldoa | -0.307126 |
| Aldoc | 3.27763087 |
| Alg1 | 1.18455259 |
| Alg10b | 2.01623874 |
| Alg11 | 2.09009612 |
| Alg11 | 1.70627506 |
| Alg12 | 0.14854711 |
| Alg12 | 0.02273472 |
| Alg14 | -0.2729984 |
| Alg2 | -0.1090686 |
| Alg3 | 0.01206476 |
| Alg3 | -0.4919357 |
| Alg3 | -0.0981944 |
| Alg5 | 1.27592525 |
| Alg5 | -0.5432158 |
| Alg5 | 0.58277217 |
| Alg8 | -0.7059591 |
| Alg8 | -0.5376069 |
| Alg8 | -0.6395361 |
| Alg8 | -0.0621602 |
| Alg9 | 0.56241626 |
| Alg9 | 1.28959344 |
| Alg9 | 0.96976683 |
| Alk | -0.5545377 |
| Alkbh2 | -0.440859 |
| Alkbh3 | -0.606541 |
| Alkbh4 | -0.5997832 |
| Alkbh5 | -0.4886171 |
| Alkbh7 | -0.6810951 |
| Alkbh8 | 1.60838458 |
| Allc | -0.2533215 |
| Allc | 1.29743407 |
| Alox12 | -0.5196121 |
| Alox12b | 0.75610129 |
| Alox12e | 1.43344668 |
| Alox15 | 0.98854709 |
| Alox5ap | 1.47713657 |
| Alox8 | -0.6497503 |
| Aloxe3 | -0.4234192 |
| Alpk3 | 0.24616647 |
| Als2 | 2.75361063 |
| Als2 | -0.713552 |
| Als2 | -0.7507252 |
| Als2cl | -0.781226 |
| Als2cr13 | 1.13906617 |
| Als2cr2 | -0.3158606 |
| Als2cr2 | -0.4191107 |
| Als2cr4 | 0.65938169 |
| Als2cr4 | 0.53642555 |
| Als2cr4 | -0.2703851 |
| Alx3 | 1.49656017 |
| Amac1 | -0.388318 |
| Ambp | 1.34758029 |
| Ambra1 | -0.2709071 |
| Ambra1 | -0.7332473 |
| Amd2 | 0.60047138 |
| Amdhd2 | 0.25502651 |
| Amdhd2 | -0.8184617 |
| Amfr | -0.3912915 |
| Amh | -0.0416081 |
| Amhr2 | 2.65083368 |
| Amhr2 | 2.13569055 |
| Amhr2 | 0.16976121 |
| Amigo1 | 1.41708759 |
| Amigo1 | 0.1544712 |
| Amigo2 | -0.6118319 |
| Amigo3 | 1.09672149 |
| Ammecr1l | -0.3437063 |
| Amn | -0.6984287 |
| Amotl2 | 2.5493185 |
| Ampd1 | 1.94691931 |
| Ampd1 | 1.48919109 |
| Ampd2 | 1.84304911 |
| Amph | -0.7454467 |
| Amph | 1.08136584 |
| Amy1 | -0.713552 |
| Amz2 | -0.5127842 |
| Anapc10 | 2.02918119 |
| Anapc11 | -0.495934 |
| Anapc13 | -0.5842062 |
| Anapc2 | 0.60649602 |
| Anapc4 | 0.10354379 |
| Anapc5 | 0.9037929 |
| Anapc5 | -0.6616792 |
| Anapc5 | 0.22484945 |
| Anapc7 | 0.55036489 |
| Ang | 0.31997124 |
| Angel1 | -0.4979397 |
| Angel2 | -0.2646618 |
| Angpt1 | 2.17354128 |
| Angpt2 | -0.2245776 |
| Angpt2 | -0.064241 |
| Angptl1 | 0.15905245 |
| Angptl2 | 1.34436904 |
| Angptl3 | 0.68066899 |
| Angptl4 | -0.4008721 |
| Angptl7 | -0.7463243 |
| Ank | 1.4954245 |
| Ank1 | -0.2750945 |
| Ank2 | 0.79763617 |
| Ank2 | 0.05657703 |
| Ank2 | -0.3049553 |
| Ank3 | -0.7263486 |
| Ank3 | -0.5674446 |
| Ank3 | 1.75364142 |
| Ankar | -0.632531 |
| Ankar | -0.0135894 |
| Ankfy1 | 0.6389406 |
| Ankle2 | -0.7894729 |
| Ankle2 | 3.04383539 |
| Ankle2 | 0.17906957 |
| Ankmy1 | 0.2290923 |
| Ankmy1 | -0.6909598 |
| Ankmy1 | -0.5481519 |
| Ankmy2 | 1.65231011 |
| Ankmy2 | 1.40796384 |
| Ankrd10 | -0.6616792 |
| Ankrd12 | -0.3533702 |
| Ankrd12 | -0.568167 |
| Ankrd13a | 0.49870666 |
| Ankrd13b | 0.02802341 |
| Ankrd13d | -0.7419445 |
| Ankrd17 | 0.61407073 |
| Ankrd17 | -0.4644265 |
| Ankrd17 | -0.4345923 |
| Ankrd22 | -0.563122 |
| Ankrd23 | 0.98986449 |
| Ankrd24 | 0.71579366 |
| Ankrd25 | 1.17460227 |
| Ankrd27 | 2.80920803 |
| Ankrd34a | 0.11045425 |
| Ankrd35 | -0.6984287 |
| Ankrd36 | -0.8043201 |
| Ankrd37 | 0.17747142 |
| Ankrd39 | 1.14708322 |
| Ankrd40 | -0.30604 |
| Ankrd40 | -0.668912 |
| Ankrd40 | -0.104268 |
| Ankrd46 | 2.1583608 |
| Ankrd47 | 0.10838659 |
| Ankrd49 | -0.0501838 |
| Ankrd5 | 3.1245675 |
| Ankrd50 | -0.626349 |
| Ankrd53 | 0.65045908 |
| Ankrd54 | 0.40263338 |
| Ankrd54 | 0.23451864 |
| Ankrd54 | 0.18796798 |
| Ankrd56 | -0.4057003 |
| Ankrd58 | -0.6513318 |
| Ankrd6 | -0.3818102 |
| Ankrd6 | 1.4724098 |
| Ankrd6 | -0.0857403 |
| Ankrd7 | 1.8027999 |
| Ankrd9 | 0.68269707 |
| Anks1 | 2.24994159 |
| Anks3 | 4.35671403 |
| Anks3 | 1.0755104 |
| Anks6 | 2.00019169 |
| Anks6 | 1.48045395 |
| Ankzf1 | -0.6992624 |
| Ankzf1 | 0.43603262 |
| Anln | -0.5602516 |
| Anln | -0.3990681 |
| Anp32a | -0.5175584 |
| Anp32a | -0.3252333 |
| Anp32a | 0.56430468 |
| Anp32e | -0.3369456 |
| Anpep | 1.85453073 |
| Antxr1 | 2.10422492 |
| Antxr1 | 0.98709574 |
| Antxrl | 2.65711211 |
| Anxa1 | -0.6664947 |
| Anxa11 | 3.36481722 |
| Anxa11 | -0.4258904 |
| Anxa2 | -0.3516574 |
| Anxa3 | 0.32488587 |
| Anxa3 | 3.22808582 |
| Anxa4 | 0.01053027 |
| Anxa5 | -0.5327273 |
| Anxa6 | -0.6356378 |
| Anxa8 | -0.626349 |
| Anxa8 | 0.76876092 |
| Anxa9 | -0.6035314 |
| Aoah | 0.54930014 |
| Aoc2 | 1.13559263 |
| Aoc2 | 1.62273479 |
| Aoc3 | -0.3918874 |
| Aof1 | -0.7332473 |
| Aof1 | -0.7229187 |
| Aof2 | -0.6926143 |
| Aox3 | -0.4172704 |
| Aox3l1 | -0.2441466 |
| Ap1b1 | -0.2039612 |
| Ap1g1 | -0.3408833 |
| Ap1g1 | -0.6893083 |
| Ap1g2 | 1.06707994 |
| Ap1gbp1 | -0.4240364 |
| Ap1gbp1 | 1.16649823 |
| Ap1m1 | -0.1668639 |
| Ap1m2 | 0.37830115 |
| Ap1m2 | -0.4879548 |
| Ap1m2 | 1.23566595 |
| Ap1m2 | 0.42949818 |
| Ap1s1 | -0.5990353 |
| Ap1s1 | 1.02827723 |
| Ap1s1 | 1.8242571 |
| Ap1s3 | 0.59683746 |
| Ap2a1 | -0.7830523 |
| Ap2a1 | -0.5175584 |
| Ap2a2 | -0.6348601 |
| Ap2b1 | 0.81962209 |
| Ap2b1 | -0.0227013 |
| Ap2b1 | -0.20834 |
| Ap2b1 | -0.2662191 |
| Ap2m1 | 2.00917985 |
| Ap2s1 | -0.6434512 |
| Ap2s1 | 1.43296014 |
| Ap2s1 | -0.6992624 |
| Ap3b1 | -0.6481714 |
| Ap3b1 | 2.13715689 |
| Ap3b2 | 2.20999223 |
| Ap3d1 | 1.24497711 |
| Ap3m1 | 0.78520785 |
| Ap3m2 | 2.76391353 |
| Ap3s2 | -0.4377203 |
| Ap3s2 | -0.2724751 |
| Ap4b1 | 0.97097715 |
| Ap4m1 | 0.76253739 |
| Ap4m1 | -0.7358478 |
| Ap4m1 | -0.6426668 |
| Ap4s1 | 1.20163111 |
| Apaf1 | -0.1852245 |
| Apaf1 | 0.40138192 |
| Apba1 | -0.3545139 |
| Apba2 | -0.2610383 |
| Apba2 | 0.26525594 |
| Apba3 | -0.5660015 |
| Apbb1 | -0.7367163 |
| Apbb1ip | 0.50767013 |
| Apbb1ip | 0.34614884 |
| Apbb1ip | -0.5624036 |
| Apbb2 | 0.23901614 |
| Apbb3 | -0.3257877 |
| Apc | -0.764952 |
| Apc2 | -0.6884836 |
| Apcdd1 | 0.38060998 |
| Apeh | -0.6005316 |
| Apex1 | -0.5945613 |
| Aph1a | 0.32706171 |
| Aph1b | 2.73876306 |
| Apip | 1.8674353 |
| Apip | 0.66990051 |
| Apitd1 | -0.4853105 |
| Aplp1 | -0.4148224 |
| Aplp2 | 1.21469745 |
| Apoa1 | 1.59591239 |
| Apoa1bp | -0.3044134 |
| Apoa2 | -0.3291208 |
| Apoa4 | 1.66806514 |
| Apob48r | -0.6309816 |
| Apobec1 | 0.60308693 |
| Apobec3 | -0.1909489 |
| Apoc1 | -0.626349 |
| Apoc1 | -0.1776474 |
| Apoc2 | 1.40353515 |
| Apoc3 | -0.2201205 |
| Apoe | 2.37982455 |
| Apof | -0.5652807 |
| Apoh | -0.5285653 |
| Apol7c | 0.81325727 |
| Apol9b | 0.28651451 |
| App | -0.4846506 |
| Appbp1 | -0.3093018 |
| Appbp1 | 0.33840073 |
| Appbp2 | 0.68948599 |
| Appl1 | -0.4754617 |
| Appl2 | 1.72680027 |
| Aprin | 0.51367122 |
| Aprt | -0.5703377 |
| Aptx | -0.6497503 |
| Aqp1 | -0.7410711 |
| Aqp1 | -0.6984287 |
| Aqp1 | -0.6521236 |
| Aqp11 | 0.98775573 |
| Aqp11 | -0.6545032 |
| Aqp3 | -0.4793885 |
| Aqp4 | -0.6942718 |
| Aqp5 | 1.03029474 |
| Aqp7 | 2.16967 |
| Aqp7 | 1.28588596 |
| Aqp8 | -0.6286624 |
| Aqp8 | 1.35007017 |
| Aqp9 | 0.3155804 |
| Aqr | -0.6713357 |
| Aqr | -0.1904705 |
| Ar | 0.00591129 |
| Araf | 0.27162781 |
| Araf | 1.8630357 |
| Arbp | -0.1285267 |
| Arc | 1.28645732 |
| Arcn1 | 0.1547992 |
| Ard1 | -0.0827593 |
| Ard1 | -0.5652807 |
| Areg | -0.6194438 |
| Arf2 | -0.4748089 |
| Arf3 | 0.12547598 |
| Arf5 | -0.563841 |
| Arf6 | -0.8366939 |
| Arfgap1 | 1.02054441 |
| Arfgap1 | 0.15019655 |
| Arfgap1 | -0.7839667 |
| Arfgap2 | -0.5168748 |
| Arfgap2 | 1.67630846 |
| Arfgap2 | -0.3918874 |
| Arfgap2 | -0.2605218 |
| Arfgap3 | -0.7272081 |
| Arfip2 | -0.2861784 |
| Arfip2 | -0.1240679 |
| Arfip2 | -0.716947 |
| Arfrp1 | 0.12615296 |
| Arfrp1 | -0.3191576 |
| Arg1 | 1.78624613 |
| Arg2 | 0.32950336 |
| Arglu1 | 2.55132752 |
| Arhgap1 | -0.8213164 |
| Arhgap1 | 3.58061839 |
| Arhgap1 | 2.48577168 |
| Arhgap10 | 0.12310259 |
| Arhgap12 | -0.2395934 |
| Arhgap15 | -0.6942718 |
| Arhgap15 | -0.0823342 |
| Arhgap17 | -0.2345607 |
| Arhgap18 | -0.062576 |
| Arhgap19 | 0.3286902 |
| Arhgap20 | 0.10041698 |
| Arhgap22 | -0.3642933 |
| Arhgap24 | 0.73129252 |
| Arhgap24 | -0.4345923 |
| Arhgap25 | -0.4886171 |
| Arhgap26 | 1.02118071 |
| Arhgap27 | -0.3241257 |
| Arhgap28 | 0.63874739 |
| Arhgap29 | 0.15052608 |
| Arhgap30 | 1.123501 |
| Arhgap4 | 0.82231379 |
| Arhgap6 | -0.0006724 |
| Arhgap8 | 2.67962598 |
| Arhgap9 | -0.5923325 |
| Arhgdib | 1.04230724 |
| Arhgdig | 1.46429429 |
| Arhgef1 | 0.73251419 |
| Arhgef1 | 0.70758627 |
| Arhgef10 | 1.4128688 |
| Arhgef10l | -0.5012923 |
| Arhgef10l | 1.73406638 |
| Arhgef15 | -0.4345923 |
| Arhgef18 | 1.45586887 |
| Arhgef19 | 0.12310259 |
| Arhgef2 | 3.05219258 |
| Arhgef3 | -0.6194438 |
| Arhgef4 | -0.469603 |
| Arhgef6 | 1.20110874 |
| Arid2 | 3.61974561 |
| Arid3a | -0.0469074 |
| Arid3b | 1.99520373 |
| Arid4b | -0.6217397 |
| Arid5a | -0.5696135 |
| Arid5b | -0.6794613 |
| Arih1 | 0.14125408 |
| Arih2 | -0.3044134 |
| Arl1 | 3.42232986 |
| Arl11 | -0.5559628 |
| Arl16 | 2.78789315 |
| Arl2 | -0.6278906 |
| Arl2bp | -0.5739669 |
| Arl2bp | 1.3572351 |
| Arl2bp | -0.0542959 |
| Arl3 | -0.235565 |
| Arl3 | -0.1828499 |
| Arl4a | -0.4787329 |
| Arl4a | 0.69930459 |
| Arl4c | -0.7067997 |
| Arl5a | -0.7195015 |
| Arl5b | 0.86206976 |
| Arl6 | -0.2235853 |
| Arl6ip1 | 0.62158324 |
| Arl6ip2 | 0.37211562 |
| Arl6ip2 | 2.00549974 |
| Arl6ip2 | -0.7489623 |
| Arl6ip2 | 0.51522065 |
| Arl6ip4 | -0.2808836 |
| Arl6ip4 | -0.5516941 |
| Arl6ip5 | 1.00072638 |
| Arl6ip5 | -0.1762336 |
| Arl8a | 0.64663568 |
| Arl8b | -0.5559628 |
| Armc10 | 2.2593582 |
| Armc3 | 2.01297432 |
| Armc6 | 2.42384553 |
| Armc6 | -0.6521236 |
| Armc8 | 0.76000223 |
| Armc8 | -0.6080496 |
| Armc8 | 0.89024232 |
| Armc9 | -0.2698633 |
| Armc9 | -0.5717877 |
| Armcx1 | -0.5703377 |
| Armcx2 | -0.2325553 |
| Armcx3 | 0.02877644 |
| Armcx5 | 3.13530608 |
| Armcx6 | 1.02827723 |
| Armcx6 | -0.1474945 |
| Armetl1 | 0.85294159 |
| Arnt | 2.3643352 |
| Arnt | -0.6005316 |
| Arnt2 | -0.3942747 |
| Arntl | 0.40861666 |
| Arpc1a | 0.30867724 |
| Arpc1a | -0.632531 |
| Arpc1a | -0.5141457 |
| Arpc1b | -0.4833322 |
| Arpc1b | 2.85655092 |
| Arpc1b | -0.5313378 |
| Arpc3 | 0.05108408 |
| Arpc4 | -0.5175584 |
| Arpc5 | -0.3219143 |
| Arpc5 | -0.6632816 |
| Arpc5l | -0.1156557 |
| Arpp19 | 0.48623672 |
| Arpp21 | -0.0428283 |
| Arpp21 | -0.6786455 |
| Arrb1 | -0.0242952 |
| Arrb1 | -0.491271 |
| Arrb1 | -0.145671 |
| Arrb1 | -0.4296096 |
| Arrb2 | -0.6600797 |
| Arrdc1 | -0.0650747 |
| Arrdc2 | -0.0780945 |
| Arrdc3 | 0.30423173 |
| Arrdc4 | 1.1833813 |
| Ars2 | 1.9084744 |
| Ars2 | 2.15986458 |
| Ars2 | -0.38299 |
| Arsa | -0.5100672 |
| Arsb | -0.7739574 |
| Arsb | -0.261555 |
| Arsg | 0.05767167 |
| Arsi | 1.63588925 |
| Arsj | -0.5223576 |
| Arsj | 2.11344724 |
| Art1 | -0.9283662 |
| Art2b | 1.86556679 |
| Art3 | -0.5930748 |
| Art3 | -0.4586377 |
| Art4 | 0.01550798 |
| Art5 | -0.0477254 |
| Artn | 0.26525594 |
| Artn | 0.65007746 |
| Arv1 | -0.6537093 |
| As3mt | -0.5285653 |
| As3mt | 1.21767855 |
| Asah1 | 0.04111289 |
| Asah1 | 1.72838733 |
| Asah2 | -0.2688206 |
| Asah3 | 0.03664613 |
| Asah3l | 0.84757446 |
| Asb1 | -0.342576 |
| Asb1 | -0.5754227 |
| Asb1 | -0.390101 |
| Asb12 | -0.7323821 |
| Asb13 | -0.1585125 |
| Asb15 | -0.6860142 |
| Asb17 | 0.62335352 |
| Asb2 | 0.02689269 |
| Asb3 | -0.5609684 |
| Asb3 | -0.6387551 |
| Asb4 | 1.5312304 |
| Asb4 | 0.54545675 |
| Asb6 | -0.583471 |
| Asb6 | -0.6851925 |
| Asb7 | -0.8090097 |
| Asb7 | -0.6762024 |
| Asb8 | -0.4057003 |
| Asb9 | -0.6411001 |
| Ascc1 | 0.96868963 |
| Ascc1 | -0.4402304 |
| Ascc2 | 0.42071235 |
| Ascc2 | -0.3954707 |
| Ascc2 | 2.0594505 |
| Ascc2 | -0.299551 |
| Ascc3l1 | 2.78623137 |
| Ascl1 | 1.86495997 |
| Ascl2 | -0.7401985 |
| Asf1a | -0.8015179 |
| Asf1b | -0.6403178 |
| Asgr1 | -0.38299 |
| Ash1l | -0.4952664 |
| Ash1l | 1.70633523 |
| Ash1l | 0.94990563 |
| Ash2l | -0.6364161 |
| Ash2l | -0.6458083 |
| Asl | -0.7084831 |
| Asl | 1.06756377 |
| Asna1 | -0.8165634 |
| Asns | 1.63861422 |
| Asns | 0.21936528 |
| Asns | 1.24706659 |
| Asnsd1 | -0.8396062 |
| Asnsd1 | -0.4767688 |
| Asph | -0.6125903 |
| Asph | -0.6802779 |
| Asph | -0.446536 |
| Asph | 2.70524083 |
| Asph | -0.606541 |
| Asphd1 | 1.32202696 |
| Asphd2 | -0.4452714 |
| Aspm | -0.668912 |
| Asprv1 | 2.64051134 |
| Aspscr1 | 2.17357738 |
| Aspscr1 | 0.67269763 |
| Aspscr1 | 0.4367558 |
| Asrgl1 | -0.9030083 |
| Ass1 | -0.5278735 |
| Aste1 | 0.17330313 |
| Astn1 | 0.09343001 |
| Astn2 | 4.04098235 |
| Asz1 | 0.20643958 |
| Atad1 | 1.75386991 |
| Atad2 | -0.7229187 |
| Atad3a | -0.1598991 |
| Atad4 | 2.33705707 |
| Atad4 | 0.50767013 |
| Atcay | 1.8246801 |
| Ate1 | 1.33899177 |
| Ate1 | 0.24438412 |
| Atf2 | -0.345971 |
| Atf2 | -0.0785176 |
| Atf2 | 0.13020436 |
| Atf3 | -0.7349802 |
| Atf4 | -0.4240364 |
| Atf4 | 2.26525598 |
| Atf5 | -0.7297915 |
| Atf7 | -0.6465953 |
| Atf7ip | 0.27018355 |
| Atg10 | -0.3918874 |
| Atg10 | -0.4892798 |
| Atg12 | 0.06818625 |
| Atg16l1 | 0.73111787 |
| Atg2a | 0.38699301 |
| Atg2a | 1.42930282 |
| Atg2b | -0.2819401 |
| Atg2b | -0.4414881 |
| Atg3 | -0.5292576 |
| Atg3 | -0.7118593 |
| Atg4b | -0.4258904 |
| Atg4c | 0.03664613 |
| Atg4c | 1.85412132 |
| Atg4d | -0.7830523 |
| Atg5 | -0.2370736 |
| Atg5 | 0.9072575 |
| Atg7 | -0.0302975 |
| Atg9b | -0.1781191 |
| Athl1 | -0.4986092 |
| Atic | 3.20364654 |
| Atic | -0.1608247 |
| Atic | -0.7280684 |
| Atm | -0.7569222 |
| Atm | 1.38645669 |
| Atmin | 0.37778728 |
| Atmin | -0.273522 |
| Atmin | 0.00088095 |
| Atn1 | 0.19959539 |
| Atoh8 | 0.8748474 |
| Atox1 | -0.6379748 |
| Atp10a | 0.71152073 |
| Atp10d | -0.7903938 |
| Atp11c | -0.2719522 |
| Atp11c | -0.7026046 |
| Atp12a | -0.2920383 |
| Atp13a1 | -0.5652807 |
| Atp13a1 | -0.1121366 |
| Atp13a2 | -0.6697192 |
| Atp13a4 | -0.5488592 |
| Atp13a5 | 0.14191949 |
| Atp1a1 | -0.6827319 |
| Atp1a2 | 1.83357048 |
| Atp1b1 | -0.5292576 |
| Atp1b2 | 0.01741529 |
| Atp1b3 | -0.4358422 |
| Atp2a1 | 1.32777758 |
| Atp2a2 | 0.5514284 |
| Atp2a2 | 2.16333948 |
| Atp2a3 | 1.25014235 |
| Atp2a3 | 1.45166661 |
| Atp2a3 | 3.84188311 |
| Atp2b1 | -0.8357252 |
| Atp2b2 | -0.8194122 |
| Atp2b2 | -0.1952658 |
| Atp2b2 | -0.4859708 |
| Atp2b3 | -0.7237749 |
| Atp2b3 | 1.99791985 |
| Atp2b4 | 1.30109515 |
| Atp2b4 | 0.34000918 |
| Atp2c1 | 0.50073111 |
| Atp2c1 | 3.1001511 |
| Atp5a1 | 0.34428461 |
| Atp5b | 2.29212862 |
| Atp5c1 | 0.35198326 |
| Atp5d | 1.18848143 |
| Atp5e | 0.56346586 |
| Atp5f1 | -0.7349802 |
| Atp5g2 | 1.27986503 |
| Atp5g3 | 0.15019655 |
| Atp5h | -0.6843715 |
| Atp5j | -0.656093 |
| Atp5j | -0.0112273 |
| Atp5j2 | 0.24616647 |
| Atp5l | 1.68268955 |
| Atp5o | -0.7084831 |
| Atp5s | 1.03969295 |
| Atp5sl | 0.18193922 |
| Atp6ap1 | 0.30645719 |
| Atp6ap2 | -0.7280684 |
| Atp6ap2 | 2.50058822 |
| Atp6ap2 | -0.0112273 |
| Atp6ap2 | -0.4866317 |
| Atp6v0a1 | -0.8222699 |
| Atp6v0a1 | 0.74222922 |
| Atp6v0a2 | -0.7160971 |
| Atp6v0b | 1.88872726 |
| Atp6v0d1 | -0.4992793 |
| Atp6v0d1 | 0.22515316 |
| Atp6v0d1 | -0.766746 |
| Atp6v0d1 | -0.4283682 |
| Atp6v0e | -0.4228025 |
| Atp6v0e2 | -0.5960502 |
| Atp6v1a | 0.74446982 |
| Atp6v1b1 | -0.4813582 |
| Atp6v1b2 | 0.88361282 |
| Atp6v1c1 | -0.7306542 |
| Atp6v1c2 | -0.8376636 |
| Atp6v1d | -0.2893701 |
| Atp6v1e1 | -0.5053318 |
| Atp6v1e2 | 1.72456195 |
| Atp6v1g1 | 0.70919787 |
| Atp6v1g2 | 0.84248503 |
| Atp6v1g2 | 0.42901228 |
| Atp6v1h | 2.20508731 |
| Atp7a | -0.7703446 |
| Atp8a1 | 0.76624297 |
| Atp8a2 | -0.2421201 |
| Atp8b1 | 2.08003453 |
| Atp8b3 | -0.7931622 |
| Atp8b3 | -0.632531 |
| Atp8b3 | 0.24527573 |
| Atp8b3 | 0.23301455 |
| Atp9a | 0.37056268 |
| Atp9a | 3.40775551 |
| Atp9b | 2.74012461 |
| Atp9b | 1.12190702 |
| Atpaf1 | -0.4541606 |
| Atpaf2 | -0.8184617 |
| Atpaf2 | -0.1095063 |
| Atpbd1b | 0.62590456 |
| Atpbd1c | 0.36197408 |
| Atpbd3 | -0.5566762 |
| Atpif1 | 1.23737508 |
| Atrx | 0.1498669 |
| Atxn1 | 2.20243257 |
| Atxn10 | 0.9204182 |
| Atxn2 | 1.14297212 |
| Atxn2l | -0.6640838 |
| Atxn3 | -0.8203638 |
| Atxn3 | 0.21691728 |
| Atxn3 | 0.42438337 |
| Atxn7l1 | 2.62974235 |
| Atxn7l2 | -0.6133492 |
| AU014645 | -0.7658485 |
| AU017455 | 2.22959086 |
| AU017455 | -0.4142114 |
| AU018778 | 1.45539411 |
| AU018829 | -0.3164093 |
| AU019823 | 2.64561164 |
| AU021034 | -0.1294211 |
| AU021092 | 1.68201019 |
| AU021838 | -0.6778304 |
| AU022252 | 1.4366029 |
| AU022751 | -0.8632442 |
| AU022870 | 1.29375829 |
| AU023871 | -0.0274915 |
| AU040320 | 2.77906975 |
| AU040320 | -0.6255792 |
| AU040320 | -0.6917867 |
| AU040829 | 0.6138722 |
| AU042651 | -0.632531 |
| Aup1 | 1.0884851 |
| Aup1 | 0.08426644 |
| Aurka | -0.1720054 |
| Aurkaip1 | -0.3307919 |
| Aurkc | -0.4939326 |
| Auts2 | -0.3098466 |
| Auts2 | 0.46054033 |
| AV028368 | 0.38902621 |
| AV249152 | 0.29808368 |
| AV249152 | 2.07670944 |
| Aven | 1.09084593 |
| Avil | 1.23737508 |
| Avp | -0.2605218 |
| Avpi1 | 0.45015123 |
| Avpi1 | 0.63468056 |
| Avpr1a | -0.5710624 |
| AW049604 | -0.6278906 |
| AW061290 | 0.5946096 |
| AW111922 | -0.5893691 |
| AW121567 | 0.65255516 |
| AW125753 | -0.2990124 |
| AW146242 | 0.90768965 |
| AW146299 | 0.57306614 |
| AW146299 | 1.65955694 |
| AW540478 | -0.5060068 |
| AW544981 | -0.4689543 |
| AW548124 | -0.4986092 |
| AW549877 | -0.5559628 |
| AW551984 | 0.79731126 |
| AW551984 | -0.3027898 |
| AW551984 | -0.4026797 |
| AW555464 | 0.96314903 |
| Axin2 | -0.3718428 |
| Axl | 1.10162797 |
| Axud1 | 0.10354379 |
| Azgp1 | -0.2181466 |
| Azgp1 | 1.89305439 |
| Azi1 | 3.39119147 |
| Azi2 | 1.40571039 |
| Azin1 | -0.3386311 |
| B020018G12Rik | -0.1790633 |
| B230118H07Rik | 0.97084275 |
| B230120H23Rik | 0.8755935 |
| B230206H07Rik | 2.76262488 |
| B230209C24Rik | 0.41874843 |
| B230217C12Rik | -0.3202593 |
| B230219D22Rik | -0.6411001 |
| B230312A22Rik | -0.5667227 |
| B230312I18Rik | -0.7195015 |
| B230317C12Rik | -0.6012806 |
| B230317C12Rik | -0.0057392 |
| B230339H12Rik | 1.12871886 |
| B230339M05Rik | 1.56645908 |
| B230339M05Rik | 2.33666445 |
| B230340J04Rik | -0.713552 |
| B230342M21Rik | 1.99608078 |
| B230373P09Rik | -0.8080699 |
| B230378H13Rik | 0.24942512 |
| B230380D07Rik | 0.3790714 |
| B230396O12Rik | 0.02651547 |
| B230399E16Rik | -0.0650747 |
| B2m | 1.56617864 |
| B2m | 1.50266859 |
| B3galnt1 | -0.469603 |
| B3galnt2 | -0.5674446 |
| B3galt1 | 1.28197208 |
| B3galt4 | -0.4038868 |
| B3galt4 | -0.7560344 |
| B3galt5 | -0.7658485 |
| B3galt6 | -0.1724742 |
| B3gat1 | 0.98696369 |
| B3gat1 | 1.39241113 |
| B3gat1 | 0.28480913 |
| B3gat2 | -0.342576 |
| B3gat3 | 0.97191741 |
| B3gat3 | 1.55878614 |
| B3gnt1 | 0.05621185 |
| B3gnt3 | -0.1668639 |
| B3gnt5 | -0.0428283 |
| B3gnt7 | -0.6664947 |
| B3gnt8 | 0.07106568 |
| B3gnt8 | 0.5539758 |
| B430119L13Rik | 0.35778071 |
| B4galnt3 | -0.1880822 |
| B4galnt4 | 0.28224507 |
| B4galt1 | -0.7658485 |
| B4galt3 | -0.4839912 |
| B4galt3 | -0.4899431 |
| B4galt4 | 0.18954791 |
| B4galt7 | 2.55609321 |
| B630005N14Rik | 1.21015578 |
| B830045N13Rik | 2.27192692 |
| B930006L02Rik | -0.2436396 |
| B930011P16Rik | -0.1594367 |
| B930037P14Rik | -0.5798039 |
| B930041F14Rik | -0.1348057 |
| B930041G04 | 0.98669952 |
| B930076A02 | -0.5997832 |
| B9d1 | 1.87921462 |
| B9d2 | 1.85042832 |
| Baalc | 0.68122255 |
| Baalc | -0.491271 |
| Bace1 | 1.50048631 |
| Bace2 | -0.7332473 |
| Bace2 | -0.6521236 |
| Bace2 | 2.10849889 |
| Bach1 | -0.2034759 |
| Bach2 | -0.4057003 |
| Bad | 2.08684715 |
| Bag1 | -0.3689318 |
| Bag2 | 0.23511959 |
| Bag3 | 1.03281038 |
| Bag4 | -0.8213164 |
| Bag5 | 2.02303082 |
| Bahcc1 | 1.51238776 |
| Bahd1 | 0.19771987 |
| Bai1 | 0.73981012 |
| Bai2 | 0.46499032 |
| Bai2 | 3.5720073 |
| Bai3 | -0.2340589 |
| Baiap2 | -0.2543467 |
| Baiap2 | 1.34544071 |
| Banf1 | -0.4683061 |
| Banf1 | -0.3782799 |
| Banf1 | -0.607295 |
| Banf1 | -0.3375071 |
| Banf2 | 0.36328057 |
| Bap1 | 1.02105348 |
| Bard1 | 2.04031582 |
| Barx2 | 0.76169312 |
| Basp1 | 1.29063643 |
| Bat1a | -0.1701324 |
| Bat1a | -0.6278906 |
| Bat2 | -0.3516574 |
| Bat3 | -0.7017679 |
| Bat4 | 3.50875212 |
| Bat4 | -0.4966021 |
| Bat5 | -0.7110141 |
| Batf | 0.80588257 |
| Bax | -0.840579 |
| Baz1a | -0.4722022 |
| Baz1b | 2.80284195 |
| Baz2a | -0.3499477 |
| BB014433 | -0.6403178 |
| BB128963 | 0.4336179 |
| BB146404 | -0.5645606 |
| Bbc3 | 0.2805317 |
| Bbc3 | 0.95333726 |
| Bbox1 | -0.3213623 |
| Bbs1 | 0.16750011 |
| Bbs12 | 0.8300282 |
| Bbs2 | 2.54350584 |
| Bbs4 | -0.2840568 |
| Bbs5 | -0.4172704 |
| Bbs7 | -0.2931078 |
| Bbs9 | -0.4657179 |
| Bbs9 | 0.16231079 |
| Bbx | -0.2176537 |
| BC002163 | 0.3343672 |
| BC002199 | -0.3319077 |
| BC002216 | 0.1745877 |
| BC003236 | 0.18733524 |
| BC003266 | -0.4148224 |
| BC003885 | 0.53317839 |
| BC003885 | 0.5514284 |
| BC003993 | 1.38773591 |
| BC003993 | -0.4296096 |
| BC004004 | -0.5710624 |
| BC004044 | -0.4014743 |
| BC004044 | 0.06385003 |
| BC004728 | 0.18098367 |
| BC004728 | -0.6027805 |
| BC005537 | 1.70120714 |
| BC005682 | -0.476115 |
| BC005764 | -0.7419445 |
| BC006662 | 1.75814361 |
| BC006779 | -0.7254899 |
| BC006779 | 1.6866322 |
| BC008155 | 1.85540013 |
| BC008163 | -0.2756193 |
| BC008163 | 0.86855605 |
| BC010462 | 0.30895437 |
| BC011248 | -0.6294348 |
| BC011248 | -0.6827319 |
| BC011487 | -0.6778304 |
| BC013491 | -0.5820024 |
| BC013491 | 0.26059381 |
| BC013529 | 0.51566286 |
| BC013712 | -0.3038719 |
| BC016201 | -0.3442719 |
| BC016423 | -0.8251366 |
| BC016495 | 0.52708602 |
| BC017158 | 1.71965388 |
| BC017612 | 1.4444061 |
| BC017643 | 0.27278158 |
| BC017647 | 1.16007223 |
| BC018242 | 2.74193039 |
| BC018242 | -0.7384557 |
| BC018465 | 1.00046616 |
| BC020002 | -0.3054975 |
| BC020535 | 0.1217436 |
| BC021381 | -0.100359 |
| BC021395 | -0.5798039 |
| BC021614 | -0.6088048 |
| BC021790 | 2.83840259 |
| BC021891 | -0.1914276 |
| BC022224 | -0.3027898 |
| BC022687 | 2.47539565 |
| BC023055 | -0.5827364 |
| BC023151 | 1.3155947 |
| BC023744 | 2.50469522 |
| BC023814 | 1.02460912 |
| BC023829 | 2.31297954 |
| BC024561 | 0.28195973 |
| BC024814 | -0.8662424 |
| BC024868 | -0.461206 |
| BC024997 | 0.11901955 |
| BC025076 | 2.2434758 |
| BC025546 | -0.3324661 |
| BC025575 | 0.54052213 |
| BC025920 | 0.4418021 |
| BC026439 | -0.424654 |
| BC026585 | 3.5852295 |
| BC026590 | -0.568167 |
| BC026590 | -0.5251114 |
| BC027072 | 0.0370192 |
| BC027231 | -0.1640717 |
| BC027231 | -0.716947 |
| BC027231 | 0.61466607 |
| BC027344 | -0.568167 |
| BC028528 | 1.23777677 |
| BC028528 | 1.33737241 |
| BC029169 | 0.81517137 |
| BC029169 | 4.23329515 |
| BC029214 | 1.97061415 |
| BC030183 | 1.42194401 |
| BC030307 | -0.1506944 |
| BC030476 | -0.7876338 |
| BC030499 | -0.0497737 |
| BC030500 | 0.92988488 |
| BC030867 | -0.5182424 |
| BC031181 | -0.858269 |
| BC031353 | 0.47127699 |
| BC031575 | -0.5411084 |
| BC031748 | -0.6868366 |
| BC031781 | 0.16846984 |
| BC031853 | -0.0973301 |
| BC032265 | 1.71370536 |
| BC034076 | -0.2068781 |
| BC037034 | 0.64414187 |
| BC037594 | -0.7542612 |
| BC038156 | -0.5341188 |
| BC038286 | -0.5588198 |
| BC038613 | -0.5026369 |
| BC038822 | 0.399627 |
| BC038822 | -0.166398 |
| BC038822 | -0.5960502 |
| BC038925 | -0.700932 |
| BC039093 | -0.1276332 |
| BC040758 | -0.152528 |
| BC043098 | -0.5362101 |
| BC043118 | 0.67232518 |
| BC043301 | -0.3252333 |
| BC046331 | 1.14497442 |
| BC046404 | 1.14341745 |
| BC048355 | -0.6364161 |
| BC048502 | -0.1603618 |
| BC048546 | 0.28452458 |
| BC048546 | 1.50769059 |
| BC048562 | 2.17035862 |
| BC048644 | -0.5595354 |
| BC048651 | -0.5982882 |
| BC048671 | -0.2117604 |
| BC049635 | 0.17233851 |
| BC049635 | 0.9794049 |
| BC049730 | -0.6020302 |
| BC049762 | 3.81389704 |
| BC049806 | 0.14092119 |
| BC049806 | -0.3319077 |
| BC049806 | -0.5299505 |
| BC050196 | 3.26424796 |
| BC050210 | -0.7950124 |
| BC050777 | 0.47382589 |
| BC050811 | -0.0359353 |
| BC050811 | 0.07106568 |
| BC051019 | -0.2819401 |
| BC051070 | 1.886556 |
| BC051142 | 1.34374331 |
| BC051227 | 1.48360587 |
| BC051628 | 2.5608815 |
| BC051665 | 1.47163258 |
| BC052484 | -0.3510871 |
| BC053749 | 1.06526326 |
| BC053749 | 2.32069695 |
| BC054059 | -0.6294348 |
| BC055107 | 0.68985152 |
| BC055111 | -0.447169 |
| BC055324 | 0.55968164 |
| BC055368 | 0.4873774 |
| BC056474 | -0.3960693 |
| BC056923 | -0.0116205 |
| BC057022 | 1.390119 |
| BC057371 | 2.02081505 |
| BC057371 | -0.3847626 |
| BC057371 | 1.40596105 |
| BC057552 | -0.7084831 |
| BC057627 | 0.38393471 |
| BC057893 | -0.6640838 |
| BC060267 | 0.70435453 |
| BC060615 | 3.08006534 |
| BC060632 | 0.93674679 |
| BC061039 | 1.21942159 |
| BC061237 | -0.0831846 |
| BC062115 | 1.32476986 |
| BC062650 | -0.5313378 |
| BC063749 | 0.5821557 |
| BC064033 | -0.2400982 |
| BC065085 | 0.46615778 |
| BC065397 | -0.2325553 |
| BC066028 | -0.6860142 |
| BC066107 | 1.7940413 |
| BC067047 | 0.77344264 |
| BC067068 | 0.00320609 |
| BC068157 | 1.57760682 |
| BC068281 | -0.0489538 |
| BC072620 | -0.3648718 |
| BC085271 | 2.42043383 |
| BC087945 | -0.3550863 |
| BC088983 | 2.12999091 |
| BC089491 | -0.7640563 |
| BC099439 | 0.91099606 |
| BC107230 | -0.3180573 |
| Bcam | 1.69367423 |
| Bcan | 1.61837383 |
| Bcan | -0.2512747 |
| Bcap29 | -0.8270527 |
| Bcap29 | -0.6202084 |
| Bcap31 | -0.4846506 |
| Bcap31 | -0.3718428 |
| Bcar1 | -0.4637815 |
| Bcar3 | -0.716947 |
| Bcas1 | 0.74378099 |
| Bcas2 | -0.8165634 |
| Bcas3 | 2.92634804 |
| Bcat1 | 0.90451578 |
| Bcat2 | -0.7748628 |
| Bccip | -0.403283 |
| Bcdin3d | -0.0934505 |
| Bcdo2 | -0.6608791 |
| Bckdha | -0.7931622 |
| Bckdhb | -0.0831846 |
| Bckdk | 0.36223553 |
| Bckdk | -0.6819131 |
| Bcl10 | 2.39287122 |
| Bcl11a | 0.49893182 |
| Bcl11b | 2.77576695 |
| Bcl2 | 0.36718891 |
| Bcl2 | -0.5667227 |
| Bcl2l1 | 0.72022351 |
| Bcl2l1 | 0.69695046 |
| Bcl2l1 | -0.6967637 |
| Bcl2l10 | -0.7903938 |
| Bcl2l11 | -0.3533702 |
| Bcl2l11 | -0.6959323 |
| Bcl2l11 | 0.35541346 |
| Bcl2l12 | 3.30909831 |
| Bcl2l13 | -0.1298686 |
| Bcl2l14 | 0.32325059 |
| Bcl2l14 | 0.45038866 |
| Bcl2l2 | -0.8090097 |
| Bcl2l2 | 0.63990603 |
| Bcl3 | -0.4283682 |
| Bcl6 | -0.388912 |
| Bcl6b | 0.84248503 |
| Bcl7a | 0.71614884 |
| Bcl7b | 0.09518176 |
| Bcl9l | -0.6364161 |
| Bclaf1 | -0.3972676 |
| Bclaf1 | -0.5376069 |
| Bclp2 | 0.16685305 |
| Bcmo1 | -0.5327273 |
| Bcor | -0.3499477 |
| Bcor | 0.4437172 |
| Bcorl1 | 0.14887724 |
| Bcs1l | 1.11916802 |
| Bdh1 | 1.58402457 |
| Bdh2 | -0.0266914 |
| Bdkrb1 | -0.4807012 |
| Bdnf | 0.33920531 |
| Bdnf | -0.4886171 |
| Becn1 | -0.55525 |
| Becn1 | -0.447169 |
| Bet1 | 0.02349213 |
| Bet1l | 1.69811643 |
| Bex2 | 3.33816569 |
| Bex2 | -0.4377203 |
| Bex2 | 1.06901376 |
| Bex4 | -0.1086311 |
| Bex4 | 2.27709992 |
| Bex6 | -0.8337908 |
| Bfar | -0.7454467 |
| Bfar | 0.87020797 |
| Bglap2 | 1.22157018 |
| Bgn | 1.44111596 |
| Bhlhb2 | 3.48309972 |
| Bhlhb5 | -0.408124 |
| Bhlhb8 | 0.22332936 |
| Bhlhb8 | 1.19407999 |
| Bhlhb9 | 1.46241121 |
| Bhmt | -0.3093018 |
| Bhmt2 | -0.5953055 |
| Bicc1 | 0.01703415 |
| Bicd1 | -0.4579968 |
| Bicd2 | 2.2242438 |
| Bicd2 | 0.9222627 |
| Bid | -0.5915907 |
| Bid | -0.07556 |
| Bik | 1.3376425 |
| Bin1 | 1.59121672 |
| Bin1 | -0.1209597 |
| Bin1 | 0.41973092 |
| Bin3 | 0.49351262 |
| Birc2 | -0.5509845 |
| Birc2 | -0.0994925 |
| Birc2 | -0.1947851 |
| Birc4 | 1.31762114 |
| Birc5 | 0.37521361 |
| Birc5 | 2.32051249 |
| Birc5 | 0.71383771 |
| Blcap | 1.4470457 |
| Blm | -0.3510871 |
| Blm | 3.4332105 |
| Blmh | 0.18384733 |
| Blnk | -0.6868366 |
| Bloc1s1 | -0.5404071 |
| Bloc1s3 | -0.8602559 |
| Blvra | 0.06529772 |
| Blvrb | 0.85553717 |
| Bmf | 1.86617321 |
| Bmi1 | 1.46366703 |
| Bmp1 | -0.6271195 |
| Bmp1 | 0.30116287 |
| Bmp1 | 0.1498669 |
| Bmp1 | 1.43974828 |
| Bmp15 | 1.75518264 |
| Bmp2k | 1.37589908 |
| Bmp2k | -0.1402223 |
| Bmp3 | 0.60127696 |
| Bmp4 | 1.3653929 |
| Bmp6 | -0.5230453 |
| Bmp7 | 2.32226333 |
| Bmp8a | 1.0272668 |
| Bmper | -0.8127788 |
| Bmpr1a | 1.95977883 |
| Bmpr1a | 0.99942453 |
| Bmpr1b | 1.52918828 |
| Bmpr1b | 1.73131908 |
| Bmpr2 | -0.8043201 |
| Bms1 | -0.7721492 |
| Bnc1 | 1.63977958 |
| Bnc2 | -0.0327095 |
| Bnc2 | 0.48486602 |
| Bnip1 | -0.5114247 |
| Bnip2 | 1.35864378 |
| Bnip3l | 1.54997833 |
| Bnipl | 2.21845292 |
| Bnipl | -0.5196121 |
| Bok | 0.20519898 |
| Bok | -0.6901337 |
| Bola1 | 1.22248946 |
| Bola2 | 2.36122326 |
| Bola3 | -0.7821387 |
| Boll | 1.37053082 |
| Bop1 | -0.0780945 |
| Bpi | -0.0600841 |
| Bpil1 | 1.80841337 |
| Bpil3 | -0.6240415 |
| Bpnt1 | -0.590109 |
| Bpnt1 | -0.707641 |
| Bpnt1 | -0.4184969 |
| Bpnt1 | -0.5285653 |
| Brap | -0.6545032 |
| Brca2 | 0.21476989 |
| Brca2 | -0.7587005 |
| Brcc3 | -0.403283 |
| Brcc3 | -0.766746 |
| Brcc3 | 1.99949327 |
| Brd2 | 2.36081146 |
| Brd2 | -0.6648867 |
| Brd2 | 0.95292614 |
| Brd4 | 1.97636484 |
| Brd4 | 0.96097771 |
| Brd8 | -0.6802779 |
| Brd8 | -0.6860142 |
| Brd9 | 2.15516164 |
| Brd9 | -0.3197083 |
| Brdt | -0.6364161 |
| Brdt | -0.743694 |
| Bre | 0.4635874 |
| Bre | -0.1790633 |
| Bre | -0.4952664 |
| Brf1 | 1.81099263 |
| Brf2 | 1.64268651 |
| Bri3 | 3.0026898 |
| Brip1 | -0.5975416 |
| Brms1 | 1.50776534 |
| Brp16 | 0.70651029 |
| Brp16 | 0.15970505 |
| Brp17 | 2.22284228 |
| Brp44 | -0.461206 |
| Brpf1 | 0.14557062 |
| Brpf1 | -0.4535228 |
| Brs3 | -0.3800433 |
| Brsk1 | -0.3859462 |
| Brsk2 | -0.5397062 |
| Brsk2 | -0.7922385 |
| Brunol4 | -0.8978127 |
| Brunol4 | -0.8222699 |
| Brwd1 | -0.6951017 |
| Brwd1 | -0.5006208 |
| Brwd2 | 1.12190702 |
| Bscl2 | 0.0314072 |
| Bsdc1 | -0.5467389 |
| Bsg | 0.77560818 |
| Bsg | 1.2305193 |
| Bspry | 0.25326135 |
| Bst2 | -0.724632 |
| Btbd1 | 2.06059494 |
| Btbd10 | -0.5545377 |
| Btbd11 | 0.73233976 |
| Btbd12 | -0.0617446 |
| Btbd14a | -0.7587005 |
| Btbd14b | -0.672145 |
| Btbd16 | -0.0895872 |
| Btbd3 | 0.22302504 |
| Btbd3 | -0.2740459 |
| Btbd6 | 2.74307415 |
| Btbd9 | 1.33177256 |
| Btc | -0.6035314 |
| Btd | 2.50524813 |
| Btf3 | -0.6745773 |
| Btg1 | 1.12247662 |
| Btg1 | 0.20953373 |
| Btg3 | -0.6156299 |
| Btg3 | -0.4111627 |
| Btg4 | 0.86825538 |
| Btk | -0.4283682 |
| Btla | -0.7885529 |
| Btrc | -0.3319077 |
| Btrc | -0.495934 |
| Btrc | 1.72803488 |
| Bub1b | -0.2487226 |
| Bub3 | 1.2742867 |
| Bud13 | 0.87260545 |
| Bud31 | -0.1687302 |
| Bves | 1.08433893 |
| Bxdc1 | 0.96990139 |
| Bxdc1 | -0.3054975 |
| Bxdc2 | 0.07644032 |
| Bxdc2 | 2.42013056 |
| Bxdc5 | 1.57739904 |
| Bxdc5 | 2.34031234 |
| Bxdc5 | -0.4945993 |
| Bzrap1 | 0.51011977 |
| Bzrpl1 | -0.5645606 |
| Bzw1 | -0.4683061 |
| Bzw2 | 2.94175724 |
| Bzw2 | 0.88405629 |
| C030006K11Rik | 0.86433765 |
| C030011O14Rik | -0.5355125 |
| C030014K22Rik | -0.0311008 |
| C030018G13Rik | 1.82293398 |
| C030030A07Rik | -0.3806319 |
| C030039L03Rik | 0.52490031 |
| C030045D06Rik | 0.18543439 |
| C030045D06Rik | -0.7410711 |
| C030046I01Rik | -0.5127842 |
| C030048B08Rik | -0.5652807 |
| C030048B08Rik | -0.56889 |
| C130022K22Rik | 1.91088302 |
| C130032J12Rik | 2.06722895 |
| C130034I18Rik | -0.4358422 |
| C130038G02Rik | -0.5602516 |
| C130060K24Rik | 3.78748727 |
| C130073D16Rik | 1.81778207 |
| C130074G19Rik | -0.7721492 |
| C130090K23Rik | -0.7084831 |
| C130090K23Rik | 2.8981527 |
| C1galt1 | 0.30088337 |
| C1galt1c1 | -0.6934427 |
| C1qa | -0.1710684 |
| C1qb | 0.39408953 |
| C1qbp | 1.37035713 |
| C1qc | -0.7739574 |
| C1qdc2 | 0.75030404 |
| C1ql2 | -0.5509845 |
| C1ql3 | 0.16426024 |
| C1qtnf1 | 0.18225752 |
| C1qtnf2 | 1.0964872 |
| C1qtnf3 | 2.41999268 |
| C1qtnf3 | -0.3136691 |
| C1qtnf3 | 1.25567249 |
| C1qtnf4 | 1.10360647 |
| C1qtnf6 | -0.2963237 |
| C1qtnf7 | -0.5285653 |
| C1qtnf9 | 0.34880546 |
| C1rl | 0.70812378 |
| C1s | 4.07562136 |
| C1s | 0.07070625 |
| C2 | -0.2682997 |
| C230021P08Rik | 1.10546469 |
| C230029F24Rik | -0.6843715 |
| C230052I12Rik | -0.5761514 |
| C230052I12Rik | 0.85248278 |
| C230055K05Rik | 3.2570406 |
| C230071H18Rik | -0.2589741 |
| C230078M08Rik | -0.6489605 |
| C230078M14Rik | -0.6286624 |
| C230090D14 | -0.1819018 |
| C230093N12Rik | 0.77310903 |
| C230096C10Rik | -0.447169 |
| C2cd3 | 0.69640634 |
| C3 | -0.5481519 |
| C330001K17Rik | 1.17028905 |
| C330005M16Rik | -0.672955 |
| C330016O10Rik | -0.6992624 |
| C330019G07Rik | -0.3990681 |
| C330023M02Rik | -0.7419445 |
| C330027C09Rik | -0.4327206 |
| C430003P19Rik | -0.7839667 |
| C430004E15Rik | -0.0026179 |
| C4b | 0.34268366 |
| C530028I08Rik | 2.61684571 |
| C530028I08Rik | -0.2819401 |
| C530028O21Rik | 0.43071179 |
| C530043G21Rik | -0.5667227 |
| C530044N13Rik | 3.62414935 |
| C6 | 0.37650135 |
| C630004H02Rik | -0.4440086 |
| C630004H02Rik | -0.2793011 |
| C630007B19Rik | -0.3082133 |
| C630028N24Rik | 0.90927246 |
| C630035N08Rik | -0.4215702 |
| C630035N08Rik | -0.8146692 |
| C730025P13Rik | -0.7143996 |
| C730027P07Rik | 0.03515235 |
| C78339 | 0.03589955 |
| C79127 | 3.17831981 |
| C79267 | 0.4663911 |
| C79407 | -0.8175121 |
| C79407 | 3.07633127 |
| C80008 | 1.42995408 |
| C80913 | 0.19834547 |
| C85492 | 1.16334533 |
| C85492 | -0.7323821 |
| C85492 | -0.4926009 |
| C85627 | -0.1823757 |
| C87436 | 3.18834189 |
| C87436 | 1.871613 |
| C87499 | -0.7867157 |
| C8b | 1.88936779 |
| C8g | 2.00476194 |
| C9 | 2.66082892 |
| C9 | 1.05649263 |
| C920005C14Rik | 3.92197455 |
| Cab39l | 0.33490603 |
| Cabc1 | 0.63836085 |
| Cables2 | -0.6232736 |
| Cabp2 | -0.257429 |
| Cabp7 | 0.79487077 |
| Cabyr | 2.27043426 |
| Cabyr | 1.88150272 |
| Cachd1 | 1.32823244 |
| Cacna1a | -0.6552978 |
| Cacna1b | -0.3942747 |
| Cacna1b | 0.09026838 |
| Cacna1b | 0.52183151 |
| Cacna1d | -0.5327273 |
| Cacna1g | -0.6584829 |
| Cacna1h | 2.34454491 |
| Cacna2d1 | 0.3754713 |
| Cacna2d3 | -0.6088048 |
| Cacnb1 | -0.6481714 |
| Cacnb1 | -0.5060068 |
| Cacnb2 | 2.69320867 |
| Cacnb2 | 0.29892448 |
| Cacnb3 | -0.4813582 |
| Cacnb3 | -0.4459035 |
| Cacnb3 | 2.04081683 |
| Cacnb3 | 0.98445102 |
| Cacnb4 | 1.25852323 |
| Cacnb4 | 2.59316809 |
| Cacng2 | 1.02143511 |
| Cacng3 | -0.5960502 |
| Cacng3 | -0.3274527 |
| Cacng4 | 1.10940092 |
| Cacng5 | -0.5348154 |
| Cacng5 | -0.1991202 |
| Cacng5 | -0.469603 |
| Cacng7 | 1.8801604 |
| Cacng8 | -0.4258904 |
| Cacybp | -0.235565 |
| Cad | -0.3347031 |
| Cadm2 | -0.5768808 |
| Cadm3 | -0.4592791 |
| Cadps | -0.3706773 |
| Cadps2 | 2.29781954 |
| Cadps2 | 0.37521361 |
| Cage1 | -0.3972676 |
| Calb2 | 1.22697047 |
| Calb2 | -0.6632816 |
| Calca | 1.07009974 |
| Calca | 1.36373215 |
| Calcoco1 | -0.7578109 |
| Cald1 | 1.84599761 |
| Cald1 | -0.6664947 |
| Calm2 | 2.28230795 |
| Calm3 | -0.5080347 |
| Calml4 | -0.6387551 |
| Caln1 | 0.06890696 |
| Calr | -0.4333441 |
| Calr3 | -0.6450219 |
| Calr3 | -0.0254925 |
| Calr3 | -0.3408833 |
| Calr4 | 1.6411373 |
| Calu | -0.174822 |
| Calu | -0.0840357 |
| Calu | 1.27756087 |
| Caly | -0.6171536 |
| Camk1 | 2.1222721 |
| Camk1d | 1.0567374 |
| Camk1g | 0.43482606 |
| Camk1g | 1.5413011 |
| Camk2a | -0.6672998 |
| Camk2a | 0.48326428 |
| Camk2a | 0.46732376 |
| Camk2b | -0.4228025 |
| Camk2d | -0.1516107 |
| Camk2d | 0.9786056 |
| Camk2d | -0.5864153 |
| Camk2g | 1.01402872 |
| Camk2g | 1.091553 |
| Camk2n1 | 0.96084184 |
| Camk4 | 0.35856843 |
| Camkk1 | 1.09637002 |
| Camkk2 | 2.16756469 |
| Camkk2 | 1.46083902 |
| Camkv | 0.41208892 |
| Caml | 0.03253236 |
| Camp | 2.02998405 |
| Camsap1l1 | 2.26050797 |
| Camta2 | -0.1415814 |
| Cant1 | -0.3966683 |
| Cant1 | -0.518927 |
| Cap1 | -0.2766697 |
| Capg | -0.5155092 |
| Capg | -0.6876597 |
| Capn1 | -0.0542959 |
| Capn10 | -0.6080496 |
| Capn11 | 0.10389056 |
| Capn2 | 2.26884156 |
| Capn2 | -0.5886297 |
| Capn2 | 0.2290923 |
| Capn3 | 0.28024583 |
| Capn5 | 0.1429167 |
| Capn6 | -0.5019644 |
| Capn7 | -0.5827364 |
| Capn8 | 1.02701402 |
| Capns1 | -0.6442362 |
| Caprin1 | -0.6942718 |
| Caprin1 | -0.5334228 |
| Capsl | -0.5299505 |
| Capza1 | 1.48743504 |
| Capza3 | 0.17970805 |
| Capzb | -0.5073583 |
| Capzb | 1.47628601 |
| Capzb | 1.30930261 |
| Car11 | -0.2625894 |
| Car12 | 0.29752271 |
| Car13 | 1.31670059 |
| Car14 | 0.43265022 |
| Car15 | 0.18986357 |
| Car2 | -0.4396023 |
| Car3 | 0.45038866 |
| Car4 | 0.3389372 |
| Car5b | -0.4886171 |
| Car5b | -0.358528 |
| Car5b | -0.0124076 |
| Car7 | -0.4402304 |
| Car9 | 2.30143697 |
| Card10 | -0.2161768 |
| Card11 | -0.4383472 |
| Carhsp1 | 2.77503761 |
| Carm1 | 0.31860136 |
| Cart | 2.62415552 |
| Cartpt | 0.94742686 |
| Casc1 | 1.80970391 |
| Casc1 | 2.30703926 |
| Casc1 | 4.34604826 |
| Casc3 | -0.3120289 |
| Casc4 | 0.03328169 |
| Casc4 | -0.5348154 |
| Casc4 | -0.3158606 |
| Cask | -0.7000968 |
| Caskin1 | -0.5509845 |
| Caskin1 | 1.76753233 |
| Caskin1 | -0.5432158 |
| Caskin2 | -0.766746 |
| Casp1 | -0.1938246 |
| Casp2 | 1.89530818 |
| Casp4 | -0.6959323 |
| Casp4 | -0.3706773 |
| Casp4 | 0.40438255 |
| Casp6 | -0.6020302 |
| Casp6 | -0.4172704 |
| Casp7 | 0.2739339 |
| Casp8 | 1.39714694 |
| Casp8 | -0.709326 |
| Casp9 | 0.79340335 |
| Casq2 | -0.4093383 |
| Cass4 | 0.1813023 |
| Cast | -0.7542612 |
| Cat | 0.13758565 |
| Catsper1 | 3.69657708 |
| Catsper2 | -0.5502755 |
| Catsper3 | -0.6395361 |
| Catsper4 | 1.41336614 |
| Cav1 | -0.6851925 |
| Cav2 | 0.91984992 |
| Cbara1 | 2.00506581 |
| Cbfa2t3h | -0.3539419 |
| Cbfa2t3h | -0.1947851 |
| Cbfb | -0.7220632 |
| Cbfb | -0.2672588 |
| Cbfb | 0.3169548 |
| Cbl | 0.66859225 |
| Cblb | -0.7587005 |
| Cblc | -0.250253 |
| Cbln1 | 0.57803527 |
| Cbln2 | 0.47682911 |
| Cbln4 | 2.38270163 |
| Cbr1 | 0.24616647 |
| Cbr2 | -0.6600797 |
| Cbr3 | 0.98815149 |
| Cbr3 | -0.291504 |
| Cbs | -0.7604823 |
| Cbs | 1.41667484 |
| Cbwd1 | 1.3582038 |
| Cbx2 | 1.45965809 |
| Cbx5 | 0.76892854 |
| Cbx7 | 0.54138224 |
| Cbx8 | 0.239913 |
| Cby1 | -0.4148224 |
| Cc2d1a | -0.0709317 |
| Cc2d1b | -0.7658485 |
| Cc2d2a | 1.70645554 |
| Ccar1 | -0.5842062 |
| Ccbe1 | -0.6156299 |
| Ccbl1 | 1.5374019 |
| Ccbl1 | 1.11973933 |
| Ccbl2 | -0.1077568 |
| Ccbp2 | 0.36562758 |
| Ccdc100 | 0.14424462 |
| Ccdc101 | -0.5418104 |
| Ccdc108 | 0.39837143 |
| Ccdc109a | 0.17906957 |
| Ccdc109a | 1.45855452 |
| Ccdc109b | 1.81265455 |
| Ccdc11 | -0.7177977 |
| Ccdc110 | 0.11833727 |
| Ccdc111 | -0.5717877 |
| Ccdc113 | -0.709326 |
| Ccdc114 | 0.35778071 |
| Ccdc115 | 0.23541991 |
| Ccdc116 | -0.4702521 |
| Ccdc117 | -0.211271 |
| Ccdc12 | -0.7152479 |
| Ccdc120 | 2.21996414 |
| Ccdc123 | -0.5053318 |
| Ccdc124 | 0.48623672 |
| Ccdc125 | 0.19395727 |
| Ccdc126 | -0.5827364 |
| Ccdc127 | 0.22697333 |
| Ccdc127 | 2.89986817 |
| Ccdc13 | -0.4528854 |
| Ccdc130 | 0.09658075 |
| Ccdc131 | 0.47659844 |
| Ccdc132 | 1.28331041 |
| Ccdc134 | -0.3147642 |
| Ccdc137 | 2.71619953 |
| Ccdc137 | -0.5155092 |
| Ccdc18 | -0.4592791 |
| Ccdc21 | 2.53440068 |
| Ccdc23 | -0.3666096 |
| Ccdc25 | 0.16588161 |
| Ccdc27 | -0.6271195 |
| Ccdc27 | 0.56639836 |
| Ccdc27 | -0.7658485 |
| Ccdc28a | -0.0555332 |
| Ccdc28b | -0.2000863 |
| Ccdc28b | -0.8260942 |
| Ccdc3 | 1.01914298 |
| Ccdc32 | 0.25326135 |
| Ccdc38 | -0.6340831 |
| Ccdc39 | 0.24616647 |
| Ccdc40 | -0.4478024 |
| Ccdc41 | 2.81043275 |
| Ccdc42 | -0.563122 |
| Ccdc43 | 0.53469516 |
| Ccdc45 | -0.3313496 |
| Ccdc46 | 0.51654665 |
| Ccdc46 | -0.495934 |
| Ccdc47 | 2.02566569 |
| Ccdc49 | -0.4748089 |
| Ccdc5 | -0.5251114 |
| Ccdc50 | -0.5710624 |
| Ccdc51 | 2.76495449 |
| Ccdc53 | 2.38206945 |
| Ccdc53 | -0.2990124 |
| Ccdc53 | 0.54930014 |
| Ccdc54 | 0.66934006 |
| Ccdc56 | 0.1193605 |
| Ccdc57 | 0.3910549 |
| Ccdc58 | -0.4528854 |
| Ccdc59 | -0.7885529 |
| Ccdc59 | -0.2688206 |
| Ccdc64 | -0.4497052 |
| Ccdc65 | 2.08906721 |
| Ccdc65 | 0.65654349 |
| Ccdc66 | 0.67900631 |
| Ccdc67 | -0.7748628 |
| Ccdc68 | 0.11697118 |
| Ccdc70 | -0.6608791 |
| Ccdc71 | -0.6340831 |
| Ccdc73 | -0.4203397 |
| Ccdc77 | 1.42881407 |
| Ccdc79 | 2.29640013 |
| Ccdc80 | -0.7507252 |
| Ccdc82 | 0.86328 |
| Ccdc84 | 2.03663329 |
| Ccdc85a | -0.6827319 |
| Ccdc85b | 0.41307857 |
| Ccdc86 | 0.99262489 |
| Ccdc87 | -0.6705271 |
| Ccdc88a | -0.9050948 |
| Ccdc88b | 1.21993361 |
| Ccdc88c | -0.3147642 |
| Ccdc90b | 0.69658775 |
| Ccdc91 | 1.02852966 |
| Ccdc92 | -0.7766763 |
| Ccdc93 | -0.6411001 |
| Ccdc94 | 2.90846226 |
| Ccdc95 | 1.09425835 |
| Ccdc96 | 2.96209538 |
| Ccdc97 | 1.27003206 |
| Ccdc98 | 0.64452597 |
| Ccdc99 | 0.34401798 |
| Cchcr1 | -0.8222699 |
| Ccin | 0.08284848 |
| Cck | 0.65673298 |
| Cckbr | -0.5732399 |
| Ccl11 | -0.3936773 |
| Ccl17 | -0.7160971 |
| Ccl19 | -0.8328252 |
| Ccl21b | -0.5776107 |
| Ccl21c | 1.83042402 |
| Ccl25 | -0.5930748 |
| Ccl25 | -0.0151676 |
| Ccl27 | 0.03178241 |
| Ccl27 | 0.88035417 |
| Ccl28 | 0.99890327 |
| Ccl3 | -0.4271285 |
| Ccl4 | 1.37330547 |
| Ccl4 | -0.0139837 |
| Ccl5 | 2.56071599 |
| Ccl6 | -0.8813824 |
| Ccl7 | -0.5516941 |
| Ccl9 | -0.5006208 |
| Ccm2 | -0.3006294 |
| Ccna1 | 1.58560483 |
| Ccnb1 | 2.46371825 |
| Ccnb1 | -0.6278906 |
| Ccnb3 | 0.22211147 |
| Ccnc | -0.3153122 |
| Ccnd1 | -0.5893691 |
| Ccnd1 | 0.00049286 |
| Ccnd1 | 1.25636141 |
| Ccnd2 | -0.6348601 |
| Ccnd3 | -0.4624928 |
| Ccnd3 | -0.8682466 |
| Ccndbp1 | 1.61445773 |
| Ccne1 | -0.2025062 |
| Ccne2 | 1.46703351 |
| Ccnf | -0.563841 |
| Ccng1 | -0.6868366 |
| Ccng1 | -0.4683061 |
| Ccng1 | -0.7454467 |
| Ccng2 | 1.04640038 |
| Ccnk | 0.31585544 |
| Ccnl1 | -0.709326 |
| Ccno | 0.04555795 |
| Ccr4 | 1.32312511 |
| Ccr5 | -0.4289887 |
| Ccr5 | -0.5886297 |
| Ccr5 | 1.09613563 |
| Ccr6 | 1.30976674 |
| Ccr6 | 1.95845531 |
| Ccrk | 1.18572239 |
| Ccrk | 2.15483006 |
| Ccrk | -0.1566669 |
| Ccrk | 2.15449835 |
| Ccrl2 | -0.7977948 |
| Ccrn4l | 0.03888224 |
| Ccrn4l | 0.59582547 |
| Ccs | 2.07767215 |
| Cct2 | -0.4283682 |
| Cct3 | 1.29338045 |
| Cct3 | -0.7757691 |
| Cct4 | -0.1776474 |
| Cct5 | -0.469603 |
| Cct6a | -0.4715517 |
| Cct6b | -0.766746 |
| Cct7 | 0.05181838 |
| Cct8 | -0.3978674 |
| Cd109 | 0.16296107 |
| Cd109 | 2.8892296 |
| Cd14 | 0.2816743 |
| Cd151 | -0.01754 |
| Cd151 | -0.2132302 |
| Cd151 | -0.9113831 |
| Cd160 | -0.0403896 |
| Cd163 | -0.8052561 |
| Cd164 | -0.6371951 |
| Cd177 | 0.97111153 |
| Cd180 | 2.07280811 |
| Cd1d1 | -0.261555 |
| Cd2 | 1.06744284 |
| Cd200 | -0.7323821 |
| Cd200 | -0.6513318 |
| Cd226 | -0.7480822 |
| Cd247 | -0.1384132 |
| Cd248 | 1.08990233 |
| Cd248 | -0.5285653 |
| Cd27 | -0.5717877 |
| Cd274 | -0.8005857 |
| Cd276 | -0.6186797 |
| Cd2bp2 | 1.12736055 |
| Cd300lg | -0.2260682 |
| Cd320 | -0.273522 |
| Cd34 | -0.5960502 |
| Cd3d | -0.7384557 |
| Cd3eap | 0.91185663 |
| Cd3g | -0.5624036 |
| Cd4 | 0.64510183 |
| Cd40 | 0.14490786 |
| Cd40 | 0.13054117 |
| Cd44 | 2.26643099 |
| Cd44 | -0.709326 |
| Cd44 | -0.5306439 |
| Cd44 | -0.7931622 |
| Cd47 | 0.14025506 |
| Cd52 | 0.23961414 |
| Cd53 | -0.5827364 |
| Cd55 | 2.66790212 |
| Cd59a | -0.5960502 |
| Cd59b | 1.031176 |
| Cd59b | -0.5107457 |
| Cd6 | 0.15512707 |
| Cd6 | -0.1218467 |
| Cd6 | 0.10838659 |
| Cd63 | -0.5216705 |
| Cd63 | 0.10804152 |
| Cd63 | -0.9950175 |
| Cd68 | -0.0968982 |
| Cd74 | -0.2605218 |
| Cd74 | -0.6497503 |
| Cd79b | -0.8366939 |
| Cd79b | 0.22272062 |
| Cd81 | -0.6876597 |
| Cd82 | -0.8033851 |
| Cd83 | -0.9402277 |
| Cd84 | -0.8203638 |
| Cd86 | -0.36603 |
| Cd86 | -0.8052561 |
| Cd86 | -0.5768808 |
| Cd8b1 | 2.20641179 |
| Cd9 | 0.47243646 |
| Cd93 | 0.0616742 |
| Cd96 | 0.58379869 |
| Cd97 | 1.92406571 |
| Cd97 | -0.6271195 |
| Cd97 | -0.7766763 |
| Cd99l2 | -0.2380807 |
| Cdan1 | -0.6042829 |
| Cdc123 | 1.71233171 |
| Cdc14b | -0.6697192 |
| Cdc14b | -0.6348601 |
| Cdc14b | 0.54095229 |
| Cdc16 | -0.4421176 |
| Cdc20 | -0.7830523 |
| Cdc20 | 1.77548788 |
| Cdc20 | 1.03757113 |
| Cdc20 | 0.13557847 |
| Cdc23 | -0.6340831 |
| Cdc25a | -0.2646618 |
| Cdc25b | -0.672145 |
| Cdc25c | -0.2719522 |
| Cdc25c | -0.8592619 |
| Cdc27 | 1.36460661 |
| Cdc2a | 0.810859 |
| Cdc2l1 | -0.7524915 |
| Cdc2l1 | -0.7401985 |
| Cdc2l5 | 3.52258299 |
| Cdc2l6 | 2.55652091 |
| Cdc34 | -0.1904705 |
| Cdc37 | 0.84834313 |
| Cdc37l1 | 1.45531496 |
| Cdc37l1 | 1.84749408 |
| Cdc37l1 | -0.491271 |
| Cdc37l1 | -0.0742954 |
| Cdc42 | 2.02964609 |
| Cdc42bpb | 1.34240104 |
| Cdc42ep1 | 0.4841799 |
| Cdc42ep2 | -0.4490705 |
| Cdc42ep3 | -0.6141088 |
| Cdc42ep4 | 2.9780735 |
| Cdc42ep5 | -0.8033851 |
| Cdc42se1 | 0.23030094 |
| Cdc42se1 | -0.3835805 |
| Cdc45l | 0.2770953 |
| Cdc5l | 0.54073724 |
| Cdc5l | 1.7236769 |
| Cdc5l | -0.563122 |
| Cdc5l | 0.77859818 |
| Cdc6 | 0.72638949 |
| Cdc7 | 0.45866016 |
| Cdc73 | 0.93562998 |
| Cdca2 | -0.4807012 |
| Cdca3 | -0.222594 |
| Cdca3 | -0.10732 |
| Cdca4 | -0.5306439 |
| Cdca4 | 0.99196841 |
| Cdca4 | 0.99275613 |
| Cdca5 | 3.90679302 |
| Cdca7 | 0.10354379 |
| Cdca8 | 0.1251373 |
| Cdcp1 | -0.5216705 |
| Cdgap | -0.6442362 |
| Cdh1 | -0.7578109 |
| Cdh11 | -0.7349802 |
| Cdh11 | 0.02387059 |
| Cdh12 | 1.52867704 |
| Cdh13 | 1.30632659 |
| Cdh13 | 0.12310259 |
| Cdh16 | -0.2073651 |
| Cdh22 | -0.7401985 |
| Cdh23 | -0.5481519 |
| Cdh23 | 0.2339173 |
| Cdh23 | -0.6909598 |
| Cdh24 | 0.94438811 |
| Cdh26 | 0.26670799 |
| Cdh3 | 0.88050255 |
| Cdh5 | -0.0311008 |
| Cdh6 | -0.5960502 |
| Cdh7 | -0.716947 |
| Cdh8 | 0.35330409 |
| Cdh8 | -0.7289295 |
| Cdh8 | -0.388318 |
| Cdipt | 0.00126889 |
| Cdipt | -0.3806319 |
| Cdk10 | -0.6705271 |
| Cdk10 | 0.40388313 |
| Cdk10 | -0.6529161 |
| Cdk2 | 0.88715453 |
| Cdk2 | 1.45737065 |
| Cdk2 | 0.94507962 |
| Cdk2 | -0.2909701 |
| Cdk2ap2 | -0.518927 |
| Cdk3 | 0.81133916 |
| Cdk5 | -0.6403178 |
| Cdk5r1 | -0.0861669 |
| Cdk5rap1 | -0.6489605 |
| Cdk5rap2 | 2.17620906 |
| Cdk5rap3 | 1.76747605 |
| Cdk6 | -0.563841 |
| Cdk8 | -0.7186492 |
| Cdk9 | -0.6497503 |
| Cdkal1 | 0.56660747 |
| Cdkl1 | -0.5842062 |
| Cdkl2 | -0.1809547 |
| Cdkl2 | -0.2963237 |
| Cdkl2 | -0.6835513 |
| Cdkl2 | 0.93730468 |
| Cdkl3 | 1.48130066 |
| Cdkl4 | 1.27631037 |
| Cdkn1a | 0.59501507 |
| Cdkn1a | 1.45246097 |
| Cdkn1a | -0.38299 |
| Cdkn1b | -0.1580508 |
| Cdkn1b | -0.5087117 |
| Cdkn1c | 0.14025506 |
| Cdkn2a | 1.60892011 |
| Cdkn2a | 0.1652334 |
| Cdkn2aipnl | 0.24646319 |
| Cdkn2b | -0.5121042 |
| Cdkn2c | -0.0262916 |
| Cdkn2c | -0.0904443 |
| Cdkn2d | 1.36119151 |
| Cdo1 | 1.09343583 |
| Cdr2 | 0.39232055 |
| Cdrt4 | -0.6179163 |
| Cds1 | 1.32486115 |
| Cds2 | -0.2963237 |
| Cdsn | 0.77677212 |
| Cdt1 | 1.53130325 |
| Cdv3 | -0.450976 |
| Cdv3 | 3.02913037 |
| Cdyl | 0.04998152 |
| Cdyl | 0.82720189 |
| Cdyl | -0.2137206 |
| Cdyl2 | -0.605035 |
| Ceacam1 | 0.42755305 |
| Ceacam13 | -0.0700927 |
| Ceacam2 | -0.605035 |
| Cebpa | 0.63797415 |
| Cebpb | -0.5271822 |
| Cecr5 | 1.03105016 |
| Cecr6 | 0.16717664 |
| Celsr1 | -0.3324661 |
| Celsr2 | -0.0037871 |
| Celsr3 | 1.38756545 |
| Cend1 | 0.03739211 |
| Cenpa | 1.62714066 |
| Cenpb | -0.3230193 |
| Cenph | 0.70075028 |
| Cenpi | -0.7297915 |
| Cenpj | -0.4358422 |
| Cenpk | 0.54245624 |
| Cenpl | 1.35732321 |
| Cenpl | -0.5060068 |
| Cenpm | -0.1890368 |
| Cenpm | -0.6194438 |
| Cenpm | -0.4939326 |
| Cenpn | 1.75089506 |
| Cenpp | -0.8015179 |
| Cenpq | 1.03406561 |
| Cenpt | -0.6664947 |
| Centa1 | 0.06131105 |
| Centa1 | 1.19281539 |
| Centa2 | -0.7341133 |
| Centb5 | 2.31764863 |
| Centb5 | 0.13390245 |
| Centd2 | 1.6911698 |
| Centd2 | 0.23090466 |
| Centd3 | 0.15315805 |
| Centg1 | 1.8823965 |
| Centg1 | 0.11765448 |
| Centg2 | -0.6027805 |
| Centg2 | 0.31969742 |
| Centg3 | 0.44347803 |
| Centg3 | -0.4702521 |
| Cep135 | 2.17234908 |
| Cep164 | -0.5746945 |
| Cep170 | 2.08807678 |
| Cep55 | -0.3730098 |
| Cep57 | -0.5488592 |
| Cep57 | -0.2073651 |
| Cep63 | 0.59764624 |
| Cep68 | -0.3847626 |
| Cep70 | 2.44389161 |
| Cep72 | -0.2151935 |
| Cep78 | 1.64449062 |
| Cep97 | 0.27278158 |
| Cept1 | -0.0793643 |
| Cercam | -0.2883049 |
| Ces3 | 0.21967082 |
| Cetn2 | -0.1334566 |
| Cetn3 | 2.55761301 |
| Cetn4 | -0.3202593 |
| Cetn4 | 0.53144185 |
| Cfd | 1.06307847 |
| Cfdp1 | 1.23213706 |
| Cfh | 0.98166582 |
| Cfhr1 | -0.5886297 |
| Cfi | 0.09762859 |
| Cfl1 | 0.36040381 |
| Cfl2 | -0.7507252 |
| Cfl2 | 1.29686953 |
| Cfp | 1.04627662 |
| Cftr | 0.26786798 |
| Cga | 0.68196011 |
| Cggbp1 | 0.11389 |
| Cgnl1 | 2.15486691 |
| Cgrrf1 | 2.34367598 |
| Ch25h | 0.5720275 |
| Chac1 | 1.75592378 |
| Chad | 1.26906233 |
| Chaf1a | 0.54866069 |
| Chaf1b | 1.08160404 |
| Chat | 1.51216463 |
| Chchd1 | 0.59400106 |
| Chchd2 | -0.21274 |
| Chchd4 | -0.5703377 |
| Chchd5 | 0.81787618 |
| Chchd6 | 2.22687015 |
| Chchd7 | -0.2631071 |
| Chchd8 | 0.03328169 |
| Chd1 | 0.940367 |
| Chd1 | -0.6240415 |
| Chd1l | -0.4452714 |
| Chd1l | 0.109421 |
| Chd4 | -0.5066823 |
| Chd4 | 0.6261005 |
| Chd4 | 2.3605172 |
| Chd5 | -0.5790722 |
| Chd8 | 0.6110882 |
| Chdh | 0.1462329 |
| Chdh | 2.79309895 |
| Chek1 | -0.590109 |
| Chek1 | -0.4289887 |
| Chek2 | -0.2385846 |
| Chek2 | 0.06998696 |
| Cherp | -0.3602538 |
| Chfr | -0.7017679 |
| Chfr | -0.7775845 |
| Chfr | -0.6163914 |
| Chga | 1.68626329 |
| Chga | 0.86795461 |
| Chga | 0.10111275 |
| Chgb | -0.7297915 |
| Chi3l1 | 3.3266186 |
| Chi3l3 | 2.10512022 |
| Chi3l3 | 1.41783007 |
| Chia | 2.25968686 |
| Chia | 0.74326403 |
| Chic1 | 4.32947585 |
| Chic2 | -0.0904443 |
| Chic2 | 0.1452393 |
| Chit1 | 1.25152802 |
| Chka | 1.42661146 |
| Chka | -0.672145 |
| Chka | 2.48505277 |
| Chkb | -0.4478024 |
| Chl1 | -0.7613745 |
| Chl1 | -0.5251114 |
| Chl1 | 0.81644522 |
| Chm | -0.6876597 |
| Chmp1b | -0.56889 |
| Chmp2a | 2.24113152 |
| Chmp2a | 1.21931915 |
| Chmp2b | -0.7410711 |
| Chmp4b | -0.1876053 |
| Chmp5 | 0.30060379 |
| Chmp7 | 2.3766782 |
| Chmp7 | -0.7323821 |
| Chmp7 | -0.626349 |
| Chn2 | 0.47729026 |
| Chodl | 1.58009645 |
| Chordc1 | 1.70615473 |
| Chpf | 1.95484012 |
| Chpt1 | -0.7839667 |
| Chpt1 | -0.2315543 |
| Chpt1 | -0.5667227 |
| Chrac1 | 1.52420981 |
| Chrd | 1.88304147 |
| Chrm1 | -0.1443058 |
| Chrm2 | 0.43289223 |
| Chrm3 | 0.75422972 |
| Chrna1 | -0.2899031 |
| Chrna4 | -0.4478024 |
| Chrna4 | 1.00824079 |
| Chrna4 | -0.4111627 |
| Chrna5 | -0.5053318 |
| Chrna6 | -0.7560344 |
| Chrna6 | 0.39206756 |
| Chrna7 | -0.6584829 |
| Chrna9 | -0.1012262 |
| Chrnb1 | 0.28906665 |
| Chrnb1 | 1.42251849 |
| Chrnb2 | -0.7821387 |
| Chrnb2 | -0.5945613 |
| Chrnb3 | -0.4160456 |
| Chrnb3 | -0.7332473 |
| Chrnb3 | 0.64644409 |
| Chrne | -0.1729433 |
| Chst1 | 0.42949818 |
| Chst10 | 1.41112598 |
| Chst12 | 0.92438635 |
| Chst14 | -0.6481714 |
| Chst2 | 1.66756315 |
| Chst3 | 0.53447863 |
| Chst3 | -0.2186397 |
| Chst7 | 1.991599 |
| Chst8 | 1.85718747 |
| Chtf18 | -0.631756 |
| Chuk | -0.2877728 |
| Churc1 | 1.6215475 |
| Ciao1 | 3.14392212 |
| Ciapin1 | 1.86834293 |
| Cib1 | 0.14258442 |
| Cib2 | 2.80017022 |
| Cib2 | -0.412381 |
| Cic | 1.25271407 |
| Cidea | -0.0096559 |
| Cideb | -0.3642933 |
| Cidec | 2.38304627 |
| Ciita | 1.13503115 |
| Cirbp | 3.26942848 |
| Cirh1a | -0.4826737 |
| Cisd1 | -0.6984287 |
| Cish | -0.7118593 |
| Cit | 2.76937012 |
| Cited1 | 3.41495087 |
| Cited2 | -0.3006294 |
| Cited4 | -0.430231 |
| Ciz1 | 0.72022351 |
| Ciz1 | -0.7017679 |
| Ckap2l | -0.4820158 |
| Ckap5 | -0.1790633 |
| Ckb | 1.61518912 |
| Cklf | -0.7059591 |
| Cklf | -0.0827593 |
| Ckmt1 | 0.33759545 |
| Ckmt2 | 0.05218531 |
| Ckmt2 | -0.4748089 |
| Ckt2 | -0.5674446 |
| Ckt2 | 0.9969459 |
| Clca5 | 2.02456167 |
| Clcc1 | -0.1160965 |
| Clcf1 | 0.95018063 |
| Clcf1 | -0.0650747 |
| Clcf1 | 0.61168549 |
| Clcn1 | -0.403283 |
| Clcn2 | -0.5703377 |
| Clcn3 | 1.82610627 |
| Clcn3 | -0.4389745 |
| Clcn3 | 2.66059574 |
| Clcn4-2 | -0.6348601 |
| Clcn7 | -0.5717877 |
| Clcnka | -0.3033307 |
| Cldn1 | -0.6125903 |
| Cldn10 | 1.13469409 |
| Cldn10 | 0.80330346 |
| Cldn10 | 0.59095228 |
| Cldn11 | 1.95648932 |
| Cldn12 | -0.545328 |
| Cldn15 | 0.15709187 |
| Cldn15 | -0.5566762 |
| Cldn17 | -0.6442362 |
| Cldn2 | -0.4728532 |
| Cldn23 | 0.63137507 |
| Cldn3 | -0.2672588 |
| Cldn4 | 1.61112583 |
| Cldn5 | -0.477423 |
| Cldn6 | -0.1603618 |
| Cldn9 | 0.54908704 |
| Cldnd1 | -0.4650719 |
| Cldnd2 | -0.174352 |
| Clec11a | 0.3397413 |
| Clec16a | -0.7950124 |
| Clec1a | -0.7622676 |
| Clec2d | 1.35308451 |
| Clec2e | -0.4283682 |
| Clec3b | 0.28452458 |
| Clec4a1 | 1.95369309 |
| Clec4a3 | 1.97106473 |
| Clec4b1 | 0.48349327 |
| Clec4b1 | 1.79911084 |
| Clec4d | -0.6020302 |
| Clec4g | 0.25149278 |
| Clec4n | 0.6608918 |
| Clec7a | 1.3409671 |
| Clecsf12 | -0.4787329 |
| Clgn | 0.30701271 |
| Clic1 | 1.50671833 |
| Clic3 | -0.5871528 |
| Clic4 | 0.12242335 |
| Clic6 | 2.397377 |
| Clip1 | 0.01969884 |
| Clip1 | 2.94574012 |
| Clip4 | -0.7306542 |
| Clk1 | 0.77843232 |
| Clk2 | 1.50169097 |
| Clk2 | -0.4446398 |
| Clk2 | -0.764952 |
| Clk3 | 1.01646156 |
| Clk4 | 1.17718047 |
| Clk4 | -0.7463243 |
| Clk4 | -0.7950124 |
| Clm3 | 0.52577522 |
| Clm3 | 1.30455499 |
| Clmn | -0.6209738 |
| Clmn | -0.6934427 |
| Cln3 | -0.5761514 |
| Cln5 | 0.2947127 |
| Cln6 | 0.95539007 |
| Cln6 | -0.5053318 |
| Cln8 | -0.8251366 |
| Clns1a | -0.4945993 |
| Clns1a | -0.27457 |
| Clns1a | 1.39554289 |
| Clock | -0.1996031 |
| Clock | -0.2487226 |
| Clp1 | -0.3516574 |
| Clpb | 1.72885704 |
| Clpp | -0.0717714 |
| Clptm1 | 1.59625172 |
| Clptm1l | -0.7604823 |
| Clpx | 1.83079166 |
| Clpx | 0.1544712 |
| Clrn1 | 0.60188068 |
| Clspn | -0.656093 |
| Clspn | 0.60689626 |
| Clspn | 1.15623875 |
| Clstn1 | 2.77183285 |
| Clstn3 | -0.7766763 |
| Clstn3 | 0.47266818 |
| Clta | -0.1488646 |
| Cltb | 0.12784322 |
| Cluap1 | 1.10882312 |
| Cma1 | 1.92026415 |
| Cma1 | 0.38470024 |
| Cmas | 0.28793324 |
| Cmbl | -0.4637815 |
| Cml1 | -0.656889 |
| Cml3 | -0.5046573 |
| Cml4 | 1.73429981 |
| Cmpk | -0.6240415 |
| Cmtm2a | 2.86605094 |
| Cmtm2b | 0.74222922 |
| Cmtm4 | -0.7410711 |
| Cmtm4 | -0.7894729 |
| Cmtm5 | -0.4573563 |
| Cmtm6 | -0.5531148 |
| Cmtm7 | 4.18989819 |
| Cmtm7 | 0.51897267 |
| Cmtm7 | 3.46129623 |
| Cmtm8 | -0.8137235 |
| Cmtm8 | 1.22238736 |
| Cmya1 | -0.5820024 |
| CN716893 | 1.59814965 |
| Cnbp2 | 1.77576675 |
| Cnbp2 | -0.3142165 |
| Cndp1 | 1.25665651 |
| Cnga4 | -0.4148224 |
| Cnih | -0.3087574 |
| Cnih3 | 0.08426644 |
| Cnih4 | 1.39156286 |
| Cnksr2 | 1.41956018 |
| Cnksr2 | -0.3098466 |
| Cnksr3 | -0.1659322 |
| Cnn1 | 1.69653675 |
| Cnn2 | -0.7289295 |
| Cnn2 | 0.71703618 |
| Cnnm1 | 2.07365414 |
| Cnnm1 | -0.2777214 |
| Cnnm2 | -0.4327206 |
| Cnnm3 | -0.6020302 |
| Cno | -0.6232736 |
| Cnot10 | 0.03664613 |
| Cnot2 | 0.0547497 |
| Cnot2 | 1.04392188 |
| Cnot2 | 0.87633899 |
| Cnot2 | -0.3414472 |
| Cnot3 | 1.16899111 |
| Cnot3 | 1.34766933 |
| Cnot4 | -0.7775845 |
| Cnot4 | 1.60516485 |
| Cnot4 | -0.3044134 |
| Cnot6l | -0.2325553 |
| Cnot6l | -0.5864153 |
| Cnot7 | -0.7110141 |
| Cnp | 0.14092119 |
| Cnp | -0.1231788 |
| Cnpy2 | 2.4391507 |
| Cnpy3 | -0.6975958 |
| Cnr1 | -0.3109371 |
| Cnr1 | -0.7297915 |
| Cntd1 | -0.025892 |
| Cntd1 | 3.70444213 |
| Cntfr | -0.3324661 |
| Cntln | -0.56889 |
| Cntn1 | 1.4907915 |
| Cntn4 | 0.75047507 |
| Cntn6 | 0.98046956 |
| Cntnap1 | -0.4433778 |
| Cntnap2 | 1.06027919 |
| Cntnap4 | 2.67214087 |
| Cntnap4 | 1.33186315 |
| Coasy | 0.31337705 |
| Cobl | 1.43547115 |
| Cobll1 | -0.8642425 |
| Cobll1 | -0.100359 |
| Coch | -0.2693418 |
| Cog1 | 2.23196486 |
| Cog2 | -0.4044909 |
| Cog3 | -0.7118593 |
| Cog4 | 0.01817711 |
| Cog6 | 1.4721767 |
| Cog6 | -0.5404071 |
| Cog8 | -0.235565 |
| Coil | 2.98552075 |
| Coil | -0.1885594 |
| Col11a1 | 2.03801597 |
| Col12a1 | 1.21819156 |
| Col14a1 | 2.92937169 |
| Col15a1 | -0.4879548 |
| Col16a1 | 2.25040643 |
| Col17a1 | -0.3213623 |
| Col17a1 | -0.5134647 |
| Col17a1 | 3.19662827 |
| Col18a1 | 0.60087426 |
| Col18a1 | -0.4813582 |
| Col1a1 | -0.7516079 |
| Col1a2 | 0.46895363 |
| Col24a1 | 0.79127957 |
| Col24a1 | -0.7101696 |
| Col25a1 | 1.47574433 |
| Col25a1 | 1.81120725 |
| Col25a1 | 1.76358497 |
| Col27a1 | -0.7569222 |
| Col28a1 | -0.1539056 |
| Col2a1 | -0.8033851 |
| Col2a1 | -0.5516941 |
| Col3a1 | 1.02574907 |
| Col3a1 | -0.1543652 |
| Col4a1 | 0.16068307 |
| Col4a2 | 0.12953037 |
| Col4a3 | -0.4787329 |
| Col4a3bp | 1.77347752 |
| Col4a3bp | -0.7560344 |
| Col4a3bp | 0.08284848 |
| Col4a4 | 1.91931129 |
| Col4a4 | 0.58338822 |
| Col4a5 | -0.1785911 |
| Col4a6 | 1.47357439 |
| Col4a6 | -0.5397062 |
| Col5a1 | -0.4414881 |
| Col5a3 | 0.69984699 |
| Col6a1 | 2.51459682 |
| Col6a1 | 1.05808245 |
| Col6a1 | 1.36809608 |
| Col6a2 | -0.4631369 |
| Col7a1 | -0.5967956 |
| Col8a2 | -0.3800433 |
| Col9a2 | -0.1370587 |
| Colec12 | 0.39156137 |
| Commd1 | 0.59724194 |
| Commd1 | -0.6255792 |
| Commd10 | -0.2220988 |
| Commd2 | -0.5258011 |
| Commd2 | -0.5012923 |
| Commd4 | -0.0588407 |
| Commd5 | -0.668912 |
| Commd6 | 0.95045556 |
| Commd8 | -0.553826 |
| Commd9 | -0.5997832 |
| Comp | -0.6333067 |
| Comtd1 | 0.07429425 |
| Copa | -0.5209838 |
| Copb2 | -0.3648718 |
| Cope | -0.6411001 |
| Cope | -0.6403178 |
| Cope | -0.6624801 |
| Cope | 0.72866916 |
| Copg | 0.03515235 |
| Copg | -0.8632442 |
| Copg | -0.5369083 |
| Copg | 0.45677611 |
| Cops2 | -0.5411084 |
| Cops3 | -0.7393267 |
| Cops4 | -0.6042829 |
| Cops4 | 2.05543365 |
| Cops5 | 1.84568769 |
| Cops5 | 0.60749629 |
| Cops6 | 1.08505104 |
| Cops7a | -0.0407956 |
| Cops7a | -0.7358478 |
| Cops7a | -0.2360676 |
| Cops7b | -0.7358478 |
| Cops7b | 3.53501839 |
| Cops8 | 1.55850334 |
| Copz1 | 0.16166005 |
| Copz2 | -0.5967956 |
| Copz2 | -0.4172704 |
| Copz2 | 3.88398598 |
| Copz2 | 0.48211845 |
| Coq10b | 0.77610719 |
| Coq10b | 0.65274548 |
| Coq2 | -0.01754 |
| Coq3 | 2.35286274 |
| Coq3 | -0.6951017 |
| Coq4 | -0.2451615 |
| Coq4 | 2.1505825 |
| Coq5 | 1.11251472 |
| Coq5 | -0.5278735 |
| Coq5 | 2.19290315 |
| Coq6 | 0.28651451 |
| Coq6 | 0.1729817 |
| Coq7 | -0.4754617 |
| Coq9 | -0.6513318 |
| Corin | 0.24349164 |
| Coro1a | 1.93920924 |
| Coro1a | -0.5827364 |
| Coro1a | -0.4631369 |
| Coro1a | 2.81828236 |
| Coro1b | -0.6364161 |
| Coro1b | -0.7358478 |
| Coro1c | 0.66821812 |
| Coro2a | 1.2762141 |
| Coro2a | -0.1715368 |
| Coro2b | -0.0147728 |
| Coro2b | 1.80312469 |
| Coro6 | 2.2169392 |
| Coro7 | 2.01692451 |
| Cort | -0.5827364 |
| Cort | -0.3666096 |
| Cotl1 | -0.6884836 |
| Cova1 | -0.4879548 |
| Cox10 | -0.4926009 |
| Cox11 | 1.68736955 |
| Cox15 | -0.6156299 |
| Cox17 | -0.5100672 |
| Cox18 | -0.7254899 |
| Cox18 | -0.5573902 |
| Cox19 | 2.25823989 |
| Cox4i1 | 1.00020586 |
| Cox4nb | -0.8533207 |
| Cox4nb | 0.81612692 |
| Cox5a | -0.3671896 |
| Cox5b | 1.91309337 |
| Cox6a1 | -0.174822 |
| Cox6a1 | -0.8184617 |
| Cox6a2 | 3.22351351 |
| Cox6b1 | -0.4172704 |
| Cox6b1 | 1.76239743 |
| Cox6b2 | 0.18923214 |
| Cox6b2 | -0.6901337 |
| Cox6c | 0.57265082 |
| Cox7a1 | 0.21261745 |
| Cox7a2 | -0.5761514 |
| Cox7a2l | 0.33947335 |
| Cox7b | -0.6125903 |
| Cox7b2 | -0.6411001 |
| Cox8b | -0.5019644 |
| Cox8c | 1.20642302 |
| Cp | -0.6458083 |
| Cpa1 | -0.2925729 |
| Cpa2 | -0.0977622 |
| Cpa2 | 2.44601167 |
| Cpa3 | 0.15839939 |
| Cpa3 | 0.23150798 |
| Cpa3 | -0.5886297 |
| Cpa4 | 1.37425731 |
| Cpa5 | -0.7739574 |
| Cpa6 | -0.6042829 |
| Cpb2 | -0.0469074 |
| Cpeb1 | 2.56576244 |
| Cpeb2 | 0.22545677 |
| Cpeb3 | -0.3033307 |
| Cpeb4 | 1.4765954 |
| Cphx | 2.51018435 |
| Cplx1 | -0.3533702 |
| Cplx2 | 0.87275509 |
| Cpn1 | 0.33004506 |
| Cpne2 | -0.6080496 |
| Cpne4 | 2.08728366 |
| Cpne5 | -0.6819131 |
| Cpne6 | 0.41233643 |
| Cpne7 | 2.28461315 |
| Cpne8 | 2.22475602 |
| Cpne8 | -0.3591029 |
| Cpne9 | -0.2963237 |
| Cpsf1 | 1.22401954 |
| Cpsf1 | -0.4807012 |
| Cpsf2 | 0.06203719 |
| Cpsf3l | 0.80427147 |
| Cpsf4 | 1.46726792 |
| Cpsf4l | 0.23451864 |
| Cpsf4l | -0.6745773 |
| Cpsf6 | -0.0135894 |
| Cpsf6 | -0.6810951 |
| Cpt1b | 2.08139313 |
| Cpt1b | -0.4117716 |
| Cpt1c | -0.1800085 |
| Cpt2 | -0.0469074 |
| Cpxcr1 | -0.5754227 |
| Cpxm1 | 0.12208354 |
| Cpxm2 | -0.6403178 |
| Cpz | -0.6465953 |
| Crabp2 | -0.5320323 |
| Crat | 1.86308639 |
| Crat | -0.2117604 |
| Crat | -0.7367163 |
| Crb1 | 1.235364 |
| Crbn | -0.4020768 |
| Crbn | -0.0033972 |
| Crbn | 4.17133959 |
| Crcp | -0.36603 |
| Creb1 | -0.6584829 |
| Creb1 | -0.6909598 |
| Creb3 | 2.01688166 |
| Creb3l1 | -0.6005316 |
| Creb3l2 | 0.08391216 |
| Creb3l2 | 3.39546514 |
| Creb3l3 | -0.6171536 |
| Creb3l4 | 1.77966201 |
| Creb5 | 1.35237614 |
| Crebl1 | -0.0367436 |
| Crebl2 | 1.10916984 |
| Creg2 | 2.55521343 |
| Creld1 | -0.7152479 |
| Creld2 | 0.32461353 |
| Creld2 | 0.41283126 |
| Crem | 0.86644928 |
| Crem | 2.31117976 |
| Crem | 1.21593219 |
| Crem | 1.03306156 |
| Crhbp | -0.3545139 |
| Crhr1 | -0.7254899 |
| Crim1 | 0.9329721 |
| Crim2 | -0.0654918 |
| Crip2 | -0.7498434 |
| Crip3 | 1.33023111 |
| Crip3 | 1.27890567 |
| Cript | -0.7794034 |
| Cript | -0.2941785 |
| Crisp2 | -0.5923325 |
| Crispld2 | -0.30009 |
| Crk | -0.1952658 |
| Crk | 3.04892543 |
| Crk | -0.7220632 |
| Crkl | 1.21582936 |
| Crkrs | 0.47659844 |
| Crkrs | 4.3984041 |
| Crlf3 | 1.04056521 |
| Crlf3 | -0.4446398 |
| Crls1 | -0.6309816 |
| Crls1 | 0.67418595 |
| Crls1 | -0.7839667 |
| Crls1 | -0.7839667 |
| Crmp1 | 1.46217556 |
| Crmp1 | 0.27191639 |
| Crnkl1 | -0.672955 |
| Crocc | 0.25267221 |
| Crry | 2.93800749 |
| Crry | 0.51919289 |
| Crtac1 | 1.46999827 |
| Crtam | 1.31854077 |
| Crtap | -0.0857403 |
| Crtap | 0.12072303 |
| Crtc1 | -0.7857984 |
| Crtc3 | -0.1612879 |
| Crxos1 | 0.55206592 |
| Cry2 | 1.64436187 |
| Cryab | -0.152528 |
| Cryba2 | -0.2340589 |
| Cryba4 | -0.6080496 |
| Crybb1 | -0.3448379 |
| Crybb2 | 0.64950473 |
| Crybb3 | -0.6664947 |
| Crygn | -0.7152479 |
| Crygn | -0.7229187 |
| Cryl1 | -0.605035 |
| Crym | 0.85584204 |
| Cryz | 0.40463216 |
| Cryz | 1.6191021 |
| Cryz | 0.4194854 |
| Cryzl1 | -0.5244222 |
| Cryzl1 | -0.1165375 |
| Cs | 2.17786349 |
| Csad | -0.5967956 |
| Csda | -0.5313378 |
| Csda | 1.09143519 |
| Csda | -0.1348057 |
| Csdc2 | 0.88671257 |
| Csde1 | 1.7950793 |
| Csde1 | -0.0112273 |
| Csde1 | 0.75115888 |
| Cse1l | -0.668912 |
| Csf1 | 1.48964863 |
| Csf1r | -0.3724261 |
| Csf1r | 0.12378133 |
| Csf2ra | -0.4728532 |
| Csgalnact1 | -0.6745773 |
| Csgalnact2 | -0.1397697 |
| Csk | 2.21704249 |
| Csl | -0.355659 |
| Csl | -0.2579438 |
| Csmd1 | 0.26902649 |
| Csnk1d | -0.3006294 |
| Csnk1d | 1.2862669 |
| Csnk1d | 1.35758747 |
| Csnk1d | 1.88714867 |
| Csnk1d | -0.1957467 |
| Csnk1e | 0.94949297 |
| Csnk1e | -0.1433968 |
| Csnk1g1 | 0.60469279 |
| Csnk1g1 | -0.5376069 |
| Csnk1g1 | -0.5842062 |
| Csnk1g2 | -0.672955 |
| Csnk1g2 | 1.30043911 |
| Csnk1g2 | 1.11962509 |
| Csnk1g3 | -0.3505173 |
| Csnk2a1 | -0.0819094 |
| Csnk2a2 | 2.32296887 |
| Csnk2a2 | 0.20612959 |
| Csnrp2 | -0.5460332 |
| Csnrp3 | 0.9530632 |
| Cspg4 | -0.1603618 |
| Cspg5 | 1.43732972 |
| Cspp1 | 0.86373343 |
| Csprs | 0.96002614 |
| Csrp1 | 0.89302708 |
| Csrp2 | -0.6942718 |
| Csrp2bp | 1.02041709 |
| Cst11 | 0.0089932 |
| Cst3 | 1.24068373 |
| Cst8 | 2.36031116 |
| Cst9 | 0.75388903 |
| Cstad | 3.12957167 |
| Cstb | -0.631756 |
| Cstf1 | 3.8276774 |
| Cstf2 | 0.56493329 |
| Cstf3 | -0.4296096 |
| Cstf3 | 0.10631424 |
| Cstl1 | 0.15839939 |
| Ctage5 | 0.10423719 |
| Ctcf | 1.15963493 |
| Ctdp1 | -0.8712613 |
| Ctdp1 | -0.5299505 |
| Ctdp1 | 0.39358445 |
| Ctdsp1 | 1.53457543 |
| Ctdsp1 | 1.93930252 |
| Ctdsp1 | -0.7384557 |
| Ctdsp2 | -0.7349802 |
| Ctdspl | -0.5202977 |
| Ctdspl2 | -0.5313378 |
| Ctdspl2 | 0.07178413 |
| Ctf1 | -0.3476732 |
| Ctgf | -0.4383472 |
| Ctla4 | -0.476115 |
| Ctnna1 | -0.295787 |
| Ctnna3 | -0.2049324 |
| Ctnnal1 | -0.4105541 |
| Ctnnb1 | -0.6371951 |
| Ctnnb1 | 0.16717664 |
| Ctnnb1 | 0.83206401 |
| Ctnnbip1 | -0.1294211 |
| Ctnnbl1 | 0.72428011 |
| Ctnnbl1 | -0.4793885 |
| Ctnnd2 | -0.4345923 |
| Ctnnd2 | 0.09972068 |
| Ctnnd2 | -0.6271195 |
| Ctns | -0.4657179 |
| Ctns | -0.1529869 |
| Ctps | -0.5871528 |
| Ctps | -0.2385846 |
| Ctps | 0.53750537 |
| Ctps2 | -0.6110742 |
| Ctps2 | 1.07766573 |
| Ctps2 | 0.39661072 |
| Ctr9 | -0.5216705 |
| Ctrb1 | -0.5982882 |
| Ctrl | 1.63426339 |
| Ctsa | -0.3197083 |
| Ctsa | -0.346538 |
| Ctsa | -0.4136009 |
| Ctsa | -0.3219143 |
| Ctsb | 1.01786709 |
| Ctsc | 1.75757493 |
| Ctsc | 2.78310274 |
| Ctsc | -0.5237335 |
| Ctse | -0.6876597 |
| Ctsf | 1.2730317 |
| Ctsh | -0.2295555 |
| Ctsk | 0.13825373 |
| Ctsl | -0.7631615 |
| Ctso | 0.50543748 |
| Ctsr | -0.1411281 |
| Ctss | -0.6934427 |
| Ctsw | -0.2211091 |
| Ctsz | -0.115215 |
| Ctsz | -0.2196266 |
| Cttn | -0.6458083 |
| Cttn | 1.20631911 |
| Cttnbp2nl | -0.4172704 |
| Ctxn1 | -0.0776716 |
| Cuedc1 | 0.24170407 |
| Cuedc2 | 0.44943857 |
| Cugbp1 | 1.58807283 |
| Cugbp1 | -0.4663642 |
| Cugbp1 | 1.87291776 |
| Cugbp1 | -0.5923325 |
| Cugbp2 | -0.4308528 |
| Cugbp2 | -0.0994925 |
| Cul1 | 1.62207537 |
| Cul2 | 1.04056521 |
| Cul2 | -0.568167 |
| Cul3 | -0.607295 |
| Cul4a | 0.55651612 |
| Cul4a | -0.0395781 |
| Cul4a | -0.5652807 |
| Cul4b | 1.50821369 |
| Cul5 | -0.0049579 |
| Cul7 | 0.16555756 |
| Cuta | 0.63040058 |
| Cuta | -0.5805361 |
| Cuta | -0.5376069 |
| Cutc | -0.1236232 |
| Cutc | 0.10389056 |
| Cux1 | 0.60929401 |
| Cux1 | -0.3442719 |
| Cux1 | -0.5960502 |
| Cux1 | 2.20089209 |
| Cux1 | 1.68564812 |
| Cux2 | 3.50354386 |
| Cux2 | 0.3490707 |
| Cuzd1 | -0.2973982 |
| Cwf19l2 | 0.33652063 |
| Cx3cl1 | -0.2461775 |
| Cx3cr1 | 2.55988798 |
| Cxadr | 0.59074866 |
| Cxadr | -0.5060068 |
| Cxadr | -0.2729984 |
| Cxcl1 | 1.4598944 |
| Cxcl12 | -0.7551474 |
| Cxcl12 | -0.7560344 |
| Cxcl12 | 1.7408698 |
| Cxcl13 | -0.1165375 |
| Cxcl14 | 0.13222335 |
| Cxcl14 | -0.6868366 |
| Cxcl15 | 0.9685549 |
| Cxcl16 | -0.2260682 |
| Cxcl17 | 1.11699382 |
| Cxcl4 | -0.5509845 |
| Cxcl9 | -0.5754227 |
| Cxcr3 | -0.9009265 |
| Cxcr4 | 0.59663515 |
| Cxcr5 | -0.5732399 |
| Cxcr7 | -0.2814117 |
| Cxx1a | -0.5667227 |
| Cxx1b | -0.4663642 |
| Cxx1c | -0.4586377 |
| Cxxc1 | -0.491271 |
| Cxxc1 | -0.5864153 |
| Cxxc4 | -0.2360676 |
| Cxxc4 | 3.01205066 |
| Cyb5 | 0.53577704 |
| Cyb561 | -0.1852245 |
| Cyb561d1 | 1.30902402 |
| Cyb561d1 | 1.89677505 |
| Cyb561d2 | 0.96179256 |
| Cyb5b | 0.57886084 |
| Cyb5d1 | 2.12946067 |
| Cyb5d2 | 1.52728795 |
| Cyb5d2 | -0.6926143 |
| Cyb5r1 | 0.62000678 |
| Cyb5r2 | -0.2600056 |
| Cyb5r3 | -0.6884836 |
| Cyb5r3 | 0.75712053 |
| Cyb5r3 | 2.98672392 |
| Cyb5r4 | 0.4091135 |
| Cyba | 0.12547598 |
| Cybasc3 | -0.5327273 |
| Cyc1 | -0.6705271 |
| Cycs | 2.61100029 |
| Cycs | -0.6640838 |
| Cyfip1 | 0.86554489 |
| Cyfip2 | 1.16931577 |
| Cyfip2 | 0.88228114 |
| Cygb | -0.553826 |
| Cyhr1 | 0.39635891 |
| Cyhr1 | 3.07453942 |
| Cyhr1 | -0.4567163 |
| Cyhr1 | -0.7042804 |
| Cyld | -0.5908496 |
| Cyln2 | 3.78218229 |
| Cyp11a1 | -0.3527989 |
| Cyp11b2 | -0.1668639 |
| Cyp17a1 | -0.7272081 |
| Cyp19a1 | -0.2656997 |
| Cyp1b1 | -0.4002704 |
| Cyp20a1 | -0.56889 |
| Cyp24a1 | -0.5545377 |
| Cyp26a1 | 1.1646513 |
| Cyp26b1 | -0.7857984 |
| Cyp27a1 | -0.4215702 |
| Cyp27a1 | -0.1795358 |
| Cyp27a1 | -0.4644265 |
| Cyp2a12 | -0.7428188 |
| Cyp2a5 | 2.04871325 |
| Cyp2a5 | -0.5602516 |
| Cyp2b10 | 0.26000937 |
| Cyp2c55 | 3.38729066 |
| Cyp2d10 | 1.01837766 |
| Cyp2d22 | -0.3608298 |
| Cyp2d26 | 0.14557062 |
| Cyp2d26 | -0.2646618 |
| Cyp2d26 | 1.24108396 |
| Cyp2e1 | 2.26234461 |
| Cyp2f2 | -0.4979397 |
| Cyp2j11 | 1.52918828 |
| Cyp2j9 | -0.3109371 |
| Cyp2s1 | -0.4396023 |
| Cyp2u1 | -0.5768808 |
| Cyp39a1 | -0.1947851 |
| Cyp3a13 | 0.36380265 |
| Cyp3a13 | -0.7401985 |
| Cyp46a1 | -0.2579438 |
| Cyp4b1 | 0.63971303 |
| Cyp4f13 | 1.00176652 |
| Cyp4f14 | 1.11286005 |
| Cyp4f15 | 0.95798371 |
| Cyp4f16 | -0.5278735 |
| Cyp4f16 | -0.6302079 |
| Cyp4f16 | 1.38688329 |
| Cyp4f18 | 2.38697177 |
| Cyp4f39 | -0.4780777 |
| Cyp4v3 | 1.09917802 |
| Cyp51 | 0.13020436 |
| Cyp7b1 | -0.7143996 |
| Cypt10 | -0.6489605 |
| Cypt2 | -0.2390889 |
| Cypt3 | 1.20392603 |
| Cypt6 | 1.98581061 |
| Cypt8 | 0.06240005 |
| Cypt9 | 0.24349164 |
| Cyr61 | -0.0663266 |
| Cyr61 | -0.5488592 |
| Cysltr1 | -0.5341188 |
| Cytl1 | 0.62453181 |
| D030011O10Rik | 1.34159473 |
| D030013I16Rik | 2.03411395 |
| D030018L15Rik | 2.78663786 |
| D030028O16Rik | -0.3516574 |
| D030051N19Rik | 0.22727634 |
| D030056L22Rik | -0.6745773 |
| D030070L09Rik | 0.25119769 |
| D030070L09Rik | -0.4014743 |
| D030070L09Rik | 0.0062971 |
| D030070L09Rik | 0.18002712 |
| D030074E01Rik | 2.03150332 |
| D0H4S114 | 0.27795561 |
| D0H8S2298E | -0.012014 |
| D0HXS9928E | -0.2861784 |
| D0HXS9928E | -0.6608791 |
| D10627 | -0.6942718 |
| D10Bwg1364e | -0.6395361 |
| D10Bwg1379e | 0.0462967 |
| D10Ertd322e | 0.5720275 |
| D10Ertd610e | 0.88612295 |
| D10Ertd610e | -0.6302079 |
| D10Ertd610e | 1.11009379 |
| D10Ertd641e | -0.4364678 |
| D10Jhu81e | 3.39073342 |
| D10Wsu102e | 1.08860329 |
| D10Wsu52e | -0.5093892 |
| D11Bwg0517e | 1.78817365 |
| D11Wsu47e | -0.0764041 |
| D11Wsu47e | 0.61327634 |
| D12Ertd551e | 3.24253185 |
| D12Ertd551e | -0.3363845 |
| D12Ertd553e | -0.6217397 |
| D12Ertd647e | -0.1767046 |
| D12Ertd647e | -0.6967637 |
| D130040H23Rik | 0.34481764 |
| D130043K22Rik | -0.7533759 |
| D130059P03Rik | 1.87020579 |
| D13Ertd608e | -0.7463243 |
| D14Ertd171e | 0.7902977 |
| D14Ertd231e | -0.4846506 |
| D14Ertd449e | -0.781226 |
| D14Ertd500e | -0.6951017 |
| D14Ertd668e | -0.7177977 |
| D14Ertd668e | 2.2153195 |
| D15Ertd621e | -0.5292576 |
| D15Ertd682e | -0.5652807 |
| D15Mgi27 | -0.8702553 |
| D15Wsu169e | 0.40363331 |
| D15Wsu75e | 0.9545695 |
| D16Ertd472e | -0.4899431 |
| D16H22S680E | 0.82057301 |
| D17H6S56E-5 | 0.59400106 |
| D17Wsu104e | 0.79519655 |
| D17Wsu92e | -0.7587005 |
| D19Bwg1357e | -0.6967637 |
| D19Ertd721e | -0.6584829 |
| D19Ertd737e | -0.5327273 |
| D19Wsu162e | -0.1156557 |
| D1Bwg0212e | 1.28550485 |
| D1Bwg0212e | 1.99098214 |
| D1Pas1 | -0.3403197 |
| D230007K08Rik | 0.0242489 |
| D230014K01Rik | -0.6860142 |
| D2Ertd750e | 0.94825387 |
| D2Ertd750e | 0.21814209 |
| D330014H01Rik | -0.4560767 |
| D330017J20Rik | -0.4136009 |
| D330028D13Rik | 1.01454143 |
| D330045A20Rik | 0.14191949 |
| D330045A20Rik | 0.90941622 |
| D330045A20Rik | 0.71721355 |
| D3Bwg0562e | -0.6240415 |
| D3Ertd300e | -0.6656904 |
| D3Ertd751e | 1.72638836 |
| D3Ucla1 | 1.96255655 |
| D430015B01Rik | -0.6080496 |
| D430028G21Rik | -0.5652807 |
| D430039N05Rik | -0.5652807 |
| D430041B17Rik | -0.5223576 |
| D430042O09Rik | -0.6125903 |
| D4Bwg0951e | 1.35007017 |
| D4Bwg1540e | 0.10631424 |
| D4Ertd22e | -0.3800433 |
| D4Ertd22e | -0.6827319 |
| D4Wsu114e | -0.3782799 |
| D4Wsu132e | 3.40975834 |
| D4Wsu132e | -0.3386311 |
| D530004J12Rik | -0.7640563 |
| D530033C11Rik | 1.66630697 |
| D5Ertd135e | 3.60395928 |
| D5Ertd579e | 3.7595401 |
| D5Wsu178e | -0.2360676 |
| D630003M21Rik | -0.6942718 |
| D630003M21Rik | 0.53836832 |
| D630014A15Rik | -0.6458083 |
| D630023F18Rik | 0.31640528 |
| D630042P16Rik | -0.6860142 |
| D630048P19Rik | -0.2584588 |
| D6Ertd474e | -0.5696135 |
| D6Mm5e | 2.01469389 |
| D6Wsu116e | 0.12581453 |
| D6Wsu163e | -0.5141457 |
| D6Wsu163e | -0.6505407 |
| D6Wsu163e | -0.261555 |
| D6Wsu176e | 2.27755117 |
| D6Wsu176e | -0.352228 |
| D730039F16Rik | 0.17554995 |
| D730039F16Rik | 0.97753877 |
| D7Bwg0611e | -0.6584829 |
| D830014E11Rik | -0.6705271 |
| D830030K20Rik | 0.11628737 |
| D830030K20Rik | -0.4160456 |
| D830050J10Rik | 0.69459026 |
| D8Ertd738e | 1.79250948 |
| D8Ertd82e | 0.22332936 |
| D930001I22Rik | -0.5908496 |
| D930005D10Rik | -0.0853138 |
| D930014E17Rik | 1.58230378 |
| D930015E06Rik | -0.5376069 |
| D930020B18Rik | 2.83712737 |
| D930028F11Rik | -0.4787329 |
| D930049A15Rik | 1.22116131 |
| D9Ertd280e | 0.14755603 |
| D9Ertd392e | 1.97506511 |
| D9Ertd402e | -0.4952664 |
| D9Wsu20e | 1.33728236 |
| Daam1 | -0.6379748 |
| Daam2 | 0.82310396 |
| Dab1 | -0.632531 |
| Dab1 | 1.33267808 |
| Dab1 | 1.19144343 |
| Dab2 | -0.6450219 |
| Dab2 | 0.1145756 |
| Dab2ip | 0.49010924 |
| Dact1 | 1.6487932 |
| Dact2 | 0.69131221 |
| Dad1 | 1.41320039 |
| Daf2 | 0.79519655 |
| Dag1 | 1.21665168 |
| Dag1 | -0.6802779 |
| Dagla | -0.246686 |
| Daglb | 1.20110874 |
| Dak | -0.6035314 |
| Dalrd3 | 1.90446203 |
| Dap | -0.7507252 |
| Dap3 | 1.29006768 |
| Dap3 | -0.0049579 |
| Dap3 | 0.16361088 |
| Dap3 | 0.2969614 |
| Dapk1 | -0.6148691 |
| Dapk1 | 0.94341912 |
| Dapk2 | -0.3235723 |
| Dapk2 | -0.2761443 |
| Dapl1 | 3.19379161 |
| Dapp1 | -0.8328252 |
| Dapp1 | -0.5355125 |
| Darc | -0.6294348 |
| Dars | -0.6356378 |
| Dars | -0.6194438 |
| Daxx | -0.7766763 |
| Dazap1 | 1.89667733 |
| Dazap1 | -0.4166578 |
| Dazap2 | 0.56577076 |
| Dazl | 0.10838659 |
| Dbc1 | 1.82198015 |
| Dbf4 | -0.2724751 |
| Dbi | 2.53668548 |
| Dbi | -0.7401985 |
| Dbil5 | -0.5864153 |
| Dbn1 | -0.0399837 |
| Dbndd1 | -0.3397565 |
| Dbndd2 | -0.6786455 |
| Dbndd2 | 0.21661082 |
| Dbndd2 | -0.6851925 |
| Dbnl | 0.58952603 |
| Dbp | -0.7220632 |
| Dbpht2 | 1.89496557 |
| Dbr1 | 0.791116 |
| Dbt | -0.030699 |
| Dcakd | -0.424654 |
| Dcbld1 | 1.31171352 |
| Dcbld2 | 0.94632303 |
| Dci | 0.0219767 |
| Dck | 0.80071705 |
| Dclk1 | -0.2740459 |
| Dclk2 | 0.93828016 |
| Dclk3 | 0.34188214 |
| Dclre1b | 0.9627423 |
| Dclre1b | -0.6333067 |
| Dclre1c | -0.632531 |
| Dclre1c | -0.6012806 |
| Dclre1c | -0.3730098 |
| Dcn | -0.6481714 |
| Dcn | 0.72638949 |
| Dcp1a | 1.27909762 |
| Dcp1a | 1.1650862 |
| Dcp1a | 1.12452423 |
| Dcp1a | -0.4421176 |
| Dcp1b | 0.39434196 |
| Dcp1b | 1.23596779 |
| Dcpp1 | -0.5369083 |
| Dcpp2 | -0.764952 |
| Dcpp2 | -0.0246942 |
| Dcpp3 | -0.656093 |
| Dcps | -0.5953055 |
| Dcps | -0.7237749 |
| Dct | 0.81053876 |
| Dctd | 2.6555572 |
| Dctn1 | 0.93618856 |
| Dctn2 | 0.25060722 |
| Dctn2 | 1.30072033 |
| Dctn2 | 0.5813331 |
| Dctn3 | -0.5768808 |
| Dctn3 | -0.7289295 |
| Dctn4 | 0.12919319 |
| Dctn4 | -0.2507637 |
| Dctn5 | 0.73199082 |
| Dctn6 | -0.3082133 |
| Dcun1d2 | -0.222594 |
| Dcun1d3 | -0.713552 |
| Dcun1d4 | -0.032307 |
| Dcun1d4 | 2.07449939 |
| Dcun1d5 | 1.80965018 |
| Dcx | 0.40338343 |
| Dcx | 0.60268502 |
| Dcxr | 1.21952402 |
| Dcxr | 0.35013089 |
| Ddah1 | -0.6156299 |
| Ddah2 | 0.1193605 |
| Ddb1 | 0.09518176 |
| Ddb2 | -0.8241801 |
| Ddb2 | 0.3397413 |
| Ddb2 | -0.4754617 |
| Ddc | -0.5652807 |
| Ddc8 | 1.3837217 |
| Ddef2 | -0.5893691 |
| Ddef2 | 0.09202619 |
| Ddefl1 | 0.31420393 |
| Ddhd1 | 0.59602796 |
| Ddhd1 | -0.7143996 |
| Ddi2 | 0.12986742 |
| Ddit3 | -0.3408833 |
| Ddit3 | 0.60087426 |
| Ddit4 | 1.87412051 |
| Ddit4l | -0.5223576 |
| Ddo | 1.7830983 |
| Ddo | 0.56094479 |
| Ddo | 2.80628369 |
| Ddost | 3.03696217 |
| Ddr1 | -0.4020768 |
| Ddr2 | 1.20569543 |
| Ddt | 1.04813135 |
| Ddx1 | -0.2729984 |
| Ddx1 | 1.73429981 |
| Ddx17 | 0.57472552 |
| Ddx17 | -0.4026797 |
| Ddx17 | 0.25971701 |
| Ddx19a | -0.3912915 |
| Ddx19b | -0.724632 |
| Ddx19b | -0.0274915 |
| Ddx20 | -0.2350627 |
| Ddx21 | -0.3380689 |
| Ddx24 | -0.5545377 |
| Ddx24 | 2.94622125 |
| Ddx24 | 0.1851172 |
| Ddx25 | 0.69858089 |
| Ddx26b | -0.6884836 |
| Ddx27 | -0.197191 |
| Ddx28 | 0.16911575 |
| Ddx3x | 0.09062021 |
| Ddx3y | -0.6909598 |
| Ddx4 | -0.6179163 |
| Ddx4 | 0.67400004 |
| Ddx41 | 0.82247188 |
| Ddx46 | 0.60107563 |
| Ddx47 | -0.390101 |
| Ddx47 | -0.2265655 |
| Ddx47 | 1.32065242 |
| Ddx49 | 3.07926071 |
| Ddx5 | -0.6171536 |
| Ddx50 | -0.6095606 |
| Ddx50 | 3.00206233 |
| Ddx51 | 0.97285671 |
| Ddx52 | -0.5732399 |
| Ddx54 | 2.13985782 |
| Ddx58 | -0.6984287 |
| Ddx6 | 0.80973766 |
| Ddx6 | -0.3648718 |
| Ddx6 | -0.5093892 |
| Ddx6 | -0.581269 |
| Deadc1 | -0.7472028 |
| Deb1 | 0.13691708 |
| Decr1 | 1.27505815 |
| Decr2 | 1.38355049 |
| Decr2 | 0.07214314 |
| Dedd | 0.54545675 |
| Dedd | -0.6681055 |
| Dedd2 | -0.5202977 |
| Def6 | 0.86312881 |
| Def8 | 0.64854938 |
| Defb11 | 0.63332092 |
| Defb14 | -0.3859462 |
| Defb15 | -0.1438512 |
| Defb19 | 1.00500938 |
| Defb20 | 1.07838305 |
| Defb21 | -0.3153122 |
| Defb25 | -0.3347031 |
| Defb26 | 0.86418663 |
| Defb30 | -0.3712599 |
| Defb36 | 0.45133777 |
| Defb36 | -0.5216705 |
| Defb4 | -0.656889 |
| Defcr20 | 2.4530078 |
| Defcr6 | -0.5667227 |
| Defcr-rs7 | 1.12076677 |
| Degs1 | 1.00462085 |
| Degs2 | -0.6529161 |
| Dek | 0.238717 |
| Dek | 0.39887386 |
| Dennd1a | -0.6810951 |
| Dennd1c | 0.0692671 |
| Dennd2a | 0.72866916 |
| Dennd3 | -0.2714295 |
| Denr | -0.6465953 |
| Denr | 1.67119635 |
| Depdc1b | -0.1645364 |
| Depdc5 | 1.63008796 |
| Depdc5 | -0.6403178 |
| Depdc6 | -0.656093 |
| Depdc6 | 0.78075156 |
| Dera | -0.5320323 |
| Derl2 | 0.94963054 |
| Derl3 | 1.53159458 |
| Des | -0.1785911 |
| Det1 | 2.07079061 |
| Dexi | -0.062576 |
| Dexi | 2.59248315 |
| Dffa | 1.65492304 |
| Dfna5h | 0.22969682 |
| Dgat1 | 0.98365612 |
| Dgat2 | -0.6411001 |
| Dgcr2 | 0.06710412 |
| Dgcr2 | 2.01056148 |
| Dgcr6 | 0.1281809 |
| Dgcr6 | 2.34433525 |
| Dgcr8 | 2.51419883 |
| Dgka | -0.8318606 |
| Dgka | -0.0053484 |
| Dgkb | -0.6202084 |
| Dgke | -0.6909598 |
| Dgkg | -0.3806319 |
| Dgkh | -0.707641 |
| Dgki | 0.45960073 |
| Dgkk | 1.56645908 |
| Dgkq | 0.2969614 |
| Dgkz | 2.43329854 |
| Dgkz | 0.61128734 |
| Dgkz | 2.63101331 |
| Dguok | 3.26832707 |
| Dhcr24 | -0.2171612 |
| Dhcr24 | 0.37778728 |
| Dhcr24 | 2.53848015 |
| Dhcr7 | -0.1566669 |
| Dhdds | -0.4846506 |
| Dhdds | 0.97540137 |
| Dhdh | 2.93549908 |
| Dhdh | 0.57348126 |
| Dhdh | 2.51292907 |
| Dhh | 0.72128349 |
| Dhodh | -0.3208107 |
| Dhps | 0.80797276 |
| Dhrs1 | 0.32133906 |
| Dhrs4 | 0.18733524 |
| Dhrs7 | -0.7358478 |
| Dhrs7 | 1.71620845 |
| Dhrs7 | -0.4327206 |
| Dhrs7b | -0.0481347 |
| Dhrs7b | -0.5355125 |
| Dhrs7b | 0.39635891 |
| Dhrsx | -0.5285653 |
| Dhrsx | 0.08744884 |
| Dhtkd1 | 3.06510488 |
| Dhx15 | 0.40088086 |
| Dhx15 | 0.94147804 |
| Dhx16 | -0.5531148 |
| Dhx29 | -0.3918874 |
| Dhx30 | 0.63176457 |
| Dhx32 | 0.54460046 |
| Dhx33 | -0.0930204 |
| Dhx36 | -0.1397697 |
| Dhx37 | -0.0485442 |
| Dhx38 | -0.4377203 |
| Dhx57 | -0.5107457 |
| Dhx58 | -0.5039833 |
| Dhx8 | -0.6984287 |
| Dhx9 | 1.22391761 |
| Diablo | -0.6917867 |
| Diap1 | -0.7507252 |
| Diap3 | 2.74882935 |
| Dicer1 | -0.1986375 |
| Dido1 | -0.3380689 |
| Dido1 | 1.80057739 |
| Dido1 | 0.72181302 |
| Dio1 | -0.6020302 |
| Dio1 | 1.66680965 |
| Dio3 | -0.4172704 |
| Dip2b | -0.0195216 |
| Dip2b | 0.76050983 |
| Diras1 | 1.21654893 |
| Diras2 | -0.1861761 |
| Dirc2 | -0.476115 |
| Dis3 | 0.34774374 |
| Dis3l | 0.63293208 |
| Dis3l2 | 0.40113143 |
| Disp1 | 1.37460319 |
| Dixdc1 | 1.78503246 |
| Dkk3 | 0.2008436 |
| Dkk3 | -0.5717877 |
| Dkk3 | -0.2522975 |
| Dkkl1 | -0.6156299 |
| Dkkl1 | 0.79438189 |
| Dlat | 1.81847422 |
| Dlc1 | -0.257429 |
| Dld | -0.6118319 |
| Dleu7 | -0.6992624 |
| Dlg1 | 0.95361123 |
| Dlg2 | 1.45626431 |
| Dlg3 | 2.43313544 |
| Dlg4 | -0.5674446 |
| Dlg7 | -0.5990353 |
| Dlgap1 | -0.388318 |
| Dlgap2 | -0.5805361 |
| Dlgap2 | -0.7968664 |
| Dlgap2 | 0.13858759 |
| Dlgap3 | 0.18034608 |
| Dlgap4 | -0.7730528 |
| Dlgap4 | 0.04850937 |
| Dlgap4 | 0.75218364 |
| Dlgap4 | 0.48165972 |
| Dlk1 | -0.5531148 |
| Dlk2 | 0.18225752 |
| Dll1 | 0.47243646 |
| Dlst | 0.42949818 |
| Dlx1 | -0.6125903 |
| Dlx1as | 0.24527573 |
| Dlx2 | 0.03477853 |
| Dlx3 | 3.0686271 |
| Dmap1 | 1.58800436 |
| Dmbt1 | 0.45748309 |
| Dmkn | -0.4605633 |
| Dmkn | 1.36940121 |
| Dmkn | -0.6648867 |
| Dmn | 1.81607609 |
| Dmn | -0.7195015 |
| Dmpk | 1.11159322 |
| Dmrt2 | 0.11901955 |
| Dmrt3 | -0.250253 |
| Dmrta1 | 0.49735453 |
| Dmrta2 | 0.29499408 |
| Dmrtc1a | 1.61989592 |
| Dmrtc1a | 1.12065267 |
| Dmrtc1a | 0.74222922 |
| Dmrtc1b | -0.1033979 |
| Dmrtc2 | -0.8203638 |
| Dmtf1 | -0.7203546 |
| Dmtf1 | -0.7560344 |
| Dmtf1 | 2.18861605 |
| Dmwd | 0.25737477 |
| Dmwd | 2.22829953 |
| Dmwd | 0.37521361 |
| Dnahc11 | 0.11114243 |
| Dnahc2 | -0.4886171 |
| Dnahc5 | 1.39045894 |
| Dnahc8 | 0.00126889 |
| Dnaic1 | 0.01665284 |
| Dnaic1 | 1.26847999 |
| Dnaja1 | -0.5967956 |
| Dnaja2 | 0.42901228 |
| Dnaja2 | -0.8061931 |
| Dnaja3 | -0.781226 |
| Dnajb1 | -0.5237335 |
| Dnajb10 | -0.6005316 |
| Dnajb12 | 0.22788206 |
| Dnajb13 | 0.70525338 |
| Dnajb3 | 1.2641982 |
| Dnajb4 | -0.4129907 |
| Dnajb4 | 3.16811369 |
| Dnajb5 | 1.68250432 |
| Dnajb6 | -0.1280799 |
| Dnajb6 | 0.46778973 |
| Dnajb7 | 1.30586072 |
| Dnajb8 | 4.14843974 |
| Dnajb9 | 0.63603818 |
| Dnajc10 | 1.00293528 |
| Dnajc10 | 1.09002033 |
| Dnajc12 | 0.42267206 |
| Dnajc14 | -0.3631373 |
| Dnajc15 | -0.659281 |
| Dnajc16 | 0.14920724 |
| Dnajc17 | -0.6843715 |
| Dnajc18 | 0.95634648 |
| Dnajc19 | -0.6125903 |
| Dnajc19 | -0.4258904 |
| Dnajc2 | 1.61405854 |
| Dnajc2 | -0.0772489 |
| Dnajc3a | -0.5908496 |
| Dnajc4 | 0.15709187 |
| Dnajc5 | -0.450976 |
| Dnajc5b | -0.1673302 |
| Dnajc5g | -0.3027898 |
| Dnajc6 | 0.49328612 |
| Dnajc6 | -0.5134647 |
| Dnajc7 | -0.2487226 |
| Dnajc8 | -0.0473163 |
| Dnalc1 | 0.89156247 |
| Dnali1 | 1.64455499 |
| Dnase1l1 | -0.2625894 |
| Dnase1l2 | -0.5488592 |
| Dnase1l2 | -0.2385846 |
| Dnase2a | 3.44262043 |
| Dnd1 | 0.2488335 |
| Dner | -0.5209838 |
| Dnm | 0.22757925 |
| Dnm1 | 0.63758728 |
| Dnm1l | -0.656889 |
| Dnm1l | 0.68177578 |
| Dnmbp | -0.5348154 |
| Dnmt1 | 0.00088095 |
| Dnmt3b | 0.31997124 |
| Dnmt3l | -0.6884836 |
| Dnmt3l | -0.0563589 |
| Dnttip1 | -0.2656997 |
| Doc2a | -0.2558866 |
| Doc2b | 1.10162797 |
| Doc2b | -0.6426668 |
| Dock1 | -0.6255792 |
| Dock3 | -0.3906961 |
| Dock4 | -0.7375856 |
| Dock6 | 0.1652334 |
| Dock8 | 0.25060722 |
| Dock9 | -0.0747168 |
| Dok1 | -0.2564004 |
| Dok1 | -0.4026797 |
| Dok2 | -0.6465953 |
| Dok3 | 1.40470708 |
| Dok4 | -0.6225063 |
| Dok5 | 1.72178592 |
| Dok5 | -0.1645364 |
| Dok7 | 0.46755677 |
| Dolk | 0.1452393 |
| Dolpp1 | 0.03365613 |
| Dolpp1 | 1.61903592 |
| Dolpp1 | 2.01284522 |
| Dom3z | -0.3625599 |
| Dom3z | -0.8127788 |
| Donson | -0.7757691 |
| Donson | 2.5997855 |
| Dos | -0.2275611 |
| Dos | -0.4567163 |
| Dot1l | -0.1650015 |
| Dpagt1 | 0.93086831 |
| Dpep1 | 0.80023129 |
| Dpep2 | 0.66521954 |
| Dpep2 | 1.93603204 |
| Dpep3 | 1.86470701 |
| Dpf1 | 0.08320318 |
| Dpf1 | 1.2689653 |
| Dpf3 | 0.79649849 |
| Dph2 | -0.7613745 |
| Dph2 | -0.2250742 |
| Dph2 | -0.3966683 |
| Dph3 | 2.04635147 |
| Dph3 | -0.1397697 |
| Dph3 | -0.5341188 |
| Dph4 | 0.08568221 |
| Dpm1 | -0.5717877 |
| Dpm1 | 1.37546732 |
| Dpm2 | 0.0462967 |
| Dpm3 | 0.1247985 |
| Dpp10 | -0.6934427 |
| Dpp3 | -0.5258011 |
| Dpp3 | -0.6027805 |
| Dpp4 | -0.433968 |
| Dpp6 | 3.30888957 |
| Dpp7 | 0.76741888 |
| Dpp8 | -0.4926009 |
| Dpp9 | -0.459921 |
| Dpp9 | -0.4057003 |
| Dppa3 | 0.55778365 |
| Dppa4 | 0.91443353 |
| Dppa5 | 1.05857107 |
| Dppa5 | 2.09742324 |
| Dpt | 1.30995233 |
| Dpy30 | -0.6225063 |
| Dpyd | -0.6917867 |
| Dpysl2 | -0.3614061 |
| Dpysl2 | 1.10836062 |
| Dpysl2 | 1.23355025 |
| Dpysl3 | -0.3280084 |
| Dpysl4 | 1.23465908 |
| Dpysl5 | 1.15382089 |
| Drbp1 | -0.4722022 |
| Drctnnb1a | -0.6942718 |
| Drd1a | -0.6163914 |
| Drd2 | -0.0514155 |
| Drd4 | -0.6893083 |
| Drd5 | 0.76472887 |
| Drg1 | 1.72738839 |
| Drg1 | -0.6893083 |
| Drg2 | -0.4573563 |
| Drp2 | 3.70250935 |
| Dscam | -0.4663642 |
| Dscam | 1.39908492 |
| Dscc1 | 0.75661105 |
| Dscr1 | 0.55036489 |
| Dscr1l2 | -0.5161918 |
| Dscr2 | 3.30999538 |
| Dscr3 | -0.6497503 |
| Dscr3 | 1.08540689 |
| Dscr6 | 0.05985704 |
| Dsg2 | 0.24646319 |
| Dsg2 | -0.1047034 |
| Dsn1 | -0.7110141 |
| Dsp | -0.5087117 |
| Dst | -0.6967637 |
| Dst | 0.80797276 |
| Dst | 0.9995548 |
| Dstn | 0.47336298 |
| Dtd1 | 0.04296763 |
| Dtl | 0.18954791 |
| Dtna | -0.5856783 |
| Dtna | 1.14752655 |
| Dtna | -0.587891 |
| Dtna | 0.89682412 |
| Dtnb | 2.3214344 |
| Dtnbp1 | -0.2206147 |
| Dtwd1 | 1.32622939 |
| Dtx1 | -0.5864153 |
| Dtx2 | 0.7392909 |
| Dtx2 | -0.5488592 |
| Dtx2 | -0.3841713 |
| Dtx4 | 0.33004506 |
| Dtx4 | -0.3302345 |
| Dtymk | -0.055946 |
| Dus1l | 1.82314581 |
| Dus1l | -0.5066823 |
| Dus2l | -0.5161918 |
| Dus3l | -0.3966683 |
| Dus3l | 1.74825536 |
| Dus3l | 0.69459026 |
| Dus4l | 0.47336298 |
| Dusp1 | 2.55027573 |
| Dusp11 | 1.36678909 |
| Dusp11 | 0.37082168 |
| Dusp13 | 0.95948194 |
| Dusp13 | 0.19144024 |
| Dusp13 | 0.04555795 |
| Dusp14 | -0.3049553 |
| Dusp15 | 0.08249365 |
| Dusp15 | 3.08272896 |
| Dusp16 | 1.70573341 |
| Dusp16 | 0.83954185 |
| Dusp16 | 0.54844745 |
| Dusp18 | 0.18162082 |
| Dusp19 | -0.08361 |
| Dusp2 | 2.55518964 |
| Dusp2 | 0.18543439 |
| Dusp21 | -0.3246793 |
| Dusp22 | 0.91128301 |
| Dusp23 | -0.4044909 |
| Dusp26 | 0.82199753 |
| Dusp28 | 1.25763947 |
| Dusp3 | 1.49071535 |
| Dusp3 | -0.3482413 |
| Dusp3 | -0.4657179 |
| Dusp4 | -0.5746945 |
| Dusp4 | -0.3006294 |
| Dusp4 | -0.4522485 |
| Dusp6 | -0.1285267 |
| Dusp6 | 0.61128734 |
| Dusp6 | -0.3759339 |
| Dusp7 | -0.1580508 |
| Dusp8 | 0.69422662 |
| Dut | 0.5078931 |
| Duxbl | 3.00614051 |
| Duxbl | -0.345971 |
| Dvl1 | -0.3759339 |
| Dvl1 | -0.4676583 |
| Dvl2 | -0.3954707 |
| DXBwg1396e | -0.7703446 |
| Dydc2 | -0.3853542 |
| Dym | -0.6529161 |
| Dync1h1 | 0.61605369 |
| Dync1i1 | -0.5960502 |
| Dync1i1 | -0.0187284 |
| Dync1i2 | 0.53858393 |
| Dync1li1 | -0.5842062 |
| Dync1li2 | 2.32865459 |
| Dync2li1 | -0.0030074 |
| Dynll1 | 0.10423719 |
| Dynll2 | 0.40263338 |
| Dynlrb1 | -0.5278735 |
| Dynlrb2 | -0.0700927 |
| Dynlt3 | 1.10916984 |
| Dyrk1a | -0.4787329 |
| Dyrk1b | 0.05034919 |
| Dyrk1b | 0.08603581 |
| Dyrk2 | 4.18701236 |
| Dyrk2 | 0.06203719 |
| Dyrk3 | -0.8415528 |
| Dyrk4 | 0.38112226 |
| Dysf | 0.7232236 |
| Dysfip1 | -0.5953055 |
| Dzip1 | 2.08760099 |
| E030010A14Rik | -0.668912 |
| E030034P13Rik | -0.2176537 |
| E130009J12Rik | -0.4839912 |
| E130012A19Rik | -0.2920383 |
| E130014J05Rik | 2.48561768 |
| E130016E03Rik | 0.45181195 |
| E130203B14Rik | -0.3930803 |
| E130203B14Rik | 0.70507368 |
| E130303B06Rik | -0.8241801 |
| E130306D19Rik | 0.55841675 |
| E130308A19Rik | -0.6537093 |
| E130309D02Rik | -0.6371951 |
| E130309D02Rik | -0.149779 |
| E130309D14Rik | 1.04168547 |
| E130309F12Rik | -0.7375856 |
| E130309F12Rik | 1.84055877 |
| E130309F12Rik | -0.4813582 |
| E230008N13Rik | 2.85528362 |
| E2f1 | 1.0494891 |
| E2f2 | 0.94756475 |
| E2f2 | -0.6778304 |
| E2f3 | -0.5127842 |
| E2f4 | -0.3510871 |
| E2f5 | 3.13819131 |
| E2f6 | -0.3835805 |
| E2f6 | 0.5567275 |
| E2f6 | -0.9177149 |
| E330016A19Rik | -0.147951 |
| E330017A01Rik | 1.35749939 |
| E330018D03Rik | -0.6552978 |
| E330021D16Rik | 0.41060242 |
| E330034G19Rik | -0.668912 |
| E330034G19Rik | -0.6521236 |
| E330036I19Rik | 2.06124827 |
| E430002D04Rik | -0.5019644 |
| E430002G05Rik | -0.6753895 |
| E430018J23Rik | -0.6027805 |
| E430025E21Rik | 1.12815314 |
| E430028B21Rik | -0.7507252 |
| E4f1 | 0.96599109 |
| Eaf1 | -0.2325553 |
| Ear11 | 0.57844815 |
| Ear2 | -0.1271868 |
| Ear4 | -0.1095063 |
| Ears2 | 2.41087412 |
| Ebag9 | 0.58441405 |
| Ebf2 | 0.196154 |
| Ebf3 | -0.2610383 |
| Ebf4 | 1.67213363 |
| Ebi2 | 0.77377613 |
| Ebi3 | -0.659281 |
| Ebna1bp2 | 2.33500151 |
| Ebna1bp2 | 0.76439206 |
| Ebp | -0.4240364 |
| Ebpl | -0.2280593 |
| Ebpl | 0.90682514 |
| Ecd | 0.2544385 |
| Ecd | -0.0030074 |
| Ece1 | 0.91343226 |
| Ece1 | 1.92879556 |
| Ece2 | -0.584942 |
| Ecel1 | 1.86667826 |
| Ech1 | -0.8165634 |
| Echdc1 | 0.98867891 |
| Echdc2 | 1.42824353 |
| Echdc2 | -0.583471 |
| Echdc2 | 0.238717 |
| Echdc3 | 0.35488658 |
| Echs1 | 1.48483295 |
| Ecm1 | 0.29976452 |
| Ecm1 | -0.5039833 |
| Ecsit | 0.34508403 |
| Eda2r | 1.26604962 |
| Edaradd | 0.34694664 |
| Edc4 | 0.43168152 |
| Edem1 | 2.0781932 |
| Edem2 | -0.5674446 |
| Edem2 | 1.031176 |
| Edem2 | 1.12019611 |
| Edem2 | -0.647383 |
| Edf1 | -0.7289295 |
| Edg2 | -0.346538 |
| Edg5 | 1.27486534 |
| Edg7 | 1.47466 |
| Edg8 | 0.35251182 |
| Edg8 | -0.019125 |
| Edil3 | 1.41411164 |
| Edn1 | 0.37958455 |
| Edn2 | -0.2909701 |
| Ednra | -0.459921 |
| Ednrb | 2.11236707 |
| Ednrb | 1.01978026 |
| Ednrb | -0.8642425 |
| Eed | 0.77327585 |
| Eef1a1 | -0.6656904 |
| Eef1a1 | 1.2280873 |
| Eef1a2 | 0.79078877 |
| Eef1a2 | -0.5524041 |
| Eef1a2 | 1.27254855 |
| Eef1b2 | -0.5856783 |
| Eef1b2 | 0.75098798 |
| Eef1b2 | 0.97807234 |
| Eef1d | 2.60552267 |
| Eef1d | 0.85584204 |
| Eef1d | 0.52686769 |
| Eef1d | 1.32046899 |
| Eef1e1 | 0.98617096 |
| Eef1g | -0.7297915 |
| Eef2 | 0.05328522 |
| Eef2 | 0.56827859 |
| Eef2 | 0.18321174 |
| Eef2k | 0.07070625 |
| Eefsec | 0.07858136 |
| Efcab1 | -0.0772489 |
| Efcab2 | -0.2973982 |
| Efcab2 | 2.53503322 |
| Efcab3 | -0.6942718 |
| Efcab3 | 0.50252687 |
| Efcab3 | 0.16393562 |
| Efcab4a | 0.91743076 |
| Efcab4a | -0.6088048 |
| Efcab5 | -0.062992 |
| Efemp2 | 0.82625784 |
| Efha1 | 1.9987069 |
| Efha1 | -0.8357252 |
| Efhc2 | 0.80120256 |
| Efhd1 | 0.70201338 |
| Efna1 | 1.43482381 |
| Efna3 | -0.8957427 |
| Efna4 | -0.6762024 |
| Efna4 | -0.1608247 |
| Efna5 | 1.36276929 |
| Efnb1 | 0.73076845 |
| Efnb2 | -0.0262916 |
| Efnb3 | -0.419725 |
| Efr3a | -0.7839667 |
| Efs | -0.1033979 |
| Eftud1 | 0.91300282 |
| Eftud2 | 0.67697002 |
| Eftud2 | -0.6080496 |
| Eftud2 | -0.1138945 |
| EG194588 | 0.06674312 |
| EG208426 | -0.7507252 |
| EG210155 | 0.42242733 |
| EG212225 | 1.81206519 |
| EG214321 | 0.6799304 |
| EG232599 | 0.86795461 |
| EG237009 | -0.2620721 |
| EG237009 | -0.6340831 |
| EG237300 | 0.33328858 |
| EG238829 | 1.31991849 |
| EG240916 | 2.09510539 |
| EG245297 | -0.4573563 |
| EG245297 | 2.72566707 |
| EG245376 | -0.7127053 |
| EG245376 | -0.5087117 |
| EG272350 | -0.5202977 |
| EG277333 | 1.11020922 |
| EG317677 | 0.03739211 |
| EG328264 | -0.5474451 |
| EG328280 | 0.09343001 |
| EG328644 | -0.7393267 |
| EG328839 | 0.93030648 |
| EG330305 | -0.724632 |
| EG330503 | -0.7017679 |
| EG330513 | -0.5376069 |
| EG333669 | -0.1165375 |
| EG368203 | -0.4839912 |
| EG380907 | -0.5264914 |
| EG381438 | 1.67891583 |
| EG382109 | 1.68305989 |
| EG382109 | -0.4637815 |
| EG382109 | -0.5306439 |
| EG384639 | 0.09937233 |
| EG384813 | 3.41863391 |
| EG384813 | 1.83936308 |
| EG384814 | -0.8523343 |
| EG385328 | 0.5563047 |
| EG408196 | -0.27457 |
| EG432825 | 0.47590609 |
| EG432879 | 1.00280549 |
| EG432939 | 1.97259503 |
| EG433016 | 1.89163066 |
| EG433182 | -0.1795358 |
| EG433224 | 2.06564604 |
| EG433923 | 2.22315011 |
| EG434171 | 0.89667837 |
| EG434197 | 0.96693649 |
| EG434280 | -0.6876597 |
| EG434459 | 2.47891913 |
| EG434459 | -0.3812209 |
| EG434858 | 0.6885715 |
| EG435391 | -0.4846506 |
| EG436332 | -0.3718428 |
| EG545047 | 2.03099718 |
| EG545056 | 0.10041698 |
| EG545253 | 1.63224329 |
| EG545370 | 1.3380025 |
| EG545758 | 1.98987957 |
| EG545861 | -0.7748628 |
| EG545963 | 0.05730693 |
| EG546166 | 2.47099821 |
| EG546282 | 1.10174448 |
| EG546325 | 1.53181302 |
| EG546347 | -0.3666096 |
| EG547109 | -0.7059591 |
| EG574403 | 1.54072449 |
| EG622320 | -0.1640717 |
| EG622339 | -0.6835513 |
| EG624866 | 1.31208387 |
| EG625054 | -0.5953055 |
| EG625424 | 0.08000595 |
| EG630499 | 0.45228589 |
| EG633640 | 0.79909683 |
| EG638695 | 2.16818227 |
| EG654453 | -0.5559628 |
| EG665186 | 0.24438412 |
| EG665378 | -0.3782799 |
| EG667977 | 2.8266489 |
| EG668668 | -0.2610383 |
| Egf | 0.98854709 |
| Egfbp2 | -0.7885529 |
| Egfl7 | 1.8163962 |
| Egfl7 | 0.23301455 |
| Egflam | 1.43133647 |
| Egflam | 1.65606783 |
| Egfr | -0.5827364 |
| Egfr | -0.403283 |
| Egfr | 0.45583263 |
| Egfr | -0.5121042 |
| Egln1 | 0.28395524 |
| Egln1 | -0.4605633 |
| Egln1 | -0.7560344 |
| Egln2 | 0.2805317 |
| Egln3 | 1.2448775 |
| Egr1 | 0.08850719 |
| Egr2 | -0.2156851 |
| Egr3 | 2.1434467 |
| Egr4 | -0.6893083 |
| Ehbp1 | 1.60550077 |
| Ehbp1l1 | -0.5425128 |
| Ehd1 | -0.6521236 |
| Ehd2 | 0.42949818 |
| Ehd3 | 0.32651824 |
| Ehd4 | 1.05256743 |
| Ehmt1 | -0.6442362 |
| Ehmt2 | -0.3391936 |
| Ehmt2 | -0.6442362 |
| Ei24 | -0.2507637 |
| Eid1 | 0.99733771 |
| Eid2 | 2.67010788 |
| Eid3 | -0.8156158 |
| Eif1a | -0.0391726 |
| Eif1b | 0.52730431 |
| Eif2a | 1.75186867 |
| Eif2ak1 | -0.6333067 |
| Eif2ak2 | -0.4452714 |
| Eif2b1 | 1.70295995 |
| Eif2b1 | 2.38267291 |
| Eif2b2 | 0.79925898 |
| Eif2b4 | 1.53747426 |
| Eif2b5 | -0.4899431 |
| Eif2b5 | 1.03306156 |
| Eif2b5 | -0.4221862 |
| Eif2c2 | 0.47982249 |
| Eif2c3 | -0.4859708 |
| Eif2c3 | -0.5460332 |
| Eif2c3 | -0.4807012 |
| Eif2c3 | 0.68765611 |
| Eif2c4 | 1.13323208 |
| Eif2s2 | 0.10596839 |
| Eif2s2 | -0.8184617 |
| Eif2s3x | 0.48303523 |
| Eif2s3y | -0.7177977 |
| Eif3b | 0.04185524 |
| Eif3b | -0.4644265 |
| Eif3d | 0.45015123 |
| Eif3eip | -0.0379573 |
| Eif3eip | 0.20146707 |
| Eif3f | -0.5725135 |
| Eif3g | 0.88568049 |
| Eif3i | 0.10527631 |
| Eif3i | 1.33746245 |
| Eif3i | 1.8734191 |
| Eif3k | -0.0717714 |
| Eif3s1 | -0.477423 |
| Eif3s10 | -0.3730098 |
| Eif3s10 | -0.3924836 |
| Eif3s4 | 1.17234036 |
| Eif3s8 | 2.74906035 |
| Eif4a1 | 0.16393562 |
| Eif4a1 | 0.8581254 |
| Eif4a2 | -0.5566762 |
| Eif4a3 | -0.4459035 |
| Eif4b | 1.67643278 |
| Eif4e | -0.1833244 |
| Eif4e1b | -0.4939326 |
| Eif4e1b | 0.50319937 |
| Eif4e2 | -0.7885529 |
| Eif4e2 | -0.5674446 |
| Eif4e3 | 0.05985704 |
| Eif4e3 | 0.48532315 |
| Eif4e3 | 0.98815149 |
| Eif4ebp1 | 0.04998152 |
| Eif4ebp2 | 2.50041143 |
| Eif4el3 | 1.45261976 |
| Eif4enif1 | 0.13121441 |
| Eif4enif1 | -0.2421201 |
| Eif4g1 | 0.20736892 |
| Eif4g2 | -0.1668639 |
| Eif4g2 | -0.0412018 |
| Eif4h | -0.6664947 |
| Eif5 | -0.3437063 |
| Eif5 | -0.563841 |
| Eif5 | 0.9177157 |
| Eif5a | -0.4160456 |
| Eif5a | -0.656093 |
| Eif6 | 0.15381486 |
| Ela1 | 2.97362708 |
| Ela2 | -0.1923856 |
| Ela2a | 1.55800822 |
| Ela3b | -0.6141088 |
| Elac2 | 3.18198455 |
| Elac2 | 2.75636434 |
| Elac2 | 0.92297113 |
| Elavl1 | -0.5019644 |
| Elavl2 | -0.4689543 |
| Elavl2 | -0.7341133 |
| Elavl3 | 1.920407 |
| Elavl4 | 3.65897827 |
| Elavl4 | -0.2703851 |
| Elf1 | 1.79965428 |
| Elf2 | 0.22241609 |
| Elf2 | 0.77394284 |
| Elf2 | -0.8396062 |
| Elf2 | -0.0395781 |
| Elf2 | 2.54181758 |
| Elf3 | -0.4541606 |
| Elf3 | 1.2332476 |
| Elf3 | 1.23867992 |
| Elf5 | -0.7968664 |
| Elfn1 | -0.3076695 |
| Elfn2 | -0.4728532 |
| Elk3 | 2.02409418 |
| Elk3 | 1.82446863 |
| Elk3 | 0.54780741 |
| Ell | 1.25439168 |
| Ell3 | 0.21076847 |
| Elmo1 | 0.91629011 |
| Elmo1 | -0.4484362 |
| Elmo1 | 0.68875447 |
| Elmo1 | -0.1804815 |
| Elmo1 | -0.2181466 |
| Elmo1 | 1.27293509 |
| Elmo1 | 2.29022375 |
| Elmo1 | 0.07715456 |
| Elmo1 | -0.4952664 |
| Elmo1 | 0.76152417 |
| Elmo1 | -0.6434512 |
| Elmo1 | -0.491271 |
| Elmo1 | -0.2305544 |
| Elmo2 | -0.2335575 |
| Elmo2 | -0.4283682 |
| Elmo3 | 0.98458344 |
| Elmo3 | -0.6103171 |
| Elmod1 | -0.8213164 |
| Eln | -0.6148691 |
| Elof1 | -0.4767688 |
| Elovl1 | -0.7059591 |
| Elovl1 | 0.42730962 |
| Elovl1 | -0.1996031 |
| Elovl1 | -0.2761443 |
| Elovl2 | -0.7848821 |
| Elovl4 | 0.11628737 |
| Elovl5 | 0.95798371 |
| Elovl6 | 0.71774544 |
| Elp2 | 1.11309019 |
| Elp3 | -0.7186492 |
| Elp3 | 1.39156286 |
| Eltd1 | 1.06816821 |
| Emb | -0.4919357 |
| Emcn | 1.49633315 |
| Emd | -0.1706003 |
| Emd | -0.469603 |
| Emg1 | 2.16388694 |
| Emg1 | 0.12106335 |
| Emid1 | -0.5053318 |
| Emid2 | -0.1357063 |
| Emilin1 | 1.15161728 |
| Emilin2 | 0.14788651 |
| Eml1 | -0.6458083 |
| Eml3 | -0.2600056 |
| Eml3 | -0.1280799 |
| Eml4 | -0.5495671 |
| Eml5 | 1.43886221 |
| Emp1 | -0.5761514 |
| Emp2 | -0.5915907 |
| Emp3 | -0.7272081 |
| Emr1 | -0.1580508 |
| Emr1 | 2.22761915 |
| Emr1 | 1.25093442 |
| En2 | 3.52589919 |
| Enah | 0.72761771 |
| Enc1 | 0.48623672 |
| Endod1 | 1.31568691 |
| Endogl1 | -0.5073583 |
| Endogl1 | 0.46241665 |
| Eng | 3.98051085 |
| Eno1 | -0.2034759 |
| Eno2 | -0.3285644 |
| Eno3 | 1.717398 |
| Enoph1 | 0.35672935 |
| Enox1 | 0.08709579 |
| Enpp1 | -0.0262916 |
| Enpp1 | 2.07321108 |
| Enpp2 | 2.42683405 |
| Enpp4 | -0.0112273 |
| Enpp5 | -0.1904705 |
| Enpp6 | 0.33328858 |
| Ensa | 2.52150917 |
| Ensa | -0.2620721 |
| ENSMUSG00000043795 | -0.5258011 |
| ENSMUSG00000053178 | -0.8773205 |
| ENSMUSG00000068790 | -0.6179163 |
| Entpd2 | 0.55079044 |
| Entpd3 | 1.93593842 |
| Entpd4 | 0.81469322 |
| Entpd5 | 0.09972068 |
| Entpd5 | 1.22034303 |
| Entpd5 | 0.2363203 |
| Entpd6 | 1.19754866 |
| Entpd7 | 1.86708209 |
| Ep300 | -0.3324661 |
| Ep400 | -0.3087574 |
| Epas1 | -0.6827319 |
| Epb4.1 | 1.05133734 |
| Epb4.1l1 | 0.83206401 |
| Epb4.1l1 | 0.79682369 |
| Epb4.1l1 | 1.84862771 |
| Epb4.1l2 | 0.7415387 |
| Epb4.1l3 | 0.44419535 |
| Epb4.1l3 | 0.22424171 |
| Epb4.1l3 | 1.08029315 |
| Epb4.1l4a | -0.6356378 |
| Epb4.1l5 | -0.3268974 |
| Epb4.1l5 | 0.22576029 |
| Epb4.9 | -0.6278906 |
| Epb4.9 | 0.64279623 |
| Epc1 | 1.02878202 |
| Epc1 | 0.33652063 |
| Epc1 | 1.48191595 |
| Epdr1 | -0.7472028 |
| Epgn | 1.12588676 |
| Epha1 | 1.35166723 |
| Epha1 | -0.6632816 |
| Epha3 | 0.39206756 |
| Epha3 | -0.5383062 |
| Epha3 | 1.38773591 |
| Epha4 | -0.6465953 |
| Epha5 | 0.60989246 |
| Epha6 | 0.11286063 |
| Epha7 | -0.0505942 |
| Epha7 | -0.1890368 |
| Epha7 | 2.80891962 |
| Ephb1 | -0.3706773 |
| Ephb2 | 2.00058433 |
| Ephb4 | 0.74084766 |
| Ephb6 | 0.77743656 |
| Ephx1 | -0.4490705 |
| Ephx2 | -0.3853542 |
| Epm2a | 0.10804152 |
| Epm2aip1 | 1.49496983 |
| Epm2aip1 | 0.68416922 |
| Epm2aip1 | -0.3482413 |
| Epn1 | -0.6876597 |
| Epn2 | 0.88005736 |
| Epn2 | -0.6951017 |
| Epn2 | 1.11355031 |
| Epn2 | -0.6255792 |
| Epn2 | 0.83736712 |
| Epor | 0.77394284 |
| Eprs | -0.5432158 |
| Eprs | -0.6951017 |
| Eps15 | 0.39661072 |
| Eps15l1 | -0.4579968 |
| Eps8 | 1.50379532 |
| Eps8l1 | -0.590109 |
| Eps8l2 | -0.0355315 |
| Eps8l2 | -0.5168748 |
| Eps8l3 | 2.04210986 |
| Epsti1 | -0.766746 |
| Epsti1 | 1.87266699 |
| Eraf | 0.60629584 |
| Eral1 | 0.27249327 |
| Ercc1 | 1.63471892 |
| Ercc2 | 0.20488857 |
| Ercc3 | -0.1254033 |
| Ercc3 | 1.93500175 |
| Ercc4 | -0.7922385 |
| Ercc4 | 3.4903706 |
| Ercc5 | -0.1047034 |
| Ercc5 | -0.5383062 |
| Ercc8 | -0.2904364 |
| Ercc8 | 0.70291453 |
| Erdr1 | -0.5046573 |
| Erf | -0.6664947 |
| Erf | -0.0387673 |
| Ergic1 | 1.13503115 |
| Ergic2 | 0.88980184 |
| Ergic2 | 0.25766788 |
| Ergic3 | 0.2488335 |
| Ergic3 | 2.03065959 |
| Erh | -0.3596782 |
| Erich1 | -0.7315178 |
| Erlin1 | 0.80684786 |
| Erlin2 | -0.1312124 |
| Erlin2 | 2.31774116 |
| Ermap | -0.631756 |
| Ermp1 | -0.6371951 |
| Ern1 | 1.2407838 |
| Ern2 | 1.46531267 |
| Ero1lb | -0.5616857 |
| Erp29 | -0.2808836 |
| Errfi1 | 1.95795279 |
| Es22 | -0.5975416 |
| Esam1 | 1.68349177 |
| Esco2 | 0.50028162 |
| Esd | -0.0159577 |
| Esd | 1.69049672 |
| Esd | 1.93631282 |
| Esd | -0.7658485 |
| Esm1 | -0.3936773 |
| Espl1 | -0.2054185 |
| Espn | 1.11504409 |
| Espn | 1.8260007 |
| Espn | 0.00937771 |
| Espn | -0.6333067 |
| Esr1 | 0.11526069 |
| Esr2 | 0.83237681 |
| Esr2 | -0.3539419 |
| Esr2 | -0.3695132 |
| Esrra | -0.6278906 |
| Esrrb | 0.40088086 |
| Etaa1 | 0.81117914 |
| Etf1 | 2.46490059 |
| Etfa | -0.469603 |
| Etfa | 1.70416684 |
| Etfa | -0.4277481 |
| Etfb | 0.63720025 |
| Etfb | 1.41626191 |
| Etfdh | 0.2039567 |
| Ethe1 | -0.060914 |
| Etnk2 | -0.10732 |
| Ets1 | 1.35379232 |
| Ets2 | -0.3990681 |
| Etsrp71 | -0.2385846 |
| Etv1 | -0.5362101 |
| Etv1 | 3.05474557 |
| Etv4 | -0.3175076 |
| Etv5 | -0.7739574 |
| Etv6 | -0.7569222 |
| Evc | -0.3794551 |
| Evc2 | -0.1438512 |
| Evi1 | 0.92452775 |
| Evi1 | 0.31061538 |
| Evi2a | -0.6035314 |
| Evi2a | -0.6110742 |
| Evi5 | 0.66239943 |
| Evi5l | -0.7237749 |
| Evl | -0.5348154 |
| Evx1 | -0.1752923 |
| Exdl1 | -0.5524041 |
| Exdl2 | -0.7913157 |
| Exdl2 | 1.13940162 |
| Exdl2 | 1.65847991 |
| Exoc2 | 0.04703485 |
| Exoc3 | -0.1249579 |
| Exoc3l | -0.255373 |
| Exoc4 | 1.28550485 |
| Exoc7 | -0.5341188 |
| Exoc8 | -0.7160971 |
| Exod1 | -0.2688206 |
| Exosc1 | 0.15315805 |
| Exosc10 | 1.19429059 |
| Exosc2 | 0.12140354 |
| Exosc2 | -0.7775845 |
| Exosc4 | -0.1303163 |
| Exosc5 | -0.419725 |
| Exosc6 | 1.69914783 |
| Exosc7 | -0.3642933 |
| Exosc8 | -0.2245776 |
| Ext1 | -0.8251366 |
| Ext2 | 0.06022076 |
| Extl1 | 1.99230348 |
| Extl2 | 0.32297776 |
| Extl3 | 1.50514556 |
| Extl3 | 2.75851945 |
| Eya1 | -0.3476732 |
| Eya1 | 0.07393608 |
| Eya2 | -0.4383472 |
| Eya3 | -0.5341188 |
| Eya3 | 1.32850524 |
| Eya3 | -0.8052561 |
| Eya4 | -0.4136009 |
| Ezh1 | 2.85901199 |
| Ezh2 | 1.10337393 |
| Ezh2 | -0.7263486 |
| F10 | -0.38299 |
| F11 | -0.5805361 |
| F11 | -0.5134647 |
| F11r | -0.656889 |
| F13a1 | -0.5278735 |
| F2r | 0.64510183 |
| F2rl1 | 0.17201674 |
| F2rl3 | -0.5467389 |
| F430201B04Rik | 0.45299634 |
| F5 | 1.54883564 |
| F630043A04Rik | -0.5645606 |
| F730014I05Rik | 0.15414309 |
| F730047E07Rik | 0.53339522 |
| F730047E07Rik | 0.04887763 |
| F730047E07Rik | -0.0810602 |
| F8 | -0.0810602 |
| F8 | 1.61226004 |
| F8 | -0.659281 |
| F8a | 1.60058408 |
| Fa2h | -0.3142165 |
| Faah | 0.02500505 |
| Faah | 0.35541346 |
| Fabp3 | -0.7332473 |
| Fabp5 | 1.28598121 |
| Fabp9 | 2.27835642 |
| Fadd | 2.10822751 |
| Fadd | 1.90325003 |
| Fadd | 1.79376795 |
| Fads1 | 0.65749053 |
| Fads2 | 0.81612692 |
| Fads3 | -0.6171536 |
| Fads6 | 0.14490786 |
| Faf1 | 1.02739317 |
| Faf1 | -0.5997832 |
| Fah | -0.672955 |
| Fahd1 | 0.62590456 |
| Fahd2a | 0.08036175 |
| Fahd2a | -0.3516574 |
| Fahd2a | 1.01056036 |
| Fahd2a | 0.96219972 |
| Faim | -0.4044909 |
| Faim | -0.584942 |
| Faim2 | -0.0717714 |
| Faim2 | 1.66674683 |
| Faim3 | 2.48825783 |
| Fancc | 1.21201646 |
| Fancc | -0.7867157 |
| Fancd2 | 0.3728911 |
| Fancg | -0.7876338 |
| Fanci | 0.17137287 |
| Fancl | 0.26844742 |
| Fancl | -0.4050954 |
| Fank1 | 0.25031184 |
| Fank1 | 0.0284 |
| Fap | -0.1909489 |
| Fars2 | -0.0700927 |
| Farsa | -0.56889 |
| Farsb | -0.491271 |
| Farsb | -0.2325553 |
| Fas | -0.0395781 |
| Fas | 0.07286074 |
| Fasl | -0.4748089 |
| Fastk | 0.89463544 |
| Fastk | -0.6042829 |
| Fastkd1 | 0.33247877 |
| Fastkd5 | 0.64644409 |
| Fat1 | 0.99576947 |
| Fat4 | 0.15545483 |
| Fbf1 | -0.0469074 |
| Fblim1 | -0.7152479 |
| Fbln1 | 0.06602071 |
| Fbln1 | 0.41899415 |
| Fbln1 | -0.5761514 |
| Fbln2 | 0.16943854 |
| Fbln2 | -0.4352171 |
| Fbn1 | -0.6225063 |
| Fbp1 | 2.0343662 |
| Fbp2 | 0.1745877 |
| Fbxl10 | 0.17072855 |
| Fbxl12 | 0.92861892 |
| Fbxl12 | 0.8566038 |
| Fbxl12 | 0.50655449 |
| Fbxl12 | 1.73348254 |
| Fbxl13 | 0.07893772 |
| Fbxl13 | -0.1594367 |
| Fbxl14 | 0.11286063 |
| Fbxl15 | 0.80733013 |
| Fbxl18 | -0.0274915 |
| Fbxl18 | 1.10928539 |
| Fbxl21 | 0.70597183 |
| Fbxl22 | 0.47266818 |
| Fbxl4 | -0.7721492 |
| Fbxl5 | -0.4364678 |
| Fbxl6 | -0.5595354 |
| Fbxl6 | -0.5864153 |
| Fbxl8 | 0.0547497 |
| Fbxo15 | 0.27191639 |
| Fbxo16 | -0.6095606 |
| Fbxo18 | -0.1852245 |
| Fbxo18 | 0.1359133 |
| Fbxo2 | 0.70668971 |
| Fbxo21 | 0.77510889 |
| Fbxo25 | -0.3208107 |
| Fbxo27 | 0.04666585 |
| Fbxo28 | 0.18384733 |
| Fbxo3 | -0.5237335 |
| Fbxo3 | 0.85278868 |
| Fbxo30 | 1.96880963 |
| Fbxo31 | 1.19807307 |
| Fbxo31 | 0.29386801 |
| Fbxo32 | 1.25073647 |
| Fbxo32 | -0.3358237 |
| Fbxo32 | -0.6664947 |
| Fbxo33 | 0.0696271 |
| Fbxo34 | -0.707641 |
| Fbxo34 | -0.6664947 |
| Fbxo34 | 0.57638189 |
| Fbxo36 | 0.05034919 |
| Fbxo38 | 0.33247877 |
| Fbxo38 | -0.3972676 |
| Fbxo4 | -0.257429 |
| Fbxo42 | -0.0764041 |
| Fbxo44 | 2.16158539 |
| Fbxo6 | -0.4813582 |
| Fbxo7 | -0.7857984 |
| Fbxo8 | -0.5595354 |
| Fbxo9 | 0.34054471 |
| Fbxo9 | 0.09972068 |
| Fbxw12 | 1.63458879 |
| Fbxw13 | -0.6753895 |
| Fbxw15 | 0.33947335 |
| Fbxw16 | 0.32161238 |
| Fbxw17 | 0.81181907 |
| Fbxw19 | -0.459921 |
| Fbxw2 | 2.68325262 |
| Fbxw4 | 0.96504472 |
| Fbxw4 | -0.192865 |
| Fbxw5 | -0.4715517 |
| Fbxw7 | 0.27938767 |
| Fbxw7 | -0.2088279 |
| Fbxw7 | 1.05575794 |
| Fbxw8 | -0.4605633 |
| Fcer1g | 0.8153307 |
| Fcer2a | -0.1885594 |
| Fcf1 | -0.5915907 |
| Fcgr2b | 2.90022719 |
| Fcgr2b | 0.13390245 |
| Fcgr2b | -0.56889 |
| Fcgr3 | -0.6737658 |
| Fcgr4 | 2.531207 |
| Fcgrt | -0.3978674 |
| Fcho1 | -0.7375856 |
| Fcho2 | -0.0139837 |
| Fchsd1 | 0.05511546 |
| Fchsd2 | 0.74739158 |
| Fcna | 0.60869516 |
| Fcrla | 2.13256203 |
| Fcrla | 0.16198548 |
| Fcrla | 2.79929613 |
| Fdps | 1.08907588 |
| Fdxr | -0.2451615 |
| Fdxr | 2.03537449 |
| Fem1b | 2.80713307 |
| Fem1c | -0.2245776 |
| Fem1c | 1.35547119 |
| Fen1 | -0.7375856 |
| Fermt2 | 1.63458879 |
| Fermt3 | 0.49464427 |
| Fert2 | 1.71465972 |
| Fert2 | -0.4683061 |
| Fes | 2.21200225 |
| Fetub | -0.4966021 |
| Fev | 0.37444009 |
| Fez1 | 0.41208892 |
| Fez1 | 1.17352587 |
| Fez2 | -0.605035 |
| Fezf2 | 2.67033865 |
| Ffar2 | -0.5348154 |
| Fgd2 | -0.6917867 |
| Fgd4 | 2.98653093 |
| Fgd4 | -0.7730528 |
| Fgd5 | -0.568167 |
| Fgd6 | 0.68765611 |
| Fgf1 | -0.5581047 |
| Fgf1 | 3.12088286 |
| Fgf10 | 0.01283104 |
| Fgf10 | -0.7748628 |
| Fgf12 | 1.30193794 |
| Fgf12 | 0.62551255 |
| Fgf13 | 0.20891574 |
| Fgf13 | 1.78552916 |
| Fgf17 | 1.31633211 |
| Fgf21 | 2.15427715 |
| Fgf22 | 1.18178166 |
| Fgf22 | 0.74944839 |
| Fgf5 | 0.69076473 |
| Fgf7 | -0.6217397 |
| Fgf8 | 0.68066899 |
| Fgf9 | 1.01594992 |
| Fgfbp3 | 0.03178241 |
| Fgfr1 | -0.3103917 |
| Fgfr1op | -0.4008721 |
| Fgfr1op2 | -0.6465953 |
| Fgfr1op2 | 0.60929401 |
| Fgfr2 | 0.01703415 |
| Fgfr2 | -0.3871314 |
| Fgfr3 | -0.5100672 |
| Fgfrl1 | -0.6395361 |
| Fgl2 | 1.11813878 |
| Fh1 | -0.10732 |
| Fhad1 | 2.19442184 |
| Fhit | 2.90873711 |
| Fhl1 | 2.19938412 |
| Fhl1 | 1.01107502 |
| Fhl2 | 1.27293509 |
| Fhl4 | -0.7428188 |
| Fhl5 | 1.68373847 |
| Fhod1 | 2.73583862 |
| Fhod3 | -0.3341433 |
| Fibcd1 | 1.75341287 |
| Fibp | 0.42487173 |
| Figf | -0.8803652 |
| Figla | -0.5362101 |
| Fign | -0.7203546 |
| Fignl1 | -0.5355125 |
| Fip1l1 | -0.1847491 |
| Fis1 | 1.73814288 |
| Fis1 | -0.5990353 |
| Fiz1 | -0.235565 |
| Fjx1 | 0.98020351 |
| Fkbp10 | 2.1027438 |
| Fkbp11 | 0.52686769 |
| Fkbp14 | -0.6505407 |
| Fkbp1a | 1.25970025 |
| Fkbp1b | 0.56577076 |
| Fkbp2 | -0.4289887 |
| Fkbp2 | -0.1562061 |
| Fkbp4 | -0.3648718 |
| Fkbp5 | 0.06022076 |
| Fkbp6 | 1.34374331 |
| Fkbp7 | 1.97690211 |
| Fkbp8 | 0.7589862 |
| Fkbp9 | -0.3966683 |
| Fkbpl | 0.46030552 |
| Fkbpl | -0.4702521 |
| Fkhl18 | -0.0530604 |
| Fkrp | 1.23132854 |
| Fktn | -0.1470383 |
| Flad1 | -0.0367436 |
| Flcn | 2.07695021 |
| Flcn | -0.4172704 |
| Fli1 | -0.3263423 |
| Flii | 1.35131257 |
| Flna | 2.71924697 |
| Flot1 | 0.37572892 |
| Flot1 | 0.21138521 |
| Flot2 | -0.5425128 |
| Flot2 | 1.78855867 |
| Flot2 | 0.89156247 |
| Flrt1 | 1.48590529 |
| Flrt2 | -0.2335575 |
| Flrt3 | -0.7059591 |
| Flt1 | 0.34668079 |
| Flt4 | 1.24208379 |
| Flt4 | -0.5945613 |
| Flvcr2 | 1.4760539 |
| Flywch2 | 0.51787073 |
| Fmn2 | -0.1701324 |
| Fmn2 | -0.2345607 |
| Fmnl1 | 0.07321933 |
| Fmnl3 | 1.59570873 |
| Fmnl3 | 0.62315699 |
| Fmo1 | -0.553826 |
| Fmo2 | 1.19754866 |
| Fmo5 | -0.1012262 |
| Fmod | -0.5141457 |
| Fmr1nb | 0.75030404 |
| Fmr1nb | -0.840579 |
| Fn3k | 1.55864475 |
| Fn3k | -0.053472 |
| Fnbp1 | 0.91557649 |
| Fnbp1 | 1.71245124 |
| Fnbp1 | 1.04417003 |
| Fnbp1l | 2.59241463 |
| Fnbp4 | -0.6917867 |
| Fndc3 | -0.0485442 |
| Fndc3a | 1.63054557 |
| Fndc3b | 0.52926653 |
| Fndc4 | 3.26902515 |
| Fndc5 | 1.96342002 |
| Fndc5 | -0.469603 |
| Fndc7 | -0.6156299 |
| Fndc7 | -0.2872411 |
| Fndc8 | -0.1622149 |
| Fndc8 | -0.3683507 |
| Fnip1 | -0.7254899 |
| Fnip1 | -0.7059591 |
| Fnta | -0.4926009 |
| Fnta | -0.1402223 |
| Fntb | -0.2610383 |
| Folr1 | 0.89871674 |
| Folr1 | 0.72004673 |
| Folr2 | 1.95144073 |
| Fos | 1.29602206 |
| Fosb | -0.5616857 |
| Foxa1 | 2.14011998 |
| Foxa2 | -0.5209838 |
| Foxa3 | -0.219133 |
| Foxc1 | -0.4567163 |
| Foxd2 | 2.09439708 |
| Foxg1 | 0.26409263 |
| Foxh1 | 0.79324016 |
| Foxh1 | 0.60769621 |
| Foxj1 | -0.2132302 |
| Foxj2 | -0.7551474 |
| Foxj3 | 1.42276459 |
| Foxj3 | -0.6465953 |
| Foxj3 | 2.3820982 |
| Foxj3 | 0.6106898 |
| Foxj3 | 0.67139337 |
| Foxk1 | -0.2610383 |
| Foxk1 | 0.08603581 |
| Foxk1 | 1.41914855 |
| Foxl2 | -0.6209738 |
| Foxm1 | -0.3302345 |
| Foxm1 | -0.3771061 |
| Foxn2 | 3.04730137 |
| Foxn3 | -0.4979397 |
| Foxo1 | 1.72721199 |
| Foxo6 | -0.6600797 |
| Foxo6 | -0.590109 |
| Foxp1 | -0.5313378 |
| Foxp2 | 0.54738046 |
| Foxp2 | -0.590109 |
| Foxp2 | 0.239913 |
| Foxp3 | 0.43071179 |
| Foxp4 | 1.6191021 |
| Foxq1 | 1.45174608 |
| Foxred1 | -0.6753895 |
| Foxred1 | -0.0151676 |
| Foxred1 | -0.0734533 |
| Foxred1 | 0.86675054 |
| Foxred1 | -0.0780945 |
| Foxred2 | 0.47705971 |
| Fpgs | 0.58911812 |
| Fpgs | 0.43940257 |
| Fpgs | -0.2216038 |
| Fpr2 | -0.5012923 |
| Frag1 | 0.1851172 |
| Frag1 | 1.69860194 |
| Frag1 | -0.6819131 |
| Frap1 | 3.62979715 |
| Fras1 | 2.53086503 |
| Fras1 | -0.4579968 |
| Frat1 | 0.71526063 |
| Frat1 | -0.4209547 |
| Frat2 | 0.77743656 |
| Freq | 1.71233171 |
| Freq | 0.3061793 |
| Freq | -0.4780777 |
| Frg1 | -0.6794613 |
| Frk | -0.3164093 |
| Frmd3 | -0.713552 |
| Frmd6 | -0.5341188 |
| Frmd8 | 2.25146804 |
| Frmpd4 | -0.6967637 |
| Frrs1 | -0.1904705 |
| Frs2 | 1.40771373 |
| Frs3 | 1.29479655 |
| Fryl | -0.1108205 |
| Fryl | -0.1724742 |
| Frzb | 0.40562991 |
| Fscn1 | 3.74040331 |
| Fscn2 | -0.248213 |
| Fscn3 | -0.7212085 |
| Fsd1 | 0.55503512 |
| Fsd2 | 1.06913449 |
| Fshb | 0.24468142 |
| Fshr | 3.23641703 |
| Fshr | -0.4289887 |
| Fshr | -0.459921 |
| Fsip1 | 0.61684568 |
| Fst | -0.4939326 |
| Fstl1 | -0.8834201 |
| Fstl4 | 0.04148414 |
| Fstl5 | -0.5026369 |
| Fth1 | -0.7533759 |
| Fth1 | 0.73547431 |
| Ftmt | 0.87574265 |
| Fto | -0.0339178 |
| Ftsj1 | -0.3906961 |
| Ftsj3 | -0.6513318 |
| Fubp1 | -0.6600797 |
| Fubp3 | 0.50207826 |
| Fuk | 3.11134679 |
| Fuk | 1.42481281 |
| Fundc1 | -0.3420114 |
| Fundc1 | 2.08127333 |
| Fundc2 | 1.36644024 |
| Furin | -0.7152479 |
| Furin | -0.7685435 |
| Fus | 1.84164913 |
| Fusip1 | 2.45023711 |
| Fusip1 | -0.6042829 |
| Fusip1 | -0.4522485 |
| Fut10 | 1.31568691 |
| Fut10 | 0.62512038 |
| Fut2 | -0.4433778 |
| Fut4 | 0.28906665 |
| Fut8 | 0.4413227 |
| Fut8 | -0.2610383 |
| Fut8 | 0.47890249 |
| Fvt1 | -0.6255792 |
| Fxc1 | -0.0968982 |
| Fxn | -0.5006208 |
| Fxn | -0.5258011 |
| Fxyd1 | 2.33139311 |
| Fxyd2 | 3.65269142 |
| Fxyd2 | 0.21323295 |
| Fxyd2 | -0.8415528 |
| Fxyd3 | -0.607295 |
| Fxyd4 | -0.4258904 |
| Fxyd4 | -0.606541 |
| Fxyd5 | 1.99594928 |
| Fxyd5 | 0.08249365 |
| Fxyd5 | -0.0359353 |
| Fxyd6 | 0.73373422 |
| Fxyd7 | -0.6843715 |
| Fyb | -0.6762024 |
| Fyco1 | -0.5805361 |
| Fyn | 2.82179105 |
| Fyttd1 | -0.0014501 |
| Fzd1 | -0.034321 |
| Fzd10 | -0.672145 |
| Fzd10 | -0.5244222 |
| Fzd2 | -0.5754227 |
| Fzd3 | 1.1225905 |
| Fzd4 | -0.656093 |
| Fzd5 | -0.6156299 |
| Fzd6 | -0.2290565 |
| Fzd7 | -0.584942 |
| Fzd8 | 1.03142763 |
| Fzr1 | 1.62714066 |
| Fzr1 | 0.65692243 |
| Fzr1 | 0.65597479 |
| G0s2 | -0.7560344 |
| G3bp1 | -0.273522 |
| G3bp1 | -0.7463243 |
| G3bp2 | 0.13892132 |
| G3bp2 | 0.11765448 |
| G3bp2 | 1.57295522 |
| G430022H21Rik | 1.18773943 |
| G430055L02Rik | -0.7867157 |
| G6pc | -0.1762336 |
| G6pc2 | 0.3703036 |
| G6pc2 | -0.6664947 |
| G6pc3 | 0.64510183 |
| G6pd2 | 0.61585559 |
| G6pd2 | -0.4044909 |
| G6pdx | -0.587891 |
| Gaa | -0.3741783 |
| Gab1 | 1.81356465 |
| Gab2 | 1.00811175 |
| Gab2 | 1.68009292 |
| Gab2 | 1.56273624 |
| Gab3 | -0.7026046 |
| Gab3 | -0.3319077 |
| Gab3 | -0.430231 |
| Gabarap | -0.0290939 |
| Gabarapl1 | 0.86463961 |
| Gabarapl2 | 0.61009186 |
| Gabbr1 | -0.469603 |
| Gabpa | -0.545328 |
| Gabpa | -0.6745773 |
| Gabpb1 | -0.1557456 |
| Gabpb1 | -0.0551206 |
| Gabpb2 | -0.447169 |
| Gabra3 | 0.26380157 |
| Gabra4 | 2.76063149 |
| Gabrb1 | -0.2411086 |
| Gabrb2 | -0.6005316 |
| Gabrb3 | -0.7118593 |
| Gabrb3 | -0.1222905 |
| Gabrd | -0.5624036 |
| Gabrd | -0.0022284 |
| Gabrg1 | 2.00062794 |
| Gabrg2 | -0.0367436 |
| Gabrg2 | 0.03850993 |
| Gabrg2 | -0.4528854 |
| Gabrg2 | -0.2925729 |
| Gabrp | -0.518927 |
| Gabrp | -0.632531 |
| Gad1 | -0.6934427 |
| Gad1 | -0.4722022 |
| Gadd45a | 1.56855965 |
| Gadd45a | 0.90567119 |
| Gadd45b | -0.1420349 |
| Gadd45g | 1.56252506 |
| Gadd45g | 1.02638177 |
| Gak | 1.02991679 |
| Gal | 0.51919289 |
| Gal3st1 | -0.0947418 |
| Gal3st2 | 0.11662934 |
| Gal3st3 | 0.60368946 |
| Galc | -0.4554375 |
| Galc | 0.2260637 |
| Gale | -0.6608791 |
| Gale | -0.7968664 |
| Galk1 | -0.3252333 |
| Galm | 2.57113197 |
| Galns | 0.9786056 |
| Galnt1 | 0.44729725 |
| Galnt1 | -0.1687302 |
| Galnt1 | 1.04093878 |
| Galnt1 | -0.2431328 |
| Galnt10 | 0.69767545 |
| Galnt10 | 0.45536053 |
| Galnt10 | 0.74032903 |
| Galnt11 | -0.4166578 |
| Galnt12 | -0.4846506 |
| Galnt13 | -0.766746 |
| Galnt2 | 1.38243685 |
| Galnt2 | 1.65231011 |
| Galnt2 | -0.4807012 |
| Galnt3 | 1.11435496 |
| Galnt9 | -0.352228 |
| Galntl2 | 0.80507738 |
| Galntl4 | -0.4129907 |
| Galntl5 | 2.95013843 |
| Galt | 1.09987868 |
| Galt | -0.0793643 |
| Gamt | 2.92763154 |
| Gan | -0.6465953 |
| Ganab | -0.5012923 |
| Ganab | 1.03744617 |
| Ganab | 1.31393335 |
| Ganc | 1.70229549 |
| Gap43 | -0.2782477 |
| Gapdh | 0.87872048 |
| Gapdhs | -0.2646618 |
| Gapvd1 | 2.50399103 |
| Gapvd1 | 0.7699336 |
| Garnl3 | -0.0514155 |
| Garnl4 | -0.4111627 |
| Gars | 1.95194685 |
| Gart | -0.6705271 |
| Gart | -0.7922385 |
| Gas2l1 | 0.32106566 |
| Gas2l1 | 3.11639783 |
| Gas2l1 | 1.73243068 |
| Gas2l1 | 2.19955959 |
| Gas6 | -0.713552 |
| Gas7 | 0.87395128 |
| Gas7 | -0.3689318 |
| Gas8 | -0.3448379 |
| Gata2 | -0.0493636 |
| Gata4 | -0.5710624 |
| Gata6 | -0.5768808 |
| Gatad1 | -0.6640838 |
| Gatad1 | -0.0747168 |
| Gatad2b | 1.45902764 |
| Gats | -0.4490705 |
| Gats | 3.86904415 |
| Gba | -0.1488646 |
| Gba2 | 1.44256187 |
| Gbas | 2.00123834 |
| Gbf1 | -0.5856783 |
| Gbl | -0.3631373 |
| Gbl | -0.4258904 |
| Gbl | -0.2395934 |
| Gbp2 | -0.2888374 |
| Gbp3 | -0.6909598 |
| Gbp3 | -0.2931078 |
| Gbp6 | 1.62805861 |
| Gbp6 | 2.65134782 |
| Gbx2 | 2.20445925 |
| Gca | -0.145671 |
| Gca | -0.4402304 |
| Gcap14 | 0.90509366 |
| Gcap14 | 0.83440741 |
| Gcc1 | -0.5827364 |
| Gcc2 | -0.6648867 |
| Gcdh | 1.43385193 |
| Gch1 | -0.7694436 |
| Gch1 | 2.77432641 |
| Gchfr | 1.01466957 |
| Gck | -0.7160971 |
| Gclm | 0.32706171 |
| Gcn5l2 | 1.37814095 |
| Gcnt1 | 3.82885489 |
| Gcnt1 | -0.7315178 |
| Gcnt2 | 2.00058433 |
| Gcnt2 | -0.2416142 |
| Gcs1 | -0.668912 |
| Gda | 0.49283295 |
| Gdap1 | -0.7323821 |
| Gdap1 | 0.66013706 |
| Gdap1l1 | 1.5694684 |
| Gdap1l1 | -0.7177977 |
| Gde1 | 1.76024463 |
| Gde1 | 1.32622939 |
| Gdf10 | -0.7101696 |
| Gdf15 | 0.84865041 |
| Gdf3 | 0.58071566 |
| Gdf9 | 2.31133505 |
| Gdi1 | 0.62805759 |
| Gdi1 | 0.91899683 |
| Gdi1 | 1.16247364 |
| Gdi1 | 0.6765993 |
| Gdi1 | 3.9386249 |
| Gdi2 | -0.4892798 |
| Gdnf | -0.3313496 |
| Gdpd1 | 1.2020488 |
| Gdpd2 | 1.34927059 |
| Gdpd2 | 0.42803972 |
| Gdpd3 | -0.0481347 |
| Gdpd4 | -0.3386311 |
| Gdpd5 | -0.4265092 |
| Gemin4 | -0.4008721 |
| Gemin4 | 0.53252757 |
| Gemin4 | 0.24764912 |
| Gemin5 | 1.14519663 |
| Gemin6 | -0.7631615 |
| Gemin6 | -0.7237749 |
| Gemin7 | 0.16879285 |
| Gemin7 | -0.6672998 |
| Gfap | 0.07929393 |
| Gfer | -0.5930748 |
| Gfi1 | -0.3175076 |
| Gfm1 | -0.0223032 |
| Gfod1 | -0.5334228 |
| Gfod2 | 0.80411021 |
| Gfpt2 | -0.1402223 |
| Gfra1 | -0.2558866 |
| Gfra4 | -0.4105541 |
| Gga1 | 1.65364938 |
| Gga1 | 0.20333493 |
| Gga1 | -0.5306439 |
| Gga3 | -0.0776716 |
| Ggcx | -0.3912915 |
| Ggcx | 0.1217436 |
| Ggcx | -0.8562865 |
| Ggn | -0.447169 |
| Ggn | 2.32017422 |
| Ggnbp1 | -0.6521236 |
| Ggnbp2 | 1.23294486 |
| Ggt5 | 0.84433901 |
| Ggt7 | -0.6497503 |
| Ggta1 | 0.36796857 |
| Ggtla1 | 0.02538289 |
| Gh | -0.6411001 |
| Ghitm | -0.7739574 |
| Ghr | 0.03365613 |
| Ghr | 0.61168549 |
| Ghrh | -0.4093383 |
| Ghrhr | 1.31402573 |
| Ghrl | -0.6545032 |
| Ghrl | -0.5908496 |
| Ghsr | 0.87693495 |
| Ghsr | -0.1943047 |
| Gif | -0.495934 |
| Gigyf1 | 3.61685014 |
| Gimap1 | 0.5693215 |
| Gimap4 | -0.4490705 |
| Gimap5 | -0.8156158 |
| Gimap6 | 2.79093193 |
| Gimap8 | -0.2740459 |
| Gimap9 | -0.6434512 |
| Gins3 | 1.78260029 |
| Gins4 | -0.4020768 |
| Gipc1 | -0.1847491 |
| Gipc1 | 0.15052608 |
| Gipc1 | 0.91686061 |
| Gipc2 | -0.3706773 |
| Git1 | -0.3966683 |
| Git2 | -0.5258011 |
| Git2 | 0.31282538 |
| Git2 | 0.61842759 |
| Gja1 | 0.02726975 |
| Gja4 | 0.8886262 |
| Gja5 | -0.424654 |
| Gja5 | 3.16791888 |
| Gja6 | 0.31860136 |
| Gja7 | -0.7445699 |
| Gjb1 | 0.24764912 |
| Gjb2 | -0.7237749 |
| Gjb2 | -0.6080496 |
| Gjb3 | 1.35255328 |
| Gjb6 | 1.84945128 |
| Gjc2 | 1.26077786 |
| Gjc2 | 0.12072303 |
| Gjd2 | 1.24786132 |
| Gjd2 | 0.37675868 |
| Gjd3 | -0.5820024 |
| Gk2 | -0.491271 |
| Gk5 | -0.6035314 |
| Gkap1 | 1.67910179 |
| Gkap1 | 1.58807283 |
| Glb1 | 1.42456726 |
| Glb1l2 | -0.7748628 |
| Glcci1 | -0.425272 |
| Gldc | 0.77444276 |
| Gldc | -0.6133492 |
| Gldn | -0.1580508 |
| Gle1 | -0.157128 |
| Glg1 | 0.50945234 |
| Glipr2 | 1.66296972 |
| Glis1 | 1.89692161 |
| Glo1 | -0.6371951 |
| Gloxd1 | 2.06978018 |
| Glp2r | 0.00937771 |
| Glra2 | 2.12980157 |
| Glrb | 0.77810052 |
| Glrp1 | -0.7830523 |
| Glrx | -0.6697192 |
| Glrx2 | 0.96369106 |
| Glrx2 | -0.7177977 |
| Glrx2 | -0.7195015 |
| Glrx3 | 0.1652334 |
| Glrx5 | -0.5369083 |
| Gls2 | 1.74635697 |
| Gls2 | 1.64635541 |
| Glt25d1 | 0.32106566 |
| Glt25d2 | -0.6133492 |
| Glt25d2 | 0.50005679 |
| Glt6d1 | 1.24278301 |
| Glt8d1 | -0.1701324 |
| Glt8d1 | -0.4986092 |
| Glt8d1 | 0.3169548 |
| Glt8d2 | 1.54489672 |
| Glt8d3 | -0.2893701 |
| Gltp | 0.99629251 |
| Gltscr1 | -0.7000968 |
| Glud1 | 3.47874051 |
| Glud1 | 0.10111275 |
| Glul | -0.6697192 |
| Glyctk | 0.50833887 |
| Glyctk | -0.6379748 |
| Glyctk | -0.6786455 |
| Gm1006 | -0.3759339 |
| Gm1006 | -0.5531148 |
| Gm1019 | -0.5588198 |
| Gm1040 | 0.78207421 |
| Gm1070 | 3.84436316 |
| Gm1082 | -0.1957467 |
| Gm114 | -0.3654507 |
| Gm114 | 0.60849546 |
| Gm1141 | -0.672955 |
| Gm128 | 2.06844471 |
| Gm129 | -0.1720054 |
| Gm129 | -0.1169788 |
| Gm1322 | -0.7051194 |
| Gm136 | 1.25053847 |
| Gm136 | 0.83690043 |
| Gm1381 | -0.7428188 |
| Gm1527 | -0.5271822 |
| Gm1568 | 0.92438635 |
| Gm1574 | 0.70651029 |
| Gm1574 | -0.5805361 |
| Gm1604 | -0.6225063 |
| Gm1604 | -0.8015179 |
| Gm166 | -0.7766763 |
| Gm166 | -0.5997832 |
| Gm1661 | 0.01321394 |
| Gm1673 | -0.3408833 |
| Gm1673 | -0.1981551 |
| Gm1698 | 2.90239466 |
| Gm1821 | -0.6450219 |
| Gm1943 | -0.7042804 |
| Gm1965 | -0.36603 |
| Gm1967 | -0.3865386 |
| Gm1967 | 0.98590657 |
| Gm216 | -0.8396062 |
| Gm239 | 2.331788 |
| Gm249 | 1.22279568 |
| Gm266 | 1.95024335 |
| Gm2a | 0.00513919 |
| Gm347 | -0.0696734 |
| Gm382 | -0.3966683 |
| Gm410 | 0.239913 |
| Gm428 | -0.0477254 |
| Gm443 | 0.20364587 |
| Gm484 | -0.7332473 |
| Gm50 | -0.563122 |
| Gm525 | -0.6521236 |
| Gm525 | 0.24527573 |
| Gm527 | -0.2872411 |
| Gm527 | 0.6931348 |
| Gm561 | -0.5581047 |
| Gm561 | -0.8752963 |
| Gm597 | -0.5967956 |
| Gm603 | 2.06271654 |
| Gm609 | -0.7803143 |
| Gm614 | -0.0675802 |
| Gm614 | -0.9030083 |
| Gm648 | 1.20069062 |
| Gm648 | 1.14918716 |
| Gm71 | -0.4846506 |
| Gm711 | 1.04640038 |
| Gm732 | 1.35015897 |
| Gm732 | -0.461206 |
| Gm767 | 0.91942347 |
| Gm773 | 1.5293343 |
| Gm773 | -0.8194122 |
| Gm784 | 1.65746507 |
| Gm813 | -0.6286624 |
| Gm813 | -0.3631373 |
| Gm817 | -0.7375856 |
| Gm867 | -0.6624801 |
| Gm884 | -0.3730098 |
| Gm889 | 0.80668705 |
| Gm906 | -0.8005857 |
| Gm93 | -0.1447607 |
| Gm93 | -0.5602516 |
| Gm944 | -0.451612 |
| Gm949 | 0.25678827 |
| Gm962 | 1.69592845 |
| Gm962 | 2.54895929 |
| Gm97 | -0.1957467 |
| Gm97 | 1.22473271 |
| Gmcl1 | 0.63137507 |
| Gmcl1 | -0.8232245 |
| Gmcl1 | 1.79584339 |
| Gmds | -0.2528094 |
| Gmeb2 | 1.62339373 |
| Gmfg | -0.7604823 |
| Gmfg | -0.7118593 |
| Gmfg | 1.32138576 |
| Gmip | 1.18199511 |
| Gmip | -0.7203546 |
| Gmppa | 1.33322097 |
| Gmppa | -0.5474451 |
| Gmppb | 1.32248468 |
| Gmpr | 2.46392855 |
| Gmpr2 | -0.5100672 |
| Gna11 | -0.5404071 |
| Gna13 | -0.0069124 |
| Gna14 | -0.4663642 |
| Gnai2 | 1.73167025 |
| Gnao1 | -0.4075175 |
| Gnao1 | -0.5768808 |
| Gnaq | 0.46077508 |
| Gnaq | -0.7127053 |
| Gna-rs1 | -0.6379748 |
| Gnas | -0.5856783 |
| Gnas | -0.3330248 |
| Gnas | -0.0530604 |
| Gnas | -0.0999256 |
| Gnas | -0.3782799 |
| Gnat1 | -0.7026046 |
| Gnat2 | 2.76531382 |
| Gnaz | 2.2679302 |
| Gnb1 | -0.4999498 |
| Gnb1l | 1.98087711 |
| Gnb2 | -0.4383472 |
| Gnb2l1 | -0.4972706 |
| Gnb3 | -0.0195216 |
| Gnb4 | 0.20860658 |
| Gnb4 | 1.77749384 |
| Gnb5 | 0.7878384 |
| Gnb5 | 1.15557997 |
| Gne | -0.4780777 |
| Gng10 | 1.52670243 |
| Gng11 | 1.7019933 |
| Gng13 | 2.34142491 |
| Gng2 | -0.273522 |
| Gng2 | 0.66070317 |
| Gng3 | 0.87499667 |
| Gng4 | -0.631756 |
| Gng7 | -0.8203638 |
| Gng8 | -0.4333441 |
| Gngt2 | 0.45889539 |
| Gnl2 | -0.7784935 |
| Gnl2 | 0.20115539 |
| Gnl3 | -0.1134547 |
| Gnl3 | -0.2777214 |
| Gnl3 | -0.6340831 |
| Gnl3l | 1.36556754 |
| Gnl3l | -0.7685435 |
| Gnmt | -0.6125903 |
| Gnmt | -0.0335149 |
| Gnpat | -0.4972706 |
| Gnpda1 | -0.2315543 |
| Gnpda1 | -0.7569222 |
| Gnpda1 | -0.3022492 |
| Gnpda2 | 1.14974003 |
| Gnptab | -0.656889 |
| Gnptg | 1.32403922 |
| Gnptg | 0.62217371 |
| Gnrhr | -0.4117716 |
| Gns | 1.52134223 |
| Golga1 | 0.70776547 |
| Golga2 | -0.5930748 |
| Golga2 | -0.587891 |
| Golga2 | -0.4932665 |
| Golga2 | 1.13334462 |
| Golga2 | 1.39444376 |
| Golga3 | 0.8549271 |
| Golga4 | -0.7903938 |
| Golga5 | 0.60047138 |
| Golga7 | 0.66578252 |
| Golim4 | -0.5703377 |
| Golm1 | -0.2216038 |
| Golm1 | -0.0124076 |
| Golph3l | -0.2390889 |
| Golt1a | -0.3197083 |
| Golt1b | 0.67956087 |
| Gopc | -0.5893691 |
| Gorasp1 | -0.5467389 |
| Gorasp2 | 1.21983123 |
| Gorasp2 | 0.01474394 |
| Gorasp2 | -0.4296096 |
| Gorasp2 | 0.57927335 |
| Gosr1 | -0.4866317 |
| Gosr2 | 1.78993241 |
| Gosr2 | 2.06629577 |
| Gosr2 | 1.0714253 |
| Gosr2 | 2.57962578 |
| Got1l1 | -0.5285653 |
| Got2 | 0.70038907 |
| Gp1bb | -0.7739574 |
| Gp38 | 1.0678056 |
| Gp5 | 0.10838659 |
| Gp9 | -0.388318 |
| Gpaa1 | 1.5166169 |
| Gpam | -0.1996031 |
| Gpatch1 | 1.60496324 |
| Gpatch2 | 0.16035718 |
| Gpatch3 | -0.3268974 |
| Gpatch4 | -0.8090097 |
| Gpbp1 | 0.44156243 |
| Gpbp1 | -0.8299344 |
| Gpbp1l1 | 2.93293656 |
| Gpc1 | -0.8415528 |
| Gpc3 | 0.20302388 |
| Gpc3 | 1.26974124 |
| Gpc4 | 1.49322485 |
| Gpc5 | 0.60949354 |
| Gpc5 | 2.83998977 |
| Gpc5 | 0.7909524 |
| Gpd1 | -0.7489623 |
| Gpd2 | 2.16832752 |
| Gpd2 | 1.42881407 |
| Gper | -0.764952 |
| Gpha2 | 0.1031969 |
| Gphb5 | 0.32024496 |
| Gphn | 0.61426922 |
| Gphn | 0.61486443 |
| Gphn | -0.4172704 |
| Gpihbp1 | 0.21354055 |
| Gpkow | -0.6521236 |
| Gpld1 | -0.1038329 |
| Gpld1 | 0.23541991 |
| Gpm6a | 0.6891203 |
| Gpm6a | -0.7685435 |
| Gpm6b | 0.84726682 |
| Gpnmb | 0.02121804 |
| Gpnmb | -0.7703446 |
| Gpr1 | -0.183799 |
| Gpr1 | 2.84743533 |
| Gpr103 | 1.7551256 |
| Gpr107 | -0.6778304 |
| Gpr108 | 0.45630449 |
| Gpr108 | -0.5006208 |
| Gpr108 | -0.3158606 |
| Gpr109a | -0.4377203 |
| Gpr113 | -0.2798283 |
| Gpr113 | 1.46429429 |
| Gpr114 | -0.7748628 |
| Gpr12 | 2.383936 |
| Gpr12 | -0.6379748 |
| Gpr120 | -0.0246942 |
| Gpr123 | -0.6348601 |
| Gpr124 | -0.1474945 |
| Gpr128 | 2.74582108 |
| Gpr135 | 0.46311928 |
| Gpr137 | 1.46460776 |
| Gpr137b | 1.02587564 |
| Gpr141 | -0.4008721 |
| Gpr146 | -0.4446398 |
| Gpr146 | 0.35804336 |
| Gpr149 | 0.55439968 |
| Gpr151 | -0.4586377 |
| Gpr153 | -0.5710624 |
| Gpr156 | 1.81227954 |
| Gpr156 | -0.5893691 |
| Gpr158 | -0.4899431 |
| Gpr162 | -0.1055748 |
| Gpr162 | -0.5495671 |
| Gpr17 | 2.1922313 |
| Gpr172b | 0.92070221 |
| Gpr172b | 0.11423286 |
| Gpr176 | -0.016353 |
| Gpr177 | 0.0543838 |
| Gpr18 | 2.99817114 |
| Gpr180 | 1.07479082 |
| Gpr182 | 2.14739314 |
| Gpr182 | -0.3153122 |
| Gpr19 | 1.42219026 |
| Gpr20 | -0.4148224 |
| Gpr21 | -0.390101 |
| Gpr22 | 0.51300637 |
| Gpr23 | 1.20434266 |
| Gpr26 | -0.6786455 |
| Gpr27 | 1.28397884 |
| Gpr27 | 2.44416025 |
| Gpr27 | -0.6529161 |
| Gpr34 | 0.43313419 |
| Gpr34 | 2.39573595 |
| Gpr37l1 | -0.5502755 |
| Gpr45 | -0.4939326 |
| Gpr6 | -0.2270632 |
| Gpr64 | 0.12242335 |
| Gpr65 | 1.13940162 |
| Gpr68 | -0.5674446 |
| Gpr75 | 1.6592403 |
| Gpr81 | 2.41913735 |
| Gpr83 | 0.39459432 |
| Gpr85 | -0.4899431 |
| Gpr85 | -0.7237749 |
| Gpr89 | 0.86960762 |
| Gpr89 | -0.4866317 |
| Gpr97 | -0.6217397 |
| Gprasp1 | -0.7712464 |
| Gprasp1 | 1.34356446 |
| Gprc2a-rs5 | 1.03644582 |
| Gprc2a-rs5 | 0.27364595 |
| Gprc5a | -0.6450219 |
| Gprc5b | 2.52308746 |
| Gprc5c | -0.4209547 |
| Gprk6 | -0.4573563 |
| Gprk6 | 0.74464195 |
| Gps1 | -0.3175076 |
| Gps1 | -0.5313378 |
| Gps1 | -0.6664947 |
| Gps1 | -0.3120289 |
| Gps2 | 0.65635396 |
| Gpsm1 | 1.14130018 |
| Gpsm2 | -0.5616857 |
| Gpsm3 | -0.2151935 |
| Gpsn2 | -0.3076695 |
| Gpsn2 | 0.12378133 |
| Gpsn2 | 0.52970201 |
| Gpt1 | -0.6656904 |
| Gpt2 | 0.96219972 |
| Gpx1 | 1.88734615 |
| Gpx2 | 0.92112806 |
| Gpx3 | -0.3246793 |
| Gpx4 | 0.71632637 |
| Gpx4 | 1.77247068 |
| Gpx6 | -0.5776107 |
| Gpx7 | -0.6110742 |
| Gramd1a | -0.3695132 |
| Gramd1b | -0.3562321 |
| Gramd2 | -0.4689543 |
| Gramd2 | -0.210293 |
| Gramd3 | -0.5383062 |
| Gramd4 | -0.1562061 |
| Grap2 | -0.2117604 |
| Grasp | -0.6125903 |
| Grasp | 0.13490843 |
| Grb10 | 0.02121804 |
| Grb14 | -0.5531148 |
| Grb14 | 0.95018063 |
| Grb2 | -0.4945993 |
| Grb7 | 0.3627582 |
| Grcc10 | 1.59496159 |
| Greb1 | 0.28708227 |
| Greb1 | 1.22034303 |
| Grem1 | -0.1543652 |
| Grem2 | -0.4741565 |
| Grhl1 | 1.13973695 |
| Grhl2 | -0.100359 |
| Grhpr | -0.3730098 |
| Gria1 | -0.5768808 |
| Gria1 | -0.5930748 |
| Gria1 | -0.6442362 |
| Gria2 | 2.22829953 |
| Gria2 | -0.4184969 |
| Gria3 | -0.5531148 |
| Gria3 | 0.31392838 |
| Gria3 | -0.4002704 |
| Grid2 | 2.14478891 |
| Grik1 | -0.2201205 |
| Grik2 | 1.27167824 |
| Grik4 | 2.61622279 |
| Grik5 | -0.5066823 |
| Grin1 | 1.84444695 |
| Grin1 | 1.41079363 |
| Grin2a | 2.04439944 |
| Grin2b | 1.60395453 |
| Grin2c | 0.67157981 |
| Grin3b | -0.6884836 |
| Grina | -0.5975416 |
| Grip1 | 2.91450596 |
| Grip1 | 0.14125408 |
| Grip1 | -0.5039833 |
| Gripap1 | -0.5006208 |
| Gripap1 | -0.7212085 |
| Grit | -0.5488592 |
| Grk4 | 1.04454213 |
| Grk4 | -0.6103171 |
| Grk4 | 1.79250948 |
| Grk5 | -0.5732399 |
| Grlf1 | -0.5425128 |
| Grm1 | 2.43742063 |
| Grm7 | -0.3966683 |
| Grm8 | 0.04111289 |
| Grn | 1.21377031 |
| Grp | -0.4892798 |
| Grpel1 | 0.42779642 |
| Grpr | -0.8270527 |
| Grsf1 | 1.12224882 |
| Grtp1 | -0.5053318 |
| Grwd1 | -0.1876053 |
| Grwd1 | -0.1914276 |
| Gsbs | 0.10180799 |
| Gsc | -0.7987241 |
| Gsdma1 | -0.3759339 |
| Gsdmdc1 | 1.03857025 |
| Gsdmdc1 | -0.1828499 |
| Gsdmdc1 | -0.1298686 |
| Gse1 | -0.7263486 |
| Gsg1 | -0.647383 |
| Gsg1 | 0.70651029 |
| Gsg1 | -0.285117 |
| Gsg2 | -0.2579438 |
| Gsk3a | 0.86433765 |
| Gsk3b | -0.2059047 |
| Gsn | 1.56631887 |
| Gspt1 | -0.5446234 |
| Gspt2 | -0.4826737 |
| Gss | -0.807131 |
| Gss | -0.6458083 |
| Gss | 1.28397884 |
| Gsta2 | -0.477423 |
| Gsta3 | 1.57399868 |
| Gsta3 | -0.0518265 |
| Gsta4 | -0.2446539 |
| Gstcd | 0.60067284 |
| Gstk1 | -0.7059591 |
| Gstm1 | 0.34401798 |
| Gstm1 | 0.00860853 |
| Gstm2 | -0.3437063 |
| Gstm2 | 0.37958455 |
| Gstm2 | 3.22537949 |
| Gstm4 | 1.81270811 |
| Gstm5 | 0.28566222 |
| Gstm6 | -0.4663642 |
| Gstm6 | -0.7280684 |
| Gsto1 | 3.65687556 |
| Gsto2 | -0.3996691 |
| Gsto2 | -0.0973301 |
| Gstp1 | -0.5805361 |
| Gstp1 | -0.5087117 |
| Gstp2 | -0.4020768 |
| Gstt1 | 0.71490511 |
| Gstt2 | 0.81819387 |
| Gstt3 | -0.1622149 |
| Gstt4 | 0.54352898 |
| Gstz1 | 0.41604114 |
| Gtdc1 | -0.7803143 |
| Gtf2a1 | 1.00940131 |
| Gtf2e1 | 0.39156137 |
| Gtf2e2 | -0.4709017 |
| Gtf2e2 | 1.85140431 |
| Gtf2f1 | 0.84372143 |
| Gtf2f2 | -0.2142113 |
| Gtf2h1 | 1.16345423 |
| Gtf2h1 | 1.17932344 |
| Gtf2h2 | 0.99890327 |
| Gtf2h3 | -0.5355125 |
| Gtf2h4 | 0.03215746 |
| Gtf2h5 | 1.62108535 |
| Gtf2ird1 | -0.495934 |
| Gtf2ird1 | 1.57809144 |
| Gtf2ird2 | 1.33701216 |
| Gtf2ird2 | 0.37314945 |
| Gtf2ird2 | 0.30395317 |
| Gtf3a | -0.115215 |
| Gtf3a | 0.50364743 |
| Gtf3c1 | 0.34880546 |
| Gtf3c2 | 0.53966122 |
| Gtf3c3 | 0.15315805 |
| Gtf3c4 | 1.3482034 |
| Gtf3c4 | -0.3335839 |
| Gtf3c5 | -0.4605633 |
| Gtf3c5 | -0.263625 |
| Gtf3c5 | 0.24200224 |
| Gtl3 | -0.1724742 |
| Gtpbp1 | 1.48091589 |
| Gtpbp2 | 0.19144024 |
| Gtpbp3 | -0.631756 |
| Gtpbp4 | -0.5746945 |
| Gtpbp5 | 0.63390387 |
| Gtpbp6 | -0.668912 |
| Gtpbp8 | 1.77793901 |
| Gtpbp8 | -0.0215076 |
| Gtrgeo22 | 1.77832836 |
| Gtse1 | -0.3631373 |
| Gtse1 | -0.1981551 |
| Gtse1 | -0.5975416 |
| Gtsf1l | 0.18986357 |
| Guca1a | -0.3936773 |
| Guca1b | -0.5046573 |
| Guca2a | 2.90169446 |
| Guca2b | 0.51123108 |
| Gucy1a2 | 1.93002133 |
| Gucy1a3 | -0.6926143 |
| Gucy1a3 | 0.39987791 |
| Gucy1a3 | -0.250253 |
| Gucy1b3 | 1.52391611 |
| Guk1 | 1.8475972 |
| Gulo | 2.4039961 |
| Gulp1 | -0.477423 |
| Gusb | -0.5053318 |
| Gusb | 0.21415542 |
| Gusb | -0.7341133 |
| Gvin1 | 1.33728236 |
| Gyg | 1.29959494 |
| Gyg | 0.85949268 |
| Gykl1 | 1.16388971 |
| Gyltl1b | -0.7042804 |
| Gyltl1b | 0.80298055 |
| Gypa | 0.54417202 |
| Gypc | -0.7017679 |
| Gys1 | -0.0705121 |
| Gys3 | 1.37538095 |
| Gzf1 | -0.7613745 |
| Gzf1 | -0.4547988 |
| Gzmd | 2.8886845 |
| Gzmd | 0.85873333 |
| Gzmk | -0.3700951 |
| Gzmm | -0.743694 |
| Gzmn | 1.07106398 |
| H13 | -0.4866317 |
| H13 | 2.55252127 |
| H13 | -0.4452714 |
| H13 | -0.5624036 |
| H19 | 1.5740682 |
| H1f0 | -0.6458083 |
| H1fnt | 0.20953373 |
| H1foo | 0.21538394 |
| H1fx | -0.6088048 |
| H1fx | -0.4748089 |
| H2-Aa | 0.60188068 |
| H2-Ab1 | 0.89711554 |
| H2-Ab1 | 1.21695987 |
| H2-Ab1 | 0.2782422 |
| H2afj | -0.1952658 |
| H2afx | 2.33269863 |
| H2afy | -0.5488592 |
| H2afz | 0.60509382 |
| H2-Bl | 0.01588976 |
| H2-DMa | 1.07407068 |
| H2-DMb1 | -0.7685435 |
| H2-Eb1 | -0.1529869 |
| H2-K1 | -0.6819131 |
| H2-Ke6 | -0.1465823 |
| H2-M10.3 | 0.31006205 |
| H2-M2 | 0.83378309 |
| H2-M3 | 0.29189403 |
| H2-Oa | 1.92987997 |
| H2-Ob | 0.85903715 |
| H2-Q1 | 1.13783513 |
| H2-Q2 | -0.30604 |
| H2-Q7 | 0.05621185 |
| H2-Q8 | -0.4886171 |
| H2-T10 | -0.6745773 |
| H2-T23 | 0.38060998 |
| H2-T23 | -0.6705271 |
| H2-T24 | 3.41576854 |
| H2-Tw3 | -0.1334566 |
| H3f3a | 0.37855797 |
| H3f3a | -0.4497052 |
| H3f3b | 1.02054441 |
| H47 | 2.33285031 |
| H47 | -0.5660015 |
| H6pd | -0.0977622 |
| Habp4 | -0.6860142 |
| Habp4 | 2.56364349 |
| Hace1 | -0.8280123 |
| Hacl1 | -0.7332473 |
| Hadhb | -0.6489605 |
| Hagh | -0.301169 |
| Haghl | 2.89377597 |
| Haghl | 4.05970256 |
| Hal | 2.63847192 |
| Hamp2 | -0.7480822 |
| Hao1 | -0.7959389 |
| Hao3 | -0.5739669 |
| Hap1 | 0.15643741 |
| Hap1 | 0.8985713 |
| Hap1 | 0.97218588 |
| Hapln1 | -0.0250933 |
| Hapln2 | -0.3877245 |
| Hapln4 | 0.92410348 |
| Harbi1 | -0.5168748 |
| Hars | 1.12781354 |
| Has1 | -0.6434512 |
| Hat1 | -0.6118319 |
| Havcr1 | -0.5886297 |
| Havcr2 | 1.6614545 |
| Hax1 | -0.5930748 |
| Hba-a1 | -0.781226 |
| Hbb-b1 | -0.6232736 |
| Hbegf | -0.3683507 |
| Hbp1 | -0.4631369 |
| Hbp1 | 1.21880678 |
| Hbs1l | -0.6232736 |
| Hbs1l | -0.7297915 |
| Hbs1l | -0.0250933 |
| Hbs1l | 0.57638189 |
| Hc | -0.346538 |
| Hccs | -0.3666096 |
| Hcfc1 | -0.447169 |
| Hcfc1r1 | 1.77179883 |
| Hcfc2 | 3.05652155 |
| Hcls1 | -0.5369083 |
| Hcn2 | 2.02998405 |
| Hcn3 | 1.48483295 |
| Hcn3 | 1.08005461 |
| Hcr | -0.7410711 |
| Hcst | -0.2059047 |
| Hdac10 | -0.5886297 |
| Hdac11 | 0.03253236 |
| Hdac2 | -0.3352632 |
| Hdac3 | -0.6664947 |
| Hdac4 | -0.1976729 |
| Hdac5 | -0.1178619 |
| Hdac6 | 0.05767167 |
| Hdac7 | 2.55096909 |
| Hdac8 | 0.05328522 |
| Hdac9 | -0.7542612 |
| Hdc | -0.6489605 |
| Hddc2 | -0.408124 |
| Hddc2 | -0.424654 |
| Hdgf | -0.2395934 |
| Hdgf | -0.672145 |
| Hdgf | -0.5039833 |
| Hdgf | 0.21015131 |
| Hdgfl1 | -0.1622149 |
| Hdgfrp2 | -0.7017679 |
| Hdhd1a | 2.83960631 |
| Hdhd2 | -0.1183038 |
| Hdhd2 | 0.99994549 |
| Hdhd2 | -0.0375526 |
| Hdlbp | -0.1415814 |
| Hdlbp | 1.7227319 |
| Heatr1 | 2.24847912 |
| Heatr1 | -0.5216705 |
| Heatr3 | -0.2845868 |
| Heatr5a | -0.5495671 |
| Hebp1 | 3.10277004 |
| Hebp2 | -0.7375856 |
| Heca | 1.40763034 |
| Hectd2 | -0.3224667 |
| Hectd2 | 2.30956315 |
| Hectd3 | 0.33490603 |
| Hecw2 | 0.37778728 |
| Hecw2 | -0.0721916 |
| Hecw2 | -0.7428188 |
| Heg1 | 2.49496482 |
| Hel308 | 0.92946309 |
| Helb | -0.3076695 |
| Helb | -0.2340589 |
| Helz | -0.3794551 |
| Hemgn | 1.02460912 |
| Hemk1 | -0.6576856 |
| Hemt1 | 0.43795983 |
| Hepacam | 1.4450467 |
| Heph | -0.1240679 |
| Heph | 0.30701271 |
| Heph | -0.1631428 |
| Heph | -0.3835805 |
| Heph | 2.03023744 |
| Herc2 | -0.4826737 |
| Herc3 | -0.3257877 |
| Herc3 | 0.69731303 |
| Herc4 | -0.553826 |
| Herc4 | 0.90089569 |
| Herpud1 | 0.46171348 |
| Herpud1 | 0.8729047 |
| Herpud2 | -0.0274915 |
| Herpud2 | -0.7613745 |
| Herpud2 | 1.10127838 |
| Herpud2 | 0.0962312 |
| Hes1 | -0.6035314 |
| Hes5 | -0.5975416 |
| Hes5 | -0.4787329 |
| Hes6 | -0.7894729 |
| Hes6 | 0.70363482 |
| Hes6 | 1.06149732 |
| Hexa | -0.6035314 |
| Hexb | 0.59400106 |
| Hexdc | 0.91099606 |
| Hexdc | 2.479462 |
| Hexim1 | 3.40475241 |
| Hexim2 | 2.02841994 |
| Hey1 | 3.38866932 |
| Hey2 | 1.20839498 |
| Hey2 | 1.81916585 |
| Heyl | 0.74704833 |
| Hfe | -0.1947851 |
| Hfe | 0.10562242 |
| Hgfac | -0.4396023 |
| Hgs | 0.49915692 |
| Hgs | 0.3887723 |
| Hgsnat | 1.17039713 |
| Hhat | 2.65414911 |
| Hhat | -0.606541 |
| Hhat | -0.2589741 |
| Hhatl | -0.4592791 |
| Hhex | -0.2861784 |
| Hhip | -0.273522 |
| Hibadh | 0.85064525 |
| Hic1 | -0.2370736 |
| Hic2 | -0.6103171 |
| Hif1a | 2.21852166 |
| Hif3a | -0.6489605 |
| Hif3a | 2.71218053 |
| Higd1a | 0.12851845 |
| Higd1b | -0.07556 |
| Higd2a | 1.01901547 |
| Hils1 | -0.4478024 |
| Hint1 | 2.30472739 |
| Hint2 | -0.5060068 |
| Hint3 | 0.67251143 |
| Hint3 | -0.3263423 |
| Hip1 | 0.43071179 |
| Hip1r | -0.2782477 |
| Hip2 | 0.34401798 |
| Hipk1 | 1.79578883 |
| Hipk2 | -0.7694436 |
| Hipk3 | 2.21607802 |
| Hipk4 | 0.27938767 |
| Hira | -0.4333441 |
| Hira | 2.03784849 |
| Hirip3 | 0.61148643 |
| Hisppd1 | -0.4178834 |
| Hist1h1b | -0.5856783 |
| Hist1h1c | -0.5012923 |
| Hist1h2aa | -0.6967637 |
| Hist1h2ac | -0.1659322 |
| Hist1h2ad | 2.0389366 |
| Hist1h2ae | -0.5609684 |
| Hist1h2af | 2.06137071 |
| Hist1h2ag | -0.6171536 |
| Hist1h2ah | 1.07634919 |
| Hist1h2ak | -0.568167 |
| Hist1h2an | -0.1081939 |
| Hist1h2ao | -0.3689318 |
| Hist1h2bc | -0.700932 |
| Hist1h2be | -0.6802779 |
| Hist1h2bf | 1.99454539 |
| Hist1h2bg | 1.14608492 |
| Hist1h2bh | 1.6918424 |
| Hist1h2bj | -0.4271285 |
| Hist1h2bk | -0.2548597 |
| Hist1h2bm | 1.04627662 |
| Hist1h2bn | 1.17438709 |
| Hist1h3a | 2.31557986 |
| Hist1h3d | -0.6005316 |
| Hist1h3e | -0.2300548 |
| Hist1h3f | -0.431475 |
| Hist1h4c | 1.33863216 |
| Hist1h4d | 0.29076412 |
| Hist1h4f | 0.47405726 |
| Hist1h4h | 1.85540013 |
| Hist1h4i | 2.65938647 |
| Hist1h4j | -0.2345607 |
| Hist1h4k | -0.4624928 |
| Hist1h4m | 1.04379778 |
| Hist2h2aa1 | 0.33220868 |
| Hist2h2ab | -0.0730326 |
| Hist2h2ac | -0.4172704 |
| Hist2h2bb | 0.01817711 |
| Hist2h2be | 0.42462758 |
| Hist2h3b | -0.4637815 |
| Hist2h3c1 | -0.430231 |
| Hist2h3c1 | -0.3990681 |
| Hist2h4 | 0.56304617 |
| Hist3h2a | -0.7393267 |
| Hist3h2bb | -0.4986092 |
| Hist4h4 | 1.46335324 |
| Hk1 | -0.4296096 |
| Hk3 | -0.6959323 |
| Hkdc1 | -0.6934427 |
| Hlcs | 0.76775457 |
| Hlf | 0.60849546 |
| Hltf | 2.50018408 |
| Hlx | 0.56430468 |
| Hmg20a | 2.510684 |
| Hmg20a | -0.6827319 |
| Hmg20b | -0.3022492 |
| Hmga1 | -0.5573902 |
| Hmgb2 | 0.61287888 |
| Hmgb2l1 | 1.93598523 |
| Hmgb2l1 | 0.86373343 |
| Hmgb4 | 0.01321394 |
| Hmgcl | -0.4008721 |
| Hmgcl | 0.6404848 |
| Hmgcl | 2.65074796 |
| Hmgcll1 | -0.5362101 |
| Hmgcs2 | -0.6118319 |
| Hmgn1 | 1.46851709 |
| Hmgn2 | -0.6371951 |
| Hmgn3 | 0.2076785 |
| Hmgn3 | 1.21067301 |
| Hmha1 | -0.4952664 |
| Hmox1 | 0.72234224 |
| Hmox2 | -0.3568055 |
| Hmx1 | -0.36603 |
| Hmx1 | 0.07822487 |
| Hn1 | 1.27679153 |
| Hn1 | 1.0426801 |
| Hn1l | -0.0538839 |
| Hnf1b | 1.02941262 |
| Hnf1b | -0.6819131 |
| Hnf4a | 1.45246097 |
| Hnrnpc | 1.77515313 |
| Hnrpa1 | 0.8865652 |
| Hnrpa1 | -0.1589745 |
| Hnrpab | 1.93176286 |
| Hnrpc | 3.74665432 |
| Hnrpdl | -0.4396023 |
| Hnrpdl | -0.4560767 |
| Hnrpf | -0.5148272 |
| Hnrph1 | -0.2766697 |
| Hnrph1 | -0.5923325 |
| Hnrph1 | 1.02928654 |
| Hnrph1 | 0.33947335 |
| Hnrph1 | -0.2015375 |
| Hnrph2 | -0.5827364 |
| Hnrph3 | 0.30339579 |
| Hnrpk | 1.86617321 |
| Hnrpl | 0.2290923 |
| Hnrpll | 0.23811841 |
| Hnrpll | -0.3186073 |
| Hnrpm | -0.7042804 |
| Hnrpm | -0.6186797 |
| Hnrpm | 2.77563608 |
| Hnrpul1 | 0.81517137 |
| Hnrpul1 | 0.53361201 |
| Hnrpul1 | -0.2709071 |
| Hnrpul1 | 0.87350289 |
| Hnrpul1 | -0.3136691 |
| Hnt | -0.1352559 |
| Homer1 | 1.29696364 |
| Homer1 | -0.6156299 |
| Homer1 | -0.301169 |
| Homer1 | -0.7332473 |
| Homez | -0.1060108 |
| Homez | 1.54568586 |
| Hook1 | 0.09307926 |
| Hook1 | -0.6202084 |
| Hook1 | 3.37463295 |
| Hook1 | 0.6300105 |
| Hook2 | 0.07644032 |
| Hormad2 | -0.563841 |
| Hoxa13 | -0.518927 |
| Hoxa2 | -0.7349802 |
| Hoxa2 | 0.02462705 |
| Hoxa4 | -0.2662191 |
| Hoxa5 | 0.13054117 |
| Hoxa7 | 1.52969924 |
| Hoxa9 | 1.36861836 |
| Hoxa9 | 1.77392465 |
| Hoxb2 | -0.5093892 |
| Hoxb3 | 2.81406126 |
| Hoxb3 | -0.4644265 |
| Hoxb4 | -0.7516079 |
| Hoxb5 | -0.5107457 |
| Hoxb6 | -0.6537093 |
| Hoxb7 | -0.4414881 |
| Hoxc10 | 1.16866634 |
| Hoxc10 | 1.82060063 |
| Hoxc6 | 2.48461602 |
| Hoxc9 | -0.433968 |
| Hoxd10 | -0.5237335 |
| Hoxd3 | 2.78745031 |
| Hoxd4 | 0.1851172 |
| Hoxd8 | 0.57348126 |
| Hoxd9 | 0.70020841 |
| Hp | 1.84475729 |
| Hp | 0.15085549 |
| Hp | 0.62158324 |
| Hp1bp3 | 2.33741934 |
| Hpca | -0.38299 |
| Hpcal1 | -0.5545377 |
| Hpcal1 | 1.55744205 |
| Hpcal4 | -0.563841 |
| Hpgd | -0.7017679 |
| Hpn | -0.1196308 |
| Hpn | -0.0819094 |
| Hprt1 | 1.89236735 |
| Hprt1 | 2.2666919 |
| Hprt1 | -0.5237335 |
| Hps1 | 1.70723721 |
| Hps3 | -0.2059047 |
| Hps3 | -0.433968 |
| Hps4 | -0.606541 |
| Hps5 | -0.0053484 |
| Hps6 | -0.7839667 |
| Hpse | -0.583471 |
| Hpx | 2.14575706 |
| Hr | -0.3847626 |
| Hr | -0.3038719 |
| Hras1 | 1.63132953 |
| Hrasls | -0.5107457 |
| Hrasls5 | 3.3653375 |
| Hrasls5 | -0.2569146 |
| Hrb | -0.7289295 |
| Hrb | 0.08000595 |
| Hrbl | -0.62481 |
| Hrbl | 1.46578231 |
| Hrc | 0.70991322 |
| Hrg | -0.581269 |
| Hrh3 | 0.76456048 |
| Hrk | 1.73278143 |
| Hrmt1l2 | 2.2018727 |
| Hrsp12 | 0.34161481 |
| Hrsp12 | -0.6395361 |
| Hrsp12 | 0.24527573 |
| Hs1bp3 | 2.11982503 |
| Hs2st1 | 2.18726554 |
| Hs3st1 | -0.6209738 |
| Hs3st3a1 | 0.67269763 |
| Hs3st3b1 | 2.56019561 |
| Hs3st6 | 1.15645824 |
| Hs6st1 | -0.2714295 |
| Hs6st2 | -0.5581047 |
| Hs6st2 | 0.82294599 |
| Hs6st3 | 2.33805297 |
| Hsbp1 | 0.64029192 |
| Hsd11b1 | -0.5313378 |
| Hsd11b1 | -0.4160456 |
| Hsd11b1 | -0.3408833 |
| Hsd11b1 | 0.27967381 |
| Hsd11b2 | -0.062992 |
| Hsd17b1 | -0.5624036 |
| Hsd17b10 | -0.6959323 |
| Hsd17b11 | -0.5893691 |
| Hsd17b11 | -0.6521236 |
| Hsd17b11 | 2.30403894 |
| Hsd17b12 | 0.48303523 |
| Hsd17b2 | -0.5856783 |
| Hsd17b3 | -0.4148224 |
| Hsd17b4 | -0.7830523 |
| Hsd17b7 | 1.88857938 |
| Hsd3b1 | -0.6156299 |
| Hsd3b2 | -0.7323821 |
| Hsd3b2 | -0.5299505 |
| Hsd3b4 | 0.36640858 |
| Hsd3b7 | -0.5545377 |
| Hsd3b7 | -0.2952505 |
| Hsdl1 | -0.5341188 |
| Hsf1 | 0.04777241 |
| Hsf4 | -0.5725135 |
| Hsf5 | -0.5725135 |
| Hsn2 | 1.37044398 |
| Hsp105 | 0.06565929 |
| Hsp90aa1 | -0.5573902 |
| Hsp90ab1 | 0.5944068 |
| Hsp90ab1 | 1.45134868 |
| Hsp90ab1 | 1.6105248 |
| Hsp90b1 | -0.7533759 |
| Hspa12b | 1.99515985 |
| Hspa1a | 1.00526832 |
| Hspa1l | 1.44328397 |
| Hspa2 | -0.7034421 |
| Hspa2 | 1.25596781 |
| Hspa4 | -0.7026046 |
| Hspa4l | -0.5990353 |
| Hspa5 | 0.89842582 |
| Hspa9 | -0.4433778 |
| Hspa9 | 1.12202097 |
| Hspb1 | -0.5703377 |
| Hspb2 | 1.44143747 |
| Hspb3 | -0.4111627 |
| Hspb6 | -0.590109 |
| Hspb7 | -0.0768264 |
| Hspb8 | -0.3274527 |
| Hspbap1 | -0.5595354 |
| Hspd1 | 0.40037952 |
| Hspd1 | -0.3671896 |
| Hspe1 | 2.55960391 |
| Hspg2 | -0.1271868 |
| Htatip | 0.76405513 |
| Htatip | 1.25478597 |
| Htatip2 | 0.17169487 |
| Htatip2 | -0.3954707 |
| Htatsf1 | -0.1438512 |
| Htf9c | 1.73936229 |
| Htr1a | -0.6868366 |
| Htr1b | -0.6835513 |
| Htr1d | -0.3386311 |
| Htr1f | -0.4767688 |
| Htr1f | -0.2507637 |
| Htr2b | 1.58985113 |
| Htr3a | 0.19332866 |
| Htr5b | 0.25796089 |
| Htr7 | -0.5960502 |
| Htra1 | 2.6632632 |
| Htra1 | 1.45744962 |
| Htra2 | -0.034321 |
| Htra3 | 1.95323388 |
| Htra3 | 0.74584593 |
| Htt | 2.15913134 |
| Hus1 | 1.39790577 |
| Hus1 | 1.41137515 |
| Hus1 | 0.15019655 |
| Huwe1 | 2.20463375 |
| Hvcn1 | 0.25414436 |
| Hvcn1 | 0.3624969 |
| Hyal1 | 0.76809015 |
| Hyal2 | -0.607295 |
| Hyal3 | -0.4579968 |
| Hyal5 | 0.16426024 |
| Hyal6 | -0.6697192 |
| Hydin | -0.3263423 |
| Hyi | -0.2808836 |
| Hyou1 | -0.4148224 |
| Iah1 | 2.28955611 |
| Iap | -0.0789409 |
| Iars | -0.8061931 |
| Iars | 0.5539758 |
| Iars2 | 0.55291525 |
| Ibtk | 2.45351308 |
| Ica1 | -0.7323821 |
| Ica1l | -0.409946 |
| Icam1 | 0.63565049 |
| Icam2 | -0.6616792 |
| Icam4 | 2.02034565 |
| Icam5 | -0.4637815 |
| Ick | 0.00088095 |
| Icmt | -0.6705271 |
| Ict1 | 2.19173593 |
| Id1 | -0.3654507 |
| Id2 | -0.7110141 |
| Id2 | 1.33935124 |
| Id3 | 1.5316674 |
| Id4 | -0.2216038 |
| Ide | -0.3505173 |
| Idh1 | 1.58574212 |
| Idh2 | 1.94404724 |
| Idh3a | -0.4945993 |
| Idh3b | -0.2025062 |
| Idh3g | 1.55026379 |
| Idh3g | 2.33784181 |
| Idi2 | -0.6042829 |
| Ids | -0.0947418 |
| Ids | 1.73114344 |
| Ids | 0.94507962 |
| Idua | 1.41931322 |
| Ier3 | -0.672145 |
| Ier3ip1 | -0.5798039 |
| Ier5l | 1.68293646 |
| Ifi202b | -0.7454467 |
| Ifi202b | 0.88538539 |
| Ifi27 | 0.44920089 |
| Ifi30 | -0.4972706 |
| Ifi30 | -0.6294348 |
| Ifi35 | 0.83518721 |
| Ifi47 | 3.74475692 |
| Ifit2 | 1.57899078 |
| Ifit2 | -0.0428283 |
| Ifit3 | -0.5908496 |
| Ifit3 | 1.70903851 |
| Ifitm1 | -0.55525 |
| Ifitm1 | 0.4091135 |
| Ifitm1 | -0.6020302 |
| Ifitm2 | 0.81453378 |
| Ifitm3 | 1.60831762 |
| Ifitm5 | -0.1086311 |
| Ifitm6 | -0.656093 |
| Ifna1 | -0.3054975 |
| Ifna6 | -0.6481714 |
| Ifnar2 | 1.00591533 |
| Ifnar2 | 2.53369465 |
| Ifnar2 | 3.02108799 |
| Ifngr1 | 0.26641776 |
| Ifngr2 | -0.2270632 |
| Ifngr2 | -0.440859 |
| Ifngr2 | -0.0092635 |
| Ifnz | -0.4592791 |
| Ifrd1 | 0.14158685 |
| Ifrg15 | -0.1156557 |
| Ift122 | -0.1852245 |
| Ift140 | -0.3747631 |
| Ift140 | 0.36145096 |
| Ift140 | -0.4657179 |
| Ift172 | -0.2436396 |
| Ift172 | 1.73581568 |
| Ift172 | -0.6762024 |
| Ift20 | -0.4490705 |
| Ift20 | 1.6263531 |
| Ift20 | -0.5827364 |
| Ift52 | 1.53304987 |
| Ift80 | 2.06933524 |
| Ift81 | 0.46265092 |
| Ift81 | 1.4366029 |
| Ift81 | -0.3579535 |
| Ift88 | 1.16203749 |
| Igbp1 | 0.75405939 |
| Igbp1b | -0.2310542 |
| Igf1 | -0.3208107 |
| Igf1 | 0.69204168 |
| Igf1r | 0.93953284 |
| Igf2 | -0.5087117 |
| Igf2bp1 | -0.6171536 |
| Igf2bp2 | -0.626349 |
| Igf2bp3 | 1.55346898 |
| Igf2bp3 | 3.47616193 |
| Igf2r | -0.1474945 |
| Igf2r | -0.0171441 |
| Igfals | 0.12547598 |
| Igfbp2 | -0.4780777 |
| Igfbp2 | 0.5813331 |
| Igfbp3 | -0.6786455 |
| Igfbp4 | 0.52314797 |
| Igfbp4 | -0.5093892 |
| Igfbp5 | 1.793221 |
| Igfbp6 | -0.8299344 |
| Igfbp7 | -0.7931622 |
| Igfbpl1 | 0.57989176 |
| Igsf1 | -0.6810951 |
| Igsf1 | -0.7604823 |
| Igsf1 | 2.01585277 |
| Igsf1 | 0.19175524 |
| Igsf11 | 0.58564353 |
| Igsf11 | -0.3175076 |
| Igsf11 | -0.5285653 |
| Igsf21 | -0.7748628 |
| Igsf3 | -0.5348154 |
| Igsf4a | 0.35699231 |
| Igsf5 | -0.5251114 |
| Igsf8 | -0.3103917 |
| Igsf9 | -0.3984676 |
| Igtp | 1.51231339 |
| Ihh | 0.84480192 |
| Ihpk1 | 1.46020938 |
| Ihpk2 | 0.31227338 |
| Ihpk2 | 0.92240443 |
| Iigp2 | 0.3188755 |
| Ik | 0.97847231 |
| Ikbkap | -0.55525 |
| Ikbkb | -0.7959389 |
| Ikbke | 0.89082929 |
| Ikbkg | 1.83220834 |
| Ikbkg | -0.8454587 |
| Il10ra | 0.14191949 |
| Il10rb | -0.6403178 |
| Il11 | 1.2448775 |
| Il11ra1 | 0.94715103 |
| Il12a | 0.70147226 |
| Il13 | -0.4919357 |
| Il13ra1 | -0.5439193 |
| Il13ra2 | -0.6778304 |
| Il15 | -0.016353 |
| Il15 | 1.13581713 |
| Il15 | -0.3186073 |
| Il15ra | -0.4754617 |
| Il15ra | 0.34960095 |
| Il15ra | 1.28206774 |
| Il16 | 0.59521774 |
| Il17d | 0.85584204 |
| Il17d | -0.6309816 |
| Il17ra | -0.3948725 |
| Il17rb | -0.8165634 |
| Il17rc | -0.7803143 |
| Il17rc | -0.5990353 |
| Il17rc | -0.5390059 |
| Il17rd | 2.09883464 |
| Il17rd | 1.97989613 |
| Il17re | 0.62512038 |
| Il17re | 0.28452458 |
| Il17re | 1.80166222 |
| Il18 | 2.50426774 |
| Il18bp | -0.5893691 |
| Il18r1 | 2.18162875 |
| Il1b | -0.5717877 |
| Il1r1 | 1.30193794 |
| Il1rap | -0.8146692 |
| Il1rap | 1.23556531 |
| Il1rap | -0.6333067 |
| Il1rap | 1.63757709 |
| Il1rap | 0.08744884 |
| Il1rl1l | 1.06368588 |
| Il1rl2 | 0.69984699 |
| Il20ra | -0.476115 |
| Il20rb | -0.632531 |
| Il20rb | -0.6027805 |
| Il23a | 0.74739158 |
| Il27ra | -0.7410711 |
| Il28ra | -0.6810951 |
| Il28ra | 0.08071741 |
| Il28ra | 1.55538672 |
| Il31ra | 0.58543873 |
| Il33 | -0.459921 |
| Il3ra | -0.4624928 |
| Il4i1 | 1.6451984 |
| Il6ra | -0.1923856 |
| Il6st | 0.70651029 |
| Il7 | 2.18744335 |
| Ildr1 | 0.78273482 |
| Ildr1 | 0.19834547 |
| Ilf2 | 0.68159141 |
| Ilf3 | -0.4972706 |
| Ilf3 | 0.05694205 |
| Ilf3 | 0.38750173 |
| Ilf3 | 1.00436173 |
| Ilk | 1.34168435 |
| Ilkap | -0.4528854 |
| Ilvbl | 0.82704462 |
| Ilvbl | -0.3307919 |
| Ilvbl | -0.2594897 |
| Ilvbl | -0.6442362 |
| Immp2l | 0.07178413 |
| Immt | 3.01255588 |
| Imp3 | 0.53966122 |
| Imp4 | -0.145671 |
| Imp4 | -0.1397697 |
| Impa2 | 3.31680928 |
| Impact | 1.08303196 |
| Impad1 | 0.91400455 |
| Impdh1 | 0.22757925 |
| Impdh2 | 0.42365034 |
| Ina | -0.0994925 |
| Inadl | -0.3912915 |
| Inadl | 0.46428913 |
| Incenp | -0.0497737 |
| Indo | 1.92714289 |
| Ing2 | -0.2512747 |
| Ing3 | -0.4605633 |
| Ing3 | 0.71508289 |
| Ing3 | 0.40138192 |
| Ing4 | 2.16421526 |
| Ing4 | -0.7784935 |
| Ing4 | -0.5930748 |
| Ing5 | 0.18384733 |
| Inha | -0.5502755 |
| Inhba | -0.0473163 |
| Inmt | 1.74405059 |
| Inoc1 | -0.4932665 |
| Inpp4a | -0.3942747 |
| Inpp4a | -0.059255 |
| Inpp4a | 0.38673854 |
| Inpp4b | 1.0779049 |
| Inpp5a | -0.4497052 |
| Inpp5d | -0.5320323 |
| Inpp5e | 0.46545749 |
| Inpp5f | -0.4906068 |
| Inppl1 | 3.71174295 |
| Inppl1 | 0.26176157 |
| Inppl1 | -0.6656904 |
| Ins2 | -0.5355125 |
| Insc | -0.096035 |
| Insig1 | -0.7604823 |
| Insig1 | 2.13805808 |
| Insig1 | 0.30950838 |
| Insig2 | 0.60389021 |
| Insig2 | -0.3197083 |
| Insl3 | 1.48345237 |
| Insl5 | -0.1461266 |
| Insl6 | -0.2098043 |
| Insm1 | 0.49419177 |
| Ints1 | -0.2625894 |
| Ints12 | -0.743694 |
| Ints3 | 0.30756789 |
| Ints3 | 1.5066435 |
| Ints4 | 1.10870752 |
| Ints5 | -0.3414472 |
| Ints7 | 1.10151146 |
| Ints9 | -0.4605633 |
| Ints9 | -0.4939326 |
| Ipmk | -0.7604823 |
| Ipmk | 2.32664112 |
| Ipo13 | 0.16426024 |
| Ipo4 | -0.6179163 |
| Ipo5 | 2.45098389 |
| Ipp | -0.6110742 |
| Ippk | -0.5945613 |
| Iqcb1 | 0.1193605 |
| Iqcb1 | 0.49509653 |
| Iqcd | 0.08249365 |
| Iqce | -0.1205165 |
| Iqcf3 | 0.28821672 |
| Iqcf4 | -0.5997832 |
| Iqgap1 | 0.67882138 |
| Iqgap2 | 0.89886217 |
| Iqgap3 | 2.29788258 |
| Iqsec2 | 0.69894281 |
| Iqsec3 | -0.3125753 |
| Irak1 | -0.7604823 |
| Irak1bp1 | -0.3562321 |
| Irak2 | 1.02966474 |
| Irak2 | 0.36171255 |
| Irak3 | 0.93479149 |
| Irak4 | 0.3895338 |
| Irak4 | -0.2335575 |
| Irak4 | -0.5945613 |
| Ireb2 | -0.5369083 |
| Irf1 | -0.7051194 |
| Irf1 | -0.7212085 |
| Irf1 | -0.5776107 |
| Irf1 | 1.05844894 |
| Irf2 | -0.5967956 |
| Irf2bp1 | -0.5674446 |
| Irf3 | 0.81993918 |
| Irf3 | 1.25063747 |
| Irf3 | 2.29412447 |
| Irf4 | -0.5856783 |
| Irf5 | 1.562877 |
| Irf6 | -0.3954707 |
| Irf6 | 1.76217106 |
| Irf7 | 0.98999613 |
| Irf8 | 0.17394564 |
| Irf9 | 0.05694205 |
| Irf9 | 2.51636084 |
| Irgm | 2.72750158 |
| Irgm | -0.724632 |
| Irgq | 1.10836062 |
| Irgq | -0.6348601 |
| Irgq | -0.2497426 |
| Irs2 | 0.1359133 |
| Irs3 | -0.0551206 |
| Irx2 | -0.7384557 |
| Irx3 | -0.4932665 |
| Irx5 | 0.80443271 |
| Isca1 | -0.5411084 |
| Iscu | -0.4228025 |
| Isg20 | 0.00552533 |
| Isg20l1 | -0.0567721 |
| Isg20l2 | -0.7384557 |
| Isl1 | -0.3614061 |
| Islr2 | 2.32440892 |
| Isy1 | 0.17233851 |
| Isyna1 | 2.16992377 |
| Itfg2 | 0.24170407 |
| Itfg3 | 2.15280108 |
| Itga1 | -0.5923325 |
| Itga11 | 0.2859464 |
| Itga11 | 0.90696928 |
| Itga2b | 1.41120904 |
| Itga3 | -0.5997832 |
| Itga5 | -0.4044909 |
| Itga5 | -0.3114829 |
| Itga6 | 1.95047374 |
| Itga7 | -0.250253 |
| Itgad | 1.34704587 |
| Itgad | -0.7306542 |
| Itgad | -0.4767688 |
| Itgae | 1.34311716 |
| Itgae | -0.5418104 |
| Itgae | 0.78306494 |
| Itgav | 1.6556864 |
| Itgb1 | 2.08811641 |
| Itgb1 | 0.50230259 |
| Itgb1bp1 | 0.43579144 |
| Itgb3bp | -0.5155092 |
| Itgb4 | 1.09613563 |
| Itgb4 | -0.5209838 |
| Itgb5 | -0.2132302 |
| Itgb6 | 4.27494816 |
| Itgb6 | 0.80120256 |
| Itgb6 | 1.58450581 |
| Itgb7 | -0.590109 |
| Itgbl1 | 0.03552603 |
| Itih2 | -0.1682633 |
| Itih3 | 1.32677611 |
| Itih5 | 0.12547598 |
| Itih5 | -0.7272081 |
| Itk | -0.6411001 |
| Itlnb | 0.50319937 |
| Itm2a | 1.07334997 |
| Itm2b | -0.1762336 |
| Itm2b | 0.35435938 |
| Itpk1 | -0.6901337 |
| Itpka | -0.4999498 |
| Itpkc | -0.5531148 |
| Itpr1 | -0.7622676 |
| Itpr2 | -0.5967956 |
| Itpr3 | -0.4522485 |
| Itsn1 | 1.95626044 |
| Itsn2 | -0.5827364 |
| Ivd | -0.0319048 |
| Ivns1abp | -0.1720054 |
| Ivns1abp | 0.44848749 |
| Iws1 | -0.4702521 |
| Iyd | 1.09108168 |
| Izumo1 | 1.45808116 |
| Jag1 | -0.553826 |
| Jag2 | 1.12871886 |
| Jagn1 | -0.2548597 |
| Jak1 | -0.3706773 |
| Jak2 | 1.03293598 |
| Jak2 | -0.2756193 |
| Jak3 | 2.03856009 |
| Jak3 | 0.08815454 |
| Jakmip1 | 0.06638199 |
| Jam2 | -0.4807012 |
| Jam2 | 0.5803038 |
| Jam2 | 2.3907666 |
| Jam2 | 3.0433332 |
| Jam3 | 0.0474037 |
| Jam4 | 0.42144773 |
| Jarid1b | 0.79893465 |
| Jarid1c | -0.2677791 |
| Jarid1d | 3.22573394 |
| Jazf1 | -0.5754227 |
| Jdp2 | -0.5046573 |
| Jmjd1a | -0.2005698 |
| Jmjd1a | -0.3579535 |
| Jmjd1a | -0.6125903 |
| Jmjd2a | -0.345971 |
| Jmjd2b | 0.10180799 |
| Jmjd2b | -0.4859708 |
| Jmjd2c | -0.5121042 |
| Jmjd2d | -0.6884836 |
| Jmjd3 | 0.20021971 |
| Jmjd3 | -0.0659091 |
| Jmjd5 | -0.5982882 |
| Jmjd6 | -0.5938178 |
| Jmy | 2.78343647 |
| Josd1 | -0.1904705 |
| Josd2 | -0.5148272 |
| Josd3 | -0.3735939 |
| Josd3 | 2.90114043 |
| Jph1 | -0.7160971 |
| Jph2 | -0.3488098 |
| Jph3 | 2.22996432 |
| Jph4 | 0.55968164 |
| Jph4 | -0.5264914 |
| Jtb | 0.82578545 |
| Jtv1 | -0.7051194 |
| Jtv1 | -0.631756 |
| Jun | 2.22479016 |
| Junb | 0.05912916 |
| Jund1 | 2.03897843 |
| Jup | -0.358528 |
| Kal1 | 2.78286163 |
| Kalrn | -0.2220988 |
| Kank1 | -0.3027898 |
| Kank3 | -0.1654667 |
| Kank4 | 0.28850012 |
| Kank4 | 1.40353515 |
| Kank4 | -0.0522376 |
| Kars | 0.62178011 |
| Katna1 | -0.6745773 |
| Katnal1 | 1.81810159 |
| Katnb1 | -0.5915907 |
| Kazald1 | 1.04714263 |
| Kbtbd2 | -0.105139 |
| Kbtbd2 | -0.55525 |
| Kbtbd4 | -0.4358422 |
| Kbtbd4 | -0.7118593 |
| Kbtbd7 | 0.10700554 |
| Kbtbd7 | -0.4709017 |
| Kbtbd8 | 0.68618956 |
| Kcmf1 | -0.248213 |
| Kcna1 | -0.4283682 |
| Kcna2 | 3.10896015 |
| Kcna3 | 0.93786223 |
| Kcna5 | -0.0853138 |
| Kcna6 | 1.94214301 |
| Kcnab1 | -0.7160971 |
| Kcnab1 | -0.6984287 |
| Kcnab1 | 1.29545664 |
| Kcnab2 | -0.5990353 |
| Kcnab3 | 1.40662913 |
| Kcnb1 | -0.6584829 |
| Kcnc1 | -0.5588198 |
| Kcnc1 | 0.08638927 |
| Kcnc4 | -0.4992793 |
| Kcnc4 | 0.28281548 |
| Kcnd2 | -0.2295555 |
| Kcne1 | -0.7613745 |
| Kcne1l | -0.5223576 |
| Kcne2 | -0.2487226 |
| Kcne4 | -0.7894729 |
| Kcnf1 | -0.285117 |
| Kcng4 | -0.6088048 |
| Kcnh1 | 2.44190127 |
| Kcnh2 | 1.76352845 |
| Kcnh2 | -0.6786455 |
| Kcnh3 | 0.31199726 |
| Kcnh5 | -0.1909489 |
| Kcnip1 | -0.5432158 |
| Kcnip2 | -0.6125903 |
| Kcnip2 | 0.8920021 |
| Kcnip2 | 0.02235579 |
| Kcnip3 | 2.82624211 |
| Kcnip4 | -0.4715517 |
| Kcnj12 | -0.3065828 |
| Kcnj16 | 2.2894925 |
| Kcnj2 | 1.44304333 |
| Kcnj3 | -0.8015179 |
| Kcnj4 | 0.23662023 |
| Kcnj6 | 0.46428913 |
| Kcnj6 | 1.77476243 |
| Kcnj8 | 1.52845785 |
| Kcnj9 | 2.1207052 |
| Kcnk1 | 0.29527539 |
| Kcnk10 | 0.65141244 |
| Kcnk12 | -0.6179163 |
| Kcnk13 | -0.1654667 |
| Kcnk2 | -0.7195015 |
| Kcnk2 | 1.18306159 |
| Kcnk3 | -0.3224667 |
| Kcnk4 | 1.3720059 |
| Kcnk5 | 0.55609323 |
| Kcnk5 | 0.44824956 |
| Kcnk6 | 0.14722542 |
| Kcnk6 | 0.06638199 |
| Kcnk9 | 1.71053689 |
| Kcnma1 | -0.115215 |
| Kcnmb1 | -0.3103917 |
| Kcnmb4 | 1.69288089 |
| Kcnmb4 | 0.42413909 |
| Kcnn1 | 1.83393687 |
| Kcnn2 | -0.4148224 |
| Kcnn4 | 0.51522065 |
| Kcnq1 | 0.67306994 |
| Kcnq2 | -0.0717714 |
| Kcnq2 | 1.65026556 |
| Kcnq3 | 0.49396544 |
| Kcnq5 | -0.4057003 |
| Kcnrg | -0.388318 |
| Kcnrg | 0.00976205 |
| Kcns1 | 0.15512707 |
| Kcns2 | 2.52952034 |
| Kcnt1 | -0.2803558 |
| Kcnu1 | 0.04111289 |
| Kcnv1 | 0.61763698 |
| Kcnv2 | 0.15348652 |
| Kctd10 | -0.6141088 |
| Kctd11 | 0.40188271 |
| Kctd12 | -0.6057877 |
| Kctd12b | 1.49580321 |
| Kctd12b | 0.27105038 |
| Kctd14 | -0.6917867 |
| Kctd14 | -0.8080699 |
| Kctd15 | -0.4111627 |
| Kctd18 | 1.15711639 |
| Kctd19 | 0.59257956 |
| Kctd2 | 0.59359514 |
| Kctd20 | -0.5053318 |
| Kctd20 | -0.7757691 |
| Kctd3 | 0.24349164 |
| Kctd4 | -0.6035314 |
| Kctd5 | 0.05730693 |
| Kctd5 | 1.0873024 |
| Kctd6 | 1.30352776 |
| Kctd6 | 0.79242376 |
| Kctd9 | -0.345971 |
| Kdelc1 | -0.5953055 |
| Kdelc1 | 1.93598523 |
| Kdelr2 | -0.8241801 |
| Kdelr3 | 0.77826644 |
| Kdr | -0.6984287 |
| Keap1 | -0.2176537 |
| Keap1 | -0.1307643 |
| Keap1 | 0.25620139 |
| Keap1 | -0.6035314 |
| Kera | -0.0448656 |
| Khdc1c | 3.29303266 |
| Khdrbs1 | -0.4014743 |
| Khdrbs2 | -0.3527989 |
| Khdrbs3 | 0.54502871 |
| Khk | -0.3082133 |
| Khk | 0.84433901 |
| Khsrp | 0.65767983 |
| Kif11 | -0.7445699 |
| Kif13a | 2.27066156 |
| Kif14 | -0.3180573 |
| Kif15 | -0.1038329 |
| Kif17 | 0.71259083 |
| Kif17 | -0.355659 |
| Kif18a | -0.4866317 |
| Kif1a | 1.10997835 |
| Kif1b | -0.5566762 |
| Kif1b | 2.7080029 |
| Kif1b | 0.70615135 |
| Kif1b | 0.54887389 |
| Kif1b | -0.5053318 |
| Kif1b | 1.35086905 |
| Kif1c | 2.02188099 |
| Kif21a | -0.5334228 |
| Kif21b | -0.7542612 |
| Kif22 | -0.4283682 |
| Kif23 | 0.42828296 |
| Kif24 | 3.53529554 |
| Kif27 | 0.05218531 |
| Kif2a | -0.3573793 |
| Kif2b | 1.83800954 |
| Kif2c | -0.5251114 |
| Kif3a | -0.291504 |
| Kif3a | 3.86079574 |
| Kif3b | 0.54844745 |
| Kif3c | -0.1461266 |
| Kif3c | -0.7848821 |
| Kif4 | 0.21814209 |
| Kif4 | 0.42389475 |
| Kif4 | 0.80893585 |
| Kif5a | -0.6418832 |
| Kif5a | -0.253834 |
| Kif5a | 2.66554343 |
| Kif5b | 1.43393296 |
| Kif5c | 0.5889141 |
| Kif9 | -0.7341133 |
| Kifap3 | 1.65173554 |
| Kifap3 | 0.65597479 |
| Kifap3 | 0.46802263 |
| Kifc2 | -0.7212085 |
| Kifc2 | 0.38240172 |
| Kifc3 | 0.585029 |
| Kin | -0.584942 |
| Kirrel3 | -0.656889 |
| Kiss1 | -0.563122 |
| Kiss1r | 0.15381486 |
| Kitl | -0.7830523 |
| Kl | -0.3550863 |
| Klb | -0.6340831 |
| Klc1 | 1.85785045 |
| Klc1 | 0.67622843 |
| Klc1 | -0.5182424 |
| Klc2 | 0.30004436 |
| Klc2 | 0.27565965 |
| Klc4 | 2.51812146 |
| Klf1 | 1.07814401 |
| Klf10 | -0.1420349 |
| Klf12 | -0.219133 |
| Klf12 | 0.24705635 |
| Klf12 | 1.5225933 |
| Klf13 | -0.6959323 |
| Klf16 | -0.4370939 |
| Klf17 | -0.1539056 |
| Klf17 | 1.8944759 |
| Klf2 | 0.71986991 |
| Klf4 | -0.0742954 |
| Klf4 | 0.64394976 |
| Klf4 | 0.20829733 |
| Klf5 | -0.487293 |
| Klf6 | 0.18891626 |
| Klf6 | -0.4105541 |
| Klf7 | -0.6843715 |
| Klf9 | 1.04936575 |
| Klhdc1 | 1.48214658 |
| Klhdc1 | 0.56325604 |
| Klhdc1 | -0.848399 |
| Klhdc2 | -0.7867157 |
| Klhdc3 | -0.7212085 |
| Klhdc4 | 1.98470178 |
| Klhdc4 | 1.55744205 |
| Klhdc4 | 0.24170407 |
| Klhdc8b | -0.7578109 |
| Klhdc9 | 0.19017912 |
| Klhl10 | -0.8895607 |
| Klhl11 | -0.5251114 |
| Klhl12 | -0.6942718 |
| Klhl14 | -0.3197083 |
| Klhl15 | 0.16133451 |
| Klhl15 | 0.63603818 |
| Klhl17 | 1.10627648 |
| Klhl2 | -0.6395361 |
| Klhl21 | 3.09611639 |
| Klhl22 | -0.3996691 |
| Klhl22 | 0.80652621 |
| Klhl23 | 1.15172759 |
| Klhl24 | 0.89112263 |
| Klhl24 | 0.83752263 |
| Klhl25 | -0.409946 |
| Klhl26 | -0.647383 |
| Klhl26 | 0.27795561 |
| Klhl30 | 2.0243917 |
| Klhl31 | -0.5717877 |
| Klhl32 | 1.03054661 |
| Klhl5 | 0.78915085 |
| Klhl5 | -0.6664947 |
| Klhl6 | 3.64055344 |
| Klhl6 | -0.6156299 |
| Klhl7 | -0.7026046 |
| Klhl8 | 0.00937771 |
| Klhl9 | 0.78553708 |
| Klhl9 | -0.5060068 |
| Klk1 | -0.7489623 |
| Klk11 | 1.35148992 |
| Klk11 | -0.7152479 |
| Klk15 | -0.1976729 |
| Klk1b1 | -0.6403178 |
| Klk1b11 | 0.15414309 |
| Klk1b16 | 0.13624802 |
| Klk1b21 | 1.46531267 |
| Klk1b21 | -0.1677966 |
| Klk1b22 | -0.152528 |
| Klk1b24 | -0.4289887 |
| Klk1b26 | 1.43458094 |
| Klk1b27 | -0.659281 |
| Klk1b27 | -0.6209738 |
| Klk1b4 | -0.8289728 |
| Klk1b4 | 1.8319986 |
| Klk1b5 | -0.7489623 |
| Klk1b8 | -0.2548597 |
| Klk1b9 | 0.60368946 |
| Klk6 | -0.3169583 |
| Klk8 | -0.5474451 |
| Klkb1 | -0.2558866 |
| Klra17 | -0.5418104 |
| Klra4 | -0.7152479 |
| Klrd1 | -0.3335839 |
| Kndc1 | 1.07957733 |
| Kng1 | -0.0692544 |
| Kng1 | -0.6379748 |
| Kng2 | 1.10430375 |
| Kns2 | 1.20715003 |
| Kns2 | -0.4358422 |
| Kntc1 | 1.31513352 |
| Kpna1 | 0.33571368 |
| Kpna2 | -0.766746 |
| Kpna3 | -0.6640838 |
| Kpna6 | 0.43119678 |
| Kpnb1 | 0.17105077 |
| Kptn | -0.1047034 |
| Kras | 1.81831454 |
| Kras | -0.6656904 |
| Krba1 | -0.6521236 |
| Krba1 | 1.98926154 |
| Krba1 | -0.8118351 |
| Krcc1 | -0.4926009 |
| Kremen | 3.71260279 |
| Kremen1 | -0.7931622 |
| Kremen2 | -0.6616792 |
| Krit1 | -0.6434512 |
| Krit1 | -0.1626787 |
| Krr1 | 0.09867523 |
| Krr1 | 0.20457805 |
| Krr1 | -0.3197083 |
| Krt10 | 3.12169015 |
| Krt12 | -0.3076695 |
| Krt13 | 1.01274567 |
| Krt14 | -0.2777214 |
| Krt17 | 1.83472148 |
| Krt18 | -0.4670111 |
| Krt19 | 0.39585509 |
| Krt2 | -0.8118351 |
| Krt20 | 1.36853133 |
| Krt222 | -0.7384557 |
| Krt23 | -0.1200735 |
| Krt27 | -0.0514155 |
| Krt32 | 2.00857473 |
| Krt4 | 2.05703431 |
| Krt7 | -0.3076695 |
| Krt73 | 2.26819068 |
| Krt75 | 0.09343001 |
| Krt78 | -0.759591 |
| Krt79 | -0.5127842 |
| Krt8 | -0.6959323 |
| Krt84 | -0.3493786 |
| Krt86 | -0.4979397 |
| Krt86 | 1.4444862 |
| Krt9 | -0.6035314 |
| Krtap16-4 | -0.0065212 |
| Krtap8-2 | -0.5805361 |
| Krtcap2 | 0.22058682 |
| Krtcap3 | 1.5725375 |
| Ksr2 | -0.440859 |
| Ktelc1 | 0.52029324 |
| Kti12 | 1.47295346 |
| Ktn1 | 1.14785892 |
| Ktn1 | -0.8435037 |
| Ky | -0.4184969 |
| L1cam | 2.64159429 |
| L2hgdh | 0.86418663 |
| L3mbtl2 | 1.39334331 |
| L3mbtl3 | -0.724632 |
| l7Rn6 | 1.35802775 |
| Lace1 | 1.36835726 |
| Lad1 | -0.7748628 |
| Lag3 | 2.89106075 |
| Lage3 | 0.53382874 |
| Lama1 | -0.4484362 |
| Lama2 | 0.50677773 |
| Lamb1-1 | -0.8194122 |
| Lamb2 | 2.18402328 |
| Lamb3 | -0.6080496 |
| Lamc1 | 1.96786083 |
| Lamc2 | -0.4063056 |
| Lamc2 | 1.69397918 |
| Lamc2 | 0.239913 |
| Lamc3 | 0.25001636 |
| Lamp1 | 0.46685755 |
| Lamp2 | -0.062992 |
| Lamp2 | -0.724632 |
| Lamp2 | -0.1589745 |
| Lamp2 | -0.3823999 |
| Lancl1 | -0.5938178 |
| Lancl1 | -0.0908732 |
| Lancl1 | 0.36848798 |
| Laptm4a | -0.7384557 |
| Laptm4b | -0.7059591 |
| Laptm4b | -0.4715517 |
| Laptm5 | 0.49667772 |
| Large | -0.6148691 |
| Larp2 | 1.65549562 |
| Larp5 | 0.73006922 |
| Larp6 | -0.0604989 |
| Larp6 | 0.40737336 |
| Lars | 0.56115514 |
| Lars2 | -0.713552 |
| Las1l | -0.6278906 |
| Lasp1 | 0.82578545 |
| Lasp1 | -0.2211091 |
| Lasp1 | -0.6819131 |
| Lass1 | 0.79405583 |
| Lass2 | -0.1156557 |
| Lass2 | -0.4421176 |
| Lass2 | -0.677016 |
| Lass4 | -0.4358422 |
| Lass4 | -0.4377203 |
| Lass5 | -0.1847491 |
| Lass6 | -0.0159577 |
| Lat2 | -0.491271 |
| Lat2 | -0.5446234 |
| Lats2 | -0.766746 |
| Lbh | -0.1552852 |
| Lbh | -0.4866317 |
| Lbp | -0.7748628 |
| Lbr | -0.6545032 |
| Lbx2 | -0.6095606 |
| Lca5 | 1.28864424 |
| Lca5 | -0.3038719 |
| Lca5 | 0.69604341 |
| Lcat | -0.27457 |
| Lcat | 0.37624395 |
| Lce1c | 0.10354379 |
| Lck | -0.4932665 |
| Lcmt1 | -0.1055748 |
| Lcmt1 | 1.30958112 |
| Lcmt1 | -0.6632816 |
| Lcn10 | -0.6371951 |
| Lcn12 | 1.56589811 |
| Lcn13 | 1.03394017 |
| Lcn2 | -0.1506944 |
| Lcn3 | -0.5871528 |
| Lcn8 | -0.0510048 |
| Lcor | 0.68196011 |
| Lcp1 | -0.5039833 |
| Ldb1 | -0.5404071 |
| Ldb2 | 0.97258843 |
| Ldb2 | 0.57368876 |
| Ldha | -0.3782799 |
| Ldhc | -0.6481714 |
| Ldhc | -0.246686 |
| Ldhc | -0.7341133 |
| Ldhd | 0.88272525 |
| Ldhd | -0.425272 |
| Ldoc1 | 2.22567729 |
| Ldoc1l | 3.02914454 |
| Lef1 | -0.5746945 |
| Lef1 | 0.02007888 |
| Lefty1 | 0.51831166 |
| Lefty2 | -0.4370939 |
| Lemd2 | 2.79946008 |
| Leng1 | 2.9461747 |
| Leng8 | 1.98620946 |
| Leng9 | -0.6984287 |
| Leo1 | -0.1575893 |
| Lep | 1.60684321 |
| Lepre1 | -0.3724261 |
| Lepre1 | -0.6278906 |
| Lepre1 | 2.04265155 |
| Leprel1 | -0.4240364 |
| Leprel2 | -0.1673302 |
| Leprotl1 | 1.83121165 |
| Letm1 | 1.18731516 |
| Letm1 | 3.03221464 |
| Letm1 | 1.6839851 |
| Letmd1 | 0.84325798 |
| Lgals1 | -0.0764041 |
| Lgals2 | 2.62110579 |
| Lgals3 | -0.7143996 |
| Lgals3bp | 0.71116375 |
| Lgals4 | -0.5990353 |
| Lgals4 | 2.35671275 |
| Lgals6 | -0.6810951 |
| Lgals7 | -0.6434512 |
| Lgals8 | -0.4452714 |
| Lgals8 | 0.59542036 |
| Lgals9 | 3.66655064 |
| Lgals9 | 0.12547598 |
| Lgi1 | 1.77000483 |
| Lgi1 | 1.27322489 |
| Lgi2 | -0.6616792 |
| Lgi3 | -0.6819131 |
| Lgi4 | -0.6810951 |
| Lgmn | 1.98974717 |
| Lgr5 | 2.33206128 |
| Lgtn | 0.18543439 |
| Lgtn | -0.5019644 |
| Lhb | -0.459921 |
| Lhcgr | 1.01158939 |
| Lhcgr | -0.2528094 |
| Lhfp | 2.55247355 |
| Lhfp | -0.403283 |
| Lhfp | 0.34081235 |
| Lhfpl1 | 1.10662416 |
| Lhfpl2 | -0.7229187 |
| Lhx1 | 2.44927607 |
| Lhx2 | -0.5893691 |
| Lhx3 | -0.5710624 |
| Lhx4 | -0.7739574 |
| Lhx9 | -0.8052561 |
| Lhx9 | 0.18860028 |
| Lias | 2.10508131 |
| Lias | 1.54847826 |
| Lias | 2.95293058 |
| Lifr | -0.1962279 |
| Lig1 | -1.027865 |
| Lig1 | 0.29976452 |
| Lig1 | 0.86252379 |
| Lig3 | -0.6133492 |
| Lig4 | -0.0199185 |
| Lima1 | -0.5244222 |
| Lima1 | 0.93016597 |
| Limd1 | -0.3654507 |
| Limd2 | -0.8444807 |
| Lime1 | 0.26902649 |
| Limk1 | -0.4478024 |
| Limk2 | -0.5739669 |
| Limk2 | 2.0174385 |
| Lims2 | 1.79622519 |
| Lims2 | -0.0436427 |
| Lims2 | -0.3960693 |
| Lin54 | -0.6027805 |
| Lin7b | 1.49504562 |
| Lin7b | 1.10581269 |
| Lin7b | -0.0878755 |
| Lin7b | -0.3865386 |
| Lin7c | -0.5717877 |
| Lincr | 1.78938316 |
| Lincr | -0.6088048 |
| Lingo1 | -0.7569222 |
| Lingo2 | -0.848399 |
| Lingo2 | 1.26604962 |
| Lingo4 | -0.6057877 |
| Lip1 | -0.1919065 |
| Lipe | -0.3380689 |
| Lipf | -0.3812209 |
| Liph | 1.37443027 |
| Liph | -0.5006208 |
| Litaf | 2.03381957 |
| Lix1 | -0.1452157 |
| Lix1 | 0.37881472 |
| Lix1 | -0.4800446 |
| Lix1 | 1.42423977 |
| Llgl1 | 0.81229872 |
| Llgl2 | 0.1247985 |
| Lman1 | 0.86554489 |
| Lman2 | 0.26263642 |
| Lman2l | -0.7784935 |
| Lman2l | 1.40520887 |
| Lmbr1 | -0.6545032 |
| Lmbr1l | -0.6893083 |
| Lmcd1 | -0.7551474 |
| Lmcd1 | -0.6835513 |
| Lmcd1 | -0.6088048 |
| Lmf1 | -0.1790633 |
| Lmf2 | -0.5945613 |
| Lmna | 3.2414088 |
| Lmna | 0.48646497 |
| Lmna | -0.080636 |
| Lmna | -0.6537093 |
| Lmna | -0.6156299 |
| Lmnb1 | -0.677016 |
| Lmnb1 | -0.7401985 |
| Lmnb2 | -0.6458083 |
| Lmo2 | 1.0272668 |
| Lmo3 | 0.02651547 |
| Lmo3 | -0.55525 |
| Lmo4 | -0.6959323 |
| Lmod1 | 1.8614629 |
| Lmtk2 | 0.27479718 |
| Lmtk3 | 0.71312542 |
| Lmx1a | -0.4008721 |
| Lmx1b | -0.3972676 |
| Lnx1 | -0.1271868 |
| Lnx2 | 0.3813783 |
| Lnx2 | -0.6426668 |
| LOC100038882 | -0.4853105 |
| LOC100038984 | -0.1285267 |
| LOC100039087 | -0.2507637 |
| LOC100039227 | 2.63226064 |
| LOC100039479 | -0.4069114 |
| LOC100039532 | -0.2522975 |
| LOC100039571 | -0.5975416 |
| LOC100039728 | -0.6481714 |
| LOC100040573 | -0.3895063 |
| LOC100040592 | 1.95941391 |
| LOC100040601 | -0.4421176 |
| LOC100040799 | -0.192865 |
| LOC100040899 | 0.53577704 |
| LOC100040919 | -0.2533215 |
| LOC100041103 | -0.6348601 |
| LOC100041290 | 0.32297776 |
| LOC100041463 | 0.00165665 |
| LOC100041483 | 1.4450467 |
| LOC100041500 | -0.5710624 |
| LOC100041504 | 0.65274548 |
| LOC100041703 | 0.05511546 |
| LOC100041722 | 1.93429862 |
| LOC100041725 | -0.5930748 |
| LOC100041835 | 2.58438497 |
| LOC100042179 | 0.36822832 |
| LOC100042427 | -0.5271822 |
| LOC100042777 | -0.7739574 |
| LOC100043257 | -0.5418104 |
| LOC100043391 | -0.5616857 |
| LOC100043671 | 3.10872634 |
| LOC100043822 | 3.04404456 |
| LOC100043986 | 0.0370192 |
| LOC100044087 | 0.03589955 |
| LOC100044089 | 3.43544028 |
| LOC100044101 | -0.0444578 |
| LOC100044103 | -0.7101696 |
| LOC100044103 | -0.6819131 |
| LOC100044122 | -0.4592791 |
| LOC100044133 | 1.17814543 |
| LOC100044159 | -0.0840357 |
| LOC100044170 | 2.68931321 |
| LOC100044172 | -0.3476732 |
| LOC100044177 | -0.713552 |
| LOC100044190 | -0.3335839 |
| LOC100044204 | -0.0930204 |
| LOC100044210 | -0.012014 |
| LOC100044221 | -0.6901337 |
| LOC100044257 | 1.7964433 |
| LOC100044275 | -0.6217397 |
| LOC100044289 | 3.03399257 |
| LOC100044292 | -0.080636 |
| LOC100044294 | 0.05912916 |
| LOC100044298 | 0.70937676 |
| LOC100044322 | 0.48806112 |
| LOC100044324 | 1.11182368 |
| LOC100044324 | -0.6810951 |
| LOC100044363 | -0.0542959 |
| LOC100044363 | -0.3324661 |
| LOC100044439 | 1.4120393 |
| LOC100044468 | 0.31914956 |
| LOC100044475 | 1.58354307 |
| LOC100044538 | 1.27631037 |
| LOC100044566 | -0.147951 |
| LOC100044576 | -0.5739669 |
| LOC100044636 | 1.18252851 |
| LOC100044692 | 0.05328522 |
| LOC100044708 | 2.17804314 |
| LOC100044736 | 0.40388313 |
| LOC100044756 | -0.3782799 |
| LOC100044776 | 0.61921753 |
| LOC100044779 | 1.8642515 |
| LOC100044812 | -0.626349 |
| LOC100044829 | 0.25796089 |
| LOC100044862 | 0.90408212 |
| LOC100044873 | 0.51765018 |
| LOC100044934 | 1.64648388 |
| LOC100045005 | -0.0069124 |
| LOC100045019 | -0.8503645 |
| LOC100045040 | -0.20834 |
| LOC100045098 | 2.79522425 |
| LOC100045228 | 1.80285404 |
| LOC100045241 | 0.27018355 |
| LOC100045250 | -0.7289295 |
| LOC100045280 | 1.01146082 |
| LOC100045300 | -0.2340589 |
| LOC100045304 | -0.5502755 |
| LOC100045343 | -0.2492324 |
| LOC100045359 | 0.22211147 |
| LOC100045359 | -0.518927 |
| LOC100045403 | 0.10250271 |
| LOC100045413 | 2.6704645 |
| LOC100045419 | 1.46929696 |
| LOC100045430 | -0.5237335 |
| LOC100045439 | 1.90392895 |
| LOC100045491 | 0.23901614 |
| LOC100045505 | -0.0207127 |
| LOC100045522 | 0.22332936 |
| LOC100045542 | 0.84495617 |
| LOC100045551 | -0.5842062 |
| LOC100045567 | -0.388912 |
| LOC100045607 | 1.61012389 |
| LOC100045617 | -0.606541 |
| LOC100045644 | 0.3703036 |
| LOC100045697 | 0.3397413 |
| LOC100045780 | 0.0543838 |
| LOC100045782 | 1.7268591 |
| LOC100045877 | 0.27047258 |
| LOC100045882 | -0.6379748 |
| LOC100045887 | -0.6778304 |
| LOC100045950 | 0.16846984 |
| LOC100045963 | 1.09789209 |
| LOC100045981 | 0.29892448 |
| LOC100046003 | 0.8087754 |
| LOC100046035 | -0.1919065 |
| LOC100046035 | 2.15405589 |
| LOC100046039 | 0.21169343 |
| LOC100046044 | -0.7203546 |
| LOC100046049 | 0.68820545 |
| LOC100046056 | 0.58277217 |
| LOC100046068 | 0.91070902 |
| LOC100046080 | -0.6240415 |
| LOC100046081 | 0.03178241 |
| LOC100046120 | -0.477423 |
| LOC100046128 | 0.7392909 |
| LOC100046129 | 0.91271641 |
| LOC100046163 | 0.07751147 |
| LOC100046163 | -0.6835513 |
| LOC100046207 | -0.647383 |
| LOC100046207 | -0.7694436 |
| LOC100046207 | 0.07644032 |
| LOC100046232 | -0.5334228 |
| LOC100046254 | 1.30586072 |
| LOC100046320 | 1.28778911 |
| LOC100046393 | -0.3358237 |
| LOC100046406 | -0.3978674 |
| LOC100046457 | 0.40762216 |
| LOC100046518 | -0.7177977 |
| LOC100046568 | 1.32723146 |
| LOC100046586 | 3.20521446 |
| LOC100046608 | 1.47883534 |
| LOC100046608 | 0.16685305 |
| LOC100046608 | 0.45936568 |
| LOC100046608 | 1.69336918 |
| LOC100046616 | -0.2380807 |
| LOC100046641 | 0.66334043 |
| LOC100046650 | 0.97392901 |
| LOC100046690 | 3.84862637 |
| LOC100046690 | 0.10041698 |
| LOC100046741 | 0.42535983 |
| LOC100046744 | -0.6600797 |
| LOC100046744 | -0.8232245 |
| LOC100046746 | 1.57399868 |
| LOC100046775 | 1.5316674 |
| LOC100046781 | -0.7349802 |
| LOC100046781 | -0.1529869 |
| LOC100046792 | -0.6110742 |
| LOC100046796 | 0.02349213 |
| LOC100046802 | 1.37615804 |
| LOC100046841 | -0.6348601 |
| LOC100046855 | -0.7766763 |
| LOC100046883 | 0.58153882 |
| LOC100046891 | 0.89915295 |
| LOC100046898 | -0.6340831 |
| LOC100046918 | -0.7143996 |
| LOC100046953 | -0.5609684 |
| LOC100046959 | -0.9413137 |
| LOC100046996 | 1.06853068 |
| LOC100047009 | -0.7569222 |
| LOC100047009 | 0.47890249 |
| LOC100047012 | -0.7551474 |
| LOC100047028 | -0.5667227 |
| LOC100047046 | 1.64429749 |
| LOC100047052 | 1.24388072 |
| LOC100047052 | 1.43125521 |
| LOC100047155 | -0.3608298 |
| LOC100047155 | -0.4800446 |
| LOC100047167 | -0.0171441 |
| LOC100047173 | -0.2543467 |
| LOC100047184 | 1.67406763 |
| LOC100047200 | -0.1200735 |
| LOC100047214 | 0.87797693 |
| LOC100047214 | 0.20736892 |
| LOC100047238 | 2.41123615 |
| LOC100047261 | 2.22921724 |
| LOC100047323 | -0.8612509 |
| LOC100047323 | 1.88319025 |
| LOC100047339 | 0.15905245 |
| LOC100047353 | 3.53007269 |
| LOC100047368 | 1.40445608 |
| LOC100047369 | 1.24676838 |
| LOC100047369 | -0.446536 |
| LOC100047391 | 0.47959258 |
| LOC100047393 | -0.4919357 |
| LOC100047427 | -0.5660015 |
| LOC100047480 | 1.50251825 |
| LOC100047490 | 2.06308324 |
| LOC100047579 | 0.74205664 |
| LOC100047583 | -0.590109 |
| LOC100047606 | -0.5602516 |
| LOC100047615 | 0.00591129 |
| LOC100047619 | -0.4522485 |
| LOC100047634 | 1.99151091 |
| LOC100047651 | -0.7254899 |
| LOC100047653 | 1.84862771 |
| LOC100047659 | -0.4663642 |
| LOC100047674 | 0.17970805 |
| LOC100047707 | 0.1107984 |
| LOC100047717 | 1.59278462 |
| LOC100047749 | 1.26604962 |
| LOC100047762 | -0.6632816 |
| LOC100047776 | -0.8156158 |
| LOC100047794 | -0.0282924 |
| LOC100047800 | 0.87275509 |
| LOC100047810 | -0.476115 |
| LOC100047815 | -0.904051 |
| LOC100047816 | -0.6934427 |
| LOC100047834 | -0.3065828 |
| LOC100047837 | 1.01325911 |
| LOC100047837 | 0.04259698 |
| LOC100047841 | -0.5754227 |
| LOC100047856 | 0.52926653 |
| LOC100047870 | -0.3363845 |
| LOC100047911 | -0.352228 |
| LOC100047934 | 2.15361321 |
| LOC100047935 | -0.8080699 |
| LOC100047937 | 2.41418282 |
| LOC100047937 | 0.34614884 |
| LOC100047958 | 0.27306979 |
| LOC100047963 | -0.55525 |
| LOC100047963 | 0.39484661 |
| LOC100047998 | -0.766746 |
| LOC100048046 | -0.2147023 |
| LOC100048046 | -0.2709071 |
| LOC100048049 | -0.440859 |
| LOC100048049 | -0.8366939 |
| LOC100048049 | 0.84911114 |
| LOC100048056 | 2.52007648 |
| LOC100048071 | -0.5754227 |
| LOC100048076 | -0.5827364 |
| LOC100048083 | 0.10596839 |
| LOC100048105 | 2.26692013 |
| LOC100048105 | 0.5512158 |
| LOC100048187 | 1.94014161 |
| LOC100048251 | -0.4452714 |
| LOC100048295 | 0.27335792 |
| LOC100048299 | -0.1099441 |
| LOC100048301 | 1.19796821 |
| LOC100048313 | -0.7280684 |
| LOC100048331 | 0.35013089 |
| LOC100048332 | 2.02464664 |
| LOC100048346 | 0.93171041 |
| LOC100048372 | 2.7841962 |
| LOC100048384 | -0.1307643 |
| LOC100048413 | -0.1348057 |
| LOC100048436 | -0.3936773 |
| LOC100048445 | 1.20880959 |
| LOC100048461 | -0.4289887 |
| LOC100048480 | 2.30212737 |
| LOC100048483 | -0.7445699 |
| LOC100048504 | 0.02651547 |
| LOC100048530 | -0.1991202 |
| LOC100048554 | 2.77529949 |
| LOC100048556 | -0.5348154 |
| LOC100048589 | 1.58016551 |
| LOC100048613 | 0.58707584 |
| LOC100048622 | 2.75274839 |
| LOC100048645 | 0.4194854 |
| LOC100048684 | 0.58174449 |
| LOC100048703 | 2.18184344 |
| LOC100048710 | 2.76489774 |
| LOC100048721 | -0.5893691 |
| LOC100048721 | -0.4637815 |
| LOC100048726 | 0.55461154 |
| LOC100048733 | -0.8232245 |
| LOC100048816 | -0.4433778 |
| LOC100048832 | 0.82641525 |
| LOC100048832 | -0.3313496 |
| LOC100048858 | 0.08071741 |
| LOC192758 | 0.75337774 |
| LOC194227 | -0.6348601 |
| LOC212390 | -0.8926465 |
| LOC216443 | -0.2360676 |
| LOC216963 | -0.6835513 |
| LOC217341 | 2.8306012 |
| LOC218963 | -0.5754227 |
| LOC225922 | -0.7341133 |
| LOC233529 | -0.8280123 |
| LOC234374 | -0.5230453 |
| LOC235497 | -0.4952664 |
| LOC236749 | -0.4567163 |
| LOC237408 | 0.91386151 |
| LOC237877 | 0.7515006 |
| LOC238829 | -0.5460332 |
| LOC241593 | -0.5930748 |
| LOC244595 | 1.15305023 |
| LOC245263 | -0.677016 |
| LOC329575 | 0.14092119 |
| LOC329575 | 0.72831881 |
| LOC333331 | 2.82832718 |
| LOC381066 | -0.7237749 |
| LOC381302 | 1.09296548 |
| LOC381375 | -0.2646618 |
| LOC381483 | 0.3389372 |
| LOC381629 | 2.21310934 |
| LOC381801 | 0.71650388 |
| LOC381936 | 0.37958455 |
| LOC382010 | 1.20631911 |
| LOC382044 | 1.66353728 |
| LOC382106 | -0.352228 |
| LOC382106 | -0.5609684 |
| LOC382133 | -0.1321094 |
| LOC384806 | -0.6255792 |
| LOC384806 | -0.7341133 |
| LOC386486 | 1.48183906 |
| LOC433479 | -0.7885529 |
| LOC433801 | 3.15575857 |
| LOC433886 | -0.7987241 |
| LOC434172 | 0.54481461 |
| LOC434172 | -0.5783412 |
| LOC434864 | -0.8118351 |
| LOC434960 | 3.16299823 |
| LOC435023 | -0.5244222 |
| LOC435337 | -0.8061931 |
| LOC435565 | -0.553826 |
| LOC436177 | -0.7821387 |
| LOC544988 | -0.3114829 |
| LOC544988 | 0.23692006 |
| LOC545013 | 0.87380184 |
| LOC545013 | -0.7622676 |
| LOC545056 | -0.3093018 |
| LOC545238 | -0.2803558 |
| LOC545703 | 1.02168943 |
| LOC545732 | 2.7457245 |
| LOC545952 | 0.00937771 |
| LOC546015 | -0.1804815 |
| LOC546015 | -0.3527989 |
| LOC546723 | 1.66906829 |
| LOC546840 | 1.55410867 |
| LOC547323 | 0.48919953 |
| LOC547343 | -0.4038868 |
| LOC620807 | -0.7867157 |
| LOC621823 | -0.0571854 |
| LOC621824 | 0.55333961 |
| LOC622404 | -0.8523343 |
| LOC622665 | 2.33409317 |
| LOC624855 | -0.581269 |
| LOC625480 | 0.65179351 |
| LOC626152 | -0.7401985 |
| LOC628053 | 1.46773656 |
| LOC631002 | 0.07501016 |
| LOC631784 | -0.7263486 |
| LOC632431 | 1.92050221 |
| LOC639396 | -0.2456694 |
| LOC640963 | -0.5161918 |
| LOC641240 | -0.55525 |
| LOC654426 | 1.45855452 |
| LOC654467 | 1.05539037 |
| LOC654494 | -0.5645606 |
| LOC654842 | -0.3505173 |
| LOC665250 | -0.4503404 |
| LOC666676 | -0.5155092 |
| LOC666904 | 2.43671685 |
| LOC667250 | 0.88523781 |
| LOC667370 | 1.25862137 |
| LOC667609 | 0.87693495 |
| LOC668492 | 0.29245845 |
| LOC668837 | 2.52057077 |
| LOC669168 | -0.1294211 |
| LOC669660 | 0.30840003 |
| LOC670044 | -0.3568055 |
| LOC671564 | 1.17964445 |
| LOC672705 | 2.98069217 |
| LOC674706 | 0.68618956 |
| LOC675228 | 0.65141244 |
| LOC675567 | -0.299551 |
| LOC675567 | 2.58895904 |
| LOC675813 | 2.47614812 |
| LOC676420 | 0.68196011 |
| LOC676420 | -0.5761514 |
| LOC677144 | -0.1710684 |
| LOC677205 | 1.46672087 |
| LOC677317 | -0.3380689 |
| LOC677319 | 1.1651949 |
| LOC677528 | 1.11802435 |
| LOC732482 | 0.73477867 |
| Loh11cr2a | 0.43868148 |
| Loh12cr1 | 1.90489796 |
| Loh12cr1 | -0.059255 |
| Lonp2 | -0.3966683 |
| Lonrf1 | 1.87801856 |
| Lonrf2 | -0.5609684 |
| Lor | -0.2990124 |
| Lox | 0.85141134 |
| Loxl1 | -0.5602516 |
| Lpar2 | 0.04998152 |
| Lpar2 | 1.2628316 |
| Lpar3 | -0.1060108 |
| Lpcat2 | -0.5820024 |
| Lpcat2 | 2.94544511 |
| Lpcat2 | -0.7445699 |
| Lpgat1 | 1.93453306 |
| Lphn1 | -0.6294348 |
| Lphn3 | -0.2698633 |
| Lpin1 | -0.477423 |
| Lpin1 | -0.7272081 |
| Lpin2 | 1.95208483 |
| Lpin3 | 1.5890992 |
| Lpl | -0.7152479 |
| Lpxn | -0.8998874 |
| Lrat | -0.8415528 |
| Lrba | 2.38899954 |
| Lrba | 0.95961802 |
| Lrch1 | 0.37958455 |
| Lrch4 | 2.37670711 |
| Lrch4 | 1.80852099 |
| Lrdd | -0.5990353 |
| Lrfn1 | -0.7516079 |
| Lrfn2 | -0.2305544 |
| Lrfn3 | -0.424654 |
| Lrfn4 | 2.31480672 |
| Lrg1 | -0.8533207 |
| Lrig1 | 0.26351042 |
| Lrig3 | 2.04991252 |
| Lrmp | -0.7349802 |
| Lrp1 | -0.27457 |
| Lrp10 | -0.5502755 |
| Lrp11 | -0.7084831 |
| Lrp12 | 1.53239528 |
| Lrp12 | -0.9009265 |
| Lrp4 | -0.5960502 |
| Lrp5 | 1.0647782 |
| Lrp8 | -0.2235853 |
| Lrp8 | -0.5566762 |
| Lrpap1 | -0.8099505 |
| Lrpprc | -0.6537093 |
| Lrpprc | -0.647383 |
| Lrpprc | -0.3596782 |
| Lrrc1 | -0.3812209 |
| Lrrc1 | -0.6552978 |
| Lrrc1 | 1.01594992 |
| Lrrc10 | -0.7524915 |
| Lrrc15 | 0.90335891 |
| Lrrc16b | 1.35688259 |
| Lrrc16b | -0.3730098 |
| Lrrc18 | 2.40922922 |
| Lrrc2 | 1.8152219 |
| Lrrc20 | 1.76680053 |
| Lrrc20 | -0.1465823 |
| Lrrc23 | -0.7830523 |
| Lrrc23 | 0.22332936 |
| Lrrc24 | 0.78685283 |
| Lrrc26 | 0.81453378 |
| Lrrc28 | -0.0061301 |
| Lrrc29 | 0.68875447 |
| Lrrc3 | 0.88568049 |
| Lrrc30 | 1.67618411 |
| Lrrc33 | -0.631756 |
| Lrrc34 | 1.31909211 |
| Lrrc34 | 1.17341816 |
| Lrrc36 | 0.60829571 |
| Lrrc39 | -0.7393267 |
| Lrrc39 | 0.7449861 |
| Lrrc4 | -0.2147023 |
| Lrrc40 | -0.4075175 |
| Lrrc41 | -0.6171536 |
| Lrrc43 | 1.23375195 |
| Lrrc44 | -0.8232245 |
| Lrrc45 | -0.5141457 |
| Lrrc46 | -0.0347243 |
| Lrrc48 | -0.6893083 |
| Lrrc48 | 2.09789395 |
| Lrrc48 | 0.49010924 |
| Lrrc49 | 1.60307941 |
| Lrrc49 | 3.1437596 |
| Lrrc49 | 2.90454076 |
| Lrrc4c | 2.28923801 |
| Lrrc50 | 3.45140768 |
| Lrrc50 | -0.4939326 |
| Lrrc52 | -0.5915907 |
| Lrrc52 | 1.05293613 |
| Lrrc56 | 0.43699673 |
| Lrrc57 | -0.1125758 |
| Lrrc59 | -0.3695132 |
| Lrrc6 | -0.6505407 |
| Lrrc7 | 1.35069158 |
| Lrrc8 | -0.5306439 |
| Lrrc8a | 2.1896099 |
| Lrrc8b | -0.0246942 |
| Lrrc8c | 0.50230259 |
| Lrrc8d | -0.2543467 |
| Lrrc9 | 0.2739339 |
| Lrrfip1 | 0.5539758 |
| Lrrfip1 | 1.61791009 |
| Lrriq2 | -0.425272 |
| Lrriq2 | 1.95941391 |
| Lrrk1 | 0.82483994 |
| Lrrk2 | -0.3302345 |
| Lrrn1 | 1.41295171 |
| Lrrn2 | -0.4136009 |
| Lrrn3 | 1.03218212 |
| Lrrtm1 | -0.5080347 |
| Lrrtm2 | -0.4484362 |
| Lrrtm3 | 1.24398045 |
| Lrsam1 | -0.3082133 |
| Lrsam1 | -0.5209838 |
| Lrtm1 | 0.51100893 |
| Lrtm1 | 0.35541346 |
| Lsamp | -0.2829979 |
| Lsamp | -0.3033307 |
| Lsg1 | -0.6348601 |
| Lsg1 | -0.0844615 |
| Lsm10 | 0.94549428 |
| Lsm11 | 1.09917802 |
| Lsm12 | 3.61675322 |
| Lsm12 | -0.6156299 |
| Lsm12 | 0.51875238 |
| Lsm14a | 1.23757595 |
| Lsm14b | -0.6103171 |
| Lsm14b | -0.1962279 |
| Lsm14b | 0.37366592 |
| Lsm2 | 1.92230945 |
| Lsm2 | 1.29959494 |
| Lsm3 | -0.4541606 |
| Lsm4 | 2.24904445 |
| Lsm5 | -0.2990124 |
| Lsm6 | 1.31356376 |
| Lsm6 | 1.0494891 |
| Lsm8 | -0.3369456 |
| Lsr | -0.802451 |
| Lst1 | -0.5923325 |
| Ltb4dh | -0.3054975 |
| Ltb4r1 | -0.7489623 |
| Ltb4r2 | 1.35776361 |
| Ltbp1 | 0.26554653 |
| Ltbp1 | 0.86176695 |
| Ltbp2 | -0.3175076 |
| Ltbp3 | 2.50477063 |
| Ltbp3 | -0.5667227 |
| Ltbp4 | -0.5046573 |
| Ltbp4 | -0.7220632 |
| Ltbp4 | 2.11859903 |
| Ltbr | 2.29662107 |
| Ltc4s | -0.563122 |
| Ltf | 0.41258388 |
| Ltf | -0.5209838 |
| Ltk | 0.23811841 |
| Ltk | -0.0355315 |
| Ltv1 | -0.2528094 |
| Luc7l | -0.105139 |
| Luc7l | -0.0084791 |
| Luc7l2 | 0.16231079 |
| Luc7l2 | -0.1033979 |
| Lum | 1.66548952 |
| Luzp1 | 2.12204297 |
| Luzp1 | -0.7272081 |
| Luzp1 | -0.6827319 |
| Luzp2 | 0.32488587 |
| Lxn | -0.3625599 |
| Ly6a | 1.87846726 |
| Ly6c1 | -0.6656904 |
| Ly6d | 1.11458472 |
| Ly6e | -0.5299505 |
| Ly6f | 0.23361649 |
| Ly6g6c | 0.27537225 |
| Ly6g6d | 0.18225752 |
| Ly6g6e | 0.26321918 |
| Ly6k | -0.7821387 |
| Ly86 | 0.38444514 |
| Ly9 | -0.6255792 |
| Ly9 | 2.00632364 |
| Ly96 | 0.69713176 |
| Lyar | -0.6225063 |
| Lyl1 | 2.20627246 |
| Lynx1 | 0.45983572 |
| Lynx1 | 1.41956018 |
| Lypd1 | 0.16782347 |
| Lypd3 | -0.7739574 |
| Lypd4 | 0.15282947 |
| Lypd5 | -0.4396023 |
| Lypd6 | 0.90277992 |
| Lypla1 | -0.7349802 |
| Lypla1 | 0.25913201 |
| Lypla2 | 0.47359447 |
| Lypla3 | -0.7578109 |
| Lyplal1 | -0.4892798 |
| Lyrm1 | -0.5938178 |
| Lyrm2 | -0.409946 |
| Lyrm2 | 1.00656188 |
| Lyrm5 | -0.6042829 |
| Lysmd1 | -0.5746945 |
| Lysmd1 | -0.3718428 |
| Lysmd2 | 0.07429425 |
| Lysmd4 | 0.65635396 |
| Lyst | -0.6442362 |
| Lyve1 | -0.1303163 |
| Lyz | 0.24853755 |
| Lyz1 | 1.17868107 |
| Lyz1 | -0.4093383 |
| Lyz2 | 3.70153123 |
| Lyzl1 | 2.77875304 |
| Lyzl4 | 1.9312925 |
| Lyzl6 | 3.87203856 |
| Lyzs | -0.032307 |
| Lzic | -0.487293 |
| Lztr1 | -0.7516079 |
| Lzts2 | -0.3700951 |
| M6pr | 0.74429766 |
| M6pr | -0.4605633 |
| M6prbp1 | 0.72004673 |
| Mab21l2 | 0.53944586 |
| Macrod1 | -0.5397062 |
| Mad1l1 | -0.6271195 |
| Mad2l1 | 1.12679399 |
| Mad2l1bp | -0.8454587 |
| Mad2l1bp | -0.6186797 |
| Mad2l1bp | 2.96703801 |
| Mad2l2 | -0.7821387 |
| Madcam1 | 0.54738046 |
| Madd | 0.39459432 |
| Maea | 0.9530632 |
| Maf | 0.15872598 |
| Maf1 | -0.4352171 |
| Mafg | 1.58182139 |
| Mag | -0.5285653 |
| Magea2 | 1.9358448 |
| Magea5 | -0.55525 |
| Mageb3 | -0.5938178 |
| Mageb3 | -0.3960693 |
| Maged1 | 0.83829978 |
| Maged2 | 0.20426743 |
| Magee1 | 0.10111275 |
| Mageh1 | -0.4676583 |
| Magi1 | -0.4767688 |
| Magi1 | 1.04205858 |
| Magi1 | -0.6020302 |
| Magi2 | -0.5425128 |
| Magi3 | -0.2340589 |
| Magi3 | -0.766746 |
| Magix | -0.5710624 |
| Magmas | 3.10889521 |
| Magoh | 0.79470784 |
| Magoh | -0.5237335 |
| Magohb | 1.56925877 |
| Mak | -0.3859462 |
| Mak10 | 0.80636534 |
| Mak10 | -0.2330563 |
| Mal | 1.87766941 |
| Mal | 0.6625877 |
| Mall | -0.6827319 |
| Malt1 | 0.45536053 |
| Mamdc2 | 0.5233672 |
| Maml1 | -0.6202084 |
| Maml3 | 1.0012466 |
| Man1a | 0.82041459 |
| Man1a2 | -0.6640838 |
| Man1a2 | 1.68349177 |
| Man1b1 | 2.69226708 |
| Man1c1 | 0.383424 |
| Man1c1 | -0.5481519 |
| Man2a1 | 3.31569096 |
| Man2a2 | 1.82298694 |
| Man2a2 | 1.84408475 |
| Man2a2 | 0.47544423 |
| Man2b1 | 0.03813748 |
| Man2b2 | 1.08777566 |
| Man2c1 | -0.6356378 |
| Manba | -0.6379748 |
| Manba | 2.08039435 |
| Manbal | 0.05365156 |
| Manea | -0.4741565 |
| Mansc1 | 0.86990784 |
| Maob | 1.89780047 |
| Map1lc3a | -0.3257877 |
| Map1lc3a | 0.31805284 |
| Map1lc3b | 1.7740364 |
| Map2k1 | 1.47046552 |
| Map2k2 | 0.27680835 |
| Map2k2 | -0.476115 |
| Map2k3 | 0.35620322 |
| Map2k4 | -0.7332473 |
| Map2k6 | 0.79649849 |
| Map2k6 | 0.0849746 |
| Map2k6 | -0.0319048 |
| Map2k7 | 0.84016225 |
| Map2k7 | 0.05401775 |
| Map2k7 | -0.7152479 |
| Map3k1 | 0.43627375 |
| Map3k11 | 0.37855797 |
| Map3k12 | 3.60773948 |
| Map3k12 | 0.6931348 |
| Map3k12 | 0.88183682 |
| Map3k14 | 2.69969156 |
| Map3k3 | 0.58564353 |
| Map3k6 | 2.71611972 |
| Map3k7 | 0.50989735 |
| Map3k7ip1 | 0.57906712 |
| Map3k7ip1 | -0.5060068 |
| Map3k7ip1 | -0.6810951 |
| Map3k7ip2 | -0.7127053 |
| Map3k7ip3 | -0.3782799 |
| Map3k8 | 1.92425538 |
| Map3k9 | 0.19834547 |
| Map4k2 | 3.06559526 |
| Map4k4 | -0.5923325 |
| Map4k5 | 0.74257429 |
| Map4k5 | 0.8249976 |
| Mapbpip | -0.2729984 |
| Mapk1 | 1.64892133 |
| Mapk1 | -0.3877245 |
| Mapk1 | -0.632531 |
| Mapk10 | 0.90740157 |
| Mapk10 | 0.06312533 |
| Mapk11 | -0.0956038 |
| Mapk12 | 0.01512604 |
| Mapk12 | -0.584942 |
| Mapk12 | 0.30228001 |
| Mapk13 | -0.7237749 |
| Mapk13 | -0.7186492 |
| Mapk14 | 0.02349213 |
| Mapk15 | 0.30701271 |
| Mapk15 | -0.1781191 |
| Mapk15 | -0.659281 |
| Mapk1ip1 | 1.74951878 |
| Mapk1ip1 | -0.583471 |
| Mapk1ip1 | 2.19488048 |
| Mapk3 | -0.672955 |
| Mapk3 | 1.23082285 |
| Mapk4 | 1.04056521 |
| Mapk6 | 1.14730491 |
| Mapk6 | 0.60248401 |
| Mapk6 | -0.5856783 |
| Mapk8 | -0.6302079 |
| Mapk8ip1 | -0.4433778 |
| Mapk8ip1 | 2.91747287 |
| Mapk8ip2 | -0.1701324 |
| Mapk8ip3 | 2.37676494 |
| Mapk8ip3 | 0.1813023 |
| Mapk9 | 0.82908707 |
| Mapk9 | -0.2340589 |
| Mapk9 | 0.90118583 |
| Mapkap1 | 0.78421944 |
| Mapkap1 | -0.5516941 |
| Mapkapk2 | 0.08568221 |
| Mapkapk3 | 0.20736892 |
| Mapkbp1 | 0.2509025 |
| Mapre1 | -0.6513318 |
| Mapre2 | -0.7323821 |
| Mapre2 | -0.709326 |
| Mapre3 | -0.7472028 |
| Mapt | -0.4926009 |
| Mapt | 0.34721242 |
| Mapt | -0.709326 |
| Mapt | -0.5746945 |
| March2 | -0.3960693 |
| March3 | -0.7739574 |
| March3 | 0.76489722 |
| March5 | -0.2166689 |
| March5 | -0.1909489 |
| March7 | -0.2793011 |
| March8 | -0.6513318 |
| March9 | 3.05568217 |
| March11 | -0.3871314 |
| Marcks | 0.66728212 |
| Marcksl1 | -0.0956038 |
| Marcksl1 | 0.01703415 |
| Marcksl1 | -0.1280799 |
| Mare | 0.1544712 |
| Mark1 | 2.24944329 |
| Mark2 | -0.0266914 |
| Mark2 | 0.86070633 |
| Mark3 | 0.0047529 |
| Mark3 | -0.461206 |
| Mark4 | 0.40811954 |
| Mars | 0.50185387 |
| Mars2 | -0.3408833 |
| Marveld3 | -0.6209738 |
| Marveld3 | 2.46232374 |
| Mas1 | -0.6576856 |
| Masp1 | -0.6876597 |
| Masp2 | -0.6434512 |
| Mast1 | -0.2600056 |
| Mast1 | 0.01474394 |
| Mast2 | 0.1359133 |
| Mast2 | 0.56073439 |
| Mat1a | -0.197191 |
| Mat2a | 0.26989442 |
| Mat2a | 1.5496928 |
| Mat2a | 0.92240443 |
| Mat2a | -0.487293 |
| Mat2b | -0.5717877 |
| Mat2b | -0.8108923 |
| Matk | 0.40413287 |
| Matk | 0.60248401 |
| Matn4 | -0.3972676 |
| Matn4 | -0.6418832 |
| Matn4 | 1.90977584 |
| Matr3 | 0.55693883 |
| Max | -0.4327206 |
| Maz | -0.62481 |
| Mb | 0.63370959 |
| Mbc2 | -0.5425128 |
| Mbd1 | -0.6648867 |
| Mbd1 | -0.4979397 |
| Mbd3 | 1.2926243 |
| Mbd3l2 | -0.2147023 |
| Mbd4 | 0.55270299 |
| Mbd5 | -0.6186797 |
| Mbd6 | 2.38574149 |
| Mbip | -0.210293 |
| Mbl2 | 0.17105077 |
| Mbnl1 | -0.8118351 |
| Mbnl2 | 0.386484 |
| Mbnl2 | -0.631756 |
| Mboat1 | -0.1033979 |
| Mboat2 | -0.3180573 |
| Mboat2 | 1.41120904 |
| Mboat2 | 2.05077997 |
| Mboat2 | -0.7059591 |
| Mboat5 | -0.7730528 |
| Mboat5 | 1.34114647 |
| Mbp | -0.6497503 |
| Mbp | -0.6186797 |
| Mbp | -0.1895145 |
| Mbtd1 | 0.81229872 |
| Mbtps1 | -0.6513318 |
| Mbtps1 | -0.5573902 |
| Mbtps1 | 1.59672657 |
| Mbtps2 | 0.67956087 |
| Mc3r | -0.0359353 |
| Mc4r | 0.37572892 |
| Mc5r | 0.21076847 |
| Mcam | -0.713552 |
| Mcam | 1.94028139 |
| Mcam | 0.33355835 |
| Mcat | 0.49577452 |
| Mcc | -0.5776107 |
| Mccc1 | -0.2005698 |
| Mcee | -0.476115 |
| Mcf2l | -0.0870208 |
| Mcf2l | 3.64822877 |
| Mcf2l | -0.1433968 |
| Mcfd2 | -0.7084831 |
| Mchr1 | -0.4396023 |
| Mcl1 | -0.6713357 |
| Mcm10 | -0.1552852 |
| Mcm10 | -0.7160971 |
| Mcm2 | 2.5807832 |
| Mcm3 | 3.06790744 |
| Mcm4 | 1.46296084 |
| Mcm5 | -0.7118593 |
| Mcm6 | -0.8572772 |
| Mcm6 | -0.034321 |
| Mcm6 | 0.8082939 |
| Mcm7 | 0.39206756 |
| Mcoln1 | 0.46077508 |
| Mcoln2 | 1.81099263 |
| Mcoln3 | 0.26525594 |
| Mcph1 | -0.3865386 |
| Mcph1 | 0.95470631 |
| Mcph1 | 1.61485675 |
| Mcph1 | 0.69112975 |
| Mcpt4 | 0.88479492 |
| Mcrs1 | -0.6505407 |
| Mcrs1 | -0.5645606 |
| Mcrs1 | 3.20787611 |
| Mcts1 | -0.6364161 |
| Mdfic | -0.5141457 |
| Mdga1 | 1.47721386 |
| Mdga2 | -0.4999498 |
| Mdga2 | 1.17589228 |
| Mdh1 | -0.3591029 |
| Mdh1 | 0.45606859 |
| Mdh1b | -0.5871528 |
| Mdh2 | -0.7712464 |
| Mdk | 0.99759882 |
| Mdk | 3.59352644 |
| Mdm1 | 0.42413909 |
| Mdm1 | -0.7631615 |
| Mdm1 | 2.40844755 |
| Mdm1 | -0.6371951 |
| Mdm1 | -0.6450219 |
| Mdm1 | -0.5100672 |
| Mdm1 | -0.7685435 |
| Mdm1 | 1.38909844 |
| Mdm1 | -0.3930803 |
| Mdm2 | -0.0179359 |
| Mdm2 | -0.4892798 |
| Mdm4 | 0.05621185 |
| Mdn1 | 0.52730431 |
| Me2 | -0.2625894 |
| Me3 | 2.31266948 |
| Mea1 | -0.6465953 |
| Mecp2 | -0.1236232 |
| Mecr | 0.42949818 |
| Med1 | -0.0073039 |
| Med1 | -0.6624801 |
| Med10 | 1.35281893 |
| Med11 | 0.91899683 |
| Med11 | 0.2482415 |
| Med12 | 1.06283539 |
| Med15 | 1.04056521 |
| Med15 | 1.65326693 |
| Med15 | -0.0026179 |
| Med16 | 1.18231518 |
| Med18 | -0.3510871 |
| Med18 | 1.52156313 |
| Med19 | -0.545328 |
| Med21 | -0.6745773 |
| Med22 | -0.285117 |
| Med22 | 0.22211147 |
| Med23 | -0.4063056 |
| Med23 | 2.27796999 |
| Med24 | 1.79119443 |
| Med25 | -0.4522485 |
| Med25 | -0.3296775 |
| Med26 | -0.0497737 |
| Med27 | -0.0464987 |
| Med28 | -0.5251114 |
| Med30 | 1.25409586 |
| Med31 | 2.15645001 |
| Med4 | -0.2054185 |
| Med6 | 0.21076847 |
| Med7 | 1.46146822 |
| Med8 | -0.6379748 |
| Med8 | 2.17976599 |
| Med9 | -0.8127788 |
| Mef2b | 0.90797764 |
| Mef2b | -0.6294348 |
| Mef2c | -0.5502755 |
| Mef2c | 1.45886996 |
| Mef2c | -0.4637815 |
| Megf10 | -0.6884836 |
| Megf11 | -0.6917867 |
| Megf11 | -0.4026797 |
| Megf11 | -0.7367163 |
| Megf11 | 2.76309886 |
| Megf6 | -0.4972706 |
| Megf9 | -0.5856783 |
| Mei1 | 2.6177793 |
| Meis1 | 1.08813045 |
| Meis2 | -0.8090097 |
| Meis3 | 1.44568686 |
| Melk | 1.95291229 |
| Memo1 | 0.09062021 |
| Men1 | 2.3140639 |
| Men1 | -0.6529161 |
| Meox1 | -0.6762024 |
| Meox2 | 1.38628599 |
| Mep1b | -0.4020768 |
| Mepce | -0.2049324 |
| Mertk | -0.6697192 |
| Mesdc1 | -0.2548597 |
| Mesp1 | -0.7922385 |
| Mest | -0.3038719 |
| Mest | -0.8543082 |
| Mest | -0.38299 |
| Metap1 | 2.11614209 |
| Metap2 | -0.7017679 |
| Metap2 | 0.74790622 |
| Metapl1 | -0.6537093 |
| Metrn | -0.6294348 |
| Metrnl | -0.0387673 |
| Metrnl | -0.3403197 |
| Mett11d1 | -0.21225 |
| Mettl1 | -0.7766763 |
| Mettl1 | 0.6738141 |
| Mettl2 | -0.7229187 |
| Mettl3 | -0.563122 |
| Mettl4 | -0.6545032 |
| Mettl6 | -0.7367163 |
| Mettl8 | 0.81373617 |
| Mettl9 | -0.3408833 |
| Mettl9 | -0.0904443 |
| Mex3a | 3.14775187 |
| Mex3a | 0.07715456 |
| Mex3c | 0.19959539 |
| Mfap1b | -0.5334228 |
| Mfap2 | 1.88843148 |
| Mfap2 | 0.77077031 |
| Mfap2 | -0.2533215 |
| Mfap3 | 3.25103341 |
| Mfap3 | 2.27027186 |
| Mfap3l | -0.4377203 |
| Mfap3l | 0.01244798 |
| Mfap4 | 0.94852937 |
| Mfap5 | 2.97334093 |
| Mfge8 | 1.723913 |
| Mfge8 | 0.12004202 |
| Mfi2 | -0.0139837 |
| Mfn1 | -0.6095606 |
| Mfn1 | 0.86690114 |
| Mfn2 | -0.568167 |
| Mfng | -0.6951017 |
| Mfrp | -0.5168748 |
| Mfsd1 | -0.631756 |
| Mfsd10 | -0.5930748 |
| Mfsd10 | 2.52589206 |
| Mfsd11 | 1.48682345 |
| Mfsd2 | 4.59439488 |
| Mfsd3 | -0.5856783 |
| Mfsd4 | 0.82751636 |
| Mfsd7 | -0.677016 |
| Mfsd8 | 1.03130182 |
| Mfsd9 | 0.76203092 |
| Mga | 0.10354379 |
| Mgat1 | 0.34986596 |
| Mgat3 | 1.27976914 |
| Mgat4b | 0.60829571 |
| Mgat4b | -0.4605633 |
| Mgat5 | -0.6302079 |
| MGC107098 | 1.01914298 |
| MGC117608 | -0.607295 |
| MGC118210 | 0.96002614 |
| MGC18837 | 0.55778365 |
| MGC41689 | -0.6810951 |
| MGC67181 | -0.6737658 |
| Mgea6 | 0.35409568 |
| Mgl1 | -0.7569222 |
| Mgl2 | 0.29527539 |
| Mgll | 1.93317248 |
| Mgll | -0.5425128 |
| Mgll | -0.5093892 |
| Mgmt | 0.46918623 |
| Mgp | 0.71756817 |
| Mgrn1 | 3.13180388 |
| Mgst1 | -0.6186797 |
| Mgst1 | -0.1809547 |
| Mgst2 | 1.04627662 |
| Mgst3 | 1.2304181 |
| Mgst3 | -0.7931622 |
| Mia1 | -0.2719522 |
| Mia3 | -0.1108205 |
| Mib1 | 2.28128155 |
| Mib2 | 0.75115888 |
| Mic2l1 | 0.47913258 |
| Mical1 | 1.10824496 |
| Mical1 | -0.0464987 |
| Mical1 | -0.5990353 |
| Mical2 | 0.60869516 |
| Mical3 | 1.53457543 |
| Micall2 | -0.6745773 |
| Micall2 | -0.111259 |
| Mid1 | 1.69519796 |
| Mid1 | 0.16976121 |
| Mid1ip1 | -0.1307643 |
| Midn | -0.4154338 |
| Midn | 1.23767636 |
| Mier1 | 0.74378099 |
| Mier1 | -0.38299 |
| Mif | -0.0100485 |
| Mif4gd | -0.4057003 |
| Mif4gd | -0.4741565 |
| Mill2 | 1.84862771 |
| Mill2 | -0.3608298 |
| Mina | 0.30311697 |
| Mink1 | -0.3082133 |
| Mink1 | -0.6340831 |
| Minpp1 | 1.12441059 |
| Minpp1 | -0.7375856 |
| Miox | -0.4105541 |
| Mipep | 3.50669424 |
| Mir16 | -0.7959389 |
| Mist1 | -0.5341188 |
| Mitf | -0.4579968 |
| Mizf | -0.3730098 |
| Mki67ip | -0.4452714 |
| Mkks | 0.21691728 |
| Mkks | 1.97394317 |
| Mkl1 | 1.845791 |
| Mkl2 | 0.87095786 |
| Mkl2 | -0.2688206 |
| Mkl2 | -0.446536 |
| Mkln1 | 1.05869318 |
| Mknk1 | -0.7848821 |
| Mknk2 | 0.82783072 |
| Mkrn1 | 1.20715003 |
| Mkrn2 | -0.451612 |
| Mkrn3 | 0.51698822 |
| Mkx | 0.85294159 |
| Mlana | 0.3155804 |
| Mlf1 | -0.3437063 |
| Mlf1 | -0.3488098 |
| Mlf2 | 2.2261887 |
| Mlf2 | -0.5390059 |
| Mlh1 | -0.7315178 |
| Mlh3 | 1.3789157 |
| Mlkl | -0.4142114 |
| Mllt1 | -0.3313496 |
| Mllt1 | 0.3910549 |
| Mllt1 | -0.5652807 |
| Mllt10 | -0.1452157 |
| Mllt11 | -0.0793643 |
| Mllt3 | -0.2206147 |
| Mllt3 | -0.7051194 |
| Mllt3 | -0.5107457 |
| Mlp | -0.6156299 |
| Mlstd2 | 1.14863396 |
| Mlx | 0.40188271 |
| Mlx | -0.7830523 |
| Mlxipl | -0.6753895 |
| Mlycd | -0.3109371 |
| Mmab | -0.3516574 |
| Mmab | 0.35699231 |
| Mmd | 0.35066052 |
| Mmd2 | 1.36748638 |
| Mme | -0.2446539 |
| Mme | 2.04406677 |
| Mmel1 | 0.79828564 |
| Mmp10 | -0.2451615 |
| Mmp11 | -0.1262946 |
| Mmp12 | 3.03630153 |
| Mmp13 | 0.97953805 |
| Mmp13 | 1.20267497 |
| Mmp14 | 1.94460383 |
| Mmp15 | -0.6171536 |
| Mmp16 | -0.8061931 |
| Mmp17 | -0.4327206 |
| Mmp1a | 0.14490786 |
| Mmp1a | 0.15151395 |
| Mmp2 | 0.31805284 |
| Mmp23 | -0.659281 |
| Mmp24 | 1.66574112 |
| Mmp3 | 2.11205822 |
| Mmp3 | -0.4497052 |
| Mmp3 | 0.97647069 |
| Mmp9 | -0.6333067 |
| Mmrn2 | 0.04777241 |
| Mnat1 | 0.78190898 |
| Mnat1 | -0.7472028 |
| Mnd1 | 0.49577452 |
| Mns1 | -0.4767688 |
| Mnt | 0.11423286 |
| Mobk1b | 1.30446165 |
| Mobkl2a | -0.4735046 |
| Mobkl2b | -0.848399 |
| Mobkl2c | -0.5990353 |
| Mobkl3 | -0.6521236 |
| Mobp | 0.26030164 |
| Mobp | -0.5915907 |
| Mobp | 2.72836825 |
| Mocos | -0.4820158 |
| Mocs1 | 0.76674712 |
| Mocs2 | -0.1312124 |
| Mod1 | -0.7480822 |
| Mog | 1.62148149 |
| Mog | -0.250253 |
| Mon1a | 0.78421944 |
| Mon1a | 1.6513523 |
| Mon1b | 0.30060379 |
| Mon1b | -0.6202084 |
| Mon1b | 0.24110743 |
| Morc2a | 0.07858136 |
| Morc2b | 0.61724141 |
| Morc3 | -0.8742859 |
| Morf4l1 | -0.7551474 |
| Morf4l1 | 0.49396544 |
| Morf4l2 | -0.2512747 |
| Morn2 | 1.87971252 |
| Morn3 | 1.10581269 |
| Morn3 | 0.06493601 |
| Mos | -0.3812209 |
| Mosc2 | -0.6005316 |
| Mospd1 | -0.3841713 |
| Mospd1 | 1.53522847 |
| Mospd1 | -0.518927 |
| Mospd3 | -0.5432158 |
| Mospd3 | -0.3562321 |
| Mov10 | 1.38799153 |
| Mov10l1 | 1.69489342 |
| Moxd1 | 0.76590672 |
| Moxd1 | -0.6992624 |
| Mpdu1 | 0.34614884 |
| Mpdz | -0.55525 |
| Mpdz | -0.7034421 |
| Mpg | -0.3906961 |
| Mpg | 2.13674354 |
| Mphosph6 | -0.4228025 |
| Mpnd | 0.07501016 |
| Mpo | 0.63700667 |
| Mpo | 0.93479149 |
| Mpp1 | -0.659281 |
| Mpp2 | 0.51300637 |
| Mpp3 | 1.06526326 |
| Mpp3 | 2.24086321 |
| Mpp5 | 2.15217303 |
| Mpp6 | 2.2960844 |
| Mppe1 | 0.18954791 |
| Mpped1 | -0.1325583 |
| Mpst | 1.05747131 |
| Mpv17 | 0.19301419 |
| Mpv17 | -0.7384557 |
| Mpv17 | 0.92678721 |
| Mpv17 | -0.6967637 |
| Mpv17l | 0.09413111 |
| Mpv17l | 0.43747841 |
| Mpv17l | 3.16987732 |
| Mpz | 1.10011211 |
| Mr1 | 0.7927504 |
| Mrap | 2.23460294 |
| Mras | -0.301169 |
| Mrc1 | -0.6951017 |
| Mrc2 | -0.0416081 |
| Mrc2 | 1.02028976 |
| Mre11a | 0.0261381 |
| Mre11a | -0.553826 |
| Mre11a | -0.4820158 |
| Mreg | 0.63758728 |
| Mrgpra2 | -0.5223576 |
| Mrgpra2 | 2.27179722 |
| Mrgprb2 | -0.2265655 |
| Mrgpre | -0.6035314 |
| Mrgprf | -0.7272081 |
| Mrgprg | 0.2544385 |
| Mrm1 | -0.6917867 |
| Mro | -0.7177977 |
| Mrp63 | -0.0030074 |
| Mrpl1 | 0.85401125 |
| Mrpl1 | -0.8875092 |
| Mrpl10 | 0.11491821 |
| Mrpl10 | 2.58535225 |
| Mrpl11 | -0.4683061 |
| Mrpl12 | -0.672955 |
| Mrpl13 | 0.21814209 |
| Mrpl13 | 0.92481049 |
| Mrpl14 | -0.6118319 |
| Mrpl15 | 0.89813481 |
| Mrpl16 | -0.6802779 |
| Mrpl17 | 1.32650279 |
| Mrpl18 | 0.37418211 |
| Mrpl2 | -0.2861784 |
| Mrpl20 | -0.6794613 |
| Mrpl22 | -0.4050954 |
| Mrpl23 | 0.43046919 |
| Mrpl24 | -0.3966683 |
| Mrpl27 | -0.7237749 |
| Mrpl28 | -0.8299344 |
| Mrpl3 | 0.46732376 |
| Mrpl3 | -0.6713357 |
| Mrpl30 | 0.47544423 |
| Mrpl33 | -0.3527989 |
| Mrpl33 | -0.5362101 |
| Mrpl34 | 1.61119259 |
| Mrpl36 | -0.8175121 |
| Mrpl37 | 0.5728585 |
| Mrpl37 | -0.0505942 |
| Mrpl38 | 0.8564515 |
| Mrpl39 | -0.0600841 |
| Mrpl40 | -0.3017089 |
| Mrpl43 | 1.73213828 |
| Mrpl43 | 0.93730468 |
| Mrpl45 | -0.4820158 |
| Mrpl46 | 0.12615296 |
| Mrpl47 | -0.7367163 |
| Mrpl47 | 0.60909443 |
| Mrpl48 | -0.1222905 |
| Mrpl48 | 0.58482406 |
| Mrpl48 | -0.1160965 |
| Mrpl49 | 1.87040695 |
| Mrpl49 | -0.5923325 |
| Mrpl49 | 2.30030609 |
| Mrpl49 | 1.14819116 |
| Mrpl50 | -0.5376069 |
| Mrpl51 | 0.03888224 |
| Mrpl51 | -0.4014743 |
| Mrpl51 | -0.5446234 |
| Mrpl52 | -0.5121042 |
| Mrpl53 | 0.28281548 |
| Mrpl54 | 0.11731289 |
| Mrpl55 | -0.3448379 |
| Mrpl9 | -0.4892798 |
| Mrps10 | 0.7695987 |
| Mrps10 | 2.46807224 |
| Mrps11 | -0.3741783 |
| Mrps11 | 0.54738046 |
| Mrps12 | -0.5053318 |
| Mrps14 | 0.5329615 |
| Mrps15 | -0.759591 |
| Mrps16 | -0.3505173 |
| Mrps17 | -0.5121042 |
| Mrps18a | -0.4308528 |
| Mrps18b | 0.40338343 |
| Mrps18b | 1.12031027 |
| Mrps18c | -0.5886297 |
| Mrps2 | -0.62481 |
| Mrps21 | -0.1612879 |
| Mrps22 | 1.13345716 |
| Mrps23 | 2.32825834 |
| Mrps24 | 1.23596779 |
| Mrps25 | -0.5148272 |
| Mrps25 | 0.43892191 |
| Mrps25 | 1.28397884 |
| Mrps26 | 0.29808368 |
| Mrps26 | -0.3437063 |
| Mrps27 | 2.51407442 |
| Mrps28 | -0.6584829 |
| Mrps30 | 0.68472067 |
| Mrps31 | 1.88571572 |
| Mrps31 | -0.5313378 |
| Mrps31 | -0.6278906 |
| Mrps33 | 0.39635891 |
| Mrps33 | -0.7560344 |
| Mrps34 | 0.53685763 |
| Mrps35 | 1.57836826 |
| Mrps5 | 3.10356733 |
| Mrps6 | -0.7110141 |
| Mrps7 | -0.6302079 |
| Mrps7 | 1.10812928 |
| Mrps9 | -0.1388651 |
| Mrrf | 0.36952592 |
| Mrrf | -0.2436396 |
| Mrto4 | -0.5710624 |
| Mrvi1 | -0.5182424 |
| Ms4a1 | 3.71033052 |
| Ms4a10 | -0.5460332 |
| Ms4a13 | 1.5740682 |
| Ms4a5 | 0.99942453 |
| Ms4a6d | -0.5768808 |
| Ms4a6d | 0.47498214 |
| Ms4a7 | -0.6110742 |
| Ms4a8a | 1.84651391 |
| Msc | -0.4644265 |
| Msh2 | 0.2782422 |
| Msh3 | 1.390119 |
| Msh3 | -0.4735046 |
| Msh4 | 1.04689528 |
| Msh5 | 0.78570165 |
| Msh5 | -0.5856783 |
| Msh6 | 0.41480791 |
| Msh6 | 0.79372965 |
| Msi2 | -0.1617512 |
| Msi2h | 0.41382012 |
| Msln | -0.2441466 |
| Msn | -0.4459035 |
| Msr2 | -0.659281 |
| Msrb2 | -0.5595354 |
| Msrb2 | 3.75623001 |
| Msrb3 | 1.52904224 |
| Mst1r | -0.7367163 |
| Msto1 | -0.6576856 |
| Msx1 | 2.76506798 |
| Mt1 | -0.2522975 |
| Mt3 | 0.89126927 |
| Mta1 | 0.47959258 |
| Mta2 | 0.87454879 |
| Mta3 | 1.19523767 |
| Mta3 | 0.68269707 |
| Mtag2 | 2.04402518 |
| Mtap | -0.6984287 |
| Mtap | -0.2380807 |
| Mtap1b | -0.0080872 |
| Mtap1b | -0.4478024 |
| Mtap1b | 1.55425077 |
| Mtap1b | 1.27842561 |
| Mtap1s | -0.62481 |
| Mtap2 | 1.21890928 |
| Mtap2 | 0.20984257 |
| Mtap2 | -0.2904364 |
| Mtap4 | 1.0714253 |
| Mtap6 | 1.55047781 |
| Mtap6 | -0.7507252 |
| Mtap7 | 3.00477343 |
| Mtap7d1 | -0.6992624 |
| Mtap7d1 | -0.6576856 |
| Mtap7d2 | -0.7560344 |
| Mtap7d3 | 1.9813672 |
| Mtbp | 0.44538963 |
| Mtbp | 1.88358686 |
| Mtbp | 0.02387059 |
| Mtch1 | 2.70390722 |
| Mtch1 | 0.23662023 |
| Mtch2 | -0.8464377 |
| Mtch2 | -0.1465823 |
| Mtch2 | -0.3341433 |
| Mtcp1 | 0.45252277 |
| Mtdh | 1.11607682 |
| Mterf | 3.86748091 |
| Mterf | -0.6309816 |
| Mterfd1 | 0.03850993 |
| Mterfd2 | -0.3972676 |
| Mterfd2 | 2.32134225 |
| Mterfd2 | 3.43110316 |
| Mterfd3 | -0.3324661 |
| Mterfd3 | -0.1379615 |
| Mtf1 | 0.11697118 |
| Mtf1 | 1.907316 |
| Mtf2 | -0.6356378 |
| Mtfmt | 0.63312652 |
| Mtfmt | -0.3142165 |
| Mtg1 | 0.15282947 |
| Mthfd1 | 0.36952592 |
| Mthfd2 | -0.1636071 |
| Mthfs | -0.4377203 |
| Mthfsd | -0.5039833 |
| Mthfsd | -0.1986375 |
| Mtif3 | -0.5278735 |
| Mtl5 | 0.45015123 |
| Mtl5 | 1.75226927 |
| Mtmr1 | -0.2034759 |
| Mtmr11 | -0.6434512 |
| Mtmr11 | -0.6992624 |
| Mtmr12 | 0.04148414 |
| Mtmr14 | 0.48942704 |
| Mtmr14 | 1.78569466 |
| Mtmr14 | -0.4452714 |
| Mtmr14 | -0.7613745 |
| Mtmr2 | -0.6110742 |
| Mtmr2 | -0.7160971 |
| Mtmr2 | 0.04666585 |
| Mtmr3 | -0.0857403 |
| Mtmr4 | 0.24051041 |
| Mtmr9 | 0.79291368 |
| Mto1 | 0.34614884 |
| Mtpn | -0.5134647 |
| Mtrf1 | -0.6778304 |
| Mtrf1 | 0.27652131 |
| Mtrf1l | -0.6705271 |
| Mtrr | -0.5481519 |
| Mtss1 | -0.7272081 |
| Mttp | 2.27639037 |
| Mtus1 | -0.2714295 |
| Mtus1 | -0.5182424 |
| Mtvr2 | 0.64106321 |
| Mtvr2 | -0.5087117 |
| Mtx1 | 0.37056268 |
| Mtx1 | -0.3153122 |
| Mtx1 | 3.46605567 |
| Mtx2 | -0.0571854 |
| Muc1 | 3.26973359 |
| Muc1 | -0.1800085 |
| Muc1 | -0.3677699 |
| Muc1 | 0.07214314 |
| Muc13 | 0.20457805 |
| Muc5ac | 3.49658141 |
| Mug2 | 0.31089192 |
| Mug2 | 0.73024408 |
| Mug4 | -0.7306542 |
| Mum1 | -0.3352632 |
| Mup1 | 2.4258481 |
| Mup2 | -0.4129907 |
| Mup2 | 1.80642021 |
| Mup3 | 0.44586691 |
| Mupcdh | 0.34694664 |
| Mus81 | -0.5411084 |
| Mus81 | 1.99018842 |
| Mustn1 | -0.6240415 |
| Mustn1 | 3.35951903 |
| Muted | 2.09722704 |
| Muted | 0.62296042 |
| Mutyh | 0.58379869 |
| Mvd | -0.7195015 |
| Mvp | -0.1923856 |
| Mx2 | -0.6171536 |
| Mxd1 | 2.70060525 |
| Mxd4 | -0.6975958 |
| Mxi1 | -0.2824688 |
| Mxra7 | 1.67972137 |
| Mxra8 | 1.03707117 |
| Mxra8 | -0.3847626 |
| Myadm | 1.59862352 |
| Mybbp1a | -0.2662191 |
| Mybbp1a | 0.48051189 |
| Mybbp1a | 1.83832207 |
| Mybl2 | 0.05218531 |
| Mybpc2 | -0.3022492 |
| Mybpc3 | -0.6426668 |
| Myc | -0.6762024 |
| Mycbp | 0.56890448 |
| Mycbp2 | -0.766746 |
| Mycbp2 | -0.2166689 |
| Mycbpap | -0.6202084 |
| Mycl1 | -0.4111627 |
| Myd116 | 0.72971941 |
| Myd88 | -0.6753895 |
| Myef2 | -0.2181466 |
| Myg1 | 2.35390087 |
| Myg1 | -0.0278919 |
| Myg1 | 2.29640013 |
| Myh10 | -0.412381 |
| Myh11 | 1.8758217 |
| Myh8 | 2.02124158 |
| Myh9 | 1.66347424 |
| Myl1 | 0.85568962 |
| Myl2 | -0.5761514 |
| Myl4 | -0.1943047 |
| Myl6 | -0.3208107 |
| Myl6b | -0.5390059 |
| Mylc2b | -0.4560767 |
| Mylc2b | -0.6348601 |
| Mylc2b | -0.6608791 |
| Mylc2pl | -0.2522975 |
| Mylk | 1.49398394 |
| Mylk | 1.0662326 |
| Mynn | -0.4541606 |
| Myo10 | -0.716947 |
| Myo10 | 1.16974846 |
| Myo15 | 0.19740691 |
| Myo18a | 1.92501364 |
| Myo1b | -0.7658485 |
| Myo1c | -0.4906068 |
| Myo1c | 0.84155662 |
| Myo1f | 0.61367362 |
| Myo1g | -0.4296096 |
| Myo3b | -0.4389745 |
| Myo5a | -0.0006724 |
| Myo5b | -0.802451 |
| Myo6 | -0.563122 |
| Myo7a | 1.73953636 |
| Myo9a | -0.0076954 |
| Myo9b | -0.6705271 |
| Myo9b | 0.47243646 |
| Myoc | -0.568167 |
| Myocd | -0.3213623 |
| Myocd | 0.98986449 |
| Myom1 | -0.4383472 |
| Myom1 | 4.60644505 |
| Myrip | -0.2931078 |
| Mysm1 | 1.80247499 |
| Myst1 | 2.1957266 |
| Myst2 | -0.4277481 |
| Myst4 | -0.5997832 |
| Myst4 | -0.6450219 |
| Myst4 | 0.15019655 |
| Myt1 | 0.80813334 |
| Myt1l | 0.0370192 |
| Myt1l | -0.0776716 |
| Mzf1 | -0.1366077 |
| Mzf1 | -0.6403178 |
| N28178 | -0.4414881 |
| N4bp2 | 0.69695046 |
| N6amt1 | -0.6552978 |
| N6amt1 | -0.3591029 |
| N6amt2 | -0.2952505 |
| Naalad2 | 0.41727272 |
| Nab1 | -0.3291208 |
| Nab1 | -0.5967956 |
| Nab1 | -0.568167 |
| Nab1 | -0.5341188 |
| Nab2 | 2.25866764 |
| Naca | -0.0262916 |
| Naca | -0.584942 |
| Naca | -0.4105541 |
| Nadk | 0.74893462 |
| Nadsyn1 | 0.15872598 |
| Nadsyn1 | -0.1307643 |
| Naga | -0.6545032 |
| Nagk | 1.70837844 |
| Nagk | 0.374698 |
| Nagk | -0.446536 |
| Nagk | -0.7569222 |
| Naglu | -0.4008721 |
| Nagpa | 3.05907621 |
| Nags | -0.6125903 |
| Nags | 0.70453437 |
| Naif1 | 1.98709517 |
| Naif1 | -0.7794034 |
| Naip5 | -0.5313378 |
| Nalp10 | -0.6778304 |
| Nampt | -0.012014 |
| Nanos1 | -0.632531 |
| Nanos2 | -0.6810951 |
| Nans | 2.12391257 |
| Nap1l1 | -0.6020302 |
| Nap1l1 | -0.4478024 |
| Nap1l3 | -0.0290939 |
| Nap1l4 | -0.0124076 |
| Nap1l5 | -0.3912915 |
| Napa | 3.31736793 |
| Napa | -0.1470383 |
| Napa | 1.81356465 |
| Napb | -0.2078525 |
| Napepld | -0.0298961 |
| Napg | 0.92070221 |
| Napg | 0.07536791 |
| Naprt1 | 0.13992181 |
| Naprt1 | -0.5383062 |
| Napsa | -0.0481347 |
| Napsa | -0.3087574 |
| Napsa | 0.8566038 |
| Narf | -0.4129907 |
| Narfl | 2.30225284 |
| Narg1 | -0.2605218 |
| Narg1 | -0.6103171 |
| Narg1l | 1.44352454 |
| Narg1l | -0.4478024 |
| Narg1l | 2.00732 |
| Narg2 | -0.4352171 |
| Nars | 0.3624969 |
| Nars2 | -0.1209597 |
| Nasp | -0.5100672 |
| Nasp | -0.2579438 |
| Nasp | -0.6118319 |
| Nasp | -0.5474451 |
| Nat1 | -0.7757691 |
| Nat10 | -0.2176537 |
| Nat14 | 1.34275917 |
| Nat2 | -0.6271195 |
| Nat5 | -0.6364161 |
| Nat5 | -0.6513318 |
| Nat6 | 1.75518264 |
| Nat6 | 3.10521199 |
| Nav1 | 1.10441991 |
| Nav1 | -0.8773205 |
| Nbl1 | 0.35039574 |
| Nbr1 | 1.10197743 |
| Nbr1 | 0.59845432 |
| Ncald | -0.2829979 |
| Ncam1 | -0.0367436 |
| Ncam1 | 0.29499408 |
| Ncam2 | 0.19269962 |
| Ncan | 1.20069062 |
| Ncapd2 | 1.96091826 |
| Ncapd2 | 0.09413111 |
| Ncapd2 | 0.40985826 |
| Ncapd3 | 1.60965594 |
| Ncapg2 | -0.5467389 |
| Ncapg2 | 2.78488126 |
| Ncaph | 0.77527535 |
| Ncaph2 | 1.21366723 |
| Ncaph2 | -0.5053318 |
| Ncaph2 | -0.6786455 |
| Ncbp2 | 0.63468056 |
| Ncbp2 | 0.12716749 |
| Ncdn | 1.08576259 |
| Ncdn | -0.6080496 |
| Ncdn | -0.7186492 |
| Ncdn | -0.7472028 |
| Ncf4 | -0.3812209 |
| Nck1 | -0.1130151 |
| Nckap1 | 0.36666876 |
| Nckap1l | 0.68122255 |
| Nckipsd | 0.96219972 |
| Ncl | -0.5595354 |
| Ncl | -0.4657179 |
| Ncoa2 | 1.20922402 |
| Ncoa2 | 3.12007487 |
| Ncoa4 | 0.20922479 |
| Ncoa5 | 0.77093756 |
| Ncoa6 | -0.5369083 |
| Ncoa6 | -0.6042829 |
| Ncoa6 | -0.6194438 |
| Ncor1 | -0.6458083 |
| Ncstn | 2.49884411 |
| Ncstn | 0.92861892 |
| Ncstn | 2.08744234 |
| Ndc80 | -0.6608791 |
| Nde1 | 1.68534039 |
| Nde1 | 0.87080793 |
| Ndel1 | 3.32377824 |
| Ndfip1 | 1.62451284 |
| Ndfip2 | -0.6148691 |
| Ndg2 | -0.5967956 |
| Ndn | -0.261555 |
| Ndor1 | -0.7803143 |
| Ndor1 | 2.20257249 |
| Ndor1 | -0.4142114 |
| Ndrg1 | -0.5732399 |
| Ndrg2 | -0.1562061 |
| Ndrg3 | -0.059255 |
| Ndrg4 | -0.5696135 |
| Ndrl | 2.02740396 |
| Ndst1 | -0.166398 |
| Ndst1 | -0.5467389 |
| Ndst4 | -0.3972676 |
| Ndufa1 | -0.5121042 |
| Ndufa10 | 1.44959805 |
| Ndufa12 | 1.3273225 |
| Ndufa13 | -0.656093 |
| Ndufa2 | 1.8541725 |
| Ndufa3 | 0.1031969 |
| Ndufa4 | -0.3510871 |
| Ndufa5 | 2.49590446 |
| Ndufa5 | 0.47821189 |
| Ndufa6 | -0.105139 |
| Ndufa7 | 0.41604114 |
| Ndufa8 | -0.724632 |
| Ndufa8 | -0.210293 |
| Ndufa9 | 0.81117914 |
| Ndufaf1 | 1.73907211 |
| Ndufb10 | -0.3818102 |
| Ndufb11 | -0.6395361 |
| Ndufb2 | 0.21507697 |
| Ndufb3 | -0.5864153 |
| Ndufb4 | 0.36328057 |
| Ndufb5 | -0.7203546 |
| Ndufb6 | 0.63487463 |
| Ndufb6 | 1.97115482 |
| Ndufb7 | 0.98762377 |
| Ndufb8 | 0.89551154 |
| Ndufb9 | -0.4184969 |
| Ndufc1 | -0.1125758 |
| Ndufc1 | -0.2703851 |
| Ndufc2 | 1.13592936 |
| Ndufs2 | 0.3790714 |
| Ndufs2 | 1.21304854 |
| Ndufs3 | 2.7030373 |
| Ndufs4 | 1.49200898 |
| Ndufs4 | -0.6505407 |
| Ndufs4 | -0.7000968 |
| Ndufs4 | 1.23727463 |
| Ndufs6 | 1.88936779 |
| Ndufs7 | 1.08943016 |
| Ndufs8 | -0.6802779 |
| Ndufv1 | -0.4846506 |
| Ndufv2 | 0.94133924 |
| Necab1 | 2.97376259 |
| Necab2 | 1.16236463 |
| Necab3 | 2.31366129 |
| Necap1 | 0.25796089 |
| Necap1 | 0.13490843 |
| Necap1 | 1.70102562 |
| Necap2 | -0.5474451 |
| Nedd1 | 2.453646 |
| Nedd4 | 0.86403559 |
| Nedd4l | 0.26554653 |
| Nedd8 | -0.3224667 |
| Nedd9 | -0.3319077 |
| Nedd9 | -0.6387551 |
| Nefm | -0.5006208 |
| Negr1 | -0.5223576 |
| Negr1 | 1.34302768 |
| Neil1 | -0.56889 |
| Neil2 | 1.57462418 |
| Nek11 | 0.87005792 |
| Nek3 | -0.3488098 |
| Nek3 | -0.5223576 |
| Nek3 | 2.59351036 |
| Nek4 | 0.77743656 |
| Nek5 | 1.51817009 |
| Nek6 | -0.6371951 |
| Nek8 | -0.3871314 |
| Nek9 | 1.16823313 |
| Nek9 | 1.5632288 |
| Nelf | -0.5012923 |
| Nelf | 0.00281897 |
| Nell2 | -0.4780777 |
| Nell2 | -0.6217397 |
| Nell2 | -0.5761514 |
| Nell2 | 1.02928654 |
| Nenf | -0.7401985 |
| Neo1 | -0.2688206 |
| Nes | 1.36530557 |
| Net1 | -0.6057877 |
| Net1 | -0.5856783 |
| Neto1 | 0.49101805 |
| Neto1 | -0.672145 |
| Neto1 | 2.14691006 |
| Neu1 | -0.3408833 |
| Neu2 | -0.4352171 |
| Neu3 | -0.4377203 |
| Neu4 | 1.31217643 |
| Neu4 | -0.7143996 |
| Neu4 | 1.54783461 |
| Neurl | -0.743694 |
| Neurod1 | 0.59339212 |
| Neurod2 | 1.6351092 |
| Neurod4 | -0.6309816 |
| Neurog1 | 1.06125382 |
| Neurog2 | 2.34316622 |
| Nf1 | -0.7186492 |
| Nfasc | -0.7721492 |
| Nfasc | 2.80327741 |
| Nfasc | -0.6926143 |
| Nfat5 | 0.95278906 |
| Nfat5 | 0.26467447 |
| Nfatc1 | -0.5230453 |
| Nfatc1 | 0.52095282 |
| Nfatc2 | 0.21107689 |
| Nfatc2 | 1.41087673 |
| Nfatc2ip | -0.5230453 |
| Nfatc3 | -0.7658485 |
| Nfatc4 | -0.4547988 |
| Nfe2 | 1.4327979 |
| Nfe2l1 | -0.4440086 |
| Nfe2l2 | -0.1156557 |
| Nfe2l3 | -0.3197083 |
| Nfib | 1.28540955 |
| Nfic | 0.28281548 |
| Nfic | 0.84000719 |
| Nfic | -0.5953055 |
| Nfix | -0.3202593 |
| Nfix | 0.36692887 |
| Nfkb1 | -0.1294211 |
| Nfkb2 | -0.4767688 |
| Nfkbia | -0.4427475 |
| Nfkbib | 0.71419365 |
| Nfkbid | 1.00241602 |
| Nfkbie | 1.93532969 |
| Nfkbil1 | -0.3978674 |
| Nfkbil2 | 1.89383894 |
| Nfkbiz | 0.83829978 |
| Nfrkb | -0.3625599 |
| Nfrkb | 1.20953471 |
| Nfs1 | 0.69513547 |
| Nfs1 | 2.18066202 |
| Nfs1 | 0.34214939 |
| Nfu1 | -0.0363394 |
| Nfx1 | -0.8652419 |
| Nfya | 1.2407838 |
| Nfyb | -0.0464987 |
| Nfyc | 1.24308252 |
| Ngb | 0.78157844 |
| Ngb | 0.41283126 |
| Ngef | 1.46248975 |
| Ngfb | -0.3505173 |
| Ngfb | -0.3527989 |
| Ngfr | -0.7084831 |
| Ngfrap1 | -0.6387551 |
| Ngp | -0.5798039 |
| Nhedc1 | -0.174822 |
| Nhej1 | -0.0878755 |
| Nhlrc1 | 0.77959268 |
| Nhlrc2 | -0.5292576 |
| Nhlrc2 | -0.7721492 |
| Nhp2l1 | 1.35943521 |
| Nhsl1 | -0.6371951 |
| Niban | -0.8005857 |
| Nicn1 | -0.4178834 |
| Nicn1 | 3.00355006 |
| Nid1 | -0.0904443 |
| Nid2 | 0.44443433 |
| Nif3l1 | -0.6786455 |
| Nin | 0.09727944 |
| Ninj1 | -0.3147642 |
| Ninj2 | -0.8251366 |
| Nip7 | -0.3683507 |
| Nipa1 | 1.36040159 |
| Nipbl | -0.6225063 |
| Nipbl | 3.30089017 |
| Nipsnap1 | -0.3442719 |
| Nipsnap3a | -0.7794034 |
| Nipsnap3a | 0.0547497 |
| Nisch | 0.76118618 |
| Nit1 | 0.30395317 |
| Nit2 | -0.225571 |
| Nit2 | -0.6348601 |
| Nkain3 | -0.0230995 |
| Nkain4 | -0.9071861 |
| Nkd2 | -0.6753895 |
| Nkg7 | 0.18321174 |
| Nkiras1 | -0.7472028 |
| Nkiras2 | 1.82557832 |
| Nkrf | 0.81771729 |
| Nktr | -0.2206147 |
| Nkx1-2 | 0.7535482 |
| Nkx2-1 | -0.4780777 |
| Nkx2-2 | -0.7703446 |
| Nkx2-2 | -0.2845868 |
| Nkx2-3 | -0.8543082 |
| Nkx2-4 | 0.95757468 |
| Nkx2-6 | 2.91012624 |
| Nkx3-1 | -0.3358237 |
| Nkx6-1 | 2.00926627 |
| Nle1 | 0.71294726 |
| Nlgn1 | 1.00007568 |
| Nlgn2 | 0.83518721 |
| Nlk | -0.4919357 |
| Nln | -0.2579438 |
| Nln | -0.5474451 |
| Nln | -0.8523343 |
| Nlrp14 | 0.52139227 |
| Nlrp4a | -0.2492324 |
| Nlrp4c | 2.35807119 |
| Nlrp5 | -0.2724751 |
| Nlrp5 | 1.74503151 |
| Nlrp5 | -0.4209547 |
| Nlrp9a | -0.2856476 |
| Nlrp9c | 0.7878384 |
| Nlrx1 | -0.1227345 |
| Nlrx1 | 0.15970505 |
| Nmb | 0.29048143 |
| Nmbr | -0.4800446 |
| Nmd3 | -0.7676443 |
| Nme1 | 0.5741036 |
| Nme2 | 2.00332804 |
| Nme2 | -0.583471 |
| Nme2 | -0.2899031 |
| Nme3 | 1.06319998 |
| Nme4 | 2.30488378 |
| Nme4 | 0.38009741 |
| Nme4 | -0.7857984 |
| Nme5 | 0.62118938 |
| Nme7 | -0.2365704 |
| Nme7 | 0.74739158 |
| Nme7 | -0.8543082 |
| Nme7 | 2.38077499 |
| Nme7 | 1.02105348 |
| Nmnat1 | 0.66597011 |
| Nmnat2 | -0.459921 |
| Nmnat3 | 1.38500474 |
| Nmral1 | 0.15512707 |
| Nmt1 | -0.1771759 |
| Nmu | -0.6395361 |
| Nmur2 | 0.08142832 |
| Nnat | -0.4265092 |
| Nnat | -0.7950124 |
| Nnmt | -0.13751 |
| Nnmt | -0.6576856 |
| Nob1 | -0.3285644 |
| Nob1 | 1.17460227 |
| Nobox | 0.62883925 |
| Noc3l | -0.2750945 |
| Noc4l | -0.285117 |
| Noc4l | -0.7472028 |
| Nod1 | 0.63176457 |
| Nog | -0.5060068 |
| Nol1 | 1.76589906 |
| Nol1 | -0.4735046 |
| Nol1 | 2.58657142 |
| Nol10 | 0.22999893 |
| Nol11 | -0.5006208 |
| Nol12 | 1.70585381 |
| Nol14 | 3.2328169 |
| Nol3 | -0.5573902 |
| Nol4 | 1.66800241 |
| Nol4 | 0.4350675 |
| Nol4 | 0.63778073 |
| Nol5 | 1.77860637 |
| Nol5a | 3.30625529 |
| Nol6 | -0.0563589 |
| Nol7 | -0.6934427 |
| Nol9 | -0.5938178 |
| Nola1 | 3.15563516 |
| Nola2 | -0.2305544 |
| Nola3 | -0.6672998 |
| Nola3 | 0.56199608 |
| Nolc1 | 1.17374125 |
| Nomo1 | -0.5127842 |
| Nono | 3.88753112 |
| Nope | -0.6434512 |
| Nos1 | 1.29536237 |
| Nos1ap | -0.5355125 |
| Nos2 | 1.72178592 |
| Nos3 | -0.0831846 |
| Nos3 | -0.6340831 |
| Nos3 | -0.8652419 |
| Nosip | 0.21905963 |
| Nosip | -0.430231 |
| Nosip | -0.4166578 |
| Nostrin | 0.72533541 |
| Notch1 | -0.7229187 |
| Notch3 | 0.61724141 |
| Notch4 | -0.4414881 |
| Notch4 | 1.09425835 |
| Notum | -0.7034421 |
| Nova2 | 1.03607041 |
| Nox4 | 0.85507966 |
| Noxo1 | -0.4215702 |
| Noxo1 | 2.43755591 |
| Npal2 | 0.66690745 |
| Npas1 | 0.6327376 |
| Npas2 | -0.3568055 |
| Npas4 | -0.0913022 |
| Npb | -0.4741565 |
| Npc2 | 1.4823003 |
| Npcd | 1.1833813 |
| Npcd | -0.7367163 |
| Npdc1 | 0.39585509 |
| Npepl1 | 2.65866439 |
| Npff | -0.5127842 |
| Nphp1 | -0.4345923 |
| Nphp1 | -0.7658485 |
| Nphp3 | -0.257429 |
| Nphp4 | -0.7922385 |
| Nphs2 | -0.7000968 |
| Nploc4 | 0.72445608 |
| Npm1 | -0.6537093 |
| Npm2 | -0.3788673 |
| Npm3 | -0.3539419 |
| Npm3 | 0.74188403 |
| Npm3-ps1 | 1.0012466 |
| Npr1 | 2.16286474 |
| Npr2 | -0.3230193 |
| Npr3 | -0.2651806 |
| Npsr1 | -0.4421176 |
| Nptn | -0.6012806 |
| Nptx1 | -0.5161918 |
| Nptx2 | 0.30534514 |
| Nptxr | 2.1853073 |
| Npy | 0.01893829 |
| Npy1r | -0.5348154 |
| Npy2r | -0.1209597 |
| Npy5r | -0.0037871 |
| Nqo1 | -0.5703377 |
| Nqo2 | 1.39908492 |
| Nqo2 | -0.7685435 |
| Nqo2 | -0.5908496 |
| Nr1d1 | 0.40811954 |
| Nr1d2 | -0.6371951 |
| Nr1h2 | -0.6876597 |
| Nr1h2 | -0.0420147 |
| Nr1h3 | -0.5864153 |
| Nr1h4 | -0.7533759 |
| Nr1h5 | 0.59562294 |
| Nr1i2 | -0.3153122 |
| Nr2c1 | 2.10204139 |
| Nr2c2ap | 1.39756859 |
| Nr2e1 | 3.53070363 |
| Nr2f2 | -0.6465953 |
| Nr2f2 | -0.3471054 |
| Nr2f6 | 0.0696271 |
| Nr2f6 | 0.77177335 |
| Nr4a2 | 0.60208183 |
| Nr4a3 | 0.99432954 |
| Nr4a3 | -0.2360676 |
| Nr5a1 | -0.5609684 |
| Nr5a2 | 0.77975832 |
| Nr5a2 | 2.39822466 |
| Nr5a2 | 0.39837143 |
| Nr6a1 | -0.2477037 |
| Nradd | 0.48395108 |
| Nrap | -0.4166578 |
| Nrarp | 0.34027698 |
| Nras | -0.4478024 |
| Nrbf2 | -0.700932 |
| Nrbp1 | -0.1038329 |
| Nrbp2 | 3.36407063 |
| Nrcam | 1.2163434 |
| Nrd1 | -0.4939326 |
| Nrf1 | -0.724632 |
| Nrg1 | -0.3158606 |
| Nrg3 | -0.7177977 |
| Nrg4 | 0.22818477 |
| Nrgn | 1.68595576 |
| Nrip2 | -0.5418104 |
| Nrip3 | 0.97245427 |
| Nrl | -0.2548597 |
| Nrm | 0.25414436 |
| Nrn1 | -0.7152479 |
| Nrn1l | -0.6917867 |
| Nrp1 | -0.4105541 |
| Nrp1 | 1.20735764 |
| Nrp2 | 0.10907632 |
| Nrp2 | -0.4277481 |
| Nrsn2 | -0.1626787 |
| Nrsn2 | -0.1280799 |
| Nrtn | -0.1321094 |
| Nrxn1 | 0.51566286 |
| Nrxn1 | 2.98575854 |
| Nrxn1 | 1.23264202 |
| Nrxn2 | 0.85049195 |
| Nrxn3 | -0.3642933 |
| Nrxn3 | -0.1996031 |
| Nsbp1 | 0.30950838 |
| Nsd1 | -0.4421176 |
| Nsd1 | -0.6217397 |
| Nsdhl | -0.8270527 |
| Nsdhl | 4.61587764 |
| Nsdhl | -0.7067997 |
| Nsf | -0.5938178 |
| Nsfl1c | -0.4057003 |
| Nsg1 | 0.67195257 |
| Nsg2 | -0.0238965 |
| Nsl1 | -0.0538839 |
| Nsmaf | -0.6202084 |
| Nsmaf | -0.5581047 |
| Nsmce1 | -0.6458083 |
| Nsmce2 | -0.4063056 |
| Nsmce2 | 2.32088137 |
| Nsmce2 | -0.2421201 |
| Nsun2 | -0.5783412 |
| Nsun4 | -0.4886171 |
| Nsun5 | 2.37137133 |
| Nsun5 | 0.74309164 |
| Nsun5 | -0.5820024 |
| Nsun5 | -0.6125903 |
| Nt5c | -0.7332473 |
| Nt5c1b | -0.4377203 |
| Nt5c2 | 0.85324734 |
| Nt5c2 | -0.3386311 |
| Nt5c2 | -0.5915907 |
| Nt5c3 | 0.68582256 |
| Nt5c3l | -0.4919357 |
| Nt5c3l | -0.8146692 |
| Nt5dc3 | -0.0900157 |
| Nt5dc3 | -0.7640563 |
| Nt5dc3 | 1.57149238 |
| Nt5e | -0.5609684 |
| Nt5e | -0.7940868 |
| Nt5m | -0.5264914 |
| Nt5m | 0.63195926 |
| Ntan1 | -0.3147642 |
| Ntf3 | 0.02802341 |
| Nthl1 | 0.11045425 |
| Ntn2l | -0.4370939 |
| Ntn2l | 0.14953713 |
| Ntn4 | -0.0250933 |
| Ntng1 | -0.4490705 |
| Ntng1 | -0.4826737 |
| Ntng1 | 0.76270615 |
| Ntrk2 | 0.11628737 |
| Ntrk2 | -0.209316 |
| Ntrk2 | -0.4972706 |
| Ntrk3 | 1.24855615 |
| Ntrk3 | 1.25704982 |
| Ntrk3 | 0.61882264 |
| Nts | 0.56178592 |
| Ntsr1 | -0.1819018 |
| Ntsr2 | 2.86012073 |
| Nuak1 | -0.6705271 |
| Nuak1 | -0.4573563 |
| Nuak2 | 1.49398394 |
| Nub1 | -0.1125758 |
| Nubp1 | -0.0199185 |
| Nubp2 | 0.14788651 |
| Nubp2 | 1.15448094 |
| Nubp2 | 1.390119 |
| Nucb1 | 0.33058645 |
| Nucb1 | 0.50900711 |
| Nucks1 | 0.11628737 |
| Nudc | -0.6340831 |
| Nudc | -0.3823999 |
| Nudcd2 | -0.3539419 |
| Nudcd3 | 0.99864253 |
| Nudc-ps1 | 1.0088857 |
| Nudt1 | 0.95059299 |
| Nudt10 | 0.91671802 |
| Nudt11 | 1.14163481 |
| Nudt12 | 0.61803237 |
| Nudt13 | 0.9966846 |
| Nudt14 | 0.96545043 |
| Nudt16l1 | -0.4919357 |
| Nudt18 | 1.08505104 |
| Nudt19 | -0.5820024 |
| Nudt19 | 0.9154337 |
| Nudt2 | -0.2290565 |
| Nudt21 | -0.4433778 |
| Nudt22 | 0.58461908 |
| Nudt4 | 1.43644131 |
| Nudt5 | 1.19102087 |
| Nudt5 | -0.2325553 |
| Nudt6 | 0.49532259 |
| Nudt7 | -0.5127842 |
| Nudt8 | -0.1585125 |
| Nudt9 | -0.4986092 |
| Nufip1 | -0.6411001 |
| Numa1 | -0.0604989 |
| Numb | 0.52292869 |
| Numb | 0.05108408 |
| Numb | -0.545328 |
| Numb | 1.1598536 |
| Numbl | 0.00976205 |
| Nup107 | -0.0819094 |
| Nup133 | -0.38299 |
| Nup133 | 1.75306994 |
| Nup155 | 0.88213306 |
| Nup155 | 0.12547598 |
| Nup160 | -0.5982882 |
| Nup210 | 0.31282538 |
| Nup37 | 2.18865157 |
| Nup43 | 2.0748614 |
| Nup50 | -0.6286624 |
| Nup50 | -0.2132302 |
| Nup50 | 1.43003545 |
| Nup62 | 1.37425731 |
| Nup85 | 0.00397982 |
| Nup88 | 2.12372197 |
| Nup88 | -0.4853105 |
| Nup88 | -0.1249579 |
| Nup93 | -0.6118319 |
| Nup98 | 1.26097366 |
| Nup98 | -0.1352559 |
| Nupl1 | -0.0139837 |
| Nupl1 | -0.5827364 |
| Nupl1 | -0.5502755 |
| Nupl2 | -0.4748089 |
| Nupr1 | 0.2523775 |
| Nus1 | 1.99040897 |
| Nusap1 | -0.0436427 |
| Nut | 0.73998312 |
| Nutf2 | -0.285117 |
| Nvl | -0.5390059 |
| Nwd1 | 0.61862514 |
| Nxf1 | -0.7393267 |
| Nxf2 | -0.713552 |
| Nxf3 | -0.4320976 |
| Nxph1 | 1.81345762 |
| Nxph2 | -0.1156557 |
| Nxph3 | 1.66353728 |
| Nxt1 | 0.41554805 |
| Nxt1 | 0.98537755 |
| Nxt2 | -0.1598991 |
| Nyx | -0.3700951 |
| Oaf | -0.4215702 |
| Oas1b | 0.61188449 |
| Oas1b | -0.6217397 |
| Oas1c | -0.2814117 |
| Oas1d | 0.20736892 |
| Oas1d | 0.06782568 |
| Oas1e | 0.51344965 |
| Oas1g | -0.6110742 |
| Oas1g | 1.33773251 |
| Oas2 | 0.08320318 |
| Oasl1 | 0.75064607 |
| Oasl2 | 0.09937233 |
| Oasl2 | -0.5502755 |
| Oat | 0.14887724 |
| Oaz2 | -0.299551 |
| Oaz3 | -0.6209738 |
| Obfc1 | 0.18923214 |
| Obfc2a | -0.6737658 |
| Obfc2b | -0.2947144 |
| Obfc2b | 1.49572748 |
| Obox1 | -0.5039833 |
| Obox5 | -0.5148272 |
| Obrgrp | 0.45583263 |
| Obsl1 | -0.1420349 |
| Oc90 | -0.5710624 |
| Oca2 | 0.16100885 |
| Ociad1 | -0.2920383 |
| Ociad1 | 0.31640528 |
| Ociad2 | -0.764952 |
| Ocln | 1.08100842 |
| Ocrl | 0.13892132 |
| Odc1 | 2.48320155 |
| Odf1 | -0.4754617 |
| Odf2 | -0.6843715 |
| Odf2 | -0.2610383 |
| Odf2 | -0.6737658 |
| Odf2 | -0.0151676 |
| Odf3 | -0.6340831 |
| Odf3l1 | -0.5019644 |
| Odf4 | 2.85781646 |
| Odf4 | -0.3700951 |
| Odz1 | -0.7839667 |
| Odz3 | -0.1857002 |
| Odz3 | -0.1603618 |
| Odz4 | 0.02007888 |
| Og9x | -0.2117604 |
| Ogdh | -0.631756 |
| Ogfod1 | -0.7220632 |
| Ogfod2 | 0.68472067 |
| Ogfr | 1.11733746 |
| Ogfrl1 | -0.5871528 |
| Ogg1 | -0.0262916 |
| Ogn | 2.50076497 |
| Ogn | 0.41456106 |
| Ogn | -0.8474179 |
| Ogt | -0.3782799 |
| Ogt | 0.18416497 |
| Ogt | 1.09765809 |
| Oit1 | 1.06441424 |
| Oit3 | -0.5739669 |
| Ola1 | 0.7927504 |
| Olfm1 | 0.96990139 |
| Olfm1 | -0.5488592 |
| Olfm2 | -0.568167 |
| Olfm2 | 0.66145745 |
| Olfm3 | 0.22424171 |
| Olfm3 | -0.0505942 |
| Olfm4 | 1.94534542 |
| Olfml1 | -0.5790722 |
| Olfml2b | -0.6762024 |
| Olfml3 | 1.44376505 |
| Olfr100 | -0.2610383 |
| Olfr1006 | 2.00163052 |
| Olfr1012 | -0.2771954 |
| Olfr1020 | -0.4702521 |
| Olfr1065 | 1.21273904 |
| Olfr1094 | 0.61446767 |
| Olfr1099 | -0.590109 |
| Olfr1105 | 3.22560818 |
| Olfr1112 | 1.61419162 |
| Olfr1113 | -0.012014 |
| Olfr1189 | -0.0143782 |
| Olfr1197 | -0.3853542 |
| Olfr1198 | 1.40896359 |
| Olfr1249 | -0.2569146 |
| Olfr1280 | -0.3924836 |
| Olfr133 | -0.0526489 |
| Olfr1330 | -0.5908496 |
| Olfr134 | 0.33085702 |
| Olfr1340 | -0.450976 |
| Olfr1384 | 3.19540013 |
| Olfr1386 | -0.7428188 |
| Olfr1390 | -0.0270914 |
| Olfr1394 | -0.7472028 |
| Olfr1412 | 2.19896284 |
| Olfr1437 | 0.72040026 |
| Olfr144 | 1.79959995 |
| Olfr1443 | 1.51794837 |
| Olfr1443 | -0.5997832 |
| Olfr1459 | -0.2073651 |
| Olfr1500 | 3.7056269 |
| Olfr1508 | -0.5230453 |
| Olfr166 | -0.6465953 |
| Olfr168 | 1.72190422 |
| Olfr206 | 1.02460912 |
| Olfr213 | -0.2421201 |
| Olfr215 | 0.08249365 |
| Olfr232 | 0.22788206 |
| Olfr257 | -0.3380689 |
| Olfr267 | -0.5121042 |
| Olfr272 | -0.2487226 |
| Olfr303 | 2.14389432 |
| Olfr310 | 3.76709299 |
| Olfr352 | -0.5967956 |
| Olfr371 | 1.16779971 |
| Olfr378 | 1.06937589 |
| Olfr419 | 1.29912562 |
| Olfr437 | -0.632531 |
| Olfr464 | -0.553826 |
| Olfr47 | 0.87275509 |
| Olfr49 | -0.7894729 |
| Olfr503 | -0.743694 |
| Olfr51 | 2.43386915 |
| Olfr536 | 1.64802408 |
| Olfr538 | -0.7622676 |
| Olfr54 | -0.8222699 |
| Olfr550 | 0.45701183 |
| Olfr574 | -0.7848821 |
| Olfr591 | 3.24497534 |
| Olfr593 | -0.2431328 |
| Olfr608 | 1.80690544 |
| Olfr638 | -0.4735046 |
| Olfr65 | 1.99835718 |
| Olfr655 | 1.15007159 |
| Olfr658 | -0.4992793 |
| Olfr665 | -0.0621602 |
| Olfr675 | 1.25960223 |
| Olfr691 | 0.49010924 |
| Olfr692 | 0.75830821 |
| Olfr702 | 0.26117788 |
| Olfr724 | -0.0452736 |
| Olfr725 | -0.3619828 |
| Olfr748 | 0.1908099 |
| Olfr786 | 0.71205594 |
| Olfr802 | 1.29639881 |
| Olfr824 | 0.10284987 |
| Olfr91 | 1.0569821 |
| Olfr910 | 2.63688498 |
| Olfr920 | -0.7272081 |
| Olfr924 | 1.83063412 |
| Olfr952 | 1.37226596 |
| Olfr965 | 0.0024317 |
| Olfr982 | -0.0477254 |
| Olfr983 | 0.43795983 |
| Olfr984 | -0.5739669 |
| Olig1 | -0.2400982 |
| Olig3 | -0.6387551 |
| Oma1 | -0.4142114 |
| Omd | 0.42413909 |
| Omg | -0.3408833 |
| Omt2b | -0.7445699 |
| Onecut2 | -0.7101696 |
| Onecut3 | -0.700932 |
| Onecut3 | 1.58862037 |
| Oog4 | -0.1976729 |
| Oosp1 | -0.3136691 |
| Opa1 | 0.30950838 |
| Opa1 | 0.99065403 |
| Ophn1 | 1.22849308 |
| Oplah | 1.60677614 |
| Opn1mw | -0.0238965 |
| Opn3 | -0.4396023 |
| Oprl1 | 2.77498148 |
| Oprl1 | -0.7315178 |
| Oprl1 | 1.23122742 |
| Oprs1 | -0.5320323 |
| Optc | -0.0514155 |
| Optn | -0.5856783 |
| Optn | 0.31365276 |
| Orai1 | -0.5674446 |
| Orai3 | -0.4866317 |
| Oraov1 | 0.2239377 |
| Orc2l | 1.32914147 |
| Orc3l | -0.7237749 |
| Orc5l | -0.6442362 |
| Orc5l | -0.7569222 |
| Orc6l | 1.57947468 |
| ORF19 | 2.00106398 |
| ORF28 | -0.7026046 |
| ORF34 | -0.0646578 |
| ORF61 | 0.54267089 |
| ORF63 | 0.02538289 |
| ORF9 | -0.2888374 |
| ORF9 | -0.0968982 |
| ORF9 | -0.6202084 |
| Orm1 | 1.30920976 |
| Ormdl2 | -0.0650747 |
| Ormdl3 | 0.73024408 |
| Os9 | -0.5871528 |
| Osbp2 | 0.07929393 |
| Osbpl10 | 2.95276114 |
| Osbpl11 | 0.86870635 |
| Osbpl2 | -0.235565 |
| Osbpl3 | 0.06276276 |
| Osbpl3 | 0.09693016 |
| Osbpl6 | 2.2828848 |
| Osbpl8 | -0.1361569 |
| Osbpl9 | -0.5805361 |
| Oscar | -0.4979397 |
| Osgep | 0.53793695 |
| Osgepl1 | 1.32977725 |
| Osgin1 | 1.0902563 |
| Osm | -0.8464377 |
| Osmr | 1.57629011 |
| Osr1 | 2.06304251 |
| Osr2 | -0.2709071 |
| Ostf1 | 2.92207257 |
| Ostm1 | -0.7349802 |
| Otof | 0.74790622 |
| Otop1 | 0.03739211 |
| Otop2 | -0.4541606 |
| Ott | 0.89551154 |
| Ott | 2.02026027 |
| OTTMUSG00000000421 | 1.82684489 |
| OTTMUSG00000000971 | -0.0709317 |
| OTTMUSG00000000990 | 0.9335323 |
| OTTMUSG00000001305 | -0.2054185 |
| OTTMUSG00000002038 | 1.1602908 |
| OTTMUSG00000004461 | -0.6810951 |
| OTTMUSG00000004551 | -0.7903938 |
| OTTMUSG00000005148 | 2.48124342 |
| OTTMUSG00000005491 | -0.3771061 |
| OTTMUSG00000008540 | 1.52384267 |
| OTTMUSG00000010009 | 2.5817543 |
| OTTMUSG00000010328 | 1.85181494 |
| OTTMUSG00000015762 | -0.7280684 |
| OTTMUSG00000015859 | 1.12076677 |
| OTTMUSG00000016293 | -0.032307 |
| OTTMUSG00000016790 | -0.7026046 |
| OTTMUSG00000016790 | 0.98232973 |
| OTTMUSG00000017677 | -0.4383472 |
| OTTMUSG00000020946 | 0.56514273 |
| Otub1 | -0.5390059 |
| Otub2 | -0.1303163 |
| Otub2 | -0.6843715 |
| Otub2 | -0.5805361 |
| Otud5 | -0.4057003 |
| Otud6b | 0.52555657 |
| Otud7b | 0.15545483 |
| Otud7b | 1.62615611 |
| Otx1 | -0.390101 |
| Otx2 | -0.4184969 |
| Ovca2 | -0.7186492 |
| Ovgp1 | 1.66410449 |
| Ovgp1 | 0.16620554 |
| Ovol1 | -0.3082133 |
| Ovol2 | 1.15854076 |
| Ovol2 | 0.13155085 |
| Oxct1 | -0.3930803 |
| Oxct2a | -0.6529161 |
| Oxct2b | 2.0061936 |
| Oxgr1 | 2.03331471 |
| Oxnad1 | -0.6118319 |
| Oxr1 | 0.07250201 |
| Oxr1 | -0.7730528 |
| Oxt | -0.5114247 |
| P140 | -0.0613292 |
| P2rx2 | -0.2211091 |
| P2rx3 | 2.70786198 |
| P2rx4 | 0.3826574 |
| P2rx4 | -0.631756 |
| P2rx5 | 0.12716749 |
| P2rx7 | -0.8299344 |
| P2rx7 | 2.87293529 |
| P2ry1 | 1.15547013 |
| P2ry12 | -0.5369083 |
| P2ry13 | 2.72604216 |
| P2ry14 | -0.6632816 |
| P2ry14 | -0.4807012 |
| P2ry5 | -0.7263486 |
| P2ry6 | 0.48532315 |
| P42pop | -0.0104413 |
| P4ha1 | 0.30004436 |
| P4ha2 | -0.2230895 |
| P4hb | 1.30948829 |
| Pa2g4 | -0.5237335 |
| Pabpc1 | 1.34624364 |
| Pabpc1 | -0.5798039 |
| Pabpc1 | 0.19740691 |
| Pabpc2 | 0.11970133 |
| Pabpc4 | -0.2507637 |
| Pabpn1 | -0.2631071 |
| Pabpnl1 | 0.20115539 |
| Pacrg | 1.87482138 |
| Pacs1 | -0.408124 |
| Pacs2 | 1.81628951 |
| Pacsin1 | 0.16814671 |
| Pacsin1 | 2.30996757 |
| Pacsin1 | -0.4203397 |
| Pacsin2 | 1.91362114 |
| Pacsin2 | -0.7059591 |
| Pacsin3 | 0.41456106 |
| Pacsin3 | 0.69839987 |
| Padi1 | 1.84847321 |
| Padi2 | -0.1804815 |
| Padi6 | 0.196154 |
| Paf1 | -0.4370939 |
| Paf1 | 0.65673298 |
| Pafah1b1 | -0.2646618 |
| Pafah1b1 | 0.19678067 |
| Pafah1b3 | -0.263625 |
| Pafah2 | -0.0908732 |
| Pafah2 | -0.6753895 |
| Paip1 | 0.0459274 |
| Paip2 | 1.94051429 |
| Paip2b | -0.7712464 |
| Paip2b | 1.08160404 |
| Pak1 | -0.6713357 |
| Pak3 | 1.67879184 |
| Pak4 | 0.27278158 |
| Pak6 | 2.64399386 |
| Pak7 | -0.7220632 |
| Palld | 0.68324941 |
| Palm | 1.66674683 |
| Palm | -0.5411084 |
| Palm2 | -0.6163914 |
| Palmd | -0.4966021 |
| Palmd | 1.71727911 |
| Palmd | 1.59237586 |
| Pam | 2.94483927 |
| Pank1 | 0.01931864 |
| Pank1 | -0.7876338 |
| Pank2 | 2.10332874 |
| Pank3 | 1.54776307 |
| Pank4 | 0.70919787 |
| Panx1 | 1.31060159 |
| Panx3 | -0.5573902 |
| Paox | -0.7748628 |
| Paox | 1.05244449 |
| Paox | 1.72083896 |
| Paox | 3.39200205 |
| Papd1 | -0.6640838 |
| Papd1 | -0.3753483 |
| Papd4 | -0.3093018 |
| Papln | 1.12634049 |
| Papola | 1.26058203 |
| Papolb | -0.7480822 |
| Papolg | -0.2814117 |
| Papss1 | -0.6387551 |
| Papss1 | -0.6552978 |
| Papss2 | 0.38444514 |
| Paqr3 | 1.00306505 |
| Paqr6 | -0.2320547 |
| Paqr7 | -0.4364678 |
| Paqr9 | -0.6794613 |
| Paqr9 | -0.4228025 |
| Paqr9 | 1.69452783 |
| Pard3 | 0.18162082 |
| Pard3 | -0.6737658 |
| Pard6a | -0.7177977 |
| Pard6a | 1.10928539 |
| Pard6b | -0.3280084 |
| Pard6g | -0.3408833 |
| Park7 | -0.5776107 |
| Parl | 1.32895973 |
| Parp1 | 0.81612692 |
| Parp1 | -0.2370736 |
| Parp1 | 2.32563272 |
| Parp12 | 1.3791738 |
| Parp14 | -0.246686 |
| Parp16 | -0.5495671 |
| Parp2 | -0.2867096 |
| Parp2 | -0.7885529 |
| Parp3 | 0.60829571 |
| Parp6 | -0.7885529 |
| Parp8 | 0.17490857 |
| Pars2 | -0.0654918 |
| Pars2 | -0.6827319 |
| Parva | -0.3700951 |
| Parvg | -0.6975958 |
| Pask | 0.39054815 |
| Patl1 | -0.5327273 |
| Patz1 | -0.447169 |
| Pawr | -0.5481519 |
| Pax8 | -0.5930748 |
| Paxip1 | -0.7498434 |
| Pbk | 2.21272893 |
| Pbld | 0.39711412 |
| Pbp2 | 0.39333181 |
| Pbrm1 | 0.22788206 |
| Pbrm1 | -0.7857984 |
| Pbx1 | -0.7996544 |
| Pbx1 | 0.03403042 |
| Pbx2 | 0.82278798 |
| Pbx2 | 0.45606859 |
| Pbx2 | -0.2512747 |
| Pbx3 | -0.298474 |
| Pbx3 | 1.03782101 |
| Pbx4 | 1.49920462 |
| Pcaf | -0.0542959 |
| Pcaf | 0.40587918 |
| Pcbd1 | 2.17303568 |
| Pcbd2 | -0.607295 |
| Pcbp1 | -0.6545032 |
| Pcbp2 | -0.647383 |
| Pcbp3 | -0.6217397 |
| Pcca | 0.76084807 |
| Pcca | -0.450976 |
| Pccb | -0.1267406 |
| Pccb | 0.10146044 |
| Pcdh1 | 0.67530059 |
| Pcdh10 | -0.7000968 |
| Pcdh10 | 2.35564821 |
| Pcdh10 | 3.38474811 |
| Pcdh15 | 1.32367369 |
| Pcdh17 | -0.3954707 |
| Pcdh20 | 1.60945532 |
| Pcdh21 | -0.95776 |
| Pcdh7 | 3.95139014 |
| Pcdh7 | 1.50881116 |
| Pcdh8 | -0.5938178 |
| Pcdh9 | 0.53426206 |
| Pcdha10 | 0.12885588 |
| Pcdha11 | 0.92325435 |
| Pcdha2 | 0.46918623 |
| Pcdha6 | 4.57553316 |
| Pcdhac1 | 2.02676839 |
| Pcdhac2 | 1.70861852 |
| Pcdhb1 | -0.4728532 |
| Pcdhb10 | 2.38928478 |
| Pcdhb12 | 1.28197208 |
| Pcdhb14 | 0.72989433 |
| Pcdhb19 | 0.6178347 |
| Pcdhb20 | -0.707641 |
| Pcdhb22 | -0.8926465 |
| Pcdhb3 | -0.5087117 |
| Pcdhb7 | -0.5141457 |
| Pcdhb8 | -0.0262916 |
| Pcdhga10 | -0.0956038 |
| Pcdhga12 | -0.6179163 |
| Pcdhga4 | 0.08036175 |
| Pcdhga7 | -0.6371951 |
| Pcdhga8 | 2.12143166 |
| Pcdhgb2 | 0.12445957 |
| Pcdhgb6 | 1.222081 |
| Pcdhgc3 | 1.90692954 |
| Pcdhgc4 | -0.440859 |
| Pcdhgc5 | 2.56085786 |
| Pcf11 | 1.51787445 |
| Pcgf1 | -0.8552968 |
| Pcgf2 | 0.29443122 |
| Pcgf3 | 1.43676447 |
| Pcgf5 | -0.6497503 |
| Pcgf6 | -0.7203546 |
| Pck1 | 0.36640858 |
| Pclo | 0.50319937 |
| Pclo | 3.15522779 |
| Pcmt1 | 0.28452458 |
| Pcmt1 | -0.2714295 |
| Pcmt1 | -0.4535228 |
| Pcmtd1 | -0.2698633 |
| Pcmtd2 | 0.21045994 |
| Pcna | -0.2492324 |
| Pcnt | 0.61088902 |
| Pcnx | -0.5306439 |
| Pcnxl2 | -0.5783412 |
| Pcnxl2 | 0.27652131 |
| Pcolce | 2.14199041 |
| Pcolce2 | 0.3389372 |
| Pcolce2 | 0.33301872 |
| Pcsk1n | 2.88492602 |
| Pcsk2 | 1.22391761 |
| Pcsk2 | -0.700932 |
| Pcsk2 | 1.42840658 |
| Pcsk2 | 1.47852671 |
| Pcsk4 | -0.3169583 |
| Pcsk4 | -0.4926009 |
| Pcsk4 | 0.17072855 |
| Pcsk4 | -0.4277481 |
| Pcsk4 | 2.05875496 |
| Pcsk9 | 1.28598121 |
| Pctk1 | -0.3562321 |
| Pcx | -0.1218467 |
| Pcyox1 | -0.431475 |
| Pcyox1l | -0.4728532 |
| Pcyt1a | -0.7480822 |
| Pcyt1b | -0.6458083 |
| Pcyt1b | 0.73963708 |
| Pcyt2 | 0.27220488 |
| Pcyt2 | -0.0981944 |
| Pcyt2 | 0.19489937 |
| Pdcd10 | -0.3230193 |
| Pdcd2 | -0.5439193 |
| Pdcd2l | 1.13221854 |
| Pdcd4 | 1.61425816 |
| Pdcd5 | 0.56094479 |
| Pdcd5 | -0.4846506 |
| Pdcd6ip | 1.5045457 |
| Pdcl | -0.5524041 |
| Pdcl3 | 0.9530632 |
| Pddc1 | 0.20021971 |
| Pde10a | -0.5524041 |
| Pde1a | -0.5467389 |
| Pde1a | 0.14258442 |
| Pde1a | 1.04044065 |
| Pde1b | 2.00857473 |
| Pde1c | 0.09062021 |
| Pde2a | 0.13255941 |
| Pde3a | -0.8347575 |
| Pde4a | 2.70505909 |
| Pde4a | -0.2814117 |
| Pde4a | -0.7042804 |
| Pde4a | -0.563122 |
| Pde4b | 0.94355761 |
| Pde4d | -0.4859708 |
| Pde4dip | 1.15711639 |
| Pde4dip | -0.5264914 |
| Pde4dip | -0.5244222 |
| Pde4dip | 0.33490603 |
| Pde4dip | -0.4129907 |
| Pde5a | 0.75830821 |
| Pde6a | -0.6395361 |
| Pde6d | -0.4754617 |
| Pde6d | -0.6584829 |
| Pde6d | -0.5645606 |
| Pde6d | 2.60711995 |
| Pde6h | -0.2059047 |
| Pde7a | -0.2714295 |
| Pdgfa | -0.6616792 |
| Pdgfb | 2.63022465 |
| Pdgfc | -0.2350627 |
| Pdgfra | 3.01684624 |
| Pdgfrb | -0.5805361 |
| Pdgfrl | 1.33556975 |
| Pdha1 | 1.16061856 |
| Pdhb | -0.3442719 |
| Pdhb | -0.4939326 |
| Pdia3 | -0.6545032 |
| Pdia4 | 0.14887724 |
| Pdia5 | -0.6624801 |
| Pdia5 | -0.2300548 |
| Pdia6 | -0.0563589 |
| Pdik1l | 0.18066493 |
| Pdk1 | 1.97998535 |
| Pdk2 | -0.707641 |
| Pdk3 | -0.38299 |
| Pdk4 | 0.04407866 |
| Pdlim1 | 0.63797415 |
| Pdlim2 | 0.68177578 |
| Pdlim2 | -0.8241801 |
| Pdlim2 | 2.15098965 |
| Pdlim2 | -0.6302079 |
| Pdlim3 | 1.1598536 |
| Pdlim3 | -0.1169788 |
| Pdlim4 | -0.553826 |
| Pdlim5 | 2.55173357 |
| Pdlim5 | -0.4166578 |
| Pdlim7 | -0.8251366 |
| Pdpk1 | -0.2385846 |
| Pdpk1 | 2.64533143 |
| Pdrg1 | -0.8967771 |
| Pdss1 | -0.3352632 |
| Pdxdc1 | 1.35264184 |
| Pdxdc1 | 2.0573623 |
| Pdxk | -0.0994925 |
| Pdxp | -0.7622676 |
| Pdyn | -0.5223576 |
| Pdyn | 1.12531928 |
| Pdzd11 | -0.3476732 |
| Pdzd11 | -0.1090686 |
| Pdzd11 | 0.04887763 |
| Pdzd8 | 1.28187641 |
| Pdzk1 | 0.01014624 |
| Pdzk1ip1 | -0.2507637 |
| Pdzrn3 | -0.5066823 |
| Pdzrn3 | 1.23183395 |
| Pea15 | -0.6672998 |
| Pear1 | 0.53620943 |
| Pear1 | 0.54609844 |
| Pebp1 | -0.5652807 |
| Pecam1 | -0.2952505 |
| Pecam1 | -0.7821387 |
| Peci | 0.43916227 |
| Pef1 | 0.82594294 |
| Peg10 | -0.4780777 |
| Peg3 | 0.35039574 |
| Peli1 | -0.7721492 |
| Peli1 | 0.14557062 |
| Peli2 | -0.1294211 |
| Peli3 | 0.38902621 |
| Pelo | 0.08249365 |
| Pelp1 | -0.6387551 |
| Pemt | 1.46484279 |
| Peo1 | 2.84788463 |
| Pepd | 1.32841432 |
| Per1 | -0.5285653 |
| Per1 | -0.6171536 |
| Per2 | 0.53923046 |
| Per2 | -0.6893083 |
| Per2 | 0.84572703 |
| Per3 | 0.33463665 |
| Perld1 | 0.68802237 |
| Pes1 | -0.4787329 |
| Pet112l | -0.1981551 |
| Pet112l | 0.32651824 |
| Pet112l | 0.79909683 |
| Pet112l | 0.39987791 |
| Pex11a | 0.43844099 |
| Pex11a | -0.759591 |
| Pex11a | -0.6403178 |
| Pex11b | -0.7703446 |
| Pex11c | -0.2290565 |
| Pex14 | -0.412381 |
| Pex16 | 1.62595907 |
| Pex19 | -0.1325583 |
| Pex19 | -0.659281 |
| Pex2 | 0.93758349 |
| Pex26 | 0.93590931 |
| Pex3 | 1.04924239 |
| Pex3 | 2.52350623 |
| Pex5 | -0.0041772 |
| Pex5 | -0.0262916 |
| Pex5 | -0.8523343 |
| Pex5l | -0.3695132 |
| Pex6 | 0.3728911 |
| Pex6 | 0.86358231 |
| Pex6 | 0.03328169 |
| Pex7 | -0.388318 |
| Pfdn1 | -0.6697192 |
| Pfdn2 | 1.04156106 |
| Pfdn4 | 0.1247985 |
| Pfdn5 | -0.5776107 |
| Pfdn5 | 1.62891017 |
| Pfkfb2 | 2.77471951 |
| Pfkfb2 | -0.659281 |
| Pfkfb4 | 0.40037952 |
| Pfkl | 1.43449997 |
| Pfkl | -0.0802119 |
| Pfkm | -0.2078525 |
| Pfkm | 3.46745236 |
| Pfkm | -0.037148 |
| Pfkp | -0.7177977 |
| Pfkp | 0.19771987 |
| Pfkp | 1.11504409 |
| Pfn1 | 3.42358309 |
| Pfn1 | -0.7263486 |
| Pfn2 | 0.59501507 |
| Pfn2 | 1.67568656 |
| Pfn2 | -0.0493636 |
| Pfn3 | 1.15172759 |
| Pfn4 | -0.3017089 |
| Pftk1 | -0.4637815 |
| Pga5 | 1.46343169 |
| Pgam1 | -0.5545377 |
| Pgam2 | 1.25310908 |
| Pgam5 | 1.38799153 |
| Pgbd5 | -0.0738743 |
| Pgcp | -0.6255792 |
| Pgd | 0.54738046 |
| Pgk1 | -0.1116977 |
| Pgk2 | -0.5997832 |
| Pgls | 1.89192541 |
| Pglyrp1 | -0.1388651 |
| Pglyrp4 | -0.4528854 |
| Pgm2 | -0.3859462 |
| Pgm2l1 | -0.197191 |
| Pgm3 | -0.5790722 |
| Pgm5 | 0.52117257 |
| Pgm5 | 0.07393608 |
| Pgpep1 | -0.840579 |
| Pgpep1 | -0.0266914 |
| Pgr | 0.08107293 |
| Pgrmc1 | -0.6827319 |
| Pgs1 | -0.1379615 |
| Phactr1 | -0.5595354 |
| Phactr1 | -0.5046573 |
| Phactr1 | -0.5509845 |
| Phactr4 | -0.6951017 |
| Phb2 | 0.61902011 |
| Phc1 | -0.7703446 |
| Phc1 | -0.5087117 |
| Phc2 | 1.61425816 |
| Phca | 0.22058682 |
| Phca | 2.78634225 |
| Phex | 1.09425835 |
| Phf1 | 1.3091169 |
| Phf11 | 1.94311884 |
| Phf11 | -0.6156299 |
| Phf12 | 0.93828016 |
| Phf12 | 0.49938197 |
| Phf13 | 0.06818625 |
| Phf13 | -0.6333067 |
| Phf14 | 0.29132925 |
| Phf16 | -0.5285653 |
| Phf17 | -0.3533702 |
| Phf19 | 1.54331639 |
| Phf2 | 0.8167634 |
| Phf20l1 | 2.37812282 |
| Phf21a | 0.18289378 |
| Phf5a | 1.8484217 |
| Phf6 | 1.17814543 |
| Phf7 | 2.19396297 |
| Phgdh | 2.12326436 |
| Phka1 | 2.11329301 |
| Phka1 | -0.2260682 |
| Phka2 | -0.0538839 |
| Phka2 | -0.5703377 |
| Phka2 | -0.3285644 |
| Phkb | -0.1738822 |
| Phkg1 | 1.22136577 |
| Phkg2 | 0.14391282 |
| Phlda1 | 1.15678738 |
| Phlda2 | 1.20663079 |
| Phlda3 | 0.6885715 |
| Phlda3 | 0.65749053 |
| Phldb1 | -0.430231 |
| Phldb1 | -0.1447607 |
| Phlpp | -0.5046573 |
| Phospho1 | 1.49761887 |
| Phospho2 | -0.2176537 |
| Phox2a | 0.0089932 |
| Phox2b | 2.40814029 |
| Phpt1 | 0.95716546 |
| Phtf1 | -0.8127788 |
| Phtf2 | -0.3202593 |
| Phyhip | -0.668912 |
| Pi16 | -0.6909598 |
| Pi4k2b | 0.47127699 |
| Pi4k2b | 2.67931445 |
| Pi4ka | 1.62956468 |
| Pi4kb | -0.2290565 |
| Pi4kb | 1.76651892 |
| Pias1 | 0.52904871 |
| Pias2 | 0.31777845 |
| Pias3 | 0.82845911 |
| Pias3 | 1.57330317 |
| Pias4 | 0.4365148 |
| Pibf1 | -0.6225063 |
| Pibf1 | -0.6876597 |
| Picalm | -0.012014 |
| Pick1 | 1.80846718 |
| Pick1 | 0.94480308 |
| Pif1 | -0.0596694 |
| Piga | 1.25685319 |
| Piga | 2.65034069 |
| Pigb | 1.61232671 |
| Pigc | -0.3871314 |
| Pigf | -0.8662424 |
| Pigh | 0.05840071 |
| Pigk | -0.56889 |
| Pigk | 0.07679751 |
| Pigl | 0.12851845 |
| Pign | -0.4433778 |
| Pigo | 2.38685739 |
| Pigo | -0.677016 |
| Pigo | 0.53728951 |
| Pigp | -0.6778304 |
| Pigp | 2.91942418 |
| Pigq | -0.6762024 |
| Pigt | 1.26107154 |
| Pigx | -0.5768808 |
| Pigy | -0.7507252 |
| Pigyl | 1.434095 |
| Pigz | -0.1262946 |
| Pih1d1 | 0.68948599 |
| Pik3ap1 | 0.90999102 |
| Pik3c2a | -0.4820158 |
| Pik3c3 | -0.7127053 |
| Pik3ca | 0.43603262 |
| Pik3cb | -0.4258904 |
| Pik3cd | 1.59373768 |
| Pik3cg | -0.5595354 |
| Pik3ip1 | -0.5148272 |
| Pik3r1 | -0.7694436 |
| Pik3r1 | 0.21905963 |
| Pik3r1 | 0.00320609 |
| Pik3r2 | 0.70237395 |
| Pik3r2 | 0.89492757 |
| Pik3r3 | -0.3516574 |
| Pik3r4 | 1.7898775 |
| Pik3r6 | 2.11898233 |
| Pik3r6 | -0.7034421 |
| Pim1 | 0.05840071 |
| Pim2 | 0.13557847 |
| Pim2 | 1.51617265 |
| Pim3 | -0.0041772 |
| Pip4k2a | 2.11074443 |
| Pip4k2b | 2.48232606 |
| Pip4k2b | 0.8721564 |
| Pip4k2c | -0.2390889 |
| Pip5k1a | -0.7551474 |
| Pip5k1a | -0.4414881 |
| Pip5k1b | -0.4503404 |
| Pip5kl1 | -0.447169 |
| Pipox | -0.4459035 |
| Pir | 2.02621721 |
| Pira11 | 1.29158358 |
| Pira4 | 1.87371977 |
| Pisd | -0.6202084 |
| Pit1 | -0.2320547 |
| Pitpna | 0.98246246 |
| Pitpnb | 1.14286075 |
| Pitpnc1 | 3.53083468 |
| Pitpnm1 | -0.2029909 |
| Pitpnm2 | 2.17299956 |
| Pitpnm3 | 0.99629251 |
| Pitx1 | 0.38750173 |
| Pitx2 | -0.7578109 |
| Pitx2 | -0.3038719 |
| Pitx2 | -0.5975416 |
| Pitx2 | 3.78355025 |
| Piwil1 | -0.5967956 |
| Piwil2 | -0.3476732 |
| Piwil4 | 1.11389524 |
| Pja1 | -0.5039833 |
| Pja2 | 1.13660244 |
| Pja2 | 2.01396347 |
| Pkd1 | -0.1833244 |
| Pkd1l3 | 2.46063697 |
| Pkd2 | -0.5545377 |
| Pkd2l1 | -0.6753895 |
| Pkd2l2 | -0.7931622 |
| Pkdrej | 0.08568221 |
| Pkhd1l1 | 1.51780053 |
| Pkhd1l1 | 1.38149348 |
| Pkia | 0.29499408 |
| Pkib | 2.35057468 |
| Pkib | -0.7622676 |
| Pkig | -0.5019644 |
| Pkig | -0.5299505 |
| Pkig | -0.4879548 |
| Pkm2 | 0.47289984 |
| Pkmyt1 | -0.21225 |
| Pkn1 | -0.5502755 |
| Pkn1 | 1.29300245 |
| Pkn2 | 1.5001849 |
| Pkn2 | 0.16620554 |
| Pkn2 | 1.4591853 |
| Pkn3 | 0.24497862 |
| Pknox1 | 2.92949812 |
| Pknox1 | 2.5413588 |
| Pknox2 | -0.0588407 |
| Pknox2 | 1.13828297 |
| Pknox2 | 1.03318713 |
| Pknox2 | -0.5182424 |
| Pknox2 | 1.32932317 |
| Pkp1 | 1.03657093 |
| Pkp2 | -0.6868366 |
| Pkp2 | -0.1933447 |
| Pkp2 | 1.32513496 |
| Pkp3 | -0.7358478 |
| Pla1a | -0.4154338 |
| Pla1a | -0.6967637 |
| Pla1a | -0.5915907 |
| Pla2g10 | -0.6497503 |
| Pla2g12a | -0.7472028 |
| Pla2g1b | -0.2698633 |
| Pla2g2c | -0.6672998 |
| Pla2g2d | 0.63156984 |
| Pla2g2f | -0.4166578 |
| Pla2g4c | -0.388912 |
| Pla2g4d | -0.4793885 |
| Pla2g4e | -0.6163914 |
| Pla2g4f | 2.07401649 |
| Pla2g5 | 1.97142503 |
| Pla2g6 | 0.6424114 |
| Pla2g7 | 0.28793324 |
| Plaa | 0.75269559 |
| Plac1 | -0.6240415 |
| Plac1l | -0.433968 |
| Plac8 | 0.2782422 |
| Plag1 | 0.72551117 |
| Plag1 | 3.08098978 |
| Plagl2 | -0.707641 |
| Plau | -0.060914 |
| Plb1 | 0.71348163 |
| Plcb2 | 1.78464596 |
| Plcb3 | 1.34606527 |
| Plcb4 | 0.24557274 |
| Plcd1 | -0.5667227 |
| Plcd3 | -0.6080496 |
| Plcd4 | -0.7803143 |
| Plcd4 | -0.2719522 |
| Plcd4 | -0.6356378 |
| Plce1 | 2.26502733 |
| Plcg2 | 0.77909556 |
| Plch2 | 0.27306979 |
| Plcl2 | 0.40562991 |
| Plcxd1 | 0.26902649 |
| Plcxd1 | -0.4105541 |
| Plcz1 | 3.04939685 |
| Pld1 | 1.29828024 |
| Pld2 | -0.6513318 |
| Pld3 | 0.34880546 |
| Pld3 | 0.49260629 |
| Pld4 | 0.60509382 |
| Pld5 | -0.4839912 |
| Pldn | -0.059255 |
| Plec1 | 0.58379869 |
| Plec1 | -0.1715368 |
| Plec1 | 0.12649126 |
| Plec1 | -0.0319048 |
| Plek | 2.52795312 |
| Plek2 | -0.5460332 |
| Plekha1 | 1.60267522 |
| Plekha1 | 0.55567013 |
| Plekha2 | 1.05035208 |
| Plekha3 | -0.4793885 |
| Plekha4 | 2.73163056 |
| Plekha4 | -0.6552978 |
| Plekha5 | -0.5334228 |
| Plekha6 | -0.5425128 |
| Plekha7 | -0.5306439 |
| Plekha8 | -0.8232245 |
| Plekhb1 | 0.51011977 |
| Plekhb2 | -0.0331121 |
| Plekhf1 | -0.0045674 |
| Plekhf1 | -0.2651806 |
| Plekhf2 | -0.6364161 |
| Plekhf2 | 2.21051248 |
| Plekhg2 | 1.05244449 |
| Plekhg2 | -0.1819018 |
| Plekhg2 | 1.44616668 |
| Plekhg3 | 2.0182945 |
| Plekhg3 | 0.4896545 |
| Plekhg4 | -0.0981944 |
| Plekhg5 | -0.5148272 |
| Plekhg6 | -0.5073583 |
| Plekhj1 | 2.41451581 |
| Plekhm1 | 0.19740691 |
| Plekhm3 | -0.5320323 |
| Plekho2 | 1.18837546 |
| Plin | -0.7977948 |
| Plk1 | 0.20581949 |
| Plk2 | -0.2029909 |
| Plk3 | 1.12053855 |
| Plk4 | 0.33301872 |
| Pllp | -0.4478024 |
| Plod1 | 2.59166055 |
| Plod2 | -0.1280799 |
| Plod3 | 0.03850993 |
| Plp1 | -0.6148691 |
| Plp2 | 1.18922283 |
| Plrg1 | -0.1543652 |
| Pls3 | -0.7885529 |
| Plscr1 | 1.69568502 |
| Plscr1 | 0.65026829 |
| Plscr3 | -0.7931622 |
| Plscr4 | 0.42609148 |
| Pltp | -0.487293 |
| Plunc | 0.78570165 |
| Plvap | -0.4926009 |
| Plxdc1 | 1.03769608 |
| Plxdc1 | 1.6875538 |
| Plxna2 | -0.7212085 |
| Plxnb1 | -0.5216705 |
| Plxnb3 | -0.6513318 |
| Plxnc1 | 0.67585741 |
| Plxnd1 | 0.29920458 |
| Pmaip1 | -0.4154338 |
| Pmch | -0.1125758 |
| Pmepa1 | -0.0088712 |
| Pmf1 | 2.27597082 |
| Pmfbp1 | -0.5033099 |
| Pml | 1.19995846 |
| Pml | -0.553826 |
| Pmm1 | -0.5134647 |
| Pmm1 | 0.36380265 |
| Pmm2 | -0.2295555 |
| Pmm2 | -0.1952658 |
| Pmm2 | 0.7477347 |
| Pmm2 | 1.18550981 |
| Pmp2 | 0.24942512 |
| Pmp22 | -0.5502755 |
| Pmp22 | -0.781226 |
| Pmpca | 1.98603221 |
| Pmpcb | 0.26931589 |
| Pms2 | -0.7254899 |
| Pmvk | -0.4879548 |
| Pnck | 2.09506606 |
| Pnkd | -0.6648867 |
| Pnkd | 2.0594914 |
| Pnkp | 0.26786798 |
| Pnkp | 3.38974973 |
| Pnliprp1 | -0.2973982 |
| Pnma1 | -0.5602516 |
| Pnma2 | 0.52380549 |
| Pno1 | 1.66850415 |
| Pnpla2 | 0.83159461 |
| Pnpla6 | 0.84202094 |
| Pnpla7 | -0.5923325 |
| Pnpla8 | 0.85110498 |
| Pnpla8 | -0.1169788 |
| Pnpo | 1.66296972 |
| Pnpo | 0.03253236 |
| Pnrc2 | 0.94921776 |
| Podn | -0.5559628 |
| Podxl | -0.5278735 |
| Podxl2 | -0.1771759 |
| Pof1b | 0.56199608 |
| Pofut1 | 0.26380157 |
| Pofut1 | -0.7127053 |
| Pofut2 | -0.632531 |
| Pogz | -0.7489623 |
| Pogz | 0.56136545 |
| Pola2 | -0.2211091 |
| Polb | 1.36905336 |
| Pold1 | -0.5798039 |
| Pold2 | -0.6984287 |
| Pold3 | 1.25261529 |
| Pold4 | -0.5609684 |
| Poldip2 | -0.5660015 |
| Poldip3 | -0.6917867 |
| Poldip3 | 0.35488658 |
| Pole | 0.12682944 |
| Pole3 | -0.3771061 |
| Pole3 | -0.7220632 |
| Pole4 | -0.2761443 |
| Polg | 1.60422363 |
| Polg2 | 2.24764713 |
| Polh | -0.7401985 |
| Poli | -0.4389745 |
| Polk | -0.709326 |
| Poll | 2.61187278 |
| Poll | 1.80814428 |
| Polm | 3.07806588 |
| Polm | -0.1752923 |
| Poln | 2.22116446 |
| Polq | -0.6697192 |
| Polr1e | 0.15282947 |
| Polr2d | 1.32495243 |
| Polr2d | -0.5033099 |
| Polr2d | -0.7839667 |
| Polr2d | -0.8080699 |
| Polr2e | -0.7280684 |
| Polr2e | -0.3087574 |
| Polr2f | 1.04379778 |
| Polr2g | -0.672145 |
| Polr2h | 0.54310004 |
| Polr2j | -0.7551474 |
| Polr3a | -0.3573793 |
| Polr3a | -0.7280684 |
| Polr3b | -0.5080347 |
| Polr3d | 2.11302304 |
| Polr3e | 3.1373481 |
| Polr3g | 0.31392838 |
| Polr3h | -0.4026797 |
| Polr3h | 0.2482415 |
| Polr3h | -0.8318606 |
| Polr3k | -0.7839667 |
| Polr3k | 1.54761997 |
| Polrmt | 0.83190757 |
| Pols | -0.2305544 |
| Pom121 | -0.7821387 |
| Pomc | -0.6403178 |
| Pomgnt1 | -0.2107818 |
| Pomgnt1 | 2.66032008 |
| Pomp | 1.91174858 |
| Pomt1 | 2.56052679 |
| Pomt1 | -0.6118319 |
| Pomt2 | -0.7604823 |
| Pon1 | 0.98999613 |
| Pon2 | 1.06611149 |
| Pon3 | 0.19521319 |
| Pon3 | -0.184274 |
| Pop4 | 1.05219858 |
| Pop5 | 0.2509025 |
| Pop5 | -0.5581047 |
| Pop7 | 0.5266493 |
| Popdc2 | -0.7640563 |
| Popdc2 | 1.68521726 |
| Popdc3 | -0.7341133 |
| Por | 0.30840003 |
| Por | -0.3347031 |
| Porcn | 0.34401798 |
| Porcn | -0.4670111 |
| Pou2af1 | 0.95525336 |
| Pou2f1 | -0.5761514 |
| Pou2f1 | -0.3735939 |
| Pou2f2 | -0.4952664 |
| Pou3f1 | -0.431475 |
| Pou3f3 | 0.00937771 |
| Pou5f1 | 1.48398951 |
| Pou5f1 | -0.037148 |
| Pou6f1 | 0.52205105 |
| Ppa1 | 1.42464912 |
| Ppa2 | 1.34177397 |
| Ppa2 | 2.04439944 |
| Ppa2 | -0.7560344 |
| Ppap2a | -0.6418832 |
| Ppap2a | 2.10126029 |
| Ppap2a | -0.8503645 |
| Ppap2b | -0.8464377 |
| Ppap2b | 1.44464638 |
| Ppap2c | -0.6163914 |
| Ppapdc1 | 1.35873175 |
| Ppapdc1a | 1.53239528 |
| Ppapdc2 | -0.6125903 |
| Ppapdc3 | 0.95210335 |
| Ppapdc3 | 2.70870721 |
| Pparbp | -0.7694436 |
| Pparg | -0.7959389 |
| Ppargc1a | 3.70884849 |
| Ppargc1b | 1.60550077 |
| Ppat | 0.16035718 |
| Ppcs | -0.1187459 |
| Ppef2 | -0.0772489 |
| Ppfia3 | 2.66169754 |
| Ppfia3 | -0.2181466 |
| Ppfibp1 | -0.7569222 |
| Ppfibp2 | 1.53928136 |
| Ppfibp2 | 1.65936697 |
| Ppia | 0.42901228 |
| Ppic | -0.1612879 |
| Ppic | -0.7959389 |
| Ppid | 0.31640528 |
| Ppie | -0.1258488 |
| Ppie | -0.433968 |
| Ppif | -0.8005857 |
| Ppil2 | -0.4105541 |
| Ppil3 | -0.1986375 |
| Ppl | 0.92141185 |
| Ppm1a | -0.7757691 |
| Ppm1a | 0.21323295 |
| Ppm1a | -0.2856476 |
| Ppm1b | 0.14191949 |
| Ppm1b | -0.5645606 |
| Ppm1b | 0.23301455 |
| Ppm1e | -0.7848821 |
| Ppm1e | -0.8108923 |
| Ppm1f | 2.15821396 |
| Ppm1f | -0.6926143 |
| Ppm1f | 4.18126154 |
| Ppm1g | 0.34295068 |
| Ppm1h | 0.64567732 |
| Ppm1j | -0.6364161 |
| Ppm1k | 0.9511425 |
| Ppm1l | -0.459921 |
| Ppm1l | -0.7676443 |
| Ppm1m | -0.4421176 |
| Ppm1m | 0.45086334 |
| Ppm2c | 0.56346586 |
| Ppme1 | -0.6648867 |
| Ppnr | 1.53660557 |
| Ppox | 0.58707584 |
| Ppox | -0.3296775 |
| Ppp1ca | -0.581269 |
| Ppp1ca | 0.4048817 |
| Ppp1ca | 0.17330313 |
| Ppp1cb | 0.11731289 |
| Ppp1cb | 0.02083848 |
| Ppp1cc | -0.6584829 |
| Ppp1r10 | 1.1761071 |
| Ppp1r10 | 0.68251289 |
| Ppp1r11 | 1.05869318 |
| Ppp1r12c | -0.2497426 |
| Ppp1r13b | 1.90567243 |
| Ppp1r13b | 0.02726975 |
| Ppp1r13b | 2.43996055 |
| Ppp1r14a | 1.18007229 |
| Ppp1r14a | -0.8523343 |
| Ppp1r14c | 1.8382179 |
| Ppp1r14d | 2.00145624 |
| Ppp1r15b | -0.285117 |
| Ppp1r16a | -0.7587005 |
| Ppp1r1a | -0.7160971 |
| Ppp1r1b | 0.96828536 |
| Ppp1r1b | 0.87574265 |
| Ppp1r2 | -1.0207263 |
| Ppp1r3c | -0.3420114 |
| Ppp1r3f | -0.3853542 |
| Ppp1r8 | -0.5739669 |
| Ppp1r9a | -0.3654507 |
| Ppp1r9b | 0.11765448 |
| Ppp2ca | -0.342576 |
| Ppp2cb | -0.587891 |
| Ppp2cz | -0.4142114 |
| Ppp2r1a | -0.3841713 |
| Ppp2r1b | -0.2528094 |
| Ppp2r2b | 0.66126894 |
| Ppp2r2b | 3.5443543 |
| Ppp2r2b | 1.03732118 |
| Ppp2r2c | -0.4389745 |
| Ppp2r2c | 1.02473585 |
| Ppp2r2d | 0.46988368 |
| Ppp2r3c | 0.2544385 |
| Ppp2r4 | -0.8813824 |
| Ppp2r5b | 1.39410531 |
| Ppp2r5c | -0.225571 |
| Ppp2r5c | 0.74015609 |
| Ppp2r5c | -0.4478024 |
| Ppp2r5d | 0.46241665 |
| Ppp2r5d | -0.8957427 |
| Ppp2r5d | -0.1677966 |
| Ppp2r5d | 0.98140012 |
| Ppp2r5e | -0.5725135 |
| Ppp2r5e | 0.44682072 |
| Ppp3ca | -0.4421176 |
| Ppp3ca | -0.8260942 |
| Ppp3ca | -0.2814117 |
| Ppp3cb | -1.0242887 |
| Ppp3cc | -0.5761514 |
| Ppp3r1 | -0.409946 |
| Ppp4c | -0.8099505 |
| Ppp4r1 | -1.0350598 |
| Ppp4r1 | 0.96084184 |
| Ppp4r1 | -0.5474451 |
| Ppp4r1l | 0.07142497 |
| Ppp5c | -0.5006208 |
| Ppp5c | -0.7358478 |
| Ppp6c | 0.39686245 |
| Pps | 0.46382137 |
| Ppt1 | 0.01244798 |
| Ppt2 | -0.6278906 |
| Pptc7 | -0.4221862 |
| Pqbp1 | 0.20581949 |
| Pqlc1 | -0.1121366 |
| Pqlc2 | -0.8523343 |
| Pqlc3 | 1.12565981 |
| Pqlc3 | 0.62296042 |
| Pqlc3 | 1.3993374 |
| Pqlc3 | -0.7186492 |
| Pramef12 | 0.98643528 |
| Prap1 | 1.1833813 |
| Prc1 | -0.8280123 |
| Prcp | -0.07556 |
| Prdm10 | 1.05942553 |
| Prdm4 | -0.5182424 |
| Prdm8 | -0.235565 |
| Prdm9 | -0.5012923 |
| Prdx1 | 1.35776361 |
| Prdx2 | -0.1312124 |
| Prdx2 | 2.29006483 |
| Prdx2 | -0.1589745 |
| Prdx3 | 0.19269962 |
| Prdx4 | -0.5893691 |
| Prdx5 | 0.41184133 |
| Prdx5 | -1.0042828 |
| Prdx6 | -0.6387551 |
| Prdx6-rs1 | -0.3924836 |
| Prdx6-rs1 | 1.21108659 |
| Preb | 0.21015131 |
| Preb | 2.03914571 |
| Prei3 | 0.19959539 |
| Prei4 | 0.97767219 |
| Prei4 | 0.05912916 |
| Prelp | 1.69489342 |
| Prep | -0.2861784 |
| Prep | -0.6442362 |
| Prf1 | -0.2073651 |
| Prickle1 | 0.1251373 |
| Prickle2 | -0.1205165 |
| Prickle3 | -0.2477037 |
| Prickle4 | 0.51256287 |
| Prim2 | -0.053472 |
| Prim2 | -0.2782477 |
| Prima1 | 2.04539674 |
| Prkaa2 | 1.08883961 |
| Prkaa2 | -0.2703851 |
| Prkab1 | 0.20426743 |
| Prkab2 | -0.106447 |
| Prkaca | 1.92468198 |
| Prkaca | -0.8562865 |
| Prkacb | 0.8303417 |
| Prkag1 | -0.4754617 |
| Prkag2 | -0.9061399 |
| Prkar1a | 0.08921207 |
| Prkar1b | -0.6884836 |
| Prkar2b | 0.29555661 |
| Prkca | 0.57782876 |
| Prkcb1 | -1.0031195 |
| Prkcbp1 | 1.18391389 |
| Prkcbp1 | -0.8251366 |
| Prkcc | -0.2250742 |
| Prkcd | 1.04466614 |
| Prkcdbp | 3.27937899 |
| Prkcdbp | -0.6118319 |
| Prkce | -0.3972676 |
| Prkch | -0.2584588 |
| Prkcq | -0.8357252 |
| Prkcsh | -0.13751 |
| Prkcz | 0.13892132 |
| Prkcz | -0.2300548 |
| Prkcz | -0.4670111 |
| Prkd1 | -0.7894729 |
| Prkd1 | -0.6057877 |
| Prkd2 | 0.26496525 |
| Prkd3 | -0.4228025 |
| Prkd3 | 3.5866792 |
| Prkd3 | 0.89798927 |
| Prkg1 | -0.7084831 |
| Prkg2 | -0.1047034 |
| Prkra | 0.1251373 |
| Prkrip1 | -0.7143996 |
| Prkrip1 | -0.3596782 |
| Prkrir | 1.45452307 |
| Prkx | 0.85507966 |
| Prkx | 1.12962329 |
| Prl | -0.248213 |
| Prlr | -0.6171536 |
| Prlr | -0.1285267 |
| Prm1 | 2.25262776 |
| Prm3 | 2.50788242 |
| Prmt2 | -0.6656904 |
| Prmt2 | -0.1406751 |
| Prmt3 | -0.1734126 |
| Prmt5 | -0.4402304 |
| Prmt5 | -0.251786 |
| Prmt5 | -0.358528 |
| Prmt7 | 0.43192379 |
| Prmt7 | 0.13992181 |
| Prmt7 | -0.6163914 |
| Prmt7 | 0.31089192 |
| Prmt8 | -0.1016601 |
| Prnp | 0.00552533 |
| Prnpip1 | -0.6426668 |
| Prnpip1 | -0.3153122 |
| Prnpip1 | -0.7306542 |
| Proc | 0.97593619 |
| Proc | 2.54401171 |
| Procr | 2.2307108 |
| Prodh | 0.81421482 |
| Prok2 | -0.6403178 |
| Prok2 | -0.6042829 |
| Prokr1 | 0.33004506 |
| Prokr2 | -0.0551206 |
| Prom1 | -0.6537093 |
| Prom2 | 0.52402456 |
| Prom2 | 2.09021476 |
| Pros1 | -0.4446398 |
| Prosapip1 | 0.4071245 |
| Prosapip1 | -0.9145435 |
| Prosapip1 | -0.5566762 |
| Prosc | -0.2240813 |
| Prosc | -0.2295555 |
| Prosc | -0.2662191 |
| Prox1 | -0.7034421 |
| Prox1 | 2.12421745 |
| Prpf18 | -0.4484362 |
| Prpf19 | -0.0436427 |
| Prpf19 | -0.7428188 |
| Prpf19 | 3.31822593 |
| Prpf3 | -0.6608791 |
| Prpf3 | 0.44419535 |
| Prpf3 | 2.39957929 |
| Prpf3 | 0.34214939 |
| Prpf3 | -0.6217397 |
| Prpf31 | 0.77210745 |
| Prpf38a | -0.8376636 |
| Prpf38a | -0.8682466 |
| Prpf38a | 0.06203719 |
| Prpf38b | 3.43210726 |
| Prpf39 | 0.96693649 |
| Prpf40a | -0.5292576 |
| Prpf40a | 1.7034429 |
| Prpf40b | -0.3158606 |
| Prpf40b | -0.7604823 |
| Prpf40b | -0.152528 |
| Prpf6 | 0.99472247 |
| Prpf8 | -0.587891 |
| Prpf8 | -0.3302345 |
| Prph2 | 0.16263599 |
| Prps1 | -0.6294348 |
| Prps1l1 | -0.8885344 |
| Prps2 | -0.27457 |
| Prpsap2 | -0.709326 |
| Prpsap2 | 1.28416973 |
| Prr11 | -0.8127788 |
| Prr13 | -0.6294348 |
| Prr14 | -0.225571 |
| Prr15 | 2.42543697 |
| Prr16 | -0.419725 |
| Prr18 | 0.52139227 |
| Prr19 | 1.35299599 |
| Prr3 | 1.26857708 |
| Prr7 | 0.21691728 |
| Prrc1 | 2.51866632 |
| Prrg2 | 0.11251724 |
| Prrt1 | -0.8213164 |
| Prrt1 | -0.5066823 |
| Prrt1 | -0.4490705 |
| Prrt3 | -0.6876597 |
| Prrx1 | 0.1452393 |
| Prrx1 | -0.1222905 |
| Prrx1 | -0.2798283 |
| Prss21 | -0.8061931 |
| Prss23 | 0.46194793 |
| Prss27 | -0.0002838 |
| Prss28 | -0.1285267 |
| Prss29 | 1.83042402 |
| Prss29 | 0.29836403 |
| Prss34 | -0.4966021 |
| Prss35 | 1.70029919 |
| Prss36 | -0.5488592 |
| Prss7 | -0.4427475 |
| Prss8 | 0.23030094 |
| Prss8 | 1.33989018 |
| Prtn3 | 1.07718721 |
| Prune | 0.64797569 |
| Prune | 0.21722363 |
| Prune | 1.92373371 |
| Prune | 2.35818922 |
| Psap | -0.7186492 |
| Psat1 | 1.07286918 |
| Psca | 0.62237045 |
| Pscd1 | -0.700932 |
| Pscd2 | 2.4493829 |
| Pscd3 | 0.54993914 |
| Pscdbp | -0.4258904 |
| Psd2 | 0.18891626 |
| Psd3 | 1.23898077 |
| Psd4 | 0.38699301 |
| Psen1 | -0.7410711 |
| Psen2 | 0.69966622 |
| Psenen | -0.3147642 |
| Psg19 | 1.67941163 |
| Psg23 | -0.6762024 |
| Psg27 | 0.46171348 |
| Psip1 | -0.7524915 |
| Psip1 | 2.85511222 |
| Pskh1 | 2.52289032 |
| Psma2 | 0.93632814 |
| Psma4 | 0.42340587 |
| Psma5 | 0.72796833 |
| Psma7 | 3.05278662 |
| Psma7 | -0.1116977 |
| Psmb1 | -0.5761514 |
| Psmb10 | 1.58457453 |
| Psmb10 | -0.5306439 |
| Psmb2 | -0.7775845 |
| Psmb2 | 0.01550798 |
| Psmb3 | -0.019125 |
| Psmb4 | 0.2544385 |
| Psmb5 | -0.3454043 |
| Psmb6 | -0.1236232 |
| Psmb7 | -0.563122 |
| Psmb7 | -0.7876338 |
| Psmb7 | -0.3806319 |
| Psmb8 | -0.0908732 |
| Psmb9 | 1.5717015 |
| Psmc1 | 0.92579938 |
| Psmc2 | -0.781226 |
| Psmc3 | -0.8337908 |
| Psmc3ip | -0.7237749 |
| Psmc4 | 1.41220526 |
| Psmc5 | -0.8213164 |
| Psmc5 | 1.193764 |
| Psmd1 | 0.26438359 |
| Psmd10 | -0.7143996 |
| Psmd10 | -0.3033307 |
| Psmd10 | -0.2406032 |
| Psmd11 | 0.15611 |
| Psmd11 | 1.6581629 |
| Psmd12 | 1.75899596 |
| Psmd12 | 1.62457863 |
| Psmd13 | 0.80152609 |
| Psmd14 | 2.00688693 |
| Psmd2 | 1.12395591 |
| Psmd2 | 0.34800929 |
| Psmd4 | -0.1366077 |
| Psmd5 | -0.6035314 |
| Psmd6 | 1.09601842 |
| Psmd6 | 0.62355001 |
| Psmd7 | -0.6926143 |
| Psmd7 | 1.64307341 |
| Psmd8 | 0.32597444 |
| Psmd9 | 1.31734507 |
| Psmd9 | 0.23572014 |
| Psme1 | -0.104268 |
| Psme1 | 0.51078672 |
| Psme2 | 1.12338723 |
| Psme3 | 0.69930459 |
| Psme4 | -1.018359 |
| Psme4 | -0.6786455 |
| Psmf1 | 0.77810052 |
| Psmf1 | -0.5299505 |
| Psmg2 | 2.47381084 |
| Psmg2 | 2.0785137 |
| Psmg3 | 2.19049635 |
| Psors1c2 | 2.13052085 |
| Psp | 0.77260837 |
| Pspc1 | -0.0883031 |
| Pstpip1 | 0.585029 |
| Pstpip2 | 0.87320384 |
| Ptbp1 | -0.8052561 |
| Ptbp1 | -0.0870208 |
| Ptcd3 | 2.71713675 |
| Ptch1 | 1.49716529 |
| Ptch2 | -0.8213164 |
| Ptdss2 | 0.78157844 |
| Pten | 3.34866982 |
| Pter | -0.700932 |
| Ptgds | 1.52134223 |
| Ptgds2 | 2.38241433 |
| Ptgds2 | 0.81165912 |
| Ptger2 | -0.6537093 |
| Ptger2 | -0.6656904 |
| Ptger3 | -0.2421201 |
| Ptges | -0.430231 |
| Ptges | 0.01014624 |
| Ptges2 | -0.2968608 |
| Ptges3 | 0.9147194 |
| Ptgfr | -0.1086311 |
| Ptgir | -0.7867157 |
| Ptgis | -0.6545032 |
| Ptgs1 | -0.9700114 |
| Ptgs1 | -0.9177149 |
| Pth2 | -0.3591029 |
| Pth2r | -0.724632 |
| Pthlh | 0.36040381 |
| Pthr1 | -1.0089508 |
| Pthr1 | -0.8357252 |
| Pthr1 | 0.68324941 |
| Ptk2 | 1.23132854 |
| Ptk2b | 2.51606293 |
| Ptk7 | -0.8156158 |
| Ptms | 0.40438255 |
| Ptn | -0.7341133 |
| Ptp4a2 | -0.1415814 |
| Ptp4a2 | 1.89040153 |
| Ptp4a2 | -0.8493812 |
| Ptp4a2 | -0.7051194 |
| Ptp4a3 | -0.4289887 |
| Ptp4a3 | -0.0895872 |
| Ptpdc1 | 1.15974427 |
| Ptpdc1 | 0.48143027 |
| Ptpdc1 | -0.0246942 |
| Ptpla | -0.6450219 |
| Ptpla | -0.0234979 |
| Ptplad1 | -0.2771954 |
| Ptplad2 | 0.36145096 |
| Ptplb | -0.4228025 |
| Ptpn1 | -0.7516079 |
| Ptpn11 | -0.4160456 |
| Ptpn11 | -0.1060108 |
| Ptpn11 | -0.3082133 |
| Ptpn12 | -0.4389745 |
| Ptpn12 | -0.4741565 |
| Ptpn12 | -0.4879548 |
| Ptpn12 | -0.6240415 |
| Ptpn12 | -0.0424214 |
| Ptpn14 | 1.13581713 |
| Ptpn18 | 2.4904564 |
| Ptpn20 | 1.83142157 |
| Ptpn21 | -0.4670111 |
| Ptpn22 | -1.0219122 |
| Ptpn5 | 2.00411044 |
| Ptpn6 | -0.9402277 |
| Ptpn6 | -0.2015375 |
| Ptpra | -0.184274 |
| Ptprb | 0.16361088 |
| Ptprd | 0.9995548 |
| Ptprd | -0.5588198 |
| Ptpre | -0.1249579 |
| Ptpre | -0.3493786 |
| Ptprf | 0.61049053 |
| Ptprf | -0.8936774 |
| Ptprg | 0.39987791 |
| Ptprj | -0.5930748 |
| Ptprk | 1.08231828 |
| Ptprk | 0.11765448 |
| Ptprn | 1.46413751 |
| Ptprn2 | -0.3568055 |
| Ptpro | 0.91928128 |
| Ptpro | -0.8533207 |
| Ptprq | 1.04640038 |
| Ptprr | 1.74283741 |
| Ptprr | 1.39706259 |
| Ptprs | 0.61704357 |
| Ptprs | -0.7766763 |
| Ptprt | -0.3296775 |
| Ptpru | 0.91671802 |
| Ptprv | -0.6110742 |
| Ptrf | -0.7445699 |
| Ptrh1 | 3.19353115 |
| Ptrh1 | -0.5953055 |
| Ptrh2 | 1.35237614 |
| Pts | -0.5746945 |
| Pts | -0.6851925 |
| Pttg1 | 0.68380141 |
| Pttg1ip | 1.95327981 |
| Ptx3 | 1.10151146 |
| Ptx3 | -0.2416142 |
| Puf60 | 1.62359132 |
| Puf60 | -0.8328252 |
| Pum1 | 0.14656386 |
| Pum2 | -0.4992793 |
| Pum2 | 0.15709187 |
| Punc | 1.95827261 |
| Purb | -0.7118593 |
| Purg | 1.29658713 |
| Pus1 | 0.2969614 |
| Pus1 | -0.8213164 |
| Pus10 | -0.0319048 |
| Pus10 | 0.69003424 |
| Pus10 | 0.67956087 |
| Pus3 | -0.7784935 |
| Pus7l | -0.0734533 |
| Pvalb | -0.5411084 |
| Pvr | 0.837989 |
| Pvr | -0.7323821 |
| Pvrl1 | 1.7561517 |
| Pvrl2 | 0.11970133 |
| Pvrl2 | 2.36988686 |
| Pvrl2 | 0.9932809 |
| Pvrl3 | -0.4522485 |
| Pvrl3 | 1.50948284 |
| Pvrl3 | -0.4979397 |
| Pwp1 | 2.41357203 |
| Pwp2 | 0.02802341 |
| Pwwp2b | -0.5355125 |
| Pxdn | 1.45507746 |
| Pxmp2 | 0.8523298 |
| Pxmp4 | -0.1790633 |
| Pxn | -0.6600797 |
| Pxt1 | 0.06312533 |
| Pycr2 | 1.73814288 |
| Pycrl | 2.31903549 |
| Pygb | -0.4683061 |
| Pygl | -0.235565 |
| Pygl | 0.0370192 |
| Pygm | -0.4142114 |
| Pygo1 | -0.6426668 |
| Pygo2 | 0.68490441 |
| Pyroxd1 | -0.6426668 |
| Pyy | -0.8165634 |
| Qars | 0.16814671 |
| Qdpr | 0.57451826 |
| Qpct | 0.4612444 |
| Qprt | -0.166398 |
| Qrich2 | 1.38799153 |
| Qrich2 | 1.62897565 |
| Qrsl1 | 1.20548745 |
| Qsox1 | -0.5842062 |
| Qtrt1 | 0.64701874 |
| R3hdm1 | 3.78066892 |
| R3hdm1 | -0.5886297 |
| R3hdm2 | 0.22484945 |
| R74862 | -0.2829979 |
| Rab1 | 0.11697118 |
| Rab10 | 1.22391761 |
| Rab10 | -0.8957427 |
| Rab11a | 0.19897064 |
| Rab11b | 0.81724047 |
| Rab11fip2 | 1.02296001 |
| Rab11fip3 | -0.5674446 |
| Rab11fip5 | 1.02118071 |
| Rab11fip5 | 1.40185836 |
| Rab11fip5 | 0.66934006 |
| Rab12 | 0.62825307 |
| Rab13 | 1.11848199 |
| Rab13 | -0.0327095 |
| Rab14 | -0.5886297 |
| Rab15 | 0.32570242 |
| Rab17 | 3.27320247 |
| Rab17 | -0.8080699 |
| Rab18 | 0.32896133 |
| Rab19 | -0.5495671 |
| Rab19 | 1.1842333 |
| Rab1b | 0.8193049 |
| Rab1b | -0.6529161 |
| Rab1b | 0.21967082 |
| Rab21 | 1.00436173 |
| Rab22a | 1.79562514 |
| Rab22a | -0.4846506 |
| Rab23 | 1.23203603 |
| Rab24 | -0.6497503 |
| Rab24 | -0.032307 |
| Rab24 | -0.1134547 |
| Rab25 | 1.52809242 |
| Rab27a | 2.56909573 |
| Rab27b | 1.52728795 |
| Rab28 | -0.4624928 |
| Rab28 | 1.14007215 |
| Rab2a | 2.10846013 |
| Rab31 | 2.35789412 |
| Rab32 | -0.1762336 |
| Rab33a | -0.6240415 |
| Rab33b | -0.1138945 |
| Rab34 | -0.1511525 |
| Rab34 | 2.89474492 |
| Rab34 | 0.21691728 |
| Rab35 | 0.20612959 |
| Rab36 | -0.7212085 |
| Rab38 | -0.0793643 |
| Rab38 | -0.4979397 |
| Rab39 | -0.656889 |
| Rab39b | -0.4289887 |
| Rab3a | -0.3666096 |
| Rab3b | -0.1626787 |
| Rab3d | -0.5369083 |
| Rab3gap1 | -0.7254899 |
| Rab3gap2 | -0.647383 |
| Rab3il1 | -0.5460332 |
| Rab3il1 | -0.1470383 |
| Rab3ip | -0.5652807 |
| Rab40b | -0.6240415 |
| Rab40c | -0.0073039 |
| Rab43 | -0.7867157 |
| Rab4a | 0.16879285 |
| Rab4b | 0.77975832 |
| Rab5a | 2.11174926 |
| Rab5a | -0.477423 |
| Rab5b | -0.6156299 |
| Rab5c | 0.03888224 |
| Rab6 | -0.6537093 |
| Rab6 | -0.025892 |
| Rab6 | -0.4446398 |
| Rab6b | -0.5175584 |
| Rab6ip1 | 0.57265082 |
| Rab7 | 0.30701271 |
| Rab7 | 2.15758961 |
| Rab7 | -0.6893083 |
| Rab7l1 | -0.355659 |
| Rab8a | -0.100359 |
| Rab8a | -0.4820158 |
| Rab8b | -0.225571 |
| Rab8b | 0.02273472 |
| Rab9 | -0.5516941 |
| Rab9 | 0.26205328 |
| Rab9b | 1.53812523 |
| Rabac1 | 0.65045908 |
| Rabac1 | 2.16056062 |
| Rabep1 | -0.5087117 |
| Rabep1 | -0.4389745 |
| Rabep2 | -0.3847626 |
| Rabgap1 | 0.40463216 |
| Rabgap1 | -0.285117 |
| Rabgap1l | 0.50722404 |
| Rabgap1l | 0.02083848 |
| Rabgef1 | -0.2054185 |
| Rabggta | 0.61446767 |
| Rabggtb | -0.6713357 |
| Rabif | 0.73790489 |
| Rabif | -0.4136009 |
| Rabl2a | 2.31276251 |
| Rabl2a | -0.590109 |
| Rabl3 | -0.5418104 |
| Rabl4 | -0.5953055 |
| Rabl4 | 0.45015123 |
| Rabl5 | 0.57782876 |
| Rac1 | 1.2008997 |
| Rac2 | -0.6753895 |
| Rac3 | 1.4413571 |
| Rac3 | 0.23481917 |
| Rad1 | -0.6095606 |
| Rad17 | 1.30362119 |
| Rad18 | -0.2290565 |
| Rad23a | -0.3806319 |
| Rad23a | -0.3865386 |
| Rad23a | -0.358528 |
| Rad23b | 0.15052608 |
| Rad50 | 1.80782126 |
| Rad51 | -0.4258904 |
| Rad51 | -0.6333067 |
| Rad51ap1 | 0.6765993 |
| Rad51c | 0.71739088 |
| Rad51l3 | 0.42438337 |
| Rad51l3 | 2.05777213 |
| Rad51l3 | -0.5502755 |
| Rad51l3 | 2.09918715 |
| Rad52 | -0.4277481 |
| Rad54l | 0.07858136 |
| Rad9 | -0.1438512 |
| Rad9 | -0.4333441 |
| Rad9 | -0.5114247 |
| Rad9b | -0.4787329 |
| Rae1 | -0.5141457 |
| Raet1b | -0.7143996 |
| Raet1b | 0.30311697 |
| Raf1 | -0.2068781 |
| Rag1 | 0.46405528 |
| Rag1ap1 | -0.7067997 |
| Rage | -0.6442362 |
| Rage | -0.6403178 |
| Rai1 | -0.1366077 |
| Rai12 | -0.0990596 |
| Rai12 | 0.55355173 |
| Rai12 | 0.04333812 |
| Rai12 | 2.68665891 |
| Rai14 | -0.2963237 |
| Rala | 2.04035758 |
| Ralb | 0.07429425 |
| Ralbp1 | -0.4624928 |
| Ralgds | 1.50626924 |
| Ralgps1 | 1.81179717 |
| Ralgps1 | 0.50856167 |
| Ralgps1 | -0.0844615 |
| Ralgps2 | 0.1429167 |
| Ralgps2 | -0.0717714 |
| Raly | 1.76296312 |
| Raly | -0.6868366 |
| Ralyl | 2.01301735 |
| Ramp1 | -0.1814281 |
| Ramp2 | -0.5609684 |
| Ramp3 | 1.81158269 |
| Ramp3 | 0.19395727 |
| Ran | -0.6465953 |
| Ranbp1 | 0.30423173 |
| Ranbp10 | 0.63487463 |
| Ranbp17 | -0.3960693 |
| Ranbp2 | -0.8127788 |
| Ranbp3l | 0.39937603 |
| Ranbp6 | 2.11321588 |
| Ranbp9 | 2.38212694 |
| Rangap1 | -0.6194438 |
| Rangrf | 1.19796821 |
| Rap1b | -0.6302079 |
| Rap1gap | 3.06832844 |
| Rap2a | -0.6497503 |
| Rap2b | -0.7428188 |
| Rap2c | 0.07465228 |
| Rap2c | -0.8090097 |
| Rap2c | 0.08391216 |
| Rap2ip | 1.67437919 |
| Rapgef1 | 0.23572014 |
| Rapgef1 | -0.5271822 |
| Rapgef1 | -0.1520692 |
| Rapgef1 | -0.5886297 |
| Rapgef3 | -0.6450219 |
| Rapgef4 | 0.68710643 |
| Rapgef5 | 2.27603538 |
| Rapgef6 | -0.5588198 |
| Rapgefl1 | 0.8082939 |
| Rapgefl1 | -0.553826 |
| Rapsn | -0.6810951 |
| Rapsn | -0.0742954 |
| Rara | 0.95961802 |
| Rarb | -0.7604823 |
| Rarb | 0.33947335 |
| Rarg | -0.6513318 |
| Rarg | -0.2677791 |
| Rarg | -0.4833322 |
| Rarres2 | 2.12581639 |
| Rars | -0.7721492 |
| Rars | -0.7507252 |
| Rars2 | -0.6403178 |
| Rars2 | 1.62943382 |
| Rars2 | -0.1598991 |
| Rasa1 | -0.5114247 |
| Rasa2 | -0.5390059 |
| Rasal1 | -0.253834 |
| Rasd1 | 1.4576865 |
| Rasef | -0.672955 |
| Rasgef1b | -0.2416142 |
| Rasgef1b | -0.584942 |
| Rasgef1c | -0.2761443 |
| Rasgef1c | -0.6697192 |
| Rasgrf1 | -0.6794613 |
| Rasgrf1 | 0.35092522 |
| Rasgrf1 | -0.7445699 |
| Rasgrf1 | -0.0900157 |
| Rasgrp1 | -0.4728532 |
| Rasgrp1 | -0.6513318 |
| Rasgrp1 | -0.3125753 |
| Rasgrp3 | 0.19427141 |
| Rasip1 | 0.76557034 |
| Rasl10a | -0.2610383 |
| Rasl10b | 1.25379994 |
| Rasl10b | -0.7160971 |
| Rasl11a | 0.39837143 |
| Rasl11b | 0.92636398 |
| Rasl11b | 1.71370536 |
| Rasl12 | 1.05905943 |
| Rasl2-9 | -0.5798039 |
| Rassf1 | 1.78762335 |
| Rassf1 | -0.5960502 |
| Rassf10 | -0.2651806 |
| Rassf2 | -0.3954707 |
| Rassf2 | -0.2461775 |
| Rassf3 | 2.27419371 |
| Rassf4 | 0.00204426 |
| Rassf4 | 0.34933586 |
| Rassf4 | -0.2714295 |
| Rassf4 | -0.5306439 |
| Rassf5 | 0.33786395 |
| Rassf7 | -0.6648867 |
| Rassf7 | 2.37169119 |
| Raver1 | 0.21997625 |
| Raver2 | 0.24586965 |
| Rb1 | 0.56304617 |
| Rb1 | 0.60167949 |
| Rbak | -0.2380807 |
| Rbak | -0.4427475 |
| Rbbp5 | -0.1334566 |
| Rbbp5 | -0.2147023 |
| Rbbp6 | -0.4892798 |
| Rbbp7 | -0.2920383 |
| Rbbp7 | 0.98020351 |
| Rbbp9 | -0.563841 |
| Rbck1 | -0.255373 |
| Rbed1 | -0.7560344 |
| Rbed1 | -0.3654507 |
| Rbj | -0.0436427 |
| Rbks | -0.2856476 |
| Rbl1 | 0.98603877 |
| Rbl2 | 0.3169548 |
| Rbm10 | 1.74272179 |
| Rbm10 | -0.7152479 |
| Rbm10 | 2.27283431 |
| Rbm11 | -0.8251366 |
| Rbm12 | 3.16389153 |
| Rbm12 | 2.20999223 |
| Rbm12 | 1.76222766 |
| Rbm12 | 0.01779628 |
| Rbm13 | -0.5609684 |
| Rbm13 | 1.46515607 |
| Rbm14 | -0.4308528 |
| Rbm14 | 0.52446254 |
| Rbm15 | 1.25468741 |
| Rbm16 | 1.07622941 |
| Rbm18 | -0.6286624 |
| Rbm18 | -0.1227345 |
| Rbm19 | 0.20053171 |
| Rbm24 | 1.22064997 |
| Rbm26 | -0.430231 |
| Rbm28 | 1.74503151 |
| Rbm28 | 0.69094726 |
| Rbm3 | -0.3471054 |
| Rbm35a | -0.5474451 |
| Rbm38 | 0.20177865 |
| Rbm38 | -0.3448379 |
| Rbm38 | -0.3136691 |
| Rbm4 | 0.91700318 |
| Rbm41 | 0.28651451 |
| Rbm42 | 0.01206476 |
| Rbm42 | 0.22788206 |
| Rbm43 | -0.4748089 |
| Rbm45 | -0.5516941 |
| Rbm47 | 0.38724741 |
| Rbm47 | -0.5820024 |
| Rbm47 | 1.13323208 |
| Rbm47 | 2.76463287 |
| Rbm4b | -0.7739574 |
| Rbm5 | 0.03850993 |
| Rbm5 | 2.08143306 |
| Rbm6 | 0.26380157 |
| Rbm6 | 2.29111318 |
| Rbm6 | 2.43381482 |
| Rbm8a | -0.451612 |
| Rbm9 | -0.3307919 |
| Rbm9 | -0.1406751 |
| Rbms1 | 0.52621236 |
| Rbms1 | -0.5202977 |
| Rbms2 | 1.96600556 |
| Rbms2 | -0.7516079 |
| Rbms3 | 0.90249029 |
| Rbmx | 1.29846817 |
| Rbmy1a1 | 1.08718404 |
| Rbp1 | -0.3224667 |
| Rbp7 | 2.72773803 |
| Rbpms | 2.3438858 |
| Rbpms | 0.97245427 |
| Rbpms | -0.6458083 |
| Rbpms2 | 2.32425583 |
| Rbx1 | -0.1909489 |
| Rbx1 | -0.5334228 |
| Rc3h1 | 1.26536795 |
| Rcan1 | 0.14887724 |
| Rcan2 | -0.4069114 |
| Rcan2 | 1.51964687 |
| Rcan2 | -0.7480822 |
| Rcan3 | -0.709326 |
| Rcbtb2 | -0.8005857 |
| Rcbtb2 | -0.6778304 |
| Rcc1 | 0.29976452 |
| Rccd1 | 0.81882891 |
| Rce1 | -0.1420349 |
| Rchy1 | 0.22757925 |
| Rcl1 | 1.75421254 |
| Rcl1 | -0.6552978 |
| Rcl1 | 0.64452597 |
| Rcn3 | -0.034321 |
| Rcor1 | 0.26117788 |
| Rcor2 | 0.238717 |
| Rcsd1 | 2.02068706 |
| Rdbp | -0.3835805 |
| Rdbp | 0.08249365 |
| Rdh11 | -0.5717877 |
| Rdh12 | 1.0272668 |
| Rdh14 | -0.3942747 |
| Rdh14 | -0.2305544 |
| Rdh5 | 0.03028066 |
| Rdh7 | 0.44991374 |
| Rdh9 | 2.0987171 |
| Rdm1 | -0.8357252 |
| Rec8 | 1.87472129 |
| Reck | 0.53426206 |
| Recql | 1.23445757 |
| Recql4 | -0.8033851 |
| Recql5 | 1.23203603 |
| Reep1 | 1.94909054 |
| Reep2 | -0.3984676 |
| Reep3 | 3.09244738 |
| Reep4 | -0.3918874 |
| Reep5 | -0.1682633 |
| Reep6 | 1.45555239 |
| Reep6 | 1.8260007 |
| Refbp2 | -0.55525 |
| Reg1 | -0.2579438 |
| Rela | -0.6505407 |
| Relb | 0.5310072 |
| Rell1 | 1.45357191 |
| Rell2 | 2.38925626 |
| Rell2 | 1.38440621 |
| Reln | 2.12987731 |
| Relt | 0.12208354 |
| Relt | 0.78603071 |
| Rem1 | 0.02349213 |
| Rem2 | 2.44732418 |
| Renbp | 1.23072168 |
| Repin1 | -0.7410711 |
| Repin1 | 0.03215746 |
| Reps1 | -0.487293 |
| Reps1 | -0.0159577 |
| Rer1 | 0.53317839 |
| Rer1 | 0.41307857 |
| Rerg | 0.31777845 |
| Resp18 | 1.47054337 |
| Rest | -0.3788673 |
| Ret | 1.97560315 |
| Retnla | -0.7315178 |
| Retnlg | -0.6909598 |
| Retsat | -0.6762024 |
| Retsat | -0.4952664 |
| Rev1 | 1.58051073 |
| Rev3l | 0.651603 |
| Rex3 | 2.51233092 |
| Rexo1 | -0.235565 |
| Rexo1 | -0.6713357 |
| Rexo2 | 3.20572872 |
| Rfc1 | 2.68595805 |
| Rfc2 | 1.97178519 |
| Rfc3 | -0.7110141 |
| Rfc4 | -0.0819094 |
| Rfc4 | -0.7084831 |
| Rfc5 | 0.62276381 |
| Rfesd | 1.45950052 |
| Rffl | 1.2231018 |
| Rffl | -0.5953055 |
| Rffl | 2.64239485 |
| Rffl | -0.7712464 |
| Rffl | -0.4605633 |
| Rfng | 3.40171113 |
| Rfpl4 | 2.35910344 |
| Rfpl4 | -0.4289887 |
| Rft1 | 0.18352959 |
| Rftn1 | 0.36328057 |
| Rftn2 | 1.28369243 |
| Rftn2 | 2.19724062 |
| Rfwd2 | -0.7229187 |
| Rfwd3 | 0.55545851 |
| Rfwd3 | 1.45499829 |
| Rfx1 | 1.40059875 |
| Rfx1 | 1.07443082 |
| Rfx2 | 1.4182423 |
| Rfx3 | -0.7419445 |
| Rfx4 | -0.4283682 |
| Rfx4 | 0.41184133 |
| Rfx4 | 0.10631424 |
| Rfxank | 2.53488729 |
| Rfxap | 2.74388748 |
| Rfxdc1 | 1.04367366 |
| Rg9mtd1 | -0.6851925 |
| Rg9mtd2 | 1.38926861 |
| Rgag4 | -0.0379573 |
| Rgl1 | 1.74779551 |
| Rgl2 | -0.4358422 |
| Rgl2 | -0.5230453 |
| Rgl3 | 1.469219 |
| Rgma | 1.01799476 |
| Rgmb | 2.69453753 |
| Rgmb | 2.41587425 |
| Rgnef | 2.08565562 |
| Rgs1 | -0.3812209 |
| Rgs1 | -0.3545139 |
| Rgs1 | -0.8722684 |
| Rgs10 | -0.581269 |
| Rgs12 | 2.79039858 |
| Rgs14 | 2.35375264 |
| Rgs16 | 0.48074157 |
| Rgs17 | 1.40252945 |
| Rgs2 | 1.65955694 |
| Rgs3 | 0.39686245 |
| Rgs3 | 1.04342537 |
| Rgs3 | -0.3414472 |
| Rgs3 | 1.24637063 |
| Rgs3 | -0.8978127 |
| Rgs3 | -0.8742859 |
| Rgs3 | -0.6271195 |
| Rgs4 | 3.61496205 |
| Rgs5 | -0.0844615 |
| Rgs6 | 0.04887763 |
| Rgs7 | 1.02384836 |
| Rgs7bp | 1.40796384 |
| Rgs8 | 0.00860853 |
| Rgs9 | 2.30143697 |
| Rgs9bp | -0.8988495 |
| Rhag | 3.23181814 |
| Rhbdd1 | 1.76944349 |
| Rhbdd3 | -0.5481519 |
| Rhbdf1 | 2.51103359 |
| Rhbdf2 | -0.2088279 |
| Rhbdl1 | -0.3213623 |
| Rhbdl2 | 2.36013452 |
| Rhbdl3 | -0.0399837 |
| Rhbdl7 | 0.57596808 |
| Rhcg | 1.74791049 |
| Rheb | 0.62335352 |
| Rhebl1 | -0.6186797 |
| Rhob | -0.3280084 |
| Rhobtb2 | 1.35890767 |
| Rhobtb2 | 1.01863284 |
| Rhobtb2 | -0.9071861 |
| Rhobtb2 | 1.57843745 |
| Rhobtb3 | 0.87797693 |
| Rhoc | -0.9019668 |
| Rhoc | 0.48395108 |
| Rhod | 3.47129758 |
| Rhof | 0.22241609 |
| Rhog | -0.1276332 |
| Rhoj | -0.9061399 |
| Rhot1 | 1.25675485 |
| Rhot2 | 1.75055118 |
| Rhot2 | 0.16003117 |
| Rhot2 | 0.01359668 |
| Rhou | -0.4020768 |
| Rhov | -0.2861784 |
| Rhox11 | -0.700932 |
| Rhox12 | 0.88803782 |
| Rhox3a | -0.4044909 |
| Rhox3f | 1.84066267 |
| Rhox4d | -0.5717877 |
| Rhox5 | -0.7931622 |
| Rhox8 | -0.8444807 |
| Rhpn1 | 0.32651824 |
| Rhpn1 | -0.0853138 |
| Rhpn1 | 0.48440866 |
| Ribc1 | -0.166398 |
| Ric3 | 1.65784577 |
| Ric3 | -0.355659 |
| Ric3 | 2.2541828 |
| Ric8b | -0.8052561 |
| Ric8b | 0.12750542 |
| Rif1 | 0.42974103 |
| Rilp | -0.7341133 |
| Rilpl1 | 0.05255209 |
| Rilpl1 | -0.5524041 |
| Rilpl2 | 0.47982249 |
| Rims1 | 0.37727312 |
| Rims2 | -0.4567163 |
| Rims3 | 2.25962113 |
| Rin1 | -0.3420114 |
| Rin2 | -0.5271822 |
| Rin2 | -0.7604823 |
| Rin2 | 0.94673712 |
| Rin3 | -0.6884836 |
| Riok1 | 0.17394564 |
| Riok1 | 1.50184144 |
| Riok3 | -0.3093018 |
| Riok3 | 0.01474394 |
| Ripk1 | -0.0999256 |
| Ripk2 | 0.20426743 |
| Ripk4 | 2.45566403 |
| Ripk5 | 1.08374509 |
| Rit1 | -0.197191 |
| Rit2 | -0.3954707 |
| Rlbp1 | -0.4258904 |
| Rlbp1l1 | 1.5694684 |
| Rlbp1l1 | -0.7067997 |
| Rln1 | 1.02334083 |
| Rltpr | 0.84757446 |
| Rmnd1 | 1.50522051 |
| Rmnd5b | -0.5258011 |
| Rmnd5b | 0.58666684 |
| Rnase1 | 1.24954785 |
| Rnase4 | -0.6179163 |
| Rnase4 | 1.3739113 |
| Rnase6 | -0.5768808 |
| Rnaseh1 | 0.2805317 |
| Rnaseh1 | -0.3841713 |
| Rnaseh2c | 0.39004112 |
| Rnasek | -0.4209547 |
| Rnasen | 1.02143511 |
| Rnaset2 | 0.04296763 |
| Rnaset2b | -0.5141457 |
| Rnd2 | 0.3370582 |
| Rnd3 | 1.45626431 |
| Rnf10 | -0.0230995 |
| Rnf103 | -0.7384557 |
| Rnf11 | -0.7885529 |
| Rnf11 | -0.261555 |
| Rnf11 | -0.5495671 |
| Rnf111 | -0.5418104 |
| Rnf113a1 | 1.62055691 |
| Rnf113a2 | 3.69794266 |
| Rnf12 | -0.5754227 |
| Rnf121 | -0.5467389 |
| Rnf123 | -0.8090097 |
| Rnf125 | -0.0938807 |
| Rnf125 | -0.5725135 |
| Rnf128 | -0.4722022 |
| Rnf133 | 2.89013738 |
| Rnf135 | 2.43779936 |
| Rnf135 | 0.38571996 |
| Rnf135 | -0.6035314 |
| Rnf138 | 1.7896029 |
| Rnf138 | 0.02159745 |
| Rnf138 | -0.4866317 |
| Rnf138 | 1.45547325 |
| Rnf139 | 0.20891574 |
| Rnf139 | -0.4644265 |
| Rnf14 | -0.5754227 |
| Rnf14 | -0.6975958 |
| Rnf14 | -0.1996031 |
| Rnf141 | 4.06233606 |
| Rnf141 | 1.8636438 |
| Rnf144a | -0.2688206 |
| Rnf145 | -0.724632 |
| Rnf150 | -0.668912 |
| Rnf166 | 0.0962312 |
| Rnf167 | -0.518927 |
| Rnf167 | -0.3403197 |
| Rnf167 | -0.297936 |
| Rnf167 | -0.7280684 |
| Rnf167 | 0.22150192 |
| Rnf168 | -0.1771759 |
| Rnf17 | 0.66990051 |
| Rnf170 | -0.6868366 |
| Rnf181 | 2.77880894 |
| Rnf182 | 0.68765611 |
| Rnf182 | 1.52465017 |
| Rnf183 | -0.563841 |
| Rnf185 | 0.42340587 |
| Rnf185 | -0.1289738 |
| Rnf185 | 1.83325632 |
| Rnf185 | 0.22058682 |
| Rnf186 | 2.52451543 |
| Rnf19a | -0.8366939 |
| Rnf2 | 0.94894246 |
| Rnf20 | 1.05869318 |
| Rnf207 | 1.41807743 |
| Rnf208 | 3.28639179 |
| Rnf214 | 0.42046709 |
| Rnf215 | -0.301169 |
| Rnf216 | 2.21776518 |
| Rnf216 | -0.4327206 |
| Rnf219 | 0.0543838 |
| Rnf24 | -0.7748628 |
| Rnf24 | 0.59339212 |
| Rnf25 | -0.5355125 |
| Rnf25 | -0.5509845 |
| Rnf25 | 0.16879285 |
| Rnf26 | -0.8357252 |
| Rnf31 | 1.57239822 |
| Rnf32 | 1.26780014 |
| Rnf34 | -0.237577 |
| Rnf34 | -0.1249579 |
| Rnf34 | 3.76831944 |
| Rnf38 | 1.73026473 |
| Rnf38 | 2.24143326 |
| Rnf38 | 3.37376793 |
| Rnf4 | -0.7676443 |
| Rnf4 | -0.2132302 |
| Rnf40 | -0.9208973 |
| Rnf41 | -0.3942747 |
| Rnf41 | -0.4414881 |
| Rnf41 | -0.3579535 |
| Rnf44 | -0.8280123 |
| Rnf5 | -0.5341188 |
| Rnf6 | -0.3990681 |
| Rnf6 | -0.6843715 |
| Rnf8 | -0.3307919 |
| Rnf8 | -0.7604823 |
| Rngtt | 0.01627138 |
| Rnmt | 1.4251401 |
| Rnmtl1 | 0.54245624 |
| Rnpc2 | -0.0124076 |
| Rnpep | -0.7604823 |
| Rnpepl1 | 0.56535212 |
| Rnps1 | -0.3054975 |
| Rnps1 | 0.35515006 |
| Rnu6 | -0.6835513 |
| Rnuxa | 0.19049456 |
| Rnuxa | -0.581269 |
| Robo1 | -0.7775845 |
| Robo2 | -0.5717877 |
| Robo4 | -0.1174202 |
| Rock1 | -0.3637151 |
| Rock2 | 0.78372483 |
| Rock2 | -0.019125 |
| Rod1 | 0.60949354 |
| Rogdi | -0.0171441 |
| Rom1 | 0.65994827 |
| Ropn1 | 0.29920458 |
| Ror1 | -0.495934 |
| Ror2 | 1.6228007 |
| Rora | 1.54783461 |
| Rorb | -0.8415528 |
| Rorb | -0.581269 |
| Rorc | -0.6232736 |
| Rorc | 2.85790191 |
| Ros1 | -0.13751 |
| Rp23-297j14.5 | 2.01932065 |
| Rp23-438h3.2 | 0.73582193 |
| rp9 | -0.1289738 |
| rp9 | 0.85690832 |
| Rpa1 | -0.3818102 |
| Rpa2 | -0.4002704 |
| Rpa2 | -0.6426668 |
| Rpa3 | 0.74481404 |
| Rpain | 0.97232008 |
| Rpap1 | -0.6876597 |
| Rpap1 | 1.00384328 |
| Rpap2 | -0.8464377 |
| Rpap2 | 0.54138224 |
| Rpap3 | 1.42268256 |
| Rpe | -0.409946 |
| Rpgr | -0.4333441 |
| Rpgrip1 | 1.29762218 |
| Rpgrip1l | 3.4835483 |
| Rph3a | -0.1790633 |
| Rph3al | 1.12962329 |
| Rpia | 0.2947127 |
| Rpl12 | -0.2756193 |
| Rpl13a | -0.4979397 |
| Rpl18 | 0.82940089 |
| Rpl19 | 0.87305428 |
| Rpl21 | -0.6489605 |
| Rpl21 | 1.0921418 |
| Rpl22 | 0.51123108 |
| Rpl22l1 | -0.6333067 |
| Rpl23 | 0.82373561 |
| Rpl23 | 0.46499032 |
| Rpl23 | 0.11594527 |
| Rpl24 | 0.64337317 |
| Rpl27 | -0.6027805 |
| Rpl27a | -0.6917867 |
| Rpl28 | -0.3648718 |
| Rpl29 | -0.5820024 |
| Rpl29 | -0.1585125 |
| Rpl3 | -0.8194122 |
| Rpl30 | 1.07022032 |
| Rpl31 | -0.632531 |
| Rpl31 | -0.4663642 |
| Rpl34 | 2.71763488 |
| Rpl35 | -0.4722022 |
| Rpl36a | -0.3164093 |
| Rpl36al | -0.3136691 |
| Rpl37 | -0.668912 |
| Rpl38 | -0.012014 |
| Rpl38 | 0.16458474 |
| Rpl39 | 0.21844804 |
| Rpl39l | 2.06030897 |
| Rpl3l | 1.84398124 |
| Rpl4 | -0.6505407 |
| Rpl41 | 0.75405939 |
| Rpl7a | -0.4702521 |
| Rpl7l1 | 0.58071566 |
| Rpl7l1 | 1.32995882 |
| Rpl9 | -0.5390059 |
| Rplp1 | -0.9230251 |
| Rpn2 | 0.46100977 |
| Rpo1-1 | 0.19865811 |
| Rpo1-3 | 0.29386801 |
| Rpo1-3 | 1.8534044 |
| Rpo1-4 | 2.62682122 |
| Rpo1-4 | 0.3343672 |
| Rpo1-4 | -0.4886171 |
| Rpo2tc1 | 2.00749317 |
| Rpp21 | -0.1687302 |
| Rpp25 | -0.183799 |
| Rpp30 | -0.5148272 |
| Rpp38 | 2.04920969 |
| Rpp40 | -0.1696648 |
| Rprm | 3.05834899 |
| Rprml | -0.7472028 |
| Rps10 | -0.9082336 |
| Rps11 | 1.10221033 |
| Rps12 | 0.386484 |
| Rps13 | 0.13624802 |
| Rps14 | 1.70072301 |
| Rps15 | -0.5645606 |
| Rps15a | -0.8773205 |
| Rps16 | -0.7315178 |
| Rps19 | -0.4839912 |
| Rps19 | -0.1771759 |
| Rps19bp1 | -0.8213164 |
| Rps2 | 1.19723387 |
| Rps21 | 0.28736601 |
| Rps24 | 3.64330979 |
| Rps24 | 0.38393471 |
| Rps25 | 0.6389406 |
| Rps26 | -0.606541 |
| Rps27a | 0.47613693 |
| Rps27a | 0.73998312 |
| Rps27l | 0.20053171 |
| Rps3 | 0.48257695 |
| Rps3 | 2.18420172 |
| Rps3a | -0.6209738 |
| Rps4x | -0.6497503 |
| Rps4y2 | 0.16393562 |
| Rps5 | 0.10700554 |
| Rps6 | 0.76624297 |
| Rps6ka1 | 1.63054557 |
| Rps6ka3 | 3.30159542 |
| Rps6ka4 | -0.6967637 |
| Rps6ka5 | -0.034321 |
| Rps6kb1 | 2.20745608 |
| Rps6kl1 | -0.2166689 |
| Rps7 | 1.29167824 |
| Rps8 | 0.83049841 |
| Rps9 | 0.03028066 |
| Rps9 | -0.3806319 |
| Rpsa | -0.9061399 |
| Rpusd1 | 0.85263574 |
| Rpusd4 | 0.50275109 |
| Rqcd1 | -0.6584829 |
| Rrad | 2.2793861 |
| Rragb | 3.02231671 |
| Rragc | -0.862247 |
| Rragd | -0.6851925 |
| Rras | -0.476115 |
| Rras2 | -0.4939326 |
| Rrbp1 | -0.1452157 |
| Rrbp1 | 0.14125408 |
| Rreb1 | -0.4215702 |
| Rreb1 | -0.5566762 |
| Rrh | 0.71739088 |
| Rrm1 | -0.3625599 |
| Rrm2 | 1.90446203 |
| Rrm2 | -0.7084831 |
| Rrm2b | 0.84987852 |
| Rrn3 | 1.5552448 |
| Rrp1 | -0.6027805 |
| Rrp12 | -0.1566669 |
| Rrp15 | 1.01107502 |
| Rrp1b | -0.2600056 |
| Rrp1b | -0.0179359 |
| Rrp1b | 1.19386934 |
| Rrp9 | -0.451612 |
| Rs1 | -0.563122 |
| Rsad1 | -0.6851925 |
| Rsad2 | 1.14152328 |
| Rsf1 | 2.49618362 |
| Rshl1 | -0.5383062 |
| Rshl2a | 0.10076493 |
| Rsl1d1 | 0.15545483 |
| Rsl1d1 | 0.10354379 |
| Rsn | 0.26117788 |
| Rsph1 | 0.26263642 |
| Rspo1 | -0.1236232 |
| Rspo2 | -0.6827319 |
| Rspo3 | 1.61319304 |
| Rspo4 | -0.2275611 |
| Rspry1 | -0.5107457 |
| Rsrc1 | 1.51669092 |
| Rsrc1 | -0.3006294 |
| Rsrc2 | 3.54838037 |
| Rsu1 | -0.6309816 |
| Rtbdn | -0.355659 |
| Rtel1 | -0.3044134 |
| Rtel1 | -0.7110141 |
| Rtkn | 0.71703618 |
| Rtn1 | 0.52686769 |
| Rtn1 | -0.4358422 |
| Rtn1 | 0.13222335 |
| Rtn1 | 2.1678917 |
| Rtn2 | 0.55291525 |
| Rtn3 | 0.06493601 |
| Rtn4 | -0.8572772 |
| Rtn4 | -0.9240908 |
| Rtn4 | -0.2724751 |
| Rtn4ip1 | 0.17715146 |
| Rtn4r | 0.51720893 |
| Rtn4rl1 | -0.5209838 |
| Rtn4rl2 | 3.02435617 |
| Rtp1 | -0.5710624 |
| Rtp4 | -0.6917867 |
| Rttn | -0.5768808 |
| Rufy1 | 0.67530059 |
| Rufy2 | 1.3053946 |
| Rufy3 | 2.163449 |
| Rufy3 | 0.9523777 |
| Rufy3 | 0.41727272 |
| Rufy4 | -0.5908496 |
| Rundc1 | -0.6753895 |
| Rundc3b | -0.5107457 |
| Runx1 | -0.447169 |
| Runx1t1 | 0.36796857 |
| Runx1t1 | 0.01359668 |
| Runx2 | 2.56834563 |
| Rusc1 | 0.78372483 |
| Rusc2 | -0.6088048 |
| Rusc2 | -0.5502755 |
| Ruvbl1 | 0.32461353 |
| Ruvbl1 | -0.5237335 |
| Ruvbl1 | 1.65358565 |
| Ruvbl2 | 1.35864378 |
| Rwdd1 | -0.5746945 |
| Rwdd1 | 2.3871433 |
| Rwdd3 | 0.07679751 |
| Rwdd3 | 1.53355867 |
| Rwdd4a | -0.6616792 |
| Rxfp1 | 1.32294217 |
| Rxfp3 | 0.12750542 |
| Rxra | 0.09588152 |
| Rxrb | -0.1433968 |
| Rxrb | 0.00397982 |
| Rxrg | -0.713552 |
| Rya3 | 0.41480791 |
| Ryk | -0.1952658 |
| Ryk | -0.6513318 |
| Ryr1 | -0.0999256 |
| Ryr1 | 0.94646108 |
| Ryr1 | 0.98999613 |
| Ryr3 | -0.764952 |
| S100a1 | 0.12242335 |
| S100a10 | -0.7507252 |
| S100a11 | 0.04333812 |
| S100a13 | 0.3389372 |
| S100a16 | -0.4490705 |
| S100a4 | -0.672145 |
| S100a6 | 0.52511912 |
| S100a8 | -0.4166578 |
| S100a9 | 0.20519898 |
| S100b | 1.44392536 |
| S100pbp | 0.35171886 |
| S100pbp | -0.4203397 |
| S3-12 | 2.02405167 |
| Saa3 | -0.5006208 |
| Sac3d1 | -0.115215 |
| Sacm1l | -0.2250742 |
| Sacs | -0.764952 |
| Sae1 | 0.39535099 |
| Sae1 | 0.46988368 |
| Sae1 | -0.5652807 |
| Sag | 0.19865811 |
| Sall1 | -0.848399 |
| Sall1 | -0.3747631 |
| Sall2 | -0.7489623 |
| Sall4 | 0.60308693 |
| Sall4 | 2.1425508 |
| Samd10 | -0.5908496 |
| Samd11 | 0.30283807 |
| Samd12 | -0.7237749 |
| Samd14 | 0.63623196 |
| Samd4 | -0.4421176 |
| Samd4 | 3.32403495 |
| Samd4b | 0.55227832 |
| Samd5 | -0.6909598 |
| Samd7 | -0.7757691 |
| Samd8 | 0.11765448 |
| Samd9l | -0.9155994 |
| Samhd1 | 0.21538394 |
| Samm50 | 1.27254855 |
| Samsn1 | -0.5446234 |
| Sap130 | -0.6810951 |
| Sap30 | 1.47357439 |
| Sap30bp | 2.76799488 |
| Sap30l | -0.672145 |
| Saps1 | -0.0033972 |
| Saps2 | 1.87901538 |
| Saps3 | -0.3706773 |
| Saps3 | 1.42284661 |
| Sar1a | -0.743694 |
| Sar1b | 0.53685763 |
| Sar1b | -0.5093892 |
| Sarm1 | -0.5990353 |
| Sars | 0.39383703 |
| Sars | 2.40514603 |
| Sars2 | -0.677016 |
| Sart1 | 1.23657118 |
| Sart3 | -0.5588198 |
| Sas | 0.92466913 |
| Sass6 | -0.5502755 |
| Sat2 | -0.6232736 |
| Satb1 | 1.35978674 |
| Sav1 | 3.27592268 |
| Sbds | 1.0954321 |
| Sbf1 | -0.6893083 |
| Sbk | 2.8365678 |
| Sbno1 | 0.03328169 |
| Sbno2 | 0.40363331 |
| Sc4mol | -0.3516574 |
| Sc5d | -0.7516079 |
| Scaf1 | -0.7516079 |
| Scamp1 | 1.95822692 |
| Scamp2 | -0.6278906 |
| Scamp3 | 1.74647214 |
| Scamp4 | -0.3782799 |
| Scamp5 | 2.28195526 |
| Scamp5 | 3.92927851 |
| Scand1 | 0.72445608 |
| Scap | 0.42218253 |
| Scaper | 0.2363203 |
| Scara3 | -0.3800433 |
| Scara5 | 2.06161556 |
| Scarb1 | 1.55439284 |
| Scarb2 | -0.545328 |
| Scarf1 | -0.2808836 |
| Scarf2 | 0.31503006 |
| Sccpdh | -0.7578109 |
| Scd1 | -0.0621602 |
| Scd2 | 0.32270485 |
| Scd4 | -0.5761514 |
| Scel | -0.6713357 |
| Scg2 | 0.02121804 |
| Scg3 | -0.2176537 |
| Scg5 | -0.6835513 |
| Scgn | 0.61088902 |
| Scgn | -0.8033851 |
| Scgn | 1.42079397 |
| Schip1 | 1.61797635 |
| Schip1 | 0.8303417 |
| Scin | -0.3550863 |
| scl0003251.1_21 | 1.48368261 |
| Sclt1 | -0.5066823 |
| Scly | -0.6600797 |
| Scmh1 | -0.7358478 |
| Scmh1 | -0.1465823 |
| Scml2 | 1.29432476 |
| Scml4 | -0.5155092 |
| Scn10a | 1.30277995 |
| Scn11a | -0.6110742 |
| Scn1a | 0.23060285 |
| Scn2b | 1.08445766 |
| Scn3b | -0.4541606 |
| Scn3b | 1.22146798 |
| Scn3b | -0.5761514 |
| Scn3b | -0.7315178 |
| Scn4b | -0.6975958 |
| Scn5a | 0.65369646 |
| Scn7a | -0.6545032 |
| Scn8a | 0.07178413 |
| Scnm1 | 0.60328782 |
| Scnm1 | 0.81197898 |
| Scnn1a | -0.8043201 |
| Scnn1b | 0.69803773 |
| Scnn1g | -0.3369456 |
| Scoc | 2.64276244 |
| Scoc | 1.27928953 |
| Scotin | -0.3695132 |
| Scotin | -0.7152479 |
| Scotin | -0.3408833 |
| Scp2 | 0.3690071 |
| Scp2 | 1.24656953 |
| Scp2 | -0.8376636 |
| Scrg1 | -0.5425128 |
| Scrib | 1.95804417 |
| Scrib | 1.2651731 |
| Scrn1 | -0.096035 |
| Scrn1 | -0.1819018 |
| Scrn1 | -0.2698633 |
| Scrn2 | -0.7428188 |
| Sct | -0.4780777 |
| Sctr | 0.84526459 |
| Scube1 | 1.42750946 |
| Scube2 | 1.06489949 |
| Scube3 | 0.33571368 |
| Scube3 | -0.709326 |
| Scx | 0.33058645 |
| Scye1 | -0.3871314 |
| Scyl1 | 1.28645732 |
| Scyl1bp1 | 0.94715103 |
| Scyl3 | 3.31233818 |
| Scyl3 | -0.5710624 |
| Sdad1 | -0.3741783 |
| Sdc1 | 1.53580856 |
| Sdc1 | 0.00552533 |
| Sdc2 | 0.21691728 |
| Sdc3 | -0.5033099 |
| Sdc4 | 4.46643639 |
| Sdcbp | 1.93392339 |
| Sdcbp | 3.42338969 |
| Sdcbp2 | -0.1771759 |
| Sdcbp2 | 0.26641776 |
| Sdccag1 | 0.39686245 |
| Sdccag1 | -0.6992624 |
| Sdccag3 | -0.5397062 |
| Sdccag3 | 0.5990599 |
| Sdf2 | -0.6552978 |
| Sdf2 | -0.8015179 |
| Sdf4 | -0.4573563 |
| Sdhb | -0.2039612 |
| Sdhb | 2.65675025 |
| Sdhb | -0.5674446 |
| Sdhc | -0.5418104 |
| Sdhd | 0.70291453 |
| Sdk1 | 0.67845141 |
| Sdpr | -0.5990353 |
| Sds | -0.5467389 |
| Sdsl | -0.6697192 |
| Sec1 | -0.7676443 |
| Sec11a | -0.6917867 |
| Sec11c | 1.08966628 |
| Sec13 | 2.48615658 |
| Sec14l1 | 0.76034066 |
| Sec14l1 | 1.13604157 |
| Sec14l1 | 0.03925439 |
| Sec14l2 | 2.70228811 |
| Sec14l3 | 0.18796798 |
| Sec14l3 | -0.7160971 |
| Sec14l4 | 0.16263599 |
| Sec15l1 | 0.16296107 |
| Sec16a | 0.0062971 |
| Sec16b | -0.2166689 |
| Sec22a | -0.4075175 |
| Sec22b | -0.5588198 |
| Sec22c | 1.64003835 |
| Sec22c | -0.6778304 |
| Sec23a | 2.43960971 |
| Sec23ip | 0.11799594 |
| Sec24b | 0.04370847 |
| Sec24c | 0.12649126 |
| Sec24d | 2.91259241 |
| Sec61a1 | -0.7042804 |
| Sec61a2 | -0.4787329 |
| Sec61a2 | 1.16909934 |
| Sec61b | 0.65274548 |
| Sec61b | 2.81183557 |
| Sec61g | -0.096035 |
| Sec63 | 0.59237631 |
| Sec63 | -0.4547988 |
| Secisbp2 | -0.7026046 |
| Sectm1b | -0.5602516 |
| Seh1l | -0.1316608 |
| Seh1l | 1.3553829 |
| Sel1l | -0.5251114 |
| Sel1l | -0.4277481 |
| Sel1l | 0.44491211 |
| Sel1l2 | 1.24368124 |
| Sel1l2 | 1.51290819 |
| Selenbp2 | 0.4896545 |
| Selk | 2.29187487 |
| Sell | 3.30652744 |
| Selp | -0.3818102 |
| Selplg | -0.7839667 |
| Sema3a | -0.5006208 |
| Sema3b | 0.11594527 |
| Sema3b | 0.24497862 |
| Sema3b | 2.29873311 |
| Sema3e | -0.6545032 |
| Sema3f | 1.18932869 |
| Sema3f | 0.4841799 |
| Sema3g | -0.6133492 |
| Sema4a | -0.250253 |
| Sema4b | -0.607295 |
| Sema4d | 1.1861474 |
| Sema4f | -0.7067997 |
| Sema4g | 2.08175242 |
| Sema5a | -0.6608791 |
| Sema5a | -0.4879548 |
| Sema5b | 2.80109803 |
| Sema6b | -0.7524915 |
| Sema6b | 1.08552547 |
| Sema6b | -0.1757628 |
| Sema6d | 0.41776489 |
| Sema6d | -0.6225063 |
| Sema7a | 0.87782815 |
| Sema7a | -0.5790722 |
| Senp1 | -0.6458083 |
| Senp2 | -0.3800433 |
| Senp3 | 0.07965001 |
| Senp5 | -0.3006294 |
| Senp5 | -0.6163914 |
| Senp5 | 0.52817692 |
| Senp6 | -0.2330563 |
| Senp6 | 0.7878384 |
| Senp7 | 0.84248503 |
| Senp7 | -0.5696135 |
| Senp7 | 1.14907655 |
| Senp8 | 2.4517567 |
| Sephs1 | -0.7867157 |
| Sephs2 | 0.61088902 |
| Sepm | -0.7059591 |
| Sepn1 | 0.42876924 |
| Sepp1 | -0.0679985 |
| Sepp1 | 0.23150798 |
| Sept3 | -0.2594897 |
| Sept4 | 3.15124327 |
| Sept4 | -0.3654507 |
| Sept6 | -0.1267406 |
| Sept7 | 1.36346965 |
| Sept7 | -0.6271195 |
| Sept8 | 0.39661072 |
| Sept9 | -0.8203638 |
| Sept10 | 0.15839939 |
| Sept11 | 0.74412547 |
| Sept11 | -0.5376069 |
| Sept13 | -0.2370736 |
| Sept16 | 1.27976914 |
| Sepsecs | -0.7463243 |
| Sepw1 | -0.4389745 |
| Sepx1 | -0.5495671 |
| Sepx1 | 0.51367122 |
| Serac1 | -0.0238965 |
| Serbp1 | 0.15315805 |
| Serbp1 | 1.30874535 |
| Serbp1 | -0.0045674 |
| Serf1 | -0.8692504 |
| Serf2 | 1.15139663 |
| Sergef | -0.2421201 |
| Serhl | -0.5006208 |
| Serhl | 0.81117914 |
| Serinc1 | 1.27563631 |
| Serinc2 | 1.66956946 |
| Serinc3 | -0.4370939 |
| Serpina1a | 2.01867944 |
| Serpina1a | 0.21107689 |
| Serpina1b | -0.2431328 |
| Serpina1b | -0.1967093 |
| Serpina1d | 0.91285962 |
| Serpina1e | -0.0274915 |
| Serpina3b | -0.5439193 |
| Serpina3c | 1.10731914 |
| Serpina3g | 0.17554995 |
| Serpina3h | 1.94987461 |
| Serpina3k | 1.15382089 |
| Serpina3n | -0.8328252 |
| Serpina5 | -0.5196121 |
| Serpina5 | 2.83585036 |
| Serpina7 | -0.7297915 |
| Serpina9 | -0.0580127 |
| Serpinb11 | 0.96016214 |
| Serpinb12 | 1.00202638 |
| Serpinb1a | 0.88759628 |
| Serpinb3a | -0.192865 |
| Serpinb6a | -0.631756 |
| Serpinb6b | 0.24438412 |
| Serpinb6c | -0.459921 |
| Serpind1 | -0.5930748 |
| Serpine2 | 0.37366592 |
| Serpinf1 | -0.5645606 |
| Serping1 | -0.8435037 |
| Serpinh1 | -0.716947 |
| Serpini1 | 0.8724558 |
| Sertad1 | -0.1636071 |
| Sertad2 | 0.45157489 |
| Sertad2 | -0.3654507 |
| Sertad3 | -0.6521236 |
| Sertad4 | 3.61536537 |
| Sesn1 | 0.05584654 |
| Sesn1 | 0.24438412 |
| Sesn2 | -0.6737658 |
| Sesn3 | -0.0207127 |
| Setd1b | -0.2920383 |
| Setd2 | -0.5725135 |
| Setd3 | 1.18082053 |
| Setd4 | 1.11996775 |
| Setd4 | 1.04044065 |
| Setd5 | 2.54427658 |
| Setd6 | -0.7419445 |
| Setd6 | 0.30088337 |
| Setd7 | 1.56750997 |
| Setd7 | -0.5930748 |
| Setd8 | 1.16149201 |
| Setd8 | -0.3948725 |
| Setd8 | -0.5754227 |
| Setdb1 | 0.6431809 |
| Setdb2 | -0.2925729 |
| Setmar | -0.605035 |
| Setx | -0.3972676 |
| Sez6 | -0.4644265 |
| Sez6l | 0.6891203 |
| Sez6l2 | -0.6505407 |
| Sez6l2 | -0.5488592 |
| Sf1 | 3.02597878 |
| Sf1 | -0.1880822 |
| Sf3a1 | -0.028693 |
| Sf3a2 | 1.64872913 |
| Sf3a3 | 0.98206423 |
| Sf3b1 | -0.6616792 |
| Sf3b2 | -0.6110742 |
| Sf3b3 | 1.33773251 |
| Sf3b4 | 0.25178778 |
| Sf3b4 | -0.5516941 |
| Sf3b5 | -0.3631373 |
| Sf4 | 0.40861666 |
| Sfi1 | 0.96342009 |
| Sfi1 | -0.6426668 |
| Sfmbt1 | 0.75081704 |
| Sfmbt1 | 0.48303523 |
| Sfmbt1 | 1.3082807 |
| Sfmbt2 | -0.4966021 |
| Sfrp1 | -0.6057877 |
| Sfrp2 | 0.17938887 |
| Sfrp4 | 1.75421254 |
| Sfrp5 | -0.5524041 |
| Sfrs1 | 2.90588763 |
| Sfrs1 | -0.713552 |
| Sfrs11 | 1.11848199 |
| Sfrs12 | 0.12851845 |
| Sfrs14 | 0.80459392 |
| Sfrs16 | 0.05218531 |
| Sfrs2 | 0.978339 |
| Sfrs2 | 2.12748849 |
| Sfrs2 | -0.6762024 |
| Sfrs3 | 0.62355001 |
| Sfrs3 | -0.6209738 |
| Sfrs4 | 1.97138 |
| Sfrs5 | -0.5725135 |
| Sfrs5 | -0.6194438 |
| Sfrs6 | 3.72625811 |
| Sfrs7 | -0.5783412 |
| Sfrs8 | 2.14728168 |
| Sfrs9 | -0.7332473 |
| Sft2d2 | 0.53231053 |
| Sft2d2 | -0.5588198 |
| Sft2d2 | -0.700932 |
| Sftpc | 0.04370847 |
| Sftpd | -0.6681055 |
| Sfxn1 | 2.11717942 |
| Sfxn1 | 0.02387059 |
| Sfxn3 | -0.8099505 |
| Sfxn3 | -0.6843715 |
| Sfxn4 | 1.71888276 |
| Sfxn4 | -0.4919357 |
| Sfxn5 | 0.74893462 |
| Sgca | -0.5404071 |
| Sgca | 0.71951617 |
| Sgcb | 1.71721966 |
| Sgcb | -0.7587005 |
| Sgcz | -0.6786455 |
| Sgip1 | 1.44568686 |
| Sgip1 | -0.0895872 |
| Sgip1 | 1.01158939 |
| Sgip1 | -0.7042804 |
| Sgip1 | -0.3648718 |
| Sgk1 | -0.6917867 |
| Sgk2 | 1.66151769 |
| Sgk2 | -0.6458083 |
| Sgk3 | 1.71775456 |
| Sgk3 | 0.38546514 |
| Sgk3 | -0.6537093 |
| Sgk3 | -0.759591 |
| Sgk3 | 0.87618994 |
| Sgms2 | 0.03813748 |
| Sgol1 | -0.6278906 |
| Sgpl1 | 1.90096711 |
| Sgpl1 | -0.6584829 |
| Sgpp1 | 1.50154047 |
| Sgpp2 | -0.55525 |
| Sgsm1 | -0.4859708 |
| Sgsm2 | 0.15315805 |
| Sgsm3 | 0.99968505 |
| Sgta | -0.1904705 |
| Sgtb | -0.2814117 |
| Sgtb | 1.16541225 |
| Sh2b1 | 1.91275736 |
| Sh2b1 | 0.7373846 |
| Sh2b3 | -0.5967956 |
| Sh2d2a | -0.6271195 |
| Sh3bgrl | -0.7051194 |
| Sh3bgrl3 | 1.49867634 |
| Sh3bp2 | 0.26670799 |
| Sh3bp5 | 1.68736955 |
| Sh3bp5l | -0.8396062 |
| Sh3gl1 | 2.27622903 |
| Sh3gl2 | -0.7026046 |
| Sh3gl2 | -0.235565 |
| Sh3gl2 | -0.5026369 |
| Sh3gl3 | 0.50722404 |
| Sh3glb1 | -0.6103171 |
| Sh3glb2 | -0.2662191 |
| Sh3kbp1 | 0.03365613 |
| Sh3kbp1 | 0.21507697 |
| Sh3kbp1 | -0.781226 |
| Sh3kbp1 | 1.74243266 |
| Sh3md4 | 0.87797693 |
| Sh3md4 | -0.7026046 |
| Sh3pxd2a | 0.45818951 |
| Sh3pxd2b | -0.2390889 |
| Sh3rf1 | -0.62481 |
| Sh3rf2 | -0.345971 |
| Sh3tc1 | -0.6901337 |
| Sh3tc2 | -0.4945993 |
| Sh3yl1 | -0.656093 |
| Shank2 | -0.6489605 |
| Sharpin | 0.02500505 |
| Shbg | -0.4320976 |
| Shc1 | 1.17481739 |
| Shc1 | 1.3022187 |
| Shc3 | 0.08213867 |
| Shcbp1 | -0.2872411 |
| Shd | -0.5446234 |
| She | -0.2507637 |
| Shf | -0.4780777 |
| Shf | 1.10558071 |
| Shf | -0.3516574 |
| Shfm1 | -0.343141 |
| Shh | 0.96124941 |
| Shisa2 | 0.01359668 |
| Shisa3 | 0.4153014 |
| Shisa4 | -0.1086311 |
| Shkbp1 | 2.32664112 |
| Shkbp1 | -0.6819131 |
| Shmt1 | -0.2365704 |
| Shmt2 | -0.4026797 |
| Shoc2 | 1.83936308 |
| Shox2 | -0.3263423 |
| Shprh | 0.940367 |
| Shq1 | -0.5674446 |
| Shroom3 | -0.5616857 |
| Shroom3 | -0.1880822 |
| Si | -0.3936773 |
| Si | 0.51610486 |
| Siah1a | 2.88567232 |
| Siah1b | 0.55503512 |
| Siah1b | -0.4459035 |
| Siah1b | -0.6942718 |
| Siah1b | 0.99301855 |
| Siah2 | 0.42876924 |
| Siat7f | 0.14092119 |
| Siat9 | 0.94203306 |
| Sidt1 | 0.53339522 |
| Sidt2 | -0.4320976 |
| Sidt2 | -0.3562321 |
| Sidt2 | -0.0600841 |
| Sigirr | -0.2750945 |
| Siglec1 | -0.1214031 |
| Siglech | 0.33301872 |
| Siglech | -0.0061301 |
| Sil1 | -0.2798283 |
| Sin3a | -0.7830523 |
| Sin3b | -0.0155626 |
| Sip1 | 0.87827443 |
| Sip1 | 0.05255209 |
| Sipa1 | -0.0238965 |
| Sipa1l1 | 0.14490786 |
| Sirpa | -0.4748089 |
| Sirpa | -0.192865 |
| Sirpb1 | -0.4748089 |
| Sirt1 | -0.4296096 |
| Sirt2 | -0.5012923 |
| Sirt3 | 0.48532315 |
| Sirt5 | -0.5990353 |
| Sirt5 | 1.4120393 |
| Sirt6 | -0.6600797 |
| Sirt7 | -0.0171441 |
| Siva1 | -0.0696734 |
| Six1 | 0.37159826 |
| Six2 | 0.08320318 |
| Six3 | 2.28732711 |
| Six5 | -0.5418104 |
| Six6os1 | 2.1854855 |
| Skap2 | -0.6418832 |
| Skil | -0.5161918 |
| Skiv2l | 0.32297776 |
| Skiv2l | 0.40886511 |
| Skiv2l2 | -0.2941785 |
| Skp1a | 2.26874396 |
| Skp2 | 1.42366637 |
| Skp2 | 0.14887724 |
| Skp2 | 4.18261226 |
| Sla | 0.49441805 |
| Slain1 | -0.3712599 |
| Slain2 | -0.2824688 |
| Slamf7 | 1.16454254 |
| Slamf8 | 2.04680773 |
| Slamf9 | -0.0203155 |
| Slc10a3 | 4.02894512 |
| Slc10a4 | 0.82436682 |
| Slc10a6 | -0.6608791 |
| Slc10a6 | -0.111259 |
| Slc10a7 | 1.25478597 |
| Slc10a7 | -0.4952664 |
| Slc10a7 | -0.743694 |
| Slc11a1 | -0.5776107 |
| Slc11a1 | 1.81185078 |
| Slc11a1 | 1.53580856 |
| Slc11a1 | 1.31504125 |
| Slc11a2 | 1.52391611 |
| Slc12a2 | 0.30784535 |
| Slc12a2 | 2.47099821 |
| Slc12a3 | -0.5182424 |
| Slc12a5 | -0.6926143 |
| Slc12a6 | -0.7306542 |
| Slc12a7 | -0.1814281 |
| Slc12a8 | -0.713552 |
| Slc12a9 | 0.01244798 |
| Slc12a9 | -0.5960502 |
| Slc13a2 | -0.802451 |
| Slc13a3 | -0.6450219 |
| Slc13a3 | 0.07465228 |
| Slc13a4 | -0.4859708 |
| Slc14a1 | 2.67656844 |
| Slc14a1 | 0.47590609 |
| Slc14a1 | -0.656889 |
| Slc14a2 | -0.8357252 |
| Slc14a2 | -0.7730528 |
| Slc15a2 | -0.7631615 |
| Slc15a3 | -0.2899031 |
| Slc15a4 | -0.1178619 |
| Slc15a4 | -0.743694 |
| Slc16a10 | -0.6110742 |
| Slc16a11 | 1.78657685 |
| Slc16a4 | 1.8487307 |
| Slc16a6 | -0.2973982 |
| Slc16a6 | -0.7195015 |
| Slc16a6 | 1.58526153 |
| Slc16a8 | 0.69767545 |
| Slc16a9 | -0.6868366 |
| Slc17a2 | 0.31668008 |
| Slc17a5 | -0.5495671 |
| Slc17a6 | -0.5320323 |
| Slc17a7 | 0.12208354 |
| Slc17a8 | -0.6616792 |
| Slc18a2 | 1.05722674 |
| Slc19a1 | 0.0474037 |
| Slc19a2 | -0.8099505 |
| Slc1a1 | 1.66441946 |
| Slc1a1 | -0.0359353 |
| Slc1a2 | 1.86450458 |
| Slc1a2 | -0.2564004 |
| Slc1a2 | 1.49170476 |
| Slc1a2 | 0.28395524 |
| Slc1a3 | 0.98471584 |
| Slc1a4 | -0.4503404 |
| Slc1a6 | 0.107351 |
| Slc1a7 | 0.44276015 |
| Slc20a1 | -0.545328 |
| Slc22a1 | -0.183799 |
| Slc22a12 | -0.4748089 |
| Slc22a13 | -0.0810602 |
| Slc22a14 | 0.6404848 |
| Slc22a17 | -0.6681055 |
| Slc22a17 | 0.7695987 |
| Slc22a18 | -0.390101 |
| Slc22a21 | -0.0861669 |
| Slc22a3 | 2.51484548 |
| Slc22a4 | -0.6118319 |
| Slc22a5 | 0.64067764 |
| Slc22a5 | -0.4383472 |
| Slc22a5 | -0.6984287 |
| Slc22a6 | 0.18575148 |
| Slc22a7 | 0.10804152 |
| Slc22a8 | -0.0298961 |
| Slc23a2 | 0.84588113 |
| Slc23a3 | 2.3511398 |
| Slc24a1 | 1.02079898 |
| Slc24a3 | -0.2620721 |
| Slc24a4 | -0.5202977 |
| Slc24a5 | 0.39029467 |
| Slc24a6 | 1.7157322 |
| Slc25a1 | 0.01206476 |
| Slc25a1 | -0.6778304 |
| Slc25a10 | 2.83346652 |
| Slc25a11 | -0.677016 |
| Slc25a11 | -0.2137206 |
| Slc25a11 | 0.587689 |
| Slc25a12 | 1.44344436 |
| Slc25a14 | -0.6209738 |
| Slc25a15 | 0.7810824 |
| Slc25a15 | 0.71169917 |
| Slc25a16 | 0.45818951 |
| Slc25a17 | -0.5251114 |
| Slc25a18 | 0.95388513 |
| Slc25a19 | -0.2044467 |
| Slc25a19 | 3.13058698 |
| Slc25a20 | 1.13356967 |
| Slc25a20 | -0.4522485 |
| Slc25a23 | -0.7186492 |
| Slc25a24 | -0.568167 |
| Slc25a24 | -0.0617446 |
| Slc25a25 | 0.01014624 |
| Slc25a26 | -0.3022492 |
| Slc25a27 | 1.0477607 |
| Slc25a28 | -0.7237749 |
| Slc25a29 | 1.61259336 |
| Slc25a3 | 0.18986357 |
| Slc25a3 | -0.2416142 |
| Slc25a30 | -0.7542612 |
| Slc25a32 | -0.6442362 |
| Slc25a33 | -0.7821387 |
| Slc25a34 | -0.1325583 |
| Slc25a35 | -0.4452714 |
| Slc25a36 | 0.7480777 |
| Slc25a37 | -0.5060068 |
| Slc25a38 | -0.6403178 |
| Slc25a38 | 3.2057404 |
| Slc25a38 | 2.10857642 |
| Slc25a39 | -0.2235853 |
| Slc25a41 | -0.2345607 |
| Slc25a42 | 0.3627582 |
| Slc25a44 | 1.24218371 |
| Slc25a45 | 1.93096311 |
| Slc25a46 | 0.49622623 |
| Slc25a5 | -0.3516574 |
| Slc26a4 | -0.3666096 |
| Slc26a6 | -0.4154338 |
| Slc26a7 | -0.3054975 |
| Slc26a8 | -0.8916167 |
| Slc27a1 | -0.5141457 |
| Slc27a2 | 0.43892191 |
| Slc27a3 | -0.5168748 |
| Slc27a4 | -0.0037871 |
| Slc27a6 | -0.2421201 |
| Slc29a1 | -0.8916167 |
| Slc29a2 | -0.5674446 |
| Slc29a3 | -0.6497503 |
| Slc29a4 | 1.39452836 |
| Slc2a1 | 1.16214655 |
| Slc2a10 | -0.1861761 |
| Slc2a10 | -0.6005316 |
| Slc2a13 | -0.1603618 |
| Slc2a3 | 1.31863269 |
| Slc2a4 | -0.5087117 |
| Slc2a5 | -0.7160971 |
| Slc2a6 | -0.7463243 |
| Slc2a8 | -0.358528 |
| Slc2a8 | -0.2861784 |
| Slc2a8 | 1.10616055 |
| Slc2a9 | -0.2646618 |
| Slc30a1 | 0.6749292 |
| Slc30a10 | 0.34321762 |
| Slc30a3 | 0.08603581 |
| Slc30a5 | 0.96369106 |
| Slc30a5 | -0.5717877 |
| Slc30a6 | -0.759591 |
| Slc30a7 | 2.94659356 |
| Slc30a7 | 1.45681764 |
| Slc30a9 | -0.568167 |
| Slc31a1 | 0.17683138 |
| Slc31a2 | 1.11973933 |
| Slc32a1 | 0.2039567 |
| Slc34a2 | -0.4966021 |
| Slc35a1 | -0.3038719 |
| Slc35a2 | 0.88892025 |
| Slc35a3 | -0.3906961 |
| Slc35a4 | -0.2171612 |
| Slc35a5 | 2.11259865 |
| Slc35a5 | 2.21435321 |
| Slc35b1 | 0.09867523 |
| Slc35b2 | 0.7805861 |
| Slc35b3 | 2.25794364 |
| Slc35b3 | 1.75990425 |
| Slc35b3 | -0.4215702 |
| Slc35b4 | -0.5148272 |
| Slc35c1 | -0.2446539 |
| Slc35c1 | 1.56988753 |
| Slc35c2 | -0.6217397 |
| Slc35c2 | -0.5481519 |
| Slc35c2 | -0.3414472 |
| Slc35e1 | -0.5278735 |
| Slc35e3 | -0.6027805 |
| Slc35e4 | -0.2667388 |
| Slc35f1 | -0.3562321 |
| Slc35f2 | 1.43547115 |
| Slc35f3 | -0.3724261 |
| Slc35f4 | -0.1205165 |
| Slc35f4 | -0.8184617 |
| Slc36a1 | 0.53772119 |
| Slc36a3 | 0.94355761 |
| Slc37a1 | -0.4026797 |
| Slc37a3 | -0.2667388 |
| Slc38a1 | 0.59744411 |
| Slc38a10 | 2.0307018 |
| Slc38a11 | -0.313122 |
| Slc38a2 | 0.36354165 |
| Slc38a2 | -0.4833322 |
| Slc38a2 | -0.4676583 |
| Slc38a3 | -0.5717877 |
| Slc38a4 | 1.27138795 |
| Slc38a5 | -0.1800085 |
| Slc38a8 | 0.98841524 |
| Slc38a9 | 1.053059 |
| Slc38a9 | -0.7622676 |
| Slc39a10 | -0.7694436 |
| Slc39a11 | 0.13858759 |
| Slc39a12 | 0.37159826 |
| Slc39a13 | 0.11697118 |
| Slc39a13 | -0.2166689 |
| Slc39a14 | 0.49441805 |
| Slc39a3 | 0.05730693 |
| Slc39a3 | -0.4631369 |
| Slc39a4 | -0.6884836 |
| Slc39a5 | -0.0250933 |
| Slc39a5 | -0.2512747 |
| Slc39a5 | 1.86672875 |
| Slc39a6 | -0.6042829 |
| Slc39a7 | -0.6802779 |
| Slc39a7 | -0.3191576 |
| Slc39a7 | -0.5725135 |
| Slc39a8 | -0.2952505 |
| Slc3a2 | 0.69549875 |
| Slc40a1 | -0.1068834 |
| Slc41a2 | 0.27910144 |
| Slc41a3 | 3.23578407 |
| Slc41a3 | -0.6217397 |
| Slc41a3 | -0.5251114 |
| Slc43a2 | 1.23183395 |
| Slc43a2 | -0.3865386 |
| Slc44a1 | -0.3224667 |
| Slc44a1 | 3.50433669 |
| Slc44a2 | -0.5871528 |
| Slc44a2 | 1.01454143 |
| Slc44a2 | -0.3735939 |
| Slc44a3 | -0.6529161 |
| Slc44a4 | 2.71159922 |
| Slc45a1 | -0.8146692 |
| Slc45a2 | -0.2039612 |
| Slc45a3 | -0.030699 |
| Slc45a4 | -0.1814281 |
| Slc46a1 | 0.91529088 |
| Slc46a3 | -0.3230193 |
| Slc47a1 | -0.3823999 |
| Slc4a1 | -0.6608791 |
| Slc4a1ap | -0.4352171 |
| Slc4a2 | 1.85273819 |
| Slc4a3 | -0.3164093 |
| Slc4a3 | 0.07679751 |
| Slc4a3 | -0.0823342 |
| Slc4a3 | 0.79258709 |
| Slc4a8 | -0.4979397 |
| Slc4a8 | 0.28281548 |
| Slc5a4a | -0.5053318 |
| Slc5a4b | -0.4683061 |
| Slc5a5 | 0.34694664 |
| Slc5a6 | -0.3285644 |
| Slc5a7 | 0.96666648 |
| Slc5a8 | 1.81083164 |
| Slc5a9 | 0.55439968 |
| Slc6a1 | -0.4142114 |
| Slc6a11 | 0.2260637 |
| Slc6a12 | -0.4184969 |
| Slc6a13 | -0.6917867 |
| Slc6a15 | 1.45047379 |
| Slc6a15 | 0.39206756 |
| Slc6a17 | -0.5856783 |
| Slc6a2 | 0.86328 |
| Slc6a4 | -0.0705121 |
| Slc6a6 | -0.1191882 |
| Slc6a7 | -0.7017679 |
| Slc6a8 | -0.5460332 |
| Slc6a9 | -0.147951 |
| Slc7a1 | 0.61268009 |
| Slc7a10 | -0.2054185 |
| Slc7a11 | -0.807131 |
| Slc7a14 | 2.32740432 |
| Slc7a14 | 0.71027069 |
| Slc7a14 | 0.9154337 |
| Slc7a2 | 2.0857351 |
| Slc7a3 | -0.5856783 |
| Slc7a4 | -0.7533759 |
| Slc7a6 | 0.17201674 |
| Slc7a6os | -0.7084831 |
| Slc7a7 | 0.84418465 |
| Slc7a7 | 2.01799499 |
| Slc7a8 | 0.49305956 |
| Slc7a8 | 2.70050377 |
| Slc8a1 | 0.11286063 |
| Slc8a2 | -0.6458083 |
| Slc8a2 | 0.71597126 |
| Slc9a1 | 1.81991009 |
| Slc9a10 | -0.656093 |
| Slc9a3r1 | -0.219133 |
| Slc9a3r2 | 0.74119325 |
| Slc9a4 | -0.9082336 |
| Slc9a6 | -0.9434895 |
| Slc9a6 | 2.04792667 |
| Slc9a8 | -0.7821387 |
| Slc9a8 | 1.18699682 |
| Slc9a8 | -0.7987241 |
| Slc9a8 | 3.20928551 |
| Slc9a9 | 0.02273472 |
| Slco1a4 | -0.2548597 |
| Slco1c1 | 1.15623875 |
| Slco2a1 | -0.8043201 |
| Slco2b1 | -0.7676443 |
| Slco3a1 | -0.5175584 |
| Slco3a1 | 0.2816743 |
| Slco3a1 | -0.8061931 |
| Slco4a1 | 1.38517568 |
| Slfnl1 | -0.9733813 |
| Slit1 | 0.35330409 |
| Slit2 | -0.7051194 |
| Slit3 | 0.66013706 |
| Slitrk1 | 0.68232867 |
| Slitrk3 | -0.3823999 |
| Slitrk4 | 2.10476999 |
| Slitrk5 | 0.4612444 |
| Slitrk5 | -0.7542612 |
| Slitrk6 | 0.27076153 |
| Slk | -0.4002704 |
| Slk | 1.63484903 |
| Slmap | 1.09484542 |
| Slmap | -0.2054185 |
| Slmap | -0.6387551 |
| Slmo1 | -0.6901337 |
| Slmo2 | -0.3442719 |
| Sln | -0.7332473 |
| Slpi | 0.24616647 |
| Sltm | 0.26234489 |
| Sltm | 2.63597069 |
| Slu7 | 1.05158349 |
| Slu7 | 1.44055304 |
| Sly | 3.06545907 |
| Smad1 | -0.7524915 |
| Smad1 | -0.802451 |
| Smad2 | 1.4745825 |
| Smad3 | 1.71811098 |
| Smad5 | 0.31640528 |
| Smad5 | 1.53515593 |
| Smad6 | -0.7472028 |
| Smap2 | -0.4452714 |
| Smap2 | -0.6851925 |
| Smap2 | 1.5595634 |
| Smarca1 | -0.6088048 |
| Smarca2 | -0.5761514 |
| Smarca2 | -0.8425277 |
| Smarca4 | 2.26339246 |
| Smarcad1 | 0.7804206 |
| Smarcal1 | 0.14887724 |
| Smarcb1 | -0.2137206 |
| Smarcc1 | 0.41653397 |
| Smarcc1 | -0.6465953 |
| Smarcc2 | 0.82735914 |
| Smarcd1 | 1.9449747 |
| Smarcd1 | 0.66615765 |
| Smarcd1 | 3.59818725 |
| Smarcd2 | 1.34195318 |
| Smarcd3 | -0.4787329 |
| Smarce1 | 0.25355578 |
| Smarce1 | 0.85736491 |
| Smc1a | 0.95552676 |
| Smc1a | -0.3625599 |
| Smc1a | 0.06854667 |
| Smc1a | -0.6992624 |
| Smc1b | -0.606541 |
| Smc3 | 1.96541652 |
| Smc5l1 | 0.55609323 |
| Smc6 | 0.85964447 |
| Smchd1 | 0.08249365 |
| Smcp | -0.6934427 |
| Smcr7 | 2.10157281 |
| Smcr7l | 1.10685588 |
| Smek1 | 0.50543748 |
| Smek2 | -0.2245776 |
| Smek2 | 2.65355108 |
| Smg5 | -0.0522376 |
| Smg6 | -0.1866523 |
| Smn1 | -0.0139837 |
| Smo | 0.60368946 |
| Smo | -0.8090097 |
| Smo | -0.6340831 |
| Smoc1 | 1.62359132 |
| Smox | -0.27457 |
| Smox | 0.36301942 |
| Smox | 1.95520687 |
| Smox | 1.77699276 |
| Smox | 2.26721349 |
| Smpd1 | 3.3798639 |
| Smpd1 | 2.21040846 |
| Smpd1 | 0.16846984 |
| Smpd2 | 1.66856685 |
| Smpd3 | 0.42389475 |
| Smpd4 | 0.50744711 |
| Smpdl3a | 0.7460178 |
| Smpdl3b | -0.2667388 |
| Smpx | 1.83910294 |
| Smtn | 1.72125337 |
| Smtn | -0.419725 |
| Smtn | 1.33674188 |
| Smtnl1 | 2.38534058 |
| Smtnl2 | 0.36744887 |
| Smu1 | -0.4333441 |
| Smu1 | 0.66409254 |
| Smug1 | 1.38500474 |
| Smurf1 | 0.18352959 |
| Smyd1 | 0.42779642 |
| Smyd2 | -0.7110141 |
| Smyd2 | 1.56953827 |
| Smyd3 | 1.94941347 |
| Smyd4 | 0.07429425 |
| Smyd5 | -0.3268974 |
| Smyd5 | 0.35145439 |
| Snai1 | 0.21354055 |
| Snai3 | 1.51794837 |
| Snap23 | 0.47844215 |
| Snap29 | -0.1608247 |
| Snap91 | -0.4741565 |
| Snap91 | -0.4966021 |
| Snapc1 | -0.5776107 |
| Snapc3 | -0.4644265 |
| Snapc3 | 0.93311219 |
| Snapc3 | 0.08815454 |
| Snapc3 | -0.0347243 |
| Snapc4 | 1.60851849 |
| Snapin | 0.35198326 |
| Snca | 0.92565818 |
| Snca | 0.81389575 |
| Snca | -0.2176537 |
| Sncaip | -0.581269 |
| Sncg | 0.5558817 |
| Sncg | 4.01768593 |
| Snd1 | -0.3642933 |
| Snf1lk | 0.86569568 |
| Snf1lk2 | 0.0692671 |
| Snf8 | 0.25678827 |
| Snip1 | -0.7254899 |
| Snip1 | -0.5674446 |
| Snn | 2.06397901 |
| Snora65 | -0.3823999 |
| Snph | -0.7051194 |
| Snrk | -0.5790722 |
| Snrk | -0.251786 |
| Snrk | -0.0684169 |
| Snrp70 | 0.03365613 |
| Snrpa | -0.1720054 |
| Snrpb | -0.4541606 |
| Snrpb | -0.6917867 |
| Snrpd1 | -0.6217397 |
| Snrpd1 | -0.7160971 |
| Snrpd2 | -0.6810951 |
| Snrpd3 | -0.1121366 |
| Snrpg | -0.062576 |
| Snta1 | -0.5299505 |
| Sntb2 | -0.6901337 |
| Sntb2 | 1.14464101 |
| Sntg2 | -0.2088279 |
| Snupn | 2.49236946 |
| Snurf | 2.50753162 |
| Snurf | 1.61824136 |
| Snx1 | -0.7472028 |
| Snx10 | -0.7794034 |
| Snx11 | -0.1866523 |
| Snx12 | 0.01855778 |
| Snx13 | 1.10255957 |
| Snx14 | 1.1225905 |
| Snx14 | -0.7569222 |
| Snx14 | -0.5460332 |
| Snx15 | -0.8137235 |
| Snx16 | -0.1947851 |
| Snx17 | -0.3912915 |
| Snx17 | 0.1958405 |
| Snx18 | -0.0112273 |
| Snx19 | 1.48068495 |
| Snx19 | -0.7445699 |
| Snx2 | 1.4200539 |
| Snx2 | -0.7613745 |
| Snx20 | -0.4560767 |
| Snx21 | -0.7084831 |
| Snx22 | 2.68467861 |
| Snx24 | -0.7017679 |
| Snx25 | 1.24656953 |
| Snx25 | -0.5073583 |
| Snx26 | 1.77943987 |
| Snx29 | -0.5856783 |
| Snx3 | -0.2528094 |
| Snx30 | -0.7950124 |
| Snx33 | 1.11424005 |
| Snx4 | 1.2040302 |
| Snx6 | -0.352228 |
| Snx7 | -0.0088712 |
| Snx8 | -0.4767688 |
| Snx9 | -0.6186797 |
| Soat1 | 0.99903361 |
| Socs2 | 1.75009248 |
| Socs3 | -0.7127053 |
| Socs4 | -0.2600056 |
| Socs5 | -0.5313378 |
| Socs6 | -0.0538839 |
| Socs7 | -0.4560767 |
| Socs7 | -0.1895145 |
| Sod1 | -0.1298686 |
| Sod2 | 1.04961243 |
| Sod3 | 0.7372111 |
| Sohlh1 | 1.05269035 |
| Sohlh1 | -0.5609684 |
| Son | -0.0481347 |
| Sorbs1 | -0.2275611 |
| Sorbs2 | 3.00966512 |
| Sorbs3 | 0.76641105 |
| Sorcs1 | 1.41733515 |
| Sorcs1 | -0.700932 |
| Sorcs2 | 1.84744252 |
| Sorcs3 | 2.6806014 |
| Sorcs3 | -0.6042829 |
| Sord | 1.14018386 |
| Sorl1 | 0.25942456 |
| Sort1 | -0.4839912 |
| Sort1 | -0.707641 |
| Sort1 | -0.5502755 |
| Sort1 | 1.10244317 |
| Sostdc1 | 1.3155947 |
| Sox12 | 0.97593619 |
| Sox13 | -0.764952 |
| Sox17 | 1.36879238 |
| Sox17 | -0.7784935 |
| Sox18 | 0.54567069 |
| Sox2 | 1.20652691 |
| Sox2 | 0.5678611 |
| Sox21 | 0.52314797 |
| Sox21 | 0.10284987 |
| Sox30 | 1.55538672 |
| Sox5 | -0.5196121 |
| Sox5 | -0.7059591 |
| Sox5 | -0.5244222 |
| Sox5 | 0.20240148 |
| Sox7 | -0.6271195 |
| Sox7 | 0.13958843 |
| Sox8 | -0.5783412 |
| Sox9 | 0.91042189 |
| Sp100 | 1.86748575 |
| Sp140 | 0.3389372 |
| Sp2 | 2.58886732 |
| Sp4 | -0.4333441 |
| Sp5 | -0.7341133 |
| Sp6 | 0.60188068 |
| Sp7 | 2.39465918 |
| Sp8 | -0.7143996 |
| Sp9 | -0.0440502 |
| Spaca3 | 1.277657 |
| Spaca4 | 1.20600733 |
| Spag1 | 3.51732842 |
| Spag1 | 0.90927246 |
| Spag16 | 0.13992181 |
| Spag16 | 0.53491164 |
| Spag4 | 1.40093482 |
| Spag4 | -0.7332473 |
| Spag4l | 0.53144185 |
| Spag4l | -0.6465953 |
| Spag5 | -0.166398 |
| Spag5 | -0.1343558 |
| Spag6 | -0.7280684 |
| Spag6 | -0.6608791 |
| Spag7 | 0.30451021 |
| Spag8 | 1.44280263 |
| Spag9 | 0.80055516 |
| Spag9 | 1.24138402 |
| Spag9 | -0.1899924 |
| Spag9 | -0.3637151 |
| Spam1 | -0.3006294 |
| Spam1 | -0.028693 |
| Sparc | -0.0131953 |
| Sparc | -0.6552978 |
| Sparcl1 | -0.235565 |
| Spast | -0.5292576 |
| Spast | -0.7051194 |
| Spast | -0.4063056 |
| Spata16 | -0.5327273 |
| Spata16 | 2.24257229 |
| Spata17 | -0.584942 |
| Spata17 | -0.1470383 |
| Spata19 | 0.20984257 |
| Spata2 | 0.93157011 |
| Spata20 | -0.4793885 |
| Spata20 | -0.6934427 |
| Spata21 | -0.5776107 |
| Spata22 | 0.87200667 |
| Spata2L | 2.06536164 |
| Spata3 | 2.02973059 |
| Spata3 | -0.4709017 |
| Spata3 | 0.06782568 |
| Spata3 | 3.58037791 |
| Spata4 | -0.6959323 |
| Spata6 | 0.30534514 |
| Spata7 | -0.1715368 |
| Spata7 | 0.96314903 |
| Spatc1 | -0.4605633 |
| Spatc1 | -0.30009 |
| Spats1 | -0.7118593 |
| Spc24 | -0.5320323 |
| Spc24 | 0.49147211 |
| Spc25 | -0.6672998 |
| Spc25 | -0.3291208 |
| Spcs1 | -0.5509845 |
| Spcs1 | -0.0440502 |
| Spcs3 | 0.24912936 |
| Spdef | -0.0688356 |
| Specc1l | 0.52948429 |
| Speer1-ps1 | 0.51211915 |
| Speer1-ps1 | -0.1543652 |
| Speer2 | 0.44419535 |
| Speer2 | 3.3664262 |
| Speer3 | 0.3240686 |
| Speer4b | 0.04333812 |
| Speer4d | -0.7367163 |
| Speer4f | 1.37330547 |
| Speer6-ps1 | 0.2969614 |
| Spef1 | -0.1388651 |
| Spen | 0.78256971 |
| Spert | -0.4833322 |
| Spesp1 | -0.8127788 |
| Spg20 | -0.0010612 |
| Spg21 | 0.24942512 |
| Spg7 | 2.70534179 |
| Spg7 | 0.5045429 |
| Sphk1 | -0.1474945 |
| Sphk2 | 0.37521361 |
| Sphk2 | -0.5107457 |
| Spic | 1.21273904 |
| Spin | -0.8376636 |
| Spin2 | 0.25355578 |
| Spink10 | -0.8137235 |
| Spink2 | 2.26037664 |
| Spink4 | 0.07501016 |
| Spink8 | 0.83268951 |
| Spinlw1 | -0.3771061 |
| Spinlw1 | -0.5271822 |
| Spinlw1 | 1.40937983 |
| Spint1 | -0.8251366 |
| Spint2 | 0.66803099 |
| Spire1 | -0.4735046 |
| Spire1 | -0.6984287 |
| Spire1 | -0.2034759 |
| Spire2 | 1.21036271 |
| Spna1 | -0.6020302 |
| Spnb1 | -0.7026046 |
| Spnb2 | 1.43506662 |
| Spnb2 | 0.3243411 |
| Spnb2 | -0.6458083 |
| Spnb2 | 1.33701216 |
| Spnb3 | -0.4992793 |
| Spnb4 | -0.5886297 |
| Spns1 | -0.5264914 |
| Spo11 | 0.77594088 |
| Spo11 | 1.24736469 |
| Spock1 | 0.86900687 |
| Spock2 | 0.52970201 |
| Spock3 | 1.42054735 |
| Spon1 | 0.40587918 |
| Spon2 | -0.5033099 |
| Spop | 2.24223743 |
| Spp1 | -0.4586377 |
| Spp2 | -0.27457 |
| Sppl3 | -0.6942718 |
| Spr | 1.57309442 |
| Spred1 | 1.90213357 |
| Spred2 | 1.15996292 |
| Spred2 | 2.54160029 |
| Sprn | 0.03589955 |
| Sprn | 0.1045837 |
| Sprr2a | 0.3910549 |
| Sprr2a | 0.53944586 |
| Sprr2f | -0.7152479 |
| Spry3 | 0.98272785 |
| Spry4 | 1.89750761 |
| Spryd4 | 1.30883825 |
| Spsb1 | -0.7533759 |
| Spsb2 | 0.14158685 |
| Spsb3 | -0.2063913 |
| Spsb3 | -0.1914276 |
| Spsb4 | 0.75405939 |
| Sptlc1 | -0.6584829 |
| Sptlc2 | -0.6489605 |
| Spz1 | 0.39333181 |
| Sqle | -0.1429426 |
| Sqle | 1.80274575 |
| Sqrdl | -0.626349 |
| Sqstm1 | -0.8127788 |
| Sra1 | -0.5080347 |
| Sra1 | 1.09178856 |
| Src | 1.9798069 |
| Srd5a1 | -0.709326 |
| Srd5a2 | -0.1214031 |
| Srd5a3 | 0.67511491 |
| Srd5a3 | 1.03016877 |
| Srd5a3 | -0.7940868 |
| Srebf1 | -0.5093892 |
| Srebf2 | 1.59584451 |
| Srf | 0.72831881 |
| Srf | 2.52586749 |
| Srfbp1 | -0.2656997 |
| Srgn | -0.0262916 |
| Srgn | 0.77610719 |
| Sri | -0.6148691 |
| Sri | -0.7160971 |
| Sri | -0.1967093 |
| Srm | -0.6450219 |
| Srm | 0.71365969 |
| Srm | 1.50903511 |
| Srp14 | 3.46051826 |
| Srp19 | -0.2088279 |
| Srp19 | -0.7349802 |
| Srp54 | 0.33947335 |
| Srp68 | 2.02545349 |
| Srp72 | 0.95278906 |
| Srp9 | -0.7349802 |
| Srp9 | -0.2166689 |
| Srpk1 | 0.87395128 |
| Srpk2 | 2.26096748 |
| Srpk3 | 0.45630449 |
| Srpr | 1.44368489 |
| Srprb | 0.85614682 |
| Srprb | -0.672145 |
| Srpx | -0.568167 |
| Srpx | 0.8741007 |
| Srpx2 | 0.87753051 |
| Srpx2 | 1.62477596 |
| Srpx2 | -0.5264914 |
| Srr | 0.47659844 |
| Srrd | -0.6202084 |
| Srrm1 | 0.16685305 |
| Srrm2 | -0.6737658 |
| Srrm2 | 0.94383453 |
| Srrp | 1.40051471 |
| Srxn1 | -0.2461775 |
| Srxn1 | 3.32488672 |
| Srxn1 | 0.28024583 |
| Ss18 | 2.25232969 |
| Ss18 | -0.3038719 |
| Ss18l1 | -0.5516941 |
| Ss18l1 | 2.53496026 |
| Ss18l1 | 1.17728774 |
| Ssbp1 | 2.22765318 |
| Ssbp2 | -0.5425128 |
| Ssbp2 | 0.27938767 |
| Ssbp3 | -0.3527989 |
| Ssbp3 | 0.8340953 |
| Ssbp4 | 1.74888729 |
| Ssfa2 | -0.6917867 |
| Ssh2 | 2.54287913 |
| Ssh3 | 0.19175524 |
| Ssh3 | 0.65673298 |
| Ssna1 | -0.1720054 |
| Sspn | 2.46532062 |
| Ssr2 | -0.7177977 |
| Ssr3 | 0.06348775 |
| Ssr4 | 0.17169487 |
| Ssrp1 | 0.89126927 |
| Sssca1 | 0.83565477 |
| Sssca1 | 0.03065633 |
| Sst | 0.59298593 |
| Sstr1 | 0.4365148 |
| Sstr5 | 2.09443645 |
| Ssty2 | -0.7640563 |
| Ssu72 | -0.6786455 |
| Ssx2ip | 1.09496278 |
| Ssxb2 | -0.7127053 |
| Ssxb5 | -0.0073039 |
| St13 | 1.51595044 |
| St14 | 0.03328169 |
| St14 | 2.99768724 |
| St18 | -0.6125903 |
| St3gal1 | -0.5100672 |
| St3gal2 | 0.15118478 |
| St3gal2 | -0.2265655 |
| St3gal3 | 0.70363482 |
| St3gal3 | -0.8454587 |
| St3gal4 | 1.06997914 |
| St3gal5 | 0.6625877 |
| St3gal6 | -0.7613745 |
| St5 | 0.52248998 |
| St5 | -0.7857984 |
| St6gal1 | 1.00811175 |
| St6galnac2 | -0.2845868 |
| St6galnac3 | 0.55249068 |
| St6galnac4 | -0.343141 |
| St6galnac4 | 2.04202649 |
| St6galnac4 | 0.03962639 |
| St6galnac5 | -0.352228 |
| St6galnac6 | 0.29189403 |
| St6galnac6 | -0.7110141 |
| St7 | 0.48828892 |
| St7l | 0.82262994 |
| St7l | 0.57265082 |
| St8sia1 | -0.7867157 |
| St8sia1 | 0.88390849 |
| St8sia2 | -0.2693418 |
| St8sia3 | -0.8033851 |
| St8sia4 | 1.14018386 |
| St8sia4 | -0.3800433 |
| St8sia5 | -0.2625894 |
| Stab1 | -0.3625599 |
| Stab2 | -0.0407956 |
| Stac | -0.5320323 |
| Stac2 | -0.5938178 |
| Stac3 | -0.3164093 |
| Stag1 | -0.5026369 |
| Stag2 | 1.12747382 |
| Stag2 | -0.388318 |
| Stag3 | 3.32590166 |
| Stam | -0.5121042 |
| Stam | 2.17746814 |
| Stam | -0.0617446 |
| Stam2 | 0.03850993 |
| Stam2 | -0.3454043 |
| Stambp | -0.299551 |
| Stambpl1 | 0.72761771 |
| Stap1 | 2.85572908 |
| Stap2 | -0.6005316 |
| Star | 1.61032436 |
| Stard10 | -0.6348601 |
| Stard10 | 3.44102268 |
| Stard13 | -0.2856476 |
| Stard3 | -0.6608791 |
| Stard3nl | -0.5467389 |
| Stard4 | 0.38240172 |
| Stard5 | 0.46452292 |
| Stard5 | -0.3224667 |
| Stard6 | 2.63435758 |
| Stard7 | -0.5710624 |
| Stard7 | 0.25178778 |
| Stard8 | 2.58884439 |
| Stard8 | -0.7454467 |
| Stat1 | -0.3319077 |
| Stat1 | 0.27249327 |
| Stat2 | 0.61980953 |
| Stat3 | -0.0049579 |
| Stat3 | -0.6309816 |
| Stat3 | -0.105139 |
| Stat4 | -0.4452714 |
| Stat4 | 2.04373397 |
| Stat5a | 0.63739379 |
| Stat5a | 0.43530887 |
| Stat6 | -0.5134647 |
| Statip1 | -0.6240415 |
| Stau1 | 2.90270388 |
| Stau2 | 0.72234224 |
| Stbd1 | -0.3252333 |
| Stc2 | 1.56589811 |
| Steap1 | 1.75774557 |
| Steap2 | -0.7118593 |
| Steap3 | -0.4541606 |
| Stfa1 | -0.6387551 |
| Stfa2 | -0.7489623 |
| Stim1 | -0.6426668 |
| Stip1 | -0.2280593 |
| Stk11 | 0.26554653 |
| Stk11ip | -0.590109 |
| Stk17b | 1.32759558 |
| Stk19 | 0.90999102 |
| Stk19 | 1.7096981 |
| Stk19 | -0.3093018 |
| Stk22b | 1.43926508 |
| Stk22s1 | -0.858269 |
| Stk24 | -0.4452714 |
| Stk25 | 1.05379587 |
| Stk25 | 2.89874122 |
| Stk3 | -0.104268 |
| Stk31 | -0.5251114 |
| Stk32a | 2.17768381 |
| Stk32b | 0.49441805 |
| Stk32c | 0.52248998 |
| Stk35 | 2.07236464 |
| Stk35 | 0.80733013 |
| Stk36 | -0.4735046 |
| Stk38l | 0.76792238 |
| Stk39 | -0.6909598 |
| Stk4 | 0.06818625 |
| Stk40 | 0.08568221 |
| Stmn1 | -0.7280684 |
| Stmn2 | -0.545328 |
| Stmn3 | 0.1468947 |
| Stmn4 | -0.4377203 |
| Stmn4 | -0.2416142 |
| Stoml1 | 1.20932759 |
| Stoml1 | -0.2867096 |
| Stoml1 | -0.1729433 |
| Stoml2 | 0.42120267 |
| Stoml3 | -0.263625 |
| Stra13 | 0.382913 |
| Stra13 | 0.33328858 |
| Stra13 | 0.63758728 |
| Stra6 | -0.6851925 |
| Strap | 0.82136469 |
| Strbp | -0.6186797 |
| Strn4 | 0.28708227 |
| Strn4 | -0.6465953 |
| Stt3a | 0.90740157 |
| Stt3b | -0.659281 |
| Stx12 | -0.7803143 |
| Stx17 | 2.83297495 |
| Stx18 | 0.73199082 |
| Stx1a | -0.4579968 |
| Stx2 | 0.24170407 |
| Stx3 | -0.2390889 |
| Stx3 | -0.7332473 |
| Stx4a | -0.0282924 |
| Stx5a | -0.2117604 |
| Stx5a | 0.26438359 |
| Stx6 | -0.5495671 |
| Stx7 | -0.5805361 |
| Stx8 | -0.6202084 |
| Stx8 | -0.0895872 |
| Stx8 | -0.5602516 |
| Stx8 | 0.7392909 |
| Stxbp1 | 2.52261918 |
| Stxbp1 | -0.4846506 |
| Stxbp1 | 0.50387138 |
| Stxbp2 | 1.35140125 |
| Stxbp4 | -0.6762024 |
| Stxbp4 | 1.93157474 |
| Stxbp5l | -0.6232736 |
| Stxbp5l | -0.5588198 |
| Styx | -0.7775845 |
| Styxl1 | -0.7000968 |
| Styxl1 | 0.109421 |
| Styxl1 | 1.74526217 |
| Suclg1 | 0.4451509 |
| Suclg1 | 0.15282947 |
| Suclg2 | 2.63564394 |
| Suclg2 | -0.6917867 |
| Suds3 | -0.2471947 |
| Sufu | -0.8493812 |
| Suhw2 | 0.26699812 |
| Suhw4 | 1.74005838 |
| Sulf1 | 1.23847929 |
| Sult1a1 | -0.10732 |
| Sult1c2 | 2.42466903 |
| Sult1d1 | 0.39560307 |
| Sult1d1 | -0.6697192 |
| Sult2b1 | -0.4383472 |
| Sult2b1 | 0.13390245 |
| Sult4a1 | -0.0254925 |
| Sult5a1 | 0.83969699 |
| Sult5a1 | 2.13459867 |
| Sumf2 | 0.92650508 |
| Sumo1 | 0.12004202 |
| Sumo1 | -0.1388651 |
| Sumo1 | 0.98299316 |
| Sumo2 | 0.2782422 |
| Sumo3 | -0.1271868 |
| Sumo3 | 0.99655392 |
| Sunc1 | 1.50003415 |
| Suox | -0.3924836 |
| Supt3h | -0.5299505 |
| Supt4h1 | 1.01697291 |
| Supt4h2 | 0.72533541 |
| Supt5h | -0.1156557 |
| Supt5h | 0.07786824 |
| Supt6h | -0.6255792 |
| Supv3l1 | 1.97578243 |
| Surf1 | -0.2931078 |
| Surf1 | 0.23511959 |
| Surf2 | 2.22884347 |
| Surf4 | -0.5997832 |
| Susd2 | -0.3573793 |
| Susd3 | 1.60617224 |
| Susd4 | 1.66429348 |
| Suv39h1 | -0.5746945 |
| Suv420h1 | 0.2008436 |
| Suv420h1 | 0.92481049 |
| Suv420h1 | 2.9810059 |
| Suv420h2 | 2.26887409 |
| Suz12 | 1.35502968 |
| Sv2a | -0.8251366 |
| Sv2a | 4.3363949 |
| Sv2b | 0.33759545 |
| Sval2 | 0.68765611 |
| Svep1 | 0.23180949 |
| Svil | 0.32024496 |
| Svil | 1.85621763 |
| Svop | -0.713552 |
| Svopl | 0.15118478 |
| Svs4 | 0.59115585 |
| Svs5 | -0.6819131 |
| Svs6 | 2.26322882 |
| Swap70 | -0.4541606 |
| Swap70 | -0.3648718 |
| Swap70 | 0.83143809 |
| Syap1 | 2.09179524 |
| Syap1 | -0.6967637 |
| Sybl1 | -0.2290565 |
| Syce2 | -0.5842062 |
| Sycp1 | -0.6434512 |
| Syf2 | -0.6762024 |
| Syf2 | 0.87454879 |
| Syn1 | -0.3619828 |
| Syn1 | -0.016353 |
| Syn2 | -0.4320976 |
| Sync | -0.0789409 |
| Syncrip | -0.700932 |
| Syne1 | 1.34544071 |
| Syne1 | -0.5783412 |
| Syngr1 | -0.5696135 |
| Syngr1 | 2.67908593 |
| Syngr1 | 1.47186581 |
| Syngr1 | 2.42568367 |
| Syngr2 | -0.5953055 |
| Syngr2 | 0.35961767 |
| Syngr2 | -0.104268 |
| Syngr3 | 0.74446982 |
| Syngr4 | 2.84632841 |
| Syngr4 | -0.6992624 |
| Syngr4 | -0.6545032 |
| Synj2 | -0.2963237 |
| Synj2bp | 2.35807119 |
| Synj2bp | 1.07022032 |
| Synpo2l | -0.7127053 |
| Syp | -0.5820024 |
| Sypl | -0.3591029 |
| Sypl | 1.34998136 |
| Sypl2 | 1.86849412 |
| Sys1 | -0.5244222 |
| Syt1 | 0.74859195 |
| Syt11 | -0.6513318 |
| Syt11 | -0.4767688 |
| Syt12 | -0.1534461 |
| Syt13 | 0.39610703 |
| Syt15 | 0.32379601 |
| Syt16 | -0.7739574 |
| Syt3 | 0.4418021 |
| Syt4 | 1.46311782 |
| Syt5 | 1.09237721 |
| Syt9 | 0.34535035 |
| Sytl2 | -0.5871528 |
| Sytl3 | -0.0671622 |
| Sytl3 | -0.032307 |
| Sytl3 | 1.06186244 |
| Sytl4 | 0.61664775 |
| Sytl5 | 0.5889141 |
| Syvn1 | -0.5516941 |
| Taar2 | -0.0339178 |
| Taar3 | 1.49739211 |
| Taar4 | 0.82798785 |
| Tac1 | -0.0403896 |
| Tacc1 | -0.0734533 |
| Tacc1 | 0.49577452 |
| Tacr3 | 0.81803504 |
| Tacr3 | -0.3730098 |
| Tacstd2 | -0.5908496 |
| Tada1l | 1.33068475 |
| Tada2l | 0.14887724 |
| Tada2l | -0.4826737 |
| Tada3l | 0.35515006 |
| Tada3l | 2.27784114 |
| Taf10 | -0.2451615 |
| Taf11 | 1.42087617 |
| Taf12 | -0.6217397 |
| Taf12 | -0.6778304 |
| Taf12 | 2.50371424 |
| Taf13 | -0.3347031 |
| Taf1c | 0.42535983 |
| Taf3 | 0.18002712 |
| Taf5 | -0.5930748 |
| Taf5 | -0.21274 |
| Taf5 | -0.4142114 |
| Taf5l | -0.3906961 |
| Taf6 | 0.23511959 |
| Taf6 | 0.08142832 |
| Taf6l | -0.3895063 |
| Taf7l | 0.75491074 |
| Taf9 | -0.1608247 |
| Taf9 | -0.6884836 |
| Taf9 | -0.0351278 |
| Taf9b | -0.6513318 |
| Tagap | 2.69938679 |
| Tagap | 2.02477409 |
| Tagln | 2.47570709 |
| Tagln3 | 0.21722363 |
| Tal1 | -0.5581047 |
| Tal2 | 1.08742074 |
| Taldo1 | 3.31405244 |
| Tank | -0.2147023 |
| Tank | -0.3936773 |
| Tank | 0.7771044 |
| Tap1 | 0.85355298 |
| Tap2 | -0.5093892 |
| Tapbp | 1.7285635 |
| Tapbp | -0.2589741 |
| Tapbpl | 2.05719832 |
| Tapbpl | -0.5609684 |
| Tapt1 | -0.2651806 |
| Tarbp2 | -0.6005316 |
| Tarbp2 | -0.5856783 |
| Tardbp | -0.6893083 |
| Tardbp | 2.62430995 |
| Tardbp | 0.99118001 |
| Tars | -0.7524915 |
| Tarsl2 | 1.10453605 |
| Tarsl2 | 1.27071025 |
| Tas1r1 | 1.01786709 |
| Tasp1 | -0.568167 |
| Tatdn2 | 1.12031027 |
| Tax1bp1 | 0.13289536 |
| Tax1bp3 | 1.25152802 |
| Tax1bp3 | 0.89536558 |
| Taz | -0.4932665 |
| Taz | -0.3800433 |
| Tbc1d1 | 0.46685755 |
| Tbc1d10a | -0.6302079 |
| Tbc1d10a | 0.49192595 |
| Tbc1d10c | 2.23649226 |
| Tbc1d12 | 3.6085072 |
| Tbc1d13 | -0.0315027 |
| Tbc1d14 | 0.31668008 |
| Tbc1d15 | -0.5761514 |
| Tbc1d16 | -0.6395361 |
| Tbc1d17 | 0.72128349 |
| Tbc1d19 | -0.6481714 |
| Tbc1d2 | 1.71436159 |
| Tbc1d20 | -0.6697192 |
| Tbc1d22a | -0.5404071 |
| Tbc1d22b | -0.5481519 |
| Tbc1d23 | 1.99485267 |
| Tbc1d24 | -0.7143996 |
| Tbc1d25 | 0.41628759 |
| Tbc1d2b | -0.6537093 |
| Tbc1d5 | -0.5805361 |
| Tbc1d5 | -0.6778304 |
| Tbc1d7 | -0.4333441 |
| Tbc1d7 | -0.4178834 |
| Tbc1d8 | -0.553826 |
| Tbc1d9 | -0.3065828 |
| Tbc1d9 | -0.4142114 |
| Tbc1d9b | -0.0675802 |
| Tbca | 4.45960352 |
| Tbcb | 0.68930316 |
| Tbcc | -0.3142165 |
| Tbcd | 0.37701594 |
| Tbcel | -0.2952505 |
| Tbk1 | 1.83241803 |
| Tbkbp1 | -0.2132302 |
| Tbl1x | 1.98532289 |
| Tbl1xr1 | -0.6705271 |
| Tbl2 | 2.70267291 |
| Tbl3 | -0.6951017 |
| Tbp | -0.025892 |
| Tbpl1 | -0.6616792 |
| Tbr1 | -0.5467389 |
| Tbrg1 | -0.4452714 |
| Tbrg1 | -0.3252333 |
| Tbrg1 | -0.6125903 |
| Tbrg4 | 1.83602768 |
| Tbrg4 | -0.4807012 |
| Tbx1 | 0.79389275 |
| Tbx19 | -0.8099505 |
| Tbx2 | 1.42243645 |
| Tbx22 | -0.583471 |
| Tbx3 | 2.39627386 |
| Tbx3 | 0.99720712 |
| Tbx5 | 0.59013755 |
| Tbx6 | -0.5141457 |
| Tbxa2r | 1.39139312 |
| Tbxa2r | -0.7748628 |
| Tcam1 | 1.04553367 |
| Tcam1 | -0.668912 |
| Tcam1 | -0.6975958 |
| Tcap | 0.93213117 |
| Tcea2 | 0.51477822 |
| Tcea3 | 0.17970805 |
| Tceal1 | 0.37133948 |
| Tceal1 | 0.29639974 |
| Tceal3 | -0.672955 |
| Tceal5 | -0.3319077 |
| Tceal6 | 0.67046062 |
| Tceal6 | -0.307126 |
| Tceal8 | -0.2245776 |
| Tceb1 | 0.44012309 |
| Tceb2 | -0.1876053 |
| Tceb2 | 1.5376913 |
| Tceb3 | -0.0037871 |
| Tcerg1 | -0.4433778 |
| Tcf12 | 1.27332147 |
| Tcf12 | 2.16781904 |
| Tcf15 | 1.47675005 |
| Tcf19 | -0.3493786 |
| Tcf20 | -0.0179359 |
| Tcf20 | 0.80298055 |
| Tcf20 | 0.8418662 |
| Tcf20 | -0.3573793 |
| Tcf20 | -0.8552968 |
| Tcf21 | -0.4069114 |
| Tcf23 | -0.8803652 |
| Tcf25 | -0.862247 |
| Tcf25 | 0.12208354 |
| Tcf25 | -0.3788673 |
| Tcf3 | 0.39459432 |
| Tcf3 | 0.89624098 |
| Tcf4 | -0.766746 |
| Tcf7 | -0.6465953 |
| Tcf7 | -0.1724742 |
| Tcf7l2 | 2.82516237 |
| Tcf7l2 | 1.73825908 |
| Tcfap2b | -0.5645606 |
| Tcfap2b | 0.6883885 |
| Tcfap2c | -0.5805361 |
| Tcfap2c | 1.19292084 |
| Tcfcp2 | -0.6489605 |
| Tcfe3 | 0.87767934 |
| Tcfl5 | 1.18071368 |
| Tchp | 2.27690646 |
| Tcirg1 | -0.3591029 |
| Tcirg1 | 2.48943447 |
| Tcirg1 | -0.6103171 |
| Tcirg1 | -0.5446234 |
| Tcl1 | 3.03590773 |
| Tcl1 | 0.30200085 |
| Tcl1b2 | 0.34694664 |
| Tcl1b2 | -0.477423 |
| Tcl1b3 | 0.39862268 |
| Tcl1b4 | 0.70937676 |
| Tcn2 | -0.0359353 |
| Tcof1 | -0.4952664 |
| Tcp1 | 0.42169273 |
| Tcp1 | -0.4780777 |
| Tcp10a | 0.75457029 |
| Tcp10a | 1.48964863 |
| Tcp11 | 0.03178241 |
| Tcstv1 | -0.7323821 |
| Tcte3 | -0.7042804 |
| Tctex1d1 | 0.81437432 |
| Tctex1d2 | -0.2625894 |
| Tctex1d4 | -0.5524041 |
| Tctn3 | -0.3912915 |
| Tctn3 | -0.3753483 |
| Tctn3 | 1.23757595 |
| Tdg | -0.2235853 |
| Tdp1 | -0.3296775 |
| Tdpoz1 | 1.47093252 |
| Tdpoz2 | 1.31143567 |
| Tdrd1 | -0.8270527 |
| Tdrd12 | 0.62315699 |
| Tdrd3 | 0.62217371 |
| Tdrd3 | -0.1029632 |
| Tdrd3 | 0.42340587 |
| Tdrd6 | -0.5871528 |
| Tdrd7 | 2.30647745 |
| Tdrkh | 0.4456283 |
| Tead2 | -0.4780777 |
| Tec | 0.92551695 |
| Tecta | 2.24491288 |
| Tef | 2.27590626 |
| Tef | -0.8967771 |
| Tegt | 0.70417466 |
| Tek | 1.13772314 |
| Tekt1 | -0.9113831 |
| Tekt1 | -0.1012262 |
| Tekt3 | -0.8194122 |
| Tenc1 | 3.33688086 |
| Tenc1 | -0.3695132 |
| Terf2 | -0.5012923 |
| Terf2 | -0.3158606 |
| Terf2ip | 1.18635983 |
| Terf2ip | 0.40012875 |
| Terf2ip | 1.15426098 |
| Terf2ip | -0.1280799 |
| Tes | -0.7237749 |
| Tes | -0.8241801 |
| Tes | -0.8280123 |
| Tesc | 0.54567069 |
| Tesk1 | -0.6286624 |
| Tesp1 | 0.8153307 |
| Tesp2 | 0.70417466 |
| Tessp2 | 1.67119635 |
| Tessp3 | -0.2594897 |
| Tex10 | -0.3806319 |
| Tex10 | 0.82767355 |
| Tex101 | -0.0785176 |
| Tex11 | 2.19688875 |
| Tex13 | -0.6294348 |
| Tex14 | -0.147951 |
| Tex16 | -0.1552852 |
| Tex19 | 0.5512158 |
| Tex2 | -0.6576856 |
| Tex2 | -0.6027805 |
| Tex22 | 1.49739211 |
| Tex24 | -0.6648867 |
| Tex261 | -0.5645606 |
| Tex261 | 0.90955995 |
| Tex264 | 1.53870348 |
| Tex264 | -0.8513489 |
| Tex9 | 0.30367452 |
| Tfam | 0.59521774 |
| Tfam | 0.52490031 |
| Tfb1m | -0.7220632 |
| Tfb2m | 2.71240093 |
| Tfdp1 | 2.11093775 |
| Tfdp2 | 0.78570165 |
| Tfdp2 | 2.78772711 |
| Tfg | 3.36750381 |
| Tfip11 | 0.71579366 |
| Tfpi | 0.13992181 |
| Tfpt | 1.29762218 |
| Tfpt | -0.2631071 |
| Tfpt | 1.83660182 |
| Tfrc | 0.98339099 |
| Tfrc | -0.7280684 |
| Tg | -0.1227345 |
| Tgfb1 | -0.713552 |
| Tgfb1i1 | 1.29818626 |
| Tgfb1i1 | -0.7306542 |
| Tgfb2 | -0.1612879 |
| Tgfb3 | -0.2787742 |
| Tgfbi | -0.6012806 |
| Tgfbi | 0.75746002 |
| Tgfbr1 | -0.0432354 |
| Tgfbr1 | -0.5893691 |
| Tgfbr1 | -0.8318606 |
| Tgfbr2 | 2.39994582 |
| Tgfbr2 | -0.6529161 |
| Tgfbr3 | 1.18582866 |
| Tgfbr3 | 1.34275917 |
| Tgfbrap1 | -0.5864153 |
| Tgif1 | -0.7959389 |
| Tgif1 | -0.7051194 |
| Tgif1 | 0.39509884 |
| Tgif2 | 0.51742958 |
| Tgif2lx | 2.14370784 |
| Tgm2 | -0.6616792 |
| Tgm4 | 0.49554858 |
| Tgoln1 | -0.2315543 |
| Tgs1 | -0.5827364 |
| Tgs1 | -0.0604989 |
| Th1l | 1.12395591 |
| Tha1 | -0.6309816 |
| Thada | 2.76482207 |
| Thada | 0.32814769 |
| Thap1 | -0.4008721 |
| Thap1 | -0.2952505 |
| Thap1 | -0.8493812 |
| Thap11 | 0.80652621 |
| Thap3 | -0.6697192 |
| Thap4 | -0.6552978 |
| Thap7 | -0.7026046 |
| Thbd | 0.42584766 |
| Thbs1 | -0.7410711 |
| Thbs2 | 0.56010288 |
| Thbs3 | 0.33301872 |
| Thbs3 | -0.3906961 |
| Theg | -0.2641433 |
| Theg | 0.11594527 |
| Theg | 0.53728951 |
| Them2 | 0.52139227 |
| Them4 | -0.6893083 |
| Them5 | -0.5093892 |
| Thg1l | 0.33193851 |
| Thnsl1 | 0.92622287 |
| Thnsl2 | -0.6827319 |
| Thnsl2 | 0.74979075 |
| Thoc1 | 1.55083442 |
| Thoc3 | -0.5161918 |
| Thoc4 | 0.06240005 |
| Thoc4 | 0.30255908 |
| Thoc5 | -0.235565 |
| Thoc6 | 2.19113406 |
| Thop1 | 0.54310004 |
| Thra | 0.57265082 |
| Thra | 1.08588113 |
| Thrap2 | 1.49550025 |
| Thrap3 | 0.95607332 |
| Thrsp | -0.0195216 |
| Thsd1 | 0.2770953 |
| Thsd4 | 2.60116661 |
| Thsd4 | -0.7026046 |
| Thsd7b | 0.93841944 |
| Thsd7b | 0.12581453 |
| Thsd7b | -0.4289887 |
| Thtpa | -0.4787329 |
| Thumpd1 | 2.70697566 |
| Thumpd3 | -0.6851925 |
| Thy1 | 0.06058433 |
| Thyn1 | -0.6442362 |
| Tia1 | -0.4986092 |
| Tial1 | -0.8376636 |
| Tial1 | 0.32079218 |
| Tiam1 | -0.0705121 |
| Tiam2 | -0.3488098 |
| Ticam1 | 0.5803038 |
| Tie1 | 2.29254082 |
| Tigd2 | 1.42938425 |
| Tigd3 | 1.9297386 |
| Timeless | -0.8783343 |
| Timm10 | 0.06131105 |
| Timm13 | 2.64769973 |
| Timm17a | -0.4057003 |
| Timm17b | 0.24557274 |
| Timm22 | -0.1967093 |
| Timm44 | 1.8066359 |
| Timm44 | 3.45045137 |
| Timm44 | -0.6232736 |
| Timm50 | 3.46057133 |
| Timm8a1 | -0.632531 |
| Timm8a2 | 1.56139794 |
| Timm8a2 | -0.5369083 |
| Timm8b | -0.6286624 |
| Timm8b | -0.3918874 |
| Timm9 | 1.24477787 |
| Timm9 | 1.17470984 |
| Timm9 | -0.4820158 |
| Timp1 | 0.09413111 |
| Timp1 | 0.27249327 |
| Timp2 | -0.5196121 |
| Timp3 | 0.82908707 |
| Timp3 | -0.2156851 |
| Timp4 | -0.4839912 |
| Tinagl | -0.6194438 |
| Tipin | -0.088731 |
| Tipin | -0.5046573 |
| Tirap | -0.5595354 |
| Tirap | 2.59508319 |
| Tirap | -0.5012923 |
| Tjap1 | -0.5278735 |
| Tjap1 | 1.12236273 |
| Tjap1 | -0.253834 |
| Tjp1 | 4.04722626 |
| Tjp2 | -0.6103171 |
| Tjp3 | 0.6424114 |
| Tjp3 | 0.44658236 |
| Tk1 | -0.6186797 |
| Tk2 | 3.75770761 |
| Tkt | 2.17028616 |
| Tkt | 1.98643096 |
| Tkt | 2.97334093 |
| Tktl1 | -0.3671896 |
| Tktl1 | -0.2824688 |
| Tktl2 | 1.17632188 |
| Tlcd1 | 0.86191837 |
| Tle1 | -0.8146692 |
| Tle1 | -0.6681055 |
| Tle2 | 1.04044065 |
| Tle3 | -0.4014743 |
| Tle3 | -0.7757691 |
| Tle3 | 0.10284987 |
| Tle4 | 0.27537225 |
| Tle4 | 1.66712368 |
| Tle4 | 1.64461935 |
| Tle6 | 2.79190589 |
| Tlk1 | -0.4631369 |
| Tlk2 | 0.82357774 |
| Tln1 | -0.5446234 |
| Tln2 | -0.2310542 |
| Tlr13 | 3.28840086 |
| Tlr2 | 0.40113143 |
| Tlr3 | -0.7367163 |
| Tlr4 | 0.47682911 |
| Tlr6 | 0.26000937 |
| Tlr7 | 2.61374917 |
| Tm2d2 | -0.2078525 |
| Tm2d3 | 1.51794837 |
| Tm2d3 | -0.4573563 |
| Tm4sf4 | -0.7254899 |
| Tm4sf5 | 1.71029731 |
| Tm6sf1 | 0.12412051 |
| Tm6sf2 | 0.31089192 |
| Tm9sf2 | 1.65892355 |
| Tm9sf2 | -0.6202084 |
| Tm9sf2 | 2.1991384 |
| Tm9sf3 | -0.4657179 |
| Tm9sf4 | 0.78504319 |
| Tm9sf4 | 2.28961972 |
| Tmbim1 | -0.6309816 |
| Tmbim4 | -0.6827319 |
| Tmc1 | -0.5559628 |
| Tmc4 | -0.0659091 |
| Tmc5 | -0.2310542 |
| Tmc6 | 1.44712557 |
| Tmc6 | 0.10976554 |
| Tmc6 | 2.85270937 |
| Tmc7 | 1.27476893 |
| Tmcc2 | 3.0176646 |
| Tmcc3 | 1.40763034 |
| Tmco3 | -0.4899431 |
| Tmco3 | -0.1795358 |
| Tmco4 | -0.6148691 |
| Tmco4 | -0.7393267 |
| Tmco5 | -0.409946 |
| Tmco6 | 1.08528829 |
| Tmco6 | -0.293643 |
| Tmco7 | -0.7195015 |
| Tmed1 | 0.66446836 |
| Tmed1 | -0.7560344 |
| Tmed10 | -0.6450219 |
| Tmed2 | -0.1603618 |
| Tmed3 | -0.5761514 |
| Tmed3 | 2.39661343 |
| Tmed4 | 1.26954732 |
| Tmed4 | -0.7272081 |
| Tmed4 | 2.20001567 |
| Tmed4 | -0.5595354 |
| Tmed8 | 1.38645669 |
| Tmed8 | 1.97913738 |
| Tmed9 | -0.3341433 |
| Tmed9 | -0.1271868 |
| Tmeff2 | -0.5073583 |
| Tmem10 | -0.2290565 |
| Tmem100 | 1.59550502 |
| Tmem101 | -0.7507252 |
| Tmem102 | -0.4945993 |
| Tmem102 | -0.581269 |
| Tmem106a | -0.7384557 |
| Tmem106c | 1.4026133 |
| Tmem107 | 2.39247342 |
| Tmem107 | -0.4722022 |
| Tmem107 | -0.1397697 |
| Tmem108 | 1.05734903 |
| Tmem109 | 3.7696452 |
| Tmem11 | 1.2100523 |
| Tmem110 | -0.6348601 |
| Tmem111 | 0.89973423 |
| Tmem111 | -0.0802119 |
| Tmem111 | 2.10776209 |
| Tmem115 | 0.01283104 |
| Tmem116 | -0.100359 |
| Tmem116 | -0.7640563 |
| Tmem117 | -0.3579535 |
| Tmem118 | -0.7332473 |
| Tmem119 | 1.1814614 |
| Tmem120a | 1.25714812 |
| Tmem120b | 1.4444862 |
| Tmem121 | 0.72287116 |
| Tmem125 | -0.2883049 |
| Tmem126a | 1.36652746 |
| Tmem126b | 0.07429425 |
| Tmem127 | -0.5667227 |
| Tmem128 | 0.28024583 |
| Tmem130 | 0.35646633 |
| Tmem131 | 0.77077031 |
| Tmem132a | 0.65559546 |
| Tmem132a | 0.01779628 |
| Tmem132c | 0.63487463 |
| Tmem132d | 1.23697322 |
| Tmem132e | -0.7959389 |
| Tmem134 | -0.2206147 |
| Tmem134 | 1.29006768 |
| Tmem135 | -0.7876338 |
| Tmem136 | 1.35688259 |
| Tmem138 | 1.63861422 |
| Tmem139 | -0.1852245 |
| Tmem140 | -0.4644265 |
| Tmem141 | -0.149779 |
| Tmem143 | -0.1923856 |
| Tmem143 | 0.93828016 |
| Tmem143 | -0.7358478 |
| Tmem143 | -0.2300548 |
| Tmem144 | 0.54759396 |
| Tmem146 | 2.45468213 |
| Tmem147 | -0.476115 |
| Tmem14c | 0.49735453 |
| Tmem14c | 0.01014624 |
| Tmem14c | 1.70747759 |
| Tmem150 | 1.64403993 |
| Tmem150 | 0.34455116 |
| Tmem151a | 0.04222618 |
| Tmem154 | -0.563841 |
| Tmem158 | -0.6992624 |
| Tmem159 | 1.54460959 |
| Tmem159 | -0.4939326 |
| Tmem160 | -0.5073583 |
| Tmem160 | -0.4184969 |
| Tmem161a | -0.7730528 |
| Tmem162 | -0.2641433 |
| Tmem164 | 1.60368535 |
| Tmem166 | -0.1682633 |
| Tmem167 | -0.0999256 |
| Tmem168 | -0.3330248 |
| Tmem168 | -0.6681055 |
| Tmem168 | -0.4793885 |
| Tmem169 | 2.46765348 |
| Tmem16a | 1.33511853 |
| Tmem16b | 1.47419489 |
| Tmem16d | 0.03065633 |
| Tmem16e | 0.13892132 |
| Tmem16g | -0.1650015 |
| Tmem16j | 1.78497726 |
| Tmem16k | -0.3352632 |
| Tmem17 | 1.29338045 |
| Tmem17 | -0.3213623 |
| Tmem170 | 1.76516596 |
| Tmem176a | -0.6745773 |
| Tmem176a | 2.50940934 |
| Tmem176b | -0.4945993 |
| Tmem177 | 0.71419365 |
| Tmem178 | 1.90654292 |
| Tmem179b | -0.6118319 |
| Tmem180 | 0.82547039 |
| Tmem183a | 1.10882312 |
| Tmem183a | 0.84665121 |
| Tmem184a | -0.7237749 |
| Tmem184a | 0.66446836 |
| Tmem184b | 0.52007328 |
| Tmem185b | 0.26263642 |
| Tmem186 | -0.7445699 |
| Tmem19 | 0.32106566 |
| Tmem192 | -0.307126 |
| Tmem198 | 1.15492071 |
| Tmem199 | 1.67500198 |
| Tmem2 | 2.25791072 |
| Tmem2 | 0.58441405 |
| Tmem2 | 0.63118025 |
| Tmem201 | -0.5502755 |
| Tmem204 | -0.668912 |
| Tmem205 | -0.409946 |
| Tmem208 | -0.4820158 |
| Tmem209 | -0.5776107 |
| Tmem209 | -0.3642933 |
| Tmem23 | 0.30840003 |
| Tmem25 | -0.0448656 |
| Tmem25 | -0.0432354 |
| Tmem25 | -0.4522485 |
| Tmem27 | 0.1958405 |
| Tmem32 | 0.56388537 |
| Tmem33 | -0.5355125 |
| Tmem33 | 0.64759303 |
| Tmem34 | 0.79649849 |
| Tmem35 | 0.72128349 |
| Tmem38a | -0.4579968 |
| Tmem38b | 0.76152417 |
| Tmem38b | 0.02349213 |
| Tmem39a | 1.28302379 |
| Tmem40 | 0.66013706 |
| Tmem41a | 0.21997625 |
| Tmem41b | 1.58615385 |
| Tmem42 | -0.5397062 |
| Tmem43 | -0.7867157 |
| Tmem43 | 2.67710998 |
| Tmem44 | -0.4136009 |
| Tmem45a | 1.26292928 |
| Tmem45b | -0.7229187 |
| Tmem47 | 1.71519612 |
| Tmem47 | 3.52306219 |
| Tmem48 | 0.07214314 |
| Tmem49 | -0.1231788 |
| Tmem49 | -0.6901337 |
| Tmem49 | -0.3960693 |
| Tmem49 | 0.40637751 |
| Tmem5 | -0.6576856 |
| Tmem50a | -0.2829979 |
| Tmem50b | -0.7931622 |
| Tmem50b | -0.495934 |
| Tmem51 | 0.47590609 |
| Tmem53 | 0.29019865 |
| Tmem53 | -0.0128014 |
| Tmem53 | 0.2239377 |
| Tmem54 | 0.55778365 |
| Tmem55a | -0.2564004 |
| Tmem55b | -0.7323821 |
| Tmem56 | 2.1093513 |
| Tmem57 | -0.3153122 |
| Tmem59 | 1.19439587 |
| Tmem59l | -0.4277481 |
| Tmem60 | 0.82405127 |
| Tmem62 | -0.6713357 |
| Tmem63a | 0.24349164 |
| Tmem63b | -0.1957467 |
| Tmem63b | 0.2008436 |
| Tmem63c | -0.5073583 |
| Tmem65 | 3.13504104 |
| Tmem66 | 0.01893829 |
| Tmem66 | -0.5230453 |
| Tmem66 | -0.4676583 |
| Tmem68 | 1.43837855 |
| Tmem69 | 0.06131105 |
| Tmem70 | 0.50967487 |
| Tmem71 | 2.37430406 |
| Tmem74 | -0.13751 |
| Tmem74 | -0.6737658 |
| Tmem74 | 1.90692954 |
| Tmem77 | -0.0391726 |
| Tmem77 | 2.81545853 |
| Tmem77 | -0.759591 |
| Tmem79 | -0.6576856 |
| Tmem8 | 0.1452393 |
| Tmem8 | -0.5066823 |
| Tmem8 | 0.9336723 |
| Tmem81 | -0.2829979 |
| Tmem82 | 0.17233851 |
| Tmem85 | 0.01969884 |
| Tmem85 | 1.9465955 |
| Tmem86a | 0.70848195 |
| Tmem86b | -0.807131 |
| Tmem87a | -0.5376069 |
| Tmem88 | -0.6057877 |
| Tmem9 | -0.626349 |
| Tmem90a | 0.27335792 |
| Tmem91 | -0.5645606 |
| Tmem93 | 0.95155442 |
| Tmem97 | 1.32312511 |
| Tmem98 | 3.74540494 |
| Tmem98 | 0.92155371 |
| Tmem9b | 2.57255699 |
| Tmem9b | 1.84965706 |
| Tmem9b | -0.3741783 |
| Tmie | 2.73843209 |
| Tmod1 | -0.5127842 |
| Tmod2 | -0.3823999 |
| Tmod2 | -0.3510871 |
| Tmod3 | -0.5842062 |
| Tmod4 | 3.00480254 |
| Tmod4 | 0.86870635 |
| Tmod4 | 2.61648981 |
| Tmpo | -0.4440086 |
| Tmpo | 2.32529634 |
| Tmpo | 2.68228002 |
| Tmprss11a | -0.6884836 |
| Tmprss11c | -0.4452714 |
| Tmprss13 | -0.4446398 |
| Tmprss13 | -0.4986092 |
| Tmprss2 | 0.08071741 |
| Tmprss4 | -0.4972706 |
| Tmprss4 | -0.4459035 |
| Tmprss4 | 2.4742268 |
| Tmprss7 | -0.6286624 |
| Tmprss9 | 0.63720025 |
| Tmsb10 | -0.545328 |
| Tmsb10 | 0.16361088 |
| Tmsb10 | 1.10372272 |
| Tmsb4x | 0.1544712 |
| Tmtc2 | -0.4389745 |
| Tmtc4 | -0.6505407 |
| Tmub1 | 0.20736892 |
| Tmub1 | 0.50230259 |
| Tnc | 0.08249365 |
| Tnc | 1.38841742 |
| Tnc | 2.33148425 |
| Tnc | -0.1819018 |
| Tnfaip1 | 1.33213488 |
| Tnfaip2 | -0.2522975 |
| Tnfaip2 | -0.4038868 |
| Tnfaip2 | 1.12600021 |
| Tnfaip3 | -0.469603 |
| Tnfaip8 | 1.11435496 |
| Tnfaip8l2 | 2.39434725 |
| Tnfaip8l2 | 1.57947468 |
| Tnfaip8l3 | -0.6005316 |
| Tnfrsf10b | 1.13446932 |
| Tnfrsf10b | -0.7384557 |
| Tnfrsf10b | 2.25424891 |
| Tnfrsf11a | 1.65116061 |
| Tnfrsf11b | 0.53382874 |
| Tnfrsf12a | -0.7220632 |
| Tnfrsf13c | -0.6348601 |
| Tnfrsf19 | 0.60549467 |
| Tnfrsf19 | 1.83178881 |
| Tnfrsf1a | -0.1303163 |
| Tnfrsf1a | -0.403283 |
| Tnfrsf1a | -0.5327273 |
| Tnfrsf1b | 1.4058775 |
| Tnfrsf21 | 0.79454488 |
| Tnfrsf21 | -0.7987241 |
| Tnfrsf22 | -0.0002838 |
| Tnfrsf22 | -0.6088048 |
| Tnfrsf25 | 1.68287474 |
| Tnfrsf4 | -0.5746945 |
| Tnfsf13b | -0.2073651 |
| Tnfsf13b | 1.02422882 |
| Tnip1 | 0.19207014 |
| Tnip1 | -0.8108923 |
| Tnip1 | -0.1303163 |
| Tnip1 | 2.73466622 |
| Tnip2 | 3.07386489 |
| Tnks | 0.28537794 |
| Tnks1bp1 | -0.4722022 |
| Tnnc1 | -0.0395781 |
| Tnnc2 | 0.11491821 |
| Tnni2 | 1.51706094 |
| Tnni3 | 0.95182893 |
| Tnnt1 | 1.54288491 |
| Tnp1 | 0.86990784 |
| Tnp2 | 3.5424559 |
| Tnpo1 | 0.49283295 |
| Tnpo2 | -0.4979397 |
| Tnr | 0.52817692 |
| Tnrc15 | -0.6271195 |
| Tnrc18 | -0.6225063 |
| Tnrc18 | -0.6624801 |
| Tnrc18 | -0.7177977 |
| Tnrc6a | 1.96205627 |
| Tnrc6a | 0.37650135 |
| Tnrc6b | -0.7803143 |
| Tnrc6b | -0.7631615 |
| Tnrc6c | -0.1996031 |
| Tns4 | 2.67291515 |
| Tnxb | 0.88774348 |
| Tnxb | -0.5982882 |
| Tnxb | 4.0778222 |
| Tob1 | -0.1659322 |
| Tob2 | 0.77992394 |
| Toe1 | -0.4178834 |
| Toe1 | -0.4986092 |
| Tollip | 3.05296613 |
| Tollip | 0.95702902 |
| Tom1 | 1.57816066 |
| Tom1l2 | 1.71864536 |
| Tom1l2 | -0.343141 |
| Tom1l2 | -0.5982882 |
| Tomm22 | 2.62457462 |
| Tomm34 | -0.3642933 |
| Tomm34 | 1.39096861 |
| Tomm7 | 0.68306533 |
| Tomm70a | -0.4522485 |
| Top1 | -0.6616792 |
| Top1mt | -0.3120289 |
| Top2a | -0.7280684 |
| Top3a | 1.25842508 |
| Top3b | -0.2005698 |
| Topors | 2.48789942 |
| Tor1a | -0.4644265 |
| Tor1a | -0.3776928 |
| Tor1aip1 | -0.0865937 |
| Tor1aip2 | -0.1461266 |
| Tor1aip2 | -0.5066823 |
| Tor1b | 2.08450232 |
| Tor3a | 0.31777845 |
| Tox3 | 1.01223195 |
| Tpbg | 1.82779367 |
| Tpcn1 | 1.71620845 |
| Tpcn1 | -0.5798039 |
| Tpcn2 | 0.76017146 |
| Tpd52 | -0.848399 |
| Tpd52l1 | -0.5141457 |
| Tpd52l1 | -0.7903938 |
| Tpd52l2 | -0.5369083 |
| Tph2 | 0.06348775 |
| Tpi1 | 1.46429429 |
| Tpi1 | 1.89054912 |
| Tpi1 | 0.26380157 |
| Tpk1 | -0.3087574 |
| Tpk1 | 0.01129783 |
| Tpk1 | -0.5871528 |
| Tpm1 | 1.50439567 |
| Tpm2 | -0.4002704 |
| Tpm2 | 1.56765 |
| Tpm3 | -0.7950124 |
| Tpm3 | 1.25192354 |
| Tpm4 | 0.15545483 |
| Tpo | 1.37943184 |
| Tpp1 | 0.2739339 |
| Tpp2 | 1.22167236 |
| Tppp | 0.20021971 |
| Tppp | -0.7152479 |
| Tppp3 | -0.6012806 |
| Tpr | -0.7254899 |
| Tpra40 | -0.6110742 |
| Tprgl | -0.431475 |
| Tprgl | 1.90943861 |
| Tprkb | -0.5093892 |
| Tprkb | 2.88593752 |
| Tpsab1 | 2.32456199 |
| Tpst1 | 1.75175418 |
| Tpst2 | -0.6926143 |
| Tpte | -0.5967956 |
| Tpte | -0.2320547 |
| Tpx2 | -0.5216705 |
| Tra2a | -0.4820158 |
| Trabd | 0.07679751 |
| Trabd | -0.7613745 |
| Traf1 | -0.5923325 |
| Traf2 | -0.8652419 |
| Traf3 | 1.21036271 |
| Traf3 | -0.2201205 |
| Traf3ip1 | -0.5768808 |
| Traf3ip2 | 1.54021965 |
| Traf4 | 0.13289536 |
| Traf4 | 2.28352531 |
| Traf4 | 0.16198548 |
| Traf4 | 3.03272297 |
| Traf5 | 0.92763307 |
| Traf6 | -0.4689543 |
| Traf6 | -0.6371951 |
| Traf6 | -0.5033099 |
| Traf7 | 1.93138659 |
| Trafd1 | -0.7101696 |
| Trafd1 | -0.6442362 |
| Trafd1 | -0.8396062 |
| Traip | 0.96219972 |
| Trak1 | -0.2709071 |
| Trak2 | -0.672955 |
| Trak2 | 0.36614832 |
| Trak2 | 0.92410348 |
| Tram1 | -0.7685435 |
| Tram1 | -0.0567721 |
| Tram1 | -0.6240415 |
| Tram2 | -0.5121042 |
| Tram2 | -0.3759339 |
| Trap1 | -0.7263486 |
| Trappc1 | 0.39004112 |
| Trappc1 | 0.84865041 |
| Trappc2 | 1.28693318 |
| Trappc2l | 0.13121441 |
| Trappc3 | 2.39068118 |
| Trappc3 | -0.7143996 |
| Trappc3 | -0.3448379 |
| Trappc3 | 0.96124941 |
| Trappc4 | -0.5161918 |
| Trappc4 | -0.3871314 |
| Trappc5 | -0.5768808 |
| Trappc6a | 0.66239943 |
| Trat1 | 2.35072343 |
| Trcg1 | -0.184274 |
| Trdmt1 | -0.4853105 |
| Trdmt1 | 1.61405854 |
| Trem2 | -0.5616857 |
| Treml1 | 0.42120267 |
| Treml2 | 1.5546059 |
| Treml4 | -0.5175584 |
| Trerf1 | 3.69559943 |
| Trerf1 | 0.93185068 |
| Trex1 | -0.707641 |
| Trex1 | 0.3981201 |
| Trex2 | -0.3169583 |
| Trf | 0.5567275 |
| Trfp | -0.5046573 |
| Trfr2 | -0.7604823 |
| Trfr2 | 0.34348449 |
| Trh | 1.04404596 |
| Trhde | -0.2390889 |
| Trhr | -0.5439193 |
| Triap1 | -0.2240813 |
| Trib2 | 0.98815149 |
| Trib3 | -0.0399837 |
| Trib3 | -0.6458083 |
| Trim11 | 3.33197789 |
| Trim16 | 1.78293233 |
| Trim17 | -0.5923325 |
| Trim2 | -0.7051194 |
| Trim2 | -0.8090097 |
| Trim21 | 0.58625765 |
| Trim21 | 1.58855194 |
| Trim23 | 0.32024496 |
| Trim23 | -0.5790722 |
| Trim23 | -0.4459035 |
| Trim25 | -0.7631615 |
| Trim26 | -0.7658485 |
| Trim28 | -0.8328252 |
| Trim3 | 2.27639037 |
| Trim3 | -0.3545139 |
| Trim30 | -0.7026046 |
| Trim32 | -0.7480822 |
| Trim32 | 0.80700865 |
| Trim33 | 1.28540955 |
| Trim33 | 0.63468056 |
| Trim33 | -0.0485442 |
| Trim35 | -0.225571 |
| Trim36 | -0.3442719 |
| Trim36 | -0.7578109 |
| Trim36 | 0.46054033 |
| Trim37 | 0.29132925 |
| Trim38 | 1.17438709 |
| Trim38 | -0.6778304 |
| Trim39 | 2.20870766 |
| Trim40 | 1.22157018 |
| Trim41 | 1.18911695 |
| Trim42 | -0.4221862 |
| Trim44 | 0.63234851 |
| Trim45 | -0.6705271 |
| Trim45 | 1.39984215 |
| Trim46 | 1.51750476 |
| Trim47 | 0.51100893 |
| Trim54 | -0.4787329 |
| Trim56 | -0.6810951 |
| Trim59 | 0.21967082 |
| Trim61 | 0.34800929 |
| Trim62 | 0.55567013 |
| Trim67 | -0.6442362 |
| Trim68 | 1.98713943 |
| Trim69 | -0.1762336 |
| Trim72 | -0.4631369 |
| Trim8 | -0.0351278 |
| Trim9 | -0.4986092 |
| Trim9 | -0.6020302 |
| Trim9 | 0.81389575 |
| Trim9 | -0.6379748 |
| Triml1 | -0.0742954 |
| Triobp | 0.03664613 |
| Triobp | -0.6959323 |
| Trip10 | 0.34401798 |
| Trip12 | -0.2507637 |
| Trip12 | 3.08382428 |
| Trip12 | 1.11481444 |
| Trip6 | -0.607295 |
| Trip6 | 0.2488335 |
| Trit1 | -0.5306439 |
| Trmt1 | 1.8958463 |
| Trmt12 | 1.45808116 |
| Trmu | -0.7306542 |
| Tro | -0.6186797 |
| Tro | 0.50342343 |
| Tro | 0.06276276 |
| Trove2 | 0.58952603 |
| Trp53 | 0.14854711 |
| Trp53bp1 | -0.7987241 |
| Trp53bp1 | -0.6020302 |
| Trp53bp2 | -0.5893691 |
| Trp53i11 | 0.0849746 |
| Trp53i11 | -0.2793011 |
| Trp53inp1 | -0.4986092 |
| Trp53inp1 | 1.37607173 |
| Trp53inp2 | 0.46218232 |
| Trp53rk | 0.06022076 |
| Trp63 | -0.6819131 |
| Trpc1 | -0.3169583 |
| Trpc1 | -0.5196121 |
| Trpc2 | 0.6958619 |
| Trpc3 | -0.4154338 |
| Trpc4 | 0.08744884 |
| Trpc4ap | -0.5997832 |
| Trpc4ap | -0.6835513 |
| Trpc5 | -0.5141457 |
| Trpc6 | 0.10180799 |
| Trpc7 | 1.39824283 |
| Trpd52l3 | -0.6537093 |
| Trpm1 | -0.1165375 |
| Trpm2 | -0.581269 |
| Trpm3 | 0.17747142 |
| Trpm3 | -0.3186073 |
| Trpm3 | -0.4919357 |
| Trpm3 | -0.6294348 |
| Trpm3 | 2.00045347 |
| Trpm4 | -0.7254899 |
| Trpm6 | -0.4780777 |
| Trpm6 | -0.21274 |
| Trpm6 | 1.06344296 |
| Trpm8 | 0.21045994 |
| Trps1 | -0.8425277 |
| Trpt1 | 2.02115629 |
| Trpt1 | 0.47798157 |
| Trpt1 | -0.5264914 |
| Trpv2 | 1.14630686 |
| Trpv4 | -0.3568055 |
| Trpv6 | 0.44634394 |
| Trrap | 1.51424506 |
| Trub1 | 3.31308662 |
| Trub1 | 4.24947093 |
| Trub1 | -0.4414881 |
| Trub2 | 1.78828367 |
| Tsc1 | -0.3224667 |
| Tsc2 | -0.5244222 |
| Tsc2 | 0.28708227 |
| Tsc22d1 | 0.82357774 |
| Tsc22d1 | 0.44467325 |
| Tsc22d2 | -0.7498434 |
| Tsc22d3 | 0.69459026 |
| Tsc22d3 | -0.7640563 |
| Tsc22d4 | -0.4044909 |
| Tsen2 | 1.43773326 |
| Tsen34 | -0.2406032 |
| Tsen54 | -0.6762024 |
| Tsfm | -0.3448379 |
| Tsg101 | -0.7703446 |
| Tsga10 | 2.72446178 |
| Tsga10 | -0.9009265 |
| Tsga14 | -0.7254899 |
| Tsga8 | -0.848399 |
| Tshb | 1.5721196 |
| Tshr | 0.53426206 |
| Tshz3 | -0.5938178 |
| Tsku | -0.2872411 |
| Tsn | -0.656093 |
| Tsnaxip1 | 1.28655251 |
| Tsnaxip1 | -0.5348154 |
| Tspan1 | 0.08426644 |
| Tspan11 | -0.7739574 |
| Tspan12 | -0.5244222 |
| Tspan13 | -0.8213164 |
| Tspan14 | 0.09658075 |
| Tspan17 | 1.34811441 |
| Tspan17 | 0.22272062 |
| Tspan18 | 0.55057769 |
| Tspan18 | 1.26993513 |
| Tspan2 | 0.21538394 |
| Tspan3 | -0.1316608 |
| Tspan31 | 0.51720893 |
| Tspan33 | -0.545328 |
| Tspan6 | 0.5045429 |
| Tspan6 | 1.92907857 |
| Tspan7 | -0.0776716 |
| Tspan8 | -0.6640838 |
| Tspo | -0.080636 |
| Tspo | 2.61122408 |
| Tspo | -0.6959323 |
| Tspyl1 | 3.02806685 |
| Tspyl3 | 0.41134597 |
| Tsr1 | 1.25989628 |
| Tsr2 | -0.2250742 |
| Tsr2 | 1.35432282 |
| Tsr2 | 0.4048817 |
| Tssc1 | -0.2340589 |
| Tssc1 | -0.7272081 |
| Tssc1 | 0.02952886 |
| Tssc1 | 1.51394814 |
| Tssc1 | 0.36484591 |
| Tssc4 | 1.35317301 |
| Tssc4 | -0.6426668 |
| Tssk4 | -0.3794551 |
| Tssk5 | 1.07058198 |
| Tssk6 | -0.5155092 |
| Tst | -0.2117604 |
| Tsta3 | -0.2931078 |
| Tsx | -0.261555 |
| Ttbk2 | -0.6545032 |
| Ttc1 | -0.6640838 |
| Ttc12 | -0.6681055 |
| Ttc13 | 0.68361744 |
| Ttc14 | -0.6271195 |
| Ttc16 | -0.1890368 |
| Ttc17 | 0.58646227 |
| Ttc19 | 3.89622997 |
| Ttc19 | 0.65597479 |
| Ttc21a | 1.11401019 |
| Ttc22 | 0.6573012 |
| Ttc23 | -0.0785176 |
| Ttc24 | 0.70543305 |
| Ttc24 | 1.17363356 |
| Ttc26 | 0.51344965 |
| Ttc27 | 2.44211665 |
| Ttc29 | 1.84237531 |
| Ttc29 | -0.6080496 |
| Ttc3 | 2.12383633 |
| Ttc3 | -0.5127842 |
| Ttc3 | -0.1484077 |
| Ttc3 | -0.6095606 |
| Ttc3 | 0.18606845 |
| Ttc3 | 1.67294516 |
| Ttc30a1 | 0.07286074 |
| Ttc30a2 | -0.0084791 |
| Ttc33 | 0.56952993 |
| Ttc33 | 0.04444871 |
| Ttc33 | 1.74699021 |
| Ttc35 | 1.90663959 |
| Ttc4 | 2.8809521 |
| Ttc5 | -0.5856783 |
| Ttc5 | 0.9335323 |
| Ttc5 | 0.3169548 |
| Ttc7 | -0.2385846 |
| Ttc8 | -0.5033099 |
| Ttc8 | 1.2020488 |
| Ttf2 | 0.09867523 |
| Ttk | -0.8194122 |
| Ttl | -0.1343558 |
| Ttll1 | 0.84526459 |
| Ttll1 | 0.28081749 |
| Ttll1 | 0.51522065 |
| Ttll10 | -0.0968982 |
| Ttll11 | -0.1095063 |
| Ttll13 | -0.4184969 |
| Ttll4 | 3.33078625 |
| Ttll5 | -0.7712464 |
| Ttll5 | 1.24994422 |
| Ttll6 | 2.87592261 |
| Ttll8 | 2.83817563 |
| Ttll9 | 0.98511292 |
| Ttn | 1.52969924 |
| Ttn | -0.0908732 |
| Ttr | -0.0917315 |
| Ttyh1 | 0.50028162 |
| Ttyh1 | 2.69582366 |
| Ttyh2 | -0.3380689 |
| Ttyh2 | -0.1303163 |
| Ttyh3 | -0.6951017 |
| Tub | -0.4605633 |
| Tub | -0.6967637 |
| Tuba1a | 0.96138523 |
| Tuba1b | -0.7676443 |
| Tuba3a | -0.7051194 |
| Tuba3a | 1.47976061 |
| Tuba3a | -0.7587005 |
| Tuba3a | -0.1461266 |
| Tuba3b | 3.43088393 |
| Tuba4a | 1.13435691 |
| Tuba6 | -0.477423 |
| Tuba8 | 3.29145942 |
| Tubb2b | -0.5087117 |
| Tubb2b | 0.11765448 |
| Tubb2b | -0.1529869 |
| Tubb2b | 1.72697674 |
| Tubb2b | 2.58519111 |
| Tubb2c | -0.743694 |
| Tubb4 | -0.7587005 |
| Tubb5 | -0.5060068 |
| Tubb6 | 1.80760586 |
| Tubd1 | 2.24354269 |
| Tubd1 | -0.5251114 |
| Tube1 | -0.8612509 |
| Tubg1 | -0.6403178 |
| Tubgcp2 | 1.26175641 |
| Tubgcp4 | 1.1900694 |
| Tufm | -0.6186797 |
| Tuft1 | -0.5768808 |
| Tug1 | 0.72004673 |
| Tulp1 | -0.4853105 |
| Tulp2 | 2.12353133 |
| Tulp3 | 2.85351674 |
| Tulp4 | 1.26595227 |
| Tusc2 | -0.5237335 |
| Tusc2 | 1.05563543 |
| Tusc4 | 0.30923142 |
| Tut1 | -0.7463243 |
| Twf2 | 0.52073301 |
| Twist1 | -0.7757691 |
| Twist2 | -0.0159577 |
| Twistnb | 0.91856998 |
| Twistnb | 1.32750456 |
| Twsg1 | -0.7067997 |
| Txk | -0.6600797 |
| Txlna | -0.6884836 |
| Txn1 | 1.60301205 |
| Txn2 | 0.37958455 |
| Txndc1 | -0.4839912 |
| Txndc11 | 1.04392188 |
| Txndc11 | -0.409946 |
| Txndc11 | 3.62256526 |
| Txndc12 | 1.64751097 |
| Txndc13 | 0.02651547 |
| Txndc13 | 2.77529949 |
| Txndc15 | 1.8218741 |
| Txndc2 | -0.7143996 |
| Txndc5 | -0.7578109 |
| Txndc8 | -0.6552978 |
| Txndc9 | 0.09237735 |
| Txnip | 1.41708759 |
| Txnl2 | 0.42706612 |
| Txnl4 | -0.677016 |
| Txnl4a | 2.13297727 |
| Txnl4a | -0.6552978 |
| Txnl4a | 1.13503115 |
| Txnl4b | 0.01091413 |
| Txnrd2 | -0.7922385 |
| Txnrd3 | -0.7042804 |
| Tyk2 | 1.16779971 |
| Tyk2 | 1.82499722 |
| Tyk2 | 1.41154123 |
| Tyki | 0.0219767 |
| Tyms | -0.0207127 |
| Tyms | 0.00088095 |
| Tyms | -0.7613745 |
| Tyms-ps | 0.47220469 |
| Tyro3 | -0.5732399 |
| Tyrobp | 1.52509033 |
| Tyw1 | -0.1090686 |
| Tyw1 | 0.36666876 |
| U2af1l4 | 2.98094615 |
| U2af2 | 0.16588161 |
| U2af2 | -0.5073583 |
| Uaca | -0.0387673 |
| Uap1 | 0.29217628 |
| Uap1l1 | -0.4014743 |
| Uba1 | 0.20550929 |
| Uba1 | -0.6179163 |
| Uba2 | -0.6992624 |
| Uba5 | 0.0231135 |
| Ubac1 | -0.6395361 |
| Ubac1 | 1.61432469 |
| Ubac1 | -0.6294348 |
| Ubac2 | 0.24616647 |
| Ubap1 | 1.84050681 |
| Ubap2 | -0.8005857 |
| Ubap2l | -0.100359 |
| Ubap2l | 0.76590672 |
| Ubap2l | -0.9240908 |
| Ubb | 3.48333267 |
| Ubd | 0.26467447 |
| Ube1c | -0.7489623 |
| Ube1l | -0.2861784 |
| Ube1x | 0.28537794 |
| Ube2a | 1.92837087 |
| Ube2b | 1.56414286 |
| Ube2d1 | -0.5997832 |
| Ube2d2 | -0.6035314 |
| Ube2d3 | -0.6302079 |
| Ube2e1 | -0.3257877 |
| Ube2e2 | -0.3677699 |
| Ube2e3 | 3.13127169 |
| Ube2f | -0.8005857 |
| Ube2f | -0.1904705 |
| Ube2f | 0.5558817 |
| Ube2g1 | 1.15393093 |
| Ube2g1 | -0.6088048 |
| Ube2g1 | -0.4879548 |
| Ube2g2 | -0.5369083 |
| Ube2i | 0.17811102 |
| Ube2j1 | -0.3516574 |
| Ube2j2 | 2.33521332 |
| Ube2j2 | 0.56031343 |
| Ube2j2 | 0.69021691 |
| Ube2k | 1.6594303 |
| Ube2k | 1.09929484 |
| Ube2l3 | 1.83665399 |
| Ube2l3 | 0.30895437 |
| Ube2l6 | 0.84757446 |
| Ube2m | 0.03215746 |
| Ube2n | -0.607295 |
| Ube2n | -0.7026046 |
| Ube2o | -0.1631428 |
| Ube2q1 | -0.6697192 |
| Ube2q1 | -0.7622676 |
| Ube2q2 | -0.4741565 |
| Ube2q2 | 1.71900143 |
| Ube2q2 | -0.7306542 |
| Ube2r2 | 1.69000689 |
| Ube2s | 1.59509748 |
| Ube2t | 0.22576029 |
| Ube2u | 1.48322207 |
| Ube2u | -0.8280123 |
| Ube3a | 0.21323295 |
| Ube3a | -0.2305544 |
| Ube3b | -0.6489605 |
| Ube3b | -0.0696734 |
| Ube3c | -0.6648867 |
| Ube4a | -0.3654507 |
| Ube4b | 0.53057234 |
| Ubfd1 | 0.89156247 |
| Ubiad1 | 0.94133924 |
| Ubie | -0.8203638 |
| Ubl3 | 1.64629117 |
| Ubl4 | -0.5432158 |
| Ubl4b | -0.7685435 |
| Ubl5 | -0.5053318 |
| Ubl7 | -0.6827319 |
| Ublcp1 | -0.6672998 |
| Ubox5 | -0.3471054 |
| Ubox5 | -0.7931622 |
| Ubp1 | -0.4820158 |
| Ubp1 | -0.5886297 |
| Ubqln1 | -0.5134647 |
| Ubqln1 | -0.4105541 |
| Ubqln1 | 1.01492578 |
| Ubqln3 | -0.4670111 |
| Ubqln4 | -0.6521236 |
| Ubqlnl | -0.6827319 |
| Ubr1 | 1.82589512 |
| Ubr5 | -0.1411281 |
| Ubtd1 | -0.7551474 |
| Ubtd2 | 1.06538448 |
| Ubtd2 | -0.5066823 |
| Ubtd2 | 0.17072855 |
| Ubtf | -0.1594367 |
| Ubxd1 | -0.1520692 |
| Ubxd1 | -0.7195015 |
| Ubxd1 | 0.05876501 |
| Ubxd3 | 0.85994799 |
| Ubxd4 | -0.5202977 |
| Ubxd5 | 1.1383949 |
| Ubxd8 | -0.7410711 |
| Uchl1 | -0.1981551 |
| Uchl3 | -0.4715517 |
| Uchl5 | -0.3865386 |
| Uchl5 | 0.15118478 |
| Uchl5 | 0.46895363 |
| Uck1 | 0.15118478 |
| Uck1 | 1.24128401 |
| Uck1 | 1.02207079 |
| Uck1 | 0.06602071 |
| Uck2 | -0.0223032 |
| Uck2 | -0.4265092 |
| Uck2 | 0.27594696 |
| Uevld | 0.26496525 |
| Ufc1 | -0.459921 |
| Ufsp1 | 1.48712929 |
| Ufsp2 | 0.80636534 |
| Ugcg | -0.1298686 |
| Ugp2 | 0.73146715 |
| Ugt1a10 | -0.6012806 |
| Ugt1a6a | 2.08565562 |
| Ugt1a6a | 0.60167949 |
| Ugt1a6a | 0.18891626 |
| Ugt3a2 | -0.0921609 |
| Ugt8a | 0.75098798 |
| Uhmk1 | -0.1876053 |
| Uhrf1 | 1.03054661 |
| Uhrf1bp1 | -0.5761514 |
| Uhrf1bp1l | -0.7229187 |
| Uhrf2 | -0.5383062 |
| Uhrf2 | 0.19489937 |
| Uhrf2 | -0.3257877 |
| Uhrf2 | 1.04156106 |
| Uimc1 | 1.86085334 |
| Ulk1 | -0.672955 |
| Ulk1 | 0.66952691 |
| Ulk2 | 1.34499433 |
| Ulk2 | -0.5066823 |
| Umodl1 | -0.5341188 |
| Umps | -0.6387551 |
| Umps | -0.6141088 |
| Umps | -0.6608791 |
| Unc119 | 0.75405939 |
| Unc119 | -0.295787 |
| Unc119b | -0.5790722 |
| Unc119b | -0.0956038 |
| Unc119b | 0.1435809 |
| Unc13b | 1.60670906 |
| Unc13c | -0.5573902 |
| Unc13d | -0.5334228 |
| Unc45a | -0.3076695 |
| Unc45a | 2.15755287 |
| Unc45b | -0.3302345 |
| Unc5a | -0.4722022 |
| Unc5a | 1.74266397 |
| Unc5b | 1.92026415 |
| Unc5c | 1.28044016 |
| Unc84a | -0.8376636 |
| Unc84a | 1.16573818 |
| Unc84b | -0.4859708 |
| Unc93a | -0.3614061 |
| Unc93b1 | -0.5467389 |
| Unc93b1 | -0.3776928 |
| Ung | -0.2441466 |
| Ung | 1.67437919 |
| Unk | -0.0147728 |
| Unkl | -0.2782477 |
| Uox | 0.1107984 |
| Upb1 | -0.10732 |
| Upb1 | -0.5271822 |
| Upb1 | 0.07465228 |
| Upf1 | -0.7885529 |
| Upf1 | -0.5953055 |
| Upf1 | -0.1871287 |
| Upf2 | 0.62492423 |
| Upf3a | 1.00189646 |
| Upk1a | -0.6576856 |
| Upk1a | -0.7375856 |
| Upk1b | -0.5495671 |
| Upk2 | -0.563122 |
| Upk3a | 0.62296042 |
| Upk3b | -0.342576 |
| Upk3b | 1.80371984 |
| Upk3b | 1.43482381 |
| Upp1 | 2.55022789 |
| Upp1 | -0.0934505 |
| Upp1 | -0.0022284 |
| Upp2 | 1.21562366 |
| Upp2 | 2.77528078 |
| Upp2 | 1.27274184 |
| Upp2 | 0.41480791 |
| Uqcc | 2.25814116 |
| Uqcr | 1.00552718 |
| Uqcrc1 | -0.7578109 |
| Uqcrc2 | -0.0234979 |
| Uqcrh | 0.46802263 |
| Urm1 | -0.8357252 |
| Urod | 1.50056165 |
| Uros | -0.388318 |
| Uros | -0.3033307 |
| Use1 | 0.33840073 |
| Use1 | -0.6371951 |
| Use1 | -0.5087117 |
| Usf1 | 1.10232676 |
| Usf1 | -0.388318 |
| Usf2 | 0.79226039 |
| Usf2 | -0.6035314 |
| Ush1c | -0.4754617 |
| Ush1g | 0.74446982 |
| Ush2a | -0.8533207 |
| Ush3a | -0.4352171 |
| Ushbp1 | 2.02085771 |
| Usmg5 | -0.5660015 |
| Uso1 | 1.03969295 |
| Usp1 | 1.68299818 |
| Usp1 | -0.8052561 |
| Usp1 | -0.56889 |
| Usp10 | -0.2151935 |
| Usp10 | 0.90869721 |
| Usp10 | -0.36603 |
| Usp11 | 0.55757251 |
| Usp11 | 1.03857025 |
| Usp12 | -0.7950124 |
| Usp12 | -0.3054975 |
| Usp13 | 0.46825547 |
| Usp14 | -0.4427475 |
| Usp15 | -0.7341133 |
| Usp16 | 0.95798371 |
| Usp18 | -0.5982882 |
| Usp18 | 3.16906283 |
| Usp19 | 1.20683852 |
| Usp2 | -0.8543082 |
| Usp2 | 0.82672999 |
| Usp2 | 1.33971057 |
| Usp20 | -0.2005698 |
| Usp21 | 0.97740532 |
| Usp22 | -0.5039833 |
| Usp25 | 0.57679552 |
| Usp28 | 1.90358955 |
| Usp29 | 0.23961414 |
| Usp3 | -0.2528094 |
| Usp3 | -0.7551474 |
| Usp33 | 1.28588596 |
| Usp37 | -0.6333067 |
| Usp38 | -0.3573793 |
| Usp38 | 1.25685319 |
| Usp39 | -0.4136009 |
| Usp39 | -0.1724742 |
| Usp39 | -0.7341133 |
| Usp39 | -0.5573902 |
| Usp4 | 0.4336179 |
| Usp4 | -0.5209838 |
| Usp43 | 2.02400916 |
| Usp45 | 0.58236123 |
| Usp46 | 1.2951738 |
| Usp47 | 1.18593492 |
| Usp48 | -0.3213623 |
| Usp49 | 0.26612744 |
| Usp5 | 0.61446767 |
| Usp50 | 1.50311945 |
| Usp52 | 0.2739339 |
| Usp52 | -0.01754 |
| Usp52 | -0.2365704 |
| Usp53 | 2.0748614 |
| Usp6nl | 2.72351224 |
| Usp6nl | -0.563841 |
| Usp7 | 0.3343672 |
| Usp8 | 0.63390387 |
| Usp8 | 0.74670495 |
| Usp9x | 3.03983813 |
| Usp9y | 0.38035373 |
| Uspl1 | -0.7059591 |
| Utp11l | 1.10824496 |
| Utp15 | -0.5182424 |
| Utp6 | 0.19489937 |
| Utx | -0.0968982 |
| Utx | 0.21323295 |
| Uty | 1.54654597 |
| Uvrag | -0.4657179 |
| Uxt | 1.04417003 |
| Uxt | 2.461876 |
| V1ra2 | 0.99603103 |
| V1rd2 | 0.0888597 |
| V1rd20 | 1.69684075 |
| V1rd21 | -0.5595354 |
| V2r8 | 1.40202618 |
| Vac14 | 3.52734728 |
| Vamp1 | -0.6020302 |
| Vamp1 | -0.6088048 |
| Vamp2 | -0.430231 |
| Vamp3 | 0.54631223 |
| Vamp3 | -0.0319048 |
| Vamp4 | -0.7428188 |
| Vamp5 | -0.3948725 |
| Vamp8 | 0.30004436 |
| Vamp8 | -0.5341188 |
| Vangl2 | -0.5223576 |
| Vapa | -0.425272 |
| Vapb | 3.07987765 |
| Vars | -0.7186492 |
| Vars | 1.71751686 |
| Vars | 1.390204 |
| Vars2 | 0.82767355 |
| Vash1 | -0.4586377 |
| Vash1 | -0.6778304 |
| Vasn | -0.4670111 |
| Vasp | 0.22363358 |
| Vat1 | 0.03253236 |
| Vav1 | 0.52029324 |
| Vav2 | 2.18982272 |
| Vav3 | 0.81053876 |
| Vax1 | -0.1529869 |
| Vbp1 | -0.0870208 |
| Vcam1 | 0.58297756 |
| Vcl | -0.7017679 |
| Vcp | -0.700932 |
| Vcpip1 | 2.0727275 |
| Vdac1 | -0.7186492 |
| Vdac2 | -0.3136691 |
| Vdac3 | 0.6625877 |
| Vdr | -0.1594367 |
| Vdr | 0.61228238 |
| Vdr | 0.6625877 |
| Vegfa | 0.70002772 |
| Vegfa | -0.461206 |
| Vegfb | 2.58237804 |
| Vegfc | -0.2803558 |
| Vezf1 | -0.6975958 |
| Vezt | 0.42462758 |
| Vgf | 1.93514231 |
| Vgll2 | -0.2682997 |
| Vgll4 | 1.88635836 |
| Vhlh | 1.71781397 |
| Vil1 | 1.13962518 |
| Vill | -0.5053318 |
| Vim | -0.910332 |
| Vip | 1.95263655 |
| Vit | -0.2325553 |
| Vkorc1 | -0.5652807 |
| Vkorc1 | 0.23331557 |
| Vkorc1 | 0.00049286 |
| Vkorc1l1 | 1.98105535 |
| Vkorc1l1 | 0.50811601 |
| Vkorc1l1 | 0.54652598 |
| Vldlr | -0.6737658 |
| Vldlr | -0.1214031 |
| Vldlr | 0.11662934 |
| Vldlr | -0.5278735 |
| Vldlr | 0.97727186 |
| Vmn2r81 | 0.44920089 |
| Vmo1 | -0.0759819 |
| Vnn3 | 0.09378063 |
| Vnn3 | 0.69821882 |
| Vpreb1 | -0.5148272 |
| Vpreb3 | 0.14887724 |
| Vps11 | 0.01244798 |
| Vps13a | -0.6371951 |
| Vps16 | 1.1448633 |
| Vps16 | -0.5717877 |
| Vps16 | -0.5467389 |
| Vps16 | -0.7419445 |
| Vps18 | 0.79747373 |
| Vps24 | 0.74636143 |
| Vps25 | -0.1165375 |
| Vps25 | -0.7026046 |
| Vps26 | -0.5006208 |
| Vps26a | -0.4879548 |
| Vps26b | -0.4879548 |
| Vps28 | 1.24925045 |
| Vps29 | 0.54138224 |
| Vps29 | -0.8299344 |
| Vps29 | -0.6125903 |
| Vps33a | 2.8709327 |
| Vps33a | 0.01627138 |
| Vps33b | 0.60729632 |
| Vps33b | 2.70659267 |
| Vps35 | 0.42852613 |
| Vps36 | 0.65881475 |
| Vps37b | -0.724632 |
| Vps37c | -0.2216038 |
| Vps37d | 1.47566692 |
| Vps39 | -0.2245776 |
| Vps39 | -0.5237335 |
| Vps41 | 1.56806995 |
| Vps41 | -0.028693 |
| Vps45 | -0.6387551 |
| Vps4a | -0.4932665 |
| Vps4b | 0.62766651 |
| Vps53 | -0.5997832 |
| Vps53 | 0.73547431 |
| Vps53 | 0.81069889 |
| Vps54 | -0.1357063 |
| Vps72 | 0.45630449 |
| Vrk1 | 1.6044254 |
| Vrk1 | -0.647383 |
| Vrk2 | 2.68921047 |
| Vrk3 | -0.5488592 |
| Vsig2 | -0.2698633 |
| Vsig4 | -0.9434895 |
| Vsnl1 | 3.57862285 |
| Vstm2a | 1.29224599 |
| Vstm2a | -0.433968 |
| Vstm2a | 0.46685755 |
| Vstm2b | 0.20271273 |
| Vsx2 | -0.7463243 |
| Vti1a | -0.4014743 |
| Vti1a | 2.36028172 |
| Vti1a | 0.00165665 |
| Vti1b | 1.60885318 |
| Vti1b | -0.7959389 |
| Vtn | -0.0014501 |
| Vwa2 | 2.40118531 |
| Vwa3a | -0.6163914 |
| Vwf | -0.6057877 |
| Wars | -0.7977948 |
| Wars | -0.5871528 |
| Wars | -0.4859708 |
| Wars | -0.1334566 |
| Wars2 | 2.38805761 |
| Wars2 | -0.7419445 |
| Was | -0.7950124 |
| Wasf1 | -0.3759339 |
| Wasf2 | -0.1357063 |
| Wasf3 | 0.08780176 |
| Wasl | 2.04974719 |
| Wbp1 | -0.2782477 |
| Wbp1 | -0.3202593 |
| Wbp11 | 0.93897631 |
| Wbp2 | 2.66434068 |
| Wbp2nl | 0.32923239 |
| Wbp4 | 0.38086616 |
| Wbp5 | 0.12140354 |
| Wbp7 | -0.6835513 |
| Wbscr16 | 1.19481687 |
| Wbscr18 | -0.5161918 |
| Wbscr22 | -0.1209597 |
| Wbscr22 | 0.41480791 |
| Wbscr22 | -0.5746945 |
| Wbscr27 | 1.39537388 |
| Wbscr28 | -0.3369456 |
| Wbscr28 | 0.05291873 |
| Wdfy1 | 0.41923981 |
| Wdfy3 | 0.03999825 |
| Wdhd1 | 0.00010459 |
| Wdr1 | -0.6255792 |
| Wdr1 | 1.56146842 |
| Wdr13 | -0.5390059 |
| Wdr13 | -0.7922385 |
| Wdr16 | -0.5053318 |
| Wdr17 | 1.21767855 |
| Wdr18 | -0.7220632 |
| Wdr20a | 0.67789617 |
| Wdr20b | 0.56577076 |
| Wdr21 | -0.183799 |
| Wdr22 | -0.5886297 |
| Wdr23 | 1.95740426 |
| Wdr24 | -0.2883049 |
| Wdr27 | 1.08813045 |
| Wdr3 | 0.45204895 |
| Wdr31 | -0.6095606 |
| Wdr31 | -0.1130151 |
| Wdr31 | 0.39560307 |
| Wdr32 | 0.8729047 |
| Wdr33 | 1.40621164 |
| Wdr34 | -0.5856783 |
| Wdr37 | 0.780917 |
| Wdr37 | -0.419725 |
| Wdr37 | -0.5488592 |
| Wdr37 | 0.39610703 |
| Wdr38 | 0.18321174 |
| Wdr41 | 0.24912936 |
| Wdr45 | 0.56806987 |
| Wdr45l | -0.3499477 |
| Wdr45l | 1.66963209 |
| Wdr46 | -0.2305544 |
| Wdr46 | -0.5997832 |
| Wdr47 | -0.1361569 |
| Wdr5 | -0.8080699 |
| Wdr51b | -0.4676583 |
| Wdr53 | -0.7289295 |
| Wdr54 | -0.1249579 |
| Wdr55 | -0.3978674 |
| Wdr59 | -0.6133492 |
| Wdr5b | 0.92819654 |
| Wdr6 | 0.06058433 |
| Wdr60 | -0.2492324 |
| Wdr61 | -0.7306542 |
| Wdr62 | -0.0659091 |
| Wdr63 | 0.15937881 |
| Wdr66 | -0.1757628 |
| Wdr67 | 1.28854926 |
| Wdr68 | 0.79925898 |
| Wdr69 | 1.56855965 |
| Wdr70 | -0.5864153 |
| Wdr70 | -0.5710624 |
| Wdr74 | 0.33571368 |
| Wdr75 | -0.7419445 |
| Wdr75 | 1.80846718 |
| Wdr76 | -0.2845868 |
| Wdr77 | -0.345971 |
| Wdr78 | 0.35987979 |
| Wdr8 | 1.43198628 |
| Wdr8 | -0.6387551 |
| Wdr9 | 0.43916227 |
| Wdr93 | -0.1474945 |
| Wdsof1 | -0.5376069 |
| Wfdc1 | -0.7569222 |
| Wfdc10 | 1.58944106 |
| Wfdc10 | 0.68655642 |
| Wfdc12 | 0.82578545 |
| Wfdc15b | -0.5710624 |
| Wfdc15b | -0.6340831 |
| Wfdc2 | -0.4663642 |
| Wfdc3 | -0.5066823 |
| Wfdc6a | -0.6379748 |
| Wfdc6b | 0.35541346 |
| Wfdc6b | -0.807131 |
| Wfikkn2 | -0.3718428 |
| Wfs1 | -0.3996691 |
| Whdc1 | 0.43555019 |
| Whrn | -0.7622676 |
| Whrn | -0.1603618 |
| Whsc2 | -0.3202593 |
| Wif1 | -0.3823999 |
| Wipf1 | 1.74121733 |
| Wipf2 | -0.4258904 |
| Wipi1 | 1.63990897 |
| Wipi1 | 0.31365276 |
| Wipi1 | -0.6133492 |
| Wipi2 | -0.0151676 |
| Wipi2 | -0.6141088 |
| Wipi2 | 0.6261005 |
| Wisp1 | 0.57472552 |
| Wisp1 | 1.37607173 |
| Wisp2 | 1.29940724 |
| Wiz | 4.61594769 |
| Wiz | 0.22119699 |
| Wnk1 | -0.858269 |
| Wnk1 | 0.31227338 |
| Wnk2 | -0.1267406 |
| Wnk4 | 0.58092152 |
| Wnt10a | -0.3257877 |
| Wnt10b | 1.0390694 |
| Wnt16 | -0.100359 |
| Wnt16 | -0.4939326 |
| Wnt16 | 1.42062956 |
| Wnt2 | -0.2436396 |
| Wnt3a | 0.04481861 |
| Wnt4 | -0.5348154 |
| Wnt4 | 0.26931589 |
| Wnt5a | -0.3912915 |
| Wnt5a | 0.94203306 |
| Wnt5b | 2.17097432 |
| Wnt6 | 0.98498058 |
| Wnt7a | 1.30474164 |
| Wnt7b | 1.52008944 |
| Wnt7b | 0.16198548 |
| Wrb | 0.0284 |
| Wrn | -0.6217397 |
| Wsb1 | 0.58871003 |
| Wsb2 | -0.1016601 |
| Wscd1 | 1.45737065 |
| Wscd2 | 2.03872745 |
| Wtap | 2.78057723 |
| Wtip | -0.0663266 |
| Wwc1 | -0.5168748 |
| Wwc1 | -0.4866317 |
| Wwc2 | 3.86333332 |
| Wwc2 | -0.6080496 |
| Wwox | -0.5230453 |
| Wwox | -0.7143996 |
| Wwp1 | 2.37820943 |
| Wwp2 | 1.17104533 |
| Wwp2 | -0.1470383 |
| Wwp2 | 0.09588152 |
| Wwtr1 | 1.03318713 |
| X99384 | 0.98973284 |
| Xab1 | -0.4807012 |
| Xab1 | 0.53274456 |
| Xbp1 | -0.6656904 |
| Xdh | 0.49215278 |
| Xist | 0.4413227 |
| Xist | 0.12615296 |
| Xk | -0.5432158 |
| Xkr4 | 1.65250156 |
| Xkr5 | -0.5488592 |
| Xkr6 | 0.74944839 |
| Xkr8 | 0.41997637 |
| Xkrx | 0.27133914 |
| Xlr4a | 2.06686391 |
| Xpc | 2.76135341 |
| Xpnpep1 | 3.17320072 |
| Xpnpep1 | 0.63448645 |
| Xpnpep1 | 0.31392838 |
| Xpnpep2 | 0.92706925 |
| Xpnpep2 | 1.89231825 |
| Xpnpep3 | -0.6697192 |
| Xpo4 | -0.7186492 |
| Xpo4 | -0.7784935 |
| Xpo5 | -0.5754227 |
| Xpo6 | -0.4846506 |
| Xpo7 | 1.91160437 |
| Xpo7 | -0.6778304 |
| Xpr1 | 0.29611878 |
| Xrcc1 | 1.61205998 |
| Xrcc2 | 1.27003206 |
| Xrcc2 | -0.0088712 |
| Xrcc3 | 0.6431809 |
| Xrcc6 | 2.54610448 |
| Xrcc6 | -0.183799 |
| Xrcc6 | 2.04256823 |
| Xrn1 | 2.01306038 |
| Xrn2 | 1.46531267 |
| Xylb | -0.6584829 |
| Xylt2 | -0.6042829 |
| Yaf2 | 0.55057769 |
| Yap1 | 0.49147211 |
| Ybx2 | 1.11067078 |
| Ybx2 | -0.6294348 |
| Yeats2 | 3.04021624 |
| Yeats4 | 0.89126927 |
| Yeats4 | 0.18796798 |
| Yif1a | -0.4459035 |
| Yif1a | -0.656093 |
| Yif1a | 1.61019072 |
| Yif1b | 1.34633281 |
| Yipf1 | 2.30763191 |
| Yipf2 | 1.89882474 |
| Yipf3 | 0.44252073 |
| Yipf3 | 0.268737 |
| Yipf3 | 3.29628749 |
| Yipf4 | 1.60637358 |
| Yipf6 | -0.6697192 |
| Yipf6 | 0.20643958 |
| Yipf6 | -0.6255792 |
| Yipf6 | -0.6294348 |
| Yipf7 | -0.3735939 |
| Ykt6 | 0.11594527 |
| Ykt6 | 2.31260746 |
| Ylpm1 | 1.56097491 |
| Yme1l1 | -0.4709017 |
| Yme1l1 | -0.0262916 |
| Yme1l1 | -0.7542612 |
| Yod1 | 0.96949765 |
| Ypel1 | -0.7676443 |
| Ypel3 | -0.709326 |
| Ypel3 | -0.7160971 |
| Ypel4 | 2.70451365 |
| Ypel4 | -0.518927 |
| Ypel5 | 0.24051041 |
| Yrdc | -0.3666096 |
| Ythdf1 | 1.54324449 |
| Ythdf2 | -0.9881296 |
| Ythdf2 | -0.36603 |
| Ythdf3 | 0.26321918 |
| Ythdf3 | -0.3936773 |
| Ywhab | -0.2877728 |
| Ywhab | -0.8203638 |
| Ywhae | -0.5754227 |
| Ywhag | 0.70848195 |
| Ywhah | 1.30651288 |
| Ywhaq | -0.4945993 |
| Ywhaz | -0.5230453 |
| Yy2 | 0.32297776 |
| Zadh1 | -0.7848821 |
| Zadh2 | 1.24118399 |
| Zan | -0.6217397 |
| Zap70 | 0.65179351 |
| Zap70 | 2.53274429 |
| Zbbx | -0.0555332 |
| Zbed4 | 0.11491821 |
| Zbp1 | 1.24128401 |
| Zbtb17 | 0.54374338 |
| Zbtb2 | 1.58498679 |
| Zbtb22 | -0.6217397 |
| Zbtb24 | -0.5046573 |
| Zbtb3 | -0.8386344 |
| Zbtb32 | 0.68618956 |
| Zbtb33 | -0.9124354 |
| Zbtb38 | 0.23180949 |
| Zbtb39 | -0.6810951 |
| Zbtb40 | -0.0045674 |
| Zbtb41 | 0.93702577 |
| Zbtb43 | 0.13658261 |
| Zbtb45 | -0.4528854 |
| Zbtb46 | 0.49893182 |
| Zbtb5 | -0.5645606 |
| Zbtb7a | 0.61961224 |
| Zbtb7c | -0.8127788 |
| Zbtb8 | 1.30399482 |
| Zbtb9 | 0.60047138 |
| Zbtb9 | -0.7127053 |
| Zc3h10 | -0.4234192 |
| Zc3h11a | -0.6624801 |
| Zc3h12a | 1.98952646 |
| Zc3h12b | -0.7341133 |
| Zc3h13 | -0.4932665 |
| Zc3h14 | -0.4676583 |
| Zc3h14 | 2.67671427 |
| Zc3h15 | -0.3735939 |
| Zc3h18 | -0.7143996 |
| Zc3h3 | 2.21968956 |
| Zc3h3 | -0.7051194 |
| Zc3h3 | 0.13725142 |
| Zc3h6 | 2.17386615 |
| Zc3h7a | -0.6458083 |
| Zc3h7a | 0.55376379 |
| Zc3h8 | 3.14150627 |
| Zc3hc1 | -0.1348057 |
| Zc3hc1 | -0.563841 |
| Zc3hc1 | -0.461206 |
| Zc3hc1 | -0.1298686 |
| Zcchc12 | -0.6737658 |
| Zcchc14 | 2.82822128 |
| Zcchc16 | -0.3274527 |
| Zcchc17 | -0.7463243 |
| Zcchc17 | 2.58719188 |
| Zcchc18 | -0.6975958 |
| Zcchc18 | 0.97232008 |
| Zcchc3 | 2.20379583 |
| Zcchc3 | 2.89715462 |
| Zcchc3 | -0.7968664 |
| Zcchc5 | -0.2171612 |
| Zcchc6 | -0.5975416 |
| Zcchc8 | -0.581269 |
| Zcchc8 | 0.86297759 |
| Zcchc8 | 0.6931348 |
| Zcchc9 | -0.8337908 |
| Zcrb1 | -0.2605218 |
| Zcwpw1 | 0.90104077 |
| Zdhhc1 | 0.12919319 |
| Zdhhc12 | 0.18098367 |
| Zdhhc12 | -0.0128014 |
| Zdhhc13 | 1.08386389 |
| Zdhhc13 | -0.7323821 |
| Zdhhc13 | -0.5703377 |
| Zdhhc14 | -0.5960502 |
| Zdhhc15 | -0.7101696 |
| Zdhhc16 | -0.7542612 |
| Zdhhc16 | -0.8156158 |
| Zdhhc17 | -0.5886297 |
| Zdhhc19 | 0.83643351 |
| Zdhhc19 | -0.7393267 |
| Zdhhc21 | 2.62521392 |
| Zdhhc24 | 1.20277929 |
| Zdhhc3 | 0.63506865 |
| Zdhhc3 | 0.32270485 |
| Zdhhc4 | 0.00126889 |
| Zdhhc6 | 1.30184433 |
| Zdhhc7 | 1.19281539 |
| Zdhhc9 | -0.5783412 |
| Zeb1 | -0.257429 |
| Zeb2 | -0.6802779 |
| Zer1 | 0.56806987 |
| Zf | 0.55461154 |
| Zfa | -0.6632816 |
| Zfand2a | -0.766746 |
| Zfand2b | 0.24497862 |
| Zfand2b | 2.03217775 |
| Zfand2b | 2.48211994 |
| Zfand2b | 0.22818477 |
| Zfand3 | -0.7821387 |
| Zfand6 | 1.62207537 |
| Zfand6 | -0.7384557 |
| Zfat | 3.08358395 |
| Zfh4 | 2.23016797 |
| Zfhx2 | 1.38423513 |
| Zfhx2 | -0.3296775 |
| Zfhx3 | 0.22484945 |
| Zfhx3 | 2.15567721 |
| Zfml | 1.48076194 |
| Zfp1 | -0.3291208 |
| Zfp101 | 2.44743124 |
| Zfp105 | -0.7263486 |
| Zfp106 | 0.50945234 |
| Zfp106 | 1.00202638 |
| Zfp109 | 0.37753024 |
| Zfp11 | 0.37211562 |
| Zfp119 | -0.4820158 |
| Zfp12 | -0.6294348 |
| Zfp128 | -0.7289295 |
| Zfp13 | -0.3505173 |
| Zfp131 | 0.07608299 |
| Zfp142 | 0.28395524 |
| Zfp143 | 1.41931322 |
| Zfp148 | -0.7177977 |
| Zfp160 | -0.0026179 |
| Zfp161 | -0.4886171 |
| Zfp161 | -0.2620721 |
| Zfp169 | -0.0481347 |
| Zfp179 | -0.2211091 |
| Zfp182 | -0.6762024 |
| Zfp184 | -0.2904364 |
| Zfp185 | 0.17875017 |
| Zfp187 | -0.055946 |
| Zfp187 | -0.3408833 |
| Zfp189 | -0.1650015 |
| Zfp191 | 1.14408505 |
| Zfp2 | -0.3230193 |
| Zfp2 | -0.1249579 |
| Zfp202 | -0.080636 |
| Zfp207 | 2.4910435 |
| Zfp212 | -0.4221862 |
| Zfp212 | -0.1814281 |
| Zfp212 | 1.50154047 |
| Zfp212 | -0.553826 |
| Zfp213 | -0.1952658 |
| Zfp217 | 0.62197693 |
| Zfp219 | -0.5327273 |
| Zfp235 | -0.1645364 |
| Zfp236 | -0.6656904 |
| Zfp236 | 0.61426922 |
| Zfp238 | -0.1967093 |
| Zfp238 | 0.71330354 |
| Zfp238 | -0.0219053 |
| Zfp238 | -0.1187459 |
| Zfp238 | -0.6209738 |
| Zfp239 | -0.6411001 |
| Zfp251 | -0.8682466 |
| Zfp259 | -0.3437063 |
| Zfp26 | -0.4271285 |
| Zfp263 | 0.45110059 |
| Zfp27 | -0.9198353 |
| Zfp27 | -0.4452714 |
| Zfp275 | 1.24348171 |
| Zfp276 | -0.5893691 |
| Zfp277 | -0.4866317 |
| Zfp277 | 0.613475 |
| Zfp280c | 1.44584683 |
| Zfp281 | 2.28946069 |
| Zfp282 | -0.3442719 |
| Zfp286 | -0.7730528 |
| Zfp288 | -0.1388651 |
| Zfp292 | 1.22707205 |
| Zfp292 | -0.6118319 |
| Zfp295 | 0.28850012 |
| Zfp296 | -0.6681055 |
| Zfp296 | 2.24427817 |
| Zfp3 | 3.08183303 |
| Zfp30 | 3.30086911 |
| Zfp313 | 0.03028066 |
| Zfp313 | -0.5502755 |
| Zfp316 | 0.07321933 |
| Zfp316 | -0.7280684 |
| Zfp318 | -0.4702521 |
| Zfp322a | -0.3625599 |
| Zfp324 | 2.98635274 |
| Zfp326 | 2.62860136 |
| Zfp326 | 0.57534701 |
| Zfp326 | 0.72057697 |
| Zfp329 | -0.4484362 |
| Zfp329 | 2.86240221 |
| Zfp330 | -0.285117 |
| Zfp330 | 2.15102665 |
| Zfp334 | 2.42910319 |
| Zfp335 | 0.77143912 |
| Zfp339 | -0.5230453 |
| Zfp346 | 1.27679153 |
| Zfp346 | -0.5667227 |
| Zfp35 | 0.98920603 |
| Zfp354a | -0.5264914 |
| Zfp36 | 0.50364743 |
| Zfp365 | -0.8444807 |
| Zfp367 | 0.45370624 |
| Zfp367 | 0.25883937 |
| Zfp36l1 | -0.8793492 |
| Zfp36l1 | -0.299551 |
| Zfp36l1 | -0.7712464 |
| Zfp36l2 | 0.54352898 |
| Zfp37 | 0.97993739 |
| Zfp371 | -0.0895872 |
| Zfp382 | 0.88449954 |
| Zfp385a | -0.8895607 |
| Zfp385a | -0.1169788 |
| Zfp385c | -0.8203638 |
| Zfp386 | 0.26989442 |
| Zfp386 | -0.7712464 |
| Zfp39 | 0.1342379 |
| Zfp393 | 1.34490503 |
| Zfp395 | 0.00088095 |
| Zfp397 | 1.59380572 |
| Zfp398 | 0.21015131 |
| Zfp398 | -0.7280684 |
| Zfp398 | -0.606541 |
| Zfp41 | 1.59087551 |
| Zfp41 | -0.1562061 |
| Zfp410 | -0.9113831 |
| Zfp410 | -0.6753895 |
| Zfp410 | -0.5645606 |
| Zfp414 | -0.5161918 |
| Zfp418 | -0.2677791 |
| Zfp42 | -0.1012262 |
| Zfp423 | 0.13155085 |
| Zfp423 | 0.51011977 |
| Zfp428 | 0.57824173 |
| Zfp428 | -0.5285653 |
| Zfp446 | 0.48600841 |
| Zfp446 | -0.1952658 |
| Zfp449 | -0.7118593 |
| Zfp451 | 1.57413772 |
| Zfp457 | 0.88950807 |
| Zfp46 | 1.2628316 |
| Zfp467 | 2.74555062 |
| Zfp472 | -0.2646618 |
| Zfp473 | -0.3579535 |
| Zfp473 | 2.07115409 |
| Zfp474 | -0.5674446 |
| Zfp474 | -0.2265655 |
| Zfp512 | 1.82710854 |
| Zfp513 | 0.83222043 |
| Zfp516 | 0.32051861 |
| Zfp52 | 1.50063698 |
| Zfp521 | -0.5114247 |
| Zfp523 | -0.0802119 |
| Zfp523 | 0.49079093 |
| Zfp524 | 0.10700554 |
| Zfp526 | 1.23596779 |
| Zfp532 | -0.1267406 |
| Zfp532 | 2.01129462 |
| Zfp536 | 0.88627039 |
| Zfp551 | 1.05133734 |
| Zfp553 | -0.2029909 |
| Zfp553 | -0.0493636 |
| Zfp560 | 0.63816752 |
| Zfp566 | 1.58649682 |
| Zfp57 | -0.3044134 |
| Zfp574 | 0.15184301 |
| Zfp579 | 1.25675485 |
| Zfp592 | -0.7323821 |
| Zfp593 | 1.35969887 |
| Zfp595 | 0.18193922 |
| Zfp598 | 1.86075171 |
| Zfp60 | 0.37133948 |
| Zfp606 | 0.7377315 |
| Zfp607 | 1.71757629 |
| Zfp608 | 1.16530358 |
| Zfp609 | -0.0638245 |
| Zfp61 | 0.72901938 |
| Zfp612 | -0.5559628 |
| Zfp617 | 1.26341754 |
| Zfp619 | 1.32339944 |
| Zfp62 | -0.0203155 |
| Zfp622 | 0.29217628 |
| Zfp623 | -0.2579438 |
| Zfp629 | 1.8102948 |
| Zfp637 | 0.32215878 |
| Zfp639 | -0.6333067 |
| Zfp64 | 0.02915273 |
| Zfp64 | -0.3197083 |
| Zfp64 | -0.3082133 |
| Zfp641 | 0.48806112 |
| Zfp644 | 0.26554653 |
| Zfp646 | 0.82924399 |
| Zfp653 | 3.6045564 |
| Zfp654 | 2.27925746 |
| Zfp654 | 0.53664161 |
| Zfp655 | -0.656889 |
| Zfp661 | -0.7220632 |
| Zfp667 | -0.424654 |
| Zfp668 | 2.57101507 |
| Zfp67 | -0.6876597 |
| Zfp672 | 1.82621182 |
| Zfp68 | 2.46416509 |
| Zfp68 | -0.4879548 |
| Zfp68 | 1.84744252 |
| Zfp687 | -0.3230193 |
| Zfp688 | -0.5292576 |
| Zfp689 | -0.9666538 |
| Zfp69 | -0.6632816 |
| Zfp691 | 0.74653321 |
| Zfp692 | -0.7152479 |
| Zfp692 | 1.77598979 |
| Zfp704 | 2.65414911 |
| Zfp704 | 1.15437097 |
| Zfp706 | 1.41527011 |
| Zfp709 | 1.11446985 |
| Zfp709 | 0.2076785 |
| Zfp710 | -0.3573793 |
| Zfp711 | 0.15217195 |
| Zfp715 | -0.7127053 |
| Zfp71-rs1 | -0.3341433 |
| Zfp74 | 1.12713397 |
| Zfp75 | 2.0434843 |
| Zfp75 | 1.76826353 |
| Zfp75 | 2.59373847 |
| Zfp750 | -0.6624801 |
| Zfp758 | 1.5766368 |
| Zfp760 | -0.3683507 |
| Zfp764 | -0.7084831 |
| Zfp768 | -0.2181466 |
| Zfp770 | 2.56383204 |
| Zfp771 | -0.8602559 |
| Zfp771 | 0.92466913 |
| Zfp78 | -0.6934427 |
| Zfp78 | 0.9177157 |
| Zfp780b | -0.5348154 |
| Zfp786 | 1.705613 |
| Zfp787 | -0.6529161 |
| Zfp787 | -0.6876597 |
| Zfp790 | -0.3291208 |
| Zfp804a | 0.26583703 |
| Zfp809 | -0.862247 |
| Zfp810 | 0.55206592 |
| Zfp811 | 1.49671149 |
| Zfp817 | 0.89958895 |
| Zfp819 | 0.20798796 |
| Zfp82 | 1.19912099 |
| Zfp825 | 1.46586056 |
| Zfp828 | 1.21685715 |
| Zfp85-rs1 | -0.1804815 |
| Zfp87 | 2.11306161 |
| Zfp87 | -0.584942 |
| Zfp90 | -0.6186797 |
| Zfp91 | -0.210293 |
| Zfp91-cntf | 1.24038343 |
| Zfp92 | -0.2290565 |
| Zfp93 | 0.76169312 |
| Zfp93 | 1.82049442 |
| Zfp94 | 2.44125482 |
| Zfp97 | 2.10137749 |
| Zfpl1 | 2.84843732 |
| Zfpm2 | -0.412381 |
| Zfr | -0.5481519 |
| Zfx | -0.5960502 |
| Zfx | 0.42120267 |
| Zfy1 | 1.50821369 |
| Zfyve1 | -0.4618492 |
| Zfyve16 | -0.8127788 |
| Zfyve19 | 0.69785661 |
| Zfyve20 | -0.5418104 |
| Zfyve21 | 0.84310344 |
| Zfyve26 | 0.83143809 |
| Zfyve27 | -0.1814281 |
| Zgpat | 0.32161238 |
| Zgpat | 1.48735861 |
| Zhx1 | -0.8712613 |
| Zhx1 | -0.6713357 |
| Zhx1 | -0.4573563 |
| Zhx2 | -0.5006208 |
| Zhx3 | -0.7280684 |
| Zic1 | -0.3912915 |
| Zic2 | 3.77367378 |
| Zic3 | -0.2512747 |
| Zic4 | -0.8682466 |
| Zkscan1 | -0.7775845 |
| Zkscan14 | 0.35488658 |
| Zkscan17 | -0.7977948 |
| Zkscan3 | 0.7858662 |
| Zkscan6 | -0.7341133 |
| Zkscan6 | 1.2887392 |
| Zmat1 | -0.7212085 |
| Zmat2 | -0.583471 |
| Zmat3 | 1.00241602 |
| Zmat4 | 1.22513998 |
| Zmat5 | 1.55977527 |
| Zmiz1 | -0.7332473 |
| Zmiz1 | -0.7177977 |
| Zmiz2 | 0.69821882 |
| Zmiz2 | -0.584942 |
| Zmpste24 | 0.32188562 |
| Zmpste24 | 0.94949297 |
| Zmpste24 | 0.14656386 |
| Zmym3 | -0.1895145 |
| Zmym3 | 0.04036995 |
| Zmym5 | 0.1870187 |
| Zmym6 | 3.59063874 |
| Zmynd10 | -0.5864153 |
| Zmynd11 | 0.71312542 |
| Zmynd11 | 2.80554209 |
| Zmynd11 | -0.5595354 |
| Zmynd12 | 0.09378063 |
| Zmynd15 | -0.7384557 |
| Znhit2 | -0.1007925 |
| Znhit3 | -0.7867157 |
| Znhit4 | -0.1231788 |
| Znrd1 | -0.0789409 |
| Znrd1 | -0.6309816 |
| Znrd1 | 0.27306979 |
| Znrf1 | 1.45007583 |
| Znrf2 | -0.4966021 |
| Znrf3 | 0.52511912 |
| Znrf4 | -0.0112273 |
| Zp1 | 3.99023845 |
| Zp3 | -0.3853542 |
| Zp3r | -0.38299 |
| Zpbp | -0.358528 |
| Zpbp2 | -0.5127842 |
| Zpbp2 | 3.39505672 |
| Zranb1 | 2.20912448 |
| Zranb2 | -0.7463243 |
| Zranb3 | 1.21397642 |
| Zrsr2 | -0.1289738 |
| Zscan10 | -0.3847626 |
| Zscan12 | -0.0917315 |
| Zscan2 | -0.5100672 |
| Zscan2 | -0.3984676 |
| Zscan2 | 0.07214314 |
| Zscan20 | 1.79606158 |
| Zscan20 | 1.10011211 |
| Zscan21 | 0.14125408 |
| Zscan21 | -0.166398 |
| Zswim1 | 0.18638531 |
| Zswim2 | 0.11148633 |
| Zswim3 | -0.9082336 |
| Zswim4 | -0.3835805 |
| Zswim5 | 2.33324457 |
| Zw10 | 0.11183009 |
| Zw10 | 0.63254307 |
| Zw10 | -0.6133492 |
| Zwilch | 1.27003206 |
| Zxda | 0.06782568 |
| Zxdc | 1.99726347 |
| Zxdc | 0.81117914 |
| Zxdc | -0.0977622 |
| Zyg11b | 0.83081174 |
| Zyx | -0.3087574 |
| Zzz3 | 1.59496159 |
| Zzz3 | -0.8080699 |
| Zzz3 | 1.53479316 |
| Zzz3 | 0.08744884 |

| **Gene Symbol** | **HET4** |
| --- | --- |
| 0610006I08Rik | -0.3848113 |
| 0610006I08Rik | 0.06855476 |
| 0610007C21Rik | -0.547164 |
| 0610007C21Rik | 0.13903969 |
| 0610007P08Rik | 1.15670035 |
| 0610007P14Rik | -0.5897103 |
| 0610007P22Rik | -0.6334806 |
| 0610009B22Rik | 0.03239797 |
| 0610009D07Rik | -0.353923 |
| 0610009O20Rik | -0.0553667 |
| 0610010E21Rik | 1.56274801 |
| 0610010K06Rik | 0.1469759 |
| 0610010K14Rik | -0.4343993 |
| 0610012A05Rik | -0.4331273 |
| 0610012D14Rik | -0.5711356 |
| 0610012G03Rik | -0.6622096 |
| 0610012H03Rik | 1.29444057 |
| 0610025P10Rik | -0.6397897 |
| 0610031J06Rik | -0.3302625 |
| 0610031J06Rik | 1.36237376 |
| 0610031J06Rik | 3.16625595 |
| 0610037D15Rik | 0.04281587 |
| 0610037L13Rik | -0.4274244 |
| 0610037L13Rik | 0.3291931 |
| 0610038F07Rik | -0.0656422 |
| 0610040J01Rik | 1.8771485 |
| 0710008K08Rik | 0.26047057 |
| 0910001A06Rik | 1.68019298 |
| 0910001A06Rik | -0.5485996 |
| 0910001L09Rik | 0.95186958 |
| 1110001A07Rik | 0.12261155 |
| 1110001J03Rik | 1.90248816 |
| 1110002B05Rik | -0.7346045 |
| 1110002N22Rik | 0.3294763 |
| 1110003E01Rik | 1.85364447 |
| 1110006G06Rik | -0.1497874 |
| 1110007A13Rik | -0.7585291 |
| 1110007A13Rik | -0.2701027 |
| 1110007C09Rik | 1.55767953 |
| 1110007L15Rik | 2.41817158 |
| 1110007L15Rik | 1.40795033 |
| 1110007L15Rik | -0.5294008 |
| 1110007L15Rik | -0.2589405 |
| 1110007M04Rik | -0.3830015 |
| 1110008F13Rik | 0.7323566 |
| 1110008J03Rik | 2.02364757 |
| 1110008J03Rik | -0.650933 |
| 1110008J03Rik | -0.4363106 |
| 1110008P14Rik | -0.4667703 |
| 1110012D08Rik | -0.555087 |
| 1110012J17Rik | -0.4874129 |
| 1110012L19Rik | -0.435036 |
| 1110012L19Rik | 0.0976011 |
| 1110012M11Rik | 2.14795387 |
| 1110013L07Rik | -0.432492 |
| 1110017D15Rik | -0.7666407 |
| 1110017D15Rik | 0.33230363 |
| 1110017D15Rik | -0.5837265 |
| 1110018G07Rik | -0.311082 |
| 1110018J18Rik | -0.294476 |
| 1110018J18Rik | 0.6785754 |
| 1110019N10Rik | 0.17173134 |
| 1110019N10Rik | 0.40560371 |
| 1110020C03Rik | 1.66244525 |
| 1110020P15Rik | 1.89540973 |
| 1110020P15Rik | -0.7639291 |
| 1110031B06Rik | -0.4433518 |
| 1110031I02Rik | 1.22331994 |
| 1110032A13Rik | -0.6010334 |
| 1110032A13Rik | -0.6926778 |
| 1110032E23Rik | 1.15516775 |
| 1110034A24Rik | 0.49190749 |
| 1110036O03Rik | 0.01279635 |
| 1110038D17Rik | 0.84176456 |
| 1110038F14Rik | 0.89547508 |
| 1110038F14Rik | 0.1212001 |
| 1110039B18Rik | -0.5414433 |
| 1110039B18Rik | -0.6437543 |
| 1110039B18Rik | -0.284651 |
| 1110048D14Rik | 1.98548695 |
| 1110049B09Rik | 1.10314208 |
| 1110049B09Rik | 0.85223372 |
| 1110049F12Rik | -0.0372318 |
| 1110049F12Rik | -0.4874129 |
| 1110057K04Rik | -0.5428702 |
| 1110057K04Rik | 1.09889344 |
| 1110057K04Rik | 2.29256539 |
| 1110058L19Rik | -0.5897103 |
| 1110059E24Rik | -0.6670841 |
| 1110059G10Rik | 0.79055836 |
| 1110067D22Rik | -0.787693 |
| 1110067I12Rik | -0.1339193 |
| 1190002A17Rik | 0.06180959 |
| 1190002H23Rik | 1.50527506 |
| 1190002N15Rik | -0.6240955 |
| 1190003J15Rik | -0.4337631 |
| 1190003M12Rik | 0.82917959 |
| 1190005F20Rik | -0.7250289 |
| 1190005I06Rik | 1.26055337 |
| 1190005P17Rik | 0.2543578 |
| 1190005P17Rik | 1.09625969 |
| 1190007F08Rik | -0.6752645 |
| 1190017O12Rik | 3.33334197 |
| 1190020J12Rik | -0.5587105 |
| 1200002N14Rik | -0.4874129 |
| 1200003C05Rik | 0.67426319 |
| 1200003C05Rik | 0.70954678 |
| 1200003C05Rik | 0.03045604 |
| 1200003I07Rik | -0.5740831 |
| 1200009F10Rik | -0.5182055 |
| 1200009I06Rik | 2.51618249 |
| 1200009I06Rik | 2.52784396 |
| 1200009O22Rik | -0.1818606 |
| 1200011O22Rik | 2.78200235 |
| 1200013B08Rik | 0.10121488 |
| 1200013P24Rik | 0.10445549 |
| 1200013P24Rik | -0.424271 |
| 1200014J11Rik | 2.97102563 |
| 1200015F23Rik | -0.0882974 |
| 1200015F23Rik | 0.59691542 |
| 1200015N20Rik | 1.32439536 |
| 1200016B10Rik | -0.6843456 |
| 1200016B10Rik | -0.6678989 |
| 1200016D23Rik | 2.40553235 |
| 1300001I01Rik | 1.15670035 |
| 1300007L22Rik | 3.46800937 |
| 1300010M03Rik | 0.07599325 |
| 1300013J15Rik | 0.05766366 |
| 1300013J15Rik | -0.5733453 |
| 1300014I06Rik | 0.36231063 |
| 1300017J02Rik | 0.11837087 |
| 1300018I05Rik | -0.6421665 |
| 1300018J18Rik | -0.699397 |
| 1500001L15Rik | -0.5237865 |
| 1500001M20Rik | -0.3388501 |
| 1500001M20Rik | 1.89540973 |
| 1500001M20Rik | -0.6485341 |
| 1500002O20Rik | -0.4647964 |
| 1500003O03Rik | 1.44032917 |
| 1500005A01Rik | 1.22068194 |
| 1500010J02Rik | -0.780317 |
| 1500010J02Rik | 0.31808146 |
| 1500011H22Rik | -0.3633198 |
| 1500012F01Rik | -0.4740434 |
| 1500015O10Rik | 0.06892808 |
| 1500019C06Rik | 0.5617207 |
| 1500019G21Rik | 2.81290381 |
| 1500031L02Rik | 0.11197388 |
| 1500032D16Rik | -0.5667315 |
| 1500032D16Rik | -0.2277576 |
| 1500032D16Rik | 0.99156633 |
| 1500032L24Rik | -0.121049 |
| 1500041J02Rik | 2.4048426 |
| 1500041N16Rik | -0.431857 |
| 1520401A03Rik | 0.30596685 |
| 1600002K03Rik | 0.5338653 |
| 1600012H06Rik | -0.5587105 |
| 1600012H06Rik | 1.24652157 |
| 1600014C10Rik | -0.5189013 |
| 1600016N20Rik | -0.6613997 |
| 1600021P15Rik | 1.81593571 |
| 1600027N09Rik | -0.0052003 |
| 1600029D21Rik | 2.64365349 |
| 1700001C02Rik | -0.4968855 |
| 1700001C19Rik | -0.0161655 |
| 1700001E04Rik | -0.4472147 |
| 1700001J03Rik | 1.83380134 |
| 1700001O22Rik | 1.65552928 |
| 1700001O22Rik | -0.3127588 |
| 1700001P01Rik | -0.7241631 |
| 1700003F12Rik | 1.07915053 |
| 1700003M02Rik | 0.99198725 |
| 1700007G11Rik | 1.66842117 |
| 1700007I06Rik | -0.0173917 |
| 1700007K09Rik | 0.67426319 |
| 1700008F21Rik | 1.95511076 |
| 1700008I05Rik | 0.24387431 |
| 1700008P20Rik | 0.15926527 |
| 1700009P17Rik | 0.2773785 |
| 1700010A17Rik | -0.5919641 |
| 1700010A17Rik | -0.406186 |
| 1700010D01Rik | 0.19160278 |
| 1700010M22Rik | 0.70860009 |
| 1700010M22Rik | 0.33989535 |
| 1700011A15Rik | -0.5587105 |
| 1700011F03Rik | -0.4727169 |
| 1700011F14Rik | 3.01375106 |
| 1700011H14Rik | -0.4012575 |
| 1700011K15Rik | -0.4267929 |
| 1700012A03Rik | 0.45098784 |
| 1700012B09Rik | 0.84979041 |
| 1700012G19Rik | -0.0515421 |
| 1700012H05Rik | -0.3775929 |
| 1700012H17Rik | -0.7258954 |
| 1700012L04Rik | -0.1592478 |
| 1700013B16Rik | 0.80537687 |
| 1700013G24Rik | -0.2803169 |
| 1700013N18Rik | -0.7914027 |
| 1700013N18Rik | 0.68286798 |
| 1700015E13Rik | -0.6876697 |
| 1700016C15Rik | -0.4948476 |
| 1700016D06Rik | -0.201542 |
| 1700016G05Rik | 0.09651426 |
| 1700016K19Rik | 0.42601609 |
| 1700016K19Rik | -0.3149992 |
| 1700017N19Rik | 3.49626338 |
| 1700018B24Rik | -0.6461412 |
| 1700018C11Rik | 1.84715191 |
| 1700018F24Rik | -0.1196804 |
| 1700019D03Rik | -0.2610564 |
| 1700019E19Rik | -0.5442993 |
| 1700019G17Rik | 0.11588831 |
| 1700019H03Rik | -0.6517341 |
| 1700019L03Rik | -0.5493182 |
| 1700019M22Rik | 2.37356194 |
| 1700019N19Rik | -0.273853 |
| 1700020C11Rik | -0.6662699 |
| 1700020D05Rik | -0.5867137 |
| 1700020L24Rik | 0.16737012 |
| 1700020N01Rik | 1.88827778 |
| 1700020O03Rik | 2.94926677 |
| 1700021C14Rik | 0.34297084 |
| 1700021F05Rik | 2.25963646 |
| 1700021K02Rik | 0.60519564 |
| 1700021K02Rik | 1.07440665 |
| 1700021K19Rik | -0.7433949 |
| 1700022C21Rik | 0.32720832 |
| 1700023B02Rik | 1.90125861 |
| 1700023B02Rik | 2.38156831 |
| 1700023E05Rik | -0.6429601 |
| 1700023E05Rik | -0.6525358 |
| 1700023I07Rik | -0.2155675 |
| 1700023M03Rik | -0.3692407 |
| 1700024G13Rik | 0.15858671 |
| 1700025D03Rik | -0.5874619 |
| 1700025E21Rik | 1.08195994 |
| 1700025F22Rik | 0.07525204 |
| 1700025G04Rik | -0.1573482 |
| 1700025K23Rik | -0.778482 |
| 1700026D08Rik | -0.5500374 |
| 1700026L06Rik | 0.36558657 |
| 1700027A23Rik | 0.31549854 |
| 1700027D21Rik | -0.6202125 |
| 1700027J05Rik | -0.5829812 |
| 1700027N10Rik | -0.73373 |
| 1700028K03Rik | -0.6350539 |
| 1700029F12Rik | 1.05058632 |
| 1700029F12Rik | -0.5726082 |
| 1700029G01Rik | 3.12841829 |
| 1700029H14Rik | 0.00842878 |
| 1700029H14Rik | 0.26077517 |
| 1700029I01Rik | -0.6728029 |
| 1700029I08Rik | 2.62959783 |
| 1700029I15Rik | -0.4485059 |
| 1700029J07Rik | -0.5565347 |
| 1700029P11Rik | -0.6868376 |
| 1700029P11Rik | 1.55505782 |
| 1700029P11Rik | -0.5098953 |
| 1700030J22Rik | 2.04547533 |
| 1700030K09Rik | 0.64776549 |
| 1700034E13Rik | 0.76610409 |
| 1700034H14Rik | 0.40246408 |
| 1700034H14Rik | 0.16400156 |
| 1700034H14Rik | 4.13635604 |
| 1700034H14Rik | -0.5126572 |
| 1700034I23Rik | 2.49410275 |
| 1700034I23Rik | 1.7338829 |
| 1700034O15Rik | 1.64337326 |
| 1700036D21Rik | -0.2770794 |
| 1700037C18Rik | 0.73530755 |
| 1700037C18Rik | 1.37264186 |
| 1700037H04Rik | 3.40465393 |
| 1700039E15Rik | -0.6695307 |
| 1700040I03Rik | 1.58425232 |
| 1700042B14Rik | 1.01361986 |
| 1700045I19Rik | -0.4043348 |
| 1700047I17Rik1 | 0.81811263 |
| 1700047I17Rik1 | -0.166406 |
| 1700049G17Rik | 0.45248024 |
| 1700049K14Rik | -0.6453449 |
| 1700049L16Rik | 0.87394322 |
| 1700052N19Rik | -0.0297428 |
| 1700052N19Rik | 0.49095356 |
| 1700054O13Rik | -0.7181253 |
| 1700055M20Rik | 1.56702728 |
| 1700057G04Rik | 1.65545986 |
| 1700057K13Rik | -0.6148029 |
| 1700058C13Rik | -0.7363561 |
| 1700060H10Rik | 2.43800008 |
| 1700061G19Rik | -0.5733453 |
| 1700063I17Rik | 3.14055829 |
| 1700065I17Rik | -0.2216428 |
| 1700065O13Rik | -0.613263 |
| 1700065O13Rik | 0.27137469 |
| 1700067C01Rik | -0.7576321 |
| 1700067K01Rik | -0.2685 |
| 1700067P10Rik | -0.3088509 |
| 1700071K01Rik | -0.8045029 |
| 1700072E05Rik | -0.7293694 |
| 1700072E05Rik | 1.60256451 |
| 1700072E05Rik | 2.41336622 |
| 1700073E17Rik | 0.62960889 |
| 1700080E11Rik | -0.7720875 |
| 1700081D17Rik | -0.0149408 |
| 1700081D17Rik | 0.36367698 |
| 1700084C01Rik | -0.2354572 |
| 1700084C01Rik | 1.9900259 |
| 1700088E04Rik | -0.20453 |
| 1700093K21Rik | 2.40462303 |
| 1700094C09Rik | 1.70468949 |
| 1700095K08Rik | 1.70679557 |
| 1700102P08Rik | 1.18283899 |
| 1700108M19Rik | -0.2130476 |
| 1700109H08Rik | 0.84848472 |
| 1700112C13Rik | -0.2695681 |
| 1700113I22Rik | -0.5837265 |
| 1700113I22Rik | 1.26329277 |
| 1700113O17Rik | 0.2800678 |
| 1700120B06Rik | -0.094935 |
| 1700123D08Rik | -0.6101907 |
| 1700123K08Rik | -0.3728111 |
| 1700123L14Rik | 1.5549806 |
| 1700123O20Rik | -0.513349 |
| 1700123O20Rik | -0.7739101 |
| 1700127D06Rik | -0.1668851 |
| 1700129C05Rik | -0.4760368 |
| 1700129I15Rik | -0.2494769 |
| 1810007M14Rik | 1.56763702 |
| 1810007P19Rik | -0.4543381 |
| 1810007P19Rik | 3.26172294 |
| 1810008A18Rik | 0.23421321 |
| 1810008A18Rik | -0.7224341 |
| 1810009A15Rik | -0.2364886 |
| 1810009A15Rik | 0.31894087 |
| 1810009N02Rik | 1.28049439 |
| 1810009O10Rik | -0.3480972 |
| 1810009O10Rik | 0.00083682 |
| 1810010M01Rik | 3.9695262 |
| 1810011O10Rik | -0.3811952 |
| 1810014F10Rik | -0.5733453 |
| 1810015A11Rik | 1.95040022 |
| 1810015C04Rik | -0.5674641 |
| 1810019J16Rik | -0.6703477 |
| 1810020D17Rik | -0.4382257 |
| 1810020G14Rik | 0.25313048 |
| 1810020G14Rik | 2.68058732 |
| 1810021J13Rik | 0.23702825 |
| 1810021J13Rik | 0.12683324 |
| 1810022C23Rik | 0.35324269 |
| 1810026J23Rik | 0.9842405 |
| 1810027O10Rik | 1.50511225 |
| 1810030J14Rik | -0.2145587 |
| 1810030N24Rik | -0.6565545 |
| 1810030N24Rik | -0.634267 |
| 1810030N24Rik | -0.0502706 |
| 1810031K17Rik | 2.17511005 |
| 1810035L17Rik | 2.42224466 |
| 1810035L17Rik | 0.77691397 |
| 1810037C20Rik | -0.5507571 |
| 1810043G02Rik | 0.94082759 |
| 1810044A24Rik | -0.7594269 |
| 1810046J19Rik | 1.34949944 |
| 1810048J11Rik | 0.35929766 |
| 1810049H13Rik | 1.24308825 |
| 1810049H19Rik | -0.196583 |
| 1810055E12Rik | -0.789546 |
| 1810055E12Rik | 1.67258871 |
| 1810055E12Rik | -0.7078638 |
| 1810055G02Rik | -0.6711654 |
| 1810063B05Rik | -0.1155865 |
| 1810064F22Rik | 0.93085289 |
| 1810065E05Rik | -0.0993861 |
| 1810073G14Rik | -0.7207082 |
| 1810074P20Rik | -0.6117256 |
| 1810074P20Rik | -0.0971579 |
| 2010001J22Rik | -0.554364 |
| 2010001J22Rik | 0.21394907 |
| 2010001M09Rik | -0.127922 |
| 2010003J03Rik | 0.83034578 |
| 2010003O18Rik | 1.05702364 |
| 2010004A03Rik | -0.3446189 |
| 2010005H15Rik | 0.0789523 |
| 2010005J08Rik | -0.3377005 |
| 2010005J08Rik | -0.3884413 |
| 2010005J08Rik | -0.2983247 |
| 2010007H12Rik | 0.2948703 |
| 2010007H12Rik | -0.0795194 |
| 2010011I20Rik | -0.8370962 |
| 2010012O05Rik | 0.79851349 |
| 2010100O12Rik | -0.7087146 |
| 2010100O12Rik | -0.1178587 |
| 2010106G01Rik | -0.3480972 |
| 2010106G01Rik | 0.93771253 |
| 2010107E04Rik | 1.40397098 |
| 2010107G12Rik | -0.3010834 |
| 2010109I03Rik | -0.7010842 |
| 2010110K16Rik | -0.7657359 |
| 2010111I01Rik | -0.682688 |
| 2010200O16Rik | -0.6178903 |
| 2010301N04Rik | -0.3562634 |
| 2010305A19Rik | 1.63162826 |
| 2010305C02Rik | -0.7531598 |
| 2010305C02Rik | 0.74465255 |
| 2010308M01Rik | 0.66953636 |
| 2010311D03Rik | -0.3388501 |
| 2010316F05Rik | 1.4337661 |
| 2010321M09Rik | 1.07825486 |
| 2010321M09Rik | -0.4975658 |
| 2200001I15Rik | -0.6935151 |
| 2210009G21Rik | -0.1755516 |
| 2210009G21Rik | -0.7207082 |
| 2210009G21Rik | 1.25589926 |
| 2210010B09Rik | -0.166406 |
| 2210010L05Rik | 0.30509554 |
| 2210012G02Rik | -0.1789435 |
| 2210012G02Rik | 1.6254855 |
| 2210012G02Rik | -0.7540526 |
| 2210012G02Rik | 0.44022265 |
| 2210016L21Rik | 0.28453299 |
| 2210016L21Rik | 4.1693414 |
| 2210016L21Rik | -0.6437543 |
| 2210021J22Rik | 3.2252054 |
| 2210023G05Rik | -0.6094242 |
| 2210023G05Rik | 1.3933071 |
| 2210407C18Rik | -0.6687145 |
| 2210412D01Rik | 0.8081083 |
| 2210417D09Rik | 0.79885785 |
| 2210418O10Rik | 1.39970167 |
| 2300002D11Rik | 0.53272466 |
| 2300002D11Rik | -0.3545075 |
| 2300002M23Rik | -0.7469341 |
| 2300005B03Rik | 1.00894716 |
| 2310001A20Rik | -0.6117256 |
| 2310002B06Rik | 0.17906624 |
| 2310003C23Rik | 0.28927268 |
| 2310003F16Rik | -0.0515421 |
| 2310003H01Rik | 1.14675318 |
| 2310003L22Rik | 1.41406741 |
| 2310003L22Rik | 2.22399772 |
| 2310004I24Rik | -0.4867399 |
| 2310005E10Rik | 0.34408672 |
| 2310005E10Rik | -0.2733163 |
| 2310005G13Rik | 0.79885785 |
| 2310005N01Rik | 1.10737167 |
| 2310005N03Rik | -0.6851756 |
| 2310005P05Rik | 0.23389991 |
| 2310006J04Rik | 0.56921692 |
| 2310007A19Rik | -0.6178903 |
| 2310007B03Rik | -0.3615507 |
| 2310007F21Rik | -0.5336335 |
| 2310007F21Rik | -0.3981901 |
| 2310008H04Rik | -0.7748227 |
| 2310008H09Rik | -0.4024872 |
| 2310008H09Rik | 2.76412997 |
| 2310008M10Rik | 2.15361996 |
| 2310009E04Rik | 0.6513955 |
| 2310010B21Rik | 0.3368098 |
| 2310010M24Rik | 2.30937236 |
| 2310011J03Rik | 1.20749172 |
| 2310014L17Rik | -0.5949775 |
| 2310016C08Rik | -0.554364 |
| 2310016C08Rik | -0.5934696 |
| 2310016C16Rik | -0.7594269 |
| 2310016C16Rik | 2.6525713 |
| 2310016C16Rik | 0.30596685 |
| 2310016E02Rik | 1.79617166 |
| 2310016M24Rik | 1.64386533 |
| 2310021P13Rik | -0.3988028 |
| 2310022A10Rik | 0.11090349 |
| 2310022B05Rik | 2.00634073 |
| 2310031L18Rik | -0.2085287 |
| 2310031L18Rik | 0.88861317 |
| 2310033E01Rik | -0.0777735 |
| 2310033K02Rik | 1.25218065 |
| 2310033P09Rik | -0.4874129 |
| 2310035C23Rik | -0.4760368 |
| 2310035C23Rik | -0.1597234 |
| 2310035K24Rik | -0.7504866 |
| 2310036D22Rik | 2.26707012 |
| 2310036O22Rik | -0.5711356 |
| 2310037I24Rik | 0.54856946 |
| 2310037I24Rik | 0.50375089 |
| 2310038H17Rik | 1.2899511 |
| 2310039E09Rik | 0.95493831 |
| 2310039H08Rik | 2.68730629 |
| 2310040C09Rik | -0.1274622 |
| 2310042D19Rik | 0.90105247 |
| 2310044G17Rik | 0.07747394 |
| 2310044H10Rik | 1.5943791 |
| 2310045A20Rik | -0.6453449 |
| 2310046K01Rik | 0.56591711 |
| 2310046O06Rik | -0.6248741 |
| 2310047C04Rik | -0.2824815 |
| 2310047D13Rik | 1.52029545 |
| 2310047D13Rik | 1.23813085 |
| 2310047M10Rik | 1.91748718 |
| 2310047M10Rik | 1.14580033 |
| 2310047O13Rik | -0.3787921 |
| 2310056P07Rik | 0.38900282 |
| 2310057J16Rik | 0.80074957 |
| 2310058J06Rik | -0.6485341 |
| 2310061C15Rik | -0.3908691 |
| 2310061F22Rik | -0.3627297 |
| 2310061F22Rik | -0.6719838 |
| 2310065K24Rik | 0.13138553 |
| 2310065K24Rik | -0.2911896 |
| 2310066E14Rik | -0.6910055 |
| 2310067B10Rik | 0.74337537 |
| 2310076L09Rik | 0.50563211 |
| 2310076L09Rik | -0.4261618 |
| 2310079F23Rik | -0.5601638 |
| 2310079N02Rik | -0.5792634 |
| 2400001E08Rik | 2.08225624 |
| 2400003C14Rik | 2.3908452 |
| 2400003C14Rik | -0.2972235 |
| 2400003C14Rik | 0.10625102 |
| 2400006N03Rik | 3.41E-05 |
| 2400010D15Rik | 1.59829631 |
| 2410001C21Rik | 0.92258512 |
| 2410001C21Rik | 1.61951884 |
| 2410001C21Rik | 0.19911052 |
| 2410002F23Rik | -0.2010449 |
| 2410002F23Rik | 3.4613208 |
| 2410002F23Rik | -0.6597819 |
| 2410002I01Rik | 2.06451759 |
| 2410002O22Rik | -0.2025369 |
| 2410003P15Rik | -0.182835 |
| 2410004A20Rik | 1.11614335 |
| 2410004B18Rik | 1.36822803 |
| 2410004L22Rik | 0.2543578 |
| 2410005O16Rik | 0.37830887 |
| 2410008K03Rik | 0.87267056 |
| 2410014A08Rik | 1.1718909 |
| 2410014A08Rik | 0.06368809 |
| 2410014A08Rik | 0.59306913 |
| 2410014A08Rik | 1.11565133 |
| 2410015M20Rik | 0.34853708 |
| 2410015N17Rik | 0.59755495 |
| 2410016F19Rik | 0.69312968 |
| 2410016F19Rik | 1.3091862 |
| 2410016O06Rik | 0.07599325 |
| 2410017P07Rik | 0.8499535 |
| 2410018C17Rik | -0.3060694 |
| 2410018G20Rik | 0.5260816 |
| 2410018M08Rik | 0.62112556 |
| 2410019A14Rik | -0.5009745 |
| 2410019G02Rik | 1.98734421 |
| 2410022L05Rik | -0.2819399 |
| 2410025L10Rik | 1.15658254 |
| 2410042D21Rik | -0.6117256 |
| 2410042D21Rik | -0.3055141 |
| 2410066E13Rik | -0.0755957 |
| 2410081M15Rik | 1.78643427 |
| 2410091C18Rik | -0.4615157 |
| 2410116G06Rik | 2.58018356 |
| 2410127E18Rik | 0.1250765 |
| 2410127L17Rik | 1.35237563 |
| 2410127L17Rik | -0.5889603 |
| 2410129H14Rik | 0.90429078 |
| 2410137M14Rik | -0.0695246 |
| 2410146L05Rik | -0.6163453 |
| 2410166I05Rik | -0.5126572 |
| 2500002L14Rik | 0.53249637 |
| 2500003M10Rik | -0.213551 |
| 2500003M10Rik | -0.5378854 |
| 2500003M10Rik | -0.9335502 |
| 2510002A14Rik | 0.38660723 |
| 2510003E04Rik | -0.6851756 |
| 2510006D16Rik | -0.4543381 |
| 2510009E07Rik | -0.4747074 |
| 2510010F15Rik | -0.5579847 |
| 2510012J08Rik | 1.4748334 |
| 2510039O18Rik | 0.24449422 |
| 2510040D07Rik | 0.50186591 |
| 2510048L02Rik | -0.2911896 |
| 2510049I19Rik | -0.6597819 |
| 2600001B17Rik | -0.3388501 |
| 2600009E05Rik | -0.7276308 |
| 2600009E05Rik | -0.73373 |
| 2600010E01Rik | -0.5400185 |
| 2600011E07Rik | -0.1823477 |
| 2600011E07Rik | 1.87103723 |
| 2610002J02Rik | -0.7766506 |
| 2610002M06Rik | -0.6056009 |
| 2610003J06Rik | 0.42191746 |
| 2610016F04Rik | 1.01635773 |
| 2610019A05Rik | 1.54034988 |
| 2610019P18Rik | -0.6851756 |
| 2610020C11Rik | 2.37177709 |
| 2610020H08Rik | -0.0178008 |
| 2610020H08Rik | 3.90508415 |
| 2610020H08Rik | -0.5385959 |
| 2610020O08Rik | 2.16509371 |
| 2610021K21Rik | 1.94270145 |
| 2610021K21Rik | 0.97970385 |
| 2610024G14Rik | -0.555087 |
| 2610024G14Rik | 2.05292894 |
| 2610024G14Rik | -0.7603256 |
| 2610027C15Rik | 1.19513281 |
| 2610027C15Rik | -0.7381108 |
| 2610027L16Rik | 0.66557917 |
| 2610028A01Rik | 0.92379222 |
| 2610028H24Rik | 0.2854235 |
| 2610029G23Rik | -0.9037738 |
| 2610029G23Rik | 1.32054175 |
| 2610034E13Rik | -0.295574 |
| 2610034E13Rik | 0.91042841 |
| 2610034M16Rik | 1.24104379 |
| 2610036D13Rik | 1.17536194 |
| 2610039C10Rik | -0.7311112 |
| 2610042L04Rik | 1.1769774 |
| 2610044O15Rik | -0.4369485 |
| 2610101N10Rik | -0.7693601 |
| 2610101N10Rik | 0.26563545 |
| 2610103J23Rik | -0.6233176 |
| 2610110G12Rik | -0.8678284 |
| 2610200G18Rik | 1.37057839 |
| 2610204K14Rik | 2.88411372 |
| 2610204L23Rik | -0.4793685 |
| 2610204M08Rik | 0.88861317 |
| 2610205E22Rik | 1.26108079 |
| 2610207I05Rik | -0.6477358 |
| 2610208M17Rik | -0.7513768 |
| 2610209A20Rik | -0.632695 |
| 2610209A20Rik | -0.4074222 |
| 2610209M04Rik | -0.0980486 |
| 2610301B20Rik | 0.69908046 |
| 2610301F02Rik | 2.89080722 |
| 2610304G08Rik | -0.5792634 |
| 2610305D13Rik | -0.8469009 |
| 2610318N02Rik | -0.536466 |
| 2610507B11Rik | 0.20462156 |
| 2610507B11Rik | -0.6868376 |
| 2610510B01Rik | 0.53500457 |
| 2610510J17Rik | 0.11339919 |
| 2610524A10Rik | -0.5030257 |
| 2610524H06Rik | 1.21449775 |
| 2610528E23Rik | 0.43441368 |
| 2610528J11Rik | 0.66577742 |
| 2610528O22Rik | 2.86887739 |
| 2700007P21Rik | -0.2080279 |
| 2700007P21Rik | -0.4388649 |
| 2700038C09Rik | 2.38368764 |
| 2700038C09Rik | 3.40267428 |
| 2700050L05Rik | -0.7215708 |
| 2700055A20Rik | 1.18695476 |
| 2700060E02Rik | -0.5329268 |
| 2700062C07Rik | -0.8254637 |
| 2700062C07Rik | 1.44163632 |
| 2700078K21Rik | -0.3884413 |
| 2700081O15Rik | 0.89205037 |
| 2700087H15Rik | 0.67072029 |
| 2700094K13Rik | 0.21170603 |
| 2700097O09Rik | 1.2731309 |
| 2810002N01Rik | 1.2282464 |
| 2810002O09Rik | -0.7540526 |
| 2810003C17Rik | -0.3279856 |
| 2810004N20Rik | -0.5414433 |
| 2810004N23Rik | 0.32237068 |
| 2810008M24Rik | 0.39562549 |
| 2810021B07Rik | 1.24018163 |
| 2810021J22Rik | 0.4772542 |
| 2810021J22Rik | -0.7793991 |
| 2810022L02Rik | -0.7693601 |
| 2810022L02Rik | -0.0971579 |
| 2810022L02Rik | -0.4793685 |
| 2810030E01Rik | 1.60922247 |
| 2810046L04Rik | -0.4105197 |
| 2810046M22Rik | -0.6397897 |
| 2810403A07Rik | 0.22508493 |
| 2810405J04Rik | -0.5195976 |
| 2810405K02Rik | 1.53397558 |
| 2810408A11Rik | 0.08042835 |
| 2810408M09Rik | -0.6622096 |
| 2810410C14Rik | -0.5529196 |
| 2810410P22Rik | 1.73796241 |
| 2810410P22Rik | -0.4700693 |
| 2810417H13Rik | -0.8759246 |
| 2810422O20Rik | 1.48727757 |
| 2810428I15Rik | 0.53066803 |
| 2810432D09Rik | -0.2500001 |
| 2810432L12Rik | 0.40534247 |
| 2810439F02Rik | 0.55972639 |
| 2810439F02Rik | -0.4928141 |
| 2810441K11Rik | 0.86708242 |
| 2810452K22Rik | -0.9834664 |
| 2810453I06Rik | -1.0100371 |
| 2810453I06Rik | 1.77781556 |
| 2810470D21Rik | 0.15450508 |
| 2810485I05Rik | 0.05879618 |
| 2900006K08Rik | 1.6414024 |
| 2900010M23Rik | 0.6813093 |
| 2900011O08Rik | 2.07391874 |
| 2900011O08Rik | 2.47269113 |
| 2900024C23Rik | -0.3639102 |
| 2900024O10Rik | -0.7078638 |
| 2900026A02Rik | 0.32237068 |
| 2900046G09Rik | -0.3486781 |
| 2900062L11Rik | -0.7232982 |
| 2900064A13Rik | 1.22606004 |
| 2900073G15Rik | 0.19911052 |
| 2900092C05Rik | -0.9303146 |
| 3000004C01Rik | -0.994927 |
| 3000004C01Rik | -0.4000293 |
| 3000004C01Rik | 0.23827669 |
| 3010003L21Rik | 0.81303895 |
| 3010026O09Rik | 1.86533078 |
| 3110001A13Rik | -0.2631772 |
| 3110001D03Rik | 0.01596012 |
| 3110004L20Rik | -0.8538252 |
| 3110007F17Rik | -0.8882006 |
| 3110009E18Rik | -0.6638316 |
| 3110023B02Rik | 0.7380657 |
| 3110035E14Rik | 0.15484589 |
| 3110035E14Rik | -0.7839978 |
| 3110037I16Rik | 0.10265652 |
| 3110040N11Rik | 0.10481487 |
| 3110040N11Rik | 0.15586759 |
| 3110043J09Rik | -0.7923324 |
| 3110043J09Rik | 2.00096584 |
| 3110043J09Rik | -0.585966 |
| 3110048E14Rik | -0.4694086 |
| 3110049J23Rik | -0.4820423 |
| 3110050N22Rik | -0.6140326 |
| 3110050N22Rik | 1.96306008 |
| 3110056O03Rik | -0.4142502 |
| 3110057O12Rik | 1.30888552 |
| 3110057O12Rik | 0.86033265 |
| 3110070M22Rik | 0.5381872 |
| 3110082I17Rik | 0.75390587 |
| 3200001I04Rik | 1.04410475 |
| 3200002M19Rik | -0.6901704 |
| 3200002M19Rik | 0.23546538 |
| 3300001G02Rik | -0.5616193 |
| 3300001P08Rik | 0.92995447 |
| 3300001P08Rik | -0.5807488 |
| 3300001P08Rik | -0.1474371 |
| 3300002I08Rik | -0.0865353 |
| 3321401G04Rik | -0.7155494 |
| 3322402L07Rik | -0.5874619 |
| 3632451O06Rik | 1.16770872 |
| 3830402I07Rik | -0.8568085 |
| 3830406C13Rik | 1.39358204 |
| 3830406C13Rik | 0.37453716 |
| 3830406C13Rik | 2.43672745 |
| 3830408P04Rik | 1.61380446 |
| 3930401K13Rik | 0.14456759 |
| 4121402D02Rik | 2.28147804 |
| 4121402D02Rik | -0.6094242 |
| 4430402I18Rik | 0.64796753 |
| 4432405B04Rik | 0.25894608 |
| 4432416J03Rik | 0.17773685 |
| 4631426J05Rik | 2.40531294 |
| 4631427C17Rik | -0.2303171 |
| 4632404H12Rik | 1.2895419 |
| 4632404H12Rik | 3.16789142 |
| 4632404H12Rik | -0.2060275 |
| 4632404H22Rik | -0.6818603 |
| 4632411B12Rik | -0.7858436 |
| 4632411B12Rik | 1.87120292 |
| 4632412N22Rik | 3.21024918 |
| 4632415K11Rik | 1.27676797 |
| 4632417K18Rik | 2.20879289 |
| 4632417N05Rik | -0.1088025 |
| 4632419K20Rik | 1.24812664 |
| 4632434I11Rik | 1.40033875 |
| 4732415M23Rik | 0.85855684 |
| 4732418C07Rik | 0.18700342 |
| 4732418C07Rik | 1.06406732 |
| 4732418C07Rik | 0.34714859 |
| 4732429D16Rik | -0.1070016 |
| 4732456N10Rik | -0.7146924 |
| 4732471D19Rik | -0.3359789 |
| 4732473B16Rik | -0.6217638 |
| 4732479N06Rik | -0.9090226 |
| 4732479N06Rik | 0.24201213 |
| 4732496O08Rik | 0.61758557 |
| 4733401H18Rik | -0.2231678 |
| 4831426I19Rik | 0.92379222 |
| 4833418A01Rik | 0.30014284 |
| 4833420G17Rik | 0.83117789 |
| 4833421E05Rik | -0.6010334 |
| 4833424O15Rik | 1.40079355 |
| 4833424O15Rik | -0.7087146 |
| 4833426J09Rik | -0.421128 |
| 4833426J09Rik | 0.24076863 |
| 4833427G06Rik | 0.82433282 |
| 4833436C18Rik | -0.4299548 |
| 4833439L19Rik | -0.2494769 |
| 4833439L19Rik | 1.06562546 |
| 4833439L19Rik | 1.4871946 |
| 4833442J19Rik | -0.0673657 |
| 4833446K15Rik | -0.8954361 |
| 4921501E09Rik | 0.00523961 |
| 4921504E06Rik | -0.364501 |
| 4921504I05Rik | 0.91317736 |
| 4921505C17Rik | -0.4111404 |
| 4921505C17Rik | 0.07339645 |
| 4921507P07Rik | 1.4748334 |
| 4921511H03Rik | -0.0764662 |
| 4921511H03Rik | 0.16433896 |
| 4921511K06Rik | 0.54564691 |
| 4921511K06Rik | -0.6178903 |
| 4921515J06Rik | 3.12400993 |
| 4921517D21Rik | -0.4142502 |
| 4921517D22Rik | 1.27073316 |
| 4921520G13Rik | 1.51362234 |
| 4921521F21Rik | 1.75953038 |
| 4921521F21Rik | 2.17065807 |
| 4921523A10Rik | 0.59670215 |
| 4921523A10Rik | -0.4921373 |
| 4921523A10Rik | 0.63781243 |
| 4921525H12Rik | -0.4955264 |
| 4921528G01Rik | -0.7061643 |
| 4921528I01Rik | -0.5182055 |
| 4921530G04Rik | -0.413005 |
| 4921530L21Rik | -0.5536415 |
| 4921530L21Rik | 1.65982315 |
| 4921536K21Rik | -0.5674641 |
| 4921537P18Rik | 0.40377353 |
| 4922502D21Rik | 1.11602037 |
| 4922503N01Rik | -0.6094242 |
| 4922503N01Rik | 0.36449585 |
| 4922505E12Rik | 0.51195366 |
| 4922505G16Rik | 2.82585786 |
| 4922505G16Rik | -0.6437543 |
| 4930401F20Rik | -0.7104187 |
| 4930402H24Rik | 2.60589876 |
| 4930403C10Rik | 0.3362477 |
| 4930403L05Rik | 0.25619579 |
| 4930403O06Rik | 0.54834497 |
| 4930404H21Rik | 1.1113352 |
| 4930404N11Rik | -0.4299548 |
| 4930408O21Rik | -0.7495972 |
| 4930408O21Rik | -0.6382084 |
| 4930412F15Rik | 0.72400722 |
| 4930415L06Rik | -0.568931 |
| 4930418G15Rik | 0.02460614 |
| 4930427A07Rik | -0.2323698 |
| 4930428D18Rik | 0.31175513 |
| 4930428E23Rik | 1.97173757 |
| 4930430D24Rik | -0.7138361 |
| 4930430E16Rik | -0.4800362 |
| 4930430E16Rik | -0.5770398 |
| 4930431L04Rik | -0.4230125 |
| 4930432H15Rik | -0.3668679 |
| 4930432K21Rik | -0.2145587 |
| 4930432O21Rik | -0.353923 |
| 4930432O21Rik | 0.46163155 |
| 4930438O05Rik | -0.2841081 |
| 4930438O05Rik | 0.00244021 |
| 4930449I24Rik | 1.1688723 |
| 4930451C15Rik | -0.0764662 |
| 4930451I11Rik | -0.7729983 |
| 4930452B06Rik | 0.72419356 |
| 4930453N24Rik | 1.72830885 |
| 4930455C21Rik | -0.7567359 |
| 4930455F23Rik | -0.2500001 |
| 4930467M19Rik | -0.3633198 |
| 4930470P17Rik | -0.1493169 |
| 4930473A06Rik | 2.09619341 |
| 4930473A06Rik | -0.3692407 |
| 4930473A06Rik | -0.63191 |
| 4930474N05Rik | -0.3562634 |
| 4930478A21Rik | 1.15386899 |
| 4930481F22Rik | 0.21522841 |
| 4930481F22Rik | 0.43567955 |
| 4930481M05 | -0.6736227 |
| 4930486L24Rik | 0.35599977 |
| 4930502E18Rik | -0.36214 |
| 4930503B20Rik | 3.46323229 |
| 4930503L19Rik | -0.1616279 |
| 4930504E06Rik | -0.5428702 |
| 4930504E06Rik | 3.83041612 |
| 4930504O13Rik | -0.1554523 |
| 4930506M07Rik | -0.7702684 |
| 4930507C10Rik | -0.6573603 |
| 4930511I11Rik | 1.73464915 |
| 4930511J11Rik | -0.3342603 |
| 4930515G01Rik | -0.3787921 |
| 4930518C23Rik | -0.3302625 |
| 4930519F16Rik | 1.33557034 |
| 4930519G04Rik | -0.2105344 |
| 4930519N13Rik | -0.514734 |
| 4930519N16Rik | -0.0613471 |
| 4930522H14Rik | -0.4414262 |
| 4930524B15Rik | 2.13797637 |
| 4930524B15Rik | 0.33540393 |
| 4930524E20Rik | -0.3451977 |
| 4930526D03Rik | -0.421128 |
| 4930535B03Rik | 2.94145111 |
| 4930535E21Rik | 0.07116491 |
| 4930538K18Rik | 1.57022404 |
| 4930539E08Rik | -0.3417301 |
| 4930540L03Rik | -0.6025534 |
| 4930544G11Rik | -0.5435845 |
| 4930544G21Rik | 1.40006577 |
| 4930547C10Rik | 2.68814281 |
| 4930547N16Rik | -0.516121 |
| 4930549C01Rik | -0.6063643 |
| 4930550C14Rik | -0.5202945 |
| 4930550L24Rik | -0.4117615 |
| 4930555G01Rik | -0.4186212 |
| 4930555I21Rik | 0.78148406 |
| 4930562D19Rik | -0.2170827 |
| 4930562D19Rik | -0.2401073 |
| 4930563D23Rik | -0.4369485 |
| 4930563P21Rik | 1.74518564 |
| 4930570C03Rik | -0.7112719 |
| 4930572J05Rik | 2.28607805 |
| 4930572J05Rik | 1.09776559 |
| 4930572J05Rik | -0.4055685 |
| 4930573I19Rik | 0.46580662 |
| 4930578I06Rik | 2.67829004 |
| 4930579C15Rik | 0.76020406 |
| 4930579E17Rik | -0.0751608 |
| 4930579E17Rik | -0.513349 |
| 4930579J09Rik | -0.2287806 |
| 4930583H14Rik | 0.65701456 |
| 4930590J08Rik | 0.21298842 |
| 4930595M18Rik | 1.31468182 |
| 4930596D02Rik | -0.6389987 |
| 4930599N23Rik | 0.94893767 |
| 4931406B18Rik | -0.747821 |
| 4931406C07Rik | -0.4773681 |
| 4931406H21Rik | -0.1838104 |
| 4931406I20Rik | 1.25664122 |
| 4931406I20Rik | 0.30451422 |
| 4931406P16Rik | 0.6801386 |
| 4931407G18Rik | -0.16497 |
| 4931408A02Rik | -0.0847764 |
| 4931408C20Rik | 2.65675869 |
| 4931408C20Rik | -0.7328563 |
| 4931409K22Rik | 1.3896335 |
| 4931417E11Rik | -0.8101734 |
| 4931417G12Rik | -0.8016804 |
| 4931428L18Rik | 1.3572451 |
| 4931432M23Rik | 0.09470007 |
| 4931433A01Rik | -0.0843372 |
| 4931440B09Rik | -0.3325449 |
| 4931440F15Rik | -0.0190292 |
| 4931440F15Rik | -0.4337631 |
| 4931440L10Rik | 1.08221493 |
| 4932409I22Rik | -0.0773375 |
| 4932411E22Rik | -0.3698348 |
| 4932411G14Rik | 0.4861694 |
| 4932412H11Rik | 0.82801196 |
| 4932413O14Rik | 2.16193406 |
| 4932414J04Rik | 0.92860522 |
| 4932414N04Rik | 0.85450844 |
| 4932418E24Rik | 2.48423647 |
| 4932431H17Rik | 0.66716404 |
| 4932431H17Rik | -0.649333 |
| 4932441K18Rik | 3.4705358 |
| 4932441K18Rik | 0.11837087 |
| 4932442K08Rik | 0.83117789 |
| 4932702K14Rik | 0.15416414 |
| 4933400A11Rik | -0.4713922 |
| 4933400C05Rik | 0.68617165 |
| 4933400E14Rik | 1.52261594 |
| 4933402J07Rik | -0.5837265 |
| 4933402N03Rik | 0.77196743 |
| 4933402P03Rik | -0.5279941 |
| 4933403G14Rik | -0.8892309 |
| 4933404M02Rik | -0.6573603 |
| 4933405O20Rik | -0.209531 |
| 4933406M09Rik | 1.29281051 |
| 4933407N01Rik | 1.49808441 |
| 4933407N01Rik | -0.4934915 |
| 4933407N01Rik | 0.70461354 |
| 4933407P14Rik | -0.3251472 |
| 4933407P14Rik | 0.6884967 |
| 4933407P14Rik | 0.90305845 |
| 4933409N07Rik | 1.726701 |
| 4933411G06Rik | -0.2642393 |
| 4933411K20Rik | -0.0359795 |
| 4933412E14Rik | -0.8459158 |
| 4933413G11Rik | -0.0523906 |
| 4933415F23Rik | -0.7087146 |
| 4933416C03Rik | -0.737233 |
| 4933417A18Rik | -0.6209878 |
| 4933417E01Rik | -0.5800058 |
| 4933421E11Rik | -0.2186003 |
| 4933421E11Rik | 1.41711113 |
| 4933421E11Rik | 0.0277306 |
| 4933422H20Rik | -0.3769938 |
| 4933424B01Rik | 0.24387431 |
| 4933424B01Rik | 0.01279635 |
| 4933424B01Rik | -0.6437543 |
| 4933425O20Rik | 1.46876076 |
| 4933426M11Rik | -0.525187 |
| 4933427D14Rik | -0.2642393 |
| 4933428G09Rik | 3.4429919 |
| 4933428G20Rik | -0.6421665 |
| 4933429E10Rik | 1.70883137 |
| 4933429E10Rik | -0.0202592 |
| 4933430I17Rik | -0.5357571 |
| 4933434E20Rik | -0.7522679 |
| 4933434E20Rik | -0.0052003 |
| 4933434E20Rik | -0.4641393 |
| 4933434I06Rik | 0.69543767 |
| 4933436I01Rik | 1.26906157 |
| 4933437F05Rik | 1.74430155 |
| 4933439C20Rik | 0.42037587 |
| 4933440H19Rik | -0.7621256 |
| 5031414D18Rik | -0.4989278 |
| 5031414D18Rik | -0.7630269 |
| 5033411D12Rik | -0.4407853 |
| 5033414D02Rik | -0.1891931 |
| 5033414K04Rik | -0.0309868 |
| 5133400G04Rik | -0.8518417 |
| 5133400G04Rik | -0.6217638 |
| 5133400G04Rik | 3.13195051 |
| 5133401N09Rik | 1.34622913 |
| 5230400G24Rik | 0.0961517 |
| 5230400G24Rik | 1.77427246 |
| 5330401P04Rik | 0.64574277 |
| 5330417C22Rik | 0.51265345 |
| 5330431N19Rik | -0.6687145 |
| 5330431N19Rik | 0.29604453 |
| 5330431N19Rik | -0.7129806 |
| 5330437I02Rik | 1.42954165 |
| 5330439C02Rik | 0.34881453 |
| 5330439J01Rik | -0.1995553 |
| 5330439J01Rik | 0.85742504 |
| 5430407P10Rik | -0.1941133 |
| 5430432M24Rik | -0.5748214 |
| 5430432M24Rik | -0.4813732 |
| 5430432M24Rik | 0.17306921 |
| 5430435G22Rik | 2.14345429 |
| 5430437P03Rik | 1.39577872 |
| 5530601I19Rik | -0.4510935 |
| 5630401D24Rik | -0.5071414 |
| 5630401D24Rik | -0.780317 |
| 5630401D24Rik | 2.698652 |
| 5730403M16Rik | -0.5652681 |
| 5730409E04Rik | 1.5579105 |
| 5730410E15Rik | 0.7772663 |
| 5730410E15Rik | 0.40115281 |
| 5730410I19Rik | -0.4641393 |
| 5730410I19Rik | 0.18831981 |
| 5730410I19Rik | -0.311082 |
| 5730419I09Rik | -0.1731362 |
| 5730437N04Rik | -0.6935151 |
| 5730446C15Rik | -0.2808576 |
| 5730449L18Rik | 1.60717887 |
| 5730449L18Rik | -0.6711654 |
| 5730449L18Rik | -0.416121 |
| 5730453I16Rik | 0.51405147 |
| 5730453I16Rik | -0.6843456 |
| 5730469M10Rik | 1.55914143 |
| 5730472N09Rik | 0.39852479 |
| 5730494M16Rik | -0.6557494 |
| 5730494N06Rik | -0.0856554 |
| 5730502D15Rik | 2.66809603 |
| 5730507A09Rik | 1.82148239 |
| 5730507A09Rik | -0.7839978 |
| 5730528L13Rik | 1.02180975 |
| 5730589K01Rik | 0.81726891 |
| 5730593F17Rik | 0.02460614 |
| 5730596K20Rik | -0.5814923 |
| 5730596K20Rik | -0.1469677 |
| 5730596K20Rik | -0.2900967 |
| 5730596K20Rik | -0.4602066 |
| 5830403L16Rik | 1.27946075 |
| 5830404H04Rik | 0.59606205 |
| 5830405N20Rik | -0.5216897 |
| 5830415F09Rik | -0.4621709 |
| 5830415L20Rik | 0.06818129 |
| 5830417C01Rik | 0.04969775 |
| 5830426I05Rik | 0.11801662 |
| 5830427D03Rik | -0.5785216 |
| 5830434P21Rik | 0.0285101 |
| 5830435K17Rik | -0.7267627 |
| 5830457O10Rik | -0.3890476 |
| 5830457O10Rik | -0.6557494 |
| 5830457O10Rik | -0.650933 |
| 5830467P10Rik | -0.6048381 |
| 5830468K18Rik | 3.81121655 |
| 5830472M02Rik | 1.16735937 |
| 5830472M02Rik | 0.38473978 |
| 5830472M02Rik | 0.0569079 |
| 5930416I19Rik | -0.4647964 |
| 5930431H10 | -0.4780344 |
| 5930434B04Rik | -0.6876697 |
| 5930434B04Rik | -0.4217558 |
| 5930434B04Rik | -0.2695681 |
| 6030405A18Rik | -0.5023415 |
| 6030408B16Rik | -0.4142502 |
| 6030408B16Rik | 0.64148088 |
| 6030419C18Rik | -0.5748214 |
| 6030429G01Rik | 0.81659339 |
| 6030443O07Rik | 0.4232002 |
| 6030446N20Rik | -0.8302931 |
| 6030458C11Rik | 1.17374371 |
| 6230409E13Rik | 2.35796887 |
| 6230409E13Rik | 0.63638196 |
| 6230416J20Rik | -0.5182055 |
| 6230427J02Rik | 1.11589737 |
| 6330403K07Rik | 0.06780767 |
| 6330406I15Rik | 0.64958224 |
| 6330406I15Rik | 0.72102064 |
| 6330407J23Rik | 0.39562549 |
| 6330408A02Rik | -0.7164073 |
| 6330409N04Rik | -0.6202125 |
| 6330416G13Rik | -0.6943531 |
| 6330439K17Rik | 2.6671228 |
| 6330442E10Rik | 0.37830887 |
| 6330442E10Rik | -0.4299548 |
| 6330500D04Rik | 2.04813429 |
| 6330503C03Rik | -0.215063 |
| 6330503K22Rik | -0.3793923 |
| 6330503K22Rik | -0.6040759 |
| 6330503K22Rik | -0.63191 |
| 6330505N24Rik | 3.04889065 |
| 6330512M04Rik | 0.0888711 |
| 6330534C20Rik | -0.294476 |
| 6330548G22Rik | -0.3257142 |
| 6330569M22Rik | -0.5322205 |
| 6330577E15Rik | -0.6687145 |
| 6330578E17Rik | 0.72493858 |
| 6332401O19Rik | -0.6086583 |
| 6430411K18Rik | 2.42381366 |
| 6430502M16Rik | 1.53720746 |
| 6430510M02Rik | 0.61758557 |
| 6430517E21Rik | -0.7693601 |
| 6430526N21Rik | -0.516121 |
| 6430527G18Rik | 0.49452583 |
| 6430527G18Rik | 0.18502537 |
| 6430531B16Rik | -0.7224341 |
| 6430537H07Rik | -0.4465698 |
| 6430537H07Rik | 0.9626391 |
| 6430548M08Rik | 2.25184869 |
| 6430550H21Rik | -0.5919641 |
| 6430571L13Rik | 0.10373631 |
| 6430573F11Rik | 1.07324912 |
| 6430598A04Rik | -0.0984942 |
| 6430604K15Rik | -0.5336335 |
| 6430706D22Rik | -0.7302399 |
| 6530404N21Rik | 1.76089707 |
| 6530406M24Rik | -0.5630771 |
| 6530418L21Rik | -0.1119623 |
| 6530418L21Rik | 1.6589936 |
| 6530418L21Rik | 3.8804368 |
| 6530421E24Rik | -0.5594369 |
| 6620401K05Rik | -0.3138783 |
| 6720456B07Rik | -0.6818603 |
| 6720456B07Rik | -0.7398689 |
| 6720456H20Rik | -0.2070272 |
| 6720456H20Rik | 1.58462656 |
| 6720458F09Rik | 0.57948221 |
| 6720463M24Rik | 0.83101153 |
| 6720467C03Rik | 1.44736636 |
| 6720467C03Rik | 0.36258406 |
| 6820408C15Rik | 0.61088582 |
| 7420416P09Rik | -0.6109578 |
| 7530404M11Rik | -0.4478601 |
| 8030411F24Rik | 0.10875905 |
| 8030451F13Rik | -0.4031026 |
| 8030451F13Rik | 2.3860902 |
| 8030451F13Rik | 1.85869461 |
| 8430408G22Rik | 0.41418419 |
| 8430410A17Rik | -0.432492 |
| 8430410K20Rik | 1.51103691 |
| 8430415E04Rik | -0.6622096 |
| 8430415E04Rik | -0.5385959 |
| 8430419L09Rik | -0.8197007 |
| 8430427H17Rik | 1.87335426 |
| 8430432M10Rik | 0.14318865 |
| 9030025P20Rik | -0.5485996 |
| 9030025P20Rik | -0.5071414 |
| 9030025P20Rik | -0.5195976 |
| 9030205A07Rik | -0.5792634 |
| 9030221M09Rik | 1.57090765 |
| 9030224M15Rik | 1.87357463 |
| 9030227G01Rik | -0.6910055 |
| 9030409G11Rik | -0.4615157 |
| 9030418K01Rik | 0.92950493 |
| 9030425E11Rik | 0.5833842 |
| 9030607L17Rik | 2.15578705 |
| 9030607L17Rik | 0.48688857 |
| 9030611O19Rik | -0.0528152 |
| 9030612M13Rik | -0.6662699 |
| 9030617O03Rik | 0.40324997 |
| 9030624J02Rik | 0.82600694 |
| 9030625A04Rik | -0.7702684 |
| 9130005N14Rik | 0.33315018 |
| 9130011E15Rik | 2.1472123 |
| 9130011J15Rik | -0.525888 |
| 9130014G24Rik | 0.68714111 |
| 9130017N09Rik | -0.0031836 |
| 9130206N08Rik | 1.47701673 |
| 9130211I03Rik | 1.31378473 |
| 9130213B05Rik | -0.6613997 |
| 9130213B05Rik | 1.36085706 |
| 9130218O11Rik | 0.85174556 |
| 9130221H12Rik | 0.13799959 |
| 9130227C08Rik | -0.5521982 |
| 9130401M01Rik | 0.74136488 |
| 9130404D08Rik | -0.4105197 |
| 9130404D08Rik | 0.55861662 |
| 9130404D14Rik | -0.4205007 |
| 9130422G05Rik | -0.2468649 |
| 9230104L09Rik | -0.2988758 |
| 9330129D05Rik | 1.28132049 |
| 9330134C04Rik | -0.1151327 |
| 9330176C04Rik | -0.3533388 |
| 9330186A19Rik | 0.88059663 |
| 9430015G10Rik | 1.10637817 |
| 9430015G10Rik | 4.38664353 |
| 9430023L20Rik | 1.62290219 |
| 9430025C20Rik | -0.6785566 |
| 9430028L06Rik | 0.64857337 |
| 9430029K10Rik | 0.50092201 |
| 9430031J16Rik | 0.82550501 |
| 9430034D17Rik | 0.29721729 |
| 9430057O19Rik | 0.7851592 |
| 9430069J07Rik | 2.93321198 |
| 9430077A04Rik | 0.06780767 |
| 9430079M16Rik | 0.84390053 |
| 9530015I07Rik | 0.73106265 |
| 9530027K23Rik | 2.15013401 |
| 9530058B02Rik | 1.2117238 |
| 9530058B02Rik | 1.81470519 |
| 9530068E07Rik | -0.5378854 |
| 9530077C05Rik | 0.23546538 |
| 9530096D07Rik | 1.04807834 |
| 9530096D07Rik | -0.455639 |
| 9630015D15Rik | 0.30712735 |
| 9630020C08Rik | 0.22381889 |
| 9630028B13Rik | -0.5792634 |
| 9630031F12Rik | -0.4205007 |
| 9630050M13Rik | -0.568931 |
| 9630058J23Rik | 0.13138553 |
| 9830163H01Rik | 0.05463696 |
| 9830169C18Rik | -0.510585 |
| 9930013L23Rik | -0.5755603 |
| 9930021D14Rik | -0.5807488 |
| 9930023K05Rik | -0.7425121 |
| 9930023K05Rik | 0.5418114 |
| 9930104L06Rik | -0.7198465 |
| a | -0.3890476 |
| A030003K21Rik | 1.70935602 |
| A030007L17Rik | 0.02068595 |
| A030007L17Rik | -0.7121258 |
| A030007L17Rik | 1.15375083 |
| A030007L17Rik | 1.48586604 |
| A030009H04Rik | 1.80016965 |
| A030010B05Rik | -0.7146924 |
| A030012M09Rik | 1.235859 |
| A130010J15Rik | 0.9029043 |
| A130038H09Rik | 1.34969146 |
| A130042E20Rik | -0.3504232 |
| A130090K04Rik | -0.7567359 |
| A130092J06Rik | -0.1731362 |
| A230050P20Rik | -0.5536415 |
| A230051G13Rik | -0.650933 |
| A230054D04Rik | 1.84215333 |
| A230067G21Rik | -0.5195976 |
| A230069A22Rik | 1.3299994 |
| A230074B11Rik | 0.08374104 |
| A230074B11Rik | 1.54629178 |
| A230074B11Rik | -0.0153488 |
| A230106M15Rik | -0.1535602 |
| A230106N23Rik | 1.62727532 |
| A230107C01Rik | 1.03490147 |
| A2bp1 | 1.85054491 |
| A2bp1 | 1.33596005 |
| A330021E22Rik | -0.5230871 |
| A330021E22Rik | 1.46171149 |
| A330049M08Rik | 2.46058892 |
| A330080J22Rik | 0.83945886 |
| A3galt2 | 0.60307943 |
| A430005L14Rik | -0.8779595 |
| A430005L14Rik | 1.07620444 |
| A430005L14Rik | 2.05192636 |
| A430005L14Rik | -0.6549449 |
| A430033K04Rik | -0.5792634 |
| A430079E08 | 0.33173884 |
| A430083B19Rik | -0.1741016 |
| A430083B19Rik | 1.45752501 |
| A430107D22Rik | 0.383671 |
| A430107P09Rik | 1.58806262 |
| A430110N23Rik | 0.26077517 |
| A4galt | -0.4382257 |
| A4gnt | 0.06593737 |
| A530050D06Rik | -0.7867678 |
| A530082C11Rik | 0.7195238 |
| A530082C11Rik | 0.53363729 |
| A530088H08Rik | -0.3138783 |
| A530088H08Rik | 1.17939541 |
| A530098C11Rik | 2.19600399 |
| A630042L21Rik | -0.2895507 |
| A630054L15Rik | 0.365314 |
| A630077B13Rik | -0.511966 |
| A630095E13Rik | 1.48611528 |
| A730008H23Rik | -0.6469381 |
| A730008L03Rik | -0.0251953 |
| A730008L03Rik | -0.6437543 |
| A730017C20Rik | -0.4934915 |
| A730024F05Rik | -0.7648321 |
| A730037C10Rik | 0.25251622 |
| A730042J05Rik | 1.02885058 |
| A730098D12Rik | 1.9211083 |
| A830006F12Rik | -0.1516719 |
| A830007P12Rik | -0.5942232 |
| A830053O21Rik | -0.562348 |
| A830059I20Rik | -0.7381108 |
| A830073O21Rik | 1.50462363 |
| A830080D01Rik | 2.28154953 |
| A830093I24Rik | -0.5378854 |
| A930001N09Rik | -0.0326481 |
| A930005I04Rik | -0.2717081 |
| A930008G19Rik | -0.3668679 |
| A930010I20Rik | 1.22408797 |
| A930016P21Rik | 0.24789648 |
| A930018P22Rik | -0.4074222 |
| A930024E05Rik | 0.72437987 |
| A930025D01Rik | 2.09675881 |
| A930025D01Rik | 2.66288584 |
| A930025D01Rik | -0.6810333 |
| A930034L06Rik | -0.6517341 |
| A930038C07Rik | 0.79073201 |
| A930041I02Rik | -0.6703477 |
| AA388235 | -0.2669 |
| AA407270 | 0.45148557 |
| AA407659 | -0.4880863 |
| AA407659 | 1.01142383 |
| AA408296 | 0.93160092 |
| AA408296 | -0.516121 |
| AA408296 | -0.7284997 |
| AA409316 | -0.5867137 |
| AA415398 | -0.1137726 |
| AA467197 | 0.42345653 |
| AA536717 | -0.7904739 |
| AA536717 | -0.5638069 |
| AA536749 | -0.7702684 |
| AA536749 | 0.00284063 |
| AA536749 | -0.6178903 |
| AA536749 | -0.7979301 |
| AA673488 | -0.0401603 |
| AA691260 | 0.35544899 |
| AA792892 | -0.0515421 |
| AA881470 | -0.5536415 |
| AA881470 | 1.15351447 |
| AA960436 | 1.75398124 |
| AA960436 | -0.3116406 |
| Aaas | -0.2626465 |
| Aaas | -0.1721718 |
| Aacs | 1.68518381 |
| Aadacl1 | -0.3951328 |
| Aak1 | -0.4305884 |
| Aak1 | 1.15327805 |
| Aamp | 0.26926429 |
| Aard | -0.6303419 |
| Aars | 0.17540592 |
| Aars2 | 0.24325399 |
| Aarsd1 | 0.77108139 |
| Aasdh | -0.6918413 |
| Aatf | -0.5050813 |
| Aatf | 1.51160308 |
| Aatk | -0.121049 |
| AB112350 | 2.62754436 |
| AB182283 | 0.04588069 |
| AB182283 | 1.29219851 |
| Abat | 3.15804992 |
| Abat | -0.4813732 |
| Abat | -0.5400185 |
| Abca14 | 1.34217361 |
| Abca14 | -0.7558406 |
| Abca15 | -0.8139728 |
| Abca15 | -0.1493169 |
| Abca15 | -0.3704293 |
| Abca15 | 2.48582088 |
| Abca17 | 0.12998716 |
| Abca2 | 0.58532916 |
| Abca3 | -0.5140412 |
| Abca4 | -0.7849203 |
| Abca4 | 0.60032124 |
| Abca5 | 0.9271038 |
| Abca6 | -0.5223881 |
| Abca6 | -0.1526156 |
| Abca7 | -0.6264331 |
| Abca7 | 0.51428427 |
| Abca8a | -0.4530389 |
| Abca8b | 0.03317363 |
| Abca9 | -0.8459158 |
| Abca9 | -0.5209919 |
| Abca9 | 0.96133585 |
| Abcb11 | -0.7830763 |
| Abcb1b | 1.79095482 |
| Abcb4 | -0.1274622 |
| Abcb6 | 1.54893793 |
| Abcb8 | -0.8054456 |
| Abcb8 | -0.3365524 |
| Abcb8 | -0.5085174 |
| Abcb9 | -0.7328563 |
| Abcb9 | -0.2917366 |
| Abcc1 | -0.6868376 |
| Abcc10 | -0.6728029 |
| Abcc10 | 1.01704096 |
| Abcc12 | 0.2722777 |
| Abcc3 | 0.56877761 |
| Abcc5 | -0.4343993 |
| Abcc6 | 2.33410189 |
| Abcc8 | -0.6109578 |
| Abcc9 | 0.02538823 |
| Abcc9 | -0.7129806 |
| Abcd1 | -0.4433518 |
| Abcd2 | -0.7078638 |
| Abcd2 | -0.8608008 |
| Abcd3 | 0.08813994 |
| Abcd4 | -0.1427539 |
| Abcd4 | 1.02044969 |
| Abce1 | -0.4478601 |
| Abcf1 | -0.8254637 |
| Abcf1 | 0.14077064 |
| Abcf1 | 2.87255203 |
| Abcf2 | 1.85992499 |
| Abcf2 | -0.7164073 |
| Abcf2 | 0.41547748 |
| Abcf2 | 0.18667403 |
| Abcf3 | -0.6885025 |
| Abcg1 | 0.28096253 |
| Abcg4 | 2.06909557 |
| Abcg5 | -0.7198465 |
| Abhd1 | 2.12650295 |
| Abhd1 | 0.31808146 |
| Abhd10 | -0.3686469 |
| Abhd11 | 0.81015169 |
| Abhd12 | -0.2536712 |
| Abhd14a | 0.15450508 |
| Abhd14b | 0.63924075 |
| Abhd2 | 3.60110142 |
| Abhd2 | 2.00432873 |
| Abhd2 | 2.11104176 |
| Abhd3 | 0.62960889 |
| Abhd4 | 0.27857469 |
| Abhd5 | -0.1441564 |
| Abhd6 | -0.607893 |
| Abhd7 | 0.58208533 |
| Abhd7 | 0.55706074 |
| Abhd8 | 1.19105265 |
| Abi1 | -0.4192473 |
| Abi1 | -0.4615157 |
| Abi2 | -0.2416624 |
| Abi2 | 0.00324089 |
| Abi2 | -0.7087146 |
| Abi3 | -0.4641393 |
| Abi3bp | 2.64993866 |
| Abi3bp | -0.4569418 |
| Abl1 | 1.68599065 |
| Ablim1 | -0.3446189 |
| Ablim2 | 0.48832527 |
| Ablim2 | -0.4167454 |
| Ablim3 | -0.7232982 |
| Ablim3 | 0.92122529 |
| Abp1 | -0.0464666 |
| Abr | -0.4747074 |
| Abr | 0.32862645 |
| Abtb1 | 1.65795564 |
| Acaa1a | 2.1158261 |
| Acaa1b | 0.18205051 |
| Acaa2 | 1.40351771 |
| Acaa2 | -0.5030257 |
| Acaa2 | 0.95945018 |
| Acaa2 | 1.44032917 |
| Acad10 | 0.34964639 |
| Acad10 | 1.16069695 |
| Acad8 | -0.3486781 |
| Acad8 | -0.0363967 |
| Acad9 | -0.0153488 |
| Acad9 | 1.58507546 |
| Acadl | -0.0456233 |
| Acadl | 1.804625 |
| Acadm | -0.5529196 |
| Acads | -0.6703477 |
| Acads | 0.22001056 |
| Acadsb | -0.4549883 |
| Acadvl | -0.3674605 |
| Acan | 4.46619334 |
| Acat2 | -0.6597819 |
| Acbd3 | -0.4874129 |
| Acbd4 | -0.198068 |
| Acbd4 | -0.6960314 |
| Acbd5 | -0.6217638 |
| Acbd6 | 2.30860855 |
| Accn1 | 1.55266108 |
| Accn1 | -0.5852189 |
| Accn2 | 1.07812684 |
| Accn3 | -0.7224341 |
| Accn3 | 1.02790583 |
| Accs | -0.6279946 |
| Acd | 1.35905281 |
| Ace | 0.92635218 |
| Ace | 1.54136891 |
| Ache | -0.3331163 |
| Acin1 | -0.5814923 |
| Acly | 4.3699549 |
| Acn9 | -0.5608913 |
| Aco1 | -0.2999789 |
| Aco2 | -0.6295588 |
| Acot1 | -0.5792634 |
| Acot1 | -0.1813738 |
| Acot10 | -0.6985544 |
| Acot11 | 0.12296408 |
| Acot11 | -0.4628266 |
| Acot2 | -0.3854153 |
| Acot2 | -0.6876697 |
| Acot3 | 0.96090103 |
| Acot5 | -0.5126572 |
| Acot7 | 0.22540117 |
| Acot7 | -0.3568494 |
| Acot7 | 0.35655022 |
| Acot7 | -0.8026203 |
| Acot8 | -0.4847238 |
| Acot8 | -0.568931 |
| Acot8 | -0.2375211 |
| Acot9 | -0.19165 |
| Acox2 | -0.3342603 |
| Acox3 | 1.35275845 |
| Acox3 | -0.3769938 |
| Acoxl | -0.7258954 |
| Acp2 | 0.21298842 |
| Acp6 | -0.6695307 |
| Acpl2 | -0.7711775 |
| Acpp | 2.25277041 |
| Acr | 1.08170488 |
| Acr | 0.79229345 |
| Acrbp | -0.2216428 |
| Acrbp | -0.5507571 |
| Acrv1 | -0.5112753 |
| Acsbg1 | -0.7284997 |
| Acsbg1 | -0.7558406 |
| Acsbg2 | 0.23421321 |
| Acsf3 | -0.4713922 |
| Acsl1 | -0.7002402 |
| Acsl3 | 3.32514994 |
| Acsl3 | 0.55861662 |
| Acsl4 | -0.3981901 |
| Acsl4 | -0.0389041 |
| Acsl5 | 0.17973022 |
| Acsl6 | 2.41798602 |
| Acsl6 | 1.16666027 |
| Acsl6 | -0.0494239 |
| Acsm3 | -0.5092061 |
| Acss1 | 3.66638106 |
| Acss2 | -0.73373 |
| Acss2 | -0.7354799 |
| Acta1 | -0.6421665 |
| Acta2 | -0.2427006 |
| Acta2 | 0.38793887 |
| Acta2 | 1.35943294 |
| Acta2 | -0.5140412 |
| Actb | 1.46230805 |
| Actb | 0.07821341 |
| Actb | 0.56016994 |
| Actb | 1.38529865 |
| Actb | 0.25067104 |
| Actg2 | 0.01793209 |
| Actl6a | -0.7276308 |
| Actl6b | -0.6094242 |
| Actl6b | 1.92872486 |
| Actl6b | -0.4867399 |
| Actl6b | 1.28430904 |
| Actl7a | -0.8410059 |
| Actl7b | 0.12261155 |
| Actn1 | -0.2191067 |
| Actn2 | 0.6152879 |
| Actn3 | -0.6171175 |
| Actn3 | 0.64897705 |
| Actn4 | 0.92815504 |
| Actn4 | -0.0096522 |
| Actn4 | 1.49931398 |
| Actr10 | -0.2950249 |
| Actr10 | 0.05045932 |
| Actr1a | -0.3388501 |
| Actr1b | -0.4674291 |
| Actr3 | 0.95625044 |
| Actr3b | -0.8216178 |
| Actr5 | 0.67524497 |
| Actr5 | -0.6025534 |
| Actr6 | 0.8186185 |
| Actrt1 | 1.91922076 |
| Actrt2 | 0.25894608 |
| Acvr1 | -0.6960314 |
| Acvr1c | 0.48592955 |
| Acvr2a | 1.60116843 |
| Acvr2b | -0.4024872 |
| Acvrl1 | 0.20332774 |
| Acy1 | -0.4472147 |
| Acy1l2 | -0.7121258 |
| Acy3 | 1.8210748 |
| Ada | -0.4955264 |
| Adal | 3.00887052 |
| Adal | 0.35462223 |
| Adam11 | -0.3189327 |
| Adam11 | -0.4887603 |
| Adam12 | 1.36028767 |
| Adam15 | -0.4700693 |
| Adam17 | 0.17306921 |
| Adam1b | -0.778482 |
| Adam1b | -0.6334806 |
| Adam21 | -0.6240955 |
| Adam22 | 2.18676171 |
| Adam22 | -0.8380721 |
| Adam22 | -0.6910055 |
| Adam23 | -0.1061025 |
| Adam24 | 1.2852346 |
| Adam26a | 0.75912736 |
| Adam28 | 1.29799655 |
| Adam29 | 0.64513511 |
| Adam29 | 2.27961741 |
| Adam3 | -0.1549789 |
| Adam30 | -0.8302931 |
| Adam32 | -0.3475166 |
| Adam33 | -0.6240955 |
| Adam39 | -0.3793923 |
| Adam4 | 0.65380777 |
| Adam4 | 0.09069653 |
| Adam4 | -0.6893361 |
| Adam5 | -0.6178903 |
| Adam6 | -0.4654539 |
| Adam8 | -0.6678989 |
| Adam8 | -0.5098953 |
| Adam9 | 2.28938047 |
| Adamdec1 | 1.82757522 |
| Adamts1 | 0.39006558 |
| Adamts13 | -0.6017931 |
| Adamts15 | -0.7469341 |
| Adamts16 | 0.30480492 |
| Adamts17 | 0.80023402 |
| Adamts18 | -0.5336335 |
| Adamts19 | 1.06976797 |
| Adamts2 | 0.47361579 |
| Adamts2 | -0.7138361 |
| Adamts2 | 1.73834397 |
| Adamts20 | -0.392694 |
| Adamts20 | 0.45347386 |
| Adamts4 | -0.1493169 |
| Adamts7 | 0.10301659 |
| Adamtsl2 | -0.4305884 |
| Adamtsl4 | 0.21905607 |
| Adamtsl5 | 0.16400156 |
| Adar | 0.71896188 |
| Adar | -0.6071283 |
| Adar | 0.70935752 |
| Adarb1 | -0.4700693 |
| Adarb1 | 0.365314 |
| Adarb1 | -0.7830763 |
| Adat1 | 0.20365136 |
| Adc | -0.2020393 |
| Adc | -0.5202945 |
| Adck1 | -0.3527549 |
| Adck2 | 2.90891797 |
| Adck4 | -0.307181 |
| Adck5 | 1.2160476 |
| Adcy1 | -0.0120894 |
| Adcy10 | 1.2653946 |
| Adcy2 | 1.24179752 |
| Adcy4 | 0.9694159 |
| Adcy4 | 0.8143946 |
| Adcy6 | 0.91363474 |
| Adcy7 | 1.81505693 |
| Adcy8 | 0.4308602 |
| Adcy9 | 1.29616944 |
| Adcyap1 | 2.43921051 |
| Adcyap1r1 | 1.37927064 |
| Add1 | -0.7821556 |
| Add1 | -0.8361214 |
| Add1 | -0.1247084 |
| Add1 | 2.25956329 |
| Add3 | -0.5244865 |
| Add3 | 3.39778351 |
| Add3 | 0.35241399 |
| Adfp | -0.7155494 |
| Adh1 | -0.1311466 |
| Adh1 | 1.72431634 |
| Adh4 | 0.79696232 |
| Adh5 | 0.48424891 |
| Adh7 | 0.27348037 |
| Adhfe1 | 0.26654397 |
| Adi1 | 1.22002128 |
| Adipoq | -0.2090297 |
| Adipor1 | -0.6453449 |
| Adipor2 | 0.07710398 |
| Adk | -0.2813986 |
| Adk | 0.06293714 |
| Adm | -0.7398689 |
| Adm2 | -0.8168324 |
| Adnp | 0.24789648 |
| Ado | -0.072989 |
| Adora1 | 0.23421321 |
| Adora1 | 1.81212173 |
| Adora1 | -0.2505237 |
| Adora2b | 0.82600694 |
| Adora3 | -0.3504232 |
| Adpgk | 1.77726664 |
| Adprh | 0.31693437 |
| Adprhl1 | -0.0092466 |
| Adprhl1 | -0.3234483 |
| Adprhl1 | 1.30083171 |
| Adprhl2 | 1.14591949 |
| Adra1a | 1.34091479 |
| Adra1a | 0.16028219 |
| Adra1d | -0.5037104 |
| Adra2a | -0.7469341 |
| Adra2c | 0.57490939 |
| Adra2c | -0.4747074 |
| Adrb1 | -0.0962681 |
| Adrb2 | -0.6557494 |
| Adrbk1 | 0.20462156 |
| Adrbk2 | -0.4753719 |
| Adrm1 | 2.04372918 |
| Adsl | -0.3510056 |
| Adss | 0.77285265 |
| Adssl1 | 1.26916613 |
| Adssl1 | 1.29809795 |
| Adssl1 | 0.05463696 |
| Aebp1 | -0.4018721 |
| Aebp2 | -0.145561 |
| Aes | 0.3710213 |
| Afap1 | -0.7684528 |
| Afap1 | 0.23264566 |
| Afap1 | 0.34964639 |
| Afap1l1 | 0.63289888 |
| Aff1 | 1.00480488 |
| Aff4 | 0.10625102 |
| Afg3l1 | -0.7487087 |
| Afg3l1 | -0.6048381 |
| Afg3l1 | 1.4509947 |
| Afg3l1 | -0.6303419 |
| Afg3l2 | 2.89862486 |
| Afmid | -0.6148029 |
| Aftph | 0.88673305 |
| Agbl1 | 1.61670215 |
| Ager | 0.22255115 |
| Ager | 1.39769662 |
| Aggf1 | -0.6744432 |
| Aggf1 | -0.7250289 |
| Aggf1 | -0.682688 |
| Agpat1 | -0.5889603 |
| Agpat2 | 1.35275845 |
| Agpat3 | 0.17673859 |
| Agpat4 | -0.7284997 |
| Agpat5 | 0.34157413 |
| Agpat6 | -0.4517415 |
| Agpat6 | -0.5740831 |
| Agpat7 | 1.22737247 |
| Agpat9 | -0.7095663 |
| Agpat9 | 1.89863379 |
| Agps | -0.6397897 |
| Agps | -0.5286972 |
| Agrn | -0.5085174 |
| Agrp | -0.3842076 |
| Agt | 2.14828327 |
| Agtpbp1 | 1.77848603 |
| Agtr1a | -0.7302399 |
| Agtrap | -0.3099658 |
| Agtrl1 | 2.10962745 |
| Agxt2l1 | -0.0708222 |
| Agxt2l1 | 0.57643613 |
| Agxt2l2 | 0.83499617 |
| Agxt2l2 | -0.6010334 |
| Agxt2l2 | 1.22988285 |
| Agxt2l2 | -0.4337631 |
| Ahctf1 | -0.3533388 |
| Ahcy | 0.00043554 |
| Ahcyl1 | -0.5493182 |
| Ahdc1 | -0.8341746 |
| Ahi1 | -0.1582975 |
| Ahnak | -0.7639291 |
| Ahnak | -0.0922744 |
| Ahnak2 | -0.3371263 |
| Ahrr | -0.4433518 |
| Ahsa1 | -0.7612252 |
| Ahsa2 | 1.8796148 |
| AI118078 | 0.1764056 |
| AI132487 | -0.0468885 |
| AI314180 | -0.6835165 |
| AI314976 | -0.5594369 |
| AI316807 | 2.28646926 |
| AI317395 | 0.49997716 |
| AI413582 | 1.87736798 |
| AI413782 | -0.6287764 |
| AI427515 | -0.3609618 |
| AI428936 | 1.29870614 |
| AI449175 | 2.03360236 |
| AI449175 | -0.6287764 |
| AI450540 | -0.8082795 |
| AI451557 | 0.20235621 |
| AI451617 | 0.40220197 |
| AI467606 | 1.11022214 |
| AI480653 | -0.3639102 |
| AI504432 | -0.6777325 |
| AI553587 | -0.1784582 |
| AI593442 | -0.5043956 |
| AI593442 | 0.83549306 |
| AI593442 | 0.21394907 |
| AI595366 | -0.4427095 |
| AI595406 | 1.61525441 |
| AI597468 | -0.7224341 |
| AI597479 | -0.3763952 |
| AI606181 | 1.04490081 |
| AI646023 | 2.26996937 |
| AI661453 | 1.42476641 |
| AI662250 | -0.3945225 |
| AI747699 | 1.05321911 |
| AI836003 | -0.3451977 |
| AI837181 | 0.47773827 |
| AI842396 | -0.3957435 |
| AI846148 | 1.09789098 |
| AI847670 | -0.3650922 |
| AI851790 | -0.6910055 |
| AI894139 | 0.9451125 |
| AI987692 | 3.15343791 |
| AI987692 | 0.45892016 |
| AI987944 | -0.4641393 |
| Aicda | 1.83397371 |
| Aif1 | -0.4055685 |
| Aif1 | -0.432492 |
| Aif1 | 1.07966195 |
| Aifm1 | -0.562348 |
| Aifm1 | 4.18201237 |
| Aifm2 | -0.7425121 |
| Aifm2 | -0.2216428 |
| Aifm2 | 1.06549572 |
| Aifm3 | 1.52469153 |
| Aig1 | 0.09470007 |
| Aig1-pending | 0.07562272 |
| Aim1 | -0.0239588 |
| Aim2 | -0.3716195 |
| Aip | -0.4948476 |
| Ak1 | -0.1316081 |
| AK122209 | -0.6225404 |
| AK122525 | 0.20365136 |
| AK129128 | -0.3133184 |
| AK129302 | -0.6413735 |
| AK129302 | -0.3650922 |
| AK129302 | -0.6033144 |
| AK129341 | 0.04319952 |
| AK162044 | -0.5407306 |
| Ak2 | -0.5638069 |
| Ak2 | 1.32892051 |
| Ak2 | 0.77814656 |
| Ak3 | 1.43481927 |
| Ak3 | 4.35552255 |
| Ak3l1 | -0.6063643 |
| Ak5 | 1.13175388 |
| Akap1 | 2.77374504 |
| Akap1 | 0.90213314 |
| Akap1 | 0.38500679 |
| Akap10 | -0.8302931 |
| Akap10 | 0.21170603 |
| Akap12 | -0.4948476 |
| Akap14 | -0.3066251 |
| Akap2 | 0.40977369 |
| Akap2 | -0.5009745 |
| Akap3 | 1.67197499 |
| Akap4 | -0.1196804 |
| Akap4 | -0.2626465 |
| Akap4 | -0.1535602 |
| Akap4 | -0.6728029 |
| Akap6 | -0.3451977 |
| Akap7 | 1.60381188 |
| Akap8 | 1.28430904 |
| Akap8l | 2.85126369 |
| Akap9 | -0.6533381 |
| Akap9 | -0.5023415 |
| Akap9 | 1.82247153 |
| Akna | -0.5529196 |
| Akp2 | -0.6194378 |
| Akp5 | 1.42121354 |
| Akr1a4 | -0.4787012 |
| Akr1a4 | -0.6769091 |
| Akr1b3 | -0.513349 |
| Akr1b8 | -0.2879146 |
| Akr1c12 | 0.47288643 |
| Akr1c14 | 0.27588113 |
| Akr1c14 | -0.3586097 |
| Akr1c18 | 1.04556368 |
| Akr1c19 | 1.40948332 |
| Akr1e1 | -0.3354057 |
| Akr7a5 | -0.6793814 |
| Akr7a5 | 0.01041655 |
| Akr7a5 | 0.92875523 |
| Akt1s1 | 0.43314612 |
| Akt3 | 0.57359879 |
| Akt3 | 2.80526258 |
| Aktip | -0.5800058 |
| Alad | -0.2615862 |
| Alas1 | -0.6662699 |
| Alas2 | -0.3550925 |
| Alcam | -0.6094242 |
| Aldh16a1 | 0.48160185 |
| Aldh18a1 | -0.6670841 |
| Aldh1a1 | 0.05349944 |
| Aldh1a2 | -0.2743899 |
| Aldh1a2 | 2.58527421 |
| Aldh1a7 | 0.15484589 |
| Aldh1b1 | 2.55409569 |
| Aldh1l1 | -0.2000516 |
| Aldh1l2 | -0.7198465 |
| Aldh1l2 | -0.7198465 |
| Aldh2 | 1.06976797 |
| Aldh3a1 | 0.08594306 |
| Aldh3a2 | 1.05518889 |
| Aldh3b1 | 0.17707146 |
| Aldh3b1 | 0.03897079 |
| Aldh3b1 | 2.6469833 |
| Aldh4a1 | 1.18649834 |
| Aldh5a1 | 0.54901828 |
| Aldh5a1 | 2.76706112 |
| Aldh6a1 | 0.20332774 |
| Aldh8a1 | 2.09836618 |
| Aldh8a1 | -0.5927165 |
| Aldoa | -0.7070137 |
| Aldoc | 0.07339645 |
| Alg1 | 1.87824543 |
| Alg10b | -0.6777325 |
| Alg11 | 2.08516585 |
| Alg11 | 2.46233156 |
| Alg12 | 0.41908928 |
| Alg12 | -0.2370047 |
| Alg14 | 0.4489943 |
| Alg2 | -0.5071414 |
| Alg3 | -0.0940473 |
| Alg3 | -0.4255311 |
| Alg3 | 0.37803997 |
| Alg5 | 1.41836158 |
| Alg5 | -0.5565347 |
| Alg5 | -0.6117256 |
| Alg8 | -0.7603256 |
| Alg8 | -0.3746015 |
| Alg8 | -0.7302399 |
| Alg8 | -0.1074515 |
| Alg9 | 0.25650178 |
| Alg9 | 1.2973879 |
| Alg9 | 0.7146426 |
| Alk | -0.4674291 |
| Alkbh2 | -0.4117615 |
| Alkbh3 | -0.5934696 |
| Alkbh4 | -0.6985544 |
| Alkbh5 | -0.4694086 |
| Alkbh7 | -0.1540329 |
| Alkbh8 | 1.55211903 |
| Allc | 1.35323677 |
| Allc | 1.12702485 |
| Alox12 | -0.391477 |
| Alox12b | -0.5740831 |
| Alox12e | 0.62734036 |
| Alox15 | 1.08945322 |
| Alox5ap | 1.5954894 |
| Alox8 | -0.0468885 |
| Aloxe3 | 0.10049323 |
| Alpk3 | -0.1247084 |
| Als2 | 0.68286798 |
| Als2 | -0.6977127 |
| Als2 | -0.6843456 |
| Als2cl | -0.6910055 |
| Als2cr13 | -0.1106068 |
| Als2cr2 | -0.6148029 |
| Als2cr2 | -0.0665036 |
| Als2cr4 | 1.59378641 |
| Als2cr4 | 0.27588113 |
| Als2cr4 | -0.5726082 |
| Alx3 | 0.99506822 |
| Amac1 | -0.1183138 |
| Ambp | 0.79402536 |
| Ambra1 | 2.20418711 |
| Ambra1 | -0.4962057 |
| Amd2 | 1.14078205 |
| Amdhd2 | 0.23421321 |
| Amdhd2 | -0.5814923 |
| Amfr | -0.7639291 |
| Amh | -0.0695246 |
| Amhr2 | 2.21847269 |
| Amhr2 | 2.1827615 |
| Amhr2 | 0.82215202 |
| Amigo1 | 1.64807259 |
| Amigo1 | 0.15518658 |
| Amigo2 | 0.44198352 |
| Amigo3 | 1.2394266 |
| Ammecr1l | -0.607893 |
| Amn | -0.2841081 |
| Amotl2 | 1.76052453 |
| Ampd1 | -0.0393227 |
| Ampd1 | 1.76652921 |
| Ampd2 | 2.486914 |
| Amph | -0.5500374 |
| Amph | 0.72568302 |
| Amy1 | -0.7258954 |
| Amz2 | -0.5336335 |
| Anapc10 | 2.24120231 |
| Anapc11 | -0.2922838 |
| Anapc13 | -0.4459253 |
| Anapc2 | 0.66080852 |
| Anapc4 | 1.79617166 |
| Anapc5 | 0.13173479 |
| Anapc5 | 2.53185464 |
| Anapc5 | -0.3951328 |
| Anapc7 | 0.50163002 |
| Ang | -0.2765409 |
| Angel1 | -0.5279941 |
| Angel2 | -0.175068 |
| Angpt1 | -0.5223881 |
| Angpt2 | -0.7381108 |
| Angpt2 | 0.10696828 |
| Angptl1 | -0.1106068 |
| Angptl2 | 1.60513083 |
| Angptl3 | 0.99184696 |
| Angptl4 | -0.4074222 |
| Angptl7 | -0.7104187 |
| Ank | 1.68807179 |
| Ank1 | -0.273853 |
| Ank2 | 1.01361986 |
| Ank2 | 0.40638699 |
| Ank2 | 1.6254855 |
| Ank3 | -0.747821 |
| Ank3 | -0.7104187 |
| Ank3 | 2.43821202 |
| Ankar | -0.4173703 |
| Ankar | -0.1882121 |
| Ankfy1 | 2.04027332 |
| Ankle2 | -0.6810333 |
| Ankle2 | 3.50275848 |
| Ankle2 | 0.83978859 |
| Ankmy1 | 0.76485565 |
| Ankmy1 | -0.4223839 |
| Ankmy1 | -0.3793923 |
| Ankmy2 | 1.70909373 |
| Ankmy2 | 1.31268712 |
| Ankrd10 | 1.47701673 |
| Ankrd12 | -0.3890476 |
| Ankrd12 | -0.6777325 |
| Ankrd13a | -0.2354572 |
| Ankrd13b | 0.25772472 |
| Ankrd13d | -0.0900629 |
| Ankrd17 | 0.63781243 |
| Ankrd17 | -0.701929 |
| Ankrd17 | -0.4420676 |
| Ankrd22 | -0.145561 |
| Ankrd23 | 0.39588943 |
| Ankrd24 | 0.6886902 |
| Ankrd25 | 1.63801418 |
| Ankrd27 | 2.41724342 |
| Ankrd34a | -0.2765409 |
| Ankrd35 | -0.546447 |
| Ankrd36 | -0.6678989 |
| Ankrd37 | -0.078646 |
| Ankrd39 | 1.42085752 |
| Ankrd40 | -0.5874619 |
| Ankrd40 | -0.6413735 |
| Ankrd40 | 0.84472077 |
| Ankrd46 | 0.71989824 |
| Ankrd47 | -0.0760309 |
| Ankrd49 | -0.1092532 |
| Ankrd5 | 1.80266724 |
| Ankrd50 | -0.4780344 |
| Ankrd53 | 0.82901288 |
| Ankrd54 | 1.52882906 |
| Ankrd54 | 0.24045749 |
| Ankrd54 | 0.06180959 |
| Ankrd56 | -0.7442784 |
| Ankrd58 | -0.0905048 |
| Ankrd6 | -0.6893361 |
| Ankrd6 | 1.63041716 |
| Ankrd6 | -0.3842076 |
| Ankrd7 | 1.95989844 |
| Ankrd9 | 0.37696357 |
| Anks1 | 2.35862787 |
| Anks3 | 4.59195317 |
| Anks3 | 1.2018941 |
| Anks6 | 2.75177083 |
| Anks6 | 1.29025788 |
| Ankzf1 | -0.4727169 |
| Ankzf1 | 0.40246408 |
| Anln | -0.6233176 |
| Anln | -0.4582464 |
| Anp32a | -0.7585291 |
| Anp32a | 0.29516399 |
| Anp32a | -0.2277576 |
| Anp32e | -0.2717081 |
| Anpep | 1.18524204 |
| Antxr1 | 2.71835875 |
| Antxr1 | 0.44198352 |
| Antxrl | 2.62282676 |
| Anxa1 | -0.1621046 |
| Anxa11 | 4.18155475 |
| Anxa11 | 0.93145136 |
| Anxa2 | 0.50210174 |
| Anxa3 | 2.17527009 |
| Anxa3 | 3.05249625 |
| Anxa4 | 1.89858014 |
| Anxa5 | -0.2760027 |
| Anxa6 | -0.8400269 |
| Anxa8 | -0.4274244 |
| Anxa8 | 0.15382309 |
| Anxa9 | -0.5098953 |
| Aoah | 0.90659707 |
| Aoc2 | 2.33589193 |
| Aoc2 | 0.54946689 |
| Aoc3 | -0.6382084 |
| Aof1 | -0.2201203 |
| Aof1 | -0.747821 |
| Aof2 | -0.3133184 |
| Aox3 | -0.8082795 |
| Aox3l1 | -0.2267357 |
| Ap1b1 | -0.4186212 |
| Ap1g1 | -0.6662699 |
| Ap1g1 | -0.7363561 |
| Ap1g2 | 1.24244311 |
| Ap1gbp1 | 0.25527725 |
| Ap1gbp1 | 1.05071813 |
| Ap1m1 | -0.607893 |
| Ap1m2 | 0.07599325 |
| Ap1m2 | -0.5659995 |
| Ap1m2 | 0.76289047 |
| Ap1m2 | 1.11170593 |
| Ap1s1 | -0.6397897 |
| Ap1s1 | 0.17740422 |
| Ap1s1 | 1.51587879 |
| Ap1s3 | 0.42958784 |
| Ap2a1 | -0.7207082 |
| Ap2a1 | -0.3945225 |
| Ap2a2 | -0.5949775 |
| Ap2b1 | 2.32765672 |
| Ap2b1 | -0.4369485 |
| Ap2b1 | -0.0380676 |
| Ap2b1 | -0.3842076 |
| Ap2m1 | 1.76541783 |
| Ap2s1 | -0.6517341 |
| Ap2s1 | 1.77286374 |
| Ap2s1 | 0.3764249 |
| Ap3b1 | -0.6810333 |
| Ap3b1 | 2.72561879 |
| Ap3b2 | 2.38484163 |
| Ap3d1 | 1.61126166 |
| Ap3m1 | -0.1029622 |
| Ap3m2 | 2.89305279 |
| Ap3s2 | -0.0953792 |
| Ap3s2 | 0.2737808 |
| Ap4b1 | -0.3144386 |
| Ap4m1 | 2.06918511 |
| Ap4m1 | -0.4249008 |
| Ap4m1 | -0.5587105 |
| Ap4s1 | 0.98494739 |
| Apaf1 | 1.06159498 |
| Apaf1 | 1.00078331 |
| Apba1 | -0.7757362 |
| Apba2 | 0.08923647 |
| Apba2 | 0.2954576 |
| Apba3 | -0.6033144 |
| Apbb1 | -0.311082 |
| Apbb1ip | 2.33680253 |
| Apbb1ip | 0.22096408 |
| Apbb1ip | -0.650933 |
| Apbb2 | 0.19160278 |
| Apbb3 | -0.5230871 |
| Apc | -0.5652681 |
| Apc2 | 0.44173216 |
| Apcdd1 | -0.4887603 |
| Apeh | -0.6901704 |
| Apex1 | -0.590461 |
| Aph1a | 0.14007864 |
| Aph1b | 2.4989123 |
| Apip | 1.71862039 |
| Apip | -0.4485059 |
| Apitd1 | -0.5064542 |
| Aplp1 | -0.0068167 |
| Aplp2 | 1.44433203 |
| Apoa1 | 1.62182371 |
| Apoa1bp | -0.4286887 |
| Apoa2 | -0.7398689 |
| Apoa4 | -0.2626465 |
| Apob48r | -0.4727169 |
| Apobec1 | 0.93354304 |
| Apobec3 | -0.3811952 |
| Apoc1 | -0.5800058 |
| Apoc1 | -0.3740043 |
| Apoc2 | 1.63055973 |
| Apoc3 | -0.2803169 |
| Apoe | 0.00803072 |
| Apof | -0.2966733 |
| Apoh | -0.4105197 |
| Apol7c | 0.78253557 |
| Apol9b | 0.32179991 |
| App | 0.08337354 |
| Appbp1 | -0.3055141 |
| Appbp1 | 0.37372697 |
| Appbp2 | -0.1002789 |
| Appl1 | -0.5279941 |
| Appl2 | -0.5126572 |
| Aprin | 0.78060692 |
| Aprt | -0.2105344 |
| Aptx | -0.6851756 |
| Aqp1 | -0.8668213 |
| Aqp1 | -0.6461412 |
| Aqp1 | -0.699397 |
| Aqp11 | 1.05387616 |
| Aqp11 | -0.6240955 |
| Aqp3 | -0.7766506 |
| Aqp4 | -0.8158783 |
| Aqp5 | 1.63963922 |
| Aqp7 | 1.31108831 |
| Aqp7 | 0.8998159 |
| Aqp8 | 0.29192833 |
| Aqp8 | 0.34881453 |
| Aqp9 | 1.15008057 |
| Aqr | -0.4773681 |
| Aqr | 0.2282426 |
| Ar | 0.04243207 |
| Araf | 0.10481487 |
| Araf | 2.60539177 |
| Arbp | 1.84027201 |
| Arc | 1.51523464 |
| Arcn1 | -0.3302625 |
| Ard1 | 0.95143038 |
| Ard1 | -0.1270027 |
| Areg | -0.5949775 |
| Arf2 | -0.7381108 |
| Arf3 | 0.7623538 |
| Arf5 | -0.7381108 |
| Arf6 | -0.778482 |
| Arfgap1 | -0.7087146 |
| Arfgap1 | 0.22413556 |
| Arfgap1 | -0.8035611 |
| Arfgap2 | -0.2653027 |
| Arfgap2 | 1.88240217 |
| Arfgap2 | -0.3710242 |
| Arfgap2 | 0.33849408 |
| Arfgap3 | -0.1765195 |
| Arfip2 | -0.514734 |
| Arfip2 | -0.3498411 |
| Arfip2 | -0.7914027 |
| Arfrp1 | 0.86836263 |
| Arfrp1 | -0.8332027 |
| Arg1 | 0.48880369 |
| Arg2 | 0.32179991 |
| Arglu1 | 2.43445063 |
| Arhgap1 | -0.8187437 |
| Arhgap1 | 2.88294313 |
| Arhgap1 | 2.18932716 |
| Arhgap10 | 1.488024 |
| Arhgap12 | -0.1151327 |
| Arhgap15 | -0.4747074 |
| Arhgap15 | -0.0397414 |
| Arhgap17 | 1.11503595 |
| Arhgap18 | 0.416769 |
| Arhgap19 | 0.03742836 |
| Arhgap20 | 0.31808146 |
| Arhgap22 | 0.13208393 |
| Arhgap24 | -0.5223881 |
| Arhgap24 | -0.4962057 |
| Arhgap25 | -0.5371754 |
| Arhgap26 | -0.0931604 |
| Arhgap27 | 0.20429828 |
| Arhgap28 | 1.67183855 |
| Arhgap29 | -0.094935 |
| Arhgap30 | 1.72747311 |
| Arhgap4 | 1.43455609 |
| Arhgap6 | 0.24882224 |
| Arhgap8 | 2.78674236 |
| Arhgap9 | -0.1592478 |
| Arhgdib | 3.91715149 |
| Arhgdig | -0.5748214 |
| Arhgef1 | -0.4343993 |
| Arhgef1 | 1.57166664 |
| Arhgef10 | 2.11973802 |
| Arhgef10l | -0.4800362 |
| Arhgef10l | -0.7104187 |
| Arhgef15 | -0.5223881 |
| Arhgef18 | -0.0682287 |
| Arhgef19 | 0.6886902 |
| Arhgef2 | 2.21199206 |
| Arhgef3 | -0.778482 |
| Arhgef4 | -0.4608609 |
| Arhgef6 | 0.22159923 |
| Arid2 | 3.5838369 |
| Arid3a | -0.0301573 |
| Arid3b | 1.84283652 |
| Arid4b | -0.547164 |
| Arid5a | -0.5949775 |
| Arid5b | -0.6533381 |
| Arih1 | -0.809226 |
| Arih2 | -0.7112719 |
| Arl1 | -0.0376496 |
| Arl11 | -0.5357571 |
| Arl16 | 2.93405346 |
| Arl2 | -0.5565347 |
| Arl2bp | -0.2339122 |
| Arl2bp | 1.02898546 |
| Arl2bp | -0.4727169 |
| Arl3 | -0.0536649 |
| Arl3 | -0.4510935 |
| Arl4a | -0.4395046 |
| Arl4a | 0.15348191 |
| Arl4c | -0.3183697 |
| Arl5a | -0.7729983 |
| Arl5b | 1.33955725 |
| Arl6 | 0.33709072 |
| Arl6ip1 | 1.15398713 |
| Arl6ip2 | 0.60751801 |
| Arl6ip2 | 2.66066456 |
| Arl6ip2 | -0.3121995 |
| Arl6ip2 | 1.03597313 |
| Arl6ip4 | 1.98239972 |
| Arl6ip4 | -0.3988028 |
| Arl6ip5 | 1.1554037 |
| Arl6ip5 | 0.79609946 |
| Arl8a | 1.71868531 |
| Arl8b | -0.6662699 |
| Armc10 | 1.39358204 |
| Armc3 | 1.23499209 |
| Armc6 | 1.74758114 |
| Armc6 | 0.15382309 |
| Armc8 | 0.77515032 |
| Armc8 | -0.1906665 |
| Armc8 | 1.3780604 |
| Armc9 | -0.0764662 |
| Armc9 | -0.7189855 |
| Armcx1 | -0.5882108 |
| Armcx2 | 0.00123792 |
| Armcx3 | -0.3908691 |
| Armcx5 | 3.35185128 |
| Armcx6 | 1.04011435 |
| Armcx6 | 1.2711506 |
| Armetl1 | 1.33965428 |
| Arnt | 2.36040488 |
| Arnt | -0.0600624 |
| Arnt2 | -0.1297633 |
| Arntl | 0.36395002 |
| Arpc1a | 0.67012851 |
| Arpc1a | -0.4472147 |
| Arpc1a | -0.2857376 |
| Arpc1b | 0.18733269 |
| Arpc1b | 3.48338736 |
| Arpc1b | -0.5638069 |
| Arpc3 | -0.3656837 |
| Arpc4 | -0.5844724 |
| Arpc5 | -0.6194378 |
| Arpc5 | -0.4530389 |
| Arpc5l | 1.00535824 |
| Arpp19 | 1.35342803 |
| Arpp21 | 0.04893557 |
| Arpp21 | -0.1215057 |
| Arrb1 | 1.80498052 |
| Arrb1 | -0.392694 |
| Arrb1 | 0.03084475 |
| Arrb1 | -0.1201364 |
| Arrb2 | -0.6703477 |
| Arrdc1 | -0.2505237 |
| Arrdc2 | 0.08263811 |
| Arrdc3 | 0.19585363 |
| Arrdc4 | -0.7078638 |
| Ars2 | 2.67699829 |
| Ars2 | 2.69173603 |
| Ars2 | -0.5912122 |
| Arsa | -0.4793685 |
| Arsb | 0.13347919 |
| Arsb | 0.06368809 |
| Arsg | 0.0366562 |
| Arsi | 0.29163362 |
| Arsj | -0.6405813 |
| Arsj | 1.98617163 |
| Art1 | -0.4955264 |
| Art2b | -0.424271 |
| Art3 | 0.13556822 |
| Art3 | -0.5964878 |
| Art4 | -0.0104639 |
| Art5 | 0.07784375 |
| Artn | 0.1843651 |
| Artn | 0.85677768 |
| Arv1 | -0.7036209 |
| As3mt | -0.6876697 |
| As3mt | 1.7128245 |
| Asah1 | 0.08667592 |
| Asah1 | 0.68091923 |
| Asah2 | -0.0962681 |
| Asah3 | -0.3234483 |
| Asah3l | 0.73438637 |
| Asb1 | -0.3279856 |
| Asb1 | -0.1137726 |
| Asb1 | -0.2589405 |
| Asb12 | -0.6977127 |
| Asb13 | -0.0751608 |
| Asb15 | -0.7812359 |
| Asb17 | 0.83681685 |
| Asb2 | 0.50398625 |
| Asb3 | -0.5414433 |
| Asb3 | -0.340001 |
| Asb4 | -0.632695 |
| Asb4 | 1.3182617 |
| Asb6 | 0.04549813 |
| Asb6 | -0.5237865 |
| Asb7 | -0.7567359 |
| Asb7 | -0.7129806 |
| Asb8 | -0.3920853 |
| Asb9 | -0.054941 |
| Ascc1 | 0.40951361 |
| Ascc1 | -0.7766506 |
| Ascc2 | 2.98391191 |
| Ascc2 | -0.8111218 |
| Ascc2 | 0.14869236 |
| Ascc2 | -0.4236415 |
| Ascc3l1 | 1.72179624 |
| Ascl1 | 1.46740596 |
| Ascl2 | -0.7558406 |
| Asf1a | -0.7302399 |
| Asf1b | -0.701929 |
| Asgr1 | -0.5740831 |
| Ash1l | -0.7061643 |
| Ash1l | 0.56965603 |
| Ash1l | 1.61438471 |
| Ash2l | -0.5912122 |
| Ash2l | -0.5308096 |
| Asl | -0.1755516 |
| Asl | 1.34796188 |
| Asna1 | -0.7612252 |
| Asns | 0.11197388 |
| Asns | 0.11978655 |
| Asns | 0.50704056 |
| Asnsd1 | 0.39905098 |
| Asnsd1 | -0.4280564 |
| Asph | -0.2196134 |
| Asph | -0.4921373 |
| Asph | -0.3574358 |
| Asph | -0.8035611 |
| Asph | -0.7389895 |
| Asphd1 | 0.39799831 |
| Asphd2 | 0.06030409 |
| Aspm | -0.4369485 |
| Asprv1 | 1.95531284 |
| Aspscr1 | -0.0447808 |
| Aspscr1 | 1.52181642 |
| Aspscr1 | -0.0918316 |
| Asrgl1 | -0.8668213 |
| Ass1 | -0.3217524 |
| Aste1 | 0.79177326 |
| Astn1 | -0.7302399 |
| Astn2 | 1.31288678 |
| Asz1 | 0.27947084 |
| Atad1 | 1.73993211 |
| Atad2 | 0.81049183 |
| Atad3a | 0.06855476 |
| Atad4 | 2.05955553 |
| Atad4 | 0.70973601 |
| Atcay | 2.07592114 |
| Ate1 | 1.03704357 |
| Ate1 | 0.56260571 |
| Atf2 | -0.2339122 |
| Atf2 | -0.1119623 |
| Atf2 | 1.01348276 |
| Atf3 | -0.2841081 |
| Atf4 | -0.6581668 |
| Atf4 | 2.12705047 |
| Atf5 | -0.2599979 |
| Atf7 | -0.5322205 |
| Atf7ip | 0.44474397 |
| Atg10 | -0.290643 |
| Atg10 | -0.7433949 |
| Atg12 | 0.64877523 |
| Atg16l1 | 1.26612913 |
| Atg2a | 0.1306866 |
| Atg2a | 1.53381764 |
| Atg2b | -0.4388649 |
| Atg2b | -0.5478815 |
| Atg3 | -0.6501327 |
| Atg3 | -0.3740043 |
| Atg4b | -0.4485059 |
| Atg4c | -0.5329268 |
| Atg4c | 1.78516462 |
| Atg4d | -0.7639291 |
| Atg5 | -0.4661118 |
| Atg5 | -0.0769018 |
| Atg7 | -0.290643 |
| Atg9b | -0.0435184 |
| Athl1 | -0.4497988 |
| Atic | 2.82197619 |
| Atic | 0.05955043 |
| Atic | -0.9006385 |
| Atm | -0.4024872 |
| Atm | 1.28358853 |
| Atmin | -0.5308096 |
| Atmin | 0.71124845 |
| Atmin | -0.5414433 |
| Atn1 | 1.40795033 |
| Atoh8 | 0.84913779 |
| Atox1 | -0.0219017 |
| Atp10a | 0.56260571 |
| Atp10d | -0.5748214 |
| Atp11c | 0.06780767 |
| Atp11c | -0.7036209 |
| Atp12a | -0.3896544 |
| Atp13a1 | -0.6670841 |
| Atp13a1 | 0.46507119 |
| Atp13a2 | -0.658974 |
| Atp13a4 | -0.5711356 |
| Atp13a5 | -0.7793991 |
| Atp1a1 | -0.6744432 |
| Atp1a2 | 1.3029525 |
| Atp1b1 | -0.650933 |
| Atp1b2 | 0.9897402 |
| Atp1b3 | -0.562348 |
| Atp2a1 | 1.00867157 |
| Atp2a2 | 1.98008996 |
| Atp2a2 | 2.30763554 |
| Atp2a3 | 1.548627 |
| Atp2a3 | 1.39092089 |
| Atp2a3 | 3.6165292 |
| Atp2b1 | -0.8628034 |
| Atp2b2 | -0.5521982 |
| Atp2b2 | -0.5964878 |
| Atp2b2 | -0.5711356 |
| Atp2b3 | 0.45892016 |
| Atp2b3 | 2.48146457 |
| Atp2b4 | -0.0007693 |
| Atp2b4 | 0.67994334 |
| Atp2c1 | 0.96104599 |
| Atp2c1 | 2.73750788 |
| Atp5a1 | 0.39033108 |
| Atp5b | 2.40449754 |
| Atp5c1 | 0.57206745 |
| Atp5d | 0.26472605 |
| Atp5e | -0.0043931 |
| Atp5f1 | -0.8588025 |
| Atp5g2 | 0.60095846 |
| Atp5g3 | 0.68442409 |
| Atp5h | -0.6835165 |
| Atp5j | 0.5418114 |
| Atp5j | -0.1867426 |
| Atp5j2 | -0.747821 |
| Atp5l | -0.5837265 |
| Atp5o | -0.4205007 |
| Atp5s | 1.00853375 |
| Atp5sl | 0.29222294 |
| Atp6ap1 | 1.05295616 |
| Atp6ap2 | -0.8073339 |
| Atp6ap2 | 2.71430111 |
| Atp6ap2 | -0.1833226 |
| Atp6ap2 | -0.0976032 |
| Atp6v0a1 | -0.6703477 |
| Atp6v0a1 | 0.81134163 |
| Atp6v0a2 | -0.5681973 |
| Atp6v0b | 1.34458971 |
| Atp6v0d1 | -0.7988662 |
| Atp6v0d1 | 0.38420554 |
| Atp6v0d1 | -0.6719838 |
| Atp6v0d1 | -0.3178071 |
| Atp6v0e | -0.6002742 |
| Atp6v0e2 | 0.19911052 |
| Atp6v1a | 0.28304694 |
| Atp6v1b1 | 0.16400156 |
| Atp6v1b2 | 1.27521094 |
| Atp6v1c1 | -0.0161655 |
| Atp6v1c2 | -0.7858436 |
| Atp6v1d | -0.8322319 |
| Atp6v1e1 | 0.14215312 |
| Atp6v1e2 | 1.54066354 |
| Atp6v1g1 | 1.80781987 |
| Atp6v1g2 | 0.71577128 |
| Atp6v1g2 | 1.36085706 |
| Atp6v1h | 0.82517025 |
| Atp7a | -0.7693601 |
| Atp8a1 | 0.80092136 |
| Atp8a2 | 0.17906624 |
| Atp8b1 | 2.86094356 |
| Atp8b3 | 2.83066264 |
| Atp8b3 | -0.7585291 |
| Atp8b3 | 0.44949308 |
| Atp8b3 | 0.41211123 |
| Atp9a | 0.52147277 |
| Atp9a | 3.42487423 |
| Atp9b | 3.66689136 |
| Atp9b | 1.07787075 |
| Atpaf1 | -0.6687145 |
| Atpaf2 | -0.8668213 |
| Atpaf2 | 0.02967815 |
| Atpbd1b | 1.65504325 |
| Atpbd1c | 0.43314612 |
| Atpbd3 | 0.19291277 |
| Atpif1 | 1.93988157 |
| Atrx | 0.41521896 |
| Atxn1 | 2.49814555 |
| Atxn10 | 1.3933071 |
| Atxn2 | 0.48424891 |
| Atxn2l | -0.6728029 |
| Atxn3 | -0.8351475 |
| Atxn3 | 0.43238476 |
| Atxn3 | 0.4216607 |
| Atxn7l1 | 2.16852492 |
| Atxn7l2 | -0.6171175 |
| AU014645 | -0.7558406 |
| AU017455 | 1.7661589 |
| AU017455 | -0.3451977 |
| AU018778 | 1.54870474 |
| AU018829 | -0.3884413 |
| AU019823 | 3.31421104 |
| AU021034 | 0.21362896 |
| AU021092 | 1.21327822 |
| AU021838 | -0.6240955 |
| AU022252 | 0.95886921 |
| AU022751 | -0.2206276 |
| AU022870 | 1.82322722 |
| AU023871 | 0.46971929 |
| AU040320 | 2.4817826 |
| AU040320 | -0.5315148 |
| AU040320 | -0.6217638 |
| AU040829 | 0.53750611 |
| AU042651 | -0.5521982 |
| Aup1 | 0.6453377 |
| Aup1 | -0.112867 |
| Aurka | -0.2186003 |
| Aurkaip1 | -0.4989278 |
| Aurkc | -0.6287764 |
| Auts2 | 1.17073104 |
| Auts2 | 0.54991528 |
| AV028368 | 0.78707857 |
| AV249152 | 1.91922076 |
| AV249152 | 0.74428782 |
| Aven | 0.16231276 |
| Avil | 1.73605234 |
| Avp | 0.27016932 |
| Avpi1 | 0.79989017 |
| Avpi1 | 1.10426352 |
| Avpr1a | -0.7129806 |
| AW049604 | -0.7138361 |
| AW061290 | 0.28779409 |
| AW111922 | -0.5098953 |
| AW121567 | 1.25822918 |
| AW125753 | -0.5092061 |
| AW146242 | 0.99744209 |
| AW146299 | -0.6389987 |
| AW146299 | 1.97871123 |
| AW540478 | -0.5601638 |
| AW544981 | -0.5785216 |
| AW548124 | -0.4661118 |
| AW549877 | -0.5016577 |
| AW551984 | 1.16093151 |
| AW551984 | 0.30886543 |
| AW551984 | -0.3274173 |
| AW555464 | 0.91226193 |
| Axin2 | -0.6525358 |
| Axl | -0.7241631 |
| Axud1 | -0.391477 |
| Azgp1 | -0.1174038 |
| Azgp1 | 1.85454425 |
| Azi1 | 3.65830308 |
| Azi2 | 1.04024764 |
| Azin1 | 0.30973327 |
| B020018G12Rik | -0.4674291 |
| B230118H07Rik | 0.15279917 |
| B230120H23Rik | 0.9815495 |
| B230206H07Rik | 2.99998468 |
| B230209C24Rik | 0.57752515 |
| B230217C12Rik | 0.14560048 |
| B230219D22Rik | -0.0031836 |
| B230312A22Rik | -0.613263 |
| B230312I18Rik | -0.5616193 |
| B230317C12Rik | -0.73373 |
| B230317C12Rik | -0.1745847 |
| B230339H12Rik | 2.61698405 |
| B230339M05Rik | 1.02194564 |
| B230339M05Rik | 1.97357253 |
| B230340J04Rik | -0.2999789 |
| B230342M21Rik | 1.90749667 |
| B230373P09Rik | -0.1169491 |
| B230378H13Rik | 0.5377332 |
| B230380D07Rik | 0.40847255 |
| B230396O12Rik | 0.12998716 |
| B230399E16Rik | 0.20881427 |
| B2m | 0.06743391 |
| B2m | 1.52516986 |
| B3galnt1 | -0.5195976 |
| B3galnt2 | -0.4867399 |
| B3galt1 | 2.18359463 |
| B3galt4 | -0.5897103 |
| B3galt4 | -0.4874129 |
| B3galt5 | -0.7729983 |
| B3galt6 | -0.7232982 |
| B3gat1 | 1.12410286 |
| B3gat1 | -0.5009745 |
| B3gat1 | 0.63063825 |
| B3gat2 | -0.4092794 |
| B3gat3 | 1.42236969 |
| B3gat3 | 0.57228637 |
| B3gnt1 | -0.3763952 |
| B3gnt3 | -0.3668679 |
| B3gnt5 | -0.0289143 |
| B3gnt7 | -0.613263 |
| B3gnt8 | 0.26077517 |
| B3gnt8 | 1.8207253 |
| B430119L13Rik | 0.20784838 |
| B4galnt3 | -0.1357724 |
| B4galnt4 | -0.3933031 |
| B4galt1 | -0.7112719 |
| B4galt3 | -0.7657359 |
| B4galt3 | -0.6752645 |
| B4galt4 | -0.0178008 |
| B4galt7 | 1.98910036 |
| B630005N14Rik | 1.19162039 |
| B830045N13Rik | 1.98622052 |
| B930006L02Rik | 0.37965227 |
| B930011P16Rik | -0.4459253 |
| B930037P14Rik | -0.5674641 |
| B930041F14Rik | 0.74081581 |
| B930041G04 | 0.8662814 |
| B930076A02 | -0.7567359 |
| B9d1 | 1.30325508 |
| B9d2 | 2.87453651 |
| Baalc | 0.64958224 |
| Baalc | -0.455639 |
| Bace1 | 2.48886697 |
| Bace2 | -0.5500374 |
| Bace2 | -0.1838104 |
| Bace2 | 1.9325102 |
| Bach1 | -0.3908691 |
| Bach2 | -0.1079016 |
| Bad | 2.45033394 |
| Bag1 | -0.1545058 |
| Bag2 | 1.04622608 |
| Bag3 | 0.6869473 |
| Bag4 | -0.7821556 |
| Bag5 | 1.82653353 |
| Bahcc1 | 1.96280954 |
| Bahd1 | 0.53636987 |
| Bai1 | 2.85210343 |
| Bai2 | 0.59178353 |
| Bai2 | 3.09784051 |
| Bai3 | -0.4773681 |
| Baiap2 | 0.1271842 |
| Baiap2 | 0.85984867 |
| Banf1 | -0.0003675 |
| Banf1 | 0.51544742 |
| Banf1 | -0.6086583 |
| Banf1 | -0.525187 |
| Banf2 | 1.6580941 |
| Bap1 | 1.37329748 |
| Bard1 | -0.3550925 |
| Barx2 | 0.94452264 |
| Basp1 | 0.78533385 |
| Bat1a | 2.55122659 |
| Bat1a | -0.7522679 |
| Bat2 | 0.03006717 |
| Bat3 | -0.3698348 |
| Bat4 | 3.48500526 |
| Bat4 | -0.6581668 |
| Bat5 | -0.6437543 |
| Batf | 0.93696933 |
| Bax | -0.814925 |
| Baz1a | -0.5002918 |
| Baz1b | 2.1081251 |
| Baz2a | -0.3296928 |
| BB014433 | -0.5428702 |
| BB128963 | 1.08818736 |
| BB146404 | -0.340001 |
| Bbc3 | -0.4840527 |
| Bbc3 | 0.30101874 |
| Bbox1 | -0.198068 |
| Bbs1 | 0.79989017 |
| Bbs12 | 1.03771199 |
| Bbs2 | 2.73918158 |
| Bbs4 | -0.5329268 |
| Bbs5 | -0.340001 |
| Bbs7 | -0.2631772 |
| Bbs9 | -0.4395046 |
| Bbs9 | -0.4847238 |
| Bbx | 1.03878046 |
| BC002163 | 1.0937445 |
| BC002199 | 1.85583619 |
| BC002216 | 1.2079381 |
| BC003236 | -0.2999789 |
| BC003266 | -0.4874129 |
| BC003885 | -0.1119623 |
| BC003885 | 0.90659707 |
| BC003993 | 1.49092118 |
| BC003993 | -0.2722439 |
| BC004004 | -0.7858436 |
| BC004044 | -0.7442784 |
| BC004044 | 0.01477495 |
| BC004728 | 0.26107967 |
| BC004728 | -0.5957324 |
| BC005537 | 2.30645268 |
| BC005682 | -0.6303419 |
| BC005764 | -0.6968717 |
| BC006662 | 1.42281399 |
| BC006779 | -0.4800362 |
| BC006779 | 0.63228288 |
| BC008155 | 2.57642591 |
| BC008163 | -0.1823477 |
| BC008163 | 0.70841063 |
| BC010462 | 0.3750769 |
| BC011248 | -0.4874129 |
| BC011248 | -0.7621256 |
| BC011487 | -0.7036209 |
| BC013491 | -0.5230871 |
| BC013491 | -0.1774883 |
| BC013529 | -0.6630203 |
| BC013712 | 0.05728586 |
| BC016201 | -0.1164947 |
| BC016423 | 1.04450287 |
| BC016495 | 0.38286863 |
| BC017158 | 1.67667005 |
| BC017612 | 1.0918537 |
| BC017643 | 0.50492709 |
| BC017647 | 1.74310039 |
| BC018242 | 2.53846515 |
| BC018242 | -0.7576321 |
| BC018465 | -0.3365524 |
| BC020002 | -0.0695246 |
| BC020535 | -0.1020669 |
| BC021381 | -0.2647709 |
| BC021395 | -0.6662699 |
| BC021614 | -0.4880863 |
| BC021790 | 1.50029647 |
| BC021891 | -0.139024 |
| BC022224 | -0.6960314 |
| BC022687 | 2.23005697 |
| BC023055 | -0.6557494 |
| BC023151 | 2.25177491 |
| BC023744 | 0.56855788 |
| BC023814 | 0.98791053 |
| BC023829 | 1.5519641 |
| BC024561 | 0.6513955 |
| BC024814 | -0.4497988 |
| BC024868 | -0.5071414 |
| BC024997 | 0.6119358 |
| BC025076 | -0.6662699 |
| BC025546 | -0.8073339 |
| BC025575 | -0.6910055 |
| BC025920 | -0.8648102 |
| BC026439 | -0.0652118 |
| BC026585 | 3.72195422 |
| BC026590 | -0.6728029 |
| BC026590 | -0.6557494 |
| BC027072 | 0.82383001 |
| BC027231 | 0.37777098 |
| BC027231 | -0.6140326 |
| BC027231 | 0.17173134 |
| BC027344 | -0.4996095 |
| BC028528 | 2.03229973 |
| BC028528 | 1.1559933 |
| BC029169 | 2.01301657 |
| BC029169 | 4.1272131 |
| BC029214 | 2.84971895 |
| BC030183 | -0.7904739 |
| BC030307 | -0.4018721 |
| BC030476 | -0.5964878 |
| BC030499 | -0.0047966 |
| BC030500 | 0.1710617 |
| BC030867 | -0.4043348 |
| BC031181 | -0.6910055 |
| BC031353 | 0.55193042 |
| BC031575 | -0.536466 |
| BC031748 | -0.6843456 |
| BC031781 | 0.65159676 |
| BC031853 | 0.19193045 |
| BC032265 | 1.66650201 |
| BC034076 | 0.10983188 |
| BC037034 | 0.66993117 |
| BC037594 | -0.7078638 |
| BC038156 | -0.7867678 |
| BC038286 | -0.7053157 |
| BC038613 | -0.4472147 |
| BC038822 | 0.31290853 |
| BC038822 | -0.2505237 |
| BC038822 | -0.4982465 |
| BC038925 | -0.6063643 |
| BC039093 | -0.6622096 |
| BC040758 | -0.1852753 |
| BC043098 | -0.4472147 |
| BC043118 | 0.93934551 |
| BC043301 | -0.2669 |
| BC046331 | -0.7241631 |
| BC046404 | 1.68120677 |
| BC048355 | 0.03935601 |
| BC048502 | -0.1137726 |
| BC048546 | -0.2065272 |
| BC048546 | 1.56817023 |
| BC048562 | 0.99156633 |
| BC048644 | -0.5755603 |
| BC048651 | -0.5704001 |
| BC048671 | -0.455639 |
| BC049635 | 0.10121488 |
| BC049635 | 1.11244695 |
| BC049730 | -0.7748227 |
| BC049762 | 2.10031739 |
| BC049806 | 0.50068588 |
| BC049806 | -0.4582464 |
| BC049806 | -0.4921373 |
| BC050196 | 3.47161938 |
| BC050210 | -0.290643 |
| BC050777 | 1.31138829 |
| BC050811 | 1.11565133 |
| BC050811 | -0.2500001 |
| BC051019 | -0.033896 |
| BC051070 | 2.57671348 |
| BC051142 | 1.19840682 |
| BC051227 | 1.45220105 |
| BC051628 | 2.29581001 |
| BC051665 | 1.55806446 |
| BC052484 | -0.6565545 |
| BC053749 | 1.3029525 |
| BC053749 | 1.45666834 |
| BC054059 | -0.6233176 |
| BC055107 | 0.19454771 |
| BC055111 | -0.6140326 |
| BC055324 | 1.03463336 |
| BC055368 | -0.8206588 |
| BC056474 | -0.295574 |
| BC056923 | 0.37345675 |
| BC057022 | 2.29686563 |
| BC057371 | 2.6481714 |
| BC057371 | -0.7027746 |
| BC057371 | 1.20603953 |
| BC057552 | 0.09723895 |
| BC057627 | -0.1205926 |
| BC057893 | -0.4179955 |
| BC060267 | 0.67209967 |
| BC060615 | 3.35685973 |
| BC060632 | 0.66060921 |
| BC061039 | 0.78847203 |
| BC061237 | 0.13033695 |
| BC062115 | -0.1002789 |
| BC062650 | -0.525888 |
| BC063749 | 1.07221901 |
| BC064033 | 0.14007864 |
| BC065085 | 1.10662665 |
| BC065397 | 0.23671589 |
| BC066028 | -0.6101907 |
| BC066107 | 0.39668081 |
| BC067047 | 0.72176817 |
| BC067068 | -0.3848113 |
| BC068157 | 2.70190797 |
| BC068281 | -0.1002789 |
| BC072620 | -0.809226 |
| BC085271 | 0.93234837 |
| BC087945 | 0.24758769 |
| BC088983 | 2.62444333 |
| BC089491 | 0.32037147 |
| BC099439 | -0.3615507 |
| BC107230 | -0.3854153 |
| Bcam | 1.55428534 |
| Bcan | 1.72888701 |
| Bcan | 0.09940972 |
| Bcap29 | -0.7594269 |
| Bcap29 | 0.25313048 |
| Bcap31 | -0.1804009 |
| Bcap31 | -0.5844724 |
| Bcar1 | -0.511966 |
| Bcar3 | -0.5957324 |
| Bcas1 | -0.4375869 |
| Bcas2 | -0.3896544 |
| Bcas3 | 0.0277306 |
| Bcat1 | 0.68228378 |
| Bcat2 | -0.7433949 |
| Bccip | 0.01477495 |
| Bcdin3d | -0.2226592 |
| Bcdo2 | -0.6793814 |
| Bckdha | -0.5400185 |
| Bckdhb | -0.4941693 |
| Bckdk | -0.0660728 |
| Bckdk | -0.7648321 |
| Bcl10 | -0.2972235 |
| Bcl11a | 0.63679089 |
| Bcl11b | 3.01903178 |
| Bcl2 | 0.47580052 |
| Bcl2 | -0.0314019 |
| Bcl2l1 | 0.56348988 |
| Bcl2l1 | 0.9530398 |
| Bcl2l1 | -0.7558406 |
| Bcl2l10 | 1.09701294 |
| Bcl2l11 | -0.5500374 |
| Bcl2l11 | -0.3963545 |
| Bcl2l11 | 0.91027545 |
| Bcl2l12 | 2.07463119 |
| Bcl2l13 | -0.0760309 |
| Bcl2l14 | 0.08667592 |
| Bcl2l14 | 1.15740686 |
| Bcl2l2 | -0.7164073 |
| Bcl2l2 | -0.5711356 |
| Bcl3 | -0.3394254 |
| Bcl6 | -0.7757362 |
| Bcl6b | 0.46507119 |
| Bcl7a | 0.9093572 |
| Bcl7b | -0.7346045 |
| Bcl9l | -0.6485341 |
| Bclaf1 | -0.7146924 |
| Bclaf1 | 0.2435642 |
| Bclp2 | 0.31865449 |
| Bcmo1 | -0.4491521 |
| Bcor | -0.4452813 |
| Bcor | -0.6248741 |
| Bcorl1 | 0.50422155 |
| Bcs1l | 1.17674679 |
| Bdh1 | 1.79533411 |
| Bdh2 | 0.57359879 |
| Bdkrb1 | -0.3633198 |
| Bdnf | -0.7639291 |
| Bdnf | -0.4747074 |
| Becn1 | -0.7129806 |
| Becn1 | 0.23640341 |
| Bet1 | -0.3088509 |
| Bet1l | 1.42724548 |
| Bex2 | 2.92155131 |
| Bex2 | -0.3650922 |
| Bex2 | 0.86932165 |
| Bex4 | -0.5897103 |
| Bex4 | 2.35029061 |
| Bex6 | -0.8995958 |
| Bfar | -0.5098953 |
| Bfar | 0.95654177 |
| Bglap2 | 1.45786746 |
| Bgn | 0.90536775 |
| Bhlhb2 | 0.69697319 |
| Bhlhb5 | -0.6461412 |
| Bhlhb8 | 0.21873769 |
| Bhlhb8 | 0.09832496 |
| Bhlhb9 | 2.01553157 |
| Bhmt | -0.7104187 |
| Bhmt2 | -0.7389895 |
| Bicc1 | -0.3331163 |
| Bicd1 | -0.6926778 |
| Bicd2 | 2.11052771 |
| Bicd2 | 0.50469197 |
| Bid | 0.28779409 |
| Bid | -0.0834594 |
| Bik | 2.06846853 |
| Bin1 | 2.14159039 |
| Bin1 | -0.0936037 |
| Bin1 | 0.70270925 |
| Bin3 | 0.67367363 |
| Birc2 | -0.6040759 |
| Birc2 | 0.46187766 |
| Birc2 | 0.81015169 |
| Birc4 | 0.40010248 |
| Birc5 | 0.33004244 |
| Birc5 | 1.47584172 |
| Birc5 | 1.03797922 |
| Blcap | 1.64786267 |
| Blm | 1.26044785 |
| Blm | 3.97275635 |
| Blmh | 0.1693855 |
| Blnk | -0.7867678 |
| Bloc1s1 | -0.5529196 |
| Bloc1s3 | -0.7241631 |
| Blvra | -0.3781923 |
| Blvrb | 1.71751606 |
| Bmf | 1.235859 |
| Bmi1 | 1.66835269 |
| Bmp1 | -0.8073339 |
| Bmp1 | 0.88437761 |
| Bmp1 | 0.35434649 |
| Bmp1 | 0.4689869 |
| Bmp15 | 0.73585983 |
| Bmp2k | 1.35132204 |
| Bmp2k | 1.15646472 |
| Bmp3 | 0.74008324 |
| Bmp4 | 0.68325725 |
| Bmp6 | -0.525888 |
| Bmp7 | 1.99080459 |
| Bmp8a | 2.02256633 |
| Bmper | -0.2385547 |
| Bmpr1a | 1.5073079 |
| Bmpr1a | 1.44268074 |
| Bmpr1b | 3.55512596 |
| Bmpr1b | 1.57477213 |
| Bmpr2 | -0.6597819 |
| Bms1 | -0.2690339 |
| Bnc1 | 1.65795564 |
| Bnc2 | -0.6728029 |
| Bnc2 | -0.8498622 |
| Bnip1 | -0.1931272 |
| Bnip2 | -0.7416302 |
| Bnip3l | 1.58485104 |
| Bnipl | 2.82525554 |
| Bnipl | 0.94127176 |
| Bok | -0.5457305 |
| Bok | -0.6910055 |
| Bola1 | 1.39860854 |
| Bola2 | 2.43769723 |
| Bola3 | 0.17906624 |
| Boll | 1.67578726 |
| Bop1 | 0.7772663 |
| Bpi | 0.07228134 |
| Bpil1 | 2.42871912 |
| Bpil3 | -0.5867137 |
| Bpnt1 | -0.3872297 |
| Bpnt1 | -0.5294008 |
| Bpnt1 | -0.3716195 |
| Bpnt1 | -0.5762997 |
| Brap | -0.6002742 |
| Brca2 | 0.59478053 |
| Brca2 | -0.4575939 |
| Brcc3 | 0.13695834 |
| Brcc3 | -0.8206588 |
| Brcc3 | 2.32424675 |
| Brd2 | 1.635465 |
| Brd2 | -0.5995157 |
| Brd2 | -0.2642393 |
| Brd4 | 1.1618692 |
| Brd4 | -0.7002402 |
| Brd8 | 0.01437956 |
| Brd8 | -0.5485996 |
| Brd9 | 2.62158135 |
| Brd9 | -0.213551 |
| Brdt | -0.3763952 |
| Brdt | -0.5980006 |
| Bre | 0.18996272 |
| Bre | 0.50821267 |
| Bre | 0.01556522 |
| Brf1 | 3.25731263 |
| Brf2 | 1.63432981 |
| Bri3 | 1.51418696 |
| Brip1 | -0.5711356 |
| Brms1 | 1.97129068 |
| Brp16 | 0.65581329 |
| Brp16 | 0.1212001 |
| Brp17 | 2.59921003 |
| Brp44 | 0.57600017 |
| Brpf1 | -0.1951004 |
| Brpf1 | -0.6461412 |
| Brs3 | -0.6295588 |
| Brsk1 | -0.0971579 |
| Brsk2 | -0.92709 |
| Brsk2 | -0.8759246 |
| Brunol4 | -0.3580226 |
| Brunol4 | -0.1741016 |
| Brwd1 | -0.4807045 |
| Brwd1 | -0.2879146 |
| Brwd2 | -0.3933031 |
| Bscl2 | -0.7276308 |
| Bsdc1 | -0.6063643 |
| Bsg | 0.9846647 |
| Bsg | 1.20301629 |
| Bspry | -0.6935151 |
| Bst2 | -0.7513768 |
| Btbd1 | 1.73745344 |
| Btbd10 | -0.6565545 |
| Btbd11 | -0.2939275 |
| Btbd12 | -0.1721718 |
| Btbd14a | -0.4536883 |
| Btbd14b | -0.6835165 |
| Btbd16 | -0.1535602 |
| Btbd3 | 0.50422155 |
| Btbd3 | 0.31060031 |
| Btbd6 | 2.72192853 |
| Btbd9 | 0.17407136 |
| Btc | -0.7469341 |
| Btd | 0.27827579 |
| Btf3 | -0.7002402 |
| Btg1 | 1.4570111 |
| Btg1 | 0.82047104 |
| Btg3 | -0.4780344 |
| Btg3 | -0.1736188 |
| Btg4 | 1.84158363 |
| Btk | 0.48039618 |
| Btla | 0.38554057 |
| Btrc | 0.5152149 |
| Btrc | 0.92620179 |
| Btrc | 1.72425181 |
| Bub1b | -0.1833226 |
| Bub3 | 1.38705344 |
| Bud13 | 0.75786964 |
| Bud31 | -0.1779731 |
| Bves | 1.28121726 |
| Bxdc1 | -0.554364 |
| Bxdc1 | -0.2631772 |
| Bxdc2 | -0.0202592 |
| Bxdc2 | 0.31406052 |
| Bxdc5 | 2.18806504 |
| Bxdc5 | 2.72459145 |
| Bxdc5 | -0.5645372 |
| Bzrap1 | 1.52588691 |
| Bzrpl1 | -0.6295588 |
| Bzw1 | -0.6303419 |
| Bzw2 | 3.12668368 |
| Bzw2 | 2.66424469 |
| C030006K11Rik | 3.69858352 |
| C030011O14Rik | -0.5244865 |
| C030014K22Rik | -0.7998033 |
| C030018G13Rik | 2.71955018 |
| C030030A07Rik | -0.4602066 |
| C030039L03Rik | 0.55550229 |
| C030045D06Rik | -0.0268464 |
| C030045D06Rik | -0.7904739 |
| C030046I01Rik | -0.5777804 |
| C030048B08Rik | -0.5964878 |
| C030048B08Rik | -0.5912122 |
| C130022K22Rik | 1.17304933 |
| C130032J12Rik | -0.239072 |
| C130034I18Rik | -0.5037104 |
| C130038G02Rik | -0.6893361 |
| C130060K24Rik | 2.53119652 |
| C130073D16Rik | 1.06211601 |
| C130074G19Rik | -0.8628034 |
| C130090K23Rik | -0.8035611 |
| C130090K23Rik | 2.55562053 |
| C1galt1 | 0.66835095 |
| C1galt1c1 | -0.7389895 |
| C1qa | 0.56326892 |
| C1qb | 0.8081083 |
| C1qbp | 1.38177926 |
| C1qc | -0.6334806 |
| C1qdc2 | 1.07466369 |
| C1ql2 | -0.5175101 |
| C1ql3 | -0.1507292 |
| C1qtnf1 | 0.55750554 |
| C1qtnf2 | 1.2064866 |
| C1qtnf3 | 1.4448529 |
| C1qtnf3 | -0.649333 |
| C1qtnf3 | 1.99952091 |
| C1qtnf4 | 1.57356145 |
| C1qtnf6 | -0.4504459 |
| C1qtnf7 | -0.4753719 |
| C1qtnf9 | 0.7535447 |
| C1rl | 0.58035072 |
| C1s | 3.23768701 |
| C1s | -0.5507571 |
| C2 | -0.0913891 |
| C230021P08Rik | 0.43009699 |
| C230029F24Rik | -0.7998033 |
| C230052I12Rik | -0.2983247 |
| C230052I12Rik | -0.7585291 |
| C230055K05Rik | 3.06577132 |
| C230071H18Rik | -0.4575939 |
| C230078M08Rik | 0.30973327 |
| C230078M14Rik | -0.7495972 |
| C230090D14 | 0.26835839 |
| C230093N12Rik | 1.09663639 |
| C230096C10Rik | -0.3890476 |
| C2cd3 | 0.150748 |
| C3 | -0.3763952 |
| C330001K17Rik | 0.21873769 |
| C330005M16Rik | -0.6397897 |
| C330016O10Rik | -0.6413735 |
| C330019G07Rik | -0.5711356 |
| C330023M02Rik | 0.05615152 |
| C330027C09Rik | -0.4173703 |
| C430003P19Rik | 0.47020724 |
| C430004E15Rik | 0.10373631 |
| C4b | -0.7346045 |
| C530028I08Rik | 2.65339098 |
| C530028I08Rik | -0.0314019 |
| C530028O21Rik | 1.09437393 |
| C530043G21Rik | -0.5844724 |
| C530044N13Rik | 3.62075541 |
| C6 | -0.7504866 |
| C630004H02Rik | -0.7328563 |
| C630004H02Rik | 0.32151439 |
| C630007B19Rik | -0.1872322 |
| C630028N24Rik | -0.0545154 |
| C630035N08Rik | 0.68481271 |
| C630035N08Rik | -0.8469009 |
| C730025P13Rik | -0.0690924 |
| C730027P07Rik | 0.02577903 |
| C78339 | 0.62174888 |
| C79127 | 3.46506857 |
| C79267 | 0.30886543 |
| C79407 | -0.8264276 |
| C79407 | 2.46912362 |
| C80008 | 1.95682704 |
| C80913 | -0.1755516 |
| C85492 | 0.36121612 |
| C85492 | -0.4249008 |
| C85492 | -0.5189013 |
| C85627 | -0.2186003 |
| C87436 | 1.9951244 |
| C87436 | 1.8492457 |
| C87499 | -0.8400269 |
| C8b | 1.69940361 |
| C8g | 1.01375694 |
| C9 | 2.97135252 |
| C9 | 1.56481365 |
| C920005C14Rik | 2.26117171 |
| Cab39l | -0.7044679 |
| Cabc1 | 1.14173998 |
| Cables2 | -0.6549449 |
| Cabp2 | -0.1673645 |
| Cabp7 | 0.47991348 |
| Cabyr | 1.99841164 |
| Cabyr | 1.98048351 |
| Cachd1 | 2.28899046 |
| Cacna1a | -0.6868376 |
| Cacna1b | -0.3890476 |
| Cacna1b | -0.1155865 |
| Cacna1b | 0.74483486 |
| Cacna1d | -0.2717081 |
| Cacna1g | -0.511966 |
| Cacna1h | 1.94474698 |
| Cacna2d1 | 1.04926716 |
| Cacna2d3 | -0.5949775 |
| Cacnb1 | -0.6272135 |
| Cacnb1 | -0.5407306 |
| Cacnb2 | 1.9441849 |
| Cacnb2 | -0.3591972 |
| Cacnb3 | -0.7258954 |
| Cacnb3 | -0.267433 |
| Cacnb3 | 1.63942742 |
| Cacnb3 | 1.71575946 |
| Cacnb4 | 1.53885853 |
| Cacnb4 | 1.89379356 |
| Cacng2 | 0.32065733 |
| Cacng3 | -0.4760368 |
| Cacng3 | -0.3049591 |
| Cacng4 | 1.08476101 |
| Cacng5 | -0.1872322 |
| Cacng5 | -0.3423071 |
| Cacng5 | -0.3099658 |
| Cacng7 | 1.05150863 |
| Cacng8 | -0.4800362 |
| Cacybp | -0.2005481 |
| Cad | 0.02734061 |
| Cadm2 | -0.1712084 |
| Cadm3 | 0.88720343 |
| Cadps | -0.4198738 |
| Cadps2 | 2.17981985 |
| Cadps2 | 0.76521251 |
| Cage1 | -0.4982465 |
| Calb2 | 0.64452705 |
| Calb2 | -0.5837265 |
| Calca | 1.25505058 |
| Calca | 0.86884226 |
| Calcoco1 | -0.7666407 |
| Cald1 | 1.82884685 |
| Cald1 | -0.7311112 |
| Calm2 | 2.19058759 |
| Calm3 | -0.6541412 |
| Calml4 | -0.6695307 |
| Caln1 | 0.02068595 |
| Calr | 0.05955043 |
| Calr3 | -0.6445493 |
| Calr3 | 1.09914389 |
| Calr3 | 1.00452808 |
| Calr4 | 1.84129865 |
| Calu | -0.0301573 |
| Calu | 0.33032539 |
| Calu | 1.53783678 |
| Caly | -0.7129806 |
| Camk1 | 2.77444475 |
| Camk1d | 1.43534541 |
| Camk1g | 1.16104878 |
| Camk1g | 1.47927869 |
| Camk2a | 0.65420922 |
| Camk2a | 0.3603944 |
| Camk2a | 0.41908928 |
| Camk2b | -0.1833226 |
| Camk2d | -0.6525358 |
| Camk2d | -0.3580226 |
| Camk2d | -0.2313429 |
| Camk2g | 0.7677068 |
| Camk2g | 1.17766889 |
| Camk2n1 | 0.61737692 |
| Camk4 | -0.0435184 |
| Camkk1 | 0.97628699 |
| Camkk2 | 1.569692 |
| Camkk2 | 1.72631471 |
| Camkv | -0.4867399 |
| Caml | 0.96379604 |
| Camp | 1.58798805 |
| Camsap1l1 | 1.1643262 |
| Camta2 | -0.1237922 |
| Cant1 | -0.6654564 |
| Cant1 | -0.5652681 |
| Cap1 | -0.2972235 |
| Capg | -0.2841081 |
| Capg | 0.07710398 |
| Capn1 | -0.1784582 |
| Capn10 | -0.3365524 |
| Capn11 | 0.27047081 |
| Capn2 | -0.4968855 |
| Capn2 | -0.2303171 |
| Capn2 | 0.07969062 |
| Capn3 | 0.25005517 |
| Capn5 | -0.6901704 |
| Capn6 | -0.5536415 |
| Capn7 | -0.5874619 |
| Capn8 | 1.90366275 |
| Capns1 | -0.6140326 |
| Caprin1 | -0.7044679 |
| Caprin1 | -0.4092794 |
| Capsl | -0.7711775 |
| Capza1 | 0.75155579 |
| Capza3 | -0.1432211 |
| Capzb | -0.5329268 |
| Capzb | -0.4941693 |
| Capzb | 1.2785295 |
| Car11 | -0.5435845 |
| Car12 | -0.5414433 |
| Car13 | 0.69120204 |
| Car14 | 1.51812986 |
| Car15 | 0.17673859 |
| Car2 | -0.4562902 |
| Car3 | 1.77604567 |
| Car4 | 0.32265593 |
| Car5b | -0.4588993 |
| Car5b | -0.7979301 |
| Car5b | -0.2749272 |
| Car7 | -0.5016577 |
| Car9 | 2.95009405 |
| Card10 | -0.1975727 |
| Card11 | -0.4901096 |
| Carhsp1 | 2.85261082 |
| Carm1 | 0.57490939 |
| Cart | 2.21958028 |
| Cartpt | 2.56328754 |
| Casc1 | 0.36395002 |
| Casc1 | 2.20247842 |
| Casc1 | 4.08136132 |
| Casc3 | -0.6630203 |
| Casc4 | -0.7155494 |
| Casc4 | -0.7567359 |
| Casc4 | -0.5785216 |
| Cask | -0.6002742 |
| Caskin1 | -0.6445493 |
| Caskin1 | 2.08296243 |
| Caskin1 | -0.1043068 |
| Caskin2 | -0.7923324 |
| Casp1 | 0.92620179 |
| Casp2 | 0.3921875 |
| Casp4 | -0.6918413 |
| Casp4 | -0.8658152 |
| Casp4 | 0.47652764 |
| Casp6 | 0.30218534 |
| Casp6 | -0.3510056 |
| Casp7 | 0.04664534 |
| Casp8 | 1.44502645 |
| Casp8 | -0.3527549 |
| Casp9 | 1.0727342 |
| Casq2 | -0.4914609 |
| Cass4 | 0.65981158 |
| Cast | -0.7998033 |
| Cat | -0.6719838 |
| Catsper1 | 3.23876291 |
| Catsper2 | -0.7293694 |
| Catsper3 | -0.7311112 |
| Catsper4 | 1.92335793 |
| Cav1 | -0.4395046 |
| Cav2 | -0.6477358 |
| Cbara1 | 1.09976973 |
| Cbfa2t3h | 0.52654125 |
| Cbfa2t3h | -0.2065272 |
| Cbfb | -0.6835165 |
| Cbfb | -0.3172449 |
| Cbfb | 0.29692424 |
| Cbl | 0.46089285 |
| Cblb | -0.7549462 |
| Cblc | -0.413005 |
| Cbln1 | -0.5272915 |
| Cbln2 | 0.79678981 |
| Cbln4 | 1.91790774 |
| Cbr1 | 0.61025527 |
| Cbr2 | -0.4941693 |
| Cbr3 | 1.45443734 |
| Cbr3 | -0.0477329 |
| Cbs | -0.7603256 |
| Cbs | 0.7915998 |
| Cbwd1 | 2.10343097 |
| Cbx2 | 1.48984612 |
| Cbx5 | 1.70981485 |
| Cbx7 | -0.1770038 |
| Cbx8 | -0.5572594 |
| Cby1 | 0.71275847 |
| Cc2d1a | 1.35514762 |
| Cc2d1b | -0.7104187 |
| Cc2d2a | 1.55335753 |
| Ccar1 | -0.2835656 |
| Ccbe1 | -0.3890476 |
| Ccbl1 | 2.08212378 |
| Ccbl1 | 1.13573693 |
| Ccbl2 | 0.20784838 |
| Ccbp2 | -0.0738571 |
| Ccdc100 | 0.74901836 |
| Ccdc101 | -0.3908691 |
| Ccdc108 | 0.57904766 |
| Ccdc109a | -0.7639291 |
| Ccdc109a | 0.20041014 |
| Ccdc109b | 0.3209431 |
| Ccdc11 | -0.392694 |
| Ccdc110 | 0.47580052 |
| Ccdc111 | -0.4433518 |
| Ccdc113 | -0.5175101 |
| Ccdc114 | 0.89671735 |
| Ccdc115 | 0.52239634 |
| Ccdc116 | -0.2922838 |
| Ccdc117 | -0.5071414 |
| Ccdc12 | -0.7585291 |
| Ccdc120 | 1.84998026 |
| Ccdc123 | -0.3206235 |
| Ccdc124 | -0.7104187 |
| Ccdc125 | 0.23295937 |
| Ccdc126 | -0.4337631 |
| Ccdc127 | -0.1601991 |
| Ccdc127 | 2.48050985 |
| Ccdc13 | -0.3836043 |
| Ccdc130 | 0.47507284 |
| Ccdc131 | 0.83466476 |
| Ccdc132 | 0.90966338 |
| Ccdc134 | -0.4760368 |
| Ccdc137 | 3.70852432 |
| Ccdc137 | -0.5740831 |
| Ccdc18 | -0.5696653 |
| Ccdc21 | 1.92189367 |
| Ccdc23 | -0.4595527 |
| Ccdc25 | 0.58511325 |
| Ccdc27 | -0.5645372 |
| Ccdc27 | 1.32901864 |
| Ccdc27 | -0.6217638 |
| Ccdc28a | -0.7121258 |
| Ccdc28b | -0.1731362 |
| Ccdc28b | -0.2489539 |
| Ccdc3 | 1.24459186 |
| Ccdc32 | -0.1654484 |
| Ccdc38 | 0.28986347 |
| Ccdc39 | 0.19454771 |
| Ccdc40 | -0.4401447 |
| Ccdc41 | 2.48079926 |
| Ccdc42 | -0.6389987 |
| Ccdc43 | 0.46507119 |
| Ccdc45 | -0.1931272 |
| Ccdc46 | -0.2328837 |
| Ccdc46 | -0.1178587 |
| Ccdc47 | 0.8499535 |
| Ccdc49 | -0.2621162 |
| Ccdc5 | -0.6389987 |
| Ccdc50 | -0.5244865 |
| Ccdc51 | 3.74348613 |
| Ccdc53 | 2.48449592 |
| Ccdc53 | -0.3527549 |
| Ccdc53 | -0.030572 |
| Ccdc54 | 1.46358511 |
| Ccdc56 | 0.22476858 |
| Ccdc57 | 1.3876069 |
| Ccdc58 | -0.7684528 |
| Ccdc59 | -0.4793685 |
| Ccdc59 | -0.5777804 |
| Ccdc64 | -0.8293253 |
| Ccdc65 | 2.4831691 |
| Ccdc65 | 0.50210174 |
| Ccdc66 | 0.98650067 |
| Ccdc67 | -0.4433518 |
| Ccdc68 | 0.33989535 |
| Ccdc70 | -0.2489539 |
| Ccdc71 | -0.415497 |
| Ccdc73 | 0.35103121 |
| Ccdc77 | -0.1692845 |
| Ccdc79 | 2.48010452 |
| Ccdc80 | -0.6573603 |
| Ccdc82 | 1.33790619 |
| Ccdc84 | 2.00748852 |
| Ccdc85a | 0.39878792 |
| Ccdc85b | 1.01238522 |
| Ccdc86 | 2.1691694 |
| Ccdc87 | -0.7630269 |
| Ccdc88a | -0.9857474 |
| Ccdc88b | 1.86009265 |
| Ccdc88c | -0.8638062 |
| Ccdc90b | 0.12823629 |
| Ccdc91 | 1.20144485 |
| Ccdc92 | -0.5696653 |
| Ccdc93 | -0.4674291 |
| Ccdc94 | 2.93887298 |
| Ccdc95 | 1.34091479 |
| Ccdc96 | 2.88394389 |
| Ccdc97 | 0.58468128 |
| Ccdc98 | 1.09512869 |
| Ccdc99 | 0.51218698 |
| Cchcr1 | -0.7576321 |
| Ccin | 0.43162279 |
| Cck | 1.07389237 |
| Cckbr | -0.5980006 |
| Ccl11 | -0.5536415 |
| Ccl17 | -0.8254637 |
| Ccl19 | -0.4459253 |
| Ccl21b | -0.0219017 |
| Ccl21c | 1.69595184 |
| Ccl25 | -0.7198465 |
| Ccl25 | 0.1428436 |
| Ccl27 | 0.15211594 |
| Ccl27 | 0.33905484 |
| Ccl28 | 1.04847478 |
| Ccl3 | 0.70118304 |
| Ccl4 | 1.60432502 |
| Ccl4 | -0.0931604 |
| Ccl5 | 2.28133504 |
| Ccl6 | -0.8197007 |
| Ccl7 | -0.6736227 |
| Ccl9 | -0.5442993 |
| Ccm2 | 0.22381889 |
| Ccna1 | 1.30617504 |
| Ccnb1 | 0.65561293 |
| Ccnb1 | -0.1450926 |
| Ccnb3 | 0.52906531 |
| Ccnc | -0.8197007 |
| Ccnd1 | -0.8063893 |
| Ccnd1 | 1.0729917 |
| Ccnd1 | -0.7576321 |
| Ccnd2 | -0.612494 |
| Ccnd3 | -0.4123831 |
| Ccnd3 | 0.26714916 |
| Ccndbp1 | 0.47093868 |
| Ccne1 | -0.7224341 |
| Ccne2 | 0.85774855 |
| Ccnf | -0.0665036 |
| Ccng1 | -0.554364 |
| Ccng1 | -0.4667703 |
| Ccng1 | -0.7224341 |
| Ccng2 | 1.12714641 |
| Ccnk | -0.7433949 |
| Ccnl1 | -0.5704001 |
| Ccno | 0.9200149 |
| Ccr4 | 0.86097757 |
| Ccr5 | 0.44499452 |
| Ccr5 | -0.0905048 |
| Ccr5 | 1.55906454 |
| Ccr6 | 1.78727975 |
| Ccr6 | 0.90197884 |
| Ccrk | -0.857805 |
| Ccrk | 2.18244393 |
| Ccrk | -0.3510056 |
| Ccrk | 1.80858739 |
| Ccrl2 | -0.7354799 |
| Ccrn4l | -0.7666407 |
| Ccrn4l | 0.79402536 |
| Ccs | 1.62770437 |
| Cct2 | 0.61821124 |
| Cct3 | 1.88240217 |
| Cct3 | -0.7487087 |
| Cct4 | -0.0515421 |
| Cct5 | -0.6287764 |
| Cct6a | -0.4472147 |
| Cct6b | -0.9016825 |
| Cct7 | 0.20170797 |
| Cct8 | -0.6670841 |
| Cd109 | 0.91591833 |
| Cd109 | 3.14607722 |
| Cd14 | -0.5792634 |
| Cd151 | -0.5140412 |
| Cd151 | 1.78709864 |
| Cd151 | -0.7979301 |
| Cd160 | -0.3319738 |
| Cd163 | -0.2977739 |
| Cd164 | 0.56260571 |
| Cd177 | -0.6256532 |
| Cd180 | 1.79431609 |
| Cd1d1 | 0.9846647 |
| Cd2 | 1.12714641 |
| Cd200 | 0.44824563 |
| Cd200 | -0.5529196 |
| Cd226 | -0.6086583 |
| Cd247 | -0.2241858 |
| Cd248 | 1.34574724 |
| Cd248 | -0.0023782 |
| Cd27 | -0.7567359 |
| Cd274 | -0.8216178 |
| Cd276 | -0.7886191 |
| Cd2bp2 | 1.59556337 |
| Cd300lg | -0.6951919 |
| Cd320 | -0.3434623 |
| Cd34 | -0.19165 |
| Cd3d | -0.7389895 |
| Cd3eap | 1.05649979 |
| Cd3g | -0.4459253 |
| Cd4 | -0.0039898 |
| Cd40 | 0.00563884 |
| Cd40 | -0.2427006 |
| Cd44 | 2.2243016 |
| Cd44 | -0.6094242 |
| Cd44 | -0.4446377 |
| Cd44 | -0.9455102 |
| Cd47 | 0.00324089 |
| Cd52 | -0.2070272 |
| Cd53 | -0.7189855 |
| Cd55 | 2.17866454 |
| Cd59a | -0.6885025 |
| Cd59b | 1.3517053 |
| Cd59b | -0.5572594 |
| Cd6 | 0.93607671 |
| Cd6 | 0.01517017 |
| Cd6 | -1.0065286 |
| Cd63 | -0.3884413 |
| Cd63 | 1.13211667 |
| Cd63 | -0.994927 |
| Cd68 | 0.2737808 |
| Cd74 | -0.7469341 |
| Cd74 | -0.4874129 |
| Cd79b | -0.6248741 |
| Cd79b | 1.23129882 |
| Cd81 | 0.18897732 |
| Cd82 | 0.08740822 |
| Cd83 | -0.7302399 |
| Cd84 | -0.482712 |
| Cd86 | -0.6334806 |
| Cd86 | -0.6926778 |
| Cd86 | -0.3325449 |
| Cd8b1 | 2.03749949 |
| Cd9 | 0.0896017 |
| Cd93 | 1.28605655 |
| Cd96 | 0.99646534 |
| Cd97 | -0.873894 |
| Cd97 | -0.6117256 |
| Cd97 | -0.9576238 |
| Cd99l2 | -0.4142502 |
| Cdan1 | -0.6977127 |
| Cdc123 | 1.61872524 |
| Cdc14b | -0.3365524 |
| Cdc14b | -0.3308326 |
| Cdc14b | 1.12288268 |
| Cdc16 | -0.8718678 |
| Cdc20 | -0.7923324 |
| Cdc20 | 1.6839049 |
| Cdc20 | 0.45743792 |
| Cdc20 | 0.07265319 |
| Cdc23 | -0.6960314 |
| Cdc25a | -0.2933793 |
| Cdc25b | 0.47531547 |
| Cdc25c | -0.0231354 |
| Cdc25c | -0.831262 |
| Cdc27 | 1.30285162 |
| Cdc2a | 1.7577881 |
| Cdc2l1 | -0.8789786 |
| Cdc2l1 | -0.4847238 |
| Cdc2l5 | 3.59300268 |
| Cdc2l6 | 2.84422349 |
| Cdc34 | -0.1592478 |
| Cdc37 | 1.82757522 |
| Cdc37l1 | 1.09613409 |
| Cdc37l1 | 2.01695219 |
| Cdc37l1 | -0.5770398 |
| Cdc37l1 | -0.4217558 |
| Cdc42 | 2.39636785 |
| Cdc42bpb | 1.64316229 |
| Cdc42ep1 | 0.63617742 |
| Cdc42ep2 | 0.98452332 |
| Cdc42ep3 | -0.632695 |
| Cdc42ep4 | 2.48883828 |
| Cdc42ep5 | -0.4549883 |
| Cdc42se1 | 2.13947351 |
| Cdc42se1 | -0.7138361 |
| Cdc45l | -0.0363967 |
| Cdc5l | 0.6096243 |
| Cdc5l | 2.57517002 |
| Cdc5l | -0.5350487 |
| Cdc5l | 1.7052823 |
| Cdc6 | 0.57097211 |
| Cdc7 | 0.11268681 |
| Cdc73 | 1.36672073 |
| Cdca2 | 0.38900282 |
| Cdca3 | -0.4615157 |
| Cdca3 | -0.0334799 |
| Cdca4 | -0.2939275 |
| Cdca4 | 1.46494541 |
| Cdca4 | 1.03275448 |
| Cdca5 | 1.63048845 |
| Cdca7 | -0.8598012 |
| Cdca8 | -0.7027746 |
| Cdcp1 | -0.6295588 |
| Cdgap | -0.3860197 |
| Cdh1 | 0.64168426 |
| Cdh11 | -0.5279941 |
| Cdh11 | -0.8964743 |
| Cdh12 | -0.5154273 |
| Cdh13 | 1.71099369 |
| Cdh13 | 0.32749212 |
| Cdh16 | -0.3411534 |
| Cdh22 | 0.47991348 |
| Cdh23 | -0.4968855 |
| Cdh23 | 0.19487436 |
| Cdh23 | -0.7036209 |
| Cdh24 | 0.70803161 |
| Cdh26 | 0.21138516 |
| Cdh3 | 0.48975979 |
| Cdh5 | -0.5140412 |
| Cdh6 | -0.6638316 |
| Cdh7 | -0.413005 |
| Cdh8 | 0.35407066 |
| Cdh8 | -0.547164 |
| Cdh8 | -0.239072 |
| Cdipt | 0.19552732 |
| Cdipt | -0.5616193 |
| Cdk10 | -0.613263 |
| Cdk10 | 0.59925824 |
| Cdk10 | -0.7267627 |
| Cdk2 | 1.13718115 |
| Cdk2 | -0.0129032 |
| Cdk2 | 1.80929532 |
| Cdk2 | -0.3377005 |
| Cdk2ap2 | -0.4807045 |
| Cdk3 | 1.29484765 |
| Cdk5 | -0.7053157 |
| Cdk5r1 | 1.72166682 |
| Cdk5rap1 | -0.5085174 |
| Cdk5rap2 | 2.96567606 |
| Cdk5rap3 | 0.96898479 |
| Cdk6 | -0.3127588 |
| Cdk8 | -0.7241631 |
| Cdk9 | -0.555087 |
| Cdkal1 | 0.62548021 |
| Cdkl1 | -0.514734 |
| Cdkl2 | -0.3510056 |
| Cdkl2 | 0.12296408 |
| Cdkl2 | -0.4582464 |
| Cdkl2 | 0.69850622 |
| Cdkl3 | 0.67445962 |
| Cdkl4 | 0.41496038 |
| Cdkn1a | -0.027673 |
| Cdkn1a | 1.42236969 |
| Cdkn1a | 0.05007861 |
| Cdkn1b | 1.71177876 |
| Cdkn1b | 0.42037587 |
| Cdkn1c | 0.49595095 |
| Cdkn2a | 1.00756842 |
| Cdkn2a | -0.3830015 |
| Cdkn2aipnl | 0.72846942 |
| Cdkn2b | -0.2160723 |
| Cdkn2c | 0.20945765 |
| Cdkn2c | 2.6282376 |
| Cdkn2d | -0.1891931 |
| Cdo1 | 0.51218698 |
| Cdr2 | -0.7684528 |
| Cdrt4 | -0.7748227 |
| Cds1 | 1.60087426 |
| Cds2 | -0.6101907 |
| Cdsn | 0.8036657 |
| Cdt1 | 2.02388246 |
| Cdv3 | 0.22666508 |
| Cdv3 | 2.176869 |
| Cdyl | -0.6638316 |
| Cdyl | 0.99058347 |
| Cdyl | 0.8988874 |
| Cdyl2 | 0.66538087 |
| Ceacam1 | -0.3722151 |
| Ceacam13 | -0.260527 |
| Ceacam2 | -0.1702459 |
| Cebpa | 0.27287923 |
| Cebpb | -0.5457305 |
| Cecr5 | 0.53705178 |
| Cecr6 | 0.65018705 |
| Celsr1 | -0.4363106 |
| Celsr2 | 0.06743391 |
| Celsr3 | 1.03637469 |
| Cend1 | 0.02304002 |
| Cenpa | 1.12031516 |
| Cenpb | -0.45239 |
| Cenph | 0.51055253 |
| Cenpi | -0.5064542 |
| Cenpj | -0.6646437 |
| Cenpk | -0.5237865 |
| Cenpl | -0.6760864 |
| Cenpl | -0.3480972 |
| Cenpm | -0.2637081 |
| Cenpm | -0.650933 |
| Cenpm | 1.15975809 |
| Cenpn | 0.82717705 |
| Cenpp | -0.5777804 |
| Cenpq | 1.41110378 |
| Cenpt | -0.7558406 |
| Centa1 | -0.2505237 |
| Centa1 | 1.23271266 |
| Centa2 | -0.6868376 |
| Centb5 | 1.79623145 |
| Centb5 | 0.34129454 |
| Centd2 | 1.80260785 |
| Centd2 | 0.21937434 |
| Centd3 | 0.39588943 |
| Centg1 | 1.6677362 |
| Centg1 | 0.25741914 |
| Centg2 | -0.5521982 |
| Centg2 | 0.74428782 |
| Centg3 | -0.5558106 |
| Centg3 | -0.4621709 |
| Cep135 | 0.44072609 |
| Cep164 | -0.4687483 |
| Cep170 | 3.3260007 |
| Cep55 | -0.6469381 |
| Cep57 | -0.6389987 |
| Cep57 | -0.1784582 |
| Cep63 | 0.28631317 |
| Cep68 | 0.0191133 |
| Cep70 | 1.00092227 |
| Cep72 | -0.0856554 |
| Cep78 | 1.86821602 |
| Cep97 | 2.2657262 |
| Cept1 | -0.2364886 |
| Cercam | 0.04473255 |
| Ces3 | 0.29045389 |
| Cetn2 | -0.2879146 |
| Cetn3 | 2.49549976 |
| Cetn4 | -0.4006432 |
| Cetn4 | 0.33427773 |
| Cfd | 1.79161602 |
| Cfdp1 | 1.81581859 |
| Cfh | 0.54767118 |
| Cfhr1 | -0.7267627 |
| Cfi | 1.82066703 |
| Cfl1 | -0.112867 |
| Cfl2 | -0.7684528 |
| Cfl2 | 1.00632583 |
| Cfp | 2.12633441 |
| Cftr | -0.0060082 |
| Cga | 0.12648216 |
| Cggbp1 | 0.2622967 |
| Cgnl1 | 2.28700247 |
| Cgrrf1 | 2.40088249 |
| Ch25h | 0.1404247 |
| Chac1 | 1.59386052 |
| Chad | 0.30247676 |
| Chaf1a | -0.6264331 |
| Chaf1b | 2.32796301 |
| Chat | 2.21682799 |
| Chchd1 | 0.64168426 |
| Chchd2 | 0.0569079 |
| Chchd4 | -0.5230871 |
| Chchd5 | 0.55728317 |
| Chchd6 | 0.70689363 |
| Chchd7 | -0.4395046 |
| Chchd8 | 0.78951577 |
| Chd1 | -0.5587105 |
| Chd1 | 0.1843651 |
| Chd1l | -0.3060694 |
| Chd1l | 0.07636364 |
| Chd4 | -0.416121 |
| Chd4 | 0.71238119 |
| Chd4 | -0.7346045 |
| Chd5 | -0.6501327 |
| Chd8 | 0.68481271 |
| Chdh | 0.08153388 |
| Chdh | -0.8101734 |
| Chek1 | 1.06471686 |
| Chek1 | -0.8016804 |
| Chek2 | 0.42652717 |
| Chek2 | 0.43238476 |
| Cherp | -0.3200596 |
| Chfr | -0.7121258 |
| Chfr | 1.5044607 |
| Chfr | 0.24480403 |
| Chga | 2.51918781 |
| Chga | -0.699397 |
| Chga | -0.0039898 |
| Chgb | -0.7129806 |
| Chi3l1 | 4.12521592 |
| Chi3l3 | 1.15917083 |
| Chi3l3 | 1.98919783 |
| Chia | 2.15778617 |
| Chia | 1.00991108 |
| Chic1 | 4.12817731 |
| Chic2 | -0.0669345 |
| Chic2 | 0.05577311 |
| Chit1 | 1.0837434 |
| Chka | 1.9911937 |
| Chka | -0.4853953 |
| Chka | -0.1702459 |
| Chkb | 0.44148075 |
| Chl1 | 3.91702526 |
| Chl1 | -0.2999789 |
| Chl1 | 1.15019919 |
| Chm | -0.8045029 |
| Chmp1b | -0.5867137 |
| Chmp2a | 2.1454797 |
| Chmp2a | 1.30113497 |
| Chmp2b | -0.814925 |
| Chmp4b | -0.0202592 |
| Chmp5 | 0.35130792 |
| Chmp7 | 1.75373107 |
| Chmp7 | -0.5043956 |
| Chmp7 | -0.2972235 |
| Chn2 | 0.64330976 |
| Chodl | 3.00343268 |
| Chordc1 | 1.59718932 |
| Chpf | 1.03262013 |
| Chpt1 | -0.8139728 |
| Chpt1 | 0.6152879 |
| Chpt1 | -0.5272915 |
| Chrac1 | -0.6071283 |
| Chrd | 1.82421453 |
| Chrm1 | 1.02641931 |
| Chrm2 | -0.4478601 |
| Chrm3 | 1.5106323 |
| Chrna1 | -0.0596346 |
| Chrna4 | -0.3866245 |
| Chrna4 | 0.89267397 |
| Chrna4 | -0.2272465 |
| Chrna5 | -0.3860197 |
| Chrna6 | -0.8187437 |
| Chrna6 | -0.0460448 |
| Chrna7 | 0.11695306 |
| Chrna9 | -0.5216897 |
| Chrnb1 | 0.5207795 |
| Chrnb1 | 0.7404496 |
| Chrnb2 | -0.392694 |
| Chrnb2 | -0.2370047 |
| Chrnb3 | 0.88939545 |
| Chrnb3 | -0.455639 |
| Chrnb3 | 0.86371357 |
| Chrne | 0.07004716 |
| Chst1 | 0.84701366 |
| Chst10 | 1.51192646 |
| Chst12 | 0.97485963 |
| Chst14 | -0.6769091 |
| Chst2 | 0.8478312 |
| Chst3 | 0.44799594 |
| Chst3 | -0.4024872 |
| Chst7 | 1.91780262 |
| Chst8 | 1.76837862 |
| Chtf18 | 1.46367019 |
| Chuk | -0.2453011 |
| Churc1 | 2.5260253 |
| Ciao1 | 2.63305141 |
| Ciapin1 | 2.33359466 |
| Cib1 | 0.36640377 |
| Cib2 | 2.4953858 |
| Cib2 | -0.4485059 |
| Cic | 1.14460792 |
| Cidea | -0.205528 |
| Cideb | -0.168324 |
| Cidec | 2.00365711 |
| Ciita | -0.2323698 |
| Cirbp | 1.85026262 |
| Cirh1a | -0.4887603 |
| Cisd1 | -0.7224341 |
| Cish | -0.2241858 |
| Cit | 2.24176173 |
| Cited1 | 3.41120494 |
| Cited2 | 0.82600694 |
| Cited4 | -0.5630771 |
| Ciz1 | 1.53555349 |
| Ciz1 | -0.7172659 |
| Ckap2l | -0.5209919 |
| Ckap5 | 0.48880369 |
| Ckb | 1.46638858 |
| Cklf | 0.03317363 |
| Cklf | -0.5652681 |
| Ckmt1 | 1.20849577 |
| Ckmt2 | -0.2196134 |
| Ckmt2 | -0.3428845 |
| Ckt2 | -0.1578227 |
| Ckt2 | 1.27500315 |
| Clca5 | 1.9699985 |
| Clcc1 | -0.6835165 |
| Clcf1 | 1.1020193 |
| Clcf1 | -0.0725553 |
| Clcf1 | 0.81591738 |
| Clcn1 | 0.14937807 |
| Clcn2 | -0.5987578 |
| Clcn3 | -0.8101734 |
| Clcn3 | 0.32550375 |
| Clcn3 | 3.27285927 |
| Clcn4-2 | -0.6893361 |
| Clcn7 | -0.415497 |
| Clcnka | -0.3830015 |
| Cldn1 | -0.7711775 |
| Cldn10 | 1.60812824 |
| Cldn10 | 1.60035923 |
| Cldn10 | 0.81794395 |
| Cldn11 | -0.5964878 |
| Cldn12 | -0.0953792 |
| Cldn15 | -0.0821442 |
| Cldn15 | -0.4753719 |
| Cldn17 | -0.5844724 |
| Cldn2 | -0.5407306 |
| Cldn23 | 0.56393166 |
| Cldn3 | -0.510585 |
| Cldn4 | 1.5003783 |
| Cldn5 | 0.3710213 |
| Cldn6 | 0.11411104 |
| Cldn9 | 0.19846004 |
| Cldnd1 | -0.4621709 |
| Cldnd2 | 0.01753803 |
| Clec11a | 0.68364635 |
| Clec16a | -0.8063893 |
| Clec1a | -0.1224196 |
| Clec2d | 3.2043824 |
| Clec2e | 1.56641714 |
| Clec3b | 0.28215419 |
| Clec4a1 | 1.85673388 |
| Clec4a3 | 0.05615152 |
| Clec4b1 | 0.74099887 |
| Clec4b1 | 0.95552171 |
| Clec4d | -0.5762997 |
| Clec4g | -0.4123831 |
| Clec4n | 0.45098784 |
| Clec7a | 1.08907364 |
| Clecsf12 | 1.3900015 |
| Clgn | -0.2781573 |
| Clic1 | 1.71393434 |
| Clic3 | 0.07451024 |
| Clic4 | 0.61130595 |
| Clic6 | 2.16286686 |
| Clip1 | -0.0936037 |
| Clip1 | 0.2528234 |
| Clip4 | -0.789546 |
| Clk1 | 0.83250774 |
| Clk2 | 2.24704117 |
| Clk2 | -0.5493182 |
| Clk2 | -0.5043956 |
| Clk3 | 1.17304933 |
| Clk4 | 1.28790334 |
| Clk4 | -0.6968717 |
| Clk4 | -0.7812359 |
| Clm3 | 0.67877094 |
| Clm3 | 1.25833495 |
| Clmn | -0.5800058 |
| Clmn | -0.5807488 |
| Cln3 | -0.3234483 |
| Cln5 | 0.35103121 |
| Cln6 | 1.93116374 |
| Cln6 | -0.1293026 |
| Cln8 | -0.8902622 |
| Clns1a | 1.39101278 |
| Clns1a | -0.3027425 |
| Clns1a | 1.48694564 |
| Clock | -0.5189013 |
| Clock | 0.07932153 |
| Clp1 | -0.4395046 |
| Clpb | 1.98284138 |
| Clpp | 0.58208533 |
| Clptm1 | 2.11973802 |
| Clptm1l | -0.1901751 |
| Clpx | 1.41308057 |
| Clpx | 0.93279655 |
| Clrn1 | 1.71184415 |
| Clspn | -0.6622096 |
| Clspn | -0.2447805 |
| Clspn | 1.3561016 |
| Clstn1 | -0.680207 |
| Clstn3 | 1.06692195 |
| Clstn3 | 0.0896017 |
| Clta | 1.0003663 |
| Cltb | -0.3656837 |
| Cluap1 | 1.80770173 |
| Cma1 | 2.58620625 |
| Cma1 | 0.84406464 |
| Cmas | 1.13441111 |
| Cmbl | -0.5016577 |
| Cml1 | -0.4305884 |
| Cml3 | -0.7812359 |
| Cml4 | 1.66828421 |
| Cmpk | -0.7702684 |
| Cmtm2a | 2.66028188 |
| Cmtm2b | -0.6968717 |
| Cmtm4 | -0.7603256 |
| Cmtm4 | -0.7302399 |
| Cmtm5 | -0.3245806 |
| Cmtm6 | -0.5785216 |
| Cmtm7 | 4.49670521 |
| Cmtm7 | 0.68442409 |
| Cmtm7 | 2.89398687 |
| Cmtm8 | -0.8273925 |
| Cmtm8 | -0.682688 |
| Cmya1 | -0.5674641 |
| CN716893 | 2.88575388 |
| Cnbp2 | 1.82566457 |
| Cnbp2 | -0.5601638 |
| Cndp1 | 1.65636187 |
| Cnga4 | -0.5521982 |
| Cnih | -0.2030348 |
| Cnih3 | 2.33379759 |
| Cnih4 | 1.31228767 |
| Cnksr2 | 1.34796188 |
| Cnksr2 | 0.00803072 |
| Cnksr3 | 0.40298808 |
| Cnn1 | 1.42901226 |
| Cnn2 | -0.6217638 |
| Cnn2 | -0.1688041 |
| Cnnm1 | 1.25430736 |
| Cnnm1 | -0.2873699 |
| Cnnm2 | -0.4787012 |
| Cnnm3 | -0.4867399 |
| Cno | -0.1640139 |
| Cnot10 | 1.35714986 |
| Cnot2 | -0.6248741 |
| Cnot2 | 0.79885785 |
| Cnot2 | 0.80195142 |
| Cnot2 | 1.01361986 |
| Cnot3 | 1.3274473 |
| Cnot3 | -0.7495972 |
| Cnot4 | -0.5393069 |
| Cnot4 | 1.63347753 |
| Cnot4 | -0.5942232 |
| Cnot6l | -0.3866245 |
| Cnot6l | -0.3504232 |
| Cnot7 | 0.61004499 |
| Cnp | 0.52883613 |
| Cnp | 0.81777523 |
| Cnpy2 | 1.13970322 |
| Cnpy3 | -0.6760864 |
| Cnr1 | -0.3077373 |
| Cnr1 | -0.7766506 |
| Cntd1 | -0.7172659 |
| Cntd1 | 3.14474703 |
| Cntfr | -0.3550925 |
| Cntln | -0.3686469 |
| Cntn1 | 0.80212299 |
| Cntn4 | 1.29951644 |
| Cntn6 | 0.18171939 |
| Cntnap1 | -0.5478815 |
| Cntnap2 | 1.85144765 |
| Cntnap4 | 2.17606988 |
| Cntnap4 | 1.48203596 |
| Coasy | -0.1659271 |
| Cobl | -0.6101907 |
| Cobll1 | -0.8882006 |
| Cobll1 | 2.43311233 |
| Coch | 0.65921292 |
| Cog1 | 2.26630759 |
| Cog2 | -0.7302399 |
| Cog3 | -0.6968717 |
| Cog4 | -0.0812684 |
| Cog6 | 1.46884537 |
| Cog6 | -0.6769091 |
| Cog8 | -0.6752645 |
| Coil | 3.62503158 |
| Coil | -0.0468885 |
| Col11a1 | 2.82042307 |
| Col12a1 | 1.02547212 |
| Col14a1 | 0.59606205 |
| Col15a1 | 0.14249842 |
| Col16a1 | 0.50445679 |
| Col17a1 | 0.0789523 |
| Col17a1 | -0.7061643 |
| Col17a1 | 2.57239067 |
| Col18a1 | 1.88681231 |
| Col18a1 | -0.1353088 |
| Col1a1 | -0.6272135 |
| Col1a2 | 0.45372211 |
| Col24a1 | -0.3200596 |
| Col24a1 | -0.8449318 |
| Col25a1 | 1.48170218 |
| Col25a1 | 1.57831955 |
| Col25a1 | 2.0392572 |
| Col27a1 | -0.6186637 |
| Col28a1 | -0.0583522 |
| Col2a1 | 0.46187766 |
| Col2a1 | -0.2040313 |
| Col3a1 | 0.88122784 |
| Col3a1 | 0.41470172 |
| Col4a1 | -0.2727799 |
| Col4a2 | 0.64594524 |
| Col4a3 | -0.613263 |
| Col4a3bp | 1.36879265 |
| Col4a3bp | -0.5949775 |
| Col4a3bp | -0.0145329 |
| Col4a4 | 2.33047766 |
| Col4a4 | 0.52401041 |
| Col4a5 | -0.5711356 |
| Col4a6 | 1.60498437 |
| Col4a6 | 0.77338338 |
| Col5a1 | -0.5964878 |
| Col5a3 | 0.90736459 |
| Col6a1 | -0.3769938 |
| Col6a1 | 1.10861206 |
| Col6a1 | 0.67661774 |
| Col6a2 | -0.6389987 |
| Col7a1 | -0.2349419 |
| Col8a2 | -0.6421665 |
| Col9a2 | 0.18831981 |
| Colec12 | 0.66001105 |
| Commd1 | 0.39668081 |
| Commd1 | -0.007626 |
| Commd10 | -0.140421 |
| Commd2 | -0.6002742 |
| Commd2 | -0.4807045 |
| Commd4 | 0.06930125 |
| Commd5 | -0.1483765 |
| Commd6 | -0.5822365 |
| Commd8 | -0.5308096 |
| Commd9 | -0.5393069 |
| Comp | -0.3775929 |
| Comtd1 | -0.2140547 |
| Copa | -0.5400185 |
| Copb2 | -0.382399 |
| Cope | -0.1625815 |
| Cope | -0.6389987 |
| Cope | -0.5807488 |
| Cope | 1.58671957 |
| Copg | 0.31175513 |
| Copg | -0.5814923 |
| Copg | -0.5867137 |
| Copg | -0.5755603 |
| Cops2 | -0.5336335 |
| Cops3 | -0.9400551 |
| Cops4 | -0.0742915 |
| Cops4 | 0.70347143 |
| Cops5 | -0.7711775 |
| Cops5 | 0.44424267 |
| Cops6 | -0.4452813 |
| Cops7a | -0.510585 |
| Cops7a | -0.6533381 |
| Cops7a | -0.4031026 |
| Cops7b | -0.8370962 |
| Cops7b | 4.04766359 |
| Cops8 | 2.10945587 |
| Copz1 | -0.4628266 |
| Copz2 | -0.5659995 |
| Copz2 | -0.5385959 |
| Copz2 | 2.29970992 |
| Copz2 | 0.97685733 |
| Coq10b | -0.7276308 |
| Coq10b | 1.55312544 |
| Coq2 | 0.20462156 |
| Coq3 | -0.855813 |
| Coq3 | -0.6397897 |
| Coq4 | 0.09033173 |
| Coq4 | -0.6469381 |
| Coq5 | 1.59837006 |
| Coq5 | -0.7812359 |
| Coq5 | 2.7857795 |
| Coq6 | 1.18295356 |
| Coq6 | 1.1762854 |
| Coq7 | -0.6605904 |
| Coq9 | -0.1483765 |
| Corin | 0.24418432 |
| Coro1a | 1.79461562 |
| Coro1a | -0.4018721 |
| Coro1a | 0.09033173 |
| Coro1a | 2.83938855 |
| Coro1b | -0.5428702 |
| Coro1b | -0.7495972 |
| Coro1c | 1.70304083 |
| Coro2a | 1.56404913 |
| Coro2a | -0.7258954 |
| Coro2b | 1.19162039 |
| Coro2b | 2.69069425 |
| Coro6 | -0.7904739 |
| Coro7 | 0.40220197 |
| Cort | -0.5536415 |
| Cort | -0.7346045 |
| Cotl1 | -0.2578842 |
| Cova1 | -0.7276308 |
| Cox10 | -0.2406254 |
| Cox11 | 2.46970938 |
| Cox15 | -0.5844724 |
| Cox17 | -0.555087 |
| Cox18 | -0.4055685 |
| Cox18 | -0.4024872 |
| Cox19 | 1.23877895 |
| Cox4i1 | 1.2579118 |
| Cox4nb | -0.6662699 |
| Cox4nb | -0.0243708 |
| Cox5a | 0.98621845 |
| Cox5b | 2.44520941 |
| Cox6a1 | -0.2873699 |
| Cox6a1 | 0.13313057 |
| Cox6a2 | -0.3920853 |
| Cox6b1 | -0.3016361 |
| Cox6b1 | 1.14484653 |
| Cox6b2 | -0.307181 |
| Cox6b2 | -0.737233 |
| Cox6c | 1.13030132 |
| Cox7a1 | 0.63043247 |
| Cox7a2 | -0.4217558 |
| Cox7a2l | 0.28393885 |
| Cox7b | -0.6893361 |
| Cox7b2 | -0.5972439 |
| Cox8b | -0.3734075 |
| Cox8c | 1.25674717 |
| Cp | -0.6248741 |
| Cpa1 | -0.2873699 |
| Cpa2 | -0.5957324 |
| Cpa2 | -0.7398689 |
| Cpa3 | 0.31002237 |
| Cpa3 | -0.7469341 |
| Cpa3 | -0.5050813 |
| Cpa4 | 1.24983572 |
| Cpa5 | -0.4641393 |
| Cpa6 | -0.2733163 |
| Cpb2 | -0.5030257 |
| Cpeb1 | 1.71972343 |
| Cpeb2 | -0.3568494 |
| Cpeb3 | -0.4687483 |
| Cpeb4 | 0.25680766 |
| Cphx | -0.5230871 |
| Cplx1 | 0.12402088 |
| Cplx2 | 0.33032539 |
| Cpn1 | -0.7232982 |
| Cpne2 | -0.6565545 |
| Cpne4 | 2.57488198 |
| Cpne5 | -0.6711654 |
| Cpne6 | 0.78847203 |
| Cpne7 | 2.40640948 |
| Cpne8 | 3.54881494 |
| Cpne8 | -0.1951004 |
| Cpne9 | -0.6240955 |
| Cpsf1 | 1.18729693 |
| Cpsf1 | -0.5964878 |
| Cpsf2 | 0.28245187 |
| Cpsf3l | 0.4847294 |
| Cpsf4 | 1.23444987 |
| Cpsf4l | 1.32842969 |
| Cpsf4l | -0.2526209 |
| Cpsf6 | -0.0962681 |
| Cpsf6 | -0.4727169 |
| Cpt1b | 2.18041688 |
| Cpt1b | 0.28868152 |
| Cpt1c | -0.1367003 |
| Cpt2 | -0.5182055 |
| Cpxcr1 | -0.6194378 |
| Cpxm1 | 0.83101153 |
| Cpxm2 | 0.06818129 |
| Cpz | -0.5601638 |
| Crabp2 | -0.5972439 |
| Crat | 0.95113746 |
| Crat | -0.0280866 |
| Crat | -0.3044045 |
| Crb1 | -0.7095663 |
| Crbn | -0.4727169 |
| Crbn | 0.87505538 |
| Crbn | 3.77469133 |
| Crcp | 0.19552732 |
| Creb1 | -0.6366299 |
| Creb1 | -0.7363561 |
| Creb3 | 1.89492517 |
| Creb3l1 | 0.54744648 |
| Creb3l2 | -0.3854153 |
| Creb3l2 | 3.23238032 |
| Creb3l3 | -0.6646437 |
| Creb3l4 | 1.57310704 |
| Creb5 | 1.17789927 |
| Crebl1 | -0.0268464 |
| Crebl2 | 1.03033327 |
| Creg2 | 2.58002729 |
| Creld1 | -0.4401447 |
| Creld2 | 0.04626309 |
| Creld2 | 1.30143813 |
| Crem | -0.6968717 |
| Crem | 0.77709015 |
| Crem | 1.05005888 |
| Crem | 1.20357688 |
| Crhbp | -0.3627297 |
| Crhr1 | -0.0699569 |
| Crim1 | 0.5779604 |
| Crim2 | -0.3550925 |
| Crip2 | -0.3603732 |
| Crip3 | 1.52723985 |
| Crip3 | 0.67190274 |
| Cript | -0.7363561 |
| Cript | -0.7328563 |
| Crisp2 | -0.4608609 |
| Crispld2 | -0.127922 |
| Crk | -0.0613471 |
| Crk | 2.64379957 |
| Crk | -0.7354799 |
| Crkl | 1.29362589 |
| Crkrs | 1.57409131 |
| Crkrs | -0.7693601 |
| Crlf3 | 1.35275845 |
| Crlf3 | -0.5572594 |
| Crls1 | 0.25925118 |
| Crls1 | -0.2841081 |
| Crls1 | -0.8035611 |
| Crls1 | -0.7250289 |
| Crmp1 | 1.54050672 |
| Crmp1 | 0.87901673 |
| Crnkl1 | -0.2349419 |
| Crocc | 0.94038321 |
| Crry | 2.56973478 |
| Crry | 0.83813878 |
| Crtac1 | 1.25770016 |
| Crtam | 1.56984404 |
| Crtap | 0.01635484 |
| Crtap | 0.83929395 |
| Crtc1 | -0.4934915 |
| Crtc3 | 0.16871418 |
| Crxos1 | -0.5030257 |
| Cry2 | -0.1601991 |
| Cryab | 0.61549701 |
| Cryba2 | -0.6943531 |
| Cryba4 | 0.23952348 |
| Crybb1 | -0.7969948 |
| Crybb2 | 0.6925518 |
| Crybb3 | 0.34352894 |
| Crygn | -0.6287764 |
| Crygn | -0.5740831 |
| Cryl1 | 0.3898 |
| Crym | 1.4163064 |
| Cryz | 0.43238476 |
| Cryz | 2.52660434 |
| Cryz | 0.34352894 |
| Cryzl1 | -0.8035611 |
| Cryzl1 | -0.1357724 |
| Cs | -0.4098994 |
| Csad | -0.6533381 |
| Csda | -0.5972439 |
| Csda | 1.18569907 |
| Csda | -0.2110365 |
| Csdc2 | -0.7319833 |
| Csde1 | 2.30725301 |
| Csde1 | 0.03084475 |
| Csde1 | 0.53203962 |
| Cse1l | -0.7720875 |
| Csf1 | 2.59869943 |
| Csf1r | 0.78742713 |
| Csf1r | -0.5168153 |
| Csf2ra | 0.04127972 |
| Csgalnact1 | -0.7354799 |
| Csgalnact2 | -0.5098953 |
| Csk | 3.262696 |
| Csl | 0.26745161 |
| Csl | -0.4840527 |
| Csmd1 | 0.32008552 |
| Csnk1d | -0.2911896 |
| Csnk1d | 1.50884993 |
| Csnk1d | 0.83632065 |
| Csnk1d | 1.26065888 |
| Csnk1d | -0.0790826 |
| Csnk1e | 1.50486797 |
| Csnk1e | 0.14766286 |
| Csnk1g1 | 3.85471685 |
| Csnk1g1 | -0.5667315 |
| Csnk1g1 | -0.4117615 |
| Csnk1g2 | -0.8120712 |
| Csnk1g2 | 0.40220197 |
| Csnk1g2 | 2.28835192 |
| Csnk1g3 | -0.4948476 |
| Csnk2a1 | 0.21554798 |
| Csnk2a2 | 2.09043315 |
| Csnk2a2 | 0.94038321 |
| Csnrp2 | -0.5237865 |
| Csnrp3 | 2.05470362 |
| Cspg4 | -0.6960314 |
| Cspg5 | 1.53279041 |
| Cspp1 | 0.54519648 |
| Csprs | 1.6149646 |
| Csrp1 | 1.26150252 |
| Csrp2 | 0.59840698 |
| Csrp2bp | 0.99548757 |
| Cst11 | 0.42907842 |
| Cst3 | 0.00244021 |
| Cst8 | 3.3867521 |
| Cst9 | 0.3294763 |
| Cstad | 2.39952721 |
| Cstb | -0.6334806 |
| Cstf1 | 4.17943537 |
| Cstf2 | 0.56216331 |
| Cstf3 | -0.6366299 |
| Cstf3 | -0.6311256 |
| Cstl1 | 0.38179772 |
| Ctage5 | 0.29133883 |
| Ctcf | -0.6893361 |
| Ctdp1 | 1.74858795 |
| Ctdp1 | 0.39588943 |
| Ctdp1 | 0.35296653 |
| Ctdsp1 | 1.31198797 |
| Ctdsp1 | 1.90648645 |
| Ctdsp1 | -0.5421565 |
| Ctdsp2 | -0.6968717 |
| Ctdspl | -0.7442784 |
| Ctdspl2 | -0.5009745 |
| Ctdspl2 | 0.5180013 |
| Ctf1 | -0.2950249 |
| Ctgf | -0.3377005 |
| Ctla4 | -0.4807045 |
| Ctnna1 | -0.3994159 |
| Ctnna3 | 0.25680766 |
| Ctnnal1 | -0.6785566 |
| Ctnnb1 | -0.7121258 |
| Ctnnb1 | -0.4989278 |
| Ctnnb1 | 0.68306263 |
| Ctnnbip1 | 2.02425816 |
| Ctnnbl1 | 0.84127095 |
| Ctnnbl1 | -0.1906665 |
| Ctnnd2 | -0.5182055 |
| Ctnnd2 | -0.3228827 |
| Ctnnd2 | -0.5748214 |
| Ctns | 0.93085289 |
| Ctns | -0.0953792 |
| Ctps | -0.6194378 |
| Ctps | -0.0686604 |
| Ctps | 1.35419268 |
| Ctps2 | -0.6033144 |
| Ctps2 | 1.70006597 |
| Ctps2 | 0.73879985 |
| Ctr9 | -0.5078292 |
| Ctrb1 | -0.5529196 |
| Ctrl | 0.10085412 |
| Ctsa | -0.0096522 |
| Ctsa | -0.2884597 |
| Ctsa | -0.424271 |
| Ctsa | -0.7027746 |
| Ctsb | 0.83846897 |
| Ctsc | 1.48644751 |
| Ctsc | 2.43687905 |
| Ctsc | -0.6366299 |
| Ctse | -0.6033144 |
| Ctsf | -0.7812359 |
| Ctsh | 0.82817885 |
| Ctsk | -0.6382084 |
| Ctsl | -0.2573566 |
| Ctso | -0.3842076 |
| Ctsr | -0.294476 |
| Ctss | -0.7469341 |
| Ctsw | 0.23264566 |
| Ctsz | 0.81185115 |
| Ctsz | -0.4820423 |
| Cttn | -0.5565347 |
| Cttn | 2.18735436 |
| Cttnbp2nl | -0.510585 |
| Ctxn1 | 0.94230735 |
| Cuedc1 | 0.45198304 |
| Cuedc2 | 0.77338338 |
| Cugbp1 | 2.09060822 |
| Cugbp1 | -0.5493182 |
| Cugbp1 | 1.79772513 |
| Cugbp1 | -0.7558406 |
| Cugbp2 | 1.41568002 |
| Cugbp2 | 0.150748 |
| Cul1 | 2.542973 |
| Cul2 | -0.4661118 |
| Cul2 | -0.1630587 |
| Cul3 | -0.0173917 |
| Cul4a | 0.52101064 |
| Cul4a | 0.63043247 |
| Cul4a | -0.5308096 |
| Cul4b | 0.01398401 |
| Cul5 | 0.9880514 |
| Cul7 | 0.0277306 |
| Cuta | 0.72586903 |
| Cuta | -0.4098994 |
| Cuta | -0.6638316 |
| Cutc | -0.2292925 |
| Cutc | 0.00324089 |
| Cux1 | -0.7979301 |
| Cux1 | -0.5085174 |
| Cux1 | 0.05955043 |
| Cux1 | 1.78691749 |
| Cux1 | 1.60622855 |
| Cux2 | 4.11992703 |
| Cux2 | 0.29663109 |
| Cuzd1 | -0.2578842 |
| Cwf19l2 | 0.68520118 |
| Cx3cl1 | -0.8111218 |
| Cx3cr1 | 2.47000213 |
| Cxadr | -0.4833821 |
| Cxadr | 0.80691427 |
| Cxadr | 1.07633272 |
| Cxcl1 | 0.57991656 |
| Cxcl12 | -0.6202125 |
| Cxcl12 | -0.7657359 |
| Cxcl12 | 1.9538468 |
| Cxcl13 | -0.3044045 |
| Cxcl14 | -0.4510935 |
| Cxcl14 | -0.3094082 |
| Cxcl15 | 0.92605137 |
| Cxcl16 | -0.0878566 |
| Cxcl17 | -0.1270027 |
| Cxcl4 | -0.2427006 |
| Cxcl9 | -0.5814923 |
| Cxcr3 | -0.8871715 |
| Cxcr4 | 0.21394907 |
| Cxcr5 | -0.2776182 |
| Cxcr7 | -0.1549789 |
| Cxx1a | 0.09868668 |
| Cxx1b | -0.7821556 |
| Cxx1c | -0.2626465 |
| Cxxc1 | -0.7720875 |
| Cxxc1 | -0.1002789 |
| Cxxc4 | -0.05324 |
| Cxxc4 | 3.04426865 |
| Cyb5 | 1.40514844 |
| Cyb561 | -0.2647709 |
| Cyb561d1 | 1.49174731 |
| Cyb561d1 | 2.04184155 |
| Cyb561d2 | 0.97813933 |
| Cyb5b | 0.96495157 |
| Cyb5d1 | 2.40559503 |
| Cyb5d2 | 1.24093606 |
| Cyb5d2 | -0.7830763 |
| Cyb5r1 | -0.5829812 |
| Cyb5r2 | 1.14209895 |
| Cyb5r3 | -0.7138361 |
| Cyb5r3 | 0.55817235 |
| Cyb5r3 | -0.5852189 |
| Cyb5r4 | 0.06593737 |
| Cyba | 0.88861317 |
| Cybasc3 | -0.3951328 |
| Cyc1 | -0.4000293 |
| Cycs | -0.6533381 |
| Cycs | -0.7513768 |
| Cyfip1 | 0.83466476 |
| Cyfip2 | 1.87136858 |
| Cyfip2 | 0.44198352 |
| Cygb | -0.6264331 |
| Cyhr1 | 1.15126609 |
| Cyhr1 | 3.29719583 |
| Cyhr1 | -0.3354057 |
| Cyhr1 | -0.6010334 |
| Cyld | -0.7078638 |
| Cyln2 | 4.12737471 |
| Cyp11a1 | -0.3698348 |
| Cyp11b2 | 0.06556286 |
| Cyp17a1 | -0.7648321 |
| Cyp19a1 | 0.23264566 |
| Cyp1b1 | -0.4867399 |
| Cyp20a1 | -0.4928141 |
| Cyp24a1 | -0.63191 |
| Cyp26a1 | 1.30577282 |
| Cyp26b1 | -0.7621256 |
| Cyp27a1 | 0.33145632 |
| Cyp27a1 | -0.0936037 |
| Cyp27a1 | -0.5168153 |
| Cyp2a12 | -0.1034102 |
| Cyp2a5 | 1.65954671 |
| Cyp2a5 | -0.5867137 |
| Cyp2b10 | -0.170727 |
| Cyp2c55 | 3.78938776 |
| Cyp2d10 | 0.73696341 |
| Cyp2d22 | -0.166406 |
| Cyp2d26 | 0.00163885 |
| Cyp2d26 | -0.106552 |
| Cyp2d26 | 1.33078327 |
| Cyp2e1 | 2.66543506 |
| Cyp2f2 | -0.513349 |
| Cyp2j11 | 1.97895758 |
| Cyp2j9 | -0.3533388 |
| Cyp2s1 | -0.7793991 |
| Cyp2u1 | -0.5182055 |
| Cyp39a1 | 0.10875905 |
| Cyp3a13 | 0.13903969 |
| Cyp3a13 | -0.6622096 |
| Cyp46a1 | -0.8130215 |
| Cyp4b1 | 1.20054571 |
| Cyp4f13 | 0.96119093 |
| Cyp4f14 | 1.42130253 |
| Cyp4f15 | 0.95216227 |
| Cyp4f16 | -0.3077373 |
| Cyp4f16 | -0.63191 |
| Cyp4f16 | 1.91374653 |
| Cyp4f18 | 2.58270635 |
| Cyp4f39 | 0.38554057 |
| Cyp4v3 | 3.07535612 |
| Cyp51 | -0.2803169 |
| Cyp7b1 | -0.7232982 |
| Cypt10 | 0.25374434 |
| Cypt2 | -0.6429601 |
| Cypt3 | 1.07376375 |
| Cypt6 | -0.8245007 |
| Cypt8 | -0.1205926 |
| Cypt9 | 1.16350791 |
| Cyr61 | -0.2287806 |
| Cyr61 | -0.5421565 |
| Cysltr1 | 0.04319952 |
| Cytl1 | 0.95959536 |
| D030011O10Rik | 0.12788572 |
| D030013I16Rik | 1.79329697 |
| D030018L15Rik | 2.45194644 |
| D030028O16Rik | -0.4641393 |
| D030051N19Rik | -0.6225404 |
| D030056L22Rik | -0.7250289 |
| D030070L09Rik | -0.6413735 |
| D030070L09Rik | -0.2526209 |
| D030070L09Rik | -0.6202125 |
| D030070L09Rik | 0.22571731 |
| D030074E01Rik | 2.29799033 |
| D0H4S114 | -0.0686604 |
| D0H8S2298E | -0.0112763 |
| D0HXS9928E | 0.70784204 |
| D0HXS9928E | -0.4536883 |
| D10627 | -0.6968717 |
| D10Bwg1364e | -0.6573603 |
| D10Bwg1379e | -0.7002402 |
| D10Ertd322e | 1.8516732 |
| D10Ertd610e | 0.48136084 |
| D10Ertd610e | -0.6437543 |
| D10Ertd610e | 0.54474584 |
| D10Ertd641e | -0.5023415 |
| D10Jhu81e | 3.32341058 |
| D10Wsu102e | 0.9679781 |
| D10Wsu52e | -0.6382084 |
| D11Bwg0517e | 2.19510341 |
| D11Wsu47e | 0.10085412 |
| D11Wsu47e | 1.00438965 |
| D12Ertd551e | -0.6194378 |
| D12Ertd551e | -0.392694 |
| D12Ertd553e | -0.4452813 |
| D12Ertd647e | 0.04127972 |
| D12Ertd647e | -0.4123831 |
| D130040H23Rik | 1.22835559 |
| D130043K22Rik | -0.7720875 |
| D130059P03Rik | 1.08945322 |
| D13Ertd608e | -0.8073339 |
| D14Ertd171e | 1.06963879 |
| D14Ertd231e | -0.5371754 |
| D14Ertd449e | -0.7904739 |
| D14Ertd500e | 0.19617982 |
| D14Ertd668e | -0.8508514 |
| D14Ertd668e | -0.6461412 |
| D15Ertd621e | -0.5098953 |
| D15Ertd682e | -0.5393069 |
| D15Mgi27 | -0.300531 |
| D15Wsu169e | 1.07620444 |
| D15Wsu75e | 0.89547508 |
| D16Ertd472e | -0.391477 |
| D16H22S680E | 1.61118891 |
| D17H6S56E-5 | 1.38298474 |
| D17Wsu104e | 1.35495671 |
| D17Wsu92e | -0.0452019 |
| D19Bwg1357e | -0.2685 |
| D19Ertd721e | -0.6140326 |
| D19Ertd737e | -0.2303171 |
| D19Wsu162e | 0.1840348 |
| D1Bwg0212e | 1.19286823 |
| D1Bwg0212e | 1.6504484 |
| D1Pas1 | -0.3878353 |
| D230007K08Rik | 0.36830785 |
| D230014K01Rik | -0.7078638 |
| D2Ertd750e | 2.10975611 |
| D2Ertd750e | 0.77356022 |
| D330014H01Rik | -0.4459253 |
| D330017J20Rik | -0.5912122 |
| D330028D13Rik | -0.5329268 |
| D330045A20Rik | -0.1380938 |
| D330045A20Rik | 1.6982102 |
| D330045A20Rik | 3.12653777 |
| D3Bwg0562e | -0.5078292 |
| D3Ertd300e | -0.8101734 |
| D3Ertd751e | -0.0186196 |
| D3Ucla1 | 0.11411104 |
| D430015B01Rik | -0.5343408 |
| D430028G21Rik | -0.7053157 |
| D430039N05Rik | -0.5336335 |
| D430041B17Rik | -0.4800362 |
| D430042O09Rik | -0.5272915 |
| D4Bwg0951e | -0.7053157 |
| D4Bwg1540e | 0.95084445 |
| D4Ertd22e | -0.4299548 |
| D4Ertd22e | -0.6256532 |
| D4Wsu114e | -0.4305884 |
| D4Wsu132e | 3.22580978 |
| D4Wsu132e | 0.05463696 |
| D530004J12Rik | -0.7078638 |
| D530033C11Rik | 1.58477622 |
| D5Ertd135e | 0.57206745 |
| D5Ertd579e | -0.3094082 |
| D5Wsu178e | -0.4753719 |
| D630003M21Rik | -0.7027746 |
| D630003M21Rik | 1.04794616 |
| D630014A15Rik | -0.482712 |
| D630023F18Rik | 0.2854235 |
| D630042P16Rik | -0.4874129 |
| D630048P19Rik | -0.2160723 |
| D6Ertd474e | -0.3568494 |
| D6Mm5e | 1.80415076 |
| D6Wsu116e | 0.25864089 |
| D6Wsu163e | -0.5659995 |
| D6Wsu163e | -0.6573603 |
| D6Wsu163e | -0.0647816 |
| D6Wsu176e | 1.87627003 |
| D6Wsu176e | 0.20977918 |
| D730039F16Rik | -0.0088412 |
| D730039F16Rik | 0.57294281 |
| D7Bwg0611e | -0.7061643 |
| D830014E11Rik | -0.7104187 |
| D830030K20Rik | 1.12702485 |
| D830030K20Rik | -0.2160723 |
| D830050J10Rik | 0.93204946 |
| D8Ertd738e | 2.58801591 |
| D8Ertd82e | 1.4070474 |
| D930001I22Rik | -0.5777804 |
| D930005D10Rik | 0.19160278 |
| D930014E17Rik | 1.25122207 |
| D930015E06Rik | -0.4446377 |
| D930020B18Rik | 2.60404694 |
| D930028F11Rik | -0.4549883 |
| D930049A15Rik | 1.268957 |
| D9Ertd280e | 0.7546278 |
| D9Ertd392e | 1.23401587 |
| D9Ertd402e | -0.3866245 |
| D9Wsu20e | 1.01649442 |
| Daam1 | -0.3836043 |
| Daam2 | 1.10799207 |
| Dab1 | -0.5565347 |
| Dab1 | -0.0264333 |
| Dab1 | 0.78916799 |
| Dab2 | -0.5645372 |
| Dab2 | 0.62361639 |
| Dab2ip | 0.71802458 |
| Dact1 | 1.67067803 |
| Dact2 | -0.2100326 |
| Dad1 | 1.56044753 |
| Daf2 | 1.24437721 |
| Dag1 | 1.35055506 |
| Dag1 | -0.7311112 |
| Dagla | -0.0971579 |
| Daglb | 0.41029365 |
| Dak | -0.525888 |
| Dalrd3 | -0.6056009 |
| Dap | -0.7425121 |
| Dap3 | -0.6217638 |
| Dap3 | 0.19356709 |
| Dap3 | 0.11197388 |
| Dap3 | -0.4230125 |
| Dapk1 | -0.6835165 |
| Dapk1 | 2.02683705 |
| Dapk2 | 0.31146655 |
| Dapk2 | -0.5092061 |
| Dapl1 | 2.25177491 |
| Dapp1 | -0.432492 |
| Dapp1 | -0.5478815 |
| Darc | -0.7181253 |
| Dars | 1.2756264 |
| Dars | -0.6033144 |
| Daxx | -0.5521982 |
| Dazap1 | 2.07101886 |
| Dazap1 | -0.4433518 |
| Dazap2 | 0.33737156 |
| Dazl | -0.7451628 |
| Dbc1 | 1.64119099 |
| Dbf4 | -0.1205926 |
| Dbi | -0.4853953 |
| Dbi | -0.7711775 |
| Dbil5 | 0.33849408 |
| Dbn1 | 0.84570412 |
| Dbndd1 | -0.2939275 |
| Dbndd2 | 0.56855788 |
| Dbndd2 | 0.136611 |
| Dbndd2 | -0.4223839 |
| Dbnl | -0.1215057 |
| Dbp | -0.7121258 |
| Dbpht2 | -0.5733453 |
| Dbr1 | 0.87219287 |
| Dbt | 0.07747394 |
| Dcakd | -0.746048 |
| Dcbld1 | 1.24972899 |
| Dcbld2 | 0.95886921 |
| Dci | 0.1690499 |
| Dck | 0.72176817 |
| Dclk1 | -0.0553667 |
| Dclk2 | 0.96596149 |
| Dclk3 | -0.4707305 |
| Dclre1b | 1.76714608 |
| Dclre1b | -0.6233176 |
| Dclre1c | -0.6711654 |
| Dclre1c | 0.87996499 |
| Dclre1c | 0.06256144 |
| Dcn | -0.3200596 |
| Dcn | -0.6613997 |
| Dcp1a | 1.08348883 |
| Dcp1a | 0.77920178 |
| Dcp1a | 1.45829535 |
| Dcp1a | -0.5500374 |
| Dcp1b | 0.24882224 |
| Dcp1b | 0.74099887 |
| Dcpp1 | -0.4955264 |
| Dcpp2 | -0.5393069 |
| Dcpp2 | 1.07144568 |
| Dcpp3 | -0.2994272 |
| Dcps | -0.6541412 |
| Dcps | -0.7675463 |
| Dct | 0.43669102 |
| Dctd | 2.15827511 |
| Dctn1 | -0.2030348 |
| Dctn2 | 0.63474446 |
| Dctn2 | 1.57809351 |
| Dctn2 | 1.28327957 |
| Dctn3 | -0.5521982 |
| Dctn3 | -0.7232982 |
| Dctn4 | -0.0808309 |
| Dctn4 | -0.170727 |
| Dctn5 | 0.12402088 |
| Dctn6 | 1.25970887 |
| Dcun1d2 | 0.52975252 |
| Dcun1d3 | -0.787693 |
| Dcun1d4 | -0.074726 |
| Dcun1d4 | 2.57558884 |
| Dcun1d5 | 1.9190108 |
| Dcx | 1.19456718 |
| Dcx | 0.61361334 |
| Dcxr | 1.83695633 |
| Dcxr | 0.3209431 |
| Ddah1 | -0.6573603 |
| Ddah2 | -0.536466 |
| Ddb1 | 1.07915053 |
| Ddb2 | -0.7363561 |
| Ddb2 | 0.07376786 |
| Ddb2 | -0.5608913 |
| Ddc | -0.7258954 |
| Ddc8 | 0.81456392 |
| Ddef2 | -0.5407306 |
| Ddef2 | -0.3348328 |
| Ddefl1 | -0.1530878 |
| Ddhd1 | 0.63043247 |
| Ddhd1 | -0.7328563 |
| Ddi2 | -0.5209919 |
| Ddit3 | -0.1789435 |
| Ddit3 | 0.85027958 |
| Ddit4 | 1.40106632 |
| Ddit4l | -0.6638316 |
| Ddo | 1.58985039 |
| Ddo | 0.2800678 |
| Ddo | 2.50081227 |
| Ddost | -0.0380676 |
| Ddr1 | -0.2868255 |
| Ddr2 | 1.31855941 |
| Ddt | 1.67958419 |
| Ddx1 | 0.6221642 |
| Ddx1 | -0.5078292 |
| Ddx17 | 0.72884032 |
| Ddx17 | -0.6171175 |
| Ddx17 | 0.33117371 |
| Ddx19a | -0.406186 |
| Ddx19b | -0.7311112 |
| Ddx19b | 0.12296408 |
| Ddx20 | -0.6366299 |
| Ddx21 | -0.6033144 |
| Ddx24 | -0.3434623 |
| Ddx24 | 2.23753926 |
| Ddx24 | 0.51428427 |
| Ddx25 | -0.4148734 |
| Ddx26b | -0.6469381 |
| Ddx27 | -0.4117615 |
| Ddx28 | -0.1774883 |
| Ddx3x | 0.63781243 |
| Ddx3y | -0.7720875 |
| Ddx4 | -0.5995157 |
| Ddx4 | -0.1038584 |
| Ddx41 | 0.96869727 |
| Ddx46 | 0.89904222 |
| Ddx47 | -0.3799929 |
| Ddx47 | 0.23421321 |
| Ddx47 | -0.6736227 |
| Ddx49 | 2.52525268 |
| Ddx5 | -0.4491521 |
| Ddx50 | -0.4687483 |
| Ddx50 | 3.11790356 |
| Ddx51 | 1.21958059 |
| Ddx52 | 0.87012009 |
| Ddx54 | 1.64252911 |
| Ddx58 | -0.5457305 |
| Ddx6 | 1.11515906 |
| Ddx6 | 0.87107733 |
| Ddx6 | 0.55683826 |
| Ddx6 | -0.4427095 |
| Deadc1 | -0.7693601 |
| Deb1 | 0.76521251 |
| Decr1 | 1.86821602 |
| Decr2 | 1.7263791 |
| Decr2 | 0.66577742 |
| Dedd | 1.09538014 |
| Dedd | -0.6687145 |
| Dedd2 | -0.3836043 |
| Def6 | 1.57386427 |
| Def8 | 1.59932833 |
| Defb11 | 0.65400851 |
| Defb14 | 0.15109017 |
| Defb15 | 0.49452583 |
| Defb19 | -0.0830208 |
| Defb20 | 0.1846953 |
| Defb21 | -0.2030348 |
| Defb25 | -0.6272135 |
| Defb26 | 0.40481978 |
| Defb30 | -0.5572594 |
| Defb36 | 0.40977369 |
| Defb36 | -0.1559259 |
| Defb4 | -0.482712 |
| Defcr20 | 2.49939474 |
| Defcr6 | -0.7258954 |
| Defcr-rs7 | 1.55536664 |
| Degs1 | 2.06864773 |
| Degs2 | -0.5919641 |
| Dek | -0.4031026 |
| Dek | 0.12472476 |
| Dennd1a | -0.7407492 |
| Dennd1c | 0.84028297 |
| Dennd2a | -0.7495972 |
| Dennd3 | -0.3049591 |
| Denr | -0.7311112 |
| Denr | 1.81029731 |
| Depdc1b | -0.0351455 |
| Depdc5 | 2.36201446 |
| Depdc5 | -0.6405813 |
| Depdc6 | -0.0622046 |
| Depdc6 | 0.94067949 |
| Dera | 0.03819989 |
| Derl2 | 1.30567224 |
| Derl3 | 0.67935732 |
| Des | 0.60793964 |
| Det1 | 0.57512765 |
| Dexi | -0.0464666 |
| Dexi | 1.16514378 |
| Dffa | 1.7856485 |
| Dfna5h | 0.0277306 |
| Dgat1 | 1.03288881 |
| Dgat2 | -0.5733453 |
| Dgcr2 | 0.57861291 |
| Dgcr2 | -0.6311256 |
| Dgcr6 | 0.58985185 |
| Dgcr6 | 2.57076121 |
| Dgcr8 | 1.33400988 |
| Dgka | -0.2717081 |
| Dgka | 0.33315018 |
| Dgkb | -0.5874619 |
| Dgke | -0.4634827 |
| Dgkg | -0.5493182 |
| Dgkh | -0.554364 |
| Dgki | 0.59178353 |
| Dgkk | 1.92919237 |
| Dgkq | 0.10193598 |
| Dgkz | 2.67786748 |
| Dgkz | 0.7594864 |
| Dgkz | 2.43575668 |
| Dguok | 2.14341291 |
| Dhcr24 | 1.73106796 |
| Dhcr24 | -0.1002789 |
| Dhcr24 | 0.27648036 |
| Dhcr7 | 0.41780094 |
| Dhdds | -0.632695 |
| Dhdds | 1.22627891 |
| Dhdh | 3.6569985 |
| Dhdh | 0.56216331 |
| Dhdh | 2.42841328 |
| Dhh | 0.2528234 |
| Dhodh | -0.613263 |
| Dhps | 1.58027623 |
| Dhrs1 | 0.5147497 |
| Dhrs4 | 1.10600535 |
| Dhrs7 | -0.3194959 |
| Dhrs7 | 1.0855235 |
| Dhrs7 | -0.3586097 |
| Dhrs7b | -0.3257142 |
| Dhrs7b | -0.3854153 |
| Dhrs7b | 0.1846953 |
| Dhrsx | -0.4941693 |
| Dhrsx | -0.2879146 |
| Dhtkd1 | 1.83224866 |
| Dhx15 | 1.15658254 |
| Dhx15 | 1.43086382 |
| Dhx16 | -0.1882121 |
| Dhx29 | -0.1007256 |
| Dhx30 | 1.22244143 |
| Dhx32 | 0.20105927 |
| Dhx33 | 0.01950671 |
| Dhx36 | 0.71633511 |
| Dhx37 | -0.0639218 |
| Dhx38 | -0.5638069 |
| Dhx57 | -0.5009745 |
| Dhx58 | -0.5696653 |
| Dhx8 | -0.3633198 |
| Dhx9 | 1.62455345 |
| Diablo | -0.5704001 |
| Diap1 | -0.5414433 |
| Diap3 | 2.80121263 |
| Dicer1 | -0.5315148 |
| Dido1 | -0.4707305 |
| Dido1 | 1.2342329 |
| Dido1 | 0.54609712 |
| Dio1 | -0.2241858 |
| Dio1 | 2.12189961 |
| Dio3 | -0.5659995 |
| Dip2b | -0.1526156 |
| Dip2b | 0.96350694 |
| Diras1 | 0.90413683 |
| Diras2 | 0.72828391 |
| Dirc2 | 0.42294379 |
| Dis3 | 0.01121048 |
| Dis3l | 0.17139658 |
| Dis3l2 | 0.66478573 |
| Disp1 | 1.20804966 |
| Dixdc1 | 1.71790597 |
| Dkk3 | 0.21138516 |
| Dkk3 | -0.6860062 |
| Dkk3 | 0.12893703 |
| Dkkl1 | -0.6186637 |
| Dkkl1 | 1.53350169 |
| Dlat | 2.01016128 |
| Dlc1 | -0.2463433 |
| Dld | -0.6769091 |
| Dleu7 | -0.7002402 |
| Dlg1 | 0.83863402 |
| Dlg2 | 1.38307741 |
| Dlg3 | 1.56702728 |
| Dlg4 | -0.3457769 |
| Dlg7 | -0.2568292 |
| Dlgap1 | -0.0210801 |
| Dlgap2 | 0.92920512 |
| Dlgap2 | -0.6728029 |
| Dlgap2 | 0.20655898 |
| Dlgap3 | 0.78864607 |
| Dlgap4 | -0.7189855 |
| Dlgap4 | 0.52424077 |
| Dlgap4 | 0.51172029 |
| Dlgap4 | -0.4700693 |
| Dlk1 | -0.2727799 |
| Dlk2 | 0.14422304 |
| Dll1 | 0.14525631 |
| Dlst | -0.0318171 |
| Dlx1 | -0.4989278 |
| Dlx1as | 0.26563545 |
| Dlx2 | 0.04204811 |
| Dlx3 | 0.36585905 |
| Dmap1 | 2.17278641 |
| Dmbt1 | 0.3033505 |
| Dmkn | -0.056645 |
| Dmkn | 3.35605924 |
| Dmkn | -0.5726082 |
| Dmn | 1.50560061 |
| Dmn | -0.6533381 |
| Dmpk | 0.48760719 |
| Dmrt2 | 1.84420141 |
| Dmrt3 | -0.4720543 |
| Dmrta1 | 0.88154329 |
| Dmrta2 | 0.58403296 |
| Dmrtc1a | 0.96422953 |
| Dmrtc1a | 1.2581234 |
| Dmrtc1a | 0.24076863 |
| Dmrtc1b | -0.4465698 |
| Dmrtc2 | -0.6793814 |
| Dmtf1 | -0.6670841 |
| Dmtf1 | -0.7311112 |
| Dmtf1 | 2.35297849 |
| Dmwd | 0.20526781 |
| Dmwd | -0.7442784 |
| Dmwd | 1.15670035 |
| Dnahc11 | -0.2110365 |
| Dnahc2 | -0.5478815 |
| Dnahc5 | 2.37999179 |
| Dnahc8 | 0.76610409 |
| Dnaic1 | 4.13389181 |
| Dnaic1 | 0.94761533 |
| Dnaja1 | -0.0812684 |
| Dnaja2 | 2.09775831 |
| Dnaja2 | -0.5867137 |
| Dnaja3 | 0.03433591 |
| Dnajb1 | -0.511966 |
| Dnajb10 | -0.5867137 |
| Dnajb12 | 0.75101263 |
| Dnajb13 | 0.17039157 |
| Dnajb3 | 1.19422763 |
| Dnajb4 | -0.7061643 |
| Dnajb4 | 3.36955334 |
| Dnajb5 | 1.34401041 |
| Dnajb6 | -0.6148029 |
| Dnajb6 | 0.67798853 |
| Dnajb7 | 1.18111877 |
| Dnajb8 | 4.36722547 |
| Dnajb9 | -0.1265434 |
| Dnajc10 | 1.62419473 |
| Dnajc10 | 3.80860039 |
| Dnajc12 | 1.46867614 |
| Dnajc14 | -0.4575939 |
| Dnajc15 | -0.1192246 |
| Dnajc16 | 0.00603791 |
| Dnajc17 | -0.6670841 |
| Dnajc18 | 1.15975809 |
| Dnajc19 | -0.4687483 |
| Dnajc19 | -0.4588993 |
| Dnajc2 | 1.73541478 |
| Dnajc2 | -0.2458221 |
| Dnajc3a | 0.13903969 |
| Dnajc4 | -0.3077373 |
| Dnajc5 | -0.4588993 |
| Dnajc5b | 0.94452264 |
| Dnajc5g | 0.81710008 |
| Dnajc6 | 2.50086893 |
| Dnajc6 | -0.5493182 |
| Dnajc7 | -0.6960314 |
| Dnajc8 | -0.2180941 |
| Dnalc1 | 1.78480155 |
| Dnali1 | 1.74770705 |
| Dnase1l1 | 0.48952086 |
| Dnase1l2 | -0.7010842 |
| Dnase1l2 | 0.59627546 |
| Dnase2a | 3.58526456 |
| Dnd1 | -0.2313429 |
| Dner | -0.5140412 |
| Dnm | 0.80571873 |
| Dnm1 | 0.90136136 |
| Dnm1l | -0.5807488 |
| Dnm1l | 1.10314208 |
| Dnmbp | 0.91210928 |
| Dnmt1 | 1.49529146 |
| Dnmt3b | 1.82235521 |
| Dnmt3l | -0.7146924 |
| Dnmt3l | -0.6835165 |
| Dnttip1 | 0.92092284 |
| Doc2a | -0.6217638 |
| Doc2b | 0.90552151 |
| Doc2b | -0.7398689 |
| Dock1 | -0.7172659 |
| Dock3 | -0.5057675 |
| Dock4 | -0.6186637 |
| Dock6 | 1.00798225 |
| Dock8 | 1.03114103 |
| Dock9 | -0.6951919 |
| Dok1 | -0.4608609 |
| Dok1 | -0.3377005 |
| Dok2 | -0.7594269 |
| Dok3 | -0.4975658 |
| Dok4 | -0.5652681 |
| Dok5 | 2.3070095 |
| Dok5 | -0.6630203 |
| Dok7 | 0.93607671 |
| Dolk | 0.31549854 |
| Dolpp1 | -0.1779731 |
| Dolpp1 | -0.4700693 |
| Dolpp1 | 0.62320171 |
| Dom3z | 0.33793299 |
| Dom3z | -0.5315148 |
| Donson | -0.6605904 |
| Donson | -0.7398689 |
| Dos | -0.3805938 |
| Dos | -0.4667703 |
| Dot1l | 0.28096253 |
| Dpagt1 | -0.2813986 |
| Dpep1 | 0.6007461 |
| Dpep2 | 1.6402038 |
| Dpep2 | 3.15703325 |
| Dpep3 | 1.65906276 |
| Dpf1 | -0.311082 |
| Dpf1 | 0.99869643 |
| Dpf3 | 0.34520128 |
| Dph2 | -0.2125444 |
| Dph2 | 1.63169945 |
| Dph2 | -0.5216897 |
| Dph3 | 2.66071239 |
| Dph3 | 0.19683188 |
| Dph3 | -0.0562187 |
| Dph4 | 0.64695692 |
| Dpm1 | -0.6148029 |
| Dpm1 | 1.62376408 |
| Dpm2 | -0.3099658 |
| Dpm3 | -0.7164073 |
| Dpp10 | -0.6256532 |
| Dpp3 | 0.84881132 |
| Dpp3 | -0.4179955 |
| Dpp4 | -0.6148029 |
| Dpp6 | 0.73971673 |
| Dpp7 | -0.6769091 |
| Dpp8 | -0.2589405 |
| Dpp9 | -0.5536415 |
| Dpp9 | -0.0596346 |
| Dppa3 | 0.83516183 |
| Dppa4 | 0.8945423 |
| Dppa5 | 1.5127344 |
| Dppa5 | 2.83367315 |
| Dpt | 1.85150404 |
| Dpy30 | -0.5435845 |
| Dpyd | -0.7061643 |
| Dpysl2 | -0.2110365 |
| Dpysl2 | 0.25405112 |
| Dpysl2 | 1.52835258 |
| Dpysl3 | 0.28897715 |
| Dpysl4 | 0.98720586 |
| Dpysl5 | 1.39523003 |
| Drbp1 | -0.1726539 |
| Drctnnb1a | -0.4491521 |
| Drd1a | -0.6086583 |
| Drd2 | -0.4433518 |
| Drd4 | 0.52216553 |
| Drd5 | 0.85693956 |
| Drg1 | 1.84050025 |
| Drg1 | -0.682688 |
| Drg2 | -0.260527 |
| Drp2 | 2.56627883 |
| Dscam | -0.6389987 |
| Dscam | 1.59962299 |
| Dscc1 | 1.65323583 |
| Dscr1 | 0.47288643 |
| Dscr1l2 | -0.3854153 |
| Dscr2 | 3.08682513 |
| Dscr3 | -0.6622096 |
| Dscr3 | -0.4641393 |
| Dscr6 | 0.94422757 |
| Dsg2 | -0.2262252 |
| Dsg2 | -0.0096522 |
| Dsn1 | -0.7053157 |
| Dsp | -0.019439 |
| Dst | -0.2030348 |
| Dst | 1.38511375 |
| Dst | 1.13597778 |
| Dstn | 0.46015358 |
| Dtd1 | 0.55817235 |
| Dtl | 1.51103691 |
| Dtna | -0.5733453 |
| Dtna | -0.5182055 |
| Dtna | -0.6217638 |
| Dtna | 2.43869626 |
| Dtnb | 1.59755846 |
| Dtnbp1 | 0.11197388 |
| Dtwd1 | 1.72224906 |
| Dtx1 | -0.4647964 |
| Dtx2 | 1.61206156 |
| Dtx2 | -0.6217638 |
| Dtx2 | -0.4369485 |
| Dtx4 | 0.89345289 |
| Dtx4 | -0.6851756 |
| Dtymk | -0.2211351 |
| Dus1l | 1.79179626 |
| Dus1l | -0.6202125 |
| Dus2l | -0.1568738 |
| Dus3l | -0.1872322 |
| Dus3l | 1.82578048 |
| Dus3l | 0.56149932 |
| Dus4l | 0.7444702 |
| Dusp1 | 3.37797399 |
| Dusp11 | 2.23929781 |
| Dusp11 | 0.12823629 |
| Dusp13 | -0.5696653 |
| Dusp13 | 1.54605794 |
| Dusp13 | -0.3296928 |
| Dusp14 | -0.7886191 |
| Dusp15 | 0.01793209 |
| Dusp15 | 2.21774631 |
| Dusp16 | 0.67779282 |
| Dusp16 | 1.30868501 |
| Dusp16 | 0.6418876 |
| Dusp18 | -0.0958235 |
| Dusp19 | 1.27956416 |
| Dusp2 | 2.54042301 |
| Dusp2 | -0.2040313 |
| Dusp21 | -0.2868255 |
| Dusp22 | 0.42294379 |
| Dusp23 | 0.84685007 |
| Dusp26 | 0.98706487 |
| Dusp28 | 1.03731099 |
| Dusp3 | 1.32528243 |
| Dusp3 | -0.4236415 |
| Dusp3 | -0.5209919 |
| Dusp4 | -0.1688041 |
| Dusp4 | -0.3908691 |
| Dusp4 | -0.311082 |
| Dusp6 | 0.19323999 |
| Dusp6 | 1.46935287 |
| Dusp6 | -0.5435845 |
| Dusp7 | -0.0019757 |
| Dusp8 | 1.93457793 |
| Dut | 0.18766184 |
| Duxbl | 1.32478972 |
| Duxbl | -0.3331163 |
| Dvl1 | -0.106552 |
| Dvl1 | -0.5400185 |
| Dvl2 | -0.4111404 |
| DXBwg1396e | -0.359785 |
| Dydc2 | -0.3860197 |
| Dym | -0.5807488 |
| Dync1h1 | 1.05702364 |
| Dync1i1 | -0.6202125 |
| Dync1i1 | -0.1990593 |
| Dync1i2 | -0.7998033 |
| Dync1li1 | 0.32777583 |
| Dync1li2 | 2.52334604 |
| Dync2li1 | 0.25190156 |
| Dynll1 | 2.30603484 |
| Dynll2 | 0.95988567 |
| Dynlrb1 | -0.4217558 |
| Dynlrb2 | 0.22159923 |
| Dynlt3 | 1.74075688 |
| Dyrk1a | -0.613263 |
| Dyrk1b | 0.77779455 |
| Dyrk1b | -0.4375869 |
| Dyrk2 | 1.95566637 |
| Dyrk2 | -0.1493169 |
| Dyrk3 | -0.2339122 |
| Dyrk4 | 0.3091548 |
| Dysf | 0.82967956 |
| Dysfip1 | -0.5844724 |
| Dzip1 | 1.90232788 |
| E030010A14Rik | -0.3144386 |
| E030034P13Rik | -0.3319738 |
| E130009J12Rik | -0.5949775 |
| E130012A19Rik | -0.6334806 |
| E130014J05Rik | 2.03239284 |
| E130016E03Rik | 1.26371351 |
| E130203B14Rik | -0.6646437 |
| E130203B14Rik | -0.5852189 |
| E130303B06Rik | -0.8111218 |
| E130306D19Rik | 0.96422953 |
| E130308A19Rik | -0.3133184 |
| E130309D02Rik | -0.63191 |
| E130309D02Rik | -0.5308096 |
| E130309D14Rik | 1.86599739 |
| E130309F12Rik | -0.4192473 |
| E130309F12Rik | 1.75247924 |
| E130309F12Rik | -0.7346045 |
| E230008N13Rik | 2.16848462 |
| E2f1 | -0.6533381 |
| E2f2 | -0.658974 |
| E2f2 | -0.6901704 |
| E2f3 | -0.5912122 |
| E2f4 | -0.6264331 |
| E2f5 | 3.36139817 |
| E2f6 | 0.42345653 |
| E2f6 | 1.74265747 |
| E2f6 | -0.5829812 |
| E330016A19Rik | 0.05992733 |
| E330017A01Rik | 1.60863904 |
| E330018D03Rik | -0.1142258 |
| E330021D16Rik | 0.49023748 |
| E330034G19Rik | -0.0830208 |
| E330034G19Rik | -0.2494769 |
| E330036I19Rik | -0.6117256 |
| E430002D04Rik | -0.4807045 |
| E430002G05Rik | -0.1043068 |
| E430018J23Rik | -0.6233176 |
| E430025E21Rik | 0.76127954 |
| E430028B21Rik | -0.7258954 |
| E4f1 | 0.86194412 |
| Eaf1 | -0.1867426 |
| Ear11 | -0.3049591 |
| Ear2 | 0.38740644 |
| Ear4 | 0.00364097 |
| Ears2 | -0.7979301 |
| Ebag9 | 1.04091379 |
| Ebf2 | -0.6063643 |
| Ebf3 | 1.09097002 |
| Ebf4 | 0.30393254 |
| Ebi2 | 1.51176478 |
| Ebi3 | -0.4588993 |
| Ebna1bp2 | 2.27552545 |
| Ebna1bp2 | 1.73106796 |
| Ebp | -0.7354799 |
| Ebpl | 1.89686192 |
| Ebpl | 2.00101396 |
| Ecd | 1.41711113 |
| Ecd | 0.85710142 |
| Ece1 | -0.4753719 |
| Ece1 | 2.16812189 |
| Ece2 | -0.7232982 |
| Ecel1 | 2.83516487 |
| Ech1 | -0.7951271 |
| Echdc1 | 1.69001453 |
| Echdc2 | 1.64288093 |
| Echdc2 | -0.6056009 |
| Echdc2 | -0.2489539 |
| Echdc3 | 0.39245241 |
| Echs1 | 0.10301659 |
| Ecm1 | 1.91559237 |
| Ecm1 | -0.5995157 |
| Ecsit | -0.2536712 |
| Eda2r | 1.20827274 |
| Edaradd | 0.81286935 |
| Edc4 | 0.40664794 |
| Edem1 | 1.50282986 |
| Edem2 | -0.6295588 |
| Edem2 | 2.54921032 |
| Edem2 | 1.82165702 |
| Edem2 | -0.6613997 |
| Edf1 | -0.6744432 |
| Edg2 | 2.60493525 |
| Edg5 | 1.08691979 |
| Edg7 | 0.44097771 |
| Edg8 | 0.40272612 |
| Edg8 | -0.5493182 |
| Edil3 | 1.36549427 |
| Edn1 | 0.11411104 |
| Edn2 | -0.3860197 |
| Ednra | -0.5037104 |
| Ednrb | 2.33318868 |
| Ednrb | 1.48336993 |
| Ednrb | -0.8638062 |
| Eed | -0.6851756 |
| Eef1a1 | -0.634267 |
| Eef1a1 | 0.45173434 |
| Eef1a2 | 1.17223858 |
| Eef1a2 | -0.5807488 |
| Eef1a2 | 0.84734076 |
| Eef1b2 | -0.0231354 |
| Eef1b2 | 1.14126114 |
| Eef1b2 | 0.76646048 |
| Eef1d | 2.13434845 |
| Eef1d | -0.7839978 |
| Eef1d | -0.1196804 |
| Eef1d | 2.51979883 |
| Eef1e1 | -0.4928141 |
| Eef1g | -0.5450146 |
| Eef2 | -0.2727799 |
| Eef2 | 1.41478446 |
| Eef2 | 0.01200375 |
| Eef2k | 0.15892605 |
| Eefsec | 0.43542651 |
| Efcab1 | 0.08996678 |
| Efcab2 | -0.7250289 |
| Efcab2 | 1.04490081 |
| Efcab3 | -0.6148029 |
| Efcab3 | 0.53203962 |
| Efcab3 | 0.2512865 |
| Efcab4a | 1.00812016 |
| Efcab4a | -0.2231678 |
| Efcab5 | 0.59776803 |
| Efemp2 | 0.94761533 |
| Efha1 | 1.95156738 |
| Efha1 | -0.8054456 |
| Efhc2 | 1.12288268 |
| Efhd1 | 0.7714359 |
| Efna1 | 2.46342276 |
| Efna3 | -0.2568292 |
| Efna4 | -0.4760368 |
| Efna4 | -0.6977127 |
| Efna5 | 1.23358167 |
| Efnb1 | 0.92755447 |
| Efnb2 | 0.17573926 |
| Efnb3 | -0.3633198 |
| Efr3a | -0.5064542 |
| Efs | 0.73346429 |
| Eftud1 | 1.70948714 |
| Eftud2 | 0.47943053 |
| Eftud2 | -0.4293216 |
| Eftud2 | -0.6951919 |
| EG194588 | 0.29575111 |
| EG208426 | -0.3609618 |
| EG210155 | 2.96338893 |
| EG212225 | 2.55289012 |
| EG214321 | 0.41081331 |
| EG232599 | 2.90436738 |
| EG237009 | -0.4012575 |
| EG237009 | -0.6695307 |
| EG237300 | 1.20760333 |
| EG238829 | 0.57969941 |
| EG240916 | 0.49642552 |
| EG245297 | 0.79661727 |
| EG245297 | 2.82505468 |
| EG245376 | -0.5189013 |
| EG245376 | -0.8419858 |
| EG272350 | -0.3094082 |
| EG277333 | -0.7250289 |
| EG317677 | 0.4308602 |
| EG328264 | -0.585966 |
| EG328280 | -0.5078292 |
| EG328644 | -0.789546 |
| EG328839 | 2.50862703 |
| EG330305 | -0.7002402 |
| EG330503 | -0.3836043 |
| EG330513 | -0.0677971 |
| EG333669 | -0.2895507 |
| EG368203 | -0.3836043 |
| EG380907 | -0.1297633 |
| EG381438 | 2.03518171 |
| EG382109 | 0.69582178 |
| EG382109 | 0.13417605 |
| EG382109 | -0.6711654 |
| EG384639 | 0.74974404 |
| EG384813 | 3.83884673 |
| EG384813 | 1.77702257 |
| EG384814 | -0.8638062 |
| EG385328 | 0.85661578 |
| EG408196 | -0.1052042 |
| EG432825 | 0.09832496 |
| EG432879 | 1.70909373 |
| EG432939 | 2.35503072 |
| EG433016 | 1.4461538 |
| EG433182 | 0.12998716 |
| EG433224 | 2.53001624 |
| EG433923 | 1.18752498 |
| EG434171 | 0.29074896 |
| EG434197 | -0.45239 |
| EG434280 | -0.7053157 |
| EG434459 | 2.99188996 |
| EG434459 | 0.32436565 |
| EG434858 | -0.7250289 |
| EG435391 | -0.3686469 |
| EG436332 | 0.41780094 |
| EG545047 | 2.54513778 |
| EG545056 | 0.59755495 |
| EG545253 | 1.19705344 |
| EG545370 | 1.87258255 |
| EG545758 | -0.73373 |
| EG545861 | -0.7215708 |
| EG545963 | 0.49950438 |
| EG546166 | 2.37089966 |
| EG546282 | 0.63924075 |
| EG546325 | 1.11232349 |
| EG546347 | -0.0515421 |
| EG547109 | -0.6178903 |
| EG574403 | 0.42627167 |
| EG622320 | -0.3354057 |
| EG622339 | -0.4074222 |
| EG624866 | 1.46367019 |
| EG625054 | -0.6163453 |
| EG625424 | -0.290643 |
| EG630499 | -0.2547227 |
| EG633640 | -0.3440404 |
| EG638695 | -0.6654564 |
| EG654453 | -0.6445493 |
| EG665186 | 0.62568708 |
| EG665378 | -0.7839978 |
| EG667977 | 3.20094992 |
| EG668668 | 0.90552151 |
| Egf | -0.7979301 |
| Egfbp2 | 0.67288698 |
| Egfl7 | 1.8188007 |
| Egfl7 | -0.3296928 |
| Egflam | 1.81341434 |
| Egflam | 0.96307312 |
| Egfr | -0.682688 |
| Egfr | -0.2515717 |
| Egfr | 0.38179772 |
| Egfr | -0.3463564 |
| Egln1 | 0.34324993 |
| Egln1 | -0.5889603 |
| Egln1 | -0.6272135 |
| Egln2 | 0.14387837 |
| Egln3 | 1.19851951 |
| Egr1 | -0.5762997 |
| Egr2 | -0.4989278 |
| Egr3 | 0.42294379 |
| Egr4 | -0.205528 |
| Ehbp1 | 1.69342138 |
| Ehbp1l1 | -0.2287806 |
| Ehd1 | -0.3172449 |
| Ehd2 | 1.72952899 |
| Ehd3 | -0.4820423 |
| Ehd4 | 0.98931829 |
| Ehmt1 | -0.8101734 |
| Ehmt2 | -0.5711356 |
| Ehmt2 | -0.5457305 |
| Ei24 | -0.4198738 |
| Eid1 | 1.56496648 |
| Eid2 | 2.25802541 |
| Eid3 | -0.7010842 |
| Eif1a | 0.05045932 |
| Eif1b | -0.7276308 |
| Eif2a | 2.5668601 |
| Eif2ak1 | -0.6549449 |
| Eif2ak2 | -0.139024 |
| Eif2b1 | -0.7155494 |
| Eif2b1 | 2.68278558 |
| Eif2b2 | 0.75028793 |
| Eif2b4 | 1.47508558 |
| Eif2b5 | -0.7425121 |
| Eif2b5 | 0.71011435 |
| Eif2b5 | -0.0376496 |
| Eif2c2 | 0.59989618 |
| Eif2c3 | -0.7540526 |
| Eif2c3 | -0.5407306 |
| Eif2c3 | -0.9335502 |
| Eif2c3 | 1.03181364 |
| Eif2c4 | 1.08970619 |
| Eif2s2 | 1.47424472 |
| Eif2s2 | -0.8120712 |
| Eif2s3x | 0.45941372 |
| Eif2s3y | -0.6094242 |
| Eif3b | 0.32749212 |
| Eif3b | -0.5350487 |
| Eif3d | 1.6592702 |
| Eif3eip | -0.8139728 |
| Eif3eip | 0.34213306 |
| Eif3f | -0.4536883 |
| Eif3g | 1.36643784 |
| Eif3i | 0.32037147 |
| Eif3i | 1.0525616 |
| Eif3i | -0.6533381 |
| Eif3k | 0.21010059 |
| Eif3s1 | -0.5378854 |
| Eif3s10 | -0.3016361 |
| Eif3s10 | -0.6358416 |
| Eif3s4 | 0.53523226 |
| Eif3s8 | -0.6670841 |
| Eif4a1 | 0.09868668 |
| Eif4a1 | 0.88876968 |
| Eif4a2 | -0.7988662 |
| Eif4a3 | -0.4382257 |
| Eif4b | 2.1431232 |
| Eif4e | -0.1427539 |
| Eif4e1b | -0.421128 |
| Eif4e1b | -0.7215708 |
| Eif4e2 | -0.6951919 |
| Eif4e2 | -0.4337631 |
| Eif4e3 | 0.86804274 |
| Eif4e3 | 0.74811048 |
| Eif4e3 | 0.26502928 |
| Eif4ebp1 | 1.90206067 |
| Eif4ebp2 | -0.6910055 |
| Eif4el3 | 1.56687478 |
| Eif4enif1 | 0.52562173 |
| Eif4enif1 | -0.45239 |
| Eif4g1 | -0.2515717 |
| Eif4g2 | -0.4740434 |
| Eif4g2 | -0.4167454 |
| Eif4h | 0.13313057 |
| Eif5 | 0.85904148 |
| Eif5 | -0.4840527 |
| Eif5 | -0.0498472 |
| Eif5a | -0.1721718 |
| Eif5a | 1.05071813 |
| Eif6 | 0.37588593 |
| Ela1 | 0.35599977 |
| Ela2 | 1.43525774 |
| Ela2a | -0.5112753 |
| Ela3b | -0.4813732 |
| Elac2 | 0.09251843 |
| Elac2 | 2.43939194 |
| Elac2 | -0.3388501 |
| Elavl1 | -0.6835165 |
| Elavl2 | -0.6469381 |
| Elavl2 | -0.7121258 |
| Elavl3 | 1.3572451 |
| Elavl4 | 3.456777 |
| Elavl4 | -0.1219625 |
| Elf1 | 1.29230053 |
| Elf2 | -0.127922 |
| Elf2 | 1.54730442 |
| Elf2 | -0.4921373 |
| Elf2 | -0.030572 |
| Elf2 | 1.55312544 |
| Elf3 | -0.5154273 |
| Elf3 | 1.00949809 |
| Elf3 | 1.59814879 |
| Elf5 | -0.787693 |
| Elfn1 | -0.4602066 |
| Elfn2 | 0.51031881 |
| Elk3 | 1.75959255 |
| Elk3 | 1.90446277 |
| Elk3 | -0.658974 |
| Ell | 1.0000882 |
| Ell3 | 0.445245 |
| Elmo1 | 1.74556428 |
| Elmo1 | -0.1891931 |
| Elmo1 | 0.62403089 |
| Elmo1 | -0.5572594 |
| Elmo1 | -0.4375869 |
| Elmo1 | 1.45761063 |
| Elmo1 | 1.0100487 |
| Elmo1 | 0.56061327 |
| Elmo1 | -0.6868376 |
| Elmo1 | 0.46971929 |
| Elmo1 | -0.5350487 |
| Elmo1 | -0.6622096 |
| Elmo1 | 0.12542811 |
| Elmo2 | -0.4582464 |
| Elmo2 | -0.5244865 |
| Elmo3 | 1.25960326 |
| Elmo3 | -0.6549449 |
| Elmod1 | -0.7612252 |
| Eln | -0.7398689 |
| Elof1 | -0.0896212 |
| Elovl1 | -0.3257142 |
| Elovl1 | 0.4123706 |
| Elovl1 | 1.17547742 |
| Elovl1 | -0.0927173 |
| Elovl2 | -0.8120712 |
| Elovl4 | 0.94127176 |
| Elovl5 | 0.57447273 |
| Elovl6 | 0.65260241 |
| Elp2 | -0.5837265 |
| Elp3 | -0.3746015 |
| Elp3 | 1.73904308 |
| Eltd1 | -0.0878566 |
| Emb | -0.5536415 |
| Emcn | 2.15631785 |
| Emd | -0.7129806 |
| Emd | -0.4136274 |
| Emg1 | 3.40448006 |
| Emg1 | 0.17373743 |
| Emid1 | -0.699397 |
| Emid2 | -0.7129806 |
| Emilin1 | 1.32153135 |
| Emilin2 | -0.7241631 |
| Eml1 | -0.6769091 |
| Eml3 | -0.4588993 |
| Eml3 | -0.4975658 |
| Eml4 | -0.7567359 |
| Eml5 | -0.6421665 |
| Emp1 | -0.6901704 |
| Emp2 | -0.6233176 |
| Emp3 | -0.6752645 |
| Emr1 | -0.20453 |
| Emr1 | 1.62038383 |
| Emr1 | -0.6868376 |
| En2 | 0.49023748 |
| Enah | 0.65741464 |
| Enc1 | 0.19454771 |
| Endod1 | 0.22223395 |
| Endogl1 | -0.3371263 |
| Endogl1 | 0.61424164 |
| Eng | 4.32676309 |
| Eno1 | -0.1215057 |
| Eno2 | -0.6901704 |
| Eno3 | 1.93116374 |
| Enoph1 | 0.59135461 |
| Enox1 | 0.60244364 |
| Enpp1 | 1.15563958 |
| Enpp1 | 2.36761013 |
| Enpp2 | -0.6048381 |
| Enpp4 | -0.1432211 |
| Enpp5 | -0.284651 |
| Enpp6 | 0.28601671 |
| Ensa | 2.31086347 |
| Ensa | -0.3504232 |
| ENSMUSG00000043795 | -0.2862814 |
| ENSMUSG00000053178 | -0.0626336 |
| ENSMUSG00000068790 | 0.27528151 |
| Entpd2 | 0.55505655 |
| Entpd3 | 2.32482732 |
| Entpd4 | 0.92092284 |
| Entpd5 | 0.9581425 |
| Entpd5 | 0.69716496 |
| Entpd5 | 0.42601609 |
| Entpd6 | 0.80023402 |
| Entpd7 | 1.05767805 |
| Ep300 | -0.4968855 |
| Ep400 | 0.21298842 |
| Epas1 | 1.30537043 |
| Epb4.1 | 0.45941372 |
| Epb4.1l1 | -0.239072 |
| Epb4.1l1 | 0.79627209 |
| Epb4.1l1 | 1.69168609 |
| Epb4.1l2 | 0.1151778 |
| Epb4.1l3 | 1.15363266 |
| Epb4.1l3 | -0.7594269 |
| Epb4.1l3 | 1.14532355 |
| Epb4.1l4a | -0.6010334 |
| Epb4.1l5 | -0.7363561 |
| Epb4.1l5 | -0.1106068 |
| Epb4.9 | -0.5949775 |
| Epb4.9 | 1.97198574 |
| Epc1 | 1.09500293 |
| Epc1 | 0.31290853 |
| Epc1 | 2.58922902 |
| Epdr1 | -0.7849203 |
| Epgn | -0.5272915 |
| Epha1 | 1.87797131 |
| Epha1 | -0.2050289 |
| Epha3 | 2.06041448 |
| Epha3 | -0.4055685 |
| Epha3 | 1.74234097 |
| Epha4 | -0.7585291 |
| Epha5 | 1.23151647 |
| Epha6 | 0.33371412 |
| Epha7 | -0.0716883 |
| Epha7 | -0.699397 |
| Epha7 | 2.26830339 |
| Ephb1 | -0.658974 |
| Ephb2 | 0.89718278 |
| Ephb4 | 1.37357832 |
| Ephb6 | 1.40894255 |
| Ephx1 | 0.38687371 |
| Ephx2 | -0.6695307 |
| Epm2a | 0.37372697 |
| Epm2aip1 | 1.89562501 |
| Epm2aip1 | 0.14903528 |
| Epm2aip1 | -0.4055685 |
| Epn1 | 0.18601491 |
| Epn2 | 1.02167383 |
| Epn2 | -0.6851756 |
| Epn2 | 1.09751478 |
| Epn2 | -0.6581668 |
| Epn2 | 1.0319481 |
| Epor | 0.71595926 |
| Eprs | -0.4800362 |
| Eprs | -0.7389895 |
| Eps15 | 1.15504975 |
| Eps15l1 | -0.5002918 |
| Eps8 | 1.64252911 |
| Eps8l1 | -0.6217638 |
| Eps8l2 | 0.22666508 |
| Eps8l2 | -0.5800058 |
| Eps8l3 | 2.04831739 |
| Epsti1 | -0.3981901 |
| Epsti1 | 1.63055973 |
| Eraf | 0.4288236 |
| Eral1 | 0.30770707 |
| Ercc1 | 1.90456939 |
| Ercc2 | 1.34988344 |
| Ercc3 | 0.11126042 |
| Ercc3 | 1.62333323 |
| Ercc4 | -0.8410059 |
| Ercc4 | 1.89890196 |
| Ercc5 | -0.2070272 |
| Ercc5 | -0.4780344 |
| Ercc8 | -0.3896544 |
| Ercc8 | 0.40977369 |
| Erdr1 | -0.513349 |
| Erf | -0.7319833 |
| Erf | 0.18864862 |
| Ergic1 | 1.67890728 |
| Ergic2 | 0.80109311 |
| Ergic2 | 0.26107967 |
| Ergic3 | 0.42780366 |
| Ergic3 | 1.44015474 |
| Erh | -0.3781923 |
| Erich1 | -0.3251472 |
| Erlin1 | 0.93771253 |
| Erlin2 | 2.59560443 |
| Erlin2 | 0.34714859 |
| Ermap | -0.5807488 |
| Ermp1 | -0.6366299 |
| Ern1 | 1.17847499 |
| Ern2 | -0.7425121 |
| Ero1lb | -0.6163453 |
| Erp29 | 0.27528151 |
| Errfi1 | 2.1232111 |
| Es22 | -0.6605904 |
| Esam1 | -0.1559259 |
| Esco2 | 1.23238658 |
| Esd | 0.57119128 |
| Esd | 1.52779638 |
| Esd | 2.40094548 |
| Esd | -0.6951919 |
| Esm1 | -0.289005 |
| Espl1 | 0.19258545 |
| Espn | 0.67837981 |
| Espn | 1.69953612 |
| Espn | -0.6793814 |
| Espn | -0.7302399 |
| Esr1 | -0.4337631 |
| Esr2 | -0.7407492 |
| Esr2 | -0.3951328 |
| Esr2 | -0.4667703 |
| Esrra | 3.66689136 |
| Esrrb | -0.4989278 |
| Etaa1 | 1.73688051 |
| Etf1 | 3.15907959 |
| Etfa | -0.5733453 |
| Etfa | -0.198068 |
| Etfa | -0.6350539 |
| Etfb | 1.01169861 |
| Etfb | 1.47163327 |
| Etfdh | -0.1512005 |
| Ethe1 | 0.32664047 |
| Etnk2 | 0.50914931 |
| Ets1 | 0.6869473 |
| Ets2 | -0.7354799 |
| Etsrp71 | 1.60717887 |
| Etv1 | 0.12014014 |
| Etv1 | 1.65009939 |
| Etv4 | -0.7095663 |
| Etv5 | -0.7821556 |
| Etv6 | -0.6117256 |
| Evc | -0.4740434 |
| Evc2 | 0.0648134 |
| Evi1 | 0.76859602 |
| Evi1 | 0.01556522 |
| Evi2a | -0.3021891 |
| Evi2a | -0.7711775 |
| Evi5 | 2.13497495 |
| Evi5l | -0.7812359 |
| Evl | -0.5308096 |
| Evx1 | -0.3189327 |
| Exdl1 | -0.634267 |
| Exdl2 | -0.5414433 |
| Exdl2 | 0.68208896 |
| Exdl2 | 1.55737147 |
| Exoc2 | 0.84946416 |
| Exoc3 | -0.3854153 |
| Exoc3l | 1.63914494 |
| Exoc4 | 1.73828038 |
| Exoc7 | -0.4996095 |
| Exoc8 | -0.699397 |
| Exod1 | 0.57425432 |
| Exosc1 | 0.31750809 |
| Exosc10 | 1.23173407 |
| Exosc2 | 0.26835839 |
| Exosc2 | -0.6670841 |
| Exosc4 | 0.01989995 |
| Exosc5 | -0.3763952 |
| Exosc6 | 1.55990994 |
| Exosc7 | 0.12753503 |
| Exosc8 | -0.4621709 |
| Ext1 | -0.8187437 |
| Ext2 | -0.1380938 |
| Extl1 | 1.58215383 |
| Extl2 | 1.635465 |
| Extl3 | 1.22627891 |
| Extl3 | 2.69986857 |
| Eya1 | -0.4236415 |
| Eya1 | -0.6295588 |
| Eya2 | 0.08042835 |
| Eya3 | -0.1233344 |
| Eya3 | 2.5654327 |
| Eya3 | -0.789546 |
| Eya4 | -0.2873699 |
| Ezh1 | -0.6233176 |
| Ezh2 | 1.43770942 |
| Ezh2 | -0.4356731 |
| F10 | -0.7198465 |
| F11 | -0.4608609 |
| F11 | -0.5919641 |
| F11r | -0.7155494 |
| F13a1 | -0.6943531 |
| F2r | 0.91226193 |
| F2rl1 | -0.2211351 |
| F2rl3 | -0.2191067 |
| F430201B04Rik | 0.55036346 |
| F5 | 1.30526981 |
| F630043A04Rik | 0.31290853 |
| F730014I05Rik | -0.0068167 |
| F730047E07Rik | 1.38187205 |
| F730047E07Rik | 0.66438877 |
| F730047E07Rik | -0.632695 |
| F8 | -0.1371645 |
| F8 | 1.61749745 |
| F8 | -0.658974 |
| F8a | 0.85239638 |
| Fa2h | -0.1029622 |
| Faah | 0.16197463 |
| Faah | 0.39773496 |
| Fabp3 | -0.6793814 |
| Fabp5 | 1.49824845 |
| Fabp9 | 2.20728591 |
| Fadd | 2.22642609 |
| Fadd | 0.74319278 |
| Fadd | 1.32014561 |
| Fads1 | 0.7200854 |
| Fads2 | 0.79834126 |
| Fads3 | -0.5126572 |
| Fads6 | 0.16568734 |
| Faf1 | 0.99170666 |
| Faf1 | 2.68488551 |
| Fah | -0.7425121 |
| Fahd1 | 0.9728577 |
| Fahd2a | -0.7078638 |
| Fahd2a | -0.4388649 |
| Fahd2a | 1.21272336 |
| Fahd2a | 0.51985434 |
| Faim | -0.5064542 |
| Faim | -0.6033144 |
| Faim2 | 1.72907965 |
| Faim2 | 2.11117023 |
| Faim3 | 0.72307493 |
| Fancc | 1.49133434 |
| Fancc | -0.3319738 |
| Fancd2 | 0.89017709 |
| Fancg | -0.650933 |
| Fanci | -0.7002402 |
| Fancl | 1.34574724 |
| Fancl | -0.5762997 |
| Fank1 | 0.20300401 |
| Fank1 | 0.16804239 |
| Fap | -0.14182 |
| Fars2 | -0.7207082 |
| Farsa | -0.5126572 |
| Farsb | -0.2808576 |
| Farsb | -0.4280564 |
| Fas | -0.7793991 |
| Fas | 0.67994334 |
| Fasl | -0.5216897 |
| Fastk | 0.61067568 |
| Fastk | -0.6397897 |
| Fastkd1 | 1.33127286 |
| Fastkd5 | 0.76663863 |
| Fat1 | 1.11970281 |
| Fat4 | -0.1265434 |
| Fbf1 | -0.1784582 |
| Fblim1 | -0.7433949 |
| Fbln1 | 0.11126042 |
| Fbln1 | 0.43719635 |
| Fbln1 | -0.6695307 |
| Fbln2 | 0.02225598 |
| Fbln2 | 0.30393254 |
| Fbn1 | -0.6272135 |
| Fbp1 | 1.99080459 |
| Fbp2 | 0.10481487 |
| Fbxl10 | 0.48952086 |
| Fbxl12 | 0.53454903 |
| Fbxl12 | -0.7319833 |
| Fbxl12 | -0.525888 |
| Fbxl12 | -0.6777325 |
| Fbxl13 | 0.62485935 |
| Fbxl13 | 0.2269808 |
| Fbxl14 | 0.68267328 |
| Fbxl15 | 0.86097757 |
| Fbxl18 | 0.32550375 |
| Fbxl18 | 0.18072533 |
| Fbxl21 | 2.00977992 |
| Fbxl22 | -0.5572594 |
| Fbxl4 | -0.8598012 |
| Fbxl5 | -0.4989278 |
| Fbxl6 | -0.4286887 |
| Fbxl6 | -0.6287764 |
| Fbxl8 | 0.90843806 |
| Fbxo15 | 0.42831377 |
| Fbxo16 | -0.5030257 |
| Fbxo18 | 0.3027681 |
| Fbxo18 | 0.27917222 |
| Fbxo2 | 0.01596012 |
| Fbxo21 | 1.36558867 |
| Fbxo25 | -0.406186 |
| Fbxo27 | 0.78288581 |
| Fbxo28 | -0.0219017 |
| Fbxo3 | -0.392694 |
| Fbxo3 | 0.9988357 |
| Fbxo30 | 2.35470001 |
| Fbxo31 | 0.92107407 |
| Fbxo31 | -0.8073339 |
| Fbxo32 | 0.22603334 |
| Fbxo32 | -0.1990593 |
| Fbxo32 | -0.4459253 |
| Fbxo33 | 0.0277306 |
| Fbxo34 | -0.4274244 |
| Fbxo34 | -0.8254637 |
| Fbxo34 | 0.87917484 |
| Fbxo36 | 1.11552829 |
| Fbxo38 | 0.3710213 |
| Fbxo38 | 0.24851375 |
| Fbxo4 | -0.1215057 |
| Fbxo42 | -0.2824815 |
| Fbxo44 | 2.2228953 |
| Fbxo6 | -0.364501 |
| Fbxo7 | -0.6728029 |
| Fbxo8 | -0.3527549 |
| Fbxo9 | 0.75840887 |
| Fbxo9 | -0.2647709 |
| Fbxw12 | 2.20313896 |
| Fbxw13 | 1.25313827 |
| Fbxw15 | 0.31779482 |
| Fbxw16 | 1.3893574 |
| Fbxw17 | 0.40925345 |
| Fbxw19 | -0.2781573 |
| Fbxw2 | -0.699397 |
| Fbxw4 | 0.48664891 |
| Fbxw4 | 1.14030272 |
| Fbxw5 | 0.59606205 |
| Fbxw7 | 0.12472476 |
| Fbxw7 | -0.1545058 |
| Fbxw7 | 1.036107 |
| Fbxw8 | -0.2966733 |
| Fcer1g | -0.0380676 |
| Fcer2a | -0.6985544 |
| Fcf1 | -0.7381108 |
| Fcgr2b | 2.90672832 |
| Fcgr2b | -0.0562187 |
| Fcgr2b | -0.7087146 |
| Fcgr3 | -0.2421814 |
| Fcgr4 | 0.52516164 |
| Fcgrt | -0.2939275 |
| Fcho1 | -0.4641393 |
| Fcho2 | -0.3793923 |
| Fchsd1 | -0.4452813 |
| Fchsd2 | 0.84979041 |
| Fcna | 0.38340362 |
| Fcrla | 1.57143901 |
| Fcrla | -0.2631772 |
| Fcrla | 3.53344277 |
| Fdps | 1.44137503 |
| Fdxr | -0.0256078 |
| Fdxr | 1.99206861 |
| Fem1b | 1.86865912 |
| Fem1c | -0.4667703 |
| Fem1c | -0.7839978 |
| Fen1 | -0.6163453 |
| Fermt2 | 2.20635733 |
| Fermt3 | 0.31088915 |
| Fert2 | 2.71092904 |
| Fert2 | -0.2584122 |
| Fes | 2.30526833 |
| Fetub | -0.3099658 |
| Fev | 0.64432428 |
| Fez1 | 0.00603791 |
| Fez1 | 0.59861987 |
| Fez2 | -0.6445493 |
| Fezf2 | 1.74726629 |
| Ffar2 | 0.40115281 |
| Fgd2 | -0.6613997 |
| Fgd4 | -0.5638069 |
| Fgd4 | -0.6711654 |
| Fgd5 | -0.3228827 |
| Fgd6 | 0.70175566 |
| Fgf1 | -0.5934696 |
| Fgf1 | 3.11694586 |
| Fgf10 | -0.0874159 |
| Fgf10 | -0.2359727 |
| Fgf12 | 1.793177 |
| Fgf12 | 0.58424912 |
| Fgf13 | 0.51055253 |
| Fgf13 | 1.84720856 |
| Fgf17 | 1.61300603 |
| Fgf21 | 2.45376467 |
| Fgf22 | 1.14496581 |
| Fgf22 | 0.2954576 |
| Fgf5 | -0.0124962 |
| Fgf7 | -0.6148029 |
| Fgf8 | 0.507744 |
| Fgf9 | 0.82901288 |
| Fgfbp3 | -0.8273925 |
| Fgfr1 | -0.5565347 |
| Fgfr1op | -0.6901704 |
| Fgfr1op2 | -0.6960314 |
| Fgfr1op2 | 0.67170577 |
| Fgfr2 | -0.0372318 |
| Fgfr2 | -0.2473867 |
| Fgfr3 | -0.4068039 |
| Fgfrl1 | -0.6541412 |
| Fgl2 | 0.54564691 |
| Fh1 | -0.1187691 |
| Fhad1 | -0.5777804 |
| Fhit | 1.61605094 |
| Fhl1 | 2.09405924 |
| Fhl1 | -0.0570715 |
| Fhl2 | 0.77779455 |
| Fhl4 | -0.8245007 |
| Fhl5 | 2.04863773 |
| Fhod1 | 2.56158913 |
| Fhod3 | -0.5030257 |
| Fibcd1 | 2.35668249 |
| Fibp | 0.06293714 |
| Figf | -0.7585291 |
| Figla | -0.4707305 |
| Fign | -0.6777325 |
| Fignl1 | -0.6818603 |
| Fip1l1 | -0.1371645 |
| Fis1 | 2.00212018 |
| Fis1 | -0.516121 |
| Fiz1 | -0.1146791 |
| Fjx1 | 0.57053362 |
| Fkbp10 | 2.98904116 |
| Fkbp11 | 0.42473718 |
| Fkbp14 | -0.289005 |
| Fkbp1a | 1.0000882 |
| Fkbp1b | 0.30247676 |
| Fkbp2 | -0.7232982 |
| Fkbp2 | 0.97828166 |
| Fkbp4 | -0.5681973 |
| Fkbp5 | 0.17306921 |
| Fkbp6 | 0.98494739 |
| Fkbp7 | 0.07190934 |
| Fkbp8 | 0.00123792 |
| Fkbp9 | -0.5558106 |
| Fkbpl | -0.0825824 |
| Fkbpl | -0.699397 |
| Fkhl18 | 0.38018909 |
| Fkrp | 0.85758681 |
| Fktn | -0.2030348 |
| Flad1 | 2.37634605 |
| Flcn | 2.26819464 |
| Flcn | -0.5329268 |
| Fli1 | 0.02656014 |
| Flii | 1.21094563 |
| Flna | 2.09309946 |
| Flot1 | 0.44349022 |
| Flot1 | -0.7389895 |
| Flot2 | -0.3492595 |
| Flot2 | 2.2933423 |
| Flot2 | -0.1288422 |
| Flrt1 | 1.22922861 |
| Flrt2 | -0.4000293 |
| Flrt3 | -0.4388649 |
| Flt1 | 0.24635152 |
| Flt4 | 0.56016994 |
| Flt4 | -0.406186 |
| Flvcr2 | 0.98748779 |
| Flywch2 | -0.6397897 |
| Fmn2 | -0.0498472 |
| Fmn2 | -0.3348328 |
| Fmnl1 | 0.73953343 |
| Fmnl3 | -0.6048381 |
| Fmnl3 | 0.71933653 |
| Fmo1 | -0.6311256 |
| Fmo2 | 1.17720795 |
| Fmo5 | -0.612494 |
| Fmod | -0.482712 |
| Fmr1nb | -0.2819399 |
| Fmr1nb | -0.6703477 |
| Fn3k | 2.02800695 |
| Fn3k | -0.2473867 |
| Fnbp1 | 0.87980701 |
| Fnbp1 | 1.22310039 |
| Fnbp1 | 2.08004601 |
| Fnbp1l | 2.52649408 |
| Fnbp4 | -0.63191 |
| Fndc3 | -0.0808309 |
| Fndc3a | 0.78968962 |
| Fndc3b | 1.0430423 |
| Fndc4 | 0.62091769 |
| Fndc5 | -0.7576321 |
| Fndc5 | 0.45198304 |
| Fndc7 | -0.6565545 |
| Fndc7 | -0.4800362 |
| Fndc8 | 1.2668631 |
| Fndc8 | -0.6010334 |
| Fnip1 | -0.8528329 |
| Fnip1 | -0.5645372 |
| Fnta | -0.4543381 |
| Fnta | -0.5608913 |
| Fntb | 0.97970385 |
| Folr1 | -0.0604905 |
| Folr1 | -0.2851941 |
| Folr2 | 1.46409545 |
| Fos | 1.71913962 |
| Fosb | 0.0191133 |
| Foxa1 | 2.17786694 |
| Foxa2 | -0.4395046 |
| Foxa3 | 0.33652879 |
| Foxc1 | -0.6240955 |
| Foxd2 | 2.90351747 |
| Foxg1 | 1.07427811 |
| Foxh1 | 0.73935009 |
| Foxh1 | 0.4818428 |
| Foxj1 | -0.196583 |
| Foxj2 | -0.8332027 |
| Foxj3 | 2.02618128 |
| Foxj3 | -0.585966 |
| Foxj3 | 1.04171254 |
| Foxj3 | 0.77462059 |
| Foxj3 | 2.80806883 |
| Foxk1 | 0.04166399 |
| Foxk1 | -0.0268464 |
| Foxk1 | 1.75491878 |
| Foxl2 | -0.4820423 |
| Foxm1 | -0.3394254 |
| Foxm1 | -0.5016577 |
| Foxn2 | 3.12770442 |
| Foxn3 | -0.4167454 |
| Foxo1 | 0.0896017 |
| Foxo6 | -0.5980006 |
| Foxo6 | -0.3359789 |
| Foxp1 | -0.3746015 |
| Foxp2 | 1.10650242 |
| Foxp2 | -0.6437543 |
| Foxp2 | -0.4536883 |
| Foxp3 | 0.52101064 |
| Foxp4 | 1.56603561 |
| Foxq1 | 2.136185 |
| Foxred1 | -0.6350539 |
| Foxred1 | -0.2621162 |
| Foxred1 | 0.6223718 |
| Foxred1 | 1.3933071 |
| Foxred1 | -0.0326481 |
| Foxred2 | 1.21969078 |
| Fpgs | 0.73143253 |
| Fpgs | 0.33933509 |
| Fpgs | 0.49095356 |
| Fpr2 | 1.07312042 |
| Frag1 | 0.23577816 |
| Frag1 | 0.81168134 |
| Frag1 | -0.3740043 |
| Frap1 | 2.44949674 |
| Fras1 | 2.24770829 |
| Fras1 | -0.4491521 |
| Frat1 | 1.04529859 |
| Frat1 | -0.6622096 |
| Frat2 | 0.82466788 |
| Freq | 1.93318271 |
| Freq | -0.1760354 |
| Freq | -0.6233176 |
| Frg1 | -0.5630771 |
| Frk | -0.780317 |
| Frmd3 | -0.5428702 |
| Frmd6 | 0.11339919 |
| Frmd8 | 2.43344709 |
| Frmpd4 | -0.8293253 |
| Frrs1 | -0.1155865 |
| Frs2 | 1.37488779 |
| Frs3 | 1.38613027 |
| Fryl | -0.4530389 |
| Fryl | -0.8820426 |
| Frzb | -0.6793814 |
| Fscn1 | 3.92498838 |
| Fscn2 | -0.4661118 |
| Fscn3 | -0.4459253 |
| Fsd1 | 1.27010666 |
| Fsd2 | 1.02547212 |
| Fshb | 0.48232451 |
| Fshr | 2.04326913 |
| Fshr | -0.406186 |
| Fshr | -0.546447 |
| Fsip1 | 1.11577436 |
| Fst | -0.4787012 |
| Fstl1 | -0.8628034 |
| Fstl4 | -0.1224196 |
| Fstl5 | -0.6163453 |
| Fth1 | -0.2432202 |
| Fth1 | 1.40088448 |
| Ftmt | 1.20782652 |
| Fto | -0.778482 |
| Ftsj1 | 1.09097002 |
| Ftsj3 | -0.6711654 |
| Fubp1 | -0.5972439 |
| Fubp3 | 0.72846942 |
| Fuk | 3.36280771 |
| Fuk | 0.51707342 |
| Fundc1 | -0.0660728 |
| Fundc1 | 1.47676506 |
| Fundc2 | 3.75507523 |
| Furin | -0.7585291 |
| Furin | -0.6736227 |
| Fus | 0.86964111 |
| Fusip1 | 3.36248961 |
| Fusip1 | -0.5182055 |
| Fusip1 | -0.4628266 |
| Fut10 | 0.20945765 |
| Fut10 | -0.2669 |
| Fut2 | -0.1946067 |
| Fut4 | 0.1565481 |
| Fut8 | -0.4887603 |
| Fut8 | 0.02656014 |
| Fut8 | -0.6581668 |
| Fvt1 | -0.2983247 |
| Fxc1 | -0.375199 |
| Fxn | -0.7433949 |
| Fxn | -0.4186212 |
| Fxyd1 | 2.15312861 |
| Fxyd2 | 2.75474058 |
| Fxyd2 | 0.66240142 |
| Fxyd2 | -0.8139728 |
| Fxyd3 | -0.7044679 |
| Fxyd4 | -0.375199 |
| Fxyd4 | -0.5972439 |
| Fxyd5 | 1.42848257 |
| Fxyd5 | 0.04855425 |
| Fxyd5 | -0.1697651 |
| Fxyd6 | -0.1348454 |
| Fxyd7 | -0.6453449 |
| Fyb | -0.6885025 |
| Fyco1 | -0.7002402 |
| Fyn | 2.22456743 |
| Fyttd1 | -0.831262 |
| Fzd1 | -0.0773375 |
| Fzd10 | -0.168324 |
| Fzd10 | -0.607893 |
| Fzd2 | -0.0887385 |
| Fzd3 | 1.06575519 |
| Fzd4 | -0.6033144 |
| Fzd5 | -0.5002918 |
| Fzd6 | 0.52262709 |
| Fzd7 | -0.5822365 |
| Fzd8 | -0.6225404 |
| Fzr1 | 1.36549427 |
| Fzr1 | 2.89121931 |
| Fzr1 | 0.26654397 |
| G0s2 | -0.5050813 |
| G3bp1 | -0.168324 |
| G3bp1 | -0.1151327 |
| G3bp2 | 0.18963437 |
| G3bp2 | 0.40612597 |
| G3bp2 | 1.84482632 |
| G430022H21Rik | 0.7827107 |
| G430055L02Rik | -0.8478869 |
| G6pc | -0.0477329 |
| G6pc2 | 0.27857469 |
| G6pc2 | -0.2701027 |
| G6pc3 | 0.63740395 |
| G6pd2 | 1.13814273 |
| G6pd2 | -0.1644918 |
| G6pdx | -0.6943531 |
| Gaa | 1.44128792 |
| Gab1 | 1.22868307 |
| Gab2 | 1.32872421 |
| Gab2 | 1.28018442 |
| Gab2 | 1.82037566 |
| Gab3 | -0.5536415 |
| Gab3 | -0.455639 |
| Gab3 | 0.66676807 |
| Gabarap | -0.1070016 |
| Gabarapl1 | 1.16491026 |
| Gabarapl2 | 0.25067104 |
| Gabbr1 | -0.6405813 |
| Gabpa | -0.7284997 |
| Gabpa | -0.5385959 |
| Gabpb1 | -0.2857376 |
| Gabpb1 | -0.7904739 |
| Gabpb2 | -0.4793685 |
| Gabra3 | -0.1799149 |
| Gabra4 | -0.7603256 |
| Gabrb1 | -0.6744432 |
| Gabrb2 | -0.6117256 |
| Gabrb3 | -0.7112719 |
| Gabrb3 | -0.6893361 |
| Gabrd | -0.7319833 |
| Gabrd | 0.09651426 |
| Gabrg1 | 1.36000285 |
| Gabrg2 | 2.00571843 |
| Gabrg2 | 0.41055352 |
| Gabrg2 | -0.8026203 |
| Gabrg2 | -0.4894347 |
| Gabrp | -0.5792634 |
| Gabrp | -0.7241631 |
| Gad1 | -0.6256532 |
| Gad1 | -0.4661118 |
| Gadd45a | 0.82767809 |
| Gadd45a | 0.83665148 |
| Gadd45b | 0.30422342 |
| Gadd45g | 1.54699295 |
| Gadd45g | 1.72043647 |
| Gak | -0.5587105 |
| Gal | 0.2348395 |
| Gal3st1 | -0.2781573 |
| Gal3st2 | 0.57359879 |
| Gal3st3 | 1.7801297 |
| Galc | -0.778482 |
| Galc | 0.56613745 |
| Gale | 0.13033695 |
| Gale | -0.8528329 |
| Galk1 | -0.2100326 |
| Galm | 2.66721779 |
| Galns | 1.31118832 |
| Galnt1 | 0.4940503 |
| Galnt1 | 0.12366874 |
| Galnt1 | -0.205528 |
| Galnt1 | 0.22476858 |
| Galnt10 | 1.44241974 |
| Galnt10 | 1.20693345 |
| Galnt10 | 0.40925345 |
| Galnt11 | -0.4575939 |
| Galnt12 | -0.1074515 |
| Galnt13 | -0.8518417 |
| Galnt2 | 0.93324451 |
| Galnt2 | 0.76253272 |
| Galnt2 | -0.5718716 |
| Galnt3 | 1.41764724 |
| Galnt9 | -0.8101734 |
| Galntl2 | 0.93130178 |
| Galntl4 | -0.2536712 |
| Galntl5 | 2.6130687 |
| Galt | 0.97199843 |
| Galt | 0.38500679 |
| Gamt | 3.01205563 |
| Gan | -0.4055685 |
| Ganab | -0.1450926 |
| Ganab | 1.50714544 |
| Ganab | 0.82784504 |
| Ganc | 1.4204123 |
| Gap43 | 0.68675345 |
| Gapdh | -0.6769091 |
| Gapdhs | 0.13556822 |
| Gapvd1 | 2.41854259 |
| Gapvd1 | 0.93399065 |
| Garnl3 | -0.8628034 |
| Garnl4 | -0.16497 |
| Gars | 2.87081219 |
| Gart | -0.4853953 |
| Gart | 0.28897715 |
| Gas2l1 | 0.99450879 |
| Gas2l1 | 2.83713034 |
| Gas2l1 | 1.89729177 |
| Gas2l1 | 2.28447594 |
| Gas6 | -0.6358416 |
| Gas7 | 0.77356022 |
| Gas7 | -0.746048 |
| Gas8 | -0.5294008 |
| Gata2 | 0.07041989 |
| Gata4 | -0.5400185 |
| Gata6 | -0.6094242 |
| Gatad1 | -0.7416302 |
| Gatad1 | -0.1668851 |
| Gatad2b | 1.28018442 |
| Gats | -0.6256532 |
| Gats | 4.00948392 |
| Gba | 0.28601671 |
| Gba2 | 1.3178646 |
| Gbas | 2.1858127 |
| Gbf1 | -0.3674605 |
| Gbl | -0.3908691 |
| Gbl | -0.4343993 |
| Gbl | 0.57338018 |
| Gbp2 | -0.1293026 |
| Gbp3 | -0.6711654 |
| Gbp3 | 1.07376375 |
| Gbp6 | -0.6040759 |
| Gbp6 | 2.08639752 |
| Gbx2 | 1.88300229 |
| Gca | -0.5202945 |
| Gca | 0.06556286 |
| Gcap14 | -0.416121 |
| Gcap14 | 0.29604453 |
| Gcc1 | -0.1867426 |
| Gcc2 | -0.432492 |
| Gcdh | 1.81017948 |
| Gch1 | -0.7104187 |
| Gch1 | 2.96864795 |
| Gchfr | 1.313685 |
| Gck | -0.7172659 |
| Gclm | -0.2505237 |
| Gcn5l2 | 2.1643655 |
| Gcnt1 | 3.73407749 |
| Gcnt1 | -0.7164073 |
| Gcnt2 | 1.43936944 |
| Gcnt2 | 0.19780911 |
| Gcs1 | -0.0852158 |
| Gda | 2.18391182 |
| Gdap1 | 1.25875791 |
| Gdap1 | 0.40977369 |
| Gdap1l1 | 1.3315665 |
| Gdap1l1 | -0.8177876 |
| Gde1 | 1.48328661 |
| Gde1 | -0.7504866 |
| Gdf10 | 0.34603633 |
| Gdf15 | 1.06873405 |
| Gdf3 | -0.6818603 |
| Gdf9 | 2.93796451 |
| Gdi1 | 1.27573024 |
| Gdi1 | 0.42652717 |
| Gdi1 | 1.44779903 |
| Gdi1 | 0.97300084 |
| Gdi1 | 3.71521627 |
| Gdi2 | -0.6752645 |
| Gdnf | -0.1056532 |
| Gdpd1 | 1.983577 |
| Gdpd2 | 1.28821079 |
| Gdpd2 | 1.18741096 |
| Gdpd3 | 0.03394864 |
| Gdpd4 | -0.5493182 |
| Gdpd5 | -0.4536883 |
| Gemin4 | -0.3781923 |
| Gemin4 | 1.10264323 |
| Gemin4 | 0.17607249 |
| Gemin5 | 1.02153789 |
| Gemin6 | -0.8216178 |
| Gemin6 | -0.7433949 |
| Gemin7 | 0.45198304 |
| Gemin7 | -0.7146924 |
| Gfap | 0.10445549 |
| Gfer | -0.7621256 |
| Gfi1 | -0.3734075 |
| Gfm1 | -0.3545075 |
| Gfod1 | -0.5785216 |
| Gfod2 | 0.66637194 |
| Gfpt2 | -0.2125444 |
| Gfra1 | -0.3469363 |
| Gfra4 | 0.13973245 |
| Gga1 | 1.67156561 |
| Gga1 | -0.4375869 |
| Gga1 | -0.4853953 |
| Gga3 | -0.0464666 |
| Ggcx | 0.08005956 |
| Ggcx | -0.5755603 |
| Ggcx | -0.6240955 |
| Ggn | -0.7189855 |
| Ggn | 2.22475725 |
| Ggnbp1 | -0.6477358 |
| Ggnbp2 | 1.5677894 |
| Ggt5 | 0.92650255 |
| Ggt7 | -0.7585291 |
| Ggta1 | 0.46971929 |
| Ggtla1 | -0.1224196 |
| Gh | -0.546447 |
| Ghitm | -0.6736227 |
| Ghr | -0.6711654 |
| Ghr | -0.2879146 |
| Ghrh | -0.4337631 |
| Ghrhr | 1.35734033 |
| Ghrl | -0.267433 |
| Ghrl | -0.4667703 |
| Ghsr | 0.7444702 |
| Ghsr | -0.4186212 |
| Gif | 0.00882667 |
| Gigyf1 | -0.168324 |
| Gimap1 | 0.26835839 |
| Gimap4 | -0.2292925 |
| Gimap5 | -0.8478869 |
| Gimap6 | 2.43530129 |
| Gimap8 | -0.3099658 |
| Gimap9 | -0.4395046 |
| Gins3 | 2.3056865 |
| Gins4 | -0.3615507 |
| Gipc1 | -0.746048 |
| Gipc1 | 0.3961533 |
| Gipc1 | 1.00880938 |
| Gipc2 | 0.36203712 |
| Git1 | 0.0072341 |
| Git2 | -0.6868376 |
| Git2 | 0.83549306 |
| Git2 | 0.90352076 |
| Gja1 | 0.8004059 |
| Gja4 | 0.41288912 |
| Gja5 | -0.4996095 |
| Gja5 | 2.04377518 |
| Gja6 | 0.20235621 |
| Gja7 | -0.3105237 |
| Gjb1 | 0.41728511 |
| Gjb2 | -0.3878353 |
| Gjb2 | -0.6985544 |
| Gjb3 | 0.66498416 |
| Gjb6 | 1.37264186 |
| Gjc2 | 1.21891916 |
| Gjc2 | 0.82550501 |
| Gjd2 | 1.57234921 |
| Gjd2 | -0.7319833 |
| Gjd3 | -0.8054456 |
| Gk2 | -0.05324 |
| Gk5 | -0.6613997 |
| Gkap1 | 0.73512339 |
| Gkap1 | 2.1428334 |
| Glb1 | 1.52716032 |
| Glb1l2 | -0.5378854 |
| Glcci1 | -0.4491521 |
| Gldc | 1.05689271 |
| Gldc | -0.5092061 |
| Gldn | -0.0256078 |
| Gle1 | -0.2115389 |
| Glg1 | 0.3764249 |
| Glipr2 | 1.88262044 |
| Glis1 | 1.53807267 |
| Glo1 | -0.8508514 |
| Gloxd1 | 1.38122237 |
| Glp2r | -0.2547227 |
| Glra2 | 2.36204728 |
| Glrb | 0.75318339 |
| Glrp1 | -0.5829812 |
| Glrx | 0.19029096 |
| Glrx2 | 1.08132217 |
| Glrx2 | -0.3365524 |
| Glrx2 | -0.3866245 |
| Glrx3 | 0.33427773 |
| Glrx5 | 1.06821666 |
| Gls2 | 2.03304431 |
| Gls2 | 1.11552829 |
| Glt25d1 | -0.0019757 |
| Glt25d2 | -0.406186 |
| Glt25d2 | 0.2512865 |
| Glt6d1 | -0.5882108 |
| Glt8d1 | 1.79413633 |
| Glt8d1 | -0.3908691 |
| Glt8d1 | 0.65741464 |
| Glt8d2 | 1.67944884 |
| Glt8d3 | -0.4530389 |
| Gltp | 1.18843661 |
| Gltscr1 | -0.7293694 |
| Glud1 | 2.27062047 |
| Glud1 | -0.0116828 |
| Glul | -0.6597819 |
| Glyctk | 0.34964639 |
| Glyctk | -0.6793814 |
| Glyctk | -0.7549462 |
| Gm1006 | 0.85272162 |
| Gm1006 | -0.5829812 |
| Gm1019 | -0.054941 |
| Gm1040 | 1.89384748 |
| Gm1070 | 3.46229236 |
| Gm1082 | -0.2165773 |
| Gm114 | -0.2186003 |
| Gm114 | 0.25527725 |
| Gm1141 | -0.6279946 |
| Gm128 | 0.90182451 |
| Gm129 | -0.396966 |
| Gm129 | -0.3457769 |
| Gm1322 | -0.5711356 |
| Gm136 | 1.2071568 |
| Gm136 | 1.30627556 |
| Gm1381 | -0.24374 |
| Gm1527 | -0.5478815 |
| Gm1568 | -0.4720543 |
| Gm1574 | 0.81659339 |
| Gm1574 | -0.5792634 |
| Gm1604 | -0.6728029 |
| Gm1604 | -0.8954361 |
| Gm166 | -0.8168324 |
| Gm166 | -0.7558406 |
| Gm1661 | 0.0896017 |
| Gm1673 | -0.6477358 |
| Gm1673 | -0.0773375 |
| Gm1698 | 2.40781744 |
| Gm1821 | -0.7998033 |
| Gm1943 | -0.8120712 |
| Gm1965 | -0.3302625 |
| Gm1967 | -0.778482 |
| Gm1967 | 1.36738047 |
| Gm216 | -0.7381108 |
| Gm239 | 2.40562637 |
| Gm249 | 1.08628537 |
| Gm266 | 1.90483588 |
| Gm2a | 0.01240013 |
| Gm347 | -0.5674641 |
| Gm382 | 0.13765263 |
| Gm410 | 0.11907897 |
| Gm428 | -0.4654539 |
| Gm443 | -0.3787921 |
| Gm484 | -0.5822365 |
| Gm50 | 0.00842878 |
| Gm525 | -0.4173703 |
| Gm525 | 0.3710213 |
| Gm527 | 0.02343179 |
| Gm527 | 1.44719323 |
| Gm561 | 0.5388678 |
| Gm561 | -0.8429668 |
| Gm597 | -0.5126572 |
| Gm603 | 1.33936316 |
| Gm609 | -0.8054456 |
| Gm614 | -0.6533381 |
| Gm614 | -0.8332027 |
| Gm648 | 1.60571644 |
| Gm648 | 1.65982315 |
| Gm71 | -0.5286972 |
| Gm711 | 0.7643201 |
| Gm732 | 1.38242856 |
| Gm732 | -0.6240955 |
| Gm767 | 1.28059769 |
| Gm773 | -0.6295588 |
| Gm773 | -0.7793991 |
| Gm784 | 1.5484715 |
| Gm813 | -0.7729983 |
| Gm813 | -0.4407853 |
| Gm817 | -0.6071283 |
| Gm867 | -0.6477358 |
| Gm884 | -0.5897103 |
| Gm889 | 0.58877699 |
| Gm906 | -0.8351475 |
| Gm93 | 0.6188365 |
| Gm93 | -0.4694086 |
| Gm944 | 0.87012009 |
| Gm949 | 0.58273499 |
| Gm962 | 1.23791472 |
| Gm962 | 2.5087394 |
| Gm97 | 0.27016932 |
| Gm97 | 0.85060555 |
| Gmcl1 | 0.8850063 |
| Gmcl1 | -0.6469381 |
| Gmcl1 | 2.16173116 |
| Gmds | -0.1862533 |
| Gmeb2 | 0.60223161 |
| Gmfg | -0.7155494 |
| Gmfg | 0.3710213 |
| Gmfg | 0.24232275 |
| Gmip | 1.26150252 |
| Gmip | -0.5223881 |
| Gmppa | 1.16502703 |
| Gmppa | 0.72586903 |
| Gmppb | 1.14245778 |
| Gmpr | 1.41155341 |
| Gmpr2 | 0.12472476 |
| Gna11 | -0.737233 |
| Gna13 | -0.364501 |
| Gna14 | -0.3365524 |
| Gnai2 | -0.7036209 |
| Gnao1 | -0.1540329 |
| Gnao1 | -0.413005 |
| Gnaq | 0.78847203 |
| Gnaq | -0.7407492 |
| Gna-rs1 | -0.6868376 |
| Gnas | -0.8101734 |
| Gnas | 1.33868352 |
| Gnas | 0.69716496 |
| Gnas | 0.69158788 |
| Gnas | -0.2808576 |
| Gnat1 | -0.7276308 |
| Gnat2 | 3.01580454 |
| Gnaz | 2.75120617 |
| Gnb1 | -0.6695307 |
| Gnb1l | 2.3967159 |
| Gnb2 | -0.3206235 |
| Gnb2l1 | 0.095789 |
| Gnb3 | 0.58532916 |
| Gnb4 | -0.0712552 |
| Gnb4 | 1.68880916 |
| Gnb5 | 0.19422095 |
| Gnb5 | -0.2776182 |
| Gne | 0.00643681 |
| Gng10 | -0.4517415 |
| Gng11 | 0.96017588 |
| Gng13 | 3.06344954 |
| Gng2 | -0.0481554 |
| Gng2 | 2.49467321 |
| Gng3 | 0.82917959 |
| Gng4 | -0.658974 |
| Gng7 | -0.5972439 |
| Gng8 | -0.4223839 |
| Gngt2 | 0.4232002 |
| Gnl2 | 0.66953636 |
| Gnl2 | -0.8588025 |
| Gnl3 | 0.14766286 |
| Gnl3 | -0.0447808 |
| Gnl3 | -0.121049 |
| Gnl3l | 1.64013324 |
| Gnl3l | -0.7433949 |
| Gnmt | -0.5874619 |
| Gnmt | 0.11054642 |
| Gnpat | -0.5726082 |
| Gnpda1 | -0.6382084 |
| Gnpda1 | 0.36066838 |
| Gnpda1 | 1.69661663 |
| Gnpda2 | 1.69041597 |
| Gnptab | -0.555087 |
| Gnptg | 0.96336236 |
| Gnptg | 1.76213779 |
| Gnrhr | 0.32351118 |
| Gns | 2.18601049 |
| Golga1 | 0.88814349 |
| Golga2 | -0.5608913 |
| Golga2 | -0.701929 |
| Golga2 | -0.5407306 |
| Golga2 | -0.63191 |
| Golga2 | 0.81015169 |
| Golga3 | 0.22128171 |
| Golga4 | -0.7433949 |
| Golga5 | -0.0447808 |
| Golga7 | 0.6730837 |
| Golim4 | -0.6094242 |
| Golm1 | 1.12993783 |
| Golm1 | 0.47822208 |
| Golph3l | -0.1192246 |
| Golt1a | -0.658974 |
| Golt1b | 0.59798106 |
| Gopc | -0.6728029 |
| Gorasp1 | -0.5009745 |
| Gorasp2 | 1.74524875 |
| Gorasp2 | -0.6086583 |
| Gorasp2 | -0.5085174 |
| Gorasp2 | 0.03472303 |
| Gosr1 | -0.3674605 |
| Gosr2 | 1.5579105 |
| Gosr2 | 2.02505602 |
| Gosr2 | 1.56740842 |
| Gosr2 | 3.17415538 |
| Got1l1 | -0.2216428 |
| Got2 | 1.81581859 |
| Gp1bb | -0.8101734 |
| Gp38 | -0.8361214 |
| Gp5 | 0.12788572 |
| Gp9 | -0.4401447 |
| Gpaa1 | 1.36199481 |
| Gpam | -0.1549789 |
| Gpatch1 | -0.7693601 |
| Gpatch2 | 0.23858855 |
| Gpatch3 | -0.511966 |
| Gpatch4 | -0.7821556 |
| Gpbp1 | 0.62610067 |
| Gpbp1 | -0.8026203 |
| Gpbp1l1 | -0.6910055 |
| Gpc1 | -0.6413735 |
| Gpc3 | 0.5861923 |
| Gpc3 | 1.0475495 |
| Gpc4 | -0.0007693 |
| Gpc5 | 0.6588136 |
| Gpc5 | 2.37177709 |
| Gpc5 | -0.2473867 |
| Gpd1 | -0.7112719 |
| Gpd2 | 1.87857426 |
| Gpd2 | 1.45340587 |
| Gper | -0.5092061 |
| Gpha2 | 1.90845272 |
| Gphb5 | 0.6119358 |
| Gphn | 1.95253048 |
| Gphn | -0.2895507 |
| Gphn | -0.5696653 |
| Gpihbp1 | -0.4205007 |
| Gpkow | -0.0447808 |
| Gpld1 | 1.20592773 |
| Gpld1 | -0.4588993 |
| Gpm6a | -0.7276308 |
| Gpm6a | 0.65501159 |
| Gpm6b | 1.23053664 |
| Gpnmb | 0.76217484 |
| Gpnmb | 0.81962943 |
| Gpr1 | -0.4793685 |
| Gpr1 | 1.32301382 |
| Gpr103 | 2.33798163 |
| Gpr107 | -0.6605904 |
| Gpr108 | 0.25497086 |
| Gpr108 | -0.5785216 |
| Gpr108 | -0.3371263 |
| Gpr109a | -0.0699569 |
| Gpr113 | 0.25650178 |
| Gpr113 | -0.7576321 |
| Gpr114 | 1.18843661 |
| Gpr12 | 2.52101969 |
| Gpr12 | -0.6549449 |
| Gpr120 | 0.8191241 |
| Gpr123 | -0.3457769 |
| Gpr124 | -0.1016196 |
| Gpr128 | 2.91574857 |
| Gpr135 | 2.26794083 |
| Gpr137 | 1.86299393 |
| Gpr137b | 0.33343219 |
| Gpr141 | -0.2303171 |
| Gpr146 | 0.35599977 |
| Gpr146 | 0.43770141 |
| Gpr149 | 0.63043247 |
| Gpr151 | -0.6860062 |
| Gpr153 | -0.5829812 |
| Gpr156 | 1.80870542 |
| Gpr156 | -0.6264331 |
| Gpr158 | 0.00643681 |
| Gpr162 | 0.74974404 |
| Gpr162 | -0.7010842 |
| Gpr17 | 1.65205218 |
| Gpr172b | 1.0306026 |
| Gpr172b | -0.5002918 |
| Gpr176 | -0.043939 |
| Gpr177 | 0.50092201 |
| Gpr18 | 3.27789392 |
| Gpr180 | 1.60219732 |
| Gpr182 | 2.19983161 |
| Gpr182 | -0.5770398 |
| Gpr19 | 1.9634608 |
| Gpr20 | -0.5189013 |
| Gpr21 | -0.3799929 |
| Gpr22 | 1.07092978 |
| Gpr23 | -0.5733453 |
| Gpr26 | -0.8419858 |
| Gpr27 | 1.37076616 |
| Gpr27 | 2.70565444 |
| Gpr27 | -0.7442784 |
| Gpr34 | 0.12261155 |
| Gpr34 | 1.7244454 |
| Gpr37l1 | -0.1960886 |
| Gpr45 | -0.4375869 |
| Gpr6 | -0.7648321 |
| Gpr64 | 1.08565052 |
| Gpr65 | 3.602186 |
| Gpr68 | -0.634267 |
| Gpr75 | 1.92330568 |
| Gpr81 | 1.64961056 |
| Gpr83 | 0.17673859 |
| Gpr85 | -0.5357571 |
| Gpr85 | -0.6918413 |
| Gpr89 | 1.17801444 |
| Gpr89 | -0.4840527 |
| Gpr97 | -0.7363561 |
| Gprasp1 | -0.6943531 |
| Gprasp1 | 0.65841411 |
| Gprc2a-rs5 | 0.64270053 |
| Gprc2a-rs5 | 0.3490919 |
| Gprc5a | -0.7095663 |
| Gprc5b | 3.02916564 |
| Gprc5c | -0.4733799 |
| Gprk6 | -0.0905048 |
| Gprk6 | 1.64358418 |
| Gps1 | -0.4760368 |
| Gps1 | -0.4427095 |
| Gps1 | -0.6217638 |
| Gps1 | -0.4249008 |
| Gps2 | 0.59606205 |
| Gpsm1 | 1.63503946 |
| Gpsm2 | -0.2201203 |
| Gpsm3 | 0.10301659 |
| Gpsn2 | -0.5329268 |
| Gpsn2 | -0.5336335 |
| Gpsn2 | 1.34091479 |
| Gpt1 | -0.5837265 |
| Gpt2 | 0.67642176 |
| Gpx1 | 2.12304198 |
| Gpx2 | 2.33040979 |
| Gpx3 | 0.13313057 |
| Gpx4 | 0.59071087 |
| Gpx4 | 0.35874881 |
| Gpx6 | -0.6926778 |
| Gpx7 | -0.6405813 |
| Gramd1a | -0.3591972 |
| Gramd1b | -0.375199 |
| Gramd2 | -0.0359795 |
| Gramd2 | -0.353923 |
| Gramd3 | -0.3890476 |
| Gramd4 | 0.0648134 |
| Grap2 | -0.5829812 |
| Grasp | -0.6397897 |
| Grasp | -0.63191 |
| Grb10 | 0.107685 |
| Grb14 | 0.12331648 |
| Grb14 | 1.0025882 |
| Grb2 | 0.28304694 |
| Grb7 | -0.7164073 |
| Grcc10 | 3.12916071 |
| Greb1 | 0.0819021 |
| Greb1 | 1.27010666 |
| Grem1 | 0.32379609 |
| Grem2 | -0.4006432 |
| Grhl1 | 1.50665787 |
| Grhl2 | 0.88484917 |
| Grhpr | -0.5718716 |
| Gria1 | -0.6186637 |
| Gria1 | -0.5755603 |
| Gria1 | -0.699397 |
| Gria2 | 2.81155985 |
| Gria2 | 0.51125336 |
| Gria3 | -0.6760864 |
| Gria3 | 0.3209431 |
| Gria3 | -0.613263 |
| Grid2 | 2.25802541 |
| Grik1 | -0.3805938 |
| Grik2 | -0.6101907 |
| Grik4 | 2.37902526 |
| Grik5 | -0.5064542 |
| Grin1 | 2.56363215 |
| Grin1 | 1.42308047 |
| Grin2a | 1.9180654 |
| Grin2b | 2.4199017 |
| Grin2c | -0.4867399 |
| Grin3b | -0.6910055 |
| Grina | -0.510585 |
| Grip1 | 2.41352162 |
| Grip1 | 0.34742645 |
| Grip1 | -0.6926778 |
| Gripap1 | -0.3251472 |
| Gripap1 | -0.4037185 |
| Grit | 0.46286143 |
| Grk4 | 0.63104967 |
| Grk4 | -0.6040759 |
| Grk4 | -0.634267 |
| Grk5 | -0.6178903 |
| Grlf1 | -0.5071414 |
| Grm1 | 1.86243668 |
| Grm7 | 1.50258499 |
| Grm8 | -0.1833226 |
| Grn | 1.46418048 |
| Grp | -0.4536883 |
| Grpel1 | 0.48352772 |
| Grpr | -0.6477358 |
| Grsf1 | 1.24823355 |
| Grtp1 | -0.5286972 |
| Grwd1 | 0.0171438 |
| Grwd1 | -0.2669 |
| Gsbs | 0.3294763 |
| Gsc | -0.7558406 |
| Gsdma1 | -0.7504866 |
| Gsdmdc1 | 1.06769899 |
| Gsdmdc1 | 0.0651882 |
| Gsdmdc1 | 0.82164804 |
| Gse1 | -0.8322319 |
| Gsg1 | 0.16400156 |
| Gsg1 | 0.63964844 |
| Gsg1 | -0.421128 |
| Gsg2 | -0.4867399 |
| Gsk3a | 1.14556197 |
| Gsk3b | -0.432492 |
| Gsn | 1.42706862 |
| Gspt1 | 0.12402088 |
| Gspt2 | -0.3434623 |
| Gss | -0.1376291 |
| Gss | -0.7027746 |
| Gss | 1.21924993 |
| Gsta2 | 0.18864862 |
| Gsta3 | 0.21394907 |
| Gsta3 | 0.13869311 |
| Gsta4 | -0.3933031 |
| Gstcd | 1.89011996 |
| Gstk1 | -0.6501327 |
| Gstm1 | -0.3580226 |
| Gstm1 | 2.58179684 |
| Gstm2 | 0.0116072 |
| Gstm2 | -0.2642393 |
| Gstm2 | -0.416121 |
| Gstm4 | 1.76040032 |
| Gstm5 | 0.0108136 |
| Gstm6 | -0.5565347 |
| Gstm6 | -0.2313429 |
| Gsto1 | 3.2108899 |
| Gsto2 | -0.5064542 |
| Gsto2 | -0.6140326 |
| Gstp1 | -0.5565347 |
| Gstp1 | -0.7311112 |
| Gstp2 | -0.555087 |
| Gstt1 | 0.60793964 |
| Gstt2 | 0.73585983 |
| Gstt3 | -0.5740831 |
| Gstt4 | 1.13018017 |
| Gstz1 | 0.31060031 |
| Gtdc1 | -0.6719838 |
| Gtf2a1 | 1.22846476 |
| Gtf2e1 | 1.35026728 |
| Gtf2e2 | -0.6295588 |
| Gtf2e2 | 1.99177706 |
| Gtf2f1 | 0.97998803 |
| Gtf2f2 | -0.1784582 |
| Gtf2h1 | 1.94065147 |
| Gtf2h1 | 0.77249866 |
| Gtf2h2 | 0.73217186 |
| Gtf2h3 | 0.61946134 |
| Gtf2h4 | -0.6605904 |
| Gtf2h5 | 1.39559586 |
| Gtf2ird1 | -0.511966 |
| Gtf2ird1 | -0.4414262 |
| Gtf2ird2 | 1.18558483 |
| Gtf2ird2 | 0.02538823 |
| Gtf2ird2 | 0.08005956 |
| Gtf3a | -0.5301049 |
| Gtf3a | 0.5779604 |
| Gtf3c1 | -0.5209919 |
| Gtf3c2 | 0.18006204 |
| Gtf3c3 | -0.6445493 |
| Gtf3c4 | 0.52906531 |
| Gtf3c4 | -0.1348454 |
| Gtf3c5 | -0.536466 |
| Gtf3c5 | -0.0751608 |
| Gtf3c5 | -0.0506942 |
| Gtl3 | 0.12084691 |
| Gtpbp1 | 1.7044259 |
| Gtpbp2 | 0.34520128 |
| Gtpbp3 | -0.6851756 |
| Gtpbp4 | -0.7044679 |
| Gtpbp5 | 0.50704056 |
| Gtpbp6 | 0.70613421 |
| Gtpbp8 | 0.11197388 |
| Gtpbp8 | -0.1224196 |
| Gtrgeo22 | 1.35113036 |
| Gtse1 | -0.0600624 |
| Gtse1 | -0.0068167 |
| Gtse1 | -0.2851941 |
| Gtsf1l | 0.08813994 |
| Guca1a | -0.5919641 |
| Guca1b | -0.4255311 |
| Guca2a | 1.94882479 |
| Guca2b | 0.23921194 |
| Gucy1a2 | 1.42839426 |
| Gucy1a3 | -0.6109578 |
| Gucy1a3 | 0.21426907 |
| Gucy1a3 | -0.3245806 |
| Gucy1b3 | 0.94171572 |
| Guk1 | 1.20949876 |
| Gulo | 1.72721581 |
| Gulp1 | -0.6605904 |
| Gusb | -0.2786968 |
| Gusb | 0.66775768 |
| Gusb | -0.3155601 |
| Gvin1 | 1.84516701 |
| Gyg | 0.68558949 |
| Gyg | 1.18318266 |
| Gykl1 | 0.9678342 |
| Gyltl1b | -0.5322205 |
| Gyltl1b | 0.94952478 |
| Gypa | 0.52952351 |
| Gypc | 0.62651408 |
| Gys1 | 0.11054642 |
| Gys3 | 0.27558137 |
| Gzf1 | -0.6711654 |
| Gzf1 | -0.4446377 |
| Gzmd | 2.68807313 |
| Gzmd | 0.62403089 |
| Gzmk | -0.4363106 |
| Gzmm | -0.6670841 |
| Gzmn | 0.92845519 |
| H13 | -0.3116406 |
| H13 | 1.8059871 |
| H13 | -0.3371263 |
| H13 | 0.19780911 |
| H19 | 2.49947986 |
| H1f0 | -0.4363106 |
| H1fnt | -0.023547 |
| H1foo | -0.0677971 |
| H1fx | -0.5777804 |
| H1fx | -0.2206276 |
| H2-Aa | 2.28536634 |
| H2-Ab1 | -0.7129806 |
| H2-Ab1 | 1.38483633 |
| H2-Ab1 | 0.71933653 |
| H2afj | -0.2900967 |
| H2afx | 3.09012204 |
| H2afy | -0.6605904 |
| H2afz | 0.04319952 |
| H2-Bl | -0.0409987 |
| H2-DMa | 1.69615133 |
| H2-DMb1 | -0.7129806 |
| H2-Eb1 | 1.83684179 |
| H2-K1 | -0.5987578 |
| H2-Ke6 | -0.209531 |
| H2-M10.3 | 1.88262044 |
| H2-M2 | 0.22286825 |
| H2-M3 | -0.1007256 |
| H2-Oa | 1.57446961 |
| H2-Ob | 0.69389964 |
| H2-Q1 | 1.50470508 |
| H2-Q2 | -0.2328837 |
| H2-Q7 | -0.290643 |
| H2-Q8 | -0.5385959 |
| H2-T10 | 0.26472605 |
| H2-T23 | 0.29604453 |
| H2-T23 | -0.7146924 |
| H2-T24 | 3.80062954 |
| H2-Tw3 | -0.2303171 |
| H3f3a | 0.06967428 |
| H3f3a | 0.05007861 |
| H3f3b | 1.02438846 |
| H47 | 0.63453958 |
| H47 | -0.5350487 |
| H6pd | 0.06256144 |
| Habp4 | -0.6613997 |
| Habp4 | 0.48328721 |
| Hace1 | -0.9620676 |
| Hacl1 | -0.8130215 |
| Hadhb | -0.3963545 |
| Hagh | -0.8975136 |
| Haghl | 2.62672668 |
| Haghl | 2.31442564 |
| Hal | -0.0141252 |
| Hamp2 | -0.5601638 |
| Hao1 | -0.7849203 |
| Hao3 | -0.1034102 |
| Hap1 | 0.30828643 |
| Hap1 | 1.66148006 |
| Hap1 | 0.69293709 |
| Hapln1 | 0.12084691 |
| Hapln2 | -0.4975658 |
| Hapln4 | 0.62423807 |
| Harbi1 | -0.0116828 |
| Hars | 2.16759771 |
| Has1 | -0.3698348 |
| Hat1 | 0.54316689 |
| Havcr1 | -0.4530389 |
| Havcr2 | 1.53917269 |
| Hax1 | -0.5638069 |
| Hba-a1 | -0.8016804 |
| Hbb-b1 | -0.2835656 |
| Hbegf | -0.5023415 |
| Hbp1 | -0.431857 |
| Hbp1 | 1.39915526 |
| Hbs1l | -0.568931 |
| Hbs1l | -0.607893 |
| Hbs1l | -0.0686604 |
| Hbs1l | 0.55438753 |
| Hc | -0.4407853 |
| Hccs | -0.6025534 |
| Hcfc1 | -0.3077373 |
| Hcfc1r1 | -0.6501327 |
| Hcfc2 | 3.20786936 |
| Hcls1 | -0.4024872 |
| Hcn2 | 2.72107661 |
| Hcn3 | 1.39650981 |
| Hcn3 | 0.75822916 |
| Hcr | -0.3521715 |
| Hcst | -0.0363967 |
| Hdac10 | -0.4433518 |
| Hdac11 | 0.38660723 |
| Hdac2 | 0.0366562 |
| Hdac3 | -0.5748214 |
| Hdac4 | 0.11624336 |
| Hdac5 | -0.0473106 |
| Hdac6 | 1.97802111 |
| Hdac7 | 0.48208369 |
| Hdac8 | -0.2196134 |
| Hdac9 | -0.7407492 |
| Hdc | -0.6851756 |
| Hddc2 | -0.5521982 |
| Hddc2 | -0.5837265 |
| Hdgf | 1.03262013 |
| Hdgf | -0.6094242 |
| Hdgf | -0.525187 |
| Hdgf | 0.17840178 |
| Hdgfl1 | 1.33527795 |
| Hdgfrp2 | -0.607893 |
| Hdhd1a | 2.42279873 |
| Hdhd2 | -0.3680535 |
| Hdhd2 | 0.90475249 |
| Hdhd2 | 0.39086186 |
| Hdlbp | 1.25377614 |
| Hdlbp | 1.70403037 |
| Heatr1 | 0.79229345 |
| Heatr1 | -0.2344269 |
| Heatr3 | -0.3245806 |
| Heatr5a | -0.3728111 |
| Hebp1 | 3.72958416 |
| Hebp2 | -0.7078638 |
| Heca | 1.16561066 |
| Hectd2 | -0.3639102 |
| Hectd2 | 2.62984493 |
| Hectd3 | -0.5740831 |
| Hecw2 | 0.26016587 |
| Hecw2 | 0.0131924 |
| Hecw2 | -0.6893361 |
| Heg1 | 2.60361517 |
| Hel308 | 1.45735374 |
| Helb | -0.5800058 |
| Helb | -0.4800362 |
| Helz | -0.4504459 |
| Hemgn | 1.44276772 |
| Hemk1 | -0.6186637 |
| Hemt1 | -0.7757362 |
| Hepacam | 1.75616738 |
| Heph | 0.41986145 |
| Heph | 0.6406669 |
| Heph | 1.58724209 |
| Heph | -0.416121 |
| Heph | 2.4026446 |
| Herc2 | -0.6382084 |
| Herc3 | -0.3933031 |
| Herc3 | 0.29222294 |
| Herc4 | -0.0622046 |
| Herc4 | 0.60857174 |
| Herpud1 | 1.23650866 |
| Herpud1 | 0.99827851 |
| Herpud2 | -0.7293694 |
| Herpud2 | -0.8120712 |
| Herpud2 | 2.54516481 |
| Herpud2 | -0.0656422 |
| Hes1 | -0.6148029 |
| Hes5 | -0.8273925 |
| Hes5 | 0.58727011 |
| Hes6 | -0.7328563 |
| Hes6 | 0.09868668 |
| Hes6 | 1.70211633 |
| Hexa | 0.19748348 |
| Hexb | -0.7504866 |
| Hexdc | -0.5755603 |
| Hexdc | 0.34742645 |
| Hexim1 | 1.69575231 |
| Hexim2 | 2.0083962 |
| Hey1 | -0.1353088 |
| Hey2 | 0.70328094 |
| Hey2 | 1.96571169 |
| Heyl | 0.75733011 |
| Hfe | 0.6981232 |
| Hfe | 0.29633785 |
| Hgfac | -0.4293216 |
| Hgs | 0.44273717 |
| Hgs | 0.74701987 |
| Hgsnat | 1.57507456 |
| Hhat | 1.87862905 |
| Hhat | -0.5777804 |
| Hhat | -0.2125444 |
| Hhatl | -0.5934696 |
| Hhex | -0.2473867 |
| Hhip | -0.3656837 |
| Hibadh | 0.40743035 |
| Hic1 | 1.15893583 |
| Hic2 | -0.6256532 |
| Hif1a | 1.97228347 |
| Hif3a | -0.8429668 |
| Hif3a | 2.79579931 |
| Higd1a | -0.2536712 |
| Higd1b | 0.52814825 |
| Higd2a | 1.13597778 |
| Hils1 | -0.1288422 |
| Hint1 | 1.46740596 |
| Hint2 | -0.6405813 |
| Hint3 | -0.0596346 |
| Hint3 | -0.3704293 |
| Hip1 | -0.612494 |
| Hip1r | -0.4874129 |
| Hip2 | -0.2552489 |
| Hipk1 | 1.32774213 |
| Hipk2 | -0.3848113 |
| Hipk3 | 2.75064118 |
| Hipk4 | -0.1985635 |
| Hira | -0.6397897 |
| Hira | 2.72840487 |
| Hirip3 | 0.73824929 |
| Hisppd1 | -0.3121995 |
| Hist1h1b | -0.5957324 |
| Hist1h1c | -0.3662756 |
| Hist1h2aa | -0.5934696 |
| Hist1h2ac | 0.86852254 |
| Hist1h2ad | 2.29960473 |
| Hist1h2ae | -0.2339122 |
| Hist1h2af | 2.05165275 |
| Hist1h2ag | -0.632695 |
| Hist1h2ah | 1.01156123 |
| Hist1h2ak | -0.3217524 |
| Hist1h2an | 0.63964844 |
| Hist1h2ao | 0.27197679 |
| Hist1h2bc | -0.7988662 |
| Hist1h2be | -0.340577 |
| Hist1h2bf | 2.95900714 |
| Hist1h2bg | 1.90834654 |
| Hist1h2bh | 1.99148543 |
| Hist1h2bj | -0.6760864 |
| Hist1h2bk | -0.05324 |
| Hist1h2bm | 0.34825954 |
| Hist1h2bn | 1.44467931 |
| Hist1h3a | -0.7164073 |
| Hist1h3d | -0.2999789 |
| Hist1h3e | -0.3446189 |
| Hist1h3f | -0.4186212 |
| Hist1h4c | 1.21294534 |
| Hist1h4d | 1.51861153 |
| Hist1h4f | 0.48039618 |
| Hist1h4h | 1.45598244 |
| Hist1h4i | -0.5272915 |
| Hist1h4j | 1.52021534 |
| Hist1h4k | -0.7354799 |
| Hist1h4m | -0.4293216 |
| Hist2h2aa1 | -0.536466 |
| Hist2h2ab | 0.36613145 |
| Hist2h2ac | -0.650933 |
| Hist2h2bb | 0.16231276 |
| Hist2h2be | 1.013894 |
| Hist2h3b | -0.4569418 |
| Hist2h3c1 | -0.6048381 |
| Hist2h3c1 | -0.3200596 |
| Hist2h4 | 1.90211412 |
| Hist3h2a | -0.7087146 |
| Hist3h2bb | 0.12893703 |
| Hist4h4 | 0.47507284 |
| Hk1 | -0.2385547 |
| Hk3 | 2.94469487 |
| Hkdc1 | -0.7070137 |
| Hlcs | 1.69348806 |
| Hlf | -0.6926778 |
| Hltf | -0.4747074 |
| Hlx | -0.5852189 |
| Hmg20a | 3.21383429 |
| Hmg20a | -0.6605904 |
| Hmg20b | -0.406186 |
| Hmga1 | -0.5927165 |
| Hmgb2 | -0.1192246 |
| Hmgb2l1 | 1.97004823 |
| Hmgb2l1 | 1.95657484 |
| Hmgb4 | -0.3639102 |
| Hmgcl | -0.2983247 |
| Hmgcl | 0.45471442 |
| Hmgcl | 2.87997237 |
| Hmgcll1 | -0.5995157 |
| Hmgcs2 | -0.5329268 |
| Hmgn1 | 2.18794663 |
| Hmgn2 | 0.64796753 |
| Hmgn3 | -0.0393227 |
| Hmgn3 | 1.03583924 |
| Hmha1 | -0.5195976 |
| Hmox1 | 0.24820516 |
| Hmox2 | -0.5357571 |
| Hmx1 | -0.3245806 |
| Hmx1 | -0.1563997 |
| Hn1 | 1.85936593 |
| Hn1 | 2.25295465 |
| Hn1l | -0.6413735 |
| Hnf1b | -0.0414182 |
| Hnf1b | -0.511966 |
| Hnf4a | 3.2793966 |
| Hnrnpc | 1.88463704 |
| Hnrpa1 | 0.51101981 |
| Hnrpa1 | 0.88532049 |
| Hnrpab | -0.2781573 |
| Hnrpc | 3.6351445 |
| Hnrpdl | -0.7720875 |
| Hnrpdl | -0.4595527 |
| Hnrpf | 0.21362896 |
| Hnrph1 | -0.2819399 |
| Hnrph1 | -0.650933 |
| Hnrph1 | 1.05348198 |
| Hnrph1 | 0.52975252 |
| Hnrph1 | 0.02147129 |
| Hnrph2 | -0.6910055 |
| Hnrph3 | 2.35997739 |
| Hnrpk | 1.60557007 |
| Hnrpl | 0.44624627 |
| Hnrpll | 1.44302862 |
| Hnrpll | -0.5837265 |
| Hnrpm | 0.21490874 |
| Hnrpm | -0.4356731 |
| Hnrpm | 2.9566863 |
| Hnrpul1 | 1.71881513 |
| Hnrpul1 | 0.69139498 |
| Hnrpul1 | -0.375199 |
| Hnrpul1 | 0.10445549 |
| Hnrpul1 | -0.3268492 |
| Hnt | 0.31204361 |
| Homer1 | 1.21350007 |
| Homer1 | -0.24374 |
| Homer1 | -0.4767022 |
| Homer1 | -0.3279856 |
| Homez | -0.3811952 |
| Homez | 1.92147489 |
| Hook1 | 0.17840178 |
| Hook1 | -0.4299548 |
| Hook1 | 3.36632236 |
| Hook1 | 0.76983952 |
| Hook2 | 0.76859602 |
| Hormad2 | -0.455639 |
| Hoxa13 | 1.06523617 |
| Hoxa2 | -0.7969948 |
| Hoxa2 | 0.150748 |
| Hoxa4 | -0.6094242 |
| Hoxa5 | -0.0326481 |
| Hoxa7 | 1.45915055 |
| Hoxa9 | 1.30123604 |
| Hoxa9 | 2.03374182 |
| Hoxb2 | 1.82293664 |
| Hoxb3 | 2.36715325 |
| Hoxb3 | -0.5428702 |
| Hoxb4 | -0.2080279 |
| Hoxb5 | -0.4968855 |
| Hoxb6 | -0.6687145 |
| Hoxb7 | -0.4255311 |
| Hoxc10 | 1.46553992 |
| Hoxc10 | 1.66972123 |
| Hoxc6 | 0.11411104 |
| Hoxc9 | -0.6163453 |
| Hoxd10 | -0.6117256 |
| Hoxd3 | 1.24780585 |
| Hoxd4 | 0.107685 |
| Hoxd8 | 0.41960413 |
| Hoxd9 | -0.4840527 |
| Hp | 0.78638107 |
| Hp | 0.0979631 |
| Hp | 0.3209431 |
| Hp1bp3 | -0.737233 |
| Hpca | -0.3890476 |
| Hpcal1 | -0.1808872 |
| Hpcal1 | 1.74411199 |
| Hpcal4 | -0.6687145 |
| Hpgd | -0.8073339 |
| Hpn | -0.1432211 |
| Hpn | -0.1823477 |
| Hprt1 | 1.85280015 |
| Hprt1 | 1.73286025 |
| Hprt1 | -0.1702459 |
| Hps1 | 0.94849709 |
| Hps3 | -0.1124145 |
| Hps3 | -0.6429601 |
| Hps4 | -0.1838104 |
| Hps5 | -0.4261618 |
| Hps6 | -0.6851756 |
| Hpse | -0.5371754 |
| Hpx | 1.7560426 |
| Hr | 1.77255722 |
| Hr | -0.4582464 |
| Hras1 | 0.19323999 |
| Hrasls | 0.09687667 |
| Hrasls5 | 2.61625742 |
| Hrasls5 | -0.2427006 |
| Hrb | -0.5064542 |
| Hrb | -0.4941693 |
| Hrbl | -0.5770398 |
| Hrbl | 1.12043758 |
| Hrc | -0.1857642 |
| Hrg | -0.4000293 |
| Hrh3 | 0.71652298 |
| Hrk | 1.68073381 |
| Hrmt1l2 | 2.77013224 |
| Hrsp12 | 1.20827274 |
| Hrsp12 | -0.3428845 |
| Hrsp12 | -0.6777325 |
| Hs1bp3 | 1.6474427 |
| Hs2st1 | 1.11220001 |
| Hs3st1 | -0.3308326 |
| Hs3st3a1 | 1.19343489 |
| Hs3st3b1 | 3.69585048 |
| Hs3st6 | 1.04913514 |
| Hs6st1 | -0.1512005 |
| Hs6st2 | -0.658974 |
| Hs6st2 | -0.0721217 |
| Hs6st3 | 2.26295969 |
| Hsbp1 | 1.18524204 |
| Hsd11b1 | -0.4192473 |
| Hsd11b1 | -0.1479067 |
| Hsd11b1 | -0.0913891 |
| Hsd11b1 | 0.10481487 |
| Hsd11b2 | -0.0755957 |
| Hsd17b1 | -0.7398689 |
| Hsd17b10 | -0.5223881 |
| Hsd17b11 | -0.4280564 |
| Hsd17b11 | -0.1597234 |
| Hsd17b11 | 3.09612404 |
| Hsd17b12 | 0.83117789 |
| Hsd17b2 | 0.64715913 |
| Hsd17b3 | -0.0634922 |
| Hsd17b4 | -0.6517341 |
| Hsd17b7 | 1.57416698 |
| Hsd3b1 | -0.4363106 |
| Hsd3b2 | -0.6287764 |
| Hsd3b2 | -0.6752645 |
| Hsd3b4 | 0.45198304 |
| Hsd3b7 | -0.4382257 |
| Hsd3b7 | -0.1784582 |
| Hsdl1 | -0.2711727 |
| Hsf1 | -0.5608913 |
| Hsf4 | -0.4833821 |
| Hsf5 | -0.63191 |
| Hsn2 | 2.08723238 |
| Hsp105 | -0.5704001 |
| Hsp90aa1 | -0.2873699 |
| Hsp90ab1 | 0.58813148 |
| Hsp90ab1 | 1.55821838 |
| Hsp90ab1 | 1.43534541 |
| Hsp90b1 | -0.7293694 |
| Hspa12b | 2.75024983 |
| Hspa1a | -0.3556778 |
| Hspa1l | 1.35524307 |
| Hspa2 | -0.3262816 |
| Hspa2 | 3.23121876 |
| Hspa4 | -0.6366299 |
| Hspa4l | -0.4813732 |
| Hspa5 | 0.67269021 |
| Hspa9 | -0.5594369 |
| Hspa9 | 2.00317709 |
| Hspb1 | -0.4382257 |
| Hspb2 | 1.77610676 |
| Hspb3 | -0.4760368 |
| Hspb6 | -0.6217638 |
| Hspb7 | 1.28389738 |
| Hspb8 | 0.28274945 |
| Hspbap1 | -0.6056009 |
| Hspd1 | -0.6752645 |
| Hspd1 | -0.3811952 |
| Hspe1 | 2.49982023 |
| Hspg2 | 0.61130595 |
| Htatip | 1.09475138 |
| Htatip | -0.510585 |
| Htatip2 | -0.6769091 |
| Htatip2 | 1.33566778 |
| Htatsf1 | -0.4602066 |
| Htf9c | -0.7070137 |
| Htr1a | -0.6960314 |
| Htr1b | -0.6366299 |
| Htr1d | 0.18897732 |
| Htr1f | -0.6557494 |
| Htr1f | 0.27016932 |
| Htr2b | 1.37908455 |
| Htr3a | 0.27438137 |
| Htr5b | -0.2421814 |
| Htr7 | -0.5852189 |
| Htra1 | 3.03912774 |
| Htra1 | 1.41961038 |
| Htra2 | 0.21106419 |
| Htra3 | 3.47122458 |
| Htra3 | 0.86129986 |
| Htt | 2.91315401 |
| Hus1 | 1.93116374 |
| Hus1 | 1.79035333 |
| Hus1 | -0.5272915 |
| Huwe1 | 2.61768508 |
| Hvcn1 | -0.2303171 |
| Hvcn1 | 0.22350212 |
| Hyal1 | 1.31766599 |
| Hyal2 | -0.632695 |
| Hyal3 | -0.1174038 |
| Hyal5 | 0.13799959 |
| Hyal6 | -0.5957324 |
| Hydin | -0.2025369 |
| Hyi | -0.6217638 |
| Hyou1 | -0.0695246 |
| Iah1 | 1.36095193 |
| Iap | -0.4674291 |
| Iars | -0.6977127 |
| Iars | 0.45048985 |
| Iars2 | -0.6810333 |
| Ibtk | 2.28251411 |
| Ica1 | -0.7104187 |
| Ica1l | -0.4674291 |
| Icam1 | 1.41056394 |
| Icam2 | -0.3896544 |
| Icam4 | 2.00130267 |
| Icam5 | -0.1659271 |
| Ick | 0.18238152 |
| Icmt | 0.10911679 |
| Ict1 | 1.78079853 |
| Id1 | -0.3698348 |
| Id2 | -0.7433949 |
| Id2 | 1.59022241 |
| Id3 | 1.04423748 |
| Id4 | -0.3872297 |
| Ide | 0.10229632 |
| Idh1 | 0.52631145 |
| Idh2 | 2.26881074 |
| Idh3a | -0.4012575 |
| Idh3b | -0.5189013 |
| Idh3g | 2.22801632 |
| Idh3g | 2.55831641 |
| Idi2 | -0.5616193 |
| Ids | -0.6868376 |
| Ids | 2.08903141 |
| Ids | 1.27021111 |
| Idua | 1.6149646 |
| Ier3 | -0.1274622 |
| Ier3ip1 | -0.562348 |
| Ier5l | 1.82444668 |
| Ifi202b | 0.899197 |
| Ifi202b | 0.98324997 |
| Ifi27 | 0.82967956 |
| Ifi30 | -0.7129806 |
| Ifi30 | -0.6622096 |
| Ifi35 | 1.12690328 |
| Ifi47 | 4.03007513 |
| Ifit2 | 1.31208788 |
| Ifit2 | -0.5064542 |
| Ifit3 | -0.7389895 |
| Ifit3 | -0.0852158 |
| Ifitm1 | -0.5844724 |
| Ifitm1 | 1.40133901 |
| Ifitm1 | -0.6421665 |
| Ifitm2 | 0.80349442 |
| Ifitm3 | 1.74802175 |
| Ifitm5 | -0.8799988 |
| Ifitm6 | -0.4654539 |
| Ifna1 | -0.5308096 |
| Ifna6 | -0.6918413 |
| Ifnar2 | 1.20927596 |
| Ifnar2 | 2.94138009 |
| Ifnar2 | 0.08300589 |
| Ifngr1 | -0.1516719 |
| Ifngr2 | 0.14007864 |
| Ifngr2 | -0.1464986 |
| Ifngr2 | -0.7513768 |
| Ifnz | -0.3161214 |
| Ifrd1 | 0.25955617 |
| Ifrg15 | -0.5371754 |
| Ift122 | -0.5579847 |
| Ift140 | -0.5867137 |
| Ift140 | 0.23076115 |
| Ift140 | -0.4820423 |
| Ift172 | -0.2626465 |
| Ift172 | 1.72218439 |
| Ift172 | -0.7095663 |
| Ift20 | 0.14422304 |
| Ift20 | 2.15308765 |
| Ift20 | 0.32890982 |
| Ift52 | 1.49463309 |
| Ift80 | -0.9132427 |
| Ift81 | 0.45669593 |
| Ift81 | 1.3068785 |
| Ift81 | -0.4948476 |
| Ift88 | 1.08068397 |
| Igbp1 | 0.09433681 |
| Igbp1b | -0.8111218 |
| Igf1 | -0.3951328 |
| Igf1 | 0.66874625 |
| Igf1r | 0.69505339 |
| Igf2 | -0.5393069 |
| Igf2bp1 | -0.5897103 |
| Igf2bp2 | -0.7095663 |
| Igf2bp3 | 1.40957342 |
| Igf2bp3 | 4.27571646 |
| Igf2r | -0.2370047 |
| Igf2r | -0.6785566 |
| Igfals | -0.0622046 |
| Igfbp2 | -0.1371645 |
| Igfbp2 | 0.54744648 |
| Igfbp3 | -0.5244865 |
| Igfbp4 | 0.02421485 |
| Igfbp4 | 0.01871973 |
| Igfbp5 | 0.56149932 |
| Igfbp6 | -0.6264331 |
| Igfbp7 | -0.831262 |
| Igfbpl1 | 0.12998716 |
| Igsf1 | -0.7675463 |
| Igsf1 | -0.6485341 |
| Igsf1 | 2.10872633 |
| Igsf1 | 0.14560048 |
| Igsf11 | -0.2749272 |
| Igsf11 | -0.7941946 |
| Igsf11 | -0.3896544 |
| Igsf21 | -0.8789786 |
| Igsf3 | -0.5371754 |
| Igsf4a | 0.24045749 |
| Igsf5 | -0.4747074 |
| Igsf8 | 0.1404247 |
| Igsf9 | -0.4880863 |
| Igtp | 1.24480647 |
| Ihh | -0.2442601 |
| Ihpk1 | 1.19264148 |
| Ihpk2 | -0.1106068 |
| Ihpk2 | 0.7131356 |
| Iigp2 | 0.23076115 |
| Ik | 0.4689869 |
| Ikbkap | -0.5478815 |
| Ikbkb | -0.8158783 |
| Ikbke | 0.72735584 |
| Ikbkg | 2.53048304 |
| Ikbkg | -0.8026203 |
| Il10ra | 0.09179009 |
| Il10rb | -0.4760368 |
| Il11 | 0.85076849 |
| Il11ra1 | 2.08243284 |
| Il12a | 1.16689336 |
| Il13 | -0.5829812 |
| Il13ra1 | 0.59925824 |
| Il13ra2 | -0.8628034 |
| Il15 | 0.26381577 |
| Il15 | 1.65253975 |
| Il15 | 0.28572015 |
| Il15ra | -0.5343408 |
| Il15ra | 1.13934334 |
| Il15ra | 1.35132204 |
| Il16 | 0.46727578 |
| Il17d | 1.43455609 |
| Il17d | -0.7112719 |
| Il17ra | 0.41960413 |
| Il17rb | -0.7711775 |
| Il17rc | -0.5674641 |
| Il17rc | -0.7998033 |
| Il17rc | -0.6557494 |
| Il17rd | 1.85443183 |
| Il17rd | 1.77243459 |
| Il17re | 0.44474397 |
| Il17re | 1.42165838 |
| Il17re | 1.99026931 |
| Il18 | 4.04356071 |
| Il18bp | 0.27558137 |
| Il18r1 | 1.88480036 |
| Il1b | -0.455639 |
| Il1r1 | 1.71965858 |
| Il1rap | -0.3077373 |
| Il1rap | 0.37911514 |
| Il1rap | -0.273853 |
| Il1rap | -0.6851756 |
| Il1rap | -0.2359727 |
| Il1rl1l | 1.64175464 |
| Il1rl2 | 0.62610067 |
| Il20ra | -0.4293216 |
| Il20rb | -0.4569418 |
| Il20rb | -0.4249008 |
| Il23a | 0.87442002 |
| Il27ra | -0.2727799 |
| Il28ra | -0.3805938 |
| Il28ra | 2.24232081 |
| Il28ra | 2.27678364 |
| Il31ra | -0.364501 |
| Il33 | -0.6695307 |
| Il3ra | -0.0056042 |
| Il4i1 | 1.20279196 |
| Il6ra | -0.4080408 |
| Il6st | 1.41594852 |
| Il7 | 2.7963379 |
| Ildr1 | 0.25680766 |
| Ildr1 | 0.31088915 |
| Ilf2 | 0.26381577 |
| Ilf3 | -0.4305884 |
| Ilf3 | -0.072989 |
| Ilf3 | 0.48760719 |
| Ilf3 | 1.81657963 |
| Ilk | 1.14209895 |
| Ilkap | -0.4914609 |
| Ilvbl | 1.42014507 |
| Ilvbl | -0.7267627 |
| Ilvbl | -0.1101554 |
| Ilvbl | -0.63191 |
| Immp2l | -0.1007256 |
| Immt | -0.7469341 |
| Imp3 | 0.87901673 |
| Imp4 | -0.4530389 |
| Imp4 | -0.1741016 |
| Impa2 | 3.56394059 |
| Impact | 1.55010299 |
| Impad1 | 0.9454073 |
| Impdh1 | 0.18733269 |
| Impdh2 | 0.1404247 |
| Ina | 0.40638699 |
| Inadl | -0.6148029 |
| Inadl | 1.05532006 |
| Incenp | -0.1842984 |
| Indo | 2.02096569 |
| Ing2 | -0.2401073 |
| Ing3 | -0.6010334 |
| Ing3 | 0.45644847 |
| Ing3 | 0.61214565 |
| Ing4 | 1.31627456 |
| Ing4 | -0.8478869 |
| Ing4 | 1.34081789 |
| Ing5 | -0.2282689 |
| Inha | -0.5536415 |
| Inhba | 0.75840887 |
| Inmt | 0.83632065 |
| Inoc1 | -0.5972439 |
| Inpp4a | -0.6605904 |
| Inpp4a | 0.36121612 |
| Inpp4a | 0.04051071 |
| Inpp4b | -0.6233176 |
| Inpp5a | 0.29163362 |
| Inpp5d | -0.0976032 |
| Inpp5e | 0.49808461 |
| Inpp5f | -0.2781573 |
| Inppl1 | -0.7793991 |
| Inppl1 | 0.10660972 |
| Inppl1 | -0.6109578 |
| Ins2 | 0.14834932 |
| Insc | -0.1270027 |
| Insig1 | -0.4236415 |
| Insig1 | 2.18952421 |
| Insig1 | 0.69562974 |
| Insig2 | 1.10364066 |
| Insig2 | -0.3308326 |
| Insl3 | 1.03019858 |
| Insl5 | -0.0931604 |
| Insl6 | -0.7104187 |
| Insm1 | 0.14318865 |
| Ints1 | -0.3121995 |
| Ints12 | -0.3285543 |
| Ints3 | -0.7087146 |
| Ints3 | 2.15411106 |
| Ints4 | 1.16631053 |
| Ints5 | -0.6769091 |
| Ints7 | 1.2018941 |
| Ints9 | 0.21905607 |
| Ints9 | -0.3860197 |
| Ipmk | -0.7603256 |
| Ipmk | 2.92341732 |
| Ipo13 | 0.17673859 |
| Ipo4 | -0.295574 |
| Ipo5 | 3.35204661 |
| Ipp | 0.84570412 |
| Ippk | -0.6358416 |
| Iqcb1 | 2.69407008 |
| Iqcb1 | -0.3021891 |
| Iqcd | 0.40089034 |
| Iqce | -0.7146924 |
| Iqcf3 | 0.87521416 |
| Iqcf4 | -0.5002918 |
| Iqgap1 | -0.6109578 |
| Iqgap2 | 1.10588104 |
| Iqgap3 | 2.3696308 |
| Iqsec2 | 0.41831649 |
| Iqsec3 | -0.4948476 |
| Irak1 | -0.7513768 |
| Irak1bp1 | -0.7087146 |
| Irak2 | 1.00770639 |
| Irak2 | 1.2658144 |
| Irak3 | 0.73677957 |
| Irak4 | 0.70575427 |
| Irak4 | -0.3423071 |
| Irak4 | -0.8264276 |
| Ireb2 | 0.6221642 |
| Irf1 | -0.5777804 |
| Irf1 | -0.7164073 |
| Irf1 | -0.5674641 |
| Irf1 | 1.76325304 |
| Irf2 | -0.490785 |
| Irf2bp1 | 0.43111446 |
| Irf3 | -0.1110584 |
| Irf3 | 1.65163407 |
| Irf3 | 2.55289012 |
| Irf4 | -0.6382084 |
| Irf5 | -0.0363967 |
| Irf6 | -0.2145587 |
| Irf6 | 2.63786527 |
| Irf7 | 1.23206038 |
| Irf8 | 0.34798193 |
| Irf9 | 0.03935601 |
| Irf9 | 2.27184911 |
| Irgm | 2.12932141 |
| Irgm | -0.6155738 |
| Irgq | 0.15824725 |
| Irgq | 0.97771219 |
| Irgq | -0.6918413 |
| Irs2 | 1.06899264 |
| Irs3 | 0.7915998 |
| Irx2 | 0.1764056 |
| Irx3 | -0.1975727 |
| Irx5 | 0.13799959 |
| Isca1 | -0.5500374 |
| Iscu | -0.4713922 |
| Isg20 | -0.094935 |
| Isg20l1 | 0.13903969 |
| Isg20l2 | -0.6311256 |
| Isl1 | -0.4536883 |
| Islr2 | 2.62942482 |
| Isy1 | 1.3470959 |
| Isyna1 | -0.5608913 |
| Itfg2 | 0.15756796 |
| Itfg3 | 2.38445712 |
| Itga1 | -0.6818603 |
| Itga11 | 0.14903528 |
| Itga11 | 0.48760719 |
| Itga2b | 1.48245303 |
| Itga3 | -0.4173703 |
| Itga5 | -0.4813732 |
| Itga5 | -0.7567359 |
| Itga6 | -0.4921373 |
| Itga7 | 0.50563211 |
| Itgad | 1.38576076 |
| Itgad | -0.682688 |
| Itgad | -0.3440404 |
| Itgae | 0.7146426 |
| Itgae | -0.554364 |
| Itgae | 0.71971104 |
| Itgav | 2.43265566 |
| Itgb1 | 1.54870474 |
| Itgb1 | 0.94275082 |
| Itgb1bp1 | -0.746048 |
| Itgb3bp | 0.58705465 |
| Itgb4 | 0.98211666 |
| Itgb4 | 0.08740822 |
| Itgb5 | -0.0708222 |
| Itgb6 | 2.29277734 |
| Itgb6 | 0.82383001 |
| Itgb6 | 2.02599383 |
| Itgb7 | -0.2835656 |
| Itgbl1 | 0.47361579 |
| Itih2 | 0.13765263 |
| Itih3 | 1.66258305 |
| Itih5 | 0.0210787 |
| Itih5 | -0.6622096 |
| Itk | -0.4562902 |
| Itlnb | 0.62050182 |
| Itm2a | 0.77125866 |
| Itm2b | -0.3094082 |
| Itm2b | 0.57359879 |
| Itpk1 | -0.6670841 |
| Itpka | -0.6810333 |
| Itpkc | -0.0755957 |
| Itpr1 | -0.3342603 |
| Itpr2 | -0.6445493 |
| Itpr3 | -0.7215708 |
| Itsn1 | 1.83540884 |
| Itsn2 | -0.682688 |
| Ivd | -0.0186196 |
| Ivns1abp | -0.7407492 |
| Ivns1abp | -0.7207082 |
| Iws1 | -0.5927165 |
| Iyd | 0.94643836 |
| Izumo1 | 2.8425883 |
| Jag1 | 0.69062297 |
| Jag2 | 2.00269682 |
| Jagn1 | -0.4111404 |
| Jak1 | -0.300531 |
| Jak2 | 2.62665231 |
| Jak2 | -0.5659995 |
| Jak3 | 1.78255952 |
| Jak3 | -0.4575939 |
| Jakmip1 | 0.43719635 |
| Jam2 | -0.3988028 |
| Jam2 | 0.79437136 |
| Jam2 | -0.6477358 |
| Jam2 | 2.43265566 |
| Jam3 | -0.2432202 |
| Jam4 | 0.59947093 |
| Jarid1b | 0.54000104 |
| Jarid1c | -0.2637081 |
| Jarid1d | 2.8586589 |
| Jazf1 | -0.5500374 |
| Jdp2 | -0.2701027 |
| Jmjd1a | 0.46138538 |
| Jmjd1a | -0.2754648 |
| Jmjd1a | -0.6485341 |
| Jmjd2a | -0.4894347 |
| Jmjd2b | 0.91834807 |
| Jmjd2b | -0.2862814 |
| Jmjd2c | -0.6501327 |
| Jmjd2d | -0.7576321 |
| Jmjd3 | -0.1540329 |
| Jmjd3 | 2.4191915 |
| Jmjd5 | -0.5098953 |
| Jmjd6 | -0.5322205 |
| Jmy | 1.62634504 |
| Josd1 | -0.0003675 |
| Josd2 | -0.5630771 |
| Josd3 | -0.3440404 |
| Josd3 | 3.24780112 |
| Jph1 | -0.6295588 |
| Jph2 | 0.48880369 |
| Jph3 | 1.50617005 |
| Jph4 | 0.72064666 |
| Jph4 | -0.6233176 |
| Jtb | 0.61486953 |
| Jtv1 | -0.547164 |
| Jtv1 | -0.5897103 |
| Jun | 0.50657131 |
| Junb | -0.4996095 |
| Jund1 | 1.69561927 |
| Jup | -0.2411438 |
| Kal1 | 2.27610083 |
| Kalrn | -0.3781923 |
| Kank1 | -0.2868255 |
| Kank3 | -0.1833226 |
| Kank4 | 0.60815039 |
| Kank4 | 1.45185654 |
| Kank4 | -0.340001 |
| Kars | 1.33459536 |
| Katna1 | -0.5336335 |
| Katnal1 | 2.05932936 |
| Katnb1 | 0.2800678 |
| Kazald1 | 1.50755155 |
| Kbtbd2 | -0.3423071 |
| Kbtbd2 | -0.6272135 |
| Kbtbd4 | -0.2868255 |
| Kbtbd4 | -0.7729983 |
| Kbtbd7 | 0.18072533 |
| Kbtbd7 | -0.1302241 |
| Kbtbd8 | 1.01690435 |
| Kcmf1 | -0.511966 |
| Kcna1 | -0.0967129 |
| Kcna2 | -0.6622096 |
| Kcna3 | 1.07620444 |
| Kcna5 | 0.64472978 |
| Kcna6 | -0.5558106 |
| Kcnab1 | -0.6397897 |
| Kcnab1 | -0.6405813 |
| Kcnab1 | 1.93048979 |
| Kcnab2 | -0.382399 |
| Kcnab3 | 1.3803864 |
| Kcnb1 | -0.7451628 |
| Kcnc1 | 0.09033173 |
| Kcnc1 | 0.82951294 |
| Kcnc4 | -0.5733453 |
| Kcnc4 | 0.62981485 |
| Kcnd2 | -0.5237865 |
| Kcne1 | -0.6581668 |
| Kcne1l | -0.6405813 |
| Kcne2 | -0.5442993 |
| Kcne4 | -0.7766506 |
| Kcnf1 | -0.3757969 |
| Kcng4 | -0.6860062 |
| Kcnh1 | 2.58680123 |
| Kcnh2 | 1.83764329 |
| Kcnh2 | -0.6025534 |
| Kcnh3 | 0.32065733 |
| Kcnh5 | -0.1573482 |
| Kcnip1 | -0.5265895 |
| Kcnip2 | -0.340001 |
| Kcnip2 | -0.3763952 |
| Kcnip2 | -0.3698348 |
| Kcnip3 | 2.34161073 |
| Kcnip4 | -0.510585 |
| Kcnj12 | -0.0133104 |
| Kcnj16 | 2.41177974 |
| Kcnj2 | 0.52700067 |
| Kcnj3 | -0.7612252 |
| Kcnj4 | 0.50492709 |
| Kcnj6 | 0.02889961 |
| Kcnj6 | 2.10593167 |
| Kcnj8 | 2.57869795 |
| Kcnj9 | 2.39039944 |
| Kcnk1 | 0.55795013 |
| Kcnk10 | 1.46435052 |
| Kcnk12 | -0.5294008 |
| Kcnk13 | -0.4820423 |
| Kcnk2 | -0.7061643 |
| Kcnk2 | 0.91226193 |
| Kcnk3 | -0.3336881 |
| Kcnk4 | 0.52791885 |
| Kcnk5 | 0.27438137 |
| Kcnk5 | 0.3691227 |
| Kcnk6 | 0.57425432 |
| Kcnk6 | -0.0967129 |
| Kcnk9 | 2.26146386 |
| Kcnma1 | -0.0318171 |
| Kcnmb1 | -0.196583 |
| Kcnmb4 | 2.18620824 |
| Kcnmb4 | 0.8745789 |
| Kcnn1 | 2.12679781 |
| Kcnn2 | -0.6943531 |
| Kcnn4 | 0.66755984 |
| Kcnq1 | 0.84390053 |
| Kcnq2 | -0.0104639 |
| Kcnq2 | -0.4833821 |
| Kcnq3 | 1.3520884 |
| Kcnq5 | -0.6094242 |
| Kcnrg | 0.11268681 |
| Kcnrg | -0.5995157 |
| Kcns1 | -1.0088661 |
| Kcns2 | 2.5335253 |
| Kcnt1 | -0.1371645 |
| Kcnu1 | 1.3156776 |
| Kcnv1 | 0.66260034 |
| Kcnv2 | -0.0617758 |
| Kctd10 | 0.03819989 |
| Kctd11 | 1.67197499 |
| Kctd12 | -0.5450146 |
| Kctd12b | 2.46032281 |
| Kctd12b | 0.81676232 |
| Kctd14 | -0.5579847 |
| Kctd14 | -0.8618016 |
| Kctd15 | 0.61841971 |
| Kctd18 | 1.00050532 |
| Kctd19 | 1.1704989 |
| Kctd2 | 0.31521112 |
| Kctd20 | -0.300531 |
| Kctd20 | -0.5343408 |
| Kctd3 | 0.53340921 |
| Kctd4 | -0.1329942 |
| Kctd5 | 0.71671081 |
| Kctd5 | 1.01826952 |
| Kctd6 | 1.71562921 |
| Kctd6 | 1.09926909 |
| Kctd9 | -0.8082795 |
| Kdelc1 | -0.2125444 |
| Kdelc1 | 1.76455251 |
| Kdelr2 | -0.5514774 |
| Kdelr3 | 1.63255335 |
| Kdr | -0.7711775 |
| Keap1 | -0.1882121 |
| Keap1 | -0.3793923 |
| Keap1 | 0.28868152 |
| Keap1 | -0.6769091 |
| Kera | 0.57294281 |
| Khdc1c | 2.86154285 |
| Khdrbs1 | -0.4148734 |
| Khdrbs2 | -0.5030257 |
| Khdrbs3 | 1.16420935 |
| Khk | -0.290643 |
| Khk | 1.88327494 |
| Khsrp | 0.5107862 |
| Kif11 | -0.6597819 |
| Kif13a | -0.7469341 |
| Kif14 | -0.2505237 |
| Kif15 | 0.16095953 |
| Kif17 | 1.220792 |
| Kif17 | -0.0157571 |
| Kif18a | -0.5294008 |
| Kif1a | 0.48039618 |
| Kif1b | -0.3836043 |
| Kif1b | 2.18220568 |
| Kif1b | 0.88264654 |
| Kif1b | 0.77691397 |
| Kif1b | -0.7224341 |
| Kif1b | 1.45666834 |
| Kif1c | 1.94459372 |
| Kif21a | -0.5098953 |
| Kif21b | -0.7648321 |
| Kif22 | -0.5168153 |
| Kif23 | 0.39165747 |
| Kif24 | 2.04749316 |
| Kif27 | -0.1702459 |
| Kif2a | 1.00756842 |
| Kif2b | 1.88103684 |
| Kif2c | -0.562348 |
| Kif3a | 1.7661589 |
| Kif3a | 3.20500026 |
| Kif3b | 1.10662665 |
| Kif3c | 0.56260571 |
| Kif3c | -0.8206588 |
| Kif4 | 0.91119281 |
| Kif4 | 0.73161742 |
| Kif4 | 0.83698219 |
| Kif5a | -0.6910055 |
| Kif5a | -0.525888 |
| Kif5a | 2.64552644 |
| Kif5b | 1.24384035 |
| Kif5c | 0.97870855 |
| Kif9 | -0.7250289 |
| Kifap3 | 1.72244306 |
| Kifap3 | 1.42156943 |
| Kifap3 | 0.09651426 |
| Kifc2 | 0.48160185 |
| Kifc2 | -0.0905048 |
| Kifc3 | 1.54590201 |
| Kin | -0.8293253 |
| Kirrel3 | -0.5696653 |
| Kiss1 | -0.5681973 |
| Kiss1r | 0.49357454 |
| Kitl | -0.747821 |
| Kl | 0.10696828 |
| Klb | -0.525888 |
| Klc1 | -0.5421565 |
| Klc1 | 0.76378424 |
| Klc1 | -0.4740434 |
| Klc2 | -0.273853 |
| Klc2 | -0.0219017 |
| Klc4 | 2.38208252 |
| Klf1 | -0.259469 |
| Klf10 | -0.2857376 |
| Klf12 | 0.19650591 |
| Klf12 | -0.4747074 |
| Klf12 | 1.01498979 |
| Klf13 | -0.3890476 |
| Klf16 | -0.4582464 |
| Klf17 | 0.38660723 |
| Klf17 | 0.5107862 |
| Klf2 | 2.28208553 |
| Klf4 | -0.2370047 |
| Klf4 | 1.50787631 |
| Klf4 | -0.4921373 |
| Klf5 | 2.19807596 |
| Klf6 | -0.7576321 |
| Klf6 | -0.1985635 |
| Klf7 | -0.4465698 |
| Klf9 | 1.71119001 |
| Klhdc1 | 1.79341692 |
| Klhdc1 | 1.20256757 |
| Klhdc1 | -0.8459158 |
| Klhdc2 | -0.3382751 |
| Klhdc3 | -0.747821 |
| Klhdc4 | 1.8602044 |
| Klhdc4 | 1.95425145 |
| Klhdc4 | -0.0887385 |
| Klhdc8b | -0.3580226 |
| Klhdc9 | 0.43643825 |
| Klhl10 | -0.8810202 |
| Klhl11 | -0.1842984 |
| Klhl12 | -0.4092794 |
| Klhl14 | -0.6662699 |
| Klhl15 | -0.1507292 |
| Klhl15 | -0.4006432 |
| Klhl17 | -0.6094242 |
| Klhl2 | 1.1081161 |
| Klhl21 | 2.80802792 |
| Klhl22 | -0.2873699 |
| Klhl22 | -0.3793923 |
| Klhl23 | 1.13874323 |
| Klhl24 | 0.93488524 |
| Klhl24 | 0.18039375 |
| Klhl25 | -0.5230871 |
| Klhl26 | -0.3138783 |
| Klhl26 | 0.03162168 |
| Klhl30 | 2.22007637 |
| Klhl31 | -0.3302625 |
| Klhl32 | 1.28276442 |
| Klhl5 | 0.60730712 |
| Klhl5 | -0.5209919 |
| Klhl6 | 3.21650426 |
| Klhl6 | -0.6760864 |
| Klhl7 | -0.6793814 |
| Klhl8 | 1.30717983 |
| Klhl9 | 0.85904148 |
| Klhl9 | -0.3734075 |
| Klk1 | -0.2463433 |
| Klk11 | 1.92815315 |
| Klk11 | -0.7164073 |
| Klk15 | -0.5230871 |
| Klk1b1 | -0.7407492 |
| Klk1b11 | 0.28215419 |
| Klk1b16 | 0.09142571 |
| Klk1b21 | 0.75876818 |
| Klk1b21 | 0.17673859 |
| Klk1b22 | 0.26684661 |
| Klk1b24 | -0.431857 |
| Klk1b26 | 1.8444287 |
| Klk1b27 | -0.3994159 |
| Klk1b27 | -0.1731362 |
| Klk1b4 | -0.7381108 |
| Klk1b4 | 2.21828159 |
| Klk1b5 | -0.809226 |
| Klk1b8 | 0.09433681 |
| Klk1b9 | 1.16268891 |
| Klk6 | -0.1625815 |
| Klk8 | -0.6334806 |
| Klkb1 | 0.0277306 |
| Klra17 | 0.28245187 |
| Klra4 | -0.7720875 |
| Klrd1 | 0.76717286 |
| Kndc1 | 1.14484653 |
| Kng1 | -0.1247084 |
| Kng1 | -0.8120712 |
| Kng2 | 0.6119358 |
| Kns2 | -0.0669345 |
| Kns2 | -0.525888 |
| Kntc1 | 1.26843396 |
| Kpna1 | 0.28096253 |
| Kpna2 | -0.1862533 |
| Kpna3 | -0.7087146 |
| Kpna6 | 0.48760719 |
| Kpnb1 | 0.00763249 |
| Kptn | -0.0795194 |
| Kras | 1.23748231 |
| Kras | -0.5645372 |
| Krba1 | -0.5587105 |
| Krba1 | 1.73228453 |
| Krba1 | -0.1847868 |
| Krcc1 | -0.1526156 |
| Kremen | 2.84646479 |
| Kremen1 | -0.7621256 |
| Kremen2 | -0.5202945 |
| Krit1 | -0.4173703 |
| Krit1 | 1.77836416 |
| Krr1 | 0.50516215 |
| Krr1 | 0.50304446 |
| Krr1 | -0.511966 |
| Krt10 | 2.13547585 |
| Krt12 | -0.5852189 |
| Krt13 | 2.75731257 |
| Krt14 | -0.121049 |
| Krt17 | 1.71797094 |
| Krt18 | -0.6485341 |
| Krt19 | 1.00355864 |
| Krt2 | -0.7224341 |
| Krt20 | 2.26652552 |
| Krt222 | -0.6926778 |
| Krt23 | -0.0124962 |
| Krt27 | 0.73843285 |
| Krt32 | 2.1135224 |
| Krt4 | 1.70961824 |
| Krt7 | -0.7540526 |
| Krt73 | 0.72027252 |
| Krt75 | 0.15586759 |
| Krt78 | 2.88422693 |
| Krt79 | -0.5638069 |
| Krt8 | -0.5755603 |
| Krt84 | -0.4968855 |
| Krt86 | -0.364501 |
| Krt86 | -0.1371645 |
| Krt9 | -0.7531598 |
| Krtap16-4 | -0.1568738 |
| Krtap8-2 | -0.3787921 |
| Krtcap2 | -0.0958235 |
| Krtcap3 | 2.65656657 |
| Ksr2 | -0.5350487 |
| Ktelc1 | 0.41780094 |
| Kti12 | 1.45932149 |
| Ktn1 | 1.35495671 |
| Ktn1 | -0.747821 |
| Ky | -0.260527 |
| L1cam | 2.85852319 |
| L2hgdh | 0.96379604 |
| L3mbtl2 | 1.08894708 |
| L3mbtl3 | 1.4976742 |
| l7Rn6 | 1.48910113 |
| Lace1 | 1.7767174 |
| Lad1 | -0.8177876 |
| Lag3 | 2.58294008 |
| Lage3 | -0.0643516 |
| Lama1 | -0.260527 |
| Lama2 | 0.54969111 |
| Lamb1-1 | -0.6876697 |
| Lamb2 | 2.3533098 |
| Lamb3 | -0.2494769 |
| Lamc1 | 1.96775783 |
| Lamc2 | -0.5071414 |
| Lamc2 | 0.41418419 |
| Lamc2 | -0.5844724 |
| Lamc3 | 0.6287846 |
| Lamp1 | 0.13973245 |
| Lamp2 | -0.2458221 |
| Lamp2 | -0.5616193 |
| Lamp2 | -0.1921421 |
| Lamp2 | -0.4000293 |
| Lancl1 | -0.7104187 |
| Lancl1 | 0.64046329 |
| Lancl1 | 0.57119128 |
| Laptm4a | -0.6421665 |
| Laptm4b | -0.562348 |
| Laptm4b | -0.2385547 |
| Laptm5 | -0.7839978 |
| Large | -0.0251953 |
| Larp2 | 1.84884994 |
| Larp5 | 0.0116072 |
| Larp6 | -0.3492595 |
| Larp6 | 0.49023748 |
| Lars | 1.05558235 |
| Lars2 | -0.431857 |
| Las1l | -0.585966 |
| Lasp1 | 1.45495265 |
| Lasp1 | -0.5659995 |
| Lasp1 | -0.4595527 |
| Lass1 | 1.0512452 |
| Lass2 | 0.0648134 |
| Lass2 | -0.6303419 |
| Lass2 | -0.546447 |
| Lass4 | -0.482712 |
| Lass4 | 0.48376818 |
| Lass5 | 0.17005633 |
| Lass6 | 0.50210174 |
| Lat2 | -0.5800058 |
| Lat2 | -0.5175101 |
| Lats2 | -0.5949775 |
| Lbh | -0.4478601 |
| Lbh | -0.5748214 |
| Lbp | -0.8478869 |
| Lbr | -0.0334799 |
| Lbx2 | -0.7036209 |
| Lca5 | 2.24258161 |
| Lca5 | -0.0790826 |
| Lca5 | -0.2792365 |
| Lcat | -0.2489539 |
| Lcat | 1.17559289 |
| Lce1c | 0.73475495 |
| Lck | -0.3757969 |
| Lcmt1 | -0.1765195 |
| Lcmt1 | 0.83101153 |
| Lcmt1 | -0.4414262 |
| Lcn10 | -0.7276308 |
| Lcn12 | 1.6191582 |
| Lcn13 | 1.87599534 |
| Lcn2 | -0.308294 |
| Lcn3 | -0.6163453 |
| Lcn8 | -0.5829812 |
| Lcor | 1.0155372 |
| Lcp1 | -0.4018721 |
| Ldb1 | -0.273853 |
| Ldb2 | 0.92514836 |
| Ldb2 | -0.4407853 |
| Ldha | -0.6048381 |
| Ldhc | 0.0048402 |
| Ldhc | -0.3172449 |
| Ldhc | 2.47298295 |
| Ldhd | 1.10451255 |
| Ldhd | -0.5315148 |
| Ldoc1 | 2.46286257 |
| Ldoc1l | 2.67974407 |
| Lef1 | -0.2484312 |
| Lef1 | -0.0007693 |
| Lefty1 | 1.16770872 |
| Lefty2 | -0.8789786 |
| Lemd2 | 2.28760641 |
| Leng1 | 1.416038 |
| Leng8 | 0.59028146 |
| Leng9 | -0.6565545 |
| Leo1 | -0.3842076 |
| Lep | 0.78672988 |
| Lepre1 | -0.73373 |
| Lepre1 | -0.6048381 |
| Lepre1 | 0.45867328 |
| Leprel1 | 0.26714916 |
| Leprel2 | -0.2479088 |
| Leprotl1 | 2.18644548 |
| Letm1 | 1.07940627 |
| Letm1 | 2.69968504 |
| Letm1 | 2.68441927 |
| Letmd1 | 3.51231625 |
| Lgals1 | -0.2966733 |
| Lgals2 | -0.8235388 |
| Lgals3 | 0.16770632 |
| Lgals3bp | 0.54339262 |
| Lgals4 | -0.4634827 |
| Lgals4 | 4.58013793 |
| Lgals6 | -0.3291234 |
| Lgals7 | -0.5674641 |
| Lgals8 | 0.0976011 |
| Lgals8 | 0.35572442 |
| Lgals9 | 1.22321017 |
| Lgals9 | 0.29751025 |
| Lgi1 | 0.99660494 |
| Lgi1 | 1.45812422 |
| Lgi2 | -0.6985544 |
| Lgi3 | -0.0639218 |
| Lgi4 | -0.5733453 |
| Lgmn | 2.0856939 |
| Lgr5 | 3.31235552 |
| Lgtn | 0.09069653 |
| Lgtn | -0.3434623 |
| Lhb | -0.3032961 |
| Lhcgr | 0.01871973 |
| Lhcgr | 0.05992733 |
| Lhfp | -0.649333 |
| Lhfp | 0.1015755 |
| Lhfp | 0.57009493 |
| Lhfpl1 | 1.65205218 |
| Lhfpl2 | -0.8528329 |
| Lhx1 | 3.04574825 |
| Lhx2 | -0.6501327 |
| Lhx3 | -0.6025534 |
| Lhx4 | -0.6501327 |
| Lhx9 | 0.00842878 |
| Lhx9 | 0.50821267 |
| Lias | 2.34539296 |
| Lias | 2.15827511 |
| Lias | 2.51134837 |
| Lifr | -0.6063643 |
| Lig1 | -0.4813732 |
| Lig1 | 0.70480376 |
| Lig1 | 1.25356356 |
| Lig3 | -0.9006385 |
| Lig4 | 1.37694187 |
| Lima1 | -0.3746015 |
| Lima1 | 1.23271266 |
| Limd1 | 0.09723895 |
| Limd2 | -0.7941946 |
| Lime1 | 1.34661446 |
| Limk1 | 1.57733968 |
| Limk2 | -0.2489539 |
| Limk2 | 2.38127894 |
| Lims2 | 2.86022778 |
| Lims2 | 0.69197357 |
| Lims2 | -0.8293253 |
| Lin54 | -0.7027746 |
| Lin7b | 1.36057241 |
| Lin7b | -0.7729983 |
| Lin7b | -0.0596346 |
| Lin7b | -0.6985544 |
| Lin7c | -0.3884413 |
| Lincr | 1.11982532 |
| Lincr | -0.7433949 |
| Lingo1 | -0.2776182 |
| Lingo2 | -0.6350539 |
| Lingo2 | -0.6140326 |
| Lingo4 | -0.1774883 |
| Lip1 | -0.6736227 |
| Lipe | -0.8892309 |
| Lipf | -0.5572594 |
| Liph | 0.8957858 |
| Liph | -0.7112719 |
| Litaf | 0.68364635 |
| Lix1 | 0.11161722 |
| Lix1 | 0.62506635 |
| Lix1 | 0.03510998 |
| Lix1 | 0.96711437 |
| Llgl1 | 1.03624085 |
| Llgl2 | 0.05274032 |
| Lman1 | 0.81032178 |
| Lman2 | 0.68772232 |
| Lman2l | -0.680207 |
| Lman2l | 1.38917329 |
| Lmbr1 | -0.7407492 |
| Lmbr1l | -0.6769091 |
| Lmcd1 | -0.590461 |
| Lmcd1 | -0.6662699 |
| Lmcd1 | 1.33761452 |
| Lmf1 | 0.40586488 |
| Lmf2 | 0.38580735 |
| Lmna | 3.04463479 |
| Lmna | -0.6549449 |
| Lmna | 1.48835556 |
| Lmna | -0.5521982 |
| Lmna | -0.7267627 |
| Lmnb1 | -0.7639291 |
| Lmnb1 | -0.6951919 |
| Lmnb2 | -0.3359789 |
| Lmo2 | 1.13657966 |
| Lmo3 | 1.2495155 |
| Lmo3 | -0.7389895 |
| Lmo4 | 0.03974106 |
| Lmod1 | 0.32322618 |
| Lmtk2 | -0.1568738 |
| Lmtk3 | 1.01485289 |
| Lmx1a | -0.1847868 |
| Lmx1b | -0.7693601 |
| Lnx1 | -0.1688041 |
| Lnx2 | -0.2961235 |
| Lnx2 | -0.3279856 |
| LOC100038882 | -0.5308096 |
| LOC100038984 | -0.3021891 |
| LOC100039087 | -0.3055141 |
| LOC100039227 | -0.2552489 |
| LOC100039479 | -0.4043348 |
| LOC100039532 | 0.40324997 |
| LOC100039571 | -0.7224341 |
| LOC100039728 | -0.6405813 |
| LOC100040573 | -0.5216897 |
| LOC100040592 | 0.77196743 |
| LOC100040601 | -0.6056009 |
| LOC100040799 | -0.2515717 |
| LOC100040899 | 0.17573926 |
| LOC100040919 | -0.1654484 |
| LOC100041103 | -0.5681973 |
| LOC100041290 | 0.30654728 |
| LOC100041463 | 0.08813994 |
| LOC100041483 | 1.63319326 |
| LOC100041500 | 0.34520128 |
| LOC100041504 | 0.73457068 |
| LOC100041703 | 1.37320385 |
| LOC100041722 | 2.20852258 |
| LOC100041725 | -0.295574 |
| LOC100041835 | 2.4800466 |
| LOC100042179 | 0.136611 |
| LOC100042427 | -0.5844724 |
| LOC100042777 | 1.38779132 |
| LOC100043257 | -0.6597819 |
| LOC100043391 | -0.4255311 |
| LOC100043671 | 2.35902305 |
| LOC100043822 | 3.04603436 |
| LOC100043986 | 0.21554798 |
| LOC100044087 | 0.3490919 |
| LOC100044089 | -0.1251668 |
| LOC100044101 | 0.21650602 |
| LOC100044103 | -0.7302399 |
| LOC100044103 | -0.5587105 |
| LOC100044122 | -0.0821442 |
| LOC100044133 | 0.84848472 |
| LOC100044159 | -0.3099658 |
| LOC100044170 | 3.38915211 |
| LOC100044172 | -0.3365524 |
| LOC100044177 | -0.5308096 |
| LOC100044190 | -0.5030257 |
| LOC100044204 | 0.31204361 |
| LOC100044210 | 0.33905484 |
| LOC100044221 | -0.6613997 |
| LOC100044257 | -0.5800058 |
| LOC100044275 | -0.6860062 |
| LOC100044289 | 2.35470001 |
| LOC100044292 | 0.34297084 |
| LOC100044294 | -0.3149992 |
| LOC100044298 | 0.84979041 |
| LOC100044322 | 0.81202092 |
| LOC100044324 | 1.34796188 |
| LOC100044324 | -0.1133197 |
| LOC100044363 | 0.05955043 |
| LOC100044363 | 0.13452429 |
| LOC100044439 | -0.1488466 |
| LOC100044468 | 0.4123706 |
| LOC100044475 | 0.91971206 |
| LOC100044538 | -0.1038584 |
| LOC100044566 | 0.02186372 |
| LOC100044576 | -0.5822365 |
| LOC100044636 | 1.03114103 |
| LOC100044692 | 0.11837087 |
| LOC100044708 | 2.69614506 |
| LOC100044736 | 3.35421555 |
| LOC100044756 | -0.482712 |
| LOC100044776 | 0.48448919 |
| LOC100044779 | -0.5659995 |
| LOC100044812 | -0.4401447 |
| LOC100044829 | 2.79954236 |
| LOC100044862 | 1.02641931 |
| LOC100044873 | 0.50797837 |
| LOC100044934 | 2.64075161 |
| LOC100045005 | -0.0604905 |
| LOC100045019 | -0.8830662 |
| LOC100045040 | -0.5558106 |
| LOC100045098 | 2.69616809 |
| LOC100045228 | 1.30838416 |
| LOC100045241 | 0.61946134 |
| LOC100045250 | -0.7576321 |
| LOC100045280 | -0.680207 |
| LOC100045300 | -0.3609618 |
| LOC100045304 | -0.9058698 |
| LOC100045343 | -0.4452813 |
| LOC100045359 | 0.42191746 |
| LOC100045359 | -0.5442993 |
| LOC100045403 | 0.19813463 |
| LOC100045413 | 2.87016131 |
| LOC100045419 | 1.9446959 |
| LOC100045430 | -0.4887603 |
| LOC100045439 | 2.20220629 |
| LOC100045491 | 1.87522579 |
| LOC100045505 | 1.05203526 |
| LOC100045522 | 0.38420554 |
| LOC100045542 | 1.06289702 |
| LOC100045551 | -0.0190292 |
| LOC100045567 | -0.5216897 |
| LOC100045607 | -0.4312225 |
| LOC100045617 | -0.3746015 |
| LOC100045644 | 0.09033173 |
| LOC100045697 | 0.61466028 |
| LOC100045780 | -0.5822365 |
| LOC100045782 | 1.71060091 |
| LOC100045877 | 1.32143244 |
| LOC100045882 | -0.6557494 |
| LOC100045887 | -0.6501327 |
| LOC100045950 | 0.38714011 |
| LOC100045963 | 0.96307312 |
| LOC100045981 | 0.3387745 |
| LOC100046003 | -0.1160405 |
| LOC100046035 | 0.06331269 |
| LOC100046035 | 1.71855546 |
| LOC100046039 | -0.9389678 |
| LOC100046044 | -0.6719838 |
| LOC100046049 | 1.02979438 |
| LOC100046056 | 1.47449706 |
| LOC100046068 | 0.84127095 |
| LOC100046080 | 0.0976011 |
| LOC100046081 | 0.95945018 |
| LOC100046120 | -0.5126572 |
| LOC100046128 | 1.08004534 |
| LOC100046129 | 1.01238522 |
| LOC100046163 | -0.2819399 |
| LOC100046163 | -0.4485059 |
| LOC100046207 | -0.6557494 |
| LOC100046207 | -0.4092794 |
| LOC100046207 | 0.26593838 |
| LOC100046232 | -0.5565347 |
| LOC100046254 | 1.51821016 |
| LOC100046320 | 0.75552943 |
| LOC100046393 | -0.2797766 |
| LOC100046406 | 0.08484269 |
| LOC100046457 | -0.0860952 |
| LOC100046518 | -0.5681973 |
| LOC100046568 | 1.1598755 |
| LOC100046586 | 1.7416443 |
| LOC100046608 | -0.2463433 |
| LOC100046608 | 0.13973245 |
| LOC100046608 | -0.4941693 |
| LOC100046608 | 2.36352299 |
| LOC100046616 | -0.3178071 |
| LOC100046641 | 2.11057056 |
| LOC100046650 | 1.32123458 |
| LOC100046690 | 3.41170118 |
| LOC100046690 | 1.92815315 |
| LOC100046741 | 0.65260241 |
| LOC100046744 | -0.4780344 |
| LOC100046744 | -0.5428702 |
| LOC100046746 | 1.27707907 |
| LOC100046775 | -0.7830763 |
| LOC100046781 | -0.6893361 |
| LOC100046781 | -0.1540329 |
| LOC100046792 | -0.607893 |
| LOC100046796 | 0.52308842 |
| LOC100046802 | 1.29076896 |
| LOC100046841 | -0.7036209 |
| LOC100046855 | -0.7576321 |
| LOC100046883 | 0.5714104 |
| LOC100046891 | 1.11318739 |
| LOC100046898 | -0.5659995 |
| LOC100046918 | 0.52654125 |
| LOC100046953 | -0.3633198 |
| LOC100046959 | -0.8658152 |
| LOC100046996 | 1.41352926 |
| LOC100047009 | -0.9260175 |
| LOC100047009 | 1.21027812 |
| LOC100047012 | -0.7121258 |
| LOC100047028 | -0.7164073 |
| LOC100047046 | 1.89756033 |
| LOC100047052 | 0.07673389 |
| LOC100047052 | 0.77779455 |
| LOC100047155 | -0.0967129 |
| LOC100047155 | -0.3262816 |
| LOC100047167 | 0.7823604 |
| LOC100047173 | -0.2427006 |
| LOC100047184 | 1.21316727 |
| LOC100047200 | -0.0426778 |
| LOC100047214 | 0.84340804 |
| LOC100047214 | 2.78309508 |
| LOC100047238 | 2.0230836 |
| LOC100047261 | 0.8995065 |
| LOC100047323 | -1.0581084 |
| LOC100047323 | 1.73209254 |
| LOC100047339 | 0.22159923 |
| LOC100047353 | -0.392694 |
| LOC100047368 | -0.008436 |
| LOC100047369 | 1.38140803 |
| LOC100047369 | -0.2922838 |
| LOC100047391 | 0.50257322 |
| LOC100047393 | -0.3860197 |
| LOC100047427 | -0.6311256 |
| LOC100047480 | 1.03851345 |
| LOC100047490 | 1.86516405 |
| LOC100047579 | 0.15824725 |
| LOC100047583 | -0.4267929 |
| LOC100047606 | -0.5209919 |
| LOC100047615 | 0.06855476 |
| LOC100047619 | -0.1804009 |
| LOC100047634 | 2.75489206 |
| LOC100047651 | -0.3615507 |
| LOC100047653 | 1.67565137 |
| LOC100047659 | 0.74374046 |
| LOC100047674 | -0.3365524 |
| LOC100047707 | 1.75660401 |
| LOC100047717 | 2.09880013 |
| LOC100047749 | -0.7839978 |
| LOC100047762 | -0.6374188 |
| LOC100047776 | 0.27857469 |
| LOC100047794 | 0.46971929 |
| LOC100047800 | 0.61987767 |
| LOC100047810 | -0.5659995 |
| LOC100047815 | -0.6818603 |
| LOC100047816 | -0.0660728 |
| LOC100047834 | 0.20688149 |
| LOC100047837 | 1.98489969 |
| LOC100047837 | 0.39192252 |
| LOC100047841 | -0.9422333 |
| LOC100047856 | 0.68772232 |
| LOC100047870 | -0.267433 |
| LOC100047911 | 1.22288079 |
| LOC100047934 | 2.77535571 |
| LOC100047935 | -0.4674291 |
| LOC100047937 | 2.3934836 |
| LOC100047937 | 0.26986774 |
| LOC100047958 | 0.42294379 |
| LOC100047963 | -0.0202592 |
| LOC100047963 | 0.61570607 |
| LOC100047998 | -0.3668679 |
| LOC100048046 | 0.31722128 |
| LOC100048046 | -0.267433 |
| LOC100048049 | -0.4136274 |
| LOC100048049 | -0.5140412 |
| LOC100048049 | 0.98126579 |
| LOC100048056 | 2.31124454 |
| LOC100048071 | -0.5594369 |
| LOC100048076 | -0.4517415 |
| LOC100048083 | -0.3428845 |
| LOC100048105 | 1.51281516 |
| LOC100048105 | -0.0330639 |
| LOC100048187 | 2.02730519 |
| LOC100048251 | -0.6056009 |
| LOC100048295 | 0.31290853 |
| LOC100048299 | -0.4407853 |
| LOC100048301 | 1.20043326 |
| LOC100048313 | -0.5874619 |
| LOC100048331 | 0.60201954 |
| LOC100048332 | 2.63061055 |
| LOC100048346 | 1.17778409 |
| LOC100048372 | -0.7639291 |
| LOC100048384 | 1.28090753 |
| LOC100048413 | -0.857805 |
| LOC100048436 | -0.4198738 |
| LOC100048445 | 1.88125543 |
| LOC100048461 | -0.4975658 |
| LOC100048480 | 2.34899371 |
| LOC100048483 | 0.47701208 |
| LOC100048504 | -0.1887025 |
| LOC100048530 | 0.01200375 |
| LOC100048554 | 2.18940599 |
| LOC100048556 | -0.2966733 |
| LOC100048589 | 1.32350746 |
| LOC100048613 | 0.32237068 |
| LOC100048622 | 2.02500911 |
| LOC100048645 | 1.45469503 |
| LOC100048684 | 0.52054829 |
| LOC100048703 | 1.34207684 |
| LOC100048710 | -0.7250289 |
| LOC100048721 | -0.5681973 |
| LOC100048721 | -0.650933 |
| LOC100048726 | 0.54812043 |
| LOC100048733 | -0.4401447 |
| LOC100048816 | -0.5450146 |
| LOC100048832 | 1.12031516 |
| LOC100048832 | 1.63312218 |
| LOC100048858 | 0.68247855 |
| LOC192758 | 1.53791541 |
| LOC194227 | 0.00643681 |
| LOC212390 | -0.9665325 |
| LOC216443 | 0.26107967 |
| LOC216963 | -0.4948476 |
| LOC217341 | 3.72050165 |
| LOC218963 | 0.03549677 |
| LOC225922 | -0.0622046 |
| LOC233529 | -0.8820426 |
| LOC234374 | -0.2760027 |
| LOC235497 | -0.5844724 |
| LOC236749 | -0.2658348 |
| LOC237408 | -0.0080309 |
| LOC237877 | 0.58165197 |
| LOC238829 | -0.7693601 |
| LOC241593 | -0.7522679 |
| LOC244595 | 0.22950271 |
| LOC245263 | -0.8708563 |
| LOC329575 | 0.85710142 |
| LOC329575 | 0.96046601 |
| LOC333331 | -0.6279946 |
| LOC381066 | -0.3440404 |
| LOC381302 | -0.1862533 |
| LOC381375 | -0.4740434 |
| LOC381483 | 1.41370868 |
| LOC381629 | 0.98537126 |
| LOC381801 | 0.08630956 |
| LOC381936 | 0.39059651 |
| LOC382010 | 1.70758415 |
| LOC382044 | 1.61670215 |
| LOC382106 | -0.2663672 |
| LOC382106 | -0.5371754 |
| LOC382133 | 0.2583356 |
| LOC384806 | -0.4562902 |
| LOC384806 | -0.3446189 |
| LOC386486 | 2.16662921 |
| LOC433479 | -0.2541968 |
| LOC433801 | 2.92147877 |
| LOC433886 | -0.8187437 |
| LOC434172 | -0.839049 |
| LOC434172 | -0.6646437 |
| LOC434864 | -0.6605904 |
| LOC434960 | 3.64607591 |
| LOC435023 | -0.6525358 |
| LOC435337 | -0.8469009 |
| LOC435565 | -0.182835 |
| LOC436177 | -0.3568494 |
| LOC544988 | -0.8419858 |
| LOC544988 | 0.3921875 |
| LOC545013 | 1.25218065 |
| LOC545013 | -0.6048381 |
| LOC545056 | -0.5009745 |
| LOC545238 | -0.0435184 |
| LOC545703 | 1.30486721 |
| LOC545732 | 2.55302415 |
| LOC545952 | 0.16231276 |
| LOC546015 | 0.30305934 |
| LOC546015 | -0.5223881 |
| LOC546723 | -0.789546 |
| LOC546840 | -0.6769091 |
| LOC547323 | 0.51125336 |
| LOC547343 | 1.29107547 |
| LOC620807 | -0.7389895 |
| LOC621823 | 0.63269359 |
| LOC621824 | 0.63597285 |
| LOC622404 | -0.8459158 |
| LOC622665 | 2.76013583 |
| LOC624855 | -0.6272135 |
| LOC625480 | 0.96437398 |
| LOC626152 | -0.8498622 |
| LOC628053 | 1.63354858 |
| LOC631002 | -0.5154273 |
| LOC631784 | -0.6646437 |
| LOC632431 | 2.48995657 |
| LOC639396 | -0.5500374 |
| LOC640963 | 0.95537589 |
| LOC641240 | -0.6202125 |
| LOC654426 | 2.10170247 |
| LOC654467 | 0.9266529 |
| LOC654494 | -0.5315148 |
| LOC654842 | -0.0562187 |
| LOC665250 | -0.5711356 |
| LOC666676 | 0.0116072 |
| LOC666904 | 2.7438128 |
| LOC667250 | -0.814925 |
| LOC667370 | -0.3325449 |
| LOC667609 | 0.63002077 |
| LOC668492 | 0.5338653 |
| LOC668837 | 2.90950592 |
| LOC669168 | -0.4111404 |
| LOC669660 | 0.39139234 |
| LOC670044 | -0.2479088 |
| LOC671564 | -0.225205 |
| LOC672705 | 3.38696249 |
| LOC674706 | 0.93115217 |
| LOC675228 | 0.30567651 |
| LOC675567 | 0.47628533 |
| LOC675567 | 2.54972151 |
| LOC675813 | 2.34789494 |
| LOC676420 | 1.02750065 |
| LOC676420 | -0.4068039 |
| LOC677144 | 0.08740822 |
| LOC677205 | -0.9466051 |
| LOC677317 | -0.4337631 |
| LOC677319 | 0.89516426 |
| LOC677528 | 1.48128478 |
| LOC732482 | 0.40351179 |
| Loh11cr2a | 0.73971673 |
| Loh12cr1 | 2.04639306 |
| Loh12cr1 | 0.50398625 |
| Lonp2 | -0.4427095 |
| Lonrf1 | 1.28759579 |
| Lonrf2 | 0.02225598 |
| Lor | -0.4874129 |
| Lox | 1.57060389 |
| Loxl1 | -0.2160723 |
| Lpar2 | -0.8177876 |
| Lpar2 | 1.09676193 |
| Lpar3 | 0.27797679 |
| Lpcat2 | 1.21183492 |
| Lpcat2 | -0.6048381 |
| Lpcat2 | -0.7748227 |
| Lpgat1 | 0.80520589 |
| Lphn1 | 0.1909471 |
| Lphn3 | -0.1911581 |
| Lpin1 | -0.510585 |
| Lpin1 | 0.0841084 |
| Lpin2 | 1.18432731 |
| Lpin3 | 1.28348555 |
| Lpl | -0.4382257 |
| Lpxn | -0.8370962 |
| Lrat | -0.7639291 |
| Lrba | 2.05397595 |
| Lrba | 0.89205037 |
| Lrch1 | 1.07915053 |
| Lrch4 | -0.7328563 |
| Lrch4 | 1.27437948 |
| Lrdd | -0.6033144 |
| Lrfn1 | -0.4833821 |
| Lrfn2 | 0.47918897 |
| Lrfn3 | 0.60307943 |
| Lrfn4 | 3.06408899 |
| Lrg1 | -0.6630203 |
| Lrig1 | -0.0604905 |
| Lrig3 | 1.83666995 |
| Lrmp | -0.7657359 |
| Lrp1 | -0.0326481 |
| Lrp10 | -0.6868376 |
| Lrp11 | -0.3627297 |
| Lrp12 | 2.04409706 |
| Lrp12 | -0.6202125 |
| Lrp4 | 0.97085151 |
| Lrp5 | 0.47312961 |
| Lrp8 | -0.406186 |
| Lrp8 | -0.5393069 |
| Lrpap1 | -0.7567359 |
| Lrpprc | -0.5934696 |
| Lrpprc | -0.6646437 |
| Lrpprc | -0.239072 |
| Lrrc1 | -0.5897103 |
| Lrrc1 | -0.7757362 |
| Lrrc1 | 0.70860009 |
| Lrrc10 | -0.8410059 |
| Lrrc15 | 0.98098199 |
| Lrrc16b | 1.45047722 |
| Lrrc16b | 0.15892605 |
| Lrrc18 | 1.45598244 |
| Lrrc2 | -0.2922838 |
| Lrrc20 | 2.04882074 |
| Lrrc20 | -0.8158783 |
| Lrrc23 | -0.3475166 |
| Lrrc23 | -0.6573603 |
| Lrrc24 | 0.68927044 |
| Lrrc26 | 0.8982679 |
| Lrrc28 | -0.2658348 |
| Lrrc29 | 0.92875523 |
| Lrrc3 | 0.8702797 |
| Lrrc30 | 1.85218049 |
| Lrrc33 | -0.5638069 |
| Lrrc34 | 1.22999185 |
| Lrrc34 | 3.17401663 |
| Lrrc36 | 0.13487239 |
| Lrrc39 | -0.1521437 |
| Lrrc39 | 0.6349493 |
| Lrrc4 | -0.1862533 |
| Lrrc40 | 2.50118048 |
| Lrrc41 | -0.5521982 |
| Lrrc43 | 1.26549957 |
| Lrrc44 | -0.4853953 |
| Lrrc45 | -0.5016577 |
| Lrrc46 | 0.65521208 |
| Lrrc48 | -0.0414182 |
| Lrrc48 | 1.28800584 |
| Lrrc48 | 0.81743772 |
| Lrrc49 | 2.5991845 |
| Lrrc49 | 2.40966027 |
| Lrrc49 | 2.49686626 |
| Lrrc4c | 0.7535447 |
| Lrrc50 | 1.16933733 |
| Lrrc50 | -0.2669 |
| Lrrc52 | -0.6597819 |
| Lrrc52 | 0.29045389 |
| Lrrc56 | -0.396966 |
| Lrrc57 | -0.8206588 |
| Lrrc59 | -0.513349 |
| Lrrc6 | 0.13869311 |
| Lrrc7 | 0.69850622 |
| Lrrc8 | -0.4274244 |
| Lrrc8a | 1.64933112 |
| Lrrc8b | -0.1020669 |
| Lrrc8c | 0.505867 |
| Lrrc8d | 0.18799088 |
| Lrrc9 | 1.2243073 |
| Lrrfip1 | 0.56965603 |
| Lrrfip1 | 2.07275986 |
| Lrriq2 | -0.5400185 |
| Lrriq2 | 1.6265598 |
| Lrrk1 | -0.1394894 |
| Lrrk2 | -0.5071414 |
| Lrrn1 | 1.10588104 |
| Lrrn2 | -0.4787012 |
| Lrrn3 | 0.97771219 |
| Lrrtm1 | -0.4261618 |
| Lrrtm2 | -0.6063643 |
| Lrrtm3 | -0.2933793 |
| Lrsam1 | -0.1187691 |
| Lrsam1 | -0.6597819 |
| Lrtm1 | -0.0169828 |
| Lrtm1 | 0.09288238 |
| Lsamp | -0.5294008 |
| Lsamp | -0.4388649 |
| Lsg1 | -0.6968717 |
| Lsg1 | 0.79609946 |
| Lsm10 | 0.16535043 |
| Lsm11 | 1.68066622 |
| Lsm12 | 3.3378431 |
| Lsm12 | -0.511966 |
| Lsm12 | 0.23076115 |
| Lsm14a | 0.9064435 |
| Lsm14b | -0.1960886 |
| Lsm14b | 0.59456677 |
| Lsm14b | 0.49689984 |
| Lsm2 | 0.02225598 |
| Lsm2 | 0.11730771 |
| Lsm3 | -0.7389895 |
| Lsm4 | 2.84989506 |
| Lsm5 | -0.2025369 |
| Lsm6 | -0.8861435 |
| Lsm6 | 1.5038087 |
| Lsm8 | -0.8498622 |
| Lsr | -0.8206588 |
| Lst1 | -0.7095663 |
| Ltb4dh | -0.0178008 |
| Ltb4r1 | -0.4720543 |
| Ltb4r2 | 1.40224741 |
| Ltbp1 | 0.66260034 |
| Ltbp1 | 1.77976469 |
| Ltbp2 | 0.10625102 |
| Ltbp3 | 2.03624859 |
| Ltbp3 | -0.6549449 |
| Ltbp4 | -0.455639 |
| Ltbp4 | -0.7775659 |
| Ltbp4 | 2.35622029 |
| Ltbr | 1.69442106 |
| Ltc4s | 0.71011435 |
| Ltf | -0.1469677 |
| Ltf | -0.201542 |
| Ltk | -0.7639291 |
| Ltk | 0.11411104 |
| Ltv1 | 0.17340338 |
| Luc7l | -0.8488741 |
| Luc7l | -0.8718678 |
| Luc7l2 | 0.8496273 |
| Luc7l2 | -0.3172449 |
| Lum | 1.40659562 |
| Luzp1 | 2.60928855 |
| Luzp1 | -0.7504866 |
| Luzp1 | -0.6695307 |
| Luzp2 | 0.32008552 |
| Lxn | -0.359785 |
| Ly6a | 2.39820157 |
| Ly6c1 | -0.1731362 |
| Ly6d | 0.6785754 |
| Ly6e | -0.4661118 |
| Ly6f | 1.32498683 |
| Ly6g6c | -0.7867678 |
| Ly6g6d | 1.60754412 |
| Ly6g6e | 0.72568302 |
| Ly6k | -0.8698459 |
| Ly86 | 0.62734036 |
| Ly9 | -0.7363561 |
| Ly9 | 0.16636082 |
| Ly96 | -0.6178903 |
| Lyar | -0.3710242 |
| Lyl1 | 1.78920944 |
| Lynx1 | 0.4940503 |
| Lynx1 | 1.74183435 |
| Lypd1 | 0.39720803 |
| Lypd3 | -0.5674641 |
| Lypd4 | 0.20559077 |
| Lypd5 | -0.0838982 |
| Lypd6 | 0.81541006 |
| Lypla1 | -0.8177876 |
| Lypla1 | 2.14972304 |
| Lypla2 | 0.71821212 |
| Lypla3 | -0.6148029 |
| Lyplal1 | 0.25925118 |
| Lyrm1 | -0.547164 |
| Lyrm2 | -0.316683 |
| Lyrm2 | 1.28667255 |
| Lyrm5 | 1.65274862 |
| Lysmd1 | -0.7328563 |
| Lysmd1 | -0.0064124 |
| Lysmd2 | 0.95683302 |
| Lysmd4 | -0.4773681 |
| Lyst | -0.2085287 |
| Lyve1 | -0.4562902 |
| Lyz | 0.58662357 |
| Lyz1 | 1.81123939 |
| Lyz1 | -0.4031026 |
| Lyz2 | 3.0704133 |
| Lyzl1 | 2.25795211 |
| Lyzl4 | 1.21682157 |
| Lyzl6 | 3.10045436 |
| Lyzs | -0.2813986 |
| Lzic | -0.0773375 |
| Lztr1 | -0.1702459 |
| Lzts2 | 0.29897371 |
| M6pr | 1.50828211 |
| M6pr | -0.4968855 |
| M6prbp1 | 0.96162561 |
| Mab21l2 | 0.24014626 |
| Macrod1 | -0.4928141 |
| Mad1l1 | -0.284651 |
| Mad2l1 | 1.77909513 |
| Mad2l1bp | -0.7311112 |
| Mad2l1bp | -0.585966 |
| Mad2l1bp | -0.4602066 |
| Mad2l2 | -0.4707305 |
| Madcam1 | 0.66597564 |
| Madd | 1.76263362 |
| Maea | 1.28338257 |
| Maf | 0.70213721 |
| Maf1 | -0.1224196 |
| Mafg | 1.13657966 |
| Mag | -0.5755603 |
| Magea2 | 2.74080839 |
| Magea5 | -0.63191 |
| Mageb3 | -0.1774883 |
| Mageb3 | -0.6148029 |
| Maged1 | 1.42236969 |
| Maged2 | -0.6935151 |
| Magee1 | 0.57556401 |
| Mageh1 | -0.3279856 |
| Magi1 | -0.6163453 |
| Magi1 | 1.82919335 |
| Magi1 | -0.6117256 |
| Magi2 | -0.5521982 |
| Magi3 | 1.13862316 |
| Magi3 | -0.6605904 |
| Magix | -0.5829812 |
| Magmas | 0.39404028 |
| Magoh | 0.07302489 |
| Magoh | -0.5770398 |
| Magohb | 2.10791028 |
| Mak | 0.84717723 |
| Mak10 | 1.00147791 |
| Mak10 | -0.0812684 |
| Mal | 3.22724035 |
| Mal | 1.08679294 |
| Mall | -0.7407492 |
| Malt1 | 0.68111428 |
| Mamdc2 | -0.4055685 |
| Maml1 | -0.6202125 |
| Maml3 | 1.92471558 |
| Man1a | 0.35627503 |
| Man1a2 | -0.6654564 |
| Man1a2 | 0.15109017 |
| Man1b1 | 0.81371702 |
| Man1c1 | -0.0587794 |
| Man1c1 | -0.7284997 |
| Man2a1 | 3.52744249 |
| Man2a2 | 0.25036316 |
| Man2a2 | 2.02800695 |
| Man2a2 | 0.54497119 |
| Man2b1 | 1.13585736 |
| Man2b2 | 1.36190005 |
| Man2c1 | 2.46430659 |
| Manba | -0.482712 |
| Manba | 2.41996343 |
| Manbal | 0.15348191 |
| Manea | -0.4006432 |
| Mansc1 | 1.86171177 |
| Maob | 1.61329645 |
| Map1lc3a | 0.17707146 |
| Map1lc3a | 1.3282333 |
| Map1lc3b | 2.9658318 |
| Map2k1 | 0.98084006 |
| Map2k2 | -0.6233176 |
| Map2k2 | -0.187722 |
| Map2k3 | 1.9961408 |
| Map2k4 | -0.7886191 |
| Map2k6 | 0.8465228 |
| Map2k6 | -0.0927173 |
| Map2k6 | -0.2862814 |
| Map2k7 | 0.095789 |
| Map2k7 | 0.16703381 |
| Map2k7 | -0.612494 |
| Map3k1 | 0.50328 |
| Map3k11 | -0.0958235 |
| Map3k12 | 2.45899113 |
| Map3k12 | 0.82114379 |
| Map3k12 | 0.99380904 |
| Map3k14 | 2.09200762 |
| Map3k3 | 0.9140919 |
| Map3k6 | 3.17194686 |
| Map3k7 | 0.31290853 |
| Map3k7ip1 | 0.2000854 |
| Map3k7ip1 | -0.4274244 |
| Map3k7ip1 | 0.93904882 |
| Map3k7ip2 | -0.5265895 |
| Map3k7ip3 | 0.49119213 |
| Map3k8 | 1.81985097 |
| Map3k9 | 0.51148685 |
| Map4k2 | 3.23395723 |
| Map4k4 | -0.6295588 |
| Map4k5 | 0.48544967 |
| Map4k5 | 0.5698755 |
| Mapbpip | -0.2349419 |
| Mapk1 | 1.64695249 |
| Mapk1 | -0.4217558 |
| Mapk1 | -0.3799929 |
| Mapk10 | -0.7053157 |
| Mapk10 | 0.4861694 |
| Mapk11 | 0.81642443 |
| Mapk12 | -0.056645 |
| Mapk12 | -0.6421665 |
| Mapk12 | 0.06743391 |
| Mapk13 | -0.7603256 |
| Mapk13 | 0.13695834 |
| Mapk14 | -0.0562187 |
| Mapk15 | 0.33032539 |
| Mapk15 | -0.6366299 |
| Mapk15 | -0.6926778 |
| Mapk1ip1 | 2.58566267 |
| Mapk1ip1 | -0.3734075 |
| Mapk1ip1 | 2.29619721 |
| Mapk3 | -0.6630203 |
| Mapk3 | -0.0347289 |
| Mapk4 | 1.80426934 |
| Mapk6 | -0.6977127 |
| Mapk6 | 0.87091785 |
| Mapk6 | -0.6240955 |
| Mapk8 | -0.6397897 |
| Mapk8ip1 | -0.5995157 |
| Mapk8ip1 | -0.7594269 |
| Mapk8ip2 | -0.4111404 |
| Mapk8ip3 | 2.22790282 |
| Mapk8ip3 | 1.48003147 |
| Mapk9 | 0.73916671 |
| Mapk9 | 0.52331901 |
| Mapk9 | 1.65030881 |
| Mapkap1 | 1.73986863 |
| Mapkap1 | -0.6033144 |
| Mapkapk2 | -0.7389895 |
| Mapkapk3 | 0.19748348 |
| Mapkbp1 | 0.01832599 |
| Mapre1 | -0.5078292 |
| Mapre2 | -0.6501327 |
| Mapre2 | -0.6240955 |
| Mapre3 | -0.5777804 |
| Mapt | -0.6622096 |
| Mapt | 0.52516164 |
| Mapt | -0.6951919 |
| Mapt | -0.5301049 |
| March2 | -0.5126572 |
| March3 | -0.6736227 |
| March3 | 0.33821357 |
| March5 | -0.4510935 |
| March5 | -0.7540526 |
| March7 | -0.3206235 |
| March8 | -0.5949775 |
| March9 | 2.69083322 |
| March11 | 0.00364097 |
| Marcks | 2.02228406 |
| Marcksl1 | 0.09142571 |
| Marcksl1 | 0.445245 |
| Marcksl1 | -0.2797766 |
| Mare | -0.6485341 |
| Mark1 | 1.32054175 |
| Mark2 | -0.2075274 |
| Mark2 | 0.94776235 |
| Mark3 | 0.42448119 |
| Mark3 | -0.1297633 |
| Mark4 | -0.4117615 |
| Mars | 0.20365136 |
| Mars2 | 0.24573283 |
| Marveld3 | -0.6469381 |
| Marveld3 | 1.17420634 |
| Mas1 | -0.4975658 |
| Masp1 | -0.6613997 |
| Masp2 | -0.6793814 |
| Mast1 | -0.3591972 |
| Mast1 | 0.05160051 |
| Mast2 | 0.99576704 |
| Mast2 | 1.47154891 |
| Mat1a | -0.239072 |
| Mat2a | 0.22064635 |
| Mat2a | 2.07720933 |
| Mat2a | 1.81359047 |
| Mat2a | -0.5807488 |
| Mat2b | -0.7284997 |
| Mat2b | -0.8799988 |
| Matk | 1.20927596 |
| Matk | 1.39202297 |
| Matn4 | -0.2637081 |
| Matn4 | -0.6525358 |
| Matn4 | 2.3228451 |
| Matr3 | 0.99086439 |
| Max | -0.5400185 |
| Maz | -0.7469341 |
| Mb | -0.4388649 |
| Mbc2 | 1.50242171 |
| Mbd1 | -0.8861435 |
| Mbd1 | -0.0695246 |
| Mbd3 | 1.76991702 |
| Mbd3l2 | 0.3526903 |
| Mbd4 | 0.74118189 |
| Mbd5 | 0.15586759 |
| Mbd6 | 0.42933316 |
| Mbip | -0.2642393 |
| Mbl2 | 0.6582143 |
| Mbnl1 | 1.12080475 |
| Mbnl2 | -0.0660728 |
| Mbnl2 | -0.7070137 |
| Mboat1 | 0.18766184 |
| Mboat2 | -0.421128 |
| Mboat2 | 2.08044423 |
| Mboat2 | 0.33286808 |
| Mboat2 | -0.7036209 |
| Mboat5 | -0.6086583 |
| Mboat5 | 2.3344061 |
| Mbp | 0.12893703 |
| Mbp | -0.7354799 |
| Mbp | 1.18615588 |
| Mbtd1 | 1.45064975 |
| Mbtps1 | -0.6703477 |
| Mbtps1 | -0.5912122 |
| Mbtps1 | 1.52293556 |
| Mbtps2 | -0.0351455 |
| Mc3r | -0.1882121 |
| Mc4r | 0.2986812 |
| Mc5r | 0.6188365 |
| Mcam | -0.650933 |
| Mcam | 1.92830911 |
| Mcam | -0.2432202 |
| Mcat | 0.55170673 |
| Mcc | -0.1061025 |
| Mccc1 | 0.18634453 |
| Mcee | 0.80708494 |
| Mcf2l | -0.2201203 |
| Mcf2l | 2.30415228 |
| Mcf2l | 0.89749294 |
| Mcfd2 | 0.07525204 |
| Mchr1 | -0.5934696 |
| Mcl1 | 2.26193841 |
| Mcm10 | -0.5897103 |
| Mcm10 | 1.5049494 |
| Mcm2 | -0.6163453 |
| Mcm3 | 2.47665219 |
| Mcm4 | 1.40015678 |
| Mcm5 | -0.5209919 |
| Mcm6 | -0.0817062 |
| Mcm6 | 0.06780767 |
| Mcm6 | -0.6868376 |
| Mcm7 | -0.8187437 |
| Mcoln1 | 0.51195366 |
| Mcoln2 | 1.95934532 |
| Mcoln3 | 1.07414954 |
| Mcph1 | -0.1862533 |
| Mcph1 | 0.89173841 |
| Mcph1 | 2.01372904 |
| Mcph1 | 1.82815345 |
| Mcpt4 | -0.3591972 |
| Mcrs1 | -0.2065272 |
| Mcrs1 | -0.4968855 |
| Mcrs1 | 3.17209982 |
| Mcts1 | -0.3440404 |
| Mdfic | -0.5964878 |
| Mdga1 | 0.96984681 |
| Mdga2 | -0.6549449 |
| Mdga2 | 2.13843408 |
| Mdh1 | -0.3189327 |
| Mdh1 | -0.0178008 |
| Mdh1b | -0.4628266 |
| Mdh2 | -0.6501327 |
| Mdk | 1.04264357 |
| Mdk | 2.13464087 |
| Mdm1 | 0.27588113 |
| Mdm1 | -0.6662699 |
| Mdm1 | -0.2760027 |
| Mdm1 | -0.4880863 |
| Mdm1 | -0.2421814 |
| Mdm1 | 0.03742836 |
| Mdm1 | -0.7389895 |
| Mdm1 | 1.53468596 |
| Mdm1 | 0.3362477 |
| Mdm2 | 1.13018017 |
| Mdm2 | -0.511966 |
| Mdm4 | 0.33989535 |
| Mdn1 | 0.69042987 |
| Me2 | -0.2447805 |
| Me3 | 2.10554406 |
| Mea1 | -0.5126572 |
| Mecp2 | -0.8548186 |
| Mecr | 0.03084475 |
| Med1 | -0.2277576 |
| Med1 | 0.46776499 |
| Med10 | 1.13344571 |
| Med11 | 0.54924261 |
| Med11 | -0.0712552 |
| Med12 | 1.08412513 |
| Med15 | 0.86146097 |
| Med15 | 1.22123213 |
| Med15 | -0.1784582 |
| Med16 | 0.25772472 |
| Med18 | -0.5980006 |
| Med18 | -0.1311466 |
| Med19 | -0.431857 |
| Med21 | -0.525187 |
| Med22 | -0.3884413 |
| Med22 | 0.41211123 |
| Med23 | -0.6525358 |
| Med23 | 1.12836108 |
| Med24 | 1.44528673 |
| Med25 | -0.2484312 |
| Med25 | -0.2421814 |
| Med26 | 0.365314 |
| Med27 | -0.8943991 |
| Med28 | -0.4031026 |
| Med30 | 0.9515768 |
| Med31 | 2.42942219 |
| Med4 | -0.5755603 |
| Med6 | -0.7138361 |
| Med7 | 1.08030084 |
| Med8 | -0.7513768 |
| Med8 | -0.4840527 |
| Med9 | -0.8158783 |
| Mef2b | 0.94614388 |
| Mef2b | -0.6469381 |
| Mef2c | 1.30315423 |
| Mef2c | 0.64513511 |
| Mef2c | 0.70765244 |
| Megf10 | 0.42473718 |
| Megf11 | -0.5098953 |
| Megf11 | -0.3469363 |
| Megf11 | -0.6240955 |
| Megf11 | 2.87976388 |
| Megf6 | -0.4968855 |
| Megf9 | -0.6171175 |
| Mei1 | -0.2416624 |
| Meis1 | 1.28410323 |
| Meis2 | -0.7044679 |
| Meis3 | 2.17926229 |
| Melk | 1.18180723 |
| Memo1 | -0.8168324 |
| Men1 | 0.41029365 |
| Men1 | -0.5230871 |
| Meox1 | -0.3066251 |
| Meox2 | 1.40822104 |
| Mep1b | -0.1563997 |
| Mepce | -0.5667315 |
| Mertk | -0.3603732 |
| Mesdc1 | 0.05501583 |
| Mesp1 | -0.4894347 |
| Mest | -0.1545058 |
| Mest | -0.612494 |
| Mest | -0.2994272 |
| Metap1 | 1.7052823 |
| Metap2 | -0.5980006 |
| Metap2 | 1.16805794 |
| Metapl1 | -0.5912122 |
| Metrn | -0.6835165 |
| Metrnl | -0.1931272 |
| Metrnl | 0.63002077 |
| Mett11d1 | 0.75300268 |
| Mettl1 | -0.6248741 |
| Mettl1 | 0.78638107 |
| Mettl2 | -0.4740434 |
| Mettl3 | -0.4105197 |
| Mettl4 | -0.5407306 |
| Mettl6 | -0.455639 |
| Mettl8 | 1.05545121 |
| Mettl9 | -0.2401073 |
| Mettl9 | 0.18502537 |
| Mex3a | 2.53295049 |
| Mex3a | 1.69195327 |
| Mex3c | 1.17235444 |
| Mfap1b | -0.6793814 |
| Mfap2 | 0.73069263 |
| Mfap2 | 0.76092119 |
| Mfap2 | 0.32777583 |
| Mfap3 | 3.73791632 |
| Mfap3 | -0.7988662 |
| Mfap3l | -0.3545075 |
| Mfap3l | 0.53318108 |
| Mfap4 | 1.14544277 |
| Mfap5 | 1.29158611 |
| Mfge8 | 1.35543392 |
| Mfge8 | 0.35929766 |
| Mfi2 | 0.07413912 |
| Mfn1 | -0.7346045 |
| Mfn1 | 1.21560505 |
| Mfn2 | -0.4894347 |
| Mfng | -0.562348 |
| Mfrp | -0.6017931 |
| Mfsd1 | -0.6461412 |
| Mfsd10 | 0.44974237 |
| Mfsd10 | 2.51871538 |
| Mfsd11 | 2.14395071 |
| Mfsd2 | 4.46134683 |
| Mfsd3 | -0.5322205 |
| Mfsd4 | 1.3091862 |
| Mfsd7 | -0.6565545 |
| Mfsd8 | 1.39468102 |
| Mfsd9 | -0.4694086 |
| Mga | -0.3094082 |
| Mgat1 | 0.00083682 |
| Mgat3 | 1.47407646 |
| Mgat4b | 1.38594553 |
| Mgat4b | 0.12613094 |
| Mgat5 | 1.21671104 |
| MGC107098 | 1.11318739 |
| MGC117608 | -0.6421665 |
| MGC118210 | 0.7404496 |
| MGC18837 | 0.11837087 |
| MGC41689 | -0.6002742 |
| MGC67181 | -0.7469341 |
| Mgea6 | 0.65360698 |
| Mgl1 | -0.5357571 |
| Mgl2 | 0.20300401 |
| Mgll | 2.58558499 |
| Mgll | -0.5175101 |
| Mgll | -0.5428702 |
| Mgmt | 0.4040352 |
| Mgp | 2.10014411 |
| Mgrn1 | -0.6630203 |
| Mgst1 | -0.6695307 |
| Mgst1 | 0.28066438 |
| Mgst2 | 1.58791348 |
| Mgst3 | 0.25588971 |
| Mgst3 | -0.7319833 |
| Mia1 | -0.2065272 |
| Mia3 | -0.2563021 |
| Mib1 | 0.58511325 |
| Mib2 | -0.3866245 |
| Mic2l1 | -0.1160405 |
| Mical1 | 0.78916799 |
| Mical1 | 0.06218559 |
| Mical1 | -0.510585 |
| Mical2 | 1.3733911 |
| Mical3 | 1.69375472 |
| Micall2 | 1.60776321 |
| Micall2 | 0.14834932 |
| Mid1 | 1.47902763 |
| Mid1 | -0.019439 |
| Mid1ip1 | 0.16062093 |
| Midn | -0.308294 |
| Midn | 2.29718209 |
| Mier1 | 0.39006558 |
| Mier1 | 0.06030409 |
| Mif | 0.87743419 |
| Mif4gd | -0.359785 |
| Mif4gd | -0.3144386 |
| Mill2 | 0.73971673 |
| Mill2 | -0.2155675 |
| Mina | 0.81066185 |
| Mink1 | -0.3957435 |
| Mink1 | -0.612494 |
| Minpp1 | 0.84324382 |
| Minpp1 | -0.7258954 |
| Miox | 0.15722813 |
| Mipep | -0.7070137 |
| Mir16 | 1.4322721 |
| Mist1 | -0.8429668 |
| Mitf | 0.00123792 |
| Mizf | -0.7904739 |
| Mki67ip | 1.30234704 |
| Mkks | -0.6086583 |
| Mkks | 2.00801413 |
| Mkl1 | 1.55544382 |
| Mkl2 | 1.09613409 |
| Mkl2 | -0.5957324 |
| Mkl2 | 0.0171438 |
| Mkln1 | 0.69004356 |
| Mknk1 | -0.6155738 |
| Mknk2 | 0.61403225 |
| Mkrn1 | 1.3561016 |
| Mkrn2 | -0.3027425 |
| Mkrn3 | 0.80092136 |
| Mkx | -0.1348454 |
| Mlana | 0.21394907 |
| Mlf1 | -0.5652681 |
| Mlf1 | -0.780317 |
| Mlf2 | 1.99430079 |
| Mlf2 | -0.6217638 |
| Mlh1 | -0.6630203 |
| Mlh3 | 0.43770141 |
| Mlkl | -0.6421665 |
| Mllt1 | -0.3662756 |
| Mllt1 | 0.74319278 |
| Mllt1 | -0.6010334 |
| Mllt10 | -0.1985635 |
| Mllt11 | -0.2115389 |
| Mllt3 | 0.3134847 |
| Mllt3 | -0.5057675 |
| Mllt3 | -0.3830015 |
| Mlp | -0.7181253 |
| Mlstd2 | 1.10923165 |
| Mlx | 1.0779988 |
| Mlx | -0.7830763 |
| Mlxipl | -0.4018721 |
| Mlycd | -0.3428845 |
| Mmab | -0.5867137 |
| Mmab | 0.24387431 |
| Mmd | -0.7867678 |
| Mmd2 | 1.05702364 |
| Mme | -0.2241858 |
| Mme | 1.67136086 |
| Mmel1 | 0.89407556 |
| Mmp10 | -0.0896212 |
| Mmp11 | -0.2706375 |
| Mmp12 | 0.92995447 |
| Mmp13 | 0.16028219 |
| Mmp13 | 0.44122926 |
| Mmp14 | 2.15423379 |
| Mmp15 | 1.2016695 |
| Mmp16 | 0.03626988 |
| Mmp17 | -0.5674641 |
| Mmp1a | 0.70841063 |
| Mmp1a | 0.36966555 |
| Mmp2 | -0.0976032 |
| Mmp23 | -0.6760864 |
| Mmp24 | 2.1711002 |
| Mmp3 | -0.4921373 |
| Mmp3 | 0.21170603 |
| Mmp3 | 0.95040476 |
| Mmp9 | -0.4847238 |
| Mmrn2 | 0.53203962 |
| Mnat1 | -0.0264333 |
| Mnat1 | -0.8120712 |
| Mnd1 | 0.47555803 |
| Mns1 | -0.5874619 |
| Mnt | -0.2110365 |
| Mobk1b | 1.01772369 |
| Mobkl2a | -0.435036 |
| Mobkl2b | -0.7189855 |
| Mobkl2c | -0.3480972 |
| Mobkl3 | -0.6501327 |
| Mobp | -0.0570715 |
| Mobp | 0.31951337 |
| Mobp | 2.12915338 |
| Mocos | -0.4713922 |
| Mocs1 | 1.07376375 |
| Mocs2 | -0.121049 |
| Mod1 | -0.9281636 |
| Mog | 1.01567401 |
| Mog | -0.1469677 |
| Mon1a | 2.00077331 |
| Mon1a | 1.69946987 |
| Mon1b | -0.1274622 |
| Mon1b | 0.32975941 |
| Mon1b | -0.7748227 |
| Morc2a | 0.01279635 |
| Morc2b | 0.14800615 |
| Morc3 | -0.8120712 |
| Morf4l1 | 0.28690582 |
| Morf4l1 | 0.06293714 |
| Morf4l2 | -0.5485996 |
| Morn2 | 1.51111782 |
| Morn3 | 0.39006558 |
| Morn3 | 0.20559077 |
| Mos | -0.4667703 |
| Mosc2 | -0.4111404 |
| Mospd1 | 1.12019272 |
| Mospd1 | 1.42121354 |
| Mospd1 | -0.6541412 |
| Mospd3 | 2.18842018 |
| Mospd3 | -0.4928141 |
| Mov10 | 1.54473175 |
| Mov10l1 | 1.41191297 |
| Moxd1 | 0.57316152 |
| Moxd1 | -0.3805938 |
| Mpdu1 | 0.8437364 |
| Mpdz | -0.145561 |
| Mpdz | -0.6678989 |
| Mpg | -0.5023415 |
| Mpg | 0.7776185 |
| Mphosph6 | -0.6525358 |
| Mpnd | 0.39456898 |
| Mpo | 0.76806259 |
| Mpo | 1.1244686 |
| Mpp1 | 1.2502625 |
| Mpp2 | 0.7087895 |
| Mpp3 | -0.8459158 |
| Mpp3 | 2.53759369 |
| Mpp5 | 2.69837634 |
| Mpp6 | 2.6358543 |
| Mppe1 | 0.11624336 |
| Mpped1 | -0.2711727 |
| Mpst | 0.34213306 |
| Mpv17 | 0.11054642 |
| Mpv17 | -0.4343993 |
| Mpv17 | 0.47918897 |
| Mpv17 | -0.6366299 |
| Mpv17l | -0.0570715 |
| Mpv17l | 1.25992007 |
| Mpv17l | 3.05274876 |
| Mpz | 1.31228767 |
| Mr1 | 0.24604222 |
| Mrap | -0.4142502 |
| Mras | -0.3268492 |
| Mrc1 | -0.7603256 |
| Mrc2 | -0.2328837 |
| Mrc2 | 1.00078331 |
| Mre11a | -0.0096522 |
| Mre11a | -0.5800058 |
| Mre11a | -0.3285543 |
| Mreg | -0.7433949 |
| Mrgpra2 | -0.4647964 |
| Mrgpra2 | 2.53707588 |
| Mrgprb2 | -0.3325449 |
| Mrgpre | 0.26260071 |
| Mrgprf | -0.6140326 |
| Mrgprg | 0.41418419 |
| Mrm1 | -0.7775659 |
| Mro | -0.382399 |
| Mrp63 | -0.6860062 |
| Mrpl1 | 1.69168609 |
| Mrpl1 | -0.5800058 |
| Mrpl10 | -0.8035611 |
| Mrpl10 | 2.59841849 |
| Mrpl11 | -0.5814923 |
| Mrpl12 | -0.5189013 |
| Mrpl13 | 0.19323999 |
| Mrpl13 | 0.99016193 |
| Mrpl14 | -0.5457305 |
| Mrpl15 | 0.81625545 |
| Mrpl16 | -0.2895507 |
| Mrpl17 | 0.4772542 |
| Mrpl18 | 1.07427811 |
| Mrpl2 | -0.6910055 |
| Mrpl20 | -0.6785566 |
| Mrpl22 | 0.0238234 |
| Mrpl23 | -0.4312225 |
| Mrpl24 | -0.1516719 |
| Mrpl27 | -0.6935151 |
| Mrpl28 | -0.7585291 |
| Mrpl3 | 0.49190749 |
| Mrpl3 | -0.8498622 |
| Mrpl30 | -0.2115389 |
| Mrpl33 | -0.6703477 |
| Mrpl33 | -0.4530389 |
| Mrpl34 | 1.59200609 |
| Mrpl36 | -0.4356731 |
| Mrpl37 | 0.0976011 |
| Mrpl37 | -0.0852158 |
| Mrpl38 | -0.0355624 |
| Mrpl39 | 0.3209431 |
| Mrpl40 | 0.06892808 |
| Mrpl43 | 2.87094614 |
| Mrpl43 | -0.9303146 |
| Mrpl45 | -0.2020393 |
| Mrpl46 | 0.58035072 |
| Mrpl47 | 3.59600572 |
| Mrpl47 | 1.15681814 |
| Mrpl48 | -0.2626465 |
| Mrpl48 | 0.51381861 |
| Mrpl48 | 0.15620791 |
| Mrpl49 | 1.29779371 |
| Mrpl49 | -0.5030257 |
| Mrpl49 | 2.38156831 |
| Mrpl49 | 1.07863882 |
| Mrpl50 | -0.3121995 |
| Mrpl51 | 0.83283991 |
| Mrpl51 | -0.0980486 |
| Mrpl51 | -0.5230871 |
| Mrpl52 | -0.3296928 |
| Mrpl53 | -0.043098 |
| Mrpl54 | -0.5521982 |
| Mrpl55 | -0.514734 |
| Mrpl9 | 0.12014014 |
| Mrps10 | 0.91591833 |
| Mrps10 | 1.98053269 |
| Mrps11 | -0.5493182 |
| Mrps11 | 1.01279695 |
| Mrps12 | -0.4472147 |
| Mrps14 | 0.30160222 |
| Mrps15 | 0.15586759 |
| Mrps16 | -0.3428845 |
| Mrps17 | 0.26775397 |
| Mrps18a | -0.4478601 |
| Mrps18b | 0.92890522 |
| Mrps18b | 1.33936316 |
| Mrps18c | -0.5638069 |
| Mrps2 | -0.4037185 |
| Mrps21 | -0.1554523 |
| Mrps22 | 1.32892051 |
| Mrps23 | 1.30416224 |
| Mrps24 | 1.53649897 |
| Mrps25 | -0.5279941 |
| Mrps25 | 1.76757765 |
| Mrps25 | 2.00853944 |
| Mrps26 | -0.8332027 |
| Mrps26 | -0.6810333 |
| Mrps27 | 0.26956607 |
| Mrps28 | -0.6186637 |
| Mrps30 | 0.50961728 |
| Mrps31 | 1.42698017 |
| Mrps31 | -0.6389987 |
| Mrps31 | -0.5822365 |
| Mrps33 | 0.77691397 |
| Mrps33 | -0.8322319 |
| Mrps34 | 0.58035072 |
| Mrps35 | 1.86960002 |
| Mrps5 | 3.09630487 |
| Mrps6 | 0.93964211 |
| Mrps7 | -0.546447 |
| Mrps7 | 1.07581949 |
| Mrps9 | 0.77409055 |
| Mrrf | 0.12823629 |
| Mrrf | -0.2075274 |
| Mrto4 | -0.6148029 |
| Mrvi1 | -0.6670841 |
| Ms4a1 | 3.46480355 |
| Ms4a10 | -0.5934696 |
| Ms4a13 | 1.68565455 |
| Ms4a5 | 1.95667573 |
| Ms4a6d | -0.6025534 |
| Ms4a6d | -0.5175101 |
| Ms4a7 | -0.7630269 |
| Ms4a8a | 1.55821838 |
| Msc | -0.63191 |
| Msh2 | 0.7931598 |
| Msh3 | 0.95785166 |
| Msh3 | -0.6233176 |
| Msh4 | 1.09336664 |
| Msh5 | -0.2706375 |
| Msh5 | -0.5630771 |
| Msh6 | -0.5016577 |
| Msh6 | 0.83499617 |
| Msi2 | 0.22476858 |
| Msi2h | 0.0648134 |
| Msln | -0.1469677 |
| Msn | -0.3686469 |
| Msr2 | -0.7398689 |
| Msrb2 | -0.5942232 |
| Msrb2 | 2.91229367 |
| Msrb3 | 2.18025771 |
| Mst1r | -0.4369485 |
| Msto1 | -0.4223839 |
| Msx1 | 2.86335773 |
| Mt1 | 0.03006717 |
| Mt3 | 2.72481488 |
| Mta1 | 0.35434649 |
| Mta2 | 1.5508789 |
| Mta3 | 1.20827274 |
| Mta3 | 0.7546278 |
| Mtag2 | 1.43130414 |
| Mtap | -0.6557494 |
| Mtap | -0.0647816 |
| Mtap1b | 0.4162526 |
| Mtap1b | -0.5704001 |
| Mtap1b | -0.4388649 |
| Mtap1b | 0.23796474 |
| Mtap1s | -0.7585291 |
| Mtap2 | 1.13718115 |
| Mtap2 | 0.71181499 |
| Mtap2 | -0.1479067 |
| Mtap4 | 1.40596275 |
| Mtap6 | 1.75279235 |
| Mtap6 | -0.2770794 |
| Mtap7 | 3.0324443 |
| Mtap7d1 | -0.3296928 |
| Mtap7d1 | -0.2808576 |
| Mtap7d2 | -0.6109578 |
| Mtap7d3 | 1.6000648 |
| Mtbp | 0.92695352 |
| Mtbp | 2.17598993 |
| Mtbp | -0.307181 |
| Mtch1 | 2.06068556 |
| Mtch1 | 0.30828643 |
| Mtch2 | -0.6951919 |
| Mtch2 | 0.05122027 |
| Mtch2 | -0.4305884 |
| Mtcp1 | 0.06331269 |
| Mtdh | 1.06198578 |
| Mterf | -0.3348328 |
| Mterf | -0.7044679 |
| Mterfd1 | -0.4420676 |
| Mterfd2 | -0.5565347 |
| Mterfd2 | 1.69348806 |
| Mterfd2 | 2.95827486 |
| Mterfd3 | -0.4312225 |
| Mterfd3 | -0.0936037 |
| Mtf1 | 0.22128171 |
| Mtf1 | 1.93478446 |
| Mtf2 | -0.3854153 |
| Mtfmt | -0.0072213 |
| Mtfmt | 1.64112051 |
| Mtg1 | 0.32065733 |
| Mthfd1 | -0.340577 |
| Mthfd2 | 0.4328924 |
| Mthfs | -0.6926778 |
| Mthfsd | -0.7087146 |
| Mthfsd | -0.2961235 |
| Mtif3 | -0.6501327 |
| Mtl5 | 1.11835425 |
| Mtl5 | 1.14913108 |
| Mtmr1 | -0.0936037 |
| Mtmr11 | 0.50821267 |
| Mtmr11 | -0.547164 |
| Mtmr12 | -0.554364 |
| Mtmr14 | 0.62154115 |
| Mtmr14 | -0.7112719 |
| Mtmr14 | 0.33004244 |
| Mtmr14 | -0.7513768 |
| Mtmr2 | -0.5807488 |
| Mtmr2 | -0.6525358 |
| Mtmr2 | -0.3716195 |
| Mtmr3 | 0.10696828 |
| Mtmr4 | -0.1316081 |
| Mtmr9 | 1.86432998 |
| Mto1 | 0.09904827 |
| Mtpn | -0.5168153 |
| Mtrf1 | 2.98645463 |
| Mtrf1 | 0.33427773 |
| Mtrf1l | -0.5428702 |
| Mtrr | -0.5536415 |
| Mtss1 | -0.5037104 |
| Mttp | 2.37463124 |
| Mtus1 | 1.9725315 |
| Mtus1 | -0.4356731 |
| Mtvr2 | 0.47604296 |
| Mtvr2 | -0.5987578 |
| Mtx1 | 0.64310673 |
| Mtx1 | 2.93007221 |
| Mtx1 | 3.09171319 |
| Mtx2 | 0.01635484 |
| Muc1 | 3.69412532 |
| Muc1 | -0.7567359 |
| Muc1 | -0.4800362 |
| Muc1 | 0.36640377 |
| Muc13 | 0.50186591 |
| Muc5ac | 3.27267182 |
| Mug2 | -0.5514774 |
| Mug2 | -0.5995157 |
| Mug4 | -0.2000516 |
| Mum1 | -0.0703895 |
| Mup1 | 1.62512713 |
| Mup2 | -0.4111404 |
| Mup2 | 1.34439665 |
| Mup3 | 0.03858542 |
| Mupcdh | 0.80092136 |
| Mus81 | -0.5414433 |
| Mus81 | 2.86192928 |
| Mustn1 | 1.00770639 |
| Mustn1 | 4.24197207 |
| Muted | 2.44680069 |
| Muted | 0.6840353 |
| Mutyh | 1.05885484 |
| Mvd | -0.7621256 |
| Mvp | 0.68169921 |
| Mx2 | -0.5587105 |
| Mxd1 | 2.34204663 |
| Mxd4 | -0.7594269 |
| Mxi1 | 0.16871418 |
| Mxra7 | 1.7309398 |
| Mxra8 | 1.17119516 |
| Mxra8 | -0.3308326 |
| Myadm | 1.48445239 |
| Mybbp1a | 0.34241241 |
| Mybbp1a | 0.37426718 |
| Mybbp1a | 2.31546064 |
| Mybl2 | -0.359785 |
| Mybpc2 | -0.4230125 |
| Mybpc3 | -0.6777325 |
| Myc | -0.4820423 |
| Mycbp | 1.20559225 |
| Mycbp2 | -0.7224341 |
| Mycbp2 | 0.58273499 |
| Mycbpap | -0.5078292 |
| Mycl1 | 0.52193467 |
| Myd116 | 0.60159525 |
| Myd88 | -0.7172659 |
| Myef2 | -0.0418379 |
| Myg1 | 1.78098085 |
| Myg1 | 0.37696357 |
| Myg1 | 2.72920573 |
| Myh10 | -0.2380377 |
| Myh11 | -0.1921421 |
| Myh8 | 1.69108467 |
| Myh9 | 2.02388246 |
| Myl1 | 0.34742645 |
| Myl2 | -0.3920853 |
| Myl4 | 0.49143064 |
| Myl6 | 1.80545434 |
| Myl6b | 0.20623635 |
| Mylc2b | -0.6209878 |
| Mylc2b | -0.0596346 |
| Mylc2b | -0.4510935 |
| Mylc2pl | -0.3740043 |
| Mylk | 2.22581958 |
| Mylk | 0.86226609 |
| Mynn | 0.22223395 |
| Myo10 | -0.7407492 |
| Myo10 | 1.58529982 |
| Myo15 | 0.68655955 |
| Myo18a | 1.65386187 |
| Myo1b | -0.0708222 |
| Myo1c | -0.547164 |
| Myo1c | -0.7319833 |
| Myo1f | 0.29575111 |
| Myo1g | -0.2030348 |
| Myo3b | -0.3866245 |
| Myo5a | -0.2000516 |
| Myo5b | -0.7766506 |
| Myo6 | -0.513349 |
| Myo7a | 1.98749068 |
| Myo9a | 0.41496038 |
| Myo9b | -0.7044679 |
| Myo9b | 0.71068157 |
| Myoc | 0.34547971 |
| Myocd | -0.4595527 |
| Myocd | 2.09327404 |
| Myom1 | -0.7522679 |
| Myom1 | 3.88959764 |
| Myrip | 0.13173479 |
| Mysm1 | -0.2658348 |
| Myst1 | 2.17523008 |
| Myst2 | -0.3021891 |
| Myst4 | -0.6453449 |
| Myst4 | -0.6646437 |
| Myst4 | 0.36012033 |
| Myt1 | 0.72884032 |
| Myt1l | -0.0777735 |
| Myt1l | 0.2512865 |
| Mzf1 | 0.10911679 |
| Mzf1 | -0.4853953 |
| N28178 | -0.6638316 |
| N4bp2 | 0.99632572 |
| N6amt1 | -0.4080408 |
| N6amt1 | 0.56877761 |
| N6amt2 | -0.0285004 |
| Naalad2 | -0.1469677 |
| Nab1 | -0.1741016 |
| Nab1 | -0.2754648 |
| Nab1 | -0.4530389 |
| Nab1 | -0.6541412 |
| Nab2 | 1.75141387 |
| Naca | -0.4414262 |
| Naca | -0.1270027 |
| Naca | -0.5442993 |
| Nadk | 0.58920708 |
| Nadsyn1 | 0.08557641 |
| Nadsyn1 | -0.0359795 |
| Naga | 0.05577311 |
| Nagk | 0.99114523 |
| Nagk | 0.48208369 |
| Nagk | -0.1635362 |
| Nagk | -0.5071414 |
| Naglu | -0.555087 |
| Nagpa | 3.47418665 |
| Nags | -0.7821556 |
| Nags | 0.46776499 |
| Naif1 | 2.31804319 |
| Naif1 | -0.6622096 |
| Naip5 | 0.12683324 |
| Nalp10 | -0.4934915 |
| Nampt | 0.28126059 |
| Nanos1 | 0.50914931 |
| Nanos2 | 0.34297084 |
| Nans | 2.62010966 |
| Nap1l1 | -0.4439945 |
| Nap1l1 | -0.1852753 |
| Nap1l3 | -0.307181 |
| Nap1l4 | -0.6413735 |
| Nap1l5 | -0.3268492 |
| Napa | 3.03754149 |
| Napa | -0.3094082 |
| Napa | 1.42005597 |
| Napb | 0.47701208 |
| Napepld | 0.28334434 |
| Napg | 1.00812016 |
| Napg | 0.33371412 |
| Naprt1 | 0.25619579 |
| Naprt1 | -0.5696653 |
| Napsa | -0.0795194 |
| Napsa | -0.7381108 |
| Napsa | 0.3027681 |
| Narf | -0.6477358 |
| Narfl | 2.799563 |
| Narg1 | -0.5098953 |
| Narg1 | -0.4223839 |
| Narg1l | 1.86588632 |
| Narg1l | -0.5514774 |
| Narg1l | 2.08811039 |
| Narg2 | -0.3183697 |
| Nars | 1.03971438 |
| Nars2 | -0.5514774 |
| Nasp | -0.5837265 |
| Nasp | -0.3060694 |
| Nasp | -0.5514774 |
| Nasp | -0.7198465 |
| Nat1 | -0.5085174 |
| Nat10 | -0.2950249 |
| Nat14 | 0.78690424 |
| Nat2 | -0.4439945 |
| Nat5 | -0.6501327 |
| Nat5 | -0.649333 |
| Nat6 | 2.12278824 |
| Nat6 | -0.7172659 |
| Nav1 | 1.00355864 |
| Nav1 | -0.9598431 |
| Nbl1 | 0.46849833 |
| Nbr1 | 0.49666271 |
| Nbr1 | 0.41960413 |
| Ncald | 0.18205051 |
| Ncam1 | -0.2257149 |
| Ncam1 | 0.12753503 |
| Ncam2 | 0.1151778 |
| Ncan | -0.7416302 |
| Ncapd2 | 2.05019213 |
| Ncapd2 | -0.6358416 |
| Ncapd2 | 0.3163603 |
| Ncapd3 | 2.23780138 |
| Ncapg2 | -0.2447805 |
| Ncapg2 | 1.9017934 |
| Ncaph | 1.15893583 |
| Ncaph2 | 1.27229759 |
| Ncaph2 | -0.7267627 |
| Ncaph2 | -0.6033144 |
| Ncbp2 | 0.58165197 |
| Ncbp2 | 0.30014284 |
| Ncdn | 1.55967945 |
| Ncdn | -0.0922744 |
| Ncdn | -0.6736227 |
| Ncdn | 0.06818129 |
| Ncf4 | -0.3988028 |
| Nck1 | 0.0976011 |
| Nckap1 | 2.00332112 |
| Nckap1l | 0.71802458 |
| Nckipsd | 1.22737247 |
| Ncl | -0.6557494 |
| Ncl | 0.45297718 |
| Ncoa2 | 0.39668081 |
| Ncoa2 | 3.05561629 |
| Ncoa4 | -0.3021891 |
| Ncoa5 | 1.19750478 |
| Ncoa6 | -0.5195976 |
| Ncoa6 | -0.2323698 |
| Ncoa6 | -0.6334806 |
| Ncor1 | -0.7363561 |
| Ncstn | 2.42427463 |
| Ncstn | 1.04543114 |
| Ncstn | 2.40869245 |
| Ndc80 | -0.6366299 |
| Nde1 | 1.87092675 |
| Nde1 | 0.62403089 |
| Ndel1 | 1.56557756 |
| Ndfip1 | 0.5954215 |
| Ndfip2 | -0.8007414 |
| Ndg2 | -0.3574358 |
| Ndn | 0.45570569 |
| Ndor1 | -0.5837265 |
| Ndor1 | 2.74477569 |
| Ndor1 | -0.1970777 |
| Ndrg1 | -0.3545075 |
| Ndrg2 | -0.0703895 |
| Ndrg3 | 0.59435297 |
| Ndrg4 | -0.5378854 |
| Ndrl | 1.21936016 |
| Ndst1 | 1.60417844 |
| Ndst1 | -0.5837265 |
| Ndst4 | -0.3121995 |
| Ndufa1 | 0.00523961 |
| Ndufa10 | -0.7002402 |
| Ndufa12 | 1.8481145 |
| Ndufa13 | -0.699397 |
| Ndufa2 | 1.20860727 |
| Ndufa3 | 0.44022265 |
| Ndufa4 | 0.92379222 |
| Ndufa5 | 0.72083367 |
| Ndufa5 | 0.23421321 |
| Ndufa6 | -0.3757969 |
| Ndufa7 | 0.42114698 |
| Ndufa8 | -0.36214 |
| Ndufa8 | -0.7027746 |
| Ndufa9 | 0.69524555 |
| Ndufaf1 | 1.93772252 |
| Ndufb10 | -0.3550925 |
| Ndufb11 | -0.5844724 |
| Ndufb2 | -0.6876697 |
| Ndufb3 | -0.8197007 |
| Ndufb4 | 0.5861923 |
| Ndufb5 | -0.7585291 |
| Ndufb6 | -0.7720875 |
| Ndufb6 | 1.7559178 |
| Ndufb7 | 0.06443844 |
| Ndufb8 | 0.82063928 |
| Ndufb9 | -0.4198738 |
| Ndufc1 | -0.7198465 |
| Ndufc1 | -0.4497988 |
| Ndufc2 | 1.35333241 |
| Ndufs2 | 0.69965436 |
| Ndufs2 | 2.20871566 |
| Ndufs3 | 2.18129183 |
| Ndufs4 | 0.78498452 |
| Ndufs4 | -0.6010334 |
| Ndufs4 | -0.2669 |
| Ndufs4 | 1.89239064 |
| Ndufs6 | 1.97920386 |
| Ndufs7 | 0.50304446 |
| Ndufs8 | -0.5652681 |
| Ndufv1 | -0.6662699 |
| Ndufv2 | 0.79125278 |
| Necab1 | 2.67396161 |
| Necab2 | 0.41857416 |
| Necab3 | 3.31826847 |
| Necap1 | 0.95945018 |
| Necap1 | 1.72263702 |
| Necap1 | 1.43007074 |
| Necap2 | -0.3308326 |
| Nedd1 | 2.09257554 |
| Nedd4 | 1.22288079 |
| Nedd4l | 0.34185364 |
| Nedd8 | -0.5874619 |
| Nedd9 | -0.5230871 |
| Nedd9 | -0.5919641 |
| Nefm | -0.6071283 |
| Negr1 | -0.1362362 |
| Negr1 | 2.15676674 |
| Neil1 | -0.7711775 |
| Neil2 | 1.85088354 |
| Nek11 | 1.569692 |
| Nek3 | -0.1601991 |
| Nek3 | -0.5493182 |
| Nek3 | 2.35314416 |
| Nek4 | 1.01512667 |
| Nek5 | 1.01142383 |
| Nek6 | -0.6155738 |
| Nek8 | -0.6109578 |
| Nek9 | 1.40288278 |
| Nek9 | 2.0076319 |
| Nelf | -0.2125444 |
| Nelf | 0.82181607 |
| Nell2 | 0.02029303 |
| Nell2 | -0.3933031 |
| Nell2 | -0.8638062 |
| Nell2 | 1.55080134 |
| Nenf | -0.8130215 |
| Neo1 | -0.1070016 |
| Nes | 0.56679819 |
| Net1 | -0.6163453 |
| Net1 | 0.04702743 |
| Neto1 | -0.1951004 |
| Neto1 | -0.8206588 |
| Neto1 | 1.46281908 |
| Neu1 | -0.307181 |
| Neu2 | -0.7381108 |
| Neu3 | -0.4497988 |
| Neu4 | 2.26833964 |
| Neu4 | -0.5652681 |
| Neu4 | 0.50938333 |
| Neurl | -0.5726082 |
| Neurod1 | -0.0435184 |
| Neurod2 | 2.12540694 |
| Neurod4 | -0.3793923 |
| Neurog1 | 1.28595385 |
| Neurog2 | 2.88262191 |
| Nf1 | 0.12331648 |
| Nfasc | -0.7087146 |
| Nfasc | 1.77714461 |
| Nfasc | -0.6272135 |
| Nfat5 | 2.50963793 |
| Nfat5 | -0.3451977 |
| Nfatc1 | -0.4860674 |
| Nfatc1 | 0.89516426 |
| Nfatc2 | 0.44499452 |
| Nfatc2 | 1.84726521 |
| Nfatc2ip | -0.7241631 |
| Nfatc3 | -0.8187437 |
| Nfatc4 | -0.5265895 |
| Nfe2 | 1.43341479 |
| Nfe2l1 | -0.0998324 |
| Nfe2l2 | -0.0587794 |
| Nfe2l3 | -0.0502706 |
| Nfib | -0.1847868 |
| Nfic | 0.57053362 |
| Nfic | -0.0039898 |
| Nfic | -0.4018721 |
| Nfix | 0.27137469 |
| Nfix | 0.33793299 |
| Nfkb1 | -0.1169491 |
| Nfkb2 | -0.649333 |
| Nfkbia | -0.680207 |
| Nfkbib | 0.88107008 |
| Nfkbid | 1.27229759 |
| Nfkbie | 1.50682042 |
| Nfkbil1 | 0.04243207 |
| Nfkbil2 | 0.90475249 |
| Nfkbiz | 2.14568612 |
| Nfrkb | -0.4569418 |
| Nfrkb | 1.99981008 |
| Nfs1 | 1.41227238 |
| Nfs1 | 2.46118 |
| Nfs1 | -0.8063893 |
| Nfu1 | -0.3325449 |
| Nfx1 | -0.0007693 |
| Nfya | 0.72846942 |
| Nfyb | -0.0716883 |
| Nfyc | 0.36749229 |
| Ngb | 1.05793968 |
| Ngb | 0.9550842 |
| Ngef | 1.65913191 |
| Ngfb | -0.8749087 |
| Ngfb | 0.30422342 |
| Ngfr | -0.4628266 |
| Ngfrap1 | -0.5852189 |
| Ngp | -0.5230871 |
| Nhedc1 | -0.424271 |
| Nhej1 | -0.2500001 |
| Nhlrc1 | -0.2105344 |
| Nhlrc2 | -0.6695307 |
| Nhlrc2 | -0.839049 |
| Nhp2l1 | 2.02973575 |
| Nhsl1 | -0.1479067 |
| Niban | -0.6109578 |
| Nicn1 | -0.2030348 |
| Nicn1 | 3.54633103 |
| Nid1 | -0.8054456 |
| Nid2 | -0.196583 |
| Nif3l1 | -0.6429601 |
| Nin | 0.68675345 |
| Ninj1 | -0.4820423 |
| Ninj2 | -0.8923284 |
| Nip7 | 0.08923647 |
| Nipa1 | -0.1654484 |
| Nipbl | -0.1174038 |
| Nipbl | 2.04753897 |
| Nipsnap1 | -0.0318171 |
| Nipsnap3a | -0.6760864 |
| Nipsnap3a | 0.58576083 |
| Nisch | 0.27917222 |
| Nit1 | 0.20203214 |
| Nit2 | -0.2473867 |
| Nit2 | -0.7558406 |
| Nkain3 | -0.7389895 |
| Nkain4 | -0.7998033 |
| Nkd2 | -0.1101554 |
| Nkg7 | 0.00962194 |
| Nkiras1 | -0.7867678 |
| Nkiras2 | 1.61872524 |
| Nkrf | 1.34728841 |
| Nktr | 2.81682292 |
| Nkx1-2 | 0.48592955 |
| Nkx2-1 | -0.2972235 |
| Nkx2-2 | -0.7675463 |
| Nkx2-2 | 0.30857597 |
| Nkx2-3 | -0.5770398 |
| Nkx2-4 | 0.23295937 |
| Nkx2-6 | 2.7380808 |
| Nkx3-1 | 0.59199792 |
| Nkx6-1 | 2.57354536 |
| Nle1 | 0.91454884 |
| Nlgn1 | 1.12593017 |
| Nlgn2 | 1.66698217 |
| Nlk | -0.5407306 |
| Nln | 0.53932126 |
| Nln | -0.4305884 |
| Nln | -0.8120712 |
| Nlrp14 | 0.57665403 |
| Nlrp4a | -0.2201203 |
| Nlrp4c | 2.50149193 |
| Nlrp5 | 0.0976011 |
| Nlrp5 | 1.8969694 |
| Nlrp5 | -0.8054456 |
| Nlrp9a | -0.5844724 |
| Nlrp9c | -0.7739101 |
| Nlrx1 | -0.9292385 |
| Nlrx1 | 2.564321 |
| Nmb | -0.4049514 |
| Nmbr | -0.3562634 |
| Nmd3 | 1.443724 |
| Nme1 | 0.16602414 |
| Nme2 | 1.14532355 |
| Nme2 | -0.4186212 |
| Nme2 | -0.5343408 |
| Nme3 | 0.80674358 |
| Nme4 | 2.7934757 |
| Nme4 | 0.19029096 |
| Nme4 | -0.8361214 |
| Nme5 | 0.95099096 |
| Nme7 | -0.4773681 |
| Nme7 | 1.24512829 |
| Nme7 | -0.6818603 |
| Nme7 | 1.93782545 |
| Nme7 | 0.83731279 |
| Nmnat1 | 2.21862554 |
| Nmnat2 | -0.2658348 |
| Nmnat3 | 1.4976742 |
| Nmral1 | 0.06406334 |
| Nmt1 | -0.455639 |
| Nmu | -0.1493169 |
| Nmur2 | -0.7504866 |
| Nnat | 0.40925345 |
| Nnat | -0.3038502 |
| Nnmt | 1.40804058 |
| Nnmt | -0.0280866 |
| Nob1 | -0.4921373 |
| Nob1 | 0.99324886 |
| Nobox | 0.74374046 |
| Noc3l | -0.2515717 |
| Noc4l | -0.4012575 |
| Noc4l | -0.7988662 |
| Nod1 | 0.42140388 |
| Nog | 0.74974404 |
| Nol1 | 2.370607 |
| Nol1 | -0.1083519 |
| Nol1 | 3.54865639 |
| Nol10 | 0.41340736 |
| Nol11 | 0.73032246 |
| Nol12 | -0.7702684 |
| Nol14 | -0.5435845 |
| Nol3 | 0.06631172 |
| Nol4 | 1.924037 |
| Nol4 | -0.3155601 |
| Nol4 | 1.07581949 |
| Nol5 | 2.44259147 |
| Nol5a | 3.90752912 |
| Nol6 | -0.4331273 |
| Nol7 | -0.3044045 |
| Nol9 | -0.5536415 |
| Nola1 | 3.20749446 |
| Nola2 | -0.3088509 |
| Nola3 | -0.5949775 |
| Nola3 | -0.5223881 |
| Nolc1 | 0.63638196 |
| Nomo1 | -0.5762997 |
| Nono | -0.1765195 |
| Nope | -0.4388649 |
| Nos1 | 0.18205051 |
| Nos1ap | -0.6835165 |
| Nos2 | -0.4485059 |
| Nos3 | -0.1606751 |
| Nos3 | -0.7241631 |
| Nos3 | -0.5718716 |
| Nosip | 1.19694057 |
| Nosip | -0.5493182 |
| Nosip | -0.5659995 |
| Nostrin | 0.84619541 |
| Notch1 | -0.2313429 |
| Notch3 | 0.48039618 |
| Notch4 | -0.7207082 |
| Notch4 | -0.3842076 |
| Notum | -0.5980006 |
| Nova2 | 0.11446676 |
| Nox4 | 0.92152764 |
| Noxo1 | -0.3121995 |
| Noxo1 | 2.93851682 |
| Npal2 | 0.51172029 |
| Npas1 | 1.19366145 |
| Npas2 | 0.01793209 |
| Npas4 | -0.0210801 |
| Npb | -0.6541412 |
| Npc2 | 1.51989479 |
| Npcd | 1.87048472 |
| Npcd | -0.7839978 |
| Npdc1 | -0.5175101 |
| Npepl1 | 1.82607019 |
| Npff | -0.562348 |
| Nphp1 | 0.07190934 |
| Nphp1 | -0.7914027 |
| Nphp3 | -0.1813738 |
| Nphp4 | -0.3094082 |
| Nphs2 | -0.7487087 |
| Nploc4 | 0.4216607 |
| Npm1 | 0.09723895 |
| Npm2 | 1.97074421 |
| Npm3 | -0.6033144 |
| Npm3 | 1.25717084 |
| Npm3-ps1 | 1.59940201 |
| Npr1 | 1.9079217 |
| Npr2 | -0.6719838 |
| Npr3 | -0.1521437 |
| Npsr1 | -0.4080408 |
| Nptn | 0.07858293 |
| Nptx1 | -0.364501 |
| Nptx2 | 0.8004059 |
| Nptxr | 1.55844923 |
| Npy | 0.52262709 |
| Npy1r | -0.284651 |
| Npy2r | 0.12542811 |
| Npy5r | -0.2155675 |
| Nqo1 | -0.4517415 |
| Nqo2 | 1.23661689 |
| Nqo2 | -0.2803169 |
| Nqo2 | -0.2917366 |
| Nr1d1 | 0.20300401 |
| Nr1d2 | 1.59814879 |
| Nr1h2 | -0.7302399 |
| Nr1h2 | -0.1882121 |
| Nr1h3 | -0.5450146 |
| Nr1h4 | -0.6926778 |
| Nr1h5 | -0.649333 |
| Nr1i2 | -0.1224196 |
| Nr2c1 | 1.36719202 |
| Nr2c2ap | 1.15422337 |
| Nr2e1 | 4.32648509 |
| Nr2f2 | -0.5126572 |
| Nr2f2 | 0.59028146 |
| Nr2f6 | -0.0268464 |
| Nr2f6 | 0.67387019 |
| Nr4a2 | 0.191275 |
| Nr4a3 | 0.93384147 |
| Nr4a3 | -0.4962057 |
| Nr5a1 | -0.1479067 |
| Nr5a2 | -0.6303419 |
| Nr5a2 | 2.38214678 |
| Nr5a2 | 0.32208533 |
| Nr6a1 | -0.525187 |
| Nradd | 0.93815817 |
| Nrap | -0.5064542 |
| Nrarp | -0.1119623 |
| Nras | -0.5442993 |
| Nrbf2 | -0.7416302 |
| Nrbp1 | -0.391477 |
| Nrbp2 | 3.63655611 |
| Nrcam | 1.66746208 |
| Nrd1 | -0.5414433 |
| Nrf1 | -0.8187437 |
| Nrg1 | 0.63719964 |
| Nrg3 | -0.6960314 |
| Nrg4 | -0.5980006 |
| Nrgn | -0.2463433 |
| Nrip2 | -0.3556778 |
| Nrip3 | 1.32911676 |
| Nrl | -0.4217558 |
| Nrm | 0.04434952 |
| Nrn1 | -0.7104187 |
| Nrn1l | -0.5315148 |
| Nrp1 | 0.03162168 |
| Nrp1 | 0.20073476 |
| Nrp2 | 1.01073652 |
| Nrp2 | -0.6389987 |
| Nrsn2 | -0.4536883 |
| Nrsn2 | -0.1936201 |
| Nrtn | -0.3510056 |
| Nrxn1 | 1.15917083 |
| Nrxn1 | 2.41516722 |
| Nrxn1 | 2.11343696 |
| Nrxn2 | 0.23796474 |
| Nrxn3 | -0.2180941 |
| Nrxn3 | -0.4733799 |
| Nsbp1 | 0.25986107 |
| Nsd1 | -0.19165 |
| Nsd1 | -0.7164073 |
| Nsdhl | -0.016574 |
| Nsdhl | 4.38983753 |
| Nsdhl | -0.6109578 |
| Nsf | -0.1526156 |
| Nsfl1c | -0.1635362 |
| Nsg1 | 0.85402146 |
| Nsg2 | 0.75336406 |
| Nsl1 | -0.0384858 |
| Nsmaf | -0.7311112 |
| Nsmaf | -0.5085174 |
| Nsmce1 | -0.6752645 |
| Nsmce2 | -0.239072 |
| Nsmce2 | 2.31159083 |
| Nsmce2 | -0.514734 |
| Nsun2 | -0.632695 |
| Nsun4 | -0.5098953 |
| Nsun5 | -0.5726082 |
| Nsun5 | 1.03490147 |
| Nsun5 | -0.0716883 |
| Nsun5 | -0.0869755 |
| Nt5c | -0.7189855 |
| Nt5c1b | -0.6943531 |
| Nt5c2 | 1.0733778 |
| Nt5c2 | -0.5329268 |
| Nt5c2 | -0.525187 |
| Nt5c3 | 0.8077673 |
| Nt5c3l | -0.1985635 |
| Nt5c3l | -0.3463564 |
| Nt5dc3 | -0.3359789 |
| Nt5dc3 | -0.6935151 |
| Nt5dc3 | 2.51637757 |
| Nt5e | -0.5733453 |
| Nt5e | -0.7648321 |
| Nt5m | -0.3574358 |
| Nt5m | 0.55260118 |
| Ntan1 | -0.7121258 |
| Ntf3 | 0.38233333 |
| Nthl1 | -0.5987578 |
| Ntn2l | -0.5442993 |
| Ntn2l | -0.273853 |
| Ntn4 | -0.1408871 |
| Ntng1 | -0.7241631 |
| Ntng1 | -0.4261618 |
| Ntng1 | 0.77214454 |
| Ntrk2 | 0.12613094 |
| Ntrk2 | -0.0536649 |
| Ntrk2 | -0.2287806 |
| Ntrk3 | 1.35876761 |
| Ntrk3 | -0.5755603 |
| Ntrk3 | 1.09600847 |
| Nts | -0.1640139 |
| Ntsr1 | -0.6256532 |
| Ntsr2 | 0.80503489 |
| Nuak1 | -0.650933 |
| Nuak1 | -0.6040759 |
| Nuak2 | 1.53523812 |
| Nub1 | -0.0490009 |
| Nubp1 | -0.2221509 |
| Nubp2 | 0.12155316 |
| Nubp2 | -0.6744432 |
| Nubp2 | 0.94141977 |
| Nucb1 | 0.80554782 |
| Nucb1 | 0.25894608 |
| Nucks1 | 0.83117789 |
| Nudc | -0.6793814 |
| Nudc | -0.128382 |
| Nudcd2 | -0.0931604 |
| Nudcd3 | 1.53207859 |
| Nudc-ps1 | 1.37123544 |
| Nudt1 | 2.45727197 |
| Nudt10 | -0.1784582 |
| Nudt11 | 1.16782514 |
| Nudt12 | 1.15658254 |
| Nudt13 | 1.17720795 |
| Nudt14 | 0.92454587 |
| Nudt16l1 | -0.650933 |
| Nudt18 | 0.80469278 |
| Nudt19 | -0.6109578 |
| Nudt19 | 1.62891895 |
| Nudt2 | -0.0015734 |
| Nudt21 | -0.2568292 |
| Nudt22 | 0.00203962 |
| Nudt4 | 0.59648883 |
| Nudt5 | 1.35819696 |
| Nudt5 | 1.85824681 |
| Nudt6 | 0.86596081 |
| Nudt7 | -0.6622096 |
| Nudt8 | -0.0773375 |
| Nudt9 | -0.431857 |
| Nufip1 | -0.5770398 |
| Numa1 | 0.73069263 |
| Numb | -0.0481554 |
| Numb | 0.0819021 |
| Numb | -0.5949775 |
| Numb | 1.25494444 |
| Numbl | 0.37399711 |
| Nup107 | 0.2528234 |
| Nup133 | -0.1247084 |
| Nup133 | 1.36888672 |
| Nup155 | 0.83565864 |
| Nup155 | 0.39324668 |
| Nup160 | -0.4928141 |
| Nup210 | -0.613263 |
| Nup37 | 1.93900827 |
| Nup43 | 1.65982315 |
| Nup50 | -0.4934915 |
| Nup50 | -0.3457769 |
| Nup50 | 1.31378473 |
| Nup62 | 0.21458896 |
| Nup85 | -0.4989278 |
| Nup88 | 0.86307052 |
| Nup88 | -0.7250289 |
| Nup88 | -0.4055685 |
| Nup93 | 0.56569671 |
| Nup98 | -0.2359727 |
| Nup98 | -0.128382 |
| Nupl1 | -0.2020393 |
| Nupl1 | -0.2722439 |
| Nupl1 | -0.5202945 |
| Nupl2 | -0.3656837 |
| Nupr1 | -0.316683 |
| Nus1 | 1.60028563 |
| Nusap1 | -0.4043348 |
| Nut | 1.78006888 |
| Nutf2 | 0.17440518 |
| Nvl | -0.6202125 |
| Nwd1 | 0.49357454 |
| Nxf1 | 1.55490338 |
| Nxf2 | -0.8688366 |
| Nxf3 | -0.3854153 |
| Nxph1 | 1.11269383 |
| Nxph2 | 1.35199264 |
| Nxph3 | 1.39724033 |
| Nxt1 | 0.95915974 |
| Nxt1 | 0.41055352 |
| Nxt2 | 0.36476865 |
| Nyx | -0.5800058 |
| Oaf | -0.1016196 |
| Oas1b | -0.0243708 |
| Oas1b | -0.1233344 |
| Oas1c | -0.3451977 |
| Oas1d | -0.0219017 |
| Oas1d | 0.60329127 |
| Oas1e | 0.24820516 |
| Oas1g | -0.5529196 |
| Oas1g | 1.28441192 |
| Oas2 | 0.18963437 |
| Oasl1 | 1.21150153 |
| Oasl2 | 0.28986347 |
| Oasl2 | -0.5980006 |
| Oat | 1.29911138 |
| Oaz2 | 0.13522037 |
| Oaz3 | 0.62029381 |
| Obfc1 | -0.6071283 |
| Obfc2a | -0.7630269 |
| Obfc2b | 0.03472303 |
| Obfc2b | 2.43091829 |
| Obox1 | -0.4727169 |
| Obox5 | -0.514734 |
| Obrgrp | -0.3451977 |
| Obsl1 | -0.1002789 |
| Oc90 | -0.5536415 |
| Oca2 | 0.92092284 |
| Ociad1 | -0.6581668 |
| Ociad1 | 0.2788735 |
| Ociad2 | -0.6893361 |
| Ocln | 1.41809377 |
| Ocrl | 0.0191133 |
| Odc1 | 1.70922489 |
| Odf1 | -0.3811952 |
| Odf2 | 0.27197679 |
| Odf2 | -0.316683 |
| Odf2 | -0.0821442 |
| Odf2 | 0.96840967 |
| Odf3 | 1.26034231 |
| Odf3l1 | -0.3836043 |
| Odf4 | -0.7549462 |
| Odf4 | -0.4080408 |
| Odz1 | -0.8478869 |
| Odz3 | 0.40010248 |
| Odz3 | -0.4914609 |
| Odz4 | 0.24851375 |
| Og9x | -0.0137177 |
| Ogdh | -0.6777325 |
| Ogfod1 | -0.4337631 |
| Ogfod2 | -0.0721217 |
| Ogfr | 0.66319686 |
| Ogfrl1 | -0.2110365 |
| Ogg1 | 0.8115115 |
| Ogn | 1.6677362 |
| Ogn | 0.57053362 |
| Ogn | -0.3492595 |
| Ogt | -0.5315148 |
| Ogt | 0.09723895 |
| Ogt | 0.92785481 |
| Oit1 | 1.44892307 |
| Oit3 | 0.20365136 |
| Ola1 | 0.64330976 |
| Olfm1 | -0.6240955 |
| Olfm1 | -0.6109578 |
| Olfm2 | -0.0703895 |
| Olfm2 | -0.1311466 |
| Olfm3 | 0.15484589 |
| Olfm3 | -0.2895507 |
| Olfm4 | 2.12582863 |
| Olfml1 | -0.8419858 |
| Olfml2b | -0.4536883 |
| Olfml3 | 1.84726521 |
| Olfr100 | -0.1115102 |
| Olfr1006 | 2.07311659 |
| Olfr1012 | -0.7129806 |
| Olfr1020 | -0.3515883 |
| Olfr1065 | 1.48345324 |
| Olfr1094 | 0.94053136 |
| Olfr1099 | -0.5718716 |
| Olfr1105 | 3.17519534 |
| Olfr1112 | 1.56251821 |
| Olfr1113 | 0.13903969 |
| Olfr1189 | 0.53705178 |
| Olfr1197 | -0.4894347 |
| Olfr1198 | 1.79742658 |
| Olfr1249 | -0.5852189 |
| Olfr1280 | -0.6860062 |
| Olfr133 | -0.3365524 |
| Olfr1330 | -0.5478815 |
| Olfr134 | 1.01403105 |
| Olfr1340 | -0.6358416 |
| Olfr1384 | 2.94907305 |
| Olfr1386 | -0.510585 |
| Olfr1390 | 0.87775091 |
| Olfr1394 | -0.5371754 |
| Olfr1412 | 1.81306199 |
| Olfr1437 | 0.96898479 |
| Olfr144 | 1.90238131 |
| Olfr1443 | 1.54901564 |
| Olfr1443 | 0.17072669 |
| Olfr1459 | -0.215063 |
| Olfr1500 | 0.45570569 |
| Olfr1508 | -0.6654564 |
| Olfr166 | -0.0268464 |
| Olfr168 | 2.07649882 |
| Olfr206 | 2.05788059 |
| Olfr213 | -0.3533388 |
| Olfr215 | 0.1250765 |
| Olfr232 | 0.32635642 |
| Olfr257 | 0.54969111 |
| Olfr267 | -0.4760368 |
| Olfr272 | -0.6233176 |
| Olfr303 | 2.25018732 |
| Olfr310 | 3.5848338 |
| Olfr352 | -0.6477358 |
| Olfr371 | 1.98220337 |
| Olfr378 | 1.96651071 |
| Olfr419 | 2.02959569 |
| Olfr437 | -0.814925 |
| Olfr464 | -0.5942232 |
| Olfr47 | 0.99072394 |
| Olfr49 | -0.8054456 |
| Olfr503 | -0.7830763 |
| Olfr51 | 2.77806222 |
| Olfr536 | 1.93787691 |
| Olfr538 | -0.7839978 |
| Olfr54 | -0.8638062 |
| Olfr550 | 1.37544844 |
| Olfr574 | -0.4820423 |
| Olfr591 | 3.46266027 |
| Olfr593 | 0.32890982 |
| Olfr608 | -0.7748227 |
| Olfr638 | -0.1838104 |
| Olfr65 | 2.51640543 |
| Olfr655 | 0.82717705 |
| Olfr658 | -0.45239 |
| Olfr665 | -0.789546 |
| Olfr675 | -0.0068167 |
| Olfr691 | 0.43492023 |
| Olfr692 | 0.49190749 |
| Olfr702 | -0.4707305 |
| Olfr724 | 0.83383572 |
| Olfr725 | -0.2879146 |
| Olfr748 | 0.67779282 |
| Olfr786 | -0.0047966 |
| Olfr802 | -0.6868376 |
| Olfr824 | -0.3521715 |
| Olfr91 | 1.49800239 |
| Olfr910 | 2.10191871 |
| Olfr920 | -0.6225404 |
| Olfr924 | 1.91173838 |
| Olfr952 | 2.21862554 |
| Olfr965 | 0.15416414 |
| Olfr982 | 0.04817278 |
| Olfr983 | 0.53682453 |
| Olfr984 | -0.7585291 |
| Olig1 | -0.6985544 |
| Olig3 | -0.4773681 |
| Oma1 | -0.3417301 |
| Omd | 0.5630479 |
| Omg | 0.13973245 |
| Omt2b | -0.5587105 |
| Onecut2 | -0.6573603 |
| Onecut3 | -0.7914027 |
| Onecut3 | 1.07863882 |
| Oog4 | -0.0596346 |
| Oosp1 | -0.6025534 |
| Opa1 | -0.0852158 |
| Opa1 | 1.96725918 |
| Ophn1 | 0.03084475 |
| Oplah | 2.32287931 |
| Opn1mw | 3.41E-05 |
| Opn3 | -0.3302625 |
| Oprl1 | -0.0953792 |
| Oprl1 | -0.7932631 |
| Oprl1 | 0.90120693 |
| Oprs1 | -0.6071283 |
| Optc | 0.27498156 |
| Optn | 0.53705178 |
| Optn | 0.06818129 |
| Orai1 | -0.4687483 |
| Orai3 | -0.4369485 |
| Oraov1 | 0.33989535 |
| Orc2l | 2.07146558 |
| Orc3l | -0.6033144 |
| Orc5l | -0.4369485 |
| Orc5l | -0.7181253 |
| Orc6l | 0.59755495 |
| ORF19 | 1.77842509 |
| ORF28 | -0.4080408 |
| ORF34 | 0.15790767 |
| ORF61 | 0.73420203 |
| ORF63 | -0.2000516 |
| ORF9 | -0.3032961 |
| ORF9 | -0.8293253 |
| ORF9 | -0.585966 |
| Orm1 | 0.6582143 |
| Ormdl2 | 0.34687065 |
| Ormdl3 | -0.2983247 |
| Os9 | -0.5897103 |
| Osbp2 | -0.1931272 |
| Osbpl10 | -0.189684 |
| Osbpl11 | 1.34004231 |
| Osbpl2 | 0.13730555 |
| Osbpl3 | 0.2954576 |
| Osbpl3 | -0.1174038 |
| Osbpl6 | -0.7087146 |
| Osbpl8 | -0.6885025 |
| Osbpl9 | -0.4982465 |
| Oscar | -0.2972235 |
| Osgep | 0.24076863 |
| Osgepl1 | 1.21127921 |
| Osgin1 | 0.83681685 |
| Osm | -0.7812359 |
| Osmr | 1.67517561 |
| Osr1 | 1.49594937 |
| Osr2 | -0.5009745 |
| Ostf1 | 0.0789523 |
| Ostm1 | -0.7267627 |
| Otof | 0.36585905 |
| Otop1 | 0.31549854 |
| Otop2 | 0.07525204 |
| Ott | -0.9090226 |
| Ott | 1.39092089 |
| OTTMUSG00000000421 | 1.52509016 |
| OTTMUSG00000000971 | -0.7250289 |
| OTTMUSG00000000990 | 1.47626153 |
| OTTMUSG00000001305 | -0.8478869 |
| OTTMUSG00000002038 | 1.21847795 |
| OTTMUSG00000004461 | -0.1770038 |
| OTTMUSG00000004551 | -0.6148029 |
| OTTMUSG00000005148 | 2.21973295 |
| OTTMUSG00000005491 | -0.0414182 |
| OTTMUSG00000008540 | 2.52129693 |
| OTTMUSG00000010009 | -0.1765195 |
| OTTMUSG00000010328 | 2.0625359 |
| OTTMUSG00000015762 | -0.6186637 |
| OTTMUSG00000015859 | 0.84750427 |
| OTTMUSG00000016293 | -0.1460297 |
| OTTMUSG00000016790 | -0.6101907 |
| OTTMUSG00000016790 | -0.0896212 |
| OTTMUSG00000017677 | 0.04396634 |
| OTTMUSG00000020946 | 0.90490635 |
| Otub1 | -0.5294008 |
| Otub2 | 0.05083987 |
| Otub2 | 0.25588971 |
| Otub2 | -0.5949775 |
| Otud5 | 0.38500679 |
| Otud6b | 0.51381861 |
| Otud7b | 0.50938333 |
| Otud7b | 2.22676708 |
| Otx1 | -0.3172449 |
| Otx2 | -0.4948476 |
| Ovca2 | -0.6048381 |
| Ovgp1 | 1.13078575 |
| Ovgp1 | 1.8857253 |
| Ovol1 | 0.87314801 |
| Ovol2 | 1.77280245 |
| Ovol2 | 0.01635484 |
| Oxct1 | -0.4123831 |
| Oxct2a | -0.4305884 |
| Oxct2b | 2.65010795 |
| Oxgr1 | 0.70232793 |
| Oxnad1 | -0.3240143 |
| Oxr1 | 0.58511325 |
| Oxr1 | 0.12437288 |
| Oxt | -0.4274244 |
| P140 | 0.38286863 |
| P2rx2 | -0.3382751 |
| P2rx3 | -0.1133197 |
| P2rx4 | 0.17707146 |
| P2rx4 | -0.7469341 |
| P2rx5 | 0.58770089 |
| P2rx7 | -0.7181253 |
| P2rx7 | 1.9767875 |
| P2ry1 | 3.93269861 |
| P2ry12 | -0.3609618 |
| P2ry13 | 2.80782336 |
| P2ry14 | -0.2879146 |
| P2ry14 | -0.4439945 |
| P2ry5 | -0.5286972 |
| P2ry6 | 0.76556924 |
| P42pop | 0.14077064 |
| P4ha1 | -0.6541412 |
| P4ha2 | -0.3609618 |
| P4hb | 1.35352365 |
| Pa2g4 | -0.2359727 |
| Pabpc1 | -0.4249008 |
| Pabpc1 | -0.1712084 |
| Pabpc1 | -0.1061025 |
| Pabpc2 | -0.1926345 |
| Pabpc4 | -0.2722439 |
| Pabpn1 | -0.7558406 |
| Pabpnl1 | -0.855813 |
| Pacrg | 2.93410714 |
| Pacs1 | -0.36214 |
| Pacs2 | 0.64310673 |
| Pacsin1 | 0.12472476 |
| Pacsin1 | 0.98013009 |
| Pacsin1 | -0.7250289 |
| Pacsin2 | 1.25218065 |
| Pacsin2 | 0.2174631 |
| Pacsin3 | 0.50844692 |
| Pacsin3 | 0.59712865 |
| Padi1 | 1.89578644 |
| Padi2 | -0.0738571 |
| Padi6 | -0.0231354 |
| Paf1 | -0.1088025 |
| Paf1 | 0.64493246 |
| Pafah1b1 | -0.4497988 |
| Pafah1b1 | -0.0769018 |
| Pafah1b3 | 0.80878992 |
| Pafah2 | -0.1020669 |
| Pafah2 | -0.7666407 |
| Paip1 | 0.94805631 |
| Paip2 | 1.95420088 |
| Paip2b | -0.6469381 |
| Paip2b | 0.58877699 |
| Pak1 | -0.6163453 |
| Pak3 | 1.08463387 |
| Pak4 | 0.58748553 |
| Pak6 | 1.62856188 |
| Pak7 | -0.3044045 |
| Palld | 1.31875783 |
| Palm | 1.30788254 |
| Palm | -0.4382257 |
| Palm2 | -0.5529196 |
| Palmd | -0.5237865 |
| Palmd | 3.15335282 |
| Palmd | 2.96840641 |
| Pam | 2.8350655 |
| Pank1 | -0.3325449 |
| Pank1 | -0.634267 |
| Pank2 | 2.49098767 |
| Pank3 | 1.6791104 |
| Pank4 | 1.59667227 |
| Panx1 | -0.6769091 |
| Panx3 | -0.737233 |
| Paox | -0.5493182 |
| Paox | 1.50274824 |
| Paox | 0.43921496 |
| Paox | 2.09279388 |
| Papd1 | -0.7711775 |
| Papd1 | -0.5572594 |
| Papd4 | -0.0665036 |
| Papln | 0.1292872 |
| Papola | 0.97585901 |
| Papolb | -0.3161214 |
| Papolg | -0.3359789 |
| Papss1 | -0.2246953 |
| Papss1 | -0.5934696 |
| Papss2 | 1.06068249 |
| Paqr3 | 1.30768182 |
| Paqr6 | 0.03626988 |
| Paqr7 | 0.19650591 |
| Paqr9 | -0.5428702 |
| Paqr9 | 0.04931674 |
| Paqr9 | 1.80456573 |
| Pard3 | 0.62795959 |
| Pard3 | -0.5957324 |
| Pard6a | -0.699397 |
| Pard6a | 0.70727311 |
| Pard6b | 0.08996678 |
| Pard6g | -0.3348328 |
| Park7 | -0.4111404 |
| Parl | 0.6188365 |
| Parp1 | 0.9498182 |
| Parp1 | -0.3860197 |
| Parp1 | 3.65329933 |
| Parp12 | -0.6264331 |
| Parp14 | -0.3722151 |
| Parp16 | -0.6171175 |
| Parp2 | -0.4459253 |
| Parp2 | -0.8429668 |
| Parp3 | 0.31031138 |
| Parp6 | -0.8264276 |
| Parp8 | 0.86387427 |
| Pars2 | -0.8177876 |
| Pars2 | -0.5223881 |
| Parva | -0.5385959 |
| Parvg | -0.6581668 |
| Pask | -0.1219625 |
| Patl1 | -0.3044045 |
| Patz1 | -0.789546 |
| Pawr | -0.0243708 |
| Pax8 | -0.3811952 |
| Paxip1 | -0.5995157 |
| Pbk | 1.7934769 |
| Pbld | 0.38953435 |
| Pbp2 | 0.59371127 |
| Pbrm1 | 0.35792494 |
| Pbrm1 | -0.5897103 |
| Pbx1 | -0.6728029 |
| Pbx1 | -0.3527549 |
| Pbx2 | 0.53432117 |
| Pbx2 | 1.44710665 |
| Pbx2 | -0.3480972 |
| Pbx3 | -0.0035866 |
| Pbx3 | 1.26276658 |
| Pbx4 | 1.8478881 |
| Pcaf | 0.16703381 |
| Pcaf | -0.778482 |
| Pcbd1 | 2.34472464 |
| Pcbd2 | -0.4382257 |
| Pcbp1 | -0.6893361 |
| Pcbp2 | -0.4031026 |
| Pcbp3 | -0.5934696 |
| Pcca | 0.73493919 |
| Pcca | -0.3533388 |
| Pccb | -0.5957324 |
| Pccb | 0.10481487 |
| Pcdh1 | 0.59114008 |
| Pcdh10 | -0.7748227 |
| Pcdh10 | 2.88216823 |
| Pcdh10 | 0.81878707 |
| Pcdh15 | 1.33293557 |
| Pcdh17 | -0.5223881 |
| Pcdh20 | 1.76905582 |
| Pcdh21 | -0.9335502 |
| Pcdh7 | 3.20618113 |
| Pcdh7 | 2.32253714 |
| Pcdh8 | -0.7407492 |
| Pcdh9 | 1.59984392 |
| Pcdha10 | 0.65079147 |
| Pcdha11 | 0.26320844 |
| Pcdha2 | -0.2100326 |
| Pcdha6 | 4.75809112 |
| Pcdhac1 | 1.42370195 |
| Pcdhac2 | 0.47894734 |
| Pcdhb1 | -0.2615862 |
| Pcdhb10 | 3.21440611 |
| Pcdhb12 | -0.1621046 |
| Pcdhb14 | 0.63638196 |
| Pcdhb19 | 1.17766889 |
| Pcdhb20 | -0.778482 |
| Pcdhb22 | -0.3556778 |
| Pcdhb3 | -0.2857376 |
| Pcdhb7 | -0.1867426 |
| Pcdhb8 | -0.3510056 |
| Pcdhga10 | -0.7540526 |
| Pcdhga12 | -0.4446377 |
| Pcdhga4 | 0.18535534 |
| Pcdhga7 | -0.7711775 |
| Pcdhga8 | 2.51621036 |
| Pcdhgb2 | -0.5350487 |
| Pcdhgb6 | 2.12515384 |
| Pcdhgc3 | 2.81700528 |
| Pcdhgc4 | -0.5814923 |
| Pcdhgc5 | 2.56243872 |
| Pcf11 | 1.48976337 |
| Pcgf1 | -0.6350539 |
| Pcgf2 | 1.42901226 |
| Pcgf3 | 1.93731072 |
| Pcgf5 | -0.4621709 |
| Pcgf6 | -0.6287764 |
| Pck1 | 0.29398867 |
| Pclo | 0.2072039 |
| Pclo | 3.21956343 |
| Pcmt1 | -0.1092532 |
| Pcmt1 | -0.3574358 |
| Pcmt1 | -0.3463564 |
| Pcmtd1 | -0.3866245 |
| Pcmtd2 | -0.0971579 |
| Pcna | 0.00803072 |
| Pcnt | 1.35943294 |
| Pcnx | -0.5485996 |
| Pcnxl2 | -0.5223881 |
| Pcnxl2 | 0.52883613 |
| Pcolce | 2.36466918 |
| Pcolce2 | 1.04543114 |
| Pcolce2 | 0.39245241 |
| Pcsk1n | 3.25372357 |
| Pcsk2 | 1.17281776 |
| Pcsk2 | -0.6295588 |
| Pcsk2 | 1.03409692 |
| Pcsk2 | 1.48644751 |
| Pcsk4 | 0.46825394 |
| Pcsk4 | -0.6835165 |
| Pcsk4 | -0.1274622 |
| Pcsk4 | 0.10517411 |
| Pcsk4 | 2.16812189 |
| Pcsk9 | 0.77885017 |
| Pctk1 | -0.5667315 |
| Pcx | -0.2813986 |
| Pcyox1 | 1.18398406 |
| Pcyox1l | -0.2884597 |
| Pcyt1a | -0.2262252 |
| Pcyt1b | -0.4914609 |
| Pcyt1b | 0.5418114 |
| Pcyt2 | 0.28304694 |
| Pcyt2 | -0.9048212 |
| Pcyt2 | 0.99226775 |
| Pdcd10 | -0.4713922 |
| Pdcd2 | -0.6793814 |
| Pdcd2l | 2.43259475 |
| Pdcd4 | 1.21083441 |
| Pdcd5 | 1.37170448 |
| Pdcd5 | -0.5507571 |
| Pdcd6ip | 1.66196278 |
| Pdcl | -0.5057675 |
| Pdcl3 | 1.1986322 |
| Pddc1 | -0.0293285 |
| Pde10a | -0.5301049 |
| Pde1a | -0.3981901 |
| Pde1a | 0.63964844 |
| Pde1a | 0.64635004 |
| Pde1b | 1.92278296 |
| Pde1c | -0.1297633 |
| Pde2a | 0.78306088 |
| Pde3a | -0.6279946 |
| Pde4a | 0.72772718 |
| Pde4a | -0.780317 |
| Pde4a | -0.3545075 |
| Pde4a | -0.7027746 |
| Pde4b | 1.69368806 |
| Pde4d | -0.4867399 |
| Pde4dip | 0.21873769 |
| Pde4dip | -0.2030348 |
| Pde4dip | 0.04434952 |
| Pde4dip | 1.6936214 |
| Pde4dip | -0.2328837 |
| Pde5a | -0.6703477 |
| Pde6a | -0.4595527 |
| Pde6d | -0.6613997 |
| Pde6d | -0.6334806 |
| Pde6d | -0.6744432 |
| Pde6d | 2.68999909 |
| Pde6h | -0.1408871 |
| Pde7a | 0.61716823 |
| Pdgfa | -0.6437543 |
| Pdgfb | 2.60966725 |
| Pdgfc | -0.1794291 |
| Pdgfra | -0.536466 |
| Pdgfrb | -0.3223174 |
| Pdgfrl | 0.88311896 |
| Pdha1 | 0.53454903 |
| Pdhb | -0.6295588 |
| Pdhb | 0.23890029 |
| Pdia3 | -0.4117615 |
| Pdia4 | -0.6695307 |
| Pdia5 | -0.3308326 |
| Pdia5 | -0.3365524 |
| Pdia6 | 0.02695046 |
| Pdik1l | 0.47410173 |
| Pdk1 | 2.2224768 |
| Pdk2 | -0.7594269 |
| Pdk3 | -0.5711356 |
| Pdk4 | 0.73069263 |
| Pdlim1 | 1.04011435 |
| Pdlim2 | 0.34853708 |
| Pdlim2 | -0.590461 |
| Pdlim2 | 0.97571631 |
| Pdlim2 | -0.7241631 |
| Pdlim3 | 1.23748231 |
| Pdlim3 | 1.17605462 |
| Pdlim4 | -0.4853953 |
| Pdlim5 | 2.35658347 |
| Pdlim5 | -0.5521982 |
| Pdlim7 | -0.746048 |
| Pdpk1 | -0.5748214 |
| Pdpk1 | 3.39895392 |
| Pdrg1 | -0.8688366 |
| Pdss1 | -0.699397 |
| Pdxdc1 | 1.05348198 |
| Pdxdc1 | 1.49125172 |
| Pdxk | -0.0068167 |
| Pdxp | -0.7540526 |
| Pdyn | 0.2435642 |
| Pdyn | 1.23292999 |
| Pdzd11 | -0.2505237 |
| Pdzd11 | -0.3331163 |
| Pdzd11 | 0.69908046 |
| Pdzd8 | 2.075521 |
| Pdzk1 | 0.01240013 |
| Pdzk1ip1 | 0.65340615 |
| Pdzrn3 | -0.7138361 |
| Pdzrn3 | 0.78148406 |
| Pea15 | 0.7714359 |
| Pear1 | 0.2800678 |
| Pear1 | 0.4743446 |
| Pebp1 | -0.6101907 |
| Pecam1 | -0.4996095 |
| Pecam1 | -0.8361214 |
| Peci | 0.92755447 |
| Pef1 | 1.47222358 |
| Peg10 | -0.7612252 |
| Peg3 | -0.0011713 |
| Peli1 | -0.8168324 |
| Peli1 | 1.52946399 |
| Peli2 | 1.32508537 |
| Peli3 | -0.106552 |
| Pelo | -0.3556778 |
| Pelp1 | -0.6086583 |
| Pemt | 0.92333974 |
| Peo1 | 3.47198367 |
| Pepd | -0.2000516 |
| Per1 | -0.0096522 |
| Per1 | -0.5209919 |
| Per2 | 0.90675063 |
| Per2 | -0.5957324 |
| Per2 | 1.5824539 |
| Per3 | -0.1311466 |
| Perld1 | 0.46187766 |
| Pes1 | -0.8995958 |
| Pet112l | 0.22540117 |
| Pet112l | 0.40508116 |
| Pet112l | 0.150748 |
| Pet112l | 1.77970384 |
| Pex11a | 0.5418114 |
| Pex11a | -0.6918413 |
| Pex11a | 0.8335039 |
| Pex11b | -0.5822365 |
| Pex11c | -0.359785 |
| Pex14 | 0.89966121 |
| Pex16 | 0.75209863 |
| Pex19 | 0.12190609 |
| Pex19 | 0.63576823 |
| Pex2 | -0.8016804 |
| Pex26 | 0.68675345 |
| Pex3 | 0.31836802 |
| Pex3 | 3.09583766 |
| Pex5 | 0.07413912 |
| Pex5 | -0.2385547 |
| Pex5 | 2.46118 |
| Pex5l | -0.5175101 |
| Pex6 | 0.5338653 |
| Pex6 | 1.64646203 |
| Pex6 | -0.4800362 |
| Pex7 | -0.340001 |
| Pfdn1 | -0.6926778 |
| Pfdn2 | 0.81202092 |
| Pfdn4 | -0.0129032 |
| Pfdn5 | 0.05425794 |
| Pfdn5 | 1.33058737 |
| Pfkfb2 | 1.69674953 |
| Pfkfb2 | -0.7630269 |
| Pfkfb4 | 0.4680095 |
| Pfkl | 1.28008107 |
| Pfkl | -0.4621709 |
| Pfkm | 0.35379475 |
| Pfkm | 1.7327963 |
| Pfkm | 1.42795259 |
| Pfkp | -0.6469381 |
| Pfkp | 1.01320849 |
| Pfkp | 0.9697032 |
| Pfn1 | 3.16293437 |
| Pfn1 | -0.525888 |
| Pfn2 | 0.89795799 |
| Pfn2 | 0.96307312 |
| Pfn2 | -0.0490009 |
| Pfn3 | 0.4275485 |
| Pfn4 | 0.23890029 |
| Pftk1 | -0.6477358 |
| Pga5 | 1.32064075 |
| Pgam1 | -0.0984942 |
| Pgam2 | 1.70317283 |
| Pgam5 | 1.38539109 |
| Pgbd5 | -0.2494769 |
| Pgcp | -0.3981901 |
| Pgd | -0.6240955 |
| Pgk1 | 0.49571358 |
| Pgk2 | -0.7693601 |
| Pgls | 1.81739853 |
| Pglyrp1 | -0.0953792 |
| Pglyrp4 | -0.2479088 |
| Pgm2 | -0.4941693 |
| Pgm2l1 | -0.5792634 |
| Pgm3 | -0.4674291 |
| Pgm5 | -0.0909468 |
| Pgm5 | -0.311082 |
| Pgpep1 | -0.8273925 |
| Pgpep1 | 0.2196925 |
| Pgr | 0.5258517 |
| Pgrmc1 | -0.7381108 |
| Pgs1 | 0.50891524 |
| Phactr1 | -0.6835165 |
| Phactr1 | -0.391477 |
| Phactr1 | -0.5785216 |
| Phactr4 | 0.38393831 |
| Phb2 | 0.25619579 |
| Phc1 | -0.8759246 |
| Phc1 | -0.2830234 |
| Phc2 | 1.54011456 |
| Phca | 0.23421321 |
| Phca | 3.00721596 |
| Phex | 1.72050127 |
| Phf1 | 1.02248904 |
| Phf11 | 1.58252891 |
| Phf11 | -0.6155738 |
| Phf12 | 0.78568305 |
| Phf12 | 0.90367482 |
| Phf13 | 0.23640341 |
| Phf13 | -0.5912122 |
| Phf14 | 0.45570569 |
| Phf16 | -0.4608609 |
| Phf17 | -0.5308096 |
| Phf19 | 1.7865551 |
| Phf2 | 0.05652979 |
| Phf20l1 | 2.78433338 |
| Phf21a | 0.45867328 |
| Phf5a | 2.04611784 |
| Phf6 | -0.3939126 |
| Phf7 | 0.73217186 |
| Phgdh | 1.48253642 |
| Phka1 | 0.02225598 |
| Phka1 | 0.90151577 |
| Phka2 | -0.6094242 |
| Phka2 | -0.6868376 |
| Phka2 | -0.4661118 |
| Phkb | 1.43068764 |
| Phkg1 | 1.36265787 |
| Phkg2 | 0.19715773 |
| Phlda1 | 1.29819934 |
| Phlda2 | 1.35381044 |
| Phlda3 | 0.82851255 |
| Phlda3 | -0.6835165 |
| Phldb1 | -0.2364886 |
| Phldb1 | 0.01001933 |
| Phlpp | -0.585966 |
| Phospho1 | 1.96616122 |
| Phospho2 | 0.09069653 |
| Phox2a | 0.00364097 |
| Phox2b | 0.87964901 |
| Phpt1 | 0.88075447 |
| Phtf1 | -0.9174818 |
| Phtf2 | -0.8264276 |
| Phyhip | -0.3994159 |
| Pi16 | -0.5696653 |
| Pi4k2b | 1.04741724 |
| Pi4k2b | -0.4167454 |
| Pi4ka | 1.56435501 |
| Pi4kb | -0.746048 |
| Pi4kb | 1.30194319 |
| Pias1 | -0.7594269 |
| Pias2 | 0.54316689 |
| Pias3 | 1.20536854 |
| Pias3 | 3.70599367 |
| Pias4 | 0.35462223 |
| Pibf1 | -0.8488741 |
| Pibf1 | -0.6977127 |
| Picalm | 0.64330976 |
| Pick1 | 0.78095787 |
| Pick1 | 1.50966052 |
| Pif1 | 0.45892016 |
| Piga | 0.97128177 |
| Piga | 2.94856213 |
| Pigb | 1.26318755 |
| Pigc | -0.5175101 |
| Pigf | -0.8498622 |
| Pigh | 0.02186372 |
| Pigk | -0.5718716 |
| Pigk | 0.3362477 |
| Pigl | 0.52331901 |
| Pign | -0.6687145 |
| Pigo | 3.38114308 |
| Pigo | -0.5762997 |
| Pigo | -0.5400185 |
| Pigp | -0.7036209 |
| Pigp | 1.71164796 |
| Pigq | -0.5711356 |
| Pigt | 1.2869804 |
| Pigx | -0.4280564 |
| Pigy | -0.6040759 |
| Pigyl | -0.585966 |
| Pigz | -0.3211878 |
| Pih1d1 | 0.98211666 |
| Pik3ap1 | 0.89469783 |
| Pik3c2a | -0.4962057 |
| Pik3c3 | -0.1201364 |
| Pik3ca | 0.26016587 |
| Pik3cb | -0.6785566 |
| Pik3cd | 1.61474718 |
| Pik3cg | -0.1625815 |
| Pik3ip1 | 0.64776549 |
| Pik3r1 | -0.2211351 |
| Pik3r1 | 0.09179009 |
| Pik3r1 | 0.22350212 |
| Pik3r2 | 0.56921692 |
| Pik3r2 | -0.3325449 |
| Pik3r3 | 0.08153388 |
| Pik3r4 | 1.92872486 |
| Pik3r6 | 2.44325416 |
| Pik3r6 | -0.7146924 |
| Pim1 | -0.2862814 |
| Pim2 | -0.0141252 |
| Pim2 | 1.01416807 |
| Pim3 | 0.66795548 |
| Pip4k2a | -0.431857 |
| Pip4k2b | -0.3799929 |
| Pip4k2b | 0.86660189 |
| Pip4k2c | -0.3556778 |
| Pip5k1a | -0.7932631 |
| Pip5k1a | 0.02304002 |
| Pip5k1b | -0.2950249 |
| Pip5kl1 | 0.19976055 |
| Pipox | -0.8322319 |
| Pir | 2.46109136 |
| Pira11 | 1.11281724 |
| Pira4 | -0.0712552 |
| Pisd | -0.0169828 |
| Pit1 | -0.4267929 |
| Pitpna | 2.53968929 |
| Pitpnb | 2.13601819 |
| Pitpnc1 | 2.39816998 |
| Pitpnm1 | -0.746048 |
| Pitpnm2 | 1.89141816 |
| Pitpnm3 | 0.80126484 |
| Pitx1 | 0.54767118 |
| Pitx2 | -0.7070137 |
| Pitx2 | -0.213551 |
| Pitx2 | -0.4485059 |
| Pitx2 | 0.13765263 |
| Piwil1 | 0.03433591 |
| Piwil2 | -0.6374188 |
| Piwil4 | -0.5435845 |
| Pja1 | -0.6056009 |
| Pja2 | 1.54886021 |
| Pja2 | 2.39981104 |
| Pkd1 | -0.0962681 |
| Pkd1l3 | -0.5800058 |
| Pkd2 | -0.3603732 |
| Pkd2l1 | -0.0251953 |
| Pkd2l2 | -0.7469341 |
| Pkdrej | -0.0422577 |
| Pkhd1l1 | 2.02341262 |
| Pkhd1l1 | 1.41451563 |
| Pkia | 0.11375518 |
| Pkib | 1.56328398 |
| Pkib | -0.6935151 |
| Pkig | -0.1535602 |
| Pkig | -0.2020393 |
| Pkig | -0.2267357 |
| Pkm2 | 0.42217415 |
| Pkmyt1 | 0.69158788 |
| Pkn1 | -0.5500374 |
| Pkn1 | 1.47154891 |
| Pkn2 | 2.54886039 |
| Pkn2 | -0.4407853 |
| Pkn2 | 1.49693538 |
| Pkn3 | -0.6429601 |
| Pknox1 | 2.37128973 |
| Pknox1 | 0.46947522 |
| Pknox2 | -0.8598012 |
| Pknox2 | 1.30617504 |
| Pknox2 | 1.06315721 |
| Pknox2 | -0.2221509 |
| Pknox2 | 1.6791781 |
| Pkp1 | 1.29870614 |
| Pkp2 | -0.680207 |
| Pkp2 | -0.4707305 |
| Pkp2 | 1.29759083 |
| Pkp3 | -0.2318562 |
| Pla1a | -0.1995553 |
| Pla1a | -0.8054456 |
| Pla1a | -0.6885025 |
| Pla2g10 | -0.391477 |
| Pla2g12a | -0.7666407 |
| Pla2g1b | 1.0918537 |
| Pla2g2c | -0.5478815 |
| Pla2g2d | 0.30799679 |
| Pla2g2f | 0.2435642 |
| Pla2g4c | 0.1690499 |
| Pla2g4d | -0.3757969 |
| Pla2g4e | -0.6437543 |
| Pla2g4f | 1.25780599 |
| Pla2g5 | 1.93287238 |
| Pla2g6 | -0.205528 |
| Pla2g7 | 0.14594452 |
| Plaa | 0.44824563 |
| Plac1 | -0.435036 |
| Plac1l | -0.6835165 |
| Plac8 | 0.42140388 |
| Plag1 | 1.74556428 |
| Plag1 | 3.59705188 |
| Plagl2 | -0.4562902 |
| Plau | -0.1568738 |
| Plb1 | 0.59520789 |
| Plcb2 | 1.44952777 |
| Plcb3 | 1.54574606 |
| Plcb4 | 0.54429498 |
| Plcd1 | -0.5579847 |
| Plcd3 | -0.6943531 |
| Plcd4 | -0.6744432 |
| Plcd4 | 0.13626354 |
| Plcd4 | -0.555087 |
| Plce1 | 1.99031798 |
| Plcg2 | 0.46138538 |
| Plch2 | 0.60751801 |
| Plcl2 | 2.63509293 |
| Plcxd1 | -0.3257142 |
| Plcxd1 | -0.510585 |
| Plcz1 | 1.91690862 |
| Pld1 | 0.91561417 |
| Pld2 | -0.6350539 |
| Pld3 | 0.79471723 |
| Pld3 | 0.00203962 |
| Pld4 | 0.2528234 |
| Pld5 | -0.4452813 |
| Pldn | 0.37857771 |
| Plec1 | 0.3526903 |
| Plec1 | -0.1736188 |
| Plec1 | 0.48760719 |
| Plec1 | 0.90244168 |
| Plek | 1.78104162 |
| Plek2 | -0.699397 |
| Plekha1 | 1.73745344 |
| Plekha1 | 0.59371127 |
| Plekha2 | 1.27167213 |
| Plekha3 | -0.5942232 |
| Plekha4 | 3.10504858 |
| Plekha4 | -0.4641393 |
| Plekha5 | -0.2323698 |
| Plekha6 | 0.6616053 |
| Plekha7 | -0.5414433 |
| Plekha8 | -0.8341746 |
| Plekhb1 | 0.32237068 |
| Plekhb2 | -0.0182101 |
| Plekhf1 | -0.5740831 |
| Plekhf1 | -0.3650922 |
| Plekhf2 | -0.1371645 |
| Plekhf2 | -0.3872297 |
| Plekhg2 | 1.74688833 |
| Plekhg2 | 0.03742836 |
| Plekhg2 | 0.63638196 |
| Plekhg3 | 2.09797545 |
| Plekhg3 | 1.53917269 |
| Plekhg4 | -0.0764662 |
| Plekhg5 | -0.3183697 |
| Plekhg6 | -0.4847238 |
| Plekhj1 | 2.58166684 |
| Plekhm1 | -0.0334799 |
| Plekhm3 | -0.5286972 |
| Plekho2 | 1.90483588 |
| Plin | -0.7442784 |
| Plk1 | 2.12726097 |
| Plk2 | -0.3722151 |
| Plk3 | 0.74063272 |
| Plk4 | -0.0918316 |
| Pllp | -0.634267 |
| Plod1 | 3.59551771 |
| Plod2 | -0.1842984 |
| Plod3 | 0.63433464 |
| Plp1 | -0.6549449 |
| Plp2 | 2.16392021 |
| Plrg1 | -0.1483765 |
| Pls3 | -0.6140326 |
| Plscr1 | 1.38806788 |
| Plscr1 | 1.16479348 |
| Plscr3 | -0.5897103 |
| Plscr4 | 0.7971348 |
| Pltp | -0.6711654 |
| Plunc | -0.2879146 |
| Plvap | -0.5594369 |
| Plxdc1 | -0.5822365 |
| Plxdc1 | 1.35524307 |
| Plxna2 | -0.2510476 |
| Plxnb1 | -0.5980006 |
| Plxnb3 | -0.6736227 |
| Plxnc1 | -0.1343822 |
| Plxnd1 | 0.69158788 |
| Pmaip1 | 0.11624336 |
| Pmch | -0.0847764 |
| Pmepa1 | -0.3354057 |
| Pmf1 | 2.39017648 |
| Pmfbp1 | 0.18006204 |
| Pml | 0.98254181 |
| Pml | 1.01526354 |
| Pmm1 | -0.3933031 |
| Pmm1 | -0.0665036 |
| Pmm2 | 0.17473888 |
| Pmm2 | 0.13173479 |
| Pmm2 | 1.07851085 |
| Pmm2 | 0.45048985 |
| Pmp2 | -0.1493169 |
| Pmp22 | 0.01477495 |
| Pmp22 | -0.5897103 |
| Pmpca | 1.45495265 |
| Pmpcb | 0.36722028 |
| Pms2 | -0.5844724 |
| Pmvk | -0.2776182 |
| Pnck | 2.52101969 |
| Pnkd | -0.4840527 |
| Pnkd | 1.41308057 |
| Pnkp | -0.2851941 |
| Pnkp | -0.1872322 |
| Pnliprp1 | -0.1587726 |
| Pnma1 | 1.22966482 |
| Pnma2 | 0.4926223 |
| Pno1 | 1.57477213 |
| Pnpla2 | 1.99812205 |
| Pnpla6 | -0.2287806 |
| Pnpla7 | -0.658974 |
| Pnpla8 | 0.40481978 |
| Pnpla8 | -0.2917366 |
| Pnpo | 1.64765271 |
| Pnpo | 0.48592955 |
| Pnrc2 | 0.82416525 |
| Podn | -0.5964878 |
| Podxl | 0.7732065 |
| Podxl2 | -0.1530878 |
| Pof1b | 0.51614462 |
| Pofut1 | -0.1479067 |
| Pofut1 | 0.1565481 |
| Pofut2 | 0.15040571 |
| Pogz | -0.746048 |
| Pogz | 1.00397424 |
| Pola2 | -0.3121995 |
| Polb | 2.7688325 |
| Pold1 | 1.01826952 |
| Pold2 | -0.4536883 |
| Pold3 | 1.99985826 |
| Pold4 | -0.4713922 |
| Poldip2 | -0.6851756 |
| Poldip3 | -0.5972439 |
| Poldip3 | 2.142792 |
| Pole | 0.11126042 |
| Pole3 | -0.4975658 |
| Pole3 | -0.5659995 |
| Pole4 | 0.1015755 |
| Polg | 1.66058293 |
| Polg2 | 2.72416678 |
| Polh | -0.7757362 |
| Poli | -0.5428702 |
| Polk | -0.2813986 |
| Poll | 4.04650338 |
| Poll | 0.82817885 |
| Polm | 1.49742799 |
| Polm | 0.16636082 |
| Poln | 2.03229973 |
| Polq | -0.4261618 |
| Polr1e | 0.25313048 |
| Polr2d | 1.48328661 |
| Polr2d | -0.4948476 |
| Polr2d | -0.0725553 |
| Polr2d | -0.746048 |
| Polr2e | -0.7407492 |
| Polr2e | -0.6303419 |
| Polr2f | 1.57862084 |
| Polr2g | -0.6638316 |
| Polr2h | 0.76414151 |
| Polr2j | -0.6943531 |
| Polr3a | -0.4031026 |
| Polr3a | -0.0953792 |
| Polr3b | -0.3206235 |
| Polr3d | 0.36121612 |
| Polr3e | 3.39649108 |
| Polr3g | -0.2557754 |
| Polr3h | -0.2010449 |
| Polr3h | -0.4018721 |
| Polr3h | -0.6517341 |
| Polr3k | -0.5638069 |
| Polr3k | 2.6785951 |
| Polrmt | 3.73881641 |
| Pols | 0.10553322 |
| Pom121 | -0.5792634 |
| Pomc | -0.6202125 |
| Pomgnt1 | -0.2808576 |
| Pomgnt1 | 0.74465255 |
| Pomp | 1.62806176 |
| Pomt1 | 0.4042968 |
| Pomt1 | 0.04588069 |
| Pomt2 | -0.7469341 |
| Pon1 | 3.15485504 |
| Pon2 | 2.09148309 |
| Pon3 | 0.15109017 |
| Pon3 | -0.1274622 |
| Pop4 | 1.90749667 |
| Pop5 | 0.14594452 |
| Pop5 | -0.4433518 |
| Pop7 | 0.84472077 |
| Popdc2 | -0.5652681 |
| Popdc2 | 1.89228264 |
| Popdc3 | -0.7657359 |
| Por | 0.71275847 |
| Por | -0.6101907 |
| Porcn | -0.3342603 |
| Porcn | 0.11801662 |
| Pou2af1 | -0.5301049 |
| Pou2f1 | -0.2950249 |
| Pou2f1 | 0.09977103 |
| Pou2f2 | -0.4928141 |
| Pou3f1 | -0.353923 |
| Pou3f3 | -0.289005 |
| Pou5f1 | 1.62763287 |
| Pou5f1 | -0.3206235 |
| Pou6f1 | 0.95260114 |
| Ppa1 | 1.37815355 |
| Ppa2 | -0.649333 |
| Ppa2 | 2.36721854 |
| Ppa2 | -0.9576238 |
| Ppap2a | -0.2743899 |
| Ppap2a | -0.6358416 |
| Ppap2a | -0.7904739 |
| Ppap2b | -0.8158783 |
| Ppap2b | 1.51612024 |
| Ppap2c | -0.5681973 |
| Ppapdc1 | 2.15558282 |
| Ppapdc1a | 2.10537174 |
| Ppapdc2 | -0.4914609 |
| Ppapdc3 | 0.30654728 |
| Ppapdc3 | 1.45786746 |
| Pparbp | -0.8111218 |
| Pparg | -0.7867678 |
| Ppargc1a | 2.91532881 |
| Ppargc1b | 1.79173618 |
| Ppat | -0.9422333 |
| Ppcs | -0.3223174 |
| Ppef2 | 0.87346617 |
| Ppfia3 | 2.77698478 |
| Ppfia3 | -0.0799563 |
| Ppfibp1 | 1.35294981 |
| Ppfibp2 | 1.78486207 |
| Ppfibp2 | 2.54045017 |
| Ppia | 1.34265732 |
| Ppic | -0.7044679 |
| Ppic | -0.5972439 |
| Ppid | -0.1760354 |
| Ppie | -0.2776182 |
| Ppie | -0.396966 |
| Ppif | -0.9228076 |
| Ppil2 | 0.06180959 |
| Ppil3 | -0.3848113 |
| Ppl | 0.97956173 |
| Ppm1a | -0.6769091 |
| Ppm1a | 1.4845356 |
| Ppm1a | -0.7513768 |
| Ppm1b | -0.9238763 |
| Ppm1b | -0.205528 |
| Ppm1b | 0.05804132 |
| Ppm1e | -0.6935151 |
| Ppm1e | -0.6893361 |
| Ppm1f | 2.35035707 |
| Ppm1f | -0.4173703 |
| Ppm1f | 0.5163769 |
| Ppm1g | -0.747821 |
| Ppm1h | 1.00203322 |
| Ppm1j | -0.6926778 |
| Ppm1k | 0.90813148 |
| Ppm1l | -0.2988758 |
| Ppm1l | -0.4018721 |
| Ppm1m | -0.3860197 |
| Ppm1m | 0.84685007 |
| Ppm2c | 1.43262385 |
| Ppme1 | -0.8026203 |
| Ppnr | -0.2349419 |
| Ppox | 0.90275012 |
| Ppox | -0.6687145 |
| Ppp1ca | -0.6140326 |
| Ppp1ca | -0.7830763 |
| Ppp1ca | 0.23827669 |
| Ppp1cb | -0.0931604 |
| Ppp1cb | -0.0989401 |
| Ppp1cc | -0.7232982 |
| Ppp1r10 | 1.10538364 |
| Ppp1r10 | 1.00355864 |
| Ppp1r11 | 1.24780585 |
| Ppp1r12c | -0.3055141 |
| Ppp1r13b | 2.57278447 |
| Ppp1r13b | -0.7267627 |
| Ppp1r13b | 2.9581702 |
| Ppp1r14a | 1.87357463 |
| Ppp1r14a | -0.5972439 |
| Ppp1r14c | 0.49214582 |
| Ppp1r14d | 1.38029347 |
| Ppp1r15b | 0.37696357 |
| Ppp1r16a | -0.7858436 |
| Ppp1r1a | 0.01041655 |
| Ppp1r1b | 2.35678151 |
| Ppp1r1b | 1.16491026 |
| Ppp1r2 | -0.8264276 |
| Ppp1r3c | -0.094491 |
| Ppp1r3f | -0.9389678 |
| Ppp1r8 | -0.7328563 |
| Ppp1r9a | -0.7558406 |
| Ppp1r9b | 2.82374805 |
| Ppp2ca | 0.53841412 |
| Ppp2cb | -0.5189013 |
| Ppp2cz | -0.3088509 |
| Ppp2r1a | -0.3650922 |
| Ppp2r1b | -0.5785216 |
| Ppp2r2b | -0.4661118 |
| Ppp2r2b | 2.77312972 |
| Ppp2r2b | 1.45940695 |
| Ppp2r2c | 1.49166473 |
| Ppp2r2c | 1.1262952 |
| Ppp2r2d | -0.9777879 |
| Ppp2r3c | -0.0931604 |
| Ppp2r4 | -0.6860062 |
| Ppp2r5b | -0.7675463 |
| Ppp2r5c | 0.28215419 |
| Ppp2r5c | 0.92484716 |
| Ppp2r5c | 0.15143222 |
| Ppp2r5d | 0.42448119 |
| Ppp2r5d | -0.6094242 |
| Ppp2r5d | 0.03200991 |
| Ppp2r5d | 0.81591738 |
| Ppp2r5e | -0.4261618 |
| Ppp2r5e | 0.02029303 |
| Ppp3ca | -0.0751608 |
| Ppp3ca | -0.9090226 |
| Ppp3ca | -0.3200596 |
| Ppp3cb | 0.05007861 |
| Ppp3cc | 0.14937807 |
| Ppp3r1 | -0.5057675 |
| Ppp4c | 0.45421839 |
| Ppp4r1 | 0.1810568 |
| Ppp4r1 | 2.5362577 |
| Ppp4r1 | 0.15518658 |
| Ppp4r1l | -0.1573482 |
| Ppp5c | -0.6777325 |
| Ppp5c | -0.7112719 |
| Ppp6c | 0.28927268 |
| Pps | 0.86868241 |
| Ppt1 | 1.06731055 |
| Ppt2 | -0.8528329 |
| Pptc7 | 1.0837434 |
| Pqbp1 | 0.49286045 |
| Pqlc1 | -0.121049 |
| Pqlc2 | -0.7215708 |
| Pqlc3 | 1.48411945 |
| Pqlc3 | 1.2581234 |
| Pqlc3 | 1.25642929 |
| Pqlc3 | -0.7293694 |
| Pramef12 | 2.1911385 |
| Prap1 | 1.55667797 |
| Prc1 | -0.9800552 |
| Prcp | -0.8158783 |
| Prdm10 | 1.10799207 |
| Prdm4 | -0.5740831 |
| Prdm8 | -0.1592478 |
| Prdm9 | -0.2879146 |
| Prdx1 | 2.11557041 |
| Prdx2 | -0.5450146 |
| Prdx2 | 1.82060877 |
| Prdx2 | -0.3302625 |
| Prdx3 | 0.72270175 |
| Prdx4 | -0.6272135 |
| Prdx5 | 0.87695891 |
| Prdx5 | -0.8902622 |
| Prdx6 | -0.0838982 |
| Prdx6-rs1 | -0.5500374 |
| Prdx6-rs1 | 1.09976973 |
| Preb | 2.06707861 |
| Preb | 2.15116062 |
| Prei3 | 0.48544967 |
| Prei4 | 0.16535043 |
| Prei4 | 0.09179009 |
| Prelp | 1.72695844 |
| Prep | -0.1545058 |
| Prep | -0.0686604 |
| Prf1 | 0.46947522 |
| Prickle1 | -0.0112763 |
| Prickle2 | 0.25711345 |
| Prickle3 | 0.16770632 |
| Prickle4 | 0.67190274 |
| Prim2 | 0.1292872 |
| Prim2 | -0.5057675 |
| Prima1 | 2.00873039 |
| Prkaa2 | 1.63610294 |
| Prkaa2 | 1.51869179 |
| Prkab1 | -0.3951328 |
| Prkab2 | 0.11197388 |
| Prkaca | 0.16197463 |
| Prkaca | -0.6678989 |
| Prkacb | 0.45941372 |
| Prkag1 | -0.1668851 |
| Prkag2 | -0.8508514 |
| Prkar1a | 1.58012586 |
| Prkar1b | 1.45889407 |
| Prkar2b | -0.2318562 |
| Prkca | -0.3515883 |
| Prkcb1 | -0.969895 |
| Prkcbp1 | 1.75529353 |
| Prkcbp1 | -0.9755261 |
| Prkcc | -0.3365524 |
| Prkcd | 0.85353423 |
| Prkcdbp | 2.34375474 |
| Prkcdbp | 0.34520128 |
| Prkce | -0.3963545 |
| Prkch | 0.095789 |
| Prkcq | -0.8975136 |
| Prkcsh | -0.0401603 |
| Prkcz | 0.30886543 |
| Prkcz | -0.2282689 |
| Prkcz | -0.6901704 |
| Prkd1 | 0.76681674 |
| Prkd1 | -0.6711654 |
| Prkd2 | -0.8912947 |
| Prkd3 | -0.7666407 |
| Prkd3 | 1.66985797 |
| Prkd3 | -0.6374188 |
| Prkg1 | 1.8900117 |
| Prkg2 | 0.60456128 |
| Prkra | 0.87648339 |
| Prkrip1 | -0.5478815 |
| Prkrip1 | 2.96628156 |
| Prkrir | 1.58895693 |
| Prkx | 1.79850091 |
| Prkx | 1.41128366 |
| Prl | 0.32720832 |
| Prlr | -0.5350487 |
| Prlr | 0.33540393 |
| Prm1 | 1.97471139 |
| Prm3 | 2.84607197 |
| Prmt2 | 0.01200375 |
| Prmt2 | -0.3434623 |
| Prmt3 | -0.612494 |
| Prmt5 | 1.5484715 |
| Prmt5 | -0.7241631 |
| Prmt5 | 0.09470007 |
| Prmt7 | 0.85060555 |
| Prmt7 | 0.24387431 |
| Prmt7 | -0.7258954 |
| Prmt7 | 1.19230124 |
| Prmt8 | 0.6884967 |
| Prnp | 0.00083682 |
| Prnpip1 | -0.6485341 |
| Prnpip1 | -0.2411438 |
| Prnpip1 | -0.7155494 |
| Proc | 1.0809393 |
| Proc | 2.59593743 |
| Procr | 1.27687168 |
| Prodh | 0.68946378 |
| Prok2 | 0.47239987 |
| Prok2 | -0.7328563 |
| Prokr1 | 0.54519648 |
| Prokr2 | 0.98296677 |
| Prom1 | -0.213551 |
| Prom2 | -0.0962681 |
| Prom2 | 2.35360787 |
| Pros1 | 0.95347826 |
| Prosapip1 | 1.20077058 |
| Prosapip1 | -0.382399 |
| Prosapip1 | -0.8749087 |
| Prosc | 1.46562482 |
| Prosc | -0.4024872 |
| Prosc | 0.01753803 |
| Prox1 | -0.5995157 |
| Prox1 | 1.6736788 |
| Prpf18 | -0.3656837 |
| Prpf19 | 0.02499726 |
| Prpf19 | -0.8273925 |
| Prpf19 | 3.23799824 |
| Prpf3 | -0.340001 |
| Prpf3 | 0.29633785 |
| Prpf3 | 0.95216227 |
| Prpf3 | 0.00962194 |
| Prpf3 | -0.4713922 |
| Prpf31 | 0.74538159 |
| Prpf38a | -0.4261618 |
| Prpf38a | -0.9260175 |
| Prpf38a | -0.632695 |
| Prpf38b | 2.69494674 |
| Prpf39 | 0.86916188 |
| Prpf40a | -0.4820423 |
| Prpf40a | -0.0309868 |
| Prpf40b | 0.18238152 |
| Prpf40b | -0.4562902 |
| Prpf40b | -0.0755957 |
| Prpf6 | 0.60878235 |
| Prpf8 | -0.290643 |
| Prpf8 | 0.24480403 |
| Prph2 | 0.21330875 |
| Prps1 | -0.3830015 |
| Prps1l1 | -0.649333 |
| Prps2 | 0.03356122 |
| Prpsap2 | -0.7407492 |
| Prpsap2 | 1.09336664 |
| Prr11 | -0.831262 |
| Prr13 | -0.4280564 |
| Prr14 | -0.9743972 |
| Prr15 | 2.67044191 |
| Prr16 | 0.01437956 |
| Prr18 | 0.6869473 |
| Prr19 | 1.03019858 |
| Prr3 | 1.35294981 |
| Prr7 | 0.22792731 |
| Prrc1 | 2.2042259 |
| Prrg2 | 0.2282426 |
| Prrt1 | -0.5681973 |
| Prrt1 | -0.7960605 |
| Prrt1 | -0.4582464 |
| Prrt3 | 1.36426621 |
| Prrx1 | 0.18171939 |
| Prrx1 | 0.50868111 |
| Prrx1 | -0.4049514 |
| Prss21 | -0.8698459 |
| Prss23 | 1.19388797 |
| Prss27 | 0.84472077 |
| Prss28 | 0.11268681 |
| Prss29 | 2.36617348 |
| Prss29 | -0.2621162 |
| Prss34 | -0.1427539 |
| Prss35 | 1.55552101 |
| Prss36 | 0.28453299 |
| Prss7 | -0.3787921 |
| Prss8 | 1.04064738 |
| Prss8 | 1.81996759 |
| Prtn3 | 0.50422155 |
| Prune | 1.43165624 |
| Prune | 1.69920482 |
| Prune | 2.25553018 |
| Prune | 1.93406143 |
| Psap | -0.607893 |
| Psat1 | 0.00203962 |
| Psca | 1.35524307 |
| Pscd1 | -0.7941946 |
| Pscd2 | -0.4148734 |
| Pscd3 | 1.55575251 |
| Pscdbp | -0.525888 |
| Psd2 | -0.7078638 |
| Psd3 | 2.05665645 |
| Psd4 | 0.46752041 |
| Psen1 | -0.7666407 |
| Psen2 | 0.62651408 |
| Psenen | -0.3262816 |
| Psg19 | 0.89702767 |
| Psg23 | -0.7181253 |
| Psg27 | -0.2364886 |
| Psip1 | -0.7381108 |
| Psip1 | 2.41062709 |
| Pskh1 | 2.37167964 |
| Psma2 | 0.60244364 |
| Psma4 | -0.7311112 |
| Psma5 | 1.90701828 |
| Psma7 | 2.52207279 |
| Psma7 | 0.2512865 |
| Psmb1 | -0.6209878 |
| Psmb10 | 1.28266135 |
| Psmb10 | -0.3308326 |
| Psmb2 | -0.699397 |
| Psmb2 | 0.48856451 |
| Psmb3 | 0.32664047 |
| Psmb4 | 0.20752619 |
| Psmb5 | -0.215063 |
| Psmb6 | -0.7739101 |
| Psmb7 | 0.28334434 |
| Psmb7 | -0.649333 |
| Psmb7 | -0.4043348 |
| Psmb8 | -0.3144386 |
| Psmb9 | 1.67354261 |
| Psmc1 | 1.66016858 |
| Psmc2 | -0.7594269 |
| Psmc3 | -0.3981901 |
| Psmc3ip | -0.4465698 |
| Psmc4 | 1.47230788 |
| Psmc5 | -0.391477 |
| Psmc5 | 0.99939257 |
| Psmd1 | 1.29413515 |
| Psmd10 | -0.9006385 |
| Psmd10 | 0.04358301 |
| Psmd10 | 0.30712735 |
| Psmd11 | -0.8912947 |
| Psmd11 | 0.66617381 |
| Psmd12 | 1.81458791 |
| Psmd12 | 1.74512251 |
| Psmd13 | 0.4985581 |
| Psmd14 | 2.26036784 |
| Psmd2 | 1.48361984 |
| Psmd2 | 0.20655898 |
| Psmd4 | 0.23921194 |
| Psmd5 | -0.787693 |
| Psmd6 | -0.6851756 |
| Psmd6 | 0.12648216 |
| Psmd7 | 0.18963437 |
| Psmd7 | 1.02696013 |
| Psmd8 | -0.8995958 |
| Psmd9 | 0.87298889 |
| Psmd9 | 0.72456615 |
| Psme1 | -0.2282689 |
| Psme1 | 0.23295937 |
| Psme2 | -0.9324704 |
| Psme3 | 0.02967815 |
| Psme4 | -0.9378818 |
| Psme4 | -0.8933632 |
| Psmf1 | -0.1741016 |
| Psmf1 | -0.6654564 |
| Psmg2 | 1.44207163 |
| Psmg2 | 1.73266838 |
| Psmg3 | 2.42599361 |
| Psors1c2 | 1.41871855 |
| Psp | 0.67642176 |
| Pspc1 | 1.5400361 |
| Pstpip1 | 0.59989618 |
| Pstpip2 | 1.31398416 |
| Ptbp1 | 0.05007861 |
| Ptbp1 | -0.1862533 |
| Ptcd3 | 2.30930295 |
| Ptch1 | 2.01992854 |
| Ptch2 | -0.7657359 |
| Ptdss2 | 0.5861923 |
| Pten | 3.02728698 |
| Pter | -0.6960314 |
| Ptgds | -0.3285543 |
| Ptgds2 | 3.13448612 |
| Ptgds2 | 0.64513511 |
| Ptger2 | -0.4331273 |
| Ptger2 | -0.7155494 |
| Ptger3 | -0.0389041 |
| Ptges | -0.513349 |
| Ptges | -0.0596346 |
| Ptges2 | -0.14182 |
| Ptges3 | 1.26276658 |
| Ptgfr | 1.38751468 |
| Ptgir | -0.8206588 |
| Ptgis | 0.03123329 |
| Ptgs1 | -0.9335502 |
| Ptgs1 | -0.7250289 |
| Pth2 | 0.07636364 |
| Pth2r | -0.8508514 |
| Pthlh | -0.8912947 |
| Pthr1 | -0.9687728 |
| Pthr1 | -0.3325449 |
| Pthr1 | 1.43867084 |
| Ptk2 | 1.15917083 |
| Ptk2b | 3.02753012 |
| Ptk7 | 0.0116072 |
| Ptms | 0.1633264 |
| Ptn | -0.1726539 |
| Ptp4a2 | 0.58316785 |
| Ptp4a2 | 2.360372 |
| Ptp4a2 | -0.5536415 |
| Ptp4a2 | 1.29362589 |
| Ptp4a3 | -0.1654484 |
| Ptp4a3 | -0.1678441 |
| Ptpdc1 | 0.46654149 |
| Ptpdc1 | 0.25466438 |
| Ptpdc1 | 0.69716496 |
| Ptpla | -0.5175101 |
| Ptpla | 0.334841 |
| Ptplad1 | -0.1469677 |
| Ptplad2 | -0.0397414 |
| Ptplb | -0.6777325 |
| Ptpn1 | -0.4928141 |
| Ptpn11 | 0.42652717 |
| Ptpn11 | 0.06743391 |
| Ptpn11 | 0.56789838 |
| Ptpn12 | -0.4375869 |
| Ptpn12 | -0.4363106 |
| Ptpn12 | 0.26563545 |
| Ptpn12 | -0.5711356 |
| Ptpn12 | -0.431857 |
| Ptpn14 | 1.22780954 |
| Ptpn18 | 2.40744219 |
| Ptpn20 | 2.21014326 |
| Ptpn21 | -0.4504459 |
| Ptpn22 | -0.0088412 |
| Ptpn5 | 1.93048979 |
| Ptpn6 | -0.9444167 |
| Ptpn6 | 0.16366404 |
| Ptpra | 0.17306921 |
| Ptprb | -0.2685 |
| Ptprd | 1.12459048 |
| Ptprd | -0.2983247 |
| Ptpre | 0.64310673 |
| Ptpre | 0.65541253 |
| Ptprf | -0.4962057 |
| Ptprf | -0.5785216 |
| Ptprg | 1.19127979 |
| Ptprj | -0.649333 |
| Ptprk | 1.19150687 |
| Ptprk | -0.0725553 |
| Ptprn | 1.76948652 |
| Ptprn2 | 0.00962194 |
| Ptpro | 1.01676773 |
| Ptpro | -0.6695307 |
| Ptprq | -0.1985635 |
| Ptprr | 1.75254187 |
| Ptprr | 1.92810116 |
| Ptprs | 1.29809795 |
| Ptprs | 0.15858671 |
| Ptprt | -0.7442784 |
| Ptpru | 1.02316784 |
| Ptprv | -0.4674291 |
| Ptrf | -0.7657359 |
| Ptrh1 | 2.88812424 |
| Ptrh1 | -0.7576321 |
| Ptrh2 | 1.78407499 |
| Pts | -0.1061025 |
| Pts | -0.9185445 |
| Pttg1 | -0.780317 |
| Pttg1ip | 2.1242252 |
| Ptx3 | -0.7181253 |
| Ptx3 | -0.1357724 |
| Puf60 | 1.62297404 |
| Puf60 | -0.8923284 |
| Pum1 | -0.2663672 |
| Pum2 | -0.0769018 |
| Pum2 | 0.46678631 |
| Punc | 1.18008513 |
| Purb | -0.8351475 |
| Purg | 0.35434649 |
| Pus1 | -0.7729983 |
| Pus1 | -0.8332027 |
| Pus10 | -0.2803169 |
| Pus10 | 0.33540393 |
| Pus10 | -0.2333978 |
| Pus3 | 0.36449585 |
| Pus7l | -0.4167454 |
| Pvalb | -0.6678989 |
| Pvr | -0.5500374 |
| Pvr | -0.9281636 |
| Pvrl1 | 1.31088827 |
| Pvrl2 | -0.2489539 |
| Pvrl2 | 1.0575472 |
| Pvrl2 | 0.94776235 |
| Pvrl3 | 0.41651084 |
| Pvrl3 | 2.41277549 |
| Pvrl3 | -0.0622046 |
| Pwp1 | 2.74488505 |
| Pwp2 | -0.3394254 |
| Pwwp2b | -0.3842076 |
| Pxdn | 2.08031151 |
| Pxmp2 | 0.87362522 |
| Pxmp4 | -0.260527 |
| Pxn | -0.0380676 |
| Pxt1 | 0.43263861 |
| Pycr2 | 2.03527453 |
| Pycrl | 2.47702995 |
| Pygb | -0.6793814 |
| Pygl | 0.00244021 |
| Pygl | 0.25313048 |
| Pygm | -0.4510935 |
| Pygo1 | -0.3704293 |
| Pygo2 | 1.18478478 |
| Pyroxd1 | -0.5748214 |
| Pyy | -0.4982465 |
| Qars | 0.34492276 |
| Qdpr | 0.45743792 |
| Qpct | 0.76681674 |
| Qprt | 0.39483322 |
| Qrich2 | 0.49500111 |
| Qrich2 | 1.83918712 |
| Qrsl1 | 1.60907665 |
| Qsox1 | -0.5704001 |
| Qtrt1 | 0.49333657 |
| R3hdm1 | 3.6399066 |
| R3hdm1 | -0.2961235 |
| R3hdm2 | 0.03123329 |
| R74862 | -0.6101907 |
| Rab1 | 0.17473888 |
| Rab10 | 1.31278695 |
| Rab10 | -0.9937747 |
| Rab11a | 0.14353357 |
| Rab11b | 0.92333974 |
| Rab11fip2 | 1.21072318 |
| Rab11fip3 | -0.6926778 |
| Rab11fip5 | 1.25058247 |
| Rab11fip5 | 2.84901418 |
| Rab11fip5 | 1.07761458 |
| Rab12 | 0.84160005 |
| Rab13 | 2.52516987 |
| Rab13 | -0.1990593 |
| Rab14 | -0.73373 |
| Rab15 | 0.34770423 |
| Rab17 | 1.49792035 |
| Rab17 | -0.6868376 |
| Rab18 | -0.4375869 |
| Rab19 | -0.6086583 |
| Rab19 | 0.95843326 |
| Rab1b | -0.8370962 |
| Rab1b | -0.6461412 |
| Rab1b | 0.01279635 |
| Rab21 | 1.8200842 |
| Rab22a | 2.89441624 |
| Rab22a | -0.4582464 |
| Rab23 | 1.33303329 |
| Rab24 | -0.6501327 |
| Rab24 | 0.1840348 |
| Rab24 | -0.0129032 |
| Rab25 | 1.40288278 |
| Rab27a | 3.05665402 |
| Rab27b | 2.14927078 |
| Rab28 | 0.24666071 |
| Rab28 | 1.14066224 |
| Rab2a | 1.82282038 |
| Rab31 | 0.85353423 |
| Rab32 | -0.3817969 |
| Rab33a | 0.47894734 |
| Rab33b | -0.5608913 |
| Rab34 | -0.7914027 |
| Rab34 | -0.4549883 |
| Rab34 | 0.08740822 |
| Rab35 | -0.1265434 |
| Rab36 | 0.13033695 |
| Rab38 | -0.7112719 |
| Rab38 | -0.6094242 |
| Rab39 | -0.5587105 |
| Rab39b | -0.5428702 |
| Rab3a | -0.6752645 |
| Rab3b | -0.2647709 |
| Rab3d | -0.1441564 |
| Rab3gap1 | -0.7146924 |
| Rab3gap2 | -0.6843456 |
| Rab3il1 | -0.331403 |
| Rab3il1 | -0.3740043 |
| Rab3ip | 0.51031881 |
| Rab40b | -0.612494 |
| Rab40c | -0.0825824 |
| Rab43 | -0.4055685 |
| Rab4a | -0.5500374 |
| Rab4b | 0.12823629 |
| Rab5a | 1.35543392 |
| Rab5a | -0.3574358 |
| Rab5b | -0.612494 |
| Rab5c | -0.7061643 |
| Rab6 | -0.3245806 |
| Rab6 | 0.04626309 |
| Rab6 | -0.3734075 |
| Rab6b | -0.6389987 |
| Rab6ip1 | 1.64034489 |
| Rab7 | 0.28690582 |
| Rab7 | 3.0381667 |
| Rab7 | -0.6719838 |
| Rab7l1 | -0.7302399 |
| Rab8a | -0.6002742 |
| Rab8a | -0.7684528 |
| Rab8b | -0.0574982 |
| Rab8b | 0.35737529 |
| Rab9 | -0.5912122 |
| Rab9 | 0.37696357 |
| Rab9b | 0.61214565 |
| Rabac1 | -0.8158783 |
| Rabac1 | -0.4356731 |
| Rabep1 | -0.6670841 |
| Rabep1 | -0.4485059 |
| Rabep2 | -0.0865353 |
| Rabgap1 | 1.8263598 |
| Rabgap1 | -0.1115102 |
| Rabgap1l | 0.95055135 |
| Rabgap1l | 0.37992072 |
| Rabgef1 | 0.72939639 |
| Rabggta | 0.59178353 |
| Rabggtb | -0.6017931 |
| Rabif | 2.30149638 |
| Rabif | -0.198068 |
| Rabl2a | 2.54934487 |
| Rabl2a | 0.17273492 |
| Rabl3 | -0.490785 |
| Rabl4 | -0.2473867 |
| Rabl4 | 0.87330711 |
| Rabl5 | 0.45198304 |
| Rac1 | 2.22850799 |
| Rac2 | -0.4968855 |
| Rac3 | 0.21810061 |
| Rac3 | 0.21554798 |
| Rad1 | -0.3010834 |
| Rad17 | 1.18364068 |
| Rad18 | 0.72176817 |
| Rad23a | -0.4375869 |
| Rad23a | -0.6445493 |
| Rad23a | -0.5265895 |
| Rad23b | 0.90429078 |
| Rad50 | 1.93023045 |
| Rad51 | -0.562348 |
| Rad51 | -0.5244865 |
| Rad51ap1 | 0.88783024 |
| Rad51c | 1.03167916 |
| Rad51l3 | 0.32975941 |
| Rad51l3 | 1.52922594 |
| Rad51l3 | -0.5889603 |
| Rad51l3 | 1.96586156 |
| Rad52 | -0.5301049 |
| Rad54l | 0.55572508 |
| Rad9 | 0.86387427 |
| Rad9 | 0.55125919 |
| Rad9 | -0.0330639 |
| Rad9b | -0.6719838 |
| Rae1 | -0.2851941 |
| Raet1b | -0.4407853 |
| Raet1b | 0.03588341 |
| Raf1 | -0.1970777 |
| Rag1 | 0.29015873 |
| Rag1ap1 | -0.746048 |
| Rage | -0.2313429 |
| Rage | -0.273853 |
| Rai1 | -0.2328837 |
| Rai12 | 0.72698436 |
| Rai12 | 0.27107349 |
| Rai12 | -0.2282689 |
| Rai12 | 2.84357369 |
| Rai14 | 0.68091923 |
| Rala | 2.55556707 |
| Ralb | -0.7112719 |
| Ralbp1 | -0.5792634 |
| Ralgds | 0.48952086 |
| Ralgps1 | 2.05443081 |
| Ralgps1 | 0.50210174 |
| Ralgps1 | -0.3627297 |
| Ralgps2 | 0.29369461 |
| Ralgps2 | -0.7198465 |
| Raly | 1.10176961 |
| Raly | -0.6769091 |
| Ralyl | -0.3951328 |
| Ramp1 | -0.5696653 |
| Ramp2 | -0.2442601 |
| Ramp3 | 2.27466171 |
| Ramp3 | 1.45623971 |
| Ran | -0.5652681 |
| Ranbp1 | 1.10501041 |
| Ranbp10 | -0.7433949 |
| Ranbp17 | -0.2781573 |
| Ranbp2 | -0.6287764 |
| Ranbp3l | 0.28927268 |
| Ranbp6 | 2.80411309 |
| Ranbp9 | 1.87780681 |
| Rangap1 | -0.4847238 |
| Rangrf | 1.37582202 |
| Rap1b | -0.4793685 |
| Rap1gap | 2.47181513 |
| Rap2a | 1.4163064 |
| Rap2b | -0.6835165 |
| Rap2c | -0.1192246 |
| Rap2c | -0.7495972 |
| Rap2c | 0.25036316 |
| Rap2ip | 1.81017948 |
| Rapgef1 | -0.4621709 |
| Rapgef1 | -0.6453449 |
| Rapgef1 | 0.38740644 |
| Rapgef1 | -0.3291234 |
| Rapgef3 | -0.3274173 |
| Rapgef4 | -0.4205007 |
| Rapgef5 | 2.6133956 |
| Rapgef6 | -0.7198465 |
| Rapgefl1 | 0.84357223 |
| Rapgefl1 | -0.4312225 |
| Rapsn | -0.6926778 |
| Rapsn | -0.1092532 |
| Rara | 0.80246603 |
| Rarb | -0.6605904 |
| Rarb | 1.25748847 |
| Rarg | -0.4261618 |
| Rarg | -0.0502706 |
| Rarg | -0.6461412 |
| Rarres2 | 1.52906721 |
| Rars | -0.7129806 |
| Rars | -0.514734 |
| Rars2 | 0.08923647 |
| Rars2 | 1.71952887 |
| Rars2 | -0.1765195 |
| Rasa1 | -0.6868376 |
| Rasa2 | 1.87599534 |
| Rasal1 | -0.4331273 |
| Rasd1 | -0.294476 |
| Rasef | -0.6272135 |
| Rasgef1b | 0.34492276 |
| Rasgef1b | 0.05083987 |
| Rasgef1c | 0.24076863 |
| Rasgef1c | -0.1578227 |
| Rasgrf1 | 0.18535534 |
| Rasgrf1 | 0.68247855 |
| Rasgrf1 | -0.8007414 |
| Rasgrf1 | -0.267433 |
| Rasgrp1 | 0.84258666 |
| Rasgrp1 | -0.1640139 |
| Rasgrp1 | -0.2939275 |
| Rasgrp3 | -0.1493169 |
| Rasip1 | 1.08437954 |
| Rasl10a | 0.54969111 |
| Rasl10b | 1.74625806 |
| Rasl10b | 0.74901836 |
| Rasl11a | 1.34401041 |
| Rasl11b | 1.95763362 |
| Rasl11b | 1.66801025 |
| Rasl12 | 2.38243588 |
| Rasl2-9 | -0.4293216 |
| Rassf1 | 1.67299764 |
| Rassf1 | -0.6876697 |
| Rassf10 | 0.53318108 |
| Rassf2 | -0.4549883 |
| Rassf2 | 0.20849242 |
| Rassf3 | 0.61277494 |
| Rassf4 | 0.44749637 |
| Rassf4 | 0.07488121 |
| Rassf4 | -0.3498411 |
| Rassf4 | -0.5329268 |
| Rassf5 | -0.63191 |
| Rassf7 | -0.4031026 |
| Rassf7 | -0.5565347 |
| Raver1 | 0.27498156 |
| Raver2 | -0.0056042 |
| Rb1 | 0.39799831 |
| Rb1 | 0.41986145 |
| Rbak | -0.5740831 |
| Rbak | -0.4293216 |
| Rbbp5 | -0.4198738 |
| Rbbp5 | -0.1640139 |
| Rbbp6 | -0.6264331 |
| Rbbp7 | -0.4880863 |
| Rbbp7 | 0.40455833 |
| Rbbp9 | -0.1376291 |
| Rbck1 | -0.0447808 |
| Rbed1 | -0.5927165 |
| Rbed1 | -0.2364886 |
| Rbj | -0.1011725 |
| Rbks | -0.1721718 |
| Rbl1 | 2.62722233 |
| Rbl2 | 0.3294763 |
| Rbm10 | 1.75554329 |
| Rbm10 | 0.53909456 |
| Rbm10 | 2.33342552 |
| Rbm11 | -0.7923324 |
| Rbm12 | 2.19971467 |
| Rbm12 | 2.03043571 |
| Rbm12 | 1.69628429 |
| Rbm12 | -0.3336881 |
| Rbm13 | -0.4478601 |
| Rbm13 | 0.96017588 |
| Rbm14 | 0.14937807 |
| Rbm14 | 0.5329529 |
| Rbm15 | 0.31521112 |
| Rbm16 | 0.5909255 |
| Rbm18 | -0.5450146 |
| Rbm18 | 0.136611 |
| Rbm19 | -0.3060694 |
| Rbm24 | 2.28222841 |
| Rbm26 | -0.4465698 |
| Rbm28 | 1.77415005 |
| Rbm28 | 1.37001483 |
| Rbm3 | -0.0764662 |
| Rbm35a | -0.5279941 |
| Rbm38 | -0.3533388 |
| Rbm38 | 0.21937434 |
| Rbm38 | -0.2765409 |
| Rbm4 | 0.97343011 |
| Rbm41 | 0.7441054 |
| Rbm42 | -0.4800362 |
| Rbm42 | 0.26290462 |
| Rbm43 | -0.5558106 |
| Rbm45 | -0.5601638 |
| Rbm47 | 0.53954791 |
| Rbm47 | -0.19165 |
| Rbm47 | 0.40141521 |
| Rbm47 | 1.75015895 |
| Rbm4b | -0.8073339 |
| Rbm5 | -0.6719838 |
| Rbm5 | 2.21483521 |
| Rbm6 | 0.18370437 |
| Rbm6 | 2.63221385 |
| Rbm6 | 1.93297584 |
| Rbm8a | -0.6810333 |
| Rbm9 | -0.1507292 |
| Rbm9 | 1.74638415 |
| Rbms1 | 0.45098784 |
| Rbms1 | -0.6581668 |
| Rbms2 | -0.3099658 |
| Rbms2 | -0.6910055 |
| Rbms3 | -0.7328563 |
| Rbmx | 1.48628141 |
| Rbmy1a1 | 1.86003676 |
| Rbp1 | 0.37696357 |
| Rbp7 | 2.72503826 |
| Rbpms | 1.87720337 |
| Rbpms | 0.7623538 |
| Rbpms | -0.5616193 |
| Rbpms2 | 2.15136581 |
| Rbx1 | 0.18733269 |
| Rbx1 | -0.5957324 |
| Rc3h1 | 1.57416698 |
| Rcan1 | -0.1616279 |
| Rcan2 | 0.51148685 |
| Rcan2 | 1.40106632 |
| Rcan2 | -0.3291234 |
| Rcan3 | -0.1688041 |
| Rcbtb2 | -0.8618016 |
| Rcbtb2 | -0.554364 |
| Rcc1 | -0.3686469 |
| Rccd1 | 0.94746829 |
| Rce1 | -0.1931272 |
| Rchy1 | -0.0104639 |
| Rcl1 | 2.61055028 |
| Rcl1 | -0.6453449 |
| Rcl1 | -0.0540901 |
| Rcn3 | 0.1015755 |
| Rcor1 | 0.96524023 |
| Rcor2 | 0.26835839 |
| Rcsd1 | 0.46433518 |
| Rdbp | -0.1124145 |
| Rdbp | 0.91180389 |
| Rdh11 | -0.3627297 |
| Rdh12 | -0.7036209 |
| Rdh14 | -0.6711654 |
| Rdh14 | -0.3746015 |
| Rdh5 | 0.0048402 |
| Rdh7 | 1.27770096 |
| Rdh9 | 1.69121835 |
| Rdm1 | -0.1297633 |
| Rec8 | 1.88778954 |
| Reck | 0.46947522 |
| Recql | 0.76983952 |
| Recql4 | -0.1813738 |
| Recql5 | -0.5704001 |
| Reep1 | 1.1660773 |
| Reep2 | -0.6148029 |
| Reep3 | 3.01093464 |
| Reep4 | -0.1192246 |
| Reep5 | 0.59776803 |
| Reep6 | 0.94805631 |
| Reep6 | 1.75448138 |
| Refbp2 | -0.7302399 |
| Reg1 | 0.00043554 |
| Rela | -0.7189855 |
| Relb | 0.06593737 |
| Rell1 | 1.89487132 |
| Rell2 | 1.81657963 |
| Rell2 | -0.7839978 |
| Reln | 0.22064635 |
| Relt | 0.38660723 |
| Relt | 1.00743044 |
| Rem1 | -0.225205 |
| Rem2 | 2.06985641 |
| Renbp | -0.0682287 |
| Repin1 | -0.6968717 |
| Repin1 | -0.3336881 |
| Reps1 | -0.7095663 |
| Reps1 | 0.48208369 |
| Rer1 | 0.13278182 |
| Rer1 | 0.9699904 |
| Rerg | 0.04319952 |
| Resp18 | 2.72197335 |
| Rest | -0.514734 |
| Ret | 1.8649973 |
| Retnla | -0.6063643 |
| Retnlg | -0.7932631 |
| Retsat | -0.7442784 |
| Retsat | -0.6202125 |
| Rev1 | 1.62397943 |
| Rev3l | 0.87964901 |
| Rex3 | 2.30299998 |
| Rexo1 | -0.4602066 |
| Rexo1 | -0.4928141 |
| Rexo2 | 0.89298561 |
| Rfc1 | 2.86243142 |
| Rfc2 | 0.74063272 |
| Rfc3 | -0.3486781 |
| Rfc4 | -0.6926778 |
| Rfc4 | -0.6943531 |
| Rfc5 | 0.8478312 |
| Rfesd | 1.40885239 |
| Rffl | 1.31108831 |
| Rffl | -0.701929 |
| Rffl | 1.443724 |
| Rffl | -0.4569418 |
| Rffl | -0.5536415 |
| Rfng | 3.61522635 |
| Rfpl4 | 0.73198708 |
| Rfpl4 | -0.2803169 |
| Rft1 | -0.4192473 |
| Rftn1 | 0.07710398 |
| Rftn2 | 1.70922489 |
| Rftn2 | 1.18146306 |
| Rfwd2 | -0.63191 |
| Rfwd3 | 0.47943053 |
| Rfwd3 | 1.6816795 |
| Rfx1 | 1.93209611 |
| Rfx1 | 1.0575472 |
| Rfx2 | 1.12884652 |
| Rfx3 | -0.7867678 |
| Rfx4 | -0.7044679 |
| Rfx4 | -0.5718716 |
| Rfx4 | 0.82617419 |
| Rfxank | 2.32441755 |
| Rfxap | 2.90486588 |
| Rfxdc1 | 1.04237765 |
| Rg9mtd1 | -0.2442601 |
| Rg9mtd2 | 1.30516918 |
| Rgag4 | 1.17096313 |
| Rgl1 | 0.9988357 |
| Rgl2 | -0.0830208 |
| Rgl2 | -0.778482 |
| Rgl3 | 0.97956173 |
| Rgma | 1.39036936 |
| Rgmb | 1.65719374 |
| Rgmb | 2.50640486 |
| Rgnef | 0.90197884 |
| Rgs1 | -0.4793685 |
| Rgs1 | 2.19235723 |
| Rgs1 | -0.8779595 |
| Rgs10 | -0.0690924 |
| Rgs12 | 3.34096373 |
| Rgs14 | 2.93986951 |
| Rgs16 | -0.5718716 |
| Rgs17 | 1.32990137 |
| Rgs2 | 2.07534311 |
| Rgs3 | 0.42217415 |
| Rgs3 | 2.21318415 |
| Rgs3 | 0.25036316 |
| Rgs3 | 1.71894494 |
| Rgs3 | -0.7666407 |
| Rgs3 | 0.45248024 |
| Rgs3 | 0.06256144 |
| Rgs4 | 3.42224363 |
| Rgs5 | 0.31262031 |
| Rgs6 | 1.36992088 |
| Rgs7 | 1.57242502 |
| Rgs7bp | -0.8206588 |
| Rgs8 | 0.55237764 |
| Rgs9 | -0.8728804 |
| Rgs9bp | -0.9100758 |
| Rhag | 3.52218113 |
| Rhbdd1 | 1.4213915 |
| Rhbdd3 | -0.7138361 |
| Rhbdf1 | -0.5286972 |
| Rhbdf2 | 0.29955846 |
| Rhbdl1 | -0.2653027 |
| Rhbdl2 | 0.9626391 |
| Rhbdl3 | 0.10840117 |
| Rhbdl7 | 0.80930082 |
| Rhcg | 1.90034879 |
| Rheb | 0.28304694 |
| Rhebl1 | -0.8254637 |
| Rhob | -0.4439945 |
| Rhobtb2 | 0.06406334 |
| Rhobtb2 | 1.04556368 |
| Rhobtb2 | -0.5092061 |
| Rhobtb2 | 2.07862873 |
| Rhobtb3 | 0.65340615 |
| Rhoc | 0.08996678 |
| Rhoc | 0.21810061 |
| Rhod | 2.83315547 |
| Rhof | 0.10337652 |
| Rhog | 0.97257136 |
| Rhoj | -0.8912947 |
| Rhot1 | 0.8957858 |
| Rhot2 | 2.07995749 |
| Rhot2 | 0.00284063 |
| Rhot2 | -0.1020669 |
| Rhou | 0.42678261 |
| Rhov | -0.2236767 |
| Rhox11 | -0.7087146 |
| Rhox12 | 1.05321911 |
| Rhox3a | -0.6217638 |
| Rhox3f | 0.17673859 |
| Rhox4d | -0.3066251 |
| Rhox5 | -0.9281636 |
| Rhox8 | 0.2854235 |
| Rhpn1 | -0.2241858 |
| Rhpn1 | -0.2669 |
| Rhpn1 | 0.25619579 |
| Ribc1 | -0.5322205 |
| Ric3 | 0.50563211 |
| Ric3 | -0.3609618 |
| Ric3 | 1.02520132 |
| Ric8b | -0.8139728 |
| Ric8b | 1.02533673 |
| Rif1 | 0.43542651 |
| Rilp | -0.7442784 |
| Rilpl1 | -0.6086583 |
| Rilpl1 | -0.5565347 |
| Rilpl2 | 1.70580893 |
| Rims1 | -0.2241858 |
| Rims2 | -0.3945225 |
| Rims3 | 2.6089097 |
| Rin1 | -0.6968717 |
| Rin2 | -0.5514774 |
| Rin2 | -0.6240955 |
| Rin2 | 1.22693519 |
| Rin3 | -0.5016577 |
| Riok1 | 0.43136866 |
| Riok1 | 2.16675032 |
| Riok3 | -0.7070137 |
| Riok3 | 0.46040007 |
| Ripk1 | 0.16602414 |
| Ripk2 | -0.0878566 |
| Ripk4 | 3.41528574 |
| Ripk5 | 1.32331004 |
| Rit1 | -0.0243708 |
| Rit2 | -0.6272135 |
| Rlbp1 | -0.6048381 |
| Rlbp1l1 | 0.86387427 |
| Rlbp1l1 | -0.2427006 |
| Rln1 | -0.8216178 |
| Rltpr | -0.2463433 |
| Rmnd1 | -0.7328563 |
| Rmnd5b | -0.4186212 |
| Rmnd5b | 1.2986048 |
| Rnase1 | 1.06523617 |
| Rnase4 | -0.4293216 |
| Rnase4 | 1.14042258 |
| Rnase6 | -0.814925 |
| Rnaseh1 | 0.38607405 |
| Rnaseh1 | -0.4948476 |
| Rnaseh2c | 0.47288643 |
| Rnasek | -0.6613997 |
| Rnasen | 1.09713843 |
| Rnaset2 | -0.8459158 |
| Rnaset2b | 0.38045739 |
| Rnd2 | -0.4086599 |
| Rnd3 | 1.63893302 |
| Rnf10 | -0.1187691 |
| Rnf103 | -0.6695307 |
| Rnf11 | -0.7886191 |
| Rnf11 | -0.1446244 |
| Rnf11 | -0.6163453 |
| Rnf111 | 0.78463506 |
| Rnf113a1 | 1.65816332 |
| Rnf113a2 | 1.0418456 |
| Rnf12 | 1.06315721 |
| Rnf121 | -0.7164073 |
| Rnf123 | -0.8035611 |
| Rnf125 | -0.6303419 |
| Rnf125 | -0.5126572 |
| Rnf128 | -0.9292385 |
| Rnf133 | 0.26533241 |
| Rnf135 | 0.69601378 |
| Rnf135 | 0.11090349 |
| Rnf135 | -0.1074515 |
| Rnf138 | 2.28586459 |
| Rnf138 | 0.11446676 |
| Rnf138 | 1.33088121 |
| Rnf138 | 0.9180447 |
| Rnf139 | 0.28334434 |
| Rnf139 | -0.7433949 |
| Rnf14 | -0.4337631 |
| Rnf14 | -0.5336335 |
| Rnf14 | -0.3908691 |
| Rnf141 | 3.36912468 |
| Rnf141 | 2.35493152 |
| Rnf144a | -0.7146924 |
| Rnf145 | -0.1659271 |
| Rnf150 | 0.04243207 |
| Rnf166 | -0.1891931 |
| Rnf167 | -0.112867 |
| Rnf167 | -0.3515883 |
| Rnf167 | -0.1142258 |
| Rnf167 | -0.7138361 |
| Rnf167 | -0.5601638 |
| Rnf168 | -0.7711775 |
| Rnf17 | 0.79038468 |
| Rnf170 | -0.7027746 |
| Rnf181 | 2.66555401 |
| Rnf182 | -0.8054456 |
| Rnf182 | 1.43517006 |
| Rnf183 | -0.6413735 |
| Rnf185 | -0.787693 |
| Rnf185 | 1.20860727 |
| Rnf185 | 1.50291147 |
| Rnf185 | -0.7224341 |
| Rnf186 | -0.307181 |
| Rnf19a | -0.6541412 |
| Rnf2 | 0.84635912 |
| Rnf20 | 0.73457068 |
| Rnf207 | 1.52771689 |
| Rnf208 | 3.89272442 |
| Rnf214 | 0.54294111 |
| Rnf215 | -0.4382257 |
| Rnf216 | 2.53781163 |
| Rnf216 | -0.3440404 |
| Rnf219 | 0.52768939 |
| Rnf24 | -0.7293694 |
| Rnf24 | 0.7389833 |
| Rnf25 | -0.7757362 |
| Rnf25 | -0.17169 |
| Rnf25 | -0.1578227 |
| Rnf26 | -0.8130215 |
| Rnf31 | 1.13970322 |
| Rnf32 | 1.02099395 |
| Rnf34 | 0.30189382 |
| Rnf34 | -0.0936037 |
| Rnf34 | 1.165844 |
| Rnf38 | 0.61256522 |
| Rnf38 | 0.76145867 |
| Rnf38 | 3.91171489 |
| Rnf4 | -0.73373 |
| Rnf4 | -0.73373 |
| Rnf40 | -0.5681973 |
| Rnf41 | -0.5371754 |
| Rnf41 | -0.6148029 |
| Rnf41 | -0.536466 |
| Rnf44 | -0.4249008 |
| Rnf5 | -0.6225404 |
| Rnf6 | -0.4860674 |
| Rnf6 | -0.7451628 |
| Rnf8 | -0.2679663 |
| Rnf8 | -0.7495972 |
| Rngtt | -0.4261618 |
| Rnmt | 2.3277248 |
| Rnmtl1 | 1.78158835 |
| Rnpc2 | -0.4049514 |
| Rnpep | -0.7078638 |
| Rnpepl1 | 1.00355864 |
| Rnps1 | 0.36422297 |
| Rnps1 | 0.65841411 |
| Rnu6 | -0.5987578 |
| Rnuxa | -0.2370047 |
| Rnuxa | -0.7241631 |
| Robo1 | -0.511966 |
| Robo2 | -0.6985544 |
| Robo4 | -0.2328837 |
| Rock1 | -0.3178071 |
| Rock2 | 0.91834807 |
| Rock2 | -0.2797766 |
| Rod1 | -0.0931604 |
| Rogdi | -0.4049514 |
| Rom1 | -0.6935151 |
| Ropn1 | -0.3504232 |
| Ror1 | -0.6155738 |
| Ror2 | 1.5853746 |
| Rora | 1.57862084 |
| Rorb | -0.8235388 |
| Rorb | -0.3746015 |
| Rorc | -0.6264331 |
| Rorc | 2.75789515 |
| Ros1 | -0.1951004 |
| Rp23-297j14.5 | 2.69936377 |
| Rp23-438h3.2 | 1.0027269 |
| rp9 | -0.3480972 |
| rp9 | 1.23921077 |
| Rpa1 | -0.73373 |
| Rpa2 | -0.7657359 |
| Rpa2 | -0.7241631 |
| Rpa3 | 0.73438637 |
| Rpain | 1.69228713 |
| Rpap1 | -0.5733453 |
| Rpap1 | 0.39773496 |
| Rpap2 | -0.649333 |
| Rpap2 | 0.57687189 |
| Rpap3 | 1.12921043 |
| Rpe | 0.40977369 |
| Rpgr | -0.2505237 |
| Rpgrip1 | 1.54566808 |
| Rpgrip1l | 3.02446197 |
| Rph3a | 0.39192252 |
| Rph3al | 1.59430504 |
| Rpia | 0.33230363 |
| Rpl12 | 0.2954576 |
| Rpl13a | -0.4549883 |
| Rpl18 | 0.75264117 |
| Rpl19 | -0.4901096 |
| Rpl21 | -0.8130215 |
| Rpl21 | 1.48445239 |
| Rpl22 | 0.73641178 |
| Rpl22l1 | -0.6977127 |
| Rpl23 | 1.05610671 |
| Rpl23 | 0.43035146 |
| Rpl23 | 0.32208533 |
| Rpl24 | 0.2788735 |
| Rpl27 | -0.4439945 |
| Rpl27a | -0.4989278 |
| Rpl28 | -0.6163453 |
| Rpl29 | -0.4337631 |
| Rpl29 | 0.19454771 |
| Rpl3 | -0.4043348 |
| Rpl30 | 1.05623776 |
| Rpl31 | -0.6793814 |
| Rpl31 | -0.4934915 |
| Rpl34 | 2.87224636 |
| Rpl35 | -0.1469677 |
| Rpl36a | 0.68091923 |
| Rpl36al | -0.6421665 |
| Rpl37 | -0.7363561 |
| Rpl38 | 0.19976055 |
| Rpl38 | -0.3793923 |
| Rpl39 | 0.83383572 |
| Rpl39l | -0.7258954 |
| Rpl3l | 1.99783237 |
| Rpl4 | -0.7036209 |
| Rpl41 | 0.58963698 |
| Rpl7a | -0.2359727 |
| Rpl7l1 | 0.65781456 |
| Rpl7l1 | 0.706514 |
| Rpl9 | -0.585966 |
| Rplp1 | -0.7748227 |
| Rpn2 | 0.66279922 |
| Rpo1-1 | 0.03239797 |
| Rpo1-3 | 0.75046916 |
| Rpo1-3 | 1.09008552 |
| Rpo1-4 | 3.12292659 |
| Rpo1-4 | 0.84192903 |
| Rpo1-4 | -0.7675463 |
| Rpo2tc1 | 2.15945565 |
| Rpp21 | 0.24201213 |
| Rpp25 | -0.0092466 |
| Rpp30 | 0.54045396 |
| Rpp38 | 2.43213783 |
| Rpp40 | 2.05729139 |
| Rprm | -0.0149408 |
| Rprml | -0.413005 |
| Rps10 | 0.01279635 |
| Rps11 | 1.82142418 |
| Rps12 | 0.05160051 |
| Rps13 | 0.21682516 |
| Rps14 | 0.92920512 |
| Rps15 | -0.1582975 |
| Rps15a | -0.6517341 |
| Rps16 | -0.6876697 |
| Rps19 | -0.1549789 |
| Rps19 | 1.43683455 |
| Rps19bp1 | -0.8130215 |
| Rps2 | 2.01789808 |
| Rps21 | 0.33737156 |
| Rps24 | 4.15531711 |
| Rps24 | 0.71483081 |
| Rps25 | -0.8769415 |
| Rps26 | -0.6960314 |
| Rps27a | -0.2196134 |
| Rps27a | 0.15211594 |
| Rps27l | -0.1297633 |
| Rps3 | 0.45694332 |
| Rps3 | 1.9476033 |
| Rps3a | -0.6194378 |
| Rps4x | -0.3650922 |
| Rps4y2 | 1.60615541 |
| Rps5 | -0.5189013 |
| Rps6 | -0.0579251 |
| Rps6ka1 | 2.42329095 |
| Rps6ka3 | 3.62591715 |
| Rps6ka4 | -0.2615862 |
| Rps6ka5 | -0.3568494 |
| Rps6kb1 | 1.98156493 |
| Rps6kl1 | 0.18072533 |
| Rps7 | 1.42352443 |
| Rps8 | -0.4375869 |
| Rps9 | -0.3627297 |
| Rps9 | -0.1043068 |
| Rpsa | 0.11801662 |
| Rpusd1 | 0.96610568 |
| Rpusd4 | 0.2174631 |
| Rqcd1 | -0.8508514 |
| Rrad | 3.12583708 |
| Rragb | 0.98169132 |
| Rragc | -0.8538252 |
| Rragd | -0.5216897 |
| Rras | -0.5244865 |
| Rras2 | -0.7711775 |
| Rrbp1 | 0.35765015 |
| Rrbp1 | 1.56603561 |
| Rreb1 | -0.260527 |
| Rreb1 | -0.273853 |
| Rrh | -0.4280564 |
| Rrm1 | -0.4465698 |
| Rrm2 | 1.80864641 |
| Rrm2 | -0.6581668 |
| Rrm2b | -0.4807045 |
| Rrn3 | 1.2071568 |
| Rrp1 | -0.4485059 |
| Rrp12 | 0.11090349 |
| Rrp15 | 0.10804315 |
| Rrp1b | -0.5748214 |
| Rrp1b | -0.3475166 |
| Rrp1b | 1.12896784 |
| Rrp9 | -0.3257142 |
| Rs1 | -0.4700693 |
| Rsad1 | -0.6943531 |
| Rsad2 | 1.17859009 |
| Rsf1 | 1.79263695 |
| Rshl1 | -0.634267 |
| Rshl2a | -0.3609618 |
| Rsl1d1 | 0.77054936 |
| Rsl1d1 | 0.04779115 |
| Rsn | 0.69428438 |
| Rsph1 | -0.1394894 |
| Rspo1 | -0.2743899 |
| Rspo2 | 0.00244021 |
| Rspo3 | 1.73266838 |
| Rspo4 | -0.7381108 |
| Rspry1 | -0.4192473 |
| Rsrc1 | 1.21869858 |
| Rsrc1 | 0.2184192 |
| Rsrc2 | 0.44974237 |
| Rsu1 | -0.6581668 |
| Rtbdn | -0.4136274 |
| Rtel1 | -0.1256254 |
| Rtel1 | -0.3716195 |
| Rtkn | 0.6791619 |
| Rtn1 | 0.24107966 |
| Rtn1 | -0.3710242 |
| Rtn1 | 0.71746176 |
| Rtn1 | 2.09619341 |
| Rtn2 | -0.5748214 |
| Rtn3 | -0.0574982 |
| Rtn4 | -0.7328563 |
| Rtn4 | -0.8923284 |
| Rtn4 | -0.2857376 |
| Rtn4ip1 | 0.94348948 |
| Rtn4r | 0.24604222 |
| Rtn4rl1 | -0.5168153 |
| Rtn4rl2 | 0.4784639 |
| Rtp1 | -0.5092061 |
| Rtp4 | -0.5964878 |
| Rttn | -0.5435845 |
| Rufy1 | 0.82834572 |
| Rufy2 | 0.96133585 |
| Rufy3 | 2.19039076 |
| Rufy3 | 0.52539171 |
| Rufy3 | 1.15952323 |
| Rufy4 | -0.5385959 |
| Rundc1 | -0.5681973 |
| Rundc3b | -0.2900967 |
| Runx1 | -0.2685 |
| Runx1t1 | 0.49808461 |
| Runx1t1 | -0.6613997 |
| Runx2 | 2.5734929 |
| Rusc1 | -0.6295588 |
| Rusc2 | -0.3138783 |
| Rusc2 | -0.5616193 |
| Ruvbl1 | 0.0961517 |
| Ruvbl1 | -0.5126572 |
| Ruvbl1 | -0.3586097 |
| Ruvbl2 | 1.5073079 |
| Rwdd1 | -0.658974 |
| Rwdd1 | 2.0978886 |
| Rwdd3 | -0.1985635 |
| Rwdd3 | 0.98069811 |
| Rwdd4a | -0.8130215 |
| Rxfp1 | 0.72195496 |
| Rxfp3 | 0.12683324 |
| Rxra | 0.2000854 |
| Rxrb | -0.1408871 |
| Rxrb | -0.4472147 |
| Rxrg | -0.3434623 |
| Rya3 | -0.5927165 |
| Ryk | 0.84979041 |
| Ryk | -0.6525358 |
| Ryr1 | 0.64127745 |
| Ryr1 | 3.43767698 |
| Ryr1 | 0.96003079 |
| Ryr3 | -0.516121 |
| S100a1 | -0.3088509 |
| S100a10 | -0.8111218 |
| S100a11 | 0.44022265 |
| S100a13 | 0.4861694 |
| S100a16 | -0.2563021 |
| S100a4 | -0.4000293 |
| S100a6 | 0.3844727 |
| S100a8 | -0.3457769 |
| S100a9 | -0.3775929 |
| S100b | -0.7284997 |
| S100pbp | 0.24107966 |
| S100pbp | 0.25405112 |
| S3-12 | 1.67592313 |
| Saa3 | -0.6605904 |
| Sac3d1 | -0.2010449 |
| Sacm1l | -0.0660728 |
| Sacs | -0.6951919 |
| Sae1 | 0.71746176 |
| Sae1 | 0.38072561 |
| Sae1 | -0.2308299 |
| Sag | 0.33173884 |
| Sall1 | -0.3746015 |
| Sall1 | -0.3627297 |
| Sall2 | -0.6885025 |
| Sall4 | -0.4024872 |
| Sall4 | 2.25033512 |
| Samd10 | -0.6818603 |
| Samd11 | 0.23983492 |
| Samd12 | -0.5726082 |
| Samd14 | 1.58999921 |
| Samd4 | -0.4530389 |
| Samd4 | 1.7970682 |
| Samd4b | 0.29074896 |
| Samd5 | -0.4934915 |
| Samd7 | -0.7666407 |
| Samd8 | 0.29516399 |
| Samd9l | -0.6048381 |
| Samhd1 | -0.0506942 |
| Samm50 | 1.25621731 |
| Samsn1 | -0.0860952 |
| Sap130 | -0.7087146 |
| Sap30 | 1.80016965 |
| Sap30bp | 3.20019445 |
| Sap30l | -0.6517341 |
| Saps1 | 0.75101263 |
| Saps2 | 0.60772885 |
| Saps3 | 0.28304694 |
| Saps3 | 1.99362199 |
| Sar1a | -0.7311112 |
| Sar1b | 0.66240142 |
| Sar1b | -0.4031026 |
| Sarm1 | -0.5435845 |
| Sars | -0.5514774 |
| Sars | 2.2262745 |
| Sars2 | -0.7711775 |
| Sart1 | -0.4497988 |
| Sart3 | -0.6581668 |
| Sas | 2.15807142 |
| Sass6 | -0.5934696 |
| Sat2 | -0.7284997 |
| Satb1 | 1.72457444 |
| Sav1 | 2.63646786 |
| Sbds | -0.1731362 |
| Sbf1 | -0.2494769 |
| Sbk | -0.2515717 |
| Sbno1 | 0.24014626 |
| Sbno2 | 0.69332223 |
| Sc4mol | -0.3704293 |
| Sc5d | -0.5407306 |
| Scaf1 | -0.5265895 |
| Scamp1 | 0.78341092 |
| Scamp2 | -0.5536415 |
| Scamp3 | -0.680207 |
| Scamp4 | -0.5182055 |
| Scamp5 | 2.25195934 |
| Scamp5 | 0.27257851 |
| Scand1 | 0.86226609 |
| Scap | 0.7851592 |
| Scaper | -0.1549789 |
| Scara3 | -0.3446189 |
| Scara5 | 1.03503549 |
| Scarb1 | 2.19659086 |
| Scarb2 | 0.507744 |
| Scarf1 | -0.3674605 |
| Scarf2 | 0.73935009 |
| Sccpdh | -0.5867137 |
| Scd1 | 0.10265652 |
| Scd2 | 1.07002628 |
| Scd4 | -0.340001 |
| Scel | -0.747821 |
| Scg2 | 0.22729641 |
| Scg3 | -0.1545058 |
| Scg5 | 1.17432197 |
| Scgn | 1.73432995 |
| Scgn | -0.7960605 |
| Scgn | 2.2505937 |
| Schip1 | 1.95748244 |
| Schip1 | 0.68422971 |
| Scin | -0.0557926 |
| scl0003251.1_21 | 2.06073074 |
| Sclt1 | -0.6654564 |
| Scly | -0.7657359 |
| Scmh1 | -0.4807045 |
| Scmh1 | 0.44022265 |
| Scml2 | 1.24759192 |
| Scml4 | 0.52031703 |
| Scn10a | 2.27836299 |
| Scn11a | -0.6638316 |
| Scn1a | 1.24201277 |
| Scn2b | 1.16805794 |
| Scn3b | -0.585966 |
| Scn3b | 0.73512339 |
| Scn3b | -0.1554523 |
| Scn3b | 2.08199129 |
| Scn4b | -0.8073339 |
| Scn5a | -0.3609618 |
| Scn7a | -0.3291234 |
| Scn8a | 1.53940825 |
| Scnm1 | 0.34853708 |
| Scnm1 | 1.13054357 |
| Scnn1a | -0.7821556 |
| Scnn1b | 1.38317006 |
| Scnn1g | -0.1353088 |
| Scoc | -0.4274244 |
| Scoc | 0.93025404 |
| Scotin | -0.2526209 |
| Scotin | -0.5601638 |
| Scotin | -0.1025145 |
| Scp2 | -0.3217524 |
| Scp2 | 2.02345961 |
| Scp2 | -0.2663672 |
| Scrg1 | -0.4962057 |
| Scrib | 1.902221 |
| Scrib | 0.57883031 |
| Scrn1 | -0.0852158 |
| Scrn1 | -0.0699569 |
| Scrn1 | -0.3359789 |
| Scrn2 | -0.778482 |
| Sct | -0.7010842 |
| Sctr | 3.35687116 |
| Scube1 | 1.20447313 |
| Scube2 | 1.05859346 |
| Scube3 | 0.40508116 |
| Scube3 | -0.201542 |
| Scx | -0.7969948 |
| Scye1 | 0.34073511 |
| Scyl1 | 1.59208034 |
| Scyl1bp1 | 1.48785818 |
| Scyl3 | 4.1445686 |
| Scyl3 | -0.2615862 |
| Sdad1 | -0.4549883 |
| Sdc1 | 1.56044753 |
| Sdc1 | -0.2236767 |
| Sdc2 | 0.50938333 |
| Sdc3 | -0.4068039 |
| Sdc4 | 4.50434142 |
| Sdcbp | 2.81398166 |
| Sdcbp | 3.32286564 |
| Sdcbp2 | 0.16197463 |
| Sdcbp2 | 0.3362477 |
| Sdccag1 | 0.85580582 |
| Sdccag1 | 0.25741914 |
| Sdccag3 | -0.4760368 |
| Sdccag3 | 0.31060031 |
| Sdf2 | -0.585966 |
| Sdf2 | 0.39878792 |
| Sdf4 | -0.607893 |
| Sdhb | 0.56723842 |
| Sdhb | 2.24970682 |
| Sdhb | -0.5762997 |
| Sdhc | -0.4517415 |
| Sdhd | -0.3055141 |
| Sdk1 | 0.85174556 |
| Sdpr | -0.4363106 |
| Sds | -0.4602066 |
| Sdsl | -0.7425121 |
| Sec1 | -0.7675463 |
| Sec11a | -0.5050813 |
| Sec11c | 3.15701913 |
| Sec13 | 1.91906329 |
| Sec14l1 | 1.09462558 |
| Sec14l1 | 0.55125919 |
| Sec14l1 | -0.4647964 |
| Sec14l2 | 2.85669898 |
| Sec14l3 | 0.37318646 |
| Sec14l3 | -0.7549462 |
| Sec14l4 | -0.3105237 |
| Sec15l1 | 0.76735088 |
| Sec16a | 0.32237068 |
| Sec16b | 0.14663223 |
| Sec22a | -0.3574358 |
| Sec22b | -0.7328563 |
| Sec22c | 1.92669632 |
| Sec22c | -0.4198738 |
| Sec23a | -0.0755957 |
| Sec23ip | -0.2216428 |
| Sec24b | 0.39006558 |
| Sec24c | 0.45421839 |
| Sec24d | 2.81755215 |
| Sec61a1 | -0.3746015 |
| Sec61a2 | -0.4820423 |
| Sec61a2 | -0.6389987 |
| Sec61b | 1.56106154 |
| Sec61b | -0.1025145 |
| Sec61g | 0.04740937 |
| Sec63 | 0.26502928 |
| Sec63 | 2.08048847 |
| Secisbp2 | 1.41397774 |
| Sectm1b | -0.8235388 |
| Seh1l | -0.7675463 |
| Seh1l | 0.92529892 |
| Sel1l | 0.02734061 |
| Sel1l | -0.2145587 |
| Sel1l | 0.58877699 |
| Sel1l2 | 1.7318365 |
| Sel1l2 | 1.02614878 |
| Selenbp2 | 0.93384147 |
| Selk | 1.32073975 |
| Sell | 0.9496715 |
| Selp | -0.24374 |
| Selplg | -0.7693601 |
| Sema3a | -0.3591972 |
| Sema3b | 1.33907194 |
| Sema3b | 0.61716823 |
| Sema3b | 1.91348255 |
| Sema3e | -0.658974 |
| Sema3f | 0.01950671 |
| Sema3f | 0.84766775 |
| Sema3g | -0.7495972 |
| Sema4a | 1.16245478 |
| Sema4b | -0.6413735 |
| Sema4d | 1.24919518 |
| Sema4f | 0.04089529 |
| Sema4g | 1.90270183 |
| Sema5a | -0.7594269 |
| Sema5a | -0.6117256 |
| Sema5b | 2.23438815 |
| Sema6b | -0.7416302 |
| Sema6b | 0.94082759 |
| Sema6b | -0.554364 |
| Sema6d | 0.39059651 |
| Sema6d | -0.6785566 |
| Sema7a | -0.7469341 |
| Sema7a | -0.8749087 |
| Senp1 | -0.7198465 |
| Senp2 | 0.57621818 |
| Senp3 | 0.44724648 |
| Senp5 | -0.6960314 |
| Senp5 | 0.17407136 |
| Senp5 | 0.16400156 |
| Senp6 | 0.08484269 |
| Senp6 | -0.6334806 |
| Senp7 | 0.47020724 |
| Senp7 | 0.44749637 |
| Senp7 | 1.96140531 |
| Senp8 | -0.8158783 |
| Sephs1 | 0.20397488 |
| Sephs2 | -0.0393227 |
| Sepm | -0.0557926 |
| Sepn1 | 1.49996909 |
| Sepp1 | 1.1232489 |
| Sepp1 | -0.2835656 |
| Sept3 | 0.02029303 |
| Sept4 | 2.54810627 |
| Sept4 | -0.3027425 |
| Sept6 | -0.4136274 |
| Sept7 | 0.59499424 |
| Sept7 | -0.3365524 |
| Sept8 | 0.17673859 |
| Sept9 | -0.7215708 |
| Sept10 | -0.1274622 |
| Sept11 | 0.80554782 |
| Sept11 | -0.2547227 |
| Sept13 | -0.4111404 |
| Sept16 | -0.1450926 |
| Sepsecs | -0.7648321 |
| Sepw1 | -0.5414433 |
| Sepx1 | -0.2631772 |
| Sepx1 | -0.63191 |
| Serac1 | 0.38099375 |
| Serbp1 | 1.94643122 |
| Serbp1 | -0.8016804 |
| Serbp1 | -0.8410059 |
| Serf1 | -0.5507571 |
| Serf2 | -0.7830763 |
| Sergef | -0.5357571 |
| Serhl | -0.2370047 |
| Serhl | 0.34101487 |
| Serinc1 | -0.7095663 |
| Serinc2 | 2.10997051 |
| Serinc3 | 0.55304808 |
| Serpina1a | 2.52505943 |
| Serpina1a | -0.4720543 |
| Serpina1b | 0.21810061 |
| Serpina1b | -0.6002742 |
| Serpina1d | 0.9428986 |
| Serpina1e | -0.056645 |
| Serpina3b | -0.6002742 |
| Serpina3c | 0.60498424 |
| Serpina3g | -0.0604905 |
| Serpina3h | 1.27125493 |
| Serpina3k | -0.175068 |
| Serpina3n | 1.24834045 |
| Serpina5 | 0.08374104 |
| Serpina5 | 3.12405383 |
| Serpina7 | -0.6793814 |
| Serpina9 | -0.36214 |
| Serpinb11 | -0.2695681 |
| Serpinb12 | 1.21671104 |
| Serpinb1a | 0.56194203 |
| Serpinb3a | -0.2160723 |
| Serpinb6a | -0.4080408 |
| Serpinb6b | 0.70061008 |
| Serpinb6c | 0.33202128 |
| Serpind1 | -0.3417301 |
| Serpine2 | 0.62981485 |
| Serpinf1 | -0.3021891 |
| Serping1 | -0.1946067 |
| Serpinh1 | 0.64877523 |
| Serpini1 | 1.23780664 |
| Sertad1 | -0.1142258 |
| Sertad2 | 0.99604642 |
| Sertad2 | -0.340001 |
| Sertad3 | -0.63191 |
| Sertad4 | 1.49693538 |
| Sesn1 | 0.03549677 |
| Sesn1 | 0.57730744 |
| Sesn2 | -0.6397897 |
| Sesn3 | -0.2201203 |
| Setd1b | -0.73373 |
| Setd2 | -0.5294008 |
| Setd3 | 0.94467014 |
| Setd4 | -0.431857 |
| Setd4 | -0.7002402 |
| Setd5 | 3.49179606 |
| Setd6 | -0.3811952 |
| Setd6 | -0.0161655 |
| Setd7 | -0.1625815 |
| Setd7 | -0.4236415 |
| Setd8 | 1.21527299 |
| Setd8 | -1.1074619 |
| Setd8 | -0.6810333 |
| Setdb1 | 0.52631145 |
| Setdb2 | -0.2090297 |
| Setmar | -0.4395046 |
| Setx | -0.3268492 |
| Sez6 | -0.8975136 |
| Sez6l | -0.632695 |
| Sez6l2 | -0.7010842 |
| Sez6l2 | 0.40089034 |
| Sf1 | 2.62821285 |
| Sf1 | -0.2080279 |
| Sf3a1 | -0.2615862 |
| Sf3a2 | 1.5494818 |
| Sf3a3 | 2.70626981 |
| Sf3b1 | -0.3285543 |
| Sf3b2 | -0.6350539 |
| Sf3b3 | 2.24138882 |
| Sf3b4 | 0.94835019 |
| Sf3b4 | -0.0817062 |
| Sf3b5 | -0.1288422 |
| Sf4 | 1.20009582 |
| Sfi1 | 0.81337804 |
| Sfi1 | -0.5092061 |
| Sfmbt1 | 1.12848247 |
| Sfmbt1 | 1.30436371 |
| Sfmbt1 | -0.4395046 |
| Sfmbt2 | -0.6155738 |
| Sfrp1 | -0.2370047 |
| Sfrp2 | 0.6582143 |
| Sfrp4 | 1.84868027 |
| Sfrp5 | -0.2115389 |
| Sfrs1 | 2.74911835 |
| Sfrs1 | -0.5987578 |
| Sfrs11 | 1.32626708 |
| Sfrs12 | 0.41055352 |
| Sfrs14 | 0.37669427 |
| Sfrs16 | 0.03935601 |
| Sfrs2 | 0.46163155 |
| Sfrs2 | 2.06045967 |
| Sfrs2 | -0.7276308 |
| Sfrs3 | 0.75552943 |
| Sfrs3 | -0.6901704 |
| Sfrs4 | 1.50787631 |
| Sfrs5 | -0.7207082 |
| Sfrs5 | -0.2928314 |
| Sfrs6 | 4.24011429 |
| Sfrs7 | -0.2025369 |
| Sfrs8 | 0.15279917 |
| Sfrs9 | -0.8035611 |
| Sft2d2 | 0.94452264 |
| Sft2d2 | -0.7104187 |
| Sft2d2 | -0.7612252 |
| Sftpc | 0.11482235 |
| Sftpd | -0.4901096 |
| Sfxn1 | 2.31176393 |
| Sfxn1 | -0.1488466 |
| Sfxn3 | -0.7487087 |
| Sfxn3 | -0.5755603 |
| Sfxn4 | -0.6033144 |
| Sfxn4 | 0.47749627 |
| Sfxn5 | 0.41908928 |
| Sgca | 1.24480647 |
| Sgca | 0.52952351 |
| Sgcb | 2.17738806 |
| Sgcb | 0.13834641 |
| Sgcz | -0.7425121 |
| Sgip1 | 1.3520884 |
| Sgip1 | 0.72735584 |
| Sgip1 | -0.2005481 |
| Sgip1 | -0.3060694 |
| Sgip1 | -0.121049 |
| Sgk1 | -0.6533381 |
| Sgk2 | 1.39202297 |
| Sgk2 | -0.7189855 |
| Sgk3 | 1.03583924 |
| Sgk3 | 0.72531087 |
| Sgk3 | -0.8235388 |
| Sgk3 | -0.8245007 |
| Sgk3 | 1.5927483 |
| Sgms2 | -0.6477358 |
| Sgol1 | -0.4395046 |
| Sgpl1 | 1.8010624 |
| Sgpl1 | -0.1413534 |
| Sgpp1 | 2.37954087 |
| Sgpp2 | -0.6194378 |
| Sgsm1 | -0.7748227 |
| Sgsm2 | -0.4230125 |
| Sgsm3 | 1.05950798 |
| Sgta | 0.15040571 |
| Sgtb | -0.1891931 |
| Sgtb | 1.56756083 |
| Sh2b1 | 0.63166648 |
| Sh2b1 | 1.02085792 |
| Sh2b3 | -0.5421565 |
| Sh2d2a | -0.6002742 |
| Sh3bgrl | 2.20678304 |
| Sh3bgrl3 | -0.7044679 |
| Sh3bp2 | 0.60582958 |
| Sh3bp5 | -0.3099658 |
| Sh3bp5l | -0.7504866 |
| Sh3gl1 | 2.0504205 |
| Sh3gl2 | -0.7621256 |
| Sh3gl2 | 0.47215649 |
| Sh3gl2 | -0.5016577 |
| Sh3gl3 | 0.57097211 |
| Sh3glb1 | -0.2479088 |
| Sh3glb2 | 0.01279635 |
| Sh3kbp1 | 1.21327822 |
| Sh3kbp1 | -0.182835 |
| Sh3kbp1 | -0.8063893 |
| Sh3kbp1 | 1.71777601 |
| Sh3md4 | 0.32351118 |
| Sh3md4 | -0.510585 |
| Sh3pxd2a | 0.84028297 |
| Sh3pxd2b | -0.4337631 |
| Sh3rf1 | -0.7684528 |
| Sh3rf2 | -0.2411438 |
| Sh3tc1 | -0.3331163 |
| Sh3tc2 | -0.05324 |
| Sh3yl1 | -0.4286887 |
| Shank2 | -0.7442784 |
| Sharpin | 0.30567651 |
| Shbg | -0.6851756 |
| Shc1 | 1.28307354 |
| Shc1 | 1.58686889 |
| Shc3 | -0.307181 |
| Shcbp1 | 2.0428549 |
| Shd | -0.6303419 |
| She | -0.7224341 |
| Shf | -0.6033144 |
| Shf | 1.92278296 |
| Shf | -0.5630771 |
| Shfm1 | -0.4941693 |
| Shh | 0.51753748 |
| Shisa2 | -0.1901751 |
| Shisa3 | 0.55750554 |
| Shisa4 | -0.2500001 |
| Shkbp1 | 1.72024206 |
| Shkbp1 | -0.8954361 |
| Shmt1 | -0.3510056 |
| Shmt2 | -0.340577 |
| Shoc2 | 2.15161198 |
| Shox2 | 0.03588341 |
| Shprh | 1.20346479 |
| Shq1 | -0.4934915 |
| Shroom3 | -0.2180941 |
| Shroom3 | -0.682688 |
| Si | -0.7070137 |
| Si | 0.55550229 |
| Siah1a | -0.5223881 |
| Siah1b | -0.5154273 |
| Siah1b | -0.6086583 |
| Siah1b | -0.7070137 |
| Siah1b | 1.66210065 |
| Siah2 | -0.3274173 |
| Siat7f | 0.45743792 |
| Siat9 | 0.80537687 |
| Sidt1 | 0.36885116 |
| Sidt2 | -0.4707305 |
| Sidt2 | -0.2770794 |
| Sidt2 | 0.51753748 |
| Sigirr | -0.2663672 |
| Siglec1 | 0.24014626 |
| Siglech | -0.546447 |
| Siglech | -0.048578 |
| Sil1 | 0.14353357 |
| Sin3a | -0.5762997 |
| Sin3b | -0.3692407 |
| Sip1 | 0.90659707 |
| Sip1 | -0.16497 |
| Sipa1 | -0.391477 |
| Sipa1l1 | 0.28066438 |
| Sirpa | -0.4694086 |
| Sirpa | 0.1094744 |
| Sirpb1 | -0.7053157 |
| Sirt1 | -0.3674605 |
| Sirt2 | -0.4255311 |
| Sirt3 | 1.38520621 |
| Sirt5 | -0.4280564 |
| Sirt5 | 1.93163004 |
| Sirt6 | -0.6233176 |
| Sirt7 | -0.421128 |
| Siva1 | 0.50563211 |
| Six1 | 0.59306913 |
| Six2 | 0.26290462 |
| Six3 | 2.13864206 |
| Six5 | -0.555087 |
| Six6os1 | 2.04428095 |
| Skap2 | -0.5755603 |
| Skil | -0.2277576 |
| Skiv2l | 0.15892605 |
| Skiv2l | -0.0967129 |
| Skiv2l2 | 0.07562272 |
| Skp1a | 1.44476611 |
| Skp2 | 0.64046329 |
| Skp2 | 1.971092 |
| Skp2 | 4.43612217 |
| Sla | 0.5487939 |
| Slain1 | -0.5987578 |
| Slain2 | -0.1521437 |
| Slamf7 | 1.46749069 |
| Slamf8 | -0.0161655 |
| Slamf9 | -0.3394254 |
| Slc10a3 | 1.84431506 |
| Slc10a4 | 1.23575068 |
| Slc10a6 | -0.5500374 |
| Slc10a6 | 0.02421485 |
| Slc10a7 | 1.04860689 |
| Slc10a7 | -0.6622096 |
| Slc10a7 | -0.4654539 |
| Slc11a1 | -0.514734 |
| Slc11a1 | 1.66471643 |
| Slc11a1 | 1.01663108 |
| Slc11a1 | 2.02233111 |
| Slc11a2 | 0.38420554 |
| Slc12a2 | 0.17973022 |
| Slc12a2 | 2.58586978 |
| Slc12a3 | -0.05324 |
| Slc12a5 | -0.8082795 |
| Slc12a6 | -0.7612252 |
| Slc12a7 | -0.3133184 |
| Slc12a8 | -0.6071283 |
| Slc12a9 | 0.43035146 |
| Slc12a9 | -0.525187 |
| Slc13a2 | -0.6703477 |
| Slc13a3 | -0.0494239 |
| Slc13a3 | 0.30538607 |
| Slc13a4 | 0.16129802 |
| Slc14a1 | 2.67396161 |
| Slc14a1 | 0.57338018 |
| Slc14a1 | -0.2427006 |
| Slc14a2 | -0.5536415 |
| Slc14a2 | -0.7739101 |
| Slc15a2 | -0.8598012 |
| Slc15a3 | -0.6437543 |
| Slc15a4 | -0.4312225 |
| Slc15a4 | 0.81726891 |
| Slc16a10 | -0.4928141 |
| Slc16a11 | 1.84981079 |
| Slc16a4 | 1.19241466 |
| Slc16a6 | -0.431857 |
| Slc16a6 | -0.7495972 |
| Slc16a6 | 2.33501422 |
| Slc16a8 | 0.03200991 |
| Slc16a9 | -0.8351475 |
| Slc17a2 | 1.16922109 |
| Slc17a5 | -0.7215708 |
| Slc17a6 | -0.1654484 |
| Slc17a7 | 1.30234704 |
| Slc17a8 | -0.6086583 |
| Slc18a2 | 1.64849229 |
| Slc19a1 | -0.36214 |
| Slc19a2 | -0.7215708 |
| Slc1a1 | 1.89686192 |
| Slc1a1 | 1.37432681 |
| Slc1a2 | -0.7585291 |
| Slc1a2 | -0.4860674 |
| Slc1a2 | 1.45846645 |
| Slc1a2 | 0.07784375 |
| Slc1a3 | 1.70138929 |
| Slc1a4 | -0.5800058 |
| Slc1a6 | -0.4286887 |
| Slc1a7 | 0.6795527 |
| Slc20a1 | 0.23577816 |
| Slc22a1 | 0.68286798 |
| Slc22a12 | -0.2717081 |
| Slc22a13 | -0.0359795 |
| Slc22a14 | -0.6785566 |
| Slc22a17 | -0.6793814 |
| Slc22a17 | 0.60032124 |
| Slc22a18 | 1.82229705 |
| Slc22a21 | -0.0617758 |
| Slc22a3 | 2.66847556 |
| Slc22a4 | -0.3633198 |
| Slc22a5 | -0.7155494 |
| Slc22a5 | -0.5385959 |
| Slc22a5 | -0.5837265 |
| Slc22a6 | 1.00383572 |
| Slc22a7 | -0.2500001 |
| Slc22a8 | 0.24604222 |
| Slc23a2 | 0.05879618 |
| Slc23a3 | 1.73311602 |
| Slc24a1 | -0.5814923 |
| Slc24a3 | -0.1160405 |
| Slc24a4 | -0.5696653 |
| Slc24a5 | -0.1325319 |
| Slc24a6 | 0.98621845 |
| Slc25a1 | 1.20682176 |
| Slc25a1 | -0.650933 |
| Slc25a10 | 2.48498579 |
| Slc25a11 | -0.6163453 |
| Slc25a11 | -0.3860197 |
| Slc25a11 | 0.51498233 |
| Slc25a12 | 1.69508692 |
| Slc25a14 | -0.2917366 |
| Slc25a15 | 0.65761462 |
| Slc25a15 | 0.80006211 |
| Slc25a16 | -0.3586097 |
| Slc25a17 | -0.8449318 |
| Slc25a18 | -0.7969948 |
| Slc25a19 | 0.78253557 |
| Slc25a19 | 2.00519155 |
| Slc25a20 | 1.91964054 |
| Slc25a20 | -0.1808872 |
| Slc25a23 | -0.7720875 |
| Slc25a24 | -0.1146791 |
| Slc25a24 | 0.21937434 |
| Slc25a25 | -0.3878353 |
| Slc25a26 | -0.7215708 |
| Slc25a27 | 0.45719065 |
| Slc25a28 | -0.5450146 |
| Slc25a29 | 1.39101278 |
| Slc25a3 | 0.72307493 |
| Slc25a3 | -0.0725553 |
| Slc25a30 | -0.6810333 |
| Slc25a32 | -0.0545154 |
| Slc25a33 | -0.5822365 |
| Slc25a34 | 0.07265319 |
| Slc25a35 | -0.2733163 |
| Slc25a36 | 1.36445524 |
| Slc25a37 | -0.7389895 |
| Slc25a38 | -0.5704001 |
| Slc25a38 | 3.69695103 |
| Slc25a38 | 1.80722904 |
| Slc25a39 | -0.196583 |
| Slc25a41 | -0.4968855 |
| Slc25a42 | 0.77090408 |
| Slc25a44 | 1.74669929 |
| Slc25a45 | 1.64062701 |
| Slc25a46 | 0.77108139 |
| Slc25a5 | -0.7969948 |
| Slc26a4 | -0.3939126 |
| Slc26a6 | -0.45239 |
| Slc26a7 | -0.546447 |
| Slc26a8 | -0.8902622 |
| Slc27a1 | -0.2110365 |
| Slc27a2 | 1.73432995 |
| Slc27a3 | -0.5814923 |
| Slc27a4 | -0.2988758 |
| Slc27a6 | 0.14903528 |
| Slc29a1 | -0.7951271 |
| Slc29a2 | -0.4343993 |
| Slc29a3 | -0.4647964 |
| Slc29a4 | 2.0453376 |
| Slc2a1 | 0.80109311 |
| Slc2a10 | -0.391477 |
| Slc2a10 | -0.3591972 |
| Slc2a13 | -0.0592069 |
| Slc2a3 | 1.57067984 |
| Slc2a4 | -0.5770398 |
| Slc2a5 | -0.6171175 |
| Slc2a6 | -0.9111303 |
| Slc2a8 | 0.11233041 |
| Slc2a8 | 0.90043439 |
| Slc2a8 | 1.65830175 |
| Slc2a9 | 0.36476865 |
| Slc30a1 | 0.68597763 |
| Slc30a10 | -0.1376291 |
| Slc30a3 | 0.62133338 |
| Slc30a5 | 0.88091229 |
| Slc30a5 | -0.5667315 |
| Slc30a6 | -0.546447 |
| Slc30a7 | 2.98355538 |
| Slc30a7 | 1.21327822 |
| Slc30a9 | 0.24666071 |
| Slc31a1 | -0.0219017 |
| Slc31a2 | 0.73935009 |
| Slc32a1 | 0.47069493 |
| Slc34a2 | 0.14560048 |
| Slc35a1 | 0.17740422 |
| Slc35a2 | 0.39957688 |
| Slc35a3 | -0.3902615 |
| Slc35a4 | 0.58748553 |
| Slc35a5 | 2.34929315 |
| Slc35a5 | 1.2485542 |
| Slc35b1 | 0.14903528 |
| Slc35b2 | 0.25466438 |
| Slc35b3 | 0.87394322 |
| Slc35b3 | -0.7675463 |
| Slc35b3 | -0.7451628 |
| Slc35b4 | -0.2541968 |
| Slc35c1 | -0.5927165 |
| Slc35c1 | 2.14291621 |
| Slc35c2 | -0.7793991 |
| Slc35c2 | 0.26472605 |
| Slc35c2 | -0.4588993 |
| Slc35e1 | -0.2075274 |
| Slc35e3 | -0.6256532 |
| Slc35e4 | 0.11978655 |
| Slc35f1 | 0.51172029 |
| Slc35f2 | 1.91052084 |
| Slc35f3 | -0.2900967 |
| Slc35f4 | -0.5064542 |
| Slc35f4 | -0.6549449 |
| Slc36a1 | 0.54789583 |
| Slc36a3 | 0.79281336 |
| Slc37a1 | 0.18667403 |
| Slc37a3 | -0.2977739 |
| Slc38a1 | 2.01439353 |
| Slc38a10 | -0.6279946 |
| Slc38a11 | -0.1155865 |
| Slc38a2 | 0.32436565 |
| Slc38a2 | -0.391477 |
| Slc38a2 | -0.7522679 |
| Slc38a3 | 0.0366562 |
| Slc38a4 | 0.23702825 |
| Slc38a5 | -0.0293285 |
| Slc38a8 | 0.5610564 |
| Slc38a9 | 0.93458714 |
| Slc38a9 | -0.6860062 |
| Slc39a10 | -0.8026203 |
| Slc39a11 | 0.61361334 |
| Slc39a12 | 0.24045749 |
| Slc39a13 | -0.4485059 |
| Slc39a13 | -0.1348454 |
| Slc39a14 | -0.0031836 |
| Slc39a3 | 0.29340046 |
| Slc39a3 | -0.6605904 |
| Slc39a4 | -0.7711775 |
| Slc39a5 | -0.094935 |
| Slc39a5 | -0.0878566 |
| Slc39a5 | 0.73604385 |
| Slc39a6 | -0.5209919 |
| Slc39a7 | -0.6017931 |
| Slc39a7 | -0.2201203 |
| Slc39a7 | -0.6311256 |
| Slc39a8 | -0.6033144 |
| Slc3a2 | 0.99506822 |
| Slc40a1 | -0.2308299 |
| Slc41a2 | -0.0161655 |
| Slc41a3 | 1.6447785 |
| Slc41a3 | -0.413005 |
| Slc41a3 | 0.12998716 |
| Slc43a2 | 1.28687779 |
| Slc43a2 | -0.6040759 |
| Slc44a1 | -0.1142258 |
| Slc44a1 | 3.42964803 |
| Slc44a2 | -0.2380377 |
| Slc44a2 | 0.87123677 |
| Slc44a2 | -0.1061025 |
| Slc44a3 | -0.2541968 |
| Slc44a4 | 2.9349657 |
| Slc45a1 | -0.8322319 |
| Slc45a2 | 0.3710213 |
| Slc45a3 | -0.6868376 |
| Slc45a4 | -0.3475166 |
| Slc46a1 | 0.87171494 |
| Slc46a3 | -0.5301049 |
| Slc47a1 | -0.4012575 |
| Slc4a1 | -0.7969948 |
| Slc4a1ap | -0.1251668 |
| Slc4a2 | 1.61749745 |
| Slc4a3 | -0.5638069 |
| Slc4a3 | -0.340577 |
| Slc4a3 | -0.3055141 |
| Slc4a3 | 1.14472723 |
| Slc4a8 | -0.3872297 |
| Slc4a8 | -0.5594369 |
| Slc5a4a | -0.4813732 |
| Slc5a4b | 0.49309854 |
| Slc5a5 | 0.79367923 |
| Slc5a6 | -0.5829812 |
| Slc5a7 | 1.34594002 |
| Slc5a8 | 2.09240083 |
| Slc5a9 | 0.49523866 |
| Slc6a1 | -0.1052042 |
| Slc6a11 | -0.4236415 |
| Slc6a12 | -0.2589405 |
| Slc6a13 | -0.809226 |
| Slc6a15 | -0.4092794 |
| Slc6a15 | 0.81371702 |
| Slc6a17 | -0.7104187 |
| Slc6a2 | 1.32980333 |
| Slc6a4 | 0.17072669 |
| Slc6a6 | -0.3722151 |
| Slc6a7 | -0.5092061 |
| Slc6a8 | -0.0452019 |
| Slc6a9 | -0.2349419 |
| Slc7a1 | 0.10840117 |
| Slc7a10 | -0.5674641 |
| Slc7a11 | -0.3945225 |
| Slc7a14 | 3.01886823 |
| Slc7a14 | 0.76199585 |
| Slc7a14 | 0.8995065 |
| Slc7a2 | 2.94531399 |
| Slc7a3 | -0.2489539 |
| Slc7a4 | -0.490785 |
| Slc7a6 | 0.03510998 |
| Slc7a6os | -0.737233 |
| Slc7a7 | -0.0330639 |
| Slc7a7 | 0.78341092 |
| Slc7a8 | 0.22855779 |
| Slc7a8 | 2.0861777 |
| Slc8a1 | 0.14007864 |
| Slc8a2 | -0.7224341 |
| Slc8a2 | 1.06627393 |
| Slc9a1 | 2.03169433 |
| Slc9a10 | -0.7988662 |
| Slc9a3r1 | -0.3257142 |
| Slc9a3r2 | 0.38340362 |
| Slc9a4 | -0.9532009 |
| Slc9a6 | -0.9857474 |
| Slc9a6 | 1.45538187 |
| Slc9a8 | -0.6744432 |
| Slc9a8 | 1.83086635 |
| Slc9a8 | -0.4068039 |
| Slc9a8 | 2.51073185 |
| Slc9a9 | 0.39905098 |
| Slco1a4 | -0.6155738 |
| Slco1c1 | 2.04666821 |
| Slco2a1 | -0.4760368 |
| Slco2b1 | -0.9249463 |
| Slco3a1 | -0.5565347 |
| Slco3a1 | -0.5393069 |
| Slco3a1 | -0.7433949 |
| Slco4a1 | 1.53995764 |
| Slfnl1 | -0.9281636 |
| Slit1 | 0.17240051 |
| Slit2 | -0.8245007 |
| Slit3 | 0.01398401 |
| Slitrk1 | 0.9093572 |
| Slitrk3 | -0.7960605 |
| Slitrk4 | 0.73272597 |
| Slitrk5 | 0.19878534 |
| Slitrk5 | -0.7164073 |
| Slitrk6 | 0.48160185 |
| Slk | -0.3394254 |
| Slk | 1.63723599 |
| Slmap | 1.38705344 |
| Slmap | -0.4491521 |
| Slmap | -0.5912122 |
| Slmo1 | 1.44849091 |
| Slmo2 | 0.15450508 |
| Sln | -0.3365524 |
| Slpi | 1.18604169 |
| Sltm | 0.29310622 |
| Sltm | -0.7513768 |
| Slu7 | -0.2211351 |
| Slu7 | 2.17442958 |
| Sly | 3.16917544 |
| Smad1 | -0.8322319 |
| Smad1 | -0.1755516 |
| Smad2 | 1.69781206 |
| Smad3 | 2.5671506 |
| Smad5 | 0.55772786 |
| Smad5 | 1.34246387 |
| Smad6 | -0.273853 |
| Smap2 | -0.5085174 |
| Smap2 | -0.5002918 |
| Smap2 | 2.1534562 |
| Smarca1 | -0.5400185 |
| Smarca2 | 1.38548352 |
| Smarca2 | -0.8638062 |
| Smarca4 | 1.14257736 |
| Smarcad1 | -0.3615507 |
| Smarcal1 | 0.00203962 |
| Smarcb1 | -0.5740831 |
| Smarcc1 | 0.47191305 |
| Smarcc1 | -0.1164947 |
| Smarcc2 | 0.73087766 |
| Smarcd1 | 1.74151758 |
| Smarcd1 | 0.9764296 |
| Smarcd1 | 3.59149756 |
| Smarcd2 | 1.40786008 |
| Smarcd3 | 1.19411442 |
| Smarce1 | -0.7181253 |
| Smarce1 | 0.92605137 |
| Smc1a | 0.99226775 |
| Smc1a | -0.6186637 |
| Smc1a | -0.8302931 |
| Smc1a | -0.6985544 |
| Smc1b | -0.2515717 |
| Smc3 | 2.89977616 |
| Smc5l1 | -0.6777325 |
| Smc6 | 0.28986347 |
| Smchd1 | 0.43643825 |
| Smcp | -0.4833821 |
| Smcr7 | 2.29383636 |
| Smcr7l | 1.39915526 |
| Smek1 | 0.32521935 |
| Smek2 | -0.6477358 |
| Smek2 | 3.14245414 |
| Smg5 | 0.74118189 |
| Smg6 | -0.701929 |
| Smn1 | 0.58424912 |
| Smo | 1.39650981 |
| Smo | -0.5762997 |
| Smo | -0.6405813 |
| Smoc1 | 2.0163842 |
| Smox | -0.3308326 |
| Smox | -0.1731362 |
| Smox | 1.10102015 |
| Smox | 2.02453983 |
| Smox | 1.53128705 |
| Smpd1 | 3.83907299 |
| Smpd1 | 2.34043616 |
| Smpd1 | 0.61633299 |
| Smpd2 | 0.76966198 |
| Smpd3 | 1.80343896 |
| Smpd4 | 0.46408971 |
| Smpdl3a | 0.77462059 |
| Smpdl3b | -0.340577 |
| Smpx | 1.72708714 |
| Smtn | 2.04593431 |
| Smtn | -0.0314019 |
| Smtn | 1.92700868 |
| Smtnl1 | 2.64245968 |
| Smtnl2 | -0.0149408 |
| Smu1 | -0.4707305 |
| Smu1 | 0.85936444 |
| Smug1 | 0.1001322 |
| Smurf1 | 0.41754306 |
| Smyd1 | 0.92905518 |
| Smyd2 | -0.4337631 |
| Smyd2 | -0.5807488 |
| Smyd3 | 2.36692473 |
| Smyd4 | 0.35902327 |
| Smyd5 | -0.3866245 |
| Smyd5 | -0.1192246 |
| Snai1 | -0.513349 |
| Snai3 | 1.67958419 |
| Snap23 | 1.05071813 |
| Snap29 | 0.78393575 |
| Snap91 | -0.4853953 |
| Snap91 | 0.20688149 |
| Snapc1 | 0.17072669 |
| Snapc3 | -0.8082795 |
| Snapc3 | 2.3860902 |
| Snapc3 | 0.04740937 |
| Snapc3 | 0.90905092 |
| Snapc4 | 1.55729444 |
| Snapin | -0.8439488 |
| Snca | 1.11675801 |
| Snca | 1.34091479 |
| Snca | -0.2552489 |
| Sncaip | -0.027673 |
| Sncg | -0.4414262 |
| Sncg | 3.85369816 |
| Snd1 | 0.30973327 |
| Snf1lk | 0.61151595 |
| Snf1lk2 | 0.79730725 |
| Snf8 | 0.33032539 |
| Snip1 | -0.3799929 |
| Snip1 | -0.4996095 |
| Snn | 2.40220438 |
| Snora65 | -0.5393069 |
| Snph | -0.8810202 |
| Snrk | -0.4452813 |
| Snrk | 0.05539455 |
| Snrk | 2.56193437 |
| Snrp70 | 0.44724648 |
| Snrpa | 0.49214582 |
| Snrpb | -0.546447 |
| Snrpb | -0.8245007 |
| Snrpd1 | -0.5995157 |
| Snrpd1 | -0.6549449 |
| Snrpd2 | -0.7675463 |
| Snrpd3 | 0.10229632 |
| Snrpg | 0.11978655 |
| Snta1 | -0.6217638 |
| Sntb2 | -0.634267 |
| Sntb2 | 0.0976011 |
| Sntg2 | -0.8439488 |
| Snupn | 2.17706867 |
| Snurf | 2.47481935 |
| Snurf | 1.03019858 |
| Snx1 | -0.4608609 |
| Snx10 | -0.3615507 |
| Snx11 | -0.4293216 |
| Snx12 | -0.3121995 |
| Snx13 | 0.42422513 |
| Snx14 | 1.29971891 |
| Snx14 | -0.5645372 |
| Snx14 | -0.778482 |
| Snx15 | -0.5587105 |
| Snx16 | 0.38045739 |
| Snx17 | -0.3533388 |
| Snx17 | 0.33343219 |
| Snx18 | 0.24789648 |
| Snx19 | 1.19298159 |
| Snx19 | -0.7513768 |
| Snx2 | 1.56542483 |
| Snx2 | -0.5189013 |
| Snx20 | -0.6240955 |
| Snx21 | -0.5016577 |
| Snx22 | 3.12194465 |
| Snx24 | -0.6225404 |
| Snx25 | 0.78603213 |
| Snx25 | -0.5645372 |
| Snx26 | 1.7168008 |
| Snx29 | -0.5378854 |
| Snx3 | -0.1119623 |
| Snx30 | -0.375199 |
| Snx33 | 0.0366562 |
| Snx4 | 0.63964844 |
| Snx6 | -0.5638069 |
| Snx7 | 0.31060031 |
| Snx8 | -0.6549449 |
| Snx9 | 0.09977103 |
| Soat1 | 1.93535217 |
| Socs2 | 2.39816998 |
| Socs3 | -0.5594369 |
| Socs4 | -0.2994272 |
| Socs5 | -0.2663672 |
| Socs6 | 0.40925345 |
| Socs7 | -0.0742915 |
| Socs7 | -0.1343822 |
| Sod1 | 0.0715372 |
| Sod2 | -0.8016804 |
| Sod3 | 0.57294281 |
| Sohlh1 | 1.57477213 |
| Sohlh1 | -0.4031026 |
| Son | 0.2707722 |
| Sorbs1 | -0.5889603 |
| Sorbs2 | 3.0366591 |
| Sorbs3 | 0.00603791 |
| Sorcs1 | 1.50152315 |
| Sorcs1 | -0.5927165 |
| Sorcs2 | -0.5175101 |
| Sorcs3 | 2.6573828 |
| Sorcs3 | -0.7172659 |
| Sord | 1.20704513 |
| Sorl1 | 0.82231995 |
| Sort1 | -0.4694086 |
| Sort1 | -0.6303419 |
| Sort1 | -0.5822365 |
| Sort1 | 1.30113497 |
| Sostdc1 | 1.61597856 |
| Sox12 | 1.37833982 |
| Sox13 | -0.7748227 |
| Sox17 | 1.75172733 |
| Sox17 | -0.6810333 |
| Sox18 | 0.46605164 |
| Sox2 | 0.80964126 |
| Sox2 | 1.03637469 |
| Sox21 | 0.9679781 |
| Sox21 | 0.41780094 |
| Sox30 | 0.64917882 |
| Sox5 | 0.08520962 |
| Sox5 | -0.2552489 |
| Sox5 | -0.4369485 |
| Sox5 | 0.26926429 |
| Sox7 | -0.5485996 |
| Sox7 | 0.3091548 |
| Sox8 | 0.22950271 |
| Sox9 | 1.6652662 |
| Sp100 | 2.49581309 |
| Sp140 | 2.21992376 |
| Sp2 | 2.77085591 |
| Sp4 | 1.52954333 |
| Sp5 | -0.7354799 |
| Sp6 | 0.76199585 |
| Sp7 | 2.35019091 |
| Sp8 | -0.6622096 |
| Sp9 | 0.55950454 |
| Spaca3 | 2.65601402 |
| Spaca4 | 1.15410526 |
| Spag1 | 3.19414235 |
| Spag1 | 1.09210602 |
| Spag16 | 0.10193598 |
| Spag16 | 0.42140388 |
| Spag4 | 1.50029647 |
| Spag4 | -0.6630203 |
| Spag4l | 1.02871567 |
| Spag4l | 0.04434952 |
| Spag5 | -0.2050289 |
| Spag5 | -0.4647964 |
| Spag6 | 0.23013214 |
| Spag6 | -0.6002742 |
| Spag7 | 0.66299806 |
| Spag8 | -0.074726 |
| Spag9 | 0.75840887 |
| Spag9 | -0.5829812 |
| Spag9 | 0.09179009 |
| Spag9 | -0.4186212 |
| Spam1 | 0.14318865 |
| Spam1 | 0.06331269 |
| Sparc | -0.289005 |
| Sparc | -0.7757362 |
| Sparcl1 | -0.3639102 |
| Spast | -0.5182055 |
| Spast | -0.7224341 |
| Spast | 0.28779409 |
| Spata16 | -0.4968855 |
| Spata16 | 1.05989964 |
| Spata17 | -0.4621709 |
| Spata17 | 0.19748348 |
| Spata19 | 0.37830887 |
| Spata2 | 0.85629188 |
| Spata20 | -0.0652118 |
| Spata20 | -0.7070137 |
| Spata21 | -0.0725553 |
| Spata22 | 1.52133638 |
| Spata2L | 2.99449522 |
| Spata3 | 1.23097225 |
| Spata3 | -0.8419858 |
| Spata3 | 0.08153388 |
| Spata3 | 1.37507471 |
| Spata4 | -0.778482 |
| Spata6 | 0.46703108 |
| Spata7 | 0.00803072 |
| Spata7 | 0.76663863 |
| Spatc1 | 0.04779115 |
| Spatc1 | -0.455639 |
| Spats1 | -0.7104187 |
| Spc24 | 0.03200991 |
| Spc24 | 0.17573926 |
| Spc25 | -0.2125444 |
| Spc25 | -0.6350539 |
| Spcs1 | -0.0553667 |
| Spcs1 | 0.19291277 |
| Spcs3 | 0.65621388 |
| Spdef | 0.19585363 |
| Specc1l | 1.56450792 |
| Speer1-ps1 | 0.06180959 |
| Speer1-ps1 | 0.45347386 |
| Speer2 | 0.64917882 |
| Speer2 | 0.61779418 |
| Speer3 | 1.36454975 |
| Speer4b | -0.2272465 |
| Speer4d | -0.63191 |
| Speer4f | 1.63169945 |
| Speer6-ps1 | 0.13591594 |
| Spef1 | 1.0200413 |
| Spen | -0.0760309 |
| Spert | -0.6405813 |
| Spesp1 | -0.4536883 |
| Spg20 | -0.2928314 |
| Spg21 | 0.9271038 |
| Spg7 | 2.75124962 |
| Spg7 | 1.54160392 |
| Sphk1 | 1.53041559 |
| Sphk2 | 0.53614246 |
| Sphk2 | -0.4989278 |
| Spic | 1.92382811 |
| Spin | -0.6101907 |
| Spin2 | 0.17005633 |
| Spink10 | -0.7267627 |
| Spink2 | -0.6769091 |
| Spink4 | -0.6935151 |
| Spink8 | 1.43490698 |
| Spinlw1 | -0.8668213 |
| Spinlw1 | -0.2851941 |
| Spinlw1 | 1.82832684 |
| Spint1 | 2.15941497 |
| Spint2 | 0.45743792 |
| Spire1 | 0.20235621 |
| Spire1 | -0.525187 |
| Spire1 | 0.72679856 |
| Spire2 | 0.96017588 |
| Spna1 | -0.3088509 |
| Spnb1 | -0.1611514 |
| Spnb2 | 1.1199478 |
| Spnb2 | 1.02939001 |
| Spnb2 | -0.7104187 |
| Spnb2 | 1.39183938 |
| Spnb3 | -0.5393069 |
| Spnb4 | -0.7757362 |
| Spns1 | -0.1678441 |
| Spo11 | 1.48611528 |
| Spo11 | 2.83633671 |
| Spock1 | 0.53112544 |
| Spock2 | -0.4887603 |
| Spock3 | 1.49479773 |
| Spon1 | 0.63310412 |
| Spon2 | -0.5057675 |
| Spop | 1.94065147 |
| Spp1 | -0.6670841 |
| Spp2 | -0.5279941 |
| Sppl3 | -0.6445493 |
| Spr | 1.10947937 |
| Spred1 | 2.1051132 |
| Spred2 | 1.13754186 |
| Spred2 | 2.57141859 |
| Sprn | 0.80452168 |
| Sprn | 0.82867936 |
| Sprr2a | 1.64849229 |
| Sprr2a | 1.67870412 |
| Sprr2f | -0.6918413 |
| Spry3 | 0.36203712 |
| Spry4 | 2.30874747 |
| Spryd4 | 1.21038941 |
| Spsb1 | -0.7639291 |
| Spsb2 | -0.1187691 |
| Spsb3 | -0.4787012 |
| Spsb3 | -0.3556778 |
| Spsb4 | -0.2416624 |
| Sptlc1 | -0.4280564 |
| Sptlc2 | -0.682688 |
| Spz1 | -0.3650922 |
| Sqle | 0.61403225 |
| Sqle | 1.94897737 |
| Sqrdl | -0.6056009 |
| Sqstm1 | 0.05804132 |
| Sra1 | -0.6248741 |
| Sra1 | -0.4086599 |
| Src | 1.63098729 |
| Srd5a1 | -0.5980006 |
| Srd5a2 | 1.33547288 |
| Srd5a3 | 1.61387701 |
| Srd5a3 | 0.86948139 |
| Srd5a3 | -0.634267 |
| Srebf1 | -0.1926345 |
| Srebf2 | 1.73605234 |
| Srf | 0.86371357 |
| Srf | 2.06073074 |
| Srfbp1 | -0.413005 |
| Srgn | 0.4621237 |
| Srgn | 0.06631172 |
| Sri | -0.145561 |
| Sri | -0.6033144 |
| Sri | -0.1325319 |
| Srm | -0.1097042 |
| Srm | 0.79090564 |
| Srm | 1.53657771 |
| Srp14 | 3.48870997 |
| Srp19 | -0.424271 |
| Srp19 | -0.8073339 |
| Srp54 | 1.16840704 |
| Srp68 | 2.45060288 |
| Srp72 | -0.4305884 |
| Srp9 | 0.21810061 |
| Srp9 | -0.4312225 |
| Srpk1 | 0.48328721 |
| Srpk2 | 0.81946102 |
| Srpk3 | -0.2354572 |
| Srpr | 0.12963725 |
| Srprb | 0.87426111 |
| Srprb | -0.6437543 |
| Srpx | -0.5748214 |
| Srpx | 0.69562974 |
| Srpx2 | 0.32237068 |
| Srpx2 | 1.28882538 |
| Srpx2 | -0.7044679 |
| Srr | 1.78243818 |
| Srrd | -0.5645372 |
| Srrm1 | 1.1243467 |
| Srrm2 | -0.6985544 |
| Srrm2 | 0.26047057 |
| Srrp | 1.96150568 |
| Srxn1 | 0.1846953 |
| Srxn1 | 1.36464424 |
| Srxn1 | -0.2631772 |
| Ss18 | 2.40040994 |
| Ss18 | 0.3691227 |
| Ss18l1 | -0.3016361 |
| Ss18l1 | 2.30586069 |
| Ss18l1 | 1.22057186 |
| Ssbp1 | 1.02750065 |
| Ssbp2 | -0.1808872 |
| Ssbp2 | 0.53591499 |
| Ssbp3 | 0.25251622 |
| Ssbp3 | 1.67951652 |
| Ssbp4 | -0.7267627 |
| Ssfa2 | -0.6389987 |
| Ssh2 | 2.36816462 |
| Ssh3 | 0.33737156 |
| Ssh3 | 1.27956416 |
| Ssna1 | 0.21937434 |
| Sspn | 1.33117497 |
| Ssr2 | -0.6389987 |
| Ssr3 | -0.2939275 |
| Ssr4 | -0.3981901 |
| Ssrp1 | -0.2928314 |
| Sssca1 | -0.415497 |
| Sssca1 | -0.0993861 |
| Sst | -0.3240143 |
| Sstr1 | -0.613263 |
| Sstr5 | 2.24592826 |
| Ssty2 | -0.7155494 |
| Ssu72 | -0.1502582 |
| Ssx2ip | -0.7198465 |
| Ssxb2 | -0.7693601 |
| Ssxb5 | 0.81405587 |
| St13 | -0.295574 |
| St14 | -0.513349 |
| St14 | 3.29182266 |
| St18 | -0.5608913 |
| St3gal1 | -0.4427095 |
| St3gal2 | 0.42448119 |
| St3gal2 | -0.3032961 |
| St3gal3 | 1.13187483 |
| St3gal3 | -0.4223839 |
| St3gal4 | 0.43263861 |
| St3gal5 | 0.92228311 |
| St3gal6 | -0.5987578 |
| St5 | -0.6646437 |
| St5 | -0.2610564 |
| St6gal1 | 0.34742645 |
| St6galnac2 | -0.0096522 |
| St6galnac3 | 0.3764249 |
| St6galnac4 | -0.1818606 |
| St6galnac4 | 1.7794604 |
| St6galnac4 | 0.30770707 |
| St6galnac5 | 0.06030409 |
| St6galnac6 | -0.0068167 |
| St6galnac6 | -0.7941946 |
| St7 | -0.1507292 |
| St7l | 0.20105927 |
| St7l | 0.36585905 |
| St8sia1 | -0.6581668 |
| St8sia1 | -0.7146924 |
| St8sia2 | 0.31002237 |
| St8sia3 | -0.4934915 |
| St8sia4 | 0.47894734 |
| St8sia4 | -0.3038502 |
| St8sia5 | -0.3878353 |
| Stab1 | 0.59328322 |
| Stab2 | 0.64046329 |
| Stac | -0.5265895 |
| Stac2 | -0.2401073 |
| Stac3 | 0.12963725 |
| Stag1 | -0.6678989 |
| Stag2 | -0.6086583 |
| Stag2 | -0.3342603 |
| Stag3 | 0.28155855 |
| Stam | -0.5964878 |
| Stam | 2.21556383 |
| Stam | 1.42290282 |
| Stam2 | 0.52470132 |
| Stam2 | -0.4588993 |
| Stambp | 1.36000285 |
| Stambpl1 | 0.27047081 |
| Stap1 | 2.65901313 |
| Stap2 | 1.07324912 |
| Star | 1.44511322 |
| Stard10 | -0.6374188 |
| Stard10 | 2.87995342 |
| Stard13 | -0.6437543 |
| Stard3 | -0.6777325 |
| Stard3nl | -0.6010334 |
| Stard4 | 0.48808597 |
| Stard5 | 1.46892997 |
| Stard5 | -0.0401603 |
| Stard6 | 2.74696043 |
| Stard7 | -0.649333 |
| Stard7 | 0.5397745 |
| Stard8 | 2.52924689 |
| Stard8 | -0.2272465 |
| Stat1 | -0.0251953 |
| Stat1 | -0.0182101 |
| Stat2 | 1.13934334 |
| Stat3 | -0.2427006 |
| Stat3 | -0.1460297 |
| Stat3 | -0.0626336 |
| Stat4 | -0.514734 |
| Stat4 | 1.8750058 |
| Stat5a | -0.3094082 |
| Stat5a | 0.45297718 |
| Stat6 | -0.607893 |
| Statip1 | 0.93771253 |
| Stau1 | 2.78282202 |
| Stau2 | 1.66340945 |
| Stbd1 | -0.4249008 |
| Stc2 | 2.77789329 |
| Steap1 | 0.29955846 |
| Steap2 | -0.2966733 |
| Steap3 | -0.5322205 |
| Stfa1 | -0.6071283 |
| Stfa2 | -0.2262252 |
| Stim1 | -0.8638062 |
| Stip1 | -0.2175883 |
| Stk11 | 0.03781421 |
| Stk11ip | -0.0656422 |
| Stk17b | 1.63454276 |
| Stk19 | 0.32834299 |
| Stk19 | 1.61576137 |
| Stk19 | 0.04012596 |
| Stk22b | 1.7070585 |
| Stk22s1 | -0.6736227 |
| Stk24 | -0.0506942 |
| Stk25 | 0.93741532 |
| Stk25 | 2.65877355 |
| Stk3 | 0.1633264 |
| Stk31 | -0.2065272 |
| Stk32a | 1.66896878 |
| Stk32b | 0.5630479 |
| Stk32c | -0.747821 |
| Stk35 | 2.38240377 |
| Stk35 | 0.26168838 |
| Stk36 | -0.4747074 |
| Stk38l | -0.3704293 |
| Stk39 | -0.5844724 |
| Stk4 | 0.21810061 |
| Stk40 | -0.054941 |
| Stmn1 | -0.7748227 |
| Stmn2 | -0.6178903 |
| Stmn3 | 0.64493246 |
| Stmn4 | -0.5711356 |
| Stmn4 | 0.7462921 |
| Stoml1 | 1.15280503 |
| Stoml1 | 0.81422525 |
| Stoml1 | -0.680207 |
| Stoml2 | 0.82700998 |
| Stoml3 | 0.04012596 |
| Stra13 | 1.89831188 |
| Stra13 | 0.36667602 |
| Stra13 | 0.86692227 |
| Stra6 | -0.7293694 |
| Strap | 1.6643039 |
| Strbp | -0.6217638 |
| Strn4 | 0.42089001 |
| Strn4 | -0.2115389 |
| Stt3a | -0.7531598 |
| Stt3b | 0.11624336 |
| Stx12 | -0.5696653 |
| Stx17 | 2.06249081 |
| Stx18 | -0.6256532 |
| Stx1a | -0.6469381 |
| Stx2 | 0.28423597 |
| Stx3 | 0.36776422 |
| Stx3 | -0.022724 |
| Stx4a | -0.2695681 |
| Stx5a | -0.2432202 |
| Stx5a | 0.34269166 |
| Stx6 | -0.6256532 |
| Stx7 | -0.7138361 |
| Stx8 | -0.3510056 |
| Stx8 | 0.23170387 |
| Stx8 | 0.46236967 |
| Stx8 | 1.68350067 |
| Stxbp1 | -0.0452019 |
| Stxbp1 | -0.3957435 |
| Stxbp1 | 0.20235621 |
| Stxbp2 | 2.48901041 |
| Stxbp4 | -0.6719838 |
| Stxbp4 | 2.31065555 |
| Stxbp5l | -0.6311256 |
| Stxbp5l | -0.585966 |
| Styx | 0.18601491 |
| Styxl1 | -0.3830015 |
| Styxl1 | -0.0769018 |
| Styxl1 | 1.65670857 |
| Suclg1 | 0.44122926 |
| Suclg1 | -0.1265434 |
| Suclg2 | 2.9317062 |
| Suclg2 | -0.6926778 |
| Suds3 | 0.01793209 |
| Sufu | -0.7711775 |
| Suhw2 | 0.31922716 |
| Suhw4 | 0.69351474 |
| Sulf1 | 1.71210568 |
| Sult1a1 | 1.2287922 |
| Sult1c2 | 2.5621733 |
| Sult1d1 | 0.70594426 |
| Sult1d1 | -0.5301049 |
| Sult2b1 | -0.4707305 |
| Sult2b1 | 0.48160185 |
| Sult4a1 | -0.2862814 |
| Sult5a1 | 0.65340615 |
| Sult5a1 | 1.75222867 |
| Sumf2 | 0.39641709 |
| Sumo1 | 0.05122027 |
| Sumo1 | 0.44774619 |
| Sumo1 | 1.38640732 |
| Sumo2 | 1.37787407 |
| Sumo3 | -0.0141252 |
| Sumo3 | 2.29302455 |
| Sunc1 | 2.91135922 |
| Suox | -0.3710242 |
| Supt3h | -0.5002918 |
| Supt4h1 | 0.22634926 |
| Supt4h2 | 0.82299139 |
| Supt5h | 0.7971348 |
| Supt5h | -0.0596346 |
| Supt6h | -0.3434623 |
| Supv3l1 | 1.81147476 |
| Surf1 | -0.4485059 |
| Surf1 | -0.1530878 |
| Surf2 | 2.01610007 |
| Surf4 | -0.4369485 |
| Susd2 | -0.3178071 |
| Susd3 | 2.0298291 |
| Susd4 | 0.95828789 |
| Suv39h1 | -0.4588993 |
| Suv420h1 | 0.04893557 |
| Suv420h1 | 0.20655898 |
| Suv420h1 | 2.40575171 |
| Suv420h2 | 2.30739213 |
| Suz12 | 1.56603561 |
| Sv2a | -1.0265848 |
| Sv2a | 4.34706723 |
| Sv2b | -0.9444167 |
| Sval2 | 0.58899206 |
| Svep1 | 0.27468151 |
| Svil | 0.3721045 |
| Svil | 1.49996909 |
| Svop | 0.12014014 |
| Svopl | -0.1726539 |
| Svs4 | 0.52424077 |
| Svs5 | -0.7513768 |
| Svs6 | 2.69189798 |
| Swap70 | -0.1654484 |
| Swap70 | -0.6678989 |
| Swap70 | -0.2727799 |
| Syap1 | 2.16327213 |
| Syap1 | -0.1760354 |
| Sybl1 | -0.2526209 |
| Syce2 | 1.65643122 |
| Sycp1 | 0.10696828 |
| Syf2 | -0.7487087 |
| Syf2 | 0.77054936 |
| Syn1 | 0.33145632 |
| Syn1 | 0.3368098 |
| Syn2 | -0.1659271 |
| Sync | -0.2155675 |
| Syncrip | -0.6893361 |
| Syne1 | 0.5147497 |
| Syne1 | -0.6086583 |
| Syngr1 | -0.20453 |
| Syngr1 | 0.68558949 |
| Syngr1 | 1.27573024 |
| Syngr1 | 2.33339169 |
| Syngr2 | -0.3359789 |
| Syngr2 | 0.6925518 |
| Syngr2 | 0.11375518 |
| Syngr3 | 1.22911953 |
| Syngr4 | 2.24246985 |
| Syngr4 | -0.7044679 |
| Syngr4 | -0.2922838 |
| Synj2 | 0.77161311 |
| Synj2bp | 2.81716735 |
| Synj2bp | 0.7765615 |
| Synpo2l | -0.7027746 |
| Syp | -0.3291234 |
| Sypl | 3.06085611 |
| Sypl | 1.50144142 |
| Sypl2 | 1.55266108 |
| Sys1 | -0.554364 |
| Syt1 | -0.0643516 |
| Syt11 | 0.16703381 |
| Syt11 | -0.4136274 |
| Syt12 | -0.0343123 |
| Syt13 | 0.57162947 |
| Syt15 | -0.1196804 |
| Syt16 | -0.4901096 |
| Syt3 | 0.11588831 |
| Syt4 | 1.73509585 |
| Syt5 | 1.77616784 |
| Syt9 | 1.2785295 |
| Sytl2 | -0.6860062 |
| Sytl3 | 0.29163362 |
| Sytl3 | -0.5822365 |
| Sytl3 | 1.12714641 |
| Sytl4 | 0.58985185 |
| Sytl5 | 0.99604642 |
| Syvn1 | -0.6272135 |
| Taar2 | -0.4086599 |
| Taar3 | 2.2282811 |
| Taar4 | 1.4436371 |
| Tac1 | -0.187722 |
| Tacc1 | -0.6613997 |
| Tacc1 | -0.3044045 |
| Tacr3 | 1.26392381 |
| Tacr3 | -0.4720543 |
| Tacstd2 | -0.3805938 |
| Tada1l | 0.7776185 |
| Tada2l | -0.6295588 |
| Tada2l | -0.3933031 |
| Tada3l | -0.4092794 |
| Tada3l | 2.40468577 |
| Taf10 | 0.30712735 |
| Taf11 | 0.04664534 |
| Taf12 | -0.6048381 |
| Taf12 | -0.5785216 |
| Taf12 | 1.36095193 |
| Taf13 | 0.03045604 |
| Taf1c | 0.76806259 |
| Taf3 | -0.2615862 |
| Taf5 | -0.6728029 |
| Taf5 | -0.6171175 |
| Taf5 | -0.5050813 |
| Taf5l | -0.308294 |
| Taf6 | 0.07784375 |
| Taf6 | 1.44181047 |
| Taf6l | -0.5189013 |
| Taf7l | 0.48880369 |
| Taf9 | -0.7112719 |
| Taf9 | -0.4608609 |
| Taf9 | 0.24604222 |
| Taf9b | -0.6025534 |
| Tagap | 2.77998167 |
| Tagap | 2.05292894 |
| Tagln | 1.8263598 |
| Tagln3 | 0.35186112 |
| Tal1 | 1.38871286 |
| Tal2 | 1.60094781 |
| Taldo1 | 3.34843213 |
| Tank | -0.0334799 |
| Tank | -0.5829812 |
| Tank | -0.2722439 |
| Tap1 | 2.05083143 |
| Tap2 | -0.7969948 |
| Tapbp | 0.67602966 |
| Tapbp | -0.5329268 |
| Tapbpl | 1.21139038 |
| Tapbpl | -0.6311256 |
| Tapt1 | -0.0322325 |
| Tarbp2 | -0.3133184 |
| Tarbp2 | -0.6350539 |
| Tardbp | -0.6876697 |
| Tardbp | 2.29196462 |
| Tardbp | 1.49315008 |
| Tars | -0.5867137 |
| Tarsl2 | -0.6835165 |
| Tarsl2 | -0.7720875 |
| Tas1r1 | 0.8437364 |
| Tasp1 | -0.6040759 |
| Tatdn2 | 0.88970819 |
| Tax1bp1 | 0.01041655 |
| Tax1bp3 | 2.00744073 |
| Tax1bp3 | 1.2863646 |
| Taz | -0.1911581 |
| Taz | -0.5223881 |
| Tbc1d1 | 0.47142599 |
| Tbc1d10a | -0.6186637 |
| Tbc1d10a | -0.0186196 |
| Tbc1d10c | 2.25199623 |
| Tbc1d12 | 0.05615152 |
| Tbc1d13 | 2.73946758 |
| Tbc1d14 | -0.7941946 |
| Tbc1d15 | -0.6209878 |
| Tbc1d16 | -0.7603256 |
| Tbc1d17 | 0.26624122 |
| Tbc1d19 | -0.5927165 |
| Tbc1d2 | -0.4452813 |
| Tbc1d20 | -0.5874619 |
| Tbc1d22a | -0.6233176 |
| Tbc1d22b | 0.0885056 |
| Tbc1d23 | 2.42715828 |
| Tbc1d24 | 1.2668631 |
| Tbc1d25 | -0.3257142 |
| Tbc1d2b | -0.7495972 |
| Tbc1d5 | -0.4667703 |
| Tbc1d5 | -0.4833821 |
| Tbc1d7 | -0.3504232 |
| Tbc1d7 | -0.6256532 |
| Tbc1d8 | -0.6202125 |
| Tbc1d9 | 0.19846004 |
| Tbc1d9 | -0.4975658 |
| Tbc1d9b | 0.7821852 |
| Tbca | 3.34268264 |
| Tbcb | 0.69466897 |
| Tbcc | -0.0998324 |
| Tbcd | 0.74611007 |
| Tbcel | -0.5889603 |
| Tbk1 | 1.61713603 |
| Tbkbp1 | -0.4996095 |
| Tbl1x | 0.0885056 |
| Tbl1xr1 | -0.5934696 |
| Tbl2 | 2.78883212 |
| Tbl3 | -0.7129806 |
| Tbp | -0.3830015 |
| Tbpl1 | -0.7138361 |
| Tbr1 | -0.5748214 |
| Tbrg1 | 0.03356122 |
| Tbrg1 | -0.2323698 |
| Tbrg1 | -0.4388649 |
| Tbrg4 | 0.87394322 |
| Tbrg4 | -0.5428702 |
| Tbx1 | -0.6876697 |
| Tbx19 | -0.613263 |
| Tbx2 | 0.34045528 |
| Tbx22 | -0.5785216 |
| Tbx3 | 2.19105982 |
| Tbx3 | 0.13138553 |
| Tbx5 | 0.74738355 |
| Tbx6 | -0.8120712 |
| Tbxa2r | -0.308294 |
| Tbxa2r | -0.5844724 |
| Tcam1 | 1.15858321 |
| Tcam1 | -0.682688 |
| Tcam1 | -0.6703477 |
| Tcap | 1.66732498 |
| Tcea2 | 1.71667069 |
| Tcea3 | 0.87648339 |
| Tceal1 | 1.81792459 |
| Tceal1 | 0.59328322 |
| Tceal3 | -0.7181253 |
| Tceal5 | 1.08387066 |
| Tceal6 | -0.6951919 |
| Tceal6 | -0.6453449 |
| Tceal8 | 0.29222294 |
| Tceb1 | 0.67622573 |
| Tceb2 | -0.1362362 |
| Tceb2 | 1.88349301 |
| Tceb3 | 0.30770707 |
| Tcerg1 | -0.3920853 |
| Tcf12 | 1.21958059 |
| Tcf12 | 2.46618934 |
| Tcf15 | 0.62816591 |
| Tcf19 | -0.074726 |
| Tcf20 | -0.0468885 |
| Tcf20 | 0.23327299 |
| Tcf20 | -0.6736227 |
| Tcf20 | 0.61424164 |
| Tcf20 | -0.8082795 |
| Tcf21 | -0.5874619 |
| Tcf23 | -0.8528329 |
| Tcf25 | -0.7215708 |
| Tcf25 | 1.56374314 |
| Tcf25 | -0.7207082 |
| Tcf3 | 0.6754412 |
| Tcf3 | 1.48411945 |
| Tcf4 | -0.7232982 |
| Tcf7 | -0.7886191 |
| Tcf7 | -0.6810333 |
| Tcf7l2 | 2.81981726 |
| Tcf7l2 | 1.78474103 |
| Tcfap2b | -0.4459253 |
| Tcfap2b | -0.0251953 |
| Tcfap2c | -0.4975658 |
| Tcfap2c | 1.058332 |
| Tcfcp2 | -0.6818603 |
| Tcfe3 | 1.15858321 |
| Tcfl5 | 0.72549696 |
| Tchp | 2.1390163 |
| Tcirg1 | -0.3498411 |
| Tcirg1 | 0.92755447 |
| Tcirg1 | -0.4820423 |
| Tcirg1 | -0.6549449 |
| Tcl1 | 3.63389269 |
| Tcl1 | -0.4167454 |
| Tcl1b2 | 0.90659707 |
| Tcl1b2 | -0.5050813 |
| Tcl1b3 | 0.47701208 |
| Tcl1b4 | 0.52031703 |
| Tcn2 | 1.18283899 |
| Tcof1 | -0.7549462 |
| Tcp1 | 0.34936919 |
| Tcp1 | 0.52883613 |
| Tcp10a | 0.66478573 |
| Tcp10a | 2.26895564 |
| Tcp11 | 0.2641193 |
| Tcstv1 | -0.7328563 |
| Tcte3 | -0.4230125 |
| Tctex1d1 | 1.15457762 |
| Tctex1d2 | 0.49071493 |
| Tctex1d4 | -0.4874129 |
| Tctn3 | -0.5748214 |
| Tctn3 | -0.5112753 |
| Tctn3 | 0.38473978 |
| Tdg | 0.31664738 |
| Tdp1 | -0.6056009 |
| Tdpoz1 | 1.94928245 |
| Tdpoz2 | 0.95274738 |
| Tdrd1 | -0.8380721 |
| Tdrd12 | 0.45990702 |
| Tdrd3 | 0.79834126 |
| Tdrd3 | 1.14365291 |
| Tdrd3 | 0.77744242 |
| Tdrd6 | -0.4018721 |
| Tdrd7 | -0.6557494 |
| Tdrkh | -0.5608913 |
| Tead2 | 0.10121488 |
| Tec | -0.1007256 |
| Tecta | 1.2731309 |
| Tef | 2.704354 |
| Tef | -0.5630771 |
| Tegt | 0.08116551 |
| Tek | -0.5594369 |
| Tekt1 | -0.6926778 |
| Tekt1 | 0.9665381 |
| Tekt3 | -0.8830662 |
| Tenc1 | 3.16102301 |
| Tenc1 | -0.1611514 |
| Terf2 | -0.0003675 |
| Terf2 | 0.90352076 |
| Terf2ip | 0.98621845 |
| Terf2ip | 0.34157413 |
| Terf2ip | 0.11233041 |
| Terf2ip | -0.0223128 |
| Tes | -0.4800362 |
| Tes | -0.4446377 |
| Tes | -0.8322319 |
| Tesc | 0.37750192 |
| Tesk1 | -0.7095663 |
| Tesp1 | -0.2563021 |
| Tesp2 | 1.29036012 |
| Tessp2 | 1.03503549 |
| Tessp3 | -0.1450926 |
| Tex10 | 1.08869391 |
| Tex10 | 1.90307564 |
| Tex101 | -0.1164947 |
| Tex11 | 2.52406494 |
| Tex13 | -0.6893361 |
| Tex14 | 0.02967815 |
| Tex16 | 1.6839049 |
| Tex19 | -0.6695307 |
| Tex2 | -0.7558406 |
| Tex2 | -0.6885025 |
| Tex22 | 0.54564691 |
| Tex24 | -0.6437543 |
| Tex261 | -0.6163453 |
| Tex261 | 1.47819027 |
| Tex264 | 1.72405818 |
| Tex264 | -0.8158783 |
| Tex9 | -0.6793814 |
| Tfam | 0.60201954 |
| Tfam | 0.50563211 |
| Tfb1m | -0.1436887 |
| Tfb2m | 1.84232417 |
| Tfdp1 | 0.7008011 |
| Tfdp2 | -0.8216178 |
| Tfdp2 | 1.26360834 |
| Tfg | 1.19626306 |
| Tfip11 | 0.91622239 |
| Tfpi | -0.5202945 |
| Tfpt | 0.81591738 |
| Tfpt | -0.2226592 |
| Tfpt | 1.85622903 |
| Tfrc | 1.71367331 |
| Tfrc | -0.839049 |
| Tg | -0.7630269 |
| Tgfb1 | -0.6533381 |
| Tgfb1i1 | -0.0334799 |
| Tgfb1i1 | -0.787693 |
| Tgfb2 | 0.35213759 |
| Tgfb3 | -0.2706375 |
| Tgfbi | -0.4694086 |
| Tgfbi | -0.5972439 |
| Tgfbr1 | 0.191275 |
| Tgfbr1 | -0.5457305 |
| Tgfbr1 | -0.7513768 |
| Tgfbr2 | 1.87775197 |
| Tgfbr2 | -0.4472147 |
| Tgfbr3 | 2.15087328 |
| Tgfbr3 | 2.29721724 |
| Tgfbrap1 | -0.5558106 |
| Tgif1 | -0.7711775 |
| Tgif1 | -0.6581668 |
| Tgif1 | 0.79142631 |
| Tgif2 | 1.09701294 |
| Tgif2lx | 1.8201425 |
| Tgm2 | -0.6445493 |
| Tgm4 | 0.57926496 |
| Tgoln1 | -0.2813986 |
| Tgs1 | -0.3781923 |
| Tgs1 | 0.1306866 |
| Th1l | 1.1219054 |
| Tha1 | -0.3469363 |
| Thada | 2.6871203 |
| Thada | -0.2453011 |
| Thap1 | -0.0838982 |
| Thap1 | 0.4489943 |
| Thap1 | -0.8478869 |
| Thap11 | 0.79937415 |
| Thap3 | -0.8749087 |
| Thap4 | -0.4439945 |
| Thap7 | -0.4955264 |
| Thbd | 0.04817278 |
| Thbs1 | -0.8439488 |
| Thbs2 | 0.59242655 |
| Thbs3 | 0.26745161 |
| Thbs3 | 0.40115281 |
| Theg | 0.01832599 |
| Theg | 0.44624627 |
| Theg | -0.043098 |
| Them2 | 0.6188365 |
| Them4 | -0.5085174 |
| Them5 | -0.1325319 |
| Thg1l | 0.40377353 |
| Thnsl1 | 0.88610551 |
| Thnsl2 | -0.4012575 |
| Thnsl2 | 0.73143253 |
| Thoc1 | 0.91607037 |
| Thoc3 | -0.6413735 |
| Thoc4 | -0.3533388 |
| Thoc4 | 0.02029303 |
| Thoc5 | 0.23546538 |
| Thoc6 | 2.53778439 |
| Thop1 | 0.88532049 |
| Thra | 0.35792494 |
| Thra | 1.0475495 |
| Thrap2 | 1.2899511 |
| Thrap3 | -0.3988028 |
| Thrsp | -0.4602066 |
| Thsd1 | 0.48712817 |
| Thsd4 | 2.99829773 |
| Thsd4 | -0.7675463 |
| Thsd7b | -0.340577 |
| Thsd7b | 0.35929766 |
| Thsd7b | -0.6272135 |
| Thtpa | -0.5322205 |
| Thumpd1 | 2.80667699 |
| Thumpd3 | 0.61445099 |
| Thy1 | -0.6048381 |
| Thyn1 | 3.73210613 |
| Tia1 | -0.7442784 |
| Tial1 | -0.873894 |
| Tial1 | 0.32550375 |
| Tiam1 | -0.4667703 |
| Tiam2 | -0.4012575 |
| Ticam1 | 1.52851143 |
| Tie1 | 0.14731944 |
| Tigd2 | 1.31248742 |
| Tigd3 | 1.03396276 |
| Timeless | -0.7729983 |
| Timm10 | -0.2395895 |
| Timm13 | 2.17438953 |
| Timm17a | -0.516121 |
| Timm17b | 0.58446523 |
| Timm22 | 0.24542333 |
| Timm44 | 1.97708371 |
| Timm44 | 2.04313107 |
| Timm44 | -0.5844724 |
| Timm50 | -0.8449318 |
| Timm8a1 | -0.8026203 |
| Timm8a2 | 1.3838184 |
| Timm8a2 | -0.4575939 |
| Timm8b | -0.6017931 |
| Timm8b | -0.6453449 |
| Timm9 | 2.11905885 |
| Timm9 | 0.80417938 |
| Timm9 | -0.1288422 |
| Timp1 | 0.94467014 |
| Timp1 | -0.7451628 |
| Timp2 | -0.2972235 |
| Timp3 | 1.52045567 |
| Timp3 | 0.33230363 |
| Timp4 | -0.3757969 |
| Tinagl | -0.6071283 |
| Tipin | 0.40246408 |
| Tipin | -0.5294008 |
| Tirap | -0.0878566 |
| Tirap | 2.76761636 |
| Tirap | -0.5934696 |
| Tjap1 | -0.5733453 |
| Tjap1 | 1.82717026 |
| Tjap1 | -0.0703895 |
| Tjp1 | 3.36672967 |
| Tjp2 | -0.7078638 |
| Tjp3 | 0.15552715 |
| Tjp3 | 0.49428809 |
| Tk1 | -0.6517341 |
| Tk2 | 3.99566113 |
| Tkt | 2.06712348 |
| Tkt | 2.1799791 |
| Tkt | 0.26866045 |
| Tktl1 | -0.7087146 |
| Tktl1 | -0.6605904 |
| Tktl2 | 1.76188977 |
| Tlcd1 | 1.07889471 |
| Tle1 | -0.7621256 |
| Tle1 | -0.7657359 |
| Tle2 | 0.88075447 |
| Tle3 | -0.6002742 |
| Tle3 | -0.7849203 |
| Tle3 | -0.7914027 |
| Tle4 | 0.13033695 |
| Tle4 | 2.16152821 |
| Tle4 | 0.02499726 |
| Tle6 | 1.29025788 |
| Tlk1 | -0.340577 |
| Tlk2 | 1.53665646 |
| Tln1 | -0.6654564 |
| Tln2 | 0.62050182 |
| Tlr13 | 3.30032015 |
| Tlr2 | 1.59991756 |
| Tlr3 | -0.7639291 |
| Tlr4 | 0.54022753 |
| Tlr6 | 0.02029303 |
| Tlr7 | 2.93226218 |
| Tm2d2 | 0.97813933 |
| Tm2d3 | 1.9980255 |
| Tm2d3 | -0.4595527 |
| Tm4sf4 | 0.52424077 |
| Tm4sf5 | 0.83582418 |
| Tm6sf1 | -0.4312225 |
| Tm6sf2 | 0.55683826 |
| Tm9sf2 | 2.55235382 |
| Tm9sf2 | -0.4634827 |
| Tm9sf2 | 3.07123374 |
| Tm9sf3 | -0.4186212 |
| Tm9sf4 | 0.68052899 |
| Tm9sf4 | 2.18041688 |
| Tmbim1 | -0.8302931 |
| Tmbim4 | -0.855813 |
| Tmc1 | -0.6760864 |
| Tmc4 | 0.47361579 |
| Tmc5 | -0.307181 |
| Tmc6 | 0.88091229 |
| Tmc6 | -0.4894347 |
| Tmc6 | 2.46026366 |
| Tmc7 | -0.0027808 |
| Tmcc2 | 1.92534152 |
| Tmcc3 | 0.63883288 |
| Tmco3 | -0.5500374 |
| Tmco3 | 1.35218415 |
| Tmco4 | -0.6525358 |
| Tmco4 | -0.7087146 |
| Tmco5 | -0.5919641 |
| Tmco6 | 1.56037075 |
| Tmco6 | 0.54767118 |
| Tmco7 | -0.8678284 |
| Tmed1 | 1.29809795 |
| Tmed1 | -0.6728029 |
| Tmed10 | -0.4249008 |
| Tmed2 | -0.5400185 |
| Tmed3 | -0.4299548 |
| Tmed3 | 2.97004427 |
| Tmed4 | 1.54535607 |
| Tmed4 | -0.1083519 |
| Tmed4 | 1.06120402 |
| Tmed4 | -0.6295588 |
| Tmed8 | 2.08784707 |
| Tmed8 | -0.6477358 |
| Tmed9 | -0.2206276 |
| Tmed9 | -0.3562634 |
| Tmeff2 | 0.3345594 |
| Tmem10 | -0.2442601 |
| Tmem100 | 2.36884912 |
| Tmem101 | -0.6687145 |
| Tmem102 | -0.6240955 |
| Tmem102 | -0.6573603 |
| Tmem106a | -0.5652681 |
| Tmem106c | 1.35876761 |
| Tmem107 | 2.28959313 |
| Tmem107 | -0.701929 |
| Tmem107 | 1.03262013 |
| Tmem108 | 1.06289702 |
| Tmem109 | 2.58817086 |
| Tmem11 | 1.35381044 |
| Tmem110 | -0.5385959 |
| Tmem111 | 1.4826198 |
| Tmem111 | -0.1311466 |
| Tmem111 | 2.08252113 |
| Tmem115 | -0.4549883 |
| Tmem116 | -0.6303419 |
| Tmem116 | -0.8478869 |
| Tmem117 | -0.2999789 |
| Tmem118 | -0.7639291 |
| Tmem119 | 0.79264009 |
| Tmem120a | 1.63006066 |
| Tmem120b | 1.3737655 |
| Tmem121 | 1.01403105 |
| Tmem125 | -0.3274173 |
| Tmem126a | 2.03383478 |
| Tmem126b | 0.49903136 |
| Tmem127 | -0.7451628 |
| Tmem128 | 1.11244695 |
| Tmem130 | -0.7181253 |
| Tmem131 | 0.61904482 |
| Tmem132a | 0.09288238 |
| Tmem132a | -0.5718716 |
| Tmem132c | 0.68714111 |
| Tmem132d | 0.90089799 |
| Tmem132e | -0.8618016 |
| Tmem134 | 0.01517017 |
| Tmem134 | 1.92388034 |
| Tmem135 | -0.6597819 |
| Tmem136 | 1.56999606 |
| Tmem138 | 1.46358511 |
| Tmem139 | 0.38045739 |
| Tmem140 | -0.382399 |
| Tmem141 | -0.6654564 |
| Tmem143 | -0.2900967 |
| Tmem143 | 2.20022131 |
| Tmem143 | -0.1483765 |
| Tmem143 | -0.7293694 |
| Tmem144 | 1.39979271 |
| Tmem146 | 2.33400047 |
| Tmem147 | 0.08227017 |
| Tmem14c | 0.15688818 |
| Tmem14c | -0.1007256 |
| Tmem14c | -0.391477 |
| Tmem150 | 2.14448821 |
| Tmem150 | 0.94864397 |
| Tmem151a | 1.17663146 |
| Tmem154 | -0.2308299 |
| Tmem158 | -0.6501327 |
| Tmem159 | 2.2340499 |
| Tmem159 | -0.5202945 |
| Tmem160 | -0.525888 |
| Tmem160 | -0.3674605 |
| Tmem161a | -0.7044679 |
| Tmem162 | -0.1110584 |
| Tmem164 | 1.90536864 |
| Tmem166 | -0.1343822 |
| Tmem167 | 0.71577128 |
| Tmem168 | 0.42933316 |
| Tmem168 | -0.4274244 |
| Tmem168 | -0.4894347 |
| Tmem169 | 1.54870474 |
| Tmem16a | 1.85031909 |
| Tmem16b | 1.15681814 |
| Tmem16d | -0.5294008 |
| Tmem16e | 0.18568518 |
| Tmem16g | 0.23796474 |
| Tmem16j | 1.27261017 |
| Tmem16k | -0.6695307 |
| Tmem17 | 2.46833226 |
| Tmem17 | -0.3149992 |
| Tmem170 | -0.4700693 |
| Tmem176a | -0.6233176 |
| Tmem176a | 0.58273499 |
| Tmem176b | -0.6638316 |
| Tmem177 | 0.60201954 |
| Tmem178 | 2.01818166 |
| Tmem179b | -0.5822365 |
| Tmem180 | 2.61337046 |
| Tmem183a | 0.95055135 |
| Tmem183a | -0.5674641 |
| Tmem184a | -0.4092794 |
| Tmem184a | 0.18337383 |
| Tmem184b | 0.61987767 |
| Tmem185b | -0.1906665 |
| Tmem186 | -0.5428702 |
| Tmem19 | 1.63340647 |
| Tmem192 | -0.4363106 |
| Tmem198 | 1.37833982 |
| Tmem199 | 1.8021326 |
| Tmem2 | 2.64489443 |
| Tmem2 | 0.97941959 |
| Tmem2 | 0.89485333 |
| Tmem201 | -0.5057675 |
| Tmem204 | -0.6885025 |
| Tmem205 | -0.6209878 |
| Tmem208 | -0.5594369 |
| Tmem209 | -0.632695 |
| Tmem209 | -0.2808576 |
| Tmem23 | 0.1151778 |
| Tmem25 | 0.29457652 |
| Tmem25 | -0.0874159 |
| Tmem25 | -0.4901096 |
| Tmem27 | -0.546447 |
| Tmem32 | 1.53452814 |
| Tmem33 | -0.2917366 |
| Tmem33 | 1.03677608 |
| Tmem34 | 0.65420922 |
| Tmem35 | 1.20760333 |
| Tmem38a | -0.510585 |
| Tmem38b | 1.56014037 |
| Tmem38b | -0.5829812 |
| Tmem39a | 0.46089285 |
| Tmem40 | 0.94835019 |
| Tmem41a | -0.4773681 |
| Tmem41b | -0.6638316 |
| Tmem42 | -0.7053157 |
| Tmem43 | -0.8698459 |
| Tmem43 | 1.89653941 |
| Tmem44 | -0.6461412 |
| Tmem45a | 1.37020272 |
| Tmem45b | -0.7172659 |
| Tmem47 | -0.4267929 |
| Tmem47 | 0.92137648 |
| Tmem48 | 0.49452583 |
| Tmem49 | 0.17540592 |
| Tmem49 | -0.6272135 |
| Tmem49 | -0.6071283 |
| Tmem49 | 1.21449775 |
| Tmem5 | -0.3417301 |
| Tmem50a | -0.6086583 |
| Tmem50b | -0.7328563 |
| Tmem50b | -0.4793685 |
| Tmem51 | 0.11837087 |
| Tmem53 | 0.35324269 |
| Tmem53 | 0.08557641 |
| Tmem53 | 0.60667417 |
| Tmem54 | -0.7621256 |
| Tmem55a | -0.1092532 |
| Tmem55b | -0.8216178 |
| Tmem56 | 0.95959536 |
| Tmem57 | 0.01871973 |
| Tmem59 | 1.57839488 |
| Tmem59l | -0.5807488 |
| Tmem60 | -0.4049514 |
| Tmem62 | -0.019849 |
| Tmem63a | 0.37264564 |
| Tmem63b | -0.3963545 |
| Tmem63b | -0.1488466 |
| Tmem63c | -0.5023415 |
| Tmem65 | 2.90394252 |
| Tmem66 | -0.1872322 |
| Tmem66 | -0.5536415 |
| Tmem66 | -0.4433518 |
| Tmem68 | 0.48520964 |
| Tmem69 | -0.2277576 |
| Tmem70 | 0.6981232 |
| Tmem71 | 2.22892382 |
| Tmem74 | 0.05841882 |
| Tmem74 | -0.5565347 |
| Tmem74 | -0.7164073 |
| Tmem77 | 0.0277306 |
| Tmem77 | 1.86042787 |
| Tmem77 | -0.3189327 |
| Tmem79 | -0.6056009 |
| Tmem8 | 0.21394907 |
| Tmem8 | -0.2653027 |
| Tmem8 | 1.10749578 |
| Tmem81 | 1.60987841 |
| Tmem82 | -0.413005 |
| Tmem85 | -0.7381108 |
| Tmem85 | 2.03907233 |
| Tmem86a | 1.39211475 |
| Tmem86b | 2.35803479 |
| Tmem87a | 0.77179029 |
| Tmem88 | -0.5638069 |
| Tmem9 | -0.2631772 |
| Tmem90a | 0.58877699 |
| Tmem91 | 0.07747394 |
| Tmem93 | 1.72341247 |
| Tmem97 | 0.53043923 |
| Tmem98 | 1.46409545 |
| Tmem98 | 0.87426111 |
| Tmem9b | 2.82005963 |
| Tmem9b | 2.42580958 |
| Tmem9b | -0.4375869 |
| Tmie | 2.37767047 |
| Tmod1 | -0.4602066 |
| Tmod2 | -0.2344269 |
| Tmod2 | -0.4996095 |
| Tmod3 | -0.3933031 |
| Tmod4 | 3.31354108 |
| Tmod4 | 0.74301015 |
| Tmod4 | -0.6613997 |
| Tmpo | -0.4680885 |
| Tmpo | -0.634267 |
| Tmpo | 2.99091324 |
| Tmprss11a | -0.7198465 |
| Tmprss11c | -0.5279941 |
| Tmprss13 | -0.4914609 |
| Tmprss13 | -0.3423071 |
| Tmprss2 | 0.24418432 |
| Tmprss4 | -0.3775929 |
| Tmprss4 | -0.382399 |
| Tmprss4 | 0.74428782 |
| Tmprss7 | -0.5202945 |
| Tmprss9 | 0.83615519 |
| Tmsb10 | -0.6525358 |
| Tmsb10 | 0.29133883 |
| Tmsb10 | 0.11126042 |
| Tmsb4x | 0.48376818 |
| Tmtc2 | 0.17173134 |
| Tmtc4 | -0.6155738 |
| Tmub1 | 0.03356122 |
| Tmub1 | 0.19356709 |
| Tnc | -0.6248741 |
| Tnc | 1.21505156 |
| Tnc | 2.5711031 |
| Tnc | -0.3291234 |
| Tnfaip1 | 0.92995447 |
| Tnfaip2 | 0.3134847 |
| Tnfaip2 | -0.2145587 |
| Tnfaip2 | 1.14018285 |
| Tnfaip3 | -0.6977127 |
| Tnfaip8 | 0.17473888 |
| Tnfaip8l2 | 2.39608299 |
| Tnfaip8l2 | 0.95974053 |
| Tnfaip8l3 | -0.2370047 |
| Tnfrsf10b | 1.4461538 |
| Tnfrsf10b | -0.5558106 |
| Tnfrsf10b | 2.43094879 |
| Tnfrsf11a | 1.96661054 |
| Tnfrsf11b | 0.82617419 |
| Tnfrsf12a | -0.7207082 |
| Tnfrsf13c | -0.7630269 |
| Tnfrsf19 | 0.16669737 |
| Tnfrsf19 | 0.64330976 |
| Tnfrsf1a | -0.7433949 |
| Tnfrsf1a | -0.63191 |
| Tnfrsf1a | -0.3245806 |
| Tnfrsf1b | 1.30597395 |
| Tnfrsf21 | 1.44111367 |
| Tnfrsf21 | -0.1573482 |
| Tnfrsf22 | 0.07190934 |
| Tnfrsf22 | -0.5755603 |
| Tnfrsf25 | 1.81306199 |
| Tnfrsf4 | -0.5579847 |
| Tnfsf13b | -0.6703477 |
| Tnfsf13b | 2.33393284 |
| Tnip1 | 1.5964506 |
| Tnip1 | -0.7087146 |
| Tnip1 | -0.5889603 |
| Tnip1 | 2.65920475 |
| Tnip2 | 3.6484619 |
| Tnks | -0.6885025 |
| Tnks1bp1 | -0.3417301 |
| Tnnc1 | 0.13033695 |
| Tnnc2 | 0.24480403 |
| Tnni2 | 1.24866106 |
| Tnni3 | 0.85612988 |
| Tnnt1 | 0.22286825 |
| Tnp1 | 0.83084513 |
| Tnp2 | -0.6194378 |
| Tnpo1 | 0.82013449 |
| Tnpo2 | -0.5050813 |
| Tnr | -0.2085287 |
| Tnrc15 | -0.6893361 |
| Tnrc18 | -0.510585 |
| Tnrc18 | 0.93294589 |
| Tnrc18 | -0.5594369 |
| Tnrc6a | 1.76776255 |
| Tnrc6a | 0.33652879 |
| Tnrc6b | -0.3274173 |
| Tnrc6b | -0.7010842 |
| Tnrc6c | 0.54090666 |
| Tns4 | 3.36134129 |
| Tnxb | 0.88012294 |
| Tnxb | -0.7398689 |
| Tnxb | 3.82190947 |
| Tob1 | -0.8168324 |
| Tob2 | 1.58582313 |
| Toe1 | 0.68772232 |
| Toe1 | -0.5949775 |
| Tollip | 2.87579372 |
| Tollip | 1.60087426 |
| Tom1 | 1.78854656 |
| Tom1l2 | 1.55443989 |
| Tom1l2 | -0.0019757 |
| Tom1l2 | -0.359785 |
| Tomm22 | 2.97289942 |
| Tomm34 | 0.27947084 |
| Tomm34 | 2.6275939 |
| Tomm7 | 2.98158355 |
| Tomm70a | 0.26866045 |
| Top1 | -0.6287764 |
| Top1mt | 0.40586488 |
| Top2a | -0.8361214 |
| Top3a | -0.6901704 |
| Top3b | -0.1233344 |
| Topors | 3.31977957 |
| Tor1a | -0.5874619 |
| Tor1a | -0.5272915 |
| Tor1aip1 | 0.17206599 |
| Tor1aip2 | 0.45867328 |
| Tor1aip2 | -0.6445493 |
| Tor1b | 2.50705236 |
| Tor3a | 0.66657003 |
| Tox3 | 1.64069752 |
| Tpbg | 1.38242856 |
| Tpcn1 | 1.99691447 |
| Tpcn1 | -0.5594369 |
| Tpcn2 | 1.06055206 |
| Tpd52 | -0.9079705 |
| Tpd52l1 | -0.3486781 |
| Tpd52l1 | -0.5711356 |
| Tpd52l2 | -0.6985544 |
| Tph2 | 0.78078241 |
| Tpi1 | 1.62763287 |
| Tpi1 | 1.56717975 |
| Tpi1 | 0.16231276 |
| Tpk1 | -0.128382 |
| Tpk1 | -0.2830234 |
| Tpk1 | -0.3251472 |
| Tpm1 | 1.78945038 |
| Tpm2 | 0.34853708 |
| Tpm2 | 1.84004372 |
| Tpm3 | -0.6573603 |
| Tpm3 | 2.60971773 |
| Tpm4 | 0.05463696 |
| Tpo | 2.4788005 |
| Tpp1 | 0.78060692 |
| Tpp2 | 0.18667403 |
| Tppp | 0.35407066 |
| Tppp | -0.5428702 |
| Tppp3 | -0.5837265 |
| Tpr | -0.7146924 |
| Tpra40 | 1.84312103 |
| Tprgl | -0.6613997 |
| Tprgl | 1.86343949 |
| Tprkb | -0.7302399 |
| Tprkb | 1.91316566 |
| Tpsab1 | 3.48282744 |
| Tpst1 | -0.6303419 |
| Tpst2 | -0.7112719 |
| Tpte | -0.0604905 |
| Tpte | 0.07599325 |
| Tpx2 | 0.76806259 |
| Tra2a | -0.4465698 |
| Trabd | 0.41805875 |
| Trabd | -0.7549462 |
| Traf1 | -0.7739101 |
| Traf2 | -0.4117615 |
| Traf3 | 0.21905607 |
| Traf3 | -0.0617758 |
| Traf3ip1 | -0.4654539 |
| Traf3ip2 | 0.14456759 |
| Traf4 | -0.3319738 |
| Traf4 | 1.69980108 |
| Traf4 | -0.1521437 |
| Traf4 | 2.02425816 |
| Traf5 | 1.49603158 |
| Traf6 | -0.1201364 |
| Traf6 | -0.7155494 |
| Traf6 | 1.02533673 |
| Traf7 | 1.15150301 |
| Trafd1 | -0.8283584 |
| Trafd1 | -0.6240955 |
| Trafd1 | 2.03323036 |
| Traip | 1.37245446 |
| Trak1 | 0.85158279 |
| Trak2 | 0.04549813 |
| Trak2 | 1.39486406 |
| Trak2 | 0.30451422 |
| Tram1 | -0.7104187 |
| Tram1 | -0.514734 |
| Tram1 | -0.6374188 |
| Tram2 | -0.7648321 |
| Tram2 | 0.01398401 |
| Trap1 | -0.311082 |
| Trappc1 | 0.18502537 |
| Trappc1 | 0.53545989 |
| Trappc2 | 0.60329127 |
| Trappc2l | -0.0309868 |
| Trappc3 | 2.02805372 |
| Trappc3 | 0.31117789 |
| Trappc3 | -0.2873699 |
| Trappc3 | -0.6818603 |
| Trappc4 | -0.658974 |
| Trappc4 | 0.14353357 |
| Trappc5 | 0.08374104 |
| Trappc6a | 1.19105265 |
| Trat1 | 2.50061395 |
| Trcg1 | -0.2658348 |
| Trdmt1 | -0.6117256 |
| Trdmt1 | 3.45963097 |
| Trem2 | -0.2727799 |
| Treml1 | 0.6152879 |
| Treml2 | 1.59726316 |
| Treml4 | 0.25955617 |
| Trerf1 | 3.19530976 |
| Trerf1 | 0.86146097 |
| Trex1 | -0.189684 |
| Trex1 | -0.4395046 |
| Trex2 | 0.73585983 |
| Trf | 0.56569671 |
| Trfp | -0.2563021 |
| Trfr2 | -0.4894347 |
| Trfr2 | 0.03742836 |
| Trh | 1.42874745 |
| Trhde | -0.4230125 |
| Trhr | -0.5064542 |
| Triap1 | -0.3933031 |
| Trib2 | 1.14687222 |
| Trib3 | -0.5016577 |
| Trib3 | -0.4117615 |
| Trim11 | 1.34536155 |
| Trim16 | 1.87440057 |
| Trim17 | 0.01517017 |
| Trim2 | -0.7363561 |
| Trim2 | -0.6525358 |
| Trim21 | 0.10337652 |
| Trim21 | 1.97178721 |
| Trim23 | 0.78463506 |
| Trim23 | -0.4588993 |
| Trim23 | -0.5485996 |
| Trim25 | -0.6002742 |
| Trim26 | -0.5202945 |
| Trim28 | -0.7867678 |
| Trim3 | 2.34208015 |
| Trim3 | 0.50210174 |
| Trim30 | -0.6453449 |
| Trim32 | 2.45984954 |
| Trim32 | 0.64087046 |
| Trim33 | 2.56214676 |
| Trim33 | 0.75715019 |
| Trim33 | -0.1970777 |
| Trim35 | 0.2472788 |
| Trim36 | -0.4037185 |
| Trim36 | -0.5897103 |
| Trim36 | 1.02533673 |
| Trim37 | 0.84061241 |
| Trim38 | -0.0322325 |
| Trim38 | -0.6366299 |
| Trim39 | 3.0529381 |
| Trim40 | 2.74850853 |
| Trim41 | 1.47558974 |
| Trim42 | -0.0799563 |
| Trim44 | 0.49761088 |
| Trim45 | -0.2510476 |
| Trim45 | 1.86771728 |
| Trim46 | 1.50625137 |
| Trim47 | 0.73383323 |
| Trim54 | -0.513349 |
| Trim56 | -0.1270027 |
| Trim59 | -0.6101907 |
| Trim61 | 0.30393254 |
| Trim62 | 0.23983492 |
| Trim67 | -0.5726082 |
| Trim68 | 0.4216607 |
| Trim69 | 0.93428895 |
| Trim72 | -0.5175101 |
| Trim8 | 0.17139658 |
| Trim9 | -0.3200596 |
| Trim9 | -0.3228827 |
| Trim9 | 0.63433464 |
| Trim9 | -0.5485996 |
| Triml1 | -0.4255311 |
| Triobp | 1.1883227 |
| Triobp | -0.7328563 |
| Trip10 | 0.4489943 |
| Trip12 | -0.4407853 |
| Trip12 | -0.6148029 |
| Trip12 | 1.33186004 |
| Trip6 | -0.649333 |
| Trip6 | 0.91789297 |
| Trit1 | -0.5638069 |
| Trmt1 | 1.80022919 |
| Trmt12 | 1.86488611 |
| Trmu | -0.649333 |
| Tro | -0.4439945 |
| Tro | 0.33540393 |
| Tro | -0.0256078 |
| Trove2 | 0.64148088 |
| Trp53 | -0.4975658 |
| Trp53bp1 | -0.7639291 |
| Trp53bp1 | -0.4517415 |
| Trp53bp2 | -0.5064542 |
| Trp53i11 | -0.7585291 |
| Trp53i11 | 0.2983886 |
| Trp53inp1 | -0.3728111 |
| Trp53inp1 | 1.37245446 |
| Trp53inp2 | 0.22950271 |
| Trp53rk | 0.67485238 |
| Trp63 | -0.7960605 |
| Trpc1 | 0.74173074 |
| Trpc1 | -0.7061643 |
| Trpc2 | 0.24882224 |
| Trpc3 | 0.38873695 |
| Trpc4 | 0.65781456 |
| Trpc4ap | -0.3475166 |
| Trpc4ap | -0.7181253 |
| Trpc5 | -0.6194378 |
| Trpc6 | 0.92469653 |
| Trpc7 | 0.92107407 |
| Trpd52l3 | -0.5016577 |
| Trpm1 | -0.1469677 |
| Trpm2 | -0.4369485 |
| Trpm3 | 2.58197881 |
| Trpm3 | -0.4006432 |
| Trpm3 | -0.1936201 |
| Trpm3 | 0.17873407 |
| Trpm3 | -0.2857376 |
| Trpm4 | -0.3836043 |
| Trpm6 | -0.8871715 |
| Trpm6 | 0.68052899 |
| Trpm6 | 0.75876818 |
| Trpm8 | 0.89205037 |
| Trps1 | -0.4800362 |
| Trpt1 | 1.12921043 |
| Trpt1 | 0.8036657 |
| Trpt1 | -0.6421665 |
| Trpv2 | 0.20913602 |
| Trpv4 | -0.1804009 |
| Trpv6 | 1.95934532 |
| Trrap | 0.99044298 |
| Trub1 | 1.04370647 |
| Trub1 | 4.51865406 |
| Trub1 | -0.5507571 |
| Trub2 | 1.66113511 |
| Tsc1 | -0.2642393 |
| Tsc2 | 0.22634926 |
| Tsc2 | -0.5867137 |
| Tsc22d1 | 1.40740868 |
| Tsc22d1 | 0.44248602 |
| Tsc22d2 | -0.7766506 |
| Tsc22d3 | 1.06315721 |
| Tsc22d3 | -0.7693601 |
| Tsc22d4 | -0.2610564 |
| Tsen2 | 1.60234421 |
| Tsen34 | -0.7363561 |
| Tsen54 | -0.7487087 |
| Tsfm | -0.455639 |
| Tsg101 | 0.89173841 |
| Tsga10 | 2.43100981 |
| Tsga10 | 1.90797482 |
| Tsga14 | -0.7487087 |
| Tsga8 | -0.7969948 |
| Tshb | 1.75191537 |
| Tshr | 0.93503426 |
| Tshz3 | -0.3504232 |
| Tsku | -0.4694086 |
| Tsn | -0.2344269 |
| Tsnaxip1 | 0.00922439 |
| Tsnaxip1 | -0.6086583 |
| Tspan1 | -0.1823477 |
| Tspan11 | -0.6951919 |
| Tspan12 | -0.3234483 |
| Tspan13 | -0.5343408 |
| Tspan14 | -0.555087 |
| Tspan17 | 1.09487717 |
| Tspan17 | 0.61737692 |
| Tspan18 | -0.7215708 |
| Tspan18 | 1.7463211 |
| Tspan2 | 0.04281587 |
| Tspan3 | 0.20977918 |
| Tspan31 | -0.3639102 |
| Tspan33 | 0.36313068 |
| Tspan6 | 0.86852254 |
| Tspan6 | 1.9345263 |
| Tspan7 | -0.0120894 |
| Tspan8 | 0.00203962 |
| Tspo | -0.353923 |
| Tspo | -0.7053157 |
| Tspo | -0.2364886 |
| Tspyl1 | 3.78604332 |
| Tspyl3 | 1.53468596 |
| Tsr1 | -0.3116406 |
| Tsr2 | -0.0622046 |
| Tsr2 | 1.34969146 |
| Tsr2 | 0.8698008 |
| Tssc1 | -0.0182101 |
| Tssc1 | -0.4068039 |
| Tssc1 | -0.7398689 |
| Tssc1 | 1.57764129 |
| Tssc1 | 0.19911052 |
| Tssc4 | 1.83661266 |
| Tssc4 | 0.22413556 |
| Tssk4 | -0.2749272 |
| Tssk5 | 1.11417374 |
| Tssk6 | -0.2589405 |
| Tst | 0.63063825 |
| Tsta3 | 0.0072341 |
| Tsx | -0.6760864 |
| Ttbk2 | -0.3527549 |
| Ttc1 | -0.6071283 |
| Ttc12 | -0.5071414 |
| Ttc13 | 1.21825727 |
| Ttc14 | -0.5867137 |
| Ttc16 | -0.094491 |
| Ttc17 | 0.11978655 |
| Ttc19 | 2.9712321 |
| Ttc19 | -0.4179955 |
| Ttc21a | 1.29444057 |
| Ttc22 | 0.46138538 |
| Ttc23 | 0.18568518 |
| Ttc24 | 0.68052899 |
| Ttc24 | 1.72740879 |
| Ttc26 | 2.04290093 |
| Ttc27 | 2.2564116 |
| Ttc29 | 0.85125715 |
| Ttc29 | -0.1219625 |
| Ttc3 | 1.89249863 |
| Ttc3 | -0.6860062 |
| Ttc3 | -0.1265434 |
| Ttc3 | -0.7121258 |
| Ttc3 | 0.15756796 |
| Ttc3 | 2.42128969 |
| Ttc30a1 | -0.2669 |
| Ttc30a2 | 0.01121048 |
| Ttc33 | 0.04931674 |
| Ttc33 | -0.3200596 |
| Ttc33 | 1.6774842 |
| Ttc35 | 1.13054357 |
| Ttc4 | 2.98636998 |
| Ttc5 | -0.3662756 |
| Ttc5 | 3.49440786 |
| Ttc5 | 0.11872499 |
| Ttc7 | -0.4517415 |
| Ttc8 | 1.47272927 |
| Ttc8 | 1.31905538 |
| Ttf2 | -0.4433518 |
| Ttk | -0.2797766 |
| Ttl | 0.14007864 |
| Ttll1 | -0.7594269 |
| Ttll1 | -0.516121 |
| Ttll1 | 0.52814825 |
| Ttll10 | -0.5733453 |
| Ttll11 | 0.71238119 |
| Ttll13 | -0.5168153 |
| Ttll4 | 3.02459204 |
| Ttll5 | -0.8548186 |
| Ttll5 | -0.7250289 |
| Ttll6 | 2.18786768 |
| Ttll8 | -0.4713922 |
| Ttll9 | 2.63977418 |
| Ttn | 2.75402609 |
| Ttn | -0.1146791 |
| Ttr | 0.2000854 |
| Ttyh1 | -0.4312225 |
| Ttyh1 | 2.1780664 |
| Ttyh2 | -0.016574 |
| Ttyh2 | 0.2072039 |
| Ttyh3 | -0.6295588 |
| Tub | -0.482712 |
| Tub | -0.3021891 |
| Tuba1a | -0.8177876 |
| Tuba1b | -0.7951271 |
| Tuba3a | -0.4136274 |
| Tuba3a | 1.40632444 |
| Tuba3a | -0.6622096 |
| Tuba3a | -0.3440404 |
| Tuba3b | 0.85239638 |
| Tuba4a | -0.0247829 |
| Tuba6 | -0.585966 |
| Tuba8 | 0.7138894 |
| Tubb2b | -0.2862814 |
| Tubb2b | -0.7675463 |
| Tubb2b | 1.03409692 |
| Tubb2b | 1.60747108 |
| Tubb2b | 2.07720933 |
| Tubb2c | -0.809226 |
| Tubb4 | -0.7513768 |
| Tubb5 | -0.4840527 |
| Tubb6 | 2.18145082 |
| Tubd1 | 2.56699216 |
| Tubd1 | -0.290643 |
| Tube1 | -0.5043956 |
| Tubg1 | -0.4982465 |
| Tubgcp2 | -0.0699569 |
| Tubgcp4 | 0.25772472 |
| Tufm | -0.5371754 |
| Tuft1 | -0.6977127 |
| Tug1 | -0.6968717 |
| Tulp1 | -0.1842984 |
| Tulp2 | 1.62016766 |
| Tulp3 | 1.8200259 |
| Tulp4 | 1.60600911 |
| Tusc2 | -0.4249008 |
| Tusc2 | 0.36313068 |
| Tusc4 | 0.29163362 |
| Tut1 | -0.8177876 |
| Twf2 | 1.47491747 |
| Twist1 | -0.9016825 |
| Twist2 | 3.15929102 |
| Twistnb | 1.26675828 |
| Twistnb | -0.8158783 |
| Twsg1 | -0.4286887 |
| Txk | 0.04051071 |
| Txlna | -0.7061643 |
| Txn1 | 0.80640209 |
| Txn2 | 0.57730744 |
| Txndc1 | -0.4654539 |
| Txndc11 | 0.79627209 |
| Txndc11 | -0.2760027 |
| Txndc11 | 2.33295178 |
| Txndc12 | 2.11514411 |
| Txndc13 | 0.96495157 |
| Txndc13 | 3.02216652 |
| Txndc15 | 2.00284093 |
| Txndc2 | -0.6010334 |
| Txndc5 | -0.7711775 |
| Txndc8 | 1.11577436 |
| Txndc9 | 0.17840178 |
| Txnip | 1.02965961 |
| Txnl2 | 0.14869236 |
| Txnl4 | -0.6810333 |
| Txnl4a | 2.93378498 |
| Txnl4a | -0.4780344 |
| Txnl4a | -0.5645372 |
| Txnl4b | -0.1474371 |
| Txnrd2 | -0.6910055 |
| Txnrd3 | -0.73373 |
| Tyk2 | 2.2261987 |
| Tyk2 | 1.20927596 |
| Tyk2 | -0.6918413 |
| Tyki | -0.3734075 |
| Tyms | 0.25864089 |
| Tyms | -0.5733453 |
| Tyms | -0.7630269 |
| Tyms-ps | 0.47045111 |
| Tyro3 | -0.5230871 |
| Tyrobp | 1.63879172 |
| Tyw1 | 0.48400858 |
| Tyw1 | 0.43643825 |
| U2af1l4 | 1.47584172 |
| U2af2 | 0.42907842 |
| U2af2 | -0.5507571 |
| Uaca | 0.35351876 |
| Uap1 | 0.21202679 |
| Uap1l1 | -0.3545075 |
| Uba1 | 0.20332774 |
| Uba1 | -0.613263 |
| Uba2 | -0.5507571 |
| Uba5 | 0.31175513 |
| Ubac1 | -0.4497988 |
| Ubac1 | 2.21755507 |
| Ubac1 | -0.6910055 |
| Ubac2 | -0.8054456 |
| Ubap1 | 2.4796121 |
| Ubap2 | -0.6163453 |
| Ubap2l | 0.33821357 |
| Ubap2l | 0.82583966 |
| Ubap2l | -0.7487087 |
| Ubb | 1.24201277 |
| Ubd | 0.42473718 |
| Ube1c | -0.7416302 |
| Ube1l | -0.1079016 |
| Ube1x | 3.1212112 |
| Ube2a | 1.88103684 |
| Ube2b | 1.82653353 |
| Ube2d1 | -0.5009745 |
| Ube2d2 | -0.6646437 |
| Ube2d3 | -0.7775659 |
| Ube2e1 | -0.5638069 |
| Ube2e2 | -0.019849 |
| Ube2e3 | 3.41001723 |
| Ube2f | -0.2994272 |
| Ube2f | -0.20453 |
| Ube2f | 0.35130792 |
| Ube2g1 | 1.24972899 |
| Ube2g1 | -0.513349 |
| Ube2g1 | -0.0401603 |
| Ube2g2 | -0.3388501 |
| Ube2i | 1.08336152 |
| Ube2j1 | 0.83648608 |
| Ube2j2 | 2.32294773 |
| Ube2j2 | -0.7684528 |
| Ube2j2 | 0.94614388 |
| Ube2k | 0.55148298 |
| Ube2k | 0.53454903 |
| Ube2l3 | 2.29154031 |
| Ube2l3 | -0.1857642 |
| Ube2l6 | 1.4734031 |
| Ube2m | -0.2354572 |
| Ube2n | -0.514734 |
| Ube2n | -0.6760864 |
| Ube2o | 0.16568734 |
| Ube2q1 | 0.15348191 |
| Ube2q1 | -0.0825824 |
| Ube2q2 | -0.4179955 |
| Ube2q2 | 1.86516405 |
| Ube2q2 | -0.632695 |
| Ube2r2 | 2.31511577 |
| Ube2s | 1.05032263 |
| Ube2t | 0.34687065 |
| Ube2u | 2.28948681 |
| Ube2u | -0.809226 |
| Ube3a | -0.3411534 |
| Ube3a | -0.6171175 |
| Ube3b | -0.6140326 |
| Ube3b | -0.5085174 |
| Ube3c | 0.26654397 |
| Ube4a | -0.4491521 |
| Ube4b | 0.7776185 |
| Ubfd1 | 0.34492276 |
| Ubiad1 | 0.40141521 |
| Ubie | -0.8007414 |
| Ubl3 | 2.12075586 |
| Ubl4 | -0.4123831 |
| Ubl4b | -0.7363561 |
| Ubl5 | -0.7070137 |
| Ubl7 | -0.7061643 |
| Ublcp1 | 0.35710034 |
| Ubox5 | -0.4401447 |
| Ubox5 | -0.1721718 |
| Ubp1 | -0.209531 |
| Ubp1 | -0.6893361 |
| Ubqln1 | -0.0843372 |
| Ubqln1 | -0.128382 |
| Ubqln1 | 1.02493044 |
| Ubqln3 | -0.6461412 |
| Ubqln4 | -0.7044679 |
| Ubqlnl | -0.4472147 |
| Ubr1 | 1.45666834 |
| Ubr5 | -0.2191067 |
| Ubtd1 | -0.6744432 |
| Ubtd2 | 0.33989535 |
| Ubtd2 | -0.5964878 |
| Ubtd2 | 0.4847294 |
| Ubtf | -0.2395895 |
| Ubxd1 | -0.2473867 |
| Ubxd1 | -0.8370962 |
| Ubxd1 | 1.03087186 |
| Ubxd3 | -0.4793685 |
| Ubxd4 | -0.4049514 |
| Ubxd5 | 1.82988599 |
| Ubxd8 | -0.6901704 |
| Uchl1 | -0.1789435 |
| Uchl3 | -0.4575939 |
| Uchl5 | -0.094491 |
| Uchl5 | 0.8191241 |
| Uchl5 | -0.8177876 |
| Uck1 | -0.0256078 |
| Uck1 | 1.33975131 |
| Uck1 | 0.15211594 |
| Uck1 | -0.1891931 |
| Uck2 | 0.73861637 |
| Uck2 | -0.3896544 |
| Uck2 | 1.02357489 |
| Uevld | 1.9840181 |
| Ufc1 | 1.87136858 |
| Ufsp1 | 1.85280015 |
| Ufsp2 | -0.5792634 |
| Ugcg | -0.1339193 |
| Ugp2 | 0.88484917 |
| Ugt1a10 | -0.7960605 |
| Ugt1a6a | 2.02402336 |
| Ugt1a6a | 0.5004497 |
| Ugt1a6a | -0.8273925 |
| Ugt3a2 | -0.3336881 |
| Ugt8a | -0.0777735 |
| Uhmk1 | 0.39826159 |
| Uhrf1 | 1.35045914 |
| Uhrf1bp1 | -0.6002742 |
| Uhrf1bp1l | -0.7839978 |
| Uhrf2 | -0.1260843 |
| Uhrf2 | 0.63330932 |
| Uhrf2 | -0.4497988 |
| Uhrf2 | 1.33703092 |
| Uimc1 | 2.01254129 |
| Ulk1 | -0.8245007 |
| Ulk1 | -0.7739101 |
| Ulk2 | 2.0347639 |
| Ulk2 | -0.3734075 |
| Umodl1 | 0.34687065 |
| Umps | -0.4136274 |
| Umps | -0.3574358 |
| Umps | -0.5867137 |
| Unc119 | 0.77037195 |
| Unc119 | -0.4928141 |
| Unc119b | -0.5565347 |
| Unc119b | -0.4860674 |
| Unc119b | 0.24294368 |
| Unc13b | 1.77537347 |
| Unc13c | -0.140421 |
| Unc13d | -0.5357571 |
| Unc45a | -0.027673 |
| Unc45a | 1.96125473 |
| Unc45b | -0.7215708 |
| Unc5a | -0.4000293 |
| Unc5a | 1.53799404 |
| Unc5b | 1.83414604 |
| Unc5c | 0.45048985 |
| Unc84a | -0.4615157 |
| Unc84a | 1.9162244 |
| Unc84b | -0.5980006 |
| Unc93a | 0.42473718 |
| Unc93b1 | -0.4080408 |
| Unc93b1 | -0.5154273 |
| Ung | -0.1353088 |
| Ung | 1.78164907 |
| Unk | 0.31922716 |
| Unkl | 0.70935752 |
| Uox | 0.32635642 |
| Upb1 | -0.3533388 |
| Upb1 | -0.225205 |
| Upb1 | 0.65941252 |
| Upf1 | -0.5995157 |
| Upf1 | -0.6086583 |
| Upf1 | 0.70954678 |
| Upf2 | -0.5601638 |
| Upf3a | 1.03530348 |
| Upk1a | -0.6033144 |
| Upk1a | -0.5897103 |
| Upk1b | -0.5400185 |
| Upk2 | -0.364501 |
| Upk3a | 0.82884613 |
| Upk3b | -0.4497988 |
| Upk3b | 0.75083151 |
| Upk3b | -0.0506942 |
| Upp1 | 2.12890128 |
| Upp1 | -0.3010834 |
| Upp1 | -0.6025534 |
| Upp2 | 1.20916455 |
| Upp2 | 3.21740582 |
| Upp2 | 1.48295327 |
| Upp2 | -0.0347289 |
| Uqcc | 2.49378885 |
| Uqcr | 0.64917882 |
| Uqcrc1 | -0.9389678 |
| Uqcrc2 | -0.5837265 |
| Uqcrh | -0.5770398 |
| Urm1 | -0.7540526 |
| Urod | 2.27386926 |
| Uros | -0.2994272 |
| Uros | -0.6148029 |
| Use1 | 0.68772232 |
| Use1 | -0.7389895 |
| Use1 | -0.4407853 |
| Usf1 | 0.38260101 |
| Usf1 | -0.7112719 |
| Usf2 | -0.5457305 |
| Usf2 | -0.9357135 |
| Ush1c | -0.5608913 |
| Ush1g | 1.74505939 |
| Ush2a | -0.780317 |
| Ush3a | -0.3811952 |
| Ushbp1 | 2.28183543 |
| Usmg5 | -0.6163453 |
| Uso1 | 1.85751866 |
| Usp1 | 1.81915092 |
| Usp1 | -0.7693601 |
| Usp1 | -0.5889603 |
| Usp10 | 1.7986202 |
| Usp10 | -0.0790826 |
| Usp10 | 0.39086186 |
| Usp11 | 1.1998708 |
| Usp11 | 1.13005901 |
| Usp12 | -0.3521715 |
| Usp12 | 0.84750427 |
| Usp13 | -0.0998324 |
| Usp14 | 0.1531406 |
| Usp15 | -0.8769415 |
| Usp16 | -0.4414262 |
| Usp18 | -0.5889603 |
| Usp18 | 3.13805339 |
| Usp19 | 1.37086003 |
| Usp2 | 0.40062779 |
| Usp2 | 0.58316785 |
| Usp2 | 1.10575671 |
| Usp20 | -0.2547227 |
| Usp21 | 1.24233554 |
| Usp22 | -0.3504232 |
| Usp25 | 1.22682584 |
| Usp28 | 2.32380255 |
| Usp29 | -0.074726 |
| Usp3 | -0.3639102 |
| Usp3 | -0.0574982 |
| Usp33 | 1.30062948 |
| Usp37 | -0.9016825 |
| Usp38 | -0.7181253 |
| Usp38 | 0.26502928 |
| Usp39 | -0.0120894 |
| Usp39 | -0.1960886 |
| Usp39 | -0.5957324 |
| Usp39 | 0.43871071 |
| Usp4 | -0.7425121 |
| Usp4 | -0.6662699 |
| Usp43 | 2.33183414 |
| Usp45 | 0.44799594 |
| Usp46 | -0.6148029 |
| Usp47 | -0.4840527 |
| Usp48 | -0.5272915 |
| Usp49 | 0.56921692 |
| Usp5 | 1.13802259 |
| Usp50 | 2.22760009 |
| Usp52 | 0.04166399 |
| Usp52 | 0.26199259 |
| Usp52 | 0.23640341 |
| Usp53 | -0.4433518 |
| Usp6nl | 2.81341238 |
| Usp6nl | -0.4249008 |
| Usp7 | 1.38085092 |
| Usp8 | -0.1654484 |
| Usp8 | 1.00812016 |
| Usp9x | 1.28872298 |
| Usp9y | 0.3921875 |
| Uspl1 | -0.6063643 |
| Utp11l | 0.86772274 |
| Utp15 | -0.6389987 |
| Utp6 | 1.16537725 |
| Utx | -0.7232982 |
| Utx | -0.0256078 |
| Uty | 1.65253975 |
| Uvrag | -0.5272915 |
| Uxt | 0.9738592 |
| Uxt | 0.99142599 |
| V1ra2 | 1.31627456 |
| V1rd2 | 2.61107971 |
| V1rd20 | 1.60013842 |
| V1rd21 | 0.22413556 |
| V2r8 | 1.2157157 |
| Vac14 | 0.03626988 |
| Vamp1 | -0.0137177 |
| Vamp1 | 0.69888909 |
| Vamp2 | 0.19389408 |
| Vamp3 | 0.07079247 |
| Vamp3 | 0.25864089 |
| Vamp4 | -0.7164073 |
| Vamp5 | -0.6926778 |
| Vamp8 | 0.06930125 |
| Vamp8 | -0.5329268 |
| Vangl2 | 0.45048985 |
| Vapa | -0.4962057 |
| Vapb | -0.5092061 |
| Vars | -0.4331273 |
| Vars | 3.8782954 |
| Vars | 1.86960002 |
| Vars2 | -0.6421665 |
| Vash1 | 0.11943283 |
| Vash1 | -0.7138361 |
| Vasn | 0.00922439 |
| Vasp | 0.5648146 |
| Vat1 | -0.0289143 |
| Vav1 | 0.84504867 |
| Vav2 | 1.75279235 |
| Vav3 | 0.92213206 |
| Vax1 | -0.3388501 |
| Vbp1 | 0.13869311 |
| Vcam1 | 4.10714646 |
| Vcl | -0.6760864 |
| Vcp | -0.6217638 |
| Vcpip1 | 2.29942938 |
| Vdac1 | -0.6860062 |
| Vdac2 | -0.7821556 |
| Vdac3 | -0.787693 |
| Vdr | -0.3710242 |
| Vdr | 0.67818419 |
| Vdr | 0.92785481 |
| Vegfa | 0.67091747 |
| Vegfa | -0.3149992 |
| Vegfb | 1.80231084 |
| Vegfc | 0.57512765 |
| Vezf1 | -0.516121 |
| Vezt | 0.42063298 |
| Vgf | 2.11172673 |
| Vgll2 | -0.658974 |
| Vgll4 | -0.0206696 |
| Vhlh | 1.33381465 |
| Vil1 | -0.5457305 |
| Vill | -0.3211878 |
| Vim | -0.7416302 |
| Vip | 1.78073774 |
| Vit | -0.1755516 |
| Vkorc1 | -0.6670841 |
| Vkorc1 | 0.17373743 |
| Vkorc1 | 0.24511373 |
| Vkorc1l1 | 0.87251136 |
| Vkorc1l1 | 1.03583924 |
| Vkorc1l1 | 0.28393885 |
| Vldlr | 0.06331269 |
| Vldlr | 0.6693389 |
| Vldlr | 0.46187766 |
| Vldlr | -0.6477358 |
| Vldlr | -0.406186 |
| Vmn2r81 | -0.5889603 |
| Vmo1 | 0.22729641 |
| Vnn3 | 1.16642712 |
| Vnn3 | -0.737233 |
| Vpreb1 | -0.701929 |
| Vpreb3 | 0.42140388 |
| Vps11 | -0.353923 |
| Vps13a | -0.7311112 |
| Vps16 | 2.10928427 |
| Vps16 | -0.6413735 |
| Vps16 | -0.5740831 |
| Vps16 | 0.47239987 |
| Vps18 | 1.04144636 |
| Vps24 | 2.16908886 |
| Vps25 | -0.5154273 |
| Vps25 | -0.7720875 |
| Vps26 | -0.4733799 |
| Vps26a | -0.5435845 |
| Vps26b | -0.259469 |
| Vps28 | 1.03878046 |
| Vps29 | 1.55829534 |
| Vps29 | -0.7181253 |
| Vps29 | -0.7164073 |
| Vps33a | 1.62247095 |
| Vps33a | 0.44649642 |
| Vps33b | -0.2110365 |
| Vps33b | -0.7061643 |
| Vps35 | 0.41444299 |
| Vps36 | -0.0716883 |
| Vps37b | -0.778482 |
| Vps37c | -0.6752645 |
| Vps37d | 1.78637385 |
| Vps39 | -0.4136274 |
| Vps39 | -0.094491 |
| Vps41 | 3.40310972 |
| Vps41 | 3.07749511 |
| Vps45 | -0.6264331 |
| Vps4a | -0.3817969 |
| Vps4b | -0.6968717 |
| Vps53 | 0.10625102 |
| Vps53 | 0.5338653 |
| Vps53 | 0.86868241 |
| Vps54 | -0.2406254 |
| Vps72 | 0.45496234 |
| Vrk1 | 1.6677362 |
| Vrk1 | -0.7666407 |
| Vrk2 | 2.69708882 |
| Vrk3 | 0.23107549 |
| Vsig2 | -0.0397414 |
| Vsig4 | 0.52239634 |
| Vsnl1 | 3.27914839 |
| Vstm2a | 0.84357223 |
| Vstm2a | -0.8302931 |
| Vstm2a | 0.0648134 |
| Vstm2b | 0.6246523 |
| Vsx2 | 0.69062297 |
| Vti1a | -0.7155494 |
| Vti1a | 2.67894697 |
| Vti1a | -0.2308299 |
| Vti1b | 1.6754475 |
| Vti1b | 0.00523961 |
| Vtn | 1.29698192 |
| Vwa2 | 2.5870081 |
| Vwa3a | -0.8264276 |
| Vwf | -0.3325449 |
| Wars | -0.6810333 |
| Wars | -0.7061643 |
| Wars | -0.300531 |
| Wars | -0.1137726 |
| Wars2 | 2.32830322 |
| Wars2 | -0.4105197 |
| Was | -0.7302399 |
| Wasf1 | -0.4588993 |
| Wasf2 | 1.24844733 |
| Wasf3 | -0.3446189 |
| Wasl | 1.63617379 |
| Wbp1 | -0.3285543 |
| Wbp1 | 0.26168838 |
| Wbp11 | 0.27947084 |
| Wbp2 | 2.68744577 |
| Wbp2nl | 0.06706 |
| Wbp4 | -0.682688 |
| Wbp5 | 0.14663223 |
| Wbp7 | -0.5762997 |
| Wbscr16 | -0.7319833 |
| Wbscr18 | -0.4641393 |
| Wbscr22 | -0.0652118 |
| Wbscr22 | -0.2231678 |
| Wbscr22 | -0.4414262 |
| Wbscr27 | 1.32980333 |
| Wbscr28 | 0.10265652 |
| Wbscr28 | -0.0052003 |
| Wdfy1 | 0.31750809 |
| Wdfy3 | -0.0422577 |
| Wdhd1 | 0.42191746 |
| Wdr1 | -0.6910055 |
| Wdr1 | 1.93416475 |
| Wdr13 | -0.6876697 |
| Wdr13 | -0.2206276 |
| Wdr16 | -0.6248741 |
| Wdr17 | 0.31951337 |
| Wdr18 | -0.5630771 |
| Wdr20a | 1.03033327 |
| Wdr20b | -0.73373 |
| Wdr21 | 0.52768939 |
| Wdr22 | -0.6140326 |
| Wdr23 | 2.11420556 |
| Wdr24 | -0.5882108 |
| Wdr27 | 1.24384035 |
| Wdr3 | -0.5897103 |
| Wdr31 | -0.4975658 |
| Wdr31 | 0.0191133 |
| Wdr31 | -0.5800058 |
| Wdr32 | 0.74154783 |
| Wdr33 | -0.7630269 |
| Wdr34 | -0.1228769 |
| Wdr37 | -0.7078638 |
| Wdr37 | -0.4105197 |
| Wdr37 | -0.2170827 |
| Wdr37 | 0.40847255 |
| Wdr38 | 0.24139058 |
| Wdr41 | 0.25036316 |
| Wdr45 | 0.70366188 |
| Wdr45l | -0.6295588 |
| Wdr45l | 1.13126994 |
| Wdr46 | -0.4880863 |
| Wdr46 | -0.536466 |
| Wdr47 | -0.2717081 |
| Wdr5 | -0.737233 |
| Wdr51b | 0.66835095 |
| Wdr53 | -0.3533388 |
| Wdr54 | -0.7104187 |
| Wdr55 | 0.17507246 |
| Wdr59 | -0.2510476 |
| Wdr5b | -0.4740434 |
| Wdr6 | -0.8380721 |
| Wdr60 | -0.4680885 |
| Wdr61 | -0.789546 |
| Wdr62 | -0.8361214 |
| Wdr63 | 0.55661573 |
| Wdr66 | 2.49905422 |
| Wdr67 | 1.72082518 |
| Wdr68 | 2.72976149 |
| Wdr69 | 1.69953612 |
| Wdr70 | 0.35957196 |
| Wdr70 | -0.6303419 |
| Wdr74 | 0.27618079 |
| Wdr75 | -0.73373 |
| Wdr75 | 0.88359115 |
| Wdr76 | -0.3486781 |
| Wdr77 | -0.3574358 |
| Wdr78 | -0.8658152 |
| Wdr8 | 1.902221 |
| Wdr8 | -0.4236415 |
| Wdr9 | 0.72307493 |
| Wdr93 | 0.91134562 |
| Wdsof1 | -0.2895507 |
| Wfdc1 | 0.20105927 |
| Wfdc10 | 1.71007694 |
| Wfdc10 | 0.55861662 |
| Wfdc12 | 0.66240142 |
| Wfdc15b | -0.406186 |
| Wfdc15b | -0.2453011 |
| Wfdc2 | -0.8045029 |
| Wfdc3 | -0.5092061 |
| Wfdc6a | -0.6951919 |
| Wfdc6b | 1.69161928 |
| Wfdc6b | -0.9303146 |
| Wfikkn2 | -0.3060694 |
| Wfs1 | 0.34825954 |
| Whdc1 | 0.71087057 |
| Whrn | -0.510585 |
| Whrn | 1.77090029 |
| Whsc2 | -0.513349 |
| Wif1 | 0.02068595 |
| Wipf1 | 2.01025659 |
| Wipf2 | 2.31432208 |
| Wipi1 | 1.15858321 |
| Wipi1 | 0.99100482 |
| Wipi1 | -0.4517415 |
| Wipi2 | 0.22159923 |
| Wipi2 | -0.7036209 |
| Wipi2 | 0.48328721 |
| Wisp1 | 0.60983467 |
| Wisp1 | 1.23737418 |
| Wisp2 | 0.1015755 |
| Wiz | 4.35958971 |
| Wiz | 0.40664794 |
| Wnk1 | -0.4615157 |
| Wnk1 | 0.58532916 |
| Wnk2 | 0.68500697 |
| Wnk4 | 1.24233554 |
| Wnt10a | 0.04664534 |
| Wnt10b | -0.4853953 |
| Wnt16 | -0.0891797 |
| Wnt16 | -0.5478815 |
| Wnt16 | 1.60498437 |
| Wnt2 | -0.3521715 |
| Wnt3a | -0.2547227 |
| Wnt4 | -0.590461 |
| Wnt4 | 0.8263414 |
| Wnt5a | 1.70580893 |
| Wnt5a | 1.30335592 |
| Wnt5b | 1.19547203 |
| Wnt6 | 1.77854695 |
| Wnt7a | -0.2035329 |
| Wnt7b | -0.3279856 |
| Wnt7b | 0.24045749 |
| Wrb | 0.44148075 |
| Wrn | -0.6295588 |
| Wsb1 | 0.40141521 |
| Wsb2 | 0.37129222 |
| Wscd1 | 1.14758613 |
| Wscd2 | 0.45372211 |
| Wtap | 2.88874407 |
| Wtip | -0.3533388 |
| Wwc1 | -0.413005 |
| Wwc1 | -0.4727169 |
| Wwc2 | 4.53481064 |
| Wwc2 | -0.7576321 |
| Wwox | -0.6109578 |
| Wwox | -0.8341746 |
| Wwp1 | 3.26807394 |
| Wwp2 | -0.4700693 |
| Wwp2 | -0.5400185 |
| Wwp2 | 0.12049359 |
| Wwtr1 | 1.18134831 |
| X99384 | 3.01146233 |
| Xab1 | -0.1697651 |
| Xab1 | -0.649333 |
| Xbp1 | -0.7061643 |
| Xdh | -0.3975779 |
| Xist | -0.7666407 |
| Xist | -0.2005481 |
| Xk | 0.7200854 |
| Xkr4 | -0.4037185 |
| Xkr5 | -0.3975779 |
| Xkr6 | 0.14249842 |
| Xkr8 | 0.64290365 |
| Xkrx | 0.36504137 |
| Xlr4a | 2.09558414 |
| Xpc | -0.4420676 |
| Xpnpep1 | 1.95171952 |
| Xpnpep1 | -0.2557754 |
| Xpnpep1 | -0.2642393 |
| Xpnpep2 | 1.75535598 |
| Xpnpep2 | 0.8995065 |
| Xpnpep3 | -0.6413735 |
| Xpo4 | -0.5493182 |
| Xpo4 | -0.7675463 |
| Xpo5 | -0.6711654 |
| Xpo6 | -0.2541968 |
| Xpo7 | 0.65038857 |
| Xpo7 | -0.562348 |
| Xpr1 | 0.78951577 |
| Xrcc1 | -0.5638069 |
| Xrcc2 | 1.10885994 |
| Xrcc2 | -0.7198465 |
| Xrcc3 | 1.48702863 |
| Xrcc6 | 1.0916013 |
| Xrcc6 | -0.634267 |
| Xrcc6 | 1.50844438 |
| Xrn1 | 2.49794666 |
| Xrn2 | 1.80876442 |
| Xylb | -0.5608913 |
| Xylt2 | 0.07451024 |
| Yaf2 | -0.7648321 |
| Yap1 | 0.44949308 |
| Ybx2 | 1.55714036 |
| Ybx2 | 0.0108136 |
| Yeats2 | 2.92462915 |
| Yeats4 | 0.99016193 |
| Yeats4 | -0.6810333 |
| Yif1a | -0.3251472 |
| Yif1a | -0.6533381 |
| Yif1a | 1.64154331 |
| Yif1b | 1.89330817 |
| Yipf1 | 2.43724277 |
| Yipf2 | 1.41648529 |
| Yipf3 | -0.1697651 |
| Yipf3 | -0.787693 |
| Yipf3 | 0.1909471 |
| Yipf4 | -0.007626 |
| Yipf6 | -0.0847764 |
| Yipf6 | -0.1362362 |
| Yipf6 | -0.2599979 |
| Yipf6 | -0.547164 |
| Yipf7 | -0.311082 |
| Ykt6 | 0.00324089 |
| Ykt6 | 2.47807527 |
| Ylpm1 | 2.20987334 |
| Yme1l1 | -0.4510935 |
| Yme1l1 | 0.00763249 |
| Yme1l1 | -0.5230871 |
| Yod1 | 1.59037118 |
| Ypel1 | -0.6885025 |
| Ypel3 | -0.8120712 |
| Ypel3 | -0.0043931 |
| Ypel4 | -0.5630771 |
| Ypel4 | 0.0285101 |
| Ypel5 | -0.0962681 |
| Yrdc | -0.316683 |
| Ythdf1 | 1.10923165 |
| Ythdf2 | -0.170727 |
| Ythdf2 | -0.4414262 |
| Ythdf3 | -0.5272915 |
| Ythdf3 | -0.4299548 |
| Ywhab | 0.29045389 |
| Ywhab | -0.7666407 |
| Ywhae | -0.5112753 |
| Ywhag | 1.04873898 |
| Ywhah | -0.4198738 |
| Ywhaq | -0.4123831 |
| Ywhaz | -0.8351475 |
| Yy2 | 0.97941959 |
| Zadh1 | -0.7702684 |
| Zadh2 | 0.34045528 |
| Zan | -0.7250289 |
| Zap70 | 1.27645677 |
| Zap70 | 1.80917737 |
| Zbbx | -0.2473867 |
| Zbed4 | 0.41986145 |
| Zbp1 | 1.38557594 |
| Zbtb17 | 0.73272597 |
| Zbtb2 | 1.06834604 |
| Zbtb22 | -0.3049591 |
| Zbtb24 | -0.5112753 |
| Zbtb3 | -0.8351475 |
| Zbtb32 | -0.4217558 |
| Zbtb33 | -0.4148734 |
| Zbtb38 | 0.28126059 |
| Zbtb39 | -0.4472147 |
| Zbtb40 | 0.57752515 |
| Zbtb41 | 0.61277494 |
| Zbtb43 | 0.1764056 |
| Zbtb45 | -0.1215057 |
| Zbtb46 | 0.46825394 |
| Zbtb5 | -0.5934696 |
| Zbtb7a | 1.30647659 |
| Zbtb7c | -0.737233 |
| Zbtb8 | 1.41594852 |
| Zbtb9 | 0.43162279 |
| Zbtb9 | -0.6785566 |
| Zc3h10 | 0.05879618 |
| Zc3h11a | -0.7010842 |
| Zc3h12a | 1.56801791 |
| Zc3h12b | -0.6769091 |
| Zc3h13 | -0.3746015 |
| Zc3h14 | 0.22413556 |
| Zc3h14 | 0.5703143 |
| Zc3h15 | 0.70328094 |
| Zc3h18 | -0.4621709 |
| Zc3h3 | 1.26002565 |
| Zc3h3 | -0.4517415 |
| Zc3h3 | 0.29251746 |
| Zc3h6 | 2.62185548 |
| Zc3h7a | 0.11482235 |
| Zc3h7a | -0.6063643 |
| Zc3h8 | 2.7505977 |
| Zc3hc1 | 0.27016932 |
| Zc3hc1 | -0.4293216 |
| Zc3hc1 | -0.1119623 |
| Zc3hc1 | 0.93413981 |
| Zcchc12 | -0.7451628 |
| Zcchc14 | 2.52505943 |
| Zcchc16 | -0.3591972 |
| Zcchc17 | -0.5414433 |
| Zcchc17 | 3.33283791 |
| Zcchc18 | -0.7830763 |
| Zcchc18 | 1.45271759 |
| Zcchc3 | 0.36830785 |
| Zcchc3 | 2.06833411 |
| Zcchc3 | -0.8206588 |
| Zcchc5 | -0.2669 |
| Zcchc6 | -0.5085174 |
| Zcchc8 | -0.3371263 |
| Zcchc8 | 0.82198406 |
| Zcchc8 | 1.40948332 |
| Zcchc9 | -0.0821442 |
| Zcrb1 | -0.2727799 |
| Zcwpw1 | -0.6960314 |
| Zdhhc1 | -0.027673 |
| Zdhhc12 | 0.47458741 |
| Zdhhc12 | 0.99618608 |
| Zdhhc13 | 0.87059883 |
| Zdhhc13 | -0.2879146 |
| Zdhhc13 | -0.6002742 |
| Zdhhc14 | -0.6272135 |
| Zdhhc15 | -0.1946067 |
| Zdhhc16 | -0.2105344 |
| Zdhhc16 | -0.7433949 |
| Zdhhc17 | -0.5601638 |
| Zdhhc19 | 0.13452429 |
| Zdhhc19 | -0.4641393 |
| Zdhhc21 | 2.99530055 |
| Zdhhc24 | -0.7821556 |
| Zdhhc3 | 0.75912736 |
| Zdhhc3 | 0.97371619 |
| Zdhhc4 | 0.82030278 |
| Zdhhc6 | 1.3093866 |
| Zdhhc7 | 1.716085 |
| Zdhhc9 | -0.554364 |
| Zeb1 | 0.34101487 |
| Zeb2 | -0.6525358 |
| Zer1 | 0.74683798 |
| Zf | -0.0545154 |
| Zfa | -0.7311112 |
| Zfand2a | -0.780317 |
| Zfand2b | -0.2005481 |
| Zfand2b | 0.39562549 |
| Zfand2b | 1.30214514 |
| Zfand2b | 0.24604222 |
| Zfand3 | -0.7585291 |
| Zfand6 | 0.84603168 |
| Zfand6 | -0.0443598 |
| Zfat | 2.69718085 |
| Zfh4 | 1.93761959 |
| Zfhx2 | 0.54226345 |
| Zfhx2 | -0.511966 |
| Zfhx3 | -0.043939 |
| Zfhx3 | 2.20037715 |
| Zfml | 0.97600169 |
| Zfp1 | 0.22729641 |
| Zfp101 | 2.42871912 |
| Zfp105 | -0.5168153 |
| Zfp106 | 1.17974033 |
| Zfp106 | -0.3686469 |
| Zfp109 | -0.2401073 |
| Zfp11 | 0.24696981 |
| Zfp119 | -0.6461412 |
| Zfp12 | 0.04204811 |
| Zfp128 | -0.632695 |
| Zfp13 | 1.96110413 |
| Zfp131 | 0.43441368 |
| Zfp142 | -0.4280564 |
| Zfp143 | 1.21825727 |
| Zfp148 | -0.7095663 |
| Zfp160 | 1.52229621 |
| Zfp161 | -0.4517415 |
| Zfp161 | -0.1931272 |
| Zfp169 | 0.78025583 |
| Zfp179 | -0.364501 |
| Zfp182 | 0.00683554 |
| Zfp184 | -0.331403 |
| Zfp185 | -0.0355624 |
| Zfp187 | -0.2658348 |
| Zfp187 | -0.5674641 |
| Zfp189 | 1.25324461 |
| Zfp191 | 0.54609712 |
| Zfp2 | -0.3811952 |
| Zfp2 | 1.52906721 |
| Zfp202 | 0.31979949 |
| Zfp207 | 1.49849445 |
| Zfp212 | -0.3562634 |
| Zfp212 | 1.6191582 |
| Zfp212 | 1.03208255 |
| Zfp212 | -0.3149992 |
| Zfp213 | 0.6064631 |
| Zfp217 | 2.11181231 |
| Zfp219 | -0.0326481 |
| Zfp235 | 0.59478053 |
| Zfp236 | -0.7657359 |
| Zfp236 | 0.15211594 |
| Zfp238 | -0.1673645 |
| Zfp238 | -0.2328837 |
| Zfp238 | 0.78882007 |
| Zfp238 | -0.9554098 |
| Zfp238 | -0.4074222 |
| Zfp239 | 0.35682532 |
| Zfp251 | -1.0065286 |
| Zfp259 | -0.0145329 |
| Zfp26 | -0.6171175 |
| Zfp263 | 0.8998159 |
| Zfp27 | -0.3793923 |
| Zfp27 | -0.6501327 |
| Zfp275 | 1.7440488 |
| Zfp276 | -0.340577 |
| Zfp277 | -0.7594269 |
| Zfp277 | 1.05479526 |
| Zfp280c | 1.86405179 |
| Zfp281 | 2.71579094 |
| Zfp282 | -0.4142502 |
| Zfp286 | -0.9346313 |
| Zfp288 | -0.7319833 |
| Zfp292 | 0.70309041 |
| Zfp292 | -0.0104639 |
| Zfp295 | -0.590461 |
| Zfp296 | -0.5209919 |
| Zfp296 | 1.98122097 |
| Zfp3 | 3.44666758 |
| Zfp30 | 3.20896643 |
| Zfp313 | -0.0243708 |
| Zfp313 | -0.513349 |
| Zfp316 | 1.85212414 |
| Zfp316 | -0.8538252 |
| Zfp318 | -0.300531 |
| Zfp322a | 0.22350212 |
| Zfp324 | 0.09651426 |
| Zfp326 | 1.4486638 |
| Zfp326 | 0.63556356 |
| Zfp326 | 1.84459913 |
| Zfp329 | -0.6217638 |
| Zfp329 | 0.51753748 |
| Zfp330 | -0.3527549 |
| Zfp330 | 1.20413714 |
| Zfp334 | -0.7311112 |
| Zfp335 | -0.8302931 |
| Zfp339 | -0.7830763 |
| Zfp346 | 0.93726668 |
| Zfp346 | -0.6810333 |
| Zfp35 | 0.7978244 |
| Zfp354a | 0.24294368 |
| Zfp36 | 0.7399 |
| Zfp365 | -0.8548186 |
| Zfp367 | 0.60434973 |
| Zfp367 | -0.2599979 |
| Zfp36l1 | -0.7540526 |
| Zfp36l1 | 0.22064635 |
| Zfp36l1 | -0.4293216 |
| Zfp36l2 | -0.510585 |
| Zfp37 | 1.25505058 |
| Zfp371 | -0.3521715 |
| Zfp382 | 0.91834807 |
| Zfp385a | -0.7858436 |
| Zfp385a | 0.06068069 |
| Zfp385c | 0.42268731 |
| Zfp386 | -0.0755957 |
| Zfp386 | 1.37385907 |
| Zfp39 | 0.2512865 |
| Zfp393 | 1.51668336 |
| Zfp395 | 0.43795383 |
| Zfp397 | 0.55861662 |
| Zfp398 | 1.52540894 |
| Zfp398 | -0.6358416 |
| Zfp398 | 1.87362972 |
| Zfp41 | 1.39523003 |
| Zfp41 | 0.15211594 |
| Zfp410 | -0.6662699 |
| Zfp410 | -0.5182055 |
| Zfp410 | -0.1521437 |
| Zfp414 | -0.514734 |
| Zfp418 | -0.3609618 |
| Zfp42 | 0.17139658 |
| Zfp423 | 0.16095953 |
| Zfp423 | 0.05236054 |
| Zfp428 | -0.4773681 |
| Zfp428 | -0.6549449 |
| Zfp446 | 0.67935732 |
| Zfp446 | -0.0003675 |
| Zfp449 | -0.632695 |
| Zfp451 | 2.69508508 |
| Zfp457 | 3.34876653 |
| Zfp46 | 1.20503286 |
| Zfp467 | 1.52213631 |
| Zfp472 | 0.93622554 |
| Zfp473 | -0.7914027 |
| Zfp473 | 0.50328 |
| Zfp474 | 0.47870565 |
| Zfp474 | 0.40847255 |
| Zfp512 | 2.28874219 |
| Zfp513 | 0.91226193 |
| Zfp516 | -0.0447808 |
| Zfp52 | 1.72231374 |
| Zfp521 | -0.5009745 |
| Zfp523 | -0.2711727 |
| Zfp523 | 1.78055537 |
| Zfp524 | -0.128382 |
| Zfp526 | 0.80452168 |
| Zfp532 | -0.1659271 |
| Zfp532 | 1.58701819 |
| Zfp536 | 0.46947522 |
| Zfp551 | 1.30113497 |
| Zfp553 | -0.0494239 |
| Zfp553 | -0.1196804 |
| Zfp560 | 0.88767358 |
| Zfp566 | 1.57530132 |
| Zfp57 | -0.128382 |
| Zfp574 | -0.8245007 |
| Zfp579 | -0.3692407 |
| Zfp592 | -0.294476 |
| Zfp593 | 1.45185654 |
| Zfp595 | 0.66180439 |
| Zfp598 | 1.87676427 |
| Zfp60 | 0.85499516 |
| Zfp606 | 0.86708242 |
| Zfp607 | -0.5182055 |
| Zfp608 | 1.47508558 |
| Zfp609 | 0.96408506 |
| Zfp61 | 0.31808146 |
| Zfp612 | 0.22223395 |
| Zfp617 | 0.45198304 |
| Zfp619 | -0.14182 |
| Zfp62 | -0.0141252 |
| Zfp622 | 0.75894778 |
| Zfp623 | -0.4439945 |
| Zfp629 | 2.15517423 |
| Zfp637 | 0.75083151 |
| Zfp639 | -0.3371263 |
| Zfp64 | 1.13573693 |
| Zfp64 | 0.0108136 |
| Zfp64 | -0.3144386 |
| Zfp641 | 0.41780094 |
| Zfp644 | -0.6010334 |
| Zfp646 | 0.64249736 |
| Zfp653 | 3.41804292 |
| Zfp654 | 1.60674038 |
| Zfp654 | 0.82717705 |
| Zfp655 | -0.7232982 |
| Zfp661 | 0.14146213 |
| Zfp667 | -0.209531 |
| Zfp668 | 3.04261129 |
| Zfp67 | 0.39694446 |
| Zfp672 | 0.63924075 |
| Zfp68 | 2.51492744 |
| Zfp68 | -0.3674605 |
| Zfp68 | 1.63127222 |
| Zfp687 | 0.19487436 |
| Zfp688 | -0.9378818 |
| Zfp689 | -0.8459158 |
| Zfp69 | -0.6843456 |
| Zfp691 | 0.5128866 |
| Zfp692 | -0.8779595 |
| Zfp692 | 0.55349478 |
| Zfp704 | 1.20704513 |
| Zfp704 | 0.15756796 |
| Zfp706 | 1.55374423 |
| Zfp709 | 1.88125543 |
| Zfp709 | 1.5494818 |
| Zfp710 | -0.7585291 |
| Zfp711 | -0.9174818 |
| Zfp715 | -0.5286972 |
| Zfp71-rs1 | -0.4595527 |
| Zfp74 | 0.91088712 |
| Zfp75 | 2.07088481 |
| Zfp75 | -0.0540901 |
| Zfp75 | 3.31634933 |
| Zfp750 | -0.4628266 |
| Zfp758 | 1.08412513 |
| Zfp760 | -0.2851941 |
| Zfp764 | -0.7155494 |
| Zfp768 | 0.19715773 |
| Zfp770 | -0.1526156 |
| Zfp771 | -0.969895 |
| Zfp771 | 1.18535631 |
| Zfp78 | -0.8283584 |
| Zfp78 | 1.0695096 |
| Zfp780b | -0.5949775 |
| Zfp786 | 2.28126354 |
| Zfp787 | 0.58856187 |
| Zfp787 | 0.01001933 |
| Zfp790 | -0.6202125 |
| Zfp804a | -0.1568738 |
| Zfp809 | -1.0100371 |
| Zfp810 | 0.21170603 |
| Zfp811 | 1.22024155 |
| Zfp817 | 1.06328728 |
| Zfp819 | 0.53432117 |
| Zfp82 | 1.92361917 |
| Zfp825 | -0.3492595 |
| Zfp828 | 1.52013521 |
| Zfp85-rs1 | 0.97671477 |
| Zfp87 | 1.83523674 |
| Zfp87 | -0.406186 |
| Zfp90 | -0.4569418 |
| Zfp91 | 0.46580662 |
| Zfp91-cntf | -0.9732696 |
| Zfp92 | -0.0443598 |
| Zfp93 | -0.4375869 |
| Zfp93 | 1.59755846 |
| Zfp94 | 2.02247225 |
| Zfp97 | 2.88581039 |
| Zfpl1 | 2.91660577 |
| Zfpm2 | 0.15211594 |
| Zfr | -0.747821 |
| Zfx | -0.2824815 |
| Zfx | -0.6358416 |
| Zfy1 | 0.61340381 |
| Zfyve1 | -0.2201203 |
| Zfyve16 | -0.5674641 |
| Zfyve19 | 0.77179029 |
| Zfyve20 | -0.5350487 |
| Zfyve21 | 0.56525576 |
| Zfyve26 | 0.68810959 |
| Zfyve27 | -0.0528152 |
| Zgpat | -0.7044679 |
| Zgpat | 2.00882585 |
| Zhx1 | -0.1741016 |
| Zhx1 | -0.590461 |
| Zhx1 | -0.6389987 |
| Zhx2 | 0.14560048 |
| Zhx3 | -0.9079705 |
| Zic1 | -0.7603256 |
| Zic2 | 2.77681566 |
| Zic3 | 0.07339645 |
| Zic4 | -0.7549462 |
| Zkscan1 | -0.4720543 |
| Zkscan14 | 0.46507119 |
| Zkscan17 | -0.7711775 |
| Zkscan3 | 1.23314726 |
| Zkscan6 | -0.6248741 |
| Zkscan6 | -0.8678284 |
| Zmat1 | -0.73373 |
| Zmat2 | -0.5378854 |
| Zmat3 | 1.11982532 |
| Zmat4 | -0.2120415 |
| Zmat5 | 1.30436371 |
| Zmiz1 | -0.5912122 |
| Zmiz1 | -0.5237865 |
| Zmiz2 | 0.10589219 |
| Zmiz2 | -0.7354799 |
| Zmpste24 | 1.32251993 |
| Zmpste24 | 1.30153917 |
| Zmpste24 | -0.6413735 |
| Zmym3 | -0.1960886 |
| Zmym3 | 1.16327399 |
| Zmym5 | -0.043098 |
| Zmym6 | 2.83647564 |
| Zmynd10 | -0.6935151 |
| Zmynd11 | 1.72728014 |
| Zmynd11 | -0.1702459 |
| Zmynd11 | -0.554364 |
| Zmynd12 | 1.13910334 |
| Zmynd15 | -0.8245007 |
| Znhit2 | 0.69004356 |
| Znhit3 | -0.8225778 |
| Znhit4 | -0.0178008 |
| Znrd1 | 0.01871973 |
| Znrd1 | -0.3988028 |
| Znrd1 | 0.27588113 |
| Znrf1 | 1.65823254 |
| Znrf2 | -0.5755603 |
| Znrf3 | 0.65240136 |
| Znrf4 | -0.1721718 |
| Zp1 | -0.3105237 |
| Zp3 | -0.7250289 |
| Zp3r | 1.2986048 |
| Zpbp | -0.1549789 |
| Zpbp2 | -0.1436887 |
| Zpbp2 | 2.76962108 |
| Zranb1 | 1.49874038 |
| Zranb2 | -0.5421565 |
| Zranb3 | 1.91569774 |
| Zrsr2 | -0.7328563 |
| Zscan10 | 0.97099495 |
| Zscan12 | -0.7720875 |
| Zscan2 | 0.01556522 |
| Zscan2 | 0.03045604 |
| Zscan2 | 0.59968358 |
| Zscan20 | 1.87026362 |
| Zscan20 | -0.3394254 |
| Zscan21 | 0.2622967 |
| Zscan21 | 0.72232843 |
| Zswim1 | 0.58273499 |
| Zswim2 | 0.05274032 |
| Zswim3 | -0.0843372 |
| Zswim4 | -0.4575939 |
| Zswim5 | 1.91848569 |
| Zw10 | -0.4793685 |
| Zw10 | 0.24943891 |
| Zw10 | -0.3021891 |
| Zwilch | 1.13958328 |
| Zxda | 0.70118304 |
| Zxdc | 2.49244647 |
| Zxdc | 0.40612597 |
| Zxdc | 0.80964126 |
| Zyg11b | -0.3884413 |
| Zyx | -0.8035611 |
| Zzz3 | 1.69541968 |
| Zzz3 | -0.2065272 |
| Zzz3 | 0.50304446 |
| Zzz3 | 0.12402088 |

| **Gene Symbol** | **HET5** |
| --- | --- |
| 0610006I08Rik | -0.388178 |
| 0610006I08Rik | -0.1448996 |
| 0610007C21Rik | -0.6932909 |
| 0610007C21Rik | -0.1046466 |
| 0610007P08Rik | 1.1521128 |
| 0610007P14Rik | -0.5973594 |
| 0610007P22Rik | -0.5328847 |
| 0610009B22Rik | 0.04057522 |
| 0610009D07Rik | -0.2976907 |
| 0610009O20Rik | -0.1636462 |
| 0610010E21Rik | 1.36024241 |
| 0610010K06Rik | 0.12956894 |
| 0610010K14Rik | -0.4747119 |
| 0610012A05Rik | -0.4431257 |
| 0610012D14Rik | -0.5559602 |
| 0610012G03Rik | -0.557519 |
| 0610012H03Rik | 1.13722087 |
| 0610025P10Rik | -0.4782883 |
| 0610031J06Rik | -0.408573 |
| 0610031J06Rik | 1.15636002 |
| 0610031J06Rik | 3.3306392 |
| 0610037D15Rik | -0.129971 |
| 0610037L13Rik | -0.28362 |
| 0610037L13Rik | 0.72858184 |
| 0610038F07Rik | -0.1221055 |
| 0610040J01Rik | 1.66399722 |
| 0710008K08Rik | 0.33247306 |
| 0910001A06Rik | 1.51604809 |
| 0910001A06Rik | -0.653556 |
| 0910001L09Rik | 0.84144893 |
| 1110001A07Rik | 0.81164985 |
| 1110001J03Rik | 1.03246315 |
| 1110002B05Rik | -0.7644906 |
| 1110002N22Rik | 0.28588434 |
| 1110003E01Rik | 1.9085447 |
| 1110006G06Rik | -0.3054017 |
| 1110007A13Rik | -0.6941934 |
| 1110007A13Rik | -0.3295351 |
| 1110007C09Rik | 1.83053392 |
| 1110007L15Rik | 2.65922687 |
| 1110007L15Rik | 0.97434004 |
| 1110007L15Rik | -0.5957327 |
| 1110007L15Rik | -0.3449412 |
| 1110007M04Rik | -0.5973594 |
| 1110008F13Rik | 0.82773172 |
| 1110008J03Rik | 1.78770637 |
| 1110008J03Rik | -0.5397477 |
| 1110008J03Rik | -0.3356671 |
| 1110008P14Rik | -0.4521415 |
| 1110012D08Rik | -0.5389827 |
| 1110012J17Rik | -0.4876516 |
| 1110012L19Rik | -0.557519 |
| 1110012L19Rik | -0.0370062 |
| 1110012M11Rik | 1.98481479 |
| 1110013L07Rik | -0.233216 |
| 1110017D15Rik | -0.780197 |
| 1110017D15Rik | 0.32885443 |
| 1110017D15Rik | -0.6449509 |
| 1110018G07Rik | 0.01090568 |
| 1110018J18Rik | -0.29651 |
| 1110018J18Rik | 0.54070239 |
| 1110019N10Rik | 0.1956129 |
| 1110019N10Rik | 0.365588 |
| 1110020C03Rik | 1.7647212 |
| 1110020P15Rik | 2.93212239 |
| 1110020P15Rik | -0.7693705 |
| 1110031B06Rik | -0.4424358 |
| 1110031I02Rik | 1.30098528 |
| 1110032A13Rik | -0.5852279 |
| 1110032A13Rik | -0.7991979 |
| 1110032E23Rik | 1.16294243 |
| 1110034A24Rik | 0.87471764 |
| 1110036O03Rik | 0.03314203 |
| 1110038D17Rik | 1.24895696 |
| 1110038F14Rik | 0.81056782 |
| 1110038F14Rik | 0.05080198 |
| 1110039B18Rik | -0.7471307 |
| 1110039B18Rik | -0.7395175 |
| 1110039B18Rik | -0.1580335 |
| 1110048D14Rik | 2.05150904 |
| 1110049B09Rik | 1.41587066 |
| 1110049B09Rik | 0.87218714 |
| 1110049F12Rik | -0.0053695 |
| 1110049F12Rik | -0.4876516 |
| 1110057K04Rik | -0.697812 |
| 1110057K04Rik | 0.89356362 |
| 1110057K04Rik | 2.11107054 |
| 1110058L19Rik | -0.4804408 |
| 1110059E24Rik | -0.6834208 |
| 1110059G10Rik | 0.50885486 |
| 1110067D22Rik | -0.7851599 |
| 1110067I12Rik | -0.4119046 |
| 1190002A17Rik | 0.04385997 |
| 1190002H23Rik | 1.62088217 |
| 1190002N15Rik | -0.3301465 |
| 1190003J15Rik | -0.6406778 |
| 1190003M12Rik | 0.85771677 |
| 1190005F20Rik | -0.7500017 |
| 1190005I06Rik | 1.31444698 |
| 1190005P17Rik | 0.04467936 |
| 1190005P17Rik | 1.1812031 |
| 1190007F08Rik | -0.5772269 |
| 1190017O12Rik | 3.31950095 |
| 1190020J12Rik | -0.6552866 |
| 1200002N14Rik | -0.3986488 |
| 1200003C05Rik | 0.84719229 |
| 1200003C05Rik | 0.63566414 |
| 1200003C05Rik | 0.89257244 |
| 1200003I07Rik | -0.300649 |
| 1200009F10Rik | -0.4396812 |
| 1200009I06Rik | 2.34104326 |
| 1200009I06Rik | 2.14512705 |
| 1200009O22Rik | -0.1980493 |
| 1200011O22Rik | 2.7680228 |
| 1200013B08Rik | 0.18896901 |
| 1200013P24Rik | -0.0688263 |
| 1200013P24Rik | -0.6754221 |
| 1200014J11Rik | 2.86654345 |
| 1200015F23Rik | 0.36267856 |
| 1200015F23Rik | 0.52784916 |
| 1200015N20Rik | 1.48201811 |
| 1200016B10Rik | -0.5559602 |
| 1200016B10Rik | -0.6104755 |
| 1200016D23Rik | 2.37426466 |
| 1300001I01Rik | 1.27708728 |
| 1300007L22Rik | 3.36006574 |
| 1300010M03Rik | 0.26185133 |
| 1300013J15Rik | -0.0383479 |
| 1300013J15Rik | -0.6587574 |
| 1300014I06Rik | 0.23317599 |
| 1300017J02Rik | 0.0772749 |
| 1300018I05Rik | -0.5653524 |
| 1300018J18Rik | -0.697812 |
| 1500001L15Rik | -0.603075 |
| 1500001M20Rik | -0.4287431 |
| 1500001M20Rik | 1.93751563 |
| 1500001M20Rik | -0.5238117 |
| 1500002O20Rik | -0.5520745 |
| 1500003O03Rik | 1.69104091 |
| 1500005A01Rik | 1.10288614 |
| 1500010J02Rik | -0.6923893 |
| 1500010J02Rik | 0.23083007 |
| 1500011H22Rik | -0.2499042 |
| 1500012F01Rik | -0.4092383 |
| 1500015O10Rik | 0.05607599 |
| 1500019C06Rik | 0.43309821 |
| 1500019G21Rik | 2.66107196 |
| 1500031L02Rik | 0.12807193 |
| 1500032D16Rik | -0.6279747 |
| 1500032D16Rik | -0.3301465 |
| 1500032D16Rik | 0.94947964 |
| 1500032L24Rik | -0.2119576 |
| 1500041J02Rik | 2.43145667 |
| 1500041N16Rik | -0.5949204 |
| 1520401A03Rik | 0.80858084 |
| 1600002K03Rik | 0.56542134 |
| 1600012H06Rik | -0.653556 |
| 1600012H06Rik | 1.31276166 |
| 1600014C10Rik | -0.5366913 |
| 1600016N20Rik | -0.6381234 |
| 1600021P15Rik | 1.79478269 |
| 1600027N09Rik | -0.0321029 |
| 1600029D21Rik | 2.64440961 |
| 1700001C02Rik | -0.5374544 |
| 1700001C19Rik | -0.1718708 |
| 1700001E04Rik | -0.4747119 |
| 1700001J03Rik | 1.53999622 |
| 1700001O22Rik | 1.70410145 |
| 1700001O22Rik | -0.5497508 |
| 1700001P01Rik | -0.7683925 |
| 1700003F12Rik | 1.15436374 |
| 1700003M02Rik | 1.21820536 |
| 1700007G11Rik | 1.85428004 |
| 1700007I06Rik | 0.17232827 |
| 1700007K09Rik | 0.64475817 |
| 1700008F21Rik | 1.05342952 |
| 1700008I05Rik | 0.08750115 |
| 1700008P20Rik | 0.00835469 |
| 1700009P17Rik | -0.0828023 |
| 1700010A17Rik | -0.7471307 |
| 1700010A17Rik | -0.3246583 |
| 1700010D01Rik | 0.08082744 |
| 1700010M22Rik | 0.59542801 |
| 1700010M22Rik | 0.45579138 |
| 1700011A15Rik | -0.1838219 |
| 1700011F03Rik | -0.3631363 |
| 1700011F14Rik | 3.20784926 |
| 1700011H14Rik | -0.1874898 |
| 1700011K15Rik | -0.4424358 |
| 1700012A03Rik | 0.17944569 |
| 1700012B09Rik | 0.79436884 |
| 1700012G19Rik | -0.1314532 |
| 1700012H05Rik | -0.3587119 |
| 1700012H17Rik | -0.6807469 |
| 1700012L04Rik | -0.0716048 |
| 1700013B16Rik | 1.16219958 |
| 1700013G24Rik | -0.3739695 |
| 1700013N18Rik | -0.7282128 |
| 1700013N18Rik | 1.00179695 |
| 1700015E13Rik | -0.780197 |
| 1700016C15Rik | -0.4920049 |
| 1700016D06Rik | -0.2918021 |
| 1700016G05Rik | 0.26799839 |
| 1700016K19Rik | 0.6185368 |
| 1700016K19Rik | -0.3688559 |
| 1700017N19Rik | 3.45440025 |
| 1700018B24Rik | -0.6022564 |
| 1700018C11Rik | 1.82244181 |
| 1700018F24Rik | -0.3644043 |
| 1700019D03Rik | 0.02523121 |
| 1700019E19Rik | -0.5528504 |
| 1700019G17Rik | 0.23284122 |
| 1700019H03Rik | -0.5780239 |
| 1700019L03Rik | -0.5900616 |
| 1700019M22Rik | 2.41714396 |
| 1700019N19Rik | -0.3746107 |
| 1700020C11Rik | -0.572459 |
| 1700020D05Rik | -0.7235426 |
| 1700020L24Rik | 0.27057458 |
| 1700020N01Rik | 1.90659216 |
| 1700020O03Rik | 2.96198237 |
| 1700021C14Rik | 0.30498543 |
| 1700021F05Rik | 2.80230866 |
| 1700021K02Rik | 1.44304078 |
| 1700021K02Rik | 1.40351549 |
| 1700021K19Rik | -0.4898257 |
| 1700022C21Rik | 0.36819877 |
| 1700023B02Rik | 1.7308339 |
| 1700023B02Rik | 2.3439855 |
| 1700023E05Rik | -0.6526919 |
| 1700023E05Rik | -0.5528504 |
| 1700023I07Rik | -0.3599739 |
| 1700023M03Rik | -0.403267 |
| 1700024G13Rik | 0.1917722 |
| 1700025D03Rik | -0.6692478 |
| 1700025E21Rik | 1.0486865 |
| 1700025F22Rik | 0.01471915 |
| 1700025G04Rik | -0.3455627 |
| 1700025K23Rik | -0.6674912 |
| 1700026D08Rik | -0.6674912 |
| 1700026L06Rik | 0.78773584 |
| 1700027A23Rik | 0.66141582 |
| 1700027D21Rik | -0.6279747 |
| 1700027J05Rik | -0.1041662 |
| 1700027N10Rik | -0.7703496 |
| 1700028K03Rik | -0.6432393 |
| 1700029F12Rik | 1.07325492 |
| 1700029F12Rik | -0.5397477 |
| 1700029G01Rik | 3.4671136 |
| 1700029H14Rik | -0.0688263 |
| 1700029H14Rik | -0.3077872 |
| 1700029I01Rik | -0.6987189 |
| 1700029I08Rik | 2.52129498 |
| 1700029I15Rik | -0.5740456 |
| 1700029J07Rik | -0.0993763 |
| 1700029P11Rik | -0.7078361 |
| 1700029P11Rik | 1.58691302 |
| 1700029P11Rik | -0.5306082 |
| 1700030J22Rik | 2.07986402 |
| 1700030K09Rik | 0.50735844 |
| 1700034E13Rik | 0.47691562 |
| 1700034H14Rik | 0.71728551 |
| 1700034H14Rik | -0.1245564 |
| 1700034H14Rik | 1.61304028 |
| 1700034H14Rik | -0.5629955 |
| 1700034I23Rik | 2.45532106 |
| 1700034I23Rik | 1.86695079 |
| 1700034O15Rik | 1.86321437 |
| 1700036D21Rik | 0.0776703 |
| 1700037C18Rik | 0.60873491 |
| 1700037C18Rik | 1.34671754 |
| 1700037H04Rik | 3.55171449 |
| 1700039E15Rik | -0.5716667 |
| 1700040I03Rik | 1.64623682 |
| 1700042B14Rik | 1.17572565 |
| 1700045I19Rik | -0.356822 |
| 1700047I17Rik1 | 0.44250929 |
| 1700047I17Rik1 | -0.3125765 |
| 1700049G17Rik | 0.43714314 |
| 1700049K14Rik | -0.6570204 |
| 1700049L16Rik | 0.96826288 |
| 1700052N19Rik | 0.1064747 |
| 1700052N19Rik | 0.64152045 |
| 1700054O13Rik | -0.782179 |
| 1700055M20Rik | 1.81026358 |
| 1700057G04Rik | 1.77100507 |
| 1700057K13Rik | -0.6063569 |
| 1700058C13Rik | -0.7811875 |
| 1700060H10Rik | 2.37402764 |
| 1700061G19Rik | -0.0965141 |
| 1700063I17Rik | 3.10285813 |
| 1700065I17Rik | -0.1196611 |
| 1700065O13Rik | -0.8618703 |
| 1700065O13Rik | 0.35624572 |
| 1700067C01Rik | -0.7615748 |
| 1700067K01Rik | -0.2566638 |
| 1700067P10Rik | -0.4321475 |
| 1700071K01Rik | -0.7713297 |
| 1700072E05Rik | -0.6896897 |
| 1700072E05Rik | 1.69307834 |
| 1700072E05Rik | 2.70832964 |
| 1700073E17Rik | 0.52858144 |
| 1700080E11Rik | -0.8310535 |
| 1700081D17Rik | -0.0254579 |
| 1700081D17Rik | 0.5063595 |
| 1700084C01Rik | -0.2092668 |
| 1700084C01Rik | 2.11196752 |
| 1700088E04Rik | -0.2409668 |
| 1700093K21Rik | 2.33491898 |
| 1700094C09Rik | 1.87726483 |
| 1700095K08Rik | 1.71465323 |
| 1700102P08Rik | 1.22344008 |
| 1700108M19Rik | -0.3758944 |
| 1700109H08Rik | 0.58560898 |
| 1700112C13Rik | -0.2617658 |
| 1700113I22Rik | -0.5692938 |
| 1700113I22Rik | 1.39070386 |
| 1700113O17Rik | 0.52246149 |
| 1700120B06Rik | 0.2880962 |
| 1700123D08Rik | -0.7433164 |
| 1700123K08Rik | -0.2923893 |
| 1700123L14Rik | 1.54994625 |
| 1700123O20Rik | -0.3234431 |
| 1700123O20Rik | -0.780197 |
| 1700127D06Rik | -0.2304634 |
| 1700129C05Rik | -0.5200571 |
| 1700129I15Rik | -0.105608 |
| 1810007M14Rik | 1.32927253 |
| 1810007P19Rik | -0.2555338 |
| 1810007P19Rik | 3.42069605 |
| 1810008A18Rik | 0.11220675 |
| 1810008A18Rik | -0.8320994 |
| 1810009A15Rik | -0.2017736 |
| 1810009A15Rik | 0.08984524 |
| 1810009N02Rik | 1.48684356 |
| 1810009O10Rik | -0.260062 |
| 1810009O10Rik | -0.0307701 |
| 1810010M01Rik | 2.97558405 |
| 1810011O10Rik | -0.2900428 |
| 1810014F10Rik | -0.566927 |
| 1810015A11Rik | 2.06579407 |
| 1810015C04Rik | -0.5512993 |
| 1810019J16Rik | -0.6639879 |
| 1810020D17Rik | -0.3778232 |
| 1810020G14Rik | 0.70261595 |
| 1810020G14Rik | 2.58834453 |
| 1810021J13Rik | -0.0162335 |
| 1810021J13Rik | 0.20979079 |
| 1810022C23Rik | 0.25762203 |
| 1810026J23Rik | 0.84597695 |
| 1810027O10Rik | 1.65541498 |
| 1810030J14Rik | -0.2017736 |
| 1810030N24Rik | -0.6754221 |
| 1810030N24Rik | -0.6622411 |
| 1810030N24Rik | 0.13404565 |
| 1810031K17Rik | 2.013193 |
| 1810035L17Rik | 2.34573245 |
| 1810035L17Rik | 0.68874085 |
| 1810037C20Rik | -0.6254547 |
| 1810043G02Rik | 1.43632195 |
| 1810044A24Rik | -0.8521972 |
| 1810046J19Rik | 1.22933754 |
| 1810048J11Rik | 0.25402837 |
| 1810049H13Rik | 1.29981013 |
| 1810049H19Rik | -0.2691849 |
| 1810055E12Rik | -0.6736538 |
| 1810055E12Rik | 1.67149643 |
| 1810055E12Rik | -0.6960009 |
| 1810055G02Rik | -0.7841652 |
| 1810063B05Rik | -0.2012406 |
| 1810064F22Rik | 0.86897198 |
| 1810065E05Rik | -0.2515895 |
| 1810073G14Rik | -0.758668 |
| 1810074P20Rik | -0.5567393 |
| 1810074P20Rik | -0.1060891 |
| 2010001J22Rik | -0.3424591 |
| 2010001J22Rik | -0.0790549 |
| 2010001M09Rik | -0.1906451 |
| 2010003J03Rik | 0.84788606 |
| 2010003O18Rik | 1.13019779 |
| 2010004A03Rik | -0.3350521 |
| 2010005H15Rik | -0.2387457 |
| 2010005J08Rik | -0.3688559 |
| 2010005J08Rik | -0.4500532 |
| 2010005J08Rik | -0.2994645 |
| 2010007H12Rik | 0.29626616 |
| 2010007H12Rik | -0.2271709 |
| 2010011I20Rik | -0.8575588 |
| 2010012O05Rik | 0.50210203 |
| 2010100O12Rik | -0.7703496 |
| 2010100O12Rik | 0.26412074 |
| 2010106G01Rik | -0.5520745 |
| 2010106G01Rik | 0.88593761 |
| 2010107E04Rik | 1.2739055 |
| 2010107G12Rik | -0.3993072 |
| 2010109I03Rik | -0.8727368 |
| 2010110K16Rik | -0.6950967 |
| 2010111I01Rik | -0.7151945 |
| 2010200O16Rik | -0.6347282 |
| 2010301N04Rik | 0.12507068 |
| 2010305A19Rik | 1.36214437 |
| 2010305C02Rik | -0.6683691 |
| 2010305C02Rik | 0.87032708 |
| 2010308M01Rik | 0.71648764 |
| 2010311D03Rik | -0.43488 |
| 2010316F05Rik | 1.15959492 |
| 2010321M09Rik | 1.03232084 |
| 2010321M09Rik | -0.5505247 |
| 2200001I15Rik | -0.6458078 |
| 2210009G21Rik | -0.3375145 |
| 2210009G21Rik | -0.7198231 |
| 2210009G21Rik | 0.96719531 |
| 2210010B09Rik | -0.0688263 |
| 2210010L05Rik | 0.38740863 |
| 2210012G02Rik | -0.1225952 |
| 2210012G02Rik | 1.77287558 |
| 2210012G02Rik | -0.8002088 |
| 2210012G02Rik | 0.24613937 |
| 2210016L21Rik | 0.49504717 |
| 2210016L21Rik | 4.21982209 |
| 2210016L21Rik | -0.6932909 |
| 2210021J22Rik | 3.64824854 |
| 2210023G05Rik | -0.4934605 |
| 2210023G05Rik | 1.43881263 |
| 2210407C18Rik | -0.6104755 |
| 2210412D01Rik | 0.52368869 |
| 2210417D09Rik | 0.70504326 |
| 2210418O10Rik | 1.09732671 |
| 2300002D11Rik | 0.45579138 |
| 2300002D11Rik | -0.4739983 |
| 2300002M23Rik | -0.7557703 |
| 2300005B03Rik | 1.00590535 |
| 2310001A20Rik | -0.4668917 |
| 2310002B06Rik | 0.3130097 |
| 2310003C23Rik | 0.47717345 |
| 2310003F16Rik | -0.1791272 |
| 2310003H01Rik | 1.09533315 |
| 2310003L22Rik | 1.18180973 |
| 2310003L22Rik | 2.16870897 |
| 2310004I24Rik | -0.5405134 |
| 2310005E10Rik | 0.36471612 |
| 2310005E10Rik | 0.14404053 |
| 2310005G13Rik | 0.88726835 |
| 2310005N01Rik | 1.06132744 |
| 2310005N03Rik | -0.7282128 |
| 2310005P05Rik | 0.08710989 |
| 2310006J04Rik | 0.10186361 |
| 2310007A19Rik | -0.5892543 |
| 2310007B03Rik | -0.22389 |
| 2310007F21Rik | -0.401285 |
| 2310007F21Rik | -0.3270935 |
| 2310008H04Rik | -0.8500612 |
| 2310008H09Rik | -0.6014384 |
| 2310008H09Rik | 3.26107175 |
| 2310008M10Rik | 2.11066662 |
| 2310009E04Rik | 0.40873135 |
| 2310010B21Rik | 0.55859404 |
| 2310010M24Rik | 2.4897582 |
| 2310011J03Rik | 0.87184922 |
| 2310014L17Rik | -0.5892543 |
| 2310016C08Rik | -0.4905515 |
| 2310016C08Rik | -0.6501044 |
| 2310016C16Rik | -0.5505247 |
| 2310016C16Rik | 2.95132556 |
| 2310016C16Rik | 0.43687402 |
| 2310016E02Rik | 1.48806789 |
| 2310016M24Rik | 1.50048251 |
| 2310021P13Rik | -0.5780239 |
| 2310022A10Rik | 0.41866426 |
| 2310022B05Rik | 1.95119205 |
| 2310031L18Rik | -0.3953641 |
| 2310031L18Rik | 0.32915652 |
| 2310033E01Rik | -0.1595609 |
| 2310033K02Rik | 1.08664493 |
| 2310033P09Rik | -0.6022564 |
| 2310035C23Rik | -0.425351 |
| 2310035C23Rik | 0.02271908 |
| 2310035K24Rik | -0.8405091 |
| 2310036D22Rik | 2.14200681 |
| 2310036O22Rik | -0.5268262 |
| 2310037I24Rik | 0.46523893 |
| 2310037I24Rik | 0.48231521 |
| 2310038H17Rik | 0.9045609 |
| 2310039E09Rik | 0.81056782 |
| 2310039H08Rik | 2.65087859 |
| 2310040C09Rik | -0.3319831 |
| 2310042D19Rik | 0.81811585 |
| 2310044G17Rik | 0.09877686 |
| 2310044H10Rik | 1.50409765 |
| 2310045A20Rik | -0.7060055 |
| 2310046K01Rik | 0.57707667 |
| 2310046O06Rik | -0.6509661 |
| 2310047C04Rik | -0.3065937 |
| 2310047D13Rik | 1.89139101 |
| 2310047D13Rik | 1.21855525 |
| 2310047M10Rik | 1.88346775 |
| 2310047M10Rik | 1.4095234 |
| 2310047O13Rik | -0.3059975 |
| 2310056P07Rik | 0.12882073 |
| 2310057J16Rik | 0.93663929 |
| 2310058J06Rik | -0.6710077 |
| 2310061C15Rik | -0.3511754 |
| 2310061F22Rik | -0.5590805 |
| 2310061F22Rik | -0.5764306 |
| 2310065K24Rik | 0.06413218 |
| 2310065K24Rik | -0.3593426 |
| 2310066E14Rik | -0.7272769 |
| 2310067B10Rik | 0.46314771 |
| 2310076L09Rik | 0.42577293 |
| 2310076L09Rik | -0.4825982 |
| 2310079F23Rik | -0.5590805 |
| 2310079N02Rik | -0.6950967 |
| 2400001E08Rik | 2.23561042 |
| 2400003C14Rik | 2.54494538 |
| 2400003C14Rik | -0.5298506 |
| 2400003C14Rik | -0.1147913 |
| 2400006N03Rik | 0.18896901 |
| 2400010D15Rik | 1.29970322 |
| 2410001C21Rik | 0.77451447 |
| 2410001C21Rik | 1.47946371 |
| 2410001C21Rik | 0.27057458 |
| 2410002F23Rik | -0.157525 |
| 2410002F23Rik | 3.45387786 |
| 2410002F23Rik | -0.6570204 |
| 2410002I01Rik | 1.93740757 |
| 2410002O22Rik | -0.1384013 |
| 2410003P15Rik | -0.0347742 |
| 2410004A20Rik | 1.14973092 |
| 2410004B18Rik | 1.1084127 |
| 2410004L22Rik | 0.42113107 |
| 2410005O16Rik | 0.66500725 |
| 2410008K03Rik | 0.86948037 |
| 2410014A08Rik | 1.3112845 |
| 2410014A08Rik | 0.7244359 |
| 2410014A08Rik | 0.52564887 |
| 2410014A08Rik | 1.21703809 |
| 2410015M20Rik | 0.41343505 |
| 2410015N17Rik | 0.79326661 |
| 2410016F19Rik | 1.04082161 |
| 2410016F19Rik | 1.3245997 |
| 2410016O06Rik | -0.2560986 |
| 2410017P07Rik | 0.85891695 |
| 2410018C17Rik | 0.03603974 |
| 2410018G20Rik | 0.85599955 |
| 2410018M08Rik | 0.2261205 |
| 2410019A14Rik | -0.2623344 |
| 2410019G02Rik | 2.11335613 |
| 2410022L05Rik | -0.1177101 |
| 2410025L10Rik | 1.13275775 |
| 2410042D21Rik | -0.6389741 |
| 2410042D21Rik | -0.593298 |
| 2410066E13Rik | -0.3107777 |
| 2410081M15Rik | 1.46640707 |
| 2410091C18Rik | -0.3095803 |
| 2410116G06Rik | 2.39323371 |
| 2410127E18Rik | 1.31149567 |
| 2410127L17Rik | 1.0687486 |
| 2410127L17Rik | -0.4725727 |
| 2410129H14Rik | 0.94480239 |
| 2410137M14Rik | 0.04549803 |
| 2410146L05Rik | -0.6789686 |
| 2410166I05Rik | -0.5482049 |
| 2500002L14Rik | 0.55100212 |
| 2500003M10Rik | -0.1379033 |
| 2500003M10Rik | -0.2195333 |
| 2500003M10Rik | -0.8237648 |
| 2510002A14Rik | 0.25827393 |
| 2510003E04Rik | -0.8405091 |
| 2510006D16Rik | -0.6212697 |
| 2510009E07Rik | -0.397991 |
| 2510010F15Rik | -0.4790053 |
| 2510012J08Rik | 1.79967938 |
| 2510039O18Rik | 0.23451387 |
| 2510040D07Rik | 0.67653294 |
| 2510048L02Rik | -0.3125765 |
| 2510049I19Rik | -0.9163665 |
| 2600001B17Rik | -0.3350521 |
| 2600009E05Rik | -0.6648625 |
| 2600009E05Rik | -0.7423652 |
| 2600010E01Rik | -0.5998047 |
| 2600011E07Rik | -0.0432841 |
| 2600011E07Rik | 1.80236791 |
| 2610002J02Rik | -0.7105887 |
| 2610002M06Rik | -0.7528816 |
| 2610003J06Rik | 0.67131161 |
| 2610016F04Rik | 1.31854227 |
| 2610019A05Rik | 1.46524357 |
| 2610019P18Rik | -0.8206584 |
| 2610020C11Rik | 2.59210645 |
| 2610020H08Rik | 0.47433338 |
| 2610020H08Rik | 4.09597932 |
| 2610020H08Rik | -0.6212697 |
| 2610020O08Rik | 2.36288756 |
| 2610021K21Rik | 1.91315876 |
| 2610021K21Rik | 0.94464608 |
| 2610024G14Rik | -0.5163176 |
| 2610024G14Rik | 1.92839466 |
| 2610024G14Rik | -0.7931558 |
| 2610027C15Rik | 0.52024847 |
| 2610027C15Rik | -0.7891493 |
| 2610027L16Rik | 0.71688666 |
| 2610028A01Rik | 1.09053119 |
| 2610028H24Rik | 0.07251709 |
| 2610029G23Rik | -0.9061158 |
| 2610029G23Rik | 1.53202592 |
| 2610034E13Rik | -0.2162791 |
| 2610034E13Rik | 0.96978588 |
| 2610034M16Rik | 1.34407048 |
| 2610036D13Rik | 0.97267276 |
| 2610039C10Rik | -0.7041785 |
| 2610042L04Rik | 1.78153418 |
| 2610044O15Rik | -0.497841 |
| 2610101N10Rik | -0.8373466 |
| 2610101N10Rik | 0.50535948 |
| 2610103J23Rik | -0.5474329 |
| 2610110G12Rik | -0.9525607 |
| 2610200G18Rik | 1.82756076 |
| 2610204K14Rik | 2.87684263 |
| 2610204L23Rik | -0.5125929 |
| 2610204M08Rik | 1.47264727 |
| 2610205E22Rik | 1.65263434 |
| 2610207I05Rik | -0.7433164 |
| 2610208M17Rik | -0.7115081 |
| 2610209A20Rik | -0.5022422 |
| 2610209A20Rik | -0.2443083 |
| 2610209M04Rik | 0.29938954 |
| 2610301B20Rik | 0.67465662 |
| 2610301F02Rik | 2.81534477 |
| 2610304G08Rik | -0.5812188 |
| 2610305D13Rik | -0.8586348 |
| 2610318N02Rik | -0.3113769 |
| 2610507B11Rik | -0.0140505 |
| 2610507B11Rik | -0.7567352 |
| 2610510B01Rik | 0.47381607 |
| 2610510J17Rik | 0.24283253 |
| 2610524A10Rik | -0.5756349 |
| 2610524H06Rik | 1.02460346 |
| 2610528E23Rik | 0.60492011 |
| 2610528J11Rik | 0.70180545 |
| 2610528O22Rik | 2.95043046 |
| 2700007P21Rik | -0.2288157 |
| 2700007P21Rik | -0.6492435 |
| 2700038C09Rik | 2.49518796 |
| 2700038C09Rik | 2.49560433 |
| 2700050L05Rik | -0.7226113 |
| 2700055A20Rik | 1.11090212 |
| 2700060E02Rik | -0.5260717 |
| 2700062C07Rik | -0.8196252 |
| 2700062C07Rik | 1.64815332 |
| 2700078K21Rik | -0.5051879 |
| 2700081O15Rik | 0.69181929 |
| 2700087H15Rik | 0.70463915 |
| 2700094K13Rik | 0.3981308 |
| 2700097O09Rik | 1.36034261 |
| 2810002N01Rik | 2.039912 |
| 2810002O09Rik | -0.6843138 |
| 2810003C17Rik | -0.4287431 |
| 2810004N20Rik | -0.4912779 |
| 2810004N23Rik | 0.34621614 |
| 2810008M24Rik | 0.4578991 |
| 2810021B07Rik | 1.20006579 |
| 2810021J22Rik | 0.60671725 |
| 2810021J22Rik | -0.8394537 |
| 2810022L02Rik | -0.6763074 |
| 2810022L02Rik | -0.0131788 |
| 2810022L02Rik | -0.6458078 |
| 2810030E01Rik | 1.55841448 |
| 2810046L04Rik | -0.5096238 |
| 2810046M22Rik | -0.6639879 |
| 2810403A07Rik | 0.42984962 |
| 2810405J04Rik | -0.5820193 |
| 2810405K02Rik | 1.07230085 |
| 2810408A11Rik | 0.0906253 |
| 2810408M09Rik | -0.4704383 |
| 2810410C14Rik | 0.81416972 |
| 2810410P22Rik | 1.57185398 |
| 2810410P22Rik | -0.6171033 |
| 2810417H13Rik | -0.8586348 |
| 2810422O20Rik | 1.50675702 |
| 2810428I15Rik | 0.56612477 |
| 2810432D09Rik | 0.06292817 |
| 2810432L12Rik | 0.39728877 |
| 2810439F02Rik | 0.62119229 |
| 2810439F02Rik | -0.4905515 |
| 2810441K11Rik | -0.4927324 |
| 2810452K22Rik | -0.9573347 |
| 2810453I06Rik | -0.8352442 |
| 2810453I06Rik | 1.29927548 |
| 2810470D21Rik | 0.5738278 |
| 2810485I05Rik | -0.0127432 |
| 2900006K08Rik | 1.58210467 |
| 2900010M23Rik | 0.68420737 |
| 2900011O08Rik | 2.26578913 |
| 2900011O08Rik | 2.18547441 |
| 2900024C23Rik | -0.4514449 |
| 2900024O10Rik | -0.7490437 |
| 2900026A02Rik | 0.40650963 |
| 2900046G09Rik | -0.5133367 |
| 2900062L11Rik | -0.8426233 |
| 2900064A13Rik | 1.05939377 |
| 2900073G15Rik | 0.24216976 |
| 2900092C05Rik | -0.9976797 |
| 3000004C01Rik | -0.8716444 |
| 3000004C01Rik | -0.6449509 |
| 3000004C01Rik | 0.10762393 |
| 3010003L21Rik | 0.10070727 |
| 3010026O09Rik | 1.86782436 |
| 3110001A13Rik | -0.1329377 |
| 3110001D03Rik | 0.13143685 |
| 3110004L20Rik | -0.9419075 |
| 3110007F17Rik | -0.8848378 |
| 3110009E18Rik | -0.8237648 |
| 3110023B02Rik | 0.85186529 |
| 3110035E14Rik | 0.23351064 |
| 3110035E14Rik | -0.522308 |
| 3110037I16Rik | 0.01302619 |
| 3110040N11Rik | 0.02564924 |
| 3110040N11Rik | 0.26541508 |
| 3110043J09Rik | -0.8175623 |
| 3110043J09Rik | 2.04834737 |
| 3110043J09Rik | -0.6657379 |
| 3110048E14Rik | -0.2795556 |
| 3110049J23Rik | -0.388829 |
| 3110050N22Rik | -0.4854825 |
| 3110050N22Rik | 1.88134731 |
| 3110056O03Rik | -0.3726885 |
| 3110057O12Rik | 1.23198504 |
| 3110057O12Rik | 0.94870172 |
| 3110070M22Rik | 0.39700793 |
| 3110082I17Rik | 0.46654357 |
| 3200001I04Rik | 0.97494559 |
| 3200002M19Rik | -0.7161184 |
| 3200002M19Rik | 0.44384603 |
| 3300001G02Rik | -0.7654645 |
| 3300001P08Rik | 0.82418181 |
| 3300001P08Rik | -0.4732852 |
| 3300001P08Rik | -0.2732043 |
| 3300002I08Rik | -0.1022473 |
| 3321401G04Rik | -0.7742761 |
| 3322402L07Rik | -0.6321899 |
| 3632451O06Rik | 1.24284162 |
| 3830402I07Rik | -0.8268816 |
| 3830406C13Rik | 1.25559311 |
| 3830406C13Rik | 0.21765105 |
| 3830406C13Rik | 2.4629576 |
| 3830408P04Rik | 1.7085988 |
| 3930401K13Rik | 0.42413726 |
| 4121402D02Rik | 2.22891025 |
| 4121402D02Rik | -0.5828204 |
| 4430402I18Rik | 0.22443274 |
| 4432405B04Rik | 0.18439582 |
| 4432416J03Rik | 0.01598686 |
| 4631426J05Rik | 2.78960927 |
| 4631427C17Rik | -0.272629 |
| 4632404H12Rik | 1.23919874 |
| 4632404H12Rik | 3.01175512 |
| 4632404H12Rik | -0.6423847 |
| 4632404H22Rik | -0.7480867 |
| 4632411B12Rik | -0.5489775 |
| 4632411B12Rik | 2.39832896 |
| 4632412N22Rik | 3.74975824 |
| 4632415K11Rik | 1.42311242 |
| 4632417K18Rik | 2.0838916 |
| 4632417N05Rik | -0.2703315 |
| 4632419K20Rik | 1.18362726 |
| 4632434I11Rik | 1.64874222 |
| 4732415M23Rik | 0.96978588 |
| 4732418C07Rik | 0.05648045 |
| 4732418C07Rik | 1.22910696 |
| 4732418C07Rik | 0.57336275 |
| 4732429D16Rik | 0.14145951 |
| 4732456N10Rik | -0.4335127 |
| 4732471D19Rik | -0.1329377 |
| 4732473B16Rik | -0.7742761 |
| 4732479N06Rik | -0.8352442 |
| 4732479N06Rik | 0.2808089 |
| 4732496O08Rik | 0.91236073 |
| 4733401H18Rik | -0.1494251 |
| 4831426I19Rik | 0.89306816 |
| 4833418A01Rik | 0.40121177 |
| 4833420G17Rik | 0.78976759 |
| 4833421E05Rik | -0.721681 |
| 4833424O15Rik | 1.61632209 |
| 4833424O15Rik | -0.7841652 |
| 4833426J09Rik | -0.2743559 |
| 4833426J09Rik | 0.44143852 |
| 4833427G06Rik | 0.7272019 |
| 4833436C18Rik | -0.5298506 |
| 4833439L19Rik | -0.0757882 |
| 4833439L19Rik | 1.31075638 |
| 4833439L19Rik | 1.37525218 |
| 4833442J19Rik | -0.0842115 |
| 4833446K15Rik | -0.8926208 |
| 4921501E09Rik | 0.78902929 |
| 4921504E06Rik | -0.4718606 |
| 4921504I05Rik | 0.87975817 |
| 4921505C17Rik | -0.557519 |
| 4921505C17Rik | -0.1621122 |
| 4921507P07Rik | 1.42461019 |
| 4921511H03Rik | 0.10032151 |
| 4921511H03Rik | 0.0273195 |
| 4921511K06Rik | 0.74091042 |
| 4921511K06Rik | -0.5397477 |
| 4921515J06Rik | 3.52301382 |
| 4921517D21Rik | -0.4654769 |
| 4921517D22Rik | 1.24579104 |
| 4921520G13Rik | 1.37544938 |
| 4921521F21Rik | 1.57615866 |
| 4921521F21Rik | 2.13109102 |
| 4921523A10Rik | 0.50810695 |
| 4921523A10Rik | -0.5351668 |
| 4921523A10Rik | 0.53490395 |
| 4921525H12Rik | -0.4369346 |
| 4921528G01Rik | -0.7078361 |
| 4921528I01Rik | -0.4619491 |
| 4921530G04Rik | -0.3474299 |
| 4921530L21Rik | -0.4927324 |
| 4921530L21Rik | 1.71937317 |
| 4921536K21Rik | -0.260062 |
| 4921537P18Rik | 0.31515835 |
| 4922502D21Rik | 1.91791568 |
| 4922503N01Rik | -0.5764306 |
| 4922503N01Rik | 0.35418959 |
| 4922505E12Rik | 0.7411048 |
| 4922505G16Rik | 3.03696513 |
| 4922505G16Rik | -0.6196009 |
| 4930401F20Rik | -0.6834208 |
| 4930402H24Rik | 2.37009099 |
| 4930403C10Rik | 0.11296837 |
| 4930403L05Rik | 0.50059475 |
| 4930403O06Rik | 0.62273785 |
| 4930404H21Rik | 1.23118007 |
| 4930404N11Rik | -0.3688559 |
| 4930408O21Rik | -0.8124248 |
| 4930408O21Rik | -0.5528504 |
| 4930412F15Rik | 0.80350393 |
| 4930415L06Rik | -0.4059162 |
| 4930418G15Rik | -0.0523264 |
| 4930427A07Rik | -0.3137777 |
| 4930428D18Rik | 0.41729098 |
| 4930428E23Rik | 1.73915444 |
| 4930430D24Rik | -0.7723108 |
| 4930430E16Rik | -0.5567393 |
| 4930430E16Rik | -0.6229414 |
| 4930431L04Rik | -0.5458908 |
| 4930432H15Rik | -0.2168207 |
| 4930432K21Rik | -0.388829 |
| 4930432O21Rik | -0.2589279 |
| 4930432O21Rik | 0.37512539 |
| 4930438O05Rik | -0.1996436 |
| 4930438O05Rik | 0.46602193 |
| 4930449I24Rik | 0.9229803 |
| 4930451C15Rik | -0.2882867 |
| 4930451I11Rik | -0.7282128 |
| 4930452B06Rik | 0.75824523 |
| 4930453N24Rik | 0.8897584 |
| 4930455C21Rik | -0.7528816 |
| 4930455F23Rik | -0.5466615 |
| 4930467M19Rik | -0.4026059 |
| 4930470P17Rik | 0.0933504 |
| 4930473A06Rik | 1.98532826 |
| 4930473A06Rik | -0.7032664 |
| 4930473A06Rik | -0.6657379 |
| 4930474N05Rik | -0.3682187 |
| 4930478A21Rik | 0.96917698 |
| 4930481F22Rik | 0.01344972 |
| 4930481F22Rik | 0.2425012 |
| 4930481M05 | -0.7385702 |
| 4930486L24Rik | 0.43525769 |
| 4930502E18Rik | 0.32703984 |
| 4930503B20Rik | 3.45558253 |
| 4930503L19Rik | -0.3277033 |
| 4930504E06Rik | -0.4739983 |
| 4930504E06Rik | 3.92605348 |
| 4930504O13Rik | -0.1230851 |
| 4930506M07Rik | -0.6296585 |
| 4930507C10Rik | -0.5998047 |
| 4930511I11Rik | 1.79175654 |
| 4930511J11Rik | -0.233216 |
| 4930515G01Rik | -0.3246583 |
| 4930518C23Rik | -0.1916993 |
| 4930519F16Rik | 1.31612927 |
| 4930519G04Rik | -0.4424358 |
| 4930519N13Rik | -0.3914376 |
| 4930519N16Rik | -0.1626232 |
| 4930522H14Rik | -0.4026059 |
| 4930524B15Rik | 2.00241882 |
| 4930524B15Rik | 0.37195706 |
| 4930524E20Rik | -0.469018 |
| 4930526D03Rik | -0.4633586 |
| 4930535B03Rik | 2.96391189 |
| 4930535E21Rik | 0.3230961 |
| 4930538K18Rik | 1.63674404 |
| 4930539E08Rik | -0.4479696 |
| 4930540L03Rik | -0.6596271 |
| 4930544G11Rik | -0.4389938 |
| 4930544G21Rik | 1.82115767 |
| 4930547C10Rik | 2.69466111 |
| 4930547N16Rik | -0.5614275 |
| 4930549C01Rik | -0.5957327 |
| 4930550C14Rik | -0.4362492 |
| 4930550L24Rik | -0.0781204 |
| 4930555G01Rik | -0.3637701 |
| 4930555I21Rik | 0.83091515 |
| 4930562D19Rik | -0.1796477 |
| 4930562D19Rik | -0.0809267 |
| 4930563D23Rik | -0.4192762 |
| 4930563P21Rik | 1.61120355 |
| 4930570C03Rik | -0.6492435 |
| 4930572J05Rik | 2.06960122 |
| 4930572J05Rik | 1.08812093 |
| 4930572J05Rik | -0.4718606 |
| 4930573I19Rik | 0.12507068 |
| 4930578I06Rik | 2.58581015 |
| 4930579C15Rik | 0.66395238 |
| 4930579E17Rik | 0.01175446 |
| 4930579E17Rik | -0.7005352 |
| 4930579J09Rik | -0.129971 |
| 4930583H14Rik | 0.49858126 |
| 4930590J08Rik | 0.42577293 |
| 4930595M18Rik | 1.31990339 |
| 4930596D02Rik | -0.6389741 |
| 4930599N23Rik | 1.09971337 |
| 4931406B18Rik | -0.7674155 |
| 4931406C07Rik | -0.4584345 |
| 4931406H21Rik | -0.3246583 |
| 4931406I20Rik | 1.28276557 |
| 4931406I20Rik | 0.18510085 |
| 4931406P16Rik | 0.70383039 |
| 4931407G18Rik | -0.1838219 |
| 4931408A02Rik | 0.53247727 |
| 4931408C20Rik | 2.6017906 |
| 4931408C20Rik | -0.721681 |
| 4931409K22Rik | 1.11625152 |
| 4931417E11Rik | -0.7310262 |
| 4931417G12Rik | -0.6763074 |
| 4931428L18Rik | 1.20303457 |
| 4931432M23Rik | -0.1080159 |
| 4931433A01Rik | -0.2988729 |
| 4931440B09Rik | -0.399966 |
| 4931440F15Rik | 0.18404311 |
| 4931440F15Rik | -0.4424358 |
| 4931440L10Rik | 1.03118153 |
| 4932409I22Rik | -0.3198068 |
| 4932411E22Rik | -0.4683087 |
| 4932411G14Rik | 0.32703984 |
| 4932412H11Rik | 0.8659158 |
| 4932413O14Rik | 2.15249614 |
| 4932414J04Rik | 0.81074825 |
| 4932414N04Rik | 0.92393976 |
| 4932418E24Rik | 2.78320519 |
| 4932431H17Rik | 0.54718291 |
| 4932431H17Rik | -0.7871525 |
| 4932441K18Rik | 3.3813616 |
| 4932441K18Rik | 0.155022 |
| 4932442K08Rik | 0.64948662 |
| 4932702K14Rik | 0.39193823 |
| 4933400A11Rik | -0.4059162 |
| 4933400C05Rik | 0.6269201 |
| 4933400E14Rik | 1.57009452 |
| 4933402J07Rik | -0.6179351 |
| 4933402N03Rik | 1.05356866 |
| 4933402P03Rik | -0.5924879 |
| 4933403G14Rik | -0.8479301 |
| 4933404M02Rik | -0.721681 |
| 4933405O20Rik | 0.02313824 |
| 4933406M09Rik | 1.01898989 |
| 4933407N01Rik | 1.43862836 |
| 4933407N01Rik | -0.6204349 |
| 4933407N01Rik | 0.54094321 |
| 4933407P14Rik | -0.3252665 |
| 4933407P14Rik | 0.74032701 |
| 4933407P14Rik | 0.60334478 |
| 4933409N07Rik | 1.62941039 |
| 4933411G06Rik | -0.3204119 |
| 4933411K20Rik | -0.1418948 |
| 4933412E14Rik | -0.6923893 |
| 4933413G11Rik | -0.1206381 |
| 4933415F23Rik | -0.6771936 |
| 4933416C03Rik | -0.8394537 |
| 4933417A18Rik | -0.696906 |
| 4933417E01Rik | -0.5313664 |
| 4933421E11Rik | -0.2617658 |
| 4933421E11Rik | 1.45053081 |
| 4933421E11Rik | 0.15356524 |
| 4933422H20Rik | -0.0823331 |
| 4933424B01Rik | 0.2408428 |
| 4933424B01Rik | -0.0536903 |
| 4933424B01Rik | -0.6483834 |
| 4933425O20Rik | 1.33041123 |
| 4933426M11Rik | -0.3707702 |
| 4933427D14Rik | -0.1177101 |
| 4933428G09Rik | 3.11784367 |
| 4933428G20Rik | -0.7179689 |
| 4933429E10Rik | 1.8134773 |
| 4933429E10Rik | -0.178607 |
| 4933430I17Rik | -0.5074032 |
| 4933434E20Rik | -0.8196252 |
| 4933434E20Rik | 0.41039418 |
| 4933434E20Rik | -0.6038944 |
| 4933434I06Rik | 0.61298016 |
| 4933436I01Rik | 0.78495814 |
| 4933437F05Rik | 2.20669561 |
| 4933439C20Rik | 0.35888269 |
| 4933440H19Rik | -0.9152219 |
| 5031414D18Rik | -0.7244748 |
| 5031414D18Rik | -0.7272769 |
| 5033411D12Rik | -0.6526919 |
| 5033414D02Rik | -0.354936 |
| 5033414K04Rik | -0.0144867 |
| 5133400G04Rik | -0.5732519 |
| 5133400G04Rik | -0.5852279 |
| 5133400G04Rik | 2.99992231 |
| 5133401N09Rik | 0.64734029 |
| 5230400G24Rik | 0.25631684 |
| 5230400G24Rik | 2.42130632 |
| 5330401P04Rik | 0.53514627 |
| 5330417C22Rik | 0.67736566 |
| 5330431N19Rik | -0.7538435 |
| 5330431N19Rik | 0.3494728 |
| 5330431N19Rik | -0.7207516 |
| 5330437I02Rik | 1.31854227 |
| 5330439C02Rik | 0.152836 |
| 5330439J01Rik | -0.4125723 |
| 5330439J01Rik | 0.91736375 |
| 5430407P10Rik | -0.2824569 |
| 5430432M24Rik | -0.5965457 |
| 5430432M24Rik | -0.8405091 |
| 5430432M24Rik | 0.16551615 |
| 5430435G22Rik | 2.14209362 |
| 5430437P03Rik | 0.93852942 |
| 5530601I19Rik | -0.5208068 |
| 5630401D24Rik | -0.3277033 |
| 5630401D24Rik | -0.6552866 |
| 5630401D24Rik | 2.86560333 |
| 5730403M16Rik | -0.4825982 |
| 5730409E04Rik | 1.51341506 |
| 5730410E15Rik | 0.84458605 |
| 5730410E15Rik | 0.53804923 |
| 5730410I19Rik | -0.0157965 |
| 5730410I19Rik | 0.46888734 |
| 5730410I19Rik | -0.5029777 |
| 5730419I09Rik | -0.3077872 |
| 5730437N04Rik | -0.7509607 |
| 5730446C15Rik | -0.3204119 |
| 5730449L18Rik | 1.38808142 |
| 5730449L18Rik | -0.5482049 |
| 5730449L18Rik | -0.4612451 |
| 5730453I16Rik | 0.90423449 |
| 5730453I16Rik | -0.5772269 |
| 5730469M10Rik | 1.42825097 |
| 5730472N09Rik | 0.46000209 |
| 5730494M16Rik | -0.8155039 |
| 5730494N06Rik | -0.1590515 |
| 5730502D15Rik | 2.56619408 |
| 5730507A09Rik | 1.73345884 |
| 5730507A09Rik | -0.5780239 |
| 5730528L13Rik | 1.10420496 |
| 5730589K01Rik | 1.11077126 |
| 5730593F17Rik | 0.47070621 |
| 5730596K20Rik | -0.3868774 |
| 5730596K20Rik | -0.1544793 |
| 5730596K20Rik | -0.2674675 |
| 5730596K20Rik | -0.5981737 |
| 5830403L16Rik | 1.5644741 |
| 5830404H04Rik | 0.61119504 |
| 5830405N20Rik | -0.4206227 |
| 5830415F09Rik | -0.5029777 |
| 5830415L20Rik | 0.01556448 |
| 5830417C01Rik | 0.32855224 |
| 5830426I05Rik | 0.08003915 |
| 5830427D03Rik | -0.5344055 |
| 5830434P21Rik | -0.1443982 |
| 5830435K17Rik | -0.6701274 |
| 5830457O10Rik | -0.4927324 |
| 5830457O10Rik | -0.6852076 |
| 5830457O10Rik | -0.6683691 |
| 5830467P10Rik | -0.5788216 |
| 5830468K18Rik | 3.86517036 |
| 5830472M02Rik | 1.0368642 |
| 5830472M02Rik | 0.6297709 |
| 5830472M02Rik | 0.71608844 |
| 5930416I19Rik | -0.4790053 |
| 5930431H10 | -0.4431257 |
| 5930434B04Rik | -0.7811875 |
| 5930434B04Rik | -0.5567393 |
| 5930434B04Rik | -0.3077872 |
| 6030405A18Rik | -0.4619491 |
| 6030408B16Rik | -0.4403691 |
| 6030408B16Rik | 0.91591402 |
| 6030419C18Rik | -0.7404658 |
| 6030429G01Rik | 0.78607021 |
| 6030443O07Rik | 0.42113107 |
| 6030446N20Rik | -0.7347906 |
| 6030458C11Rik | 1.24703608 |
| 6230409E13Rik | 2.48031557 |
| 6230409E13Rik | 0.65291053 |
| 6230416J20Rik | -0.4747119 |
| 6230427J02Rik | 1.1304541 |
| 6330403K07Rik | -0.2098044 |
| 6330406I15Rik | 0.54622567 |
| 6330406I15Rik | 0.54094321 |
| 6330407J23Rik | 0.27153883 |
| 6330408A02Rik | -0.6254547 |
| 6330409N04Rik | -0.5852279 |
| 6330416G13Rik | -0.8331465 |
| 6330439K17Rik | 2.88185164 |
| 6330442E10Rik | 0.53659888 |
| 6330442E10Rik | -0.388829 |
| 6330500D04Rik | 1.97692335 |
| 6330503C03Rik | -0.5876416 |
| 6330503K22Rik | -0.2640423 |
| 6330503K22Rik | -0.6271339 |
| 6330503K22Rik | -0.741415 |
| 6330505N24Rik | 3.04216514 |
| 6330512M04Rik | 0.23250633 |
| 6330534C20Rik | -0.3973336 |
| 6330548G22Rik | -0.3089822 |
| 6330569M22Rik | -0.5435824 |
| 6330577E15Rik | -0.8970975 |
| 6330578E17Rik | 0.62801759 |
| 6332401O19Rik | -0.5653524 |
| 6430411K18Rik | 2.39190572 |
| 6430502M16Rik | 1.50555695 |
| 6430510M02Rik | 0.61986549 |
| 6430517E21Rik | -0.7263419 |
| 6430526N21Rik | -0.486928 |
| 6430527G18Rik | 0.53563072 |
| 6430527G18Rik | 0.09567989 |
| 6430531B16Rik | -0.7300875 |
| 6430537H07Rik | -0.2012406 |
| 6430537H07Rik | 0.94182798 |
| 6430548M08Rik | 2.1453866 |
| 6430550H21Rik | -0.5732519 |
| 6430571L13Rik | 0.21013392 |
| 6430573F11Rik | 0.9465201 |
| 6430598A04Rik | -0.0001993 |
| 6430604K15Rik | -0.6279747 |
| 6430706D22Rik | -0.7674155 |
| 6530404N21Rik | 2.1803638 |
| 6530406M24Rik | -0.5559602 |
| 6530418L21Rik | -0.2249824 |
| 6530418L21Rik | 1.71273107 |
| 6530418L21Rik | 4.11667465 |
| 6530421E24Rik | -0.5366913 |
| 6620401K05Rik | -0.3746107 |
| 6720456B07Rik | -0.7207516 |
| 6720456B07Rik | -0.7023551 |
| 6720456H20Rik | -0.3077872 |
| 6720456H20Rik | 0.98891509 |
| 6720458F09Rik | 0.53732434 |
| 6720463M24Rik | 0.82967843 |
| 6720467C03Rik | 1.43020553 |
| 6720467C03Rik | 0.40956314 |
| 6820408C15Rik | 0.39785021 |
| 7420416P09Rik | -0.6657379 |
| 7530404M11Rik | -0.5661393 |
| 8030411F24Rik | 0.72423802 |
| 8030451F13Rik | -0.401285 |
| 8030451F13Rik | 2.70019553 |
| 8030451F13Rik | 1.76796559 |
| 8430408G22Rik | 0.34235267 |
| 8430410A17Rik | -0.3412205 |
| 8430410K20Rik | 1.60821115 |
| 8430415E04Rik | -0.7615748 |
| 8430415E04Rik | -0.4675999 |
| 8430419L09Rik | -0.903853 |
| 8430427H17Rik | 2.13687044 |
| 8430432M10Rik | 0.2125323 |
| 9030025P20Rik | -0.4132405 |
| 9030025P20Rik | -0.5892543 |
| 9030025P20Rik | -0.603075 |
| 9030205A07Rik | -0.6432393 |
| 9030221M09Rik | 1.33947124 |
| 9030224M15Rik | 1.7466729 |
| 9030227G01Rik | -0.6355758 |
| 9030409G11Rik | -0.3739695 |
| 9030418K01Rik | 0.64368017 |
| 9030425E11Rik | 0.49327511 |
| 9030607L17Rik | 2.1109808 |
| 9030607L17Rik | 0.93126306 |
| 9030611O19Rik | 0.1495474 |
| 9030612M13Rik | -0.7170432 |
| 9030617O03Rik | 0.41838976 |
| 9030624J02Rik | 0.80821912 |
| 9030625A04Rik | -0.7423652 |
| 9130005N14Rik | 0.08867393 |
| 9130011E15Rik | 2.10273247 |
| 9130011J15Rik | -0.5111072 |
| 9130014G24Rik | 0.73798976 |
| 9130017N09Rik | 0.50935313 |
| 9130206N08Rik | 1.53477339 |
| 9130211I03Rik | 0.92218 |
| 9130213B05Rik | -0.7124283 |
| 9130213B05Rik | 1.33320036 |
| 9130218O11Rik | 0.951344 |
| 9130221H12Rik | 1.56841194 |
| 9130227C08Rik | -0.4854825 |
| 9130401M01Rik | 0.96887238 |
| 9130404D08Rik | -0.5435824 |
| 9130404D08Rik | 0.62823694 |
| 9130404D14Rik | -0.2953308 |
| 9130422G05Rik | -0.3198068 |
| 9230104L09Rik | -0.3947085 |
| 9330129D05Rik | 1.23071978 |
| 9330134C04Rik | -0.0546007 |
| 9330176C04Rik | -0.0993763 |
| 9330186A19Rik | 1.09706114 |
| 9430015G10Rik | 0.98802011 |
| 9430015G10Rik | 4.51732996 |
| 9430023L20Rik | 2.56611136 |
| 9430025C20Rik | -0.7014447 |
| 9430028L06Rik | 1.03246315 |
| 9430029K10Rik | 0.5334487 |
| 9430031J16Rik | 0.77244925 |
| 9430034D17Rik | 0.48103242 |
| 9430057O19Rik | 0.93931585 |
| 9430069J07Rik | 2.64397843 |
| 9430077A04Rik | 0.45842529 |
| 9430079M16Rik | 1.00531954 |
| 9530015I07Rik | 0.58606797 |
| 9530027K23Rik | 2.92249318 |
| 9530058B02Rik | 0.61097166 |
| 9530058B02Rik | 1.87835907 |
| 9530068E07Rik | -0.6014384 |
| 9530077C05Rik | 0.0976167 |
| 9530096D07Rik | 1.24340954 |
| 9530096D07Rik | -0.2343194 |
| 9630015D15Rik | 0.23116556 |
| 9630020C08Rik | 0.42658969 |
| 9630028B13Rik | -0.5283372 |
| 9630031F12Rik | -0.3901324 |
| 9630050M13Rik | -0.6878943 |
| 9630058J23Rik | 0.11790385 |
| 9830163H01Rik | -0.0646739 |
| 9830169C18Rik | -0.5528504 |
| 9930013L23Rik | -0.4920049 |
| 9930021D14Rik | -0.6458078 |
| 9930023K05Rik | -0.7282128 |
| 9930023K05Rik | 0.58054511 |
| 9930104L06Rik | -0.6338813 |
| a | -0.1812108 |
| A030003K21Rik | 1.8276823 |
| A030007L17Rik | 0.03272733 |
| A030007L17Rik | -0.7161184 |
| A030007L17Rik | 0.95999552 |
| A030007L17Rik | 1.20882616 |
| A030009H04Rik | 1.68348483 |
| A030010B05Rik | -0.6950967 |
| A030012M09Rik | 0.90014472 |
| A130010J15Rik | 0.86574571 |
| A130038H09Rik | 1.02789828 |
| A130042E20Rik | -0.1051272 |
| A130090K04Rik | -0.7005352 |
| A130092J06Rik | -0.1012893 |
| A230050P20Rik | -0.5283372 |
| A230051G13Rik | -0.672771 |
| A230054D04Rik | 1.74726912 |
| A230067G21Rik | -0.3319831 |
| A230069A22Rik | 1.54057474 |
| A230074B11Rik | 0.1521062 |
| A230074B11Rik | 2.02167442 |
| A230074B11Rik | -0.0101341 |
| A230106M15Rik | -0.2443083 |
| A230106N23Rik | 1.39283524 |
| A230107C01Rik | 1.0697063 |
| A2bp1 | 1.76550088 |
| A2bp1 | 1.62436323 |
| A330021E22Rik | -0.522308 |
| A330021E22Rik | 1.56801088 |
| A330049M08Rik | 2.10399923 |
| A330080J22Rik | 0.65972094 |
| A3galt2 | 0.55076388 |
| A430005L14Rik | -0.8362948 |
| A430005L14Rik | 0.77282508 |
| A430005L14Rik | 2.27456929 |
| A430005L14Rik | -0.469018 |
| A430033K04Rik | -0.4675999 |
| A430079E08 | 0.44704641 |
| A430083B19Rik | -0.0851521 |
| A430083B19Rik | 1.53726409 |
| A430107D22Rik | 0.57753987 |
| A430107P09Rik | 1.44102101 |
| A430110N23Rik | 0.61498436 |
| A4galt | -0.4840392 |
| A4gnt | -0.0482465 |
| A530050D06Rik | -0.8002088 |
| A530082C11Rik | 0.76452161 |
| A530082C11Rik | 0.53853219 |
| A530088H08Rik | -0.6763074 |
| A530088H08Rik | 1.19708754 |
| A530098C11Rik | 2.42906511 |
| A630042L21Rik | -0.1494251 |
| A630054L15Rik | 0.42794937 |
| A630077B13Rik | -0.5298506 |
| A630095E13Rik | 1.66276564 |
| A730008H23Rik | -0.6798573 |
| A730008L03Rik | 0.85531177 |
| A730008L03Rik | -0.7404658 |
| A730017C20Rik | -0.3455627 |
| A730024F05Rik | -0.6657379 |
| A730037C10Rik | 0.53053139 |
| A730042J05Rik | 0.99485985 |
| A730098D12Rik | 1.69909438 |
| A830006F12Rik | -0.0893967 |
| A830007P12Rik | -0.5037139 |
| A830053O21Rik | -0.4934605 |
| A830059I20Rik | -0.5405134 |
| A830073O21Rik | 2.59422258 |
| A830080D01Rik | 2.34276069 |
| A830093I24Rik | -0.4431257 |
| A930001N09Rik | -0.0860937 |
| A930005I04Rik | -0.3829863 |
| A930008G19Rik | -0.4112373 |
| A930010I20Rik | 1.13160664 |
| A930016P21Rik | 0.44170633 |
| A930018P22Rik | -0.0767204 |
| A930024E05Rik | 0.85272811 |
| A930025D01Rik | 2.27656358 |
| A930025D01Rik | 2.49331199 |
| A930025D01Rik | -0.593298 |
| A930034L06Rik | -0.7423652 |
| A930038C07Rik | 0.62030796 |
| A930041I02Rik | -0.6389741 |
| AA388235 | -0.3246583 |
| AA407270 | 0.61208802 |
| AA407659 | -0.6038944 |
| AA407659 | 1.00429349 |
| AA408296 | 0.8880991 |
| AA408296 | -0.4941892 |
| AA408296 | -0.7606049 |
| AA409316 | -0.6604976 |
| AA415398 | 0.09490404 |
| AA467197 | 0.54622567 |
| AA536717 | -0.7723108 |
| AA536717 | -0.6923893 |
| AA536749 | -0.6475241 |
| AA536749 | 0.06172261 |
| AA536749 | -0.4898257 |
| AA536749 | -0.7991979 |
| AA673488 | 0.10186361 |
| AA691260 | 0.36122043 |
| AA792892 | -0.1880149 |
| AA881470 | -0.6415309 |
| AA881470 | 1.24873118 |
| AA960436 | 1.66789855 |
| AA960436 | -0.0450856 |
| Aaas | -0.2343194 |
| Aaas | -0.3270935 |
| Aacs | 1.8766884 |
| Aadacl1 | -0.5037139 |
| Aak1 | -0.4833184 |
| Aak1 | 1.12092344 |
| Aamp | 0.14991337 |
| Aard | -0.5520745 |
| Aars | 0.56565588 |
| Aars2 | 0.22814179 |
| Aarsd1 | 0.81883154 |
| Aasdh | -0.7133495 |
| Aatf | -0.7142716 |
| Aatf | 1.41237079 |
| Aatk | 0.23351064 |
| AB112350 | 2.76012528 |
| AB182283 | 0.02020018 |
| AB182283 | 1.28515758 |
| Abat | 2.92638303 |
| Abat | -0.5208068 |
| Abat | -0.4654769 |
| Abca14 | 1.20800098 |
| Abca14 | -0.7557703 |
| Abca15 | -0.7742761 |
| Abca15 | 0.04262954 |
| Abca15 | -0.4591364 |
| Abca15 | 2.15570996 |
| Abca17 | 0.12956894 |
| Abca2 | 0.5071088 |
| Abca3 | -0.519308 |
| Abca4 | -0.9015958 |
| Abca4 | 0.71528954 |
| Abca5 | 0.69570415 |
| Abca6 | -0.5359287 |
| Abca6 | -0.1364108 |
| Abca7 | -0.7263419 |
| Abca7 | 0.58100659 |
| Abca8a | -0.3804012 |
| Abca8b | 0.07410572 |
| Abca9 | -0.7931558 |
| Abca9 | -0.4754261 |
| Abca9 | 0.63173945 |
| Abcb11 | -0.9198085 |
| Abcb1b | 1.80074321 |
| Abcb4 | 0.45552758 |
| Abcb6 | 1.2142307 |
| Abcb8 | -0.8196252 |
| Abcb8 | -0.3947085 |
| Abcb8 | -0.5103652 |
| Abcb9 | -0.7713297 |
| Abcb9 | -0.0118727 |
| Abcc1 | -0.5828204 |
| Abcc10 | -0.7366785 |
| Abcc10 | 1.05036326 |
| Abcc12 | -0.0272253 |
| Abcc3 | 0.59520083 |
| Abcc5 | -0.068364 |
| Abcc6 | 2.29413879 |
| Abcc8 | -0.5397477 |
| Abcc9 | -0.1221055 |
| Abcc9 | -0.6622411 |
| Abcd1 | -0.4119046 |
| Abcd2 | -0.5505247 |
| Abcd2 | -0.7615748 |
| Abcd3 | 0.15720289 |
| Abcd4 | -0.1610908 |
| Abcd4 | 1.08058233 |
| Abce1 | -0.2001757 |
| Abcf1 | -0.7366785 |
| Abcf1 | 0.20876064 |
| Abcf1 | 2.91346748 |
| Abcf2 | 1.93274908 |
| Abcf2 | -0.7891493 |
| Abcf2 | 0.56024658 |
| Abcf2 | -0.1032063 |
| Abcf3 | -0.7087528 |
| Abcg1 | 0.89058694 |
| Abcg4 | 2.60571034 |
| Abcg5 | -0.7911504 |
| Abhd1 | 1.90530684 |
| Abhd1 | -0.3113769 |
| Abhd10 | -0.4920049 |
| Abhd11 | 0.5789281 |
| Abhd12 | -0.2200768 |
| Abhd14a | 0.24580922 |
| Abhd14b | 0.5789281 |
| Abhd2 | 3.7511325 |
| Abhd2 | 1.9358934 |
| Abhd2 | 1.88146205 |
| Abhd3 | 0.67340364 |
| Abhd4 | 0.4023296 |
| Abhd5 | -0.117223 |
| Abhd6 | -0.7606049 |
| Abhd7 | 0.57591765 |
| Abhd7 | 0.46602193 |
| Abhd8 | 0.7254247 |
| Abi1 | -0.5973594 |
| Abi1 | -0.5645661 |
| Abi2 | -0.0201757 |
| Abi2 | 0.05850015 |
| Abi2 | -0.5374544 |
| Abi3 | -0.3739695 |
| Abi3bp | 2.76021489 |
| Abi3bp | -0.4389938 |
| Abl1 | 1.5728921 |
| Ablim1 | -0.4438162 |
| Ablim2 | 0.42277201 |
| Ablim2 | -0.1294775 |
| Ablim3 | -0.7557703 |
| Ablim3 | 1.13760243 |
| Abp1 | -0.0140505 |
| Abr | -0.317994 |
| Abr | 0.59769679 |
| Abtb1 | 1.54904606 |
| Acaa1a | 1.98814731 |
| Acaa1b | 0.21047692 |
| Acaa2 | 1.29186592 |
| Acaa2 | -0.3524273 |
| Acaa2 | 0.98263213 |
| Acaa2 | 1.54781714 |
| Acad10 | 0.30962322 |
| Acad10 | 1.40083287 |
| Acad8 | -0.2087296 |
| Acad8 | -0.0729972 |
| Acad9 | 0.36964606 |
| Acad9 | 1.65519575 |
| Acadl | -0.0893967 |
| Acadl | 1.8345237 |
| Acadm | -0.5290936 |
| Acads | -0.7991979 |
| Acads | 0.55361881 |
| Acadsb | -0.5412797 |
| Acadvl | -0.4556324 |
| Acan | 4.53717804 |
| Acat2 | -0.741415 |
| Acbd3 | -0.6212697 |
| Acbd4 | -0.2173626 |
| Acbd4 | -0.5965457 |
| Acbd5 | -0.7723108 |
| Acbd6 | 2.43761462 |
| Accn1 | 1.65358654 |
| Accn1 | -0.6552866 |
| Accn2 | 1.12299238 |
| Accn3 | -0.6987189 |
| Accn3 | 0.74556456 |
| Accs | -0.3752523 |
| Acd | 1.55541053 |
| Ace | 0.87353759 |
| Ace | 1.38944212 |
| Ache | -0.4507488 |
| Acin1 | -0.6932909 |
| Acly | 4.36580418 |
| Acn9 | -0.4782883 |
| Aco1 | -0.29651 |
| Aco2 | -0.6914886 |
| Acot1 | -0.4993058 |
| Acot1 | -0.1801684 |
| Acot10 | -1.0731054 |
| Acot11 | -0.0075322 |
| Acot11 | -0.6745375 |
| Acot2 | -0.4640641 |
| Acot2 | -0.859712 |
| Acot3 | 1.07936508 |
| Acot5 | -0.5497508 |
| Acot7 | 0.6064928 |
| Acot7 | -0.3875275 |
| Acot7 | -0.3077872 |
| Acot7 | -0.9489961 |
| Acot8 | -0.6254547 |
| Acot8 | -0.6509661 |
| Acot8 | 0.60266882 |
| Acot9 | 0.05486155 |
| Acox2 | -0.1041662 |
| Acox3 | 1.07175522 |
| Acox3 | -0.5245644 |
| Acoxl | -0.8782178 |
| Acp2 | 0.01090568 |
| Acp6 | -0.661369 |
| Acpl2 | -0.7683925 |
| Acpp | 2.35486801 |
| Acr | 0.9167197 |
| Acr | 0.46471656 |
| Acrbp | -0.1739383 |
| Acrbp | -0.6146124 |
| Acrv1 | -0.5458908 |
| Acsbg1 | -0.6187676 |
| Acsbg1 | -0.5924879 |
| Acsbg2 | 0.04139749 |
| Acsf3 | -0.4739983 |
| Acsl1 | -0.7041785 |
| Acsl3 | 3.16954103 |
| Acsl3 | 0.68338074 |
| Acsl4 | -0.425351 |
| Acsl4 | -0.3993072 |
| Acsl5 | -0.0032117 |
| Acsl6 | 2.14815067 |
| Acsl6 | 1.45380024 |
| Acsl6 | -0.0832718 |
| Acsm3 | -0.6771936 |
| Acss1 | 3.66233782 |
| Acss2 | -0.6570204 |
| Acss2 | -0.6187676 |
| Acta1 | -0.6798573 |
| Acta2 | -0.0019195 |
| Acta2 | 0.27314369 |
| Acta2 | 1.19302184 |
| Acta2 | -0.5836222 |
| Actb | 0.42930708 |
| Actb | -0.0842115 |
| Actb | 0.72483155 |
| Actb | 1.07175522 |
| Actb | 0.32885443 |
| Actg2 | 0.3086975 |
| Actl6a | -0.7981882 |
| Actl6b | -0.5359287 |
| Actl6b | 1.92034012 |
| Actl6b | -0.4790053 |
| Actl6b | 1.29369693 |
| Actl7a | -0.5989888 |
| Actl7b | 0.18404311 |
| Actn1 | -0.0550562 |
| Actn2 | 1.16763352 |
| Actn3 | -0.5836222 |
| Actn3 | 0.40623154 |
| Actn4 | 0.60266882 |
| Actn4 | 0.20600803 |
| Actn4 | 1.68071549 |
| Actr10 | -0.3048063 |
| Actr10 | 0.44411314 |
| Actr1a | -0.3580815 |
| Actr1b | -0.6113014 |
| Actr3 | 0.93363874 |
| Actr3b | -0.8216927 |
| Actr5 | 0.75748158 |
| Actr5 | -0.5981737 |
| Actr6 | 0.55717524 |
| Actrt1 | 2.17144707 |
| Actrt2 | 0.27602552 |
| Acvr1 | -0.7577011 |
| Acvr1c | 0.78736596 |
| Acvr2a | 1.48236989 |
| Acvr2b | -0.0716048 |
| Acvrl1 | 0.25925095 |
| Acy1 | -0.3855785 |
| Acy1l2 | -0.8383995 |
| Acy3 | 1.81248964 |
| Ada | -0.5497508 |
| Adal | 2.92687047 |
| Adal | 0.45868828 |
| Adam11 | -0.1964578 |
| Adam11 | -0.5965457 |
| Adam12 | 1.21843864 |
| Adam15 | -0.497841 |
| Adam17 | -0.1041662 |
| Adam1b | -0.8093558 |
| Adam1b | -0.7366785 |
| Adam21 | -0.4260284 |
| Adam22 | 2.09404519 |
| Adam22 | -0.7395175 |
| Adam22 | -0.6330353 |
| Adam23 | 0.07092575 |
| Adam24 | 0.97358256 |
| Adam26a | 0.92856331 |
| Adam28 | 1.20077915 |
| Adam29 | 0.61654021 |
| Adam29 | 2.46738104 |
| Adam3 | -0.1948689 |
| Adam30 | -0.7423652 |
| Adam32 | -0.4355644 |
| Adam33 | -0.6389741 |
| Adam39 | -0.3113769 |
| Adam4 | 0.55788491 |
| Adam4 | -0.1662087 |
| Adam4 | -0.6509661 |
| Adam5 | -0.3234431 |
| Adam6 | -0.519308 |
| Adam8 | -0.5884476 |
| Adam8 | -0.6047145 |
| Adam9 | 2.1623595 |
| Adamdec1 | 1.12505674 |
| Adamts1 | 0.50560959 |
| Adamts13 | -0.5344055 |
| Adamts15 | -0.7087528 |
| Adamts16 | 0.22375678 |
| Adamts17 | 1.09493393 |
| Adamts18 | -0.4619491 |
| Adamts19 | 1.03558861 |
| Adamts2 | 0.3375756 |
| Adamts2 | -0.6905887 |
| Adamts2 | 1.88019912 |
| Adamts20 | -0.5582994 |
| Adamts20 | 0.49706829 |
| Adamts4 | -0.1464057 |
| Adamts7 | 0.08789224 |
| Adamtsl2 | -0.5283372 |
| Adamtsl4 | 0.06091803 |
| Adamtsl5 | -0.1344246 |
| Adar | 1.01841223 |
| Adar | -0.7366785 |
| Adar | 0.5588303 |
| Adarb1 | -0.5716667 |
| Adarb1 | 0.46210035 |
| Adarb1 | -0.7124283 |
| Adat1 | 0.04016381 |
| Adc | -0.4542345 |
| Adc | -0.4704383 |
| Adck1 | -0.6692478 |
| Adck2 | 2.94902214 |
| Adck4 | -0.5007728 |
| Adck5 | 0.85668682 |
| Adcy1 | -0.0356664 |
| Adcy10 | 1.2542472 |
| Adcy2 | 1.02316729 |
| Adcy4 | 1.10762519 |
| Adcy4 | 0.7735763 |
| Adcy6 | 0.90145539 |
| Adcy7 | 1.65402569 |
| Adcy8 | 0.48154574 |
| Adcy9 | 1.48789308 |
| Adcyap1 | 2.54562205 |
| Adcyap1r1 | 1.33206505 |
| Add1 | -0.6674912 |
| Add1 | -0.8586348 |
| Add1 | -0.1022473 |
| Add1 | 2.17819478 |
| Add3 | -0.7329065 |
| Add3 | 3.40852917 |
| Add3 | 0.21492454 |
| Adfp | -0.6639879 |
| Adh1 | -0.281876 |
| Adh1 | 1.62715384 |
| Adh4 | 0.8360213 |
| Adh5 | 0.45209138 |
| Adh7 | 0.54118397 |
| Adhfe1 | 0.24415667 |
| Adi1 | 0.94198476 |
| Adipoq | -0.2611975 |
| Adipor1 | -0.5389827 |
| Adipor2 | -0.1806895 |
| Adk | -0.4219712 |
| Adk | 1.18386933 |
| Adm | -0.6861023 |
| Adm2 | -0.7096703 |
| Adnp | 0.31638394 |
| Ado | -0.1089808 |
| Adora1 | -0.1123659 |
| Adora1 | 1.39351239 |
| Adora1 | -0.2871179 |
| Adora2b | 0.56846574 |
| Adora3 | -0.3012417 |
| Adpgk | 1.72941741 |
| Adprh | 0.44970506 |
| Adprhl1 | -0.1504338 |
| Adprhl1 | -0.5428142 |
| Adprhl1 | 1.00238497 |
| Adprhl2 | 1.22760685 |
| Adra1a | 1.62209445 |
| Adra1a | 0.21424167 |
| Adra1d | -0.5245644 |
| Adra2a | -0.8155039 |
| Adra2c | 0.67611631 |
| Adra2c | -0.5351668 |
| Adrb1 | 0.11029997 |
| Adrb2 | -0.5606445 |
| Adrbk1 | 0.38939303 |
| Adrbk2 | -0.4403691 |
| Adrm1 | 2.09773919 |
| Adsl | -0.3656741 |
| Adss | 0.79858208 |
| Adssl1 | 0.9011279 |
| Adssl1 | 1.24884408 |
| Adssl1 | -0.226076 |
| Aebp1 | -0.2146562 |
| Aebp2 | -0.0053695 |
| Aes | 0.61787174 |
| Afap1 | -0.8052794 |
| Afap1 | 0.1737561 |
| Afap1 | 0.66289631 |
| Afap1l1 | 0.68523964 |
| Aff1 | 0.96520942 |
| Aff4 | 0.42140476 |
| Afg3l1 | -0.859712 |
| Afg3l1 | -0.6683691 |
| Afg3l1 | 1.65116752 |
| Afg3l1 | -0.7404658 |
| Afg3l2 | 2.95119774 |
| Afmid | -0.5812188 |
| Aftph | 0.82257994 |
| Agbl1 | 1.65607234 |
| Ager | 0.32734252 |
| Ager | 0.69140942 |
| Aggf1 | -0.7096703 |
| Aggf1 | -0.7509607 |
| Aggf1 | -0.7151945 |
| Agpat1 | -0.4376205 |
| Agpat2 | 1.40265406 |
| Agpat3 | 0.57638143 |
| Agpat4 | -0.7096703 |
| Agpat5 | -0.3325961 |
| Agpat6 | -0.5551818 |
| Agpat6 | -0.4790053 |
| Agpat7 | 1.29906154 |
| Agpat9 | -0.721681 |
| Agpat9 | 1.58604777 |
| Agps | -0.6674912 |
| Agps | -0.6113014 |
| Agrn | -0.5512993 |
| Agrp | -0.5590805 |
| Agt | 2.08725771 |
| Agtpbp1 | 0.84510788 |
| Agtr1a | -0.6932909 |
| Agtrap | 0.1064747 |
| Agtrl1 | 1.64298454 |
| Agxt2l1 | -0.061457 |
| Agxt2l1 | 0.42441007 |
| Agxt2l2 | 0.84684521 |
| Agxt2l2 | -0.6113014 |
| Agxt2l2 | 1.23347815 |
| Agxt2l2 | -0.5007728 |
| Ahctf1 | -0.3637701 |
| Ahcy | 0.09296159 |
| Ahcyl1 | -0.5321252 |
| Ahdc1 | -0.6104755 |
| Ahi1 | -0.1314532 |
| Ahnak | -0.8926208 |
| Ahnak | -0.0795225 |
| Ahnak2 | -0.233216 |
| Ahrr | -0.4718606 |
| Ahsa1 | -0.7772319 |
| Ahsa2 | 1.7339292 |
| AI118078 | 0.37627485 |
| AI132487 | -0.1895922 |
| AI314180 | -0.5876416 |
| AI314976 | -0.6587574 |
| AI316807 | 0.90341797 |
| AI317395 | 0.78291589 |
| AI413582 | 1.92297782 |
| AI413782 | -0.6330353 |
| AI427515 | -0.4172599 |
| AI428936 | 1.23976887 |
| AI449175 | 2.65975085 |
| AI449175 | -0.6406778 |
| AI450540 | -0.7528816 |
| AI451557 | 0.27985418 |
| AI451617 | 0.31821932 |
| AI467606 | 1.14216908 |
| AI480653 | -0.3733288 |
| AI504432 | -0.672771 |
| AI553587 | -0.0473423 |
| AI593442 | -0.5700841 |
| AI593442 | 1.19326149 |
| AI593442 | -0.062375 |
| AI595366 | -0.4920049 |
| AI595406 | 1.72427304 |
| AI597468 | -0.7811875 |
| AI597479 | -0.3855785 |
| AI606181 | 1.12299238 |
| AI646023 | 2.27671392 |
| AI661453 | 1.41898115 |
| AI662250 | -0.136908 |
| AI747699 | 1.73821873 |
| AI836003 | -0.1464057 |
| AI837181 | 0.95876448 |
| AI842396 | -0.486928 |
| AI846148 | 1.4838635 |
| AI847670 | -0.0114377 |
| AI851790 | -0.6330353 |
| AI894139 | 0.88410475 |
| AI987692 | 3.00610715 |
| AI987692 | 0.34235267 |
| AI987944 | -0.4739983 |
| Aicda | 1.98167651 |
| Aif1 | -0.3393657 |
| Aif1 | -0.5044506 |
| Aif1 | 1.23244472 |
| Aifm1 | -0.6321899 |
| Aifm1 | 4.19180723 |
| Aifm2 | -0.7951655 |
| Aifm2 | -0.1230851 |
| Aifm2 | 1.06806393 |
| Aifm3 | 1.04532394 |
| Aig1 | -0.0432841 |
| Aig1-pending | 0.11486972 |
| Aim1 | 0.01471915 |
| Aim2 | -0.4119046 |
| Aip | -0.5074032 |
| Ak1 | -0.1687781 |
| AK122209 | -0.697812 |
| AK122525 | 0.28271542 |
| AK129128 | -0.2714795 |
| AK129302 | -0.4362492 |
| AK129302 | -0.3688559 |
| AK129302 | -0.5397477 |
| AK129341 | 0.10991815 |
| AK162044 | -0.3894805 |
| Ak2 | -0.6878943 |
| Ak2 | 1.05579203 |
| Ak2 | 0.77282508 |
| Ak3 | 1.67479429 |
| Ak3 | 4.16414912 |
| Ak3l1 | -0.7041785 |
| Ak5 | 1.18966028 |
| Akap1 | 3.12036123 |
| Akap1 | 1.31371003 |
| Akap1 | 0.32431134 |
| Akap10 | -0.8012207 |
| Akap10 | 0.58767268 |
| Akap12 | -0.4410575 |
| Akap14 | -0.4704383 |
| Akap2 | 0.6620506 |
| Akap2 | -0.5215571 |
| Akap3 | 1.50589999 |
| Akap4 | 0.92713086 |
| Akap4 | -0.3283135 |
| Akap4 | -0.0383479 |
| Akap4 | -0.7282128 |
| Akap6 | -0.43488 |
| Akap7 | 1.5835261 |
| Akap8 | 1.11664174 |
| Akap8l | 2.89175089 |
| Akap9 | -0.6088259 |
| Akap9 | -0.5645661 |
| Akap9 | 1.80741143 |
| Akna | -0.4883757 |
| Akp2 | -0.5567393 |
| Akp5 | 1.22146596 |
| Akr1a4 | -0.277241 |
| Akr1a4 | -0.796172 |
| Akr1b3 | -0.5029777 |
| Akr1b8 | -0.3059975 |
| Akr1c12 | 0.01471915 |
| Akr1c14 | 0.28525141 |
| Akr1c14 | -0.3953641 |
| Akr1c18 | 1.23071978 |
| Akr1c19 | 1.41492604 |
| Akr1e1 | 0.73290556 |
| Akr7a5 | -0.7300875 |
| Akr7a5 | -0.0546007 |
| Akr7a5 | 1.06586959 |
| Akt1s1 | 0.45288547 |
| Akt3 | 0.47096576 |
| Akt3 | 2.82247663 |
| Aktip | -0.6834208 |
| Alad | -0.272629 |
| Alas1 | -0.57962 |
| Alas2 | -0.1319478 |
| Alcam | -0.4811594 |
| Aldh16a1 | 0.29907767 |
| Aldh18a1 | -0.6825286 |
| Aldh1a1 | 0.19317064 |
| Aldh1a2 | -0.0931859 |
| Aldh1a2 | 1.06765288 |
| Aldh1a7 | -0.1265217 |
| Aldh1b1 | 2.30848802 |
| Aldh1l1 | -0.3210173 |
| Aldh1l2 | -0.5382182 |
| Aldh1l2 | -0.7161184 |
| Aldh2 | 0.90292771 |
| Aldh3a1 | 0.12205976 |
| Aldh3a2 | 0.97176208 |
| Aldh3b1 | -0.0258994 |
| Aldh3b1 | 0.14661444 |
| Aldh3b1 | 3.24439528 |
| Aldh4a1 | 1.58699164 |
| Aldh5a1 | 0.53441912 |
| Aldh5a1 | 2.90044805 |
| Aldh6a1 | 0.24811788 |
| Aldh8a1 | 2.61171686 |
| Aldh8a1 | -0.5941089 |
| Aldoa | -0.5756349 |
| Aldoc | 0.31852486 |
| Alg1 | 1.38574416 |
| Alg10b | -0.6578885 |
| Alg11 | 2.03870071 |
| Alg11 | 2.56484213 |
| Alg12 | 0.41811519 |
| Alg12 | -0.0988987 |
| Alg14 | 0.4499705 |
| Alg2 | -0.4790053 |
| Alg3 | -0.0514182 |
| Alg3 | -0.5044506 |
| Alg3 | 0.59064556 |
| Alg5 | 1.45280246 |
| Alg5 | -0.5661393 |
| Alg5 | -0.6038944 |
| Alg8 | -0.759636 |
| Alg8 | -0.2912153 |
| Alg8 | -0.6932909 |
| Alg8 | -0.1349208 |
| Alg9 | 0.19665763 |
| Alg9 | 1.40475839 |
| Alg9 | 0.40400386 |
| Alk | -0.4493581 |
| Alkbh2 | -0.2504656 |
| Alkbh3 | -0.5208068 |
| Alkbh4 | -0.7452216 |
| Alkbh5 | -0.4711492 |
| Alkbh7 | 0.00665015 |
| Alkbh8 | 1.8543391 |
| Allc | 2.09764815 |
| Allc | 0.96582091 |
| Alox12 | -0.4971095 |
| Alox12b | -0.5732519 |
| Alox12e | 0.2785797 |
| Alox15 | 1.1446965 |
| Alox5ap | 1.44642786 |
| Alox8 | 0.34473208 |
| Aloxe3 | 0.46654357 |
| Alpk3 | -0.0762542 |
| Als2 | 0.18439582 |
| Als2 | -0.6578885 |
| Als2 | -0.7282128 |
| Als2cl | -0.7105887 |
| Als2cr13 | 0.13478969 |
| Als2cr2 | -0.5989888 |
| Als2cr2 | -0.5359287 |
| Als2cr4 | 1.62933526 |
| Als2cr4 | 0.43255756 |
| Als2cr4 | -0.4825982 |
| Alx3 | 1.03785513 |
| Amac1 | 0.45526371 |
| Ambp | 0.72522703 |
| Ambra1 | 1.57033464 |
| Ambra1 | -0.5215571 |
| Amd2 | 0.77113264 |
| Amdhd2 | 0.4109478 |
| Amdhd2 | -0.5081428 |
| Amfr | -0.8447424 |
| Amh | 0.53756603 |
| Amhr2 | 2.34023603 |
| Amhr2 | 2.01964787 |
| Amhr2 | 0.55480579 |
| Amigo1 | 1.61441546 |
| Amigo1 | 0.15064488 |
| Amigo2 | 0.83637242 |
| Amigo3 | 0.95475237 |
| Ammecr1l | -0.7300875 |
| Amn | -0.105608 |
| Amotl2 | 1.55817127 |
| Ampd1 | -0.0254579 |
| Ampd1 | 1.5187583 |
| Ampd2 | 2.39396331 |
| Amph | -0.6146124 |
| Amph | 0.7056491 |
| Amy1 | -0.7713297 |
| Amz2 | -0.5397477 |
| Anapc10 | 2.0770297 |
| Anapc11 | -0.4059162 |
| Anapc13 | -0.3966766 |
| Anapc2 | 0.85376243 |
| Anapc4 | 0.50460877 |
| Anapc5 | 0.33907106 |
| Anapc5 | 2.54429644 |
| Anapc5 | -0.2244361 |
| Anapc7 | 0.38939303 |
| Ang | -0.2288157 |
| Angel1 | -0.5178116 |
| Angel2 | -0.1806895 |
| Angpt1 | -0.2510274 |
| Angpt2 | -0.8532671 |
| Angpt2 | 0.0273195 |
| Angptl1 | -0.0998542 |
| Angptl2 | 1.47778635 |
| Angptl3 | 0.92553667 |
| Angptl4 | -0.6221052 |
| Angptl7 | -0.7674155 |
| Ank | 1.73922125 |
| Ank1 | -0.2691849 |
| Ank2 | 0.72641245 |
| Ank2 | 0.4320166 |
| Ank2 | 1.42010967 |
| Ank3 | -0.6923893 |
| Ank3 | -0.5653524 |
| Ank3 | 2.6047584 |
| Ankar | -0.5133367 |
| Ankar | -0.0505109 |
| Ankfy1 | 2.11585937 |
| Ankle2 | -0.4704383 |
| Ankle2 | 3.51726204 |
| Ankle2 | 0.79142665 |
| Ankmy1 | 0.72562234 |
| Ankmy1 | -0.403267 |
| Ankmy1 | -0.1393981 |
| Ankmy2 | 2.00302345 |
| Ankmy2 | 1.29165027 |
| Ankrd10 | 2.70064929 |
| Ankrd12 | -0.3778232 |
| Ankrd12 | -0.6544209 |
| Ankrd13a | -0.4711492 |
| Ankrd13b | 0.03769155 |
| Ankrd13d | -0.5428142 |
| Ankrd17 | 0.66563961 |
| Ankrd17 | -0.7480867 |
| Ankrd17 | -0.4704383 |
| Ankrd22 | -0.1216161 |
| Ankrd23 | 0.24316374 |
| Ankrd24 | 0.71788347 |
| Ankrd25 | 1.51230868 |
| Ankrd27 | 2.57775712 |
| Ankrd34a | 0.14808207 |
| Ankrd35 | -0.4956482 |
| Ankrd36 | -0.6544209 |
| Ankrd37 | -0.0032117 |
| Ankrd39 | 1.48894143 |
| Ankrd40 | -0.5238117 |
| Ankrd40 | -0.7395175 |
| Ankrd40 | 1.19050181 |
| Ankrd46 | 0.82578094 |
| Ankrd47 | 0.00792884 |
| Ankrd49 | -0.0637537 |
| Ankrd5 | 1.81846137 |
| Ankrd50 | -0.3555643 |
| Ankrd53 | 0.67611631 |
| Ankrd54 | 1.71657145 |
| Ankrd54 | 0.21731068 |
| Ankrd54 | 0.06172261 |
| Ankrd56 | -0.8447424 |
| Ankrd58 | 0.04713322 |
| Ankrd6 | -0.7050916 |
| Ankrd6 | 1.69511134 |
| Ankrd6 | -0.4072436 |
| Ankrd7 | 1.92757594 |
| Ankrd9 | 0.3071526 |
| Anks1 | 2.48474804 |
| Anks3 | 4.64467324 |
| Anks3 | 1.14318087 |
| Anks6 | 2.27309907 |
| Anks6 | 0.82293615 |
| Ankzf1 | -0.4139092 |
| Ankzf1 | 0.39756953 |
| Anln | -0.5941089 |
| Anln | -0.3791113 |
| Anp32a | -0.6736538 |
| Anp32a | -0.1036861 |
| Anp32a | -0.2266233 |
| Anp32e | -0.1590515 |
| Anpep | 0.99826103 |
| Antxr1 | 2.8634194 |
| Antxr1 | 0.40511836 |
| Antxrl | 2.65095413 |
| Anxa1 | 0.23584985 |
| Anxa11 | 3.88653525 |
| Anxa11 | 0.8009551 |
| Anxa2 | 0.55219243 |
| Anxa3 | 2.08652097 |
| Anxa3 | 3.10416137 |
| Anxa4 | 1.72766068 |
| Anxa5 | -0.4985731 |
| Anxa6 | -0.6834208 |
| Anxa8 | -0.3107777 |
| Anxa8 | 0.33487773 |
| Anxa9 | -0.378467 |
| Aoah | 0.94823466 |
| Aoc2 | 2.50783386 |
| Aoc2 | 0.62582132 |
| Aoc3 | -0.566927 |
| Aof1 | 0.34205482 |
| Aof1 | -0.7851599 |
| Aof2 | -0.3036166 |
| Aox3 | -0.6279747 |
| Aox3l1 | -0.1459034 |
| Ap1b1 | -0.2471021 |
| Ap1g1 | -0.7050916 |
| Ap1g1 | -0.6798573 |
| Ap1g2 | 1.34844426 |
| Ap1gbp1 | 0.41039418 |
| Ap1gbp1 | 0.97992645 |
| Ap1m1 | -0.6055354 |
| Ap1m2 | 0.02690222 |
| Ap1m2 | -0.5788216 |
| Ap1m2 | 0.80204831 |
| Ap1m2 | 0.91381598 |
| Ap1s1 | -0.5466615 |
| Ap1s1 | 0.15465802 |
| Ap1s1 | 1.52910309 |
| Ap1s3 | 0.23384517 |
| Ap2a1 | -0.7635176 |
| Ap2a1 | -0.1938112 |
| Ap2a2 | -0.5884476 |
| Ap2b1 | 2.28494558 |
| Ap2b1 | -0.5884476 |
| Ap2b1 | -0.0762542 |
| Ap2b1 | -0.554404 |
| Ap2m1 | 1.7176658 |
| Ap2s1 | -0.4683087 |
| Ap2s1 | 1.86420832 |
| Ap2s1 | 0.28366723 |
| Ap3b1 | -0.7060055 |
| Ap3b1 | 2.7516406 |
| Ap3b2 | 2.23246338 |
| Ap3d1 | 1.82968541 |
| Ap3m1 | 0.15756588 |
| Ap3m2 | 2.97794722 |
| Ap3s2 | -0.1656956 |
| Ap3s2 | -0.117223 |
| Ap4b1 | -0.277241 |
| Ap4m1 | 2.33417712 |
| Ap4m1 | -0.6807469 |
| Ap4m1 | -0.6014384 |
| Ap4s1 | 0.98966026 |
| Apaf1 | 0.96765299 |
| Apaf1 | 1.054125 |
| Apba1 | -0.7664395 |
| Apba2 | -0.0114377 |
| Apba2 | 0.29438712 |
| Apba3 | -0.6816374 |
| Apbb1 | -0.5290936 |
| Apbb1ip | 2.51581157 |
| Apbb1ip | 0.48128911 |
| Apbb1ip | -0.572459 |
| Apbb2 | 0.35094935 |
| Apbb3 | -0.4563321 |
| Apc | -0.6162723 |
| Apc2 | 1.13352437 |
| Apcdd1 | -0.5081428 |
| Apeh | -0.5868364 |
| Apex1 | -0.5428142 |
| Aph1a | -0.1980493 |
| Aph1b | 2.59977135 |
| Apip | 1.79163024 |
| Apip | -0.2381913 |
| Apitd1 | -0.5836222 |
| Aplp1 | -0.0049375 |
| Aplp2 | 1.40733451 |
| Apoa1 | 1.74302103 |
| Apoa1bp | -0.4383069 |
| Apoa2 | -0.804263 |
| Apoa4 | -0.3071903 |
| Apob48r | -0.6221052 |
| Apobec1 | 0.79234708 |
| Apobec3 | -0.2583614 |
| Apoc1 | -0.4927324 |
| Apoc1 | -0.1469083 |
| Apoc2 | 1.32188916 |
| Apoc3 | 0.10263371 |
| Apoe | 0.60985395 |
| Apof | -0.0423847 |
| Apoh | -0.3587119 |
| Apol7c | 0.79967807 |
| Apol9b | 0.10570776 |
| App | 0.03148215 |
| Appbp1 | -0.2255291 |
| Appbp1 | 0.26799839 |
| Appbp2 | 0.02940314 |
| Appl1 | -0.5215571 |
| Appl2 | -0.5582994 |
| Aprin | 0.89554282 |
| Aprt | -0.4039286 |
| Aptx | -0.6014384 |
| Aqp1 | -0.7124283 |
| Aqp1 | -0.6596271 |
| Aqp1 | -0.7310262 |
| Aqp11 | 1.42142456 |
| Aqp11 | -0.6763074 |
| Aqp3 | -0.7625457 |
| Aqp4 | -0.7509607 |
| Aqp5 | 1.617312 |
| Aqp7 | 1.31812307 |
| Aqp7 | 0.66458545 |
| Aqp8 | 0.90879389 |
| Aqp8 | 0.56565588 |
| Aqp9 | 1.23152515 |
| Aqr | -0.5037139 |
| Aqr | 0.37885598 |
| Ar | -0.0321029 |
| Araf | 0.04672469 |
| Araf | 2.26704308 |
| Arbp | 1.60119138 |
| Arc | 1.19098234 |
| Arcn1 | -0.1906451 |
| Ard1 | 0.99485985 |
| Ard1 | -0.1128505 |
| Areg | -0.6771936 |
| Arf2 | -0.696906 |
| Arf3 | 0.69079427 |
| Arf5 | -0.7941601 |
| Arf6 | -0.7198231 |
| Arfgap1 | -0.7105887 |
| Arfgap1 | 0.13180998 |
| Arfgap1 | -0.7615748 |
| Arfgap2 | -0.2476618 |
| Arfgap2 | 1.82109648 |
| Arfgap2 | -0.397991 |
| Arfgap2 | 0.58721447 |
| Arfgap3 | -0.2381913 |
| Arfip2 | -0.5435824 |
| Arfip2 | -0.4072436 |
| Arfip2 | -0.7772319 |
| Arfrp1 | 0.54214639 |
| Arfrp1 | -0.7901493 |
| Arg1 | 0.63805448 |
| Arg2 | 0.01260247 |
| Arglu1 | 2.38851076 |
| Arhgap1 | -0.7557703 |
| Arhgap1 | 2.96198237 |
| Arhgap1 | 2.082551 |
| Arhgap10 | 0.36732932 |
| Arhgap12 | -0.3155824 |
| Arhgap15 | -0.3675819 |
| Arhgap15 | -0.0711412 |
| Arhgap17 | 1.47601745 |
| Arhgap18 | 0.23451387 |
| Arhgap19 | 0.01767447 |
| Arhgap20 | 0.27346433 |
| Arhgap22 | 0.25337349 |
| Arhgap24 | -0.5981737 |
| Arhgap24 | -0.4052532 |
| Arhgap25 | -0.5989888 |
| Arhgap26 | -0.1084983 |
| Arhgap27 | 0.0933504 |
| Arhgap28 | 1.88151942 |
| Arhgap29 | -0.1932828 |
| Arhgap30 | 2.14638088 |
| Arhgap4 | 1.17156573 |
| Arhgap6 | 0.17125597 |
| Arhgap8 | 3.14890252 |
| Arhgap9 | -0.1869649 |
| Arhgdib | 4.41996753 |
| Arhgdig | -0.4739983 |
| Arhgef1 | -0.3054017 |
| Arhgef1 | 1.36274418 |
| Arhgef10 | 2.07628484 |
| Arhgef10l | -0.6330353 |
| Arhgef10l | -0.7385702 |
| Arhgef15 | -0.5215571 |
| Arhgef18 | 0.16515623 |
| Arhgef19 | 0.60311951 |
| Arhgef2 | 2.12434711 |
| Arhgef3 | -0.7480867 |
| Arhgef4 | 0.17232827 |
| Arhgef6 | 0.26928738 |
| Arid2 | 3.4178742 |
| Arid3a | 0.29689167 |
| Arid3b | 1.67643886 |
| Arid4b | -0.5397477 |
| Arid5a | -0.6406778 |
| Arid5b | -0.6212697 |
| Arih1 | -0.7198231 |
| Arih2 | -0.6807469 |
| Arl1 | -0.0066665 |
| Arl11 | -0.7282128 |
| Arl16 | 2.77948203 |
| Arl2 | -0.6736538 |
| Arl2bp | 0.03314203 |
| Arl2bp | 1.07366351 |
| Arl2bp | -0.34432 |
| Arl3 | -0.2714795 |
| Arl3 | -0.4192762 |
| Arl4a | -0.2611975 |
| Arl4a | 0.12994282 |
| Arl4c | -0.2023068 |
| Arl5a | -0.7693705 |
| Arl5b | 1.34793673 |
| Arl6 | 0.15574953 |
| Arl6ip1 | 0.95087826 |
| Arl6ip2 | 0.49352847 |
| Arl6ip2 | 2.67736899 |
| Arl6ip2 | -0.5435824 |
| Arl6ip2 | 0.87151118 |
| Arl6ip4 | 2.79210237 |
| Arl6ip4 | -0.4782883 |
| Arl6ip5 | 1.03941017 |
| Arl6ip5 | 0.70443702 |
| Arl8a | 1.99049854 |
| Arl8b | -0.5253177 |
| Armc10 | 1.34895151 |
| Armc3 | 0.73290556 |
| Armc6 | 1.87351165 |
| Armc6 | -0.0014891 |
| Armc8 | 0.67819763 |
| Armc8 | -0.4431257 |
| Armc8 | 1.27708728 |
| Armc9 | -0.175492 |
| Armc9 | -0.6440947 |
| Armcx1 | -0.7151945 |
| Armcx2 | -0.0632939 |
| Armcx3 | -0.3486767 |
| Armcx5 | 3.13346456 |
| Armcx6 | 1.12299238 |
| Armcx6 | 1.5519889 |
| Armetl1 | 1.17913761 |
| Arnt | 2.48984796 |
| Arnt | 0.00365971 |
| Arnt2 | -0.2365299 |
| Arntl | 0.38114435 |
| Arpc1a | 0.7244359 |
| Arpc1a | -0.5957327 |
| Arpc1a | -0.3071903 |
| Arpc1b | -0.1032063 |
| Arpc1b | 3.58158059 |
| Arpc1b | -0.6121281 |
| Arpc3 | -0.2982816 |
| Arpc4 | -0.5037139 |
| Arpc5 | -0.6807469 |
| Arpc5 | -0.5812188 |
| Arpc5l | 0.74653127 |
| Arpp19 | 1.13696641 |
| Arpp21 | -0.2028404 |
| Arpp21 | 0.58560898 |
| Arrb1 | 1.61928867 |
| Arrb1 | -0.3338233 |
| Arrb1 | -0.0049375 |
| Arrb1 | -0.2049776 |
| Arrb2 | -0.6080022 |
| Arrdc1 | -0.4052532 |
| Arrdc2 | -0.0813953 |
| Arrdc3 | 0.30900618 |
| Arrdc4 | -0.7338481 |
| Ars2 | 2.46132424 |
| Ars2 | 2.8663835 |
| Ars2 | -0.5096238 |
| Arsa | -0.5306082 |
| Arsb | 0.07370882 |
| Arsb | 0.00665015 |
| Arsg | 0.07806552 |
| Arsi | -0.0105684 |
| Arsj | -0.4577332 |
| Arsj | 1.82299161 |
| Art1 | -0.4119046 |
| Art2b | -0.3089822 |
| Art3 | -0.0179835 |
| Art3 | -0.7319659 |
| Art4 | 0.11638802 |
| Art5 | 0.08161506 |
| Artn | 0.21390004 |
| Artn | 0.79308278 |
| Arv1 | -0.7041785 |
| As3mt | -0.6389741 |
| As3mt | 1.65942498 |
| Asah1 | 0.23884882 |
| Asah1 | 0.89554282 |
| Asah2 | -0.0974672 |
| Asah3 | -0.4711492 |
| Asah3l | 0.74730391 |
| Asb1 | -0.4507488 |
| Asb1 | 0.3300622 |
| Asb1 | -0.4341961 |
| Asb12 | -0.4833184 |
| Asb13 | -0.0123079 |
| Asb15 | -0.8749254 |
| Asb17 | 1.44157229 |
| Asb2 | 0.66984439 |
| Asb3 | -0.4985731 |
| Asb3 | -0.1022473 |
| Asb4 | -0.3714092 |
| Asb4 | 1.18435328 |
| Asb6 | 1.5840783 |
| Asb6 | -0.3119765 |
| Asb7 | -0.741415 |
| Asb7 | -0.7703496 |
| Asb8 | -0.4219712 |
| Asb9 | -0.1775675 |
| Ascc1 | 0.24976343 |
| Ascc1 | -0.7782192 |
| Ascc2 | 3.60275185 |
| Ascc2 | -0.7548064 |
| Ascc2 | 0.57033429 |
| Ascc2 | -0.4790053 |
| Ascc3l1 | 1.8523882 |
| Ascl1 | 1.14947984 |
| Ascl2 | -0.7179689 |
| Asf1a | -0.6923893 |
| Asf1b | -0.7151945 |
| Asgr1 | -0.5466615 |
| Ash1l | -0.7811875 |
| Ash1l | 0.7498106 |
| Ash1l | 1.3712995 |
| Ash2l | -0.4486636 |
| Ash2l | -0.5260717 |
| Asl | 0.02940314 |
| Asl | 1.34407048 |
| Asna1 | -0.8022337 |
| Asns | -0.1959279 |
| Asns | 0.10109288 |
| Asns | 0.8215105 |
| Asnsd1 | 0.55575429 |
| Asnsd1 | -0.4971095 |
| Asph | -0.1539726 |
| Asph | -0.4341961 |
| Asph | -0.2448664 |
| Asph | -0.8672877 |
| Asph | -0.7762456 |
| Asphd1 | 0.35536507 |
| Asphd2 | 0.09296159 |
| Aspm | -0.4165888 |
| Asprv1 | 2.01144751 |
| Aspscr1 | -0.1354172 |
| Aspscr1 | 1.21867186 |
| Aspscr1 | 0.00792884 |
| Asrgl1 | -0.7625457 |
| Ass1 | -0.2611975 |
| Aste1 | 0.88377111 |
| Astn1 | -0.7732929 |
| Astn2 | 0.89554282 |
| Asz1 | 1.1714431 |
| Atad1 | 2.00669324 |
| Atad2 | 0.66395238 |
| Atad3a | -0.1084983 |
| Atad4 | 1.93111855 |
| Atad4 | 0.32157484 |
| Atcay | 2.09226138 |
| Ate1 | 1.05717896 |
| Ate1 | 0.39532108 |
| Atf2 | -0.5567393 |
| Atf2 | -0.232114 |
| Atf2 | 1.34885008 |
| Atf3 | -0.0767204 |
| Atf4 | -0.5844247 |
| Atf4 | 2.32308528 |
| Atf5 | -0.1413949 |
| Atf7 | -0.6288162 |
| Atf7ip | 0.35771162 |
| Atg10 | -0.4963786 |
| Atg10 | -0.429423 |
| Atg12 | 0.51159208 |
| Atg16l1 | 1.24511123 |
| Atg2a | -0.0729972 |
| Atg2a | 1.54617606 |
| Atg2b | -0.4052532 |
| Atg2b | -0.5820193 |
| Atg3 | -0.6570204 |
| Atg3 | 0.44277679 |
| Atg4b | -0.3375145 |
| Atg4c | -0.2994645 |
| Atg4c | 1.70963358 |
| Atg4d | -0.804263 |
| Atg5 | -0.3707702 |
| Atg5 | 0.07964476 |
| Atg7 | -0.1744559 |
| Atg9b | -0.0669785 |
| Athl1 | -0.5653524 |
| Atic | 2.74837933 |
| Atic | -0.1240657 |
| Atic | -0.8012207 |
| Atm | -0.5965457 |
| Atm | 1.4136966 |
| Atmin | -0.5051879 |
| Atmin | 0.18721274 |
| Atmin | -0.557519 |
| Atn1 | 0.64927221 |
| Atoh8 | 0.74459686 |
| Atox1 | -0.3656741 |
| Atp10a | 0.61275721 |
| Atp10d | -0.5428142 |
| Atp11c | 0.32431134 |
| Atp11c | -0.5780239 |
| Atp12a | -0.3612376 |
| Atp13a1 | -0.6364242 |
| Atp13a1 | 0.73035305 |
| Atp13a2 | -0.7654645 |
| Atp13a4 | -0.408573 |
| Atp13a5 | -0.7792076 |
| Atp1a1 | -0.5582994 |
| Atp1a2 | 1.23828579 |
| Atp1b1 | -0.5497508 |
| Atp1b2 | 0.84892576 |
| Atp1b3 | -0.4847606 |
| Atp2a1 | 1.16442638 |
| Atp2a2 | 1.90597766 |
| Atp2a2 | 1.94799203 |
| Atp2a3 | 1.41558737 |
| Atp2a3 | 1.50177524 |
| Atp2a3 | 4.01044563 |
| Atp2b1 | -0.8289652 |
| Atp2b2 | -0.6950967 |
| Atp2b2 | -0.7861557 |
| Atp2b2 | -0.4898257 |
| Atp2b3 | 0.18580534 |
| Atp2b3 | 2.49259635 |
| Atp2b4 | -0.0276677 |
| Atp2b4 | 0.89141474 |
| Atp2c1 | 0.85943084 |
| Atp2c1 | 2.62945668 |
| Atp5a1 | 0.44197406 |
| Atp5b | 2.3602793 |
| Atp5c1 | 0.43795003 |
| Atp5d | 0.34145883 |
| Atp5e | 0.08789224 |
| Atp5f1 | -0.8352442 |
| Atp5g2 | 0.57313014 |
| Atp5g3 | 0.81936794 |
| Atp5h | -0.7851599 |
| Atp5j | 1.17596977 |
| Atp5j | -0.1275059 |
| Atp5j2 | -0.6923893 |
| Atp5l | -0.6622411 |
| Atp5o | -0.6398256 |
| Atp5s | 1.25008506 |
| Atp5sl | 0.54022056 |
| Atp6ap1 | 1.35087657 |
| Atp6ap2 | -0.8310535 |
| Atp6ap2 | 2.71885541 |
| Atp6ap2 | 0.06493399 |
| Atp6ap2 | -0.1245564 |
| Atp6v0a1 | -0.6449509 |
| Atp6v0a1 | 0.66921488 |
| Atp6v0a2 | -0.5389827 |
| Atp6v0b | 1.31170678 |
| Atp6v0d1 | -0.7050916 |
| Atp6v0d1 | 0.18404311 |
| Atp6v0d1 | -0.7170432 |
| Atp6v0d1 | -0.3048063 |
| Atp6v0e | -0.6754221 |
| Atp6v0e2 | 0.06373101 |
| Atp6v1a | 0.11410964 |
| Atp6v1b1 | 0.25631684 |
| Atp6v1b2 | 1.36394266 |
| Atp6v1c1 | -0.6406778 |
| Atp6v1c2 | -0.877119 |
| Atp6v1d | -0.8300088 |
| Atp6v1e1 | 0.40789882 |
| Atp6v1e2 | 1.20030364 |
| Atp6v1g1 | 1.99487851 |
| Atp6v1g2 | 0.6622621 |
| Atp6v1g2 | 1.95395657 |
| Atp6v1h | 1.07841723 |
| Atp7a | -0.8248026 |
| Atp8a1 | 0.65844776 |
| Atp8a2 | 0.34740169 |
| Atp8b1 | 2.81875971 |
| Atp8b3 | 3.55535688 |
| Atp8b3 | -0.7207516 |
| Atp8b3 | 0.4751088 |
| Atp8b3 | 0.64862868 |
| Atp9a | 0.53272022 |
| Atp9a | 3.29932525 |
| Atp9b | 4.06037169 |
| Atp9b | 1.0287559 |
| Atpaf1 | -0.6381234 |
| Atpaf2 | -0.7752603 |
| Atpaf2 | -0.0898695 |
| Atpbd1b | 1.77171502 |
| Atpbd1c | 0.14661444 |
| Atpbd3 | 0.22104805 |
| Atpif1 | 2.03966987 |
| Atrx | 0.23250633 |
| Atxn1 | 2.51949974 |
| Atxn10 | 1.27982154 |
| Atxn2 | 0.57126717 |
| Atxn2l | -0.6347282 |
| Atxn3 | -0.8002088 |
| Atxn3 | 0.35653908 |
| Atxn3 | 0.32066089 |
| Atxn7l1 | 2.03364482 |
| Atxn7l2 | -0.566927 |
| AU014645 | -0.848995 |
| AU017455 | 1.5595486 |
| AU017455 | -0.2859504 |
| AU018778 | 1.64194735 |
| AU018829 | -0.4577332 |
| AU019823 | 3.20958211 |
| AU021034 | 0.11448976 |
| AU021092 | 0.99055367 |
| AU021838 | -0.5981737 |
| AU022252 | 1.10209396 |
| AU022751 | -0.7841652 |
| AU022870 | 1.42386161 |
| AU023871 | 0.54334803 |
| AU040320 | 2.30765216 |
| AU040320 | -0.6288162 |
| AU040320 | -0.554404 |
| AU040829 | 0.3303639 |
| AU042651 | -0.3947085 |
| Aup1 | 0.58354071 |
| Aup1 | -0.1008107 |
| Aurka | -0.0988987 |
| Aurkaip1 | -0.4059162 |
| Aurkc | -0.5772269 |
| Auts2 | 1.08098773 |
| Auts2 | 0.53028786 |
| AV028368 | 0.63479341 |
| AV249152 | 2.15618039 |
| AV249152 | 0.88059562 |
| Aven | 0.19909081 |
| Avil | 1.60405193 |
| Avp | 0.28525141 |
| Avpi1 | 0.80386748 |
| Avpi1 | 1.16244726 |
| Avpr1a | -0.5482049 |
| AW049604 | -0.7723108 |
| AW061290 | 0.18263092 |
| AW111922 | -0.5268262 |
| AW121567 | 1.09480082 |
| AW125753 | -0.4410575 |
| AW146242 | 0.85651505 |
| AW146299 | -0.6398256 |
| AW146299 | 1.72277858 |
| AW540478 | -0.6187676 |
| AW544981 | -0.6509661 |
| AW548124 | -0.5351668 |
| AW549877 | -0.4493581 |
| AW551984 | 1.0747522 |
| AW551984 | 0.489212 |
| AW551984 | -0.3024284 |
| AW555464 | 0.95320465 |
| Axin2 | -0.7567352 |
| Axl | -0.5908697 |
| Axud1 | -0.4486636 |
| Azgp1 | -0.1099467 |
| Azgp1 | 1.78840375 |
| Azi1 | 3.54337962 |
| Azi2 | 1.05315118 |
| Azin1 | 0.81020687 |
| B020018G12Rik | -0.6347282 |
| B230118H07Rik | 0.1276973 |
| B230120H23Rik | 0.96856768 |
| B230206H07Rik | 3.1457196 |
| B230209C24Rik | 0.52024847 |
| B230217C12Rik | 0.0218802 |
| B230219D22Rik | 0.39700793 |
| B230312A22Rik | -0.5700841 |
| B230312I18Rik | -0.6798573 |
| B230317C12Rik | -0.6355758 |
| B230317C12Rik | -0.022813 |
| B230339H12Rik | 2.42775512 |
| B230339M05Rik | 1.07922973 |
| B230339M05Rik | 2.02152628 |
| B230340J04Rik | -0.0258994 |
| B230342M21Rik | 1.89936023 |
| B230373P09Rik | -0.3054017 |
| B230378H13Rik | 0.16046472 |
| B230380D07Rik | 0.1917722 |
| B230396O12Rik | 0.28588434 |
| B230399E16Rik | -0.0716048 |
| B2m | 0.12919491 |
| B2m | 1.29498724 |
| B3galnt1 | -0.6718889 |
| B3galnt2 | -0.5876416 |
| B3galt1 | 2.36944461 |
| B3galt4 | -0.6221052 |
| B3galt4 | -0.5756349 |
| B3galt5 | -0.6372734 |
| B3galt6 | -0.603075 |
| B3gat1 | 0.97826911 |
| B3gat1 | -0.4591364 |
| B3gat1 | 0.51728959 |
| B3gat2 | -0.2912153 |
| B3gat3 | 1.3870109 |
| B3gat3 | 0.39419482 |
| B3gnt1 | -0.6279747 |
| B3gnt3 | -0.3778232 |
| B3gnt5 | -0.0884518 |
| B3gnt7 | -0.7014447 |
| B3gnt8 | 0.06493399 |
| B3gnt8 | 1.92122018 |
| B430119L13Rik | 0.10991815 |
| B4galnt3 | -0.4092383 |
| B4galnt4 | -0.4105705 |
| B4galt1 | -0.6552866 |
| B4galt3 | -0.7519206 |
| B4galt3 | -0.6006212 |
| B4galt4 | 0.20220989 |
| B4galt7 | 1.77878495 |
| B630005N14Rik | 1.35239355 |
| B830045N13Rik | 1.96151709 |
| B930006L02Rik | 0.84562942 |
| B930011P16Rik | -0.3682187 |
| B930037P14Rik | -0.6113014 |
| B930041F14Rik | 0.7244359 |
| B930041G04 | 0.80948454 |
| B930076A02 | -0.721681 |
| B9d1 | 1.09878595 |
| B9d2 | 3.05949847 |
| Baalc | 0.44678013 |
| Baalc | -0.5489775 |
| Bace1 | 2.92461863 |
| Bace2 | -0.5981737 |
| Bace2 | -0.1413949 |
| Bace2 | 1.7969831 |
| Bach1 | -0.4206227 |
| Bach2 | 0.00622353 |
| Bad | 1.92495121 |
| Bag1 | -0.4145784 |
| Bag2 | 1.01927859 |
| Bag3 | 0.71768419 |
| Bag4 | -0.8206584 |
| Bag5 | 1.67242961 |
| Bahcc1 | 1.62171578 |
| Bahd1 | 0.49226099 |
| Bai1 | 3.05852219 |
| Bai2 | 0.59178646 |
| Bai2 | 3.19058202 |
| Bai3 | -0.425351 |
| Baiap2 | 0.21355829 |
| Baiap2 | 1.0195672 |
| Banf1 | 0.06893277 |
| Banf1 | 0.44143852 |
| Banf1 | -0.6338813 |
| Banf1 | -0.5989888 |
| Banf2 | 1.30130552 |
| Bap1 | 1.86426675 |
| Bard1 | -0.1980493 |
| Barx2 | 0.85582765 |
| Basp1 | 1.0273261 |
| Bat1a | 3.25179821 |
| Bat1a | -0.6552866 |
| Bat2 | -0.1349208 |
| Bat3 | -0.4307842 |
| Bat4 | 3.26915027 |
| Bat4 | -0.7226113 |
| Bat5 | -0.6509661 |
| Batf | 0.51159208 |
| Bax | -0.6288162 |
| Baz1a | -0.5155714 |
| Baz1b | 2.01726972 |
| Baz2a | -0.4267064 |
| BB014433 | -0.3543082 |
| BB128963 | 0.66858494 |
| BB146404 | -0.4811594 |
| Bbc3 | -0.4697279 |
| Bbc3 | 0.54694369 |
| Bbox1 | -0.3505501 |
| Bbs1 | 0.65950886 |
| Bbs12 | 0.93695458 |
| Bbs2 | 2.61205818 |
| Bbs4 | -0.5125929 |
| Bbs5 | -0.2299139 |
| Bbs7 | -0.3107777 |
| Bbs9 | -0.5029777 |
| Bbs9 | -0.4993058 |
| Bbx | 1.47335779 |
| BC002163 | 1.14507502 |
| BC002199 | 2.04165351 |
| BC002216 | 1.13301336 |
| BC003236 | -0.4971095 |
| BC003266 | -0.500039 |
| BC003885 | -0.0514182 |
| BC003885 | 1.13683915 |
| BC003993 | 1.30290507 |
| BC003993 | -0.1880149 |
| BC004004 | -0.6262939 |
| BC004044 | -0.6798573 |
| BC004044 | 0.07608772 |
| BC004728 | 0.32400768 |
| BC004728 | -0.6798573 |
| BC005537 | 2.17031068 |
| BC005682 | -0.6440947 |
| BC005764 | -0.8662017 |
| BC006662 | 1.35370628 |
| BC006779 | -0.3960201 |
| BC006779 | 0.82826304 |
| BC008155 | 2.69727668 |
| BC008163 | -0.1570167 |
| BC008163 | 0.61542915 |
| BC010462 | 0.55314359 |
| BC011248 | -0.5482049 |
| BC011248 | -0.7557703 |
| BC011487 | -0.6807469 |
| BC013491 | -0.4804408 |
| BC013491 | -0.3499252 |
| BC013529 | -0.7060055 |
| BC013712 | 0.14404053 |
| BC016201 | -0.0950863 |
| BC016423 | 0.64540437 |
| BC016495 | 0.47871895 |
| BC017158 | 1.49321001 |
| BC017612 | 1.10050758 |
| BC017643 | 0.50760801 |
| BC017647 | 1.60157845 |
| BC018242 | 2.51371417 |
| BC018242 | -0.721681 |
| BC018465 | -0.388178 |
| BC020002 | -0.2039084 |
| BC020535 | -0.0550562 |
| BC021381 | -0.1827766 |
| BC021395 | -0.6246162 |
| BC021614 | -0.6347282 |
| BC021790 | 1.58919031 |
| BC021891 | -0.2184473 |
| BC022224 | -0.7078361 |
| BC022687 | 2.12160037 |
| BC023055 | -0.5559602 |
| BC023151 | 2.12994842 |
| BC023744 | 0.38342713 |
| BC023814 | 1.06036111 |
| BC023829 | 1.31602422 |
| BC024561 | 0.62955192 |
| BC024814 | -0.4654769 |
| BC024868 | -0.4840392 |
| BC024997 | 0.51233721 |
| BC025076 | -0.6022564 |
| BC025546 | -0.8543382 |
| BC025575 | -0.6423847 |
| BC025920 | -0.8310535 |
| BC026439 | -0.1991119 |
| BC026585 | 3.62051435 |
| BC026590 | -0.5536269 |
| BC026590 | -0.6923893 |
| BC027072 | 0.73896433 |
| BC027231 | 0.45552758 |
| BC027231 | -0.5653524 |
| BC027231 | -0.0387956 |
| BC027344 | -0.5007728 |
| BC028528 | 2.02192126 |
| BC028528 | 1.30205232 |
| BC029169 | 1.89744478 |
| BC029169 | 4.21093263 |
| BC029214 | 2.7652863 |
| BC030183 | -0.7235426 |
| BC030307 | -0.5155714 |
| BC030476 | -0.8073153 |
| BC030499 | -0.121127 |
| BC030500 | 0.33577788 |
| BC030867 | -0.4246741 |
| BC031181 | -0.4883757 |
| BC031353 | 0.46758597 |
| BC031575 | -0.4507488 |
| BC031748 | -0.4818785 |
| BC031781 | 0.49757288 |
| BC031853 | 0.0776703 |
| BC032265 | 1.36633501 |
| BC034076 | 0.37426237 |
| BC037034 | 1.02014414 |
| BC037594 | -0.8165325 |
| BC038156 | -0.7161184 |
| BC038286 | -0.6718889 |
| BC038613 | -0.4905515 |
| BC038822 | 0.59451895 |
| BC038822 | -0.2900428 |
| BC038822 | -0.5466615 |
| BC038925 | -0.7188956 |
| BC039093 | -0.5916784 |
| BC040758 | 0.23951395 |
| BC043098 | -0.4697279 |
| BC043118 | 0.73447288 |
| BC043301 | -0.2947418 |
| BC046331 | -0.7471307 |
| BC046404 | 1.58265771 |
| BC048355 | 0.06292817 |
| BC048502 | -0.2906289 |
| BC048546 | -0.2271709 |
| BC048546 | 1.3598415 |
| BC048562 | 1.35652745 |
| BC048644 | -0.5677153 |
| BC048651 | -0.6262939 |
| BC048671 | -0.5037139 |
| BC049635 | -0.2266233 |
| BC049635 | 1.13645728 |
| BC049730 | -0.7782192 |
| BC049762 | 2.52484634 |
| BC049806 | 0.07806552 |
| BC049806 | -0.5405134 |
| BC049806 | -0.6096504 |
| BC050196 | 3.25711804 |
| BC050210 | 0.2514061 |
| BC050777 | 1.2670665 |
| BC050811 | 1.24045258 |
| BC050811 | -0.2499042 |
| BC051019 | 0.00878033 |
| BC051070 | 2.32594192 |
| BC051142 | 1.2617372 |
| BC051227 | 1.5411529 |
| BC051628 | 2.15008927 |
| BC051665 | 1.59067932 |
| BC052484 | -0.6288162 |
| BC053749 | 1.22239548 |
| BC053749 | 1.6144918 |
| BC054059 | -0.7347906 |
| BC055107 | 0.05728886 |
| BC055111 | -0.6096504 |
| BC055324 | 0.8880991 |
| BC055368 | -0.7519206 |
| BC056474 | -0.2611975 |
| BC056923 | 0.4478448 |
| BC057022 | 2.40182001 |
| BC057371 | 2.3999773 |
| BC057371 | -0.6932909 |
| BC057371 | 1.29466483 |
| BC057552 | -0.2135758 |
| BC057627 | -0.4507488 |
| BC057893 | -0.2365299 |
| BC060267 | 0.40956314 |
| BC060615 | 3.26770675 |
| BC060632 | 0.77413931 |
| BC061039 | 0.67047348 |
| BC061237 | 0.02523121 |
| BC062115 | -0.4605417 |
| BC062650 | -0.7207516 |
| BC063749 | 1.01999994 |
| BC064033 | -0.3018349 |
| BC065085 | 0.96352575 |
| BC065397 | -0.006234 |
| BC066028 | -0.4675999 |
| BC066107 | 0.19421811 |
| BC067047 | 0.7451776 |
| BC067068 | -0.4199492 |
| BC068157 | 2.67088424 |
| BC068281 | -0.0651344 |
| BC072620 | -0.8258415 |
| BC085271 | 0.92153927 |
| BC087945 | -0.4825982 |
| BC088983 | 2.72880072 |
| BC089491 | 1.31012224 |
| BC099439 | -0.4267064 |
| BC107230 | -0.4619491 |
| Bcam | 1.46917573 |
| Bcan | 1.46479569 |
| Bcan | 0.12130552 |
| Bcap29 | -0.6296585 |
| Bcap29 | 0.10301852 |
| Bcap31 | -0.1901185 |
| Bcap31 | -0.4971095 |
| Bcar1 | -0.4833184 |
| Bcar3 | -0.4768561 |
| Bcas1 | -0.4591364 |
| Bcas2 | -0.3511754 |
| Bcas3 | 0.14698156 |
| Bcat1 | 0.53635693 |
| Bcat2 | -0.7606049 |
| Bccip | -0.0688263 |
| Bcdin3d | -0.2135758 |
| Bcdo2 | -0.6578885 |
| Bckdha | -0.6905887 |
| Bckdhb | -0.6896897 |
| Bckdk | -0.068364 |
| Bckdk | -0.7471307 |
| Bcl10 | 0.75000314 |
| Bcl11a | 0.731532 |
| Bcl11b | 3.04052353 |
| Bcl2 | 0.36326117 |
| Bcl2 | -0.0149231 |
| Bcl2l1 | 0.66900495 |
| Bcl2l1 | 0.97570198 |
| Bcl2l1 | -0.7133495 |
| Bcl2l10 | -0.2293646 |
| Bcl2l11 | -0.5582994 |
| Bcl2l11 | -0.3173905 |
| Bcl2l11 | 0.64583492 |
| Bcl2l12 | 2.03525205 |
| Bcl2l13 | -0.2365299 |
| Bcl2l14 | -0.0162335 |
| Bcl2l14 | 0.77620081 |
| Bcl2l2 | -0.7423652 |
| Bcl2l2 | -0.3018349 |
| Bcl3 | -0.4239976 |
| Bcl6 | -0.759636 |
| Bcl6b | 0.6522695 |
| Bcl7a | 0.73837971 |
| Bcl7b | -0.6570204 |
| Bcl9l | -0.6843138 |
| Bclaf1 | -0.7683925 |
| Bclaf1 | 0.2689653 |
| Bclp2 | 0.10532406 |
| Bcmo1 | -0.4267064 |
| Bcor | -0.4905515 |
| Bcor | -0.5692938 |
| Bcorl1 | 0.34740169 |
| Bcs1l | 1.26839409 |
| Bdh1 | 1.75143206 |
| Bdh2 | 0.71528954 |
| Bdkrb1 | -0.1932828 |
| Bdnf | -0.7272769 |
| Bdnf | -0.5283372 |
| Becn1 | -0.7254079 |
| Becn1 | 0.61498436 |
| Bet1 | -0.3283135 |
| Bet1l | 1.33649525 |
| Bex2 | 2.57666721 |
| Bex2 | -0.0171081 |
| Bex2 | 0.90651697 |
| Bex4 | -0.5458908 |
| Bex4 | 2.27030305 |
| Bex6 | -0.8738304 |
| Bfar | -0.4920049 |
| Bfar | 1.04672648 |
| Bglap2 | 1.59645612 |
| Bgn | 1.05773316 |
| Bhlhb2 | 0.81327055 |
| Bhlhb5 | -0.6237784 |
| Bhlhb8 | 0.32096564 |
| Bhlhb8 | 0.58377074 |
| Bhlhb9 | 2.1832757 |
| Bhmt | -0.7635176 |
| Bhmt2 | -0.6996266 |
| Bicc1 | -0.4287431 |
| Bicd1 | -0.7577011 |
| Bicd2 | 1.88243679 |
| Bicd2 | 0.56330785 |
| Bid | 0.70685961 |
| Bid | -0.0875078 |
| Bik | 1.74335361 |
| Bin1 | 2.37541508 |
| Bin1 | -0.242636 |
| Bin1 | 0.68172527 |
| Bin3 | 0.73054965 |
| Birc2 | -0.7615748 |
| Birc2 | 0.26152668 |
| Birc2 | 0.80912317 |
| Birc4 | 0.41564039 |
| Birc5 | -0.1991119 |
| Birc5 | 1.41360197 |
| Birc5 | 0.78607021 |
| Blcap | 1.67558118 |
| Blm | 1.57560176 |
| Blm | 3.99529219 |
| Blmh | 0.59837636 |
| Blnk | -0.7170432 |
| Bloc1s1 | -0.5066642 |
| Bloc1s3 | -0.8694635 |
| Blvra | -0.2634727 |
| Blvrb | 1.67815186 |
| Bmf | 1.04952526 |
| Bmi1 | 1.44496469 |
| Bmp1 | -0.6466656 |
| Bmp1 | 1.00517303 |
| Bmp1 | 0.10724101 |
| Bmp1 | 0.27121752 |
| Bmp15 | 0.70484122 |
| Bmp2k | 1.35642684 |
| Bmp2k | 1.35249459 |
| Bmp3 | 0.5858385 |
| Bmp4 | 0.55266813 |
| Bmp6 | -0.6321899 |
| Bmp7 | 2.02093351 |
| Bmp8a | 1.80883859 |
| Bmper | 0.11562918 |
| Bmpr1a | 1.53269272 |
| Bmpr1a | 1.55353833 |
| Bmpr1b | 3.56178262 |
| Bmpr1b | 1.50478466 |
| Bmpr2 | -0.6674912 |
| Bms1 | -0.3137777 |
| Bnc1 | 1.67558118 |
| Bnc2 | -0.6137835 |
| Bnc2 | -0.7519206 |
| Bnip1 | -0.2674675 |
| Bnip2 | -0.7881504 |
| Bnip3l | 1.52968839 |
| Bnipl | 2.63154141 |
| Bnipl | 1.16763352 |
| Bok | -0.4963786 |
| Bok | -0.5772269 |
| Bola1 | 1.41907524 |
| Bola2 | 2.16206213 |
| Bola3 | 0.38171557 |
| Boll | 1.52633888 |
| Bop1 | 1.0567631 |
| Bpi | 0.17089826 |
| Bpil1 | 2.16167967 |
| Bpil3 | -0.6221052 |
| Bpnt1 | -0.1534663 |
| Bpnt1 | -0.4105705 |
| Bpnt1 | -0.4598388 |
| Bpnt1 | -0.2515895 |
| Brap | -0.2646123 |
| Brca2 | 0.86165445 |
| Brca2 | -0.5088831 |
| Brcc3 | -0.2277189 |
| Brcc3 | -0.7772319 |
| Brcc3 | 2.31759753 |
| Brd2 | 1.78998675 |
| Brd2 | -0.6754221 |
| Brd2 | 0.1455122 |
| Brd4 | 1.24431749 |
| Brd4 | -0.8310535 |
| Brd8 | 0.33787488 |
| Brd8 | -0.5374544 |
| Brd9 | 2.37541508 |
| Brd9 | -0.3234431 |
| Brdt | -0.4125723 |
| Brdt | -0.8300088 |
| Bre | 0.14771538 |
| Bre | 0.53296311 |
| Bre | 0.11941723 |
| Brf1 | 3.08342128 |
| Brf2 | 1.58612646 |
| Bri3 | 1.4275053 |
| Brip1 | -0.6347282 |
| Brms1 | 2.04671445 |
| Brp16 | 0.81614487 |
| Brp16 | 0.02940314 |
| Brp17 | 2.40533081 |
| Brp44 | 0.6926385 |
| Brpf1 | -0.2130361 |
| Brpf1 | -0.6587574 |
| Brs3 | -0.6204349 |
| Brsk1 | -0.2812953 |
| Brsk2 | -0.7442685 |
| Brsk2 | -0.7423652 |
| Brunol4 | -0.1687781 |
| Brunol4 | -0.1595609 |
| Brwd1 | -0.5598622 |
| Brwd1 | -0.2651827 |
| Brwd2 | -0.061457 |
| Bscl2 | -0.5973594 |
| Bsdc1 | -0.6987189 |
| Bsg | 0.83847639 |
| Bsg | 1.16108419 |
| Bspry | -0.7452216 |
| Bst2 | -0.7226113 |
| Btbd1 | 1.9522563 |
| Btbd10 | -0.5804191 |
| Btbd11 | -0.4761409 |
| Btbd12 | -0.3486767 |
| Btbd14a | -0.5637805 |
| Btbd14b | -0.6639879 |
| Btbd16 | -0.2076561 |
| Btbd3 | 0.51803019 |
| Btbd3 | 0.51134357 |
| Btbd6 | 2.58259184 |
| Btbd9 | 0.39869173 |
| Btc | -0.7124283 |
| Btd | 0.24017861 |
| Btf3 | -0.6355758 |
| Btg1 | 1.34253991 |
| Btg1 | 0.73623297 |
| Btg3 | -0.4486636 |
| Btg3 | -0.1008107 |
| Btg4 | 1.71149331 |
| Btk | 0.64884324 |
| Btla | 0.56659344 |
| Btrc | 0.52197016 |
| Btrc | 0.80604584 |
| Btrc | 1.66421438 |
| Bub1b | -0.1980493 |
| Bub3 | 1.22887633 |
| Bud13 | 0.76414242 |
| Bud31 | -0.1428953 |
| Bves | 1.43011255 |
| Bxdc1 | -0.6389741 |
| Bxdc1 | -0.2454248 |
| Bxdc2 | 0.06172261 |
| Bxdc2 | 0.55100212 |
| Bxdc5 | 2.16308127 |
| Bxdc5 | 2.58242928 |
| Bxdc5 | -0.6146124 |
| Bzrap1 | 1.10037526 |
| Bzrpl1 | -0.6022564 |
| Bzw1 | -0.322836 |
| Bzw2 | 3.09238201 |
| Bzw2 | 2.32007639 |
| C030006K11Rik | 4.00639837 |
| C030011O14Rik | -0.4934605 |
| C030014K22Rik | -0.7557703 |
| C030018G13Rik | 2.40614919 |
| C030030A07Rik | -0.4376205 |
| C030039L03Rik | 0.47070621 |
| C030045D06Rik | 0.01556448 |
| C030045D06Rik | -0.7528816 |
| C030046I01Rik | -0.7871525 |
| C030048B08Rik | -0.696906 |
| C030048B08Rik | -0.4179315 |
| C130022K22Rik | 1.26960939 |
| C130032J12Rik | -0.3406018 |
| C130034I18Rik | -0.557519 |
| C130038G02Rik | -0.6146124 |
| C130060K24Rik | 2.16756924 |
| C130073D16Rik | 1.02503388 |
| C130074G19Rik | -0.7772319 |
| C130090K23Rik | -0.6780807 |
| C130090K23Rik | 2.53342346 |
| C1galt1 | 0.07450246 |
| C1galt1c1 | -0.7244748 |
| C1qa | 0.33247306 |
| C1qb | 0.45182654 |
| C1qbp | 1.28211214 |
| C1qc | -0.5804191 |
| C1qdc2 | 1.0010614 |
| C1ql2 | -0.5412797 |
| C1ql3 | 0.18404311 |
| C1qtnf1 | 0.44143852 |
| C1qtnf2 | 1.2571609 |
| C1qtnf3 | 1.43206322 |
| C1qtnf3 | -0.6501044 |
| C1qtnf3 | 1.6568022 |
| C1qtnf4 | 1.46488528 |
| C1qtnf6 | -0.661369 |
| C1qtnf7 | -0.4521415 |
| C1qtnf9 | 0.71668717 |
| C1rl | 0.41067104 |
| C1s | 3.12782814 |
| C1s | -0.5344055 |
| C2 | -0.2135758 |
| C230021P08Rik | 0.47949074 |
| C230029F24Rik | -0.8002088 |
| C230052I12Rik | -0.0651344 |
| C230052I12Rik | -0.7625457 |
| C230055K05Rik | 3.16603604 |
| C230071H18Rik | -0.5148259 |
| C230078M08Rik | 0.14477666 |
| C230078M14Rik | -0.7282128 |
| C230090D14 | 0.12356644 |
| C230093N12Rik | 1.11560079 |
| C230096C10Rik | -0.5989888 |
| C2cd3 | 0.1276973 |
| C3 | -0.3406018 |
| C330001K17Rik | 0.19978484 |
| C330005M16Rik | -0.2859504 |
| C330016O10Rik | -0.7347906 |
| C330019G07Rik | -0.7014447 |
| C330023M02Rik | -0.021054 |
| C330027C09Rik | -0.3986488 |
| C430003P19Rik | 0.46367095 |
| C430004E15Rik | 0.23718391 |
| C4b | -0.8694635 |
| C530028I08Rik | 2.71432526 |
| C530028I08Rik | -0.2471021 |
| C530028O21Rik | 1.22517849 |
| C530043G21Rik | -0.5812188 |
| C530044N13Rik | 3.61686695 |
| C6 | -0.8331465 |
| C630004H02Rik | -0.7032664 |
| C630004H02Rik | 0.53853219 |
| C630007B19Rik | -0.2249824 |
| C630028N24Rik | -0.0596237 |
| C630035N08Rik | 0.38797603 |
| C630035N08Rik | -0.7557703 |
| C730025P13Rik | 0.3902422 |
| C730027P07Rik | -0.0370062 |
| C78339 | 0.51753652 |
| C79127 | 3.44937753 |
| C79267 | 0.28525141 |
| C79407 | -0.8870549 |
| C79407 | 2.56310103 |
| C80008 | 1.78332003 |
| C80913 | 0.04794975 |
| C85492 | 0.52123268 |
| C85492 | -0.1289842 |
| C85492 | -0.5949204 |
| C85627 | -0.4139092 |
| C87436 | 1.96708218 |
| C87436 | 1.68043099 |
| C87499 | -0.7772319 |
| C8b | 1.90396375 |
| C8g | 1.17205609 |
| C9 | 2.79452406 |
| C9 | 1.48474097 |
| C920005C14Rik | 2.31500386 |
| Cab39l | -0.780197 |
| Cabc1 | 1.0331744 |
| Cables2 | -0.6763074 |
| Cabp2 | -0.1344246 |
| Cabp7 | 0.63370385 |
| Cabyr | 1.98881238 |
| Cabyr | 1.7466729 |
| Cachd1 | 2.48263996 |
| Cacna1a | -0.6754221 |
| Cacna1b | -0.4739983 |
| Cacna1b | 0.04303987 |
| Cacna1b | 1.01566345 |
| Cacna1d | -0.1408953 |
| Cacna1g | -0.4876516 |
| Cacna1h | 2.04671445 |
| Cacna2d1 | 1.01493874 |
| Cacna2d3 | -0.6423847 |
| Cacnb1 | -0.6754221 |
| Cacnb1 | -0.5420466 |
| Cacnb2 | 1.69244651 |
| Cacnb2 | -0.1864404 |
| Cacnb3 | -0.6432393 |
| Cacnb3 | -0.260062 |
| Cacnb3 | 1.66840309 |
| Cacnb3 | 2.28438663 |
| Cacnb4 | 1.29197373 |
| Cacnb4 | 1.84906823 |
| Cacng2 | 0.38939303 |
| Cacng3 | -0.4697279 |
| Cacng3 | -0.3901324 |
| Cacng4 | 1.12968496 |
| Cacng5 | -0.6047145 |
| Cacng5 | -0.572459 |
| Cacng5 | -0.3418396 |
| Cacng7 | 1.16158008 |
| Cacng8 | -0.5412797 |
| Cacybp | -0.0790549 |
| Cad | 0.12994282 |
| Cadm2 | -0.2393005 |
| Cadm3 | 0.84075036 |
| Cadps | -0.3289241 |
| Cadps2 | 2.07077314 |
| Cadps2 | 0.85565572 |
| Cage1 | -0.5916784 |
| Calb2 | 0.96475054 |
| Calb2 | -0.5238117 |
| Calca | 1.28678498 |
| Calca | 0.86284958 |
| Calcoco1 | -0.7841652 |
| Cald1 | 1.50787 |
| Cald1 | -0.6432393 |
| Calm2 | 2.21732845 |
| Calm3 | -0.6372734 |
| Calml4 | -0.6154419 |
| Caln1 | -0.0075322 |
| Calr | 0.34175687 |
| Calr3 | -0.3036166 |
| Calr3 | 1.58115585 |
| Calr3 | 0.0040875 |
| Calr4 | 1.88980035 |
| Calu | -0.2293646 |
| Calu | 0.34265043 |
| Calu | 1.62572187 |
| Caly | -0.6544209 |
| Camk1 | 2.73129948 |
| Camk1d | 1.46407862 |
| Camk1g | 1.21668763 |
| Camk1g | 1.43816754 |
| Camk2a | 1.32220231 |
| Camk2a | 0.25599026 |
| Camk2a | 0.34858574 |
| Camk2b | -0.2092668 |
| Camk2d | -0.6338813 |
| Camk2d | -0.2532779 |
| Camk2d | -0.2124967 |
| Camk2g | 1.05870221 |
| Camk2g | 0.96275944 |
| Camk2n1 | 0.68976812 |
| Camk4 | 0.19630952 |
| Camkk1 | 0.83970152 |
| Camkk2 | 1.64704813 |
| Camkk2 | 1.83271227 |
| Camkv | -0.4563321 |
| Caml | 0.90096411 |
| Camp | 1.7560359 |
| Camsap1l1 | 1.06861171 |
| Camta2 | 0.31208733 |
| Cant1 | -0.6666141 |
| Cant1 | -0.4369346 |
| Cap1 | -0.0720687 |
| Capg | -0.1489212 |
| Capg | 0.37224554 |
| Capn1 | -0.0984213 |
| Capn10 | -0.4092383 |
| Capn11 | 0.29720427 |
| Capn2 | -0.4941892 |
| Capn2 | -0.2482219 |
| Capn2 | -0.1104301 |
| Capn3 | 0.14219768 |
| Capn5 | -0.5804191 |
| Capn6 | -0.6622411 |
| Capn7 | -0.6055354 |
| Capn8 | 1.77993677 |
| Capns1 | -0.3198068 |
| Caprin1 | -0.6683691 |
| Caprin1 | -0.3356671 |
| Capsl | -0.6816374 |
| Capza1 | 0.72423802 |
| Capza3 | -0.1621122 |
| Capzb | -0.5645661 |
| Capzb | -0.5981737 |
| Capzb | 1.15473836 |
| Car11 | -0.5489775 |
| Car12 | -0.5764306 |
| Car13 | 0.72660988 |
| Car14 | 1.82329691 |
| Car15 | 0.27057458 |
| Car2 | -0.5852279 |
| Car3 | 1.6376381 |
| Car4 | 0.23150094 |
| Car5b | -0.4862049 |
| Car5b | -0.8144764 |
| Car5b | -0.0674401 |
| Car7 | -0.5629955 |
| Car9 | 2.70026719 |
| Card10 | -0.0258994 |
| Card11 | -0.6055354 |
| Carhsp1 | 2.81798085 |
| Carm1 | -0.1027267 |
| Cart | 2.2987017 |
| Cartpt | 2.46067627 |
| Casc1 | 0.1521062 |
| Casc1 | 2.18335876 |
| Casc1 | 3.96251514 |
| Casc3 | -0.8216927 |
| Casc4 | -0.5185595 |
| Casc4 | -0.6129554 |
| Casc4 | -0.4818785 |
| Cask | -0.6825286 |
| Caskin1 | -0.5949204 |
| Caskin1 | 1.80560885 |
| Caskin1 | -0.1975185 |
| Caskin2 | -0.8586348 |
| Casp1 | 1.38953924 |
| Casp2 | 0.40344611 |
| Casp4 | -0.6271339 |
| Casp4 | -0.6887916 |
| Casp4 | 0.48051881 |
| Casp6 | 0.44009834 |
| Casp6 | -0.2629034 |
| Casp7 | 0.11600867 |
| Casp8 | 1.48140217 |
| Casp8 | -0.4458906 |
| Casp9 | 1.29906154 |
| Casq2 | -0.4099042 |
| Cass4 | 0.18545316 |
| Cast | -0.7404658 |
| Cat | -0.6171033 |
| Catsper1 | 3.18320306 |
| Catsper2 | -0.5685042 |
| Catsper3 | -0.6843138 |
| Catsper4 | 2.14343821 |
| Cav1 | -0.6406778 |
| Cav2 | -0.7395175 |
| Cbara1 | 1.26640199 |
| Cbfa2t3h | 0.43687402 |
| Cbfa2t3h | -0.3986488 |
| Cbfb | -0.6692478 |
| Cbfb | -0.0941356 |
| Cbfb | 0.37022435 |
| Cbl | 0.3200511 |
| Cblb | -0.7041785 |
| Cblc | -0.5804191 |
| Cbln1 | -0.4971095 |
| Cbln2 | 0.69977611 |
| Cbln4 | 1.74322059 |
| Cbr1 | 0.69631608 |
| Cbr2 | -0.4862049 |
| Cbr3 | 1.57161425 |
| Cbr3 | -0.0405884 |
| Cbs | -0.6483834 |
| Cbs | 0.56893323 |
| Cbwd1 | 2.05542234 |
| Cbx2 | 1.5306909 |
| Cbx5 | 1.40102474 |
| Cbx7 | -0.0536903 |
| Cbx8 | -0.3580815 |
| Cby1 | 0.50410796 |
| Cc2d1a | 0.93173868 |
| Cc2d1b | -0.3907847 |
| Cc2d2a | 1.37535078 |
| Ccar1 | -0.3449412 |
| Ccbe1 | -0.3778232 |
| Ccbl1 | 2.00528735 |
| Ccbl1 | 1.21657078 |
| Ccbl2 | 0.22915081 |
| Ccbp2 | -0.0053695 |
| Ccdc100 | 0.44864251 |
| Ccdc101 | -0.4934605 |
| Ccdc108 | 0.56377793 |
| Ccdc109a | -0.7151945 |
| Ccdc109a | 0.11600867 |
| Ccdc109b | 0.26670763 |
| Ccdc11 | -0.0450856 |
| Ccdc110 | 0.8064084 |
| Ccdc111 | -0.4438162 |
| Ccdc113 | -0.5185595 |
| Ccdc114 | 0.79289891 |
| Ccdc115 | 0.20082492 |
| Ccdc116 | -0.2760859 |
| Ccdc117 | -0.486928 |
| Ccdc12 | -0.7831716 |
| Ccdc120 | 1.7085988 |
| Ccdc123 | -0.4039286 |
| Ccdc124 | -0.6905887 |
| Ccdc125 | 0.48103242 |
| Ccdc126 | -0.4633586 |
| Ccdc127 | 0.30653392 |
| Ccdc127 | 3.08489433 |
| Ccdc13 | -0.5103652 |
| Ccdc130 | 0.5853794 |
| Ccdc131 | 0.70706121 |
| Ccdc132 | 0.89471866 |
| Ccdc134 | -0.4125723 |
| Ccdc137 | 3.61896952 |
| Ccdc137 | -0.6347282 |
| Ccdc18 | -0.5412797 |
| Ccdc21 | 1.94980672 |
| Ccdc23 | -0.4335127 |
| Ccdc25 | 0.57103404 |
| Ccdc27 | -0.5860318 |
| Ccdc27 | 1.33731716 |
| Ccdc27 | -0.8062968 |
| Ccdc28a | -0.6552866 |
| Ccdc28b | -0.1927546 |
| Ccdc28b | -0.4675999 |
| Ccdc3 | 1.12865844 |
| Ccdc32 | 0.03272733 |
| Ccdc38 | 0.489212 |
| Ccdc39 | 0.13069013 |
| Ccdc40 | -0.3406018 |
| Ccdc41 | 2.58872141 |
| Ccdc42 | -0.6561531 |
| Ccdc43 | 0.33607774 |
| Ccdc45 | -0.277241 |
| Ccdc46 | -0.1911721 |
| Ccdc46 | -0.2244361 |
| Ccdc47 | 1.47813973 |
| Ccdc49 | -0.4362492 |
| Ccdc5 | -0.5941089 |
| Ccdc50 | -0.4079081 |
| Ccdc51 | 3.67386118 |
| Ccdc53 | 2.21118098 |
| Ccdc53 | -0.2732043 |
| Ccdc53 | -0.0468905 |
| Ccdc54 | 1.21023905 |
| Ccdc56 | 0.16874913 |
| Ccdc57 | 1.05495889 |
| Ccdc58 | -0.7782192 |
| Ccdc59 | -0.4307842 |
| Ccdc59 | -0.365039 |
| Ccdc64 | -0.8062968 |
| Ccdc65 | 2.38235514 |
| Ccdc65 | 0.61587373 |
| Ccdc66 | 1.14935428 |
| Ccdc67 | -0.4898257 |
| Ccdc68 | 0.20082492 |
| Ccdc70 | -0.4186036 |
| Ccdc71 | -0.6852076 |
| Ccdc73 | 0.33367617 |
| Ccdc77 | -0.2888717 |
| Ccdc79 | 2.49298406 |
| Ccdc80 | -0.4328299 |
| Ccdc82 | 1.22529427 |
| Ccdc84 | 1.89445109 |
| Ccdc85a | 0.8121904 |
| Ccdc85b | 0.73447288 |
| Ccdc86 | 2.01244533 |
| Ccdc87 | -0.7991979 |
| Ccdc88a | -0.6171033 |
| Ccdc88b | 1.53327577 |
| Ccdc88c | -0.868375 |
| Ccdc90b | 0.06011277 |
| Ccdc91 | 1.09799028 |
| Ccdc92 | -0.5780239 |
| Ccdc93 | -0.6347282 |
| Ccdc94 | 2.79889744 |
| Ccdc95 | 1.16689439 |
| Ccdc96 | 2.95591544 |
| Ccdc97 | 0.57150025 |
| Ccdc98 | 1.01174333 |
| Ccdc99 | 0.53320594 |
| Cchcr1 | -0.7991979 |
| Ccin | 0.75346217 |
| Cck | 0.55764842 |
| Cckbr | -0.6254547 |
| Ccl11 | -0.3823393 |
| Ccl17 | -0.7500017 |
| Ccl19 | -0.9186598 |
| Ccl21b | -0.0032117 |
| Ccl21c | 1.8449016 |
| Ccl25 | -0.7654645 |
| Ccl25 | 0.01175446 |
| Ccl27 | 0.67757372 |
| Ccl27 | 0.47614169 |
| Ccl28 | 1.17731132 |
| Ccl3 | 0.88610406 |
| Ccl4 | 1.31212888 |
| Ccl4 | -0.1235753 |
| Ccl5 | 2.21114067 |
| Ccl6 | -0.8436823 |
| Ccl7 | -0.6096504 |
| Ccl9 | -0.4079081 |
| Ccm2 | 0.48436401 |
| Ccna1 | 1.3187518 |
| Ccnb1 | 0.71428996 |
| Ccnb1 | 0.01809589 |
| Ccnb3 | 0.66458545 |
| Ccnc | -0.7792076 |
| Ccnd1 | -0.8216927 |
| Ccnd1 | 1.32282831 |
| Ccnd1 | -0.6701274 |
| Ccnd2 | -0.6254547 |
| Ccnd3 | -0.5482049 |
| Ccnd3 | 0.3230961 |
| Ccndbp1 | 0.60311951 |
| Ccne1 | -0.7752603 |
| Ccne2 | 1.03345875 |
| Ccnf | 1.1724237 |
| Ccng1 | -0.4125723 |
| Ccng1 | -0.4854825 |
| Ccng1 | -0.7115081 |
| Ccng2 | 1.02918441 |
| Ccnk | -0.5764306 |
| Ccnl1 | -0.3543082 |
| Ccno | 0.40734339 |
| Ccr4 | 0.82026127 |
| Ccr5 | 0.49200728 |
| Ccr5 | -0.1585424 |
| Ccr5 | 1.76394087 |
| Ccr6 | 1.66775434 |
| Ccr6 | 0.83567005 |
| Ccrk | -0.9501828 |
| Ccrk | 2.29528161 |
| Ccrk | -0.4307842 |
| Ccrk | 1.73741594 |
| Ccrl2 | -0.7577011 |
| Ccrn4l | -0.6692478 |
| Ccrn4l | 0.91688075 |
| Ccs | 1.66508255 |
| Cct2 | 0.89570756 |
| Cct3 | 1.71698198 |
| Cct3 | -0.6825286 |
| Cct4 | -0.2634727 |
| Cct5 | -0.4584345 |
| Cct6a | -0.3940534 |
| Cct6b | -0.8227282 |
| Cct7 | 0.21355829 |
| Cct8 | -0.5780239 |
| Cd109 | 1.01522869 |
| Cd109 | 2.82044151 |
| Cd14 | -0.4941892 |
| Cd151 | -0.6639879 |
| Cd151 | 2.42775512 |
| Cd151 | -0.8289652 |
| Cd160 | -0.3125765 |
| Cd163 | -0.4898257 |
| Cd164 | 0.62472125 |
| Cd177 | -0.4577332 |
| Cd180 | 1.33618685 |
| Cd1d1 | 0.83654793 |
| Cd2 | 0.96978588 |
| Cd200 | 0.84788606 |
| Cd200 | -0.572459 |
| Cd226 | 0.02355721 |
| Cd247 | -0.3927446 |
| Cd248 | 1.71101151 |
| Cd248 | 0.1882669 |
| Cd27 | -0.6055354 |
| Cd274 | -0.8903904 |
| Cd276 | -0.5443512 |
| Cd2bp2 | 1.38252086 |
| Cd300lg | -0.759636 |
| Cd320 | -0.3119765 |
| Cd34 | -0.2017736 |
| Cd3d | -0.808335 |
| Cd3eap | 0.84406392 |
| Cd3g | -0.4438162 |
| Cd4 | -0.1250473 |
| Cd40 | 0.03106672 |
| Cd40 | -0.356822 |
| Cd44 | 2.05370632 |
| Cd44 | -0.7762456 |
| Cd44 | -0.5622112 |
| Cd44 | -0.9865026 |
| Cd47 | -0.0179835 |
| Cd52 | -0.0387956 |
| Cd53 | -0.7713297 |
| Cd55 | 2.18331723 |
| Cd59a | -0.6423847 |
| Cd59b | 1.2617372 |
| Cd59b | -0.5844247 |
| Cd6 | 0.73818475 |
| Cd6 | 0.26087703 |
| Cd6 | -0.7891493 |
| Cd63 | -0.3486767 |
| Cd63 | 1.10604818 |
| Cd63 | -0.908384 |
| Cd68 | 0.24844722 |
| Cd74 | -0.7115081 |
| Cd74 | -0.6518286 |
| Cd79b | -0.7188956 |
| Cd79b | 1.0709362 |
| Cd81 | 0.57313014 |
| Cd82 | 0.46628278 |
| Cd83 | -0.7423652 |
| Cd84 | -0.5059257 |
| Cd86 | -0.7023551 |
| Cd86 | -0.7376239 |
| Cd86 | -0.3192022 |
| Cd8b1 | 2.20129273 |
| Cd9 | -0.3054017 |
| Cd93 | 1.13849214 |
| Cd96 | 0.8900899 |
| Cd97 | -0.8002088 |
| Cd97 | -0.5374544 |
| Cd97 | -0.7615748 |
| Cd99l2 | -0.3375145 |
| Cdan1 | -0.8511286 |
| Cdc123 | 1.38847039 |
| Cdc14b | -0.3119765 |
| Cdc14b | -0.3054017 |
| Cdc14b | 0.89075256 |
| Cdc16 | -0.6440947 |
| Cdc20 | -0.908384 |
| Cdc20 | 1.63248555 |
| Cdc20 | 0.22881459 |
| Cdc20 | -0.0527808 |
| Cdc23 | -0.8405091 |
| Cdc25a | -0.3855785 |
| Cdc25b | 0.93189717 |
| Cdc25c | -0.1240657 |
| Cdc25c | -0.8073153 |
| Cdc27 | 1.21458208 |
| Cdc2a | 1.58478779 |
| Cdc2l1 | -0.7881504 |
| Cdc2l1 | -0.5884476 |
| Cdc2l5 | 3.63067494 |
| Cdc2l6 | 2.84882303 |
| Cdc34 | -0.3344375 |
| Cdc37 | 2.19557155 |
| Cdc37l1 | 0.92951705 |
| Cdc37l1 | 1.62133696 |
| Cdc37l1 | -0.5200571 |
| Cdc37l1 | -0.5290936 |
| Cdc42 | 2.23851203 |
| Cdc42bpb | 1.53701532 |
| Cdc42ep1 | 0.3882596 |
| Cdc42ep2 | 0.43471829 |
| Cdc42ep3 | -0.4840392 |
| Cdc42ep4 | 2.70901612 |
| Cdc42ep5 | -0.3338233 |
| Cdc42se1 | 2.32111627 |
| Cdc42se1 | -0.5328847 |
| Cdc45l | -0.2244361 |
| Cdc5l | 0.67277653 |
| Cdc5l | 2.4854096 |
| Cdc5l | -0.6237784 |
| Cdc5l | 1.58455135 |
| Cdc6 | 0.5404615 |
| Cdc7 | 0.01005612 |
| Cdc73 | 1.37169552 |
| Cdca2 | 0.08945497 |
| Cdca3 | -0.3933988 |
| Cdca3 | 0.24712915 |
| Cdca4 | -0.2691849 |
| Cdca4 | 1.30162565 |
| Cdca4 | 0.96275944 |
| Cdca5 | 2.49399731 |
| Cdca7 | -0.6834208 |
| Cdca8 | -0.6604976 |
| Cdcp1 | -0.7538435 |
| Cdgap | -0.1964578 |
| Cdh1 | -0.1344246 |
| Cdh11 | -0.4212967 |
| Cdh11 | -0.7881504 |
| Cdh12 | -0.4119046 |
| Cdh13 | 1.59552177 |
| Cdh13 | 0.3224879 |
| Cdh16 | -0.3173905 |
| Cdh22 | 0.63020871 |
| Cdh23 | -0.5536269 |
| Cdh23 | 0.20082492 |
| Cdh23 | -0.6923893 |
| Cdh24 | 0.61275721 |
| Cdh26 | 0.45182654 |
| Cdh3 | 0.40984023 |
| Cdh5 | -0.5973594 |
| Cdh6 | -0.6587574 |
| Cdh7 | -0.6305016 |
| Cdh8 | 0.29156145 |
| Cdh8 | -0.5764306 |
| Cdh8 | -0.232114 |
| Cdipt | 0.19212201 |
| Cdipt | -0.6440947 |
| Cdk10 | -0.429423 |
| Cdk10 | 0.38085861 |
| Cdk10 | -0.6622411 |
| Cdk2 | 1.13480068 |
| Cdk2 | 0.11448976 |
| Cdk2 | 2.10666288 |
| Cdk2 | -0.4328299 |
| Cdk2ap2 | -0.5007728 |
| Cdk3 | 1.59005266 |
| Cdk5 | -0.5606445 |
| Cdk5r1 | 2.31993286 |
| Cdk5rap1 | -0.557519 |
| Cdk5rap2 | 2.97467628 |
| Cdk5rap3 | 0.60334478 |
| Cdk6 | 0.12807193 |
| Cdk8 | -0.7207516 |
| Cdk9 | -0.129971 |
| Cdkal1 | 0.65802298 |
| Cdkl1 | -0.5474329 |
| Cdkl2 | -0.4451986 |
| Cdkl2 | 0.28398428 |
| Cdkl2 | -0.4898257 |
| Cdkl2 | 0.65781052 |
| Cdkl3 | 0.14404053 |
| Cdkl4 | 0.40065235 |
| Cdkn1a | -0.1022473 |
| Cdkn1a | 1.26983018 |
| Cdkn1a | 0.0306511 |
| Cdkn1b | 1.38953924 |
| Cdkn1b | -0.0396916 |
| Cdkn1c | 0.93868676 |
| Cdkn2a | 0.99914627 |
| Cdkn2a | -0.4369346 |
| Cdkn2aipnl | 0.89850378 |
| Cdkn2b | -0.117223 |
| Cdkn2c | 0.27153883 |
| Cdkn2c | 2.51659689 |
| Cdkn2d | -0.3418396 |
| Cdo1 | 0.4208573 |
| Cdr2 | -0.8373466 |
| Cdrt4 | -0.758668 |
| Cds1 | 1.52230356 |
| Cds2 | -0.6229414 |
| Cdsn | 0.67465662 |
| Cdt1 | 1.70638743 |
| Cdv3 | 0.51555924 |
| Cdv3 | 2.21355637 |
| Cdyl | -0.6204349 |
| Cdyl | 0.90602834 |
| Cdyl | 1.08691346 |
| Cdyl2 | 0.65781052 |
| Ceacam1 | -0.4732852 |
| Ceacam13 | -0.3953641 |
| Ceacam2 | 0.05890356 |
| Cebpa | 0.23384517 |
| Cebpb | -0.6254547 |
| Cecr5 | 0.93221406 |
| Cecr6 | 0.96168556 |
| Celsr1 | -0.4598388 |
| Celsr2 | -0.0144867 |
| Celsr3 | 1.20256019 |
| Cend1 | -0.0267831 |
| Cenpa | 1.14671352 |
| Cenpb | -0.4369346 |
| Cenph | 0.90537643 |
| Cenpi | -0.5868364 |
| Cenpj | -0.572459 |
| Cenpk | -0.4704383 |
| Cenpl | -0.7198231 |
| Cenpl | -0.2663244 |
| Cenpm | -0.2953308 |
| Cenpm | -0.7385702 |
| Cenpm | 1.49806462 |
| Cenpn | 0.32945851 |
| Cenpp | -0.5908697 |
| Cenpq | 1.80883859 |
| Cenpt | -0.6825286 |
| Centa1 | -0.2437505 |
| Centa1 | 1.27916605 |
| Centa2 | -0.6492435 |
| Centb5 | 1.8134773 |
| Centb5 | 0.43282793 |
| Centd2 | 2.20353079 |
| Centd2 | 0.08358121 |
| Centd3 | 0.2261205 |
| Centg1 | 1.70097518 |
| Centg1 | 0.46888734 |
| Centg2 | -0.5614275 |
| Centg2 | 0.9944153 |
| Centg3 | -0.3914376 |
| Centg3 | -0.4314656 |
| Cep135 | 0.24811788 |
| Cep164 | -0.5306082 |
| Cep170 | 1.39341568 |
| Cep55 | -0.7005352 |
| Cep57 | -0.6313454 |
| Cep57 | -0.300649 |
| Cep63 | 0.23884882 |
| Cep68 | -0.0785875 |
| Cep70 | 0.95861049 |
| Cep72 | -0.12603 |
| Cep78 | 1.77615604 |
| Cep97 | 1.49477317 |
| Cept1 | -0.1985805 |
| Cercam | 0.11676721 |
| Ces3 | 0.52442424 |
| Cetn2 | -0.3313705 |
| Cetn3 | 2.47455519 |
| Cetn4 | -0.4486636 |
| Cetn4 | 0.19212201 |
| Cfd | 1.67772391 |
| Cfdp1 | 1.68772941 |
| Cfh | 0.4128829 |
| Cfhr1 | -0.759636 |
| Cfi | 1.71855402 |
| Cfl1 | -0.0753224 |
| Cfl2 | -0.8331465 |
| Cfl2 | 0.92057734 |
| Cfp | 1.93274908 |
| Cftr | -0.0893967 |
| Cga | 0.13813055 |
| Cggbp1 | 0.04958066 |
| Cgnl1 | 2.31109981 |
| Cgrrf1 | 2.31489563 |
| Ch25h | -0.0988987 |
| Chac1 | 1.71417306 |
| Chad | 0.26799839 |
| Chaf1a | -0.5170643 |
| Chaf1b | 2.4125731 |
| Chat | 2.08412254 |
| Chchd1 | 0.47794652 |
| Chchd2 | -0.0674401 |
| Chchd4 | -0.6014384 |
| Chchd5 | 0.19491577 |
| Chchd6 | 0.69488763 |
| Chchd7 | -0.466184 |
| Chchd8 | 0.78958307 |
| Chd1 | -0.6047145 |
| Chd1 | 0.42167837 |
| Chd1l | -0.3424591 |
| Chd1l | 0.15356524 |
| Chd4 | -0.2783976 |
| Chd4 | 0.87117302 |
| Chd4 | -0.5957327 |
| Chd5 | -0.6398256 |
| Chd8 | 0.88560463 |
| Chdh | 0.20531861 |
| Chdh | -0.6492435 |
| Chek1 | 2.18298492 |
| Chek1 | -0.6807469 |
| Chek2 | 0.18721274 |
| Chek2 | 0.41948725 |
| Cherp | -0.3746107 |
| Chfr | -0.6710077 |
| Chfr | 1.86869711 |
| Chfr | 0.58123725 |
| Chga | 3.12066593 |
| Chga | -0.8237648 |
| Chga | -0.1008107 |
| Chgb | -0.7115081 |
| Chi3l1 | 4.08437971 |
| Chi3l3 | 1.14633566 |
| Chi3l3 | 1.75334212 |
| Chia | 1.87020795 |
| Chia | 0.77900463 |
| Chic1 | 4.22015093 |
| Chic2 | -0.1099467 |
| Chic2 | -0.119173 |
| Chit1 | 1.13658459 |
| Chka | 1.93935076 |
| Chka | -0.5268262 |
| Chka | -0.3137777 |
| Chkb | 0.42331835 |
| Chl1 | 3.54823137 |
| Chl1 | -0.286534 |
| Chl1 | 1.0985208 |
| Chm | -0.6604976 |
| Chmp1b | 0.58077588 |
| Chmp2a | 2.17840356 |
| Chmp2a | 1.38671873 |
| Chmp2b | -0.5163176 |
| Chmp4b | -0.0739266 |
| Chmp5 | 0.34116069 |
| Chmp7 | 1.87368521 |
| Chmp7 | -0.81345 |
| Chmp7 | -0.3252665 |
| Chn2 | 0.63239471 |
| Chodl | 2.9358982 |
| Chordc1 | 1.34854573 |
| Chpf | 1.12428314 |
| Chpt1 | -0.8032478 |
| Chpt1 | 0.61164164 |
| Chpt1 | -0.5716667 |
| Chrac1 | -0.7480867 |
| Chrd | 1.73888719 |
| Chrm1 | 0.91752469 |
| Chrm2 | -0.4825982 |
| Chrm3 | 1.2768682 |
| Chrna1 | -0.1916993 |
| Chrna4 | -0.5096238 |
| Chrna4 | 0.99144623 |
| Chrna4 | -0.3449412 |
| Chrna5 | -0.5420466 |
| Chrna6 | -0.6347282 |
| Chrna6 | -0.1964578 |
| Chrna7 | 0.66816475 |
| Chrna9 | -0.4431257 |
| Chrnb1 | 0.2830328 |
| Chrnb1 | 0.50510931 |
| Chrnb2 | 0.353307 |
| Chrnb2 | 0.06533464 |
| Chrnb3 | 1.05329036 |
| Chrnb3 | -0.3307583 |
| Chrnb3 | 0.65950886 |
| Chrne | -0.0352202 |
| Chst1 | 0.74923273 |
| Chst10 | 1.64874222 |
| Chst12 | 1.11247097 |
| Chst14 | -0.6347282 |
| Chst2 | 0.63631666 |
| Chst3 | 0.45605511 |
| Chst3 | -0.356193 |
| Chst7 | 2.05551758 |
| Chst8 | 1.63121171 |
| Chtf18 | 1.59661176 |
| Chuk | -0.2976907 |
| Churc1 | 2.34370569 |
| Ciao1 | 2.60600101 |
| Ciapin1 | 2.40745712 |
| Cib1 | 0.41067104 |
| Cib2 | 2.60346114 |
| Cib2 | -0.5088831 |
| Cic | 1.13785672 |
| Cidea | -0.1770482 |
| Cideb | -0.117223 |
| Cidec | 1.69958236 |
| Ciita | 0.18086269 |
| Cirbp | 1.43011255 |
| Cirh1a | -0.4711492 |
| Cisd1 | -0.6789686 |
| Cish | 0.00707657 |
| Cit | 2.18638556 |
| Cited1 | 3.49978105 |
| Cited2 | 1.16058803 |
| Cited4 | -0.6271339 |
| Ciz1 | 1.33587834 |
| Ciz1 | -0.6137835 |
| Ckap2l | -0.2959202 |
| Ckap5 | 0.2294869 |
| Ckb | 1.26429461 |
| Cklf | -0.0101341 |
| Cklf | -0.4732852 |
| Ckmt1 | 0.93837206 |
| Ckmt2 | -0.252152 |
| Ckmt2 | -0.2906289 |
| Ckt2 | -0.0049375 |
| Ckt2 | 1.16380835 |
| Clca5 | 1.99034538 |
| Clcc1 | -0.6798573 |
| Clcf1 | 1.02861302 |
| Clcf1 | -0.1008107 |
| Clcf1 | 0.55004878 |
| Clcn1 | 0.16982431 |
| Clcn2 | -0.6587574 |
| Clcn3 | -0.6869979 |
| Clcn3 | 0.44864251 |
| Clcn3 | 3.12355563 |
| Clcn4-2 | -0.7338481 |
| Clcn7 | -0.2853673 |
| Clcnka | -0.3149804 |
| Cldn1 | -0.5268262 |
| Cldn10 | 1.4359524 |
| Cldn10 | 1.37377188 |
| Cldn10 | 0.78310174 |
| Cldn11 | -0.6932909 |
| Cldn12 | -0.2629034 |
| Cldn15 | 0.06973047 |
| Cldn15 | -0.4267064 |
| Cldn17 | -0.6229414 |
| Cldn2 | -0.5732519 |
| Cldn23 | 0.54190588 |
| Cldn3 | -0.7050916 |
| Cldn4 | 1.43632195 |
| Cldn5 | 1.05189765 |
| Cldn6 | 0.13961156 |
| Cldn9 | 0.29938954 |
| Cldnd1 | -0.34432 |
| Cldnd2 | 0.19002119 |
| Clec11a | 0.37684905 |
| Clec16a | -0.8629512 |
| Clec1a | 0.41067104 |
| Clec2d | 3.5834484 |
| Clec2e | 1.27082311 |
| Clec3b | 0.2049737 |
| Clec4a1 | 1.71828082 |
| Clec4a3 | 0.00750281 |
| Clec4b1 | 0.75977065 |
| Clec4b1 | 0.8395266 |
| Clec4d | -0.5505247 |
| Clec4g | -0.2674675 |
| Clec4n | 0.64454267 |
| Clec7a | 0.94182798 |
| Clecsf12 | 2.10607663 |
| Clgn | -0.3455627 |
| Clic1 | 1.71073609 |
| Clic3 | 0.79032094 |
| Clic4 | 0.49631088 |
| Clic6 | 2.15322558 |
| Clip1 | 0.03645297 |
| Clip1 | 0.25696966 |
| Clip4 | -0.9186598 |
| Clk1 | 0.92473856 |
| Clk2 | 2.58017761 |
| Clk2 | -0.57962 |
| Clk2 | -0.5949204 |
| Clk3 | 0.94480239 |
| Clk4 | 1.03983383 |
| Clk4 | -0.8155039 |
| Clk4 | -0.8694635 |
| Clm3 | 0.62845623 |
| Clm3 | 1.31075638 |
| Clmn | -0.6146124 |
| Clmn | -0.5892543 |
| Cln3 | -0.3113769 |
| Cln5 | 0.27985418 |
| Cln6 | 1.98038728 |
| Cln6 | -0.0577941 |
| Cln8 | -0.6381234 |
| Clns1a | 1.55565445 |
| Clns1a | -0.3746107 |
| Clns1a | 1.49616046 |
| Clock | -0.5981737 |
| Clock | 0.05283397 |
| Clp1 | -0.5629955 |
| Clpb | 1.64756405 |
| Clpp | 0.65504412 |
| Clptm1 | 2.00594035 |
| Clptm1l | 0.84005126 |
| Clpx | 1.3852565 |
| Clpx | 0.6064928 |
| Clrn1 | 1.60482356 |
| Clspn | -0.5892543 |
| Clspn | -0.1744559 |
| Clspn | 0.8730314 |
| Clstn1 | -0.603075 |
| Clstn3 | 0.24514855 |
| Clstn3 | 0.24382581 |
| Clta | 0.83443962 |
| Cltb | -0.399966 |
| Cluap1 | 1.81551106 |
| Cma1 | 2.87307811 |
| Cma1 | 0.95768599 |
| Cmas | 1.60920968 |
| Cmbl | -0.5614275 |
| Cml1 | -0.4246741 |
| Cml3 | -0.6631141 |
| Cml4 | 1.54880041 |
| Cmpk | -0.8521972 |
| Cmtm2a | 2.7558363 |
| Cmtm2b | -0.721681 |
| Cmtm4 | -0.6372734 |
| Cmtm4 | -0.8114006 |
| Cmtm5 | -0.2572293 |
| Cmtm6 | -0.4179315 |
| Cmtm7 | 4.50847111 |
| Cmtm7 | 0.85771677 |
| Cmtm7 | 2.91401871 |
| Cmtm8 | -0.7347906 |
| Cmtm8 | -0.6905887 |
| Cmya1 | -0.4233217 |
| CN716893 | 2.73712702 |
| Cnbp2 | 1.84573642 |
| Cnbp2 | -0.6047145 |
| Cndp1 | 1.46165447 |
| Cnga4 | -0.6449509 |
| Cnih | -0.3381312 |
| Cnih3 | 0.40706555 |
| Cnih4 | 1.18966028 |
| Cnksr2 | 1.38554913 |
| Cnksr2 | -0.1749738 |
| Cnksr3 | 0.48334017 |
| Cnn1 | 1.3844757 |
| Cnn2 | -0.7615748 |
| Cnn2 | -0.2882867 |
| Cnnm1 | 1.19314167 |
| Cnnm1 | -0.2812953 |
| Cnnm2 | -0.5637805 |
| Cnnm3 | -0.4396812 |
| Cno | -0.1549862 |
| Cnot10 | 1.39002467 |
| Cnot2 | -0.7347906 |
| Cnot2 | 0.9121989 |
| Cnot2 | 1.00003066 |
| Cnot2 | 1.80765979 |
| Cnot3 | 1.44469009 |
| Cnot3 | -0.6006212 |
| Cnot4 | -0.6834208 |
| Cnot4 | 1.68681139 |
| Cnot4 | -0.6221052 |
| Cnot6l | -0.0432841 |
| Cnot6l | -0.4199492 |
| Cnot7 | 0.52564887 |
| Cnp | 0.36964606 |
| Cnp | 0.67985935 |
| Cnpy2 | 0.94589584 |
| Cnpy3 | -0.5965457 |
| Cnr1 | -0.3933988 |
| Cnr1 | -0.5892543 |
| Cntd1 | -0.6789686 |
| Cntd1 | 3.29070483 |
| Cntfr | -0.2028404 |
| Cntln | -0.2527148 |
| Cntn1 | 0.95567977 |
| Cntn4 | 1.23187009 |
| Cntn6 | 0.06813438 |
| Cntnap1 | -0.3901324 |
| Cntnap2 | 1.6384569 |
| Cntnap4 | 2.35773266 |
| Cntnap4 | 1.30948767 |
| Coasy | -0.3726885 |
| Cobl | -0.5685042 |
| Cobll1 | -0.7404658 |
| Cobll1 | 2.63149 |
| Coch | 0.36935678 |
| Cog1 | 2.27211764 |
| Cog2 | -0.6154419 |
| Cog3 | -0.6896897 |
| Cog4 | -0.3095803 |
| Cog6 | 1.44952953 |
| Cog6 | -0.447276 |
| Cog8 | -0.5582994 |
| Coil | 3.47121554 |
| Coil | 0.0983903 |
| Col11a1 | 2.56724118 |
| Col12a1 | 1.07230085 |
| Col14a1 | 0.91913259 |
| Col15a1 | 0.32855224 |
| Col16a1 | 0.44943953 |
| Col17a1 | 0.16803166 |
| Col17a1 | -0.6526919 |
| Col17a1 | 2.40457726 |
| Col18a1 | 2.41811361 |
| Col18a1 | 0.02439458 |
| Col1a1 | -0.2611975 |
| Col1a2 | 0.28335007 |
| Col24a1 | 0.39137323 |
| Col24a1 | -0.7226113 |
| Col25a1 | 1.44148043 |
| Col25a1 | 1.46730108 |
| Col25a1 | 2.02748253 |
| Col27a1 | -0.6221052 |
| Col28a1 | -0.1279984 |
| Col2a1 | 0.27153883 |
| Col2a1 | -0.1708388 |
| Col3a1 | 0.69753873 |
| Col3a1 | 0.37713602 |
| Col4a1 | 0.17624956 |
| Col4a2 | 0.63783743 |
| Col4a3 | -0.7404658 |
| Col4a3bp | 1.19863741 |
| Col4a3bp | -0.5629955 |
| Col4a3bp | 0.06172261 |
| Col4a4 | 2.20681712 |
| Col4a4 | 0.48895747 |
| Col4a5 | -0.5336448 |
| Col4a6 | 1.69518136 |
| Col4a6 | 0.63064632 |
| Col5a1 | -0.7528816 |
| Col5a3 | 0.68089643 |
| Col6a1 | -0.1744559 |
| Col6a1 | 0.91122731 |
| Col6a1 | 0.74343429 |
| Col6a2 | -0.6096504 |
| Col7a1 | 0.01344972 |
| Col8a2 | -0.6162723 |
| Col9a2 | 0.08515116 |
| Colec12 | 0.8428445 |
| Commd1 | 0.56518674 |
| Commd1 | -0.0019195 |
| Commd10 | -0.2393005 |
| Commd2 | -0.514081 |
| Commd2 | -0.4739983 |
| Commd4 | 0.11258764 |
| Commd5 | -0.4493581 |
| Commd6 | -0.4092383 |
| Commd8 | -0.5155714 |
| Commd9 | -0.3816928 |
| Comp | -0.5007728 |
| Comtd1 | -0.233216 |
| Copa | -0.6047145 |
| Copb2 | -0.3644043 |
| Cope | 0.0389285 |
| Cope | -0.7124283 |
| Cope | -0.5590805 |
| Cope | 1.45543068 |
| Copg | 0.30777087 |
| Copg | -0.5512993 |
| Copg | -0.4891004 |
| Copg | -0.4697279 |
| Cops2 | -0.5536269 |
| Cops3 | -0.8362948 |
| Cops4 | 0.30125856 |
| Cops4 | 0.78847517 |
| Cops5 | -0.7762456 |
| Cops5 | 0.158654 |
| Cops6 | -0.3966766 |
| Cops7a | -0.2686121 |
| Cops7a | -0.6162723 |
| Cops7a | -0.5924879 |
| Cops7b | -0.7981882 |
| Cops7b | 4.02889125 |
| Cops8 | 2.17982201 |
| Copz1 | -0.4563321 |
| Copz2 | -0.697812 |
| Copz2 | -0.7732929 |
| Copz2 | 2.25053177 |
| Copz2 | 1.01261589 |
| Coq10b | -0.9337049 |
| Coq10b | 1.69167369 |
| Coq2 | 0.0593068 |
| Coq3 | -0.7500017 |
| Coq3 | -0.7664395 |
| Coq4 | 0.2572959 |
| Coq4 | -0.7060055 |
| Coq5 | 1.56487669 |
| Coq5 | -0.6950967 |
| Coq5 | 2.5231734 |
| Coq6 | 1.14254863 |
| Coq6 | 1.37722227 |
| Coq7 | -0.572459 |
| Coq9 | -0.3368983 |
| Corin | 0.09567989 |
| Coro1a | 1.76828941 |
| Coro1a | -0.4403691 |
| Coro1a | 0.08984524 |
| Coro1a | 2.80164456 |
| Coro1b | -0.5275814 |
| Coro1b | -0.8185932 |
| Coro1c | 1.48885411 |
| Coro2a | 1.65636435 |
| Coro2a | -0.6096504 |
| Coro2b | 1.59333796 |
| Coro2b | 2.74837933 |
| Coro6 | -0.7831716 |
| Coro7 | 0.45895119 |
| Cort | -0.5876416 |
| Cort | -0.7188956 |
| Cotl1 | 0.08945497 |
| Cova1 | -0.7291497 |
| Cox10 | -0.242636 |
| Cox11 | 2.22116514 |
| Cox15 | -0.4898257 |
| Cox17 | -0.4768561 |
| Cox18 | -0.2589279 |
| Cox18 | -0.5044506 |
| Cox19 | 1.20196689 |
| Cox4i1 | 1.47149153 |
| Cox4nb | -0.7300875 |
| Cox4nb | 0.17089826 |
| Cox5a | 1.24579104 |
| Cox5b | 2.31853236 |
| Cox6a1 | -0.3663096 |
| Cox6a1 | 0.67590792 |
| Cox6a2 | -0.3234431 |
| Cox6b1 | -0.2189902 |
| Cox6b1 | 1.13531071 |
| Cox6b2 | -0.4458906 |
| Cox6b2 | -0.8362948 |
| Cox6c | 0.85840278 |
| Cox7a1 | 0.80477575 |
| Cox7a2 | -0.4307842 |
| Cox7a2l | 0.57289748 |
| Cox7b | -0.7528816 |
| Cox7b2 | -0.5788216 |
| Cox8b | -0.2337675 |
| Cox8c | 0.97434004 |
| Cp | -0.5828204 |
| Cpa1 | -0.2487823 |
| Cpa2 | -0.3036166 |
| Cpa2 | -0.661369 |
| Cpa3 | 0.51778338 |
| Cpa3 | -0.8062968 |
| Cpa3 | -0.4199492 |
| Cpa4 | 1.19170267 |
| Cpa5 | 0.1455122 |
| Cpa6 | -0.1474111 |
| Cpb2 | -0.4854825 |
| Cpeb1 | 1.7463415 |
| Cpeb2 | -0.4396812 |
| Cpeb3 | -0.4445071 |
| Cpeb4 | 0.11486972 |
| Cphx | -0.6038944 |
| Cplx1 | -0.2646123 |
| Cplx2 | 0.30591483 |
| Cpn1 | -0.7762456 |
| Cpne2 | -0.8268816 |
| Cpne4 | 2.46646122 |
| Cpne5 | -0.7625457 |
| Cpne6 | 0.69896313 |
| Cpne7 | 2.22048687 |
| Cpne8 | 3.41936918 |
| Cpne8 | -0.0776535 |
| Cpne9 | -0.4417464 |
| Cpsf1 | 1.10143329 |
| Cpsf1 | -0.5163176 |
| Cpsf2 | 0.43120457 |
| Cpsf3l | 0.4208573 |
| Cpsf4 | 1.23577059 |
| Cpsf4l | 1.39583032 |
| Cpsf4l | -0.8185932 |
| Cpsf6 | -0.0473423 |
| Cpsf6 | -0.6204349 |
| Cpt1b | 2.28725237 |
| Cpt1b | 0.61809348 |
| Cpt1c | 0.0516153 |
| Cpt2 | -0.4768561 |
| Cpxcr1 | -0.5118498 |
| Cpxm1 | 0.80186619 |
| Cpxm2 | 0.1228134 |
| Cpz | -0.5973594 |
| Crabp2 | -0.5780239 |
| Crat | 1.03771363 |
| Crat | -0.3240505 |
| Crat | -0.2087296 |
| Crb1 | -0.7404658 |
| Crbn | -0.4246741 |
| Crbn | 0.85617141 |
| Crbn | 3.554371 |
| Crcp | 0.15101043 |
| Creb1 | -0.7971795 |
| Creb1 | -0.6389741 |
| Creb3 | 1.5837628 |
| Creb3l1 | 0.94370766 |
| Creb3l2 | -0.3289241 |
| Creb3l2 | 3.19707538 |
| Creb3l3 | -0.7615748 |
| Creb3l4 | 1.55833342 |
| Creb5 | 1.01841223 |
| Crebl1 | -0.2431931 |
| Crebl2 | 1.0035599 |
| Creg2 | 2.71876175 |
| Creld1 | -0.4619491 |
| Creld2 | 0.08632689 |
| Creld2 | 0.96887238 |
| Crem | -0.7901493 |
| Crem | 0.45050118 |
| Crem | 0.93363874 |
| Crem | 0.97570198 |
| Crhbp | -0.4179315 |
| Crhr1 | -0.0245754 |
| Crim1 | 0.41893867 |
| Crim2 | -0.28362 |
| Crip2 | -0.2760859 |
| Crip3 | 1.55589831 |
| Crip3 | 0.6926385 |
| Cript | -0.6449509 |
| Cript | -0.7105887 |
| Crisp2 | -0.4854825 |
| Crispld2 | -0.1230851 |
| Crk | 0.16154947 |
| Crk | 2.71305382 |
| Crk | -0.782179 |
| Crkl | 1.40428055 |
| Crkrs | 1.30736931 |
| Crkrs | -0.6657379 |
| Crlf3 | 1.37169552 |
| Crlf3 | -0.5965457 |
| Crls1 | 0.20290161 |
| Crls1 | -0.388178 |
| Crls1 | -0.7723108 |
| Crls1 | -0.780197 |
| Crmp1 | 1.33988098 |
| Crmp1 | 0.98218173 |
| Crnkl1 | -0.277241 |
| Crocc | 0.69814945 |
| Crry | 2.62837388 |
| Crry | 0.64776995 |
| Crtac1 | 1.22541004 |
| Crtam | 1.24238703 |
| Crtap | 0.17873638 |
| Crtap | 1.16158008 |
| Crtc1 | -0.354936 |
| Crtc3 | 0.23250633 |
| Crxos1 | -0.3412205 |
| Cry2 | -0.1906451 |
| Cryab | 0.62339945 |
| Cryba2 | -0.1152771 |
| Cryba4 | 0.3200511 |
| Crybb1 | -0.6932909 |
| Crybb2 | 0.46393247 |
| Crybb3 | 0.10032151 |
| Crygn | -0.7263419 |
| Crygn | -0.5253177 |
| Cryl1 | 0.47122524 |
| Crym | 1.19110243 |
| Cryz | 0.58675604 |
| Cryz | 2.55350851 |
| Cryz | 0.25173429 |
| Cryzl1 | -0.7105887 |
| Cryzl1 | -0.117223 |
| Cs | -0.2691849 |
| Csad | -0.6861023 |
| Csda | -0.514081 |
| Csda | 1.25132439 |
| Csda | -0.2906289 |
| Csdc2 | -0.7254079 |
| Csde1 | 2.00201551 |
| Csde1 | 0.20393823 |
| Csde1 | 0.50960217 |
| Cse1l | -0.7376239 |
| Csf1 | 2.6656688 |
| Csf1r | 1.56157039 |
| Csf1r | -0.3252665 |
| Csf2ra | 0.49656342 |
| Csgalnact1 | -0.7500017 |
| Csgalnact2 | -0.3940534 |
| Csk | 3.44476928 |
| Csl | 0.19804879 |
| Csl | -0.1780871 |
| Csmd1 | 0.53393403 |
| Csnk1d | -0.2092668 |
| Csnk1d | 1.59310368 |
| Csnk1d | 0.82737733 |
| Csnk1d | 1.41756857 |
| Csnk1d | -0.2055127 |
| Csnk1e | 1.34090454 |
| Csnk1e | 0.28208035 |
| Csnk1g1 | 3.87630413 |
| Csnk1g1 | -0.6204349 |
| Csnk1g1 | -0.2381913 |
| Csnk1g2 | -0.8165325 |
| Csnk1g2 | 0.63718597 |
| Csnk1g2 | 2.09964886 |
| Csnk1g3 | -0.5283372 |
| Csnk2a1 | 0.10724101 |
| Csnk2a2 | 2.1313984 |
| Csnk2a2 | 0.8900899 |
| Csnrp2 | -0.5868364 |
| Csnrp3 | 2.07502656 |
| Cspg4 | -0.8575588 |
| Cspg5 | 1.44432383 |
| Cspp1 | 0.46706491 |
| Csprs | 2.00839755 |
| Csrp1 | 1.36693214 |
| Csrp2 | 0.60108968 |
| Csrp2bp | 1.13454556 |
| Cst11 | 0.60761453 |
| Cst3 | 0.09451587 |
| Cst8 | 3.31411698 |
| Cst9 | 0.34976829 |
| Cstad | 2.29833455 |
| Cstb | -0.5306082 |
| Cstf1 | 4.14523301 |
| Cstf2 | 0.40789882 |
| Cstf3 | -0.6321899 |
| Cstf3 | -0.6604976 |
| Cstl1 | 0.14698156 |
| Ctage5 | 0.4009321 |
| Ctcf | -0.7509607 |
| Ctdp1 | 1.8281683 |
| Ctdp1 | 0.3468091 |
| Ctdp1 | 0.38542 |
| Ctdsp1 | 1.37810746 |
| Ctdsp1 | 1.81501843 |
| Ctdsp1 | -0.5125929 |
| Ctdsp2 | -0.6279747 |
| Ctdspl | -0.5629955 |
| Ctdspl2 | -0.5559602 |
| Ctdspl2 | 1.13988855 |
| Ctf1 | -0.4825982 |
| Ctgf | -0.3688559 |
| Ctla4 | -0.5336448 |
| Ctnna1 | -0.4718606 |
| Ctnna3 | 0.51975597 |
| Ctnnal1 | -0.6262939 |
| Ctnnb1 | -0.6544209 |
| Ctnnb1 | -0.4985731 |
| Ctnnb1 | 0.70342576 |
| Ctnnbip1 | 2.18048878 |
| Ctnnbl1 | 0.94932411 |
| Ctnnbl1 | -0.3270935 |
| Ctnnd2 | -0.4891004 |
| Ctnnd2 | -0.3258751 |
| Ctnnd2 | -0.5685042 |
| Ctns | 1.88809307 |
| Ctns | -0.1489212 |
| Ctps | -0.5941089 |
| Ctps | -0.2071198 |
| Ctps | 0.98876599 |
| Ctps2 | -0.5748399 |
| Ctps2 | 1.7186906 |
| Ctps2 | 1.03983383 |
| Ctr9 | -0.5170643 |
| Ctrb1 | -0.514081 |
| Ctrl | -0.2044428 |
| Ctsa | -0.0343285 |
| Ctsa | -0.4026059 |
| Ctsa | -0.4079081 |
| Ctsa | -0.759636 |
| Ctsb | 0.91865051 |
| Ctsc | 1.4935576 |
| Ctsc | 2.73891635 |
| Ctsc | -0.7538435 |
| Ctse | -0.6423847 |
| Ctsf | -0.6246162 |
| Ctsh | 1.28483175 |
| Ctsk | -0.7050916 |
| Ctsl | -0.4092383 |
| Ctso | -0.5125929 |
| Ctsr | -0.173421 |
| Ctss | -0.7105887 |
| Ctsw | 0.21424167 |
| Ctsz | 0.81363032 |
| Ctsz | -0.5029777 |
| Cttn | -0.5629955 |
| Cttn | 2.03646782 |
| Cttnbp2nl | -0.3536808 |
| Ctxn1 | 1.20800098 |
| Cuedc1 | 0.1495474 |
| Cuedc2 | 1.25278694 |
| Cugbp1 | 1.9537973 |
| Cugbp1 | -0.6038944 |
| Cugbp1 | 1.68730582 |
| Cugbp1 | -0.4971095 |
| Cugbp2 | 1.6559263 |
| Cugbp2 | -0.2049776 |
| Cul1 | 3.27494091 |
| Cul2 | -0.447276 |
| Cul2 | -0.0396916 |
| Cul3 | -0.0468905 |
| Cul4a | 0.66816475 |
| Cul4a | 0.48513116 |
| Cul4a | -0.5133367 |
| Cul4b | 0.66120412 |
| Cul5 | 1.27401539 |
| Cul7 | 0.18227754 |
| Cuta | 0.71668717 |
| Cuta | -0.5732519 |
| Cuta | -0.6063569 |
| Cutc | -0.2233443 |
| Cutc | 0.23384517 |
| Cux1 | -0.7567352 |
| Cux1 | -0.5374544 |
| Cux1 | 0.15792873 |
| Cux1 | 2.38252295 |
| Cux1 | 1.88968663 |
| Cux2 | 3.4217893 |
| Cux2 | 0.13031655 |
| Cuzd1 | -0.4403691 |
| Cwf19l2 | 0.10455618 |
| Cx3cl1 | -0.7124283 |
| Cx3cr1 | 2.37477238 |
| Cxadr | -0.0619159 |
| Cxadr | 1.41907524 |
| Cxadr | 0.69407039 |
| Cxcl1 | 0.66984439 |
| Cxcl12 | -0.6483834 |
| Cxcl12 | -0.7772319 |
| Cxcl12 | 2.16278414 |
| Cxcl13 | -0.3418396 |
| Cxcl14 | -0.2611975 |
| Cxcl14 | -0.6154419 |
| Cxcl15 | 1.00517303 |
| Cxcl16 | -0.178607 |
| Cxcl17 | 0.3297604 |
| Cxcl4 | -0.4260284 |
| Cxcl9 | -0.7032664 |
| Cxcr3 | -0.7971795 |
| Cxcr4 | 0.01893817 |
| Cxcr5 | -0.1744559 |
| Cxcr7 | -0.1514435 |
| Cxx1a | 0.00237515 |
| Cxx1b | -0.8012207 |
| Cxx1c | 0.19665763 |
| Cxxc1 | -0.6692478 |
| Cxxc1 | 0.40316711 |
| Cxxc4 | -0.0040742 |
| Cxxc4 | 2.78719656 |
| Cyb5 | 1.1206645 |
| Cyb561 | -0.2033742 |
| Cyb561d1 | 1.57185398 |
| Cyb561d1 | 2.03598171 |
| Cyb561d2 | 1.09599814 |
| Cyb5b | 0.96260611 |
| Cyb5d1 | 2.45507268 |
| Cyb5d2 | 1.10709982 |
| Cyb5d2 | -0.7198231 |
| Cyb5r1 | -0.5275814 |
| Cyb5r2 | 1.24465775 |
| Cyb5r3 | -0.5661393 |
| Cyb5r3 | 0.75058054 |
| Cyb5r3 | -0.272629 |
| Cyb5r4 | 0.06573512 |
| Cyba | 0.9932288 |
| Cybasc3 | -0.4521415 |
| Cyc1 | -0.3338233 |
| Cycs | -0.6905887 |
| Cycs | -0.7683925 |
| Cyfip1 | 0.71628806 |
| Cyfip2 | 1.84680865 |
| Cyfip2 | 0.60739029 |
| Cygb | -0.6789686 |
| Cyhr1 | 1.52885214 |
| Cyhr1 | 3.28987907 |
| Cyhr1 | -0.6047145 |
| Cyhr1 | -0.5283372 |
| Cyld | -0.6475241 |
| Cyln2 | 4.11242557 |
| Cyp11a1 | -0.1235753 |
| Cyp11b2 | -0.1027267 |
| Cyp17a1 | -0.8738304 |
| Cyp19a1 | 0.83777559 |
| Cyp1b1 | -0.4598388 |
| Cyp20a1 | -0.4369346 |
| Cyp24a1 | -0.6887916 |
| Cyp26a1 | 1.3731791 |
| Cyp26b1 | -0.7319659 |
| Cyp27a1 | 0.50460877 |
| Cyp27a1 | -0.1423949 |
| Cyp27a1 | -0.4528386 |
| Cyp2a12 | 0.08358121 |
| Cyp2a5 | 1.77609183 |
| Cyp2a5 | -0.4811594 |
| Cyp2b10 | -0.1240657 |
| Cyp2c55 | 3.75413794 |
| Cyp2d10 | 0.92377992 |
| Cyp2d22 | -0.447276 |
| Cyp2d26 | -0.046439 |
| Cyp2d26 | -0.1359139 |
| Cyp2d26 | 1.47539754 |
| Cyp2e1 | 2.4430405 |
| Cyp2f2 | -0.3804012 |
| Cyp2j11 | 1.88649678 |
| Cyp2j9 | -0.4825982 |
| Cyp2s1 | -0.7480867 |
| Cyp2u1 | -0.5497508 |
| Cyp39a1 | 0.46210035 |
| Cyp3a13 | 0.46628278 |
| Cyp3a13 | -0.4993058 |
| Cyp46a1 | -0.7395175 |
| Cyp4b1 | 1.25031051 |
| Cyp4f13 | 0.99648804 |
| Cyp4f14 | 1.52768012 |
| Cyp4f15 | 0.84753924 |
| Cyp4f16 | -0.3234431 |
| Cyp4f16 | -0.5949204 |
| Cyp4f16 | 2.00864792 |
| Cyp4f18 | 2.47242396 |
| Cyp4f39 | 0.31239488 |
| Cyp4v3 | 2.72752563 |
| Cyp51 | -0.4206227 |
| Cyp7b1 | -0.7625457 |
| Cypt10 | 0.43417857 |
| Cypt2 | -0.7272769 |
| Cypt3 | 1.11625152 |
| Cypt6 | -0.7881504 |
| Cypt8 | -0.1817325 |
| Cypt9 | 0.88643686 |
| Cyr61 | -0.2714795 |
| Cyr61 | -0.5111072 |
| Cysltr1 | -0.2304634 |
| Cytl1 | 1.00955836 |
| D030011O10Rik | -0.1600706 |
| D030013I16Rik | 1.7442842 |
| D030018L15Rik | 2.51237169 |
| D030028O16Rik | -0.4139092 |
| D030051N19Rik | -0.5059257 |
| D030056L22Rik | -0.5125929 |
| D030070L09Rik | -0.7951655 |
| D030070L09Rik | -0.2847845 |
| D030070L09Rik | -0.7772319 |
| D030070L09Rik | 0.37368657 |
| D030074E01Rik | 2.08670521 |
| D0H4S114 | -0.0716048 |
| D0H8S2298E | -0.0075322 |
| D0HXS9928E | 0.98218173 |
| D0HXS9928E | -0.2233443 |
| D10627 | -0.7105887 |
| D10Bwg1364e | -0.7310262 |
| D10Bwg1379e | -0.8511286 |
| D10Ertd322e | 1.54461436 |
| D10Ertd610e | 0.46471656 |
| D10Ertd610e | -0.6648625 |
| D10Ertd610e | 0.5816984 |
| D10Ertd641e | -0.4206227 |
| D10Jhu81e | 3.33394282 |
| D10Wsu102e | 0.65866008 |
| D10Wsu52e | -0.6771936 |
| D11Bwg0517e | 2.09696512 |
| D11Wsu47e | -0.0541454 |
| D11Wsu47e | 1.04110364 |
| D12Ertd551e | -0.6047145 |
| D12Ertd551e | -0.4065797 |
| D12Ertd553e | -0.4079081 |
| D12Ertd647e | 0.57753987 |
| D12Ertd647e | -0.0988987 |
| D130040H23Rik | 0.98980922 |
| D130043K22Rik | -0.7235426 |
| D130059P03Rik | 1.17132045 |
| D13Ertd608e | -0.7124283 |
| D14Ertd171e | 0.96183904 |
| D14Ertd231e | -0.5200571 |
| D14Ertd449e | -0.6941934 |
| D14Ertd500e | 0.50260392 |
| D14Ertd668e | -0.6570204 |
| D14Ertd668e | -0.8093558 |
| D15Ertd621e | -0.6825286 |
| D15Ertd682e | -0.6113014 |
| D15Mgi27 | 0.44277679 |
| D15Wsu169e | 1.18628659 |
| D15Wsu75e | 0.72166169 |
| D16Ertd472e | -0.5037139 |
| D16H22S680E | 1.5740088 |
| D17H6S56E-5 | 1.22006996 |
| D17Wsu104e | 1.39467207 |
| D17Wsu92e | 0.26152668 |
| D19Bwg1357e | 0.01598686 |
| D19Ertd721e | -0.5812188 |
| D19Ertd737e | -0.5118498 |
| D19Wsu162e | -0.0837415 |
| D1Bwg0212e | 0.92457885 |
| D1Bwg0212e | 1.84233287 |
| D1Pas1 | -0.5037139 |
| D230007K08Rik | 0.46602193 |
| D230014K01Rik | -0.6063569 |
| D2Ertd750e | 2.10255136 |
| D2Ertd750e | 0.83109169 |
| D330014H01Rik | -0.466184 |
| D330017J20Rik | -0.519308 |
| D330028D13Rik | -0.4335127 |
| D330045A20Rik | 0.02648475 |
| D330045A20Rik | 1.81106804 |
| D330045A20Rik | 1.56157039 |
| D3Bwg0562e | -0.5645661 |
| D3Ertd300e | -0.6666141 |
| D3Ertd751e | 0.41866426 |
| D3Ucla1 | 0.09218347 |
| D430015B01Rik | -0.519308 |
| D430028G21Rik | -0.6570204 |
| D430039N05Rik | -0.6449509 |
| D430041B17Rik | -0.5428142 |
| D430042O09Rik | -0.7385702 |
| D4Bwg0951e | -0.7981882 |
| D4Bwg1540e | 0.99114881 |
| D4Ertd22e | -0.4654769 |
| D4Ertd22e | -0.5200571 |
| D4Wsu114e | -0.0776535 |
| D4Wsu132e | 3.32110028 |
| D4Wsu132e | 0.06693552 |
| D530004J12Rik | -0.672771 |
| D530033C11Rik | 1.50392582 |
| D5Ertd135e | 0.67403034 |
| D5Ertd579e | -0.3387482 |
| D5Wsu178e | -0.5366913 |
| D630003M21Rik | -0.1123659 |
| D630003M21Rik | 0.78421602 |
| D630014A15Rik | -0.6055354 |
| D630023F18Rik | 0.25468279 |
| D630042P16Rik | -0.4535363 |
| D630048P19Rik | -0.2882867 |
| D6Ertd474e | -0.2935648 |
| D6Mm5e | 1.73096869 |
| D6Wsu116e | -0.2760859 |
| D6Wsu163e | -0.5653524 |
| D6Wsu163e | -0.7041785 |
| D6Wsu163e | 0.12092817 |
| D6Wsu176e | 1.81526478 |
| D6Wsu176e | 0.42304522 |
| D730039F16Rik | 0.08279525 |
| D730039F16Rik | 0.26120191 |
| D7Bwg0611e | -0.5622112 |
| D830014E11Rik | -0.6212697 |
| D830030K20Rik | 0.92330023 |
| D830030K20Rik | -0.3675819 |
| D830050J10Rik | 0.74904003 |
| D8Ertd738e | 2.54871721 |
| D8Ertd82e | 0.77769722 |
| D930001I22Rik | -0.7142716 |
| D930005D10Rik | 0.3777097 |
| D930014E17Rik | 1.17572565 |
| D930015E06Rik | -0.365039 |
| D930020B18Rik | 2.52126605 |
| D930028F11Rik | -0.6338813 |
| D930049A15Rik | 1.27335582 |
| D9Ertd280e | 0.86523528 |
| D9Ertd392e | 1.24929554 |
| D9Ertd402e | -0.3493008 |
| D9Wsu20e | 1.1340351 |
| Daam1 | -0.3131769 |
| Daam2 | 0.89471866 |
| Dab1 | -0.4165888 |
| Dab1 | 0.11296837 |
| Dab1 | 0.74227027 |
| Dab2 | -0.6080022 |
| Dab2 | 0.02313824 |
| Dab2ip | 0.88527153 |
| Dact1 | 1.73587533 |
| Dact2 | -0.1438969 |
| Dad1 | 1.45750988 |
| Daf2 | 1.14419156 |
| Dag1 | 0.53635693 |
| Dag1 | -0.6492435 |
| Dagla | -0.2906289 |
| Daglb | 0.32582815 |
| Dak | -0.5405134 |
| Dalrd3 | -0.7170432 |
| Dap | -0.6905887 |
| Dap3 | -0.5756349 |
| Dap3 | 0.09140471 |
| Dap3 | 0.02229973 |
| Dap3 | -0.2271709 |
| Dapk1 | -0.5015072 |
| Dapk1 | 1.74984836 |
| Dapk2 | 0.40316711 |
| Dapk2 | -0.5458908 |
| Dapl1 | 2.3598668 |
| Dapp1 | -0.3119765 |
| Dapp1 | -0.5374544 |
| Darc | -0.6432393 |
| Dars | 1.3252241 |
| Dars | -0.6071792 |
| Daxx | -0.4383069 |
| Dazap1 | 2.24007672 |
| Dazap1 | -0.5015072 |
| Dazap2 | 0.37022435 |
| Dazl | -0.6229414 |
| Dbc1 | 1.66818689 |
| Dbf4 | -0.1080159 |
| Dbi | -0.5828204 |
| Dbi | -0.7548064 |
| Dbil5 | 0.16982431 |
| Dbn1 | 0.88909503 |
| Dbndd1 | -0.2778192 |
| Dbndd2 | 0.90423449 |
| Dbndd2 | -0.1080159 |
| Dbndd2 | -0.6914886 |
| Dbnl | 0.03189739 |
| Dbp | -0.759636 |
| Dbpht2 | -0.5916784 |
| Dbr1 | 0.71108408 |
| Dbt | 0.01048099 |
| Dcakd | -0.6179351 |
| Dcbld1 | 1.13518323 |
| Dcbld2 | 0.82507055 |
| Dci | 0.08436651 |
| Dck | 0.75652614 |
| Dclk1 | 0.12243666 |
| Dclk2 | 0.85703026 |
| Dclk3 | -0.4633586 |
| Dclre1b | 1.63143663 |
| Dclre1b | -0.7170432 |
| Dclre1c | -0.6313454 |
| Dclre1c | 0.85891695 |
| Dclre1c | 0.3300622 |
| Dcn | -0.3849298 |
| Dcn | -0.7851599 |
| Dcp1a | 1.17046147 |
| Dcp1a | 0.81291064 |
| Dcp1a | 1.41672 |
| Dcp1a | -0.6692478 |
| Dcp1b | 0.13998144 |
| Dcp1b | 0.57568568 |
| Dcpp1 | -0.5489775 |
| Dcpp2 | -0.7272769 |
| Dcpp2 | 1.21294115 |
| Dcpp3 | -0.2988729 |
| Dcps | -0.6204349 |
| Dcps | -0.6843138 |
| Dct | 0.50385745 |
| Dctd | 2.2688261 |
| Dctn1 | 0.04180835 |
| Dctn2 | 0.81596548 |
| Dctn2 | 1.84412575 |
| Dctn2 | 1.36633501 |
| Dctn3 | -0.5598622 |
| Dctn3 | -0.7291497 |
| Dctn4 | -0.0564241 |
| Dctn4 | -0.3240505 |
| Dctn5 | 0.08554323 |
| Dctn6 | 1.99817539 |
| Dcun1d2 | 0.2500922 |
| Dcun1d3 | -0.7235426 |
| Dcun1d4 | -0.1459034 |
| Dcun1d4 | 2.56495257 |
| Dcun1d5 | 1.75898358 |
| Dcx | 1.68836444 |
| Dcx | 0.49656342 |
| Dcxr | 1.75044256 |
| Dcxr | 0.35653908 |
| Ddah1 | -0.7078361 |
| Ddah2 | -0.6960009 |
| Ddb1 | 0.85445135 |
| Ddb2 | -0.8749254 |
| Ddb2 | -0.0049375 |
| Ddb2 | -0.5716667 |
| Ddc | -0.7951655 |
| Ddc8 | 0.65461779 |
| Ddef2 | -0.57962 |
| Ddef2 | -0.3024284 |
| Ddefl1 | -0.2206206 |
| Ddhd1 | 0.4290357 |
| Ddhd1 | -0.5957327 |
| Ddi2 | -0.5306082 |
| Ddit3 | -0.1959279 |
| Ddit3 | 1.00311948 |
| Ddit4 | 1.07120928 |
| Ddit4l | -0.6914886 |
| Ddo | 1.55402709 |
| Ddo | 0.47096576 |
| Ddo | 2.31381269 |
| Ddost | 0.20704121 |
| Ddr1 | -0.2760859 |
| Ddr2 | 1.18435328 |
| Ddt | 1.64859503 |
| Ddx1 | 0.09529204 |
| Ddx1 | -0.4790053 |
| Ddx17 | 0.75518689 |
| Ddx17 | -0.7385702 |
| Ddx17 | 0.25631684 |
| Ddx19a | -0.4912779 |
| Ddx19b | -0.6288162 |
| Ddx19b | 0.04713322 |
| Ddx20 | -0.653556 |
| Ddx21 | -0.5148259 |
| Ddx24 | -0.3375145 |
| Ddx24 | 2.12106781 |
| Ddx24 | 0.61141836 |
| Ddx25 | -0.3842815 |
| Ddx26b | -0.7693705 |
| Ddx27 | -0.3823393 |
| Ddx28 | -0.3270935 |
| Ddx3x | 0.88693585 |
| Ddx3y | -0.7654645 |
| Ddx4 | -0.6869979 |
| Ddx4 | -0.0079653 |
| Ddx41 | 0.91800735 |
| Ddx46 | 0.95629753 |
| Ddx47 | -0.4768561 |
| Ddx47 | 0.02815352 |
| Ddx47 | -0.5443512 |
| Ddx49 | 2.29983896 |
| Ddx5 | -0.2044428 |
| Ddx50 | -0.6047145 |
| Ddx50 | 3.15185368 |
| Ddx51 | 1.36921762 |
| Ddx52 | 0.78884462 |
| Ddx54 | 1.68398103 |
| Ddx58 | -0.5772269 |
| Ddx6 | 1.00502649 |
| Ddx6 | 0.88660322 |
| Ddx6 | 1.24022473 |
| Ddx6 | -0.4711492 |
| Deadc1 | -0.7881504 |
| Deb1 | 0.92729013 |
| Decr1 | 2.0627277 |
| Decr2 | 1.06270618 |
| Decr2 | 0.6675341 |
| Dedd | 1.06049921 |
| Dedd | -0.6398256 |
| Dedd2 | -0.5298506 |
| Def6 | 0.57359531 |
| Def8 | 1.95782378 |
| Defb11 | 0.50335624 |
| Defb14 | 0.0049425 |
| Defb15 | 0.35800452 |
| Defb19 | -0.1760104 |
| Defb20 | 0.37856954 |
| Defb21 | -0.1349208 |
| Defb25 | -0.500039 |
| Defb26 | 0.47794652 |
| Defb30 | -0.3024284 |
| Defb36 | 0.36413441 |
| Defb36 | -0.136908 |
| Defb4 | -0.5374544 |
| Defcr20 | 2.74152538 |
| Defcr6 | -0.7244748 |
| Defcr-rs7 | 1.49546707 |
| Degs1 | 0.93046983 |
| Degs2 | -0.7078361 |
| Dek | -0.2431931 |
| Dek | 0.48051881 |
| Dennd1a | -0.6718889 |
| Dennd1c | 0.77657514 |
| Dennd2a | -0.5163176 |
| Dennd3 | -0.3714092 |
| Denr | -0.6518286 |
| Denr | 1.47486586 |
| Depdc1b | 0.01598686 |
| Depdc5 | 2.23068851 |
| Depdc5 | -0.5820193 |
| Depdc6 | -0.3752523 |
| Depdc6 | 1.09119961 |
| Dera | 0.11334895 |
| Derl2 | 1.12299238 |
| Derl3 | 0.68462042 |
| Des | 0.71348952 |
| Det1 | 0.66879497 |
| Dexi | -0.1534663 |
| Dexi | -0.43488 |
| Dffa | 1.67936334 |
| Dfna5h | 0.19978484 |
| Dgat1 | 0.1284464 |
| Dgat2 | -0.6843138 |
| Dgcr2 | 0.88109773 |
| Dgcr2 | -0.6254547 |
| Dgcr6 | 0.5404615 |
| Dgcr6 | 2.57434693 |
| Dgcr8 | 1.30215896 |
| Dgka | -0.6187676 |
| Dgka | 0.18615739 |
| Dgkb | -0.5756349 |
| Dgke | -0.3894805 |
| Dgkg | -0.5614275 |
| Dgkh | -0.4732852 |
| Dgki | 0.56589035 |
| Dgkk | 1.98378701 |
| Dgkq | 0.18192403 |
| Dgkz | 2.51269289 |
| Dgkz | 0.48077565 |
| Dgkz | 2.3735534 |
| Dguok | 2.48483828 |
| Dhcr24 | 1.74740156 |
| Dhcr24 | -0.1662087 |
| Dhcr24 | 0.10991815 |
| Dhcr7 | 0.32582815 |
| Dhdds | -0.4260284 |
| Dhdds | 1.15348902 |
| Dhdh | 3.53299258 |
| Dhdh | -0.0541454 |
| Dhdh | 2.29657021 |
| Dhh | 0.32613122 |
| Dhodh | -0.7170432 |
| Dhps | 1.35229249 |
| Dhrs1 | 0.43633556 |
| Dhrs4 | 1.46309176 |
| Dhrs7 | -0.3344375 |
| Dhrs7 | 1.43918106 |
| Dhrs7 | -0.2493431 |
| Dhrs7b | -0.1838219 |
| Dhrs7b | -0.2409668 |
| Dhrs7b | -0.062375 |
| Dhrsx | -0.519308 |
| Dhrsx | -0.4493581 |
| Dhtkd1 | 1.65876998 |
| Dhx15 | 1.66167761 |
| Dhx15 | 1.20315312 |
| Dhx16 | -0.1864404 |
| Dhx29 | -0.1364108 |
| Dhx30 | 1.280913 |
| Dhx32 | 0.41398687 |
| Dhx33 | -0.0637537 |
| Dhx36 | 1.05106102 |
| Dhx37 | 0.13850102 |
| Dhx38 | -0.6338813 |
| Dhx57 | -0.4424358 |
| Dhx58 | -0.4159182 |
| Dhx8 | -0.5482049 |
| Dhx9 | 1.63472928 |
| Diablo | -0.6246162 |
| Diap1 | -0.4956482 |
| Diap3 | 2.61405108 |
| Dicer1 | -0.4833184 |
| Dido1 | -0.4246741 |
| Dido1 | 1.21106226 |
| Dido1 | 0.67632465 |
| Dio1 | -0.0860937 |
| Dio1 | 1.76485119 |
| Dio3 | -0.6338813 |
| Dip2b | -0.1022473 |
| Dip2b | 0.78086918 |
| Diras1 | 0.83391179 |
| Diras2 | 0.99352557 |
| Dirc2 | 0.66479637 |
| Dis3 | -0.0559679 |
| Dis3l | 0.08984524 |
| Dis3l2 | 0.56799801 |
| Disp1 | 1.06311941 |
| Dixdc1 | 1.65935223 |
| Dkk3 | 0.08554323 |
| Dkk3 | -0.6987189 |
| Dkk3 | 0.19247168 |
| Dkkl1 | -0.6718889 |
| Dkkl1 | 2.30024217 |
| Dlat | 1.79308169 |
| Dlc1 | -0.1953982 |
| Dld | -0.7115081 |
| Dleu7 | -0.7376239 |
| Dlg1 | 0.68750662 |
| Dlg2 | 1.31938012 |
| Dlg3 | 1.92664719 |
| Dlg4 | -0.0865648 |
| Dlg7 | -0.2233443 |
| Dlgap1 | 0.0306511 |
| Dlgap2 | 1.06956955 |
| Dlgap2 | -0.7023551 |
| Dlgap2 | 0.38655687 |
| Dlgap3 | 0.76546891 |
| Dlgap4 | -0.7567352 |
| Dlgap4 | 0.44917393 |
| Dlgap4 | 0.36034446 |
| Dlgap4 | -0.3493008 |
| Dlk1 | -0.0276677 |
| Dlk2 | -0.1662087 |
| Dll1 | 0.16407566 |
| Dlst | -0.0496045 |
| Dlx1 | -0.5860318 |
| Dlx1as | 0.28049077 |
| Dlx2 | 0.08710989 |
| Dlx3 | 0.65589619 |
| Dmap1 | 2.0997397 |
| Dmbt1 | 0.32643419 |
| Dmkn | 0.00878033 |
| Dmkn | 2.56085771 |
| Dmkn | -0.5512993 |
| Dmn | 1.06105145 |
| Dmn | -0.6657379 |
| Dmpk | 0.30001296 |
| Dmrt2 | 2.13621549 |
| Dmrt3 | -0.5443512 |
| Dmrta1 | 0.73662366 |
| Dmrta2 | 0.43795003 |
| Dmrtc1a | 0.85393471 |
| Dmrtc1a | 1.19050181 |
| Dmrtc1a | 0.38485104 |
| Dmrtc1b | -0.6096504 |
| Dmrtc2 | -0.7300875 |
| Dmtf1 | -0.7105887 |
| Dmtf1 | -0.6905887 |
| Dmtf1 | 2.3323376 |
| Dmwd | 0.15792873 |
| Dmwd | -0.6692478 |
| Dmwd | 0.91236073 |
| Dnahc11 | -0.0998542 |
| Dnahc2 | -0.6162723 |
| Dnahc5 | 2.47646915 |
| Dnahc8 | 0.55670183 |
| Dnaic1 | 4.12867546 |
| Dnaic1 | 1.04938552 |
| Dnaja1 | -0.1329377 |
| Dnaja2 | 1.65716691 |
| Dnaja2 | -0.4782883 |
| Dnaja3 | 1.60095906 |
| Dnajb1 | -0.808335 |
| Dnajb10 | -0.519308 |
| Dnajb12 | 0.52466929 |
| Dnajb13 | 0.06893277 |
| Dnajb3 | 1.16454993 |
| Dnajb4 | -0.7300875 |
| Dnajb4 | 3.25885395 |
| Dnajb5 | 1.52044819 |
| Dnajb6 | -0.6622411 |
| Dnajb6 | 0.73231714 |
| Dnajb7 | 1.25883776 |
| Dnajb8 | 4.55841381 |
| Dnajb9 | -0.1284912 |
| Dnajc10 | 1.52188221 |
| Dnajc10 | 4.00514589 |
| Dnajc12 | 1.23014409 |
| Dnajc14 | -0.4287431 |
| Dnajc15 | 0.4655 |
| Dnajc16 | 0.19386908 |
| Dnajc17 | -0.603075 |
| Dnajc18 | 1.05259426 |
| Dnajc19 | -0.3574516 |
| Dnajc19 | -0.3393657 |
| Dnajc2 | 1.80921054 |
| Dnajc2 | -0.2795556 |
| Dnajc3a | 0.35183417 |
| Dnajc4 | -0.3216232 |
| Dnajc5 | -0.5788216 |
| Dnajc5b | 0.63239471 |
| Dnajc5g | 1.50863974 |
| Dnajc6 | 1.57735089 |
| Dnajc6 | -0.5298506 |
| Dnajc7 | -0.7347906 |
| Dnajc8 | -0.3973336 |
| Dnalc1 | 1.54822696 |
| Dnali1 | 1.6179967 |
| Dnase1l1 | 0.52564887 |
| Dnase1l2 | -0.6763074 |
| Dnase1l2 | 0.58100659 |
| Dnase2a | 3.5123766 |
| Dnd1 | -0.1384013 |
| Dner | -0.6561531 |
| Dnm | 1.08193298 |
| Dnm1 | 0.8458032 |
| Dnm1l | -0.7941601 |
| Dnm1l | 1.08314689 |
| Dnmbp | 0.75000314 |
| Dnmt1 | 2.13599707 |
| Dnmt3b | 1.84089608 |
| Dnmt3l | -0.5653524 |
| Dnmt3l | -0.7032664 |
| Dnttip1 | 1.48631836 |
| Doc2a | -0.6458078 |
| Doc2b | 0.98443158 |
| Doc2b | -0.7032664 |
| Dock1 | -0.6657379 |
| Dock3 | -0.7014447 |
| Dock4 | -0.5148259 |
| Dock6 | 0.7775103 |
| Dock8 | 0.69407039 |
| Dock9 | -0.7635176 |
| Dok1 | -0.4260284 |
| Dok1 | -0.3393657 |
| Dok2 | -0.7509607 |
| Dok3 | -0.7713297 |
| Dok4 | -0.6196009 |
| Dok5 | 2.31511207 |
| Dok5 | -0.5748399 |
| Dok7 | 0.62538145 |
| Dolk | 0.25664331 |
| Dolpp1 | -0.1996436 |
| Dolpp1 | -0.4697279 |
| Dolpp1 | 0.62185499 |
| Dom3z | -0.4119046 |
| Dom3z | -0.252152 |
| Donson | -0.6432393 |
| Donson | -0.7577011 |
| Dos | -0.4199492 |
| Dos | -0.3524273 |
| Dot1l | 0.04385997 |
| Dpagt1 | -0.3618701 |
| Dpep1 | 0.63870533 |
| Dpep2 | 1.52541566 |
| Dpep2 | 2.97201911 |
| Dpep3 | 1.95411581 |
| Dpf1 | 0.19491577 |
| Dpf1 | 0.77694932 |
| Dpf3 | 0.38740863 |
| Dph2 | 0.35035901 |
| Dph2 | 2.33095558 |
| Dph2 | -0.5981737 |
| Dph3 | 2.65750316 |
| Dph3 | 0.12092817 |
| Dph3 | 0.02355721 |
| Dph4 | 1.03658089 |
| Dpm1 | -0.6330353 |
| Dpm1 | 1.79333389 |
| Dpm2 | -0.4159182 |
| Dpm3 | -0.6288162 |
| Dpp10 | -0.6501044 |
| Dpp3 | 1.14608367 |
| Dpp3 | -0.157525 |
| Dpp4 | -0.6657379 |
| Dpp6 | 1.12942843 |
| Dpp7 | -0.6604976 |
| Dpp8 | 0.32035604 |
| Dpp9 | -0.5685042 |
| Dpp9 | 0.01851713 |
| Dppa3 | 0.90537643 |
| Dppa4 | 1.1492287 |
| Dppa5 | 1.46658594 |
| Dppa5 | 2.75990121 |
| Dpt | 1.68057325 |
| Dpy30 | -0.6229414 |
| Dpyd | -0.7664395 |
| Dpysl2 | -0.3307583 |
| Dpysl2 | 0.13701826 |
| Dpysl2 | 1.22853028 |
| Dpysl3 | 0.24976343 |
| Dpysl4 | 0.45394321 |
| Dpysl5 | 1.05161885 |
| Drbp1 | -0.2049776 |
| Drctnnb1a | -0.5283372 |
| Drd1a | -0.566927 |
| Drd2 | -0.4328299 |
| Drd4 | 0.47897628 |
| Drd5 | 1.20693897 |
| Drg1 | 1.80753562 |
| Drg1 | -0.6861023 |
| Drg2 | -0.1250473 |
| Drp2 | 2.59748114 |
| Dscam | -0.6432393 |
| Dscam | 1.48166619 |
| Dscc1 | 2.24925545 |
| Dscr1 | -0.1080159 |
| Dscr1l2 | -0.0870362 |
| Dscr2 | 3.24078557 |
| Dscr3 | -0.5428142 |
| Dscr3 | -0.4376205 |
| Dscr6 | 1.62232157 |
| Dsg2 | -0.2912153 |
| Dsg2 | 0.2808089 |
| Dsn1 | -0.8165325 |
| Dsp | -0.4172599 |
| Dst | -0.3173905 |
| Dst | 1.11781142 |
| Dst | 0.88393794 |
| Dstn | 0.14330383 |
| Dtd1 | 0.5043584 |
| Dtl | 1.37199242 |
| Dtna | -0.5868364 |
| Dtna | -0.5163176 |
| Dtna | -0.5313664 |
| Dtna | 2.2028396 |
| Dtnb | 1.58730605 |
| Dtnbp1 | 0.136276 |
| Dtwd1 | 1.3591395 |
| Dtx1 | -0.6423847 |
| Dtx2 | 1.50727087 |
| Dtx2 | -0.6162723 |
| Dtx2 | -0.4963786 |
| Dtx4 | 0.74788297 |
| Dtx4 | -0.4321475 |
| Dtymk | -0.3543082 |
| Dus1l | 1.77923305 |
| Dus1l | -0.5443512 |
| Dus2l | -0.4711492 |
| Dus3l | 0.21150517 |
| Dus3l | 1.56905328 |
| Dus3l | 0.57522156 |
| Dus4l | 0.54142467 |
| Dusp1 | 3.57259847 |
| Dusp11 | 2.12972853 |
| Dusp11 | 0.01005612 |
| Dusp13 | -0.486928 |
| Dusp13 | 1.51519954 |
| Dusp13 | -0.4026059 |
| Dusp14 | -0.6347282 |
| Dusp15 | 0.36063654 |
| Dusp15 | 2.04940245 |
| Dusp16 | 0.5879017 |
| Dusp16 | 1.39486521 |
| Dusp16 | 0.58238969 |
| Dusp18 | 0.00323172 |
| Dusp19 | 1.54978264 |
| Dusp2 | 2.38841072 |
| Dusp2 | -0.0568806 |
| Dusp21 | -0.4019452 |
| Dusp22 | 0.47252155 |
| Dusp23 | 1.49815109 |
| Dusp26 | 0.89405881 |
| Dusp28 | 0.9723693 |
| Dusp3 | 1.46443722 |
| Dusp3 | -0.4898257 |
| Dusp3 | -0.5344055 |
| Dusp4 | 0.01851713 |
| Dusp4 | -0.3455627 |
| Dusp4 | -0.514081 |
| Dusp6 | 0.18545316 |
| Dusp6 | 1.60266141 |
| Dusp6 | -0.5359287 |
| Dusp7 | 0.08436651 |
| Dusp8 | 2.04748324 |
| Dut | 0.19282123 |
| Duxbl | 1.41851053 |
| Duxbl | -0.4192762 |
| Dvl1 | -0.3375145 |
| Dvl1 | -0.5949204 |
| Dvl2 | -0.4963786 |
| DXBwg1396e | -0.1911721 |
| Dydc2 | -0.2606296 |
| Dym | -0.6330353 |
| Dync1h1 | 1.0257508 |
| Dync1i1 | -0.5606445 |
| Dync1i1 | -0.1662087 |
| Dync1i2 | -0.7357341 |
| Dync1li1 | 0.38485104 |
| Dync1li2 | 2.37389217 |
| Dync2li1 | -0.2859504 |
| Dynll1 | 2.8638205 |
| Dynll2 | 0.88210115 |
| Dynlrb1 | -0.3185979 |
| Dynlrb2 | 0.09877686 |
| Dynlt3 | 1.78255508 |
| Dyrk1a | -0.4797227 |
| Dyrk1b | 0.79821646 |
| Dyrk1b | -0.2200768 |
| Dyrk2 | 2.21820908 |
| Dyrk2 | -0.2217091 |
| Dyrk3 | -0.4438162 |
| Dyrk4 | 0.40149135 |
| Dysf | 0.48819346 |
| Dysfip1 | -0.5804191 |
| Dzip1 | 1.85286152 |
| E030010A14Rik | -0.3933988 |
| E030034P13Rik | -0.2119576 |
| E130009J12Rik | -0.6121281 |
| E130012A19Rik | -0.6869979 |
| E130014J05Rik | 1.90435568 |
| E130016E03Rik | 0.92073773 |
| E130203B14Rik | -0.6578885 |
| E130203B14Rik | -0.7235426 |
| E130303B06Rik | -0.8352442 |
| E130306D19Rik | 1.17828579 |
| E130308A19Rik | -0.1723873 |
| E130309D02Rik | -0.4697279 |
| E130309D02Rik | -0.7693705 |
| E130309D14Rik | 1.63106173 |
| E130309F12Rik | -0.6162723 |
| E130309F12Rik | 1.61273441 |
| E130309F12Rik | -0.7941601 |
| E230008N13Rik | 2.18248622 |
| E2f1 | -0.5344055 |
| E2f2 | -0.4768561 |
| E2f2 | -0.7752603 |
| E2f3 | -0.6692478 |
| E2f4 | -0.5868364 |
| E2f5 | 3.19697712 |
| E2f6 | -0.3726885 |
| E2f6 | 1.82804682 |
| E2f6 | -0.5074032 |
| E330016A19Rik | 0.02020018 |
| E330017A01Rik | 1.27225548 |
| E330018D03Rik | -0.2130361 |
| E330021D16Rik | 0.23217132 |
| E330034G19Rik | 0.18263092 |
| E330034G19Rik | -0.1060891 |
| E330036I19Rik | -0.6587574 |
| E430002D04Rik | -0.5382182 |
| E430002G05Rik | -0.0019195 |
| E430018J23Rik | -0.7654645 |
| E430025E21Rik | 0.5789281 |
| E430028B21Rik | -0.672771 |
| E4f1 | 0.7444032 |
| Eaf1 | -0.1279984 |
| Ear11 | -0.1975185 |
| Ear2 | 0.4623623 |
| Ear4 | 0.02857025 |
| Ears2 | -0.9372115 |
| Ebag9 | 1.00209101 |
| Ebf2 | -0.6288162 |
| Ebf3 | 1.03969263 |
| Ebf4 | 0.72759638 |
| Ebi2 | 1.54716106 |
| Ebi3 | -0.5497508 |
| Ebna1bp2 | 2.03904003 |
| Ebna1bp2 | 1.61043718 |
| Ebp | -0.808335 |
| Ebpl | 2.302475 |
| Ebpl | 2.04353649 |
| Ecd | 1.44414064 |
| Ecd | 0.90309116 |
| Ece1 | -0.0804584 |
| Ece1 | 2.10693334 |
| Ece2 | -0.7548064 |
| Ecel1 | 2.87355447 |
| Ech1 | -0.7548064 |
| Echdc1 | 1.59793361 |
| Echdc2 | 1.45334683 |
| Echdc2 | -0.6229414 |
| Echdc2 | -0.1364108 |
| Echdc3 | 0.60311951 |
| Echs1 | -0.1672356 |
| Ecm1 | 2.38373029 |
| Ecm1 | -0.3107777 |
| Ecsit | -0.2611975 |
| Eda2r | 1.17682371 |
| Edaradd | 0.56940048 |
| Edc4 | 0.136276 |
| Edem1 | 1.64009235 |
| Edem2 | -0.6288162 |
| Edem2 | 2.51111501 |
| Edem2 | 1.77955299 |
| Edem2 | -0.4528386 |
| Edf1 | -0.5820193 |
| Edg2 | 2.71988513 |
| Edg5 | 1.06710454 |
| Edg7 | 0.4290357 |
| Edg8 | 0.49883318 |
| Edg8 | -0.5740456 |
| Edil3 | 1.34407048 |
| Edn1 | 0.0218802 |
| Edn2 | -0.3258751 |
| Ednra | -0.3270935 |
| Ednrb | 2.28068913 |
| Ednrb | 1.47699076 |
| Ednrb | -0.8206584 |
| Eed | -0.6458078 |
| Eef1a1 | -0.7461757 |
| Eef1a1 | 0.86319077 |
| Eef1a2 | 1.21832201 |
| Eef1a2 | -0.6055354 |
| Eef1a2 | 1.00722208 |
| Eef1b2 | 0.10340317 |
| Eef1b2 | 1.33247805 |
| Eef1b2 | 0.7519264 |
| Eef1d | 2.29748955 |
| Eef1d | -0.6587574 |
| Eef1d | 0.08043338 |
| Eef1d | 1.75268392 |
| Eef1e1 | -0.6146124 |
| Eef1g | -0.4521415 |
| Eef2 | -0.2888717 |
| Eef2 | 1.65153444 |
| Eef2 | 0.10186361 |
| Eef2k | 0.20151766 |
| Eefsec | 0.65440455 |
| Efcab1 | 0.16874913 |
| Efcab2 | -0.7226113 |
| Efcab2 | 1.11949838 |
| Efcab3 | -0.4591364 |
| Efcab3 | 0.08475892 |
| Efcab3 | 0.28935778 |
| Efcab4a | 1.19122251 |
| Efcab4a | -0.2515895 |
| Efcab5 | 0.5931537 |
| Efemp2 | 0.96902469 |
| Efha1 | 2.16099934 |
| Efha1 | -0.7226113 |
| Efhc2 | 1.31969411 |
| Efhd1 | 0.66183905 |
| Efna1 | 2.57968858 |
| Efna3 | -0.2663244 |
| Efna4 | -0.2766633 |
| Efna4 | -0.7480867 |
| Efna5 | 1.15398896 |
| Efnb1 | 0.9016191 |
| Efnb2 | 0.1896706 |
| Efnb3 | -0.500039 |
| Efr3a | -0.3137777 |
| Efs | 0.38598861 |
| Eftud1 | 1.55027337 |
| Eftud2 | 0.64238494 |
| Eftud2 | -0.5820193 |
| Eftud2 | -0.8926208 |
| EG194588 | 0.20324728 |
| EG208426 | -0.5685042 |
| EG210155 | 3.26482605 |
| EG212225 | 2.54087502 |
| EG214321 | 0.84840606 |
| EG232599 | 3.13615022 |
| EG237009 | -0.3580815 |
| EG237009 | -0.6501044 |
| EG237300 | 0.84458605 |
| EG238829 | 0.45973947 |
| EG240916 | 0.41866426 |
| EG245297 | 1.27610099 |
| EG245297 | 2.78233049 |
| EG245376 | -0.4862049 |
| EG245376 | -0.7404658 |
| EG272350 | -0.4362492 |
| EG277333 | -0.9004692 |
| EG317677 | 0.03686601 |
| EG328264 | -0.5924879 |
| EG328280 | -0.5708751 |
| EG328644 | -0.8511286 |
| EG328839 | 2.49369941 |
| EG330305 | -0.7782192 |
| EG330503 | -0.4260284 |
| EG330513 | -0.0637537 |
| EG333669 | -0.2146562 |
| EG368203 | -0.4179315 |
| EG380907 | -0.4006253 |
| EG381438 | 1.9304111 |
| EG382109 | 0.96091774 |
| EG382109 | 0.5187702 |
| EG382109 | -0.6798573 |
| EG384639 | 0.87151118 |
| EG384813 | 3.90554352 |
| EG384813 | 1.69790823 |
| EG384814 | -0.7244748 |
| EG385328 | 0.83056197 |
| EG408196 | -0.3771798 |
| EG432825 | 0.04221904 |
| EG432879 | 0.74961801 |
| EG432939 | 2.05427868 |
| EG433016 | 1.33185849 |
| EG433182 | 0.20944753 |
| EG433224 | 2.57805662 |
| EG433923 | 1.31476264 |
| EG434171 | 0.73368955 |
| EG434197 | -0.1499293 |
| EG434280 | -0.6692478 |
| EG434459 | 2.94397502 |
| EG434459 | 0.84354151 |
| EG434858 | -0.8672877 |
| EG435391 | -0.3030223 |
| EG436332 | 0.40873135 |
| EG545047 | 2.44768421 |
| EG545056 | 0.43848757 |
| EG545253 | 1.43169198 |
| EG545370 | 2.38040635 |
| EG545758 | -0.6996266 |
| EG545861 | -0.8144764 |
| EG545963 | 1.01898989 |
| EG546166 | 2.19573549 |
| EG546282 | 0.36384342 |
| EG546325 | 1.06504539 |
| EG546347 | 0.17125597 |
| EG547109 | -0.5772269 |
| EG574403 | 0.376562 |
| EG622320 | -0.2299139 |
| EG622339 | -0.4725727 |
| EG624866 | 1.25402269 |
| EG625054 | -0.6047145 |
| EG625424 | -0.3933988 |
| EG630499 | -0.0804584 |
| EG633640 | -0.3493008 |
| EG638695 | -0.6246162 |
| EG654453 | -0.6622411 |
| EG665186 | 0.47407476 |
| EG665378 | -0.8012207 |
| EG667977 | 3.01367007 |
| EG668668 | 0.69038395 |
| Egf | -0.7376239 |
| Egfbp2 | 0.75461234 |
| Egfl7 | 1.62926012 |
| Egfl7 | -0.2606296 |
| Egflam | 1.48289732 |
| Egflam | 1.41634261 |
| Egfr | -0.672771 |
| Egfr | -0.3810468 |
| Egfr | 0.23317599 |
| Egfr | -0.4883757 |
| Egln1 | 0.20324728 |
| Egln1 | -0.6932909 |
| Egln1 | -0.6657379 |
| Egln2 | 0.39532108 |
| Egln3 | 1.26140311 |
| Egr1 | -0.3791113 |
| Egr2 | -0.286534 |
| Egr3 | 0.58077588 |
| Egr4 | 0.19282123 |
| Ehbp1 | 1.71985068 |
| Ehbp1l1 | -0.1744559 |
| Ehd1 | -0.522308 |
| Ehd2 | 1.80517322 |
| Ehd3 | -0.4458906 |
| Ehd4 | 1.10367766 |
| Ehmt1 | -0.7310262 |
| Ehmt2 | -0.5148259 |
| Ehmt2 | -0.4451986 |
| Ei24 | -0.4493581 |
| Eid1 | 1.85616807 |
| Eid2 | 2.4819161 |
| Eid3 | -0.6262939 |
| Eif1a | -0.0365594 |
| Eif1b | -0.8705534 |
| Eif2a | 2.46421919 |
| Eif2ak1 | -0.5772269 |
| Eif2ak2 | 0.0197797 |
| Eif2b1 | -0.6816374 |
| Eif2b1 | 2.30397276 |
| Eif2b2 | 0.80350393 |
| Eif2b4 | 1.42563851 |
| Eif2b5 | -0.7272769 |
| Eif2b5 | 0.60559444 |
| Eif2b5 | -0.0632939 |
| Eif2c2 | 0.56729599 |
| Eif2c3 | -0.6570204 |
| Eif2c3 | -0.5606445 |
| Eif2c3 | -0.9106578 |
| Eif2c3 | 1.0682009 |
| Eif2c4 | 1.20800098 |
| Eif2s2 | 1.99609718 |
| Eif2s2 | -0.7871525 |
| Eif2s3x | 0.74653127 |
| Eif2s3y | -0.5645661 |
| Eif3b | 0.25074938 |
| Eif3b | -0.3966766 |
| Eif3d | 1.90558641 |
| Eif3eip | -0.6254547 |
| Eif3eip | 0.3160777 |
| Eif3f | -0.5200571 |
| Eif3g | 0.76053243 |
| Eif3i | 0.33996719 |
| Eif3i | 0.8215105 |
| Eif3i | -0.7911504 |
| Eif3k | 0.06292817 |
| Eif3s1 | -0.7452216 |
| Eif3s10 | -0.4079081 |
| Eif3s10 | -0.6923893 |
| Eif3s4 | 0.13367341 |
| Eif3s8 | -0.7366785 |
| Eif4a1 | -0.1529602 |
| Eif4a1 | 0.89356362 |
| Eif4a2 | -0.697812 |
| Eif4a3 | -0.1890661 |
| Eif4b | 1.85975658 |
| Eif4e | -0.3185979 |
| Eif4e1b | -0.4389938 |
| Eif4e1b | -0.6518286 |
| Eif4e2 | -0.5661393 |
| Eif4e2 | -0.6121281 |
| Eif4e3 | 1.166278 |
| Eif4e3 | 0.82329222 |
| Eif4e3 | -0.0637537 |
| Eif4ebp1 | 1.82494385 |
| Eif4ebp2 | -0.7891493 |
| Eif4el3 | 1.57512416 |
| Eif4enif1 | 0.48308403 |
| Eif4enif1 | -0.5321252 |
| Eif4g1 | -0.2830383 |
| Eif4g2 | -0.5482049 |
| Eif4g2 | -0.4542345 |
| Eif4h | 0.54861695 |
| Eif5 | 0.7542291 |
| Eif5 | -0.5298506 |
| Eif5 | -0.1128505 |
| Eif5a | -0.1739383 |
| Eif5a | 0.69509182 |
| Eif6 | 0.51753652 |
| Ela1 | 1.28352733 |
| Ela2 | 1.34325449 |
| Ela2a | -0.4920049 |
| Ela3b | -0.3836337 |
| Elac2 | 0.20186384 |
| Elac2 | 2.84154114 |
| Elac2 | -0.140396 |
| Elavl1 | -0.7041785 |
| Elavl2 | -0.6648625 |
| Elavl2 | -0.8500612 |
| Elavl3 | 1.37830405 |
| Elavl4 | 3.45963991 |
| Elavl4 | -0.0922371 |
| Elf1 | 1.12840164 |
| Elf2 | -0.1703232 |
| Elf2 | 1.35229249 |
| Elf2 | -0.2365299 |
| Elf2 | -0.0040742 |
| Elf2 | 1.51281948 |
| Elf3 | -0.4905515 |
| Elf3 | 0.97434004 |
| Elf3 | 1.20563931 |
| Elf5 | -0.8073153 |
| Elfn1 | -0.4092383 |
| Elfn2 | 0.63173945 |
| Elk3 | 1.56535955 |
| Elk3 | 1.77916905 |
| Elk3 | -0.6080022 |
| Ell | 0.68832962 |
| Ell3 | 0.30807985 |
| Elmo1 | 2.25903456 |
| Elmo1 | 0.31054802 |
| Elmo1 | 1.20929736 |
| Elmo1 | -0.5756349 |
| Elmo1 | -0.3325961 |
| Elmo1 | 1.30375705 |
| Elmo1 | 1.34874864 |
| Elmo1 | 0.47200324 |
| Elmo1 | -0.6552866 |
| Elmo1 | 0.39644598 |
| Elmo1 | -0.446583 |
| Elmo1 | -0.9267309 |
| Elmo1 | 0.2744256 |
| Elmo2 | 0.42304522 |
| Elmo2 | -0.5645661 |
| Elmo3 | 1.0010614 |
| Elmo3 | -0.7762456 |
| Elmod1 | -0.7452216 |
| Eln | -0.7023551 |
| Elof1 | -0.0628343 |
| Elovl1 | -0.5780239 |
| Elovl1 | 0.29187583 |
| Elovl1 | 1.45714861 |
| Elovl1 | 0.25304588 |
| Elovl2 | -0.486928 |
| Elovl4 | 0.63848843 |
| Elovl5 | 0.45658233 |
| Elovl6 | 0.47639974 |
| Elp2 | -0.5253177 |
| Elp3 | -0.4654769 |
| Elp3 | 1.80093082 |
| Eltd1 | 0.12394272 |
| Emb | -0.3612376 |
| Emcn | 1.99070271 |
| Emd | -0.6710077 |
| Emd | -0.4711492 |
| Emg1 | 3.50044074 |
| Emg1 | 0.09101509 |
| Emid1 | -0.7376239 |
| Emid2 | -0.7742761 |
| Emilin1 | 1.32021721 |
| Emilin2 | -0.7291497 |
| Eml1 | -0.5111072 |
| Eml3 | -0.5170643 |
| Eml3 | -0.6372734 |
| Eml4 | -0.7693705 |
| Eml5 | -0.8103776 |
| Emp1 | -0.5474329 |
| Emp2 | -0.721681 |
| Emp3 | -0.6518286 |
| Emr1 | -0.2437505 |
| Emr1 | 1.63128669 |
| Emr1 | -0.6578885 |
| En2 | 0.36326117 |
| Enah | 0.96826288 |
| Enc1 | 0.13106356 |
| Endod1 | 0.6622621 |
| Endogl1 | -0.1133353 |
| Endogl1 | 0.56869951 |
| Eng | 4.2691873 |
| Eno1 | -0.0739266 |
| Eno2 | -0.5981737 |
| Eno3 | 1.93508123 |
| Enoph1 | 0.54478795 |
| Enox1 | 0.6100776 |
| Enpp1 | 1.0624306 |
| Enpp1 | 2.30462956 |
| Enpp2 | -0.6204349 |
| Enpp4 | -0.1980493 |
| Enpp5 | -0.4486636 |
| Enpp6 | 0.35418959 |
| Ensa | 2.09404519 |
| Ensa | -0.3240505 |
| ENSMUSG00000043795 | -0.4905515 |
| ENSMUSG00000053178 | -0.2959202 |
| ENSMUSG00000068790 | -0.1838219 |
| Entpd2 | 0.73015642 |
| Entpd3 | 2.18894859 |
| Entpd4 | 0.63978907 |
| Entpd5 | 1.09732671 |
| Entpd5 | 0.65696019 |
| Entpd5 | 0.13031655 |
| Entpd6 | 1.28732682 |
| Entpd7 | 1.08664493 |
| Ep300 | -0.5606445 |
| Ep400 | 0.3025025 |
| Epas1 | 0.25271816 |
| Epb4.1 | 0.40623154 |
| Epb4.1l1 | 0.04262954 |
| Epb4.1l1 | 0.82293615 |
| Epb4.1l1 | 1.98383843 |
| Epb4.1l2 | 0.08436651 |
| Epb4.1l3 | 1.1063112 |
| Epb4.1l3 | -0.8543382 |
| Epb4.1l3 | 1.24918269 |
| Epb4.1l4a | -0.6212697 |
| Epb4.1l5 | -0.6038944 |
| Epb4.1l5 | -0.222799 |
| Epb4.9 | -0.5677153 |
| Epb4.9 | 1.47937551 |
| Epc1 | 0.91413906 |
| Epc1 | 0.19037165 |
| Epc1 | 2.78808846 |
| Epdr1 | -0.6736538 |
| Epgn | -0.4417464 |
| Epha1 | 1.82992792 |
| Epha1 | -0.379756 |
| Epha3 | 2.38964378 |
| Epha3 | -0.6666141 |
| Epha3 | 1.97909627 |
| Epha4 | -0.6288162 |
| Epha5 | 1.62745503 |
| Epha6 | 0.61320306 |
| Epha7 | 0.08200862 |
| Epha7 | -0.7087528 |
| Epha7 | 1.94338614 |
| Ephb1 | -0.5275814 |
| Ephb2 | 0.97887212 |
| Ephb4 | 1.53410808 |
| Ephb6 | 0.94135747 |
| Ephx1 | 0.53925615 |
| Ephx2 | -0.486928 |
| Epm2a | 0.15792873 |
| Epm2aip1 | 1.55247847 |
| Epm2aip1 | 0.1882669 |
| Epm2aip1 | -0.272629 |
| Epn1 | -0.0254579 |
| Epn2 | 0.83197388 |
| Epn2 | -0.696906 |
| Epn2 | 1.09066492 |
| Epn2 | -0.6355758 |
| Epn2 | 0.93094585 |
| Epor | 0.58904595 |
| Eprs | -0.3669455 |
| Eprs | -0.7861557 |
| Eps15 | 0.9737341 |
| Eps15l1 | -0.5458908 |
| Eps8 | 1.57647674 |
| Eps8l1 | -0.6440947 |
| Eps8l2 | 0.16154947 |
| Eps8l2 | -0.4993058 |
| Eps8l3 | 2.03500871 |
| Epsti1 | -0.3914376 |
| Epsti1 | 2.03115545 |
| Eraf | 0.44651378 |
| Eral1 | 0.51926321 |
| Ercc1 | 1.76815989 |
| Ercc2 | 1.34110912 |
| Ercc3 | 0.14771538 |
| Ercc3 | 1.49068606 |
| Ercc4 | -0.81345 |
| Ercc4 | 1.87136847 |
| Ercc5 | -0.3455627 |
| Ercc5 | -0.5283372 |
| Ercc8 | -0.3125765 |
| Ercc8 | 0.33847316 |
| Erdr1 | -0.6509661 |
| Erf | -0.7991979 |
| Erf | 0.04098644 |
| Ergic1 | 1.5569543 |
| Ergic2 | 0.67026383 |
| Ergic2 | 0.2747458 |
| Ergic3 | 0.40009259 |
| Ergic3 | 1.37020957 |
| Erh | -0.5298506 |
| Erich1 | -0.3277033 |
| Erlin1 | 0.88260245 |
| Erlin2 | 2.80179456 |
| Erlin2 | 0.70847118 |
| Ermap | -0.5344055 |
| Ermp1 | -0.7423652 |
| Ern1 | 1.12557212 |
| Ern2 | -0.7981882 |
| Ero1lb | -0.5876416 |
| Erp29 | 0.58077588 |
| Errfi1 | 2.23988128 |
| Es22 | -0.6878943 |
| Esam1 | -0.1565086 |
| Esco2 | 0.92362005 |
| Esd | 0.59383658 |
| Esd | 1.8451402 |
| Esd | 2.32537129 |
| Esd | -0.7319659 |
| Esm1 | -0.2830383 |
| Espl1 | 0.0772749 |
| Espn | 0.72936946 |
| Espn | 1.49190537 |
| Espn | -0.7291497 |
| Espn | -0.721681 |
| Esr1 | -0.3907847 |
| Esr2 | -0.7442685 |
| Esr2 | -0.3574516 |
| Esr2 | -0.5306082 |
| Esrra | 3.23953899 |
| Esrrb | -0.0232533 |
| Etaa1 | 1.6121989 |
| Etf1 | 3.17816047 |
| Etfa | -0.5185595 |
| Etfa | -0.0781204 |
| Etfa | -0.7096703 |
| Etfb | 0.96260611 |
| Etfb | 1.56921355 |
| Etfdh | -0.2982816 |
| Ethe1 | 0.40567512 |
| Etnk2 | 0.38028687 |
| Ets1 | 0.87049633 |
| Ets2 | -0.7792076 |
| Etsrp71 | 1.57177408 |
| Etv1 | 0.5618962 |
| Etv1 | 1.87945204 |
| Etv4 | -0.6338813 |
| Etv5 | -0.7366785 |
| Etv6 | -0.5876416 |
| Evc | -0.4697279 |
| Evc2 | 0.3757003 |
| Evi1 | 0.92009601 |
| Evi1 | 0.17553782 |
| Evi2a | -0.3940534 |
| Evi2a | -0.5836222 |
| Evi5 | 2.21150342 |
| Evi5l | -0.7461757 |
| Evl | -0.5260717 |
| Evx1 | -0.2135758 |
| Exdl1 | -0.7096703 |
| Exdl2 | -0.4410575 |
| Exdl2 | 0.64927221 |
| Exdl2 | 1.58289462 |
| Exoc2 | 0.85427916 |
| Exoc3 | -0.4818785 |
| Exoc3l | 2.25769311 |
| Exoc4 | 1.61966832 |
| Exoc7 | -0.5497508 |
| Exoc8 | -0.7198231 |
| Exod1 | 0.76148378 |
| Exosc1 | 0.21833144 |
| Exosc10 | 1.13620261 |
| Exosc2 | 0.465761 |
| Exosc2 | -0.6683691 |
| Exosc4 | -0.081864 |
| Exosc5 | -0.3849298 |
| Exosc6 | 1.65840588 |
| Exosc7 | 0.22881459 |
| Exosc8 | -0.4192762 |
| Ext1 | -0.7452216 |
| Ext2 | -0.1901185 |
| Extl1 | 1.51494482 |
| Extl2 | 1.56768991 |
| Extl3 | 1.12402513 |
| Extl3 | 2.59529221 |
| Eya1 | -0.2039084 |
| Eya1 | -0.6389741 |
| Eya2 | 0.29061765 |
| Eya3 | -0.2376372 |
| Eya3 | 2.15566718 |
| Eya3 | -0.7752603 |
| Eya4 | -0.2714795 |
| Ezh1 | -0.6279747 |
| Ezh2 | 1.37781249 |
| Ezh2 | -0.3746107 |
| F10 | -0.6736538 |
| F11 | -0.5622112 |
| F11 | -0.6296585 |
| F11r | -0.7635176 |
| F13a1 | -0.7772319 |
| F2r | 0.86250827 |
| F2rl1 | -0.1304648 |
| F2rl3 | -0.1162496 |
| F430201B04Rik | 0.56118958 |
| F5 | 1.27093337 |
| F630043A04Rik | 0.75115757 |
| F730014I05Rik | 0.00665015 |
| F730047E07Rik | 1.33988098 |
| F730047E07Rik | 0.78736596 |
| F730047E07Rik | -0.8458038 |
| F8 | -0.1250473 |
| F8 | 1.42114296 |
| F8 | -0.7096703 |
| F8a | 0.48410816 |
| Fa2h | -0.2033742 |
| Faah | 0.21628879 |
| Faah | 0.46966729 |
| Fabp3 | -0.7423652 |
| Fabp5 | 1.62805711 |
| Fabp9 | 2.330388 |
| Fadd | 2.22617701 |
| Fadd | 0.83215022 |
| Fadd | 1.39050986 |
| Fads1 | 0.49073774 |
| Fads2 | 0.61986549 |
| Fads3 | -0.4362492 |
| Fads6 | 0.20082492 |
| Faf1 | 1.19708754 |
| Faf1 | 1.33505517 |
| Fah | -0.7861557 |
| Fahd1 | 0.85238308 |
| Fahd2a | -0.7244748 |
| Fahd2a | -0.4165888 |
| Fahd2a | 1.09066492 |
| Fahd2a | 0.58354071 |
| Faim | -0.4542345 |
| Faim | -0.5536269 |
| Faim2 | 2.30779758 |
| Faim2 | 1.99863097 |
| Faim3 | 0.8602867 |
| Fancc | 1.46730108 |
| Fancc | -0.3474299 |
| Fancd2 | 0.69651996 |
| Fancg | -0.7032664 |
| Fanci | -0.7124283 |
| Fancl | 0.92234012 |
| Fancl | -0.6771936 |
| Fank1 | 0.32582815 |
| Fank1 | 0.04180835 |
| Fap | -0.048699 |
| Fars2 | -0.7366785 |
| Farsa | -0.3920908 |
| Farsb | -0.2749322 |
| Farsb | -0.4825982 |
| Fas | -0.7235426 |
| Fas | 0.57684498 |
| Fasl | -0.4754261 |
| Fastk | 0.71448996 |
| Fastk | -0.6887916 |
| Fastkd1 | 1.27181499 |
| Fastkd5 | 0.55266813 |
| Fat1 | 1.28211214 |
| Fat4 | -0.0014891 |
| Fbf1 | -0.1113975 |
| Fblim1 | -0.7901493 |
| Fbln1 | 0.10186361 |
| Fbln1 | 0.39841131 |
| Fbln1 | -0.5998047 |
| Fbln2 | 0.06011277 |
| Fbln2 | 0.6345756 |
| Fbn1 | -0.6639879 |
| Fbp1 | 1.92839466 |
| Fbp2 | 0.06051549 |
| Fbxl10 | 0.9781183 |
| Fbxl12 | 0.47871895 |
| Fbxl12 | -0.808335 |
| Fbxl12 | -0.4403691 |
| Fbxl12 | -0.5582994 |
| Fbxl13 | 0.52736065 |
| Fbxl13 | 0.3479939 |
| Fbxl14 | 0.63086505 |
| Fbxl15 | 0.88776689 |
| Fbxl18 | 0.3142381 |
| Fbxl18 | 0.50260392 |
| Fbxl21 | 1.66746587 |
| Fbxl22 | -0.5412797 |
| Fbxl4 | -0.7606049 |
| Fbxl5 | -0.3065937 |
| Fbxl6 | -0.4891004 |
| Fbxl6 | -0.6736538 |
| Fbxl8 | 1.17633584 |
| Fbxo15 | 0.35183417 |
| Fbxo16 | -0.3606055 |
| Fbxo18 | 0.2418382 |
| Fbxo18 | 0.16191077 |
| Fbxo2 | -0.0298827 |
| Fbxo21 | 1.16009161 |
| Fbxo25 | -0.4424358 |
| Fbxo27 | 0.56963402 |
| Fbxo28 | -0.1585424 |
| Fbxo3 | -0.3960201 |
| Fbxo3 | 0.93679695 |
| Fbxo30 | 2.32529994 |
| Fbxo31 | 0.7675496 |
| Fbxo31 | -0.7282128 |
| Fbxo32 | 0.23451387 |
| Fbxo32 | -0.1060891 |
| Fbxo32 | -0.447276 |
| Fbxo33 | 0.00835469 |
| Fbxo34 | -0.3739695 |
| Fbxo34 | -0.7981882 |
| Fbxo34 | 0.46862721 |
| Fbxo36 | 0.4246828 |
| Fbxo38 | 0.3661688 |
| Fbxo38 | 0.1325558 |
| Fbxo4 | 0.63348579 |
| Fbxo42 | -0.4451986 |
| Fbxo44 | 2.23917734 |
| Fbxo6 | -0.4626536 |
| Fbxo7 | -0.7263419 |
| Fbxo8 | -0.4396812 |
| Fbxo9 | 0.62141324 |
| Fbxo9 | -0.3065937 |
| Fbxw12 | 2.51187506 |
| Fbxw13 | 1.40552242 |
| Fbxw15 | 0.54382825 |
| Fbxw16 | 1.28927485 |
| Fbxw17 | 0.4748504 |
| Fbxw19 | -0.3701317 |
| Fbxw2 | -0.6861023 |
| Fbxw4 | 0.35624572 |
| Fbxw4 | 1.72671338 |
| Fbxw5 | 0.53393403 |
| Fbxw7 | -0.0245754 |
| Fbxw7 | -0.0762542 |
| Fbxw7 | 1.14280158 |
| Fbxw8 | -0.5949204 |
| Fcer1g | 0.1118257 |
| Fcer2a | -0.3455627 |
| Fcf1 | -0.7078361 |
| Fcgr2b | 3.26892933 |
| Fcgr2b | 0.03231246 |
| Fcgr2b | -0.6330353 |
| Fcgr3 | -0.2293646 |
| Fcgr4 | 0.64626527 |
| Fcgrt | -0.466184 |
| Fcho1 | -0.6923893 |
| Fcho2 | -0.4165888 |
| Fchsd1 | -0.2028404 |
| Fchsd2 | 0.82435963 |
| Fcna | 0.31852486 |
| Fcrla | 1.94026697 |
| Fcrla | -0.2370834 |
| Fcrla | 3.46018027 |
| Fdps | 1.53892088 |
| Fdxr | 0.1366472 |
| Fdxr | 1.95480555 |
| Fem1b | 2.35804276 |
| Fem1c | -0.4521415 |
| Fem1c | -0.7471307 |
| Fen1 | -0.2760859 |
| Fermt2 | 2.2541578 |
| Fermt3 | 0.27953573 |
| Fert2 | 2.58424295 |
| Fert2 | -0.4260284 |
| Fes | 1.95650119 |
| Fetub | -0.3555643 |
| Fev | 0.71248801 |
| Fez1 | 0.21287442 |
| Fez1 | 0.94088672 |
| Fez2 | -0.5957327 |
| Fezf2 | 1.77132784 |
| Ffar2 | 0.78718096 |
| Fgd2 | -0.7901493 |
| Fgd4 | -0.5876416 |
| Fgd4 | -0.5804191 |
| Fgd5 | -0.4145784 |
| Fgd6 | 0.53417661 |
| Fgf1 | -0.7226113 |
| Fgf1 | 3.08342128 |
| Fgf10 | 0.0273195 |
| Fgf10 | -0.0396916 |
| Fgf12 | 2.02581271 |
| Fgf12 | 0.50960217 |
| Fgf13 | 0.36935678 |
| Fgf13 | 1.83813604 |
| Fgf17 | 1.59489834 |
| Fgf21 | 2.38366326 |
| Fgf22 | 1.28786835 |
| Fgf22 | 0.27282294 |
| Fgf5 | 0.09800358 |
| Fgf7 | -0.5900616 |
| Fgf8 | 0.55504301 |
| Fgf9 | 0.50585962 |
| Fgfbp3 | -0.7263419 |
| Fgfr1 | -0.5614275 |
| Fgfr1op | -0.7207516 |
| Fgfr1op2 | -0.6683691 |
| Fgfr1op2 | 0.65077205 |
| Fgfr2 | 0.06292817 |
| Fgfr2 | -0.3656741 |
| Fgfr3 | -0.5275814 |
| Fgfrl1 | -0.5852279 |
| Fgl2 | 0.46862721 |
| Fh1 | 0.0142962 |
| Fhad1 | -0.2589279 |
| Fhit | 1.53285934 |
| Fhl1 | 1.93918898 |
| Fhl1 | -0.061457 |
| Fhl2 | 0.77301294 |
| Fhl4 | -0.7347906 |
| Fhl5 | 2.11937936 |
| Fhod1 | 2.56132897 |
| Fhod3 | -0.5828204 |
| Fibcd1 | 2.39528839 |
| Fibp | 0.22881459 |
| Figf | -0.877119 |
| Figla | -0.4833184 |
| Fign | -0.6080022 |
| Fignl1 | -0.7713297 |
| Fip1l1 | -0.0860937 |
| Fis1 | 1.92495121 |
| Fis1 | -0.6055354 |
| Fiz1 | -0.1314532 |
| Fjx1 | 0.52809331 |
| Fkbp10 | 2.95480646 |
| Fkbp11 | 0.36935678 |
| Fkbp14 | -0.3418396 |
| Fkbp1a | 0.9100925 |
| Fkbp1b | 0.34384051 |
| Fkbp2 | -0.6552866 |
| Fkbp2 | 1.31843749 |
| Fkbp4 | -0.5245644 |
| Fkbp5 | 0.07489903 |
| Fkbp6 | 1.00414682 |
| Fkbp7 | 0.59338138 |
| Fkbp8 | -0.1354172 |
| Fkbp9 | -0.6996266 |
| Fkbpl | 0.14404053 |
| Fkbpl | -0.7548064 |
| Fkhl18 | 0.353307 |
| Fkrp | 0.62560141 |
| Fktn | -0.1554934 |
| Flad1 | 1.33546684 |
| Flcn | 2.14061668 |
| Flcn | -0.653556 |
| Fli1 | -0.0828023 |
| Flii | 0.88059562 |
| Flna | 2.36247621 |
| Flot1 | 0.46654357 |
| Flot1 | -0.7310262 |
| Flot2 | -0.4747119 |
| Flot2 | 2.52953192 |
| Flot2 | -0.0771868 |
| Flrt1 | 1.31054505 |
| Flrt2 | -0.3295351 |
| Flrt3 | -0.6834208 |
| Flt1 | 0.17589376 |
| Flt4 | 0.38399696 |
| Flt4 | -0.300649 |
| Flvcr2 | 1.3058836 |
| Flywch2 | -0.6483834 |
| Fmn2 | -0.1687781 |
| Fmn2 | -0.3289241 |
| Fmnl1 | 0.98233189 |
| Fmnl3 | -0.6596271 |
| Fmnl3 | 0.76357335 |
| Fmo1 | -0.697812 |
| Fmo2 | 1.13173461 |
| Fmo5 | -0.6869979 |
| Fmod | -0.5489775 |
| Fmr1nb | -0.3663096 |
| Fmr1nb | -0.7023551 |
| Fn3k | 1.89614681 |
| Fn3k | -0.3071903 |
| Fnbp1 | 0.9157528 |
| Fnbp1 | 1.03032615 |
| Fnbp1 | 1.97256228 |
| Fnbp1l | 2.76760061 |
| Fnbp4 | -0.7005352 |
| Fndc3 | -0.1744559 |
| Fndc3a | 1.0228798 |
| Fndc3b | 0.96734789 |
| Fndc4 | 1.21715489 |
| Fndc5 | -0.6154419 |
| Fndc5 | 0.50735844 |
| Fndc7 | -0.5844247 |
| Fndc7 | -0.6096504 |
| Fndc8 | 1.70347704 |
| Fndc8 | -0.5185595 |
| Fnip1 | -0.5965457 |
| Fnip1 | -0.5756349 |
| Fnta | -0.3350521 |
| Fnta | -0.5567393 |
| Fntb | 1.00326631 |
| Folr1 | 0.07331174 |
| Folr1 | -0.2566638 |
| Folr2 | 1.60382032 |
| Fos | 1.81242787 |
| Fosb | -0.2959202 |
| Foxa1 | 2.07142877 |
| Foxa2 | -0.1906451 |
| Foxa3 | 0.31116405 |
| Foxc1 | -0.6869979 |
| Foxd2 | 3.08758103 |
| Foxg1 | 1.02503388 |
| Foxh1 | 0.74168772 |
| Foxh1 | 0.27698416 |
| Foxj1 | -0.3264841 |
| Foxj2 | -0.7841652 |
| Foxj3 | 1.79578924 |
| Foxj3 | -0.5916784 |
| Foxj3 | 1.16516746 |
| Foxj3 | 0.58054511 |
| Foxj3 | 2.63531298 |
| Foxk1 | -0.0334376 |
| Foxk1 | -0.1265217 |
| Foxk1 | 1.63629669 |
| Foxl2 | -0.12603 |
| Foxm1 | -0.401285 |
| Foxm1 | -0.3804012 |
| Foxn2 | 2.854774 |
| Foxn3 | -0.6022564 |
| Foxo1 | -0.2130361 |
| Foxo6 | -0.6596271 |
| Foxo6 | -0.566927 |
| Foxp1 | -0.4486636 |
| Foxp2 | 1.32875448 |
| Foxp2 | -0.6518286 |
| Foxp2 | -0.4396812 |
| Foxp3 | 0.39193823 |
| Foxp4 | 1.36034261 |
| Foxq1 | 1.65519575 |
| Foxred1 | -0.3849298 |
| Foxred1 | -0.3920908 |
| Foxred1 | 0.51778338 |
| Foxred1 | 1.38992761 |
| Foxred1 | 0.04754157 |
| Foxred2 | 1.33752253 |
| Fpgs | 0.72404009 |
| Fpgs | 0.36471612 |
| Fpgs | 0.56916688 |
| Fpr2 | 1.20018472 |
| Frag1 | 0.09606757 |
| Frag1 | 0.63566414 |
| Frag1 | -0.4396812 |
| Frap1 | 2.43482635 |
| Fras1 | 2.12017953 |
| Fras1 | -0.2594948 |
| Frat1 | 0.84458605 |
| Frat1 | -0.5916784 |
| Frat2 | 0.91252254 |
| Freq | 1.75031055 |
| Freq | 0.11941723 |
| Freq | -0.557519 |
| Frg1 | -0.6162723 |
| Frk | -0.6578885 |
| Frmd3 | -0.5382182 |
| Frmd6 | -0.4052532 |
| Frmd8 | 2.57254133 |
| Frmpd4 | -0.7310262 |
| Frrs1 | 0.0049425 |
| Frs2 | 1.11103295 |
| Frs3 | 0.9797759 |
| Fryl | -0.3295351 |
| Fryl | -0.8012207 |
| Frzb | -0.6364242 |
| Fscn1 | 3.8433126 |
| Fscn2 | -0.4321475 |
| Fscn3 | -0.4479696 |
| Fsd1 | 1.36901911 |
| Fsd2 | 1.15198759 |
| Fshb | 0.47355731 |
| Fshr | 2.377543 |
| Fshr | -0.4500532 |
| Fshr | -0.6296585 |
| Fsip1 | 1.18362726 |
| Fst | -0.6113014 |
| Fstl1 | -0.8760216 |
| Fstl4 | 0.0459071 |
| Fstl5 | -0.6246162 |
| Fth1 | -0.3663096 |
| Fth1 | 0.86744514 |
| Ftmt | 1.0978576 |
| Fto | -0.8500612 |
| Ftsj1 | 1.29530955 |
| Ftsj3 | -0.7087528 |
| Fubp1 | -0.4920049 |
| Fubp3 | 0.90619124 |
| Fuk | 3.30297939 |
| Fuk | 0.01090568 |
| Fundc1 | 0.04098644 |
| Fundc1 | 1.55898171 |
| Fundc2 | 3.69033363 |
| Furin | -0.7329065 |
| Furin | -0.6923893 |
| Fus | 0.98936226 |
| Fusip1 | 3.4062091 |
| Fusip1 | -0.672771 |
| Fusip1 | -0.6639879 |
| Fut10 | 0.2808089 |
| Fut10 | -0.277241 |
| Fut2 | -0.184345 |
| Fut4 | 0.16982431 |
| Fut8 | -0.4521415 |
| Fut8 | -0.0032117 |
| Fut8 | -0.7170432 |
| Fvt1 | -0.3449412 |
| Fxc1 | -0.4145784 |
| Fxn | -0.7871525 |
| Fxn | -0.4605417 |
| Fxyd1 | 2.15202385 |
| Fxyd2 | 2.85307179 |
| Fxyd2 | 0.74013247 |
| Fxyd2 | -0.6807469 |
| Fxyd3 | -0.7319659 |
| Fxyd4 | -0.2566638 |
| Fxyd4 | -0.5328847 |
| Fxyd5 | 1.39380244 |
| Fxyd5 | -0.0720687 |
| Fxyd5 | -0.1801684 |
| Fxyd6 | -0.1474111 |
| Fxyd7 | -0.5451207 |
| Fyb | -0.865117 |
| Fyco1 | -0.6798573 |
| Fyn | 2.70709743 |
| Fyttd1 | -0.6406778 |
| Fzd1 | -0.0729972 |
| Fzd10 | 0.12882073 |
| Fzd10 | -0.5685042 |
| Fzd2 | 0.08906453 |
| Fzd3 | 1.02889876 |
| Fzd4 | -0.6221052 |
| Fzd5 | -0.3083845 |
| Fzd6 | 0.4320166 |
| Fzd7 | -0.5852279 |
| Fzd8 | -0.6047145 |
| Fzr1 | 1.45723894 |
| Fzr1 | 2.97505017 |
| Fzr1 | -0.0410372 |
| G0s2 | -0.6338813 |
| G3bp1 | -0.1651829 |
| G3bp1 | -0.2482219 |
| G3bp2 | 0.03769155 |
| G3bp2 | -0.2465426 |
| G3bp2 | 1.74680542 |
| G430022H21Rik | 0.7735763 |
| G430055L02Rik | -0.7198231 |
| G6pc | -0.1459034 |
| G6pc2 | 0.16587592 |
| G6pc2 | -0.3543082 |
| G6pc3 | 0.70079133 |
| G6pd2 | 0.98547966 |
| G6pd2 | -0.2807151 |
| G6pdx | -0.7674155 |
| Gaa | 1.46908655 |
| Gab1 | 1.30141224 |
| Gab2 | 1.18966028 |
| Gab2 | 1.23851411 |
| Gab2 | 1.62443876 |
| Gab3 | -0.4675999 |
| Gab3 | -0.4956482 |
| Gab3 | 0.8073142 |
| Gabarap | -0.0988987 |
| Gabarapl1 | 0.91236073 |
| Gabarapl2 | 0.24382581 |
| Gabbr1 | -0.5155714 |
| Gabpa | -0.8062968 |
| Gabpa | -0.5458908 |
| Gabpb1 | -0.0884518 |
| Gabpb1 | -0.7395175 |
| Gabpb2 | -0.4424358 |
| Gabra3 | -0.2381913 |
| Gabra4 | -0.7901493 |
| Gabrb1 | -0.7124283 |
| Gabrb2 | -0.5836222 |
| Gabrb3 | -0.6878943 |
| Gabrb3 | -0.6509661 |
| Gabrd | -0.6330353 |
| Gabrd | -0.0856228 |
| Gabrg1 | 1.58012687 |
| Gabrg2 | 1.29067924 |
| Gabrg2 | 0.30094732 |
| Gabrg2 | -0.8352442 |
| Gabrg2 | -0.4725727 |
| Gabrp | -0.5622112 |
| Gabrp | -0.4383069 |
| Gad1 | -0.6674912 |
| Gad1 | -0.6398256 |
| Gadd45a | 0.8885972 |
| Gadd45a | 0.84684521 |
| Gadd45b | 0.07450246 |
| Gadd45g | 1.61441546 |
| Gadd45g | 1.51460509 |
| Gak | -0.8289652 |
| Gal | 0.17161354 |
| Gal3st1 | -0.3606055 |
| Gal3st2 | 0.56518674 |
| Gal3st3 | 1.75813298 |
| Galc | -0.8793179 |
| Galc | 0.84944517 |
| Gale | 0.33547793 |
| Gale | -0.8032478 |
| Galk1 | -0.0692888 |
| Galm | 2.63192697 |
| Galns | 1.53044038 |
| Galnt1 | 0.25925095 |
| Galnt1 | 0.15792873 |
| Galnt1 | -0.1806895 |
| Galnt1 | 0.3500637 |
| Galnt10 | 1.10222604 |
| Galnt10 | 0.83549437 |
| Galnt10 | 0.45921402 |
| Galnt11 | -0.5066642 |
| Galnt12 | -0.105608 |
| Galnt13 | -0.6789686 |
| Galnt2 | 0.81237051 |
| Galnt2 | 1.43039146 |
| Galnt2 | -0.5029777 |
| Galnt3 | 1.43400983 |
| Galnt9 | -0.7693705 |
| Galntl2 | 0.89636625 |
| Galntl4 | -0.3375145 |
| Galntl5 | 2.49286478 |
| Galt | 0.9136544 |
| Galt | 0.60176678 |
| Gamt | 2.90684641 |
| Gan | -0.3993072 |
| Ganab | -0.0969905 |
| Ganab | 1.57917603 |
| Ganab | 0.90014472 |
| Ganc | 1.68497263 |
| Gap43 | 0.79308278 |
| Gapdh | -0.7032664 |
| Gapdhs | 0.37282222 |
| Gapvd1 | 2.04058964 |
| Gapvd1 | 0.98067881 |
| Garnl3 | -0.9372115 |
| Garnl4 | -0.2017736 |
| Gars | 2.8930927 |
| Gart | -0.6701274 |
| Gart | -0.5015072 |
| Gas2l1 | 0.50510931 |
| Gas2l1 | 2.96257788 |
| Gas2l1 | 1.61913677 |
| Gas2l1 | 2.21908889 |
| Gas6 | -0.5685042 |
| Gas7 | 0.74691767 |
| Gas7 | -0.6745375 |
| Gas8 | -0.5482049 |
| Gata2 | -0.1423949 |
| Gata4 | -0.4521415 |
| Gata6 | -0.554404 |
| Gatad1 | -0.8426233 |
| Gatad1 | -0.2732043 |
| Gatad2b | 1.09706114 |
| Gats | -0.7901493 |
| Gats | 3.70941333 |
| Gba | 0.28620064 |
| Gba2 | 1.26507157 |
| Gbas | 2.23882517 |
| Gbf1 | -0.2760859 |
| Gbl | -0.4273848 |
| Gbl | -0.4754261 |
| Gbl | 0.42495545 |
| Gbp2 | -0.1610908 |
| Gbp3 | -0.6055354 |
| Gbp3 | 0.89455374 |
| Gbp6 | -0.6763074 |
| Gbp6 | 2.12712991 |
| Gbx2 | 1.80423905 |
| Gca | -0.4626536 |
| Gca | -0.2783976 |
| Gcap14 | -0.5260717 |
| Gcap14 | 0.43821884 |
| Gcc1 | -0.3933988 |
| Gcc2 | -0.4052532 |
| Gcdh | 2.3462559 |
| Gch1 | -0.5949204 |
| Gch1 | 2.95382365 |
| Gchfr | 1.00955836 |
| Gck | -0.741415 |
| Gclm | -0.2277189 |
| Gcn5l2 | 2.33017508 |
| Gcnt1 | 3.68440294 |
| Gcnt1 | -0.6113014 |
| Gcnt2 | 1.40341982 |
| Gcnt2 | 0.08436651 |
| Gcs1 | -0.3131769 |
| Gda | 1.07066303 |
| Gdap1 | -0.3599739 |
| Gdap1 | 0.64260094 |
| Gdap1l1 | 1.10670559 |
| Gdap1l1 | -0.8258415 |
| Gde1 | 1.44642786 |
| Gde1 | -0.741415 |
| Gdf10 | 0.57266475 |
| Gdf15 | 0.9016191 |
| Gdf3 | -0.6816374 |
| Gdf9 | 2.8127224 |
| Gdi1 | 1.03175136 |
| Gdi1 | 0.48615304 |
| Gdi1 | 1.40914309 |
| Gdi1 | 0.91510764 |
| Gdi1 | 3.5753181 |
| Gdi2 | -0.7291497 |
| Gdnf | -0.081864 |
| Gdpd1 | 2.08826979 |
| Gdpd2 | 1.07801071 |
| Gdpd2 | 1.28222108 |
| Gdpd3 | -0.0321029 |
| Gdpd4 | -0.6154419 |
| Gdpd5 | -0.4445071 |
| Gemin4 | -0.3399835 |
| Gemin4 | 0.92425936 |
| Gemin4 | 0.19351993 |
| Gemin5 | 1.0472869 |
| Gemin6 | -0.8575588 |
| Gemin6 | -0.6718889 |
| Gemin7 | 0.32582815 |
| Gemin7 | -0.7851599 |
| Gfap | 0.16659505 |
| Gfer | -0.6852076 |
| Gfi1 | -0.4431257 |
| Gfm1 | -0.2222539 |
| Gfod1 | -0.388178 |
| Gfod2 | 0.48205879 |
| Gfpt2 | -0.2033742 |
| Gfra1 | -0.2222539 |
| Gfra4 | 0.00707657 |
| Gga1 | 1.63897758 |
| Gga1 | -0.2255291 |
| Gga1 | -0.5321252 |
| Gga3 | -0.0804584 |
| Ggcx | -0.0688263 |
| Ggcx | -0.5029777 |
| Ggcx | -0.6683691 |
| Ggn | -0.8175623 |
| Ggn | 2.13630284 |
| Ggnbp1 | -0.5208068 |
| Ggnbp2 | 1.63734017 |
| Ggt5 | 0.85531177 |
| Ggt7 | -0.7161184 |
| Ggta1 | 0.54646507 |
| Ggtla1 | -0.235424 |
| Gh | -0.4833184 |
| Ghitm | -0.7291497 |
| Ghr | -0.4570324 |
| Ghr | -0.4797227 |
| Ghrh | -0.4493581 |
| Ghrhr | 1.78217237 |
| Ghrl | -0.2206206 |
| Ghrl | -0.5804191 |
| Ghsr | 0.60963025 |
| Ghsr | -0.4139092 |
| Gif | 0.17873638 |
| Gigyf1 | -0.1499293 |
| Gimap1 | 0.45842529 |
| Gimap4 | -0.3855785 |
| Gimap5 | -0.7329065 |
| Gimap6 | 2.38010359 |
| Gimap8 | -0.3307583 |
| Gimap9 | -0.5812188 |
| Gins3 | 1.94991336 |
| Gins4 | -0.3000566 |
| Gipc1 | -0.7480867 |
| Gipc1 | 0.50835632 |
| Gipc1 | 0.90358133 |
| Gipc2 | 0.14109021 |
| Git1 | 0.10609131 |
| Git2 | -0.8032478 |
| Git2 | 0.70423486 |
| Git2 | 0.78755092 |
| Gja1 | 0.9360084 |
| Gja4 | 0.25696966 |
| Gja5 | -0.4683087 |
| Gja5 | 2.15064856 |
| Gja6 | 0.04835774 |
| Gja7 | -0.388829 |
| Gjb1 | 0.38513556 |
| Gjb2 | -0.269758 |
| Gjb2 | -0.6104755 |
| Gjb3 | 0.7036281 |
| Gjb6 | 1.15623538 |
| Gjc2 | 1.34345855 |
| Gjc2 | 0.66984439 |
| Gjd2 | 1.62963574 |
| Gjd2 | -0.6869979 |
| Gjd3 | -0.7105887 |
| Gk2 | 0.52123268 |
| Gk5 | -0.6398256 |
| Gkap1 | 0.54094321 |
| Gkap1 | 1.8533346 |
| Glb1 | 1.56318468 |
| Glb1l2 | -0.5306082 |
| Glcci1 | -0.5567393 |
| Gldc | 0.92760859 |
| Gldc | -0.6071792 |
| Gldn | -0.1948689 |
| Gle1 | -0.1075338 |
| Glg1 | 1.01145229 |
| Glipr2 | 1.77622025 |
| Glis1 | 1.32927253 |
| Glo1 | -0.7635176 |
| Gloxd1 | 1.27060255 |
| Glp2r | 0.11790385 |
| Glra2 | 2.18874215 |
| Glrb | 0.5858385 |
| Glrp1 | -0.6483834 |
| Glrx | 0.59497359 |
| Glrx2 | 0.85754519 |
| Glrx2 | -0.5606445 |
| Glrx2 | -0.5708751 |
| Glrx3 | 0.3491772 |
| Glrx5 | 1.00707586 |
| Gls2 | 1.90865615 |
| Gls2 | 1.07434409 |
| Glt25d1 | 0.06373101 |
| Glt25d2 | -0.4212967 |
| Glt25d2 | 0.55599127 |
| Glt6d1 | -0.6683691 |
| Glt8d1 | 1.462014 |
| Glt8d1 | -0.3701317 |
| Glt8d1 | 0.62538145 |
| Glt8d2 | 1.74594367 |
| Glt8d3 | 0.00579671 |
| Gltp | 1.08972846 |
| Gltscr1 | -0.8216927 |
| Glud1 | 2.23604231 |
| Glud1 | -0.1590515 |
| Glul | -0.8237648 |
| Glyctk | 0.53174804 |
| Glyctk | -0.6887916 |
| Glyctk | -0.7782192 |
| Gm1006 | 0.97887212 |
| Gm1006 | -0.5567393 |
| Gm1019 | -0.2906289 |
| Gm1040 | 0.73427711 |
| Gm1070 | 3.60825537 |
| Gm1082 | -0.3264841 |
| Gm114 | -0.2459836 |
| Gm114 | 0.17161354 |
| Gm1141 | -0.5451207 |
| Gm128 | 1.14039581 |
| Gm129 | -0.354936 |
| Gm129 | -0.1953982 |
| Gm1322 | -0.6834208 |
| Gm136 | 1.02689674 |
| Gm136 | 1.1492287 |
| Gm1381 | -0.6561531 |
| Gm1527 | -0.5133367 |
| Gm1568 | -0.3920908 |
| Gm1574 | 0.74052152 |
| Gm1574 | -0.5185595 |
| Gm1604 | -0.5283372 |
| Gm1604 | -0.8543382 |
| Gm166 | -0.7023551 |
| Gm166 | -0.7752603 |
| Gm1661 | 0.1521062 |
| Gm1673 | -0.6113014 |
| Gm1673 | -0.2801352 |
| Gm1698 | 2.16206213 |
| Gm1821 | -0.6372734 |
| Gm1943 | -0.8405091 |
| Gm1965 | -0.4563321 |
| Gm1967 | -0.7319659 |
| Gm1967 | 1.35511793 |
| Gm216 | -0.865117 |
| Gm239 | 2.31055629 |
| Gm249 | 1.00634446 |
| Gm266 | 1.70201843 |
| Gm2a | -0.0523264 |
| Gm347 | -0.4145784 |
| Gm382 | 0.14145951 |
| Gm410 | 0.1542939 |
| Gm428 | -0.6389741 |
| Gm443 | -0.3350521 |
| Gm484 | -0.5981737 |
| Gm50 | 0.13516149 |
| Gm525 | -0.4396812 |
| Gm525 | 0.49302168 |
| Gm527 | -0.095562 |
| Gm527 | 1.55288625 |
| Gm561 | 0.43633556 |
| Gm561 | -0.7752603 |
| Gm597 | -0.4369346 |
| Gm603 | 1.42853044 |
| Gm609 | -0.7142716 |
| Gm614 | -0.7538435 |
| Gm614 | -0.8002088 |
| Gm648 | 1.76712313 |
| Gm648 | 1.28167627 |
| Gm71 | -0.572459 |
| Gm711 | 0.89191108 |
| Gm732 | 1.13594787 |
| Gm732 | -0.5606445 |
| Gm767 | 1.59114904 |
| Gm773 | -0.5133367 |
| Gm773 | -0.7577011 |
| Gm784 | 1.93062884 |
| Gm813 | -0.8447424 |
| Gm813 | -0.5283372 |
| Gm817 | -0.6458078 |
| Gm867 | -0.7452216 |
| Gm884 | -0.5088831 |
| Gm889 | 0.67985935 |
| Gm906 | -0.8279228 |
| Gm93 | 0.38257174 |
| Gm93 | -0.3030223 |
| Gm944 | 1.14947984 |
| Gm949 | 0.52049462 |
| Gm962 | 1.28298328 |
| Gm962 | 2.52536492 |
| Gm97 | 0.52613827 |
| Gm97 | 0.77900463 |
| Gmcl1 | 1.07570378 |
| Gmcl1 | -0.6121281 |
| Gmcl1 | 1.88404002 |
| Gmds | -0.1539726 |
| Gmeb2 | 0.32522172 |
| Gmfg | -0.6587574 |
| Gmfg | 0.64734029 |
| Gmfg | 0.20566338 |
| Gmip | 1.24839241 |
| Gmip | -0.401285 |
| Gmppa | 1.27214538 |
| Gmppa | 0.64734029 |
| Gmppb | 1.2414772 |
| Gmpr | 1.39843114 |
| Gmpr2 | 0.46966729 |
| Gna11 | -0.8144764 |
| Gna13 | -0.4626536 |
| Gna14 | -0.4112373 |
| Gnai2 | -0.7452216 |
| Gnao1 | -0.0505109 |
| Gnao1 | -0.6905887 |
| Gnaq | 0.62273785 |
| Gnaq | -0.8144764 |
| Gna-rs1 | -0.7161184 |
| Gnas | -0.7811875 |
| Gnas | 1.01696651 |
| Gnas | 1.07107275 |
| Gnas | 0.75938953 |
| Gnas | -0.2249824 |
| Gnat1 | -0.7811875 |
| Gnat2 | 3.00324441 |
| Gnaz | 2.50034971 |
| Gnb1 | -0.6570204 |
| Gnb1l | 2.26939443 |
| Gnb2 | -0.343079 |
| Gnb2l1 | 0.1917722 |
| Gnb3 | 0.52221586 |
| Gnb4 | -0.1631346 |
| Gnb4 | 1.48316092 |
| Gnb5 | 0.2351821 |
| Gnb5 | -0.0582511 |
| Gne | -0.200708 |
| Gng10 | -0.5260717 |
| Gng11 | 0.82008268 |
| Gng13 | 3.03457974 |
| Gng2 | -0.0127432 |
| Gng2 | 1.28602588 |
| Gng3 | 0.81110899 |
| Gng4 | -0.3682187 |
| Gng7 | -0.8196252 |
| Gng8 | -0.6088259 |
| Gngt2 | 0.51382568 |
| Gnl2 | 1.31812307 |
| Gnl2 | -0.81345 |
| Gnl3 | 0.17089826 |
| Gnl3 | -0.1008107 |
| Gnl3 | -0.1104301 |
| Gnl3l | 1.83035216 |
| Gnl3l | -0.7931558 |
| Gnmt | -0.4570324 |
| Gnmt | 1.60782681 |
| Gnpat | -0.5949204 |
| Gnpda1 | -0.661369 |
| Gnpda1 | 0.24844722 |
| Gnpda1 | 2.20710058 |
| Gnpda2 | 1.42301873 |
| Gnptab | -0.4963786 |
| Gnptg | 1.10657414 |
| Gnptg | 1.65687515 |
| Gnrhr | 0.2049737 |
| Gns | 2.12779126 |
| Golga1 | 0.74401575 |
| Golga2 | -0.6674912 |
| Golga2 | -0.7752603 |
| Golga2 | -0.4854825 |
| Golga2 | -0.446583 |
| Golga2 | 0.62251721 |
| Golga3 | 0.38967617 |
| Golga4 | -0.6789686 |
| Golga5 | 0.14440867 |
| Golga7 | 0.40595337 |
| Golim4 | -0.572459 |
| Golm1 | 1.17474851 |
| Golm1 | 0.46836701 |
| Golph3l | -0.0846817 |
| Golt1a | -0.6807469 |
| Golt1b | 0.68565223 |
| Gopc | -0.6843138 |
| Gorasp1 | -0.5321252 |
| Gorasp2 | 1.74965021 |
| Gorasp2 | -0.5505247 |
| Gorasp2 | -0.6415309 |
| Gorasp2 | -0.143396 |
| Gosr1 | -0.3449412 |
| Gosr2 | 1.62783137 |
| Gosr2 | 2.11871202 |
| Gosr2 | 1.61852891 |
| Gosr2 | 3.21048192 |
| Got1l1 | -0.2830383 |
| Got2 | 1.78134263 |
| Gp1bb | -0.7538435 |
| Gp38 | -0.8500612 |
| Gp5 | 0.04713322 |
| Gp9 | -0.6710077 |
| Gpaa1 | 1.27313585 |
| Gpam | -0.166722 |
| Gpatch1 | -0.8144764 |
| Gpatch2 | 0.10070727 |
| Gpatch3 | -0.232114 |
| Gpatch4 | -0.8575588 |
| Gpbp1 | 0.62384026 |
| Gpbp1 | -0.908384 |
| Gpbp1l1 | -0.6561531 |
| Gpc1 | -0.856484 |
| Gpc3 | -0.0123079 |
| Gpc3 | 1.06353245 |
| Gpc4 | -0.0365594 |
| Gpc5 | 0.6936615 |
| Gpc5 | 2.4760138 |
| Gpc5 | -0.1953982 |
| Gpd1 | -0.6121281 |
| Gpd2 | 1.94322506 |
| Gpd2 | 1.43881263 |
| Gper | -0.6905887 |
| Gpha2 | 0.96689006 |
| Gphb5 | 0.72384212 |
| Gphn | 2.06781708 |
| Gphn | -0.0596237 |
| Gphn | -0.5359287 |
| Gpihbp1 | -0.0804584 |
| Gpkow | 1.46309176 |
| Gpld1 | 1.63300957 |
| Gpld1 | -0.2493431 |
| Gpm6a | -0.7606049 |
| Gpm6a | 1.02746918 |
| Gpm6b | 1.01826776 |
| Gpnmb | 0.6866829 |
| Gpnmb | 1.42778499 |
| Gpr1 | -0.6596271 |
| Gpr1 | 1.22344008 |
| Gpr103 | 2.43808906 |
| Gpr107 | -0.7263419 |
| Gpr108 | 0.37022435 |
| Gpr108 | -0.6887916 |
| Gpr108 | -0.3412205 |
| Gpr109a | -0.1279984 |
| Gpr113 | 0.14881502 |
| Gpr113 | -0.7538435 |
| Gpr114 | 1.6010365 |
| Gpr12 | 2.49492019 |
| Gpr12 | -0.6526919 |
| Gpr120 | 0.46732548 |
| Gpr123 | -0.1304648 |
| Gpr124 | 0.10532406 |
| Gpr128 | 2.91720565 |
| Gpr135 | 2.85600819 |
| Gpr137 | 1.8237852 |
| Gpr137b | 0.49175351 |
| Gpr141 | -0.1874898 |
| Gpr146 | 0.32613122 |
| Gpr146 | 0.4748504 |
| Gpr149 | 0.51407353 |
| Gpr151 | -0.6570204 |
| Gpr153 | -0.6254547 |
| Gpr156 | 1.82895751 |
| Gpr156 | -0.696906 |
| Gpr158 | -0.0842115 |
| Gpr162 | 0.57266475 |
| Gpr162 | -0.6950967 |
| Gpr17 | 1.42208131 |
| Gpr172b | 1.17731132 |
| Gpr172b | -0.4993058 |
| Gpr176 | 0.0520217 |
| Gpr177 | 0.52024847 |
| Gpr18 | 3.30669421 |
| Gpr180 | 1.66558861 |
| Gpr182 | 1.97349851 |
| Gpr182 | -0.6237784 |
| Gpr19 | 1.94590613 |
| Gpr20 | -0.5366913 |
| Gpr21 | -0.3714092 |
| Gpr22 | 0.87471764 |
| Gpr23 | -0.7376239 |
| Gpr26 | -0.7713297 |
| Gpr27 | 1.41142262 |
| Gpr27 | 2.63022935 |
| Gpr27 | -0.8629512 |
| Gpr34 | 0.10263371 |
| Gpr34 | 1.6955314 |
| Gpr37l1 | 0.00065967 |
| Gpr45 | -0.5148259 |
| Gpr6 | -0.7762456 |
| Gpr64 | 0.82257994 |
| Gpr65 | 3.82126968 |
| Gpr68 | -0.6570204 |
| Gpr75 | 1.62798187 |
| Gpr81 | 1.60382032 |
| Gpr83 | 0.23884882 |
| Gpr85 | -0.5637805 |
| Gpr85 | -0.672771 |
| Gpr89 | 0.93711218 |
| Gpr89 | -0.5366913 |
| Gpr97 | -0.6666141 |
| Gprasp1 | -0.7376239 |
| Gprasp1 | 0.7882904 |
| Gprc2a-rs5 | 0.79858208 |
| Gprc2a-rs5 | 0.22003025 |
| Gprc5a | -0.7310262 |
| Gprc5b | 3.1602721 |
| Gprc5c | -0.4782883 |
| Gprk6 | -0.0790549 |
| Gprk6 | 1.46569124 |
| Gps1 | -0.6080022 |
| Gps1 | -0.5170643 |
| Gps1 | -0.5328847 |
| Gps1 | -0.5328847 |
| Gps2 | 0.69509182 |
| Gpsm1 | 1.31139009 |
| Gpsm2 | -0.3036166 |
| Gpsm3 | -0.1474111 |
| Gpsn2 | -0.6080022 |
| Gpsn2 | -0.5253177 |
| Gpsn2 | 1.3792864 |
| Gpt1 | -0.6807469 |
| Gpt2 | 0.49175351 |
| Gpx1 | 2.12066819 |
| Gpx2 | 2.06617078 |
| Gpx3 | 0.1865093 |
| Gpx4 | 0.51901674 |
| Gpx4 | 0.42631751 |
| Gpx6 | -0.5836222 |
| Gpx7 | -0.5081428 |
| Gramd1a | -0.4768561 |
| Gramd1b | -0.4287431 |
| Gramd2 | 0.31883031 |
| Gramd2 | -0.1874898 |
| Gramd3 | -0.6080022 |
| Gramd4 | 0.05567135 |
| Grap2 | -0.5389827 |
| Grasp | -0.6798573 |
| Grasp | -0.7142716 |
| Grb10 | 0.11903912 |
| Grb14 | 0.00280353 |
| Grb14 | 0.90341797 |
| Grb2 | 0.30838873 |
| Grb7 | -0.9372115 |
| Grcc10 | 2.57644908 |
| Greb1 | 0.09684246 |
| Greb1 | 1.3500665 |
| Grem1 | 0.21184767 |
| Grem2 | -0.4341961 |
| Grhl1 | 1.4409291 |
| Grhl2 | 1.30917022 |
| Grhpr | -0.43488 |
| Gria1 | -0.6221052 |
| Gria1 | -0.557519 |
| Gria1 | -0.6204349 |
| Gria2 | 2.63022935 |
| Gria2 | 0.58652674 |
| Gria3 | -0.7105887 |
| Gria3 | 0.15829143 |
| Gria3 | -0.5812188 |
| Grid2 | 2.21323464 |
| Grik1 | -0.3986488 |
| Grik2 | -0.7069204 |
| Grik4 | 2.19261553 |
| Grik5 | -0.4761409 |
| Grin1 | 2.49714922 |
| Grin1 | 1.27049225 |
| Grin2a | 1.71800753 |
| Grin2b | 2.43269787 |
| Grin2c | -0.5230596 |
| Grin3b | -0.7005352 |
| Grina | -0.2778192 |
| Grip1 | 2.62615279 |
| Grip1 | 0.85599955 |
| Grip1 | -0.6950967 |
| Gripap1 | -0.3771798 |
| Gripap1 | -0.5590805 |
| Grit | 0.36935678 |
| Grk4 | 0.69407039 |
| Grk4 | -0.6843138 |
| Grk4 | -0.7041785 |
| Grk5 | -0.6692478 |
| Grlf1 | -0.5876416 |
| Grm1 | 1.91088237 |
| Grm7 | 2.07194359 |
| Grm8 | 0.0002303 |
| Grn | 1.51528443 |
| Grp | -0.4949184 |
| Grpel1 | 0.3142381 |
| Grpr | -0.5260717 |
| Grsf1 | 1.22309201 |
| Grtp1 | -0.522308 |
| Grwd1 | 0.24448742 |
| Grwd1 | -0.2282672 |
| Gsbs | 0.07569166 |
| Gsc | -0.7452216 |
| Gsdma1 | -0.5163176 |
| Gsdmdc1 | 1.18132446 |
| Gsdmdc1 | -0.1032063 |
| Gsdmdc1 | 0.89323334 |
| Gse1 | -0.6666141 |
| Gsg1 | 0.04998794 |
| Gsg1 | 0.62867547 |
| Gsg1 | -0.3855785 |
| Gsg2 | -0.3083845 |
| Gsk3a | 1.45316541 |
| Gsk3b | -0.3855785 |
| Gsn | 1.23405179 |
| Gspt1 | 0.33637751 |
| Gspt2 | -0.1539726 |
| Gss | 0.21321642 |
| Gss | -0.6432393 |
| Gss | 1.37663168 |
| Gsta2 | 0.3160777 |
| Gsta3 | 0.23785023 |
| Gsta3 | -0.0316585 |
| Gsta4 | -0.5336448 |
| Gstcd | 2.30104806 |
| Gstk1 | -0.2471021 |
| Gstm1 | -0.3894805 |
| Gstm1 | 2.73604718 |
| Gstm2 | 0.02898679 |
| Gstm2 | -0.0206147 |
| Gstm2 | -0.2871179 |
| Gstm4 | 1.73882037 |
| Gstm5 | 0.0197797 |
| Gstm6 | -0.4854825 |
| Gstm6 | -0.3277033 |
| Gsto1 | 3.22413531 |
| Gsto2 | -0.5836222 |
| Gsto2 | -0.6878943 |
| Gstp1 | -0.4389938 |
| Gstp1 | -0.804263 |
| Gstp2 | -0.4941892 |
| Gstt1 | 0.67110215 |
| Gstt2 | 0.74653127 |
| Gstt3 | -0.3344375 |
| Gstt4 | 1.11599128 |
| Gstz1 | 0.46262418 |
| Gtdc1 | -0.7567352 |
| Gtf2a1 | 1.06545758 |
| Gtf2e1 | 1.23611397 |
| Gtf2e2 | -0.6657379 |
| Gtf2e2 | 1.6505067 |
| Gtf2f1 | 0.83830124 |
| Gtf2f2 | -0.062375 |
| Gtf2h1 | 2.24526056 |
| Gtf2h1 | 0.61986549 |
| Gtf2h2 | 0.8231142 |
| Gtf2h3 | 0.5068591 |
| Gtf2h4 | -0.6137835 |
| Gtf2h5 | 1.52188221 |
| Gtf2ird1 | -0.4862049 |
| Gtf2ird1 | -0.5700841 |
| Gtf2ird2 | 1.07202807 |
| Gtf2ird2 | 0.03727887 |
| Gtf2ird2 | -0.1423949 |
| Gtf3a | -0.5520745 |
| Gtf3a | 0.27826081 |
| Gtf3c1 | -0.429423 |
| Gtf3c2 | -0.0946108 |
| Gtf3c3 | -0.5466615 |
| Gtf3c4 | 0.7254247 |
| Gtf3c4 | -0.0365594 |
| Gtf3c5 | -0.3524273 |
| Gtf3c5 | 0.15320069 |
| Gtf3c5 | -0.0842115 |
| Gtl3 | 0.19386908 |
| Gtpbp1 | 1.69075954 |
| Gtpbp2 | 0.5844605 |
| Gtpbp3 | -0.6701274 |
| Gtpbp4 | -0.6313454 |
| Gtpbp5 | 0.45605511 |
| Gtpbp6 | 0.22847825 |
| Gtpbp8 | 0.02313824 |
| Gtpbp8 | -0.1484176 |
| Gtrgeo22 | 1.55418995 |
| Gtse1 | 0.17767141 |
| Gtse1 | -0.0950863 |
| Gtse1 | -0.3663096 |
| Gtsf1l | 0.01514191 |
| Guca1a | -0.7319659 |
| Guca1b | -0.4226462 |
| Guca2a | 2.10787932 |
| Guca2b | 0.42413726 |
| Gucy1a2 | 2.03032419 |
| Gucy1a3 | -0.6987189 |
| Gucy1a3 | 0.10724101 |
| Gucy1a3 | -0.3387482 |
| Gucy1b3 | 1.18556207 |
| Guk1 | 1.48780567 |
| Gulo | 1.80118091 |
| Gulp1 | -0.4833184 |
| Gusb | -0.3726885 |
| Gusb | 0.1495474 |
| Gusb | -0.4704383 |
| Gvin1 | 1.73063167 |
| Gyg | 0.74479048 |
| Gyg | 1.14935428 |
| Gykl1 | 1.27192513 |
| Gyltl1b | -0.5148259 |
| Gyltl1b | 1.25961926 |
| Gypa | 0.69753873 |
| Gypc | 0.10224874 |
| Gys1 | 0.26509167 |
| Gys3 | 0.14072077 |
| Gzf1 | -0.6780807 |
| Gzf1 | -0.3511754 |
| Gzmd | 2.6254285 |
| Gzmd | 0.6413042 |
| Gzmk | -0.3530538 |
| Gzmm | -0.6587574 |
| Gzmn | 0.94292492 |
| H13 | -0.2033742 |
| H13 | 2.21596584 |
| H13 | -0.3771798 |
| H13 | 0.69570415 |
| H19 | 1.63091173 |
| H1f0 | -0.3593426 |
| H1fnt | 0.11448976 |
| H1foo | -0.1595609 |
| H1fx | -0.6381234 |
| H1fx | 0.54478795 |
| H2-Aa | 2.86784206 |
| H2-Ab1 | -0.6088259 |
| H2-Ab1 | 1.31244532 |
| H2-Ab1 | 0.79565314 |
| H2afj | 0.01175446 |
| H2afx | 3.14123032 |
| H2afy | -0.6950967 |
| H2afz | -0.0573372 |
| H2-Bl | 0.06413218 |
| H2-DMa | 1.63285988 |
| H2-DMb1 | -0.7347906 |
| H2-Eb1 | 1.68291743 |
| H2-K1 | -0.6666141 |
| H2-Ke6 | -0.0739266 |
| H2-M10.3 | 1.73278648 |
| H2-M2 | 0.19804879 |
| H2-M3 | -0.3368983 |
| H2-Oa | 1.43890475 |
| H2-Ob | 0.61986549 |
| H2-Q1 | 1.76315989 |
| H2-Q2 | -0.2249824 |
| H2-Q7 | -0.2680396 |
| H2-Q8 | -0.5653524 |
| H2-T10 | 0.53028786 |
| H2-T23 | 0.30343438 |
| H2-T23 | -0.7078361 |
| H2-T24 | 3.8709893 |
| H2-Tw3 | -0.1504338 |
| H3f3a | -0.4355644 |
| H3f3a | 0.04713322 |
| H3f3b | 0.92041692 |
| H47 | 0.98547966 |
| H47 | -0.6950967 |
| H6pd | 0.10877174 |
| Habp4 | -0.6807469 |
| Habp4 | 0.3375756 |
| Hace1 | -0.8405091 |
| Hacl1 | -0.7105887 |
| Hadhb | -0.4840392 |
| Hagh | -0.8881654 |
| Haghl | 2.82555238 |
| Haghl | 2.38951055 |
| Hal | -0.0979441 |
| Hamp2 | -0.7170432 |
| Hao1 | -0.8500612 |
| Hao3 | -0.0079653 |
| Hap1 | 0.1847484 |
| Hap1 | 2.00211636 |
| Hap1 | 0.600638 |
| Hapln1 | 0.23718391 |
| Hapln2 | -0.43488 |
| Hapln4 | 0.69079427 |
| Harbi1 | -0.1113975 |
| Hars | 2.30561389 |
| Has1 | -0.4898257 |
| Hat1 | 0.21492454 |
| Havcr1 | -0.5351668 |
| Havcr2 | 1.38115003 |
| Hax1 | -0.4747119 |
| Hba-a1 | -0.8352442 |
| Hbb-b1 | -0.0523264 |
| Hbegf | -0.4971095 |
| Hbp1 | -0.3836337 |
| Hbp1 | 1.29884754 |
| Hbs1l | -0.5260717 |
| Hbs1l | -0.4192762 |
| Hbs1l | 0.09993559 |
| Hbs1l | 0.30591483 |
| Hc | -0.4528386 |
| Hccs | -0.4535363 |
| Hcfc1 | -0.2611975 |
| Hcfc1r1 | -0.7732929 |
| Hcfc2 | 3.08193002 |
| Hcls1 | -0.3511754 |
| Hcn2 | 2.25114996 |
| Hcn3 | 1.26129172 |
| Hcn3 | 0.59905543 |
| Hcr | -0.4417464 |
| Hcst | 0.14293525 |
| Hdac10 | -0.4963786 |
| Hdac11 | 0.30219167 |
| Hdac2 | 0.01556448 |
| Hdac3 | -0.5924879 |
| Hdac4 | -0.2282672 |
| Hdac5 | -0.0860937 |
| Hdac6 | 2.08624454 |
| Hdac7 | 0.35800452 |
| Hdac8 | -0.2824569 |
| Hdac9 | -0.7069204 |
| Hdc | -0.6604976 |
| Hddc2 | -0.4647702 |
| Hddc2 | -0.5374544 |
| Hdgf | 1.13722087 |
| Hdgf | -0.6544209 |
| Hdgf | -0.4985731 |
| Hdgf | 0.08867393 |
| Hdgfl1 | 1.43696832 |
| Hdgfrp2 | -0.5155714 |
| Hdhd1a | 2.26419076 |
| Hdhd2 | -0.2376372 |
| Hdhd2 | 0.9290403 |
| Hdhd2 | 0.09800358 |
| Hdlbp | 1.47539754 |
| Hdlbp | 1.58762036 |
| Heatr1 | 1.17633584 |
| Heatr1 | -0.2398556 |
| Heatr3 | -0.3555643 |
| Heatr5a | -0.4260284 |
| Hebp1 | 3.74831971 |
| Hebp2 | -0.6561531 |
| Heca | 1.11494961 |
| Hectd2 | -0.3940534 |
| Hectd2 | 2.59020051 |
| Hectd3 | -0.4782883 |
| Hecw2 | 0.41122449 |
| Hecw2 | 0.62141324 |
| Hecw2 | -0.6381234 |
| Heg1 | 2.68305512 |
| Hel308 | 1.45343753 |
| Helb | -0.6406778 |
| Helb | -0.500039 |
| Helz | -0.5748399 |
| Hemgn | 1.43512036 |
| Hemk1 | -0.661369 |
| Hemt1 | -0.7207516 |
| Hepacam | 1.6765103 |
| Heph | 0.85823132 |
| Heph | 0.85823132 |
| Heph | 2.00141023 |
| Heph | -0.5804191 |
| Heph | 2.30382674 |
| Herc2 | -0.4956482 |
| Herc3 | -0.2801352 |
| Herc3 | 0.34354313 |
| Herc4 | -0.0692888 |
| Herc4 | 0.46052709 |
| Herpud1 | 1.25121179 |
| Herpud1 | 0.88527153 |
| Herpud2 | -0.7329065 |
| Herpud2 | -0.7713297 |
| Herpud2 | 1.81569573 |
| Herpud2 | -0.0036429 |
| Hes1 | -0.7096703 |
| Hes5 | -0.7263419 |
| Hes5 | 0.04549803 |
| Hes6 | -0.6466656 |
| Hes6 | -0.0753224 |
| Hes6 | 1.56777016 |
| Hexa | 0.26767587 |
| Hexb | -0.6648625 |
| Hexdc | -0.4619491 |
| Hexdc | 0.1987436 |
| Hexim1 | 1.32595203 |
| Hexim2 | 1.7969203 |
| Hey1 | -0.6364242 |
| Hey2 | 0.7132893 |
| Hey2 | 1.84251231 |
| Heyl | 0.59497359 |
| Hfe | 0.52197016 |
| Hfe | 0.05890356 |
| Hgfac | -0.6905887 |
| Hgs | 0.53199118 |
| Hgs | 0.78681086 |
| Hgsnat | 1.42703895 |
| Hhat | 1.9096586 |
| Hhat | -0.6432393 |
| Hhat | -0.2476618 |
| Hhatl | -0.7490437 |
| Hhex | -0.2560986 |
| Hhip | -0.2680396 |
| Hibadh | 0.2125323 |
| Hic1 | -0.0927114 |
| Hic2 | -0.8155039 |
| Hif1a | 1.79478269 |
| Hif3a | -0.7723108 |
| Hif3a | 2.88960778 |
| Higd1a | -0.2315635 |
| Higd1b | 0.52246149 |
| Higd2a | 1.24748841 |
| Hils1 | -0.1349208 |
| Hint1 | 1.4409291 |
| Hint2 | -0.6154419 |
| Hint3 | -0.0767204 |
| Hint3 | -0.3204119 |
| Hip1 | -0.6196009 |
| Hip1r | -0.3107777 |
| Hip2 | -0.2493431 |
| Hipk1 | 1.28689338 |
| Hipk2 | -0.425351 |
| Hipk3 | 2.75175364 |
| Hipk4 | -0.1359139 |
| Hira | -0.5653524 |
| Hira | 3.04156852 |
| Hirip3 | 0.65141411 |
| Hisppd1 | -0.4239976 |
| Hist1h1b | -0.5520745 |
| Hist1h1c | -0.2293646 |
| Hist1h2aa | -0.6006212 |
| Hist1h2ac | 1.07828174 |
| Hist1h2ad | 2.33028155 |
| Hist1h2ae | -0.3024284 |
| Hist1h2af | 1.9402131 |
| Hist1h2ag | -0.6526919 |
| Hist1h2ah | 0.96856768 |
| Hist1h2ak | -0.2549693 |
| Hist1h2an | 0.65419127 |
| Hist1h2ao | 0.48513116 |
| Hist1h2bc | -0.7713297 |
| Hist1h2be | -0.1708388 |
| Hist1h2bf | 2.82886784 |
| Hist1h2bg | 1.23680035 |
| Hist1h2bh | 1.97950959 |
| Hist1h2bj | -0.5155714 |
| Hist1h2bk | -0.0753224 |
| Hist1h2bm | 0.46497778 |
| Hist1h2bn | 1.64844782 |
| Hist1h3a | -0.5740456 |
| Hist1h3d | -0.3325961 |
| Hist1h3e | -0.2493431 |
| Hist1h3f | -0.446583 |
| Hist1h4c | 1.1276308 |
| Hist1h4d | 1.29358932 |
| Hist1h4f | 0.24943455 |
| Hist1h4h | 1.57830358 |
| Hist1h4i | -0.4556324 |
| Hist1h4j | 1.51765823 |
| Hist1h4k | -0.7404658 |
| Hist1h4m | -0.4847606 |
| Hist2h2aa1 | -0.6415309 |
| Hist2h2ab | 0.37022435 |
| Hist2h2ac | -0.6587574 |
| Hist2h2bb | -0.0875078 |
| Hist2h2be | 1.10380951 |
| Hist2h3b | -0.4956482 |
| Hist2h3c1 | -0.7060055 |
| Hist2h3c1 | -0.362503 |
| Hist2h4 | 1.49901525 |
| Hist3h2a | -0.6587574 |
| Hist3h2bb | 0.06091803 |
| Hist4h4 | 0.84476002 |
| Hk1 | -0.4052532 |
| Hk3 | 0.71628806 |
| Hkdc1 | -0.8237648 |
| Hlcs | 1.94756453 |
| Hlf | -0.6701274 |
| Hltf | -0.3240505 |
| Hlx | -0.6544209 |
| Hmg20a | 3.3189467 |
| Hmg20a | -0.6313454 |
| Hmg20b | -0.5382182 |
| Hmga1 | -0.557519 |
| Hmgb2 | -0.0131788 |
| Hmgb2l1 | 1.62993613 |
| Hmgb2l1 | 2.0437294 |
| Hmgb4 | -0.2888717 |
| Hmgcl | -0.3511754 |
| Hmgcl | 0.73505995 |
| Hmgcl | 2.82613709 |
| Hmgcll1 | -0.6509661 |
| Hmgcs2 | -0.6196009 |
| Hmgn1 | 2.21375739 |
| Hmgn2 | 1.1487262 |
| Hmgn3 | 0.17232827 |
| Hmgn3 | 0.97887212 |
| Hmha1 | -0.5366913 |
| Hmox1 | 0.30931475 |
| Hmox2 | -0.4804408 |
| Hmx1 | -0.5148259 |
| Hmx1 | -0.1349208 |
| Hn1 | 1.74740156 |
| Hn1 | 2.07063258 |
| Hn1l | -0.6212697 |
| Hnf1b | 0.09722966 |
| Hnf1b | -0.5900616 |
| Hnf4a | 3.08734428 |
| Hnrnpc | 1.86997567 |
| Hnrpa1 | 0.71148542 |
| Hnrpa1 | 1.02818424 |
| Hnrpab | -0.0131788 |
| Hnrpc | 3.66181723 |
| Hnrpdl | -0.7235426 |
| Hnrpdl | -0.6212697 |
| Hnrpf | 0.82043984 |
| Hnrph1 | -0.2098044 |
| Hnrph1 | -0.7096703 |
| Hnrph1 | 0.75211851 |
| Hnrph1 | 0.53441912 |
| Hnrph1 | 0.24415667 |
| Hnrph2 | -0.5748399 |
| Hnrph3 | 2.68208142 |
| Hnrpk | 1.69797805 |
| Hnrpl | 0.53077484 |
| Hnrpll | 1.17156573 |
| Hnrpll | -0.5051879 |
| Hnrpm | 0.55741186 |
| Hnrpm | -0.3277033 |
| Hnrpm | 3.00856624 |
| Hnrpul1 | 1.92209941 |
| Hnrpul1 | 0.47665771 |
| Hnrpul1 | -0.2982816 |
| Hnrpul1 | 0.19491577 |
| Hnrpul1 | -0.3065937 |
| Hnt | 0.10762393 |
| Homer1 | 1.11403719 |
| Homer1 | 0.12994282 |
| Homer1 | -0.4065797 |
| Homer1 | -0.2266233 |
| Homez | -0.4818785 |
| Homez | 2.04005725 |
| Hook1 | 0.19002119 |
| Hook1 | -0.4949184 |
| Hook1 | 3.01764132 |
| Hook1 | 1.16046395 |
| Hook2 | 1.18507874 |
| Hormad2 | -0.4280637 |
| Hoxa13 | 1.45180361 |
| Hoxa2 | -0.7851599 |
| Hoxa2 | 0.05890356 |
| Hoxa4 | -0.3688559 |
| Hoxa5 | 0.1977012 |
| Hoxa7 | 1.33433429 |
| Hoxa9 | 1.32168033 |
| Hoxa9 | 2.03976673 |
| Hoxb2 | 2.70079254 |
| Hoxb3 | 2.33724671 |
| Hoxb3 | -0.2510274 |
| Hoxb4 | 0.01260247 |
| Hoxb5 | -0.4761409 |
| Hoxb6 | -0.6789686 |
| Hoxb7 | -0.2538414 |
| Hoxc10 | 1.51358516 |
| Hoxc10 | 1.4736241 |
| Hoxc6 | 0.38399696 |
| Hoxc9 | -0.6296585 |
| Hoxd10 | -0.6544209 |
| Hoxd3 | 1.05953203 |
| Hoxd4 | 0.12130552 |
| Hoxd8 | 0.28935778 |
| Hoxd9 | -0.5645661 |
| Hp | 1.15561192 |
| Hp | 0.24349484 |
| Hp | 0.19735347 |
| Hp1bp3 | -0.6816374 |
| Hpca | -0.500039 |
| Hpcal1 | -0.2947418 |
| Hpcal1 | 2.04358472 |
| Hpcal4 | -0.4556324 |
| Hpgd | -0.7179689 |
| Hpn | -0.1065704 |
| Hpn | -0.1201495 |
| Hprt1 | 1.60405193 |
| Hprt1 | 1.65146107 |
| Hprt1 | -0.0518722 |
| Hps1 | 0.95845647 |
| Hps3 | 1.35279766 |
| Hps3 | -0.6896897 |
| Hps4 | -0.3927446 |
| Hps5 | -0.397991 |
| Hps6 | -0.6771936 |
| Hpse | -0.7078361 |
| Hpx | 1.78351117 |
| Hr | 2.4095786 |
| Hr | -0.2651827 |
| Hras1 | 0.00750281 |
| Hrasls | -0.1880149 |
| Hrasls5 | 2.5811549 |
| Hrasls5 | -0.0716048 |
| Hrb | -0.4993058 |
| Hrb | -0.5512993 |
| Hrbl | -0.6104755 |
| Hrbl | 1.19804161 |
| Hrc | -0.1294775 |
| Hrg | -0.1284912 |
| Hrh3 | 0.57196622 |
| Hrk | 1.76731761 |
| Hrmt1l2 | 2.55774692 |
| Hrsp12 | 1.14494887 |
| Hrsp12 | -0.3940534 |
| Hrsp12 | -0.6423847 |
| Hs1bp3 | 1.7203961 |
| Hs2st1 | 1.71684515 |
| Hs3st1 | -0.2549693 |
| Hs3st3a1 | 1.18713116 |
| Hs3st3b1 | 3.8935497 |
| Hs3st6 | 1.35592363 |
| Hs6st1 | -0.2168207 |
| Hs6st2 | -0.4934605 |
| Hs6st2 | -0.0379005 |
| Hs6st3 | 2.16231702 |
| Hsbp1 | 1.07882357 |
| Hsd11b1 | -0.4683087 |
| Hsd11b1 | -0.2959202 |
| Hsd11b1 | -0.2709053 |
| Hsd11b1 | 0.15683976 |
| Hsd11b2 | -0.2288157 |
| Hsd17b1 | -0.7115081 |
| Hsd17b10 | -0.7452216 |
| Hsd17b11 | -0.3543082 |
| Hsd17b11 | 0.0049425 |
| Hsd17b11 | 3.06410574 |
| Hsd17b12 | 0.75958011 |
| Hsd17b2 | 0.85221051 |
| Hsd17b3 | -0.4059162 |
| Hsd17b4 | -0.7319659 |
| Hsd17b7 | 1.14658759 |
| Hsd3b1 | -0.5965457 |
| Hsd3b2 | -0.6501044 |
| Hsd3b2 | -0.696906 |
| Hsd3b4 | 0.71588878 |
| Hsd3b7 | -0.4718606 |
| Hsd3b7 | -0.242636 |
| Hsdl1 | 0.0906253 |
| Hsf1 | -0.6570204 |
| Hsf4 | -0.5653524 |
| Hsf5 | -0.6313454 |
| Hsn2 | 1.90261872 |
| Hsp105 | -0.466184 |
| Hsp90aa1 | -0.3907847 |
| Hsp90ab1 | 0.31270234 |
| Hsp90ab1 | 1.61814879 |
| Hsp90ab1 | 1.40590421 |
| Hsp90b1 | -0.6745375 |
| Hspa12b | 2.88656001 |
| Hspa1a | -0.2640423 |
| Hspa1l | 1.55792801 |
| Hspa2 | -0.277241 |
| Hspa2 | 3.13340446 |
| Hspa4 | -0.6296585 |
| Hspa4l | -0.5981737 |
| Hspa5 | 0.19700562 |
| Hspa9 | -0.4883757 |
| Hspa9 | 2.11581472 |
| Hspb1 | -0.43488 |
| Hspb2 | 1.49858327 |
| Hspb3 | -0.4647702 |
| Hspb6 | -0.6055354 |
| Hspb7 | 1.03103902 |
| Hspb8 | 0.85771677 |
| Hspbap1 | -0.6578885 |
| Hspd1 | -0.5037139 |
| Hspd1 | -0.5328847 |
| Hspe1 | 2.35175182 |
| Hspg2 | 0.54118397 |
| Htatip | 1.18277952 |
| Htatip | -0.5973594 |
| Htatip2 | -0.7635176 |
| Htatip2 | 1.03983383 |
| Htatsf1 | -0.3810468 |
| Htf9c | -0.6745375 |
| Htr1a | -0.7693705 |
| Htr1b | -0.6381234 |
| Htr1d | 0.09101509 |
| Htr1f | -0.4898257 |
| Htr1f | 0.01471915 |
| Htr2b | 1.2267981 |
| Htr3a | 0.25337349 |
| Htr5b | -0.1245564 |
| Htr7 | -0.4389938 |
| Htra1 | 2.94830802 |
| Htra1 | 1.27258572 |
| Htra2 | -0.0214934 |
| Htra3 | 3.89312325 |
| Htra3 | 0.72877881 |
| Htt | 2.93102212 |
| Hus1 | 1.7647212 |
| Hus1 | 1.81741754 |
| Hus1 | -0.3436993 |
| Huwe1 | 2.90694215 |
| Hvcn1 | -0.2566638 |
| Hvcn1 | 0.15974085 |
| Hyal1 | 1.09652979 |
| Hyal2 | -0.5892543 |
| Hyal3 | -0.3574516 |
| Hyal5 | 0.06453317 |
| Hyal6 | -0.5559602 |
| Hydin | -0.2877021 |
| Hyi | -0.6692478 |
| Hyou1 | -0.0600817 |
| Iah1 | 1.33700903 |
| Iap | -0.3192022 |
| Iars | -0.7272769 |
| Iars | 0.3958837 |
| Iars2 | -0.6088259 |
| Ibtk | 2.11093592 |
| Ica1 | -0.6338813 |
| Ica1l | -0.3461847 |
| Icam1 | 1.33154855 |
| Icam2 | -0.34432 |
| Icam4 | 2.55602042 |
| Icam5 | -0.3778232 |
| Ick | 0.13516149 |
| Icmt | 0.37455013 |
| Ict1 | 1.9509258 |
| Id1 | -0.4542345 |
| Id2 | -0.7452216 |
| Id2 | 1.6335333 |
| Id3 | 0.99870375 |
| Id4 | -0.3933988 |
| Ide | -0.2222539 |
| Idh1 | 0.52344338 |
| Idh2 | 1.70942672 |
| Idh3a | -0.5336448 |
| Idh3b | -0.7891493 |
| Idh3g | 2.01969735 |
| Idh3g | 2.54519919 |
| Idi2 | -0.6622411 |
| Ids | -0.7207516 |
| Ids | 2.25730948 |
| Ids | 1.04223092 |
| Idua | 1.41009357 |
| Ier3 | -0.1027267 |
| Ier3ip1 | -0.6381234 |
| Ier5l | 1.64490771 |
| Ifi202b | 1.53817569 |
| Ifi202b | 0.71608844 |
| Ifi27 | 0.81452915 |
| Ifi30 | -0.7254079 |
| Ifi30 | -0.566927 |
| Ifi35 | 1.04756699 |
| Ifi47 | 4.02907567 |
| Ifit2 | 1.22702924 |
| Ifit2 | -0.3973336 |
| Ifit3 | -0.7654645 |
| Ifit3 | -0.1094637 |
| Ifitm1 | -0.697812 |
| Ifitm1 | 1.24940837 |
| Ifitm1 | -0.5622112 |
| Ifitm2 | 0.79234708 |
| Ifitm3 | 1.48456554 |
| Ifitm5 | -0.856484 |
| Ifitm6 | -0.4233217 |
| Ifna1 | -0.5148259 |
| Ifna6 | -0.7376239 |
| Ifnar2 | 0.9684153 |
| Ifnar2 | 2.98286758 |
| Ifnar2 | 0.07806552 |
| Ifngr1 | -0.260062 |
| Ifngr2 | -0.0600817 |
| Ifngr2 | -0.3669455 |
| Ifngr2 | -0.8511286 |
| Ifnz | -0.1464057 |
| Ifrd1 | 0.04345001 |
| Ifrg15 | -0.3914376 |
| Ift122 | -0.4941892 |
| Ift140 | -0.4768561 |
| Ift140 | 0.34443498 |
| Ift140 | -0.5606445 |
| Ift172 | -0.4260284 |
| Ift172 | 1.51290459 |
| Ift172 | -0.7188956 |
| Ift20 | 0.11334895 |
| Ift20 | 1.99645232 |
| Ift20 | 0.61275721 |
| Ift52 | 1.45515913 |
| Ift80 | -0.8716444 |
| Ift81 | 0.19247168 |
| Ift81 | 1.08152799 |
| Ift81 | -0.6843138 |
| Ift88 | 1.0228798 |
| Igbp1 | 0.2224034 |
| Igbp1b | -0.7723108 |
| Igf1 | -0.514081 |
| Igf1 | 0.6607806 |
| Igf1r | 0.80803821 |
| Igf2 | -0.6313454 |
| Igf2bp1 | -0.6381234 |
| Igf2bp2 | -0.6596271 |
| Igf2bp3 | 1.37889359 |
| Igf2bp3 | 4.33433797 |
| Igf2r | -0.3137777 |
| Igf2r | -0.5215571 |
| Igfals | -0.1123659 |
| Igfbp2 | -0.0669785 |
| Igfbp2 | 0.65291053 |
| Igfbp3 | -0.6347282 |
| Igfbp4 | -0.0396916 |
| Igfbp4 | 0.27186002 |
| Igfbp5 | 1.03146649 |
| Igfbp6 | -0.6475241 |
| Igfbp7 | -0.7742761 |
| Igfbpl1 | 0.24646941 |
| Igsf1 | -0.7198231 |
| Igsf1 | -0.5924879 |
| Igsf1 | 2.08116215 |
| Igsf1 | 0.02146048 |
| Igsf11 | -0.3493008 |
| Igsf11 | -0.7433164 |
| Igsf11 | -0.4514449 |
| Igsf21 | -0.8103776 |
| Igsf3 | -0.5103652 |
| Igsf4a | 0.1118257 |
| Igsf5 | -0.4079081 |
| Igsf8 | 0.2294869 |
| Igsf9 | -0.5852279 |
| Igtp | 1.27302585 |
| Ihh | -0.3071903 |
| Ihpk1 | 1.28700176 |
| Ihpk2 | -0.3119765 |
| Ihpk2 | 0.37512539 |
| Iigp2 | 0.4219519 |
| Ik | 0.35242358 |
| Ikbkap | -0.6718889 |
| Ikbkb | -0.6381234 |
| Ikbke | 0.51704259 |
| Ikbkg | 2.03787614 |
| Ikbkg | -0.9198085 |
| Il10ra | 0.2049737 |
| Il10rb | -0.354936 |
| Il11 | 0.96291276 |
| Il11ra1 | 2.21073745 |
| Il12a | 0.86727533 |
| Il13 | -0.3599739 |
| Il13ra1 | 0.53199118 |
| Il13ra2 | -0.7851599 |
| Il15 | 0.50335624 |
| Il15 | 1.85292067 |
| Il15 | 0.2997013 |
| Il15ra | -0.4854825 |
| Il15ra | 1.09119961 |
| Il15ra | 1.32989381 |
| Il16 | 0.42686178 |
| Il17d | 1.75649504 |
| Il17d | -0.6552866 |
| Il17ra | 0.22172597 |
| Il17rb | -0.8175623 |
| Il17rc | -0.6279747 |
| Il17rc | -0.5957327 |
| Il17rc | -0.5804191 |
| Il17rd | 1.64815332 |
| Il17rd | 1.78108718 |
| Il17re | 0.28525141 |
| Il17re | 1.97723407 |
| Il17re | 2.2217632 |
| Il18 | 3.64994464 |
| Il18bp | 1.33227158 |
| Il18r1 | 1.7647862 |
| Il1b | -0.4535363 |
| Il1r1 | 1.66058831 |
| Il1rap | -0.3368983 |
| Il1rap | 0.5947463 |
| Il1rap | -0.3694936 |
| Il1rap | -0.6789686 |
| Il1rap | -0.2766633 |
| Il1rl1l | 1.39225444 |
| Il1rl2 | 0.75211851 |
| Il20ra | -0.235424 |
| Il20rb | -0.4521415 |
| Il20rb | -0.3947085 |
| Il23a | 0.7411048 |
| Il27ra | -0.1294775 |
| Il28ra | -0.3524273 |
| Il28ra | 2.75480041 |
| Il28ra | 2.26810571 |
| Il31ra | -0.1123659 |
| Il33 | -0.6825286 |
| Il3ra | 0.39165577 |
| Il4i1 | 1.53577047 |
| Il6ra | -0.4782883 |
| Il6st | 1.12182916 |
| Il7 | 2.68981863 |
| Ildr1 | 0.25271816 |
| Ildr1 | 0.15611308 |
| Ilf2 | 0.06373101 |
| Ilf3 | -0.477572 |
| Ilf3 | -0.1682637 |
| Ilf3 | 0.43039183 |
| Ilf3 | 1.94954008 |
| Ilk | 1.11507988 |
| Ilkap | -0.500039 |
| Ilvbl | 1.5226405 |
| Ilvbl | -0.721681 |
| Ilvbl | -0.252152 |
| Ilvbl | -0.782179 |
| Immp2l | 0.15465802 |
| Immt | -0.6941934 |
| Imp3 | 0.76868257 |
| Imp4 | -0.4818785 |
| Imp4 | -0.3368983 |
| Impa2 | 3.20512566 |
| Impact | 1.40657195 |
| Impad1 | 0.91881123 |
| Impdh1 | -0.000629 |
| Impdh2 | 0.353307 |
| Ina | 0.59224243 |
| Inadl | -0.4725727 |
| Inadl | 1.14356001 |
| Incenp | -0.2853673 |
| Indo | 1.68298838 |
| Ing2 | -0.2959202 |
| Ing3 | -0.5949204 |
| Ing3 | 0.55124031 |
| Ing3 | 1.12325067 |
| Ing4 | 1.56809111 |
| Ing4 | -0.7500017 |
| Ing4 | 1.27104362 |
| Ing5 | -0.3234431 |
| Inha | -0.4883757 |
| Inhba | 0.44277679 |
| Inmt | 0.8991605 |
| Inoc1 | -0.6552866 |
| Inpp4a | -0.6364242 |
| Inpp4a | 0.48972086 |
| Inpp4a | 0.02940314 |
| Inpp4b | -0.6834208 |
| Inpp5a | 0.43309821 |
| Inpp5d | 0.25762203 |
| Inpp5e | 0.70079133 |
| Inpp5f | -0.1595609 |
| Inppl1 | -0.8073153 |
| Inppl1 | 0.06693552 |
| Inppl1 | -0.6648625 |
| Ins2 | 0.2871489 |
| Insc | -0.2124967 |
| Insig1 | -0.4833184 |
| Insig1 | 2.08305973 |
| Insig1 | 0.89504841 |
| Insig2 | 1.29401967 |
| Insig2 | -0.3131769 |
| Insl3 | 1.15573664 |
| Insl5 | 0.02648475 |
| Insl6 | -0.759636 |
| Insm1 | 0.15720289 |
| Ints1 | -0.2538414 |
| Ints12 | -0.2640423 |
| Ints3 | -0.6834208 |
| Ints3 | 2.08042058 |
| Ints4 | 1.16269488 |
| Ints5 | -0.5074032 |
| Ints7 | 1.1693559 |
| Ints9 | -0.3283135 |
| Ints9 | -0.4219712 |
| Ipmk | -0.8155039 |
| Ipmk | 2.66044902 |
| Ipo13 | 0.07450246 |
| Ipo4 | 0.29281835 |
| Ipo5 | 3.3371744 |
| Ipp | 0.28746477 |
| Ippk | -0.5820193 |
| Iqcb1 | 3.38681971 |
| Iqcb1 | -0.3468071 |
| Iqcd | 0.35094935 |
| Iqce | -0.859712 |
| Iqcf3 | 0.60671725 |
| Iqcf4 | -0.4535363 |
| Iqgap1 | -0.6923893 |
| Iqgap2 | 1.08489755 |
| Iqgap3 | 2.51014944 |
| Iqsec2 | 0.62185499 |
| Iqsec3 | -0.4985731 |
| Irak1 | -0.7023551 |
| Irak1bp1 | -0.6950967 |
| Irak2 | 1.12105288 |
| Irak2 | 1.18652797 |
| Irak3 | 0.84840606 |
| Irak4 | 0.77169713 |
| Irak4 | -0.3907847 |
| Irak4 | -0.5497508 |
| Ireb2 | 0.79766777 |
| Irf1 | -0.6121281 |
| Irf1 | -0.893738 |
| Irf1 | -0.7105887 |
| Irf1 | 0.96689006 |
| Irf2 | -0.3733288 |
| Irf2bp1 | -0.0546007 |
| Irf3 | -0.1796477 |
| Irf3 | 1.70756287 |
| Irf3 | 2.47303338 |
| Irf4 | -0.5868364 |
| Irf5 | -0.175492 |
| Irf6 | -0.0245754 |
| Irf6 | 1.6085185 |
| Irf7 | 1.19672952 |
| Irf8 | 0.30188074 |
| Irf9 | -0.0423847 |
| Irf9 | 2.31138956 |
| Irgm | 1.95777091 |
| Irgm | -0.6736538 |
| Irgq | 0.22206474 |
| Irgq | 1.30821723 |
| Irgq | -0.6648625 |
| Irs2 | 0.77413931 |
| Irs3 | 0.68172527 |
| Irx2 | -0.3606055 |
| Irx3 | -0.0823331 |
| Irx5 | 0.08710989 |
| Isca1 | -0.6347282 |
| Iscu | -0.5451207 |
| Isg20 | -0.2365299 |
| Isg20l1 | -0.0232533 |
| Isg20l2 | -0.8124248 |
| Isl1 | -0.5313664 |
| Islr2 | 2.53832202 |
| Isy1 | 1.39399576 |
| Isyna1 | -0.2720541 |
| Itfg2 | 0.40845392 |
| Itfg3 | 2.43393743 |
| Itga1 | -0.5828204 |
| Itga11 | 0.10724101 |
| Itga11 | 0.49681589 |
| Itga2b | 1.42610556 |
| Itga3 | -0.5118498 |
| Itga5 | -0.5820193 |
| Itga5 | -0.6509661 |
| Itga6 | -0.4500532 |
| Itga7 | 1.00370667 |
| Itgad | 1.20906179 |
| Itgad | -0.6372734 |
| Itgad | -0.2877021 |
| Itgae | 0.66141582 |
| Itgae | -0.6271339 |
| Itgae | 0.62273785 |
| Itgav | 2.09184924 |
| Itgb1 | 1.53568742 |
| Itgb1 | 0.88343736 |
| Itgb1bp1 | -0.7557703 |
| Itgb3bp | 0.6526969 |
| Itgb4 | 0.86097082 |
| Itgb4 | 0.330967 |
| Itgb5 | -0.2157378 |
| Itgb6 | 2.42871383 |
| Itgb6 | 0.97887212 |
| Itgb6 | 1.94563836 |
| Itgb7 | -0.3000566 |
| Itgbl1 | 0.68812394 |
| Itih2 | -0.1319478 |
| Itih3 | 1.52339818 |
| Itih5 | -0.0804584 |
| Itih5 | -0.6313454 |
| Itk | -0.4486636 |
| Itlnb | 0.43444847 |
| Itm2a | 0.58560898 |
| Itm2b | -0.3042113 |
| Itm2b | 0.47381607 |
| Itpk1 | -0.5306082 |
| Itpka | -0.6096504 |
| Itpkc | 0.11029997 |
| Itpr1 | -0.3637701 |
| Itpr2 | -0.5908697 |
| Itpr3 | -0.8227282 |
| Itsn1 | 1.88432602 |
| Itsn2 | -0.7347906 |
| Ivd | -0.2842021 |
| Ivns1abp | -0.6657379 |
| Ivns1abp | -0.6798573 |
| Iws1 | -0.6271339 |
| Iyd | 1.04840675 |
| Izumo1 | 2.77472993 |
| Jag1 | 0.80477575 |
| Jag2 | 2.4008333 |
| Jagn1 | -0.4654769 |
| Jak1 | -0.3765369 |
| Jak2 | 2.51438468 |
| Jak2 | -0.5788216 |
| Jak3 | 1.4038981 |
| Jak3 | -0.4212967 |
| Jakmip1 | 0.38939303 |
| Jam2 | -0.5118498 |
| Jam2 | 0.53174804 |
| Jam2 | -0.6137835 |
| Jam2 | 2.27105956 |
| Jam3 | -0.2640423 |
| Jam4 | 0.24679934 |
| Jarid1b | 0.72463375 |
| Jarid1c | -0.2299139 |
| Jarid1d | 2.70243829 |
| Jazf1 | -0.5629955 |
| Jdp2 | -0.4654769 |
| Jmjd1a | 0.70423486 |
| Jmjd1a | -0.1339287 |
| Jmjd1a | -0.7115081 |
| Jmjd2a | -0.5328847 |
| Jmjd2b | 1.66493792 |
| Jmjd2b | -0.2923893 |
| Jmjd2c | -0.6596271 |
| Jmjd2d | -0.5215571 |
| Jmjd3 | -0.3012417 |
| Jmjd3 | 2.19610428 |
| Jmjd5 | -0.672771 |
| Jmjd6 | -0.6578885 |
| Jmy | 1.97878616 |
| Josd1 | 0.28683292 |
| Josd2 | -0.6262939 |
| Josd3 | -0.272629 |
| Josd3 | 3.13561071 |
| Jph1 | -0.6905887 |
| Jph2 | 0.61831516 |
| Jph3 | 1.72975486 |
| Jph4 | 1.03203614 |
| Jph4 | -0.6415309 |
| Jtb | 0.53101824 |
| Jtv1 | -0.5283372 |
| Jtv1 | -0.5645661 |
| Jun | 0.77769722 |
| Junb | -0.5520745 |
| Jund1 | 1.49849685 |
| Jup | -0.3631363 |
| Kal1 | 2.55068153 |
| Kalrn | -0.4139092 |
| Kank1 | -0.2830383 |
| Kank3 | -0.2348715 |
| Kank4 | 0.70927593 |
| Kank4 | 1.34590386 |
| Kank4 | -0.5351668 |
| Kars | 1.27653947 |
| Katna1 | -0.6552866 |
| Katnal1 | 1.83247049 |
| Katnb1 | 0.04426976 |
| Kazald1 | 1.25413495 |
| Kbtbd2 | -0.4059162 |
| Kbtbd2 | -0.6780807 |
| Kbtbd4 | -0.200708 |
| Kbtbd4 | -0.7772319 |
| Kbtbd7 | 0.13069013 |
| Kbtbd7 | -0.00235 |
| Kbtbd8 | 0.93679695 |
| Kcmf1 | -0.466184 |
| Kcna1 | -0.0846817 |
| Kcna2 | -0.6406778 |
| Kcna3 | 0.74633801 |
| Kcna5 | 0.25239031 |
| Kcna6 | -0.4396812 |
| Kcnab1 | -0.7087528 |
| Kcnab1 | -0.6941934 |
| Kcnab1 | 1.56592259 |
| Kcnab2 | -0.3264841 |
| Kcnab3 | 1.37850061 |
| Kcnb1 | -0.6896897 |
| Kcnc1 | 0.06051549 |
| Kcnc1 | 0.55528016 |
| Kcnc4 | -0.5051879 |
| Kcnc4 | 0.53901489 |
| Kcnd2 | -0.6896897 |
| Kcne1 | -0.8300088 |
| Kcne1l | -0.5748399 |
| Kcne2 | -0.6014384 |
| Kcne4 | -0.6932909 |
| Kcnf1 | -0.4549332 |
| Kcng4 | -0.5298506 |
| Kcnh1 | 2.62075891 |
| Kcnh2 | 1.70347704 |
| Kcnh2 | -0.721681 |
| Kcnh3 | 0.30777087 |
| Kcnh5 | -0.2049776 |
| Kcnip1 | -0.5015072 |
| Kcnip2 | -0.2611975 |
| Kcnip2 | -0.603075 |
| Kcnip2 | -0.5163176 |
| Kcnip3 | 2.50827397 |
| Kcnip4 | -0.4883757 |
| Kcnj12 | 0.0906253 |
| Kcnj16 | 2.25331041 |
| Kcnj2 | 0.63064632 |
| Kcnj3 | -0.7871525 |
| Kcnj4 | 0.5747572 |
| Kcnj6 | 0.1366472 |
| Kcnj6 | 2.09691956 |
| Kcnj8 | 2.65183503 |
| Kcnj9 | 2.31770544 |
| Kcnk1 | 0.38143001 |
| Kcnk10 | 1.53651758 |
| Kcnk12 | -0.5957327 |
| Kcnk13 | -0.5645661 |
| Kcnk2 | -0.696906 |
| Kcnk2 | 0.87184922 |
| Kcnk3 | -0.403267 |
| Kcnk4 | 0.54670441 |
| Kcnk5 | 0.27826081 |
| Kcnk5 | 0.2808089 |
| Kcnk6 | 0.46966729 |
| Kcnk6 | -0.0428343 |
| Kcnk9 | 2.42133851 |
| Kcnma1 | -0.048699 |
| Kcnmb1 | -0.143396 |
| Kcnmb4 | 1.89167478 |
| Kcnmb4 | 0.3456228 |
| Kcnn1 | 2.07544617 |
| Kcnn2 | -0.7567352 |
| Kcnn4 | 0.73015642 |
| Kcnq1 | 0.70907481 |
| Kcnq2 | 0.18896901 |
| Kcnq2 | -0.4840392 |
| Kcnq3 | 0.64970098 |
| Kcnq5 | -0.6631141 |
| Kcnrg | -0.0136146 |
| Kcnrg | -0.7606049 |
| Kcns1 | -0.7404658 |
| Kcns2 | 2.46802438 |
| Kcnt1 | 0.01133016 |
| Kcnu1 | 1.92823097 |
| Kcnv1 | 0.75096527 |
| Kcnv2 | 0.02481299 |
| Kctd10 | -0.3089822 |
| Kctd11 | 1.72277858 |
| Kctd12 | -0.6196009 |
| Kctd12b | 2.26696713 |
| Kctd12b | 1.12840164 |
| Kctd14 | -0.6212697 |
| Kctd14 | -0.8500612 |
| Kctd15 | 0.40456128 |
| Kctd18 | 1.23885649 |
| Kctd19 | 1.1332689 |
| Kctd2 | 0.24316374 |
| Kctd20 | -0.2657534 |
| Kctd20 | -0.5088831 |
| Kctd3 | 0.2644445 |
| Kctd4 | -0.0401399 |
| Kctd5 | 0.80259443 |
| Kctd5 | 0.85169264 |
| Kctd6 | 1.68348483 |
| Kctd6 | 1.19194266 |
| Kctd9 | -0.8447424 |
| Kdelc1 | -0.3733288 |
| Kdelc1 | 1.67572419 |
| Kdelr2 | -0.1012893 |
| Kdelr3 | 1.38134599 |
| Kdr | -0.7567352 |
| Keap1 | -0.317994 |
| Keap1 | -0.3543082 |
| Keap1 | 0.43606621 |
| Keap1 | -0.8468663 |
| Kera | 0.40928596 |
| Khdc1c | 2.77853794 |
| Khdrbs1 | -0.3914376 |
| Khdrbs2 | -0.5900616 |
| Khdrbs3 | 1.32595203 |
| Khk | -0.4410575 |
| Khk | 1.66797064 |
| Khsrp | 0.21799131 |
| Kif11 | -0.6271339 |
| Kif13a | -0.572459 |
| Kif14 | -0.425351 |
| Kif15 | 0.00194658 |
| Kif17 | 0.82329222 |
| Kif17 | 0.31239488 |
| Kif18a | -0.6432393 |
| Kif1a | 0.68068911 |
| Kif1b | -0.4376205 |
| Kif1b | 2.27615002 |
| Kif1b | 0.65461779 |
| Kif1b | 0.94402057 |
| Kif1b | -0.8022337 |
| Kif1b | 1.5331925 |
| Kif1c | 1.97930295 |
| Kif21a | -0.5374544 |
| Kif21b | -0.8341948 |
| Kif22 | -0.4754261 |
| Kif23 | 0.2721811 |
| Kif24 | 2.32194018 |
| Kif27 | 0.25500983 |
| Kif2a | 1.33608402 |
| Kif2b | 1.85510652 |
| Kif2c | -0.4500532 |
| Kif3a | 1.44331587 |
| Kif3a | 3.28027224 |
| Kif3b | 1.0709362 |
| Kif3c | 0.48000491 |
| Kif3c | -0.8618703 |
| Kif4 | 0.69284319 |
| Kif4 | 0.46052709 |
| Kif4 | 0.94198476 |
| Kif5a | -0.6683691 |
| Kif5a | -0.4507488 |
| Kif5a | 2.56889212 |
| Kif5b | 1.06449551 |
| Kif5c | 0.76357335 |
| Kif9 | -0.8196252 |
| Kifap3 | 1.56809111 |
| Kifap3 | 1.33649525 |
| Kifap3 | 0.10263371 |
| Kifc2 | 0.55812135 |
| Kifc2 | -0.2017736 |
| Kifc3 | 1.78516607 |
| Kin | -0.7357341 |
| Kirrel3 | -0.3486767 |
| Kiss1 | -0.5066642 |
| Kiss1r | 0.46026463 |
| Kitl | -0.7811875 |
| Kl | 0.33247306 |
| Klb | -0.3669455 |
| Klc1 | -0.4301033 |
| Klc1 | 0.72285165 |
| Klc1 | -0.566927 |
| Klc2 | -0.1349208 |
| Klc2 | 0.08122133 |
| Klc4 | 1.88243679 |
| Klf1 | -0.4912779 |
| Klf10 | -0.2657534 |
| Klf12 | 0.08397394 |
| Klf12 | -0.4711492 |
| Klf12 | 1.31717917 |
| Klf13 | -0.6347282 |
| Klf16 | -0.5700841 |
| Klf17 | 0.27953573 |
| Klf17 | 0.45023588 |
| Klf2 | 2.06936666 |
| Klf4 | -0.252152 |
| Klf4 | 1.62873399 |
| Klf4 | -0.5582994 |
| Klf5 | 1.04532394 |
| Klf6 | -0.6212697 |
| Klf6 | -0.3350521 |
| Klf7 | -0.3829863 |
| Klf9 | 1.9866107 |
| Klhdc1 | 2.04107337 |
| Klhdc1 | 1.45841244 |
| Klhdc1 | -0.8165325 |
| Klhdc2 | -0.4431257 |
| Klhdc3 | -0.7310262 |
| Klhdc4 | 1.81827725 |
| Klhdc4 | 2.18356639 |
| Klhdc4 | -0.0437342 |
| Klhdc8b | -0.1744559 |
| Klhdc9 | 0.37051336 |
| Klhl10 | -0.9290497 |
| Klhl11 | -0.2381913 |
| Klhl12 | -0.0370062 |
| Klhl14 | -0.6466656 |
| Klhl15 | -0.1605806 |
| Klhl15 | -0.240411 |
| Klhl17 | -0.6415309 |
| Klhl2 | 1.57488526 |
| Klhl21 | 2.85562395 |
| Klhl22 | -0.0281102 |
| Klhl22 | -0.1448996 |
| Klhl23 | 1.02818424 |
| Klhl24 | 0.78607021 |
| Klhl24 | 0.43093374 |
| Klhl25 | -0.6834208 |
| Klhl26 | -0.1718708 |
| Klhl26 | 0.11410964 |
| Klhl30 | 2.15253907 |
| Klhl31 | -0.1853921 |
| Klhl32 | 1.30343765 |
| Klhl5 | 0.47562539 |
| Klhl5 | -0.4458906 |
| Klhl6 | 3.08040457 |
| Klhl6 | -0.6313454 |
| Klhl7 | -0.7133495 |
| Klhl8 | 1.03118153 |
| Klhl9 | 0.94573971 |
| Klhl9 | 0.16010285 |
| Klk1 | -0.1544793 |
| Klk11 | 1.98188263 |
| Klk11 | -0.6229414 |
| Klk15 | -0.6246162 |
| Klk1b1 | -0.7385702 |
| Klk1b11 | 0.52613827 |
| Klk1b16 | -0.0049375 |
| Klk1b21 | 0.68420737 |
| Klk1b21 | 0.29313232 |
| Klk1b22 | -0.1250473 |
| Klk1b24 | -0.6330353 |
| Klk1b26 | 1.69244651 |
| Klk1b27 | -0.3947085 |
| Klk1b27 | 0.01344972 |
| Klk1b4 | -0.7366785 |
| Klk1b4 | 2.36405206 |
| Klk1b5 | -0.7713297 |
| Klk1b8 | 0.00280353 |
| Klk1b9 | 1.09984579 |
| Klk6 | -0.1848684 |
| Klk8 | -0.5957327 |
| Klkb1 | -0.2271709 |
| Klra17 | 0.34710544 |
| Klra4 | -0.804263 |
| Klrd1 | 1.15822768 |
| Kndc1 | 1.15023287 |
| Kng1 | -0.0131788 |
| Kng1 | -0.8093558 |
| Kng2 | 0.8710039 |
| Kns2 | 0.07012907 |
| Kns2 | -0.6262939 |
| Kntc1 | 1.23209998 |
| Kpna1 | 0.28493479 |
| Kpna2 | -0.0162335 |
| Kpna3 | -0.7105887 |
| Kpna6 | 0.54478795 |
| Kpnb1 | -0.2124967 |
| Kptn | 0.16191077 |
| Kras | 1.41643697 |
| Kras | -0.5505247 |
| Krba1 | -0.5998047 |
| Krba1 | 1.54961901 |
| Krba1 | -0.3313705 |
| Krcc1 | 0.12092817 |
| Kremen | 2.86137108 |
| Kremen1 | -0.7395175 |
| Kremen2 | -0.3618701 |
| Krit1 | -0.2114188 |
| Krit1 | 1.56817133 |
| Krr1 | 0.43093374 |
| Krr1 | -0.0281102 |
| Krr1 | -0.5412797 |
| Krt10 | 2.40119521 |
| Krt12 | -0.6355758 |
| Krt13 | 3.08874813 |
| Krt14 | -0.0969905 |
| Krt17 | 1.57687418 |
| Krt18 | -0.7654645 |
| Krt19 | 0.87218714 |
| Krt2 | -0.6080022 |
| Krt20 | 2.16409931 |
| Krt222 | -0.8405091 |
| Krt23 | 1.23393709 |
| Krt27 | 0.60221791 |
| Krt32 | 2.01354171 |
| Krt4 | 1.1994709 |
| Krt7 | -0.782179 |
| Krt73 | 0.33727622 |
| Krt75 | 0.16946606 |
| Krt78 | 2.95535202 |
| Krt79 | -0.486928 |
| Krt8 | -0.6254547 |
| Krt84 | -0.4280637 |
| Krt86 | -0.466184 |
| Krt86 | -0.0823331 |
| Krt9 | -0.8062968 |
| Krtap16-4 | -0.2071198 |
| Krtap8-2 | -0.3381312 |
| Krtcap2 | -0.1509385 |
| Krtcap3 | 2.68149671 |
| Ksr2 | -0.6179351 |
| Ktelc1 | 0.37253392 |
| Kti12 | 1.44715859 |
| Ktn1 | 1.16331364 |
| Ktn1 | -0.8618703 |
| Ky | -0.1801684 |
| L1cam | 2.81350565 |
| L2hgdh | 0.5931537 |
| L3mbtl2 | 0.72897573 |
| L3mbtl3 | 0.02815352 |
| l7Rn6 | 1.49207942 |
| Lace1 | 1.80958233 |
| Lad1 | -0.6718889 |
| Lag3 | 2.52227801 |
| Lage3 | -0.1418948 |
| Lama1 | -0.2830383 |
| Lama2 | 0.32066089 |
| Lamb1-1 | -0.780197 |
| Lamb2 | 2.02068641 |
| Lamb3 | -0.1812108 |
| Lamc1 | 1.9864569 |
| Lamc2 | -0.4092383 |
| Lamc2 | 0.37856954 |
| Lamc2 | -0.7226113 |
| Lamc3 | 0.72818778 |
| Lamp1 | 0.07885548 |
| Lamp2 | -0.1230851 |
| Lamp2 | -0.4556324 |
| Lamp2 | -0.0254579 |
| Lamp2 | -0.4445071 |
| Lancl1 | -0.6212697 |
| Lancl1 | 0.45605511 |
| Lancl1 | 0.72285165 |
| Laptm4a | -0.6129554 |
| Laptm4b | -0.5973594 |
| Laptm4b | 0.15683976 |
| Laptm5 | -0.8216927 |
| Large | 0.21424167 |
| Larp2 | 1.6610968 |
| Larp5 | -0.1544793 |
| Larp6 | -0.4199492 |
| Larp6 | 0.78235814 |
| Lars | 1.05287277 |
| Lars2 | -0.4605417 |
| Las1l | -0.4790053 |
| Lasp1 | 1.45705827 |
| Lasp1 | -0.5852279 |
| Lasp1 | -0.4445071 |
| Lass1 | 0.94151433 |
| Lass2 | 0.02020018 |
| Lass2 | -0.6570204 |
| Lass2 | -0.4754261 |
| Lass4 | -0.5653524 |
| Lass4 | 0.17446919 |
| Lass5 | 0.15974085 |
| Lass6 | 0.61498436 |
| Lat2 | -0.4963786 |
| Lat2 | -0.5949204 |
| Lats2 | -0.6237784 |
| Lbh | -0.5125929 |
| Lbh | -0.5238117 |
| Lbp | -0.7366785 |
| Lbr | -0.362503 |
| Lbx2 | -0.8093558 |
| Lca5 | 2.23490326 |
| Lca5 | -0.1489212 |
| Lca5 | -0.2732043 |
| Lcat | -0.1060891 |
| Lcat | 1.44523921 |
| Lce1c | 0.25566356 |
| Lck | -0.554404 |
| Lcmt1 | -0.2028404 |
| Lcmt1 | 0.87824886 |
| Lcmt1 | -0.3505501 |
| Lcn10 | -0.6701274 |
| Lcn12 | 1.62466532 |
| Lcn13 | 1.778913 |
| Lcn2 | -0.5606445 |
| Lcn3 | -0.5306082 |
| Lcn8 | -0.554404 |
| Lcor | 0.77226128 |
| Lcp1 | -0.5884476 |
| Ldb1 | 0.08397394 |
| Ldb2 | 0.81416972 |
| Ldb2 | -0.3543082 |
| Ldha | -0.3289241 |
| Ldhc | 0.33457748 |
| Ldhc | -0.1560009 |
| Ldhc | 2.29210776 |
| Ldhd | 1.25413495 |
| Ldhd | -0.5238117 |
| Ldoc1 | 2.3498771 |
| Ldoc1l | 2.73487402 |
| Lef1 | -0.3765369 |
| Lef1 | 0.88276949 |
| Lefty1 | 1.0653202 |
| Lefty2 | -0.8468663 |
| Lemd2 | 1.96130643 |
| Leng1 | 1.60890255 |
| Leng8 | 1.11116377 |
| Leng9 | -0.6604976 |
| Leo1 | -0.3966766 |
| Lep | 0.73701417 |
| Lepre1 | -0.8073153 |
| Lepre1 | -0.6440947 |
| Lepre1 | 0.45605511 |
| Leprel1 | 0.53393403 |
| Leprel2 | -0.3399835 |
| Leprotl1 | 2.33265624 |
| Letm1 | 1.19649075 |
| Letm1 | 2.54795844 |
| Letm1 | 0.85617141 |
| Letmd1 | 3.89090791 |
| Lgals1 | -0.2894571 |
| Lgals2 | -0.7442685 |
| Lgals3 | -0.0541454 |
| Lgals3bp | 1.49987862 |
| Lgals4 | -0.2157378 |
| Lgals4 | 4.61270129 |
| Lgals6 | 0.25990172 |
| Lgals7 | -0.5313664 |
| Lgals8 | 0.36151224 |
| Lgals8 | 0.29124695 |
| Lgals9 | 0.62008675 |
| Lgals9 | 0.75843604 |
| Lgi1 | 0.38655687 |
| Lgi1 | 1.32709478 |
| Lgi2 | -0.6834208 |
| Lgi3 | -0.3894805 |
| Lgi4 | -0.6432393 |
| Lgmn | 2.07833177 |
| Lgr5 | 3.11568691 |
| Lgtn | -0.0096999 |
| Lgtn | -0.3362825 |
| Lhb | -0.343079 |
| Lhcgr | 0.15683976 |
| Lhcgr | -0.2277189 |
| Lhfp | -0.5981737 |
| Lhfp | 0.31821932 |
| Lhfp | 0.47407476 |
| Lhfpl1 | 1.55833342 |
| Lhfpl2 | -0.8532671 |
| Lhx1 | 3.25479846 |
| Lhx2 | -0.6509661 |
| Lhx3 | -0.7087528 |
| Lhx4 | -0.5253177 |
| Lhx9 | 0.35741862 |
| Lhx9 | 0.43821884 |
| Lias | 2.18032213 |
| Lias | 1.43632195 |
| Lias | 2.42149946 |
| Lifr | -0.5428142 |
| Lig1 | -0.0550562 |
| Lig1 | 1.002238 |
| Lig1 | 1.72250661 |
| Lig3 | -0.865117 |
| Lig4 | 1.25244963 |
| Lima1 | -0.3914376 |
| Lima1 | 1.11833081 |
| Limd1 | -0.3065937 |
| Limd2 | -0.8310535 |
| Lime1 | 2.43593632 |
| Limk1 | 1.08435923 |
| Limk2 | -0.3720487 |
| Limk2 | 2.66261504 |
| Lims2 | 3.07395754 |
| Lims2 | 0.76433203 |
| Lims2 | -0.89822 |
| Lin54 | -0.6526919 |
| Lin7b | 1.35863775 |
| Lin7b | -0.7519206 |
| Lin7b | -0.2179048 |
| Lin7b | -0.7105887 |
| Lin7c | -0.1152771 |
| Lincr | 1.05842544 |
| Lincr | -0.5941089 |
| Lingo1 | -0.1379033 |
| Lingo2 | -0.4192762 |
| Lingo2 | -0.5230596 |
| Lingo4 | -0.011003 |
| Lip1 | -0.6038944 |
| Lipe | -0.8436823 |
| Lipf | -0.603075 |
| Liph | 0.87875224 |
| Liph | -0.5998047 |
| Litaf | 0.66099238 |
| Lix1 | 0.29124695 |
| Lix1 | 0.68296714 |
| Lix1 | -0.2877021 |
| Lix1 | 1.11742169 |
| Llgl1 | 0.97947474 |
| Llgl2 | 0.18050864 |
| Lman1 | 0.13404565 |
| Lman2 | 0.58652674 |
| Lman2l | -0.7615748 |
| Lman2l | 1.32750998 |
| Lmbr1 | -0.6604976 |
| Lmbr1l | -0.8124248 |
| Lmcd1 | -0.8093558 |
| Lmcd1 | -0.6423847 |
| Lmcd1 | 1.96104305 |
| Lmf1 | 0.19491577 |
| Lmf2 | 0.27121752 |
| Lmna | 2.94946132 |
| Lmna | -0.7161184 |
| Lmna | -0.121127 |
| Lmna | -0.317994 |
| Lmna | -0.5957327 |
| Lmnb1 | -0.8185932 |
| Lmnb1 | -0.5551818 |
| Lmnb2 | -0.5622112 |
| Lmo2 | 1.1276308 |
| Lmo3 | 1.81612648 |
| Lmo3 | -0.7060055 |
| Lmo4 | 0.16731363 |
| Lmod1 | 0.02815352 |
| Lmtk2 | 0.06091803 |
| Lmtk3 | 0.88427152 |
| Lmx1a | 0.22409482 |
| Lmx1b | -0.7625457 |
| Lnx1 | -0.2988729 |
| Lnx2 | -0.1985805 |
| Lnx2 | -0.4883757 |
| LOC100038882 | -0.5505247 |
| LOC100038984 | -0.4542345 |
| LOC100039087 | -0.3486767 |
| LOC100039227 | -0.2577952 |
| LOC100039479 | -0.6014384 |
| LOC100039532 | 0.4320166 |
| LOC100039571 | -0.7461757 |
| LOC100039728 | -0.7207516 |
| LOC100040573 | -0.4718606 |
| LOC100040592 | 0.6967238 |
| LOC100040601 | -0.5466615 |
| LOC100040799 | -0.3350521 |
| LOC100040899 | 0.24976343 |
| LOC100040919 | -0.4369346 |
| LOC100041103 | -0.7941601 |
| LOC100041290 | 0.1455122 |
| LOC100041463 | 0.24117471 |
| LOC100041483 | 1.64888939 |
| LOC100041500 | 0.76792741 |
| LOC100041504 | 0.70200814 |
| LOC100041703 | 1.36563788 |
| LOC100041722 | 2.0123456 |
| LOC100041725 | -0.1245564 |
| LOC100041835 | 2.3098311 |
| LOC100042179 | 0.16227193 |
| LOC100042427 | -0.5443512 |
| LOC100042777 | 1.18640729 |
| LOC100043257 | -0.7366785 |
| LOC100043391 | -0.4612451 |
| LOC100043671 | 2.49572326 |
| LOC100043822 | 3.03806403 |
| LOC100043986 | 0.02146048 |
| LOC100044087 | 0.28493479 |
| LOC100044089 | 0.10762393 |
| LOC100044101 | 0.22713169 |
| LOC100044103 | -0.8165325 |
| LOC100044103 | -0.4577332 |
| LOC100044122 | 0.03314203 |
| LOC100044133 | 0.62251721 |
| LOC100044159 | -0.269758 |
| LOC100044170 | 3.38414635 |
| LOC100044172 | -0.4132405 |
| LOC100044177 | -0.2304634 |
| LOC100044190 | -0.4912779 |
| LOC100044204 | 0.25827393 |
| LOC100044210 | 0.22814179 |
| LOC100044221 | -0.7338481 |
| LOC100044257 | -0.4956482 |
| LOC100044275 | -0.6683691 |
| LOC100044289 | 2.39426162 |
| LOC100044292 | 0.19700562 |
| LOC100044294 | -0.5692938 |
| LOC100044298 | 0.58238969 |
| LOC100044322 | 0.92713086 |
| LOC100044324 | 1.27467451 |
| LOC100044324 | -0.4389938 |
| LOC100044363 | -0.1041662 |
| LOC100044363 | 0.14109021 |
| LOC100044439 | -0.2732043 |
| LOC100044468 | 0.79473597 |
| LOC100044475 | 0.94495868 |
| LOC100044538 | -0.2065838 |
| LOC100044566 | -0.3089822 |
| LOC100044576 | -0.741415 |
| LOC100044636 | 0.94229825 |
| LOC100044692 | 0.21355829 |
| LOC100044708 | 2.47734886 |
| LOC100044736 | 3.26192335 |
| LOC100044756 | -0.447276 |
| LOC100044776 | 0.34175687 |
| LOC100044779 | -0.5828204 |
| LOC100044812 | -0.5022422 |
| LOC100044829 | 2.704319 |
| LOC100044862 | 1.18156713 |
| LOC100044873 | -0.2282672 |
| LOC100044934 | 2.71705068 |
| LOC100045005 | -0.1770482 |
| LOC100045019 | -0.8458038 |
| LOC100045040 | -0.4825982 |
| LOC100045098 | 2.54376002 |
| LOC100045228 | 1.37209137 |
| LOC100045241 | 0.69079427 |
| LOC100045250 | -0.7041785 |
| LOC100045280 | -0.5661393 |
| LOC100045300 | -0.2847845 |
| LOC100045304 | -0.8052794 |
| LOC100045343 | -0.2703315 |
| LOC100045359 | 0.61275721 |
| LOC100045359 | -0.4747119 |
| LOC100045403 | 0.2744256 |
| LOC100045413 | 3.30451965 |
| LOC100045419 | 1.85315722 |
| LOC100045430 | -0.5443512 |
| LOC100045439 | 2.50560078 |
| LOC100045491 | 1.6167791 |
| LOC100045505 | 1.34069992 |
| LOC100045522 | 0.4126067 |
| LOC100045542 | 1.13950792 |
| LOC100045551 | -0.1109136 |
| LOC100045567 | -0.4668917 |
| LOC100045607 | -0.3720487 |
| LOC100045617 | -0.1964578 |
| LOC100045644 | -0.1359139 |
| LOC100045697 | 0.49048362 |
| LOC100045780 | -0.5260717 |
| LOC100045782 | 1.59746729 |
| LOC100045877 | 1.11299333 |
| LOC100045882 | -0.6458078 |
| LOC100045887 | -0.6869979 |
| LOC100045950 | 0.22679475 |
| LOC100045963 | 1.14746879 |
| LOC100045981 | 0.40316711 |
| LOC100046003 | -0.1927546 |
| LOC100046035 | -0.1167362 |
| LOC100046035 | 1.77884898 |
| LOC100046039 | -0.7142716 |
| LOC100046044 | -0.5435824 |
| LOC100046049 | 1.10024292 |
| LOC100046056 | 1.15785445 |
| LOC100046068 | 0.6194228 |
| LOC100046080 | 0.39503964 |
| LOC100046081 | 1.00400012 |
| LOC100046120 | -0.300649 |
| LOC100046128 | 0.93742731 |
| LOC100046129 | 1.01174333 |
| LOC100046163 | -0.3252665 |
| LOC100046163 | 0.08789224 |
| LOC100046207 | -0.7198231 |
| LOC100046207 | -0.3644043 |
| LOC100046207 | 0.27410529 |
| LOC100046232 | -0.6834208 |
| LOC100046254 | 1.61837688 |
| LOC100046320 | 1.09026369 |
| LOC100046393 | -0.4445071 |
| LOC100046406 | -0.1801684 |
| LOC100046457 | -0.0936606 |
| LOC100046518 | -0.5780239 |
| LOC100046568 | 1.40418495 |
| LOC100046586 | 1.97469344 |
| LOC100046608 | 0.04958066 |
| LOC100046608 | -0.1324426 |
| LOC100046608 | -0.4396812 |
| LOC100046608 | 2.11237087 |
| LOC100046616 | -0.5081428 |
| LOC100046641 | 1.67185545 |
| LOC100046650 | 1.35239355 |
| LOC100046690 | 3.27669562 |
| LOC100046690 | 2.41788744 |
| LOC100046741 | 0.62273785 |
| LOC100046744 | -0.6154419 |
| LOC100046744 | -0.7811875 |
| LOC100046746 | 1.94478101 |
| LOC100046775 | -0.8383995 |
| LOC100046781 | -0.6204349 |
| LOC100046781 | -0.2387457 |
| LOC100046792 | -0.6710077 |
| LOC100046796 | 0.31147191 |
| LOC100046802 | 1.37623774 |
| LOC100046841 | -0.7442685 |
| LOC100046855 | -0.7901493 |
| LOC100046883 | 0.55433118 |
| LOC100046891 | 1.04462188 |
| LOC100046898 | -0.5435824 |
| LOC100046918 | 0.64991529 |
| LOC100046953 | -0.1469083 |
| LOC100046959 | -0.9313744 |
| LOC100046996 | 1.41822804 |
| LOC100047009 | -0.9027237 |
| LOC100047009 | 1.06435799 |
| LOC100047012 | -0.6869979 |
| LOC100047028 | -0.6372734 |
| LOC100047046 | 1.87241171 |
| LOC100047052 | 0.17304246 |
| LOC100047052 | 0.82844008 |
| LOC100047155 | -0.1186851 |
| LOC100047155 | -0.3765369 |
| LOC100047167 | 1.03714743 |
| LOC100047173 | -0.469018 |
| LOC100047184 | 1.1588494 |
| LOC100047200 | -0.0250166 |
| LOC100047214 | 0.94605195 |
| LOC100047214 | 2.66673403 |
| LOC100047238 | 2.43145667 |
| LOC100047261 | 0.4884482 |
| LOC100047323 | -0.8426233 |
| LOC100047323 | 1.76192199 |
| LOC100047339 | 0.2125323 |
| LOC100047353 | -0.2510274 |
| LOC100047368 | 0.03231246 |
| LOC100047369 | 1.2768682 |
| LOC100047369 | -0.12603 |
| LOC100047391 | 0.49302168 |
| LOC100047393 | -0.3791113 |
| LOC100047427 | -0.697812 |
| LOC100047480 | 1.05731754 |
| LOC100047490 | 1.92231909 |
| LOC100047579 | 0.20117136 |
| LOC100047583 | -0.5133367 |
| LOC100047606 | -0.6381234 |
| LOC100047615 | 0.03231246 |
| LOC100047619 | -0.107052 |
| LOC100047634 | 2.97421315 |
| LOC100047651 | -0.1109136 |
| LOC100047653 | 1.62345638 |
| LOC100047659 | 1.0983882 |
| LOC100047674 | -0.4112373 |
| LOC100047707 | 1.97873447 |
| LOC100047717 | 1.85834635 |
| LOC100047749 | -0.8248026 |
| LOC100047762 | -0.4314656 |
| LOC100047776 | 0.15647649 |
| LOC100047794 | 0.41729098 |
| LOC100047800 | 0.62735925 |
| LOC100047810 | -0.697812 |
| LOC100047815 | -0.672771 |
| LOC100047816 | 0.20462867 |
| LOC100047834 | 0.08200862 |
| LOC100047837 | 1.86321437 |
| LOC100047837 | 0.30653392 |
| LOC100047841 | -0.7752603 |
| LOC100047856 | 0.63152092 |
| LOC100047870 | -0.1895922 |
| LOC100047911 | 1.90982558 |
| LOC100047934 | 2.68626116 |
| LOC100047935 | -0.7151945 |
| LOC100047937 | 2.49895825 |
| LOC100047937 | 0.49403498 |
| LOC100047958 | 0.44598084 |
| LOC100047963 | 0.04057522 |
| LOC100047963 | 0.88343736 |
| LOC100047998 | -0.2168207 |
| LOC100048046 | 0.2514061 |
| LOC100048046 | 0.08003915 |
| LOC100048049 | -0.260062 |
| LOC100048049 | -0.486928 |
| LOC100048049 | 1.0955992 |
| LOC100048056 | 2.20867829 |
| LOC100048071 | -0.5230596 |
| LOC100048076 | -0.4920049 |
| LOC100048083 | -0.356822 |
| LOC100048105 | 1.28776007 |
| LOC100048105 | 0.17696075 |
| LOC100048187 | 1.90781995 |
| LOC100048251 | -0.5614275 |
| LOC100048295 | 0.31208733 |
| LOC100048299 | -0.4697279 |
| LOC100048301 | 1.25019779 |
| LOC100048313 | -0.5374544 |
| LOC100048331 | 0.54022056 |
| LOC100048332 | 2.79969236 |
| LOC100048346 | 0.96993805 |
| LOC100048372 | -0.6389741 |
| LOC100048384 | 1.0321785 |
| LOC100048413 | -0.8103776 |
| LOC100048436 | -0.5059257 |
| LOC100048445 | 1.8834105 |
| LOC100048461 | -0.5582994 |
| LOC100048480 | 2.62847706 |
| LOC100048483 | -0.1838219 |
| LOC100048504 | -0.2028404 |
| LOC100048530 | -0.0423847 |
| LOC100048554 | 2.45993518 |
| LOC100048556 | -0.4633586 |
| LOC100048589 | 0.70927593 |
| LOC100048613 | 0.54574667 |
| LOC100048622 | 1.91354686 |
| LOC100048645 | 1.15748107 |
| LOC100048684 | 0.53563072 |
| LOC100048703 | 1.31885655 |
| LOC100048710 | -0.7762456 |
| LOC100048721 | -0.4905515 |
| LOC100048721 | -0.6483834 |
| LOC100048726 | 0.57684498 |
| LOC100048733 | -0.4479696 |
| LOC100048816 | -0.6466656 |
| LOC100048832 | 0.96597372 |
| LOC100048832 | 1.70901285 |
| LOC100048858 | 0.3375756 |
| LOC192758 | 1.44459853 |
| LOC194227 | -0.0410372 |
| LOC212390 | -0.8970975 |
| LOC216443 | 0.31913566 |
| LOC216963 | -0.5230596 |
| LOC217341 | 3.91527893 |
| LOC218963 | -0.2315635 |
| LOC225922 | -0.3077872 |
| LOC233529 | -0.8175623 |
| LOC234374 | -0.5748399 |
| LOC235497 | -0.7161184 |
| LOC236749 | -0.1911721 |
| LOC237408 | 0.03189739 |
| LOC237877 | 0.74807591 |
| LOC238829 | -0.7841652 |
| LOC241593 | -0.6466656 |
| LOC244595 | 0.39841131 |
| LOC245263 | -0.808335 |
| LOC329575 | 0.93726976 |
| LOC329575 | 0.79289891 |
| LOC333331 | -0.7179689 |
| LOC381066 | -0.2766633 |
| LOC381302 | -0.1932828 |
| LOC381375 | -0.6055354 |
| LOC381483 | 1.93334623 |
| LOC381629 | 0.80532028 |
| LOC381801 | 0.0277366 |
| LOC381936 | 0.49099179 |
| LOC382010 | 1.81575727 |
| LOC382044 | 1.69720979 |
| LOC382106 | -0.1143057 |
| LOC382106 | -0.4287431 |
| LOC382133 | 0.22814179 |
| LOC384806 | -0.4797227 |
| LOC384806 | -0.4389938 |
| LOC386486 | 2.13446669 |
| LOC433479 | -0.3344375 |
| LOC433801 | 2.92820026 |
| LOC433886 | -0.7519206 |
| LOC434172 | -0.758668 |
| LOC434172 | -0.6432393 |
| LOC434864 | -0.300649 |
| LOC434960 | 3.76357147 |
| LOC435023 | -0.6204349 |
| LOC435337 | -0.8352442 |
| LOC435565 | -0.0379005 |
| LOC436177 | -0.4321475 |
| LOC544988 | -0.7490437 |
| LOC544988 | 0.7244359 |
| LOC545013 | 1.03912763 |
| LOC545013 | -0.6423847 |
| LOC545056 | -0.5836222 |
| LOC545238 | -0.0568806 |
| LOC545703 | 1.19278213 |
| LOC545732 | 2.68402781 |
| LOC545952 | 0.08122133 |
| LOC546015 | 0.50385745 |
| LOC546015 | -0.5290936 |
| LOC546723 | -0.8093558 |
| LOC546840 | -0.603075 |
| LOC547323 | 0.41453837 |
| LOC547343 | 1.45389089 |
| LOC620807 | -0.7931558 |
| LOC621823 | 0.89768223 |
| LOC621824 | 0.53997955 |
| LOC622404 | -0.9049837 |
| LOC622665 | 2.80658189 |
| LOC624855 | -0.5957327 |
| LOC625480 | 0.77376401 |
| LOC626152 | -0.8032478 |
| LOC628053 | 1.41935747 |
| LOC631002 | -0.4052532 |
| LOC631784 | -0.7338481 |
| LOC632431 | 2.40033955 |
| LOC639396 | -0.4417464 |
| LOC640963 | 0.94605195 |
| LOC641240 | -0.5685042 |
| LOC654426 | 2.13564749 |
| LOC654467 | 1.03558861 |
| LOC654494 | -0.7226113 |
| LOC654842 | -0.0505109 |
| LOC665250 | -0.6063569 |
| LOC666676 | 0.04672469 |
| LOC666904 | 2.88334519 |
| LOC667250 | -0.8629512 |
| LOC667370 | -0.3907847 |
| LOC667609 | 0.40344611 |
| LOC668492 | 0.4392933 |
| LOC668837 | 2.95088725 |
| LOC669168 | -0.4542345 |
| LOC669660 | 0.16407566 |
| LOC670044 | -0.1621122 |
| LOC671564 | -0.2918021 |
| LOC672705 | 3.1673121 |
| LOC674706 | 0.95180951 |
| LOC675228 | 0.49933683 |
| LOC675567 | 0.58331062 |
| LOC675567 | 2.59194553 |
| LOC675813 | 2.30130434 |
| LOC676420 | 1.00370667 |
| LOC676420 | -0.403267 |
| LOC677144 | -0.2087296 |
| LOC677205 | -0.8415656 |
| LOC677317 | -0.3675819 |
| LOC677319 | 0.82489287 |
| LOC677528 | 1.42123683 |
| LOC732482 | 0.18192403 |
| Loh11cr2a | 0.80876165 |
| Loh12cr1 | 2.04175017 |
| Loh12cr1 | 0.08397394 |
| Lonp2 | -0.3933988 |
| Lonrf1 | 1.30418275 |
| Lonrf2 | 0.05970987 |
| Lor | -0.6780807 |
| Lox | 1.57663573 |
| Loxl1 | -0.3524273 |
| Lpar2 | -0.8672877 |
| Lpar2 | 1.10749388 |
| Lpar3 | 0.31085609 |
| Lpcat2 | 1.21598631 |
| Lpcat2 | -0.5268262 |
| Lpcat2 | -0.7490437 |
| Lpgat1 | 1.35662805 |
| Lphn1 | 0.0015178 |
| Lphn3 | -0.235424 |
| Lpin1 | -0.5283372 |
| Lpin1 | 0.55764842 |
| Lpin2 | 1.42189372 |
| Lpin3 | 1.55938666 |
| Lpl | -0.4125723 |
| Lpxn | -0.8383995 |
| Lrat | -0.7723108 |
| Lrba | 2.00292271 |
| Lrba | 1.17840753 |
| Lrch1 | 1.06641866 |
| Lrch4 | -0.8662017 |
| Lrch4 | 1.28721848 |
| Lrdd | -0.6246162 |
| Lrfn1 | -0.4355644 |
| Lrfn2 | 0.65866008 |
| Lrfn3 | 0.48768378 |
| Lrfn4 | 2.80483223 |
| Lrg1 | -0.7319659 |
| Lrig1 | -0.0739266 |
| Lrig3 | 2.19282111 |
| Lrmp | -0.7901493 |
| Lrp1 | -0.1760104 |
| Lrp10 | -0.7069204 |
| Lrp11 | -0.486928 |
| Lrp12 | 2.27851611 |
| Lrp12 | -0.300649 |
| Lrp4 | 1.87553447 |
| Lrp5 | 0.49200728 |
| Lrp8 | -0.4172599 |
| Lrp8 | -0.6683691 |
| Lrpap1 | -0.8175623 |
| Lrpprc | -0.6146124 |
| Lrpprc | -0.6389741 |
| Lrpprc | -0.3277033 |
| Lrrc1 | -0.7366785 |
| Lrrc1 | -0.8848378 |
| Lrrc1 | 0.66858494 |
| Lrrc10 | -0.804263 |
| Lrrc15 | 0.94261164 |
| Lrrc16b | 1.30556493 |
| Lrrc16b | 0.02146048 |
| Lrrc18 | 1.41502054 |
| Lrrc2 | -0.2686121 |
| Lrrc20 | 2.03208364 |
| Lrrc20 | -0.7911504 |
| Lrrc23 | -0.2189902 |
| Lrrc23 | -0.5740456 |
| Lrrc24 | 0.58606797 |
| Lrrc26 | 0.96658471 |
| Lrrc28 | -0.2060481 |
| Lrrc29 | 0.79363416 |
| Lrrc3 | 0.85082887 |
| Lrrc30 | 1.77068219 |
| Lrrc33 | -0.4633586 |
| Lrrc34 | 0.99367392 |
| Lrrc34 | 3.25339866 |
| Lrrc36 | 0.03727887 |
| Lrrc39 | 0.06091803 |
| Lrrc39 | 0.49706829 |
| Lrrc4 | -0.3461847 |
| Lrrc40 | 2.12425863 |
| Lrrc41 | -0.5389827 |
| Lrrc43 | 1.08583885 |
| Lrrc44 | -0.4675999 |
| Lrrc45 | -0.5268262 |
| Lrrc46 | 0.48154574 |
| Lrrc48 | 0.26799839 |
| Lrrc48 | 1.72434092 |
| Lrrc48 | 0.80767627 |
| Lrrc49 | 2.90050589 |
| Lrrc49 | 2.52475988 |
| Lrrc49 | 2.60356711 |
| Lrrc4c | 0.64368017 |
| Lrrc50 | 1.24873118 |
| Lrrc50 | -0.4675999 |
| Lrrc52 | -0.5606445 |
| Lrrc52 | 0.62955192 |
| Lrrc56 | -0.3804012 |
| Lrrc57 | -0.7490437 |
| Lrrc59 | -0.5645661 |
| Lrrc6 | 0.61186486 |
| Lrrc7 | 0.73564664 |
| Lrrc8 | -0.6544209 |
| Lrrc8a | 1.76640969 |
| Lrrc8b | -0.1895922 |
| Lrrc8c | 0.4208573 |
| Lrrc8d | 0.1228134 |
| Lrrc9 | 1.41719152 |
| Lrrfip1 | 0.49073774 |
| Lrrfip1 | 2.21576529 |
| Lrriq2 | -0.401285 |
| Lrriq2 | 1.53651758 |
| Lrrk1 | 0.21390004 |
| Lrrk2 | -0.4654769 |
| Lrrn1 | 0.81488844 |
| Lrrn2 | -0.5245644 |
| Lrrn3 | 0.76565826 |
| Lrrtm1 | -0.4818785 |
| Lrrtm2 | -0.5788216 |
| Lrrtm3 | -0.5230596 |
| Lrsam1 | -0.1775675 |
| Lrsam1 | -0.5044506 |
| Lrtm1 | 0.02648475 |
| Lrtm1 | 0.16335459 |
| Lsamp | -0.4920049 |
| Lsamp | -0.447276 |
| Lsg1 | -0.8479301 |
| Lsg1 | 0.55290589 |
| Lsm10 | 0.19142226 |
| Lsm11 | 1.66196787 |
| Lsm12 | 3.35305986 |
| Lsm12 | -0.4833184 |
| Lsm12 | 0.26022694 |
| Lsm14a | 0.94542738 |
| Lsm14b | -0.3436993 |
| Lsm14b | 0.50260392 |
| Lsm14b | 0.73231714 |
| Lsm2 | 0.08003915 |
| Lsm2 | 0.194567 |
| Lsm3 | -0.603075 |
| Lsm4 | 2.64458709 |
| Lsm5 | -0.3325961 |
| Lsm6 | -0.9117967 |
| Lsm6 | 1.38749765 |
| Lsm8 | -0.8022337 |
| Lsr | -0.8705534 |
| Lst1 | -0.6196009 |
| Ltb4dh | -0.2044428 |
| Ltb4r1 | -0.322836 |
| Ltb4r2 | 1.56503767 |
| Ltbp1 | 0.79216307 |
| Ltbp1 | 1.56374902 |
| Ltbp2 | 0.48205879 |
| Ltbp3 | 1.94279537 |
| Ltbp3 | -0.7115081 |
| Ltbp4 | -0.4152481 |
| Ltbp4 | -1.0411532 |
| Ltbp4 | 2.2930315 |
| Ltbr | 2.15916851 |
| Ltc4s | 0.71868016 |
| Ltf | -0.3675819 |
| Ltf | -0.4570324 |
| Ltk | -0.8114006 |
| Ltk | 0.3306655 |
| Ltv1 | -0.0419353 |
| Luc7l | -1.0346318 |
| Luc7l | -0.8268816 |
| Luc7l2 | 0.92888133 |
| Luc7l2 | -0.3474299 |
| Lum | 1.36981292 |
| Luzp1 | 2.76970964 |
| Luzp1 | -0.7207516 |
| Luzp1 | -0.7226113 |
| Luzp2 | 0.62757875 |
| Lxn | -0.184345 |
| Ly6a | 2.22768326 |
| Ly6c1 | 0.02815352 |
| Ly6d | 0.96536233 |
| Ly6e | -0.4206227 |
| Ly6f | 1.82938219 |
| Ly6g6c | -0.7500017 |
| Ly6g6d | 1.57560176 |
| Ly6g6e | 0.79931288 |
| Ly6k | -0.8618703 |
| Ly86 | 0.73466861 |
| Ly9 | -0.6834208 |
| Ly9 | -0.2119576 |
| Ly96 | -0.7329065 |
| Lyar | -0.2714795 |
| Lyl1 | 1.64623682 |
| Lynx1 | 0.40288802 |
| Lynx1 | 1.60644189 |
| Lypd1 | 0.17304246 |
| Lypd3 | -0.4059162 |
| Lypd4 | 0.17696075 |
| Lypd5 | 0.10877174 |
| Lypd6 | 0.86114178 |
| Lypla1 | -0.8394537 |
| Lypla1 | 2.66485048 |
| Lypla2 | 0.8428445 |
| Lypla3 | -0.7300875 |
| Lyplal1 | 0.46992713 |
| Lyrm1 | -0.6171033 |
| Lyrm2 | -0.2544052 |
| Lyrm2 | 1.54527224 |
| Lyrm5 | 1.29831234 |
| Lysmd1 | -0.6047145 |
| Lysmd1 | -0.1201495 |
| Lysmd2 | 0.97115446 |
| Lysmd4 | -0.5804191 |
| Lyst | 0.02523121 |
| Lyve1 | -0.4854825 |
| Lyz | 0.71628806 |
| Lyz1 | 1.39244808 |
| Lyz1 | -0.4112373 |
| Lyz2 | 2.9835205 |
| Lyzl1 | 2.21548444 |
| Lyzl4 | 0.96398523 |
| Lyzl6 | 3.02829529 |
| Lyzs | -0.2572293 |
| Lzic | -0.067902 |
| Lztr1 | -0.5780239 |
| Lzts2 | 0.2514061 |
| M6pr | 1.43696832 |
| M6pr | -0.3947085 |
| M6prbp1 | 0.86489484 |
| Mab21l2 | 0.09101509 |
| Macrod1 | -0.4920049 |
| Mad1l1 | -0.1796477 |
| Mad2l1 | 1.94306395 |
| Mad2l1bp | -0.7538435 |
| Mad2l1bp | -0.5788216 |
| Mad2l1bp | -0.1895922 |
| Mad2l2 | -0.5208068 |
| Madcam1 | 0.63978907 |
| Madd | 1.66616663 |
| Maea | 1.12917184 |
| Maf | 0.78458715 |
| Maf1 | -0.1084983 |
| Mafg | 0.71508971 |
| Mag | -0.4212967 |
| Magea2 | 2.79422169 |
| Magea5 | -0.6492435 |
| Mageb3 | 0.06973047 |
| Mageb3 | -0.5780239 |
| Maged1 | 1.31833269 |
| Maged2 | -0.6869979 |
| Magee1 | 0.39306721 |
| Mageh1 | -0.3424591 |
| Magi1 | -0.5844247 |
| Magi1 | 1.67135278 |
| Magi1 | -0.6096504 |
| Magi2 | -0.5170643 |
| Magi3 | 0.99648804 |
| Magi3 | -0.7366785 |
| Magix | -0.6347282 |
| Magmas | 1.36742946 |
| Magoh | -0.0875078 |
| Magoh | -0.6330353 |
| Magohb | 2.14226723 |
| Mak | 0.7444032 |
| Mak10 | 1.1280163 |
| Mak10 | -0.172904 |
| Mal | 2.93450497 |
| Mal | 0.80713311 |
| Mall | -0.7161184 |
| Malt1 | 0.83197388 |
| Mamdc2 | -0.4577332 |
| Maml1 | -0.5965457 |
| Maml3 | 1.97723407 |
| Man1a | 0.71288874 |
| Man1a2 | -0.5059257 |
| Man1a2 | 0.08632689 |
| Man1b1 | 0.90863144 |
| Man1c1 | -0.1379033 |
| Man1c1 | -0.6229414 |
| Man2a1 | 3.7660105 |
| Man2a2 | 0.31515835 |
| Man2a2 | 1.99746628 |
| Man2a2 | 0.25827393 |
| Man2b1 | 0.87218714 |
| Man2b2 | 0.56753006 |
| Man2c1 | 0.98248202 |
| Manba | -0.5096238 |
| Manba | 2.53584747 |
| Manbal | -0.0655951 |
| Manea | -0.4883757 |
| Mansc1 | 2.01637635 |
| Maob | 1.43122772 |
| Map1lc3a | 0.36063654 |
| Map1lc3a | 1.84948387 |
| Map1lc3b | 1.88398281 |
| Map2k1 | 0.84388982 |
| Map2k2 | -0.4782883 |
| Map2k2 | -0.172904 |
| Map2k3 | 1.87582308 |
| Map2k4 | -0.7105887 |
| Map2k6 | 0.89323334 |
| Map2k6 | -0.2795556 |
| Map2k6 | -0.3855785 |
| Map2k7 | 0.11714624 |
| Map2k7 | 0.2997013 |
| Map2k7 | -0.5389827 |
| Map3k1 | 0.45842529 |
| Map3k11 | -0.1744559 |
| Map3k12 | 2.54753663 |
| Map3k12 | 0.63283129 |
| Map3k12 | 0.86625586 |
| Map3k14 | 1.96974796 |
| Map3k3 | 0.70383039 |
| Map3k6 | 2.93469086 |
| Map3k7 | 0.43660483 |
| Map3k7ip1 | 0.37195706 |
| Map3k7ip1 | -0.6423847 |
| Map3k7ip1 | 0.99129753 |
| Map3k7ip2 | -0.6736538 |
| Map3k7ip3 | 0.63522888 |
| Map3k8 | 1.6916034 |
| Map3k9 | 0.36005229 |
| Map4k2 | 3.15870856 |
| Map4k4 | -0.6364242 |
| Map4k5 | 0.31116405 |
| Map4k5 | 0.57359531 |
| Mapbpip | -0.2646123 |
| Mapk1 | 1.53119176 |
| Mapk1 | -0.3986488 |
| Mapk1 | -0.4747119 |
| Mapk10 | -0.5428142 |
| Mapk10 | 0.46497778 |
| Mapk11 | 0.94776736 |
| Mapk12 | -0.0053695 |
| Mapk12 | -0.4307842 |
| Mapk12 | -0.0428343 |
| Mapk13 | -0.7941601 |
| Mapk13 | 1.70139262 |
| Mapk14 | 0.02355721 |
| Mapk15 | 0.40205027 |
| Mapk15 | -0.4172599 |
| Mapk15 | -0.6047145 |
| Mapk1ip1 | 2.456655 |
| Mapk1ip1 | -0.4912779 |
| Mapk1ip1 | 2.36285329 |
| Mapk3 | -0.7050916 |
| Mapk3 | -0.1089808 |
| Mapk4 | 1.82524852 |
| Mapk6 | -0.5941089 |
| Mapk6 | 0.81327055 |
| Mapk6 | -0.6526919 |
| Mapk8 | -0.5973594 |
| Mapk8ip1 | -0.4172599 |
| Mapk8ip1 | -0.7050916 |
| Mapk8ip2 | -0.4570324 |
| Mapk8ip3 | 2.229108 |
| Mapk8ip3 | 1.54978264 |
| Mapk9 | 0.70907481 |
| Mapk9 | 0.64497362 |
| Mapk9 | 1.49832398 |
| Mapkap1 | 1.46380958 |
| Mapkap1 | -0.6121281 |
| Mapkapk2 | -0.8155039 |
| Mapkapk3 | 0.20393823 |
| Mapkbp1 | 0.08279525 |
| Mapre1 | -0.5290936 |
| Mapre2 | -0.7300875 |
| Mapre2 | -0.7161184 |
| Mapre3 | -0.5290936 |
| Mapt | -0.6825286 |
| Mapt | 0.34443498 |
| Mapt | -0.6449509 |
| Mapt | -0.5653524 |
| March2 | -0.5892543 |
| March3 | -0.7032664 |
| March3 | 0.40650963 |
| March5 | -0.566927 |
| March5 | -0.8073153 |
| March7 | -0.0912894 |
| March8 | -0.5298506 |
| March9 | 2.4949797 |
| March11 | 0.08515116 |
| Marcks | 1.99090684 |
| Marcksl1 | -0.0468905 |
| Marcksl1 | 0.67507391 |
| Marcksl1 | -0.000629 |
| Mare | -0.7124283 |
| Mark1 | 1.64631061 |
| Mark2 | -0.4782883 |
| Mark2 | 0.82400396 |
| Mark3 | 0.45235616 |
| Mark3 | -0.0153597 |
| Mark4 | -0.2663244 |
| Mars | 0.25859972 |
| Mars2 | 0.42140476 |
| Marveld3 | -0.6096504 |
| Marveld3 | 0.68234641 |
| Mas1 | -0.4383069 |
| Masp1 | -0.7703496 |
| Masp2 | -0.7161184 |
| Mast1 | -0.3393657 |
| Mast1 | -0.1012893 |
| Mast2 | 1.11638161 |
| Mast2 | 1.91249307 |
| Mat1a | -0.3283135 |
| Mat2a | 0.08318831 |
| Mat2a | 1.84853356 |
| Mat2a | 1.71382993 |
| Mat2a | -0.5732519 |
| Mat2b | -0.7023551 |
| Mat2b | -0.7115081 |
| Matk | 1.2142307 |
| Matk | 1.51909652 |
| Matn4 | -0.2119576 |
| Matn4 | -0.6271339 |
| Matn4 | 2.04136349 |
| Matr3 | 1.06888548 |
| Max | -0.5015072 |
| Maz | -0.7528816 |
| Mb | 0.03479896 |
| Mbc2 | 1.32115805 |
| Mbd1 | -0.6852076 |
| Mbd1 | -0.1359139 |
| Mbd3 | 1.65927946 |
| Mbd3l2 | 0.25599026 |
| Mbd4 | 0.58996035 |
| Mbd5 | 0.51531178 |
| Mbd6 | 0.64991529 |
| Mbip | -0.233216 |
| Mbl2 | 0.49933683 |
| Mbnl1 | 1.67207081 |
| Mbnl2 | -0.1113975 |
| Mbnl2 | -0.557519 |
| Mboat1 | -0.0179835 |
| Mboat2 | -0.3960201 |
| Mboat2 | 1.83975705 |
| Mboat2 | 0.41784053 |
| Mboat2 | -0.8607905 |
| Mboat5 | -0.6372734 |
| Mboat5 | 2.14892659 |
| Mbp | 0.19839626 |
| Mbp | -0.7347906 |
| Mbp | 1.46219371 |
| Mbtd1 | 1.6206547 |
| Mbtps1 | -0.6622411 |
| Mbtps1 | -0.5622112 |
| Mbtps1 | 1.78465719 |
| Mbtps2 | -0.0941356 |
| Mc3r | -0.2141159 |
| Mc4r | 0.4578991 |
| Mc5r | 0.53877357 |
| Mcam | -0.8052794 |
| Mcam | 1.9447274 |
| Mcam | -0.1927546 |
| Mcat | -0.061457 |
| Mcc | -0.0387956 |
| Mccc1 | 0.2689653 |
| Mcee | 0.23818321 |
| Mcf2l | -0.0316585 |
| Mcf2l | 2.41319034 |
| Mcf2l | 0.94901296 |
| Mcfd2 | 0.30063597 |
| Mchr1 | -0.5185595 |
| Mcl1 | 0.49276818 |
| Mcm10 | -0.477572 |
| Mcm10 | 1.32802873 |
| Mcm2 | -0.7151945 |
| Mcm3 | 2.56773677 |
| Mcm4 | 1.27566231 |
| Mcm5 | -0.7782192 |
| Mcm6 | 0.54718291 |
| Mcm6 | -0.0998542 |
| Mcm6 | -0.7347906 |
| Mcm7 | -0.796172 |
| Mcoln1 | 0.5207407 |
| Mcoln2 | 1.90793148 |
| Mcoln3 | 0.85788832 |
| Mcph1 | -0.2465426 |
| Mcph1 | 0.25631684 |
| Mcph1 | 1.83897691 |
| Mcph1 | 1.90441166 |
| Mcpt4 | 0.69631608 |
| Mcrs1 | -0.1859161 |
| Mcrs1 | -0.4647702 |
| Mcrs1 | 3.24227125 |
| Mcts1 | -0.4883757 |
| Mdfic | -0.6338813 |
| Mdga1 | 0.73818475 |
| Mdga2 | -0.5336448 |
| Mdga2 | 2.39406276 |
| Mdh1 | -0.2732043 |
| Mdh1 | 0.23116556 |
| Mdh1b | -0.3192022 |
| Mdh2 | -0.6246162 |
| Mdk | 1.01043296 |
| Mdk | 2.16176467 |
| Mdm1 | 0.45895119 |
| Mdm1 | -0.7635176 |
| Mdm1 | -0.2504656 |
| Mdm1 | -0.5389827 |
| Mdm1 | -0.0739266 |
| Mdm1 | -0.1827766 |
| Mdm1 | -0.7005352 |
| Mdm1 | 1.56584217 |
| Mdm1 | 0.26638466 |
| Mdm2 | 1.07868814 |
| Mdm2 | -0.5828204 |
| Mdm4 | 0.1495474 |
| Mdn1 | 0.58100659 |
| Me2 | -0.3368983 |
| Me3 | 2.05988824 |
| Mea1 | -0.5700841 |
| Mecp2 | -0.8426233 |
| Mecr | 0.11296837 |
| Med1 | -0.4314656 |
| Med1 | -0.0771868 |
| Med10 | 1.15648465 |
| Med11 | 0.59633618 |
| Med11 | 0.0015178 |
| Med12 | 0.86931094 |
| Med15 | 0.94636407 |
| Med15 | 1.41142262 |
| Med15 | -0.3555643 |
| Med16 | 0.37253392 |
| Med18 | -0.6381234 |
| Med18 | -0.5474329 |
| Med19 | -0.5268262 |
| Med21 | -0.6710077 |
| Med22 | -0.5590805 |
| Med22 | 0.38995923 |
| Med23 | -0.6137835 |
| Med23 | 0.97947474 |
| Med24 | 1.44450697 |
| Med25 | -0.1718708 |
| Med25 | -0.4328299 |
| Med26 | 0.21355829 |
| Med27 | -0.7244748 |
| Med28 | -0.5756349 |
| Med30 | 1.08274243 |
| Med31 | 2.3499466 |
| Med4 | -0.4493581 |
| Med6 | -0.6905887 |
| Med7 | 1.18941971 |
| Med8 | -0.7471307 |
| Med8 | -0.4633586 |
| Med9 | -0.893738 |
| Mef2b | 1.05022364 |
| Mef2b | -0.6896897 |
| Mef2c | 1.18362726 |
| Mef2c | 0.90260073 |
| Mef2c | 0.86998849 |
| Megf10 | 1.19827998 |
| Megf11 | -0.5290936 |
| Megf11 | -0.1801684 |
| Megf11 | -0.5466615 |
| Megf11 | 3.33184297 |
| Megf6 | -0.4633586 |
| Megf9 | -0.5351668 |
| Mei1 | 0.83285525 |
| Meis1 | 1.08220287 |
| Meis2 | -0.7891493 |
| Meis3 | 1.51350012 |
| Melk | 1.03189376 |
| Memo1 | -0.8926208 |
| Men1 | 0.3995325 |
| Men1 | -0.5374544 |
| Meox1 | -0.1177101 |
| Meox2 | 1.36693214 |
| Mep1b | -0.0771868 |
| Mepce | -0.5836222 |
| Mertk | 0.04467936 |
| Mesdc1 | -0.1364108 |
| Mesp1 | -0.5740456 |
| Mest | -0.0771868 |
| Mest | 0.74304644 |
| Mest | -0.2409668 |
| Metap1 | 1.54814501 |
| Metap2 | -0.4549332 |
| Metap2 | 0.17802653 |
| Metapl1 | -0.5973594 |
| Metrn | -0.5482049 |
| Metrnl | -0.2200768 |
| Metrnl | 0.35595226 |
| Mett11d1 | 0.92553667 |
| Mettl1 | -0.7188956 |
| Mettl1 | 0.48000491 |
| Mettl2 | -0.425351 |
| Mettl3 | -0.4019452 |
| Mettl4 | -0.5653524 |
| Mettl6 | -0.5118498 |
| Mettl8 | 1.08691346 |
| Mettl9 | -0.4226462 |
| Mettl9 | 0.07529543 |
| Mex3a | 2.95252985 |
| Mex3a | 1.40895288 |
| Mex3c | 1.28765178 |
| Mfap1b | -0.6288162 |
| Mfap2 | 0.86795436 |
| Mfap2 | 0.86455428 |
| Mfap2 | 0.4576359 |
| Mfap3 | 3.37321302 |
| Mfap3 | -0.7683925 |
| Mfap3l | -0.5015072 |
| Mfap3l | 0.63696872 |
| Mfap4 | 1.38798415 |
| Mfap5 | 1.23164014 |
| Mfge8 | 1.46093499 |
| Mfge8 | 0.08710989 |
| Mfi2 | -0.0596237 |
| Mfn1 | -0.6196009 |
| Mfn1 | 1.29121883 |
| Mfn2 | -0.6288162 |
| Mfng | -0.6657379 |
| Mfrp | -0.6355758 |
| Mfsd1 | -0.6914886 |
| Mfsd10 | 0.44491404 |
| Mfsd10 | 2.47525439 |
| Mfsd11 | 1.96356848 |
| Mfsd2 | 4.46951698 |
| Mfsd3 | -0.6006212 |
| Mfsd4 | 1.19672952 |
| Mfsd7 | -0.4985731 |
| Mfsd8 | 1.52491169 |
| Mfsd9 | -0.5208068 |
| Mga | -0.3486767 |
| Mgat1 | 0.04057522 |
| Mgat3 | 1.4899886 |
| Mgat4b | 1.52516371 |
| Mgat4b | 0.46680428 |
| Mgat5 | 1.11286277 |
| MGC107098 | 1.15236317 |
| MGC117608 | -0.5590805 |
| MGC118210 | 0.58721447 |
| MGC18837 | -0.0688263 |
| MGC41689 | -0.5567393 |
| MGC67181 | -0.6869979 |
| Mgea6 | 0.91752469 |
| Mgl1 | -0.5253177 |
| Mgl2 | 0.04345001 |
| Mgll | 2.34111342 |
| Mgll | -0.2959202 |
| Mgll | -0.6154419 |
| Mgmt | 0.20566338 |
| Mgp | 2.13188123 |
| Mgrn1 | -0.8114006 |
| Mgst1 | -0.3295351 |
| Mgst1 | 0.2351821 |
| Mgst2 | 1.41085326 |
| Mgst3 | 0.37971478 |
| Mgst3 | -0.8331465 |
| Mia1 | -0.2108804 |
| Mia3 | -0.173421 |
| Mib1 | 0.58400072 |
| Mib2 | -0.4179315 |
| Mic2l1 | -0.2119576 |
| Mical1 | 0.91494628 |
| Mical1 | -0.061457 |
| Mical1 | -0.4633586 |
| Mical2 | 1.23702904 |
| Mical3 | 1.61472078 |
| Micall2 | 0.99530418 |
| Micall2 | 0.05405104 |
| Mid1 | 1.45814177 |
| Mid1 | -0.105608 |
| Mid1ip1 | 0.07489903 |
| Midn | -0.2504656 |
| Midn | 2.03724509 |
| Mier1 | 0.41811519 |
| Mier1 | 0.03106672 |
| Mif | 0.9684153 |
| Mif4gd | -0.3688559 |
| Mif4gd | -0.0870362 |
| Mill2 | 0.65098612 |
| Mill2 | -0.1027267 |
| Mina | 0.94088672 |
| Mink1 | -0.356193 |
| Mink1 | -0.3313705 |
| Minpp1 | 0.79546978 |
| Minpp1 | -0.6896897 |
| Miox | 0.12544636 |
| Mipep | -0.7901493 |
| Mir16 | 0.73015642 |
| Mist1 | -0.6458078 |
| Mitf | 0.2572959 |
| Mizf | -0.6692478 |
| Mki67ip | 1.71616073 |
| Mkks | -0.8155039 |
| Mkks | 1.90603354 |
| Mkl1 | 0.32157484 |
| Mkl2 | 0.70139993 |
| Mkl2 | -0.5359287 |
| Mkl2 | 0.21833144 |
| Mkln1 | 0.95506161 |
| Mknk1 | -0.5133367 |
| Mknk2 | 0.66942476 |
| Mkrn1 | 1.23794321 |
| Mkrn2 | -0.4445071 |
| Mkrn3 | 0.52638287 |
| Mkx | 0.1812166 |
| Mlana | 0.14661444 |
| Mlf1 | -0.4619491 |
| Mlf1 | -0.6096504 |
| Mlf2 | 1.91802602 |
| Mlf2 | -0.6179351 |
| Mlh1 | -0.697812 |
| Mlh3 | 0.49832927 |
| Mlkl | -0.5103652 |
| Mllt1 | -0.4804408 |
| Mllt1 | 1.10328198 |
| Mllt1 | -0.6440947 |
| Mllt10 | -0.1065704 |
| Mllt11 | -0.1428953 |
| Mllt3 | 0.21355829 |
| Mllt3 | -0.4912779 |
| Mllt3 | -0.2359768 |
| Mlp | -0.6022564 |
| Mlstd2 | 0.99678378 |
| Mlx | 1.25973085 |
| Mlx | -0.8575588 |
| Mlxipl | -0.2709053 |
| Mlycd | -0.4172599 |
| Mmab | -0.4668917 |
| Mmab | 0.31577135 |
| Mmd | -0.7723108 |
| Mmd2 | 0.96183904 |
| Mme | -0.4192762 |
| Mme | 1.52322986 |
| Mmel1 | 0.56776406 |
| Mmp10 | -0.0036429 |
| Mmp11 | -0.1147913 |
| Mmp12 | 1.08058233 |
| Mmp13 | 0.34116069 |
| Mmp13 | 0.7486545 |
| Mmp14 | 1.91465482 |
| Mmp15 | 1.64453818 |
| Mmp16 | 0.67507391 |
| Mmp17 | -0.5685042 |
| Mmp1a | 0.7348643 |
| Mmp1a | 0.11448976 |
| Mmp2 | -0.2646123 |
| Mmp23 | -0.7170432 |
| Mmp24 | 2.16223207 |
| Mmp3 | -0.2994645 |
| Mmp3 | 0.89801094 |
| Mmp3 | 1.14216908 |
| Mmp9 | -0.6088259 |
| Mmrn2 | 0.6868889 |
| Mnat1 | 0.14991337 |
| Mnat1 | -0.8727368 |
| Mnd1 | 0.49908504 |
| Mns1 | -0.6162723 |
| Mnt | -0.2894571 |
| Mobk1b | 1.02517731 |
| Mobkl2a | -0.4811594 |
| Mobkl2b | -0.6914886 |
| Mobkl2c | -0.2266233 |
| Mobkl3 | -0.5820193 |
| Mobp | 0.14109021 |
| Mobp | 0.11714624 |
| Mobp | 2.3036807 |
| Mocos | -0.1279984 |
| Mocs1 | 1.0287559 |
| Mocs2 | 0.08906453 |
| Mod1 | -0.7133495 |
| Mog | 0.99352557 |
| Mog | -0.4768561 |
| Mon1a | 0.6794442 |
| Mon1a | 1.79005002 |
| Mon1b | -0.0568806 |
| Mon1b | 0.51555924 |
| Mon1b | -0.6950967 |
| Morc2a | 0.0776703 |
| Morc2b | 0.03851637 |
| Morc3 | -0.7951655 |
| Morf4l1 | 0.21526579 |
| Morf4l1 | -0.1240657 |
| Morf4l2 | -0.6330353 |
| Morn2 | 1.42713224 |
| Morn3 | 0.53199118 |
| Morn3 | 0.18015446 |
| Mos | -0.4438162 |
| Mosc2 | -0.4314656 |
| Mospd1 | 1.31149567 |
| Mospd1 | 1.27478431 |
| Mospd1 | -0.5200571 |
| Mospd3 | 2.58126343 |
| Mospd3 | -0.5559602 |
| Mov10 | 1.44487316 |
| Mov10l1 | 1.43632195 |
| Moxd1 | 0.57707667 |
| Moxd1 | -0.4500532 |
| Mpdu1 | 0.76262412 |
| Mpdz | 0.10532406 |
| Mpdz | -0.557519 |
| Mpg | 0.2625003 |
| Mpg | 0.4009321 |
| Mphosph6 | -0.6852076 |
| Mpnd | 0.31852486 |
| Mpo | 0.58973183 |
| Mpo | 1.17070697 |
| Mpp1 | 0.95552527 |
| Mpp2 | 1.34895151 |
| Mpp3 | -0.7664395 |
| Mpp3 | 2.44580441 |
| Mpp5 | 2.386274 |
| Mpp6 | 2.63431384 |
| Mppe1 | 0.41343505 |
| Mpped1 | -0.3771798 |
| Mpst | 0.27538588 |
| Mpv17 | 0.1952644 |
| Mpv17 | -0.3277033 |
| Mpv17 | 0.37137985 |
| Mpv17 | -0.7385702 |
| Mpv17l | 0.50335624 |
| Mpv17l | 1.77313329 |
| Mpv17l | 3.01370423 |
| Mpz | 1.29003132 |
| Mr1 | 0.38085861 |
| Mrap | -0.4139092 |
| Mras | -0.1853921 |
| Mrc1 | -0.8607905 |
| Mrc2 | -0.2651827 |
| Mrc2 | 1.17986713 |
| Mre11a | 0.21799131 |
| Mre11a | -0.5528504 |
| Mre11a | -0.2830383 |
| Mreg | -0.7951655 |
| Mrgpra2 | -0.4854825 |
| Mrgpra2 | 2.27143758 |
| Mrgprb2 | -0.4206227 |
| Mrgpre | -0.1806895 |
| Mrgprf | -0.4019452 |
| Mrgprg | 0.30993159 |
| Mrm1 | -0.7357341 |
| Mro | -0.4486636 |
| Mrp63 | -0.4704383 |
| Mrpl1 | 1.88512636 |
| Mrpl1 | -0.4438162 |
| Mrpl10 | -0.6196009 |
| Mrpl10 | 2.45445143 |
| Mrpl11 | -0.4307842 |
| Mrpl12 | -0.3733288 |
| Mrpl13 | 0.19037165 |
| Mrpl13 | 0.86438395 |
| Mrpl14 | -0.5366913 |
| Mrpl15 | 0.45341449 |
| Mrpl16 | -0.429423 |
| Mrpl17 | 0.59087385 |
| Mrpl18 | 1.11768153 |
| Mrpl2 | -0.5780239 |
| Mrpl20 | -0.6701274 |
| Mrpl22 | -0.1524544 |
| Mrpl23 | -0.2487823 |
| Mrpl24 | -0.1374055 |
| Mrpl27 | -0.6570204 |
| Mrpl28 | -0.9198085 |
| Mrpl3 | 0.66900495 |
| Mrpl3 | -0.7811875 |
| Mrpl30 | -0.4052532 |
| Mrpl33 | -0.7263419 |
| Mrpl33 | -0.4307842 |
| Mrpl34 | 1.70818457 |
| Mrpl36 | -0.4840392 |
| Mrpl37 | 0.04057522 |
| Mrpl37 | -0.0410372 |
| Mrpl38 | -0.1484176 |
| Mrpl39 | 0.09373905 |
| Mrpl40 | 0.04345001 |
| Mrpl43 | 2.53125077 |
| Mrpl43 | -0.7713297 |
| Mrpl45 | 0.10915403 |
| Mrpl46 | 0.57452494 |
| Mrpl47 | 2.67211587 |
| Mrpl47 | 0.91041687 |
| Mrpl48 | -0.1255385 |
| Mrpl48 | 0.56565588 |
| Mrpl48 | 0.15101043 |
| Mrpl49 | 1.11872016 |
| Mrpl49 | -0.4092383 |
| Mrpl49 | 2.2541963 |
| Mrpl49 | 1.05245498 |
| Mrpl50 | -0.5268262 |
| Mrpl51 | 0.72285165 |
| Mrpl51 | -0.0149231 |
| Mrpl51 | -0.5876416 |
| Mrpl52 | -0.1885404 |
| Mrpl53 | 0.28239794 |
| Mrpl54 | -0.5708751 |
| Mrpl55 | -0.5268262 |
| Mrpl9 | 0.34591951 |
| Mrps10 | 0.9535144 |
| Mrps10 | 1.99386175 |
| Mrps11 | -0.6381234 |
| Mrps11 | 0.66690303 |
| Mrps12 | -0.6071792 |
| Mrps14 | 0.35976003 |
| Mrps15 | 0.32885443 |
| Mrps16 | -0.4431257 |
| Mrps17 | 0.49251462 |
| Mrps18a | -0.4920049 |
| Mrps18b | 0.85272811 |
| Mrps18b | 1.3532016 |
| Mrps18c | -0.6372734 |
| Mrps2 | -0.497841 |
| Mrps21 | -0.1474111 |
| Mrps22 | 1.27796312 |
| Mrps23 | 1.65891557 |
| Mrps24 | 1.44852717 |
| Mrps25 | -0.7124283 |
| Mrps25 | 1.642392 |
| Mrps25 | 1.95119205 |
| Mrps26 | -0.782179 |
| Mrps26 | -0.8144764 |
| Mrps27 | 0.08710989 |
| Mrps28 | -0.6561531 |
| Mrps30 | 0.51531178 |
| Mrps31 | 1.46327127 |
| Mrps31 | -0.6121281 |
| Mrps31 | -0.6570204 |
| Mrps33 | 1.08691346 |
| Mrps33 | -0.7433164 |
| Mrps34 | 0.49302168 |
| Mrps35 | 1.94761798 |
| Mrps5 | 2.97964927 |
| Mrps6 | 0.82026127 |
| Mrps7 | -0.6129554 |
| Mrps7 | 1.17913761 |
| Mrps9 | 0.58331062 |
| Mrrf | -0.0879797 |
| Mrrf | -0.1641582 |
| Mrto4 | -0.6896897 |
| Mrvi1 | -0.6692478 |
| Ms4a1 | 3.42198793 |
| Ms4a10 | -0.6501044 |
| Ms4a13 | 1.64475992 |
| Ms4a5 | 1.53327577 |
| Ms4a6d | -0.6905887 |
| Ms4a6d | -0.3739695 |
| Ms4a7 | -0.7480867 |
| Ms4a8a | 1.40006498 |
| Msc | -0.5772269 |
| Msh2 | 1.0697063 |
| Msh3 | 0.90260073 |
| Msh3 | -0.4152481 |
| Msh4 | 1.0293272 |
| Msh5 | 0.40009259 |
| Msh5 | -0.4956482 |
| Msh6 | -0.0743916 |
| Msh6 | 0.60018611 |
| Msi2 | 0.52246149 |
| Msi2h | 0.0705275 |
| Msln | -0.2001757 |
| Msn | -0.3720487 |
| Msr2 | -0.7423652 |
| Msrb2 | -0.557519 |
| Msrb2 | 2.76120005 |
| Msrb3 | 2.18364943 |
| Mst1r | -0.3264841 |
| Msto1 | -0.4179315 |
| Msx1 | 2.85933842 |
| Mt1 | -0.1853921 |
| Mt3 | 2.88830002 |
| Mta1 | 0.82168883 |
| Mta2 | 1.95623645 |
| Mta3 | 1.08247269 |
| Mta3 | 0.58881721 |
| Mtag2 | 1.61043718 |
| Mtap | -0.5868364 |
| Mtap | -0.1384013 |
| Mtap1b | 0.45947678 |
| Mtap1b | -0.5051879 |
| Mtap1b | -0.7060055 |
| Mtap1b | 0.28746477 |
| Mtap1s | -0.6941934 |
| Mtap2 | 1.3175988 |
| Mtap2 | 0.87151118 |
| Mtap2 | -0.2515895 |
| Mtap4 | 1.61601729 |
| Mtap6 | 1.74348661 |
| Mtap6 | -0.0984213 |
| Mtap7 | 3.13016822 |
| Mtap7d1 | -0.2988729 |
| Mtap7d1 | 0.15611308 |
| Mtap7d2 | -0.6710077 |
| Mtap7d3 | 1.43853622 |
| Mtbp | 0.80749525 |
| Mtbp | 2.04527126 |
| Mtbp | -0.4105705 |
| Mtch1 | 1.81846137 |
| Mtch1 | 0.14991337 |
| Mtch2 | -0.7921526 |
| Mtch2 | 0.14440867 |
| Mtch2 | -0.3862277 |
| Mtcp1 | 0.02940314 |
| Mtdh | 0.77132084 |
| Mterf | -0.6852076 |
| Mterf | -0.5074032 |
| Mterfd1 | -0.1932828 |
| Mterfd2 | -0.6364242 |
| Mterfd2 | 1.52457557 |
| Mterfd2 | 3.16433676 |
| Mterfd3 | -0.5230596 |
| Mterfd3 | 0.34235267 |
| Mtf1 | 0.26638466 |
| Mtf1 | 1.77390599 |
| Mtf2 | -0.4328299 |
| Mtfmt | -0.1428953 |
| Mtfmt | 2.54823954 |
| Mtg1 | 0.1987436 |
| Mthfd1 | -0.226076 |
| Mthfd2 | 0.43956172 |
| Mthfs | -0.7376239 |
| Mthfsd | -0.7782192 |
| Mthfsd | -0.2459836 |
| Mtif3 | -0.6288162 |
| Mtl5 | 1.46434759 |
| Mtl5 | 1.26218247 |
| Mtmr1 | -0.0568806 |
| Mtmr11 | 0.59792337 |
| Mtmr11 | -0.6798573 |
| Mtmr12 | -0.5344055 |
| Mtmr14 | 0.26314881 |
| Mtmr14 | -0.7050916 |
| Mtmr14 | 0.11866085 |
| Mtmr14 | -0.8124248 |
| Mtmr2 | -0.5892543 |
| Mtmr2 | -0.603075 |
| Mtmr2 | -0.3295351 |
| Mtmr3 | 0.17589376 |
| Mtmr4 | -0.0734618 |
| Mtmr9 | 1.80933448 |
| Mto1 | -0.0392435 |
| Mtpn | -0.519308 |
| Mtrf1 | 3.21281739 |
| Mtrf1 | 0.27953573 |
| Mtrf1l | -0.5614275 |
| Mtrr | -0.5892543 |
| Mtss1 | -0.5732519 |
| Mttp | 2.29077597 |
| Mtus1 | 0.94761155 |
| Mtus1 | -0.3204119 |
| Mtvr2 | 0.54718291 |
| Mtvr2 | -0.603075 |
| Mtx1 | 0.68276028 |
| Mtx1 | 1.97837252 |
| Mtx1 | 3.02034166 |
| Mtx2 | 0.01217856 |
| Muc1 | 3.76498673 |
| Muc1 | -0.7881504 |
| Muc1 | -0.554404 |
| Muc1 | 0.38712479 |
| Muc13 | 0.37224554 |
| Muc5ac | 3.41428758 |
| Mug2 | -0.0702145 |
| Mug2 | -0.6492435 |
| Mug4 | -0.5275814 |
| Mum1 | -0.1692929 |
| Mup1 | 1.91576147 |
| Mup2 | -0.5382182 |
| Mup2 | 1.10709982 |
| Mup3 | -0.009266 |
| Mupcdh | 0.91349279 |
| Mus81 | -0.4912779 |
| Mus81 | 2.74109105 |
| Mustn1 | 1.35209035 |
| Mustn1 | 3.27829264 |
| Muted | 2.39412905 |
| Muted | 0.64841407 |
| Mutyh | 0.95506161 |
| Mvd | -0.7752603 |
| Mvp | 0.39503964 |
| Mx2 | -0.4949184 |
| Mxd1 | 2.03056875 |
| Mxd4 | -0.7723108 |
| Mxi1 | -0.0045058 |
| Mxra7 | 1.83945707 |
| Mxra8 | 1.14204253 |
| Mxra8 | -0.4105705 |
| Myadm | 1.61822483 |
| Mybbp1a | 0.53393403 |
| Mybbp1a | 0.38684088 |
| Mybbp1a | 2.42833047 |
| Mybl2 | -0.3319831 |
| Mybpc2 | -0.3752523 |
| Mybpc3 | -0.7291497 |
| Myc | -0.4782883 |
| Mycbp | 0.87740931 |
| Mycbp2 | -0.7732929 |
| Mycbp2 | 0.22138707 |
| Mycbpap | -0.7005352 |
| Mycl1 | 0.7519264 |
| Myd116 | 1.01580832 |
| Myd88 | -0.8144764 |
| Myef2 | -0.0936606 |
| Myg1 | 1.67879343 |
| Myg1 | 0.4748504 |
| Myg1 | 2.72689904 |
| Myh10 | -0.0842115 |
| Myh11 | 0.04180835 |
| Myh8 | 1.61571239 |
| Myh9 | 1.87697666 |
| Myl1 | 0.34502908 |
| Myl2 | -0.4362492 |
| Myl4 | 0.75499541 |
| Myl6 | 1.23725766 |
| Myl6b | 0.64087156 |
| Mylc2b | -0.6587574 |
| Mylc2b | -0.269758 |
| Mylc2b | -0.497841 |
| Mylc2pl | -0.2217091 |
| Mylk | 1.86834811 |
| Mylk | 0.80113739 |
| Mynn | -0.0564241 |
| Myo10 | -0.6449509 |
| Myo10 | 1.44157229 |
| Myo15 | 0.72739916 |
| Myo18a | 1.71369265 |
| Myo1b | 0.17553782 |
| Myo1c | -0.4633586 |
| Myo1c | -0.6896897 |
| Myo1f | -0.0329925 |
| Myo1g | -0.3493008 |
| Myo3b | -0.3940534 |
| Myo5a | -0.1384013 |
| Myo5b | -0.7538435 |
| Myo6 | -0.6816374 |
| Myo7a | 1.95209674 |
| Myo9a | 0.40316711 |
| Myo9b | -0.6221052 |
| Myo9b | 0.3148517 |
| Myoc | 0.41838976 |
| Myocd | -0.408573 |
| Myocd | 1.83861663 |
| Myom1 | -0.8352442 |
| Myom1 | 4.18011478 |
| Myrip | -0.1438969 |
| Mysm1 | -0.3048063 |
| Myst1 | 2.08000319 |
| Myst2 | -0.3486767 |
| Myst4 | -0.5981737 |
| Myst4 | -0.6736538 |
| Myst4 | 0.63413983 |
| Myt1 | 0.60424529 |
| Myt1l | 0.08789224 |
| Myt1l | 0.33847316 |
| Mzf1 | 0.23150094 |
| Mzf1 | -0.5716667 |
| N28178 | -0.6063569 |
| N4bp2 | 0.98082921 |
| N6amt1 | -0.4985731 |
| N6amt1 | 0.01767447 |
| N6amt2 | -0.2028404 |
| Naalad2 | -0.0032117 |
| Nab1 | -0.0401399 |
| Nab1 | 0.158654 |
| Nab1 | -0.6296585 |
| Nab1 | -0.5482049 |
| Nab2 | 1.67300342 |
| Naca | -0.4417464 |
| Naca | -0.055512 |
| Naca | -0.6121281 |
| Nadk | 0.59406409 |
| Nadsyn1 | -0.062375 |
| Nadsyn1 | 0.64948662 |
| Naga | -0.1089808 |
| Nagk | 1.25177465 |
| Nagk | 0.56799801 |
| Nagk | -0.2222539 |
| Nagk | -0.4971095 |
| Naglu | -0.557519 |
| Nagpa | 3.65012618 |
| Nags | -0.7713297 |
| Nags | 0.53417661 |
| Naif1 | 2.52539373 |
| Naif1 | -0.7347906 |
| Naip5 | 0.38598861 |
| Nalp10 | -0.1765292 |
| Nampt | 0.56424777 |
| Nanos1 | 0.60221791 |
| Nanos2 | 0.4023296 |
| Nans | 2.3715522 |
| Nap1l1 | -0.3468071 |
| Nap1l1 | -0.3823393 |
| Nap1l3 | 0.01090568 |
| Nap1l4 | -0.6321899 |
| Nap1l5 | -0.4605417 |
| Napa | 3.08478354 |
| Napa | -0.4725727 |
| Napa | 1.30524614 |
| Napb | 0.49681589 |
| Napepld | 0.20220989 |
| Napg | 0.83567005 |
| Napg | 0.51901674 |
| Naprt1 | 0.58996035 |
| Naprt1 | -0.5074032 |
| Napsa | -0.1017682 |
| Napsa | -0.7732929 |
| Napsa | 0.67736566 |
| Narf | -0.653556 |
| Narfl | 2.74380802 |
| Narg1 | -0.4854825 |
| Narg1 | -0.3656741 |
| Narg1l | 1.74726912 |
| Narg1l | -0.5163176 |
| Narg1l | 2.19055722 |
| Narg2 | -0.4335127 |
| Nars | 0.79050532 |
| Nars2 | -0.572459 |
| Nasp | -0.572459 |
| Nasp | -0.1479142 |
| Nasp | -0.3234431 |
| Nasp | -0.6129554 |
| Nat1 | -0.5716667 |
| Nat10 | -0.4927324 |
| Nat14 | 0.82061837 |
| Nat2 | -0.5812188 |
| Nat5 | -0.6923893 |
| Nat5 | -0.6861023 |
| Nat6 | 2.24128756 |
| Nat6 | -0.7244748 |
| Nav1 | 1.06201706 |
| Nav1 | -0.7762456 |
| Nbl1 | 0.16731363 |
| Nbr1 | 0.51901674 |
| Nbr1 | 0.43795003 |
| Ncald | -0.1723873 |
| Ncam1 | -0.1186851 |
| Ncam1 | 0.06132041 |
| Ncam2 | 0.47897628 |
| Ncan | -0.7385702 |
| Ncapd2 | 2.22633567 |
| Ncapd2 | -0.8331465 |
| Ncapd2 | 0.34828987 |
| Ncapd3 | 2.18394001 |
| Ncapg2 | -0.3295351 |
| Ncapg2 | 1.87136847 |
| Ncaph | 1.0713458 |
| Ncaph2 | 1.19994684 |
| Ncaph2 | -0.5788216 |
| Ncaph2 | -0.5949204 |
| Ncbp2 | 0.75671731 |
| Ncbp2 | 0.35771162 |
| Ncdn | 1.47042341 |
| Ncdn | 0.155022 |
| Ncdn | -0.5622112 |
| Ncdn | 0.24811788 |
| Ncf4 | -0.2859504 |
| Nck1 | -0.0692888 |
| Nckap1 | 2.09189505 |
| Nckap1l | 0.94245496 |
| Nckipsd | 1.0767901 |
| Ncl | -0.6129554 |
| Ncl | 0.14440867 |
| Ncoa2 | 0.53732434 |
| Ncoa2 | 1.71780251 |
| Ncoa4 | -0.317994 |
| Ncoa5 | 0.94932411 |
| Ncoa6 | -0.5716667 |
| Ncoa6 | 0.07806552 |
| Ncoa6 | -0.7347906 |
| Ncor1 | -0.6798573 |
| Ncstn | 2.40490496 |
| Ncstn | 0.98921323 |
| Ncstn | 2.12880443 |
| Ndc80 | -0.7921526 |
| Nde1 | 1.72495155 |
| Nde1 | 0.7542291 |
| Ndel1 | 1.49181833 |
| Ndfip1 | 1.26184854 |
| Ndfip2 | -0.7500017 |
| Ndg2 | -0.4563321 |
| Ndn | 0.35153932 |
| Ndor1 | -0.5629955 |
| Ndor1 | 2.88214661 |
| Ndor1 | -0.1801684 |
| Ndrg1 | -0.2092668 |
| Ndrg2 | -0.1985805 |
| Ndrg3 | 1.01348764 |
| Ndrg4 | -0.6843138 |
| Ndrl | 1.05856383 |
| Ndst1 | 1.86782436 |
| Ndst1 | -0.5366913 |
| Ndst4 | -0.2732043 |
| Ndufa1 | 0.16946606 |
| Ndufa10 | -0.780197 |
| Ndufa12 | 1.74740156 |
| Ndufa13 | -0.7404658 |
| Ndufa2 | 1.20976833 |
| Ndufa3 | 0.06573512 |
| Ndufa4 | 1.12814476 |
| Ndufa5 | 0.99293195 |
| Ndufa5 | 0.30529534 |
| Ndufa6 | -0.2877021 |
| Ndufa7 | 0.53466156 |
| Ndufa8 | -0.0577941 |
| Ndufa8 | -0.8618703 |
| Ndufa9 | 0.59133026 |
| Ndufaf1 | 1.98784019 |
| Ndufb10 | -0.466184 |
| Ndufb11 | -0.6561531 |
| Ndufb2 | -0.6631141 |
| Ndufb3 | -0.6080022 |
| Ndufb4 | 0.53684076 |
| Ndufb5 | -0.804263 |
| Ndufb6 | -0.6526919 |
| Ndufb6 | 1.52633888 |
| Ndufb7 | -0.356822 |
| Ndufb8 | 0.83443962 |
| Ndufb9 | -0.3986488 |
| Ndufc1 | -0.6789686 |
| Ndufc1 | -0.2504656 |
| Ndufc2 | 1.06435799 |
| Ndufs2 | 0.4115011 |
| Ndufs2 | 2.15579551 |
| Ndufs3 | 2.21355637 |
| Ndufs4 | 0.92665289 |
| Ndufs4 | -0.5238117 |
| Ndufs4 | -0.3733288 |
| Ndufs4 | 1.71197486 |
| Ndufs6 | 2.03149752 |
| Ndufs7 | 0.43282793 |
| Ndufs8 | -0.4732852 |
| Ndufv1 | -0.6440947 |
| Ndufv2 | 0.83882659 |
| Necab1 | 2.63720584 |
| Necab2 | 0.47303958 |
| Necab3 | 3.54082767 |
| Necap1 | 1.13938101 |
| Necap1 | 1.76660432 |
| Necap1 | 1.34793673 |
| Necap2 | -0.2510274 |
| Nedd1 | 2.05613641 |
| Nedd4 | 1.31265622 |
| Nedd4l | 0.33187093 |
| Nedd8 | -0.4186036 |
| Nedd9 | -0.5740456 |
| Nedd9 | -0.5344055 |
| Nefm | -0.5852279 |
| Negr1 | 0.14771538 |
| Negr1 | 2.22981956 |
| Neil1 | -0.7366785 |
| Neil2 | 1.85475241 |
| Nek11 | 1.54994625 |
| Nek3 | -0.0642137 |
| Nek3 | -0.5981737 |
| Nek3 | 2.17480606 |
| Nek4 | 0.81237051 |
| Nek5 | 0.81560661 |
| Nek6 | -0.6113014 |
| Nek8 | -0.6950967 |
| Nek9 | 1.55190727 |
| Nek9 | 1.91088237 |
| Nelf | -0.3393657 |
| Nelf | 0.71308904 |
| Nell2 | 0.27889849 |
| Nell2 | -0.388178 |
| Nell2 | -0.4563321 |
| Nell2 | 1.72284657 |
| Nenf | -0.7198231 |
| Neo1 | -0.0651344 |
| Nes | 0.80821912 |
| Net1 | -0.6146124 |
| Net1 | 0.22375678 |
| Neto1 | 0.19037165 |
| Neto1 | -0.6736538 |
| Neto1 | 1.40542695 |
| Neu1 | -0.1359139 |
| Neu2 | -0.7538435 |
| Neu3 | -0.4891004 |
| Neu4 | 2.15938158 |
| Neu4 | -0.5088831 |
| Neu4 | 0.5588303 |
| Neurl | -0.81345 |
| Neurod1 | 0.07489903 |
| Neurod2 | 1.99100889 |
| Neurod4 | 0.53223426 |
| Neurog1 | 1.47584037 |
| Neurog2 | 2.99914199 |
| Nf1 | -0.095562 |
| Nfasc | -0.6745375 |
| Nfasc | 1.72853946 |
| Nfasc | -0.6355758 |
| Nfat5 | 2.90835793 |
| Nfat5 | -0.3907847 |
| Nfatc1 | -0.5096238 |
| Nfatc1 | 0.69529598 |
| Nfatc2 | 0.39869173 |
| Nfatc2 | 1.86233649 |
| Nfatc2ip | -0.7235426 |
| Nfatc3 | -0.8206584 |
| Nfatc4 | -0.5685042 |
| Nfe2 | 1.31476264 |
| Nfe2l1 | -0.1324426 |
| Nfe2l2 | -0.1046466 |
| Nfe2l3 | -0.3493008 |
| Nfib | -0.1938112 |
| Nfic | 0.54286756 |
| Nfic | 0.2425012 |
| Nfic | -0.5088831 |
| Nfix | 0.21628879 |
| Nfix | 0.19386908 |
| Nfkb1 | -0.0541454 |
| Nfkb2 | -0.6321899 |
| Nfkbia | -0.6570204 |
| Nfkbib | 0.73447288 |
| Nfkbid | 1.0687486 |
| Nfkbie | 1.57830358 |
| Nfkbil1 | 0.29281835 |
| Nfkbil2 | 0.792715 |
| Nfkbiz | 1.8619851 |
| Nfrkb | -0.5298506 |
| Nfrkb | 1.96661095 |
| Nfs1 | 1.12865844 |
| Nfs1 | 2.19532557 |
| Nfs1 | -0.7226113 |
| Nfu1 | -0.4697279 |
| Nfx1 | -0.2001757 |
| Nfya | 0.62097129 |
| Nfyb | 0.09645509 |
| Nfyc | 0.5682319 |
| Ngb | 1.16393199 |
| Ngb | 1.19230252 |
| Ngef | 1.72515501 |
| Ngfb | -0.8521972 |
| Ngfb | 0.0306511 |
| Ngfr | -0.2703315 |
| Ngfrap1 | -0.4790053 |
| Ngp | -0.4079081 |
| Nhedc1 | -0.2795556 |
| Nhej1 | -0.1953982 |
| Nhlrc1 | -0.1428953 |
| Nhlrc2 | -0.6987189 |
| Nhlrc2 | -0.8554105 |
| Nhp2l1 | 1.95331933 |
| Nhsl1 | -0.1906451 |
| Niban | -0.388178 |
| Nicn1 | -0.2941531 |
| Nicn1 | 3.56806067 |
| Nid1 | -0.7762456 |
| Nid2 | -0.105608 |
| Nif3l1 | -0.4993058 |
| Nin | 0.67235821 |
| Ninj1 | -0.5351668 |
| Ninj2 | -1.0014352 |
| Nip7 | -0.2370834 |
| Nipa1 | -0.3173905 |
| Nipbl | -0.1544793 |
| Nipbl | 1.97531627 |
| Nipsnap1 | 0.02606709 |
| Nipsnap3a | -0.5622112 |
| Nipsnap3a | 0.65887235 |
| Nisch | 0.55906649 |
| Nit1 | 0.16910766 |
| Nit2 | -0.300649 |
| Nit2 | -0.7654645 |
| Nkain3 | -0.7452216 |
| Nkain4 | -0.9383833 |
| Nkd2 | -0.3375145 |
| Nkg7 | -0.0988987 |
| Nkiras1 | -0.7179689 |
| Nkiras2 | 1.50160298 |
| Nkrf | 1.31465743 |
| Nktr | 3.33517868 |
| Nkx1-2 | 1.00766057 |
| Nkx2-1 | -0.2760859 |
| Nkx2-2 | -0.7901493 |
| Nkx2-2 | 0.81883154 |
| Nkx2-3 | -0.7235426 |
| Nkx2-4 | -0.1162496 |
| Nkx2-6 | 2.7902169 |
| Nkx3-1 | 1.1491031 |
| Nkx6-1 | 2.78372962 |
| Nle1 | 0.89653085 |
| Nlgn1 | 0.41674111 |
| Nlgn2 | 1.5597105 |
| Nlk | -0.4410575 |
| Nln | 0.14991337 |
| Nln | -0.1408953 |
| Nln | -0.7782192 |
| Nlrp14 | 0.62735925 |
| Nlrp4a | -0.2824569 |
| Nlrp4c | 2.34608145 |
| Nlrp5 | 0.16910766 |
| Nlrp5 | 2.06499307 |
| Nlrp5 | -0.7142716 |
| Nlrp9a | -0.7461757 |
| Nlrp9c | -1.0204429 |
| Nlrx1 | -0.7509607 |
| Nlrx1 | 2.74029044 |
| Nmb | -0.2795556 |
| Nmbr | -0.3791113 |
| Nmd3 | 1.71403583 |
| Nme1 | 0.23751713 |
| Nme2 | 1.01145229 |
| Nme2 | -0.4883757 |
| Nme2 | -0.5103652 |
| Nme3 | 1.10854389 |
| Nme4 | 2.88869059 |
| Nme4 | 0.17838152 |
| Nme4 | -0.9466273 |
| Nme5 | 1.07066303 |
| Nme7 | -0.3656741 |
| Nme7 | 1.18628659 |
| Nme7 | -0.6415309 |
| Nme7 | 1.73164236 |
| Nme7 | 0.60041209 |
| Nmnat1 | 2.13095925 |
| Nmnat2 | -0.2663244 |
| Nmnat3 | 1.43825972 |
| Nmral1 | 0.46497778 |
| Nmt1 | -0.5155714 |
| Nmu | 0.06132041 |
| Nmur2 | -0.7490437 |
| Nnat | 0.68400078 |
| Nnat | 0.194567 |
| Nnmt | 1.65351333 |
| Nnmt | -0.2947418 |
| Nob1 | -0.4493581 |
| Nob1 | 1.03018351 |
| Nobox | 0.79876483 |
| Noc3l | -0.3436993 |
| Noc4l | -0.5844247 |
| Noc4l | -0.7347906 |
| Nod1 | 0.47717345 |
| Nog | 1.12853005 |
| Nol1 | 2.39945015 |
| Nol1 | 0.04303987 |
| Nol1 | 3.82132725 |
| Nol10 | 0.42113107 |
| Nol11 | 0.22578319 |
| Nol12 | -0.7347906 |
| Nol14 | -0.4179315 |
| Nol3 | 0.13775994 |
| Nol4 | 2.22542297 |
| Nol4 | -0.5474329 |
| Nol4 | 1.2101214 |
| Nol5 | 2.25435028 |
| Nol5a | 3.51503729 |
| Nol6 | -0.5860318 |
| Nol7 | -0.354936 |
| Nol9 | -0.5336448 |
| Nola1 | 2.8929761 |
| Nola2 | -0.3499252 |
| Nola3 | -0.6022564 |
| Nola3 | -0.6355758 |
| Nolc1 | 0.61320306 |
| Nomo1 | -0.572459 |
| Nono | 0.21731068 |
| Nope | -0.343079 |
| Nos1 | 0.03106672 |
| Nos1ap | -0.554404 |
| Nos2 | -0.3927446 |
| Nos3 | 0.31054802 |
| Nos3 | -0.6834208 |
| Nos3 | -0.5614275 |
| Nosip | 1.33680355 |
| Nosip | -0.6296585 |
| Nosip | -0.6526919 |
| Nostrin | 0.72146322 |
| Notch1 | -0.2560986 |
| Notch3 | 0.76603683 |
| Notch4 | -0.7891493 |
| Notch4 | -0.3295351 |
| Notum | -0.5567393 |
| Nova2 | 0.2261205 |
| Nox4 | 1.20941513 |
| Noxo1 | -0.5374544 |
| Noxo1 | 2.79106258 |
| Npal2 | 0.48205879 |
| Npas1 | 0.84684521 |
| Npas2 | -0.1344246 |
| Npas4 | -0.1514435 |
| Npb | -0.6196009 |
| Npc2 | 1.25727278 |
| Npcd | 1.60366588 |
| Npcd | -0.8012207 |
| Npdc1 | -0.4039286 |
| Npepl1 | 1.79232469 |
| Npff | -0.6364242 |
| Nphp1 | 0.33577788 |
| Nphp1 | -0.6701274 |
| Nphp3 | -0.2555338 |
| Nphp4 | -0.2044428 |
| Nphs2 | -0.3350521 |
| Nploc4 | 0.41205406 |
| Npm1 | 0.1952644 |
| Npm2 | 2.1339852 |
| Npm3 | -0.6271339 |
| Npm3 | 1.54230815 |
| Npm3-ps1 | 1.8134156 |
| Npr1 | 1.70174035 |
| Npr2 | -0.4493581 |
| Npr3 | -0.140396 |
| Npsr1 | -0.4668917 |
| Nptn | -0.3593426 |
| Nptx1 | -0.2640423 |
| Nptx2 | 0.90390797 |
| Nptxr | 1.41293922 |
| Npy | 0.52098672 |
| Npy1r | -0.0851521 |
| Npy2r | 0.29595325 |
| Npy5r | -0.3656741 |
| Nqo1 | -0.5559602 |
| Nqo2 | 1.25850263 |
| Nqo2 | -0.4314656 |
| Nqo2 | 0.00792884 |
| Nr1d1 | 0.27057458 |
| Nr1d2 | 1.85805228 |
| Nr1h2 | -0.7347906 |
| Nr1h2 | -0.0984213 |
| Nr1h3 | -0.7069204 |
| Nr1h4 | -0.9573347 |
| Nr1h5 | -0.741415 |
| Nr1i2 | -0.3746107 |
| Nr2c1 | 1.32875448 |
| Nr2c2ap | 1.06614417 |
| Nr2e1 | 4.31702412 |
| Nr2f2 | -0.4761409 |
| Nr2f2 | 0.35624572 |
| Nr2f6 | 0.3456228 |
| Nr2f6 | 1.09373524 |
| Nr4a2 | 0.13516149 |
| Nr4a3 | 0.74459686 |
| Nr4a3 | -0.4570324 |
| Nr5a1 | -0.3816928 |
| Nr5a2 | -0.7404658 |
| Nr5a2 | 2.45637596 |
| Nr5a2 | 0.39447652 |
| Nr6a1 | -0.5941089 |
| Nradd | 1.26584788 |
| Nrap | -0.4369346 |
| Nrarp | -0.1765292 |
| Nras | -0.3993072 |
| Nrbf2 | -0.7096703 |
| Nrbp1 | -0.3505501 |
| Nrbp2 | 3.51832765 |
| Nrcam | 1.51985708 |
| Nrd1 | -0.4172599 |
| Nrf1 | -0.8405091 |
| Nrg1 | -0.2431931 |
| Nrg3 | -0.6780807 |
| Nrg4 | -0.4768561 |
| Nrgn | -0.1250473 |
| Nrip2 | -0.12603 |
| Nrip3 | 1.3818357 |
| Nrl | -0.4934605 |
| Nrm | 0.0520217 |
| Nrn1 | -0.7179689 |
| Nrn1l | -0.2060481 |
| Nrp1 | 0.27089611 |
| Nrp1 | 0.27378486 |
| Nrp2 | 0.98518033 |
| Nrp2 | -0.7207516 |
| Nrsn2 | -0.4273848 |
| Nrsn2 | -0.3173905 |
| Nrtn | -0.3393657 |
| Nrxn1 | 1.14734295 |
| Nrxn1 | 2.30728851 |
| Nrxn1 | 2.21267134 |
| Nrxn2 | 0.16731363 |
| Nrxn3 | -0.4092383 |
| Nrxn3 | -0.1723873 |
| Nsbp1 | 0.23751713 |
| Nsd1 | -0.3543082 |
| Nsd1 | -0.697812 |
| Nsdhl | 0.02857025 |
| Nsdhl | 4.13938797 |
| Nsdhl | -0.6834208 |
| Nsf | 0.28017253 |
| Nsfl1c | 0.14182867 |
| Nsg1 | 0.53659888 |
| Nsg2 | 0.96091774 |
| Nsl1 | 0.07648362 |
| Nsmaf | -0.6237784 |
| Nsmaf | -0.5716667 |
| Nsmce1 | -0.7951655 |
| Nsmce2 | -0.4403691 |
| Nsmce2 | 1.96246444 |
| Nsmce2 | -0.5306082 |
| Nsun2 | -0.5590805 |
| Nsun4 | -0.4099042 |
| Nsun5 | -0.5382182 |
| Nsun5 | 1.27894745 |
| Nsun5 | 0.0218802 |
| Nsun5 | -0.3332094 |
| Nt5c | -0.7357341 |
| Nt5c1b | -0.6905887 |
| Nt5c2 | 0.90096411 |
| Nt5c2 | -0.5998047 |
| Nt5c2 | -0.3842815 |
| Nt5c3 | 0.771509 |
| Nt5c3l | -0.388829 |
| Nt5c3l | -0.0325476 |
| Nt5dc3 | -0.2755089 |
| Nt5dc3 | -0.6745375 |
| Nt5dc3 | 2.53056361 |
| Nt5e | -0.5756349 |
| Nt5e | -0.6807469 |
| Nt5m | -0.3914376 |
| Nt5m | 0.56635913 |
| Ntan1 | -0.8216927 |
| Ntf3 | 0.2634729 |
| Nthl1 | -0.4854825 |
| Ntn2l | -0.5844247 |
| Ntn2l | -0.379756 |
| Ntn4 | -0.0912894 |
| Ntng1 | -0.6825286 |
| Ntng1 | -0.5998047 |
| Ntng1 | 0.86608585 |
| Ntrk2 | 0.31852486 |
| Ntrk2 | -0.3424591 |
| Ntrk2 | -0.4605417 |
| Ntrk3 | 1.31843749 |
| Ntrk3 | -0.759636 |
| Ntrk3 | 1.19362085 |
| Nts | 0.04303987 |
| Ntsr1 | -0.5155714 |
| Ntsr2 | 1.12040549 |
| Nuak1 | -0.6229414 |
| Nuak1 | -0.5916784 |
| Nuak2 | 1.38124802 |
| Nub1 | -0.0739266 |
| Nubp1 | -0.240411 |
| Nubp2 | 0.09645509 |
| Nubp2 | -0.7161184 |
| Nubp2 | 1.19863741 |
| Nucb1 | 0.79124246 |
| Nucb1 | 0.12243666 |
| Nucks1 | 0.47226243 |
| Nudc | -0.7423652 |
| Nudc | 0.06973047 |
| Nudcd2 | 0.02062046 |
| Nudcd3 | 1.5375128 |
| Nudc-ps1 | 0.87707327 |
| Nudt1 | 2.36391513 |
| Nudt10 | -0.1703232 |
| Nudt11 | 0.72818778 |
| Nudt12 | 0.98203155 |
| Nudt13 | 1.15561192 |
| Nudt14 | 0.94854606 |
| Nudt16l1 | -0.7282128 |
| Nudt18 | 0.88992416 |
| Nudt19 | -0.6736538 |
| Nudt19 | 1.58651982 |
| Nudt2 | -0.0908158 |
| Nudt21 | -0.3511754 |
| Nudt22 | 0.0053697 |
| Nudt4 | 0.67590792 |
| Nudt5 | 1.45243936 |
| Nudt5 | 0.68151813 |
| Nudt6 | 0.76660441 |
| Nudt7 | -0.6710077 |
| Nudt8 | -0.2049776 |
| Nudt9 | -0.6063569 |
| Nufip1 | -0.5567393 |
| Numa1 | 1.02388565 |
| Numb | 0.03314203 |
| Numb | -0.0804584 |
| Numb | -0.6381234 |
| Numb | 1.36553824 |
| Numbl | 0.18791564 |
| Nup107 | 0.06332968 |
| Nup133 | -0.2124967 |
| Nup133 | 1.03813806 |
| Nup155 | 1.16207571 |
| Nup155 | 0.28588434 |
| Nup160 | -0.5614275 |
| Nup210 | -0.6631141 |
| Nup37 | 1.77788811 |
| Nup43 | 1.67565269 |
| Nup50 | -0.469018 |
| Nup50 | -0.365039 |
| Nup50 | 1.41596507 |
| Nup62 | 0.38143001 |
| Nup85 | -0.4287431 |
| Nup88 | 1.00736826 |
| Nup88 | -0.7991979 |
| Nup88 | -0.2634727 |
| Nup93 | 1.20327166 |
| Nup98 | -0.2499042 |
| Nup98 | -0.0541454 |
| Nupl1 | -0.2906289 |
| Nupl1 | -0.2310133 |
| Nupl1 | -0.6129554 |
| Nupl2 | -0.446583 |
| Nupr1 | -0.2737799 |
| Nus1 | 1.59738955 |
| Nusap1 | -0.3185979 |
| Nut | 1.81415571 |
| Nutf2 | 0.15538583 |
| Nvl | -0.5606445 |
| Nwd1 | 0.87774522 |
| Nxf1 | 2.35410724 |
| Nxf2 | -0.7772319 |
| Nxf3 | -0.4739983 |
| Nxph1 | 1.02172892 |
| Nxph2 | 1.59785591 |
| Nxph3 | 1.24940837 |
| Nxt1 | 1.11690179 |
| Nxt1 | 0.59928167 |
| Nxt2 | -0.0352202 |
| Nyx | -0.4045907 |
| Oaf | -0.2971002 |
| Oas1b | 0.38000087 |
| Oas1b | -0.2941531 |
| Oas1c | -0.2493431 |
| Oas1d | 0.02355721 |
| Oas1d | 0.57985245 |
| Oas1e | 0.84075036 |
| Oas1g | -0.554404 |
| Oas1g | 1.42526471 |
| Oas2 | 0.29782916 |
| Oasl1 | 1.05231568 |
| Oasl2 | 0.15320069 |
| Oasl2 | -0.6526919 |
| Oat | 1.88512636 |
| Oaz2 | -0.0352202 |
| Oaz3 | 0.82382608 |
| Obfc1 | -0.6745375 |
| Obfc2a | -0.782179 |
| Obfc2b | 0.04876556 |
| Obfc2b | 2.30181668 |
| Obox1 | -0.425351 |
| Obox5 | -0.7050916 |
| Obrgrp | -0.5118498 |
| Obsl1 | 0.16839046 |
| Oc90 | -0.3746107 |
| Oca2 | 0.6967238 |
| Ociad1 | -0.5582994 |
| Ociad1 | 0.42550051 |
| Ociad2 | -0.5677153 |
| Ocln | 1.32000801 |
| Ocrl | 0.03272733 |
| Odc1 | 1.33680355 |
| Odf1 | -0.3101788 |
| Odf2 | 0.03727887 |
| Odf2 | -0.2017736 |
| Odf2 | -0.2634727 |
| Odf2 | 1.15797887 |
| Odf3 | 0.30622443 |
| Odf3l1 | -0.2959202 |
| Odf4 | -0.6923893 |
| Odf4 | -0.3277033 |
| Odz1 | -0.7742761 |
| Odz3 | 0.32035604 |
| Odz3 | -0.2499042 |
| Odz4 | 0.22003025 |
| Og9x | 0.58262001 |
| Ogdh | -0.4825982 |
| Ogfod1 | -0.4335127 |
| Ogfod2 | -0.22389 |
| Ogfr | 0.67819763 |
| Ogfrl1 | -0.379756 |
| Ogg1 | 0.63435774 |
| Ogn | 1.95866925 |
| Ogn | 0.50510931 |
| Ogn | -0.4486636 |
| Ogt | -0.4949184 |
| Ogt | 0.06613542 |
| Ogt | 1.44249037 |
| Oit1 | 1.09719394 |
| Oit3 | 0.27921716 |
| Ola1 | 0.66837487 |
| Olfm1 | -0.6561531 |
| Olfm1 | -0.7461757 |
| Olfm2 | 0.16946606 |
| Olfm2 | 0.08043338 |
| Olfm3 | 0.43471829 |
| Olfm3 | -0.3071903 |
| Olfm4 | 2.01259491 |
| Olfml1 | -0.7772319 |
| Olfml2b | -0.6080022 |
| Olfml3 | 1.79503443 |
| Olfr100 | -0.0014891 |
| Olfr1006 | 1.68412275 |
| Olfr1012 | -0.7606049 |
| Olfr1020 | -0.2510274 |
| Olfr1065 | 1.30290507 |
| Olfr1094 | 0.99515609 |
| Olfr1099 | -0.5820193 |
| Olfr1105 | 3.11291098 |
| Olfr1112 | 1.45769046 |
| Olfr1113 | -0.3192022 |
| Olfr1189 | 0.44063464 |
| Olfr1197 | -0.3836337 |
| Olfr1198 | 1.67772391 |
| Olfr1249 | -0.6096504 |
| Olfr1280 | -0.6372734 |
| Olfr133 | -0.3192022 |
| Olfr1330 | -0.5111072 |
| Olfr134 | 1.03274772 |
| Olfr1340 | -0.5973594 |
| Olfr1384 | 3.01561519 |
| Olfr1386 | -0.7723108 |
| Olfr1390 | 0.65781052 |
| Olfr1394 | -0.5598622 |
| Olfr1412 | 1.96414626 |
| Olfr1437 | 0.90145539 |
| Olfr144 | 1.9101038 |
| Olfr1443 | 1.72032794 |
| Olfr1443 | 0.18931987 |
| Olfr1459 | -0.140396 |
| Olfr1500 | 0.59882912 |
| Olfr1508 | -0.343079 |
| Olfr166 | -0.4761409 |
| Olfr168 | 2.11977953 |
| Olfr206 | 2.1185785 |
| Olfr213 | -0.1651829 |
| Olfr215 | -0.1250473 |
| Olfr232 | 0.2887272 |
| Olfr257 | -0.0329925 |
| Olfr267 | -0.4528386 |
| Olfr272 | -0.5088831 |
| Olfr303 | 2.11304275 |
| Olfr310 | 3.62897001 |
| Olfr352 | -0.4797227 |
| Olfr371 | 1.69546141 |
| Olfr378 | 2.0166246 |
| Olfr419 | 2.07953921 |
| Olfr437 | -0.7452216 |
| Olfr464 | -0.6692478 |
| Olfr47 | 0.98398204 |
| Olfr49 | -0.7490437 |
| Olfr503 | -0.7674155 |
| Olfr51 | 2.89404441 |
| Olfr536 | 1.91021506 |
| Olfr538 | -0.7664395 |
| Olfr54 | -0.8331465 |
| Olfr550 | 1.52482767 |
| Olfr574 | -0.5908697 |
| Olfr591 | 3.6440209 |
| Olfr593 | 0.14182867 |
| Olfr608 | -0.7263419 |
| Olfr638 | -0.3222294 |
| Olfr65 | 1.72833673 |
| Olfr655 | 0.69855638 |
| Olfr658 | -0.3947085 |
| Olfr665 | -0.8352442 |
| Olfr675 | 0.4175658 |
| Olfr691 | 0.58881721 |
| Olfr692 | 0.21321642 |
| Olfr702 | -0.3543082 |
| Olfr724 | 0.83355974 |
| Olfr725 | -0.1334331 |
| Olfr748 | 0.75595241 |
| Olfr786 | 0.1284464 |
| Olfr802 | -0.8268816 |
| Olfr824 | -0.3399835 |
| Olfr91 | 1.40685799 |
| Olfr910 | 2.22637534 |
| Olfr920 | -0.7179689 |
| Olfr924 | 2.03032419 |
| Olfr952 | 2.14026883 |
| Olfr965 | 0.24051076 |
| Olfr982 | 0.07885548 |
| Olfr983 | 0.52662741 |
| Olfr984 | -0.6415309 |
| Olig1 | -0.7124283 |
| Olig3 | -0.3149804 |
| Oma1 | -0.5245644 |
| Omd | 0.64432712 |
| Omg | -0.0757882 |
| Omt2b | -0.5949204 |
| Onecut2 | -0.6657379 |
| Onecut3 | -0.8837312 |
| Onecut3 | 1.09984579 |
| Oog4 | -0.1713547 |
| Oosp1 | -0.5374544 |
| Opa1 | -0.3461847 |
| Opa1 | 1.86566808 |
| Ophn1 | 0.25892539 |
| Oplah | 2.50336236 |
| Opn1mw | -0.2001757 |
| Opn3 | -0.4369346 |
| Oprl1 | 0.14771538 |
| Oprl1 | -0.81345 |
| Oprl1 | 1.03374301 |
| Oprs1 | -0.5283372 |
| Optc | 0.11790385 |
| Optn | 0.23284122 |
| Optn | 0.25402837 |
| Orai1 | -0.4612451 |
| Orai3 | -0.5716667 |
| Oraov1 | 0.419213 |
| Orc2l | 2.55334081 |
| Orc3l | -0.5780239 |
| Orc5l | -0.4045907 |
| Orc5l | -0.6254547 |
| Orc6l | 0.80948454 |
| ORF19 | 1.6326353 |
| ORF28 | -0.403267 |
| ORF34 | 0.08710989 |
| ORF61 | 0.63435774 |
| ORF63 | -0.3071903 |
| ORF9 | -0.4079081 |
| ORF9 | -0.8062968 |
| ORF9 | -0.6843138 |
| Orm1 | 0.70119711 |
| Ormdl2 | 0.56142518 |
| Ormdl3 | -0.4026059 |
| Os9 | -0.6878943 |
| Osbp2 | -0.2288157 |
| Osbpl10 | 0.25957639 |
| Osbpl11 | 1.20953287 |
| Osbpl2 | -0.2001757 |
| Osbpl3 | 0.34473208 |
| Osbpl3 | -0.3264841 |
| Osbpl6 | -0.6475241 |
| Osbpl8 | -0.6578885 |
| Osbpl9 | -0.3842815 |
| Oscar | -0.2900428 |
| Osgep | 0.35212892 |
| Osgepl1 | 1.27357573 |
| Osgin1 | 0.86829369 |
| Osm | -0.859712 |
| Osmr | 1.79836357 |
| Osr1 | 1.52322986 |
| Osr2 | -0.5096238 |
| Ostf1 | -0.1464057 |
| Ostm1 | -0.7500017 |
| Otof | 0.55147843 |
| Otop1 | 0.22274193 |
| Otop2 | 0.43848757 |
| Ott | -0.8760216 |
| Ott | 1.58691302 |
| OTTMUSG00000000421 | 1.39737242 |
| OTTMUSG00000000971 | -0.7282128 |
| OTTMUSG00000000990 | 1.67900719 |
| OTTMUSG00000001305 | -0.6825286 |
| OTTMUSG00000002038 | 1.1957741 |
| OTTMUSG00000004461 | -0.0001993 |
| OTTMUSG00000004551 | -0.3966766 |
| OTTMUSG00000005148 | 2.16337832 |
| OTTMUSG00000005491 | -0.3012417 |
| OTTMUSG00000008540 | 2.22442988 |
| OTTMUSG00000010009 | -0.4119046 |
| OTTMUSG00000010328 | 2.11133973 |
| OTTMUSG00000015762 | -0.6047145 |
| OTTMUSG00000015859 | 0.97706193 |
| OTTMUSG00000016293 | -0.3283135 |
| OTTMUSG00000016790 | -0.1094637 |
| OTTMUSG00000016790 | 0.1118257 |
| OTTMUSG00000017677 | 0.11106315 |
| OTTMUSG00000020946 | 0.77263719 |
| Otub1 | -0.5582994 |
| Otub2 | 0.0729145 |
| Otub2 | -0.1524544 |
| Otub2 | -0.5820193 |
| Otud5 | 0.26541508 |
| Otud6b | 0.46732548 |
| Otud7b | 0.39363119 |
| Otud7b | 2.22803965 |
| Otx1 | -0.2877021 |
| Otx2 | -0.3920908 |
| Ovca2 | -0.6896897 |
| Ovgp1 | 1.16021574 |
| Ovgp1 | 1.51799685 |
| Ovol1 | 1.02589412 |
| Ovol2 | 1.92478692 |
| Ovol2 | 0.20979079 |
| Oxct1 | -0.3823393 |
| Oxct2a | -0.5328847 |
| Oxct2b | 2.51656782 |
| Oxgr1 | 0.53296311 |
| Oxnad1 | 0.14771538 |
| Oxr1 | 0.49530005 |
| Oxr1 | 0.23618354 |
| Oxt | -0.5637805 |
| P140 | 0.35829734 |
| P2rx2 | -0.3739695 |
| P2rx3 | 0.54813918 |
| P2rx4 | 0.05970987 |
| P2rx4 | -0.759636 |
| P2rx5 | 0.98667602 |
| P2rx7 | -0.796172 |
| P2rx7 | 2.28110069 |
| P2ry1 | 4.42433031 |
| P2ry12 | -0.447276 |
| P2ry13 | 2.72673652 |
| P2ry14 | -0.3258751 |
| P2ry14 | -0.3593426 |
| P2ry5 | -0.3752523 |
| P2ry6 | 0.89653085 |
| P42pop | 0.20151766 |
| P4ha1 | -0.5884476 |
| P4ha2 | 0.01005612 |
| P4hb | 1.32916894 |
| Pa2g4 | -0.2795556 |
| Pabpc1 | -0.4535363 |
| Pabpc1 | -0.1339287 |
| Pabpc1 | -0.1718708 |
| Pabpc2 | -0.1590515 |
| Pabpc4 | -0.3849298 |
| Pabpn1 | -0.758668 |
| Pabpnl1 | -0.8237648 |
| Pacrg | 2.64468849 |
| Pacs1 | -0.4493581 |
| Pacs2 | 0.62998983 |
| Pacsin1 | 0.01175446 |
| Pacsin1 | 0.82613593 |
| Pacsin1 | -0.8458038 |
| Pacsin2 | 1.24443093 |
| Pacsin2 | 0.44090268 |
| Pacsin3 | 0.4290357 |
| Pacsin3 | 0.59905543 |
| Padi1 | 1.9562894 |
| Padi2 | 0.07687934 |
| Padi6 | -0.1294775 |
| Paf1 | -0.362503 |
| Paf1 | 0.63173945 |
| Pafah1b1 | -0.5148259 |
| Pafah1b1 | -0.0706777 |
| Pafah1b3 | 0.73192465 |
| Pafah2 | 0.21424167 |
| Pafah2 | -0.7291497 |
| Paip1 | 1.17328088 |
| Paip2 | 1.92275829 |
| Paip2b | -0.6458078 |
| Paip2b | 0.67193971 |
| Pak1 | -0.6825286 |
| Pak3 | 1.15273861 |
| Pak4 | 0.81273063 |
| Pak6 | 1.56688699 |
| Pak7 | 0.35771162 |
| Palld | 1.27368566 |
| Palm | 1.44706728 |
| Palm | -0.3816928 |
| Palm2 | -0.6736538 |
| Palmd | -0.5653524 |
| Palmd | 3.31712151 |
| Palmd | 2.88794837 |
| Pam | 2.87039407 |
| Pank1 | -0.3663096 |
| Pank1 | -0.6146124 |
| Pank2 | 2.11187786 |
| Pank3 | 1.52348233 |
| Pank4 | 1.38095404 |
| Panx1 | -0.661369 |
| Panx3 | -0.7310262 |
| Paox | -0.6736538 |
| Paox | 1.5163873 |
| Paox | 0.34740169 |
| Paox | 1.95039307 |
| Papd1 | -0.6771936 |
| Papd1 | -0.5397477 |
| Papd4 | -0.0743916 |
| Papln | 0.14440867 |
| Papola | 1.10103667 |
| Papolb | -0.4396812 |
| Papolg | -0.3399835 |
| Papss1 | -0.0083987 |
| Papss1 | -0.7041785 |
| Papss2 | 1.38973344 |
| Paqr3 | 1.24726227 |
| Paqr6 | 0.10991815 |
| Paqr7 | 0.29595325 |
| Paqr9 | -0.6622411 |
| Paqr9 | 0.02815352 |
| Paqr9 | 1.69363961 |
| Pard3 | 0.66310762 |
| Pard3 | -0.6088259 |
| Pard6a | -0.7811875 |
| Pard6a | 0.49428813 |
| Pard6b | 0.330967 |
| Pard6g | -0.3192022 |
| Park7 | -0.3192022 |
| Parl | 0.4802619 |
| Parp1 | 0.8933985 |
| Parp1 | -0.3480531 |
| Parp1 | 3.77570347 |
| Parp12 | -0.6113014 |
| Parp14 | -0.3656741 |
| Parp16 | -0.6887916 |
| Parp2 | -0.4314656 |
| Parp2 | -0.8426233 |
| Parp3 | 0.05039505 |
| Parp6 | -0.8521972 |
| Parp8 | 0.67715755 |
| Pars2 | -0.7142716 |
| Pars2 | -0.4934605 |
| Parva | -0.6212697 |
| Parvg | -0.6914886 |
| Pask | -0.0370062 |
| Patl1 | 0.00835469 |
| Patz1 | -0.6648625 |
| Pawr | -0.1641582 |
| Pax8 | -0.4683087 |
| Paxip1 | -0.6960009 |
| Pbk | 1.68871704 |
| Pbld | -0.0294392 |
| Pbp2 | 0.23417958 |
| Pbrm1 | 0.29501389 |
| Pbrm1 | -0.5804191 |
| Pbx1 | -0.477572 |
| Pbx1 | -0.4647702 |
| Pbx2 | 0.85823132 |
| Pbx2 | 1.42685235 |
| Pbx2 | -0.0619159 |
| Pbx3 | 1.04826684 |
| Pbx3 | 0.48436401 |
| Pbx4 | 1.7485927 |
| Pcaf | 0.13553314 |
| Pcaf | -0.8320994 |
| Pcbd1 | 2.30659718 |
| Pcbd2 | -0.3107777 |
| Pcbp1 | -0.6449509 |
| Pcbp2 | -0.6262939 |
| Pcbp3 | -0.6631141 |
| Pcca | 0.79289891 |
| Pcca | -0.4445071 |
| Pccb | -0.4934605 |
| Pccb | -0.0245754 |
| Pcdh1 | 0.68997344 |
| Pcdh10 | -0.7772319 |
| Pcdh10 | 2.93042482 |
| Pcdh10 | 0.2997013 |
| Pcdh15 | 1.5519889 |
| Pcdh17 | -0.1089808 |
| Pcdh20 | 1.72820156 |
| Pcdh21 | -0.6825286 |
| Pcdh7 | 3.07882884 |
| Pcdh7 | 2.11263968 |
| Pcdh8 | -0.7376239 |
| Pcdh9 | 2.01488551 |
| Pcdha10 | 0.23417958 |
| Pcdha11 | 0.30622443 |
| Pcdha2 | -0.0298827 |
| Pcdha6 | 4.76613534 |
| Pcdhac1 | 1.20089799 |
| Pcdhac2 | 0.13516149 |
| Pcdhb1 | -0.2923893 |
| Pcdhb10 | 3.28536901 |
| Pcdhb12 | -0.4112373 |
| Pcdhb14 | 0.55764842 |
| Pcdhb19 | 1.1226048 |
| Pcdhb20 | -0.7752603 |
| Pcdhb22 | -0.175492 |
| Pcdhb3 | -0.3301465 |
| Pcdhb7 | -0.107052 |
| Pcdhb8 | -0.4280637 |
| Pcdhga10 | -0.6305016 |
| Pcdhga12 | -0.3656741 |
| Pcdhga4 | 0.25173429 |
| Pcdhga7 | -0.7519206 |
| Pcdhga8 | 2.39756974 |
| Pcdhgb2 | -0.3947085 |
| Pcdhgb6 | 2.09322232 |
| Pcdhgc3 | 2.90370074 |
| Pcdhgc4 | -0.5892543 |
| Pcdhgc5 | 2.4084695 |
| Pcf11 | 1.27137428 |
| Pcgf1 | -0.5780239 |
| Pcgf2 | 1.40408934 |
| Pcgf3 | 1.80423905 |
| Pcgf5 | -0.3210173 |
| Pcgf6 | -0.6179351 |
| Pck1 | 0.33126841 |
| Pclo | 0.17161354 |
| Pclo | 3.06846493 |
| Pcmt1 | -0.0879797 |
| Pcmt1 | -0.401285 |
| Pcmt1 | -0.4549332 |
| Pcmtd1 | -0.3399835 |
| Pcmtd2 | -0.105608 |
| Pcna | -0.0219331 |
| Pcnt | 1.60936321 |
| Pcnx | -0.4079081 |
| Pcnxl2 | -0.6458078 |
| Pcnxl2 | 0.44304421 |
| Pcolce | 2.17539264 |
| Pcolce2 | 0.82932479 |
| Pcolce2 | 0.42767759 |
| Pcsk1n | 3.08299318 |
| Pcsk2 | 1.28645973 |
| Pcsk2 | -0.6288162 |
| Pcsk2 | 0.98413191 |
| Pcsk2 | 1.35562157 |
| Pcsk4 | 0.88510494 |
| Pcsk4 | -0.7742761 |
| Pcsk4 | 0.03397086 |
| Pcsk4 | -0.0267831 |
| Pcsk4 | 1.88157678 |
| Pcsk9 | 0.60289419 |
| Pctk1 | -0.6179351 |
| Pcx | -0.067902 |
| Pcyox1 | 1.4423986 |
| Pcyox1l | -0.2293646 |
| Pcyt1a | -0.2173626 |
| Pcyt1b | -0.6798573 |
| Pcyt1b | 0.34473208 |
| Pcyt2 | 0.12807193 |
| Pcyt2 | -0.8640335 |
| Pcyt2 | 1.03587223 |
| Pdcd10 | -0.6631141 |
| Pdcd2 | -0.6544209 |
| Pdcd2l | 2.38540456 |
| Pdcd4 | 1.24805351 |
| Pdcd5 | 1.15536241 |
| Pdcd5 | -0.5111072 |
| Pdcd6ip | 1.88780822 |
| Pdcl | -0.6415309 |
| Pdcl3 | 0.93268919 |
| Pddc1 | -0.3599739 |
| Pde10a | -0.6932909 |
| Pde1a | -0.6080022 |
| Pde1a | 0.73662366 |
| Pde1a | 0.52540407 |
| Pde1b | 1.90675968 |
| Pde1c | -0.119173 |
| Pde2a | 0.37368657 |
| Pde3a | -0.7941601 |
| Pde4a | 1.17389267 |
| Pde4a | -0.759636 |
| Pde4a | -0.4092383 |
| Pde4a | -0.7272769 |
| Pde4b | 1.63972091 |
| Pde4d | -0.4993058 |
| Pde4dip | 0.34354313 |
| Pde4dip | -0.3048063 |
| Pde4dip | 0.11866085 |
| Pde4dip | 2.22653397 |
| Pde4dip | -0.2853673 |
| Pde5a | -0.5489775 |
| Pde6a | -0.0813953 |
| Pde6d | -0.3059975 |
| Pde6d | -0.7161184 |
| Pde6d | -0.7357341 |
| Pde6d | 2.68977032 |
| Pde6h | -0.1448996 |
| Pde7a | 0.48257155 |
| Pdgfa | -0.4862049 |
| Pdgfb | 2.61316004 |
| Pdgfc | 0.09412754 |
| Pdgfra | -0.7142716 |
| Pdgfrb | -0.4280637 |
| Pdgfrl | 0.97902281 |
| Pdha1 | 0.70241339 |
| Pdhb | -0.57962 |
| Pdhb | -0.0321029 |
| Pdia3 | -0.2812953 |
| Pdia4 | -0.6262939 |
| Pdia5 | -0.34432 |
| Pdia5 | -0.356193 |
| Pdia6 | 0.1114445 |
| Pdik1l | 0.48997518 |
| Pdk1 | 2.12182218 |
| Pdk2 | -0.6237784 |
| Pdk3 | -0.6154419 |
| Pdk4 | 0.32491836 |
| Pdlim1 | 0.89174566 |
| Pdlim2 | 0.66690303 |
| Pdlim2 | -0.6834208 |
| Pdlim2 | 1.20042254 |
| Pdlim2 | -0.804263 |
| Pdlim3 | 1.09706114 |
| Pdlim3 | 1.30173233 |
| Pdlim4 | -0.3530538 |
| Pdlim5 | 2.25176774 |
| Pdlim5 | -0.5836222 |
| Pdlim7 | -0.7254079 |
| Pdpk1 | -0.4876516 |
| Pdpk1 | 3.42903417 |
| Pdrg1 | -0.7291497 |
| Pdss1 | -0.7625457 |
| Pdxdc1 | 0.86353183 |
| Pdxdc1 | 1.69005573 |
| Pdxk | -0.1964578 |
| Pdxp | -0.7548064 |
| Pdyn | 0.38028687 |
| Pdyn | 1.35783439 |
| Pdzd11 | -0.1959279 |
| Pdzd11 | -0.4417464 |
| Pdzd11 | 0.20255582 |
| Pdzd8 | 2.08398398 |
| Pdzk1 | 0.20220989 |
| Pdzk1ip1 | 0.51531178 |
| Pdzrn3 | -0.5536269 |
| Pdzrn3 | 0.72581993 |
| Pea15 | 0.78180006 |
| Pear1 | 0.26638466 |
| Pear1 | 0.22308033 |
| Pebp1 | -0.5812188 |
| Pecam1 | -0.5780239 |
| Pecam1 | -0.7654645 |
| Peci | 0.85703026 |
| Pef1 | 1.40131247 |
| Peg10 | -0.8782178 |
| Peg3 | -0.011003 |
| Peli1 | -0.8341948 |
| Peli1 | 1.95591866 |
| Peli2 | 1.43687601 |
| Peli3 | -0.0984213 |
| Pelo | -0.3637701 |
| Pelp1 | -0.5125929 |
| Pemt | 0.99010707 |
| Peo1 | 3.60104981 |
| Pepd | 0.13813055 |
| Per1 | 0.23083007 |
| Per1 | -0.5716667 |
| Per2 | 1.02402926 |
| Per2 | -0.5989888 |
| Per2 | 1.62933526 |
| Per3 | -0.1927546 |
| Perld1 | 0.465761 |
| Pes1 | -0.8310535 |
| Pet112l | -0.5505247 |
| Pet112l | 0.39278509 |
| Pet112l | 0.2747458 |
| Pet112l | 1.55027337 |
| Pex11a | 0.72818778 |
| Pex11a | -0.7911504 |
| Pex11a | 1.09293526 |
| Pex11b | -0.6666141 |
| Pex11c | -0.3862277 |
| Pex14 | 0.5588303 |
| Pex16 | 0.75019565 |
| Pex19 | -0.0118727 |
| Pex19 | 1.16553779 |
| Pex2 | -0.500039 |
| Pex26 | 0.41067104 |
| Pex3 | 0.37022435 |
| Pex3 | 3.49675061 |
| Pex5 | 0.11979519 |
| Pex5 | -0.0893967 |
| Pex5 | 2.56002547 |
| Pex5l | -0.3344375 |
| Pex6 | 0.33307481 |
| Pex6 | 1.37830405 |
| Pex6 | -0.2255291 |
| Pex7 | -0.4335127 |
| Pfdn1 | -0.6552866 |
| Pfdn2 | 1.00253192 |
| Pfdn4 | 0.0389285 |
| Pfdn5 | 0.5404615 |
| Pfdn5 | 1.34874864 |
| Pfkfb2 | 1.37139852 |
| Pfkfb2 | -0.8352442 |
| Pfkfb4 | 0.73427711 |
| Pfkl | 1.23451044 |
| Pfkl | -0.5081428 |
| Pfkm | 0.03603974 |
| Pfkm | 2.29155308 |
| Pfkm | 0.15064488 |
| Pfkp | -0.5559602 |
| Pfkp | 0.67861334 |
| Pfkp | 0.837425 |
| Pfn1 | 3.10290471 |
| Pfn1 | -0.3095803 |
| Pfn2 | 0.84632435 |
| Pfn2 | 0.9781183 |
| Pfn2 | 0.330967 |
| Pfn3 | 0.64605012 |
| Pfn4 | 0.14808207 |
| Pftk1 | -0.6869979 |
| Pga5 | 1.39515485 |
| Pgam1 | -0.2044428 |
| Pgam2 | 1.5705747 |
| Pgam5 | 1.1588494 |
| Pgbd5 | -0.0550562 |
| Pgcp | -0.4898257 |
| Pgd | -0.4219712 |
| Pgk1 | 0.13775994 |
| Pgk2 | -0.6432393 |
| Pgls | 1.84257212 |
| Pglyrp1 | -0.1499293 |
| Pglyrp4 | -0.0316585 |
| Pgm2 | -0.4768561 |
| Pgm2l1 | -0.4006253 |
| Pgm3 | -0.5336448 |
| Pgm5 | -0.0523264 |
| Pgm5 | -0.2437505 |
| Pgpep1 | -0.9267309 |
| Pgpep1 | 0.2294869 |
| Pgr | 0.51580663 |
| Pgrmc1 | -0.6305016 |
| Pgs1 | 0.53538853 |
| Phactr1 | -0.6710077 |
| Phactr1 | -0.4711492 |
| Phactr1 | -0.5606445 |
| Phactr4 | 0.33517788 |
| Phb2 | 0.50360688 |
| Phc1 | -0.7635176 |
| Phc1 | -0.4079081 |
| Phc2 | 1.52985555 |
| Phca | 0.11828243 |
| Phca | 2.98002118 |
| Phex | 1.51536931 |
| Phf1 | 0.98173111 |
| Phf11 | 1.65380614 |
| Phf11 | -0.6330353 |
| Phf12 | 0.81038736 |
| Phf12 | 0.85013728 |
| Phf13 | 0.70706121 |
| Phf13 | -0.5772269 |
| Phf14 | 0.58858842 |
| Phf16 | -0.5336448 |
| Phf17 | -0.6055354 |
| Phf19 | 1.4686405 |
| Phf2 | 0.00108883 |
| Phf20l1 | 2.77033035 |
| Phf21a | 0.25631684 |
| Phf5a | 2.23451016 |
| Phf6 | -0.7069204 |
| Phf7 | 0.42876424 |
| Phgdh | 1.48342445 |
| Phka1 | -0.7142716 |
| Phka1 | 0.85737358 |
| Phka2 | -0.7142716 |
| Phka2 | -0.7951655 |
| Phka2 | -0.4905515 |
| Phkb | 1.2332486 |
| Phkg1 | 1.98553357 |
| Phkg2 | 0.10109288 |
| Phlda1 | 1.04349748 |
| Phlda2 | 1.18205227 |
| Phlda3 | 0.71528954 |
| Phlda3 | -0.6745375 |
| Phldb1 | -0.0573372 |
| Phldb1 | -0.0514182 |
| Phlpp | -0.5551818 |
| Phospho1 | 1.84782019 |
| Phospho2 | 0.29061765 |
| Phox2a | 0.0491732 |
| Phox2b | 0.9303111 |
| Phpt1 | 0.94025869 |
| Phtf1 | -0.8500612 |
| Phtf2 | -0.7901493 |
| Phyhip | -0.425351 |
| Pi16 | -0.4458906 |
| Pi4k2b | 1.4612948 |
| Pi4k2b | -0.3791113 |
| Pi4ka | 1.47946371 |
| Pi4kb | -0.6578885 |
| Pi4kb | 1.44138857 |
| Pias1 | -0.8216927 |
| Pias2 | 0.25859972 |
| Pias3 | 1.11220967 |
| Pias3 | 2.3043377 |
| Pias4 | 0.2744256 |
| Pibf1 | -0.9175124 |
| Pibf1 | -0.6179351 |
| Picalm | 0.62251721 |
| Pick1 | 0.55835772 |
| Pick1 | 1.40980853 |
| Pif1 | 0.22578319 |
| Piga | 0.83760031 |
| Piga | 3.04820918 |
| Pigb | 1.45588307 |
| Pigc | -0.2994645 |
| Pigf | -0.8093558 |
| Pigh | 0.04467936 |
| Pigk | -0.5637805 |
| Pigk | 0.28398428 |
| Pigl | 0.43633556 |
| Pign | -0.6561531 |
| Pigo | 3.42141398 |
| Pigo | -0.5366913 |
| Pigo | -0.3289241 |
| Pigp | -0.4891004 |
| Pigp | 1.49155716 |
| Pigq | -0.5804191 |
| Pigt | 1.47893435 |
| Pigx | -0.5466615 |
| Pigy | -0.6246162 |
| Pigyl | -0.5677153 |
| Pigz | -0.1692929 |
| Pih1d1 | 0.79546978 |
| Pik3ap1 | 0.68709485 |
| Pik3c2a | -0.4898257 |
| Pik3c3 | -0.0014891 |
| Pik3ca | 0.37397452 |
| Pik3cb | -0.721681 |
| Pik3cd | 1.56720824 |
| Pik3cg | -0.0468905 |
| Pik3ip1 | 0.76224416 |
| Pik3r1 | -0.0753224 |
| Pik3r1 | -0.1084983 |
| Pik3r1 | 0.33367617 |
| Pik3r2 | 0.57313014 |
| Pik3r2 | -0.260062 |
| Pik3r3 | 0.13961156 |
| Pik3r4 | 1.69846661 |
| Pik3r6 | 2.30302324 |
| Pik3r6 | -0.7338481 |
| Pim1 | -0.4619491 |
| Pim2 | 0.20220989 |
| Pim2 | 0.80858084 |
| Pim3 | 0.56916688 |
| Pip4k2a | -0.4192762 |
| Pip4k2b | -0.6692478 |
| Pip4k2b | 0.67444791 |
| Pip4k2c | -0.3714092 |
| Pip5k1a | -0.8093558 |
| Pip5k1a | -0.1113975 |
| Pip5k1b | -0.0281102 |
| Pip5kl1 | -0.028996 |
| Pipox | -0.8575588 |
| Pir | 2.18456236 |
| Pira11 | 1.09213459 |
| Pira4 | -0.166722 |
| Pisd | -0.0842115 |
| Pit1 | -0.4019452 |
| Pitpna | 2.3104838 |
| Pitpnb | 1.97630158 |
| Pitpnc1 | 2.64105645 |
| Pitpnm1 | -0.6834208 |
| Pitpnm2 | 2.25784651 |
| Pitpnm3 | 0.83020865 |
| Pitx1 | 0.59520083 |
| Pitx2 | -0.804263 |
| Pitx2 | 0.0593068 |
| Pitx2 | -0.4683087 |
| Pitx2 | 0.28746477 |
| Piwil1 | 0.01090568 |
| Piwil2 | -0.7005352 |
| Piwil4 | -0.5989888 |
| Pja1 | -0.7124283 |
| Pja2 | 0.94947964 |
| Pja2 | 2.40293702 |
| Pkd1 | 0.04345001 |
| Pkd1l3 | -0.6843138 |
| Pkd2 | -0.399966 |
| Pkd2l1 | 0.2049737 |
| Pkd2l2 | -0.7991979 |
| Pkdrej | -0.0423847 |
| Pkhd1l1 | 2.00604077 |
| Pkhd1l1 | 1.35863775 |
| Pkia | 0.08828317 |
| Pkib | 1.13377977 |
| Pkib | -0.8532671 |
| Pkig | -0.1423949 |
| Pkig | -0.4577332 |
| Pkig | 0.25664331 |
| Pkm2 | 0.35917523 |
| Pkmyt1 | 0.75173425 |
| Pkn1 | -0.4619491 |
| Pkn1 | 1.58675576 |
| Pkn2 | 2.56567008 |
| Pkn2 | 0.00280353 |
| Pkn2 | 1.34427437 |
| Pkn3 | -0.5405134 |
| Pknox1 | 2.32974907 |
| Pknox1 | 0.67507391 |
| Pknox2 | -0.6807469 |
| Pknox2 | 1.1725462 |
| Pknox2 | 0.92393976 |
| Pknox2 | -0.3270935 |
| Pknox2 | 1.77326212 |
| Pkp1 | 1.34793673 |
| Pkp2 | -0.7170432 |
| Pkp2 | -0.5567393 |
| Pkp2 | 1.45389089 |
| Pkp3 | 0.20013166 |
| Pla1a | -0.3048063 |
| Pla1a | -0.7151945 |
| Pla1a | -0.7078361 |
| Pla2g10 | -0.4072436 |
| Pla2g12a | -0.7931558 |
| Pla2g1b | 1.24227335 |
| Pla2g2c | -0.6578885 |
| Pla2g2d | 0.58331062 |
| Pla2g2f | 0.41122449 |
| Pla2g4c | 0.07489903 |
| Pla2g4d | -0.2611975 |
| Pla2g4e | -0.6622411 |
| Pla2g4f | 1.17974558 |
| Pla2g5 | 2.01169706 |
| Pla2g6 | 0.14404053 |
| Pla2g7 | 0.18721274 |
| Plaa | 0.27378486 |
| Plac1 | -0.4301033 |
| Plac1l | -0.7272769 |
| Plac8 | 0.56940048 |
| Plag1 | 1.85475241 |
| Plag1 | 3.69261612 |
| Plagl2 | -0.4912779 |
| Plau | -0.2310133 |
| Plb1 | 0.92665289 |
| Plcb2 | 1.53568742 |
| Plcb3 | 1.40590421 |
| Plcb4 | 0.63957242 |
| Plcd1 | -0.5118498 |
| Plcd3 | -0.804263 |
| Plcd4 | -0.5420466 |
| Plcd4 | 0.02397599 |
| Plcd4 | -0.5582994 |
| Plce1 | 2.01418896 |
| Plcg2 | 0.38485104 |
| Plch2 | 0.82808597 |
| Plcl2 | 2.10774423 |
| Plcxd1 | -0.3042113 |
| Plcxd1 | -0.3644043 |
| Plcz1 | 1.85669839 |
| Pld1 | 0.48666357 |
| Pld2 | -0.7713297 |
| Pld3 | 0.88143232 |
| Pld3 | -0.0514182 |
| Pld4 | 0.3015697 |
| Pld5 | -0.6129554 |
| Pldn | 0.4655 |
| Plec1 | 0.34205482 |
| Plec1 | -0.1677495 |
| Plec1 | 0.65419127 |
| Plec1 | 0.75824523 |
| Plek | 1.56479618 |
| Plek2 | -0.7124283 |
| Plekha1 | 2.21980811 |
| Plekha1 | 0.80658963 |
| Plekha2 | 1.16898708 |
| Plekha3 | -0.5253177 |
| Plekha4 | 2.83789942 |
| Plekha4 | -0.4704383 |
| Plekha5 | -0.3234431 |
| Plekha6 | 1.25906111 |
| Plekha7 | -0.6014384 |
| Plekha8 | -0.8415656 |
| Plekhb1 | 0.51034888 |
| Plekhb2 | -0.0379005 |
| Plekhf1 | -0.5351668 |
| Plekhf1 | -0.3511754 |
| Plekhf2 | 0.43444847 |
| Plekhf2 | -0.2935648 |
| Plekhg2 | 1.9394586 |
| Plekhg2 | -0.0936606 |
| Plekhg2 | 1.19982787 |
| Plekhg3 | 2.01682314 |
| Plekhg3 | 0.80568313 |
| Plekhg4 | 0.06973047 |
| Plekhg5 | -0.2151968 |
| Plekhg6 | -0.6262939 |
| Plekhj1 | 2.65780316 |
| Plekhm1 | -0.0828023 |
| Plekhm3 | -0.5081428 |
| Plekho2 | 1.69097058 |
| Plin | -0.8511286 |
| Plk1 | 1.00238497 |
| Plk2 | -0.3474299 |
| Plk3 | 0.53756603 |
| Plk4 | -0.0105684 |
| Pllp | -0.5366913 |
| Plod1 | 3.79701156 |
| Plod2 | -0.1499293 |
| Plod3 | 1.1491031 |
| Plp1 | -0.6604976 |
| Plp2 | 2.05675484 |
| Plrg1 | -0.2663244 |
| Pls3 | -0.5037139 |
| Plscr1 | 1.49650696 |
| Plscr1 | 1.27741582 |
| Plscr3 | -0.3842815 |
| Plscr4 | 0.89965274 |
| Pltp | -0.7792076 |
| Plunc | -0.2387457 |
| Plvap | -0.6330353 |
| Plxdc1 | -0.2749322 |
| Plxdc1 | 1.38398738 |
| Plxna2 | -0.354936 |
| Plxnb1 | -0.5622112 |
| Plxnb3 | -0.6458078 |
| Plxnc1 | -0.081864 |
| Plxnd1 | 0.59201447 |
| Pmaip1 | -0.0075322 |
| Pmch | 0.11790385 |
| Pmepa1 | -0.3746107 |
| Pmf1 | 2.29483939 |
| Pmfbp1 | 0.36122043 |
| Pml | 1.10169762 |
| Pml | 0.39644598 |
| Pmm1 | -0.1443982 |
| Pmm1 | -0.1384013 |
| Pmm2 | 0.10609131 |
| Pmm2 | 0.21867145 |
| Pmm2 | 1.13160664 |
| Pmm2 | 0.63805448 |
| Pmp2 | -0.0936606 |
| Pmp22 | -0.1206381 |
| Pmp22 | -0.000629 |
| Pmpca | 1.55516654 |
| Pmpcb | 0.3303639 |
| Pms2 | -0.4818785 |
| Pmvk | -0.4039286 |
| Pnck | 2.5565777 |
| Pnkd | -0.2830383 |
| Pnkd | 1.67214258 |
| Pnkp | -0.1874898 |
| Pnkp | -0.4493581 |
| Pnliprp1 | 0.05648045 |
| Pnma1 | -0.1075338 |
| Pnma2 | 0.39363119 |
| Pno1 | 1.69783841 |
| Pnpla2 | 1.87518801 |
| Pnpla6 | -0.0860937 |
| Pnpla7 | -0.7087528 |
| Pnpla8 | 0.39560243 |
| Pnpla8 | -0.3816928 |
| Pnpo | 1.79352299 |
| Pnpo | 0.61141836 |
| Pnrc2 | 0.84493397 |
| Podn | -0.5298506 |
| Podxl | 1.21376197 |
| Podxl2 | -0.1230851 |
| Pof1b | 0.44651378 |
| Pofut1 | 0.29814144 |
| Pofut1 | -0.0070992 |
| Pofut2 | -0.062375 |
| Pogz | -0.8032478 |
| Pogz | 0.98203155 |
| Pola2 | -0.2634727 |
| Polb | 2.87759372 |
| Pold1 | 1.33392211 |
| Pold2 | -0.469018 |
| Pold3 | 1.78954378 |
| Pold4 | -0.514081 |
| Poldip2 | -0.6432393 |
| Poldip3 | -0.7207516 |
| Poldip3 | 1.66674429 |
| Pole | 0.12055066 |
| Pole3 | -0.5567393 |
| Pole3 | -0.6271339 |
| Pole4 | 0.21424167 |
| Polg | 1.57782736 |
| Polg2 | 2.7313919 |
| Polh | -0.8662017 |
| Poli | -0.5748399 |
| Polk | -0.3574516 |
| Poll | 4.0274377 |
| Poll | 1.0747522 |
| Polm | 1.42647903 |
| Polm | 0.56846574 |
| Poln | 1.87835907 |
| Polq | -0.6162723 |
| Polr1e | 0.09722966 |
| Polr2d | 1.52608718 |
| Polr2d | -0.4577332 |
| Polr2d | -0.1539726 |
| Polr2d | -0.7871525 |
| Polr2e | -0.7087528 |
| Polr2e | -0.672771 |
| Polr2f | 1.67350521 |
| Polr2g | -0.5590805 |
| Polr2h | 0.83443962 |
| Polr2j | -0.721681 |
| Polr3a | -0.365039 |
| Polr3a | 0.05728886 |
| Polr3b | -0.3119765 |
| Polr3d | 0.35565871 |
| Polr3e | 3.45540156 |
| Polr3g | -0.3894805 |
| Polr3h | -0.233216 |
| Polr3h | -0.3089822 |
| Polr3h | -0.3765369 |
| Polr3k | -0.3973336 |
| Polr3k | 2.5964407 |
| Polrmt | 3.90096803 |
| Pols | -0.0665172 |
| Pom121 | -0.6347282 |
| Pomc | -0.6887916 |
| Pomgnt1 | -0.4179315 |
| Pomgnt1 | 0.88210115 |
| Pomp | 1.31707423 |
| Pomt1 | 0.33156972 |
| Pomt1 | 0.34591951 |
| Pomt2 | -0.7142716 |
| Pon1 | 3.5896476 |
| Pon2 | 1.41208644 |
| Pon3 | 0.08671848 |
| Pon3 | -0.1838219 |
| Pop4 | 2.05494598 |
| Pop5 | 0.16443599 |
| Pop5 | -0.4697279 |
| Pop7 | 0.72601748 |
| Popdc2 | -0.6055354 |
| Popdc2 | 1.70069678 |
| Popdc3 | -0.7151945 |
| Por | 0.60492011 |
| Por | -0.7635176 |
| Porcn | 0.10570776 |
| Porcn | 0.60783871 |
| Pou2af1 | -0.5756349 |
| Pou2f1 | -0.5420466 |
| Pou2f1 | -0.0984213 |
| Pou2f2 | -0.4818785 |
| Pou3f1 | -0.3107777 |
| Pou3f3 | -0.3252665 |
| Pou5f1 | 1.59614477 |
| Pou5f1 | -0.2651827 |
| Pou6f1 | 0.90781875 |
| Ppa1 | 1.31444698 |
| Ppa2 | -0.7096703 |
| Ppa2 | 2.40628007 |
| Ppa2 | -0.9049837 |
| Ppap2a | -0.3089822 |
| Ppap2a | -0.7060055 |
| Ppap2a | -0.9815777 |
| Ppap2b | -0.7005352 |
| Ppap2b | 1.98033568 |
| Ppap2c | -0.6338813 |
| Ppapdc1 | 2.19269776 |
| Ppapdc1a | 2.0046339 |
| Ppapdc2 | -0.2076561 |
| Ppapdc3 | 0.07012907 |
| Ppapdc3 | 1.94799203 |
| Pparbp | -0.81345 |
| Pparg | -0.868375 |
| Ppargc1a | 2.88898341 |
| Ppargc1b | 1.70970253 |
| Ppat | -0.7713297 |
| Ppcs | -0.028996 |
| Ppef2 | 0.53538853 |
| Ppfia3 | 2.61724134 |
| Ppfia3 | -0.3077872 |
| Ppfibp1 | 1.2739055 |
| Ppfibp2 | 1.52078581 |
| Ppfibp2 | 2.16613205 |
| Ppia | 1.29057129 |
| Ppic | -0.6914886 |
| Ppic | -0.4768561 |
| Ppid | -0.1196611 |
| Ppie | -0.3486767 |
| Ppie | -0.4226462 |
| Ppif | -0.7941601 |
| Ppil2 | 0.31913566 |
| Ppil3 | -0.378467 |
| Ppl | 1.00575893 |
| Ppm1a | -0.7782192 |
| Ppm1a | 1.99223261 |
| Ppm1a | -0.868375 |
| Ppm1b | -0.9939391 |
| Ppm1b | -0.2081927 |
| Ppm1b | 0.0306511 |
| Ppm1e | -0.7423652 |
| Ppm1e | -0.9175124 |
| Ppm1f | 2.19925347 |
| Ppm1f | -0.6544209 |
| Ppm1f | 0.57150025 |
| Ppm1g | -0.8185932 |
| Ppm1h | 0.85169264 |
| Ppm1j | -0.7179689 |
| Ppm1k | 0.87522292 |
| Ppm1l | -0.3036166 |
| Ppm1l | -0.8618703 |
| Ppm1m | -0.3973336 |
| Ppm1m | 0.5217244 |
| Ppm2c | 1.39534789 |
| Ppme1 | -0.8629512 |
| Ppnr | -0.1094637 |
| Ppox | 0.54070239 |
| Ppox | -0.6296585 |
| Ppp1ca | -0.5860318 |
| Ppp1ca | -0.7811875 |
| Ppp1ca | 0.20324728 |
| Ppp1cb | -0.1499293 |
| Ppp1cb | 0.07608772 |
| Ppp1cc | -0.7272769 |
| Ppp1r10 | 0.79381789 |
| Ppp1r10 | 1.13722087 |
| Ppp1r11 | 1.15822768 |
| Ppp1r12c | -0.5382182 |
| Ppp1r13b | 2.48939908 |
| Ppp1r13b | -0.6896897 |
| Ppp1r13b | 2.97656203 |
| Ppp1r14a | 1.90312333 |
| Ppp1r14a | -0.697812 |
| Ppp1r14c | 0.77807095 |
| Ppp1r14d | 1.38095404 |
| Ppp1r15b | 0.44970506 |
| Ppp1r16a | -0.6154419 |
| Ppp1r1a | -0.2853673 |
| Ppp1r1b | 2.53050633 |
| Ppp1r1b | 0.79711876 |
| Ppp1r2 | -0.9360411 |
| Ppp1r3c | 0.05323984 |
| Ppp1r3f | -0.8640335 |
| Ppp1r8 | -0.8458038 |
| Ppp1r9a | -0.6923893 |
| Ppp1r9b | 1.98558489 |
| Ppp2ca | 0.64216889 |
| Ppp2cb | -0.7198231 |
| Ppp2cz | -0.3543082 |
| Ppp2r1a | -0.4704383 |
| Ppp2r1b | -0.8554105 |
| Ppp2r2b | -0.8279228 |
| Ppp2r2b | 2.85794722 |
| Ppp2r2b | 1.45216695 |
| Ppp2r2c | 1.75708503 |
| Ppp2r2c | 1.10156546 |
| Ppp2r2d | -0.9693776 |
| Ppp2r3c | -0.1494251 |
| Ppp2r4 | -0.6338813 |
| Ppp2r5b | -0.6104755 |
| Ppp2r5c | 0.24216976 |
| Ppp2r5c | 0.82097532 |
| Ppp2r5c | 0.15683976 |
| Ppp2r5d | 0.22003025 |
| Ppp2r5d | 0.04262954 |
| Ppp2r5d | -0.0851521 |
| Ppp2r5d | 0.81668282 |
| Ppp2r5e | -0.5653524 |
| Ppp2r5e | -0.1713547 |
| Ppp3ca | -0.3387482 |
| Ppp3ca | -0.8881654 |
| Ppp3ca | -0.3155824 |
| Ppp3cb | -0.3258751 |
| Ppp3cc | -0.2703315 |
| Ppp3r1 | -0.4654769 |
| Ppp4c | 0.26638466 |
| Ppp4r1 | 0.61298016 |
| Ppp4r1 | 2.43732983 |
| Ppp4r1 | 0.39363119 |
| Ppp4r1l | 0.21287442 |
| Ppp5c | -0.6466656 |
| Ppp5c | -0.7871525 |
| Ppp6c | 0.05890356 |
| Pps | 1.0210089 |
| Ppt1 | 0.81739961 |
| Ppt2 | -0.8341948 |
| Pptc7 | 1.42198752 |
| Pqbp1 | 0.48205879 |
| Pqlc1 | -0.1118815 |
| Pqlc2 | -0.804263 |
| Pqlc3 | 0.83637242 |
| Pqlc3 | 1.6134989 |
| Pqlc3 | 1.34661586 |
| Pqlc3 | -0.6080022 |
| Pramef12 | 2.30725214 |
| Prap1 | 1.37189346 |
| Prc1 | -0.9645418 |
| Prcp | -0.4993058 |
| Prdm10 | 0.65355112 |
| Prdm4 | -0.7050916 |
| Prdm8 | -0.1848684 |
| Prdm9 | -0.3131769 |
| Prdx1 | 2.49812831 |
| Prdx2 | -0.5374544 |
| Prdx2 | 1.74022269 |
| Prdx2 | -0.286534 |
| Prdx3 | 0.54406827 |
| Prdx4 | -0.7291497 |
| Prdx5 | 1.03912763 |
| Prdx5 | -0.6754221 |
| Prdx6 | -0.0201757 |
| Prdx6-rs1 | -0.403267 |
| Prdx6-rs1 | 1.0287559 |
| Preb | 2.09244449 |
| Preb | 2.20997084 |
| Prei3 | 0.23651712 |
| Prei4 | 0.20669694 |
| Prei4 | 0.0792502 |
| Prelp | 1.57862093 |
| Prep | 0.06373101 |
| Prep | -0.3059975 |
| Prf1 | 0.67611631 |
| Prickle1 | -0.0898695 |
| Prickle2 | 0.79216307 |
| Prickle3 | 0.20359282 |
| Prickle4 | 0.52442424 |
| Prim2 | 0.09529204 |
| Prim2 | -0.3362825 |
| Prima1 | 2.02418914 |
| Prkaa2 | 1.16380835 |
| Prkaa2 | 0.60221791 |
| Prkab1 | -0.2504656 |
| Prkab2 | 0.09993559 |
| Prkaca | 0.37311043 |
| Prkaca | -0.7762456 |
| Prkacb | 0.29250429 |
| Prkag1 | -0.0931859 |
| Prkag2 | -0.8062968 |
| Prkar1a | 2.03520339 |
| Prkar1b | 0.18015446 |
| Prkar2b | -0.3474299 |
| Prkca | -0.4301033 |
| Prkcb1 | -0.8543382 |
| Prkcbp1 | 1.49364448 |
| Prkcbp1 | -0.9278896 |
| Prkcc | -0.3714092 |
| Prkcd | 0.79821646 |
| Prkcdbp | 2.38831067 |
| Prkcdbp | 0.46966729 |
| Prkce | -0.4577332 |
| Prkch | -0.081864 |
| Prkcq | -0.951371 |
| Prkcsh | -0.2119576 |
| Prkcz | 0.53877357 |
| Prkcz | 0.63413983 |
| Prkcz | -0.7124283 |
| Prkd1 | 1.28036743 |
| Prkd1 | -0.6162723 |
| Prkd2 | -0.8114006 |
| Prkd3 | -0.6449509 |
| Prkd3 | 0.08710989 |
| Prkd3 | -0.5044506 |
| Prkg1 | 1.15498803 |
| Prkg2 | 0.52295257 |
| Prkra | 1.07202807 |
| Prkrip1 | -0.5230596 |
| Prkrip1 | 3.37218943 |
| Prkrir | 1.17792048 |
| Prkx | 1.91653535 |
| Prkx | 1.29283573 |
| Prl | 0.33847316 |
| Prlr | -0.5916784 |
| Prlr | 0.25402837 |
| Prm1 | 2.075586 |
| Prm3 | 2.91951318 |
| Prmt2 | 0.18192403 |
| Prmt2 | -0.5489775 |
| Prmt3 | -0.5876416 |
| Prmt5 | 1.7501125 |
| Prmt5 | -0.6950967 |
| Prmt5 | 0.06733531 |
| Prmt7 | 0.62229652 |
| Prmt7 | 0.89422382 |
| Prmt7 | -0.8804193 |
| Prmt7 | 1.01116117 |
| Prmt8 | 1.42769177 |
| Prnp | -0.0832718 |
| Prnpip1 | -0.6288162 |
| Prnpip1 | -0.1474111 |
| Prnpip1 | -0.4818785 |
| Proc | 0.99634014 |
| Proc | 2.5889636 |
| Procr | 1.73002474 |
| Prodh | 0.85238308 |
| Prok2 | 0.98742298 |
| Prok2 | -0.7548064 |
| Prokr1 | 0.56307273 |
| Prokr2 | 0.91057901 |
| Prom1 | 0.90341797 |
| Prom2 | 0.16443599 |
| Prom2 | 2.20194436 |
| Pros1 | 1.03175136 |
| Prosapip1 | 0.90944342 |
| Prosapip1 | -0.1636462 |
| Prosapip1 | -0.8394537 |
| Prosc | 1.82828976 |
| Prosc | -0.272629 |
| Prosc | 0.18298417 |
| Prox1 | -0.5344055 |
| Prox1 | 1.73634448 |
| Prpf18 | -0.4179315 |
| Prpf19 | 0.09412754 |
| Prpf19 | -0.8426233 |
| Prpf19 | 3.27910298 |
| Prpf3 | -0.2206206 |
| Prpf3 | 0.60086387 |
| Prpf3 | 1.29487979 |
| Prpf3 | 0.50235301 |
| Prpf3 | -0.6096504 |
| Prpf31 | 0.77713635 |
| Prpf38a | -0.4949184 |
| Prpf38a | -0.8258415 |
| Prpf38a | -0.5451207 |
| Prpf38b | 2.89487879 |
| Prpf39 | 1.13658459 |
| Prpf40a | -0.477572 |
| Prpf40a | 0.19421811 |
| Prpf40b | 0.28176265 |
| Prpf40b | -0.5884476 |
| Prpf40b | 0.03727887 |
| Prpf6 | 0.91623637 |
| Prpf8 | -0.3726885 |
| Prpf8 | 0.35712554 |
| Prph2 | 0.01935903 |
| Prps1 | -0.6475241 |
| Prps1l1 | -0.8300088 |
| Prps2 | -0.1713547 |
| Prpsap2 | -0.8405091 |
| Prpsap2 | 1.00663709 |
| Prr11 | -0.8227282 |
| Prr13 | -0.5637805 |
| Prr14 | -0.9537519 |
| Prr15 | 2.70690771 |
| Prr16 | 0.06172261 |
| Prr18 | 0.71168602 |
| Prr19 | 1.03997501 |
| Prr3 | 1.51647208 |
| Prr7 | 0.20566338 |
| Prrc1 | 2.39987849 |
| Prrg2 | 0.44063464 |
| Prrt1 | -0.3823393 |
| Prrt1 | -0.7871525 |
| Prrt1 | -0.2923893 |
| Prrt3 | 0.42930708 |
| Prrx1 | 0.24613937 |
| Prrx1 | 0.39756953 |
| Prrx1 | -0.2871179 |
| Prss21 | -0.7674155 |
| Prss23 | 1.03813806 |
| Prss27 | 1.03983383 |
| Prss28 | 0.28430122 |
| Prss29 | 2.46833058 |
| Prss29 | -0.3746107 |
| Prss34 | -0.2081927 |
| Prss35 | 1.69644091 |
| Prss36 | 0.56024658 |
| Prss7 | -0.6022564 |
| Prss8 | 0.73466861 |
| Prss8 | 1.91321421 |
| Prtn3 | 0.54358817 |
| Prune | 1.3302043 |
| Prune | 1.9313905 |
| Prune | 2.30123113 |
| Prune | 2.04575257 |
| Psap | -0.5268262 |
| Psat1 | 0.03397086 |
| Psca | 1.32397489 |
| Pscd1 | -0.9198085 |
| Pscd2 | -0.2119576 |
| Pscd3 | 1.96938251 |
| Pscdbp | -0.5253177 |
| Psd2 | -0.7170432 |
| Psd3 | 1.92231909 |
| Psd4 | 0.62075023 |
| Psen1 | -0.804263 |
| Psen2 | 0.53028786 |
| Psenen | -0.4445071 |
| Psg19 | 0.76053243 |
| Psg23 | -0.6509661 |
| Psg27 | -0.0468905 |
| Psip1 | -0.8738304 |
| Psip1 | 2.49673354 |
| Pskh1 | 2.5223647 |
| Psma2 | 0.86114178 |
| Psma4 | -0.8394537 |
| Psma5 | 1.84287109 |
| Psma7 | 2.37889156 |
| Psma7 | 0.40428261 |
| Psmb1 | -0.5756349 |
| Psmb10 | 1.46917573 |
| Psmb10 | -0.2510274 |
| Psmb2 | -0.446583 |
| Psmb2 | 0.42577293 |
| Psmb3 | 0.31208733 |
| Psmb4 | 0.08475892 |
| Psmb5 | -0.157525 |
| Psmb6 | -0.9360411 |
| Psmb7 | -0.000629 |
| Psmb7 | -0.7792076 |
| Psmb7 | -0.4092383 |
| Psmb8 | -0.4045907 |
| Psmb9 | 1.60320241 |
| Psmc1 | 1.35310063 |
| Psmc2 | -0.8165325 |
| Psmc3 | -0.3849298 |
| Psmc3ip | -0.4697279 |
| Psmc4 | 1.94085934 |
| Psmc5 | -0.1177101 |
| Psmc5 | 1.01450365 |
| Psmd1 | 1.52633888 |
| Psmd10 | -0.9255736 |
| Psmd10 | 0.11296837 |
| Psmd10 | 0.57010093 |
| Psmd11 | -0.9669566 |
| Psmd11 | 0.4507664 |
| Psmd12 | 1.72772831 |
| Psmd12 | 1.5963783 |
| Psmd13 | 0.72838483 |
| Psmd14 | 2.12682112 |
| Psmd2 | 1.49564047 |
| Psmd2 | 0.28049077 |
| Psmd4 | 0.17944569 |
| Psmd5 | -0.8373466 |
| Psmd6 | -0.6262939 |
| Psmd6 | 0.19909081 |
| Psmd7 | 0.48819346 |
| Psmd7 | 0.89323334 |
| Psmd8 | -0.951371 |
| Psmd9 | 1.05481996 |
| Psmd9 | 0.73603757 |
| Psme1 | -0.1339287 |
| Psme1 | 0.36326117 |
| Psme2 | -0.89822 |
| Psme3 | -0.3030223 |
| Psme4 | -0.9015958 |
| Psme4 | -0.9573347 |
| Psmf1 | -0.3580815 |
| Psmf1 | -0.6657379 |
| Psmg2 | 1.93383453 |
| Psmg2 | 1.75846023 |
| Psmg3 | 2.38941062 |
| Psors1c2 | 1.36174428 |
| Psp | 0.58308047 |
| Pspc1 | 1.89150453 |
| Pstpip1 | 0.33247306 |
| Pstpip2 | 1.26872569 |
| Ptbp1 | -0.2572293 |
| Ptbp1 | -0.2959202 |
| Ptcd3 | 2.10512883 |
| Ptch1 | 2.26419076 |
| Ptch2 | -0.9267309 |
| Ptdss2 | 0.82861709 |
| Pten | 2.85240222 |
| Pter | -0.6861023 |
| Ptgds | -0.1916993 |
| Ptgds2 | 2.81149328 |
| Ptgds2 | 0.83056197 |
| Ptger2 | -0.5957327 |
| Ptger2 | -0.5957327 |
| Ptger3 | -0.2179048 |
| Ptges | -0.3289241 |
| Ptges | -0.0419353 |
| Ptges2 | 0.24679934 |
| Ptges3 | 0.97039439 |
| Ptgfr | 1.29799107 |
| Ptgir | -0.7674155 |
| Ptgis | -0.2778192 |
| Ptgs1 | -0.8738304 |
| Ptgs1 | -0.7664395 |
| Pth2 | -0.1996436 |
| Pth2r | -0.7664395 |
| Pthlh | -0.808335 |
| Pthr1 | -0.8662017 |
| Pthr1 | -0.5103652 |
| Pthr1 | 1.6366695 |
| Ptk2 | 1.23347815 |
| Ptk2b | 3.17763035 |
| Ptk7 | -0.3192022 |
| Ptms | -0.0559679 |
| Ptn | -0.2033742 |
| Ptp4a2 | 0.99914627 |
| Ptp4a2 | 2.56586317 |
| Ptp4a2 | -0.6254547 |
| Ptp4a2 | 1.68906951 |
| Ptp4a3 | -0.3406018 |
| Ptp4a3 | -0.0564241 |
| Ptpdc1 | 0.5738278 |
| Ptpdc1 | 0.34443498 |
| Ptpdc1 | 0.73231714 |
| Ptpla | -0.4355644 |
| Ptpla | 0.09529204 |
| Ptplad1 | -0.34432 |
| Ptplad2 | -0.1284912 |
| Ptplb | -0.6406778 |
| Ptpn1 | -0.2393005 |
| Ptpn11 | 0.59996008 |
| Ptpn11 | -0.1060891 |
| Ptpn11 | 0.89091815 |
| Ptpn12 | -0.4500532 |
| Ptpn12 | -0.5268262 |
| Ptpn12 | -0.429423 |
| Ptpn12 | -0.3675819 |
| Ptpn12 | -0.5505247 |
| Ptpn14 | 1.16763352 |
| Ptpn18 | 1.90474745 |
| Ptpn20 | 2.12770311 |
| Ptpn21 | -0.4739983 |
| Ptpn22 | 0.23884882 |
| Ptpn5 | 1.89127749 |
| Ptpn6 | -0.8892773 |
| Ptpn6 | 0.28112693 |
| Ptpra | 0.46210035 |
| Ptprb | -0.5844247 |
| Ptprd | 1.15148658 |
| Ptprd | -0.4668917 |
| Ptpre | 0.44143852 |
| Ptpre | 0.64281688 |
| Ptprf | -0.6923893 |
| Ptprf | -0.6196009 |
| Ptprg | 1.11807115 |
| Ptprj | -0.6022564 |
| Ptprk | 1.35330256 |
| Ptprk | 0.02815352 |
| Ptprn | 1.98620051 |
| Ptprn2 | -0.1610908 |
| Ptpro | 1.06929598 |
| Ptpro | -0.5482049 |
| Ptprq | 0.45235616 |
| Ptprr | 1.70174035 |
| Ptprr | 1.70825362 |
| Ptprs | 1.12634466 |
| Ptprs | 0.74595136 |
| Ptprt | -0.758668 |
| Ptpru | 0.61698427 |
| Ptprv | -0.4647702 |
| Ptrf | -0.5740456 |
| Ptrh1 | 3.04753406 |
| Ptrh1 | -0.8175623 |
| Ptrh2 | 1.86601809 |
| Pts | -0.0281102 |
| Pts | -0.8279228 |
| Pttg1 | -0.6475241 |
| Pttg1ip | 2.23080694 |
| Ptx3 | -0.8575588 |
| Ptx3 | -0.2448664 |
| Puf60 | 1.66349029 |
| Puf60 | -0.8881654 |
| Pum1 | 0.03106672 |
| Pum2 | -0.0988987 |
| Pum2 | 0.61809348 |
| Punc | 1.15198759 |
| Purb | -0.7235426 |
| Purg | 0.21047692 |
| Pus1 | -0.8320994 |
| Pus1 | -0.8532671 |
| Pus10 | -0.0045058 |
| Pus10 | 0.27025295 |
| Pus10 | -0.1869649 |
| Pus3 | 0.73388544 |
| Pus7l | -0.5238117 |
| Pvalb | -0.5653524 |
| Pvr | -0.4675999 |
| Pvr | -0.8073153 |
| Pvrl1 | 1.29444983 |
| Pvrl2 | -0.4431257 |
| Pvrl2 | 0.93553495 |
| Pvrl2 | 1.14001539 |
| Pvrl3 | 0.58675604 |
| Pvrl3 | 2.61368428 |
| Pvrl3 | 0.31023986 |
| Pwp1 | 2.82588654 |
| Pwp2 | -0.1459034 |
| Pwwp2b | -0.1580335 |
| Pxdn | 1.76615012 |
| Pxmp2 | 0.72877881 |
| Pxmp4 | -0.3143789 |
| Pxn | -0.2577952 |
| Pxt1 | 0.37051336 |
| Pycr2 | 1.96430377 |
| Pycrl | 2.41000232 |
| Pygb | -0.5780239 |
| Pygl | -0.1349208 |
| Pygl | 0.51084636 |
| Pygm | -0.4985731 |
| Pygo1 | -0.4206227 |
| Pygo2 | 1.1013011 |
| Pyroxd1 | -0.4563321 |
| Pyy | -0.6137835 |
| Qars | 0.59429155 |
| Qdpr | 0.34413779 |
| Qpct | 1.57933457 |
| Qprt | 0.53659888 |
| Qrich2 | 0.43983007 |
| Qrich2 | 1.85256572 |
| Qrsl1 | 1.42404881 |
| Qsox1 | -0.7069204 |
| Qtrt1 | 0.69550009 |
| R3hdm1 | 3.65053236 |
| R3hdm1 | -0.2277189 |
| R3hdm2 | -0.1494251 |
| R74862 | -0.5551818 |
| Rab1 | 0.4884482 |
| Rab10 | 1.12299238 |
| Rab10 | -0.9939391 |
| Rab11a | 0.37713602 |
| Rab11b | 0.6136487 |
| Rab11fip2 | 1.23198504 |
| Rab11fip3 | -0.593298 |
| Rab11fip5 | 1.36703163 |
| Rab11fip5 | 2.73698924 |
| Rab11fip5 | 1.1332689 |
| Rab12 | 1.1643028 |
| Rab13 | 2.90287421 |
| Rab13 | 0.01260247 |
| Rab14 | -0.7792076 |
| Rab15 | 0.29532711 |
| Rab17 | 1.30726327 |
| Rab17 | -0.6807469 |
| Rab18 | -0.6137835 |
| Rab19 | -0.6596271 |
| Rab19 | 0.98203155 |
| Rab1b | -0.7538435 |
| Rab1b | -0.782179 |
| Rab1b | -0.2195333 |
| Rab21 | 1.96577264 |
| Rab22a | 2.40830629 |
| Rab22a | -0.4383069 |
| Rab23 | 1.14734295 |
| Rab24 | -0.6798573 |
| Rab24 | 0.36587844 |
| Rab24 | 0.16839046 |
| Rab25 | 1.43816754 |
| Rab27a | 3.25333258 |
| Rab27b | 2.03359607 |
| Rab28 | 0.19142226 |
| Rab28 | 1.15810328 |
| Rab2a | 1.61105033 |
| Rab31 | 0.92872233 |
| Rab32 | -0.5170643 |
| Rab33a | 0.37195706 |
| Rab33b | -0.6137835 |
| Rab34 | -0.6639879 |
| Rab34 | -0.4619491 |
| Rab34 | 0.1847484 |
| Rab35 | 0.02397599 |
| Rab36 | -0.447276 |
| Rab38 | -0.8073153 |
| Rab38 | -0.7452216 |
| Rab39 | -0.4612451 |
| Rab39b | -0.5661393 |
| Rab3a | -0.4985731 |
| Rab3b | -0.1938112 |
| Rab3d | -0.0767204 |
| Rab3gap1 | -0.7254079 |
| Rab3gap2 | -0.5366913 |
| Rab3il1 | -0.3675819 |
| Rab3il1 | -0.1656956 |
| Rab3ip | 0.6526969 |
| Rab40b | -0.6330353 |
| Rab40c | 0.01767447 |
| Rab43 | -0.1201495 |
| Rab4a | -0.6296585 |
| Rab4b | 0.05688474 |
| Rab5a | 1.44249037 |
| Rab5a | -0.3381312 |
| Rab5b | -0.6869979 |
| Rab5c | -0.5884476 |
| Rab6 | 0.01893817 |
| Rab6 | -0.1240657 |
| Rab6 | -0.4804408 |
| Rab6b | -0.7366785 |
| Rab6ip1 | 1.40923818 |
| Rab7 | 0.23217132 |
| Rab7 | 3.00909848 |
| Rab7 | -0.7235426 |
| Rab7l1 | -0.5290936 |
| Rab8a | -0.6578885 |
| Rab8a | -0.6648625 |
| Rab8b | -0.1162496 |
| Rab8b | 0.34324566 |
| Rab9 | -0.5748399 |
| Rab9 | 0.136276 |
| Rab9b | 0.45341449 |
| Rabac1 | -0.7014447 |
| Rabac1 | -0.4458906 |
| Rabep1 | -0.7170432 |
| Rabep1 | -0.429423 |
| Rabep2 | -0.1162496 |
| Rabgap1 | 1.32074002 |
| Rabgap1 | -0.0272253 |
| Rabgap1l | 0.77263719 |
| Rabgap1l | 0.4576359 |
| Rabgef1 | 0.88527153 |
| Rabggta | 0.62361988 |
| Rabggtb | -0.5208068 |
| Rabif | 2.22934525 |
| Rabif | -0.1479142 |
| Rabl2a | 2.40359346 |
| Rabl2a | 0.24349484 |
| Rabl3 | -0.4747119 |
| Rabl4 | -0.0532355 |
| Rabl4 | 0.71927723 |
| Rabl5 | 0.4802619 |
| Rac1 | 2.22462858 |
| Rac2 | -0.5275814 |
| Rac3 | 0.44063464 |
| Rac3 | 0.18050864 |
| Rad1 | -0.2812953 |
| Rad17 | 1.03898633 |
| Rad18 | 0.92729013 |
| Rad23a | -0.388178 |
| Rad23a | -0.5528504 |
| Rad23a | -0.5916784 |
| Rad23b | 0.85393471 |
| Rad50 | 1.69209531 |
| Rad51 | -0.7713297 |
| Rad51 | -0.5482049 |
| Rad51ap1 | 0.75900825 |
| Rad51c | 1.11534037 |
| Rad51l3 | 0.48359624 |
| Rad51l3 | 1.42853044 |
| Rad51l3 | -0.6355758 |
| Rad51l3 | 1.66225805 |
| Rad52 | -0.4287431 |
| Rad54l | 0.17161354 |
| Rad9 | 1.2836361 |
| Rad9 | 0.49782508 |
| Rad9 | -0.4314656 |
| Rad9b | -0.6415309 |
| Rae1 | -0.356822 |
| Raet1b | -0.5397477 |
| Raet1b | 0.87049633 |
| Raf1 | -0.3940534 |
| Rag1 | 0.23851607 |
| Rag1ap1 | -0.7404658 |
| Rage | -0.5074032 |
| Rage | -0.2326649 |
| Rai1 | -0.2141159 |
| Rai12 | 0.91155128 |
| Rai12 | 0.34888152 |
| Rai12 | -0.2959202 |
| Rai12 | 2.6231485 |
| Rai14 | 0.30374479 |
| Rala | 2.45519688 |
| Ralb | -0.8073153 |
| Ralbp1 | -0.6449509 |
| Ralgds | 1.11116377 |
| Ralgps1 | 1.93171674 |
| Ralgps1 | 0.46758597 |
| Ralgps1 | -0.5924879 |
| Ralgps2 | 0.09800358 |
| Ralgps2 | -0.7105887 |
| Raly | 1.4315063 |
| Raly | -0.7703496 |
| Ralyl | -0.5941089 |
| Ramp1 | -0.6789686 |
| Ramp2 | -0.2420793 |
| Ramp3 | 2.25226939 |
| Ramp3 | 0.92473856 |
| Ran | -0.6798573 |
| Ranbp1 | 1.21563545 |
| Ranbp10 | -0.7577011 |
| Ranbp17 | -0.2577952 |
| Ranbp2 | -0.593298 |
| Ranbp3l | 0.39560243 |
| Ranbp6 | 2.12682112 |
| Ranbp9 | 1.85404377 |
| Rangap1 | -0.1585424 |
| Rangrf | 1.42825097 |
| Rap1b | -0.3940534 |
| Rap1gap | 2.3431108 |
| Rap2a | 0.77000263 |
| Rap2b | -0.7032664 |
| Rap2c | 0.02146048 |
| Rap2c | -0.7931558 |
| Rap2c | 0.06853366 |
| Rap2ip | 1.54263795 |
| Rapgef1 | -0.3593426 |
| Rapgef1 | -0.6736538 |
| Rapgef1 | 0.49403498 |
| Rapgef1 | -0.4549332 |
| Rapgef3 | -0.2092668 |
| Rapgef4 | -0.2409668 |
| Rapgef5 | 2.52421213 |
| Rapgef6 | -0.782179 |
| Rapgefl1 | 1.03175136 |
| Rapgefl1 | -0.1017682 |
| Rapsn | -0.8002088 |
| Rapsn | -0.2443083 |
| Rara | 0.73545112 |
| Rarb | -0.6466656 |
| Rarb | 1.5486366 |
| Rarg | -0.235424 |
| Rarg | 0.07529543 |
| Rarg | -0.6552866 |
| Rarres2 | 1.40361116 |
| Rars | -0.7606049 |
| Rars | -0.3791113 |
| Rars2 | 1.32532812 |
| Rars2 | 2.00141023 |
| Rars2 | -0.2211647 |
| Rasa1 | -0.6449509 |
| Rasa2 | 1.71616073 |
| Rasal1 | -0.3036166 |
| Rasd1 | -0.0889241 |
| Rasef | -0.5567393 |
| Rasgef1b | 0.41067104 |
| Rasgef1b | 0.08750115 |
| Rasgef1c | 0.11638802 |
| Rasgef1c | -0.2651827 |
| Rasgrf1 | -0.1631346 |
| Rasgrf1 | 0.7486545 |
| Rasgrf1 | -0.6415309 |
| Rasgrf1 | -0.3089822 |
| Rasgrp1 | 0.40623154 |
| Rasgrp1 | -0.6372734 |
| Rasgrp1 | -0.4328299 |
| Rasgrp3 | 0.26799839 |
| Rasip1 | 0.84040088 |
| Rasl10a | 1.075432 |
| Rasl10b | 1.51985708 |
| Rasl10b | 1.01566345 |
| Rasl11a | 1.36424204 |
| Rasl11b | 2.02468142 |
| Rasl11b | 1.62141274 |
| Rasl12 | 2.16786486 |
| Rasl2-9 | -0.3953641 |
| Rassf1 | 1.63562526 |
| Rassf1 | -0.6622411 |
| Rassf10 | 0.44571425 |
| Rassf2 | -0.5230596 |
| Rassf2 | 0.11562918 |
| Rassf3 | 1.06036111 |
| Rassf4 | 0.36993525 |
| Rassf4 | 0.120173 |
| Rassf4 | 0.00707657 |
| Rassf4 | -0.5716667 |
| Rassf5 | -0.6639879 |
| Rassf7 | -0.5163176 |
| Rassf7 | -0.7244748 |
| Raver1 | 0.30001296 |
| Raver2 | 0.02606709 |
| Rb1 | 0.44864251 |
| Rb1 | 0.51778338 |
| Rbak | -0.388829 |
| Rbak | -0.5170643 |
| Rbbp5 | -0.3036166 |
| Rbbp5 | -0.2555338 |
| Rbbp6 | -0.6657379 |
| Rbbp7 | -0.4905515 |
| Rbbp7 | 0.48282783 |
| Rbbp9 | -0.1354172 |
| Rbck1 | -0.1036861 |
| Rbed1 | -0.554404 |
| Rbed1 | -0.2617658 |
| Rbj | -0.0692888 |
| Rbks | -0.3804012 |
| Rbl1 | 2.86704313 |
| Rbl2 | 0.38712479 |
| Rbm10 | 1.76653944 |
| Rbm10 | 0.39925233 |
| Rbm10 | 2.40814304 |
| Rbm11 | -0.8289652 |
| Rbm12 | 2.08693546 |
| Rbm12 | 2.02650064 |
| Rbm12 | 1.56189347 |
| Rbm12 | -0.3524273 |
| Rbm13 | -0.6137835 |
| Rbm13 | 0.87454915 |
| Rbm14 | 0.90602834 |
| Rbm14 | 0.49555286 |
| Rbm15 | 0.75000314 |
| Rbm16 | 0.73779472 |
| Rbm18 | -0.477572 |
| Rbm18 | 0.3136241 |
| Rbm19 | -0.3940534 |
| Rbm24 | 2.19347866 |
| Rbm26 | -0.554404 |
| Rbm28 | 1.53867255 |
| Rbm28 | 1.36633501 |
| Rbm3 | 0.120173 |
| Rbm35a | -0.557519 |
| Rbm38 | -0.2686121 |
| Rbm38 | 0.16191077 |
| Rbm38 | -0.2720541 |
| Rbm4 | 0.91961442 |
| Rbm41 | 0.60469523 |
| Rbm42 | -0.3663096 |
| Rbm42 | 0.04754157 |
| Rbm43 | -0.5155714 |
| Rbm45 | -0.6798573 |
| Rbm47 | 0.50985114 |
| Rbm47 | -0.5512993 |
| Rbm47 | 0.2808089 |
| Rbm47 | 1.84668957 |
| Rbm4b | -0.8268816 |
| Rbm5 | -0.6364242 |
| Rbm5 | 1.36742946 |
| Rbm6 | 0.26541508 |
| Rbm6 | 2.35676725 |
| Rbm6 | 2.15929636 |
| Rbm8a | -0.5908697 |
| Rbm9 | -0.1109136 |
| Rbm9 | 0.94245496 |
| Rbms1 | 0.47044659 |
| Rbms1 | -0.6604976 |
| Rbms2 | -0.12603 |
| Rbms2 | -0.7032664 |
| Rbms3 | -0.7831716 |
| Rbmx | 1.45660645 |
| Rbmy1a1 | 1.710185 |
| Rbp1 | 0.52098672 |
| Rbp7 | 2.72975011 |
| Rbpms | 1.99105991 |
| Rbpms | 0.64583492 |
| Rbpms | -0.661369 |
| Rbpms2 | 2.08416872 |
| Rbx1 | -0.0263412 |
| Rbx1 | -0.4438162 |
| Rc3h1 | 1.45434403 |
| Rcan1 | 0.20359282 |
| Rcan2 | 0.44357883 |
| Rcan2 | 1.65585327 |
| Rcan2 | -0.0688263 |
| Rcan3 | -0.2233443 |
| Rcbtb2 | -0.8165325 |
| Rcbtb2 | -0.572459 |
| Rcc1 | -0.3688559 |
| Rccd1 | 0.77788411 |
| Rce1 | -0.3131769 |
| Rchy1 | -0.0917631 |
| Rcl1 | 3.25022153 |
| Rcl1 | -0.7557703 |
| Rcl1 | 0.32431134 |
| Rcn3 | -0.0898695 |
| Rcor1 | 1.03587223 |
| Rcor2 | 0.33547793 |
| Rcsd1 | 0.61875837 |
| Rdbp | -0.0716048 |
| Rdbp | 0.49149967 |
| Rdh11 | -0.3289241 |
| Rdh12 | -0.4920049 |
| Rdh14 | -0.6055354 |
| Rdh14 | -0.4563321 |
| Rdh5 | 0.12431886 |
| Rdh7 | 1.09506702 |
| Rdh9 | 1.91808118 |
| Rdm1 | 0.21013392 |
| Rec8 | 1.7999924 |
| Reck | 0.52368869 |
| Recql | 0.85307301 |
| Recql4 | -0.1089808 |
| Recql5 | -0.7310262 |
| Reep1 | 1.35923982 |
| Reep2 | -0.6657379 |
| Reep3 | 3.10572595 |
| Reep4 | -0.140396 |
| Reep5 | 0.55219243 |
| Reep6 | 1.0257508 |
| Reep6 | 1.91177138 |
| Refbp2 | -0.6905887 |
| Reg1 | 0.12130552 |
| Rela | -0.8331465 |
| Relb | 0.21219005 |
| Rell1 | 1.70527978 |
| Rell2 | 1.8900846 |
| Rell2 | -0.7792076 |
| Reln | 0.55670183 |
| Relt | 0.4126067 |
| Relt | 0.80785726 |
| Rem1 | -0.34432 |
| Rem2 | 2.08167163 |
| Renbp | -0.0729972 |
| Repin1 | -0.7226113 |
| Repin1 | -0.2211647 |
| Reps1 | -0.6423847 |
| Reps1 | 0.43336842 |
| Rer1 | 0.25500983 |
| Rer1 | 0.66099238 |
| Rerg | -0.0352202 |
| Resp18 | 2.67305082 |
| Rest | -0.6171033 |
| Ret | 2.05045634 |
| Retnla | -0.6596271 |
| Retnlg | -0.8760216 |
| Retsat | -0.7319659 |
| Retsat | -0.603075 |
| Rev1 | 1.74122306 |
| Rev3l | 1.06477049 |
| Rex3 | 2.59547927 |
| Rexo1 | -0.5382182 |
| Rexo1 | -0.1443982 |
| Rexo2 | 0.52466929 |
| Rfc1 | 2.85242252 |
| Rfc2 | 0.44197406 |
| Rfc3 | -0.4045907 |
| Rfc4 | -0.6578885 |
| Rfc4 | -0.6372734 |
| Rfc5 | 0.77488947 |
| Rfesd | 1.28450582 |
| Rffl | 1.37406813 |
| Rffl | -0.8289652 |
| Rffl | 1.82895751 |
| Rffl | 0.25042085 |
| Rffl | -0.557519 |
| Rfng | 3.16000926 |
| Rfpl4 | 0.83830124 |
| Rfpl4 | -0.3155824 |
| Rft1 | -0.3714092 |
| Rftn1 | 0.60311951 |
| Rftn2 | 1.68362663 |
| Rftn2 | 1.24170474 |
| Rfwd2 | -0.6526919 |
| Rfwd3 | 0.32340006 |
| Rfwd3 | 1.82537036 |
| Rfx1 | 1.81938144 |
| Rfx1 | 1.08597324 |
| Rfx2 | 0.82079686 |
| Rfx3 | -0.8216927 |
| Rfx4 | -0.6483834 |
| Rfx4 | -0.4898257 |
| Rfx4 | 0.71068257 |
| Rfxank | 2.30038875 |
| Rfxap | 2.91483545 |
| Rfxdc1 | 1.02804127 |
| Rg9mtd1 | -0.3301465 |
| Rg9mtd2 | 1.4567872 |
| Rgag4 | 1.10275416 |
| Rgl1 | 1.3414159 |
| Rgl2 | -0.1289842 |
| Rgl2 | -0.7480867 |
| Rgl3 | 0.66479637 |
| Rgma | 1.17889431 |
| Rgmb | 1.64512936 |
| Rgmb | 2.60877111 |
| Rgnef | 1.0364392 |
| Rgs1 | -0.8073153 |
| Rgs1 | 3.03743149 |
| Rgs1 | -0.8749254 |
| Rgs10 | -0.4301033 |
| Rgs12 | 3.33528765 |
| Rgs14 | 3.04072267 |
| Rgs16 | -0.6544209 |
| Rgs17 | 1.30694506 |
| Rgs2 | 1.59645612 |
| Rgs3 | 0.44224171 |
| Rgs3 | 2.14013835 |
| Rgs3 | 0.63740317 |
| Rgs3 | 1.59122731 |
| Rgs3 | -0.7244748 |
| Rgs3 | -0.3739695 |
| Rgs3 | -0.178607 |
| Rgs4 | 3.59343014 |
| Rgs5 | 0.34828987 |
| Rgs6 | 1.68546804 |
| Rgs7 | 1.81483363 |
| Rgs7bp | -0.7871525 |
| Rgs8 | 0.36471612 |
| Rgs9 | -0.6483834 |
| Rgs9bp | -0.893738 |
| Rhag | 3.46059328 |
| Rhbdd1 | 1.3545132 |
| Rhbdd3 | -0.5275814 |
| Rhbdf1 | -0.6914886 |
| Rhbdf2 | 0.34324566 |
| Rhbdl1 | -0.1235753 |
| Rhbdl2 | 0.98802011 |
| Rhbdl3 | -0.0184215 |
| Rhbdl7 | 1.01566345 |
| Rhcg | 1.77968094 |
| Rheb | 0.27057458 |
| Rhebl1 | -0.7881504 |
| Rhob | -0.3468071 |
| Rhobtb2 | 0.13478969 |
| Rhobtb2 | 1.21878844 |
| Rhobtb2 | 0.86659581 |
| Rhobtb2 | 1.97079129 |
| Rhobtb3 | 0.61119504 |
| Rhoc | 0.92808607 |
| Rhoc | 0.13404565 |
| Rhod | 2.99065516 |
| Rhof | 0.09645509 |
| Rhog | 1.72141791 |
| Rhoj | -0.7891493 |
| Rhot1 | 0.84214698 |
| Rhot2 | 1.99553882 |
| Rhot2 | 0.23818321 |
| Rhot2 | 0.01683104 |
| Rhou | 0.59678994 |
| Rhov | -0.362503 |
| Rhox11 | -0.8175623 |
| Rhox12 | 0.92840425 |
| Rhox3a | -0.782179 |
| Rhox3f | 0.44491404 |
| Rhox4d | -0.5037139 |
| Rhox5 | -0.782179 |
| Rhox8 | -0.6204349 |
| Rhpn1 | -0.0716048 |
| Rhpn1 | -0.2200768 |
| Rhpn1 | 0.27282294 |
| Ribc1 | -0.4438162 |
| Ric3 | -0.0879797 |
| Ric3 | 0.02229973 |
| Ric3 | 1.0228798 |
| Ric8b | -0.8320994 |
| Ric8b | 1.30237219 |
| Rif1 | 0.31883031 |
| Rilp | -0.780197 |
| Rilpl1 | -0.0450856 |
| Rilpl1 | -0.5692938 |
| Rilpl2 | 1.88723825 |
| Rims1 | -0.3907847 |
| Rims2 | -0.2657534 |
| Rims3 | 2.79043381 |
| Rin1 | -0.7078361 |
| Rin2 | -0.6526919 |
| Rin2 | -0.8032478 |
| Rin2 | 1.14885185 |
| Rin3 | -0.3836337 |
| Riok1 | 0.35683236 |
| Riok1 | 2.26140613 |
| Riok3 | -0.6171033 |
| Riok3 | 0.22814179 |
| Ripk1 | 0.05769279 |
| Ripk2 | -0.0365594 |
| Ripk4 | 3.45390985 |
| Ripk5 | 1.24873118 |
| Rit1 | -0.0083987 |
| Rit2 | -0.6071792 |
| Rlbp1 | -0.5804191 |
| Rlbp1l1 | 1.21516743 |
| Rlbp1l1 | -0.2179048 |
| Rln1 | -0.7115081 |
| Rltpr | -0.2623344 |
| Rmnd1 | -0.7272769 |
| Rmnd5b | -0.3953641 |
| Rmnd5b | 1.12195848 |
| Rnase1 | 1.01884551 |
| Rnase4 | -0.5328847 |
| Rnase4 | 0.92776777 |
| Rnase6 | -0.8279228 |
| Rnaseh1 | 0.25827393 |
| Rnaseh1 | -0.5852279 |
| Rnaseh2c | 0.35035901 |
| Rnasek | -0.759636 |
| Rnasen | 1.00663709 |
| Rnaset2 | -0.7752603 |
| Rnaset2b | 0.1847484 |
| Rnd2 | -0.3167874 |
| Rnd3 | 1.55329385 |
| Rnf10 | -0.1580335 |
| Rnf103 | -0.6648625 |
| Rnf11 | -0.8258415 |
| Rnf11 | -0.2437505 |
| Rnf11 | -0.4192762 |
| Rnf111 | 1.37967906 |
| Rnf113a1 | 1.56173194 |
| Rnf113a2 | 0.74904003 |
| Rnf12 | 1.66087891 |
| Rnf121 | -0.6544209 |
| Rnf123 | -0.8352442 |
| Rnf125 | -0.7272769 |
| Rnf125 | -0.5389827 |
| Rnf128 | -0.8237648 |
| Rnf133 | -0.0184215 |
| Rnf135 | 0.74691767 |
| Rnf135 | 0.12243666 |
| Rnf135 | -0.1027267 |
| Rnf138 | 2.40030662 |
| Rnf138 | 0.1284464 |
| Rnf138 | 0.76868257 |
| Rnf138 | 1.26994056 |
| Rnf139 | 0.41122449 |
| Rnf139 | -0.7188956 |
| Rnf14 | -0.5637805 |
| Rnf14 | -0.4725727 |
| Rnf14 | -0.466184 |
| Rnf141 | 3.28172214 |
| Rnf141 | 2.38727619 |
| Rnf144a | -0.6843138 |
| Rnf145 | -0.1443982 |
| Rnf150 | 0.79436884 |
| Rnf166 | 0.69427476 |
| Rnf167 | -0.1636462 |
| Rnf167 | -0.3362825 |
| Rnf167 | -0.1838219 |
| Rnf167 | -0.6639879 |
| Rnf167 | -0.6080022 |
| Rnf168 | -0.7831716 |
| Rnf17 | 1.00795279 |
| Rnf170 | -0.6843138 |
| Rnf181 | 2.99893379 |
| Rnf182 | -0.6038944 |
| Rnf182 | 1.32657552 |
| Rnf183 | -0.6104755 |
| Rnf185 | -0.7841652 |
| Rnf185 | 1.02775527 |
| Rnf185 | 1.38788687 |
| Rnf185 | -0.7263419 |
| Rnf186 | 0.34591951 |
| Rnf19a | -0.8607905 |
| Rnf2 | 0.79105823 |
| Rnf20 | 0.64065516 |
| Rnf207 | 1.59785591 |
| Rnf208 | 3.70716342 |
| Rnf214 | 0.489212 |
| Rnf215 | -0.5590805 |
| Rnf216 | 2.44461191 |
| Rnf216 | 0.17660522 |
| Rnf219 | 0.12619728 |
| Rnf24 | -0.6509661 |
| Rnf24 | 0.76527953 |
| Rnf25 | -0.6518286 |
| Rnf25 | -0.3362825 |
| Rnf25 | -0.0655951 |
| Rnf26 | -0.808335 |
| Rnf31 | 1.00032528 |
| Rnf32 | 1.36224436 |
| Rnf34 | 0.16659505 |
| Rnf34 | -0.1610908 |
| Rnf34 | 1.07325492 |
| Rnf38 | 0.54526744 |
| Rnf38 | 1.05565323 |
| Rnf38 | 3.72040596 |
| Rnf4 | -0.7060055 |
| Rnf4 | -0.8227282 |
| Rnf40 | -0.5629955 |
| Rnf41 | -0.4052532 |
| Rnf41 | -0.6718889 |
| Rnf41 | -0.6631141 |
| Rnf44 | -0.3024284 |
| Rnf5 | -0.6121281 |
| Rnf6 | -0.4971095 |
| Rnf6 | -0.6187676 |
| Rnf8 | -0.3381312 |
| Rnf8 | -0.7723108 |
| Rngtt | -0.2971002 |
| Rnmt | 2.91278275 |
| Rnmtl1 | 1.94649493 |
| Rnpc2 | -0.3758944 |
| Rnpep | -0.8227282 |
| Rnpepl1 | 0.99840863 |
| Rnps1 | 0.54262723 |
| Rnps1 | 0.81722046 |
| Rnu6 | -0.6587574 |
| Rnuxa | -0.2824569 |
| Rnuxa | -0.6604976 |
| Robo1 | -0.2173626 |
| Robo2 | -0.7625457 |
| Robo4 | -0.2623344 |
| Rock1 | -0.3264841 |
| Rock2 | 0.71508971 |
| Rock2 | -0.3083845 |
| Rod1 | 0.17054042 |
| Rogdi | -0.4927324 |
| Rom1 | -0.8196252 |
| Ropn1 | -0.4633586 |
| Ror1 | -0.6381234 |
| Ror2 | 1.32366232 |
| Rora | 1.63218598 |
| Rorb | -0.7732929 |
| Rorb | -0.5185595 |
| Rorc | -0.6816374 |
| Rorc | 2.66937945 |
| Ros1 | -0.2315635 |
| Rp23-297j14.5 | 2.81420367 |
| Rp23-438h3.2 | 1.0321785 |
| rp9 | -0.2431931 |
| rp9 | 0.90814391 |
| Rpa1 | -0.6745375 |
| Rpa2 | -0.6296585 |
| Rpa2 | -0.5692938 |
| Rpa3 | 0.79858208 |
| Rpain | 1.65475715 |
| Rpap1 | -0.6364242 |
| Rpap1 | -0.0642137 |
| Rpap2 | -0.7357341 |
| Rpap2 | 0.69936971 |
| Rpap3 | 1.0331744 |
| Rpe | 0.39363119 |
| Rpgr | -0.1656956 |
| Rpgrip1 | 1.62201873 |
| Rpgrip1l | 3.032657 |
| Rph3a | 0.69977611 |
| Rph3al | 1.50709962 |
| Rpia | -0.0720687 |
| Rpl12 | 0.10953617 |
| Rpl13a | -0.6113014 |
| Rpl18 | 0.70261595 |
| Rpl19 | -0.3474299 |
| Rpl21 | -0.893738 |
| Rpl21 | 1.53999622 |
| Rpl22 | 0.5879017 |
| Rpl22l1 | -0.3752523 |
| Rpl23 | 1.09865338 |
| Rpl23 | 0.40956314 |
| Rpl23 | 0.18721274 |
| Rpl24 | 0.376562 |
| Rpl27 | -0.4683087 |
| Rpl27a | -0.5567393 |
| Rpl28 | -0.5590805 |
| Rpl29 | -0.4514449 |
| Rpl29 | 0.28112693 |
| Rpl3 | -0.5836222 |
| Rpl30 | 0.87757728 |
| Rpl31 | -0.6596271 |
| Rpl31 | -0.3849298 |
| Rpl34 | 2.56710347 |
| Rpl35 | -0.3461847 |
| Rpl36a | 0.87841669 |
| Rpl36al | -0.4697279 |
| Rpl37 | -0.7319659 |
| Rpl38 | 0.21560692 |
| Rpl38 | -0.4927324 |
| Rpl39 | 0.91187515 |
| Rpl39l | -0.8662017 |
| Rpl3l | 2.07628484 |
| Rpl4 | -0.804263 |
| Rpl41 | 0.60604373 |
| Rpl7a | -0.2108804 |
| Rpl7l1 | 0.53973848 |
| Rpl7l1 | 0.50485908 |
| Rpl9 | -0.519308 |
| Rplp1 | -0.7060055 |
| Rpn2 | 0.45447163 |
| Rpo1-1 | 0.16118802 |
| Rpo1-3 | 0.83285525 |
| Rpo1-3 | 0.57452494 |
| Rpo1-4 | 3.21543612 |
| Rpo1-4 | 1.25883776 |
| Rpo1-4 | -0.7891493 |
| Rpo2tc1 | 2.35542092 |
| Rpp21 | 0.26476814 |
| Rpp25 | -0.1118815 |
| Rpp30 | 0.46680428 |
| Rpp38 | 2.626799 |
| Rpp40 | 2.73339974 |
| Rprm | 1.42918222 |
| Rprml | -0.222799 |
| Rps10 | 1.01768965 |
| Rps11 | 1.89252554 |
| Rps12 | 0.0976167 |
| Rps13 | 0.45579138 |
| Rps14 | 0.85977328 |
| Rps15 | -0.34432 |
| Rps15a | -0.7772319 |
| Rps16 | -0.7404658 |
| Rps19 | 0.27314369 |
| Rps19 | 1.23965487 |
| Rps19bp1 | -0.7901493 |
| Rps2 | 2.30452012 |
| Rps21 | 0.22409482 |
| Rps24 | 4.21674891 |
| Rps24 | 0.81273063 |
| Rps25 | -0.7319659 |
| Rps26 | -0.6852076 |
| Rps27a | -0.3966766 |
| Rps27a | 0.31393115 |
| Rps27l | -0.1817325 |
| Rps3 | 0.48154574 |
| Rps3 | 1.65607234 |
| Rps3a | -0.5756349 |
| Rps4x | -0.446583 |
| Rps4y2 | 1.68716458 |
| Rps5 | -0.5044506 |
| Rps6 | 0.71288874 |
| Rps6ka1 | 2.2695838 |
| Rps6ka3 | 3.76558279 |
| Rps6ka4 | -0.0405884 |
| Rps6ka5 | -0.5812188 |
| Rps6kb1 | 2.03247418 |
| Rps6kl1 | 0.24580922 |
| Rps7 | 1.19589358 |
| Rps8 | -0.046439 |
| Rps9 | -0.466184 |
| Rps9 | -0.3240505 |
| Rpsa | 0.1502792 |
| Rpusd1 | 0.91155128 |
| Rpusd4 | 0.04508879 |
| Rqcd1 | -0.7772319 |
| Rrad | 2.99378351 |
| Rragb | 1.22053552 |
| Rragc | -0.7921526 |
| Rragd | -0.6055354 |
| Rras | -0.6162723 |
| Rras2 | -0.7703496 |
| Rrbp1 | 0.48487551 |
| Rrbp1 | 1.13250207 |
| Rreb1 | -0.317994 |
| Rreb1 | -0.0851521 |
| Rrh | -0.5155714 |
| Rrm1 | -0.3669455 |
| Rrm2 | 1.90026026 |
| Rrm2 | -0.7683925 |
| Rrm2b | -0.3350521 |
| Rrn3 | 1.20018472 |
| Rrp1 | -0.3185979 |
| Rrp12 | -0.0263412 |
| Rrp15 | 0.396727 |
| Rrp1b | -0.5637805 |
| Rrp1b | -0.322836 |
| Rrp1b | 1.03288997 |
| Rrp9 | -0.2594948 |
| Rs1 | -0.3966766 |
| Rsad1 | -0.6869979 |
| Rsad2 | 1.0364392 |
| Rsf1 | 2.00186423 |
| Rshl1 | -0.7851599 |
| Rshl2a | -0.2755089 |
| Rsl1d1 | 0.78681086 |
| Rsl1d1 | -0.0988987 |
| Rsn | 0.6064928 |
| Rsph1 | -0.0828023 |
| Rspo1 | -0.2532779 |
| Rspo2 | 0.61476188 |
| Rspo3 | 1.57750974 |
| Rspo4 | -0.6179351 |
| Rspry1 | -0.6104755 |
| Rsrc1 | 1.07529608 |
| Rsrc1 | 0.13961156 |
| Rsrc2 | 0.55504301 |
| Rsu1 | -0.7404658 |
| Rtbdn | -0.3155824 |
| Rtel1 | -0.1008107 |
| Rtel1 | 0.0520217 |
| Rtkn | 0.68997344 |
| Rtn1 | 0.37483781 |
| Rtn1 | -0.4383069 |
| Rtn1 | 0.64260094 |
| Rtn1 | 2.01124781 |
| Rtn2 | -0.6449509 |
| Rtn3 | -0.0577941 |
| Rtn4 | -0.7931558 |
| Rtn4 | -0.8629512 |
| Rtn4 | -0.0776535 |
| Rtn4ip1 | 0.98622757 |
| Rtn4r | 0.2408428 |
| Rtn4rl1 | -0.6987189 |
| Rtn4rl2 | 0.07211951 |
| Rtp1 | -0.3682187 |
| Rtp4 | -0.7078361 |
| Rttn | -0.5466615 |
| Rufy1 | 0.77993737 |
| Rufy2 | 0.9667374 |
| Rufy3 | 2.26487611 |
| Rufy3 | 0.62295844 |
| Rufy3 | 1.13352437 |
| Rufy4 | -0.5820193 |
| Rundc1 | -0.6544209 |
| Rundc3b | -0.3149804 |
| Runx1 | -0.3024284 |
| Runx1t1 | 0.489212 |
| Runx1t1 | -0.6458078 |
| Runx2 | 2.53937243 |
| Rusc1 | -0.6146124 |
| Rusc2 | -0.4159182 |
| Rusc2 | -0.5844247 |
| Ruvbl1 | -0.0352202 |
| Ruvbl1 | -0.6415309 |
| Ruvbl1 | -0.2387457 |
| Ruvbl2 | 1.40361116 |
| Rwdd1 | -0.6313454 |
| Rwdd1 | 1.99335295 |
| Rwdd3 | 0.00920579 |
| Rwdd3 | 1.28374486 |
| Rwdd4a | -0.3726885 |
| Rxfp1 | 0.85462349 |
| Rxfp3 | 0.10301852 |
| Rxra | -0.0776535 |
| Rxrb | -0.2783976 |
| Rxrb | -0.4132405 |
| Rxrg | -0.5103652 |
| Rya3 | -0.6121281 |
| Ryk | 1.48850476 |
| Ryk | -0.6604976 |
| Ryr1 | 0.62801759 |
| Ryr1 | 3.22858365 |
| Ryr1 | 0.83285525 |
| Ryr3 | -0.3986488 |
| S100a1 | -0.34432 |
| S100a10 | -0.7841652 |
| S100a11 | 0.55266813 |
| S100a13 | 0.52147857 |
| S100a16 | -0.4697279 |
| S100a4 | -0.6406778 |
| S100a6 | 0.24514855 |
| S100a8 | -0.4065797 |
| S100a9 | -0.2566638 |
| S100b | -0.7357341 |
| S100pbp | 0.41122449 |
| S100pbp | 0.27953573 |
| S3-12 | 1.80685238 |
| Saa3 | -0.7235426 |
| Sac3d1 | -0.1662087 |
| Sacm1l | -0.2824569 |
| Sacs | -0.2982816 |
| Sae1 | 0.76148378 |
| Sae1 | 0.19804879 |
| Sae1 | -0.3682187 |
| Sag | 0.31270234 |
| Sall1 | -0.4335127 |
| Sall1 | -0.4905515 |
| Sall2 | -0.7490437 |
| Sall4 | -0.3511754 |
| Sall4 | 2.18003043 |
| Samd10 | -0.7366785 |
| Samd11 | 0.09490404 |
| Samd12 | -0.6171033 |
| Samd14 | 1.43530532 |
| Samd4 | -0.3424591 |
| Samd4 | 1.98177958 |
| Samd4b | 0.54310782 |
| Samd5 | -0.5059257 |
| Samd7 | -0.8543382 |
| Samd8 | 0.17696075 |
| Samd9l | -0.5685042 |
| Samhd1 | -0.1284912 |
| Samm50 | 1.317389 |
| Samsn1 | 0.3339767 |
| Sap130 | -0.7087528 |
| Sap30 | 1.5460118 |
| Sap30bp | 3.01510371 |
| Sap30l | -0.5812188 |
| Saps1 | 0.83777559 |
| Saps2 | 0.23015873 |
| Saps3 | 0.4219519 |
| Saps3 | 1.75123424 |
| Sar1a | -0.6449509 |
| Sar1b | 0.52931315 |
| Sar1b | -0.5059257 |
| Sarm1 | -0.6381234 |
| Sars | -0.0267831 |
| Sars | 2.12310765 |
| Sars2 | -0.7567352 |
| Sart1 | -0.3393657 |
| Sart3 | -0.4654769 |
| Sas | 2.00654271 |
| Sass6 | -0.6896897 |
| Sat2 | -0.6914886 |
| Satb1 | 2.36425741 |
| Sav1 | 2.6968932 |
| Sbds | -0.1832991 |
| Sbf1 | -0.2482219 |
| Sbk | -0.2420793 |
| Sbno1 | 0.12356644 |
| Sbno2 | 0.57939039 |
| Sc4mol | -0.3682187 |
| Sc5d | -0.5716667 |
| Scaf1 | -0.4424358 |
| Scamp1 | 1.2325596 |
| Scamp2 | -0.4790053 |
| Scamp3 | -0.6996266 |
| Scamp4 | -0.5474329 |
| Scamp5 | 2.10051148 |
| Scamp5 | 0.32673707 |
| Scand1 | 1.15173712 |
| Scap | 0.92505789 |
| Scaper | -0.1499293 |
| Scara3 | -0.322836 |
| Scara5 | 1.42161225 |
| Scarb1 | 2.49441421 |
| Scarb2 | 1.19768395 |
| Scarf1 | -0.4431257 |
| Scarf2 | 0.62801759 |
| Sccpdh | -0.5685042 |
| Scd1 | 0.0729145 |
| Scd2 | 0.81002634 |
| Scd4 | -0.3631363 |
| Scel | -0.7376239 |
| Scg2 | 0.18686109 |
| Scg3 | -0.240411 |
| Scg5 | 1.41341266 |
| Scgn | 1.77229548 |
| Scgn | -0.7366785 |
| Scgn | 2.15296819 |
| Schip1 | 2.16934155 |
| Schip1 | 0.80894243 |
| Scin | 0.11258764 |
| scl0003251.1_21 | 1.9307921 |
| Sclt1 | -0.7115081 |
| Scly | -0.8012207 |
| Scmh1 | -0.4640641 |
| Scmh1 | 0.84040088 |
| Scml2 | 1.15909798 |
| Scml4 | -0.1975185 |
| Scn10a | 2.52663136 |
| Scn11a | -0.6763074 |
| Scn1a | 1.54625819 |
| Scn2b | 1.41794547 |
| Scn3b | -0.4179315 |
| Scn3b | 0.58077588 |
| Scn3b | -0.1901185 |
| Scn3b | 1.07257354 |
| Scn4b | -0.7971795 |
| Scn5a | -0.1294775 |
| Scn7a | -0.3277033 |
| Scn8a | 1.45253015 |
| Scnm1 | 0.46758597 |
| Scnm1 | 1.20315312 |
[truncated: 5,818,852 more chars]
