# Supplementary material for: Plurigon: three dimensional visualization and classification of high-dimensionality data
Source: Front Physiol. 2013 Jul 22;4:190. doi: 10.3389/fphys.2013.00190 (PMC3717481; doi:10.3389/fphys.2013.00190)
Supplement: Table S10 — Standard PCA analysis of WT and HET genotypic datasets. PCA was applied to reduce data dimensionality to three primary dimensions, D1, D2 and D3. Using these three dimensions the two genotype groups appeared non-separable. [file 46440_Maudsley_Data_Sheet_5.ZIP › 46440_Maudsley_Table_10.DOC]

**Table S10. Standard PCA analysis of WT and HET genotypic datasets.** PCA was applied to reduce data dimensionality to three primary dimensions, D1, D2 and D3. Using these three dimensions the two genotype groups appeared non-separable.

| **WT** |  |  |
| --- | --- | --- |
| **D1** | **D2** | **D3** |
| -125.5518694 | -4.592075776 | -0.467230349 |
| -124.7447701 | -4.382992397 | -0.583799219 |
| -125.2161348 | -4.391145176 | -0.476372002 |
| -121.410174 | -4.492812146 | 0.213109223 |
| -120.093382 | -4.277192325 | 0.08448007 |
| -134.8674605 | -5.435706524 | 1.231476157 |
| -132.929635 | -5.317195244 | 0.847633613 |
| -133.8011299 | -5.285666863 | 1.115464571 |
| 5.312740772 | -125.0400912 | -0.14137914 |
| 5.560727214 | -123.6532295 | -0.146236285 |
| 5.322870461 | -126.1029604 | 3.877234352 |
| 4.136278687 | -113.1060795 | -69.90508336 |
| 4.045171926 | -113.242867 | -69.72696152 |
| 4.019627584 | -114.7475366 | -69.3432922 |
| 6.079534979 | -122.2598028 | 61.563456 |
| 5.879464074 | -122.8974167 | 61.60261516 |
| 5.997186202 | -123.3151675 | 60.57250778 |
|  |  |  |
| **HET** |  |  |
| **D1** | **D2** | **D3** |
| -125.4358271 | -4.557393634 | -0.702886758 |
| -125.4324609 | -4.694715386 | -0.515794368 |
| -124.6813627 | -4.515141137 | -0.622456617 |
| -121.3354593 | -3.953776937 | 0.174149986 |
| -119.7656987 | -4.088795076 | 0.304484993 |
| -119.4094309 | -3.987151902 | 0.415063172 |
| -131.4849676 | -5.190832229 | 0.880753841 |
| -133.548697 | -5.299862041 | 0.925053427 |
| -133.2778547 | -5.504964383 | 0.930206108 |
| -133.8744988 | -5.430734267 | 1.018580456 |
| 5.390838321 | -126.3572609 | 4.604566043 |
| 5.43404762 | -125.1215183 | 2.913907036 |
| 5.456618522 | -126.4292806 | 4.721952267 |
| 3.909383644 | -115.4640799 | -68.164045 |
| 3.942034263 | -114.0380776 | -67.18664582 |
| 3.963454368 | -113.8717292 | -67.60533893 |
| 6.095140249 | -122.0686726 | 60.10848813 |
| 6.02900415 | -122.2161425 | 62.14569221 |
| 6.116778935 | -122.7200955 | 60.85076129 |
