# Supplementary material for: Plurigon: three dimensional visualization and classification of high-dimensionality data
Source: Front Physiol. 2013 Jul 22;4:190. doi: 10.3389/fphys.2013.00190 (PMC3717481; doi:10.3389/fphys.2013.00190)
Supplement: Table S10 — Standard PCA analysis of WT and HET genotypic datasets. PCA was applied to reduce data dimensionality to three primary dimensions, D1, D2 and D3. Using these three dimensions the two genotype groups appeared non-separable. [file 46440_Maudsley_Data_Sheet_5.ZIP › 46440_Maudsley_Table_12.DOC]

**Table S12. Human neuronal cell (SH-SY5Y) transcriptomic effects induced by amitriptyline exposure.** For each amitriptyline (AMI) regulated transcript the official Gene Symbol, z-ratio and transcript description are indicated.

| **Gene Symbol** | **z-ratio** | **Description** |
| --- | --- | --- |
| HSPA1B | 7.33 | Homo sapiens heat shock 70kDa protein 1B (HSPA1B), mRNA. |
| NEUROG2 | 7.28 | Homo sapiens neurogenin 2 (NEUROG2), mRNA. |
| SLC25A22 | 6.67 | Homo sapiens solute carrier family 25 (mitochondrial carrier: glutamate), member 22 (SLC25A22), nuclear gene encoding mitochondrial protein, mRNA. |
| ACCN2 | 6.48 | Homo sapiens amiloride-sensitive cation channel 2, neuronal (ACCN2), transcript variant 2, mRNA. |
| CLK1 | 6.44 | Homo sapiens CDC-like kinase 1 (CLK1), mRNA. |
| MAP6 | 6.43 | Homo sapiens microtubule-associated protein 6 (MAP6), transcript variant 1, mRNA. |
| LOC100130123 | 6.11 | PREDICTED: Homo sapiens similar to PRO2870 (LOC100130123), mRNA. |
| DPP7 | 5.91 | Homo sapiens dipeptidyl-peptidase 7 (DPP7), mRNA. |
| TXNIP | 5.81 | Homo sapiens thioredoxin interacting protein (TXNIP), mRNA. |
| MOBKL2C | 5.71 | Homo sapiens MOB1, Mps One Binder kinase activator-like 2C (yeast) (MOBKL2C), transcript variant 1, mRNA. |
| TINF2 | 5.18 | Homo sapiens TERF1 (TRF1)-interacting nuclear factor 2 (TINF2), mRNA. |
| ZNF672 | 5.09 | Homo sapiens zinc finger protein 672 (ZNF672), mRNA. |
| ORAOV1 | 5.07 | Homo sapiens oral cancer overexpressed 1 (ORAOV1), mRNA. |
| GAL3ST3 | 5.01 | Homo sapiens galactose-3-O-sulfotransferase 3 (GAL3ST3), mRNA. |
| GFOD2 | 4.95 | Homo sapiens glucose-fructose oxidoreductase domain containing 2 (GFOD2), mRNA. |
| LOC389286 | 4.95 | Homo sapiens similar to FKSG62 (LOC389286), mRNA. |
| C8orf55 | 4.89 | Homo sapiens chromosome 8 open reading frame 55 (C8orf55), mRNA. |
| HSPA5 | 4.83 | Homo sapiens heat shock 70kDa protein 5 (glucose-regulated protein, 78kDa) (HSPA5), mRNA. |
|  | 4.77 | Homo sapiens cDNA FLJ38396 fis, clone FEBRA2007957 |
| CCDC136 | 4.75 | Homo sapiens coiled-coil domain containing 136 (CCDC136), mRNA. |
| GCH1 | 4.74 | Homo sapiens GTP cyclohydrolase 1 (GCH1), transcript variant 3, mRNA. |
| CCND3 | 4.7 | Homo sapiens cyclin D3 (CCND3), mRNA. |
| LOC148413 | 4.69 | Homo sapiens hypothetical LOC148413 (LOC148413), non-coding RNA. |
| GRAMD1A | 4.67 | Homo sapiens GRAM domain containing 1A (GRAMD1A), mRNA. |
| SIPA1 | 4.67 | Homo sapiens signal-induced proliferation-associated gene 1 (SIPA1), transcript variant 2, mRNA. |
| GADD45G | 4.65 | Homo sapiens growth arrest and DNA-damage-inducible, gamma (GADD45G), mRNA. |
| MAT2B | 4.59 | Homo sapiens methionine adenosyltransferase II, beta (MAT2B), transcript variant 1, mRNA. |
| ZNF200 | 4.54 | Homo sapiens zinc finger protein 200 (ZNF200), transcript variant 3, mRNA. |
| HSPA1A | 4.53 | Homo sapiens heat shock 70kDa protein 1A (HSPA1A), mRNA. |
| SLC26A6 | 4.48 | Homo sapiens solute carrier family 26, member 6 (SLC26A6), transcript variant 3, mRNA. |
| HNRNPA1 | 4.45 | Homo sapiens heterogeneous nuclear ribonucleoprotein A1 (HNRNPA1), transcript variant 2, mRNA. |
| SC4MOL | 4.44 | Homo sapiens sterol-C4-methyl oxidase-like (SC4MOL), transcript variant 1, mRNA. |
| ASCC2 | 4.41 | Homo sapiens activating signal cointegrator 1 complex subunit 2 (ASCC2), mRNA. |
| LOC401805 | 4.36 | PREDICTED: Homo sapiens misc_RNA (LOC401805), partial miscRNA. |
| B4GALT7 | 4.33 | Homo sapiens xylosylprotein beta 1,4-galactosyltransferase, polypeptide 7 (galactosyltransferase I) (B4GALT7), mRNA. |
| SDF2L1 | 4.33 | Homo sapiens stromal cell-derived factor 2-like 1 (SDF2L1), mRNA. |
| FGF14 | 4.32 | Homo sapiens fibroblast growth factor 14 (FGF14), transcript variant 2, mRNA. |
| LOC100133312 | 4.28 | PREDICTED: Homo sapiens misc_RNA (LOC100133312), miscRNA. |
| SAFB | 4.27 | Homo sapiens scaffold attachment factor B (SAFB), mRNA. |
| CLK3 | 4.25 | Homo sapiens CDC-like kinase 3 (CLK3), transcript variant phclk3, mRNA. |
| DEDD2 | 4.21 | Homo sapiens death effector domain containing 2 (DEDD2), mRNA. |
| SDHAP2 | 4.16 | Homo sapiens succinate dehydrogenase complex, subunit A, flavoprotein pseudogene 2 (SDHAP2), non-coding RNA. |
| VPS24 | 4.15 | Homo sapiens vacuolar protein sorting 24 homolog (S. cerevisiae) (VPS24), transcript variant 2, mRNA. |
| PSPH | 4.15 | Homo sapiens phosphoserine phosphatase (PSPH), mRNA. |
| ZNF250 | 4.13 | Homo sapiens zinc finger protein 250 (ZNF250), mRNA. |
| CPT1C | 4.11 | Homo sapiens carnitine palmitoyltransferase 1C (CPT1C), transcript variant 2, mRNA. |
| ARMET | 4.1 | Homo sapiens arginine-rich, mutated in early stage tumors (ARMET), mRNA. |
| ADCK2 | 4.09 | Homo sapiens aarF domain containing kinase 2 (ADCK2), mRNA. |
| KCNQ2 | 4.09 | Homo sapiens potassium voltage-gated channel, KQT-like subfamily, member 2 (KCNQ2), transcript variant 5, mRNA. |
| FAM131A | 4.08 | Homo sapiens family with sequence similarity 131, member A (FAM131A), mRNA. |
| ALDOC | 4.07 | Homo sapiens aldolase C, fructose-bisphosphate (ALDOC), mRNA. |
| MFSD3 | 4.06 | Homo sapiens major facilitator superfamily domain containing 3 (MFSD3), mRNA. |
| C1orf63 | 4.06 | Homo sapiens chromosome 1 open reading frame 63 (C1orf63), transcript variant 1, mRNA. |
| CCDC106 | 4.04 | Homo sapiens coiled-coil domain containing 106 (CCDC106), mRNA. |
| DBH | 4.04 | Homo sapiens dopamine beta-hydroxylase (dopamine beta-monooxygenase) (DBH), mRNA. |
| LOC729423 | 4.03 | PREDICTED: Homo sapiens similar to Heterogeneous nuclear ribonucleoprotein A1 (Helix-destabilizing protein) (Single-strand RNA-binding protein) (hnRNP core protein A1) (HDP) (LOC729423), mRNA. |
| ZNF585A | 4.01 | Homo sapiens zinc finger protein 585A (ZNF585A), transcript variant 2, mRNA. |
| RBM4 | 4.01 | Homo sapiens RNA binding motif protein 4 (RBM4), mRNA. |
| HPS6 | 4 | Homo sapiens Hermansky-Pudlak syndrome 6 (HPS6), mRNA. |
| WDR86 | 3.99 | Homo sapiens WD repeat domain 86 (WDR86), mRNA. |
| RTKN | 3.99 | Homo sapiens rhotekin (RTKN), transcript variant 2, mRNA. |
| BTBD11 | 3.98 | Homo sapiens BTB (POZ) domain containing 11 (BTBD11), transcript variant 1, mRNA. |
| DDIT4 | 3.95 | Homo sapiens DNA-damage-inducible transcript 4 (DDIT4), mRNA. |
| UBE2L3 | 3.93 | Homo sapiens ubiquitin-conjugating enzyme E2L 3 (UBE2L3), transcript variant 2, mRNA. |
| FBXL20 | 3.91 | Homo sapiens F-box and leucine-rich repeat protein 20 (FBXL20), mRNA. |
| ARMCX3 | 3.9 | Homo sapiens armadillo repeat containing, X-linked 3 (ARMCX3), transcript variant 2, mRNA. |
| TMEM79 | 3.9 | Homo sapiens transmembrane protein 79 (TMEM79), mRNA. |
| CHGB | 3.89 | Homo sapiens chromogranin B (secretogranin 1) (CHGB), mRNA. |
| MYCN | 3.89 | Homo sapiens v-myc myelocytomatosis viral related oncogene, neuroblastoma derived (avian) (MYCN), mRNA. |
| RASSF7 | 3.87 | Homo sapiens Ras association (RalGDS/AF-6) domain family (N-terminal) member 7 (RASSF7), mRNA. |
| SBDSP | 3.86 | Homo sapiens Shwachman-Bodian-Diamond syndrome pseudogene (SBDSP) on chromosome 7. |
| ENO3 | 3.86 | Homo sapiens enolase 3 (beta, muscle) (ENO3), transcript variant 1, mRNA. |
| REXO4 | 3.86 | Homo sapiens REX4, RNA exonuclease 4 homolog (S. cerevisiae) (REXO4), mRNA. |
| CLK1 | 3.85 | Homo sapiens CDC-like kinase 1 (CLK1), mRNA. |
| DNAJB1 | 3.85 | Homo sapiens DnaJ (Hsp40) homolog, subfamily B, member 1 (DNAJB1), mRNA. |
| HNRNPA1 | 3.84 | Homo sapiens heterogeneous nuclear ribonucleoprotein A1 (HNRNPA1), transcript variant 2, mRNA. |
| SPSB3 | 3.83 | Homo sapiens splA/ryanodine receptor domain and SOCS box containing 3 (SPSB3), mRNA. |
| DPYSL4 | 3.81 | Homo sapiens dihydropyrimidinase-like 4 (DPYSL4), mRNA. |
| VPS37D | 3.78 | Homo sapiens vacuolar protein sorting 37 homolog D (S. cerevisiae) (VPS37D), mRNA. |
| HIST1H2AC | 3.77 | Homo sapiens histone cluster 1, H2ac (HIST1H2AC), mRNA. |
| HPCAL1 | 3.76 | Homo sapiens hippocalcin-like 1 (HPCAL1), transcript variant 2, mRNA. |
| N6AMT1 | 3.75 | Homo sapiens N-6 adenine-specific DNA methyltransferase 1 (putative) (N6AMT1), transcript variant 2, mRNA. |
| DTX2 | 3.73 | Homo sapiens deltex homolog 2 (Drosophila) (DTX2), mRNA. |
| NAMPT | 3.72 | Homo sapiens nicotinamide phosphoribosyltransferase (NAMPT), mRNA. |
| LOC100132439 | 3.72 | PREDICTED: Homo sapiens similar to Protein FAM27E3 (LOC100132439), mRNA. |
| PTGES2 | 3.71 | Homo sapiens prostaglandin E synthase 2 (PTGES2), transcript variant 2, mRNA. |
| ZNF556 | 3.71 | Homo sapiens zinc finger protein 556 (ZNF556), mRNA. |
| LRWD1 | 3.7 | Homo sapiens leucine-rich repeats and WD repeat domain containing 1 (LRWD1), mRNA. |
| ZNF615 | 3.7 | Homo sapiens zinc finger protein 615 (ZNF615), mRNA. |
| SLC25A3 | 3.7 | Homo sapiens solute carrier family 25 (mitochondrial carrier; phosphate carrier), member 3 (SLC25A3), nuclear gene encoding mitochondrial protein, transcript variant 3, mRNA. |
| RCOR2 | 3.69 | Homo sapiens REST corepressor 2 (RCOR2), mRNA. |
| EHD1 | 3.68 | Homo sapiens EH-domain containing 1 (EHD1), mRNA. |
| GNL1 | 3.67 | Homo sapiens guanine nucleotide binding protein-like 1 (GNL1), mRNA. |
| FADD | 3.66 | Homo sapiens Fas (TNFRSF6)-associated via death domain (FADD), mRNA. |
| MRFAP1L1 | 3.66 | Homo sapiens Morf4 family associated protein 1-like 1 (MRFAP1L1), transcript variant 1, mRNA. |
| SPOCK1 | 3.64 | Homo sapiens sparc/osteonectin, cwcv and kazal-like domains proteoglycan (testican) 1 (SPOCK1), mRNA. |
| KCNJ8 | 3.59 | Homo sapiens potassium inwardly-rectifying channel, subfamily J, member 8 (KCNJ8), mRNA. |
| ZSCAN2 | 3.58 | Homo sapiens zinc finger and SCAN domain containing 2 (ZSCAN2), transcript variant 1, mRNA. |
| PICK1 | 3.58 | Homo sapiens protein interacting with PRKCA 1 (PICK1), transcript variant 1, mRNA. |
| FAM53C | 3.57 | Homo sapiens family with sequence similarity 53, member C (FAM53C), mRNA. |
| LOC441711 | 3.56 | PREDICTED: Homo sapiens misc_RNA (LOC441711), miscRNA. |
| LOC255783 | 3.56 | Homo sapiens hypothetical protein LOC255783 (LOC255783), non-coding RNA. |
| LRSAM1 | 3.55 | Homo sapiens leucine rich repeat and sterile alpha motif containing 1 (LRSAM1), transcript variant 1, mRNA. |
| PDXK | 3.54 | Homo sapiens pyridoxal (pyridoxine, vitamin B6) kinase (PDXK), mRNA. |
| LOC729992 | 3.53 | PREDICTED: Homo sapiens misc_RNA (LOC729992), miscRNA. |
| IFNAR1 | 3.53 | Homo sapiens interferon (alpha, beta and omega) receptor 1 (IFNAR1), mRNA. |
| LOC113386 | 3.52 | Homo sapiens similar to envelope protein (LOC113386), mRNA. |
| ZBTB45 | 3.51 | Homo sapiens zinc finger and BTB domain containing 45 (ZBTB45), mRNA. |
| CHRM3 | 3.51 | Homo sapiens cholinergic receptor, muscarinic 3 (CHRM3), mRNA. |
| RPS6KB2 | 3.5 | Homo sapiens ribosomal protein S6 kinase, 70kDa, polypeptide 2 (RPS6KB2), mRNA. |
| LOC158318 | 3.49 | Homo sapiens similar to hypothetical protein MGC42630 (LOC158318), mRNA. |
| NDST2 | 3.48 | Homo sapiens N-deacetylase/N-sulfotransferase (heparan glucosaminyl) 2 (NDST2), mRNA. |
| FRMD8 | 3.47 | Homo sapiens FERM domain containing 8 (FRMD8), mRNA. |
| CRABP2 | 3.47 | Homo sapiens cellular retinoic acid binding protein 2 (CRABP2), mRNA. |
| RXRB | 3.47 | Homo sapiens retinoid X receptor, beta (RXRB), mRNA. |
| TPRG1L | 3.46 | Homo sapiens tumor protein p63 regulated 1-like (TPRG1L), mRNA. |
| C9orf114 | 3.45 | Homo sapiens chromosome 9 open reading frame 114 (C9orf114), mRNA. |
| LOC100131737 | 3.45 | PREDICTED: Homo sapiens misc_RNA (LOC100131737), miscRNA. |
| IMP4 | 3.45 | Homo sapiens IMP4, U3 small nucleolar ribonucleoprotein, homolog (yeast) (IMP4), mRNA. |
| CRELD2 | 3.45 | Homo sapiens cysteine-rich with EGF-like domains 2 (CRELD2), mRNA. |
| CHRNA3 | 3.45 | Homo sapiens cholinergic receptor, nicotinic, alpha 3 (CHRNA3), mRNA. |
| TMEM115 | 3.44 | Homo sapiens transmembrane protein 115 (TMEM115), mRNA. |
| C20orf27 | 3.44 | Homo sapiens chromosome 20 open reading frame 27 (C20orf27), mRNA. |
| PLEKHB2 | 3.43 | Homo sapiens pleckstrin homology domain containing, family B (evectins) member 2 (PLEKHB2), transcript variant 1, mRNA. |
| TPD52L2 | 3.43 | Homo sapiens tumor protein D52-like 2 (TPD52L2), transcript variant 5, mRNA. |
| SPHK2 | 3.42 | Homo sapiens sphingosine kinase 2 (SPHK2), mRNA. |
| SLC2A8 | 3.42 | Homo sapiens solute carrier family 2 (facilitated glucose transporter), member 8 (SLC2A8), mRNA. |
| TRABD | 3.4 | Homo sapiens TraB domain containing (TRABD), mRNA. |
| ATP8B2 | 3.4 | Homo sapiens ATPase, class I, type 8B, member 2 (ATP8B2), transcript variant 1, mRNA. |
| CCDC92 | 3.4 | Homo sapiens coiled-coil domain containing 92 (CCDC92), mRNA. |
| LOC653994 | 3.39 | PREDICTED: Homo sapiens similar to Eukaryotic translation initiation factor 4H (eIF-4H) (Williams-Beuren syndrome chromosome region 1 protein homolog), transcript variant 2 (LOC653994), mRNA. |
| KLHDC8B | 3.39 | Homo sapiens kelch domain containing 8B (KLHDC8B), mRNA. |
| MAP6 | 3.38 | Homo sapiens microtubule-associated protein 6 (MAP6), transcript variant 2, mRNA. |
| C17orf70 | 3.36 | Homo sapiens chromosome 17 open reading frame 70 (C17orf70), mRNA. |
| PEX16 | 3.34 | Homo sapiens peroxisomal biogenesis factor 16 (PEX16), transcript variant 1, mRNA. |
| CSTF3 | 3.33 | Homo sapiens cleavage stimulation factor, 3' pre-RNA, subunit 3, 77kDa (CSTF3), transcript variant 3, mRNA. |
| TAF1A | 3.32 | Homo sapiens TATA box binding protein (TBP)-associated factor, RNA polymerase I, A, 48kDa (TAF1A), transcript variant 1, mRNA. |
| SLC9A3R1 | 3.32 | Homo sapiens solute carrier family 9 (sodium/hydrogen exchanger), member 3 regulator 1 (SLC9A3R1), mRNA. |
| TP53I13 | 3.32 | Homo sapiens tumor protein p53 inducible protein 13 (TP53I13), mRNA. |
| ZNF696 | 3.31 | Homo sapiens zinc finger protein 696 (ZNF696), mRNA. |
| SLC25A28 | 3.31 | Homo sapiens solute carrier family 25, member 28 (SLC25A28), mRNA. |
| CCDC130 | 3.29 | Homo sapiens coiled-coil domain containing 130 (CCDC130), mRNA. |
| PPP2R2C | 3.29 | Homo sapiens protein phosphatase 2 (formerly 2A), regulatory subunit B, gamma isoform (PPP2R2C), transcript variant 2, mRNA. |
| ZBTB48 | 3.27 | Homo sapiens zinc finger and BTB domain containing 48 (ZBTB48), mRNA. |
| LOC643300 | 3.27 | PREDICTED: Homo sapiens similar to 60 kDa heat shock protein, mitochondrial precursor (Hsp60) (60 kDa chaperonin) (CPN60) (Heat shock protein 60) (HSP-60) (Mitochondrial matrix protein P1) (P60 lymphocyte protein) (HuCHA60) (LOC643300), mRNA. |
| EFNB3 | 3.27 | Homo sapiens ephrin-B3 (EFNB3), mRNA. |
| ZNF175 | 3.26 | Homo sapiens zinc finger protein 175 (ZNF175), mRNA. |
| BCDIN3D | 3.26 | Homo sapiens BCDIN3 domain containing (BCDIN3D), mRNA. |
| ARL2 | 3.26 | Homo sapiens ADP-ribosylation factor-like 2 (ARL2), mRNA. |
| HERPUD1 | 3.26 | Homo sapiens homocysteine-inducible, endoplasmic reticulum stress-inducible, ubiquitin-like domain member 1 (HERPUD1), transcript variant 3, mRNA. |
| MAPKAPK3 | 3.25 | Homo sapiens mitogen-activated protein kinase-activated protein kinase 3 (MAPKAPK3), mRNA. |
| ZNRD1 | 3.24 | Homo sapiens zinc ribbon domain containing 1 (ZNRD1), transcript variant a, mRNA. |
| JSRP1 | 3.23 | Homo sapiens junctional sarcoplasmic reticulum protein 1 (JSRP1), mRNA. |
| LOC728969 | 3.22 | PREDICTED: Homo sapiens similar to heparan sulfate 6-O-sulfotransferase 1 (LOC728969), mRNA. |
| METTL13 | 3.22 | Homo sapiens methyltransferase like 13 (METTL13), transcript variant 3, mRNA. |
| LRRN2 | 3.22 | Homo sapiens leucine rich repeat neuronal 2 (LRRN2), transcript variant 2, mRNA. |
| DHX34 | 3.22 | Homo sapiens DEAH (Asp-Glu-Ala-His) box polypeptide 34 (DHX34), mRNA. |
| FXR1 | 3.22 | Homo sapiens fragile X mental retardation, autosomal homolog 1 (FXR1), transcript variant 1, mRNA. |
| TOB1 | 3.21 | Homo sapiens transducer of ERBB2, 1 (TOB1), mRNA. |
| THOC3 | 3.21 | Homo sapiens THO complex 3 (THOC3), mRNA. |
| SBDSP | 3.2 | Homo sapiens Shwachman-Bodian-Diamond syndrome pseudogene (SBDSP) on chromosome 7. |
| SLC2A6 | 3.2 | Homo sapiens solute carrier family 2 (facilitated glucose transporter), member 6 (SLC2A6), mRNA. |
| ZFP82 | 3.19 | Homo sapiens zinc finger protein 82 homolog (mouse) (ZFP82), mRNA. |
| HERPUD1 | 3.18 | Homo sapiens homocysteine-inducible, endoplasmic reticulum stress-inducible, ubiquitin-like domain member 1 (HERPUD1), transcript variant 3, mRNA. |
| TMEM50B | 3.18 | Homo sapiens transmembrane protein 50B (TMEM50B), mRNA. |
| BCKDK | 3.18 | Homo sapiens branched chain ketoacid dehydrogenase kinase (BCKDK), mRNA. |
| CACYBP | 3.18 | Homo sapiens calcyclin binding protein (CACYBP), transcript variant 1, mRNA. |
| CCDC93 | 3.17 | Homo sapiens coiled-coil domain containing 93 (CCDC93), mRNA. |
| UROS | 3.17 | Homo sapiens uroporphyrinogen III synthase (congenital erythropoietic porphyria) (UROS), mRNA. |
| RFTN1 | 3.17 | Homo sapiens raftlin, lipid raft linker 1 (RFTN1), mRNA. |
| HNRNPA3 | 3.17 | Homo sapiens heterogeneous nuclear ribonucleoprotein A3 (HNRNPA3), mRNA. |
| ADPRHL2 | 3.16 | Homo sapiens ADP-ribosylhydrolase like 2 (ADPRHL2), mRNA. |
| TCEAL3 | 3.16 | Homo sapiens transcription elongation factor A (SII)-like 3 (TCEAL3), transcript variant 2, mRNA. |
| DBNDD1 | 3.15 | Homo sapiens dysbindin (dystrobrevin binding protein 1) domain containing 1 (DBNDD1), transcript variant 1, mRNA. |
|  | 3.15 | Homo sapiens cDNA clone MGC:13035 IMAGE:3613409, complete cds |
| HS6ST2 | 3.15 | Homo sapiens heparan sulfate 6-O-sulfotransferase 2 (HS6ST2), transcript variant S, mRNA. |
| SELS | 3.15 | Homo sapiens selenoprotein S (SELS), transcript variant 2, mRNA. |
| VPS16 | 3.15 | Homo sapiens vacuolar protein sorting 16 homolog (S. cerevisiae) (VPS16), transcript variant 1, mRNA. |
| LOC643387 | 3.14 | PREDICTED: Homo sapiens misc_RNA (LOC643387), miscRNA. |
| EIF4G2 | 3.14 | Homo sapiens eukaryotic translation initiation factor 4 gamma, 2 (EIF4G2), transcript variant 1, mRNA. |
| NELF | 3.14 | Homo sapiens nasal embryonic LHRH factor (NELF), mRNA. |
| FAM48A | 3.14 | Homo sapiens family with sequence similarity 48, member A (FAM48A), transcript variant 1, mRNA. |
| CPSF2 | 3.13 | Homo sapiens cleavage and polyadenylation specific factor 2, 100kDa (CPSF2), mRNA. |
| RET | 3.13 | Homo sapiens ret proto-oncogene (RET), transcript variant 4, mRNA. |
| TSSC4 | 3.12 | Homo sapiens tumor suppressing subtransferable candidate 4 (TSSC4), mRNA. |
| PARD6A | 3.11 | Homo sapiens par-6 partitioning defective 6 homolog alpha (C. elegans) (PARD6A), transcript variant 2, mRNA. |
| LOC651621 | 3.11 | PREDICTED: Homo sapiens similar to hydroxysteroid (17-beta) dehydrogenase 7 (LOC651621), mRNA. |
| PTCD1 | 3.11 | Homo sapiens pentatricopeptide repeat domain 1 (PTCD1), mRNA. |
| C12orf10 | 3.1 | Homo sapiens chromosome 12 open reading frame 10 (C12orf10), mRNA. |
| LOC388275 | 3.09 | PREDICTED: Homo sapiens similar to Heterogeneous nuclear ribonucleoprotein A1 (Helix-destabilizing protein) (Single-strand binding protein) (hnRNP core protein A1) (HDP-1) (Topoisomerase-inhibitor suppressed) (LOC388275), mRNA. |
| XPO5 | 3.08 | Homo sapiens exportin 5 (XPO5), mRNA. |
| EFNA1 | 3.08 | Homo sapiens ephrin-A1 (EFNA1), transcript variant 1, mRNA. |
| ADAMTSL2 | 3.07 | Homo sapiens ADAMTS-like 2 (ADAMTSL2), mRNA. |
| SLC27A3 | 3.07 | Homo sapiens solute carrier family 27 (fatty acid transporter), member 3 (SLC27A3), mRNA. |
| CD24 | 3.07 | Homo sapiens CD24 molecule (CD24), mRNA. |
| PSMD10 | 3.06 | Homo sapiens proteasome (prosome, macropain) 26S subunit, non-ATPase, 10 (PSMD10), transcript variant 1, mRNA. |
| C9orf37 | 3.06 | Homo sapiens chromosome 9 open reading frame 37 (C9orf37), mRNA. |
| DNLZ | 3.06 | Homo sapiens DNL-type zinc finger (DNLZ), mRNA. |
| KAT2A | 3.06 | Homo sapiens K(lysine) acetyltransferase 2A (KAT2A), mRNA. |
| TUSC4 | 3.06 | Homo sapiens tumor suppressor candidate 4 (TUSC4), mRNA. |
|  | 3.06 | Homo sapiens mRNA; cDNA DKFZp547E107 (from clone DKFZp547E107) |
| SNORA25 | 3.05 | Homo sapiens small nucleolar RNA, H/ACA box 25 (SNORA25), small nucleolar RNA. |
| CDR2L | 3.05 | Homo sapiens cerebellar degeneration-related protein 2-like (CDR2L), mRNA. |
| FKBP4 | 3.04 | Homo sapiens FK506 binding protein 4, 59kDa (FKBP4), mRNA. |
| SRPRB | 3.04 | Homo sapiens signal recognition particle receptor, B subunit (SRPRB), mRNA. |
| NAPEPLD | 3.04 | Homo sapiens N-acyl phosphatidylethanolamine phospholipase D (NAPEPLD), mRNA. |
| ZNF2 | 3.04 | Homo sapiens zinc finger protein 2 (ZNF2), transcript variant 1, mRNA. |
| NELL1 | 3.03 | Homo sapiens NEL-like 1 (chicken) (NELL1), mRNA. |
| C10orf137 | 3.03 | Homo sapiens chromosome 10 open reading frame 137 (C10orf137), mRNA. |
| LOC390557 | 3.03 | PREDICTED: Homo sapiens hypothetical LOC390557 (LOC390557), mRNA. |
| FAM189B | 3.03 | Homo sapiens family with sequence similarity 189, member B (FAM189B), transcript variant 1, mRNA. |
| CS | 3.02 | Homo sapiens citrate synthase (CS), nuclear gene encoding mitochondrial protein, mRNA. |
| IRF2BP2 | 3.01 | Homo sapiens interferon regulatory factor 2 binding protein 2 (IRF2BP2), transcript variant 1, mRNA. |
| C7orf52 | 3.01 | Homo sapiens chromosome 7 open reading frame 52 (C7orf52), mRNA. |
| LOC646808 | 3.01 | PREDICTED: Homo sapiens misc_RNA (LOC646808), miscRNA. |
| C3orf37 | 3.01 | Homo sapiens chromosome 3 open reading frame 37 (C3orf37), transcript variant 2, mRNA. |
| TPM3 | 3 | Homo sapiens tropomyosin 3 (TPM3), transcript variant 1, mRNA. |
| NOP2 | 3 | Homo sapiens NOP2 nucleolar protein homolog (yeast) (NOP2), transcript variant 2, mRNA. |
| OPRL1 | 3 | Homo sapiens opiate receptor-like 1 (OPRL1), transcript variant 2, mRNA. |
|  | 2.99 | Homo sapiens mRNA; cDNA DKFZp762M127 (from clone DKFZp762M127) |
| VSNL1 | 2.99 | Homo sapiens visinin-like 1 (VSNL1), mRNA. |
| AMD1 | 2.98 | Homo sapiens adenosylmethionine decarboxylase 1 (AMD1), transcript variant 1, mRNA. |
| ZNF823 | 2.98 | Homo sapiens zinc finger protein 823 (ZNF823), mRNA. |
| MFSD10 | 2.98 | Homo sapiens major facilitator superfamily domain containing 10 (MFSD10), mRNA. |
| G3BP1 | 2.97 | Homo sapiens GTPase activating protein (SH3 domain) binding protein 1 (G3BP1), transcript variant 2, mRNA. |
| SLC25A19 | 2.97 | Homo sapiens solute carrier family 25 (mitochondrial thiamine pyrophosphate carrier), member 19 (SLC25A19), nuclear gene encoding mitochondrial protein, mRNA. |
| LOC729779 | 2.97 | PREDICTED: Homo sapiens misc_RNA (LOC729779), miscRNA. |
| RAE1 | 2.96 | Homo sapiens RAE1 RNA export 1 homolog (S. pombe) (RAE1), transcript variant 1, mRNA. |
| PACSIN2 | 2.96 | Homo sapiens protein kinase C and casein kinase substrate in neurons 2 (PACSIN2), mRNA. |
| C20orf100 | 2.96 | Homo sapiens chromosome 20 open reading frame 100 (C20orf100), mRNA. |
| NCBP2 | 2.96 | Homo sapiens nuclear cap binding protein subunit 2, 20kDa (NCBP2), transcript variant 1, mRNA. |
| KLHL28 | 2.96 | Homo sapiens kelch-like 28 (Drosophila) (KLHL28), mRNA. |
| SMAP2 | 2.95 | Homo sapiens small ArfGAP2 (SMAP2), mRNA. |
| DNAJB12 | 2.95 | Homo sapiens DnaJ (Hsp40) homolog, subfamily B, member 12 (DNAJB12), transcript variant 1, mRNA. |
| DUSP4 | 2.94 | Homo sapiens dual specificity phosphatase 4 (DUSP4), transcript variant 1, mRNA. |
| STX5 | 2.94 | Homo sapiens syntaxin 5 (STX5), mRNA. |
| RAB11FIP5 | 2.94 | Homo sapiens RAB11 family interacting protein 5 (class I) (RAB11FIP5), mRNA. |
| FANCE | 2.94 | Homo sapiens Fanconi anemia, complementation group E (FANCE), mRNA. |
| RRP8 | 2.94 | Homo sapiens ribosomal RNA processing 8, methyltransferase, homolog (yeast) (RRP8), mRNA. |
| LOC644330 | 2.93 | PREDICTED: Homo sapiens similar to tropomyosin 3 isoform 2 (LOC644330), mRNA. |
| MGC57346 | 2.93 | PREDICTED: Homo sapiens hypothetical LOC401884 (MGC57346), mRNA. |
| TRIB3 | 2.93 | Homo sapiens tribbles homolog 3 (Drosophila) (TRIB3), mRNA. |
| PDE9A | 2.92 | Homo sapiens phosphodiesterase 9A (PDE9A), transcript variant 2, mRNA. |
| CCNC | 2.92 | Homo sapiens cyclin C (CCNC), transcript variant 1, mRNA. |
| HIST3H2A | 2.92 | Homo sapiens histone cluster 3, H2a (HIST3H2A), mRNA. |
| IRF7 | 2.91 | Homo sapiens interferon regulatory factor 7 (IRF7), transcript variant b, mRNA. |
| LIX1L | 2.91 | Homo sapiens Lix1 homolog (mouse)-like (LIX1L), mRNA. |
| SQSTM1 | 2.91 | Homo sapiens sequestosome 1 (SQSTM1), mRNA. |
| FAM39E | 2.91 | Homo sapiens family with sequence similarity 39, member E (FAM39E), mRNA. |
| C1orf77 | 2.91 | Homo sapiens chromosome 1 open reading frame 77 (C1orf77), mRNA. |
| BSN | 2.91 | Homo sapiens bassoon (presynaptic cytomatrix protein) (BSN), mRNA. |
| C9orf40 | 2.9 | Homo sapiens chromosome 9 open reading frame 40 (C9orf40), mRNA. |
| FAM65A | 2.9 | Homo sapiens family with sequence similarity 65, member A (FAM65A), mRNA. |
| HNRPA1P4 | 2.89 | PREDICTED: Homo sapiens heterogeneous nuclear ribonucleoprotein A1 pseudogene 4 (HNRPA1P4), mRNA. |
| MANEAL | 2.89 | Homo sapiens mannosidase, endo-alpha-like (MANEAL), transcript variant 1, mRNA. |
| LOC345041 | 2.89 | PREDICTED: Homo sapiens misc_RNA (LOC345041), miscRNA. |
| LOC644063 | 2.89 | PREDICTED: Homo sapiens similar to heterogeneous nuclear ribonucleoprotein K (LOC644063), mRNA. |
| NKIRAS1 | 2.88 | Homo sapiens NFKB inhibitor interacting Ras-like 1 (NKIRAS1), mRNA. |
| HSPD1 | 2.88 | Homo sapiens heat shock 60kDa protein 1 (chaperonin) (HSPD1), nuclear gene encoding mitochondrial protein, transcript variant 1, mRNA. |
| KRTCAP2 | 2.88 | Homo sapiens keratinocyte associated protein 2 (KRTCAP2), mRNA. |
| LOC100130887 | 2.88 | PREDICTED: Homo sapiens hypothetical protein LOC100130887 (LOC100130887), mRNA. |
| MBD6 | 2.87 | Homo sapiens methyl-CpG binding domain protein 6 (MBD6), mRNA. |
| FAIM2 | 2.87 | Homo sapiens Fas apoptotic inhibitory molecule 2 (FAIM2), mRNA. |
| BSCL2 | 2.87 | Homo sapiens Bernardinelli-Seip congenital lipodystrophy 2 (seipin) (BSCL2), mRNA. |
| EXOSC3 | 2.87 | Homo sapiens exosome component 3 (EXOSC3), transcript variant 1, mRNA. |
| SLC38A1 | 2.87 | Homo sapiens solute carrier family 38, member 1 (SLC38A1), transcript variant 1, mRNA. |
| VPS24 | 2.86 | Homo sapiens vacuolar protein sorting 24 homolog (S. cerevisiae) (VPS24), transcript variant 2, mRNA. |
| DCUN1D3 | 2.86 | Homo sapiens DCN1, defective in cullin neddylation 1, domain containing 3 (S. cerevisiae) (DCUN1D3), mRNA. |
| NDEL1 | 2.86 | Homo sapiens nudE nuclear distribution gene E homolog (A. nidulans)-like 1 (NDEL1), transcript variant 2, mRNA. |
| PHRF1 | 2.86 | Homo sapiens PHD and ring finger domains 1 (PHRF1), mRNA. |
| NME1 | 2.85 | Homo sapiens non-metastatic cells 1, protein (NM23A) expressed in (NME1), transcript variant 1, mRNA. |
| NENF | 2.85 | Homo sapiens neuron derived neurotrophic factor (NENF), mRNA. |
| NAT10 | 2.85 | Homo sapiens N-acetyltransferase 10 (GCN5-related) (NAT10), mRNA. |
|  | 2.84 | zh47e08.r1 Soares_fetal_liver_spleen_1NFLS_S1 Homo sapiens cDNA clone IMAGE:415238 5, mRNA sequence |
| PRDX2 | 2.83 | Homo sapiens peroxiredoxin 2 (PRDX2), nuclear gene encoding mitochondrial protein, transcript variant 3, mRNA. |
| LOC729423 | 2.83 | PREDICTED: Homo sapiens similar to Heterogeneous nuclear ribonucleoprotein A1 (Helix-destabilizing protein) (Single-strand RNA-binding protein) (hnRNP core protein A1) (HDP) (LOC729423), mRNA. |
| MRPS25 | 2.83 | Homo sapiens mitochondrial ribosomal protein S25 (MRPS25), nuclear gene encoding mitochondrial protein, mRNA. |
| ELAVL3 | 2.83 | Homo sapiens ELAV (embryonic lethal, abnormal vision, Drosophila)-like 3 (Hu antigen C) (ELAVL3), transcript variant 1, mRNA. |
| RPS3 | 2.82 | Homo sapiens ribosomal protein S3 (RPS3), mRNA. |
| ZADH2 | 2.82 | Homo sapiens zinc binding alcohol dehydrogenase domain containing 2 (ZADH2), mRNA. |
| CXXC1 | 2.81 | Homo sapiens CXXC finger 1 (PHD domain) (CXXC1), mRNA. |
| CACNB3 | 2.81 | Homo sapiens calcium channel, voltage-dependent, beta 3 subunit (CACNB3), mRNA. |
| PLEKHA6 | 2.81 | Homo sapiens pleckstrin homology domain containing, family A member 6 (PLEKHA6), mRNA. |
| LOC402112 | 2.8 | PREDICTED: Homo sapiens misc_RNA (LOC402112), miscRNA. |
| TRPT1 | 2.8 | Homo sapiens tRNA phosphotransferase 1 (TRPT1), transcript variant 2, mRNA. |
| LOC401720 | 2.8 | Homo sapiens similar to fidgetin (LOC401720), mRNA. |
| INPP1 | 2.79 | Homo sapiens inositol polyphosphate-1-phosphatase (INPP1), mRNA. |
| YIF1A | 2.79 | Homo sapiens Yip1 interacting factor homolog A (S. cerevisiae) (YIF1A), mRNA. |
| SPATA20 | 2.78 | Homo sapiens spermatogenesis associated 20 (SPATA20), mRNA. |
| LOC729660 | 2.78 | PREDICTED: Homo sapiens misc_RNA (LOC729660), miscRNA. |
| DEXI | 2.78 | Homo sapiens Dexi homolog (mouse) (DEXI), mRNA. |
| LOC100133836 | 2.78 | PREDICTED: Homo sapiens hypothetical protein LOC100133836, transcript variant 1 (LOC100133836), mRNA. |
| ZNF317 | 2.77 | Homo sapiens zinc finger protein 317 (ZNF317), mRNA. |
| ZNF490 | 2.77 | Homo sapiens zinc finger protein 490 (ZNF490), mRNA. |
| POLG2 | 2.77 | Homo sapiens polymerase (DNA directed), gamma 2, accessory subunit (POLG2), mRNA. |
| ZMYM2 | 2.76 | Homo sapiens zinc finger, MYM-type 2 (ZMYM2), mRNA. |
| RNF146 | 2.76 | Homo sapiens ring finger protein 146 (RNF146), mRNA. |
| COX15 | 2.76 | Homo sapiens COX15 homolog, cytochrome c oxidase assembly protein (yeast) (COX15), nuclear gene encoding mitochondrial protein, transcript variant 1, mRNA. |
| GALT | 2.75 | Homo sapiens galactose-1-phosphate uridylyltransferase (GALT), mRNA. |
| C5orf54 | 2.75 | Homo sapiens chromosome 5 open reading frame 54 (C5orf54), mRNA. |
| LOC100131866 | 2.75 | PREDICTED: Homo sapiens misc_RNA (LOC100131866), miscRNA. |
| PLEKHA3 | 2.75 | Homo sapiens pleckstrin homology domain containing, family A (phosphoinositide binding specific) member 3 (PLEKHA3), mRNA. |
| IRF9 | 2.74 | Homo sapiens interferon regulatory factor 9 (IRF9), mRNA. |
| C17orf45 | 2.74 | Homo sapiens chromosome 17 open reading frame 45 (C17orf45), mRNA. |
| STMN3 | 2.74 | Homo sapiens stathmin-like 3 (STMN3), mRNA. |
| DBNL | 2.74 | Homo sapiens drebrin-like (DBNL), transcript variant 2, mRNA. |
| GAR1 | 2.74 | Homo sapiens GAR1 ribonucleoprotein homolog (yeast) (GAR1), transcript variant 1, mRNA. |
| C6orf47 | 2.73 | Homo sapiens chromosome 6 open reading frame 47 (C6orf47), mRNA. |
| LOC100129890 | 2.73 | PREDICTED: Homo sapiens similar to hCG1750329 (LOC100129890), mRNA. |
| PMS2 | 2.72 | Homo sapiens PMS2 postmeiotic segregation increased 2 (S. cerevisiae) (PMS2), transcript variant 1, mRNA. |
| PRPF4B | 2.72 | Homo sapiens PRP4 pre-mRNA processing factor 4 homolog B (yeast) (PRPF4B), transcript variant 1, mRNA. |
| TOR3A | 2.71 | Homo sapiens torsin family 3, member A (TOR3A), mRNA. |
| ZNF202 | 2.71 | Homo sapiens zinc finger protein 202 (ZNF202), mRNA. |
| MXD1 | 2.71 | Homo sapiens MAX dimerization protein 1 (MXD1), mRNA. |
| LEMD2 | 2.71 | Homo sapiens LEM domain containing 2 (LEMD2), mRNA. |
| LRCH4 | 2.7 | Homo sapiens leucine-rich repeats and calponin homology (CH) domain containing 4 (LRCH4), mRNA. |
| C11orf75 | 2.7 | Homo sapiens chromosome 11 open reading frame 75 (C11orf75), mRNA. |
| LOC100128689 | 2.7 | PREDICTED: Homo sapiens misc_RNA (LOC100128689), miscRNA. |
| RP9 | 2.7 | Homo sapiens retinitis pigmentosa 9 (autosomal dominant) (RP9), mRNA. |
| ARHGEF18 | 2.69 | Homo sapiens rho/rac guanine nucleotide exchange factor (GEF) 18 (ARHGEF18), mRNA. |
| REPIN1 | 2.68 | Homo sapiens replication initiator 1 (REPIN1), transcript variant 2, mRNA. |
| LOC728417 | 2.68 | PREDICTED: Homo sapiens hypothetical protein LOC728417 (LOC728417), mRNA. |
| SCAP | 2.68 | Homo sapiens SREBF chaperone (SCAP), mRNA. |
| ZKSCAN4 | 2.68 | Homo sapiens zinc finger with KRAB and SCAN domains 4 (ZKSCAN4), mRNA. |
| CDC37 | 2.68 | Homo sapiens cell division cycle 37 homolog (S. cerevisiae) (CDC37), mRNA. |
| RNF144A | 2.68 | Homo sapiens ring finger protein 144A (RNF144A), mRNA. |
| INTS6 | 2.67 | Homo sapiens integrator complex subunit 6 (INTS6), transcript variant 2, mRNA. |
| ZNF213 | 2.67 | Homo sapiens zinc finger protein 213 (ZNF213), mRNA. |
| LIG3 | 2.66 | Homo sapiens ligase III, DNA, ATP-dependent (LIG3), nuclear gene encoding mitochondrial protein, transcript variant alpha, mRNA. |
| U2AF1L2 | 2.66 | Homo sapiens U2(RNU2) small nuclear RNA auxiliary factor 1-like 2 (U2AF1L2), mRNA. |
| GAPVD1 | 2.66 | Homo sapiens GTPase activating protein and VPS9 domains 1 (GAPVD1), mRNA. |
| TCF25 | 2.65 | Homo sapiens transcription factor 25 (basic helix-loop-helix) (TCF25), mRNA. |
| BMP7 | 2.65 | Homo sapiens bone morphogenetic protein 7 (osteogenic protein 1) (BMP7), mRNA. |
| LOC389816 | 2.65 | Homo sapiens cytokeratin associated protein (LOC389816), mRNA. |
| CKS2 | 2.65 | Homo sapiens CDC28 protein kinase regulatory subunit 2 (CKS2), mRNA. |
| TOP1MT | 2.64 | Homo sapiens topoisomerase (DNA) I, mitochondrial (TOP1MT), nuclear gene encoding mitochondrial protein, mRNA. |
| ZNF322A | 2.64 | Homo sapiens zinc finger protein 322A (ZNF322A), mRNA. |
| DLK1 | 2.64 | Homo sapiens delta-like 1 homolog (Drosophila) (DLK1), mRNA. |
| LOC401357 | 2.63 | Homo sapiens hypothetical LOC401357 (LOC401357), mRNA. |
| EGLN2 | 2.63 | Homo sapiens egl nine homolog 2 (C. elegans) (EGLN2), transcript variant 3, mRNA. |
| SERF2 | 2.63 | Homo sapiens small EDRK-rich factor 2 (SERF2), mRNA. |
| NSDHL | 2.63 | Homo sapiens NAD(P) dependent steroid dehydrogenase-like (NSDHL), mRNA. |
| ACTR3B | 2.62 | Homo sapiens ARP3 actin-related protein 3 homolog B (yeast) (ACTR3B), transcript variant 1, mRNA. |
| GLDC | 2.62 | Homo sapiens glycine dehydrogenase (decarboxylating) (GLDC), mRNA. |
| ASB3 | 2.62 | Homo sapiens ankyrin repeat and SOCS box-containing 3 (ASB3), transcript variant 1, mRNA. |
| C16orf72 | 2.62 | Homo sapiens chromosome 16 open reading frame 72 (C16orf72), mRNA. |
| LOC440991 | 2.62 | PREDICTED: Homo sapiens misc_RNA (LOC440991), miscRNA. |
| C17orf90 | 2.62 | Homo sapiens chromosome 17 open reading frame 90 (C17orf90), mRNA. |
| C1orf74 | 2.62 | Homo sapiens chromosome 1 open reading frame 74 (C1orf74), mRNA. |
| C3orf38 | 2.61 | Homo sapiens chromosome 3 open reading frame 38 (C3orf38), mRNA. |
| HSP90B1 | 2.61 | Homo sapiens heat shock protein 90kDa beta (Grp94), member 1 (HSP90B1), mRNA. |
| SHD | 2.61 | Homo sapiens Src homology 2 domain containing transforming protein D (SHD), mRNA. |
| LOC651149 | 2.6 | PREDICTED: Homo sapiens similar to 60S ribosomal protein L3 (L4) (LOC651149), mRNA. |
| PAOX | 2.6 | Homo sapiens polyamine oxidase (exo-N4-amino) (PAOX), transcript variant 5, mRNA. |
| SCAND3 | 2.6 | Homo sapiens SCAN domain containing 3 (SCAND3), mRNA. |
| C3orf38 | 2.59 | Homo sapiens chromosome 3 open reading frame 38 (C3orf38), mRNA. |
| SORCS1 | 2.59 | Homo sapiens sortilin-related VPS10 domain containing receptor 1 (SORCS1), transcript variant 2, mRNA. |
| BBS10 | 2.59 | Homo sapiens Bardet-Biedl syndrome 10 (BBS10), mRNA. |
| PLSCR3 | 2.59 | Homo sapiens phospholipid scramblase 3 (PLSCR3), mRNA. |
| MYOZ3 | 2.59 | Homo sapiens myozenin 3 (MYOZ3), mRNA. |
| LMNA | 2.59 | Homo sapiens lamin A/C (LMNA), transcript variant 2, mRNA. |
| HINFP | 2.59 | Homo sapiens histone H4 transcription factor (HINFP), transcript variant 1, mRNA. |
| NOMO3 | 2.58 | Homo sapiens NODAL modulator 3 (NOMO3), mRNA. |
| CHORDC1 | 2.58 | Homo sapiens cysteine and histidine-rich domain (CHORD)-containing 1 (CHORDC1), mRNA. |
| ATP6AP1 | 2.58 | Homo sapiens ATPase, H+ transporting, lysosomal accessory protein 1 (ATP6AP1), mRNA. |
| CLK1 | 2.58 | Homo sapiens CDC-like kinase 1 (CLK1), transcript variant 2, mRNA. |
| DNAJA1 | 2.58 | Homo sapiens DnaJ (Hsp40) homolog, subfamily A, member 1 (DNAJA1), mRNA. |
| FARP1 | 2.58 | Homo sapiens FERM, RhoGEF (ARHGEF) and pleckstrin domain protein 1 (chondrocyte-derived) (FARP1), transcript variant 1, mRNA. |
| CLDN15 | 2.58 | Homo sapiens claudin 15 (CLDN15), mRNA. |
| ZNF613 | 2.58 | Homo sapiens zinc finger protein 613 (ZNF613), transcript variant 2, mRNA. |
| ECD | 2.57 | Homo sapiens ecdysoneless homolog (Drosophila) (ECD), mRNA. |
| LOC728554 | 2.57 | PREDICTED: Homo sapiens similar to THO complex 3 (LOC728554), mRNA. |
| TRK1 | 2.57 | Homo sapiens tRNA lysine 1 (TRK1) on chromosome 17. |
| LOC654254 | 2.56 | PREDICTED: Homo sapiens similar to zinc finger protein 595 (LOC654254), mRNA. |
| NCL | 2.56 | Homo sapiens nucleolin (NCL), mRNA. |
| EXO1 | 2.56 | Homo sapiens exonuclease 1 (EXO1), transcript variant 1, mRNA. |
| WFS1 | 2.55 | Homo sapiens Wolfram syndrome 1 (wolframin) (WFS1), mRNA. |
| MAD2L1BP | 2.55 | Homo sapiens MAD2L1 binding protein (MAD2L1BP), transcript variant 2, mRNA. |
| MTP18 | 2.55 | Homo sapiens mitochondrial protein 18 kDa (MTP18), nuclear gene encoding mitochondrial protein, transcript variant 1, mRNA. |
| ZNF35 | 2.55 | Homo sapiens zinc finger protein 35 (ZNF35), mRNA. |
| CASP2 | 2.55 | Homo sapiens caspase 2, apoptosis-related cysteine peptidase (CASP2), transcript variant 1, mRNA. |
| ZNF285A | 2.55 | Homo sapiens zinc finger protein 285A (ZNF285A), mRNA. |
| LOC652685 | 2.55 | PREDICTED: Homo sapiens similar to PMS1 protein homolog 2 (DNA mismatch repair protein PMS2) (LOC652685), mRNA. |
| COPS7B | 2.55 | Homo sapiens COP9 constitutive photomorphogenic homolog subunit 7B (Arabidopsis) (COPS7B), mRNA. |
| NPPA | 2.55 | Homo sapiens natriuretic peptide precursor A (NPPA), mRNA. |
| TOMM34 | 2.54 | Homo sapiens translocase of outer mitochondrial membrane 34 (TOMM34), nuclear gene encoding mitochondrial protein, mRNA. |
| NHP2L1 | 2.54 | Homo sapiens NHP2 non-histone chromosome protein 2-like 1 (S. cerevisiae) (NHP2L1), transcript variant 2, mRNA. |
| CRMP1 | 2.54 | Homo sapiens collapsin response mediator protein 1 (CRMP1), transcript variant 2, mRNA. |
| RBM3 | 2.53 | Homo sapiens RNA binding motif (RNP1, RRM) protein 3 (RBM3), mRNA. |
| U1SNRNPBP | 2.53 | Homo sapiens U11/U12 snRNP 35K (U1SNRNPBP), transcript variant 4, mRNA. |
| HEY1 | 2.53 | Homo sapiens hairy/enhancer-of-split related with YRPW motif 1 (HEY1), transcript variant 2, mRNA. |
| PUS1 | 2.53 | Homo sapiens pseudouridylate synthase 1 (PUS1), transcript variant 2, mRNA. |
| RAMP1 | 2.53 | Homo sapiens receptor (G protein-coupled) activity modifying protein 1 (RAMP1), mRNA. |
| CSTF3 | 2.52 | Homo sapiens cleavage stimulation factor, 3' pre-RNA, subunit 3, 77kDa (CSTF3), transcript variant 2, mRNA. |
| UBA52 | 2.52 | Homo sapiens ubiquitin A-52 residue ribosomal protein fusion product 1 (UBA52), transcript variant 2, mRNA. |
| LOC100132119 | 2.52 | PREDICTED: Homo sapiens misc_RNA (LOC100132119), miscRNA. |
| GDAP1 | 2.51 | Homo sapiens ganglioside-induced differentiation-associated protein 1 (GDAP1), transcript variant 1, mRNA. |
| CCNE1 | 2.51 | Homo sapiens cyclin E1 (CCNE1), transcript variant 1, mRNA. |
| NXT1 | 2.51 | Homo sapiens NTF2-like export factor 1 (NXT1), mRNA. |
| MRRF | 2.51 | Homo sapiens mitochondrial ribosome recycling factor (MRRF), nuclear gene encoding mitochondrial protein, transcript variant 2, mRNA. |
| ANKRD41 | 2.51 | Homo sapiens ankyrin repeat domain 41 (ANKRD41), mRNA. |
| LOC100132797 | 2.51 | PREDICTED: Homo sapiens misc_RNA (LOC100132797), miscRNA. |
| GAMT | 2.51 | Homo sapiens guanidinoacetate N-methyltransferase (GAMT), transcript variant 1, mRNA. |
| LOC643980 | 2.51 | PREDICTED: Homo sapiens similar to FRG1 protein (FSHD region gene 1 protein), transcript variant 2 (LOC643980), mRNA. |
| SAMM50 | 2.51 | Homo sapiens sorting and assembly machinery component 50 homolog (S. cerevisiae) (SAMM50), mRNA. |
| PDIA3P | 2.5 | Homo sapiens protein disulfide isomerase family A, member 3 pseudogene (PDIA3P), non-coding RNA. |
| MED8 | 2.5 | Homo sapiens mediator of RNA polymerase II transcription, subunit 8 homolog (S. cerevisiae) (MED8), transcript variant 5, mRNA. |
| PHF23 | 2.5 | Homo sapiens PHD finger protein 23 (PHF23), mRNA. |
| TUBG1 | 2.49 | Homo sapiens tubulin, gamma 1 (TUBG1), mRNA. |
| SEH1L | 2.49 | Homo sapiens SEH1-like (S. cerevisiae) (SEH1L), transcript variant 1, mRNA. |
| LOC730413 | 2.49 | PREDICTED: Homo sapiens similar to cholinergic receptor, muscarinic 3 (LOC730413), mRNA. |
| DBT | 2.48 | Homo sapiens dihydrolipoamide branched chain transacylase E2 (DBT), nuclear gene encoding mitochondrial protein, mRNA. |
| NAGK | 2.48 | Homo sapiens N-acetylglucosamine kinase (NAGK), mRNA. |
| IP6K1 | 2.48 | Homo sapiens inositol hexakisphosphate kinase 1 (IP6K1), transcript variant 2, mRNA. |
| CDK10 | 2.48 | Homo sapiens cyclin-dependent kinase (CDC2-like) 10 (CDK10), transcript variant 3, mRNA. |
| SAMD14 | 2.48 | Homo sapiens sterile alpha motif domain containing 14 (SAMD14), mRNA. |
| NCKAP1 | 2.48 | Homo sapiens NCK-associated protein 1 (NCKAP1), transcript variant 2, mRNA. |
| H19 | 2.48 | Homo sapiens H19, imprinted maternally expressed transcript (non-protein coding) (H19), non-coding RNA. |
| EXO1 | 2.48 | Homo sapiens exonuclease 1 (EXO1), transcript variant 1, mRNA. |
| PLD3 | 2.47 | Homo sapiens phospholipase D family, member 3 (PLD3), transcript variant 1, mRNA. |
| ZNF197 | 2.47 | Homo sapiens zinc finger protein 197 (ZNF197), transcript variant 1, mRNA. |
| C17orf80 | 2.47 | Homo sapiens chromosome 17 open reading frame 80 (C17orf80), mRNA. |
| PIAS4 | 2.47 | Homo sapiens protein inhibitor of activated STAT, 4 (PIAS4), mRNA. |
| CENPV | 2.46 | Homo sapiens centromere protein V (CENPV), mRNA. |
| LOC642755 | 2.46 | PREDICTED: Homo sapiens similar to DEXI, transcript variant 1 (LOC642755), mRNA. |
| XBP1 | 2.46 | Homo sapiens X-box binding protein 1 (XBP1), transcript variant 1, mRNA. |
| ANKFY1 | 2.46 | Homo sapiens ankyrin repeat and FYVE domain containing 1 (ANKFY1), transcript variant 1, mRNA. |
| TMEM100 | 2.46 | Homo sapiens transmembrane protein 100 (TMEM100), transcript variant 2, mRNA. |
| C2orf56 | 2.46 | Homo sapiens chromosome 2 open reading frame 56 (C2orf56), transcript variant 1, mRNA. |
| LOC653566 | 2.46 | PREDICTED: Homo sapiens similar to Signal peptidase complex subunit 2 (Microsomal signal peptidase 25 kDa subunit) (SPase 25 kDa subunit), transcript variant 3 (LOC653566), mRNA. |
| IDH3A | 2.46 | Homo sapiens isocitrate dehydrogenase 3 (NAD+) alpha (IDH3A), nuclear gene encoding mitochondrial protein, mRNA. |
| MTERFD1 | 2.46 | Homo sapiens MTERF domain containing 1 (MTERFD1), mRNA. |
| LONP2 | 2.46 | Homo sapiens lon peptidase 2, peroxisomal (LONP2), mRNA. |
| GRB2 | 2.46 | Homo sapiens growth factor receptor-bound protein 2 (GRB2), transcript variant 1, mRNA. |
| EIF3CL | 2.45 | Homo sapiens eukaryotic translation initiation factor 3, subunit C-like (EIF3CL), mRNA. |
| WWC1 | 2.45 | Homo sapiens WW and C2 domain containing 1 (WWC1), mRNA. |
| ZNF434 | 2.45 | Homo sapiens zinc finger protein 434 (ZNF434), mRNA. |
| ABHD8 | 2.45 | Homo sapiens abhydrolase domain containing 8 (ABHD8), mRNA. |
| CAMKV | 2.45 | Homo sapiens CaM kinase-like vesicle-associated (CAMKV), mRNA. |
| ZNF514 | 2.45 | Homo sapiens zinc finger protein 514 (ZNF514), mRNA. |
| TICAM2 | 2.45 | Homo sapiens toll-like receptor adaptor molecule 2 (TICAM2), mRNA. |
| SNX6 | 2.45 | Homo sapiens sorting nexin 6 (SNX6), transcript variant 2, mRNA. |
| KIAA0363 | 2.44 | PREDICTED: Homo sapiens KIAA0363 protein (KIAA0363), mRNA. |
| FIBP | 2.44 | Homo sapiens fibroblast growth factor (acidic) intracellular binding protein (FIBP), transcript variant 2, mRNA. |
| SLC25A26 | 2.44 | Homo sapiens solute carrier family 25, member 26 (SLC25A26), transcript variant 1, mRNA. |
| TMEM44 | 2.44 | Homo sapiens transmembrane protein 44 (TMEM44), transcript variant 1, mRNA. |
| PRR14 | 2.44 | Homo sapiens proline rich 14 (PRR14), mRNA. |
| LOC652771 | 2.44 | PREDICTED: Homo sapiens similar to general transcription factor II, i isoform 1 (LOC652771), mRNA. |
| CEP170 | 2.44 | Homo sapiens centrosomal protein 170kDa (CEP170), transcript variant beta, mRNA. |
| LOC730052 | 2.44 | PREDICTED: Homo sapiens misc_RNA (LOC730052), miscRNA. |
| EIF2B5 | 2.44 | Homo sapiens eukaryotic translation initiation factor 2B, subunit 5 epsilon, 82kDa (EIF2B5), mRNA. |
| METTL13 | 2.43 | Homo sapiens methyltransferase like 13 (METTL13), transcript variant 1, mRNA. |
| TPST2 | 2.43 | Homo sapiens tyrosylprotein sulfotransferase 2 (TPST2), transcript variant 1, mRNA. |
| GFOD1 | 2.43 | Homo sapiens glucose-fructose oxidoreductase domain containing 1 (GFOD1), mRNA. |
| IMPDH1 | 2.43 | Homo sapiens IMP (inosine monophosphate) dehydrogenase 1 (IMPDH1), transcript variant 1, mRNA. |
| RALGDS | 2.43 | Homo sapiens ral guanine nucleotide dissociation stimulator (RALGDS), transcript variant 1, mRNA. |
| NUP37 | 2.43 | Homo sapiens nucleoporin 37kDa (NUP37), mRNA. |
| XPOT | 2.43 | Homo sapiens exportin, tRNA (nuclear export receptor for tRNAs) (XPOT), mRNA. |
| C7orf13 | 2.43 | Homo sapiens chromosome 7 open reading frame 13 (C7orf13), mRNA. |
| BANP | 2.43 | Homo sapiens BTG3 associated nuclear protein (BANP), transcript variant 2, mRNA. |
| SF3A3 | 2.42 | Homo sapiens splicing factor 3a, subunit 3, 60kDa (SF3A3), mRNA. |
| SELI | 2.42 | Homo sapiens selenoprotein I (SELI), mRNA. |
| ARS2 | 2.42 | Homo sapiens arsenate resistance protein 2 (ARS2), transcript variant 1, mRNA. |
| DNAJC27 | 2.41 | Homo sapiens DnaJ (Hsp40) homolog, subfamily C, member 27 (DNAJC27), mRNA. |
| CLUAP1 | 2.41 | Homo sapiens clusterin associated protein 1 (CLUAP1), transcript variant 1, mRNA. |
| 5-Sep | 2.41 | Homo sapiens septin 5 (SEPT5), mRNA. |
| MATR3 | 2.41 | Homo sapiens matrin 3 (MATR3), transcript variant 1, mRNA. |
| DPH5 | 2.41 | Homo sapiens DPH5 homolog (S. cerevisiae) (DPH5), transcript variant 3, mRNA. |
| LOC100133372 | 2.41 | PREDICTED: Homo sapiens misc_RNA (LOC100133372), miscRNA. |
| PSMC4 | 2.41 | Homo sapiens proteasome (prosome, macropain) 26S subunit, ATPase, 4 (PSMC4), transcript variant 1, mRNA. |
| C7orf41 | 2.4 | Homo sapiens chromosome 7 open reading frame 41 (C7orf41), mRNA. |
| HAGH | 2.4 | Homo sapiens hydroxyacylglutathione hydrolase (HAGH), nuclear gene encoding mitochondrial protein, transcript variant 1, mRNA. |
| EEF1A2 | 2.4 | Homo sapiens eukaryotic translation elongation factor 1 alpha 2 (EEF1A2), mRNA. |
| MRPL35 | 2.4 | Homo sapiens mitochondrial ribosomal protein L35 (MRPL35), nuclear gene encoding mitochondrial protein, transcript variant 1, mRNA. |
| ZP3 | 2.4 | Homo sapiens zona pellucida glycoprotein 3 (sperm receptor) (ZP3), mRNA. |
| LOC644877 | 2.4 | PREDICTED: Homo sapiens misc_RNA (LOC644877), miscRNA. |
| SF3B1 | 2.39 | Homo sapiens splicing factor 3b, subunit 1, 155kDa (SF3B1), transcript variant 1, mRNA. |
| CHCHD4 | 2.39 | Homo sapiens coiled-coil-helix-coiled-coil-helix domain containing 4 (CHCHD4), nuclear gene encoding mitochondrial protein, transcript variant 2, mRNA. |
| BANP | 2.39 | Homo sapiens BTG3 associated nuclear protein (BANP), transcript variant 2, mRNA. |
| PPP2R2C | 2.39 | Homo sapiens protein phosphatase 2 (formerly 2A), regulatory subunit B, gamma isoform (PPP2R2C), transcript variant 2, mRNA. |
| MAT2B | 2.39 | Homo sapiens methionine adenosyltransferase II, beta (MAT2B), transcript variant 2, mRNA. |
| ACTN1 | 2.39 | Homo sapiens actinin, alpha 1 (ACTN1), mRNA. |
| MOCS2 | 2.39 | Homo sapiens molybdenum cofactor synthesis 2 (MOCS2), transcript variant 1, mRNA. |
| DNAJB6 | 2.38 | Homo sapiens DnaJ (Hsp40) homolog, subfamily B, member 6 (DNAJB6), transcript variant 2, mRNA. |
| ZNF443 | 2.38 | Homo sapiens zinc finger protein 443 (ZNF443), mRNA. |
| NAPEPLD | 2.38 | Homo sapiens N-acyl phosphatidylethanolamine phospholipase D (NAPEPLD), mRNA. |
| TMEM22 | 2.38 | Homo sapiens transmembrane protein 22 (TMEM22), transcript variant 2, mRNA. |
| ZNF69 | 2.38 | Homo sapiens zinc finger protein 69 (ZNF69), mRNA. |
| PDIA6 | 2.38 | Homo sapiens protein disulfide isomerase family A, member 6 (PDIA6), mRNA. |
| PUF60 | 2.38 | Homo sapiens poly-U binding splicing factor 60KDa (PUF60), transcript variant 2, mRNA. |
| MRPL52 | 2.37 | Homo sapiens mitochondrial ribosomal protein L52 (MRPL52), nuclear gene encoding mitochondrial protein, transcript variant 6, mRNA. |
| VIL2 | 2.37 | Homo sapiens villin 2 (ezrin) (VIL2), mRNA. |
| MRPL18 | 2.37 | Homo sapiens mitochondrial ribosomal protein L18 (MRPL18), nuclear gene encoding mitochondrial protein, mRNA. |
|  | 2.37 | 17000532640995 GRN_ES Homo sapiens cDNA 5, mRNA sequence |
| FKBP1A | 2.37 | Homo sapiens FK506 binding protein 1A, 12kDa (FKBP1A), transcript variant 12A, mRNA. |
| GDAP1 | 2.37 | Homo sapiens ganglioside-induced differentiation-associated protein 1 (GDAP1), mRNA. |
| FLJ25404 | 2.36 | PREDICTED: Homo sapiens hypothetical protein FLJ25404, transcript variant 2 (FLJ25404), mRNA. |
| FLJ30058 | 2.36 | Homo sapiens hypothetical protein FLJ30058 (FLJ30058), mRNA. |
| MAX | 2.36 | Homo sapiens MYC associated factor X (MAX), transcript variant 1, mRNA. |
| PPFIBP2 | 2.36 | Homo sapiens PTPRF interacting protein, binding protein 2 (liprin beta 2) (PPFIBP2), mRNA. |
| CAPRIN1 | 2.36 | Homo sapiens cell cycle associated protein 1 (CAPRIN1), transcript variant 1, mRNA. |
| C6orf64 | 2.36 | Homo sapiens chromosome 6 open reading frame 64 (C6orf64), mRNA. |
| ACTR3B | 2.36 | Homo sapiens ARP3 actin-related protein 3 homolog B (yeast) (ACTR3B), transcript variant 2, mRNA. |
| LOC644330 | 2.36 | PREDICTED: Homo sapiens similar to tropomyosin 3 isoform 2 (LOC644330), mRNA. |
| SLC39A3 | 2.36 | Homo sapiens solute carrier family 39 (zinc transporter), member 3 (SLC39A3), transcript variant 1, mRNA. |
| MRPS12 | 2.36 | Homo sapiens mitochondrial ribosomal protein S12 (MRPS12), nuclear gene encoding mitochondrial protein, transcript variant 3, mRNA. |
| FLOT2 | 2.35 | Homo sapiens flotillin 2 (FLOT2), mRNA. |
| IFFO1 | 2.35 | Homo sapiens intermediate filament family orphan 1 (IFFO1), transcript variant 2, mRNA. |
| MKNK2 | 2.35 | Homo sapiens MAP kinase interacting serine/threonine kinase 2 (MKNK2), transcript variant 1, mRNA. |
| LOC152217 | 2.35 | Homo sapiens hypothetical LOC152217 (LOC152217), non-coding RNA. |
| SAR1B | 2.34 | Homo sapiens SAR1 homolog B (S. cerevisiae) (SAR1B), transcript variant 1, mRNA. |
| AZIN1 | 2.34 | Homo sapiens antizyme inhibitor 1 (AZIN1), transcript variant 1, mRNA. |
| LOC728873 | 2.34 | PREDICTED: Homo sapiens misc_RNA (LOC728873), miscRNA. |
| TMED3 | 2.34 | Homo sapiens transmembrane emp24 protein transport domain containing 3 (TMED3), mRNA. |
| SCO2 | 2.33 | Homo sapiens SCO cytochrome oxidase deficient homolog 2 (yeast) (SCO2), nuclear gene encoding mitochondrial protein, mRNA. |
| TMEM205 | 2.33 | Homo sapiens transmembrane protein 205 (TMEM205), mRNA. |
| RSBN1 | 2.33 | Homo sapiens round spermatid basic protein 1 (RSBN1), mRNA. |
| CYGB | 2.33 | Homo sapiens cytoglobin (CYGB), mRNA. |
| HS6ST2 | 2.33 | Homo sapiens heparan sulfate 6-O-sulfotransferase 2 (HS6ST2), transcript variant L, mRNA. |
| RAB40C | 2.33 | Homo sapiens RAB40C, member RAS oncogene family (RAB40C), mRNA. |
| RCCD1 | 2.32 | Homo sapiens RCC1 domain containing 1 (RCCD1), transcript variant 2, mRNA. |
| TRIM13 | 2.32 | Homo sapiens tripartite motif-containing 13 (TRIM13), transcript variant 4, mRNA. |
| ISY1 | 2.32 | Homo sapiens ISY1 splicing factor homolog (S. cerevisiae) (ISY1), mRNA. |
| TOP1MT | 2.32 | PREDICTED: Homo sapiens topoisomerase (DNA) I, mitochondrial (TOP1MT), mRNA. |
| RPS9 | 2.31 | Homo sapiens ribosomal protein S9 (RPS9), mRNA. |
| CCDC126 | 2.31 | Homo sapiens coiled-coil domain containing 126 (CCDC126), mRNA. |
| NBPF1 | 2.31 | Homo sapiens neuroblastoma breakpoint family, member 1 (NBPF1), mRNA. XM_934962 XM_934964 XM_934966 XM_934967 XM_934969 XM_934972 XM_934973 XM_934974 XM_934975 XM_934976 XM_934978 |
| C2orf49 | 2.31 | Homo sapiens chromosome 2 open reading frame 49 (C2orf49), mRNA. |
| AMD1 | 2.31 | Homo sapiens adenosylmethionine decarboxylase 1 (AMD1), transcript variant 2, mRNA. |
| TMEM189-UBE2V1 | 2.31 | Homo sapiens TMEM189-UBE2V1 readthrough transcript (TMEM189-UBE2V1), transcript variant 2, mRNA. |
| TCEA2 | 2.31 | Homo sapiens transcription elongation factor A (SII), 2 (TCEA2), transcript variant 2, mRNA. |
| ZNF189 | 2.31 | Homo sapiens zinc finger protein 189 (ZNF189), transcript variant 1, mRNA. |
| C1orf86 | 2.3 | Homo sapiens chromosome 1 open reading frame 86 (C1orf86), mRNA. |
| IPO7 | 2.3 | Homo sapiens importin 7 (IPO7), mRNA. |
| ORC5L | 2.3 | Homo sapiens origin recognition complex, subunit 5-like (yeast) (ORC5L), transcript variant 1, mRNA. |
| CLIP3 | 2.3 | Homo sapiens CAP-GLY domain containing linker protein 3 (CLIP3), mRNA. |
| EEF1G | 2.3 | Homo sapiens eukaryotic translation elongation factor 1 gamma (EEF1G), mRNA. XM_935976 XM_935977 XM_935978 XM_935979 |
| TRIM4 | 2.3 | Homo sapiens tripartite motif-containing 4 (TRIM4), transcript variant beta, mRNA. |
| C11orf17 | 2.3 | Homo sapiens chromosome 11 open reading frame 17 (C11orf17), transcript variant 1, mRNA. |
| NAT9 | 2.3 | Homo sapiens N-acetyltransferase 9 (GCN5-related, putative) (NAT9), mRNA. |
| ASTE1 | 2.29 | Homo sapiens asteroid homolog 1 (Drosophila) (ASTE1), mRNA. |
| LOC652481 | 2.29 | PREDICTED: Homo sapiens similar to Mitochondrial import inner membrane translocase subunit Tim23 (LOC652481), mRNA. |
| LETMD1 | 2.29 | Homo sapiens LETM1 domain containing 1 (LETMD1), transcript variant 1, mRNA. |
| LOC100128771 | 2.29 | PREDICTED: Homo sapiens misc_RNA (LOC100128771), miscRNA. |
| ARF4 | 2.29 | Homo sapiens ADP-ribosylation factor 4 (ARF4), mRNA. |
| C21orf59 | 2.29 | Homo sapiens chromosome 21 open reading frame 59 (C21orf59), transcript variant 1, mRNA. |
| CCND1 | 2.29 | Homo sapiens cyclin D1 (CCND1), mRNA. |
| ZDHHC6 | 2.29 | Homo sapiens zinc finger, DHHC-type containing 6 (ZDHHC6), mRNA. |
| ESRRG | 2.28 | Homo sapiens estrogen-related receptor gamma (ESRRG), transcript variant 1, mRNA. |
| ADAM15 | 2.28 | Homo sapiens ADAM metallopeptidase domain 15 (ADAM15), transcript variant 4, mRNA. |
| ACYP1 | 2.28 | Homo sapiens acylphosphatase 1, erythrocyte (common) type (ACYP1), transcript variant 1, mRNA. |
| BTBD1 | 2.28 | Homo sapiens BTB (POZ) domain containing 1 (BTBD1), transcript variant 1, mRNA. |
| ECHS1 | 2.28 | Homo sapiens enoyl Coenzyme A hydratase, short chain, 1, mitochondrial (ECHS1), nuclear gene encoding mitochondrial protein, mRNA. |
| MRRF | 2.28 | Homo sapiens mitochondrial ribosome recycling factor (MRRF), nuclear gene encoding mitochondrial protein, transcript variant 3, mRNA. |
| SMCR7L | 2.28 | Homo sapiens Smith-Magenis syndrome chromosome region, candidate 7-like (SMCR7L), mRNA. |
| YWHAZ | 2.27 | Homo sapiens tyrosine 3-monooxygenase/tryptophan 5-monooxygenase activation protein, zeta polypeptide (YWHAZ), transcript variant 1, mRNA. |
| SNAP25 | 2.27 | Homo sapiens synaptosomal-associated protein, 25kDa (SNAP25), transcript variant 2, mRNA. |
| LOC401431 | 2.27 | Homo sapiens hypothetical gene LOC401431 (LOC401431), mRNA. |
| BCAS4 | 2.27 | Homo sapiens breast carcinoma amplified sequence 4 (BCAS4), transcript variant 1, mRNA. |
| PSMC3IP | 2.27 | Homo sapiens PSMC3 interacting protein (PSMC3IP), transcript variant 2, mRNA. |
| DPH5 | 2.27 | Homo sapiens DPH5 homolog (S. cerevisiae) (DPH5), transcript variant 1, mRNA. |
| C1orf229 | 2.26 | Homo sapiens chromosome 1 open reading frame 229 (C1orf229), mRNA. |
|  | 2.26 | Homo sapiens clone 25194 mRNA sequence |
| LOC728732 | 2.26 | PREDICTED: Homo sapiens misc_RNA (LOC728732), miscRNA. |
| SUOX | 2.26 | Homo sapiens sulfite oxidase (SUOX), nuclear gene encoding mitochondrial protein, transcript variant 1, mRNA. |
| FAM18B2 | 2.26 | Homo sapiens family with sequence similarity 18, member B2 (FAM18B2), mRNA. |
| GTF2E2 | 2.26 | Homo sapiens general transcription factor IIE, polypeptide 2, beta 34kDa (GTF2E2), mRNA. |
| LOC642755 | 2.26 | PREDICTED: Homo sapiens similar to DEXI, transcript variant 1 (LOC642755), mRNA. |
| CSNK1E | 2.26 | Homo sapiens casein kinase 1, epsilon (CSNK1E), transcript variant 1, mRNA. |
| GABARAPL2 | 2.26 | Homo sapiens GABA(A) receptor-associated protein-like 2 (GABARAPL2), mRNA. |
| LOC100132528 | 2.25 | PREDICTED: Homo sapiens misc_RNA (LOC100132528), miscRNA. |
| C16orf48 | 2.25 | Homo sapiens chromosome 16 open reading frame 48 (C16orf48), mRNA. |
| ZFC3H1 | 2.25 | Homo sapiens zinc finger, C3H1-type containing (ZFC3H1), mRNA. |
| C22orf13 | 2.25 | Homo sapiens chromosome 22 open reading frame 13 (C22orf13), mRNA. |
| C12orf47 | 2.25 | PREDICTED: Homo sapiens chromosome 12 open reading frame 47 (C12orf47), misc RNA. |
| FAM108B1 | 2.25 | Homo sapiens family with sequence similarity 108, member B1 (FAM108B1), transcript variant 1, mRNA. |
| FBXL18 | 2.25 | Homo sapiens F-box and leucine-rich repeat protein 18 (FBXL18), mRNA. |
| HNRNPH2 | 2.25 | Homo sapiens heterogeneous nuclear ribonucleoprotein H2 (H') (HNRNPH2), transcript variant 1, mRNA. |
| DNAJA3 | 2.25 | Homo sapiens DnaJ (Hsp40) homolog, subfamily A, member 3 (DNAJA3), mRNA. |
| SDCCAG3 | 2.25 | Homo sapiens serologically defined colon cancer antigen 3 (SDCCAG3), transcript variant 3, mRNA. |
| SRF | 2.25 | Homo sapiens serum response factor (c-fos serum response element-binding transcription factor) (SRF), mRNA. |
| TAGLN3 | 2.24 | Homo sapiens transgelin 3 (TAGLN3), transcript variant 3, mRNA. |
| LOC730746 | 2.24 | PREDICTED: Homo sapiens similar to Heterogeneous nuclear ribonucleoprotein A1 (Helix-destabilizing protein) (Single-strand RNA-binding protein) (hnRNP core protein A1) (HDP) (LOC730746), mRNA. |
| SLC9A1 | 2.24 | Homo sapiens solute carrier family 9 (sodium/hydrogen exchanger), member 1 (SLC9A1), mRNA. |
| BRWD1 | 2.24 | Homo sapiens bromodomain and WD repeat domain containing 1 (BRWD1), transcript variant 3, mRNA. |
| PHB | 2.24 | Homo sapiens prohibitin (PHB), mRNA. |
| CSAG1 | 2.24 | Homo sapiens chondrosarcoma associated gene 1 (CSAG1), transcript variant b, mRNA. |
| CCRK | 2.24 | Homo sapiens cell cycle related kinase (CCRK), transcript variant 2, mRNA. |
| CCDC128 | 2.23 | Homo sapiens coiled-coil domain containing 128 (CCDC128), mRNA. |
| CCDC16 | 2.23 | Homo sapiens coiled-coil domain containing 16 (CCDC16), mRNA. |
| AP3S2 | 2.23 | Homo sapiens adaptor-related protein complex 3, sigma 2 subunit (AP3S2), mRNA. |
| PSMA1 | 2.23 | Homo sapiens proteasome (prosome, macropain) subunit, alpha type, 1 (PSMA1), transcript variant 2, mRNA. |
| C21orf57 | 2.23 | Homo sapiens chromosome 21 open reading frame 57 (C21orf57), transcript variant 1, mRNA. |
|  | 2.23 | Homo sapiens cDNA FLJ34755 fis, clone NHNPC1000034 |
| PLEKHF1 | 2.23 | Homo sapiens pleckstrin homology domain containing, family F (with FYVE domain) member 1 (PLEKHF1), mRNA. |
| SPOP | 2.22 | Homo sapiens speckle-type POZ protein (SPOP), transcript variant 5, mRNA. |
| RBM18 | 2.22 | Homo sapiens RNA binding motif protein 18 (RBM18), mRNA. |
| LOC100129273 | 2.22 | PREDICTED: Homo sapiens similar to PLEKHA6 protein (LOC100129273), mRNA. |
| TCEAL3 | 2.22 | Homo sapiens transcription elongation factor A (SII)-like 3 (TCEAL3), transcript variant 1, mRNA. |
| ATF2 | 2.22 | Homo sapiens activating transcription factor 2 (ATF2), mRNA. |
| C8orf33 | 2.22 | Homo sapiens chromosome 8 open reading frame 33 (C8orf33), mRNA. |
| RPE | 2.22 | Homo sapiens ribulose-5-phosphate-3-epimerase (RPE), transcript variant 1, mRNA. |
| SPG7 | 2.22 | Homo sapiens spastic paraplegia 7 (pure and complicated autosomal recessive) (SPG7), nuclear gene encoding mitochondrial protein, transcript variant 1, mRNA. |
| OAZ2 | 2.22 | Homo sapiens ornithine decarboxylase antizyme 2 (OAZ2), mRNA. |
| CLIP4 | 2.22 | Homo sapiens CAP-GLY domain containing linker protein family, member 4 (CLIP4), mRNA. |
| AGPAT5 | 2.22 | Homo sapiens 1-acylglycerol-3-phosphate O-acyltransferase 5 (lysophosphatidic acid acyltransferase, epsilon) (AGPAT5), mRNA. |
| HEATR1 | 2.22 | Homo sapiens HEAT repeat containing 1 (HEATR1), mRNA. |
| ATP2C1 | 2.22 | Homo sapiens ATPase, Ca++ transporting, type 2C, member 1 (ATP2C1), transcript variant 4, mRNA. |
| TYW3 | 2.22 | Homo sapiens tRNA-yW synthesizing protein 3 homolog (S. cerevisiae) (TYW3), mRNA. |
| DGCR6 | 2.21 | Homo sapiens DiGeorge syndrome critical region gene 6 (DGCR6), mRNA. |
| PRDX2 | 2.21 | Homo sapiens peroxiredoxin 2 (PRDX2), nuclear gene encoding mitochondrial protein, transcript variant 3, mRNA. |
| BCAS4 | 2.21 | Homo sapiens breast carcinoma amplified sequence 4 (BCAS4), transcript variant 1, mRNA. |
| PALM | 2.21 | Homo sapiens paralemmin (PALM), transcript variant 1, mRNA. |
| TNS3 | 2.21 | Homo sapiens tensin 3 (TNS3), mRNA. |
| CCT6A | 2.21 | Homo sapiens chaperonin containing TCP1, subunit 6A (zeta 1) (CCT6A), transcript variant 1, mRNA. |
| GPR89A | 2.21 | Homo sapiens G protein-coupled receptor 89A (GPR89A), mRNA. |
| TMEM39A | 2.21 | Homo sapiens transmembrane protein 39A (TMEM39A), mRNA. |
| FKBP7 | 2.21 | Homo sapiens FK506 binding protein 7 (FKBP7), mRNA. |
| C18orf19 | 2.2 | Homo sapiens chromosome 18 open reading frame 19 (C18orf19), mRNA. |
| NUDT9 | 2.2 | Homo sapiens nudix (nucleoside diphosphate linked moiety X)-type motif 9 (NUDT9), transcript variant 3, mRNA. |
| GLRX2 | 2.2 | Homo sapiens glutaredoxin 2 (GLRX2), transcript variant 2, mRNA. |
| ZNF816A | 2.2 | Homo sapiens zinc finger protein 816A (ZNF816A), mRNA. |
| FAM18B | 2.2 | Homo sapiens family with sequence similarity 18, member B (FAM18B), mRNA. |
| PFDN6 | 2.2 | Homo sapiens prefoldin subunit 6 (PFDN6), mRNA. |
| NCRNA00094 | 2.2 | Homo sapiens non-protein coding RNA 94 (NCRNA00094), non-coding RNA. |
| RBM4B | 2.2 | Homo sapiens RNA binding motif protein 4B (RBM4B), mRNA. |
| SLC12A9 | 2.19 | Homo sapiens solute carrier family 12 (potassium/chloride transporters), member 9 (SLC12A9), mRNA. |
| RNF149 | 2.19 | Homo sapiens ring finger protein 149 (RNF149), mRNA. |
| SLC25A20 | 2.19 | Homo sapiens solute carrier family 25 (carnitine/acylcarnitine translocase), member 20 (SLC25A20), nuclear gene encoding mitochondrial protein, mRNA. |
| ZNF322A | 2.19 | Homo sapiens zinc finger protein 322A (ZNF322A), mRNA. |
| BTBD2 | 2.19 | Homo sapiens BTB (POZ) domain containing 2 (BTBD2), mRNA. |
| INTS4 | 2.19 | Homo sapiens integrator complex subunit 4 (INTS4), mRNA. |
| TRIM39 | 2.18 | Homo sapiens tripartite motif-containing 39 (TRIM39), transcript variant 1, mRNA. |
| ZNF746 | 2.18 | Homo sapiens zinc finger protein 746 (ZNF746), mRNA. |
| MBTPS2 | 2.18 | Homo sapiens membrane-bound transcription factor peptidase, site 2 (MBTPS2), mRNA. |
| NDUFS7 | 2.18 | Homo sapiens NADH dehydrogenase (ubiquinone) Fe-S protein 7, 20kDa (NADH-coenzyme Q reductase) (NDUFS7), mRNA. |
| C1orf85 | 2.18 | Homo sapiens chromosome 1 open reading frame 85 (C1orf85), mRNA. |
| RPS9 | 2.18 | Homo sapiens ribosomal protein S9 (RPS9), mRNA. |
| PDXP | 2.18 | Homo sapiens pyridoxal (pyridoxine, vitamin B6) phosphatase (PDXP), mRNA. |
| PAQR4 | 2.18 | Homo sapiens progestin and adipoQ receptor family member IV (PAQR4), mRNA. |
| LOC731777 | 2.17 | PREDICTED: Homo sapiens similar to heat shock 70kD protein binding protein (LOC731777), mRNA. |
| MAP3K7 | 2.17 | Homo sapiens mitogen-activated protein kinase kinase kinase 7 (MAP3K7), transcript variant D, mRNA. |
| ZNF622 | 2.17 | Homo sapiens zinc finger protein 622 (ZNF622), mRNA. |
| ARMCX3 | 2.17 | Homo sapiens armadillo repeat containing, X-linked 3 (ARMCX3), transcript variant 2, mRNA. |
| CICE | 2.17 | PREDICTED: Homo sapiens cell death-inducing CIDE-like effector pseudogene (CICE), misc RNA. |
| RRAGC | 2.17 | Homo sapiens Ras-related GTP binding C (RRAGC), mRNA. |
| HSZFP36 | 2.17 | Homo sapiens ZFP-36 for a zinc finger protein (HSZFP36), mRNA. |
| MYL6B | 2.17 | Homo sapiens myosin, light chain 6B, alkali, smooth muscle and non-muscle (MYL6B), mRNA. |
| BMS1 | 2.16 | Homo sapiens BMS1 homolog, ribosome assembly protein (yeast) (BMS1), mRNA. |
| THOC3 | 2.16 | Homo sapiens THO complex 3 (THOC3), mRNA. |
| TXNL4B | 2.16 | Homo sapiens thioredoxin-like 4B (TXNL4B), mRNA. |
| MYST1 | 2.16 | Homo sapiens MYST histone acetyltransferase 1 (MYST1), transcript variant 1, mRNA. |
| GTPBP6 | 2.16 | Homo sapiens GTP binding protein 6 (putative) (GTPBP6), mRNA. |
| PMEPA1 | 2.16 | Homo sapiens prostate transmembrane protein, androgen induced 1 (PMEPA1), transcript variant 2, mRNA. |
| C1orf174 | 2.16 | Homo sapiens chromosome 1 open reading frame 174 (C1orf174), mRNA. |
| AIMP2 | 2.15 | Homo sapiens aminoacyl tRNA synthetase complex-interacting multifunctional protein 2 (AIMP2), mRNA. |
| AP1S1 | 2.15 | Homo sapiens adaptor-related protein complex 1, sigma 1 subunit (AP1S1), transcript variant 1, mRNA. |
| LACTB | 2.15 | Homo sapiens lactamase, beta (LACTB), nuclear gene encoding mitochondrial protein, transcript variant 1, mRNA. |
| LOC100129086 | 2.15 | PREDICTED: Homo sapiens similar to HIG1 domain family, member 1A (LOC100129086), mRNA. |
| PNCK | 2.15 | Homo sapiens pregnancy upregulated non-ubiquitously expressed CaM kinase (PNCK), mRNA. |
| ZNF248 | 2.15 | Homo sapiens zinc finger protein 248 (ZNF248), mRNA. |
| ZW10 | 2.15 | Homo sapiens ZW10, kinetochore associated, homolog (Drosophila) (ZW10), mRNA. |
| INTS2 | 2.15 | Homo sapiens integrator complex subunit 2 (INTS2), mRNA. |
| C2orf44 | 2.15 | Homo sapiens chromosome 2 open reading frame 44 (C2orf44), mRNA. |
| TACC2 | 2.15 | Homo sapiens transforming, acidic coiled-coil containing protein 2 (TACC2), transcript variant 2, mRNA. |
| RNF144 | 2.14 | Homo sapiens ring finger protein 144 (RNF144), mRNA. |
| STT3B | 2.14 | Homo sapiens STT3, subunit of the oligosaccharyltransferase complex, homolog B (S. cerevisiae) (STT3B), mRNA. |
| BOP1 | 2.14 | Homo sapiens block of proliferation 1 (BOP1), mRNA. |
| LOC100132288 | 2.14 | Homo sapiens hypothetical protein LOC100132288 (LOC100132288), mRNA. |
| C12orf52 | 2.14 | Homo sapiens chromosome 12 open reading frame 52 (C12orf52), mRNA. |
| ZMYM6 | 2.14 | Homo sapiens zinc finger, MYM-type 6 (ZMYM6), mRNA. |
| ALKBH1 | 2.14 | Homo sapiens alkB, alkylation repair homolog 1 (E. coli) (ALKBH1), mRNA. |
| CCT7 | 2.14 | Homo sapiens chaperonin containing TCP1, subunit 7 (eta) (CCT7), transcript variant 1, mRNA. |
| PPM1A | 2.14 | Homo sapiens protein phosphatase 1A (formerly 2C), magnesium-dependent, alpha isoform (PPM1A), transcript variant 3, mRNA. |
| LOC399804 | 2.14 | PREDICTED: Homo sapiens misc_RNA (LOC399804), miscRNA. |
| APM-1 | 2.14 | PREDICTED: Homo sapiens BTB/POZ-zinc finger protein-like (APM-1), mRNA. |
| NICN1 | 2.14 | Homo sapiens nicolin 1 (NICN1), mRNA. |
| UBL5 | 2.14 | Homo sapiens ubiquitin-like 5 (UBL5), mRNA. |
| NR2C1 | 2.13 | Homo sapiens nuclear receptor subfamily 2, group C, member 1 (NR2C1), transcript variant 1, mRNA. |
| ZNF211 | 2.13 | Homo sapiens zinc finger protein 211 (ZNF211), transcript variant 1, mRNA. |
| WDR33 | 2.12 | Homo sapiens WD repeat domain 33 (WDR33), transcript variant 2, mRNA. |
| GNPDA1 | 2.12 | Homo sapiens glucosamine-6-phosphate deaminase 1 (GNPDA1), mRNA. |
| RNF14 | 2.12 | Homo sapiens ring finger protein 14 (RNF14), transcript variant 4, mRNA. |
| ZNF326 | 2.12 | Homo sapiens zinc finger protein 326 (ZNF326), transcript variant 1, mRNA. |
| TMEM41A | 2.12 | Homo sapiens transmembrane protein 41A (TMEM41A), mRNA. |
| GPN2 | 2.12 | Homo sapiens GPN-loop GTPase 2 (GPN2), mRNA. |
| GPT2 | 2.11 | Homo sapiens glutamic pyruvate transaminase (alanine aminotransferase) 2 (GPT2), mRNA. |
| SIP1 | 2.11 | Homo sapiens survival of motor neuron protein interacting protein 1 (SIP1), transcript variant beta, mRNA. |
| SMG5 | 2.11 | Homo sapiens Smg-5 homolog, nonsense mediated mRNA decay factor (C. elegans) (SMG5), mRNA. |
| MRPL43 | 2.11 | Homo sapiens mitochondrial ribosomal protein L43 (MRPL43), nuclear gene encoding mitochondrial protein, transcript variant 2, mRNA. |
| C1orf216 | 2.11 | Homo sapiens chromosome 1 open reading frame 216 (C1orf216), mRNA. |
| VASH1 | 2.11 | Homo sapiens vasohibin 1 (VASH1), mRNA. |
| UBE2E1 | 2.11 | Homo sapiens ubiquitin-conjugating enzyme E2E 1 (UBC4/5 homolog, yeast) (UBE2E1), transcript variant 1, mRNA. |
| SNRPN | 2.1 | Homo sapiens small nuclear ribonucleoprotein polypeptide N (SNRPN), transcript variant 2, mRNA. |
| RASSF1 | 2.1 | Homo sapiens Ras association (RalGDS/AF-6) domain family member 1 (RASSF1), transcript variant C, mRNA. |
| LYSMD4 | 2.1 | Homo sapiens LysM, putative peptidoglycan-binding, domain containing 4 (LYSMD4), mRNA. |
| NR2F6 | 2.1 | Homo sapiens nuclear receptor subfamily 2, group F, member 6 (NR2F6), mRNA. |
| IQCC | 2.1 | Homo sapiens IQ motif containing C (IQCC), mRNA. |
| PJA1 | 2.1 | Homo sapiens praja ring finger 1 (PJA1), transcript variant 2, mRNA. |
| PAFAH1B3 | 2.1 | Homo sapiens platelet-activating factor acetylhydrolase, isoform Ib, gamma subunit 29kDa (PAFAH1B3), mRNA. |
| SPNS1 | 2.09 | Homo sapiens spinster homolog 1 (Drosophila) (SPNS1), mRNA. |
| CBX6 | 2.09 | Homo sapiens chromobox homolog 6 (CBX6), mRNA. |
| RPL9 | 2.09 | Homo sapiens ribosomal protein L9 (RPL9), transcript variant 2, mRNA. |
| LOC387867 | 2.09 | PREDICTED: Homo sapiens similar to 40S ribosomal protein SA (p40) (34/67 kDa laminin receptor) (Colon carcinoma laminin-binding protein) (NEM/1CHD4) (Multidrug resistance-associated protein MGr1-Ag) (LOC387867), mRNA. |
| NFKB1 | 2.09 | Homo sapiens nuclear factor of kappa light polypeptide gene enhancer in B-cells 1 (NFKB1), mRNA. |
| SIVA | 2.09 | Homo sapiens CD27-binding (Siva) protein (SIVA), transcript variant 2, mRNA. |
| SFRS17A | 2.09 | Homo sapiens splicing factor, arginine/serine-rich 17A (SFRS17A), transcript variant 1, mRNA. |
| AKT1 | 2.09 | Homo sapiens v-akt murine thymoma viral oncogene homolog 1 (AKT1), transcript variant 2, mRNA. |
| AP1S1 | 2.08 | Homo sapiens adaptor-related protein complex 1, sigma 1 subunit (AP1S1), transcript variant 2, mRNA. |
| PIK3R2 | 2.08 | Homo sapiens phosphoinositide-3-kinase, regulatory subunit 2 (beta) (PIK3R2), mRNA. |
| MRPS33 | 2.08 | Homo sapiens mitochondrial ribosomal protein S33 (MRPS33), nuclear gene encoding mitochondrial protein, transcript variant 1, mRNA. |
| RABL3 | 2.08 | Homo sapiens RAB, member of RAS oncogene family-like 3 (RABL3), mRNA. |
| ATP6V0A1 | 2.08 | Homo sapiens ATPase, H+ transporting, lysosomal V0 subunit a1 (ATP6V0A1), transcript variant 3, mRNA. |
| LOC728505 | 2.08 | PREDICTED: Homo sapiens similar to CG17293-PA (LOC728505), mRNA. |
| TSC22D1 | 2.08 | Homo sapiens TSC22 domain family, member 1 (TSC22D1), transcript variant 2, mRNA. |
| SFMBT1 | 2.08 | Homo sapiens Scm-like with four mbt domains 1 (SFMBT1), transcript variant 2, mRNA. |
| MRPL52 | 2.08 | Homo sapiens mitochondrial ribosomal protein L52 (MRPL52), nuclear gene encoding mitochondrial protein, transcript variant 2, mRNA. |
| TRUB1 | 2.08 | Homo sapiens TruB pseudouridine (psi) synthase homolog 1 (E. coli) (TRUB1), mRNA. |
| PABPC1L | 2.07 | PREDICTED: Homo sapiens poly(A) binding protein, cytoplasmic 1-like (PABPC1L), mRNA. |
| RPIA | 2.07 | Homo sapiens ribose 5-phosphate isomerase A (RPIA), mRNA. |
| CECR5 | 2.07 | Homo sapiens cat eye syndrome chromosome region, candidate 5 (CECR5), transcript variant 2, mRNA. |
| RPRC1 | 2.07 | Homo sapiens arginine/proline rich coiled-coil 1 (RPRC1), mRNA. |
| CUTA | 2.07 | Homo sapiens cutA divalent cation tolerance homolog (E. coli) (CUTA), transcript variant 2, mRNA. |
| BRWD1 | 2.07 | Homo sapiens bromodomain and WD repeat domain containing 1 (BRWD1), transcript variant 3, mRNA. |
| TMEM126B | 2.07 | Homo sapiens transmembrane protein 126B (TMEM126B), mRNA. |
| SYF2 | 2.06 | Homo sapiens SYF2 homolog, RNA splicing factor (S. cerevisiae) (SYF2), transcript variant 1, mRNA. |
| TNFSF12 | 2.06 | Homo sapiens tumor necrosis factor (ligand) superfamily, member 12 (TNFSF12), mRNA. |
| HDAC1 | 2.06 | Homo sapiens histone deacetylase 1 (HDAC1), mRNA. |
| CCDC56 | 2.06 | Homo sapiens coiled-coil domain containing 56 (CCDC56), mRNA. |
|  | 2.06 | full-length cDNA clone CS0DF005YI08 of Fetal brain of Homo sapiens (human) |
| EVL | 2.06 | Homo sapiens Enah/Vasp-like (EVL), mRNA. |
| TCEB3 | 2.06 | Homo sapiens transcription elongation factor B (SIII), polypeptide 3 (110kDa, elongin A) (TCEB3), mRNA. |
| EXOSC3 | 2.06 | Homo sapiens exosome component 3 (EXOSC3), transcript variant 1, mRNA. |
| TRIM23 | 2.06 | Homo sapiens tripartite motif-containing 23 (TRIM23), transcript variant alpha, mRNA. |
| ALKBH1 | 2.06 | Homo sapiens alkB, alkylation repair homolog 1 (E. coli) (ALKBH1), mRNA. |
| NGRN | 2.06 | Homo sapiens neugrin, neurite outgrowth associated (NGRN), transcript variant 1, mRNA. |
| ATP6V1E1 | 2.06 | Homo sapiens ATPase, H+ transporting, lysosomal 31kDa, V1 subunit E1 (ATP6V1E1), transcript variant 3, mRNA. |
| PIPSL | 2.05 | Homo sapiens PIP5K1A and PSMD4-like (PIPSL), non-coding RNA. |
| LOC652773 | 2.05 | PREDICTED: Homo sapiens similar to Shwachman-Bodian-Diamond syndrome (predicted) (LOC652773), mRNA. |
| SLC25A39 | 2.05 | Homo sapiens solute carrier family 25, member 39 (SLC25A39), mRNA. |
| DBR1 | 2.05 | Homo sapiens debranching enzyme homolog 1 (S. cerevisiae) (DBR1), mRNA. |
| SUV420H1 | 2.04 | Homo sapiens suppressor of variegation 4-20 homolog 1 (Drosophila) (SUV420H1), transcript variant 1, mRNA. |
| ATP5F1 | 2.04 | Homo sapiens ATP synthase, H+ transporting, mitochondrial F0 complex, subunit B1 (ATP5F1), nuclear gene encoding mitochondrial protein, mRNA. |
| SRP9 | 2.04 | Homo sapiens signal recognition particle 9kDa (SRP9), mRNA. |
| PWWP2A | 2.04 | Homo sapiens PWWP domain containing 2A (PWWP2A), mRNA. |
| ZNF263 | 2.04 | Homo sapiens zinc finger protein 263 (ZNF263), mRNA. |
| LOC653226 | 2.04 | PREDICTED: Homo sapiens similar to Signal recognition particle 9 kDa protein (SRP9) (LOC653226), mRNA. |
| NR2C1 | 2.04 | Homo sapiens nuclear receptor subfamily 2, group C, member 1 (NR2C1), transcript variant 2, mRNA. |
| MTERFD1 | 2.04 | Homo sapiens MTERF domain containing 1 (MTERFD1), mRNA. |
| LCMT1 | 2.04 | Homo sapiens leucine carboxyl methyltransferase 1 (LCMT1), transcript variant 1, mRNA. |
| C3orf75 | 2.04 | Homo sapiens chromosome 3 open reading frame 75 (C3orf75), mRNA. |
| HES6 | 2.04 | Homo sapiens hairy and enhancer of split 6 (Drosophila) (HES6), mRNA. |
| BEXL1 | 2.03 | PREDICTED: Homo sapiens brain expressed X-linked-like 1 (BEXL1), mRNA. |
| C10orf6 | 2.03 | Homo sapiens chromosome 10 open reading frame 6 (C10orf6), mRNA. |
| RHBDL3 | 2.03 | Homo sapiens rhomboid, veinlet-like 3 (Drosophila) (RHBDL3), mRNA. |
| PPIA | 2.03 | Homo sapiens peptidylprolyl isomerase A (cyclophilin A) (PPIA), mRNA. |
| LOC100134018 | 2.03 | PREDICTED: Homo sapiens similar to hCG1774568 (LOC100134018), mRNA. |
| DDIT3 | 2.03 | Homo sapiens DNA-damage-inducible transcript 3 (DDIT3), mRNA. |
| DOK6 | 2.03 | Homo sapiens docking protein 6 (DOK6), mRNA. |
| PAF1 | 2.03 | Homo sapiens Paf1, RNA polymerase II associated factor, homolog (S. cerevisiae) (PAF1), mRNA. |
| FBP1 | 2.03 | Homo sapiens fructose-1,6-bisphosphatase 1 (FBP1), mRNA. |
| LOC284023 | 2.02 | PREDICTED: Homo sapiens hypothetical protein LOC284023, transcript variant 3 (LOC284023), mRNA. |
| AMMECR1L | 2.02 | Homo sapiens AMME chromosomal region gene 1-like (AMMECR1L), mRNA. |
| ZNF322B | 2.02 | Homo sapiens zinc finger protein 322B (ZNF322B), mRNA. |
| HSPA8 | 2.02 | Homo sapiens heat shock 70kDa protein 8 (HSPA8), transcript variant 2, mRNA. |
| B3GNT1 | 2.02 | Homo sapiens UDP-GlcNAc:betaGal beta-1,3-N-acetylglucosaminyltransferase 1 (B3GNT1), mRNA. |
| EIF4E3 | 2.02 | Homo sapiens eukaryotic translation initiation factor 4E family member 3 (EIF4E3), mRNA. |
| PGAM5 | 2.02 | PREDICTED: Homo sapiens phosphoglycerate mutase family member 5 (PGAM5), mRNA. |
| TSPAN17 | 2.02 | Homo sapiens tetraspanin 17 (TSPAN17), transcript variant 1, mRNA. |
| SPTLC1 | 2.02 | Homo sapiens serine palmitoyltransferase, long chain base subunit 1 (SPTLC1), transcript variant 2, mRNA. |
| LOC441455 | 2.02 | PREDICTED: Homo sapiens misc_RNA (LOC441455), miscRNA. |
| KCNQ2 | 2.02 | Homo sapiens potassium voltage-gated channel, KQT-like subfamily, member 2 (KCNQ2), transcript variant 3, mRNA. |
| PMEPA1 | 2.01 | Homo sapiens prostate transmembrane protein, androgen induced 1 (PMEPA1), transcript variant 2, mRNA. |
|  | 2.01 | Homo sapiens cDNA FLJ45619 fis, clone BRTHA3027318 |
| 6-Sep | 2.01 | Homo sapiens septin 6 (SEPT6), transcript variant III, mRNA. |
| SLC25A14 | 2.01 | Homo sapiens solute carrier family 25 (mitochondrial carrier, brain), member 14 (SLC25A14), nuclear gene encoding mitochondrial protein, transcript variant long, mRNA. |
| TTC9C | 2.01 | Homo sapiens tetratricopeptide repeat domain 9C (TTC9C), mRNA. |
| LOC100132717 | 2.01 | PREDICTED: Homo sapiens hypothetical protein LOC100132717 (LOC100132717), mRNA. |
| TTC4 | 2.01 | Homo sapiens tetratricopeptide repeat domain 4 (TTC4), mRNA. |
| RPL6 | 2.01 | Homo sapiens ribosomal protein L6 (RPL6), transcript variant 1, mRNA. |
| SON | 2.01 | Homo sapiens SON DNA binding protein (SON), transcript variant f, mRNA. |
| PDRG1 | 2.01 | Homo sapiens p53 and DNA-damage regulated 1 (PDRG1), mRNA. |
| HSPA4 | 2.01 | Homo sapiens heat shock 70kDa protein 4 (HSPA4), mRNA. |
| MAP6D1 | 2.01 | Homo sapiens MAP6 domain containing 1 (MAP6D1), mRNA. |
| C14orf109 | 2 | Homo sapiens chromosome 14 open reading frame 109 (C14orf109), transcript variant 2, mRNA. |
| LOC643888 | 2 | PREDICTED: Homo sapiens hypothetical LOC643888 (LOC643888), mRNA. |
| PMVK | 2 | Homo sapiens phosphomevalonate kinase (PMVK), mRNA. |
| GBA2 | 2 | Homo sapiens glucosidase, beta (bile acid) 2 (GBA2), mRNA. |
| DBN1 | 2 | Homo sapiens drebrin 1 (DBN1), transcript variant 1, mRNA. |
|  | 2 | UI-E-EJ0-ahj-d-03-0-UI.r2 UI-E-EJ0 Homo sapiens cDNA clone UI-E-EJ0-ahj-d-03-0-UI 5, mRNA sequence |
| VEZF1 | 2 | Homo sapiens vascular endothelial zinc finger 1 (VEZF1), mRNA. |
| COPB2 | 2 | Homo sapiens coatomer protein complex, subunit beta 2 (beta prime) (COPB2), mRNA. |
| TAF15 | 2 | Homo sapiens TAF15 RNA polymerase II, TATA box binding protein (TBP)-associated factor, 68kDa (TAF15), transcript variant 2, mRNA. |
| LOC100131609 | 2 | PREDICTED: Homo sapiens misc_RNA (LOC100131609), miscRNA. |
| ABCC5 | 1.99 | Homo sapiens ATP-binding cassette, sub-family C (CFTR/MRP), member 5 (ABCC5), transcript variant 1, mRNA. |
| UBL4A | 1.99 | Homo sapiens ubiquitin-like 4A (UBL4A), mRNA. |
| DPAGT1 | 1.99 | Homo sapiens dolichyl-phosphate (UDP-N-acetylglucosamine) N-acetylglucosaminephosphotransferase 1 (GlcNAc-1-P transferase) (DPAGT1), transcript variant 1, mRNA. |
| PTRH1 | 1.99 | Homo sapiens peptidyl-tRNA hydrolase 1 homolog (S. cerevisiae) (PTRH1), mRNA. |
| RAB7A | 1.99 | Homo sapiens RAB7A, member RAS oncogene family (RAB7A), mRNA. |
| C22orf9 | 1.99 | Homo sapiens chromosome 22 open reading frame 9 (C22orf9), transcript variant 1, mRNA. |
| MYB | 1.99 | Homo sapiens v-myb myeloblastosis viral oncogene homolog (avian) (MYB), transcript variant 2, mRNA. |
| TBCC | 1.99 | Homo sapiens tubulin folding cofactor C (TBCC), mRNA. |
| STMN1 | 1.99 | Homo sapiens stathmin 1 (STMN1), transcript variant 3, mRNA. |
| SDCBP | 1.99 | Homo sapiens syndecan binding protein (syntenin) (SDCBP), transcript variant 2, mRNA. |
|  | 1.99 | BX447862 Homo sapiens T CELLS (JURKAT CELL LINE) Homo sapiens cDNA clone CS0DH002YG04 3-PRIME, mRNA sequence |
| LOC646791 | 1.98 | PREDICTED: Homo sapiens misc_RNA (LOC646791), miscRNA. |
| APBB3 | 1.98 | Homo sapiens amyloid beta (A4) precursor protein-binding, family B, member 3 (APBB3), transcript variant 2, mRNA. |
| SCAMP1 | 1.98 | Homo sapiens secretory carrier membrane protein 1 (SCAMP1), mRNA. |
| BAT1 | 1.98 | Homo sapiens HLA-B associated transcript 1 (BAT1), transcript variant 1, mRNA. |
| SDHAF1 | 1.98 | Homo sapiens succinate dehydrogenase complex assembly factor 1 (SDHAF1), mRNA. |
| DPAGT1 | 1.98 | Homo sapiens dolichyl-phosphate (UDP-N-acetylglucosamine) N-acetylglucosaminephosphotransferase 1 (GlcNAc-1-P transferase) (DPAGT1), transcript variant 1, mRNA. |
| BRMS1 | 1.98 | Homo sapiens breast cancer metastasis suppressor 1 (BRMS1), transcript variant 3, mRNA. |
| CTDSPL | 1.98 | Homo sapiens CTD (carboxy-terminal domain, RNA polymerase II, polypeptide A) small phosphatase-like (CTDSPL), transcript variant 2, mRNA. |
| HSPBAP1 | 1.98 | Homo sapiens HSPB (heat shock 27kDa) associated protein 1 (HSPBAP1), mRNA. |
| ZBTB34 | 1.97 | Homo sapiens zinc finger and BTB domain containing 34 (ZBTB34), mRNA. |
| SCNM1 | 1.97 | Homo sapiens sodium channel modifier 1 (SCNM1), mRNA. |
| C5orf22 | 1.97 | Homo sapiens chromosome 5 open reading frame 22 (C5orf22), mRNA. |
| RBM14 | 1.97 | Homo sapiens RNA binding motif protein 14 (RBM14), mRNA. |
| MAD2L1BP | 1.97 | Homo sapiens MAD2L1 binding protein (MAD2L1BP), transcript variant 2, mRNA. |
| UCHL5IP | 1.97 | Homo sapiens UCHL5 interacting protein (UCHL5IP), transcript variant 1, mRNA. |
| PKNOX1 | 1.97 | Homo sapiens PBX/knotted 1 homeobox 1 (PKNOX1), mRNA. |
| MAGED2 | 1.96 | Homo sapiens melanoma antigen family D, 2 (MAGED2), transcript variant 2, mRNA. |
| LOC100128737 | 1.96 | PREDICTED: Homo sapiens hypothetical protein LOC100128737 (LOC100128737), mRNA. |
| RPL12 | 1.96 | Homo sapiens ribosomal protein L12 (RPL12), mRNA. |
| TMEM216 | 1.96 | Homo sapiens transmembrane protein 216 (TMEM216), mRNA. |
| LOC729102 | 1.96 | PREDICTED: Homo sapiens misc_RNA (LOC729102), miscRNA. |
| TMEM14A | 1.96 | Homo sapiens transmembrane protein 14A (TMEM14A), mRNA. |
| MED8 | 1.96 | Homo sapiens mediator of RNA polymerase II transcription, subunit 8 homolog (S. cerevisiae) (MED8), transcript variant 3, mRNA. |
| LOC650029 | 1.96 | PREDICTED: Homo sapiens similar to RNA-binding protein 4 (RNA-binding motif protein 4) (Lark homolog) (Hlark) (RNA-binding motif protein 4a), transcript variant 1 (LOC650029), mRNA. |
| MATR3 | 1.96 | Homo sapiens matrin 3 (MATR3), transcript variant 2, mRNA. |
| PPRC1 | 1.96 | Homo sapiens peroxisome proliferator-activated receptor gamma, coactivator-related 1 (PPRC1), mRNA. |
| PHF13 | 1.95 | Homo sapiens PHD finger protein 13 (PHF13), mRNA. |
| PRSS3 | 1.95 | Homo sapiens protease, serine, 3 (mesotrypsin) (PRSS3), mRNA. |
| SLC45A4 | 1.95 | PREDICTED: Homo sapiens solute carrier family 45, member 4, transcript variant 2 (SLC45A4), mRNA. |
|  | 1.95 | BX417162 Homo sapiens PLACENTA Homo sapiens cDNA clone CS0DE005YO02 5-PRIME, mRNA sequence |
| LOC646567 | 1.95 | PREDICTED: Homo sapiens similar to HSPC307 (LOC646567), mRNA. |
| POLR1C | 1.95 | Homo sapiens polymerase (RNA) I polypeptide C, 30kDa (POLR1C), transcript variant 1, mRNA. |
| LOC653566 | 1.95 | Homo sapiens similar to Signal peptidase complex subunit 2 (Microsomal signal peptidase 25 kDa subunit) (SPase 25 kDa subunit) (LOC653566), mRNA. |
| DLK1 | 1.94 | Homo sapiens delta-like 1 homolog (Drosophila) (DLK1), mRNA. |
| DACT3 | 1.94 | Homo sapiens dapper, antagonist of beta-catenin, homolog 3 (Xenopus laevis) (DACT3), mRNA. |
|  | 1.94 | Homo sapiens cDNA clone IMAGE:6250506 |
| AARS | 1.94 | Homo sapiens alanyl-tRNA synthetase (AARS), mRNA. |
| LOC400506 | 1.93 | Homo sapiens similar to TSG118.1 (LOC400506), mRNA. |
| LOC100131713 | 1.93 | PREDICTED: Homo sapiens similar to ribosomal protein L29, transcript variant 2 (LOC100131713), mRNA. |
| MFAP3 | 1.93 | Homo sapiens microfibrillar-associated protein 3 (MFAP3), mRNA. |
| GLTSCR2 | 1.93 | Homo sapiens glioma tumor suppressor candidate region gene 2 (GLTSCR2), mRNA. |
| REC8 | 1.93 | Homo sapiens REC8 homolog (yeast) (REC8), transcript variant 1, mRNA. |
| LOC652455 | 1.93 | PREDICTED: Homo sapiens similar to Transcriptional regulator ATRX (ATP-dependent helicase ATRX) (X-linked helicase II) (X-linked nuclear protein) (XNP) (Znf-HX) (LOC652455), mRNA. |
| HIATL1 | 1.93 | Homo sapiens hippocampus abundant transcript-like 1 (HIATL1), mRNA. |
| LYSMD2 | 1.93 | Homo sapiens LysM, putative peptidoglycan-binding, domain containing 2 (LYSMD2), mRNA. |
| LOC644214 | 1.93 | PREDICTED: Homo sapiens misc_RNA (LOC644214), miscRNA. |
| ZDHHC13 | 1.93 | Homo sapiens zinc finger, DHHC-type containing 13 (ZDHHC13), transcript variant 1, mRNA. |
| PDCL3 | 1.93 | Homo sapiens phosducin-like 3 (PDCL3), mRNA. |
| DIDO1 | 1.92 | Homo sapiens death inducer-obliterator 1 (DIDO1), transcript variant 3, mRNA. |
| C1orf109 | 1.92 | Homo sapiens chromosome 1 open reading frame 109 (C1orf109), mRNA. |
| ARHGAP17 | 1.92 | Homo sapiens Rho GTPase activating protein 17 (ARHGAP17), transcript variant 2, mRNA. |
| POLR1C | 1.92 | Homo sapiens polymerase (RNA) I polypeptide C, 30kDa (POLR1C), transcript variant 1, mRNA. |
| C20orf20 | 1.92 | Homo sapiens chromosome 20 open reading frame 20 (C20orf20), mRNA. |
| LOC100131360 | 1.92 | PREDICTED: Homo sapiens misc_RNA (LOC100131360), miscRNA. |
| IP6K2 | 1.92 | Homo sapiens inositol hexakisphosphate kinase 2 (IP6K2), transcript variant 3, mRNA. |
| DNAJB9 | 1.92 | Homo sapiens DnaJ (Hsp40) homolog, subfamily B, member 9 (DNAJB9), mRNA. |
| PTCD2 | 1.92 | Homo sapiens pentatricopeptide repeat domain 2 (PTCD2), mRNA. |
| DCTPP1 | 1.91 | Homo sapiens dCTP pyrophosphatase 1 (DCTPP1), mRNA. |
| ZNF30 | 1.91 | Homo sapiens zinc finger protein 30 (ZNF30), transcript variant 2, mRNA. |
| CDCA1 | 1.91 | Homo sapiens cell division cycle associated 1 (CDCA1), transcript variant 1, mRNA. |
| HNRNPA1L2 | 1.91 | Homo sapiens heterogeneous nuclear ribonucleoprotein A1-like 2 (HNRNPA1L2), transcript variant 1, mRNA. |
| LIN7C | 1.91 | Homo sapiens lin-7 homolog C (C. elegans) (LIN7C), mRNA. |
| POU3F2 | 1.91 | Homo sapiens POU class 3 homeobox 2 (POU3F2), mRNA. |
| ATP9A | 1.91 | Homo sapiens ATPase, class II, type 9A (ATP9A), mRNA. |
| C7orf59 | 1.9 | Homo sapiens chromosome 7 open reading frame 59 (C7orf59), mRNA. |
| RPL28 | 1.9 | Homo sapiens ribosomal protein L28 (RPL28), mRNA. |
| KBTBD11 | 1.9 | Homo sapiens kelch repeat and BTB (POZ) domain containing 11 (KBTBD11), mRNA. |
| NCBP2 | 1.9 | Homo sapiens nuclear cap binding protein subunit 2, 20kDa (NCBP2), transcript variant 1, mRNA. |
| HGS | 1.9 | Homo sapiens hepatocyte growth factor-regulated tyrosine kinase substrate (HGS), mRNA. |
| DDX31 | 1.9 | Homo sapiens DEAD (Asp-Glu-Ala-Asp) box polypeptide 31 (DDX31), transcript variant 1, mRNA. |
| ARMC10 | 1.9 | Homo sapiens armadillo repeat containing 10 (ARMC10), mRNA. |
| LOC728554 | 1.9 | PREDICTED: Homo sapiens similar to THO complex 3 (LOC728554), mRNA. |
| KLHL35 | 1.89 | Homo sapiens kelch-like 35 (Drosophila) (KLHL35), mRNA. |
| KLHL12 | 1.89 | Homo sapiens kelch-like 12 (Drosophila) (KLHL12), mRNA. |
| SLC35F3 | 1.89 | PREDICTED: Homo sapiens solute carrier family 35, member F3 (SLC35F3), mRNA. |
| RHBDD1 | 1.89 | Homo sapiens rhomboid domain containing 1 (RHBDD1), mRNA. |
| MFAP1 | 1.89 | Homo sapiens microfibrillar-associated protein 1 (MFAP1), mRNA. |
| LOC653199 | 1.89 | PREDICTED: Homo sapiens hypothetical LOC653199 (LOC653199), mRNA. |
| MAGED2 | 1.89 | Homo sapiens melanoma antigen family D, 2 (MAGED2), transcript variant 1, mRNA. |
| DNMT3A | 1.89 | Homo sapiens DNA (cytosine-5-)-methyltransferase 3 alpha (DNMT3A), transcript variant 2, mRNA. |
| C6orf170 | 1.89 | Homo sapiens chromosome 6 open reading frame 170 (C6orf170), mRNA. |
| OAZ1 | 1.89 | Homo sapiens ornithine decarboxylase antizyme 1 (OAZ1), mRNA. |
| C17orf69 | 1.89 | Homo sapiens chromosome 17 open reading frame 69 (C17orf69), mRNA. |
| STX16 | 1.89 | Homo sapiens syntaxin 16 (STX16), transcript variant 1, mRNA. |
| HEY1 | 1.89 | Homo sapiens hairy/enhancer-of-split related with YRPW motif 1 (HEY1), transcript variant 2, mRNA. |
| C1orf203 | 1.88 | Homo sapiens chromosome 1 open reading frame 203 (C1orf203), non-coding RNA. |
| ZFP14 | 1.88 | Homo sapiens zinc finger protein 14 homolog (mouse) (ZFP14), mRNA. |
| CCM2 | 1.88 | Homo sapiens cerebral cavernous malformation 2 (CCM2), transcript variant 1, mRNA. |
| DKFZp686I15217 | 1.88 | Homo sapiens hypothetical protein DKFZp686I15217 (DKFZp686I15217), mRNA. |
| DCTPP1 | 1.88 | Homo sapiens dCTP pyrophosphatase 1 (DCTPP1), mRNA. |
| TAX1BP3 | 1.88 | Homo sapiens Tax1 (human T-cell leukemia virus type I) binding protein 3 (TAX1BP3), mRNA. |
| TCEB2 | 1.88 | Homo sapiens transcription elongation factor B (SIII), polypeptide 2 (18kDa, elongin B) (TCEB2), transcript variant 1, mRNA. |
| TGIF1 | 1.87 | Homo sapiens TGFB-induced factor homeobox 1 (TGIF1), transcript variant 1, mRNA. |
| MAK16 | 1.87 | Homo sapiens MAK16 homolog (S. cerevisiae) (MAK16), mRNA. |
| ZNF264 | 1.87 | Homo sapiens zinc finger protein 264 (ZNF264), mRNA. |
| ZRANB2 | 1.87 | Homo sapiens zinc finger, RAN-binding domain containing 2 (ZRANB2), transcript variant 2, mRNA. |
| ATP6V1F | 1.87 | Homo sapiens ATPase, H+ transporting, lysosomal 14kDa, V1 subunit F (ATP6V1F), mRNA. |
| SLC25A42 | 1.87 | Homo sapiens solute carrier family 25, member 42 (SLC25A42), mRNA. |
| KIAA1826 | 1.87 | Homo sapiens KIAA1826 (KIAA1826), mRNA. |
| RUFY3 | 1.87 | Homo sapiens RUN and FYVE domain containing 3 (RUFY3), transcript variant 1, mRNA. |
| LSM12 | 1.87 | Homo sapiens LSM12 homolog (S. cerevisiae) (LSM12), mRNA. |
| SERTAD3 | 1.87 | Homo sapiens SERTA domain containing 3 (SERTAD3), transcript variant 1, mRNA. |
| SPG21 | 1.87 | Homo sapiens spastic paraplegia 21 (autosomal recessive, Mast syndrome) (SPG21), transcript variant 1, mRNA. XM_945612 XM_945613 XM_945615 XM_945617 XM_945619 XM_945622 |
| ZAK | 1.87 | Homo sapiens sterile alpha motif and leucine zipper containing kinase AZK (ZAK), transcript variant 1, mRNA. |
| KCTD15 | 1.87 | Homo sapiens potassium channel tetramerisation domain containing 15 (KCTD15), mRNA. |
| CTXN1 | 1.87 | Homo sapiens cortexin 1 (CTXN1), mRNA. |
| ANKLE2 | 1.87 | Homo sapiens ankyrin repeat and LEM domain containing 2 (ANKLE2), mRNA. |
| FAM163A | 1.87 | Homo sapiens family with sequence similarity 163, member A (FAM163A), mRNA. |
| XBP1 | 1.87 | Homo sapiens X-box binding protein 1 (XBP1), transcript variant 2, mRNA. |
| SETDB2 | 1.86 | Homo sapiens SET domain, bifurcated 2 (SETDB2), mRNA. |
| DCAKD | 1.86 | Homo sapiens dephospho-CoA kinase domain containing (DCAKD), mRNA. |
| PANK1 | 1.86 | Homo sapiens pantothenate kinase 1 (PANK1), transcript variant gamma, mRNA. |
| PNMA2 | 1.86 | Homo sapiens paraneoplastic antigen MA2 (PNMA2), mRNA. |
| TTC13 | 1.86 | Homo sapiens tetratricopeptide repeat domain 13 (TTC13), mRNA. |
| LOC641710 | 1.86 | PREDICTED: Homo sapiens hypothetical protein LOC641710, transcript variant 1 (LOC641710), mRNA. |
| RASSF1 | 1.86 | Homo sapiens Ras association (RalGDS/AF-6) domain family 1 (RASSF1), transcript variant B, mRNA. |
| AIFM1 | 1.86 | Homo sapiens apoptosis-inducing factor, mitochondrion-associated, 1 (AIFM1), nuclear gene encoding mitochondrial protein, transcript variant 1, mRNA. |
| TMOD3 | 1.86 | Homo sapiens tropomodulin 3 (ubiquitous) (TMOD3), mRNA. |
| DAND5 | 1.86 | Homo sapiens DAN domain family, member 5 (DAND5), mRNA. |
| RTCD1 | 1.86 | Homo sapiens RNA terminal phosphate cyclase domain 1 (RTCD1), mRNA. |
| PRKAG2 | 1.86 | Homo sapiens protein kinase, AMP-activated, gamma 2 non-catalytic subunit (PRKAG2), transcript variant b, mRNA. |
| DPH2 | 1.86 | Homo sapiens DPH2 homolog (S. cerevisiae) (DPH2), transcript variant 1, mRNA. |
| FNTA | 1.86 | Homo sapiens farnesyltransferase, CAAX box, alpha (FNTA), transcript variant 1, mRNA. |
| DNAJC12 | 1.86 | Homo sapiens DnaJ (Hsp40) homolog, subfamily C, member 12 (DNAJC12), transcript variant 2, mRNA. |
| MXD4 | 1.85 | Homo sapiens MAX dimerization protein 4 (MXD4), mRNA. |
| C11orf61 | 1.85 | Homo sapiens chromosome 11 open reading frame 61 (C11orf61), mRNA. |
| USPL1 | 1.85 | Homo sapiens ubiquitin specific peptidase like 1 (USPL1), mRNA. |
| THAP2 | 1.85 | Homo sapiens THAP domain containing, apoptosis associated protein 2 (THAP2), mRNA. |
| ZNF182 | 1.85 | Homo sapiens zinc finger protein 182 (ZNF182), transcript variant 2, mRNA. |
| THAP1 | 1.85 | Homo sapiens THAP domain containing, apoptosis associated protein 1 (THAP1), transcript variant 1, mRNA. |
| ZC3H14 | 1.85 | Homo sapiens zinc finger CCCH-type containing 14 (ZC3H14), transcript variant 2, mRNA. |
| NOP16 | 1.84 | Homo sapiens NOP16 nucleolar protein homolog (yeast) (NOP16), mRNA. |
| UBE2M | 1.84 | Homo sapiens ubiquitin-conjugating enzyme E2M (UBC12 homolog, yeast) (UBE2M), mRNA. |
| SNRPB | 1.84 | Homo sapiens small nuclear ribonucleoprotein polypeptides B and B1 (SNRPB), transcript variant 2, mRNA. |
| AUH | 1.84 | Homo sapiens AU RNA binding protein/enoyl-Coenzyme A hydratase (AUH), nuclear gene encoding mitochondrial protein, mRNA. |
| FBXO28 | 1.84 | Homo sapiens F-box protein 28 (FBXO28), mRNA. |
| PPWD1 | 1.84 | Homo sapiens peptidylprolyl isomerase domain and WD repeat containing 1 (PPWD1), mRNA. |
| MGC42630 | 1.84 | Homo sapiens hypothetical protein MGC42630 (MGC42630), mRNA. |
| NUFIP1 | 1.84 | Homo sapiens nuclear fragile X mental retardation protein interacting protein 1 (NUFIP1), mRNA. |
| PPM1G | 1.84 | Homo sapiens protein phosphatase 1G (formerly 2C), magnesium-dependent, gamma isoform (PPM1G), transcript variant 2, mRNA. |
| ALAD | 1.83 | Homo sapiens aminolevulinate, delta-, dehydratase (ALAD), transcript variant 1, mRNA. |
| SLC37A3 | 1.83 | Homo sapiens solute carrier family 37 (glycerol-3-phosphate transporter), member 3 (SLC37A3), transcript variant 1, mRNA. |
| RPL14L | 1.83 | PREDICTED: Homo sapiens misc_RNA (RPL14L), miscRNA. |
| LOC388237 | 1.83 | PREDICTED: Homo sapiens similar to kidney-specific protein (KS), transcript variant 6 (LOC388237), mRNA. |
| DDX47 | 1.83 | Homo sapiens DEAD (Asp-Glu-Ala-Asp) box polypeptide 47 (DDX47), transcript variant 1, mRNA. |
| RBM39 | 1.83 | Homo sapiens RNA binding motif protein 39 (RBM39), transcript variant 1, mRNA. |
| C20orf45 | 1.83 | Homo sapiens chromosome 20 open reading frame 45 (C20orf45), mRNA. |
| PREPL | 1.83 | Homo sapiens prolyl endopeptidase-like (PREPL), transcript variant C, mRNA. |
| MTERF | 1.83 | Homo sapiens mitochondrial transcription termination factor (MTERF), nuclear gene encoding mitochondrial protein, mRNA. |
| NHP2L1 | 1.83 | Homo sapiens NHP2 non-histone chromosome protein 2-like 1 (S. cerevisiae) (NHP2L1), transcript variant 2, mRNA. |
| CHMP5 | 1.83 | Homo sapiens chromatin modifying protein 5 (CHMP5), mRNA. |
| GIT2 | 1.83 | Homo sapiens G protein-coupled receptor kinase interactor 2 (GIT2), transcript variant 3, mRNA. |
| FKBP1A | 1.83 | Homo sapiens FK506 binding protein 1A, 12kDa (FKBP1A), transcript variant 12A, mRNA. |
| ZC3HC1 | 1.82 | Homo sapiens zinc finger, C3HC-type containing 1 (ZC3HC1), mRNA. |
| LOC341965 | 1.82 | PREDICTED: Homo sapiens misc_RNA (LOC341965), miscRNA. |
| ERCC8 | 1.82 | Homo sapiens excision repair cross-complementing rodent repair deficiency, complementation group 8 (ERCC8), transcript variant 3, mRNA. |
| PLEKHA9 | 1.82 | Homo sapiens pleckstrin homology domain containing, family A (phosphoinositide binding specific) member 9 (PLEKHA9), mRNA. |
| NFIL3 | 1.82 | Homo sapiens nuclear factor, interleukin 3 regulated (NFIL3), mRNA. |
| WDR18 | 1.82 | Homo sapiens WD repeat domain 18 (WDR18), mRNA. |
| KCMF1 | 1.82 | PREDICTED: Homo sapiens potassium channel modulatory factor 1 (KCMF1), mRNA. |
| TRAPPC1 | 1.82 | Homo sapiens trafficking protein particle complex 1 (TRAPPC1), mRNA. |
| SNORA63 | 1.82 | Homo sapiens small nucleolar RNA, H/ACA box 63 (SNORA63), small nucleolar RNA. |
| UBE2G1 | 1.82 | Homo sapiens ubiquitin-conjugating enzyme E2G 1 (UBC7 homolog, yeast) (UBE2G1), mRNA. |
| PCYT2 | 1.82 | Homo sapiens phosphate cytidylyltransferase 2, ethanolamine (PCYT2), mRNA. |
| BIRC2 | 1.82 | Homo sapiens baculoviral IAP repeat-containing 2 (BIRC2), mRNA. |
| EIF2B4 | 1.81 | Homo sapiens eukaryotic translation initiation factor 2B, subunit 4 delta, 67kDa (EIF2B4), transcript variant 1, mRNA. |
| ZNF717 | 1.81 | PREDICTED: Homo sapiens zinc finger protein 717, transcript variant 3 (ZNF717), mRNA. |
| LOC100128086 | 1.81 | PREDICTED: Homo sapiens misc_RNA (LOC100128086), miscRNA. |
| LOC648249 | 1.81 | PREDICTED: Homo sapiens similar to 40S ribosomal protein SA (p40) (34/67 kDa laminin receptor) (Colon carcinoma laminin-binding protein) (NEM/1CHD4) (Multidrug resistance-associated protein MGr1-Ag), transcript variant 3 (LOC648249), mRNA. |
| C15orf57 | 1.81 | Homo sapiens chromosome 15 open reading frame 57 (C15orf57), transcript variant 2, mRNA. |
| CPEB4 | 1.81 | Homo sapiens cytoplasmic polyadenylation element binding protein 4 (CPEB4), mRNA. |
| CBX5 | 1.81 | Homo sapiens chromobox homolog 5 (HP1 alpha homolog, Drosophila) (CBX5), mRNA. |
| GSPT2 | 1.81 | Homo sapiens G1 to S phase transition 2 (GSPT2), mRNA. |
| LOC643167 | 1.81 | PREDICTED: Homo sapiens misc_RNA (LOC643167), miscRNA. |
| LBR | 1.81 | Homo sapiens lamin B receptor (LBR), transcript variant 1, mRNA. |
| FBXO38 | 1.8 | Homo sapiens F-box protein 38 (FBXO38), transcript variant 1, mRNA. |
| POLR3F | 1.8 | Homo sapiens polymerase (RNA) III (DNA directed) polypeptide F, 39 kDa (POLR3F), mRNA. |
| PTPN2 | 1.8 | Homo sapiens protein tyrosine phosphatase, non-receptor type 2 (PTPN2), transcript variant 2, mRNA. |
| ATP6V1E1 | 1.8 | Homo sapiens ATPase, H+ transporting, lysosomal 31kDa, V1 subunit E1 (ATP6V1E1), transcript variant 3, mRNA. |
| PPM1A | 1.8 | Homo sapiens protein phosphatase 1A (formerly 2C), magnesium-dependent, alpha isoform (PPM1A), transcript variant 2, mRNA. |
| LOC730996 | 1.8 | PREDICTED: Homo sapiens similar to chromosome 1 open reading frame 80 (LOC730996), mRNA. |
| BCCIP | 1.8 | Homo sapiens BRCA2 and CDKN1A interacting protein (BCCIP), transcript variant A, mRNA. |
| YWHAZ | 1.8 | Homo sapiens tyrosine 3-monooxygenase/tryptophan 5-monooxygenase activation protein, zeta polypeptide (YWHAZ), transcript variant 1, mRNA. |
| FAM127A | 1.8 | Homo sapiens family with sequence similarity 127, member A (FAM127A), mRNA. |
| METTL14 | 1.8 | Homo sapiens methyltransferase like 14 (METTL14), mRNA. |
| SON | 1.8 | Homo sapiens SON DNA binding protein (SON), transcript variant f, mRNA. |
| CDC2L2 | 1.8 | Homo sapiens cell division cycle 2-like 2 (PITSLRE proteins) (CDC2L2), transcript variant 1, mRNA. XM_001713745 XM_001713746 XM_001713747 |
| CDK7 | 1.79 | Homo sapiens cyclin-dependent kinase 7 (MO15 homolog, Xenopus laevis, cdk-activating kinase) (CDK7), mRNA. |
| EZR | 1.79 | Homo sapiens ezrin (EZR), transcript variant 1, mRNA. |
| GRK4 | 1.79 | Homo sapiens G protein-coupled receptor kinase 4 (GRK4), transcript variant 3, mRNA. |
| FBXL6 | 1.79 | Homo sapiens F-box and leucine-rich repeat protein 6 (FBXL6), transcript variant 1, mRNA. |
| PLCH2 | 1.79 | Homo sapiens phospholipase C, eta 2 (PLCH2), mRNA. |
| C17orf28 | 1.79 | Homo sapiens chromosome 17 open reading frame 28 (C17orf28), mRNA. |
| LRRC28 | 1.79 | Homo sapiens leucine rich repeat containing 28 (LRRC28), mRNA. |
| RAD51L3 | 1.79 | Homo sapiens RAD51-like 3 (S. cerevisiae) (RAD51L3), transcript variant 1, mRNA. |
| NUP35 | 1.78 | Homo sapiens nucleoporin 35kDa (NUP35), mRNA. |
| CTNS | 1.78 | Homo sapiens cystinosis, nephropathic (CTNS), transcript variant 2, mRNA. |
| BP75 | 1.78 | Homo sapiens bromodomain containing protein 75 kDa pseudogene (BP75) on chromosome 6. |
| ANAPC7 | 1.78 | Homo sapiens anaphase promoting complex subunit 7 (ANAPC7), mRNA. |
| MOBKL2A | 1.77 | Homo sapiens MOB1, Mps One Binder kinase activator-like 2A (yeast) (MOBKL2A), mRNA. |
| GEMIN6 | 1.77 | Homo sapiens gem (nuclear organelle) associated protein 6 (GEMIN6), mRNA. |
| CHIC2 | 1.77 | Homo sapiens cysteine-rich hydrophobic domain 2 (CHIC2), mRNA. |
| LOC645001 | 1.77 | PREDICTED: Homo sapiens misc_RNA (LOC645001), miscRNA. |
| LOC643507 | 1.77 | PREDICTED: Homo sapiens misc_RNA (LOC643507), miscRNA. |
| ZFP90 | 1.77 | Homo sapiens zinc finger protein 90 homolog (mouse) (ZFP90), mRNA. |
| PBRM1 | 1.77 | Homo sapiens polybromo 1 (PBRM1), transcript variant 4, mRNA. |
| TCP1 | 1.77 | Homo sapiens t-complex 1 (TCP1), transcript variant 1, mRNA. |
| LOC645676 | 1.76 | PREDICTED: Homo sapiens hypothetical protein LOC645676, transcript variant 1 (LOC645676), mRNA. |
| C1orf91 | 1.76 | Homo sapiens chromosome 1 open reading frame 91 (C1orf91), mRNA. |
| POM121C | 1.76 | Homo sapiens POM121 membrane glycoprotein C (POM121C), mRNA. |
| LSM10 | 1.76 | Homo sapiens LSM10, U7 small nuclear RNA associated (LSM10), mRNA. |
| GNAQ | 1.76 | Homo sapiens guanine nucleotide binding protein (G protein), q polypeptide (GNAQ), mRNA. |
| ZNF192 | 1.76 | Homo sapiens zinc finger protein 192 (ZNF192), mRNA. |
| FAM165B | 1.76 | Homo sapiens family with sequence similarity 165, member B (FAM165B), mRNA. |
| ZDHHC13 | 1.76 | Homo sapiens zinc finger, DHHC-type containing 13 (ZDHHC13), transcript variant 2, mRNA. |
| SIN3A | 1.76 | Homo sapiens SIN3 homolog A, transcription regulator (yeast) (SIN3A), mRNA. |
| C15orf17 | 1.76 | Homo sapiens chromosome 15 open reading frame 17 (C15orf17), mRNA. |
| LOC652388 | 1.76 | PREDICTED: Homo sapiens similar to nodal modulator 2 isoform 2 (LOC652388), mRNA. |
| LOC100134393 | 1.76 | PREDICTED: Homo sapiens misc_RNA (LOC100134393), miscRNA. |
| ETV4 | 1.75 | Homo sapiens ets variant gene 4 (E1A enhancer binding protein, E1AF) (ETV4), mRNA. |
| STYXL1 | 1.75 | Homo sapiens serine/threonine/tyrosine interacting-like 1 (STYXL1), mRNA. |
| CRELD1 | 1.75 | Homo sapiens cysteine-rich with EGF-like domains 1 (CRELD1), transcript variant 1, mRNA. |
| EIF3B | 1.75 | Homo sapiens eukaryotic translation initiation factor 3, subunit B (EIF3B), transcript variant 2, mRNA. |
| C8orf41 | 1.75 | Homo sapiens chromosome 8 open reading frame 41 (C8orf41), mRNA. |
| INTS4 | 1.75 | Homo sapiens integrator complex subunit 4 (INTS4), mRNA. |
| ZC3H10 | 1.75 | Homo sapiens zinc finger CCCH-type containing 10 (ZC3H10), mRNA. |
| NUDT3 | 1.75 | Homo sapiens nudix (nucleoside diphosphate linked moiety X)-type motif 3 (NUDT3), mRNA. |
| ZNF485 | 1.74 | Homo sapiens zinc finger protein 485 (ZNF485), mRNA. |
| MKKS | 1.74 | Homo sapiens McKusick-Kaufman syndrome (MKKS), transcript variant 2, mRNA. |
| SPIN3 | 1.74 | Homo sapiens spindlin family, member 3 (SPIN3), mRNA. |
| NAV2 | 1.74 | Homo sapiens neuron navigator 2 (NAV2), transcript variant 2, mRNA. |
| SLC35A2 | 1.74 | Homo sapiens solute carrier family 35 (UDP-galactose transporter), member A2 (SLC35A2), transcript variant 2, mRNA. |
| ATXN3 | 1.74 | Homo sapiens ataxin 3 (ATXN3), transcript variant 2, mRNA. |
| ME2 | 1.74 | Homo sapiens malic enzyme 2, NAD(+)-dependent, mitochondrial (ME2), nuclear gene encoding mitochondrial protein, mRNA. |
| TUBD1 | 1.74 | Homo sapiens tubulin, delta 1 (TUBD1), mRNA. |
| WDR33 | 1.74 | Homo sapiens WD repeat domain 33 (WDR33), transcript variant 2, mRNA. |
| LOC654174 | 1.73 | PREDICTED: Homo sapiens similar to lethal (2) k00619 CG4775-PA (LOC654174), mRNA. |
| TMEM98 | 1.73 | Homo sapiens transmembrane protein 98 (TMEM98), transcript variant 2, mRNA. |
| FBXO28 | 1.73 | Homo sapiens F-box protein 28 (FBXO28), mRNA. |
| SKP2 | 1.73 | Homo sapiens S-phase kinase-associated protein 2 (p45) (SKP2), transcript variant 1, mRNA. |
| KLC1 | 1.73 | Homo sapiens kinesin light chain 1 (KLC1), transcript variant 1, mRNA. |
| RNFT1 | 1.73 | Homo sapiens ring finger protein, transmembrane 1 (RNFT1), mRNA. |
| C21orf33 | 1.73 | Homo sapiens chromosome 21 open reading frame 33 (C21orf33), nuclear gene encoding mitochondrial protein, transcript variant 1, mRNA. |
| DYRK3 | 1.73 | Homo sapiens dual-specificity tyrosine-(Y)-phosphorylation regulated kinase 3 (DYRK3), transcript variant 1, mRNA. |
| ARMCX2 | 1.73 | Homo sapiens armadillo repeat containing, X-linked 2 (ARMCX2), mRNA. |
| SUMO2 | 1.73 | Homo sapiens SMT3 suppressor of mif two 3 homolog 2 (S. cerevisiae) (SUMO2), transcript variant 2, mRNA. |
| UQCC | 1.73 | Homo sapiens ubiquinol-cytochrome c reductase complex chaperone (UQCC), nuclear gene encoding mitochondrial protein, transcript variant 1, mRNA. |
| BCS1L | 1.73 | Homo sapiens BCS1-like (yeast) (BCS1L), nuclear gene encoding mitochondrial protein, transcript variant 2, mRNA. |
| EPS15L1 | 1.73 | Homo sapiens epidermal growth factor receptor pathway substrate 15-like 1 (EPS15L1), mRNA. |
| WDR1 | 1.73 | Homo sapiens WD repeat domain 1 (WDR1), transcript variant 1, mRNA. |
| RAB28 | 1.73 | Homo sapiens RAB28, member RAS oncogene family (RAB28), transcript variant 2, mRNA. |
| CTGLF7 | 1.72 | PREDICTED: Homo sapiens centaurin, gamma-like family, member 7 (CTGLF7), mRNA. |
| HIST1H2BD | 1.72 | Homo sapiens histone cluster 1, H2bd (HIST1H2BD), transcript variant 2, mRNA. |
| LOC653566 | 1.72 | PREDICTED: Homo sapiens similar to Signal peptidase complex subunit 2 (Microsomal signal peptidase 25 kDa subunit) (SPase 25 kDa subunit), transcript variant 3 (LOC653566), mRNA. |
| RHOT1 | 1.72 | Homo sapiens ras homolog gene family, member T1 (RHOT1), transcript variant 2, mRNA. |
| TMEM150A | 1.72 | Homo sapiens transmembrane protein 150A (TMEM150A), transcript variant 1, mRNA. |
| PTCD2 | 1.72 | Homo sapiens pentatricopeptide repeat domain 2 (PTCD2), mRNA. |
| KIFAP3 | 1.72 | Homo sapiens kinesin-associated protein 3 (KIFAP3), mRNA. |
| ZKSCAN3 | 1.71 | Homo sapiens zinc finger with KRAB and SCAN domains 3 (ZKSCAN3), mRNA. |
| BET1 | 1.71 | Homo sapiens blocked early in transport 1 homolog (S. cerevisiae) (BET1), mRNA. |
| NUP50 | 1.71 | Homo sapiens nucleoporin 50kDa (NUP50), transcript variant 2, mRNA. |
| FBXO3 | 1.71 | Homo sapiens F-box protein 3 (FBXO3), transcript variant 2, mRNA. |
| LOC645515 | 1.71 | PREDICTED: Homo sapiens misc_RNA (LOC645515), miscRNA. |
| DMAP1 | 1.71 | Homo sapiens DNA methyltransferase 1 associated protein 1 (DMAP1), transcript variant 2, mRNA. |
| FBXO45 | 1.71 | Homo sapiens F-box protein 45 (FBXO45), mRNA. |
| QARS | 1.71 | Homo sapiens glutaminyl-tRNA synthetase (QARS), mRNA. |
| CCDC5 | 1.71 | Homo sapiens coiled-coil domain containing 5 (spindle associated) (CCDC5), mRNA. |
| IRX3 | 1.71 | Homo sapiens iroquois homeobox 3 (IRX3), mRNA. |
| HDAC8 | 1.71 | Homo sapiens histone deacetylase 8 (HDAC8), mRNA. |
| GOSR2 | 1.7 | Homo sapiens golgi SNAP receptor complex member 2 (GOSR2), transcript variant A, mRNA. |
| ACAA1 | 1.7 | Homo sapiens acetyl-Coenzyme A acyltransferase 1 (peroxisomal 3-oxoacyl-Coenzyme A thiolase) (ACAA1), nuclear gene encoding mitochondrial protein, mRNA. |
|  | 1.7 | Homo sapiens cDNA clone IMAGE:30332316 |
| LRRC26 | 1.7 | Homo sapiens leucine rich repeat containing 26 (LRRC26), mRNA. |
| C15orf57 | 1.7 | Homo sapiens chromosome 15 open reading frame 57 (C15orf57), transcript variant 3, mRNA. |
| PPP1R11 | 1.7 | Homo sapiens protein phosphatase 1, regulatory (inhibitor) subunit 11 (PPP1R11), mRNA. |
| EIF2AK1 | 1.7 | Homo sapiens eukaryotic translation initiation factor 2-alpha kinase 1 (EIF2AK1), mRNA. |
| LOC644877 | 1.7 | PREDICTED: Homo sapiens misc_RNA (LOC644877), miscRNA. |
| ZNF187 | 1.7 | Homo sapiens zinc finger protein 187 (ZNF187), transcript variant 1, mRNA. |
| CBWD5 | 1.7 | Homo sapiens COBW domain containing 5 (CBWD5), mRNA. |
| HRB | 1.7 | Homo sapiens HIV-1 Rev binding protein (HRB), mRNA. |
| ATOX1 | 1.7 | Homo sapiens ATX1 antioxidant protein 1 homolog (yeast) (ATOX1), mRNA. |
|  | 1.7 | AGENCOURT_15364206 Human Anterior Horn Homo sapiens cDNA clone IMAGE:30513831 5, mRNA sequence |
| RFC3 | 1.7 | Homo sapiens replication factor C (activator 1) 3, 38kDa (RFC3), transcript variant 1, mRNA. |
| NUDC | 1.7 | Homo sapiens nuclear distribution gene C homolog (A. nidulans) (NUDC), mRNA. |
| NDUFAF3 | 1.7 | Homo sapiens NADH dehydrogenase (ubiquinone) 1 alpha subcomplex, assembly factor 3 (NDUFAF3), nuclear gene encoding mitochondrial protein, transcript variant 1, mRNA. |
| DGUOK | 1.7 | Homo sapiens deoxyguanosine kinase (DGUOK), nuclear gene encoding mitochondrial protein, transcript variant 1, mRNA. |
| LOC653820 | 1.7 | PREDICTED: Homo sapiens similar to family with sequence similarity 72, member A, transcript variant 1 (LOC653820), mRNA. |
| SGSM2 | 1.7 | Homo sapiens small G protein signaling modulator 2 (SGSM2), transcript variant 1, mRNA. |
| TIPARP | 1.69 | Homo sapiens TCDD-inducible poly(ADP-ribose) polymerase (TIPARP), mRNA. |
| APTX | 1.69 | Homo sapiens aprataxin (APTX), transcript variant 2, mRNA. |
| ZNF385D | 1.69 | Homo sapiens zinc finger protein 385D (ZNF385D), mRNA. |
| LOC91561 | 1.69 | PREDICTED: Homo sapiens similar to ribosomal protein S2, transcript variant 3 (LOC91561), mRNA. |
| MOCS1 | 1.69 | Homo sapiens molybdenum cofactor synthesis 1 (MOCS1), transcript variant 4, mRNA. |
| ATRX | 1.69 | Homo sapiens alpha thalassemia/mental retardation syndrome X-linked (RAD54 homolog, S. cerevisiae) (ATRX), transcript variant 1, mRNA. |
| CCDC117 | 1.69 | Homo sapiens coiled-coil domain containing 117 (CCDC117), mRNA. |
| LOC401115 | 1.69 | PREDICTED: Homo sapiens hypothetical gene supported by BC038466; BC062790 (LOC401115), mRNA. |
| CBX2 | 1.69 | Homo sapiens chromobox homolog 2 (Pc class homolog, Drosophila) (CBX2), transcript variant 1, mRNA. |
| IMP3 | 1.69 | Homo sapiens IMP3, U3 small nucleolar ribonucleoprotein, homolog (yeast) (IMP3), mRNA. |
| PLD5 | 1.68 | Homo sapiens phospholipase D family, member 5 (PLD5), mRNA. |
| PDLIM7 | 1.68 | Homo sapiens PDZ and LIM domain 7 (enigma) (PDLIM7), transcript variant 4, mRNA. |
| SEC61A1 | 1.68 | Homo sapiens Sec61 alpha 1 subunit (S. cerevisiae) (SEC61A1), mRNA. |
| SLC35F2 | 1.68 | Homo sapiens solute carrier family 35, member F2 (SLC35F2), mRNA. |
| CYB561 | 1.68 | Homo sapiens cytochrome b-561 (CYB561), transcript variant 3, mRNA. |
| MSL3 | 1.68 | Homo sapiens male-specific lethal 3 homolog (Drosophila) (MSL3), transcript variant 2, mRNA. |
| PNPO | 1.68 | Homo sapiens pyridoxamine 5'-phosphate oxidase (PNPO), mRNA. |
| HSPC111 | 1.68 | Homo sapiens hypothetical protein HSPC111 (HSPC111), mRNA. |
| RPUSD2 | 1.68 | Homo sapiens RNA pseudouridylate synthase domain containing 2 (RPUSD2), mRNA. |
| LRRC49 | 1.68 | Homo sapiens leucine rich repeat containing 49 (LRRC49), mRNA. |
| HNRPA1L-2 | 1.68 | Homo sapiens heterogeneous nuclear ribonucleoprotein A1 pseudogene (HNRPA1L-2), non-coding RNA. |
| PARG | 1.68 | Homo sapiens poly (ADP-ribose) glycohydrolase (PARG), mRNA. |
| ARMC7 | 1.68 | Homo sapiens armadillo repeat containing 7 (ARMC7), mRNA. |
| WBSCR22 | 1.67 | Homo sapiens Williams Beuren syndrome chromosome region 22 (WBSCR22), mRNA. |
| THOC4 | 1.67 | Homo sapiens THO complex 4 (THOC4), mRNA. |
| ALDH7A1 | 1.67 | Homo sapiens aldehyde dehydrogenase 7 family, member A1 (ALDH7A1), mRNA. |
| RASSF1 | 1.67 | Homo sapiens Ras association (RalGDS/AF-6) domain family member 1 (RASSF1), transcript variant A, mRNA. |
| NSMCE2 | 1.67 | Homo sapiens non-SMC element 2, MMS21 homolog (S. cerevisiae) (NSMCE2), mRNA. |
| PPP1CC | 1.67 | Homo sapiens protein phosphatase 1, catalytic subunit, gamma isoform (PPP1CC), mRNA. |
| FIP1L1 | 1.67 | Homo sapiens FIP1 like 1 (S. cerevisiae) (FIP1L1), mRNA. |
| C1orf25 | 1.67 | Homo sapiens chromosome 1 open reading frame 25 (C1orf25), mRNA. |
| MORF4L2 | 1.67 | Homo sapiens mortality factor 4 like 2 (MORF4L2), mRNA. |
| DPY30 | 1.67 | Homo sapiens dpy-30 homolog (C. elegans) (DPY30), mRNA. |
| HSD17B7P2 | 1.67 | Homo sapiens hydroxysteroid (17-beta) dehydrogenase 7 pseudogene 2 (HSD17B7P2), non-coding RNA. |
| WDR57 | 1.66 | Homo sapiens WD repeat domain 57 (U5 snRNP specific) (WDR57), mRNA. |
| CRK | 1.66 | Homo sapiens v-crk sarcoma virus CT10 oncogene homolog (avian) (CRK), transcript variant II, mRNA. |
| DNAJB11 | 1.66 | Homo sapiens DnaJ (Hsp40) homolog, subfamily B, member 11 (DNAJB11), mRNA. |
| IP6K2 | 1.66 | Homo sapiens inositol hexakisphosphate kinase 2 (IP6K2), transcript variant 2, mRNA. |
| CAT | 1.66 | Homo sapiens catalase (CAT), mRNA. |
| CARM1 | 1.66 | Homo sapiens coactivator-associated arginine methyltransferase 1 (CARM1), mRNA. |
| NLN | 1.66 | Homo sapiens neurolysin (metallopeptidase M3 family) (NLN), mRNA. |
| CHST12 | 1.66 | Homo sapiens carbohydrate (chondroitin 4) sulfotransferase 12 (CHST12), mRNA. |
| CRLF3 | 1.66 | Homo sapiens cytokine receptor-like factor 3 (CRLF3), mRNA. |
| 9-Sep | 1.66 | Homo sapiens septin 9 (SEPT9), mRNA. |
| CNKSR3 | 1.65 | Homo sapiens CNKSR family member 3 (CNKSR3), mRNA. |
| SNORA18 | 1.65 | Homo sapiens small nucleolar RNA, H/ACA box 18 (SNORA18), small nucleolar RNA. |
| LOC205251 | 1.65 | PREDICTED: Homo sapiens misc_RNA (LOC205251), miscRNA. |
| C21orf33 | 1.65 | Homo sapiens chromosome 21 open reading frame 33 (C21orf33), nuclear gene encoding mitochondrial protein, transcript variant 2, mRNA. |
| PSMD4 | 1.65 | Homo sapiens proteasome (prosome, macropain) 26S subunit, non-ATPase, 4 (PSMD4), mRNA. |
| PDDC1 | 1.65 | Homo sapiens Parkinson disease 7 domain containing 1 (PDDC1), mRNA. |
| KIAA0494 | 1.65 | Homo sapiens KIAA0494 (KIAA0494), mRNA. |
| SSH2 | 1.65 | Homo sapiens slingshot homolog 2 (Drosophila) (SSH2), mRNA. |
| CHMP2A | 1.65 | Homo sapiens chromatin modifying protein 2A (CHMP2A), transcript variant 2, mRNA. |
| HNRPDL | 1.65 | Homo sapiens heterogeneous nuclear ribonucleoprotein D-like (HNRPDL), transcript variant 3, transcribed RNA. |
| CNTN1 | 1.65 | Homo sapiens contactin 1 (CNTN1), transcript variant 1, mRNA. |
| THYN1 | 1.64 | Homo sapiens thymocyte nuclear protein 1 (THYN1), transcript variant 2, mRNA. |
| LOC727865 | 1.64 | PREDICTED: Homo sapiens misc_RNA (LOC727865), miscRNA. |
| C9orf3 | 1.64 | Homo sapiens chromosome 9 open reading frame 3 (C9orf3), mRNA. |
| TSPAN9 | 1.64 | Homo sapiens tetraspanin 9 (TSPAN9), mRNA. |
| NGRN | 1.64 | Homo sapiens neugrin, neurite outgrowth associated (NGRN), transcript variant 1, mRNA. |
| LOC100128805 | 1.64 | PREDICTED: Homo sapiens hypothetical protein LOC100128805 (LOC100128805), mRNA. |
| AGAP3 | 1.64 | Homo sapiens ArfGAP with GTPase domain, ankyrin repeat and PH domain 3 (AGAP3), transcript variant 2, mRNA. |
| SNORD89 | 1.64 | Homo sapiens small nucleolar RNA, C/D box 89 (SNORD89), small nucleolar RNA. |
| CNIH | 1.64 | Homo sapiens cornichon homolog (Drosophila) (CNIH), mRNA. |
| SUGT1 | 1.64 | Homo sapiens SGT1, suppressor of G2 allele of SKP1 (S. cerevisiae) (SUGT1), mRNA. |
| LGR5 | 1.64 | Homo sapiens leucine-rich repeat-containing G protein-coupled receptor 5 (LGR5), mRNA. |
| NME3 | 1.64 | Homo sapiens non-metastatic cells 3, protein expressed in (NME3), mRNA. |
| UBN2 | 1.64 | Homo sapiens ubinuclein 2 (UBN2), mRNA. |
|  | 1.63 | UI-E-EJ0-aho-m-08-0-UI.s1 UI-E-EJ0 Homo sapiens cDNA clone UI-E-EJ0-aho-m-08-0-UI 3, mRNA sequence |
| G3BP2 | 1.63 | Homo sapiens GTPase activating protein (SH3 domain) binding protein 2 (G3BP2), transcript variant 3, mRNA. |
| SH3PXD2A | 1.63 | Homo sapiens SH3 and PX domains 2A (SH3PXD2A), mRNA. |
| EXOSC7 | 1.63 | Homo sapiens exosome component 7 (EXOSC7), mRNA. |
| PRDM4 | 1.63 | Homo sapiens PR domain containing 4 (PRDM4), mRNA. |
| HARS | 1.63 | Homo sapiens histidyl-tRNA synthetase (HARS), mRNA. |
| CDC42EP3 | 1.63 | Homo sapiens CDC42 effector protein (Rho GTPase binding) 3 (CDC42EP3), mRNA. |
| GSR | 1.63 | Homo sapiens glutathione reductase (GSR), mRNA. |
| SLC39A4 | 1.63 | Homo sapiens solute carrier family 39 (zinc transporter), member 4 (SLC39A4), transcript variant 1, mRNA. |
| MGC10997 | 1.63 | Homo sapiens pseudogene MGC10997 (MGC10997) on chromosome 15. |
| NAV2 | 1.63 | Homo sapiens neuron navigator 2 (NAV2), transcript variant 2, mRNA. |
| LOC653450 | 1.63 | PREDICTED: Homo sapiens hypothetical LOC653450 (LOC653450), mRNA. |
| SLC25A4 | 1.63 | Homo sapiens solute carrier family 25 (mitochondrial carrier; adenine nucleotide translocator), member 4 (SLC25A4), nuclear gene encoding mitochondrial protein, mRNA. |
| C2orf63 | 1.63 | Homo sapiens chromosome 2 open reading frame 63 (C2orf63), mRNA. |
| ZNF428 | 1.62 | Homo sapiens zinc finger protein 428 (ZNF428), mRNA. |
| ELMOD2 | 1.62 | Homo sapiens ELMO/CED-12 domain containing 2 (ELMOD2), mRNA. |
|  | 1.62 | 602971152F1 NIH_MGC_12 Homo sapiens cDNA clone IMAGE:5110692 5, mRNA sequence |
| SUMF2 | 1.62 | Homo sapiens sulfatase modifying factor 2 (SUMF2), transcript variant 4, mRNA. |
| UBE2A | 1.62 | Homo sapiens ubiquitin-conjugating enzyme E2A (RAD6 homolog) (UBE2A), transcript variant 2, mRNA. |
| RBM4 | 1.62 | Homo sapiens RNA binding motif protein 4 (RBM4), mRNA. |
| RPL32 | 1.62 | Homo sapiens ribosomal protein L32 (RPL32), transcript variant 2, mRNA. |
| PLAGL2 | 1.62 | Homo sapiens pleiomorphic adenoma gene-like 2 (PLAGL2), mRNA. |
| GNL3 | 1.61 | Homo sapiens guanine nucleotide binding protein-like 3 (nucleolar) (GNL3), transcript variant 3, mRNA. |
| ARID4B | 1.61 | Homo sapiens AT rich interactive domain 4B (RBP1-like) (ARID4B), transcript variant 1, mRNA. |
| CNOT4 | 1.61 | Homo sapiens CCR4-NOT transcription complex, subunit 4 (CNOT4), transcript variant 1, mRNA. |
| KPNA2 | 1.61 | Homo sapiens karyopherin alpha 2 (RAG cohort 1, importin alpha 1) (KPNA2), mRNA. XM_001133262 XM_001133265 XM_001133267 XM_001133271 |
| RND3 | 1.61 | Homo sapiens Rho family GTPase 3 (RND3), mRNA. |
| ZFP161 | 1.61 | Homo sapiens zinc finger protein 161 homolog (mouse) (ZFP161), mRNA. |
| C22orf28 | 1.61 | Homo sapiens chromosome 22 open reading frame 28 (C22orf28), mRNA. |
| FBXO21 | 1.61 | Homo sapiens F-box protein 21 (FBXO21), transcript variant 1, mRNA. |
| MGC16384 | 1.61 | PREDICTED: Homo sapiens hypothetical protein MGC16384 (MGC16384), mRNA. |
| NAT5 | 1.61 | Homo sapiens N-acetyltransferase 5 (GCN5-related, putative) (NAT5), transcript variant 3, mRNA. |
| OSBP | 1.61 | Homo sapiens oxysterol binding protein (OSBP), mRNA. |
| LOC730167 | 1.61 | PREDICTED: Homo sapiens similar to protein tyrosine phosphatase 4a1, transcript variant 1 (LOC730167), mRNA. |
| FRMD3 | 1.6 | Homo sapiens FERM domain containing 3 (FRMD3), mRNA. |
| HINFP | 1.6 | Homo sapiens histone H4 transcription factor (HINFP), transcript variant 2, mRNA. |
| COMMD7 | 1.6 | Homo sapiens COMM domain containing 7 (COMMD7), transcript variant 2, mRNA. |
| HSPA4 | 1.6 | Homo sapiens heat shock 70kDa protein 4 (HSPA4), transcript variant 2, mRNA. |
| MED20 | 1.6 | Homo sapiens mediator complex subunit 20 (MED20), mRNA. |
| LOC442041 | 1.6 | PREDICTED: Homo sapiens misc_RNA (LOC442041), partial miscRNA. |
| ZNF193 | 1.6 | Homo sapiens zinc finger protein 193 (ZNF193), mRNA. |
| SETD3 | 1.6 | Homo sapiens SET domain containing 3 (SETD3), transcript variant 1, mRNA. |
| SPTLC1 | 1.6 | Homo sapiens serine palmitoyltransferase, long chain base subunit 1 (SPTLC1), transcript variant 1, mRNA. |
| PSEN2 | 1.6 | Homo sapiens presenilin 2 (Alzheimer disease 4) (PSEN2), transcript variant 2, mRNA. |
| YIPF6 | 1.6 | Homo sapiens Yip1 domain family, member 6 (YIPF6), mRNA. |
| POLR3K | 1.59 | Homo sapiens polymerase (RNA) III (DNA directed) polypeptide K, 12.3 kDa (POLR3K), mRNA. |
| SLC7A6OS | 1.59 | Homo sapiens solute carrier family 7, member 6 opposite strand (SLC7A6OS), mRNA. |
| ZNF691 | 1.59 | Homo sapiens zinc finger protein 691 (ZNF691), mRNA. |
| LOC653210 | 1.59 | PREDICTED: Homo sapiens similar to melanoma antigen family D, 4 isoform 1, transcript variant 2 (LOC653210), mRNA. |
| UBL7 | 1.59 | Homo sapiens ubiquitin-like 7 (bone marrow stromal cell-derived) (UBL7), transcript variant 1, mRNA. |
| AFTPH | 1.59 | Homo sapiens aftiphilin (AFTPH), transcript variant 2, mRNA. |
| OGT | 1.59 | Homo sapiens O-linked N-acetylglucosamine (GlcNAc) transferase (UDP-N-acetylglucosamine:polypeptide-N-acetylglucosaminyl transferase) (OGT), transcript variant 1, mRNA. |
| TMEM5 | 1.59 | Homo sapiens transmembrane protein 5 (TMEM5), mRNA. |
| FAM50A | 1.59 | Homo sapiens family with sequence similarity 50, member A (FAM50A), mRNA. |
| PXDN | 1.59 | Homo sapiens peroxidasin homolog (Drosophila) (PXDN), mRNA. XM_935183 XM_935184 XM_936786 XM_942966 XM_942982 XM_942986 XM_942994 XM_943000 |
| SNHG11 | 1.59 | Homo sapiens small nucleolar RNA host gene 11 (non-protein coding) (SNHG11), non-coding RNA. |
| RBM4 | 1.59 | Homo sapiens RNA binding motif protein 4 (RBM4), mRNA. |
| KATNA1 | 1.58 | Homo sapiens katanin p60 (ATPase-containing) subunit A 1 (KATNA1), mRNA. |
| C15orf44 | 1.58 | Homo sapiens chromosome 15 open reading frame 44 (C15orf44), transcript variant 2, mRNA. |
| ACVR1B | 1.58 | Homo sapiens activin A receptor, type IB (ACVR1B), transcript variant 1, mRNA. |
| LOC728006 | 1.58 | PREDICTED: Homo sapiens hypothetical protein LOC728006 (LOC728006), mRNA. |
| SELK | 1.58 | Homo sapiens selenoprotein K (SELK), mRNA. |
| KIAA1949 | 1.58 | Homo sapiens KIAA1949 (KIAA1949), mRNA. |
| EIF3H | 1.58 | Homo sapiens eukaryotic translation initiation factor 3, subunit H (EIF3H), mRNA. |
| SNAPC2 | 1.58 | Homo sapiens small nuclear RNA activating complex, polypeptide 2, 45kDa (SNAPC2), mRNA. |
| C12orf11 | 1.58 | Homo sapiens chromosome 12 open reading frame 11 (C12orf11), mRNA. |
| PCBD1 | 1.58 | Homo sapiens pterin-4 alpha-carbinolamine dehydratase/dimerization cofactor of hepatocyte nuclear factor 1 alpha (PCBD1), mRNA. |
| COG2 | 1.57 | Homo sapiens component of oligomeric golgi complex 2 (COG2), mRNA. |
| LOC649970 | 1.57 | PREDICTED: Homo sapiens similar to creatine kinase, mitochondrial 1B precursor (LOC649970), mRNA. |
| FXC1 | 1.57 | Homo sapiens fracture callus 1 homolog (rat) (FXC1), nuclear gene encoding mitochondrial protein, mRNA. |
| ACYP1 | 1.57 | Homo sapiens acylphosphatase 1, erythrocyte (common) type (ACYP1), transcript variant 1, mRNA. |
| MAP1B | 1.57 | Homo sapiens microtubule-associated protein 1B (MAP1B), mRNA. |
| LOC441506 | 1.57 | PREDICTED: Homo sapiens misc_RNA (LOC441506), miscRNA. |
| SNORD80 | 1.57 | Homo sapiens small nucleolar RNA, C/D box 80 (SNORD80), small nucleolar RNA. |
| TCEAL8 | 1.57 | Homo sapiens transcription elongation factor A (SII)-like 8 (TCEAL8), transcript variant 1, mRNA. |
| PSMD10 | 1.57 | Homo sapiens proteasome (prosome, macropain) 26S subunit, non-ATPase, 10 (PSMD10), transcript variant 1, mRNA. |
| MRPS36 | 1.56 | Homo sapiens mitochondrial ribosomal protein S36 (MRPS36), nuclear gene encoding mitochondrial protein, mRNA. |
| C11orf74 | 1.56 | Homo sapiens chromosome 11 open reading frame 74 (C11orf74), mRNA. |
| XPO7 | 1.56 | Homo sapiens exportin 7 (XPO7), mRNA. |
| TWF1 | 1.56 | Homo sapiens twinfilin, actin-binding protein, homolog 1 (Drosophila) (TWF1), mRNA. |
| KIAA1409 | 1.56 | Homo sapiens KIAA1409 (KIAA1409), mRNA. |
| LOC387856 | 1.56 | Homo sapiens similar to expressed sequence AI836003 (LOC387856), mRNA. |
| ZNF664 | 1.56 | Homo sapiens zinc finger protein 664 (ZNF664), mRNA. |
|  | 1.56 | Homo sapiens cDNA FLJ37828 fis, clone BRSSN2006575 |
| TASP1 | 1.56 | Homo sapiens taspase, threonine aspartase, 1 (TASP1), mRNA. |
| NAMPT | 1.56 | Homo sapiens nicotinamide phosphoribosyltransferase (NAMPT), mRNA. |
| PMS2L4 | 1.56 | Homo sapiens postmeiotic segregation increased 2-like 4 pseudogene (PMS2L4), non-coding RNA. |
| ZNF614 | 1.56 | Homo sapiens zinc finger protein 614 (ZNF614), mRNA. |
| ACP1 | 1.56 | Homo sapiens acid phosphatase 1, soluble (ACP1), transcript variant 2, mRNA. |
| ZFAND6 | 1.56 | Homo sapiens zinc finger, AN1-type domain 6 (ZFAND6), mRNA. |
| LOC389322 | 1.55 | PREDICTED: Homo sapiens misc_RNA (LOC389322), miscRNA. |
| NME1 | 1.55 | Homo sapiens non-metastatic cells 1, protein (NM23A) expressed in (NME1), transcript variant 2, mRNA. |
| PIM2 | 1.55 | Homo sapiens pim-2 oncogene (PIM2), mRNA. |
| GTF2B | 1.55 | Homo sapiens general transcription factor IIB (GTF2B), mRNA. |
| D2HGDH | 1.55 | Homo sapiens D-2-hydroxyglutarate dehydrogenase (D2HGDH), nuclear gene encoding mitochondrial protein, mRNA. |
| NHP2L1 | 1.55 | Homo sapiens NHP2 non-histone chromosome protein 2-like 1 (S. cerevisiae) (NHP2L1), transcript variant 1, mRNA. |
| MAEA | 1.55 | Homo sapiens macrophage erythroblast attacher (MAEA), transcript variant 1, mRNA. |
| HS6ST1 | 1.55 | Homo sapiens heparan sulfate 6-O-sulfotransferase 1 (HS6ST1), mRNA. |
| SRP14P1 | 1.55 | Homo sapiens signal recognition particle 14kDa (homologous Alu RNA binding protein) pseudogene 1 (SRP14P1), non-coding RNA. |
| ATP6V1D | 1.54 | Homo sapiens ATPase, H+ transporting, lysosomal 34kDa, V1 subunit D (ATP6V1D), mRNA. |
| CNPY4 | 1.54 | Homo sapiens canopy 4 homolog (zebrafish) (CNPY4), mRNA. |
| MED31 | 1.54 | Homo sapiens mediator complex subunit 31 (MED31), mRNA. |
|  | 1.54 | BX093763 Soares_fetal_heart_NbHH19W Homo sapiens cDNA clone IMAGp998N10870, mRNA sequence |
| RUNDC1 | 1.54 | Homo sapiens RUN domain containing 1 (RUNDC1), mRNA. |
| LOC728620 | 1.54 | PREDICTED: Homo sapiens misc_RNA (LOC728620), miscRNA. |
| DPM2 | 1.54 | Homo sapiens dolichyl-phosphate mannosyltransferase polypeptide 2, regulatory subunit (DPM2), transcript variant 2, mRNA. |
| HNRPK | 1.54 | Homo sapiens heterogeneous nuclear ribonucleoprotein K (HNRPK), transcript variant 3, mRNA. |
| FARSA | 1.54 | Homo sapiens phenylalanyl-tRNA synthetase, alpha subunit (FARSA), mRNA. |
| RAP1GAP | 1.54 | Homo sapiens RAP1 GTPase activating protein (RAP1GAP), mRNA. |
| MRPS26 | 1.54 | Homo sapiens mitochondrial ribosomal protein S26 (MRPS26), nuclear gene encoding mitochondrial protein, mRNA. |
| ERP29 | 1.54 | Homo sapiens endoplasmic reticulum protein 29 (ERP29), transcript variant 2, mRNA. |
| GCH1 | 1.54 | Homo sapiens GTP cyclohydrolase 1 (GCH1), transcript variant 4, mRNA. |
| ELAVL1 | 1.54 | Homo sapiens ELAV (embryonic lethal, abnormal vision, Drosophila)-like 1 (Hu antigen R) (ELAVL1), mRNA. |
| AMACR | 1.54 | Homo sapiens alpha-methylacyl-CoA racemase (AMACR), transcript variant 1, mRNA. |
| NUDT16L1 | 1.53 | Homo sapiens nudix (nucleoside diphosphate linked moiety X)-type motif 16-like 1 (NUDT16L1), mRNA. |
| EIF4A2 | 1.53 | Homo sapiens eukaryotic translation initiation factor 4A, isoform 2 (EIF4A2), mRNA. |
| LOC652826 | 1.53 | PREDICTED: Homo sapiens similar to 26S protease regulatory subunit 6B (MIP224) (MB67-interacting protein) (TAT-binding protein 7) (TBP-7) (LOC652826), mRNA. |
| ZNF137 | 1.53 | Homo sapiens zinc finger protein 137 (ZNF137), mRNA. |
| STAMBPL1 | 1.53 | Homo sapiens STAM binding protein-like 1 (STAMBPL1), mRNA. |
| MBD5 | 1.53 | Homo sapiens methyl-CpG binding domain protein 5 (MBD5), mRNA. |
| SLC25A25 | 1.53 | Homo sapiens solute carrier family 25 (mitochondrial carrier; phosphate carrier), member 25 (SLC25A25), nuclear gene encoding mitochondrial protein, transcript variant 1, mRNA. |
| XRCC1 | 1.53 | Homo sapiens X-ray repair complementing defective repair in Chinese hamster cells 1 (XRCC1), mRNA. |
| KLHL8 | 1.53 | Homo sapiens kelch-like 8 (Drosophila) (KLHL8), mRNA. |
| TAF1L | 1.53 | Homo sapiens TAF1 RNA polymerase II, TATA box binding protein (TBP)-associated factor, 210kDa-like (TAF1L), mRNA. |
| C14orf124 | 1.53 | Homo sapiens chromosome 14 open reading frame 124 (C14orf124), mRNA. |
| SLC39A6 | 1.53 | Homo sapiens solute carrier family 39 (zinc transporter), member 6 (SLC39A6), transcript variant 1, mRNA. |
| ARMC8 | 1.52 | Homo sapiens armadillo repeat containing 8 (ARMC8), transcript variant 2, mRNA. |
| MAPBPIP | 1.52 | Homo sapiens mitogen-activated protein-binding protein-interacting protein (MAPBPIP), mRNA. |
| MRPL45 | 1.52 | Homo sapiens mitochondrial ribosomal protein L45 (MRPL45), nuclear gene encoding mitochondrial protein, mRNA. |
| ZNF330 | 1.52 | Homo sapiens zinc finger protein 330 (ZNF330), mRNA. |
| ACOT8 | 1.52 | Homo sapiens acyl-CoA thioesterase 8 (ACOT8), transcript variant 1, mRNA. |
| KIAA1009 | 1.52 | Homo sapiens KIAA1009 (KIAA1009), mRNA. |
| ARAP3 | 1.52 | Homo sapiens ArfGAP with RhoGAP domain, ankyrin repeat and PH domain 3 (ARAP3), mRNA. |
| HSPA12A | 1.52 | Homo sapiens heat shock 70kDa protein 12A (HSPA12A), mRNA. |
| UGCGL1 | 1.52 | Homo sapiens UDP-glucose ceramide glucosyltransferase-like 1 (UGCGL1), transcript variant 1, mRNA. |
| POU3F2 | 1.52 | Homo sapiens POU class 3 homeobox 2 (POU3F2), mRNA. |
| KIAA1908 | 1.52 | Homo sapiens KIAA1908 protein (KIAA1908), non-coding RNA. |
| LOC643668 | 1.52 | PREDICTED: Homo sapiens similar to peptidase (prosome, macropain) 26S subunit, ATPase 1 (LOC643668), mRNA. |
| PLEKHB2 | 1.52 | Homo sapiens pleckstrin homology domain containing, family B (evectins) member 2 (PLEKHB2), transcript variant 2, mRNA. |
| MAGED1 | 1.52 | Homo sapiens melanoma antigen family D, 1 (MAGED1), transcript variant 3, mRNA. |
| LOC649553 | 1.51 | PREDICTED: Homo sapiens misc_RNA (LOC649553), miscRNA. |
| KCMF1 | 1.51 | PREDICTED: Homo sapiens potassium channel modulatory factor 1 (KCMF1), mRNA. |
| GSTM3 | 1.51 | Homo sapiens glutathione S-transferase M3 (brain) (GSTM3), mRNA. |
| RDBP | 1.51 | Homo sapiens RD RNA binding protein (RDBP), mRNA. |
| SNRPB2 | 1.51 | Homo sapiens small nuclear ribonucleoprotein polypeptide B'' (SNRPB2), transcript variant 1, mRNA. |
| ACOX1 | 1.51 | Homo sapiens acyl-Coenzyme A oxidase 1, palmitoyl (ACOX1), transcript variant 2, mRNA. |
| CCNE1 | 1.51 | Homo sapiens cyclin E1 (CCNE1), transcript variant 2, mRNA. |
| SAP18 | 1.51 | Homo sapiens Sin3A-associated protein, 18kDa (SAP18), mRNA. |
| SLC25A26 | 1.51 | Homo sapiens solute carrier family 25, member 26 (SLC25A26), nuclear gene encoding mitochondrial protein, mRNA. |
| COX4I1 | 1.51 | Homo sapiens cytochrome c oxidase subunit IV isoform 1 (COX4I1), mRNA. |
| PAAF1 | 1.51 | Homo sapiens proteasomal ATPase-associated factor 1 (PAAF1), mRNA. |
| WDR74 | 1.51 | PREDICTED: Homo sapiens WD repeat domain 74 (WDR74), mRNA. |
| FZD3 | 1.51 | Homo sapiens frizzled homolog 3 (Drosophila) (FZD3), mRNA. |
| PREI3 | 1.51 | Homo sapiens preimplantation protein 3 (PREI3), transcript variant 1, mRNA. |
| VPS54 | 1.51 | Homo sapiens vacuolar protein sorting 54 homolog (S. cerevisiae) (VPS54), transcript variant 2, mRNA. |
| KLRAQ1 | 1.51 | Homo sapiens KLRAQ motif containing 1 (KLRAQ1), transcript variant 1, mRNA. |
| MBOAT2 | 1.5 | PREDICTED: Homo sapiens membrane bound O-acyltransferase domain containing 2 (MBOAT2), mRNA. |
| DIABLO | 1.5 | Homo sapiens diablo homolog (Drosophila) (DIABLO), nuclear gene encoding mitochondrial protein, transcript variant 2, mRNA. |
| ARSK | 1.5 | Homo sapiens arylsulfatase K (ARSK), mRNA. |
| DRD1IP | 1.5 | Homo sapiens dopamine receptor D1 interacting protein (DRD1IP), mRNA. |
| CUGBP1 | 1.5 | Homo sapiens CUG triplet repeat, RNA binding protein 1 (CUGBP1), transcript variant 2, mRNA. |
| ISYNA1 | 1.5 | Homo sapiens inositol-3-phosphate synthase 1 (ISYNA1), mRNA. |
| LOC653354 | 1.5 | PREDICTED: Homo sapiens similar to amrmadillo repeat containing, X-linked 6 (H. sapiens)-like (LOC653354), mRNA. |
| LOC730060 | -1.5 | PREDICTED: Homo sapiens hypothetical LOC730060 (LOC730060), mRNA. |
| ATRN | -1.5 | Homo sapiens attractin (ATRN), transcript variant 1, mRNA. |
| DHX36 | -1.5 | Homo sapiens DEAH (Asp-Glu-Ala-His) box polypeptide 36 (DHX36), mRNA. |
| HERC1 | -1.5 | Homo sapiens hect (homologous to the E6-AP (UBE3A) carboxyl terminus) domain and RCC1 (CHC1)-like domain (RLD) 1 (HERC1), mRNA. |
| PTPN13 | -1.5 | Homo sapiens protein tyrosine phosphatase, non-receptor type 13 (APO-1/CD95 (Fas)-associated phosphatase) (PTPN13), transcript variant 3, mRNA. |
| SNORD12C | -1.5 | Homo sapiens small nucleolar RNA, C/D box 12C (SNORD12C), small nucleolar RNA. |
| NPY | -1.51 | Homo sapiens neuropeptide Y (NPY), mRNA. |
| LOC641746 | -1.51 | PREDICTED: Homo sapiens misc_RNA (LOC641746), miscRNA. |
| NET1 | -1.51 | Homo sapiens neuroepithelial cell transforming 1 (NET1), transcript variant 1, mRNA. |
| LOC137107 | -1.51 | PREDICTED: Homo sapiens similar to ribosomal protein L10a (LOC137107), mRNA. |
| LEF1 | -1.51 | Homo sapiens lymphoid enhancer-binding factor 1 (LEF1), mRNA. |
| ZFP91 | -1.51 | Homo sapiens zinc finger protein 91 homolog (mouse) (ZFP91), mRNA. |
| SP3 | -1.51 | Homo sapiens Sp3 transcription factor (SP3), transcript variant 2, mRNA. |
| IL10 | -1.51 | Homo sapiens interleukin 10 (IL10), mRNA. |
| MED25 | -1.51 | Homo sapiens mediator complex subunit 25 (MED25), mRNA. |
| ADAM15 | -1.51 | Homo sapiens ADAM metallopeptidase domain 15 (ADAM15), transcript variant 4, mRNA. |
| LOC100133517 | -1.51 | PREDICTED: Homo sapiens hypothetical protein LOC100133517 (LOC100133517), mRNA. |
| TLK1 | -1.51 | Homo sapiens tousled-like kinase 1 (TLK1), mRNA. |
| KCTD17 | -1.51 | Homo sapiens potassium channel tetramerisation domain containing 17 (KCTD17), mRNA. |
| COQ2 | -1.52 | Homo sapiens coenzyme Q2 homolog, prenyltransferase (yeast) (COQ2), mRNA. |
| MAP1B | -1.52 | Homo sapiens microtubule-associated protein 1B (MAP1B), transcript variant 2, mRNA. |
| TMEM18 | -1.52 | Homo sapiens transmembrane protein 18 (TMEM18), mRNA. |
| TSHZ1 | -1.52 | Homo sapiens teashirt zinc finger homeobox 1 (TSHZ1), mRNA. |
| SLC10A4 | -1.52 | Homo sapiens solute carrier family 10 (sodium/bile acid cotransporter family), member 4 (SLC10A4), mRNA. |
| ZNF318 | -1.52 | Homo sapiens zinc finger protein 318 (ZNF318), mRNA. |
| CEP152 | -1.52 | Homo sapiens centrosomal protein 152kDa (CEP152), mRNA. |
| C1QTNF1 | -1.52 | Homo sapiens C1q and tumor necrosis factor related protein 1 (C1QTNF1), mRNA. |
| GALNT1 | -1.52 | Homo sapiens UDP-N-acetyl-alpha-D-galactosamine:polypeptide N-acetylgalactosaminyltransferase 1 (GalNAc-T1) (GALNT1), mRNA. |
| LOC100133430 | -1.52 | PREDICTED: Homo sapiens similar to hCG1640620 (LOC100133430), mRNA. |
| DOLK | -1.52 | Homo sapiens dolichol kinase (DOLK), mRNA. |
| CBFB | -1.52 | Homo sapiens core-binding factor, beta subunit (CBFB), transcript variant 2, mRNA. |
| LOC100132457 | -1.52 | PREDICTED: Homo sapiens similar to Sm protein G (LOC100132457), mRNA. |
| PAN3 | -1.53 | Homo sapiens PAN3 polyA specific ribonuclease subunit homolog (S. cerevisiae) (PAN3), mRNA. |
| APOBEC3F | -1.53 | Homo sapiens apolipoprotein B mRNA editing enzyme, catalytic polypeptide-like 3F (APOBEC3F), transcript variant 2, mRNA. |
|  | -1.53 | Homo sapiens cDNA FLJ38153 fis, clone DFNES1000083 |
| SBF2 | -1.53 | Homo sapiens SET binding factor 2 (SBF2), mRNA. |
| FZD1 | -1.53 | Homo sapiens frizzled homolog 1 (Drosophila) (FZD1), mRNA. |
| PIGO | -1.53 | Homo sapiens phosphatidylinositol glycan anchor biosynthesis, class O (PIGO), transcript variant 2, mRNA. |
| PPCS | -1.53 | Homo sapiens phosphopantothenoylcysteine synthetase (PPCS), transcript variant 2, mRNA. |
| MMGT1 | -1.53 | Homo sapiens membrane magnesium transporter 1 (MMGT1), mRNA. |
| RAD51AP1 | -1.53 | Homo sapiens RAD51 associated protein 1 (RAD51AP1), mRNA. |
| BBC3 | -1.53 | Homo sapiens BCL2 binding component 3 (BBC3), mRNA. |
| ELAC1 | -1.53 | Homo sapiens elaC homolog 1 (E. coli) (ELAC1), mRNA. |
| DUSP12 | -1.53 | Homo sapiens dual specificity phosphatase 12 (DUSP12), mRNA. |
| KPNA3 | -1.53 | Homo sapiens karyopherin alpha 3 (importin alpha 4) (KPNA3), mRNA. |
| LOC100133185 | -1.53 | PREDICTED: Homo sapiens misc_RNA (LOC100133185), miscRNA. |
| SS18 | -1.53 | Homo sapiens synovial sarcoma translocation, chromosome 18 (SS18), transcript variant 2, mRNA. |
| RNF123 | -1.53 | Homo sapiens ring finger protein 123 (RNF123), mRNA. |
| KIF26A | -1.53 | Homo sapiens kinesin family member 26A (KIF26A), mRNA. |
| TSPAN7 | -1.53 | Homo sapiens tetraspanin 7 (TSPAN7), mRNA. |
| LOC150568 | -1.53 | Homo sapiens hypothetical LOC150568 (LOC150568), non-coding RNA. |
| LOC654074 | -1.53 | PREDICTED: Homo sapiens similar to heterogeneous nuclear ribonucleoprotein C isoform b, transcript variant 1 (LOC654074), mRNA. |
| PATE2 | -1.54 | Homo sapiens prostate and testis expressed 2 (PATE2), mRNA. |
| FABP5L2 | -1.54 | PREDICTED: Homo sapiens fatty acid binding protein 5-like 2 (FABP5L2), mRNA. |
| LOC401076 | -1.54 | PREDICTED: Homo sapiens misc_RNA (LOC401076), miscRNA. |
| C19orf28 | -1.54 | Homo sapiens chromosome 19 open reading frame 28 (C19orf28), transcript variant 3, mRNA. |
|  | -1.54 | BX107250 Soares_pregnant_uterus_NbHPU Homo sapiens cDNA clone IMAGp998M081119, mRNA sequence |
| KIAA0564 | -1.54 | Homo sapiens KIAA0564 (KIAA0564), transcript variant 1, mRNA. |
| RXRA | -1.54 | Homo sapiens retinoid X receptor, alpha (RXRA), mRNA. |
| ASNS | -1.54 | Homo sapiens asparagine synthetase (ASNS), transcript variant 1, mRNA. |
| TNPO1 | -1.54 | Homo sapiens transportin 1 (TNPO1), transcript variant 2, mRNA. |
| ITM2A | -1.54 | Homo sapiens integral membrane protein 2A (ITM2A), mRNA. |
| MAST3 | -1.54 | Homo sapiens microtubule associated serine/threonine kinase 3 (MAST3), mRNA. |
| MIR1185-1 | -1.54 | Homo sapiens microRNA 1185-1 (MIR1185-1), microRNA. |
| KIAA0564 | -1.54 | Homo sapiens KIAA0564 (KIAA0564), transcript variant 1, mRNA. |
| FDPS | -1.55 | Homo sapiens farnesyl diphosphate synthase (farnesyl pyrophosphate synthetase, dimethylallyltranstransferase, geranyltranstransferase) (FDPS), mRNA. |
| B3GAT3 | -1.55 | Homo sapiens beta-1,3-glucuronyltransferase 3 (glucuronosyltransferase I) (B3GAT3), mRNA. |
| SNW1 | -1.55 | Homo sapiens SNW domain containing 1 (SNW1), mRNA. |
| 5-Mar | -1.55 | Homo sapiens membrane-associated ring finger (C3HC4) 5 (MARCH5), mRNA. |
| NACAD | -1.55 | PREDICTED: Homo sapiens NAC alpha domain containing (NACAD), mRNA. |
| TSTD1 | -1.55 | Homo sapiens thiosulfate sulfurtransferase (rhodanese)-like domain containing 1 (TSTD1), transcript variant 2, mRNA. |
| MGC2752 | -1.55 | Homo sapiens hypothetical protein MGC2752 (MGC2752), mRNA. |
| IGSF3 | -1.55 | Homo sapiens immunoglobulin superfamily, member 3 (IGSF3), transcript variant 1, mRNA. |
| ANKRD20A1 | -1.55 | Homo sapiens ankyrin repeat domain 20 family, member A1 (ANKRD20A1), mRNA. |
| LSM12 | -1.55 | Homo sapiens LSM12 homolog (S. cerevisiae) (LSM12), mRNA. |
| ZNF618 | -1.55 | Homo sapiens zinc finger protein 618 (ZNF618), mRNA. |
| C10orf76 | -1.55 | Homo sapiens chromosome 10 open reading frame 76 (C10orf76), mRNA. |
| VPS52 | -1.56 | Homo sapiens vacuolar protein sorting 52 homolog (S. cerevisiae) (VPS52), mRNA. |
| RUFY3 | -1.56 | Homo sapiens RUN and FYVE domain containing 3 (RUFY3), transcript variant 2, mRNA. |
| PARP2 | -1.56 | Homo sapiens poly (ADP-ribose) polymerase family, member 2 (PARP2), mRNA. |
| C21orf66 | -1.56 | Homo sapiens chromosome 21 open reading frame 66 (C21orf66), transcript variant 2, mRNA. |
| FTHL11 | -1.56 | Homo sapiens ferritin, heavy polypeptide-like 11 (FTHL11) on chromosome 8. |
| C11orf57 | -1.56 | Homo sapiens chromosome 11 open reading frame 57 (C11orf57), transcript variant 1, mRNA. |
| TMTC2 | -1.56 | Homo sapiens transmembrane and tetratricopeptide repeat containing 2 (TMTC2), mRNA. |
| MYD88 | -1.56 | Homo sapiens myeloid differentiation primary response gene (88) (MYD88), mRNA. |
| CCNA2 | -1.56 | Homo sapiens cyclin A2 (CCNA2), mRNA. |
| ZNF536 | -1.56 | Homo sapiens zinc finger protein 536 (ZNF536), mRNA. |
| AFF4 | -1.56 | Homo sapiens AF4/FMR2 family, member 4 (AFF4), mRNA. |
| POPDC2 | -1.56 | Homo sapiens popeye domain containing 2 (POPDC2), mRNA. |
| ST6GALNAC4 | -1.56 | Homo sapiens ST6 (alpha-N-acetyl-neuraminyl-2,3-beta-galactosyl-1, 3)-N-acetylgalactosaminide alpha-2,6-sialyltransferase 4 (ST6GALNAC4), transcript variant 1, mRNA. |
| NUP160 | -1.56 | Homo sapiens nucleoporin 160kDa (NUP160), mRNA. |
| MAP3K1 | -1.56 | Homo sapiens mitogen-activated protein kinase kinase kinase 1 (MAP3K1), mRNA. |
| BEND6 | -1.57 | Homo sapiens BEN domain containing 6 (BEND6), mRNA. |
| FAM195A | -1.57 | Homo sapiens family with sequence similarity 195, member A (FAM195A), mRNA. |
| ADAM19 | -1.57 | Homo sapiens ADAM metallopeptidase domain 19 (meltrin beta) (ADAM19), mRNA. |
| NGFRAP1 | -1.57 | Homo sapiens nerve growth factor receptor (TNFRSF16) associated protein 1 (NGFRAP1), transcript variant 3, mRNA. |
| DCLRE1C | -1.57 | Homo sapiens DNA cross-link repair 1C (PSO2 homolog, S. cerevisiae) (DCLRE1C), transcript variant b, mRNA. |
| LOC645466 | -1.57 | PREDICTED: Homo sapiens similar to coiled-coil domain containing 55 (LOC645466), mRNA. |
| LOC729964 | -1.57 | PREDICTED: Homo sapiens hypothetical LOC729964 (LOC729964), mRNA. |
| ZNF75A | -1.57 | Homo sapiens zinc finger protein 75a (ZNF75A), mRNA. |
| EPB41L4A | -1.57 | Homo sapiens erythrocyte membrane protein band 4.1 like 4A (EPB41L4A), mRNA. |
| TOE1 | -1.57 | Homo sapiens target of EGR1, member 1 (nuclear) (TOE1), mRNA. |
| LOC347376 | -1.57 | PREDICTED: Homo sapiens similar to H3 histone, family 3B (LOC347376), mRNA. |
| KIAA0368 | -1.57 | Homo sapiens KIAA0368 (KIAA0368), mRNA. |
| NLGN4X | -1.58 | Homo sapiens neuroligin 4, X-linked (NLGN4X), transcript variant 1, mRNA. |
| SEC24D | -1.58 | Homo sapiens SEC24 related gene family, member D (S. cerevisiae) (SEC24D), mRNA. |
| JPH2 | -1.58 | Homo sapiens junctophilin 2 (JPH2), transcript variant 2, mRNA. |
| ABCB1 | -1.58 | Homo sapiens ATP-binding cassette, sub-family B (MDR/TAP), member 1 (ABCB1), mRNA. |
| ASF1A | -1.58 | Homo sapiens ASF1 anti-silencing function 1 homolog A (S. cerevisiae) (ASF1A), mRNA. |
| UBR4 | -1.58 | Homo sapiens ubiquitin protein ligase E3 component n-recognin 4 (UBR4), mRNA. |
| SMCR5 | -1.58 | Homo sapiens Smith-Magenis syndrome chromosome region, candidate 5 (non-protein coding) (SMCR5), non-coding RNA. |
| AGPAT2 | -1.58 | Homo sapiens 1-acylglycerol-3-phosphate O-acyltransferase 2 (lysophosphatidic acid acyltransferase, beta) (AGPAT2), transcript variant 2, mRNA. |
| SNORD14A | -1.58 | Homo sapiens small nucleolar RNA, C/D box 14A (SNORD14A), small nucleolar RNA. |
| RAPGEF4 | -1.58 | Homo sapiens Rap guanine nucleotide exchange factor (GEF) 4 (RAPGEF4), mRNA. |
| DDX21 | -1.58 | Homo sapiens DEAD (Asp-Glu-Ala-Asp) box polypeptide 21 (DDX21), mRNA. |
| DRAP1 | -1.58 | Homo sapiens DR1-associated protein 1 (negative cofactor 2 alpha) (DRAP1), mRNA. |
| UBXN6 | -1.58 | Homo sapiens UBX domain protein 6 (UBXN6), mRNA. |
| STOM | -1.59 | Homo sapiens stomatin (STOM), transcript variant 1, mRNA. |
| PXN | -1.59 | Homo sapiens paxillin (PXN), mRNA. |
| FLNB | -1.59 | Homo sapiens filamin B, beta (actin binding protein 278) (FLNB), mRNA. |
| AKAP8 | -1.59 | Homo sapiens A kinase (PRKA) anchor protein 8 (AKAP8), mRNA. |
| FLJ10986 | -1.59 | Homo sapiens hypothetical protein FLJ10986 (FLJ10986), mRNA. |
| TCTEX1D2 | -1.59 | Homo sapiens Tctex1 domain containing 2 (TCTEX1D2), mRNA. |
| LOC100132740 | -1.59 | PREDICTED: Homo sapiens hypothetical protein LOC100132740 (LOC100132740), mRNA. |
| SOCS2 | -1.59 | Homo sapiens suppressor of cytokine signaling 2 (SOCS2), mRNA. |
| CHPT1 | -1.59 | Homo sapiens choline phosphotransferase 1 (CHPT1), mRNA. |
| LAPTM4A | -1.59 | Homo sapiens lysosomal-associated protein transmembrane 4 alpha (LAPTM4A), mRNA. |
| ALDH5A1 | -1.59 | Homo sapiens aldehyde dehydrogenase 5 family, member A1 (ALDH5A1), nuclear gene encoding mitochondrial protein, transcript variant 2, mRNA. |
| NARFL | -1.59 | Homo sapiens nuclear prelamin A recognition factor-like (NARFL), mRNA. |
| LOC100130003 | -1.59 | PREDICTED: Homo sapiens misc_RNA (LOC100130003), miscRNA. |
| KIAA0913 | -1.59 | Homo sapiens KIAA0913 (KIAA0913), mRNA. |
| TUBB6 | -1.59 | PREDICTED: Homo sapiens tubulin, beta 6 (TUBB6), mRNA. |
| GARS | -1.59 | Homo sapiens glycyl-tRNA synthetase (GARS), mRNA. |
| MTHFD1L | -1.59 | Homo sapiens methylenetetrahydrofolate dehydrogenase (NADP+ dependent) 1-like (MTHFD1L), mRNA. |
| ERCC3 | -1.59 | Homo sapiens excision repair cross-complementing rodent repair deficiency, complementation group 3 (xeroderma pigmentosum group B complementing) (ERCC3), mRNA. |
| ELF3 | -1.59 | Homo sapiens E74-like factor 3 (ets domain transcription factor, epithelial-specific ) (ELF3), mRNA. |
| SLC25A13 | -1.6 | Homo sapiens solute carrier family 25, member 13 (citrin) (SLC25A13), mRNA. |
| SEZ6 | -1.6 | Homo sapiens seizure related 6 homolog (mouse) (SEZ6), transcript variant 2, mRNA. |
| DYNC1I1 | -1.6 | Homo sapiens dynein, cytoplasmic 1, intermediate chain 1 (DYNC1I1), mRNA. |
| PTPRF | -1.6 | Homo sapiens protein tyrosine phosphatase, receptor type, F (PTPRF), transcript variant 2, mRNA. |
| NBEA | -1.6 | Homo sapiens neurobeachin (NBEA), mRNA. |
| GNG11 | -1.6 | Homo sapiens guanine nucleotide binding protein (G protein), gamma 11 (GNG11), mRNA. |
| BRD8 | -1.6 | Homo sapiens bromodomain containing 8 (BRD8), transcript variant 3, mRNA. |
| LOC100131139 | -1.6 | PREDICTED: Homo sapiens misc_RNA (LOC100131139), miscRNA. |
| NKAP | -1.61 | Homo sapiens NF-kappaB activating protein (NKAP), mRNA. |
| RBMS1 | -1.61 | Homo sapiens RNA binding motif, single stranded interacting protein 1 (RBMS1), transcript variant 3, mRNA. |
|  | -1.61 | Homo sapiens cDNA FLJ26539 fis, clone KDN09310 |
| TIPIN | -1.61 | Homo sapiens TIMELESS interacting protein (TIPIN), mRNA. |
| HMBOX1 | -1.61 | Homo sapiens homeobox containing 1 (HMBOX1), mRNA. |
| LOC100130775 | -1.61 | PREDICTED: Homo sapiens misc_RNA (LOC100130775), miscRNA. |
|  | -1.61 | qm23d08.x1 NCI_CGAP_Lu5 Homo sapiens cDNA clone IMAGE:1882671 3, mRNA sequence |
| NRIP3 | -1.61 | Homo sapiens nuclear receptor interacting protein 3 (NRIP3), mRNA. |
| CPVL | -1.61 | Homo sapiens carboxypeptidase, vitellogenic-like (CPVL), transcript variant 2, mRNA. |
| C13orf25 | -1.61 | PREDICTED: Homo sapiens chromosome 13 open reading frame 25 (C13orf25), mRNA. |
| 3-Sep | -1.61 | Homo sapiens septin 3 (SEPT3), transcript variant B, mRNA. |
| RNF10 | -1.61 | Homo sapiens ring finger protein 10 (RNF10), mRNA. |
| UBAP2 | -1.61 | Homo sapiens ubiquitin associated protein 2 (UBAP2), mRNA. |
| LSP1 | -1.61 | Homo sapiens lymphocyte-specific protein 1 (LSP1), transcript variant 4, mRNA. |
| LOC728115 | -1.61 | PREDICTED: Homo sapiens misc_RNA (LOC728115), miscRNA. |
| BTG2 | -1.61 | Homo sapiens BTG family, member 2 (BTG2), mRNA. |
| C1orf122 | -1.61 | Homo sapiens chromosome 1 open reading frame 122 (C1orf122), mRNA. |
| PSMD14 | -1.61 | Homo sapiens proteasome (prosome, macropain) 26S subunit, non-ATPase, 14 (PSMD14), mRNA. |
|  | -1.61 | CR738291 Homo sapiens library (Ebert L) Homo sapiens cDNA clone IMAGp971D2453 ; IMAGE:811744 5, mRNA sequence |
|  | -1.62 | full-length cDNA clone CS0DM012YE14 of Fetal liver of Homo sapiens (human) |
| MCM3 | -1.62 | Homo sapiens minichromosome maintenance complex component 3 (MCM3), mRNA. |
| GPS1 | -1.62 | Homo sapiens G protein pathway suppressor 1 (GPS1), transcript variant 2, mRNA. |
| C14orf143 | -1.62 | Homo sapiens chromosome 14 open reading frame 143 (C14orf143), mRNA. |
| DNASE1L1 | -1.62 | Homo sapiens deoxyribonuclease I-like 1 (DNASE1L1), transcript variant 4, mRNA. |
| FAM39DP | -1.62 | Homo sapiens family with sequence similarity 39, member D pseudogene (FAM39DP) on chromosome 15. |
| TMEM185B | -1.62 | Homo sapiens transmembrane protein 185B (pseudogene) (TMEM185B), non-coding RNA. |
| CYTH2 | -1.62 | Homo sapiens cytohesin 2 (CYTH2), transcript variant 1, mRNA. |
| N-PAC | -1.62 | Homo sapiens cytokine-like nuclear factor n-pac (N-PAC), mRNA. |
| USP16 | -1.62 | Homo sapiens ubiquitin specific peptidase 16 (USP16), transcript variant 1, mRNA. |
| ME1 | -1.62 | Homo sapiens malic enzyme 1, NADP(+)-dependent, cytosolic (ME1), mRNA. |
| FTHL2 | -1.63 | Homo sapiens ferritin, heavy polypeptide-like 2 (FTHL2) on chromosome 1. |
| MAFB | -1.63 | Homo sapiens v-maf musculoaponeurotic fibrosarcoma oncogene homolog B (avian) (MAFB), mRNA. |
| SERINC1 | -1.63 | Homo sapiens serine incorporator 1 (SERINC1), mRNA. |
| CUX2 | -1.63 | Homo sapiens cut-like homeobox 2 (CUX2), mRNA. |
| GATA3 | -1.63 | Homo sapiens GATA binding protein 3 (GATA3), transcript variant 2, mRNA. |
| C14orf138 | -1.63 | Homo sapiens chromosome 14 open reading frame 138 (C14orf138), transcript variant 1, mRNA. |
| VEGFB | -1.63 | Homo sapiens vascular endothelial growth factor B (VEGFB), mRNA. |
| KIAA0247 | -1.64 | Homo sapiens KIAA0247 (KIAA0247), mRNA. |
| GNA13 | -1.64 | Homo sapiens guanine nucleotide binding protein (G protein), alpha 13 (GNA13), mRNA. |
| LPAR1 | -1.64 | Homo sapiens lysophosphatidic acid receptor 1 (LPAR1), transcript variant 2, mRNA. |
| MRPL2 | -1.64 | Homo sapiens mitochondrial ribosomal protein L2 (MRPL2), nuclear gene encoding mitochondrial protein, mRNA. |
| SEPN1 | -1.64 | Homo sapiens selenoprotein N, 1 (SEPN1), transcript variant 1, mRNA. |
| RUNDC2C | -1.64 | Homo sapiens RUN domain containing 2C (RUNDC2C), non-coding RNA. |
| NAT14 | -1.64 | Homo sapiens N-acetyltransferase 14 (GCN5-related, putative) (NAT14), mRNA. |
| NEK2 | -1.64 | Homo sapiens NIMA (never in mitosis gene a)-related kinase 2 (NEK2), mRNA. |
| AFG3L1 | -1.64 | Homo sapiens AFG3 ATPase family gene 3-like 1 (yeast) (AFG3L1), transcript variant 2, mRNA. |
| SIGMAR1 | -1.64 | Homo sapiens sigma non-opioid intracellular receptor 1 (SIGMAR1), transcript variant 2, mRNA. |
| ALDOA | -1.64 | Homo sapiens aldolase A, fructose-bisphosphate (ALDOA), transcript variant 3, mRNA. |
| ZNF33A | -1.64 | Homo sapiens zinc finger protein 33A (ZNF33A), transcript variant 2, mRNA. |
| LOC728411 | -1.64 | PREDICTED: Homo sapiens similar to Beta-glucuronidase precursor (LOC728411), mRNA. |
| PLP2 | -1.64 | Homo sapiens proteolipid protein 2 (colonic epithelium-enriched) (PLP2), mRNA. |
| SHANK3 | -1.64 | Homo sapiens SH3 and multiple ankyrin repeat domains 3 (SHANK3), mRNA. |
| GTPBP8 | -1.64 | Homo sapiens GTP-binding protein 8 (putative) (GTPBP8), transcript variant 1, mRNA. |
| DYRK2 | -1.64 | Homo sapiens dual-specificity tyrosine-(Y)-phosphorylation regulated kinase 2 (DYRK2), transcript variant 1, mRNA. |
| OSBPL7 | -1.64 | Homo sapiens oxysterol binding protein-like 7 (OSBPL7), transcript variant 1, mRNA. |
| RG9MTD1 | -1.64 | Homo sapiens RNA (guanine-9-) methyltransferase domain containing 1 (RG9MTD1), mRNA. |
|  | -1.64 | CM3-MT0357-260101-690-b10 MT0357 Homo sapiens cDNA, mRNA sequence |
| SLC30A7 | -1.64 | Homo sapiens solute carrier family 30 (zinc transporter), member 7 (SLC30A7), mRNA. |
| CENPE | -1.64 | Homo sapiens centromere protein E, 312kDa (CENPE), mRNA. |
| SORD | -1.65 | Homo sapiens sorbitol dehydrogenase (SORD), mRNA. |
| C13orf27 | -1.65 | Homo sapiens chromosome 13 open reading frame 27 (C13orf27), mRNA. |
| PABPC3 | -1.65 | Homo sapiens poly(A) binding protein, cytoplasmic 3 (PABPC3), mRNA. |
| SCML1 | -1.65 | Homo sapiens sex comb on midleg-like 1 (Drosophila) (SCML1), transcript variant 1, mRNA. |
| ARHGEF17 | -1.65 | Homo sapiens Rho guanine nucleotide exchange factor (GEF) 17 (ARHGEF17), mRNA. |
| LZTS1 | -1.65 | Homo sapiens leucine zipper, putative tumor suppressor 1 (LZTS1), mRNA. |
| CTSL2 | -1.65 | Homo sapiens cathepsin L2 (CTSL2), mRNA. |
| FVT1 | -1.65 | Homo sapiens follicular lymphoma variant translocation 1 (FVT1), mRNA. |
| C6orf125 | -1.65 | Homo sapiens chromosome 6 open reading frame 125 (C6orf125), mRNA. |
| CDCA8 | -1.65 | Homo sapiens cell division cycle associated 8 (CDCA8), mRNA. |
| POLE | -1.65 | Homo sapiens polymerase (DNA directed), epsilon (POLE), mRNA. |
| NCRNA00095 | -1.65 | Homo sapiens non-protein coding RNA 95 (NCRNA00095), non-coding RNA. |
| CCNY | -1.65 | Homo sapiens cyclin Y (CCNY), transcript variant 1, mRNA. |
| SAPS1 | -1.65 | Homo sapiens SAPS domain family, member 1 (SAPS1), mRNA. |
| LOC130773 | -1.66 | PREDICTED: Homo sapiens similar to 60S ribosomal protein L23a (LOC130773), mRNA. |
| MKI67 | -1.66 | Homo sapiens antigen identified by monoclonal antibody Ki-67 (MKI67), mRNA. |
| GPX4 | -1.66 | Homo sapiens glutathione peroxidase 4 (phospholipid hydroperoxidase) (GPX4), transcript variant 2, mRNA. |
| LOC729255 | -1.66 | PREDICTED: Homo sapiens misc_RNA (LOC729255), miscRNA. |
| LOC644330 | -1.66 | PREDICTED: Homo sapiens similar to tropomyosin 3 isoform 2 (LOC644330), mRNA. |
| DENR | -1.66 | Homo sapiens density-regulated protein (DENR), mRNA. |
| LPHN2 | -1.66 | Homo sapiens latrophilin 2 (LPHN2), mRNA. |
| C12orf32 | -1.66 | Homo sapiens chromosome 12 open reading frame 32 (C12orf32), mRNA. |
| FEZ2 | -1.66 | Homo sapiens fasciculation and elongation protein zeta 2 (zygin II) (FEZ2), transcript variant 1, mRNA. |
| MOBKL3 | -1.66 | Homo sapiens MOB1, Mps One Binder kinase activator-like 3 (yeast) (MOBKL3), transcript variant 2, mRNA. |
| ATP11C | -1.66 | Homo sapiens ATPase, class VI, type 11C (ATP11C), transcript variant 2, mRNA. |
| MCM10 | -1.66 | Homo sapiens minichromosome maintenance complex component 10 (MCM10), transcript variant 2, mRNA. |
| MAP2K4 | -1.66 | Homo sapiens mitogen-activated protein kinase kinase 4 (MAP2K4), mRNA. |
| OLFM1 | -1.67 | Homo sapiens olfactomedin 1 (OLFM1), transcript variant 2, mRNA. |
| SH2B3 | -1.67 | Homo sapiens SH2B adaptor protein 3 (SH2B3), mRNA. |
| ERC1 | -1.67 | Homo sapiens ELKS/RAB6-interacting/CAST family member 1 (ERC1), transcript variant beta, mRNA. |
| LOC92249 | -1.67 | Homo sapiens hypothetical LOC92249 (LOC92249), non-coding RNA. |
| PIP5K1C | -1.67 | Homo sapiens phosphatidylinositol-4-phosphate 5-kinase, type I, gamma (PIP5K1C), mRNA. |
| LOC731878 | -1.67 | PREDICTED: Homo sapiens similar to MAPK-interacting and spindle-stabilizing protein (LOC731878), mRNA. |
| ZNF24 | -1.67 | Homo sapiens zinc finger protein 24 (ZNF24), mRNA. |
| RBM16 | -1.67 | Homo sapiens RNA binding motif protein 16 (RBM16), mRNA. |
| CRY2 | -1.67 | Homo sapiens cryptochrome 2 (photolyase-like) (CRY2), mRNA. |
| FBXW5 | -1.67 | Homo sapiens F-box and WD repeat domain containing 5 (FBXW5), mRNA. |
| CDKN1B | -1.67 | Homo sapiens cyclin-dependent kinase inhibitor 1B (p27, Kip1) (CDKN1B), mRNA. |
| FNDC4 | -1.67 | Homo sapiens fibronectin type III domain containing 4 (FNDC4), mRNA. |
| LOC389787 | -1.67 | PREDICTED: Homo sapiens similar to Translationally-controlled tumor protein (TCTP) (p23) (Histamine-releasing factor) (HRF) (Fortilin) (LOC389787), mRNA. |
| RPS6KB1 | -1.67 | Homo sapiens ribosomal protein S6 kinase, 70kDa, polypeptide 1 (RPS6KB1), mRNA. |
| CDK10 | -1.67 | Homo sapiens cyclin-dependent kinase 10 (CDK10), transcript variant c, mRNA. |
| XPA | -1.67 | Homo sapiens xeroderma pigmentosum, complementation group A (XPA), mRNA. |
| DHX30 | -1.67 | Homo sapiens DEAH (Asp-Glu-Ala-His) box polypeptide 30 (DHX30), transcript variant 2, mRNA. |
| YDJC | -1.67 | Homo sapiens YdjC homolog (bacterial) (YDJC), mRNA. |
| VAC14 | -1.67 | Homo sapiens Vac14 homolog (S. cerevisiae) (VAC14), mRNA. |
| BRD1 | -1.67 | Homo sapiens bromodomain containing 1 (BRD1), mRNA. |
| MBD3 | -1.68 | Homo sapiens methyl-CpG binding domain protein 3 (MBD3), mRNA. |
| SIGMAR1 | -1.68 | Homo sapiens sigma non-opioid intracellular receptor 1 (SIGMAR1), transcript variant 2, mRNA. |
| PRIM1 | -1.68 | Homo sapiens primase, DNA, polypeptide 1 (49kDa) (PRIM1), mRNA. |
| TPI1 | -1.68 | Homo sapiens triosephosphate isomerase 1 (TPI1), mRNA. |
| SPARC | -1.68 | Homo sapiens secreted protein, acidic, cysteine-rich (osteonectin) (SPARC), mRNA. |
| NRXN2 | -1.68 | Homo sapiens neurexin 2 (NRXN2), transcript variant beta, mRNA. |
| SLC29A1 | -1.68 | Homo sapiens solute carrier family 29 (nucleoside transporters), member 1 (SLC29A1), nuclear gene encoding mitochondrial protein, transcript variant 4, mRNA. |
| SHISA5 | -1.68 | Homo sapiens shisa homolog 5 (Xenopus laevis) (SHISA5), mRNA. |
| DHTKD1 | -1.68 | Homo sapiens dehydrogenase E1 and transketolase domain containing 1 (DHTKD1), mRNA. |
| DLGAP5 | -1.68 | Homo sapiens discs, large (Drosophila) homolog-associated protein 5 (DLGAP5), mRNA. |
| CREB3L4 | -1.68 | Homo sapiens cAMP responsive element binding protein 3-like 4 (CREB3L4), mRNA. |
| LCOR | -1.68 | Homo sapiens ligand dependent nuclear receptor corepressor (LCOR), mRNA. |
| BCOR | -1.69 | Homo sapiens BCL6 co-repressor (BCOR), transcript variant 1, mRNA. |
| ABCB6 | -1.69 | Homo sapiens ATP-binding cassette, sub-family B (MDR/TAP), member 6 (ABCB6), nuclear gene encoding mitochondrial protein, mRNA. |
| BTBD3 | -1.69 | Homo sapiens BTB (POZ) domain containing 3 (BTBD3), transcript variant 1, mRNA. |
| EHMT2 | -1.69 | Homo sapiens euchromatic histone-lysine N-methyltransferase 2 (EHMT2), transcript variant NG36/G9a-SPI, mRNA. |
| PABPC5 | -1.69 | Homo sapiens poly(A) binding protein, cytoplasmic 5 (PABPC5), mRNA. |
| MGC40489 | -1.69 | PREDICTED: Homo sapiens hypothetical protein MGC40489 (MGC40489), misc RNA. |
| ZCCHC24 | -1.7 | Homo sapiens zinc finger, CCHC domain containing 24 (ZCCHC24), mRNA. |
| ZNF502 | -1.7 | Homo sapiens zinc finger protein 502 (ZNF502), mRNA. |
| MBNL1 | -1.7 | Homo sapiens muscleblind-like (Drosophila) (MBNL1), transcript variant 5, mRNA. |
| MED9 | -1.7 | Homo sapiens mediator complex subunit 9 (MED9), mRNA. |
| FCGR3B | -1.7 | Homo sapiens Fc fragment of IgG, low affinity IIIb, receptor (CD16b) (FCGR3B), mRNA. |
| SMARCA5 | -1.7 | Homo sapiens SWI/SNF related, matrix associated, actin dependent regulator of chromatin, subfamily a, member 5 (SMARCA5), mRNA. |
| HLA-F | -1.7 | Homo sapiens major histocompatibility complex, class I, F (HLA-F), mRNA. |
| PCDHB6 | -1.7 | Homo sapiens protocadherin beta 6 (PCDHB6), mRNA. |
| CHPT1 | -1.7 | Homo sapiens choline phosphotransferase 1 (CHPT1), mRNA. |
| RERG | -1.7 | Homo sapiens RAS-like, estrogen-regulated, growth inhibitor (RERG), mRNA. |
| C2orf24 | -1.7 | Homo sapiens chromosome 2 open reading frame 24 (C2orf24), mRNA. |
| RNF112 | -1.7 | Homo sapiens ring finger protein 112 (RNF112), mRNA. |
| TADA3 | -1.7 | Homo sapiens transcriptional adaptor 3 (TADA3), transcript variant 1, mRNA. |
| ZFHX3 | -1.71 | Homo sapiens zinc finger homeobox 3 (ZFHX3), transcript variant A, mRNA. |
| TUBGCP6 | -1.71 | Homo sapiens tubulin, gamma complex associated protein 6 (TUBGCP6), mRNA. |
| RDH11 | -1.71 | Homo sapiens retinol dehydrogenase 11 (all-trans/9-cis/11-cis) (RDH11), mRNA. |
| CACHD1 | -1.71 | Homo sapiens cache domain containing 1 (CACHD1), mRNA. |
| LOC729208 | -1.71 | PREDICTED: Homo sapiens misc_RNA (LOC729208), miscRNA. |
| ST6GALNAC3 | -1.71 | Homo sapiens ST6 (alpha-N-acetyl-neuraminyl-2,3-beta-galactosyl-1, 3)-N-acetylgalactosaminide alpha-2,6-sialyltransferase 3 (ST6GALNAC3), mRNA. |
| MYO1C | -1.71 | Homo sapiens myosin IC (MYO1C), transcript variant 3, mRNA. |
| NOC2L | -1.71 | Homo sapiens nucleolar complex associated 2 homolog (S. cerevisiae) (NOC2L), mRNA. |
| CPSF3L | -1.71 | Homo sapiens cleavage and polyadenylation specific factor 3-like (CPSF3L), mRNA. |
| TM9SF4 | -1.71 | Homo sapiens transmembrane 9 superfamily protein member 4 (TM9SF4), mRNA. |
| FGGY | -1.71 | Homo sapiens FGGY carbohydrate kinase domain containing (FGGY), transcript variant 1, mRNA. |
| C5orf41 | -1.71 | Homo sapiens chromosome 5 open reading frame 41 (C5orf41), mRNA. |
| ODZ3 | -1.71 | Homo sapiens odz, odd Oz/ten-m homolog 3 (Drosophila) (ODZ3), mRNA. |
| WDYHV1 | -1.71 | Homo sapiens WDYHV motif containing 1 (WDYHV1), mRNA. |
| PHAX | -1.71 | Homo sapiens phosphorylated adaptor for RNA export (PHAX), mRNA. |
| C7orf36 | -1.71 | Homo sapiens chromosome 7 open reading frame 36 (C7orf36), mRNA. |
| PPP1R12C | -1.71 | Homo sapiens protein phosphatase 1, regulatory (inhibitor) subunit 12C (PPP1R12C), mRNA. |
| NDUFA13 | -1.71 | Homo sapiens NADH dehydrogenase (ubiquinone) 1 alpha subcomplex, 13 (NDUFA13), mRNA. |
| CCDC74A | -1.71 | Homo sapiens coiled-coil domain containing 74A (CCDC74A), mRNA. |
| SYT4 | -1.71 | Homo sapiens synaptotagmin IV (SYT4), mRNA. |
| LOC100132547 | -1.72 | PREDICTED: Homo sapiens misc_RNA (LOC100132547), miscRNA. |
| SLC9A5 | -1.72 | Homo sapiens solute carrier family 9 (sodium/hydrogen exchanger), member 5 (SLC9A5), mRNA. |
| TRIM9 | -1.72 | Homo sapiens tripartite motif-containing 9 (TRIM9), transcript variant 1, mRNA. |
| RAB11FIP2 | -1.72 | Homo sapiens RAB11 family interacting protein 2 (class I) (RAB11FIP2), mRNA. |
| AMOT | -1.72 | Homo sapiens angiomotin (AMOT), transcript variant 2, mRNA. |
| FTHL12 | -1.72 | Homo sapiens ferritin, heavy polypeptide-like 12 (FTHL12) on chromosome 9. |
| FAM171A1 | -1.72 | Homo sapiens family with sequence similarity 171, member A1 (FAM171A1), mRNA. |
| ZNF281 | -1.72 | Homo sapiens zinc finger protein 281 (ZNF281), mRNA. |
| DSTN | -1.72 | Homo sapiens destrin (actin depolymerizing factor) (DSTN), transcript variant 1, mRNA. |
| OCIAD1 | -1.72 | Homo sapiens OCIA domain containing 1 (OCIAD1), mRNA. |
| NAV1 | -1.72 | Homo sapiens neuron navigator 1 (NAV1), mRNA. |
| MAD1L1 | -1.73 | Homo sapiens MAD1 mitotic arrest deficient-like 1 (yeast) (MAD1L1), transcript variant 2, mRNA. |
| ELOVL5 | -1.73 | Homo sapiens ELOVL family member 5, elongation of long chain fatty acids (FEN1/Elo2, SUR4/Elo3-like, yeast) (ELOVL5), mRNA. |
| R3HDM1 | -1.73 | Homo sapiens R3H domain containing 1 (R3HDM1), mRNA. |
| PPM2C | -1.73 | Homo sapiens protein phosphatase 2C, magnesium-dependent, catalytic subunit (PPM2C), nuclear gene encoding mitochondrial protein, mRNA. |
| CYTH3 | -1.73 | Homo sapiens cytohesin 3 (CYTH3), mRNA. |
| DEDD | -1.73 | Homo sapiens death effector domain containing (DEDD), transcript variant 4, mRNA. |
| OGDHL | -1.73 | Homo sapiens oxoglutarate dehydrogenase-like (OGDHL), mRNA. |
| LOC653375 | -1.73 | PREDICTED: Homo sapiens RCC1-like G exchanging factor-like (LOC653375), mRNA. |
| ING3 | -1.74 | Homo sapiens inhibitor of growth family, member 3 (ING3), transcript variant 1, mRNA. |
| PDPR | -1.74 | PREDICTED: Homo sapiens pyruvate dehydrogenase phosphatase regulatory subunit (PDPR), mRNA. |
| C1orf97 | -1.74 | Homo sapiens chromosome 1 open reading frame 97 (C1orf97), mRNA. |
| 6-Mar | -1.74 | Homo sapiens membrane-associated ring finger (C3HC4) 6 (MARCH6), mRNA. |
| FAM49B | -1.74 | Homo sapiens family with sequence similarity 49, member B (FAM49B), mRNA. |
| HMGXB4 | -1.74 | Homo sapiens HMG box domain containing 4 (HMGXB4), transcript variant 1, mRNA. |
| FRMD6 | -1.74 | Homo sapiens FERM domain containing 6 (FRMD6), mRNA. |
| HUWE1 | -1.74 | Homo sapiens HECT, UBA and WWE domain containing 1 (HUWE1), mRNA. |
| ICA1 | -1.74 | Homo sapiens islet cell autoantigen 1, 69kDa (ICA1), transcript variant 3, mRNA. |
| PCNA | -1.74 | Homo sapiens proliferating cell nuclear antigen (PCNA), transcript variant 2, mRNA. |
| TBC1D22A | -1.74 | Homo sapiens TBC1 domain family, member 22A (TBC1D22A), mRNA. |
| NBPF3 | -1.74 | Homo sapiens neuroblastoma breakpoint family, member 3 (NBPF3), mRNA. |
| SERPINB1 | -1.74 | Homo sapiens serpin peptidase inhibitor, clade B (ovalbumin), member 1 (SERPINB1), mRNA. |
| LOC127295 | -1.74 | PREDICTED: Homo sapiens similar to 60S ribosomal protein L36 (LOC127295), mRNA. |
| SPIRE2 | -1.74 | Homo sapiens spire homolog 2 (Drosophila) (SPIRE2), mRNA. |
| FAM119A | -1.74 | Homo sapiens family with sequence similarity 119, member A (FAM119A), mRNA. |
| TOR1A | -1.74 | Homo sapiens torsin family 1, member A (torsin A) (TOR1A), mRNA. |
| LOC402644 | -1.75 | PREDICTED: Homo sapiens similar to peptidylprolyl isomerase A isoform 1 (LOC402644), mRNA. |
| RIMS2 | -1.75 | Homo sapiens regulating synaptic membrane exocytosis 2 (RIMS2), transcript variant 2, mRNA. |
| CDC7 | -1.75 | Homo sapiens cell division cycle 7 homolog (S. cerevisiae) (CDC7), mRNA. |
| RPN2 | -1.75 | Homo sapiens ribophorin II (RPN2), mRNA. |
| CYFIP1 | -1.75 | Homo sapiens cytoplasmic FMR1 interacting protein 1 (CYFIP1), transcript variant 2, mRNA. |
| MCM5 | -1.75 | Homo sapiens minichromosome maintenance complex component 5 (MCM5), mRNA. |
| LOC654260 | -1.75 | PREDICTED: Homo sapiens similar to beta-tubulin cofactor D isoform 1 (LOC654260), mRNA. |
| PTPRN2 | -1.75 | Homo sapiens protein tyrosine phosphatase, receptor type, N polypeptide 2 (PTPRN2), transcript variant 2, mRNA. |
| RPS6KA5 | -1.75 | Homo sapiens ribosomal protein S6 kinase, 90kDa, polypeptide 5 (RPS6KA5), transcript variant 1, mRNA. |
| ING2 | -1.75 | Homo sapiens inhibitor of growth family, member 2 (ING2), mRNA. |
| MGC70857 | -1.75 | Homo sapiens similar to RIKEN cDNA C030006K11 gene (MGC70857), mRNA. |
| LOC727761 | -1.75 | PREDICTED: Homo sapiens similar to Deoxythymidylate kinase (thymidylate kinase), transcript variant 4 (LOC727761), mRNA. |
|  | -1.75 | AV700714 GKC Homo sapiens cDNA clone GKCAEH11 3, mRNA sequence |
| SLC25A29 | -1.76 | Homo sapiens solute carrier family 25, member 29 (SLC25A29), nuclear gene encoding mitochondrial protein, mRNA. |
| RPL26L1 | -1.76 | Homo sapiens ribosomal protein L26-like 1 (RPL26L1), mRNA. |
| AGXT2L2 | -1.76 | Homo sapiens alanine-glyoxylate aminotransferase 2-like 2 (AGXT2L2), mRNA. |
| AURKA | -1.76 | Homo sapiens aurora kinase A (AURKA), transcript variant 3, mRNA. |
| NPLOC4 | -1.76 | Homo sapiens nuclear protein localization 4 homolog (S. cerevisiae) (NPLOC4), mRNA. |
| ALDH16A1 | -1.76 | Homo sapiens aldehyde dehydrogenase 16 family, member A1 (ALDH16A1), mRNA. |
| PIAS3 | -1.76 | Homo sapiens protein inhibitor of activated STAT, 3 (PIAS3), mRNA. |
| ARHGEF1 | -1.76 | Homo sapiens Rho guanine nucleotide exchange factor (GEF) 1 (ARHGEF1), transcript variant 1, mRNA. |
| VLDLR | -1.76 | Homo sapiens very low density lipoprotein receptor (VLDLR), transcript variant 2, mRNA. |
| TNFRSF1A | -1.76 | Homo sapiens tumor necrosis factor receptor superfamily, member 1A (TNFRSF1A), mRNA. |
| LOC100130353 | -1.76 | PREDICTED: Homo sapiens hypothetical protein LOC100130353 (LOC100130353), mRNA. |
| LOC387934 | -1.76 | PREDICTED: Homo sapiens similar to Fatty acid-binding protein, epidermal (E-FABP) (Psoriasis-associated fatty acid-binding protein homolog) (PA-FABP) (LOC387934), mRNA. |
| C10orf32 | -1.76 | Homo sapiens chromosome 10 open reading frame 32 (C10orf32), mRNA. |
| USP13 | -1.77 | Homo sapiens ubiquitin specific peptidase 13 (isopeptidase T-3) (USP13), mRNA. |
|  | -1.77 | AV737943 CB Homo sapiens cDNA clone CBDAEG06 5, mRNA sequence |
| HMMR | -1.77 | Homo sapiens hyaluronan-mediated motility receptor (RHAMM) (HMMR), transcript variant 1, mRNA. |
| C17orf96 | -1.77 | Homo sapiens chromosome 17 open reading frame 96 (C17orf96), mRNA. |
| FAT1 | -1.77 | Homo sapiens FAT tumor suppressor homolog 1 (Drosophila) (FAT1), mRNA. |
| LOC651576 | -1.77 | PREDICTED: Homo sapiens similar to tubulin, alpha 8 like, transcript variant 2 (LOC651576), mRNA. |
| MGC26356 | -1.77 | PREDICTED: Homo sapiens misc_RNA (MGC26356), miscRNA. |
| PCDHB10 | -1.77 | Homo sapiens protocadherin beta 10 (PCDHB10), mRNA. |
| C6orf62 | -1.77 | Homo sapiens chromosome 6 open reading frame 62 (C6orf62), mRNA. |
| SLC12A2 | -1.77 | Homo sapiens solute carrier family 12 (sodium/potassium/chloride transporters), member 2 (SLC12A2), mRNA. |
| GTF3A | -1.77 | Homo sapiens general transcription factor IIIA (GTF3A), mRNA. |
| PRAF2 | -1.77 | Homo sapiens PRA1 domain family, member 2 (PRAF2), mRNA. |
| NUMA1 | -1.77 | Homo sapiens nuclear mitotic apparatus protein 1 (NUMA1), mRNA. |
| TSPO | -1.77 | Homo sapiens translocator protein (18kDa) (TSPO), transcript variant PBR-S, mRNA. |
| PIK3C2A | -1.77 | Homo sapiens phosphoinositide-3-kinase, class 2, alpha polypeptide (PIK3C2A), mRNA. |
| LOC91431 | -1.77 | Homo sapiens prematurely terminated mRNA decay factor-like (LOC91431), mRNA. |
| BUB3 | -1.77 | Homo sapiens BUB3 budding uninhibited by benzimidazoles 3 homolog (yeast) (BUB3), transcript variant 2, mRNA. |
| C6orf170 | -1.77 | Homo sapiens chromosome 6 open reading frame 170 (C6orf170), mRNA. |
| MYST2 | -1.77 | Homo sapiens MYST histone acetyltransferase 2 (MYST2), mRNA. |
| TIMM9 | -1.77 | Homo sapiens translocase of inner mitochondrial membrane 9 homolog (yeast) (TIMM9), nuclear gene encoding mitochondrial protein, mRNA. |
| RHBDD3 | -1.78 | Homo sapiens rhomboid domain containing 3 (RHBDD3), mRNA. |
| OIP5 | -1.78 | Homo sapiens Opa interacting protein 5 (OIP5), mRNA. |
| LOC643949 | -1.78 | PREDICTED: Homo sapiens similar to 60S acidic ribosomal protein P2 (LOC643949), mRNA. |
| TUSC1 | -1.78 | Homo sapiens tumor suppressor candidate 1 (TUSC1), mRNA. |
| C14orf100 | -1.78 | Homo sapiens chromosome 14 open reading frame 100 (C14orf100), mRNA. |
| ARAP3 | -1.78 | Homo sapiens ArfGAP with RhoGAP domain, ankyrin repeat and PH domain 3 (ARAP3), mRNA. |
| ADRM1 | -1.78 | Homo sapiens adhesion regulating molecule 1 (ADRM1), transcript variant 2, mRNA. |
| PRMT6 | -1.78 | Homo sapiens protein arginine methyltransferase 6 (PRMT6), mRNA. |
| PON2 | -1.78 | Homo sapiens paraoxonase 2 (PON2), transcript variant 1, mRNA. |
| RERE | -1.78 | Homo sapiens arginine-glutamic acid dipeptide (RE) repeats (RERE), transcript variant 3, mRNA. |
| C6orf111 | -1.78 | Homo sapiens chromosome 6 open reading frame 111 (C6orf111), mRNA. |
| TRMT6 | -1.79 | Homo sapiens tRNA methyltransferase 6 homolog (S. cerevisiae) (TRMT6), mRNA. |
| BRPF1 | -1.79 | Homo sapiens bromodomain and PHD finger containing, 1 (BRPF1), transcript variant 1, mRNA. |
| SEMA6C | -1.79 | Homo sapiens sema domain, transmembrane domain (TM), and cytoplasmic domain, (semaphorin) 6C (SEMA6C), mRNA. |
| UBL7 | -1.79 | Homo sapiens ubiquitin-like 7 (bone marrow stromal cell-derived) (UBL7), transcript variant 2, mRNA. |
| BRCA1 | -1.79 | Homo sapiens breast cancer 1, early onset (BRCA1), transcript variant BRCA1-delta14-17, mRNA. |
| LOC338799 | -1.79 | PREDICTED: Homo sapiens hypothetical locus LOC338799, transcript variant 3 (LOC338799), misc RNA. |
| KIAA1545 | -1.79 | PREDICTED: Homo sapiens KIAA1545 protein (KIAA1545), mRNA. |
| SLC36A4 | -1.79 | Homo sapiens solute carrier family 36 (proton/amino acid symporter), member 4 (SLC36A4), mRNA. |
| SLC9A8 | -1.79 | Homo sapiens solute carrier family 9 (sodium/hydrogen exchanger), member 8 (SLC9A8), mRNA. |
| LRP10 | -1.79 | Homo sapiens low density lipoprotein receptor-related protein 10 (LRP10), mRNA. |
| NOL8 | -1.79 | Homo sapiens nucleolar protein 8 (NOL8), mRNA. |
| YPEL1 | -1.79 | Homo sapiens yippee-like 1 (Drosophila) (YPEL1), mRNA. |
| IER5 | -1.79 | Homo sapiens immediate early response 5 (IER5), mRNA. |
| KIFC2 | -1.79 | Homo sapiens kinesin family member C2 (KIFC2), mRNA. |
| ROD1 | -1.8 | Homo sapiens ROD1 regulator of differentiation 1 (S. pombe) (ROD1), mRNA. |
| ZNF804A | -1.8 | Homo sapiens zinc finger protein 804A (ZNF804A), mRNA. |
| PHKB | -1.8 | Homo sapiens phosphorylase kinase, beta (PHKB), transcript variant 1, mRNA. |
| FAM110A | -1.8 | Homo sapiens family with sequence similarity 110, member A (FAM110A), transcript variant 3, mRNA. |
| SETDB1 | -1.8 | Homo sapiens SET domain, bifurcated 1 (SETDB1), mRNA. |
| NCAN | -1.8 | Homo sapiens neurocan (NCAN), mRNA. |
| MCF2L | -1.8 | Homo sapiens MCF.2 cell line derived transforming sequence-like (MCF2L), transcript variant 1, mRNA. |
| MAZ | -1.8 | Homo sapiens MYC-associated zinc finger protein (purine-binding transcription factor) (MAZ), transcript variant 2, mRNA. |
| GMNN | -1.81 | Homo sapiens geminin, DNA replication inhibitor (GMNN), mRNA. |
| MT1F | -1.81 | Homo sapiens metallothionein 1F (MT1F), mRNA. |
|  | -1.81 | BX393727 Homo sapiens NEUROBLASTOMA COT 25-NORMALIZED Homo sapiens cDNA clone CS0DC001YP02 5-PRIME, mRNA sequence |
| PPP1R2 | -1.81 | Homo sapiens protein phosphatase 1, regulatory (inhibitor) subunit 2 (PPP1R2), mRNA. |
| HDAC6 | -1.81 | Homo sapiens histone deacetylase 6 (HDAC6), mRNA. |
| CCDC25 | -1.81 | Homo sapiens coiled-coil domain containing 25 (CCDC25), mRNA. |
| GALNT3 | -1.81 | Homo sapiens UDP-N-acetyl-alpha-D-galactosamine:polypeptide N-acetylgalactosaminyltransferase 3 (GalNAc-T3) (GALNT3), mRNA. |
| KIAA1522 | -1.81 | Homo sapiens KIAA1522 (KIAA1522), mRNA. |
| RPGR | -1.81 | Homo sapiens retinitis pigmentosa GTPase regulator (RPGR), transcript variant B, mRNA. |
| RAB11FIP2 | -1.81 | Homo sapiens RAB11 family interacting protein 2 (class I) (RAB11FIP2), mRNA. |
| SIRT2 | -1.81 | Homo sapiens sirtuin (silent mating type information regulation 2 homolog) 2 (S. cerevisiae) (SIRT2), transcript variant 1, mRNA. |
| MVK | -1.81 | Homo sapiens mevalonate kinase (mevalonic aciduria) (MVK), mRNA. |
| R3HDM2 | -1.81 | Homo sapiens R3H domain containing 2 (R3HDM2), mRNA. |
| ZNF738 | -1.81 | PREDICTED: Homo sapiens misc_RNA (ZNF738), partial miscRNA. |
| CALD1 | -1.81 | Homo sapiens caldesmon 1 (CALD1), transcript variant 1, mRNA. |
| CD99 | -1.81 | Homo sapiens CD99 molecule (CD99), transcript variant 1, mRNA. |
| COL4A2 | -1.81 | Homo sapiens collagen, type IV, alpha 2 (COL4A2), mRNA. |
| SEMA5A | -1.81 | Homo sapiens sema domain, seven thrombospondin repeats (type 1 and type 1-like), transmembrane domain (TM) and short cytoplasmic domain, (semaphorin) 5A (SEMA5A), mRNA. |
| COL8A2 | -1.81 | Homo sapiens collagen, type VIII, alpha 2 (COL8A2), mRNA. |
| DDX28 | -1.82 | Homo sapiens DEAD (Asp-Glu-Ala-Asp) box polypeptide 28 (DDX28), nuclear gene encoding mitochondrial protein, mRNA. |
| SLK | -1.82 | Homo sapiens STE20-like kinase (yeast) (SLK), mRNA. |
| C19orf22 | -1.82 | Homo sapiens chromosome 19 open reading frame 22 (C19orf22), mRNA. |
| LOC390298 | -1.82 | PREDICTED: Homo sapiens similar to translocase of inner mitochondrial membrane 17 homolog B (LOC390298), miscRNA. |
| MRP63 | -1.82 | Homo sapiens mitochondrial ribosomal protein 63 (MRP63), nuclear gene encoding mitochondrial protein, mRNA. |
| WDR74 | -1.82 | Homo sapiens WD repeat domain 74 (WDR74), mRNA. |
| PGAP1 | -1.82 | Homo sapiens post-GPI attachment to proteins 1 (PGAP1), mRNA. |
| HERC2 | -1.82 | Homo sapiens hect domain and RLD 2 (HERC2), mRNA. |
| IMPDH1 | -1.82 | Homo sapiens IMP (inosine monophosphate) dehydrogenase 1 (IMPDH1), transcript variant 2, mRNA. |
| METRNL | -1.82 | PREDICTED: Homo sapiens meteorin, glial cell differentiation regulator-like (METRNL), mRNA. |
| UNC93B1 | -1.82 | Homo sapiens unc-93 homolog B1 (C. elegans) (UNC93B1), mRNA. |
| GABARAPL2 | -1.82 | Homo sapiens GABA(A) receptor-associated protein-like 2 (GABARAPL2), mRNA. |
| ARVCF | -1.83 | Homo sapiens armadillo repeat gene deletes in velocardiofacial syndrome (ARVCF), mRNA. |
| CAMK1 | -1.83 | Homo sapiens calcium/calmodulin-dependent protein kinase I (CAMK1), mRNA. |
| NECAB3 | -1.83 | Homo sapiens N-terminal EF-hand calcium binding protein 3 (NECAB3), transcript variant 1, mRNA. |
| PTPLB | -1.83 | Homo sapiens protein tyrosine phosphatase-like (proline instead of catalytic arginine), member b (PTPLB), mRNA. |
| FTHL8 | -1.83 | Homo sapiens ferritin, heavy polypeptide-like 8 (FTHL8) on chromosome X. |
| LOC646527 | -1.83 | PREDICTED: Homo sapiens misc_RNA (LOC646527), miscRNA. |
| GABARAP | -1.83 | Homo sapiens GABA(A) receptor-associated protein (GABARAP), mRNA. |
| FKBP9L | -1.83 | Homo sapiens FK506 binding protein 9-like (FKBP9L), mRNA. |
|  | -1.83 | full-length cDNA clone CS0DA009YB08 of Neuroblastoma of Homo sapiens (human) |
| NPAL3 | -1.84 | Homo sapiens NIPA-like domain containing 3 (NPAL3), mRNA. |
| KAT5 | -1.84 | Homo sapiens K(lysine) acetyltransferase 5 (KAT5), transcript variant 1, mRNA. |
| NMU | -1.84 | Homo sapiens neuromedin U (NMU), mRNA. |
| MAPK3 | -1.84 | Homo sapiens mitogen-activated protein kinase 3 (MAPK3), transcript variant 2, mRNA. |
| TMEM184B | -1.84 | Homo sapiens transmembrane protein 184B (TMEM184B), mRNA. |
| CDC42SE1 | -1.84 | Homo sapiens CDC42 small effector 1 (CDC42SE1), transcript variant 1, mRNA. |
| COL1A1 | -1.84 | Homo sapiens collagen, type I, alpha 1 (COL1A1), mRNA. |
| DOK4 | -1.84 | Homo sapiens docking protein 4 (DOK4), mRNA. |
| P4HB | -1.84 | Homo sapiens prolyl 4-hydroxylase, beta polypeptide (P4HB), mRNA. |
| LOC728484 | -1.84 | PREDICTED: Homo sapiens misc_RNA (LOC728484), miscRNA. |
| APEH | -1.84 | Homo sapiens N-acylaminoacyl-peptide hydrolase (APEH), mRNA. |
| SENP2 | -1.84 | Homo sapiens SUMO1/sentrin/SMT3 specific peptidase 2 (SENP2), mRNA. |
| PC | -1.84 | Homo sapiens pyruvate carboxylase (PC), nuclear gene encoding mitochondrial protein, transcript variant 2, mRNA. |
| BTN3A2 | -1.85 | Homo sapiens butyrophilin, subfamily 3, member A2 (BTN3A2), mRNA. |
| BUB1 | -1.85 | Homo sapiens BUB1 budding uninhibited by benzimidazoles 1 homolog (yeast) (BUB1), mRNA. |
| SGOL2 | -1.85 | Homo sapiens shugoshin-like 2 (S. pombe) (SGOL2), mRNA. |
| WDR24 | -1.85 | Homo sapiens WD repeat domain 24 (WDR24), mRNA. |
| LOC613037 | -1.85 | Homo sapiens nuclear pore complex interacting protein pseudogene (LOC613037), non-coding RNA. |
| LOC730990 | -1.85 | PREDICTED: Homo sapiens hypothetical LOC730990 (LOC730990), mRNA. |
| AP2B1 | -1.85 | Homo sapiens adaptor-related protein complex 2, beta 1 subunit (AP2B1), transcript variant 2, mRNA. |
| LIMCH1 | -1.85 | Homo sapiens LIM and calponin homology domains 1 (LIMCH1), mRNA. |
| LOC645937 | -1.85 | PREDICTED: Homo sapiens similar to zinc finger protein 598 (LOC645937), mRNA. |
| LOC643911 | -1.85 | PREDICTED: Homo sapiens hypothetical LOC643911 (LOC643911), mRNA. |
| ATXN7L2 | -1.85 | Homo sapiens ataxin 7-like 2 (ATXN7L2), mRNA. |
| SLC35A5 | -1.86 | Homo sapiens solute carrier family 35, member A5 (SLC35A5), mRNA. |
| SLC30A9 | -1.86 | Homo sapiens solute carrier family 30 (zinc transporter), member 9 (SLC30A9), mRNA. |
| WDR34 | -1.86 | Homo sapiens WD repeat domain 34 (WDR34), mRNA. |
| LOC645452 | -1.86 | PREDICTED: Homo sapiens similar to hCG1782414 (LOC645452), mRNA. |
| ATG9A | -1.86 | Homo sapiens ATG9 autophagy related 9 homolog A (S. cerevisiae) (ATG9A), transcript variant 1, mRNA. |
| PLCG1 | -1.86 | Homo sapiens phospholipase C, gamma 1 (PLCG1), transcript variant 2, mRNA. |
| CCNL1 | -1.86 | Homo sapiens cyclin L1 (CCNL1), mRNA. |
| FAM72D | -1.86 | Homo sapiens family with sequence similarity 72, member D (FAM72D), mRNA. |
| ZIK1 | -1.86 | Homo sapiens zinc finger protein interacting with K protein 1 homolog (mouse) (ZIK1), mRNA. |
| SV2A | -1.86 | Homo sapiens synaptic vesicle glycoprotein 2A (SV2A), mRNA. |
| ARSD | -1.86 | Homo sapiens arylsulfatase D (ARSD), transcript variant 1, mRNA. |
| SULF2 | -1.87 | Homo sapiens sulfatase 2 (SULF2), transcript variant 1, mRNA. |
| ASF1B | -1.87 | Homo sapiens ASF1 anti-silencing function 1 homolog B (S. cerevisiae) (ASF1B), mRNA. |
| PPAP2B | -1.87 | Homo sapiens phosphatidic acid phosphatase type 2B (PPAP2B), transcript variant 2, mRNA. |
| LOC100129685 | -1.87 | PREDICTED: Homo sapiens hypothetical protein LOC100129685 (LOC100129685), mRNA. |
| SCLY | -1.87 | Homo sapiens selenocysteine lyase (SCLY), mRNA. |
| LIN54 | -1.87 | Homo sapiens lin-54 homolog (C. elegans) (LIN54), mRNA. |
| LOC388279 | -1.87 | PREDICTED: Homo sapiens hypothetical LOC388279 (LOC388279), mRNA. |
| ASAP2 | -1.87 | Homo sapiens ArfGAP with SH3 domain, ankyrin repeat and PH domain 2 (ASAP2), transcript variant 1, mRNA. |
| KNTC1 | -1.87 | Homo sapiens kinetochore associated 1 (KNTC1), mRNA. |
| IMMT | -1.87 | Homo sapiens inner membrane protein, mitochondrial (mitofilin) (IMMT), nuclear gene encoding mitochondrial protein, transcript variant 2, mRNA. |
| DDR2 | -1.88 | Homo sapiens discoidin domain receptor tyrosine kinase 2 (DDR2), transcript variant 2, mRNA. |
| CCDC59 | -1.88 | Homo sapiens coiled-coil domain containing 59 (CCDC59), mRNA. |
| DTWD2 | -1.88 | Homo sapiens DTW domain containing 2 (DTWD2), mRNA. |
| SFRS6 | -1.88 | Homo sapiens splicing factor, arginine/serine-rich 6 (SFRS6), mRNA. |
|  | -1.88 | Homo sapiens cDNA FLJ25252 fis, clone STM03814 |
| ZBTB4 | -1.88 | Homo sapiens zinc finger and BTB domain containing 4 (ZBTB4), mRNA. |
| ARHGAP28 | -1.88 | Homo sapiens Rho GTPase activating protein 28 (ARHGAP28), transcript variant 1, mRNA. |
| FLJ43681 | -1.88 | PREDICTED: Homo sapiens similar to 60S ribosomal protein L23a (FLJ43681), miscRNA. |
| DDX51 | -1.88 | Homo sapiens DEAD (Asp-Glu-Ala-Asp) box polypeptide 51 (DDX51), mRNA. |
| TP53INP2 | -1.89 | Homo sapiens tumor protein p53 inducible nuclear protein 2 (TP53INP2), mRNA. |
| PPAP2A | -1.89 | Homo sapiens phosphatidic acid phosphatase type 2A (PPAP2A), transcript variant 2, mRNA. |
| LOC652837 | -1.89 | PREDICTED: Homo sapiens similar to C-terminal binding protein 2 (LOC652837), mRNA. |
| TTLL3 | -1.89 | Homo sapiens tubulin tyrosine ligase-like family, member 3 (TTLL3), transcript variant 2, mRNA. |
| MMAB | -1.89 | Homo sapiens methylmalonic aciduria (cobalamin deficiency) cblB type (MMAB), mRNA. |
| RALGAPA1 | -1.89 | Homo sapiens Ral GTPase activating protein, alpha subunit 1 (catalytic) (RALGAPA1), transcript variant 1, mRNA. |
| PHAX | -1.89 | Homo sapiens phosphorylated adaptor for RNA export (PHAX), mRNA. |
| PLCG1 | -1.89 | Homo sapiens phospholipase C, gamma 1 (PLCG1), transcript variant 2, mRNA. |
| PTPLB | -1.89 | Homo sapiens protein tyrosine phosphatase-like (proline instead of catalytic arginine), member b (PTPLB), mRNA. |
| HHAT | -1.89 | Homo sapiens hedgehog acyltransferase (HHAT), mRNA. |
| LOC728640 | -1.9 | PREDICTED: Homo sapiens misc_RNA (LOC728640), miscRNA. |
| BAT3 | -1.9 | Homo sapiens HLA-B associated transcript 3 (BAT3), transcript variant 3, mRNA. |
| DRG2 | -1.9 | Homo sapiens developmentally regulated GTP binding protein 2 (DRG2), mRNA. |
| DYRK2 | -1.9 | Homo sapiens dual-specificity tyrosine-(Y)-phosphorylation regulated kinase 2 (DYRK2), transcript variant 1, mRNA. |
| LOC440737 | -1.9 | PREDICTED: Homo sapiens similar to 60S ribosomal protein L35 (LOC440737), mRNA. |
| RBM27 | -1.9 | Homo sapiens RNA binding motif protein 27 (RBM27), mRNA. |
| LUM | -1.9 | Homo sapiens lumican (LUM), mRNA. |
| OCIAD1 | -1.9 | Homo sapiens OCIA domain containing 1 (OCIAD1), transcript variant 5, mRNA. |
| C17orf58 | -1.9 | Homo sapiens chromosome 17 open reading frame 58 (C17orf58), transcript variant 2, mRNA. |
| FRMD6 | -1.9 | Homo sapiens FERM domain containing 6 (FRMD6), transcript variant 2, mRNA. |
| ZBTB8OS | -1.9 | Homo sapiens zinc finger and BTB domain containing 8 opposite strand (ZBTB8OS), mRNA. |
| SUPT6H | -1.9 | Homo sapiens suppressor of Ty 6 homolog (S. cerevisiae) (SUPT6H), mRNA. |
| NSFL1C | -1.91 | Homo sapiens NSFL1 (p97) cofactor (p47) (NSFL1C), transcript variant 1, mRNA. |
| C21orf81 | -1.91 | Homo sapiens chromosome 21 open reading frame 81 (C21orf81), mRNA. |
| PI4KAP2 | -1.91 | Homo sapiens phosphatidylinositol 4-kinase, catalytic, alpha polypeptide pseudogene 2 (PI4KAP2), mRNA. |
| DLEU2 | -1.91 | PREDICTED: Homo sapiens deleted in lymphocytic leukemia, 2 (DLEU2), misc RNA. |
| NOL8 | -1.91 | Homo sapiens nucleolar protein 8 (NOL8), mRNA. |
| KIF4A | -1.91 | Homo sapiens kinesin family member 4A (KIF4A), mRNA. |
| TMEM39B | -1.91 | Homo sapiens transmembrane protein 39B (TMEM39B), mRNA. |
| FBXO2 | -1.91 | Homo sapiens F-box protein 2 (FBXO2), mRNA. |
| SNORA62 | -1.91 | Homo sapiens small nucleolar RNA, H/ACA box 62 (SNORA62), small nucleolar RNA. |
| IL27RA | -1.91 | Homo sapiens interleukin 27 receptor, alpha (IL27RA), mRNA. |
| TTC15 | -1.91 | Homo sapiens tetratricopeptide repeat domain 15 (TTC15), mRNA. |
| GARNL4 | -1.92 | Homo sapiens GTPase activating Rap/RanGAP domain-like 4 (GARNL4), mRNA. |
| GOLPH4 | -1.92 | Homo sapiens golgi phosphoprotein 4 (GOLPH4), mRNA. |
| ENC1 | -1.92 | Homo sapiens ectodermal-neural cortex (with BTB-like domain) (ENC1), mRNA. |
| NUP214 | -1.92 | Homo sapiens nucleoporin 214kDa (NUP214), mRNA. |
| TBC1D17 | -1.92 | Homo sapiens TBC1 domain family, member 17 (TBC1D17), mRNA. |
| KIAA0460 | -1.92 | Homo sapiens KIAA0460 (KIAA0460), mRNA. |
| AHCTF1 | -1.92 | Homo sapiens AT hook containing transcription factor 1 (AHCTF1), mRNA. |
| MND1 | -1.92 | Homo sapiens meiotic nuclear divisions 1 homolog (S. cerevisiae) (MND1), mRNA. |
| CCNB1 | -1.93 | Homo sapiens cyclin B1 (CCNB1), mRNA. |
| SCARNA16 | -1.93 | Homo sapiens small Cajal body-specific RNA 16 (SCARNA16), guide RNA. |
| EP400 | -1.93 | Homo sapiens E1A binding protein p400 (EP400), mRNA. |
| MEX3A | -1.94 | Homo sapiens mex-3 homolog A (C. elegans) (MEX3A), mRNA. |
| ITM2B | -1.94 | Homo sapiens integral membrane protein 2B (ITM2B), mRNA. |
| DNPEP | -1.94 | Homo sapiens aspartyl aminopeptidase (DNPEP), mRNA. |
| NAP1L1 | -1.94 | Homo sapiens nucleosome assembly protein 1-like 1 (NAP1L1), transcript variant 1, mRNA. |
| CD151 | -1.94 | Homo sapiens CD151 molecule (Raph blood group) (CD151), transcript variant 5, mRNA. |
| BNC2 | -1.94 | Homo sapiens basonuclin 2 (BNC2), mRNA. |
| LOC100134634 | -1.94 | PREDICTED: Homo sapiens hypothetical protein LOC100134634 (LOC100134634), mRNA. |
| FLJ31306 | -1.94 | PREDICTED: Homo sapiens hypothetical protein FLJ31306 (FLJ31306), mRNA. |
| PRKDC | -1.94 | Homo sapiens protein kinase, DNA-activated, catalytic polypeptide (PRKDC), transcript variant 1, mRNA. |
| CEP164 | -1.94 | Homo sapiens centrosomal protein 164kDa (CEP164), mRNA. |
| TUBGCP2 | -1.94 | Homo sapiens tubulin, gamma complex associated protein 2 (TUBGCP2), mRNA. |
| C7orf11 | -1.94 | Homo sapiens chromosome 7 open reading frame 11 (C7orf11), mRNA. |
| FLJ31306 | -1.94 | PREDICTED: Homo sapiens hypothetical protein FLJ31306 (FLJ31306), mRNA. |
| FAM122B | -1.95 | Homo sapiens family with sequence similarity 122B (FAM122B), mRNA. |
| NAE1 | -1.95 | Homo sapiens NEDD8 activating enzyme E1 subunit 1 (NAE1), transcript variant 3, mRNA. |
| ARID5B | -1.95 | Homo sapiens AT rich interactive domain 5B (MRF1-like) (ARID5B), mRNA. |
| UBE2C | -1.95 | Homo sapiens ubiquitin-conjugating enzyme E2C (UBE2C), transcript variant 6, mRNA. |
| BCYRN1 | -1.95 | Homo sapiens brain cytoplasmic RNA 1 (non-protein coding) (BCYRN1), non-coding RNA. |
| TBC1D9B | -1.96 | Homo sapiens TBC1 domain family, member 9B (with GRAM domain) (TBC1D9B), transcript variant 2, mRNA. |
| RPL9 | -1.96 | Homo sapiens ribosomal protein L9 (RPL9), transcript variant 2, mRNA. |
| VRK1 | -1.96 | Homo sapiens vaccinia related kinase 1 (VRK1), mRNA. |
| TTK | -1.96 | Homo sapiens TTK protein kinase (TTK), mRNA. |
| SPN | -1.96 | Homo sapiens sialophorin (SPN), transcript variant 1, mRNA. |
| TMEM19 | -1.96 | Homo sapiens transmembrane protein 19 (TMEM19), mRNA. |
| ACTRT1 | -1.96 | Homo sapiens actin-related protein T1 (ACTRT1), mRNA. |
| MAP3K10 | -1.96 | Homo sapiens mitogen-activated protein kinase kinase kinase 10 (MAP3K10), mRNA. |
| TMEM109 | -1.97 | Homo sapiens transmembrane protein 109 (TMEM109), mRNA. |
| NFYC | -1.97 | Homo sapiens nuclear transcription factor Y, gamma (NFYC), mRNA. |
| DAG1 | -1.97 | Homo sapiens dystroglycan 1 (dystrophin-associated glycoprotein 1) (DAG1), mRNA. |
| CWC22 | -1.97 | Homo sapiens CWC22 spliceosome-associated protein homolog (S. cerevisiae) (CWC22), mRNA. |
| GPR124 | -1.97 | Homo sapiens G protein-coupled receptor 124 (GPR124), mRNA. |
| TMEM209 | -1.97 | Homo sapiens transmembrane protein 209 (TMEM209), mRNA. |
| LOC390735 | -1.97 | PREDICTED: Homo sapiens hCG1642987 (LOC390735), mRNA. |
| TMEM18 | -1.97 | Homo sapiens transmembrane protein 18 (TMEM18), mRNA. |
| FLJ44342 | -1.97 | PREDICTED: Homo sapiens hypothetical LOC645460 (FLJ44342), miscRNA. |
| ARHGAP19 | -1.97 | Homo sapiens Rho GTPase activating protein 19 (ARHGAP19), mRNA. |
| DNM2 | -1.98 | Homo sapiens dynamin 2 (DNM2), transcript variant 2, mRNA. |
| LOC441066 | -1.98 | PREDICTED: Homo sapiens similar to Beta-glucuronidase precursor (LOC441066), mRNA. |
| TBC1D10B | -1.98 | Homo sapiens TBC1 domain family, member 10B (TBC1D10B), mRNA. |
| ETS1 | -1.98 | Homo sapiens v-ets erythroblastosis virus E26 oncogene homolog 1 (avian) (ETS1), mRNA. |
| SLC39A11 | -1.98 | Homo sapiens solute carrier family 39 (metal ion transporter), member 11 (SLC39A11), mRNA. |
| CBX3 | -1.98 | Homo sapiens chromobox homolog 3 (HP1 gamma homolog, Drosophila) (CBX3), transcript variant 2, mRNA. |
| LOC441642 | -1.98 | PREDICTED: Homo sapiens similar to hCG27427 (LOC441642), mRNA. |
| ANKRD44 | -1.98 | Homo sapiens ankyrin repeat domain 44 (ANKRD44), mRNA. |
| BAZ1A | -1.98 | Homo sapiens bromodomain adjacent to zinc finger domain, 1A (BAZ1A), transcript variant 1, mRNA. |
| LOC649009 | -1.98 | PREDICTED: Homo sapiens similar to paraspeckle protein 1, transcript variant 1 (LOC649009), mRNA. |
| RAC3 | -1.99 | Homo sapiens ras-related C3 botulinum toxin substrate 3 (rho family, small GTP binding protein Rac3) (RAC3), mRNA. |
| LOC643384 | -1.99 | PREDICTED: Homo sapiens hypothetical LOC643384 (LOC643384), mRNA. |
| ZNHIT2 | -1.99 | Homo sapiens zinc finger, HIT type 2 (ZNHIT2), mRNA. |
| LOC440595 | -1.99 | PREDICTED: Homo sapiens misc_RNA (LOC440595), miscRNA. |
| DNM1 | -1.99 | Homo sapiens dynamin 1 (DNM1), transcript variant 2, mRNA. |
| ALG5 | -1.99 | Homo sapiens asparagine-linked glycosylation 5 homolog (S. cerevisiae, dolichyl-phosphate beta-glucosyltransferase) (ALG5), mRNA. |
| GTPBP8 | -1.99 | Homo sapiens GTP-binding protein 8 (putative) (GTPBP8), transcript variant 1, mRNA. |
| FHL1 | -1.99 | Homo sapiens four and a half LIM domains 1 (FHL1), mRNA. |
| TDG | -2 | Homo sapiens thymine-DNA glycosylase (TDG), mRNA. |
| TIMP2 | -2 | Homo sapiens TIMP metallopeptidase inhibitor 2 (TIMP2), mRNA. |
| RGL2 | -2 | Homo sapiens ral guanine nucleotide dissociation stimulator-like 2 (RGL2), mRNA. |
| ADAMTS19 | -2 | Homo sapiens ADAM metallopeptidase with thrombospondin type 1 motif, 19 (ADAMTS19), mRNA. |
| LOC730286 | -2 | PREDICTED: Homo sapiens misc_RNA (LOC730286), miscRNA. |
|  | -2 | Homo sapiens cDNA: FLJ21199 fis, clone COL00235 |
|  | -2 | Homo sapiens cDNA clone IMAGE:5922621, partial cds |
| LOC653377 | -2 | PREDICTED: Homo sapiens similar to family with sequence similarity 36, member A (LOC653377), mRNA. |
| MFF | -2 | Homo sapiens mitochondrial fission factor (MFF), nuclear gene encoding mitochondrial protein, mRNA. |
| AEBP1 | -2 | Homo sapiens AE binding protein 1 (AEBP1), mRNA. |
| LOC100128196 | -2 | PREDICTED: Homo sapiens misc_RNA (LOC100128196), miscRNA. |
| EPOR | -2.01 | Homo sapiens erythropoietin receptor (EPOR), mRNA. |
| ESRRA | -2.01 | Homo sapiens estrogen-related receptor alpha (ESRRA), mRNA. |
| LOC730107 | -2.01 | PREDICTED: Homo sapiens similar to Glycine cleavage system H protein, mitochondrial (LOC730107), mRNA. |
| CTSK | -2.01 | Homo sapiens cathepsin K (CTSK), mRNA. |
| C1orf159 | -2.01 | Homo sapiens chromosome 1 open reading frame 159 (C1orf159), mRNA. |
| RNF182 | -2.01 | Homo sapiens ring finger protein 182 (RNF182), mRNA. |
| SSPN | -2.01 | Homo sapiens sarcospan (Kras oncogene-associated gene) (SSPN), mRNA. |
| HSF4 | -2.01 | Homo sapiens heat shock transcription factor 4 (HSF4), transcript variant 2, mRNA. |
| NEK1 | -2.02 | Homo sapiens NIMA (never in mitosis gene a)-related kinase 1 (NEK1), mRNA. |
| ZNF768 | -2.02 | Homo sapiens zinc finger protein 768 (ZNF768), mRNA. |
| TMEM151A | -2.02 | Homo sapiens transmembrane protein 151A (TMEM151A), mRNA. |
| LOC642333 | -2.02 | PREDICTED: Homo sapiens similar to M-phase phosphoprotein, mpp8 (LOC642333), mRNA. |
| LOC388796 | -2.03 | Homo sapiens hypothetical LOC388796 (LOC388796), non-coding RNA. |
|  | -2.03 | UI-E-CK1-agb-m-11-0-UI.s1 UI-E-CK1 Homo sapiens cDNA clone UI-E-CK1-agb-m-11-0-UI 3, mRNA sequence |
| GMIP | -2.03 | Homo sapiens GEM interacting protein (GMIP), mRNA. |
| GPER | -2.03 | Homo sapiens G protein-coupled estrogen receptor 1 (GPER), transcript variant 3, mRNA. |
| FAM64A | -2.03 | Homo sapiens family with sequence similarity 64, member A (FAM64A), mRNA. |
| CEP152 | -2.03 | Homo sapiens centrosomal protein 152kDa (CEP152), mRNA. |
| ATP2B4 | -2.04 | Homo sapiens ATPase, Ca++ transporting, plasma membrane 4 (ATP2B4), transcript variant 1, mRNA. |
| RICS | -2.04 | Homo sapiens Rho GTPase-activating protein (RICS), mRNA. |
| HCFC1R1 | -2.04 | Homo sapiens host cell factor C1 regulator 1 (XPO1 dependent) (HCFC1R1), transcript variant 3, mRNA. |
| ENDOG | -2.04 | Homo sapiens endonuclease G (ENDOG), nuclear gene encoding mitochondrial protein, mRNA. |
| CTR9 | -2.04 | Homo sapiens Ctr9, Paf1/RNA polymerase II complex component, homolog (S. cerevisiae) (CTR9), mRNA. |
| LOC728728 | -2.04 | PREDICTED: Homo sapiens misc_RNA (LOC728728), miscRNA. |
| FAM125B | -2.04 | Homo sapiens family with sequence similarity 125, member B (FAM125B), transcript variant 1, mRNA. |
| VCAN | -2.04 | Homo sapiens versican (VCAN), mRNA. |
| SKA2 | -2.04 | Homo sapiens spindle and kinetochore associated complex subunit 2 (SKA2), transcript variant 1, mRNA. |
| SKA2 | -2.05 | Homo sapiens spindle and kinetochore associated complex subunit 2 (SKA2), transcript variant 1, mRNA. |
| JAGN1 | -2.05 | Homo sapiens jagunal homolog 1 (Drosophila) (JAGN1), mRNA. |
| OFD1 | -2.05 | Homo sapiens oral-facial-digital syndrome 1 (OFD1), mRNA. |
| IL11RA | -2.05 | Homo sapiens interleukin 11 receptor, alpha (IL11RA), transcript variant 1, mRNA. |
| NPB | -2.05 | Homo sapiens neuropeptide B (NPB), mRNA. |
| LOC648659 | -2.06 | PREDICTED: Homo sapiens similar to ribosomal protein S3a, transcript variant 4 (LOC648659), mRNA. |
| SULT1A1 | -2.06 | Homo sapiens sulfotransferase family, cytosolic, 1A, phenol-preferring, member 1 (SULT1A1), transcript variant 3, mRNA. |
| LOC729843 | -2.06 | PREDICTED: Homo sapiens similar to WW domain binding protein 1 (LOC729843), mRNA. |
| KIAA0427 | -2.06 | Homo sapiens KIAA0427 (KIAA0427), mRNA. |
| C18orf56 | -2.06 | Homo sapiens chromosome 18 open reading frame 56 (C18orf56), mRNA. |
| CPE | -2.06 | Homo sapiens carboxypeptidase E (CPE), mRNA. |
| BCHE | -2.06 | Homo sapiens butyrylcholinesterase (BCHE), mRNA. |
| ARMC1 | -2.06 | Homo sapiens armadillo repeat containing 1 (ARMC1), mRNA. |
|  | -2.06 | Homo sapiens mRNA; cDNA DKFZp762F0616 (from clone DKFZp762F0616) |
| LGALS9B | -2.07 | Homo sapiens lectin, galactoside-binding, soluble, 9B (LGALS9B), mRNA. |
| TRIO | -2.07 | Homo sapiens triple functional domain (PTPRF interacting) (TRIO), mRNA. |
| WBP11 | -2.07 | Homo sapiens WW domain binding protein 11 (WBP11), mRNA. |
| PIM1 | -2.07 | Homo sapiens pim-1 oncogene (PIM1), mRNA. |
| UPF3A | -2.07 | Homo sapiens UPF3 regulator of nonsense transcripts homolog A (yeast) (UPF3A), transcript variant 2, mRNA. |
| RTN4 | -2.07 | Homo sapiens reticulon 4 (RTN4), transcript variant 3, mRNA. |
| HBEGF | -2.07 | Homo sapiens heparin-binding EGF-like growth factor (HBEGF), mRNA. |
| FABP5 | -2.08 | Homo sapiens fatty acid binding protein 5 (psoriasis-associated) (FABP5), mRNA. |
| LOC645609 | -2.08 | PREDICTED: Homo sapiens similar to TBC1 domain family member 3 (Rab GTPase-activating protein PRC17) (Prostate cancer gene 17 protein) (TRE17 alpha protein) (LOC645609), mRNA. |
| PLS3 | -2.08 | Homo sapiens plastin 3 (T isoform) (PLS3), mRNA. |
| C9orf46 | -2.08 | Homo sapiens chromosome 9 open reading frame 46 (C9orf46), mRNA. |
| TECR | -2.08 | Homo sapiens trans-2,3-enoyl-CoA reductase (TECR), mRNA. |
| MTMR3 | -2.08 | Homo sapiens myotubularin related protein 3 (MTMR3), transcript variant 3, mRNA. |
| LSM5 | -2.08 | Homo sapiens LSM5 homolog, U6 small nuclear RNA associated (S. cerevisiae) (LSM5), mRNA. |
| LOC441154 | -2.08 | PREDICTED: Homo sapiens misc_RNA (LOC441154), miscRNA. |
| SMARCC2 | -2.09 | Homo sapiens SWI/SNF related, matrix associated, actin dependent regulator of chromatin, subfamily c, member 2 (SMARCC2), transcript variant 2, mRNA. |
| LOC613037 | -2.09 | Homo sapiens nuclear pore complex interacting protein pseudogene (LOC613037), non-coding RNA. |
| LOC653147 | -2.09 | PREDICTED: Homo sapiens similar to 60S ribosomal protein L26-like 1 (LOC653147), mRNA. |
| ATP2B4 | -2.09 | Homo sapiens ATPase, Ca++ transporting, plasma membrane 4 (ATP2B4), transcript variant 2, mRNA. |
| GPR125 | -2.09 | PREDICTED: Homo sapiens G protein-coupled receptor 125, transcript variant 4 (GPR125), mRNA. |
| TROAP | -2.09 | Homo sapiens trophinin associated protein (tastin) (TROAP), mRNA. |
| RAMP2 | -2.09 | Homo sapiens receptor (G protein-coupled) activity modifying protein 2 (RAMP2), mRNA. |
| METTL7A | -2.09 | Homo sapiens methyltransferase like 7A (METTL7A), mRNA. |
| PTPRE | -2.1 | Homo sapiens protein tyrosine phosphatase, receptor type, E (PTPRE), transcript variant 2, mRNA. |
| SHC3 | -2.1 | Homo sapiens SHC (Src homology 2 domain containing) transforming protein 3 (SHC3), mRNA. |
| ICA1L | -2.1 | Homo sapiens islet cell autoantigen 1,69kDa-like (ICA1L), transcript variant 1, mRNA. |
| ATP2A2 | -2.1 | Homo sapiens ATPase, Ca++ transporting, cardiac muscle, slow twitch 2 (ATP2A2), transcript variant 2, mRNA. |
| TRAK1 | -2.1 | Homo sapiens trafficking protein, kinesin binding 1 (TRAK1), transcript variant 1, mRNA. |
|  | -2.11 | Homo sapiens cDNA FLJ32174 fis, clone PLACE6001064 |
| SF3B4 | -2.11 | Homo sapiens splicing factor 3b, subunit 4, 49kDa (SF3B4), mRNA. |
| C9orf82 | -2.11 | Homo sapiens chromosome 9 open reading frame 82 (C9orf82), mRNA. |
| SLC4A8 | -2.11 | Homo sapiens solute carrier family 4, sodium bicarbonate cotransporter, member 8 (SLC4A8), transcript variant 2, mRNA. |
| RERE | -2.11 | Homo sapiens arginine-glutamic acid dipeptide (RE) repeats (RERE), transcript variant 1, mRNA. |
| NT5C3 | -2.11 | Homo sapiens 5'-nucleotidase, cytosolic III (NT5C3), transcript variant 1, mRNA. |
| FKBP10 | -2.11 | Homo sapiens FK506 binding protein 10, 65 kDa (FKBP10), mRNA. |
| ADAMTS1 | -2.11 | Homo sapiens ADAM metallopeptidase with thrombospondin type 1 motif, 1 (ADAMTS1), mRNA. |
| STIM1 | -2.11 | Homo sapiens stromal interaction molecule 1 (STIM1), mRNA. |
| CDKN1B | -2.11 | Homo sapiens cyclin-dependent kinase inhibitor 1B (p27, Kip1) (CDKN1B), mRNA. |
| CDKN3 | -2.11 | Homo sapiens cyclin-dependent kinase inhibitor 3 (CDK2-associated dual specificity phosphatase) (CDKN3), mRNA. |
| LOC100131582 | -2.12 | PREDICTED: Homo sapiens hypothetical protein LOC100131582 (LOC100131582), mRNA. |
| FAM101B | -2.12 | Homo sapiens family with sequence similarity 101, member B (FAM101B), mRNA. |
| LOC652755 | -2.12 | PREDICTED: Homo sapiens similar to Baculoviral IAP repeat-containing protein 1 (Neuronal apoptosis inhibitory protein) (LOC652755), mRNA. |
| C16orf42 | -2.12 | Homo sapiens chromosome 16 open reading frame 42 (C16orf42), mRNA. |
| PSD3 | -2.12 | Homo sapiens pleckstrin and Sec7 domain containing 3 (PSD3), transcript variant 1, mRNA. |
| GBAS | -2.12 | Homo sapiens glioblastoma amplified sequence (GBAS), mRNA. |
| TRIM41 | -2.12 | Homo sapiens tripartite motif-containing 41 (TRIM41), transcript variant 2, mRNA. |
| LOC642989 | -2.13 | PREDICTED: Homo sapiens similar to 40S ribosomal protein S25 (LOC642989), mRNA. |
| NAGLU | -2.13 | Homo sapiens N-acetylglucosaminidase, alpha- (NAGLU), mRNA. |
| TUBG1 | -2.13 | Homo sapiens tubulin, gamma 1 (TUBG1), mRNA. |
| SCARB1 | -2.13 | Homo sapiens scavenger receptor class B, member 1 (SCARB1), mRNA. |
| CCDC125 | -2.13 | Homo sapiens coiled-coil domain containing 125 (CCDC125), mRNA. |
| MAB21L1 | -2.13 | Homo sapiens mab-21-like 1 (C. elegans) (MAB21L1), mRNA. |
| NUBPL | -2.14 | Homo sapiens nucleotide binding protein-like (NUBPL), mRNA. |
| NT5C3 | -2.14 | Homo sapiens 5'-nucleotidase, cytosolic III (NT5C3), transcript variant 1, mRNA. |
| ILF3 | -2.14 | Homo sapiens interleukin enhancer binding factor 3, 90kDa (ILF3), transcript variant 1, mRNA. |
| CALCOCO1 | -2.14 | Homo sapiens calcium binding and coiled-coil domain 1 (CALCOCO1), mRNA. |
| HLA-A | -2.14 | Homo sapiens major histocompatibility complex, class I, A (HLA-A), mRNA. |
| SDF4 | -2.14 | Homo sapiens stromal cell derived factor 4 (SDF4), mRNA. |
| GOLIM4 | -2.14 | Homo sapiens golgi integral membrane protein 4 (GOLIM4), mRNA. |
| SMARCA1 | -2.14 | Homo sapiens SWI/SNF related, matrix associated, actin dependent regulator of chromatin, subfamily a, member 1 (SMARCA1), transcript variant 1, mRNA. |
| ESPL1 | -2.14 | Homo sapiens extra spindle pole bodies homolog 1 (S. cerevisiae) (ESPL1), mRNA. |
| DGKD | -2.14 | Homo sapiens diacylglycerol kinase, delta 130kDa (DGKD), transcript variant 1, mRNA. |
| LOC644928 | -2.15 | Homo sapiens hCG2033311 (LOC644928), mRNA. |
| LOC729009 | -2.15 | PREDICTED: Homo sapiens misc_RNA (LOC729009), miscRNA. |
| HJURP | -2.15 | Homo sapiens Holliday junction recognition protein (HJURP), mRNA. |
| LOC728809 | -2.15 | PREDICTED: Homo sapiens hypothetical LOC728809 (LOC728809), mRNA. |
| LOC100131526 | -2.15 | PREDICTED: Homo sapiens misc_RNA (LOC100131526), miscRNA. |
| MYLIP | -2.15 | Homo sapiens myosin regulatory light chain interacting protein (MYLIP), mRNA. |
| SYDE1 | -2.15 | Homo sapiens synapse defective 1, Rho GTPase, homolog 1 (C. elegans) (SYDE1), mRNA. |
| LOC100132564 | -2.15 | PREDICTED: Homo sapiens hypothetical protein LOC100132564 (LOC100132564), mRNA. |
| GFRA2 | -2.15 | Homo sapiens GDNF family receptor alpha 2 (GFRA2), transcript variant 1, mRNA. |
| CDCA3 | -2.15 | Homo sapiens cell division cycle associated 3 (CDCA3), mRNA. |
| TM4SF1 | -2.15 | Homo sapiens transmembrane 4 L six family member 1 (TM4SF1), mRNA. |
| TMUB1 | -2.16 | Homo sapiens transmembrane and ubiquitin-like domain containing 1 (TMUB1), mRNA. |
| LOC100170939 | -2.16 | Homo sapiens glucuronidase, beta pseudogene (LOC100170939), non-coding RNA. |
| CEP350 | -2.16 | Homo sapiens centrosomal protein 350kDa (CEP350), mRNA. |
| BCAT1 | -2.16 | Homo sapiens branched chain aminotransferase 1, cytosolic (BCAT1), mRNA. |
| LTV1 | -2.16 | Homo sapiens LTV1 homolog (S. cerevisiae) (LTV1), mRNA. |
| TSEN34 | -2.17 | Homo sapiens tRNA splicing endonuclease 34 homolog (S. cerevisiae) (TSEN34), transcript variant 2, mRNA. |
| HSDL2 | -2.17 | Homo sapiens hydroxysteroid dehydrogenase like 2 (HSDL2), mRNA. |
| SH3KBP1 | -2.17 | Homo sapiens SH3-domain kinase binding protein 1 (SH3KBP1), transcript variant 1, mRNA. |
| LOC643778 | -2.17 | PREDICTED: Homo sapiens misc_RNA (LOC643778), miscRNA. |
| RORB | -2.17 | Homo sapiens RAR-related orphan receptor B (RORB), mRNA. |
| WDR81 | -2.17 | Homo sapiens WD repeat domain 81 (WDR81), mRNA. |
| GNG5 | -2.17 | Homo sapiens guanine nucleotide binding protein (G protein), gamma 5 (GNG5), mRNA. |
| SUMF2 | -2.17 | Homo sapiens sulfatase modifying factor 2 (SUMF2), transcript variant 4, mRNA. |
| NES | -2.18 | Homo sapiens nestin (NES), mRNA. |
| ABCB1 | -2.18 | Homo sapiens ATP-binding cassette, sub-family B (MDR/TAP), member 1 (ABCB1), mRNA. |
| LOC100133277 | -2.18 | PREDICTED: Homo sapiens misc_RNA (LOC100133277), miscRNA. |
| LOC643308 | -2.18 | PREDICTED: Homo sapiens similar to hCG1810802 (LOC643308), mRNA. |
| LYN | -2.18 | Homo sapiens v-yes-1 Yamaguchi sarcoma viral related oncogene homolog (LYN), mRNA. |
|  | -2.19 | Homo sapiens mRNA; cDNA DKFZp779F0411 (from clone DKFZp779F0411) |
| LOC653829 | -2.19 | PREDICTED: Homo sapiens similar to Williams Beuren syndrome chromosome region 19, transcript variant 6 (LOC653829), mRNA. |
| FOXP1 | -2.19 | Homo sapiens forkhead box P1 (FOXP1), transcript variant 1, mRNA. |
| SLC35E3 | -2.19 | Homo sapiens solute carrier family 35, member E3 (SLC35E3), mRNA. |
| LOC729342 | -2.19 | PREDICTED: Homo sapiens misc_RNA (LOC729342), miscRNA. |
| LOC643995 | -2.19 | PREDICTED: Homo sapiens similar to Importin alpha-2 subunit (Karyopherin alpha-2 subunit) (SRP1-alpha) (RAG cohort protein 1), transcript variant 2 (LOC643995), mRNA. |
| CALD1 | -2.19 | Homo sapiens caldesmon 1 (CALD1), transcript variant 5, mRNA. |
| TMX4 | -2.2 | Homo sapiens thioredoxin-related transmembrane protein 4 (TMX4), mRNA. |
| GAS6 | -2.2 | Homo sapiens growth arrest-specific 6 (GAS6), mRNA. |
|  | -2.2 | BX114764 NCI_CGAP_GCB1 Homo sapiens cDNA clone IMAGp998M182012, mRNA sequence |
| FSTL5 | -2.2 | Homo sapiens follistatin-like 5 (FSTL5), mRNA. |
| LOC643911 | -2.2 | PREDICTED: Homo sapiens hCG1815491 (LOC643911), miscRNA. |
| TFAP4 | -2.2 | Homo sapiens transcription factor AP-4 (activating enhancer binding protein 4) (TFAP4), mRNA. |
| SLC7A2 | -2.2 | Homo sapiens solute carrier family 7 (cationic amino acid transporter, y+ system), member 2 (SLC7A2), transcript variant 2, mRNA. |
| LOC729686 | -2.2 | PREDICTED: Homo sapiens misc_RNA (LOC729686), miscRNA. |
| CD276 | -2.2 | Homo sapiens CD276 molecule (CD276), transcript variant 1, mRNA. XM_945872 XM_945874 |
| YPEL3 | -2.2 | Homo sapiens yippee-like 3 (Drosophila) (YPEL3), transcript variant 1, mRNA. |
| SPC25 | -2.2 | Homo sapiens SPC25, NDC80 kinetochore complex component, homolog (S. cerevisiae) (SPC25), mRNA. |
| LOC646044 | -2.21 | PREDICTED: Homo sapiens misc_RNA (LOC646044), miscRNA. |
| RNASEH2B | -2.21 | Homo sapiens ribonuclease H2, subunit B (RNASEH2B), mRNA. |
| RNU1-3 | -2.21 | Homo sapiens RNA, U1 small nuclear 3 (RNU1-3), small nuclear RNA. |
| SNORD4A | -2.21 | Homo sapiens small nucleolar RNA, C/D box 4A (SNORD4A), small nucleolar RNA. |
| DEPDC1 | -2.21 | Homo sapiens DEP domain containing 1 (DEPDC1), mRNA. |
| ACTR1B | -2.22 | Homo sapiens ARP1 actin-related protein 1 homolog B, centractin beta (yeast) (ACTR1B), mRNA. |
| MIIP | -2.22 | Homo sapiens migration and invasion inhibitory protein (MIIP), mRNA. |
| BAMBI | -2.22 | Homo sapiens BMP and activin membrane-bound inhibitor homolog (Xenopus laevis) (BAMBI), mRNA. |
| NAIF1 | -2.22 | Homo sapiens nuclear apoptosis inducing factor 1 (NAIF1), mRNA. |
| DENND4B | -2.22 | Homo sapiens DENN/MADD domain containing 4B (DENND4B), mRNA. |
| LOC100134530 | -2.22 | PREDICTED: Homo sapiens hypothetical protein LOC100134530 (LOC100134530), mRNA. |
| C5orf41 | -2.22 | Homo sapiens chromosome 5 open reading frame 41 (C5orf41), mRNA. |
|  | -2.22 | Homo sapiens cDNA FLJ26784 fis, clone PRS04220 |
| PKD2 | -2.23 | Homo sapiens polycystic kidney disease 2 (autosomal dominant) (PKD2), mRNA. |
| BASP1 | -2.23 | Homo sapiens brain abundant, membrane attached signal protein 1 (BASP1), mRNA. |
| EIF5 | -2.23 | Homo sapiens eukaryotic translation initiation factor 5 (EIF5), transcript variant 1, mRNA. |
| TMEM219 | -2.23 | Homo sapiens transmembrane protein 219 (TMEM219), transcript variant 1, mRNA. |
| ASPM | -2.23 | Homo sapiens asp (abnormal spindle) homolog, microcephaly associated (Drosophila) (ASPM), mRNA. |
| LOC729495 | -2.23 | PREDICTED: Homo sapiens similar to hCG1999863 (LOC729495), mRNA. |
| RNF126 | -2.24 | Homo sapiens ring finger protein 126 (RNF126), mRNA. |
| PRMT6 | -2.24 | Homo sapiens protein arginine methyltransferase 6 (PRMT6), mRNA. |
| UBE2O | -2.24 | Homo sapiens ubiquitin-conjugating enzyme E2O (UBE2O), mRNA. |
| BUB1B | -2.24 | Homo sapiens BUB1 budding uninhibited by benzimidazoles 1 homolog beta (yeast) (BUB1B), mRNA. |
| LOC440895 | -2.24 | PREDICTED: Homo sapiens LIM and senescent cell antigen-like domains 3-like, transcript variant 1 (LOC440895), mRNA. |
| TPM4 | -2.24 | Homo sapiens tropomyosin 4 (TPM4), mRNA. |
| KHDRBS3 | -2.24 | Homo sapiens KH domain containing, RNA binding, signal transduction associated 3 (KHDRBS3), mRNA. |
| LOC644101 | -2.24 | PREDICTED: Homo sapiens misc_RNA (LOC644101), miscRNA. |
| SMC4 | -2.24 | Homo sapiens structural maintenance of chromosomes 4 (SMC4), transcript variant 2, mRNA. |
| NR1D2 | -2.24 | PREDICTED: Homo sapiens nuclear receptor subfamily 1, group D, member 2 (NR1D2), mRNA. |
| DDX11 | -2.24 | Homo sapiens DEAD/H (Asp-Glu-Ala-Asp/His) box polypeptide 11 (CHL1-like helicase homolog, S. cerevisiae) (DDX11), transcript variant 1, mRNA. |
| ZSCAN2 | -2.24 | Homo sapiens zinc finger and SCAN domain containing 2 (ZSCAN2), transcript variant 3, mRNA. |
| LOC653972 | -2.24 | PREDICTED: Homo sapiens similar to chromobox homolog 3 (LOC653972), mRNA. |
| AKT1 | -2.24 | Homo sapiens v-akt murine thymoma viral oncogene homolog 1 (AKT1), transcript variant 3, mRNA. |
| LOC400304 | -2.25 | PREDICTED: Homo sapiens similar to Golgi autoantigen, golgin subfamily A member 2 (Golgi matrix protein GM130), transcript variant 1 (LOC400304), mRNA. |
| GRINA | -2.25 | Homo sapiens glutamate receptor, ionotropic, N-methyl D-aspartate-associated protein 1 (glutamate binding) (GRINA), transcript variant 2, mRNA. |
| MTA2 | -2.25 | Homo sapiens metastasis associated 1 family, member 2 (MTA2), mRNA. |
| LOC100133478 | -2.25 | PREDICTED: Homo sapiens misc_RNA (LOC100133478), miscRNA. |
| YWHAG | -2.25 | Homo sapiens tyrosine 3-monooxygenase/tryptophan 5-monooxygenase activation protein, gamma polypeptide (YWHAG), mRNA. |
| AGL | -2.25 | Homo sapiens amylo-1, 6-glucosidase, 4-alpha-glucanotransferase (AGL), transcript variant 5, mRNA. |
| FAM96A | -2.25 | Homo sapiens family with sequence similarity 96, member A (FAM96A), transcript variant 2, mRNA. |
| CBLL1 | -2.25 | Homo sapiens Cas-Br-M (murine) ecotropic retroviral transforming sequence-like 1 (CBLL1), mRNA. |
| FLJ10324 | -2.26 | Homo sapiens hypothetical protein FLJ10324 (FLJ10324), mRNA. |
| UBE3B | -2.26 | Homo sapiens ubiquitin protein ligase E3B (UBE3B), transcript variant 1, mRNA. |
| FAM181B | -2.26 | Homo sapiens family with sequence similarity 181, member B (FAM181B), mRNA. |
| USP5 | -2.26 | Homo sapiens ubiquitin specific peptidase 5 (isopeptidase T) (USP5), transcript variant 2, mRNA. |
| MLST8 | -2.26 | Homo sapiens MTOR associated protein, LST8 homolog (S. cerevisiae) (MLST8), mRNA. |
| C11orf84 | -2.26 | Homo sapiens chromosome 11 open reading frame 84 (C11orf84), mRNA. |
| MACROD1 | -2.26 | Homo sapiens MACRO domain containing 1 (MACROD1), mRNA. |
| TWIST1 | -2.26 | Homo sapiens twist homolog 1 (Drosophila) (TWIST1), mRNA. |
| VSTM2L | -2.27 | Homo sapiens V-set and transmembrane domain containing 2 like (VSTM2L), mRNA. |
|  | -2.27 | Homo sapiens cDNA FLJ31750 fis, clone NT2RI2007406 |
| FEZ2 | -2.27 | Homo sapiens fasciculation and elongation protein zeta 2 (zygin II) (FEZ2), transcript variant 1, mRNA. |
| LOC647302 | -2.27 | PREDICTED: Homo sapiens misc_RNA (LOC647302), miscRNA. |
| LOC100131139 | -2.27 | PREDICTED: Homo sapiens misc_RNA (LOC100131139), miscRNA. |
| TYMS | -2.27 | Homo sapiens thymidylate synthetase (TYMS), mRNA. |
| CABIN1 | -2.28 | Homo sapiens calcineurin binding protein 1 (CABIN1), mRNA. |
| LOC100132658 | -2.28 | PREDICTED: Homo sapiens misc_RNA (LOC100132658), miscRNA. |
| FKRP | -2.28 | Homo sapiens fukutin related protein (FKRP), transcript variant 1, mRNA. |
| 11-Mar | -2.28 | Homo sapiens membrane-associated ring finger (C3HC4) 11 (MARCH11), mRNA. |
| FILIP1L | -2.28 | Homo sapiens filamin A interacting protein 1-like (FILIP1L), transcript variant 2, mRNA. |
| ARHGAP11B | -2.28 | Homo sapiens Rho GTPase activating protein 11B (ARHGAP11B), mRNA. XM_931045 XM_931050 |
| LOC100132324 | -2.28 | PREDICTED: Homo sapiens misc_RNA (LOC100132324), miscRNA. |
| LOC729646 | -2.28 | PREDICTED: Homo sapiens misc_RNA (LOC729646), miscRNA. |
| MAP3K7IP1 | -2.29 | Homo sapiens mitogen-activated protein kinase kinase kinase 7 interacting protein 1 (MAP3K7IP1), transcript variant alpha, mRNA. |
| NUP188 | -2.29 | Homo sapiens nucleoporin 188kDa (NUP188), mRNA. |
| RNU1F1 | -2.29 | Homo sapiens RNA, U1F1 small nuclear (RNU1F1), small nuclear RNA. |
| PLK4 | -2.29 | Homo sapiens polo-like kinase 4 (Drosophila) (PLK4), mRNA. |
| C1orf86 | -2.29 | Homo sapiens chromosome 1 open reading frame 86 (C1orf86), transcript variant 2, mRNA. |
| DDX3X | -2.29 | Homo sapiens DEAD (Asp-Glu-Ala-Asp) box polypeptide 3, X-linked (DDX3X), mRNA. |
| GSTTP2 | -2.29 | Homo sapiens glutathione S-transferase theta pseudogene 2 (GSTTP2), non-coding RNA. XM_941198 XM_945014 XM_945016 |
| ZNF195 | -2.3 | Homo sapiens zinc finger protein 195 (ZNF195), mRNA. |
| ZNF462 | -2.3 | Homo sapiens zinc finger protein 462 (ZNF462), mRNA. |
| GLRX | -2.3 | Homo sapiens glutaredoxin (thioltransferase) (GLRX), mRNA. |
| RNU1-5 | -2.3 | Homo sapiens RNA, U1 small nuclear 5 (RNU1-5), small nuclear RNA. |
| ADPRHL1 | -2.3 | Homo sapiens ADP-ribosylhydrolase like 1 (ADPRHL1), transcript variant 2, mRNA. |
| GAA | -2.3 | Homo sapiens glucosidase, alpha; acid (GAA), transcript variant 3, mRNA. |
| CCDC90A | -2.3 | Homo sapiens coiled-coil domain containing 90A (CCDC90A), mRNA. |
| METRN | -2.3 | Homo sapiens meteorin, glial cell differentiation regulator (METRN), mRNA. |
| LOC732360 | -2.3 | PREDICTED: Homo sapiens misc_RNA (LOC732360), miscRNA. |
| JUND | -2.3 | Homo sapiens jun D proto-oncogene (JUND), mRNA. |
| TSNAX | -2.3 | Homo sapiens translin-associated factor X (TSNAX), mRNA. |
| CADM4 | -2.31 | Homo sapiens cell adhesion molecule 4 (CADM4), mRNA. |
|  | -2.31 | Homo sapiens cDNA FLJ31093 fis, clone IMR321000161 |
| MAD2L1 | -2.31 | Homo sapiens MAD2 mitotic arrest deficient-like 1 (yeast) (MAD2L1), mRNA. |
| C16orf7 | -2.31 | Homo sapiens chromosome 16 open reading frame 7 (C16orf7), mRNA. |
| FXR2 | -2.31 | Homo sapiens fragile X mental retardation, autosomal homolog 2 (FXR2), mRNA. |
| LOC729623 | -2.32 | PREDICTED: Homo sapiens similar to protein kinase, cAMP-dependent, regulatory, type I, beta (LOC729623), mRNA. |
| LOC90586 | -2.32 | Homo sapiens AOC3 pseudogene (LOC90586), non-coding RNA. |
| CABYR | -2.32 | Homo sapiens calcium binding tyrosine-(Y)-phosphorylation regulated (CABYR), transcript variant 2, mRNA. |
| CDCA4 | -2.32 | Homo sapiens cell division cycle associated 4 (CDCA4), transcript variant 14, mRNA. |
| CPSF3L | -2.32 | Homo sapiens cleavage and polyadenylation specific factor 3-like (CPSF3L), mRNA. |
| MAPK12 | -2.32 | Homo sapiens mitogen-activated protein kinase 12 (MAPK12), mRNA. |
| LOC100133600 | -2.33 | PREDICTED: Homo sapiens hypothetical protein LOC100133600 (LOC100133600), mRNA. |
| SMYD3 | -2.33 | Homo sapiens SET and MYND domain containing 3 (SMYD3), mRNA. |
| PDZD4 | -2.33 | Homo sapiens PDZ domain containing 4 (PDZD4), mRNA. |
| PEX14 | -2.33 | Homo sapiens peroxisomal biogenesis factor 14 (PEX14), mRNA. |
| ACIN1 | -2.33 | Homo sapiens apoptotic chromatin condensation inducer 1 (ACIN1), mRNA. |
| GIT1 | -2.33 | Homo sapiens G protein-coupled receptor kinase interacting ArfGAP 1 (GIT1), transcript variant 2, mRNA. |
| ANXA2P1 | -2.33 | Homo sapiens annexin A2 pseudogene 1 (ANXA2P1) on chromosome 4. |
| LOC728452 | -2.33 | PREDICTED: Homo sapiens similar to nuclear pore membrane protein 121 (LOC728452), mRNA. |
| PVRL3 | -2.33 | Homo sapiens poliovirus receptor-related 3 (PVRL3), mRNA. |
| SNORD36C | -2.33 | Homo sapiens small nucleolar RNA, C/D box 36C (SNORD36C), small nucleolar RNA. |
| C14orf102 | -2.33 | Homo sapiens chromosome 14 open reading frame 102 (C14orf102), transcript variant 1, mRNA. |
| PCYOX1L | -2.33 | Homo sapiens prenylcysteine oxidase 1 like (PCYOX1L), mRNA. |
| LOC728953 | -2.33 | PREDICTED: Homo sapiens similar to S19 ribosomal protein (LOC728953), mRNA. |
| TAPBP | -2.34 | Homo sapiens TAP binding protein (tapasin) (TAPBP), transcript variant 1, mRNA. |
| SLC18A1 | -2.34 | Homo sapiens solute carrier family 18 (vesicular monoamine), member 1 (SLC18A1), mRNA. |
| C18orf32 | -2.34 | Homo sapiens chromosome 18 open reading frame 32 (C18orf32), mRNA. |
| MIR130A | -2.34 | Homo sapiens microRNA 130a (MIR130A), microRNA. |
| LOC645233 | -2.34 | Homo sapiens thymine-DNA glycosylase pseudogene (LOC645233), non-coding RNA. |
| C16orf79 | -2.34 | Homo sapiens chromosome 16 open reading frame 79 (C16orf79), mRNA. |
| FAM53B | -2.34 | Homo sapiens family with sequence similarity 53, member B (FAM53B), mRNA. |
| LRRC45 | -2.34 | Homo sapiens leucine rich repeat containing 45 (LRRC45), mRNA. |
| PIF1 | -2.35 | Homo sapiens PIF1 5'-to-3' DNA helicase homolog (S. cerevisiae) (PIF1), mRNA. |
| LOC390705 | -2.35 | PREDICTED: Homo sapiens similar to protein phosphatase 2A 48 kDa regulatory subunit isoform 1; serine/threonine protein phosphatase 2A, 48kDa regulatory subunit; PP2A, subunit B, PR48 isoform; PP2A B subunit PR48; NY-REN-8 antigen (LOC390705), misc RNA. |
|  | -2.35 | Homo sapiens cDNA: FLJ21333 fis, clone COL02535 |
| LOC100128056 | -2.35 | PREDICTED: Homo sapiens similar to hCG2013701 (LOC100128056), mRNA. |
| KIAA0492 | -2.35 | PREDICTED: Homo sapiens KIAA0492 protein (KIAA0492), mRNA. |
| LOC645381 | -2.35 | PREDICTED: Homo sapiens misc_RNA (LOC645381), miscRNA. |
| HMGB2 | -2.35 | Homo sapiens high-mobility group box 2 (HMGB2), mRNA. |
| UBAP2L | -2.35 | Homo sapiens ubiquitin associated protein 2-like (UBAP2L), mRNA. |
|  | -2.35 | Homo sapiens mRNA; cDNA DKFZp686F09166 (from clone DKFZp686F09166) |
| LOC100130178 | -2.35 | PREDICTED: Homo sapiens misc_RNA (LOC100130178), miscRNA. |
| WASF1 | -2.35 | Homo sapiens WAS protein family, member 1 (WASF1), transcript variant 4, mRNA. |
| AATK | -2.35 | Homo sapiens apoptosis-associated tyrosine kinase (AATK), mRNA. |
| BMPR2 | -2.35 | Homo sapiens bone morphogenetic protein receptor, type II (serine/threonine kinase) (BMPR2), mRNA. |
| CLSTN1 | -2.36 | Homo sapiens calsyntenin 1 (CLSTN1), transcript variant 1, mRNA. |
| TTC9B | -2.36 | Homo sapiens tetratricopeptide repeat domain 9B (TTC9B), mRNA. |
| RASA4P | -2.36 | Homo sapiens RAS p21 protein activator 4 pseudogene (RASA4P), non-coding RNA. |
| SUV420H1 | -2.37 | Homo sapiens suppressor of variegation 4-20 homolog 1 (Drosophila) (SUV420H1), transcript variant 2, mRNA. |
| MYH9 | -2.37 | Homo sapiens myosin, heavy chain 9, non-muscle (MYH9), mRNA. |
| EPB49 | -2.37 | Homo sapiens erythrocyte membrane protein band 4.9 (dematin) (EPB49), mRNA. |
| SPATS2L | -2.38 | Homo sapiens spermatogenesis associated, serine-rich 2-like (SPATS2L), transcript variant 2, mRNA. |
| FOXRED2 | -2.38 | Homo sapiens FAD-dependent oxidoreductase domain containing 2 (FOXRED2), mRNA. |
| SEMA5A | -2.38 | Homo sapiens sema domain, seven thrombospondin repeats (type 1 and type 1-like), transmembrane domain (TM) and short cytoplasmic domain, (semaphorin) 5A (SEMA5A), mRNA. |
| GPC1 | -2.38 | Homo sapiens glypican 1 (GPC1), mRNA. |
| BIN1 | -2.38 | Homo sapiens bridging integrator 1 (BIN1), transcript variant 1, mRNA. |
| DDX51 | -2.38 | Homo sapiens DEAD (Asp-Glu-Ala-Asp) box polypeptide 51 (DDX51), mRNA. |
| ATF3 | -2.38 | Homo sapiens activating transcription factor 3 (ATF3), transcript variant 4, mRNA. |
| RNU1G2 | -2.38 | Homo sapiens RNA, U1G2 small nuclear (RNU1G2), small nuclear RNA. |
| AAK1 | -2.38 | Homo sapiens AP2 associated kinase 1 (AAK1), mRNA. |
| RPLP1 | -2.39 | Homo sapiens ribosomal protein, large, P1 (RPLP1), transcript variant 1, mRNA. |
| MCART1 | -2.39 | Homo sapiens mitochondrial carrier triple repeat 1 (MCART1), mRNA. |
| CSF2RA | -2.39 | PREDICTED: Homo sapiens colony stimulating factor 2 receptor, alpha, low-affinity (granulocyte-macrophage) (CSF2RA), mRNA. |
| MAN2B1 | -2.39 | Homo sapiens mannosidase, alpha, class 2B, member 1 (MAN2B1), mRNA. |
| PRR16 | -2.39 | Homo sapiens proline rich 16 (PRR16), mRNA. |
| C20orf29 | -2.39 | Homo sapiens chromosome 20 open reading frame 29 (C20orf29), mRNA. |
| PCBP3 | -2.39 | Homo sapiens poly(rC) binding protein 3 (PCBP3), mRNA. |
| MT1A | -2.4 | Homo sapiens metallothionein 1A (MT1A), mRNA. |
| NLGN4X | -2.4 | Homo sapiens neuroligin 4, X-linked (NLGN4X), transcript variant 1, mRNA. |
| MKNK2 | -2.4 | Homo sapiens MAP kinase interacting serine/threonine kinase 2 (MKNK2), transcript variant 2, mRNA. |
| SC65 | -2.4 | Homo sapiens synaptonemal complex protein SC65 (SC65), mRNA. |
| C19orf63 | -2.4 | Homo sapiens chromosome 19 open reading frame 63 (C19orf63), transcript variant HSS1, mRNA. |
| FSTL1 | -2.4 | Homo sapiens follistatin-like 1 (FSTL1), mRNA. |
| ISOC1 | -2.41 | Homo sapiens isochorismatase domain containing 1 (ISOC1), mRNA. |
| MAPKAPK2 | -2.41 | Homo sapiens mitogen-activated protein kinase-activated protein kinase 2 (MAPKAPK2), transcript variant 2, mRNA. |
| CBL | -2.41 | Homo sapiens Cas-Br-M (murine) ecotropic retroviral transforming sequence (CBL), mRNA. |
| LAMB1 | -2.41 | Homo sapiens laminin, beta 1 (LAMB1), mRNA. |
| PKN3 | -2.41 | Homo sapiens protein kinase N3 (PKN3), mRNA. |
| SCG2 | -2.41 | Homo sapiens secretogranin II (chromogranin C) (SCG2), mRNA. |
| RELL1 | -2.41 | Homo sapiens RELT-like 1 (RELL1), transcript variant 1, mRNA. |
| LOC651453 | -2.41 | PREDICTED: Homo sapiens similar to ribosomal protein L36 (LOC651453), mRNA. |
| ENHO | -2.41 | Homo sapiens energy homeostasis associated (ENHO), mRNA. |
| NFATC2IP | -2.41 | Homo sapiens nuclear factor of activated T-cells, cytoplasmic, calcineurin-dependent 2 interacting protein (NFATC2IP), mRNA. |
| CANT1 | -2.41 | Homo sapiens calcium activated nucleotidase 1 (CANT1), mRNA. |
| RPS6KC1 | -2.42 | Homo sapiens ribosomal protein S6 kinase, 52kDa, polypeptide 1 (RPS6KC1), mRNA. |
| CYP1B1 | -2.42 | Homo sapiens cytochrome P450, family 1, subfamily B, polypeptide 1 (CYP1B1), mRNA. |
| PTK7 | -2.42 | Homo sapiens PTK7 protein tyrosine kinase 7 (PTK7), transcript variant PTK7-4, mRNA. |
| SLC38A2 | -2.42 | Homo sapiens solute carrier family 38, member 2 (SLC38A2), mRNA. |
| BRMS1L | -2.42 | Homo sapiens breast cancer metastasis-suppressor 1-like (BRMS1L), mRNA. |
| NR2F1 | -2.42 | Homo sapiens nuclear receptor subfamily 2, group F, member 1 (NR2F1), mRNA. |
| DNAJC2 | -2.42 | Homo sapiens DnaJ (Hsp40) homolog, subfamily C, member 2 (DNAJC2), transcript variant 1, mRNA. |
| PNPLA8 | -2.42 | Homo sapiens patatin-like phospholipase domain containing 8 (PNPLA8), mRNA. |
| SHANK3 | -2.42 | Homo sapiens SH3 and multiple ankyrin repeat domains 3 (SHANK3), mRNA. |
| ATP1A3 | -2.42 | Homo sapiens ATPase, Na+/K+ transporting, alpha 3 polypeptide (ATP1A3), mRNA. |
| C1QL1 | -2.42 | Homo sapiens complement component 1, q subcomponent-like 1 (C1QL1), mRNA. |
| CES2 | -2.42 | Homo sapiens carboxylesterase 2 (intestine, liver) (CES2), transcript variant 1, mRNA. |
| SPTAN1 | -2.42 | Homo sapiens spectrin, alpha, non-erythrocytic 1 (alpha-fodrin) (SPTAN1), mRNA. |
| SLC30A1 | -2.43 | Homo sapiens solute carrier family 30 (zinc transporter), member 1 (SLC30A1), mRNA. |
| MTDH | -2.43 | Homo sapiens metadherin (MTDH), mRNA. |
| LHFP | -2.43 | Homo sapiens lipoma HMGIC fusion partner (LHFP), mRNA. |
| CCDC3 | -2.43 | Homo sapiens coiled-coil domain containing 3 (CCDC3), mRNA. |
| SMC3 | -2.43 | Homo sapiens structural maintenance of chromosomes 3 (SMC3), mRNA. |
| LOC100008589 | -2.43 | Homo sapiens 28S ribosomal RNA (LOC100008589), non-coding RNA. |
| ACVR2B | -2.43 | Homo sapiens activin A receptor, type IIB (ACVR2B), mRNA. |
| BMP1 | -2.43 | Homo sapiens bone morphogenetic protein 1 (BMP1), transcript variant BMP1-3, mRNA. |
| TSGA14 | -2.43 | Homo sapiens testis specific, 14 (TSGA14), mRNA. |
| METTL9 | -2.44 | Homo sapiens methyltransferase like 9 (METTL9), transcript variant 1, mRNA. |
| RTN4 | -2.44 | Homo sapiens reticulon 4 (RTN4), transcript variant 1, mRNA. |
| CYB5R3 | -2.44 | Homo sapiens cytochrome b5 reductase 3 (CYB5R3), transcript variant M, mRNA. |
| LOC730993 | -2.44 | PREDICTED: Homo sapiens misc_RNA (LOC730993), miscRNA. |
| FBLN2 | -2.44 | Homo sapiens fibulin 2 (FBLN2), transcript variant 2, mRNA. |
| NBN | -2.44 | Homo sapiens nibrin (NBN), mRNA. |
| P2RX7 | -2.44 | Homo sapiens purinergic receptor P2X, ligand-gated ion channel, 7 (P2RX7), mRNA. |
| BCL7C | -2.44 | Homo sapiens B-cell CLL/lymphoma 7C (BCL7C), mRNA. |
| BRF1 | -2.44 | Homo sapiens BRF1 homolog, subunit of RNA polymerase III transcription initiation factor IIIB (S. cerevisiae) (BRF1), transcript variant 1, mRNA. |
| LOC729120 | -2.45 | PREDICTED: Homo sapiens hypothetical LOC729120 (LOC729120), mRNA. |
| MEGF8 | -2.45 | Homo sapiens multiple EGF-like-domains 8 (MEGF8), mRNA. |
| ZBTB7A | -2.45 | Homo sapiens zinc finger and BTB domain containing 7A (ZBTB7A), mRNA. |
| TBCD | -2.45 | Homo sapiens tubulin folding cofactor D (TBCD), mRNA. |
| IPO4 | -2.45 | Homo sapiens importin 4 (IPO4), mRNA. |
| KIF23 | -2.46 | Homo sapiens kinesin family member 23 (KIF23), transcript variant 2, mRNA. |
| LOC400061 | -2.46 | PREDICTED: Homo sapiens misc_RNA (LOC400061), miscRNA. |
| MID1IP1 | -2.46 | Homo sapiens MID1 interacting protein 1 (gastrulation specific G12 homolog (zebrafish)) (MID1IP1), transcript variant 1, mRNA. |
| MEGF6 | -2.46 | Homo sapiens multiple EGF-like-domains 6 (MEGF6), mRNA. |
| SLC19A1 | -2.46 | Homo sapiens solute carrier family 19 (folate transporter), member 1 (SLC19A1), mRNA. |
| LOC391825 | -2.46 | PREDICTED: Homo sapiens similar to hCG1643032 (LOC391825), mRNA. |
| LOC645979 | -2.46 | PREDICTED: Homo sapiens similar to ribosomal protein S26 (LOC645979), mRNA. |
| JAM2 | -2.46 | Homo sapiens junctional adhesion molecule 2 (JAM2), mRNA. |
| LEP | -2.46 | Homo sapiens leptin (obesity homolog, mouse) (LEP), mRNA. |
| LMO4 | -2.46 | Homo sapiens LIM domain only 4 (LMO4), mRNA. |
| SUMO2 | -2.47 | Homo sapiens SMT3 suppressor of mif two 3 homolog 2 (S. cerevisiae) (SUMO2), transcript variant 2, mRNA. |
| FADS1 | -2.47 | Homo sapiens fatty acid desaturase 1 (FADS1), mRNA. |
| CTDSP1 | -2.47 | Homo sapiens CTD (carboxy-terminal domain, RNA polymerase II, polypeptide A) small phosphatase 1 (CTDSP1), transcript variant 1, mRNA. |
| C14orf43 | -2.47 | Homo sapiens chromosome 14 open reading frame 43 (C14orf43), transcript variant 1, mRNA. |
| SLC35C2 | -2.47 | Homo sapiens solute carrier family 35, member C2 (SLC35C2), transcript variant 2, mRNA. |
| TMEM70 | -2.47 | Homo sapiens transmembrane protein 70 (TMEM70), transcript variant 1, mRNA. |
| TMEM26 | -2.47 | Homo sapiens transmembrane protein 26 (TMEM26), mRNA. |
| SMO | -2.47 | Homo sapiens smoothened homolog (Drosophila) (SMO), mRNA. |
| DYNLT3 | -2.48 | Homo sapiens dynein, light chain, Tctex-type 3 (DYNLT3), mRNA. |
| MFSD11 | -2.48 | Homo sapiens major facilitator superfamily domain containing 11 (MFSD11), mRNA. |
| NCOA5 | -2.48 | Homo sapiens nuclear receptor coactivator 5 (NCOA5), mRNA. |
| TAZ | -2.48 | Homo sapiens tafazzin (cardiomyopathy, dilated 3A (X-linked); endocardial fibroelastosis 2; Barth syndrome) (TAZ), transcript variant 2, mRNA. |
| AGAP8 | -2.49 | Homo sapiens ArfGAP with GTPase domain, ankyrin repeat and PH domain 8 (AGAP8), mRNA. |
| NUCB2 | -2.49 | Homo sapiens nucleobindin 2 (NUCB2), mRNA. |
| LOC643007 | -2.49 | PREDICTED: Homo sapiens similar to large subunit ribosomal protein L36a (LOC643007), mRNA. |
| KIF15 | -2.49 | Homo sapiens kinesin family member 15 (KIF15), mRNA. |
| S100A4 | -2.49 | Homo sapiens S100 calcium binding protein A4 (S100A4), transcript variant 2, mRNA. |
| GAS6 | -2.49 | Homo sapiens growth arrest-specific 6 (GAS6), mRNA. |
| SLC6A8 | -2.5 | Homo sapiens solute carrier family 6 (neurotransmitter transporter, creatine), member 8 (SLC6A8), mRNA. |
| C19orf29 | -2.5 | Homo sapiens chromosome 19 open reading frame 29 (C19orf29), transcript variant 1, mRNA. |
| C7orf26 | -2.5 | Homo sapiens chromosome 7 open reading frame 26 (C7orf26), mRNA. |
| LOC100131971 | -2.5 | PREDICTED: Homo sapiens similar to 40S ribosomal protein S26 (LOC100131971), mRNA. |
| ADM | -2.5 | Homo sapiens adrenomedullin (ADM), mRNA. |
| CHERP | -2.5 | Homo sapiens calcium homeostasis endoplasmic reticulum protein (CHERP), mRNA. |
| LASS6 | -2.5 | Homo sapiens LAG1 homolog, ceramide synthase 6 (LASS6), mRNA. |
| ZNF860 | -2.5 | Homo sapiens zinc finger protein 860 (ZNF860), mRNA. XM_001725699 XM_496634 |
| IRAK1 | -2.5 | Homo sapiens interleukin-1 receptor-associated kinase 1 (IRAK1), transcript variant 3, mRNA. |
| CAPZB | -2.5 | Homo sapiens capping protein (actin filament) muscle Z-line, beta (CAPZB), mRNA. |
| LOC648581 | -2.51 | PREDICTED: Homo sapiens similar to alveolar soft part sarcoma chromosome region, candidate 1 (LOC648581), mRNA. |
| RBM42 | -2.51 | Homo sapiens RNA binding motif protein 42 (RBM42), mRNA. |
| ZDHHC12 | -2.51 | Homo sapiens zinc finger, DHHC-type containing 12 (ZDHHC12), mRNA. |
| CYTSB | -2.51 | Homo sapiens cytospin B (CYTSB), transcript variant NSP5alpha3beta, mRNA. |
| PRDM8 | -2.51 | Homo sapiens PR domain containing 8 (PRDM8), transcript variant 1, mRNA. |
| SLC39A8 | -2.51 | Homo sapiens solute carrier family 39 (zinc transporter), member 8 (SLC39A8), transcript variant 1, mRNA. |
| DNMT3B | -2.51 | Homo sapiens DNA (cytosine-5-)-methyltransferase 3 beta (DNMT3B), transcript variant 1, mRNA. |
| FAM133B | -2.51 | Homo sapiens family with sequence similarity 133, member B (FAM133B), transcript variant 1, mRNA. |
| RAB5C | -2.51 | Homo sapiens RAB5C, member RAS oncogene family (RAB5C), transcript variant 1, mRNA. |
|  | -2.51 | UI-E-EJ0-ahk-m-09-0-UI.r2 UI-E-EJ0 Homo sapiens cDNA clone UI-E-EJ0-ahk-m-09-0-UI 5, mRNA sequence |
| RPS26P10 | -2.51 | PREDICTED: Homo sapiens ribosomal protein S26 pseudogene 10 (RPS26P10), mRNA. |
| KTI12 | -2.52 | Homo sapiens KTI12 homolog, chromatin associated (S. cerevisiae) (KTI12), mRNA. |
| GATAD2B | -2.52 | Homo sapiens GATA zinc finger domain containing 2B (GATAD2B), mRNA. |
| VIM | -2.52 | Homo sapiens vimentin (VIM), mRNA. |
| MESDC1 | -2.52 | Homo sapiens mesoderm development candidate 1 (MESDC1), mRNA. |
| LPHN1 | -2.52 | Homo sapiens latrophilin 1 (LPHN1), transcript variant 2, mRNA. |
| ZNF256 | -2.52 | Homo sapiens zinc finger protein 256 (ZNF256), mRNA. |
| PYGO2 | -2.53 | Homo sapiens pygopus homolog 2 (Drosophila) (PYGO2), mRNA. |
| FUZ | -2.54 | Homo sapiens fuzzy homolog (Drosophila) (FUZ), mRNA. |
| CAPZA1 | -2.54 | Homo sapiens capping protein (actin filament) muscle Z-line, alpha 1 (CAPZA1), mRNA. |
| LOC100128525 | -2.54 | PREDICTED: Homo sapiens similar to hCG1641472 (LOC100128525), mRNA. |
| PRPF4B | -2.54 | Homo sapiens PRP4 pre-mRNA processing factor 4 homolog B (yeast) (PRPF4B), transcript variant 2, mRNA. |
| C3orf39 | -2.55 | Homo sapiens chromosome 3 open reading frame 39 (C3orf39), mRNA. |
| LOC100131672 | -2.55 | PREDICTED: Homo sapiens misc_RNA (LOC100131672), miscRNA. |
| C9orf140 | -2.55 | Homo sapiens chromosome 9 open reading frame 140 (C9orf140), mRNA. |
| CYLN2 | -2.55 | Homo sapiens cytoplasmic linker 2 (CYLN2), transcript variant 2, mRNA. |
| PRSS35 | -2.56 | Homo sapiens protease, serine, 35 (PRSS35), mRNA. |
| DUSP5 | -2.56 | Homo sapiens dual specificity phosphatase 5 (DUSP5), mRNA. |
| IQGAP1 | -2.56 | Homo sapiens IQ motif containing GTPase activating protein 1 (IQGAP1), mRNA. |
| CCDC88C | -2.56 | Homo sapiens coiled-coil domain containing 88C (CCDC88C), mRNA. |
| SNORA45 | -2.56 | Homo sapiens small nucleolar RNA, H/ACA box 45 (SNORA45), small nucleolar RNA. |
| LPPR4 | -2.56 | Homo sapiens plasticity related gene 1 (LPPR4), mRNA. |
| DUXAP3 | -2.57 | Homo sapiens double homeobox A pseudogene 3 (DUXAP3) on chromosome 10. |
| ANLN | -2.57 | Homo sapiens anillin, actin binding protein (ANLN), mRNA. |
| LOC647000 | -2.57 | PREDICTED: Homo sapiens similar to tubulin, beta 5 (LOC647000), mRNA. |
| LOC100129094 | -2.57 | PREDICTED: Homo sapiens similar to hCG2036765 (LOC100129094), mRNA. |
| POLR2A | -2.57 | Homo sapiens polymerase (RNA) II (DNA directed) polypeptide A, 220kDa (POLR2A), mRNA. |
| MAD2L1 | -2.58 | Homo sapiens MAD2 mitotic arrest deficient-like 1 (yeast) (MAD2L1), mRNA. |
| LOC728819 | -2.58 | Homo sapiens hCG1645220 (LOC728819), mRNA. |
| LOC645231 | -2.58 | PREDICTED: Homo sapiens misc_RNA (LOC645231), miscRNA. |
| KLHL17 | -2.58 | Homo sapiens kelch-like 17 (Drosophila) (KLHL17), mRNA. |
| RHBDL1 | -2.58 | Homo sapiens rhomboid, veinlet-like 1 (Drosophila) (RHBDL1), mRNA. |
| HDGF | -2.58 | Homo sapiens hepatoma-derived growth factor (high-mobility group protein 1-like) (HDGF), mRNA. |
| IRS2 | -2.58 | Homo sapiens insulin receptor substrate 2 (IRS2), mRNA. |
| SYNE2 | -2.58 | Homo sapiens spectrin repeat containing, nuclear envelope 2 (SYNE2), transcript variant 1, mRNA. |
| SREBF1 | -2.58 | Homo sapiens sterol regulatory element binding transcription factor 1 (SREBF1), transcript variant 1, mRNA. |
| CHES1 | -2.59 | Homo sapiens checkpoint suppressor 1 (CHES1), mRNA. |
| PKD2 | -2.59 | Homo sapiens polycystic kidney disease 2 (autosomal dominant) (PKD2), mRNA. |
| PLXNB2 | -2.59 | Homo sapiens plexin B2 (PLXNB2), mRNA. |
| ZNF384 | -2.59 | Homo sapiens zinc finger protein 384 (ZNF384), transcript variant 2, mRNA. |
| ALPP | -2.59 | Homo sapiens alkaline phosphatase, placental (Regan isozyme) (ALPP), mRNA. |
| MYO9B | -2.59 | Homo sapiens myosin IXB (MYO9B), mRNA. |
| LOC100131718 | -2.59 | PREDICTED: Homo sapiens misc_RNA (LOC100131718), miscRNA. |
| GGCT | -2.6 | Homo sapiens gamma-glutamyl cyclotransferase (GGCT), mRNA. |
| APLP1 | -2.6 | Homo sapiens amyloid beta (A4) precursor-like protein 1 (APLP1), transcript variant 2, mRNA. |
| LOC100134053 | -2.6 | PREDICTED: Homo sapiens similar to POLR2J4 protein (LOC100134053), mRNA. |
| SYP | -2.6 | Homo sapiens synaptophysin (SYP), mRNA. |
| LOC100130764 | -2.6 | PREDICTED: Homo sapiens p150-like (LOC100130764), mRNA. |
| LOC400406 | -2.6 | PREDICTED: Homo sapiens similar to ADAM metallopeptidase with thrombospondin type 1 motif, 7 preproprotein (LOC400406), mRNA. |
| DHX30 | -2.61 | Homo sapiens DEAH (Asp-Glu-Ala-His) box polypeptide 30 (DHX30), transcript variant 1, mRNA. |
| PLEC1 | -2.61 | Homo sapiens plectin 1, intermediate filament binding protein 500kDa (PLEC1), transcript variant 1, mRNA. |
| LOC644852 | -2.62 | PREDICTED: Homo sapiens hypothetical protein LOC644852, transcript variant 2 (LOC644852), mRNA. |
| LOC100131541 | -2.62 | PREDICTED: Homo sapiens similar to MRSS6228 (LOC100131541), mRNA. |
| SON | -2.62 | Homo sapiens SON DNA binding protein (SON), transcript variant b, mRNA. |
| VANGL2 | -2.62 | Homo sapiens vang-like 2 (van gogh, Drosophila) (VANGL2), mRNA. |
| PCGF1 | -2.63 | Homo sapiens polycomb group ring finger 1 (PCGF1), mRNA. |
| LOC727762 | -2.63 | PREDICTED: Homo sapiens similar to NADH dehydrogenase (ubiquinone) 1 beta subcomplex, 4, 15kDa, transcript variant 1 (LOC727762), mRNA. |
| RDH10 | -2.63 | Homo sapiens retinol dehydrogenase 10 (all-trans) (RDH10), mRNA. |
| LOC646966 | -2.64 | PREDICTED: Homo sapiens misc_RNA (LOC646966), miscRNA. |
| ACTN4 | -2.64 | Homo sapiens actinin, alpha 4 (ACTN4), mRNA. |
| MIR25 | -2.64 | Homo sapiens microRNA 25 (MIR25), microRNA. |
| SLC5A8 | -2.64 | Homo sapiens solute carrier family 5 (iodide transporter), member 8 (SLC5A8), mRNA. |
| CREB5 | -2.64 | Homo sapiens cAMP responsive element binding protein 5 (CREB5), transcript variant 1, mRNA. |
| PRRT1 | -2.64 | Homo sapiens proline-rich transmembrane protein 1 (PRRT1), mRNA. |
| KTN1 | -2.64 | Homo sapiens kinectin 1 (kinesin receptor) (KTN1), transcript variant 1, mRNA. |
| CST3 | -2.64 | Homo sapiens cystatin C (CST3), mRNA. |
| C14orf82 | -2.65 | PREDICTED: Homo sapiens chromosome 14 open reading frame 82 (C14orf82), mRNA. |
| MBOAT1 | -2.65 | Homo sapiens membrane bound O-acyltransferase domain containing 1 (MBOAT1), mRNA. |
| SF1 | -2.65 | Homo sapiens splicing factor 1 (SF1), transcript variant 1, mRNA. |
| JUP | -2.65 | Homo sapiens junction plakoglobin (JUP), transcript variant 1, mRNA. |
| AP1G2 | -2.66 | Homo sapiens adaptor-related protein complex 1, gamma 2 subunit (AP1G2), mRNA. |
| CYTH1 | -2.66 | Homo sapiens cytohesin 1 (CYTH1), transcript variant 2, mRNA. |
| LOC399491 | -2.67 | PREDICTED: Homo sapiens misc_RNA (LOC399491), miscRNA. |
| LOC728715 | -2.67 | PREDICTED: Homo sapiens similar to hCG38149 (LOC728715), mRNA. |
| PRR7 | -2.67 | Homo sapiens proline rich 7 (synaptic) (PRR7), mRNA. |
| YEATS4 | -2.68 | Homo sapiens YEATS domain containing 4 (YEATS4), mRNA. |
| PRO1853 | -2.68 | Homo sapiens hypothetical protein PRO1853 (PRO1853), transcript variant 1, mRNA. |
| TRAF7 | -2.68 | Homo sapiens TNF receptor-associated factor 7 (TRAF7), mRNA. |
| WBP2 | -2.69 | Homo sapiens WW domain binding protein 2 (WBP2), mRNA. |
| POLD1 | -2.69 | Homo sapiens polymerase (DNA directed), delta 1, catalytic subunit 125kDa (POLD1), mRNA. |
| GDPD1 | -2.69 | Homo sapiens glycerophosphodiester phosphodiesterase domain containing 1 (GDPD1), mRNA. |
| LOC727821 | -2.69 | PREDICTED: Homo sapiens misc_RNA (LOC727821), miscRNA. |
| LOC644191 | -2.69 | PREDICTED: Homo sapiens similar to hCG15685, transcript variant 1 (LOC644191), mRNA. |
| ASPSCR1 | -2.69 | PREDICTED: Homo sapiens alveolar soft part sarcoma chromosome region, candidate 1 (ASPSCR1), mRNA. |
| LOC441061 | -2.69 | PREDICTED: Homo sapiens similar to membrane-associated ring finger (C3HC4) 4 (LOC441061), mRNA. |
| SLC38A10 | -2.69 | Homo sapiens solute carrier family 38, member 10 (SLC38A10), transcript variant 2, mRNA. |
| LOC649841 | -2.69 | PREDICTED: Homo sapiens similar to protein immuno-reactive with anti-PTH polyclonal antibodies (LOC649841), mRNA. |
| MBTPS1 | -2.69 | Homo sapiens membrane-bound transcription factor peptidase, site 1 (MBTPS1), mRNA. |
| FAM107B | -2.69 | Homo sapiens family with sequence similarity 107, member B (FAM107B), mRNA. |
| FAM128A | -2.7 | Homo sapiens family with sequence similarity 128, member A (FAM128A), mRNA. |
| FAM100B | -2.7 | Homo sapiens family with sequence similarity 100, member B (FAM100B), mRNA. |
| NR1D2 | -2.7 | PREDICTED: Homo sapiens nuclear receptor subfamily 1, group D, member 2 (NR1D2), mRNA. |
| CENPT | -2.7 | Homo sapiens centromere protein T (CENPT), mRNA. |
| LY6E | -2.7 | Homo sapiens lymphocyte antigen 6 complex, locus E (LY6E), mRNA. |
| MDK | -2.7 | Homo sapiens midkine (neurite growth-promoting factor 2) (MDK), transcript variant 1, mRNA. |
| POMT2 | -2.7 | Homo sapiens protein-O-mannosyltransferase 2 (POMT2), mRNA. |
| C9orf140 | -2.71 | Homo sapiens chromosome 9 open reading frame 140 (C9orf140), mRNA. |
| MEIS3 | -2.71 | Homo sapiens Meis homeobox 3 (MEIS3), transcript variant 2, mRNA. |
| PKN1 | -2.71 | Homo sapiens protein kinase N1 (PKN1), transcript variant 1, mRNA. |
| DMC1 | -2.71 | Homo sapiens DMC1 dosage suppressor of mck1 homolog, meiosis-specific homologous recombination (yeast) (DMC1), mRNA. |
| KLF6 | -2.72 | Homo sapiens Kruppel-like factor 6 (KLF6), transcript variant 1, mRNA. |
| SFRP1 | -2.72 | Homo sapiens secreted frizzled-related protein 1 (SFRP1), mRNA. |
| C9orf7 | -2.72 | Homo sapiens chromosome 9 open reading frame 7 (C9orf7), mRNA. |
| ZFPM1 | -2.72 | Homo sapiens zinc finger protein, multitype 1 (ZFPM1), mRNA. |
| KIAA0195 | -2.72 | Homo sapiens KIAA0195 (KIAA0195), mRNA. |
| GTPBP8 | -2.72 | Homo sapiens GTP-binding protein 8 (putative) (GTPBP8), transcript variant 1, mRNA. |
| LOC389049 | -2.73 | PREDICTED: Homo sapiens misc_RNA (LOC389049), miscRNA. |
| RNF26 | -2.73 | Homo sapiens ring finger protein 26 (RNF26), mRNA. |
| ADAM23 | -2.73 | Homo sapiens ADAM metallopeptidase domain 23 (ADAM23), mRNA. |
| LOC653171 | -2.73 | PREDICTED: Homo sapiens similar to MAPK-interacting and spindle-stabilizing protein (LOC653171), mRNA. |
| SLC30A1 | -2.74 | Homo sapiens solute carrier family 30 (zinc transporter), member 1 (SLC30A1), mRNA. |
| OGDH | -2.74 | Homo sapiens oxoglutarate (alpha-ketoglutarate) dehydrogenase (lipoamide) (OGDH), nuclear gene encoding mitochondrial protein, transcript variant 1, mRNA. |
| USP16 | -2.74 | Homo sapiens ubiquitin specific peptidase 16 (USP16), transcript variant 1, mRNA. |
| SMARCB1 | -2.74 | Homo sapiens SWI/SNF related, matrix associated, actin dependent regulator of chromatin, subfamily b, member 1 (SMARCB1), transcript variant 2, mRNA. |
| DULLARD | -2.74 | Homo sapiens dullard homolog (Xenopus laevis) (DULLARD), mRNA. |
| C14orf102 | -2.74 | Homo sapiens chromosome 14 open reading frame 102 (C14orf102), transcript variant 2, mRNA. |
| TERF1 | -2.75 | Homo sapiens telomeric repeat binding factor (NIMA-interacting) 1 (TERF1), transcript variant 1, mRNA. |
| SMG7 | -2.75 | Homo sapiens Smg-7 homolog, nonsense mediated mRNA decay factor (C. elegans) (SMG7), transcript variant 1, mRNA. |
| ROCK2 | -2.76 | Homo sapiens Rho-associated, coiled-coil containing protein kinase 2 (ROCK2), mRNA. |
| NDC80 | -2.76 | Homo sapiens NDC80 homolog, kinetochore complex component (S. cerevisiae) (NDC80), mRNA. |
| ALCAM | -2.76 | Homo sapiens activated leukocyte cell adhesion molecule (ALCAM), mRNA. |
| CTNNA2 | -2.77 | Homo sapiens catenin (cadherin-associated protein), alpha 2 (CTNNA2), mRNA. |
| MOV10 | -2.77 | Homo sapiens Mov10, Moloney leukemia virus 10, homolog (mouse) (MOV10), mRNA. |
| USP49 | -2.77 | Homo sapiens ubiquitin specific peptidase 49 (USP49), mRNA. |
| LOC730288 | -2.77 | PREDICTED: Homo sapiens similar to 40S ribosomal protein S28 (LOC730288), mRNA. |
| DTL | -2.78 | Homo sapiens denticleless homolog (Drosophila) (DTL), mRNA. |
| TMEM70 | -2.78 | Homo sapiens transmembrane protein 70 (TMEM70), transcript variant 1, mRNA. |
| PPP2R3B | -2.79 | Homo sapiens protein phosphatase 2 (formerly 2A), regulatory subunit B'', beta (PPP2R3B), transcript variant 1, mRNA. |
| PDZRN3 | -2.79 | PREDICTED: Homo sapiens PDZ domain containing RING finger 3 (PDZRN3), mRNA. |
| FCGRT | -2.79 | Homo sapiens Fc fragment of IgG, receptor, transporter, alpha (FCGRT), transcript variant 1, mRNA. |
| IGFBP3 | -2.79 | Homo sapiens insulin-like growth factor binding protein 3 (IGFBP3), transcript variant 1, mRNA. |
| FAM108A2 | -2.79 | PREDICTED: Homo sapiens family with sequence similarity 108, member A2 (FAM108A2), mRNA. |
| LOC388076 | -2.79 | PREDICTED: Homo sapiens hypothetical LOC388076 (LOC388076), mRNA. |
| NEURL1B | -2.79 | Homo sapiens neuralized homolog 1B (Drosophila) (NEURL1B), mRNA. |
| DKFZp547K054 | -2.8 | PREDICTED: Homo sapiens hypothetical protein DKFZp547K054 (DKFZp547K054), mRNA. |
| NOTCH3 | -2.8 | Homo sapiens Notch homolog 3 (Drosophila) (NOTCH3), mRNA. |
| CENTG2 | -2.8 | Homo sapiens centaurin, gamma 2 (CENTG2), mRNA. |
| CCL2 | -2.8 | Homo sapiens chemokine (C-C motif) ligand 2 (CCL2), mRNA. |
| VAMP2 | -2.81 | Homo sapiens vesicle-associated membrane protein 2 (synaptobrevin 2) (VAMP2), mRNA. |
| OIP5 | -2.81 | Homo sapiens Opa interacting protein 5 (OIP5), mRNA. |
| FAM57B | -2.81 | Homo sapiens family with sequence similarity 57, member B (FAM57B), mRNA. |
| ARHGDIA | -2.82 | Homo sapiens Rho GDP dissociation inhibitor (GDI) alpha (ARHGDIA), mRNA. |
| GPRIN1 | -2.82 | Homo sapiens G protein regulated inducer of neurite outgrowth 1 (GPRIN1), mRNA. |
| C9orf30 | -2.82 | Homo sapiens chromosome 9 open reading frame 30 (C9orf30), mRNA. |
| BNIP2 | -2.82 | Homo sapiens BCL2/adenovirus E1B 19kDa interacting protein 2 (BNIP2), mRNA. |
| KIF11 | -2.82 | Homo sapiens kinesin family member 11 (KIF11), mRNA. |
| LOC387882 | -2.82 | Homo sapiens hypothetical protein (LOC387882), mRNA. |
| HCG2P7 | -2.82 | Homo sapiens HLA complex group 2 pseudogene 7 (HCG2P7), non-coding RNA. |
| CSNK1E | -2.82 | Homo sapiens casein kinase 1, epsilon (CSNK1E), transcript variant 1, mRNA. |
| LOC653438 | -2.83 | PREDICTED: Homo sapiens similar to CG33096-PB, isoform B, transcript variant 1 (LOC653438), mRNA. |
| LOC644988 | -2.83 | PREDICTED: Homo sapiens misc_RNA (LOC644988), miscRNA. |
| SPRY2 | -2.83 | Homo sapiens sprouty homolog 2 (Drosophila) (SPRY2), mRNA. |
| EPHB4 | -2.83 | Homo sapiens EPH receptor B4 (EPHB4), mRNA. |
| DUSP8 | -2.83 | Homo sapiens dual specificity phosphatase 8 (DUSP8), mRNA. |
| LOC100131989 | -2.83 | PREDICTED: Homo sapiens hypothetical protein LOC100131989 (LOC100131989), mRNA. |
| S100A10 | -2.84 | Homo sapiens S100 calcium binding protein A10 (S100A10), mRNA. |
| C9orf25 | -2.84 | Homo sapiens chromosome 9 open reading frame 25 (C9orf25), mRNA. |
| UPF3A | -2.84 | Homo sapiens UPF3 regulator of nonsense transcripts homolog A (yeast) (UPF3A), transcript variant 1, mRNA. |
| SLC22A17 | -2.84 | Homo sapiens solute carrier family 22, member 17 (SLC22A17), transcript variant 2, mRNA. |
| LOC644934 | -2.84 | PREDICTED: Homo sapiens similar to 40S ribosomal protein S26, transcript variant 1 (LOC644934), mRNA. |
| VPS4A | -2.84 | Homo sapiens vacuolar protein sorting 4 homolog A (S. cerevisiae) (VPS4A), mRNA. |
| EBF4 | -2.85 | Homo sapiens early B-cell factor 4 (EBF4), mRNA. |
| TTC32 | -2.85 | Homo sapiens tetratricopeptide repeat domain 32 (TTC32), mRNA. |
| RPS26L | -2.85 | Homo sapiens 40S ribosomal protein S26-like (RPS26L), non-coding RNA. |
| CTSF | -2.85 | Homo sapiens cathepsin F (CTSF), mRNA. |
| LOC730525 | -2.86 | PREDICTED: Homo sapiens hypothetical protein LOC730525 (LOC730525), mRNA. |
| TNFRSF6B | -2.87 | Homo sapiens tumor necrosis factor receptor superfamily, member 6b, decoy (TNFRSF6B), transcript variant M68C, mRNA. |
| LOC100129441 | -2.87 | PREDICTED: Homo sapiens hypothetical protein LOC100129441 (LOC100129441), mRNA. |
|  | -2.87 | AGENCOURT_14354957 NIH_MGC_191 Homo sapiens cDNA clone IMAGE:30413554 5, mRNA sequence |
| PCNXL3 | -2.88 | PREDICTED: Homo sapiens pecanex-like 3 (Drosophila), transcript variant 1 (PCNXL3), mRNA. |
| ST5 | -2.88 | Homo sapiens suppression of tumorigenicity 5 (ST5), transcript variant 3, mRNA. |
| MIER2 | -2.88 | Homo sapiens mesoderm induction early response 1, family member 2 (MIER2), mRNA. |
| DUS1L | -2.88 | Homo sapiens dihydrouridine synthase 1-like (S. cerevisiae) (DUS1L), mRNA. |
| SAV1 | -2.88 | Homo sapiens salvador homolog 1 (Drosophila) (SAV1), mRNA. |
| PTPN12 | -2.88 | Homo sapiens protein tyrosine phosphatase, non-receptor type 12 (PTPN12), mRNA. |
| LOC729495 | -2.88 | PREDICTED: Homo sapiens similar to hCG1999863 (LOC729495), mRNA. |
| LOC727962 | -2.88 | PREDICTED: Homo sapiens hypothetical LOC727962 (LOC727962), mRNA. |
| DUSP1 | -2.89 | Homo sapiens dual specificity phosphatase 1 (DUSP1), mRNA. |
| PYGL | -2.89 | Homo sapiens phosphorylase, glycogen, liver (PYGL), mRNA. |
| IKZF4 | -2.9 | Homo sapiens IKAROS family zinc finger 4 (Eos) (IKZF4), mRNA. |
| SFRS16 | -2.9 | Homo sapiens splicing factor, arginine/serine-rich 16 (SFRS16), mRNA. |
| KCTD12 | -2.9 | Homo sapiens potassium channel tetramerisation domain containing 12 (KCTD12), mRNA. |
| LOC100129502 | -2.9 | PREDICTED: Homo sapiens hypothetical protein LOC100129502 (LOC100129502), mRNA. |
| BLZF1 | -2.9 | Homo sapiens basic leucine zipper nuclear factor 1 (BLZF1), mRNA. |
| IGFBP4 | -2.9 | Homo sapiens insulin-like growth factor binding protein 4 (IGFBP4), mRNA. |
| TPM2 | -2.9 | Homo sapiens tropomyosin 2 (beta) (TPM2), transcript variant 2, mRNA. |
| SBNO2 | -2.9 | Homo sapiens strawberry notch homolog 2 (Drosophila) (SBNO2), transcript variant 1, mRNA. |
| CCDC74B | -2.9 | Homo sapiens coiled-coil domain containing 74B (CCDC74B), mRNA. |
| LOC648149 | -2.9 | PREDICTED: Homo sapiens hypothetical protein LOC648149 (LOC648149), mRNA. |
| SH3BP1 | -2.9 | Homo sapiens SH3-domain binding protein 1 (SH3BP1), mRNA. |
| GAS1 | -2.92 | Homo sapiens growth arrest-specific 1 (GAS1), mRNA. |
| LOC143543 | -2.92 | Homo sapiens RNA binding motif protein, X-linked pseudogene (LOC143543) on chromosome 11. |
| NUP62 | -2.92 | Homo sapiens nucleoporin 62kDa (NUP62), transcript variant 1, mRNA. |
| FOXM1 | -2.92 | Homo sapiens forkhead box M1 (FOXM1), transcript variant 3, mRNA. |
| TOP2A | -2.92 | Homo sapiens topoisomerase (DNA) II alpha 170kDa (TOP2A), mRNA. |
| ZBED3 | -2.92 | Homo sapiens zinc finger, BED-type containing 3 (ZBED3), mRNA. |
| SF1 | -2.92 | Homo sapiens splicing factor 1 (SF1), transcript variant 4, mRNA. |
| GAP43 | -2.93 | Homo sapiens growth associated protein 43 (GAP43), mRNA. |
| LSS | -2.93 | Homo sapiens lanosterol synthase (2,3-oxidosqualene-lanosterol cyclase) (LSS), transcript variant 1, mRNA. |
| AGRN | -2.93 | Homo sapiens agrin (AGRN), mRNA. |
| PLTP | -2.93 | Homo sapiens phospholipid transfer protein (PLTP), transcript variant 2, mRNA. |
| GRIK5 | -2.93 | Homo sapiens glutamate receptor, ionotropic, kainate 5 (GRIK5), mRNA. |
| NUMA1 | -2.93 | Homo sapiens nuclear mitotic apparatus protein 1 (NUMA1), mRNA. |
| C21orf45 | -2.94 | Homo sapiens chromosome 21 open reading frame 45 (C21orf45), mRNA. |
| ERGIC1 | -2.94 | Homo sapiens endoplasmic reticulum-golgi intermediate compartment (ERGIC) 1 (ERGIC1), transcript variant 2, mRNA. |
| ZNF682 | -2.95 | Homo sapiens zinc finger protein 682 (ZNF682), transcript variant 1, mRNA. |
| UQCRH | -2.95 | Homo sapiens ubiquinol-cytochrome c reductase hinge protein (UQCRH), mRNA. |
| SNORD13 | -2.95 | Homo sapiens small nucleolar RNA, C/D box 13 (SNORD13), small nucleolar RNA. |
| ANKRD13B | -2.95 | Homo sapiens ankyrin repeat domain 13B (ANKRD13B), mRNA. |
| NYNRIN | -2.95 | Homo sapiens NYN domain and retroviral integrase containing (NYNRIN), mRNA. |
| GDE1 | -2.95 | Homo sapiens glycerophosphodiester phosphodiesterase 1 (GDE1), mRNA. |
| LOC389342 | -2.96 | PREDICTED: Homo sapiens similar to QM protein, transcript variant 11 (LOC389342), mRNA. |
| IL11RA | -2.96 | Homo sapiens interleukin 11 receptor, alpha (IL11RA), transcript variant 1, mRNA. |
| ZNF385A | -2.96 | Homo sapiens zinc finger protein 385A (ZNF385A), transcript variant 3, mRNA. |
| APC2 | -2.96 | Homo sapiens adenomatosis polyposis coli 2 (APC2), mRNA. |
| COL3A1 | -2.96 | Homo sapiens collagen, type III, alpha 1 (COL3A1), mRNA. |
| RAD23B | -2.97 | Homo sapiens RAD23 homolog B (S. cerevisiae) (RAD23B), mRNA. |
| BUB3 | -2.97 | Homo sapiens BUB3 budding uninhibited by benzimidazoles 3 homolog (yeast) (BUB3), transcript variant 1, mRNA. |
| LOC440704 | -2.98 | PREDICTED: Homo sapiens hypothetical gene supported by BC042042 (LOC440704), mRNA. |
| NLRP8 | -2.98 | Homo sapiens NLR family, pyrin domain containing 8 (NLRP8), mRNA. |
| GRK6 | -2.98 | Homo sapiens G protein-coupled receptor kinase 6 (GRK6), transcript variant 2, mRNA. |
| TSC22D4 | -2.99 | Homo sapiens TSC22 domain family, member 4 (TSC22D4), mRNA. |
| PPP2R3B | -2.99 | Homo sapiens protein phosphatase 2 (formerly 2A), regulatory subunit B'', beta (PPP2R3B), transcript variant 1, mRNA. |
| PCDH17 | -3 | Homo sapiens protocadherin 17 (PCDH17), mRNA. |
| USF2 | -3.01 | Homo sapiens upstream transcription factor 2, c-fos interacting (USF2), transcript variant 2, mRNA. |
| SMTN | -3.01 | Homo sapiens smoothelin (SMTN), transcript variant 3, mRNA. |
| HMGB2 | -3.01 | Homo sapiens high-mobility group box 2 (HMGB2), mRNA. |
| BEGAIN | -3.01 | Homo sapiens brain-enriched guanylate kinase-associated homolog (rat) (BEGAIN), mRNA. |
| TOR1AIP1 | -3.01 | Homo sapiens torsin A interacting protein 1 (TOR1AIP1), mRNA. |
| UCKL1 | -3.01 | Homo sapiens uridine-cytidine kinase 1-like 1 (UCKL1), mRNA. |
| MIB2 | -3.01 | Homo sapiens mindbomb homolog 2 (Drosophila) (MIB2), mRNA. |
| DUSP10 | -3.01 | Homo sapiens dual specificity phosphatase 10 (DUSP10), transcript variant 3, mRNA. |
| C8orf45 | -3.02 | Homo sapiens chromosome 8 open reading frame 45 (C8orf45), mRNA. |
| GJA1 | -3.02 | Homo sapiens gap junction protein, alpha 1, 43kDa (GJA1), mRNA. |
| LOC147727 | -3.03 | Homo sapiens hypothetical LOC147727 (LOC147727), non-coding RNA. |
| UNC5B | -3.03 | Homo sapiens unc-5 homolog B (C. elegans) (UNC5B), mRNA. |
| CPT1B | -3.03 | Homo sapiens carnitine palmitoyltransferase 1B (muscle) (CPT1B), nuclear gene encoding mitochondrial protein, transcript variant 3, mRNA. |
| C20orf117 | -3.03 | Homo sapiens chromosome 20 open reading frame 117 (C20orf117), transcript variant 2, mRNA. |
| S100A10 | -3.04 | Homo sapiens S100 calcium binding protein A10 (annexin II ligand, calpactin I, light polypeptide (p11)) (S100A10), mRNA. |
| H1F0 | -3.04 | Homo sapiens H1 histone family, member 0 (H1F0), mRNA. |
| ARHGEF11 | -3.04 | Homo sapiens Rho guanine nucleotide exchange factor (GEF) 11 (ARHGEF11), transcript variant 1, mRNA. |
| SSTR2 | -3.04 | Homo sapiens somatostatin receptor 2 (SSTR2), mRNA. |
| IGSF1 | -3.05 | Homo sapiens immunoglobulin superfamily, member 1 (IGSF1), transcript variant 1, mRNA. |
| MGP | -3.05 | Homo sapiens matrix Gla protein (MGP), mRNA. |
| GGH | -3.05 | Homo sapiens gamma-glutamyl hydrolase (conjugase, folylpolygammaglutamyl hydrolase) (GGH), mRNA. |
| TANC1 | -3.05 | Homo sapiens tetratricopeptide repeat, ankyrin repeat and coiled-coil containing 1 (TANC1), mRNA. |
| LOC641768 | -3.05 | PREDICTED: Homo sapiens similar to ribosomal protein S26, transcript variant 2 (LOC641768), mRNA. |
| PRRG4 | -3.06 | Homo sapiens proline rich Gla (G-carboxyglutamic acid) 4 (transmembrane) (PRRG4), mRNA. |
| LOC399900 | -3.06 | Homo sapiens hypothetical gene supported by AK093779 (LOC399900), mRNA. |
| NFIA | -3.06 | Homo sapiens nuclear factor I/A (NFIA), mRNA. |
| RGS2 | -3.06 | Homo sapiens regulator of G-protein signalling 2, 24kDa (RGS2), mRNA. |
| KIF11 | -3.06 | Homo sapiens kinesin family member 11 (KIF11), mRNA. |
| FLJ40722 | -3.06 | PREDICTED: Homo sapiens hypothetical protein FLJ40722, transcript variant 3 (FLJ40722), mRNA. |
| LOC652330 | -3.07 | PREDICTED: Homo sapiens similar to neurofibromin 1 (neurofibromatosis, von Recklinghausen disease, Watson disease) (LOC652330), mRNA. |
| LOC100132394 | -3.07 | PREDICTED: Homo sapiens hypothetical protein LOC100132394 (LOC100132394), mRNA. |
| LOC100134364 | -3.07 | PREDICTED: Homo sapiens hypothetical protein LOC100134364 (LOC100134364), mRNA. |
| PRKCSH | -3.07 | Homo sapiens protein kinase C substrate 80K-H (PRKCSH), transcript variant 2, mRNA. |
| CSAD | -3.08 | Homo sapiens cysteine sulfinic acid decarboxylase (CSAD), mRNA. |
| FOXM1 | -3.08 | Homo sapiens forkhead box M1 (FOXM1), transcript variant 2, mRNA. |
|  | -3.09 | te46f04.x1 Soares_NhHMPu_S1 Homo sapiens cDNA clone IMAGE:2089759 3, mRNA sequence |
| ITGAV | -3.09 | Homo sapiens integrin, alpha V (vitronectin receptor, alpha polypeptide, antigen CD51) (ITGAV), mRNA. |
| DMWD | -3.09 | Homo sapiens dystrophia myotonica, WD repeat containing (DMWD), mRNA. |
| ZNF91 | -3.09 | Homo sapiens zinc finger protein 91 (ZNF91), mRNA. |
| PTOV1 | -3.09 | Homo sapiens prostate tumor overexpressed 1 (PTOV1), mRNA. |
| LMO4 | -3.1 | Homo sapiens LIM domain only 4 (LMO4), mRNA. |
| STAG2 | -3.1 | Homo sapiens stromal antigen 2 (STAG2), transcript variant 2, mRNA. |
| PPM1K | -3.11 | Homo sapiens protein phosphatase 1K (PP2C domain containing) (PPM1K), mRNA. |
| FLJ46309 | -3.11 | Homo sapiens hypothetical protein LOC649598 (FLJ46309), mRNA. |
| NRP1 | -3.12 | Homo sapiens neuropilin 1 (NRP1), transcript variant 1, mRNA. |
| LOC123688 | -3.12 | Homo sapiens similar to RIKEN cDNA C630028N24 gene (LOC123688), mRNA. |
| BIRC3 | -3.12 | Homo sapiens baculoviral IAP repeat-containing 3 (BIRC3), transcript variant 2, mRNA. |
| CCNK | -3.12 | Homo sapiens cyclin K (CCNK), transcript variant 2, mRNA. |
| FAM116A | -3.12 | PREDICTED: Homo sapiens family with sequence similarity 116, member A (FAM116A), mRNA. |
| COBRA1 | -3.12 | Homo sapiens cofactor of BRCA1 (COBRA1), mRNA. |
| LSM3 | -3.13 | Homo sapiens LSM3 homolog, U6 small nuclear RNA associated (S. cerevisiae) (LSM3), mRNA. |
| PTGFRN | -3.13 | Homo sapiens prostaglandin F2 receptor negative regulator (PTGFRN), mRNA. |
| CHST14 | -3.15 | Homo sapiens carbohydrate (N-acetylgalactosamine 4-0) sulfotransferase 14 (CHST14), mRNA. |
| COL6A2 | -3.15 | Homo sapiens collagen, type VI, alpha 2 (COL6A2), transcript variant 2C2, mRNA. |
| NFIB | -3.15 | Homo sapiens nuclear factor I/B (NFIB), mRNA. |
| RAD23A | -3.16 | Homo sapiens RAD23 homolog A (S. cerevisiae) (RAD23A), mRNA. |
| DUSP6 | -3.16 | Homo sapiens dual specificity phosphatase 6 (DUSP6), transcript variant 2, mRNA. |
| PCDHB3 | -3.16 | Homo sapiens protocadherin beta 3 (PCDHB3), mRNA. |
| RBM3 | -3.16 | Homo sapiens RNA binding motif (RNP1, RRM) protein 3 (RBM3), transcript variant 2, mRNA. |
| LOC645452 | -3.17 | PREDICTED: Homo sapiens similar to hCG1782414 (LOC645452), mRNA. |
| C7 | -3.18 | Homo sapiens complement component 7 (C7), mRNA. |
| RADIL | -3.18 | Homo sapiens Ras association and DIL domains (RADIL), mRNA. |
| EP300 | -3.18 | Homo sapiens E1A binding protein p300 (EP300), mRNA. |
| LOC728823 | -3.19 | PREDICTED: Homo sapiens misc_RNA (LOC728823), miscRNA. |
| SFRS8 | -3.19 | Homo sapiens splicing factor, arginine/serine-rich 8 (suppressor-of-white-apricot homolog, Drosophila) (SFRS8), mRNA. |
| LOC652608 | -3.19 | PREDICTED: Homo sapiens similar to 60S ribosomal protein L6 (TAX-responsive enhancer element-binding protein 107) (TAXREB107) (Neoplasm-related protein C140) (LOC652608), mRNA. |
| LRRC37B2 | -3.2 | Homo sapiens leucine rich repeat containing 37, member B2 (LRRC37B2), non-coding RNA. |
| HAT1 | -3.2 | Homo sapiens histone acetyltransferase 1 (HAT1), transcript variant 1, mRNA. |
| LPPR2 | -3.2 | Homo sapiens lipid phosphate phosphatase-related protein type 2 (LPPR2), mRNA. |
| UBE2E3 | -3.21 | Homo sapiens ubiquitin-conjugating enzyme E2E 3 (UBC4/5 homolog, yeast) (UBE2E3), transcript variant 1, mRNA. |
| LOC402057 | -3.21 | Homo sapiens similar to 40S ribosomal protein S17 (LOC402057), mRNA. |
| VASN | -3.22 | Homo sapiens vasorin (VASN), mRNA. |
| FAM108A3 | -3.22 | Homo sapiens family with sequence similarity 108, member A3 (FAM108A3), mRNA. |
| CCDC97 | -3.22 | Homo sapiens coiled-coil domain containing 97 (CCDC97), mRNA. |
| INTS1 | -3.22 | PREDICTED: Homo sapiens integrator complex subunit 1, transcript variant 11 (INTS1), mRNA. |
| HTRA1 | -3.23 | Homo sapiens HtrA serine peptidase 1 (HTRA1), mRNA. |
| RNU1A3 | -3.24 | Homo sapiens RNA, U1A3 small nuclear (RNU1A3), small nuclear RNA. |
| GOLPH4 | -3.24 | Homo sapiens golgi phosphoprotein 4 (GOLPH4), mRNA. |
| LOC730313 | -3.25 | PREDICTED: Homo sapiens hypothetical LOC730313 (LOC730313), mRNA. |
| TUBA3D | -3.25 | Homo sapiens tubulin, alpha 3d (TUBA3D), mRNA. |
| PHOX2B | -3.26 | Homo sapiens paired-like homeobox 2b (PHOX2B), mRNA. |
| PTMA | -3.26 | Homo sapiens prothymosin, alpha (PTMA), transcript variant 1, mRNA. |
| TMEM106A | -3.26 | Homo sapiens transmembrane protein 106A (TMEM106A), mRNA. |
| MTA1 | -3.26 | Homo sapiens metastasis associated 1 (MTA1), mRNA. |
| AKAP1 | -3.27 | Homo sapiens A kinase (PRKA) anchor protein 1 (AKAP1), nuclear gene encoding mitochondrial protein, mRNA. |
| PBRM1 | -3.27 | Homo sapiens polybromo 1 (PBRM1), transcript variant 4, mRNA. |
| UBE1 | -3.27 | Homo sapiens ubiquitin-activating enzyme E1 (UBE1), transcript variant 1, mRNA. |
| TFPI2 | -3.27 | Homo sapiens tissue factor pathway inhibitor 2 (TFPI2), mRNA. |
| SPRY1 | -3.27 | Homo sapiens sprouty homolog 1, antagonist of FGF signaling (Drosophila) (SPRY1), transcript variant 2, mRNA. |
| LOC255167 | -3.28 | Homo sapiens hypothetical LOC255167 (LOC255167), non-coding RNA. |
| FKBP8 | -3.28 | Homo sapiens FK506 binding protein 8, 38kDa (FKBP8), mRNA. |
| GAA | -3.28 | Homo sapiens glucosidase, alpha; acid (GAA), transcript variant 1, mRNA. |
| TCF3 | -3.29 | Homo sapiens transcription factor 3 (E2A immunoglobulin enhancer binding factors E12/E47) (TCF3), mRNA. |
| GDI1 | -3.29 | Homo sapiens GDP dissociation inhibitor 1 (GDI1), mRNA. |
| LOC441377 | -3.3 | PREDICTED: Homo sapiens similar to 40S ribosomal protein S26 (LOC441377), mRNA. |
| LOC647030 | -3.3 | PREDICTED: Homo sapiens misc_RNA (LOC647030), miscRNA. |
| ROBO3 | -3.3 | Homo sapiens roundabout, axon guidance receptor, homolog 3 (Drosophila) (ROBO3), mRNA. |
| LRDD | -3.31 | Homo sapiens leucine-rich repeats and death domain containing (LRDD), transcript variant 3, mRNA. |
| ADAM15 | -3.31 | Homo sapiens ADAM metallopeptidase domain 15 (ADAM15), transcript variant 2, mRNA. |
| KIAA0408 | -3.32 | Homo sapiens KIAA0408 (KIAA0408), mRNA. |
| COL6A1 | -3.32 | Homo sapiens collagen, type VI, alpha 1 (COL6A1), mRNA. |
| SP4 | -3.32 | Homo sapiens Sp4 transcription factor (SP4), mRNA. |
| TM4SF4 | -3.32 | Homo sapiens transmembrane 4 L six family member 4 (TM4SF4), mRNA. |
| PHOX2A | -3.32 | Homo sapiens paired-like homeobox 2a (PHOX2A), mRNA. |
| TAGLN | -3.32 | Homo sapiens transgelin (TAGLN), transcript variant 2, mRNA. |
| DCTN1 | -3.32 | Homo sapiens dynactin 1 (p150, glued homolog, Drosophila) (DCTN1), transcript variant 1, mRNA. |
| GNB2 | -3.33 | Homo sapiens guanine nucleotide binding protein (G protein), beta polypeptide 2 (GNB2), mRNA. |
| CNOT3 | -3.34 | Homo sapiens CCR4-NOT transcription complex, subunit 3 (CNOT3), mRNA. |
| CRABP1 | -3.34 | Homo sapiens cellular retinoic acid binding protein 1 (CRABP1), mRNA. |
| HMGB1 | -3.34 | Homo sapiens high-mobility group box 1 (HMGB1), mRNA. |
| SPRY1 | -3.35 | Homo sapiens sprouty homolog 1, antagonist of FGF signaling (Drosophila) (SPRY1), transcript variant 1, mRNA. |
| EYA4 | -3.35 | Homo sapiens eyes absent homolog 4 (Drosophila) (EYA4), transcript variant 1, mRNA. |
| SSBP4 | -3.35 | Homo sapiens single stranded DNA binding protein 4 (SSBP4), transcript variant 1, mRNA. |
| LOC100133772 | -3.36 | PREDICTED: Homo sapiens similar to MCT (LOC100133772), mRNA. |
| SPTLC1 | -3.37 | Homo sapiens serine palmitoyltransferase, long chain base subunit 1 (SPTLC1), transcript variant 2, mRNA. |
| SFPQ | -3.37 | Homo sapiens splicing factor proline/glutamine-rich (polypyrimidine tract binding protein associated) (SFPQ), mRNA. |
| SYN2 | -3.37 | Homo sapiens synapsin II (SYN2), transcript variant IIa, mRNA. |
| IDUA | -3.38 | Homo sapiens iduronidase, alpha-L- (IDUA), mRNA. |
| LOC100130276 | -3.38 | PREDICTED: Homo sapiens hypothetical protein LOC100130276 (LOC100130276), mRNA. |
| EMILIN1 | -3.39 | Homo sapiens elastin microfibril interfacer 1 (EMILIN1), mRNA. |
| RAVER1 | -3.39 | Homo sapiens RAVER1 (RAVER1), mRNA. |
| LOC100008589 | -3.4 | Homo sapiens 28S ribosomal RNA (LOC100008589), non-coding RNA. |
| RHOB | -3.4 | Homo sapiens ras homolog gene family, member B (RHOB), mRNA. |
| BCL2L1 | -3.4 | Homo sapiens BCL2-like 1 (BCL2L1), nuclear gene encoding mitochondrial protein, transcript variant 1, mRNA. |
| AGPAT1 | -3.4 | Homo sapiens 1-acylglycerol-3-phosphate O-acyltransferase 1 (lysophosphatidic acid acyltransferase, alpha) (AGPAT1), transcript variant 1, mRNA. |
| LOC100127975 | -3.41 | PREDICTED: Homo sapiens misc_RNA (LOC100127975), partial miscRNA. |
| MGAT4B | -3.41 | Homo sapiens mannosyl (alpha-1,3-)-glycoprotein beta-1,4-N-acetylglucosaminyltransferase, isozyme B (MGAT4B), transcript variant 2, mRNA. |
| LTBP3 | -3.41 | Homo sapiens latent transforming growth factor beta binding protein 3 (LTBP3), mRNA. |
| SNAI2 | -3.41 | Homo sapiens snail homolog 2 (Drosophila) (SNAI2), mRNA. |
| FLJ22184 | -3.41 | PREDICTED: Homo sapiens hypothetical protein FLJ22184 (FLJ22184), mRNA. |
| SLITRK6 | -3.42 | Homo sapiens SLIT and NTRK-like family, member 6 (SLITRK6), mRNA. |
| LOC440926 | -3.43 | Homo sapiens H3 histone, family 3A pseudogene (LOC440926), non-coding RNA. |
| BLOC1S1 | -3.43 | Homo sapiens biogenesis of lysosome-related organelles complex-1, subunit 1 (BLOC1S1), mRNA. |
| IL17RD | -3.43 | Homo sapiens interleukin 17 receptor D (IL17RD), transcript variant 1, mRNA. |
| C21orf24 | -3.44 | Homo sapiens chromosome 21 open reading frame 24 (C21orf24), mRNA. |
| IGFBP3 | -3.44 | Homo sapiens insulin-like growth factor binding protein 3 (IGFBP3), transcript variant 2, mRNA. |
| GNAI2 | -3.44 | Homo sapiens guanine nucleotide binding protein (G protein), alpha inhibiting activity polypeptide 2 (GNAI2), transcript variant 1, mRNA. |
| TSPAN8 | -3.44 | Homo sapiens tetraspanin 8 (TSPAN8), mRNA. |
| ZNF444 | -3.45 | Homo sapiens zinc finger protein 444 (ZNF444), mRNA. |
| GIPC1 | -3.45 | Homo sapiens GIPC PDZ domain containing family, member 1 (GIPC1), transcript variant 3, mRNA. |
| AHNAK | -3.46 | Homo sapiens AHNAK nucleoprotein (AHNAK), transcript variant 1, mRNA. |
| SLC35E1 | -3.48 | Homo sapiens solute carrier family 35, member E1 (SLC35E1), mRNA. |
| LOC388344 | -3.48 | PREDICTED: Homo sapiens similar to ribosomal protein L13, transcript variant 1 (LOC388344), mRNA. |
| S1PR3 | -3.48 | Homo sapiens sphingosine-1-phosphate receptor 3 (S1PR3), mRNA. |
| ASAP1 | -3.48 | Homo sapiens ArfGAP with SH3 domain, ankyrin repeat and PH domain 1 (ASAP1), mRNA. |
| MT2A | -3.48 | Homo sapiens metallothionein 2A (MT2A), mRNA. |
| PODXL2 | -3.49 | Homo sapiens podocalyxin-like 2 (PODXL2), mRNA. |
| ATN1 | -3.5 | Homo sapiens atrophin 1 (ATN1), transcript variant 1, mRNA. |
| TTC32 | -3.51 | Homo sapiens tetratricopeptide repeat domain 32 (TTC32), mRNA. |
| FBLN7 | -3.51 | Homo sapiens fibulin 7 (FBLN7), mRNA. |
| TBX3 | -3.51 | Homo sapiens T-box 3 (TBX3), transcript variant 1, mRNA. |
| LOC100128084 | -3.52 | PREDICTED: Homo sapiens hypothetical protein LOC100128084 (LOC100128084), mRNA. |
| CHTF18 | -3.52 | Homo sapiens CTF18, chromosome transmission fidelity factor 18 homolog (S. cerevisiae) (CHTF18), mRNA. |
| LOC730004 | -3.52 | PREDICTED: Homo sapiens misc_RNA (LOC730004), miscRNA. |
| LOC346950 | -3.52 | PREDICTED: Homo sapiens similar to ribosomal protein L37 (LOC346950), mRNA. |
| ZNF423 | -3.52 | Homo sapiens zinc finger protein 423 (ZNF423), mRNA. |
| PLDN | -3.53 | Homo sapiens pallidin homolog (mouse) (PLDN), mRNA. |
| SRRM2 | -3.53 | Homo sapiens serine/arginine repetitive matrix 2 (SRRM2), mRNA. |
| CORO6 | -3.53 | Homo sapiens coronin 6 (CORO6), mRNA. |
| CUL9 | -3.53 | Homo sapiens cullin 9 (CUL9), mRNA. |
| ZNF579 | -3.53 | Homo sapiens zinc finger protein 579 (ZNF579), mRNA. |
| SLC35E1 | -3.54 | Homo sapiens solute carrier family 35, member E1 (SLC35E1), mRNA. |
| MBOAT7 | -3.54 | Homo sapiens membrane bound O-acyltransferase domain containing 7 (MBOAT7), mRNA. |
| IRF2BP2 | -3.56 | Homo sapiens interferon regulatory factor 2 binding protein 2 (IRF2BP2), transcript variant 1, mRNA. |
| LOC338799 | -3.56 | Homo sapiens hypothetical locus LOC338799 (LOC338799), non-coding RNA. |
| PABPC1 | -3.56 | Homo sapiens poly(A) binding protein, cytoplasmic 1 (PABPC1), mRNA. |
| KDELC2 | -3.56 | Homo sapiens KDEL (Lys-Asp-Glu-Leu) containing 2 (KDELC2), mRNA. |
| PTN | -3.56 | Homo sapiens pleiotrophin (PTN), mRNA. |
| LOC728908 | -3.58 | PREDICTED: Homo sapiens misc_RNA (LOC728908), miscRNA. |
| MAP1S | -3.58 | Homo sapiens microtubule-associated protein 1S (MAP1S), mRNA. |
| LOC647307 | -3.59 | PREDICTED: Homo sapiens misc_RNA (LOC647307), miscRNA. |
| NUF2 | -3.59 | Homo sapiens NUF2, NDC80 kinetochore complex component, homolog (S. cerevisiae) (NUF2), transcript variant 2, mRNA. |
| LOC730130 | -3.6 | PREDICTED: Homo sapiens hypothetical protein LOC730130 (LOC730130), mRNA. |
| RHBDL2 | -3.6 | Homo sapiens rhomboid, veinlet-like 2 (Drosophila) (RHBDL2), mRNA. |
| GAS1 | -3.6 | Homo sapiens growth arrest-specific 1 (GAS1), mRNA. |
| TSC2 | -3.6 | Homo sapiens tuberous sclerosis 2 (TSC2), transcript variant 4, mRNA. |
| HMGB1 | -3.6 | Homo sapiens high-mobility group box 1 (HMGB1), mRNA. |
| LOC153561 | -3.62 | Homo sapiens hypothetical protein LOC153561 (LOC153561), mRNA. |
| MLF1IP | -3.62 | Homo sapiens MLF1 interacting protein (MLF1IP), mRNA. |
| XAB2 | -3.62 | Homo sapiens XPA binding protein 2 (XAB2), mRNA. |
| LOC100129539 | -3.63 | PREDICTED: Homo sapiens misc_RNA (LOC100129539), miscRNA. |
| FBRS | -3.63 | Homo sapiens fibrosin (FBRS), mRNA. |
| JOSD2 | -3.64 | Homo sapiens Josephin domain containing 2 (JOSD2), mRNA. |
| KCTD13 | -3.64 | Homo sapiens potassium channel tetramerisation domain containing 13 (KCTD13), mRNA. |
| C15orf63 | -3.65 | Homo sapiens chromosome 15 open reading frame 63 (C15orf63), mRNA. |
| ZNF549 | -3.66 | Homo sapiens zinc finger protein 549 (ZNF549), mRNA. |
| LOC389765 | -3.67 | PREDICTED: Homo sapiens similar to KIF27C (LOC389765), mRNA. |
| MGP | -3.67 | Homo sapiens matrix Gla protein (MGP), mRNA. |
| PKM2 | -3.67 | Homo sapiens pyruvate kinase, muscle (PKM2), transcript variant 3, mRNA. |
| LOC728620 | -3.67 | PREDICTED: Homo sapiens misc_RNA (LOC728620), miscRNA. |
| MBD4 | -3.68 | Homo sapiens methyl-CpG binding domain protein 4 (MBD4), mRNA. |
| CCBE1 | -3.7 | Homo sapiens collagen and calcium binding EGF domains 1 (CCBE1), mRNA. |
| KIDINS220 | -3.7 | Homo sapiens kinase D-interacting substrate of 220 kDa (KIDINS220), mRNA. |
| HSD17B7 | -3.71 | Homo sapiens hydroxysteroid (17-beta) dehydrogenase 7 (HSD17B7), mRNA. |
| LOC347292 | -3.72 | PREDICTED: Homo sapiens similar to ribosomal protein L36 (LOC347292), mRNA. |
| ZYX | -3.72 | Homo sapiens zyxin (ZYX), transcript variant 1, mRNA. |
| MNT | -3.73 | Homo sapiens MAX binding protein (MNT), mRNA. |
| LOC400713 | -3.73 | PREDICTED: Homo sapiens zinc finger-like, transcript variant 3 (LOC400713), misc RNA. |
| MIB2 | -3.73 | Homo sapiens mindbomb homolog 2 (Drosophila) (MIB2), mRNA. |
| DKK1 | -3.76 | Homo sapiens dickkopf homolog 1 (Xenopus laevis) (DKK1), mRNA. |
| INSIG1 | -3.76 | Homo sapiens insulin induced gene 1 (INSIG1), transcript variant 2, mRNA. |
| IGFBP2 | -3.77 | Homo sapiens insulin-like growth factor binding protein 2, 36kDa (IGFBP2), mRNA. |
| FAM73A | -3.78 | Homo sapiens family with sequence similarity 73, member A (FAM73A), mRNA. |
| PCDH20 | -3.79 | Homo sapiens protocadherin 20 (PCDH20), mRNA. |
| PNPLA2 | -3.79 | Homo sapiens patatin-like phospholipase domain containing 2 (PNPLA2), mRNA. |
| LIMA1 | -3.8 | Homo sapiens LIM domain and actin binding 1 (LIMA1), mRNA. |
| LOC441087 | -3.81 | Homo sapiens hypothetical gene supported by AK125735 (LOC441087), mRNA. |
| TBX2 | -3.83 | Homo sapiens T-box 2 (TBX2), mRNA. |
| CDC42BPB | -3.84 | Homo sapiens CDC42 binding protein kinase beta (DMPK-like) (CDC42BPB), mRNA. |
| POFUT1 | -3.85 | Homo sapiens protein O-fucosyltransferase 1 (POFUT1), transcript variant 1, mRNA. |
| CATSPER2 | -3.85 | Homo sapiens cation channel, sperm associated 2 (CATSPER2), transcript variant 4, mRNA. |
| SLC4A2 | -3.85 | Homo sapiens solute carrier family 4, anion exchanger, member 2 (erythrocyte membrane protein band 3-like 1) (SLC4A2), mRNA. |
| HIATL2 | -3.87 | Homo sapiens hippocampus abundant transcript-like 2 (HIATL2), non-coding RNA. |
| LRRC17 | -3.87 | Homo sapiens leucine rich repeat containing 17 (LRRC17), transcript variant 2, mRNA. |
| FBXL16 | -3.89 | Homo sapiens F-box and leucine-rich repeat protein 16 (FBXL16), mRNA. |
| LOC647389 | -3.89 | PREDICTED: Homo sapiens hypothetical protein LOC647389 (LOC647389), mRNA. |
| KLF6 | -3.89 | Homo sapiens Kruppel-like factor 6 (KLF6), transcript variant 2, mRNA. |
| YTHDF2 | -3.89 | Homo sapiens YTH domain family, member 2 (YTHDF2), mRNA. |
| LRRN3 | -3.9 | Homo sapiens leucine rich repeat neuronal 3 (LRRN3), transcript variant 1, mRNA. |
| IRF2BP1 | -3.9 | Homo sapiens interferon regulatory factor 2 binding protein 1 (IRF2BP1), mRNA. |
| PELP1 | -3.92 | Homo sapiens proline, glutamic acid and leucine rich protein 1 (PELP1), mRNA. |
| NCOR2 | -3.93 | Homo sapiens nuclear receptor co-repressor 2 (NCOR2), transcript variant 2, mRNA. |
| LMOD3 | -3.95 | Homo sapiens leiomodin 3 (fetal) (LMOD3), mRNA. |
| LOC644380 | -3.95 | PREDICTED: Homo sapiens similar to High mobility group protein 1-like 10 (HMG-1L10), transcript variant 1 (LOC644380), mRNA. |
| LOC731314 | -3.95 | PREDICTED: Homo sapiens similar to H2A histone family, member X (LOC731314), mRNA. |
| ZNF14 | -3.96 | Homo sapiens zinc finger protein 14 (ZNF14), mRNA. |
| ID3 | -3.97 | Homo sapiens inhibitor of DNA binding 3, dominant negative helix-loop-helix protein (ID3), mRNA. |
| TDRD1 | -3.97 | Homo sapiens tudor domain containing 1 (TDRD1), mRNA. |
| SIX3 | -4 | Homo sapiens sine oculis homeobox homolog 3 (Drosophila) (SIX3), mRNA. |
| LMF2 | -4.02 | Homo sapiens lipase maturation factor 2 (LMF2), mRNA. |
| LOC387763 | -4.03 | PREDICTED: Homo sapiens hypothetical LOC387763 (LOC387763), mRNA. |
| CNTFR | -4.03 | Homo sapiens ciliary neurotrophic factor receptor (CNTFR), transcript variant 2, mRNA. |
| MGRN1 | -4.06 | Homo sapiens mahogunin, ring finger 1 (MGRN1), mRNA. |
| AP2A1 | -4.06 | Homo sapiens adaptor-related protein complex 2, alpha 1 subunit (AP2A1), transcript variant 2, mRNA. |
| C14orf85 | -4.07 | Homo sapiens chromosome 14 open reading frame 85 (C14orf85), non-coding RNA. |
| CENTG3 | -4.07 | Homo sapiens centaurin, gamma 3 (CENTG3), mRNA. |
| SAC3D1 | -4.07 | Homo sapiens SAC3 domain containing 1 (SAC3D1), mRNA. |
| LOC729859 | -4.08 | PREDICTED: Homo sapiens misc_RNA (LOC729859), miscRNA. |
| NCOR2 | -4.08 | Homo sapiens nuclear receptor co-repressor 2 (NCOR2), transcript variant 1, mRNA. |
| CLPTM1 | -4.09 | Homo sapiens cleft lip and palate associated transmembrane protein 1 (CLPTM1), mRNA. |
| ITFG3 | -4.13 | Homo sapiens integrin alpha FG-GAP repeat containing 3 (ITFG3), mRNA. |
| SSBP3 | -4.13 | Homo sapiens single stranded DNA binding protein 3 (SSBP3), transcript variant 2, mRNA. |
| YRDC | -4.13 | Homo sapiens yrdC domain containing (E. coli) (YRDC), nuclear gene encoding mitochondrial protein, mRNA. |
| TRAPPC9 | -4.14 | Homo sapiens trafficking protein particle complex 9 (TRAPPC9), mRNA. |
| DCN | -4.14 | Homo sapiens decorin (DCN), transcript variant C, mRNA. |
| LAMB1 | -4.15 | Homo sapiens laminin, beta 1 (LAMB1), mRNA. |
| TNFSF15 | -4.15 | Homo sapiens tumor necrosis factor (ligand) superfamily, member 15 (TNFSF15), mRNA. |
| PHAX | -4.15 | Homo sapiens phosphorylated adaptor for RNA export (PHAX), mRNA. |
| LRDD | -4.15 | Homo sapiens leucine-rich repeats and death domain containing (LRDD), transcript variant 2, mRNA. |
| HNRPUL1 | -4.15 | Homo sapiens heterogeneous nuclear ribonucleoprotein U-like 1 (HNRPUL1), transcript variant 4, mRNA. |
| NUCKS1 | -4.16 | Homo sapiens nuclear casein kinase and cyclin-dependent kinase substrate 1 (NUCKS1), mRNA. |
| MAGT1 | -4.16 | Homo sapiens magnesium transporter 1 (MAGT1), mRNA. |
| C14orf153 | -4.16 | Homo sapiens chromosome 14 open reading frame 153 (C14orf153), mRNA. |
| MXD3 | -4.17 | Homo sapiens MAX dimerization protein 3 (MXD3), mRNA. |
| TDRD1 | -4.17 | Homo sapiens tudor domain containing 1 (TDRD1), mRNA. |
| PKMYT1 | -4.2 | Homo sapiens protein kinase, membrane associated tyrosine/threonine 1 (PKMYT1), transcript variant 2, mRNA. |
| TRIM28 | -4.21 | Homo sapiens tripartite motif-containing 28 (TRIM28), mRNA. |
| GRINA | -4.21 | Homo sapiens glutamate receptor, ionotropic, N-methyl D-aspartate-associated protein 1 (glutamate binding) (GRINA), transcript variant 1, mRNA. |
| LRRC17 | -4.23 | Homo sapiens leucine rich repeat containing 17 (LRRC17), transcript variant 1, mRNA. |
| SP2 | -4.26 | Homo sapiens Sp2 transcription factor (SP2), mRNA. |
| SLC38A10 | -4.26 | Homo sapiens solute carrier family 38, member 10 (SLC38A10), transcript variant 1, mRNA. |
| LOC100128126 | -4.28 | PREDICTED: Homo sapiens hypothetical protein LOC100128126 (LOC100128126), mRNA. |
| FLNA | -4.29 | Homo sapiens filamin A, alpha (actin binding protein 280) (FLNA), mRNA. |
| LOC642828 | -4.29 | PREDICTED: Homo sapiens misc_RNA (LOC642828), miscRNA. |
| ZNF577 | -4.29 | Homo sapiens zinc finger protein 577 (ZNF577), mRNA. |
| FAM175A | -4.31 | Homo sapiens family with sequence similarity 175, member A (FAM175A), mRNA. |
| APLP1 | -4.31 | Homo sapiens amyloid beta (A4) precursor-like protein 1 (APLP1), transcript variant 1, mRNA. |
| PTGR2 | -4.32 | Homo sapiens prostaglandin reductase 2 (PTGR2), mRNA. |
| C11orf63 | -4.32 | Homo sapiens chromosome 11 open reading frame 63 (C11orf63), transcript variant 2, mRNA. |
| TRIM13 | -4.33 | Homo sapiens tripartite motif-containing 13 (TRIM13), transcript variant 4, mRNA. |
| SETD1A | -4.33 | Homo sapiens SET domain containing 1A (SETD1A), mRNA. |
| FUT6 | -4.33 | Homo sapiens fucosyltransferase 6 (alpha (1,3) fucosyltransferase) (FUT6), transcript variant 1, mRNA. |
| ANO8 | -4.33 | Homo sapiens anoctamin 8 (ANO8), mRNA. |
| C1orf173 | -4.35 | Homo sapiens chromosome 1 open reading frame 173 (C1orf173), mRNA. |
| RASSF6 | -4.36 | Homo sapiens Ras association (RalGDS/AF-6) domain family member 6 (RASSF6), transcript variant 1, mRNA. |
| MAPK8IP3 | -4.37 | Homo sapiens mitogen-activated protein kinase 8 interacting protein 3 (MAPK8IP3), transcript variant 2, mRNA. |
| LRAP | -4.37 | Homo sapiens leukocyte-derived arginine aminopeptidase (LRAP), mRNA. |
| SEPN1 | -4.37 | Homo sapiens selenoprotein N, 1 (SEPN1), transcript variant 2, mRNA. |
| TUBB4 | -4.38 | Homo sapiens tubulin, beta 4 (TUBB4), mRNA. |
| RNF13 | -4.38 | Homo sapiens ring finger protein 13 (RNF13), transcript variant 4, mRNA. |
| ELN | -4.38 | Homo sapiens elastin (ELN), transcript variant 4, mRNA. |
| CYCSL1 | -4.39 | Homo sapiens cytochrome c, somatic-like 1 (CYCSL1) on chromosome 6. |
| BAT2 | -4.44 | Homo sapiens HLA-B associated transcript 2 (BAT2), mRNA. |
| GPR1 | -4.46 | Homo sapiens G protein-coupled receptor 1 (GPR1), mRNA. |
| DCAF15 | -4.48 | Homo sapiens DDB1 and CUL4 associated factor 15 (DCAF15), mRNA. |
| ZMAT3 | -4.49 | Homo sapiens zinc finger, matrin type 3 (ZMAT3), transcript variant 2, mRNA. |
| LOC401098 | -4.49 | PREDICTED: Homo sapiens misc_RNA (LOC401098), miscRNA. |
| LOC100132585 | -4.5 | PREDICTED: Homo sapiens hypothetical protein LOC100132585 (LOC100132585), mRNA. |
| FLJ36131 | -4.5 | PREDICTED: Homo sapiens hypothetical protein FLJ36131, transcript variant 2 (FLJ36131), mRNA. |
| SOX4 | -4.52 | Homo sapiens SRY (sex determining region Y)-box 4 (SOX4), mRNA. |
| ID1 | -4.53 | Homo sapiens inhibitor of DNA binding 1, dominant negative helix-loop-helix protein (ID1), transcript variant 2, mRNA. |
| FAM63A | -4.53 | Homo sapiens family with sequence similarity 63, member A (FAM63A), transcript variant 1, mRNA. |
| OLFML3 | -4.56 | Homo sapiens olfactomedin-like 3 (OLFML3), mRNA. |
| EHBP1L1 | -4.62 | Homo sapiens EH domain binding protein 1-like 1 (EHBP1L1), mRNA. |
| FAM100A | -4.67 | Homo sapiens family with sequence similarity 100, member A (FAM100A), mRNA. |
| TMEM17 | -4.69 | Homo sapiens transmembrane protein 17 (TMEM17), mRNA. |
| ABCA2 | -4.7 | Homo sapiens ATP-binding cassette, sub-family A (ABC1), member 2 (ABCA2), transcript variant 2, mRNA. |
| BLZF1 | -4.71 | Homo sapiens basic leucine zipper nuclear factor 1 (BLZF1), mRNA. |
| TMEM132A | -4.72 | Homo sapiens transmembrane protein 132A (TMEM132A), transcript variant 2, mRNA. |
| DCHS1 | -4.74 | Homo sapiens dachsous 1 (Drosophila) (DCHS1), mRNA. |
| ZNF423 | -4.75 | Homo sapiens zinc finger protein 423 (ZNF423), mRNA. |
| CREB1 | -4.81 | Homo sapiens cAMP responsive element binding protein 1 (CREB1), transcript variant A, mRNA. |
| KLHL28 | -4.81 | Homo sapiens kelch-like 28 (Drosophila) (KLHL28), mRNA. |
| AMH | -4.81 | Homo sapiens anti-Mullerian hormone (AMH), mRNA. |
| LOC100129362 | -4.84 | PREDICTED: Homo sapiens hypothetical protein LOC100129362 (LOC100129362), mRNA. |
| LOC100130516 | -4.85 | PREDICTED: Homo sapiens hypothetical protein LOC100130516 (LOC100130516), mRNA. |
| LOC648852 | -4.86 | PREDICTED: Homo sapiens hypothetical protein LOC648852 (LOC648852), mRNA. |
| CHRNA5 | -4.87 | Homo sapiens cholinergic receptor, nicotinic, alpha 5 (CHRNA5), mRNA. |
| BMS1P5 | -4.88 | Homo sapiens BMS1 pseudogene 5 (BMS1P5), non-coding RNA. |
| QRFPR | -4.92 | Homo sapiens pyroglutamylated RFamide peptide receptor (QRFPR), mRNA. |
| FAM119A | -4.96 | Homo sapiens family with sequence similarity 119, member A (FAM119A), mRNA. |
| DUSP19 | -4.99 | Homo sapiens dual specificity phosphatase 19 (DUSP19), mRNA. |
| HSPC268 | -5 | Homo sapiens hypothetical protein HSPC268 (HSPC268), mRNA. |
| LOC100132391 | -5.02 | PREDICTED: Homo sapiens hypothetical protein LOC100132391 (LOC100132391), mRNA. |
| LOC644914 | -5.04 | PREDICTED: Homo sapiens similar to H3 histone, family 3B (LOC644914), mRNA. |
| C3orf34 | -5.04 | Homo sapiens chromosome 3 open reading frame 34 (C3orf34), mRNA. |
| TTYH3 | -5.05 | Homo sapiens tweety homolog 3 (Drosophila) (TTYH3), mRNA. |
| TACC3 | -5.1 | Homo sapiens transforming, acidic coiled-coil containing protein 3 (TACC3), mRNA. |
| MYO3B | -5.11 | Homo sapiens myosin IIIB (MYO3B), mRNA. |
| LOC100129269 | -5.13 | PREDICTED: Homo sapiens hypothetical protein LOC100129269 (LOC100129269), mRNA. |
| ARMC5 | -5.17 | Homo sapiens armadillo repeat containing 5 (ARMC5), mRNA. |
| LOC100128288 | -5.2 | Homo sapiens hypothetical protein LOC100128288 (LOC100128288), non-coding RNA. |
| EID2B | -5.21 | Homo sapiens EP300 interacting inhibitor of differentiation 2B (EID2B), mRNA. |
| SHROOM4 | -5.21 | Homo sapiens shroom family member 4 (SHROOM4), mRNA. |
| LOC100130835 | -5.22 | PREDICTED: Homo sapiens misc_RNA (LOC100130835), miscRNA. |
| PCDH9 | -5.25 | Homo sapiens protocadherin 9 (PCDH9), transcript variant 1, mRNA. |
| H1FX | -5.25 | Homo sapiens H1 histone family, member X (H1FX), mRNA. |
|  | -5.26 | xj89b12.x1 Soares_NFL_T_GBC_S1 Homo sapiens cDNA clone IMAGE:2664383 3, mRNA sequence |
| ZNF580 | -5.3 | Homo sapiens zinc finger protein 580 (ZNF580), transcript variant 1, mRNA. |
| LRRN3 | -5.34 | Homo sapiens leucine rich repeat neuronal 3 (LRRN3), mRNA. |
| SGK1 | -5.34 | Homo sapiens serum/glucocorticoid regulated kinase 1 (SGK1), transcript variant 1, mRNA. |
| ZNF394 | -5.35 | Homo sapiens zinc finger protein 394 (ZNF394), mRNA. |
| LOC100128510 | -5.36 | PREDICTED: Homo sapiens hypothetical protein LOC100128510 (LOC100128510), mRNA. |
| LOC100133516 | -5.43 | PREDICTED: Homo sapiens hypothetical protein LOC100133516 (LOC100133516), mRNA. |
| KIAA1751 | -5.44 | Homo sapiens KIAA1751 (KIAA1751), mRNA. |
| LOC100128098 | -5.45 | PREDICTED: Homo sapiens hypothetical protein LOC100128098 (LOC100128098), mRNA. |
| LOC728903 | -5.45 | PREDICTED: Homo sapiens hypothetical LOC728903, transcript variant 1 (LOC728903), mRNA. |
| GRIPAP1 | -5.47 | Homo sapiens GRIP1 associated protein 1 (GRIPAP1), transcript variant 2, mRNA. |
| FKBP14 | -5.47 | Homo sapiens FK506 binding protein 14, 22 kDa (FKBP14), mRNA. |
| ZNF69 | -5.49 | Homo sapiens zinc finger protein 69 (ZNF69), mRNA. |
| DEM1 | -5.5 | Homo sapiens defects in morphology 1 homolog (S. cerevisiae) (DEM1), mRNA. |
| LOC100129211 | -5.51 | PREDICTED: Homo sapiens hypothetical protein LOC100129211 (LOC100129211), mRNA. |
|  | -5.52 | FNPANH10 FNP Homo sapiens cDNA, mRNA sequence |
| HNRNPU | -5.63 | Homo sapiens heterogeneous nuclear ribonucleoprotein U (scaffold attachment factor A) (HNRNPU), transcript variant 2, mRNA. |
| SNORA12 | -5.68 | Homo sapiens small nucleolar RNA, H/ACA box 12 (SNORA12), small nucleolar RNA. |
| KIAA0319L | -5.69 | Homo sapiens KIAA0319-like (KIAA0319L), transcript variant 1, mRNA. |
| IGFBP5 | -5.79 | Homo sapiens insulin-like growth factor binding protein 5 (IGFBP5), mRNA. |
| FCAR | -5.83 | Homo sapiens Fc fragment of IgA, receptor for (FCAR), transcript variant 9, mRNA. |
| SGK1 | -5.92 | Homo sapiens serum/glucocorticoid regulated kinase 1 (SGK1), transcript variant 1, mRNA. |
| H3F3B | -5.97 | Homo sapiens H3 histone, family 3B (H3.3B) (H3F3B), mRNA. |
| XRCC2 | -6.07 | Homo sapiens X-ray repair complementing defective repair in Chinese hamster cells 2 (XRCC2), mRNA. |
| ZNF503 | -6.1 | Homo sapiens zinc finger protein 503 (ZNF503), mRNA. |
| TDP1 | -6.11 | Homo sapiens tyrosyl-DNA phosphodiesterase 1 (TDP1), transcript variant 1, mRNA. |
| ZNF483 | -6.17 | Homo sapiens zinc finger protein 483 (ZNF483), transcript variant 2, mRNA. |
| MIR1978 | -6.21 | Homo sapiens microRNA 1978 (MIR1978), microRNA. |
|  | -6.23 | zr87e09.r1 NCI_CGAP_GCB1 Homo sapiens cDNA clone IMAGE:682696 5, mRNA sequence |
| NFIX | -6.31 | Homo sapiens nuclear factor I/X (CCAAT-binding transcription factor) (NFIX), mRNA. |
| ID2 | -6.49 | Homo sapiens inhibitor of DNA binding 2, dominant negative helix-loop-helix protein (ID2), mRNA. |
|  | -6.58 | xr14b10.x1 NCI_CGAP_Lu28 Homo sapiens cDNA clone IMAGE:2760091 3, mRNA sequence |
| MBTD1 | -6.62 | Homo sapiens mbt domain containing 1 (MBTD1), mRNA. |
|  | -6.67 | MR1-GN0172-061100-005-h03 GN0172 Homo sapiens cDNA, mRNA sequence |
| SEMA3E | -6.74 | Homo sapiens sema domain, immunoglobulin domain (Ig), short basic domain, secreted, (semaphorin) 3E (SEMA3E), mRNA. |
| SGK | -6.88 | Homo sapiens serum/glucocorticoid regulated kinase (SGK), mRNA. |
| ID2 | -7.06 | Homo sapiens inhibitor of DNA binding 2, dominant negative helix-loop-helix protein (ID2), mRNA. |
| LRRFIP1 | -7.34 | Homo sapiens leucine rich repeat (in FLII) interacting protein 1 (LRRFIP1), mRNA. |
| IGFBP5 | -8.34 | Homo sapiens insulin-like growth factor binding protein 5 (IGFBP5), mRNA. |
|  | -8.86 | AGENCOURT_10229596 NIH_MGC_141 Homo sapiens cDNA clone IMAGE:6563923 5, mRNA sequence |
| CYR61 | -17.65 | Homo sapiens cysteine-rich, angiogenic inducer, 61 (CYR61), mRNA. |
| FOS | -18.99 | Homo sapiens v-fos FBJ murine osteosarcoma viral oncogene homolog (FOS), mRNA. |
| EGR1 | -23.42 | Homo sapiens early growth response 1 (EGR1), mRNA. |
