# Supplementary material for: Plurigon: three dimensional visualization and classification of high-dimensionality data
Source: Front Physiol. 2013 Jul 22;4:190. doi: 10.3389/fphys.2013.00190 (PMC3717481; doi:10.3389/fphys.2013.00190)
Supplement: Table S10 — Standard PCA analysis of WT and HET genotypic datasets. PCA was applied to reduce data dimensionality to three primary dimensions, D1, D2 and D3. Using these three dimensions the two genotype groups appeared non-separable. [file 46440_Maudsley_Data_Sheet_5.ZIP › 46440_Maudsley_Table_13.DOC]

**Table S13. Human neuronal cell (SH-SY5Y) transcriptomic effects induced by brain-derived neurotrophic factor exposure.** For each brain-derived neurotrophic factor (BDNF) regulated transcript the official Gene Symbol, z-ratio and transcript description are indicated.

| **Gene Symbol** | **z-ratio** | **Description** |
| --- | --- | --- |
| SLC25A22 | 6.07 | Homo sapiens solute carrier family 25 (mitochondrial carrier: glutamate), member 22 (SLC25A22), nuclear gene encoding mitochondrial protein, mRNA. |
| MAP6 | 5.82 | Homo sapiens microtubule-associated protein 6 (MAP6), transcript variant 1, mRNA. |
| CLK1 | 5.66 | Homo sapiens CDC-like kinase 1 (CLK1), mRNA. |
| LOC730235 | 5.39 | PREDICTED: Homo sapiens hypothetical LOC730235 (LOC730235), mRNA. |
| LOC100130123 | 5.35 | PREDICTED: Homo sapiens similar to PRO2870 (LOC100130123), mRNA. |
| C19orf44 | 5.23 | Homo sapiens chromosome 19 open reading frame 44 (C19orf44), mRNA. |
| GADD45G | 5.2 | Homo sapiens growth arrest and DNA-damage-inducible, gamma (GADD45G), mRNA. |
| ACCN2 | 5.16 | Homo sapiens amiloride-sensitive cation channel 2, neuronal (ACCN2), transcript variant 2, mRNA. |
| ORAOV1 | 4.91 | Homo sapiens oral cancer overexpressed 1 (ORAOV1), mRNA. |
| DPP7 | 4.79 | Homo sapiens dipeptidyl-peptidase 7 (DPP7), mRNA. |
| B4GALT7 | 4.77 | Homo sapiens xylosylprotein beta 1,4-galactosyltransferase, polypeptide 7 (galactosyltransferase I) (B4GALT7), mRNA. |
| GFOD2 | 4.59 | Homo sapiens glucose-fructose oxidoreductase domain containing 2 (GFOD2), mRNA. |
| HSPA1B | 4.44 | Homo sapiens heat shock 70kDa protein 1B (HSPA1B), mRNA. |
| GAL3ST3 | 4.38 | Homo sapiens galactose-3-O-sulfotransferase 3 (GAL3ST3), mRNA. |
| CLK1 | 4.34 | Homo sapiens CDC-like kinase 1 (CLK1), mRNA. |
| SLC43A2 | 4.19 | Homo sapiens solute carrier family 43, member 2 (SLC43A2), mRNA. |
| LOC148413 | 4.16 | Homo sapiens hypothetical LOC148413 (LOC148413), non-coding RNA. |
| MOBKL2C | 4.12 | Homo sapiens MOB1, Mps One Binder kinase activator-like 2C (yeast) (MOBKL2C), transcript variant 1, mRNA. |
| C8orf55 | 4.08 | Homo sapiens chromosome 8 open reading frame 55 (C8orf55), mRNA. |
| ANKRD33 | 4.06 | Homo sapiens ankyrin repeat domain 33 (ANKRD33), mRNA. |
| ASCC2 | 4.03 | Homo sapiens activating signal cointegrator 1 complex subunit 2 (ASCC2), mRNA. |
| SPSB3 | 4.02 | Homo sapiens splA/ryanodine receptor domain and SOCS box containing 3 (SPSB3), mRNA. |
| NDST2 | 4.01 | Homo sapiens N-deacetylase/N-sulfotransferase (heparan glucosaminyl) 2 (NDST2), mRNA. |
| LOC100129645 | 4 | PREDICTED: Homo sapiens misc_RNA (LOC100129645), miscRNA. |
| SH3GL1 | 3.97 | Homo sapiens SH3-domain GRB2-like 1 (SH3GL1), mRNA. |
| GRAMD1A | 3.96 | Homo sapiens GRAM domain containing 1A (GRAMD1A), mRNA. |
| TINF2 | 3.95 | Homo sapiens TERF1 (TRF1)-interacting nuclear factor 2 (TINF2), mRNA. |
| CLK3 | 3.94 | Homo sapiens CDC-like kinase 3 (CLK3), transcript variant phclk3, mRNA. |
| EHD1 | 3.94 | Homo sapiens EH-domain containing 1 (EHD1), mRNA. |
| ZNF200 | 3.93 | Homo sapiens zinc finger protein 200 (ZNF200), transcript variant 3, mRNA. |
| ZNF672 | 3.92 | Homo sapiens zinc finger protein 672 (ZNF672), mRNA. |
| GJC2 | 3.91 | Homo sapiens gap junction protein, gamma 2, 47kDa (GJC2), mRNA. |
| CCDC106 | 3.89 | Homo sapiens coiled-coil domain containing 106 (CCDC106), mRNA. |
| SLC26A6 | 3.86 | Homo sapiens solute carrier family 26, member 6 (SLC26A6), transcript variant 3, mRNA. |
| DTX2 | 3.86 | Homo sapiens deltex homolog 2 (Drosophila) (DTX2), mRNA. |
| KCNQ2 | 3.84 | Homo sapiens potassium voltage-gated channel, KQT-like subfamily, member 2 (KCNQ2), transcript variant 5, mRNA. |
| CCND3 | 3.79 | Homo sapiens cyclin D3 (CCND3), mRNA. |
| LOC100131866 | 3.78 | PREDICTED: Homo sapiens misc_RNA (LOC100131866), miscRNA. |
| DEDD2 | 3.76 | Homo sapiens death effector domain containing 2 (DEDD2), mRNA. |
| LOC100129890 | 3.74 | PREDICTED: Homo sapiens similar to hCG1750329 (LOC100129890), mRNA. |
| CCS | 3.73 | Homo sapiens copper chaperone for superoxide dismutase (CCS), mRNA. |
| RXRB | 3.71 | Homo sapiens retinoid X receptor, beta (RXRB), mRNA. |
| HPS6 | 3.7 | Homo sapiens Hermansky-Pudlak syndrome 6 (HPS6), mRNA. |
| CLK1 | 3.7 | Homo sapiens CDC-like kinase 1 (CLK1), transcript variant 2, mRNA. |
| SAFB | 3.7 | Homo sapiens scaffold attachment factor B (SAFB), mRNA. |
| HSPA5 | 3.69 | Homo sapiens heat shock 70kDa protein 5 (glucose-regulated protein, 78kDa) (HSPA5), mRNA. |
| HCFC1R1 | 3.67 | Homo sapiens host cell factor C1 regulator 1 (XPO1 dependent) (HCFC1R1), transcript variant 3, mRNA. |
| NEUROG2 | 3.63 | Homo sapiens neurogenin 2 (NEUROG2), mRNA. |
| FAM53C | 3.63 | Homo sapiens family with sequence similarity 53, member C (FAM53C), mRNA. |
| C14orf80 | 3.62 | Homo sapiens chromosome 14 open reading frame 80 (C14orf80), mRNA. |
| NR2F2 | 3.61 | Homo sapiens nuclear receptor subfamily 2, group F, member 2 (NR2F2), mRNA. |
| RPS6KB2 | 3.6 | Homo sapiens ribosomal protein S6 kinase, 70kDa, polypeptide 2 (RPS6KB2), mRNA. |
| LOC255783 | 3.59 | Homo sapiens hypothetical protein LOC255783 (LOC255783), non-coding RNA. |
| RASSF7 | 3.57 | Homo sapiens Ras association (RalGDS/AF-6) domain family (N-terminal) member 7 (RASSF7), mRNA. |
| LOC643534 | 3.57 | PREDICTED: Homo sapiens similar to hCG37526 (LOC643534), mRNA. |
| TXNIP | 3.56 | Homo sapiens thioredoxin interacting protein (TXNIP), mRNA. |
| SIPA1 | 3.56 | Homo sapiens signal-induced proliferation-associated gene 1 (SIPA1), transcript variant 2, mRNA. |
| MED16 | 3.55 | Homo sapiens mediator complex subunit 16 (MED16), mRNA. |
| DKFZp434K191 | 3.54 | Homo sapiens hypothetical protein DKFZp434K191 (DKFZp434K191), mRNA. |
| NFX1 | 3.53 | Homo sapiens nuclear transcription factor, X-box binding 1 (NFX1), transcript variant 1, mRNA. |
| C20orf27 | 3.53 | Homo sapiens chromosome 20 open reading frame 27 (C20orf27), mRNA. |
| MANEAL | 3.52 | Homo sapiens mannosidase, endo-alpha-like (MANEAL), transcript variant 1, mRNA. |
| PTGES2 | 3.51 | Homo sapiens prostaglandin E synthase 2 (PTGES2), transcript variant 2, mRNA. |
| RTKN | 3.51 | Homo sapiens rhotekin (RTKN), transcript variant 2, mRNA. |
| LOC729423 | 3.5 | PREDICTED: Homo sapiens similar to Heterogeneous nuclear ribonucleoprotein A1 (Helix-destabilizing protein) (Single-strand RNA-binding protein) (hnRNP core protein A1) (HDP) (LOC729423), mRNA. |
| SC4MOL | 3.5 | Homo sapiens sterol-C4-methyl oxidase-like (SC4MOL), transcript variant 1, mRNA. |
| SLC2A8 | 3.49 | Homo sapiens solute carrier family 2 (facilitated glucose transporter), member 8 (SLC2A8), mRNA. |
| ARMCX3 | 3.47 | Homo sapiens armadillo repeat containing, X-linked 3 (ARMCX3), transcript variant 2, mRNA. |
| PICK1 | 3.45 | Homo sapiens protein interacting with PRKCA 1 (PICK1), transcript variant 1, mRNA. |
| HS6ST2 | 3.44 | Homo sapiens heparan sulfate 6-O-sulfotransferase 2 (HS6ST2), transcript variant S, mRNA. |
|  | 3.42 | Homo sapiens cDNA clone MGC:13035 IMAGE:3613409, complete cds |
| GNL1 | 3.4 | Homo sapiens guanine nucleotide binding protein-like 1 (GNL1), mRNA. |
| REXO4 | 3.36 | Homo sapiens REX4, RNA exonuclease 4 homolog (S. cerevisiae) (REXO4), mRNA. |
| C9orf37 | 3.34 | Homo sapiens chromosome 9 open reading frame 37 (C9orf37), mRNA. |
| CCDC136 | 3.34 | Homo sapiens coiled-coil domain containing 136 (CCDC136), mRNA. |
| CCDC92 | 3.34 | Homo sapiens coiled-coil domain containing 92 (CCDC92), mRNA. |
| LOC728772 | 3.33 | PREDICTED: Homo sapiens similar to transmembrane protein 106A, transcript variant 1 (LOC728772), mRNA. |
| ARFGAP2 | 3.33 | Homo sapiens ADP-ribosylation factor GTPase activating protein 2 (ARFGAP2), mRNA. |
| IRF7 | 3.31 | Homo sapiens interferon regulatory factor 7 (IRF7), transcript variant b, mRNA. |
| RER1 | 3.31 | Homo sapiens RER1 retention in endoplasmic reticulum 1 homolog (S. cerevisiae) (RER1), mRNA. |
| ZBTB45 | 3.31 | Homo sapiens zinc finger and BTB domain containing 45 (ZBTB45), mRNA. |
| LOC100131096 | 3.31 | PREDICTED: Homo sapiens hypothetical protein LOC100131096 (LOC100131096), mRNA. |
| TRK1 | 3.31 | Homo sapiens tRNA lysine 1 (TRK1) on chromosome 17. |
| XAF1 | 3.3 | Homo sapiens XIAP associated factor 1 (XAF1), transcript variant 2, mRNA. |
| GNG3 | 3.28 | Homo sapiens guanine nucleotide binding protein (G protein), gamma 3 (GNG3), mRNA. |
| STK32C | 3.27 | Homo sapiens serine/threonine kinase 32C (STK32C), mRNA. |
| HNRNPA1 | 3.26 | Homo sapiens heterogeneous nuclear ribonucleoprotein A1 (HNRNPA1), transcript variant 2, mRNA. |
| LHPP | 3.26 | Homo sapiens phospholysine phosphohistidine inorganic pyrophosphate phosphatase (LHPP), mRNA. |
| VPS24 | 3.25 | Homo sapiens vacuolar protein sorting 24 homolog (S. cerevisiae) (VPS24), transcript variant 2, mRNA. |
| ATPBD3 | 3.24 | Homo sapiens ATP binding domain 3 (ATPBD3), mRNA. |
| IFNAR1 | 3.24 | Homo sapiens interferon (alpha, beta and omega) receptor 1 (IFNAR1), mRNA. |
| ZNF615 | 3.22 | Homo sapiens zinc finger protein 615 (ZNF615), mRNA. |
| LRCH4 | 3.21 | Homo sapiens leucine-rich repeats and calponin homology (CH) domain containing 4 (LRCH4), mRNA. |
| PPAP2C | 3.21 | Homo sapiens phosphatidic acid phosphatase type 2C (PPAP2C), transcript variant 2, mRNA. |
| TMUB2 | 3.21 | Homo sapiens transmembrane and ubiquitin-like domain containing 2 (TMUB2), transcript variant 1, mRNA. |
| PTRF | 3.2 | Homo sapiens polymerase I and transcript release factor (PTRF), mRNA. |
| DPYSL4 | 3.2 | Homo sapiens dihydropyrimidinase-like 4 (DPYSL4), mRNA. |
| FADD | 3.19 | Homo sapiens Fas (TNFRSF6)-associated via death domain (FADD), mRNA. |
| MPST | 3.19 | Homo sapiens mercaptopyruvate sulfurtransferase (MPST), nuclear gene encoding mitochondrial protein, transcript variant 1, mRNA. |
| IMP4 | 3.17 | Homo sapiens IMP4, U3 small nucleolar ribonucleoprotein, homolog (yeast) (IMP4), mRNA. |
| DBH | 3.17 | Homo sapiens dopamine beta-hydroxylase (dopamine beta-monooxygenase) (DBH), mRNA. |
| PARD6A | 3.16 | Homo sapiens par-6 partitioning defective 6 homolog alpha (C. elegans) (PARD6A), transcript variant 2, mRNA. |
| IP6K1 | 3.16 | Homo sapiens inositol hexakisphosphate kinase 1 (IP6K1), transcript variant 2, mRNA. |
| LOC91561 | 3.16 | PREDICTED: Homo sapiens similar to ribosomal protein S2, transcript variant 3 (LOC91561), mRNA. |
| TP53I13 | 3.16 | Homo sapiens tumor protein p53 inducible protein 13 (TP53I13), mRNA. |
| CPT1C | 3.16 | Homo sapiens carnitine palmitoyltransferase 1C (CPT1C), transcript variant 2, mRNA. |
| C8orf77 | 3.15 | PREDICTED: Homo sapiens chromosome 8 open reading frame 77 (C8orf77), misc RNA. |
| PEX16 | 3.15 | Homo sapiens peroxisomal biogenesis factor 16 (PEX16), transcript variant 1, mRNA. |
| UROS | 3.14 | Homo sapiens uroporphyrinogen III synthase (congenital erythropoietic porphyria) (UROS), mRNA. |
| SPHK2 | 3.13 | Homo sapiens sphingosine kinase 2 (SPHK2), mRNA. |
| CLOCK | 3.13 | Homo sapiens clock homolog (mouse) (CLOCK), mRNA. |
| FRS3 | 3.13 | Homo sapiens fibroblast growth factor receptor substrate 3 (FRS3), mRNA. |
| RBM3 | 3.12 | Homo sapiens RNA binding motif (RNP1, RRM) protein 3 (RBM3), mRNA. |
| WDR86 | 3.11 | Homo sapiens WD repeat domain 86 (WDR86), mRNA. |
| FAM131A | 3.11 | Homo sapiens family with sequence similarity 131, member A (FAM131A), mRNA. |
| LOC645001 | 3.11 | PREDICTED: Homo sapiens misc_RNA (LOC645001), miscRNA. |
| VPS37D | 3.11 | Homo sapiens vacuolar protein sorting 37 homolog D (S. cerevisiae) (VPS37D), mRNA. |
| BCDIN3D | 3.1 | Homo sapiens BCDIN3 domain containing (BCDIN3D), mRNA. |
| LOC389286 | 3.1 | Homo sapiens similar to FKSG62 (LOC389286), mRNA. |
|  | 3.09 | NISC_na09g08.y1 COGENE 8.5 EAT Homo sapiens cDNA clone IMAGE:5794742 5, mRNA sequence |
| AIFM1 | 3.08 | Homo sapiens apoptosis-inducing factor, mitochondrion-associated, 1 (AIFM1), nuclear gene encoding mitochondrial protein, transcript variant 2, mRNA. |
| ALDOC | 3.08 | Homo sapiens aldolase C, fructose-bisphosphate (ALDOC), mRNA. |
| FBXW7 | 3.07 | Homo sapiens F-box and WD repeat domain containing 7 (FBXW7), transcript variant 1, mRNA. |
| DNLZ | 3.07 | Homo sapiens DNL-type zinc finger (DNLZ), mRNA. |
| MAPKAPK3 | 3.06 | Homo sapiens mitogen-activated protein kinase-activated protein kinase 3 (MAPKAPK3), mRNA. |
| C17orf70 | 3.06 | Homo sapiens chromosome 17 open reading frame 70 (C17orf70), mRNA. |
| MAT2B | 3.05 | Homo sapiens methionine adenosyltransferase II, beta (MAT2B), transcript variant 1, mRNA. |
| RAB11FIP5 | 3.05 | Homo sapiens RAB11 family interacting protein 5 (class I) (RAB11FIP5), mRNA. |
| MYCN | 3.04 | Homo sapiens v-myc myelocytomatosis viral related oncogene, neuroblastoma derived (avian) (MYCN), mRNA. |
| ZNF585A | 3.03 | Homo sapiens zinc finger protein 585A (ZNF585A), transcript variant 2, mRNA. |
| KCNJ8 | 3.03 | Homo sapiens potassium inwardly-rectifying channel, subfamily J, member 8 (KCNJ8), mRNA. |
| LOC401720 | 3.03 | Homo sapiens similar to fidgetin (LOC401720), mRNA. |
| LOC727818 | 3.02 | PREDICTED: Homo sapiens similar to PRP38 pre-mRNA processing factor 38 (yeast) domain containing B (LOC727818), miscRNA. |
| PSPH | 3.02 | Homo sapiens phosphoserine phosphatase (PSPH), mRNA. |
| FGF14 | 3.02 | Homo sapiens fibroblast growth factor 14 (FGF14), transcript variant 2, mRNA. |
| SLC25A19 | 3.01 | Homo sapiens solute carrier family 25 (mitochondrial thiamine pyrophosphate carrier), member 19 (SLC25A19), nuclear gene encoding mitochondrial protein, mRNA. |
| ATP10A | 3.01 | Homo sapiens ATPase, class V, type 10A (ATP10A), mRNA. |
| MIR2116 | 2.99 | Homo sapiens microRNA 2116 (MIR2116), microRNA. |
| HERPUD1 | 2.99 | Homo sapiens homocysteine-inducible, endoplasmic reticulum stress-inducible, ubiquitin-like domain member 1 (HERPUD1), transcript variant 3, mRNA. |
| MFSD3 | 2.97 | Homo sapiens major facilitator superfamily domain containing 3 (MFSD3), mRNA. |
| C1orf63 | 2.97 | Homo sapiens chromosome 1 open reading frame 63 (C1orf63), transcript variant 1, mRNA. |
| LOC440487 | 2.97 | PREDICTED: Homo sapiens hypothetical LOC440487 (LOC440487), mRNA. |
| TSSC4 | 2.95 | Homo sapiens tumor suppressing subtransferable candidate 4 (TSSC4), mRNA. |
| BTBD11 | 2.95 | Homo sapiens BTB (POZ) domain containing 11 (BTBD11), transcript variant 1, mRNA. |
| LMNA | 2.95 | Homo sapiens lamin A/C (LMNA), transcript variant 2, mRNA. |
| LOC100133740 | 2.93 | PREDICTED: Homo sapiens misc_RNA (LOC100133740), miscRNA. |
| LRWD1 | 2.93 | Homo sapiens leucine-rich repeats and WD repeat domain containing 1 (LRWD1), mRNA. |
| LOC286016 | 2.93 | Homo sapiens triosephosphate isomerase 1 pseudogene (LOC286016), non-coding RNA. |
| CCDC130 | 2.91 | Homo sapiens coiled-coil domain containing 130 (CCDC130), mRNA. |
| HOXC13 | 2.91 | Homo sapiens homeobox C13 (HOXC13), mRNA. |
| IFFO1 | 2.91 | Homo sapiens intermediate filament family orphan 1 (IFFO1), transcript variant 2, mRNA. |
| C9orf40 | 2.91 | Homo sapiens chromosome 9 open reading frame 40 (C9orf40), mRNA. |
| FAM65A | 2.9 | Homo sapiens family with sequence similarity 65, member A (FAM65A), mRNA. |
| ZNF653 | 2.9 | Homo sapiens zinc finger protein 653 (ZNF653), mRNA. |
| FAM153B | 2.89 | Homo sapiens family with sequence similarity 153, member B (FAM153B), mRNA. |
| DHX34 | 2.89 | Homo sapiens DEAH (Asp-Glu-Ala-His) box polypeptide 34 (DHX34), mRNA. |
| ALS2CR14 | 2.88 | Homo sapiens amyotrophic lateral sclerosis 2 (juvenile) chromosome region, candidate 14 (ALS2CR14), mRNA. |
| ZNF823 | 2.87 | Homo sapiens zinc finger protein 823 (ZNF823), mRNA. |
| LOC100133329 | 2.86 | PREDICTED: Homo sapiens misc_RNA (LOC100133329), miscRNA. |
| NAPEPLD | 2.86 | Homo sapiens N-acyl phosphatidylethanolamine phospholipase D (NAPEPLD), mRNA. |
| RAE1 | 2.85 | Homo sapiens RAE1 RNA export 1 homolog (S. pombe) (RAE1), transcript variant 1, mRNA. |
| FAM39E | 2.85 | Homo sapiens family with sequence similarity 39, member E (FAM39E), mRNA. |
| FAM129B | 2.85 | Homo sapiens family with sequence similarity 129, member B (FAM129B), transcript variant 1, mRNA. |
| ENO3 | 2.85 | Homo sapiens enolase 3 (beta, muscle) (ENO3), transcript variant 1, mRNA. |
| LOC649209 | 2.85 | PREDICTED: Homo sapiens similar to 40S ribosomal protein S2 (LOC649209), mRNA. |
| TRIB3 | 2.84 | Homo sapiens tribbles homolog 3 (Drosophila) (TRIB3), mRNA. |
| LOC391169 | 2.83 | PREDICTED: Homo sapiens misc_RNA (LOC391169), miscRNA. |
| LOC126235 | 2.83 | PREDICTED: Homo sapiens misc_RNA (LOC126235), miscRNA. |
| R3HCC1 | 2.83 | PREDICTED: Homo sapiens R3H domain and coiled-coil containing 1 (R3HCC1), mRNA. |
| CPSF1 | 2.83 | Homo sapiens cleavage and polyadenylation specific factor 1, 160kDa (CPSF1), mRNA. |
| EPHA10 | 2.82 | Homo sapiens EPH receptor A10 (EPHA10), transcript variant 2, mRNA. |
| HNRNPA3 | 2.82 | Homo sapiens heterogeneous nuclear ribonucleoprotein A3 (HNRNPA3), mRNA. |
| LOC728969 | 2.81 | PREDICTED: Homo sapiens similar to heparan sulfate 6-O-sulfotransferase 1 (LOC728969), mRNA. |
| EYS | 2.81 | Homo sapiens eyes shut homolog (Drosophila) (EYS), transcript variant 3, mRNA. |
| EMILIN2 | 2.81 | Homo sapiens elastin microfibril interfacer 2 (EMILIN2), mRNA. |
| TPD52L2 | 2.81 | Homo sapiens tumor protein D52-like 2 (TPD52L2), transcript variant 5, mRNA. |
| CENPB | 2.8 | Homo sapiens centromere protein B, 80kDa (CENPB), mRNA. |
| WDR74 | 2.8 | PREDICTED: Homo sapiens WD repeat domain 74 (WDR74), mRNA. |
| BSN | 2.8 | Homo sapiens bassoon (presynaptic cytomatrix protein) (BSN), mRNA. |
| LOC389672 | 2.8 | PREDICTED: Homo sapiens similar to 40S ribosomal protein SA (p40) (34/67 kDa laminin receptor) (Colon carcinoma laminin-binding protein) (NEM/1CHD4) (Multidrug resistance-associated protein MGr1-Ag), transcript variant 3 (LOC389672), mRNA. |
| CS | 2.79 | Homo sapiens citrate synthase (CS), nuclear gene encoding mitochondrial protein, mRNA. |
| UBE2L3 | 2.79 | Homo sapiens ubiquitin-conjugating enzyme E2L 3 (UBE2L3), transcript variant 2, mRNA. |
| PES1 | 2.79 | Homo sapiens pescadillo homolog 1, containing BRCT domain (zebrafish) (PES1), mRNA. |
| TMEM59L | 2.77 | Homo sapiens transmembrane protein 59-like (TMEM59L), mRNA. |
| STMN3 | 2.77 | Homo sapiens stathmin-like 3 (STMN3), mRNA. |
| C1orf63 | 2.77 | Homo sapiens chromosome 1 open reading frame 63 (C1orf63), mRNA. |
| PLEKHA6 | 2.76 | Homo sapiens pleckstrin homology domain containing, family A member 6 (PLEKHA6), mRNA. |
| RFTN1 | 2.76 | Homo sapiens raftlin, lipid raft linker 1 (RFTN1), mRNA. |
| SDHAP2 | 2.75 | Homo sapiens succinate dehydrogenase complex, subunit A, flavoprotein pseudogene 2 (SDHAP2), non-coding RNA. |
| C9orf114 | 2.73 | Homo sapiens chromosome 9 open reading frame 114 (C9orf114), mRNA. |
| TRABD | 2.73 | Homo sapiens TraB domain containing (TRABD), mRNA. |
| ABTB1 | 2.73 | Homo sapiens ankyrin repeat and BTB (POZ) domain containing 1 (ABTB1), transcript variant 1, mRNA. |
| C19orf12 | 2.72 | Homo sapiens chromosome 19 open reading frame 12 (C19orf12), transcript variant 1, mRNA. |
| TJAP1 | 2.72 | Homo sapiens tight junction associated protein 1 (peripheral) (TJAP1), mRNA. |
| TMEM79 | 2.72 | Homo sapiens transmembrane protein 79 (TMEM79), mRNA. |
| FMO2 | 2.71 | Homo sapiens flavin containing monooxygenase 2 (non-functional) (FMO2), mRNA. |
| NOP2 | 2.71 | Homo sapiens NOP2 nucleolar protein homolog (yeast) (NOP2), transcript variant 2, mRNA. |
| KBTBD4 | 2.71 | Homo sapiens kelch repeat and BTB (POZ) domain containing 4 (KBTBD4), transcript variant 1, mRNA. |
| PLSCR3 | 2.7 | Homo sapiens phospholipid scramblase 3 (PLSCR3), mRNA. |
| CSDE1 | 2.7 | Homo sapiens cold shock domain containing E1, RNA-binding (CSDE1), transcript variant 1, mRNA. |
| CKS2 | 2.7 | Homo sapiens CDC28 protein kinase regulatory subunit 2 (CKS2), mRNA. |
| ZBTB48 | 2.69 | Homo sapiens zinc finger and BTB domain containing 48 (ZBTB48), mRNA. |
| BBS10 | 2.69 | Homo sapiens Bardet-Biedl syndrome 10 (BBS10), mRNA. |
| LOC729423 | 2.69 | PREDICTED: Homo sapiens similar to Heterogeneous nuclear ribonucleoprotein A1 (Helix-destabilizing protein) (Single-strand RNA-binding protein) (hnRNP core protein A1) (HDP) (LOC729423), mRNA. |
| LOC100131737 | 2.69 | PREDICTED: Homo sapiens misc_RNA (LOC100131737), miscRNA. |
| ZNF821 | 2.69 | Homo sapiens zinc finger protein 821 (ZNF821), mRNA. |
| BCKDK | 2.69 | Homo sapiens branched chain ketoacid dehydrogenase kinase (BCKDK), mRNA. |
| PRPF4B | 2.69 | Homo sapiens PRP4 pre-mRNA processing factor 4 homolog B (yeast) (PRPF4B), transcript variant 1, mRNA. |
| VSNL1 | 2.69 | Homo sapiens visinin-like 1 (VSNL1), mRNA. |
| SDF2L1 | 2.69 | Homo sapiens stromal cell-derived factor 2-like 1 (SDF2L1), mRNA. |
| NELF | 2.68 | Homo sapiens nasal embryonic LHRH factor (NELF), mRNA. |
| CHGB | 2.67 | Homo sapiens chromogranin B (secretogranin 1) (CHGB), mRNA. |
| FASTK | 2.67 | Homo sapiens Fas-activated serine/threonine kinase (FASTK), transcript variant 4, mRNA. |
| SIVA | 2.67 | Homo sapiens CD27-binding (Siva) protein (SIVA), transcript variant 2, mRNA. |
| NSDHL | 2.67 | Homo sapiens NAD(P) dependent steroid dehydrogenase-like (NSDHL), mRNA. |
| AMY1A | 2.66 | Homo sapiens amylase, alpha 1A (salivary) (AMY1A), transcript variant 1, mRNA. |
| TMEM50B | 2.66 | Homo sapiens transmembrane protein 50B (TMEM50B), mRNA. |
| C16orf93 | 2.66 | Homo sapiens chromosome 16 open reading frame 93 (C16orf93), mRNA. |
| SLC25A28 | 2.66 | Homo sapiens solute carrier family 25, member 28 (SLC25A28), mRNA. |
| SBDSP | 2.65 | Homo sapiens Shwachman-Bodian-Diamond syndrome pseudogene (SBDSP) on chromosome 7. |
| FBXL6 | 2.65 | Homo sapiens F-box and leucine-rich repeat protein 6 (FBXL6), transcript variant 1, mRNA. |
| C6orf47 | 2.65 | Homo sapiens chromosome 6 open reading frame 47 (C6orf47), mRNA. |
| SNAP47 | 2.64 | Homo sapiens synaptosomal-associated protein, 47kDa (SNAP47), mRNA. |
| LOC100130818 | 2.64 | PREDICTED: Homo sapiens hypothetical protein LOC100130818 (LOC100130818), mRNA. |
| DBNL | 2.64 | Homo sapiens drebrin-like (DBNL), transcript variant 2, mRNA. |
| LOC729660 | 2.64 | PREDICTED: Homo sapiens misc_RNA (LOC729660), miscRNA. |
| PIAS4 | 2.64 | Homo sapiens protein inhibitor of activated STAT, 4 (PIAS4), mRNA. |
| TRPT1 | 2.64 | Homo sapiens tRNA phosphotransferase 1 (TRPT1), transcript variant 2, mRNA. |
| PCDHGB6 | 2.63 | Homo sapiens protocadherin gamma subfamily B, 6 (PCDHGB6), transcript variant 1, mRNA. |
| ZMYM2 | 2.63 | Homo sapiens zinc finger, MYM-type 2 (ZMYM2), mRNA. |
| LOC284023 | 2.62 | PREDICTED: Homo sapiens hypothetical protein LOC284023, transcript variant 3 (LOC284023), mRNA. |
| C21orf126 | 2.62 | PREDICTED: Homo sapiens chromosome 21 open reading frame 126 (C21orf126), mRNA. |
| FAM174B | 2.62 | Homo sapiens family with sequence similarity 174, member B (FAM174B), mRNA. |
| HS6ST2 | 2.62 | Homo sapiens heparan sulfate 6-O-sulfotransferase 2 (HS6ST2), transcript variant L, mRNA. |
| ULK1 | 2.62 | Homo sapiens unc-51-like kinase 1 (C. elegans) (ULK1), mRNA. |
| MFSD10 | 2.62 | Homo sapiens major facilitator superfamily domain containing 10 (MFSD10), mRNA. |
| LOC100130715 | 2.61 | PREDICTED: Homo sapiens misc_RNA (LOC100130715), miscRNA. |
| LOC644330 | 2.61 | PREDICTED: Homo sapiens similar to tropomyosin 3 isoform 2 (LOC644330), mRNA. |
|  | 2.61 | 602812728F1 NCI_CGAP_Brn67 Homo sapiens cDNA clone IMAGE:4944798 5, mRNA sequence |
| TCEAL3 | 2.6 | Homo sapiens transcription elongation factor A (SII)-like 3 (TCEAL3), transcript variant 2, mRNA. |
| TPRG1L | 2.6 | Homo sapiens tumor protein p63 regulated 1-like (TPRG1L), mRNA. |
| PTCD1 | 2.6 | Homo sapiens pentatricopeptide repeat domain 1 (PTCD1), mRNA. |
| ZNF322A | 2.59 | Homo sapiens zinc finger protein 322A (ZNF322A), mRNA. |
| ADPRHL2 | 2.59 | Homo sapiens ADP-ribosylhydrolase like 2 (ADPRHL2), mRNA. |
| CXXC1 | 2.59 | Homo sapiens CXXC finger 1 (PHD domain) (CXXC1), mRNA. |
| NCKIPSD | 2.59 | Homo sapiens NCK interacting protein with SH3 domain (NCKIPSD), transcript variant 2, mRNA. |
| LOC100131835 | 2.59 | PREDICTED: Homo sapiens hypothetical protein LOC100131835 (LOC100131835), mRNA. |
| SNORA25 | 2.59 | Homo sapiens small nucleolar RNA, H/ACA box 25 (SNORA25), small nucleolar RNA. |
| PPP1R16A | 2.59 | Homo sapiens protein phosphatase 1, regulatory (inhibitor) subunit 16A (PPP1R16A), mRNA. |
| LOC441013 | 2.58 | PREDICTED: Homo sapiens misc_RNA (LOC441013), miscRNA. |
| GCH1 | 2.58 | Homo sapiens GTP cyclohydrolase 1 (GCH1), transcript variant 3, mRNA. |
| EIF4G2 | 2.58 | Homo sapiens eukaryotic translation initiation factor 4 gamma, 2 (EIF4G2), transcript variant 1, mRNA. |
| CHRNA3 | 2.58 | Homo sapiens cholinergic receptor, nicotinic, alpha 3 (CHRNA3), mRNA. |
| UBN2 | 2.58 | Homo sapiens ubinuclein 2 (UBN2), mRNA. |
| LOC390557 | 2.57 | PREDICTED: Homo sapiens hypothetical LOC390557 (LOC390557), mRNA. |
| SLC9A3R1 | 2.57 | Homo sapiens solute carrier family 9 (sodium/hydrogen exchanger), member 3 regulator 1 (SLC9A3R1), mRNA. |
| C16orf72 | 2.57 | Homo sapiens chromosome 16 open reading frame 72 (C16orf72), mRNA. |
| ELAVL3 | 2.57 | Homo sapiens ELAV (embryonic lethal, abnormal vision, Drosophila)-like 3 (Hu antigen C) (ELAVL3), transcript variant 1, mRNA. |
| DCUN1D3 | 2.56 | Homo sapiens DCN1, defective in cullin neddylation 1, domain containing 3 (S. cerevisiae) (DCUN1D3), mRNA. |
| SORCS1 | 2.56 | Homo sapiens sortilin-related VPS10 domain containing receptor 1 (SORCS1), transcript variant 2, mRNA. |
| ARS2 | 2.56 | Homo sapiens arsenate resistance protein 2 (ARS2), transcript variant 1, mRNA. |
| BZW1 | 2.56 | Homo sapiens basic leucine zipper and W2 domains 1 (BZW1), mRNA. XM_943165 |
| LOC646808 | 2.56 | PREDICTED: Homo sapiens misc_RNA (LOC646808), miscRNA. |
| LOC100132369 | 2.56 | PREDICTED: Homo sapiens hypothetical LOC100132369 (LOC100132369), mRNA. |
| POLG2 | 2.56 | Homo sapiens polymerase (DNA directed), gamma 2, accessory subunit (POLG2), mRNA. |
| LOC643387 | 2.55 | PREDICTED: Homo sapiens misc_RNA (LOC643387), miscRNA. |
| LOC100130562 | 2.55 | PREDICTED: Homo sapiens hypothetical protein LOC100130562, transcript variant 1 (LOC100130562), mRNA. |
| ARSA | 2.55 | Homo sapiens arylsulfatase A (ARSA), mRNA. |
| TOMM34 | 2.54 | Homo sapiens translocase of outer mitochondrial membrane 34 (TOMM34), nuclear gene encoding mitochondrial protein, mRNA. |
| NUFIP1 | 2.54 | Homo sapiens nuclear fragile X mental retardation protein interacting protein 1 (NUFIP1), mRNA. |
| LOC401056 | 2.54 | PREDICTED: Homo sapiens hypothetical LOC401056 (LOC401056), mRNA. |
| PHF23 | 2.53 | Homo sapiens PHD finger protein 23 (PHF23), mRNA. |
| HPCAL1 | 2.53 | Homo sapiens hippocalcin-like 1 (HPCAL1), transcript variant 2, mRNA. |
| ZNF322B | 2.52 | Homo sapiens zinc finger protein 322B (ZNF322B), mRNA. |
| CARD11 | 2.52 | Homo sapiens caspase recruitment domain family, member 11 (CARD11), mRNA. |
| LOC400652 | 2.52 | PREDICTED: Homo sapiens similar to 40S ribosomal protein S3a, transcript variant 1 (LOC400652), mRNA. |
| ZNF696 | 2.52 | Homo sapiens zinc finger protein 696 (ZNF696), mRNA. |
| LOC100128627 | 2.52 | PREDICTED: Homo sapiens misc_RNA (LOC100128627), miscRNA. |
| BSCL2 | 2.52 | Homo sapiens Bernardinelli-Seip congenital lipodystrophy 2 (seipin) (BSCL2), mRNA. |
| PLEKHA3 | 2.52 | Homo sapiens pleckstrin homology domain containing, family A (phosphoinositide binding specific) member 3 (PLEKHA3), mRNA. |
| ARHGEF18 | 2.51 | Homo sapiens rho/rac guanine nucleotide exchange factor (GEF) 18 (ARHGEF18), mRNA. |
| ZNF175 | 2.5 | Homo sapiens zinc finger protein 175 (ZNF175), mRNA. |
| LOC728457 | 2.5 | PREDICTED: Homo sapiens misc_RNA (LOC728457), miscRNA. |
| SRPRB | 2.5 | Homo sapiens signal recognition particle receptor, B subunit (SRPRB), mRNA. |
| SLC2A6 | 2.5 | Homo sapiens solute carrier family 2 (facilitated glucose transporter), member 6 (SLC2A6), mRNA. |
| RDH5 | 2.5 | Homo sapiens retinol dehydrogenase 5 (11-cis/9-cis) (RDH5), mRNA. |
|  | 2.5 | BX099310 Soares_NFL_T_GBC_S1 Homo sapiens cDNA clone IMAGp998P164537, mRNA sequence |
|  | 2.49 | Homo sapiens clone 25194 mRNA sequence |
| RSBN1 | 2.49 | Homo sapiens round spermatid basic protein 1 (RSBN1), mRNA. |
| MTERFD1 | 2.49 | Homo sapiens MTERF domain containing 1 (MTERFD1), mRNA. |
| CD82 | 2.49 | Homo sapiens CD82 molecule (CD82), transcript variant 1, mRNA. |
| LOC729298 | 2.49 | PREDICTED: Homo sapiens misc_RNA (LOC729298), miscRNA. |
| ATP13A1 | 2.48 | Homo sapiens ATPase type 13A1 (ATP13A1), mRNA. |
| LOC648740 | 2.48 | Homo sapiens ACTB pseudogene (LOC648740), non-coding RNA. |
| LOC113386 | 2.48 | Homo sapiens similar to envelope protein (LOC113386), mRNA. |
| NDEL1 | 2.48 | Homo sapiens nudE nuclear distribution gene E homolog (A. nidulans)-like 1 (NDEL1), transcript variant 2, mRNA. |
| C1orf77 | 2.48 | Homo sapiens chromosome 1 open reading frame 77 (C1orf77), mRNA. |
| SPATA2L | 2.47 | Homo sapiens spermatogenesis associated 2-like (SPATA2L), mRNA. |
| C12orf10 | 2.47 | Homo sapiens chromosome 12 open reading frame 10 (C12orf10), mRNA. |
| CCDC101 | 2.47 | Homo sapiens coiled-coil domain containing 101 (CCDC101), mRNA. |
| AP1B1 | 2.47 | Homo sapiens adaptor-related protein complex 1, beta 1 subunit (AP1B1), transcript variant 2, mRNA. |
| ZNF250 | 2.46 | Homo sapiens zinc finger protein 250 (ZNF250), mRNA. |
| LOC402112 | 2.46 | PREDICTED: Homo sapiens misc_RNA (LOC402112), miscRNA. |
| CDC20 | 2.46 | Homo sapiens cell division cycle 20 homolog (S. cerevisiae) (CDC20), mRNA. |
| LOC645968 | 2.46 | PREDICTED: Homo sapiens similar to 40S ribosomal protein S3a (V-fos transformation effector protein) (LOC645968), mRNA. |
| SKP1 | 2.46 | Homo sapiens S-phase kinase-associated protein 1 (SKP1), transcript variant 1, mRNA. |
| PDXK | 2.46 | Homo sapiens pyridoxal (pyridoxine, vitamin B6) kinase (PDXK), mRNA. |
| LOC647104 | 2.46 | PREDICTED: Homo sapiens hypothetical LOC647104 (LOC647104), mRNA. |
| SLC27A3 | 2.46 | Homo sapiens solute carrier family 27 (fatty acid transporter), member 3 (SLC27A3), mRNA. |
| MAPK4 | 2.45 | Homo sapiens mitogen-activated protein kinase 4 (MAPK4), mRNA. |
| IL1RL1 | 2.44 | Homo sapiens interleukin 1 receptor-like 1 (IL1RL1), transcript variant 2, mRNA. |
| TOR3A | 2.44 | Homo sapiens torsin family 3, member A (TOR3A), mRNA. |
| SOBP | 2.44 | Homo sapiens sine oculis binding protein homolog (Drosophila) (SOBP), mRNA. |
|  | 2.44 | full-length cDNA clone XCL0BB001ZD04 of Neuroblastoma of Homo sapiens (human) |
| BRI3P1 | 2.44 | PREDICTED: Homo sapiens misc_RNA (BRI3P1), miscRNA. |
| DNAJC27 | 2.43 | Homo sapiens DnaJ (Hsp40) homolog, subfamily C, member 27 (DNAJC27), mRNA. |
| MOBKL2A | 2.43 | Homo sapiens MOB1, Mps One Binder kinase activator-like 2A (yeast) (MOBKL2A), mRNA. |
| CPSF2 | 2.43 | Homo sapiens cleavage and polyadenylation specific factor 2, 100kDa (CPSF2), mRNA. |
| NUMBL | 2.43 | Homo sapiens numb homolog (Drosophila)-like (NUMBL), mRNA. |
| U2AF1L2 | 2.43 | Homo sapiens U2(RNU2) small nuclear RNA auxiliary factor 1-like 2 (U2AF1L2), mRNA. |
| SNRPB | 2.43 | Homo sapiens small nuclear ribonucleoprotein polypeptides B and B1 (SNRPB), transcript variant 2, mRNA. |
| FLJ32011 | 2.43 | Homo sapiens hypothetical protein FLJ32011 (FLJ32011), mRNA. |
| VPS24 | 2.42 | Homo sapiens vacuolar protein sorting 24 homolog (S. cerevisiae) (VPS24), transcript variant 2, mRNA. |
| ZYX | 2.42 | Homo sapiens zyxin (ZYX), transcript variant 1, mRNA. |
| TMEM115 | 2.42 | Homo sapiens transmembrane protein 115 (TMEM115), mRNA. |
| TCF25 | 2.42 | Homo sapiens transcription factor 25 (basic helix-loop-helix) (TCF25), mRNA. |
| LOC729687 | 2.42 | PREDICTED: Homo sapiens hypothetical LOC729687 (LOC729687), mRNA. |
| TST | 2.42 | Homo sapiens thiosulfate sulfurtransferase (rhodanese) (TST), nuclear gene encoding mitochondrial protein, mRNA. |
| DBNDD1 | 2.41 | Homo sapiens dysbindin (dystrobrevin binding protein 1) domain containing 1 (DBNDD1), transcript variant 1, mRNA. |
| OTUB1 | 2.41 | Homo sapiens OTU domain, ubiquitin aldehyde binding 1 (OTUB1), transcript variant 2, transcribed RNA. |
| RCOR2 | 2.41 | Homo sapiens REST corepressor 2 (RCOR2), mRNA. |
| PAQR4 | 2.41 | Homo sapiens progestin and adipoQ receptor family member IV (PAQR4), mRNA. |
| PLD3 | 2.4 | Homo sapiens phospholipase D family, member 3 (PLD3), transcript variant 1, mRNA. |
| JSRP1 | 2.4 | Homo sapiens junctional sarcoplasmic reticulum protein 1 (JSRP1), mRNA. |
| LOC642082 | 2.4 | PREDICTED: Homo sapiens hypothetical protein LOC642082 (LOC642082), mRNA. |
| SPATA20 | 2.39 | Homo sapiens spermatogenesis associated 20 (SPATA20), mRNA. |
| ANKFY1 | 2.38 | Homo sapiens ankyrin repeat and FYVE domain containing 1 (ANKFY1), transcript variant 1, mRNA. |
| ALKBH1 | 2.38 | Homo sapiens alkB, alkylation repair homolog 1 (E. coli) (ALKBH1), mRNA. |
| LOC644642 | 2.38 | PREDICTED: Homo sapiens hypothetical protein LOC644642 (LOC644642), mRNA. |
| ATP8B2 | 2.38 | Homo sapiens ATPase, class I, type 8B, member 2 (ATP8B2), transcript variant 1, mRNA. |
| SUV420H1 | 2.37 | Homo sapiens suppressor of variegation 4-20 homolog 1 (Drosophila) (SUV420H1), transcript variant 1, mRNA. |
| ALG3 | 2.37 | Homo sapiens asparagine-linked glycosylation 3 homolog (S. cerevisiae, alpha-1,3-mannosyltransferase) (ALG3), mRNA. |
| RBBP4 | 2.37 | Homo sapiens retinoblastoma binding protein 4 (RBBP4), mRNA. |
| RRAGC | 2.37 | Homo sapiens Ras-related GTP binding C (RRAGC), mRNA. |
| ECHS1 | 2.37 | Homo sapiens enoyl Coenzyme A hydratase, short chain, 1, mitochondrial (ECHS1), nuclear gene encoding mitochondrial protein, mRNA. |
| KLHL28 | 2.37 | Homo sapiens kelch-like 28 (Drosophila) (KLHL28), mRNA. |
| CSNK1E | 2.37 | Homo sapiens casein kinase 1, epsilon (CSNK1E), transcript variant 1, mRNA. |
| NPPA | 2.37 | Homo sapiens natriuretic peptide precursor A (NPPA), mRNA. |
| ZNF197 | 2.36 | Homo sapiens zinc finger protein 197 (ZNF197), transcript variant 1, mRNA. |
| SNORA18 | 2.36 | Homo sapiens small nucleolar RNA, H/ACA box 18 (SNORA18), small nucleolar RNA. |
| PSMD8 | 2.36 | Homo sapiens proteasome (prosome, macropain) 26S subunit, non-ATPase, 8 (PSMD8), mRNA. |
| KRBA1 | 2.36 | Homo sapiens KRAB-A domain containing 1 (KRBA1), mRNA. |
| ORC5L | 2.36 | Homo sapiens origin recognition complex, subunit 5-like (yeast) (ORC5L), transcript variant 1, mRNA. |
| SPG7 | 2.36 | Homo sapiens spastic paraplegia 7 (pure and complicated autosomal recessive) (SPG7), nuclear gene encoding mitochondrial protein, transcript variant 1, mRNA. |
|  | 2.35 | ov58f12.s1 Soares_testis_NHT Homo sapiens cDNA clone IMAGE:1641551 3, mRNA sequence |
| C17orf97 | 2.35 | Homo sapiens chromosome 17 open reading frame 97 (C17orf97), mRNA. |
| G3BP2 | 2.35 | Homo sapiens GTPase activating protein (SH3 domain) binding protein 2 (G3BP2), transcript variant 3, mRNA. |
| FIBP | 2.35 | Homo sapiens fibroblast growth factor (acidic) intracellular binding protein (FIBP), transcript variant 2, mRNA. |
| SC4MOL | 2.35 | Homo sapiens sterol-C4-methyl oxidase-like (SC4MOL), transcript variant 2, mRNA. |
| LOC729992 | 2.35 | PREDICTED: Homo sapiens misc_RNA (LOC729992), miscRNA. |
| SLC38A9 | 2.35 | Homo sapiens solute carrier family 38, member 9 (SLC38A9), mRNA. |
| FAM86D | 2.35 | Homo sapiens family with sequence similarity 86, member D (FAM86D), non-coding RNA. |
| LOC728732 | 2.34 | PREDICTED: Homo sapiens misc_RNA (LOC728732), miscRNA. |
| MAP6 | 2.34 | Homo sapiens microtubule-associated protein 6 (MAP6), transcript variant 2, mRNA. |
| ARMET | 2.34 | Homo sapiens arginine-rich, mutated in early stage tumors (ARMET), mRNA. |
| PRNPIP | 2.34 | PREDICTED: Homo sapiens prion protein interacting protein (PRNPIP), mRNA. |
| TMEM150A | 2.34 | Homo sapiens transmembrane protein 150A (TMEM150A), transcript variant 1, mRNA. |
| SNORA76 | 2.34 | Homo sapiens small nucleolar RNA, H/ACA box 76 (SNORA76), small nucleolar RNA. |
| RET | 2.34 | Homo sapiens ret proto-oncogene (RET), transcript variant 4, mRNA. |
| ATG7 | 2.34 | Homo sapiens ATG7 autophagy related 7 homolog (S. cerevisiae) (ATG7), mRNA. |
| KBTBD8 | 2.34 | Homo sapiens kelch repeat and BTB (POZ) domain containing 8 (KBTBD8), mRNA. |
| YIF1A | 2.34 | Homo sapiens Yip1 interacting factor homolog A (S. cerevisiae) (YIF1A), mRNA. |
| C1orf74 | 2.34 | Homo sapiens chromosome 1 open reading frame 74 (C1orf74), mRNA. |
| CD163L1 | 2.34 | Homo sapiens CD163 molecule-like 1 (CD163L1), mRNA. |
| TIMM22 | 2.33 | Homo sapiens translocase of inner mitochondrial membrane 22 homolog (yeast) (TIMM22), nuclear gene encoding mitochondrial protein, mRNA. |
| FGL1 | 2.33 | PREDICTED: Homo sapiens fibrinogen-like 1, transcript variant 5 (FGL1), mRNA. |
| IFT122 | 2.33 | Homo sapiens intraflagellar transport 122 homolog (Chlamydomonas) (IFT122), transcript variant 2, mRNA. |
| C10orf137 | 2.33 | Homo sapiens chromosome 10 open reading frame 137 (C10orf137), mRNA. |
| C3orf54 | 2.33 | Homo sapiens chromosome 3 open reading frame 54 (C3orf54), mRNA. |
| ADAMTSL2 | 2.33 | Homo sapiens ADAMTS-like 2 (ADAMTSL2), mRNA. |
| C11orf2 | 2.33 | Homo sapiens chromosome 11 open reading frame 2 (C11orf2), mRNA. |
| N6AMT1 | 2.33 | Homo sapiens N-6 adenine-specific DNA methyltransferase 1 (putative) (N6AMT1), transcript variant 2, mRNA. |
| CSTF3 | 2.33 | Homo sapiens cleavage stimulation factor, 3' pre-RNA, subunit 3, 77kDa (CSTF3), transcript variant 3, mRNA. |
| CDC37 | 2.33 | Homo sapiens cell division cycle 37 homolog (S. cerevisiae) (CDC37), mRNA. |
| FGF23 | 2.32 | Homo sapiens fibroblast growth factor 23 (FGF23), mRNA. |
| INPP1 | 2.32 | Homo sapiens inositol polyphosphate-1-phosphatase (INPP1), mRNA. |
| LOC100128689 | 2.32 | PREDICTED: Homo sapiens misc_RNA (LOC100128689), miscRNA. |
| CRABP2 | 2.32 | Homo sapiens cellular retinoic acid binding protein 2 (CRABP2), mRNA. |
| PUS1 | 2.32 | Homo sapiens pseudouridylate synthase 1 (PUS1), transcript variant 2, mRNA. |
| ABHD8 | 2.32 | Homo sapiens abhydrolase domain containing 8 (ABHD8), mRNA. |
| RNF213 | 2.32 | Homo sapiens ring finger protein 213 (RNF213), transcript variant 2, mRNA. |
| ZSCAN2 | 2.32 | Homo sapiens zinc finger and SCAN domain containing 2 (ZSCAN2), transcript variant 1, mRNA. |
| ZNF17 | 2.32 | Homo sapiens zinc finger protein 17 (ZNF17), mRNA. |
| CEP170 | 2.32 | Homo sapiens centrosomal protein 170kDa (CEP170), transcript variant beta, mRNA. |
| LOC100133836 | 2.32 | PREDICTED: Homo sapiens hypothetical protein LOC100133836, transcript variant 1 (LOC100133836), mRNA. |
| CCDC51 | 2.31 | Homo sapiens coiled-coil domain containing 51 (CCDC51), mRNA. |
| AMD1 | 2.31 | Homo sapiens adenosylmethionine decarboxylase 1 (AMD1), transcript variant 2, mRNA. |
|  | 2.31 | Homo sapiens mRNA; cDNA DKFZp547E107 (from clone DKFZp547E107) |
| C17orf90 | 2.31 | Homo sapiens chromosome 17 open reading frame 90 (C17orf90), mRNA. |
| C3orf38 | 2.3 | Homo sapiens chromosome 3 open reading frame 38 (C3orf38), mRNA. |
| ZBTB6 | 2.3 | Homo sapiens zinc finger and BTB domain containing 6 (ZBTB6), mRNA. |
| MIF4GD | 2.3 | Homo sapiens MIF4G domain containing (MIF4GD), mRNA. |
|  | 2.3 | 602696288F1 NIH_MGC_97 Homo sapiens cDNA clone IMAGE:4828652 5, mRNA sequence |
| ERI3 | 2.3 | Homo sapiens ERI1 exoribonuclease family member 3 (ERI3), mRNA. |
| COPS7B | 2.3 | Homo sapiens COP9 constitutive photomorphogenic homolog subunit 7B (Arabidopsis) (COPS7B), mRNA. |
| MRPS12 | 2.3 | Homo sapiens mitochondrial ribosomal protein S12 (MRPS12), nuclear gene encoding mitochondrial protein, transcript variant 3, mRNA. |
| SHD | 2.29 | Homo sapiens Src homology 2 domain containing transforming protein D (SHD), mRNA. |
| PHRF1 | 2.29 | Homo sapiens PHD and ring finger domains 1 (PHRF1), mRNA. |
| MAP3K14 | 2.29 | Homo sapiens mitogen-activated protein kinase kinase kinase 14 (MAP3K14), mRNA. |
| DEXI | 2.29 | Homo sapiens Dexi homolog (mouse) (DEXI), mRNA. |
| TICAM2 | 2.29 | Homo sapiens toll-like receptor adaptor molecule 2 (TICAM2), mRNA. |
| LOC100130562 | 2.28 | PREDICTED: Homo sapiens hypothetical protein LOC100130562, transcript variant 1 (LOC100130562), mRNA. |
| MRRF | 2.28 | Homo sapiens mitochondrial ribosome recycling factor (MRRF), nuclear gene encoding mitochondrial protein, transcript variant 2, mRNA. |
| MAT2B | 2.28 | Homo sapiens methionine adenosyltransferase II, beta (MAT2B), transcript variant 2, mRNA. |
| DDX46 | 2.28 | Homo sapiens DEAD (Asp-Glu-Ala-Asp) box polypeptide 46 (DDX46), mRNA. |
| DNAJA3 | 2.28 | Homo sapiens DnaJ (Hsp40) homolog, subfamily A, member 3 (DNAJA3), mRNA. |
| MRPS25 | 2.28 | Homo sapiens mitochondrial ribosomal protein S25 (MRPS25), nuclear gene encoding mitochondrial protein, mRNA. |
| FAM19A3 | 2.28 | Homo sapiens family with sequence similarity 19 (chemokine (C-C motif)-like), member A3 (FAM19A3), transcript variant 1, mRNA. |
| ST3GAL3 | 2.28 | Homo sapiens ST3 beta-galactoside alpha-2,3-sialyltransferase 3 (ST3GAL3), transcript variant 5, mRNA. |
| C5orf54 | 2.27 | Homo sapiens chromosome 5 open reading frame 54 (C5orf54), mRNA. |
| LYSMD4 | 2.27 | Homo sapiens LysM, putative peptidoglycan-binding, domain containing 4 (LYSMD4), mRNA. |
| LOC648099 | 2.27 | PREDICTED: Homo sapiens similar to positive cofactor 2, glutamine/Q-rich-associated protein isoform b (LOC648099), mRNA. |
| TMEM39A | 2.27 | Homo sapiens transmembrane protein 39A (TMEM39A), mRNA. |
| ALKBH1 | 2.27 | Homo sapiens alkB, alkylation repair homolog 1 (E. coli) (ALKBH1), mRNA. |
| C16orf48 | 2.26 | Homo sapiens chromosome 16 open reading frame 48 (C16orf48), mRNA. |
| LOC651621 | 2.26 | PREDICTED: Homo sapiens similar to hydroxysteroid (17-beta) dehydrogenase 7 (LOC651621), mRNA. |
| LRCH4 | 2.26 | Homo sapiens leucine-rich repeats and calponin homology (CH) domain containing 4 (LRCH4), mRNA. |
| C6orf64 | 2.26 | Homo sapiens chromosome 6 open reading frame 64 (C6orf64), mRNA. |
| INTS6 | 2.25 | Homo sapiens integrator complex subunit 6 (INTS6), transcript variant 2, mRNA. |
| CCDC93 | 2.25 | Homo sapiens coiled-coil domain containing 93 (CCDC93), mRNA. |
| DGCR6 | 2.25 | Homo sapiens DiGeorge syndrome critical region gene 6 (DGCR6), mRNA. |
| CCNC | 2.25 | Homo sapiens cyclin C (CCNC), transcript variant 1, mRNA. |
| TMEM44 | 2.25 | Homo sapiens transmembrane protein 44 (TMEM44), transcript variant 1, mRNA. |
| APM-1 | 2.25 | PREDICTED: Homo sapiens BTB/POZ-zinc finger protein-like (APM-1), mRNA. |
| DNAJB1 | 2.25 | Homo sapiens DnaJ (Hsp40) homolog, subfamily B, member 1 (DNAJB1), mRNA. |
| C10orf28 | 2.25 | Homo sapiens chromosome 10 open reading frame 28 (C10orf28), mRNA. |
| DGCR6 | 2.24 | Homo sapiens DiGeorge syndrome critical region gene 6 (DGCR6), mRNA. |
| PRR3 | 2.24 | Homo sapiens proline rich 3 (PRR3), transcript variant 2, mRNA. |
| BRMS1 | 2.24 | Homo sapiens breast cancer metastasis suppressor 1 (BRMS1), transcript variant 3, mRNA. |
| KIAA1826 | 2.24 | Homo sapiens KIAA1826 (KIAA1826), mRNA. |
| ZNF69 | 2.24 | Homo sapiens zinc finger protein 69 (ZNF69), mRNA. |
| EDC4 | 2.24 | Homo sapiens enhancer of mRNA decapping 4 (EDC4), mRNA. |
| PSMD5 | 2.24 | Homo sapiens proteasome (prosome, macropain) 26S subunit, non-ATPase, 5 (PSMD5), mRNA. |
| KLHL35 | 2.23 | Homo sapiens kelch-like 35 (Drosophila) (KLHL35), mRNA. |
| SPOCK1 | 2.23 | Homo sapiens sparc/osteonectin, cwcv and kazal-like domains proteoglycan (testican) 1 (SPOCK1), mRNA. |
|  | 2.23 | cs23b05.x1 Human Retinal pigment epithelium/choroid cDNA (Un-normalized, unamplified): cs Homo sapiens cDNA clone cs23b05 3, mRNA sequence |
| LOC645969 | 2.23 | PREDICTED: Homo sapiens misc_RNA (LOC645969), miscRNA. |
| LOC645691 | 2.23 | PREDICTED: Homo sapiens similar to heterogeneous nuclear ribonucleoprotein A1 (LOC645691), mRNA. |
| LOC643287 | 2.23 | PREDICTED: Homo sapiens similar to prothymosin alpha, transcript variant 1 (LOC643287), mRNA. |
| HSPBAP1 | 2.22 | Homo sapiens HSPB (heat shock 27kDa) associated protein 1 (HSPBAP1), mRNA. |
| LOC442232 | 2.21 | PREDICTED: Homo sapiens misc_RNA (LOC442232), miscRNA. |
| ATG4C | 2.21 | Homo sapiens ATG4 autophagy related 4 homolog C (S. cerevisiae) (ATG4C), transcript variant 7, mRNA. |
| RNF19A | 2.21 | Homo sapiens ring finger protein 19A (RNF19A), transcript variant 2, mRNA. |
| LOC401805 | 2.21 | PREDICTED: Homo sapiens misc_RNA (LOC401805), partial miscRNA. |
| INSIG1 | 2.21 | Homo sapiens insulin induced gene 1 (INSIG1), transcript variant 2, mRNA. |
| SAT1 | 2.21 | Homo sapiens spermidine/spermine N1-acetyltransferase 1 (SAT1), mRNA. |
| SNHG11 | 2.21 | Homo sapiens small nucleolar RNA host gene 11 (non-protein coding) (SNHG11), non-coding RNA. |
| MAP6D1 | 2.21 | Homo sapiens MAP6 domain containing 1 (MAP6D1), mRNA. |
| WFS1 | 2.2 | Homo sapiens Wolfram syndrome 1 (wolframin) (WFS1), mRNA. |
| CLK2 | 2.2 | PREDICTED: Homo sapiens CDC-like kinase 2, transcript variant 4 (CLK2), mRNA. |
| DBN1 | 2.2 | Homo sapiens drebrin 1 (DBN1), transcript variant 1, mRNA. |
| ZFP36L1 | 2.2 | Homo sapiens zinc finger protein 36, C3H type-like 1 (ZFP36L1), mRNA. |
| MAP2K3 | 2.2 | Homo sapiens mitogen-activated protein kinase kinase 3 (MAP2K3), transcript variant A, mRNA. |
| PHB | 2.2 | Homo sapiens prohibitin (PHB), mRNA. |
| C3orf37 | 2.2 | Homo sapiens chromosome 3 open reading frame 37 (C3orf37), transcript variant 2, mRNA. |
| KCNQ1OT1 | 2.19 | Homo sapiens KCNQ1 overlapping transcript 1 (non-protein coding) (KCNQ1OT1), non-coding RNA. |
| MSL1 | 2.19 | Homo sapiens male-specific lethal 1 homolog (Drosophila) (MSL1), mRNA. |
| ZNF434 | 2.19 | Homo sapiens zinc finger protein 434 (ZNF434), mRNA. |
| BTBD1 | 2.19 | Homo sapiens BTB (POZ) domain containing 1 (BTBD1), transcript variant 1, mRNA. |
| C8orf33 | 2.19 | Homo sapiens chromosome 8 open reading frame 33 (C8orf33), mRNA. |
| FAM158A | 2.19 | Homo sapiens family with sequence similarity 158, member A (FAM158A), mRNA. |
| PIB5PA | 2.19 | Homo sapiens phosphatidylinositol (4,5) bisphosphate 5-phosphatase, A (PIB5PA), transcript variant 1, mRNA. |
| RFP | 2.18 | Homo sapiens ret finger protein (RFP), transcript variant beta, mRNA. |
| NKIRAS1 | 2.18 | Homo sapiens NFKB inhibitor interacting Ras-like 1 (NKIRAS1), mRNA. |
| MAD2L1BP | 2.18 | Homo sapiens MAD2L1 binding protein (MAD2L1BP), transcript variant 2, mRNA. |
| NIT1 | 2.18 | Homo sapiens nitrilase 1 (NIT1), mRNA. |
| LOC392522 | 2.18 | PREDICTED: Homo sapiens misc_RNA (LOC392522), miscRNA. |
| STMN1 | 2.18 | Homo sapiens stathmin 1 (STMN1), transcript variant 3, mRNA. |
| METTL13 | 2.18 | Homo sapiens methyltransferase like 13 (METTL13), transcript variant 3, mRNA. |
| C1orf96 | 2.18 | Homo sapiens chromosome 1 open reading frame 96 (C1orf96), mRNA. |
| NHP2L1 | 2.18 | Homo sapiens NHP2 non-histone chromosome protein 2-like 1 (S. cerevisiae) (NHP2L1), transcript variant 2, mRNA. |
| TTC13 | 2.18 | Homo sapiens tetratricopeptide repeat domain 13 (TTC13), mRNA. |
| LOC644937 | 2.18 | PREDICTED: Homo sapiens similar to ribosomal protein L10 (LOC644937), mRNA. |
| SMAP2 | 2.17 | Homo sapiens small ArfGAP2 (SMAP2), mRNA. |
| COLQ | 2.17 | Homo sapiens collagen-like tail subunit (single strand of homotrimer) of asymmetric acetylcholinesterase (COLQ), transcript variant VIII, mRNA. |
| HYDIN | 2.17 | Homo sapiens hydrocephalus inducing homolog (mouse) (HYDIN), transcript variant 1, mRNA. |
| FAM179B | 2.17 | Homo sapiens family with sequence similarity 179, member B (FAM179B), mRNA. |
| CHML | 2.17 | Homo sapiens choroideremia-like (Rab escort protein 2) (CHML), mRNA. |
| LOC645332 | 2.16 | Homo sapiens family with sequence similarity 86, member A pseudogene (LOC645332), non-coding RNA. |
| CCDC5 | 2.16 | Homo sapiens coiled-coil domain containing 5 (spindle associated) (CCDC5), mRNA. |
| PIK3R3 | 2.16 | Homo sapiens phosphoinositide-3-kinase, regulatory subunit 3 (p55, gamma) (PIK3R3), mRNA. |
| COQ10B | 2.16 | Homo sapiens coenzyme Q10 homolog B (S. cerevisiae) (COQ10B), mRNA. |
| ZNF556 | 2.16 | Homo sapiens zinc finger protein 556 (ZNF556), mRNA. |
| ZC3H5 | 2.16 | PREDICTED: Homo sapiens zinc finger CCCH-type containing 5 (ZC3H5), mRNA. |
| EXOSC3 | 2.16 | Homo sapiens exosome component 3 (EXOSC3), transcript variant 1, mRNA. |
| LRRC58 | 2.16 | PREDICTED: Homo sapiens leucine rich repeat containing 58 (LRRC58), mRNA. |
|  | 2.16 | UI-E-CL1-aff-f-07-0-UI.s2 UI-E-CL1 Homo sapiens cDNA clone UI-E-CL1-aff-f-07-0-UI 3, mRNA sequence |
| LOC729779 | 2.15 | PREDICTED: Homo sapiens misc_RNA (LOC729779), miscRNA. |
| CCDC28B | 2.15 | Homo sapiens coiled-coil domain containing 28B (CCDC28B), mRNA. |
| LOC100128115 | 2.15 | PREDICTED: Homo sapiens misc_RNA (LOC100128115), miscRNA. |
| ARL2 | 2.15 | Homo sapiens ADP-ribosylation factor-like 2 (ARL2), mRNA. |
| RRP8 | 2.15 | Homo sapiens ribosomal RNA processing 8, methyltransferase, homolog (yeast) (RRP8), mRNA. |
| CLRN1 | 2.15 | Homo sapiens clarin 1 (CLRN1), transcript variant 4, mRNA. |
| MTCH2 | 2.15 | Homo sapiens mitochondrial carrier homolog 2 (C. elegans) (MTCH2), nuclear gene encoding mitochondrial protein, mRNA. |
| IMP3 | 2.15 | Homo sapiens IMP3, U3 small nucleolar ribonucleoprotein, homolog (yeast) (IMP3), mRNA. |
| TMEM126B | 2.15 | Homo sapiens transmembrane protein 126B (TMEM126B), mRNA. |
| SLC12A9 | 2.14 | Homo sapiens solute carrier family 12 (potassium/chloride transporters), member 9 (SLC12A9), mRNA. |
| FAM18B2 | 2.14 | Homo sapiens family with sequence similarity 18, member B2 (FAM18B2), mRNA. |
| GALT | 2.14 | Homo sapiens galactose-1-phosphate uridylyltransferase (GALT), mRNA. |
| TMEM165 | 2.14 | Homo sapiens transmembrane protein 165 (TMEM165), mRNA. |
| RNF144 | 2.13 | Homo sapiens ring finger protein 144 (RNF144), mRNA. |
| ARMCX3 | 2.13 | Homo sapiens armadillo repeat containing, X-linked 3 (ARMCX3), transcript variant 2, mRNA. |
| MAX | 2.13 | Homo sapiens MYC associated factor X (MAX), transcript variant 1, mRNA. |
| FAM54A | 2.13 | Homo sapiens family with sequence similarity 54, member A (FAM54A), mRNA. |
| RDBP | 2.13 | Homo sapiens RD RNA binding protein (RDBP), mRNA. |
| VAX2 | 2.13 | Homo sapiens ventral anterior homeobox 2 (VAX2), mRNA. |
| ZNF613 | 2.13 | Homo sapiens zinc finger protein 613 (ZNF613), transcript variant 2, mRNA. |
| LOC642755 | 2.12 | PREDICTED: Homo sapiens similar to DEXI, transcript variant 1 (LOC642755), mRNA. |
| LOC643224 | 2.12 | PREDICTED: Homo sapiens similar to tubulin, beta 8, transcript variant 2 (LOC643224), mRNA. |
| BMP7 | 2.12 | Homo sapiens bone morphogenetic protein 7 (osteogenic protein 1) (BMP7), mRNA. |
| NPW | 2.12 | Homo sapiens neuropeptide W (NPW), mRNA. |
| CTXN1 | 2.12 | Homo sapiens cortexin 1 (CTXN1), mRNA. |
| ZFP82 | 2.11 | Homo sapiens zinc finger protein 82 homolog (mouse) (ZFP82), mRNA. |
| BAI1 | 2.11 | PREDICTED: Homo sapiens brain-specific angiogenesis inhibitor 1, transcript variant 1 (BAI1), mRNA. |
| MBD6 | 2.11 | Homo sapiens methyl-CpG binding domain protein 6 (MBD6), mRNA. |
| AMD1 | 2.11 | Homo sapiens adenosylmethionine decarboxylase 1 (AMD1), transcript variant 1, mRNA. |
| SLC41A3 | 2.11 | Homo sapiens solute carrier family 41, member 3 (SLC41A3), transcript variant 1, mRNA. |
| PHCA | 2.11 | Homo sapiens phytoceramidase, alkaline (PHCA), mRNA. |
| MYB | 2.11 | Homo sapiens v-myb myeloblastosis viral oncogene homolog (avian) (MYB), transcript variant 2, mRNA. |
| C1orf85 | 2.11 | Homo sapiens chromosome 1 open reading frame 85 (C1orf85), mRNA. |
| CLDN15 | 2.11 | Homo sapiens claudin 15 (CLDN15), mRNA. |
| SLC39A3 | 2.11 | Homo sapiens solute carrier family 39 (zinc transporter), member 3 (SLC39A3), transcript variant 1, mRNA. |
| DHX37 | 2.11 | Homo sapiens DEAH (Asp-Glu-Ala-His) box polypeptide 37 (DHX37), mRNA. |
| C20orf100 | 2.1 | Homo sapiens chromosome 20 open reading frame 100 (C20orf100), mRNA. |
| FLOT2 | 2.1 | Homo sapiens flotillin 2 (FLOT2), mRNA. |
| SLC4A5 | 2.1 | Homo sapiens solute carrier family 4, sodium bicarbonate cotransporter, member 5 (SLC4A5), transcript variant c, mRNA. |
| LOC100131381 | 2.1 | PREDICTED: Homo sapiens hypothetical LOC100131381 (LOC100131381), mRNA. |
| NCKAP1 | 2.1 | Homo sapiens NCK-associated protein 1 (NCKAP1), transcript variant 2, mRNA. |
| LOC100133312 | 2.1 | PREDICTED: Homo sapiens misc_RNA (LOC100133312), miscRNA. |
| SBDSP | 2.09 | Homo sapiens Shwachman-Bodian-Diamond syndrome pseudogene (SBDSP) on chromosome 7. |
| C9orf3 | 2.09 | Homo sapiens chromosome 9 open reading frame 3 (C9orf3), mRNA. |
| LOC100127999 | 2.09 | PREDICTED: Homo sapiens misc_RNA (LOC100127999), miscRNA. |
| ZW10 | 2.09 | Homo sapiens ZW10, kinetochore associated, homolog (Drosophila) (ZW10), mRNA. |
| DNAJB12 | 2.09 | Homo sapiens DnaJ (Hsp40) homolog, subfamily B, member 12 (DNAJB12), transcript variant 1, mRNA. |
| ZNF689 | 2.09 | Homo sapiens zinc finger protein 689 (ZNF689), mRNA. |
| TCEB3 | 2.09 | Homo sapiens transcription elongation factor B (SIII), polypeptide 3 (110kDa, elongin A) (TCEB3), mRNA. |
| LOC389322 | 2.08 | PREDICTED: Homo sapiens misc_RNA (LOC389322), miscRNA. |
| SF3A3 | 2.08 | Homo sapiens splicing factor 3a, subunit 3, 60kDa (SF3A3), mRNA. |
| SYN2 | 2.08 | Homo sapiens synapsin II (SYN2), transcript variant IIb, mRNA. |
| MSL3 | 2.08 | Homo sapiens male-specific lethal 3 homolog (Drosophila) (MSL3), transcript variant 4, mRNA. |
| LOC100132918 | 2.08 | PREDICTED: Homo sapiens misc_RNA (LOC100132918), miscRNA. |
| LRRC26 | 2.08 | Homo sapiens leucine rich repeat containing 26 (LRRC26), mRNA. |
| APEG1 | 2.08 | Homo sapiens aortic preferentially expressed gene 1 (APEG1), mRNA. |
| LOC646278 | 2.08 | PREDICTED: Homo sapiens similar to programmed cell death 6 interacting protein (LOC646278), miscRNA. |
| PDCL3 | 2.08 | Homo sapiens phosducin-like 3 (PDCL3), mRNA. |
| LOC391352 | 2.07 | PREDICTED: Homo sapiens similar to peptidylprolyl isomerase A isoform 1 (LOC391352), mRNA. |
| ZNF213 | 2.07 | Homo sapiens zinc finger protein 213 (ZNF213), mRNA. |
| LOC392501 | 2.07 | PREDICTED: Homo sapiens misc_RNA (LOC392501), miscRNA. |
| AKAP13 | 2.07 | Homo sapiens A kinase (PRKA) anchor protein 13 (AKAP13), transcript variant 2, mRNA. |
| ZKSCAN4 | 2.07 | Homo sapiens zinc finger with KRAB and SCAN domains 4 (ZKSCAN4), mRNA. |
| C19orf54 | 2.07 | Homo sapiens chromosome 19 open reading frame 54 (C19orf54), mRNA. |
| FBXO28 | 2.07 | Homo sapiens F-box protein 28 (FBXO28), mRNA. |
| RAB40C | 2.07 | Homo sapiens RAB40C, member RAS oncogene family (RAB40C), mRNA. |
| RNF146 | 2.06 | Homo sapiens ring finger protein 146 (RNF146), mRNA. |
| LOC730746 | 2.06 | PREDICTED: Homo sapiens similar to Heterogeneous nuclear ribonucleoprotein A1 (Helix-destabilizing protein) (Single-strand RNA-binding protein) (hnRNP core protein A1) (HDP) (LOC730746), mRNA. |
| LOC100130171 | 2.06 | PREDICTED: Homo sapiens misc_RNA (LOC100130171), miscRNA. |
| EGLN2 | 2.06 | Homo sapiens egl nine homolog 2 (C. elegans) (EGLN2), transcript variant 3, mRNA. |
|  | 2.06 | hd40e02.x1 Soares_NFL_T_GBC_S1 Homo sapiens cDNA clone IMAGE:2911994 3, mRNA sequence |
| MKLN1 | 2.06 | Homo sapiens muskelin 1, intracellular mediator containing kelch motifs (MKLN1), mRNA. |
| TMEM100 | 2.06 | Homo sapiens transmembrane protein 100 (TMEM100), transcript variant 2, mRNA. |
| SLC9A1 | 2.06 | Homo sapiens solute carrier family 9 (sodium/hydrogen exchanger), member 1 (SLC9A1), mRNA. |
| LOC441711 | 2.06 | PREDICTED: Homo sapiens misc_RNA (LOC441711), miscRNA. |
| C1orf151 | 2.06 | Homo sapiens chromosome 1 open reading frame 151 (C1orf151), mRNA. |
| DNAJC17 | 2.06 | Homo sapiens DnaJ (Hsp40) homolog, subfamily C, member 17 (DNAJC17), mRNA. |
| IL25 | 2.06 | Homo sapiens interleukin 25 (IL25), transcript variant 1, mRNA. |
| C17orf53 | 2.06 | Homo sapiens chromosome 17 open reading frame 53 (C17orf53), mRNA. |
| TUSC4 | 2.06 | Homo sapiens tumor suppressor candidate 4 (TUSC4), mRNA. |
| PITPNA | 2.06 | Homo sapiens phosphatidylinositol transfer protein, alpha (PITPNA), mRNA. |
| LONP2 | 2.06 | Homo sapiens lon peptidase 2, peroxisomal (LONP2), mRNA. |
| ST3GAL2 | 2.05 | Homo sapiens ST3 beta-galactoside alpha-2,3-sialyltransferase 2 (ST3GAL2), mRNA. |
| FZD7 | 2.05 | Homo sapiens frizzled homolog 7 (Drosophila) (FZD7), mRNA. |
| C11orf24 | 2.05 | Homo sapiens chromosome 11 open reading frame 24 (C11orf24), mRNA. |
| LOC554206 | 2.05 | PREDICTED: Homo sapiens hypothetical LOC554206 (LOC554206), misc RNA. |
| SELS | 2.05 | Homo sapiens selenoprotein S (SELS), transcript variant 2, mRNA. |
| C3orf19 | 2.05 | Homo sapiens chromosome 3 open reading frame 19 (C3orf19), mRNA. |
| WDR33 | 2.04 | Homo sapiens WD repeat domain 33 (WDR33), transcript variant 2, mRNA. |
| LOC653752 | 2.04 | PREDICTED: Homo sapiens similar to dexamethasone-induced transcript (LOC653752), mRNA. |
| C1orf229 | 2.04 | Homo sapiens chromosome 1 open reading frame 229 (C1orf229), mRNA. |
|  | 2.04 | Homo sapiens mRNA; cDNA DKFZp761E1721 (from clone DKFZp761E1721) |
| CEP27 | 2.04 | Homo sapiens centrosomal protein 27kDa (CEP27), mRNA. |
| UBE2A | 2.04 | Homo sapiens ubiquitin-conjugating enzyme E2A (RAD6 homolog) (UBE2A), transcript variant 2, mRNA. |
| RALGDS | 2.04 | Homo sapiens ral guanine nucleotide dissociation stimulator (RALGDS), transcript variant 1, mRNA. |
| RP9 | 2.04 | Homo sapiens retinitis pigmentosa 9 (autosomal dominant) (RP9), mRNA. |
| PRR14 | 2.04 | Homo sapiens proline rich 14 (PRR14), mRNA. |
| NENF | 2.04 | Homo sapiens neuron derived neurotrophic factor (NENF), mRNA. |
| PDXP | 2.04 | Homo sapiens pyridoxal (pyridoxine, vitamin B6) phosphatase (PDXP), mRNA. |
| ALOX15B | 2.03 | Homo sapiens arachidonate 15-lipoxygenase, type B (ALOX15B), transcript variant b, mRNA. |
| FKBP4 | 2.03 | Homo sapiens FK506 binding protein 4, 59kDa (FKBP4), mRNA. |
| HIST1H2BK | 2.03 | Homo sapiens histone cluster 1, H2bk (HIST1H2BK), mRNA. |
| ANKRD13D | 2.03 | PREDICTED: Homo sapiens ankyrin repeat domain 13 family, member D, transcript variant 7 (ANKRD13D), mRNA. |
| NCBP2 | 2.03 | Homo sapiens nuclear cap binding protein subunit 2, 20kDa (NCBP2), transcript variant 1, mRNA. |
| HERPUD1 | 2.03 | Homo sapiens homocysteine-inducible, endoplasmic reticulum stress-inducible, ubiquitin-like domain member 1 (HERPUD1), transcript variant 3, mRNA. |
| LUZP1 | 2.03 | Homo sapiens leucine zipper protein 1 (LUZP1), mRNA. |
| SERF2 | 2.03 | Homo sapiens small EDRK-rich factor 2 (SERF2), mRNA. |
| EEF1A2 | 2.03 | Homo sapiens eukaryotic translation elongation factor 1 alpha 2 (EEF1A2), mRNA. |
| RAMP1 | 2.03 | Homo sapiens receptor (G protein-coupled) activity modifying protein 1 (RAMP1), mRNA. |
| LOC401676 | 2.03 | PREDICTED: Homo sapiens misc_RNA (LOC401676), miscRNA. |
| LOC727865 | 2.02 | PREDICTED: Homo sapiens misc_RNA (LOC727865), miscRNA. |
| CENPV | 2.02 | Homo sapiens centromere protein V (CENPV), mRNA. |
| LOC100130561 | 2.02 | PREDICTED: Homo sapiens similar to high-mobility group (nonhistone chromosomal) protein 1-like 10 (LOC100130561), mRNA. |
| LRRN2 | 2.02 | Homo sapiens leucine rich repeat neuronal 2 (LRRN2), transcript variant 2, mRNA. |
| RAB35 | 2.02 | Homo sapiens RAB35, member RAS oncogene family (RAB35), mRNA. |
| ATRX | 2.02 | Homo sapiens alpha thalassemia/mental retardation syndrome X-linked (RAD54 homolog, S. cerevisiae) (ATRX), transcript variant 1, mRNA. |
| RABL3 | 2.01 | Homo sapiens RAB, member of RAS oncogene family-like 3 (RABL3), mRNA. |
| CDC42EP3 | 2.01 | Homo sapiens CDC42 effector protein (Rho GTPase binding) 3 (CDC42EP3), mRNA. |
| SMG5 | 2.01 | Homo sapiens Smg-5 homolog, nonsense mediated mRNA decay factor (C. elegans) (SMG5), mRNA. |
| ZNF408 | 2.01 | Homo sapiens zinc finger protein 408 (ZNF408), mRNA. |
| KRTCAP2 | 2.01 | Homo sapiens keratinocyte associated protein 2 (KRTCAP2), mRNA. |
| BTBD2 | 2.01 | Homo sapiens BTB (POZ) domain containing 2 (BTBD2), mRNA. |
| ZNF512B | 2.01 | Homo sapiens zinc finger protein 512B (ZNF512B), mRNA. |
| PRR3 | 2.01 | Homo sapiens proline rich 3 (PRR3), transcript variant 1, mRNA. |
| FAM136A | 2 | Homo sapiens family with sequence similarity 136, member A (FAM136A), mRNA. |
| TPM3 | 2 | Homo sapiens tropomyosin 3 (TPM3), transcript variant 1, mRNA. |
| POMT1 | 2 | Homo sapiens protein-O-mannosyltransferase 1 (POMT1), mRNA. |
| CCT3 | 2 | Homo sapiens chaperonin containing TCP1, subunit 3 (gamma) (CCT3), transcript variant 3, mRNA. |
| TMX1 | 2 | Homo sapiens thioredoxin-related transmembrane protein 1 (TMX1), mRNA. |
| ZNF202 | 2 | Homo sapiens zinc finger protein 202 (ZNF202), mRNA. |
| PCDHB19P | 2 | Homo sapiens protocadherin beta 19 pseudogene (PCDHB19P), non-coding RNA. |
|  | 2 | Homo sapiens cDNA FLJ38396 fis, clone FEBRA2007957 |
| WDR35 | 2 | Homo sapiens WD repeat domain 35 (WDR35), transcript variant 1, mRNA. |
| HDHD1A | 2 | Homo sapiens haloacid dehalogenase-like hydrolase domain containing 1A (HDHD1A), mRNA. |
| ZNF491 | 2 | Homo sapiens zinc finger protein 491 (ZNF491), mRNA. |
| LOC440396 | 2 | Homo sapiens similar to Heterogeneous nuclear ribonucleoprotein A1 (Helix-destabilizing protein) (Single-strand binding protein) (hnRNP core protein A1) (HDP-1) (Topoisomerase-inhibitor suppressed) (LOC440396), non-coding RNA. |
| RPL23AP7 | 2 | Homo sapiens ribosomal protein L23a pseudogene 7 (RPL23AP7) on chromosome 2. |
| SNN | 1.99 | Homo sapiens stannin (SNN), mRNA. |
|  | 1.99 | Homo sapiens mRNA; cDNA DKFZp434C1613 (from clone DKFZp434C1613) |
| PRKACB | 1.99 | Homo sapiens protein kinase, cAMP-dependent, catalytic, beta (PRKACB), transcript variant 2, mRNA. |
| MGC57346 | 1.99 | PREDICTED: Homo sapiens hypothetical LOC401884 (MGC57346), mRNA. |
| FAM48A | 1.99 | Homo sapiens family with sequence similarity 48, member A (FAM48A), transcript variant 1, mRNA. |
| LOC728602 | 1.99 | PREDICTED: Homo sapiens misc_RNA (LOC728602), partial miscRNA. |
| FLVCR1 | 1.99 | Homo sapiens feline leukemia virus subgroup C cellular receptor 1 (FLVCR1), mRNA. |
| CHCHD4 | 1.98 | Homo sapiens coiled-coil-helix-coiled-coil-helix domain containing 4 (CHCHD4), nuclear gene encoding mitochondrial protein, transcript variant 2, mRNA. |
| C5orf22 | 1.98 | Homo sapiens chromosome 5 open reading frame 22 (C5orf22), mRNA. |
| MED20 | 1.98 | Homo sapiens mediator complex subunit 20 (MED20), mRNA. |
| REPIN1 | 1.98 | Homo sapiens replication initiator 1 (REPIN1), transcript variant 2, mRNA. |
| PHF2 | 1.98 | Homo sapiens PHD finger protein 2 (PHF2), mRNA. |
| POGZ | 1.98 | Homo sapiens pogo transposable element with ZNF domain (POGZ), transcript variant 3, mRNA. |
| AZIN1 | 1.98 | Homo sapiens antizyme inhibitor 1 (AZIN1), transcript variant 2, mRNA. |
| FLJ90086 | 1.98 | PREDICTED: Homo sapiens similar to AI661453 protein (FLJ90086), mRNA. |
| MXD1 | 1.98 | Homo sapiens MAX dimerization protein 1 (MXD1), mRNA. |
| SCAND3 | 1.98 | Homo sapiens SCAN domain containing 3 (SCAND3), mRNA. |
| C2orf44 | 1.98 | Homo sapiens chromosome 2 open reading frame 44 (C2orf44), mRNA. |
| ZP3 | 1.98 | Homo sapiens zona pellucida glycoprotein 3 (sperm receptor) (ZP3), mRNA. |
| GAPVD1 | 1.98 | Homo sapiens GTPase activating protein and VPS9 domains 1 (GAPVD1), mRNA. |
| MTMR14 | 1.97 | Homo sapiens myotubularin related protein 14 (MTMR14), transcript variant 2, mRNA. |
| LOC100130071 | 1.97 | PREDICTED: Homo sapiens similar to GSQS6193 (LOC100130071), mRNA. |
| CHIC2 | 1.97 | Homo sapiens cysteine-rich hydrophobic domain 2 (CHIC2), mRNA. |
| PSMA1 | 1.97 | Homo sapiens proteasome (prosome, macropain) subunit, alpha type, 1 (PSMA1), transcript variant 2, mRNA. |
| ZNF93 | 1.97 | Homo sapiens zinc finger protein 93 (HTF34) (ZNF93), transcript variant 2, mRNA. |
| LOC652685 | 1.97 | PREDICTED: Homo sapiens similar to PMS1 protein homolog 2 (DNA mismatch repair protein PMS2) (LOC652685), mRNA. |
| ATP9A | 1.97 | Homo sapiens ATPase, class II, type 9A (ATP9A), mRNA. |
| BANP | 1.97 | Homo sapiens BTG3 associated nuclear protein (BANP), transcript variant 2, mRNA. |
| DGCR11 | 1.96 | Homo sapiens DiGeorge syndrome critical region gene 11 (DGCR11), non-coding RNA. |
| CXorf64 | 1.96 | Homo sapiens chromosome X open reading frame 64 (CXorf64), mRNA. |
| LOC650832 | 1.96 | PREDICTED: Homo sapiens similar to mitogen-activated protein kinase kinase 3 isoform A (LOC650832), mRNA. |
|  | 1.96 | full-length cDNA clone CS0DF005YI08 of Fetal brain of Homo sapiens (human) |
| LUC7L2 | 1.96 | Homo sapiens LUC7-like 2 (S. cerevisiae) (LUC7L2), mRNA. |
| CDR2L | 1.96 | Homo sapiens cerebellar degeneration-related protein 2-like (CDR2L), mRNA. |
| HIST3H2A | 1.96 | Homo sapiens histone cluster 3, H2a (HIST3H2A), mRNA. |
| NR2C1 | 1.95 | Homo sapiens nuclear receptor subfamily 2, group C, member 1 (NR2C1), transcript variant 1, mRNA. |
| EIF4A1 | 1.95 | Homo sapiens eukaryotic translation initiation factor 4A, isoform 1 (EIF4A1), mRNA. |
| C11orf75 | 1.95 | Homo sapiens chromosome 11 open reading frame 75 (C11orf75), mRNA. |
| CCDC126 | 1.95 | Homo sapiens coiled-coil domain containing 126 (CCDC126), mRNA. |
|  | 1.95 | Homo sapiens cDNA FLJ26692 fis, clone MPG07890 |
| FAM188B | 1.95 | Homo sapiens family with sequence similarity 188, member B (FAM188B), mRNA. |
| SYT11 | 1.95 | Homo sapiens synaptotagmin XI (SYT11), mRNA. |
| PAFAH1B3 | 1.95 | Homo sapiens platelet-activating factor acetylhydrolase, isoform Ib, gamma subunit 29kDa (PAFAH1B3), mRNA. |
| ZNF490 | 1.95 | Homo sapiens zinc finger protein 490 (ZNF490), mRNA. |
| ETV5 | 1.95 | Homo sapiens ets variant gene 5 (ets-related molecule) (ETV5), mRNA. |
| BMS1 | 1.94 | Homo sapiens BMS1 homolog, ribosome assembly protein (yeast) (BMS1), mRNA. |
| FAM58A | 1.94 | Homo sapiens family with sequence similarity 58, member A (FAM58A), mRNA. |
| LOC646785 | 1.94 | PREDICTED: Homo sapiens misc_RNA (LOC646785), miscRNA. |
| LOC646034 | 1.94 | PREDICTED: Homo sapiens hypothetical LOC646034 (LOC646034), mRNA. |
| ATP6AP1 | 1.94 | Homo sapiens ATPase, H+ transporting, lysosomal accessory protein 1 (ATP6AP1), mRNA. |
| TAF15 | 1.94 | Homo sapiens TAF15 RNA polymerase II, TATA box binding protein (TBP)-associated factor, 68kDa (TAF15), transcript variant 2, mRNA. |
| LOC653635 | 1.94 | PREDICTED: Homo sapiens similar to CXYorf1-related protein (LOC653635), mRNA. |
| TTC4 | 1.94 | Homo sapiens tetratricopeptide repeat domain 4 (TTC4), mRNA. |
| TMED3 | 1.94 | Homo sapiens transmembrane emp24 protein transport domain containing 3 (TMED3), mRNA. |
| ITGA4 | 1.94 | Homo sapiens integrin, alpha 4 (antigen CD49D, alpha 4 subunit of VLA-4 receptor) (ITGA4), mRNA. |
| LOC651149 | 1.93 | PREDICTED: Homo sapiens similar to 60S ribosomal protein L3 (L4) (LOC651149), mRNA. |
| BEXL1 | 1.93 | PREDICTED: Homo sapiens brain expressed X-linked-like 1 (BEXL1), mRNA. |
| LOC100132032 | 1.93 | PREDICTED: Homo sapiens hypothetical protein LOC100132032 (LOC100132032), mRNA. |
| C10orf6 | 1.93 | Homo sapiens chromosome 10 open reading frame 6 (C10orf6), mRNA. |
| SCYL1 | 1.93 | Homo sapiens SCY1-like 1 (S. cerevisiae) (SCYL1), transcript variant A, mRNA. |
| LOC441484 | 1.93 | PREDICTED: Homo sapiens misc_RNA (LOC441484), miscRNA. |
| FXYD5 | 1.93 | Homo sapiens FXYD domain containing ion transport regulator 5 (FXYD5), transcript variant 2, mRNA. |
| LOC644063 | 1.93 | PREDICTED: Homo sapiens similar to heterogeneous nuclear ribonucleoprotein K (LOC644063), mRNA. |
| PUF60 | 1.93 | Homo sapiens poly-U binding splicing factor 60KDa (PUF60), transcript variant 2, mRNA. |
| KIAA0363 | 1.92 | PREDICTED: Homo sapiens KIAA0363 protein (KIAA0363), mRNA. |
| PDE9A | 1.92 | Homo sapiens phosphodiesterase 9A (PDE9A), transcript variant 2, mRNA. |
| ZNF622 | 1.92 | Homo sapiens zinc finger protein 622 (ZNF622), mRNA. |
| TMEM216 | 1.92 | Homo sapiens transmembrane protein 216 (TMEM216), mRNA. |
| LOC388275 | 1.92 | PREDICTED: Homo sapiens similar to Heterogeneous nuclear ribonucleoprotein A1 (Helix-destabilizing protein) (Single-strand binding protein) (hnRNP core protein A1) (HDP-1) (Topoisomerase-inhibitor suppressed) (LOC388275), mRNA. |
| NAV2 | 1.92 | Homo sapiens neuron navigator 2 (NAV2), transcript variant 2, mRNA. |
| CNDP2 | 1.92 | Homo sapiens CNDP dipeptidase 2 (metallopeptidase M20 family) (CNDP2), mRNA. |
| PPP1CC | 1.92 | Homo sapiens protein phosphatase 1, catalytic subunit, gamma isoform (PPP1CC), mRNA. |
| LOC727970 | 1.91 | PREDICTED: Homo sapiens misc_RNA (LOC727970), miscRNA. |
| G3BP1 | 1.91 | Homo sapiens GTPase activating protein (SH3 domain) binding protein 1 (G3BP1), transcript variant 2, mRNA. |
| LOC100129086 | 1.91 | PREDICTED: Homo sapiens similar to HIG1 domain family, member 1A (LOC100129086), mRNA. |
| ITPA | 1.91 | Homo sapiens inosine triphosphatase (nucleoside triphosphate pyrophosphatase) (ITPA), transcript variant 1, mRNA. |
| THOC5 | 1.91 | Homo sapiens THO complex 5 (THOC5), transcript variant 1, mRNA. |
| VEZF1 | 1.91 | Homo sapiens vascular endothelial zinc finger 1 (VEZF1), mRNA. |
| RPRC1 | 1.91 | Homo sapiens arginine/proline rich coiled-coil 1 (RPRC1), mRNA. |
| WDR18 | 1.91 | Homo sapiens WD repeat domain 18 (WDR18), mRNA. |
| DNAJB9 | 1.91 | Homo sapiens DnaJ (Hsp40) homolog, subfamily B, member 9 (DNAJB9), mRNA. |
| HNRNPA1 | 1.91 | Homo sapiens heterogeneous nuclear ribonucleoprotein A1 (HNRNPA1), transcript variant 2, mRNA. |
| LOC644330 | 1.91 | PREDICTED: Homo sapiens similar to tropomyosin 3 isoform 2 (LOC644330), mRNA. |
| IQCC | 1.91 | Homo sapiens IQ motif containing C (IQCC), mRNA. |
| CERK | 1.91 | Homo sapiens ceramide kinase (CERK), transcript variant 2, mRNA. |
| SMCR7L | 1.91 | Homo sapiens Smith-Magenis syndrome chromosome region, candidate 7-like (SMCR7L), mRNA. |
| MXD4 | 1.9 | Homo sapiens MAX dimerization protein 4 (MXD4), mRNA. |
| LOC100131866 | 1.9 | PREDICTED: Homo sapiens misc_RNA (LOC100131866), miscRNA. |
| LOC643888 | 1.9 | PREDICTED: Homo sapiens hypothetical LOC643888 (LOC643888), mRNA. |
| TTC13 | 1.9 | Homo sapiens tetratricopeptide repeat domain 13 (TTC13), mRNA. |
| MED22 | 1.9 | Homo sapiens mediator complex subunit 22 (MED22), transcript variant c, mRNA. |
| TRIM13 | 1.9 | Homo sapiens tripartite motif-containing 13 (TRIM13), transcript variant 4, mRNA. |
| CD24 | 1.9 | Homo sapiens CD24 molecule (CD24), mRNA. |
| SLC35F3 | 1.89 | PREDICTED: Homo sapiens solute carrier family 35, member F3 (SLC35F3), mRNA. |
| POLR3F | 1.89 | Homo sapiens polymerase (RNA) III (DNA directed) polypeptide F, 39 kDa (POLR3F), mRNA. |
| ZNF35 | 1.89 | Homo sapiens zinc finger protein 35 (ZNF35), mRNA. |
| CTNS | 1.89 | Homo sapiens cystinosis, nephropathic (CTNS), transcript variant 2, mRNA. |
| AADACL1 | 1.89 | Homo sapiens arylacetamide deacetylase-like 1 (AADACL1), mRNA. |
| C17orf45 | 1.89 | Homo sapiens chromosome 17 open reading frame 45 (C17orf45), mRNA. |
| LOC643466 | 1.89 | PREDICTED: Homo sapiens hypothetical protein LOC643466 (LOC643466), mRNA. |
| ASPSCR1 | 1.89 | Homo sapiens alveolar soft part sarcoma chromosome region, candidate 1 (ASPSCR1), mRNA. |
| COX19 | 1.88 | Homo sapiens COX19 cytochrome c oxidase assembly homolog (S. cerevisiae) (COX19), mRNA. |
| SCMH1 | 1.88 | Homo sapiens sex comb on midleg homolog 1 (Drosophila) (SCMH1), transcript variant 1, mRNA. |
| C1orf19 | 1.88 | Homo sapiens chromosome 1 open reading frame 19 (C1orf19), mRNA. |
| ITGB2 | 1.88 | Homo sapiens integrin, beta 2 (complement component 3 receptor 3 and 4 subunit) (ITGB2), mRNA. |
| LOC100130289 | 1.88 | PREDICTED: Homo sapiens misc_RNA (LOC100130289), miscRNA. |
| FBXO21 | 1.88 | Homo sapiens F-box protein 21 (FBXO21), transcript variant 1, mRNA. |
| HGS | 1.88 | Homo sapiens hepatocyte growth factor-regulated tyrosine kinase substrate (HGS), mRNA. |
| C10orf35 | 1.88 | Homo sapiens chromosome 10 open reading frame 35 (C10orf35), mRNA. |
| NAPRT1 | 1.88 | Homo sapiens nicotinate phosphoribosyltransferase domain containing 1 (NAPRT1), mRNA. |
| ABCC5 | 1.87 | Homo sapiens ATP-binding cassette, sub-family C (CFTR/MRP), member 5 (ABCC5), transcript variant 1, mRNA. |
| BAT1 | 1.87 | Homo sapiens HLA-B associated transcript 1 (BAT1), transcript variant 1, mRNA. |
| FBXL10 | 1.87 | Homo sapiens F-box and leucine-rich repeat protein 10 (FBXL10), transcript variant 1, mRNA. |
| TLN1 | 1.87 | Homo sapiens talin 1 (TLN1), mRNA. |
| FAIM2 | 1.87 | Homo sapiens Fas apoptotic inhibitory molecule 2 (FAIM2), mRNA. |
| DDIT4 | 1.87 | Homo sapiens DNA-damage-inducible transcript 4 (DDIT4), mRNA. |
| NOL12 | 1.87 | Homo sapiens nucleolar protein 12 (NOL12), mRNA. |
| CUTA | 1.87 | Homo sapiens cutA divalent cation tolerance homolog (E. coli) (CUTA), transcript variant 2, mRNA. |
| OSBP | 1.87 | Homo sapiens oxysterol binding protein (OSBP), mRNA. |
| LOC390671 | 1.87 | PREDICTED: Homo sapiens misc_RNA (LOC390671), miscRNA. |
| NMRAL1 | 1.87 | Homo sapiens NmrA-like family domain containing 1 (NMRAL1), mRNA. |
| C2orf63 | 1.87 | Homo sapiens chromosome 2 open reading frame 63 (C2orf63), mRNA. |
| CDK7 | 1.86 | Homo sapiens cyclin-dependent kinase 7 (MO15 homolog, Xenopus laevis, cdk-activating kinase) (CDK7), mRNA. |
| LOC646936 | 1.86 | PREDICTED: Homo sapiens misc_RNA (LOC646936), miscRNA. |
| NAPEPLD | 1.86 | Homo sapiens N-acyl phosphatidylethanolamine phospholipase D (NAPEPLD), mRNA. |
| MGC16121 | 1.86 | PREDICTED: Homo sapiens hypothetical protein MGC16121 (MGC16121), mRNA. |
| CAPRIN1 | 1.86 | Homo sapiens cell cycle associated protein 1 (CAPRIN1), transcript variant 1, mRNA. |
| CBWD5 | 1.86 | Homo sapiens COBW domain containing 5 (CBWD5), mRNA. |
| CUL5 | 1.86 | Homo sapiens cullin 5 (CUL5), mRNA. |
| LOC388556 | 1.86 | PREDICTED: Homo sapiens misc_RNA (LOC388556), miscRNA. |
| RNASE10 | 1.86 | Homo sapiens ribonuclease, RNase A family, 10 (non-active) (RNASE10), mRNA. |
| TMEM98 | 1.85 | Homo sapiens transmembrane protein 98 (TMEM98), transcript variant 2, mRNA. |
| BRD9 | 1.85 | Homo sapiens bromodomain containing 9 (BRD9), transcript variant 1, mRNA. |
| XBP1 | 1.85 | Homo sapiens X-box binding protein 1 (XBP1), transcript variant 1, mRNA. |
| FN3KRP | 1.85 | Homo sapiens fructosamine-3-kinase-related protein (FN3KRP), mRNA. |
| WTIP | 1.85 | PREDICTED: Homo sapiens Wilms tumor 1 interacting protein (WTIP), mRNA. |
| ABCF1 | 1.85 | Homo sapiens ATP-binding cassette, sub-family F (GCN20), member 1 (ABCF1), transcript variant 1, mRNA. |
| SLC25A39 | 1.85 | Homo sapiens solute carrier family 25, member 39 (SLC25A39), mRNA. |
| FBXL20 | 1.85 | Homo sapiens F-box and leucine-rich repeat protein 20 (FBXL20), mRNA. |
|  | 1.85 | zh47e08.r1 Soares_fetal_liver_spleen_1NFLS_S1 Homo sapiens cDNA clone IMAGE:415238 5, mRNA sequence |
| LOC286444 | 1.85 | PREDICTED: Homo sapiens misc_RNA (LOC286444), miscRNA. |
| SNX4 | 1.85 | Homo sapiens sorting nexin 4 (SNX4), mRNA. |
| FAM90A3 | 1.84 | PREDICTED: Homo sapiens family with sequence similarity 90, member A3 (FAM90A3), mRNA. |
| SCO2 | 1.84 | Homo sapiens SCO cytochrome oxidase deficient homolog 2 (yeast) (SCO2), nuclear gene encoding mitochondrial protein, mRNA. |
| GNAQ | 1.84 | Homo sapiens guanine nucleotide binding protein (G protein), q polypeptide (GNAQ), mRNA. |
| MFAP2 | 1.84 | Homo sapiens microfibrillar-associated protein 2 (MFAP2), transcript variant 2, mRNA. |
| LOC100132444 | 1.84 | PREDICTED: Homo sapiens hypothetical protein LOC100132444 (LOC100132444), mRNA. |
| IRF2BP2 | 1.84 | Homo sapiens interferon regulatory factor 2 binding protein 2 (IRF2BP2), transcript variant 1, mRNA. |
| AUH | 1.84 | Homo sapiens AU RNA binding protein/enoyl-Coenzyme A hydratase (AUH), nuclear gene encoding mitochondrial protein, mRNA. |
| C9orf130 | 1.84 | PREDICTED: Homo sapiens chromosome 9 open reading frame 130 (C9orf130), mRNA. |
| SLC25A3 | 1.84 | Homo sapiens solute carrier family 25 (mitochondrial carrier; phosphate carrier), member 3 (SLC25A3), nuclear gene encoding mitochondrial protein, transcript variant 3, mRNA. |
| UIMC1 | 1.84 | Homo sapiens ubiquitin interaction motif containing 1 (UIMC1), mRNA. |
| METTL13 | 1.83 | Homo sapiens methyltransferase like 13 (METTL13), transcript variant 1, mRNA. |
| LOC728138 | 1.83 | PREDICTED: Homo sapiens similar to KIAA2013 (LOC728138), partial mRNA. |
| ZNF251 | 1.83 | Homo sapiens zinc finger protein 251 (ZNF251), mRNA. |
| MTMR12 | 1.83 | Homo sapiens myotubularin related protein 12 (MTMR12), mRNA. |
| LOC728554 | 1.83 | PREDICTED: Homo sapiens similar to THO complex 3 (LOC728554), mRNA. |
| SLC35A2 | 1.83 | Homo sapiens solute carrier family 35 (UDP-galactose transporter), member A2 (SLC35A2), transcript variant 2, mRNA. |
| ACTN1 | 1.83 | Homo sapiens actinin, alpha 1 (ACTN1), mRNA. |
| GSPT2 | 1.83 | Homo sapiens G1 to S phase transition 2 (GSPT2), mRNA. |
| ZNF2 | 1.83 | Homo sapiens zinc finger protein 2 (ZNF2), transcript variant 1, mRNA. |
| ATP6V1E1 | 1.83 | Homo sapiens ATPase, H+ transporting, lysosomal 31kDa, V1 subunit E1 (ATP6V1E1), transcript variant 3, mRNA. |
| HLA-DRB3 | 1.83 | Homo sapiens major histocompatibility complex, class II, DR beta 3 (HLA-DRB3), mRNA. |
| HEY1 | 1.83 | Homo sapiens hairy/enhancer-of-split related with YRPW motif 1 (HEY1), transcript variant 2, mRNA. |
| ZNF576 | 1.82 | Homo sapiens zinc finger protein 576 (ZNF576), mRNA. |
| TOB1 | 1.82 | Homo sapiens transducer of ERBB2, 1 (TOB1), mRNA. |
| C1orf91 | 1.82 | Homo sapiens chromosome 1 open reading frame 91 (C1orf91), mRNA. |
| TRAF6 | 1.82 | Homo sapiens TNF receptor-associated factor 6 (TRAF6), transcript variant 2, mRNA. |
| PLK1 | 1.82 | Homo sapiens polo-like kinase 1 (Drosophila) (PLK1), mRNA. |
| MRPL17 | 1.82 | Homo sapiens mitochondrial ribosomal protein L17 (MRPL17), nuclear gene encoding mitochondrial protein, mRNA. |
| EIF3B | 1.82 | Homo sapiens eukaryotic translation initiation factor 3, subunit B (EIF3B), transcript variant 2, mRNA. |
| GAMT | 1.82 | Homo sapiens guanidinoacetate N-methyltransferase (GAMT), transcript variant 1, mRNA. |
| TOMM40 | 1.82 | Homo sapiens translocase of outer mitochondrial membrane 40 homolog (yeast) (TOMM40), nuclear gene encoding mitochondrial protein, mRNA. |
| LOC729142 | 1.82 | PREDICTED: Homo sapiens misc_RNA (LOC729142), miscRNA. |
| OPA3 | 1.82 | Homo sapiens optic atrophy 3 (autosomal recessive, with chorea and spastic paraplegia) (OPA3), transcript variant 2, mRNA. |
| DDX19B | 1.82 | Homo sapiens DEAD (Asp-Glu-Ala-As) box polypeptide 19B (DDX19B), transcript variant 2, mRNA. |
| GP1BA | 1.82 | Homo sapiens glycoprotein Ib (platelet), alpha polypeptide (GP1BA), mRNA. |
| C1orf174 | 1.82 | Homo sapiens chromosome 1 open reading frame 174 (C1orf174), mRNA. |
| LOC283953 | 1.82 | PREDICTED: Homo sapiens hypothetical LOC283953 (LOC283953), mRNA. |
| COL16A1 | 1.82 | Homo sapiens collagen, type XVI, alpha 1 (COL16A1), mRNA. |
| DAND5 | 1.82 | Homo sapiens DAN domain family, member 5 (DAND5), mRNA. |
| LOC283314 | 1.82 | PREDICTED: Homo sapiens misc_RNA (LOC283314), miscRNA. |
| LOC645351 | 1.82 | PREDICTED: Homo sapiens similar to SMT3 suppressor of mif two 3 homolog 2 (LOC645351), mRNA. |
| ZADH2 | 1.82 | Homo sapiens zinc binding alcohol dehydrogenase domain containing 2 (ZADH2), mRNA. |
| CLIP4 | 1.82 | Homo sapiens CAP-GLY domain containing linker protein family, member 4 (CLIP4), mRNA. |
| SNX6 | 1.82 | Homo sapiens sorting nexin 6 (SNX6), transcript variant 2, mRNA. |
| NXT1 | 1.81 | Homo sapiens NTF2-like export factor 1 (NXT1), mRNA. |
| LOC646949 | 1.81 | PREDICTED: Homo sapiens misc_RNA (LOC646949), miscRNA. |
| TSPAN9 | 1.81 | Homo sapiens tetraspanin 9 (TSPAN9), mRNA. |
| HNRNPA1L2 | 1.81 | Homo sapiens heterogeneous nuclear ribonucleoprotein A1-like 2 (HNRNPA1L2), transcript variant 1, mRNA. |
| TSPAN17 | 1.81 | Homo sapiens tetraspanin 17 (TSPAN17), transcript variant 1, mRNA. |
| GPR89A | 1.81 | Homo sapiens G protein-coupled receptor 89A (GPR89A), mRNA. |
| ZNF322A | 1.81 | Homo sapiens zinc finger protein 322A (ZNF322A), mRNA. |
| DDX31 | 1.81 | Homo sapiens DEAD (Asp-Glu-Ala-Asp) box polypeptide 31 (DDX31), transcript variant 1, mRNA. |
| E2F6 | 1.81 | Homo sapiens E2F transcription factor 6 (E2F6), mRNA. |
| USP41 | 1.81 | PREDICTED: Homo sapiens ubiquitin specific peptidase 41 (USP41), mRNA. |
| MGC12760 | 1.81 | PREDICTED: Homo sapiens hypothetical protein MGC12760, transcript variant 2 (MGC12760), mRNA. |
| FLJ40113 | 1.81 | Homo sapiens golgi autoantigen, golgin subfamily a-like (FLJ40113), mRNA. |
| AIMP2 | 1.8 | Homo sapiens aminoacyl tRNA synthetase complex-interacting multifunctional protein 2 (AIMP2), mRNA. |
| FBXO28 | 1.8 | Homo sapiens F-box protein 28 (FBXO28), mRNA. |
| UBTD2 | 1.8 | Homo sapiens ubiquitin domain containing 2 (UBTD2), mRNA. |
| LOC651436 | 1.8 | PREDICTED: Homo sapiens similar to ribosomal protein L9 (LOC651436), mRNA. |
| LOC441050 | 1.8 | PREDICTED: Homo sapiens similar to unactive progesterone receptor, 23 kD (LOC441050), mRNA. |
| SLC25A26 | 1.79 | Homo sapiens solute carrier family 25, member 26 (SLC25A26), transcript variant 3, mRNA. |
| SPNS1 | 1.79 | Homo sapiens spinster homolog 1 (Drosophila) (SPNS1), mRNA. |
| LOC100128737 | 1.79 | PREDICTED: Homo sapiens hypothetical protein LOC100128737 (LOC100128737), mRNA. |
| LOC643300 | 1.79 | PREDICTED: Homo sapiens similar to 60 kDa heat shock protein, mitochondrial precursor (Hsp60) (60 kDa chaperonin) (CPN60) (Heat shock protein 60) (HSP-60) (Mitochondrial matrix protein P1) (P60 lymphocyte protein) (HuCHA60) (LOC643300), mRNA. |
| ANKRD32 | 1.79 | Homo sapiens ankyrin repeat domain 32 (ANKRD32), mRNA. |
| ZNF784 | 1.79 | Homo sapiens zinc finger protein 784 (ZNF784), mRNA. |
| SERF1A | 1.79 | Homo sapiens small EDRK-rich factor 1A (telomeric) (SERF1A), mRNA. |
| ATP6V0E2 | 1.78 | Homo sapiens ATPase, H+ transporting V0 subunit e2 (ATP6V0E2), transcript variant 1, mRNA. |
| ARHGAP17 | 1.78 | Homo sapiens Rho GTPase activating protein 17 (ARHGAP17), transcript variant 2, mRNA. |
| XAGE1 | 1.78 | Homo sapiens X antigen family, member 1 (XAGE1), transcript variant 2, mRNA. |
| LOC100130332 | 1.78 | PREDICTED: Homo sapiens similar to PRO2474 (LOC100130332), mRNA. |
| TNFSF14 | 1.78 | Homo sapiens tumor necrosis factor (ligand) superfamily, member 14 (TNFSF14), transcript variant 2, mRNA. |
| EFNA1 | 1.78 | Homo sapiens ephrin-A1 (EFNA1), transcript variant 1, mRNA. |
| LOC645722 | 1.78 | PREDICTED: Homo sapiens similar to hCG2037003 (LOC645722), mRNA. |
| LOC648705 | 1.78 | PREDICTED: Homo sapiens hypothetical protein LOC648705 (LOC648705), mRNA. |
| CCNE1 | 1.77 | Homo sapiens cyclin E1 (CCNE1), transcript variant 1, mRNA. |
| LOC400446 | 1.77 | PREDICTED: Homo sapiens misc_RNA (LOC400446), miscRNA. |
| LCMT1 | 1.77 | Homo sapiens leucine carboxyl methyltransferase 1 (LCMT1), transcript variant 1, mRNA. |
| ATF2 | 1.77 | Homo sapiens activating transcription factor 2 (ATF2), mRNA. |
| JMJD8 | 1.77 | Homo sapiens jumonji domain containing 8 (JMJD8), mRNA. |
| ARMC10 | 1.77 | Homo sapiens armadillo repeat containing 10 (ARMC10), mRNA. |
| ARMC7 | 1.77 | Homo sapiens armadillo repeat containing 7 (ARMC7), mRNA. |
| CBX2 | 1.77 | Homo sapiens chromobox homolog 2 (Pc class homolog, Drosophila) (CBX2), transcript variant 1, mRNA. |
| PPM1D | 1.76 | Homo sapiens protein phosphatase 1D magnesium-dependent, delta isoform (PPM1D), mRNA. |
| STRADA | 1.76 | Homo sapiens STE20-related kinase adaptor alpha (STRADA), transcript variant 2, mRNA. |
| C12orf47 | 1.76 | PREDICTED: Homo sapiens chromosome 12 open reading frame 47 (C12orf47), misc RNA. |
| LOC391578 | 1.76 | PREDICTED: Homo sapiens misc_RNA (LOC391578), miscRNA. |
| GPT2 | 1.76 | Homo sapiens glutamic pyruvate transaminase (alanine aminotransferase) 2 (GPT2), mRNA. |
| TMEM205 | 1.76 | Homo sapiens transmembrane protein 205 (TMEM205), mRNA. |
| LYSMD2 | 1.76 | Homo sapiens LysM, putative peptidoglycan-binding, domain containing 2 (LYSMD2), mRNA. |
| OSCP1 | 1.76 | Homo sapiens organic solute carrier partner 1 (OSCP1), transcript variant 2, mRNA. |
| CD59 | 1.76 | Homo sapiens CD59 molecule, complement regulatory protein (CD59), transcript variant 2, mRNA. |
| TAOK3 | 1.76 | Homo sapiens TAO kinase 3 (TAOK3), mRNA. |
|  | 1.76 | BX447862 Homo sapiens T CELLS (JURKAT CELL LINE) Homo sapiens cDNA clone CS0DH002YG04 3-PRIME, mRNA sequence |
| MRFAP1L1 | 1.76 | Homo sapiens Morf4 family associated protein 1-like 1 (MRFAP1L1), transcript variant 1, mRNA. |
| GAR1 | 1.76 | Homo sapiens GAR1 ribonucleoprotein homolog (yeast) (GAR1), transcript variant 1, mRNA. |
| MGC4677 | 1.76 | Homo sapiens hypothetical protein MGC4677 (MGC4677), mRNA. |
| LOC649330 | 1.76 | PREDICTED: Homo sapiens similar to heterogeneous nuclear ribonucleoprotein C-like 1 (LOC649330), mRNA. |
| FAM83F | 1.75 | Homo sapiens family with sequence similarity 83, member F (FAM83F), mRNA. |
| PSMF1 | 1.75 | Homo sapiens proteasome (prosome, macropain) inhibitor subunit 1 (PI31) (PSMF1), transcript variant 3, mRNA. |
| ADAM15 | 1.75 | Homo sapiens ADAM metallopeptidase domain 15 (ADAM15), transcript variant 4, mRNA. |
| DCTPP1 | 1.75 | Homo sapiens dCTP pyrophosphatase 1 (DCTPP1), mRNA. |
| KAT5 | 1.75 | Homo sapiens K(lysine) acetyltransferase 5 (KAT5), transcript variant 3, mRNA. |
| ZSWIM1 | 1.75 | Homo sapiens zinc finger, SWIM-type containing 1 (ZSWIM1), mRNA. |
| AQP12B | 1.75 | Homo sapiens aquaporin 12B (AQP12B), mRNA. |
| CRLS1 | 1.75 | Homo sapiens cardiolipin synthase 1 (CRLS1), mRNA. |
| ZNF30 | 1.75 | Homo sapiens zinc finger protein 30 (ZNF30), transcript variant 2, mRNA. |
| RPS6KA3 | 1.75 | Homo sapiens ribosomal protein S6 kinase, 90kDa, polypeptide 3 (RPS6KA3), mRNA. |
| NDUFS7 | 1.75 | Homo sapiens NADH dehydrogenase (ubiquinone) Fe-S protein 7, 20kDa (NADH-coenzyme Q reductase) (NDUFS7), mRNA. |
| MRPL52 | 1.75 | Homo sapiens mitochondrial ribosomal protein L52 (MRPL52), nuclear gene encoding mitochondrial protein, transcript variant 2, mRNA. |
| GTPBP6 | 1.74 | Homo sapiens GTP binding protein 6 (putative) (GTPBP6), mRNA. |
| MED19 | 1.74 | Homo sapiens mediator complex subunit 19 (MED19), mRNA. |
| LOC390345 | 1.74 | PREDICTED: Homo sapiens misc_RNA (LOC390345), miscRNA. |
| PDCD10 | 1.74 | Homo sapiens programmed cell death 10 (PDCD10), transcript variant 2, mRNA. |
| BRD2 | 1.74 | Homo sapiens bromodomain containing 2 (BRD2), mRNA. |
| SIP1 | 1.74 | Homo sapiens survival of motor neuron protein interacting protein 1 (SIP1), transcript variant beta, mRNA. |
| PTBP1 | 1.74 | Homo sapiens polypyrimidine tract binding protein 1 (PTBP1), transcript variant 1, mRNA. |
| LOC401640 | 1.74 | PREDICTED: Homo sapiens misc_RNA (LOC401640), miscRNA. |
| PREI3 | 1.74 | Homo sapiens preimplantation protein 3 (PREI3), transcript variant 1, mRNA. |
| CRELD2 | 1.74 | Homo sapiens cysteine-rich with EGF-like domains 2 (CRELD2), mRNA. |
| PPM1G | 1.74 | Homo sapiens protein phosphatase 1G (formerly 2C), magnesium-dependent, gamma isoform (PPM1G), transcript variant 2, mRNA. |
| PALM | 1.73 | Homo sapiens paralemmin (PALM), transcript variant 1, mRNA. |
| SNRPN | 1.73 | Homo sapiens small nuclear ribonucleoprotein polypeptide N (SNRPN), transcript variant 2, mRNA. |
| ZNF644 | 1.73 | Homo sapiens zinc finger protein 644 (ZNF644), transcript variant 1, mRNA. |
| FKBP1A | 1.73 | Homo sapiens FK506 binding protein 1A, 12kDa (FKBP1A), transcript variant 12A, mRNA. |
| C7orf13 | 1.73 | Homo sapiens chromosome 7 open reading frame 13 (C7orf13), mRNA. |
| LGR5 | 1.73 | Homo sapiens leucine-rich repeat-containing G protein-coupled receptor 5 (LGR5), mRNA. |
| LOC440991 | 1.73 | PREDICTED: Homo sapiens misc_RNA (LOC440991), miscRNA. |
| DOPEY2 | 1.73 | Homo sapiens dopey family member 2 (DOPEY2), mRNA. |
| SYF2 | 1.72 | Homo sapiens SYF2 homolog, RNA splicing factor (S. cerevisiae) (SYF2), transcript variant 1, mRNA. |
| TUBG1 | 1.72 | Homo sapiens tubulin, gamma 1 (TUBG1), mRNA. |
| TRIM39 | 1.72 | Homo sapiens tripartite motif-containing 39 (TRIM39), transcript variant 1, mRNA. |
| FSTL4 | 1.72 | Homo sapiens follistatin-like 4 (FSTL4), mRNA. |
| LOC642755 | 1.72 | PREDICTED: Homo sapiens similar to DEXI, transcript variant 1 (LOC642755), mRNA. |
|  | 1.72 | Homo sapiens cDNA FLJ37828 fis, clone BRSSN2006575 |
| C6orf48 | 1.72 | Homo sapiens chromosome 6 open reading frame 48 (C6orf48), mRNA. |
| SLC25A44 | 1.72 | Homo sapiens solute carrier family 25, member 44 (SLC25A44), mRNA. |
| SMOX | 1.72 | Homo sapiens spermine oxidase (SMOX), transcript variant 4, mRNA. |
| LOC729500 | 1.72 | PREDICTED: Homo sapiens misc_RNA (LOC729500), partial miscRNA. |
| ZBTB42 | 1.72 | Homo sapiens zinc finger and BTB domain containing 42 (ZBTB42), mRNA. |
| MTRR | 1.72 | Homo sapiens 5-methyltetrahydrofolate-homocysteine methyltransferase reductase (MTRR), transcript variant 2, mRNA. |
| FBXL18 | 1.71 | Homo sapiens F-box and leucine-rich repeat protein 18 (FBXL18), mRNA. |
| LOC389816 | 1.71 | Homo sapiens cytokeratin associated protein (LOC389816), mRNA. |
| ADRBK1 | 1.71 | Homo sapiens adrenergic, beta, receptor kinase 1 (ADRBK1), mRNA. |
| NCAM2 | 1.71 | Homo sapiens neural cell adhesion molecule 2 (NCAM2), mRNA. |
| SETDB2 | 1.7 | Homo sapiens SET domain, bifurcated 2 (SETDB2), mRNA. |
| RPS3 | 1.7 | Homo sapiens ribosomal protein S3 (RPS3), mRNA. |
| SRP9 | 1.7 | Homo sapiens signal recognition particle 9kDa (SRP9), mRNA. |
| LOC652481 | 1.7 | PREDICTED: Homo sapiens similar to Mitochondrial import inner membrane translocase subunit Tim23 (LOC652481), mRNA. |
| SCNM1 | 1.7 | Homo sapiens sodium channel modifier 1 (SCNM1), mRNA. |
| LOC653489 | 1.7 | PREDICTED: Homo sapiens similar to Ran-binding protein 2 (RanBP2) (Nuclear pore complex protein Nup358) (Nucleoporin Nup358) (358 kDa nucleoporin) (P270), transcript variant 7 (LOC653489), mRNA. |
| MTMR9 | 1.7 | Homo sapiens myotubularin related protein 9 (MTMR9), mRNA. |
| KIAA1641 | 1.7 | PREDICTED: Homo sapiens KIAA1641, transcript variant 7 (KIAA1641), mRNA. |
| SDHALP1 | 1.7 | Homo sapiens succinate dehydrogenase complex, subunit A, flavoprotein pseudogene 1 (SDHALP1) on chromosome 3. |
| PRSS3 | 1.7 | Homo sapiens protease, serine, 3 (mesotrypsin) (PRSS3), mRNA. |
| NLN | 1.7 | Homo sapiens neurolysin (metallopeptidase M3 family) (NLN), mRNA. |
| SELS | 1.7 | Homo sapiens selenoprotein S (SELS), transcript variant 2, mRNA. |
| LOC648822 | 1.7 | PREDICTED: Homo sapiens similar to hCG2040565 (LOC648822), mRNA. |
| C17orf28 | 1.7 | Homo sapiens chromosome 17 open reading frame 28 (C17orf28), mRNA. |
| ZDHHC9 | 1.7 | Homo sapiens zinc finger, DHHC-type containing 9 (ZDHHC9), transcript variant 1, mRNA. |
| COMMD5 | 1.7 | Homo sapiens COMM domain containing 5 (COMMD5), transcript variant 1, mRNA. |
| PSAT1 | 1.7 | Homo sapiens phosphoserine aminotransferase 1 (PSAT1), transcript variant 2, mRNA. |
| C11orf17 | 1.7 | Homo sapiens chromosome 11 open reading frame 17 (C11orf17), transcript variant 1, mRNA. |
| PBRM1 | 1.7 | Homo sapiens polybromo 1 (PBRM1), transcript variant 4, mRNA. |
| LOC100130598 | 1.69 | PREDICTED: Homo sapiens hypothetical protein LOC100130598 (LOC100130598), mRNA. |
| FAM40B | 1.69 | Homo sapiens family with sequence similarity 40, member B (FAM40B), mRNA. |
| MYST1 | 1.69 | Homo sapiens MYST histone acetyltransferase 1 (MYST1), transcript variant 1, mRNA. |
| EMP3 | 1.69 | Homo sapiens epithelial membrane protein 3 (EMP3), mRNA. |
| HCG4 | 1.69 | Homo sapiens HLA complex group 4 (HCG4), non-coding RNA. |
| GLTSCR2 | 1.69 | Homo sapiens glioma tumor suppressor candidate region gene 2 (GLTSCR2), mRNA. |
| LOC652771 | 1.69 | PREDICTED: Homo sapiens similar to general transcription factor II, i isoform 1 (LOC652771), mRNA. |
| PI4K2B | 1.69 | Homo sapiens phosphatidylinositol 4-kinase type 2 beta (PI4K2B), mRNA. |
| NEDD8 | 1.69 | Homo sapiens neural precursor cell expressed, developmentally down-regulated 8 (NEDD8), mRNA. |
| TAF9 | 1.69 | Homo sapiens TAF9 RNA polymerase II, TATA box binding protein (TBP)-associated factor, 32kDa (TAF9), transcript variant 3, mRNA. |
| CCDC16 | 1.68 | Homo sapiens coiled-coil domain containing 16 (CCDC16), mRNA. |
|  | 1.68 | BX090227 NCI_CGAP_GC6 Homo sapiens cDNA clone IMAGp998D135719 ; IMAGE:2307804, mRNA sequence |
| MFN1 | 1.68 | Homo sapiens mitofusin 1 (MFN1), nuclear gene encoding mitochondrial protein, mRNA. |
| ZNF746 | 1.68 | Homo sapiens zinc finger protein 746 (ZNF746), mRNA. |
| ZNF655 | 1.68 | Homo sapiens zinc finger protein 655 (ZNF655), transcript variant 4, mRNA. |
| LOC100132086 | 1.68 | PREDICTED: Homo sapiens misc_RNA (LOC100132086), miscRNA. |
| MRPL18 | 1.68 | Homo sapiens mitochondrial ribosomal protein L18 (MRPL18), nuclear gene encoding mitochondrial protein, mRNA. |
| ASB3 | 1.68 | Homo sapiens ankyrin repeat and SOCS box-containing 3 (ASB3), transcript variant 1, mRNA. |
| EXOSC3 | 1.68 | Homo sapiens exosome component 3 (EXOSC3), transcript variant 1, mRNA. |
| LOC390578 | 1.68 | PREDICTED: Homo sapiens misc_RNA (LOC390578), miscRNA. |
| TAF1 | 1.68 | Homo sapiens TAF1 RNA polymerase II, TATA box binding protein (TBP)-associated factor, 250kDa (TAF1), transcript variant 2, mRNA. |
| SLC38A1 | 1.68 | Homo sapiens solute carrier family 38, member 1 (SLC38A1), transcript variant 1, mRNA. |
| LOC653994 | 1.68 | PREDICTED: Homo sapiens similar to Eukaryotic translation initiation factor 4H (eIF-4H) (Williams-Beuren syndrome chromosome region 1 protein homolog), transcript variant 2 (LOC653994), mRNA. |
| PDRG1 | 1.68 | Homo sapiens p53 and DNA-damage regulated 1 (PDRG1), mRNA. |
| LARP4 | 1.67 | Homo sapiens La ribonucleoprotein domain family, member 4 (LARP4), transcript variant 1, mRNA. |
| METTL1 | 1.67 | Homo sapiens methyltransferase like 1 (METTL1), transcript variant 1, mRNA. |
| 5-Sep | 1.67 | Homo sapiens septin 5 (SEPT5), mRNA. |
| TOP1P2 | 1.67 | Homo sapiens topoisomerase (DNA) I pseudogene 2 (TOP1P2), non-coding RNA. |
| NR2C1 | 1.67 | Homo sapiens nuclear receptor subfamily 2, group C, member 1 (NR2C1), transcript variant 2, mRNA. |
| HSPD1 | 1.67 | Homo sapiens heat shock 60kDa protein 1 (chaperonin) (HSPD1), nuclear gene encoding mitochondrial protein, transcript variant 1, mRNA. |
| LOC644172 | 1.67 | PREDICTED: Homo sapiens similar to C-jun-amino-terminal kinase-interacting protein 1 (JNK-interacting protein 1) (JIP-1) (JNK MAP kinase scaffold protein 1) (Islet-brain 1) (IB-1) (Mitogen-activated protein kinase 8-interacting protein 1) (LOC644172), mRNA. |
| LOC100132761 | 1.67 | PREDICTED: Homo sapiens hypothetical protein LOC100132761 (LOC100132761), mRNA. |
| LOC645693 | 1.67 | PREDICTED: Homo sapiens misc_RNA (LOC645693), miscRNA. |
| PUS7 | 1.67 | Homo sapiens pseudouridylate synthase 7 homolog (S. cerevisiae) (PUS7), mRNA. |
| ANGEL2 | 1.66 | Homo sapiens angel homolog 2 (Drosophila) (ANGEL2), mRNA. |
| TCEAL3 | 1.66 | Homo sapiens transcription elongation factor A (SII)-like 3 (TCEAL3), transcript variant 1, mRNA. |
| TEX261 | 1.66 | Homo sapiens testis expressed 261 (TEX261), mRNA. |
| NOB1 | 1.66 | Homo sapiens NIN1/RPN12 binding protein 1 homolog (S. cerevisiae) (NOB1), mRNA. |
| SSH2 | 1.66 | Homo sapiens slingshot homolog 2 (Drosophila) (SSH2), mRNA. |
| MAP1B | 1.66 | Homo sapiens microtubule-associated protein 1B (MAP1B), mRNA. |
| GPN2 | 1.66 | Homo sapiens GPN-loop GTPase 2 (GPN2), mRNA. |
| PIK3R2 | 1.65 | Homo sapiens phosphoinositide-3-kinase, regulatory subunit 2 (beta) (PIK3R2), mRNA. |
| NCL | 1.65 | Homo sapiens nucleolin (NCL), mRNA. |
| HEY1 | 1.65 | Homo sapiens hairy/enhancer-of-split related with YRPW motif 1 (HEY1), transcript variant 2, mRNA. |
| LOC388122 | 1.65 | PREDICTED: Homo sapiens hypothetical LOC388122 (LOC388122), mRNA. |
| PFDN6 | 1.65 | Homo sapiens prefoldin subunit 6 (PFDN6), mRNA. |
| HNRPA1P4 | 1.65 | PREDICTED: Homo sapiens heterogeneous nuclear ribonucleoprotein A1 pseudogene 4 (HNRPA1P4), mRNA. |
| C9orf169 | 1.65 | Homo sapiens chromosome 9 open reading frame 169 (C9orf169), mRNA. |
| LOC100130255 | 1.64 | PREDICTED: Homo sapiens hypothetical LOC100130255 (LOC100130255), mRNA. |
| MTMR2 | 1.64 | Homo sapiens myotubularin related protein 2 (MTMR2), transcript variant 3, mRNA. |
| STRA13 | 1.64 | Homo sapiens stimulated by retinoic acid 13 homolog (mouse) (STRA13), mRNA. |
| LOC728006 | 1.64 | PREDICTED: Homo sapiens hypothetical protein LOC728006 (LOC728006), mRNA. |
| MIR586 | 1.64 | Homo sapiens microRNA 586 (MIR586), microRNA. |
| HSZFP36 | 1.64 | Homo sapiens ZFP-36 for a zinc finger protein (HSZFP36), mRNA. |
| AGPAT5 | 1.64 | Homo sapiens 1-acylglycerol-3-phosphate O-acyltransferase 5 (lysophosphatidic acid acyltransferase, epsilon) (AGPAT5), mRNA. |
| LOC25845 | 1.63 | Homo sapiens hypothetical LOC25845 (LOC25845), non-coding RNA. |
| CNKSR3 | 1.63 | Homo sapiens CNKSR family member 3 (CNKSR3), mRNA. |
| NOMO3 | 1.63 | Homo sapiens NODAL modulator 3 (NOMO3), mRNA. |
| EIF3CL | 1.63 | Homo sapiens eukaryotic translation initiation factor 3, subunit C-like (EIF3CL), mRNA. |
| SLC25A14 | 1.63 | Homo sapiens solute carrier family 25 (mitochondrial carrier, brain), member 14 (SLC25A14), nuclear gene encoding mitochondrial protein, transcript variant long, mRNA. |
| LOC728476 | 1.63 | PREDICTED: Homo sapiens hypothetical LOC728476 (LOC728476), mRNA. |
| ANKRD22 | 1.63 | Homo sapiens ankyrin repeat domain 22 (ANKRD22), mRNA. |
| LOC652773 | 1.63 | PREDICTED: Homo sapiens similar to Shwachman-Bodian-Diamond syndrome (predicted) (LOC652773), mRNA. |
| ARF4 | 1.63 | Homo sapiens ADP-ribosylation factor 4 (ARF4), mRNA. |
| CBX5 | 1.63 | Homo sapiens chromobox homolog 5 (HP1 alpha homolog, Drosophila) (CBX5), mRNA. |
| TSR2 | 1.63 | Homo sapiens TSR2, 20S rRNA accumulation, homolog (S. cerevisiae) (TSR2), mRNA. |
| NBPF3 | 1.63 | Homo sapiens neuroblastoma breakpoint family, member 3 (NBPF3), mRNA. |
| SERBP1 | 1.63 | Homo sapiens SERPINE1 mRNA binding protein 1 (SERBP1), transcript variant 3, mRNA. |
| HTR1E | 1.63 | Homo sapiens 5-hydroxytryptamine (serotonin) receptor 1E (HTR1E), mRNA. |
| RFX7 | 1.63 | Homo sapiens regulatory factor X, 7 (RFX7), mRNA. |
| EIF5 | 1.63 | Homo sapiens eukaryotic translation initiation factor 5 (EIF5), transcript variant 1, mRNA. |
| ARPC2 | 1.63 | Homo sapiens actin related protein 2/3 complex, subunit 2, 34kDa (ARPC2), transcript variant 2, mRNA. |
| LOC729375 | 1.63 | Homo sapiens family with sequence similarity 86, member A pseudogene (LOC729375), non-coding RNA. |
|  | 1.63 | PT2.1_10_D05.r tumor2 Homo sapiens cDNA 3, mRNA sequence |
| ASTE1 | 1.62 | Homo sapiens asteroid homolog 1 (Drosophila) (ASTE1), mRNA. |
| TMEM87A | 1.62 | Homo sapiens transmembrane protein 87A (TMEM87A), mRNA. |
| NUFIP1 | 1.62 | Homo sapiens nuclear fragile X mental retardation protein interacting protein 1 (NUFIP1), mRNA. |
| LOC728643 | 1.62 | Homo sapiens heterogeneous nuclear ribonucleoprotein A1 pseudogene (LOC728643), non-coding RNA. |
| H2AFV | 1.62 | Homo sapiens H2A histone family, member V (H2AFV), transcript variant 2, mRNA. |
| SRA1 | 1.62 | Homo sapiens steroid receptor RNA activator 1 (SRA1), mRNA. |
| PPP1R13B | 1.62 | Homo sapiens protein phosphatase 1, regulatory (inhibitor) subunit 13B (PPP1R13B), mRNA. |
| EIF4E | 1.62 | Homo sapiens eukaryotic translation initiation factor 4E (EIF4E), mRNA. |
| LOC728732 | 1.62 | PREDICTED: Homo sapiens misc_RNA (LOC728732), miscRNA. |
| SUOX | 1.62 | Homo sapiens sulfite oxidase (SUOX), nuclear gene encoding mitochondrial protein, transcript variant 1, mRNA. |
| C5orf24 | 1.62 | Homo sapiens chromosome 5 open reading frame 24 (C5orf24), mRNA. |
| CCM2 | 1.62 | Homo sapiens cerebral cavernous malformation 2 (CCM2), transcript variant 1, mRNA. |
| ASAH1 | 1.62 | Homo sapiens N-acylsphingosine amidohydrolase (acid ceramidase) 1 (ASAH1), transcript variant 1, mRNA. |
| EVL | 1.62 | Homo sapiens Enah/Vasp-like (EVL), mRNA. |
| SDHAF2 | 1.62 | Homo sapiens succinate dehydrogenase complex assembly factor 2 (SDHAF2), mRNA. |
|  | 1.62 | 17000532640995 GRN_ES Homo sapiens cDNA 5, mRNA sequence |
| GABARAPL2 | 1.62 | Homo sapiens GABA(A) receptor-associated protein-like 2 (GABARAPL2), mRNA. |
| LOC727865 | 1.61 | PREDICTED: Homo sapiens misc_RNA (LOC727865), miscRNA. |
| PLEKHB2 | 1.61 | Homo sapiens pleckstrin homology domain containing, family B (evectins) member 2 (PLEKHB2), transcript variant 1, mRNA. |
| ZNF443 | 1.61 | Homo sapiens zinc finger protein 443 (ZNF443), mRNA. |
| PANK1 | 1.61 | Homo sapiens pantothenate kinase 1 (PANK1), transcript variant gamma, mRNA. |
| CBX6 | 1.61 | Homo sapiens chromobox homolog 6 (CBX6), mRNA. |
| MTERFD1 | 1.61 | Homo sapiens MTERF domain containing 1 (MTERFD1), mRNA. |
| LOC387867 | 1.61 | PREDICTED: Homo sapiens similar to 40S ribosomal protein SA (p40) (34/67 kDa laminin receptor) (Colon carcinoma laminin-binding protein) (NEM/1CHD4) (Multidrug resistance-associated protein MGr1-Ag) (LOC387867), mRNA. |
| SPTLC1 | 1.61 | Homo sapiens serine palmitoyltransferase, long chain base subunit 1 (SPTLC1), transcript variant 2, mRNA. |
| RBM4B | 1.61 | Homo sapiens RNA binding motif protein 4B (RBM4B), mRNA. |
| POTEE | 1.61 | Homo sapiens POTE ankyrin domain family, member E (POTEE), mRNA. |
| ZNF716 | 1.61 | PREDICTED: Homo sapiens zinc finger protein 716 (ZNF716), mRNA. |
| AKT1 | 1.61 | Homo sapiens v-akt murine thymoma viral oncogene homolog 1 (AKT1), transcript variant 2, mRNA. |
| LOC643452 | 1.6 | PREDICTED: Homo sapiens similar to bromodomain containing 7 (LOC643452), mRNA. |
| SDHAF1 | 1.6 | Homo sapiens succinate dehydrogenase complex assembly factor 1 (SDHAF1), mRNA. |
| LOC653226 | 1.6 | PREDICTED: Homo sapiens similar to Signal recognition particle 9 kDa protein (SRP9) (LOC653226), mRNA. |
| TUBD1 | 1.6 | Homo sapiens tubulin, delta 1 (TUBD1), mRNA. |
| PPM1A | 1.6 | Homo sapiens protein phosphatase 1A (formerly 2C), magnesium-dependent, alpha isoform (PPM1A), transcript variant 3, mRNA. |
| OAZ2 | 1.6 | Homo sapiens ornithine decarboxylase antizyme 2 (OAZ2), mRNA. |
| CCDC128 | 1.59 | Homo sapiens coiled-coil domain containing 128 (CCDC128), mRNA. |
| POM121C | 1.59 | Homo sapiens POM121 membrane glycoprotein C (POM121C), mRNA. |
| DACT3 | 1.59 | Homo sapiens dapper, antagonist of beta-catenin, homolog 3 (Xenopus laevis) (DACT3), mRNA. |
| LOC654254 | 1.59 | PREDICTED: Homo sapiens similar to zinc finger protein 595 (LOC654254), mRNA. |
| CECR5 | 1.59 | Homo sapiens cat eye syndrome chromosome region, candidate 5 (CECR5), transcript variant 2, mRNA. |
| APLP2 | 1.59 | Homo sapiens amyloid beta (A4) precursor-like protein 2 (APLP2), mRNA. |
| C7orf41 | 1.59 | Homo sapiens chromosome 7 open reading frame 41 (C7orf41), mRNA. |
| ZNF436 | 1.59 | Homo sapiens zinc finger protein 436 (ZNF436), mRNA. |
| LOC391670 | 1.59 | PREDICTED: Homo sapiens misc_RNA (LOC391670), miscRNA. |
| CDH2 | 1.59 | Homo sapiens cadherin 2, type 1, N-cadherin (neuronal) (CDH2), mRNA. |
| HINFP | 1.59 | Homo sapiens histone H4 transcription factor (HINFP), transcript variant 1, mRNA. |
| LOC645676 | 1.58 | PREDICTED: Homo sapiens hypothetical protein LOC645676, transcript variant 1 (LOC645676), mRNA. |
| PSMD10 | 1.58 | Homo sapiens proteasome (prosome, macropain) 26S subunit, non-ATPase, 10 (PSMD10), transcript variant 1, mRNA. |
| LOC402112 | 1.58 | PREDICTED: Homo sapiens misc_RNA (LOC402112), miscRNA. |
| GHITM | 1.58 | Homo sapiens growth hormone inducible transmembrane protein (GHITM), mRNA. |
| NBPF1 | 1.58 | Homo sapiens neuroblastoma breakpoint family, member 1 (NBPF1), mRNA. XM_934962 XM_934964 XM_934966 XM_934967 XM_934969 XM_934972 XM_934973 XM_934974 XM_934975 XM_934976 XM_934978 |
| CAMKV | 1.58 | Homo sapiens CaM kinase-like vesicle-associated (CAMKV), mRNA. |
| AMY1C | 1.58 | Homo sapiens amylase, alpha 1C (salivary) (AMY1C), mRNA. |
| PRDX2 | 1.57 | Homo sapiens peroxiredoxin 2 (PRDX2), nuclear gene encoding mitochondrial protein, transcript variant 3, mRNA. |
| ZC3HC1 | 1.57 | Homo sapiens zinc finger, C3HC-type containing 1 (ZC3HC1), mRNA. |
| ENTPD3 | 1.57 | Homo sapiens ectonucleoside triphosphate diphosphohydrolase 3 (ENTPD3), mRNA. |
|  | 1.57 | EST391198 MAGE resequences, MAGP Homo sapiens cDNA, mRNA sequence |
| MGC87895 | 1.57 | PREDICTED: Homo sapiens similar to ribosomal protein S14 (MGC87895), mRNA. |
| C11orf61 | 1.57 | Homo sapiens chromosome 11 open reading frame 61 (C11orf61), mRNA. |
| ULK1 | 1.57 | Homo sapiens unc-51-like kinase 1 (C. elegans) (ULK1), mRNA. |
| EXO1 | 1.57 | Homo sapiens exonuclease 1 (EXO1), transcript variant 1, mRNA. |
| PLEKHO1 | 1.57 | Homo sapiens pleckstrin homology domain containing, family O member 1 (PLEKHO1), mRNA. |
| FTHL16 | 1.56 | PREDICTED: Homo sapiens misc_RNA (FTHL16), miscRNA. |
| MAPK3 | 1.56 | Homo sapiens mitogen-activated protein kinase 3 (MAPK3), mRNA. |
| INTS2 | 1.56 | Homo sapiens integrator complex subunit 2 (INTS2), mRNA. |
|  | 1.56 | Homo sapiens cDNA FLJ32550 fis, clone SPLEN1000056 |
| SEC22C | 1.55 | Homo sapiens SEC22 vesicle trafficking protein homolog C (S. cerevisiae) (SEC22C), transcript variant 1, mRNA. |
| DNAJB6 | 1.55 | Homo sapiens DnaJ (Hsp40) homolog, subfamily B, member 6 (DNAJB6), transcript variant 2, mRNA. |
| KBTBD6 | 1.55 | Homo sapiens kelch repeat and BTB (POZ) domain containing 6 (KBTBD6), mRNA. |
| MGC5139 | 1.55 | PREDICTED: Homo sapiens hypothetical protein MGC5139, transcript variant 2 (MGC5139), mRNA. |
| KCTD5 | 1.55 | Homo sapiens potassium channel tetramerisation domain containing 5 (KCTD5), mRNA. |
| RNF149 | 1.55 | Homo sapiens ring finger protein 149 (RNF149), mRNA. |
| ATP6V0A1 | 1.55 | Homo sapiens ATPase, H+ transporting, lysosomal V0 subunit a1 (ATP6V0A1), transcript variant 3, mRNA. |
| DMAP1 | 1.55 | Homo sapiens DNA methyltransferase 1 associated protein 1 (DMAP1), transcript variant 2, mRNA. |
| PPM1A | 1.55 | Homo sapiens protein phosphatase 1A (formerly 2C), magnesium-dependent, alpha isoform (PPM1A), transcript variant 2, mRNA. |
| TXNDC9 | 1.55 | Homo sapiens thioredoxin domain containing 9 (TXNDC9), mRNA. |
| OGT | 1.55 | Homo sapiens O-linked N-acetylglucosamine (GlcNAc) transferase (UDP-N-acetylglucosamine:polypeptide-N-acetylglucosaminyl transferase) (OGT), transcript variant 1, mRNA. |
| ZNF32 | 1.55 | Homo sapiens zinc finger protein 32 (ZNF32), transcript variant 1, mRNA. |
| STRN3 | 1.55 | Homo sapiens striatin, calmodulin binding protein 3 (STRN3), transcript variant 2, mRNA. |
| C2orf42 | 1.55 | Homo sapiens chromosome 2 open reading frame 42 (C2orf42), mRNA. |
| HIST1H2AC | 1.55 | Homo sapiens histone cluster 1, H2ac (HIST1H2AC), mRNA. |
| CBX3 | 1.55 | Homo sapiens chromobox homolog 3 (HP1 gamma homolog, Drosophila) (CBX3), transcript variant 1, mRNA. |
| 9-Sep | 1.55 | Homo sapiens septin 9 (SEPT9), mRNA. |
| LOC152217 | 1.55 | Homo sapiens hypothetical LOC152217 (LOC152217), non-coding RNA. |
| SERP2 | 1.55 | Homo sapiens stress-associated endoplasmic reticulum protein family member 2 (SERP2), mRNA. |
| LOC390530 | 1.54 | PREDICTED: Homo sapiens similar to Ig heavy chain V-I region HG3 precursor (LOC390530), mRNA. |
| APBB3 | 1.54 | Homo sapiens amyloid beta (A4) precursor protein-binding, family B, member 3 (APBB3), transcript variant 2, mRNA. |
| LOC100131810 | 1.54 | PREDICTED: Homo sapiens misc_RNA (LOC100131810), miscRNA. |
| RPIA | 1.54 | Homo sapiens ribose 5-phosphate isomerase A (RPIA), mRNA. |
| MADD | 1.54 | Homo sapiens MAP-kinase activating death domain (MADD), transcript variant 5, mRNA. |
| UBE2L6 | 1.54 | Homo sapiens ubiquitin-conjugating enzyme E2L 6 (UBE2L6), transcript variant 1, mRNA. |
| LOC391044 | 1.54 | PREDICTED: Homo sapiens misc_RNA (LOC391044), miscRNA. |
| RABGEF1 | 1.54 | Homo sapiens RAB guanine nucleotide exchange factor (GEF) 1 (RABGEF1), mRNA. |
| IDS | 1.54 | Homo sapiens iduronate 2-sulfatase (Hunter syndrome) (IDS), transcript variant 2, mRNA. |
| TIRAP | 1.54 | Homo sapiens toll-interleukin 1 receptor (TIR) domain containing adaptor protein (TIRAP), transcript variant 1, mRNA. |
| AMMECR1L | 1.53 | Homo sapiens AMME chromosomal region gene 1-like (AMMECR1L), mRNA. |
| MFAP3 | 1.53 | Homo sapiens microfibrillar-associated protein 3 (MFAP3), mRNA. |
| ZNF816A | 1.53 | Homo sapiens zinc finger protein 816A (ZNF816A), mRNA. |
| ADAM10 | 1.53 | Homo sapiens ADAM metallopeptidase domain 10 (ADAM10), mRNA. |
| KBTBD7 | 1.53 | Homo sapiens kelch repeat and BTB (POZ) domain containing 7 (KBTBD7), mRNA. |
| FAM18B | 1.53 | Homo sapiens family with sequence similarity 18, member B (FAM18B), mRNA. |
| C3orf23 | 1.53 | Homo sapiens chromosome 3 open reading frame 23 (C3orf23), transcript variant 3, mRNA. |
| LOC642236 | 1.53 | PREDICTED: Homo sapiens similar to FRG1 protein (FSHD region gene 1 protein), transcript variant 8 (LOC642236), mRNA. |
| BRPF3 | 1.53 | Homo sapiens bromodomain and PHD finger containing, 3 (BRPF3), mRNA. |
| MCL1 | 1.53 | Homo sapiens myeloid cell leukemia sequence 1 (BCL2-related) (MCL1), transcript variant 1, mRNA. |
| LOC402251 | 1.52 | PREDICTED: Homo sapiens similar to eukaryotic translation elongation factor 1 alpha 2 (LOC402251), mRNA. |
| FLRT3 | 1.52 | Homo sapiens fibronectin leucine rich transmembrane protein 3 (FLRT3), transcript variant 2, mRNA. |
| CCNY | 1.52 | Homo sapiens cyclin Y (CCNY), transcript variant 1, mRNA. |
| BET1 | 1.52 | Homo sapiens blocked early in transport 1 homolog (S. cerevisiae) (BET1), mRNA. |
| TMEM41B | 1.52 | Homo sapiens transmembrane protein 41B (TMEM41B), mRNA. |
| LOC651380 | 1.52 | PREDICTED: Homo sapiens hypothetical protein LOC651380 (LOC651380), mRNA. |
| PPRC1 | 1.52 | Homo sapiens peroxisome proliferator-activated receptor gamma, coactivator-related 1 (PPRC1), mRNA. |
| ALS2CR4 | 1.52 | Homo sapiens amyotrophic lateral sclerosis 2 (juvenile) chromosome region, candidate 4 (ALS2CR4), transcript variant 1, mRNA. |
| TAX1BP3 | 1.52 | Homo sapiens Tax1 (human T-cell leukemia virus type I) binding protein 3 (TAX1BP3), mRNA. |
| ZNF364 | 1.52 | Homo sapiens zinc finger protein 364 (ZNF364), mRNA. |
| SLC48A1 | 1.52 | Homo sapiens solute carrier family 48 (heme transporter), member 1 (SLC48A1), mRNA. |
| C18orf25 | 1.51 | Homo sapiens chromosome 18 open reading frame 25 (C18orf25), transcript variant 2, mRNA. |
| ZSWIM3 | 1.51 | Homo sapiens zinc finger, SWIM-type containing 3 (ZSWIM3), mRNA. |
| DNAJB11 | 1.51 | Homo sapiens DnaJ (Hsp40) homolog, subfamily B, member 11 (DNAJB11), mRNA. |
| RBM14 | 1.51 | Homo sapiens RNA binding motif protein 14 (RBM14), mRNA. |
| C17orf79 | 1.51 | Homo sapiens chromosome 17 open reading frame 79 (C17orf79), mRNA. |
| DDX47 | 1.51 | Homo sapiens DEAD (Asp-Glu-Ala-Asp) box polypeptide 47 (DDX47), transcript variant 1, mRNA. |
| AK3L1 | 1.51 | Homo sapiens adenylate kinase 3-like 1 (AK3L1), nuclear gene encoding mitochondrial protein, transcript variant 6, mRNA. |
| KIAA1009 | 1.51 | Homo sapiens KIAA1009 (KIAA1009), mRNA. |
| RNF14 | 1.51 | Homo sapiens ring finger protein 14 (RNF14), transcript variant 4, mRNA. |
| CHD1 | 1.51 | Homo sapiens chromodomain helicase DNA binding protein 1 (CHD1), mRNA. |
| MTERF | 1.51 | Homo sapiens mitochondrial transcription termination factor (MTERF), nuclear gene encoding mitochondrial protein, mRNA. |
| GMFB | 1.51 | Homo sapiens glia maturation factor, beta (GMFB), mRNA. |
| C11orf61 | 1.5 | Homo sapiens chromosome 11 open reading frame 61 (C11orf61), mRNA. |
| MGC40489 | 1.5 | PREDICTED: Homo sapiens hypothetical protein MGC40489 (MGC40489), misc RNA. |
| PCNX | 1.5 | Homo sapiens pecanex homolog (Drosophila) (PCNX), mRNA. |
| ARMCX2 | 1.5 | Homo sapiens armadillo repeat containing, X-linked 2 (ARMCX2), mRNA. |
| BOK | 1.5 | Homo sapiens BCL2-related ovarian killer (BOK), mRNA. |
| ZNF80 | 1.5 | Homo sapiens zinc finger protein 80 (ZNF80), mRNA. |
| NUDT21 | 1.5 | Homo sapiens nudix (nucleoside diphosphate linked moiety X)-type motif 21 (NUDT21), mRNA. |
| SEMA4C | 1.5 | Homo sapiens sema domain, immunoglobulin domain (Ig), transmembrane domain (TM) and short cytoplasmic domain, (semaphorin) 4C (SEMA4C), mRNA. |
| CCRK | 1.5 | Homo sapiens cell cycle related kinase (CCRK), transcript variant 2, mRNA. |
| PPM2C | -1.5 | Homo sapiens protein phosphatase 2C, magnesium-dependent, catalytic subunit (PPM2C), nuclear gene encoding mitochondrial protein, mRNA. |
| SRGAP3 | -1.5 | Homo sapiens SLIT-ROBO Rho GTPase activating protein 3 (SRGAP3), transcript variant 2, mRNA. |
| LARP1 | -1.5 | Homo sapiens La ribonucleoprotein domain family, member 1 (LARP1), transcript variant 2, mRNA. |
| PKN1 | -1.5 | Homo sapiens protein kinase N1 (PKN1), transcript variant 1, mRNA. |
| TMEM19 | -1.5 | Homo sapiens transmembrane protein 19 (TMEM19), mRNA. |
| CTNNA2 | -1.51 | Homo sapiens catenin (cadherin-associated protein), alpha 2 (CTNNA2), mRNA. |
| TSHZ1 | -1.51 | Homo sapiens teashirt zinc finger homeobox 1 (TSHZ1), mRNA. |
| MCART1 | -1.51 | Homo sapiens mitochondrial carrier triple repeat 1 (MCART1), mRNA. |
| NOC2L | -1.51 | Homo sapiens nucleolar complex associated 2 homolog (S. cerevisiae) (NOC2L), mRNA. |
| EML5 | -1.51 | Homo sapiens echinoderm microtubule associated protein like 5 (EML5), mRNA. |
| MFSD11 | -1.51 | Homo sapiens major facilitator superfamily domain containing 11 (MFSD11), mRNA. |
| SNHG7 | -1.51 | Homo sapiens small nucleolar RNA host gene 7 (non-protein coding) (SNHG7), transcript variant 2, non-coding RNA. |
| LOC728411 | -1.51 | PREDICTED: Homo sapiens similar to Beta-glucuronidase precursor (LOC728411), mRNA. |
| IGSF9B | -1.51 | Homo sapiens immunoglobulin superfamily, member 9B (IGSF9B), mRNA. |
| RNPS1 | -1.51 | Homo sapiens RNA binding protein S1, serine-rich domain (RNPS1), transcript variant 2, mRNA. |
| TSSC1 | -1.51 | Homo sapiens tumor suppressing subtransferable candidate 1 (TSSC1), mRNA. |
| ARID5B | -1.51 | Homo sapiens AT rich interactive domain 5B (MRF1-like) (ARID5B), mRNA. |
| MLST8 | -1.51 | Homo sapiens MTOR associated protein, LST8 homolog (S. cerevisiae) (MLST8), mRNA. |
| TMEM151A | -1.51 | Homo sapiens transmembrane protein 151A (TMEM151A), mRNA. |
| DIAPH1 | -1.51 | Homo sapiens diaphanous homolog 1 (Drosophila) (DIAPH1), mRNA. |
| SSBP1 | -1.52 | Homo sapiens single-stranded DNA binding protein 1 (SSBP1), mRNA. |
| TTC17 | -1.52 | Homo sapiens tetratricopeptide repeat domain 17 (TTC17), mRNA. |
| GAB2 | -1.52 | Homo sapiens GRB2-associated binding protein 2 (GAB2), transcript variant 1, mRNA. |
| PTMA | -1.52 | Homo sapiens prothymosin, alpha (PTMA), transcript variant 1, mRNA. |
| CEP72 | -1.52 | Homo sapiens centrosomal protein 72kDa (CEP72), mRNA. |
| B3GAT1 | -1.52 | Homo sapiens beta-1,3-glucuronyltransferase 1 (glucuronosyltransferase P) (B3GAT1), transcript variant 1, mRNA. |
| CASC3 | -1.52 | Homo sapiens cancer susceptibility candidate 3 (CASC3), mRNA. |
| PSMG2 | -1.52 | Homo sapiens proteasome (prosome, macropain) assembly chaperone 2 (PSMG2), mRNA. |
| SCG2 | -1.52 | Homo sapiens secretogranin II (chromogranin C) (SCG2), mRNA. |
| PRAME | -1.52 | Homo sapiens preferentially expressed antigen in melanoma (PRAME), transcript variant 4, mRNA. |
| WDR27 | -1.52 | Homo sapiens WD repeat domain 27 (WDR27), mRNA. |
| RBMS1 | -1.52 | Homo sapiens RNA binding motif, single stranded interacting protein 1 (RBMS1), transcript variant 3, mRNA. |
| PSD3 | -1.52 | Homo sapiens pleckstrin and Sec7 domain containing 3 (PSD3), transcript variant 1, mRNA. |
| MYT1 | -1.52 | Homo sapiens myelin transcription factor 1 (MYT1), mRNA. |
| MLL4 | -1.52 | Homo sapiens myeloid/lymphoid or mixed-lineage leukemia 4 (MLL4), mRNA. |
| OIP5 | -1.53 | Homo sapiens Opa interacting protein 5 (OIP5), mRNA. |
| TNFRSF10B | -1.53 | Homo sapiens tumor necrosis factor receptor superfamily, member 10b (TNFRSF10B), transcript variant 1, mRNA. |
| CLN3 | -1.53 | Homo sapiens ceroid-lipofuscinosis, neuronal 3 (CLN3), transcript variant 1, mRNA. |
| TMEM143 | -1.53 | Homo sapiens transmembrane protein 143 (TMEM143), mRNA. |
| DENND5B | -1.53 | Homo sapiens DENN/MADD domain containing 5B (DENND5B), mRNA. |
| LOC100131727 | -1.53 | PREDICTED: Homo sapiens hypothetical LOC100131727 (LOC100131727), mRNA. |
| SORCS1 | -1.53 | Homo sapiens sortilin-related VPS10 domain containing receptor 1 (SORCS1), transcript variant 1, mRNA. |
| FOXD4L1 | -1.53 | Homo sapiens forkhead box D4-like 1 (FOXD4L1), mRNA. |
| ARHGAP23 | -1.53 | PREDICTED: Homo sapiens Rho GTPase activating protein 23, transcript variant 1 (ARHGAP23), mRNA. |
| C20orf29 | -1.53 | Homo sapiens chromosome 20 open reading frame 29 (C20orf29), mRNA. |
| GAL | -1.53 | Homo sapiens galanin prepropeptide (GAL), mRNA. |
| CENPV | -1.53 | Homo sapiens centromere protein V (CENPV), mRNA. |
| LOC727761 | -1.53 | PREDICTED: Homo sapiens similar to Deoxythymidylate kinase (thymidylate kinase), transcript variant 4 (LOC727761), mRNA. |
| CCDC88C | -1.53 | Homo sapiens coiled-coil domain containing 88C (CCDC88C), mRNA. |
| CENPA | -1.53 | Homo sapiens centromere protein A (CENPA), transcript variant 2, mRNA. |
| LOC729978 | -1.53 | PREDICTED: Homo sapiens similar to LOC339047 protein, transcript variant 2 (LOC729978), mRNA. |
| SCARF2 | -1.54 | Homo sapiens scavenger receptor class F, member 2 (SCARF2), transcript variant 1, mRNA. |
| ATG10 | -1.54 | Homo sapiens ATG10 autophagy related 10 homolog (S. cerevisiae) (ATG10), mRNA. |
| P4HB | -1.54 | Homo sapiens prolyl 4-hydroxylase, beta polypeptide (P4HB), mRNA. |
| RBBP7 | -1.54 | Homo sapiens retinoblastoma binding protein 7 (RBBP7), mRNA. |
| ABCB1 | -1.54 | Homo sapiens ATP-binding cassette, sub-family B (MDR/TAP), member 1 (ABCB1), mRNA. |
| LOC100132740 | -1.54 | PREDICTED: Homo sapiens hypothetical protein LOC100132740 (LOC100132740), mRNA. |
| PNMAL1 | -1.54 | Homo sapiens PNMA-like 1 (PNMAL1), transcript variant 1, mRNA. |
| USP22 | -1.54 | Homo sapiens ubiquitin specific peptidase 22 (USP22), mRNA. |
| SUSD1 | -1.54 | Homo sapiens sushi domain containing 1 (SUSD1), mRNA. |
| ZNF219 | -1.54 | Homo sapiens zinc finger protein 219 (ZNF219), mRNA. |
| FASTK | -1.54 | Homo sapiens Fas-activated serine/threonine kinase (FASTK), transcript variant 1, mRNA. |
| LOC644928 | -1.55 | Homo sapiens hCG2033311 (LOC644928), mRNA. |
| LOC400061 | -1.55 | PREDICTED: Homo sapiens misc_RNA (LOC400061), miscRNA. |
| EXT1 | -1.55 | Homo sapiens exostoses (multiple) 1 (EXT1), mRNA. |
| NPEPPS | -1.55 | Homo sapiens aminopeptidase puromycin sensitive (NPEPPS), mRNA. |
| ANLN | -1.55 | Homo sapiens anillin, actin binding protein (ANLN), mRNA. |
| RPGR | -1.55 | Homo sapiens retinitis pigmentosa GTPase regulator (RPGR), transcript variant B, mRNA. |
| LOC440704 | -1.55 | PREDICTED: Homo sapiens hypothetical gene supported by BC042042 (LOC440704), mRNA. |
| CPT2 | -1.55 | Homo sapiens carnitine palmitoyltransferase II (CPT2), nuclear gene encoding mitochondrial protein, mRNA. |
| DHCR24 | -1.55 | Homo sapiens 24-dehydrocholesterol reductase (DHCR24), mRNA. |
| FRMD6 | -1.55 | Homo sapiens FERM domain containing 6 (FRMD6), mRNA. |
| DNAJB6 | -1.55 | Homo sapiens DnaJ (Hsp40) homolog, subfamily B, member 6 (DNAJB6), transcript variant 1, mRNA. |
| SNORA62 | -1.55 | Homo sapiens small nucleolar RNA, H/ACA box 62 (SNORA62), small nucleolar RNA. |
| ELAVL4 | -1.55 | Homo sapiens ELAV (embryonic lethal, abnormal vision, Drosophila)-like 4 (Hu antigen D) (ELAVL4), mRNA. |
| NEURL1B | -1.55 | Homo sapiens neuralized homolog 1B (Drosophila) (NEURL1B), mRNA. |
| CD99 | -1.55 | Homo sapiens CD99 molecule (CD99), transcript variant 1, mRNA. |
| TAGLN | -1.55 | Homo sapiens transgelin (TAGLN), transcript variant 2, mRNA. |
| SNHG9 | -1.55 | Homo sapiens small nucleolar RNA host gene 9 (non-protein coding) (SNHG9), non-coding RNA. |
| CHD4 | -1.55 | Homo sapiens chromodomain helicase DNA binding protein 4 (CHD4), mRNA. |
| FLJ22536 | -1.56 | Homo sapiens hypothetical locus LOC401237 (FLJ22536), non-coding RNA. |
| NPAL3 | -1.56 | Homo sapiens NIPA-like domain containing 3 (NPAL3), mRNA. |
| LOC729009 | -1.56 | PREDICTED: Homo sapiens misc_RNA (LOC729009), miscRNA. |
| FAM156B | -1.56 | Homo sapiens family with sequence similarity 156, member B (FAM156B), mRNA. |
| NPLOC4 | -1.56 | Homo sapiens nuclear protein localization 4 homolog (S. cerevisiae) (NPLOC4), mRNA. |
| SLC2A10 | -1.56 | Homo sapiens solute carrier family 2 (facilitated glucose transporter), member 10 (SLC2A10), mRNA. |
| FLJ12949 | -1.56 | Homo sapiens hypothetical protein FLJ12949 (FLJ12949), transcript variant 1, mRNA. |
| ZNF609 | -1.56 | Homo sapiens zinc finger protein 609 (ZNF609), mRNA. |
| VIM | -1.56 | Homo sapiens vimentin (VIM), mRNA. |
| NFE2L1 | -1.56 | Homo sapiens nuclear factor (erythroid-derived 2)-like 1 (NFE2L1), mRNA. |
| XPC | -1.56 | Homo sapiens xeroderma pigmentosum, complementation group C (XPC), mRNA. |
| PPFIBP1 | -1.56 | Homo sapiens PTPRF interacting protein, binding protein 1 (liprin beta 1) (PPFIBP1), transcript variant 1, mRNA. |
| PVRL3 | -1.56 | Homo sapiens poliovirus receptor-related 3 (PVRL3), mRNA. |
| CYFIP1 | -1.56 | Homo sapiens cytoplasmic FMR1 interacting protein 1 (CYFIP1), transcript variant 2, mRNA. |
| TRAFD1 | -1.56 | Homo sapiens TRAF-type zinc finger domain containing 1 (TRAFD1), mRNA. |
| CUL7 | -1.56 | Homo sapiens cullin 7 (CUL7), mRNA. |
| USP13 | -1.57 | Homo sapiens ubiquitin specific peptidase 13 (isopeptidase T-3) (USP13), mRNA. |
| SFRS13A | -1.57 | Homo sapiens splicing factor, arginine/serine-rich 13A (SFRS13A), transcript variant 2, mRNA. |
| CUTL1 | -1.57 | Homo sapiens cut-like 1, CCAAT displacement protein (Drosophila) (CUTL1), transcript variant 1, mRNA. |
| PHF21B | -1.57 | Homo sapiens PHD finger protein 21B (PHF21B), mRNA. |
| PI4KAP2 | -1.57 | Homo sapiens phosphatidylinositol 4-kinase, catalytic, alpha polypeptide pseudogene 2 (PI4KAP2), mRNA. |
| LEF1 | -1.57 | Homo sapiens lymphoid enhancer-binding factor 1 (LEF1), mRNA. |
| NQO2 | -1.57 | Homo sapiens NAD(P)H dehydrogenase, quinone 2 (NQO2), mRNA. |
| NUP214 | -1.57 | Homo sapiens nucleoporin 214kDa (NUP214), mRNA. |
| STAT5B | -1.57 | Homo sapiens signal transducer and activator of transcription 5B (STAT5B), mRNA. |
| LTBP2 | -1.57 | Homo sapiens latent transforming growth factor beta binding protein 2 (LTBP2), mRNA. |
| FOXN4 | -1.57 | Homo sapiens forkhead box N4 (FOXN4), mRNA. |
| YPEL1 | -1.57 | Homo sapiens yippee-like 1 (Drosophila) (YPEL1), mRNA. |
| LOC643995 | -1.57 | PREDICTED: Homo sapiens similar to Importin alpha-2 subunit (Karyopherin alpha-2 subunit) (SRP1-alpha) (RAG cohort protein 1), transcript variant 2 (LOC643995), mRNA. |
| TRIM26 | -1.58 | Homo sapiens tripartite motif-containing 26 (TRIM26), mRNA. |
| TSGA14 | -1.58 | Homo sapiens testis specific, 14 (TSGA14), mRNA. |
| NLGN4X | -1.58 | Homo sapiens neuroligin 4, X-linked (NLGN4X), transcript variant 2, mRNA. |
| TMEM18 | -1.58 | Homo sapiens transmembrane protein 18 (TMEM18), mRNA. |
| KHDRBS3 | -1.58 | Homo sapiens KH domain containing, RNA binding, signal transduction associated 3 (KHDRBS3), mRNA. |
| NCAN | -1.58 | Homo sapiens neurocan (NCAN), mRNA. |
| NR2F1 | -1.58 | Homo sapiens nuclear receptor subfamily 2, group F, member 1 (NR2F1), mRNA. |
| TMBIM6 | -1.58 | Homo sapiens transmembrane BAX inhibitor motif containing 6 (TMBIM6), transcript variant 1, mRNA. |
| DKFZp686O24166 | -1.58 | Homo sapiens hypothetical protein DKFZp686O24166 (DKFZp686O24166), mRNA. |
| LOC345041 | -1.58 | PREDICTED: Homo sapiens misc_RNA (LOC345041), miscRNA. |
| PPP1R10 | -1.58 | Homo sapiens protein phosphatase 1, regulatory (inhibitor) subunit 10 (PPP1R10), mRNA. |
| C15orf42 | -1.58 | Homo sapiens chromosome 15 open reading frame 42 (C15orf42), mRNA. |
| CCDC125 | -1.58 | Homo sapiens coiled-coil domain containing 125 (CCDC125), mRNA. |
| LOC100134241 | -1.58 | PREDICTED: Homo sapiens hypothetical protein LOC100134241 (LOC100134241), mRNA. |
| CDC42SE1 | -1.58 | Homo sapiens CDC42 small effector 1 (CDC42SE1), transcript variant 1, mRNA. |
| BRD8 | -1.58 | Homo sapiens bromodomain containing 8 (BRD8), transcript variant 3, mRNA. |
| TOR1A | -1.58 | Homo sapiens torsin family 1, member A (torsin A) (TOR1A), mRNA. |
| BVES | -1.59 | Homo sapiens blood vessel epicardial substance (BVES), transcript variant 5, mRNA. |
| SPAG5 | -1.59 | Homo sapiens sperm associated antigen 5 (SPAG5), mRNA. |
| RGS4 | -1.59 | Homo sapiens regulator of G-protein signalling 4 (RGS4), mRNA. |
| LOC729646 | -1.59 | PREDICTED: Homo sapiens misc_RNA (LOC729646), miscRNA. |
| FAM184B | -1.59 | Homo sapiens family with sequence similarity 184, member B (FAM184B), mRNA. |
| CHPT1 | -1.59 | Homo sapiens choline phosphotransferase 1 (CHPT1), mRNA. |
| SNAP25 | -1.59 | Homo sapiens synaptosomal-associated protein, 25kDa (SNAP25), transcript variant 2, mRNA. |
| TMEM151A | -1.59 | Homo sapiens transmembrane protein 151A (TMEM151A), mRNA. |
| NAV1 | -1.59 | Homo sapiens neuron navigator 1 (NAV1), mRNA. |
| ITM2B | -1.6 | Homo sapiens integral membrane protein 2B (ITM2B), mRNA. |
| SORD | -1.6 | Homo sapiens sorbitol dehydrogenase (SORD), mRNA. |
| GPX4 | -1.6 | Homo sapiens glutathione peroxidase 4 (phospholipid hydroperoxidase) (GPX4), transcript variant 2, mRNA. |
| WBP11 | -1.6 | Homo sapiens WW domain binding protein 11 (WBP11), mRNA. |
| DYNC1H1 | -1.6 | Homo sapiens dynein, cytoplasmic 1, heavy chain 1 (DYNC1H1), mRNA. |
| LOC732360 | -1.6 | PREDICTED: Homo sapiens misc_RNA (LOC732360), miscRNA. |
| CKAP4 | -1.6 | Homo sapiens cytoskeleton-associated protein 4 (CKAP4), mRNA. |
| ING2 | -1.6 | Homo sapiens inhibitor of growth family, member 2 (ING2), mRNA. |
| MGC71993 | -1.6 | Homo sapiens similar to DNA segment, Chr 11, Brigham & Womens Genetics 0434 expressed (MGC71993), mRNA. |
| DUSP5 | -1.6 | Homo sapiens dual specificity phosphatase 5 (DUSP5), mRNA. |
| POFUT2 | -1.61 | Homo sapiens protein O-fucosyltransferase 2 (POFUT2), transcript variant 3, mRNA. |
|  | -1.61 | Homo sapiens cDNA FLJ41853 fis, clone NT2RI3004161 |
| ASTN2 | -1.61 | Homo sapiens astrotactin 2 (ASTN2), transcript variant 2, mRNA. |
| IFT172 | -1.61 | Homo sapiens intraflagellar transport 172 homolog (Chlamydomonas) (IFT172), mRNA. |
| MIR25 | -1.61 | Homo sapiens microRNA 25 (MIR25), microRNA. |
| GPR108 | -1.61 | Homo sapiens G protein-coupled receptor 108 (GPR108), transcript variant 1, mRNA. |
| ERICH1 | -1.61 | Homo sapiens glutamate-rich 1 (ERICH1), mRNA. |
| LSM12 | -1.61 | Homo sapiens LSM12 homolog (S. cerevisiae) (LSM12), mRNA. |
| NUDT14 | -1.61 | Homo sapiens nudix (nucleoside diphosphate linked moiety X)-type motif 14 (NUDT14), mRNA. |
| C19orf50 | -1.61 | Homo sapiens chromosome 19 open reading frame 50 (C19orf50), mRNA. |
| ANKRD37 | -1.61 | Homo sapiens ankyrin repeat domain 37 (ANKRD37), mRNA. |
| CES2 | -1.61 | Homo sapiens carboxylesterase 2 (intestine, liver) (CES2), transcript variant 1, mRNA. |
| LOC646044 | -1.62 | PREDICTED: Homo sapiens misc_RNA (LOC646044), miscRNA. |
| ZCCHC24 | -1.62 | Homo sapiens zinc finger, CCHC domain containing 24 (ZCCHC24), mRNA. |
| DUXAP3 | -1.62 | Homo sapiens double homeobox A pseudogene 3 (DUXAP3) on chromosome 10. |
| LOC100170939 | -1.62 | Homo sapiens glucuronidase, beta pseudogene (LOC100170939), non-coding RNA. |
| LOC613037 | -1.62 | Homo sapiens nuclear pore complex interacting protein pseudogene (LOC613037), non-coding RNA. |
| TSPAN7 | -1.62 | Homo sapiens tetraspanin 7 (TSPAN7), mRNA. |
| CDK5RAP2 | -1.62 | Homo sapiens CDK5 regulatory subunit associated protein 2 (CDK5RAP2), transcript variant 2, mRNA. |
| FAM72D | -1.62 | Homo sapiens family with sequence similarity 72, member D (FAM72D), mRNA. |
| LOC647597 | -1.62 | PREDICTED: Homo sapiens hypothetical LOC647597 (LOC647597), mRNA. |
| SEMA4D | -1.62 | Homo sapiens sema domain, immunoglobulin domain (Ig), transmembrane domain (TM) and short cytoplasmic domain, (semaphorin) 4D (SEMA4D), mRNA. |
| PILRB | -1.62 | Homo sapiens paired immunoglobin-like type 2 receptor beta (PILRB), transcript variant 1, mRNA. |
| UVRAG | -1.62 | Homo sapiens UV radiation resistance associated gene (UVRAG), mRNA. |
| ZNF775 | -1.62 | Homo sapiens zinc finger protein 775 (ZNF775), mRNA. |
| LOC729021 | -1.63 | PREDICTED: Homo sapiens hypothetical protein LOC729021 (LOC729021), mRNA. |
| FAM100B | -1.63 | Homo sapiens family with sequence similarity 100, member B (FAM100B), mRNA. |
| MAP3K7IP1 | -1.63 | Homo sapiens mitogen-activated protein kinase kinase kinase 7 interacting protein 1 (MAP3K7IP1), transcript variant alpha, mRNA. |
| LOC653583 | -1.63 | PREDICTED: Homo sapiens similar to pleckstrin homology-like domain, family B, member 1 (LOC653583), mRNA. |
| ADD1 | -1.63 | Homo sapiens adducin 1 (alpha) (ADD1), transcript variant 1, mRNA. |
| SETBP1 | -1.63 | Homo sapiens SET binding protein 1 (SETBP1), mRNA. |
| DYRK2 | -1.63 | Homo sapiens dual-specificity tyrosine-(Y)-phosphorylation regulated kinase 2 (DYRK2), transcript variant 1, mRNA. |
| UBR4 | -1.63 | Homo sapiens ubiquitin protein ligase E3 component n-recognin 4 (UBR4), mRNA. |
| NBEA | -1.63 | Homo sapiens neurobeachin (NBEA), mRNA. |
| MYRIP | -1.63 | Homo sapiens myosin VIIA and Rab interacting protein (MYRIP), mRNA. |
| C1GALT1C1 | -1.63 | Homo sapiens C1GALT1-specific chaperone 1 (C1GALT1C1), transcript variant 1, mRNA. |
| DGKD | -1.63 | Homo sapiens diacylglycerol kinase, delta 130kDa (DGKD), transcript variant 1, mRNA. |
| RPL26L1 | -1.64 | Homo sapiens ribosomal protein L26-like 1 (RPL26L1), mRNA. |
| MGP | -1.64 | Homo sapiens matrix Gla protein (MGP), mRNA. |
| C9orf16 | -1.64 | Homo sapiens chromosome 9 open reading frame 16 (C9orf16), mRNA. |
| WDYHV1 | -1.64 | Homo sapiens WDYHV motif containing 1 (WDYHV1), mRNA. |
| PTPN12 | -1.64 | Homo sapiens protein tyrosine phosphatase, non-receptor type 12 (PTPN12), mRNA. |
| FOXM1 | -1.64 | Homo sapiens forkhead box M1 (FOXM1), transcript variant 3, mRNA. |
| MCF2L | -1.64 | Homo sapiens MCF.2 cell line derived transforming sequence-like (MCF2L), transcript variant 1, mRNA. |
| C7orf36 | -1.64 | Homo sapiens chromosome 7 open reading frame 36 (C7orf36), mRNA. |
| DMC1 | -1.64 | Homo sapiens DMC1 dosage suppressor of mck1 homolog, meiosis-specific homologous recombination (yeast) (DMC1), mRNA. |
| SMARCD3 | -1.64 | Homo sapiens SWI/SNF related, matrix associated, actin dependent regulator of chromatin, subfamily d, member 3 (SMARCD3), transcript variant 1, mRNA. |
| YTHDF1 | -1.65 | Homo sapiens YTH domain family, member 1 (YTHDF1), mRNA. |
| TIMP2 | -1.65 | Homo sapiens TIMP metallopeptidase inhibitor 2 (TIMP2), mRNA. |
| THOP1 | -1.65 | Homo sapiens thimet oligopeptidase 1 (THOP1), mRNA. |
| MRPL55 | -1.65 | Homo sapiens mitochondrial ribosomal protein L55 (MRPL55), nuclear gene encoding mitochondrial protein, transcript variant 5, mRNA. |
|  | -1.65 | BX356403 Homo sapiens PLACENTA COT 25-NORMALIZED Homo sapiens cDNA clone CS0DI011YD16 5-PRIME, mRNA sequence |
| CORO1C | -1.65 | Homo sapiens coronin, actin binding protein, 1C (CORO1C), transcript variant 1, mRNA. |
| CRY2 | -1.65 | Homo sapiens cryptochrome 2 (photolyase-like) (CRY2), mRNA. |
| FAM116A | -1.65 | PREDICTED: Homo sapiens family with sequence similarity 116, member A (FAM116A), mRNA. |
| CALY | -1.65 | Homo sapiens calcyon neuron-specific vesicular protein (CALY), mRNA. |
| TRPC4AP | -1.65 | Homo sapiens transient receptor potential cation channel, subfamily C, member 4 associated protein (TRPC4AP), transcript variant 1, mRNA. |
| SMC1A | -1.65 | Homo sapiens structural maintenance of chromosomes 1A (SMC1A), mRNA. |
| ITM2A | -1.65 | Homo sapiens integral membrane protein 2A (ITM2A), mRNA. |
| PNKP | -1.65 | Homo sapiens polynucleotide kinase 3'-phosphatase (PNKP), mRNA. |
| AURKB | -1.65 | Homo sapiens aurora kinase B (AURKB), mRNA. |
| WDR82 | -1.65 | Homo sapiens WD repeat domain 82 (WDR82), mRNA. |
| AKT3 | -1.65 | Homo sapiens v-akt murine thymoma viral oncogene homolog 3 (protein kinase B, gamma) (AKT3), transcript variant 2, mRNA. |
| GTF3C5 | -1.65 | Homo sapiens general transcription factor IIIC, polypeptide 5, 63kDa (GTF3C5), mRNA. |
| OLFM1 | -1.66 | Homo sapiens olfactomedin 1 (OLFM1), transcript variant 2, mRNA. |
| EIF1AX | -1.66 | Homo sapiens eukaryotic translation initiation factor 1A, X-linked (EIF1AX), mRNA. |
| SEZ6 | -1.66 | Homo sapiens seizure related 6 homolog (mouse) (SEZ6), transcript variant 2, mRNA. |
| LOC389765 | -1.66 | PREDICTED: Homo sapiens similar to KIF27C (LOC389765), mRNA. |
| TSTD1 | -1.66 | Homo sapiens thiosulfate sulfurtransferase (rhodanese)-like domain containing 1 (TSTD1), transcript variant 2, mRNA. |
| CEP350 | -1.66 | Homo sapiens centrosomal protein 350kDa (CEP350), mRNA. |
| PLCXD1 | -1.66 | Homo sapiens phosphatidylinositol-specific phospholipase C, X domain containing 1 (PLCXD1), mRNA. |
| ESYT1 | -1.66 | Homo sapiens extended synaptotagmin-like protein 1 (ESYT1), mRNA. |
| BCKDHB | -1.66 | Homo sapiens branched chain keto acid dehydrogenase E1, beta polypeptide (maple syrup urine disease) (BCKDHB), nuclear gene encoding mitochondrial protein, transcript variant 1, mRNA. |
| FAM107B | -1.66 | Homo sapiens family with sequence similarity 107, member B (FAM107B), mRNA. |
| C17orf49 | -1.66 | Homo sapiens chromosome 17 open reading frame 49 (C17orf49), mRNA. |
| LOC100131139 | -1.66 | PREDICTED: Homo sapiens misc_RNA (LOC100131139), miscRNA. |
| PTGFRN | -1.66 | Homo sapiens prostaglandin F2 receptor negative regulator (PTGFRN), mRNA. |
| ATXN7L2 | -1.66 | Homo sapiens ataxin 7-like 2 (ATXN7L2), mRNA. |
| DDX28 | -1.67 | Homo sapiens DEAD (Asp-Glu-Ala-Asp) box polypeptide 28 (DDX28), nuclear gene encoding mitochondrial protein, mRNA. |
|  | -1.67 | Homo sapiens cDNA: FLJ21333 fis, clone COL02535 |
| GRIA2 | -1.67 | Homo sapiens glutamate receptor, ionotropic, AMPA 2 (GRIA2), mRNA. |
| LOC643778 | -1.67 | PREDICTED: Homo sapiens misc_RNA (LOC643778), miscRNA. |
| SCML1 | -1.67 | Homo sapiens sex comb on midleg-like 1 (Drosophila) (SCML1), transcript variant 1, mRNA. |
| PPM1K | -1.67 | Homo sapiens protein phosphatase 1K (PP2C domain containing) (PPM1K), mRNA. |
| IGSF3 | -1.67 | Homo sapiens immunoglobulin superfamily, member 3 (IGSF3), transcript variant 1, mRNA. |
| RSRC1 | -1.67 | Homo sapiens arginine/serine-rich coiled-coil 1 (RSRC1), mRNA. |
| MAPKAP1 | -1.67 | Homo sapiens mitogen-activated protein kinase associated protein 1 (MAPKAP1), transcript variant 1, mRNA. |
| CBX4 | -1.67 | Homo sapiens chromobox homolog 4 (Pc class homolog, Drosophila) (CBX4), mRNA. |
| ATP7B | -1.67 | Homo sapiens ATPase, Cu++ transporting, beta polypeptide (ATP7B), transcript variant 1, mRNA. |
| BRD8 | -1.67 | Homo sapiens bromodomain containing 8 (BRD8), transcript variant 3, mRNA. |
| RG9MTD1 | -1.67 | Homo sapiens RNA (guanine-9-) methyltransferase domain containing 1 (RG9MTD1), mRNA. |
| C5orf41 | -1.67 | Homo sapiens chromosome 5 open reading frame 41 (C5orf41), mRNA. |
| MXRA7 | -1.68 | Homo sapiens matrix-remodelling associated 7 (MXRA7), transcript variant 2, mRNA. |
| TPM4 | -1.68 | Homo sapiens tropomyosin 4 (TPM4), mRNA. |
| GORASP1 | -1.68 | Homo sapiens golgi reassembly stacking protein 1, 65kDa (GORASP1), mRNA. |
| MESDC1 | -1.68 | Homo sapiens mesoderm development candidate 1 (MESDC1), mRNA. |
| SYDE1 | -1.68 | Homo sapiens synapse defective 1, Rho GTPase, homolog 1 (C. elegans) (SYDE1), mRNA. |
| FLJ20464 | -1.68 | PREDICTED: Homo sapiens hypothetical protein FLJ20464 (FLJ20464), mRNA. |
| C19orf6 | -1.68 | Homo sapiens chromosome 19 open reading frame 6 (C19orf6), transcript variant 1, mRNA. |
| UBXN6 | -1.68 | Homo sapiens UBX domain protein 6 (UBXN6), mRNA. |
| TRAM1L1 | -1.69 | Homo sapiens translocation associated membrane protein 1-like 1 (TRAM1L1), mRNA. |
| TDG | -1.69 | Homo sapiens thymine-DNA glycosylase (TDG), mRNA. |
| PARP2 | -1.69 | Homo sapiens poly (ADP-ribose) polymerase family, member 2 (PARP2), mRNA. |
| 11-Mar | -1.69 | Homo sapiens membrane-associated ring finger (C3HC4) 11 (MARCH11), mRNA. |
| COL1A1 | -1.69 | Homo sapiens collagen, type I, alpha 1 (COL1A1), mRNA. |
| ORC6L | -1.69 | Homo sapiens origin recognition complex, subunit 6 like (yeast) (ORC6L), mRNA. |
| SLC8A3 | -1.69 | Homo sapiens solute carrier family 8 (sodium/calcium exchanger), member 3 (SLC8A3), transcript variant f, mRNA. |
| LOC730004 | -1.69 | PREDICTED: Homo sapiens misc_RNA (LOC730004), miscRNA. |
| SRC | -1.69 | Homo sapiens v-src sarcoma (Schmidt-Ruppin A-2) viral oncogene homolog (avian) (SRC), transcript variant 2, mRNA. |
| IQGAP1 | -1.69 | Homo sapiens IQ motif containing GTPase activating protein 1 (IQGAP1), mRNA. |
| LOC731049 | -1.69 | PREDICTED: Homo sapiens similar to Ubiquitin-conjugating enzyme E2S (Ubiquitin-conjugating enzyme E2-24 kDa) (Ubiquitin-protein ligase) (Ubiquitin carrier protein) (E2-EPF5) (LOC731049), mRNA. |
| ZNF395 | -1.69 | Homo sapiens zinc finger protein 395 (ZNF395), mRNA. |
| NPHP4 | -1.69 | Homo sapiens nephronophthisis 4 (NPHP4), mRNA. |
| IRF3 | -1.7 | Homo sapiens interferon regulatory factor 3 (IRF3), mRNA. |
| SLC5A6 | -1.7 | Homo sapiens solute carrier family 5 (sodium-dependent vitamin transporter), member 6 (SLC5A6), mRNA. |
| CAMK2B | -1.7 | Homo sapiens calcium/calmodulin-dependent protein kinase (CaM kinase) II beta (CAMK2B), transcript variant 4, mRNA. |
| LGMN | -1.7 | Homo sapiens legumain (LGMN), transcript variant 2, mRNA. |
| PCDHB6 | -1.7 | Homo sapiens protocadherin beta 6 (PCDHB6), mRNA. |
|  | -1.7 | Homo sapiens mRNA; cDNA DKFZp686F09166 (from clone DKFZp686F09166) |
| PPAP2C | -1.7 | Homo sapiens phosphatidic acid phosphatase type 2C (PPAP2C), transcript variant 2, mRNA. |
| SNRNP27 | -1.7 | Homo sapiens small nuclear ribonucleoprotein 27kDa (U4/U6.U5) (SNRNP27), mRNA. |
| DUSP10 | -1.7 | Homo sapiens dual specificity phosphatase 10 (DUSP10), transcript variant 3, mRNA. |
| CXCR4 | -1.7 | Homo sapiens chemokine (C-X-C motif) receptor 4 (CXCR4), transcript variant 1, mRNA. |
| CBARA1 | -1.7 | Homo sapiens calcium binding atopy-related autoantigen 1 (CBARA1), mRNA. |
| CKB | -1.7 | Homo sapiens creatine kinase, brain (CKB), mRNA. |
| LOC645937 | -1.7 | PREDICTED: Homo sapiens similar to zinc finger protein 598 (LOC645937), mRNA. |
| TYMS | -1.7 | Homo sapiens thymidylate synthetase (TYMS), mRNA. |
| SORBS3 | -1.7 | Homo sapiens sorbin and SH3 domain containing 3 (SORBS3), transcript variant 1, mRNA. |
| TSPO | -1.71 | Homo sapiens translocator protein (18kDa) (TSPO), transcript variant PBR, mRNA. |
| LPHN2 | -1.71 | Homo sapiens latrophilin 2 (LPHN2), mRNA. |
| CCDC74B | -1.71 | Homo sapiens coiled-coil domain containing 74B (CCDC74B), mRNA. |
| AP2S1 | -1.71 | Homo sapiens adaptor-related protein complex 2, sigma 1 subunit (AP2S1), transcript variant AP17, mRNA. |
| LOC143543 | -1.71 | Homo sapiens RNA binding motif protein, X-linked pseudogene (LOC143543) on chromosome 11. |
| RBMX2 | -1.71 | Homo sapiens RNA binding motif protein, X-linked 2 (RBMX2), mRNA. |
| ADAM15 | -1.71 | Homo sapiens ADAM metallopeptidase domain 15 (ADAM15), transcript variant 4, mRNA. |
| EFTUD2 | -1.71 | Homo sapiens elongation factor Tu GTP binding domain containing 2 (EFTUD2), mRNA. |
| RERE | -1.71 | Homo sapiens arginine-glutamic acid dipeptide (RE) repeats (RERE), transcript variant 3, mRNA. |
| GPAM | -1.71 | Homo sapiens glycerol-3-phosphate acyltransferase, mitochondrial (GPAM), nuclear gene encoding mitochondrial protein, mRNA. |
| DYRK2 | -1.71 | Homo sapiens dual-specificity tyrosine-(Y)-phosphorylation regulated kinase 2 (DYRK2), transcript variant 1, mRNA. |
| ATP2B4 | -1.72 | Homo sapiens ATPase, Ca++ transporting, plasma membrane 4 (ATP2B4), transcript variant 1, mRNA. |
| PDPR | -1.72 | PREDICTED: Homo sapiens pyruvate dehydrogenase phosphatase regulatory subunit (PDPR), mRNA. |
| SLC6A10P | -1.72 | Homo sapiens solute carrier family 6 (neurotransmitter transporter, creatine), member 10 (pseudogene) (SLC6A10P) on chromosome 16. |
| FAT1 | -1.72 | Homo sapiens FAT tumor suppressor homolog 1 (Drosophila) (FAT1), mRNA. |
| SHMT2 | -1.72 | Homo sapiens serine hydroxymethyltransferase 2 (mitochondrial) (SHMT2), nuclear gene encoding mitochondrial protein, mRNA. |
| DUSP22 | -1.72 | Homo sapiens dual specificity phosphatase 22 (DUSP22), mRNA. |
| ZNF618 | -1.72 | Homo sapiens zinc finger protein 618 (ZNF618), mRNA. |
| FAM64A | -1.72 | Homo sapiens family with sequence similarity 64, member A (FAM64A), mRNA. |
| LOC127295 | -1.72 | PREDICTED: Homo sapiens similar to 60S ribosomal protein L36 (LOC127295), mRNA. |
| HSD17B4 | -1.72 | Homo sapiens hydroxysteroid (17-beta) dehydrogenase 4 (HSD17B4), mRNA. |
| PTDSS2 | -1.72 | Homo sapiens phosphatidylserine synthase 2 (PTDSS2), mRNA. |
| IMMT | -1.72 | Homo sapiens inner membrane protein, mitochondrial (mitofilin) (IMMT), nuclear gene encoding mitochondrial protein, transcript variant 2, mRNA. |
| B3GAT3 | -1.73 | Homo sapiens beta-1,3-glucuronyltransferase 3 (glucuronosyltransferase I) (B3GAT3), mRNA. |
| ENC1 | -1.73 | Homo sapiens ectodermal-neural cortex (with BTB-like domain) (ENC1), mRNA. |
| NLGN4X | -1.73 | Homo sapiens neuroligin 4, X-linked (NLGN4X), transcript variant 1, mRNA. |
| DEGS1 | -1.73 | Homo sapiens degenerative spermatocyte homolog 1, lipid desaturase (Drosophila) (DEGS1), transcript variant 1, mRNA. |
| SPARC | -1.73 | Homo sapiens secreted protein, acidic, cysteine-rich (osteonectin) (SPARC), mRNA. |
| TCTEX1D2 | -1.73 | Homo sapiens Tctex1 domain containing 2 (TCTEX1D2), mRNA. |
| S100A4 | -1.73 | Homo sapiens S100 calcium binding protein A4 (S100A4), transcript variant 2, mRNA. |
| NDC80 | -1.73 | Homo sapiens NDC80 homolog, kinetochore complex component (S. cerevisiae) (NDC80), mRNA. |
| CREB5 | -1.73 | Homo sapiens cAMP responsive element binding protein 5 (CREB5), transcript variant 1, mRNA. |
| HAGHL | -1.73 | Homo sapiens hydroxyacylglutathione hydrolase-like (HAGHL), transcript variant 1, mRNA. |
| RNF44 | -1.73 | Homo sapiens ring finger protein 44 (RNF44), mRNA. |
| ADAM22 | -1.73 | Homo sapiens ADAM metallopeptidase domain 22 (ADAM22), transcript variant 1, mRNA. |
| MAP2K4 | -1.73 | Homo sapiens mitogen-activated protein kinase kinase 4 (MAP2K4), mRNA. |
| PRPF31 | -1.73 | Homo sapiens PRP31 pre-mRNA processing factor 31 homolog (S. cerevisiae) (PRPF31), mRNA. |
| SFRS16 | -1.74 | Homo sapiens splicing factor, arginine/serine-rich 16 (SFRS16), mRNA. |
| NRP1 | -1.74 | Homo sapiens neuropilin 1 (NRP1), transcript variant 1, mRNA. |
| MMP11 | -1.74 | Homo sapiens matrix metallopeptidase 11 (stromelysin 3) (MMP11), mRNA. |
| ZNF502 | -1.74 | Homo sapiens zinc finger protein 502 (ZNF502), mRNA. |
| FKBP14 | -1.74 | Homo sapiens FK506 binding protein 14, 22 kDa (FKBP14), mRNA. |
| TMEM134 | -1.74 | Homo sapiens transmembrane protein 134 (TMEM134), transcript variant 2, mRNA. |
| LMTK3 | -1.74 | PREDICTED: Homo sapiens lemur tyrosine kinase 3 (LMTK3), mRNA. |
| LOC645381 | -1.74 | PREDICTED: Homo sapiens misc_RNA (LOC645381), miscRNA. |
| ICT1 | -1.74 | Homo sapiens immature colon carcinoma transcript 1 (ICT1), mRNA. |
| USP11 | -1.74 | Homo sapiens ubiquitin specific peptidase 11 (USP11), mRNA. |
| SLC36A4 | -1.74 | Homo sapiens solute carrier family 36 (proton/amino acid symporter), member 4 (SLC36A4), mRNA. |
| ZNF586 | -1.74 | Homo sapiens zinc finger protein 586 (ZNF586), mRNA. |
| MDK | -1.75 | Homo sapiens midkine (neurite growth-promoting factor 2) (MDK), transcript variant 2, mRNA. |
| LOC100127975 | -1.75 | PREDICTED: Homo sapiens misc_RNA (LOC100127975), partial miscRNA. |
| SLC25A29 | -1.75 | Homo sapiens solute carrier family 25, member 29 (SLC25A29), nuclear gene encoding mitochondrial protein, mRNA. |
| SLC45A3 | -1.75 | Homo sapiens solute carrier family 45, member 3 (SLC45A3), mRNA. |
| SF3B2 | -1.75 | Homo sapiens splicing factor 3b, subunit 2, 145kDa (SF3B2), mRNA. |
| LOC654260 | -1.75 | PREDICTED: Homo sapiens similar to beta-tubulin cofactor D isoform 1 (LOC654260), mRNA. |
| BRD3 | -1.75 | Homo sapiens bromodomain containing 3 (BRD3), mRNA. |
| C14orf143 | -1.75 | Homo sapiens chromosome 14 open reading frame 143 (C14orf143), mRNA. |
| NR1D2 | -1.75 | PREDICTED: Homo sapiens nuclear receptor subfamily 1, group D, member 2 (NR1D2), mRNA. |
| RBM20 | -1.75 | PREDICTED: Homo sapiens RNA binding motif protein 20 (RBM20), mRNA. |
| PYGL | -1.75 | Homo sapiens phosphorylase, glycogen, liver (PYGL), mRNA. |
| NCOA1 | -1.75 | Homo sapiens nuclear receptor coactivator 1 (NCOA1), transcript variant 2, mRNA. |
| SCYL1 | -1.75 | Homo sapiens SCY1-like 1 (S. cerevisiae) (SCYL1), mRNA. |
| LONP1 | -1.75 | Homo sapiens lon peptidase 1, mitochondrial (LONP1), nuclear gene encoding mitochondrial protein, mRNA. |
| DUSP26 | -1.75 | Homo sapiens dual specificity phosphatase 26 (putative) (DUSP26), mRNA. |
| HEMK1 | -1.75 | Homo sapiens HemK methyltransferase family member 1 (HEMK1), mRNA. |
| TSPAN8 | -1.75 | Homo sapiens tetraspanin 8 (TSPAN8), mRNA. |
| ERCC3 | -1.75 | Homo sapiens excision repair cross-complementing rodent repair deficiency, complementation group 3 (xeroderma pigmentosum group B complementing) (ERCC3), mRNA. |
| CNPY3 | -1.76 | Homo sapiens canopy 3 homolog (zebrafish) (CNPY3), mRNA. |
| MYO19 | -1.76 | Homo sapiens myosin XIX (MYO19), transcript variant 1, mRNA. |
| FAM171A1 | -1.76 | Homo sapiens family with sequence similarity 171, member A1 (FAM171A1), mRNA. |
| LOC643384 | -1.76 | PREDICTED: Homo sapiens hypothetical LOC643384 (LOC643384), mRNA. |
| CORO2A | -1.76 | Homo sapiens coronin, actin binding protein, 2A (CORO2A), transcript variant 1, mRNA. |
| BIRC3 | -1.76 | Homo sapiens baculoviral IAP repeat-containing 3 (BIRC3), transcript variant 2, mRNA. |
| SMYD4 | -1.76 | Homo sapiens SET and MYND domain containing 4 (SMYD4), mRNA. |
| DEDD | -1.76 | Homo sapiens death effector domain containing (DEDD), transcript variant 4, mRNA. |
| CTBP1 | -1.76 | Homo sapiens C-terminal binding protein 1 (CTBP1), transcript variant 2, mRNA. |
| CEP152 | -1.76 | Homo sapiens centrosomal protein 152kDa (CEP152), mRNA. |
| FAM160B2 | -1.76 | Homo sapiens family with sequence similarity 160, member B2 (FAM160B2), mRNA. |
| METRNL | -1.76 | PREDICTED: Homo sapiens meteorin, glial cell differentiation regulator-like (METRNL), mRNA. |
| C3orf39 | -1.77 | Homo sapiens chromosome 3 open reading frame 39 (C3orf39), mRNA. |
| GCAT | -1.77 | Homo sapiens glycine C-acetyltransferase (2-amino-3-ketobutyrate coenzyme A ligase) (GCAT), nuclear gene encoding mitochondrial protein, mRNA. |
| MTDH | -1.77 | Homo sapiens metadherin (MTDH), mRNA. |
| BLZF1 | -1.77 | Homo sapiens basic leucine zipper nuclear factor 1 (BLZF1), mRNA. |
| ATP6V1C1 | -1.77 | Homo sapiens ATPase, H+ transporting, lysosomal 42kDa, V1 subunit C1 (ATP6V1C1), mRNA. |
| GTF2IRD1 | -1.77 | Homo sapiens GTF2I repeat domain containing 1 (GTF2IRD1), transcript variant 1, mRNA. |
| CYFIP1 | -1.77 | Homo sapiens cytoplasmic FMR1 interacting protein 1 (CYFIP1), transcript variant 2, mRNA. |
| EIF3B | -1.77 | Homo sapiens eukaryotic translation initiation factor 3, subunit B (EIF3B), transcript variant 2, mRNA. |
| PITRM1 | -1.77 | Homo sapiens pitrilysin metallopeptidase 1 (PITRM1), mRNA. |
| ALPP | -1.77 | Homo sapiens alkaline phosphatase, placental (Regan isozyme) (ALPP), mRNA. |
| ARHGEF17 | -1.77 | Homo sapiens Rho guanine nucleotide exchange factor (GEF) 17 (ARHGEF17), mRNA. |
| ICA1L | -1.77 | Homo sapiens islet cell autoantigen 1,69kDa-like (ICA1L), transcript variant 1, mRNA. |
| OGDHL | -1.77 | Homo sapiens oxoglutarate dehydrogenase-like (OGDHL), mRNA. |
| SYMPK | -1.77 | Homo sapiens symplekin (SYMPK), mRNA. |
| C1orf122 | -1.77 | Homo sapiens chromosome 1 open reading frame 122 (C1orf122), mRNA. |
| ANO10 | -1.77 | Homo sapiens anoctamin 10 (ANO10), mRNA. |
| BRPF1 | -1.78 | Homo sapiens bromodomain and PHD finger containing, 1 (BRPF1), transcript variant 1, mRNA. |
| TSEN34 | -1.78 | Homo sapiens tRNA splicing endonuclease 34 homolog (S. cerevisiae) (TSEN34), transcript variant 2, mRNA. |
| ELMO1 | -1.78 | Homo sapiens engulfment and cell motility 1 (ELMO1), transcript variant 1, mRNA. |
| PLD6 | -1.78 | Homo sapiens phospholipase D family, member 6 (PLD6), mRNA. |
| FSTL5 | -1.78 | Homo sapiens follistatin-like 5 (FSTL5), mRNA. |
| IL17RD | -1.78 | Homo sapiens interleukin 17 receptor D (IL17RD), transcript variant 1, mRNA. |
| MTHFD1L | -1.78 | Homo sapiens methylenetetrahydrofolate dehydrogenase (NADP+ dependent) 1-like (MTHFD1L), mRNA. |
| PSMD1 | -1.78 | Homo sapiens proteasome (prosome, macropain) 26S subunit, non-ATPase, 1 (PSMD1), mRNA. |
| TMX4 | -1.79 | Homo sapiens thioredoxin-related transmembrane protein 4 (TMX4), mRNA. |
| C21orf45 | -1.79 | Homo sapiens chromosome 21 open reading frame 45 (C21orf45), mRNA. |
| SLC39A3 | -1.79 | Homo sapiens solute carrier family 39 (zinc transporter), member 3 (SLC39A3), transcript variant 1, mRNA. |
| HLA-H | -1.79 | Homo sapiens major histocompatibility complex, class I, H (pseudogene) (HLA-H), non-coding RNA. |
| IFT122 | -1.79 | Homo sapiens intraflagellar transport 122 homolog (Chlamydomonas) (IFT122), transcript variant 2, mRNA. |
| TANC1 | -1.79 | Homo sapiens tetratricopeptide repeat, ankyrin repeat and coiled-coil containing 1 (TANC1), mRNA. |
| DEPDC1 | -1.79 | Homo sapiens DEP domain containing 1 (DEPDC1), mRNA. |
| PCOLCE | -1.8 | Homo sapiens procollagen C-endopeptidase enhancer (PCOLCE), mRNA. |
| SLC9A5 | -1.8 | Homo sapiens solute carrier family 9 (sodium/hydrogen exchanger), member 5 (SLC9A5), mRNA. |
| POLR3A | -1.8 | Homo sapiens polymerase (RNA) III (DNA directed) polypeptide A, 155kDa (POLR3A), mRNA. |
| CSK | -1.8 | Homo sapiens c-src tyrosine kinase (CSK), mRNA. |
| TRADD | -1.8 | Homo sapiens TNFRSF1A-associated via death domain (TRADD), transcript variant 1, mRNA. |
| FKBP3 | -1.8 | Homo sapiens FK506 binding protein 3, 25kDa (FKBP3), mRNA. |
| ATP13A2 | -1.8 | Homo sapiens ATPase type 13A2 (ATP13A2), mRNA. |
| LOC440353 | -1.8 | Homo sapiens nuclear pore complex interacting protein pseudogene (LOC440353), non-coding RNA. |
| SLC39A8 | -1.8 | Homo sapiens solute carrier family 39 (zinc transporter), member 8 (SLC39A8), transcript variant 1, mRNA. |
| DNAJC30 | -1.8 | Homo sapiens DnaJ (Hsp40) homolog, subfamily C, member 30 (DNAJC30), mRNA. |
| FAM133B | -1.8 | Homo sapiens family with sequence similarity 133, member B (FAM133B), transcript variant 1, mRNA. |
| DDX51 | -1.8 | Homo sapiens DEAD (Asp-Glu-Ala-Asp) box polypeptide 51 (DDX51), mRNA. |
| SV2A | -1.8 | Homo sapiens synaptic vesicle glycoprotein 2A (SV2A), mRNA. |
| LOC645726 | -1.8 | PREDICTED: Homo sapiens misc_RNA (LOC645726), miscRNA. |
| TRIM24 | -1.8 | Homo sapiens tripartite motif-containing 24 (TRIM24), transcript variant 1, mRNA. |
| THY1 | -1.81 | Homo sapiens Thy-1 cell surface antigen (THY1), mRNA. |
| EFS | -1.81 | Homo sapiens embryonal Fyn-associated substrate (EFS), transcript variant 2, mRNA. |
| C7orf26 | -1.81 | Homo sapiens chromosome 7 open reading frame 26 (C7orf26), mRNA. |
| CRAMP1L | -1.81 | Homo sapiens Crm, cramped-like (Drosophila) (CRAMP1L), mRNA. |
| SBF2 | -1.81 | Homo sapiens SET binding factor 2 (SBF2), mRNA. |
| RNU1-5 | -1.81 | Homo sapiens RNA, U1 small nuclear 5 (RNU1-5), small nuclear RNA. |
| ADPRHL1 | -1.81 | Homo sapiens ADP-ribosylhydrolase like 1 (ADPRHL1), transcript variant 2, mRNA. |
| TUBA3D | -1.81 | Homo sapiens tubulin, alpha 3d (TUBA3D), mRNA. |
| VLDLR | -1.81 | Homo sapiens very low density lipoprotein receptor (VLDLR), transcript variant 2, mRNA. |
| BMPR2 | -1.81 | Homo sapiens bone morphogenetic protein receptor, type II (serine/threonine kinase) (BMPR2), mRNA. |
| LTBP4 | -1.82 | Homo sapiens latent transforming growth factor beta binding protein 4 (LTBP4), transcript variant 3, mRNA. |
| LOC653147 | -1.82 | PREDICTED: Homo sapiens similar to 60S ribosomal protein L26-like 1 (LOC653147), mRNA. |
| AIP | -1.82 | Homo sapiens aryl hydrocarbon receptor interacting protein (AIP), mRNA. |
| GNB4 | -1.82 | Homo sapiens guanine nucleotide binding protein (G protein), beta polypeptide 4 (GNB4), mRNA. |
| PNPLA8 | -1.82 | Homo sapiens patatin-like phospholipase domain containing 8 (PNPLA8), mRNA. |
| LRP10 | -1.82 | Homo sapiens low density lipoprotein receptor-related protein 10 (LRP10), mRNA. |
| N-PAC | -1.82 | Homo sapiens cytokine-like nuclear factor n-pac (N-PAC), mRNA. |
| SNRNP70 | -1.82 | Homo sapiens small nuclear ribonucleoprotein 70kDa (U1) (SNRNP70), mRNA. |
| LOC652672 | -1.82 | PREDICTED: Homo sapiens similar to damage-specific DNA binding protein 1 (LOC652672), mRNA. |
| QRICH1 | -1.82 | Homo sapiens glutamine-rich 1 (QRICH1), transcript variant 1, mRNA. |
| SGTA | -1.83 | Homo sapiens small glutamine-rich tetratricopeptide repeat (TPR)-containing, alpha (SGTA), mRNA. |
| TRIM52 | -1.83 | Homo sapiens tripartite motif-containing 52 (TRIM52), mRNA. |
| CABYR | -1.83 | Homo sapiens calcium binding tyrosine-(Y)-phosphorylation regulated (CABYR), transcript variant 2, mRNA. |
| PNPLA6 | -1.83 | Homo sapiens patatin-like phospholipase domain containing 6 (PNPLA6), mRNA. |
| LZTS1 | -1.83 | Homo sapiens leucine zipper, putative tumor suppressor 1 (LZTS1), mRNA. |
| SLC38A2 | -1.83 | Homo sapiens solute carrier family 38, member 2 (SLC38A2), mRNA. |
| TSPAN5 | -1.83 | Homo sapiens tetraspanin 5 (TSPAN5), mRNA. |
| SLC29A3 | -1.83 | Homo sapiens solute carrier family 29 (nucleoside transporters), member 3 (SLC29A3), mRNA. |
| SRM | -1.83 | Homo sapiens spermidine synthase (SRM), mRNA. |
| POLR2A | -1.83 | Homo sapiens polymerase (RNA) II (DNA directed) polypeptide A, 220kDa (POLR2A), mRNA. |
| PIP5K1C | -1.84 | Homo sapiens phosphatidylinositol-4-phosphate 5-kinase, type I, gamma (PIP5K1C), mRNA. |
| JPH3 | -1.84 | Homo sapiens junctophilin 3 (JPH3), mRNA. |
| ZNF384 | -1.84 | Homo sapiens zinc finger protein 384 (ZNF384), transcript variant 2, mRNA. |
| PLS3 | -1.84 | Homo sapiens plastin 3 (T isoform) (PLS3), mRNA. |
| ADAMTS1 | -1.84 | Homo sapiens ADAM metallopeptidase with thrombospondin type 1 motif, 1 (ADAMTS1), mRNA. |
| YEATS4 | -1.84 | Homo sapiens YEATS domain containing 4 (YEATS4), mRNA. |
| LOC100132324 | -1.84 | PREDICTED: Homo sapiens misc_RNA (LOC100132324), miscRNA. |
| ST6GALNAC4 | -1.84 | Homo sapiens ST6 (alpha-N-acetyl-neuraminyl-2,3-beta-galactosyl-1, 3)-N-acetylgalactosaminide alpha-2,6-sialyltransferase 4 (ST6GALNAC4), transcript variant 1, mRNA. |
| FKBP9L | -1.84 | Homo sapiens FK506 binding protein 9-like (FKBP9L), mRNA. |
| MEX3A | -1.85 | Homo sapiens mex-3 homolog A (C. elegans) (MEX3A), mRNA. |
| PPAPDC3 | -1.85 | Homo sapiens phosphatidic acid phosphatase type 2 domain containing 3 (PPAPDC3), mRNA. |
| HSP90AB1 | -1.85 | Homo sapiens heat shock protein 90kDa alpha (cytosolic), class B member 1 (HSP90AB1), mRNA. |
| FBLN1 | -1.85 | Homo sapiens fibulin 1 (FBLN1), transcript variant C, mRNA. |
| MYADM | -1.85 | Homo sapiens myeloid-associated differentiation marker (MYADM), transcript variant 2, mRNA. |
| CTSA | -1.85 | Homo sapiens cathepsin A (CTSA), transcript variant 1, mRNA. |
| THEM2 | -1.85 | Homo sapiens thioesterase superfamily member 2 (THEM2), mRNA. |
| ARHGAP28 | -1.85 | Homo sapiens Rho GTPase activating protein 28 (ARHGAP28), transcript variant 1, mRNA. |
| RHOT2 | -1.85 | Homo sapiens ras homolog gene family, member T2 (RHOT2), nuclear gene encoding mitochondrial protein, mRNA. |
| FUZ | -1.86 | Homo sapiens fuzzy homolog (Drosophila) (FUZ), mRNA. |
| SH3KBP1 | -1.86 | Homo sapiens SH3-domain kinase binding protein 1 (SH3KBP1), transcript variant 1, mRNA. |
| ABCB6 | -1.86 | Homo sapiens ATP-binding cassette, sub-family B (MDR/TAP), member 6 (ABCB6), nuclear gene encoding mitochondrial protein, mRNA. |
| TBC1D9B | -1.86 | Homo sapiens TBC1 domain family, member 9B (with GRAM domain) (TBC1D9B), transcript variant 2, mRNA. |
| EIF5 | -1.86 | Homo sapiens eukaryotic translation initiation factor 5 (EIF5), transcript variant 1, mRNA. |
| MAST3 | -1.86 | Homo sapiens microtubule associated serine/threonine kinase 3 (MAST3), mRNA. |
| C11orf68 | -1.86 | Homo sapiens chromosome 11 open reading frame 68 (C11orf68), mRNA. |
| RAPGEF4 | -1.86 | Homo sapiens Rap guanine nucleotide exchange factor (GEF) 4 (RAPGEF4), mRNA. |
| LOC728823 | -1.87 | PREDICTED: Homo sapiens misc_RNA (LOC728823), miscRNA. |
| SLITRK6 | -1.87 | Homo sapiens SLIT and NTRK-like family, member 6 (SLITRK6), mRNA. |
| CCBE1 | -1.87 | Homo sapiens collagen and calcium binding EGF domains 1 (CCBE1), mRNA. |
| LOC347292 | -1.87 | PREDICTED: Homo sapiens similar to ribosomal protein L36 (LOC347292), mRNA. |
|  | -1.87 | PREDICTED: Homo sapiens LOC440151 (LOC440151), mRNA |
| BASP1 | -1.87 | Homo sapiens brain abundant, membrane attached signal protein 1 (BASP1), mRNA. |
| TIAF1 | -1.87 | Homo sapiens TGFB1-induced anti-apoptotic factor 1 (TIAF1), mRNA. |
| KIAA1602 | -1.87 | Homo sapiens KIAA1602 (KIAA1602), mRNA. |
| FADS2 | -1.87 | Homo sapiens fatty acid desaturase 2 (FADS2), mRNA. |
| DNAJC24 | -1.87 | Homo sapiens DnaJ (Hsp40) homolog, subfamily C, member 24 (DNAJC24), mRNA. |
| SNORD68 | -1.87 | Homo sapiens small nucleolar RNA, C/D box 68 (SNORD68), small nucleolar RNA. |
| NOL8 | -1.87 | Homo sapiens nucleolar protein 8 (NOL8), mRNA. |
| CALD1 | -1.87 | Homo sapiens caldesmon 1 (CALD1), transcript variant 5, mRNA. |
| KIF23 | -1.88 | Homo sapiens kinesin family member 23 (KIF23), transcript variant 2, mRNA. |
| C17orf96 | -1.88 | Homo sapiens chromosome 17 open reading frame 96 (C17orf96), mRNA. |
| CYB5R3 | -1.88 | Homo sapiens cytochrome b5 reductase 3 (CYB5R3), transcript variant M, mRNA. |
| MYCBP2 | -1.88 | Homo sapiens MYC binding protein 2 (MYCBP2), mRNA. |
| MCM5 | -1.88 | Homo sapiens minichromosome maintenance complex component 5 (MCM5), mRNA. |
| KIF4A | -1.88 | Homo sapiens kinesin family member 4A (KIF4A), mRNA. |
| TMEM39B | -1.88 | Homo sapiens transmembrane protein 39B (TMEM39B), mRNA. |
| DAK | -1.88 | Homo sapiens dihydroxyacetone kinase 2 homolog (S. cerevisiae) (DAK), mRNA. |
| LEF1 | -1.88 | Homo sapiens lymphoid enhancer-binding factor 1 (LEF1), mRNA. |
| PHKB | -1.88 | Homo sapiens phosphorylase kinase, beta (PHKB), transcript variant 2, mRNA. |
| GPR124 | -1.88 | Homo sapiens G protein-coupled receptor 124 (GPR124), mRNA. |
| ALDH1A3 | -1.88 | Homo sapiens aldehyde dehydrogenase 1 family, member A3 (ALDH1A3), mRNA. |
| LRRC37B2 | -1.89 | Homo sapiens leucine rich repeat containing 37, member B2 (LRRC37B2), non-coding RNA. |
| TARBP1 | -1.89 | Homo sapiens TAR (HIV-1) RNA binding protein 1 (TARBP1), mRNA. |
| LOC388796 | -1.89 | Homo sapiens hypothetical LOC388796 (LOC388796), non-coding RNA. |
| SMARCA5 | -1.89 | Homo sapiens SWI/SNF related, matrix associated, actin dependent regulator of chromatin, subfamily a, member 5 (SMARCA5), mRNA. |
| CADM4 | -1.89 | Homo sapiens cell adhesion molecule 4 (CADM4), mRNA. |
| RNF10 | -1.89 | Homo sapiens ring finger protein 10 (RNF10), mRNA. |
| BCHE | -1.89 | Homo sapiens butyrylcholinesterase (BCHE), mRNA. |
| RELA | -1.89 | Homo sapiens v-rel reticuloendotheliosis viral oncogene homolog A (avian) (RELA), mRNA. |
| USP14 | -1.89 | Homo sapiens ubiquitin specific peptidase 14 (tRNA-guanine transglycosylase) (USP14), transcript variant 2, mRNA. |
| TIMM9 | -1.89 | Homo sapiens translocase of inner mitochondrial membrane 9 homolog (yeast) (TIMM9), nuclear gene encoding mitochondrial protein, mRNA. |
| DVL2 | -1.9 | Homo sapiens dishevelled, dsh homolog 2 (Drosophila) (DVL2), mRNA. |
| MGP | -1.9 | Homo sapiens matrix Gla protein (MGP), mRNA. |
| ENOSF1 | -1.9 | Homo sapiens enolase superfamily member 1 (ENOSF1), mRNA. |
|  | -1.9 | Homo sapiens cDNA clone IMAGE:6254031 |
| ROR1 | -1.9 | Homo sapiens receptor tyrosine kinase-like orphan receptor 1 (ROR1), transcript variant 1, mRNA. |
| RGS2 | -1.9 | Homo sapiens regulator of G-protein signalling 2, 24kDa (RGS2), mRNA. |
| NRXN2 | -1.9 | Homo sapiens neurexin 2 (NRXN2), transcript variant beta, mRNA. |
| LOC648509 | -1.9 | PREDICTED: Homo sapiens similar to plakophilin 4 isoform a (LOC648509), mRNA. |
| PTPRA | -1.9 | Homo sapiens protein tyrosine phosphatase, receptor type, A (PTPRA), transcript variant 1, mRNA. |
| NIPSNAP1 | -1.91 | Homo sapiens nipsnap homolog 1 (C. elegans) (NIPSNAP1), mRNA. |
| DBNL | -1.91 | Homo sapiens drebrin-like (DBNL), transcript variant 1, mRNA. |
| PDGFRB | -1.91 | Homo sapiens platelet-derived growth factor receptor, beta polypeptide (PDGFRB), mRNA. |
| MARK2 | -1.91 | Homo sapiens MAP/microtubule affinity-regulating kinase 2 (MARK2), transcript variant 2, mRNA. |
| ILVBL | -1.91 | Homo sapiens ilvB (bacterial acetolactate synthase)-like (ILVBL), mRNA. |
| MAP1A | -1.91 | Homo sapiens microtubule-associated protein 1A (MAP1A), mRNA. |
| AHNAK | -1.91 | Homo sapiens AHNAK nucleoprotein (AHNAK), transcript variant 1, mRNA. |
| ARSD | -1.91 | Homo sapiens arylsulfatase D (ARSD), transcript variant 1, mRNA. |
| EPOR | -1.92 | Homo sapiens erythropoietin receptor (EPOR), mRNA. |
| ZNF682 | -1.92 | Homo sapiens zinc finger protein 682 (ZNF682), transcript variant 1, mRNA. |
| PDZRN3 | -1.92 | PREDICTED: Homo sapiens PDZ domain containing RING finger 3 (PDZRN3), mRNA. |
| KIAA0895L | -1.92 | Homo sapiens KIAA0895-like (KIAA0895L), mRNA. XM_933435 XM_933438 XM_933442 XM_933445 XM_933447 XM_941971 XM_945589 XM_945591 XM_945592 XM_945593 XM_945594 XM_945596 XM_945598 XM_945599 XM_945600 |
| CENPM | -1.92 | Homo sapiens centromere protein M (CENPM), transcript variant 1, mRNA. |
| VIM | -1.92 | Homo sapiens vimentin (VIM), mRNA. |
| SLU7 | -1.92 | Homo sapiens SLU7 splicing factor homolog (S. cerevisiae) (SLU7), mRNA. |
| BEGAIN | -1.92 | Homo sapiens brain-enriched guanylate kinase-associated homolog (rat) (BEGAIN), mRNA. |
| MYH9 | -1.92 | Homo sapiens myosin, heavy chain 9, non-muscle (MYH9), mRNA. |
| LOC729120 | -1.93 | PREDICTED: Homo sapiens hypothetical LOC729120 (LOC729120), mRNA. |
| MCM3APAS | -1.93 | Homo sapiens MCM3 minichromosome maintenance deficient 3 (S. cerevisiae) associated protein antisense (MCM3APAS) on chromosome 21. |
| ADAM19 | -1.93 | Homo sapiens ADAM metallopeptidase domain 19 (meltrin beta) (ADAM19), mRNA. |
| FAM160B2 | -1.93 | Homo sapiens family with sequence similarity 160, member B2 (FAM160B2), mRNA. |
| RHBDL2 | -1.93 | Homo sapiens rhomboid, veinlet-like 2 (Drosophila) (RHBDL2), mRNA. |
| C9orf69 | -1.93 | Homo sapiens chromosome 9 open reading frame 69 (C9orf69), mRNA. |
| ATP1B3 | -1.93 | PREDICTED: Homo sapiens ATPase, Na+/K+ transporting, beta 3 polypeptide, transcript variant 2 (ATP1B3), mRNA. |
| CD276 | -1.93 | Homo sapiens CD276 molecule (CD276), transcript variant 1, mRNA. XM_945872 XM_945874 |
| LOC730525 | -1.93 | PREDICTED: Homo sapiens hypothetical protein LOC730525 (LOC730525), mRNA. |
| SLC35C2 | -1.93 | Homo sapiens solute carrier family 35, member C2 (SLC35C2), transcript variant 2, mRNA. |
| RNU1A3 | -1.93 | Homo sapiens RNA, U1A3 small nuclear (RNU1A3), small nuclear RNA. |
| DUSP10 | -1.93 | Homo sapiens dual specificity phosphatase 10 (DUSP10), transcript variant 3, mRNA. |
| LOC100133923 | -1.93 | PREDICTED: Homo sapiens hypothetical protein LOC100133923 (LOC100133923), mRNA. |
| RFX1 | -1.94 | Homo sapiens regulatory factor X, 1 (influences HLA class II expression) (RFX1), mRNA. |
| GAS1 | -1.94 | Homo sapiens growth arrest-specific 1 (GAS1), mRNA. |
| C1QTNF1 | -1.94 | Homo sapiens C1q and tumor necrosis factor related protein 1 (C1QTNF1), mRNA. |
| NT5C3 | -1.94 | Homo sapiens 5'-nucleotidase, cytosolic III (NT5C3), transcript variant 1, mRNA. |
| STIM1 | -1.94 | Homo sapiens stromal interaction molecule 1 (STIM1), mRNA. |
| SPRY1 | -1.94 | Homo sapiens sprouty homolog 1, antagonist of FGF signaling (Drosophila) (SPRY1), transcript variant 1, mRNA. |
| DDX51 | -1.94 | Homo sapiens DEAD (Asp-Glu-Ala-Asp) box polypeptide 51 (DDX51), mRNA. |
| NKAIN1 | -1.94 | Homo sapiens Na+/K+ transporting ATPase interacting 1 (NKAIN1), mRNA. |
| ENTPD6 | -1.94 | Homo sapiens ectonucleoside triphosphate diphosphohydrolase 6 (putative function) (ENTPD6), mRNA. |
| TUBG1 | -1.94 | Homo sapiens tubulin, gamma 1 (TUBG1), mRNA. |
| UNC5A | -1.94 | Homo sapiens unc-5 homolog A (C. elegans) (UNC5A), mRNA. |
| SLIT3 | -1.94 | Homo sapiens slit homolog 3 (Drosophila) (SLIT3), mRNA. |
| SEMA4B | -1.95 | Homo sapiens sema domain, immunoglobulin domain (Ig), transmembrane domain (TM) and short cytoplasmic domain, (semaphorin) 4B (SEMA4B), transcript variant 2, mRNA. |
| RNU1F1 | -1.95 | Homo sapiens RNA, U1F1 small nuclear (RNU1F1), small nuclear RNA. |
| SHANK3 | -1.95 | Homo sapiens SH3 and multiple ankyrin repeat domains 3 (SHANK3), mRNA. |
| DULLARD | -1.95 | Homo sapiens dullard homolog (Xenopus laevis) (DULLARD), mRNA. |
| C1QL1 | -1.95 | Homo sapiens complement component 1, q subcomponent-like 1 (C1QL1), mRNA. |
| LOC647302 | -1.95 | PREDICTED: Homo sapiens misc_RNA (LOC647302), miscRNA. |
| PROX1 | -1.95 | Homo sapiens prospero homeobox 1 (PROX1), mRNA. |
| MFF | -1.96 | Homo sapiens mitochondrial fission factor (MFF), nuclear gene encoding mitochondrial protein, mRNA. |
| PRSS35 | -1.96 | Homo sapiens protease, serine, 35 (PRSS35), mRNA. |
| LOC100133185 | -1.96 | PREDICTED: Homo sapiens misc_RNA (LOC100133185), miscRNA. |
| PPAP2A | -1.97 | Homo sapiens phosphatidic acid phosphatase type 2A (PPAP2A), transcript variant 2, mRNA. |
| FAM189B | -1.97 | Homo sapiens family with sequence similarity 189, member B (FAM189B), transcript variant 2, mRNA. |
| ARFGAP1 | -1.97 | Homo sapiens ADP-ribosylation factor GTPase activating protein 1 (ARFGAP1), transcript variant 1, mRNA. |
| C5orf41 | -1.97 | Homo sapiens chromosome 5 open reading frame 41 (C5orf41), mRNA. |
| FAM181B | -1.97 | Homo sapiens family with sequence similarity 181, member B (FAM181B), mRNA. |
| C16orf7 | -1.97 | Homo sapiens chromosome 16 open reading frame 7 (C16orf7), mRNA. |
| LOC100131582 | -1.98 | PREDICTED: Homo sapiens hypothetical protein LOC100131582 (LOC100131582), mRNA. |
| IL7 | -1.98 | Homo sapiens interleukin 7 (IL7), mRNA. |
| RERE | -1.98 | Homo sapiens arginine-glutamic acid dipeptide (RE) repeats (RERE), transcript variant 1, mRNA. |
| MED24 | -1.98 | Homo sapiens mediator complex subunit 24 (MED24), transcript variant 1, mRNA. |
| ZIK1 | -1.98 | Homo sapiens zinc finger protein interacting with K protein 1 homolog (mouse) (ZIK1), mRNA. |
| FNDC4 | -1.98 | Homo sapiens fibronectin type III domain containing 4 (FNDC4), mRNA. |
| C16orf42 | -1.98 | Homo sapiens chromosome 16 open reading frame 42 (C16orf42), mRNA. |
| RAB7L1 | -1.98 | Homo sapiens RAB7, member RAS oncogene family-like 1 (RAB7L1), mRNA. |
| MKNK1 | -1.98 | Homo sapiens MAP kinase interacting serine/threonine kinase 1 (MKNK1), transcript variant 1, mRNA. |
| C14orf102 | -1.98 | Homo sapiens chromosome 14 open reading frame 102 (C14orf102), transcript variant 1, mRNA. |
| C20orf3 | -1.98 | Homo sapiens chromosome 20 open reading frame 3 (C20orf3), mRNA. |
| C22orf36 | -1.99 | Homo sapiens chromosome 22 open reading frame 36 (C22orf36), mRNA. |
| C9orf140 | -1.99 | Homo sapiens chromosome 9 open reading frame 140 (C9orf140), mRNA. |
| RASSF6 | -1.99 | Homo sapiens Ras association (RalGDS/AF-6) domain family member 6 (RASSF6), transcript variant 1, mRNA. |
| SLC39A8 | -1.99 | Homo sapiens solute carrier family 39 (zinc transporter), member 8 (SLC39A8), transcript variant 1, mRNA. |
| CEL | -1.99 | Homo sapiens carboxyl ester lipase (bile salt-stimulated lipase) (CEL), mRNA. |
| LOC90586 | -1.99 | Homo sapiens AOC3 pseudogene (LOC90586), non-coding RNA. |
| SLC19A1 | -1.99 | Homo sapiens solute carrier family 19 (folate transporter), member 1 (SLC19A1), mRNA. |
| SEZ6L2 | -1.99 | Homo sapiens seizure related 6 homolog (mouse)-like 2 (SEZ6L2), transcript variant 2, mRNA. |
| CKMT1B | -1.99 | Homo sapiens creatine kinase, mitochondrial 1B (CKMT1B), nuclear gene encoding mitochondrial protein, mRNA. |
| TUFM | -1.99 | Homo sapiens Tu translation elongation factor, mitochondrial (TUFM), nuclear gene encoding mitochondrial protein, mRNA. |
| TTC38 | -1.99 | Homo sapiens tetratricopeptide repeat domain 38 (TTC38), mRNA. |
| SHISA5 | -1.99 | Homo sapiens shisa homolog 5 (Xenopus laevis) (SHISA5), mRNA. |
| C6orf111 | -1.99 | Homo sapiens chromosome 6 open reading frame 111 (C6orf111), mRNA. |
| MBOAT2 | -1.99 | Homo sapiens membrane bound O-acyltransferase domain containing 2 (MBOAT2), mRNA. |
| JAKMIP2 | -1.99 | Homo sapiens janus kinase and microtubule interacting protein 2 (JAKMIP2), mRNA. |
| C1orf57 | -1.99 | Homo sapiens chromosome 1 open reading frame 57 (C1orf57), mRNA. |
| MAF1 | -1.99 | Homo sapiens MAF1 homolog (S. cerevisiae) (MAF1), mRNA. |
| DDX23 | -1.99 | Homo sapiens DEAD (Asp-Glu-Ala-Asp) box polypeptide 23 (DDX23), mRNA. |
| FLJ21865 | -1.99 | Homo sapiens endo-beta-N-acetylglucosaminidase (FLJ21865), mRNA. |
| SPATS2L | -2 | Homo sapiens spermatogenesis associated, serine-rich 2-like (SPATS2L), transcript variant 2, mRNA. |
| RACGAP1 | -2 | Homo sapiens Rac GTPase activating protein 1 (RACGAP1), mRNA. |
| DKFZp547K054 | -2 | PREDICTED: Homo sapiens hypothetical protein DKFZp547K054 (DKFZp547K054), mRNA. |
| MRPL2 | -2 | Homo sapiens mitochondrial ribosomal protein L2 (MRPL2), nuclear gene encoding mitochondrial protein, mRNA. |
| NAT14 | -2 | Homo sapiens N-acetyltransferase 14 (GCN5-related, putative) (NAT14), mRNA. |
| EP400 | -2 | Homo sapiens E1A binding protein p400 (EP400), mRNA. |
| BCAR1 | -2 | Homo sapiens breast cancer anti-estrogen resistance 1 (BCAR1), mRNA. |
| METTL9 | -2.01 | Homo sapiens methyltransferase like 9 (METTL9), transcript variant 1, mRNA. |
| PIR | -2.01 | Homo sapiens pirin (iron-binding nuclear protein) (PIR), transcript variant 2, mRNA. |
| VARS | -2.01 | Homo sapiens valyl-tRNA synthetase (VARS), nuclear gene encoding mitochondrial protein, mRNA. |
| NMU | -2.01 | Homo sapiens neuromedin U (NMU), mRNA. |
| LOC100134634 | -2.01 | PREDICTED: Homo sapiens hypothetical protein LOC100134634 (LOC100134634), mRNA. |
| SMC3 | -2.01 | Homo sapiens structural maintenance of chromosomes 3 (SMC3), mRNA. |
| LOC641765 | -2.01 | PREDICTED: Homo sapiens similar to General transcription factor II-I repeat domain-containing protein 1 (GTF2I repeat domain containing protein 1) (Muscle TFII-I repeat domain-containing protein 1) (General transcription factor III) (Slow-muscle-fiber enhancer binding pro... (LOC641765), mRNA. |
| KIF11 | -2.01 | Homo sapiens kinesin family member 11 (KIF11), mRNA. |
| YPEL3 | -2.01 | Homo sapiens yippee-like 3 (Drosophila) (YPEL3), transcript variant 1, mRNA. |
| SULF2 | -2.02 | Homo sapiens sulfatase 2 (SULF2), transcript variant 1, mRNA. |
| FAM195A | -2.02 | Homo sapiens family with sequence similarity 195, member A (FAM195A), mRNA. |
| ZBTB17 | -2.02 | Homo sapiens zinc finger and BTB domain containing 17 (ZBTB17), mRNA. |
| ADORA2A | -2.02 | Homo sapiens adenosine A2a receptor (ADORA2A), mRNA. |
| IGFBP3 | -2.02 | Homo sapiens insulin-like growth factor binding protein 3 (IGFBP3), transcript variant 2, mRNA. |
| MORC2 | -2.02 | Homo sapiens MORC family CW-type zinc finger 2 (MORC2), mRNA. |
| OLFML3 | -2.02 | Homo sapiens olfactomedin-like 3 (OLFML3), mRNA. |
| RECQL4 | -2.02 | Homo sapiens RecQ protein-like 4 (RECQL4), mRNA. |
| COL3A1 | -2.02 | Homo sapiens collagen, type III, alpha 1 (COL3A1), mRNA. |
| CABIN1 | -2.03 | Homo sapiens calcineurin binding protein 1 (CABIN1), mRNA. |
| WASH3P | -2.03 | Homo sapiens WAS protein family homolog 3 pseudogene (WASH3P), non-coding RNA. |
| STMN4 | -2.03 | Homo sapiens stathmin-like 4 (STMN4), mRNA. |
| GNAO1 | -2.03 | Homo sapiens guanine nucleotide binding protein (G protein), alpha activating activity polypeptide O (GNAO1), transcript variant 2, mRNA. |
| LOC100131735 | -2.03 | PREDICTED: Homo sapiens misc_RNA (LOC100131735), miscRNA. |
| SETDB1 | -2.03 | Homo sapiens SET domain, bifurcated 1 (SETDB1), mRNA. |
| C6orf125 | -2.03 | Homo sapiens chromosome 6 open reading frame 125 (C6orf125), mRNA. |
| TRAK1 | -2.03 | Homo sapiens trafficking protein, kinesin binding 1 (TRAK1), transcript variant 1, mRNA. |
| ABCB1 | -2.03 | Homo sapiens ATP-binding cassette, sub-family B (MDR/TAP), member 1 (ABCB1), mRNA. |
| CDKN3 | -2.03 | Homo sapiens cyclin-dependent kinase inhibitor 3 (CDK2-associated dual specificity phosphatase) (CDKN3), mRNA. |
| SPTAN1 | -2.03 | Homo sapiens spectrin, alpha, non-erythrocytic 1 (alpha-fodrin) (SPTAN1), mRNA. |
| GOLPH4 | -2.03 | Homo sapiens golgi phosphoprotein 4 (GOLPH4), mRNA. |
| SLC29A1 | -2.04 | Homo sapiens solute carrier family 29 (nucleoside transporters), member 1 (SLC29A1), nuclear gene encoding mitochondrial protein, transcript variant 4, mRNA. |
| CA11 | -2.04 | Homo sapiens carbonic anhydrase XI (CA11), mRNA. |
| RNF24 | -2.04 | Homo sapiens ring finger protein 24 (RNF24), mRNA. |
| LOC642333 | -2.04 | PREDICTED: Homo sapiens similar to M-phase phosphoprotein, mpp8 (LOC642333), mRNA. |
| FSTL1 | -2.04 | Homo sapiens follistatin-like 1 (FSTL1), mRNA. |
| LMO4 | -2.05 | Homo sapiens LIM domain only 4 (LMO4), mRNA. |
| UPF3A | -2.05 | Homo sapiens UPF3 regulator of nonsense transcripts homolog A (yeast) (UPF3A), transcript variant 2, mRNA. |
| CYTL1 | -2.05 | Homo sapiens cytokine-like 1 (CYTL1), mRNA. |
| LOC645979 | -2.05 | PREDICTED: Homo sapiens similar to ribosomal protein S26 (LOC645979), mRNA. |
| C10orf76 | -2.05 | Homo sapiens chromosome 10 open reading frame 76 (C10orf76), mRNA. |
| CKMT1A | -2.05 | Homo sapiens creatine kinase, mitochondrial 1A (CKMT1A), nuclear gene encoding mitochondrial protein, mRNA. |
| DPP7 | -2.05 | Homo sapiens dipeptidyl-peptidase 7 (DPP7), mRNA. |
| BFAR | -2.05 | Homo sapiens bifunctional apoptosis regulator (BFAR), mRNA. |
| YTHDF2 | -2.05 | Homo sapiens YTH domain family, member 2 (YTHDF2), mRNA. |
| SLC35E1 | -2.06 | Homo sapiens solute carrier family 35, member E1 (SLC35E1), mRNA. |
| SF3B4 | -2.06 | Homo sapiens splicing factor 3b, subunit 4, 49kDa (SF3B4), mRNA. |
| ST6GALNAC3 | -2.06 | Homo sapiens ST6 (alpha-N-acetyl-neuraminyl-2,3-beta-galactosyl-1, 3)-N-acetylgalactosaminide alpha-2,6-sialyltransferase 3 (ST6GALNAC3), mRNA. |
| LOC440341 | -2.06 | PREDICTED: Homo sapiens similar to nuclear pore complex interacting protein, transcript variant 4 (LOC440341), mRNA. |
| HSD17B7 | -2.06 | Homo sapiens hydroxysteroid (17-beta) dehydrogenase 7 (HSD17B7), mRNA. |
| GATA3 | -2.06 | Homo sapiens GATA binding protein 3 (GATA3), transcript variant 2, mRNA. |
| LOC100129424 | -2.06 | PREDICTED: Homo sapiens misc_RNA (LOC100129424), miscRNA. |
| GATA2 | -2.06 | Homo sapiens GATA binding protein 2 (GATA2), mRNA. |
| TMEM63B | -2.06 | Homo sapiens transmembrane protein 63B (TMEM63B), mRNA. |
| RPS26L | -2.06 | Homo sapiens 40S ribosomal protein S26-like (RPS26L), non-coding RNA. |
| HERC2 | -2.06 | Homo sapiens hect domain and RLD 2 (HERC2), mRNA. |
| ARMCX5 | -2.06 | Homo sapiens armadillo repeat containing, X-linked 5 (ARMCX5), mRNA. |
| PCDHB3 | -2.06 | Homo sapiens protocadherin beta 3 (PCDHB3), mRNA. |
| SNRNP70 | -2.06 | Homo sapiens small nuclear ribonucleoprotein 70kDa (U1) (SNRNP70), mRNA. |
| SMO | -2.06 | Homo sapiens smoothened homolog (Drosophila) (SMO), mRNA. |
| AKAP8L | -2.06 | Homo sapiens A kinase (PRKA) anchor protein 8-like (AKAP8L), mRNA. |
| RPS6KC1 | -2.07 | Homo sapiens ribosomal protein S6 kinase, 52kDa, polypeptide 1 (RPS6KC1), mRNA. |
| TPP1 | -2.07 | Homo sapiens tripeptidyl peptidase I (TPP1), mRNA. |
| C21orf81 | -2.07 | Homo sapiens chromosome 21 open reading frame 81 (C21orf81), mRNA. |
| NR1D2 | -2.07 | PREDICTED: Homo sapiens nuclear receptor subfamily 1, group D, member 2 (NR1D2), mRNA. |
| MLEC | -2.07 | Homo sapiens malectin (MLEC), mRNA. |
| INPP5A | -2.07 | PREDICTED: Homo sapiens inositol polyphosphate-5-phosphatase, 40kDa (INPP5A), mRNA. |
| DNAJC2 | -2.07 | Homo sapiens DnaJ (Hsp40) homolog, subfamily C, member 2 (DNAJC2), transcript variant 1, mRNA. |
| C12orf51 | -2.08 | Homo sapiens chromosome 12 open reading frame 51 (C12orf51), mRNA. |
| SERINC1 | -2.08 | Homo sapiens serine incorporator 1 (SERINC1), mRNA. |
| FBXO17 | -2.08 | Homo sapiens F-box protein 17 (FBXO17), transcript variant 2, mRNA. |
| KIAA0427 | -2.08 | Homo sapiens KIAA0427 (KIAA0427), mRNA. |
| DNM2 | -2.08 | Homo sapiens dynamin 2 (DNM2), transcript variant 2, mRNA. |
| ALDH16A1 | -2.08 | Homo sapiens aldehyde dehydrogenase 16 family, member A1 (ALDH16A1), mRNA. |
| ETS1 | -2.08 | Homo sapiens v-ets erythroblastosis virus E26 oncogene homolog 1 (avian) (ETS1), mRNA. |
| C1orf165 | -2.08 | Homo sapiens chromosome 1 open reading frame 165 (C1orf165), mRNA. |
| TBC1D24 | -2.08 | Homo sapiens TBC1 domain family, member 24 (TBC1D24), mRNA. XM_945422 |
| LOC730313 | -2.09 | PREDICTED: Homo sapiens hypothetical LOC730313 (LOC730313), mRNA. |
| LOC441377 | -2.09 | PREDICTED: Homo sapiens similar to 40S ribosomal protein S26 (LOC441377), mRNA. |
| KIAA0247 | -2.09 | Homo sapiens KIAA0247 (KIAA0247), mRNA. |
| FKRP | -2.09 | Homo sapiens fukutin related protein (FKRP), transcript variant 1, mRNA. |
| SPG11 | -2.09 | Homo sapiens spastic paraplegia 11 (autosomal recessive) (SPG11), transcript variant 1, mRNA. |
| SEMA5A | -2.09 | Homo sapiens sema domain, seven thrombospondin repeats (type 1 and type 1-like), transmembrane domain (TM) and short cytoplasmic domain, (semaphorin) 5A (SEMA5A), mRNA. |
| SP4 | -2.09 | Homo sapiens Sp4 transcription factor (SP4), mRNA. |
| IRAK1 | -2.09 | Homo sapiens interleukin-1 receptor-associated kinase 1 (IRAK1), transcript variant 3, mRNA. |
| LOC729859 | -2.09 | PREDICTED: Homo sapiens misc_RNA (LOC729859), miscRNA. |
| MED25 | -2.09 | Homo sapiens mediator complex subunit 25 (MED25), mRNA. |
| SULF2 | -2.09 | Homo sapiens sulfatase 2 (SULF2), transcript variant 1, mRNA. |
| CDK10 | -2.09 | Homo sapiens cyclin-dependent kinase 10 (CDK10), transcript variant c, mRNA. |
| TMEM18 | -2.09 | Homo sapiens transmembrane protein 18 (TMEM18), mRNA. |
| DSTN | -2.09 | Homo sapiens destrin (actin depolymerizing factor) (DSTN), transcript variant 1, mRNA. |
| ERCC5 | -2.1 | Homo sapiens excision repair cross-complementing rodent repair deficiency, complementation group 5 (ERCC5), mRNA. |
| C19orf22 | -2.1 | Homo sapiens chromosome 19 open reading frame 22 (C19orf22), mRNA. |
| PDZD4 | -2.1 | Homo sapiens PDZ domain containing 4 (PDZD4), mRNA. |
| AHCY | -2.1 | Homo sapiens S-adenosylhomocysteine hydrolase (AHCY), mRNA. |
| SLC35C1 | -2.1 | Homo sapiens solute carrier family 35, member C1 (SLC35C1), mRNA. |
| TAF6 | -2.1 | Homo sapiens TAF6 RNA polymerase II, TATA box binding protein (TBP)-associated factor, 80kDa (TAF6), transcript variant 2, mRNA. |
| GRN | -2.1 | Homo sapiens granulin (GRN), mRNA. |
| FLJ25006 | -2.1 | Homo sapiens uncharacterized serine/threonine-protein kinase SgK494 (FLJ25006), mRNA. |
| ALG6 | -2.1 | Homo sapiens asparagine-linked glycosylation 6 homolog (S. cerevisiae, alpha-1,3-glucosyltransferase) (ALG6), mRNA. |
| RBMS1 | -2.11 | Homo sapiens RNA binding motif, single stranded interacting protein 1 (RBMS1), transcript variant 3, mRNA. |
| C9orf46 | -2.11 | Homo sapiens chromosome 9 open reading frame 46 (C9orf46), mRNA. |
| NUCKS1 | -2.11 | Homo sapiens nuclear casein kinase and cyclin-dependent kinase substrate 1 (NUCKS1), mRNA. |
| LASS6 | -2.11 | Homo sapiens LAG1 homolog, ceramide synthase 6 (LASS6), mRNA. |
| SORL1 | -2.11 | Homo sapiens sortilin-related receptor, L(DLR class) A repeats-containing (SORL1), mRNA. |
| ZMYM3 | -2.12 | Homo sapiens zinc finger, MYM-type 3 (ZMYM3), transcript variant 2, mRNA. |
| MAPK3 | -2.12 | Homo sapiens mitogen-activated protein kinase 3 (MAPK3), transcript variant 2, mRNA. |
| PRDM8 | -2.12 | Homo sapiens PR domain containing 8 (PRDM8), transcript variant 1, mRNA. |
| CAMK1 | -2.12 | Homo sapiens calcium/calmodulin-dependent protein kinase I (CAMK1), mRNA. |
| CEP164 | -2.12 | Homo sapiens centrosomal protein 164kDa (CEP164), mRNA. |
| KIF11 | -2.12 | Homo sapiens kinesin family member 11 (KIF11), mRNA. |
| POLE | -2.12 | Homo sapiens polymerase (DNA directed), epsilon (POLE), mRNA. |
| DGCR8 | -2.12 | Homo sapiens DiGeorge syndrome critical region gene 8 (DGCR8), mRNA. |
| POPDC2 | -2.12 | Homo sapiens popeye domain containing 2 (POPDC2), mRNA. |
| RTN2 | -2.12 | Homo sapiens reticulon 2 (RTN2), transcript variant 3, mRNA. |
| LOC729495 | -2.12 | PREDICTED: Homo sapiens similar to hCG1999863 (LOC729495), mRNA. |
| ZNF581 | -2.12 | Homo sapiens zinc finger protein 581 (ZNF581), mRNA. |
| CACHD1 | -2.13 | Homo sapiens cache domain containing 1 (CACHD1), mRNA. |
| WASF2 | -2.13 | Homo sapiens WAS protein family, member 2 (WASF2), mRNA. |
| MEIS3 | -2.13 | Homo sapiens Meis homeobox 3 (MEIS3), transcript variant 2, mRNA. |
| LOC729580 | -2.13 | PREDICTED: Homo sapiens hypothetical LOC729580 (LOC729580), mRNA. |
| CPNE1 | -2.13 | Homo sapiens copine I (CPNE1), transcript variant 8, mRNA. |
| LOC100130178 | -2.13 | PREDICTED: Homo sapiens misc_RNA (LOC100130178), miscRNA. |
| C2orf24 | -2.13 | Homo sapiens chromosome 2 open reading frame 24 (C2orf24), mRNA. |
| VAC14 | -2.13 | Homo sapiens Vac14 homolog (S. cerevisiae) (VAC14), mRNA. |
| AGAP8 | -2.14 | Homo sapiens ArfGAP with GTPase domain, ankyrin repeat and PH domain 8 (AGAP8), mRNA. |
| LOC648581 | -2.14 | PREDICTED: Homo sapiens similar to alveolar soft part sarcoma chromosome region, candidate 1 (LOC648581), mRNA. |
| LOC147727 | -2.14 | Homo sapiens hypothetical LOC147727 (LOC147727), non-coding RNA. |
| ZFYVE1 | -2.14 | Homo sapiens zinc finger, FYVE domain containing 1 (ZFYVE1), transcript variant 1, mRNA. |
| DNASE1L1 | -2.14 | Homo sapiens deoxyribonuclease I-like 1 (DNASE1L1), transcript variant 4, mRNA. |
| BRSK1 | -2.14 | Homo sapiens BR serine/threonine kinase 1 (BRSK1), mRNA. |
| TOP2A | -2.14 | Homo sapiens topoisomerase (DNA) II alpha 170kDa (TOP2A), mRNA. |
| JUND | -2.14 | Homo sapiens jun D proto-oncogene (JUND), mRNA. |
| FAM125B | -2.14 | Homo sapiens family with sequence similarity 125, member B (FAM125B), transcript variant 1, mRNA. |
| MBTPS1 | -2.14 | Homo sapiens membrane-bound transcription factor peptidase, site 1 (MBTPS1), mRNA. |
| LOC255167 | -2.14 | Homo sapiens hypothetical LOC255167 (LOC255167), non-coding RNA. |
| PYGO2 | -2.15 | Homo sapiens pygopus homolog 2 (Drosophila) (PYGO2), mRNA. |
| LOC390298 | -2.15 | PREDICTED: Homo sapiens similar to translocase of inner mitochondrial membrane 17 homolog B (LOC390298), miscRNA. |
| FAM73B | -2.15 | Homo sapiens family with sequence similarity 73, member B (FAM73B), mRNA. |
| LOC729843 | -2.15 | PREDICTED: Homo sapiens similar to WW domain binding protein 1 (LOC729843), mRNA. |
| SCARNA22 | -2.15 | Homo sapiens small Cajal body-specific RNA 22 (SCARNA22), guide RNA. |
| MAPK11 | -2.15 | Homo sapiens mitogen-activated protein kinase 11 (MAPK11), mRNA. |
| MBD4 | -2.15 | Homo sapiens methyl-CpG binding domain protein 4 (MBD4), mRNA. |
| C14orf102 | -2.15 | Homo sapiens chromosome 14 open reading frame 102 (C14orf102), transcript variant 2, mRNA. |
| MLEC | -2.15 | Homo sapiens malectin (MLEC), mRNA. |
| EIF3B | -2.16 | Homo sapiens eukaryotic translation initiation factor 3, subunit B (EIF3B), transcript variant 1, mRNA. |
| TROAP | -2.16 | Homo sapiens trophinin associated protein (tastin) (TROAP), mRNA. |
| FRMD6 | -2.16 | Homo sapiens FERM domain containing 6 (FRMD6), transcript variant 2, mRNA. |
| MAPK12 | -2.16 | Homo sapiens mitogen-activated protein kinase 12 (MAPK12), mRNA. |
| MPST | -2.16 | Homo sapiens mercaptopyruvate sulfurtransferase (MPST), nuclear gene encoding mitochondrial protein, transcript variant 2, mRNA. |
| FCGRT | -2.17 | Homo sapiens Fc fragment of IgG, receptor, transporter, alpha (FCGRT), transcript variant 1, mRNA. |
| NPIP | -2.17 | Homo sapiens nuclear pore complex interacting protein (NPIP), mRNA. |
| SLC7A2 | -2.17 | Homo sapiens solute carrier family 7 (cationic amino acid transporter, y+ system), member 2 (SLC7A2), transcript variant 2, mRNA. |
| ZFPM1 | -2.17 | Homo sapiens zinc finger protein, multitype 1 (ZFPM1), mRNA. |
| VPS13A | -2.17 | Homo sapiens vacuolar protein sorting 13 homolog A (S. cerevisiae) (VPS13A), transcript variant D, mRNA. |
| FAM73A | -2.18 | Homo sapiens family with sequence similarity 73, member A (FAM73A), mRNA. |
| CHMP1A | -2.18 | Homo sapiens chromatin modifying protein 1A (CHMP1A), transcript variant 2, mRNA. |
| PCK2 | -2.18 | Homo sapiens phosphoenolpyruvate carboxykinase 2 (mitochondrial) (PCK2), nuclear gene encoding mitochondrial protein, transcript variant 1, mRNA. |
| ARHGAP11B | -2.18 | Homo sapiens Rho GTPase activating protein 11B (ARHGAP11B), mRNA. XM_931045 XM_931050 |
| ST7OT1 | -2.18 | Homo sapiens ST7 overlapping transcript 1 (non-protein coding) (ST7OT1), antisense RNA. |
| KCTD20 | -2.18 | Homo sapiens potassium channel tetramerisation domain containing 20 (KCTD20), mRNA. |
| NME4 | -2.18 | Homo sapiens non-metastatic cells 4, protein expressed in (NME4), nuclear gene encoding mitochondrial protein, mRNA. |
| SSPN | -2.18 | Homo sapiens sarcospan (Kras oncogene-associated gene) (SSPN), mRNA. |
| SPIRE2 | -2.18 | Homo sapiens spire homolog 2 (Drosophila) (SPIRE2), mRNA. |
| POMT2 | -2.18 | Homo sapiens protein-O-mannosyltransferase 2 (POMT2), mRNA. |
| SSBP4 | -2.18 | Homo sapiens single stranded DNA binding protein 4 (SSBP4), transcript variant 1, mRNA. |
| ASAM | -2.19 | Homo sapiens adipocyte-specific adhesion molecule (ASAM), mRNA. |
| DUSP6 | -2.19 | Homo sapiens dual specificity phosphatase 6 (DUSP6), transcript variant 2, mRNA. |
| PIM1 | -2.19 | Homo sapiens pim-1 oncogene (PIM1), mRNA. |
| BBS9 | -2.2 | Homo sapiens Bardet-Biedl syndrome 9 (BBS9), transcript variant 3, mRNA. |
| ATP6V0C | -2.2 | PREDICTED: Homo sapiens ATPase, H+ transporting, lysosomal 16kDa, V0 subunit c (ATP6V0C), mRNA. |
| GTF3A | -2.2 | Homo sapiens general transcription factor IIIA (GTF3A), mRNA. |
| SF1 | -2.2 | Homo sapiens splicing factor 1 (SF1), transcript variant 1, mRNA. |
| PRKDC | -2.2 | Homo sapiens protein kinase, DNA-activated, catalytic polypeptide (PRKDC), transcript variant 1, mRNA. |
| SCLY | -2.2 | Homo sapiens selenocysteine lyase (SCLY), mRNA. |
| ZFHX3 | -2.21 | Homo sapiens zinc finger homeobox 3 (ZFHX3), transcript variant A, mRNA. |
| SLC29A1 | -2.21 | Homo sapiens solute carrier family 29 (nucleoside transporters), member 1 (SLC29A1), nuclear gene encoding mitochondrial protein, transcript variant 4, mRNA. |
| ERC1 | -2.21 | Homo sapiens ELKS/RAB6-interacting/CAST family member 1 (ERC1), transcript variant beta, mRNA. |
| PTPLA | -2.21 | Homo sapiens protein tyrosine phosphatase-like (proline instead of catalytic arginine), member A (PTPLA), mRNA. |
| SHARPIN | -2.21 | Homo sapiens SHANK-associated RH domain interactor (SHARPIN), mRNA. |
| MPRIP | -2.21 | Homo sapiens myosin phosphatase Rho interacting protein (MPRIP), transcript variant 1, mRNA. |
| WDR24 | -2.21 | Homo sapiens WD repeat domain 24 (WDR24), mRNA. |
| MYST3 | -2.21 | Homo sapiens MYST histone acetyltransferase (monocytic leukemia) 3 (MYST3), mRNA. |
| C1orf66 | -2.21 | Homo sapiens chromosome 1 open reading frame 66 (C1orf66), mRNA. |
| ZKSCAN1 | -2.21 | Homo sapiens zinc finger with KRAB and SCAN domains 1 (ZKSCAN1), mRNA. |
| LOC100008589 | -2.21 | Homo sapiens 28S ribosomal RNA (LOC100008589), non-coding RNA. |
| MPRIP | -2.21 | Homo sapiens myosin phosphatase Rho interacting protein (MPRIP), transcript variant 2, mRNA. |
| CREBZF | -2.21 | Homo sapiens CREB/ATF bZIP transcription factor (CREBZF), mRNA. |
| RFNG | -2.21 | PREDICTED: Homo sapiens radical fringe homolog (Drosophila) (RFNG), mRNA. |
| FAM3A | -2.21 | Homo sapiens family with sequence similarity 3, member A (FAM3A), mRNA. |
| NUMA1 | -2.21 | Homo sapiens nuclear mitotic apparatus protein 1 (NUMA1), mRNA. |
| NFIB | -2.21 | Homo sapiens nuclear factor I/B (NFIB), mRNA. |
| VPS52 | -2.22 | Homo sapiens vacuolar protein sorting 52 homolog (S. cerevisiae) (VPS52), mRNA. |
| LOC644936 | -2.22 | Homo sapiens cytoplasmic beta-actin pseudogene (LOC644936), non-coding RNA. |
| LOC92249 | -2.22 | Homo sapiens hypothetical LOC92249 (LOC92249), non-coding RNA. |
| CBL | -2.22 | Homo sapiens Cas-Br-M (murine) ecotropic retroviral transforming sequence (CBL), mRNA. |
| LRRC17 | -2.22 | Homo sapiens leucine rich repeat containing 17 (LRRC17), transcript variant 1, mRNA. |
| TNFSF15 | -2.22 | Homo sapiens tumor necrosis factor (ligand) superfamily, member 15 (TNFSF15), mRNA. |
| MAGEC1 | -2.22 | Homo sapiens melanoma antigen family C, 1 (MAGEC1), mRNA. |
| C19orf29 | -2.22 | Homo sapiens chromosome 19 open reading frame 29 (C19orf29), transcript variant 1, mRNA. |
| ZYX | -2.22 | Homo sapiens zyxin (ZYX), transcript variant 1, mRNA. |
| IL27RA | -2.22 | Homo sapiens interleukin 27 receptor, alpha (IL27RA), mRNA. |
| XPO1 | -2.22 | Homo sapiens exportin 1 (CRM1 homolog, yeast) (XPO1), mRNA. |
| BMP1 | -2.22 | Homo sapiens bone morphogenetic protein 1 (BMP1), transcript variant BMP1-3, mRNA. |
| FLJ27365 | -2.22 | Homo sapiens FLJ27365 protein (FLJ27365), mRNA. |
| VSTM2L | -2.23 | Homo sapiens V-set and transmembrane domain containing 2 like (VSTM2L), mRNA. |
| GGH | -2.23 | Homo sapiens gamma-glutamyl hydrolase (conjugase, folylpolygammaglutamyl hydrolase) (GGH), mRNA. |
| TDRD1 | -2.23 | Homo sapiens tudor domain containing 1 (TDRD1), mRNA. |
| LOC647000 | -2.23 | PREDICTED: Homo sapiens similar to tubulin, beta 5 (LOC647000), mRNA. |
| GNAO1 | -2.23 | Homo sapiens guanine nucleotide binding protein (G protein), alpha activating activity polypeptide O (GNAO1), transcript variant 1, mRNA. |
| DYM | -2.24 | Homo sapiens dymeclin (DYM), mRNA. |
| RIMS2 | -2.24 | Homo sapiens regulating synaptic membrane exocytosis 2 (RIMS2), transcript variant 2, mRNA. |
| UBE2O | -2.24 | Homo sapiens ubiquitin-conjugating enzyme E2O (UBE2O), mRNA. |
| PCNXL3 | -2.24 | PREDICTED: Homo sapiens pecanex-like 3 (Drosophila), transcript variant 1 (PCNXL3), mRNA. |
| CDC42SE1 | -2.24 | Homo sapiens CDC42 small effector 1 (CDC42SE1), transcript variant 1, mRNA. |
| TBC1D10B | -2.24 | Homo sapiens TBC1 domain family, member 10B (TBC1D10B), mRNA. |
| C9orf30 | -2.24 | Homo sapiens chromosome 9 open reading frame 30 (C9orf30), mRNA. |
| OSBPL7 | -2.24 | Homo sapiens oxysterol binding protein-like 7 (OSBPL7), transcript variant 1, mRNA. |
| GMEB1 | -2.24 | Homo sapiens glucocorticoid modulatory element binding protein 1 (GMEB1), transcript variant 2, mRNA. |
| ESPL1 | -2.24 | Homo sapiens extra spindle pole bodies homolog 1 (S. cerevisiae) (ESPL1), mRNA. |
| LYN | -2.24 | Homo sapiens v-yes-1 Yamaguchi sarcoma viral related oncogene homolog (LYN), mRNA. |
| LOC653438 | -2.25 | PREDICTED: Homo sapiens similar to CG33096-PB, isoform B, transcript variant 1 (LOC653438), mRNA. |
| FLNB | -2.25 | Homo sapiens filamin B, beta (actin binding protein 278) (FLNB), mRNA. |
| ASPSCR1 | -2.25 | PREDICTED: Homo sapiens alveolar soft part sarcoma chromosome region, candidate 1 (ASPSCR1), mRNA. |
| GPR125 | -2.25 | PREDICTED: Homo sapiens G protein-coupled receptor 125, transcript variant 4 (GPR125), mRNA. |
| LOC100128196 | -2.25 | PREDICTED: Homo sapiens misc_RNA (LOC100128196), miscRNA. |
| NBL1 | -2.25 | Homo sapiens neuroblastoma, suppression of tumorigenicity 1 (NBL1), transcript variant 2, mRNA. |
| CBLL1 | -2.25 | Homo sapiens Cas-Br-M (murine) ecotropic retroviral transforming sequence-like 1 (CBLL1), mRNA. |
| RHBDD3 | -2.26 | Homo sapiens rhomboid domain containing 3 (RHBDD3), mRNA. |
| PC | -2.26 | Homo sapiens pyruvate carboxylase (PC), nuclear gene encoding mitochondrial protein, transcript variant A, mRNA. |
| LAMB1 | -2.26 | Homo sapiens laminin, beta 1 (LAMB1), mRNA. |
| CCNY | -2.26 | Homo sapiens cyclin Y (CCNY), transcript variant 1, mRNA. |
| LOC651816 | -2.26 | PREDICTED: Homo sapiens similar to Ubiquitin-conjugating enzyme E2S (Ubiquitin-conjugating enzyme E2-24 kDa) (Ubiquitin-protein ligase) (Ubiquitin carrier protein) (E2-EPF5) (LOC651816), mRNA. |
| SYNE2 | -2.26 | Homo sapiens spectrin repeat containing, nuclear envelope 2 (SYNE2), transcript variant 1, mRNA. |
| LRRC45 | -2.26 | Homo sapiens leucine rich repeat containing 45 (LRRC45), mRNA. |
| FBLN1 | -2.27 | Homo sapiens fibulin 1 (FBLN1), transcript variant A, mRNA. |
| GMIP | -2.27 | Homo sapiens GEM interacting protein (GMIP), mRNA. |
| KLF11 | -2.27 | PREDICTED: Homo sapiens Kruppel-like factor 11 (KLF11), mRNA. |
| CMBL | -2.28 | Homo sapiens carboxymethylenebutenolidase homolog (Pseudomonas) (CMBL), mRNA. |
| FBLN2 | -2.28 | Homo sapiens fibulin 2 (FBLN2), transcript variant 2, mRNA. |
| MRPL12 | -2.28 | Homo sapiens mitochondrial ribosomal protein L12 (MRPL12), nuclear gene encoding mitochondrial protein, mRNA. |
| TBC1D22A | -2.28 | Homo sapiens TBC1 domain family, member 22A (TBC1D22A), mRNA. |
| LOC648149 | -2.28 | PREDICTED: Homo sapiens hypothetical protein LOC648149 (LOC648149), mRNA. |
| TPM4 | -2.29 | Homo sapiens tropomyosin 4 (TPM4), mRNA. |
| DIAPH1 | -2.29 | Homo sapiens diaphanous homolog 1 (Drosophila) (DIAPH1), transcript variant 1, mRNA. |
| ANKRD13B | -2.29 | Homo sapiens ankyrin repeat domain 13B (ANKRD13B), mRNA. |
| ADCY1 | -2.29 | Homo sapiens adenylate cyclase 1 (brain) (ADCY1), mRNA. |
| LOC652755 | -2.29 | PREDICTED: Homo sapiens similar to Baculoviral IAP repeat-containing protein 1 (Neuronal apoptosis inhibitory protein) (LOC652755), mRNA. |
| SREBF1 | -2.29 | Homo sapiens sterol regulatory element binding transcription factor 1 (SREBF1), transcript variant 1, mRNA. |
| IKZF4 | -2.3 | Homo sapiens IKAROS family zinc finger 4 (Eos) (IKZF4), mRNA. |
| SEMA6C | -2.3 | Homo sapiens sema domain, transmembrane domain (TM), and cytoplasmic domain, (semaphorin) 6C (SEMA6C), mRNA. |
| CHES1 | -2.3 | Homo sapiens checkpoint suppressor 1 (CHES1), mRNA. |
| OTUB1 | -2.3 | Homo sapiens OTU domain, ubiquitin aldehyde binding 1 (OTUB1), mRNA. |
| ATRN | -2.3 | Homo sapiens attractin (ATRN), transcript variant 1, mRNA. |
| MYO1C | -2.3 | Homo sapiens myosin IC (MYO1C), transcript variant 3, mRNA. |
| MEGF6 | -2.3 | Homo sapiens multiple EGF-like-domains 6 (MEGF6), mRNA. |
| DENND4B | -2.3 | Homo sapiens DENN/MADD domain containing 4B (DENND4B), mRNA. |
| DNMT3B | -2.3 | Homo sapiens DNA (cytosine-5-)-methyltransferase 3 beta (DNMT3B), transcript variant 1, mRNA. |
| SAV1 | -2.3 | Homo sapiens salvador homolog 1 (Drosophila) (SAV1), mRNA. |
|  | -2.3 | AV652851 GLC Homo sapiens cDNA clone GLCDEG06 3, mRNA sequence |
| CYFIP2 | -2.3 | Homo sapiens cytoplasmic FMR1 interacting protein 2 (CYFIP2), transcript variant 1, mRNA. |
| TUBGCP2 | -2.3 | Homo sapiens tubulin, gamma complex associated protein 2 (TUBGCP2), mRNA. |
| C16orf79 | -2.3 | Homo sapiens chromosome 16 open reading frame 79 (C16orf79), mRNA. |
| PRKCSH | -2.3 | Homo sapiens protein kinase C substrate 80K-H (PRKCSH), transcript variant 2, mRNA. |
| TERF1 | -2.31 | Homo sapiens telomeric repeat binding factor (NIMA-interacting) 1 (TERF1), transcript variant 1, mRNA. |
| POFUT1 | -2.31 | Homo sapiens protein O-fucosyltransferase 1 (POFUT1), transcript variant 1, mRNA. |
| LOC100129269 | -2.31 | PREDICTED: Homo sapiens hypothetical protein LOC100129269 (LOC100129269), mRNA. |
| TMEM109 | -2.31 | Homo sapiens transmembrane protein 109 (TMEM109), mRNA. |
| PITPNC1 | -2.31 | Homo sapiens phosphatidylinositol transfer protein, cytoplasmic 1 (PITPNC1), transcript variant 2, mRNA. |
| RNF214 | -2.31 | Homo sapiens ring finger protein 214 (RNF214), transcript variant 1, mRNA. |
| STUB1 | -2.31 | Homo sapiens STIP1 homology and U-box containing protein 1 (STUB1), mRNA. |
| TMUB2 | -2.31 | Homo sapiens transmembrane and ubiquitin-like domain containing 2 (TMUB2), transcript variant 3, mRNA. |
| OIP5 | -2.32 | Homo sapiens Opa interacting protein 5 (OIP5), mRNA. |
| KPNA6 | -2.32 | Homo sapiens karyopherin alpha 6 (importin alpha 7) (KPNA6), mRNA. |
| CEP152 | -2.32 | Homo sapiens centrosomal protein 152kDa (CEP152), mRNA. |
| PHKB | -2.32 | Homo sapiens phosphorylase kinase, beta (PHKB), transcript variant 1, mRNA. |
| ADCK4 | -2.32 | Homo sapiens aarF domain containing kinase 4 (ADCK4), mRNA. |
| POR | -2.32 | Homo sapiens P450 (cytochrome) oxidoreductase (POR), mRNA. |
| ADRM1 | -2.32 | Homo sapiens adhesion regulating molecule 1 (ADRM1), transcript variant 2, mRNA. |
| STUB1 | -2.32 | Homo sapiens STIP1 homology and U-box containing protein 1 (STUB1), mRNA. |
| ST6GALNAC4 | -2.32 | Homo sapiens ST6 (alpha-N-acetyl-neuraminyl-2,3-beta-galactosyl-1, 3)-N-acetylgalactosaminide alpha-2,6-sialyltransferase 4 (ST6GALNAC4), transcript variant 1, mRNA. |
| LOC729495 | -2.32 | PREDICTED: Homo sapiens similar to hCG1999863 (LOC729495), mRNA. |
| PI4KAP1 | -2.32 | Homo sapiens phosphatidylinositol 4-kinase, catalytic, alpha pseudogene 1 (PI4KAP1), non-coding RNA. |
| SLC30A1 | -2.33 | Homo sapiens solute carrier family 30 (zinc transporter), member 1 (SLC30A1), mRNA. |
| LOC728819 | -2.34 | Homo sapiens hCG1645220 (LOC728819), mRNA. |
| CYTH3 | -2.34 | Homo sapiens cytohesin 3 (CYTH3), mRNA. |
| DLG4 | -2.34 | Homo sapiens discs, large homolog 4 (Drosophila) (DLG4), mRNA. |
| FLJ10986 | -2.34 | Homo sapiens hypothetical protein FLJ10986 (FLJ10986), mRNA. |
| FAM39DP | -2.34 | Homo sapiens family with sequence similarity 39, member D pseudogene (FAM39DP) on chromosome 15. |
| CTR9 | -2.34 | Homo sapiens Ctr9, Paf1/RNA polymerase II complex component, homolog (S. cerevisiae) (CTR9), mRNA. |
| PNPLA2 | -2.34 | Homo sapiens patatin-like phospholipase domain containing 2 (PNPLA2), mRNA. |
| CENPT | -2.35 | Homo sapiens centromere protein T (CENPT), mRNA. |
| NUF2 | -2.35 | Homo sapiens NUF2, NDC80 kinetochore complex component, homolog (S. cerevisiae) (NUF2), transcript variant 2, mRNA. |
| COBRA1 | -2.35 | Homo sapiens cofactor of BRCA1 (COBRA1), mRNA. |
| C1orf159 | -2.35 | Homo sapiens chromosome 1 open reading frame 159 (C1orf159), mRNA. |
| TDRD1 | -2.35 | Homo sapiens tudor domain containing 1 (TDRD1), mRNA. |
| INTS1 | -2.35 | PREDICTED: Homo sapiens integrator complex subunit 1, transcript variant 11 (INTS1), mRNA. |
| SCARB1 | -2.35 | Homo sapiens scavenger receptor class B, member 1 (SCARB1), mRNA. |
| SNORD3D | -2.36 | Homo sapiens small nucleolar RNA, C/D box 3D (SNORD3D), small nucleolar RNA. |
| FAM110A | -2.36 | Homo sapiens family with sequence similarity 110, member A (FAM110A), transcript variant 3, mRNA. |
| MYO9B | -2.36 | Homo sapiens myosin IXB (MYO9B), mRNA. |
|  | -2.36 | AGENCOURT_14354957 NIH_MGC_191 Homo sapiens cDNA clone IMAGE:30413554 5, mRNA sequence |
| CDCA3 | -2.36 | Homo sapiens cell division cycle associated 3 (CDCA3), mRNA. |
| SPRY2 | -2.37 | Homo sapiens sprouty homolog 2 (Drosophila) (SPRY2), mRNA. |
| FOXRED2 | -2.37 | Homo sapiens FAD-dependent oxidoreductase domain containing 2 (FOXRED2), mRNA. |
| CDC25B | -2.37 | Homo sapiens cell division cycle 25 homolog B (S. pombe) (CDC25B), transcript variant 2, mRNA. |
| CTSK | -2.37 | Homo sapiens cathepsin K (CTSK), mRNA. |
| CHERP | -2.37 | Homo sapiens calcium homeostasis endoplasmic reticulum protein (CHERP), mRNA. |
| AK5 | -2.37 | Homo sapiens adenylate kinase 5 (AK5), transcript variant 2, mRNA. |
| PPP2R1A | -2.37 | Homo sapiens protein phosphatase 2 (formerly 2A), regulatory subunit A , alpha isoform (PPP2R1A), mRNA. |
| RASA4P | -2.38 | Homo sapiens RAS p21 protein activator 4 pseudogene (RASA4P), non-coding RNA. |
| NUP188 | -2.38 | Homo sapiens nucleoporin 188kDa (NUP188), mRNA. |
| HMGB2 | -2.38 | Homo sapiens high-mobility group box 2 (HMGB2), mRNA. |
| LOC400406 | -2.38 | PREDICTED: Homo sapiens similar to ADAM metallopeptidase with thrombospondin type 1 motif, 7 preproprotein (LOC400406), mRNA. |
| LOC642828 | -2.38 | PREDICTED: Homo sapiens misc_RNA (LOC642828), miscRNA. |
| POLM | -2.38 | Homo sapiens polymerase (DNA directed), mu (POLM), mRNA. |
| NCOA5 | -2.39 | Homo sapiens nuclear receptor coactivator 5 (NCOA5), mRNA. |
| HLA-A | -2.39 | Homo sapiens major histocompatibility complex, class I, A (HLA-A), mRNA. |
| RXRA | -2.39 | Homo sapiens retinoid X receptor, alpha (RXRA), mRNA. |
| FBXL6 | -2.39 | Homo sapiens F-box and leucine-rich repeat protein 6 (FBXL6), transcript variant 2, mRNA. |
| SSTR2 | -2.39 | Homo sapiens somatostatin receptor 2 (SSTR2), mRNA. |
| ST5 | -2.4 | Homo sapiens suppression of tumorigenicity 5 (ST5), transcript variant 3, mRNA. |
| USP21 | -2.4 | Homo sapiens ubiquitin specific peptidase 21 (USP21), transcript variant 1, mRNA. |
| CYP1B1 | -2.4 | Homo sapiens cytochrome P450, family 1, subfamily B, polypeptide 1 (CYP1B1), mRNA. |
| C1orf86 | -2.4 | Homo sapiens chromosome 1 open reading frame 86 (C1orf86), transcript variant 2, mRNA. |
| SPRY1 | -2.4 | Homo sapiens sprouty homolog 1, antagonist of FGF signaling (Drosophila) (SPRY1), transcript variant 2, mRNA. |
|  | -2.41 | full-length cDNA clone CS0DF032YA11 of Fetal brain of Homo sapiens (human) |
| TADA3 | -2.41 | Homo sapiens transcriptional adaptor 3 (TADA3), transcript variant 2, mRNA. |
| BCOR | -2.41 | Homo sapiens BCL6 co-repressor (BCOR), transcript variant 1, mRNA. |
| ACIN1 | -2.41 | Homo sapiens apoptotic chromatin condensation inducer 1 (ACIN1), mRNA. |
| TBCD | -2.41 | Homo sapiens tubulin folding cofactor D (TBCD), mRNA. |
| PHAX | -2.41 | Homo sapiens phosphorylated adaptor for RNA export (PHAX), mRNA. |
| PCDH20 | -2.41 | Homo sapiens protocadherin 20 (PCDH20), mRNA. |
| MAN2C1 | -2.41 | Homo sapiens mannosidase, alpha, class 2C, member 1 (MAN2C1), mRNA. |
| CANT1 | -2.41 | Homo sapiens calcium activated nucleotidase 1 (CANT1), mRNA. |
| LOC652837 | -2.42 | PREDICTED: Homo sapiens similar to C-terminal binding protein 2 (LOC652837), mRNA. |
| FHOD1 | -2.42 | Homo sapiens formin homology 2 domain containing 1 (FHOD1), mRNA. |
| PKD2 | -2.42 | Homo sapiens polycystic kidney disease 2 (autosomal dominant) (PKD2), mRNA. |
| UPF3A | -2.42 | Homo sapiens UPF3 regulator of nonsense transcripts homolog A (yeast) (UPF3A), transcript variant 1, mRNA. |
| SMYD3 | -2.43 | Homo sapiens SET and MYND domain containing 3 (SMYD3), mRNA. |
| LSM3 | -2.43 | Homo sapiens LSM3 homolog, U6 small nuclear RNA associated (S. cerevisiae) (LSM3), mRNA. |
| RAB15 | -2.43 | Homo sapiens RAB15, member RAS onocogene family (RAB15), mRNA. |
| GNG8 | -2.43 | Homo sapiens guanine nucleotide binding protein (G protein), gamma 8 (GNG8), mRNA. |
| TSPAN7 | -2.43 | Homo sapiens tetraspanin 7 (TSPAN7), mRNA. |
| NPTX2 | -2.44 | Homo sapiens neuronal pentraxin II (NPTX2), mRNA. |
| CTSB | -2.44 | Homo sapiens cathepsin B (CTSB), transcript variant 2, mRNA. |
| PCDHB10 | -2.44 | Homo sapiens protocadherin beta 10 (PCDHB10), mRNA. |
| CCDC97 | -2.44 | Homo sapiens coiled-coil domain containing 97 (CCDC97), mRNA. |
| LOC100132658 | -2.44 | PREDICTED: Homo sapiens misc_RNA (LOC100132658), miscRNA. |
| RBM10 | -2.44 | Homo sapiens RNA binding motif protein 10 (RBM10), transcript variant 1, mRNA. |
| GATAD2B | -2.44 | Homo sapiens GATA zinc finger domain containing 2B (GATAD2B), mRNA. |
| C19orf6 | -2.44 | Homo sapiens chromosome 19 open reading frame 6 (C19orf6), transcript variant 1, mRNA. |
| SIGMAR1 | -2.44 | Homo sapiens sigma non-opioid intracellular receptor 1 (SIGMAR1), transcript variant 2, mRNA. |
| RPP25 | -2.44 | Homo sapiens ribonuclease P/MRP 25kDa subunit (RPP25), mRNA. |
| AGBL5 | -2.44 | Homo sapiens ATP/GTP binding protein-like 5 (AGBL5), transcript variant 1, mRNA. |
| KIAA0913 | -2.44 | Homo sapiens KIAA0913 (KIAA0913), mRNA. |
| CRTC2 | -2.44 | Homo sapiens CREB regulated transcription coactivator 2 (CRTC2), mRNA. |
| ENO2 | -2.44 | Homo sapiens enolase 2 (gamma, neuronal) (ENO2), mRNA. |
| RAC3 | -2.45 | Homo sapiens ras-related C3 botulinum toxin substrate 3 (rho family, small GTP binding protein Rac3) (RAC3), mRNA. |
| PTGR2 | -2.45 | Homo sapiens prostaglandin reductase 2 (PTGR2), mRNA. |
| DTL | -2.45 | Homo sapiens denticleless homolog (Drosophila) (DTL), mRNA. |
| CAMK2N2 | -2.45 | Homo sapiens calcium/calmodulin-dependent protein kinase II inhibitor 2 (CAMK2N2), mRNA. |
| CYTH1 | -2.45 | Homo sapiens cytohesin 1 (CYTH1), transcript variant 2, mRNA. |
| AHI1 | -2.45 | Homo sapiens Abelson helper integration site 1 (AHI1), mRNA. |
| MBOAT1 | -2.46 | Homo sapiens membrane bound O-acyltransferase domain containing 1 (MBOAT1), mRNA. |
| LOC648852 | -2.46 | PREDICTED: Homo sapiens hypothetical protein LOC648852 (LOC648852), mRNA. |
| ABHD12 | -2.46 | Homo sapiens abhydrolase domain containing 12 (ABHD12), transcript variant 1, mRNA. |
| AP1G2 | -2.46 | Homo sapiens adaptor-related protein complex 1, gamma 2 subunit (AP1G2), mRNA. |
| APEH | -2.46 | Homo sapiens N-acylaminoacyl-peptide hydrolase (APEH), mRNA. |
| GPS2 | -2.46 | Homo sapiens G protein pathway suppressor 2 (GPS2), mRNA. |
| PKD1 | -2.46 | Homo sapiens polycystic kidney disease 1 (autosomal dominant) (PKD1), transcript variant 1, mRNA. |
| MLF2 | -2.46 | Homo sapiens myeloid leukemia factor 2 (MLF2), mRNA. |
| RGL2 | -2.47 | Homo sapiens ral guanine nucleotide dissociation stimulator-like 2 (RGL2), mRNA. |
| CCDC86 | -2.47 | Homo sapiens coiled-coil domain containing 86 (CCDC86), mRNA. |
| SPTLC1 | -2.47 | Homo sapiens serine palmitoyltransferase, long chain base subunit 1 (SPTLC1), transcript variant 2, mRNA. |
| BIN1 | -2.47 | Homo sapiens bridging integrator 1 (BIN1), transcript variant 4, mRNA. |
| CYLN2 | -2.47 | Homo sapiens cytoplasmic linker 2 (CYLN2), transcript variant 2, mRNA. |
| HHAT | -2.47 | Homo sapiens hedgehog acyltransferase (HHAT), mRNA. |
| LOC400713 | -2.47 | PREDICTED: Homo sapiens zinc finger-like, transcript variant 3 (LOC400713), misc RNA. |
| LOC100134530 | -2.47 | PREDICTED: Homo sapiens hypothetical protein LOC100134530 (LOC100134530), mRNA. |
| EBF4 | -2.47 | PREDICTED: Homo sapiens early B-cell factor 4 (EBF4), mRNA. |
| ADAT3 | -2.48 | Homo sapiens adenosine deaminase, tRNA-specific 3, TAD3 homolog (S. cerevisiae) (ADAT3), mRNA. |
| BLOC1S1 | -2.48 | Homo sapiens biogenesis of lysosome-related organelles complex-1, subunit 1 (BLOC1S1), mRNA. |
| KLHL17 | -2.48 | Homo sapiens kelch-like 17 (Drosophila) (KLHL17), mRNA. |
| PTPRF | -2.48 | Homo sapiens protein tyrosine phosphatase, receptor type, F (PTPRF), transcript variant 2, mRNA. |
| PTPLB | -2.48 | Homo sapiens protein tyrosine phosphatase-like (proline instead of catalytic arginine), member b (PTPLB), mRNA. |
| WDR81 | -2.48 | Homo sapiens WD repeat domain 81 (WDR81), mRNA. |
| MAD1L1 | -2.49 | Homo sapiens MAD1 mitotic arrest deficient-like 1 (yeast) (MAD1L1), transcript variant 2, mRNA. |
| PACS2 | -2.49 | Homo sapiens phosphofurin acidic cluster sorting protein 2 (PACS2), mRNA. |
| HJURP | -2.49 | Homo sapiens Holliday junction recognition protein (HJURP), mRNA. |
| SFRP1 | -2.49 | Homo sapiens secreted frizzled-related protein 1 (SFRP1), mRNA. |
| TM9SF4 | -2.49 | Homo sapiens transmembrane 9 superfamily protein member 4 (TM9SF4), mRNA. |
| DDX11 | -2.49 | Homo sapiens DEAD/H (Asp-Glu-Ala-Asp/His) box polypeptide 11 (CHL1-like helicase homolog, S. cerevisiae) (DDX11), transcript variant 1, mRNA. |
| GPC1 | -2.49 | Homo sapiens glypican 1 (GPC1), mRNA. |
| CYCSL1 | -2.49 | Homo sapiens cytochrome c, somatic-like 1 (CYCSL1) on chromosome 6. |
| TMEM119 | -2.49 | Homo sapiens transmembrane protein 119 (TMEM119), mRNA. |
| SERPINB1 | -2.49 | Homo sapiens serpin peptidase inhibitor, clade B (ovalbumin), member 1 (SERPINB1), mRNA. |
| KDELC2 | -2.49 | Homo sapiens KDEL (Lys-Asp-Glu-Leu) containing 2 (KDELC2), mRNA. |
| CRAT | -2.49 | Homo sapiens carnitine acetyltransferase (CRAT), nuclear gene encoding mitochondrial protein, transcript variant 1, mRNA. |
| MACROD1 | -2.49 | Homo sapiens MACRO domain containing 1 (MACROD1), mRNA. |
| PLXNB2 | -2.5 | Homo sapiens plexin B2 (PLXNB2), mRNA. |
| TUSC1 | -2.5 | Homo sapiens tumor suppressor candidate 1 (TUSC1), mRNA. |
| KIAA1545 | -2.5 | PREDICTED: Homo sapiens KIAA1545 protein (KIAA1545), mRNA. |
| GAA | -2.5 | Homo sapiens glucosidase, alpha; acid (GAA), transcript variant 3, mRNA. |
| ATP1A3 | -2.5 | Homo sapiens ATPase, Na+/K+ transporting, alpha 3 polypeptide (ATP1A3), mRNA. |
| GTPBP8 | -2.5 | Homo sapiens GTP-binding protein 8 (putative) (GTPBP8), transcript variant 1, mRNA. |
| SNORA45 | -2.5 | Homo sapiens small nucleolar RNA, H/ACA box 45 (SNORA45), small nucleolar RNA. |
| ADA | -2.51 | Homo sapiens adenosine deaminase (ADA), mRNA. |
| RPS26 | -2.51 | Homo sapiens ribosomal protein S26 (RPS26), mRNA. |
| ALDOA | -2.51 | Homo sapiens aldolase A, fructose-bisphosphate (ALDOA), transcript variant 3, mRNA. |
| JUP | -2.51 | Homo sapiens junction plakoglobin (JUP), transcript variant 1, mRNA. |
| GARNL4 | -2.52 | Homo sapiens GTPase activating Rap/RanGAP domain-like 4 (GARNL4), mRNA. |
| GRINA | -2.52 | Homo sapiens glutamate receptor, ionotropic, N-methyl D-aspartate-associated protein 1 (glutamate binding) (GRINA), transcript variant 2, mRNA. |
| LPHN1 | -2.52 | Homo sapiens latrophilin 1 (LPHN1), transcript variant 2, mRNA. |
| LOC728452 | -2.52 | PREDICTED: Homo sapiens similar to nuclear pore membrane protein 121 (LOC728452), mRNA. |
| IRS2 | -2.52 | Homo sapiens insulin receptor substrate 2 (IRS2), mRNA. |
| CCDC90A | -2.53 | Homo sapiens coiled-coil domain containing 90A (CCDC90A), mRNA. |
| PCBP4 | -2.53 | Homo sapiens poly(rC) binding protein 4 (PCBP4), transcript variant 1, mRNA. |
| NRD1 | -2.53 | Homo sapiens nardilysin (N-arginine dibasic convertase) (NRD1), mRNA. |
| ROCK2 | -2.54 | Homo sapiens Rho-associated, coiled-coil containing protein kinase 2 (ROCK2), mRNA. |
| APOLD1 | -2.54 | Homo sapiens apolipoprotein L domain containing 1 (APOLD1), mRNA. |
| GPRIN1 | -2.54 | Homo sapiens G protein regulated inducer of neurite outgrowth 1 (GPRIN1), mRNA. |
| LOC100132247 | -2.54 | Homo sapiens similar to Uncharacterized protein KIAA0220 (LOC100132247), mRNA. |
| LOC644988 | -2.55 | PREDICTED: Homo sapiens misc_RNA (LOC644988), miscRNA. |
| CRABP1 | -2.55 | Homo sapiens cellular retinoic acid binding protein 1 (CRABP1), mRNA. |
| SDF4 | -2.55 | Homo sapiens stromal cell derived factor 4 (SDF4), mRNA. |
| PITPNM1 | -2.55 | Homo sapiens phosphatidylinositol transfer protein, membrane-associated 1 (PITPNM1), mRNA. |
| EMILIN1 | -2.55 | Homo sapiens elastin microfibril interfacer 1 (EMILIN1), mRNA. |
| KIF13B | -2.55 | Homo sapiens kinesin family member 13B (KIF13B), mRNA. |
| YRDC | -2.55 | Homo sapiens yrdC domain containing (E. coli) (YRDC), nuclear gene encoding mitochondrial protein, mRNA. |
| IGSF1 | -2.56 | Homo sapiens immunoglobulin superfamily, member 1 (IGSF1), transcript variant 1, mRNA. |
| RBM42 | -2.56 | Homo sapiens RNA binding motif protein 42 (RBM42), mRNA. |
| CLSTN1 | -2.56 | Homo sapiens calsyntenin 1 (CLSTN1), transcript variant 1, mRNA. |
| ILF3 | -2.56 | Homo sapiens interleukin enhancer binding factor 3, 90kDa (ILF3), transcript variant 1, mRNA. |
| LOC649841 | -2.56 | PREDICTED: Homo sapiens similar to protein immuno-reactive with anti-PTH polyclonal antibodies (LOC649841), mRNA. |
| LOC728728 | -2.56 | PREDICTED: Homo sapiens misc_RNA (LOC728728), miscRNA. |
| NR1H2 | -2.56 | Homo sapiens nuclear receptor subfamily 1, group H, member 2 (NR1H2), mRNA. |
| NT5DC2 | -2.57 | Homo sapiens 5'-nucleotidase domain containing 2 (NT5DC2), mRNA. |
| LOC387882 | -2.57 | Homo sapiens hypothetical protein (LOC387882), mRNA. |
| SLC6A8 | -2.58 | Homo sapiens solute carrier family 6 (neurotransmitter transporter, creatine), member 8 (SLC6A8), mRNA. |
| MDK | -2.58 | Homo sapiens midkine (neurite growth-promoting factor 2) (MDK), transcript variant 1, mRNA. |
| ADAM23 | -2.58 | Homo sapiens ADAM metallopeptidase domain 23 (ADAM23), mRNA. |
| FLJ44342 | -2.58 | PREDICTED: Homo sapiens hypothetical LOC645460 (FLJ44342), miscRNA. |
| CTDSP1 | -2.59 | Homo sapiens CTD (carboxy-terminal domain, RNA polymerase II, polypeptide A) small phosphatase 1 (CTDSP1), transcript variant 1, mRNA. |
| ENDOG | -2.59 | Homo sapiens endonuclease G (ENDOG), nuclear gene encoding mitochondrial protein, mRNA. |
| NFIA | -2.59 | Homo sapiens nuclear factor I/A (NFIA), mRNA. |
| MSI1 | -2.59 | Homo sapiens musashi homolog 1 (Drosophila) (MSI1), mRNA. |
| RNU1-3 | -2.59 | Homo sapiens RNA, U1 small nuclear 3 (RNU1-3), small nuclear RNA. |
| LOC727762 | -2.59 | PREDICTED: Homo sapiens similar to NADH dehydrogenase (ubiquinone) 1 beta subcomplex, 4, 15kDa, transcript variant 1 (LOC727762), mRNA. |
| SNORD36C | -2.59 | Homo sapiens small nucleolar RNA, C/D box 36C (SNORD36C), small nucleolar RNA. |
| CTSF | -2.59 | Homo sapiens cathepsin F (CTSF), mRNA. |
| ZNF14 | -2.6 | Homo sapiens zinc finger protein 14 (ZNF14), mRNA. |
| ST5 | -2.6 | Homo sapiens suppression of tumorigenicity 5 (ST5), transcript variant 2, mRNA. |
| MT2A | -2.6 | Homo sapiens metallothionein 2A (MT2A), mRNA. |
| TBC1D9B | -2.61 | Homo sapiens TBC1 domain family, member 9B (with GRAM domain) (TBC1D9B), transcript variant 2, mRNA. |
| CHCHD10 | -2.61 | Homo sapiens coiled-coil-helix-coiled-coil-helix domain containing 10 (CHCHD10), mRNA. |
| SLC25A1 | -2.61 | Homo sapiens solute carrier family 25 (mitochondrial carrier; citrate transporter), member 1 (SLC25A1), nuclear gene encoding mitochondrial protein, mRNA. |
| TOR1AIP1 | -2.61 | Homo sapiens torsin A interacting protein 1 (TOR1AIP1), mRNA. |
| GAA | -2.61 | Homo sapiens glucosidase, alpha; acid (GAA), transcript variant 1, mRNA. |
| TNFRSF6B | -2.62 | Homo sapiens tumor necrosis factor receptor superfamily, member 6b, decoy (TNFRSF6B), transcript variant M68C, mRNA. |
| NES | -2.62 | Homo sapiens nestin (NES), mRNA. |
| SLC35E1 | -2.62 | Homo sapiens solute carrier family 35, member E1 (SLC35E1), mRNA. |
| C14orf85 | -2.62 | Homo sapiens chromosome 14 open reading frame 85 (C14orf85), non-coding RNA. |
| TBC1D17 | -2.62 | Homo sapiens TBC1 domain family, member 17 (TBC1D17), mRNA. |
| C11orf63 | -2.62 | Homo sapiens chromosome 11 open reading frame 63 (C11orf63), transcript variant 2, mRNA. |
| NFATC2IP | -2.62 | Homo sapiens nuclear factor of activated T-cells, cytoplasmic, calcineurin-dependent 2 interacting protein (NFATC2IP), mRNA. |
| SMG7 | -2.62 | Homo sapiens Smg-7 homolog, nonsense mediated mRNA decay factor (C. elegans) (SMG7), transcript variant 1, mRNA. |
| HS1BP3 | -2.62 | Homo sapiens HCLS1 binding protein 3 (HS1BP3), mRNA. |
| SF1 | -2.62 | Homo sapiens splicing factor 1 (SF1), transcript variant 4, mRNA. |
| LGALS3BP | -2.62 | Homo sapiens lectin, galactoside-binding, soluble, 3 binding protein (LGALS3BP), mRNA. |
| MIIP | -2.63 | Homo sapiens migration and invasion inhibitory protein (MIIP), mRNA. |
| MICAL1 | -2.63 | Homo sapiens microtubule associated monoxygenase, calponin and LIM domain containing 1 (MICAL1), mRNA. |
| KLHL28 | -2.63 | Homo sapiens kelch-like 28 (Drosophila) (KLHL28), mRNA. |
| RNF182 | -2.63 | Homo sapiens ring finger protein 182 (RNF182), mRNA. |
| HYAL2 | -2.63 | Homo sapiens hyaluronoglucosaminidase 2 (HYAL2), transcript variant 1, mRNA. |
| DBNDD1 | -2.63 | Homo sapiens dysbindin (dystrobrevin binding protein 1) domain containing 1 (DBNDD1), transcript variant 1, mRNA. |
| FAM195B | -2.63 | Homo sapiens family with sequence similarity 195, member B (FAM195B), transcript variant 1, mRNA. |
| TSGA14 | -2.63 | Homo sapiens testis specific, 14 (TSGA14), mRNA. |
| AES | -2.64 | Homo sapiens amino-terminal enhancer of split (AES), transcript variant 2, mRNA. |
| FADS1 | -2.64 | Homo sapiens fatty acid desaturase 1 (FADS1), mRNA. |
| HIATL2 | -2.64 | Homo sapiens hippocampus abundant transcript-like 2 (HIATL2), non-coding RNA. |
| SUPT5H | -2.64 | Homo sapiens suppressor of Ty 5 homolog (S. cerevisiae) (SUPT5H), mRNA. |
| FAM57B | -2.64 | Homo sapiens family with sequence similarity 57, member B (FAM57B), mRNA. |
| PA2G4 | -2.64 | Homo sapiens proliferation-associated 2G4, 38kDa (PA2G4), mRNA. |
| TTC15 | -2.64 | Homo sapiens tetratricopeptide repeat domain 15 (TTC15), mRNA. |
| LOC100130835 | -2.64 | PREDICTED: Homo sapiens misc_RNA (LOC100130835), miscRNA. |
| C17orf62 | -2.65 | Homo sapiens chromosome 17 open reading frame 62 (C17orf62), transcript variant 3, mRNA. |
| TAPBP | -2.65 | Homo sapiens TAP binding protein (tapasin) (TAPBP), transcript variant 1, mRNA. |
| CCDC25 | -2.65 | Homo sapiens coiled-coil domain containing 25 (CCDC25), mRNA. |
| USF2 | -2.65 | Homo sapiens upstream transcription factor 2, c-fos interacting (USF2), transcript variant 2, mRNA. |
| SNAI2 | -2.65 | Homo sapiens snail homolog 2 (Drosophila) (SNAI2), mRNA. |
| TAZ | -2.65 | Homo sapiens tafazzin (cardiomyopathy, dilated 3A (X-linked); endocardial fibroelastosis 2; Barth syndrome) (TAZ), transcript variant 2, mRNA. |
| VASN | -2.66 | Homo sapiens vasorin (VASN), mRNA. |
| NOL8 | -2.66 | Homo sapiens nucleolar protein 8 (NOL8), mRNA. |
| EPHB4 | -2.66 | Homo sapiens EPH receptor B4 (EPHB4), mRNA. |
| LRRN3 | -2.66 | Homo sapiens leucine rich repeat neuronal 3 (LRRN3), transcript variant 1, mRNA. |
| PABPC1 | -2.66 | Homo sapiens poly(A) binding protein, cytoplasmic 1 (PABPC1), mRNA. |
| LOC389342 | -2.67 | PREDICTED: Homo sapiens similar to QM protein, transcript variant 11 (LOC389342), mRNA. |
| LOC399900 | -2.67 | Homo sapiens hypothetical gene supported by AK093779 (LOC399900), mRNA. |
| DKK1 | -2.67 | Homo sapiens dickkopf homolog 1 (Xenopus laevis) (DKK1), mRNA. |
| PPP1R12C | -2.67 | Homo sapiens protein phosphatase 1, regulatory (inhibitor) subunit 12C (PPP1R12C), mRNA. |
| LAMB2 | -2.68 | Homo sapiens laminin, beta 2 (laminin S) (LAMB2), mRNA. |
| UCP2 | -2.68 | Homo sapiens uncoupling protein 2 (mitochondrial, proton carrier) (UCP2), nuclear gene encoding mitochondrial protein, mRNA. |
| SIN3B | -2.68 | Homo sapiens SIN3 homolog B, transcription regulator (yeast) (SIN3B), mRNA. |
| USP5 | -2.68 | Homo sapiens ubiquitin specific peptidase 5 (isopeptidase T) (USP5), transcript variant 2, mRNA. |
| TECPR1 | -2.68 | Homo sapiens tectonin beta-propeller repeat containing 1 (TECPR1), mRNA. |
| HTRA1 | -2.69 | Homo sapiens HtrA serine peptidase 1 (HTRA1), mRNA. |
| CATSPER2 | -2.69 | Homo sapiens cation channel, sperm associated 2 (CATSPER2), transcript variant 4, mRNA. |
| SAMD4B | -2.69 | Homo sapiens sterile alpha motif domain containing 4B (SAMD4B), mRNA. |
| PTPRN2 | -2.69 | Homo sapiens protein tyrosine phosphatase, receptor type, N polypeptide 2 (PTPRN2), transcript variant 2, mRNA. |
| LOC649095 | -2.69 | PREDICTED: Homo sapiens hypothetical LOC649095 (LOC649095), mRNA. |
| DUS1L | -2.69 | Homo sapiens dihydrouridine synthase 1-like (S. cerevisiae) (DUS1L), mRNA. |
| C12orf11 | -2.69 | Homo sapiens chromosome 12 open reading frame 11 (C12orf11), mRNA. |
| ZNF462 | -2.7 | Homo sapiens zinc finger protein 462 (ZNF462), mRNA. |
| GRIK5 | -2.7 | Homo sapiens glutamate receptor, ionotropic, kainate 5 (GRIK5), mRNA. |
| SCRIB | -2.7 | Homo sapiens scribbled homolog (Drosophila) (SCRIB), transcript variant 1, mRNA. |
| SFRS8 | -2.7 | Homo sapiens splicing factor, arginine/serine-rich 8 (suppressor-of-white-apricot homolog, Drosophila) (SFRS8), mRNA. |
| BCL7C | -2.7 | Homo sapiens B-cell CLL/lymphoma 7C (BCL7C), mRNA. |
| PCDH17 | -2.7 | Homo sapiens protocadherin 17 (PCDH17), mRNA. |
| KIFC2 | -2.7 | Homo sapiens kinesin family member C2 (KIFC2), mRNA. |
| FLJ10324 | -2.71 | Homo sapiens hypothetical protein FLJ10324 (FLJ10324), mRNA. |
| GHDC | -2.71 | Homo sapiens GH3 domain containing (GHDC), mRNA. |
| TTC32 | -2.72 | Homo sapiens tetratricopeptide repeat domain 32 (TTC32), mRNA. |
| ATN1 | -2.72 | Homo sapiens atrophin 1 (ATN1), transcript variant 1, mRNA. |
| PKN3 | -2.72 | Homo sapiens protein kinase N3 (PKN3), mRNA. |
| GAP43 | -2.73 | Homo sapiens growth associated protein 43 (GAP43), mRNA. |
| GAS6 | -2.73 | Homo sapiens growth arrest-specific 6 (GAS6), mRNA. |
| MOV10 | -2.73 | Homo sapiens Mov10, Moloney leukemia virus 10, homolog (mouse) (MOV10), mRNA. |
| SLC38A10 | -2.73 | Homo sapiens solute carrier family 38, member 10 (SLC38A10), transcript variant 2, mRNA. |
| RNF13 | -2.73 | Homo sapiens ring finger protein 13 (RNF13), transcript variant 4, mRNA. |
| SC65 | -2.73 | Homo sapiens synaptonemal complex protein SC65 (SC65), mRNA. |
| PTK7 | -2.74 | Homo sapiens PTK7 protein tyrosine kinase 7 (PTK7), transcript variant PTK7-4, mRNA. |
| TTC9B | -2.74 | Homo sapiens tetratricopeptide repeat domain 9B (TTC9B), mRNA. |
| PLDN | -2.74 | Homo sapiens pallidin homolog (mouse) (PLDN), mRNA. |
| PCBP3 | -2.74 | Homo sapiens poly(rC) binding protein 3 (PCBP3), mRNA. |
| DRAP1 | -2.74 | Homo sapiens DR1-associated protein 1 (negative cofactor 2 alpha) (DRAP1), mRNA. |
| TWIST1 | -2.74 | Homo sapiens twist homolog 1 (Drosophila) (TWIST1), mRNA. |
| MEGF8 | -2.75 | Homo sapiens multiple EGF-like-domains 8 (MEGF8), mRNA. |
| BTN3A2 | -2.75 | Homo sapiens butyrophilin, subfamily 3, member A2 (BTN3A2), mRNA. |
| CYTSB | -2.75 | Homo sapiens cytospin B (CYTSB), transcript variant NSP5alpha3beta, mRNA. |
| NOTCH3 | -2.75 | Homo sapiens Notch homolog 3 (Drosophila) (NOTCH3), mRNA. |
| METRN | -2.75 | Homo sapiens meteorin, glial cell differentiation regulator (METRN), mRNA. |
| LOC728715 | -2.75 | PREDICTED: Homo sapiens similar to hCG38149 (LOC728715), mRNA. |
| PIF1 | -2.76 | Homo sapiens PIF1 5'-to-3' DNA helicase homolog (S. cerevisiae) (PIF1), mRNA. |
| BAT3 | -2.76 | Homo sapiens HLA-B associated transcript 3 (BAT3), transcript variant 3, mRNA. |
| SLC18A1 | -2.76 | Homo sapiens solute carrier family 18 (vesicular monoamine), member 1 (SLC18A1), mRNA. |
| TBX3 | -2.76 | Homo sapiens T-box 3 (TBX3), transcript variant 1, mRNA. |
| ZNF91 | -2.76 | Homo sapiens zinc finger protein 91 (ZNF91), mRNA. |
| LOC100132585 | -2.76 | PREDICTED: Homo sapiens hypothetical protein LOC100132585 (LOC100132585), mRNA. |
| C20orf117 | -2.76 | Homo sapiens chromosome 20 open reading frame 117 (C20orf117), transcript variant 2, mRNA. |
| PPP2R5D | -2.77 | Homo sapiens protein phosphatase 2, regulatory subunit B', delta isoform (PPP2R5D), transcript variant 2, mRNA. |
| SMARCB1 | -2.77 | Homo sapiens SWI/SNF related, matrix associated, actin dependent regulator of chromatin, subfamily b, member 1 (SMARCB1), transcript variant 2, mRNA. |
| CCDC74A | -2.77 | Homo sapiens coiled-coil domain containing 74A (CCDC74A), mRNA. |
| PC | -2.77 | Homo sapiens pyruvate carboxylase (PC), nuclear gene encoding mitochondrial protein, transcript variant 2, mRNA. |
| DYNLT3 | -2.78 | Homo sapiens dynein, light chain, Tctex-type 3 (DYNLT3), mRNA. |
| SH2D3C | -2.78 | Homo sapiens SH2 domain containing 3C (SH2D3C), transcript variant 2, mRNA. |
| EPHX2 | -2.78 | Homo sapiens epoxide hydrolase 2, cytoplasmic (EPHX2), mRNA. |
| PEX14 | -2.79 | Homo sapiens peroxisomal biogenesis factor 14 (PEX14), mRNA. |
| RAB5C | -2.79 | Homo sapiens RAB5C, member RAS oncogene family (RAB5C), transcript variant 1, mRNA. |
| LOC641768 | -2.79 | PREDICTED: Homo sapiens similar to ribosomal protein S26, transcript variant 2 (LOC641768), mRNA. |
| TMUB1 | -2.8 | Homo sapiens transmembrane and ubiquitin-like domain containing 1 (TMUB1), mRNA. |
| LOC100134364 | -2.8 | PREDICTED: Homo sapiens hypothetical protein LOC100134364 (LOC100134364), mRNA. |
| SNORD4A | -2.8 | Homo sapiens small nucleolar RNA, C/D box 4A (SNORD4A), small nucleolar RNA. |
| WBP2 | -2.81 | Homo sapiens WW domain binding protein 2 (WBP2), mRNA. |
| LPPR2 | -2.81 | Homo sapiens lipid phosphate phosphatase-related protein type 2 (LPPR2), mRNA. |
| TLX2 | -2.81 | Homo sapiens T-cell leukemia homeobox 2 (TLX2), mRNA. |
| PCYOX1L | -2.81 | Homo sapiens prenylcysteine oxidase 1 like (PCYOX1L), mRNA. |
| FAM53B | -2.81 | Homo sapiens family with sequence similarity 53, member B (FAM53B), mRNA. |
| SLC30A1 | -2.82 | Homo sapiens solute carrier family 30 (zinc transporter), member 1 (SLC30A1), mRNA. |
| IL11RA | -2.82 | Homo sapiens interleukin 11 receptor, alpha (IL11RA), transcript variant 1, mRNA. |
|  | -2.82 | Homo sapiens cDNA FLJ31750 fis, clone NT2RI2007406 |
| LOC642469 | -2.82 | PREDICTED: Homo sapiens misc_RNA (LOC642469), miscRNA. |
| VPS4A | -2.82 | Homo sapiens vacuolar protein sorting 4 homolog A (S. cerevisiae) (VPS4A), mRNA. |
| KIF15 | -2.83 | Homo sapiens kinesin family member 15 (KIF15), mRNA. |
| TMEM219 | -2.83 | Homo sapiens transmembrane protein 219 (TMEM219), transcript variant 1, mRNA. |
| EP300 | -2.83 | Homo sapiens E1A binding protein p300 (EP300), mRNA. |
| CST3 | -2.83 | Homo sapiens cystatin C (CST3), mRNA. |
| DYRK1B | -2.84 | Homo sapiens dual-specificity tyrosine-(Y)-phosphorylation regulated kinase 1B (DYRK1B), transcript variant c, mRNA. |
| MAP2K2 | -2.84 | Homo sapiens mitogen-activated protein kinase kinase 2 (MAP2K2), mRNA. |
| LMOD3 | -2.84 | Homo sapiens leiomodin 3 (fetal) (LMOD3), mRNA. |
| LOC389049 | -2.84 | PREDICTED: Homo sapiens misc_RNA (LOC389049), miscRNA. |
| FEV | -2.84 | Homo sapiens FEV (ETS oncogene family) (FEV), mRNA. |
| GDI1 | -2.84 | Homo sapiens GDP dissociation inhibitor 1 (GDI1), mRNA. |
| LOC401098 | -2.84 | PREDICTED: Homo sapiens misc_RNA (LOC401098), miscRNA. |
| AKT1 | -2.84 | Homo sapiens v-akt murine thymoma viral oncogene homolog 1 (AKT1), transcript variant 3, mRNA. |
| STRN4 | -2.84 | Homo sapiens striatin, calmodulin binding protein 4 (STRN4), transcript variant 2, mRNA. |
| S1PR3 | -2.85 | Homo sapiens sphingosine-1-phosphate receptor 3 (S1PR3), mRNA. |
| ARAP3 | -2.85 | Homo sapiens ArfGAP with RhoGAP domain, ankyrin repeat and PH domain 3 (ARAP3), mRNA. |
| TYRO3 | -2.85 | Homo sapiens TYRO3 protein tyrosine kinase (TYRO3), mRNA. |
| MAGT1 | -2.85 | Homo sapiens magnesium transporter 1 (MAGT1), mRNA. |
| LOC100132394 | -2.87 | PREDICTED: Homo sapiens hypothetical protein LOC100132394 (LOC100132394), mRNA. |
| DCN | -2.87 | Homo sapiens decorin (DCN), transcript variant C, mRNA. |
| CXXC4 | -2.87 | Homo sapiens CXXC finger 4 (CXXC4), mRNA. |
| DNM1 | -2.87 | Homo sapiens dynamin 1 (DNM1), transcript variant 2, mRNA. |
| C12orf34 | -2.87 | Homo sapiens chromosome 12 open reading frame 34 (C12orf34), mRNA. |
| HCFC1R1 | -2.88 | Homo sapiens host cell factor C1 regulator 1 (XPO1 dependent) (HCFC1R1), transcript variant 3, mRNA. |
| GMEB1 | -2.88 | Homo sapiens glucocorticoid modulatory element binding protein 1 (GMEB1), transcript variant 2, mRNA. |
| DHX30 | -2.89 | Homo sapiens DEAH (Asp-Glu-Ala-His) box polypeptide 30 (DHX30), transcript variant 1, mRNA. |
| RFNG | -2.89 | PREDICTED: Homo sapiens radical fringe homolog (Drosophila) (RFNG), mRNA. |
| KIDINS220 | -2.89 | Homo sapiens kinase D-interacting substrate of 220 kDa (KIDINS220), mRNA. |
| EBF4 | -2.9 | Homo sapiens early B-cell factor 4 (EBF4), mRNA. |
| PRPF4B | -2.9 | Homo sapiens PRP4 pre-mRNA processing factor 4 homolog B (yeast) (PRPF4B), transcript variant 2, mRNA. |
| HSF4 | -2.9 | Homo sapiens heat shock transcription factor 4 (HSF4), transcript variant 2, mRNA. |
| PHOX2B | -2.91 | Homo sapiens paired-like homeobox 2b (PHOX2B), mRNA. |
| AES | -2.91 | Homo sapiens amino-terminal enhancer of split (AES), transcript variant 3, mRNA. |
| LOC441061 | -2.91 | PREDICTED: Homo sapiens similar to membrane-associated ring finger (C3HC4) 4 (LOC441061), mRNA. |
| PLTP | -2.91 | Homo sapiens phospholipid transfer protein (PLTP), transcript variant 2, mRNA. |
| CENTG2 | -2.91 | Homo sapiens centaurin, gamma 2 (CENTG2), mRNA. |
| POLD1 | -2.92 | Homo sapiens polymerase (DNA directed), delta 1, catalytic subunit 125kDa (POLD1), mRNA. |
| FSD1 | -2.92 | Homo sapiens fibronectin type III and SPRY domain containing 1 (FSD1), mRNA. |
| SHC3 | -2.92 | Homo sapiens SHC (Src homology 2 domain containing) transforming protein 3 (SHC3), mRNA. |
| SH2B2 | -2.92 | Homo sapiens SH2B adaptor protein 2 (SH2B2), mRNA. |
| LOC647307 | -2.93 | PREDICTED: Homo sapiens misc_RNA (LOC647307), miscRNA. |
| TCF3 | -2.93 | Homo sapiens transcription factor 3 (E2A immunoglobulin enhancer binding factors E12/E47) (TCF3), mRNA. |
| PRR7 | -2.93 | Homo sapiens proline rich 7 (synaptic) (PRR7), mRNA. |
| APLP1 | -2.94 | Homo sapiens amyloid beta (A4) precursor-like protein 1 (APLP1), transcript variant 2, mRNA. |
| GNAI2 | -2.94 | Homo sapiens guanine nucleotide binding protein (G protein), alpha inhibiting activity polypeptide 2 (GNAI2), transcript variant 1, mRNA. |
| MIB2 | -2.94 | Homo sapiens mindbomb homolog 2 (Drosophila) (MIB2), mRNA. |
| WASF1 | -2.94 | Homo sapiens WAS protein family, member 1 (WASF1), transcript variant 4, mRNA. |
| TNFRSF25 | -2.96 | Homo sapiens tumor necrosis factor receptor superfamily, member 25 (TNFRSF25), transcript variant 10, mRNA. |
| GRK6 | -2.96 | Homo sapiens G protein-coupled receptor kinase 6 (GRK6), transcript variant 2, mRNA. |
| FAM175A | -2.97 | Homo sapiens family with sequence similarity 175, member A (FAM175A), mRNA. |
| PLXNA1 | -2.97 | Homo sapiens plexin A1 (PLXNA1), mRNA. |
| LOC100128126 | -2.98 | PREDICTED: Homo sapiens hypothetical protein LOC100128126 (LOC100128126), mRNA. |
| LOC643911 | -2.98 | PREDICTED: Homo sapiens hCG1815491 (LOC643911), miscRNA. |
| CCNK | -2.98 | Homo sapiens cyclin K (CCNK), transcript variant 2, mRNA. |
| SYN2 | -2.98 | Homo sapiens synapsin II (SYN2), transcript variant IIa, mRNA. |
| UBE3B | -2.98 | Homo sapiens ubiquitin protein ligase E3B (UBE3B), transcript variant 1, mRNA. |
| LY6E | -2.98 | Homo sapiens lymphocyte antigen 6 complex, locus E (LY6E), mRNA. |
| SIGMAR1 | -2.98 | Homo sapiens sigma non-opioid intracellular receptor 1 (SIGMAR1), transcript variant 1, mRNA. |
| ATF3 | -2.98 | Homo sapiens activating transcription factor 3 (ATF3), transcript variant 4, mRNA. |
| SLC39A11 | -2.99 | Homo sapiens solute carrier family 39 (metal ion transporter), member 11 (SLC39A11), mRNA. |
| UCKL1 | -2.99 | Homo sapiens uridine-cytidine kinase 1-like 1 (UCKL1), mRNA. |
| OAF | -2.99 | Homo sapiens OAF homolog (Drosophila) (OAF), mRNA. |
| WDR34 | -3 | Homo sapiens WD repeat domain 34 (WDR34), mRNA. |
| ATP2B4 | -3 | Homo sapiens ATPase, Ca++ transporting, plasma membrane 4 (ATP2B4), transcript variant 2, mRNA. |
| RHOB | -3 | Homo sapiens ras homolog gene family, member B (RHOB), mRNA. |
| AEBP1 | -3 | Homo sapiens AE binding protein 1 (AEBP1), mRNA. |
| FLNA | -3 | Homo sapiens filamin A, alpha (actin binding protein 280) (FLNA), mRNA. |
| SIGMAR1 | -3 | Homo sapiens sigma non-opioid intracellular receptor 1 (SIGMAR1), transcript variant 1, mRNA. |
| HNRNPU | -3 | Homo sapiens heterogeneous nuclear ribonucleoprotein U (scaffold attachment factor A) (HNRNPU), transcript variant 2, mRNA. |
| DOK4 | -3.01 | Homo sapiens docking protein 4 (DOK4), mRNA. |
| CUL9 | -3.01 | Homo sapiens cullin 9 (CUL9), mRNA. |
| KIF26B | -3.02 | Homo sapiens kinesin family member 26B (KIF26B), mRNA. |
| AP3B2 | -3.02 | Homo sapiens adaptor-related protein complex 3, beta 2 subunit (AP3B2), mRNA. |
| CHTF18 | -3.02 | Homo sapiens CTF18, chromosome transmission fidelity factor 18 homolog (S. cerevisiae) (CHTF18), mRNA. |
| HDGF | -3.02 | Homo sapiens hepatoma-derived growth factor (high-mobility group protein 1-like) (HDGF), mRNA. |
| SHANK3 | -3.03 | Homo sapiens SH3 and multiple ankyrin repeat domains 3 (SHANK3), mRNA. |
| TRAF7 | -3.03 | Homo sapiens TNF receptor-associated factor 7 (TRAF7), mRNA. |
| KCTD12 | -3.04 | Homo sapiens potassium channel tetramerisation domain containing 12 (KCTD12), mRNA. |
| PCGF2 | -3.04 | Homo sapiens polycomb group ring finger 2 (PCGF2), mRNA. |
| SRRM2 | -3.04 | Homo sapiens serine/arginine repetitive matrix 2 (SRRM2), mRNA. |
| FLJ22184 | -3.04 | PREDICTED: Homo sapiens hypothetical protein FLJ22184 (FLJ22184), mRNA. |
| RELL2 | -3.05 | Homo sapiens RELT-like 2 (RELL2), mRNA. |
| AAK1 | -3.05 | Homo sapiens AP2 associated kinase 1 (AAK1), mRNA. |
| AGPAT2 | -3.06 | Homo sapiens 1-acylglycerol-3-phosphate O-acyltransferase 2 (lysophosphatidic acid acyltransferase, beta) (AGPAT2), transcript variant 2, mRNA. |
| PCSK1N | -3.07 | Homo sapiens proprotein convertase subtilisin/kexin type 1 inhibitor (PCSK1N), mRNA. |
| BLZF1 | -3.08 | Homo sapiens basic leucine zipper nuclear factor 1 (BLZF1), mRNA. |
| PLEC1 | -3.08 | Homo sapiens plectin 1, intermediate filament binding protein 500kDa (PLEC1), transcript variant 1, mRNA. |
| UBAP2L | -3.08 | Homo sapiens ubiquitin associated protein 2-like (UBAP2L), mRNA. |
| C15orf63 | -3.08 | Homo sapiens chromosome 15 open reading frame 63 (C15orf63), mRNA. |
| LOC100132391 | -3.09 | PREDICTED: Homo sapiens hypothetical protein LOC100132391 (LOC100132391), mRNA. |
| GIT1 | -3.09 | Homo sapiens G protein-coupled receptor kinase interacting ArfGAP 1 (GIT1), transcript variant 2, mRNA. |
| APC2 | -3.1 | Homo sapiens adenomatosis polyposis coli 2 (APC2), mRNA. |
| TRIM13 | -3.11 | Homo sapiens tripartite motif-containing 13 (TRIM13), transcript variant 4, mRNA. |
| TECR | -3.12 | Homo sapiens trans-2,3-enoyl-CoA reductase (TECR), mRNA. |
| LSS | -3.13 | Homo sapiens lanosterol synthase (2,3-oxidosqualene-lanosterol cyclase) (LSS), transcript variant 1, mRNA. |
| CSNK1E | -3.13 | Homo sapiens casein kinase 1, epsilon (CSNK1E), transcript variant 1, mRNA. |
| KCNH2 | -3.14 | Homo sapiens potassium voltage-gated channel, subfamily H (eag-related), member 2 (KCNH2), transcript variant 2, mRNA. |
| LOC100128288 | -3.14 | Homo sapiens hypothetical protein LOC100128288 (LOC100128288), non-coding RNA. |
| VAMP2 | -3.15 | Homo sapiens vesicle-associated membrane protein 2 (synaptobrevin 2) (VAMP2), mRNA. |
| MID1IP1 | -3.15 | Homo sapiens MID1 interacting protein 1 (gastrulation specific G12 homolog (zebrafish)) (MID1IP1), transcript variant 1, mRNA. |
| VANGL2 | -3.15 | Homo sapiens vang-like 2 (van gogh, Drosophila) (VANGL2), mRNA. |
| LOC153561 | -3.17 | Homo sapiens hypothetical protein LOC153561 (LOC153561), mRNA. |
| PPP2R3B | -3.17 | Homo sapiens protein phosphatase 2 (formerly 2A), regulatory subunit B'', beta (PPP2R3B), transcript variant 1, mRNA. |
| ACTN4 | -3.17 | Homo sapiens actinin, alpha 4 (ACTN4), mRNA. |
| LOC647030 | -3.17 | PREDICTED: Homo sapiens misc_RNA (LOC647030), miscRNA. |
| LOC728908 | -3.17 | PREDICTED: Homo sapiens misc_RNA (LOC728908), miscRNA. |
| PPP2R3B | -3.17 | Homo sapiens protein phosphatase 2 (formerly 2A), regulatory subunit B'', beta (PPP2R3B), transcript variant 1, mRNA. |
| C14orf153 | -3.17 | Homo sapiens chromosome 14 open reading frame 153 (C14orf153), mRNA. |
| TSC2 | -3.17 | Homo sapiens tuberous sclerosis 2 (TSC2), transcript variant 4, mRNA. |
| SMARCC2 | -3.18 | Homo sapiens SWI/SNF related, matrix associated, actin dependent regulator of chromatin, subfamily c, member 2 (SMARCC2), transcript variant 2, mRNA. |
| COL6A1 | -3.18 | Homo sapiens collagen, type VI, alpha 1 (COL6A1), mRNA. |
| GNB2 | -3.18 | Homo sapiens guanine nucleotide binding protein (G protein), beta polypeptide 2 (GNB2), mRNA. |
| INSIG1 | -3.18 | Homo sapiens insulin induced gene 1 (INSIG1), transcript variant 2, mRNA. |
| G6PC3 | -3.19 | Homo sapiens glucose 6 phosphatase, catalytic, 3 (G6PC3), mRNA. |
| ROBO3 | -3.19 | Homo sapiens roundabout, axon guidance receptor, homolog 3 (Drosophila) (ROBO3), mRNA. |
| SYP | -3.19 | Homo sapiens synaptophysin (SYP), mRNA. |
| BIN1 | -3.19 | Homo sapiens bridging integrator 1 (BIN1), transcript variant 1, mRNA. |
| PKM2 | -3.19 | Homo sapiens pyruvate kinase, muscle (PKM2), transcript variant 3, mRNA. |
| LMF2 | -3.2 | Homo sapiens lipase maturation factor 2 (LMF2), mRNA. |
| MIR1974 | -3.2 | Homo sapiens microRNA 1974 (MIR1974), microRNA. |
| CPLX1 | -3.21 | Homo sapiens complexin 1 (CPLX1), mRNA. |
| IRF2BP2 | -3.21 | Homo sapiens interferon regulatory factor 2 binding protein 2 (IRF2BP2), transcript variant 1, mRNA. |
| AP2A1 | -3.21 | Homo sapiens adaptor-related protein complex 2, alpha 1 subunit (AP2A1), transcript variant 2, mRNA. |
| C20orf108 | -3.21 | Homo sapiens chromosome 20 open reading frame 108 (C20orf108), mRNA. |
| FAM108A2 | -3.23 | PREDICTED: Homo sapiens family with sequence similarity 108, member A2 (FAM108A2), mRNA. |
| RNF26 | -3.24 | Homo sapiens ring finger protein 26 (RNF26), mRNA. |
| TRIM46 | -3.24 | Homo sapiens tripartite motif-containing 46 (TRIM46), mRNA. |
| GOLPH4 | -3.26 | Homo sapiens golgi phosphoprotein 4 (GOLPH4), mRNA. |
| TAF6 | -3.27 | Homo sapiens TAF6 RNA polymerase II, TATA box binding protein (TBP)-associated factor, 80kDa (TAF6), transcript variant 1, mRNA. |
| LOC652330 | -3.28 | PREDICTED: Homo sapiens similar to neurofibromin 1 (neurofibromatosis, von Recklinghausen disease, Watson disease) (LOC652330), mRNA. |
| LOC100008589 | -3.28 | Homo sapiens 28S ribosomal RNA (LOC100008589), non-coding RNA. |
| NYNRIN | -3.28 | Homo sapiens NYN domain and retroviral integrase containing (NYNRIN), mRNA. |
| QRFPR | -3.29 | Homo sapiens pyroglutamylated RFamide peptide receptor (QRFPR), mRNA. |
| AKAP1 | -3.29 | Homo sapiens A kinase (PRKA) anchor protein 1 (AKAP1), nuclear gene encoding mitochondrial protein, mRNA. |
| POMZP3 | -3.29 | Homo sapiens POM (POM121 homolog, rat) and ZP3 fusion (POMZP3), transcript variant 2, mRNA. |
| MNT | -3.29 | Homo sapiens MAX binding protein (MNT), mRNA. |
| KLF6 | -3.3 | Homo sapiens Kruppel-like factor 6 (KLF6), transcript variant 1, mRNA. |
| SLC8A2 | -3.31 | Homo sapiens solute carrier family 8 (sodium/calcium exchanger), member 2 (SLC8A2), mRNA. |
| LOC653171 | -3.31 | PREDICTED: Homo sapiens similar to MAPK-interacting and spindle-stabilizing protein (LOC653171), mRNA. |
| EYA4 | -3.32 | Homo sapiens eyes absent homolog 4 (Drosophila) (EYA4), transcript variant 1, mRNA. |
| DUSP8 | -3.33 | Homo sapiens dual specificity phosphatase 8 (DUSP8), mRNA. |
| FOXM1 | -3.33 | Homo sapiens forkhead box M1 (FOXM1), transcript variant 2, mRNA. |
| CREB1 | -3.34 | Homo sapiens cAMP responsive element binding protein 1 (CREB1), transcript variant A, mRNA. |
| LOC100133516 | -3.34 | PREDICTED: Homo sapiens hypothetical protein LOC100133516 (LOC100133516), mRNA. |
| SBNO2 | -3.34 | Homo sapiens strawberry notch homolog 2 (Drosophila) (SBNO2), transcript variant 1, mRNA. |
| GOLIM4 | -3.34 | Homo sapiens golgi integral membrane protein 4 (GOLIM4), mRNA. |
| SUMF2 | -3.34 | Homo sapiens sulfatase modifying factor 2 (SUMF2), transcript variant 4, mRNA. |
| ID3 | -3.35 | Homo sapiens inhibitor of DNA binding 3, dominant negative helix-loop-helix protein (ID3), mRNA. |
| ANO8 | -3.35 | Homo sapiens anoctamin 8 (ANO8), mRNA. |
| PCGF1 | -3.36 | Homo sapiens polycomb group ring finger 1 (PCGF1), mRNA. |
| PTOV1 | -3.36 | Homo sapiens prostate tumor overexpressed 1 (PTOV1), mRNA. |
| ADAM15 | -3.36 | Homo sapiens ADAM metallopeptidase domain 15 (ADAM15), transcript variant 2, mRNA. |
| CHST14 | -3.37 | Homo sapiens carbohydrate (N-acetylgalactosamine 4-0) sulfotransferase 14 (CHST14), mRNA. |
| DTX3 | -3.37 | Homo sapiens deltex homolog 3 (Drosophila) (DTX3), mRNA. |
| GPAA1 | -3.38 | Homo sapiens glycosylphosphatidylinositol anchor attachment protein 1 homolog (yeast) (GPAA1), mRNA. |
| RADIL | -3.38 | Homo sapiens Ras association and DIL domains (RADIL), mRNA. |
| DCAF15 | -3.38 | Homo sapiens DDB1 and CUL4 associated factor 15 (DCAF15), mRNA. |
| LOC100130516 | -3.39 | PREDICTED: Homo sapiens hypothetical protein LOC100130516 (LOC100130516), mRNA. |
| SP2 | -3.39 | Homo sapiens Sp2 transcription factor (SP2), mRNA. |
| SLC22A17 | -3.39 | Homo sapiens solute carrier family 22, member 17 (SLC22A17), transcript variant 2, mRNA. |
| GTF3C1 | -3.41 | Homo sapiens general transcription factor IIIC, polypeptide 1, alpha 220kDa (GTF3C1), mRNA. |
| CXXC5 | -3.42 | Homo sapiens CXXC finger 5 (CXXC5), mRNA. |
| APLP1 | -3.44 | Homo sapiens amyloid beta (A4) precursor-like protein 1 (APLP1), transcript variant 1, mRNA. |
| KCTD13 | -3.44 | Homo sapiens potassium channel tetramerisation domain containing 13 (KCTD13), mRNA. |
| KIAA1751 | -3.45 | Homo sapiens KIAA1751 (KIAA1751), mRNA. |
| FAM119A | -3.46 | Homo sapiens family with sequence similarity 119, member A (FAM119A), mRNA. |
| LOC731314 | -3.46 | PREDICTED: Homo sapiens similar to H2A histone family, member X (LOC731314), mRNA. |
| RAD23A | -3.48 | Homo sapiens RAD23 homolog A (S. cerevisiae) (RAD23A), mRNA. |
| RAVER1 | -3.48 | Homo sapiens RAVER1 (RAVER1), mRNA. |
| TMEM17 | -3.49 | Homo sapiens transmembrane protein 17 (TMEM17), mRNA. |
| C3orf34 | -3.49 | Homo sapiens chromosome 3 open reading frame 34 (C3orf34), mRNA. |
|  | -3.49 | Homo sapiens cDNA FLJ26784 fis, clone PRS04220 |
| UBA1 | -3.5 | Homo sapiens ubiquitin-like modifier activating enzyme 1 (UBA1), transcript variant 1, mRNA. |
| KIAA0195 | -3.51 | Homo sapiens KIAA0195 (KIAA0195), mRNA. |
| CIC | -3.52 | Homo sapiens capicua homolog (Drosophila) (CIC), mRNA. |
| H3F3B | -3.52 | Homo sapiens H3 histone, family 3B (H3.3B) (H3F3B), mRNA. |
| DDR2 | -3.53 | Homo sapiens discoidin domain receptor tyrosine kinase 2 (DDR2), transcript variant 2, mRNA. |
| OGDH | -3.53 | Homo sapiens oxoglutarate (alpha-ketoglutarate) dehydrogenase (lipoamide) (OGDH), nuclear gene encoding mitochondrial protein, transcript variant 1, mRNA. |
|  | -3.53 | FNPANH10 FNP Homo sapiens cDNA, mRNA sequence |
| CPT1B | -3.53 | Homo sapiens carnitine palmitoyltransferase 1B (muscle) (CPT1B), nuclear gene encoding mitochondrial protein, transcript variant 3, mRNA. |
| L1CAM | -3.54 | Homo sapiens L1 cell adhesion molecule (L1CAM), transcript variant 1, mRNA. |
| UNC5B | -3.54 | Homo sapiens unc-5 homolog B (C. elegans) (UNC5B), mRNA. |
| SSBP3 | -3.54 | Homo sapiens single stranded DNA binding protein 3 (SSBP3), transcript variant 2, mRNA. |
| GAS6 | -3.55 | Homo sapiens growth arrest-specific 6 (GAS6), mRNA. |
| H2AFX | -3.56 | Homo sapiens H2A histone family, member X (H2AFX), mRNA. |
| LRRN3 | -3.56 | Homo sapiens leucine rich repeat neuronal 3 (LRRN3), mRNA. |
| C7 | -3.56 | Homo sapiens complement component 7 (C7), mRNA. |
| SSBP3 | -3.56 | Homo sapiens single stranded DNA binding protein 3 (SSBP3), transcript variant 2, mRNA. |
| ZNF423 | -3.56 | Homo sapiens zinc finger protein 423 (ZNF423), mRNA. |
| LOC387763 | -3.58 | PREDICTED: Homo sapiens hypothetical LOC387763 (LOC387763), mRNA. |
| INO80E | -3.59 | Homo sapiens INO80 complex subunit E (INO80E), mRNA. |
|  | -3.59 | zr87e09.r1 NCI_CGAP_GCB1 Homo sapiens cDNA clone IMAGE:682696 5, mRNA sequence |
| DCTN1 | -3.59 | Homo sapiens dynactin 1 (p150, glued homolog, Drosophila) (DCTN1), transcript variant 1, mRNA. |
| BAT2 | -3.6 | Homo sapiens HLA-B associated transcript 2 (BAT2), transcript variant 1, mRNA. |
| COBRA1 | -3.6 | Homo sapiens cofactor of BRCA1 (COBRA1), mRNA. |
| ZNF385A | -3.61 | Homo sapiens zinc finger protein 385A (ZNF385A), transcript variant 3, mRNA. |
| DEM1 | -3.61 | Homo sapiens defects in morphology 1 homolog (S. cerevisiae) (DEM1), mRNA. |
| ZNF577 | -3.61 | Homo sapiens zinc finger protein 577 (ZNF577), mRNA. |
| GIPC1 | -3.62 | Homo sapiens GIPC PDZ domain containing family, member 1 (GIPC1), transcript variant 3, mRNA. |
| LRAP | -3.62 | Homo sapiens leukocyte-derived arginine aminopeptidase (LRAP), mRNA. |
| MAP1S | -3.63 | Homo sapiens microtubule-associated protein 1S (MAP1S), mRNA. |
| B4GALNT4 | -3.64 | Homo sapiens beta-1,4-N-acetyl-galactosaminyl transferase 4 (B4GALNT4), mRNA. |
| BCL2L1 | -3.64 | Homo sapiens BCL2-like 1 (BCL2L1), nuclear gene encoding mitochondrial protein, transcript variant 1, mRNA. |
| MXD3 | -3.65 | Homo sapiens MAX dimerization protein 3 (MXD3), mRNA. |
| ZMAT3 | -3.65 | Homo sapiens zinc finger, matrin type 3 (ZMAT3), transcript variant 2, mRNA. |
| KLF6 | -3.66 | Homo sapiens Kruppel-like factor 6 (KLF6), transcript variant 2, mRNA. |
| LOC652900 | -3.67 | PREDICTED: Homo sapiens similar to SEZ6L2 protein (LOC652900), mRNA. |
| IGFBP4 | -3.68 | Homo sapiens insulin-like growth factor binding protein 4 (IGFBP4), mRNA. |
| LOC730130 | -3.68 | PREDICTED: Homo sapiens hypothetical protein LOC730130 (LOC730130), mRNA. |
| C1orf173 | -3.68 | Homo sapiens chromosome 1 open reading frame 173 (C1orf173), mRNA. |
| MIR1978 | -3.68 | Homo sapiens microRNA 1978 (MIR1978), microRNA. |
| SETD1A | -3.69 | Homo sapiens SET domain containing 1A (SETD1A), mRNA. |
| PODXL2 | -3.69 | Homo sapiens podocalyxin-like 2 (PODXL2), mRNA. |
| FAM63A | -3.69 | Homo sapiens family with sequence similarity 63, member A (FAM63A), transcript variant 1, mRNA. |
| LOC100128098 | -3.7 | PREDICTED: Homo sapiens hypothetical protein LOC100128098 (LOC100128098), mRNA. |
| ZNF394 | -3.7 | Homo sapiens zinc finger protein 394 (ZNF394), mRNA. |
| PHOX2A | -3.71 | Homo sapiens paired-like homeobox 2a (PHOX2A), mRNA. |
| CSAD | -3.72 | Homo sapiens cysteine sulfinic acid decarboxylase (CSAD), mRNA. |
| LRDD | -3.72 | Homo sapiens leucine-rich repeats and death domain containing (LRDD), transcript variant 3, mRNA. |
| EHBP1L1 | -3.72 | Homo sapiens EH domain binding protein 1-like 1 (EHBP1L1), mRNA. |
| DKFZp547K054 | -3.75 | PREDICTED: Homo sapiens hypothetical protein DKFZp547K054 (DKFZp547K054), mRNA. |
| COL6A2 | -3.76 | Homo sapiens collagen, type VI, alpha 2 (COL6A2), transcript variant 2C2, mRNA. |
| FKBP14 | -3.77 | Homo sapiens FK506 binding protein 14, 22 kDa (FKBP14), mRNA. |
| FBLN7 | -3.78 | Homo sapiens fibulin 7 (FBLN7), mRNA. |
| XRCC2 | -3.79 | Homo sapiens X-ray repair complementing defective repair in Chinese hamster cells 2 (XRCC2), mRNA. |
| NCOR2 | -3.79 | Homo sapiens nuclear receptor co-repressor 2 (NCOR2), transcript variant 2, mRNA. |
| BMS1P5 | -3.8 | Homo sapiens BMS1 pseudogene 5 (BMS1P5), non-coding RNA. |
| HNRPUL1 | -3.81 | Homo sapiens heterogeneous nuclear ribonucleoprotein U-like 1 (HNRPUL1), transcript variant 4, mRNA. |
| MTA1 | -3.83 | Homo sapiens metastasis associated 1 (MTA1), mRNA. |
| GRIPAP1 | -3.84 | Homo sapiens GRIP1 associated protein 1 (GRIPAP1), transcript variant 2, mRNA. |
| PEBP1 | -3.86 | Homo sapiens phosphatidylethanolamine binding protein 1 (PEBP1), mRNA. |
| MBTD1 | -3.88 | Homo sapiens mbt domain containing 1 (MBTD1), mRNA. |
| LTBP3 | -3.88 | Homo sapiens latent transforming growth factor beta binding protein 3 (LTBP3), mRNA. |
| MBOAT7 | -3.91 | Homo sapiens membrane bound O-acyltransferase domain containing 7 (MBOAT7), mRNA. |
| PTGFRN | -3.91 | Homo sapiens prostaglandin F2 receptor negative regulator (PTGFRN), mRNA. |
| ARHGDIA | -3.92 | Homo sapiens Rho GDP dissociation inhibitor (GDI) alpha (ARHGDIA), mRNA. |
| IDUA | -3.93 | Homo sapiens iduronidase, alpha-L- (IDUA), mRNA. |
| LAMB1 | -3.94 | Homo sapiens laminin, beta 1 (LAMB1), mRNA. |
| ID2 | -3.94 | Homo sapiens inhibitor of DNA binding 2, dominant negative helix-loop-helix protein (ID2), mRNA. |
| LOC100129362 | -3.94 | PREDICTED: Homo sapiens hypothetical protein LOC100129362 (LOC100129362), mRNA. |
| DUSP19 | -3.96 | Homo sapiens dual specificity phosphatase 19 (DUSP19), mRNA. |
| TRIM28 | -3.97 | Homo sapiens tripartite motif-containing 28 (TRIM28), mRNA. |
| DMWD | -3.97 | Homo sapiens dystrophia myotonica, WD repeat containing (DMWD), mRNA. |
| LRDD | -3.97 | Homo sapiens leucine-rich repeats and death domain containing (LRDD), transcript variant 2, mRNA. |
| FBRS | -3.97 | Homo sapiens fibrosin (FBRS), mRNA. |
| PTMS | -3.99 | Homo sapiens parathymosin (PTMS), mRNA. |
| ZNF69 | -3.99 | Homo sapiens zinc finger protein 69 (ZNF69), mRNA. |
| PCDH9 | -4 | Homo sapiens protocadherin 9 (PCDH9), transcript variant 1, mRNA. |
| CDC42BPB | -4.01 | Homo sapiens CDC42 binding protein kinase beta (DMPK-like) (CDC42BPB), mRNA. |
| DLGAP4 | -4.02 | Homo sapiens discs, large (Drosophila) homolog-associated protein 4 (DLGAP4), transcript variant 3, mRNA. |
| SLC38A10 | -4.03 | Homo sapiens solute carrier family 38, member 10 (SLC38A10), transcript variant 1, mRNA. |
| LOC100128510 | -4.04 | PREDICTED: Homo sapiens hypothetical protein LOC100128510 (LOC100128510), mRNA. |
| TDP1 | -4.04 | Homo sapiens tyrosyl-DNA phosphodiesterase 1 (TDP1), transcript variant 1, mRNA. |
| HSPC268 | -4.05 | Homo sapiens hypothetical protein HSPC268 (HSPC268), mRNA. |
| ITFG3 | -4.05 | Homo sapiens integrin alpha FG-GAP repeat containing 3 (ITFG3), mRNA. |
| PELP1 | -4.07 | Homo sapiens proline, glutamic acid and leucine rich protein 1 (PELP1), mRNA. |
| XAB2 | -4.08 | Homo sapiens XPA binding protein 2 (XAB2), mRNA. |
| SMTN | -4.09 | Homo sapiens smoothelin (SMTN), transcript variant 3, mRNA. |
| CCL2 | -4.1 | Homo sapiens chemokine (C-C motif) ligand 2 (CCL2), mRNA. |
| BSG | -4.11 | Homo sapiens basigin (Ok blood group) (BSG), transcript variant 3, mRNA. |
| H1F0 | -4.13 | Homo sapiens H1 histone family, member 0 (H1F0), mRNA. |
| TACC3 | -4.13 | Homo sapiens transforming, acidic coiled-coil containing protein 3 (TACC3), mRNA. |
| SAC3D1 | -4.14 | Homo sapiens SAC3 domain containing 1 (SAC3D1), mRNA. |
| HMGA1 | -4.15 | Homo sapiens high mobility group AT-hook 1 (HMGA1), transcript variant 1, mRNA. |
| PKMYT1 | -4.15 | Homo sapiens protein kinase, membrane associated tyrosine/threonine 1 (PKMYT1), transcript variant 2, mRNA. |
| CHRNA5 | -4.16 | Homo sapiens cholinergic receptor, nicotinic, alpha 5 (CHRNA5), mRNA. |
| SNORA12 | -4.17 | Homo sapiens small nucleolar RNA, H/ACA box 12 (SNORA12), small nucleolar RNA. |
| LOC100129211 | -4.19 | PREDICTED: Homo sapiens hypothetical protein LOC100129211 (LOC100129211), mRNA. |
| SHROOM4 | -4.19 | Homo sapiens shroom family member 4 (SHROOM4), mRNA. |
| CLPTM1 | -4.2 | Homo sapiens cleft lip and palate associated transmembrane protein 1 (CLPTM1), mRNA. |
| MAPK8IP3 | -4.2 | Homo sapiens mitogen-activated protein kinase 8 interacting protein 3 (MAPK8IP3), transcript variant 2, mRNA. |
| AGPAT1 | -4.2 | Homo sapiens 1-acylglycerol-3-phosphate O-acyltransferase 1 (lysophosphatidic acid acyltransferase, alpha) (AGPAT1), transcript variant 1, mRNA. |
| LOC407835 | -4.24 | Homo sapiens mitogen-activated protein kinase kinase 2 pseudogene (LOC407835), non-coding RNA. |
|  | -4.25 | MR1-GN0172-061100-005-h03 GN0172 Homo sapiens cDNA, mRNA sequence |
| GRINA | -4.25 | Homo sapiens glutamate receptor, ionotropic, N-methyl D-aspartate-associated protein 1 (glutamate binding) (GRINA), transcript variant 1, mRNA. |
| EID2B | -4.28 | Homo sapiens EP300 interacting inhibitor of differentiation 2B (EID2B), mRNA. |
| LOC728903 | -4.28 | PREDICTED: Homo sapiens hypothetical LOC728903, transcript variant 1 (LOC728903), mRNA. |
| TM4SF4 | -4.28 | Homo sapiens transmembrane 4 L six family member 4 (TM4SF4), mRNA. |
| UBE1 | -4.28 | Homo sapiens ubiquitin-activating enzyme E1 (UBE1), transcript variant 1, mRNA. |
| TRAPPC9 | -4.3 | Homo sapiens trafficking protein particle complex 9 (TRAPPC9), mRNA. |
| SIX3 | -4.3 | Homo sapiens sine oculis homeobox homolog 3 (Drosophila) (SIX3), mRNA. |
| ID2 | -4.31 | Homo sapiens inhibitor of DNA binding 2, dominant negative helix-loop-helix protein (ID2), mRNA. |
| ID1 | -4.31 | Homo sapiens inhibitor of DNA binding 1, dominant negative helix-loop-helix protein (ID1), transcript variant 2, mRNA. |
| FAM108A3 | -4.32 | Homo sapiens family with sequence similarity 108, member A3 (FAM108A3), mRNA. |
| NCOR2 | -4.34 | Homo sapiens nuclear receptor co-repressor 2 (NCOR2), transcript variant 1, mRNA. |
| LRRC4B | -4.36 | Homo sapiens leucine rich repeat containing 4B (LRRC4B), mRNA. |
| ZNF483 | -4.37 | Homo sapiens zinc finger protein 483 (ZNF483), transcript variant 2, mRNA. |
| TTYH3 | -4.37 | Homo sapiens tweety homolog 3 (Drosophila) (TTYH3), mRNA. |
| TMEM132A | -4.39 | Homo sapiens transmembrane protein 132A (TMEM132A), transcript variant 2, mRNA. |
| H1FX | -4.39 | Homo sapiens H1 histone family, member X (H1FX), mRNA. |
| TPM2 | -4.4 | Homo sapiens tropomyosin 2 (beta) (TPM2), transcript variant 2, mRNA. |
| ABCA2 | -4.46 | Homo sapiens ATP-binding cassette, sub-family A (ABC1), member 2 (ABCA2), transcript variant 2, mRNA. |
| ARMC5 | -4.47 | Homo sapiens armadillo repeat containing 5 (ARMC5), mRNA. |
| TBX2 | -4.52 | Homo sapiens T-box 2 (TBX2), mRNA. |
| IGFBP2 | -4.54 | Homo sapiens insulin-like growth factor binding protein 2, 36kDa (IGFBP2), mRNA. |
| BAT2 | -4.54 | Homo sapiens HLA-B associated transcript 2 (BAT2), mRNA. |
| FBXL16 | -4.55 | Homo sapiens F-box and leucine-rich repeat protein 16 (FBXL16), mRNA. |
| SOX4 | -4.55 | Homo sapiens SRY (sex determining region Y)-box 4 (SOX4), mRNA. |
|  | -4.56 | xr14b10.x1 NCI_CGAP_Lu28 Homo sapiens cDNA clone IMAGE:2760091 3, mRNA sequence |
| FAM100A | -4.6 | Homo sapiens family with sequence similarity 100, member A (FAM100A), mRNA. |
| CENTG3 | -4.6 | Homo sapiens centaurin, gamma 3 (CENTG3), mRNA. |
| LOC338799 | -4.6 | Homo sapiens hypothetical locus LOC338799 (LOC338799), non-coding RNA. |
| FCAR | -4.63 | Homo sapiens Fc fragment of IgA, receptor for (FCAR), transcript variant 9, mRNA. |
| LDOC1 | -4.63 | Homo sapiens leucine zipper, down-regulated in cancer 1 (LDOC1), mRNA. |
| TUBB4 | -4.66 | Homo sapiens tubulin, beta 4 (TUBB4), mRNA. |
| SLC4A2 | -4.67 | Homo sapiens solute carrier family 4, anion exchanger, member 2 (erythrocyte membrane protein band 3-like 1) (SLC4A2), mRNA. |
| CNTFR | -4.69 | Homo sapiens ciliary neurotrophic factor receptor (CNTFR), transcript variant 2, mRNA. |
| LRRFIP1 | -4.71 | Homo sapiens leucine rich repeat (in FLII) interacting protein 1 (LRRFIP1), mRNA. |
| PACS1 | -4.71 | Homo sapiens phosphofurin acidic cluster sorting protein 1 (PACS1), mRNA. |
| AMH | -4.71 | Homo sapiens anti-Mullerian hormone (AMH), mRNA. |
| MIB2 | -4.79 | Homo sapiens mindbomb homolog 2 (Drosophila) (MIB2), mRNA. |
| SGK1 | -4.84 | Homo sapiens serum/glucocorticoid regulated kinase 1 (SGK1), transcript variant 1, mRNA. |
| MGRN1 | -4.88 | Homo sapiens mahogunin, ring finger 1 (MGRN1), mRNA. |
| ZNF580 | -4.97 | Homo sapiens zinc finger protein 580 (ZNF580), transcript variant 1, mRNA. |
| SEPN1 | -4.99 | Homo sapiens selenoprotein N, 1 (SEPN1), transcript variant 2, mRNA. |
| ZNF423 | -5.09 | Homo sapiens zinc finger protein 423 (ZNF423), mRNA. |
| SGK1 | -5.12 | Homo sapiens serum/glucocorticoid regulated kinase 1 (SGK1), transcript variant 1, mRNA. |
| IGFBP5 | -5.12 | Homo sapiens insulin-like growth factor binding protein 5 (IGFBP5), mRNA. |
| KIAA0319L | -5.14 | Homo sapiens KIAA0319-like (KIAA0319L), transcript variant 1, mRNA. |
| YWHAE | -5.14 | Homo sapiens tyrosine 3-monooxygenase/tryptophan 5-monooxygenase activation protein, epsilon polypeptide (YWHAE), mRNA. |
| CNOT3 | -5.14 | Homo sapiens CCR4-NOT transcription complex, subunit 3 (CNOT3), mRNA. |
| CORO6 | -5.14 | Homo sapiens coronin 6 (CORO6), mRNA. |
| SEMA3E | -5.19 | Homo sapiens sema domain, immunoglobulin domain (Ig), short basic domain, secreted, (semaphorin) 3E (SEMA3E), mRNA. |
| ZNF503 | -5.53 | Homo sapiens zinc finger protein 503 (ZNF503), mRNA. |
| PALM | -5.55 | Homo sapiens paralemmin (PALM), transcript variant 1, mRNA. |
| SGK | -5.77 | Homo sapiens serum/glucocorticoid regulated kinase (SGK), mRNA. |
| NFIX | -6.41 | Homo sapiens nuclear factor I/X (CCAAT-binding transcription factor) (NFIX), mRNA. |
| IGFBP5 | -7.29 | Homo sapiens insulin-like growth factor binding protein 5 (IGFBP5), mRNA. |
| FOSB | -7.76 | Homo sapiens FBJ murine osteosarcoma viral oncogene homolog B (FOSB), mRNA. |
|  | -7.85 | AGENCOURT_10229596 NIH_MGC_141 Homo sapiens cDNA clone IMAGE:6563923 5, mRNA sequence |
| CYR61 | -15.21 | Homo sapiens cysteine-rich, angiogenic inducer, 61 (CYR61), mRNA. |
| FOS | -17.02 | Homo sapiens v-fos FBJ murine osteosarcoma viral oncogene homolog (FOS), mRNA. |
| EGR1 | -19.57 | Homo sapiens early growth response 1 (EGR1), mRNA. |
