# Supplementary material for: Plurigon: three dimensional visualization and classification of high-dimensionality data
Source: Front Physiol. 2013 Jul 22;4:190. doi: 10.3389/fphys.2013.00190 (PMC3717481; doi:10.3389/fphys.2013.00190)
Supplement: Table S10 — Standard PCA analysis of WT and HET genotypic datasets. PCA was applied to reduce data dimensionality to three primary dimensions, D1, D2 and D3. Using these three dimensions the two genotype groups appeared non-separable. [file 46440_Maudsley_Data_Sheet_5.ZIP › 46440_Maudsley_Table_14.DOC]

**Table S14. Human neuronal cell (SH-SY5Y) transcriptomic effects induced by nerve growth factor exposure.** For each nerve growth factor (NGF) regulated transcript the official Gene Symbol, z-ratio and transcript description are indicated.

| **Gene Symbol** | **z-ratio** | **Description** |
| --- | --- | --- |
| NEUROG2 | 6.9 | Homo sapiens neurogenin 2 (NEUROG2), mRNA. |
| CLK1 | 6.68 | Homo sapiens CDC-like kinase 1 (CLK1), mRNA. |
| HSPA1B | 6.63 | Homo sapiens heat shock 70kDa protein 1B (HSPA1B), mRNA. |
| ACCN2 | 5.82 | Homo sapiens amiloride-sensitive cation channel 2, neuronal (ACCN2), transcript variant 2, mRNA. |
| DPP7 | 4.99 | Homo sapiens dipeptidyl-peptidase 7 (DPP7), mRNA. |
| GFOD2 | 4.89 | Homo sapiens glucose-fructose oxidoreductase domain containing 2 (GFOD2), mRNA. |
| GADD45G | 4.8 | Homo sapiens growth arrest and DNA-damage-inducible, gamma (GADD45G), mRNA. |
| SLC25A22 | 4.71 | Homo sapiens solute carrier family 25 (mitochondrial carrier: glutamate), member 22 (SLC25A22), nuclear gene encoding mitochondrial protein, mRNA. |
| C1orf63 | 4.48 | Homo sapiens chromosome 1 open reading frame 63 (C1orf63), mRNA. |
| CLK1 | 4.37 | Homo sapiens CDC-like kinase 1 (CLK1), transcript variant 2, mRNA. |
| CCDC106 | 4.37 | Homo sapiens coiled-coil domain containing 106 (CCDC106), mRNA. |
| DEDD2 | 4.31 | Homo sapiens death effector domain containing 2 (DEDD2), mRNA. |
| HSPA1A | 4.28 | Homo sapiens heat shock 70kDa protein 1A (HSPA1A), mRNA. |
| WDR86 | 4.22 | Homo sapiens WD repeat domain 86 (WDR86), mRNA. |
| ORAOV1 | 4.05 | Homo sapiens oral cancer overexpressed 1 (ORAOV1), mRNA. |
| MOBKL2C | 4.03 | Homo sapiens MOB1, Mps One Binder kinase activator-like 2C (yeast) (MOBKL2C), transcript variant 1, mRNA. |
| ARMCX3 | 4.01 | Homo sapiens armadillo repeat containing, X-linked 3 (ARMCX3), transcript variant 2, mRNA. |
| SIPA1 | 3.97 | Homo sapiens signal-induced proliferation-associated gene 1 (SIPA1), transcript variant 2, mRNA. |
| LOC730746 | 3.96 | PREDICTED: Homo sapiens similar to Heterogeneous nuclear ribonucleoprotein A1 (Helix-destabilizing protein) (Single-strand RNA-binding protein) (hnRNP core protein A1) (HDP) (LOC730746), mRNA. |
| CHGB | 3.91 | Homo sapiens chromogranin B (secretogranin 1) (CHGB), mRNA. |
| EHD1 | 3.87 | Homo sapiens EH-domain containing 1 (EHD1), mRNA. |
| CHORDC1 | 3.86 | Homo sapiens cysteine and histidine-rich domain (CHORD)-containing 1 (CHORDC1), mRNA. |
| ZBTB11 | 3.72 | Homo sapiens zinc finger and BTB domain containing 11 (ZBTB11), mRNA. |
| LOC100129144 | 3.7 | PREDICTED: Homo sapiens similar to hCG2042189 (LOC100129144), mRNA. |
| SH3GL1 | 3.7 | Homo sapiens SH3-domain GRB2-like 1 (SH3GL1), mRNA. |
| DBNDD1 | 3.59 | Homo sapiens dysbindin (dystrobrevin binding protein 1) domain containing 1 (DBNDD1), transcript variant 1, mRNA. |
| DPYSL4 | 3.58 | Homo sapiens dihydropyrimidinase-like 4 (DPYSL4), mRNA. |
| RFTN1 | 3.58 | Homo sapiens raftlin, lipid raft linker 1 (RFTN1), mRNA. |
| SDHAP2 | 3.55 | Homo sapiens succinate dehydrogenase complex, subunit A, flavoprotein pseudogene 2 (SDHAP2), non-coding RNA. |
| B4GALT7 | 3.54 | Homo sapiens xylosylprotein beta 1,4-galactosyltransferase, polypeptide 7 (galactosyltransferase I) (B4GALT7), mRNA. |
|  | 3.53 | Homo sapiens cDNA FLJ38396 fis, clone FEBRA2007957 |
| CLK1 | 3.53 | Homo sapiens CDC-like kinase 1 (CLK1), mRNA. |
| CCDC136 | 3.49 | Homo sapiens coiled-coil domain containing 136 (CCDC136), mRNA. |
| HSPA5 | 3.48 | Homo sapiens heat shock 70kDa protein 5 (glucose-regulated protein, 78kDa) (HSPA5), mRNA. |
| RSRC2 | 3.45 | Homo sapiens arginine/serine-rich coiled-coil 2 (RSRC2), transcript variant 1, mRNA. |
| TXNIP | 3.45 | Homo sapiens thioredoxin interacting protein (TXNIP), mRNA. |
| AMY1A | 3.43 | Homo sapiens amylase, alpha 1A (salivary) (AMY1A), transcript variant 1, mRNA. |
| HCFC1R1 | 3.39 | Homo sapiens host cell factor C1 regulator 1 (XPO1 dependent) (HCFC1R1), transcript variant 3, mRNA. |
| NELF | 3.38 | Homo sapiens nasal embryonic LHRH factor (NELF), mRNA. |
| DBN1 | 3.37 | Homo sapiens drebrin 1 (DBN1), transcript variant 1, mRNA. |
| LOC645691 | 3.35 | PREDICTED: Homo sapiens similar to heterogeneous nuclear ribonucleoprotein A1 (LOC645691), mRNA. |
| TAF1A | 3.33 | Homo sapiens TATA box binding protein (TBP)-associated factor, RNA polymerase I, A, 48kDa (TAF1A), transcript variant 1, mRNA. |
| ZNF200 | 3.32 | Homo sapiens zinc finger protein 200 (ZNF200), transcript variant 3, mRNA. |
| VPS37D | 3.31 | Homo sapiens vacuolar protein sorting 37 homolog D (S. cerevisiae) (VPS37D), mRNA. |
| LOC148413 | 3.29 | Homo sapiens hypothetical LOC148413 (LOC148413), non-coding RNA. |
| C5orf54 | 3.29 | Homo sapiens chromosome 5 open reading frame 54 (C5orf54), mRNA. |
| SDF2L1 | 3.29 | Homo sapiens stromal cell-derived factor 2-like 1 (SDF2L1), mRNA. |
| LOC100130289 | 3.28 | PREDICTED: Homo sapiens misc_RNA (LOC100130289), miscRNA. |
| SPATA2L | 3.27 | Homo sapiens spermatogenesis associated 2-like (SPATA2L), mRNA. |
| LOC645691 | 3.27 | PREDICTED: Homo sapiens similar to heterogeneous nuclear ribonucleoprotein A1 (LOC645691), mRNA. |
| ZBTB45 | 3.27 | Homo sapiens zinc finger and BTB domain containing 45 (ZBTB45), mRNA. |
| SPHK2 | 3.25 | Homo sapiens sphingosine kinase 2 (SPHK2), mRNA. |
| C9orf40 | 3.25 | Homo sapiens chromosome 9 open reading frame 40 (C9orf40), mRNA. |
| ZNF2 | 3.25 | Homo sapiens zinc finger protein 2 (ZNF2), transcript variant 1, mRNA. |
| ALDOC | 3.25 | Homo sapiens aldolase C, fructose-bisphosphate (ALDOC), mRNA. |
| RPS6KB2 | 3.23 | Homo sapiens ribosomal protein S6 kinase, 70kDa, polypeptide 2 (RPS6KB2), mRNA. |
| LOC100131609 | 3.23 | PREDICTED: Homo sapiens misc_RNA (LOC100131609), miscRNA. |
| FAM129B | 3.22 | Homo sapiens family with sequence similarity 129, member B (FAM129B), transcript variant 1, mRNA. |
| ZNF615 | 3.22 | Homo sapiens zinc finger protein 615 (ZNF615), mRNA. |
| RCOR2 | 3.21 | Homo sapiens REST corepressor 2 (RCOR2), mRNA. |
| NDEL1 | 3.21 | Homo sapiens nudE nuclear distribution gene E homolog (A. nidulans)-like 1 (NDEL1), transcript variant 2, mRNA. |
| SLC25A28 | 3.2 | Homo sapiens solute carrier family 25, member 28 (SLC25A28), mRNA. |
| C16orf58 | 3.17 | Homo sapiens chromosome 16 open reading frame 58 (C16orf58), mRNA. |
| CS | 3.16 | Homo sapiens citrate synthase (CS), nuclear gene encoding mitochondrial protein, mRNA. |
| HPS6 | 3.15 | Homo sapiens Hermansky-Pudlak syndrome 6 (HPS6), mRNA. |
| KCNQ2 | 3.15 | Homo sapiens potassium voltage-gated channel, KQT-like subfamily, member 2 (KCNQ2), transcript variant 5, mRNA. |
| GAPVD1 | 3.15 | Homo sapiens GTPase activating protein and VPS9 domains 1 (GAPVD1), mRNA. |
| JSRP1 | 3.14 | Homo sapiens junctional sarcoplasmic reticulum protein 1 (JSRP1), mRNA. |
| MTP18 | 3.11 | Homo sapiens mitochondrial protein 18 kDa (MTP18), nuclear gene encoding mitochondrial protein, transcript variant 1, mRNA. |
| EFNA1 | 3.11 | Homo sapiens ephrin-A1 (EFNA1), transcript variant 1, mRNA. |
| PDIA3P | 3.1 | Homo sapiens protein disulfide isomerase family A, member 3 pseudogene (PDIA3P), non-coding RNA. |
| C1orf77 | 3.09 | Homo sapiens chromosome 1 open reading frame 77 (C1orf77), mRNA. |
| ZNF606 | 3.09 | Homo sapiens zinc finger protein 606 (ZNF606), mRNA. |
| RASSF7 | 3.07 | Homo sapiens Ras association (RalGDS/AF-6) domain family (N-terminal) member 7 (RASSF7), mRNA. |
| VSNL1 | 3.07 | Homo sapiens visinin-like 1 (VSNL1), mRNA. |
| PHF23 | 3.06 | Homo sapiens PHD finger protein 23 (PHF23), mRNA. |
| SORCS1 | 3.04 | Homo sapiens sortilin-related VPS10 domain containing receptor 1 (SORCS1), transcript variant 2, mRNA. |
| SNRPE | 3.03 | Homo sapiens small nuclear ribonucleoprotein polypeptide E (SNRPE), mRNA. |
| IMP4 | 3.03 | Homo sapiens IMP4, U3 small nucleolar ribonucleoprotein, homolog (yeast) (IMP4), mRNA. |
| TINF2 | 3.01 | Homo sapiens TERF1 (TRF1)-interacting nuclear factor 2 (TINF2), mRNA. |
| FAM131A | 3.01 | Homo sapiens family with sequence similarity 131, member A (FAM131A), mRNA. |
| ZNF696 | 3.01 | Homo sapiens zinc finger protein 696 (ZNF696), mRNA. |
| ZNF175 | 3 | Homo sapiens zinc finger protein 175 (ZNF175), mRNA. |
| DDX17 | 3 | Homo sapiens DEAD (Asp-Glu-Ala-Asp) box polypeptide 17 (DDX17), transcript variant 2, mRNA. |
| ARMCX3 | 3 | Homo sapiens armadillo repeat containing, X-linked 3 (ARMCX3), transcript variant 2, mRNA. |
| CSTF3 | 3 | Homo sapiens cleavage stimulation factor, 3' pre-RNA, subunit 3, 77kDa (CSTF3), transcript variant 3, mRNA. |
| TRK1 | 3 | Homo sapiens tRNA lysine 1 (TRK1) on chromosome 17. |
| ZNF250 | 2.99 | Homo sapiens zinc finger protein 250 (ZNF250), mRNA. |
| LOC100133329 | 2.99 | PREDICTED: Homo sapiens misc_RNA (LOC100133329), miscRNA. |
| LOC643387 | 2.97 | PREDICTED: Homo sapiens misc_RNA (LOC643387), miscRNA. |
| PEX16 | 2.97 | Homo sapiens peroxisomal biogenesis factor 16 (PEX16), transcript variant 1, mRNA. |
| MRFAP1L1 | 2.97 | Homo sapiens Morf4 family associated protein 1-like 1 (MRFAP1L1), transcript variant 1, mRNA. |
|  | 2.96 | 602971152F1 NIH_MGC_12 Homo sapiens cDNA clone IMAGE:5110692 5, mRNA sequence |
| ATP8B2 | 2.96 | Homo sapiens ATPase, class I, type 8B, member 2 (ATP8B2), transcript variant 1, mRNA. |
| CCDC92 | 2.95 | Homo sapiens coiled-coil domain containing 92 (CCDC92), mRNA. |
| ZNF613 | 2.94 | Homo sapiens zinc finger protein 613 (ZNF613), transcript variant 2, mRNA. |
| REXO4 | 2.94 | Homo sapiens REX4, RNA exonuclease 4 homolog (S. cerevisiae) (REXO4), mRNA. |
| CHRNA3 | 2.92 | Homo sapiens cholinergic receptor, nicotinic, alpha 3 (CHRNA3), mRNA. |
| GPR162 | 2.9 | Homo sapiens G protein-coupled receptor 162 (GPR162), transcript variant A-2, mRNA. |
| MAP4K5 | 2.87 | Homo sapiens mitogen-activated protein kinase kinase kinase kinase 5 (MAP4K5), transcript variant 1, mRNA. |
| TMEM100 | 2.86 | Homo sapiens transmembrane protein 100 (TMEM100), transcript variant 2, mRNA. |
| TLX2 | 2.86 | Homo sapiens T-cell leukemia homeobox 2 (TLX2), mRNA. |
| MGC42630 | 2.86 | Homo sapiens hypothetical protein MGC42630 (MGC42630), mRNA. |
| ALDH7A1 | 2.85 | Homo sapiens aldehyde dehydrogenase 7 family, member A1 (ALDH7A1), mRNA. |
| LOC401720 | 2.85 | Homo sapiens similar to fidgetin (LOC401720), mRNA. |
| R3HCC1 | 2.83 | PREDICTED: Homo sapiens R3H domain and coiled-coil containing 1 (R3HCC1), mRNA. |
| FAM98A | 2.82 | Homo sapiens family with sequence similarity 98, member A (FAM98A), mRNA. |
| C1orf174 | 2.82 | Homo sapiens chromosome 1 open reading frame 174 (C1orf174), mRNA. |
| HSP90B1 | 2.77 | Homo sapiens heat shock protein 90kDa beta (Grp94), member 1 (HSP90B1), mRNA. |
| PDIA3P | 2.77 | Homo sapiens protein disulfide isomerase family A, member 3 pseudogene (PDIA3P), non-coding RNA. |
| PARD6A | 2.76 | Homo sapiens par-6 partitioning defective 6 homolog alpha (C. elegans) (PARD6A), transcript variant 2, mRNA. |
| CSTF3 | 2.76 | Homo sapiens cleavage stimulation factor, 3' pre-RNA, subunit 3, 77kDa (CSTF3), transcript variant 2, mRNA. |
| LOC100130291 | 2.75 | PREDICTED: Homo sapiens misc_RNA (LOC100130291), miscRNA. |
| DKK3 | 2.75 | Homo sapiens dickkopf homolog 3 (Xenopus laevis) (DKK3), transcript variant 1, mRNA. |
| C20orf100 | 2.74 | Homo sapiens chromosome 20 open reading frame 100 (C20orf100), mRNA. |
| MRPL15 | 2.74 | Homo sapiens mitochondrial ribosomal protein L15 (MRPL15), nuclear gene encoding mitochondrial protein, mRNA. |
| FAM18B2 | 2.74 | Homo sapiens family with sequence similarity 18, member B2 (FAM18B2), mRNA. |
| BCDIN3D | 2.74 | Homo sapiens BCDIN3 domain containing (BCDIN3D), mRNA. |
| FADD | 2.74 | Homo sapiens Fas (TNFRSF6)-associated via death domain (FADD), mRNA. |
| ARMET | 2.73 | Homo sapiens arginine-rich, mutated in early stage tumors (ARMET), mRNA. |
| EGLN2 | 2.72 | Homo sapiens egl nine homolog 2 (C. elegans) (EGLN2), transcript variant 3, mRNA. |
| EXOSC3 | 2.71 | Homo sapiens exosome component 3 (EXOSC3), transcript variant 1, mRNA. |
| FGF14 | 2.7 | Homo sapiens fibroblast growth factor 14 (FGF14), transcript variant 2, mRNA. |
| THAP2 | 2.69 | Homo sapiens THAP domain containing, apoptosis associated protein 2 (THAP2), mRNA. |
| UBE2L3 | 2.69 | Homo sapiens ubiquitin-conjugating enzyme E2L 3 (UBE2L3), transcript variant 2, mRNA. |
| TWF1 | 2.68 | Homo sapiens twinfilin, actin-binding protein, homolog 1 (Drosophila) (TWF1), mRNA. |
| DYRK3 | 2.68 | Homo sapiens dual-specificity tyrosine-(Y)-phosphorylation regulated kinase 3 (DYRK3), transcript variant 1, mRNA. |
| IMP3 | 2.68 | Homo sapiens IMP3, U3 small nucleolar ribonucleoprotein, homolog (yeast) (IMP3), mRNA. |
| FBXL20 | 2.67 | Homo sapiens F-box and leucine-rich repeat protein 20 (FBXL20), mRNA. |
| SMG5 | 2.65 | Homo sapiens Smg-5 homolog, nonsense mediated mRNA decay factor (C. elegans) (SMG5), mRNA. |
| MRPL52 | 2.65 | Homo sapiens mitochondrial ribosomal protein L52 (MRPL52), nuclear gene encoding mitochondrial protein, transcript variant 2, mRNA. |
| TCP1 | 2.65 | Homo sapiens t-complex 1 (TCP1), transcript variant 1, mRNA. |
| SERF2 | 2.64 | Homo sapiens small EDRK-rich factor 2 (SERF2), mRNA. |
| GNL1 | 2.63 | Homo sapiens guanine nucleotide binding protein-like 1 (GNL1), mRNA. |
| LOC390557 | 2.62 | PREDICTED: Homo sapiens hypothetical LOC390557 (LOC390557), mRNA. |
| KCNJ8 | 2.62 | Homo sapiens potassium inwardly-rectifying channel, subfamily J, member 8 (KCNJ8), mRNA. |
|  | 2.62 | zh47e08.r1 Soares_fetal_liver_spleen_1NFLS_S1 Homo sapiens cDNA clone IMAGE:415238 5, mRNA sequence |
| CCAR1 | 2.61 | Homo sapiens cell division cycle and apoptosis regulator 1 (CCAR1), mRNA. |
| TUSC4 | 2.61 | Homo sapiens tumor suppressor candidate 4 (TUSC4), mRNA. |
| LOC345041 | 2.61 | PREDICTED: Homo sapiens misc_RNA (LOC345041), miscRNA. |
| LOC440991 | 2.6 | PREDICTED: Homo sapiens misc_RNA (LOC440991), miscRNA. |
| UBAC2 | 2.59 | Homo sapiens UBA domain containing 2 (UBAC2), mRNA. |
| LOC100132547 | 2.58 | PREDICTED: Homo sapiens misc_RNA (LOC100132547), miscRNA. |
| ZNF585A | 2.57 | Homo sapiens zinc finger protein 585A (ZNF585A), transcript variant 2, mRNA. |
| IFP38 | 2.55 | Homo sapiens IFP38 (IFP38), mRNA. |
| HNRNPA1 | 2.55 | Homo sapiens heterogeneous nuclear ribonucleoprotein A1 (HNRNPA1), transcript variant 2, mRNA. |
| CCDC130 | 2.54 | Homo sapiens coiled-coil domain containing 130 (CCDC130), mRNA. |
| LOC401805 | 2.54 | PREDICTED: Homo sapiens misc_RNA (LOC401805), partial miscRNA. |
| LOC729992 | 2.54 | PREDICTED: Homo sapiens misc_RNA (LOC729992), miscRNA. |
| CKS2 | 2.53 | Homo sapiens CDC28 protein kinase regulatory subunit 2 (CKS2), mRNA. |
| KLHDC8B | 2.52 | Homo sapiens kelch domain containing 8B (KLHDC8B), mRNA. |
| LRCH4 | 2.51 | Homo sapiens leucine-rich repeats and calponin homology (CH) domain containing 4 (LRCH4), mRNA. |
| PDXK | 2.51 | Homo sapiens pyridoxal (pyridoxine, vitamin B6) kinase (PDXK), mRNA. |
| FRG1 | 2.51 | Homo sapiens FSHD region gene 1 (FRG1), mRNA. |
| DNAJB1 | 2.51 | Homo sapiens DnaJ (Hsp40) homolog, subfamily B, member 1 (DNAJB1), mRNA. |
| TRPT1 | 2.49 | Homo sapiens tRNA phosphotransferase 1 (TRPT1), transcript variant 2, mRNA. |
| BTBD11 | 2.49 | Homo sapiens BTB (POZ) domain containing 11 (BTBD11), transcript variant 1, mRNA. |
| PLD3 | 2.48 | Homo sapiens phospholipase D family, member 3 (PLD3), transcript variant 1, mRNA. |
| TBPL1 | 2.48 | Homo sapiens TBP-like 1 (TBPL1), mRNA. |
| LRRN2 | 2.48 | Homo sapiens leucine rich repeat neuronal 2 (LRRN2), transcript variant 2, mRNA. |
| CROP | 2.48 | Homo sapiens cisplatin resistance-associated overexpressed protein (CROP), transcript variant 2, mRNA. |
| C6orf47 | 2.47 | Homo sapiens chromosome 6 open reading frame 47 (C6orf47), mRNA. |
| TMEM79 | 2.47 | Homo sapiens transmembrane protein 79 (TMEM79), mRNA. |
| LOC645157 | 2.45 | PREDICTED: Homo sapiens misc_RNA (LOC645157), miscRNA. |
| HSPE1 | 2.45 | Homo sapiens heat shock 10kDa protein 1 (chaperonin 10) (HSPE1), mRNA. |
| BSN | 2.44 | Homo sapiens bassoon (presynaptic cytomatrix protein) (BSN), mRNA. |
| ZNF69 | 2.44 | Homo sapiens zinc finger protein 69 (ZNF69), mRNA. |
| XPO5 | 2.43 | Homo sapiens exportin 5 (XPO5), mRNA. |
| LOC100131737 | 2.43 | PREDICTED: Homo sapiens misc_RNA (LOC100131737), miscRNA. |
| FAM120A | 2.43 | Homo sapiens family with sequence similarity 120A (FAM120A), mRNA. |
| LOC728554 | 2.43 | PREDICTED: Homo sapiens similar to THO complex 3 (LOC728554), mRNA. |
| LOC286512 | 2.42 | PREDICTED: Homo sapiens misc_RNA (LOC286512), miscRNA. |
| C1orf74 | 2.42 | Homo sapiens chromosome 1 open reading frame 74 (C1orf74), mRNA. |
| LOC643888 | 2.41 | PREDICTED: Homo sapiens hypothetical LOC643888 (LOC643888), mRNA. |
| FAM189B | 2.4 | Homo sapiens family with sequence similarity 189, member B (FAM189B), transcript variant 1, mRNA. |
| PAF1 | 2.4 | Homo sapiens Paf1, RNA polymerase II associated factor, homolog (S. cerevisiae) (PAF1), mRNA. |
| NSUN6 | 2.4 | Homo sapiens NOL1/NOP2/Sun domain family, member 6 (NSUN6), mRNA. |
| EXO1 | 2.39 | Homo sapiens exonuclease 1 (EXO1), transcript variant 1, mRNA. |
| ZNF146 | 2.38 | Homo sapiens zinc finger protein 146 (ZNF146), mRNA. |
| LOC100133312 | 2.37 | PREDICTED: Homo sapiens misc_RNA (LOC100133312), miscRNA. |
| TFDP1 | 2.37 | Homo sapiens transcription factor Dp-1 (TFDP1), mRNA. |
| DDIT4 | 2.36 | Homo sapiens DNA-damage-inducible transcript 4 (DDIT4), mRNA. |
| ASCC3 | 2.35 | Homo sapiens activating signal cointegrator 1 complex subunit 3 (ASCC3), transcript variant 1, mRNA. |
| ZNF823 | 2.35 | Homo sapiens zinc finger protein 823 (ZNF823), mRNA. |
| NAV2 | 2.35 | Homo sapiens neuron navigator 2 (NAV2), transcript variant 2, mRNA. |
| NAPEPLD | 2.34 | Homo sapiens N-acyl phosphatidylethanolamine phospholipase D (NAPEPLD), mRNA. |
| LOC100134504 | 2.34 | PREDICTED: Homo sapiens hypothetical protein LOC100134504 (LOC100134504), mRNA. |
| ANP32A | 2.34 | Homo sapiens acidic (leucine-rich) nuclear phosphoprotein 32 family, member A (ANP32A), mRNA. |
| H19 | 2.34 | Homo sapiens H19, imprinted maternally expressed transcript (non-protein coding) (H19), non-coding RNA. |
| DEXI | 2.34 | Homo sapiens Dexi homolog (mouse) (DEXI), mRNA. |
| LOC729366 | 2.33 | PREDICTED: Homo sapiens misc_RNA (LOC729366), miscRNA. |
| MRPL18 | 2.33 | Homo sapiens mitochondrial ribosomal protein L18 (MRPL18), nuclear gene encoding mitochondrial protein, mRNA. |
| LOC286444 | 2.33 | PREDICTED: Homo sapiens misc_RNA (LOC286444), miscRNA. |
| NPPA | 2.33 | Homo sapiens natriuretic peptide precursor A (NPPA), mRNA. |
| IFNAR1 | 2.32 | Homo sapiens interferon (alpha, beta and omega) receptor 1 (IFNAR1), mRNA. |
| FBXO18 | 2.3 | Homo sapiens F-box protein, helicase, 18 (FBXO18), transcript variant 2, mRNA. |
| HSPH1 | 2.28 | Homo sapiens heat shock 105kDa/110kDa protein 1 (HSPH1), mRNA. |
| LOC652685 | 2.28 | PREDICTED: Homo sapiens similar to PMS1 protein homolog 2 (DNA mismatch repair protein PMS2) (LOC652685), mRNA. |
| IQCC | 2.28 | Homo sapiens IQ motif containing C (IQCC), mRNA. |
| HARS2 | 2.28 | Homo sapiens histidyl-tRNA synthetase 2, mitochondrial (putative) (HARS2), nuclear gene encoding mitochondrial protein, mRNA. |
| LOC100131572 | 2.27 | PREDICTED: Homo sapiens similar to hCG1783679 (LOC100131572), mRNA. |
| ZNF263 | 2.26 | Homo sapiens zinc finger protein 263 (ZNF263), mRNA. |
| IRF7 | 2.25 | Homo sapiens interferon regulatory factor 7 (IRF7), transcript variant b, mRNA. |
| DCAKD | 2.25 | Homo sapiens dephospho-CoA kinase domain containing (DCAKD), mRNA. |
| C1orf203 | 2.25 | Homo sapiens chromosome 1 open reading frame 203 (C1orf203), non-coding RNA. |
| CEP170 | 2.25 | Homo sapiens centrosomal protein 170kDa (CEP170), transcript variant beta, mRNA. |
| UQCC | 2.24 | Homo sapiens ubiquinol-cytochrome c reductase complex chaperone (UQCC), nuclear gene encoding mitochondrial protein, transcript variant 1, mRNA. |
| YWHAB | 2.24 | Homo sapiens tyrosine 3-monooxygenase/tryptophan 5-monooxygenase activation protein, beta polypeptide (YWHAB), transcript variant 2, mRNA. |
| CBX1 | 2.23 | Homo sapiens chromobox homolog 1 (HP1 beta homolog Drosophila ) (CBX1), mRNA. |
| ZNF187 | 2.23 | Homo sapiens zinc finger protein 187 (ZNF187), transcript variant 1, mRNA. |
| IRF2BP2 | 2.23 | Homo sapiens interferon regulatory factor 2 binding protein 2 (IRF2BP2), transcript variant 1, mRNA. |
| GCH1 | 2.22 | Homo sapiens GTP cyclohydrolase 1 (GCH1), transcript variant 3, mRNA. |
| MOCS1 | 2.22 | Homo sapiens molybdenum cofactor synthesis 1 (MOCS1), transcript variant 4, mRNA. |
| LOC391670 | 2.22 | PREDICTED: Homo sapiens misc_RNA (LOC391670), miscRNA. |
| MBTPS2 | 2.21 | Homo sapiens membrane-bound transcription factor peptidase, site 2 (MBTPS2), mRNA. |
| METTL13 | 2.21 | Homo sapiens methyltransferase like 13 (METTL13), transcript variant 3, mRNA. |
| C17orf90 | 2.21 | Homo sapiens chromosome 17 open reading frame 90 (C17orf90), mRNA. |
| SBDSP | 2.2 | Homo sapiens Shwachman-Bodian-Diamond syndrome pseudogene (SBDSP) on chromosome 7. |
| LOC100133803 | 2.2 | PREDICTED: Homo sapiens similar to mCG50656 (LOC100133803), mRNA. |
| ZNF610 | 2.2 | Homo sapiens zinc finger protein 610 (ZNF610), mRNA. |
| RGS5 | 2.2 | Homo sapiens regulator of G-protein signaling 5 (RGS5), mRNA. |
| SAP30L | 2.2 | Homo sapiens SAP30-like (SAP30L), mRNA. |
| LOC284023 | 2.19 | PREDICTED: Homo sapiens hypothetical protein LOC284023, transcript variant 3 (LOC284023), mRNA. |
| CRELD2 | 2.18 | Homo sapiens cysteine-rich with EGF-like domains 2 (CRELD2), mRNA. |
| LOC653105 | 2.17 | PREDICTED: Homo sapiens similar to Proline-rich nuclear receptor coactivator 2, transcript variant 3 (LOC653105), mRNA. |
| TMEM44 | 2.17 | Homo sapiens transmembrane protein 44 (TMEM44), transcript variant 1, mRNA. |
| KCTD9 | 2.17 | Homo sapiens potassium channel tetramerisation domain containing 9 (KCTD9), mRNA. |
| IFFO1 | 2.15 | Homo sapiens intermediate filament family orphan 1 (IFFO1), transcript variant 2, mRNA. |
| LOC158318 | 2.15 | Homo sapiens similar to hypothetical protein MGC42630 (LOC158318), mRNA. |
| ZNF514 | 2.15 | Homo sapiens zinc finger protein 514 (ZNF514), mRNA. |
| EXOSC3 | 2.15 | Homo sapiens exosome component 3 (EXOSC3), transcript variant 1, mRNA. |
| ZNF197 | 2.14 | Homo sapiens zinc finger protein 197 (ZNF197), transcript variant 1, mRNA. |
| LOC644745 | 2.14 | PREDICTED: Homo sapiens misc_RNA (LOC644745), miscRNA. |
| DNAJB12 | 2.13 | Homo sapiens DnaJ (Hsp40) homolog, subfamily B, member 12 (DNAJB12), transcript variant 1, mRNA. |
| CDR2L | 2.13 | Homo sapiens cerebellar degeneration-related protein 2-like (CDR2L), mRNA. |
| HERC4 | 2.12 | Homo sapiens hect domain and RLD 4 (HERC4), transcript variant 2, mRNA. |
|  | 2.12 | Homo sapiens cDNA FLJ34755 fis, clone NHNPC1000034 |
| LOC644424 | 2.11 | PREDICTED: Homo sapiens hypothetical protein LOC644424 (LOC644424), mRNA. |
| C7orf41 | 2.11 | Homo sapiens chromosome 7 open reading frame 41 (C7orf41), mRNA. |
| PLEKHO1 | 2.11 | Homo sapiens pleckstrin homology domain containing, family O member 1 (PLEKHO1), mRNA. |
| CRABP2 | 2.1 | Homo sapiens cellular retinoic acid binding protein 2 (CRABP2), mRNA. |
| AMD1 | 2.1 | Homo sapiens adenosylmethionine decarboxylase 1 (AMD1), transcript variant 2, mRNA. |
| SFRS17A | 2.1 | Homo sapiens splicing factor, arginine/serine-rich 17A (SFRS17A), transcript variant 1, mRNA. |
| SBDSP | 2.09 | Homo sapiens Shwachman-Bodian-Diamond syndrome pseudogene (SBDSP) on chromosome 7. |
| C2orf56 | 2.09 | Homo sapiens chromosome 2 open reading frame 56 (C2orf56), transcript variant 1, mRNA. |
| TAF7 | 2.08 | Homo sapiens TAF7 RNA polymerase II, TATA box binding protein (TBP)-associated factor, 55kDa (TAF7), mRNA. |
| STRAP | 2.08 | Homo sapiens serine/threonine kinase receptor associated protein (STRAP), mRNA. |
| PSMC3IP | 2.08 | Homo sapiens PSMC3 interacting protein (PSMC3IP), transcript variant 2, mRNA. |
| FXR1 | 2.08 | Homo sapiens fragile X mental retardation, autosomal homolog 1 (FXR1), transcript variant 1, mRNA. |
| CERK | 2.08 | Homo sapiens ceramide kinase (CERK), transcript variant 2, mRNA. |
| NCBP2 | 2.07 | Homo sapiens nuclear cap binding protein subunit 2, 20kDa (NCBP2), transcript variant 1, mRNA. |
| RNF146 | 2.07 | Homo sapiens ring finger protein 146 (RNF146), mRNA. |
| TTC4 | 2.07 | Homo sapiens tetratricopeptide repeat domain 4 (TTC4), mRNA. |
| RNF34 | 2.06 | Homo sapiens ring finger protein 34 (RNF34), transcript variant 2, mRNA. |
| ZNRD1 | 2.06 | Homo sapiens zinc ribbon domain containing 1 (ZNRD1), transcript variant a, mRNA. |
| MRPL43 | 2.06 | Homo sapiens mitochondrial ribosomal protein L43 (MRPL43), nuclear gene encoding mitochondrial protein, transcript variant 2, mRNA. |
| DCBLD2 | 2.06 | Homo sapiens discoidin, CUB and LCCL domain containing 2 (DCBLD2), mRNA. |
| ANP32C | 2.06 | Homo sapiens acidic (leucine-rich) nuclear phosphoprotein 32 family, member C (ANP32C), mRNA. |
| IRF9 | 2.05 | Homo sapiens interferon regulatory factor 9 (IRF9), mRNA. |
| UPF3B | 2.05 | Homo sapiens UPF3 regulator of nonsense transcripts homolog B (yeast) (UPF3B), transcript variant 1, mRNA. |
| MPHOSPH6 | 2.05 | Homo sapiens M-phase phosphoprotein 6 (MPHOSPH6), mRNA. |
| C5orf22 | 2.04 | Homo sapiens chromosome 5 open reading frame 22 (C5orf22), mRNA. |
| ECHS1 | 2.04 | Homo sapiens enoyl Coenzyme A hydratase, short chain, 1, mitochondrial (ECHS1), nuclear gene encoding mitochondrial protein, mRNA. |
| OPRL1 | 2.04 | Homo sapiens opiate receptor-like 1 (OPRL1), transcript variant 2, mRNA. |
| LOC728931 | 2.03 | PREDICTED: Homo sapiens similar to hCG17429 (LOC728931), mRNA. |
| C17orf80 | 2.03 | Homo sapiens chromosome 17 open reading frame 80 (C17orf80), mRNA. |
| C9orf37 | 2.03 | Homo sapiens chromosome 9 open reading frame 37 (C9orf37), mRNA. |
| DNAJA1 | 2.03 | Homo sapiens DnaJ (Hsp40) homolog, subfamily A, member 1 (DNAJA1), mRNA. |
|  | 2.02 | DA210550 BRAWH2 Homo sapiens cDNA clone BRAWH2018679 5, mRNA sequence |
| ZC3H4 | 2.02 | Homo sapiens zinc finger CCCH-type containing 4 (ZC3H4), mRNA. |
| ATP2C1 | 2.02 | Homo sapiens ATPase, Ca++ transporting, type 2C, member 1 (ATP2C1), transcript variant 4, mRNA. |
| ZNF622 | 2.01 | Homo sapiens zinc finger protein 622 (ZNF622), mRNA. |
| GTF2E2 | 2.01 | Homo sapiens general transcription factor IIE, polypeptide 2, beta 34kDa (GTF2E2), mRNA. |
| C6orf170 | 2.01 | Homo sapiens chromosome 6 open reading frame 170 (C6orf170), mRNA. |
| CROP | 2.01 | Homo sapiens cisplatin resistance-associated overexpressed protein (CROP), transcript variant 2, mRNA. |
| PPT1 | 2.01 | Homo sapiens palmitoyl-protein thioesterase 1 (ceroid-lipofuscinosis, neuronal 1, infantile) (PPT1), mRNA. |
| PHF14 | 1.99 | Homo sapiens PHD finger protein 14 (PHF14), transcript variant 1, mRNA. |
| CCDC93 | 1.99 | Homo sapiens coiled-coil domain containing 93 (CCDC93), mRNA. |
| TRIM13 | 1.99 | Homo sapiens tripartite motif-containing 13 (TRIM13), transcript variant 4, mRNA. |
| HNRNPA1 | 1.98 | Homo sapiens heterogeneous nuclear ribonucleoprotein A1 (HNRNPA1), transcript variant 2, mRNA. |
| ZNF35 | 1.98 | Homo sapiens zinc finger protein 35 (ZNF35), mRNA. |
| CACYBP | 1.98 | Homo sapiens calcyclin binding protein (CACYBP), transcript variant 1, mRNA. |
| TMEM126B | 1.98 | Homo sapiens transmembrane protein 126B (TMEM126B), mRNA. |
| HNRNPA1L2 | 1.97 | Homo sapiens heterogeneous nuclear ribonucleoprotein A1-like 2 (HNRNPA1L2), transcript variant 1, mRNA. |
| ZNF182 | 1.97 | Homo sapiens zinc finger protein 182 (ZNF182), transcript variant 2, mRNA. |
| MBD6 | 1.96 | Homo sapiens methyl-CpG binding domain protein 6 (MBD6), mRNA. |
| ZNF485 | 1.96 | Homo sapiens zinc finger protein 485 (ZNF485), mRNA. |
| LOC644877 | 1.96 | PREDICTED: Homo sapiens misc_RNA (LOC644877), miscRNA. |
| LOC100130887 | 1.96 | PREDICTED: Homo sapiens hypothetical protein LOC100130887 (LOC100130887), mRNA. |
| ZW10 | 1.95 | Homo sapiens ZW10, kinetochore associated, homolog (Drosophila) (ZW10), mRNA. |
| CUTA | 1.95 | Homo sapiens cutA divalent cation tolerance homolog (E. coli) (CUTA), transcript variant 2, mRNA. |
| LOC653354 | 1.95 | PREDICTED: Homo sapiens similar to amrmadillo repeat containing, X-linked 6 (H. sapiens)-like (LOC653354), mRNA. |
| KIFAP3 | 1.95 | Homo sapiens kinesin-associated protein 3 (KIFAP3), mRNA. |
| TFG | 1.95 | Homo sapiens TRK-fused gene (TFG), transcript variant 1, mRNA. |
| ZNF225 | 1.95 | Homo sapiens zinc finger protein 225 (ZNF225), mRNA. |
| FBL | 1.94 | Homo sapiens fibrillarin (FBL), mRNA. |
| EIF4G2 | 1.94 | Homo sapiens eukaryotic translation initiation factor 4 gamma, 2 (EIF4G2), transcript variant 1, mRNA. |
|  | 1.94 | Homo sapiens cDNA clone IMAGE:6250506 |
| PSMC3IP | 1.94 | Homo sapiens PSMC3 interacting protein (PSMC3IP), transcript variant 2, mRNA. |
| ATP9A | 1.94 | Homo sapiens ATPase, class II, type 9A (ATP9A), mRNA. |
| EIF4A2 | 1.93 | Homo sapiens eukaryotic translation initiation factor 4A, isoform 2 (EIF4A2), mRNA. |
| DNAJB11 | 1.93 | Homo sapiens DnaJ (Hsp40) homolog, subfamily B, member 11 (DNAJB11), mRNA. |
| ZNF490 | 1.93 | Homo sapiens zinc finger protein 490 (ZNF490), mRNA. |
| LOC642755 | 1.92 | PREDICTED: Homo sapiens similar to DEXI, transcript variant 1 (LOC642755), mRNA. |
| TXNL4B | 1.92 | Homo sapiens thioredoxin-like 4B (TXNL4B), mRNA. |
| APPBP2 | 1.92 | Homo sapiens amyloid beta precursor protein (cytoplasmic tail) binding protein 2 (APPBP2), mRNA. |
| PPP1CC | 1.91 | Homo sapiens protein phosphatase 1, catalytic subunit, gamma isoform (PPP1CC), mRNA. |
| TCEAL3 | 1.9 | Homo sapiens transcription elongation factor A (SII)-like 3 (TCEAL3), transcript variant 1, mRNA. |
| RBM14 | 1.9 | Homo sapiens RNA binding motif protein 14 (RBM14), mRNA. |
| ZNF512B | 1.9 | Homo sapiens zinc finger protein 512B (ZNF512B), mRNA. |
| LOC648740 | 1.89 | Homo sapiens ACTB pseudogene (LOC648740), non-coding RNA. |
| LOC643011 | 1.89 | PREDICTED: Homo sapiens hypothetical protein LOC643011 (LOC643011), mRNA. |
| RAB2B | 1.88 | Homo sapiens RAB2B, member RAS oncogene family (RAB2B), mRNA. |
| ZNF248 | 1.88 | Homo sapiens zinc finger protein 248 (ZNF248), mRNA. |
| CECR5 | 1.87 | Homo sapiens cat eye syndrome chromosome region, candidate 5 (CECR5), transcript variant 2, mRNA. |
| ARAP3 | 1.87 | Homo sapiens ArfGAP with RhoGAP domain, ankyrin repeat and PH domain 3 (ARAP3), mRNA. |
| C16orf53 | 1.87 | Homo sapiens chromosome 16 open reading frame 53 (C16orf53), mRNA. |
| UTP6 | 1.86 | Homo sapiens UTP6, small subunit (SSU) processome component, homolog (yeast) (UTP6), mRNA. |
| LOC389816 | 1.86 | Homo sapiens cytokeratin associated protein (LOC389816), mRNA. |
| GSPT2 | 1.86 | Homo sapiens G1 to S phase transition 2 (GSPT2), mRNA. |
| ZNF491 | 1.86 | Homo sapiens zinc finger protein 491 (ZNF491), mRNA. |
| LOC652771 | 1.86 | PREDICTED: Homo sapiens similar to general transcription factor II, i isoform 1 (LOC652771), mRNA. |
| OAZ2 | 1.86 | Homo sapiens ornithine decarboxylase antizyme 2 (OAZ2), mRNA. |
| NCL | 1.85 | Homo sapiens nucleolin (NCL), mRNA. |
| MED20 | 1.85 | Homo sapiens mediator complex subunit 20 (MED20), mRNA. |
| ATF5 | 1.85 | Homo sapiens activating transcription factor 5 (ATF5), mRNA. |
| SLC39A9 | 1.84 | Homo sapiens solute carrier family 39 (zinc transporter), member 9 (SLC39A9), mRNA. |
| HEXIM2 | 1.83 | Homo sapiens hexamthylene bis-acetamide inducible 2 (HEXIM2), mRNA. |
| ZC3HC1 | 1.83 | Homo sapiens zinc finger, C3HC-type containing 1 (ZC3HC1), mRNA. |
| LOC652773 | 1.83 | PREDICTED: Homo sapiens similar to Shwachman-Bodian-Diamond syndrome (predicted) (LOC652773), mRNA. |
| NAT12 | 1.83 | Homo sapiens N-acetyltransferase 12 (NAT12), mRNA. |
| NLN | 1.83 | Homo sapiens neurolysin (metallopeptidase M3 family) (NLN), mRNA. |
| ASPHD1 | 1.83 | Homo sapiens aspartate beta-hydroxylase domain containing 1 (ASPHD1), mRNA. |
| GNL2 | 1.82 | Homo sapiens guanine nucleotide binding protein-like 2 (nucleolar) (GNL2), mRNA. |
| LOC653557 | 1.82 | PREDICTED: Homo sapiens similar to Ribosome biogenesis protein BMS1 homolog (LOC653557), mRNA. |
| FH | 1.82 | Homo sapiens fumarate hydratase (FH), nuclear gene encoding mitochondrial protein, mRNA. |
| FBXL10 | 1.81 | Homo sapiens F-box and leucine-rich repeat protein 10 (FBXL10), transcript variant 1, mRNA. |
| CNPY4 | 1.81 | Homo sapiens canopy 4 homolog (zebrafish) (CNPY4), mRNA. |
| WTIP | 1.81 | PREDICTED: Homo sapiens Wilms tumor 1 interacting protein (WTIP), mRNA. |
| THAP11 | 1.81 | Homo sapiens THAP domain containing 11 (THAP11), mRNA. |
| NME1 | 1.81 | Homo sapiens non-metastatic cells 1, protein (NM23A) expressed in (NME1), transcript variant 1, mRNA. |
| ZDHHC9 | 1.81 | Homo sapiens zinc finger, DHHC-type containing 9 (ZDHHC9), transcript variant 1, mRNA. |
| MARCKS | 1.81 | Homo sapiens myristoylated alanine-rich protein kinase C substrate (MARCKS), mRNA. |
| ACYP1 | 1.8 | Homo sapiens acylphosphatase 1, erythrocyte (common) type (ACYP1), transcript variant 1, mRNA. |
| CAB39 | 1.8 | Homo sapiens calcium binding protein 39 (CAB39), mRNA. |
| C2orf44 | 1.8 | Homo sapiens chromosome 2 open reading frame 44 (C2orf44), mRNA. |
| NCL | 1.79 | Homo sapiens nucleolin (NCL), mRNA. |
| MAX | 1.79 | Homo sapiens MYC associated factor X (MAX), transcript variant 1, mRNA. |
| GSR | 1.79 | Homo sapiens glutathione reductase (GSR), mRNA. |
| KIF21A | 1.79 | Homo sapiens kinesin family member 21A (KIF21A), mRNA. |
| UPF2 | 1.79 | Homo sapiens UPF2 regulator of nonsense transcripts homolog (yeast) (UPF2), transcript variant 1, mRNA. |
| CBX5 | 1.78 | Homo sapiens chromobox homolog 5 (HP1 alpha homolog, Drosophila) (CBX5), mRNA. |
| CCT6A | 1.78 | Homo sapiens chaperonin containing TCP1, subunit 6A (zeta 1) (CCT6A), transcript variant 1, mRNA. |
| LOC654254 | 1.77 | PREDICTED: Homo sapiens similar to zinc finger protein 595 (LOC654254), mRNA. |
| DPAGT1 | 1.77 | Homo sapiens dolichyl-phosphate (UDP-N-acetylglucosamine) N-acetylglucosaminephosphotransferase 1 (GlcNAc-1-P transferase) (DPAGT1), transcript variant 1, mRNA. |
| 9-Mar | 1.77 | Homo sapiens membrane-associated ring finger (C3HC4) 9 (MARCH9), mRNA. |
| MRPL3 | 1.77 | Homo sapiens mitochondrial ribosomal protein L3 (MRPL3), nuclear gene encoding mitochondrial protein, mRNA. |
| PCYT2 | 1.77 | Homo sapiens phosphate cytidylyltransferase 2, ethanolamine (PCYT2), mRNA. |
| SERINC3 | 1.77 | Homo sapiens serine incorporator 3 (SERINC3), transcript variant 1, mRNA. |
| IVNS1ABP | 1.76 | Homo sapiens influenza virus NS1A binding protein (IVNS1ABP), mRNA. |
| LOC440396 | 1.76 | Homo sapiens similar to Heterogeneous nuclear ribonucleoprotein A1 (Helix-destabilizing protein) (Single-strand binding protein) (hnRNP core protein A1) (HDP-1) (Topoisomerase-inhibitor suppressed) (LOC440396), non-coding RNA. |
| LOC554206 | 1.76 | PREDICTED: Homo sapiens hypothetical LOC554206 (LOC554206), misc RNA. |
| LOC727865 | 1.75 | PREDICTED: Homo sapiens misc_RNA (LOC727865), miscRNA. |
| ZNF304 | 1.75 | Homo sapiens zinc finger protein 304 (ZNF304), mRNA. |
| LOC390183 | 1.75 | PREDICTED: Homo sapiens misc_RNA (LOC390183), miscRNA. |
| BRMS1 | 1.75 | Homo sapiens breast cancer metastasis suppressor 1 (BRMS1), transcript variant 3, mRNA. |
| NUP37 | 1.75 | Homo sapiens nucleoporin 37kDa (NUP37), mRNA. |
| RIC3 | 1.74 | Homo sapiens resistance to inhibitors of cholinesterase 3 homolog (C. elegans) (RIC3), mRNA. |
| PDLIM7 | 1.74 | Homo sapiens PDZ and LIM domain 7 (enigma) (PDLIM7), transcript variant 4, mRNA. |
| C5orf34 | 1.74 | Homo sapiens chromosome 5 open reading frame 34 (C5orf34), mRNA. |
| PRDX6 | 1.74 | Homo sapiens peroxiredoxin 6 (PRDX6), mRNA. |
| SEC11C | 1.74 | Homo sapiens SEC11 homolog C (S. cerevisiae) (SEC11C), mRNA. |
| HEATR1 | 1.74 | Homo sapiens HEAT repeat containing 1 (HEATR1), mRNA. |
| VSNL1 | 1.74 | Homo sapiens visinin-like 1 (VSNL1), mRNA. |
| SYF2 | 1.72 | Homo sapiens SYF2 homolog, RNA splicing factor (S. cerevisiae) (SYF2), transcript variant 1, mRNA. |
| GAB1 | 1.72 | Homo sapiens GRB2-associated binding protein 1 (GAB1), transcript variant 1, mRNA. |
| NEBL | 1.72 | Homo sapiens nebulette (NEBL), transcript variant 1, mRNA. |
| KIAA0907 | 1.72 | Homo sapiens KIAA0907 (KIAA0907), mRNA. |
| THYN1 | 1.72 | Homo sapiens thymocyte nuclear protein 1 (THYN1), transcript variant 3, mRNA. |
| CPNE1 | 1.71 | Homo sapiens copine I (CPNE1), transcript variant 3, mRNA. |
| GPR137B | 1.71 | Homo sapiens G protein-coupled receptor 137B (GPR137B), mRNA. |
| ZFP14 | 1.71 | Homo sapiens zinc finger protein 14 homolog (mouse) (ZFP14), mRNA. |
| CKS2 | 1.71 | Homo sapiens CDC28 protein kinase regulatory subunit 2 (CKS2), mRNA. |
| LOC100128689 | 1.7 | PREDICTED: Homo sapiens misc_RNA (LOC100128689), miscRNA. |
| HNRNPA3P1 | 1.7 | Homo sapiens heterogeneous nuclear ribonucleoprotein A3 pseudogene 1 (HNRNPA3P1) on chromosome 10. XR_000879 XR_001292 XR_001293 XR_001294 XR_001295 XR_001296 XR_001297 |
| RBM34 | 1.69 | Homo sapiens RNA binding motif protein 34 (RBM34), mRNA. |
| ORC5L | 1.69 | Homo sapiens origin recognition complex, subunit 5-like (yeast) (ORC5L), transcript variant 1, mRNA. |
| MRPS25 | 1.69 | Homo sapiens mitochondrial ribosomal protein S25 (MRPS25), nuclear gene encoding mitochondrial protein, mRNA. |
| LOC654121 | 1.68 | PREDICTED: Homo sapiens similar to C49H3.3 (LOC654121), mRNA. |
| GCC1 | 1.68 | Homo sapiens GRIP and coiled-coil domain containing 1 (GCC1), mRNA. |
| SDHALP1 | 1.66 | Homo sapiens succinate dehydrogenase complex, subunit A, flavoprotein pseudogene 1 (SDHALP1) on chromosome 3. |
| LOC400506 | 1.66 | Homo sapiens similar to TSG118.1 (LOC400506), mRNA. |
| SELS | 1.66 | Homo sapiens selenoprotein S (SELS), transcript variant 2, mRNA. |
| MOBKL2A | 1.65 | Homo sapiens MOB1, Mps One Binder kinase activator-like 2A (yeast) (MOBKL2A), mRNA. |
| SPTLC1 | 1.65 | Homo sapiens serine palmitoyltransferase, long chain base subunit 1 (SPTLC1), transcript variant 2, mRNA. |
| SHFM1 | 1.64 | Homo sapiens split hand/foot malformation (ectrodactyly) type 1 (SHFM1), mRNA. |
| BRWD1 | 1.64 | Homo sapiens bromodomain and WD repeat domain containing 1 (BRWD1), transcript variant 1, mRNA. |
| FAIM2 | 1.64 | Homo sapiens Fas apoptotic inhibitory molecule 2 (FAIM2), mRNA. |
| DPH5 | 1.64 | Homo sapiens DPH5 homolog (S. cerevisiae) (DPH5), transcript variant 3, mRNA. |
| DHX34 | 1.64 | Homo sapiens DEAH (Asp-Glu-Ala-His) box polypeptide 34 (DHX34), mRNA. |
| LOC731432 | 1.64 | PREDICTED: Homo sapiens similar to Ribosome biogenesis protein BMS1 homolog (LOC731432), mRNA. |
| EID2B | 1.63 | Homo sapiens EP300 interacting inhibitor of differentiation 2B (EID2B), mRNA. |
| LOC643873 | 1.63 | PREDICTED: Homo sapiens misc_RNA (LOC643873), miscRNA. |
| POLR1C | 1.63 | Homo sapiens polymerase (RNA) I polypeptide C, 30kDa (POLR1C), transcript variant 1, mRNA. |
| RHBDD1 | 1.63 | Homo sapiens rhomboid domain containing 1 (RHBDD1), mRNA. |
| SSU72 | 1.63 | Homo sapiens SSU72 RNA polymerase II CTD phosphatase homolog (S. cerevisiae) (SSU72), mRNA. |
| DENR | 1.63 | Homo sapiens density-regulated protein (DENR), mRNA. |
| LOC100132288 | 1.62 | Homo sapiens hypothetical protein LOC100132288 (LOC100132288), mRNA. |
| TMSL3 | 1.62 | Homo sapiens thymosin-like 3 (TMSL3), mRNA. |
| PNPO | 1.62 | Homo sapiens pyridoxamine 5'-phosphate oxidase (PNPO), mRNA. |
| RABL3 | 1.61 | Homo sapiens RAB, member of RAS oncogene family-like 3 (RABL3), mRNA. |
| DIABLO | 1.6 | Homo sapiens diablo homolog (Drosophila) (DIABLO), nuclear gene encoding mitochondrial protein, transcript variant 2, mRNA. |
| PNCK | 1.6 | Homo sapiens pregnancy upregulated non-ubiquitously expressed CaM kinase (PNCK), mRNA. |
| ZNF192 | 1.6 | Homo sapiens zinc finger protein 192 (ZNF192), mRNA. |
| GTPBP3 | 1.6 | Homo sapiens GTP binding protein 3 (mitochondrial) (GTPBP3), nuclear gene encoding mitochondrial protein, transcript variant V, mRNA. |
| TMEM183B | 1.6 | Homo sapiens transmembrane protein 183B (TMEM183B), mRNA. |
| NFKB1 | 1.6 | Homo sapiens nuclear factor of kappa light polypeptide gene enhancer in B-cells 1 (NFKB1), mRNA. |
| LOC339804 | 1.6 | PREDICTED: Homo sapiens hypothetical gene supported by AK075484; BC014578 (LOC339804), mRNA. |
| C7orf13 | 1.6 | Homo sapiens chromosome 7 open reading frame 13 (C7orf13), mRNA. |
| HRAS | 1.6 | Homo sapiens v-Ha-ras Harvey rat sarcoma viral oncogene homolog (HRAS), transcript variant 1, mRNA. |
| C20orf20 | 1.58 | Homo sapiens chromosome 20 open reading frame 20 (C20orf20), mRNA. |
| UBQLN4 | 1.57 | Homo sapiens ubiquilin 4 (UBQLN4), mRNA. |
| CCDC5 | 1.57 | Homo sapiens coiled-coil domain containing 5 (spindle associated) (CCDC5), mRNA. |
| C15orf17 | 1.57 | Homo sapiens chromosome 15 open reading frame 17 (C15orf17), mRNA. |
| SRP54 | 1.57 | Homo sapiens signal recognition particle 54kDa (SRP54), mRNA. |
| LOC645001 | 1.56 | PREDICTED: Homo sapiens misc_RNA (LOC645001), miscRNA. |
| RBM4B | 1.56 | Homo sapiens RNA binding motif protein 4B (RBM4B), mRNA. |
| PHF14 | 1.56 | Homo sapiens PHD finger protein 14 (PHF14), transcript variant 1, mRNA. |
| SDHALP1 | 1.56 | Homo sapiens succinate dehydrogenase complex, subunit A, flavoprotein pseudogene 1 (SDHALP1) on chromosome 3. |
| SRP54 | 1.55 | PREDICTED: Homo sapiens signal recognition particle 54kDa (SRP54), mRNA. |
| TSR2 | 1.54 | Homo sapiens TSR2, 20S rRNA accumulation, homolog (S. cerevisiae) (TSR2), mRNA. |
| RPS15A | 1.54 | Homo sapiens ribosomal protein S15a (RPS15A), transcript variant 2, mRNA. |
| LOC729708 | 1.54 | PREDICTED: Homo sapiens similar to rcTPI1, transcript variant 1 (LOC729708), mRNA. |
| C14orf132 | 1.54 | Homo sapiens chromosome 14 open reading frame 132 (C14orf132), mRNA. |
| LOC648695 | 1.53 | PREDICTED: Homo sapiens similar to retinoblastoma binding protein 4, transcript variant 5 (LOC648695), mRNA. |
| NBPF10 | 1.53 | PREDICTED: Homo sapiens neuroblastoma breakpoint family, member 10, transcript variant 4 (NBPF10), mRNA. |
| CHCHD4 | 1.53 | Homo sapiens coiled-coil-helix-coiled-coil-helix domain containing 4 (CHCHD4), nuclear gene encoding mitochondrial protein, transcript variant 2, mRNA. |
| RAB7A | 1.53 | Homo sapiens RAB7A, member RAS oncogene family (RAB7A), mRNA. |
| CCT7 | 1.53 | Homo sapiens chaperonin containing TCP1, subunit 7 (eta) (CCT7), transcript variant 1, mRNA. |
| RABEPK | 1.53 | Homo sapiens Rab9 effector protein with kelch motifs (RABEPK), mRNA. |
| FAM48A | 1.53 | Homo sapiens family with sequence similarity 48, member A (FAM48A), transcript variant 1, mRNA. |
| LOC729964 | 1.53 | PREDICTED: Homo sapiens similar to Cks1 protein homologue (LOC729964), mRNA. |
| FAM53A | 1.52 | Homo sapiens family with sequence similarity 53, member A (FAM53A), mRNA. |
| LOC374395 | -1.5 | Homo sapiens similar to RIKEN cDNA 1810059G22 (LOC374395), mRNA. |
| MCM4 | -1.5 | Homo sapiens minichromosome maintenance complex component 4 (MCM4), transcript variant 1, mRNA. |
| PRPF31 | -1.5 | Homo sapiens PRP31 pre-mRNA processing factor 31 homolog (S. cerevisiae) (PRPF31), mRNA. |
| SULT1A3 | -1.51 | Homo sapiens sulfotransferase family, cytosolic, 1A, phenol-preferring, member 3 (SULT1A3), transcript variant 1, mRNA. |
| TRIM25 | -1.51 | Homo sapiens tripartite motif-containing 25 (TRIM25), mRNA. |
| PLIN2 | -1.51 | Homo sapiens perilipin 2 (PLIN2), mRNA. |
| PIGO | -1.51 | Homo sapiens phosphatidylinositol glycan anchor biosynthesis, class O (PIGO), transcript variant 2, mRNA. |
| LOC647691 | -1.51 | PREDICTED: Homo sapiens similar to dicer1 (LOC647691), mRNA. |
| LOC100129502 | -1.52 | PREDICTED: Homo sapiens hypothetical protein LOC100129502 (LOC100129502), mRNA. |
| HSD17B8 | -1.52 | Homo sapiens hydroxysteroid (17-beta) dehydrogenase 8 (HSD17B8), mRNA. |
| C16orf53 | -1.52 | Homo sapiens chromosome 16 open reading frame 53 (C16orf53), mRNA. |
| CKS1B | -1.52 | Homo sapiens CDC28 protein kinase regulatory subunit 1B (CKS1B), mRNA. |
| TMEM185B | -1.52 | Homo sapiens transmembrane protein 185B (pseudogene) (TMEM185B), non-coding RNA. |
| ZNF473 | -1.52 | Homo sapiens zinc finger protein 473 (ZNF473), transcript variant 1, mRNA. |
| LOC646301 | -1.52 | PREDICTED: Homo sapiens misc_RNA (LOC646301), miscRNA. |
| ACADVL | -1.52 | Homo sapiens acyl-Coenzyme A dehydrogenase, very long chain (ACADVL), nuclear gene encoding mitochondrial protein, transcript variant 1, mRNA. |
| LASS2 | -1.52 | Homo sapiens LAG1 homolog, ceramide synthase 2 (LASS2), transcript variant 2, mRNA. |
| FRAP1 | -1.52 | Homo sapiens FK506 binding protein 12-rapamycin associated protein 1 (FRAP1), mRNA. |
| MAP3K3 | -1.52 | Homo sapiens mitogen-activated protein kinase kinase kinase 3 (MAP3K3), transcript variant 1, mRNA. |
|  | -1.52 | xn24e12.x1 NCI_CGAP_Kid11 Homo sapiens cDNA clone IMAGE:2694670 3, mRNA sequence |
| SYDE1 | -1.52 | Homo sapiens synapse defective 1, Rho GTPase, homolog 1 (C. elegans) (SYDE1), mRNA. |
| FLJ40722 | -1.52 | PREDICTED: Homo sapiens hypothetical protein FLJ40722, transcript variant 3 (FLJ40722), mRNA. |
| SLC29A3 | -1.52 | Homo sapiens solute carrier family 29 (nucleoside transporters), member 3 (SLC29A3), mRNA. |
| TULP3 | -1.52 | Homo sapiens tubby like protein 3 (TULP3), mRNA. |
| ARSB | -1.52 | Homo sapiens arylsulfatase B (ARSB), transcript variant 1, mRNA. |
| LOC643911 | -1.52 | PREDICTED: Homo sapiens hypothetical LOC643911 (LOC643911), mRNA. |
| DIS3L2 | -1.52 | Homo sapiens DIS3 mitotic control homolog (S. cerevisiae)-like 2 (DIS3L2), mRNA. |
| PLEKHA4 | -1.53 | Homo sapiens pleckstrin homology domain containing, family A (phosphoinositide binding specific) member 4 (PLEKHA4), mRNA. |
| C5orf46 | -1.53 | Homo sapiens chromosome 5 open reading frame 46 (C5orf46), mRNA. |
| PCSK7 | -1.53 | Homo sapiens proprotein convertase subtilisin/kexin type 7 (PCSK7), mRNA. |
| KIAA0101 | -1.53 | Homo sapiens KIAA0101 (KIAA0101), transcript variant 1, mRNA. |
|  | -1.53 | BX114974 NCI_CGAP_Kid3 Homo sapiens cDNA clone IMAGp998C023886, mRNA sequence |
| SNORA64 | -1.53 | Homo sapiens small nucleolar RNA, H/ACA box 64 (SNORA64), small nucleolar RNA. |
|  | -1.53 | UI-E-CQ1-aew-g-07-0-UI.r1 UI-E-CQ1 Homo sapiens cDNA clone UI-E-CQ1-aew-g-07-0-UI 5, mRNA sequence |
| ELF3 | -1.53 | Homo sapiens E74-like factor 3 (ets domain transcription factor, epithelial-specific ) (ELF3), mRNA. |
| NBL1 | -1.54 | Homo sapiens neuroblastoma, suppression of tumorigenicity 1 (NBL1), transcript variant 1, mRNA. |
| C9orf142 | -1.54 | Homo sapiens chromosome 9 open reading frame 142 (C9orf142), mRNA. |
| DGCR6L | -1.54 | Homo sapiens DiGeorge syndrome critical region gene 6-like (DGCR6L), mRNA. |
| SELM | -1.54 | Homo sapiens selenoprotein M (SELM), mRNA. |
| LOC644101 | -1.54 | PREDICTED: Homo sapiens misc_RNA (LOC644101), miscRNA. |
| C11orf57 | -1.54 | Homo sapiens chromosome 11 open reading frame 57 (C11orf57), transcript variant 1, mRNA. |
| OR7E156P | -1.54 | Homo sapiens olfactory receptor, family 7, subfamily E, member 156 pseudogene (OR7E156P), non-coding RNA. |
| NOC4L | -1.54 | Homo sapiens nucleolar complex associated 4 homolog (S. cerevisiae) (NOC4L), mRNA. |
| LRPPRC | -1.54 | Homo sapiens leucine-rich PPR-motif containing (LRPPRC), mRNA. |
| ARID4B | -1.54 | Homo sapiens AT rich interactive domain 4B (RBP1-like) (ARID4B), transcript variant 1, mRNA. |
| UBE2C | -1.55 | Homo sapiens ubiquitin-conjugating enzyme E2C (UBE2C), transcript variant 3, mRNA. |
| NUTF2 | -1.55 | Homo sapiens nuclear transport factor 2 (NUTF2), mRNA. |
| LOC648980 | -1.55 | PREDICTED: Homo sapiens similar to breakpoint cluster region, transcript variant 1 (LOC648980), mRNA. |
| BIRC3 | -1.55 | Homo sapiens baculoviral IAP repeat-containing 3 (BIRC3), transcript variant 2, mRNA. |
| CTSL2 | -1.55 | Homo sapiens cathepsin L2 (CTSL2), mRNA. |
| ISCA1 | -1.55 | Homo sapiens iron-sulfur cluster assembly 1 homolog (S. cerevisiae) (ISCA1), mRNA. |
| AP2B1 | -1.55 | Homo sapiens adaptor-related protein complex 2, beta 1 subunit (AP2B1), transcript variant 2, mRNA. |
| SEMA4F | -1.55 | Homo sapiens sema domain, immunoglobulin domain (Ig), transmembrane domain (TM) and short cytoplasmic domain, (semaphorin) 4F (SEMA4F), mRNA. |
| FAM163A | -1.55 | Homo sapiens family with sequence similarity 163, member A (FAM163A), mRNA. |
| ROGDI | -1.55 | Homo sapiens rogdi homolog (Drosophila) (ROGDI), mRNA. |
| FAM156A | -1.56 | Homo sapiens family with sequence similarity 156, member A (FAM156A), mRNA. |
| LOC402221 | -1.56 | PREDICTED: Homo sapiens similar to actin alpha 1 skeletal muscle protein (LOC402221), mRNA. |
| LOC645094 | -1.56 | PREDICTED: Homo sapiens misc_RNA (LOC645094), miscRNA. |
| ARHGEF7 | -1.56 | Homo sapiens Rho guanine nucleotide exchange factor (GEF) 7 (ARHGEF7), transcript variant 1, mRNA. |
| LOC728138 | -1.56 | PREDICTED: Homo sapiens hypothetical LOC728138 (LOC728138), mRNA. |
| ZFPL1 | -1.56 | Homo sapiens zinc finger protein-like 1 (ZFPL1), mRNA. |
| FYN | -1.56 | Homo sapiens FYN oncogene related to SRC, FGR, YES (FYN), transcript variant 2, mRNA. |
| PXMP2 | -1.56 | Homo sapiens peroxisomal membrane protein 2, 22kDa (PXMP2), mRNA. |
| CDC42SE1 | -1.56 | Homo sapiens CDC42 small effector 1 (CDC42SE1), transcript variant 1, mRNA. |
| TRIM26 | -1.57 | Homo sapiens tripartite motif-containing 26 (TRIM26), mRNA. |
| DEPDC5 | -1.57 | Homo sapiens DEP domain containing 5 (DEPDC5), transcript variant 1, mRNA. |
| ORAI2 | -1.57 | Homo sapiens ORAI calcium release-activated calcium modulator 2 (ORAI2), mRNA. |
| TRIM44 | -1.57 | Homo sapiens tripartite motif-containing 44 (TRIM44), mRNA. |
| TSPYL2 | -1.57 | Homo sapiens TSPY-like 2 (TSPYL2), mRNA. |
| ACY1 | -1.57 | Homo sapiens aminoacylase 1 (ACY1), mRNA. |
| TNC | -1.57 | Homo sapiens tenascin C (TNC), mRNA. |
| IPP | -1.57 | Homo sapiens intracisternal A particle-promoted polypeptide (IPP), mRNA. |
| CALU | -1.57 | Homo sapiens calumenin (CALU), mRNA. |
| IFI27L2 | -1.57 | Homo sapiens interferon, alpha-inducible protein 27-like 2 (IFI27L2), mRNA. |
| TFG | -1.57 | Homo sapiens TRK-fused gene (TFG), transcript variant 2, mRNA. |
| RAB24 | -1.58 | Homo sapiens RAB24, member RAS oncogene family (RAB24), transcript variant 1, mRNA. |
| VARS | -1.58 | Homo sapiens valyl-tRNA synthetase (VARS), nuclear gene encoding mitochondrial protein, mRNA. |
| CAD | -1.58 | Homo sapiens carbamoyl-phosphate synthetase 2, aspartate transcarbamylase, and dihydroorotase (CAD), mRNA. |
| SAFB2 | -1.58 | Homo sapiens scaffold attachment factor B2 (SAFB2), mRNA. |
| SHMT2 | -1.58 | Homo sapiens serine hydroxymethyltransferase 2 (mitochondrial) (SHMT2), nuclear gene encoding mitochondrial protein, mRNA. |
| LOC441066 | -1.58 | PREDICTED: Homo sapiens similar to Beta-glucuronidase precursor (LOC441066), mRNA. |
| TMEM208 | -1.58 | Homo sapiens transmembrane protein 208 (TMEM208), mRNA. |
| LOC23117 | -1.58 | PREDICTED: Homo sapiens KIAA0220-like protein, transcript variant 16 (LOC23117), mRNA. |
| LOC390671 | -1.58 | PREDICTED: Homo sapiens misc_RNA (LOC390671), miscRNA. |
| TMEM147 | -1.58 | Homo sapiens transmembrane protein 147 (TMEM147), mRNA. |
| CMTM7 | -1.58 | Homo sapiens CKLF-like MARVEL transmembrane domain containing 7 (CMTM7), transcript variant 2, mRNA. |
| AGXT2L2 | -1.59 | Homo sapiens alanine-glyoxylate aminotransferase 2-like 2 (AGXT2L2), mRNA. |
| TMEM143 | -1.59 | Homo sapiens transmembrane protein 143 (TMEM143), mRNA. |
| C20orf55 | -1.59 | Homo sapiens chromosome 20 open reading frame 55 (C20orf55), transcript variant 1, mRNA. |
| NOTCH4 | -1.59 | Homo sapiens Notch homolog 4 (Drosophila) (NOTCH4), mRNA. |
| CRY2 | -1.59 | Homo sapiens cryptochrome 2 (photolyase-like) (CRY2), mRNA. |
| MED12 | -1.59 | Homo sapiens mediator of RNA polymerase II transcription, subunit 12 homolog (yeast) (MED12), mRNA. |
| C9orf100 | -1.59 | Homo sapiens chromosome 9 open reading frame 100 (C9orf100), transcript variant 2, mRNA. |
| EHMT2 | -1.59 | Homo sapiens euchromatic histone-lysine N-methyltransferase 2 (EHMT2), transcript variant NG36/G9a-SPI, mRNA. |
| CRYBB2 | -1.59 | Homo sapiens crystallin, beta B2 (CRYBB2), mRNA. |
| LOC728518 | -1.59 | PREDICTED: Homo sapiens hypothetical LOC728518 (LOC728518), mRNA. |
| BOLA2 | -1.59 | Homo sapiens bolA homolog 2 (E. coli) (BOLA2), mRNA. |
| ZNF586 | -1.59 | Homo sapiens zinc finger protein 586 (ZNF586), mRNA. |
| PEF1 | -1.6 | Homo sapiens penta-EF-hand domain containing 1 (PEF1), mRNA. |
| LOC392437 | -1.6 | PREDICTED: Homo sapiens misc_RNA (LOC392437), miscRNA. |
| COL1A1 | -1.6 | Homo sapiens collagen, type I, alpha 1 (COL1A1), mRNA. |
| HNRNPR | -1.6 | Homo sapiens heterogeneous nuclear ribonucleoprotein R (HNRNPR), transcript variant 1, mRNA. |
| SIGIRR | -1.6 | PREDICTED: Homo sapiens single immunoglobulin and toll-interleukin 1 receptor (TIR) domain (SIGIRR), mRNA. |
| RGS19 | -1.6 | Homo sapiens regulator of G-protein signaling 19 (RGS19), transcript variant 1, mRNA. |
| MIR1185-1 | -1.6 | Homo sapiens microRNA 1185-1 (MIR1185-1), microRNA. |
| TNFAIP1 | -1.6 | Homo sapiens tumor necrosis factor, alpha-induced protein 1 (endothelial) (TNFAIP1), mRNA. |
| MTHFD1 | -1.61 | Homo sapiens methylenetetrahydrofolate dehydrogenase (NADP+ dependent) 1, methenyltetrahydrofolate cyclohydrolase, formyltetrahydrofolate synthetase (MTHFD1), mRNA. |
| TP53INP2 | -1.61 | Homo sapiens tumor protein p53 inducible nuclear protein 2 (TP53INP2), mRNA. |
| ZFP106 | -1.61 | Homo sapiens zinc finger protein 106 homolog (mouse) (ZFP106), mRNA. |
| GREM1 | -1.61 | Homo sapiens gremlin 1, cysteine knot superfamily, homolog (Xenopus laevis) (GREM1), mRNA. |
| PEX16 | -1.61 | Homo sapiens peroxisomal biogenesis factor 16 (PEX16), transcript variant 1, mRNA. |
| EZH2 | -1.61 | Homo sapiens enhancer of zeste homolog 2 (Drosophila) (EZH2), transcript variant 1, mRNA. |
| NAALAD2 | -1.61 | Homo sapiens N-acetylated alpha-linked acidic dipeptidase 2 (NAALAD2), mRNA. |
| PEPD | -1.61 | Homo sapiens peptidase D (PEPD), mRNA. |
| USP22 | -1.61 | Homo sapiens ubiquitin specific peptidase 22 (USP22), mRNA. |
| TPD52L2 | -1.61 | Homo sapiens tumor protein D52-like 2 (TPD52L2), transcript variant 2, mRNA. |
| CKMT1A | -1.61 | Homo sapiens creatine kinase, mitochondrial 1A (CKMT1A), nuclear gene encoding mitochondrial protein, mRNA. |
| OTUD5 | -1.61 | Homo sapiens OTU domain containing 5 (OTUD5), mRNA. |
| ARF4 | -1.61 | Homo sapiens ADP-ribosylation factor 4 (ARF4), mRNA. |
| SNX8 | -1.62 | Homo sapiens sorting nexin 8 (SNX8), mRNA. |
| JARID1A | -1.62 | Homo sapiens Jumonji, AT rich interactive domain 1A (RBBP2-like) (JARID1A), mRNA. |
| CALML4 | -1.62 | Homo sapiens calmodulin-like 4 (CALML4), transcript variant 1, mRNA. |
| RNFT2 | -1.62 | Homo sapiens ring finger protein, transmembrane 2 (RNFT2), transcript variant 2, mRNA. |
| HTRA2 | -1.62 | Homo sapiens HtrA serine peptidase 2 (HTRA2), nuclear gene encoding mitochondrial protein, transcript variant 2, mRNA. |
| RBCK1 | -1.62 | Homo sapiens RanBP-type and C3HC4-type zinc finger containing 1 (RBCK1), transcript variant 1, mRNA. |
| LSM7 | -1.62 | Homo sapiens LSM7 homolog, U6 small nuclear RNA associated (S. cerevisiae) (LSM7), mRNA. |
| PFKP | -1.62 | Homo sapiens phosphofructokinase, platelet (PFKP), mRNA. |
| LIG1 | -1.63 | Homo sapiens ligase I, DNA, ATP-dependent (LIG1), mRNA. |
|  | -1.63 | PREDICTED: Homo sapiens hypothetical LOC388227 (LOC388227), mRNA |
| FHOD1 | -1.63 | Homo sapiens formin homology 2 domain containing 1 (FHOD1), mRNA. |
| FBXL15 | -1.63 | Homo sapiens F-box and leucine-rich repeat protein 15 (FBXL15), mRNA. |
| TBC1D3F | -1.63 | PREDICTED: Homo sapiens TBC1 domain family, member 3F, transcript variant 11 (TBC1D3F), mRNA. |
| LOC729217 | -1.63 | PREDICTED: Homo sapiens misc_RNA (LOC729217), miscRNA. |
| C7orf29 | -1.63 | Homo sapiens chromosome 7 open reading frame 29 (C7orf29), mRNA. |
| APRT | -1.63 | Homo sapiens adenine phosphoribosyltransferase (APRT), transcript variant 1, mRNA. |
| PTTG1IP | -1.63 | Homo sapiens pituitary tumor-transforming 1 interacting protein (PTTG1IP), mRNA. |
| DCTD | -1.63 | Homo sapiens dCMP deaminase (DCTD), transcript variant 2, mRNA. |
| FAM160B2 | -1.63 | Homo sapiens family with sequence similarity 160, member B2 (FAM160B2), mRNA. |
| LOC100134584 | -1.63 | PREDICTED: Homo sapiens hypothetical protein LOC100134584 (LOC100134584), mRNA. |
| CTSC | -1.64 | Homo sapiens cathepsin C (CTSC), transcript variant 1, mRNA. |
| SRGAP2 | -1.64 | Homo sapiens SLIT-ROBO Rho GTPase activating protein 2 (SRGAP2), transcript variant 1, mRNA. |
| SLC27A1 | -1.64 | Homo sapiens solute carrier family 27 (fatty acid transporter), member 1 (SLC27A1), mRNA. |
| INPP5F | -1.64 | Homo sapiens inositol polyphosphate-5-phosphatase F (INPP5F), transcript variant 2, non-coding RNA. |
| ZMAT5 | -1.64 | Homo sapiens zinc finger, matrin type 5 (ZMAT5), transcript variant 1, mRNA. |
| MGC2752 | -1.64 | Homo sapiens hypothetical protein MGC2752 (MGC2752), mRNA. |
| SIPA1 | -1.64 | Homo sapiens signal-induced proliferation-associated gene 1 (SIPA1), transcript variant 1, mRNA. |
| ZNF609 | -1.64 | Homo sapiens zinc finger protein 609 (ZNF609), mRNA. |
| LOC643856 | -1.64 | PREDICTED: Homo sapiens similar to hCG2026922 (LOC643856), miscRNA. |
| DDX39 | -1.64 | Homo sapiens DEAD (Asp-Glu-Ala-Asp) box polypeptide 39 (DDX39), mRNA. |
| NONO | -1.65 | Homo sapiens non-POU domain containing, octamer-binding (NONO), mRNA. |
| C12orf51 | -1.65 | Homo sapiens chromosome 12 open reading frame 51 (C12orf51), mRNA. |
| PC | -1.65 | Homo sapiens pyruvate carboxylase (PC), nuclear gene encoding mitochondrial protein, transcript variant A, mRNA. |
| CCDC124 | -1.65 | Homo sapiens coiled-coil domain containing 124 (CCDC124), mRNA. |
| PGAM1 | -1.65 | Homo sapiens phosphoglycerate mutase 1 (brain) (PGAM1), mRNA. |
| RNF5 | -1.65 | Homo sapiens ring finger protein 5 (RNF5), mRNA. |
| APOO | -1.65 | Homo sapiens apolipoprotein O (APOO), mRNA. |
| NRXN2 | -1.65 | Homo sapiens neurexin 2 (NRXN2), transcript variant beta, mRNA. |
| SNHG7 | -1.65 | Homo sapiens small nucleolar RNA host gene 7 (non-protein coding) (SNHG7), transcript variant 1, non-coding RNA. |
| TULP4 | -1.65 | Homo sapiens tubby like protein 4 (TULP4), transcript variant 2, mRNA. |
| FAM128A | -1.66 | Homo sapiens family with sequence similarity 128, member A (FAM128A), mRNA. |
| SEZ6 | -1.66 | Homo sapiens seizure related 6 homolog (mouse) (SEZ6), transcript variant 2, mRNA. |
| EMP3 | -1.66 | Homo sapiens epithelial membrane protein 3 (EMP3), mRNA. |
| FAM171A1 | -1.66 | Homo sapiens family with sequence similarity 171, member A1 (FAM171A1), mRNA. |
| KIAA1522 | -1.66 | Homo sapiens KIAA1522 (KIAA1522), mRNA. |
| ADIPOR1 | -1.66 | Homo sapiens adiponectin receptor 1 (ADIPOR1), mRNA. |
| NEURL4 | -1.66 | Homo sapiens neuralized homolog 4 (Drosophila) (NEURL4), transcript variant 1, mRNA. |
| LPHN2 | -1.66 | Homo sapiens latrophilin 2 (LPHN2), mRNA. |
| LOC100129580 | -1.66 | PREDICTED: Homo sapiens hypothetical protein LOC100129580 (LOC100129580), mRNA. |
| MRPL41 | -1.66 | Homo sapiens mitochondrial ribosomal protein L41 (MRPL41), nuclear gene encoding mitochondrial protein, mRNA. |
| SHB | -1.66 | Homo sapiens Src homology 2 domain containing adaptor protein B (SHB), mRNA. |
| POLD2 | -1.66 | Homo sapiens polymerase (DNA directed), delta 2, regulatory subunit 50kDa (POLD2), transcript variant 1, mRNA. |
| MIR1228 | -1.66 | Homo sapiens microRNA 1228 (MIR1228), microRNA. |
| DRAP1 | -1.66 | Homo sapiens DR1-associated protein 1 (negative cofactor 2 alpha) (DRAP1), mRNA. |
| KIF3C | -1.67 | Homo sapiens kinesin family member 3C (KIF3C), mRNA. |
| SLC9A5 | -1.67 | Homo sapiens solute carrier family 9 (sodium/hydrogen exchanger), member 5 (SLC9A5), mRNA. |
| ARHGAP17 | -1.67 | Homo sapiens Rho GTPase activating protein 17 (ARHGAP17), transcript variant 2, mRNA. |
| LOC100133298 | -1.67 | PREDICTED: Homo sapiens hypothetical protein LOC100133298 (LOC100133298), mRNA. |
| LOC100128056 | -1.67 | PREDICTED: Homo sapiens similar to hCG2013701 (LOC100128056), mRNA. |
| LOC644670 | -1.67 | PREDICTED: Homo sapiens hypothetical LOC644670 (LOC644670), mRNA. |
| LOC123688 | -1.67 | Homo sapiens similar to RIKEN cDNA C630028N24 gene (LOC123688), mRNA. |
| PSCD1 | -1.67 | Homo sapiens pleckstrin homology, Sec7 and coiled-coil domains 1(cytohesin 1) (PSCD1), transcript variant 2, mRNA. |
| CAMK2B | -1.67 | Homo sapiens calcium/calmodulin-dependent protein kinase (CaM kinase) II beta (CAMK2B), transcript variant 4, mRNA. |
| ZFYVE1 | -1.67 | Homo sapiens zinc finger, FYVE domain containing 1 (ZFYVE1), transcript variant 1, mRNA. |
| RBM16 | -1.67 | Homo sapiens RNA binding motif protein 16 (RBM16), mRNA. |
| GUSBL1 | -1.67 | Homo sapiens glucuronidase, beta-like 1 (GUSBL1), non-coding RNA. |
| ZBED1 | -1.67 | Homo sapiens zinc finger, BED-type containing 1 (ZBED1), mRNA. |
| AKIRIN1 | -1.67 | Homo sapiens akirin 1 (AKIRIN1), mRNA. |
| TSKU | -1.67 | Homo sapiens tsukushin (TSKU), mRNA. |
| SHANK3 | -1.67 | Homo sapiens SH3 and multiple ankyrin repeat domains 3 (SHANK3), mRNA. |
| C9orf89 | -1.67 | Homo sapiens chromosome 9 open reading frame 89 (C9orf89), mRNA. |
| LOC283932 | -1.67 | Homo sapiens hypothetical protein LOC283932 (LOC283932), mRNA. |
| ATP5G2 | -1.67 | Homo sapiens ATP synthase, H+ transporting, mitochondrial F0 complex, subunit C2 (subunit 9) (ATP5G2), nuclear gene encoding mitochondrial protein, transcript variant 2, mRNA. |
| UHRF1 | -1.67 | Homo sapiens ubiquitin-like with PHD and ring finger domains 1 (UHRF1), transcript variant 1, mRNA. |
| LMO1 | -1.68 | Homo sapiens LIM domain only 1 (rhombotin 1) (LMO1), mRNA. |
| GGT7 | -1.68 | Homo sapiens gamma-glutamyltransferase 7 (GGT7), mRNA. |
| GCAT | -1.68 | Homo sapiens glycine C-acetyltransferase (2-amino-3-ketobutyrate coenzyme A ligase) (GCAT), nuclear gene encoding mitochondrial protein, mRNA. |
| CALR | -1.68 | Homo sapiens calreticulin (CALR), mRNA. |
| SPSB2 | -1.68 | Homo sapiens splA/ryanodine receptor domain and SOCS box containing 2 (SPSB2), mRNA. |
| LOC552889 | -1.68 | Homo sapiens hypothetical protein LOC552889 (LOC552889), mRNA. |
| PIH1D1 | -1.68 | Homo sapiens PIH1 domain containing 1 (PIH1D1), mRNA. |
| LOC100130276 | -1.68 | PREDICTED: Homo sapiens hypothetical protein LOC100130276 (LOC100130276), mRNA. |
| CLIP2 | -1.69 | Homo sapiens CAP-GLY domain containing linker protein 2 (CLIP2), transcript variant 2, mRNA. |
| DYNLRB1 | -1.69 | Homo sapiens dynein, light chain, roadblock-type 1 (DYNLRB1), mRNA. |
| RNF212 | -1.69 | Homo sapiens ring finger protein 212 (RNF212), mRNA. |
| CASP9 | -1.69 | Homo sapiens caspase 9, apoptosis-related cysteine peptidase (CASP9), transcript variant beta, mRNA. |
| CS | -1.69 | Homo sapiens citrate synthase (CS), nuclear gene encoding mitochondrial protein, mRNA. |
| DCLK2 | -1.69 | Homo sapiens doublecortin-like kinase 2 (DCLK2), transcript variant 1, mRNA. |
| LOC651575 | -1.69 | PREDICTED: Homo sapiens similar to DALR anticodon binding domain containing 3 isoform 1 (LOC651575), mRNA. |
| EIF5 | -1.69 | Homo sapiens eukaryotic translation initiation factor 5 (EIF5), transcript variant 1, mRNA. |
| PLP2 | -1.69 | Homo sapiens proteolipid protein 2 (colonic epithelium-enriched) (PLP2), mRNA. |
| LOC390466 | -1.69 | PREDICTED: Homo sapiens similar to SMT3 suppressor of mif two 3 homolog 2 (LOC390466), mRNA. |
| MMAB | -1.7 | Homo sapiens methylmalonic aciduria (cobalamin deficiency) cblB type (MMAB), mRNA. |
| FAM174A | -1.7 | Homo sapiens family with sequence similarity 174, member A (FAM174A), mRNA. |
| MAGEA6 | -1.7 | Homo sapiens melanoma antigen family A, 6 (MAGEA6), transcript variant 2, mRNA. |
| TRIO | -1.7 | Homo sapiens triple functional domain (PTPRF interacting) (TRIO), mRNA. |
| LOC200030 | -1.7 | Homo sapiens neuroblastoma breakpoint family, member 11-like (LOC200030), mRNA. |
| CDK5 | -1.7 | Homo sapiens cyclin-dependent kinase 5 (CDK5), mRNA. |
| ARPC1A | -1.7 | Homo sapiens actin related protein 2/3 complex, subunit 1A, 41kDa (ARPC1A), mRNA. |
| CTNNA1 | -1.7 | Homo sapiens catenin (cadherin-associated protein), alpha 1, 102kDa (CTNNA1), mRNA. |
| HSPBL2 | -1.7 | Homo sapiens heat shock 27kDa protein-like 2 pseudogene (HSPBL2), non-coding RNA. |
| GPR177 | -1.7 | Homo sapiens G protein-coupled receptor 177 (GPR177), transcript variant 2, mRNA. |
| MYST2 | -1.7 | Homo sapiens MYST histone acetyltransferase 2 (MYST2), mRNA. |
| MGC3020 | -1.7 | Homo sapiens hypothetical protein MGC3020 (MGC3020), mRNA. |
| MRPS34 | -1.7 | Homo sapiens mitochondrial ribosomal protein S34 (MRPS34), nuclear gene encoding mitochondrial protein, mRNA. |
| CDCA2 | -1.71 | Homo sapiens cell division cycle associated 2 (CDCA2), mRNA. |
| USF2 | -1.71 | Homo sapiens upstream transcription factor 2, c-fos interacting (USF2), transcript variant 2, mRNA. |
| ZNF446 | -1.71 | Homo sapiens zinc finger protein 446 (ZNF446), mRNA. |
| ADAM19 | -1.71 | Homo sapiens ADAM metallopeptidase domain 19 (meltrin beta) (ADAM19), mRNA. |
| FLJ20699 | -1.71 | Homo sapiens hypothetical protein FLJ20699 (FLJ20699), mRNA. |
| TAGLN2 | -1.71 | Homo sapiens transgelin 2 (TAGLN2), mRNA. |
| DDX56 | -1.71 | Homo sapiens DEAD (Asp-Glu-Ala-Asp) box polypeptide 56 (DDX56), mRNA. |
| MYT1 | -1.71 | Homo sapiens myelin transcription factor 1 (MYT1), mRNA. |
| MGC70857 | -1.71 | Homo sapiens similar to RIKEN cDNA C030006K11 gene (MGC70857), mRNA. |
| PAK4 | -1.71 | Homo sapiens p21 protein (Cdc42/Rac)-activated kinase 4 (PAK4), transcript variant 3, mRNA. |
| SLC25A29 | -1.72 | Homo sapiens solute carrier family 25, member 29 (SLC25A29), nuclear gene encoding mitochondrial protein, mRNA. |
| LSMD1 | -1.72 | Homo sapiens LSM domain containing 1 (LSMD1), mRNA. |
| AURKA | -1.72 | Homo sapiens aurora kinase A (AURKA), transcript variant 5, mRNA. |
| ZNF24 | -1.72 | Homo sapiens zinc finger protein 24 (ZNF24), mRNA. |
| GNAO1 | -1.72 | Homo sapiens guanine nucleotide binding protein (G protein), alpha activating activity polypeptide O (GNAO1), transcript variant 2, mRNA. |
| RAE1 | -1.72 | Homo sapiens RAE1 RNA export 1 homolog (S. pombe) (RAE1), transcript variant 1, mRNA. |
| CYCSL1 | -1.72 | Homo sapiens cytochrome c, somatic-like 1 (CYCSL1) on chromosome 6. |
| C10orf2 | -1.72 | Homo sapiens chromosome 10 open reading frame 2 (C10orf2), mRNA. |
| ADRM1 | -1.72 | Homo sapiens adhesion regulating molecule 1 (ADRM1), transcript variant 2, mRNA. |
| DYNLT1 | -1.72 | Homo sapiens dynein, light chain, Tctex-type 1 (DYNLT1), mRNA. |
| DDR1 | -1.72 | Homo sapiens discoidin domain receptor tyrosine kinase 1 (DDR1), transcript variant 1, mRNA. |
| ZNF470 | -1.72 | Homo sapiens zinc finger protein 470 (ZNF470), mRNA. |
| MGC71993 | -1.72 | Homo sapiens similar to DNA segment, Chr 11, Brigham & Womens Genetics 0434 expressed (MGC71993), mRNA. |
| MSL3 | -1.72 | Homo sapiens male-specific lethal 3 homolog (Drosophila) (MSL3), transcript variant 1, mRNA. |
| LOC401002 | -1.72 | PREDICTED: Homo sapiens similar to single-stranded DNA-binding protein isoform a (LOC401002), mRNA. |
| LRRC37B2 | -1.73 | Homo sapiens leucine rich repeat containing 37, member B2 (LRRC37B2), non-coding RNA. |
| ATP5SL | -1.73 | Homo sapiens ATP5S-like (ATP5SL), mRNA. |
| GGA3 | -1.73 | Homo sapiens golgi associated, gamma adaptin ear containing, ARF binding protein 3 (GGA3), transcript variant short, mRNA. |
| POLR2J4 | -1.73 | Homo sapiens polymerase (RNA) II (DNA directed) polypeptide J, 13.3kDa pseudogene (POLR2J4) on chromosome 7. |
| PHF20 | -1.73 | Homo sapiens PHD finger protein 20 (PHF20), mRNA. |
| FOXRED2 | -1.73 | Homo sapiens FAD-dependent oxidoreductase domain containing 2 (FOXRED2), mRNA. |
| GBA | -1.73 | Homo sapiens glucosidase, beta; acid (includes glucosylceramidase) (GBA), transcript variant 3, mRNA. |
| ABCB6 | -1.73 | Homo sapiens ATP-binding cassette, sub-family B (MDR/TAP), member 6 (ABCB6), nuclear gene encoding mitochondrial protein, mRNA. |
| MTX1 | -1.73 | Homo sapiens metaxin 1 (MTX1), transcript variant 1, mRNA. |
| SRPR | -1.73 | Homo sapiens signal recognition particle receptor (docking protein) (SRPR), mRNA. |
| ADCY6 | -1.73 | Homo sapiens adenylate cyclase 6 (ADCY6), transcript variant 2, mRNA. |
| KIAA1862 | -1.73 | Homo sapiens KIAA1862 protein (KIAA1862), mRNA. |
| SLC29A1 | -1.74 | Homo sapiens solute carrier family 29 (nucleoside transporters), member 1 (SLC29A1), nuclear gene encoding mitochondrial protein, transcript variant 4, mRNA. |
| SPTBN2 | -1.74 | Homo sapiens spectrin, beta, non-erythrocytic 2 (SPTBN2), mRNA. |
| MRPL53 | -1.74 | Homo sapiens mitochondrial ribosomal protein L53 (MRPL53), nuclear gene encoding mitochondrial protein, mRNA. |
| ASTN2 | -1.74 | Homo sapiens astrotactin 2 (ASTN2), transcript variant 2, mRNA. |
| R3HDM2 | -1.74 | Homo sapiens R3H domain containing 2 (R3HDM2), mRNA. |
| CCS | -1.74 | PREDICTED: Homo sapiens copper chaperone for superoxide dismutase (CCS), mRNA. |
| ZNF860 | -1.74 | Homo sapiens zinc finger protein 860 (ZNF860), mRNA. XM_001725699 XM_496634 |
| MED24 | -1.74 | Homo sapiens mediator complex subunit 24 (MED24), transcript variant 1, mRNA. |
| DOCK6 | -1.74 | Homo sapiens dedicator of cytokinesis 6 (DOCK6), mRNA. |
| RNH1 | -1.74 | Homo sapiens ribonuclease/angiogenin inhibitor 1 (RNH1), transcript variant 4, mRNA. |
| LGMN | -1.74 | Homo sapiens legumain (LGMN), transcript variant 2, mRNA. |
| CHCHD5 | -1.74 | Homo sapiens coiled-coil-helix-coiled-coil-helix domain containing 5 (CHCHD5), mRNA. |
| CNPY3 | -1.75 | Homo sapiens canopy 3 homolog (zebrafish) (CNPY3), mRNA. |
|  | -1.75 | Homo sapiens mRNA; cDNA DKFZp586O1318 (from clone DKFZp586O1318) |
| LOC92249 | -1.75 | Homo sapiens hypothetical LOC92249 (LOC92249), non-coding RNA. |
| ZNF688 | -1.75 | Homo sapiens zinc finger protein 688 (ZNF688), transcript variant 2, mRNA. |
| ZNF692 | -1.75 | Homo sapiens zinc finger protein 692 (ZNF692), transcript variant 2, mRNA. |
| MAGEF1 | -1.75 | Homo sapiens melanoma antigen family F, 1 (MAGEF1), mRNA. |
| ERGIC1 | -1.75 | Homo sapiens endoplasmic reticulum-golgi intermediate compartment (ERGIC) 1 (ERGIC1), transcript variant 2, mRNA. |
| TNPO3 | -1.75 | Homo sapiens transportin 3 (TNPO3), mRNA. |
| COPE | -1.75 | Homo sapiens coatomer protein complex, subunit epsilon (COPE), transcript variant 3, mRNA. |
| UNC93B1 | -1.75 | Homo sapiens unc-93 homolog B1 (C. elegans) (UNC93B1), mRNA. |
| KIAA0889 | -1.75 | Homo sapiens KIAA0889 protein (KIAA0889), mRNA. |
| POLR2A | -1.75 | Homo sapiens polymerase (RNA) II (DNA directed) polypeptide A, 220kDa (POLR2A), mRNA. |
| TPP1 | -1.76 | Homo sapiens tripeptidyl peptidase I (TPP1), mRNA. |
| FAM127C | -1.76 | Homo sapiens family with sequence similarity 127, member C (FAM127C), mRNA. |
| BAP1 | -1.76 | Homo sapiens BRCA1 associated protein-1 (ubiquitin carboxy-terminal hydrolase) (BAP1), mRNA. |
| LOC391532 | -1.76 | PREDICTED: Homo sapiens misc_RNA (LOC391532), miscRNA. |
| DCTD | -1.76 | Homo sapiens dCMP deaminase (DCTD), transcript variant 2, mRNA. |
| HSD11B1L | -1.76 | Homo sapiens hydroxysteroid (11-beta) dehydrogenase 1-like (HSD11B1L), transcript variant c, mRNA. |
| SYNJ2BP | -1.76 | Homo sapiens synaptojanin 2 binding protein (SYNJ2BP), mRNA. |
| XPC | -1.76 | Homo sapiens xeroderma pigmentosum, complementation group C (XPC), mRNA. |
| LOC732075 | -1.76 | PREDICTED: Homo sapiens similar to pleckstrin homology-like domain, family B, member 1 (LOC732075), mRNA. |
| LOC728324 | -1.77 | PREDICTED: Homo sapiens misc_RNA (LOC728324), miscRNA. |
| NNAT | -1.77 | Homo sapiens neuronatin (NNAT), transcript variant 1, mRNA. |
| CSF2RA | -1.77 | PREDICTED: Homo sapiens colony stimulating factor 2 receptor, alpha, low-affinity (granulocyte-macrophage) (CSF2RA), mRNA. |
| ADORA2A | -1.77 | Homo sapiens adenosine A2a receptor (ADORA2A), mRNA. |
| RNF215 | -1.77 | Homo sapiens ring finger protein 215 (RNF215), mRNA. |
| ABCF1 | -1.77 | Homo sapiens ATP-binding cassette, sub-family F (GCN20), member 1 (ABCF1), transcript variant 1, mRNA. |
| PNKP | -1.77 | Homo sapiens polynucleotide kinase 3'-phosphatase (PNKP), mRNA. |
|  | -1.77 | UI-E-EJ0-ahk-m-09-0-UI.r2 UI-E-EJ0 Homo sapiens cDNA clone UI-E-EJ0-ahk-m-09-0-UI 5, mRNA sequence |
| KPTN | -1.77 | Homo sapiens kaptin (actin binding protein) (KPTN), mRNA. |
| PDPR | -1.77 | PREDICTED: Homo sapiens pyruvate dehydrogenase phosphatase regulatory subunit (PDPR), mRNA. |
| NBL1 | -1.77 | Homo sapiens neuroblastoma, suppression of tumorigenicity 1 (NBL1), transcript variant 2, mRNA. |
| TAZ | -1.78 | Homo sapiens tafazzin (cardiomyopathy, dilated 3A (X-linked); endocardial fibroelastosis 2; Barth syndrome) (TAZ), transcript variant 3, mRNA. |
| ATP1A1 | -1.78 | Homo sapiens ATPase, Na+/K+ transporting, alpha 1 polypeptide (ATP1A1), transcript variant 1, mRNA. |
| MRPL55 | -1.78 | Homo sapiens mitochondrial ribosomal protein L55 (MRPL55), nuclear gene encoding mitochondrial protein, transcript variant 5, mRNA. |
| LOC283174 | -1.78 | Homo sapiens hypothetical protein LOC283174 (LOC283174), mRNA. |
| ADAR | -1.78 | Homo sapiens adenosine deaminase, RNA-specific (ADAR), transcript variant 2, mRNA. |
| PGAM4 | -1.78 | Homo sapiens phosphoglycerate mutase family member 4 (PGAM4), mRNA. |
| C19orf48 | -1.78 | Homo sapiens chromosome 19 open reading frame 48 (C19orf48), mRNA. |
| CHKA | -1.78 | Homo sapiens choline kinase alpha (CHKA), transcript variant 2, mRNA. |
| MPP1 | -1.78 | Homo sapiens membrane protein, palmitoylated 1, 55kDa (MPP1), mRNA. |
| GPI | -1.78 | Homo sapiens glucose phosphate isomerase (GPI), mRNA. |
| PARP3 | -1.78 | Homo sapiens poly (ADP-ribose) polymerase family, member 3 (PARP3), transcript variant 1, mRNA. |
| TMEM134 | -1.78 | Homo sapiens transmembrane protein 134 (TMEM134), transcript variant 3, mRNA. |
| STIM1 | -1.78 | Homo sapiens stromal interaction molecule 1 (STIM1), mRNA. |
| CAMK1D | -1.78 | Homo sapiens calcium/calmodulin-dependent protein kinase ID (CAMK1D), transcript variant 2, mRNA. |
| SCAMP4 | -1.78 | Homo sapiens secretory carrier membrane protein 4 (SCAMP4), mRNA. |
| MCF2L | -1.78 | Homo sapiens MCF.2 cell line derived transforming sequence-like (MCF2L), transcript variant 1, mRNA. |
| EDC3 | -1.78 | Homo sapiens enhancer of mRNA decapping 3 homolog (S. cerevisiae) (EDC3), mRNA. |
| SNORD68 | -1.78 | Homo sapiens small nucleolar RNA, C/D box 68 (SNORD68), small nucleolar RNA. |
| METRNL | -1.78 | PREDICTED: Homo sapiens meteorin, glial cell differentiation regulator-like (METRNL), mRNA. |
| IDH2 | -1.78 | Homo sapiens isocitrate dehydrogenase 2 (NADP+), mitochondrial (IDH2), nuclear gene encoding mitochondrial protein, mRNA. |
| PAPSS1 | -1.79 | Homo sapiens 3'-phosphoadenosine 5'-phosphosulfate synthase 1 (PAPSS1), mRNA. |
| SIAH1 | -1.79 | Homo sapiens seven in absentia homolog 1 (Drosophila) (SIAH1), transcript variant 2, mRNA. |
| MRPS2 | -1.79 | Homo sapiens mitochondrial ribosomal protein S2 (MRPS2), nuclear gene encoding mitochondrial protein, mRNA. |
|  | -1.79 | PREDICTED: Homo sapiens LOC440151 (LOC440151), mRNA |
| RBED1 | -1.79 | Homo sapiens RNA binding motif and ELMO/CED-12 domain 1 (RBED1), mRNA. |
| DDX51 | -1.79 | Homo sapiens DEAD (Asp-Glu-Ala-Asp) box polypeptide 51 (DDX51), mRNA. |
| TMEM57 | -1.79 | Homo sapiens transmembrane protein 57 (TMEM57), mRNA. |
| LOC653972 | -1.79 | PREDICTED: Homo sapiens similar to chromobox homolog 3 (LOC653972), mRNA. |
| IL27RA | -1.79 | Homo sapiens interleukin 27 receptor, alpha (IL27RA), mRNA. |
| SGSM3 | -1.79 | Homo sapiens small G protein signaling modulator 3 (SGSM3), mRNA. |
| SPIRE2 | -1.79 | Homo sapiens spire homolog 2 (Drosophila) (SPIRE2), mRNA. |
| FOXRED2 | -1.8 | Homo sapiens FAD-dependent oxidoreductase domain containing 2 (FOXRED2), mRNA. |
| KIAA2013 | -1.8 | Homo sapiens KIAA2013 (KIAA2013), mRNA. |
| SETD8 | -1.8 | Homo sapiens SET domain containing (lysine methyltransferase) 8 (SETD8), mRNA. |
| RBM10 | -1.8 | Homo sapiens RNA binding motif protein 10 (RBM10), transcript variant 1, mRNA. |
|  | -1.8 | UI-E-CK1-agb-m-11-0-UI.s1 UI-E-CK1 Homo sapiens cDNA clone UI-E-CK1-agb-m-11-0-UI 3, mRNA sequence |
| TAF10 | -1.8 | Homo sapiens TAF10 RNA polymerase II, TATA box binding protein (TBP)-associated factor, 30kDa (TAF10), mRNA. |
| PPAPDC1B | -1.8 | Homo sapiens phosphatidic acid phosphatase type 2 domain containing 1B (PPAPDC1B), mRNA. |
| LOC644422 | -1.8 | PREDICTED: Homo sapiens misc_RNA (LOC644422), miscRNA. |
| GGA3 | -1.8 | Homo sapiens golgi associated, gamma adaptin ear containing, ARF binding protein 3 (GGA3), transcript variant long, mRNA. |
| HSPC171 | -1.8 | Homo sapiens HSPC171 protein (HSPC171), mRNA. |
| LOC642477 | -1.8 | PREDICTED: Homo sapiens hypothetical protein LOC642477, transcript variant 2 (LOC642477), mRNA. |
| FLJ39632 | -1.8 | PREDICTED: Homo sapiens misc_RNA (FLJ39632), miscRNA. |
|  | -1.8 | Homo sapiens cDNA: FLJ22515 fis, clone HRC12122, highly similar to AF052101 Homo sapiens clone 23872 mRNA sequence |
| FASTK | -1.8 | Homo sapiens Fas-activated serine/threonine kinase (FASTK), transcript variant 4, mRNA. |
| SUPT6H | -1.8 | Homo sapiens suppressor of Ty 6 homolog (S. cerevisiae) (SUPT6H), mRNA. |
| STAT3 | -1.81 | Homo sapiens signal transducer and activator of transcription 3 (acute-phase response factor) (STAT3), transcript variant 3, mRNA. |
| PITRM1 | -1.81 | Homo sapiens pitrilysin metallopeptidase 1 (PITRM1), mRNA. |
| MPV17 | -1.81 | Homo sapiens MpV17 mitochondrial inner membrane protein (MPV17), nuclear gene encoding mitochondrial protein, mRNA. |
| INPP5E | -1.81 | Homo sapiens inositol polyphosphate-5-phosphatase, 72 kDa (INPP5E), mRNA. |
| AGBL5 | -1.81 | Homo sapiens ATP/GTP binding protein-like 5 (AGBL5), transcript variant 1, mRNA. |
| CTCF | -1.81 | Homo sapiens CCCTC-binding factor (zinc finger protein) (CTCF), mRNA. |
| VPS26B | -1.81 | Homo sapiens vacuolar protein sorting 26 homolog B (S. pombe) (VPS26B), mRNA. |
| PLEKHJ1 | -1.81 | Homo sapiens pleckstrin homology domain containing, family J member 1 (PLEKHJ1), mRNA. |
| IFI6 | -1.81 | Homo sapiens interferon, alpha-inducible protein 6 (IFI6), transcript variant 2, mRNA. |
| UBP1 | -1.82 | Homo sapiens upstream binding protein 1 (LBP-1a) (UBP1), mRNA. |
| SULT1A1 | -1.82 | Homo sapiens sulfotransferase family, cytosolic, 1A, phenol-preferring, member 1 (SULT1A1), transcript variant 5, mRNA. |
| AURKA | -1.82 | Homo sapiens aurora kinase A (AURKA), transcript variant 3, mRNA. |
| COQ6 | -1.82 | Homo sapiens coenzyme Q6 homolog, monooxygenase (S. cerevisiae) (COQ6), transcript variant 1, mRNA. |
| ANO6 | -1.82 | Homo sapiens anoctamin 6 (ANO6), mRNA. |
| ADD1 | -1.82 | Homo sapiens adducin 1 (alpha) (ADD1), transcript variant 1, mRNA. |
| SFRS13A | -1.82 | Homo sapiens splicing factor, arginine/serine-rich 13A (SFRS13A), transcript variant 2, mRNA. |
| MAZ | -1.82 | Homo sapiens MYC-associated zinc finger protein (purine-binding transcription factor) (MAZ), transcript variant 2, mRNA. |
| BAT2L | -1.82 | Homo sapiens HLA-B associated transcript 2-like (BAT2L), mRNA. |
| ACTRT1 | -1.82 | Homo sapiens actin-related protein T1 (ACTRT1), mRNA. |
| LOC100129673 | -1.83 | PREDICTED: Homo sapiens similar to hCG2042915 (LOC100129673), mRNA. |
| PIAS1 | -1.83 | Homo sapiens protein inhibitor of activated STAT, 1 (PIAS1), mRNA. |
| RACGAP1 | -1.83 | Homo sapiens Rac GTPase activating protein 1 (RACGAP1), mRNA. |
| RBM23 | -1.83 | Homo sapiens RNA binding motif protein 23 (RBM23), transcript variant 3, mRNA. |
| CTDSP1 | -1.83 | Homo sapiens CTD (carboxy-terminal domain, RNA polymerase II, polypeptide A) small phosphatase 1 (CTDSP1), transcript variant 1, mRNA. |
| C22orf27 | -1.83 | PREDICTED: Homo sapiens misc_RNA (C22orf27), miscRNA. |
| RNF150 | -1.83 | Homo sapiens ring finger protein 150 (RNF150), mRNA. |
| NECAB3 | -1.83 | Homo sapiens N-terminal EF-hand calcium binding protein 3 (NECAB3), transcript variant 1, mRNA. |
| GOT2 | -1.83 | Homo sapiens glutamic-oxaloacetic transaminase 2, mitochondrial (aspartate aminotransferase 2) (GOT2), nuclear gene encoding mitochondrial protein, mRNA. |
| LONP1 | -1.83 | Homo sapiens lon peptidase 1, mitochondrial (LONP1), nuclear gene encoding mitochondrial protein, mRNA. |
| AAAS | -1.83 | Homo sapiens achalasia, adrenocortical insufficiency, alacrimia (Allgrove, triple-A) (AAAS), mRNA. |
| PRO1853 | -1.83 | Homo sapiens hypothetical protein PRO1853 (PRO1853), transcript variant 1, mRNA. |
| ETNK2 | -1.83 | Homo sapiens ethanolamine kinase 2 (ETNK2), mRNA. |
| LZTS2 | -1.83 | Homo sapiens leucine zipper, putative tumor suppressor 2 (LZTS2), mRNA. |
| OAT | -1.83 | Homo sapiens ornithine aminotransferase (gyrate atrophy) (OAT), nuclear gene encoding mitochondrial protein, mRNA. |
| AKAP8L | -1.83 | Homo sapiens A kinase (PRKA) anchor protein 8-like (AKAP8L), mRNA. |
| CHST10 | -1.83 | Homo sapiens carbohydrate sulfotransferase 10 (CHST10), mRNA. |
| LOC441155 | -1.84 | PREDICTED: Homo sapiens similar to Zinc finger CCCH-type domain-containing protein 11A, transcript variant 2 (LOC441155), mRNA. |
| FAM160A2 | -1.84 | Homo sapiens family with sequence similarity 160, member A2 (FAM160A2), transcript variant 2, mRNA. |
| PIP4K2A | -1.84 | Homo sapiens phosphatidylinositol-5-phosphate 4-kinase, type II, alpha (PIP4K2A), mRNA. |
| FCGR3B | -1.84 | Homo sapiens Fc fragment of IgG, low affinity IIIb, receptor (CD16b) (FCGR3B), mRNA. |
| CD320 | -1.84 | Homo sapiens CD320 molecule (CD320), mRNA. |
| ATG9A | -1.84 | Homo sapiens ATG9 autophagy related 9 homolog A (S. cerevisiae) (ATG9A), transcript variant 1, mRNA. |
| PTP4A2 | -1.84 | PREDICTED: Homo sapiens protein tyrosine phosphatase type IVA, member 2, transcript variant 5 (PTP4A2), mRNA. |
| NOMO1 | -1.84 | Homo sapiens NODAL modulator 1 (NOMO1), mRNA. |
| SCG2 | -1.84 | Homo sapiens secretogranin II (chromogranin C) (SCG2), mRNA. |
| NAT15 | -1.84 | Homo sapiens N-acetyltransferase 15 (GCN5-related, putative) (NAT15), transcript variant 1, mRNA. |
| DKFZp686O24166 | -1.84 | Homo sapiens hypothetical protein DKFZp686O24166 (DKFZp686O24166), mRNA. |
| PFDN1 | -1.84 | Homo sapiens prefoldin subunit 1 (PFDN1), mRNA. |
| BRD8 | -1.84 | Homo sapiens bromodomain containing 8 (BRD8), transcript variant 3, mRNA. |
| C19orf6 | -1.84 | Homo sapiens chromosome 19 open reading frame 6 (C19orf6), transcript variant 1, mRNA. |
| LOC648509 | -1.84 | PREDICTED: Homo sapiens similar to plakophilin 4 isoform a (LOC648509), mRNA. |
| PTDSS2 | -1.84 | Homo sapiens phosphatidylserine synthase 2 (PTDSS2), mRNA. |
| WASH2P | -1.85 | Homo sapiens WAS protein family homolog 2 pseudogene (WASH2P), non-coding RNA. |
| DYRK4 | -1.85 | Homo sapiens dual-specificity tyrosine-(Y)-phosphorylation regulated kinase 4 (DYRK4), mRNA. |
| MGC27348 | -1.85 | PREDICTED: Homo sapiens similar to ribosomal protein S2; 40S ribosomal protein S2 (MGC27348), mRNA. |
| HAUS8 | -1.85 | Homo sapiens HAUS augmin-like complex, subunit 8 (HAUS8), transcript variant 1, mRNA. |
| GRIPAP1 | -1.85 | Homo sapiens GRIP1 associated protein 1 (GRIPAP1), transcript variant 1, mRNA. |
| COLEC11 | -1.85 | Homo sapiens collectin sub-family member 11 (COLEC11), transcript variant 2, mRNA. |
| TSSC1 | -1.85 | Homo sapiens tumor suppressing subtransferable candidate 1 (TSSC1), mRNA. |
| ATXN7L2 | -1.85 | Homo sapiens ataxin 7-like 2 (ATXN7L2), mRNA. |
| CYBASC3 | -1.86 | Homo sapiens cytochrome b, ascorbate dependent 3 (CYBASC3), mRNA. |
| LHFPL2 | -1.86 | Homo sapiens lipoma HMGIC fusion partner-like 2 (LHFPL2), mRNA. |
| NUP93 | -1.86 | Homo sapiens nucleoporin 93kDa (NUP93), mRNA. |
| LAMB2 | -1.86 | Homo sapiens laminin, beta 2 (laminin S) (LAMB2), mRNA. |
| MEGF6 | -1.86 | Homo sapiens multiple EGF-like-domains 6 (MEGF6), mRNA. |
| LASS5 | -1.86 | Homo sapiens LAG1 homolog, ceramide synthase 5 (S. cerevisiae) (LASS5), mRNA. |
| C4orf48 | -1.86 | Homo sapiens chromosome 4 open reading frame 48 (C4orf48), mRNA. |
| PLS3 | -1.86 | Homo sapiens plastin 3 (T isoform) (PLS3), mRNA. |
| BOLA2 | -1.86 | Homo sapiens bolA homolog 2 (E. coli) (BOLA2), mRNA. |
| TMEM35 | -1.86 | Homo sapiens transmembrane protein 35 (TMEM35), mRNA. |
| LOC641765 | -1.86 | PREDICTED: Homo sapiens similar to General transcription factor II-I repeat domain-containing protein 1 (GTF2I repeat domain containing protein 1) (Muscle TFII-I repeat domain-containing protein 1) (General transcription factor III) (Slow-muscle-fiber enhancer binding pro... (LOC641765), mRNA. |
| UHMK1 | -1.86 | Homo sapiens U2AF homology motif (UHM) kinase 1 (UHMK1), mRNA. |
| RTN3 | -1.86 | Homo sapiens reticulon 3 (RTN3), transcript variant 4, mRNA. |
| PMF1 | -1.86 | Homo sapiens polyamine-modulated factor 1 (PMF1), mRNA. |
| RELA | -1.86 | Homo sapiens v-rel reticuloendotheliosis viral oncogene homolog A (avian) (RELA), mRNA. |
| VHL | -1.86 | Homo sapiens von Hippel-Lindau tumor suppressor (VHL), transcript variant 2, mRNA. |
| YPEL1 | -1.86 | Homo sapiens yippee-like 1 (Drosophila) (YPEL1), mRNA. |
| MED10 | -1.86 | Homo sapiens mediator complex subunit 10 (MED10), mRNA. |
| LOC100129094 | -1.86 | PREDICTED: Homo sapiens similar to hCG2036765 (LOC100129094), mRNA. |
| TRAF4 | -1.87 | Homo sapiens TNF receptor-associated factor 4 (TRAF4), mRNA. |
| MAP1B | -1.87 | Homo sapiens microtubule-associated protein 1B (MAP1B), transcript variant 2, mRNA. |
| RFX1 | -1.87 | Homo sapiens regulatory factor X, 1 (influences HLA class II expression) (RFX1), mRNA. |
| GRINA | -1.87 | Homo sapiens glutamate receptor, ionotropic, N-methyl D-aspartate-associated protein 1 (glutamate binding) (GRINA), transcript variant 2, mRNA. |
| DDX11 | -1.87 | Homo sapiens DEAD/H (Asp-Glu-Ala-Asp/His) box polypeptide 11 (CHL1-like helicase homolog, S. cerevisiae) (DDX11), transcript variant 1, mRNA. |
| PHF19 | -1.87 | Homo sapiens PHD finger protein 19 (PHF19), transcript variant 2, mRNA. |
| RGS16 | -1.87 | Homo sapiens regulator of G-protein signalling 16 (RGS16), mRNA. |
| TTC38 | -1.87 | Homo sapiens tetratricopeptide repeat domain 38 (TTC38), mRNA. |
| LOC100132564 | -1.87 | PREDICTED: Homo sapiens hypothetical protein LOC100132564 (LOC100132564), mRNA. |
| KIAA0406 | -1.87 | Homo sapiens KIAA0406 (KIAA0406), mRNA. |
| NUDT22 | -1.87 | Homo sapiens nudix (nucleoside diphosphate linked moiety X)-type motif 22 (NUDT22), mRNA. |
| SNORA45 | -1.87 | Homo sapiens small nucleolar RNA, H/ACA box 45 (SNORA45), small nucleolar RNA. |
| LOC650737 | -1.88 | PREDICTED: Homo sapiens hypothetical protein LOC650737, transcript variant 1 (LOC650737), mRNA. |
| MXRA7 | -1.88 | Homo sapiens matrix-remodelling associated 7 (MXRA7), transcript variant 2, mRNA. |
| LOC728324 | -1.88 | PREDICTED: Homo sapiens misc_RNA (LOC728324), miscRNA. |
| PDGFRB | -1.88 | Homo sapiens platelet-derived growth factor receptor, beta polypeptide (PDGFRB), mRNA. |
| MAP7D1 | -1.88 | Homo sapiens MAP7 domain containing 1 (MAP7D1), mRNA. |
| RNF4 | -1.88 | Homo sapiens ring finger protein 4 (RNF4), mRNA. |
| RNF5P1 | -1.88 | PREDICTED: Homo sapiens ring finger protein 5 pseudogene 1 (RNF5P1), misc RNA. |
| JPH2 | -1.88 | Homo sapiens junctophilin 2 (JPH2), transcript variant 2, mRNA. |
| ZNF22 | -1.88 | Homo sapiens zinc finger protein 22 (KOX 15) (ZNF22), mRNA. |
| TMEM119 | -1.88 | Homo sapiens transmembrane protein 119 (TMEM119), mRNA. |
| ST5 | -1.88 | Homo sapiens suppression of tumorigenicity 5 (ST5), transcript variant 2, mRNA. |
| DUSP5 | -1.88 | Homo sapiens dual specificity phosphatase 5 (DUSP5), mRNA. |
| SNORD35B | -1.88 | Homo sapiens small nucleolar RNA, C/D box 35B (SNORD35B), small nucleolar RNA. |
| SSNA1 | -1.89 | Homo sapiens Sjogren syndrome nuclear autoantigen 1 (SSNA1), mRNA. |
| LOC728944 | -1.89 | PREDICTED: Homo sapiens similar to THAP domain-containing protein 4 (LOC728944), mRNA. |
| C19orf60 | -1.89 | Homo sapiens chromosome 19 open reading frame 60 (C19orf60), transcript variant 1, mRNA. |
| CYFIP1 | -1.89 | Homo sapiens cytoplasmic FMR1 interacting protein 1 (CYFIP1), transcript variant 2, mRNA. |
| SGPL1 | -1.89 | Homo sapiens sphingosine-1-phosphate lyase 1 (SGPL1), mRNA. |
| BANF1 | -1.89 | Homo sapiens barrier to autointegration factor 1 (BANF1), mRNA. |
| FAM110A | -1.89 | Homo sapiens family with sequence similarity 110, member A (FAM110A), transcript variant 3, mRNA. |
| HDAC3 | -1.89 | Homo sapiens histone deacetylase 3 (HDAC3), mRNA. |
| ZNF219 | -1.89 | Homo sapiens zinc finger protein 219 (ZNF219), mRNA. |
| RPA1 | -1.89 | Homo sapiens replication protein A1, 70kDa (RPA1), mRNA. |
| SMA4 | -1.89 | Homo sapiens SMA4 (SMA4), mRNA. |
| FAM193B | -1.89 | Homo sapiens family with sequence similarity 193, member B (FAM193B), transcript variant 1, mRNA. XM_944464 XM_944467 XM_944470 |
| DCTN6 | -1.89 | Homo sapiens dynactin 6 (DCTN6), mRNA. |
|  | -1.89 | Homo sapiens cDNA FLJ32080 fis, clone OCBBF2000015 |
| LOC728312 | -1.89 | PREDICTED: Homo sapiens misc_RNA (LOC728312), miscRNA. |
| TRIM41 | -1.89 | Homo sapiens tripartite motif-containing 41 (TRIM41), transcript variant 2, mRNA. |
| TIMP2 | -1.89 | Homo sapiens TIMP metallopeptidase inhibitor 2 (TIMP2), mRNA. |
| COPE | -1.89 | Homo sapiens coatomer protein complex, subunit epsilon (COPE), transcript variant 2, mRNA. |
| MRPL55 | -1.9 | Homo sapiens mitochondrial ribosomal protein L55 (MRPL55), nuclear gene encoding mitochondrial protein, transcript variant 1, mRNA. |
| TMEM106A | -1.9 | Homo sapiens transmembrane protein 106A (TMEM106A), mRNA. |
| DCLK2 | -1.9 | Homo sapiens doublecortin-like kinase 2 (DCLK2), transcript variant 1, mRNA. |
| PLD6 | -1.9 | Homo sapiens phospholipase D family, member 6 (PLD6), mRNA. |
| PEG3 | -1.9 | Homo sapiens paternally expressed 3 (PEG3), mRNA. |
| TCTEX1D2 | -1.9 | Homo sapiens Tctex1 domain containing 2 (TCTEX1D2), mRNA. |
| LOC100129866 | -1.9 | PREDICTED: Homo sapiens misc_RNA (LOC100129866), miscRNA. |
| LOC100131735 | -1.9 | PREDICTED: Homo sapiens misc_RNA (LOC100131735), miscRNA. |
| DIAPH1 | -1.9 | Homo sapiens diaphanous homolog 1 (Drosophila) (DIAPH1), mRNA. |
| DHCR7 | -1.91 | Homo sapiens 7-dehydrocholesterol reductase (DHCR7), mRNA. |
| DERL1 | -1.91 | Homo sapiens Der1-like domain family, member 1 (DERL1), mRNA. |
| TMEM62 | -1.91 | Homo sapiens transmembrane protein 62 (TMEM62), mRNA. |
| PCGF2 | -1.91 | Homo sapiens polycomb group ring finger 2 (PCGF2), mRNA. |
| AP3B2 | -1.91 | Homo sapiens adaptor-related protein complex 3, beta 2 subunit (AP3B2), mRNA. |
| TMX4 | -1.91 | Homo sapiens thioredoxin-related transmembrane protein 4 (TMX4), mRNA. |
| SYMPK | -1.91 | Homo sapiens symplekin (SYMPK), mRNA. |
| C15orf42 | -1.91 | Homo sapiens chromosome 15 open reading frame 42 (C15orf42), mRNA. |
| TADA3 | -1.91 | Homo sapiens transcriptional adaptor 3 (TADA3), transcript variant 1, mRNA. |
| LOC643995 | -1.91 | PREDICTED: Homo sapiens similar to Importin alpha-2 subunit (Karyopherin alpha-2 subunit) (SRP1-alpha) (RAG cohort protein 1), transcript variant 2 (LOC643995), mRNA. |
| CABIN1 | -1.92 | Homo sapiens calcineurin binding protein 1 (CABIN1), mRNA. |
| CIB2 | -1.92 | Homo sapiens calcium and integrin binding family member 2 (CIB2), mRNA. |
| RALY | -1.92 | Homo sapiens RNA binding protein, autoantigenic (hnRNP-associated with lethal yellow homolog (mouse)) (RALY), transcript variant 2, mRNA. |
| TP53I3 | -1.92 | Homo sapiens tumor protein p53 inducible protein 3 (TP53I3), transcript variant 2, mRNA. |
| PPM1K | -1.92 | Homo sapiens protein phosphatase 1K (PP2C domain containing) (PPM1K), mRNA. |
| UCK2 | -1.92 | Homo sapiens uridine-cytidine kinase 2 (UCK2), mRNA. |
| ATXN2L | -1.92 | PREDICTED: Homo sapiens ataxin 2-like, transcript variant 1 (ATXN2L), mRNA. |
| FZD7 | -1.92 | Homo sapiens frizzled homolog 7 (Drosophila) (FZD7), mRNA. |
| RBMX | -1.92 | Homo sapiens RNA binding motif protein, X-linked (RBMX), mRNA. |
| CLPP | -1.92 | Homo sapiens ClpP caseinolytic peptidase, ATP-dependent, proteolytic subunit homolog (E. coli) (CLPP), nuclear gene encoding mitochondrial protein, mRNA. |
| PCBP2 | -1.92 | Homo sapiens poly(rC) binding protein 2 (PCBP2), transcript variant 1, mRNA. |
| LOC440311 | -1.92 | PREDICTED: Homo sapiens misc_RNA (LOC440311), miscRNA. |
| DVL2 | -1.93 | Homo sapiens dishevelled, dsh homolog 2 (Drosophila) (DVL2), mRNA. |
| DISP2 | -1.93 | Homo sapiens dispatched homolog 2 (Drosophila) (DISP2), mRNA. |
| LOC100131541 | -1.93 | PREDICTED: Homo sapiens similar to MRSS6228 (LOC100131541), mRNA. |
| RUVBL2 | -1.93 | Homo sapiens RuvB-like 2 (E. coli) (RUVBL2), mRNA. |
| TM7SF3 | -1.93 | Homo sapiens transmembrane 7 superfamily member 3 (TM7SF3), mRNA. |
| TM9SF4 | -1.93 | Homo sapiens transmembrane 9 superfamily protein member 4 (TM9SF4), mRNA. |
| THOC5 | -1.93 | Homo sapiens THO complex 5 (THOC5), transcript variant 1, mRNA. |
| LOC100129539 | -1.93 | PREDICTED: Homo sapiens misc_RNA (LOC100129539), miscRNA. |
| GNA11 | -1.93 | Homo sapiens guanine nucleotide binding protein (G protein), alpha 11 (Gq class) (GNA11), mRNA. |
| UPF3A | -1.93 | Homo sapiens UPF3 regulator of nonsense transcripts homolog A (yeast) (UPF3A), transcript variant 2, mRNA. |
| IDH3G | -1.93 | Homo sapiens isocitrate dehydrogenase 3 (NAD+) gamma (IDH3G), nuclear gene encoding mitochondrial protein, transcript variant 2, mRNA. |
| SLC26A10 | -1.93 | Homo sapiens solute carrier family 26, member 10 (SLC26A10), transcript variant 1, mRNA. |
| DNAJC30 | -1.93 | Homo sapiens DnaJ (Hsp40) homolog, subfamily C, member 30 (DNAJC30), mRNA. |
| CUL7 | -1.93 | Homo sapiens cullin 7 (CUL7), mRNA. |
| CDIPT | -1.93 | Homo sapiens CDP-diacylglycerol--inositol 3-phosphatidyltransferase (phosphatidylinositol synthase) (CDIPT), mRNA. |
| RASSF5 | -1.94 | Homo sapiens Ras association (RalGDS/AF-6) domain family member 5 (RASSF5), transcript variant 2, mRNA. |
| LHFPL4 | -1.94 | Homo sapiens lipoma HMGIC fusion partner-like 4 (LHFPL4), mRNA. |
| SURF4 | -1.94 | Homo sapiens surfeit 4 (SURF4), mRNA. |
| MRPL34 | -1.94 | Homo sapiens mitochondrial ribosomal protein L34 (MRPL34), nuclear gene encoding mitochondrial protein, mRNA. |
| AEBP1 | -1.94 | Homo sapiens AE binding protein 1 (AEBP1), mRNA. |
| CPSF3L | -1.94 | Homo sapiens cleavage and polyadenylation specific factor 3-like (CPSF3L), mRNA. |
| LOC127295 | -1.94 | PREDICTED: Homo sapiens similar to 60S ribosomal protein L36 (LOC127295), mRNA. |
| MBTPS1 | -1.94 | Homo sapiens membrane-bound transcription factor peptidase, site 1 (MBTPS1), mRNA. |
| RBM15B | -1.94 | Homo sapiens RNA binding motif protein 15B (RBM15B), mRNA. |
| MYBBP1A | -1.94 | Homo sapiens MYB binding protein (P160) 1a (MYBBP1A), mRNA. |
| PI4KB | -1.94 | Homo sapiens phosphatidylinositol 4-kinase, catalytic, beta (PI4KB), mRNA. |
| CTBP1 | -1.95 | Homo sapiens C-terminal binding protein 1 (CTBP1), transcript variant 1, mRNA. |
| TM9SF1 | -1.95 | Homo sapiens transmembrane 9 superfamily member 1 (TM9SF1), transcript variant 1, mRNA. |
| LRFN4 | -1.95 | Homo sapiens leucine rich repeat and fibronectin type III domain containing 4 (LRFN4), mRNA. |
| SLC5A6 | -1.95 | Homo sapiens solute carrier family 5 (sodium-dependent vitamin transporter), member 6 (SLC5A6), mRNA. |
| SAPS3 | -1.95 | Homo sapiens SAPS domain family, member 3 (SAPS3), mRNA. |
| TPST1 | -1.95 | Homo sapiens tyrosylprotein sulfotransferase 1 (TPST1), mRNA. |
| RAB4B | -1.95 | Homo sapiens RAB4B, member RAS oncogene family (RAB4B), mRNA. |
| PRMT2 | -1.95 | Homo sapiens protein arginine methyltransferase 2 (PRMT2), transcript variant 2, mRNA. |
| LOC652672 | -1.95 | PREDICTED: Homo sapiens similar to damage-specific DNA binding protein 1 (LOC652672), mRNA. |
| CDKN1C | -1.95 | Homo sapiens cyclin-dependent kinase inhibitor 1C (p57, Kip2) (CDKN1C), mRNA. |
| SLC18A1 | -1.96 | Homo sapiens solute carrier family 18 (vesicular monoamine), member 1 (SLC18A1), mRNA. |
| U2AF2 | -1.96 | Homo sapiens U2 small nuclear RNA auxiliary factor 2 (U2AF2), transcript variant 1, mRNA. |
| HIATL2 | -1.96 | Homo sapiens hippocampus abundant transcript-like 2 (HIATL2), non-coding RNA. |
| TMEM55B | -1.96 | Homo sapiens transmembrane protein 55B (TMEM55B), mRNA. |
| TAX1BP1 | -1.96 | Homo sapiens Tax1 (human T-cell leukemia virus type I) binding protein 1 (TAX1BP1), transcript variant 2, mRNA. |
| LOC728188 | -1.96 | PREDICTED: Homo sapiens similar to phosphoglycerate mutase processed protein (LOC728188), mRNA. |
| NUDT14 | -1.96 | Homo sapiens nudix (nucleoside diphosphate linked moiety X)-type motif 14 (NUDT14), mRNA. |
| C11orf68 | -1.96 | Homo sapiens chromosome 11 open reading frame 68 (C11orf68), mRNA. |
| RBM3 | -1.96 | Homo sapiens RNA binding motif (RNP1, RRM) protein 3 (RBM3), transcript variant 2, mRNA. |
| ARNT2 | -1.96 | Homo sapiens aryl-hydrocarbon receptor nuclear translocator 2 (ARNT2), mRNA. |
| DOM3Z | -1.96 | Homo sapiens dom-3 homolog Z (C. elegans) (DOM3Z), mRNA. |
| C20orf3 | -1.96 | Homo sapiens chromosome 20 open reading frame 3 (C20orf3), mRNA. |
| TRIB2 | -1.96 | Homo sapiens tribbles homolog 2 (Drosophila) (TRIB2), mRNA. |
| POMT1 | -1.96 | Homo sapiens protein-O-mannosyltransferase 1 (POMT1), transcript variant 1, mRNA. |
| ZMYM3 | -1.97 | Homo sapiens zinc finger, MYM-type 3 (ZMYM3), transcript variant 2, mRNA. |
| SON | -1.97 | Homo sapiens SON DNA binding protein (SON), transcript variant b, mRNA. |
| CDC34 | -1.97 | Homo sapiens cell division cycle 34 homolog (S. cerevisiae) (CDC34), mRNA. |
| DNAJC30 | -1.97 | Homo sapiens DnaJ (Hsp40) homolog, subfamily C, member 30 (DNAJC30), mRNA. |
| ZNF763 | -1.97 | Homo sapiens zinc finger protein 763 (ZNF763), mRNA. |
| DHX35 | -1.97 | Homo sapiens DEAH (Asp-Glu-Ala-His) box polypeptide 35 (DHX35), mRNA. |
| PPP1R10 | -1.97 | Homo sapiens protein phosphatase 1, regulatory (inhibitor) subunit 10 (PPP1R10), mRNA. |
| SLC8A2 | -1.97 | Homo sapiens solute carrier family 8 (sodium/calcium exchanger), member 2 (SLC8A2), mRNA. |
| ZNF395 | -1.97 | Homo sapiens zinc finger protein 395 (ZNF395), mRNA. |
| COMMD4 | -1.98 | Homo sapiens COMM domain containing 4 (COMMD4), mRNA. |
| PYGO2 | -1.98 | Homo sapiens pygopus homolog 2 (Drosophila) (PYGO2), mRNA. |
| BTN3A2 | -1.98 | Homo sapiens butyrophilin, subfamily 3, member A2 (BTN3A2), mRNA. |
| NCAPH2 | -1.98 | Homo sapiens non-SMC condensin II complex, subunit H2 (NCAPH2), transcript variant 2, mRNA. |
| MYD88 | -1.98 | Homo sapiens myeloid differentiation primary response gene (88) (MYD88), mRNA. |
| TLN2 | -1.98 | Homo sapiens talin 2 (TLN2), mRNA. |
| GSTT2B | -1.98 | Homo sapiens glutathione S-transferase theta 2B (gene/pseudogene) (GSTT2B), mRNA. |
| TANC2 | -1.98 | Homo sapiens tetratricopeptide repeat, ankyrin repeat and coiled-coil containing 2 (TANC2), mRNA. |
| SCAND1 | -1.98 | Homo sapiens SCAN domain containing 1 (SCAND1), transcript variant 1, mRNA. |
| NRM | -1.98 | Homo sapiens nurim (nuclear envelope membrane protein) (NRM), mRNA. |
| POPDC2 | -1.98 | Homo sapiens popeye domain containing 2 (POPDC2), mRNA. |
| IQSEC1 | -1.98 | Homo sapiens IQ motif and Sec7 domain 1 (IQSEC1), mRNA. |
| C6orf129 | -1.98 | Homo sapiens chromosome 6 open reading frame 129 (C6orf129), mRNA. |
| SYNPO2 | -1.99 | PREDICTED: Homo sapiens synaptopodin 2, transcript variant 4 (SYNPO2), mRNA. |
| LOC653829 | -1.99 | PREDICTED: Homo sapiens similar to Williams Beuren syndrome chromosome region 19, transcript variant 6 (LOC653829), mRNA. |
| PYCR2 | -1.99 | Homo sapiens pyrroline-5-carboxylate reductase family, member 2 (PYCR2), mRNA. |
| EPHB2 | -1.99 | Homo sapiens EPH receptor B2 (EPHB2), transcript variant 2, mRNA. |
| TIMM17B | -1.99 | Homo sapiens translocase of inner mitochondrial membrane 17 homolog B (yeast) (TIMM17B), nuclear gene encoding mitochondrial protein, mRNA. |
| STAG3L2 | -1.99 | Homo sapiens stromal antigen 3-like 2 (STAG3L2), mRNA. |
| KIAA1967 | -1.99 | Homo sapiens KIAA1967 (KIAA1967), transcript variant 1, mRNA. |
| TMUB2 | -1.99 | Homo sapiens transmembrane and ubiquitin-like domain containing 2 (TMUB2), transcript variant 3, mRNA. |
| BRE | -2 | Homo sapiens brain and reproductive organ-expressed (TNFRSF1A modulator) (BRE), transcript variant 3, mRNA. |
| PCDHB17 | -2 | Homo sapiens protocadherin beta 17 pseudogene (PCDHB17), non-coding RNA. |
| LHFP | -2 | Homo sapiens lipoma HMGIC fusion partner (LHFP), mRNA. |
| PIGN | -2 | Homo sapiens phosphatidylinositol glycan anchor biosynthesis, class N (PIGN), transcript variant 1, mRNA. |
| BOLA2 | -2 | Homo sapiens bolA homolog 2 (E. coli) (BOLA2), mRNA. |
| SIN3B | -2 | Homo sapiens SIN3 homolog B, transcription regulator (yeast) (SIN3B), mRNA. |
| CCNJL | -2 | Homo sapiens cyclin J-like (CCNJL), mRNA. |
| LOC729646 | -2 | PREDICTED: Homo sapiens misc_RNA (LOC729646), miscRNA. |
| PARP6 | -2 | Homo sapiens poly (ADP-ribose) polymerase family, member 6 (PARP6), mRNA. |
| VPS37B | -2 | Homo sapiens vacuolar protein sorting 37 homolog B (S. cerevisiae) (VPS37B), mRNA. |
| AP3D1 | -2 | Homo sapiens adaptor-related protein complex 3, delta 1 subunit (AP3D1), transcript variant 1, mRNA. |
| PACS2 | -2.01 | Homo sapiens phosphofurin acidic cluster sorting protein 2 (PACS2), mRNA. |
| MAP3K7IP1 | -2.01 | Homo sapiens mitogen-activated protein kinase kinase kinase 7 interacting protein 1 (MAP3K7IP1), transcript variant alpha, mRNA. |
| DHX38 | -2.01 | Homo sapiens DEAH (Asp-Glu-Ala-His) box polypeptide 38 (DHX38), mRNA. |
| GRK6 | -2.01 | Homo sapiens G protein-coupled receptor kinase 6 (GRK6), transcript variant 2, mRNA. |
| CYTSB | -2.01 | Homo sapiens cytospin B (CYTSB), transcript variant NSP5alpha3beta, mRNA. |
| ALDH16A1 | -2.01 | Homo sapiens aldehyde dehydrogenase 16 family, member A1 (ALDH16A1), mRNA. |
| FNDC4 | -2.01 | Homo sapiens fibronectin type III domain containing 4 (FNDC4), mRNA. |
| ABCB1 | -2.01 | Homo sapiens ATP-binding cassette, sub-family B (MDR/TAP), member 1 (ABCB1), mRNA. |
| DHPS | -2.01 | Homo sapiens deoxyhypusine synthase (DHPS), transcript variant 2, mRNA. |
| CYFIP1 | -2.01 | Homo sapiens cytoplasmic FMR1 interacting protein 1 (CYFIP1), transcript variant 2, mRNA. |
| BAT5 | -2.01 | Homo sapiens HLA-B associated transcript 5 (BAT5), mRNA. |
| KIF22 | -2.01 | Homo sapiens kinesin family member 22 (KIF22), mRNA. |
| DYM | -2.02 | Homo sapiens dymeclin (DYM), mRNA. |
| NPAL3 | -2.02 | Homo sapiens NIPA-like domain containing 3 (NPAL3), mRNA. |
| SARM1 | -2.02 | Homo sapiens sterile alpha and TIR motif containing 1 (SARM1), mRNA. |
| DDX49 | -2.02 | Homo sapiens DEAD (Asp-Glu-Ala-Asp) box polypeptide 49 (DDX49), mRNA. |
| CAPZB | -2.02 | Homo sapiens capping protein (actin filament) muscle Z-line, beta (CAPZB), mRNA. |
| MPRIP | -2.02 | Homo sapiens myosin phosphatase Rho interacting protein (MPRIP), transcript variant 2, mRNA. |
| SHISA5 | -2.02 | Homo sapiens shisa homolog 5 (Xenopus laevis) (SHISA5), mRNA. |
| PLXNA2 | -2.02 | Homo sapiens plexin A2 (PLXNA2), mRNA. |
| COL4A2 | -2.02 | Homo sapiens collagen, type IV, alpha 2 (COL4A2), mRNA. |
| KLF13 | -2.02 | Homo sapiens Kruppel-like factor 13 (KLF13), mRNA. |
| BBS1 | -2.02 | Homo sapiens Bardet-Biedl syndrome 1 (BBS1), mRNA. |
| SUMF2 | -2.02 | Homo sapiens sulfatase modifying factor 2 (SUMF2), transcript variant 4, mRNA. |
| TSEN34 | -2.03 | Homo sapiens tRNA splicing endonuclease 34 homolog (S. cerevisiae) (TSEN34), transcript variant 2, mRNA. |
| GATA3 | -2.03 | Homo sapiens GATA binding protein 3 (GATA3), transcript variant 2, mRNA. |
| SGTA | -2.03 | Homo sapiens small glutamine-rich tetratricopeptide repeat (TPR)-containing, alpha (SGTA), mRNA. |
| CASC3 | -2.03 | Homo sapiens cancer susceptibility candidate 3 (CASC3), mRNA. |
| TSPAN7 | -2.03 | Homo sapiens tetraspanin 7 (TSPAN7), mRNA. |
| PIGT | -2.03 | Homo sapiens phosphatidylinositol glycan anchor biosynthesis, class T (PIGT), mRNA. |
| HTT | -2.03 | Homo sapiens huntingtin (HTT), mRNA. |
| DAK | -2.03 | Homo sapiens dihydroxyacetone kinase 2 homolog (S. cerevisiae) (DAK), mRNA. |
| CALD1 | -2.03 | Homo sapiens caldesmon 1 (CALD1), transcript variant 1, mRNA. |
| LOC728037 | -2.03 | PREDICTED: Homo sapiens similar to Kinesin-like protein KIF22 (Kinesin-like DNA-binding protein) (Kinesin-like protein 4) (LOC728037), mRNA. |
| OSBPL7 | -2.03 | Homo sapiens oxysterol binding protein-like 7 (OSBPL7), transcript variant 1, mRNA. |
| LOC651453 | -2.03 | PREDICTED: Homo sapiens similar to ribosomal protein L36 (LOC651453), mRNA. |
| SAPS1 | -2.03 | Homo sapiens SAPS domain family, member 1 (SAPS1), mRNA. |
| RNF24 | -2.03 | Homo sapiens ring finger protein 24 (RNF24), mRNA. |
| SNORD3D | -2.04 | Homo sapiens small nucleolar RNA, C/D box 3D (SNORD3D), small nucleolar RNA. |
| RAB12 | -2.04 | Homo sapiens RAB12, member RAS oncogene family (RAB12), mRNA. |
| PISD | -2.04 | Homo sapiens phosphatidylserine decarboxylase (PISD), mRNA. |
| TSHZ2 | -2.04 | Homo sapiens teashirt zinc finger homeobox 2 (TSHZ2), mRNA. |
| EXT1 | -2.04 | Homo sapiens exostoses (multiple) 1 (EXT1), mRNA. |
| LOC100132299 | -2.04 | PREDICTED: Homo sapiens similar to MSTP075 (LOC100132299), mRNA. |
| HYOU1 | -2.04 | Homo sapiens hypoxia up-regulated 1 (HYOU1), mRNA. |
| JPH3 | -2.04 | Homo sapiens junctophilin 3 (JPH3), mRNA. |
| GPC1 | -2.04 | Homo sapiens glypican 1 (GPC1), mRNA. |
| RABAC1 | -2.04 | Homo sapiens Rab acceptor 1 (prenylated) (RABAC1), mRNA. |
| HOXD3 | -2.04 | Homo sapiens homeobox D3 (HOXD3), mRNA. |
| LOC100131139 | -2.04 | PREDICTED: Homo sapiens misc_RNA (LOC100131139), miscRNA. |
| FUZ | -2.05 | Homo sapiens fuzzy homolog (Drosophila) (FUZ), mRNA. |
| ZMAT5 | -2.05 | Homo sapiens zinc finger, matrin type 5 (ZMAT5), transcript variant 1, mRNA. |
| LTBP4 | -2.05 | Homo sapiens latent transforming growth factor beta binding protein 4 (LTBP4), transcript variant 3, mRNA. |
| RUNDC3A | -2.05 | Homo sapiens RUN domain containing 3A (RUNDC3A), mRNA. |
| HMGXB4 | -2.05 | Homo sapiens HMG box domain containing 4 (HMGXB4), transcript variant 1, mRNA. |
| LOC100133478 | -2.05 | PREDICTED: Homo sapiens misc_RNA (LOC100133478), miscRNA. |
| SLC35B2 | -2.05 | Homo sapiens solute carrier family 35, member B2 (SLC35B2), mRNA. |
| SLC9A8 | -2.05 | Homo sapiens solute carrier family 9 (sodium/hydrogen exchanger), member 8 (SLC9A8), mRNA. |
| MAN2C1 | -2.05 | Homo sapiens mannosidase, alpha, class 2C, member 1 (MAN2C1), mRNA. |
| HINT2 | -2.05 | Homo sapiens histidine triad nucleotide binding protein 2 (HINT2), mRNA. |
| THY1 | -2.06 | Homo sapiens Thy-1 cell surface antigen (THY1), mRNA. |
| AP3D1 | -2.06 | Homo sapiens adaptor-related protein complex 3, delta 1 subunit (AP3D1), transcript variant 2, mRNA. |
| LOC732007 | -2.06 | PREDICTED: Homo sapiens similar to Phosphoglycerate mutase 1 (Phosphoglycerate mutase isozyme B) (PGAM-B) (BPG-dependent PGAM 1) (LOC732007), mRNA. |
| PRAF2 | -2.06 | Homo sapiens PRA1 domain family, member 2 (PRAF2), mRNA. |
| PEMT | -2.06 | Homo sapiens phosphatidylethanolamine N-methyltransferase (PEMT), nuclear gene encoding mitochondrial protein, transcript variant 1, mRNA. |
| CPA4 | -2.06 | Homo sapiens carboxypeptidase A4 (CPA4), mRNA. |
| UBE3B | -2.06 | Homo sapiens ubiquitin protein ligase E3B (UBE3B), transcript variant 1, mRNA. |
| FBXW5 | -2.06 | Homo sapiens F-box and WD repeat domain containing 5 (FBXW5), mRNA. |
| C17orf101 | -2.06 | Homo sapiens chromosome 17 open reading frame 101 (C17orf101), transcript variant 2, mRNA. |
| MAPK11 | -2.06 | Homo sapiens mitogen-activated protein kinase 11 (MAPK11), mRNA. |
| RPL8 | -2.06 | Homo sapiens ribosomal protein L8 (RPL8), transcript variant 2, mRNA. |
| PTPRN2 | -2.07 | Homo sapiens protein tyrosine phosphatase, receptor type, N polypeptide 2 (PTPRN2), transcript variant 2, mRNA. |
| MPDZ | -2.07 | Homo sapiens multiple PDZ domain protein (MPDZ), mRNA. |
| DAP | -2.07 | Homo sapiens death-associated protein (DAP), mRNA. |
| SMARCC2 | -2.08 | Homo sapiens SWI/SNF related, matrix associated, actin dependent regulator of chromatin, subfamily c, member 2 (SMARCC2), transcript variant 2, mRNA. |
| NUBP2 | -2.08 | Homo sapiens nucleotide binding protein 2 (MinD homolog, E. coli) (NUBP2), mRNA. |
| FREQ | -2.08 | Homo sapiens frequenin homolog (Drosophila) (FREQ), mRNA. |
| LOC729843 | -2.08 | PREDICTED: Homo sapiens similar to WW domain binding protein 1 (LOC729843), mRNA. |
| ABHD12 | -2.08 | Homo sapiens abhydrolase domain containing 12 (ABHD12), transcript variant 1, mRNA. |
| DUSP22 | -2.08 | Homo sapiens dual specificity phosphatase 22 (DUSP22), mRNA. |
| SNRPC | -2.08 | Homo sapiens small nuclear ribonucleoprotein polypeptide C (SNRPC), mRNA. |
| PPP2R5D | -2.08 | Homo sapiens protein phosphatase 2, regulatory subunit B', delta isoform (PPP2R5D), transcript variant 3, mRNA. |
|  | -2.08 | Homo sapiens mRNA; cDNA DKFZp686F09166 (from clone DKFZp686F09166) |
| ERCC1 | -2.08 | Homo sapiens excision repair cross-complementing rodent repair deficiency, complementation group 1 (includes overlapping antisense sequence) (ERCC1), transcript variant 2, mRNA. |
| RTN2 | -2.08 | Homo sapiens reticulon 2 (RTN2), transcript variant 3, mRNA. |
| WDR81 | -2.08 | Homo sapiens WD repeat domain 81 (WDR81), mRNA. |
| PTPRA | -2.08 | Homo sapiens protein tyrosine phosphatase, receptor type, A (PTPRA), transcript variant 1, mRNA. |
| LOC729406 | -2.09 | PREDICTED: Homo sapiens misc_RNA (LOC729406), miscRNA. |
| PSMD3 | -2.09 | Homo sapiens proteasome (prosome, macropain) 26S subunit, non-ATPase, 3 (PSMD3), mRNA. |
| PYCR1 | -2.09 | Homo sapiens pyrroline-5-carboxylate reductase 1 (PYCR1), transcript variant 2, mRNA. |
| CENPM | -2.09 | Homo sapiens centromere protein M (CENPM), transcript variant 2, mRNA. |
| CAMK1 | -2.09 | Homo sapiens calcium/calmodulin-dependent protein kinase I (CAMK1), mRNA. |
| FKRP | -2.09 | Homo sapiens fukutin related protein (FKRP), transcript variant 1, mRNA. |
| CBFA2T2 | -2.09 | Homo sapiens core-binding factor, runt domain, alpha subunit 2; translocated to, 2 (CBFA2T2), transcript variant 2, mRNA. |
| AURKB | -2.09 | Homo sapiens aurora kinase B (AURKB), mRNA. |
| BAD | -2.09 | Homo sapiens BCL2-antagonist of cell death (BAD), transcript variant 2, mRNA. |
| ZMYM5 | -2.09 | Homo sapiens zinc finger, MYM-type 5 (ZMYM5), transcript variant 1, mRNA. |
| TMEM222 | -2.09 | Homo sapiens transmembrane protein 222 (TMEM222), mRNA. |
| POFUT2 | -2.1 | Homo sapiens protein O-fucosyltransferase 2 (POFUT2), transcript variant 3, mRNA. |
| TMEM185A | -2.1 | Homo sapiens transmembrane protein 185A (TMEM185A), mRNA. |
| LOC728565 | -2.1 | PREDICTED: Homo sapiens similar to Beta-glucuronidase precursor (LOC728565), mRNA. |
| LOC729580 | -2.1 | PREDICTED: Homo sapiens hypothetical LOC729580 (LOC729580), mRNA. |
| CDKN1B | -2.1 | Homo sapiens cyclin-dependent kinase inhibitor 1B (p27, Kip1) (CDKN1B), mRNA. |
| UBAP2L | -2.1 | Homo sapiens ubiquitin associated protein 2-like (UBAP2L), mRNA. |
| CD276 | -2.1 | Homo sapiens CD276 molecule (CD276), transcript variant 1, mRNA. XM_945872 XM_945874 |
| CROCC | -2.1 | Homo sapiens ciliary rootlet coiled-coil, rootletin (CROCC), mRNA. |
| PPP1CA | -2.1 | Homo sapiens protein phosphatase 1, catalytic subunit, alpha isoform (PPP1CA), transcript variant 1, mRNA. |
| IRF3 | -2.11 | Homo sapiens interferon regulatory factor 3 (IRF3), mRNA. |
| C19orf29 | -2.11 | Homo sapiens chromosome 19 open reading frame 29 (C19orf29), transcript variant 1, mRNA. |
| ATP5D | -2.11 | Homo sapiens ATP synthase, H+ transporting, mitochondrial F1 complex, delta subunit (ATP5D), nuclear gene encoding mitochondrial protein, transcript variant 1, mRNA. |
| ProSAPiP1 | -2.11 | Homo sapiens ProSAPiP1 protein (ProSAPiP1), mRNA. |
| TXNDC5 | -2.11 | Homo sapiens thioredoxin domain containing 5 (endoplasmic reticulum) (TXNDC5), transcript variant 1, mRNA. |
| SNAP47 | -2.11 | Homo sapiens synaptosomal-associated protein, 47kDa (SNAP47), mRNA. |
| BRMS1 | -2.11 | Homo sapiens breast cancer metastasis suppressor 1 (BRMS1), transcript variant 1, mRNA. |
| TMEM80 | -2.11 | Homo sapiens transmembrane protein 80 (TMEM80), transcript variant 1, mRNA. |
| ULK3 | -2.11 | Homo sapiens unc-51-like kinase 3 (C. elegans) (ULK3), mRNA. |
| ISLR2 | -2.11 | Homo sapiens immunoglobulin superfamily containing leucine-rich repeat 2 (ISLR2), mRNA. |
| NLGN2 | -2.11 | Homo sapiens neuroligin 2 (NLGN2), mRNA. |
| TARBP2 | -2.12 | Homo sapiens TAR (HIV-1) RNA binding protein 2 (TARBP2), transcript variant 2, mRNA. |
| SH2D3C | -2.12 | Homo sapiens SH2 domain containing 3C (SH2D3C), transcript variant 2, mRNA. |
| TRIM52 | -2.12 | Homo sapiens tripartite motif-containing 52 (TRIM52), mRNA. |
| PIGO | -2.12 | Homo sapiens phosphatidylinositol glycan anchor biosynthesis, class O (PIGO), transcript variant 2, mRNA. |
| RPS26P10 | -2.12 | PREDICTED: Homo sapiens ribosomal protein S26 pseudogene 10 (RPS26P10), mRNA. |
| RNU1G2 | -2.12 | Homo sapiens RNA, U1G2 small nuclear (RNU1G2), small nuclear RNA. |
| SOLH | -2.12 | Homo sapiens small optic lobes homolog (Drosophila) (SOLH), mRNA. |
| IER5 | -2.12 | Homo sapiens immediate early response 5 (IER5), mRNA. |
| DDX23 | -2.12 | Homo sapiens DEAD (Asp-Glu-Ala-Asp) box polypeptide 23 (DDX23), mRNA. |
| DHRS11 | -2.13 | Homo sapiens dehydrogenase/reductase (SDR family) member 11 (DHRS11), mRNA. |
| TTC17 | -2.13 | Homo sapiens tetratricopeptide repeat domain 17 (TTC17), mRNA. |
| ARCN1 | -2.13 | Homo sapiens archain 1 (ARCN1), mRNA. |
| TMEM177 | -2.13 | Homo sapiens transmembrane protein 177 (TMEM177), mRNA. |
| TROAP | -2.13 | Homo sapiens trophinin associated protein (tastin) (TROAP), mRNA. |
| MGAT1 | -2.13 | Homo sapiens mannosyl (alpha-1,3-)-glycoprotein beta-1,2-N-acetylglucosaminyltransferase (MGAT1), mRNA. |
| SYN2 | -2.13 | Homo sapiens synapsin II (SYN2), transcript variant IIa, mRNA. |
| PLK4 | -2.13 | Homo sapiens polo-like kinase 4 (Drosophila) (PLK4), mRNA. |
| TJP2 | -2.13 | Homo sapiens tight junction protein 2 (zona occludens 2) (TJP2), transcript variant 2, mRNA. |
| RBM9 | -2.13 | Homo sapiens RNA binding motif protein 9 (RBM9), transcript variant 3, mRNA. |
| CENPA | -2.13 | Homo sapiens centromere protein A (CENPA), transcript variant 2, mRNA. |
| AKAP8 | -2.14 | Homo sapiens A kinase (PRKA) anchor protein 8 (AKAP8), mRNA. |
| DDX41 | -2.14 | Homo sapiens DEAD (Asp-Glu-Ala-Asp) box polypeptide 41 (DDX41), mRNA. |
| MAPK3 | -2.14 | Homo sapiens mitogen-activated protein kinase 3 (MAPK3), transcript variant 2, mRNA. |
| MAGT1 | -2.14 | Homo sapiens magnesium transporter 1 (MAGT1), mRNA. |
| ZNF3 | -2.14 | Homo sapiens zinc finger protein 3 (ZNF3), transcript variant 2, mRNA. |
| ELAVL4 | -2.14 | Homo sapiens ELAV (embryonic lethal, abnormal vision, Drosophila)-like 4 (Hu antigen D) (ELAVL4), mRNA. |
| GNG8 | -2.14 | Homo sapiens guanine nucleotide binding protein (G protein), gamma 8 (GNG8), mRNA. |
| CREG1 | -2.14 | Homo sapiens cellular repressor of E1A-stimulated genes 1 (CREG1), mRNA. |
| TYMS | -2.14 | Homo sapiens thymidylate synthetase (TYMS), mRNA. |
| TMEM151A | -2.14 | Homo sapiens transmembrane protein 151A (TMEM151A), mRNA. |
| MLEC | -2.14 | Homo sapiens malectin (MLEC), mRNA. |
|  | -2.15 | Homo sapiens cDNA FLJ41853 fis, clone NT2RI3004161 |
| PGAM1 | -2.15 | Homo sapiens phosphoglycerate mutase 1 (brain) (PGAM1), mRNA. |
| SURF1 | -2.15 | Homo sapiens surfeit 1 (SURF1), nuclear gene encoding mitochondrial protein, mRNA. |
| CALY | -2.15 | Homo sapiens calcyon neuron-specific vesicular protein (CALY), mRNA. |
| CEP164 | -2.15 | Homo sapiens centrosomal protein 164kDa (CEP164), mRNA. |
| SLC29A1 | -2.15 | Homo sapiens solute carrier family 29 (nucleoside transporters), member 1 (SLC29A1), nuclear gene encoding mitochondrial protein, transcript variant 4, mRNA. |
| UBAP1 | -2.15 | Homo sapiens ubiquitin associated protein 1 (UBAP1), mRNA. |
| NCDN | -2.15 | Homo sapiens neurochondrin (NCDN), transcript variant 3, mRNA. |
| POLM | -2.15 | Homo sapiens polymerase (DNA directed), mu (POLM), mRNA. |
| EME1 | -2.16 | Homo sapiens essential meiotic endonuclease 1 homolog 1 (S. pombe) (EME1), mRNA. |
| CYBA | -2.16 | Homo sapiens cytochrome b-245, alpha polypeptide (CYBA), mRNA. |
| EXTL3 | -2.16 | Homo sapiens exostoses (multiple)-like 3 (EXTL3), mRNA. |
| CHD4 | -2.16 | Homo sapiens chromodomain helicase DNA binding protein 4 (CHD4), mRNA. |
| LOC650298 | -2.16 | PREDICTED: Homo sapiens similar to 40S ribosomal protein S26 (LOC650298), mRNA. |
| CDK10 | -2.16 | Homo sapiens cyclin-dependent kinase 10 (CDK10), transcript variant c, mRNA. |
| MAP1A | -2.16 | Homo sapiens microtubule-associated protein 1A (MAP1A), mRNA. |
| PNKD | -2.16 | Homo sapiens paroxysmal nonkinesigenic dyskinesia (PNKD), transcript variant 1, mRNA. |
| SNRPA | -2.16 | Homo sapiens small nuclear ribonucleoprotein polypeptide A (SNRPA), mRNA. |
| ZNF804A | -2.17 | Homo sapiens zinc finger protein 804A (ZNF804A), mRNA. |
| LOC730286 | -2.17 | PREDICTED: Homo sapiens misc_RNA (LOC730286), miscRNA. |
| CLCN6 | -2.17 | Homo sapiens chloride channel 6 (CLCN6), transcript variant ClC-6a, mRNA. |
| TSC22D4 | -2.17 | Homo sapiens TSC22 domain family, member 4 (TSC22D4), mRNA. |
| VIM | -2.17 | Homo sapiens vimentin (VIM), mRNA. |
| MAST3 | -2.17 | Homo sapiens microtubule associated serine/threonine kinase 3 (MAST3), mRNA. |
| LOC100134241 | -2.17 | PREDICTED: Homo sapiens hypothetical protein LOC100134241 (LOC100134241), mRNA. |
| LOC728640 | -2.18 | PREDICTED: Homo sapiens misc_RNA (LOC728640), miscRNA. |
| EBPL | -2.18 | Homo sapiens emopamil binding protein-like (EBPL), mRNA. |
| C9orf69 | -2.18 | Homo sapiens chromosome 9 open reading frame 69 (C9orf69), mRNA. |
| BBC3 | -2.18 | Homo sapiens BCL2 binding component 3 (BBC3), mRNA. |
| ATXN7L3 | -2.18 | Homo sapiens ataxin 7-like 3 (ATXN7L3), transcript variant 2, mRNA. |
| NEURL1B | -2.18 | Homo sapiens neuralized homolog 1B (Drosophila) (NEURL1B), mRNA. |
| KIF13B | -2.18 | Homo sapiens kinesin family member 13B (KIF13B), mRNA. |
| ZNF513 | -2.19 | Homo sapiens zinc finger protein 513 (ZNF513), mRNA. |
| LOC648581 | -2.19 | PREDICTED: Homo sapiens similar to alveolar soft part sarcoma chromosome region, candidate 1 (LOC648581), mRNA. |
| HDAC6 | -2.19 | Homo sapiens histone deacetylase 6 (HDAC6), mRNA. |
| C21orf81 | -2.19 | Homo sapiens chromosome 21 open reading frame 81 (C21orf81), mRNA. |
| LOC347292 | -2.19 | PREDICTED: Homo sapiens similar to ribosomal protein L36 (LOC347292), mRNA. |
| NSUN5 | -2.19 | Homo sapiens NOL1/NOP2/Sun domain family, member 5 (NSUN5), transcript variant 2, mRNA. |
| NAGLU | -2.19 | Homo sapiens N-acetylglucosaminidase, alpha- (NAGLU), mRNA. |
| PHACTR1 | -2.19 | Homo sapiens phosphatase and actin regulator 1 (PHACTR1), mRNA. |
| NDUFA13 | -2.19 | Homo sapiens NADH dehydrogenase (ubiquinone) 1 alpha subcomplex, 13 (NDUFA13), mRNA. |
| ZNF134 | -2.19 | Homo sapiens zinc finger protein 134 (ZNF134), mRNA. |
| MEA1 | -2.2 | Homo sapiens male-enhanced antigen 1 (MEA1), mRNA. |
| LOC644879 | -2.2 | PREDICTED: Homo sapiens misc_RNA (LOC644879), miscRNA. |
| C9orf140 | -2.2 | Homo sapiens chromosome 9 open reading frame 140 (C9orf140), mRNA. |
| SCARNA16 | -2.2 | Homo sapiens small Cajal body-specific RNA 16 (SCARNA16), guide RNA. |
| HSPBP1 | -2.2 | Homo sapiens hsp70-interacting protein (HSPBP1), mRNA. |
| STARD3 | -2.2 | Homo sapiens StAR-related lipid transfer (START) domain containing 3 (STARD3), mRNA. |
| SORT1 | -2.2 | Homo sapiens sortilin 1 (SORT1), mRNA. |
| C2orf24 | -2.2 | Homo sapiens chromosome 2 open reading frame 24 (C2orf24), mRNA. |
| LOC400713 | -2.2 | PREDICTED: Homo sapiens zinc finger-like, transcript variant 3 (LOC400713), misc RNA. |
| DPP3 | -2.2 | Homo sapiens dipeptidyl-peptidase 3 (DPP3), transcript variant 1, mRNA. |
| DUSP10 | -2.2 | Homo sapiens dual specificity phosphatase 10 (DUSP10), transcript variant 3, mRNA. |
| MT1X | -2.2 | Homo sapiens metallothionein 1X (MT1X), mRNA. |
| EFS | -2.21 | Homo sapiens embryonal Fyn-associated substrate (EFS), transcript variant 2, mRNA. |
| UBL7 | -2.21 | Homo sapiens ubiquitin-like 7 (bone marrow stromal cell-derived) (UBL7), transcript variant 2, mRNA. |
| AMFR | -2.21 | Homo sapiens autocrine motility factor receptor (AMFR), mRNA. |
| CDCA4 | -2.21 | Homo sapiens cell division cycle associated 4 (CDCA4), transcript variant 14, mRNA. |
| GDI1 | -2.21 | Homo sapiens GDP dissociation inhibitor 1 (GDI1), mRNA. |
| AP1G2 | -2.21 | Homo sapiens adaptor-related protein complex 1, gamma 2 subunit (AP1G2), mRNA. |
| CENPV | -2.21 | Homo sapiens centromere protein V (CENPV), mRNA. |
| RNU1A3 | -2.21 | Homo sapiens RNA, U1A3 small nuclear (RNU1A3), small nuclear RNA. |
| CTDP1 | -2.21 | Homo sapiens CTD (carboxy-terminal domain, RNA polymerase II, polypeptide A) phosphatase, subunit 1 (CTDP1), transcript variant FCP1a, mRNA. |
| SRC | -2.21 | Homo sapiens v-src sarcoma (Schmidt-Ruppin A-2) viral oncogene homolog (avian) (SRC), transcript variant 2, mRNA. |
| ARHGEF11 | -2.21 | Homo sapiens Rho guanine nucleotide exchange factor (GEF) 11 (ARHGEF11), transcript variant 1, mRNA. |
| RAMP2 | -2.21 | Homo sapiens receptor (G protein-coupled) activity modifying protein 2 (RAMP2), mRNA. |
| PPM1G | -2.21 | Homo sapiens protein phosphatase 1G (formerly 2C), magnesium-dependent, gamma isoform (PPM1G), transcript variant 1, mRNA. |
| MBD3 | -2.22 | Homo sapiens methyl-CpG binding domain protein 3 (MBD3), mRNA. |
| GPX4 | -2.22 | Homo sapiens glutathione peroxidase 4 (phospholipid hydroperoxidase) (GPX4), transcript variant 2, mRNA. |
| ADIPOR1 | -2.22 | Homo sapiens adiponectin receptor 1 (ADIPOR1), mRNA. |
| GTF2IRD1 | -2.22 | Homo sapiens GTF2I repeat domain containing 1 (GTF2IRD1), transcript variant 1, mRNA. |
| TUSC1 | -2.22 | Homo sapiens tumor suppressor candidate 1 (TUSC1), mRNA. |
| WHSC2 | -2.22 | Homo sapiens Wolf-Hirschhorn syndrome candidate 2 (WHSC2), mRNA. |
| ADAM23 | -2.22 | Homo sapiens ADAM metallopeptidase domain 23 (ADAM23), mRNA. |
| RHBDL1 | -2.22 | Homo sapiens rhomboid, veinlet-like 1 (Drosophila) (RHBDL1), mRNA. |
| LOC100008589 | -2.22 | Homo sapiens 28S ribosomal RNA (LOC100008589), non-coding RNA. |
| STUB1 | -2.22 | Homo sapiens STIP1 homology and U-box containing protein 1 (STUB1), mRNA. |
| LOC648149 | -2.22 | PREDICTED: Homo sapiens hypothetical protein LOC648149 (LOC648149), mRNA. |
| DOLK | -2.22 | Homo sapiens dolichol kinase (DOLK), mRNA. |
| MCOLN1 | -2.22 | Homo sapiens mucolipin 1 (MCOLN1), mRNA. |
| GSTT2 | -2.23 | Homo sapiens glutathione S-transferase theta 2 (GSTT2), mRNA. |
| FLJ10986 | -2.23 | Homo sapiens hypothetical protein FLJ10986 (FLJ10986), mRNA. |
| LOC654260 | -2.23 | PREDICTED: Homo sapiens similar to beta-tubulin cofactor D isoform 1 (LOC654260), mRNA. |
| LOC729406 | -2.23 | PREDICTED: Homo sapiens misc_RNA (LOC729406), miscRNA. |
|  | -2.23 | AV652851 GLC Homo sapiens cDNA clone GLCDEG06 3, mRNA sequence |
| MOSC1 | -2.24 | Homo sapiens MOCO sulphurase C-terminal domain containing 1 (MOSC1), mRNA. |
| ITPK1 | -2.24 | Homo sapiens inositol 1,3,4-triphosphate 5/6 kinase (ITPK1), mRNA. |
| DPP3 | -2.24 | Homo sapiens dipeptidyl-peptidase 3 (DPP3), transcript variant 1, mRNA. |
| FKBP1A | -2.24 | Homo sapiens FK506 binding protein 1A, 12kDa (FKBP1A), transcript variant 12B, mRNA. |
| ICA1 | -2.24 | Homo sapiens islet cell autoantigen 1, 69kDa (ICA1), transcript variant 3, mRNA. |
| LOC728728 | -2.24 | PREDICTED: Homo sapiens misc_RNA (LOC728728), miscRNA. |
| HAGHL | -2.24 | Homo sapiens hydroxyacylglutathione hydrolase-like (HAGHL), transcript variant 1, mRNA. |
| UBE2C | -2.24 | Homo sapiens ubiquitin-conjugating enzyme E2C (UBE2C), transcript variant 6, mRNA. |
| HS1BP3 | -2.24 | Homo sapiens HCLS1 binding protein 3 (HS1BP3), mRNA. |
|  | -2.24 | AV700714 GKC Homo sapiens cDNA clone GKCAEH11 3, mRNA sequence |
| BMP1 | -2.24 | Homo sapiens bone morphogenetic protein 1 (BMP1), transcript variant BMP1-3, mRNA. |
| FLOT1 | -2.25 | Homo sapiens flotillin 1 (FLOT1), mRNA. |
| SLBP | -2.25 | Homo sapiens stem-loop binding protein (SLBP), mRNA. |
| BCL7A | -2.25 | Homo sapiens B-cell CLL/lymphoma 7A (BCL7A), transcript variant 2, mRNA. |
| TTC32 | -2.25 | Homo sapiens tetratricopeptide repeat domain 32 (TTC32), mRNA. |
| NFATC2IP | -2.25 | Homo sapiens nuclear factor of activated T-cells, cytoplasmic, calcineurin-dependent 2 interacting protein (NFATC2IP), mRNA. |
| LOC400836 | -2.25 | PREDICTED: Homo sapiens misc_RNA (LOC400836), miscRNA. |
| ADCK4 | -2.25 | Homo sapiens aarF domain containing kinase 4 (ADCK4), mRNA. |
| LOC644931 | -2.25 | PREDICTED: Homo sapiens hypothetical LOC644931 (LOC644931), mRNA. |
| LOC440160 | -2.25 | PREDICTED: Homo sapiens hypothetical LOC440160 (LOC440160), mRNA. |
| GOLGA8A | -2.25 | Homo sapiens golgi autoantigen, golgin subfamily a, 8A (GOLGA8A), mRNA. |
| C19orf22 | -2.26 | Homo sapiens chromosome 19 open reading frame 22 (C19orf22), mRNA. |
| LOC389765 | -2.26 | PREDICTED: Homo sapiens similar to KIF27C (LOC389765), mRNA. |
| DHCR7 | -2.26 | Homo sapiens 7-dehydrocholesterol reductase (DHCR7), transcript variant 1, mRNA. |
| ZNF462 | -2.26 | Homo sapiens zinc finger protein 462 (ZNF462), mRNA. |
| RICS | -2.26 | Homo sapiens Rho GTPase-activating protein (RICS), mRNA. |
| PPP1CA | -2.26 | Homo sapiens protein phosphatase 1, catalytic subunit, alpha isoform (PPP1CA), transcript variant 3, mRNA. |
| DNMT3B | -2.26 | Homo sapiens DNA (cytosine-5-)-methyltransferase 3 beta (DNMT3B), transcript variant 1, mRNA. |
| RNF10 | -2.26 | Homo sapiens ring finger protein 10 (RNF10), mRNA. |
| PLDN | -2.26 | Homo sapiens pallidin homolog (mouse) (PLDN), mRNA. |
| CBX3 | -2.26 | Homo sapiens chromobox homolog 3 (HP1 gamma homolog, Drosophila) (CBX3), transcript variant 2, mRNA. |
| MKNK1 | -2.26 | Homo sapiens MAP kinase interacting serine/threonine kinase 1 (MKNK1), transcript variant 1, mRNA. |
| POFUT2 | -2.26 | Homo sapiens protein O-fucosyltransferase 2 (POFUT2), transcript variant 3, mRNA. |
| LOC100130835 | -2.26 | PREDICTED: Homo sapiens misc_RNA (LOC100130835), miscRNA. |
| STX10 | -2.27 | Homo sapiens syntaxin 10 (STX10), mRNA. |
| KIAA0427 | -2.27 | Homo sapiens KIAA0427 (KIAA0427), mRNA. |
| CTSA | -2.27 | Homo sapiens cathepsin A (CTSA), transcript variant 1, mRNA. |
| SEZ6L2 | -2.27 | Homo sapiens seizure related 6 homolog (mouse)-like 2 (SEZ6L2), transcript variant 2, mRNA. |
| PTPRF | -2.27 | Homo sapiens protein tyrosine phosphatase, receptor type, F (PTPRF), transcript variant 2, mRNA. |
| PRRT1 | -2.27 | Homo sapiens proline-rich transmembrane protein 1 (PRRT1), mRNA. |
| RNF44 | -2.27 | Homo sapiens ring finger protein 44 (RNF44), mRNA. |
| LOC727762 | -2.27 | PREDICTED: Homo sapiens similar to NADH dehydrogenase (ubiquinone) 1 beta subcomplex, 4, 15kDa, transcript variant 1 (LOC727762), mRNA. |
| TAOK1 | -2.27 | Homo sapiens TAO kinase 1 (TAOK1), mRNA. |
| PHC2 | -2.28 | Homo sapiens polyhomeotic homolog 2 (Drosophila) (PHC2), transcript variant 2, mRNA. |
| HSPBP1 | -2.28 | Homo sapiens hsp70-interacting protein (HSPBP1), mRNA. |
| BRD3 | -2.28 | Homo sapiens bromodomain containing 3 (BRD3), mRNA. |
| LOC400174 | -2.28 | PREDICTED: Homo sapiens similar to single stranded DNA binding protein 3 (LOC400174), mRNA. |
| RXRA | -2.28 | Homo sapiens retinoid X receptor, alpha (RXRA), mRNA. |
| ZFPM1 | -2.28 | Homo sapiens zinc finger protein, multitype 1 (ZFPM1), mRNA. |
| P2RX7 | -2.28 | Homo sapiens purinergic receptor P2X, ligand-gated ion channel, 7 (P2RX7), mRNA. |
| RPL32 | -2.28 | Homo sapiens ribosomal protein L32 (RPL32), transcript variant 3, mRNA. |
| LOC401098 | -2.28 | PREDICTED: Homo sapiens misc_RNA (LOC401098), miscRNA. |
| MT1E | -2.28 | Homo sapiens metallothionein 1E (MT1E), mRNA. |
| FSD1 | -2.29 | Homo sapiens fibronectin type III and SPRY domain containing 1 (FSD1), mRNA. |
| DNPEP | -2.29 | Homo sapiens aspartyl aminopeptidase (DNPEP), mRNA. |
| LOC730993 | -2.29 | PREDICTED: Homo sapiens misc_RNA (LOC730993), miscRNA. |
| VPS28 | -2.29 | Homo sapiens vacuolar protein sorting 28 homolog (S. cerevisiae) (VPS28), transcript variant 1, mRNA. |
| SEMA4D | -2.29 | Homo sapiens sema domain, immunoglobulin domain (Ig), transmembrane domain (TM) and short cytoplasmic domain, (semaphorin) 4D (SEMA4D), mRNA. |
| NFE2L1 | -2.29 | Homo sapiens nuclear factor (erythroid-derived 2)-like 1 (NFE2L1), mRNA. |
| C11orf59 | -2.3 | Homo sapiens chromosome 11 open reading frame 59 (C11orf59), mRNA. |
| UBN2 | -2.3 | Homo sapiens ubinuclein 2 (UBN2), mRNA. |
| AGPAT2 | -2.3 | Homo sapiens 1-acylglycerol-3-phosphate O-acyltransferase 2 (lysophosphatidic acid acyltransferase, beta) (AGPAT2), transcript variant 1, mRNA. |
| P4HB | -2.3 | Homo sapiens prolyl 4-hydroxylase, beta polypeptide (P4HB), mRNA. |
| TYRO3 | -2.3 | Homo sapiens TYRO3 protein tyrosine kinase (TYRO3), mRNA. |
| ARHGAP23 | -2.3 | PREDICTED: Homo sapiens Rho GTPase activating protein 23, transcript variant 1 (ARHGAP23), mRNA. |
| LOC728069 | -2.3 | PREDICTED: Homo sapiens hypothetical LOC728069 (LOC728069), mRNA. |
| GMEB1 | -2.3 | Homo sapiens glucocorticoid modulatory element binding protein 1 (GMEB1), transcript variant 2, mRNA. |
| FLJ27365 | -2.3 | Homo sapiens FLJ27365 protein (FLJ27365), mRNA. |
| SIGMAR1 | -2.31 | Homo sapiens sigma non-opioid intracellular receptor 1 (SIGMAR1), transcript variant 2, mRNA. |
| GCDH | -2.31 | Homo sapiens glutaryl-Coenzyme A dehydrogenase (GCDH), nuclear gene encoding mitochondrial protein, transcript variant 2, mRNA. |
| LOC642946 | -2.31 | PREDICTED: Homo sapiens hypothetical protein LOC642945, transcript variant 2 (LOC642946), mRNA. |
| ARMC6 | -2.31 | Homo sapiens armadillo repeat containing 6 (ARMC6), mRNA. |
| UBE2Z | -2.31 | PREDICTED: Homo sapiens ubiquitin-conjugating enzyme E2Z (putative), transcript variant 3 (UBE2Z), mRNA. |
| FKBP10 | -2.31 | Homo sapiens FK506 binding protein 10, 65 kDa (FKBP10), mRNA. |
| BCL7B | -2.31 | Homo sapiens B-cell CLL/lymphoma 7B (BCL7B), mRNA. |
| ADCY1 | -2.31 | Homo sapiens adenylate cyclase 1 (brain) (ADCY1), mRNA. |
| TUBA3D | -2.31 | Homo sapiens tubulin, alpha 3d (TUBA3D), mRNA. |
| FAM184B | -2.31 | Homo sapiens family with sequence similarity 184, member B (FAM184B), mRNA. |
| CCNY | -2.31 | Homo sapiens cyclin Y (CCNY), transcript variant 1, mRNA. |
| MYH9 | -2.31 | Homo sapiens myosin, heavy chain 9, non-muscle (MYH9), mRNA. |
| ZCCHC24 | -2.32 | Homo sapiens zinc finger, CCHC domain containing 24 (ZCCHC24), mRNA. |
| LOC220686 | -2.32 | Homo sapiens hypothetical protein LOC220686 (LOC220686), mRNA. |
| DKFZp547K054 | -2.32 | PREDICTED: Homo sapiens hypothetical protein DKFZp547K054 (DKFZp547K054), mRNA. |
| GTF2F1 | -2.32 | Homo sapiens general transcription factor IIF, polypeptide 1, 74kDa (GTF2F1), mRNA. |
| TSTD1 | -2.32 | Homo sapiens thiosulfate sulfurtransferase (rhodanese)-like domain containing 1 (TSTD1), transcript variant 2, mRNA. |
|  | -2.32 | Homo sapiens cDNA clone IMAGE:5922621, partial cds |
| IPO13 | -2.32 | Homo sapiens importin 13 (IPO13), mRNA. |
| SNORA62 | -2.32 | Homo sapiens small nucleolar RNA, H/ACA box 62 (SNORA62), small nucleolar RNA. |
| LOC728715 | -2.32 | PREDICTED: Homo sapiens similar to hCG38149 (LOC728715), mRNA. |
| FSD1 | -2.32 | Homo sapiens fibronectin type III and SPRY domain containing 1 (FSD1), mRNA. |
| LOC645726 | -2.32 | PREDICTED: Homo sapiens misc_RNA (LOC645726), miscRNA. |
| THOC6 | -2.32 | Homo sapiens THO complex 6 homolog (Drosophila) (THOC6), mRNA. |
| FLJ21865 | -2.32 | Homo sapiens endo-beta-N-acetylglucosaminidase (FLJ21865), mRNA. |
| TOR1A | -2.32 | Homo sapiens torsin family 1, member A (torsin A) (TOR1A), mRNA. |
| PIP5K1C | -2.33 | Homo sapiens phosphatidylinositol-4-phosphate 5-kinase, type I, gamma (PIP5K1C), mRNA. |
| BAT3 | -2.33 | Homo sapiens HLA-B associated transcript 3 (BAT3), transcript variant 3, mRNA. |
| DGCR14 | -2.33 | Homo sapiens DiGeorge syndrome critical region gene 14 (DGCR14), mRNA. |
| LOC100129552 | -2.33 | PREDICTED: Homo sapiens misc_RNA (LOC100129552), miscRNA. |
| NCAPD2 | -2.33 | Homo sapiens non-SMC condensin I complex, subunit D2 (NCAPD2), mRNA. |
| EPAS1 | -2.33 | Homo sapiens endothelial PAS domain protein 1 (EPAS1), mRNA. |
| MSI1 | -2.33 | Homo sapiens musashi homolog 1 (Drosophila) (MSI1), mRNA. |
| C7orf20 | -2.33 | Homo sapiens chromosome 7 open reading frame 20 (C7orf20), mRNA. XM_946081 XM_946082 XM_946083 |
| PSMG4 | -2.33 | Homo sapiens proteasome (prosome, macropain) assembly chaperone 4 (PSMG4), transcript variant 2, mRNA. |
| RNF165 | -2.34 | Homo sapiens ring finger protein 165 (RNF165), mRNA. |
| TMEM214 | -2.34 | Homo sapiens transmembrane protein 214 (TMEM214), transcript variant 1, mRNA. |
| ATRN | -2.34 | Homo sapiens attractin (ATRN), transcript variant 1, mRNA. |
| ESRRA | -2.34 | Homo sapiens estrogen-related receptor alpha (ESRRA), mRNA. |
| NLGN4X | -2.34 | Homo sapiens neuroligin 4, X-linked (NLGN4X), transcript variant 1, mRNA. |
| TNFRSF25 | -2.34 | Homo sapiens tumor necrosis factor receptor superfamily, member 25 (TNFRSF25), transcript variant 10, mRNA. |
| MRPL2 | -2.34 | Homo sapiens mitochondrial ribosomal protein L2 (MRPL2), nuclear gene encoding mitochondrial protein, mRNA. |
| C19orf6 | -2.34 | Homo sapiens chromosome 19 open reading frame 6 (C19orf6), transcript variant 1, mRNA. |
| RNU1F1 | -2.34 | Homo sapiens RNA, U1F1 small nuclear (RNU1F1), small nuclear RNA. |
| LAYN | -2.34 | Homo sapiens layilin (LAYN), mRNA. |
| FBXL11 | -2.34 | Homo sapiens F-box and leucine-rich repeat protein 11 (FBXL11), mRNA. |
| MPST | -2.34 | Homo sapiens mercaptopyruvate sulfurtransferase (MPST), nuclear gene encoding mitochondrial protein, transcript variant 1, mRNA. |
| MLST8 | -2.34 | Homo sapiens MTOR associated protein, LST8 homolog (S. cerevisiae) (MLST8), mRNA. |
| TRAK1 | -2.34 | Homo sapiens trafficking protein, kinesin binding 1 (TRAK1), transcript variant 1, mRNA. |
| LOC728452 | -2.34 | PREDICTED: Homo sapiens similar to nuclear pore membrane protein 121 (LOC728452), mRNA. |
| LOC145853 | -2.34 | PREDICTED: Homo sapiens hypothetical LOC145853 (LOC145853), mRNA. |
| ZC3H18 | -2.35 | Homo sapiens zinc finger CCCH-type containing 18 (ZC3H18), mRNA. |
| B3GAT3 | -2.35 | Homo sapiens beta-1,3-glucuronyltransferase 3 (glucuronosyltransferase I) (B3GAT3), mRNA. |
| PDZD4 | -2.35 | Homo sapiens PDZ domain containing 4 (PDZD4), mRNA. |
| C9orf16 | -2.35 | Homo sapiens chromosome 9 open reading frame 16 (C9orf16), mRNA. |
| ZNF536 | -2.35 | Homo sapiens zinc finger protein 536 (ZNF536), mRNA. |
| WDR68 | -2.35 | Homo sapiens WD repeat domain 68 (WDR68), transcript variant 2, mRNA. |
| FAM168B | -2.35 | Homo sapiens family with sequence similarity 168, member B (FAM168B), mRNA. |
| VAT1 | -2.35 | Homo sapiens vesicle amine transport protein 1 homolog (T. californica) (VAT1), mRNA. |
| YPEL3 | -2.35 | Homo sapiens yippee-like 3 (Drosophila) (YPEL3), transcript variant 1, mRNA. |
| RNF130 | -2.35 | Homo sapiens ring finger protein 130 (RNF130), mRNA. |
| ZFAND3 | -2.36 | Homo sapiens zinc finger, AN1-type domain 3 (ZFAND3), mRNA. |
| LOC728755 | -2.36 | PREDICTED: Homo sapiens similar to hCG1984907 (LOC728755), mRNA. |
| DOK4 | -2.36 | Homo sapiens docking protein 4 (DOK4), mRNA. |
| POR | -2.36 | Homo sapiens P450 (cytochrome) oxidoreductase (POR), mRNA. |
| PRR11 | -2.36 | Homo sapiens proline rich 11 (PRR11), mRNA. |
| SIGMAR1 | -2.36 | Homo sapiens sigma non-opioid intracellular receptor 1 (SIGMAR1), transcript variant 1, mRNA. |
| POMT2 | -2.36 | Homo sapiens protein-O-mannosyltransferase 2 (POMT2), mRNA. |
| MAD1L1 | -2.37 | Homo sapiens MAD1 mitotic arrest deficient-like 1 (yeast) (MAD1L1), transcript variant 2, mRNA. |
| S100A10 | -2.37 | Homo sapiens S100 calcium binding protein A10 (S100A10), mRNA. |
| LOC653994 | -2.37 | PREDICTED: Homo sapiens similar to eukaryotic translation initiation factor 4H isoform 2, transcript variant 5 (LOC653994), mRNA. |
| LOC644422 | -2.37 | PREDICTED: Homo sapiens misc_RNA (LOC644422), miscRNA. |
| MAN2B1 | -2.37 | Homo sapiens mannosidase, alpha, class 2B, member 1 (MAN2B1), mRNA. |
| ZDHHC18 | -2.37 | Homo sapiens zinc finger, DHHC-type containing 18 (ZDHHC18), mRNA. |
| UNC5B | -2.37 | Homo sapiens unc-5 homolog B (C. elegans) (UNC5B), mRNA. |
| SUV420H1 | -2.37 | Homo sapiens suppressor of variegation 4-20 homolog 1 (Drosophila) (SUV420H1), transcript variant 2, mRNA. |
| PYGB | -2.37 | Homo sapiens phosphorylase, glycogen; brain (PYGB), mRNA. |
| UBR4 | -2.37 | Homo sapiens ubiquitin protein ligase E3 component n-recognin 4 (UBR4), mRNA. |
|  | -2.37 | AGENCOURT_14354957 NIH_MGC_191 Homo sapiens cDNA clone IMAGE:30413554 5, mRNA sequence |
| RECQL4 | -2.37 | Homo sapiens RecQ protein-like 4 (RECQL4), mRNA. |
| ARSD | -2.37 | Homo sapiens arylsulfatase D (ARSD), transcript variant 1, mRNA. |
| ELAVL3 | -2.38 | Homo sapiens ELAV (embryonic lethal, abnormal vision, Drosophila)-like 3 (Hu antigen C) (ELAVL3), transcript variant 1, mRNA. |
| NUP214 | -2.38 | Homo sapiens nucleoporin 214kDa (NUP214), mRNA. |
| LGMN | -2.38 | Homo sapiens legumain (LGMN), transcript variant 2, mRNA. |
| TBC1D17 | -2.38 | Homo sapiens TBC1 domain family, member 17 (TBC1D17), mRNA. |
| YDJC | -2.38 | Homo sapiens YdjC homolog (bacterial) (YDJC), mRNA. |
| ZNF384 | -2.39 | Homo sapiens zinc finger protein 384 (ZNF384), transcript variant 2, mRNA. |
| LOC653080 | -2.39 | PREDICTED: Homo sapiens similar to Beta-glucuronidase precursor (LOC653080), misc RNA. |
| MLEC | -2.39 | Homo sapiens malectin (MLEC), mRNA. |
| ATP1A1 | -2.39 | Homo sapiens ATPase, Na+/K+ transporting, alpha 1 polypeptide (ATP1A1), transcript variant 1, mRNA. |
| LOC441124 | -2.39 | PREDICTED: Homo sapiens hypothetical LOC441124 (LOC441124), mRNA. |
| TRAP1 | -2.39 | Homo sapiens TNF receptor-associated protein 1 (TRAP1), mRNA. |
| FLJ90757 | -2.39 | PREDICTED: Homo sapiens hypothetical protein LOC440465 (FLJ90757), misc RNA. |
| CANT1 | -2.39 | Homo sapiens calcium activated nucleotidase 1 (CANT1), mRNA. |
| BCAR1 | -2.39 | Homo sapiens breast cancer anti-estrogen resistance 1 (BCAR1), mRNA. |
| LOC644852 | -2.4 | PREDICTED: Homo sapiens hypothetical protein LOC644852, transcript variant 2 (LOC644852), mRNA. |
| SEMA4B | -2.4 | Homo sapiens sema domain, immunoglobulin domain (Ig), transmembrane domain (TM) and short cytoplasmic domain, (semaphorin) 4B (SEMA4B), transcript variant 2, mRNA. |
| HAUS4 | -2.4 | Homo sapiens HAUS augmin-like complex, subunit 4 (HAUS4), mRNA. |
| MIER2 | -2.4 | Homo sapiens mesoderm induction early response 1, family member 2 (MIER2), mRNA. |
| TMEM134 | -2.4 | Homo sapiens transmembrane protein 134 (TMEM134), transcript variant 2, mRNA. |
| NUMA1 | -2.4 | Homo sapiens nuclear mitotic apparatus protein 1 (NUMA1), mRNA. |
| GPR108 | -2.4 | Homo sapiens G protein-coupled receptor 108 (GPR108), transcript variant 1, mRNA. |
| SLC38A10 | -2.4 | Homo sapiens solute carrier family 38, member 10 (SLC38A10), transcript variant 2, mRNA. |
| DGCR8 | -2.4 | Homo sapiens DiGeorge syndrome critical region gene 8 (DGCR8), mRNA. |
| KIF20A | -2.41 | Homo sapiens kinesin family member 20A (KIF20A), mRNA. |
| ZNF682 | -2.41 | Homo sapiens zinc finger protein 682 (ZNF682), transcript variant 1, mRNA. |
| TMUB1 | -2.41 | Homo sapiens transmembrane and ubiquitin-like domain containing 1 (TMUB1), mRNA. |
| EBF4 | -2.41 | Homo sapiens early B-cell factor 4 (EBF4), mRNA. |
| BVES | -2.41 | Homo sapiens blood vessel epicardial substance (BVES), transcript variant 5, mRNA. |
| ADA | -2.41 | Homo sapiens adenosine deaminase (ADA), mRNA. |
| NUP188 | -2.41 | Homo sapiens nucleoporin 188kDa (NUP188), mRNA. |
| CCDC3 | -2.41 | Homo sapiens coiled-coil domain containing 3 (CCDC3), mRNA. |
| TM7SF2 | -2.41 | Homo sapiens transmembrane 7 superfamily member 2 (TM7SF2), mRNA. |
| MKI67 | -2.42 | Homo sapiens antigen identified by monoclonal antibody Ki-67 (MKI67), mRNA. |
| KTI12 | -2.42 | Homo sapiens KTI12 homolog, chromatin associated (S. cerevisiae) (KTI12), mRNA. |
| AES | -2.42 | Homo sapiens amino-terminal enhancer of split (AES), transcript variant 3, mRNA. |
| CYTH3 | -2.42 | Homo sapiens cytohesin 3 (CYTH3), mRNA. |
| CDK5RAP2 | -2.42 | Homo sapiens CDK5 regulatory subunit associated protein 2 (CDK5RAP2), transcript variant 2, mRNA. |
| SNHG7 | -2.42 | Homo sapiens small nucleolar RNA host gene 7 (non-protein coding) (SNHG7), transcript variant 2, non-coding RNA. |
| KLHL17 | -2.42 | Homo sapiens kelch-like 17 (Drosophila) (KLHL17), mRNA. |
| LOC728640 | -2.42 | PREDICTED: Homo sapiens misc_RNA (LOC728640), miscRNA. |
| LOC645937 | -2.42 | PREDICTED: Homo sapiens similar to zinc finger protein 598 (LOC645937), mRNA. |
| NKIRAS2 | -2.43 | Homo sapiens NFKB inhibitor interacting Ras-like 2 (NKIRAS2), transcript variant 2, mRNA. |
| MESDC1 | -2.43 | Homo sapiens mesoderm development candidate 1 (MESDC1), mRNA. |
| RER1 | -2.43 | Homo sapiens RER1 retention in endoplasmic reticulum 1 homolog (S. cerevisiae) (RER1), mRNA. |
| TBC1D10B | -2.43 | Homo sapiens TBC1 domain family, member 10B (TBC1D10B), mRNA. |
| GMIP | -2.43 | Homo sapiens GEM interacting protein (GMIP), mRNA. |
| TMBIM6 | -2.43 | Homo sapiens transmembrane BAX inhibitor motif containing 6 (TMBIM6), transcript variant 1, mRNA. |
| ZNF577 | -2.43 | Homo sapiens zinc finger protein 577 (ZNF577), mRNA. |
| LOC390705 | -2.44 | PREDICTED: Homo sapiens similar to protein phosphatase 2A 48 kDa regulatory subunit isoform 1; serine/threonine protein phosphatase 2A, 48kDa regulatory subunit; PP2A, subunit B, PR48 isoform; PP2A B subunit PR48; NY-REN-8 antigen (LOC390705), misc RNA. |
| LOC100131582 | -2.44 | PREDICTED: Homo sapiens hypothetical protein LOC100131582 (LOC100131582), mRNA. |
| YIF1B | -2.44 | Homo sapiens Yip1 interacting factor homolog B (S. cerevisiae) (YIF1B), transcript variant 2, mRNA. |
| DNM2 | -2.44 | Homo sapiens dynamin 2 (DNM2), transcript variant 2, mRNA. |
| ARHGEF17 | -2.44 | Homo sapiens Rho guanine nucleotide exchange factor (GEF) 17 (ARHGEF17), mRNA. |
| MEIS3 | -2.44 | Homo sapiens Meis homeobox 3 (MEIS3), transcript variant 2, mRNA. |
|  | -2.44 | UI-H-DI0-auz-o-21-0-UI.s1 NCI_CGAP_DI0 Homo sapiens cDNA clone UI-H-DI0-auz-o-21-0-UI 3, mRNA sequence |
| MAPK12 | -2.44 | Homo sapiens mitogen-activated protein kinase 12 (MAPK12), mRNA. |
| EIF3B | -2.45 | Homo sapiens eukaryotic translation initiation factor 3, subunit B (EIF3B), transcript variant 1, mRNA. |
| CHKB | -2.45 | Homo sapiens choline kinase beta (CHKB), transcript variant 1, mRNA. |
| RANBP10 | -2.45 | Homo sapiens RAN binding protein 10 (RANBP10), mRNA. |
| FAM39DP | -2.45 | Homo sapiens family with sequence similarity 39, member D pseudogene (FAM39DP) on chromosome 15. |
| SUPT5H | -2.45 | Homo sapiens suppressor of Ty 5 homolog (S. cerevisiae) (SUPT5H), mRNA. |
| STUB1 | -2.45 | Homo sapiens STIP1 homology and U-box containing protein 1 (STUB1), mRNA. |
| RTN2 | -2.45 | Homo sapiens reticulon 2 (RTN2), transcript variant 4, mRNA. |
| ZNF282 | -2.45 | Homo sapiens zinc finger protein 282 (ZNF282), mRNA. |
| ARHGAP19 | -2.45 | Homo sapiens Rho GTPase activating protein 19 (ARHGAP19), mRNA. |
| TTC15 | -2.45 | Homo sapiens tetratricopeptide repeat domain 15 (TTC15), mRNA. |
| TPM4 | -2.46 | Homo sapiens tropomyosin 4 (TPM4), mRNA. |
| FLNB | -2.46 | Homo sapiens filamin B, beta (actin binding protein 278) (FLNB), mRNA. |
| ROR1 | -2.46 | Homo sapiens receptor tyrosine kinase-like orphan receptor 1 (ROR1), transcript variant 1, mRNA. |
| ZNHIT2 | -2.46 | Homo sapiens zinc finger, HIT type 2 (ZNHIT2), mRNA. |
| TK1 | -2.46 | Homo sapiens thymidine kinase 1, soluble (TK1), mRNA. |
| HERC1 | -2.46 | Homo sapiens hect (homologous to the E6-AP (UBE3A) carboxyl terminus) domain and RCC1 (CHC1)-like domain (RLD) 1 (HERC1), mRNA. |
| HCCA2 | -2.46 | Homo sapiens HCCA2 protein (HCCA2), mRNA. |
| TUBGCP6 | -2.47 | Homo sapiens tubulin, gamma complex associated protein 6 (TUBGCP6), mRNA. |
| LOC100127975 | -2.47 | PREDICTED: Homo sapiens misc_RNA (LOC100127975), partial miscRNA. |
| FAM101B | -2.47 | Homo sapiens family with sequence similarity 101, member B (FAM101B), mRNA. |
| SLC35A4 | -2.47 | Homo sapiens solute carrier family 35, member A4 (SLC35A4), mRNA. |
| NES | -2.47 | Homo sapiens nestin (NES), mRNA. |
| MOSPD3 | -2.47 | Homo sapiens motile sperm domain containing 3 (MOSPD3), transcript variant 1, mRNA. |
| GBA | -2.47 | Homo sapiens glucosidase, beta; acid (includes glucosylceramidase) (GBA), transcript variant 3, mRNA. |
| POLRMT | -2.47 | Homo sapiens polymerase (RNA) mitochondrial (DNA directed) (POLRMT), nuclear gene encoding mitochondrial protein, mRNA. |
| PKN3 | -2.47 | Homo sapiens protein kinase N3 (PKN3), mRNA. |
| TOE1 | -2.47 | Homo sapiens target of EGR1, member 1 (nuclear) (TOE1), mRNA. |
|  | -2.47 | Homo sapiens primary neuroblastoma cDNA, clone:Nbla10527, full insert sequence |
| COPG | -2.47 | Homo sapiens coatomer protein complex, subunit gamma (COPG), mRNA. |
| DGKD | -2.47 | Homo sapiens diacylglycerol kinase, delta 130kDa (DGKD), transcript variant 1, mRNA. |
| MDK | -2.48 | Homo sapiens midkine (neurite growth-promoting factor 2) (MDK), transcript variant 1, mRNA. |
| LOC399942 | -2.48 | PREDICTED: Homo sapiens similar to Tubulin alpha-2 chain (Alpha-tubulin 2), transcript variant 5 (LOC399942), mRNA. |
| SNORD13 | -2.48 | Homo sapiens small nucleolar RNA, C/D box 13 (SNORD13), small nucleolar RNA. |
| SH3BP5L | -2.48 | Homo sapiens SH3-binding domain protein 5-like (SH3BP5L), mRNA. |
| CBARA1 | -2.48 | Homo sapiens calcium binding atopy-related autoantigen 1 (CBARA1), mRNA. |
| AHNAK | -2.48 | Homo sapiens AHNAK nucleoprotein (AHNAK), transcript variant 1, mRNA. |
| PTP4A3 | -2.48 | Homo sapiens protein tyrosine phosphatase type IVA, member 3 (PTP4A3), transcript variant 2, mRNA. |
| C1orf122 | -2.48 | Homo sapiens chromosome 1 open reading frame 122 (C1orf122), mRNA. |
| AIP | -2.49 | Homo sapiens aryl hydrocarbon receptor interacting protein (AIP), mRNA. |
| USP1 | -2.49 | Homo sapiens ubiquitin specific peptidase 1 (USP1), transcript variant 1, mRNA. |
| MLL4 | -2.49 | Homo sapiens myeloid/lymphoid or mixed-lineage leukemia 4 (MLL4), mRNA. |
| LOC100190986 | -2.5 | Homo sapiens hypothetical LOC100190986 (LOC100190986), non-coding RNA. |
| CKAP4 | -2.5 | Homo sapiens cytoskeleton-associated protein 4 (CKAP4), mRNA. |
| RNF112 | -2.5 | Homo sapiens ring finger protein 112 (RNF112), mRNA. |
| LOC729623 | -2.51 | PREDICTED: Homo sapiens similar to protein kinase, cAMP-dependent, regulatory, type I, beta (LOC729623), mRNA. |
| FKRP | -2.51 | Homo sapiens fukutin related protein (FKRP), transcript variant 1, mRNA. |
| MCM5 | -2.51 | Homo sapiens minichromosome maintenance complex component 5 (MCM5), mRNA. |
| DRG2 | -2.51 | Homo sapiens developmentally regulated GTP binding protein 2 (DRG2), mRNA. |
| LOC100134634 | -2.51 | PREDICTED: Homo sapiens hypothetical protein LOC100134634 (LOC100134634), mRNA. |
| HLA-H | -2.51 | Homo sapiens major histocompatibility complex, class I, H (pseudogene) (HLA-H), non-coding RNA. |
| MMP11 | -2.52 | Homo sapiens matrix metallopeptidase 11 (stromelysin 3) (MMP11), mRNA. |
| HSP90AA1 | -2.52 | Homo sapiens heat shock protein 90kDa alpha (cytosolic), class A member 1 (HSP90AA1), transcript variant 1, mRNA. |
| FADS2 | -2.52 | Homo sapiens fatty acid desaturase 2 (FADS2), mRNA. |
| ST6GALNAC4 | -2.52 | Homo sapiens ST6 (alpha-N-acetyl-neuraminyl-2,3-beta-galactosyl-1, 3)-N-acetylgalactosaminide alpha-2,6-sialyltransferase 4 (ST6GALNAC4), transcript variant 1, mRNA. |
| LOC728153 | -2.53 | PREDICTED: Homo sapiens similar to FAM133B protein, transcript variant 1 (LOC728153), mRNA. |
| MARK2 | -2.53 | Homo sapiens MAP/microtubule affinity-regulating kinase 2 (MARK2), transcript variant 2, mRNA. |
| SCAMP3 | -2.53 | Homo sapiens secretory carrier membrane protein 3 (SCAMP3), transcript variant 1, mRNA. |
| FAM127B | -2.53 | Homo sapiens family with sequence similarity 127, member B (FAM127B), transcript variant 1, mRNA. |
| DEAF1 | -2.53 | Homo sapiens deformed epidermal autoregulatory factor 1 (Drosophila) (DEAF1), mRNA. |
| KIF4A | -2.53 | Homo sapiens kinesin family member 4A (KIF4A), mRNA. |
| LOC100132060 | -2.53 | PREDICTED: Homo sapiens hypothetical protein LOC100132060 (LOC100132060), mRNA. |
| MAPKAP1 | -2.53 | Homo sapiens mitogen-activated protein kinase associated protein 1 (MAPKAP1), transcript variant 1, mRNA. |
| N-PAC | -2.53 | Homo sapiens cytokine-like nuclear factor n-pac (N-PAC), mRNA. |
| FLJ44342 | -2.53 | PREDICTED: Homo sapiens hypothetical LOC645460 (FLJ44342), miscRNA. |
| AGPAT2 | -2.53 | Homo sapiens 1-acylglycerol-3-phosphate O-acyltransferase 2 (lysophosphatidic acid acyltransferase, beta) (AGPAT2), transcript variant 2, mRNA. |
| PPP1R12C | -2.53 | Homo sapiens protein phosphatase 1, regulatory (inhibitor) subunit 12C (PPP1R12C), mRNA. |
| SNORD12C | -2.53 | Homo sapiens small nucleolar RNA, C/D box 12C (SNORD12C), small nucleolar RNA. |
|  | -2.53 | Homo sapiens cDNA FLJ26784 fis, clone PRS04220 |
| SPATS2L | -2.54 | Homo sapiens spermatogenesis associated, serine-rich 2-like (SPATS2L), transcript variant 2, mRNA. |
| LOC645609 | -2.54 | PREDICTED: Homo sapiens similar to TBC1 domain family member 3 (Rab GTPase-activating protein PRC17) (Prostate cancer gene 17 protein) (TRE17 alpha protein) (LOC645609), mRNA. |
| NOC2L | -2.54 | Homo sapiens nucleolar complex associated 2 homolog (S. cerevisiae) (NOC2L), mRNA. |
| PLXNA1 | -2.54 | Homo sapiens plexin A1 (PLXNA1), mRNA. |
| CDCA8 | -2.54 | Homo sapiens cell division cycle associated 8 (CDCA8), mRNA. |
| RHOT2 | -2.54 | Homo sapiens ras homolog gene family, member T2 (RHOT2), nuclear gene encoding mitochondrial protein, mRNA. |
| KIAA0319L | -2.54 | Homo sapiens KIAA0319-like (KIAA0319L), transcript variant 1, mRNA. |
| LOC643035 | -2.55 | PREDICTED: Homo sapiens similar to CG33096-PB, isoform B, transcript variant 2 (LOC643035), mRNA. |
| EFTUD2 | -2.55 | Homo sapiens elongation factor Tu GTP binding domain containing 2 (EFTUD2), mRNA. |
| HCFC1R1 | -2.56 | Homo sapiens host cell factor C1 regulator 1 (XPO1 dependent) (HCFC1R1), transcript variant 3, mRNA. |
| ADM | -2.56 | Homo sapiens adrenomedullin (ADM), mRNA. |
| PAPD1 | -2.56 | Homo sapiens PAP associated domain containing 1 (PAPD1), mRNA. |
| GPR1 | -2.56 | Homo sapiens G protein-coupled receptor 1 (GPR1), mRNA. |
| NUP210 | -2.56 | Homo sapiens nucleoporin 210kDa (NUP210), mRNA. |
| CA11 | -2.56 | Homo sapiens carbonic anhydrase XI (CA11), mRNA. |
| KIF23 | -2.57 | Homo sapiens kinesin family member 23 (KIF23), transcript variant 2, mRNA. |
| FLJ22536 | -2.57 | Homo sapiens hypothetical locus LOC401237 (FLJ22536), non-coding RNA. |
| NSFL1C | -2.57 | Homo sapiens NSFL1 (p97) cofactor (p47) (NSFL1C), transcript variant 1, mRNA. |
| CYTSA | -2.57 | Homo sapiens cytospin A (CYTSA), mRNA. |
| MRPL4 | -2.57 | Homo sapiens mitochondrial ribosomal protein L4 (MRPL4), nuclear gene encoding mitochondrial protein, transcript variant 2, mRNA. |
| HLA-A | -2.57 | Homo sapiens major histocompatibility complex, class I, A (HLA-A), mRNA. |
| RNPS1 | -2.57 | Homo sapiens RNA binding protein S1, serine-rich domain (RNPS1), transcript variant 2, mRNA. |
| PCDHA1 | -2.57 | Homo sapiens protocadherin alpha 1 (PCDHA1), transcript variant 3, mRNA. |
| NAGPA | -2.58 | Homo sapiens N-acetylglucosamine-1-phosphodiester alpha-N-acetylglucosaminidase (NAGPA), mRNA. |
| MEN1 | -2.58 | Homo sapiens multiple endocrine neoplasia I (MEN1), transcript variant 2, mRNA. |
| CCNK | -2.58 | Homo sapiens cyclin K (CCNK), transcript variant 2, mRNA. |
| RNU1-3 | -2.58 | Homo sapiens RNA, U1 small nuclear 3 (RNU1-3), small nuclear RNA. |
| GAS2L3 | -2.58 | Homo sapiens growth arrest-specific 2 like 3 (GAS2L3), mRNA. |
| NCAN | -2.58 | Homo sapiens neurocan (NCAN), mRNA. |
| ILVBL | -2.58 | Homo sapiens ilvB (bacterial acetolactate synthase)-like (ILVBL), mRNA. |
| CD99 | -2.58 | Homo sapiens CD99 molecule (CD99), transcript variant 1, mRNA. |
| TMEM80 | -2.58 | Homo sapiens transmembrane protein 80 (TMEM80), transcript variant 1, mRNA. |
| CCS | -2.59 | Homo sapiens copper chaperone for superoxide dismutase (CCS), mRNA. |
| GPR125 | -2.59 | PREDICTED: Homo sapiens G protein-coupled receptor 125, transcript variant 4 (GPR125), mRNA. |
| LOC642489 | -2.59 | PREDICTED: Homo sapiens similar to FK506-binding protein 1A (LOC642489), mRNA. |
| LRPAP1 | -2.59 | Homo sapiens low density lipoprotein receptor-related protein associated protein 1 (LRPAP1), mRNA. |
| SNORD3A | -2.59 | Homo sapiens small nucleolar RNA, C/D box 3A (SNORD3A), small nucleolar RNA. |
| TBC1D22A | -2.59 | Homo sapiens TBC1 domain family, member 22A (TBC1D22A), mRNA. |
| LOC729120 | -2.6 | PREDICTED: Homo sapiens hypothetical LOC729120 (LOC729120), mRNA. |
| LMNA | -2.6 | Homo sapiens lamin A/C (LMNA), transcript variant 2, mRNA. |
| S100A10 | -2.6 | Homo sapiens S100 calcium binding protein A10 (annexin II ligand, calpactin I, light polypeptide (p11)) (S100A10), mRNA. |
| MBOAT1 | -2.6 | Homo sapiens membrane bound O-acyltransferase domain containing 1 (MBOAT1), mRNA. |
| WDR24 | -2.6 | Homo sapiens WD repeat domain 24 (WDR24), mRNA. |
| SORCS1 | -2.6 | Homo sapiens sortilin-related VPS10 domain containing receptor 1 (SORCS1), transcript variant 1, mRNA. |
| NKAIN1 | -2.6 | Homo sapiens Na+/K+ transporting ATPase interacting 1 (NKAIN1), mRNA. |
| C11orf84 | -2.6 | Homo sapiens chromosome 11 open reading frame 84 (C11orf84), mRNA. |
| C16orf79 | -2.6 | Homo sapiens chromosome 16 open reading frame 79 (C16orf79), mRNA. |
| PKD1 | -2.6 | Homo sapiens polycystic kidney disease 1 (autosomal dominant) (PKD1), transcript variant 1, mRNA. |
| HSF4 | -2.6 | Homo sapiens heat shock transcription factor 4 (HSF4), transcript variant 2, mRNA. |
| GNAO1 | -2.6 | Homo sapiens guanine nucleotide binding protein (G protein), alpha activating activity polypeptide O (GNAO1), transcript variant 1, mRNA. |
| SLC30A1 | -2.61 | Homo sapiens solute carrier family 30 (zinc transporter), member 1 (SLC30A1), mRNA. |
| KIAA1545 | -2.61 | PREDICTED: Homo sapiens KIAA1545 protein (KIAA1545), mRNA. |
| CTTN | -2.61 | Homo sapiens cortactin (CTTN), transcript variant 2, mRNA. |
| MMP24 | -2.61 | Homo sapiens matrix metallopeptidase 24 (membrane-inserted) (MMP24), mRNA. |
| SMO | -2.61 | Homo sapiens smoothened homolog (Drosophila) (SMO), mRNA. |
| GAMT | -2.61 | Homo sapiens guanidinoacetate N-methyltransferase (GAMT), transcript variant 1, mRNA. |
| LRRC45 | -2.61 | Homo sapiens leucine rich repeat containing 45 (LRRC45), mRNA. |
| NAV1 | -2.61 | Homo sapiens neuron navigator 1 (NAV1), mRNA. |
| SFRS16 | -2.62 | Homo sapiens splicing factor, arginine/serine-rich 16 (SFRS16), mRNA. |
| TADA3 | -2.62 | Homo sapiens transcriptional adaptor 3 (TADA3), transcript variant 2, mRNA. |
|  | -2.62 | Homo sapiens cDNA: FLJ21199 fis, clone COL00235 |
| DNM1 | -2.62 | Homo sapiens dynamin 1 (DNM1), transcript variant 2, mRNA. |
| LOC642469 | -2.62 | PREDICTED: Homo sapiens misc_RNA (LOC642469), miscRNA. |
| BAZ1A | -2.62 | Homo sapiens bromodomain adjacent to zinc finger domain, 1A (BAZ1A), transcript variant 1, mRNA. |
| CLN3 | -2.63 | Homo sapiens ceroid-lipofuscinosis, neuronal 3 (CLN3), transcript variant 1, mRNA. |
| TRIM13 | -2.63 | Homo sapiens tripartite motif-containing 13 (TRIM13), transcript variant 4, mRNA. |
| 11-Mar | -2.63 | Homo sapiens membrane-associated ring finger (C3HC4) 11 (MARCH11), mRNA. |
| ACIN1 | -2.63 | Homo sapiens apoptotic chromatin condensation inducer 1 (ACIN1), mRNA. |
| PKM2 | -2.63 | Homo sapiens pyruvate kinase, muscle (PKM2), transcript variant 1, mRNA. |
| MOSPD3 | -2.63 | Homo sapiens motile sperm domain containing 3 (MOSPD3), transcript variant 2, mRNA. |
| SMARCD3 | -2.63 | Homo sapiens SWI/SNF related, matrix associated, actin dependent regulator of chromatin, subfamily d, member 3 (SMARCD3), transcript variant 1, mRNA. |
| NRP1 | -2.64 | Homo sapiens neuropilin 1 (NRP1), transcript variant 1, mRNA. |
| GUSBL1 | -2.64 | Homo sapiens glucuronidase, beta-like 1 (GUSBL1), non-coding RNA. |
| KIAA0895L | -2.64 | Homo sapiens KIAA0895-like (KIAA0895L), mRNA. XM_933435 XM_933438 XM_933442 XM_933445 XM_933447 XM_941971 XM_945589 XM_945591 XM_945592 XM_945593 XM_945594 XM_945596 XM_945598 XM_945599 XM_945600 |
| MOV10 | -2.64 | Homo sapiens Mov10, Moloney leukemia virus 10, homolog (mouse) (MOV10), mRNA. |
| DENND4B | -2.64 | Homo sapiens DENN/MADD domain containing 4B (DENND4B), mRNA. |
| DPF1 | -2.64 | Homo sapiens D4, zinc and double PHD fingers family 1 (DPF1), mRNA. |
| ZNF384 | -2.64 | Homo sapiens zinc finger protein 384 (ZNF384), transcript variant 5, mRNA. |
| PPP2R3B | -2.64 | Homo sapiens protein phosphatase 2 (formerly 2A), regulatory subunit B'', beta (PPP2R3B), transcript variant 1, mRNA. |
| TMED4 | -2.64 | Homo sapiens transmembrane emp24 protein transport domain containing 4 (TMED4), mRNA. |
| YWHAE | -2.64 | Homo sapiens tyrosine 3-monooxygenase/tryptophan 5-monooxygenase activation protein, epsilon polypeptide (YWHAE), mRNA. |
| LOC100170939 | -2.65 | Homo sapiens glucuronidase, beta pseudogene (LOC100170939), non-coding RNA. |
| BLOC1S1 | -2.65 | Homo sapiens biogenesis of lysosome-related organelles complex-1, subunit 1 (BLOC1S1), mRNA. |
| PTK7 | -2.65 | Homo sapiens PTK7 protein tyrosine kinase 7 (PTK7), transcript variant PTK7-4, mRNA. |
| LOC730525 | -2.65 | PREDICTED: Homo sapiens hypothetical protein LOC730525 (LOC730525), mRNA. |
| BCL7C | -2.65 | Homo sapiens B-cell CLL/lymphoma 7C (BCL7C), mRNA. |
| MLF2 | -2.65 | Homo sapiens myeloid leukemia factor 2 (MLF2), mRNA. |
| PGLS | -2.65 | Homo sapiens 6-phosphogluconolactonase (PGLS), mRNA. |
| LOC100133516 | -2.66 | PREDICTED: Homo sapiens hypothetical protein LOC100133516 (LOC100133516), mRNA. |
| SLC18A1 | -2.66 | Homo sapiens solute carrier family 18 (vesicular monoamine), member 1 (SLC18A1), mRNA. |
| ST7OT1 | -2.66 | Homo sapiens ST7 overlapping transcript 1 (non-protein coding) (ST7OT1), antisense RNA. |
| TUBG1 | -2.66 | Homo sapiens tubulin, gamma 1 (TUBG1), mRNA. |
| C17orf49 | -2.66 | Homo sapiens chromosome 17 open reading frame 49 (C17orf49), mRNA. |
| FSD1 | -2.67 | Homo sapiens fibronectin type III and SPRY domain containing 1 (FSD1), mRNA. |
| RASA4P | -2.67 | Homo sapiens RAS p21 protein activator 4 pseudogene (RASA4P), non-coding RNA. |
| DHX30 | -2.67 | Homo sapiens DEAH (Asp-Glu-Ala-His) box polypeptide 30 (DHX30), transcript variant 1, mRNA. |
| PIM1 | -2.67 | Homo sapiens pim-1 oncogene (PIM1), mRNA. |
| AP2M1 | -2.67 | Homo sapiens adaptor-related protein complex 2, mu 1 subunit (AP2M1), transcript variant 1, mRNA. |
| KIAA0913 | -2.67 | Homo sapiens KIAA0913 (KIAA0913), mRNA. |
| FXR2 | -2.67 | Homo sapiens fragile X mental retardation, autosomal homolog 2 (FXR2), mRNA. |
| IGSF1 | -2.68 | Homo sapiens immunoglobulin superfamily, member 1 (IGSF1), transcript variant 1, mRNA. |
| CHMP1A | -2.68 | Homo sapiens chromatin modifying protein 1A (CHMP1A), transcript variant 2, mRNA. |
| MAPK8IP2 | -2.68 | Homo sapiens mitogen-activated protein kinase 8 interacting protein 2 (MAPK8IP2), transcript variant 2, mRNA. |
| RNU1-5 | -2.68 | Homo sapiens RNA, U1 small nuclear 5 (RNU1-5), small nuclear RNA. |
| TMEM39B | -2.68 | Homo sapiens transmembrane protein 39B (TMEM39B), mRNA. |
| RPP25 | -2.68 | Homo sapiens ribonuclease P/MRP 25kDa subunit (RPP25), mRNA. |
| PKD1 | -2.68 | Homo sapiens polycystic kidney disease 1 (autosomal dominant) (PKD1), transcript variant 1, mRNA. |
| C17orf96 | -2.69 | Homo sapiens chromosome 17 open reading frame 96 (C17orf96), mRNA. |
| LOC651576 | -2.69 | PREDICTED: Homo sapiens similar to tubulin, alpha 8 like, transcript variant 2 (LOC651576), mRNA. |
| DKFZp547K054 | -2.69 | PREDICTED: Homo sapiens hypothetical protein DKFZp547K054 (DKFZp547K054), mRNA. |
| FADS1 | -2.69 | Homo sapiens fatty acid desaturase 1 (FADS1), mRNA. |
| STK11IP | -2.69 | Homo sapiens serine/threonine kinase 11 interacting protein (STK11IP), mRNA. |
| UPF3A | -2.69 | Homo sapiens UPF3 regulator of nonsense transcripts homolog A (yeast) (UPF3A), transcript variant 1, mRNA. |
| C19orf29 | -2.69 | Homo sapiens chromosome 19 open reading frame 29 (C19orf29), transcript variant 1, mRNA. |
| ARHGEF1 | -2.69 | Homo sapiens Rho guanine nucleotide exchange factor (GEF) 1 (ARHGEF1), transcript variant 1, mRNA. |
| FAM53B | -2.69 | Homo sapiens family with sequence similarity 53, member B (FAM53B), mRNA. |
| TAF6 | -2.7 | Homo sapiens TAF6 RNA polymerase II, TATA box binding protein (TBP)-associated factor, 80kDa (TAF6), transcript variant 2, mRNA. |
| NAIF1 | -2.7 | Homo sapiens nuclear apoptosis inducing factor 1 (NAIF1), mRNA. |
| PI4KAP1 | -2.7 | Homo sapiens phosphatidylinositol 4-kinase, catalytic, alpha pseudogene 1 (PI4KAP1), non-coding RNA. |
| CHRAC1 | -2.71 | Homo sapiens chromatin accessibility complex 1 (CHRAC1), mRNA. |
| C3orf39 | -2.71 | Homo sapiens chromosome 3 open reading frame 39 (C3orf39), mRNA. |
| TBCD | -2.71 | Homo sapiens tubulin folding cofactor D (TBCD), mRNA. |
| SF1 | -2.71 | Homo sapiens splicing factor 1 (SF1), transcript variant 1, mRNA. |
| LYN | -2.71 | Homo sapiens v-yes-1 Yamaguchi sarcoma viral related oncogene homolog (LYN), mRNA. |
| MDK | -2.72 | Homo sapiens midkine (neurite growth-promoting factor 2) (MDK), transcript variant 2, mRNA. |
| KCNH2 | -2.72 | Homo sapiens potassium voltage-gated channel, subfamily H (eag-related), member 2 (KCNH2), transcript variant 2, mRNA. |
| RERE | -2.72 | Homo sapiens arginine-glutamic acid dipeptide (RE) repeats (RERE), transcript variant 1, mRNA. |
| LOC100133823 | -2.72 | PREDICTED: Homo sapiens misc_RNA (LOC100133823), miscRNA. |
| NCOA5 | -2.72 | Homo sapiens nuclear receptor coactivator 5 (NCOA5), mRNA. |
| C9orf7 | -2.72 | Homo sapiens chromosome 9 open reading frame 7 (C9orf7), mRNA. |
| SDF4 | -2.72 | Homo sapiens stromal cell derived factor 4 (SDF4), mRNA. |
| TNRC6A | -2.72 | Homo sapiens trinucleotide repeat containing 6A (TNRC6A), mRNA. |
| PPAP2C | -2.72 | Homo sapiens phosphatidic acid phosphatase type 2C (PPAP2C), transcript variant 2, mRNA. |
| TMEM219 | -2.72 | Homo sapiens transmembrane protein 219 (TMEM219), transcript variant 1, mRNA. |
| SHANK3 | -2.72 | Homo sapiens SH3 and multiple ankyrin repeat domains 3 (SHANK3), mRNA. |
| GPS2 | -2.72 | Homo sapiens G protein pathway suppressor 2 (GPS2), mRNA. |
| TMEM18 | -2.73 | Homo sapiens transmembrane protein 18 (TMEM18), mRNA. |
| LOC643384 | -2.73 | PREDICTED: Homo sapiens hypothetical LOC643384 (LOC643384), mRNA. |
| EPHX2 | -2.73 | Homo sapiens epoxide hydrolase 2, cytoplasmic (EPHX2), mRNA. |
| SHARPIN | -2.73 | Homo sapiens SHANK-associated RH domain interactor (SHARPIN), mRNA. |
| 6-Mar | -2.73 | Homo sapiens membrane-associated ring finger (C3HC4) 6 (MARCH6), mRNA. |
| TPM2 | -2.73 | Homo sapiens tropomyosin 2 (beta) (TPM2), transcript variant 2, mRNA. |
| RHBDL2 | -2.73 | Homo sapiens rhomboid, veinlet-like 2 (Drosophila) (RHBDL2), mRNA. |
| ADPRHL1 | -2.73 | Homo sapiens ADP-ribosylhydrolase like 1 (ADPRHL1), transcript variant 2, mRNA. |
| CYLN2 | -2.73 | Homo sapiens cytoplasmic linker 2 (CYLN2), transcript variant 2, mRNA. |
| C17orf101 | -2.73 | Homo sapiens chromosome 17 open reading frame 101 (C17orf101), transcript variant 1, mRNA. |
| C20orf4 | -2.73 | Homo sapiens chromosome 20 open reading frame 4 (C20orf4), mRNA. |
| TAZ | -2.73 | Homo sapiens tafazzin (cardiomyopathy, dilated 3A (X-linked); endocardial fibroelastosis 2; Barth syndrome) (TAZ), transcript variant 2, mRNA. |
| MPST | -2.73 | Homo sapiens mercaptopyruvate sulfurtransferase (MPST), nuclear gene encoding mitochondrial protein, transcript variant 2, mRNA. |
| RAB5C | -2.73 | Homo sapiens RAB5C, member RAS oncogene family (RAB5C), transcript variant 2, mRNA. |
| TNPO1 | -2.74 | Homo sapiens transportin 1 (TNPO1), transcript variant 2, mRNA. |
| RFNG | -2.74 | PREDICTED: Homo sapiens radical fringe homolog (Drosophila) (RFNG), mRNA. |
| CXCR4 | -2.74 | Homo sapiens chemokine (C-X-C motif) receptor 4 (CXCR4), transcript variant 1, mRNA. |
| GPR161 | -2.74 | Homo sapiens G protein-coupled receptor 161 (GPR161), transcript variant 1, mRNA. |
| RASSF6 | -2.75 | Homo sapiens Ras association (RalGDS/AF-6) domain family member 6 (RASSF6), transcript variant 1, mRNA. |
| CCDC86 | -2.75 | Homo sapiens coiled-coil domain containing 86 (CCDC86), mRNA. |
| IGFBP3 | -2.75 | Homo sapiens insulin-like growth factor binding protein 3 (IGFBP3), transcript variant 1, mRNA. |
| SLC19A1 | -2.75 | Homo sapiens solute carrier family 19 (folate transporter), member 1 (SLC19A1), mRNA. |
| RNF31 | -2.75 | Homo sapiens ring finger protein 31 (RNF31), mRNA. |
| DHX30 | -2.75 | Homo sapiens DEAH (Asp-Glu-Ala-His) box polypeptide 30 (DHX30), transcript variant 2, mRNA. |
| PRKAB1 | -2.75 | Homo sapiens protein kinase, AMP-activated, beta 1 non-catalytic subunit (PRKAB1), mRNA. |
| SCAMP5 | -2.75 | Homo sapiens secretory carrier membrane protein 5 (SCAMP5), mRNA. |
| FAM73A | -2.76 | Homo sapiens family with sequence similarity 73, member A (FAM73A), mRNA. |
| HSPC268 | -2.76 | Homo sapiens hypothetical protein HSPC268 (HSPC268), mRNA. |
| LOC100129269 | -2.76 | PREDICTED: Homo sapiens hypothetical protein LOC100129269 (LOC100129269), mRNA. |
| SESTD1 | -2.76 | Homo sapiens SEC14 and spectrin domains 1 (SESTD1), mRNA. |
| ZBTB17 | -2.76 | Homo sapiens zinc finger and BTB domain containing 17 (ZBTB17), mRNA. |
| PXMP2 | -2.76 | Homo sapiens peroxisomal membrane protein 2, 22kDa (PXMP2), mRNA. |
| LOC100132585 | -2.76 | PREDICTED: Homo sapiens hypothetical protein LOC100132585 (LOC100132585), mRNA. |
| CUL9 | -2.76 | Homo sapiens cullin 9 (CUL9), mRNA. |
| DIAPH1 | -2.77 | Homo sapiens diaphanous homolog 1 (Drosophila) (DIAPH1), transcript variant 1, mRNA. |
| FSD1 | -2.77 | Homo sapiens fibronectin type III and SPRY domain containing 1 (FSD1), mRNA. |
| PTGFRN | -2.77 | Homo sapiens prostaglandin F2 receptor negative regulator (PTGFRN), mRNA. |
| ALDOA | -2.78 | Homo sapiens aldolase A, fructose-bisphosphate (ALDOA), transcript variant 3, mRNA. |
| LOC100128288 | -2.78 | Homo sapiens hypothetical protein LOC100128288 (LOC100128288), non-coding RNA. |
| MCM2 | -2.78 | Homo sapiens minichromosome maintenance complex component 2 (MCM2), mRNA. |
| UBAP2 | -2.78 | Homo sapiens ubiquitin associated protein 2 (UBAP2), mRNA. |
| PTCHD2 | -2.79 | PREDICTED: Homo sapiens patched domain containing 2 (PTCHD2), mRNA. |
| PI4KAP2 | -2.79 | Homo sapiens phosphatidylinositol 4-kinase, catalytic, alpha polypeptide pseudogene 2 (PI4KAP2), mRNA. |
| JPH4 | -2.79 | Homo sapiens junctophilin 4 (JPH4), mRNA. |
| RBMS1 | -2.79 | Homo sapiens RNA binding motif, single stranded interacting protein 1 (RBMS1), transcript variant 3, mRNA. |
| C1orf166 | -2.79 | Homo sapiens chromosome 1 open reading frame 166 (C1orf166), mRNA. |
| SPTAN1 | -2.79 | Homo sapiens spectrin, alpha, non-erythrocytic 1 (alpha-fodrin) (SPTAN1), mRNA. |
| ATP2B4 | -2.8 | Homo sapiens ATPase, Ca++ transporting, plasma membrane 4 (ATP2B4), transcript variant 1, mRNA. |
| NGFRAP1 | -2.8 | Homo sapiens nerve growth factor receptor (TNFRSF16) associated protein 1 (NGFRAP1), transcript variant 3, mRNA. |
| B4GALNT4 | -2.8 | Homo sapiens beta-1,4-N-acetyl-galactosaminyl transferase 4 (B4GALNT4), mRNA. |
| LOC728411 | -2.8 | PREDICTED: Homo sapiens similar to Beta-glucuronidase precursor (LOC728411), mRNA. |
| SLC35C2 | -2.8 | Homo sapiens solute carrier family 35, member C2 (SLC35C2), transcript variant 2, mRNA. |
| VPS4A | -2.8 | Homo sapiens vacuolar protein sorting 4 homolog A (S. cerevisiae) (VPS4A), mRNA. |
| PIF1 | -2.81 | Homo sapiens PIF1 5'-to-3' DNA helicase homolog (S. cerevisiae) (PIF1), mRNA. |
| DDX28 | -2.81 | Homo sapiens DEAD (Asp-Glu-Ala-Asp) box polypeptide 28 (DDX28), nuclear gene encoding mitochondrial protein, mRNA. |
| RBM42 | -2.81 | Homo sapiens RNA binding motif protein 42 (RBM42), mRNA. |
| HLA-F | -2.81 | Homo sapiens major histocompatibility complex, class I, F (HLA-F), mRNA. |
| SLC25A1 | -2.81 | Homo sapiens solute carrier family 25 (mitochondrial carrier; citrate transporter), member 1 (SLC25A1), nuclear gene encoding mitochondrial protein, mRNA. |
| BRF1 | -2.81 | Homo sapiens BRF1 homolog, subunit of RNA polymerase III transcription initiation factor IIIB (S. cerevisiae) (BRF1), transcript variant 1, mRNA. |
| DNAJC4 | -2.82 | Homo sapiens DnaJ (Hsp40) homolog, subfamily C, member 4 (DNAJC4), mRNA. |
| MVK | -2.82 | Homo sapiens mevalonate kinase (mevalonic aciduria) (MVK), mRNA. |
| SETDB1 | -2.82 | Homo sapiens SET domain, bifurcated 1 (SETDB1), mRNA. |
| MAPKAPK2 | -2.83 | Homo sapiens mitogen-activated protein kinase-activated protein kinase 2 (MAPKAPK2), transcript variant 2, mRNA. |
| FAT1 | -2.83 | Homo sapiens FAT tumor suppressor homolog 1 (Drosophila) (FAT1), mRNA. |
| FAM100B | -2.83 | Homo sapiens family with sequence similarity 100, member B (FAM100B), mRNA. |
| APLP1 | -2.83 | Homo sapiens amyloid beta (A4) precursor-like protein 1 (APLP1), transcript variant 2, mRNA. |
|  | -2.83 | Homo sapiens cDNA FLJ31093 fis, clone IMR321000161 |
| POLR2C | -2.84 | Homo sapiens polymerase (RNA) II (DNA directed) polypeptide C, 33kDa (POLR2C), mRNA. |
| LZTS1 | -2.84 | Homo sapiens leucine zipper, putative tumor suppressor 1 (LZTS1), mRNA. |
| POLE | -2.84 | Homo sapiens polymerase (DNA directed), epsilon (POLE), mRNA. |
| ZBED3 | -2.84 | Homo sapiens zinc finger, BED-type containing 3 (ZBED3), mRNA. |
| SMG7 | -2.84 | Homo sapiens Smg-7 homolog, nonsense mediated mRNA decay factor (C. elegans) (SMG7), transcript variant 1, mRNA. |
| GSS | -2.85 | Homo sapiens glutathione synthetase (GSS), mRNA. |
| OAF | -2.85 | Homo sapiens OAF homolog (Drosophila) (OAF), mRNA. |
| CHPF | -2.85 | Homo sapiens chondroitin polymerizing factor (CHPF), mRNA. |
| LOC613037 | -2.86 | Homo sapiens nuclear pore complex interacting protein pseudogene (LOC613037), non-coding RNA. |
| ILF3 | -2.86 | Homo sapiens interleukin enhancer binding factor 3, 90kDa (ILF3), transcript variant 1, mRNA. |
| GABRB3 | -2.86 | Homo sapiens gamma-aminobutyric acid (GABA) A receptor, beta 3 (GABRB3), transcript variant 1, mRNA. |
| SCARNA13 | -2.86 | Homo sapiens small Cajal body-specific RNA 13 (SCARNA13), guide RNA. |
| CACNG4 | -2.86 | Homo sapiens calcium channel, voltage-dependent, gamma subunit 4 (CACNG4), mRNA. |
| PVRL3 | -2.86 | Homo sapiens poliovirus receptor-related 3 (PVRL3), mRNA. |
| TBC1D9B | -2.87 | Homo sapiens TBC1 domain family, member 9B (with GRAM domain) (TBC1D9B), transcript variant 2, mRNA. |
| LOC646044 | -2.87 | PREDICTED: Homo sapiens misc_RNA (LOC646044), miscRNA. |
| FCGRT | -2.87 | Homo sapiens Fc fragment of IgG, receptor, transporter, alpha (FCGRT), transcript variant 1, mRNA. |
| ADRM1 | -2.87 | Homo sapiens adhesion regulating molecule 1 (ADRM1), transcript variant 2, mRNA. |
| NCAPD3 | -2.87 | Homo sapiens non-SMC condensin II complex, subunit D3 (NCAPD3), mRNA. |
| TOP2A | -2.87 | Homo sapiens topoisomerase (DNA) II alpha 170kDa (TOP2A), mRNA. |
| GSK3B | -2.88 | Homo sapiens glycogen synthase kinase 3 beta (GSK3B), mRNA. |
| PHF12 | -2.88 | Homo sapiens PHD finger protein 12 (PHF12), transcript variant 1, mRNA. |
| NAT14 | -2.88 | Homo sapiens N-acetyltransferase 14 (GCN5-related, putative) (NAT14), mRNA. |
| ATF3 | -2.88 | Homo sapiens activating transcription factor 3 (ATF3), transcript variant 4, mRNA. |
| ENO2 | -2.88 | Homo sapiens enolase 2 (gamma, neuronal) (ENO2), mRNA. |
| FKBP9L | -2.88 | Homo sapiens FK506 binding protein 9-like (FKBP9L), mRNA. |
| UBXN6 | -2.88 | Homo sapiens UBX domain protein 6 (UBXN6), mRNA. |
| TGFBRAP1 | -2.89 | Homo sapiens transforming growth factor, beta receptor associated protein 1 (TGFBRAP1), mRNA. |
| LOC100130516 | -2.89 | PREDICTED: Homo sapiens hypothetical protein LOC100130516 (LOC100130516), mRNA. |
| CSAD | -2.89 | Homo sapiens cysteine sulfinic acid decarboxylase (CSAD), mRNA. |
| RELL2 | -2.89 | Homo sapiens RELT-like 2 (RELL2), mRNA. |
| SYP | -2.89 | Homo sapiens synaptophysin (SYP), mRNA. |
| SDF4 | -2.89 | Homo sapiens stromal cell derived factor 4 (SDF4), mRNA. |
| SNORD4A | -2.89 | Homo sapiens small nucleolar RNA, C/D box 4A (SNORD4A), small nucleolar RNA. |
| FASTK | -2.89 | Homo sapiens Fas-activated serine/threonine kinase (FASTK), transcript variant 1, mRNA. |
| C14orf102 | -2.89 | Homo sapiens chromosome 14 open reading frame 102 (C14orf102), transcript variant 1, mRNA. |
| SLIT3 | -2.89 | Homo sapiens slit homolog 3 (Drosophila) (SLIT3), mRNA. |
| CSRP1 | -2.9 | Homo sapiens cysteine and glycine-rich protein 1 (CSRP1), mRNA. |
| C1orf86 | -2.9 | Homo sapiens chromosome 1 open reading frame 86 (C1orf86), transcript variant 2, mRNA. |
| PTOV1 | -2.9 | Homo sapiens prostate tumor overexpressed 1 (PTOV1), mRNA. |
| FSTL1 | -2.9 | Homo sapiens follistatin-like 1 (FSTL1), mRNA. |
| WDR34 | -2.91 | Homo sapiens WD repeat domain 34 (WDR34), mRNA. |
| CTSD | -2.91 | Homo sapiens cathepsin D (CTSD), mRNA. |
| MPRIP | -2.91 | Homo sapiens myosin phosphatase Rho interacting protein (MPRIP), transcript variant 1, mRNA. |
| MED24 | -2.91 | Homo sapiens mediator complex subunit 24 (MED24), transcript variant 1, mRNA. |
| GMEB1 | -2.91 | Homo sapiens glucocorticoid modulatory element binding protein 1 (GMEB1), transcript variant 2, mRNA. |
| GDE1 | -2.91 | Homo sapiens glycerophosphodiester phosphodiesterase 1 (GDE1), mRNA. |
| RNF40 | -2.92 | Homo sapiens ring finger protein 40 (RNF40), mRNA. |
| LOC388796 | -2.92 | Homo sapiens hypothetical LOC388796 (LOC388796), non-coding RNA. |
| CSNK1D | -2.92 | Homo sapiens casein kinase 1, delta (CSNK1D), transcript variant 2, mRNA. |
| FAM64A | -2.92 | Homo sapiens family with sequence similarity 64, member A (FAM64A), mRNA. |
| PKN1 | -2.92 | Homo sapiens protein kinase N1 (PKN1), transcript variant 1, mRNA. |
| CBLL1 | -2.92 | Homo sapiens Cas-Br-M (murine) ecotropic retroviral transforming sequence-like 1 (CBLL1), mRNA. |
| CYB5R3 | -2.93 | Homo sapiens cytochrome b5 reductase 3 (CYB5R3), transcript variant M, mRNA. |
| LOC100128510 | -2.93 | PREDICTED: Homo sapiens hypothetical protein LOC100128510 (LOC100128510), mRNA. |
| PLEC1 | -2.93 | Homo sapiens plectin 1, intermediate filament binding protein 500kDa (PLEC1), transcript variant 1, mRNA. |
| C9orf86 | -2.93 | Homo sapiens chromosome 9 open reading frame 86 (C9orf86), mRNA. |
| C1QL1 | -2.93 | Homo sapiens complement component 1, q subcomponent-like 1 (C1QL1), mRNA. |
| BRD1 | -2.93 | Homo sapiens bromodomain containing 1 (BRD1), mRNA. |
| ZSCAN2 | -2.94 | Homo sapiens zinc finger and SCAN domain containing 2 (ZSCAN2), transcript variant 3, mRNA. |
| PCBP3 | -2.94 | Homo sapiens poly(rC) binding protein 3 (PCBP3), mRNA. |
| DUS3L | -2.94 | Homo sapiens dihydrouridine synthase 3-like (S. cerevisiae) (DUS3L), mRNA. |
| ZNF444 | -2.95 | Homo sapiens zinc finger protein 444 (ZNF444), mRNA. |
| GNL3L | -2.95 | Homo sapiens guanine nucleotide binding protein-like 3 (nucleolar)-like (GNL3L), mRNA. |
| CTNNBIP1 | -2.95 | Homo sapiens catenin, beta interacting protein 1 (CTNNBIP1), transcript variant 1, mRNA. |
| LOC646753 | -2.95 | PREDICTED: Homo sapiens similar to ribosomal protein S26 (LOC646753), mRNA. |
| PCBP4 | -2.95 | Homo sapiens poly(rC) binding protein 4 (PCBP4), transcript variant 1, mRNA. |
| HIP1R | -2.96 | Homo sapiens huntingtin interacting protein 1 related (HIP1R), mRNA. |
| KIAA1602 | -2.96 | Homo sapiens KIAA1602 (KIAA1602), mRNA. |
| NRGN | -2.96 | Homo sapiens neurogranin (protein kinase C substrate, RC3) (NRGN), mRNA. |
| C16orf7 | -2.96 | Homo sapiens chromosome 16 open reading frame 7 (C16orf7), mRNA. |
| SRM | -2.96 | Homo sapiens spermidine synthase (SRM), mRNA. |
| VPS52 | -2.97 | Homo sapiens vacuolar protein sorting 52 homolog (S. cerevisiae) (VPS52), mRNA. |
| FAM89B | -2.97 | Homo sapiens family with sequence similarity 89, member B (FAM89B), transcript variant 3, mRNA. |
| MEX3A | -2.97 | Homo sapiens mex-3 homolog A (C. elegans) (MEX3A), mRNA. |
| ALDOA | -2.97 | Homo sapiens aldolase A, fructose-bisphosphate (ALDOA), transcript variant 2, mRNA. |
| GAP43 | -2.97 | Homo sapiens growth associated protein 43 (GAP43), mRNA. |
| NT5DC2 | -2.97 | Homo sapiens 5'-nucleotidase domain containing 2 (NT5DC2), mRNA. |
| LOC649095 | -2.97 | PREDICTED: Homo sapiens hypothetical LOC649095 (LOC649095), mRNA. |
| ABHD12 | -2.97 | Homo sapiens abhydrolase domain containing 12 (ABHD12), transcript variant 1, mRNA. |
| ZNF618 | -2.97 | Homo sapiens zinc finger protein 618 (ZNF618), mRNA. |
| ANO8 | -2.97 | Homo sapiens anoctamin 8 (ANO8), mRNA. |
| LYPLA2 | -2.97 | Homo sapiens lysophospholipase II (LYPLA2), mRNA. |
| CAMKK2 | -2.97 | Homo sapiens calcium/calmodulin-dependent protein kinase kinase 2, beta (CAMKK2), transcript variant 4, mRNA. |
| CTSB | -2.98 | Homo sapiens cathepsin B (CTSB), transcript variant 2, mRNA. |
| HJURP | -2.98 | Homo sapiens Holliday junction recognition protein (HJURP), mRNA. |
| LRRN3 | -2.98 | Homo sapiens leucine rich repeat neuronal 3 (LRRN3), mRNA. |
| LOC390298 | -2.98 | PREDICTED: Homo sapiens similar to translocase of inner mitochondrial membrane 17 homolog B (LOC390298), miscRNA. |
| LOC286157 | -2.98 | PREDICTED: Homo sapiens misc_RNA (LOC286157), miscRNA. |
| BIN1 | -2.98 | Homo sapiens bridging integrator 1 (BIN1), transcript variant 1, mRNA. |
| RFNG | -2.98 | PREDICTED: Homo sapiens radical fringe homolog (Drosophila) (RFNG), mRNA. |
| COBRA1 | -2.99 | Homo sapiens cofactor of BRCA1 (COBRA1), mRNA. |
| G6PD | -2.99 | Homo sapiens glucose-6-phosphate dehydrogenase (G6PD), transcript variant 1, mRNA. |
| ZNF483 | -2.99 | Homo sapiens zinc finger protein 483 (ZNF483), transcript variant 2, mRNA. |
| IRAK1 | -2.99 | Homo sapiens interleukin-1 receptor-associated kinase 1 (IRAK1), transcript variant 3, mRNA. |
| BIN1 | -2.99 | Homo sapiens bridging integrator 1 (BIN1), transcript variant 4, mRNA. |
| MIIP | -3 | Homo sapiens migration and invasion inhibitory protein (MIIP), mRNA. |
| CMTM3 | -3 | Homo sapiens CKLF-like MARVEL transmembrane domain containing 3 (CMTM3), transcript variant 5, mRNA. |
| GOLPH4 | -3 | Homo sapiens golgi phosphoprotein 4 (GOLPH4), mRNA. |
| ERC1 | -3.01 | Homo sapiens ELKS/RAB6-interacting/CAST family member 1 (ERC1), transcript variant beta, mRNA. |
| CRAMP1L | -3.01 | Homo sapiens Crm, cramped-like (Drosophila) (CRAMP1L), mRNA. |
| MED25 | -3.01 | Homo sapiens mediator complex subunit 25 (MED25), mRNA. |
| C11orf63 | -3.01 | Homo sapiens chromosome 11 open reading frame 63 (C11orf63), transcript variant 2, mRNA. |
| LOC648852 | -3.02 | PREDICTED: Homo sapiens hypothetical protein LOC648852 (LOC648852), mRNA. |
| RERE | -3.02 | Homo sapiens arginine-glutamic acid dipeptide (RE) repeats (RERE), transcript variant 3, mRNA. |
| PA2G4 | -3.02 | Homo sapiens proliferation-associated 2G4, 38kDa (PA2G4), mRNA. |
| SCAND1 | -3.02 | Homo sapiens SCAN domain containing 1 (SCAND1), transcript variant 2, mRNA. |
| C9orf25 | -3.03 | Homo sapiens chromosome 9 open reading frame 25 (C9orf25), mRNA. |
| SIGMAR1 | -3.03 | Homo sapiens sigma non-opioid intracellular receptor 1 (SIGMAR1), transcript variant 2, mRNA. |
| GRN | -3.03 | Homo sapiens granulin (GRN), mRNA. |
| CORO6 | -3.03 | Homo sapiens coronin 6 (CORO6), mRNA. |
| TUBG1 | -3.03 | Homo sapiens tubulin, gamma 1 (TUBG1), mRNA. |
| TPM4 | -3.04 | Homo sapiens tropomyosin 4 (TPM4), mRNA. |
| KPNA6 | -3.04 | Homo sapiens karyopherin alpha 6 (importin alpha 7) (KPNA6), mRNA. |
| LEPROT | -3.04 | Homo sapiens leptin receptor overlapping transcript (LEPROT), mRNA. |
| EYA4 | -3.04 | Homo sapiens eyes absent homolog 4 (Drosophila) (EYA4), transcript variant 1, mRNA. |
| RAC3 | -3.05 | Homo sapiens ras-related C3 botulinum toxin substrate 3 (rho family, small GTP binding protein Rac3) (RAC3), mRNA. |
| BRE | -3.05 | Homo sapiens brain and reproductive organ-expressed (TNFRSF1A modulator) (BRE), transcript variant 5, mRNA. |
| C19orf6 | -3.05 | Homo sapiens chromosome 19 open reading frame 6 (C19orf6), transcript variant 1, mRNA. |
| LSM12 | -3.05 | Homo sapiens LSM12 homolog (S. cerevisiae) (LSM12), mRNA. |
| SCLY | -3.05 | Homo sapiens selenocysteine lyase (SCLY), mRNA. |
| C1orf159 | -3.06 | Homo sapiens chromosome 1 open reading frame 159 (C1orf159), mRNA. |
| CCDC74A | -3.06 | Homo sapiens coiled-coil domain containing 74A (CCDC74A), mRNA. |
| VASN | -3.07 | Homo sapiens vasorin (VASN), mRNA. |
| ZDHHC12 | -3.07 | Homo sapiens zinc finger, DHHC-type containing 12 (ZDHHC12), mRNA. |
| MICAL1 | -3.07 | Homo sapiens microtubule associated monoxygenase, calponin and LIM domain containing 1 (MICAL1), mRNA. |
| LOC440345 | -3.07 | PREDICTED: Homo sapiens hypothetical protein LOC440345, transcript variant 6 (LOC440345), mRNA. |
| LOC645233 | -3.07 | Homo sapiens thymine-DNA glycosylase pseudogene (LOC645233), non-coding RNA. |
| RHBDD3 | -3.08 | Homo sapiens rhomboid domain containing 3 (RHBDD3), mRNA. |
| LOC389342 | -3.08 | PREDICTED: Homo sapiens similar to QM protein, transcript variant 11 (LOC389342), mRNA. |
| CLSTN1 | -3.08 | Homo sapiens calsyntenin 1 (CLSTN1), transcript variant 1, mRNA. |
| MYO9B | -3.08 | Homo sapiens myosin IXB (MYO9B), mRNA. |
| FAM125B | -3.08 | Homo sapiens family with sequence similarity 125, member B (FAM125B), transcript variant 1, mRNA. |
| ZNF581 | -3.08 | Homo sapiens zinc finger protein 581 (ZNF581), mRNA. |
| CPLX1 | -3.09 | Homo sapiens complexin 1 (CPLX1), mRNA. |
| ASPSCR1 | -3.1 | PREDICTED: Homo sapiens alveolar soft part sarcoma chromosome region, candidate 1 (ASPSCR1), mRNA. |
| APEH | -3.1 | Homo sapiens N-acylaminoacyl-peptide hydrolase (APEH), mRNA. |
| ACTR1B | -3.11 | Homo sapiens ARP1 actin-related protein 1 homolog B, centractin beta (yeast) (ACTR1B), mRNA. |
| SMTN | -3.11 | Homo sapiens smoothelin (SMTN), transcript variant 3, mRNA. |
| MEIS3 | -3.11 | Homo sapiens Meis homeobox 3 (MEIS3), transcript variant 2, mRNA. |
| TRAF7 | -3.12 | Homo sapiens TNF receptor-associated factor 7 (TRAF7), mRNA. |
| WASH3P | -3.13 | Homo sapiens WAS protein family homolog 3 pseudogene (WASH3P), non-coding RNA. |
| TTC9B | -3.13 | Homo sapiens tetratricopeptide repeat domain 9B (TTC9B), mRNA. |
| GAA | -3.13 | Homo sapiens glucosidase, alpha; acid (GAA), transcript variant 3, mRNA. |
| GRB2 | -3.13 | Homo sapiens growth factor receptor-bound protein 2 (GRB2), transcript variant 1, mRNA. |
| HTRA1 | -3.14 | Homo sapiens HtrA serine peptidase 1 (HTRA1), mRNA. |
| GOLPH4 | -3.14 | Homo sapiens golgi phosphoprotein 4 (GOLPH4), mRNA. |
| WASF2 | -3.14 | Homo sapiens WAS protein family, member 2 (WASF2), mRNA. |
| AES | -3.14 | Homo sapiens amino-terminal enhancer of split (AES), transcript variant 2, mRNA. |
| NFYC | -3.14 | Homo sapiens nuclear transcription factor Y, gamma (NFYC), mRNA. |
| TECR | -3.14 | Homo sapiens trans-2,3-enoyl-CoA reductase (TECR), mRNA. |
| PRR16 | -3.14 | Homo sapiens proline rich 16 (PRR16), mRNA. |
| LPHN1 | -3.14 | Homo sapiens latrophilin 1 (LPHN1), transcript variant 2, mRNA. |
| DPP7 | -3.14 | Homo sapiens dipeptidyl-peptidase 7 (DPP7), mRNA. |
| CHD4 | -3.14 | Homo sapiens chromodomain helicase DNA binding protein 4 (CHD4), mRNA. |
| FLJ10324 | -3.15 | Homo sapiens hypothetical protein FLJ10324 (FLJ10324), mRNA. |
| C1orf173 | -3.15 | Homo sapiens chromosome 1 open reading frame 173 (C1orf173), mRNA. |
| DAG1 | -3.15 | Homo sapiens dystroglycan 1 (dystrophin-associated glycoprotein 1) (DAG1), mRNA. |
| SOX4 | -3.16 | Homo sapiens SRY (sex determining region Y)-box 4 (SOX4), mRNA. |
| CD151 | -3.16 | Homo sapiens CD151 molecule (Raph blood group) (CD151), transcript variant 5, mRNA. |
| C1orf66 | -3.16 | Homo sapiens chromosome 1 open reading frame 66 (C1orf66), mRNA. |
| CTSF | -3.16 | Homo sapiens cathepsin F (CTSF), mRNA. |
| LASS6 | -3.17 | Homo sapiens LAG1 homolog, ceramide synthase 6 (LASS6), mRNA. |
| LOC728787 | -3.18 | PREDICTED: Homo sapiens misc_RNA (LOC728787), miscRNA. |
| NPTX2 | -3.18 | Homo sapiens neuronal pentraxin II (NPTX2), mRNA. |
| VSTM2L | -3.18 | Homo sapiens V-set and transmembrane domain containing 2 like (VSTM2L), mRNA. |
| DYRK2 | -3.18 | Homo sapiens dual-specificity tyrosine-(Y)-phosphorylation regulated kinase 2 (DYRK2), transcript variant 1, mRNA. |
| CCDC74B | -3.18 | Homo sapiens coiled-coil domain containing 74B (CCDC74B), mRNA. |
| SV2A | -3.18 | Homo sapiens synaptic vesicle glycoprotein 2A (SV2A), mRNA. |
| SCARB1 | -3.18 | Homo sapiens scavenger receptor class B, member 1 (SCARB1), mRNA. |
| SERINC3 | -3.19 | Homo sapiens serine incorporator 3 (SERINC3), transcript variant 1, mRNA. |
| LOC652900 | -3.19 | PREDICTED: Homo sapiens similar to SEZ6L2 protein (LOC652900), mRNA. |
| PCDHB6 | -3.19 | Homo sapiens protocadherin beta 6 (PCDHB6), mRNA. |
| FEV | -3.19 | Homo sapiens FEV (ETS oncogene family) (FEV), mRNA. |
| TECPR1 | -3.19 | Homo sapiens tectonin beta-propeller repeat containing 1 (TECPR1), mRNA. |
| GTF3C5 | -3.19 | Homo sapiens general transcription factor IIIC, polypeptide 5, 63kDa (GTF3C5), mRNA. |
| VAMP2 | -3.2 | Homo sapiens vesicle-associated membrane protein 2 (synaptobrevin 2) (VAMP2), mRNA. |
| GPS1 | -3.2 | Homo sapiens G protein pathway suppressor 1 (GPS1), transcript variant 2, mRNA. |
| RNF157 | -3.2 | Homo sapiens ring finger protein 157 (RNF157), mRNA. |
| MAP2K2 | -3.21 | Homo sapiens mitogen-activated protein kinase kinase 2 (MAP2K2), mRNA. |
| OTUB1 | -3.21 | Homo sapiens OTU domain, ubiquitin aldehyde binding 1 (OTUB1), mRNA. |
| MID1IP1 | -3.21 | Homo sapiens MID1 interacting protein 1 (gastrulation specific G12 homolog (zebrafish)) (MID1IP1), transcript variant 1, mRNA. |
| ATN1 | -3.21 | Homo sapiens atrophin 1 (ATN1), transcript variant 1, mRNA. |
| LOC440895 | -3.22 | PREDICTED: Homo sapiens LIM and senescent cell antigen-like domains 3-like, transcript variant 1 (LOC440895), mRNA. |
| TMEM109 | -3.22 | Homo sapiens transmembrane protein 109 (TMEM109), mRNA. |
| FAM73B | -3.22 | Homo sapiens family with sequence similarity 73, member B (FAM73B), mRNA. |
| VANGL2 | -3.22 | Homo sapiens vang-like 2 (van gogh, Drosophila) (VANGL2), mRNA. |
| GRIK5 | -3.22 | Homo sapiens glutamate receptor, ionotropic, kainate 5 (GRIK5), mRNA. |
| C17orf101 | -3.22 | Homo sapiens chromosome 17 open reading frame 101 (C17orf101), transcript variant 2, mRNA. |
| CNTFR | -3.22 | Homo sapiens ciliary neurotrophic factor receptor (CNTFR), transcript variant 2, mRNA. |
| KLF6 | -3.23 | Homo sapiens Kruppel-like factor 6 (KLF6), transcript variant 1, mRNA. |
| SIGMAR1 | -3.23 | Homo sapiens sigma non-opioid intracellular receptor 1 (SIGMAR1), transcript variant 1, mRNA. |
| EPHB4 | -3.24 | Homo sapiens EPH receptor B4 (EPHB4), mRNA. |
| LOC399491 | -3.24 | PREDICTED: Homo sapiens misc_RNA (LOC399491), miscRNA. |
| GHDC | -3.24 | Homo sapiens GH3 domain containing (GHDC), mRNA. |
| SERINC2 | -3.24 | Homo sapiens serine incorporator 2 (SERINC2), mRNA. |
| DYRK2 | -3.24 | Homo sapiens dual-specificity tyrosine-(Y)-phosphorylation regulated kinase 2 (DYRK2), transcript variant 1, mRNA. |
| NFATC2IP | -3.24 | Homo sapiens nuclear factor of activated T-cells, cytoplasmic, calcineurin-dependent 2 interacting protein (NFATC2IP), mRNA. |
| LOC389049 | -3.25 | PREDICTED: Homo sapiens misc_RNA (LOC389049), miscRNA. |
|  | -3.25 | Homo sapiens cDNA FLJ31750 fis, clone NT2RI2007406 |
| GIT1 | -3.25 | Homo sapiens G protein-coupled receptor kinase interacting ArfGAP 1 (GIT1), transcript variant 2, mRNA. |
| SEMA5A | -3.25 | Homo sapiens sema domain, seven thrombospondin repeats (type 1 and type 1-like), transmembrane domain (TM) and short cytoplasmic domain, (semaphorin) 5A (SEMA5A), mRNA. |
| LOC728903 | -3.25 | PREDICTED: Homo sapiens hypothetical LOC728903, transcript variant 1 (LOC728903), mRNA. |
| PKM2 | -3.25 | Homo sapiens pyruvate kinase, muscle (PKM2), transcript variant 2, mRNA. |
| GNAI2 | -3.26 | Homo sapiens guanine nucleotide binding protein (G protein), alpha inhibiting activity polypeptide 2 (GNAI2), transcript variant 1, mRNA. |
| SRRM2 | -3.26 | Homo sapiens serine/arginine repetitive matrix 2 (SRRM2), mRNA. |
| FZD2 | -3.26 | Homo sapiens frizzled homolog 2 (Drosophila) (FZD2), mRNA. |
| SH3KBP1 | -3.27 | Homo sapiens SH3-domain kinase binding protein 1 (SH3KBP1), transcript variant 1, mRNA. |
| SGSH | -3.27 | Homo sapiens N-sulfoglucosamine sulfohydrolase (sulfamidase) (SGSH), mRNA. |
| PTPLAD1 | -3.27 | Homo sapiens protein tyrosine phosphatase-like A domain containing 1 (PTPLAD1), mRNA. |
| CENTG2 | -3.27 | Homo sapiens centaurin, gamma 2 (CENTG2), mRNA. |
| AGPAT1 | -3.27 | Homo sapiens 1-acylglycerol-3-phosphate O-acyltransferase 1 (lysophosphatidic acid acyltransferase, alpha) (AGPAT1), transcript variant 1, mRNA. |
| TWIST1 | -3.27 | Homo sapiens twist homolog 1 (Drosophila) (TWIST1), mRNA. |
| GAS6 | -3.28 | Homo sapiens growth arrest-specific 6 (GAS6), mRNA. |
| ERCC3 | -3.28 | Homo sapiens excision repair cross-complementing rodent repair deficiency, complementation group 3 (xeroderma pigmentosum group B complementing) (ERCC3), mRNA. |
| PC | -3.28 | Homo sapiens pyruvate carboxylase (PC), nuclear gene encoding mitochondrial protein, transcript variant 2, mRNA. |
| PRKCSH | -3.28 | Homo sapiens protein kinase C substrate 80K-H (PRKCSH), transcript variant 2, mRNA. |
| DYRK1B | -3.29 | Homo sapiens dual-specificity tyrosine-(Y)-phosphorylation regulated kinase 1B (DYRK1B), transcript variant c, mRNA. |
| LOC100132324 | -3.29 | PREDICTED: Homo sapiens misc_RNA (LOC100132324), miscRNA. |
| TMEM198 | -3.29 | Homo sapiens transmembrane protein 198 (TMEM198), mRNA. |
| RADIL | -3.29 | Homo sapiens Ras association and DIL domains (RADIL), mRNA. |
| FAM57B | -3.29 | Homo sapiens family with sequence similarity 57, member B (FAM57B), mRNA. |
| PPP2R3B | -3.3 | Homo sapiens protein phosphatase 2 (formerly 2A), regulatory subunit B'', beta (PPP2R3B), transcript variant 1, mRNA. |
| ST5 | -3.3 | Homo sapiens suppression of tumorigenicity 5 (ST5), transcript variant 3, mRNA. |
| ATP2B4 | -3.3 | Homo sapiens ATPase, Ca++ transporting, plasma membrane 4 (ATP2B4), transcript variant 2, mRNA. |
| ANKRD13B | -3.3 | Homo sapiens ankyrin repeat domain 13B (ANKRD13B), mRNA. |
| GAA | -3.3 | Homo sapiens glucosidase, alpha; acid (GAA), transcript variant 1, mRNA. |
| FBLN2 | -3.31 | Homo sapiens fibulin 2 (FBLN2), transcript variant 2, mRNA. |
| CPSF3L | -3.31 | Homo sapiens cleavage and polyadenylation specific factor 3-like (CPSF3L), mRNA. |
| LOC647000 | -3.31 | PREDICTED: Homo sapiens similar to tubulin, beta 5 (LOC647000), mRNA. |
| SEMA5A | -3.31 | Homo sapiens sema domain, seven thrombospondin repeats (type 1 and type 1-like), transmembrane domain (TM) and short cytoplasmic domain, (semaphorin) 5A (SEMA5A), mRNA. |
| PCGF1 | -3.32 | Homo sapiens polycomb group ring finger 1 (PCGF1), mRNA. |
| TTC32 | -3.32 | Homo sapiens tetratricopeptide repeat domain 32 (TTC32), mRNA. |
| MKNK2 | -3.32 | Homo sapiens MAP kinase interacting serine/threonine kinase 2 (MKNK2), transcript variant 2, mRNA. |
| CALCOCO1 | -3.32 | Homo sapiens calcium binding and coiled-coil domain 1 (CALCOCO1), mRNA. |
| TDG | -3.33 | Homo sapiens thymine-DNA glycosylase (TDG), mRNA. |
| SNORA67 | -3.33 | Homo sapiens small nucleolar RNA, H/ACA box 67 (SNORA67), small nucleolar RNA. |
| FOXM1 | -3.34 | Homo sapiens forkhead box M1 (FOXM1), transcript variant 2, mRNA. |
| MIB2 | -3.34 | Homo sapiens mindbomb homolog 2 (Drosophila) (MIB2), mRNA. |
| AGAP8 | -3.35 | Homo sapiens ArfGAP with GTPase domain, ankyrin repeat and PH domain 8 (AGAP8), mRNA. |
| DGCR2 | -3.35 | Homo sapiens DiGeorge syndrome critical region gene 2 (DGCR2), mRNA. |
| CADM4 | -3.35 | Homo sapiens cell adhesion molecule 4 (CADM4), mRNA. |
| S100A4 | -3.35 | Homo sapiens S100 calcium binding protein A4 (S100A4), transcript variant 2, mRNA. |
| EIF3B | -3.36 | Homo sapiens eukaryotic translation initiation factor 3, subunit B (EIF3B), transcript variant 2, mRNA. |
| RAB5C | -3.36 | Homo sapiens RAB5C, member RAS oncogene family (RAB5C), transcript variant 1, mRNA. |
| OGDHL | -3.36 | Homo sapiens oxoglutarate dehydrogenase-like (OGDHL), mRNA. |
| SCAND1 | -3.36 | Homo sapiens SCAN domain containing 1 (SCAND1), transcript variant 1, mRNA. |
| LOC100129211 | -3.37 | PREDICTED: Homo sapiens hypothetical protein LOC100129211 (LOC100129211), mRNA. |
| ESPL1 | -3.37 | Homo sapiens extra spindle pole bodies homolog 1 (S. cerevisiae) (ESPL1), mRNA. |
| ZP3 | -3.38 | Homo sapiens zona pellucida glycoprotein 3 (sperm receptor) (ZP3), mRNA. |
| APC2 | -3.38 | Homo sapiens adenomatosis polyposis coli 2 (APC2), mRNA. |
| GPRIN1 | -3.39 | Homo sapiens G protein regulated inducer of neurite outgrowth 1 (GPRIN1), mRNA. |
| PTGES2 | -3.39 | Homo sapiens prostaglandin E synthase 2 (PTGES2), transcript variant 2, mRNA. |
| NPLOC4 | -3.4 | Homo sapiens nuclear protein localization 4 homolog (S. cerevisiae) (NPLOC4), mRNA. |
| SPIN1 | -3.4 | Homo sapiens spindlin 1 (SPIN1), mRNA. |
| ODZ3 | -3.41 | Homo sapiens odz, odd Oz/ten-m homolog 3 (Drosophila) (ODZ3), mRNA. |
| DCTN1 | -3.41 | Homo sapiens dynactin 1 (p150, glued homolog, Drosophila) (DCTN1), transcript variant 1, mRNA. |
| PEX14 | -3.42 | Homo sapiens peroxisomal biogenesis factor 14 (PEX14), mRNA. |
| TAPBP | -3.42 | Homo sapiens TAP binding protein (tapasin) (TAPBP), transcript variant 1, mRNA. |
| CENPT | -3.42 | Homo sapiens centromere protein T (CENPT), mRNA. |
| BCL2L1 | -3.42 | Homo sapiens BCL2-like 1 (BCL2L1), nuclear gene encoding mitochondrial protein, transcript variant 1, mRNA. |
| NUMA1 | -3.42 | Homo sapiens nuclear mitotic apparatus protein 1 (NUMA1), mRNA. |
| BEGAIN | -3.43 | Homo sapiens brain-enriched guanylate kinase-associated homolog (rat) (BEGAIN), mRNA. |
| FAM195B | -3.43 | Homo sapiens family with sequence similarity 195, member B (FAM195B), transcript variant 1, mRNA. |
| FOXM1 | -3.44 | Homo sapiens forkhead box M1 (FOXM1), transcript variant 3, mRNA. |
| SLC25A29 | -3.45 | Homo sapiens solute carrier family 25, member 29 (SLC25A29), nuclear gene encoding mitochondrial protein, mRNA. |
| SBNO2 | -3.45 | Homo sapiens strawberry notch homolog 2 (Drosophila) (SBNO2), transcript variant 1, mRNA. |
| HDGF | -3.45 | Homo sapiens hepatoma-derived growth factor (high-mobility group protein 1-like) (HDGF), mRNA. |
| RNF126 | -3.46 | Homo sapiens ring finger protein 126 (RNF126), mRNA. |
| PACS1 | -3.46 | Homo sapiens phosphofurin acidic cluster sorting protein 1 (PACS1), mRNA. |
| DULLARD | -3.46 | Homo sapiens dullard homolog (Xenopus laevis) (DULLARD), mRNA. |
| SLC6A8 | -3.47 | Homo sapiens solute carrier family 6 (neurotransmitter transporter, creatine), member 8 (SLC6A8), mRNA. |
| TUBGCP2 | -3.47 | Homo sapiens tubulin, gamma complex associated protein 2 (TUBGCP2), mRNA. |
| PELP1 | -3.47 | Homo sapiens proline, glutamic acid and leucine rich protein 1 (PELP1), mRNA. |
| C20orf117 | -3.47 | Homo sapiens chromosome 20 open reading frame 117 (C20orf117), transcript variant 2, mRNA. |
| NR1H2 | -3.47 | Homo sapiens nuclear receptor subfamily 1, group H, member 2 (NR1H2), mRNA. |
| LRRN3 | -3.48 | Homo sapiens leucine rich repeat neuronal 3 (LRRN3), transcript variant 1, mRNA. |
| LOC652330 | -3.49 | PREDICTED: Homo sapiens similar to neurofibromin 1 (neurofibromatosis, von Recklinghausen disease, Watson disease) (LOC652330), mRNA. |
| APLP1 | -3.49 | Homo sapiens amyloid beta (A4) precursor-like protein 1 (APLP1), transcript variant 1, mRNA. |
| TSPO | -3.49 | Homo sapiens translocator protein (18kDa) (TSPO), transcript variant PBR-S, mRNA. |
| CIC | -3.49 | Homo sapiens capicua homolog (Drosophila) (CIC), mRNA. |
| AAK1 | -3.49 | Homo sapiens AP2 associated kinase 1 (AAK1), mRNA. |
| CCL2 | -3.49 | Homo sapiens chemokine (C-C motif) ligand 2 (CCL2), mRNA. |
| MAF1 | -3.49 | Homo sapiens MAF1 homolog (S. cerevisiae) (MAF1), mRNA. |
| C9orf140 | -3.5 | Homo sapiens chromosome 9 open reading frame 140 (C9orf140), mRNA. |
| MGP | -3.5 | Homo sapiens matrix Gla protein (MGP), mRNA. |
| FBLN1 | -3.5 | Homo sapiens fibulin 1 (FBLN1), transcript variant A, mRNA. |
| GAS6 | -3.5 | Homo sapiens growth arrest-specific 6 (GAS6), mRNA. |
| LOC100134530 | -3.5 | PREDICTED: Homo sapiens hypothetical protein LOC100134530 (LOC100134530), mRNA. |
| C14orf102 | -3.5 | Homo sapiens chromosome 14 open reading frame 102 (C14orf102), transcript variant 2, mRNA. |
| SHC3 | -3.51 | Homo sapiens SHC (Src homology 2 domain containing) transforming protein 3 (SHC3), mRNA. |
| ENDOG | -3.51 | Homo sapiens endonuclease G (ENDOG), nuclear gene encoding mitochondrial protein, mRNA. |
| FLJ22184 | -3.51 | PREDICTED: Homo sapiens hypothetical protein FLJ22184 (FLJ22184), mRNA. |
| C12orf34 | -3.51 | Homo sapiens chromosome 12 open reading frame 34 (C12orf34), mRNA. |
| ZNF385A | -3.52 | Homo sapiens zinc finger protein 385A (ZNF385A), transcript variant 3, mRNA. |
| LOC728908 | -3.52 | PREDICTED: Homo sapiens misc_RNA (LOC728908), miscRNA. |
| RFNG | -3.53 | PREDICTED: Homo sapiens radical fringe homolog (Drosophila) (RFNG), mRNA. |
| CHCHD10 | -3.54 | Homo sapiens coiled-coil-helix-coiled-coil-helix domain containing 10 (CHCHD10), mRNA. |
| IDUA | -3.54 | Homo sapiens iduronidase, alpha-L- (IDUA), mRNA. |
| ENTPD4 | -3.54 | Homo sapiens ectonucleoside triphosphate diphosphohydrolase 4 (ENTPD4), mRNA. |
| ROBO3 | -3.54 | Homo sapiens roundabout, axon guidance receptor, homolog 3 (Drosophila) (ROBO3), mRNA. |
| PKM2 | -3.54 | Homo sapiens pyruvate kinase, muscle (PKM2), transcript variant 3, mRNA. |
| MBTPS1 | -3.55 | Homo sapiens membrane-bound transcription factor peptidase, site 1 (MBTPS1), mRNA. |
| EP300 | -3.55 | Homo sapiens E1A binding protein p300 (EP300), mRNA. |
| PLXNB2 | -3.57 | Homo sapiens plexin B2 (PLXNB2), mRNA. |
| DUSP8 | -3.57 | Homo sapiens dual specificity phosphatase 8 (DUSP8), mRNA. |
| PRR7 | -3.57 | Homo sapiens proline rich 7 (synaptic) (PRR7), mRNA. |
| LOC732360 | -3.58 | PREDICTED: Homo sapiens misc_RNA (LOC732360), miscRNA. |
| LOC729495 | -3.58 | PREDICTED: Homo sapiens similar to hCG1999863 (LOC729495), mRNA. |
| CXXC5 | -3.58 | Homo sapiens CXXC finger 5 (CXXC5), mRNA. |
| RAVER1 | -3.59 | Homo sapiens RAVER1 (RAVER1), mRNA. |
| TSPAN5 | -3.6 | Homo sapiens tetraspanin 5 (TSPAN5), mRNA. |
| UBE1 | -3.6 | Homo sapiens ubiquitin-activating enzyme E1 (UBE1), transcript variant 1, mRNA. |
| SMARCB1 | -3.6 | Homo sapiens SWI/SNF related, matrix associated, actin dependent regulator of chromatin, subfamily b, member 1 (SMARCB1), transcript variant 2, mRNA. |
| PNPLA2 | -3.6 | Homo sapiens patatin-like phospholipase domain containing 2 (PNPLA2), mRNA. |
| HYAL2 | -3.6 | Homo sapiens hyaluronoglucosaminidase 2 (HYAL2), transcript variant 1, mRNA. |
| VAC14 | -3.6 | Homo sapiens Vac14 homolog (S. cerevisiae) (VAC14), mRNA. |
| NR1D2 | -3.61 | PREDICTED: Homo sapiens nuclear receptor subfamily 1, group D, member 2 (NR1D2), mRNA. |
| TMX4 | -3.62 | Homo sapiens thioredoxin-related transmembrane protein 4 (TMX4), mRNA. |
| UCKL1 | -3.62 | Homo sapiens uridine-cytidine kinase 1-like 1 (UCKL1), mRNA. |
| LGALS3BP | -3.62 | Homo sapiens lectin, galactoside-binding, soluble, 3 binding protein (LGALS3BP), mRNA. |
| PDPR | -3.64 | PREDICTED: Homo sapiens pyruvate dehydrogenase phosphatase regulatory subunit (PDPR), mRNA. |
| CYTH1 | -3.64 | Homo sapiens cytohesin 1 (CYTH1), transcript variant 2, mRNA. |
| KIAA0195 | -3.64 | Homo sapiens KIAA0195 (KIAA0195), mRNA. |
| TRPC4AP | -3.65 | Homo sapiens transient receptor potential cation channel, subfamily C, member 4 associated protein (TRPC4AP), transcript variant 1, mRNA. |
| GOLIM4 | -3.66 | Homo sapiens golgi integral membrane protein 4 (GOLIM4), mRNA. |
| FAM107B | -3.66 | Homo sapiens family with sequence similarity 107, member B (FAM107B), mRNA. |
| ST6GALNAC4 | -3.66 | Homo sapiens ST6 (alpha-N-acetyl-neuraminyl-2,3-beta-galactosyl-1, 3)-N-acetylgalactosaminide alpha-2,6-sialyltransferase 4 (ST6GALNAC4), transcript variant 1, mRNA. |
| TCF3 | -3.67 | Homo sapiens transcription factor 3 (E2A immunoglobulin enhancer binding factors E12/E47) (TCF3), mRNA. |
| KCTD13 | -3.67 | Homo sapiens potassium channel tetramerisation domain containing 13 (KCTD13), mRNA. |
| IL11RA | -3.68 | Homo sapiens interleukin 11 receptor, alpha (IL11RA), transcript variant 1, mRNA. |
| CRABP1 | -3.68 | Homo sapiens cellular retinoic acid binding protein 1 (CRABP1), mRNA. |
| CD151 | -3.68 | Homo sapiens CD151 molecule (Raph blood group) (CD151), transcript variant 4, mRNA. |
| ACTN4 | -3.69 | Homo sapiens actinin, alpha 4 (ACTN4), mRNA. |
| MAPK7 | -3.7 | Homo sapiens mitogen-activated protein kinase 7 (MAPK7), transcript variant 2, mRNA. |
| CHERP | -3.7 | Homo sapiens calcium homeostasis endoplasmic reticulum protein (CHERP), mRNA. |
| IPO4 | -3.7 | Homo sapiens importin 4 (IPO4), mRNA. |
| PCNXL3 | -3.71 | PREDICTED: Homo sapiens pecanex-like 3 (Drosophila), transcript variant 1 (PCNXL3), mRNA. |
| AGRN | -3.71 | Homo sapiens agrin (AGRN), mRNA. |
| LY6E | -3.71 | Homo sapiens lymphocyte antigen 6 complex, locus E (LY6E), mRNA. |
| JUP | -3.71 | Homo sapiens junction plakoglobin (JUP), transcript variant 1, mRNA. |
| RNF26 | -3.72 | Homo sapiens ring finger protein 26 (RNF26), mRNA. |
| DUS1L | -3.72 | Homo sapiens dihydrouridine synthase 1-like (S. cerevisiae) (DUS1L), mRNA. |
| OGFR | -3.72 | Homo sapiens opioid growth factor receptor (OGFR), mRNA. |
| GRK6 | -3.72 | Homo sapiens G protein-coupled receptor kinase 6 (GRK6), transcript variant 2, mRNA. |
| MEGF8 | -3.74 | Homo sapiens multiple EGF-like-domains 8 (MEGF8), mRNA. |
| POLD1 | -3.74 | Homo sapiens polymerase (DNA directed), delta 1, catalytic subunit 125kDa (POLD1), mRNA. |
| RAD23A | -3.74 | Homo sapiens RAD23 homolog A (S. cerevisiae) (RAD23A), mRNA. |
| CHST14 | -3.74 | Homo sapiens carbohydrate (N-acetylgalactosamine 4-0) sulfotransferase 14 (CHST14), mRNA. |
| NUCKS1 | -3.74 | Homo sapiens nuclear casein kinase and cyclin-dependent kinase substrate 1 (NUCKS1), mRNA. |
| PLTP | -3.74 | Homo sapiens phospholipid transfer protein (PLTP), transcript variant 2, mRNA. |
| OGDH | -3.75 | Homo sapiens oxoglutarate (alpha-ketoglutarate) dehydrogenase (lipoamide) (OGDH), nuclear gene encoding mitochondrial protein, transcript variant 1, mRNA. |
| DTX3 | -3.75 | Homo sapiens deltex homolog 3 (Drosophila) (DTX3), mRNA. |
| MTA1 | -3.75 | Homo sapiens metastasis associated 1 (MTA1), mRNA. |
| NLGN4X | -3.76 | Homo sapiens neuroligin 4, X-linked (NLGN4X), transcript variant 2, mRNA. |
| GATAD2B | -3.77 | Homo sapiens GATA zinc finger domain containing 2B (GATAD2B), mRNA. |
| SREBF1 | -3.77 | Homo sapiens sterol regulatory element binding transcription factor 1 (SREBF1), transcript variant 1, mRNA. |
| DKK1 | -3.78 | Homo sapiens dickkopf homolog 1 (Xenopus laevis) (DKK1), mRNA. |
| ZYX | -3.78 | Homo sapiens zyxin (ZYX), transcript variant 1, mRNA. |
| GIPC1 | -3.78 | Homo sapiens GIPC PDZ domain containing family, member 1 (GIPC1), transcript variant 3, mRNA. |
| C7orf26 | -3.81 | Homo sapiens chromosome 7 open reading frame 26 (C7orf26), mRNA. |
| TBX3 | -3.83 | Homo sapiens T-box 3 (TBX3), transcript variant 1, mRNA. |
| METRN | -3.83 | Homo sapiens meteorin, glial cell differentiation regulator (METRN), mRNA. |
| MT1A | -3.84 | Homo sapiens metallothionein 1A (MT1A), mRNA. |
| LPPR2 | -3.84 | Homo sapiens lipid phosphate phosphatase-related protein type 2 (LPPR2), mRNA. |
| LOC100132394 | -3.85 | PREDICTED: Homo sapiens hypothetical protein LOC100132394 (LOC100132394), mRNA. |
| NCOR2 | -3.85 | Homo sapiens nuclear receptor co-repressor 2 (NCOR2), transcript variant 1, mRNA. |
| DCN | -3.86 | Homo sapiens decorin (DCN), transcript variant C, mRNA. |
| SSBP3 | -3.86 | Homo sapiens single stranded DNA binding protein 3 (SSBP3), transcript variant 2, mRNA. |
|  | -3.87 | zr87e09.r1 NCI_CGAP_GCB1 Homo sapiens cDNA clone IMAGE:682696 5, mRNA sequence |
| SPRY1 | -3.87 | Homo sapiens sprouty homolog 1, antagonist of FGF signaling (Drosophila) (SPRY1), transcript variant 2, mRNA. |
| LOC653171 | -3.87 | PREDICTED: Homo sapiens similar to MAPK-interacting and spindle-stabilizing protein (LOC653171), mRNA. |
| SSBP4 | -3.87 | Homo sapiens single stranded DNA binding protein 4 (SSBP4), transcript variant 1, mRNA. |
| IGFBP4 | -3.88 | Homo sapiens insulin-like growth factor binding protein 4 (IGFBP4), mRNA. |
| LOC100008589 | -3.89 | Homo sapiens 28S ribosomal RNA (LOC100008589), non-coding RNA. |
| ARFGAP1 | -3.89 | Homo sapiens ADP-ribosylation factor GTPase activating protein 1 (ARFGAP1), transcript variant 1, mRNA. |
| DMWD | -3.89 | Homo sapiens dystrophia myotonica, WD repeat containing (DMWD), mRNA. |
| POMZP3 | -3.89 | Homo sapiens POM (POM121 homolog, rat) and ZP3 fusion (POMZP3), transcript variant 2, mRNA. |
| CPT1B | -3.92 | Homo sapiens carnitine palmitoyltransferase 1B (muscle) (CPT1B), nuclear gene encoding mitochondrial protein, transcript variant 3, mRNA. |
| MACROD1 | -3.92 | Homo sapiens MACRO domain containing 1 (MACROD1), mRNA. |
| LMF2 | -3.93 | Homo sapiens lipase maturation factor 2 (LMF2), mRNA. |
| MYO1C | -3.93 | Homo sapiens myosin IC (MYO1C), transcript variant 3, mRNA. |
| LOC729495 | -3.94 | PREDICTED: Homo sapiens similar to hCG1999863 (LOC729495), mRNA. |
| CSNK1E | -3.95 | Homo sapiens casein kinase 1, epsilon (CSNK1E), transcript variant 1, mRNA. |
| LSS | -3.96 | Homo sapiens lanosterol synthase (2,3-oxidosqualene-lanosterol cyclase) (LSS), transcript variant 1, mRNA. |
| FLNA | -3.97 | Homo sapiens filamin A, alpha (actin binding protein 280) (FLNA), mRNA. |
| NYNRIN | -3.97 | Homo sapiens NYN domain and retroviral integrase containing (NYNRIN), mRNA. |
| TNFRSF6B | -3.98 | Homo sapiens tumor necrosis factor receptor superfamily, member 6b, decoy (TNFRSF6B), transcript variant M68C, mRNA. |
| SFRS8 | -3.98 | Homo sapiens splicing factor, arginine/serine-rich 8 (suppressor-of-white-apricot homolog, Drosophila) (SFRS8), mRNA. |
| FBXL6 | -3.98 | Homo sapiens F-box and leucine-rich repeat protein 6 (FBXL6), transcript variant 2, mRNA. |
| PCDH17 | -3.99 | Homo sapiens protocadherin 17 (PCDH17), mRNA. |
| IRF2BP1 | -3.99 | Homo sapiens interferon regulatory factor 2 binding protein 1 (IRF2BP1), mRNA. |
| IGFBP2 | -4 | Homo sapiens insulin-like growth factor binding protein 2, 36kDa (IGFBP2), mRNA. |
| ATP6V0C | -4.03 | PREDICTED: Homo sapiens ATPase, H+ transporting, lysosomal 16kDa, V0 subunit c (ATP6V0C), mRNA. |
| TLX2 | -4.03 | Homo sapiens T-cell leukemia homeobox 2 (TLX2), mRNA. |
| MIB2 | -4.03 | Homo sapiens mindbomb homolog 2 (Drosophila) (MIB2), mRNA. |
| LOC407835 | -4.04 | Homo sapiens mitogen-activated protein kinase kinase 2 pseudogene (LOC407835), non-coding RNA. |
| TAGLN | -4.04 | Homo sapiens transgelin (TAGLN), transcript variant 2, mRNA. |
| INO80E | -4.06 | Homo sapiens INO80 complex subunit E (INO80E), mRNA. |
| GNB2 | -4.06 | Homo sapiens guanine nucleotide binding protein (G protein), beta polypeptide 2 (GNB2), mRNA. |
| TUBB4 | -4.06 | Homo sapiens tubulin, beta 4 (TUBB4), mRNA. |
| PODXL2 | -4.06 | Homo sapiens podocalyxin-like 2 (PODXL2), mRNA. |
| CST3 | -4.07 | Homo sapiens cystatin C (CST3), mRNA. |
| UCP2 | -4.08 | Homo sapiens uncoupling protein 2 (mitochondrial, proton carrier) (UCP2), nuclear gene encoding mitochondrial protein, mRNA. |
| LDOC1 | -4.08 | Homo sapiens leucine zipper, down-regulated in cancer 1 (LDOC1), mRNA. |
| C17orf62 | -4.09 | Homo sapiens chromosome 17 open reading frame 62 (C17orf62), transcript variant 3, mRNA. |
| LOC153561 | -4.1 | Homo sapiens hypothetical protein LOC153561 (LOC153561), mRNA. |
| LOC729859 | -4.11 | PREDICTED: Homo sapiens misc_RNA (LOC729859), miscRNA. |
| TBC1D24 | -4.11 | Homo sapiens TBC1 domain family, member 24 (TBC1D24), mRNA. XM_945422 |
| SSBP3 | -4.11 | Homo sapiens single stranded DNA binding protein 3 (SSBP3), transcript variant 2, mRNA. |
| NPDC1 | -4.12 | Homo sapiens neural proliferation, differentiation and control, 1 (NPDC1), mRNA. |
| PPDPF | -4.12 | Homo sapiens pancreatic progenitor cell differentiation and proliferation factor homolog (zebrafish) (PPDPF), mRNA. |
| MAP3K11 | -4.12 | Homo sapiens mitogen-activated protein kinase kinase kinase 11 (MAP3K11), mRNA. |
| NCOR2 | -4.13 | Homo sapiens nuclear receptor co-repressor 2 (NCOR2), transcript variant 2, mRNA. |
| INTS1 | -4.13 | PREDICTED: Homo sapiens integrator complex subunit 1, transcript variant 11 (INTS1), mRNA. |
| LOC644988 | -4.14 | PREDICTED: Homo sapiens misc_RNA (LOC644988), miscRNA. |
| FBRS | -4.14 | Homo sapiens fibrosin (FBRS), mRNA. |
| SEPN1 | -4.15 | Homo sapiens selenoprotein N, 1 (SEPN1), transcript variant 2, mRNA. |
| FBXL16 | -4.16 | Homo sapiens F-box and leucine-rich repeat protein 16 (FBXL16), mRNA. |
| GTF3C1 | -4.16 | Homo sapiens general transcription factor IIIC, polypeptide 1, alpha 220kDa (GTF3C1), mRNA. |
| LRRC4B | -4.17 | Homo sapiens leucine rich repeat containing 4B (LRRC4B), mRNA. |
| EMILIN1 | -4.17 | Homo sapiens elastin microfibril interfacer 1 (EMILIN1), mRNA. |
| MIR25 | -4.18 | Homo sapiens microRNA 25 (MIR25), microRNA. |
| TBX2 | -4.18 | Homo sapiens T-box 2 (TBX2), mRNA. |
| SMARCB1 | -4.19 | Homo sapiens SWI/SNF related, matrix associated, actin dependent regulator of chromatin, subfamily b, member 1 (SMARCB1), transcript variant 2, mRNA. |
| IGFBP3 | -4.19 | Homo sapiens insulin-like growth factor binding protein 3 (IGFBP3), transcript variant 2, mRNA. |
| TSC2 | -4.22 | Homo sapiens tuberous sclerosis 2 (TSC2), transcript variant 4, mRNA. |
| PHOX2A | -4.22 | Homo sapiens paired-like homeobox 2a (PHOX2A), mRNA. |
| MNT | -4.24 | Homo sapiens MAX binding protein (MNT), mRNA. |
| ASF1B | -4.25 | Homo sapiens ASF1 anti-silencing function 1 homolog B (S. cerevisiae) (ASF1B), mRNA. |
| LOC100134364 | -4.26 | PREDICTED: Homo sapiens hypothetical protein LOC100134364 (LOC100134364), mRNA. |
| SLC4A2 | -4.26 | Homo sapiens solute carrier family 4, anion exchanger, member 2 (erythrocyte membrane protein band 3-like 1) (SLC4A2), mRNA. |
| LRDD | -4.26 | Homo sapiens leucine-rich repeats and death domain containing (LRDD), transcript variant 3, mRNA. |
| G6PC3 | -4.27 | Homo sapiens glucose 6 phosphatase, catalytic, 3 (G6PC3), mRNA. |
| COBRA1 | -4.27 | Homo sapiens cofactor of BRCA1 (COBRA1), mRNA. |
| MAP1S | -4.28 | Homo sapiens microtubule-associated protein 1S (MAP1S), mRNA. |
| AP2A1 | -4.3 | Homo sapiens adaptor-related protein complex 2, alpha 1 subunit (AP2A1), transcript variant 2, mRNA. |
| COL6A2 | -4.33 | Homo sapiens collagen, type VI, alpha 2 (COL6A2), transcript variant 2C2, mRNA. |
| LOC653438 | -4.34 | PREDICTED: Homo sapiens similar to CG33096-PB, isoform B, transcript variant 1 (LOC653438), mRNA. |
| MGRN1 | -4.36 | Homo sapiens mahogunin, ring finger 1 (MGRN1), mRNA. |
| ELN | -4.36 | Homo sapiens elastin (ELN), transcript variant 4, mRNA. |
| SLC38A10 | -4.37 | Homo sapiens solute carrier family 38, member 10 (SLC38A10), transcript variant 1, mRNA. |
| LOC392437 | -4.38 | PREDICTED: Homo sapiens misc_RNA (LOC392437), miscRNA. |
| ITFG3 | -4.38 | Homo sapiens integrin alpha FG-GAP repeat containing 3 (ITFG3), mRNA. |
| CHTF18 | -4.38 | Homo sapiens CTF18, chromosome transmission fidelity factor 18 homolog (S. cerevisiae) (CHTF18), mRNA. |
| OLFML3 | -4.4 | Homo sapiens olfactomedin-like 3 (OLFML3), mRNA. |
| CENTG3 | -4.41 | Homo sapiens centaurin, gamma 3 (CENTG3), mRNA. |
| MT2A | -4.41 | Homo sapiens metallothionein 2A (MT2A), mRNA. |
| LOC731314 | -4.42 | PREDICTED: Homo sapiens similar to H2A histone family, member X (LOC731314), mRNA. |
| UBE2O | -4.43 | Homo sapiens ubiquitin-conjugating enzyme E2O (UBE2O), mRNA. |
| UNC5B | -4.44 | Homo sapiens unc-5 homolog B (C. elegans) (UNC5B), mRNA. |
| FAM108A2 | -4.44 | PREDICTED: Homo sapiens family with sequence similarity 108, member A2 (FAM108A2), mRNA. |
| ZNF423 | -4.44 | Homo sapiens zinc finger protein 423 (ZNF423), mRNA. |
| LOC338799 | -4.45 | Homo sapiens hypothetical locus LOC338799 (LOC338799), non-coding RNA. |
| PCSK1N | -4.46 | Homo sapiens proprotein convertase subtilisin/kexin type 1 inhibitor (PCSK1N), mRNA. |
| NFIX | -4.47 | Homo sapiens nuclear factor I/X (CCAAT-binding transcription factor) (NFIX), mRNA. |
| MBOAT7 | -4.47 | Homo sapiens membrane bound O-acyltransferase domain containing 7 (MBOAT7), mRNA. |
| FBLN7 | -4.48 | Homo sapiens fibulin 7 (FBLN7), mRNA. |
| MGAT4B | -4.48 | Homo sapiens mannosyl (alpha-1,3-)-glycoprotein beta-1,4-N-acetylglucosaminyltransferase, isozyme B (MGAT4B), transcript variant 2, mRNA. |
| TACC3 | -4.48 | Homo sapiens transforming, acidic coiled-coil containing protein 3 (TACC3), mRNA. |
| ARMC5 | -4.5 | Homo sapiens armadillo repeat containing 5 (ARMC5), mRNA. |
| TRAPPC9 | -4.54 | Homo sapiens trafficking protein particle complex 9 (TRAPPC9), mRNA. |
| MAPK8IP3 | -4.54 | Homo sapiens mitogen-activated protein kinase 8 interacting protein 3 (MAPK8IP3), transcript variant 2, mRNA. |
| COL6A1 | -4.6 | Homo sapiens collagen, type VI, alpha 1 (COL6A1), mRNA. |
| LTBP3 | -4.62 | Homo sapiens latent transforming growth factor beta binding protein 3 (LTBP3), mRNA. |
| IRF2BP2 | -4.64 | Homo sapiens interferon regulatory factor 2 binding protein 2 (IRF2BP2), transcript variant 1, mRNA. |
| BAT2 | -4.65 | Homo sapiens HLA-B associated transcript 2 (BAT2), transcript variant 1, mRNA. |
| PALM | -4.68 | Homo sapiens paralemmin (PALM), transcript variant 1, mRNA. |
| TRIM28 | -4.7 | Homo sapiens tripartite motif-containing 28 (TRIM28), mRNA. |
| ADAM15 | -4.7 | Homo sapiens ADAM metallopeptidase domain 15 (ADAM15), transcript variant 2, mRNA. |
| PKMYT1 | -4.72 | Homo sapiens protein kinase, membrane associated tyrosine/threonine 1 (PKMYT1), transcript variant 2, mRNA. |
| CDC42BPB | -4.73 | Homo sapiens CDC42 binding protein kinase beta (DMPK-like) (CDC42BPB), mRNA. |
| IGFBP5 | -4.73 | Homo sapiens insulin-like growth factor binding protein 5 (IGFBP5), mRNA. |
| MXD3 | -4.75 | Homo sapiens MAX dimerization protein 3 (MXD3), mRNA. |
| SETD1A | -4.75 | Homo sapiens SET domain containing 1A (SETD1A), mRNA. |
| ZNF423 | -4.78 | Homo sapiens zinc finger protein 423 (ZNF423), mRNA. |
| LRDD | -4.79 | Homo sapiens leucine-rich repeats and death domain containing (LRDD), transcript variant 2, mRNA. |
| KLF6 | -4.8 | Homo sapiens Kruppel-like factor 6 (KLF6), transcript variant 2, mRNA. |
| GPAA1 | -4.81 | Homo sapiens glycosylphosphatidylinositol anchor attachment protein 1 homolog (yeast) (GPAA1), mRNA. |
| ZNF580 | -4.81 | Homo sapiens zinc finger protein 580 (ZNF580), transcript variant 1, mRNA. |
| KCTD12 | -4.84 | Homo sapiens potassium channel tetramerisation domain containing 12 (KCTD12), mRNA. |
| DCAF15 | -4.86 | Homo sapiens DDB1 and CUL4 associated factor 15 (DCAF15), mRNA. |
| BSG | -4.88 | Homo sapiens basigin (Ok blood group) (BSG), transcript variant 3, mRNA. |
| DLGAP4 | -4.88 | Homo sapiens discs, large (Drosophila) homolog-associated protein 4 (DLGAP4), transcript variant 3, mRNA. |
| ID3 | -4.89 | Homo sapiens inhibitor of DNA binding 3, dominant negative helix-loop-helix protein (ID3), mRNA. |
| SF1 | -4.9 | Homo sapiens splicing factor 1 (SF1), transcript variant 4, mRNA. |
| LOC387763 | -4.91 | PREDICTED: Homo sapiens hypothetical LOC387763 (LOC387763), mRNA. |
| CNOT3 | -4.92 | Homo sapiens CCR4-NOT transcription complex, subunit 3 (CNOT3), mRNA. |
| GRINA | -4.92 | Homo sapiens glutamate receptor, ionotropic, N-methyl D-aspartate-associated protein 1 (glutamate binding) (GRINA), transcript variant 1, mRNA. |
| EHBP1L1 | -4.94 | Homo sapiens EH domain binding protein 1-like 1 (EHBP1L1), mRNA. |
| H3F3B | -4.97 | Homo sapiens H3 histone, family 3B (H3.3B) (H3F3B), mRNA. |
| DDR2 | -4.98 | Homo sapiens discoidin domain receptor tyrosine kinase 2 (DDR2), transcript variant 2, mRNA. |
| DCHS1 | -4.98 | Homo sapiens dachsous 1 (Drosophila) (DCHS1), mRNA. |
| ZNF503 | -4.99 | Homo sapiens zinc finger protein 503 (ZNF503), mRNA. |
| H2AFX | -5.04 | Homo sapiens H2A histone family, member X (H2AFX), mRNA. |
| CLPTM1 | -5.04 | Homo sapiens cleft lip and palate associated transmembrane protein 1 (CLPTM1), mRNA. |
| FAM100A | -5.07 | Homo sapiens family with sequence similarity 100, member A (FAM100A), mRNA. |
| ID1 | -5.14 | Homo sapiens inhibitor of DNA binding 1, dominant negative helix-loop-helix protein (ID1), transcript variant 2, mRNA. |
| BAT2 | -5.19 | Homo sapiens HLA-B associated transcript 2 (BAT2), mRNA. |
| SGK1 | -5.29 | Homo sapiens serum/glucocorticoid regulated kinase 1 (SGK1), transcript variant 1, mRNA. |
| SP2 | -5.3 | Homo sapiens Sp2 transcription factor (SP2), mRNA. |
| TMEM132A | -5.38 | Homo sapiens transmembrane protein 132A (TMEM132A), transcript variant 2, mRNA. |
| KIAA0319L | -5.44 | Homo sapiens KIAA0319-like (KIAA0319L), transcript variant 1, mRNA. |
| ABCA2 | -5.46 | Homo sapiens ATP-binding cassette, sub-family A (ABC1), member 2 (ABCA2), transcript variant 2, mRNA. |
| TTYH3 | -5.54 | Homo sapiens tweety homolog 3 (Drosophila) (TTYH3), mRNA. |
| AMH | -5.58 | Homo sapiens anti-Mullerian hormone (AMH), mRNA. |
| ID2 | -5.61 | Homo sapiens inhibitor of DNA binding 2, dominant negative helix-loop-helix protein (ID2), mRNA. |
| SGK1 | -5.67 | Homo sapiens serum/glucocorticoid regulated kinase 1 (SGK1), transcript variant 1, mRNA. |
| LOC730130 | -5.71 | PREDICTED: Homo sapiens hypothetical protein LOC730130 (LOC730130), mRNA. |
| H1FX | -5.91 | Homo sapiens H1 histone family, member X (H1FX), mRNA. |
| SGK | -6.24 | Homo sapiens serum/glucocorticoid regulated kinase (SGK), mRNA. |
| MIR1978 | -6.42 | Homo sapiens microRNA 1978 (MIR1978), microRNA. |
| LOC650909 | -6.44 | PREDICTED: Homo sapiens similar to activating signal cointegrator 1 complex subunit 3-like 1 (LOC650909), mRNA. |
| FAM108A3 | -6.58 | Homo sapiens family with sequence similarity 108, member A3 (FAM108A3), mRNA. |
|  | -6.67 | AGENCOURT_10229596 NIH_MGC_141 Homo sapiens cDNA clone IMAGE:6563923 5, mRNA sequence |
| ID2 | -7.02 | Homo sapiens inhibitor of DNA binding 2, dominant negative helix-loop-helix protein (ID2), mRNA. |
| SNORA12 | -7.23 | Homo sapiens small nucleolar RNA, H/ACA box 12 (SNORA12), small nucleolar RNA. |
| IGFBP5 | -7.98 | Homo sapiens insulin-like growth factor binding protein 5 (IGFBP5), mRNA. |
| YWHAE | -8.23 | Homo sapiens tyrosine 3-monooxygenase/tryptophan 5-monooxygenase activation protein, epsilon polypeptide (YWHAE), mRNA. |
| CYR61 | -16.42 | Homo sapiens cysteine-rich, angiogenic inducer, 61 (CYR61), mRNA. |
| FOS | -17.75 | Homo sapiens v-fos FBJ murine osteosarcoma viral oncogene homolog (FOS), mRNA. |
| EGR1 | -20.9 | Homo sapiens early growth response 1 (EGR1), mRNA. |
