# Supplementary material for: Plurigon: three dimensional visualization and classification of high-dimensionality data
Source: Front Physiol. 2013 Jul 22;4:190. doi: 10.3389/fphys.2013.00190 (PMC3717481; doi:10.3389/fphys.2013.00190)
Supplement: Table S16 — Conserved neuronal transcriptomic expression data from brain-derived neurotrophic factor-treated clonal human cells. Transcriptomic gene regulation was measured from brain-derived neurotrophic factor (BDNF)-treated SH-SY5Y neuronal cell batches (BDNF-1, BDNF-2, BDNF-3). The transcriptomic data from the three separate experiments was compared to gene expression levels in vehicle (phosphate-buffered saline)-treated control SH-SY5Y cells. [file 46440_Maudsley_Data_Sheet_6.ZIP › Table-S23.doc]

**Table S23. Conserved neuronal transcriptomic expression data from pre-peroxidated nerve growth factor-treated clonal human cells**. Transcriptomic gene regulation was measured from nerve growth factor (NGF)-treated SH-SY5Y neuronal cell batches (NGF-1, NGF-2, NGF-3) that were pre-conditioned to mimic oxidative neurodegeneration with a continuous seven day treatment with 10nM hydrogen peroxide. The transcriptomic data from the three separate experiments was compared to gene expression levels in vehicle (phosphate-buffered saline)-treated control SH-SY5Y cells that were also treated with 10nM hydrogen peroxide as well.

| **Gene Symbol** | **NGF-1** | **NGF-2** | **NGF-3** |
| --- | --- | --- | --- |
|  |  |  |  |
| ACAD11 | -2.03 | -2.21 | -1.86 |
| ACOT11 | -1.68 | -1.86 | -1.51 |
| ADAM23 | -2.38 | -2.55 | -2.22 |
| ADAMTS19 | -3 | -3.27 | -2.75 |
| ADD1 | 2.79 | 3.01 | 2.58 |
| ADNP2 | -2.1 | -2.25 | -1.96 |
| AHCTF1 | -2.04 | -2.20 | -1.89 |
| AKR1C3 | -2.15 | -2.69 | -1.72 |
| ALG14 | -2.46 | -2.66 | -2.28 |
| ARHGAP11B | -3.27 | -3.30 | -3.24 |
| ARPP19 | 2.7 | 3.70 | 1.97 |
| ASNS | 2.46 | 3.22 | 1.88 |
| ATP8B2 | 2.02 | 2.06 | 1.98 |
| AURKB | -2.16 | -2.53 | -1.85 |
| BAMBI | -1.77 | -1.89 | -1.65 |
| BAT3 | 2.35 | 2.77 | 1.99 |
| BBS10 | 1.77 | 1.79 | 1.75 |
| BIRC2 | -1.82 | -2.18 | -1.52 |
| BIRC5 | -2.33 | -2.96 | -1.83 |
| BTBD7 | -1.89 | -2.15 | -1.66 |
| C13orf25 | -1.76 | -1.95 | -1.59 |
| C14orf102 | -2.5 | -2.75 | -2.27 |
| C14orf138 | -1.92 | -2.07 | -1.78 |
| C14orf153 | -2.8 | -2.83 | -2.77 |
| C18orf56 | -2.84 | -3.18 | -2.54 |
| C1orf173 | -3.03 | -3.58 | -2.57 |
| C1orf63 | 1.96 | 1.98 | 1.94 |
| C1orf97 | -2.18 | -2.77 | -1.72 |
| C3orf17 | -4.01 | -4.61 | -3.49 |
| CABYR | -1.97 | -2.07 | -1.88 |
| CALCB | -2.02 | -2.14 | -1.91 |
| CALR | 2.71 | 3.01 | 2.44 |
| CAND1 | 2.22 | 2.38 | 2.07 |
| CAPRIN1 | -1.53 | -1.79 | -1.31 |
| CCDC34 | -1.67 | -2.09 | -1.34 |
| CCDC59 | -1.92 | -2.55 | -1.44 |
| CCDC90A | -2.36 | -2.45 | -2.27 |
| CDC25C | -1.88 | -2.14 | -1.65 |
| CDC2L2 | -1.73 | -2.09 | -1.43 |
| CDCA3 | -1.63 | -1.81 | -1.47 |
| CENPA | -1.83 | -2.73 | -1.23 |
| CENPM | -1.86 | -1.99 | -1.74 |
| CEPT1 | -2.31 | -2.61 | -2.04 |
| CHGB | 2.45 | 2.57 | 2.33 |
| COLEC11 | 1.92 | 2.00 | 1.85 |
| COMMD2 | -2.17 | -2.32 | -2.03 |
| COQ2 | -2.29 | -3.27 | -1.60 |
| CRLF3 | -2.48 | -2.60 | -2.36 |
| CUTC | -2.09 | -2.65 | -1.65 |
| CYB561 | 1.66 | 1.93 | 1.43 |
| CYR61 | -5.78 | -6.42 | -5.21 |
| DBR1 | 1.65 | 1.83 | 1.49 |
| DCN | -2.7 | -3.38 | -2.16 |
| DDX51 | -2.97 | -3.18 | -2.78 |
| DECR1 | 1.75 | 2.35 | 1.31 |
| DENND5B | -2.56 | -2.66 | -2.46 |
| DIAPH3 | -2.23 | -2.25 | -2.21 |
| DLX5 | -1.91 | -2.02 | -1.80 |
| DNPEP | 1.55 | 1.63 | 1.48 |
| DPP7 | 2.68 | 2.95 | 2.44 |
| DUSP1 | -1.57 | -1.79 | -1.38 |
| DUT | 1.66 | 1.69 | 1.63 |
| EGR1 | -6.81 | -9.19 | -5.04 |
| EIF4G2 | 1.79 | 1.99 | 1.61 |
| ELF2 | -2.2 | -2.73 | -1.77 |
| ERLIN2 | -2.03 | -2.21 | -1.86 |
| EWSR1 | 3.61 | 4.30 | 3.03 |
| EXOSC3 | 1.87 | 2.28 | 1.53 |
| EXOSC6 | -2.26 | -2.28 | -2.24 |
| FAM115A | 2.46 | 2.46 | 2.46 |
| FAM134B | -2.13 | -2.22 | -2.05 |
| FAM181B | -2.21 | -2.45 | -1.99 |
| FAM46A | -1.68 | -1.73 | -1.63 |
| FANCD2 | -1.83 | -2.05 | -1.63 |
| FCHSD2 | -2.1 | -2.77 | -1.59 |
| FEZ1 | -1.82 | -2.09 | -1.58 |
| FGL1 | 2.95 | 3.27 | 2.66 |
| FKBP1A | 1.9 | 1.92 | 1.88 |
| FOSB | -4.67 | -5.00 | -4.36 |
| GADD45A | -1.53 | -1.58 | -1.49 |
| GAR1 | -1.98 | -2.22 | -1.77 |
| GAS1 | -2.6 | -2.78 | -2.43 |
| GAS2L3 | -3.39 | -3.86 | -2.97 |
| GAS6 | -2.01 | -2.17 | -1.86 |
| GATA3 | -2.03 | -2.54 | -1.62 |
| GFPT1 | -2 | -2.22 | -1.80 |
| GGH | -1.93 | -2.08 | -1.79 |
| GINS4 | -2.04 | -2.10 | -1.98 |
| GLT8D1 | 2.24 | 2.49 | 2.02 |
| GNL3L | -2.71 | -2.82 | -2.61 |
| GOLPH4 | -3.2 | -3.33 | -3.08 |
| GPER | -1.92 | -2.19 | -1.68 |
| GPKOW | 1.6 | 1.70 | 1.51 |
| GTPBP8 | -1.52 | -1.60 | -1.45 |
| HIATL1 | -2.23 | -2.39 | -2.08 |
| HIST2H2AC | -1.75 | -1.79 | -1.72 |
| HIST2H2BE | 1.55 | 1.63 | 1.48 |
| HK2 | 2.03 | 2.38 | 1.74 |
| HMGB1L1 | 2.64 | 2.93 | 2.38 |
| HSDL2 | -1.66 | -1.73 | -1.60 |
| ICK | -1.74 | -2.05 | -1.47 |
| ID1 | -2.05 | -2.21 | -1.90 |
| ID2 | -3.12 | -3.21 | -3.03 |
| ID3 | -2.4 | -2.76 | -2.09 |
| IGFBP5 | -2.73 | -3.11 | -2.39 |
| IL11RA | -2.12 | -2.23 | -2.02 |
| INCENP | -2.34 | -3.09 | -1.77 |
| ING3 | -1.83 | -1.85 | -1.81 |
| IRF2BP2 | 1.74 | 2.14 | 1.41 |
| JARID1A | -2.04 | -2.12 | -1.96 |
| JUN | -2.38 | -2.43 | -2.33 |
| KCNT1 | -1.63 | -1.83 | -1.46 |
| KCTD12 | -2.58 | -2.79 | -2.39 |
| KIAA0101 | 1.78 | 2.15 | 1.47 |
| KIAA1279 | -1.94 | -1.96 | -1.92 |
| KIAA1967 | 1.64 | 2.28 | 1.18 |
| KIF11 | -2.03 | -2.11 | -1.95 |
| KIF15 | -2.79 | -3.74 | -2.08 |
| KLF11 | -1.52 | -1.55 | -1.49 |
| KLF6 | -1.76 | -1.94 | -1.60 |
| LINGO4 | -2.56 | -2.84 | -2.31 |
| LMO4 | -2.43 | -2.53 | -2.34 |
| LOC153561 | -2.05 | -2.42 | -1.74 |
| LOC402221 | 1.83 | 1.88 | 1.78 |
| LOC440145 | -1.57 | -1.92 | -1.29 |
| LOC642197 | -2.03 | -2.07 | -1.99 |
| LOC644033 | -2.22 | -2.31 | -2.13 |
| LOC645676 | 1.96 | 2.12 | 1.81 |
| LOC646786 | -2.4 | -2.59 | -2.22 |
| LPGAT1 | 1.89 | 2.00 | 1.78 |
| LRRC17 | -2.46 | -2.46 | -2.46 |
| LRRFIP1 | -2.51 | -2.66 | -2.37 |
| MAD2L1 | -1.7 | -1.94 | -1.49 |
| MAFB | -2.03 | -2.03 | -2.03 |
| MAGEL2 | 1.59 | 1.67 | 1.51 |
| MANEAL | 1.96 | 2.16 | 1.78 |
| MAP6 | 1.78 | 1.87 | 1.70 |
| MAT2B | 1.7 | 1.72 | 1.68 |
| MBD4 | -3.42 | -3.56 | -3.29 |
| MBOAT2 | -2.72 | -2.77 | -2.67 |
| MED30 | -2.04 | -2.69 | -1.55 |
| MGC16703 | 2.16 | 2.46 | 1.89 |
| MRPL35 | -1.62 | -2.96 | -0.89 |
| MRPL55 | -4.56 | -4.65 | -4.47 |
| MT1F | -1.93 | -2.57 | -1.45 |
| MTCP1 | -1.68 | -1.92 | -1.47 |
| MTUS1 | -2.47 | -2.91 | -2.09 |
| MXD3 | -1.83 | -1.85 | -1.81 |
| NDUFB10 | 2.31 | 2.91 | 1.83 |
| NDUFS2 | 1.68 | 1.97 | 1.44 |
| NFIX | -2.87 | -4.22 | -1.95 |
| NFU1 | -2.13 | -2.13 | -2.13 |
| NGFRAP1 | 2.13 | 2.24 | 2.03 |
| NLGN4X | -1.95 | -1.99 | -1.91 |
| NOP56 | -2.45 | -2.55 | -2.36 |
| NOP58 | 2.01 | 2.15 | 1.88 |
| NOTCH3 | -1.87 | -2.08 | -1.68 |
| NRAS | -2.87 | -3.50 | -2.35 |
| NTNG1 | -1.65 | -1.80 | -1.51 |
| NUDT9 | -1.84 | -1.97 | -1.72 |
| NUTF2 | 4.51 | 5.14 | 3.96 |
| ODZ3 | -2.6 | -2.60 | -2.60 |
| OIP5 | -2.14 | -2.40 | -1.91 |
| ONECUT2 | 1.73 | 1.82 | 1.65 |
| PANK2 | -2.38 | -2.52 | -2.25 |
| PCBP4 | -3.98 | -4.14 | -3.83 |
| PCDH17 | -2.59 | -3.26 | -2.06 |
| PCDH20 | -2.73 | -3.39 | -2.20 |
| PDIK1L | -2.2 | -2.22 | -2.18 |
| PHF17 | 2.86 | 2.92 | 2.80 |
| PIK3R1 | -2.44 | -2.95 | -2.02 |
| PIM1 | -1.89 | -1.93 | -1.85 |
| PLAA | 2.87 | 2.98 | 2.76 |
| PLEKHA1 | -2.27 | -2.34 | -2.20 |
| PLEKHA6 | 1.55 | 1.55 | 1.55 |
| PLS1 | -1.67 | -1.69 | -1.65 |
| POLG2 | 3.13 | 3.60 | 2.72 |
| POLR3A | -1.56 | -1.61 | -1.51 |
| PON2 | -1.6 | -1.73 | -1.48 |
| POPDC3 | -1.84 | -2.01 | -1.69 |
| PPAP2B | -1.64 | -1.67 | -1.61 |
| PPHLN1 | -1.74 | -2.23 | -1.36 |
| PRDM8 | -3.18 | -3.69 | -2.74 |
| PROX1 | -3.4 | -3.71 | -3.12 |
| PRPH | -1.73 | -2.20 | -1.36 |
| PRR11 | -2.58 | -2.92 | -2.28 |
| PRR3 | 2.47 | 2.49 | 2.45 |
| PTGR1 | -1.54 | -1.54 | -1.54 |
| PTRF | 2.37 | 2.49 | 2.26 |
| RAB24 | 3.01 | 3.04 | 2.98 |
| RAD21 | -2.23 | -3.17 | -1.57 |
| RAD23B | -3.28 | -3.84 | -2.80 |
| RBM18 | -1.53 | -1.77 | -1.32 |
| RFESD | -1.76 | -1.95 | -1.59 |
| RFP | 1.9 | 2.32 | 1.56 |
| RGS2 | -2.84 | -3.89 | -2.07 |
| RIF1 | -2.04 | -2.04 | -2.04 |
| RNF19A | -1.63 | -1.78 | -1.50 |
| RNF219 | 2.33 | 2.38 | 2.28 |
| RPS15 | 2.15 | 2.17 | 2.13 |
| RXRB | 3.56 | 3.74 | 3.39 |
| S100PBP | 2.78 | 2.78 | 2.78 |
| SAP30 | -2.88 | -3.46 | -2.40 |
| SEC11C | 3.32 | 3.88 | 2.84 |
| SEMA3E | -3.29 | -3.72 | -2.91 |
| SFRS14 | -2.29 | -2.40 | -2.18 |
| SGK | -3.3 | -3.37 | -3.24 |
| SIAH1 | -1.97 | -2.15 | -1.81 |
| SLC10A4 | -1.89 | -1.91 | -1.87 |
| SLC35C2 | 1.66 | 1.91 | 1.44 |
| SLC35E3 | -2.58 | -2.68 | -2.48 |
| SMC4 | -2.2 | -2.68 | -1.80 |
| SMYD3 | -1.55 | -1.64 | -1.46 |
| SPAG9 | -2.08 | -2.39 | -1.81 |
| SPC25 | -1.75 | -2.07 | -1.48 |
| SPOCK1 | 1.56 | 2.01 | 1.21 |
| SPRY1 | -2.23 | -2.41 | -2.06 |
| ST6GALNAC3 | -1.67 | -1.67 | -1.67 |
| STAG3L1 | -1.57 | -1.65 | -1.50 |
| TCTEX1D2 | -1.82 | -2.11 | -1.57 |
| TDRD1 | -3.44 | -4.44 | -2.67 |
| TERF1 | -2.32 | -2.34 | -2.30 |
| TK1 | -1.62 | -2.01 | -1.31 |
| TM4SF1 | -1.7 | -1.79 | -1.62 |
| TM4SF4 | -3.28 | -4.56 | -2.36 |
| TMED1 | 1.91 | 2.27 | 1.61 |
| TMEM18 | -1.69 | -2.08 | -1.37 |
| TMEM189-UBE2V1 | 6.54 | 6.80 | 6.29 |
| TMTC4 | -1.97 | -2.01 | -1.93 |
| TNC | 2.72 | 2.86 | 2.59 |
| TNFAIP8L1 | -2.44 | -3.12 | -1.91 |
| TRIM13 | -2.26 | -2.28 | -2.24 |
| TSPAN5 | -2.14 | -2.42 | -1.89 |
| TTC14 | -2.25 | -2.41 | -2.10 |
| TTC32 | -2.25 | -2.84 | -1.79 |
| TTK | -2.39 | -2.61 | -2.19 |
| TXNIP | 6.68 | 7.48 | 5.96 |
| TXNRD2 | 1.66 | 1.71 | 1.61 |
| UGCGL1 | -1.64 | -1.89 | -1.43 |
| UPF3A | -3.01 | -4.39 | -2.06 |
| VIP | -1.5 | -1.62 | -1.39 |
| WDSUB1 | -1.74 | -1.83 | -1.66 |
| YEATS4 | -2.06 | -3.30 | -1.29 |
| YTHDC1 | -1.59 | -1.73 | -1.46 |
| YWHAE | 2.9 | 3.65 | 2.30 |
| ZBTB8OS | 1.89 | 2.14 | 1.67 |
| ZMYM5 | -2.44 | -2.93 | -2.03 |
| ZNF248 | 1.92 | 2.04 | 1.81 |
| ZNF256 | -2.01 | -2.45 | -1.65 |
| ZNF419 | 1.99 | 2.11 | 1.88 |
| ZNF549 | -2.52 | -2.95 | -2.15 |
| ZNF562 | -1.97 | -2.78 | -1.40 |
| ZNF629 | 2.23 | 2.36 | 2.10 |
| ZNF669 | -1.55 | -1.89 | -1.27 |
| ZSCAN5A | -1.98 | -2.24 | -1.75 |
