# Supplementary material for: Plurigon: three dimensional visualization and classification of high-dimensionality data
Source: Front Physiol. 2013 Jul 22;4:190. doi: 10.3389/fphys.2013.00190 (PMC3717481; doi:10.3389/fphys.2013.00190)
Supplement: Table S16 — Conserved neuronal transcriptomic expression data from brain-derived neurotrophic factor-treated clonal human cells. Transcriptomic gene regulation was measured from brain-derived neurotrophic factor (BDNF)-treated SH-SY5Y neuronal cell batches (BDNF-1, BDNF-2, BDNF-3). The transcriptomic data from the three separate experiments was compared to gene expression levels in vehicle (phosphate-buffered saline)-treated control SH-SY5Y cells. [file 46440_Maudsley_Data_Sheet_6.ZIP › Table-S24.doc]

**Table S24. Similarity score calculations for BDNF-like AMI-regulated transcripts.** A relative similarity score for AMI-regulated transcripts that possessed a regulatory mode more akin to BDNF than compared to NGF was calculated as the follows. The modulus of the ((AMI score-NGF score)/(AMI score-BDNF score)) was obtained and transcripts possessng a modulus of over 1 were considered to be more AMI-BDNF correlated than AMI-NGF correlated.

| **Gene Symbol** | **AMI (A)** | **BDNF (B)** | **NGF (N)** | **A - B** | **A - N** | **│A - N/A - B │** |
| --- | --- | --- | --- | --- | --- | --- |
|  |  |  |  |  |  |  |
| DUT | -1.54 | -1.58 | 1.66 | 0.04 | -3.2 | 80.00 |
| CAND1 | 2.64 | 2.66 | 2.22 | -0.02 | 0.42 | 21.00 |
| BIRC5 | -1.88 | -1.85 | -2.33 | -0.03 | 0.45 | 15.00 |
| STAG3L1 | 2.14 | 1.89 | -1.57 | 0.25 | 3.71 | 14.84 |
| CYB561 | 2.25 | 2.29 | 1.66 | -0.04 | 0.59 | 14.75 |
| HIST2H2AC | -2.24 | -2.2 | -1.75 | -0.04 | -0.49 | 12.25 |
| DNPEP | 2.15 | 2.1 | 1.55 | 0.05 | 0.6 | 12.00 |
| DIAPH3 | -1.65 | -1.59 | -2.23 | -0.06 | 0.58 | 9.67 |
| C14orf102 | -1.8 | -1.71 | -2.5 | -0.09 | 0.7 | 7.78 |
| GGH | -2.48 | -2.4 | -1.93 | -0.08 | -0.55 | 6.87 |
| GAS6 | -1.7 | -1.75 | -2.01 | 0.05 | 0.31 | 6.20 |
| MTUS1 | -2.12 | -2.18 | -2.47 | 0.06 | 0.35 | 5.83 |
| ALG14 | -1.72 | -1.86 | -2.46 | 0.14 | 0.74 | 5.29 |
| YEATS4 | -1.68 | -1.59 | -2.06 | -0.09 | 0.38 | 4.22 |
| JARID1A | -1.66 | -1.75 | -2.04 | 0.09 | 0.38 | 4.22 |
| DENND5B | -1.59 | -1.82 | -2.56 | 0.23 | 0.97 | 4.22 |
| PRDM8 | -2.51 | -2.35 | -3.18 | -0.16 | 0.67 | 4.19 |
| TM4SF4 | -3.98 | -3.81 | -3.28 | -0.17 | -0.7 | 4.12 |
| TMEM18 | -2.54 | -2.33 | -1.69 | -0.21 | -0.85 | 4.05 |
| KIF11 | -2.11 | -2.13 | -2.03 | 0.02 | -0.08 | 4.00 |
| POLG2 | 3.37 | 3.31 | 3.13 | 0.06 | 0.24 | 4.00 |
| ADAMTS19 | -2.26 | -2.45 | -3 | 0.19 | 0.74 | 3.89 |
| ZNF562 | -1.55 | -1.67 | -1.97 | 0.12 | 0.42 | 3.50 |
| CCDC59 | -1.56 | -1.67 | -1.92 | 0.11 | 0.36 | 3.27 |
| TDRD1 | -2.3 | -2.65 | -3.44 | 0.35 | 1.14 | 3.26 |
| DBR1 | 2.51 | 2.24 | 1.65 | 0.27 | 0.86 | 3.19 |
| EIF4G2 | 2.16 | 2.04 | 1.79 | 0.12 | 0.37 | 3.08 |
| ADNP2 | -1.55 | -1.73 | -2.1 | 0.18 | 0.55 | 3.06 |
| CAPRIN1 | 3.05 | 1.53 | -1.53 | 1.52 | 4.58 | 3.01 |
| SMC4 | -1.79 | -1.65 | -2.2 | -0.14 | 0.41 | 2.93 |
| CCDC90A | -1.85 | -2.03 | -2.36 | 0.18 | 0.51 | 2.83 |
| ATP8B2 | 2.57 | 2.37 | 2.02 | 0.2 | 0.55 | 2.75 |
| ING3 | -1.64 | -1.71 | -1.83 | 0.07 | 0.19 | 2.71 |
| CALR | 2.21 | 2.43 | 2.71 | -0.22 | -0.5 | 2.27 |
| NOP56 | -2.95 | -2.72 | -2.45 | -0.23 | -0.5 | 2.17 |
| MBD4 | -1.84 | -2.58 | -3.42 | 0.74 | 1.58 | 2.14 |
| NDUFS2 | 2 | 1.85 | 1.68 | 0.15 | 0.32 | 2.13 |
| AKR1C3 | -2.57 | -2.37 | -2.15 | -0.2 | -0.42 | 2.10 |
| FAM115A | 3.34 | 2.89 | 2.46 | 0.45 | 0.88 | 1.96 |
| LOC646786 | -3.27 | -2.8 | -2.4 | -0.47 | -0.87 | 1.85 |
| SPC25 | -2.23 | -1.96 | -1.75 | -0.27 | -0.48 | 1.78 |
| KLF11 | -2.04 | -1.74 | -1.52 | -0.3 | -0.52 | 1.73 |
| ONECUT2 | 2.69 | 2.11 | 1.73 | 0.58 | 0.96 | 1.66 |
| ADAM23 | -2.05 | -1.85 | -2.38 | -0.2 | 0.33 | 1.65 |
| YWHAE | 6.83 | 4.35 | 2.9 | 2.48 | 3.93 | 1.58 |
| GADD45A | -3.18 | -2.11 | -1.53 | -1.07 | -1.65 | 1.54 |
| WDSUB1 | -1.54 | -1.67 | -1.74 | 0.13 | 0.2 | 1.54 |
| SAP30 | -4.08 | -3.3 | -2.88 | -0.78 | -1.2 | 1.54 |
| GINS4 | -1.62 | -1.9 | -2.04 | 0.28 | 0.42 | 1.50 |
| FAM46A | -2.22 | -1.86 | -1.68 | -0.36 | -0.54 | 1.50 |
| LPGAT1 | 2.6 | 2.12 | 1.89 | 0.48 | 0.71 | 1.48 |
| CCDC34 | -2.4 | -1.89 | -1.67 | -0.51 | -0.73 | 1.43 |
| BBS10 | 4.13 | 2.48 | 1.77 | 1.65 | 2.36 | 1.43 |
| JUN | -3.5 | -2.69 | -2.38 | -0.81 | -1.12 | 1.38 |
| RAB24 | 3.27 | 3.08 | 3.01 | 0.19 | 0.26 | 1.37 |
| YTHDC1 | -3.84 | -2.18 | -1.59 | -1.66 | -2.25 | 1.36 |
| MTCP1 | -1.56 | -1.65 | -1.68 | 0.09 | 0.12 | 1.33 |
| NFIX | -2.38 | -2 | -2.87 | -0.38 | 0.49 | 1.29 |
| FCHSD2 | -1.96 | -1.85 | -2.1 | -0.11 | 0.14 | 1.27 |
| KIF15 | -2.65 | -2.54 | -2.79 | -0.11 | 0.14 | 1.27 |
| SFRS14 | -1.57 | -2.14 | -2.29 | 0.57 | 0.72 | 1.26 |
| SMYD3 | -3.3 | -1.91 | -1.55 | -1.39 | -1.75 | 1.26 |
| PTGR1 | -3.43 | -1.9 | -1.54 | -1.53 | -1.89 | 1.24 |
| CDC25C | -2.83 | -2.06 | -1.88 | -0.77 | -0.95 | 1.23 |
| HIST2H2BE | 2.61 | 1.75 | 1.55 | 0.86 | 1.06 | 1.23 |
| MBOAT2 | 2.56 | -1.73 | -2.72 | 4.29 | 5.28 | 1.23 |
| ID3 | -5.49 | -2.97 | -2.4 | -2.52 | -3.09 | 1.23 |
| DPP7 | -1.96 | 1.85 | 2.68 | -3.81 | -4.64 | 1.22 |
| RGS2 | -4.07 | -3.06 | -2.84 | -1.01 | -1.23 | 1.22 |
| GOLPH4 | 4.39 | -2.04 | -3.2 | 6.43 | 7.59 | 1.18 |
| HK2 | 2.98 | 2.17 | 2.03 | 0.81 | 0.95 | 1.17 |
| RNF219 | 4.09 | 2.58 | 2.33 | 1.51 | 1.76 | 1.17 |
| NDUFB10 | -2.59 | 1.66 | 2.31 | -4.25 | -4.9 | 1.15 |
| GAR1 | -1.75 | -1.95 | -1.98 | 0.2 | 0.23 | 1.15 |
| PLEKHA1 | 2.64 | -1.64 | -2.27 | 4.28 | 4.91 | 1.15 |
| DLX5 | -3.97 | -2.16 | -1.91 | -1.81 | -2.06 | 1.14 |
| RIF1 | 1.98 | -1.56 | -2.04 | 3.54 | 4.02 | 1.14 |
| TRIM13 | 3.78 | -1.54 | -2.26 | 5.32 | 6.04 | 1.14 |
| HIATL1 | 2.47 | -1.69 | -2.23 | 4.16 | 4.7 | 1.13 |
| PDIK1L | 2.69 | -1.65 | -2.2 | 4.34 | 4.89 | 1.13 |
| AHCTF1 | 1.75 | -1.63 | -2.04 | 3.38 | 3.79 | 1.12 |
| PCBP4 | 2.78 | -3.27 | -3.98 | 6.05 | 6.76 | 1.12 |
| DDX51 | -3.26 | -3 | -2.97 | -0.26 | -0.29 | 1.12 |
| CRLF3 | 1.81 | -2.04 | -2.48 | 3.85 | 4.29 | 1.11 |
| RPS15 | -2.27 | 1.7 | 2.15 | -3.97 | -4.42 | 1.11 |
| GNL3L | -2.11 | -1.57 | -2.71 | -0.54 | 0.6 | 1.11 |
| GLT8D1 | -1.5 | 1.88 | 2.24 | -3.38 | -3.74 | 1.11 |
| CDCA3 | -3.77 | -1.83 | -1.63 | -1.94 | -2.14 | 1.10 |
| TTC14 | 2.42 | -1.83 | -2.25 | 4.25 | 4.67 | 1.10 |
| NGFRAP1 | -3.85 | 1.6 | 2.13 | -5.45 | -5.98 | 1.10 |
| TM4SF1 | -4.97 | -1.98 | -1.7 | -2.99 | -3.27 | 1.09 |
| TXNIP | 5.37 | 6.57 | 6.68 | -1.2 | -1.31 | 1.09 |
| KIAA1279 | 1.67 | -1.65 | -1.94 | 3.32 | 3.61 | 1.09 |
| LOC402221 | 3.7 | 1.98 | 1.83 | 1.72 | 1.87 | 1.09 |
| SGK | -7.32 | -3.61 | -3.3 | -3.71 | -4.02 | 1.08 |
| COLEC11 | -2.68 | 1.57 | 1.92 | -4.25 | -4.6 | 1.08 |
| TMED1 | -1.66 | 1.65 | 1.91 | -3.31 | -3.57 | 1.08 |
| GFPT1 | 2.13 | -1.71 | -2 | 3.84 | 4.13 | 1.08 |
| KIAA0101 | -1.8 | 1.53 | 1.78 | -3.33 | -3.58 | 1.08 |
| C1orf63 | 6.29 | 2.24 | 1.96 | 4.05 | 4.33 | 1.07 |
| ELF2 | 1.5 | -1.99 | -2.2 | 3.49 | 3.7 | 1.06 |
| CYR61 | -14.63 | -6.28 | -5.78 | -8.35 | -8.85 | 1.06 |
| PRR3 | -1.58 | 2.25 | 2.47 | -3.83 | -4.05 | 1.06 |
| KLF6 | -3.84 | -1.87 | -1.76 | -1.97 | -2.08 | 1.06 |
| NRAS | 2.49 | -2.59 | -2.87 | 5.08 | 5.36 | 1.06 |
| RXRB | 1.59 | 3.46 | 3.56 | -1.87 | -1.97 | 1.05 |
| FOSB | -12.27 | -5.05 | -4.67 | -7.22 | -7.6 | 1.05 |
| ZNF419 | 2.79 | 2.03 | 1.99 | 0.76 | 0.8 | 1.05 |
| ID1 | -5.67 | -2.23 | -2.05 | -3.44 | -3.62 | 1.05 |
| TMTC4 | 2.54 | -1.77 | -1.97 | 4.31 | 4.51 | 1.05 |
| HMGB1L1 | -2.02 | 2.45 | 2.64 | -4.47 | -4.66 | 1.04 |
| DECR1 | -2.68 | 1.57 | 1.75 | -4.25 | -4.43 | 1.04 |
| VIP | -2.98 | -1.56 | -1.5 | -1.42 | -1.48 | 1.04 |
| PLS1 | 2.07 | -1.55 | -1.67 | 3.62 | 3.74 | 1.03 |
| SPOCK1 | 5.58 | 1.68 | 1.56 | 3.9 | 4.02 | 1.03 |
| NUDT9 | -2.19 | -1.85 | -1.84 | -0.34 | -0.35 | 1.03 |
| MRPL35 | 1.62 | -1.53 | -1.62 | 3.15 | 3.24 | 1.03 |
| CENPA | -2.6 | -1.85 | -1.83 | -0.75 | -0.77 | 1.03 |
| C3orf17 | 1.94 | -3.88 | -4.01 | 5.82 | 5.95 | 1.02 |
| ASNS | -2.82 | 2.36 | 2.46 | -5.18 | -5.28 | 1.02 |
| RFP | 2.43 | 1.91 | 1.9 | 0.52 | 0.53 | 1.02 |
| GPKOW | -1.57 | 1.55 | 1.6 | -3.12 | -3.17 | 1.02 |
| EGR1 | -23.34 | -7.07 | -6.81 | -16.27 | -16.53 | 1.02 |
| C13orf25 | -4.67 | -1.8 | -1.76 | -2.87 | -2.91 | 1.01 |
| PLEKHA6 | 4.47 | 1.59 | 1.55 | 2.88 | 2.92 | 1.01 |
| DUSP1 | -3.07 | -1.58 | -1.57 | -1.49 | -1.5 | 1.01 |
| CEPT1 | 3.48 | -2.29 | -2.31 | 5.77 | 5.79 | 1.00 |
| RNF19A | 2.56 | -1.62 | -1.63 | 4.18 | 4.19 | 1.00 |
| UGCGL1 | -2.1 | -2.56 | -1.64 | 0.46 | -0.46 | 1.00 |
| PPHLN1 | -1.78 | -1.74 | -1.74 | -0.04 | -0.04 | 1.00 |
| BAMBI | -4.36 | -1.76 | -1.77 | -2.6 | -2.59 | 1.00 |
| POLR3A | 1.74 | -1.58 | -1.56 | 3.32 | 3.3 | 0.99 |
| ZBTB8OS | -2.37 | 1.92 | 1.89 | -4.29 | -4.26 | 0.99 |
| RBM18 | 2.25 | -1.56 | -1.53 | 3.81 | 3.78 | 0.99 |
| PCDH20 | -5.21 | -2.71 | -2.73 | -2.5 | -2.48 | 0.99 |
| SEC11C | -1.53 | 3.38 | 3.32 | -4.91 | -4.85 | 0.99 |
| ARPP19 | 1.56 | 2.72 | 2.7 | -1.16 | -1.14 | 0.98 |
| BTBD7 | 3.46 | -2 | -1.89 | 5.46 | 5.35 | 0.98 |
| PANK2 | -1.61 | -2.4 | -2.38 | 0.79 | 0.77 | 0.97 |
| BIRC2 | 2.37 | -1.93 | -1.82 | 4.3 | 4.19 | 0.97 |
| LOC153561 | -3.21 | -2.01 | -2.05 | -1.2 | -1.16 | 0.97 |
| SPAG9 | -1.57 | -2.1 | -2.08 | 0.53 | 0.51 | 0.96 |
| IGFBP5 | -6.84 | -2.56 | -2.73 | -4.28 | -4.11 | 0.96 |
| MANEAL | 2.43 | 1.94 | 1.96 | 0.49 | 0.47 | 0.96 |
| ACOT11 | -2.14 | -1.66 | -1.68 | -0.48 | -0.46 | 0.96 |
| C1orf97 | -3.5 | -2.11 | -2.18 | -1.39 | -1.32 | 0.95 |
| PRPH | -2.66 | -1.68 | -1.73 | -0.98 | -0.93 | 0.95 |
| MAD2L1 | -3.67 | -1.59 | -1.7 | -2.08 | -1.97 | 0.95 |
| RFESD | 1.7 | -1.97 | -1.76 | 3.67 | 3.46 | 0.94 |
| ICK | -3.05 | -1.66 | -1.74 | -1.39 | -1.31 | 0.94 |
| SPRY1 | -4.11 | -2.11 | -2.23 | -2 | -1.88 | 0.94 |
| FEZ1 | -2.41 | -1.78 | -1.82 | -0.63 | -0.59 | 0.94 |
| NFU1 | -3.13 | -2.06 | -2.13 | -1.07 | -1 | 0.93 |
| CDC2L2 | -2.24 | -1.69 | -1.73 | -0.55 | -0.51 | 0.93 |
| ZNF256 | -4.04 | -1.81 | -2.01 | -2.23 | -2.03 | 0.91 |
| PHF17 | -3.31 | 3.47 | 2.86 | -6.78 | -6.17 | 0.91 |
| EWSR1 | -2.05 | 4.17 | 3.61 | -6.22 | -5.66 | 0.91 |
| NOP58 | -3.06 | 2.52 | 2.01 | -5.58 | -5.07 | 0.91 |
| AURKB | -3.74 | -2 | -2.16 | -1.74 | -1.58 | 0.91 |
| MED30 | -1.65 | -2.08 | -2.04 | 0.43 | 0.39 | 0.91 |
| NTNG1 | -2.13 | -1.6 | -1.65 | -0.53 | -0.48 | 0.91 |
| CABYR | -2.92 | -1.87 | -1.97 | -1.05 | -0.95 | 0.90 |
| ID2 | -7.61 | -2.64 | -3.12 | -4.97 | -4.49 | 0.90 |
| DCN | -4.36 | -2.51 | -2.7 | -1.85 | -1.66 | 0.90 |
| PIM1 | -1.97 | -1.88 | -1.89 | -0.09 | -0.08 | 0.89 |
| FGL1 | -3.29 | 3.74 | 2.95 | -7.03 | -6.24 | 0.89 |
| MGC16703 | 1.93 | 2.19 | 2.16 | -0.26 | -0.23 | 0.88 |
| PPAP2B | -2.32 | -1.55 | -1.64 | -0.77 | -0.68 | 0.88 |
| FKBP1A | 2.6 | 1.8 | 1.9 | 0.8 | 0.7 | 0.88 |
| NUTF2 | 2.2 | 4.87 | 4.51 | -2.67 | -2.31 | 0.87 |
| SIAH1 | -2.27 | -1.92 | -1.97 | -0.35 | -0.3 | 0.86 |
| LINGO4 | -4.33 | -2.26 | -2.56 | -2.07 | -1.77 | 0.86 |
| CALCB | -2.72 | -1.9 | -2.02 | -0.82 | -0.7 | 0.85 |
| LOC645676 | -1.74 | 2.63 | 1.96 | -4.37 | -3.7 | 0.85 |
| PCDH17 | -4.52 | -2.24 | -2.59 | -2.28 | -1.93 | 0.85 |
| NLGN4X | -3.34 | -1.69 | -1.95 | -1.65 | -1.39 | 0.84 |
| ADD1 | -1.94 | 3.68 | 2.79 | -5.62 | -4.73 | 0.84 |
| MT1F | -2.89 | -1.74 | -1.93 | -1.15 | -0.96 | 0.83 |
| HSDL2 | -1.51 | -1.69 | -1.66 | 0.18 | 0.15 | 0.83 |
| ZNF669 | -1.7 | -1.52 | -1.55 | -0.18 | -0.15 | 0.83 |
| GATA3 | -3.62 | -1.68 | -2.03 | -1.94 | -1.59 | 0.82 |
| SLC35C2 | -1.93 | 2.55 | 1.66 | -4.48 | -3.59 | 0.80 |
| MRPL55 | -1.57 | -5.32 | -4.56 | 3.75 | 2.99 | 0.80 |
| ZNF248 | 2.25 | 1.83 | 1.92 | 0.42 | 0.33 | 0.79 |
| TMEM189-UBE2V1 | 3.66 | 7.33 | 6.54 | -3.67 | -2.88 | 0.78 |
| KCNT1 | -2.06 | -1.51 | -1.63 | -0.55 | -0.43 | 0.78 |
| C18orf56 | -3.6 | -2.62 | -2.84 | -0.98 | -0.76 | 0.78 |
| LOC644033 | -3.01 | -1.99 | -2.22 | -1.02 | -0.79 | 0.77 |
| SLC10A4 | -2.94 | -1.57 | -1.89 | -1.37 | -1.05 | 0.77 |
| GAS2L3 | -4.32 | -3.09 | -3.39 | -1.23 | -0.93 | 0.76 |
| FANCD2 | -2.59 | -1.54 | -1.83 | -1.05 | -0.76 | 0.72 |
| TNFAIP8L1 | -3.49 | -2.03 | -2.44 | -1.46 | -1.05 | 0.72 |
| GAS1 | -3.68 | -2.17 | -2.6 | -1.51 | -1.08 | 0.72 |
| PRR11 | -3.81 | -2.09 | -2.58 | -1.72 | -1.23 | 0.72 |
| ZSCAN5A | -2.4 | -1.81 | -1.98 | -0.59 | -0.42 | 0.71 |
| TK1 | -1.74 | -1.57 | -1.62 | -0.17 | -0.12 | 0.71 |
| LRRC17 | -2.79 | -2.32 | -2.46 | -0.47 | -0.33 | 0.70 |
| TTK | -3.44 | -1.9 | -2.39 | -1.54 | -1.05 | 0.68 |
| TSPAN5 | -2.96 | -1.74 | -2.14 | -1.22 | -0.82 | 0.67 |
| ERLIN2 | -1.87 | -2.11 | -2.03 | 0.24 | 0.16 | 0.67 |
| PLAA | 1.77 | 3.43 | 2.87 | -1.66 | -1.1 | 0.66 |
| TNC | 1.59 | 3.31 | 2.72 | -1.72 | -1.13 | 0.66 |
| MAP6 | 2.07 | 1.62 | 1.78 | 0.45 | 0.29 | 0.64 |
| EXOSC3 | 2.32 | 1.6 | 1.87 | 0.72 | 0.45 | 0.63 |
| C1orf173 | -3.25 | -2.89 | -3.03 | -0.36 | -0.22 | 0.61 |
| CENPM | -2.19 | -1.64 | -1.86 | -0.55 | -0.33 | 0.60 |
| TERF1 | -2.42 | -2.25 | -2.32 | -0.17 | -0.1 | 0.59 |
| ACAD11 | -1.64 | -2.31 | -2.03 | 0.67 | 0.39 | 0.58 |
| IL11RA | -2.48 | -1.84 | -2.12 | -0.64 | -0.36 | 0.56 |
| MXD3 | -2.07 | -1.63 | -1.83 | -0.44 | -0.24 | 0.55 |
| ODZ3 | -3.38 | -1.92 | -2.6 | -1.46 | -0.78 | 0.53 |
| LRRFIP1 | -3.4 | -1.72 | -2.51 | -1.68 | -0.89 | 0.53 |
| KCTD12 | -3.27 | -1.91 | -2.58 | -1.36 | -0.69 | 0.51 |
| ARHGAP11B | -3.85 | -2.7 | -3.27 | -1.15 | -0.58 | 0.50 |
| S100PBP | 3.02 | 3.52 | 2.78 | -0.5 | 0.24 | 0.48 |
| MAT2B | 1.61 | 1.8 | 1.7 | -0.19 | -0.09 | 0.47 |
| INCENP | -2.93 | -1.64 | -2.34 | -1.29 | -0.59 | 0.46 |
| C14orf138 | -1.88 | -1.97 | -1.92 | 0.09 | 0.04 | 0.44 |
| IRF2BP2 | 4.28 | -1.51 | 1.74 | 5.79 | 2.54 | 0.44 |
| EXOSC6 | -2.63 | -1.78 | -2.26 | -0.85 | -0.37 | 0.44 |
| COMMD2 | -2.35 | -1.9 | -2.17 | -0.45 | -0.18 | 0.40 |
| SLC35E3 | -3.03 | -1.79 | -2.58 | -1.24 | -0.45 | 0.36 |
| OIP5 | -2.2 | -2.37 | -2.14 | 0.17 | -0.06 | 0.35 |
| COQ2 | -2.51 | -1.83 | -2.29 | -0.68 | -0.22 | 0.32 |
| PTRF | 2.23 | 1.79 | 2.37 | 0.44 | -0.14 | 0.32 |
| TTC32 | -4.11 | 2.05 | -2.25 | -6.16 | -1.86 | 0.30 |
| PROX1 | -3.93 | -2.12 | -3.4 | -1.81 | -0.53 | 0.29 |
| TCTEX1D2 | -1.75 | -1.99 | -1.82 | 0.24 | 0.07 | 0.29 |
| MAGEL2 | 1.52 | 1.77 | 1.59 | -0.25 | -0.07 | 0.28 |
| MAFB | -2.08 | -1.9 | -2.03 | -0.18 | -0.05 | 0.28 |
| LOC642197 | -2.23 | -1.51 | -2.03 | -0.72 | -0.2 | 0.28 |
| POPDC3 | -1.79 | -1.6 | -1.84 | -0.19 | 0.05 | 0.26 |
| LMO4 | -2.69 | -1.63 | -2.43 | -1.06 | -0.26 | 0.25 |
| ZNF549 | -2.81 | -1.57 | -2.52 | -1.24 | -0.29 | 0.23 |
| PIK3R1 | -2.35 | -1.93 | -2.44 | -0.42 | 0.09 | 0.21 |
| CHGB | 2.39 | 2.68 | 2.45 | -0.29 | -0.06 | 0.21 |
| UPF3A | -3.24 | -1.95 | -3.01 | -1.29 | -0.23 | 0.18 |
| RAD23B | -3.18 | -2.6 | -3.28 | -0.58 | 0.1 | 0.17 |
| C14orf153 | -2.96 | -2.03 | -2.8 | -0.93 | -0.16 | 0.17 |
| BAT3 | 2.24 | 2.88 | 2.35 | -0.64 | -0.11 | 0.17 |
| ST6GALNAC3 | -1.63 | -1.87 | -1.67 | 0.24 | 0.04 | 0.17 |
| NOTCH3 | -1.92 | -1.58 | -1.87 | -0.34 | -0.05 | 0.15 |
| FAM134B | -2.1 | -2.37 | -2.13 | 0.27 | 0.03 | 0.11 |
| SEMA3E | -3.43 | -2.11 | -3.29 | -1.32 | -0.14 | 0.11 |
| ZNF629 | 2.28 | 2.79 | 2.23 | -0.51 | 0.05 | 0.10 |
| GTPBP8 | -1.55 | -2.07 | -1.52 | 0.52 | -0.03 | 0.06 |
| TXNRD2 | 1.67 | 1.85 | 1.66 | -0.18 | 0.01 | 0.06 |
| LOC440145 | -1.56 | -1.75 | -1.57 | 0.19 | 0.01 | 0.05 |
| PON2 | -1.61 | -1.9 | -1.6 | 0.29 | -0.01 | 0.03 |
| RAD21 | -2.24 | -1.88 | -2.23 | -0.36 | -0.01 | 0.03 |
| KIAA1967 | 1.62 | 2.35 | 1.64 | -0.73 | -0.02 | 0.03 |
| CUTC | -2.08 | -1.59 | -2.09 | -0.49 | 0.01 | 0.02 |
| ZMYM5 | -2.44 | -2.26 | -2.44 | -0.18 | 0 | 0.00 |
| GPER | -1.92 | -1.71 | -1.92 | -0.21 | 0 | 0.00 |
| FAM181B | -2.21 | -1.97 | -2.21 | -0.24 | 0 | 0.00 |
