# Supplementary material for: Plurigon: three dimensional visualization and classification of high-dimensionality data
Source: Front Physiol. 2013 Jul 22;4:190. doi: 10.3389/fphys.2013.00190 (PMC3717481; doi:10.3389/fphys.2013.00190)
Supplement: Table S16 — Conserved neuronal transcriptomic expression data from brain-derived neurotrophic factor-treated clonal human cells. Transcriptomic gene regulation was measured from brain-derived neurotrophic factor (BDNF)-treated SH-SY5Y neuronal cell batches (BDNF-1, BDNF-2, BDNF-3). The transcriptomic data from the three separate experiments was compared to gene expression levels in vehicle (phosphate-buffered saline)-treated control SH-SY5Y cells. [file 46440_Maudsley_Data_Sheet_6.ZIP › Table-S25.doc]

**Table S25. KEGG pathway analysis for AMI-regulated, BDNF-like, transcripts in peroxide-treated human neuronal cells.** Using the NIH-DAVID Bioinformatic Suite (<http://david.abcc.ncifcrf.gov/>) we annotated the AMI-regulated, BDNF-like, transcripts stimulated by AMI treatment of artificially ‘aged’ neuronal tissue. For each human signaling pathway the probability of geneset enrichment (P Value), the enrichment relative to a scaled input from the human genome (Fold Enrichment) and the specific genes in the input list that populate (n≥2) the assessed pathways. The only significantly populated signaling pathway was the hsa047223: Neurotrophin signaling pathway.

| **KEGG Pathway** | **P Value** | **Fold Enrichment** | **Genes** |
| --- | --- | --- | --- |
| hsa04722:Neurotrophin signaling pathway | 0.032599 | 5.467741935 | NRAS, JUN, NGFRAP1, YWHAE |
| hsa05216:Thyroid cancer | 0.153226 | 11.68965517 | NRAS, RXRB |
| hsa04110:Cell cycle | 0.158698 | 4.068 | CDC25C, GADD45A, YWHAE |
| hsa00250:Alanine, aspartate and glutamate metabolism | 0.162913 | 10.93548387 | GFPT1, ASNS |
| hsa03410:Base excision repair | 0.181967 | 9.685714286 | HMGB1L1, MBD4 |
| hsa04010:MAPK signaling pathway | 0.193108 | 2.539325843 | NRAS, DUSP1, JUN, GADD45A |
| hsa00520:Amino sugar and nucleotide sugar metabolism | 0.223319 | 7.704545455 | GFPT1, HK2 |
| hsa05223:Non-small cell lung cancer | 0.266899 | 6.277777778 | NRAS, RXRB |
| hsa05200:Pathways in cancer | 0.286905 | 2.067073171 | NRAS, RXRB, JUN, BIRC5 |
| hsa05211:Renal cell carcinoma | 0.331758 | 4.842857143 | NRAS, JUN |
| hsa04662:B cell receptor signaling pathway | 0.350865 | 4.52 | NRAS, JUN |
| hsa05210:Colorectal cancer | 0.383935 | 4.035714286 | JUN, BIRC5 |
| hsa04350:TGF-beta signaling pathway | 0.394593 | 3.896551724 | ID1, ID3 |
| hsa04012:ErbB signaling pathway | 0.394593 | 3.896551724 | NRAS, JUN |
| hsa04912:GnRH signaling pathway | 0.432168 | 3.459183673 | NRAS, JUN |
| hsa05322:Systemic lupus erythematosus | 0.43547 | 3.424242424 | HIST2H2BE, HIST2H2AC |
| hsa04660:T cell receptor signaling pathway | 0.464366 | 3.138888889 | NRAS, JUN |
| hsa04114:Oocyte meiosis | 0.47059 | 3.081818182 | CDC25C, YWHAE |
