# Supplementary material for: Plurigon: three dimensional visualization and classification of high-dimensionality data
Source: Front Physiol. 2013 Jul 22;4:190. doi: 10.3389/fphys.2013.00190 (PMC3717481; doi:10.3389/fphys.2013.00190)
Supplement: Table S16 — Conserved neuronal transcriptomic expression data from brain-derived neurotrophic factor-treated clonal human cells. Transcriptomic gene regulation was measured from brain-derived neurotrophic factor (BDNF)-treated SH-SY5Y neuronal cell batches (BDNF-1, BDNF-2, BDNF-3). The transcriptomic data from the three separate experiments was compared to gene expression levels in vehicle (phosphate-buffered saline)-treated control SH-SY5Y cells. [file 46440_Maudsley_Data_Sheet_6.ZIP › Table-S17.doc]

**Table S17. Conserved neuronal transcriptomic expression data from nerve growth factor-treated clonal human cells**. Transcriptomic gene regulation was measured from nerve growth factor (NGF)-treated SH-SY5Y neuronal cell batches (NGF-1, NGF-2, NGF-3). The transcriptomic data from the three separate experiments was compared to gene expression levels in vehicle (phosphate-buffered saline)-treated control SH-SY5Y cells.

| **Gene Symbol** | **NGF-1** | **NGF-2** | **NGF-3** |
| --- | --- | --- | --- |
|  |  |  |  |
| AAK1 | -3.49 | -4.15 | -2.93 |
| ABCA2 | -5.46 | -5.79 | -5.15 |
| ABCB1 | -2.01 | -2.61 | -1.55 |
| ABCB6 | -1.73 | -1.85 | -1.62 |
| ACCN2 | 5.82 | 6.05 | 5.60 |
| ACIN1 | -2.63 | -2.71 | -2.55 |
| ACTN4 | -3.69 | -4.35 | -3.13 |
| ADAM15 | -4.7 | -5.41 | -4.09 |
| ADAM19 | -1.71 | -1.78 | -1.64 |
| ADAM23 | -2.22 | -2.24 | -2.20 |
| ADPRHL1 | -2.73 | -4.01 | -1.86 |
| ADRM1 | -1.72 | -2.13 | -1.39 |
| AEBP1 | -1.94 | -2.31 | -1.63 |
| AGAP8 | -3.35 | -4.15 | -2.70 |
| AGPAT1 | -3.27 | -4.15 | -2.57 |
| AGPAT2 | -2.3 | -2.32 | -2.28 |
| AHNAK | -2.48 | -4.44 | -1.39 |
| ALDH16A1 | -2.01 | -2.45 | -1.65 |
| ALDOA | -2.78 | -3.70 | -2.09 |
| ALDOC | 3.25 | 4.16 | 2.54 |
| AMD1 | 2.1 | 2.56 | 1.72 |
| AMH | -5.58 | -5.69 | -5.47 |
| ANKRD13B | -3.3 | -3.47 | -3.14 |
| ANO8 | -2.97 | -3.09 | -2.86 |
| AP1G2 | -2.21 | -2.45 | -1.99 |
| AP2A1 | -4.3 | -4.34 | -4.26 |
| APC2 | -3.38 | -3.65 | -3.13 |
| APEH | -3.1 | -3.13 | -3.07 |
| APLP1 | -2.83 | -3.03 | -2.64 |
| ARAP3 | 1.87 | 2.09 | 1.67 |
| ARF4 | -1.61 | -2.04 | -1.27 |
| ARHGAP17 | -1.67 | -2.09 | -1.34 |
| ARHGEF17 | -2.44 | -2.66 | -2.24 |
| ARMC5 | -4.5 | -4.55 | -4.46 |
| ARMCX3 | 4.01 | 4.17 | 3.86 |
| ARMET | 2.73 | 3.06 | 2.44 |
| ARSD | -2.37 | -2.82 | -1.99 |
| ASPSCR1 | -3.1 | -3.60 | -2.67 |
| ATF3 | -2.88 | -4.41 | -1.88 |
| ATN1 | -3.21 | -3.53 | -2.92 |
| ATP2B4 | -2.8 | -3.39 | -2.31 |
| ATP8B2 | 2.96 | 2.99 | 2.93 |
| ATP9A | 1.94 | 2.58 | 1.46 |
| ATRN | -2.34 | -2.39 | -2.29 |
| ATXN7L2 | -1.85 | -2.28 | -1.50 |
| B3GAT3 | -2.35 | -2.49 | -2.22 |
| B4GALT7 | 3.54 | 4.67 | 2.68 |
| BAT2 | -4.65 | -5.44 | -3.97 |
| BAT3 | -2.33 | -2.56 | -2.12 |
| BCDIN3D | 2.74 | 2.79 | 2.69 |
| BCL2L1 | -3.42 | -5.03 | -2.33 |
| BCL7C | -2.65 | -3.37 | -2.09 |
| BEGAIN | -3.43 | -6.07 | -1.94 |
| BIN1 | -2.98 | -3.46 | -2.57 |
| BIRC3 | -1.55 | -1.61 | -1.49 |
| BLOC1S1 | -2.65 | -3.68 | -1.91 |
| BMP1 | -2.24 | -2.58 | -1.95 |
| BRD8 | -1.84 | -1.88 | -1.80 |
| BRMS1 | 1.75 | 1.77 | 1.73 |
| BSN | 2.44 | 2.54 | 2.35 |
| BTBD11 | 2.49 | 3.34 | 1.86 |
| BTN3A2 | -1.98 | -2.77 | -1.41 |
| C11orf63 | -3.01 | -4.70 | -1.93 |
| C14orf102 | -2.89 | -3.15 | -2.65 |
| C16orf7 | -2.96 | -3.14 | -2.79 |
| C16orf79 | -2.6 | -2.76 | -2.45 |
| C17orf90 | 2.21 | 2.67 | 1.83 |
| C17orf96 | -2.69 | -3.55 | -2.04 |
| C19orf22 | -2.26 | -2.58 | -1.98 |
| C19orf29 | -2.11 | -2.11 | -2.11 |
| C1orf122 | -2.48 | -2.75 | -2.23 |
| C1orf159 | -3.06 | -3.73 | -2.51 |
| C1orf173 | -3.15 | -3.18 | -3.12 |
| C1orf174 | 2.82 | 3.55 | 2.24 |
| C1orf63 | 4.48 | 4.70 | 4.27 |
| C1orf74 | 2.42 | 2.57 | 2.28 |
| C1orf77 | 3.09 | 3.83 | 2.49 |
| C1orf86 | -2.9 | -3.28 | -2.57 |
| C1QL1 | -2.93 | -4.04 | -2.12 |
| C20orf100 | 2.74 | 2.93 | 2.56 |
| C20orf117 | -3.47 | -3.96 | -3.04 |
| C21orf81 | -2.19 | -2.63 | -1.83 |
| C2orf24 | -2.2 | -2.71 | -1.79 |
| C2orf44 | 1.8 | 1.87 | 1.73 |
| C3orf39 | -2.71 | -3.52 | -2.08 |
| C5orf22 | 2.04 | 2.08 | 2.00 |
| C5orf54 | 3.29 | 3.65 | 2.96 |
| C6orf47 | 2.47 | 2.99 | 2.04 |
| C7orf13 | 1.6 | 1.60 | 1.60 |
| C7orf26 | -3.81 | -4.46 | -3.26 |
| C7orf41 | 2.11 | 2.55 | 1.74 |
| C9orf140 | -2.2 | -2.82 | -1.72 |
| C9orf37 | 2.03 | 2.23 | 1.85 |
| C9orf40 | 3.25 | 4.03 | 2.62 |
| CABIN1 | -1.92 | -1.94 | -1.90 |
| CADM4 | -3.35 | -3.52 | -3.19 |
| CALD1 | -2.03 | -2.42 | -1.71 |
| CAMK1 | -2.09 | -2.63 | -1.66 |
| CANT1 | -2.39 | -3.30 | -1.73 |
| CBLL1 | -2.92 | -3.24 | -2.63 |
| CBX3 | -2.26 | -2.60 | -1.97 |
| CBX5 | 1.78 | 1.83 | 1.73 |
| CCDC106 | 4.37 | 4.54 | 4.20 |
| CCDC130 | 2.54 | 2.97 | 2.17 |
| CCDC136 | 3.49 | 5.48 | 2.22 |
| CCDC5 | 1.57 | 1.81 | 1.37 |
| CCDC74A | -3.06 | -3.61 | -2.59 |
| CCDC74B | -3.18 | -3.91 | -2.59 |
| CCDC92 | 2.95 | 3.57 | 2.44 |
| CCDC93 | 1.99 | 2.03 | 1.95 |
| CCL2 | -3.49 | -3.59 | -3.39 |
| CCNK | -2.58 | -2.81 | -2.37 |
| CCNY | -2.31 | -2.66 | -2.01 |
| CD276 | -2.1 | -2.52 | -1.75 |
| CD99 | -2.58 | -3.15 | -2.11 |
| CDC42BPB | -4.73 | -5.01 | -4.46 |
| CDC42SE1 | -1.56 | -1.61 | -1.51 |
| CDK10 | -2.16 | -2.53 | -1.85 |
| CDR2L | 2.13 | 2.53 | 1.79 |
| CECR5 | 1.87 | 1.98 | 1.76 |
| CENPT | -3.42 | -3.69 | -3.17 |
| CENPV | -2.21 | -2.65 | -1.84 |
| CENTG2 | -3.27 | -3.92 | -2.73 |
| CENTG3 | -4.41 | -5.03 | -3.87 |
| CEP164 | -2.15 | -2.73 | -1.69 |
| CEP170 | 2.25 | 2.50 | 2.03 |
| CHCHD4 | 1.53 | 1.71 | 1.37 |
| CHERP | -3.7 | -4.26 | -3.22 |
| CHGB | 3.91 | 5.63 | 2.72 |
| CHRNA3 | 2.92 | 2.98 | 2.86 |
| CHST14 | -3.74 | -3.85 | -3.63 |
| CHTF18 | -4.38 | -4.69 | -4.09 |
| CKS2 | 2.53 | 2.63 | 2.43 |
| CLK1 | 6.68 | 7.75 | 5.76 |
| CLPTM1 | -5.04 | -6.40 | -3.97 |
| CLSTN1 | -3.08 | -3.48 | -2.73 |
| CNOT3 | -4.92 | -5.66 | -4.28 |
| CNTFR | -3.22 | -3.25 | -3.19 |
| COBRA1 | -2.99 | -3.26 | -2.74 |
| COL1A1 | -1.6 | -1.65 | -1.55 |
| COL6A1 | -4.6 | -5.01 | -4.22 |
| COL6A2 | -4.33 | -4.37 | -4.29 |
| CORO6 | -3.03 | -4.55 | -2.02 |
| CPT1B | -3.92 | -4.35 | -3.53 |
| CRABP1 | -3.68 | -4.20 | -3.23 |
| CRABP2 | 2.1 | 2.25 | 1.96 |
| CRELD2 | 2.18 | 2.18 | 2.18 |
| CRY2 | -1.59 | -2.10 | -1.20 |
| CS | 3.16 | 3.19 | 3.13 |
| CSAD | -2.89 | -3.35 | -2.49 |
| CSNK1E | -3.95 | -4.11 | -3.80 |
| CST3 | -4.07 | -4.15 | -3.99 |
| CSTF3 | 3 | 3.18 | 2.83 |
| CTDSP1 | -1.83 | -2.05 | -1.63 |
| CTSF | -3.16 | -3.48 | -2.87 |
| CUL9 | -2.76 | -3.56 | -2.14 |
| CUTA | 1.95 | 2.01 | 1.89 |
| CYB5R3 | -2.93 | -3.08 | -2.79 |
| CYCSL1 | -1.72 | -1.94 | -1.52 |
| CYFIP1 | -1.89 | -2.14 | -1.67 |
| CYLN2 | -2.73 | -3.17 | -2.35 |
| CYR61 | -16.42 | -17.41 | -15.49 |
| CYTH1 | -3.64 | -3.86 | -3.43 |
| CYTH3 | -2.42 | -2.42 | -2.42 |
| CYTSB | -2.01 | -2.11 | -1.91 |
| DBN1 | 3.37 | 3.64 | 3.12 |
| DBNDD1 | 3.59 | 3.77 | 3.42 |
| DCAF15 | -4.86 | -5.69 | -4.15 |
| DCN | -3.86 | -4.44 | -3.36 |
| DCTN1 | -3.41 | -3.58 | -3.25 |
| DDIT4 | 2.36 | 2.74 | 2.03 |
| DDR2 | -4.98 | -5.43 | -4.57 |
| DDX11 | -1.87 | -1.93 | -1.82 |
| DDX28 | -2.81 | -3.03 | -2.60 |
| DDX51 | -1.79 | -1.84 | -1.74 |
| DEDD2 | 4.31 | 4.78 | 3.88 |
| DENND4B | -2.64 | -3.48 | -2.00 |
| DEXI | 2.34 | 2.46 | 2.23 |
| DGKD | -2.47 | -2.89 | -2.11 |
| DHX30 | -2.67 | -2.67 | -2.67 |
| DHX34 | 1.64 | 1.84 | 1.46 |
| DKFZp547K054 | -2.32 | -2.51 | -2.15 |
| DKK1 | -3.78 | -4.20 | -3.41 |
| DMWD | -3.89 | -4.32 | -3.50 |
| DNAJB1 | 2.51 | 2.56 | 2.46 |
| DNAJB11 | 1.93 | 2.18 | 1.71 |
| DNAJB12 | 2.13 | 2.34 | 1.94 |
| DNM1 | -2.62 | -2.67 | -2.57 |
| DNM2 | -2.44 | -3.05 | -1.95 |
| DNMT3B | -2.26 | -2.49 | -2.05 |
| DOK4 | -2.36 | -2.60 | -2.15 |
| DPP7 | 4.99 | 5.09 | 4.89 |
| DPYSL4 | 3.58 | 4.08 | 3.14 |
| DRAP1 | -1.66 | -1.76 | -1.57 |
| DULLARD | -3.46 | -3.88 | -3.09 |
| DUS1L | -3.72 | -4.02 | -3.44 |
| DUSP10 | -2.2 | -2.60 | -1.86 |
| DUSP5 | -1.88 | -2.16 | -1.63 |
| DUSP8 | -3.57 | -3.93 | -3.25 |
| DYRK2 | -3.18 | -3.63 | -2.79 |
| EBF4 | -2.41 | -4.17 | -1.39 |
| ECHS1 | 2.04 | 2.12 | 1.96 |
| EFNA1 | 3.11 | 3.36 | 2.88 |
| EGLN2 | 2.72 | 3.02 | 2.45 |
| EGR1 | -20.9 | -23.20 | -18.83 |
| EHBP1L1 | -4.94 | -5.19 | -4.70 |
| EHD1 | 3.87 | 4.18 | 3.58 |
| EID2B | 1.63 | 1.97 | 1.35 |
| EIF3B | -2.45 | -2.77 | -2.17 |
| EIF4G2 | 1.94 | 2.02 | 1.87 |
| EIF5 | -1.69 | -1.91 | -1.50 |
| ELAVL3 | -2.38 | -3.00 | -1.89 |
| EMILIN1 | -4.17 | -4.84 | -3.59 |
| ENDOG | -3.51 | -3.97 | -3.11 |
| EP300 | -3.55 | -3.73 | -3.38 |
| EPHB4 | -3.24 | -4.18 | -2.51 |
| ERC1 | -3.01 | -3.07 | -2.95 |
| ERCC3 | -3.28 | -3.54 | -3.04 |
| ESPL1 | -3.37 | -3.77 | -3.01 |
| EXO1 | 2.39 | 2.49 | 2.30 |
| EXOSC3 | 2.71 | 3.20 | 2.30 |
| EYA4 | -3.04 | -4.10 | -2.25 |
| FADD | 2.74 | 2.93 | 2.56 |
| FADS1 | -2.69 | -2.72 | -2.66 |
| FAIM2 | 1.64 | 1.72 | 1.56 |
| FAM100A | -5.07 | -5.12 | -5.02 |
| FAM100B | -2.83 | -3.06 | -2.62 |
| FAM107B | -3.66 | -4.21 | -3.18 |
| FAM108A2 | -4.44 | -4.57 | -4.31 |
| FAM108A3 | -6.58 | -7.30 | -5.93 |
| FAM110A | -1.89 | -2.32 | -1.54 |
| FAM125B | -3.08 | -3.11 | -3.05 |
| FAM131A | 3.01 | 3.73 | 2.43 |
| FAM171A1 | -1.66 | -1.76 | -1.57 |
| FAM189B | 2.4 | 2.50 | 2.31 |
| FAM18B2 | 2.74 | 3.07 | 2.45 |
| FAM39DP | -2.45 | -2.89 | -2.08 |
| FAM48A | 1.53 | 1.53 | 1.53 |
| FAM53B | -2.69 | -3.09 | -2.34 |
| FAM57B | -3.29 | -3.59 | -3.02 |
| FAM64A | -2.92 | -3.50 | -2.43 |
| FAM73A | -2.76 | -2.82 | -2.71 |
| FAT1 | -2.83 | -3.08 | -2.60 |
| FBLN2 | -3.31 | -3.57 | -3.06 |
| FBLN7 | -4.48 | -4.93 | -4.07 |
| FBRS | -4.14 | -4.39 | -3.91 |
| FBXL16 | -4.16 | -4.20 | -4.12 |
| FBXL20 | 2.67 | 3.23 | 2.21 |
| FBXL6 | -3.98 | -4.38 | -3.62 |
| FCGRT | -2.87 | -3.82 | -2.16 |
| FGF14 | 2.7 | 2.84 | 2.57 |
| FKBP1A | -2.24 | -3.40 | -1.47 |
| FKBP9L | -2.88 | -3.05 | -2.72 |
| FKRP | -2.09 | -2.19 | -1.99 |
| FLJ10324 | -3.15 | -3.59 | -2.76 |
| FLJ10986 | -2.23 | -2.30 | -2.17 |
| FLJ22184 | -3.51 | -4.25 | -2.90 |
| FLJ44342 | -2.53 | -2.83 | -2.26 |
| FLNA | -3.97 | -5.56 | -2.84 |
| FLNB | -2.46 | -2.73 | -2.22 |
| FNDC4 | -2.01 | -2.05 | -1.97 |
| FOS | -17.75 | -19.53 | -16.14 |
| FOXM1 | -3.34 | -3.71 | -3.01 |
| FOXRED2 | -1.73 | -1.85 | -1.62 |
| FSTL1 | -2.9 | -3.34 | -2.52 |
| FUZ | -2.05 | -2.13 | -1.97 |
| GAA | -3.13 | -3.63 | -2.70 |
| GADD45G | 4.8 | 5.66 | 4.07 |
| GAMT | -2.61 | -2.98 | -2.29 |
| GAP43 | -2.97 | -3.00 | -2.94 |
| GAPVD1 | 3.15 | 3.40 | 2.92 |
| GAS6 | -3.28 | -3.41 | -3.15 |
| GATA3 | -2.03 | -2.25 | -1.83 |
| GATAD2B | -3.77 | -4.07 | -3.49 |
| GCH1 | 2.22 | 2.33 | 2.11 |
| GDI1 | -2.21 | -2.45 | -1.99 |
| GFOD2 | 4.89 | 4.89 | 4.89 |
| GIPC1 | -3.78 | -3.86 | -3.71 |
| GIT1 | -3.25 | -3.35 | -3.16 |
| GMIP | -2.43 | -2.84 | -2.08 |
| GNAI2 | -3.26 | -3.46 | -3.08 |
| GNB2 | -4.06 | -4.83 | -3.41 |
| GNL1 | 2.63 | 2.84 | 2.44 |
| GOLIM4 | -3.66 | -3.84 | -3.49 |
| GOLPH4 | -3 | -3.00 | -3.00 |
| GPC1 | -2.04 | -2.10 | -1.98 |
| GPR125 | -2.59 | -2.82 | -2.38 |
| GPRIN1 | -3.39 | -3.76 | -3.05 |
| GPX4 | -2.22 | -2.69 | -1.83 |
| GRIK5 | -3.22 | -3.32 | -3.13 |
| GRINA | -1.87 | -1.94 | -1.80 |
| GRIPAP1 | -1.85 | -1.92 | -1.78 |
| GRK6 | -2.01 | -2.47 | -1.63 |
| GSPT2 | 1.86 | 1.97 | 1.75 |
| H1FX | -5.91 | -5.91 | -5.91 |
| H3F3B | -4.97 | -5.32 | -4.64 |
| HCFC1R1 | 3.39 | 3.46 | 3.32 |
| HDGF | -3.45 | -3.55 | -3.35 |
| HIATL2 | -1.96 | -2.10 | -1.83 |
| HJURP | -2.98 | -3.10 | -2.87 |
| HLA-A | -2.57 | -3.29 | -2.01 |
| HNRNPA1 | 2.55 | 2.63 | 2.48 |
| HNRNPA1L2 | 1.97 | 2.01 | 1.93 |
| HPS6 | 3.15 | 3.94 | 2.52 |
| HSF4 | -2.6 | -2.70 | -2.50 |
| HSPA1B | 6.63 | 6.70 | 6.56 |
| HSPA5 | 3.48 | 3.65 | 3.31 |
| HSPC268 | -2.76 | -3.15 | -2.42 |
| HTRA1 | -3.14 | -3.58 | -2.75 |
| ID1 | -5.14 | -5.76 | -4.59 |
| ID2 | -5.61 | -6.62 | -4.75 |
| ID3 | -4.89 | -5.48 | -4.37 |
| IDUA | -3.54 | -4.00 | -3.13 |
| IFFO1 | 2.15 | 2.28 | 2.03 |
| IFNAR1 | 2.32 | 2.39 | 2.25 |
| IGFBP2 | -4 | -4.12 | -3.88 |
| IGFBP3 | -2.75 | -3.16 | -2.39 |
| IGFBP4 | -3.88 | -4.00 | -3.77 |
| IGFBP5 | -4.73 | -5.25 | -4.26 |
| IGSF1 | -2.68 | -3.03 | -2.37 |
| IL11RA | -3.68 | -3.68 | -3.68 |
| IL27RA | -1.79 | -1.97 | -1.63 |
| ILF3 | -2.86 | -3.23 | -2.53 |
| IMP3 | 2.68 | 3.32 | 2.16 |
| IMP4 | 3.03 | 3.30 | 2.78 |
| INTS1 | -4.13 | -4.96 | -3.44 |
| IQCC | 2.28 | 2.58 | 2.02 |
| IRAK1 | -2.99 | -3.11 | -2.88 |
| IRF2BP2 | 2.23 | 3.17 | 1.57 |
| IRF7 | 2.25 | 2.54 | 1.99 |
| ITFG3 | -4.38 | -4.60 | -4.17 |
| JSRP1 | 3.14 | 3.20 | 3.08 |
| JUP | -3.71 | -4.16 | -3.31 |
| KCNJ8 | 2.62 | 2.86 | 2.40 |
| KCNQ2 | 3.15 | 3.34 | 2.97 |
| KCTD12 | -4.84 | -5.28 | -4.44 |
| KCTD13 | -3.67 | -3.71 | -3.63 |
| KIAA0195 | -3.64 | -3.64 | -3.64 |
| KIAA0319L | -2.54 | -2.72 | -2.37 |
| KIAA0427 | -2.27 | -2.41 | -2.14 |
| KIAA0913 | -2.67 | -2.70 | -2.64 |
| KIAA1545 | -2.61 | -2.90 | -2.35 |
| KIF23 | -2.57 | -2.60 | -2.54 |
| KIF4A | -2.53 | -2.81 | -2.28 |
| KLF6 | -3.23 | -3.68 | -2.83 |
| KLHL17 | -2.42 | -2.71 | -2.16 |
| LASS6 | -3.17 | -3.90 | -2.58 |
| LMF2 | -3.93 | -4.36 | -3.54 |
| LMNA | -2.6 | -2.76 | -2.45 |
| LOC100008589 | -2.22 | -2.31 | -2.13 |
| LOC100127975 | -2.47 | -2.89 | -2.11 |
| LOC100128288 | -2.78 | -3.09 | -2.50 |
| LOC100128510 | -2.93 | -3.14 | -2.74 |
| LOC100128689 | 1.7 | 1.79 | 1.62 |
| LOC100129211 | -3.37 | -3.71 | -3.06 |
| LOC100129269 | -2.76 | -4.06 | -1.88 |
| LOC100130516 | -2.89 | -2.89 | -2.89 |
| LOC100130835 | -2.26 | -2.62 | -1.95 |
| LOC100131139 | -2.04 | -2.16 | -1.92 |
| LOC100131582 | -2.44 | -2.54 | -2.35 |
| LOC100131737 | 2.43 | 2.50 | 2.36 |
| LOC100132324 | -3.29 | -3.29 | -3.29 |
| LOC100132394 | -3.85 | -5.62 | -2.64 |
| LOC100132585 | -2.76 | -2.76 | -2.76 |
| LOC100133312 | 2.37 | 2.49 | 2.26 |
| LOC100133516 | -2.66 | -2.95 | -2.40 |
| LOC100134364 | -4.26 | -4.73 | -3.84 |
| LOC100134530 | -3.5 | -3.57 | -3.43 |
| LOC100134634 | -2.51 | -2.56 | -2.46 |
| LOC100170939 | -2.65 | -3.37 | -2.09 |
| LOC127295 | -1.94 | -2.02 | -1.87 |
| LOC148413 | 3.29 | 4.05 | 2.67 |
| LOC153561 | -4.1 | -4.55 | -3.69 |
| LOC284023 | 2.19 | 2.26 | 2.13 |
| LOC338799 | -4.45 | -4.49 | -4.41 |
| LOC345041 | 2.61 | 2.79 | 2.44 |
| LOC347292 | -2.19 | -2.56 | -1.87 |
| LOC387763 | -4.91 | -5.25 | -4.59 |
| LOC388796 | -2.92 | -4.00 | -2.13 |
| LOC389049 | -3.25 | -3.48 | -3.04 |
| LOC389342 | -3.08 | -3.48 | -2.73 |
| LOC389765 | -2.26 | -2.98 | -1.71 |
| LOC389816 | 1.86 | 2.36 | 1.46 |
| LOC390298 | -2.98 | -3.34 | -2.66 |
| LOC390557 | 2.62 | 2.80 | 2.45 |
| LOC400713 | -2.2 | -2.42 | -2.00 |
| LOC401098 | -2.28 | -2.51 | -2.07 |
| LOC401720 | 2.85 | 3.16 | 2.57 |
| LOC401805 | 2.54 | 2.74 | 2.35 |
| LOC440991 | 2.6 | 3.15 | 2.15 |
| LOC613037 | -2.86 | -3.03 | -2.70 |
| LOC642755 | 1.92 | 2.05 | 1.79 |
| LOC643384 | -2.73 | -3.11 | -2.39 |
| LOC643387 | 2.97 | 3.27 | 2.70 |
| LOC643888 | 2.41 | 2.43 | 2.39 |
| LOC643911 | -1.52 | -1.61 | -1.43 |
| LOC643995 | -1.91 | -2.06 | -1.77 |
| LOC644988 | -4.14 | -5.46 | -3.14 |
| LOC645001 | 1.56 | 1.56 | 1.56 |
| LOC645937 | -2.42 | -3.39 | -1.73 |
| LOC646044 | -2.87 | -3.07 | -2.68 |
| LOC647000 | -3.31 | -3.57 | -3.06 |
| LOC648149 | -2.22 | -2.33 | -2.11 |
| LOC648581 | -2.19 | -2.21 | -2.17 |
| LOC648852 | -3.02 | -3.05 | -2.99 |
| LOC652330 | -3.49 | -3.87 | -3.14 |
| LOC652685 | 2.28 | 2.30 | 2.26 |
| LOC652771 | 1.86 | 1.93 | 1.79 |
| LOC652773 | 1.83 | 2.64 | 1.27 |
| LOC653171 | -3.87 | -4.30 | -3.49 |
| LOC653438 | -4.34 | -4.51 | -4.17 |
| LOC653994 | -2.37 | -2.39 | -2.35 |
| LOC654254 | 1.77 | 1.81 | 1.74 |
| LOC654260 | -2.23 | -2.54 | -1.96 |
| LOC727762 | -2.27 | -2.34 | -2.20 |
| LOC727865 | 1.75 | 1.84 | 1.67 |
| LOC728411 | -2.8 | -3.05 | -2.57 |
| LOC728452 | -2.34 | -3.21 | -1.71 |
| LOC728554 | 2.43 | 2.55 | 2.31 |
| LOC728715 | -2.32 | -2.90 | -1.86 |
| LOC728728 | -2.24 | -3.47 | -1.45 |
| LOC728903 | -3.25 | -4.10 | -2.58 |
| LOC728908 | -3.52 | -3.66 | -3.38 |
| LOC729120 | -2.6 | -2.83 | -2.39 |
| LOC729495 | -3.58 | -3.87 | -3.31 |
| LOC729646 | -2 | -2.42 | -1.65 |
| LOC729843 | -2.08 | -2.60 | -1.66 |
| LOC729859 | -4.11 | -4.85 | -3.48 |
| LOC729992 | 2.54 | 3.10 | 2.08 |
| LOC730130 | -5.71 | -6.05 | -5.39 |
| LOC730525 | -2.65 | -3.39 | -2.07 |
| LOC730746 | 3.96 | 4.67 | 3.36 |
| LOC731314 | -4.42 | -4.64 | -4.21 |
| LOC732360 | -3.58 | -3.97 | -3.23 |
| LOC92249 | -1.75 | -1.84 | -1.67 |
| LPHN1 | -3.14 | -3.49 | -2.83 |
| LPHN2 | -1.66 | -2.11 | -1.31 |
| LPPR2 | -3.84 | -4.15 | -3.56 |
| LRCH4 | 2.51 | 2.56 | 2.46 |
| LRDD | -4.26 | -4.81 | -3.77 |
| LRRC37B2 | -1.73 | -1.75 | -1.71 |
| LRRC45 | -2.61 | -2.92 | -2.33 |
| LRRN2 | 2.48 | 2.88 | 2.14 |
| LRRN3 | -2.98 | -3.25 | -2.73 |
| LSM12 | -3.05 | -3.54 | -2.63 |
| LSS | -3.96 | -4.55 | -3.44 |
| LTBP3 | -4.62 | -5.31 | -4.02 |
| LY6E | -3.71 | -3.97 | -3.47 |
| LYN | -2.71 | -2.85 | -2.58 |
| LZTS1 | -2.84 | -2.87 | -2.81 |
| MACROD1 | -3.92 | -4.27 | -3.60 |
| MAD1L1 | -2.37 | -2.63 | -2.14 |
| MAGT1 | -2.14 | -3.06 | -1.50 |
| MAP1B | -1.87 | -2.08 | -1.68 |
| MAP1S | -4.28 | -4.67 | -3.93 |
| MAP3K7IP1 | -2.01 | -2.05 | -1.97 |
| MAPK12 | -2.44 | -2.64 | -2.26 |
| MAPK3 | -2.14 | -2.46 | -1.86 |
| MAPK8IP3 | -4.54 | -4.90 | -4.20 |
| MAST3 | -2.17 | -2.37 | -1.99 |
| MAX | 1.79 | 2.18 | 1.47 |
| MBD6 | 1.96 | 2.31 | 1.66 |
| MBOAT1 | -2.6 | -2.83 | -2.39 |
| MBOAT7 | -4.47 | -4.47 | -4.47 |
| MBTPS1 | -1.94 | -2.04 | -1.85 |
| MCF2L | -1.78 | -2.19 | -1.45 |
| MCM5 | -2.51 | -2.51 | -2.51 |
| MDK | -2.48 | -2.58 | -2.38 |
| MED20 | 1.85 | 2.11 | 1.62 |
| MED25 | -3.01 | -4.00 | -2.26 |
| MEGF6 | -1.86 | -2.27 | -1.52 |
| MEGF8 | -3.74 | -3.78 | -3.70 |
| MEIS3 | -2.44 | -2.64 | -2.26 |
| MESDC1 | -2.43 | -3.11 | -1.90 |
| METRN | -3.83 | -4.75 | -3.09 |
| METRNL | -1.78 | -1.80 | -1.76 |
| METTL13 | 2.21 | 2.34 | 2.08 |
| MEX3A | -2.97 | -3.77 | -2.34 |
| MGP | -3.5 | -3.82 | -3.21 |
| MGRN1 | -4.36 | -4.67 | -4.07 |
| MIB2 | -3.34 | -3.61 | -3.09 |
| MID1IP1 | -3.21 | -3.63 | -2.84 |
| MIIP | -3 | -3.18 | -2.83 |
| MIR1978 | -6.42 | -7.06 | -5.84 |
| MIR25 | -4.18 | -4.93 | -3.54 |
| MLST8 | -2.34 | -2.62 | -2.09 |
| MNT | -4.24 | -4.83 | -3.72 |
| MOBKL2A | 1.65 | 1.93 | 1.41 |
| MOBKL2C | 4.03 | 4.07 | 3.99 |
| MOV10 | -2.64 | -3.01 | -2.32 |
| MRFAP1L1 | 2.97 | 3.12 | 2.83 |
| MRPL18 | 2.33 | 2.40 | 2.26 |
| MRPL2 | -2.34 | -2.60 | -2.11 |
| MRPL52 | 2.65 | 2.89 | 2.43 |
| MRPS25 | 1.69 | 1.94 | 1.47 |
| MSL3 | -1.72 | -2.08 | -1.42 |
| MT2A | -4.41 | -4.85 | -4.01 |
| MTA1 | -3.75 | -3.98 | -3.54 |
| MXD3 | -4.75 | -5.04 | -4.48 |
| MYH9 | -2.31 | -2.47 | -2.16 |
| MYO1C | -3.93 | -4.05 | -3.82 |
| MYO9B | -3.08 | -3.63 | -2.61 |
| NAPEPLD | 2.34 | 2.46 | 2.23 |
| NAT14 | -2.88 | -3.00 | -2.77 |
| NAV1 | -2.61 | -2.61 | -2.61 |
| NAV2 | 2.35 | 2.59 | 2.14 |
| NCAN | -2.58 | -2.92 | -2.28 |
| NCBP2 | 2.07 | 2.24 | 1.92 |
| NCL | 1.85 | 2.16 | 1.58 |
| NCOA5 | -2.72 | -3.70 | -2.00 |
| NCOR2 | -3.85 | -4.47 | -3.32 |
| NDEL1 | 3.21 | 3.63 | 2.84 |
| NELF | 3.38 | 3.75 | 3.05 |
| NES | -2.47 | -2.79 | -2.19 |
| NEURL1B | -2.18 | -2.53 | -1.88 |
| NEUROG2 | 6.9 | 8.28 | 5.75 |
| NFATC2IP | -2.25 | -2.63 | -1.92 |
| NFIX | -4.47 | -4.51 | -4.43 |
| NLGN4X | -2.34 | -2.62 | -2.09 |
| NLN | 1.83 | 2.07 | 1.62 |
| NOC2L | -2.54 | -3.02 | -2.13 |
| N-PAC | -2.53 | -2.53 | -2.53 |
| NPAL3 | -2.02 | -2.46 | -1.66 |
| NPLOC4 | -3.4 | -3.64 | -3.18 |
| NPPA | 2.33 | 2.61 | 2.08 |
| NR1D2 | -3.61 | -3.68 | -3.54 |
| NRP1 | -2.64 | -3.14 | -2.22 |
| NRXN2 | -1.65 | -1.93 | -1.41 |
| NUCKS1 | -3.74 | -4.60 | -3.04 |
| NUMA1 | -2.4 | -2.52 | -2.29 |
| NUP188 | -2.41 | -3.47 | -1.67 |
| NUP214 | -2.38 | -2.83 | -2.00 |
| NYNRIN | -3.97 | -4.25 | -3.71 |
| OAZ2 | 1.86 | 1.99 | 1.74 |
| OGDH | -3.75 | -4.20 | -3.35 |
| OGDHL | -3.36 | -3.39 | -3.33 |
| OLFML3 | -4.4 | -4.62 | -4.19 |
| ORAOV1 | 4.05 | 4.25 | 3.86 |
| ORC5L | 1.69 | 1.89 | 1.51 |
| OSBPL7 | -2.03 | -2.09 | -1.97 |
| P4HB | -2.3 | -2.39 | -2.21 |
| PALM | -4.68 | -4.82 | -4.54 |
| PARD6A | 2.76 | 3.04 | 2.51 |
| PC | -1.65 | -1.86 | -1.46 |
| PCBP3 | -2.94 | -3.20 | -2.70 |
| PCDH17 | -3.99 | -4.31 | -3.69 |
| PCDHB6 | -3.19 | -4.37 | -2.33 |
| PCGF1 | -3.32 | -3.92 | -2.81 |
| PCNXL3 | -3.71 | -3.86 | -3.57 |
| PDPR | -1.77 | -2.12 | -1.48 |
| PDXK | 2.51 | 2.99 | 2.11 |
| PDZD4 | -2.35 | -3.13 | -1.77 |
| PELP1 | -3.47 | -4.37 | -2.75 |
| PEX14 | -3.42 | -3.69 | -3.17 |
| PEX16 | 2.97 | 3.42 | 2.58 |
| PHF23 | 3.06 | 3.09 | 3.03 |
| PHOX2A | -4.22 | -4.35 | -4.10 |
| PI4KAP2 | -2.79 | -2.85 | -2.74 |
| PIF1 | -2.81 | -2.84 | -2.78 |
| PIM1 | -2.67 | -2.80 | -2.54 |
| PIP5K1C | -2.33 | -3.05 | -1.78 |
| PKM2 | -2.63 | -3.34 | -2.07 |
| PKMYT1 | -4.72 | -5.76 | -3.87 |
| PKN1 | -2.92 | -3.21 | -2.65 |
| PKN3 | -2.47 | -2.64 | -2.31 |
| PLD3 | 2.48 | 3.37 | 1.82 |
| PLDN | -2.26 | -2.98 | -1.71 |
| PLEC1 | -2.93 | -3.31 | -2.59 |
| PLS3 | -1.86 | -2.34 | -1.48 |
| PLTP | -3.74 | -3.78 | -3.70 |
| PLXNB2 | -3.57 | -3.57 | -3.57 |
| PNPLA2 | -3.6 | -4.90 | -2.65 |
| PODXL2 | -4.06 | -4.47 | -3.69 |
| POLD1 | -3.74 | -4.08 | -3.43 |
| POLE | -2.84 | -3.18 | -2.54 |
| POLR2A | -1.75 | -1.93 | -1.59 |
| POMT2 | -2.36 | -2.48 | -2.25 |
| POPDC2 | -1.98 | -1.98 | -1.98 |
| PPM1G | -2.21 | -2.25 | -2.17 |
| PPM1K | -1.92 | -2.02 | -1.83 |
| PPP1CC | 1.91 | 2.10 | 1.74 |
| PPP1R12C | -2.53 | -2.78 | -2.30 |
| PPP2R3B | -2.64 | -2.72 | -2.56 |
| PRKCSH | -3.28 | -4.62 | -2.33 |
| PRR7 | -3.57 | -4.32 | -2.95 |
| PTGES2 | -3.39 | -3.97 | -2.90 |
| PTGFRN | -2.77 | -3.10 | -2.47 |
| PTK7 | -2.65 | -2.86 | -2.45 |
| PTOV1 | -2.9 | -3.10 | -2.71 |
| PTPRF | -2.27 | -2.68 | -1.92 |
| PTPRN2 | -2.07 | -2.13 | -2.01 |
| PVRL3 | -2.86 | -3.09 | -2.65 |
| PYGO2 | -1.98 | -2.04 | -1.92 |
| RAB5C | -2.73 | -3.69 | -2.02 |
| RABL3 | 1.61 | 1.74 | 1.49 |
| RAC3 | -3.05 | -3.48 | -2.68 |
| RAD23A | -3.74 | -4.45 | -3.14 |
| RADIL | -3.29 | -3.65 | -2.96 |
| RAE1 | -1.72 | -2.12 | -1.40 |
| RASA4P | -2.67 | -3.23 | -2.21 |
| RASSF6 | -2.75 | -3.33 | -2.27 |
| RASSF7 | 3.07 | 3.84 | 2.46 |
| RAVER1 | -3.59 | -3.66 | -3.52 |
| RBM14 | 1.9 | 2.05 | 1.76 |
| RBM3 | -1.96 | -2.94 | -1.31 |
| RBM42 | -2.81 | -4.02 | -1.97 |
| RBM4B | 1.56 | 1.89 | 1.29 |
| RBMS1 | -2.79 | -2.85 | -2.74 |
| RCOR2 | 3.21 | 3.24 | 3.18 |
| RERE | -2.72 | -2.80 | -2.64 |
| REXO4 | 2.94 | 3.32 | 2.60 |
| RFTN1 | 3.58 | 4.69 | 2.73 |
| RHBDD3 | -3.08 | -3.57 | -2.66 |
| RHBDL2 | -2.73 | -2.87 | -2.60 |
| RNF10 | -2.26 | -2.33 | -2.19 |
| RNF146 | 2.07 | 2.24 | 1.92 |
| RNF26 | -3.72 | -4.32 | -3.21 |
| RNU1-3 | -2.58 | -3.56 | -1.87 |
| RNU1-5 | -2.68 | -3.11 | -2.31 |
| RNU1A3 | -2.21 | -2.28 | -2.15 |
| RNU1F1 | -2.34 | -2.34 | -2.34 |
| ROBO3 | -3.54 | -5.13 | -2.44 |
| RPS6KB2 | 3.23 | 3.59 | 2.91 |
| RXRA | -2.28 | -2.51 | -2.07 |
| S100A4 | -3.35 | -3.92 | -2.86 |
| SBDSP | 2.2 | 2.20 | 2.20 |
| SBNO2 | -3.45 | -3.66 | -3.25 |
| SCARB1 | -3.18 | -4.26 | -2.37 |
| SCG2 | -1.84 | -1.97 | -1.72 |
| SCLY | -3.05 | -3.75 | -2.48 |
| SDF2L1 | 3.29 | 3.36 | 3.23 |
| SDF4 | -2.72 | -2.86 | -2.59 |
| SDHAP2 | 3.55 | 3.91 | 3.23 |
| SELS | 1.66 | 2.14 | 1.29 |
| SEMA5A | -3.25 | -3.64 | -2.90 |
| SEPN1 | -4.15 | -4.32 | -3.99 |
| SERF2 | 2.64 | 2.80 | 2.49 |
| SETD1A | -4.75 | -6.08 | -3.71 |
| SETDB1 | -2.82 | -3.02 | -2.64 |
| SEZ6 | -1.66 | -1.76 | -1.57 |
| SF1 | -2.71 | -3.55 | -2.07 |
| SFRS16 | -2.62 | -3.01 | -2.28 |
| SFRS8 | -3.98 | -4.10 | -3.86 |
| SGK | -6.24 | -6.49 | -6.00 |
| SGK1 | -5.29 | -5.77 | -4.85 |
| SH3KBP1 | -3.27 | -3.27 | -3.27 |
| SHANK3 | -1.67 | -2.14 | -1.30 |
| SHC3 | -3.51 | -4.18 | -2.95 |
| SHISA5 | -2.02 | -2.04 | -2.00 |
| SIGMAR1 | -2.31 | -2.89 | -1.85 |
| SIPA1 | 3.97 | 4.53 | 3.48 |
| SLC18A1 | -1.96 | -2.23 | -1.72 |
| SLC19A1 | -2.75 | -3.03 | -2.50 |
| SLC25A22 | 4.71 | 5.28 | 4.21 |
| SLC25A28 | 3.2 | 3.78 | 2.71 |
| SLC25A29 | -1.72 | -1.86 | -1.59 |
| SLC29A1 | -1.74 | -1.86 | -1.63 |
| SLC30A1 | -2.61 | -2.79 | -2.44 |
| SLC35C2 | -2.8 | -3.98 | -1.97 |
| SLC38A10 | -2.4 | -2.54 | -2.26 |
| SLC4A2 | -4.26 | -4.52 | -4.02 |
| SLC6A8 | -3.47 | -3.54 | -3.40 |
| SLC9A5 | -1.67 | -1.77 | -1.58 |
| SMARCB1 | -3.6 | -4.07 | -3.19 |
| SMARCC2 | -2.08 | -2.54 | -1.70 |
| SMG5 | 2.65 | 2.78 | 2.52 |
| SMG7 | -2.84 | -3.21 | -2.51 |
| SMO | -2.61 | -3.50 | -1.95 |
| SMTN | -3.11 | -4.01 | -2.41 |
| SNORA12 | -7.23 | -8.53 | -6.13 |
| SNORA45 | -1.87 | -2.39 | -1.46 |
| SNORA62 | -2.32 | -2.71 | -1.98 |
| SNORD4A | -2.89 | -2.95 | -2.83 |
| SORCS1 | 3.04 | 3.34 | 2.76 |
| SOX4 | -3.16 | -3.70 | -2.70 |
| SP2 | -5.3 | -6.20 | -4.53 |
| SPATS2L | -2.54 | -2.62 | -2.47 |
| SPHK2 | 3.25 | 4.26 | 2.48 |
| SPIRE2 | -1.79 | -1.92 | -1.67 |
| SPRY1 | -3.87 | -3.91 | -3.83 |
| SPTAN1 | -2.79 | -2.85 | -2.74 |
| SPTLC1 | 1.65 | 2.08 | 1.31 |
| SREBF1 | -3.77 | -4.37 | -3.25 |
| SRRM2 | -3.26 | -3.81 | -2.79 |
| SSBP3 | -3.86 | -4.25 | -3.51 |
| SSBP4 | -3.87 | -4.45 | -3.37 |
| ST5 | -1.88 | -2.01 | -1.76 |
| ST6GALNAC4 | -2.52 | -2.55 | -2.50 |
| STIM1 | -1.78 | -1.90 | -1.66 |
| SUMF2 | -2.02 | -2.91 | -1.40 |
| SUV420H1 | -2.37 | -2.58 | -2.17 |
| SV2A | -3.18 | -3.40 | -2.97 |
| SYDE1 | -1.52 | -2.14 | -1.08 |
| SYF2 | 1.72 | 1.87 | 1.58 |
| SYN2 | -2.13 | -2.34 | -1.94 |
| SYP | -2.89 | -3.55 | -2.35 |
| TACC3 | -4.48 | -5.11 | -3.93 |
| TADA3 | -1.91 | -2.18 | -1.68 |
| TAGLN | -4.04 | -4.93 | -3.31 |
| TAPBP | -3.42 | -4.65 | -2.51 |
| TAZ | -1.78 | -1.99 | -1.59 |
| TBC1D10B | -2.43 | -3.04 | -1.94 |
| TBC1D17 | -2.38 | -2.90 | -1.95 |
| TBC1D22A | -2.59 | -2.82 | -2.38 |
| TBC1D9B | -2.87 | -3.64 | -2.26 |
| TBCD | -2.71 | -2.90 | -2.53 |
| TBX2 | -4.18 | -4.43 | -3.94 |
| TBX3 | -3.83 | -4.21 | -3.48 |
| TCEAL3 | 1.9 | 2.01 | 1.79 |
| TCF3 | -3.67 | -4.84 | -2.78 |
| TCTEX1D2 | -1.9 | -2.07 | -1.74 |
| TDG | -3.33 | -3.50 | -3.17 |
| TECR | -3.14 | -3.33 | -2.96 |
| TIMP2 | -1.89 | -2.15 | -1.66 |
| TINF2 | 3.01 | 3.34 | 2.71 |
| TM9SF4 | -1.93 | -2.10 | -1.77 |
| TMEM100 | 2.86 | 2.86 | 2.86 |
| TMEM109 | -3.22 | -3.28 | -3.16 |
| TMEM126B | 1.98 | 2.20 | 1.78 |
| TMEM132A | -5.38 | -5.76 | -5.03 |
| TMEM151A | -2.14 | -3.45 | -1.33 |
| TMEM18 | -2.73 | -3.11 | -2.39 |
| TMEM219 | -2.72 | -2.94 | -2.52 |
| TMEM39B | -2.68 | -3.46 | -2.08 |
| TMEM44 | 2.17 | 2.60 | 1.81 |
| TMEM79 | 2.47 | 2.52 | 2.42 |
| TMUB1 | -2.41 | -2.58 | -2.25 |
| TMX4 | -1.91 | -2.01 | -1.82 |
| TNFRSF6B | -3.98 | -4.58 | -3.46 |
| TOP2A | -2.87 | -3.36 | -2.45 |
| TOR1A | -2.32 | -2.60 | -2.07 |
| TPD52L2 | -1.61 | -1.66 | -1.56 |
| TPM2 | -2.73 | -3.63 | -2.05 |
| TPM4 | -2.46 | -3.22 | -1.88 |
| TRAF7 | -3.12 | -3.31 | -2.94 |
| TRAK1 | -2.34 | -2.60 | -2.11 |
| TRAPPC9 | -4.54 | -4.54 | -4.54 |
| TRIM13 | 1.99 | 2.07 | 1.91 |
| TRIM28 | -4.7 | -4.84 | -4.56 |
| TRK1 | 3 | 3.36 | 2.68 |
| TROAP | -2.13 | -2.51 | -1.81 |
| TRPT1 | 2.49 | 3.81 | 1.63 |
| TSC2 | -4.22 | -4.77 | -3.73 |
| TSEN34 | -2.03 | -2.11 | -1.95 |
| TSPAN7 | -2.03 | -2.48 | -1.66 |
| TSPO | -3.49 | -6.11 | -1.99 |
| TSTD1 | -2.32 | -2.69 | -2.00 |
| TTC15 | -2.45 | -2.62 | -2.29 |
| TTC32 | -2.25 | -2.86 | -1.77 |
| TTC4 | 2.07 | 2.09 | 2.05 |
| TTC9B | -3.13 | -3.35 | -2.93 |
| TTYH3 | -5.54 | -6.54 | -4.69 |
| TUBA3D | -2.31 | -2.40 | -2.22 |
| TUBB4 | -4.06 | -4.67 | -3.53 |
| TUBG1 | -2.66 | -2.90 | -2.44 |
| TUBGCP2 | -3.47 | -3.75 | -3.21 |
| TUSC1 | -2.22 | -2.55 | -1.93 |
| TUSC4 | 2.61 | 3.37 | 2.02 |
| TWIST1 | -3.27 | -3.63 | -2.95 |
| TXNIP | 3.45 | 5.24 | 2.27 |
| TYMS | -2.14 | -2.44 | -1.88 |
| UBAP2L | -2.1 | -2.10 | -2.10 |
| UBE1 | -3.6 | -3.60 | -3.60 |
| UBE2L3 | 2.69 | 2.74 | 2.64 |
| UBE2O | -4.43 | -4.56 | -4.30 |
| UBE3B | -2.06 | -2.27 | -1.87 |
| UBN2 | -2.3 | -2.55 | -2.07 |
| UBR4 | -2.37 | -2.37 | -2.37 |
| UBXN6 | -2.88 | -3.83 | -2.17 |
| UCKL1 | -3.62 | -3.69 | -3.55 |
| UNC5B | -2.37 | -2.65 | -2.12 |
| UPF3A | -1.93 | -2.18 | -1.71 |
| USF2 | -1.71 | -1.92 | -1.53 |
| VAC14 | -3.6 | -4.18 | -3.10 |
| VAMP2 | -3.2 | -4.10 | -2.50 |
| VANGL2 | -3.22 | -3.48 | -2.98 |
| VASN | -3.07 | -3.25 | -2.90 |
| VIM | -2.17 | -2.91 | -1.62 |
| VPS37D | 3.31 | 3.54 | 3.09 |
| VPS4A | -2.8 | -2.86 | -2.75 |
| VPS52 | -2.97 | -3.15 | -2.80 |
| VSNL1 | 3.07 | 3.41 | 2.77 |
| VSTM2L | -3.18 | -3.34 | -3.03 |
| WDR24 | -2.6 | -3.28 | -2.06 |
| WDR34 | -2.91 | -3.23 | -2.62 |
| WDR81 | -2.08 | -2.27 | -1.91 |
| WDR86 | 4.22 | 4.43 | 4.02 |
| YPEL1 | -1.86 | -2.08 | -1.66 |
| YPEL3 | -2.35 | -2.42 | -2.28 |
| ZBTB45 | 3.27 | 3.86 | 2.77 |
| ZC3HC1 | 1.83 | 1.83 | 1.83 |
| ZCCHC24 | -2.32 | -2.32 | -2.32 |
| ZFPM1 | -2.28 | -2.46 | -2.11 |
| ZNF175 | 3 | 3.24 | 2.78 |
| ZNF197 | 2.14 | 2.78 | 1.65 |
| ZNF2 | 3.25 | 3.74 | 2.83 |
| ZNF200 | 3.32 | 3.35 | 3.29 |
| ZNF250 | 2.99 | 3.62 | 2.47 |
| ZNF35 | 1.98 | 2.24 | 1.75 |
| ZNF384 | -2.39 | -2.41 | -2.37 |
| ZNF385A | -3.52 | -4.36 | -2.84 |
| ZNF423 | -4.44 | -4.71 | -4.19 |
| ZNF462 | -2.26 | -2.35 | -2.17 |
| ZNF483 | -2.99 | -3.74 | -2.39 |
| ZNF490 | 1.93 | 2.03 | 1.84 |
| ZNF503 | -4.99 | -5.44 | -4.58 |
| ZNF577 | -2.43 | -2.92 | -2.03 |
| ZNF580 | -4.81 | -5.24 | -4.41 |
| ZNF585A | 2.57 | 2.85 | 2.32 |
| ZNF613 | 2.94 | 3.09 | 2.80 |
| ZNF615 | 3.22 | 4.31 | 2.40 |
| ZNF618 | -2.97 | -3.27 | -2.70 |
| ZNF622 | 2.01 | 2.11 | 1.91 |
| ZNF682 | -2.41 | -2.63 | -2.21 |
| ZNF69 | 2.44 | 2.81 | 2.12 |
| ZNF696 | 3.01 | 3.22 | 2.81 |
| ZNF823 | 2.35 | 2.51 | 2.20 |
| ZP3 | -3.38 | -3.99 | -2.86 |
| ZSCAN2 | -2.94 | -3.15 | -2.75 |
| ZW10 | 1.95 | 1.97 | 1.93 |
| ZYX | -3.78 | -3.86 | -3.71 |
