# Supplementary material for: Plurigon: three dimensional visualization and classification of high-dimensionality data
Source: Front Physiol. 2013 Jul 22;4:190. doi: 10.3389/fphys.2013.00190 (PMC3717481; doi:10.3389/fphys.2013.00190)
Supplement: Table S16 — Conserved neuronal transcriptomic expression data from brain-derived neurotrophic factor-treated clonal human cells. Transcriptomic gene regulation was measured from brain-derived neurotrophic factor (BDNF)-treated SH-SY5Y neuronal cell batches (BDNF-1, BDNF-2, BDNF-3). The transcriptomic data from the three separate experiments was compared to gene expression levels in vehicle (phosphate-buffered saline)-treated control SH-SY5Y cells. [file 46440_Maudsley_Data_Sheet_6.ZIP › Table-S18.doc]

**Table S18. Transcriptomic effects of amitriptyline in human neuronal cells (SH-SY5Y) cells pre-treated with chronic minimal levels of hydrogen peroxide.** For each amitriptyline (AMI) regulated transcript in the peroxide-treated SH-SY5Y cells the official Gene Symbol, z-ratio and transcript description are indicated.

| **Gene Symbol** | **z-ratio** | **Description** |
| --- | --- | --- |
| NEUROG2 | 10.72 | Homo sapiens neurogenin 2 (NEUROG2), mRNA. |
| RET | 8.9 | Homo sapiens ret proto-oncogene (RET), transcript variant 2, mRNA. |
| RET | 8.88 | Homo sapiens ret proto-oncogene (RET), transcript variant 2, mRNA. |
|  | 8.8 | Homo sapiens cDNA FLJ38396 fis, clone FEBRA2007957 |
|  | 7.48 | Homo sapiens clone 25194 mRNA sequence |
| CRABP2 | 7.35 | Homo sapiens cellular retinoic acid binding protein 2 (CRABP2), mRNA. |
| NPPA | 7.35 | Homo sapiens natriuretic peptide precursor A (NPPA), mRNA. |
| RET | 6.85 | Homo sapiens ret proto-oncogene (RET), transcript variant 4, mRNA. |
| YWHAE | 6.83 | Homo sapiens tyrosine 3-monooxygenase/tryptophan 5-monooxygenase activation protein, epsilon polypeptide (YWHAE), mRNA. |
| HSPA1B | 6.66 | Homo sapiens heat shock 70kDa protein 1B (HSPA1B), mRNA. |
| TFAP2B | 6.59 | Homo sapiens transcription factor AP-2 beta (activating enhancer binding protein 2 beta) (TFAP2B), mRNA. |
| C1orf63 | 6.29 | Homo sapiens chromosome 1 open reading frame 63 (C1orf63), transcript variant 1, mRNA. |
| LOC730413 | 5.76 | PREDICTED: Homo sapiens similar to cholinergic receptor, muscarinic 3 (LOC730413), mRNA. |
| SPOCK1 | 5.58 | Homo sapiens sparc/osteonectin, cwcv and kazal-like domains proteoglycan (testican) 1 (SPOCK1), mRNA. |
| HERPUD1 | 5.57 | Homo sapiens homocysteine-inducible, endoplasmic reticulum stress-inducible, ubiquitin-like domain member 1 (HERPUD1), transcript variant 3, mRNA. |
| LOC401720 | 5.53 | Homo sapiens similar to fidgetin (LOC401720), mRNA. |
| ZNF200 | 5.53 | Homo sapiens zinc finger protein 200 (ZNF200), transcript variant 3, mRNA. |
| TXNIP | 5.37 | Homo sapiens thioredoxin interacting protein (TXNIP), mRNA. |
| ITGB5 | 5.34 | Homo sapiens integrin, beta 5 (ITGB5), mRNA. XM_944688 XM_944693 |
| DNAJB1 | 5.34 | Homo sapiens DnaJ (Hsp40) homolog, subfamily B, member 1 (DNAJB1), mRNA. |
| KCNJ8 | 5.3 | Homo sapiens potassium inwardly-rectifying channel, subfamily J, member 8 (KCNJ8), mRNA. |
| C16orf72 | 5.23 | Homo sapiens chromosome 16 open reading frame 72 (C16orf72), mRNA. |
| ZNF823 | 5.23 | Homo sapiens zinc finger protein 823 (ZNF823), mRNA. |
| LOC643980 | 5.22 | PREDICTED: Homo sapiens similar to FRG1 protein (FSHD region gene 1 protein), transcript variant 2 (LOC643980), mRNA. |
| CHORDC1 | 5.21 | Homo sapiens cysteine and histidine-rich domain (CHORD)-containing 1 (CHORDC1), mRNA. |
| ELMOD2 | 5.16 | Homo sapiens ELMO/CED-12 domain containing 2 (ELMOD2), mRNA. |
| ZBTB41 | 5.14 | Homo sapiens zinc finger and BTB domain containing 41 (ZBTB41), mRNA. |
| HSPA1A | 5.14 | Homo sapiens heat shock 70kDa protein 1A (HSPA1A), mRNA. |
| DLK1 | 5.13 | Homo sapiens delta-like 1 homolog (Drosophila) (DLK1), mRNA. |
| TNS3 | 5.11 | Homo sapiens tensin 3 (TNS3), mRNA. |
| TRK1 | 5.09 | Homo sapiens tRNA lysine 1 (TRK1) on chromosome 17. |
| CCDC117 | 5.08 | Homo sapiens coiled-coil domain containing 117 (CCDC117), mRNA. |
| KLF10 | 5.06 | Homo sapiens Kruppel-like factor 10 (KLF10), transcript variant 1, mRNA. |
| PRPF4B | 5.05 | Homo sapiens PRP4 pre-mRNA processing factor 4 homolog B (yeast) (PRPF4B), transcript variant 1, mRNA. |
| THAP2 | 4.97 | Homo sapiens THAP domain containing, apoptosis associated protein 2 (THAP2), mRNA. |
| HSPA5 | 4.94 | Homo sapiens heat shock 70kDa protein 5 (glucose-regulated protein, 78kDa) (HSPA5), mRNA. |
|  | 4.92 | Homo sapiens cDNA FLJ43676 fis, clone SYNOV4009129 |
| C13orf7 | 4.84 | Homo sapiens chromosome 13 open reading frame 7 (C13orf7), mRNA. |
| MARS2 | 4.83 | Homo sapiens methionyl-tRNA synthetase 2, mitochondrial (MARS2), nuclear gene encoding mitochondrial protein, mRNA. |
| ZNF566 | 4.82 | Homo sapiens zinc finger protein 566 (ZNF566), mRNA. |
| IFNAR1 | 4.82 | Homo sapiens interferon (alpha, beta and omega) receptor 1 (IFNAR1), mRNA. |
| WTAP | 4.76 | Homo sapiens Wilms tumor 1 associated protein (WTAP), transcript variant 1, mRNA. |
| INTS6 | 4.75 | Homo sapiens integrator complex subunit 6 (INTS6), transcript variant 2, mRNA. |
| BTBD11 | 4.73 | Homo sapiens BTB (POZ) domain containing 11 (BTBD11), transcript variant 1, mRNA. |
| MFAP3 | 4.66 | Homo sapiens microfibrillar-associated protein 3 (MFAP3), mRNA. |
| ZNF606 | 4.66 | Homo sapiens zinc finger protein 606 (ZNF606), mRNA. |
| ZNF585A | 4.65 | Homo sapiens zinc finger protein 585A (ZNF585A), transcript variant 2, mRNA. |
|  | 4.64 | Homo sapiens cDNA clone IMAGE:5297467 |
| C5orf54 | 4.61 | Homo sapiens chromosome 5 open reading frame 54 (C5orf54), mRNA. |
| FAM122A | 4.59 | Homo sapiens family with sequence similarity 122A (FAM122A), mRNA. |
| ZNF322A | 4.58 | Homo sapiens zinc finger protein 322A (ZNF322A), mRNA. |
| NOMO1 | 4.58 | Homo sapiens NODAL modulator 1 (NOMO1), mRNA. |
| HS6ST2 | 4.56 | Homo sapiens heparan sulfate 6-O-sulfotransferase 2 (HS6ST2), transcript variant L, mRNA. |
| FBXL20 | 4.55 | Homo sapiens F-box and leucine-rich repeat protein 20 (FBXL20), mRNA. |
| PHF6 | 4.55 | Homo sapiens PHD finger protein 6 (PHF6), transcript variant 1, mRNA. |
| GCH1 | 4.5 | Homo sapiens GTP cyclohydrolase 1 (GCH1), transcript variant 3, mRNA. |
| DDIT4 | 4.49 | Homo sapiens DNA-damage-inducible transcript 4 (DDIT4), mRNA. |
| ARID3A | 4.48 | Homo sapiens AT rich interactive domain 3A (BRIGHT-like) (ARID3A), mRNA. |
| PLEKHA6 | 4.47 | Homo sapiens pleckstrin homology domain containing, family A member 6 (PLEKHA6), mRNA. |
| ZNF615 | 4.45 | Homo sapiens zinc finger protein 615 (ZNF615), mRNA. |
|  | 4.44 | BX417162 Homo sapiens PLACENTA Homo sapiens cDNA clone CS0DE005YO02 5-PRIME, mRNA sequence |
| PREPL | 4.41 | Homo sapiens prolyl endopeptidase-like (PREPL), transcript variant C, mRNA. |
| FZD7 | 4.41 | Homo sapiens frizzled homolog 7 (Drosophila) (FZD7), mRNA. |
| GCH1 | 4.41 | Homo sapiens GTP cyclohydrolase 1 (GCH1), transcript variant 4, mRNA. |
| GOLPH4 | 4.39 | Homo sapiens golgi phosphoprotein 4 (GOLPH4), mRNA. |
| HERPUD1 | 4.34 | Homo sapiens homocysteine-inducible, endoplasmic reticulum stress-inducible, ubiquitin-like domain member 1 (HERPUD1), transcript variant 3, mRNA. |
| ZNF614 | 4.31 | Homo sapiens zinc finger protein 614 (ZNF614), mRNA. |
| IGSF21 | 4.28 | Homo sapiens immunoglobin superfamily, member 21 (IGSF21), mRNA. |
| C17orf80 | 4.28 | Homo sapiens chromosome 17 open reading frame 80 (C17orf80), mRNA. |
| IRF2BP2 | 4.28 | Homo sapiens interferon regulatory factor 2 binding protein 2 (IRF2BP2), transcript variant 1, mRNA. |
| SNCAIP | 4.26 | Homo sapiens synuclein, alpha interacting protein (SNCAIP), mRNA. |
| CLK1 | 4.24 | Homo sapiens CDC-like kinase 1 (CLK1), mRNA. |
| SNORA25 | 4.23 | Homo sapiens small nucleolar RNA, H/ACA box 25 (SNORA25), small nucleolar RNA. |
| ADD3 | 4.22 | Homo sapiens adducin 3 (gamma) (ADD3), transcript variant 3, mRNA. |
| ZNF175 | 4.21 | Homo sapiens zinc finger protein 175 (ZNF175), mRNA. |
| RGS7 | 4.21 | Homo sapiens regulator of G-protein signalling 7 (RGS7), mRNA. |
| ZSWIM3 | 4.18 | Homo sapiens zinc finger, SWIM-type containing 3 (ZSWIM3), mRNA. |
| FAM91A1 | 4.18 | Homo sapiens family with sequence similarity 91, member A1 (FAM91A1), mRNA. |
| ZNF816A | 4.18 | Homo sapiens zinc finger protein 816A (ZNF816A), mRNA. |
| TMEM39A | 4.16 | Homo sapiens transmembrane protein 39A (TMEM39A), mRNA. |
| ACCN4 | 4.16 | Homo sapiens amiloride-sensitive cation channel 4, pituitary (ACCN4), transcript variant 1, mRNA. |
| KLHL28 | 4.15 | Homo sapiens kelch-like 28 (Drosophila) (KLHL28), mRNA. |
| C20orf100 | 4.14 | Homo sapiens chromosome 20 open reading frame 100 (C20orf100), mRNA. |
| BBS10 | 4.13 | Homo sapiens Bardet-Biedl syndrome 10 (BBS10), mRNA. |
| LOC650111 | 4.13 | PREDICTED: Homo sapiens similar to SHC transforming protein 1 (SH2 domain protein C1) (Src homology 2 domain containing transforming protein C1) (LOC650111), mRNA. |
| LOC646723 | 4.12 | PREDICTED: Homo sapiens similar to Keratin, type I cytoskeletal 18 (Cytokeratin-18) (CK-18) (Keratin-18) (K18) (LOC646723), mRNA. |
| SDHALP1 | 4.11 | Homo sapiens succinate dehydrogenase complex, subunit A, flavoprotein pseudogene 1 (SDHALP1) on chromosome 3. |
| RNF219 | 4.09 | Homo sapiens ring finger protein 219 (RNF219), mRNA. |
| LOC649095 | 4.09 | PREDICTED: Homo sapiens hypothetical LOC649095 (LOC649095), mRNA. |
| DDB1 | 4.08 | PREDICTED: Homo sapiens damage-specific DNA binding protein 1, 127kDa (DDB1), mRNA. |
| RAB23 | 4.08 | Homo sapiens RAB23, member RAS oncogene family (RAB23), transcript variant 1, mRNA. |
| WDR47 | 4.06 | Homo sapiens WD repeat domain 47 (WDR47), mRNA. |
| MOBKL2C | 4.03 | Homo sapiens MOB1, Mps One Binder kinase activator-like 2C (yeast) (MOBKL2C), transcript variant 1, mRNA. |
| MTERFD1 | 4.03 | Homo sapiens MTERF domain containing 1 (MTERFD1), mRNA. |
| TMEM50B | 4.03 | Homo sapiens transmembrane protein 50B (TMEM50B), mRNA. |
| ZNF613 | 4.02 | Homo sapiens zinc finger protein 613 (ZNF613), transcript variant 2, mRNA. |
| TAF1A | 4.01 | Homo sapiens TATA box binding protein (TBP)-associated factor, RNA polymerase I, A, 48kDa (TAF1A), transcript variant 1, mRNA. |
| ZNF295 | 4.01 | Homo sapiens zinc finger protein 295 (ZNF295), transcript variant 1, mRNA. |
| AGTR1 | 4 | Homo sapiens angiotensin II receptor, type 1 (AGTR1), transcript variant 3, mRNA. |
| ELAVL2 | 3.99 | Homo sapiens ELAV (embryonic lethal, abnormal vision, Drosophila)-like 2 (Hu antigen B) (ELAVL2), mRNA. |
| NAV2 | 3.99 | Homo sapiens neuron navigator 2 (NAV2), transcript variant 2, mRNA. |
| ZBTB34 | 3.98 | Homo sapiens zinc finger and BTB domain containing 34 (ZBTB34), mRNA. |
| SCAND3 | 3.98 | Homo sapiens SCAN domain containing 3 (SCAND3), mRNA. |
| GFRA3 | 3.97 | Homo sapiens GDNF family receptor alpha 3 (GFRA3), mRNA. |
| DLG2 | 3.95 | Homo sapiens discs, large homolog 2, chapsyn-110 (Drosophila) (DLG2), mRNA. |
| PTPRO | 3.95 | Homo sapiens protein tyrosine phosphatase, receptor type, O (PTPRO), transcript variant 5, mRNA. |
| ACCN2 | 3.94 | Homo sapiens amiloride-sensitive cation channel 2, neuronal (ACCN2), transcript variant 2, mRNA. |
| FZD3 | 3.94 | Homo sapiens frizzled homolog 3 (Drosophila) (FZD3), mRNA. |
| SFRS12 | 3.93 | Homo sapiens splicing factor, arginine/serine-rich 12 (SFRS12), transcript variant 1, mRNA. |
| MOSPD2 | 3.93 | Homo sapiens motile sperm domain containing 2 (MOSPD2), mRNA. |
| ATF2 | 3.92 | Homo sapiens activating transcription factor 2 (ATF2), mRNA. |
| TRIM23 | 3.92 | Homo sapiens tripartite motif-containing 23 (TRIM23), transcript variant alpha, mRNA. |
| MTERFD1 | 3.92 | Homo sapiens MTERF domain containing 1 (MTERFD1), mRNA. |
| ZKSCAN4 | 3.89 | Homo sapiens zinc finger with KRAB and SCAN domains 4 (ZKSCAN4), mRNA. |
| FAM48A | 3.88 | Homo sapiens family with sequence similarity 48, member A (FAM48A), transcript variant 1, mRNA. |
|  | 3.86 | zh47e08.r1 Soares_fetal_liver_spleen_1NFLS_S1 Homo sapiens cDNA clone IMAGE:415238 5, mRNA sequence |
| FAM135A | 3.86 | Homo sapiens family with sequence similarity 135, member A (FAM135A), mRNA. |
| USP46 | 3.84 | Homo sapiens ubiquitin specific peptidase 46 (USP46), mRNA. |
| ETV4 | 3.82 | Homo sapiens ets variant gene 4 (E1A enhancer binding protein, E1AF) (ETV4), mRNA. |
| KIAA0494 | 3.81 | Homo sapiens KIAA0494 (KIAA0494), mRNA. |
| ZNF286A | 3.8 | Homo sapiens zinc finger protein 286A (ZNF286A), mRNA. |
| RNF149 | 3.79 | Homo sapiens ring finger protein 149 (RNF149), mRNA. |
| TRIM13 | 3.78 | Homo sapiens tripartite motif-containing 13 (TRIM13), transcript variant 4, mRNA. |
| OGT | 3.75 | Homo sapiens O-linked N-acetylglucosamine (GlcNAc) transferase (UDP-N-acetylglucosamine:polypeptide-N-acetylglucosaminyl transferase) (OGT), transcript variant 1, mRNA. |
| ZNF616 | 3.75 | Homo sapiens zinc finger protein 616 (ZNF616), mRNA. |
| HSPA1L | 3.74 | Homo sapiens heat shock 70kDa protein 1-like (HSPA1L), mRNA. |
| NFX1 | 3.74 | Homo sapiens nuclear transcription factor, X-box binding 1 (NFX1), transcript variant 2, mRNA. |
| LOC402221 | 3.7 | PREDICTED: Homo sapiens similar to actin alpha 1 skeletal muscle protein (LOC402221), mRNA. |
| DLK1 | 3.69 | Homo sapiens delta-like 1 homolog (Drosophila) (DLK1), mRNA. |
|  | 3.68 | Homo sapiens mRNA; cDNA DKFZp686J12188 (from clone DKFZp686J12188) |
| ZNF197 | 3.67 | Homo sapiens zinc finger protein 197 (ZNF197), transcript variant 1, mRNA. |
| LOC653631 | 3.67 | PREDICTED: Homo sapiens hypothetical LOC653631 (LOC653631), mRNA. |
| FICD | 3.66 | Homo sapiens FIC domain containing (FICD), mRNA. |
| TMEM189-UBE2V1 | 3.66 | Homo sapiens TMEM189-UBE2V1 readthrough transcript (TMEM189-UBE2V1), transcript variant 2, mRNA. |
| HSZFP36 | 3.63 | Homo sapiens ZFP-36 for a zinc finger protein (HSZFP36), mRNA. |
| ZFX | 3.63 | Homo sapiens zinc finger protein, X-linked (ZFX), mRNA. |
| AZIN1 | 3.63 | Homo sapiens antizyme inhibitor 1 (AZIN1), transcript variant 1, mRNA. |
| C7orf13 | 3.62 | Homo sapiens chromosome 7 open reading frame 13 (C7orf13), mRNA. |
| TICAM2 | 3.6 | Homo sapiens toll-like receptor adaptor molecule 2 (TICAM2), mRNA. |
| TAF1A | 3.59 | Homo sapiens TATA box binding protein (TBP)-associated factor, RNA polymerase I, A, 48kDa (TAF1A), transcript variant 1, mRNA. |
| CEP170 | 3.59 | Homo sapiens centrosomal protein 170kDa (CEP170), transcript variant beta, mRNA. |
| CD276 | 3.58 | Homo sapiens CD276 molecule (CD276), transcript variant 2, mRNA. |
| IRF9 | 3.57 | Homo sapiens interferon regulatory factor 9 (IRF9), mRNA. |
| EDEM3 | 3.56 | Homo sapiens ER degradation enhancer, mannosidase alpha-like 3 (EDEM3), mRNA. |
| SORBS2 | 3.56 | Homo sapiens sorbin and SH3 domain containing 2 (SORBS2), transcript variant 1, mRNA. |
| PWWP2A | 3.56 | Homo sapiens PWWP domain containing 2A (PWWP2A), mRNA. |
| C1orf109 | 3.55 | Homo sapiens chromosome 1 open reading frame 109 (C1orf109), mRNA. |
| PHF13 | 3.54 | Homo sapiens PHD finger protein 13 (PHF13), mRNA. |
| RAB33B | 3.53 | Homo sapiens RAB33B, member RAS oncogene family (RAB33B), mRNA. |
| TJP1 | 3.52 | Homo sapiens tight junction protein 1 (zona occludens 1) (TJP1), transcript variant 1, mRNA. |
| CHRNA3 | 3.52 | Homo sapiens cholinergic receptor, nicotinic, alpha 3 (CHRNA3), mRNA. |
|  | 3.52 | AGENCOURT_15364206 Human Anterior Horn Homo sapiens cDNA clone IMAGE:30513831 5, mRNA sequence |
| CSNK1G3 | 3.52 | Homo sapiens casein kinase 1, gamma 3 (CSNK1G3), transcript variant 2, mRNA. |
| ZNF225 | 3.52 | Homo sapiens zinc finger protein 225 (ZNF225), mRNA. |
| TBC1D2B | 3.51 | Homo sapiens TBC1 domain family, member 2B (TBC1D2B), mRNA. |
| SHC1 | 3.51 | Homo sapiens SHC (Src homology 2 domain containing) transforming protein 1 (SHC1), transcript variant 2, mRNA. |
| DLK2 | 3.5 | Homo sapiens delta-like 2 homolog (Drosophila) (DLK2), transcript variant 2, mRNA. |
| ALKBH1 | 3.5 | Homo sapiens alkB, alkylation repair homolog 1 (E. coli) (ALKBH1), mRNA. |
| PLAGL2 | 3.49 | Homo sapiens pleiomorphic adenoma gene-like 2 (PLAGL2), mRNA. |
| GNA12 | 3.48 | Homo sapiens guanine nucleotide binding protein (G protein) alpha 12 (GNA12), mRNA. |
| CEPT1 | 3.48 | Homo sapiens choline/ethanolamine phosphotransferase 1 (CEPT1), transcript variant 1, mRNA. |
| C5orf25 | 3.48 | Homo sapiens chromosome 5 open reading frame 25 (C5orf25), mRNA. |
| PRKACB | 3.46 | Homo sapiens protein kinase, cAMP-dependent, catalytic, beta (PRKACB), transcript variant 2, mRNA. |
| BTBD7 | 3.46 | Homo sapiens BTB (POZ) domain containing 7 (BTBD7), transcript variant 1, mRNA. |
| RND3 | 3.45 | Homo sapiens Rho family GTPase 3 (RND3), mRNA. |
| PRKD2 | 3.44 | Homo sapiens protein kinase D2 (PRKD2), mRNA. |
| RIOK3 | 3.44 | Homo sapiens RIO kinase 3 (yeast) (RIOK3), transcript variant 1, mRNA. |
| SCML2 | 3.43 | Homo sapiens sex comb on midleg-like 2 (Drosophila) (SCML2), mRNA. |
| HYOU1 | 3.43 | Homo sapiens hypoxia up-regulated 1 (HYOU1), mRNA. |
| PANK1 | 3.41 | Homo sapiens pantothenate kinase 1 (PANK1), transcript variant beta, mRNA. |
|  | 3.4 | Homo sapiens mRNA; cDNA DKFZp667L2214 (from clone DKFZp667L2214) |
| GNG12 | 3.4 | Homo sapiens guanine nucleotide binding protein (G protein), gamma 12 (GNG12), mRNA. |
| SERTAD3 | 3.39 | Homo sapiens SERTA domain containing 3 (SERTAD3), transcript variant 1, mRNA. |
| ZNF691 | 3.38 | Homo sapiens zinc finger protein 691 (ZNF691), mRNA. |
| NSF | 3.38 | Homo sapiens N-ethylmaleimide-sensitive factor (NSF), mRNA. |
| MRPS12 | 3.38 | Homo sapiens mitochondrial ribosomal protein S12 (MRPS12), nuclear gene encoding mitochondrial protein, transcript variant 1, mRNA. |
| ZNF160 | 3.37 | Homo sapiens zinc finger protein 160 (ZNF160), transcript variant 1, mRNA. |
| ZNF443 | 3.37 | Homo sapiens zinc finger protein 443 (ZNF443), mRNA. |
| POLG2 | 3.37 | Homo sapiens polymerase (DNA directed), gamma 2, accessory subunit (POLG2), mRNA. |
| PRKCH | 3.37 | Homo sapiens protein kinase C, eta (PRKCH), mRNA. |
| ZNF230 | 3.36 | Homo sapiens zinc finger protein 230 (ZNF230), mRNA. |
| ZNF2 | 3.36 | Homo sapiens zinc finger protein 2 (ZNF2), transcript variant 1, mRNA. |
| CCNT2 | 3.36 | Homo sapiens cyclin T2 (CCNT2), transcript variant b, mRNA. |
| RASSF7 | 3.36 | Homo sapiens Ras association (RalGDS/AF-6) domain family (N-terminal) member 7 (RASSF7), mRNA. |
| ZC3H14 | 3.35 | Homo sapiens zinc finger CCCH-type containing 14 (ZC3H14), transcript variant 2, mRNA. |
| SH3BP4 | 3.35 | Homo sapiens SH3-domain binding protein 4 (SH3BP4), mRNA. |
| FAM115A | 3.34 | Homo sapiens family with sequence similarity 115, member A (FAM115A), mRNA. |
| RAMP1 | 3.34 | Homo sapiens receptor (G protein-coupled) activity modifying protein 1 (RAMP1), mRNA. |
| CEPT1 | 3.34 | Homo sapiens choline/ethanolamine phosphotransferase 1 (CEPT1), transcript variant 2, mRNA. |
| ZNF202 | 3.34 | Homo sapiens zinc finger protein 202 (ZNF202), mRNA. |
| EFNA1 | 3.33 | Homo sapiens ephrin-A1 (EFNA1), transcript variant 1, mRNA. |
| SDCCAG1 | 3.33 | Homo sapiens serologically defined colon cancer antigen 1 (SDCCAG1), mRNA. |
| NR2C1 | 3.33 | Homo sapiens nuclear receptor subfamily 2, group C, member 1 (NR2C1), transcript variant 2, mRNA. |
| CS | 3.33 | Homo sapiens citrate synthase (CS), nuclear gene encoding mitochondrial protein, mRNA. |
| LARP4 | 3.31 | Homo sapiens La ribonucleoprotein domain family, member 4 (LARP4), transcript variant 1, mRNA. |
| ATP2A2 | 3.3 | Homo sapiens ATPase, Ca++ transporting, cardiac muscle, slow twitch 2 (ATP2A2), transcript variant 1, mRNA. |
| ZNF250 | 3.3 | Homo sapiens zinc finger protein 250 (ZNF250), mRNA. |
| NHS | 3.3 | Homo sapiens Nance-Horan syndrome (congenital cataracts and dental anomalies) (NHS), transcript variant 1, mRNA. |
| SDF2L1 | 3.3 | Homo sapiens stromal cell-derived factor 2-like 1 (SDF2L1), mRNA. |
|  | 3.29 | in34h07.y1 Human Fetal Pancreas 1B Homo sapiens cDNA 5 similar to TR:Q14754 Q14754 ORFII. ;, mRNA sequence |
| HIF1A | 3.29 | Homo sapiens hypoxia-inducible factor 1, alpha subunit (basic helix-loop-helix transcription factor) (HIF1A), transcript variant 1, mRNA. |
| RIOK2 | 3.29 | Homo sapiens RIO kinase 2 (yeast) (RIOK2), mRNA. |
| CLIP4 | 3.28 | Homo sapiens CAP-GLY domain containing linker protein family, member 4 (CLIP4), mRNA. |
| GCH1 | 3.28 | Homo sapiens GTP cyclohydrolase 1 (GCH1), transcript variant 1, mRNA. |
| ZMYM2 | 3.27 | Homo sapiens zinc finger, MYM-type 2 (ZMYM2), mRNA. |
| C3orf38 | 3.27 | Homo sapiens chromosome 3 open reading frame 38 (C3orf38), mRNA. |
| RAB24 | 3.27 | Homo sapiens RAB24, member RAS oncogene family (RAB24), transcript variant 2, mRNA. |
| TULP4 | 3.26 | Homo sapiens tubby like protein 4 (TULP4), transcript variant 1, mRNA. |
| IRX5 | 3.26 | Homo sapiens iroquois homeobox protein 5 (IRX5), mRNA. |
| ZMYM6 | 3.26 | Homo sapiens zinc finger, MYM-type 6 (ZMYM6), mRNA. |
|  | 3.26 | Homo sapiens cDNA: FLJ23004 fis, clone LNG00391 |
| VSNL1 | 3.25 | Homo sapiens visinin-like 1 (VSNL1), mRNA. |
| ZNF35 | 3.25 | Homo sapiens zinc finger protein 35 (ZNF35), mRNA. |
| NKIRAS1 | 3.25 | Homo sapiens NFKB inhibitor interacting Ras-like 1 (NKIRAS1), mRNA. |
| CAT | 3.24 | Homo sapiens catalase (CAT), mRNA. |
| USP42 | 3.24 | Homo sapiens ubiquitin specific peptidase 42 (USP42), mRNA. |
| C9orf40 | 3.24 | Homo sapiens chromosome 9 open reading frame 40 (C9orf40), mRNA. |
| KCTD5 | 3.24 | Homo sapiens potassium channel tetramerisation domain containing 5 (KCTD5), mRNA. |
| C17orf28 | 3.24 | Homo sapiens chromosome 17 open reading frame 28 (C17orf28), mRNA. |
| STRN3 | 3.22 | Homo sapiens striatin, calmodulin binding protein 3 (STRN3), transcript variant 2, mRNA. |
| USP37 | 3.22 | Homo sapiens ubiquitin specific peptidase 37 (USP37), mRNA. |
| CASP2 | 3.22 | Homo sapiens caspase 2, apoptosis-related cysteine peptidase (CASP2), transcript variant 1, mRNA. |
| KCMF1 | 3.21 | PREDICTED: Homo sapiens potassium channel modulatory factor 1 (KCMF1), mRNA. |
| IRX3 | 3.21 | Homo sapiens iroquois homeobox 3 (IRX3), mRNA. |
| RAD54B | 3.21 | Homo sapiens RAD54 homolog B (S. cerevisiae) (RAD54B), mRNA. |
| CAMTA1 | 3.2 | Homo sapiens calmodulin binding transcription activator 1 (CAMTA1), mRNA. |
| LOC387856 | 3.2 | Homo sapiens similar to expressed sequence AI836003 (LOC387856), mRNA. |
| LOC401431 | 3.2 | Homo sapiens hypothetical gene LOC401431 (LOC401431), mRNA. |
| C14orf129 | 3.2 | Homo sapiens chromosome 14 open reading frame 129 (C14orf129), mRNA. |
| XBP1 | 3.2 | Homo sapiens X-box binding protein 1 (XBP1), transcript variant 1, mRNA. |
| RECK | 3.2 | Homo sapiens reversion-inducing-cysteine-rich protein with kazal motifs (RECK), mRNA. |
| ARFGEF1 | 3.19 | Homo sapiens ADP-ribosylation factor guanine nucleotide-exchange factor 1(brefeldin A-inhibited) (ARFGEF1), mRNA. |
| DYRK3 | 3.18 | Homo sapiens dual-specificity tyrosine-(Y)-phosphorylation regulated kinase 3 (DYRK3), transcript variant 1, mRNA. |
| KIAA0895 | 3.18 | Homo sapiens KIAA0895 (KIAA0895), transcript variant 2, mRNA. |
| ZFP3 | 3.18 | Homo sapiens zinc finger protein 3 homolog (mouse) (ZFP3), mRNA. |
| UBXN7 | 3.18 | Homo sapiens UBX domain protein 7 (UBXN7), mRNA. |
| XBP1 | 3.17 | Homo sapiens X-box binding protein 1 (XBP1), transcript variant 2, mRNA. |
| EIF4E3 | 3.17 | Homo sapiens eukaryotic translation initiation factor 4E family member 3 (EIF4E3), mRNA. |
| CYP2U1 | 3.16 | Homo sapiens cytochrome P450, family 2, subfamily U, polypeptide 1 (CYP2U1), mRNA. |
| CREG1 | 3.15 | Homo sapiens cellular repressor of E1A-stimulated genes 1 (CREG1), mRNA. |
| CASC4 | 3.15 | Homo sapiens cancer susceptibility candidate 4 (CASC4), transcript variant 2, mRNA. |
| OSGIN2 | 3.15 | Homo sapiens oxidative stress induced growth inhibitor family member 2 (OSGIN2), mRNA. |
| TRIM23 | 3.15 | Homo sapiens tripartite motif-containing 23 (TRIM23), transcript variant beta, mRNA. |
| PACSIN3 | 3.14 | Homo sapiens protein kinase C and casein kinase substrate in neurons 3 (PACSIN3), mRNA. |
| TOR1AIP2 | 3.14 | Homo sapiens torsin A interacting protein 2 (TOR1AIP2), mRNA. |
| AGTR1 | 3.13 | Homo sapiens angiotensin II receptor, type 1 (AGTR1), transcript variant 1, mRNA. |
| RNF14 | 3.13 | Homo sapiens ring finger protein 14 (RNF14), transcript variant 4, mRNA. |
| TRIM32 | 3.13 | Homo sapiens tripartite motif-containing 32 (TRIM32), transcript variant 1, mRNA. |
| TUB | 3.13 | Homo sapiens tubby homolog (mouse) (TUB), transcript variant 2, mRNA. |
| PEX11B | 3.12 | Homo sapiens peroxisomal biogenesis factor 11 beta (PEX11B), mRNA. |
| XYLT1 | 3.12 | Homo sapiens xylosyltransferase I (XYLT1), mRNA. |
| KRCC1 | 3.11 | Homo sapiens lysine-rich coiled-coil 1 (KRCC1), mRNA. |
| KCMF1 | 3.11 | PREDICTED: Homo sapiens potassium channel modulatory factor 1 (KCMF1), mRNA. |
| C3orf38 | 3.11 | Homo sapiens chromosome 3 open reading frame 38 (C3orf38), mRNA. |
| MLEC | 3.11 | Homo sapiens malectin (MLEC), mRNA. |
| SMOX | 3.09 | Homo sapiens spermine oxidase (SMOX), transcript variant 4, mRNA. |
| UHRF1 | 3.08 | Homo sapiens ubiquitin-like with PHD and ring finger domains 1 (UHRF1), transcript variant 2, mRNA. |
| FAM135A | 3.07 | Homo sapiens family with sequence similarity 135, member A (FAM135A), mRNA. |
| ACOX1 | 3.07 | Homo sapiens acyl-Coenzyme A oxidase 1, palmitoyl (ACOX1), transcript variant 2, mRNA. |
| FRMD3 | 3.07 | Homo sapiens FERM domain containing 3 (FRMD3), mRNA. |
| STX5 | 3.07 | Homo sapiens syntaxin 5 (STX5), mRNA. |
| CXorf15 | 3.07 | Homo sapiens chromosome X open reading frame 15 (CXorf15), mRNA. |
| SIAH2 | 3.07 | Homo sapiens seven in absentia homolog 2 (Drosophila) (SIAH2), mRNA. |
| CDC42SE1 | 3.06 | Homo sapiens CDC42 small effector 1 (CDC42SE1), transcript variant 1, mRNA. |
| ZFP82 | 3.06 | Homo sapiens zinc finger protein 82 homolog (mouse) (ZFP82), mRNA. |
| C5orf34 | 3.06 | Homo sapiens chromosome 5 open reading frame 34 (C5orf34), mRNA. |
| CANX | 3.06 | Homo sapiens calnexin (CANX), transcript variant 1, mRNA. |
| FAM43A | 3.06 | Homo sapiens family with sequence similarity 43, member A (FAM43A), mRNA. |
| SPIN3 | 3.06 | Homo sapiens spindlin family, member 3 (SPIN3), mRNA. |
| SLC46A1 | 3.05 | Homo sapiens solute carrier family 46 (folate transporter), member 1 (SLC46A1), mRNA. |
|  | 3.05 | full-length cDNA clone XCL0BB001ZD04 of Neuroblastoma of Homo sapiens (human) |
| ARHGAP12 | 3.05 | Homo sapiens Rho GTPase activating protein 12 (ARHGAP12), mRNA. |
| CAPRIN1 | 3.05 | Homo sapiens cell cycle associated protein 1 (CAPRIN1), transcript variant 1, mRNA. |
| USF1 | 3.04 | Homo sapiens upstream transcription factor 1 (USF1), transcript variant 1, mRNA. |
| TLX2 | 3.04 | Homo sapiens T-cell leukemia homeobox 2 (TLX2), mRNA. |
| NAV2 | 3.04 | Homo sapiens neuron navigator 2 (NAV2), transcript variant 2, mRNA. |
| OTUD4 | 3.04 | Homo sapiens OTU domain containing 4 (OTUD4), transcript variant 1, mRNA. |
| TP53BP1 | 3.03 | Homo sapiens tumor protein p53 binding protein 1 (TP53BP1), mRNA. |
| LOC158318 | 3.03 | Homo sapiens similar to hypothetical protein MGC42630 (LOC158318), mRNA. |
| TRIM26 | 3.03 | Homo sapiens tripartite motif-containing 26 (TRIM26), mRNA. |
| C10orf46 | 3.02 | Homo sapiens chromosome 10 open reading frame 46 (C10orf46), mRNA. |
| ATP2A2 | 3.02 | Homo sapiens ATPase, Ca++ transporting, cardiac muscle, slow twitch 2 (ATP2A2), transcript variant 1, mRNA. |
| S100PBP | 3.02 | Homo sapiens S100P binding protein (S100PBP), transcript variant 1, mRNA. |
| SORCS1 | 3 | Homo sapiens sortilin-related VPS10 domain containing receptor 1 (SORCS1), transcript variant 1, mRNA. |
| ZNF264 | 3 | Homo sapiens zinc finger protein 264 (ZNF264), mRNA. |
| FAM126B | 3 | Homo sapiens family with sequence similarity 126, member B (FAM126B), mRNA. |
| CNOT1 | 3 | Homo sapiens CCR4-NOT transcription complex, subunit 1 (CNOT1), transcript variant 2, mRNA. |
| CAPN7 | 3 | Homo sapiens calpain 7 (CAPN7), mRNA. |
|  | 2.99 | Homo sapiens cDNA clone IMAGE:5261213 |
| PANK1 | 2.99 | Homo sapiens pantothenate kinase 1 (PANK1), transcript variant gamma, mRNA. |
| SCAMP1 | 2.99 | Homo sapiens secretory carrier membrane protein 1 (SCAMP1), mRNA. |
| GDAP1 | 2.99 | Homo sapiens ganglioside-induced differentiation-associated protein 1 (GDAP1), mRNA. |
| KCTD3 | 2.99 | Homo sapiens potassium channel tetramerisation domain containing 3 (KCTD3), mRNA. |
| HK2 | 2.98 | Homo sapiens hexokinase 2 (HK2), mRNA. |
| TRA1P2 | 2.98 | PREDICTED: Homo sapiens tumor rejection antigen (gp96) 1 pseudogene 2 (TRA1P2), misc RNA. |
| TMEM22 | 2.97 | Homo sapiens transmembrane protein 22 (TMEM22), transcript variant 2, mRNA. |
| EHHADH | 2.97 | Homo sapiens enoyl-Coenzyme A, hydratase/3-hydroxyacyl Coenzyme A dehydrogenase (EHHADH), mRNA. |
| SLC30A6 | 2.96 | Homo sapiens solute carrier family 30 (zinc transporter), member 6 (SLC30A6), mRNA. |
|  | 2.96 | Homo sapiens cDNA FLJ45905 fis, clone OCBBF3026576 |
| UBAP2L | 2.96 | Homo sapiens ubiquitin associated protein 2-like (UBAP2L), mRNA. |
| ADD3 | 2.96 | Homo sapiens adducin 3 (gamma) (ADD3), transcript variant 1, mRNA. |
| N6AMT1 | 2.96 | Homo sapiens N-6 adenine-specific DNA methyltransferase 1 (putative) (N6AMT1), transcript variant 2, mRNA. |
| GNAQ | 2.95 | Homo sapiens guanine nucleotide binding protein (G protein), q polypeptide (GNAQ), mRNA. |
| FLJ30058 | 2.94 | Homo sapiens hypothetical protein FLJ30058 (FLJ30058), mRNA. |
| PGBD5 | 2.94 | Homo sapiens piggyBac transposable element derived 5 (PGBD5), mRNA. |
| TRIM4 | 2.94 | Homo sapiens tripartite motif-containing 4 (TRIM4), transcript variant beta, mRNA. |
| TRUB1 | 2.93 | Homo sapiens TruB pseudouridine (psi) synthase homolog 1 (E. coli) (TRUB1), mRNA. |
| KCNH2 | 2.93 | Homo sapiens potassium voltage-gated channel, subfamily H (eag-related), member 2 (KCNH2), transcript variant 2, mRNA. |
| IDH3A | 2.93 | Homo sapiens isocitrate dehydrogenase 3 (NAD+) alpha (IDH3A), nuclear gene encoding mitochondrial protein, mRNA. |
| WDR1 | 2.93 | Homo sapiens WD repeat domain 1 (WDR1), transcript variant 1, mRNA. |
| ZNF217 | 2.93 | Homo sapiens zinc finger protein 217 (ZNF217), mRNA. |
| MYCN | 2.93 | Homo sapiens v-myc myelocytomatosis viral related oncogene, neuroblastoma derived (avian) (MYCN), mRNA. |
| C17orf39 | 2.92 | Homo sapiens chromosome 17 open reading frame 39 (C17orf39), mRNA. |
| BAIAP2 | 2.92 | Homo sapiens BAI1-associated protein 2 (BAIAP2), transcript variant 3, mRNA. |
| ZNF324 | 2.92 | Homo sapiens zinc finger protein 324 (ZNF324), mRNA. |
| RPRD1A | 2.92 | Homo sapiens regulation of nuclear pre-mRNA domain containing 1A (RPRD1A), mRNA. |
| SKP2 | 2.92 | Homo sapiens S-phase kinase-associated protein 2 (p45) (SKP2), transcript variant 1, mRNA. |
| ZNF193 | 2.91 | Homo sapiens zinc finger protein 193 (ZNF193), mRNA. |
| CCDC76 | 2.91 | Homo sapiens coiled-coil domain containing 76 (CCDC76), mRNA. |
| DDB1 | 2.91 | Homo sapiens damage-specific DNA binding protein 1, 127kDa (DDB1), mRNA. |
| IGFBP4 | 2.91 | Homo sapiens insulin-like growth factor binding protein 4 (IGFBP4), mRNA. |
| CYLD | 2.91 | Homo sapiens cylindromatosis (turban tumor syndrome) (CYLD), mRNA. |
| CNKSR3 | 2.91 | Homo sapiens CNKSR family member 3 (CNKSR3), mRNA. |
| TIA1 | 2.9 | Homo sapiens TIA1 cytotoxic granule-associated RNA binding protein (TIA1), transcript variant 1, mRNA. |
| CNOT4 | 2.9 | Homo sapiens CCR4-NOT transcription complex, subunit 4 (CNOT4), transcript variant 1, mRNA. |
| MCL1 | 2.9 | Homo sapiens myeloid cell leukemia sequence 1 (BCL2-related) (MCL1), transcript variant 1, mRNA. |
| WDR91 | 2.89 | Homo sapiens WD repeat domain 91 (WDR91), mRNA. |
| TOB1 | 2.89 | Homo sapiens transducer of ERBB2, 1 (TOB1), mRNA. |
| C6orf170 | 2.89 | Homo sapiens chromosome 6 open reading frame 170 (C6orf170), mRNA. |
|  | 2.88 | CR738732 NCI_CGAP_Kid11 Homo sapiens cDNA clone IMAGp971E16101 ; IMAGE:2384145 5, mRNA sequence |
| GTF2IRD2 | 2.88 | PREDICTED: Homo sapiens GTF2I repeat domain containing 2 (GTF2IRD2), mRNA. |
| SSH2 | 2.88 | Homo sapiens slingshot homolog 2 (Drosophila) (SSH2), mRNA. |
| ZNF17 | 2.88 | Homo sapiens zinc finger protein 17 (ZNF17), mRNA. |
| GLRB | 2.87 | Homo sapiens glycine receptor, beta (GLRB), mRNA. |
|  | 2.87 | Homo sapiens cDNA clone IMAGE:5299888 |
|  | 2.86 | full-length cDNA clone CS0DC013YI10 of Neuroblastoma Cot 25-normalized of Homo sapiens (human) |
| NSUN3 | 2.86 | Homo sapiens NOP2/Sun domain family, member 3 (NSUN3), mRNA. |
| PDZD8 | 2.86 | Homo sapiens PDZ domain containing 8 (PDZD8), mRNA. |
| C2orf44 | 2.86 | Homo sapiens chromosome 2 open reading frame 44 (C2orf44), mRNA. |
| SLC39A10 | 2.86 | Homo sapiens solute carrier family 39 (zinc transporter), member 10 (SLC39A10), mRNA. |
| ZNF330 | 2.86 | Homo sapiens zinc finger protein 330 (ZNF330), mRNA. |
| ARMCX3 | 2.85 | Homo sapiens armadillo repeat containing, X-linked 3 (ARMCX3), transcript variant 2, mRNA. |
| GLUD1 | 2.85 | Homo sapiens glutamate dehydrogenase 1 (GLUD1), mRNA. |
| TTC9C | 2.84 | Homo sapiens tetratricopeptide repeat domain 9C (TTC9C), mRNA. |
| ZNF434 | 2.84 | Homo sapiens zinc finger protein 434 (ZNF434), mRNA. |
| KIAA1737 | 2.84 | Homo sapiens KIAA1737 (KIAA1737), mRNA. |
| BCDIN3D | 2.84 | Homo sapiens BCDIN3 domain containing (BCDIN3D), mRNA. |
| MGC42630 | 2.84 | Homo sapiens hypothetical protein MGC42630 (MGC42630), mRNA. |
| C14orf118 | 2.84 | Homo sapiens chromosome 14 open reading frame 118 (C14orf118), transcript variant 1, mRNA. |
| H19 | 2.84 | Homo sapiens H19, imprinted maternally expressed transcript (non-protein coding) (H19), non-coding RNA. |
| C1QL1 | 2.83 | Homo sapiens complement component 1, q subcomponent-like 1 (C1QL1), mRNA. |
| IKZF5 | 2.83 | Homo sapiens IKAROS family zinc finger 5 (Pegasus) (IKZF5), mRNA. |
| OPN3 | 2.83 | Homo sapiens opsin 3 (OPN3), mRNA. |
| HERC4 | 2.83 | Homo sapiens hect domain and RLD 4 (HERC4), transcript variant 1, mRNA. |
| FGF14 | 2.83 | Homo sapiens fibroblast growth factor 14 (FGF14), transcript variant 2, mRNA. |
| FNIP1 | 2.82 | Homo sapiens folliculin interacting protein 1 (FNIP1), transcript variant 2, mRNA. |
| ZNF77 | 2.82 | Homo sapiens zinc finger protein 77 (ZNF77), mRNA. |
| NOC3L | 2.81 | Homo sapiens nucleolar complex associated 3 homolog (S. cerevisiae) (NOC3L), mRNA. |
| HIVEP1 | 2.81 | Homo sapiens human immunodeficiency virus type I enhancer binding protein 1 (HIVEP1), mRNA. |
| ZNF189 | 2.81 | Homo sapiens zinc finger protein 189 (ZNF189), transcript variant 1, mRNA. |
| SPRY4 | 2.81 | Homo sapiens sprouty homolog 4 (Drosophila) (SPRY4), mRNA. |
| ARMCX3 | 2.81 | Homo sapiens armadillo repeat containing, X-linked 3 (ARMCX3), transcript variant 2, mRNA. |
| RFFL | 2.81 | Homo sapiens ring finger and FYVE-like domain containing 1 (RFFL), transcript variant 1, mRNA. |
| PIM2 | 2.8 | Homo sapiens pim-2 oncogene (PIM2), mRNA. |
| ZNF419 | 2.79 | Homo sapiens zinc finger protein 419 (ZNF419), transcript variant 6, mRNA. |
| CTDSPL | 2.78 | Homo sapiens CTD (carboxy-terminal domain, RNA polymerase II, polypeptide A) small phosphatase-like (CTDSPL), transcript variant 2, mRNA. |
| PCBP4 | 2.78 | Homo sapiens poly(rC) binding protein 4 (PCBP4), transcript variant 3, mRNA. |
| DUSP4 | 2.78 | Homo sapiens dual specificity phosphatase 4 (DUSP4), transcript variant 1, mRNA. |
| PHF12 | 2.78 | Homo sapiens PHD finger protein 12 (PHF12), transcript variant 1, mRNA. |
| KBTBD8 | 2.78 | Homo sapiens kelch repeat and BTB (POZ) domain containing 8 (KBTBD8), mRNA. |
| PJA1 | 2.77 | Homo sapiens praja ring finger 1 (PJA1), transcript variant 2, mRNA. |
| RNF14 | 2.77 | Homo sapiens ring finger protein 14 (RNF14), transcript variant 3, mRNA. |
| ARMET | 2.77 | Homo sapiens arginine-rich, mutated in early stage tumors (ARMET), mRNA. |
| TSPYL2 | 2.77 | Homo sapiens TSPY-like 2 (TSPYL2), mRNA. |
| SFRS17A | 2.76 | Homo sapiens splicing factor, arginine/serine-rich 17A (SFRS17A), transcript variant 1, mRNA. |
| ITGB5 | 2.76 | Homo sapiens integrin, beta 5 (ITGB5), mRNA. XM_944688 XM_944693 |
| GNG8 | 2.76 | Homo sapiens guanine nucleotide binding protein (G protein), gamma 8 (GNG8), mRNA. |
| ASCC3 | 2.76 | Homo sapiens activating signal cointegrator 1 complex subunit 3 (ASCC3), transcript variant 1, mRNA. |
| ERF | 2.76 | Homo sapiens Ets2 repressor factor (ERF), mRNA. |
| U2AF2 | 2.75 | Homo sapiens U2 small nuclear RNA auxiliary factor 2 (U2AF2), transcript variant 1, mRNA. |
| RFXDC2 | 2.75 | Homo sapiens regulatory factor X domain containing 2 (RFXDC2), mRNA. |
| SYS1 | 2.75 | Homo sapiens SYS1 Golgi-localized integral membrane protein homolog (S. cerevisiae) (SYS1), transcript variant 1, mRNA. |
| KIAA0907 | 2.75 | Homo sapiens KIAA0907 (KIAA0907), mRNA. |
| GTF2E1 | 2.74 | Homo sapiens general transcription factor IIE, polypeptide 1 (alpha subunit, 56kD) (GTF2E1), mRNA. |
|  | 2.74 | Homo sapiens mRNA; cDNA DKFZp761D112 (from clone DKFZp761D112) |
| CTNNB1 | 2.74 | Homo sapiens catenin (cadherin-associated protein), beta 1, 88kDa (CTNNB1), transcript variant 2, mRNA. |
| RNF146 | 2.74 | Homo sapiens ring finger protein 146 (RNF146), mRNA. |
| ZCCHC9 | 2.73 | Homo sapiens zinc finger, CCHC domain containing 9 (ZCCHC9), mRNA. |
| RAB23 | 2.73 | Homo sapiens RAB23, member RAS oncogene family (RAB23), transcript variant 1, mRNA. |
| TINF2 | 2.73 | Homo sapiens TERF1 (TRF1)-interacting nuclear factor 2 (TINF2), mRNA. |
| LOC650832 | 2.73 | PREDICTED: Homo sapiens similar to mitogen-activated protein kinase kinase 3 isoform A (LOC650832), mRNA. |
| ZCCHC3 | 2.72 | Homo sapiens zinc finger, CCHC domain containing 3 (ZCCHC3), mRNA. |
| CSE1L | 2.72 | Homo sapiens CSE1 chromosome segregation 1-like (yeast) (CSE1L), transcript variant 2, mRNA. |
| CCNE1 | 2.72 | Homo sapiens cyclin E1 (CCNE1), transcript variant 2, mRNA. |
| ZDHHC6 | 2.72 | Homo sapiens zinc finger, DHHC-type containing 6 (ZDHHC6), mRNA. |
| ANKRD46 | 2.71 | Homo sapiens ankyrin repeat domain 46 (ANKRD46), mRNA. |
| GTF2IP1 | 2.71 | Homo sapiens general transcription factor II, i, pseudogene 1 (GTF2IP1) on chromosome 7. |
| PPP2R5A | 2.71 | Homo sapiens protein phosphatase 2, regulatory subunit B', alpha isoform (PPP2R5A), mRNA. |
| RECK | 2.71 | Homo sapiens reversion-inducing-cysteine-rich protein with kazal motifs (RECK), mRNA. |
| NSUN5 | 2.71 | Homo sapiens NOL1/NOP2/Sun domain family, member 5 (NSUN5), transcript variant 2, mRNA. |
| DNAJB9 | 2.71 | Homo sapiens DnaJ (Hsp40) homolog, subfamily B, member 9 (DNAJB9), mRNA. |
| RNF160 | 2.7 | Homo sapiens ring finger protein 160 (RNF160), mRNA. |
| STXBP1 | 2.7 | Homo sapiens syntaxin binding protein 1 (STXBP1), transcript variant 1, mRNA. |
| PKM2 | 2.7 | Homo sapiens pyruvate kinase, muscle (PKM2), transcript variant 2, mRNA. |
| TXNDC5 | 2.7 | Homo sapiens thioredoxin domain containing 5 (endoplasmic reticulum) (TXNDC5), transcript variant 1, mRNA. |
| PPM1B | 2.7 | Homo sapiens protein phosphatase 1B (formerly 2C), magnesium-dependent, beta isoform (PPM1B), transcript variant 3, mRNA. |
| GTF2I | 2.69 | Homo sapiens general transcription factor II, i (GTF2I), transcript variant 3, mRNA. |
| ONECUT2 | 2.69 | Homo sapiens one cut homeobox 2 (ONECUT2), mRNA. |
| DNMT3A | 2.69 | Homo sapiens DNA (cytosine-5-)-methyltransferase 3 alpha (DNMT3A), transcript variant 2, mRNA. |
| PDIK1L | 2.69 | Homo sapiens PDLIM1 interacting kinase 1 like (PDIK1L), mRNA. |
| PNCK | 2.69 | Homo sapiens pregnancy upregulated non-ubiquitously expressed CaM kinase (PNCK), mRNA. |
| THYN1 | 2.69 | Homo sapiens thymocyte nuclear protein 1 (THYN1), transcript variant 1, mRNA. |
| LRCH4 | 2.68 | Homo sapiens leucine-rich repeats and calponin homology (CH) domain containing 4 (LRCH4), mRNA. |
| MKLN1 | 2.68 | Homo sapiens muskelin 1, intracellular mediator containing kelch motifs (MKLN1), mRNA. |
| LOC653199 | 2.68 | PREDICTED: Homo sapiens hypothetical LOC653199 (LOC653199), mRNA. |
| TGDS | 2.68 | Homo sapiens TDP-glucose 4,6-dehydratase (TGDS), mRNA. |
| PDE7A | 2.67 | Homo sapiens phosphodiesterase 7A (PDE7A), transcript variant 2, mRNA. |
| UBP1 | 2.67 | Homo sapiens upstream binding protein 1 (LBP-1a) (UBP1), mRNA. |
| INTS4 | 2.67 | Homo sapiens integrator complex subunit 4 (INTS4), mRNA. |
| SEH1L | 2.66 | Homo sapiens SEH1-like (S. cerevisiae) (SEH1L), transcript variant 1, mRNA. |
| NPC1 | 2.66 | Homo sapiens Niemann-Pick disease, type C1 (NPC1), mRNA. |
| STC2 | 2.66 | Homo sapiens stanniocalcin 2 (STC2), mRNA. |
| INSIG2 | 2.66 | Homo sapiens insulin induced gene 2 (INSIG2), mRNA. |
| TRIB1 | 2.66 | Homo sapiens tribbles homolog 1 (Drosophila) (TRIB1), mRNA. |
| PEX1 | 2.66 | Homo sapiens peroxisome biogenesis factor 1 (PEX1), mRNA. |
| LBR | 2.66 | Homo sapiens lamin B receptor (LBR), transcript variant 1, mRNA. |
| EFR3A | 2.66 | Homo sapiens EFR3 homolog A (S. cerevisiae) (EFR3A), mRNA. |
| DDX31 | 2.66 | Homo sapiens DEAD (Asp-Glu-Ala-Asp) box polypeptide 31 (DDX31), transcript variant 2, mRNA. |
| JAG1 | 2.65 | Homo sapiens jagged 1 (Alagille syndrome) (JAG1), mRNA. |
|  | 2.65 | 17000532640995 GRN_ES Homo sapiens cDNA 5, mRNA sequence |
| PARG | 2.65 | Homo sapiens poly (ADP-ribose) glycohydrolase (PARG), mRNA. |
| KIAA1429 | 2.65 | Homo sapiens KIAA1429 (KIAA1429), transcript variant 1, mRNA. |
| HSPA4 | 2.64 | Homo sapiens heat shock 70kDa protein 4 (HSPA4), mRNA. |
| ANKRD13D | 2.64 | PREDICTED: Homo sapiens ankyrin repeat domain 13 family, member D, transcript variant 7 (ANKRD13D), mRNA. |
| PLEKHA1 | 2.64 | Homo sapiens pleckstrin homology domain containing, family A (phosphoinositide binding specific) member 1 (PLEKHA1), transcript variant 2, mRNA. |
| GOLGA1 | 2.64 | Homo sapiens golgi autoantigen, golgin subfamily a, 1 (GOLGA1), mRNA. |
| GART | 2.64 | Homo sapiens phosphoribosylglycinamide formyltransferase, phosphoribosylglycinamide synthetase, phosphoribosylaminoimidazole synthetase (GART), transcript variant 1, mRNA. |
| RGS16 | 2.64 | Homo sapiens regulator of G-protein signalling 16 (RGS16), mRNA. |
| HIP1R | 2.64 | Homo sapiens huntingtin interacting protein 1 related (HIP1R), mRNA. |
| CAND1 | 2.64 | Homo sapiens cullin-associated and neddylation-dissociated 1 (CAND1), mRNA. |
| PHF1 | 2.64 | Homo sapiens PHD finger protein 1 (PHF1), transcript variant 2, mRNA. |
| UQCC | 2.63 | Homo sapiens ubiquinol-cytochrome c reductase complex chaperone (UQCC), nuclear gene encoding mitochondrial protein, transcript variant 2, mRNA. |
| TNFRSF25 | 2.63 | Homo sapiens tumor necrosis factor receptor superfamily, member 25 (TNFRSF25), transcript variant 10, mRNA. |
| PGAM4 | 2.63 | Homo sapiens phosphoglycerate mutase family member 4 (PGAM4), mRNA. |
| ZNF649 | 2.63 | Homo sapiens zinc finger protein 649 (ZNF649), mRNA. |
| MPP5 | 2.63 | Homo sapiens membrane protein, palmitoylated 5 (MAGUK p55 subfamily member 5) (MPP5), mRNA. |
| C12orf4 | 2.63 | Homo sapiens chromosome 12 open reading frame 4 (C12orf4), mRNA. |
| PARP9 | 2.62 | Homo sapiens poly (ADP-ribose) polymerase family, member 9 (PARP9), mRNA. |
| C1orf31 | 2.62 | Homo sapiens chromosome 1 open reading frame 31 (C1orf31), mRNA. |
| HIST2H2BE | 2.61 | Homo sapiens histone cluster 2, H2be (HIST2H2BE), mRNA. |
| MYOZ3 | 2.61 | Homo sapiens myozenin 3 (MYOZ3), mRNA. |
| MYB | 2.61 | Homo sapiens v-myb myeloblastosis viral oncogene homolog (avian) (MYB), transcript variant 2, mRNA. |
|  | 2.61 | Homo sapiens primary neuroblastoma cDNA, clone:Nbla10111, full insert sequence |
| LPGAT1 | 2.6 | Homo sapiens lysophosphatidylglycerol acyltransferase 1 (LPGAT1), mRNA. |
| RIPK5 | 2.6 | Homo sapiens receptor interacting protein kinase 5 (RIPK5), transcript variant 1, mRNA. |
| BTBD10 | 2.6 | Homo sapiens BTB (POZ) domain containing 10 (BTBD10), mRNA. |
| C1orf74 | 2.6 | Homo sapiens chromosome 1 open reading frame 74 (C1orf74), mRNA. |
| FKBP1A | 2.6 | Homo sapiens FK506 binding protein 1A, 12kDa (FKBP1A), transcript variant 12A, mRNA. |
| CCDC121 | 2.59 | Homo sapiens coiled-coil domain containing 121 (CCDC121), mRNA. |
| FTSJD1 | 2.59 | Homo sapiens FtsJ methyltransferase domain containing 1 (FTSJD1), transcript variant 1, mRNA. |
| SLC39A6 | 2.59 | Homo sapiens solute carrier family 39 (zinc transporter), member 6 (SLC39A6), transcript variant 1, mRNA. |
| PRR7 | 2.59 | Homo sapiens proline rich 7 (synaptic) (PRR7), mRNA. |
| SCLY | 2.59 | Homo sapiens selenocysteine lyase (SCLY), mRNA. |
| NUFIP2 | 2.58 | Homo sapiens nuclear fragile X mental retardation protein interacting protein 2 (NUFIP2), mRNA. |
| METTL14 | 2.58 | Homo sapiens methyltransferase like 14 (METTL14), mRNA. |
|  | 2.58 | DA830074 PLACE1 Homo sapiens cDNA clone PLACE1004374 5, mRNA sequence |
| ZNF285A | 2.57 | Homo sapiens zinc finger protein 285A (ZNF285A), mRNA. |
| CASC4 | 2.57 | Homo sapiens cancer susceptibility candidate 4 (CASC4), transcript variant 1, mRNA. |
| ATP8B2 | 2.57 | Homo sapiens ATPase, class I, type 8B, member 2 (ATP8B2), transcript variant 1, mRNA. |
| IVNS1ABP | 2.57 | Homo sapiens influenza virus NS1A binding protein (IVNS1ABP), mRNA. |
| PKM2 | 2.57 | Homo sapiens pyruvate kinase, muscle (PKM2), transcript variant 1, mRNA. |
| RIMBP2 | 2.57 | Homo sapiens RIMS binding protein 2 (RIMBP2), mRNA. |
| RNF19A | 2.56 | Homo sapiens ring finger protein 19A (RNF19A), transcript variant 2, mRNA. |
| KCTD2 | 2.56 | Homo sapiens potassium channel tetramerisation domain containing 2 (KCTD2), mRNA. |
| PI4K2A | 2.56 | Homo sapiens phosphatidylinositol 4-kinase type 2 alpha (PI4K2A), mRNA. |
| TAF5L | 2.56 | Homo sapiens TAF5-like RNA polymerase II, p300/CBP-associated factor (PCAF)-associated factor, 65kDa (TAF5L), transcript variant 1, mRNA. |
| MBOAT2 | 2.56 | PREDICTED: Homo sapiens membrane bound O-acyltransferase domain containing 2 (MBOAT2), mRNA. |
| CRMP1 | 2.56 | Homo sapiens collapsin response mediator protein 1 (CRMP1), transcript variant 2, mRNA. |
| STAC | 2.56 | Homo sapiens SH3 and cysteine rich domain (STAC), mRNA. |
| ASPHD2 | 2.55 | Homo sapiens aspartate beta-hydroxylase domain containing 2 (ASPHD2), mRNA. |
| LRRN2 | 2.55 | Homo sapiens leucine rich repeat neuronal 2 (LRRN2), transcript variant 2, mRNA. |
| SCYL1BP1 | 2.55 | Homo sapiens SCY1-like 1 binding protein 1 (SCYL1BP1), mRNA. |
| ATPIF1 | 2.54 | Homo sapiens ATPase inhibitory factor 1 (ATPIF1), nuclear gene encoding mitochondrial protein, transcript variant 3, mRNA. |
| TMTC4 | 2.54 | Homo sapiens transmembrane and tetratricopeptide repeat containing 4 (TMTC4), transcript variant 1, mRNA. |
| C11orf61 | 2.54 | Homo sapiens chromosome 11 open reading frame 61 (C11orf61), mRNA. |
| PLCH2 | 2.54 | Homo sapiens phospholipase C, eta 2 (PLCH2), mRNA. |
| EARS2 | 2.53 | Homo sapiens glutamyl-tRNA synthetase 2, mitochondrial (putative) (EARS2), transcript variant 2, non-coding RNA. |
| KIAA0133 | 2.53 | Homo sapiens KIAA0133 (KIAA0133), mRNA. |
| MIER3 | 2.53 | Homo sapiens mesoderm induction early response 1, family member 3 (MIER3), mRNA. |
| PARD6A | 2.53 | Homo sapiens par-6 partitioning defective 6 homolog alpha (C. elegans) (PARD6A), transcript variant 1, mRNA. |
| ARID4A | 2.53 | Homo sapiens AT rich interactive domain 4A (RBP1-like) (ARID4A), transcript variant 3, mRNA. |
| POU3F2 | 2.52 | Homo sapiens POU class 3 homeobox 2 (POU3F2), mRNA. |
|  | 2.51 | Homo sapiens cDNA FLJ34755 fis, clone NHNPC1000034 |
| CLK1 | 2.51 | Homo sapiens CDC-like kinase 1 (CLK1), transcript variant 2, mRNA. |
| MCCC2 | 2.51 | Homo sapiens methylcrotonoyl-Coenzyme A carboxylase 2 (beta) (MCCC2), mRNA. |
| DBR1 | 2.51 | Homo sapiens debranching enzyme homolog 1 (S. cerevisiae) (DBR1), mRNA. |
| ARMC10 | 2.51 | Homo sapiens armadillo repeat containing 10 (ARMC10), mRNA. |
| TYW3 | 2.5 | Homo sapiens tRNA-yW synthesizing protein 3 homolog (S. cerevisiae) (TYW3), mRNA. |
| C5orf51 | 2.5 | Homo sapiens chromosome 5 open reading frame 51 (C5orf51), mRNA. |
| DIXDC1 | 2.5 | Homo sapiens DIX domain containing 1 (DIXDC1), transcript variant 1, mRNA. |
| WDR44 | 2.5 | Homo sapiens WD repeat domain 44 (WDR44), mRNA. |
| RRP1B | 2.5 | Homo sapiens ribosomal RNA processing 1 homolog B (S. cerevisiae) (RRP1B), mRNA. |
| PRKAB2 | 2.5 | Homo sapiens protein kinase, AMP-activated, beta 2 non-catalytic subunit (PRKAB2), mRNA. |
| PLD5 | 2.5 | Homo sapiens phospholipase D family, member 5 (PLD5), mRNA. |
| MID1IP1 | 2.49 | Homo sapiens MID1 interacting protein 1 (gastrulation specific G12 homolog (zebrafish)) (MID1IP1), transcript variant 1, mRNA. |
| NRAS | 2.49 | Homo sapiens neuroblastoma RAS viral (v-ras) oncogene homolog (NRAS), mRNA. |
| ZNF594 | 2.49 | Homo sapiens zinc finger protein 594 (ZNF594), mRNA. |
| ZNF239 | 2.49 | Homo sapiens zinc finger protein 239 (ZNF239), transcript variant 4, mRNA. |
| FXC1 | 2.49 | Homo sapiens fracture callus 1 homolog (rat) (FXC1), nuclear gene encoding mitochondrial protein, mRNA. |
| AFTPH | 2.49 | Homo sapiens aftiphilin (AFTPH), transcript variant 1, mRNA. |
| GULP1 | 2.49 | Homo sapiens GULP, engulfment adaptor PTB domain containing 1 (GULP1), mRNA. |
| FKBP1A | 2.49 | Homo sapiens FK506 binding protein 1A, 12kDa (FKBP1A), transcript variant 12A, mRNA. |
| FGFR3 | 2.48 | Homo sapiens fibroblast growth factor receptor 3 (achondroplasia, thanatophoric dwarfism) (FGFR3), transcript variant 2, mRNA. |
| C22orf29 | 2.48 | Homo sapiens chromosome 22 open reading frame 29 (C22orf29), mRNA. |
| IPO5 | 2.48 | Homo sapiens importin 5 (IPO5), mRNA. |
| ACTA2 | 2.48 | Homo sapiens actin, alpha 2, smooth muscle, aorta (ACTA2), mRNA. |
| LOC285908 | 2.48 | Homo sapiens hypothetical protein LOC285908 (LOC285908), mRNA. |
|  | 2.47 | Homo sapiens cDNA FLJ45619 fis, clone BRTHA3027318 |
| PCNX | 2.47 | Homo sapiens pecanex homolog (Drosophila) (PCNX), mRNA. |
| HIATL1 | 2.47 | Homo sapiens hippocampus abundant transcript-like 1 (HIATL1), mRNA. |
| POLR1B | 2.47 | Homo sapiens polymerase (RNA) I polypeptide B, 128kDa (POLR1B), mRNA. |
| MGC3020 | 2.46 | Homo sapiens hypothetical protein MGC3020 (MGC3020), mRNA. |
| MTMR12 | 2.46 | Homo sapiens myotubularin related protein 12 (MTMR12), mRNA. |
| C19orf44 | 2.46 | Homo sapiens chromosome 19 open reading frame 44 (C19orf44), mRNA. |
| MLL5 | 2.46 | Homo sapiens myeloid/lymphoid or mixed-lineage leukemia 5 (trithorax homolog, Drosophila) (MLL5), transcript variant 2, mRNA. |
| ZNF280C | 2.45 | Homo sapiens zinc finger protein 280C (ZNF280C), mRNA. |
| MRAS | 2.45 | Homo sapiens muscle RAS oncogene homolog (MRAS), transcript variant 1, mRNA. |
| RUNDC1 | 2.45 | Homo sapiens RUN domain containing 1 (RUNDC1), mRNA. |
| HK2 | 2.45 | Homo sapiens hexokinase 2 (HK2), mRNA. |
| PICALM | 2.45 | Homo sapiens phosphatidylinositol binding clathrin assembly protein (PICALM), transcript variant 1, mRNA. |
| PRKAR2A | 2.45 | Homo sapiens protein kinase, cAMP-dependent, regulatory, type II, alpha (PRKAR2A), mRNA. |
| ALKBH8 | 2.44 | Homo sapiens alkB, alkylation repair homolog 8 (E. coli) (ALKBH8), mRNA. |
| SH2B2 | 2.44 | Homo sapiens SH2B adaptor protein 2 (SH2B2), mRNA. |
| TP53INP1 | 2.44 | Homo sapiens tumor protein p53 inducible nuclear protein 1 (TP53INP1), mRNA. |
| B3GAT3 | 2.44 | Homo sapiens beta-1,3-glucuronyltransferase 3 (glucuronosyltransferase I) (B3GAT3), mRNA. |
|  | 2.44 | BX093763 Soares_fetal_heart_NbHH19W Homo sapiens cDNA clone IMAGp998N10870, mRNA sequence |
| SLC4A7 | 2.44 | Homo sapiens solute carrier family 4, sodium bicarbonate cotransporter, member 7 (SLC4A7), mRNA. |
| TULP4 | 2.44 | Homo sapiens tubby like protein 4 (TULP4), transcript variant 2, mRNA. |
|  | 2.44 | full-length cDNA clone CS0DF005YI08 of Fetal brain of Homo sapiens (human) |
| MANEAL | 2.43 | Homo sapiens mannosidase, endo-alpha-like (MANEAL), transcript variant 1, mRNA. |
| HEY1 | 2.43 | Homo sapiens hairy/enhancer-of-split related with YRPW motif 1 (HEY1), transcript variant 2, mRNA. |
| ZBTB9 | 2.43 | Homo sapiens zinc finger and BTB domain containing 9 (ZBTB9), mRNA. |
| DNAJC24 | 2.43 | Homo sapiens DnaJ (Hsp40) homolog, subfamily C, member 24 (DNAJC24), mRNA. |
| RFP | 2.43 | Homo sapiens ret finger protein (RFP), transcript variant beta, mRNA. |
| ELN | 2.43 | Homo sapiens elastin (ELN), transcript variant 4, mRNA. |
| USP21 | 2.43 | Homo sapiens ubiquitin specific peptidase 21 (USP21), transcript variant 1, mRNA. |
| TTC14 | 2.42 | Homo sapiens tetratricopeptide repeat domain 14 (TTC14), transcript variant 2, mRNA. |
| NR2C1 | 2.42 | Homo sapiens nuclear receptor subfamily 2, group C, member 1 (NR2C1), transcript variant 1, mRNA. |
| KRAS | 2.42 | Homo sapiens v-Ki-ras2 Kirsten rat sarcoma viral oncogene homolog (KRAS), transcript variant a, mRNA. |
| CSTF3 | 2.41 | Homo sapiens cleavage stimulation factor, 3' pre-RNA, subunit 3, 77kDa (CSTF3), transcript variant 3, mRNA. |
| HYOU1 | 2.41 | Homo sapiens hypoxia up-regulated 1 (HYOU1), mRNA. |
| NHLRC3 | 2.41 | Homo sapiens NHL repeat containing 3 (NHLRC3), transcript variant 2, mRNA. |
| TNRC6A | 2.41 | Homo sapiens trinucleotide repeat containing 6A (TNRC6A), mRNA. |
| LOC653210 | 2.41 | PREDICTED: Homo sapiens similar to melanoma antigen family D, 4 isoform 1, transcript variant 2 (LOC653210), mRNA. |
| GARNL4 | 2.41 | Homo sapiens GTPase activating Rap/RanGAP domain-like 4 (GARNL4), mRNA. |
|  | 2.41 | Homo sapiens clone 24583 mRNA sequence |
| HDAC4 | 2.41 | Homo sapiens histone deacetylase 4 (HDAC4), mRNA. |
| PLEKHF1 | 2.41 | Homo sapiens pleckstrin homology domain containing, family F (with FYVE domain) member 1 (PLEKHF1), mRNA. |
| ALDH1B1 | 2.41 | Homo sapiens aldehyde dehydrogenase 1 family, member B1 (ALDH1B1), nuclear gene encoding mitochondrial protein, mRNA. |
| SHQ1 | 2.4 | Homo sapiens SHQ1 homolog (S. cerevisiae) (SHQ1), mRNA. |
| TBK1 | 2.4 | Homo sapiens TANK-binding kinase 1 (TBK1), mRNA. |
| UBFD1 | 2.4 | Homo sapiens ubiquitin family domain containing 1 (UBFD1), mRNA. |
| YTHDF3 | 2.4 | Homo sapiens YTH domain family, member 3 (YTHDF3), mRNA. |
| PTCHD2 | 2.4 | PREDICTED: Homo sapiens patched domain containing 2 (PTCHD2), mRNA. |
| LETM1 | 2.39 | Homo sapiens leucine zipper-EF-hand containing transmembrane protein 1 (LETM1), nuclear gene encoding mitochondrial protein, mRNA. |
| CHGB | 2.39 | Homo sapiens chromogranin B (secretogranin 1) (CHGB), mRNA. |
| ZNF10 | 2.39 | Homo sapiens zinc finger protein 10 (ZNF10), mRNA. |
| AGPAT5 | 2.39 | Homo sapiens 1-acylglycerol-3-phosphate O-acyltransferase 5 (lysophosphatidic acid acyltransferase, epsilon) (AGPAT5), mRNA. |
| CTNNB1 | 2.38 | Homo sapiens catenin (cadherin-associated protein), beta 1, 88kDa (CTNNB1), transcript variant 2, mRNA. |
| FEM1C | 2.38 | Homo sapiens fem-1 homolog c (C. elegans) (FEM1C), mRNA. |
| FIGNL1 | 2.38 | Homo sapiens fidgetin-like 1 (FIGNL1), transcript variant 1, mRNA. |
| LONRF1 | 2.38 | Homo sapiens LON peptidase N-terminal domain and ring finger 1 (LONRF1), mRNA. |
| CCNE2 | 2.38 | Homo sapiens cyclin E2 (CCNE2), transcript variant 2, mRNA. |
| PPP2R2A | 2.38 | Homo sapiens protein phosphatase 2 (formerly 2A), regulatory subunit B, alpha isoform (PPP2R2A), mRNA. |
| SUOX | 2.37 | Homo sapiens sulfite oxidase (SUOX), nuclear gene encoding mitochondrial protein, transcript variant 2, mRNA. |
| BIRC2 | 2.37 | Homo sapiens baculoviral IAP repeat-containing 2 (BIRC2), mRNA. |
| KIAA1826 | 2.37 | Homo sapiens KIAA1826 (KIAA1826), mRNA. |
| GSR | 2.37 | Homo sapiens glutathione reductase (GSR), mRNA. |
| ZNF746 | 2.37 | Homo sapiens zinc finger protein 746 (ZNF746), mRNA. |
| FAM21A | 2.36 | Homo sapiens family with sequence similarity 21, member A (FAM21A), mRNA. |
| C3orf63 | 2.36 | Homo sapiens chromosome 3 open reading frame 63 (C3orf63), mRNA. |
| KIF7 | 2.36 | Homo sapiens kinesin family member 7 (KIF7), mRNA. |
| LOC642031 | 2.36 | PREDICTED: Homo sapiens hypothetical protein LOC642031 (LOC642031), mRNA. |
| SPATA2 | 2.36 | Homo sapiens spermatogenesis associated 2 (SPATA2), mRNA. |
| CLASP2 | 2.35 | Homo sapiens cytoplasmic linker associated protein 2 (CLASP2), mRNA. |
| C7orf43 | 2.35 | Homo sapiens chromosome 7 open reading frame 43 (C7orf43), mRNA. |
| CCDC132 | 2.35 | Homo sapiens coiled-coil domain containing 132 (CCDC132), transcript variant 2, mRNA. |
| ZNF609 | 2.35 | Homo sapiens zinc finger protein 609 (ZNF609), mRNA. |
| BCOR | 2.35 | Homo sapiens BCL6 co-repressor (BCOR), transcript variant 1, mRNA. |
| ZNF416 | 2.35 | Homo sapiens zinc finger protein 416 (ZNF416), mRNA. |
| SYT7 | 2.35 | Homo sapiens synaptotagmin VII (SYT7), mRNA. |
| LACTB | 2.34 | Homo sapiens lactamase, beta (LACTB), nuclear gene encoding mitochondrial protein, transcript variant 1, mRNA. |
|  | 2.34 | Homo sapiens cDNA FLJ40776 fis, clone TRACH2005210 |
| TSPYL2 | 2.33 | Homo sapiens TSPY-like 2 (TSPYL2), mRNA. |
| MGAT4B | 2.33 | Homo sapiens mannosyl (alpha-1,3-)-glycoprotein beta-1,4-N-acetylglucosaminyltransferase, isozyme B (MGAT4B), transcript variant 2, mRNA. |
| PAN3 | 2.33 | Homo sapiens PAN3 polyA specific ribonuclease subunit homolog (S. cerevisiae) (PAN3), mRNA. |
| TM2D2 | 2.33 | Homo sapiens TM2 domain containing 2 (TM2D2), transcript variant 1, mRNA. |
| MLL | 2.33 | Homo sapiens myeloid/lymphoid or mixed-lineage leukemia (trithorax homolog, Drosophila) (MLL), mRNA. |
| ETV5 | 2.32 | Homo sapiens ets variant gene 5 (ets-related molecule) (ETV5), mRNA. |
| CSNK1A1 | 2.32 | Homo sapiens casein kinase 1, alpha 1 (CSNK1A1), transcript variant 1, mRNA. |
| C7orf70 | 2.32 | Homo sapiens chromosome 7 open reading frame 70 (C7orf70), mRNA. |
| NSF | 2.32 | Homo sapiens N-ethylmaleimide-sensitive factor (NSF), mRNA. |
| RSBN1 | 2.32 | Homo sapiens round spermatid basic protein 1 (RSBN1), mRNA. |
| URG4 | 2.32 | Homo sapiens up-regulated gene 4 (URG4), nuclear gene encoding mitochondrial protein, transcript variant 2, mRNA. |
| EXOSC3 | 2.32 | Homo sapiens exosome component 3 (EXOSC3), transcript variant 1, mRNA. |
| MED23 | 2.32 | Homo sapiens mediator complex subunit 23 (MED23), transcript variant 1, mRNA. |
| C9orf6 | 2.32 | Homo sapiens chromosome 9 open reading frame 6 (C9orf6), mRNA. |
| FRAS1 | 2.32 | Homo sapiens Fraser syndrome 1 (FRAS1), mRNA. |
| LOC402509 | 2.31 | PREDICTED: Homo sapiens similar to solute carrier family 29 (nucleoside transporters), member 4 (LOC402509), mRNA. |
| ATP6V0A2 | 2.31 | Homo sapiens ATPase, H+ transporting, lysosomal V0 subunit a2 (ATP6V0A2), mRNA. |
| SHRM | 2.31 | Homo sapiens shroom (SHRM), mRNA. |
| KIAA1737 | 2.31 | Homo sapiens KIAA1737 (KIAA1737), mRNA. |
| ADCY1 | 2.31 | Homo sapiens adenylate cyclase 1 (brain) (ADCY1), mRNA. |
| C19orf6 | 2.31 | Homo sapiens chromosome 19 open reading frame 6 (C19orf6), transcript variant 1, mRNA. |
| TES | 2.31 | Homo sapiens testis derived transcript (3 LIM domains) (TES), transcript variant 1, mRNA. |
| PITPNM1 | 2.31 | Homo sapiens phosphatidylinositol transfer protein, membrane-associated 1 (PITPNM1), mRNA. |
| ZNF514 | 2.31 | Homo sapiens zinc finger protein 514 (ZNF514), mRNA. |
| MYPOP | 2.31 | Homo sapiens Myb-related transcription factor, partner of profilin (MYPOP), mRNA. |
| UTP15 | 2.31 | Homo sapiens UTP15, U3 small nucleolar ribonucleoprotein, homolog (S. cerevisiae) (UTP15), mRNA. |
| TSC1 | 2.31 | Homo sapiens tuberous sclerosis 1 (TSC1), transcript variant 1, mRNA. |
| MLL3 | 2.3 | Homo sapiens myeloid/lymphoid or mixed-lineage leukemia 3 (MLL3), mRNA. |
| CRSP6 | 2.3 | Homo sapiens cofactor required for Sp1 transcriptional activation, subunit 6, 77kDa (CRSP6), mRNA. |
| C12orf30 | 2.3 | Homo sapiens chromosome 12 open reading frame 30 (C12orf30), mRNA. |
| CLDN12 | 2.3 | Homo sapiens claudin 12 (CLDN12), mRNA. |
| PMS2L1 | 2.29 | PREDICTED: Homo sapiens postmeiotic segregation increased 2-like 1, transcript variant 2 (PMS2L1), mRNA. |
| IFT88 | 2.29 | Homo sapiens intraflagellar transport 88 homolog (Chlamydomonas) (IFT88), transcript variant 2, mRNA. |
| MTAP | 2.29 | Homo sapiens methylthioadenosine phosphorylase (MTAP), mRNA. |
| C2orf49 | 2.29 | Homo sapiens chromosome 2 open reading frame 49 (C2orf49), mRNA. |
| NDUFAF1 | 2.29 | Homo sapiens NADH dehydrogenase (ubiquinone) 1 alpha subcomplex, assembly factor 1 (NDUFAF1), mRNA. |
| NPAT | 2.29 | Homo sapiens nuclear protein, ataxia-telangiectasia locus (NPAT), mRNA. |
| ZFP37 | 2.29 | Homo sapiens zinc finger protein 37 homolog (mouse) (ZFP37), mRNA. |
| TSC1 | 2.29 | Homo sapiens tuberous sclerosis 1 (TSC1), transcript variant 1, mRNA. |
| SOS1 | 2.29 | Homo sapiens son of sevenless homolog 1 (Drosophila) (SOS1), mRNA. |
| ZNF629 | 2.28 | Homo sapiens zinc finger protein 629 (ZNF629), mRNA. |
|  | 2.28 | Homo sapiens mRNA; cDNA DKFZp686F1546 (from clone DKFZp686F1546) |
| PROSC | 2.28 | Homo sapiens proline synthetase co-transcribed homolog (bacterial) (PROSC), mRNA. |
| MED23 | 2.28 | Homo sapiens mediator complex subunit 23 (MED23), transcript variant 2, mRNA. |
| DDX20 | 2.28 | Homo sapiens DEAD (Asp-Glu-Ala-Asp) box polypeptide 20 (DDX20), mRNA. |
| PDZK1 | 2.28 | Homo sapiens PDZ domain containing 1 (PDZK1), mRNA. |
| TIFA | 2.27 | Homo sapiens TRAF-interacting protein with forkhead-associated domain (TIFA), mRNA. |
| LOC652968 | 2.27 | Homo sapiens hypothetical protein LOC652968 (LOC652968), mRNA. |
| ZNF330 | 2.27 | Homo sapiens zinc finger protein 330 (ZNF330), mRNA. |
| PCMTD1 | 2.27 | Homo sapiens protein-L-isoaspartate (D-aspartate) O-methyltransferase domain containing 1 (PCMTD1), mRNA. |
| PITPNB | 2.26 | Homo sapiens phosphatidylinositol transfer protein, beta (PITPNB), mRNA. |
| TMEM25 | 2.26 | Homo sapiens transmembrane protein 25 (TMEM25), mRNA. |
| POLR1B | 2.26 | Homo sapiens polymerase (RNA) I polypeptide B, 128kDa (POLR1B), mRNA. |
| JAK1 | 2.26 | Homo sapiens Janus kinase 1 (JAK1), mRNA. |
| VASH1 | 2.26 | Homo sapiens vasohibin 1 (VASH1), mRNA. |
| SLC44A1 | 2.26 | Homo sapiens solute carrier family 44, member 1 (SLC44A1), mRNA. |
| CLINT1 | 2.25 | Homo sapiens clathrin interactor 1 (CLINT1), mRNA. |
| NBL1 | 2.25 | Homo sapiens neuroblastoma, suppression of tumorigenicity 1 (NBL1), transcript variant 2, mRNA. |
| WDR33 | 2.25 | Homo sapiens WD repeat domain 33 (WDR33), transcript variant 2, mRNA. |
| CYB561 | 2.25 | Homo sapiens cytochrome b-561 (CYB561), transcript variant 1, mRNA. |
| RBM18 | 2.25 | Homo sapiens RNA binding motif protein 18 (RBM18), mRNA. |
| ZNF248 | 2.25 | Homo sapiens zinc finger protein 248 (ZNF248), mRNA. |
| RABL2A | 2.25 | Homo sapiens RAB, member of RAS oncogene family-like 2A (RABL2A), transcript variant 1, mRNA. |
| PPP3CA | 2.25 | Homo sapiens protein phosphatase 3 (formerly 2B), catalytic subunit, alpha isoform (PPP3CA), mRNA. |
| NMD3 | 2.25 | Homo sapiens NMD3 homolog (S. cerevisiae) (NMD3), mRNA. |
| FLJ10781 | 2.25 | Homo sapiens hypothetical protein FLJ10781 (FLJ10781), mRNA. |
| CTSD | 2.24 | Homo sapiens cathepsin D (CTSD), mRNA. |
| C1orf25 | 2.24 | Homo sapiens chromosome 1 open reading frame 25 (C1orf25), mRNA. |
| DONSON | 2.24 | Homo sapiens downstream neighbor of SON (DONSON), mRNA. |
| AMD1 | 2.24 | Homo sapiens adenosylmethionine decarboxylase 1 (AMD1), transcript variant 2, mRNA. |
| LOC728505 | 2.24 | PREDICTED: Homo sapiens similar to CG17293-PA (LOC728505), mRNA. |
| LOC441155 | 2.24 | PREDICTED: Homo sapiens similar to Zinc finger CCCH-type domain-containing protein 11A, transcript variant 1 (LOC441155), mRNA. |
| GRINA | 2.24 | Homo sapiens glutamate receptor, ionotropic, N-methyl D-aspartate-associated protein 1 (glutamate binding) (GRINA), transcript variant 1, mRNA. |
| BAT3 | 2.24 | Homo sapiens HLA-B associated transcript 3 (BAT3), transcript variant 2, mRNA. |
| B3GAT1 | 2.24 | Homo sapiens beta-1,3-glucuronyltransferase 1 (glucuronosyltransferase P) (B3GAT1), transcript variant 1, mRNA. |
| ZNF484 | 2.23 | Homo sapiens zinc finger protein 484 (ZNF484), transcript variant 2, mRNA. |
| DVL3 | 2.23 | Homo sapiens dishevelled, dsh homolog 3 (Drosophila) (DVL3), mRNA. |
| TRIM46 | 2.23 | Homo sapiens tripartite motif-containing 46 (TRIM46), mRNA. |
| ANKRD32 | 2.23 | Homo sapiens ankyrin repeat domain 32 (ANKRD32), mRNA. |
| KHNYN | 2.23 | Homo sapiens KH and NYN domain containing (KHNYN), mRNA. |
| PTRF | 2.23 | Homo sapiens polymerase I and transcript release factor (PTRF), mRNA. |
| GEMIN5 | 2.23 | Homo sapiens gem (nuclear organelle) associated protein 5 (GEMIN5), mRNA. |
| BRWD1 | 2.23 | Homo sapiens bromodomain and WD repeat domain containing 1 (BRWD1), transcript variant 3, mRNA. |
| PSME3 | 2.23 | Homo sapiens proteasome (prosome, macropain) activator subunit 3 (PA28 gamma; Ki) (PSME3), transcript variant 1, mRNA. |
| EFCAB7 | 2.23 | Homo sapiens EF-hand calcium binding domain 7 (EFCAB7), mRNA. |
| USPL1 | 2.23 | Homo sapiens ubiquitin specific peptidase like 1 (USPL1), mRNA. |
| TMTC4 | 2.23 | Homo sapiens transmembrane and tetratricopeptide repeat containing 4 (TMTC4), transcript variant 1, mRNA. |
| ZNF26 | 2.23 | Homo sapiens zinc finger protein 26 (ZNF26), mRNA. |
| C17orf63 | 2.22 | Homo sapiens chromosome 17 open reading frame 63 (C17orf63), transcript variant 1, mRNA. |
| LOC654174 | 2.22 | PREDICTED: Homo sapiens similar to lethal (2) k00619 CG4775-PA (LOC654174), mRNA. |
| LOC653803 | 2.22 | PREDICTED: Homo sapiens similar to methyltransferase 5 domain containing 1 (LOC653803), mRNA. |
| KIAA0430 | 2.22 | Homo sapiens KIAA0430 (KIAA0430), mRNA. |
| FUBP1 | 2.22 | Homo sapiens far upstream element (FUSE) binding protein 1 (FUBP1), mRNA. |
| TAF2 | 2.22 | Homo sapiens TAF2 RNA polymerase II, TATA box binding protein (TBP)-associated factor, 150kDa (TAF2), mRNA. |
| CLK4 | 2.22 | Homo sapiens CDC-like kinase 4 (CLK4), mRNA. |
| TMED10 | 2.21 | Homo sapiens transmembrane emp24-like trafficking protein 10 (yeast) (TMED10), mRNA. |
| CTTN | 2.21 | Homo sapiens cortactin (CTTN), transcript variant 1, mRNA. |
| CALR | 2.21 | Homo sapiens calreticulin (CALR), mRNA. |
| FBXO46 | 2.21 | Homo sapiens F-box protein 46 (FBXO46), mRNA. |
| ATRN | 2.21 | Homo sapiens attractin (ATRN), transcript variant 1, mRNA. |
| FAM57A | 2.21 | Homo sapiens family with sequence similarity 57, member A (FAM57A), mRNA. |
| DNER | 2.2 | Homo sapiens delta/notch-like EGF repeat containing (DNER), mRNA. |
| PMEPA1 | 2.2 | Homo sapiens prostate transmembrane protein, androgen induced 1 (PMEPA1), transcript variant 2, mRNA. |
| CHRNA3 | 2.2 | Homo sapiens cholinergic receptor, nicotinic, alpha 3 (CHRNA3), mRNA. |
| NUTF2 | 2.2 | Homo sapiens nuclear transport factor 2 (NUTF2), mRNA. |
| CUGBP1 | 2.19 | Homo sapiens CUG triplet repeat, RNA binding protein 1 (CUGBP1), transcript variant 3, mRNA. |
| AGPAT3 | 2.19 | Homo sapiens 1-acylglycerol-3-phosphate O-acyltransferase 3 (AGPAT3), mRNA. |
| AFTPH | 2.19 | Homo sapiens aftiphilin (AFTPH), transcript variant 2, mRNA. |
| C10orf6 | 2.19 | Homo sapiens chromosome 10 open reading frame 6 (C10orf6), mRNA. |
| RBL2 | 2.19 | Homo sapiens retinoblastoma-like 2 (p130) (RBL2), mRNA. |
| SRC | 2.19 | Homo sapiens v-src sarcoma (Schmidt-Ruppin A-2) viral oncogene homolog (avian) (SRC), transcript variant 2, mRNA. |
| FNBP4 | 2.18 | Homo sapiens formin binding protein 4 (FNBP4), mRNA. |
| DGCR14 | 2.18 | Homo sapiens DiGeorge syndrome critical region gene 14 (DGCR14), mRNA. |
| NUFIP1 | 2.17 | Homo sapiens nuclear fragile X mental retardation protein interacting protein 1 (NUFIP1), mRNA. |
| MGC61598 | 2.17 | PREDICTED: Homo sapiens similar to ankyrin-repeat protein Nrarp (MGC61598), mRNA. |
| RNF38 | 2.17 | Homo sapiens ring finger protein 38 (RNF38), transcript variant 2, mRNA. |
| NDEL1 | 2.17 | Homo sapiens nudE nuclear distribution gene E homolog (A. nidulans)-like 1 (NDEL1), transcript variant 2, mRNA. |
| FAM108B1 | 2.17 | Homo sapiens family with sequence similarity 108, member B1 (FAM108B1), transcript variant 1, mRNA. |
| SLC35D2 | 2.16 | Homo sapiens solute carrier family 35, member D2 (SLC35D2), mRNA. |
| TIA1 | 2.16 | Homo sapiens TIA1 cytotoxic granule-associated RNA binding protein (TIA1), transcript variant 2, mRNA. |
| LUZP1 | 2.16 | Homo sapiens leucine zipper protein 1 (LUZP1), mRNA. |
| EIF4G2 | 2.16 | Homo sapiens eukaryotic translation initiation factor 4 gamma, 2 (EIF4G2), transcript variant 1, mRNA. |
| PPP2R2C | 2.16 | Homo sapiens protein phosphatase 2 (formerly 2A), regulatory subunit B, gamma isoform (PPP2R2C), transcript variant 2, mRNA. |
| RUNDC3A | 2.15 | Homo sapiens RUN domain containing 3A (RUNDC3A), mRNA. |
| DNPEP | 2.15 | Homo sapiens aspartyl aminopeptidase (DNPEP), mRNA. |
| UPF1 | 2.15 | Homo sapiens UPF1 regulator of nonsense transcripts homolog (yeast) (UPF1), mRNA. |
| MED22 | 2.15 | Homo sapiens mediator complex subunit 22 (MED22), transcript variant c, mRNA. |
|  | 2.15 | Homo sapiens mRNA; cDNA DKFZp313N0919 (from clone DKFZp313N0919) |
| PRUNE | 2.15 | Homo sapiens prune homolog (Drosophila) (PRUNE), mRNA. |
| SPCS3 | 2.15 | Homo sapiens signal peptidase complex subunit 3 homolog (S. cerevisiae) (SPCS3), mRNA. |
| ARID4A | 2.14 | Homo sapiens AT rich interactive domain 4A (RBP1-like) (ARID4A), transcript variant 2, mRNA. |
| ZNF767 | 2.14 | Homo sapiens zinc finger family member 767 (ZNF767), mRNA. |
| STAG3L1 | 2.14 | Homo sapiens stromal antigen 3-like 1 (STAG3L1), transcript variant 1, mRNA. |
| MYADM | 2.14 | Homo sapiens myeloid-associated differentiation marker (MYADM), transcript variant 4, mRNA. |
| GLCE | 2.14 | Homo sapiens glucuronic acid epimerase (GLCE), mRNA. |
| PPP2R5D | 2.14 | Homo sapiens protein phosphatase 2, regulatory subunit B', delta isoform (PPP2R5D), transcript variant 1, mRNA. |
| MXD1 | 2.13 | Homo sapiens MAX dimerization protein 1 (MXD1), mRNA. |
| CCDC16 | 2.13 | Homo sapiens coiled-coil domain containing 16 (CCDC16), mRNA. |
| ZNF512 | 2.13 | Homo sapiens zinc finger protein 512 (ZNF512), mRNA. |
| EPM2AIP1 | 2.13 | Homo sapiens EPM2A (laforin) interacting protein 1 (EPM2AIP1), mRNA. |
| GFPT1 | 2.13 | Homo sapiens glutamine-fructose-6-phosphate transaminase 1 (GFPT1), mRNA. |
| LOC654254 | 2.13 | PREDICTED: Homo sapiens similar to zinc finger protein 595 (LOC654254), mRNA. |
| LOC643888 | 2.13 | PREDICTED: Homo sapiens hypothetical LOC643888 (LOC643888), mRNA. |
| WBSCR16 | 2.13 | Homo sapiens Williams-Beuren syndrome chromosome region 16 (WBSCR16), mRNA. |
|  | 2.12 | Homo sapiens mRNA; cDNA DKFZp761E1721 (from clone DKFZp761E1721) |
| EIF5B | 2.12 | Homo sapiens eukaryotic translation initiation factor 5B (EIF5B), mRNA. |
| CYCS | 2.12 | Homo sapiens cytochrome c, somatic (CYCS), nuclear gene encoding mitochondrial protein, mRNA. |
| CDK8 | 2.12 | Homo sapiens cyclin-dependent kinase 8 (CDK8), mRNA. |
| MRPS14 | 2.12 | Homo sapiens mitochondrial ribosomal protein S14 (MRPS14), nuclear gene encoding mitochondrial protein, mRNA. |
| DDIT3 | 2.12 | Homo sapiens DNA-damage-inducible transcript 3 (DDIT3), mRNA. |
| FBXO38 | 2.11 | Homo sapiens F-box protein 38 (FBXO38), transcript variant 1, mRNA. |
| SYN2 | 2.11 | Homo sapiens synapsin II (SYN2), transcript variant IIb, mRNA. |
| DDX23 | 2.11 | Homo sapiens DEAD (Asp-Glu-Ala-Asp) box polypeptide 23 (DDX23), mRNA. |
| THAP10 | 2.11 | Homo sapiens THAP domain containing 10 (THAP10), mRNA. |
| KIAA1009 | 2.11 | Homo sapiens KIAA1009 (KIAA1009), mRNA. |
| SRPR | 2.11 | Homo sapiens signal recognition particle receptor (docking protein) (SRPR), mRNA. |
|  | 2.1 | Homo sapiens cDNA: FLJ23063 fis, clone LNG04745 |
| ZNF182 | 2.1 | Homo sapiens zinc finger protein 182 (ZNF182), transcript variant 2, mRNA. |
| AKIRIN1 | 2.1 | Homo sapiens akirin 1 (AKIRIN1), mRNA. |
| C19orf25 | 2.1 | Homo sapiens chromosome 19 open reading frame 25 (C19orf25), mRNA. |
| ZNF548 | 2.1 | Homo sapiens zinc finger protein 548 (ZNF548), mRNA. |
| KIAA1539 | 2.1 | Homo sapiens KIAA1539 (KIAA1539), mRNA. |
| AP3M1 | 2.1 | Homo sapiens adaptor-related protein complex 3, mu 1 subunit (AP3M1), transcript variant 2, mRNA. |
| DIDO1 | 2.1 | Homo sapiens death inducer-obliterator 1 (DIDO1), transcript variant 3, mRNA. |
| MRPS25 | 2.1 | Homo sapiens mitochondrial ribosomal protein S25 (MRPS25), nuclear gene encoding mitochondrial protein, mRNA. |
| LOC653354 | 2.1 | PREDICTED: Homo sapiens similar to amrmadillo repeat containing, X-linked 6 (H. sapiens)-like (LOC653354), mRNA. |
| RRAGD | 2.1 | Homo sapiens Ras-related GTP binding D (RRAGD), mRNA. |
| UBQLN1 | 2.1 | Homo sapiens ubiquilin 1 (UBQLN1), transcript variant 2, mRNA. |
| HELZ | 2.09 | Homo sapiens helicase with zinc finger (HELZ), mRNA. |
| CBX4 | 2.09 | Homo sapiens chromobox homolog 4 (Pc class homolog, Drosophila) (CBX4), mRNA. |
| LOC285074 | 2.09 | Homo sapiens hypothetical protein LOC285074 (LOC285074), mRNA. |
| C10orf26 | 2.09 | Homo sapiens chromosome 10 open reading frame 26 (C10orf26), transcript variant 1, mRNA. |
| ZNF20 | 2.09 | Homo sapiens zinc finger protein 20 (ZNF20), mRNA. |
| TIA1 | 2.09 | Homo sapiens TIA1 cytotoxic granule-associated RNA binding protein (TIA1), transcript variant 2, mRNA. |
| TERF2 | 2.08 | Homo sapiens telomeric repeat binding factor 2 (TERF2), mRNA. |
| MBTPS1 | 2.08 | Homo sapiens membrane-bound transcription factor peptidase, site 1 (MBTPS1), mRNA. |
| INTS4 | 2.08 | Homo sapiens integrator complex subunit 4 (INTS4), mRNA. |
| LOC730358 | 2.08 | PREDICTED: Homo sapiens hypothetical protein LOC730358 (LOC730358), mRNA. |
| SH2D5 | 2.08 | PREDICTED: Homo sapiens SH2 domain containing 5 (SH2D5), mRNA. |
| MBD6 | 2.08 | Homo sapiens methyl-CpG binding domain protein 6 (MBD6), mRNA. |
| USP34 | 2.08 | Homo sapiens ubiquitin specific peptidase 34 (USP34), mRNA. |
| PTCD2 | 2.08 | Homo sapiens pentatricopeptide repeat domain 2 (PTCD2), mRNA. |
| ZNF529 | 2.08 | Homo sapiens zinc finger protein 529 (ZNF529), mRNA. |
| NEURL4 | 2.08 | Homo sapiens neuralized homolog 4 (Drosophila) (NEURL4), transcript variant 1, mRNA. |
| ZNF706 | 2.08 | Homo sapiens zinc finger protein 706 (ZNF706), transcript variant 2, mRNA. |
| LOC642489 | 2.08 | PREDICTED: Homo sapiens similar to FK506-binding protein 1A (LOC642489), mRNA. |
| GAS8 | 2.07 | Homo sapiens growth arrest-specific 8 (GAS8), mRNA. |
| ZNF197 | 2.07 | Homo sapiens zinc finger protein 197 (ZNF197), transcript variant 2, mRNA. |
| PRPF38B | 2.07 | Homo sapiens PRP38 pre-mRNA processing factor 38 (yeast) domain containing B (PRPF38B), mRNA. |
| UNC5B | 2.07 | Homo sapiens unc-5 homolog B (C. elegans) (UNC5B), mRNA. |
| PLS1 | 2.07 | Homo sapiens plastin 1 (I isoform) (PLS1), mRNA. |
| SFRS12 | 2.07 | Homo sapiens splicing factor, arginine/serine-rich 12 (SFRS12), transcript variant 1, mRNA. |
| MAP6 | 2.07 | Homo sapiens microtubule-associated protein 6 (MAP6), transcript variant 1, mRNA. |
| SLC44A3 | 2.07 | Homo sapiens solute carrier family 44, member 3 (SLC44A3), mRNA. |
| GFM2 | 2.07 | Homo sapiens G elongation factor, mitochondrial 2 (GFM2), nuclear gene encoding mitochondrial protein, transcript variant 3, mRNA. |
| CLSTN2 | 2.07 | Homo sapiens calsyntenin 2 (CLSTN2), mRNA. |
| ZC3H14 | 2.07 | Homo sapiens zinc finger CCCH-type containing 14 (ZC3H14), transcript variant 1, mRNA. |
| AXIN2 | 2.07 | Homo sapiens axin 2 (conductin, axil) (AXIN2), mRNA. |
| YPEL2 | 2.06 | Homo sapiens yippee-like 2 (Drosophila) (YPEL2), mRNA. |
| ACPL2 | 2.06 | Homo sapiens acid phosphatase-like 2 (ACPL2), transcript variant 1, mRNA. |
| SHB | 2.06 | Homo sapiens Src homology 2 domain containing adaptor protein B (SHB), mRNA. |
| RNH1 | 2.05 | Homo sapiens ribonuclease/angiogenin inhibitor 1 (RNH1), transcript variant 4, mRNA. |
| PLEKHA3 | 2.05 | Homo sapiens pleckstrin homology domain containing, family A (phosphoinositide binding specific) member 3 (PLEKHA3), mRNA. |
| BAT1 | 2.05 | Homo sapiens HLA-B associated transcript 1 (BAT1), transcript variant 1, mRNA. |
| PPFIA1 | 2.05 | Homo sapiens protein tyrosine phosphatase, receptor type, f polypeptide (PTPRF), interacting protein (liprin), alpha 1 (PPFIA1), transcript variant 1, mRNA. |
| SFRS2IP | 2.05 | Homo sapiens splicing factor, arginine/serine-rich 2, interacting protein (SFRS2IP), mRNA. |
| SH3BP5L | 2.04 | Homo sapiens SH3-binding domain protein 5-like (SH3BP5L), mRNA. |
| UBE1 | 2.04 | Homo sapiens ubiquitin-activating enzyme E1 (UBE1), transcript variant 2, mRNA. |
| RASSF1 | 2.04 | Homo sapiens Ras association (RalGDS/AF-6) domain family member 1 (RASSF1), transcript variant C, mRNA. |
| CENTB2 | 2.03 | Homo sapiens centaurin, beta 2 (CENTB2), mRNA. |
| CSRNP3 | 2.03 | Homo sapiens cysteine-serine-rich nuclear protein 3 (CSRNP3), mRNA. |
| DNAJB2 | 2.02 | Homo sapiens DnaJ (Hsp40) homolog, subfamily B, member 2 (DNAJB2), transcript variant 2, mRNA. |
| UNC45A | 2.02 | Homo sapiens unc-45 homolog A (C. elegans) (UNC45A), transcript variant 3, mRNA. |
| IL10RB | 2.02 | Homo sapiens interleukin 10 receptor, beta (IL10RB), mRNA. |
| ATP9A | 2.02 | Homo sapiens ATPase, class II, type 9A (ATP9A), mRNA. |
| PCGF2 | 2.02 | Homo sapiens polycomb group ring finger 2 (PCGF2), mRNA. |
| RFXAP | 2.02 | Homo sapiens regulatory factor X-associated protein (RFXAP), mRNA. |
| FYN | 2.02 | Homo sapiens FYN oncogene related to SRC, FGR, YES (FYN), transcript variant 1, mRNA. |
| GPT2 | 2.02 | Homo sapiens glutamic pyruvate transaminase (alanine aminotransferase) 2 (GPT2), mRNA. |
| EIF2S3 | 2.02 | Homo sapiens eukaryotic translation initiation factor 2, subunit 3 gamma, 52kDa (EIF2S3), mRNA. |
| ATG16L1 | 2.01 | Homo sapiens ATG16 autophagy related 16-like 1 (S. cerevisiae) (ATG16L1), transcript variant 1, mRNA. |
| ZNF772 | 2.01 | Homo sapiens zinc finger protein 772 (ZNF772), mRNA. |
| NUP50 | 2.01 | Homo sapiens nucleoporin 50kDa (NUP50), transcript variant 2, mRNA. |
|  | 2.01 | Homo sapiens mRNA; cDNA DKFZp547E107 (from clone DKFZp547E107) |
| RBM38 | 2.01 | Homo sapiens RNA binding motif protein 38 (RBM38), transcript variant 2, mRNA. |
| DDX31 | 2.01 | Homo sapiens DEAD (Asp-Glu-Ala-Asp) box polypeptide 31 (DDX31), transcript variant 1, mRNA. |
| HMG20A | 2.01 | Homo sapiens high-mobility group 20A (HMG20A), mRNA. |
| LPPR3 | 2.01 | Homo sapiens lipid phosphate phosphatase-related protein type 3 (LPPR3), mRNA. |
| ZNF187 | 2.01 | Homo sapiens zinc finger protein 187 (ZNF187), transcript variant 1, mRNA. |
| KCTD18 | 2 | Homo sapiens potassium channel tetramerisation domain containing 18 (KCTD18), mRNA. |
| HIST2H4A | 2 | Homo sapiens histone cluster 2, H4a (HIST2H4A), mRNA. |
| PUM2 | 2 | Homo sapiens pumilio homolog 2 (Drosophila) (PUM2), mRNA. |
| NDUFS2 | 2 | Homo sapiens NADH dehydrogenase (ubiquinone) Fe-S protein 2, 49kDa (NADH-coenzyme Q reductase) (NDUFS2), mRNA. |
| PPP3R1 | 2 | Homo sapiens protein phosphatase 3 (formerly 2B), regulatory subunit B, alpha isoform (PPP3R1), mRNA. |
| HSPD1 | 2 | Homo sapiens heat shock 60kDa protein 1 (chaperonin) (HSPD1), nuclear gene encoding mitochondrial protein, transcript variant 1, mRNA. |
| VPS37A | 2 | Homo sapiens vacuolar protein sorting 37 homolog A (S. cerevisiae) (VPS37A), mRNA. |
| SPIN2B | 2 | Homo sapiens spindlin family, member 2B (SPIN2B), transcript variant 2, mRNA. |
| TSC22D2 | 2 | Homo sapiens TSC22 domain family, member 2 (TSC22D2), mRNA. |
| MCPH1 | 1.99 | Homo sapiens microcephalin 1 (MCPH1), mRNA. |
| MLL3 | 1.99 | Homo sapiens myeloid/lymphoid or mixed-lineage leukemia 3 (MLL3), mRNA. |
| MYT1L | 1.99 | Homo sapiens myelin transcription factor 1-like (MYT1L), mRNA. |
| MAP6 | 1.99 | Homo sapiens microtubule-associated protein 6 (MAP6), transcript variant 2, mRNA. |
| TNFAIP1 | 1.99 | Homo sapiens tumor necrosis factor, alpha-induced protein 1 (endothelial) (TNFAIP1), mRNA. |
| DACT3 | 1.99 | Homo sapiens dapper, antagonist of beta-catenin, homolog 3 (Xenopus laevis) (DACT3), mRNA. |
| WDR5B | 1.99 | Homo sapiens WD repeat domain 5B (WDR5B), mRNA. |
| GDAP2 | 1.99 | Homo sapiens ganglioside induced differentiation associated protein 2 (GDAP2), mRNA. |
| XPOT | 1.99 | Homo sapiens exportin, tRNA (nuclear export receptor for tRNAs) (XPOT), mRNA. |
| NFYA | 1.98 | Homo sapiens nuclear transcription factor Y, alpha (NFYA), transcript variant 1, mRNA. |
| ABCC5 | 1.98 | Homo sapiens ATP-binding cassette, sub-family C (CFTR/MRP), member 5 (ABCC5), transcript variant 1, mRNA. |
| RIF1 | 1.98 | Homo sapiens RAP1 interacting factor homolog (yeast) (RIF1), mRNA. |
| SLBP | 1.98 | Homo sapiens stem-loop binding protein (SLBP), mRNA. |
| ANGEL1 | 1.98 | Homo sapiens angel homolog 1 (Drosophila) (ANGEL1), mRNA. |
| STAMBPL1 | 1.98 | Homo sapiens STAM binding protein-like 1 (STAMBPL1), mRNA. |
| LOC85389 | 1.97 | Homo sapiens RNA, small nucleolar (LOC85389), non-coding RNA. |
| GRPEL2 | 1.97 | Homo sapiens GrpE-like 2, mitochondrial (E. coli) (GRPEL2), nuclear gene encoding mitochondrial protein, mRNA. |
| LRCH3 | 1.97 | Homo sapiens leucine-rich repeats and calponin homology (CH) domain containing 3 (LRCH3), mRNA. |
| THUMPD1 | 1.97 | Homo sapiens THUMP domain containing 1 (THUMPD1), mRNA. |
| OGFOD1 | 1.97 | Homo sapiens 2-oxoglutarate and iron-dependent oxygenase domain containing 1 (OGFOD1), mRNA. |
| DLG4 | 1.97 | Homo sapiens discs, large homolog 4 (Drosophila) (DLG4), mRNA. |
| LOC730746 | 1.97 | PREDICTED: Homo sapiens similar to Heterogeneous nuclear ribonucleoprotein A1 (Helix-destabilizing protein) (Single-strand RNA-binding protein) (hnRNP core protein A1) (HDP) (LOC730746), mRNA. |
| KLHL7 | 1.97 | Homo sapiens kelch-like 7 (Drosophila) (KLHL7), transcript variant 2, mRNA. |
| ZNF514 | 1.97 | Homo sapiens zinc finger protein 514 (ZNF514), mRNA. |
| STK38 | 1.96 | Homo sapiens serine/threonine kinase 38 (STK38), mRNA. |
| RBMS3 | 1.96 | Homo sapiens RNA binding motif, single stranded interacting protein (RBMS3), transcript variant 1, mRNA. |
| CHD7 | 1.96 | Homo sapiens chromodomain helicase DNA binding protein 7 (CHD7), mRNA. |
| RAP2C | 1.96 | Homo sapiens RAP2C, member of RAS oncogene family (RAP2C), mRNA. |
| AMY1A | 1.95 | Homo sapiens amylase, alpha 1A (salivary) (AMY1A), transcript variant 1, mRNA. |
| ZNF509 | 1.95 | Homo sapiens zinc finger protein 509 (ZNF509), mRNA. |
|  | 1.95 | Human HeLa mRNA isolated as a false positive in a two-hybrid-screen |
| DNAJB5 | 1.95 | Homo sapiens DnaJ (Hsp40) homolog, subfamily B, member 5 (DNAJB5), mRNA. |
| CPA4 | 1.95 | Homo sapiens carboxypeptidase A4 (CPA4), mRNA. |
| NKRF | 1.95 | Homo sapiens NFKB repressing factor (NKRF), mRNA. |
| FLJ35409 | 1.95 | PREDICTED: Homo sapiens FLJ35409 protein (FLJ35409), misc RNA. |
| LOC647970 | 1.95 | PREDICTED: Homo sapiens hypothetical protein LOC647970, transcript variant 1 (LOC647970), mRNA. |
| ALKBH1 | 1.95 | Homo sapiens alkB, alkylation repair homolog 1 (E. coli) (ALKBH1), mRNA. |
| HPCAL4 | 1.94 | Homo sapiens hippocalcin like 4 (HPCAL4), mRNA. |
| FBXO28 | 1.94 | Homo sapiens F-box protein 28 (FBXO28), mRNA. |
| RELA | 1.94 | Homo sapiens v-rel reticuloendotheliosis viral oncogene homolog A (avian) (RELA), mRNA. |
| C3orf17 | 1.94 | Homo sapiens chromosome 3 open reading frame 17 (C3orf17), transcript variant 2, mRNA. |
| NNT | 1.94 | Homo sapiens nicotinamide nucleotide transhydrogenase (NNT), nuclear gene encoding mitochondrial protein, transcript variant 1, mRNA. |
| NUP98 | 1.94 | Homo sapiens nucleoporin 98kDa (NUP98), transcript variant 1, mRNA. |
| GFOD1 | 1.93 | Homo sapiens glucose-fructose oxidoreductase domain containing 1 (GFOD1), mRNA. |
| ABCC3 | 1.93 | Homo sapiens ATP-binding cassette, sub-family C (CFTR/MRP), member 3 (ABCC3), mRNA. |
| LOC375295 | 1.93 | PREDICTED: Homo sapiens hypothetical gene supported by BC013438 (LOC375295), mRNA. |
| TRNT1 | 1.93 | Homo sapiens tRNA nucleotidyl transferase, CCA-adding, 1 (TRNT1), mRNA. |
| TMEM198 | 1.93 | Homo sapiens transmembrane protein 198 (TMEM198), mRNA. |
| MGC16703 | 1.93 | Homo sapiens tubulin, alpha pseudogene (MGC16703), non-coding RNA. |
| PMS2 | 1.93 | Homo sapiens PMS2 postmeiotic segregation increased 2 (S. cerevisiae) (PMS2), transcript variant 1, mRNA. |
| KPNA6 | 1.93 | Homo sapiens karyopherin alpha 6 (importin alpha 7) (KPNA6), mRNA. |
| SFRS13B | 1.93 | Homo sapiens splicing factor, arginine/serine-rich 13B (SFRS13B), mRNA. |
| B3GAT1 | 1.93 | Homo sapiens beta-1,3-glucuronyltransferase 1 (glucuronosyltransferase P) (B3GAT1), transcript variant 1, mRNA. |
| SKIV2L2 | 1.93 | Homo sapiens superkiller viralicidic activity 2-like 2 (S. cerevisiae) (SKIV2L2), mRNA. |
|  | 1.92 | Homo sapiens cDNA clone IMAGE:5277162 |
| HS1BP3 | 1.92 | Homo sapiens HCLS1 binding protein 3 (HS1BP3), mRNA. |
| WHSC2 | 1.92 | Homo sapiens Wolf-Hirschhorn syndrome candidate 2 (WHSC2), mRNA. |
| LSM10 | 1.92 | Homo sapiens LSM10, U7 small nuclear RNA associated (LSM10), mRNA. |
| GSPT2 | 1.92 | Homo sapiens G1 to S phase transition 2 (GSPT2), mRNA. |
| AHCYL2 | 1.92 | Homo sapiens S-adenosylhomocysteine hydrolase-like 2 (AHCYL2), mRNA. |
| LOC644979 | 1.92 | PREDICTED: Homo sapiens hypothetical LOC644979 (LOC644979), mRNA. |
| PSMC3IP | 1.92 | Homo sapiens PSMC3 interacting protein (PSMC3IP), transcript variant 2, mRNA. |
| B3GALNT2 | 1.92 | Homo sapiens beta-1,3-N-acetylgalactosaminyltransferase 2 (B3GALNT2), mRNA. |
| CYFIP2 | 1.91 | Homo sapiens cytoplasmic FMR1 interacting protein 2 (CYFIP2), transcript variant 1, mRNA. |
| MMD | 1.91 | Homo sapiens monocyte to macrophage differentiation-associated (MMD), mRNA. |
| KIAA1128 | 1.91 | Homo sapiens KIAA1128 (KIAA1128), mRNA. |
| CNTNAP5 | 1.91 | Homo sapiens contactin associated protein-like 5 (CNTNAP5), mRNA. |
| FKBP1A | 1.91 | Homo sapiens FK506 binding protein 1A, 12kDa (FKBP1A), transcript variant 12B, mRNA. |
| ZNF234 | 1.91 | Homo sapiens zinc finger protein 234 (ZNF234), mRNA. |
| LGR5 | 1.91 | Homo sapiens leucine-rich repeat-containing G protein-coupled receptor 5 (LGR5), mRNA. |
| PRDM10 | 1.9 | Homo sapiens PR domain containing 10 (PRDM10), transcript variant 1, mRNA. |
| SRGAP3 | 1.9 | Homo sapiens SLIT-ROBO Rho GTPase activating protein 3 (SRGAP3), transcript variant 2, mRNA. |
| TACC2 | 1.9 | Homo sapiens transforming, acidic coiled-coil containing protein 2 (TACC2), transcript variant 4, mRNA. |
| C7orf23 | 1.9 | Homo sapiens chromosome 7 open reading frame 23 (C7orf23), mRNA. |
| FKBP4 | 1.89 | Homo sapiens FK506 binding protein 4, 59kDa (FKBP4), mRNA. |
| SLC25A22 | 1.89 | Homo sapiens solute carrier family 25 (mitochondrial carrier: glutamate), member 22 (SLC25A22), nuclear gene encoding mitochondrial protein, mRNA. |
| CCNE1 | 1.89 | Homo sapiens cyclin E1 (CCNE1), transcript variant 2, mRNA. |
| ACBD5 | 1.89 | Homo sapiens acyl-Coenzyme A binding domain containing 5 (ACBD5), transcript variant 1, mRNA. |
| C17orf81 | 1.89 | Homo sapiens chromosome 17 open reading frame 81 (C17orf81), transcript variant 2, mRNA. |
| G3BP1 | 1.89 | Homo sapiens GTPase activating protein (SH3 domain) binding protein 1 (G3BP1), transcript variant 2, mRNA. |
| TMEM169 | 1.89 | Homo sapiens transmembrane protein 169 (TMEM169), mRNA. |
| BIVM | 1.89 | Homo sapiens basic, immunoglobulin-like variable motif containing (BIVM), mRNA. |
| ATP1B3 | 1.89 | Homo sapiens ATPase, Na+/K+ transporting, beta 3 polypeptide (ATP1B3), mRNA. XM_945518 |
| NPL | 1.89 | Homo sapiens N-acetylneuraminate pyruvate lyase (dihydrodipicolinate synthase) (NPL), mRNA. |
| KBTBD7 | 1.89 | Homo sapiens kelch repeat and BTB (POZ) domain containing 7 (KBTBD7), mRNA. |
| CTNNAL1 | 1.89 | Homo sapiens catenin (cadherin-associated protein), alpha-like 1 (CTNNAL1), mRNA. |
| STT3A | 1.88 | Homo sapiens STT3, subunit of the oligosaccharyltransferase complex, homolog A (S. cerevisiae) (STT3A), mRNA. |
| ARHGEF11 | 1.88 | Homo sapiens Rho guanine nucleotide exchange factor (GEF) 11 (ARHGEF11), transcript variant 1, mRNA. |
| AP2B1 | 1.88 | Homo sapiens adaptor-related protein complex 2, beta 1 subunit (AP2B1), transcript variant 2, mRNA. |
| DHDDS | 1.88 | Homo sapiens dehydrodolichyl diphosphate synthase (DHDDS), transcript variant 1, mRNA. |
| METTL2A | 1.88 | Homo sapiens methyltransferase like 2A (METTL2A), mRNA. |
| PDIA4 | 1.87 | Homo sapiens protein disulfide isomerase family A, member 4 (PDIA4), mRNA. |
| CCDC86 | 1.87 | Homo sapiens coiled-coil domain containing 86 (CCDC86), mRNA. |
| KIAA2010 | 1.87 | Homo sapiens KIAA2010 (KIAA2010), transcript variant 1, mRNA. |
| EFNB2 | 1.87 | Homo sapiens ephrin-B2 (EFNB2), mRNA. |
| ADCY1 | 1.87 | Homo sapiens adenylate cyclase 1 (brain) (ADCY1), mRNA. |
| GRIPAP1 | 1.87 | Homo sapiens GRIP1 associated protein 1 (GRIPAP1), transcript variant 1, mRNA. |
| SRP54 | 1.87 | Homo sapiens signal recognition particle 54kDa (SRP54), mRNA. |
| PMS2CL | 1.87 | Homo sapiens PMS2 C-terminal like pseudogene (PMS2CL), non-coding RNA. |
| RHBDD1 | 1.87 | Homo sapiens rhomboid domain containing 1 (RHBDD1), mRNA. |
| REV3L | 1.87 | Homo sapiens REV3-like, catalytic subunit of DNA polymerase zeta (yeast) (REV3L), mRNA. |
| ELMO1 | 1.86 | Homo sapiens engulfment and cell motility 1 (ELMO1), transcript variant 1, mRNA. |
| ZNF664 | 1.86 | Homo sapiens zinc finger protein 664 (ZNF664), mRNA. |
| PHF10 | 1.86 | Homo sapiens PHD finger protein 10 (PHF10), transcript variant 2, mRNA. |
| SLC25A42 | 1.86 | Homo sapiens solute carrier family 25, member 42 (SLC25A42), mRNA. |
| GBA2 | 1.86 | Homo sapiens glucosidase, beta (bile acid) 2 (GBA2), mRNA. |
| DOCK6 | 1.86 | Homo sapiens dedicator of cytokinesis 6 (DOCK6), mRNA. |
| MAPRE3 | 1.86 | Homo sapiens microtubule-associated protein, RP/EB family, member 3 (MAPRE3), mRNA. |
| GLE1 | 1.86 | Homo sapiens GLE1 RNA export mediator homolog (yeast) (GLE1), transcript variant 1, mRNA. |
| FKBP7 | 1.85 | Homo sapiens FK506 binding protein 7 (FKBP7), mRNA. |
| SLC9A6 | 1.85 | Homo sapiens solute carrier family 9 (sodium/hydrogen exchanger), member 6 (SLC9A6), transcript variant 1, mRNA. |
| MTMR2 | 1.85 | Homo sapiens myotubularin related protein 2 (MTMR2), transcript variant 3, mRNA. |
| LRRC26 | 1.85 | Homo sapiens leucine rich repeat containing 26 (LRRC26), mRNA. |
| CHPF | 1.85 | Homo sapiens chondroitin polymerizing factor (CHPF), mRNA. |
| ZDHHC6 | 1.85 | Homo sapiens zinc finger, DHHC-type containing 6 (ZDHHC6), mRNA. |
| ADSSL1 | 1.85 | Homo sapiens adenylosuccinate synthase like 1 (ADSSL1), transcript variant 2, mRNA. |
| STRN4 | 1.85 | Homo sapiens striatin, calmodulin binding protein 4 (STRN4), transcript variant 2, mRNA. |
| C12orf34 | 1.85 | Homo sapiens chromosome 12 open reading frame 34 (C12orf34), mRNA. |
| CLIC4 | 1.85 | Homo sapiens chloride intracellular channel 4 (CLIC4), nuclear gene encoding mitochondrial protein, mRNA. |
| SH3RF1 | 1.84 | Homo sapiens SH3 domain containing ring finger 1 (SH3RF1), mRNA. |
| MANBA | 1.84 | Homo sapiens mannosidase, beta A, lysosomal (MANBA), mRNA. |
| ODF2 | 1.84 | Homo sapiens outer dense fiber of sperm tails 2 (ODF2), transcript variant 1, mRNA. |
| SHC1 | 1.84 | Homo sapiens SHC (Src homology 2 domain containing) transforming protein 1 (SHC1), transcript variant 2, mRNA. |
| CAB39 | 1.84 | Homo sapiens calcium binding protein 39 (CAB39), mRNA. |
| MTF1 | 1.84 | Homo sapiens metal-regulatory transcription factor 1 (MTF1), mRNA. |
| DHX9 | 1.84 | Homo sapiens DEAH (Asp-Glu-Ala-His) box polypeptide 9 (DHX9), mRNA. |
| TMEM135 | 1.83 | Homo sapiens transmembrane protein 135 (TMEM135), mRNA. |
| SHOX2 | 1.83 | Homo sapiens short stature homeobox 2 (SHOX2), transcript variant SHOX2a, mRNA. |
| WDR43 | 1.83 | PREDICTED: Homo sapiens WD repeat domain 43, transcript variant 8 (WDR43), mRNA. |
| CLIP2 | 1.83 | Homo sapiens CAP-GLY domain containing linker protein 2 (CLIP2), transcript variant 2, mRNA. |
| CYB5D1 | 1.83 | Homo sapiens cytochrome b5 domain containing 1 (CYB5D1), mRNA. |
| LOC644278 | 1.83 | PREDICTED: Homo sapiens similar to bruno-like 4, RNA binding protein (LOC644278), mRNA. |
| TTC14 | 1.83 | Homo sapiens tetratricopeptide repeat domain 14 (TTC14), transcript variant 1, mRNA. |
| HCN3 | 1.83 | Homo sapiens hyperpolarization activated cyclic nucleotide-gated potassium channel 3 (HCN3), mRNA. |
| NFKBIE | 1.83 | Homo sapiens nuclear factor of kappa light polypeptide gene enhancer in B-cells inhibitor, epsilon (NFKBIE), mRNA. |
| ILF3 | 1.83 | Homo sapiens interleukin enhancer binding factor 3, 90kDa (ILF3), transcript variant 1, mRNA. |
| LOC400890 | 1.83 | PREDICTED: Homo sapiens hypothetical LOC400890 (LOC400890), mRNA. |
| AGPS | 1.83 | Homo sapiens alkylglycerone phosphate synthase (AGPS), mRNA. |
| MAGED1 | 1.82 | Homo sapiens melanoma antigen family D, 1 (MAGED1), transcript variant 3, mRNA. |
| C16orf87 | 1.82 | Homo sapiens chromosome 16 open reading frame 87 (C16orf87), mRNA. |
| GATAD2B | 1.82 | Homo sapiens GATA zinc finger domain containing 2B (GATAD2B), mRNA. |
| PPPDE2 | 1.82 | Homo sapiens PPPDE peptidase domain containing 2 (PPPDE2), mRNA. |
| RCAN1 | 1.82 | Homo sapiens regulator of calcineurin 1 (RCAN1), transcript variant 2, mRNA. |
| ABHD10 | 1.82 | Homo sapiens abhydrolase domain containing 10 (ABHD10), mRNA. |
| TARDBP | 1.82 | Homo sapiens TAR DNA binding protein (TARDBP), mRNA. |
| KDM3B | 1.82 | Homo sapiens lysine (K)-specific demethylase 3B (KDM3B), mRNA. |
| CHD1 | 1.82 | Homo sapiens chromodomain helicase DNA binding protein 1 (CHD1), mRNA. |
| REC8 | 1.81 | Homo sapiens REC8 homolog (yeast) (REC8), transcript variant 1, mRNA. |
| GDA | 1.81 | Homo sapiens guanine deaminase (GDA), mRNA. |
| C2CD2 | 1.81 | Homo sapiens C2 calcium-dependent domain containing 2 (C2CD2), transcript variant 2, mRNA. |
| LOC651621 | 1.81 | PREDICTED: Homo sapiens similar to hydroxysteroid (17-beta) dehydrogenase 7 (LOC651621), mRNA. |
| LZTS1 | 1.81 | Homo sapiens leucine zipper, putative tumor suppressor 1 (LZTS1), mRNA. |
| NSUN5C | 1.81 | Homo sapiens NOL1/NOP2/Sun domain family, member 5C (NSUN5C), transcript variant 4, mRNA. |
| CROP | 1.81 | Homo sapiens cisplatin resistance-associated overexpressed protein (CROP), transcript variant 2, mRNA. |
| C9orf123 | 1.81 | Homo sapiens chromosome 9 open reading frame 123 (C9orf123), mRNA. |
| SEC22C | 1.81 | Homo sapiens SEC22 vesicle trafficking protein homolog C (S. cerevisiae) (SEC22C), transcript variant 1, mRNA. |
| CRLF3 | 1.81 | Homo sapiens cytokine receptor-like factor 3 (CRLF3), mRNA. |
| AZIN1 | 1.8 | Homo sapiens antizyme inhibitor 1 (AZIN1), transcript variant 1, mRNA. |
| FBXO31 | 1.8 | Homo sapiens F-box protein 31 (FBXO31), mRNA. |
| CRELD1 | 1.8 | Homo sapiens cysteine-rich with EGF-like domains 1 (CRELD1), transcript variant 1, mRNA. |
| FAM8A1 | 1.8 | Homo sapiens family with sequence similarity 8, member A1 (FAM8A1), mRNA. |
| TOP2B | 1.8 | Homo sapiens topoisomerase (DNA) II beta 180kDa (TOP2B), mRNA. |
| UBTD2 | 1.8 | Homo sapiens ubiquitin domain containing 2 (UBTD2), mRNA. |
| CD83 | 1.8 | Homo sapiens CD83 molecule (CD83), transcript variant 1, mRNA. |
| PA2G4 | 1.8 | Homo sapiens proliferation-associated 2G4, 38kDa (PA2G4), mRNA. |
| TTLL7 | 1.8 | Homo sapiens tubulin tyrosine ligase-like family, member 7 (TTLL7), mRNA. |
| NFKB1 | 1.8 | Homo sapiens nuclear factor of kappa light polypeptide gene enhancer in B-cells 1 (NFKB1), mRNA. |
| JPH4 | 1.8 | Homo sapiens junctophilin 4 (JPH4), mRNA. |
| C3orf15 | 1.79 | Homo sapiens chromosome 3 open reading frame 15 (C3orf15), mRNA. |
| LOC730820 | 1.79 | PREDICTED: Homo sapiens similar to nuclear receptor binding factor 2 (LOC730820), mRNA. |
| CRAMP1L | 1.79 | Homo sapiens Crm, cramped-like (Drosophila) (CRAMP1L), mRNA. |
| CDC25A | 1.79 | Homo sapiens cell division cycle 25 homolog A (S. pombe) (CDC25A), transcript variant 1, mRNA. |
| ACVR2A | 1.79 | Homo sapiens activin A receptor, type IIA (ACVR2A), mRNA. |
| USP42 | 1.79 | Homo sapiens ubiquitin specific peptidase 42 (USP42), mRNA. |
| ZNF260 | 1.79 | Homo sapiens zinc finger protein 260 (ZNF260), mRNA. |
| FLJ20254 | 1.79 | Homo sapiens hypothetical protein FLJ20254 (FLJ20254), mRNA. |
| TSGA14 | 1.79 | Homo sapiens testis specific, 14 (TSGA14), mRNA. |
| NOMO1 | 1.79 | Homo sapiens NODAL modulator 1 (NOMO1), mRNA. |
| TPST2 | 1.79 | Homo sapiens tyrosylprotein sulfotransferase 2 (TPST2), transcript variant 1, mRNA. |
| C9orf6 | 1.79 | Homo sapiens chromosome 9 open reading frame 6 (C9orf6), mRNA. |
| ATF7IP2 | 1.79 | Homo sapiens activating transcription factor 7 interacting protein 2 (ATF7IP2), mRNA. |
| MASTL | 1.79 | Homo sapiens microtubule associated serine/threonine kinase-like (MASTL), mRNA. |
| APM-1 | 1.79 | PREDICTED: Homo sapiens BTB/POZ-zinc finger protein-like (APM-1), mRNA. |
| SLC41A1 | 1.79 | Homo sapiens solute carrier family 41, member 1 (SLC41A1), mRNA. |
| ZNF213 | 1.79 | Homo sapiens zinc finger protein 213 (ZNF213), mRNA. |
| CREB3L2 | 1.78 | Homo sapiens cAMP responsive element binding protein 3-like 2 (CREB3L2), mRNA. |
| KIAA1949 | 1.78 | Homo sapiens KIAA1949 (KIAA1949), mRNA. |
| TMEM41B | 1.78 | Homo sapiens transmembrane protein 41B (TMEM41B), mRNA. |
| TMCO3 | 1.78 | Homo sapiens transmembrane and coiled-coil domains 3 (TMCO3), mRNA. |
| ZNF177 | 1.78 | Homo sapiens zinc finger protein 177 (ZNF177), mRNA. |
| ZFYVE26 | 1.78 | Homo sapiens zinc finger, FYVE domain containing 26 (ZFYVE26), mRNA. |
| ASB8 | 1.78 | Homo sapiens ankyrin repeat and SOCS box-containing 8 (ASB8), mRNA. |
| COX15 | 1.78 | Homo sapiens COX15 homolog, cytochrome c oxidase assembly protein (yeast) (COX15), nuclear gene encoding mitochondrial protein, transcript variant 1, mRNA. |
| FBXO30 | 1.78 | Homo sapiens F-box protein 30 (FBXO30), mRNA. |
| S100PBP | 1.78 | Homo sapiens S100P binding protein (S100PBP), transcript variant 1, mRNA. |
| AFF3 | 1.78 | Homo sapiens AF4/FMR2 family, member 3 (AFF3), transcript variant 2, mRNA. |
| TBC1D24 | 1.78 | Homo sapiens TBC1 domain family, member 24 (TBC1D24), mRNA. XM_945422 |
| SETD4 | 1.78 | Homo sapiens SET domain containing 4 (SETD4), transcript variant 1, mRNA. |
| KIAA1598 | 1.78 | Homo sapiens KIAA1598 (KIAA1598), mRNA. |
| ZBTB48 | 1.78 | Homo sapiens zinc finger and BTB domain containing 48 (ZBTB48), mRNA. |
| ARMC7 | 1.77 | Homo sapiens armadillo repeat containing 7 (ARMC7), mRNA. |
| PLAA | 1.77 | Homo sapiens phospholipase A2-activating protein (PLAA), transcript variant 1, mRNA. |
| BET1 | 1.77 | Homo sapiens blocked early in transport 1 homolog (S. cerevisiae) (BET1), mRNA. |
| SEC23B | 1.77 | Homo sapiens Sec23 homolog B (S. cerevisiae) (SEC23B), transcript variant 2, mRNA. |
| ARID3B | 1.77 | Homo sapiens AT rich interactive domain 3B (BRIGHT-like) (ARID3B), mRNA. |
| SAP130 | 1.76 | Homo sapiens Sin3A-associated protein, 130kDa (SAP130), mRNA. |
| DCLK1 | 1.76 | Homo sapiens doublecortin-like kinase 1 (DCLK1), mRNA. |
| EFNA4 | 1.76 | Homo sapiens ephrin-A4 (EFNA4), transcript variant 1, mRNA. |
| TP53 | 1.76 | Homo sapiens tumor protein p53 (TP53), mRNA. |
| LOC401238 | 1.76 | PREDICTED: Homo sapiens similar to interferon stimulated exonuclease gene 20kDa-like 2 (LOC401238), mRNA. |
|  | 1.76 | UI-E-EJ0-aho-m-08-0-UI.s1 UI-E-EJ0 Homo sapiens cDNA clone UI-E-EJ0-aho-m-08-0-UI 3, mRNA sequence |
| LOC389816 | 1.76 | Homo sapiens cytokeratin associated protein (LOC389816), mRNA. |
| PPRC1 | 1.76 | Homo sapiens peroxisome proliferator-activated receptor gamma, coactivator-related 1 (PPRC1), mRNA. |
| ZNF423 | 1.76 | Homo sapiens zinc finger protein 423 (ZNF423), mRNA. |
| IPO8 | 1.76 | Homo sapiens importin 8 (IPO8), mRNA. |
|  | 1.76 | UI-H-CO0-ara-d-07-0-UI.s1 NCI_CGAP_Sub9 Homo sapiens cDNA clone IMAGE:3105827 3, mRNA sequence |
| CCDC109A | 1.76 | Homo sapiens coiled-coil domain containing 109A (CCDC109A), mRNA. |
| SERP1 | 1.76 | Homo sapiens stress-associated endoplasmic reticulum protein 1 (SERP1), mRNA. |
| HEATR5B | 1.76 | Homo sapiens HEAT repeat containing 5B (HEATR5B), mRNA. |
| RAI1 | 1.76 | Homo sapiens retinoic acid induced 1 (RAI1), mRNA. |
| SLC8A3 | 1.76 | Homo sapiens solute carrier family 8 (sodium/calcium exchanger), member 3 (SLC8A3), transcript variant f, mRNA. |
| NGDN | 1.76 | Homo sapiens neuroguidin, EIF4E binding protein (NGDN), transcript variant 2, mRNA. XM_941350 XM_945161 XM_945163 XM_945166 XM_945170 |
| C2orf18 | 1.76 | Homo sapiens chromosome 2 open reading frame 18 (C2orf18), mRNA. |
| NAMPT | 1.76 | Homo sapiens nicotinamide phosphoribosyltransferase (NAMPT), mRNA. |
| SYP | 1.75 | Homo sapiens synaptophysin (SYP), mRNA. |
| FBXO3 | 1.75 | Homo sapiens F-box protein 3 (FBXO3), transcript variant 1, mRNA. |
| DDX47 | 1.75 | Homo sapiens DEAD (Asp-Glu-Ala-Asp) box polypeptide 47 (DDX47), transcript variant 1, mRNA. |
| AHCTF1 | 1.75 | Homo sapiens AT hook containing transcription factor 1 (AHCTF1), mRNA. |
|  | 1.75 | Homo sapiens mRNA; cDNA DKFZp686B24166 (from clone DKFZp686B24166) |
| CXorf45 | 1.75 | Homo sapiens chromosome X open reading frame 45 (CXorf45), transcript variant 1, mRNA. |
| BTBD3 | 1.74 | Homo sapiens BTB (POZ) domain containing 3 (BTBD3), transcript variant 1, mRNA. |
| TIPARP | 1.74 | Homo sapiens TCDD-inducible poly(ADP-ribose) polymerase (TIPARP), mRNA. |
| TIAL1 | 1.74 | Homo sapiens TIA1 cytotoxic granule-associated RNA binding protein-like 1 (TIAL1), transcript variant 2, mRNA. |
| RYK | 1.74 | Homo sapiens RYK receptor-like tyrosine kinase (RYK), transcript variant 2, mRNA. |
| VRK3 | 1.74 | Homo sapiens vaccinia related kinase 3 (VRK3), transcript variant 2, mRNA. |
| HSP90B1 | 1.74 | Homo sapiens heat shock protein 90kDa beta (Grp94), member 1 (HSP90B1), mRNA. |
| TRAM1 | 1.74 | Homo sapiens translocation associated membrane protein 1 (TRAM1), mRNA. |
| SPRYD4 | 1.74 | Homo sapiens SPRY domain containing 4 (SPRYD4), mRNA. |
| C11orf54 | 1.74 | Homo sapiens chromosome 11 open reading frame 54 (C11orf54), mRNA. |
| SOCS4 | 1.74 | Homo sapiens suppressor of cytokine signaling 4 (SOCS4), transcript variant 2, mRNA. |
| POLR3A | 1.74 | Homo sapiens polymerase (RNA) III (DNA directed) polypeptide A, 155kDa (POLR3A), mRNA. |
| PHTF1 | 1.73 | Homo sapiens putative homeodomain transcription factor 1 (PHTF1), mRNA. |
| HDAC9 | 1.73 | Homo sapiens histone deacetylase 9 (HDAC9), transcript variant 5, mRNA. |
| OCRL | 1.73 | Homo sapiens oculocerebrorenal syndrome of Lowe (OCRL), transcript variant a, mRNA. |
| NUPL2 | 1.73 | Homo sapiens nucleoporin like 2 (NUPL2), mRNA. |
| RGPD1 | 1.73 | Homo sapiens RANBP2-like and GRIP domain containing 1 (RGPD1), mRNA. |
| KIAA1147 | 1.73 | Homo sapiens KIAA1147 (KIAA1147), mRNA. |
| CNTN1 | 1.73 | Homo sapiens contactin 1 (CNTN1), transcript variant 1, mRNA. |
| TRIOBP | 1.73 | Homo sapiens TRIO and F-actin binding protein (TRIOBP), transcript variant 6, mRNA. |
| MOSC1 | 1.73 | Homo sapiens MOCO sulphurase C-terminal domain containing 1 (MOSC1), mRNA. |
| LGALS8 | 1.73 | Homo sapiens lectin, galactoside-binding, soluble, 8 (LGALS8), transcript variant 1, mRNA. |
| LOC727726 | 1.73 | PREDICTED: Homo sapiens similar to poly (ADP-ribose) glycohydrolase (LOC727726), mRNA. |
| RDH14 | 1.73 | Homo sapiens retinol dehydrogenase 14 (all-trans/9-cis/11-cis) (RDH14), mRNA. |
| HSPBAP1 | 1.73 | Homo sapiens HSPB (heat shock 27kDa) associated protein 1 (HSPBAP1), mRNA. |
| CTNNBIP1 | 1.72 | Homo sapiens catenin, beta interacting protein 1 (CTNNBIP1), transcript variant 1, mRNA. |
| PPP1R3F | 1.72 | Homo sapiens protein phosphatase 1, regulatory (inhibitor) subunit 3F (PPP1R3F), mRNA. |
| WARS | 1.72 | Homo sapiens tryptophanyl-tRNA synthetase (WARS), transcript variant 2, mRNA. |
| GAPVD1 | 1.72 | Homo sapiens GTPase activating protein and VPS9 domains 1 (GAPVD1), mRNA. |
| TOP1MT | 1.72 | Homo sapiens topoisomerase (DNA) I, mitochondrial (TOP1MT), nuclear gene encoding mitochondrial protein, mRNA. |
| EIF4ENIF1 | 1.72 | Homo sapiens eukaryotic translation initiation factor 4E nuclear import factor 1 (EIF4ENIF1), mRNA. |
| SLCO3A1 | 1.72 | Homo sapiens solute carrier organic anion transporter family, member 3A1 (SLCO3A1), mRNA. |
| KCNQ2 | 1.72 | Homo sapiens potassium voltage-gated channel, KQT-like subfamily, member 2 (KCNQ2), transcript variant 3, mRNA. |
| MRFAP1L1 | 1.71 | Homo sapiens Morf4 family associated protein 1-like 1 (MRFAP1L1), transcript variant 2, mRNA. |
| SERPINH1 | 1.71 | Homo sapiens serpin peptidase inhibitor, clade H (heat shock protein 47), member 1, (collagen binding protein 1) (SERPINH1), mRNA. |
| CNPY3 | 1.71 | Homo sapiens canopy 3 homolog (zebrafish) (CNPY3), mRNA. |
| ACPT | 1.71 | Homo sapiens acid phosphatase, testicular (ACPT), mRNA. |
| RAP2A | 1.71 | Homo sapiens RAP2A, member of RAS oncogene family (RAP2A), mRNA. |
| DHX34 | 1.71 | Homo sapiens DEAH (Asp-Glu-Ala-His) box polypeptide 34 (DHX34), mRNA. |
| DDX17 | 1.71 | Homo sapiens DEAD (Asp-Glu-Ala-Asp) box polypeptide 17 (DDX17), transcript variant 1, mRNA. |
| DCUN1D4 | 1.71 | Homo sapiens DCN1, defective in cullin neddylation 1, domain containing 4 (S. cerevisiae) (DCUN1D4), transcript variant 2, mRNA. |
| AP3M1 | 1.71 | Homo sapiens adaptor-related protein complex 3, mu 1 subunit (AP3M1), transcript variant 1, mRNA. |
| CACNA2D2 | 1.7 | Homo sapiens calcium channel, voltage-dependent, alpha 2/delta subunit 2 (CACNA2D2), transcript variant 2, mRNA. |
| OGG1 | 1.7 | Homo sapiens 8-oxoguanine DNA glycosylase (OGG1), nuclear gene encoding mitochondrial protein, transcript variant 2a, mRNA. |
| IQCB1 | 1.7 | Homo sapiens IQ motif containing B1 (IQCB1), transcript variant 3, mRNA. |
| NOD1 | 1.7 | Homo sapiens nucleotide-binding oligomerization domain containing 1 (NOD1), mRNA. |
| TTLL5 | 1.7 | Homo sapiens tubulin tyrosine ligase-like family, member 5 (TTLL5), mRNA. |
| C2orf56 | 1.7 | Homo sapiens chromosome 2 open reading frame 56 (C2orf56), transcript variant 1, mRNA. |
| DET1 | 1.7 | Homo sapiens de-etiolated homolog 1 (Arabidopsis) (DET1), mRNA. |
| RFESD | 1.7 | Homo sapiens Rieske (Fe-S) domain containing (RFESD), mRNA. |
| EPB41L5 | 1.69 | Homo sapiens erythrocyte membrane protein band 4.1 like 5 (EPB41L5), mRNA. |
| EFHD2 | 1.69 | Homo sapiens EF-hand domain family, member D2 (EFHD2), mRNA. |
| KIAA0922 | 1.69 | Homo sapiens KIAA0922 (KIAA0922), mRNA. |
|  | 1.69 | Homo sapiens cDNA clone IMAGE:5262496 |
| AQR | 1.69 | Homo sapiens aquarius homolog (mouse) (AQR), mRNA. |
| FAM119B | 1.69 | Homo sapiens family with sequence similarity 119, member B (FAM119B), transcript variant 1, mRNA. |
| MORN4 | 1.69 | Homo sapiens MORN repeat containing 4 (MORN4), transcript variant 1, mRNA. |
|  | 1.69 | Homo sapiens full length insert cDNA clone YZ84G08 |
| CASK | 1.68 | Homo sapiens calcium/calmodulin-dependent serine protein kinase (MAGUK family) (CASK), mRNA. |
| LSM8 | 1.68 | Homo sapiens LSM8 homolog, U6 small nuclear RNA associated (S. cerevisiae) (LSM8), mRNA. |
| STX6 | 1.68 | Homo sapiens syntaxin 6 (STX6), mRNA. |
| ANKRD41 | 1.68 | Homo sapiens ankyrin repeat domain 41 (ANKRD41), mRNA. |
| SLC39A6 | 1.68 | Homo sapiens solute carrier family 39 (zinc transporter), member 6 (SLC39A6), mRNA. |
| SRRM4 | 1.68 | Homo sapiens serine/arginine repetitive matrix 4 (SRRM4), mRNA. |
| ZNRD1 | 1.68 | Homo sapiens zinc ribbon domain containing 1 (ZNRD1), transcript variant a, mRNA. |
| SLC38A9 | 1.68 | Homo sapiens solute carrier family 38, member 9 (SLC38A9), mRNA. |
| TXNRD2 | 1.67 | Homo sapiens thioredoxin reductase 2 (TXNRD2), nuclear gene encoding mitochondrial protein, mRNA. |
| FAM49B | 1.67 | Homo sapiens family with sequence similarity 49, member B (FAM49B), mRNA. |
| RASEF | 1.67 | Homo sapiens RAS and EF-hand domain containing (RASEF), mRNA. |
| SUGT1 | 1.67 | Homo sapiens SGT1, suppressor of G2 allele of SKP1 (S. cerevisiae) (SUGT1), mRNA. |
| MAFG | 1.67 | Homo sapiens v-maf musculoaponeurotic fibrosarcoma oncogene homolog G (avian) (MAFG), transcript variant 2, mRNA. |
| SMARCD2 | 1.67 | Homo sapiens SWI/SNF related, matrix associated, actin dependent regulator of chromatin, subfamily d, member 2 (SMARCD2), mRNA. |
| SMAD2 | 1.67 | Homo sapiens SMAD family member 2 (SMAD2), transcript variant 2, mRNA. |
| ADPGK | 1.67 | Homo sapiens ADP-dependent glucokinase (ADPGK), mRNA. |
| LMAN2L | 1.67 | Homo sapiens lectin, mannose-binding 2-like (LMAN2L), mRNA. |
| KIAA1279 | 1.67 | Homo sapiens KIAA1279 (KIAA1279), mRNA. |
| SLC9A1 | 1.66 | Homo sapiens solute carrier family 9 (sodium/hydrogen exchanger), member 1 (SLC9A1), mRNA. |
| PPWD1 | 1.66 | Homo sapiens peptidylprolyl isomerase domain and WD repeat containing 1 (PPWD1), mRNA. |
| SALL2 | 1.66 | Homo sapiens sal-like 2 (Drosophila) (SALL2), mRNA. |
| S100PBP | 1.66 | Homo sapiens S100P binding protein (S100PBP), transcript variant 1, mRNA. |
| DTX2 | 1.66 | Homo sapiens deltex homolog 2 (Drosophila) (DTX2), mRNA. |
| ZNF485 | 1.66 | Homo sapiens zinc finger protein 485 (ZNF485), mRNA. |
| CTTNBP2NL | 1.66 | Homo sapiens CTTNBP2 N-terminal like (CTTNBP2NL), mRNA. |
| MCL1 | 1.66 | Homo sapiens myeloid cell leukemia sequence 1 (BCL2-related) (MCL1), transcript variant 1, mRNA. |
| USP10 | 1.66 | Homo sapiens ubiquitin specific peptidase 10 (USP10), mRNA. |
| ELOVL6 | 1.66 | Homo sapiens ELOVL family member 6, elongation of long chain fatty acids (FEN1/Elo2, SUR4/Elo3-like, yeast) (ELOVL6), mRNA. |
| KLHL17 | 1.65 | Homo sapiens kelch-like 17 (Drosophila) (KLHL17), mRNA. |
| PPDPF | 1.65 | Homo sapiens pancreatic progenitor cell differentiation and proliferation factor homolog (zebrafish) (PPDPF), mRNA. |
| C17orf69 | 1.65 | Homo sapiens chromosome 17 open reading frame 69 (C17orf69), mRNA. |
| SIN3A | 1.65 | Homo sapiens SIN3 homolog A, transcription regulator (yeast) (SIN3A), mRNA. |
| HINFP | 1.65 | Homo sapiens histone H4 transcription factor (HINFP), transcript variant 2, mRNA. |
| CERK | 1.65 | Homo sapiens ceramide kinase (CERK), transcript variant 1, mRNA. |
| ELF1 | 1.65 | Homo sapiens E74-like factor 1 (ets domain transcription factor) (ELF1), mRNA. |
| FBXO28 | 1.65 | Homo sapiens F-box protein 28 (FBXO28), mRNA. |
| HSDL1 | 1.65 | Homo sapiens hydroxysteroid dehydrogenase like 1 (HSDL1), mRNA. |
|  | 1.65 | 601336028F1 NIH_MGC_44 Homo sapiens cDNA clone IMAGE:3689911 5, mRNA sequence |
| ATG16L1 | 1.64 | Homo sapiens ATG16 autophagy related 16-like 1 (S. cerevisiae) (ATG16L1), transcript variant 2, mRNA. |
| ZRANB2 | 1.64 | Homo sapiens zinc finger, RAN-binding domain containing 2 (ZRANB2), transcript variant 2, mRNA. |
| ASAP1 | 1.64 | Homo sapiens ArfGAP with SH3 domain, ankyrin repeat and PH domain 1 (ASAP1), mRNA. |
| RPIA | 1.64 | Homo sapiens ribose 5-phosphate isomerase A (RPIA), mRNA. |
| METTL3 | 1.64 | Homo sapiens methyltransferase like 3 (METTL3), mRNA. |
| ZNFX1 | 1.64 | Homo sapiens zinc finger, NFX1-type containing 1 (ZNFX1), mRNA. |
| C7orf52 | 1.64 | Homo sapiens chromosome 7 open reading frame 52 (C7orf52), mRNA. |
| NDRG3 | 1.64 | Homo sapiens NDRG family member 3 (NDRG3), transcript variant 2, mRNA. |
| MUTED | 1.64 | Homo sapiens muted homolog (mouse) (MUTED), mRNA. |
| ST7 | 1.64 | Homo sapiens suppression of tumorigenicity 7 (ST7), transcript variant a, mRNA. |
| CUX1 | 1.64 | Homo sapiens cut-like homeobox 1 (CUX1), transcript variant 2, mRNA. |
| ZNF791 | 1.64 | Homo sapiens zinc finger protein 791 (ZNF791), mRNA. |
| RARA | 1.64 | Homo sapiens retinoic acid receptor, alpha (RARA), transcript variant 1, mRNA. |
|  | 1.63 | DKFZp781L12160_r1 781 (synonym: hlcc4) Homo sapiens cDNA clone DKFZp781L12160 5, mRNA sequence |
| APLP2 | 1.63 | Homo sapiens amyloid beta (A4) precursor-like protein 2 (APLP2), mRNA. |
| ABI2 | 1.63 | PREDICTED: Homo sapiens abl interactor 2 (ABI2), mRNA. |
| IL13RA1 | 1.63 | Homo sapiens interleukin 13 receptor, alpha 1 (IL13RA1), mRNA. |
| DPYSL3 | 1.63 | Homo sapiens dihydropyrimidinase-like 3 (DPYSL3), mRNA. |
| RASSF1 | 1.63 | Homo sapiens Ras association (RalGDS/AF-6) domain family member 1 (RASSF1), transcript variant A, mRNA. |
| SLC31A2 | 1.63 | Homo sapiens solute carrier family 31 (copper transporters), member 2 (SLC31A2), mRNA. |
| TRIM33 | 1.63 | Homo sapiens tripartite motif-containing 33 (TRIM33), transcript variant a, mRNA. |
| TRAK1 | 1.63 | Homo sapiens trafficking protein, kinesin binding 1 (TRAK1), transcript variant 1, mRNA. |
| SLC25A24 | 1.63 | Homo sapiens solute carrier family 25 (mitochondrial carrier; phosphate carrier), member 24 (SLC25A24), nuclear gene encoding mitochondrial protein, transcript variant 1, mRNA. |
| KIAA1967 | 1.62 | Homo sapiens KIAA1967 (KIAA1967), transcript variant 1, mRNA. |
| COPB2 | 1.62 | Homo sapiens coatomer protein complex, subunit beta 2 (beta prime) (COPB2), mRNA. |
| UBE3C | 1.62 | Homo sapiens ubiquitin protein ligase E3C (UBE3C), mRNA. |
| ADAT3 | 1.62 | Homo sapiens adenosine deaminase, tRNA-specific 3, TAD3 homolog (S. cerevisiae) (ADAT3), mRNA. |
| ADIPOR1 | 1.62 | Homo sapiens adiponectin receptor 1 (ADIPOR1), mRNA. |
| MRPL35 | 1.62 | Homo sapiens mitochondrial ribosomal protein L35 (MRPL35), nuclear gene encoding mitochondrial protein, transcript variant 1, mRNA. |
| PIGF | 1.62 | Homo sapiens phosphatidylinositol glycan anchor biosynthesis, class F (PIGF), transcript variant 1, mRNA. |
| MEMO1 | 1.62 | Homo sapiens mediator of cell motility 1 (MEMO1), transcript variant 1, mRNA. |
| SLC25A3 | 1.62 | Homo sapiens solute carrier family 25 (mitochondrial carrier; phosphate carrier), member 3 (SLC25A3), nuclear gene encoding mitochondrial protein, transcript variant 3, mRNA. |
| LASS5 | 1.61 | Homo sapiens LAG1 homolog, ceramide synthase 5 (S. cerevisiae) (LASS5), mRNA. |
| BTBD3 | 1.61 | Homo sapiens BTB (POZ) domain containing 3 (BTBD3), transcript variant 1, mRNA. |
| FADD | 1.61 | Homo sapiens Fas (TNFRSF6)-associated via death domain (FADD), mRNA. |
| MAT2B | 1.61 | Homo sapiens methionine adenosyltransferase II, beta (MAT2B), transcript variant 1, mRNA. |
| HSPC047 | 1.61 | Homo sapiens HSPC047 protein (HSPC047), mRNA. |
| ANKZF1 | 1.61 | Homo sapiens ankyrin repeat and zinc finger domain containing 1 (ANKZF1), transcript variant 2, mRNA. |
| ABAT | 1.61 | Homo sapiens 4-aminobutyrate aminotransferase (ABAT), nuclear gene encoding mitochondrial protein, transcript variant 2, mRNA. |
| VCL | 1.61 | Homo sapiens vinculin (VCL), transcript variant 2, mRNA. |
| VANGL2 | 1.61 | Homo sapiens vang-like 2 (van gogh, Drosophila) (VANGL2), mRNA. |
| FAM102A | 1.61 | Homo sapiens family with sequence similarity 102, member A (FAM102A), transcript variant 1, mRNA. |
| PPM1K | 1.61 | Homo sapiens protein phosphatase 1K (PP2C domain containing) (PPM1K), mRNA. |
| TRIM37 | 1.61 | Homo sapiens tripartite motif-containing 37 (TRIM37), transcript variant 1, mRNA. |
| PSMD10 | 1.61 | Homo sapiens proteasome (prosome, macropain) 26S subunit, non-ATPase, 10 (PSMD10), transcript variant 1, mRNA. |
| CLUAP1 | 1.6 | Homo sapiens clusterin associated protein 1 (CLUAP1), transcript variant 1, mRNA. |
| EIF4EBP2 | 1.6 | Homo sapiens eukaryotic translation initiation factor 4E binding protein 2 (EIF4EBP2), mRNA. |
| UQCC | 1.6 | Homo sapiens ubiquinol-cytochrome c reductase complex chaperone (UQCC), nuclear gene encoding mitochondrial protein, transcript variant 1, mRNA. |
| EGLN2 | 1.6 | Homo sapiens egl nine homolog 2 (C. elegans) (EGLN2), transcript variant 1, mRNA. |
| LOC387856 | 1.6 | Homo sapiens similar to expressed sequence AI836003 (LOC387856), mRNA. |
| CRK | 1.6 | Homo sapiens v-crk sarcoma virus CT10 oncogene homolog (avian) (CRK), transcript variant II, mRNA. |
| ARL1 | 1.6 | Homo sapiens ADP-ribosylation factor-like 1 (ARL1), mRNA. |
| SHARPIN | 1.6 | Homo sapiens SHANK-associated RH domain interactor (SHARPIN), mRNA. |
|  | 1.6 | Homo sapiens cDNA FLJ11494 fis, clone HEMBA1001942 |
| LOC389599 | 1.6 | PREDICTED: Homo sapiens similar to amyotrophic lateral sclerosis 2 (juvenile) chromosome region, candidate 2 (LOC389599), mRNA. |
| PRPF3 | 1.6 | Homo sapiens PRP3 pre-mRNA processing factor 3 homolog (S. cerevisiae) (PRPF3), mRNA. |
| IDH1 | 1.6 | Homo sapiens isocitrate dehydrogenase 1 (NADP+), soluble (IDH1), mRNA. |
| ACN9 | 1.59 | Homo sapiens ACN9 homolog (S. cerevisiae) (ACN9), mRNA. |
| SRI | 1.59 | Homo sapiens sorcin (SRI), transcript variant 2, mRNA. |
| LOC644979 | 1.59 | PREDICTED: Homo sapiens hypothetical LOC644979 (LOC644979), mRNA. |
| TGIF1 | 1.59 | Homo sapiens TGFB-induced factor homeobox 1 (TGIF1), transcript variant 1, mRNA. |
| TNC | 1.59 | Homo sapiens tenascin C (TNC), mRNA. |
| SYNJ1 | 1.59 | Homo sapiens synaptojanin 1 (SYNJ1), transcript variant 2, mRNA. |
| PAPSS2 | 1.59 | Homo sapiens 3'-phosphoadenosine 5'-phosphosulfate synthase 2 (PAPSS2), transcript variant 1, mRNA. |
| RXRB | 1.59 | Homo sapiens retinoid X receptor, beta (RXRB), mRNA. |
| RAB11FIP5 | 1.59 | Homo sapiens RAB11 family interacting protein 5 (class I) (RAB11FIP5), mRNA. |
| TEX10 | 1.58 | Homo sapiens testis expressed 10 (TEX10), mRNA. |
| SEC31A | 1.58 | Homo sapiens SEC31 homolog A (S. cerevisiae) (SEC31A), transcript variant 1, mRNA. |
| FAIM2 | 1.58 | Homo sapiens Fas apoptotic inhibitory molecule 2 (FAIM2), mRNA. |
| ERCC1 | 1.57 | Homo sapiens excision repair cross-complementing rodent repair deficiency, complementation group 1 (includes overlapping antisense sequence) (ERCC1), transcript variant 2, mRNA. |
| ADAMTSL2 | 1.57 | Homo sapiens ADAMTS-like 2 (ADAMTSL2), mRNA. |
| SLC30A3 | 1.57 | Homo sapiens solute carrier family 30 (zinc transporter), member 3 (SLC30A3), mRNA. |
| LOC644295 | 1.57 | PREDICTED: Homo sapiens similar to T25G3.1 (LOC644295), mRNA. |
| NFE2L1 | 1.57 | Homo sapiens nuclear factor (erythroid-derived 2)-like 1 (NFE2L1), mRNA. |
| LRP5 | 1.57 | Homo sapiens low density lipoprotein receptor-related protein 5 (LRP5), mRNA. |
| SLC25A4 | 1.57 | Homo sapiens solute carrier family 25 (mitochondrial carrier; adenine nucleotide translocator), member 4 (SLC25A4), nuclear gene encoding mitochondrial protein, mRNA. |
| ZNF275 | 1.57 | Homo sapiens zinc finger protein 275 (ZNF275), mRNA. |
| OTUB2 | 1.56 | Homo sapiens OTU domain, ubiquitin aldehyde binding 2 (OTUB2), mRNA. |
| TTPAL | 1.56 | Homo sapiens tocopherol (alpha) transfer protein-like (TTPAL), transcript variant 1, mRNA. |
| ARPP19 | 1.56 | Homo sapiens cAMP-regulated phosphoprotein, 19kDa (ARPP19), mRNA. |
| RAB6B | 1.56 | Homo sapiens RAB6B, member RAS oncogene family (RAB6B), mRNA. |
| SUPT6H | 1.56 | Homo sapiens suppressor of Ty 6 homolog (S. cerevisiae) (SUPT6H), mRNA. |
| FAM48A | 1.56 | Homo sapiens family with sequence similarity 48, member A (FAM48A), transcript variant 1, mRNA. |
| PHCA | 1.56 | Homo sapiens phytoceramidase, alkaline (PHCA), mRNA. |
| HECTD3 | 1.56 | Homo sapiens HECT domain containing 3 (HECTD3), mRNA. |
| PRKAA1 | 1.56 | Homo sapiens protein kinase, AMP-activated, alpha 1 catalytic subunit (PRKAA1), transcript variant 1, mRNA. |
| VPRBP | 1.56 | Homo sapiens Vpr (HIV-1) binding protein (VPRBP), mRNA. |
| IP6K1 | 1.56 | Homo sapiens inositol hexakisphosphate kinase 1 (IP6K1), transcript variant 2, mRNA. |
| MBD1 | 1.56 | Homo sapiens methyl-CpG binding domain protein 1 (MBD1), transcript variant 3, mRNA. |
| DNAJA1 | 1.55 | Homo sapiens DnaJ (Hsp40) homolog, subfamily A, member 1 (DNAJA1), mRNA. |
| HINT3 | 1.55 | Homo sapiens histidine triad nucleotide binding protein 3 (HINT3), mRNA. |
| C20orf194 | 1.55 | Homo sapiens chromosome 20 open reading frame 194 (C20orf194), mRNA. |
| MTHFR | 1.55 | Homo sapiens 5,10-methylenetetrahydrofolate reductase (NADPH) (MTHFR), mRNA. |
| CHKA | 1.55 | Homo sapiens choline kinase alpha (CHKA), transcript variant 2, mRNA. |
| CNN2 | 1.55 | Homo sapiens calponin 2 (CNN2), transcript variant 2, mRNA. |
| ZNF473 | 1.55 | Homo sapiens zinc finger protein 473 (ZNF473), transcript variant 1, mRNA. |
| CSNK2A1 | 1.55 | Homo sapiens casein kinase 2, alpha 1 polypeptide (CSNK2A1), transcript variant 2, mRNA. |
| SH2B1 | 1.54 | Homo sapiens SH2B adaptor protein 1 (SH2B1), mRNA. |
| KIAA1370 | 1.54 | Homo sapiens KIAA1370 (KIAA1370), mRNA. |
| BCL9 | 1.54 | Homo sapiens B-cell CLL/lymphoma 9 (BCL9), mRNA. |
| SNX5 | 1.54 | Homo sapiens sorting nexin 5 (SNX5), transcript variant 2, mRNA. |
| LOC646463 | 1.54 | PREDICTED: Homo sapiens similar to Ubiquitin-conjugating enzyme E2 H (Ubiquitin-protein ligase H) (Ubiquitin carrier protein H) (UBCH2) (E2-20K) (LOC646463), mRNA. |
| SPEN | 1.54 | Homo sapiens spen homolog, transcriptional regulator (Drosophila) (SPEN), mRNA. |
| BAT2 | 1.54 | Homo sapiens HLA-B associated transcript 2 (BAT2), mRNA. |
| CRELD2 | 1.53 | Homo sapiens cysteine-rich with EGF-like domains 2 (CRELD2), mRNA. |
| KBTBD11 | 1.53 | Homo sapiens kelch repeat and BTB (POZ) domain containing 11 (KBTBD11), mRNA. |
| RYK | 1.53 | Homo sapiens RYK receptor-like tyrosine kinase (RYK), transcript variant 1, mRNA. |
| RGMB | 1.53 | Homo sapiens RGM domain family, member B (RGMB), transcript variant 1, mRNA. |
| DNAJA2 | 1.53 | Homo sapiens DnaJ (Hsp40) homolog, subfamily A, member 2 (DNAJA2), mRNA. |
| PTPRA | 1.53 | Homo sapiens protein tyrosine phosphatase, receptor type, A (PTPRA), transcript variant 1, mRNA. |
| SMG5 | 1.53 | Homo sapiens Smg-5 homolog, nonsense mediated mRNA decay factor (C. elegans) (SMG5), mRNA. |
| SLC30A7 | 1.53 | Homo sapiens solute carrier family 30 (zinc transporter), member 7 (SLC30A7), mRNA. |
| AP3S2 | 1.53 | Homo sapiens adaptor-related protein complex 3, sigma 2 subunit (AP3S2), mRNA. |
| DCUN1D3 | 1.53 | Homo sapiens DCN1, defective in cullin neddylation 1, domain containing 3 (S. cerevisiae) (DCUN1D3), mRNA. |
| OPN3 | 1.52 | Homo sapiens opsin 3 (OPN3), mRNA. |
| NANOS3 | 1.52 | Homo sapiens nanos homolog 3 (Drosophila) (NANOS3), mRNA. |
| ZNF696 | 1.52 | Homo sapiens zinc finger protein 696 (ZNF696), mRNA. |
| KHK | 1.52 | Homo sapiens ketohexokinase (fructokinase) (KHK), transcript variant a, mRNA. |
| SEMA4B | 1.52 | Homo sapiens sema domain, immunoglobulin domain (Ig), transmembrane domain (TM) and short cytoplasmic domain, (semaphorin) 4B (SEMA4B), transcript variant 2, mRNA. |
| CHST3 | 1.52 | Homo sapiens carbohydrate (chondroitin 6) sulfotransferase 3 (CHST3), mRNA. |
| TBCCD1 | 1.52 | Homo sapiens TBCC domain containing 1 (TBCCD1), mRNA. |
| MARK3 | 1.52 | Homo sapiens MAP/microtubule affinity-regulating kinase 3 (MARK3), mRNA. |
| MAGEL2 | 1.52 | Homo sapiens MAGE-like 2 (MAGEL2), mRNA. |
| CBARA1 | 1.52 | Homo sapiens calcium binding atopy-related autoantigen 1 (CBARA1), mRNA. |
| UBAP2 | 1.52 | Homo sapiens ubiquitin associated protein 2 (UBAP2), mRNA. |
| PRCC | 1.51 | Homo sapiens papillary renal cell carcinoma (translocation-associated) (PRCC), transcript variant 1, mRNA. |
| RAD17 | 1.51 | Homo sapiens RAD17 homolog (S. pombe) (RAD17), transcript variant 3, mRNA. |
| ZNF775 | 1.51 | Homo sapiens zinc finger protein 775 (ZNF775), mRNA. |
| ANKRD37 | 1.51 | Homo sapiens ankyrin repeat domain 37 (ANKRD37), mRNA. |
| ZNF234 | 1.51 | Homo sapiens zinc finger protein 234 (ZNF234), mRNA. |
| LTBP3 | 1.51 | Homo sapiens latent transforming growth factor beta binding protein 3 (LTBP3), mRNA. |
| LDOC1 | 1.51 | Homo sapiens leucine zipper, down-regulated in cancer 1 (LDOC1), mRNA. |
| PKP2 | 1.51 | Homo sapiens plakophilin 2 (PKP2), transcript variant 2a, mRNA. |
| ZBTB24 | 1.5 | Homo sapiens zinc finger and BTB domain containing 24 (ZBTB24), mRNA. |
| ELF2 | 1.5 | Homo sapiens E74-like factor 2 (ets domain transcription factor) (ELF2), transcript variant 2, mRNA. |
| TRIM13 | 1.5 | Homo sapiens tripartite motif-containing 13 (TRIM13), transcript variant 4, mRNA. |
| MAP4K4 | 1.5 | Homo sapiens mitogen-activated protein kinase kinase kinase kinase 4 (MAP4K4), transcript variant 3, mRNA. |
| RPA1 | 1.5 | Homo sapiens replication protein A1, 70kDa (RPA1), mRNA. |
| RBM28 | 1.5 | Homo sapiens RNA binding motif protein 28 (RBM28), mRNA. |
| SFRS17A | 1.5 | Homo sapiens splicing factor, arginine/serine-rich 17A (SFRS17A), transcript variant 1, mRNA. |
| LOC731999 | 1.5 | PREDICTED: Homo sapiens similar to CG1486-PA, isoform A (LOC731999), mRNA. |
| PISD | 1.5 | Homo sapiens phosphatidylserine decarboxylase (PISD), mRNA. |
| GLT8D1 | -1.5 | Homo sapiens glycosyltransferase 8 domain containing 1 (GLT8D1), transcript variant 2, mRNA. |
| PLK1 | -1.5 | Homo sapiens polo-like kinase 1 (Drosophila) (PLK1), mRNA. |
| ARHGEF19 | -1.5 | Homo sapiens Rho guanine nucleotide exchange factor (GEF) 19 (ARHGEF19), mRNA. |
| RAC1 | -1.5 | Homo sapiens ras-related C3 botulinum toxin substrate 1 (rho family, small GTP binding protein Rac1) (RAC1), transcript variant Rac1, mRNA. |
| SNRPB | -1.5 | Homo sapiens small nuclear ribonucleoprotein polypeptides B and B1 (SNRPB), transcript variant 2, mRNA. |
| DHRS7B | -1.5 | Homo sapiens dehydrogenase/reductase (SDR family) member 7B (DHRS7B), mRNA. |
| FRAT2 | -1.5 | Homo sapiens frequently rearranged in advanced T-cell lymphomas 2 (FRAT2), mRNA. |
| MTX1 | -1.51 | Homo sapiens metaxin 1 (MTX1), nuclear gene encoding mitochondrial protein, transcript variant 2, mRNA. |
| HSDL2 | -1.51 | Homo sapiens hydroxysteroid dehydrogenase like 2 (HSDL2), mRNA. |
| HDAC1 | -1.51 | Homo sapiens histone deacetylase 1 (HDAC1), mRNA. |
| IGF2R | -1.51 | Homo sapiens insulin-like growth factor 2 receptor (IGF2R), mRNA. |
| C12orf32 | -1.51 | Homo sapiens chromosome 12 open reading frame 32 (C12orf32), mRNA. |
| CICE | -1.51 | PREDICTED: Homo sapiens cell death-inducing CIDE-like effector pseudogene (CICE), misc RNA. |
| SF3B14 | -1.52 | Homo sapiens splicing factor 3B, 14 kDa subunit (SF3B14), mRNA. |
| MRFAP1L1 | -1.52 | Homo sapiens Morf4 family associated protein 1-like 1 (MRFAP1L1), transcript variant 2, mRNA. |
| PDCL3 | -1.52 | Homo sapiens phosducin-like 3 (PDCL3), mRNA. |
| CARS | -1.52 | Homo sapiens cysteinyl-tRNA synthetase (CARS), transcript variant 4, mRNA. |
| TFRC | -1.52 | Homo sapiens transferrin receptor (p90, CD71) (TFRC), mRNA. |
| DYRK4 | -1.52 | Homo sapiens dual-specificity tyrosine-(Y)-phosphorylation regulated kinase 4 (DYRK4), mRNA. |
| SEC11C | -1.53 | Homo sapiens SEC11 homolog C (S. cerevisiae) (SEC11C), mRNA. |
| CARD9 | -1.53 | Homo sapiens caspase recruitment domain family, member 9 (CARD9), mRNA. |
| WDSUB1 | -1.54 | Homo sapiens WD repeat, sterile alpha motif and U-box domain containing 1 (WDSUB1), mRNA. |
| LOC390705 | -1.54 | PREDICTED: Homo sapiens similar to protein phosphatase 2A 48 kDa regulatory subunit isoform 1; serine/threonine protein phosphatase 2A, 48kDa regulatory subunit; PP2A, subunit B, PR48 isoform; PP2A B subunit PR48; NY-REN-8 antigen (LOC390705), misc RNA. |
| COPE | -1.54 | Homo sapiens coatomer protein complex, subunit epsilon (COPE), transcript variant 3, mRNA. |
| DUT | -1.54 | Homo sapiens deoxyuridine triphosphatase (DUT), nuclear gene encoding mitochondrial protein, transcript variant 1, mRNA. |
| TEAD4 | -1.54 | Homo sapiens TEA domain family member 4 (TEAD4), transcript variant 3, mRNA. |
| COL16A1 | -1.54 | Homo sapiens collagen, type XVI, alpha 1 (COL16A1), mRNA. |
| TOMM22 | -1.54 | Homo sapiens translocase of outer mitochondrial membrane 22 homolog (yeast) (TOMM22), nuclear gene encoding mitochondrial protein, mRNA. |
| CDC25B | -1.54 | Homo sapiens cell division cycle 25 homolog B (S. pombe) (CDC25B), transcript variant 2, mRNA. |
| LANCL2 | -1.54 | Homo sapiens LanC lantibiotic synthetase component C-like 2 (bacterial) (LANCL2), mRNA. |
| NSFL1C | -1.54 | Homo sapiens NSFL1 (p97) cofactor (p47) (NSFL1C), transcript variant 3, mRNA. |
| ADNP2 | -1.55 | Homo sapiens ADNP homeobox 2 (ADNP2), mRNA. |
| ZDHHC7 | -1.55 | Homo sapiens zinc finger, DHHC-type containing 7 (ZDHHC7), mRNA. |
| NRIP3 | -1.55 | Homo sapiens nuclear receptor interacting protein 3 (NRIP3), mRNA. |
| MRPL44 | -1.55 | Homo sapiens mitochondrial ribosomal protein L44 (MRPL44), nuclear gene encoding mitochondrial protein, mRNA. |
| KIAA1958 | -1.55 | Homo sapiens KIAA1958 (KIAA1958), mRNA. |
| ZNF75A | -1.55 | Homo sapiens zinc finger protein 75a (ZNF75A), mRNA. |
| LAMP1 | -1.55 | Homo sapiens lysosomal-associated membrane protein 1 (LAMP1), mRNA. |
| ZNF562 | -1.55 | Homo sapiens zinc finger protein 562 (ZNF562), mRNA. |
| GTPBP8 | -1.55 | Homo sapiens GTP-binding protein 8 (putative) (GTPBP8), transcript variant 1, mRNA. |
| SLC44A1 | -1.55 | Homo sapiens solute carrier family 44, member 1 (SLC44A1), mRNA. |
| IRS2 | -1.55 | Homo sapiens insulin receptor substrate 2 (IRS2), mRNA. |
| CDT1 | -1.56 | Homo sapiens chromatin licensing and DNA replication factor 1 (CDT1), mRNA. |
| MTCP1 | -1.56 | Homo sapiens mature T-cell proliferation 1 (MTCP1), nuclear gene encoding mitochondrial protein, transcript variant B1, mRNA. |
| LOC440145 | -1.56 | Homo sapiens similar to RIKEN cDNA 2410129H14 (LOC440145), mRNA. |
| DHRS7 | -1.56 | Homo sapiens dehydrogenase/reductase (SDR family) member 7 (DHRS7), mRNA. |
| CCDC59 | -1.56 | Homo sapiens coiled-coil domain containing 59 (CCDC59), mRNA. |
| KTI12 | -1.57 | Homo sapiens KTI12 homolog, chromatin associated (S. cerevisiae) (KTI12), mRNA. |
| THOC4 | -1.57 | Homo sapiens THO complex 4 (THOC4), mRNA. |
| CUEDC1 | -1.57 | Homo sapiens CUE domain containing 1 (CUEDC1), mRNA. |
| SFRS14 | -1.57 | Homo sapiens splicing factor, arginine/serine-rich 14 (SFRS14), transcript variant 2, mRNA. |
| MRPL55 | -1.57 | Homo sapiens mitochondrial ribosomal protein L55 (MRPL55), nuclear gene encoding mitochondrial protein, transcript variant 1, mRNA. |
| SPAG9 | -1.57 | Homo sapiens sperm associated antigen 9 (SPAG9), mRNA. |
| FAM96A | -1.57 | Homo sapiens family with sequence similarity 96, member A (FAM96A), transcript variant 2, mRNA. |
| GPKOW | -1.57 | Homo sapiens G patch domain and KOW motifs (GPKOW), mRNA. |
| TMUB1 | -1.57 | Homo sapiens transmembrane and ubiquitin-like domain containing 1 (TMUB1), mRNA. |
| C19orf48 | -1.57 | Homo sapiens chromosome 19 open reading frame 48 (C19orf48), mRNA. |
| PRPSAP1 | -1.58 | Homo sapiens phosphoribosyl pyrophosphate synthetase-associated protein 1 (PRPSAP1), mRNA. |
| PRR3 | -1.58 | Homo sapiens proline rich 3 (PRR3), transcript variant 2, mRNA. |
| PMP22 | -1.58 | Homo sapiens peripheral myelin protein 22 (PMP22), transcript variant 2, mRNA. |
| DAZAP1 | -1.58 | Homo sapiens DAZ associated protein 1 (DAZAP1), transcript variant 1, mRNA. |
| IMP4 | -1.58 | Homo sapiens IMP4, U3 small nucleolar ribonucleoprotein, homolog (yeast) (IMP4), mRNA. |
| PPAPDC3 | -1.58 | Homo sapiens phosphatidic acid phosphatase type 2 domain containing 3 (PPAPDC3), mRNA. |
| DERA | -1.58 | Homo sapiens 2-deoxyribose-5-phosphate aldolase homolog (C. elegans) (DERA), mRNA. |
| FLAD1 | -1.58 | Homo sapiens FAD1 flavin adenine dinucleotide synthetase homolog (S. cerevisiae) (FLAD1), transcript variant 2, mRNA. |
| PTMA | -1.58 | Homo sapiens prothymosin, alpha (PTMA), transcript variant 1, mRNA. |
| KCNRG | -1.58 | Homo sapiens potassium channel regulator (KCNRG), transcript variant 2, mRNA. |
| ECHDC3 | -1.59 | Homo sapiens enoyl Coenzyme A hydratase domain containing 3 (ECHDC3), mRNA. |
| PLEKHO1 | -1.59 | Homo sapiens pleckstrin homology domain containing, family O member 1 (PLEKHO1), mRNA. |
| LEPROT | -1.59 | Homo sapiens leptin receptor overlapping transcript (LEPROT), mRNA. |
| DENND5B | -1.59 | Homo sapiens DENN/MADD domain containing 5B (DENND5B), mRNA. |
| NBPF20 | -1.59 | Homo sapiens neuroblastoma breakpoint family, member 20 (NBPF20), mRNA. |
| ZDHHC16 | -1.59 | Homo sapiens zinc finger, DHHC-type containing 16 (ZDHHC16), transcript variant 4, mRNA. |
| MRPL15 | -1.59 | Homo sapiens mitochondrial ribosomal protein L15 (MRPL15), nuclear gene encoding mitochondrial protein, mRNA. |
| TMEM14B | -1.6 | Homo sapiens transmembrane protein 14B (TMEM14B), mRNA. |
| CCDC28B | -1.6 | Homo sapiens coiled-coil domain containing 28B (CCDC28B), mRNA. |
| MGP | -1.6 | Homo sapiens matrix Gla protein (MGP), mRNA. |
| SUV420H1 | -1.6 | Homo sapiens suppressor of variegation 4-20 homolog 1 (Drosophila) (SUV420H1), transcript variant 2, mRNA. |
| TPX2 | -1.6 | Homo sapiens TPX2, microtubule-associated, homolog (Xenopus laevis) (TPX2), mRNA. |
| UCKL1 | -1.6 | Homo sapiens uridine-cytidine kinase 1-like 1 (UCKL1), mRNA. |
| CRTAP | -1.6 | Homo sapiens cartilage associated protein (CRTAP), mRNA. |
| C2orf28 | -1.61 | Homo sapiens chromosome 2 open reading frame 28 (C2orf28), transcript variant 2, mRNA. |
| LOC730130 | -1.61 | PREDICTED: Homo sapiens hypothetical protein LOC730130 (LOC730130), mRNA. |
| DEF8 | -1.61 | Homo sapiens differentially expressed in FDCP 8 homolog (mouse) (DEF8), transcript variant 2, mRNA. |
| NENF | -1.61 | Homo sapiens neuron derived neurotrophic factor (NENF), mRNA. |
| GINS2 | -1.61 | Homo sapiens GINS complex subunit 2 (Psf2 homolog) (GINS2), mRNA. |
| PANK2 | -1.61 | Homo sapiens pantothenate kinase 2 (PANK2), transcript variant 3, mRNA. |
| PON2 | -1.61 | Homo sapiens paraoxonase 2 (PON2), transcript variant 1, mRNA. |
| FZR1 | -1.62 | Homo sapiens fizzy/cell division cycle 20 related 1 (Drosophila) (FZR1), mRNA. |
| CDK5RAP1 | -1.62 | Homo sapiens CDK5 regulatory subunit associated protein 1 (CDK5RAP1), transcript variant 1, mRNA. |
| GINS4 | -1.62 | Homo sapiens GINS complex subunit 4 (Sld5 homolog) (GINS4), mRNA. |
| PECR | -1.62 | Homo sapiens peroxisomal trans-2-enoyl-CoA reductase (PECR), mRNA. |
| FOXRED1 | -1.62 | Homo sapiens FAD-dependent oxidoreductase domain containing 1 (FOXRED1), mRNA. |
| B2M | -1.62 | Homo sapiens beta-2-microglobulin (B2M), mRNA. |
|  | -1.63 | BX114974 NCI_CGAP_Kid3 Homo sapiens cDNA clone IMAGp998C023886, mRNA sequence |
| ST6GALNAC3 | -1.63 | Homo sapiens ST6 (alpha-N-acetyl-neuraminyl-2,3-beta-galactosyl-1, 3)-N-acetylgalactosaminide alpha-2,6-sialyltransferase 3 (ST6GALNAC3), mRNA. |
| IFI27L2 | -1.63 | Homo sapiens interferon, alpha-inducible protein 27-like 2 (IFI27L2), mRNA. |
| SLC25A3 | -1.63 | Homo sapiens solute carrier family 25 (mitochondrial carrier; phosphate carrier), member 3 (SLC25A3), nuclear gene encoding mitochondrial protein, transcript variant 2, mRNA. |
| RBMS1 | -1.63 | Homo sapiens RNA binding motif, single stranded interacting protein 1 (RBMS1), transcript variant 3, mRNA. |
| BUD31 | -1.63 | Homo sapiens BUD31 homolog (S. cerevisiae) (BUD31), mRNA. |
| TSPAN32 | -1.63 | Homo sapiens tetraspanin 32 (TSPAN32), transcript variant 2, mRNA. |
| MTMR14 | -1.63 | Homo sapiens myotubularin related protein 14 (MTMR14), transcript variant 2, mRNA. |
| TMSB15A | -1.64 | Homo sapiens thymosin beta 15a (TMSB15A), mRNA. |
| ING3 | -1.64 | Homo sapiens inhibitor of growth family, member 3 (ING3), transcript variant 1, mRNA. |
| CDK10 | -1.64 | Homo sapiens cyclin-dependent kinase (CDC2-like) 10 (CDK10), transcript variant 3, mRNA. |
| ING2 | -1.64 | Homo sapiens inhibitor of growth family, member 2 (ING2), mRNA. |
| ACAD11 | -1.64 | Homo sapiens acyl-Coenzyme A dehydrogenase family, member 11 (ACAD11), mRNA. |
| CAMK2N1 | -1.64 | Homo sapiens calcium/calmodulin-dependent protein kinase II inhibitor 1 (CAMK2N1), mRNA. |
| CD2BP2 | -1.65 | Homo sapiens CD2 (cytoplasmic tail) binding protein 2 (CD2BP2), mRNA. |
| MED30 | -1.65 | Homo sapiens mediator complex subunit 30 (MED30), mRNA. |
| FBLN1 | -1.65 | Homo sapiens fibulin 1 (FBLN1), transcript variant D, mRNA. |
| HJURP | -1.65 | Homo sapiens Holliday junction recognition protein (HJURP), mRNA. |
| PSMD8 | -1.65 | Homo sapiens proteasome (prosome, macropain) 26S subunit, non-ATPase, 8 (PSMD8), mRNA. |
| DIAPH3 | -1.65 | Homo sapiens diaphanous homolog 3 (Drosophila) (DIAPH3), transcript variant 1, mRNA. |
| UBE2E1 | -1.66 | Homo sapiens ubiquitin-conjugating enzyme E2E 1 (UBC4/5 homolog, yeast) (UBE2E1), transcript variant 2, mRNA. |
| JARID1A | -1.66 | Homo sapiens Jumonji, AT rich interactive domain 1A (RBBP2-like) (JARID1A), mRNA. |
| FUT4 | -1.66 | Homo sapiens fucosyltransferase 4 (alpha (1,3) fucosyltransferase, myeloid-specific) (FUT4), mRNA. |
| TRAPPC2L | -1.66 | Homo sapiens trafficking protein particle complex 2-like (TRAPPC2L), mRNA. |
| GSTT1 | -1.66 | Homo sapiens glutathione S-transferase theta 1 (GSTT1), mRNA. |
| LRRC23 | -1.66 | Homo sapiens leucine rich repeat containing 23 (LRRC23), transcript variant 1, mRNA. |
| HES4 | -1.66 | Homo sapiens hairy and enhancer of split 4 (Drosophila) (HES4), mRNA. |
| TMED1 | -1.66 | Homo sapiens transmembrane emp24 protein transport domain containing 1 (TMED1), mRNA. |
| GSTO1 | -1.67 | Homo sapiens glutathione S-transferase omega 1 (GSTO1), mRNA. |
| LOC727935 | -1.67 | PREDICTED: Homo sapiens similar to CHRNA7 (cholinergic receptor, nicotinic, alpha 7, exons 5-10) and FAM7A (family with sequence similarity 7A, exons A-E) fusion (LOC727935), mRNA. |
| UBXN2A | -1.67 | Homo sapiens UBX domain protein 2A (UBXN2A), mRNA. |
| ZNF787 | -1.67 | Homo sapiens zinc finger protein 787 (ZNF787), mRNA. |
| 4-Sep | -1.67 | Homo sapiens septin 4 (SEPT4), transcript variant 2, mRNA. |
| PPP1R9A | -1.67 | PREDICTED: Homo sapiens protein phosphatase 1, regulatory (inhibitor) subunit 9A, transcript variant 3 (PPP1R9A), mRNA. |
| CDKN1A | -1.68 | Homo sapiens cyclin-dependent kinase inhibitor 1A (p21, Cip1) (CDKN1A), transcript variant 1, mRNA. |
| ITGB1BP1 | -1.68 | Homo sapiens integrin beta 1 binding protein 1 (ITGB1BP1), transcript variant 1, mRNA. |
| CGN | -1.68 | Homo sapiens cingulin (CGN), mRNA. |
| MRPL55 | -1.68 | Homo sapiens mitochondrial ribosomal protein L55 (MRPL55), nuclear gene encoding mitochondrial protein, transcript variant 5, mRNA. |
| LOC653066 | -1.68 | PREDICTED: Homo sapiens similar to inhibitor of growth family, member 5, transcript variant 4 (LOC653066), mRNA. |
| DHRS4L2 | -1.68 | Homo sapiens dehydrogenase/reductase (SDR family) member 4 like 2 (DHRS4L2), mRNA. |
| YEATS4 | -1.68 | Homo sapiens YEATS domain containing 4 (YEATS4), mRNA. |
| GLA | -1.68 | Homo sapiens galactosidase, alpha (GLA), mRNA. |
|  | -1.68 | hp06b06.y1 Human Trabecular meshwork cDNA: hohphq Homo sapiens cDNA clone hp06b06 5, mRNA sequence |
| FAH | -1.69 | Homo sapiens fumarylacetoacetate hydrolase (fumarylacetoacetase) (FAH), mRNA. |
| OPA3 | -1.69 | Homo sapiens optic atrophy 3 (autosomal recessive, with chorea and spastic paraplegia) (OPA3), transcript variant 2, mRNA. |
| WDR70 | -1.69 | Homo sapiens WD repeat domain 70 (WDR70), mRNA. |
| C16orf58 | -1.69 | Homo sapiens chromosome 16 open reading frame 58 (C16orf58), mRNA. |
| SLC27A6 | -1.7 | Homo sapiens solute carrier family 27 (fatty acid transporter), member 6 (SLC27A6), transcript variant 1, mRNA. |
| BNIP1 | -1.7 | Homo sapiens BCL2/adenovirus E1B 19kDa interacting protein 1 (BNIP1), transcript variant BNIP1-c, mRNA. |
| GAS6 | -1.7 | Homo sapiens growth arrest-specific 6 (GAS6), mRNA. |
| IFT122 | -1.7 | Homo sapiens intraflagellar transport 122 homolog (Chlamydomonas) (IFT122), transcript variant 2, mRNA. |
| LOC388494 | -1.7 | PREDICTED: Homo sapiens hypothetical gene supported by AL365406; BC034005 (LOC388494), mRNA. |
| C20orf111 | -1.7 | Homo sapiens chromosome 20 open reading frame 111 (C20orf111), mRNA. |
| DHPS | -1.7 | Homo sapiens deoxyhypusine synthase (DHPS), transcript variant 2, mRNA. |
| ZNF669 | -1.7 | Homo sapiens zinc finger protein 669 (ZNF669), mRNA. |
| PLD3 | -1.7 | Homo sapiens phospholipase D family, member 3 (PLD3), transcript variant 1, mRNA. |
| CSNK1D | -1.7 | Homo sapiens casein kinase 1, delta (CSNK1D), transcript variant 1, mRNA. |
| CDKN1B | -1.7 | Homo sapiens cyclin-dependent kinase inhibitor 1B (p27, Kip1) (CDKN1B), mRNA. |
| ZNF16 | -1.71 | Homo sapiens zinc finger protein 16 (ZNF16), transcript variant 1, mRNA. |
| RNASEH2B | -1.71 | Homo sapiens ribonuclease H2, subunit B (RNASEH2B), mRNA. |
| TTC12 | -1.71 | Homo sapiens tetratricopeptide repeat domain 12 (TTC12), mRNA. |
| WBSCR22 | -1.72 | Homo sapiens Williams Beuren syndrome chromosome region 22 (WBSCR22), mRNA. |
| ANGPTL2 | -1.72 | Homo sapiens angiopoietin-like 2 (ANGPTL2), mRNA. |
| ALG14 | -1.72 | Homo sapiens asparagine-linked glycosylation 14 homolog (S. cerevisiae) (ALG14), mRNA. |
| CENPO | -1.72 | Homo sapiens centromere protein O (CENPO), mRNA. |
| ABHD1 | -1.72 | Homo sapiens abhydrolase domain containing 1 (ABHD1), mRNA. |
| AURKA | -1.72 | Homo sapiens aurora kinase A (AURKA), transcript variant 3, mRNA. |
| RPS26 | -1.72 | Homo sapiens ribosomal protein S26 (RPS26), mRNA. |
| GSTO1 | -1.72 | Homo sapiens glutathione S-transferase omega 1 (GSTO1), mRNA. |
| CENPP | -1.72 | Homo sapiens centromere protein P (CENPP), mRNA. |
| BEX4 | -1.72 | Homo sapiens BEX family member 4 (BEX4), mRNA. |
| DNAJC30 | -1.72 | Homo sapiens DnaJ (Hsp40) homolog, subfamily C, member 30 (DNAJC30), mRNA. |
| FUS | -1.73 | Homo sapiens fusion (involved in t(12;16) in malignant liposarcoma) (FUS), transcript variant 1, mRNA. |
| DTWD2 | -1.73 | Homo sapiens DTW domain containing 2 (DTWD2), mRNA. |
| PRDX1 | -1.73 | Homo sapiens peroxiredoxin 1 (PRDX1), transcript variant 2, mRNA. |
| DGCR6 | -1.73 | Homo sapiens DiGeorge syndrome critical region gene 6 (DGCR6), mRNA. |
| RPL7L1 | -1.73 | Homo sapiens ribosomal protein L7-like 1 (RPL7L1), mRNA. |
| HPS1 | -1.74 | Homo sapiens Hermansky-Pudlak syndrome 1 (HPS1), transcript variant 3, mRNA. |
| GUSB | -1.74 | Homo sapiens glucuronidase, beta (GUSB), mRNA. |
| EFEMP2 | -1.74 | Homo sapiens EGF-containing fibulin-like extracellular matrix protein 2 (EFEMP2), mRNA. |
| TK1 | -1.74 | Homo sapiens thymidine kinase 1, soluble (TK1), mRNA. |
| SMUG1 | -1.74 | Homo sapiens single-strand-selective monofunctional uracil-DNA glycosylase 1 (SMUG1), mRNA. |
| LOC645676 | -1.74 | PREDICTED: Homo sapiens hypothetical protein LOC645676, transcript variant 1 (LOC645676), mRNA. |
| PRSS35 | -1.75 | Homo sapiens protease, serine, 35 (PRSS35), mRNA. |
| RPL9 | -1.75 | Homo sapiens ribosomal protein L9 (RPL9), transcript variant 2, mRNA. |
| C16orf93 | -1.75 | Homo sapiens chromosome 16 open reading frame 93 (C16orf93), mRNA. |
| VARS | -1.75 | Homo sapiens valyl-tRNA synthetase (VARS), nuclear gene encoding mitochondrial protein, mRNA. |
| FOXRED2 | -1.75 | Homo sapiens FAD-dependent oxidoreductase domain containing 2 (FOXRED2), mRNA. |
| ZNF586 | -1.75 | Homo sapiens zinc finger protein 586 (ZNF586), mRNA. |
| TCTEX1D2 | -1.75 | Homo sapiens Tctex1 domain containing 2 (TCTEX1D2), mRNA. |
| GAR1 | -1.75 | Homo sapiens GAR1 ribonucleoprotein homolog (yeast) (GAR1), transcript variant 2, mRNA. |
| TTC8 | -1.75 | Homo sapiens tetratricopeptide repeat domain 8 (TTC8), transcript variant 1, mRNA. |
| TCEAL4 | -1.76 | Homo sapiens transcription elongation factor A (SII)-like 4 (TCEAL4), transcript variant 4, mRNA. |
| ZNF784 | -1.76 | Homo sapiens zinc finger protein 784 (ZNF784), mRNA. |
| PEX16 | -1.76 | Homo sapiens peroxisomal biogenesis factor 16 (PEX16), transcript variant 1, mRNA. |
| ARMCX5 | -1.77 | Homo sapiens armadillo repeat containing, X-linked 5 (ARMCX5), mRNA. |
| LPIN1 | -1.77 | Homo sapiens lipin 1 (LPIN1), mRNA. |
| SNORD16 | -1.77 | Homo sapiens small nucleolar RNA, C/D box 16 (SNORD16), small nucleolar RNA. |
| FAM36A | -1.77 | Homo sapiens family with sequence similarity 36, member A (FAM36A), mRNA. |
| C9orf90 | -1.77 | Homo sapiens chromosome 9 open reading frame 90 (C9orf90), mRNA. |
| S100A4 | -1.77 | Homo sapiens S100 calcium binding protein A4 (S100A4), transcript variant 2, mRNA. |
| ANKMY1 | -1.77 | Homo sapiens ankyrin repeat and MYND domain containing 1 (ANKMY1), transcript variant 2, mRNA. |
| PPHLN1 | -1.78 | Homo sapiens periphilin 1 (PPHLN1), transcript variant 4, mRNA. |
| WBP1 | -1.78 | Homo sapiens WW domain binding protein 1 (WBP1), mRNA. |
| NSMCE4A | -1.78 | Homo sapiens non-SMC element 4 homolog A (S. cerevisiae) (NSMCE4A), mRNA. |
| ILVBL | -1.78 | Homo sapiens ilvB (bacterial acetolactate synthase)-like (ILVBL), mRNA. |
| UNC84B | -1.78 | Homo sapiens unc-84 homolog B (C. elegans) (UNC84B), mRNA. |
| TSSC1 | -1.78 | Homo sapiens tumor suppressing subtransferable candidate 1 (TSSC1), mRNA. |
| C3orf54 | -1.79 | Homo sapiens chromosome 3 open reading frame 54 (C3orf54), mRNA. |
| POPDC3 | -1.79 | Homo sapiens popeye domain containing 3 (POPDC3), mRNA. |
| SMC4 | -1.79 | Homo sapiens structural maintenance of chromosomes 4 (SMC4), transcript variant 2, mRNA. |
| ACP1 | -1.79 | Homo sapiens acid phosphatase 1, soluble (ACP1), transcript variant 2, mRNA. |
| NDUFB3 | -1.8 | Homo sapiens NADH dehydrogenase (ubiquinone) 1 beta subcomplex, 3, 12kDa (NDUFB3), mRNA. |
| KIAA0101 | -1.8 | Homo sapiens KIAA0101 (KIAA0101), transcript variant 2, mRNA. |
| WDR46 | -1.8 | Homo sapiens WD repeat domain 46 (WDR46), mRNA. |
| C14orf102 | -1.8 | Homo sapiens chromosome 14 open reading frame 102 (C14orf102), transcript variant 2, mRNA. |
| GINS3 | -1.8 | Homo sapiens GINS complex subunit 3 (Psf3 homolog) (GINS3), mRNA. |
| SKIV2L | -1.8 | Homo sapiens superkiller viralicidic activity 2-like (S. cerevisiae) (SKIV2L), mRNA. |
| AURKA | -1.8 | Homo sapiens aurora kinase A (AURKA), transcript variant 5, mRNA. |
| PRRT3 | -1.8 | Homo sapiens proline-rich transmembrane protein 3 (PRRT3), mRNA. |
| DUT | -1.8 | Homo sapiens deoxyuridine triphosphatase (DUT), nuclear gene encoding mitochondrial protein, transcript variant 3, mRNA. |
| CYB5B | -1.81 | Homo sapiens cytochrome b5 type B (outer mitochondrial membrane) (CYB5B), nuclear gene encoding mitochondrial protein, mRNA. |
| RABAC1 | -1.81 | Homo sapiens Rab acceptor 1 (prenylated) (RABAC1), mRNA. |
| CCDC101 | -1.81 | Homo sapiens coiled-coil domain containing 101 (CCDC101), mRNA. |
| RTN4 | -1.81 | Homo sapiens reticulon 4 (RTN4), transcript variant 3, mRNA. |
| MFSD11 | -1.81 | Homo sapiens major facilitator superfamily domain containing 11 (MFSD11), mRNA. |
| VPS16 | -1.81 | Homo sapiens vacuolar protein sorting 16 homolog (S. cerevisiae) (VPS16), transcript variant 1, mRNA. |
| CXCR4 | -1.81 | Homo sapiens chemokine (C-X-C motif) receptor 4 (CXCR4), transcript variant 1, mRNA. |
| GPX4 | -1.81 | Homo sapiens glutathione peroxidase 4 (phospholipid hydroperoxidase) (GPX4), transcript variant 2, mRNA. |
| CDC2 | -1.81 | Homo sapiens cell division cycle 2, G1 to S and G2 to M (CDC2), transcript variant 1, mRNA. |
| C17orf61 | -1.82 | Homo sapiens chromosome 17 open reading frame 61 (C17orf61), mRNA. |
| DUSP5 | -1.82 | Homo sapiens dual specificity phosphatase 5 (DUSP5), mRNA. |
| ABHD12 | -1.82 | Homo sapiens abhydrolase domain containing 12 (ABHD12), transcript variant 2, mRNA. |
| SETMAR | -1.82 | Homo sapiens SET domain and mariner transposase fusion gene (SETMAR), mRNA. |
| CCDC92 | -1.82 | Homo sapiens coiled-coil domain containing 92 (CCDC92), mRNA. |
| SSU72 | -1.82 | Homo sapiens SSU72 RNA polymerase II CTD phosphatase homolog (S. cerevisiae) (SSU72), mRNA. |
| GALT | -1.83 | Homo sapiens galactose-1-phosphate uridylyltransferase (GALT), mRNA. |
| NUSAP1 | -1.83 | Homo sapiens nucleolar and spindle associated protein 1 (NUSAP1), transcript variant 2, mRNA. |
| DCLRE1C | -1.83 | Homo sapiens DNA cross-link repair 1C (PSO2 homolog, S. cerevisiae) (DCLRE1C), transcript variant b, mRNA. |
| C21orf58 | -1.83 | Homo sapiens chromosome 21 open reading frame 58 (C21orf58), transcript variant 2, mRNA. |
| ALG14 | -1.83 | Homo sapiens asparagine-linked glycosylation 14 homolog (S. cerevisiae) (ALG14), mRNA. |
| ATF3 | -1.83 | Homo sapiens activating transcription factor 3 (ATF3), transcript variant 4, mRNA. |
| DIAPH2 | -1.84 | Homo sapiens diaphanous homolog 2 (Drosophila) (DIAPH2), transcript variant 156, mRNA. |
| HAND1 | -1.84 | Homo sapiens heart and neural crest derivatives expressed 1 (HAND1), mRNA. |
| AP1S2 | -1.84 | Homo sapiens adaptor-related protein complex 1, sigma 2 subunit (AP1S2), mRNA. |
|  | -1.84 | full-length cDNA clone CS0DF032YA11 of Fetal brain of Homo sapiens (human) |
| SF3B4 | -1.84 | Homo sapiens splicing factor 3b, subunit 4, 49kDa (SF3B4), mRNA. |
| OCIAD2 | -1.84 | Homo sapiens OCIA domain containing 2 (OCIAD2), transcript variant 2, mRNA. |
| TMEM160 | -1.84 | Homo sapiens transmembrane protein 160 (TMEM160), mRNA. |
| MRPL21 | -1.84 | Homo sapiens mitochondrial ribosomal protein L21 (MRPL21), nuclear gene encoding mitochondrial protein, transcript variant 1, mRNA. |
| TRMT12 | -1.84 | Homo sapiens tRNA methyltransferase 12 homolog (S. cerevisiae) (TRMT12), mRNA. |
| LOC400214 | -1.84 | PREDICTED: Homo sapiens hypothetical gene supported by BX248296 (LOC400214), mRNA. |
| MBD4 | -1.84 | Homo sapiens methyl-CpG binding domain protein 4 (MBD4), mRNA. |
| CCDC90A | -1.85 | Homo sapiens coiled-coil domain containing 90A (CCDC90A), mRNA. |
| DHCR24 | -1.85 | Homo sapiens 24-dehydrocholesterol reductase (DHCR24), mRNA. |
| TCEB1 | -1.85 | Homo sapiens transcription elongation factor B (SIII), polypeptide 1 (15kDa, elongin C) (TCEB1), mRNA. |
| GLCCI1 | -1.85 | Homo sapiens glucocorticoid induced transcript 1 (GLCCI1), mRNA. |
| MRPL54 | -1.85 | Homo sapiens mitochondrial ribosomal protein L54 (MRPL54), nuclear gene encoding mitochondrial protein, mRNA. |
| DGUOK | -1.86 | Homo sapiens deoxyguanosine kinase (DGUOK), nuclear gene encoding mitochondrial protein, transcript variant 1, mRNA. |
| PRIM1 | -1.86 | Homo sapiens primase, DNA, polypeptide 1 (49kDa) (PRIM1), mRNA. |
| PUF60 | -1.86 | Homo sapiens poly-U binding splicing factor 60KDa (PUF60), transcript variant 2, mRNA. |
| DLGAP5 | -1.86 | Homo sapiens discs, large (Drosophila) homolog-associated protein 5 (DLGAP5), mRNA. |
| PTPN13 | -1.86 | Homo sapiens protein tyrosine phosphatase, non-receptor type 13 (APO-1/CD95 (Fas)-associated phosphatase) (PTPN13), transcript variant 3, mRNA. |
| RNF126 | -1.87 | Homo sapiens ring finger protein 126 (RNF126), mRNA. |
| PLTP | -1.87 | Homo sapiens phospholipid transfer protein (PLTP), transcript variant 2, mRNA. |
| ERLIN2 | -1.87 | Homo sapiens ER lipid raft associated 2 (ERLIN2), transcript variant 1, mRNA. |
| LMO1 | -1.87 | Homo sapiens LIM domain only 1 (rhombotin 1) (LMO1), mRNA. |
| CDCA4 | -1.88 | Homo sapiens cell division cycle associated 4 (CDCA4), transcript variant 14, mRNA. |
| LOC729446 | -1.88 | PREDICTED: Homo sapiens similar to AT rich interactive domain 1B (SWI1-like) isoform 1 (LOC729446), mRNA. |
| C14orf138 | -1.88 | Homo sapiens chromosome 14 open reading frame 138 (C14orf138), transcript variant 2, mRNA. |
| LSM3 | -1.88 | Homo sapiens LSM3 homolog, U6 small nuclear RNA associated (S. cerevisiae) (LSM3), mRNA. |
| C10orf35 | -1.88 | Homo sapiens chromosome 10 open reading frame 35 (C10orf35), mRNA. |
| BIRC5 | -1.88 | Homo sapiens baculoviral IAP repeat-containing 5 (BIRC5), transcript variant 1, mRNA. |
| GAA | -1.89 | Homo sapiens glucosidase, alpha; acid (GAA), transcript variant 3, mRNA. |
| PARL | -1.89 | Homo sapiens presenilin associated, rhomboid-like (PARL), nuclear gene encoding mitochondrial protein, transcript variant 1, mRNA. |
| LYN | -1.89 | Homo sapiens v-yes-1 Yamaguchi sarcoma viral related oncogene homolog (LYN), mRNA. |
| VIM | -1.89 | Homo sapiens vimentin (VIM), mRNA. |
| C3orf75 | -1.9 | Homo sapiens chromosome 3 open reading frame 75 (C3orf75), mRNA. |
|  | -1.9 | Homo sapiens cDNA FLJ26784 fis, clone PRS04220 |
| SFT2D1 | -1.9 | Homo sapiens SFT2 domain containing 1 (SFT2D1), mRNA. |
| PVRL3 | -1.91 | Homo sapiens poliovirus receptor-related 3 (PVRL3), mRNA. |
| C20orf94 | -1.91 | Homo sapiens chromosome 20 open reading frame 94 (C20orf94), mRNA. |
| XRCC1 | -1.91 | Homo sapiens X-ray repair complementing defective repair in Chinese hamster cells 1 (XRCC1), mRNA. |
| LSM5 | -1.91 | Homo sapiens LSM5 homolog, U6 small nuclear RNA associated (S. cerevisiae) (LSM5), mRNA. |
| ZSCAN2 | -1.91 | Homo sapiens zinc finger and SCAN domain containing 2 (ZSCAN2), transcript variant 2, mRNA. |
| DYNLL2 | -1.91 | Homo sapiens dynein, light chain, LC8-type 2 (DYNLL2), mRNA. |
| GPR177 | -1.92 | Homo sapiens G protein-coupled receptor 177 (GPR177), transcript variant 1, mRNA. |
| GPER | -1.92 | Homo sapiens G protein-coupled estrogen receptor 1 (GPER), transcript variant 3, mRNA. |
| ST5 | -1.92 | Homo sapiens suppression of tumorigenicity 5 (ST5), transcript variant 3, mRNA. |
| NOTCH3 | -1.92 | Homo sapiens Notch homolog 3 (Drosophila) (NOTCH3), mRNA. |
| MSL3L1 | -1.93 | Homo sapiens male-specific lethal 3-like 1 (Drosophila) (MSL3L1), transcript variant 1, mRNA. |
| EDF1 | -1.93 | Homo sapiens endothelial differentiation-related factor 1 (EDF1), transcript variant alpha, mRNA. |
| NT5C | -1.93 | Homo sapiens 5', 3'-nucleotidase, cytosolic (NT5C), mRNA. |
| SLC35C2 | -1.93 | Homo sapiens solute carrier family 35, member C2 (SLC35C2), transcript variant 2, mRNA. |
| RNF10 | -1.93 | Homo sapiens ring finger protein 10 (RNF10), mRNA. |
| KIAA1143 | -1.93 | Homo sapiens KIAA1143 (KIAA1143), mRNA. |
| MYL6 | -1.93 | Homo sapiens myosin, light chain 6, alkali, smooth muscle and non-muscle (MYL6), transcript variant 1, mRNA. |
| ETFB | -1.94 | Homo sapiens electron-transfer-flavoprotein, beta polypeptide (ETFB), transcript variant 2, mRNA. |
| C14orf93 | -1.94 | Homo sapiens chromosome 14 open reading frame 93 (C14orf93), mRNA. |
| C7orf50 | -1.94 | Homo sapiens chromosome 7 open reading frame 50 (C7orf50), mRNA. |
| RNF121 | -1.94 | Homo sapiens ring finger protein 121 (RNF121), transcript variant 1, mRNA. |
| CKLF | -1.94 | Homo sapiens chemokine-like factor (CKLF), transcript variant 5, mRNA. |
| PTPLAD1 | -1.94 | Homo sapiens protein tyrosine phosphatase-like A domain containing 1 (PTPLAD1), mRNA. |
| C12orf48 | -1.94 | Homo sapiens chromosome 12 open reading frame 48 (C12orf48), mRNA. |
| NDUFS8 | -1.94 | Homo sapiens NADH dehydrogenase (ubiquinone) Fe-S protein 8, 23kDa (NADH-coenzyme Q reductase) (NDUFS8), mRNA. |
| CYB561D2 | -1.94 | Homo sapiens cytochrome b-561 domain containing 2 (CYB561D2), mRNA. |
| LOC644615 | -1.94 | PREDICTED: Homo sapiens similar to Ras-related protein Rab-13 (LOC644615), mRNA. |
| ADD1 | -1.94 | Homo sapiens adducin 1 (alpha) (ADD1), transcript variant 4, mRNA. |
| C14orf80 | -1.95 | Homo sapiens chromosome 14 open reading frame 80 (C14orf80), mRNA. |
| ZNHIT3 | -1.95 | Homo sapiens zinc finger, HIT type 3 (ZNHIT3), mRNA. |
| FLJ38717 | -1.95 | Homo sapiens FLJ38717 protein (FLJ38717), mRNA. |
|  | -1.95 | CM3-MT0357-260101-690-b10 MT0357 Homo sapiens cDNA, mRNA sequence |
| BICD1 | -1.95 | Homo sapiens bicaudal D homolog 1 (Drosophila) (BICD1), transcript variant 2, mRNA. |
| FCHSD2 | -1.96 | Homo sapiens FCH and double SH3 domains 2 (FCHSD2), mRNA. |
| GEMIN4 | -1.96 | Homo sapiens gem (nuclear organelle) associated protein 4 (GEMIN4), mRNA. |
| CENPH | -1.96 | Homo sapiens centromere protein H (CENPH), mRNA. |
| CCNB1 | -1.96 | Homo sapiens cyclin B1 (CCNB1), mRNA. |
| DPP7 | -1.96 | Homo sapiens dipeptidyl-peptidase 7 (DPP7), mRNA. |
| MRPS26 | -1.96 | Homo sapiens mitochondrial ribosomal protein S26 (MRPS26), nuclear gene encoding mitochondrial protein, mRNA. |
| LOC391811 | -1.97 | PREDICTED: Homo sapiens similar to DNA polymerase delta subunit 2 (DNA polymerase delta subunit p50) (LOC391811), mRNA. |
| MRPS15 | -1.97 | Homo sapiens mitochondrial ribosomal protein S15 (MRPS15), nuclear gene encoding mitochondrial protein, mRNA. |
| PIM1 | -1.97 | Homo sapiens pim-1 oncogene (PIM1), mRNA. |
| LOC283932 | -1.97 | Homo sapiens hypothetical protein LOC283932 (LOC283932), mRNA. |
| DUSP12 | -1.97 | Homo sapiens dual specificity phosphatase 12 (DUSP12), mRNA. |
| UCHL5IP | -1.97 | Homo sapiens UCHL5 interacting protein (UCHL5IP), transcript variant 1, mRNA. |
| Magmas | -1.97 | Homo sapiens mitochondria-associated protein involved in granulocyte-macrophage colony-stimulating factor signal transduction (Magmas), nuclear gene encoding mitochondrial protein, mRNA. |
| CSNK1E | -1.98 | Homo sapiens casein kinase 1, epsilon (CSNK1E), transcript variant 1, mRNA. |
| S1PR3 | -1.99 | Homo sapiens sphingosine-1-phosphate receptor 3 (S1PR3), mRNA. |
| MRPL11 | -1.99 | Homo sapiens mitochondrial ribosomal protein L11 (MRPL11), nuclear gene encoding mitochondrial protein, transcript variant 2, mRNA. |
| GTF3A | -1.99 | Homo sapiens general transcription factor IIIA (GTF3A), mRNA. |
| CLCN7 | -1.99 | Homo sapiens chloride channel 7 (CLCN7), mRNA. |
| PAF1 | -1.99 | Homo sapiens Paf1, RNA polymerase II associated factor, homolog (S. cerevisiae) (PAF1), mRNA. |
| MFAP4 | -2 | Homo sapiens microfibrillar-associated protein 4 (MFAP4), mRNA. |
| NQO2 | -2 | Homo sapiens NAD(P)H dehydrogenase, quinone 2 (NQO2), mRNA. |
| PTPRF | -2 | Homo sapiens protein tyrosine phosphatase, receptor type, F (PTPRF), transcript variant 1, mRNA. |
| 5-Mar | -2 | Homo sapiens membrane-associated ring finger (C3HC4) 5 (MARCH5), mRNA. |
| YRDC | -2 | Homo sapiens yrdC domain containing (E. coli) (YRDC), nuclear gene encoding mitochondrial protein, mRNA. |
| PSPC1 | -2.01 | Homo sapiens paraspeckle component 1 (PSPC1), transcript variant beta, transcribed RNA. |
| STAG3L4 | -2.01 | Homo sapiens stromal antigen 3-like 4 (STAG3L4), mRNA. |
| LOC728037 | -2.01 | PREDICTED: Homo sapiens similar to Kinesin-like protein KIF22 (Kinesin-like DNA-binding protein) (Kinesin-like protein 4) (LOC728037), mRNA. |
| WWP2 | -2.02 | Homo sapiens WW domain containing E3 ubiquitin protein ligase 2 (WWP2), transcript variant 3, mRNA. |
| FANCI | -2.02 | Homo sapiens Fanconi anemia, complementation group I (FANCI), transcript variant 2, mRNA. |
| ING3 | -2.02 | Homo sapiens inhibitor of growth family, member 3 (ING3), transcript variant 3, mRNA. |
| ATF5 | -2.02 | Homo sapiens activating transcription factor 5 (ATF5), mRNA. |
| PCM1 | -2.02 | Homo sapiens pericentriolar material 1 (PCM1), mRNA. |
| HMGB1L1 | -2.02 | Homo sapiens high-mobility group box 1-like 1 (HMGB1L1), mRNA. |
| MND1 | -2.03 | Homo sapiens meiotic nuclear divisions 1 homolog (S. cerevisiae) (MND1), mRNA. |
| TOX | -2.03 | Homo sapiens thymocyte selection-associated high mobility group box (TOX), mRNA. |
| KLF11 | -2.04 | PREDICTED: Homo sapiens Kruppel-like factor 11 (KLF11), mRNA. |
| BEXL1 | -2.04 | PREDICTED: Homo sapiens brain expressed X-linked-like 1 (BEXL1), mRNA. |
| OSBPL1A | -2.04 | Homo sapiens oxysterol binding protein-like 1A (OSBPL1A), transcript variant OSBPL1B, mRNA. |
| NUBPL | -2.04 | Homo sapiens nucleotide binding protein-like (NUBPL), mRNA. |
| HMGN1 | -2.04 | Homo sapiens high-mobility group nucleosome binding domain 1 (HMGN1), mRNA. |
| LOC400713 | -2.05 | PREDICTED: Homo sapiens zinc finger-like, transcript variant 3 (LOC400713), misc RNA. |
| EWSR1 | -2.05 | Homo sapiens Ewing sarcoma breakpoint region 1 (EWSR1), transcript variant EWS, mRNA. |
| LSM3 | -2.05 | Homo sapiens LSM3 homolog, U6 small nuclear RNA associated (S. cerevisiae) (LSM3), mRNA. |
| ADAM23 | -2.05 | Homo sapiens ADAM metallopeptidase domain 23 (ADAM23), mRNA. |
| DBN1 | -2.05 | Homo sapiens drebrin 1 (DBN1), transcript variant 1, mRNA. |
| B2M | -2.05 | Homo sapiens beta-2-microglobulin (B2M), mRNA. |
| KCNT1 | -2.06 | Homo sapiens potassium channel, subfamily T, member 1 (KCNT1), mRNA. |
| NDUFC1 | -2.06 | Homo sapiens NADH dehydrogenase (ubiquinone) 1, subcomplex unknown, 1, 6kDa (NDUFC1), mRNA. |
| ZFHX3 | -2.06 | Homo sapiens zinc finger homeobox 3 (ZFHX3), transcript variant A, mRNA. |
| NFIC | -2.06 | Homo sapiens nuclear factor I/C (CCAAT-binding transcription factor) (NFIC), transcript variant 2, mRNA. |
| SFRS13A | -2.06 | Homo sapiens splicing factor, arginine/serine-rich 13A (SFRS13A), transcript variant 2, mRNA. |
| C1QTNF6 | -2.06 | Homo sapiens C1q and tumor necrosis factor related protein 6 (C1QTNF6), transcript variant 1, mRNA. |
| FBLN1 | -2.06 | Homo sapiens fibulin 1 (FBLN1), transcript variant C, mRNA. |
| ANXA11 | -2.07 | Homo sapiens annexin A11 (ANXA11), transcript variant a, mRNA. |
| TMEM93 | -2.07 | Homo sapiens transmembrane protein 93 (TMEM93), transcript variant 2, mRNA. |
| ADRM1 | -2.07 | Homo sapiens adhesion regulating molecule 1 (ADRM1), transcript variant 2, mRNA. |
| PSMB6 | -2.07 | Homo sapiens proteasome (prosome, macropain) subunit, beta type, 6 (PSMB6), mRNA. |
| SNORA64 | -2.07 | Homo sapiens small nucleolar RNA, H/ACA box 64 (SNORA64), small nucleolar RNA. |
| MXD3 | -2.07 | Homo sapiens MAX dimerization protein 3 (MXD3), mRNA. |
| PSRC1 | -2.07 | Homo sapiens proline/serine-rich coiled-coil 1 (PSRC1), transcript variant 4, mRNA. |
| BCKDHA | -2.08 | Homo sapiens branched chain keto acid dehydrogenase E1, alpha polypeptide (BCKDHA), mRNA. |
| CUTC | -2.08 | Homo sapiens cutC copper transporter homolog (E. coli) (CUTC), mRNA. |
| LOC374395 | -2.08 | Homo sapiens similar to RIKEN cDNA 1810059G22 (LOC374395), mRNA. |
| MAFB | -2.08 | Homo sapiens v-maf musculoaponeurotic fibrosarcoma oncogene homolog B (avian) (MAFB), mRNA. |
| LTV1 | -2.09 | Homo sapiens LTV1 homolog (S. cerevisiae) (LTV1), mRNA. |
| MORG1 | -2.09 | Homo sapiens mitogen-activated protein kinase organizer 1 (MORG1), mRNA. |
| C16orf35 | -2.09 | Homo sapiens chromosome 16 open reading frame 35 (C16orf35), transcript variant 2, mRNA. |
| CRTAC1 | -2.09 | Homo sapiens cartilage acidic protein 1 (CRTAC1), mRNA. |
| RANBP1 | -2.09 | Homo sapiens RAN binding protein 1 (RANBP1), mRNA. |
| UGCGL1 | -2.1 | Homo sapiens UDP-glucose ceramide glucosyltransferase-like 1 (UGCGL1), transcript variant 1, mRNA. |
| FAM134B | -2.1 | Homo sapiens family with sequence similarity 134, member B (FAM134B), transcript variant 1, mRNA. |
| SLC37A4 | -2.1 | Homo sapiens solute carrier family 37 (glucose-6-phosphate transporter), member 4 (SLC37A4), mRNA. |
| PRKRA | -2.11 | Homo sapiens protein kinase, interferon-inducible double stranded RNA dependent activator (PRKRA), mRNA. |
| CHCHD5 | -2.11 | Homo sapiens coiled-coil-helix-coiled-coil-helix domain containing 5 (CHCHD5), mRNA. |
| TCTEX1D2 | -2.11 | Homo sapiens Tctex1 domain containing 2 (TCTEX1D2), mRNA. |
| KIF11 | -2.11 | Homo sapiens kinesin family member 11 (KIF11), mRNA. |
| UCHL5IP | -2.11 | Homo sapiens UCHL5 interacting protein (UCHL5IP), transcript variant 2, mRNA. |
| GNL3L | -2.11 | Homo sapiens guanine nucleotide binding protein-like 3 (nucleolar)-like (GNL3L), mRNA. |
| PHIP | -2.12 | Homo sapiens pleckstrin homology domain interacting protein (PHIP), mRNA. |
| NDUFA11 | -2.12 | Homo sapiens NADH dehydrogenase (ubiquinone) 1 alpha subcomplex, 11, 14.7kDa (NDUFA11), nuclear gene encoding mitochondrial protein, mRNA. |
| MTUS1 | -2.12 | Homo sapiens mitochondrial tumor suppressor 1 (MTUS1), nuclear gene encoding mitochondrial protein, transcript variant 5, mRNA. |
|  | -2.12 | Homo sapiens mRNA; cDNA DKFZp762F0616 (from clone DKFZp762F0616) |
| ZNF503 | -2.12 | Homo sapiens zinc finger protein 503 (ZNF503), mRNA. |
|  | -2.13 | PREDICTED: Homo sapiens hypothetical LOC388227 (LOC388227), mRNA |
| ASCL1 | -2.13 | Homo sapiens achaete-scute complex homolog 1 (Drosophila) (ASCL1), mRNA. |
| NTNG1 | -2.13 | Homo sapiens netrin G1 (NTNG1), transcript variant 3, mRNA. |
| NUSAP1 | -2.14 | Homo sapiens nucleolar and spindle associated protein 1 (NUSAP1), transcript variant 2, mRNA. |
| ACOT11 | -2.14 | Homo sapiens acyl-CoA thioesterase 11 (ACOT11), transcript variant 2, mRNA. |
| TECR | -2.14 | Homo sapiens trans-2,3-enoyl-CoA reductase (TECR), mRNA. |
| FLJ35801 | -2.15 | Homo sapiens hypothetical protein FLJ35801 (FLJ35801), mRNA. |
| PRR16 | -2.15 | Homo sapiens proline rich 16 (PRR16), mRNA. |
| TMEM108 | -2.15 | Homo sapiens transmembrane protein 108 (TMEM108), mRNA. |
| SFRS13A | -2.15 | Homo sapiens splicing factor, arginine/serine-rich 13A (SFRS13A), transcript variant 2, mRNA. |
| C7orf27 | -2.15 | Homo sapiens chromosome 7 open reading frame 27 (C7orf27), mRNA. |
| CDCA8 | -2.16 | Homo sapiens cell division cycle associated 8 (CDCA8), mRNA. |
| LOC652481 | -2.16 | PREDICTED: Homo sapiens similar to Mitochondrial import inner membrane translocase subunit Tim23 (LOC652481), mRNA. |
| UBE2G2 | -2.16 | Homo sapiens ubiquitin-conjugating enzyme E2G 2 (UBC7 homolog, yeast) (UBE2G2), transcript variant 1, mRNA. |
| WDR68 | -2.16 | Homo sapiens WD repeat domain 68 (WDR68), transcript variant 2, mRNA. |
|  | -2.17 | BX104407 Soares fetal liver spleen 1NFLS Homo sapiens cDNA clone IMAGp998O13369, mRNA sequence |
| ST7OT1 | -2.17 | Homo sapiens ST7 overlapping transcript 1 (non-protein coding) (ST7OT1), antisense RNA. |
| IMPDH2 | -2.17 | Homo sapiens IMP (inosine monophosphate) dehydrogenase 2 (IMPDH2), mRNA. |
| CHCHD6 | -2.18 | Homo sapiens coiled-coil-helix-coiled-coil-helix domain containing 6 (CHCHD6), mRNA. |
| LOC653086 | -2.18 | PREDICTED: Homo sapiens similar to RAN-binding protein 2-like 1 isoform 2, transcript variant 10 (LOC653086), mRNA. |
| COX5A | -2.18 | Homo sapiens cytochrome c oxidase subunit Va (COX5A), nuclear gene encoding mitochondrial protein, mRNA. |
| HMMR | -2.18 | Homo sapiens hyaluronan-mediated motility receptor (RHAMM) (HMMR), transcript variant 1, mRNA. |
| CENPM | -2.19 | Homo sapiens centromere protein M (CENPM), transcript variant 2, mRNA. |
| NUDT9 | -2.19 | Homo sapiens nudix (nucleoside diphosphate linked moiety X)-type motif 9 (NUDT9), transcript variant 3, mRNA. |
| TMEM118 | -2.19 | Homo sapiens transmembrane protein 118 (TMEM118), mRNA. |
| LOC731314 | -2.19 | PREDICTED: Homo sapiens similar to H2A histone family, member X (LOC731314), mRNA. |
| ZNF577 | -2.2 | Homo sapiens zinc finger protein 577 (ZNF577), mRNA. |
| OIP5 | -2.2 | Homo sapiens Opa interacting protein 5 (OIP5), mRNA. |
| AMT | -2.2 | Homo sapiens aminomethyltransferase (AMT), mRNA. |
| DTNA | -2.2 | Homo sapiens dystrobrevin, alpha (DTNA), transcript variant 7, mRNA. |
| WDR51A | -2.21 | Homo sapiens WD repeat domain 51A (WDR51A), mRNA. |
| MRPL37 | -2.21 | Homo sapiens mitochondrial ribosomal protein L37 (MRPL37), nuclear gene encoding mitochondrial protein, mRNA. |
| FAM181B | -2.21 | Homo sapiens family with sequence similarity 181, member B (FAM181B), mRNA. |
| NDUFB7 | -2.21 | Homo sapiens NADH dehydrogenase (ubiquinone) 1 beta subcomplex, 7, 18kDa (NDUFB7), nuclear gene encoding mitochondrial protein, mRNA. |
| FAM46A | -2.22 | Homo sapiens family with sequence similarity 46, member A (FAM46A), mRNA. |
| LOC642197 | -2.23 | PREDICTED: Homo sapiens similar to Protein FAM82B (LOC642197), mRNA. |
| SPC25 | -2.23 | Homo sapiens SPC25, NDC80 kinetochore complex component, homolog (S. cerevisiae) (SPC25), mRNA. |
| CYB5D2 | -2.23 | Homo sapiens cytochrome b5 domain containing 2 (CYB5D2), mRNA. |
| UQCRC1 | -2.23 | Homo sapiens ubiquinol-cytochrome c reductase core protein I (UQCRC1), mRNA. |
| TBC1D7 | -2.23 | Homo sapiens TBC1 domain family, member 7 (TBC1D7), mRNA. |
| LAMB1 | -2.23 | Homo sapiens laminin, beta 1 (LAMB1), mRNA. |
| EXOSC8 | -2.24 | Homo sapiens exosome component 8 (EXOSC8), mRNA. |
| HIST2H2AC | -2.24 | Homo sapiens histone cluster 2, H2ac (HIST2H2AC), mRNA. |
| RAD21 | -2.24 | Homo sapiens RAD21 homolog (S. pombe) (RAD21), mRNA. |
| TCEA2 | -2.24 | Homo sapiens transcription elongation factor A (SII), 2 (TCEA2), transcript variant 2, mRNA. |
| CDC2L2 | -2.24 | Homo sapiens cell division cycle 2-like 2 (PITSLRE proteins) (CDC2L2), transcript variant 1, mRNA. XM_001713745 XM_001713746 XM_001713747 |
| ITPRIPL2 | -2.25 | Homo sapiens inositol 1,4,5-triphosphate receptor interacting protein-like 2 (ITPRIPL2), mRNA. |
| MLF1IP | -2.25 | Homo sapiens MLF1 interacting protein (MLF1IP), mRNA. |
| IGFBP3 | -2.25 | Homo sapiens insulin-like growth factor binding protein 3 (IGFBP3), transcript variant 1, mRNA. |
| KIF20A | -2.25 | Homo sapiens kinesin family member 20A (KIF20A), mRNA. |
| DGCR6 | -2.25 | Homo sapiens DiGeorge syndrome critical region gene 6 (DGCR6), mRNA. |
| ADAMTS19 | -2.26 | Homo sapiens ADAM metallopeptidase with thrombospondin type 1 motif, 19 (ADAMTS19), mRNA. |
| C20orf29 | -2.26 | Homo sapiens chromosome 20 open reading frame 29 (C20orf29), mRNA. |
| NUDT1 | -2.27 | Homo sapiens nudix (nucleoside diphosphate linked moiety X)-type motif 1 (NUDT1), transcript variant 4B, mRNA. |
| C9orf86 | -2.27 | Homo sapiens chromosome 9 open reading frame 86 (C9orf86), mRNA. |
| SIAH1 | -2.27 | Homo sapiens seven in absentia homolog 1 (Drosophila) (SIAH1), transcript variant 2, mRNA. |
| CKAP2L | -2.27 | Homo sapiens cytoskeleton associated protein 2-like (CKAP2L), mRNA. |
| HIRIP3 | -2.27 | Homo sapiens HIRA interacting protein 3 (HIRIP3), mRNA. |
| RPS15 | -2.27 | Homo sapiens ribosomal protein S15 (RPS15), mRNA. |
| EZH2 | -2.28 | Homo sapiens enhancer of zeste homolog 2 (Drosophila) (EZH2), transcript variant 1, mRNA. |
| SNX8 | -2.28 | Homo sapiens sorting nexin 8 (SNX8), mRNA. |
| PTTG3P | -2.28 | Homo sapiens pituitary tumor-transforming 3 (pseudogene) (PTTG3P), non-coding RNA. |
| DMAP1 | -2.28 | Homo sapiens DNA methyltransferase 1 associated protein 1 (DMAP1), transcript variant 2, mRNA. |
| PIK4CA | -2.29 | Homo sapiens phosphatidylinositol 4-kinase, catalytic, alpha polypeptide (PIK4CA), transcript variant 1, mRNA. |
| ADK | -2.29 | Homo sapiens adenosine kinase (ADK), transcript variant ADK-short, mRNA. |
| DYNC1I1 | -2.29 | Homo sapiens dynein, cytoplasmic 1, intermediate chain 1 (DYNC1I1), mRNA. |
| PSMD14 | -2.29 | Homo sapiens proteasome (prosome, macropain) 26S subunit, non-ATPase, 14 (PSMD14), mRNA. |
| CENPE | -2.29 | Homo sapiens centromere protein E, 312kDa (CENPE), mRNA. |
| CHMP6 | -2.29 | PREDICTED: Homo sapiens chromatin modifying protein 6 (CHMP6), mRNA. |
| BCL2L12 | -2.29 | Homo sapiens BCL2-like 12 (proline rich) (BCL2L12), transcript variant 3, mRNA. |
| PHF19 | -2.3 | Homo sapiens PHD finger protein 19 (PHF19), transcript variant 2, mRNA. |
| TDRD1 | -2.3 | Homo sapiens tudor domain containing 1 (TDRD1), mRNA. |
| KLF11 | -2.3 | PREDICTED: Homo sapiens Kruppel-like factor 11 (KLF11), mRNA. |
| SMAD9 | -2.31 | Homo sapiens SMAD family member 9 (SMAD9), mRNA. |
| DCTN3 | -2.31 | Homo sapiens dynactin 3 (p22) (DCTN3), transcript variant 1, mRNA. |
| KIAA0556 | -2.31 | Homo sapiens KIAA0556 (KIAA0556), mRNA. |
| AGXT2L2 | -2.31 | Homo sapiens alanine-glyoxylate aminotransferase 2-like 2 (AGXT2L2), mRNA. |
| PPIH | -2.32 | Homo sapiens peptidylprolyl isomerase H (cyclophilin H) (PPIH), mRNA. |
| MEIS3P1 | -2.32 | Homo sapiens Meis homeobox 3 pseudogene 1 (MEIS3P1), non-coding RNA. |
| SNORA62 | -2.32 | Homo sapiens small nucleolar RNA, H/ACA box 62 (SNORA62), small nucleolar RNA. |
| PPAP2B | -2.32 | Homo sapiens phosphatidic acid phosphatase type 2B (PPAP2B), transcript variant 1, mRNA. |
| DMC1 | -2.33 | Homo sapiens DMC1 dosage suppressor of mck1 homolog, meiosis-specific homologous recombination (yeast) (DMC1), mRNA. |
| CCDC12 | -2.33 | Homo sapiens coiled-coil domain containing 12 (CCDC12), mRNA. |
| PHGDH | -2.33 | Homo sapiens phosphoglycerate dehydrogenase (PHGDH), mRNA. |
| SNRPD3 | -2.34 | Homo sapiens small nuclear ribonucleoprotein D3 polypeptide 18kDa (SNRPD3), mRNA. |
| STMN4 | -2.35 | Homo sapiens stathmin-like 4 (STMN4), mRNA. |
| COMMD2 | -2.35 | Homo sapiens COMM domain containing 2 (COMMD2), mRNA. |
| DZIP1 | -2.35 | Homo sapiens DAZ interacting protein 1 (DZIP1), transcript variant 2, mRNA. |
| SIL1 | -2.35 | Homo sapiens SIL1 homolog, endoplasmic reticulum chaperone (S. cerevisiae) (SIL1), transcript variant 1, mRNA. |
| C6orf173 | -2.35 | Homo sapiens chromosome 6 open reading frame 173 (C6orf173), mRNA. |
| FAM110A | -2.35 | Homo sapiens family with sequence similarity 110, member A (FAM110A), transcript variant 3, mRNA. |
| PIK3R1 | -2.35 | Homo sapiens phosphoinositide-3-kinase, regulatory subunit 1 (alpha) (PIK3R1), transcript variant 1, mRNA. |
| TNFSF14 | -2.35 | Homo sapiens tumor necrosis factor (ligand) superfamily, member 14 (TNFSF14), transcript variant 2, mRNA. |
| PHF19 | -2.35 | Homo sapiens PHD finger protein 19 (PHF19), transcript variant 2, mRNA. |
| SNORD4A | -2.36 | Homo sapiens small nucleolar RNA, C/D box 4A (SNORD4A), small nucleolar RNA. |
| PRC1 | -2.36 | Homo sapiens protein regulator of cytokinesis 1 (PRC1), transcript variant 2, mRNA. |
| ZBTB8OS | -2.37 | Homo sapiens zinc finger and BTB domain containing 8 opposite strand (ZBTB8OS), mRNA. |
| E4F1 | -2.37 | Homo sapiens E4F transcription factor 1 (E4F1), mRNA. |
| PIH1D1 | -2.37 | Homo sapiens PIH1 domain containing 1 (PIH1D1), mRNA. |
| CTBS | -2.38 | Homo sapiens chitobiase, di-N-acetyl- (CTBS), mRNA. |
| NFIX | -2.38 | Homo sapiens nuclear factor I/X (CCAAT-binding transcription factor) (NFIX), mRNA. |
| EDNRA | -2.38 | Homo sapiens endothelin receptor type A (EDNRA), mRNA. |
| LOC400986 | -2.38 | PREDICTED: Homo sapiens protein immuno-reactive with anti-PTH polyclonal antibodies (LOC400986), mRNA. |
| MIF4GD | -2.39 | Homo sapiens MIF4G domain containing (MIF4GD), mRNA. |
| CENPM | -2.39 | Homo sapiens centromere protein M (CENPM), transcript variant 2, mRNA. |
| LOC651816 | -2.39 | PREDICTED: Homo sapiens similar to Ubiquitin-conjugating enzyme E2S (Ubiquitin-conjugating enzyme E2-24 kDa) (Ubiquitin-protein ligase) (Ubiquitin carrier protein) (E2-EPF5) (LOC651816), mRNA. |
| C21orf81 | -2.4 | Homo sapiens chromosome 21 open reading frame 81 (C21orf81), mRNA. |
| HIST1H2BH | -2.4 | Homo sapiens histone cluster 1, H2bh (HIST1H2BH), mRNA. |
| CCDC34 | -2.4 | Homo sapiens coiled-coil domain containing 34 (CCDC34), transcript variant 1, mRNA. |
| ZSCAN5A | -2.4 | Homo sapiens zinc finger and SCAN domain containing 5A (ZSCAN5A), mRNA. |
| PSMC3 | -2.4 | Homo sapiens proteasome (prosome, macropain) 26S subunit, ATPase, 3 (PSMC3), mRNA. |
| KIAA0101 | -2.41 | Homo sapiens KIAA0101 (KIAA0101), transcript variant 1, mRNA. |
| XRCC2 | -2.41 | Homo sapiens X-ray repair complementing defective repair in Chinese hamster cells 2 (XRCC2), mRNA. |
| FEZ1 | -2.41 | Homo sapiens fasciculation and elongation protein zeta 1 (zygin I) (FEZ1), transcript variant 1, mRNA. |
| PPP2R3C | -2.41 | Homo sapiens protein phosphatase 2 (formerly 2A), regulatory subunit B'', gamma (PPP2R3C), mRNA. |
| NR2F1 | -2.42 | Homo sapiens nuclear receptor subfamily 2, group F, member 1 (NR2F1), mRNA. |
| STUB1 | -2.42 | Homo sapiens STIP1 homology and U-box containing protein 1 (STUB1), mRNA. |
| TERF1 | -2.42 | Homo sapiens telomeric repeat binding factor (NIMA-interacting) 1 (TERF1), transcript variant 1, mRNA. |
| FBXO32 | -2.42 | Homo sapiens F-box protein 32 (FBXO32), transcript variant 2, mRNA. |
| PTTG1 | -2.43 | Homo sapiens pituitary tumor-transforming 1 (PTTG1), mRNA. |
| BID | -2.43 | Homo sapiens BH3 interacting domain death agonist (BID), transcript variant 1, mRNA. |
| SYT11 | -2.43 | Homo sapiens synaptotagmin XI (SYT11), mRNA. |
| AUP1 | -2.43 | Homo sapiens ancient ubiquitous protein 1 (AUP1), mRNA. |
| C15orf23 | -2.43 | Homo sapiens chromosome 15 open reading frame 23 (C15orf23), mRNA. |
| CENPJ | -2.44 | Homo sapiens centromere protein J (CENPJ), mRNA. |
| ZMYM5 | -2.44 | Homo sapiens zinc finger, MYM-type 5 (ZMYM5), transcript variant 1, mRNA. |
| OLFM1 | -2.45 | Homo sapiens olfactomedin 1 (OLFM1), transcript variant 1, mRNA. |
| C14orf102 | -2.45 | Homo sapiens chromosome 14 open reading frame 102 (C14orf102), transcript variant 1, mRNA. |
| TOE1 | -2.45 | Homo sapiens target of EGR1, member 1 (nuclear) (TOE1), mRNA. |
| ABHD11 | -2.45 | Homo sapiens abhydrolase domain containing 11 (ABHD11), transcript variant 1, mRNA. |
| PIN1 | -2.45 | Homo sapiens peptidylprolyl cis/trans isomerase, NIMA-interacting 1 (PIN1), mRNA. |
| EYA4 | -2.46 | Homo sapiens eyes absent homolog 4 (Drosophila) (EYA4), transcript variant 1, mRNA. |
| LIMCH1 | -2.46 | Homo sapiens LIM and calponin homology domains 1 (LIMCH1), mRNA. |
| SFRS5 | -2.46 | Homo sapiens splicing factor, arginine/serine-rich 5 (SFRS5), transcript variant 2, mRNA. |
| AEBP1 | -2.46 | Homo sapiens AE binding protein 1 (AEBP1), mRNA. |
| ZC3H5 | -2.46 | PREDICTED: Homo sapiens zinc finger CCCH-type containing 5 (ZC3H5), mRNA. |
| LOC727761 | -2.47 | PREDICTED: Homo sapiens similar to Deoxythymidylate kinase (thymidylate kinase), transcript variant 4 (LOC727761), mRNA. |
| IL11RA | -2.48 | Homo sapiens interleukin 11 receptor, alpha (IL11RA), transcript variant 1, mRNA. |
| MCM4 | -2.48 | Homo sapiens minichromosome maintenance complex component 4 (MCM4), transcript variant 2, mRNA. |
| GGH | -2.48 | Homo sapiens gamma-glutamyl hydrolase (conjugase, folylpolygammaglutamyl hydrolase) (GGH), mRNA. |
| ATP5S | -2.49 | Homo sapiens ATP synthase, H+ transporting, mitochondrial F0 complex, subunit s (factor B) (ATP5S), nuclear gene encoding mitochondrial protein, transcript variant 3, mRNA. |
| IL11RA | -2.49 | Homo sapiens interleukin 11 receptor, alpha (IL11RA), transcript variant 1, mRNA. |
| HACL1 | -2.5 | Homo sapiens 2-hydroxyacyl-CoA lyase 1 (HACL1), mRNA. |
| CISD1 | -2.5 | Homo sapiens CDGSH iron sulfur domain 1 (CISD1), mRNA. |
| CYTL1 | -2.5 | Homo sapiens cytokine-like 1 (CYTL1), mRNA. |
| DUSP23 | -2.5 | Homo sapiens dual specificity phosphatase 23 (DUSP23), mRNA. |
| TBCE | -2.5 | Homo sapiens tubulin folding cofactor E (TBCE), transcript variant 1, mRNA. |
| PCNA | -2.5 | Homo sapiens proliferating cell nuclear antigen (PCNA), transcript variant 2, mRNA. |
| HIST1H1C | -2.51 | Homo sapiens histone cluster 1, H1c (HIST1H1C), mRNA. |
| PRDM8 | -2.51 | Homo sapiens PR domain containing 8 (PRDM8), transcript variant 1, mRNA. |
| COQ2 | -2.51 | Homo sapiens coenzyme Q2 homolog, prenyltransferase (yeast) (COQ2), mRNA. |
| NDC80 | -2.52 | Homo sapiens NDC80 homolog, kinetochore complex component (S. cerevisiae) (NDC80), mRNA. |
| TCEA2 | -2.52 | Homo sapiens transcription elongation factor A (SII), 2 (TCEA2), transcript variant 1, mRNA. |
| FDFT1 | -2.52 | Homo sapiens farnesyl-diphosphate farnesyltransferase 1 (FDFT1), mRNA. |
| NLGN1 | -2.52 | Homo sapiens neuroligin 1 (NLGN1), mRNA. |
| ZNF511 | -2.53 | Homo sapiens zinc finger protein 511 (ZNF511), mRNA. |
| CKS1B | -2.53 | Homo sapiens CDC28 protein kinase regulatory subunit 1B (CKS1B), mRNA. |
| ISOC1 | -2.53 | Homo sapiens isochorismatase domain containing 1 (ISOC1), mRNA. |
| NMU | -2.53 | Homo sapiens neuromedin U (NMU), mRNA. |
| ALG5 | -2.53 | Homo sapiens asparagine-linked glycosylation 5 homolog (S. cerevisiae, dolichyl-phosphate beta-glucosyltransferase) (ALG5), mRNA. |
| FDFT1 | -2.54 | Homo sapiens farnesyl-diphosphate farnesyltransferase 1 (FDFT1), mRNA. |
| KIAA1751 | -2.54 | Homo sapiens KIAA1751 (KIAA1751), mRNA. |
| TMEM18 | -2.54 | Homo sapiens transmembrane protein 18 (TMEM18), mRNA. |
| NOL7 | -2.55 | Homo sapiens nucleolar protein 7, 27kDa (NOL7), mRNA. |
| GAP43 | -2.55 | Homo sapiens growth associated protein 43 (GAP43), mRNA. |
| C14orf82 | -2.57 | PREDICTED: Homo sapiens chromosome 14 open reading frame 82 (C14orf82), mRNA. |
| AKR1C3 | -2.57 | Homo sapiens aldo-keto reductase family 1, member C3 (3-alpha hydroxysteroid dehydrogenase, type II) (AKR1C3), mRNA. |
| LOC728554 | -2.58 | PREDICTED: Homo sapiens similar to THO complex 3 (LOC728554), mRNA. |
| NDUFB10 | -2.59 | Homo sapiens NADH dehydrogenase (ubiquinone) 1 beta subcomplex, 10, 22kDa (NDUFB10), mRNA. |
| FANCD2 | -2.59 | Homo sapiens Fanconi anemia, complementation group D2 (FANCD2), transcript variant 2, mRNA. |
| CREB1 | -2.6 | Homo sapiens cAMP responsive element binding protein 1 (CREB1), transcript variant A, mRNA. |
| H1F0 | -2.6 | Homo sapiens H1 histone family, member 0 (H1F0), mRNA. |
| PPA2 | -2.6 | Homo sapiens pyrophosphatase (inorganic) 2 (PPA2), nuclear gene encoding mitochondrial protein, transcript variant 2, mRNA. |
| CENPA | -2.6 | Homo sapiens centromere protein A (CENPA), transcript variant 2, mRNA. |
| HMMR | -2.6 | Homo sapiens hyaluronan-mediated motility receptor (RHAMM) (HMMR), transcript variant 2, mRNA. |
| LOC285074 | -2.6 | Homo sapiens hypothetical protein LOC285074 (LOC285074), mRNA. |
| INSIG1 | -2.61 | Homo sapiens insulin induced gene 1 (INSIG1), transcript variant 2, mRNA. |
| LOC91561 | -2.63 | PREDICTED: Homo sapiens similar to ribosomal protein S2, transcript variant 3 (LOC91561), mRNA. |
| TOP2A | -2.63 | Homo sapiens topoisomerase (DNA) II alpha 170kDa (TOP2A), mRNA. |
| TDRD1 | -2.63 | Homo sapiens tudor domain containing 1 (TDRD1), mRNA. |
| EXOSC6 | -2.63 | Homo sapiens exosome component 6 (EXOSC6), mRNA. |
| C20orf103 | -2.65 | Homo sapiens chromosome 20 open reading frame 103 (C20orf103), mRNA. |
| KIF15 | -2.65 | Homo sapiens kinesin family member 15 (KIF15), mRNA. |
| PRPH | -2.66 | Homo sapiens peripherin (PRPH), mRNA. |
| LOC650826 | -2.66 | PREDICTED: Homo sapiens similar to CG9804-PA, transcript variant 2 (LOC650826), mRNA. |
| STOM | -2.67 | Homo sapiens stomatin (STOM), transcript variant 1, mRNA. |
| SIPA1L2 | -2.67 | Homo sapiens signal-induced proliferation-associated 1 like 2 (SIPA1L2), mRNA. |
| NDUFB11 | -2.67 | Homo sapiens NADH dehydrogenase (ubiquinone) 1 beta subcomplex, 11, 17.3kDa (NDUFB11), mRNA. |
| PMPCB | -2.68 | Homo sapiens peptidase (mitochondrial processing) beta (PMPCB), nuclear gene encoding mitochondrial protein, mRNA. |
| COLEC11 | -2.68 | Homo sapiens collectin sub-family member 11 (COLEC11), transcript variant 1, mRNA. |
| DECR1 | -2.68 | Homo sapiens 2,4-dienoyl CoA reductase 1, mitochondrial (DECR1), nuclear gene encoding mitochondrial protein, mRNA. |
| KIF2C | -2.69 | Homo sapiens kinesin family member 2C (KIF2C), mRNA. |
| LMO4 | -2.69 | Homo sapiens LIM domain only 4 (LMO4), mRNA. |
| BAZ1A | -2.7 | Homo sapiens bromodomain adjacent to zinc finger domain, 1A (BAZ1A), transcript variant 1, mRNA. |
| OLFML3 | -2.7 | Homo sapiens olfactomedin-like 3 (OLFML3), mRNA. |
| PPP1R15A | -2.7 | Homo sapiens protein phosphatase 1, regulatory (inhibitor) subunit 15A (PPP1R15A), mRNA. |
| SPRY2 | -2.7 | Homo sapiens sprouty homolog 2 (Drosophila) (SPRY2), mRNA. |
| DDX39 | -2.7 | Homo sapiens DEAD (Asp-Glu-Ala-Asp) box polypeptide 39 (DDX39), mRNA. |
| CENPE | -2.71 | Homo sapiens centromere protein E, 312kDa (CENPE), mRNA. |
| ATXN7L2 | -2.71 | Homo sapiens ataxin 7-like 2 (ATXN7L2), mRNA. |
| UBE2D2 | -2.71 | Homo sapiens ubiquitin-conjugating enzyme E2D 2 (UBC4/5 homolog, yeast) (UBE2D2), transcript variant 2, mRNA. |
| ZFAND2A | -2.72 | Homo sapiens zinc finger, AN1-type domain 2A (ZFAND2A), mRNA. |
| CALCB | -2.72 | Homo sapiens calcitonin-related polypeptide beta (CALCB), mRNA. |
| DYRK2 | -2.73 | Homo sapiens dual-specificity tyrosine-(Y)-phosphorylation regulated kinase 2 (DYRK2), transcript variant 1, mRNA. |
| BCL2L12 | -2.73 | Homo sapiens BCL2-like 12 (proline rich) (BCL2L12), transcript variant 3, mRNA. |
| GOLPH4 | -2.73 | Homo sapiens golgi phosphoprotein 4 (GOLPH4), mRNA. |
| SOX11 | -2.73 | Homo sapiens SRY (sex determining region Y)-box 11 (SOX11), mRNA. |
| PLA2G2D | -2.74 | Homo sapiens phospholipase A2, group IID (PLA2G2D), mRNA. |
| ASPM | -2.74 | Homo sapiens asp (abnormal spindle) homolog, microcephaly associated (Drosophila) (ASPM), mRNA. |
| LOC642033 | -2.74 | PREDICTED: Homo sapiens similar to ATP-binding cassette, sub-family F, member 1 isoform b (LOC642033), mRNA. |
| EEF1B2 | -2.75 | Homo sapiens eukaryotic translation elongation factor 1 beta 2 (EEF1B2), transcript variant 3, mRNA. |
| LRAP | -2.75 | Homo sapiens leukocyte-derived arginine aminopeptidase (LRAP), mRNA. |
| CD58 | -2.75 | Homo sapiens CD58 molecule (CD58), mRNA. |
| CDC45L | -2.75 | Homo sapiens CDC45 cell division cycle 45-like (S. cerevisiae) (CDC45L), mRNA. |
| DIS3L | -2.76 | Homo sapiens DIS3 mitotic control homolog (S. cerevisiae)-like (DIS3L), mRNA. |
| ELAC1 | -2.76 | Homo sapiens elaC homolog 1 (E. coli) (ELAC1), mRNA. |
| CENPM | -2.77 | Homo sapiens centromere protein M (CENPM), transcript variant 1, mRNA. |
| GSG2 | -2.78 | Homo sapiens germ cell associated 2 (haspin) (GSG2), mRNA. |
|  | -2.78 | BX090843 Soares_testis_NHT Homo sapiens cDNA clone IMAGp998C074110 ; IMAGE:1620414, mRNA sequence |
| PON2 | -2.79 | Homo sapiens paraoxonase 2 (PON2), transcript variant 1, mRNA. |
| PRDX4 | -2.79 | Homo sapiens peroxiredoxin 4 (PRDX4), mRNA. |
| LRRC17 | -2.79 | Homo sapiens leucine rich repeat containing 17 (LRRC17), transcript variant 1, mRNA. |
| FAM64A | -2.8 | Homo sapiens family with sequence similarity 64, member A (FAM64A), mRNA. |
| GNG11 | -2.81 | Homo sapiens guanine nucleotide binding protein (G protein), gamma 11 (GNG11), mRNA. |
| MGMT | -2.81 | Homo sapiens O-6-methylguanine-DNA methyltransferase (MGMT), mRNA. |
| ZNF549 | -2.81 | Homo sapiens zinc finger protein 549 (ZNF549), mRNA. |
| ANKRD16 | -2.81 | Homo sapiens ankyrin repeat domain 16 (ANKRD16), transcript variant 1, mRNA. |
| ASNS | -2.82 | Homo sapiens asparagine synthetase (ASNS), transcript variant 1, mRNA. |
| SMC4 | -2.83 | Homo sapiens structural maintenance of chromosomes 4 (SMC4), transcript variant 2, mRNA. |
| PHOX2B | -2.83 | Homo sapiens paired-like homeobox 2b (PHOX2B), mRNA. |
| CDC25C | -2.83 | Homo sapiens cell division cycle 25 homolog C (S. pombe) (CDC25C), transcript variant 1, mRNA. |
| PTTG1 | -2.85 | Homo sapiens pituitary tumor-transforming 1 (PTTG1), mRNA. |
| EID2B | -2.85 | Homo sapiens EP300 interacting inhibitor of differentiation 2B (EID2B), mRNA. |
| CSF2RA | -2.85 | Homo sapiens colony stimulating factor 2 receptor, alpha, low-affinity (granulocyte-macrophage) (CSF2RA), transcript variant 6, mRNA. |
| TACC3 | -2.86 | Homo sapiens transforming, acidic coiled-coil containing protein 3 (TACC3), mRNA. |
| RNASET2 | -2.86 | Homo sapiens ribonuclease T2 (RNASET2), mRNA. |
| SIL1 | -2.87 | Homo sapiens SIL1 homolog, endoplasmic reticulum chaperone (S. cerevisiae) (SIL1), transcript variant 1, mRNA. |
| CIAO1 | -2.87 | Homo sapiens cytosolic iron-sulfur protein assembly 1 homolog (S. cerevisiae) (CIAO1), mRNA. |
| LOC644422 | -2.88 | PREDICTED: Homo sapiens misc_RNA (LOC644422), miscRNA. |
| SYNCRIP | -2.89 | Homo sapiens synaptotagmin binding, cytoplasmic RNA interacting protein (SYNCRIP), mRNA. |
| TRIOBP | -2.89 | Homo sapiens TRIO and F-actin binding protein (TRIOBP), transcript variant 2, mRNA. |
| MT1F | -2.89 | Homo sapiens metallothionein 1F (MT1F), mRNA. |
| SFRS5 | -2.9 | Homo sapiens splicing factor, arginine/serine-rich 5 (SFRS5), transcript variant 2, mRNA. |
| SYT4 | -2.9 | Homo sapiens synaptotagmin IV (SYT4), mRNA. |
| TNFRSF10B | -2.91 | Homo sapiens tumor necrosis factor receptor superfamily, member 10b (TNFRSF10B), transcript variant 1, mRNA. |
| PDGFRL | -2.92 | Homo sapiens platelet-derived growth factor receptor-like (PDGFRL), mRNA. |
| CABYR | -2.92 | Homo sapiens calcium binding tyrosine-(Y)-phosphorylation regulated (CABYR), transcript variant 2, mRNA. |
| ZNF593 | -2.92 | Homo sapiens zinc finger protein 593 (ZNF593), mRNA. |
| INCENP | -2.93 | Homo sapiens inner centromere protein antigens 135/155kDa (INCENP), transcript variant 1, mRNA. |
| DNAJB12 | -2.93 | Homo sapiens DnaJ (Hsp40) homolog, subfamily B, member 12 (DNAJB12), transcript variant 2, mRNA. |
| SLC10A4 | -2.94 | Homo sapiens solute carrier family 10 (sodium/bile acid cotransporter family), member 4 (SLC10A4), mRNA. |
| NOP56 | -2.95 | Homo sapiens NOP56 ribonucleoprotein homolog (yeast) (NOP56), transcript variant 1, mRNA. |
| C14orf153 | -2.96 | Homo sapiens chromosome 14 open reading frame 153 (C14orf153), mRNA. |
| TSPAN5 | -2.96 | Homo sapiens tetraspanin 5 (TSPAN5), mRNA. |
| LOC653994 | -2.97 | PREDICTED: Homo sapiens similar to eukaryotic translation initiation factor 4H isoform 2, transcript variant 5 (LOC653994), mRNA. |
| VIP | -2.98 | Homo sapiens vasoactive intestinal peptide (VIP), transcript variant 1, mRNA. |
| CXCR4 | -3 | Homo sapiens chemokine (C-X-C motif) receptor 4 (CXCR4), transcript variant 2, mRNA. |
| CX3CL1 | -3.01 | Homo sapiens chemokine (C-X3-C motif) ligand 1 (CX3CL1), mRNA. |
| LOC644033 | -3.01 | PREDICTED: Homo sapiens similar to similar to RPL23AP7 protein (LOC644033), mRNA. |
| PCDH9 | -3.02 | Homo sapiens protocadherin 9 (PCDH9), transcript variant 1, mRNA. |
| MT1A | -3.03 | Homo sapiens metallothionein 1A (MT1A), mRNA. |
| GLRX | -3.03 | Homo sapiens glutaredoxin (thioltransferase) (GLRX), mRNA. |
| SLC35E3 | -3.03 | Homo sapiens solute carrier family 35, member E3 (SLC35E3), mRNA. |
| SPC24 | -3.03 | Homo sapiens SPC24, NDC80 kinetochore complex component, homolog (S. cerevisiae) (SPC24), mRNA. |
| TFPI2 | -3.04 | Homo sapiens tissue factor pathway inhibitor 2 (TFPI2), mRNA. |
| ICK | -3.05 | Homo sapiens intestinal cell (MAK-like) kinase (ICK), transcript variant 2, mRNA. |
| OIP5 | -3.06 | Homo sapiens Opa interacting protein 5 (OIP5), mRNA. |
| NOP58 | -3.06 | Homo sapiens NOP58 ribonucleoprotein homolog (yeast) (NOP58), mRNA. |
| DUSP1 | -3.07 | Homo sapiens dual specificity phosphatase 1 (DUSP1), mRNA. |
| MYLIP | -3.11 | Homo sapiens myosin regulatory light chain interacting protein (MYLIP), mRNA. |
| NFU1 | -3.13 | Homo sapiens NFU1 iron-sulfur cluster scaffold homolog (S. cerevisiae) (NFU1), transcript variant 2, mRNA. |
| MT1E | -3.13 | Homo sapiens metallothionein 1E (MT1E), mRNA. |
| SLC30A1 | -3.14 | Homo sapiens solute carrier family 30 (zinc transporter), member 1 (SLC30A1), mRNA. |
| C7orf36 | -3.14 | Homo sapiens chromosome 7 open reading frame 36 (C7orf36), mRNA. |
| PCNA | -3.16 | Homo sapiens proliferating cell nuclear antigen (PCNA), transcript variant 2, mRNA. |
| SFRS16 | -3.17 | Homo sapiens splicing factor, arginine/serine-rich 16 (SFRS16), mRNA. |
| TROAP | -3.18 | Homo sapiens trophinin associated protein (tastin) (TROAP), mRNA. |
| GADD45A | -3.18 | Homo sapiens growth arrest and DNA-damage-inducible, alpha (GADD45A), mRNA. |
| RAD23B | -3.18 | Homo sapiens RAD23 homolog B (S. cerevisiae) (RAD23B), mRNA. |
| TAGLN | -3.18 | Homo sapiens transgelin (TAGLN), transcript variant 2, mRNA. |
| NT5C3 | -3.2 | Homo sapiens 5'-nucleotidase, cytosolic III (NT5C3), transcript variant 1, mRNA. |
| LOC153561 | -3.21 | Homo sapiens hypothetical protein LOC153561 (LOC153561), mRNA. |
| MT2A | -3.22 | Homo sapiens metallothionein 2A (MT2A), mRNA. |
| LOC643995 | -3.24 | PREDICTED: Homo sapiens similar to Importin alpha-2 subunit (Karyopherin alpha-2 subunit) (SRP1-alpha) (RAG cohort protein 1), transcript variant 2 (LOC643995), mRNA. |
| CDC25C | -3.24 | Homo sapiens cell division cycle 25 homolog C (S. pombe) (CDC25C), transcript variant 2, mRNA. |
| UPF3A | -3.24 | Homo sapiens UPF3 regulator of nonsense transcripts homolog A (yeast) (UPF3A), transcript variant 1, mRNA. |
| C1orf173 | -3.25 | Homo sapiens chromosome 1 open reading frame 173 (C1orf173), mRNA. |
| DDX51 | -3.26 | Homo sapiens DEAD (Asp-Glu-Ala-Asp) box polypeptide 51 (DDX51), mRNA. |
| KCTD12 | -3.27 | Homo sapiens potassium channel tetramerisation domain containing 12 (KCTD12), mRNA. |
| LOC646786 | -3.27 | PREDICTED: Homo sapiens similar to Afadin (AF-6 protein) (LOC646786), mRNA. |
| ZBTB8OS | -3.29 | Homo sapiens zinc finger and BTB domain containing 8 opposite strand (ZBTB8OS), mRNA. |
| FGL1 | -3.29 | Homo sapiens fibrinogen-like 1 (FGL1), transcript variant 1, mRNA. |
| SMYD3 | -3.3 | Homo sapiens SET and MYND domain containing 3 (SMYD3), mRNA. |
| CDC25C | -3.3 | Homo sapiens cell division cycle 25 homolog C (S. pombe) (CDC25C), transcript variant 1, mRNA. |
| CTSL2 | -3.31 | Homo sapiens cathepsin L2 (CTSL2), mRNA. |
| PHF17 | -3.31 | Homo sapiens PHD finger protein 17 (PHF17), transcript variant S, mRNA. |
| LRRN3 | -3.31 | Homo sapiens leucine rich repeat neuronal 3 (LRRN3), transcript variant 1, mRNA. |
| NLGN4X | -3.34 | Homo sapiens neuroligin 4, X-linked (NLGN4X), transcript variant 1, mRNA. |
| GGH | -3.36 | Homo sapiens gamma-glutamyl hydrolase (conjugase, folylpolygammaglutamyl hydrolase) (GGH), mRNA. |
| INSM2 | -3.37 | Homo sapiens insulinoma-associated 2 (INSM2), mRNA. |
| LMO4 | -3.37 | Homo sapiens LIM domain only 4 (LMO4), mRNA. |
| SNORD68 | -3.37 | Homo sapiens small nucleolar RNA, C/D box 68 (SNORD68), small nucleolar RNA. |
| ODZ3 | -3.38 | Homo sapiens odz, odd Oz/ten-m homolog 3 (Drosophila) (ODZ3), mRNA. |
| LRRFIP1 | -3.4 | Homo sapiens leucine rich repeat (in FLII) interacting protein 1 (LRRFIP1), mRNA. |
| ADM | -3.4 | Homo sapiens adrenomedullin (ADM), mRNA. |
| HSPC268 | -3.4 | Homo sapiens hypothetical protein HSPC268 (HSPC268), mRNA. |
| PIF1 | -3.41 | Homo sapiens PIF1 5'-to-3' DNA helicase homolog (S. cerevisiae) (PIF1), mRNA. |
| PTGR1 | -3.43 | Homo sapiens prostaglandin reductase 1 (PTGR1), mRNA. |
| SEMA3E | -3.43 | Homo sapiens sema domain, immunoglobulin domain (Ig), short basic domain, secreted, (semaphorin) 3E (SEMA3E), mRNA. |
| TTK | -3.44 | Homo sapiens TTK protein kinase (TTK), mRNA. |
| NTNG1 | -3.45 | Homo sapiens netrin G1 (NTNG1), transcript variant 3, mRNA. |
| CRABP1 | -3.48 | Homo sapiens cellular retinoic acid binding protein 1 (CRABP1), mRNA. |
| TNFAIP8L1 | -3.49 | Homo sapiens tumor necrosis factor, alpha-induced protein 8-like 1 (TNFAIP8L1), mRNA. |
| JUN | -3.5 | Homo sapiens jun oncogene (JUN), mRNA. |
| GTPBP8 | -3.5 | Homo sapiens GTP-binding protein 8 (putative) (GTPBP8), transcript variant 1, mRNA. |
| C1orf97 | -3.5 | Homo sapiens chromosome 1 open reading frame 97 (C1orf97), mRNA. |
| UBE2C | -3.53 | Homo sapiens ubiquitin-conjugating enzyme E2C (UBE2C), transcript variant 3, mRNA. |
| CDKN3 | -3.54 | Homo sapiens cyclin-dependent kinase inhibitor 3 (CDK2-associated dual specificity phosphatase) (CDKN3), mRNA. |
| SNORD35B | -3.58 | Homo sapiens small nucleolar RNA, C/D box 35B (SNORD35B), small nucleolar RNA. |
| SLC30A1 | -3.59 | Homo sapiens solute carrier family 30 (zinc transporter), member 1 (SLC30A1), mRNA. |
| TMEM70 | -3.59 | Homo sapiens transmembrane protein 70 (TMEM70), transcript variant 1, mRNA. |
| ICT1 | -3.59 | Homo sapiens immature colon carcinoma transcript 1 (ICT1), mRNA. |
| C18orf56 | -3.6 | Homo sapiens chromosome 18 open reading frame 56 (C18orf56), mRNA. |
| GATA3 | -3.62 | Homo sapiens GATA binding protein 3 (GATA3), transcript variant 2, mRNA. |
| FGL1 | -3.64 | Homo sapiens fibrinogen-like 1 (FGL1), transcript variant 3, mRNA. |
| NFKBIA | -3.66 | Homo sapiens nuclear factor of kappa light polypeptide gene enhancer in B-cells inhibitor, alpha (NFKBIA), mRNA. |
| LMOD3 | -3.66 | Homo sapiens leiomodin 3 (fetal) (LMOD3), mRNA. |
| MAD2L1 | -3.67 | Homo sapiens MAD2 mitotic arrest deficient-like 1 (yeast) (MAD2L1), mRNA. |
| GAS1 | -3.68 | Homo sapiens growth arrest-specific 1 (GAS1), mRNA. |
| CCNB2 | -3.72 | Homo sapiens cyclin B2 (CCNB2), mRNA. |
| UBE2C | -3.72 | Homo sapiens ubiquitin-conjugating enzyme E2C (UBE2C), transcript variant 6, mRNA. |
| AURKB | -3.74 | Homo sapiens aurora kinase B (AURKB), mRNA. |
| CDCA3 | -3.77 | Homo sapiens cell division cycle associated 3 (CDCA3), mRNA. |
| PRR11 | -3.81 | Homo sapiens proline rich 11 (PRR11), mRNA. |
| YTHDC1 | -3.84 | Homo sapiens YTH domain containing 1 (YTHDC1), transcript variant 1, mRNA. |
| KLF6 | -3.84 | Homo sapiens Kruppel-like factor 6 (KLF6), transcript variant 1, mRNA. |
| NGFRAP1 | -3.85 | Homo sapiens nerve growth factor receptor (TNFRSF16) associated protein 1 (NGFRAP1), transcript variant 3, mRNA. |
| ARHGAP11B | -3.85 | Homo sapiens Rho GTPase activating protein 11B (ARHGAP11B), mRNA. XM_931045 XM_931050 |
| LOC387763 | -3.86 | PREDICTED: Homo sapiens hypothetical LOC387763 (LOC387763), mRNA. |
| ZGPAT | -3.91 | Homo sapiens zinc finger, CCCH-type with G patch domain (ZGPAT), transcript variant 1, mRNA. |
| PRPF4B | -3.92 | Homo sapiens PRP4 pre-mRNA processing factor 4 homolog B (yeast) (PRPF4B), transcript variant 2, mRNA. |
| PROX1 | -3.93 | Homo sapiens prospero homeobox 1 (PROX1), mRNA. |
| CDC20 | -3.94 | Homo sapiens cell division cycle 20 homolog (S. cerevisiae) (CDC20), mRNA. |
| DLX5 | -3.97 | Homo sapiens distal-less homeobox 5 (DLX5), mRNA. |
| TM4SF4 | -3.98 | Homo sapiens transmembrane 4 L six family member 4 (TM4SF4), mRNA. |
| LOC440093 | -3.99 | Homo sapiens histone H3-like (LOC440093), mRNA. |
| ZSCAN2 | -4.01 | Homo sapiens zinc finger and SCAN domain containing 2 (ZSCAN2), transcript variant 3, mRNA. |
| ICA1 | -4.03 | Homo sapiens islet cell autoantigen 1, 69kDa (ICA1), transcript variant 3, mRNA. |
| ZNF256 | -4.04 | Homo sapiens zinc finger protein 256 (ZNF256), mRNA. |
| FGL1 | -4.05 | Homo sapiens fibrinogen-like 1 (FGL1), transcript variant 4, mRNA. |
| IGFBP3 | -4.06 | Homo sapiens insulin-like growth factor binding protein 3 (IGFBP3), transcript variant 2, mRNA. |
| RGS2 | -4.07 | Homo sapiens regulator of G-protein signalling 2, 24kDa (RGS2), mRNA. |
| SAP30 | -4.08 | Homo sapiens Sin3A-associated protein, 30kDa (SAP30), mRNA. |
| SPRY1 | -4.11 | Homo sapiens sprouty homolog 1, antagonist of FGF signaling (Drosophila) (SPRY1), transcript variant 1, mRNA. |
| TTC32 | -4.11 | Homo sapiens tetratricopeptide repeat domain 32 (TTC32), mRNA. |
| UBE2L3 | -4.16 | Homo sapiens ubiquitin-conjugating enzyme E2L 3 (UBE2L3), transcript variant 2, mRNA. |
| CENPA | -4.2 | Homo sapiens centromere protein A (CENPA), transcript variant 2, mRNA. |
| GAS2L3 | -4.32 | Homo sapiens growth arrest-specific 2 like 3 (GAS2L3), mRNA. |
|  | -4.33 | AGENCOURT_10229596 NIH_MGC_141 Homo sapiens cDNA clone IMAGE:6563923 5, mRNA sequence |
| LINGO4 | -4.33 | Homo sapiens leucine rich repeat and Ig domain containing 4 (LINGO4), mRNA. |
| DCN | -4.36 | Homo sapiens decorin (DCN), transcript variant C, mRNA. |
| BAMBI | -4.36 | Homo sapiens BMP and activin membrane-bound inhibitor homolog (Xenopus laevis) (BAMBI), mRNA. |
| LRRN3 | -4.44 | Homo sapiens leucine rich repeat neuronal 3 (LRRN3), mRNA. |
| PCDH17 | -4.52 | Homo sapiens protocadherin 17 (PCDH17), mRNA. |
| C13orf25 | -4.67 | PREDICTED: Homo sapiens chromosome 13 open reading frame 25 (C13orf25), mRNA. |
| HMGB2 | -4.78 | Homo sapiens high-mobility group box 2 (HMGB2), mRNA. |
| VIP | -4.83 | Homo sapiens vasoactive intestinal peptide (VIP), transcript variant 2, mRNA. |
| SCG2 | -4.95 | Homo sapiens secretogranin II (chromogranin C) (SCG2), mRNA. |
| TM4SF1 | -4.97 | Homo sapiens transmembrane 4 L six family member 1 (TM4SF1), mRNA. |
| LRRC17 | -4.98 | Homo sapiens leucine rich repeat containing 17 (LRRC17), transcript variant 2, mRNA. |
| PCDH20 | -5.21 | Homo sapiens protocadherin 20 (PCDH20), mRNA. |
| H3F3B | -5.45 | Homo sapiens H3 histone, family 3B (H3.3B) (H3F3B), mRNA. |
| KLF6 | -5.46 | Homo sapiens Kruppel-like factor 6 (KLF6), transcript variant 2, mRNA. |
| ID3 | -5.49 | Homo sapiens inhibitor of DNA binding 3, dominant negative helix-loop-helix protein (ID3), mRNA. |
| NFKBIZ | -5.64 | Homo sapiens nuclear factor of kappa light polypeptide gene enhancer in B-cells inhibitor, zeta (NFKBIZ), transcript variant 2, mRNA. |
| ID1 | -5.67 | Homo sapiens inhibitor of DNA binding 1, dominant negative helix-loop-helix protein (ID1), transcript variant 2, mRNA. |
| IGFBP5 | -6.84 | Homo sapiens insulin-like growth factor binding protein 5 (IGFBP5), mRNA. |
| SGK | -7.32 | Homo sapiens serum/glucocorticoid regulated kinase (SGK), mRNA. |
| ID2 | -7.61 | Homo sapiens inhibitor of DNA binding 2, dominant negative helix-loop-helix protein (ID2), mRNA. |
| IGFBP5 | -8.34 | Homo sapiens insulin-like growth factor binding protein 5 (IGFBP5), mRNA. |
| ID2 | -8.73 | Homo sapiens inhibitor of DNA binding 2, dominant negative helix-loop-helix protein (ID2), mRNA. |
| FOSB | -12.27 | Homo sapiens FBJ murine osteosarcoma viral oncogene homolog B (FOSB), mRNA. |
| CYR61 | -14.63 | Homo sapiens cysteine-rich, angiogenic inducer, 61 (CYR61), mRNA. |
| FOS | -16.09 | Homo sapiens v-fos FBJ murine osteosarcoma viral oncogene homolog (FOS), mRNA. |
| EGR1 | -23.34 | Homo sapiens early growth response 1 (EGR1), mRNA. |
