# Supplementary material for: Plurigon: three dimensional visualization and classification of high-dimensionality data
Source: Front Physiol. 2013 Jul 22;4:190. doi: 10.3389/fphys.2013.00190 (PMC3717481; doi:10.3389/fphys.2013.00190)
Supplement: Table S16 — Conserved neuronal transcriptomic expression data from brain-derived neurotrophic factor-treated clonal human cells. Transcriptomic gene regulation was measured from brain-derived neurotrophic factor (BDNF)-treated SH-SY5Y neuronal cell batches (BDNF-1, BDNF-2, BDNF-3). The transcriptomic data from the three separate experiments was compared to gene expression levels in vehicle (phosphate-buffered saline)-treated control SH-SY5Y cells. [file 46440_Maudsley_Data_Sheet_6.ZIP › Table-S20.doc]

**Table S20. Transcriptomic effects of nerve growth factor in human neuronal cells (SH-SY5Y) cells pre-treated with chronic minimal levels of hydrogen peroxide.** For each nerve growth factor (NGF) regulated transcript in the peroxide-treated SH-SY5Y cells the official Gene Symbol, z-ratio and transcript description are indicated.

| **Gene Symbol** | **z-ratio** | **Description** |
| --- | --- | --- |
| RPL7A | 8.17 | Homo sapiens ribosomal protein L7a (RPL7A), mRNA. |
| TXNIP | 6.68 | Homo sapiens thioredoxin interacting protein (TXNIP), mRNA. |
| TMEM189 | 6.54 | Homo sapiens TMEM189-UBE2V1 readthrough transcript (TMEM189-UBE2V1), transcript variant 2, mRNA. |
| HLA-DRB3 | 5.17 | Homo sapiens major histocompatibility complex, class II, DR beta 3 (HLA-DRB3), mRNA. |
| RPL31 | 5 | Homo sapiens ribosomal protein L31 (RPL31), transcript variant 1, mRNA. |
| FTHL2 | 4.84 | Homo sapiens ferritin, heavy polypeptide-like 2 (FTHL2) on chromosome 1. |
| LOC642530 | 4.79 | PREDICTED: Homo sapiens hypothetical protein LOC642530 (LOC642530), mRNA. |
|  | 4.71 | xs48g03.x1 NCI_CGAP_Kid11 Homo sapiens cDNA clone IMAGE:2772916 3, mRNA sequence |
| LOC283412 | 4.64 | PREDICTED: Homo sapiens similar to 60S ribosomal protein L29 (Cell surface heparin-binding protein HIP) (LOC283412), mRNA. |
| CD63 | 4.61 | Homo sapiens CD63 molecule (CD63), transcript variant 2, mRNA. |
|  | 4.59 | xm52h06.x1 NCI_CGAP_GC6 Homo sapiens cDNA clone IMAGE:2687867 3, mRNA sequence |
| RPL23 | 4.57 | Homo sapiens ribosomal protein L23 (RPL23), mRNA. |
| NUTF2 | 4.51 | Homo sapiens nuclear transport factor 2 (NUTF2), mRNA. |
| FLJ40194 | 4.48 | Homo sapiens FLJ40194 protein (FLJ40194), mRNA. |
| RPL13A | 4.47 | Homo sapiens ribosomal protein L13a (RPL13A), mRNA. |
| CCT3 | 4.47 | Homo sapiens chaperonin containing TCP1, subunit 3 (gamma) (CCT3), transcript variant 3, mRNA. |
| SNAPIN | 4.3 | Homo sapiens SNAP-associated protein (SNAPIN), mRNA. |
| RPS3A | 4.3 | Homo sapiens ribosomal protein S3A (RPS3A), mRNA. |
| FAM177A1 | 4.28 | Homo sapiens family with sequence similarity 177, member A1 (FAM177A1), transcript variant 1, mRNA. |
| RBM3 | 4.25 | Homo sapiens RNA binding motif (RNP1, RRM) protein 3 (RBM3), mRNA. |
| RPS27 | 4.15 | Homo sapiens ribosomal protein S27 (metallopanstimulin 1) (RPS27), mRNA. |
| RPS24 | 4.15 | Homo sapiens ribosomal protein S24 (RPS24), transcript variant 1, mRNA. |
| RASSF5 | 4.14 | Homo sapiens Ras association (RalGDS/AF-6) domain family member 5 (RASSF5), transcript variant 2, mRNA. |
| LOC728643 | 4.14 | Homo sapiens heterogeneous nuclear ribonucleoprotein A1 pseudogene (LOC728643), non-coding RNA. |
| LOC440927 | 4.09 | PREDICTED: Homo sapiens similar to 60S acidic ribosomal protein P1, transcript variant 4 (LOC440927), mRNA. |
| LOC728973 | 4.05 | PREDICTED: Homo sapiens similar to 40S ribosomal protein S7 (S8) (LOC728973), mRNA. |
| PES1 | 4.04 | Homo sapiens pescadillo homolog 1, containing BRCT domain (zebrafish) (PES1), mRNA. |
| RPL37A | 4.02 | Homo sapiens ribosomal protein L37a (RPL37A), mRNA. |
| HMGN2 | 4.01 | Homo sapiens high-mobility group nucleosomal binding domain 2 (HMGN2), mRNA. |
| CFDP1 | 4.01 | Homo sapiens craniofacial development protein 1 (CFDP1), mRNA. |
| NME2 | 3.99 | Homo sapiens non-metastatic cells 2, protein (NM23B) expressed in (NME2), transcript variant 1, mRNA. |
| LOC728481 | 3.99 | PREDICTED: Homo sapiens similar to similar to RPL23AP7 protein (LOC728481), mRNA. |
| MRPL16 | 3.97 | Homo sapiens mitochondrial ribosomal protein L16 (MRPL16), nuclear gene encoding mitochondrial protein, mRNA. |
| RPS3A | 3.91 | Homo sapiens ribosomal protein S3A (RPS3A), mRNA. |
| DBNDD1 | 3.9 | Homo sapiens dysbindin (dystrobrevin binding protein 1) domain containing 1 (DBNDD1), transcript variant 1, mRNA. |
| LOC645138 | 3.89 | PREDICTED: Homo sapiens similar to ribosomal protein S11 (LOC645138), mRNA. |
| SQSTM1 | 3.88 | Homo sapiens sequestosome 1 (SQSTM1), mRNA. |
| NFATC2IP | 3.87 | Homo sapiens nuclear factor of activated T-cells, cytoplasmic, calcineurin-dependent 2 interacting protein (NFATC2IP), mRNA. |
| ATP6V0B | 3.86 | Homo sapiens ATPase, H+ transporting, lysosomal 21kDa, V0 subunit b (ATP6V0B), transcript variant 2, mRNA. |
| CARD14 | 3.83 | Homo sapiens caspase recruitment domain family, member 14 (CARD14), transcript variant 1, mRNA. |
| ACYP1 | 3.81 | Homo sapiens acylphosphatase 1, erythrocyte (common) type (ACYP1), transcript variant 1, mRNA. |
| RPS15A | 3.81 | Homo sapiens ribosomal protein S15a (RPS15A), transcript variant 2, mRNA. |
| LOC651202 | 3.8 | PREDICTED: Homo sapiens similar to large subunit ribosomal protein L36a (LOC651202), mRNA. |
| LRRC41 | 3.74 | Homo sapiens leucine rich repeat containing 41 (LRRC41), mRNA. |
| ASS1 | 3.74 | Homo sapiens argininosuccinate synthetase 1 (ASS1), transcript variant 2, mRNA. |
| GNPNAT1 | 3.68 | Homo sapiens glucosamine-phosphate N-acetyltransferase 1 (GNPNAT1), mRNA. |
| RPS27A | 3.68 | Homo sapiens ribosomal protein S27a (RPS27A), mRNA. |
| LOC654161 | 3.64 | PREDICTED: Homo sapiens similar to AAA-ATPase TOB3, transcript variant 2 (LOC654161), mRNA. |
| EWSR1 | 3.61 | Homo sapiens Ewing sarcoma breakpoint region 1 (EWSR1), transcript variant EWS-b, mRNA. |
| RXRB | 3.56 | Homo sapiens retinoid X receptor, beta (RXRB), mRNA. |
| LOC158345 | 3.54 | PREDICTED: Homo sapiens similar to ribosomal protein L4 (LOC158345), mRNA. |
| LOC220433 | 3.52 | PREDICTED: Homo sapiens similar to 40S ribosomal protein S4, X isoform (LOC220433), mRNA. |
| POLR2J3 | 3.49 | Homo sapiens polymerase (RNA) II (DNA directed) polypeptide J3 (POLR2J3), mRNA. |
| WBP5 | 3.49 | Homo sapiens WW domain binding protein 5 (WBP5), transcript variant 4, mRNA. |
| SNF8 | 3.49 | Homo sapiens SNF8, ESCRT-II complex subunit, homolog (S. cerevisiae) (SNF8), mRNA. |
| EXOC4 | 3.48 | Homo sapiens exocyst complex component 4 (EXOC4), transcript variant 1, mRNA. |
| SEC63 | 3.48 | Homo sapiens SEC63 homolog (S. cerevisiae) (SEC63), mRNA. |
| HDHD2 | 3.44 | Homo sapiens haloacid dehalogenase-like hydrolase domain containing 2 (HDHD2), mRNA. |
| PEBP1 | 3.43 | Homo sapiens phosphatidylethanolamine binding protein 1 (PEBP1), mRNA. |
| GAPDH | 3.42 | Homo sapiens glyceraldehyde-3-phosphate dehydrogenase (GAPDH), mRNA. |
| LOC651894 | 3.39 | PREDICTED: Homo sapiens similar to ribosomal protein S12 (LOC651894), mRNA. |
| LOC400948 | 3.39 | PREDICTED: Homo sapiens similar to CG33774-PA (LOC400948), mRNA. |
| CSNK1G2 | 3.38 | Homo sapiens casein kinase 1, gamma 2 (CSNK1G2), mRNA. |
| SNW1 | 3.37 | Homo sapiens SNW domain containing 1 (SNW1), mRNA. |
| RPS19 | 3.35 | Homo sapiens ribosomal protein S19 (RPS19), mRNA. |
| C17orf79 | 3.34 | Homo sapiens chromosome 17 open reading frame 79 (C17orf79), mRNA. |
| FLJ10374 | 3.33 | Homo sapiens hypothetical protein FLJ10374 (FLJ10374), mRNA. |
| SEC11C | 3.32 | Homo sapiens SEC11 homolog C (S. cerevisiae) (SEC11C), mRNA. |
| RPS20 | 3.31 | Homo sapiens ribosomal protein S20 (RPS20), mRNA. |
| LOC205251 | 3.29 | PREDICTED: Homo sapiens misc_RNA (LOC205251), miscRNA. |
| CHGA | 3.27 | Homo sapiens chromogranin A (parathyroid secretory protein 1) (CHGA), mRNA. |
| PDCD7 | 3.24 | Homo sapiens programmed cell death 7 (PDCD7), mRNA. |
| PKN1 | 3.22 | Homo sapiens protein kinase N1 (PKN1), transcript variant 1, mRNA. |
| NAG18 | 3.22 | Homo sapiens NAG18 protein (NAG18) on chromosome 19. |
| IRX1 | 3.19 | Homo sapiens iroquois homeobox 1 (IRX1), mRNA. |
| POLR3F | 3.17 | Homo sapiens polymerase (RNA) III (DNA directed) polypeptide F, 39 kDa (POLR3F), mRNA. |
| RPL13 | 3.16 | Homo sapiens ribosomal protein L13 (RPL13), transcript variant 2, mRNA. |
| LOC645609 | 3.15 | PREDICTED: Homo sapiens similar to TBC1 domain family member 3 (Rab GTPase-activating protein PRC17) (Prostate cancer gene 17 protein) (TRE17 alpha protein) (LOC645609), mRNA. |
| RPS28 | 3.15 | Homo sapiens ribosomal protein S28 (RPS28), mRNA. |
| HNRPA1L-2 | 3.14 | Homo sapiens heterogeneous nuclear ribonucleoprotein A1 pseudogene (HNRPA1L-2), non-coding RNA. |
| PHF23 | 3.14 | Homo sapiens PHD finger protein 23 (PHF23), mRNA. |
| POLG2 | 3.13 | Homo sapiens polymerase (DNA directed), gamma 2, accessory subunit (POLG2), mRNA. |
| TADA3 | 3.12 | Homo sapiens transcriptional adaptor 3 (TADA3), transcript variant 1, mRNA. |
| HSPB1 | 3.09 | Homo sapiens heat shock 27kDa protein 1 (HSPB1), mRNA. |
| LOC285900 | 3.08 | PREDICTED: Homo sapiens similar to 60S ribosomal protein L6 (TAX-responsive enhancer element binding protein 107) (TAXREB107) (Neoplasm-related protein C140), transcript variant 3 (LOC285900), mRNA. |
| PRAF2 | 3.07 | Homo sapiens PRA1 domain family, member 2 (PRAF2), mRNA. |
| HNRPA1P4 | 3.06 | PREDICTED: Homo sapiens heterogeneous nuclear ribonucleoprotein A1 pseudogene 4 (HNRPA1P4), mRNA. |
| LOC402644 | 3.05 | PREDICTED: Homo sapiens similar to peptidylprolyl isomerase A isoform 1 (LOC402644), mRNA. |
| LOC731985 | 3.05 | PREDICTED: Homo sapiens hypothetical protein LOC731985 (LOC731985), mRNA. |
| KIF21A | 3.05 | Homo sapiens kinesin family member 21A (KIF21A), mRNA. |
| FKSG30 | 3.02 | Homo sapiens actin-like protein (FKSG30), mRNA. |
| RAB24 | 3.01 | Homo sapiens RAB24, member RAS oncogene family (RAB24), transcript variant 1, mRNA. |
| PIGM | 3 | Homo sapiens phosphatidylinositol glycan anchor biosynthesis, class M (PIGM), mRNA. |
| CDIPT | 3 | Homo sapiens CDP-diacylglycerol--inositol 3-phosphatidyltransferase (phosphatidylinositol synthase) (CDIPT), mRNA. |
| DUSP6 | 2.95 | Homo sapiens dual specificity phosphatase 6 (DUSP6), transcript variant 2, mRNA. |
| LOC651894 | 2.95 | PREDICTED: Homo sapiens similar to ribosomal protein S12 (LOC651894), mRNA. |
| FTHL12 | 2.95 | Homo sapiens ferritin, heavy polypeptide-like 12 (FTHL12) on chromosome 9. |
| FGL1 | 2.95 | PREDICTED: Homo sapiens fibrinogen-like 1, transcript variant 5 (FGL1), mRNA. |
| LOC441743 | 2.95 | Homo sapiens similar to C367G8.3 (novel protein similar to RPL23A (60S ribosomal protein L23A)) (LOC441743), mRNA. |
| LOC401019 | 2.94 | Homo sapiens hCG1783417 (LOC401019), mRNA. |
| LRP10 | 2.93 | Homo sapiens low density lipoprotein receptor-related protein 10 (LRP10), mRNA. |
| TUBB4Q | 2.93 | Homo sapiens tubulin, beta polypeptide 4, member Q (TUBB4Q), mRNA. |
| LOC649946 | 2.92 | Homo sapiens ribosomal protein L23a pseudogene (LOC649946), non-coding RNA. |
| YWHAE | 2.9 | Homo sapiens tyrosine 3-monooxygenase/tryptophan 5-monooxygenase activation protein, epsilon polypeptide (YWHAE), mRNA. |
| PLAA | 2.87 | Homo sapiens phospholipase A2-activating protein (PLAA), transcript variant 1, mRNA. |
| PHF17 | 2.86 | Homo sapiens PHD finger protein 17 (PHF17), transcript variant L, mRNA. |
| LOC653314 | 2.86 | Homo sapiens similar to ribosomal protein L19 (LOC653314), mRNA. |
| MAGEH1 | 2.86 | Homo sapiens melanoma antigen family H, 1 (MAGEH1), mRNA. |
| RRN3 | 2.86 | Homo sapiens RRN3 RNA polymerase I transcription factor homolog (S. cerevisiae) (RRN3), mRNA. |
| STK16 | 2.86 | Homo sapiens serine/threonine kinase 16 (STK16), transcript variant 1, mRNA. |
| CAPZB | 2.85 | Homo sapiens capping protein (actin filament) muscle Z-line, beta (CAPZB), mRNA. |
| C6orf115 | 2.85 | Homo sapiens chromosome 6 open reading frame 115 (C6orf115), mRNA. |
| S100A13 | 2.85 | Homo sapiens S100 calcium binding protein A13 (S100A13), transcript variant 3, mRNA. |
| TSPO | 2.84 | Homo sapiens translocator protein (18kDa) (TSPO), transcript variant PBR-S, mRNA. |
| LOC643949 | 2.84 | PREDICTED: Homo sapiens similar to 60S acidic ribosomal protein P2 (LOC643949), mRNA. |
| THBS3 | 2.84 | Homo sapiens thrombospondin 3 (THBS3), mRNA. |
| POLR2C | 2.84 | Homo sapiens polymerase (RNA) II (DNA directed) polypeptide C, 33kDa (POLR2C), mRNA. |
| MAPKAPK2 | 2.84 | Homo sapiens mitogen-activated protein kinase-activated protein kinase 2 (MAPKAPK2), transcript variant 2, mRNA. |
| HEXDC | 2.84 | Homo sapiens hexosaminidase (glycosyl hydrolase family 20, catalytic domain) containing (HEXDC), mRNA. |
| PSMC5 | 2.83 | Homo sapiens proteasome (prosome, macropain) 26S subunit, ATPase, 5 (PSMC5), mRNA. |
| TOMM5 | 2.83 | Homo sapiens translocase of outer mitochondrial membrane 5 homolog (yeast) (TOMM5), nuclear gene encoding mitochondrial protein, transcript variant 1, mRNA. |
| LOC653773 | 2.83 | PREDICTED: Homo sapiens similar to ribosomal protein L31 (LOC653773), mRNA. |
| LOC402057 | 2.82 | Homo sapiens similar to 40S ribosomal protein S17 (LOC402057), mRNA. |
| YBX1 | 2.82 | Homo sapiens Y box binding protein 1 (YBX1), mRNA. |
| MRPL19 | 2.81 | Homo sapiens mitochondrial ribosomal protein L19 (MRPL19), nuclear gene encoding mitochondrial protein, mRNA. |
| HNRPC | 2.8 | Homo sapiens heterogeneous nuclear ribonucleoprotein C (C1/C2) (HNRPC), transcript variant 1, mRNA. |
| ADD1 | 2.79 | Homo sapiens adducin 1 (alpha) (ADD1), transcript variant 1, mRNA. |
| S100PBP | 2.78 | Homo sapiens S100P binding protein (S100PBP), transcript variant 1, mRNA. |
| ITGB2 | 2.78 | Homo sapiens integrin, beta 2 (complement component 3 receptor 3 and 4 subunit) (ITGB2), mRNA. |
| RPLP1 | 2.76 | Homo sapiens ribosomal protein, large, P1 (RPLP1), transcript variant 2, mRNA. |
| STMN2 | 2.76 | Homo sapiens stathmin-like 2 (STMN2), mRNA. |
| LOC651149 | 2.76 | PREDICTED: Homo sapiens similar to 60S ribosomal protein L3 (L4) (LOC651149), mRNA. |
| LOC653658 | 2.76 | PREDICTED: Homo sapiens similar to ribosomal protein S23 (LOC653658), mRNA. |
| TP53I13 | 2.76 | Homo sapiens tumor protein p53 inducible protein 13 (TP53I13), mRNA. |
| RPL13L | 2.75 | Homo sapiens ribosomal protein L13-like (RPL13L), non-coding RNA. |
| LOC442454 | 2.75 | Homo sapiens ubiquinol-cytochrome c reductase binding protein pseudogene (LOC442454), non-coding RNA. |
| FTHL11 | 2.74 | Homo sapiens ferritin, heavy polypeptide-like 11 (FTHL11) on chromosome 8. |
| TNC | 2.72 | Homo sapiens tenascin C (hexabrachion) (TNC), mRNA. |
| GCN1L1 | 2.72 | Homo sapiens GCN1 general control of amino-acid synthesis 1-like 1 (yeast) (GCN1L1), mRNA. |
| PPID | 2.71 | Homo sapiens peptidylprolyl isomerase D (PPID), mRNA. |
| LOC649169 | 2.71 | PREDICTED: Homo sapiens similar to WD repeat domain 74, transcript variant 1 (LOC649169), mRNA. |
| RIC8B | 2.71 | Homo sapiens resistance to inhibitors of cholinesterase 8 homolog B (C. elegans) (RIC8B), mRNA. |
| CALR | 2.71 | Homo sapiens calreticulin (CALR), mRNA. |
| CSRP2 | 2.7 | Homo sapiens cysteine and glycine-rich protein 2 (CSRP2), mRNA. |
| ARPP19 | 2.7 | Homo sapiens cAMP-regulated phosphoprotein, 19kDa (ARPP19), mRNA. |
| CFL1 | 2.69 | Homo sapiens cofilin 1 (non-muscle) (CFL1), mRNA. |
| DPP7 | 2.68 | Homo sapiens dipeptidyl-peptidase 7 (DPP7), mRNA. |
| IQCC | 2.68 | Homo sapiens IQ motif containing C (IQCC), mRNA. |
| LOC643284 | 2.67 | PREDICTED: Homo sapiens similar to 40S ribosomal protein S29 (LOC643284), mRNA. |
| LOC643997 | 2.67 | PREDICTED: Homo sapiens similar to peptidylprolyl isomerase A isoform 1 (LOC643997), mRNA. |
| C17orf71 | 2.66 | Homo sapiens chromosome 17 open reading frame 71 (C17orf71), mRNA. |
| ZNF430 | 2.66 | Homo sapiens zinc finger protein 430 (ZNF430), mRNA. |
| WDSOF1 | 2.66 | Homo sapiens WD repeats and SOF1 domain containing (WDSOF1), mRNA. |
| AK2 | 2.66 | Homo sapiens adenylate kinase 2 (AK2), transcript variant AK2A, mRNA. |
| TUBA1A | 2.66 | Homo sapiens tubulin, alpha 1a (TUBA1A), mRNA. |
| RAB13 | 2.66 | Homo sapiens RAB13, member RAS oncogene family (RAB13), mRNA. |
| GCSH | 2.66 | Homo sapiens glycine cleavage system protein H (aminomethyl carrier) (GCSH), mRNA. |
| LOC648638 | 2.66 | PREDICTED: Homo sapiens similar to Peptidyl-prolyl cis-trans isomerase NIMA-interacting 4 (Rotamase Pin4) (PPIase Pin4) (Parvulin 14) (Par14) (Peptidyl-prolyl cis/trans isomerase EPVH) (hPar14) (LOC648638), mRNA. |
| ANKRD40 | 2.66 | Homo sapiens ankyrin repeat domain 40 (ANKRD40), mRNA. |
| ZC3H8 | 2.65 | Homo sapiens zinc finger CCCH-type containing 8 (ZC3H8), mRNA. |
| HMGB1L1 | 2.64 | Homo sapiens high-mobility group box 1-like 1 (HMGB1L1), mRNA. |
| LOC644039 | 2.63 | PREDICTED: Homo sapiens similar to 60S ribosomal protein L10 (QM protein) (Tumor suppressor QM) (Laminin receptor homolog) (LOC644039), mRNA. |
| SNHG5 | 2.62 | Homo sapiens small nucleolar RNA host gene (non-protein coding) 5 (SNHG5) on chromosome 6. |
| LOC339804 | 2.62 | PREDICTED: Homo sapiens hypothetical gene supported by AK075484; BC014578 (LOC339804), mRNA. |
| H2AFJ | 2.61 | Homo sapiens H2A histone family, member J (H2AFJ), transcript variant 1, mRNA. |
| LOC651436 | 2.6 | PREDICTED: Homo sapiens similar to ribosomal protein L9 (LOC651436), mRNA. |
| LOC402694 | 2.6 | Homo sapiens similar to ribosomal protein L15 (LOC402694), mRNA. |
| LMAN1 | 2.59 | Homo sapiens lectin, mannose-binding, 1 (LMAN1), mRNA. |
| GDI2 | 2.57 | Homo sapiens GDP dissociation inhibitor 2 (GDI2), mRNA. |
| DDX49 | 2.55 | Homo sapiens DEAD (Asp-Glu-Ala-Asp) box polypeptide 49 (DDX49), mRNA. |
| ELOF1 | 2.54 | Homo sapiens elongation factor 1 homolog (S. cerevisiae) (ELOF1), mRNA. |
| YWHAG | 2.54 | Homo sapiens tyrosine 3-monooxygenase/tryptophan 5-monooxygenase activation protein, gamma polypeptide (YWHAG), mRNA. |
| SAP30L | 2.54 | Homo sapiens SAP30-like (SAP30L), mRNA. |
| VAT1 | 2.54 | Homo sapiens vesicle amine transport protein 1 homolog (T. californica) (VAT1), mRNA. |
| PFN1 | 2.53 | Homo sapiens profilin 1 (PFN1), mRNA. |
| TRMT112 | 2.53 | Homo sapiens tRNA methyltransferase 11-2 homolog (S. cerevisiae) (TRMT112), mRNA. |
| FTHL12 | 2.53 | Homo sapiens ferritin, heavy polypeptide-like 12 (FTHL12) on chromosome 9. |
| LOC644511 | 2.52 | PREDICTED: Homo sapiens similar to ribosomal protein L13a, transcript variant 1 (LOC644511), mRNA. |
| RPL11 | 2.52 | Homo sapiens ribosomal protein L11 (RPL11), mRNA. |
| LOC730740 | 2.51 | PREDICTED: Homo sapiens similar to H3 histone, family 3B (LOC730740), mRNA. |
| RPLP1 | 2.51 | Homo sapiens ribosomal protein, large, P1 (RPLP1), transcript variant 1, mRNA. |
| LOC440737 | 2.5 | PREDICTED: Homo sapiens similar to 60S ribosomal protein L35 (LOC440737), mRNA. |
| QSOX1 | 2.49 | Homo sapiens quiescin Q6 sulfhydryl oxidase 1 (QSOX1), transcript variant 2, mRNA. |
| UBL5 | 2.49 | Homo sapiens ubiquitin-like 5 (UBL5), transcript variant 2, mRNA. |
| RPL7 | 2.49 | Homo sapiens ribosomal protein L7 (RPL7), mRNA. |
| DDAH2 | 2.48 | Homo sapiens dimethylarginine dimethylaminohydrolase 2 (DDAH2), mRNA. |
| LOC653314 | 2.48 | Homo sapiens similar to ribosomal protein L19 (LOC653314), mRNA. |
| LOC286016 | 2.47 | Homo sapiens triosephosphate isomerase 1 pseudogene (LOC286016), non-coding RNA. |
| PRR3 | 2.47 | Homo sapiens proline rich 3 (PRR3), transcript variant 2, mRNA. |
| MCM8 | 2.47 | Homo sapiens minichromosome maintenance complex component 8 (MCM8), transcript variant 1, mRNA. |
| LOC441775 | 2.47 | PREDICTED: Homo sapiens similar to 60S ribosomal protein L18 (LOC441775), mRNA. |
| ASNS | 2.46 | Homo sapiens asparagine synthetase (ASNS), transcript variant 1, mRNA. |
| FAM115A | 2.46 | Homo sapiens family with sequence similarity 115, member A (FAM115A), mRNA. |
| CHGB | 2.45 | Homo sapiens chromogranin B (secretogranin 1) (CHGB), mRNA. |
| UCHL1 | 2.44 | Homo sapiens ubiquitin carboxyl-terminal esterase L1 (ubiquitin thiolesterase) (UCHL1), mRNA. |
| RPL27A | 2.44 | Homo sapiens ribosomal protein L27a (RPL27A), mRNA. |
| HNRPK | 2.44 | Homo sapiens heterogeneous nuclear ribonucleoprotein K (HNRPK), transcript variant 3, mRNA. |
| LOC401206 | 2.43 | PREDICTED: Homo sapiens similar to 40S ribosomal protein S25 (LOC401206), mRNA. |
| GNB2L1 | 2.43 | Homo sapiens guanine nucleotide binding protein (G protein), beta polypeptide 2-like 1 (GNB2L1), mRNA. |
| LOC285176 | 2.43 | PREDICTED: Homo sapiens similar to ribosomal protein L10 (LOC285176), mRNA. |
| XRCC5 | 2.42 | Homo sapiens X-ray repair complementing defective repair in Chinese hamster cells 5 (double-strand-break rejoining; Ku autoantigen, 80kDa) (XRCC5), mRNA. |
| LASS2 | 2.41 | Homo sapiens LAG1 homolog, ceramide synthase 2 (LASS2), transcript variant 2, mRNA. |
| LAPTM4A | 2.41 | Homo sapiens lysosomal-associated protein transmembrane 4 alpha (LAPTM4A), mRNA. |
| LOC646766 | 2.4 | PREDICTED: Homo sapiens similar to 60S ribosomal protein L35 (LOC646766), mRNA. |
| BLVRA | 2.39 | Homo sapiens biliverdin reductase A (BLVRA), mRNA. |
| PAIP2 | 2.38 | Homo sapiens poly(A) binding protein interacting protein 2 (PAIP2), transcript variant 2, mRNA. |
| PRR3 | 2.38 | Homo sapiens proline rich 3 (PRR3), transcript variant 1, mRNA. |
| EXOSC9 | 2.38 | Homo sapiens exosome component 9 (EXOSC9), transcript variant 1, mRNA. |
| LOC440055 | 2.37 | PREDICTED: Homo sapiens similar to ribosomal protein S12 (LOC440055), mRNA. |
| HN1 | 2.37 | Homo sapiens hematological and neurological expressed 1 (HN1), transcript variant 1, mRNA. |
| MRPL24 | 2.37 | Homo sapiens mitochondrial ribosomal protein L24 (MRPL24), nuclear gene encoding mitochondrial protein, transcript variant 2, mRNA. |
| PTRF | 2.37 | Homo sapiens polymerase I and transcript release factor (PTRF), mRNA. |
| LOC440733 | 2.37 | PREDICTED: Homo sapiens similar to 40S ribosomal protein S15 (RIG protein) (LOC440733), mRNA. |
| MAGEA6 | 2.37 | Homo sapiens melanoma antigen family A, 6 (MAGEA6), transcript variant 2, mRNA. |
| LOC647673 | 2.36 | PREDICTED: Homo sapiens similar to Translationally-controlled tumor protein (TCTP) (p23) (Histamine-releasing factor) (HRF) (Fortilin) (LOC647673), mRNA. |
| HNRNPH2 | 2.36 | Homo sapiens heterogeneous nuclear ribonucleoprotein H2 (H') (HNRNPH2), transcript variant 1, mRNA. |
|  | 2.36 | full-length cDNA clone XCL0BB001ZD04 of Neuroblastoma of Homo sapiens (human) |
| TRAPPC6A | 2.36 | Homo sapiens trafficking protein particle complex 6A (TRAPPC6A), mRNA. |
| LOC643035 | 2.35 | PREDICTED: Homo sapiens similar to CG33096-PB, isoform B, transcript variant 2 (LOC643035), mRNA. |
| LOC285053 | 2.35 | PREDICTED: Homo sapiens similar to ribosomal protein L18a, transcript variant 1 (LOC285053), mRNA. |
| PSMA4 | 2.35 | Homo sapiens proteasome (prosome, macropain) subunit, alpha type, 4 (PSMA4), mRNA. |
| BAT3 | 2.35 | Homo sapiens HLA-B associated transcript 3 (BAT3), transcript variant 2, mRNA. |
| FTSJ3 | 2.35 | Homo sapiens FtsJ homolog 3 (E. coli) (FTSJ3), mRNA. |
| PSMD12 | 2.34 | PREDICTED: Homo sapiens proteasome (prosome, macropain) 26S subunit, non-ATPase, 12 (PSMD12), mRNA. |
| TMEM120A | 2.34 | Homo sapiens transmembrane protein 120A (TMEM120A), mRNA. |
| RNF219 | 2.33 | Homo sapiens ring finger protein 219 (RNF219), mRNA. |
| MAN2B2 | 2.33 | Homo sapiens mannosidase, alpha, class 2B, member 2 (MAN2B2), mRNA. |
| RPL12 | 2.33 | Homo sapiens ribosomal protein L12 (RPL12), mRNA. |
| LDHB | 2.32 | Homo sapiens lactate dehydrogenase B (LDHB), mRNA. |
| FBXL12 | 2.32 | Homo sapiens F-box and leucine-rich repeat protein 12 (FBXL12), mRNA. |
| HAND2 | 2.32 | Homo sapiens heart and neural crest derivatives expressed 2 (HAND2), mRNA. |
| NDUFB10 | 2.31 | Homo sapiens NADH dehydrogenase (ubiquinone) 1 beta subcomplex, 10, 22kDa (NDUFB10), mRNA. |
| MPST | 2.31 | Homo sapiens mercaptopyruvate sulfurtransferase (MPST), nuclear gene encoding mitochondrial protein, transcript variant 1, mRNA. |
| FTHL11 | 2.31 | Homo sapiens ferritin, heavy polypeptide-like 11 (FTHL11) on chromosome 8. |
| TCP1 | 2.31 | Homo sapiens t-complex 1 (TCP1), transcript variant 1, mRNA. |
| C7orf68 | 2.3 | Homo sapiens chromosome 7 open reading frame 68 (C7orf68), transcript variant 1, mRNA. |
| LOC644934 | 2.29 | PREDICTED: Homo sapiens similar to 40S ribosomal protein S26, transcript variant 1 (LOC644934), mRNA. |
| USF2 | 2.29 | Homo sapiens upstream transcription factor 2, c-fos interacting (USF2), transcript variant 2, mRNA. |
| RPS18 | 2.29 | Homo sapiens ribosomal protein S18 (RPS18), mRNA. |
| RPS14 | 2.29 | Homo sapiens ribosomal protein S14 (RPS14), transcript variant 1, mRNA. |
| RPL11 | 2.28 | Homo sapiens ribosomal protein L11 (RPL11), mRNA. |
| GANAB | 2.28 | Homo sapiens glucosidase, alpha; neutral AB (GANAB), transcript variant 2, mRNA. |
| LOC648057 | 2.28 | PREDICTED: Homo sapiens similar to Guanine nucleotide-binding protein G(s), alpha subunit (Adenylate cyclase-stimulating G alpha protein) (G-alpha-8) (LOC648057), mRNA. |
| MARS | 2.28 | Homo sapiens methionyl-tRNA synthetase (MARS), mRNA. |
| LOC654194 | 2.27 | PREDICTED: Homo sapiens similar to ribosomal protein S27 (LOC654194), mRNA. |
| SIGIRR | 2.27 | PREDICTED: Homo sapiens single immunoglobulin and toll-interleukin 1 receptor (TIR) domain (SIGIRR), mRNA. |
| PCDHGB6 | 2.25 | Homo sapiens protocadherin gamma subfamily B, 6 (PCDHGB6), transcript variant 1, mRNA. |
| TIMM8B | 2.25 | Homo sapiens translocase of inner mitochondrial membrane 8 homolog B (yeast) (TIMM8B), nuclear gene encoding mitochondrial protein, mRNA. |
| RPS3A | 2.25 | Homo sapiens ribosomal protein S3A (RPS3A), mRNA. |
| CHP | 2.24 | Homo sapiens calcium binding protein P22 (CHP), mRNA. |
| RPS29 | 2.24 | Homo sapiens ribosomal protein S29 (RPS29), transcript variant 2, mRNA. |
| DSCC1 | 2.24 | Homo sapiens defective in sister chromatid cohesion 1 homolog (S. cerevisiae) (DSCC1), mRNA. |
| GLT8D1 | 2.24 | Homo sapiens glycosyltransferase 8 domain containing 1 (GLT8D1), transcript variant 2, mRNA. |
| SMU1 | 2.24 | Homo sapiens smu-1 suppressor of mec-8 and unc-52 homolog (C. elegans) (SMU1), mRNA. |
| C16orf13 | 2.24 | Homo sapiens chromosome 16 open reading frame 13 (C16orf13), transcript variant 3, mRNA. |
| GABPB2 | 2.24 | Homo sapiens GA binding protein transcription factor, beta subunit 2 (GABPB2), mRNA. |
| C12orf35 | 2.24 | Homo sapiens chromosome 12 open reading frame 35 (C12orf35), mRNA. |
| DAPP1 | 2.23 | Homo sapiens dual adaptor of phosphotyrosine and 3-phosphoinositides (DAPP1), mRNA. |
| ZNF629 | 2.23 | PREDICTED: Homo sapiens zinc finger protein 629 (ZNF629), mRNA. |
| RPS4X | 2.23 | Homo sapiens ribosomal protein S4, X-linked (RPS4X), mRNA. |
| RPS14 | 2.23 | Homo sapiens ribosomal protein S14 (RPS14), transcript variant 2, mRNA. |
| RPS6 | 2.22 | Homo sapiens ribosomal protein S6 (RPS6), mRNA. |
| CAND1 | 2.22 | Homo sapiens cullin-associated and neddylation-dissociated 1 (CAND1), mRNA. |
| LOC646197 | 2.21 | PREDICTED: Homo sapiens similar to Heat shock protein HSP 90-beta (HSP 84) (Tumor-specific transplantation 84 kDa antigen) (TSTA) (LOC646197), mRNA. |
| IFP38 | 2.21 | Homo sapiens IFP38 (IFP38), mRNA. |
| POLR2H | 2.2 | Homo sapiens polymerase (RNA) II (DNA directed) polypeptide H (POLR2H), mRNA. |
| NHP2 | 2.2 | Homo sapiens NHP2 ribonucleoprotein homolog (yeast) (NHP2), transcript variant 2, mRNA. |
| FBXO2 | 2.2 | Homo sapiens F-box protein 2 (FBXO2), mRNA. |
| SHOC2 | 2.2 | Homo sapiens soc-2 suppressor of clear homolog (C. elegans) (SHOC2), mRNA. |
| CCDC72 | 2.19 | Homo sapiens coiled-coil domain containing 72 (CCDC72), mRNA. |
| SEMA4C | 2.19 | Homo sapiens sema domain, immunoglobulin domain (Ig), transmembrane domain (TM) and short cytoplasmic domain, (semaphorin) 4C (SEMA4C), mRNA. |
| RPS17 | 2.19 | Homo sapiens ribosomal protein S17 (RPS17), mRNA. |
| VTI1B | 2.19 | Homo sapiens vesicle transport through interaction with t-SNAREs homolog 1B (yeast) (VTI1B), mRNA. |
| C6orf203 | 2.19 | Homo sapiens chromosome 6 open reading frame 203 (C6orf203), mRNA. |
| RPS13 | 2.19 | Homo sapiens ribosomal protein S13 (RPS13), mRNA. |
| ARL6IP4 | 2.18 | Homo sapiens ADP-ribosylation-like factor 6 interacting protein 4 (ARL6IP4), transcript variant 1, mRNA. |
| SARS | 2.18 | Homo sapiens seryl-tRNA synthetase (SARS), mRNA. |
| BANP | 2.18 | Homo sapiens BTG3 associated nuclear protein (BANP), transcript variant 2, mRNA. |
| RPL3 | 2.18 | Homo sapiens ribosomal protein L3 (RPL3), transcript variant 2, mRNA. |
| CD82 | 2.18 | Homo sapiens CD82 molecule (CD82), transcript variant 2, mRNA. |
| LOC130773 | 2.17 | PREDICTED: Homo sapiens similar to 60S ribosomal protein L23a (LOC130773), mRNA. |
| AKR1D1 | 2.17 | Homo sapiens aldo-keto reductase family 1, member D1 (delta 4-3-ketosteroid-5-beta-reductase) (AKR1D1), mRNA. |
| NSUN6 | 2.17 | Homo sapiens NOL1/NOP2/Sun domain family, member 6 (NSUN6), mRNA. |
| LOC646135 | 2.17 | PREDICTED: Homo sapiens hypothetical protein LOC646135 (LOC646135), mRNA. |
| MGC16703 | 2.16 | Homo sapiens alpha tubulin-like (MGC16703), mRNA. |
| C17orf95 | 2.16 | Homo sapiens chromosome 17 open reading frame 95 (C17orf95), mRNA. |
| TNIP2 | 2.16 | Homo sapiens TNFAIP3 interacting protein 2 (TNIP2), mRNA. |
| MRPS12 | 2.16 | Homo sapiens mitochondrial ribosomal protein S12 (MRPS12), nuclear gene encoding mitochondrial protein, transcript variant 3, mRNA. |
| DYNLRB1 | 2.15 | Homo sapiens dynein, light chain, roadblock-type 1 (DYNLRB1), mRNA. |
| LOC648622 | 2.15 | PREDICTED: Homo sapiens similar to ribosomal protein S27 (LOC648622), mRNA. |
| RPS15 | 2.15 | Homo sapiens ribosomal protein S15 (RPS15), mRNA. |
| SET | 2.14 | Homo sapiens SET translocation (myeloid leukemia-associated) (SET), mRNA. |
| LOC389435 | 2.14 | Homo sapiens hCG21078 (LOC389435), mRNA. |
| NGFRAP1 | 2.13 | Homo sapiens nerve growth factor receptor (TNFRSF16) associated protein 1 (NGFRAP1), transcript variant 1, mRNA. |
| LOC134997 | 2.13 | Homo sapiens peptidylprolyl isomerase A processed pseudogene (LOC134997) on chromosome 6. |
| PTK7 | 2.13 | Homo sapiens PTK7 protein tyrosine kinase 7 (PTK7), transcript variant PTK7-2, mRNA. |
| MSH3 | 2.13 | Homo sapiens mutS homolog 3 (E. coli) (MSH3), mRNA. |
| LOC731640 | 2.13 | PREDICTED: Homo sapiens similar to 60S ribosomal protein L21, transcript variant 2 (LOC731640), mRNA. |
| PSMD13 | 2.12 | Homo sapiens proteasome (prosome, macropain) 26S subunit, non-ATPase, 13 (PSMD13), transcript variant 2, mRNA. |
| ORC6L | 2.12 | Homo sapiens origin recognition complex, subunit 6 like (yeast) (ORC6L), mRNA. |
| UBA2 | 2.12 | Homo sapiens ubiquitin-like modifier activating enzyme 2 (UBA2), mRNA. |
| UNC119 | 2.11 | Homo sapiens unc-119 homolog (C. elegans) (UNC119), transcript variant 2, mRNA. |
| C8orf41 | 2.11 | Homo sapiens chromosome 8 open reading frame 41 (C8orf41), mRNA. |
| SLC25A14 | 2.11 | Homo sapiens solute carrier family 25 (mitochondrial carrier, brain), member 14 (SLC25A14), nuclear gene encoding mitochondrial protein, transcript variant long, mRNA. |
| GRAMD1A | 2.1 | Homo sapiens GRAM domain containing 1A (GRAMD1A), mRNA. |
| RPL5 | 2.1 | Homo sapiens ribosomal protein L5 (RPL5), mRNA. |
| IL10 | 2.1 | Homo sapiens interleukin 10 (IL10), mRNA. |
| RTN2 | 2.1 | Homo sapiens reticulon 2 (RTN2), transcript variant 4, mRNA. |
| SLC35F3 | 2.09 | PREDICTED: Homo sapiens solute carrier family 35, member F3 (SLC35F3), mRNA. |
| NELF | 2.09 | Homo sapiens nasal embryonic LHRH factor (NELF), mRNA. |
| ESRRAP2 | 2.08 | Homo sapiens estrogen-related receptor alpha pseudogene 2 (ESRRAP2) on chromosome 13. |
| HLA-H | 2.08 | Homo sapiens major histocompatibility complex, class I, H (pseudogene) (HLA-H), non-coding RNA. |
| TAF15 | 2.08 | Homo sapiens TAF15 RNA polymerase II, TATA box binding protein (TBP)-associated factor, 68kDa (TAF15), transcript variant 2, mRNA. |
| RPL30 | 2.08 | Homo sapiens ribosomal protein L30 (RPL30), mRNA. |
| NEUROG2 | 2.08 | Homo sapiens neurogenin 2 (NEUROG2), mRNA. |
|  | 2.08 | 602538216F1 NIH_MGC_59 Homo sapiens cDNA clone IMAGE:4659674 5, mRNA sequence |
| TIMM17B | 2.08 | Homo sapiens translocase of inner mitochondrial membrane 17 homolog B (yeast) (TIMM17B), nuclear gene encoding mitochondrial protein, mRNA. |
| TCP1 | 2.08 | Homo sapiens t-complex 1 (TCP1), transcript variant 1, mRNA. |
| RPLP0 | 2.07 | Homo sapiens ribosomal protein, large, P0 (RPLP0), transcript variant 1, mRNA. |
| MATR3 | 2.07 | Homo sapiens matrin 3 (MATR3), transcript variant 1, mRNA. |
| RUFY3 | 2.07 | Homo sapiens RUN and FYVE domain containing 3 (RUFY3), transcript variant 1, mRNA. |
| RHOC | 2.07 | Homo sapiens ras homolog gene family, member C (RHOC), transcript variant 2, mRNA. |
| F2R | 2.06 | Homo sapiens coagulation factor II (thrombin) receptor (F2R), mRNA. |
| RPS4X | 2.06 | Homo sapiens ribosomal protein S4, X-linked (RPS4X), mRNA. |
| TMED2 | 2.05 | Homo sapiens transmembrane emp24 domain trafficking protein 2 (TMED2), mRNA. |
| FKBP4 | 2.05 | Homo sapiens FK506 binding protein 4, 59kDa (FKBP4), mRNA. |
| SLC27A3 | 2.05 | Homo sapiens solute carrier family 27 (fatty acid transporter), member 3 (SLC27A3), mRNA. |
| ITIH5 | 2.05 | Homo sapiens inter-alpha (globulin) inhibitor H5 (ITIH5), transcript variant 3, mRNA. |
| GPR89C | 2.04 | Homo sapiens G protein-coupled receptor 89C (GPR89C), mRNA. |
| ATP5J | 2.04 | Homo sapiens ATP synthase, H+ transporting, mitochondrial F0 complex, subunit F6 (ATP5J), nuclear gene encoding mitochondrial protein, transcript variant 1, mRNA. |
|  | 2.04 | UI-E-CK1-agb-m-11-0-UI.s1 UI-E-CK1 Homo sapiens cDNA clone UI-E-CK1-agb-m-11-0-UI 3, mRNA sequence |
| LOC647037 | 2.04 | PREDICTED: Homo sapiens similar to Peptidyl-prolyl cis-trans isomerase NIMA-interacting 4 (Rotamase Pin4) (PPIase Pin4) (Parvulin 14) (Par14) (Peptidyl-prolyl cis/trans isomerase EPVH) (hPar14) (LOC647037), mRNA. |
| CCR6 | 2.04 | Homo sapiens chemokine (C-C motif) receptor 6 (CCR6), transcript variant 2, mRNA. |
| FOXO4 | 2.03 | Homo sapiens forkhead box O4 (FOXO4), mRNA. |
| SHCBP1 | 2.03 | Homo sapiens SHC SH2-domain binding protein 1 (SHCBP1), mRNA. |
| VAV2 | 2.03 | Homo sapiens vav 2 guanine nucleotide exchange factor (VAV2), mRNA. |
| JPH2 | 2.03 | Homo sapiens junctophilin 2 (JPH2), transcript variant 2, mRNA. |
| MAP3K12 | 2.03 | Homo sapiens mitogen-activated protein kinase kinase kinase 12 (MAP3K12), mRNA. |
| RPLP2 | 2.03 | Homo sapiens ribosomal protein, large, P2 (RPLP2), mRNA. |
| HK2 | 2.03 | Homo sapiens hexokinase 2 (HK2), mRNA. |
| ZNF408 | 2.02 | Homo sapiens zinc finger protein 408 (ZNF408), mRNA. |
| LOC644330 | 2.02 | PREDICTED: Homo sapiens similar to tropomyosin 3 isoform 2 (LOC644330), mRNA. |
| ATP8B2 | 2.02 | Homo sapiens ATPase, class I, type 8B, member 2 (ATP8B2), transcript variant 1, mRNA. |
| RPL14 | 2.02 | Homo sapiens ribosomal protein L14 (RPL14), transcript variant 1, mRNA. |
| C1GALT1C1 | 2.01 | Homo sapiens C1GALT1-specific chaperone 1 (C1GALT1C1), transcript variant 1, mRNA. |
| LOC649049 | 2.01 | PREDICTED: Homo sapiens similar to acidic ribosomal phosphoprotein P0, transcript variant 3 (LOC649049), mRNA. |
| NOP58 | 2.01 | Homo sapiens NOP58 ribonucleoprotein homolog (yeast) (NOP58), mRNA. |
| MRPS21 | 2.01 | Homo sapiens mitochondrial ribosomal protein S21 (MRPS21), nuclear gene encoding mitochondrial protein, transcript variant 2, mRNA. |
| PSEN1 | 2.01 | Homo sapiens presenilin 1 (Alzheimer disease 3) (PSEN1), mRNA. |
| RPL35 | 2.01 | Homo sapiens ribosomal protein L35 (RPL35), mRNA. |
| RNF167 | 2.01 | Homo sapiens ring finger protein 167 (RNF167), mRNA. |
| UBE2V1 | 2 | Homo sapiens ubiquitin-conjugating enzyme E2 variant 1 (UBE2V1), transcript variant 4, mRNA. |
| LOC440396 | 2 | Homo sapiens similar to Heterogeneous nuclear ribonucleoprotein A1 (Helix-destabilizing protein) (Single-strand binding protein) (hnRNP core protein A1) (HDP-1) (Topoisomerase-inhibitor suppressed) (LOC440396), non-coding RNA. |
| METT5D1 | 2 | Homo sapiens methyltransferase 5 domain containing 1 (METT5D1), mRNA. |
| AGRN | 2 | Homo sapiens agrin (AGRN), mRNA. |
| DDOST | 1.99 | Homo sapiens dolichyl-diphosphooligosaccharide-protein glycosyltransferase (DDOST), mRNA. |
| ZNF419 | 1.99 | Homo sapiens zinc finger protein 419 (ZNF419), transcript variant 6, mRNA. |
| C16orf48 | 1.98 | Homo sapiens chromosome 16 open reading frame 48 (C16orf48), mRNA. |
| LOC653972 | 1.98 | PREDICTED: Homo sapiens similar to chromobox homolog 3 (LOC653972), mRNA. |
| RPS2 | 1.98 | Homo sapiens ribosomal protein S2 (RPS2), mRNA. |
| LOC347376 | 1.98 | PREDICTED: Homo sapiens similar to H3 histone, family 3B (LOC347376), mRNA. |
| KIAA1285 | 1.98 | Homo sapiens KIAA1285 protein (KIAA1285), mRNA. |
| RRP8 | 1.97 | Homo sapiens ribosomal RNA processing 8, methyltransferase, homolog (yeast) (RRP8), mRNA. |
| C1orf43 | 1.97 | Homo sapiens chromosome 1 open reading frame 43 (C1orf43), transcript variant 1, mRNA. |
| YIF1B | 1.97 | Homo sapiens Yip1 interacting factor homolog B (S. cerevisiae) (YIF1B), transcript variant 4, mRNA. |
| PMVK | 1.96 | Homo sapiens phosphomevalonate kinase (PMVK), mRNA. |
| SULT1A4 | 1.96 | Homo sapiens sulfotransferase family, cytosolic, 1A, phenol-preferring, member 4 (SULT1A4), transcript variant 3, mRNA. |
| PRDM4 | 1.96 | Homo sapiens PR domain containing 4 (PRDM4), mRNA. |
| MANEAL | 1.96 | Homo sapiens mannosidase, endo-alpha-like (MANEAL), transcript variant 1, mRNA. |
| HCFC1R1 | 1.96 | Homo sapiens host cell factor C1 regulator 1 (XPO1 dependent) (HCFC1R1), transcript variant 3, mRNA. |
| LOC645676 | 1.96 | PREDICTED: Homo sapiens hypothetical protein LOC645676, transcript variant 1 (LOC645676), mRNA. |
| PFN1 | 1.96 | Homo sapiens profilin 1 (PFN1), mRNA. |
| C1orf63 | 1.96 | Homo sapiens chromosome 1 open reading frame 63 (C1orf63), mRNA. |
| UBC | 1.96 | Homo sapiens ubiquitin C (UBC), mRNA. |
| LOC650152 | 1.95 | PREDICTED: Homo sapiens similar to tropomyosin 3 isoform 2 (LOC650152), mRNA. |
| LOC653232 | 1.95 | PREDICTED: Homo sapiens hypothetical LOC653232, transcript variant 4 (LOC653232), mRNA. |
| ADAM15 | 1.95 | PREDICTED: Homo sapiens ADAM metallopeptidase domain 15 (metargidin) (ADAM15), mRNA. |
| SLC33A1 | 1.95 | Homo sapiens solute carrier family 33 (acetyl-CoA transporter), member 1 (SLC33A1), mRNA. |
| PSME2 | 1.95 | Homo sapiens proteasome (prosome, macropain) activator subunit 2 (PA28 beta) (PSME2), mRNA. |
| C17orf101 | 1.94 | Homo sapiens chromosome 17 open reading frame 101 (C17orf101), transcript variant 2, mRNA. |
| NACAP1 | 1.94 | Homo sapiens nascent-polypeptide-associated complex alpha polypeptide pseudogene 1 (NACAP1), non-coding RNA. |
| KCNQ2 | 1.94 | Homo sapiens potassium voltage-gated channel, KQT-like subfamily, member 2 (KCNQ2), transcript variant 5, mRNA. |
| LOC643287 | 1.94 | PREDICTED: Homo sapiens similar to prothymosin alpha, transcript variant 1 (LOC643287), mRNA. |
| THRAP3 | 1.94 | Homo sapiens thyroid hormone receptor associated protein 3 (THRAP3), mRNA. |
| ROCK2 | 1.94 | Homo sapiens Rho-associated, coiled-coil containing protein kinase 2 (ROCK2), mRNA. |
| MAD2L1BP | 1.94 | Homo sapiens MAD2L1 binding protein (MAD2L1BP), transcript variant 2, mRNA. |
| CDK7 | 1.94 | Homo sapiens cyclin-dependent kinase 7 (MO15 homolog, Xenopus laevis, cdk-activating kinase) (CDK7), mRNA. |
| RPL18 | 1.94 | Homo sapiens ribosomal protein L18 (RPL18), mRNA. |
| NAB2 | 1.93 | Homo sapiens NGFI-A binding protein 2 (EGR1 binding protein 2) (NAB2), mRNA. |
| FRAG1 | 1.93 | Homo sapiens FGF receptor activating protein 1 (FRAG1), mRNA. |
| ZNF248 | 1.92 | Homo sapiens zinc finger protein 248 (ZNF248), mRNA. |
| ZNF513 | 1.92 | Homo sapiens zinc finger protein 513 (ZNF513), mRNA. |
| LOC641849 | 1.92 | PREDICTED: Homo sapiens similar to 40S ribosomal protein S3a (V-fos transformation effector protein) (LOC641849), mRNA. |
| MORF4L1 | 1.92 | Homo sapiens mortality factor 4 like 1 (MORF4L1), transcript variant 2, mRNA. |
| COLEC11 | 1.92 | Homo sapiens collectin sub-family member 11 (COLEC11), transcript variant 1, mRNA. |
| REEP2 | 1.92 | Homo sapiens receptor accessory protein 2 (REEP2), mRNA. |
| WSB2 | 1.91 | Homo sapiens WD repeat and SOCS box-containing 2 (WSB2), mRNA. |
| TMED1 | 1.91 | Homo sapiens transmembrane emp24 protein transport domain containing 1 (TMED1), mRNA. |
| SDC2 | 1.91 | Homo sapiens syndecan 2 (SDC2), mRNA. |
| EDF1 | 1.9 | Homo sapiens endothelial differentiation-related factor 1 (EDF1), transcript variant alpha, mRNA. |
| PRDX1 | 1.9 | Homo sapiens peroxiredoxin 1 (PRDX1), transcript variant 2, mRNA. |
| LOC652864 | 1.9 | PREDICTED: Homo sapiens similar to Mitochondrial import inner membrane translocase subunit Tim23 (LOC652864), mRNA. |
| RFP | 1.9 | Homo sapiens ret finger protein (RFP), transcript variant beta, mRNA. |
| FKBP1A | 1.9 | Homo sapiens FK506 binding protein 1A, 12kDa (FKBP1A), transcript variant 12A, mRNA. |
| LOC347544 | 1.89 | PREDICTED: Homo sapiens similar to ribosomal protein L18a (LOC347544), mRNA. |
| C7orf59 | 1.89 | Homo sapiens chromosome 7 open reading frame 59 (C7orf59), mRNA. |
| ZBTB8OS | 1.89 | Homo sapiens zinc finger and BTB domain containing 8 opposite strand (ZBTB8OS), mRNA. |
| HSPA1B | 1.89 | Homo sapiens heat shock 70kDa protein 1B (HSPA1B), mRNA. |
| LOC388654 | 1.89 | PREDICTED: Homo sapiens similar to laminin receptor 1 (ribosomal protein SA) (LOC388654), mRNA. |
| LMNA | 1.89 | Homo sapiens lamin A/C (LMNA), transcript variant 2, mRNA. |
| LPGAT1 | 1.89 | Homo sapiens lysophosphatidylglycerol acyltransferase 1 (LPGAT1), mRNA. |
| ARPC4 | 1.89 | Homo sapiens actin related protein 2/3 complex, subunit 4, 20kDa (ARPC4), transcript variant 2, mRNA. |
| MRPL38 | 1.88 | Homo sapiens mitochondrial ribosomal protein L38 (MRPL38), nuclear gene encoding mitochondrial protein, mRNA. |
| HSP90AA1 | 1.88 | Homo sapiens heat shock protein 90kDa alpha (cytosolic), class A member 1 (HSP90AA1), transcript variant 1, mRNA. |
|  | 1.87 | Homo sapiens cDNA FLJ38860 fis, clone MESAN2011977 |
| RPL21 | 1.87 | Homo sapiens ribosomal protein L21 (RPL21), mRNA. |
| EXOSC3 | 1.87 | Homo sapiens exosome component 3 (EXOSC3), transcript variant 1, mRNA. |
| ACAA2 | 1.87 | Homo sapiens acetyl-Coenzyme A acyltransferase 2 (mitochondrial 3-oxoacyl-Coenzyme A thiolase) (ACAA2), nuclear gene encoding mitochondrial protein, mRNA. |
| RPL32 | 1.87 | Homo sapiens ribosomal protein L32 (RPL32), transcript variant 3, mRNA. |
| RPS9 | 1.86 | Homo sapiens ribosomal protein S9 (RPS9), mRNA. |
| FDPS | 1.86 | Homo sapiens farnesyl diphosphate synthase (farnesyl pyrophosphate synthetase, dimethylallyltranstransferase, geranyltranstransferase) (FDPS), mRNA. |
| LOC127295 | 1.86 | PREDICTED: Homo sapiens similar to 60S ribosomal protein L36 (LOC127295), mRNA. |
| LOC642989 | 1.86 | PREDICTED: Homo sapiens similar to 40S ribosomal protein S25 (LOC642989), mRNA. |
| PRICKLE4 | 1.86 | Homo sapiens prickle homolog 4 (Drosophila) (PRICKLE4), mRNA. |
| AADACL1 | 1.86 | Homo sapiens arylacetamide deacetylase-like 1 (AADACL1), mRNA. |
| RANBP1 | 1.86 | Homo sapiens RAN binding protein 1 (RANBP1), mRNA. |
| TERF2IP | 1.86 | Homo sapiens telomeric repeat binding factor 2, interacting protein (TERF2IP), mRNA. |
| FKBP1A | 1.86 | Homo sapiens FK506 binding protein 1A, 12kDa (FKBP1A), transcript variant 12A, mRNA. |
| LOC388122 | 1.86 | PREDICTED: Homo sapiens hypothetical LOC388122 (LOC388122), mRNA. |
| SFPQ | 1.85 | Homo sapiens splicing factor proline/glutamine-rich (polypyrimidine tract binding protein associated) (SFPQ), mRNA. |
| LOC402251 | 1.85 | PREDICTED: Homo sapiens similar to eukaryotic translation elongation factor 1 alpha 2 (LOC402251), mRNA. |
| GAPDH | 1.85 | Homo sapiens glyceraldehyde-3-phosphate dehydrogenase (GAPDH), mRNA. |
| ROM1 | 1.84 | Homo sapiens retinal outer segment membrane protein 1 (ROM1), mRNA. |
| LOC645968 | 1.84 | PREDICTED: Homo sapiens similar to 40S ribosomal protein S3a (V-fos transformation effector protein) (LOC645968), mRNA. |
| HNRNPD | 1.84 | Homo sapiens heterogeneous nuclear ribonucleoprotein D (AU-rich element RNA binding protein 1, 37kDa) (HNRNPD), transcript variant 3, mRNA. |
| WBP1 | 1.84 | Homo sapiens WW domain binding protein 1 (WBP1), mRNA. |
| H2AFY | 1.84 | Homo sapiens H2A histone family, member Y (H2AFY), transcript variant 1, mRNA. |
| LOC390354 | 1.84 | PREDICTED: Homo sapiens similar to ribosomal protein L18a; 60S ribosomal protein L18a, transcript variant 36 (LOC390354), misc RNA. |
| ADPRHL2 | 1.84 | Homo sapiens ADP-ribosylhydrolase like 2 (ADPRHL2), mRNA. |
| LOC402221 | 1.83 | PREDICTED: Homo sapiens similar to actin alpha 1 skeletal muscle protein (LOC402221), mRNA. |
| C4orf41 | 1.83 | Homo sapiens chromosome 4 open reading frame 41 (C4orf41), transcript variant 2, mRNA. |
| WDR74 | 1.83 | PREDICTED: Homo sapiens WD repeat domain 74 (WDR74), mRNA. |
| FAM160B2 | 1.83 | Homo sapiens family with sequence similarity 160, member B2 (FAM160B2), mRNA. |
| GSTP1 | 1.83 | Homo sapiens glutathione S-transferase pi (GSTP1), mRNA. |
| LOC652489 | 1.83 | PREDICTED: Homo sapiens similar to SMT3 suppressor of mif two 3 homolog 2 (LOC652489), mRNA. |
| QTRTD1 | 1.82 | Homo sapiens queuine tRNA-ribosyltransferase domain containing 1 (QTRTD1), mRNA. |
| LY6E | 1.82 | Homo sapiens lymphocyte antigen 6 complex, locus E (LY6E), mRNA. |
| UQCRH | 1.82 | Homo sapiens ubiquinol-cytochrome c reductase hinge protein (UQCRH), mRNA. |
| WDR23 | 1.82 | Homo sapiens WD repeat domain 23 (WDR23), transcript variant 1, mRNA. |
| FCGR3B | 1.81 | Homo sapiens Fc fragment of IgG, low affinity IIIb, receptor (CD16b) (FCGR3B), mRNA. |
| ADI1 | 1.81 | Homo sapiens acireductone dioxygenase 1 (ADI1), mRNA. |
| RPL9 | 1.81 | Homo sapiens ribosomal protein L9 (RPL9), transcript variant 2, mRNA. |
| MED16 | 1.81 | Homo sapiens mediator complex subunit 16 (MED16), mRNA. |
| FKBP1B | 1.8 | Homo sapiens FK506 binding protein 1B, 12.6 kDa (FKBP1B), transcript variant 2, mRNA. |
| ARL6IP4 | 1.8 | Homo sapiens ADP-ribosylation-like factor 6 interacting protein 4 (ARL6IP4), transcript variant 3, mRNA. |
| BCL11A | 1.8 | Homo sapiens B-cell CLL/lymphoma 11A (zinc finger protein) (BCL11A), transcript variant 1, mRNA. |
| IER2 | 1.8 | Homo sapiens immediate early response 2 (IER2), mRNA. |
| MGC3032 | 1.8 | PREDICTED: Homo sapiens hypothetical protein MGC3032, transcript variant 3 (MGC3032), mRNA. |
| ANAPC11 | 1.79 | Homo sapiens anaphase promoting complex subunit 11 (ANAPC11), transcript variant 4, mRNA. |
| EIF4G2 | 1.79 | Homo sapiens eukaryotic translation initiation factor 4 gamma, 2 (EIF4G2), transcript variant 1, mRNA. |
| CTXN1 | 1.79 | Homo sapiens cortexin 1 (CTXN1), mRNA. |
| LOC387841 | 1.79 | PREDICTED: Homo sapiens similar to ribosomal protein L13a, transcript variant 1 (LOC387841), mRNA. |
| MAP6 | 1.78 | Homo sapiens microtubule-associated protein 6 (MAP6), transcript variant 1, mRNA. |
| ERH | 1.78 | Homo sapiens enhancer of rudimentary homolog (Drosophila) (ERH), mRNA. |
| LOC389672 | 1.78 | PREDICTED: Homo sapiens similar to 40S ribosomal protein SA (p40) (34/67 kDa laminin receptor) (Colon carcinoma laminin-binding protein) (NEM/1CHD4) (Multidrug resistance-associated protein MGr1-Ag), transcript variant 3 (LOC389672), mRNA. |
| ZNF358 | 1.78 | Homo sapiens zinc finger protein 358 (ZNF358), mRNA. |
| KIAA0101 | 1.78 | Homo sapiens KIAA0101 (KIAA0101), transcript variant 2, mRNA. |
| C11orf10 | 1.78 | Homo sapiens chromosome 11 open reading frame 10 (C11orf10), mRNA. |
| CALM3 | 1.78 | Homo sapiens calmodulin 3 (phosphorylase kinase, delta) (CALM3), mRNA. |
| CCBE1 | 1.78 | Homo sapiens collagen and calcium binding EGF domains 1 (CCBE1), mRNA. |
| CUL1 | 1.77 | Homo sapiens cullin 1 (CUL1), mRNA. |
| BBS10 | 1.77 | Homo sapiens Bardet-Biedl syndrome 10 (BBS10), mRNA. |
| YY1 | 1.76 | Homo sapiens YY1 transcription factor (YY1), mRNA. |
| RNF122 | 1.76 | Homo sapiens ring finger protein 122 (RNF122), mRNA. |
| REXO4 | 1.76 | Homo sapiens REX4, RNA exonuclease 4 homolog (S. cerevisiae) (REXO4), mRNA. |
| PI4K2B | 1.76 | Homo sapiens phosphatidylinositol 4-kinase type 2 beta (PI4K2B), mRNA. |
| SULT1A1 | 1.76 | Homo sapiens sulfotransferase family, cytosolic, 1A, phenol-preferring, member 1 (SULT1A1), transcript variant 5, mRNA. |
| PIK3C3 | 1.76 | Homo sapiens phosphoinositide-3-kinase, class 3 (PIK3C3), mRNA. |
| ATP5L | 1.76 | Homo sapiens ATP synthase, H+ transporting, mitochondrial F0 complex, subunit G (ATP5L), nuclear gene encoding mitochondrial protein, mRNA. |
| LOC91561 | 1.75 | PREDICTED: Homo sapiens similar to ribosomal protein S2, transcript variant 3 (LOC91561), mRNA. |
| ARF4 | 1.75 | Homo sapiens ADP-ribosylation factor 4 (ARF4), mRNA. |
| PFDN5 | 1.75 | Homo sapiens prefoldin subunit 5 (PFDN5), transcript variant 1, mRNA. |
| LOC642946 | 1.75 | PREDICTED: Homo sapiens hypothetical protein LOC642945, transcript variant 1 (LOC642946), mRNA. |
| DECR1 | 1.75 | Homo sapiens 2,4-dienoyl CoA reductase 1, mitochondrial (DECR1), nuclear gene encoding mitochondrial protein, mRNA. |
| RABEPK | 1.75 | Homo sapiens Rab9 effector protein with kelch motifs (RABEPK), mRNA. |
| LOC650276 | 1.74 | PREDICTED: Homo sapiens similar to 60S ribosomal protein L7 (LOC650276), mRNA. |
| SSR2 | 1.74 | Homo sapiens signal sequence receptor, beta (translocon-associated protein beta) (SSR2), mRNA. |
| PTPRU | 1.74 | Homo sapiens protein tyrosine phosphatase, receptor type, U (PTPRU), transcript variant 3, mRNA. |
| C10orf58 | 1.74 | Homo sapiens chromosome 10 open reading frame 58 (C10orf58), mRNA. |
| LOC388621 | 1.74 | PREDICTED: Homo sapiens similar to ribosomal protein L21 (LOC388621), mRNA. |
| C16orf53 | 1.74 | Homo sapiens chromosome 16 open reading frame 53 (C16orf53), mRNA. |
| SNRPB | 1.74 | Homo sapiens small nuclear ribonucleoprotein polypeptides B and B1 (SNRPB), transcript variant 2, mRNA. |
| GPS1 | 1.74 | Homo sapiens G protein pathway suppressor 1 (GPS1), transcript variant 2, mRNA. |
| SLC26A6 | 1.74 | Homo sapiens solute carrier family 26, member 6 (SLC26A6), transcript variant 3, mRNA. |
| C16orf57 | 1.74 | Homo sapiens chromosome 16 open reading frame 57 (C16orf57), mRNA. |
| IRF2BP2 | 1.74 | Homo sapiens interferon regulatory factor 2 binding protein 2 (IRF2BP2), transcript variant 1, mRNA. |
| TLE1 | 1.74 | Homo sapiens transducin-like enhancer of split 1 (E(sp1) homolog, Drosophila) (TLE1), mRNA. |
| CHCHD9 | 1.74 | PREDICTED: Homo sapiens coiled-coil-helix-coiled-coil-helix domain containing 9 (CHCHD9), mRNA. |
| ONECUT2 | 1.73 | Homo sapiens one cut homeobox 2 (ONECUT2), mRNA. |
| DBI | 1.73 | Homo sapiens diazepam binding inhibitor (GABA receptor modulator, acyl-Coenzyme A binding protein) (DBI), mRNA. |
| GPC2 | 1.73 | Homo sapiens glypican 2 (GPC2), mRNA. |
| FKTN | 1.73 | Homo sapiens fukutin (FKTN), transcript variant 2, mRNA. |
| TSPAN17 | 1.72 | Homo sapiens tetraspanin 17 (TSPAN17), transcript variant 2, mRNA. |
| LOC652071 | 1.72 | PREDICTED: Homo sapiens similar to 60S ribosomal protein L23a (LOC652071), mRNA. |
| RPL17 | 1.72 | Homo sapiens ribosomal protein L17 (RPL17), transcript variant 2, mRNA. |
| RPL4 | 1.72 | Homo sapiens ribosomal protein L4 (RPL4), mRNA. |
| PARD6A | 1.72 | Homo sapiens par-6 partitioning defective 6 homolog alpha (C. elegans) (PARD6A), transcript variant 2, mRNA. |
| DBH | 1.72 | Homo sapiens dopamine beta-hydroxylase (dopamine beta-monooxygenase) (DBH), mRNA. |
| DEDD2 | 1.71 | Homo sapiens death effector domain containing 2 (DEDD2), mRNA. |
| PPIA | 1.71 | Homo sapiens peptidylprolyl isomerase A (cyclophilin A) (PPIA), mRNA. |
| LOC387867 | 1.71 | PREDICTED: Homo sapiens similar to 40S ribosomal protein SA (p40) (34/67 kDa laminin receptor) (Colon carcinoma laminin-binding protein) (NEM/1CHD4) (Multidrug resistance-associated protein MGr1-Ag) (LOC387867), mRNA. |
| PXDN | 1.71 | Homo sapiens peroxidasin homolog (Drosophila) (PXDN), mRNA. XM_935183 XM_935184 XM_936786 XM_942966 XM_942982 XM_942986 XM_942994 XM_943000 |
| ZBED5 | 1.71 | Homo sapiens zinc finger, BED-type containing 5 (ZBED5), mRNA. |
| TRAPPC6B | 1.71 | Homo sapiens trafficking protein particle complex 6B (TRAPPC6B), transcript variant 1, mRNA. |
| MANBAL | 1.71 | Homo sapiens mannosidase, beta A, lysosomal-like (MANBAL), transcript variant 2, mRNA. |
| DHRS13 | 1.7 | Homo sapiens dehydrogenase/reductase (SDR family) member 13 (DHRS13), mRNA. |
| MAT2B | 1.7 | Homo sapiens methionine adenosyltransferase II, beta (MAT2B), transcript variant 1, mRNA. |
| NAT14 | 1.7 | Homo sapiens N-acetyltransferase 14 (GCN5-related, putative) (NAT14), mRNA. |
| LOC644889 | 1.69 | PREDICTED: Homo sapiens similar to large subunit ribosomal protein L36a (LOC644889), mRNA. |
| RPS9 | 1.69 | Homo sapiens ribosomal protein S9 (RPS9), mRNA. |
| AP1S1 | 1.69 | Homo sapiens adaptor-related protein complex 1, sigma 1 subunit (AP1S1), transcript variant 2, mRNA. |
| C8orf55 | 1.69 | Homo sapiens chromosome 8 open reading frame 55 (C8orf55), mRNA. |
| RPS26P10 | 1.69 | PREDICTED: Homo sapiens ribosomal protein S26 pseudogene 10 (RPS26P10), mRNA. |
| LOC645683 | 1.68 | PREDICTED: Homo sapiens similar to ribosomal protein L13a (LOC645683), mRNA. |
| ARSA | 1.68 | Homo sapiens arylsulfatase A (ARSA), mRNA. |
| TMEM44 | 1.68 | Homo sapiens transmembrane protein 44 (TMEM44), transcript variant 1, mRNA. |
| NDUFS2 | 1.68 | Homo sapiens NADH dehydrogenase (ubiquinone) Fe-S protein 2, 49kDa (NADH-coenzyme Q reductase) (NDUFS2), mRNA. |
| LOC650909 | 1.68 | PREDICTED: Homo sapiens similar to activating signal cointegrator 1 complex subunit 3-like 1 (LOC650909), mRNA. |
| SEPHS2 | 1.68 | Homo sapiens selenophosphate synthetase 2 (SEPHS2), mRNA. |
| ACCN2 | 1.68 | Homo sapiens amiloride-sensitive cation channel 2, neuronal (ACCN2), transcript variant 2, mRNA. |
| LOC647000 | 1.68 | PREDICTED: Homo sapiens similar to tubulin, beta 5 (LOC647000), mRNA. |
| RPL36 | 1.67 | Homo sapiens ribosomal protein L36 (RPL36), transcript variant 1, mRNA. |
| COX6A1 | 1.67 | Homo sapiens cytochrome c oxidase subunit VIa polypeptide 1 (COX6A1), nuclear gene encoding mitochondrial protein, mRNA. |
| LOC644310 | 1.67 | PREDICTED: Homo sapiens similar to ubiquinol-cytochrome c reductase complex (LOC644310), mRNA. |
| SFRS1 | 1.67 | Homo sapiens splicing factor, arginine/serine-rich 1 (SFRS1), transcript variant 2, mRNA. |
| SRP14P1 | 1.67 | Homo sapiens signal recognition particle 14kDa (homologous Alu RNA binding protein) pseudogene 1 (SRP14P1), non-coding RNA. |
| RHOB | 1.67 | Homo sapiens ras homolog gene family, member B (RHOB), mRNA. |
| GTF2A2 | 1.67 | Homo sapiens general transcription factor IIA, 2, 12kDa (GTF2A2), mRNA. |
| MCL1 | 1.66 | Homo sapiens myeloid cell leukemia sequence 1 (BCL2-related) (MCL1), transcript variant 1, mRNA. |
| TXNRD2 | 1.66 | Homo sapiens thioredoxin reductase 2 (TXNRD2), nuclear gene encoding mitochondrial protein, mRNA. |
| DUT | 1.66 | Homo sapiens deoxyuridine triphosphatase (DUT), nuclear gene encoding mitochondrial protein, transcript variant 1, mRNA. |
| SLC35C2 | 1.66 | Homo sapiens solute carrier family 35, member C2 (SLC35C2), transcript variant 2, mRNA. |
| ASPHD1 | 1.66 | Homo sapiens aspartate beta-hydroxylase domain containing 1 (ASPHD1), mRNA. |
| LOC644029 | 1.66 | PREDICTED: Homo sapiens similar to 60S ribosomal protein L7a (LOC644029), mRNA. |
| CYB561 | 1.66 | Homo sapiens cytochrome b-561 (CYB561), transcript variant 1, mRNA. |
| ARPC5 | 1.65 | Homo sapiens actin related protein 2/3 complex, subunit 5, 16kDa (ARPC5), mRNA. |
| DBR1 | 1.65 | Homo sapiens debranching enzyme homolog 1 (S. cerevisiae) (DBR1), mRNA. |
| ZNF768 | 1.65 | Homo sapiens zinc finger protein 768 (ZNF768), mRNA. |
| P704P | 1.65 | PREDICTED: Homo sapiens prostate-specific P704P (P704P), mRNA. |
| NEO1 | 1.65 | Homo sapiens neogenin homolog 1 (chicken) (NEO1), mRNA. |
| NKIRAS2 | 1.65 | Homo sapiens NFKB inhibitor interacting Ras-like 2 (NKIRAS2), transcript variant 2, mRNA. |
| LOC645895 | 1.65 | PREDICTED: Homo sapiens hypothetical protein LOC645895 (LOC645895), mRNA. |
| GMPR2 | 1.65 | Homo sapiens guanosine monophosphate reductase 2 (GMPR2), transcript variant 4, mRNA. |
| SLC44A4 | 1.65 | Homo sapiens solute carrier family 44, member 4 (SLC44A4), transcript variant 2, mRNA. |
| LOC650646 | 1.65 | PREDICTED: Homo sapiens similar to 40S ribosomal protein S26 (LOC650646), mRNA. |
| RPL23AP7 | 1.65 | Homo sapiens ribosomal protein L23a pseudogene 7 (RPL23AP7) on chromosome 2. |
| GP1BA | 1.65 | Homo sapiens glycoprotein Ib (platelet), alpha polypeptide (GP1BA), mRNA. |
| EPHB2 | 1.65 | Homo sapiens EPH receptor B2 (EPHB2), transcript variant 2, mRNA. |
| LOC390466 | 1.65 | PREDICTED: Homo sapiens similar to SMT3 suppressor of mif two 3 homolog 2 (LOC390466), mRNA. |
| TAOK2 | 1.64 | Homo sapiens TAO kinase 2 (TAOK2), transcript variant 2, mRNA. |
| C8orf37 | 1.64 | Homo sapiens chromosome 8 open reading frame 37 (C8orf37), mRNA. |
| IFI27L2 | 1.64 | Homo sapiens interferon, alpha-inducible protein 27-like 2 (IFI27L2), mRNA. |
| RPS26L | 1.64 | Homo sapiens 40S ribosomal protein S26-like (RPS26L), non-coding RNA. |
|  | 1.64 | AV700714 GKC Homo sapiens cDNA clone GKCAEH11 3, mRNA sequence |
| FAU | 1.64 | Homo sapiens Finkel-Biskis-Reilly murine sarcoma virus (FBR-MuSV) ubiquitously expressed (FAU), mRNA. |
| LOC653086 | 1.64 | PREDICTED: Homo sapiens similar to RAN-binding protein 2-like 1 isoform 2, transcript variant 10 (LOC653086), mRNA. |
| LOC648249 | 1.64 | PREDICTED: Homo sapiens similar to 40S ribosomal protein SA (p40) (34/67 kDa laminin receptor) (Colon carcinoma laminin-binding protein) (NEM/1CHD4) (Multidrug resistance-associated protein MGr1-Ag), transcript variant 3 (LOC648249), mRNA. |
| LOC391656 | 1.64 | PREDICTED: Homo sapiens similar to hCG1640454 (LOC391656), mRNA. |
| TM7SF3 | 1.64 | Homo sapiens transmembrane 7 superfamily member 3 (TM7SF3), mRNA. |
| LOC649009 | 1.64 | PREDICTED: Homo sapiens similar to paraspeckle protein 1, transcript variant 1 (LOC649009), mRNA. |
| LOC646630 | 1.64 | PREDICTED: Homo sapiens similar to Coiled-coil-helix-coiled-coil-helix domain containing protein 2 (HCV NS2 trans-regulated protein) (NS2TP) (LOC646630), mRNA. |
| KIAA1967 | 1.64 | Homo sapiens KIAA1967 (KIAA1967), transcript variant 1, mRNA. |
| RER1 | 1.63 | Homo sapiens RER1 retention in endoplasmic reticulum 1 homolog (S. cerevisiae) (RER1), mRNA. |
| ACTG1 | 1.63 | Homo sapiens actin, gamma 1 (ACTG1), mRNA. |
| SS18 | 1.63 | Homo sapiens synovial sarcoma translocation, chromosome 18 (SS18), transcript variant 2, mRNA. |
| HSPA8 | 1.63 | Homo sapiens heat shock 70kDa protein 8 (HSPA8), transcript variant 1, mRNA. |
| LOC401115 | 1.63 | PREDICTED: Homo sapiens hypothetical gene supported by BC038466; BC062790 (LOC401115), mRNA. |
| TMSB10 | 1.62 | Homo sapiens thymosin beta 10 (TMSB10), mRNA. |
| SEC61G | 1.62 | Homo sapiens Sec61 gamma subunit (SEC61G), transcript variant 2, mRNA. |
| LOC649548 | 1.62 | PREDICTED: Homo sapiens similar to 60S ribosomal protein L32 (LOC649548), mRNA. |
| NDUFB2 | 1.62 | Homo sapiens NADH dehydrogenase (ubiquinone) 1 beta subcomplex, 2, 8kDa (NDUFB2), nuclear gene encoding mitochondrial protein, mRNA. |
| ACP1 | 1.62 | Homo sapiens acid phosphatase 1, soluble (ACP1), transcript variant 4, mRNA. |
| TUBA1A | 1.62 | Homo sapiens tubulin, alpha 1a (TUBA1A), mRNA. |
| PSMB1 | 1.62 | Homo sapiens proteasome (prosome, macropain) subunit, beta type, 1 (PSMB1), mRNA. |
| LOC643319 | 1.62 | PREDICTED: Homo sapiens similar to Transgelin-2 (LOC643319), mRNA. |
| MAGEA6 | 1.62 | Homo sapiens melanoma antigen family A, 6 (MAGEA6), transcript variant 2, mRNA. |
| LOC645385 | 1.61 | PREDICTED: Homo sapiens similar to heterogeneous nuclear ribonucleoprotein A1 (LOC645385), mRNA. |
|  | 1.61 | xc76e10.x1 NCI_CGAP_Ov32 Homo sapiens cDNA clone IMAGE:2590218 3, mRNA sequence |
| NIPA1 | 1.61 | Homo sapiens non imprinted in Prader-Willi/Angelman syndrome 1 (NIPA1), mRNA. |
| PARK7 | 1.61 | Homo sapiens Parkinson disease (autosomal recessive, early onset) 7 (PARK7), mRNA. |
| TGIF2 | 1.61 | Homo sapiens TGFB-induced factor homeobox 2 (TGIF2), mRNA. |
| SHF | 1.61 | Homo sapiens Src homology 2 domain containing F (SHF), mRNA. |
| NAT15 | 1.61 | Homo sapiens N-acetyltransferase 15 (GCN5-related, putative) (NAT15), transcript variant 1, mRNA. |
| EPM2A | 1.6 | Homo sapiens epilepsy, progressive myoclonus type 2A, Lafora disease (laforin) (EPM2A), transcript variant 1, mRNA. |
| GPKOW | 1.6 | Homo sapiens G patch domain and KOW motifs (GPKOW), mRNA. |
| ZCWPW1 | 1.6 | Homo sapiens zinc finger, CW type with PWWP domain 1 (ZCWPW1), mRNA. |
| LOC642489 | 1.6 | PREDICTED: Homo sapiens similar to FK506-binding protein 1A (LOC642489), mRNA. |
| RPL35A | 1.59 | Homo sapiens ribosomal protein L35a (RPL35A), mRNA. |
| RPS15A | 1.59 | Homo sapiens ribosomal protein S15a (RPS15A), transcript variant 2, mRNA. |
| ASGR1 | 1.59 | Homo sapiens asialoglycoprotein receptor 1 (ASGR1), mRNA. |
| LOC651064 | 1.59 | PREDICTED: Homo sapiens hypothetical protein LOC651064 (LOC651064), mRNA. |
| EIF3L | 1.59 | Homo sapiens eukaryotic translation initiation factor 3, subunit L (EIF3L), mRNA. |
| MAGEL2 | 1.59 | Homo sapiens MAGE-like 2 (MAGEL2), mRNA. |
| LOC646200 | 1.58 | PREDICTED: Homo sapiens similar to 60S ribosomal protein L22 (Heparin binding protein HBp15), transcript variant 1 (LOC646200), mRNA. |
| GAB2 | 1.58 | Homo sapiens GRB2-associated binding protein 2 (GAB2), transcript variant 1, mRNA. |
| FSCN1 | 1.58 | Homo sapiens fascin homolog 1, actin-bundling protein (Strongylocentrotus purpuratus) (FSCN1), mRNA. |
| TOMM22 | 1.58 | Homo sapiens translocase of outer mitochondrial membrane 22 homolog (yeast) (TOMM22), nuclear gene encoding mitochondrial protein, mRNA. |
| PALM | 1.58 | Homo sapiens paralemmin (PALM), transcript variant 1, mRNA. |
| PSMD11 | 1.57 | Homo sapiens proteasome (prosome, macropain) 26S subunit, non-ATPase, 11 (PSMD11), mRNA. |
| PLIN5 | 1.57 | Homo sapiens perilipin 5 (PLIN5), mRNA. |
| STRAP | 1.57 | Homo sapiens serine/threonine kinase receptor associated protein (STRAP), mRNA. |
| LOC730534 | 1.57 | PREDICTED: Homo sapiens similar to D-PCa-2 protein isoform c (LOC730534), mRNA. |
| MIF | 1.57 | Homo sapiens macrophage migration inhibitory factor (glycosylation-inhibiting factor) (MIF), mRNA. |
| TAF9 | 1.57 | Homo sapiens TAF9 RNA polymerase II, TATA box binding protein (TBP)-associated factor, 32kDa (TAF9), transcript variant 3, mRNA. |
| NCL | 1.56 | Homo sapiens nucleolin (NCL), mRNA. |
| LOC644131 | 1.56 | PREDICTED: Homo sapiens similar to chaperonin containing TCP1, subunit 8 (theta) (LOC644131), mRNA. |
| RNPEP | 1.56 | Homo sapiens arginyl aminopeptidase (aminopeptidase B) (RNPEP), mRNA. |
| YIF1A | 1.56 | Homo sapiens Yip1 interacting factor homolog A (S. cerevisiae) (YIF1A), mRNA. |
| SPOCK1 | 1.56 | Homo sapiens sparc/osteonectin, cwcv and kazal-like domains proteoglycan (testican) 1 (SPOCK1), mRNA. |
| UCN | 1.56 | Homo sapiens urocortin (UCN), mRNA. |
| VAT1L | 1.56 | Homo sapiens vesicle amine transport protein 1 homolog (T. californica)-like (VAT1L), mRNA. |
| NDUFA10 | 1.55 | Homo sapiens NADH dehydrogenase (ubiquinone) 1 alpha subcomplex, 10, 42kDa (NDUFA10), nuclear gene encoding mitochondrial protein, mRNA. |
| LOC645317 | 1.55 | PREDICTED: Homo sapiens similar to Coiled-coil-helix-coiled-coil-helix domain-containing protein 2 (HCV NS2 trans-regulated protein) (NS2TP), transcript variant 3 (LOC645317), mRNA. |
| DNPEP | 1.55 | Homo sapiens aspartyl aminopeptidase (DNPEP), mRNA. |
| HIST2H2BE | 1.55 | Homo sapiens histone cluster 2, H2be (HIST2H2BE), mRNA. |
| RPS17 | 1.55 | Homo sapiens ribosomal protein S17 (RPS17), mRNA. |
| PLEKHA6 | 1.55 | Homo sapiens pleckstrin homology domain containing, family A member 6 (PLEKHA6), mRNA. |
| LOC648210 | 1.55 | PREDICTED: Homo sapiens similar to Heterogeneous nuclear ribonucleoprotein A1 (Helix-destabilizing protein) (Single-strand RNA-binding protein) (hnRNP core protein A1) (HDP) (LOC648210), mRNA. |
| ATP5B | 1.55 | Homo sapiens ATP synthase, H+ transporting, mitochondrial F1 complex, beta polypeptide (ATP5B), nuclear gene encoding mitochondrial protein, mRNA. |
| KPNA2 | 1.54 | Homo sapiens karyopherin alpha 2 (RAG cohort 1, importin alpha 1) (KPNA2), mRNA. XM_001133262 XM_001133265 XM_001133267 XM_001133271 |
| UBB | 1.54 | Homo sapiens ubiquitin B (UBB), mRNA. |
| SUMO2 | 1.54 | Homo sapiens SMT3 suppressor of mif two 3 homolog 2 (S. cerevisiae) (SUMO2), transcript variant 2, mRNA. |
| LOC647856 | 1.54 | PREDICTED: Homo sapiens similar to 40S ribosomal protein SA (p40) (34/67 kDa laminin receptor) (Colon carcinoma laminin-binding protein) (NEM/1CHD4) (Multidrug resistance-associated protein MGr1-Ag) (LOC647856), mRNA. |
| RPLP0 | 1.54 | Homo sapiens ribosomal protein, large, P0 (RPLP0), transcript variant 2, mRNA. |
| SUMO2 | 1.54 | Homo sapiens SMT3 suppressor of mif two 3 homolog 2 (S. cerevisiae) (SUMO2), transcript variant 2, mRNA. |
| MRPL32 | 1.53 | Homo sapiens mitochondrial ribosomal protein L32 (MRPL32), nuclear gene encoding mitochondrial protein, mRNA. |
| PPARBP | 1.53 | Homo sapiens PPAR binding protein (PPARBP), mRNA. |
| ARL16 | 1.53 | Homo sapiens ADP-ribosylation factor-like 16 (ARL16), mRNA. |
| GGCT | 1.53 | Homo sapiens gamma-glutamyl cyclotransferase (GGCT), mRNA. |
| FBXL20 | 1.52 | Homo sapiens F-box and leucine-rich repeat protein 20 (FBXL20), mRNA. |
| SH3GL1 | 1.52 | Homo sapiens SH3-domain GRB2-like 1 (SH3GL1), mRNA. |
| PPM1G | 1.52 | Homo sapiens protein phosphatase 1G (formerly 2C), magnesium-dependent, gamma isoform (PPM1G), transcript variant 2, mRNA. |
| MIF4GD | 1.52 | Homo sapiens MIF4G domain containing (MIF4GD), mRNA. |
| ARHGEF2 | 1.52 | Homo sapiens rho/rac guanine nucleotide exchange factor (GEF) 2 (ARHGEF2), mRNA. |
| STMN2 | 1.52 | Homo sapiens stathmin-like 2 (STMN2), mRNA. |
| CALM2 | 1.52 | Homo sapiens calmodulin 2 (phosphorylase kinase, delta) (CALM2), mRNA. |
| PIGU | 1.51 | Homo sapiens phosphatidylinositol glycan anchor biosynthesis, class U (PIGU), mRNA. |
| FOXP1 | 1.51 | Homo sapiens forkhead box P1 (FOXP1), transcript variant 1, mRNA. |
| CLOCK | 1.51 | Homo sapiens clock homolog (mouse) (CLOCK), mRNA. |
| WBP5 | 1.51 | Homo sapiens WW domain binding protein 5 (WBP5), transcript variant 2, mRNA. |
| SERF2 | 1.51 | Homo sapiens small EDRK-rich factor 2 (SERF2), mRNA. |
| APH1A | 1.5 | Homo sapiens anterior pharynx defective 1 homolog A (C. elegans) (APH1A), transcript variant 1, mRNA. |
| PPP1R16A | 1.5 | Homo sapiens protein phosphatase 1, regulatory (inhibitor) subunit 16A (PPP1R16A), mRNA. |
| RPS26L | 1.5 | PREDICTED: Homo sapiens 40S ribosomal protein S26-like (RPS26L), misc RNA. |
| COX6C | 1.5 | Homo sapiens cytochrome c oxidase subunit VIc (COX6C), mRNA. |
| NDUFB3 | 1.5 | Homo sapiens NADH dehydrogenase (ubiquinone) 1 beta subcomplex, 3, 12kDa (NDUFB3), mRNA. |
| ZNF260 | -1.5 | Homo sapiens zinc finger protein 260 (ZNF260), mRNA. |
|  | -1.5 | Homo sapiens cDNA FLJ26539 fis, clone KDN09310 |
| VIP | -1.5 | Homo sapiens vasoactive intestinal peptide (VIP), transcript variant 1, mRNA. |
| FAM108A3 | -1.5 | Homo sapiens family with sequence similarity 108, member A3 (FAM108A3), mRNA. |
| CABC1 | -1.5 | Homo sapiens chaperone, ABC1 activity of bc1 complex homolog (S. pombe) (CABC1), nuclear gene encoding mitochondrial protein, mRNA. |
| SQLE | -1.5 | Homo sapiens squalene epoxidase (SQLE), mRNA. |
| SNX2 | -1.5 | Homo sapiens sorting nexin 2 (SNX2), mRNA. |
| FAM3C | -1.5 | Homo sapiens family with sequence similarity 3, member C (FAM3C), transcript variant 2, mRNA. |
| KCNMB4 | -1.5 | Homo sapiens potassium large conductance calcium-activated channel, subfamily M, beta member 4 (KCNMB4), mRNA. |
| CHTF18 | -1.5 | Homo sapiens CTF18, chromosome transmission fidelity factor 18 homolog (S. cerevisiae) (CHTF18), mRNA. |
|  | -1.5 | Homo sapiens cDNA FLJ37425 fis, clone BRAWH2001530 |
| PVRL3 | -1.5 | Homo sapiens poliovirus receptor-related 3 (PVRL3), mRNA. |
| MRPS30 | -1.5 | Homo sapiens mitochondrial ribosomal protein S30 (MRPS30), nuclear gene encoding mitochondrial protein, mRNA. |
| LOC727761 | -1.5 | PREDICTED: Homo sapiens similar to Deoxythymidylate kinase (thymidylate kinase), transcript variant 4 (LOC727761), mRNA. |
| MED8 | -1.5 | Homo sapiens mediator of RNA polymerase II transcription, subunit 8 homolog (S. cerevisiae) (MED8), transcript variant 1, mRNA. |
| CD46 | -1.51 | Homo sapiens CD46 molecule, complement regulatory protein (CD46), transcript variant m, mRNA. |
| MSL3L1 | -1.51 | Homo sapiens male-specific lethal 3-like 1 (Drosophila) (MSL3L1), transcript variant 1, mRNA. |
| TTC39C | -1.51 | Homo sapiens tetratricopeptide repeat domain 39C (TTC39C), mRNA. |
| CHMP2B | -1.51 | Homo sapiens chromatin modifying protein 2B (CHMP2B), mRNA. |
| CCT4 | -1.51 | Homo sapiens chaperonin containing TCP1, subunit 4 (delta) (CCT4), mRNA. |
| CCNYL1 | -1.51 | Homo sapiens cyclin Y-like 1 (CCNYL1), mRNA. |
| TMEM188 | -1.51 | Homo sapiens transmembrane protein 188 (TMEM188), mRNA. |
| MAP2K5 | -1.51 | Homo sapiens mitogen-activated protein kinase kinase 5 (MAP2K5), transcript variant B, mRNA. |
| LOC643995 | -1.51 | PREDICTED: Homo sapiens similar to Importin alpha-2 subunit (Karyopherin alpha-2 subunit) (SRP1-alpha) (RAG cohort protein 1), transcript variant 2 (LOC643995), mRNA. |
| SLC30A5 | -1.51 | Homo sapiens solute carrier family 30 (zinc transporter), member 5 (SLC30A5), transcript variant 1, mRNA. |
| EFNB2 | -1.51 | Homo sapiens ephrin-B2 (EFNB2), mRNA. |
| KIAA0355 | -1.51 | Homo sapiens KIAA0355 (KIAA0355), mRNA. |
| PPP2R5E | -1.51 | Homo sapiens protein phosphatase 2, regulatory subunit B', epsilon isoform (PPP2R5E), mRNA. |
| SPARC | -1.51 | Homo sapiens secreted protein, acidic, cysteine-rich (osteonectin) (SPARC), mRNA. |
| MGC15763 | -1.51 | Homo sapiens hypothetical protein BC008322 (MGC15763), mRNA. |
| ATG12 | -1.51 | Homo sapiens ATG12 autophagy related 12 homolog (S. cerevisiae) (ATG12), mRNA. |
| PKIA | -1.51 | Homo sapiens protein kinase (cAMP-dependent, catalytic) inhibitor alpha (PKIA), transcript variant 6, mRNA. |
| RDH13 | -1.51 | Homo sapiens retinol dehydrogenase 13 (all-trans/9-cis) (RDH13), mRNA. |
| PRDM10 | -1.51 | Homo sapiens PR domain containing 10 (PRDM10), transcript variant 1, mRNA. |
| DUT | -1.51 | Homo sapiens deoxyuridine triphosphatase (DUT), nuclear gene encoding mitochondrial protein, transcript variant 3, mRNA. |
| CLN3 | -1.51 | Homo sapiens ceroid-lipofuscinosis, neuronal 3 (CLN3), transcript variant 2, mRNA. |
| KBTBD8 | -1.51 | Homo sapiens kelch repeat and BTB (POZ) domain containing 8 (KBTBD8), mRNA. |
| WNT3 | -1.52 | Homo sapiens wingless-type MMTV integration site family, member 3 (WNT3), mRNA. |
| MRPS9 | -1.52 | Homo sapiens mitochondrial ribosomal protein S9 (MRPS9), nuclear gene encoding mitochondrial protein, mRNA. |
| USP33 | -1.52 | Homo sapiens ubiquitin specific peptidase 33 (USP33), transcript variant 1, mRNA. |
| ATMIN | -1.52 | Homo sapiens ATM interactor (ATMIN), mRNA. |
| KLHL5 | -1.52 | Homo sapiens kelch-like 5 (Drosophila) (KLHL5), transcript variant 3, mRNA. |
| ADAM22 | -1.52 | Homo sapiens ADAM metallopeptidase domain 22 (ADAM22), transcript variant 1, mRNA. |
| THAP1 | -1.52 | Homo sapiens THAP domain containing, apoptosis associated protein 1 (THAP1), transcript variant 1, mRNA. |
| C20orf72 | -1.52 | Homo sapiens chromosome 20 open reading frame 72 (C20orf72), mRNA. |
| DNASE2 | -1.52 | Homo sapiens deoxyribonuclease II, lysosomal (DNASE2), mRNA. |
| BUB3 | -1.52 | Homo sapiens BUB3 budding uninhibited by benzimidazoles 3 homolog (yeast) (BUB3), transcript variant 1, mRNA. |
| VPS41 | -1.52 | Homo sapiens vacuolar protein sorting 41 (yeast) (VPS41), transcript variant 1, mRNA. |
| DAPK3 | -1.52 | Homo sapiens death-associated protein kinase 3 (DAPK3), mRNA. |
| FAM64A | -1.52 | Homo sapiens family with sequence similarity 64, member A (FAM64A), mRNA. |
| WHSC1L1 | -1.52 | Homo sapiens Wolf-Hirschhorn syndrome candidate 1-like 1 (WHSC1L1), transcript variant short, mRNA. |
| PSMD1 | -1.52 | Homo sapiens proteasome (prosome, macropain) 26S subunit, non-ATPase, 1 (PSMD1), mRNA. |
| SKP2 | -1.52 | Homo sapiens S-phase kinase-associated protein 2 (p45) (SKP2), transcript variant 1, mRNA. |
| SFRS5 | -1.52 | Homo sapiens splicing factor, arginine/serine-rich 5 (SFRS5), transcript variant 2, mRNA. |
| CEP170 | -1.52 | Homo sapiens centrosomal protein 170kDa (CEP170), transcript variant alpha, mRNA. |
| KLF11 | -1.52 | PREDICTED: Homo sapiens Kruppel-like factor 11 (KLF11), mRNA. |
| GPAM | -1.52 | Homo sapiens glycerol-3-phosphate acyltransferase, mitochondrial (GPAM), mRNA. |
| MYO9A | -1.52 | Homo sapiens myosin IXA (MYO9A), mRNA. |
| SYNPO2 | -1.52 | PREDICTED: Homo sapiens synaptopodin 2, transcript variant 4 (SYNPO2), mRNA. |
| HNRPUL2 | -1.52 | PREDICTED: Homo sapiens heterogeneous nuclear ribonucleoprotein U-like 2 (HNRPUL2), mRNA. |
| GTPBP8 | -1.52 | Homo sapiens GTP-binding protein 8 (putative) (GTPBP8), transcript variant 1, mRNA. |
| PCGF1 | -1.52 | Homo sapiens polycomb group ring finger 1 (PCGF1), mRNA. |
| SEMA4B | -1.53 | Homo sapiens sema domain, immunoglobulin domain (Ig), transmembrane domain (TM) and short cytoplasmic domain, (semaphorin) 4B (SEMA4B), transcript variant 2, mRNA. |
| HIBCH | -1.53 | Homo sapiens 3-hydroxyisobutyryl-Coenzyme A hydrolase (HIBCH), nuclear gene encoding mitochondrial protein, transcript variant 2, mRNA. |
| DYM | -1.53 | Homo sapiens dymeclin (DYM), mRNA. |
| MICAL1 | -1.53 | Homo sapiens microtubule associated monoxygenase, calponin and LIM domain containing 1 (MICAL1), mRNA. |
| GADD45A | -1.53 | Homo sapiens growth arrest and DNA-damage-inducible, alpha (GADD45A), mRNA. |
| AP3D1 | -1.53 | Homo sapiens adaptor-related protein complex 3, delta 1 subunit (AP3D1), transcript variant 1, mRNA. |
| ACSS2 | -1.53 | Homo sapiens acyl-CoA synthetase short-chain family member 2 (ACSS2), transcript variant 1, mRNA. |
| PRPF18 | -1.53 | Homo sapiens PRP18 pre-mRNA processing factor 18 homolog (S. cerevisiae) (PRPF18), mRNA. |
| CPNE3 | -1.53 | Homo sapiens copine III (CPNE3), mRNA. |
| PHF14 | -1.53 | Homo sapiens PHD finger protein 14 (PHF14), transcript variant 2, mRNA. |
| DNM1L | -1.53 | Homo sapiens dynamin 1-like (DNM1L), transcript variant 1, mRNA. |
| AES | -1.53 | Homo sapiens amino-terminal enhancer of split (AES), transcript variant 3, mRNA. |
| CAPRIN1 | -1.53 | Homo sapiens cell cycle associated protein 1 (CAPRIN1), transcript variant 1, mRNA. |
| C10orf61 | -1.53 | Homo sapiens chromosome 10 open reading frame 61 (C10orf61), transcript variant 1, mRNA. |
| MLL5 | -1.53 | Homo sapiens myeloid/lymphoid or mixed-lineage leukemia 5 (trithorax homolog, Drosophila) (MLL5), transcript variant 1, mRNA. |
| LEPROT | -1.53 | Homo sapiens leptin receptor overlapping transcript (LEPROT), mRNA. |
| RBM18 | -1.53 | Homo sapiens RNA binding motif protein 18 (RBM18), mRNA. |
| HOMER2 | -1.53 | Homo sapiens homer homolog 2 (Drosophila) (HOMER2), transcript variant 3, mRNA. |
| PGGT1B | -1.53 | Homo sapiens protein geranylgeranyltransferase type I, beta subunit (PGGT1B), mRNA. |
| HMMR | -1.53 | Homo sapiens hyaluronan-mediated motility receptor (RHAMM) (HMMR), transcript variant 2, mRNA. |
| OSBPL1A | -1.54 | Homo sapiens oxysterol binding protein-like 1A (OSBPL1A), transcript variant OSBPL1B, mRNA. |
| FBXO11 | -1.54 | Homo sapiens F-box protein 11 (FBXO11), transcript variant 1, mRNA. |
| CRY1 | -1.54 | Homo sapiens cryptochrome 1 (photolyase-like) (CRY1), mRNA. |
| LOC400713 | -1.54 | PREDICTED: Homo sapiens zinc finger-like, transcript variant 3 (LOC400713), misc RNA. |
| C1orf57 | -1.54 | Homo sapiens chromosome 1 open reading frame 57 (C1orf57), mRNA. |
| CD302 | -1.54 | Homo sapiens CD302 molecule (CD302), mRNA. |
| LDOC1L | -1.54 | Homo sapiens leucine zipper, down-regulated in cancer 1-like (LDOC1L), mRNA. |
| PTGR1 | -1.54 | Homo sapiens prostaglandin reductase 1 (PTGR1), mRNA. |
| NBN | -1.54 | Homo sapiens nibrin (NBN), mRNA. |
| CKAP5 | -1.54 | Homo sapiens cytoskeleton associated protein 5 (CKAP5), transcript variant 1, mRNA. |
| KATNAL1 | -1.54 | Homo sapiens katanin p60 subunit A-like 1 (KATNAL1), transcript variant 2, mRNA. |
| SPATA2 | -1.54 | Homo sapiens spermatogenesis associated 2 (SPATA2), mRNA. |
| PTBP2 | -1.54 | Homo sapiens polypyrimidine tract binding protein 2 (PTBP2), mRNA. |
| IRAK1BP1 | -1.54 | Homo sapiens interleukin-1 receptor-associated kinase 1 binding protein 1 (IRAK1BP1), mRNA. |
| CECR6 | -1.54 | Homo sapiens cat eye syndrome chromosome region, candidate 6 (CECR6), mRNA. |
| TROVE2 | -1.54 | Homo sapiens TROVE domain family, member 2 (TROVE2), transcript variant 3, mRNA. |
| MCEE | -1.54 | Homo sapiens methylmalonyl CoA epimerase (MCEE), mRNA. |
| KIAA1598 | -1.54 | Homo sapiens KIAA1598 (KIAA1598), mRNA. |
| IKZF5 | -1.54 | Homo sapiens IKAROS family zinc finger 5 (Pegasus) (IKZF5), mRNA. |
| ZC3HAV1 | -1.54 | Homo sapiens zinc finger CCCH-type, antiviral 1 (ZC3HAV1), transcript variant 1, mRNA. |
| JMJD1C | -1.54 | Homo sapiens jumonji domain containing 1C (JMJD1C), transcript variant 1, mRNA. |
| FAM50B | -1.54 | Homo sapiens family with sequence similarity 50, member B (FAM50B), mRNA. |
| MST4 | -1.55 | Homo sapiens serine/threonine protein kinase MST4 (MST4), transcript variant 2, mRNA. |
| C20orf177 | -1.55 | Homo sapiens chromosome 20 open reading frame 177 (C20orf177), mRNA. |
|  | -1.55 | Homo sapiens cDNA clone IMAGE:5300199 |
| POLDIP2 | -1.55 | Homo sapiens polymerase (DNA-directed), delta interacting protein 2 (POLDIP2), mRNA. |
| ZNF621 | -1.55 | Homo sapiens zinc finger protein 621 (ZNF621), transcript variant 2, mRNA. |
| LOC653994 | -1.55 | PREDICTED: Homo sapiens similar to eukaryotic translation initiation factor 4H isoform 2, transcript variant 5 (LOC653994), mRNA. |
| IREB2 | -1.55 | Homo sapiens iron-responsive element binding protein 2 (IREB2), mRNA. |
| SAP18 | -1.55 | Homo sapiens Sin3A-associated protein, 18kDa (SAP18), mRNA. |
| TBC1D15 | -1.55 | Homo sapiens TBC1 domain family, member 15 (TBC1D15), mRNA. |
| ASAP1 | -1.55 | Homo sapiens ArfGAP with SH3 domain, ankyrin repeat and PH domain 1 (ASAP1), mRNA. |
| C1orf53 | -1.55 | Homo sapiens chromosome 1 open reading frame 53 (C1orf53), mRNA. |
| SMYD3 | -1.55 | Homo sapiens SET and MYND domain containing 3 (SMYD3), mRNA. |
| CCDC49 | -1.55 | Homo sapiens coiled-coil domain containing 49 (CCDC49), mRNA. |
| TMX3 | -1.55 | Homo sapiens thioredoxin-related transmembrane protein 3 (TMX3), mRNA. |
| NBPF14 | -1.55 | Homo sapiens neuroblastoma breakpoint family, member 14 (NBPF14), mRNA. |
| MTG1 | -1.55 | Homo sapiens mitochondrial GTPase 1 homolog (S. cerevisiae) (MTG1), nuclear gene encoding mitochondrial protein, mRNA. |
| NT5C3 | -1.55 | Homo sapiens 5'-nucleotidase, cytosolic III (NT5C3), transcript variant 1, mRNA. |
| ZNF669 | -1.55 | Homo sapiens zinc finger protein 669 (ZNF669), mRNA. |
| KCTD20 | -1.55 | Homo sapiens potassium channel tetramerisation domain containing 20 (KCTD20), mRNA. |
| MNS1 | -1.55 | Homo sapiens meiosis-specific nuclear structural 1 (MNS1), mRNA. |
| SLC45A3 | -1.55 | Homo sapiens solute carrier family 45, member 3 (SLC45A3), mRNA. |
| TCF20 | -1.55 | Homo sapiens transcription factor 20 (AR1) (TCF20), transcript variant 2, mRNA. |
| CHRAC1 | -1.55 | Homo sapiens chromatin accessibility complex 1 (CHRAC1), mRNA. |
| CDS2 | -1.56 | Homo sapiens CDP-diacylglycerol synthase (phosphatidate cytidylyltransferase) 2 (CDS2), mRNA. |
| RAP1GDS1 | -1.56 | Homo sapiens RAP1, GTP-GDP dissociation stimulator 1 (RAP1GDS1), mRNA. |
| SRGAP1 | -1.56 | Homo sapiens SLIT-ROBO Rho GTPase activating protein 1 (SRGAP1), mRNA. |
| LOC643389 | -1.56 | PREDICTED: Homo sapiens hypothetical protein LOC643389 (LOC643389), mRNA. |
| ARIH1 | -1.56 | Homo sapiens ariadne homolog, ubiquitin-conjugating enzyme E2 binding protein, 1 (Drosophila) (ARIH1), mRNA. |
| MANEA | -1.56 | Homo sapiens mannosidase, endo-alpha (MANEA), mRNA. |
| SPPL2A | -1.56 | Homo sapiens signal peptide peptidase-like 2A (SPPL2A), mRNA. |
| DEF8 | -1.56 | Homo sapiens differentially expressed in FDCP 8 homolog (mouse) (DEF8), transcript variant 1, mRNA. |
| POLM | -1.56 | Homo sapiens polymerase (DNA directed), mu (POLM), mRNA. |
| OCEL1 | -1.56 | Homo sapiens occludin/ELL domain containing 1 (OCEL1), mRNA. |
| ZC3H4 | -1.56 | Homo sapiens zinc finger CCCH-type containing 4 (ZC3H4), mRNA. |
| PGM2 | -1.56 | Homo sapiens phosphoglucomutase 2 (PGM2), mRNA. |
| HIF1A | -1.56 | Homo sapiens hypoxia-inducible factor 1, alpha subunit (basic helix-loop-helix transcription factor) (HIF1A), transcript variant 1, mRNA. |
| TCF12 | -1.56 | Homo sapiens transcription factor 12 (TCF12), transcript variant 2, mRNA. |
| TLE4 | -1.56 | Homo sapiens transducin-like enhancer of split 4 (E(sp1) homolog, Drosophila) (TLE4), mRNA. |
| FAM133B | -1.56 | Homo sapiens family with sequence similarity 133, member B (FAM133B), transcript variant 2, mRNA. |
| ANKMY1 | -1.56 | Homo sapiens ankyrin repeat and MYND domain containing 1 (ANKMY1), transcript variant 2, mRNA. |
| EXTL2 | -1.56 | Homo sapiens exostoses (multiple)-like 2 (EXTL2), transcript variant 2, mRNA. |
| C19orf52 | -1.56 | Homo sapiens chromosome 19 open reading frame 52 (C19orf52), mRNA. |
| POLR3A | -1.56 | Homo sapiens polymerase (RNA) III (DNA directed) polypeptide A, 155kDa (POLR3A), mRNA. |
| COBRA1 | -1.56 | Homo sapiens cofactor of BRCA1 (COBRA1), mRNA. |
| SOCS4 | -1.56 | Homo sapiens suppressor of cytokine signaling 4 (SOCS4), transcript variant 2, mRNA. |
| YRDC | -1.56 | Homo sapiens yrdC domain containing (E. coli) (YRDC), nuclear gene encoding mitochondrial protein, mRNA. |
| RASA1 | -1.57 | Homo sapiens RAS p21 protein activator (GTPase activating protein) 1 (RASA1), transcript variant 1, mRNA. |
| CCDC111 | -1.57 | Homo sapiens coiled-coil domain containing 111 (CCDC111), mRNA. |
| ZAK | -1.57 | Homo sapiens sterile alpha motif and leucine zipper containing kinase AZK (ZAK), transcript variant 2, mRNA. |
| NUDT11 | -1.57 | Homo sapiens nudix (nucleoside diphosphate linked moiety X)-type motif 11 (NUDT11), mRNA. |
| COL4A3BP | -1.57 | Homo sapiens collagen, type IV, alpha 3 (Goodpasture antigen) binding protein (COL4A3BP), transcript variant 1, mRNA. |
|  | -1.57 | AGENCOURT_10167693 NIH_MGC_71 Homo sapiens cDNA clone IMAGE:6527009 5, mRNA sequence |
| PRPF40A | -1.57 | Homo sapiens PRP40 pre-mRNA processing factor 40 homolog A (S. cerevisiae) (PRPF40A), mRNA. |
| PIP5K1C | -1.57 | Homo sapiens phosphatidylinositol-4-phosphate 5-kinase, type I, gamma (PIP5K1C), mRNA. |
| PEX13 | -1.57 | Homo sapiens peroxisome biogenesis factor 13 (PEX13), mRNA. |
| IFT81 | -1.57 | Homo sapiens intraflagellar transport 81 homolog (Chlamydomonas) (IFT81), transcript variant 1, mRNA. |
| C6orf52 | -1.57 | PREDICTED: Homo sapiens chromosome 6 open reading frame 52 (C6orf52), mRNA. |
| ENC1 | -1.57 | Homo sapiens ectodermal-neural cortex (with BTB-like domain) (ENC1), mRNA. |
| CDCA2 | -1.57 | Homo sapiens cell division cycle associated 2 (CDCA2), mRNA. |
| WDR70 | -1.57 | Homo sapiens WD repeat domain 70 (WDR70), mRNA. |
| XPO1 | -1.57 | Homo sapiens exportin 1 (CRM1 homolog, yeast) (XPO1), mRNA. |
| ING1 | -1.57 | Homo sapiens inhibitor of growth family, member 1 (ING1), transcript variant 1, mRNA. |
| STAG3L1 | -1.57 | Homo sapiens stromal antigen 3-like 1 (STAG3L1), transcript variant 1, mRNA. |
| BID | -1.57 | Homo sapiens BH3 interacting domain death agonist (BID), transcript variant 3, mRNA. |
| LOC440145 | -1.57 | Homo sapiens similar to RIKEN cDNA 2410129H14 (LOC440145), mRNA. |
| DUSP6 | -1.57 | Homo sapiens dual specificity phosphatase 6 (DUSP6), transcript variant 1, mRNA. |
| SOX11 | -1.57 | Homo sapiens SRY (sex determining region Y)-box 11 (SOX11), mRNA. |
| DUSP1 | -1.57 | Homo sapiens dual specificity phosphatase 1 (DUSP1), mRNA. |
| ICA1L | -1.57 | Homo sapiens islet cell autoantigen 1,69kDa-like (ICA1L), transcript variant 1, mRNA. |
| HSD17B7 | -1.58 | Homo sapiens hydroxysteroid (17-beta) dehydrogenase 7 (HSD17B7), mRNA. |
| COMMD10 | -1.58 | Homo sapiens COMM domain containing 10 (COMMD10), mRNA. |
| PPEF1 | -1.58 | Homo sapiens protein phosphatase, EF-hand calcium binding domain 1 (PPEF1), transcript variant 1b, mRNA. |
| SLC9A6 | -1.58 | Homo sapiens solute carrier family 9 (sodium/hydrogen exchanger), member 6 (SLC9A6), transcript variant 1, mRNA. |
| DCUN1D1 | -1.58 | Homo sapiens DCN1, defective in cullin neddylation 1, domain containing 1 (S. cerevisiae) (DCUN1D1), mRNA. |
| TMED5 | -1.58 | Homo sapiens transmembrane emp24 protein transport domain containing 5 (TMED5), mRNA. |
| ATCAY | -1.58 | Homo sapiens ataxia, cerebellar, Cayman type (caytaxin) (ATCAY), mRNA. |
| VOPP1 | -1.58 | Homo sapiens vesicular, overexpressed in cancer, prosurvival protein 1 (VOPP1), mRNA. |
| SAR1B | -1.58 | Homo sapiens SAR1 homolog B (S. cerevisiae) (SAR1B), transcript variant 1, mRNA. |
| ICA1 | -1.58 | Homo sapiens islet cell autoantigen 1, 69kDa (ICA1), transcript variant 3, mRNA. |
| TRIM9 | -1.58 | Homo sapiens tripartite motif-containing 9 (TRIM9), transcript variant 1, mRNA. |
| C5orf28 | -1.58 | Homo sapiens chromosome 5 open reading frame 28 (C5orf28), mRNA. |
| NUP54 | -1.58 | Homo sapiens nucleoporin 54kDa (NUP54), mRNA. |
| ZNF189 | -1.58 | Homo sapiens zinc finger protein 189 (ZNF189), transcript variant 1, mRNA. |
| ZNF124 | -1.58 | Homo sapiens zinc finger protein 124 (ZNF124), mRNA. |
| TANC1 | -1.58 | Homo sapiens tetratricopeptide repeat, ankyrin repeat and coiled-coil containing 1 (TANC1), mRNA. |
| YME1L1 | -1.58 | Homo sapiens YME1-like 1 (S. cerevisiae) (YME1L1), nuclear gene encoding mitochondrial protein, transcript variant 1, mRNA. |
| TTLL7 | -1.58 | Homo sapiens tubulin tyrosine ligase-like family, member 7 (TTLL7), mRNA. |
| MON1B | -1.58 | Homo sapiens MON1 homolog B (yeast) (MON1B), mRNA. |
| LRRC8A | -1.58 | Homo sapiens leucine rich repeat containing 8 family, member A (LRRC8A), mRNA. |
| LTBP3 | -1.58 | Homo sapiens latent transforming growth factor beta binding protein 3 (LTBP3), mRNA. |
| MPP6 | -1.58 | Homo sapiens membrane protein, palmitoylated 6 (MAGUK p55 subfamily member 6) (MPP6), mRNA. |
| GNAI3 | -1.58 | Homo sapiens guanine nucleotide binding protein (G protein), alpha inhibiting activity polypeptide 3 (GNAI3), mRNA. |
| EXOSC2 | -1.59 | Homo sapiens exosome component 2 (EXOSC2), mRNA. |
| COX19 | -1.59 | Homo sapiens COX19 cytochrome c oxidase assembly homolog (S. cerevisiae) (COX19), mRNA. |
|  | -1.59 | Homo sapiens cDNA FLJ31750 fis, clone NT2RI2007406 |
| KLHL24 | -1.59 | Homo sapiens kelch-like 24 (Drosophila) (KLHL24), mRNA. |
| FAM71E1 | -1.59 | Homo sapiens family with sequence similarity 71, member E1 (FAM71E1), mRNA. |
| GALC | -1.59 | Homo sapiens galactosylceramidase (GALC), transcript variant 1, mRNA. |
| FAM177A1 | -1.59 | Homo sapiens family with sequence similarity 177, member A1 (FAM177A1), transcript variant 1, mRNA. |
| SULF2 | -1.59 | Homo sapiens sulfatase 2 (SULF2), transcript variant 1, mRNA. |
| APITD1 | -1.59 | Homo sapiens apoptosis-inducing, TAF9-like domain 1 (APITD1), transcript variant B, mRNA. |
| MAP2K6 | -1.59 | Homo sapiens mitogen-activated protein kinase kinase 6 (MAP2K6), mRNA. |
| NCKAP1 | -1.59 | Homo sapiens NCK-associated protein 1 (NCKAP1), transcript variant 1, mRNA. |
| PHACTR1 | -1.59 | Homo sapiens phosphatase and actin regulator 1 (PHACTR1), mRNA. |
| YTHDC1 | -1.59 | Homo sapiens YTH domain containing 1 (YTHDC1), transcript variant 1, mRNA. |
|  | -1.59 | Homo sapiens cDNA FLJ20769 fis, clone COL06674 |
| FAM86A | -1.59 | Homo sapiens family with sequence similarity 86, member A (FAM86A), transcript variant 2, mRNA. |
| TACSTD1 | -1.6 | Homo sapiens tumor-associated calcium signal transducer 1 (TACSTD1), mRNA. |
| RRP15 | -1.6 | Homo sapiens ribosomal RNA processing 15 homolog (S. cerevisiae) (RRP15), mRNA. |
| DKK1 | -1.6 | Homo sapiens dickkopf homolog 1 (Xenopus laevis) (DKK1), mRNA. |
| CRYZ | -1.6 | Homo sapiens crystallin, zeta (quinone reductase) (CRYZ), mRNA. |
| STX6 | -1.6 | Homo sapiens syntaxin 6 (STX6), mRNA. |
| LOC441150 | -1.6 | PREDICTED: Homo sapiens similar to RIKEN cDNA 2310039H08 (LOC441150), mRNA. |
| RAB3GAP1 | -1.6 | Homo sapiens RAB3 GTPase activating protein subunit 1 (catalytic) (RAB3GAP1), mRNA. |
| CNIH | -1.6 | Homo sapiens cornichon homolog (Drosophila) (CNIH), transcript variant 2, mRNA. |
| MCM10 | -1.6 | Homo sapiens minichromosome maintenance complex component 10 (MCM10), transcript variant 2, mRNA. |
| NAE1 | -1.6 | Homo sapiens NEDD8 activating enzyme E1 subunit 1 (NAE1), transcript variant 3, mRNA. |
| ATP11C | -1.6 | Homo sapiens ATPase, class VI, type 11C (ATP11C), transcript variant 1, mRNA. |
| PTPDC1 | -1.6 | Homo sapiens protein tyrosine phosphatase domain containing 1 (PTPDC1), transcript variant 1, mRNA. |
| PXMP3 | -1.6 | Homo sapiens peroxisomal membrane protein 3, 35kDa (PXMP3), transcript variant 1, mRNA. |
| NCAPG2 | -1.6 | Homo sapiens non-SMC condensin II complex, subunit G2 (NCAPG2), mRNA. |
| SPAST | -1.6 | Homo sapiens spastin (SPAST), transcript variant 1, mRNA. |
| PON2 | -1.6 | Homo sapiens paraoxonase 2 (PON2), transcript variant 1, mRNA. |
| C1orf115 | -1.6 | Homo sapiens chromosome 1 open reading frame 115 (C1orf115), mRNA. |
| SAMD4B | -1.6 | Homo sapiens sterile alpha motif domain containing 4B (SAMD4B), mRNA. |
| CYP2R1 | -1.6 | Homo sapiens cytochrome P450, family 2, subfamily R, polypeptide 1 (CYP2R1), mRNA. |
| GRM8 | -1.6 | Homo sapiens glutamate receptor, metabotropic 8 (GRM8), mRNA. |
| GPATCH2 | -1.61 | Homo sapiens G patch domain containing 2 (GPATCH2), mRNA. |
| ATPAF1 | -1.61 | Homo sapiens ATP synthase mitochondrial F1 complex assembly factor 1 (ATPAF1), nuclear gene encoding mitochondrial protein, transcript variant 2, mRNA. |
| RRP1B | -1.61 | Homo sapiens ribosomal RNA processing 1 homolog B (S. cerevisiae) (RRP1B), mRNA. |
| CEP170 | -1.61 | Homo sapiens centrosomal protein 170kDa (CEP170), transcript variant alpha, mRNA. |
| FHOD3 | -1.61 | Homo sapiens formin homology 2 domain containing 3 (FHOD3), mRNA. |
|  | -1.61 | BX393727 Homo sapiens NEUROBLASTOMA COT 25-NORMALIZED Homo sapiens cDNA clone CS0DC001YP02 5-PRIME, mRNA sequence |
| FSD1 | -1.61 | Homo sapiens fibronectin type III and SPRY domain containing 1 (FSD1), mRNA. |
| ASAM | -1.61 | Homo sapiens adipocyte-specific adhesion molecule (ASAM), mRNA. |
| SLC18A1 | -1.61 | Homo sapiens solute carrier family 18 (vesicular monoamine), member 1 (SLC18A1), mRNA. |
| IRS2 | -1.61 | Homo sapiens insulin receptor substrate 2 (IRS2), mRNA. |
| PCM1 | -1.61 | Homo sapiens pericentriolar material 1 (PCM1), mRNA. |
| SLC25A24 | -1.61 | Homo sapiens solute carrier family 25 (mitochondrial carrier; phosphate carrier), member 24 (SLC25A24), nuclear gene encoding mitochondrial protein, transcript variant 1, mRNA. |
| ZNF329 | -1.62 | Homo sapiens zinc finger protein 329 (ZNF329), mRNA. |
| KIAA0240 | -1.62 | Homo sapiens KIAA0240 (KIAA0240), mRNA. |
| MEX3B | -1.62 | Homo sapiens mex-3 homolog B (C. elegans) (MEX3B), mRNA. |
| RFXANK | -1.62 | Homo sapiens regulatory factor X-associated ankyrin-containing protein (RFXANK), transcript variant 1, mRNA. |
| KIAA1618 | -1.62 | Homo sapiens KIAA1618 (KIAA1618), mRNA. |
| KLHL7 | -1.62 | Homo sapiens kelch-like 7 (Drosophila) (KLHL7), transcript variant 1, mRNA. |
| MRPL35 | -1.62 | Homo sapiens mitochondrial ribosomal protein L35 (MRPL35), nuclear gene encoding mitochondrial protein, transcript variant 1, mRNA. |
| MADD | -1.62 | Homo sapiens MAP-kinase activating death domain (MADD), transcript variant 5, mRNA. |
| PTCD2 | -1.62 | Homo sapiens pentatricopeptide repeat domain 2 (PTCD2), mRNA. |
| ANO8 | -1.62 | Homo sapiens anoctamin 8 (ANO8), mRNA. |
| MYL12A | -1.62 | Homo sapiens myosin, light chain 12A, regulatory, non-sarcomeric (MYL12A), mRNA. |
| TK1 | -1.62 | Homo sapiens thymidine kinase 1, soluble (TK1), mRNA. |
| PPM1E | -1.63 | Homo sapiens protein phosphatase 1E (PP2C domain containing) (PPM1E), mRNA. |
| C1orf66 | -1.63 | Homo sapiens chromosome 1 open reading frame 66 (C1orf66), mRNA. |
| ALG6 | -1.63 | Homo sapiens asparagine-linked glycosylation 6 homolog (S. cerevisiae, alpha-1,3-glucosyltransferase) (ALG6), mRNA. |
| RNF19A | -1.63 | Homo sapiens ring finger protein 19A (RNF19A), transcript variant 2, mRNA. |
|  | -1.63 | Homo sapiens mRNA; cDNA DKFZp779F0411 (from clone DKFZp779F0411) |
| KCNT1 | -1.63 | Homo sapiens potassium channel, subfamily T, member 1 (KCNT1), mRNA. |
| ERRFI1 | -1.63 | Homo sapiens ERBB receptor feedback inhibitor 1 (ERRFI1), mRNA. |
| CDCA3 | -1.63 | Homo sapiens cell division cycle associated 3 (CDCA3), mRNA. |
| BCLAF1 | -1.63 | Homo sapiens BCL2-associated transcription factor 1 (BCLAF1), transcript variant 1, mRNA. |
| TRIM32 | -1.63 | Homo sapiens tripartite motif-containing 32 (TRIM32), transcript variant 1, mRNA. |
| RAVER2 | -1.63 | Homo sapiens ribonucleoprotein, PTB-binding 2 (RAVER2), mRNA. |
| HYLS1 | -1.63 | Homo sapiens hydrolethalus syndrome 1 (HYLS1), mRNA. |
| HMGB3 | -1.63 | Homo sapiens high-mobility group box 3 (HMGB3), mRNA. |
| KIFC1 | -1.63 | Homo sapiens kinesin family member C1 (KIFC1), mRNA. |
| EIF1AX | -1.63 | Homo sapiens eukaryotic translation initiation factor 1A, X-linked (EIF1AX), mRNA. |
| GGA2 | -1.63 | Homo sapiens golgi associated, gamma adaptin ear containing, ARF binding protein 2 (GGA2), mRNA. |
| UGCG | -1.63 | Homo sapiens UDP-glucose ceramide glucosyltransferase (UGCG), mRNA. |
| ACLY | -1.63 | Homo sapiens ATP citrate lyase (ACLY), transcript variant 1, mRNA. |
| BAT2 | -1.63 | Homo sapiens HLA-B associated transcript 2 (BAT2), transcript variant 1, mRNA. |
| TAF1B | -1.64 | Homo sapiens TATA box binding protein (TBP)-associated factor, RNA polymerase I, B, 63kDa (TAF1B), mRNA. |
| DOCK1 | -1.64 | Homo sapiens dedicator of cytokinesis 1 (DOCK1), mRNA. |
| ATP5G3 | -1.64 | Homo sapiens ATP synthase, H+ transporting, mitochondrial F0 complex, subunit C3 (subunit 9) (ATP5G3), nuclear gene encoding mitochondrial protein, transcript variant 3, mRNA. |
| ARL4A | -1.64 | Homo sapiens ADP-ribosylation factor-like 4A (ARL4A), transcript variant 3, mRNA. |
| LOC283683 | -1.64 | PREDICTED: Homo sapiens hypothetical protein LOC283683, transcript variant 3 (LOC283683), mRNA. |
| PPCS | -1.64 | Homo sapiens phosphopantothenoylcysteine synthetase (PPCS), transcript variant 1, mRNA. |
| FBXO22 | -1.64 | Homo sapiens F-box protein 22 (FBXO22), transcript variant 1, mRNA. |
| UGCGL1 | -1.64 | Homo sapiens UDP-glucose ceramide glucosyltransferase-like 1 (UGCGL1), transcript variant 1, mRNA. |
| RIMBP2 | -1.64 | Homo sapiens RIMS binding protein 2 (RIMBP2), mRNA. |
| BLM | -1.64 | Homo sapiens Bloom syndrome, RecQ helicase-like (BLM), mRNA. |
| LOC730130 | -1.64 | PREDICTED: Homo sapiens hypothetical protein LOC730130 (LOC730130), mRNA. |
| KIAA1715 | -1.64 | Homo sapiens KIAA1715 (KIAA1715), mRNA. |
| TNPO1 | -1.64 | Homo sapiens transportin 1 (TNPO1), transcript variant 1, mRNA. |
| ANKHD1 | -1.64 | Homo sapiens ankyrin repeat and KH domain containing 1 (ANKHD1), transcript variant 1, mRNA. |
| CYB561D2 | -1.64 | Homo sapiens cytochrome b-561 domain containing 2 (CYB561D2), mRNA. |
| SF3A1 | -1.64 | Homo sapiens splicing factor 3a, subunit 1, 120kDa (SF3A1), transcript variant 2, mRNA. |
| STAT1 | -1.64 | Homo sapiens signal transducer and activator of transcription 1, 91kDa (STAT1), transcript variant alpha, mRNA. |
| FILIP1L | -1.64 | Homo sapiens filamin A interacting protein 1-like (FILIP1L), transcript variant 2, mRNA. |
| PPAP2B | -1.64 | Homo sapiens phosphatidic acid phosphatase type 2B (PPAP2B), transcript variant 1, mRNA. |
| C9orf127 | -1.64 | Homo sapiens chromosome 9 open reading frame 127 (C9orf127), mRNA. |
| AASDH | -1.64 | Homo sapiens aminoadipate-semialdehyde dehydrogenase (AASDH), mRNA. |
| KIF1B | -1.64 | Homo sapiens kinesin family member 1B (KIF1B), transcript variant 1, mRNA. |
| BCOR | -1.65 | Homo sapiens BCL6 co-repressor (BCOR), transcript variant 2, mRNA. |
| NAPB | -1.65 | Homo sapiens N-ethylmaleimide-sensitive factor attachment protein, beta (NAPB), mRNA. |
| TMEM168 | -1.65 | Homo sapiens transmembrane protein 168 (TMEM168), mRNA. |
| GNA12 | -1.65 | Homo sapiens guanine nucleotide binding protein (G protein) alpha 12 (GNA12), mRNA. |
| PCK2 | -1.65 | Homo sapiens phosphoenolpyruvate carboxykinase 2 (mitochondrial) (PCK2), nuclear gene encoding mitochondrial protein, transcript variant 1, mRNA. |
| ZDHHC17 | -1.65 | Homo sapiens zinc finger, DHHC-type containing 17 (ZDHHC17), mRNA. |
| IL20RA | -1.65 | Homo sapiens interleukin 20 receptor, alpha (IL20RA), mRNA. |
| TPP1 | -1.65 | Homo sapiens tripeptidyl peptidase I (TPP1), mRNA. |
| C9orf91 | -1.65 | Homo sapiens chromosome 9 open reading frame 91 (C9orf91), mRNA. |
| C1orf163 | -1.65 | Homo sapiens chromosome 1 open reading frame 163 (C1orf163), mRNA. |
| RRAS2 | -1.65 | Homo sapiens related RAS viral (r-ras) oncogene homolog 2 (RRAS2), mRNA. |
| L2HGDH | -1.65 | Homo sapiens L-2-hydroxyglutarate dehydrogenase (L2HGDH), mRNA. |
| GSK3B | -1.65 | Homo sapiens glycogen synthase kinase 3 beta (GSK3B), mRNA. |
| EYA4 | -1.65 | Homo sapiens eyes absent homolog 4 (Drosophila) (EYA4), transcript variant 1, mRNA. |
| RBM20 | -1.65 | PREDICTED: Homo sapiens RNA binding motif protein 20 (RBM20), mRNA. |
| CYP2J2 | -1.65 | Homo sapiens cytochrome P450, family 2, subfamily J, polypeptide 2 (CYP2J2), mRNA. |
| TROAP | -1.65 | Homo sapiens trophinin associated protein (tastin) (TROAP), mRNA. |
| HIP2 | -1.65 | Homo sapiens huntingtin interacting protein 2 (HIP2), mRNA. |
| NTNG1 | -1.65 | Homo sapiens netrin G1 (NTNG1), transcript variant 3, mRNA. |
| RQCD1 | -1.65 | Homo sapiens RCD1 required for cell differentiation1 homolog (S. pombe) (RQCD1), mRNA. |
| FLJ13305 | -1.65 | Homo sapiens hypothetical protein FLJ13305 (FLJ13305), mRNA. XM_934538 XM_934541 XM_934544 XM_942148 XM_945748 XM_945749 XM_945750 XM_945751 XM_945752 XM_945754 XM_945756 |
| TIAM2 | -1.66 | Homo sapiens T-cell lymphoma invasion and metastasis 2 (TIAM2), transcript variant 2, mRNA. |
| PELI1 | -1.66 | Homo sapiens pellino homolog 1 (Drosophila) (PELI1), mRNA. |
| PDCD2 | -1.66 | Homo sapiens programmed cell death 2 (PDCD2), transcript variant 2, mRNA. |
| DCLRE1A | -1.66 | Homo sapiens DNA cross-link repair 1A (PSO2 homolog, S. cerevisiae) (DCLRE1A), mRNA. |
| OAF | -1.66 | Homo sapiens OAF homolog (Drosophila) (OAF), mRNA. |
| CIAO1 | -1.66 | Homo sapiens cytosolic iron-sulfur protein assembly 1 homolog (S. cerevisiae) (CIAO1), mRNA. |
| CEP164 | -1.66 | Homo sapiens centrosomal protein 164kDa (CEP164), mRNA. |
| CREBZF | -1.66 | Homo sapiens CREB/ATF bZIP transcription factor (CREBZF), mRNA. |
| ASF1A | -1.66 | Homo sapiens ASF1 anti-silencing function 1 homolog A (S. cerevisiae) (ASF1A), mRNA. |
| HSDL2 | -1.66 | Homo sapiens hydroxysteroid dehydrogenase like 2 (HSDL2), mRNA. |
| MANSC1 | -1.66 | Homo sapiens MANSC domain containing 1 (MANSC1), mRNA. |
| CKAP2L | -1.66 | Homo sapiens cytoskeleton associated protein 2-like (CKAP2L), mRNA. |
| MSI2 | -1.66 | Homo sapiens musashi homolog 2 (Drosophila) (MSI2), transcript variant 1, mRNA. |
| MRPL45 | -1.66 | Homo sapiens mitochondrial ribosomal protein L45 (MRPL45), nuclear gene encoding mitochondrial protein, mRNA. |
| COG5 | -1.66 | Homo sapiens component of oligomeric golgi complex 5 (COG5), transcript variant 1, mRNA. |
| C4orf27 | -1.66 | Homo sapiens chromosome 4 open reading frame 27 (C4orf27), mRNA. |
| C1orf19 | -1.67 | Homo sapiens chromosome 1 open reading frame 19 (C1orf19), mRNA. |
| TARBP1 | -1.67 | Homo sapiens TAR (HIV-1) RNA binding protein 1 (TARBP1), mRNA. |
| FLJ22184 | -1.67 | PREDICTED: Homo sapiens hypothetical protein FLJ22184 (FLJ22184), mRNA. |
|  | -1.67 | MR1-GN0172-061100-005-h03 GN0172 Homo sapiens cDNA, mRNA sequence |
| RALBP1 | -1.67 | Homo sapiens ralA binding protein 1 (RALBP1), mRNA. |
| CYTL1 | -1.67 | Homo sapiens cytokine-like 1 (CYTL1), mRNA. |
| C14orf106 | -1.67 | Homo sapiens chromosome 14 open reading frame 106 (C14orf106), mRNA. |
| KIAA1530 | -1.67 | Homo sapiens KIAA1530 (KIAA1530), mRNA. |
| COMMD10 | -1.67 | Homo sapiens COMM domain containing 10 (COMMD10), mRNA. |
| CCDC34 | -1.67 | Homo sapiens coiled-coil domain containing 34 (CCDC34), transcript variant 1, mRNA. |
| NARG2 | -1.67 | Homo sapiens NMDA receptor regulated 2 (NARG2), transcript variant 1, mRNA. |
| FUS | -1.67 | Homo sapiens fusion (involved in t(12;16) in malignant liposarcoma) (FUS), transcript variant 1, mRNA. |
| CRIPAK | -1.67 | Homo sapiens cysteine-rich PAK1 inhibitor (CRIPAK), mRNA. |
| PLS1 | -1.67 | Homo sapiens plastin 1 (I isoform) (PLS1), mRNA. |
| PLGLB1 | -1.67 | Homo sapiens plasminogen-like B1 (PLGLB1), mRNA. |
| SDCCAG8 | -1.67 | Homo sapiens serologically defined colon cancer antigen 8 (SDCCAG8), mRNA. |
| NSFL1C | -1.67 | Homo sapiens NSFL1 (p97) cofactor (p47) (NSFL1C), transcript variant 3, mRNA. |
| ST6GALNAC3 | -1.67 | Homo sapiens ST6 (alpha-N-acetyl-neuraminyl-2,3-beta-galactosyl-1, 3)-N-acetylgalactosaminide alpha-2,6-sialyltransferase 3 (ST6GALNAC3), mRNA. |
| PMS2L3 | -1.67 | Homo sapiens postmeiotic segregation increased 2-like 3 (PMS2L3), transcript variant 2, mRNA. |
| LIPA | -1.67 | Homo sapiens lipase A, lysosomal acid, cholesterol esterase (LIPA), transcript variant 2, mRNA. |
|  | -1.67 | CM3-MT0357-260101-690-b10 MT0357 Homo sapiens cDNA, mRNA sequence |
| FAM46A | -1.68 | Homo sapiens family with sequence similarity 46, member A (FAM46A), mRNA. |
| TACC1 | -1.68 | Homo sapiens transforming, acidic coiled-coil containing protein 1 (TACC1), mRNA. |
| LOC652726 | -1.68 | PREDICTED: Homo sapiens similar to ankyrin repeat domain 36 (LOC652726), mRNA. |
| TRAF7 | -1.68 | Homo sapiens TNF receptor-associated factor 7 (TRAF7), mRNA. |
| CCDC25 | -1.68 | Homo sapiens coiled-coil domain containing 25 (CCDC25), mRNA. |
| CWF19L2 | -1.68 | Homo sapiens CWF19-like 2, cell cycle control (S. pombe) (CWF19L2), mRNA. |
| ABCC5 | -1.68 | Homo sapiens ATP-binding cassette, sub-family C (CFTR/MRP), member 5 (ABCC5), transcript variant 2, mRNA. |
| ERCC5 | -1.68 | Homo sapiens excision repair cross-complementing rodent repair deficiency, complementation group 5 (ERCC5), mRNA. |
| MAP4K4 | -1.68 | Homo sapiens mitogen-activated protein kinase kinase kinase kinase 4 (MAP4K4), transcript variant 3, mRNA. |
| ACOT11 | -1.68 | Homo sapiens acyl-CoA thioesterase 11 (ACOT11), transcript variant 2, mRNA. |
| ATP6V1C1 | -1.68 | Homo sapiens ATPase, H+ transporting, lysosomal 42kDa, V1 subunit C1 (ATP6V1C1), mRNA. |
| RSBN1 | -1.68 | Homo sapiens round spermatid basic protein 1 (RSBN1), mRNA. |
| MTCP1 | -1.68 | Homo sapiens mature T-cell proliferation 1 (MTCP1), nuclear gene encoding mitochondrial protein, transcript variant B1, mRNA. |
| SNHG4 | -1.68 | Homo sapiens small nucleolar RNA host gene (non-protein coding) 4 (SNHG4) on chromosome 5. |
| CRY1 | -1.68 | Homo sapiens cryptochrome 1 (photolyase-like) (CRY1), mRNA. |
| GARNL4 | -1.68 | Homo sapiens GTPase activating Rap/RanGAP domain-like 4 (GARNL4), mRNA. |
| CPT1A | -1.68 | Homo sapiens carnitine palmitoyltransferase 1A (liver) (CPT1A), nuclear gene encoding mitochondrial protein, transcript variant 2, mRNA. |
| CCDC50 | -1.68 | Homo sapiens coiled-coil domain containing 50 (CCDC50), transcript variant 1, mRNA. |
| ATP7B | -1.68 | Homo sapiens ATPase, Cu++ transporting, beta polypeptide (ATP7B), transcript variant 1, mRNA. |
|  | -1.68 | DA371742 BRTHA2 Homo sapiens cDNA clone BRTHA2001741 5, mRNA sequence |
| RSU1 | -1.68 | Homo sapiens Ras suppressor protein 1 (RSU1), transcript variant 1, mRNA. |
| YAF2 | -1.69 | Homo sapiens YY1 associated factor 2 (YAF2), transcript variant 2, mRNA. |
| GGNBP2 | -1.69 | Homo sapiens gametogenetin binding protein 2 (GGNBP2), mRNA. |
| RAB9B | -1.69 | Homo sapiens RAB9B, member RAS oncogene family (RAB9B), mRNA. |
| ZNF234 | -1.69 | Homo sapiens zinc finger protein 234 (ZNF234), mRNA. |
| C14orf135 | -1.69 | Homo sapiens chromosome 14 open reading frame 135 (C14orf135), mRNA. |
| C17orf58 | -1.69 | Homo sapiens chromosome 17 open reading frame 58 (C17orf58), transcript variant 2, mRNA. |
| TCFL5 | -1.69 | Homo sapiens transcription factor-like 5 (basic helix-loop-helix) (TCFL5), mRNA. |
| TMEM18 | -1.69 | Homo sapiens transmembrane protein 18 (TMEM18), mRNA. |
| ZNF669 | -1.69 | Homo sapiens zinc finger protein 669 (ZNF669), mRNA. |
| VPS33A | -1.69 | Homo sapiens vacuolar protein sorting 33 homolog A (S. cerevisiae) (VPS33A), mRNA. |
| LRRC37B | -1.69 | Homo sapiens leucine rich repeat containing 37B (LRRC37B), mRNA. |
| HRSP12 | -1.69 | Homo sapiens heat-responsive protein 12 (HRSP12), mRNA. |
| PTPLAD1 | -1.69 | Homo sapiens protein tyrosine phosphatase-like A domain containing 1 (PTPLAD1), mRNA. |
| CPVL | -1.69 | Homo sapiens carboxypeptidase, vitellogenic-like (CPVL), transcript variant 1, mRNA. |
| TSHZ3 | -1.69 | Homo sapiens teashirt zinc finger homeobox 3 (TSHZ3), mRNA. |
| DKFZP564O0523 | -1.69 | Homo sapiens hypothetical protein DKFZp564O0523 (DKFZP564O0523), mRNA. |
| SPTLC2 | -1.7 | Homo sapiens serine palmitoyltransferase, long chain base subunit 2 (SPTLC2), mRNA. |
| ACTR6 | -1.7 | Homo sapiens ARP6 actin-related protein 6 homolog (yeast) (ACTR6), mRNA. |
| CROT | -1.7 | Homo sapiens carnitine O-octanoyltransferase (CROT), mRNA. |
| PPAN | -1.7 | Homo sapiens peter pan homolog (Drosophila) (PPAN), mRNA. |
| SMARCAL1 | -1.7 | Homo sapiens SWI/SNF related, matrix associated, actin dependent regulator of chromatin, subfamily a-like 1 (SMARCAL1), mRNA. |
| BARD1 | -1.7 | Homo sapiens BRCA1 associated RING domain 1 (BARD1), mRNA. |
| MAP1A | -1.7 | Homo sapiens microtubule-associated protein 1A (MAP1A), mRNA. |
| ACACA | -1.7 | Homo sapiens acetyl-Coenzyme A carboxylase alpha (ACACA), transcript variant 3, mRNA. |
| TFIP11 | -1.7 | Homo sapiens tuftelin interacting protein 11 (TFIP11), transcript variant 2, mRNA. |
| MAD2L1 | -1.7 | Homo sapiens MAD2 mitotic arrest deficient-like 1 (yeast) (MAD2L1), mRNA. |
| SYT17 | -1.7 | Homo sapiens synaptotagmin XVII (SYT17), mRNA. |
|  | -1.7 | Homo sapiens cDNA FLJ44370 fis, clone TRACH3008902 |
| TM4SF1 | -1.7 | Homo sapiens transmembrane 4 L six family member 1 (TM4SF1), mRNA. |
| UCK2 | -1.7 | Homo sapiens uridine-cytidine kinase 2 (UCK2), mRNA. |
| KLHL3 | -1.7 | Homo sapiens kelch-like 3 (Drosophila) (KLHL3), mRNA. |
| ADD3 | -1.7 | Homo sapiens adducin 3 (gamma) (ADD3), transcript variant 3, mRNA. |
| S1PR3 | -1.7 | Homo sapiens sphingosine-1-phosphate receptor 3 (S1PR3), mRNA. |
| FTO | -1.7 | Homo sapiens fat mass and obesity associated (FTO), mRNA. |
| USP48 | -1.7 | Homo sapiens ubiquitin specific peptidase 48 (USP48), transcript variant 1, mRNA. |
| PTPLB | -1.7 | Homo sapiens protein tyrosine phosphatase-like (proline instead of catalytic arginine), member b (PTPLB), mRNA. |
| GMEB2 | -1.71 | Homo sapiens glucocorticoid modulatory element binding protein 2 (GMEB2), mRNA. |
| C21orf66 | -1.71 | Homo sapiens chromosome 21 open reading frame 66 (C21orf66), transcript variant 1, mRNA. |
| TXNDC5 | -1.71 | Homo sapiens thioredoxin domain containing 5 (TXNDC5), transcript variant 2, mRNA. |
| SLC27A1 | -1.71 | Homo sapiens solute carrier family 27 (fatty acid transporter), member 1 (SLC27A1), mRNA. |
| 6-Mar | -1.71 | Homo sapiens membrane-associated ring finger (C3HC4) 6 (MARCH6), mRNA. |
| FAM156A | -1.71 | Homo sapiens family with sequence similarity 156, member A (FAM156A), mRNA. |
| NHLRC3 | -1.71 | Homo sapiens NHL repeat containing 3 (NHLRC3), transcript variant 2, mRNA. |
| CDK5RAP3 | -1.71 | Homo sapiens CDK5 regulatory subunit associated protein 3 (CDK5RAP3), transcript variant 1, mRNA. |
| AGA | -1.71 | Homo sapiens aspartylglucosaminidase (AGA), mRNA. |
| JAK1 | -1.71 | Homo sapiens Janus kinase 1 (JAK1), mRNA. |
| SEMA4D | -1.71 | Homo sapiens sema domain, immunoglobulin domain (Ig), transmembrane domain (TM) and short cytoplasmic domain, (semaphorin) 4D (SEMA4D), mRNA. |
| TELO2 | -1.71 | Homo sapiens TEL2, telomere maintenance 2, homolog (S. cerevisiae) (TELO2), mRNA. |
| HNRPUL1 | -1.71 | Homo sapiens heterogeneous nuclear ribonucleoprotein U-like 1 (HNRPUL1), transcript variant 4, mRNA. |
| VHL | -1.71 | Homo sapiens von Hippel-Lindau tumor suppressor (VHL), transcript variant 2, mRNA. |
| PALLD | -1.71 | Homo sapiens palladin, cytoskeletal associated protein (PALLD), transcript variant 2, mRNA. |
| RBM12 | -1.71 | Homo sapiens RNA binding motif protein 12 (RBM12), transcript variant 1, mRNA. |
| UBE2O | -1.71 | Homo sapiens ubiquitin-conjugating enzyme E2O (UBE2O), mRNA. |
| WDR53 | -1.71 | Homo sapiens WD repeat domain 53 (WDR53), mRNA. |
| FNDC3A | -1.71 | Homo sapiens fibronectin type III domain containing 3A (FNDC3A), transcript variant 1, mRNA. |
| MBTD1 | -1.71 | Homo sapiens mbt domain containing 1 (MBTD1), mRNA. |
| ACACB | -1.71 | Homo sapiens acetyl-Coenzyme A carboxylase beta (ACACB), mRNA. |
| DDX46 | -1.72 | Homo sapiens DEAD (Asp-Glu-Ala-Asp) box polypeptide 46 (DDX46), mRNA. |
| CMBL | -1.72 | Homo sapiens carboxymethylenebutenolidase homolog (Pseudomonas) (CMBL), mRNA. |
| UBE2C | -1.72 | Homo sapiens ubiquitin-conjugating enzyme E2C (UBE2C), transcript variant 2, mRNA. |
| ASPM | -1.72 | Homo sapiens asp (abnormal spindle) homolog, microcephaly associated (Drosophila) (ASPM), mRNA. |
| RHOBTB3 | -1.72 | Homo sapiens Rho-related BTB domain containing 3 (RHOBTB3), mRNA. |
| BAIAP2L1 | -1.72 | Homo sapiens BAI1-associated protein 2-like 1 (BAIAP2L1), mRNA. |
| MKNK1 | -1.72 | Homo sapiens MAP kinase interacting serine/threonine kinase 1 (MKNK1), transcript variant 1, mRNA. |
| BNIP1 | -1.72 | Homo sapiens BCL2/adenovirus E1B 19kDa interacting protein 1 (BNIP1), transcript variant BNIP1-b, mRNA. |
| RXRA | -1.72 | Homo sapiens retinoid X receptor, alpha (RXRA), mRNA. |
| TMEM77 | -1.72 | Homo sapiens transmembrane protein 77 (TMEM77), mRNA. |
| KLF9 | -1.72 | Homo sapiens Kruppel-like factor 9 (KLF9), mRNA. |
| MAD1L1 | -1.72 | Homo sapiens MAD1 mitotic arrest deficient-like 1 (yeast) (MAD1L1), transcript variant 2, mRNA. |
| UCHL3 | -1.72 | Homo sapiens ubiquitin carboxyl-terminal esterase L3 (ubiquitin thiolesterase) (UCHL3), mRNA. |
| SNAP91 | -1.72 | Homo sapiens synaptosomal-associated protein, 91kDa homolog (mouse) (SNAP91), mRNA. |
|  | -1.72 | Homo sapiens mRNA; cDNA DKFZp686P23119 (from clone DKFZp686P23119) |
| FBXO11 | -1.72 | Homo sapiens F-box protein 11 (FBXO11), transcript variant 2, mRNA. |
| ACSL4 | -1.72 | Homo sapiens acyl-CoA synthetase long-chain family member 4 (ACSL4), transcript variant 1, mRNA. |
| PTPN12 | -1.72 | Homo sapiens protein tyrosine phosphatase, non-receptor type 12 (PTPN12), mRNA. |
| LANCL2 | -1.72 | Homo sapiens LanC lantibiotic synthetase component C-like 2 (bacterial) (LANCL2), mRNA. |
| ELAC1 | -1.72 | Homo sapiens elaC homolog 1 (E. coli) (ELAC1), mRNA. |
| C14orf135 | -1.72 | Homo sapiens chromosome 14 open reading frame 135 (C14orf135), mRNA. |
| C1orf165 | -1.73 | Homo sapiens chromosome 1 open reading frame 165 (C1orf165), mRNA. |
| CSRNP3 | -1.73 | Homo sapiens cysteine-serine-rich nuclear protein 3 (CSRNP3), mRNA. |
| SYT1 | -1.73 | Homo sapiens synaptotagmin I (SYT1), mRNA. |
| TSPAN31 | -1.73 | Homo sapiens tetraspanin 31 (TSPAN31), mRNA. |
| ZNF789 | -1.73 | Homo sapiens zinc finger protein 789 (ZNF789), transcript variant 2, mRNA. |
| APAF1 | -1.73 | Homo sapiens apoptotic peptidase activating factor 1 (APAF1), transcript variant 1, mRNA. |
| PRPH | -1.73 | Homo sapiens peripherin (PRPH), mRNA. |
| EML5 | -1.73 | Homo sapiens echinoderm microtubule associated protein like 5 (EML5), mRNA. |
| EZH2 | -1.73 | Homo sapiens enhancer of zeste homolog 2 (Drosophila) (EZH2), transcript variant 1, mRNA. |
| EPS15 | -1.73 | Homo sapiens epidermal growth factor receptor pathway substrate 15 (EPS15), transcript variant 1, mRNA. |
| ZWILCH | -1.73 | Homo sapiens Zwilch, kinetochore associated, homolog (Drosophila) (ZWILCH), transcript variant 2, transcribed RNA. |
| PPP1R9A | -1.73 | PREDICTED: Homo sapiens protein phosphatase 1, regulatory (inhibitor) subunit 9A, transcript variant 3 (PPP1R9A), mRNA. |
| NOX4 | -1.73 | Homo sapiens NADPH oxidase 4 (NOX4), mRNA. |
| SNORD31 | -1.73 | Homo sapiens small nucleolar RNA, C/D box 31 (SNORD31), small nucleolar RNA. |
| CDC2L2 | -1.73 | Homo sapiens cell division cycle 2-like 2 (PITSLRE proteins) (CDC2L2), transcript variant 1, mRNA. XM_001713745 XM_001713746 XM_001713747 |
| FOXD4 | -1.74 | Homo sapiens forkhead box D4 (FOXD4), mRNA. |
| WDSUB1 | -1.74 | Homo sapiens WD repeat, sterile alpha motif and U-box domain containing 1 (WDSUB1), mRNA. |
| ABCB1 | -1.74 | Homo sapiens ATP-binding cassette, sub-family B (MDR/TAP), member 1 (ABCB1), mRNA. |
| ICK | -1.74 | Homo sapiens intestinal cell (MAK-like) kinase (ICK), transcript variant 2, mRNA. |
| PPHLN1 | -1.74 | Homo sapiens periphilin 1 (PPHLN1), transcript variant 4, mRNA. |
| LOC402560 | -1.74 | PREDICTED: Homo sapiens hypothetical LOC402560 (LOC402560), mRNA. |
| PRAME | -1.74 | Homo sapiens preferentially expressed antigen in melanoma (PRAME), transcript variant 2, mRNA. |
| SNRK | -1.74 | Homo sapiens SNF related kinase (SNRK), mRNA. |
| FRMD6 | -1.74 | Homo sapiens FERM domain containing 6 (FRMD6), mRNA. |
| SMC6 | -1.74 | Homo sapiens structural maintenance of chromosomes 6 (SMC6), mRNA. |
| ZBTB17 | -1.74 | Homo sapiens zinc finger and BTB domain containing 17 (ZBTB17), mRNA. |
| EXOSC8 | -1.74 | Homo sapiens exosome component 8 (EXOSC8), mRNA. |
| VPS8 | -1.74 | Homo sapiens vacuolar protein sorting 8 homolog (S. cerevisiae) (VPS8), transcript variant 2, mRNA. |
| SEC16A | -1.74 | Homo sapiens SEC16 homolog A (S. cerevisiae) (SEC16A), mRNA. |
| PER2 | -1.74 | Homo sapiens period homolog 2 (Drosophila) (PER2), mRNA. |
| PRR5 | -1.74 | Homo sapiens proline rich 5 (renal) (PRR5), transcript variant 5, mRNA. |
| NT5C3 | -1.74 | Homo sapiens 5'-nucleotidase, cytosolic III (NT5C3), transcript variant 1, mRNA. |
| CUX2 | -1.74 | Homo sapiens cut-like homeobox 2 (CUX2), mRNA. |
| CNOT6 | -1.74 | Homo sapiens CCR4-NOT transcription complex, subunit 6 (CNOT6), mRNA. |
| GPM6B | -1.75 | Homo sapiens glycoprotein M6B (GPM6B), transcript variant 1, mRNA. |
| ATP5S | -1.75 | Homo sapiens ATP synthase, H+ transporting, mitochondrial F0 complex, subunit s (factor B) (ATP5S), nuclear gene encoding mitochondrial protein, transcript variant 2, mRNA. |
| CCDC15 | -1.75 | Homo sapiens coiled-coil domain containing 15 (CCDC15), mRNA. |
| CRNKL1 | -1.75 | Homo sapiens crooked neck pre-mRNA splicing factor-like 1 (Drosophila) (CRNKL1), mRNA. |
| RPUSD1 | -1.75 | Homo sapiens RNA pseudouridylate synthase domain containing 1 (RPUSD1), mRNA. |
| SPC25 | -1.75 | Homo sapiens SPC25, NDC80 kinetochore complex component, homolog (S. cerevisiae) (SPC25), mRNA. |
| HIST2H2AC | -1.75 | Homo sapiens histone cluster 2, H2ac (HIST2H2AC), mRNA. |
| LOC728499 | -1.75 | PREDICTED: Homo sapiens similar to Nuclear envelope pore membrane protein POM 121 (Pore membrane protein of 121 kDa) (P145) (LOC728499), mRNA. |
| AKAP8L | -1.75 | Homo sapiens A kinase (PRKA) anchor protein 8-like (AKAP8L), mRNA. |
| SUPT3H | -1.75 | Homo sapiens suppressor of Ty 3 homolog (S. cerevisiae) (SUPT3H), transcript variant 2, mRNA. |
| ANAPC10 | -1.75 | Homo sapiens anaphase promoting complex subunit 10 (ANAPC10), mRNA. |
| ADAMTS9 | -1.75 | Homo sapiens ADAM metallopeptidase with thrombospondin type 1 motif, 9 (ADAMTS9), mRNA. |
| ACTR5 | -1.75 | Homo sapiens ARP5 actin-related protein 5 homolog (yeast) (ACTR5), mRNA. |
| ANKRD28 | -1.75 | Homo sapiens ankyrin repeat domain 28 (ANKRD28), mRNA. |
| C16orf7 | -1.75 | Homo sapiens chromosome 16 open reading frame 7 (C16orf7), mRNA. |
| KIAA0368 | -1.76 | Homo sapiens KIAA0368 (KIAA0368), mRNA. |
| SNORD4A | -1.76 | Homo sapiens small nucleolar RNA, C/D box 4A (SNORD4A), small nucleolar RNA. |
|  | -1.76 | Homo sapiens primary neuroblastoma cDNA, clone:Nbla10527, full insert sequence |
| UBXN11 | -1.76 | Homo sapiens UBX domain protein 11 (UBXN11), transcript variant 2, mRNA. |
| C13orf25 | -1.76 | PREDICTED: Homo sapiens chromosome 13 open reading frame 25 (C13orf25), mRNA. |
| RB1CC1 | -1.76 | Homo sapiens RB1-inducible coiled-coil 1 (RB1CC1), transcript variant 2, mRNA. |
| PDE3B | -1.76 | Homo sapiens phosphodiesterase 3B, cGMP-inhibited (PDE3B), mRNA. |
| IDS | -1.76 | Homo sapiens iduronate 2-sulfatase (Hunter syndrome) (IDS), transcript variant 2, mRNA. |
| BICD2 | -1.76 | Homo sapiens bicaudal D homolog 2 (Drosophila) (BICD2), transcript variant 1, mRNA. |
| HNRNPU | -1.76 | Homo sapiens heterogeneous nuclear ribonucleoprotein U (scaffold attachment factor A) (HNRNPU), transcript variant 2, mRNA. |
| CGN | -1.76 | Homo sapiens cingulin (CGN), mRNA. |
|  | -1.76 | Homo sapiens cDNA clone IMAGE:30332316 |
| TRIM68 | -1.76 | Homo sapiens tripartite motif-containing 68 (TRIM68), mRNA. |
| PTGES3 | -1.76 | Homo sapiens prostaglandin E synthase 3 (cytosolic) (PTGES3), mRNA. |
| USF2 | -1.76 | Homo sapiens upstream transcription factor 2, c-fos interacting (USF2), transcript variant 2, mRNA. |
| DIRC2 | -1.76 | Homo sapiens disrupted in renal carcinoma 2 (DIRC2), mRNA. |
| RFESD | -1.76 | Homo sapiens Rieske (Fe-S) domain containing (RFESD), mRNA. |
| C5orf5 | -1.76 | Homo sapiens chromosome 5 open reading frame 5 (C5orf5), mRNA. |
| KLF6 | -1.76 | Homo sapiens Kruppel-like factor 6 (KLF6), transcript variant 1, mRNA. |
| TOX | -1.76 | Homo sapiens thymocyte selection-associated high mobility group box (TOX), mRNA. |
| UBE2H | -1.76 | Homo sapiens ubiquitin-conjugating enzyme E2H (UBC8 homolog, yeast) (UBE2H), transcript variant 1, mRNA. |
| HPS5 | -1.76 | Homo sapiens Hermansky-Pudlak syndrome 5 (HPS5), transcript variant 2, mRNA. |
| C5 | -1.77 | Homo sapiens complement component 5 (C5), mRNA. |
| MRS2 | -1.77 | Homo sapiens MRS2 magnesium homeostasis factor homolog (S. cerevisiae) (MRS2), mRNA. |
| LOC283174 | -1.77 | Homo sapiens hypothetical protein LOC283174 (LOC283174), mRNA. |
| TMEM181 | -1.77 | Homo sapiens transmembrane protein 181 (TMEM181), mRNA. |
| RBM39 | -1.77 | Homo sapiens RNA binding motif protein 39 (RBM39), transcript variant 1, mRNA. |
| SGOL2 | -1.77 | Homo sapiens shugoshin-like 2 (S. pombe) (SGOL2), mRNA. |
| SNRPD3 | -1.77 | Homo sapiens small nuclear ribonucleoprotein D3 polypeptide 18kDa (SNRPD3), mRNA. |
| BAMBI | -1.77 | Homo sapiens BMP and activin membrane-bound inhibitor homolog (Xenopus laevis) (BAMBI), mRNA. |
| ADCY1 | -1.77 | Homo sapiens adenylate cyclase 1 (brain) (ADCY1), mRNA. |
| MAPK6 | -1.77 | Homo sapiens mitogen-activated protein kinase 6 (MAPK6), mRNA. |
| GNA11 | -1.77 | Homo sapiens guanine nucleotide binding protein (G protein), alpha 11 (Gq class) (GNA11), mRNA. |
| ABCE1 | -1.77 | Homo sapiens ATP-binding cassette, sub-family E (OABP), member 1 (ABCE1), transcript variant 2, mRNA. |
| ZNF83 | -1.78 | Homo sapiens zinc finger protein 83 (ZNF83), mRNA. |
| MYD88 | -1.78 | Homo sapiens myeloid differentiation primary response gene (88) (MYD88), mRNA. |
| FHL3 | -1.78 | Homo sapiens four and a half LIM domains 3 (FHL3), mRNA. |
| GAA | -1.78 | Homo sapiens glucosidase, alpha; acid (GAA), transcript variant 3, mRNA. |
| MYL5 | -1.78 | Homo sapiens myosin, light chain 5, regulatory (MYL5), mRNA. |
| ITFG3 | -1.78 | Homo sapiens integrin alpha FG-GAP repeat containing 3 (ITFG3), mRNA. |
| NXPH1 | -1.78 | Homo sapiens neurexophilin 1 (NXPH1), mRNA. |
| SULT1A4 | -1.78 | Homo sapiens sulfotransferase family, cytosolic, 1A, phenol-preferring, member 4 (SULT1A4), transcript variant 3, mRNA. |
| DVL2 | -1.78 | Homo sapiens dishevelled, dsh homolog 2 (Drosophila) (DVL2), mRNA. |
| PTEN | -1.78 | Homo sapiens phosphatase and tensin homolog (PTEN), mRNA. |
| KRR1 | -1.78 | Homo sapiens KRR1, small subunit (SSU) processome component, homolog (yeast) (KRR1), mRNA. |
| C3orf26 | -1.78 | Homo sapiens chromosome 3 open reading frame 26 (C3orf26), mRNA. |
| BPNT1 | -1.78 | Homo sapiens 3'(2'), 5'-bisphosphate nucleotidase 1 (BPNT1), mRNA. |
|  | -1.78 | FNPARC07 FNP Homo sapiens cDNA, mRNA sequence |
| NSUN5B | -1.78 | Homo sapiens NOP2/Sun domain family, member 5B (NSUN5B), transcript variant 2, mRNA. |
| CCDC53 | -1.78 | Homo sapiens coiled-coil domain containing 53 (CCDC53), mRNA. |
| PROS1 | -1.78 | Homo sapiens protein S (alpha) (PROS1), mRNA. |
| CCDC112 | -1.79 | Homo sapiens coiled-coil domain containing 112 (CCDC112), transcript variant 1, mRNA. |
| HPS1 | -1.79 | Homo sapiens Hermansky-Pudlak syndrome 1 (HPS1), transcript variant 3, mRNA. |
| VAMP7 | -1.79 | Homo sapiens vesicle-associated membrane protein 7 (VAMP7), mRNA. |
| AMH | -1.79 | Homo sapiens anti-Mullerian hormone (AMH), mRNA. |
| USP48 | -1.79 | Homo sapiens ubiquitin specific peptidase 48 (USP48), transcript variant 2, mRNA. |
| NCOR2 | -1.79 | Homo sapiens nuclear receptor co-repressor 2 (NCOR2), transcript variant 1, mRNA. |
| ZWILCH | -1.79 | Homo sapiens Zwilch, kinetochore associated, homolog (Drosophila) (ZWILCH), transcript variant 1, mRNA. |
| RRAGB | -1.79 | Homo sapiens Ras-related GTP binding B (RRAGB), transcript variant RAGBs, mRNA. |
| SH2B3 | -1.79 | Homo sapiens SH2B adaptor protein 3 (SH2B3), mRNA. |
| NIPBL | -1.79 | Homo sapiens Nipped-B homolog (Drosophila) (NIPBL), transcript variant A, mRNA. |
| CLK2 | -1.79 | PREDICTED: Homo sapiens CDC-like kinase 2, transcript variant 3 (CLK2), mRNA. |
| SLC8A3 | -1.79 | Homo sapiens solute carrier family 8 (sodium/calcium exchanger), member 3 (SLC8A3), transcript variant f, mRNA. |
| AATF | -1.79 | Homo sapiens apoptosis antagonizing transcription factor (AATF), mRNA. |
| MYST3 | -1.79 | Homo sapiens MYST histone acetyltransferase (monocytic leukemia) 3 (MYST3), mRNA. |
| FUBP1 | -1.8 | Homo sapiens far upstream element (FUSE) binding protein 1 (FUBP1), mRNA. |
| SNORA70 | -1.8 | Homo sapiens small nucleolar RNA, H/ACA box 70 (SNORA70), small nucleolar RNA. |
| SLU7 | -1.8 | Homo sapiens SLU7 splicing factor homolog (S. cerevisiae) (SLU7), mRNA. |
| TTC15 | -1.8 | Homo sapiens tetratricopeptide repeat domain 15 (TTC15), mRNA. |
| RAB28 | -1.8 | Homo sapiens RAB28, member RAS oncogene family (RAB28), transcript variant 2, mRNA. |
| SUPT5H | -1.8 | Homo sapiens suppressor of Ty 5 homolog (S. cerevisiae) (SUPT5H), mRNA. |
| IRF2BP2 | -1.8 | Homo sapiens interferon regulatory factor 2 binding protein 2 (IRF2BP2), transcript variant 1, mRNA. |
| ASB6 | -1.81 | Homo sapiens ankyrin repeat and SOCS box-containing 6 (ASB6), transcript variant 2, mRNA. |
| DLAT | -1.81 | Homo sapiens dihydrolipoamide S-acetyltransferase (DLAT), mRNA. |
| NBPF20 | -1.81 | Homo sapiens neuroblastoma breakpoint family, member 20 (NBPF20), mRNA. |
| ZFP37 | -1.81 | Homo sapiens zinc finger protein 37 homolog (mouse) (ZFP37), mRNA. |
|  | -1.81 | full-length cDNA clone CS0DI004YB08 of Placenta Cot 25-normalized of Homo sapiens (human) |
| ACY1 | -1.81 | Homo sapiens aminoacylase 1 (ACY1), mRNA. |
| FLJ25006 | -1.81 | Homo sapiens uncharacterized serine/threonine-protein kinase SgK494 (FLJ25006), mRNA. |
| CACHD1 | -1.81 | Homo sapiens cache domain containing 1 (CACHD1), mRNA. |
| PLXNA3 | -1.81 | Homo sapiens plexin A3 (PLXNA3), mRNA. |
| BMPR2 | -1.81 | Homo sapiens bone morphogenetic protein receptor, type II (serine/threonine kinase) (BMPR2), mRNA. |
| NPIP | -1.81 | Homo sapiens nuclear pore complex interacting protein (NPIP), mRNA. |
| TMEM126A | -1.81 | Homo sapiens transmembrane protein 126A (TMEM126A), mRNA. |
| TGFBRAP1 | -1.82 | Homo sapiens transforming growth factor, beta receptor associated protein 1 (TGFBRAP1), mRNA. |
| GTPBP3 | -1.82 | Homo sapiens GTP binding protein 3 (mitochondrial) (GTPBP3), nuclear gene encoding mitochondrial protein, transcript variant V, mRNA. |
| ZBTB8OS | -1.82 | Homo sapiens zinc finger and BTB domain containing 8 opposite strand (ZBTB8OS), mRNA. |
| RALGAPA1 | -1.82 | Homo sapiens Ral GTPase activating protein, alpha subunit 1 (catalytic) (RALGAPA1), transcript variant 2, mRNA. |
| ANXA7 | -1.82 | Homo sapiens annexin A7 (ANXA7), transcript variant 2, mRNA. |
| GPAM | -1.82 | Homo sapiens glycerol-3-phosphate acyltransferase, mitochondrial (GPAM), nuclear gene encoding mitochondrial protein, mRNA. |
| TMEM64 | -1.82 | Homo sapiens transmembrane protein 64 (TMEM64), mRNA. |
| BIRC2 | -1.82 | Homo sapiens baculoviral IAP repeat-containing 2 (BIRC2), mRNA. |
| CMPK1 | -1.82 | Homo sapiens cytidine monophosphate (UMP-CMP) kinase 1, cytosolic (CMPK1), mRNA. |
| TCTEX1D2 | -1.82 | Homo sapiens Tctex1 domain containing 2 (TCTEX1D2), mRNA. |
| FEZ1 | -1.82 | Homo sapiens fasciculation and elongation protein zeta 1 (zygin I) (FEZ1), transcript variant 1, mRNA. |
| ATP2C1 | -1.82 | Homo sapiens ATPase, Ca++ transporting, type 2C, member 1 (ATP2C1), transcript variant 1, mRNA. |
| 4-Mar | -1.82 | Homo sapiens membrane-associated ring finger (C3HC4) 4 (MARCH4), mRNA. |
| DECR2 | -1.83 | Homo sapiens 2,4-dienoyl CoA reductase 2, peroxisomal (DECR2), mRNA. |
| SLC35C2 | -1.83 | Homo sapiens solute carrier family 35, member C2 (SLC35C2), transcript variant 3, mRNA. |
| SDCBP | -1.83 | Homo sapiens syndecan binding protein (syntenin) (SDCBP), transcript variant 2, mRNA. |
| GRIA2 | -1.83 | Homo sapiens glutamate receptor, ionotropic, AMPA 2 (GRIA2), mRNA. |
| CDRT4 | -1.83 | Homo sapiens CMT1A duplicated region transcript 4 (CDRT4), mRNA. |
| KIDINS220 | -1.83 | Homo sapiens kinase D-interacting substrate of 220 kDa (KIDINS220), mRNA. |
| PDE4D | -1.83 | Homo sapiens phosphodiesterase 4D, cAMP-specific (phosphodiesterase E3 dunce homolog, Drosophila) (PDE4D), mRNA. |
| MCM10 | -1.83 | Homo sapiens minichromosome maintenance complex component 10 (MCM10), transcript variant 2, mRNA. |
| ZNF211 | -1.83 | Homo sapiens zinc finger protein 211 (ZNF211), transcript variant 1, mRNA. |
| FANCD2 | -1.83 | Homo sapiens Fanconi anemia, complementation group D2 (FANCD2), transcript variant 2, mRNA. |
| MXD3 | -1.83 | Homo sapiens MAX dimerization protein 3 (MXD3), mRNA. |
| CENPA | -1.83 | Homo sapiens centromere protein A (CENPA), transcript variant 2, mRNA. |
| ING3 | -1.83 | Homo sapiens inhibitor of growth family, member 3 (ING3), transcript variant 3, mRNA. |
| DIAPH1 | -1.83 | Homo sapiens diaphanous homolog 1 (Drosophila) (DIAPH1), transcript variant 1, mRNA. |
| YPEL5 | -1.83 | Homo sapiens yippee-like 5 (Drosophila) (YPEL5), mRNA. |
| KIAA0427 | -1.83 | Homo sapiens KIAA0427 (KIAA0427), mRNA. |
| ZNF365 | -1.83 | Homo sapiens zinc finger protein 365 (ZNF365), transcript variant A, mRNA. |
| FGFR1OP | -1.83 | Homo sapiens FGFR1 oncogene partner (FGFR1OP), transcript variant 1, mRNA. |
| LOC730996 | -1.84 | PREDICTED: Homo sapiens similar to chromosome 1 open reading frame 80 (LOC730996), mRNA. |
| ANKRD50 | -1.84 | Homo sapiens ankyrin repeat domain 50 (ANKRD50), mRNA. |
| RAB4A | -1.84 | Homo sapiens RAB4A, member RAS oncogene family (RAB4A), mRNA. |
| C13orf23 | -1.84 | Homo sapiens chromosome 13 open reading frame 23 (C13orf23), transcript variant 1, mRNA. |
| SMTN | -1.84 | Homo sapiens smoothelin (SMTN), transcript variant 3, mRNA. |
| DNAJC25 | -1.84 | Homo sapiens DnaJ (Hsp40) homolog, subfamily C , member 25 (DNAJC25), mRNA. |
| SC65 | -1.84 | Homo sapiens synaptonemal complex protein SC65 (SC65), mRNA. |
| ANXA11 | -1.84 | Homo sapiens annexin A11 (ANXA11), transcript variant a, mRNA. |
| ZNF543 | -1.84 | Homo sapiens zinc finger protein 543 (ZNF543), mRNA. |
| ZNF195 | -1.84 | Homo sapiens zinc finger protein 195 (ZNF195), mRNA. |
| ANKRD20A1 | -1.84 | Homo sapiens ankyrin repeat domain 20 family, member A1 (ANKRD20A1), mRNA. |
| POPDC3 | -1.84 | Homo sapiens popeye domain containing 3 (POPDC3), mRNA. |
| NUDT9 | -1.84 | Homo sapiens nudix (nucleoside diphosphate linked moiety X)-type motif 9 (NUDT9), transcript variant 3, mRNA. |
| MCM2 | -1.84 | Homo sapiens minichromosome maintenance complex component 2 (MCM2), mRNA. |
| STX4 | -1.84 | Homo sapiens syntaxin 4 (STX4), mRNA. |
| WRN | -1.85 | Homo sapiens Werner syndrome (WRN), mRNA. |
| ZNF18 | -1.85 | Homo sapiens zinc finger protein 18 (ZNF18), mRNA. |
| PHKA2 | -1.85 | Homo sapiens phosphorylase kinase, alpha 2 (liver) (PHKA2), mRNA. |
| C16orf63 | -1.85 | Homo sapiens chromosome 16 open reading frame 63 (C16orf63), mRNA. |
| CEP70 | -1.85 | Homo sapiens centrosomal protein 70kDa (CEP70), mRNA. |
| CCPG1 | -1.85 | Homo sapiens cell cycle progression 1 (CCPG1), transcript variant 2, mRNA. |
| DLGAP4 | -1.85 | Homo sapiens discs, large (Drosophila) homolog-associated protein 4 (DLGAP4), transcript variant 3, mRNA. |
| ACADVL | -1.85 | Homo sapiens acyl-Coenzyme A dehydrogenase, very long chain (ACADVL), nuclear gene encoding mitochondrial protein, transcript variant 1, mRNA. |
| UCHL5 | -1.85 | Homo sapiens ubiquitin carboxyl-terminal hydrolase L5 (UCHL5), mRNA. |
| RPAP2 | -1.85 | Homo sapiens RNA polymerase II associated protein 2 (RPAP2), mRNA. |
| ZNHIT6 | -1.85 | Homo sapiens zinc finger, HIT type 6 (ZNHIT6), mRNA. |
|  | -1.86 | BX114974 NCI_CGAP_Kid3 Homo sapiens cDNA clone IMAGp998C023886, mRNA sequence |
| CENPE | -1.86 | Homo sapiens centromere protein E, 312kDa (CENPE), mRNA. |
| DIP2B | -1.86 | Homo sapiens DIP2 disco-interacting protein 2 homolog B (Drosophila) (DIP2B), mRNA. |
| WWP2 | -1.86 | Homo sapiens WW domain containing E3 ubiquitin protein ligase 2 (WWP2), transcript variant 3, mRNA. |
| LOC731878 | -1.86 | PREDICTED: Homo sapiens similar to MAPK-interacting and spindle-stabilizing protein (LOC731878), mRNA. |
| SH3KBP1 | -1.86 | Homo sapiens SH3-domain kinase binding protein 1 (SH3KBP1), transcript variant 1, mRNA. |
| FKBP11 | -1.86 | Homo sapiens FK506 binding protein 11, 19 kDa (FKBP11), mRNA. |
| TNFRSF25 | -1.86 | Homo sapiens tumor necrosis factor receptor superfamily, member 25 (TNFRSF25), transcript variant 10, mRNA. |
| CENPM | -1.86 | Homo sapiens centromere protein M (CENPM), transcript variant 2, mRNA. |
| EIF2AK2 | -1.86 | Homo sapiens eukaryotic translation initiation factor 2-alpha kinase 2 (EIF2AK2), mRNA. |
| CENPA | -1.86 | Homo sapiens centromere protein A (CENPA), transcript variant 2, mRNA. |
| FOXJ3 | -1.86 | Homo sapiens forkhead box J3 (FOXJ3), mRNA. |
| TTC25 | -1.86 | Homo sapiens tetratricopeptide repeat domain 25 (TTC25), mRNA. |
| ARL1 | -1.86 | Homo sapiens ADP-ribosylation factor-like 1 (ARL1), mRNA. |
| ZCCHC7 | -1.86 | Homo sapiens zinc finger, CCHC domain containing 7 (ZCCHC7), mRNA. |
| SKA2 | -1.86 | Homo sapiens spindle and kinetochore associated complex subunit 2 (SKA2), transcript variant 1, mRNA. |
| PRRT2 | -1.86 | Homo sapiens proline-rich transmembrane protein 2 (PRRT2), mRNA. |
| TRIOBP | -1.87 | Homo sapiens TRIO and F-actin binding protein (TRIOBP), transcript variant 2, mRNA. |
| TNPO3 | -1.87 | Homo sapiens transportin 3 (TNPO3), mRNA. |
| KIAA2026 | -1.87 | Homo sapiens KIAA2026 (KIAA2026), mRNA. |
| GAS8 | -1.87 | Homo sapiens growth arrest-specific 8 (GAS8), mRNA. |
| ATXN7L2 | -1.87 | Homo sapiens ataxin 7-like 2 (ATXN7L2), mRNA. |
| RBM15 | -1.87 | Homo sapiens RNA binding motif protein 15 (RBM15), mRNA. |
| NOTCH3 | -1.87 | Homo sapiens Notch homolog 3 (Drosophila) (NOTCH3), mRNA. |
| MIB2 | -1.87 | Homo sapiens mindbomb homolog 2 (Drosophila) (MIB2), mRNA. |
|  | -1.87 | Homo sapiens mRNA full length insert cDNA clone EUROIMAGE 966164 |
| TMEM70 | -1.87 | Homo sapiens transmembrane protein 70 (TMEM70), transcript variant 1, mRNA. |
| GMEB1 | -1.87 | Homo sapiens glucocorticoid modulatory element binding protein 1 (GMEB1), transcript variant 2, mRNA. |
| SNX7 | -1.87 | Homo sapiens sorting nexin 7 (SNX7), transcript variant 2, mRNA. |
| MYO19 | -1.87 | Homo sapiens myosin XIX (MYO19), transcript variant 3, mRNA. |
| COX11 | -1.87 | Homo sapiens COX11 homolog, cytochrome c oxidase assembly protein (yeast) (COX11), nuclear gene encoding mitochondrial protein, mRNA. |
| CDC25C | -1.88 | Homo sapiens cell division cycle 25 homolog C (S. pombe) (CDC25C), transcript variant 2, mRNA. |
|  | -1.88 | Homo sapiens mRNA; cDNA DKFZp686E0389 (from clone DKFZp686E0389) |
| DMTF1 | -1.88 | Homo sapiens cyclin D binding myb-like transcription factor 1 (DMTF1), mRNA. |
| SH2D3C | -1.88 | Homo sapiens SH2 domain containing 3C (SH2D3C), transcript variant 2, mRNA. |
| REV1 | -1.88 | Homo sapiens REV1 homolog (S. cerevisiae) (REV1), transcript variant 1, mRNA. |
| TADA1L | -1.88 | Homo sapiens transcriptional adaptor 1 (HFI1 homolog, yeast)-like (TADA1L), mRNA. |
| FLJ39632 | -1.88 | PREDICTED: Homo sapiens misc_RNA (FLJ39632), miscRNA. |
| KIAA1012 | -1.88 | Homo sapiens KIAA1012 (KIAA1012), mRNA. |
| ZNF280D | -1.88 | Homo sapiens zinc finger protein 280D (ZNF280D), transcript variant 3, mRNA. |
| C17orf58 | -1.88 | Homo sapiens chromosome 17 open reading frame 58 (C17orf58), transcript variant 2, mRNA. |
| RCCD1 | -1.88 | Homo sapiens RCC1 domain containing 1 (RCCD1), transcript variant 2, mRNA. |
| ZNF423 | -1.88 | Homo sapiens zinc finger protein 423 (ZNF423), mRNA. |
| HTRA1 | -1.89 | Homo sapiens HtrA serine peptidase 1 (HTRA1), mRNA. |
| PPIG | -1.89 | Homo sapiens peptidylprolyl isomerase G (cyclophilin G) (PPIG), mRNA. |
| PDHX | -1.89 | Homo sapiens pyruvate dehydrogenase complex, component X (PDHX), nuclear gene encoding mitochondrial protein, mRNA. |
| GOLGA8B | -1.89 | Homo sapiens golgi autoantigen, golgin subfamily a, 8B (GOLGA8B), mRNA. |
| ITFG2 | -1.89 | Homo sapiens integrin alpha FG-GAP repeat containing 2 (ITFG2), mRNA. |
| PIM1 | -1.89 | Homo sapiens pim-1 oncogene (PIM1), mRNA. |
| BTBD7 | -1.89 | Homo sapiens BTB (POZ) domain containing 7 (BTBD7), transcript variant 1, mRNA. |
| SLC10A4 | -1.89 | Homo sapiens solute carrier family 10 (sodium/bile acid cotransporter family), member 4 (SLC10A4), mRNA. |
| PGBD1 | -1.9 | Homo sapiens piggyBac transposable element derived 1 (PGBD1), mRNA. |
| NGFRAP1 | -1.9 | Homo sapiens nerve growth factor receptor (TNFRSF16) associated protein 1 (NGFRAP1), transcript variant 3, mRNA. |
| SFRS3 | -1.9 | Homo sapiens splicing factor, arginine/serine-rich 3 (SFRS3), mRNA. |
| MLLT10 | -1.9 | Homo sapiens myeloid/lymphoid or mixed-lineage leukemia (trithorax homolog, Drosophila); translocated to, 10 (MLLT10), transcript variant 1, mRNA. |
| APOLD1 | -1.9 | Homo sapiens apolipoprotein L domain containing 1 (APOLD1), mRNA. |
| C1orf71 | -1.9 | Homo sapiens chromosome 1 open reading frame 71 (C1orf71), mRNA. |
| TIPIN | -1.9 | Homo sapiens TIMELESS interacting protein (TIPIN), mRNA. |
| NISCH | -1.9 | Homo sapiens nischarin (NISCH), mRNA. |
| CBLN4 | -1.9 | Homo sapiens cerebellin 4 precursor (CBLN4), mRNA. |
| SHPRH | -1.9 | Homo sapiens SNF2 histone linker PHD RING helicase (SHPRH), transcript variant 2, mRNA. |
| GGA3 | -1.9 | Homo sapiens golgi associated, gamma adaptin ear containing, ARF binding protein 3 (GGA3), transcript variant short, mRNA. |
| C14orf82 | -1.91 | PREDICTED: Homo sapiens chromosome 14 open reading frame 82 (C14orf82), mRNA. |
| LOC81691 | -1.91 | Homo sapiens exonuclease NEF-sp (LOC81691), mRNA. |
| TFB2M | -1.91 | Homo sapiens transcription factor B2, mitochondrial (TFB2M), nuclear gene encoding mitochondrial protein, mRNA. |
| ARHGEF3 | -1.91 | Homo sapiens Rho guanine nucleotide exchange factor (GEF) 3 (ARHGEF3), mRNA. |
| DENND5A | -1.91 | Homo sapiens DENN/MADD domain containing 5A (DENND5A), mRNA. |
| STRBP | -1.91 | Homo sapiens spermatid perinuclear RNA binding protein (STRBP), mRNA. |
| C3orf32 | -1.91 | Homo sapiens chromosome 3 open reading frame 32 (C3orf32), mRNA. |
| DLX5 | -1.91 | Homo sapiens distal-less homeobox 5 (DLX5), mRNA. |
| HNRNPU | -1.91 | Homo sapiens heterogeneous nuclear ribonucleoprotein U (scaffold attachment factor A) (HNRNPU), transcript variant 1, mRNA. |
| FAM125B | -1.91 | Homo sapiens family with sequence similarity 125, member B (FAM125B), transcript variant 1, mRNA. |
| BNIP2 | -1.91 | Homo sapiens BCL2/adenovirus E1B 19kDa interacting protein 2 (BNIP2), mRNA. |
| SPIN4 | -1.91 | Homo sapiens spindlin family, member 4 (SPIN4), mRNA. |
| C11orf17 | -1.91 | Homo sapiens chromosome 11 open reading frame 17 (C11orf17), transcript variant 1, mRNA. |
| CD47 | -1.92 | Homo sapiens CD47 molecule (CD47), transcript variant 2, mRNA. |
| WRNIP1 | -1.92 | Homo sapiens Werner helicase interacting protein 1 (WRNIP1), transcript variant 2, mRNA. |
| GPER | -1.92 | Homo sapiens G protein-coupled estrogen receptor 1 (GPER), transcript variant 3, mRNA. |
| HIGD1A | -1.92 | Homo sapiens HIG1 domain family, member 1A (HIGD1A), mRNA. |
| CCDC59 | -1.92 | Homo sapiens coiled-coil domain containing 59 (CCDC59), mRNA. |
| TMF1 | -1.92 | Homo sapiens TATA element modulatory factor 1 (TMF1), mRNA. |
| LIMA1 | -1.92 | Homo sapiens LIM domain and actin binding 1 (LIMA1), mRNA. |
| EBAG9 | -1.92 | Homo sapiens estrogen receptor binding site associated, antigen, 9 (EBAG9), transcript variant 2, mRNA. |
| C14orf138 | -1.92 | Homo sapiens chromosome 14 open reading frame 138 (C14orf138), transcript variant 2, mRNA. |
| MAGI3 | -1.93 | Homo sapiens membrane associated guanylate kinase, WW and PDZ domain containing 3 (MAGI3), transcript variant 1, mRNA. |
| C12orf43 | -1.93 | Homo sapiens chromosome 12 open reading frame 43 (C12orf43), mRNA. |
| CDC5L | -1.93 | Homo sapiens CDC5 cell division cycle 5-like (S. pombe) (CDC5L), mRNA. |
| MT1F | -1.93 | Homo sapiens metallothionein 1F (MT1F), mRNA. |
| FLJ21865 | -1.93 | Homo sapiens endo-beta-N-acetylglucosaminidase (FLJ21865), mRNA. |
| STXBP3 | -1.93 | Homo sapiens syntaxin binding protein 3 (STXBP3), mRNA. |
| LOC727762 | -1.93 | PREDICTED: Homo sapiens similar to NADH dehydrogenase (ubiquinone) 1 beta subcomplex, 4, 15kDa, transcript variant 1 (LOC727762), mRNA. |
| PCDHB4 | -1.93 | Homo sapiens protocadherin beta 4 (PCDHB4), mRNA. |
| PCYOX1 | -1.93 | Homo sapiens prenylcysteine oxidase 1 (PCYOX1), mRNA. |
| TIGD2 | -1.93 | Homo sapiens tigger transposable element derived 2 (TIGD2), mRNA. |
| GGH | -1.93 | Homo sapiens gamma-glutamyl hydrolase (conjugase, folylpolygammaglutamyl hydrolase) (GGH), mRNA. |
| C9orf82 | -1.93 | Homo sapiens chromosome 9 open reading frame 82 (C9orf82), mRNA. |
| DDX19A | -1.93 | Homo sapiens DEAD (Asp-Glu-Ala-As) box polypeptide 19A (DDX19A), mRNA. |
| SNORD14A | -1.94 | Homo sapiens small nucleolar RNA, C/D box 14A (SNORD14A), small nucleolar RNA. |
| DZIP1 | -1.94 | Homo sapiens DAZ interacting protein 1 (DZIP1), transcript variant 2, mRNA. |
| CSNK2A2 | -1.94 | Homo sapiens casein kinase 2, alpha prime polypeptide (CSNK2A2), mRNA. |
| TUBGCP4 | -1.94 | Homo sapiens tubulin, gamma complex associated protein 4 (TUBGCP4), mRNA. |
| ANKRD49 | -1.94 | Homo sapiens ankyrin repeat domain 49 (ANKRD49), mRNA. |
| LMOD3 | -1.94 | Homo sapiens leiomodin 3 (fetal) (LMOD3), mRNA. |
| BTAF1 | -1.94 | Homo sapiens BTAF1 RNA polymerase II, B-TFIID transcription factor-associated, 170kDa (Mot1 homolog, S. cerevisiae) (BTAF1), mRNA. |
| ATM | -1.94 | Homo sapiens ataxia telangiectasia mutated (ATM), transcript variant 1, mRNA. |
| KIAA1279 | -1.94 | Homo sapiens KIAA1279 (KIAA1279), mRNA. |
| TATDN1 | -1.94 | Homo sapiens TatD DNase domain containing 1 (TATDN1), mRNA. |
| PPAP2B | -1.94 | Homo sapiens phosphatidic acid phosphatase type 2B (PPAP2B), transcript variant 2, mRNA. |
| PKIA | -1.94 | Homo sapiens protein kinase (cAMP-dependent, catalytic) inhibitor alpha (PKIA), transcript variant 7, mRNA. |
| TRIM52 | -1.94 | Homo sapiens tripartite motif-containing 52 (TRIM52), mRNA. |
|  | -1.94 | Homo sapiens cDNA FLJ42306 fis, clone TRACH2001646 |
| CSTF2T | -1.94 | Homo sapiens cleavage stimulation factor, 3' pre-RNA, subunit 2, 64kDa, tau variant (CSTF2T), mRNA. |
| AFG3L1 | -1.94 | Homo sapiens AFG3 ATPase family gene 3-like 1 (yeast) (AFG3L1), transcript variant 2, mRNA. |
| VBP1 | -1.94 | Homo sapiens von Hippel-Lindau binding protein 1 (VBP1), mRNA. |
| CAMSAP1 | -1.95 | Homo sapiens calmodulin regulated spectrin-associated protein 1 (CAMSAP1), mRNA. |
| VPS54 | -1.95 | Homo sapiens vacuolar protein sorting 54 homolog (S. cerevisiae) (VPS54), transcript variant 1, mRNA. |
| GPR56 | -1.95 | Homo sapiens G protein-coupled receptor 56 (GPR56), transcript variant 2, mRNA. |
| TBC1D9 | -1.95 | Homo sapiens TBC1 domain family, member 9 (with GRAM domain) (TBC1D9), mRNA. |
| CLN5 | -1.95 | Homo sapiens ceroid-lipofuscinosis, neuronal 5 (CLN5), mRNA. |
| BAZ1A | -1.95 | Homo sapiens bromodomain adjacent to zinc finger domain, 1A (BAZ1A), transcript variant 1, mRNA. |
| SIRT1 | -1.95 | Homo sapiens sirtuin (silent mating type information regulation 2 homolog) 1 (S. cerevisiae) (SIRT1), mRNA. |
| LOC283932 | -1.95 | Homo sapiens hypothetical protein LOC283932 (LOC283932), mRNA. |
| PAK2 | -1.95 | PREDICTED: Homo sapiens p21 (CDKN1A)-activated kinase 2 (PAK2), mRNA. |
| UGP2 | -1.95 | Homo sapiens UDP-glucose pyrophosphorylase 2 (UGP2), transcript variant 1, mRNA. |
| TMTC2 | -1.95 | Homo sapiens transmembrane and tetratricopeptide repeat containing 2 (TMTC2), mRNA. |
| NLGN4X | -1.95 | Homo sapiens neuroligin 4, X-linked (NLGN4X), transcript variant 1, mRNA. |
| RBBP9 | -1.96 | Homo sapiens retinoblastoma binding protein 9 (RBBP9), mRNA. |
| USP16 | -1.96 | Homo sapiens ubiquitin specific peptidase 16 (USP16), transcript variant 1, mRNA. |
| TTC19 | -1.96 | Homo sapiens tetratricopeptide repeat domain 19 (TTC19), mRNA. |
| CREB1 | -1.96 | Homo sapiens cAMP responsive element binding protein 1 (CREB1), transcript variant A, mRNA. |
| RAB7L1 | -1.96 | Homo sapiens RAB7, member RAS oncogene family-like 1 (RAB7L1), mRNA. |
| CDC42SE2 | -1.96 | Homo sapiens CDC42 small effector 2 (CDC42SE2), transcript variant 2, mRNA. |
| SLC25A15 | -1.96 | Homo sapiens solute carrier family 25 (mitochondrial carrier; ornithine transporter) member 15 (SLC25A15), nuclear gene encoding mitochondrial protein, mRNA. |
| AGL | -1.96 | Homo sapiens amylo-1, 6-glucosidase, 4-alpha-glucanotransferase (AGL), transcript variant 5, mRNA. |
|  | -1.96 | xj89b12.x1 Soares_NFL_T_GBC_S1 Homo sapiens cDNA clone IMAGE:2664383 3, mRNA sequence |
| GABPA | -1.96 | Homo sapiens GA binding protein transcription factor, alpha subunit 60kDa (GABPA), mRNA. |
| TRPC4AP | -1.96 | Homo sapiens transient receptor potential cation channel, subfamily C, member 4 associated protein (TRPC4AP), transcript variant 1, mRNA. |
| LAMP2 | -1.97 | Homo sapiens lysosomal-associated membrane protein 2 (LAMP2), transcript variant LAMP2B, mRNA. |
| NAV1 | -1.97 | Homo sapiens neuron navigator 1 (NAV1), mRNA. |
| ZXDC | -1.97 | Homo sapiens ZXD family zinc finger C (ZXDC), mRNA. |
| PAXIP1 | -1.97 | Homo sapiens PAX interacting (with transcription-activation domain) protein 1 (PAXIP1), mRNA. |
| ZNF562 | -1.97 | Homo sapiens zinc finger protein 562 (ZNF562), mRNA. |
| PITPNC1 | -1.97 | Homo sapiens phosphatidylinositol transfer protein, cytoplasmic 1 (PITPNC1), transcript variant 2, mRNA. |
| CABYR | -1.97 | Homo sapiens calcium binding tyrosine-(Y)-phosphorylation regulated (CABYR), transcript variant 2, mRNA. |
| SIAH1 | -1.97 | Homo sapiens seven in absentia homolog 1 (Drosophila) (SIAH1), transcript variant 2, mRNA. |
| TMTC4 | -1.97 | Homo sapiens transmembrane and tetratricopeptide repeat containing 4 (TMTC4), transcript variant 1, mRNA. |
| USP6NL | -1.97 | Homo sapiens USP6 N-terminal like (USP6NL), mRNA. |
| SERPINA5 | -1.98 | Homo sapiens serpin peptidase inhibitor, clade A (alpha-1 antiproteinase, antitrypsin), member 5 (SERPINA5), mRNA. |
| GAR1 | -1.98 | Homo sapiens GAR1 ribonucleoprotein homolog (yeast) (GAR1), transcript variant 2, mRNA. |
| BAZ1A | -1.98 | Homo sapiens bromodomain adjacent to zinc finger domain, 1A (BAZ1A), transcript variant 2, mRNA. |
| TSPAN4 | -1.98 | Homo sapiens tetraspanin 4 (TSPAN4), transcript variant 6, mRNA. |
| TRAF5 | -1.98 | Homo sapiens TNF receptor-associated factor 5 (TRAF5), transcript variant 1, mRNA. |
| SLC39A11 | -1.98 | Homo sapiens solute carrier family 39 (metal ion transporter), member 11 (SLC39A11), mRNA. |
| STAM | -1.98 | Homo sapiens signal transducing adaptor molecule (SH3 domain and ITAM motif) 1 (STAM), mRNA. |
| ZSCAN5A | -1.98 | Homo sapiens zinc finger and SCAN domain containing 5A (ZSCAN5A), mRNA. |
| TMPO | -1.99 | Homo sapiens thymopoietin (TMPO), transcript variant 1, mRNA. |
| C1orf135 | -1.99 | Homo sapiens chromosome 1 open reading frame 135 (C1orf135), mRNA. |
|  | -1.99 | Human mRNA for KIAA0280 gene, partial cds |
| ANKMY2 | -1.99 | Homo sapiens ankyrin repeat and MYND domain containing 2 (ANKMY2), mRNA. |
| MAGT1 | -1.99 | Homo sapiens magnesium transporter 1 (MAGT1), mRNA. |
| TRAM2 | -1.99 | Homo sapiens translocation associated membrane protein 2 (TRAM2), mRNA. |
| CDC14B | -1.99 | Homo sapiens CDC14 cell division cycle 14 homolog B (S. cerevisiae) (CDC14B), transcript variant 2, mRNA. |
| MFSD11 | -1.99 | Homo sapiens major facilitator superfamily domain containing 11 (MFSD11), mRNA. |
| MAGEC1 | -1.99 | Homo sapiens melanoma antigen family C, 1 (MAGEC1), mRNA. |
| RBM18 | -1.99 | Homo sapiens RNA binding motif protein 18 (RBM18), mRNA. |
| MERTK | -1.99 | Homo sapiens c-mer proto-oncogene tyrosine kinase (MERTK), mRNA. |
| SLTM | -2 | Homo sapiens SAFB-like, transcription modulator (SLTM), transcript variant 1, mRNA. |
| LZTFL1 | -2 | Homo sapiens leucine zipper transcription factor-like 1 (LZTFL1), mRNA. |
| RCBTB2 | -2 | Homo sapiens regulator of chromosome condensation (RCC1) and BTB (POZ) domain containing protein 2 (RCBTB2), mRNA. |
| TYRO3 | -2 | Homo sapiens TYRO3 protein tyrosine kinase (TYRO3), mRNA. |
| LRRN3 | -2 | Homo sapiens leucine rich repeat neuronal 3 (LRRN3), mRNA. |
| TPM2 | -2 | Homo sapiens tropomyosin 2 (beta) (TPM2), transcript variant 2, mRNA. |
| GFPT1 | -2 | Homo sapiens glutamine-fructose-6-phosphate transaminase 1 (GFPT1), mRNA. |
| ANKDD1A | -2 | Homo sapiens ankyrin repeat and death domain containing 1A (ANKDD1A), mRNA. |
| RNF170 | -2.01 | Homo sapiens ring finger protein 170 (RNF170), mRNA. |
| DNAJB14 | -2.01 | Homo sapiens DnaJ (Hsp40) homolog, subfamily B, member 14 (DNAJB14), transcript variant 1, mRNA. |
| ZNF256 | -2.01 | Homo sapiens zinc finger protein 256 (ZNF256), mRNA. |
| CALD1 | -2.01 | Homo sapiens caldesmon 1 (CALD1), transcript variant 5, mRNA. |
| CCS | -2.01 | Homo sapiens copper chaperone for superoxide dismutase (CCS), mRNA. |
| USP16 | -2.01 | Homo sapiens ubiquitin specific peptidase 16 (USP16), transcript variant 1, mRNA. |
| LOC729137 | -2.01 | PREDICTED: Homo sapiens hypothetical protein LOC729137 (LOC729137), mRNA. |
| GAS6 | -2.01 | Homo sapiens growth arrest-specific 6 (GAS6), mRNA. |
| PTPN2 | -2.01 | Homo sapiens protein tyrosine phosphatase, non-receptor type 2 (PTPN2), transcript variant 2, mRNA. |
| DYRK2 | -2.01 | Homo sapiens dual-specificity tyrosine-(Y)-phosphorylation regulated kinase 2 (DYRK2), transcript variant 1, mRNA. |
| C1orf159 | -2.01 | Homo sapiens chromosome 1 open reading frame 159 (C1orf159), mRNA. |
| PDE7B | -2.01 | Homo sapiens phosphodiesterase 7B (PDE7B), mRNA. |
| SLC35F2 | -2.01 | Homo sapiens solute carrier family 35, member F2 (SLC35F2), mRNA. |
| DPY19L4 | -2.01 | Homo sapiens dpy-19-like 4 (C. elegans) (DPY19L4), mRNA. |
| GLB1 | -2.01 | Homo sapiens galactosidase, beta 1 (GLB1), transcript variant 179423, mRNA. |
| GTF2IRD1 | -2.01 | Homo sapiens GTF2I repeat domain containing 1 (GTF2IRD1), transcript variant 1, mRNA. |
| TPM2 | -2.01 | Homo sapiens tropomyosin 2 (beta) (TPM2), transcript variant 2, mRNA. |
| RAD51AP1 | -2.01 | Homo sapiens RAD51 associated protein 1 (RAD51AP1), mRNA. |
| VIP | -2.02 | Homo sapiens vasoactive intestinal peptide (VIP), transcript variant 2, mRNA. |
| MAP2K4 | -2.02 | Homo sapiens mitogen-activated protein kinase kinase 4 (MAP2K4), mRNA. |
| LACTB2 | -2.02 | Homo sapiens lactamase, beta 2 (LACTB2), mRNA. |
| ATP7A | -2.02 | Homo sapiens ATPase, Cu++ transporting, alpha polypeptide (Menkes syndrome) (ATP7A), mRNA. |
| ZNF828 | -2.02 | Homo sapiens zinc finger protein 828 (ZNF828), mRNA. |
| CALCB | -2.02 | Homo sapiens calcitonin-related polypeptide beta (CALCB), mRNA. |
| SMYD4 | -2.02 | Homo sapiens SET and MYND domain containing 4 (SMYD4), mRNA. |
| LMF2 | -2.02 | Homo sapiens lipase maturation factor 2 (LMF2), mRNA. |
| GABRB3 | -2.02 | Homo sapiens gamma-aminobutyric acid (GABA) A receptor, beta 3 (GABRB3), transcript variant 1, mRNA. |
| GATA3 | -2.03 | Homo sapiens GATA binding protein 3 (GATA3), transcript variant 2, mRNA. |
| LOC642197 | -2.03 | PREDICTED: Homo sapiens similar to Protein FAM82B (LOC642197), mRNA. |
| ERLIN2 | -2.03 | Homo sapiens ER lipid raft associated 2 (ERLIN2), transcript variant 1, mRNA. |
| KIF11 | -2.03 | Homo sapiens kinesin family member 11 (KIF11), mRNA. |
| DNAJC30 | -2.03 | Homo sapiens DnaJ (Hsp40) homolog, subfamily C, member 30 (DNAJC30), mRNA. |
| ACAD11 | -2.03 | Homo sapiens acyl-Coenzyme A dehydrogenase family, member 11 (ACAD11), mRNA. |
| CNTN4 | -2.03 | Homo sapiens contactin 4 (CNTN4), transcript variant 2, mRNA. |
| TMEFF2 | -2.03 | Homo sapiens transmembrane protein with EGF-like and two follistatin-like domains 2 (TMEFF2), mRNA. |
| C5orf35 | -2.03 | Homo sapiens chromosome 5 open reading frame 35 (C5orf35), mRNA. |
| SLC25A37 | -2.03 | Homo sapiens solute carrier family 25, member 37 (SLC25A37), nuclear gene encoding mitochondrial protein, mRNA. |
| MAFB | -2.03 | Homo sapiens v-maf musculoaponeurotic fibrosarcoma oncogene homolog B (avian) (MAFB), mRNA. |
| RAB11FIP2 | -2.03 | Homo sapiens RAB11 family interacting protein 2 (class I) (RAB11FIP2), mRNA. |
| RYBP | -2.03 | Homo sapiens RING1 and YY1 binding protein (RYBP), mRNA. |
| SLC16A10 | -2.03 | Homo sapiens solute carrier family 16, member 10 (aromatic amino acid transporter) (SLC16A10), mRNA. |
| STAT5B | -2.03 | Homo sapiens signal transducer and activator of transcription 5B (STAT5B), mRNA. |
| MED30 | -2.04 | Homo sapiens mediator complex subunit 30 (MED30), mRNA. |
| AHCTF1 | -2.04 | Homo sapiens AT hook containing transcription factor 1 (AHCTF1), mRNA. |
| JARID1A | -2.04 | Homo sapiens Jumonji, AT rich interactive domain 1A (RBBP2-like) (JARID1A), mRNA. |
| RIF1 | -2.04 | Homo sapiens RAP1 interacting factor homolog (yeast) (RIF1), mRNA. |
| ITGA3 | -2.04 | Homo sapiens integrin, alpha 3 (antigen CD49C, alpha 3 subunit of VLA-3 receptor) (ITGA3), transcript variant a, mRNA. |
| IBTK | -2.04 | Homo sapiens inhibitor of Bruton agammaglobulinemia tyrosine kinase (IBTK), mRNA. |
| PSMA2 | -2.04 | Homo sapiens proteasome (prosome, macropain) subunit, alpha type, 2 (PSMA2), mRNA. |
| ELP4 | -2.04 | Homo sapiens elongation protein 4 homolog (S. cerevisiae) (ELP4), mRNA. |
| GINS4 | -2.04 | Homo sapiens GINS complex subunit 4 (Sld5 homolog) (GINS4), mRNA. |
| EPAS1 | -2.04 | Homo sapiens endothelial PAS domain protein 1 (EPAS1), mRNA. |
| AK2 | -2.04 | Homo sapiens adenylate kinase 2 (AK2), transcript variant AK2B, mRNA. |
| DYNLT3 | -2.04 | Homo sapiens dynein, light chain, Tctex-type 3 (DYNLT3), mRNA. |
| SLC7A2 | -2.05 | Homo sapiens solute carrier family 7 (cationic amino acid transporter, y+ system), member 2 (SLC7A2), transcript variant 2, mRNA. |
| LOC153561 | -2.05 | Homo sapiens hypothetical protein LOC153561 (LOC153561), mRNA. |
| ID1 | -2.05 | Homo sapiens inhibitor of DNA binding 1, dominant negative helix-loop-helix protein (ID1), transcript variant 2, mRNA. |
| ZNF7 | -2.05 | Homo sapiens zinc finger protein 7 (ZNF7), mRNA. |
| PDE4B | -2.05 | Homo sapiens phosphodiesterase 4B, cAMP-specific (phosphodiesterase E4 dunce homolog, Drosophila) (PDE4B), transcript variant a, mRNA. |
| STAG1 | -2.05 | Homo sapiens stromal antigen 1 (STAG1), mRNA. |
| PPIL5 | -2.05 | Homo sapiens peptidylprolyl isomerase (cyclophilin)-like 5 (PPIL5), transcript variant 3, mRNA. |
| FEZ1 | -2.06 | Homo sapiens fasciculation and elongation protein zeta 1 (zygin I) (FEZ1), transcript variant 2, mRNA. |
| GPR1 | -2.06 | Homo sapiens G protein-coupled receptor 1 (GPR1), mRNA. |
| YEATS4 | -2.06 | Homo sapiens YEATS domain containing 4 (YEATS4), mRNA. |
| WDR37 | -2.06 | Homo sapiens WD repeat domain 37 (WDR37), mRNA. |
| ALKBH3 | -2.06 | Homo sapiens alkB, alkylation repair homolog 3 (E. coli) (ALKBH3), mRNA. |
| RAGE | -2.06 | Homo sapiens renal tumor antigen (RAGE), mRNA. |
| SSTR2 | -2.06 | Homo sapiens somatostatin receptor 2 (SSTR2), mRNA. |
| CUL4B | -2.06 | Homo sapiens cullin 4B (CUL4B), transcript variant 2, mRNA. |
| WASPIP | -2.07 | Homo sapiens Wiskott-Aldrich syndrome protein interacting protein (WASPIP), mRNA. |
| CCDC99 | -2.07 | Homo sapiens coiled-coil domain containing 99 (CCDC99), mRNA. |
| RWDD4A | -2.07 | Homo sapiens RWD domain containing 4A (RWDD4A), mRNA. |
| C7orf28B | -2.07 | PREDICTED: Homo sapiens chromosome 7 open reading frame 28B (C7orf28B), mRNA. |
| GMEB1 | -2.07 | Homo sapiens glucocorticoid modulatory element binding protein 1 (GMEB1), transcript variant 2, mRNA. |
| RDH10 | -2.07 | Homo sapiens retinol dehydrogenase 10 (all-trans) (RDH10), mRNA. |
| SPG11 | -2.07 | Homo sapiens spastic paraplegia 11 (autosomal recessive) (SPG11), transcript variant 1, mRNA. |
| NCOA7 | -2.07 | Homo sapiens nuclear receptor coactivator 7 (NCOA7), mRNA. |
| ESRRG | -2.08 | Homo sapiens estrogen-related receptor gamma (ESRRG), transcript variant 1, mRNA. |
| XRCC2 | -2.08 | Homo sapiens X-ray repair complementing defective repair in Chinese hamster cells 2 (XRCC2), mRNA. |
| C10orf32 | -2.08 | Homo sapiens chromosome 10 open reading frame 32 (C10orf32), mRNA. |
| SLC16A9 | -2.08 | Homo sapiens solute carrier family 16, member 9 (monocarboxylic acid transporter 9) (SLC16A9), mRNA. |
| SPAG9 | -2.08 | Homo sapiens sperm associated antigen 9 (SPAG9), mRNA. |
| SMARCC2 | -2.08 | Homo sapiens SWI/SNF related, matrix associated, actin dependent regulator of chromatin, subfamily c, member 2 (SMARCC2), transcript variant 2, mRNA. |
| C2orf30 | -2.08 | Homo sapiens chromosome 2 open reading frame 30 (C2orf30), mRNA. |
| SLC6A15 | -2.08 | Homo sapiens solute carrier family 6 (neutral amino acid transporter), member 15 (SLC6A15), transcript variant 2, mRNA. |
| NRBP2 | -2.08 | Homo sapiens nuclear receptor binding protein 2 (NRBP2), mRNA. |
|  | -2.09 | UI-E-EJ1-ajw-k-15-0-UI.r1 UI-E-EJ1 Homo sapiens cDNA clone UI-E-EJ1-ajw-k-15-0-UI 5, mRNA sequence |
| ZNF471 | -2.09 | Homo sapiens zinc finger protein 471 (ZNF471), mRNA. |
| RHBDF2 | -2.09 | Homo sapiens rhomboid 5 homolog 2 (Drosophila) (RHBDF2), transcript variant 1, mRNA. |
| NR1D2 | -2.09 | PREDICTED: Homo sapiens nuclear receptor subfamily 1, group D, member 2 (NR1D2), mRNA. |
| CUTC | -2.09 | Homo sapiens cutC copper transporter homolog (E. coli) (CUTC), mRNA. |
| ADCY3 | -2.09 | Homo sapiens adenylate cyclase 3 (ADCY3), mRNA. |
| ZFHX3 | -2.09 | Homo sapiens zinc finger homeobox 3 (ZFHX3), transcript variant A, mRNA. |
| CTSO | -2.09 | Homo sapiens cathepsin O (CTSO), mRNA. |
| FCHSD2 | -2.1 | Homo sapiens FCH and double SH3 domains 2 (FCHSD2), mRNA. |
| CACYBP | -2.1 | Homo sapiens calcyclin binding protein (CACYBP), transcript variant 2, mRNA. |
| BUB1 | -2.1 | Homo sapiens BUB1 budding uninhibited by benzimidazoles 1 homolog (yeast) (BUB1), mRNA. |
| GCA | -2.1 | Homo sapiens grancalcin, EF-hand calcium binding protein (GCA), mRNA. |
| CKAP2 | -2.1 | Homo sapiens cytoskeleton associated protein 2 (CKAP2), transcript variant 2, mRNA. |
| MAN1A1 | -2.1 | Homo sapiens mannosidase, alpha, class 1A, member 1 (MAN1A1), mRNA. |
| SEC61A2 | -2.1 | Homo sapiens Sec61 alpha 2 subunit (S. cerevisiae) (SEC61A2), mRNA. |
| ADNP2 | -2.1 | Homo sapiens ADNP homeobox 2 (ADNP2), mRNA. |
| ARID4B | -2.1 | Homo sapiens AT rich interactive domain 4B (RBP1-like) (ARID4B), transcript variant 1, mRNA. |
| TOM1L1 | -2.11 | Homo sapiens target of myb1 (chicken)-like 1 (TOM1L1), mRNA. |
| GRAMD4 | -2.11 | Homo sapiens GRAM domain containing 4 (GRAMD4), mRNA. |
| CNOT3 | -2.11 | Homo sapiens CCR4-NOT transcription complex, subunit 3 (CNOT3), mRNA. |
| PLEKHB2 | -2.11 | Homo sapiens pleckstrin homology domain containing, family B (evectins) member 2 (PLEKHB2), transcript variant 1, mRNA. |
| C15orf41 | -2.11 | Homo sapiens chromosome 15 open reading frame 41 (C15orf41), mRNA. |
| PC | -2.11 | Homo sapiens pyruvate carboxylase (PC), nuclear gene encoding mitochondrial protein, transcript variant A, mRNA. |
| WSB1 | -2.11 | Homo sapiens WD repeat and SOCS box-containing 1 (WSB1), transcript variant 3, mRNA. |
| SNTB2 | -2.11 | Homo sapiens syntrophin, beta 2 (dystrophin-associated protein A1, 59kDa, basic component 2) (SNTB2), transcript variant 1, mRNA. |
| LOC399900 | -2.11 | Homo sapiens hypothetical gene supported by AK093779 (LOC399900), mRNA. |
| PRIC285 | -2.12 | Homo sapiens peroxisomal proliferator-activated receptor A interacting complex 285 (PRIC285), transcript variant 2, mRNA. |
| PON2 | -2.12 | Homo sapiens paraoxonase 2 (PON2), transcript variant 1, mRNA. |
| IL11RA | -2.12 | Homo sapiens interleukin 11 receptor, alpha (IL11RA), transcript variant 1, mRNA. |
| SP2 | -2.12 | Homo sapiens Sp2 transcription factor (SP2), mRNA. |
| MLL3 | -2.12 | Homo sapiens myeloid/lymphoid or mixed-lineage leukemia 3 (MLL3), mRNA. |
| B4GALNT4 | -2.12 | Homo sapiens beta-1,4-N-acetyl-galactosaminyl transferase 4 (B4GALNT4), mRNA. |
| AKAP8 | -2.12 | Homo sapiens A kinase (PRKA) anchor protein 8 (AKAP8), mRNA. |
| C11orf46 | -2.12 | Homo sapiens chromosome 11 open reading frame 46 (C11orf46), mRNA. |
| ITGB3BP | -2.12 | Homo sapiens integrin beta 3 binding protein (beta3-endonexin) (ITGB3BP), mRNA. |
| PAQR3 | -2.12 | Homo sapiens progestin and adipoQ receptor family member III (PAQR3), mRNA. |
| 11-Sep | -2.13 | Homo sapiens septin 11 (SEPT11), mRNA. |
| MBNL2 | -2.13 | Homo sapiens muscleblind-like 2 (Drosophila) (MBNL2), transcript variant 3, mRNA. |
| USP1 | -2.13 | Homo sapiens ubiquitin specific peptidase 1 (USP1), transcript variant 1, mRNA. |
| FAM134B | -2.13 | Homo sapiens family with sequence similarity 134, member B (FAM134B), transcript variant 1, mRNA. |
| NFU1 | -2.13 | Homo sapiens NFU1 iron-sulfur cluster scaffold homolog (S. cerevisiae) (NFU1), transcript variant 2, mRNA. |
| SLC6A15 | -2.14 | Homo sapiens solute carrier family 6, member 15 (SLC6A15), transcript variant 1, mRNA. |
| TSPAN5 | -2.14 | Homo sapiens tetraspanin 5 (TSPAN5), mRNA. |
| C6orf111 | -2.14 | Homo sapiens chromosome 6 open reading frame 111 (C6orf111), mRNA. |
| CPT2 | -2.14 | Homo sapiens carnitine palmitoyltransferase II (CPT2), nuclear gene encoding mitochondrial protein, mRNA. |
| OIP5 | -2.14 | Homo sapiens Opa interacting protein 5 (OIP5), mRNA. |
| DDX25 | -2.14 | Homo sapiens DEAD (Asp-Glu-Ala-Asp) box polypeptide 25 (DDX25), mRNA. |
| PTPRR | -2.14 | Homo sapiens protein tyrosine phosphatase, receptor type, R (PTPRR), transcript variant 1, mRNA. |
| USP49 | -2.14 | Homo sapiens ubiquitin specific peptidase 49 (USP49), mRNA. |
| SF3B3 | -2.15 | Homo sapiens splicing factor 3b, subunit 3, 130kDa (SF3B3), mRNA. |
| SCML1 | -2.15 | Homo sapiens sex comb on midleg-like 1 (Drosophila) (SCML1), transcript variant 1, mRNA. |
| PHKB | -2.15 | Homo sapiens phosphorylase kinase, beta (PHKB), transcript variant 2, mRNA. |
| ITPRIPL2 | -2.15 | Homo sapiens inositol 1,4,5-triphosphate receptor interacting protein-like 2 (ITPRIPL2), mRNA. |
| LOC284988 | -2.15 | PREDICTED: Homo sapiens hypothetical LOC284988 (LOC284988), mRNA. |
| STX2 | -2.15 | Homo sapiens syntaxin 2 (STX2), transcript variant 1, mRNA. |
| ANKRD57 | -2.15 | Homo sapiens ankyrin repeat domain 57 (ANKRD57), mRNA. |
| ZNF800 | -2.15 | Homo sapiens zinc finger protein 800 (ZNF800), mRNA. |
| TMED7 | -2.15 | Homo sapiens transmembrane emp24 protein transport domain containing 7 (TMED7), mRNA. |
| AKR1C3 | -2.15 | Homo sapiens aldo-keto reductase family 1, member C3 (3-alpha hydroxysteroid dehydrogenase, type II) (AKR1C3), mRNA. |
| ARNTL | -2.16 | Homo sapiens aryl hydrocarbon receptor nuclear translocator-like (ARNTL), transcript variant 2, mRNA. |
| BCAR3 | -2.16 | Homo sapiens breast cancer anti-estrogen resistance 3 (BCAR3), mRNA. |
| ESPL1 | -2.16 | Homo sapiens extra spindle pole bodies homolog 1 (S. cerevisiae) (ESPL1), mRNA. |
| AURKB | -2.16 | Homo sapiens aurora kinase B (AURKB), mRNA. |
| ING3 | -2.16 | Homo sapiens inhibitor of growth family, member 3 (ING3), transcript variant 1, mRNA. |
| UTP14C | -2.16 | Homo sapiens UTP14, U3 small nucleolar ribonucleoprotein, homolog C (yeast) (UTP14C), mRNA. |
| RSRC1 | -2.16 | Homo sapiens arginine/serine-rich coiled-coil 1 (RSRC1), mRNA. |
| KIAA0562 | -2.16 | Homo sapiens KIAA0562 (KIAA0562), mRNA. |
| TMX4 | -2.16 | Homo sapiens thioredoxin-related transmembrane protein 4 (TMX4), mRNA. |
| CCNG2 | -2.16 | Homo sapiens cyclin G2 (CCNG2), mRNA. |
| PAG1 | -2.16 | Homo sapiens phosphoprotein associated with glycosphingolipid microdomains 1 (PAG1), mRNA. |
| MAN2C1 | -2.17 | Homo sapiens mannosidase, alpha, class 2C, member 1 (MAN2C1), mRNA. |
| COMMD2 | -2.17 | Homo sapiens COMM domain containing 2 (COMMD2), mRNA. |
| PPIC | -2.17 | Homo sapiens peptidylprolyl isomerase C (cyclophilin C) (PPIC), mRNA. |
| CDC25C | -2.17 | Homo sapiens cell division cycle 25 homolog C (S. pombe) (CDC25C), transcript variant 1, mRNA. |
| HERC2 | -2.17 | Homo sapiens hect domain and RLD 2 (HERC2), mRNA. |
| SNAP25 | -2.18 | Homo sapiens synaptosomal-associated protein, 25kDa (SNAP25), transcript variant 2, mRNA. |
| WRB | -2.18 | Homo sapiens tryptophan rich basic protein (WRB), mRNA. |
| C1orf97 | -2.18 | Homo sapiens chromosome 1 open reading frame 97 (C1orf97), mRNA. |
| LOC731835 | -2.19 | PREDICTED: Homo sapiens hypothetical protein LOC731835 (LOC731835), mRNA. |
| MNT | -2.19 | Homo sapiens MAX binding protein (MNT), mRNA. |
| C9orf80 | -2.19 | Homo sapiens chromosome 9 open reading frame 80 (C9orf80), mRNA. |
| ELF2 | -2.2 | Homo sapiens E74-like factor 2 (ets domain transcription factor) (ELF2), transcript variant 2, mRNA. |
| SSR1 | -2.2 | Homo sapiens signal sequence receptor, alpha (translocon-associated protein alpha) (SSR1), mRNA. |
| PDIK1L | -2.2 | Homo sapiens PDLIM1 interacting kinase 1 like (PDIK1L), mRNA. |
| ORC2L | -2.2 | Homo sapiens origin recognition complex, subunit 2-like (yeast) (ORC2L), mRNA. |
| SNRNP27 | -2.2 | Homo sapiens small nuclear ribonucleoprotein 27kDa (U4/U6.U5) (SNRNP27), mRNA. |
| SMC4 | -2.2 | Homo sapiens structural maintenance of chromosomes 4 (SMC4), transcript variant 2, mRNA. |
| PLXNA1 | -2.2 | Homo sapiens plexin A1 (PLXNA1), mRNA. |
| CDC40 | -2.2 | Homo sapiens cell division cycle 40 homolog (S. cerevisiae) (CDC40), mRNA. |
| PCGF6 | -2.21 | Homo sapiens polycomb group ring finger 6 (PCGF6), transcript variant 2, mRNA. |
| FAM181B | -2.21 | Homo sapiens family with sequence similarity 181, member B (FAM181B), mRNA. |
| ZNF426 | -2.21 | Homo sapiens zinc finger protein 426 (ZNF426), mRNA. |
| SMAP1 | -2.21 | Homo sapiens stromal membrane-associated protein 1 (SMAP1), mRNA. |
| CBR4 | -2.21 | Homo sapiens carbonyl reductase 4 (CBR4), mRNA. |
| FRMD4A | -2.22 | Homo sapiens FERM domain containing 4A (FRMD4A), mRNA. |
| FOXN4 | -2.22 | Homo sapiens forkhead box N4 (FOXN4), mRNA. |
| ELOVL5 | -2.22 | Homo sapiens ELOVL family member 5, elongation of long chain fatty acids (FEN1/Elo2, SUR4/Elo3-like, yeast) (ELOVL5), mRNA. |
| GABRB3 | -2.22 | Homo sapiens gamma-aminobutyric acid (GABA) A receptor, beta 3 (GABRB3), transcript variant 1, mRNA. |
| PLCB1 | -2.22 | Homo sapiens phospholipase C, beta 1 (phosphoinositide-specific) (PLCB1), transcript variant 1, mRNA. |
| LOC644033 | -2.22 | PREDICTED: Homo sapiens similar to similar to RPL23AP7 protein (LOC644033), mRNA. |
| LOC440704 | -2.22 | PREDICTED: Homo sapiens hypothetical gene supported by BC042042 (LOC440704), mRNA. |
| KLF6 | -2.23 | Homo sapiens Kruppel-like factor 6 (KLF6), transcript variant 2, mRNA. |
| NFXL1 | -2.23 | Homo sapiens nuclear transcription factor, X-box binding-like 1 (NFXL1), mRNA. |
| RAD21 | -2.23 | Homo sapiens RAD21 homolog (S. pombe) (RAD21), mRNA. |
| PDE5A | -2.23 | Homo sapiens phosphodiesterase 5A, cGMP-specific (PDE5A), transcript variant 1, mRNA. |
| DIAPH3 | -2.23 | Homo sapiens diaphanous homolog 3 (Drosophila) (DIAPH3), transcript variant 2, mRNA. |
| EP300 | -2.23 | Homo sapiens E1A binding protein p300 (EP300), mRNA. |
| SPRY1 | -2.23 | Homo sapiens sprouty homolog 1, antagonist of FGF signaling (Drosophila) (SPRY1), transcript variant 1, mRNA. |
| AACS | -2.23 | Homo sapiens acetoacetyl-CoA synthetase (AACS), mRNA. |
| HIATL1 | -2.23 | Homo sapiens hippocampus abundant transcript-like 1 (HIATL1), mRNA. |
| FSTL5 | -2.24 | Homo sapiens follistatin-like 5 (FSTL5), mRNA. |
| EMP3 | -2.24 | Homo sapiens epithelial membrane protein 3 (EMP3), mRNA. |
| CLPX | -2.24 | Homo sapiens ClpX caseinolytic peptidase X homolog (E. coli) (CLPX), mRNA. |
| EP400 | -2.24 | Homo sapiens E1A binding protein p400 (EP400), mRNA. |
|  | -2.24 | Homo sapiens cDNA FLJ30340 fis, clone BRACE2007411 |
| SLC10A3 | -2.25 | Homo sapiens solute carrier family 10 (sodium/bile acid cotransporter family), member 3 (SLC10A3), mRNA. |
| DUSP19 | -2.25 | Homo sapiens dual specificity phosphatase 19 (DUSP19), mRNA. |
| FBXW7 | -2.25 | Homo sapiens F-box and WD repeat domain containing 7 (FBXW7), transcript variant 1, mRNA. |
| TTC14 | -2.25 | Homo sapiens tetratricopeptide repeat domain 14 (TTC14), transcript variant 1, mRNA. |
| SEPSECS | -2.25 | Homo sapiens Sep (O-phosphoserine) tRNA:Sec (selenocysteine) tRNA synthase (SEPSECS), transcript variant 2, mRNA. |
| LYRM5 | -2.25 | Homo sapiens LYR motif containing 5 (LYRM5), mRNA. |
| TTC32 | -2.25 | Homo sapiens tetratricopeptide repeat domain 32 (TTC32), mRNA. |
| TRIM13 | -2.26 | Homo sapiens tripartite motif-containing 13 (TRIM13), transcript variant 4, mRNA. |
| LOC728715 | -2.26 | PREDICTED: Homo sapiens similar to hCG38149 (LOC728715), mRNA. |
| IL18BP | -2.26 | Homo sapiens interleukin 18 binding protein (IL18BP), transcript variant A, mRNA. |
| ERICH1 | -2.26 | Homo sapiens glutamate-rich 1 (ERICH1), mRNA. |
| LARGE | -2.26 | Homo sapiens like-glycosyltransferase (LARGE), transcript variant 1, mRNA. |
| TNFRSF1A | -2.26 | Homo sapiens tumor necrosis factor receptor superfamily, member 1A (TNFRSF1A), mRNA. |
| TSPYL2 | -2.26 | Homo sapiens TSPY-like 2 (TSPYL2), mRNA. |
| PRNP | -2.26 | Homo sapiens prion protein (PRNP), transcript variant 3, mRNA. |
| EXOSC6 | -2.26 | Homo sapiens exosome component 6 (EXOSC6), mRNA. |
| CTH | -2.27 | Homo sapiens cystathionase (cystathionine gamma-lyase) (CTH), transcript variant 2, mRNA. |
| AHCTF1 | -2.27 | Homo sapiens AT hook containing transcription factor 1 (AHCTF1), mRNA. |
| PLEKHA1 | -2.27 | Homo sapiens pleckstrin homology domain containing, family A (phosphoinositide binding specific) member 1 (PLEKHA1), transcript variant 1, mRNA. |
| SRGAP2 | -2.27 | Homo sapiens SLIT-ROBO Rho GTPase activating protein 2 (SRGAP2), transcript variant 1, mRNA. |
| DGKQ | -2.27 | Homo sapiens diacylglycerol kinase, theta 110kDa (DGKQ), mRNA. |
| BCAP29 | -2.27 | Homo sapiens B-cell receptor-associated protein 29 (BCAP29), transcript variant 1, mRNA. |
| CCNA1 | -2.28 | Homo sapiens cyclin A1 (CCNA1), mRNA. |
| AGPAT4 | -2.28 | Homo sapiens 1-acylglycerol-3-phosphate O-acyltransferase 4 (lysophosphatidic acid acyltransferase, delta) (AGPAT4), mRNA. |
| DNAJC19 | -2.28 | Homo sapiens DnaJ (Hsp40) homolog, subfamily C, member 19 (DNAJC19), mRNA. |
| MAP7 | -2.28 | Homo sapiens microtubule-associated protein 7 (MAP7), mRNA. |
| 15-Sep | -2.28 | Homo sapiens 15 kDa selenoprotein (SEP15), transcript variant 1, mRNA. |
| C5orf41 | -2.28 | Homo sapiens chromosome 5 open reading frame 41 (C5orf41), mRNA. |
| ALKBH8 | -2.29 | Homo sapiens alkB, alkylation repair homolog 8 (E. coli) (ALKBH8), mRNA. |
| SFRS14 | -2.29 | Homo sapiens splicing factor, arginine/serine-rich 14 (SFRS14), transcript variant 1, mRNA. |
| LOC441601 | -2.29 | Homo sapiens septin 7 pseudogene (LOC441601), non-coding RNA. |
| COQ2 | -2.29 | Homo sapiens coenzyme Q2 homolog, prenyltransferase (yeast) (COQ2), mRNA. |
| TRIM24 | -2.29 | Homo sapiens tripartite motif-containing 24 (TRIM24), transcript variant 1, mRNA. |
| SLC11A2 | -2.29 | Homo sapiens solute carrier family 11 (proton-coupled divalent metal ion transporters), member 2 (SLC11A2), mRNA. |
| ZFHX4 | -2.29 | Homo sapiens zinc finger homeobox 4 (ZFHX4), mRNA. |
| NKTR | -2.29 | Homo sapiens natural killer-tumor recognition sequence (NKTR), mRNA. |
| PPAP2A | -2.29 | Homo sapiens phosphatidic acid phosphatase type 2A (PPAP2A), transcript variant 2, mRNA. |
| KIAA0090 | -2.3 | Homo sapiens KIAA0090 (KIAA0090), mRNA. |
| PAK7 | -2.3 | Homo sapiens p21(CDKN1A)-activated kinase 7 (PAK7), transcript variant 2, mRNA. |
| ZNF536 | -2.3 | Homo sapiens zinc finger protein 536 (ZNF536), mRNA. |
| LARP1B | -2.3 | Homo sapiens La ribonucleoprotein domain family, member 1B (LARP1B), transcript variant 3, mRNA. |
| EFHA1 | -2.3 | Homo sapiens EF-hand domain family, member A1 (EFHA1), mRNA. |
| NLRP8 | -2.31 | Homo sapiens NLR family, pyrin domain containing 8 (NLRP8), mRNA. |
| FAM122B | -2.31 | Homo sapiens family with sequence similarity 122B (FAM122B), mRNA. |
| GGCX | -2.31 | Homo sapiens gamma-glutamyl carboxylase (GGCX), mRNA. |
| CREBBP | -2.31 | Homo sapiens CREB binding protein (CREBBP), transcript variant 2, mRNA. |
| PSD3 | -2.31 | Homo sapiens pleckstrin and Sec7 domain containing 3 (PSD3), transcript variant 1, mRNA. |
| CEPT1 | -2.31 | Homo sapiens choline/ethanolamine phosphotransferase 1 (CEPT1), transcript variant 1, mRNA. |
| GDPD1 | -2.31 | Homo sapiens glycerophosphodiester phosphodiesterase domain containing 1 (GDPD1), mRNA. |
| PLXNB1 | -2.32 | Homo sapiens plexin B1 (PLXNB1), mRNA. |
| TERF1 | -2.32 | Homo sapiens telomeric repeat binding factor (NIMA-interacting) 1 (TERF1), transcript variant 1, mRNA. |
| 7-Mar | -2.33 | Homo sapiens membrane-associated ring finger (C3HC4) 7 (MARCH7), mRNA. |
| BIRC5 | -2.33 | Homo sapiens baculoviral IAP repeat-containing 5 (BIRC5), transcript variant 3, mRNA. |
|  | -2.33 | Homo sapiens cDNA: FLJ22720 fis, clone HSI14320 |
| SF3B1 | -2.33 | Homo sapiens splicing factor 3b, subunit 1, 155kDa (SF3B1), transcript variant 1, mRNA. |
| SLC38A10 | -2.34 | Homo sapiens solute carrier family 38, member 10 (SLC38A10), transcript variant 1, mRNA. |
| INCENP | -2.34 | Homo sapiens inner centromere protein antigens 135/155kDa (INCENP), transcript variant 1, mRNA. |
| SGSM1 | -2.34 | Homo sapiens small G protein signaling modulator 1 (SGSM1), transcript variant 1, mRNA. |
| E2F3 | -2.34 | Homo sapiens E2F transcription factor 3 (E2F3), mRNA. |
| VHL | -2.35 | Homo sapiens von Hippel-Lindau tumor suppressor (VHL), transcript variant 1, mRNA. |
| STAG3L2 | -2.35 | Homo sapiens stromal antigen 3-like 2 (STAG3L2), mRNA. |
| CATSPER2 | -2.35 | Homo sapiens cation channel, sperm associated 2 (CATSPER2), transcript variant 4, mRNA. |
| KLHL20 | -2.35 | Homo sapiens kelch-like 20 (Drosophila) (KLHL20), mRNA. |
| WDR60 | -2.35 | Homo sapiens WD repeat domain 60 (WDR60), mRNA. |
| IVD | -2.36 | Homo sapiens isovaleryl Coenzyme A dehydrogenase (IVD), nuclear gene encoding mitochondrial protein, mRNA. |
| SEMA5A | -2.36 | Homo sapiens sema domain, seven thrombospondin repeats (type 1 and type 1-like), transmembrane domain (TM) and short cytoplasmic domain, (semaphorin) 5A (SEMA5A), mRNA. |
| CCDC90A | -2.36 | Homo sapiens coiled-coil domain containing 90A (CCDC90A), mRNA. |
| USP13 | -2.36 | Homo sapiens ubiquitin specific peptidase 13 (isopeptidase T-3) (USP13), mRNA. |
| C15orf63 | -2.37 | Homo sapiens chromosome 15 open reading frame 63 (C15orf63), mRNA. |
| ZNF827 | -2.37 | Homo sapiens zinc finger protein 827 (ZNF827), mRNA. |
| JUN | -2.38 | Homo sapiens jun oncogene (JUN), mRNA. |
|  | -2.38 | full-length cDNA clone CS0DF032YA11 of Fetal brain of Homo sapiens (human) |
| AHI1 | -2.38 | Homo sapiens Abelson helper integration site 1 (AHI1), mRNA. |
| NEK1 | -2.38 | Homo sapiens NIMA (never in mitosis gene a)-related kinase 1 (NEK1), mRNA. |
|  | -2.38 | xr14b10.x1 NCI_CGAP_Lu28 Homo sapiens cDNA clone IMAGE:2760091 3, mRNA sequence |
| PANK2 | -2.38 | Homo sapiens pantothenate kinase 2 (PANK2), transcript variant 3, mRNA. |
| RAPGEF4 | -2.38 | Homo sapiens Rap guanine nucleotide exchange factor (GEF) 4 (RAPGEF4), mRNA. |
| PHKB | -2.38 | Homo sapiens phosphorylase kinase, beta (PHKB), transcript variant 1, mRNA. |
| ADAM23 | -2.38 | Homo sapiens ADAM metallopeptidase domain 23 (ADAM23), mRNA. |
| CHPF2 | -2.39 | Homo sapiens chondroitin polymerizing factor 2 (CHPF2), mRNA. |
| TTK | -2.39 | Homo sapiens TTK protein kinase (TTK), mRNA. |
| ID3 | -2.4 | Homo sapiens inhibitor of DNA binding 3, dominant negative helix-loop-helix protein (ID3), mRNA. |
| C10orf104 | -2.4 | Homo sapiens chromosome 10 open reading frame 104 (C10orf104), mRNA. |
| LOC646786 | -2.4 | PREDICTED: Homo sapiens similar to Afadin (AF-6 protein) (LOC646786), mRNA. |
| FAM162A | -2.41 | Homo sapiens family with sequence similarity 162, member A (FAM162A), mRNA. |
| ATG10 | -2.41 | Homo sapiens ATG10 autophagy related 10 homolog (S. cerevisiae) (ATG10), mRNA. |
| KTN1 | -2.41 | Homo sapiens kinectin 1 (kinesin receptor) (KTN1), transcript variant 1, mRNA. |
| ZNF573 | -2.42 | Homo sapiens zinc finger protein 573 (ZNF573), mRNA. |
| C3orf31 | -2.42 | Homo sapiens chromosome 3 open reading frame 31 (C3orf31), mRNA. |
| SMAD9 | -2.42 | Homo sapiens SMAD family member 9 (SMAD9), mRNA. |
| GNB5 | -2.42 | Homo sapiens guanine nucleotide binding protein (G protein), beta 5 (GNB5), transcript variant 1, mRNA. |
| GMCL1 | -2.43 | Homo sapiens germ cell-less homolog 1 (Drosophila) (GMCL1), mRNA. |
| TTC14 | -2.43 | Homo sapiens tetratricopeptide repeat domain 14 (TTC14), transcript variant 2, mRNA. |
| NTNG1 | -2.43 | Homo sapiens netrin G1 (NTNG1), transcript variant 3, mRNA. |
| LMO4 | -2.43 | Homo sapiens LIM domain only 4 (LMO4), mRNA. |
| ZMYM5 | -2.44 | Homo sapiens zinc finger, MYM-type 5 (ZMYM5), transcript variant 2, mRNA. |
| PIK3R1 | -2.44 | Homo sapiens phosphoinositide-3-kinase, regulatory subunit 1 (alpha) (PIK3R1), transcript variant 1, mRNA. |
| TNFAIP8L1 | -2.44 | Homo sapiens tumor necrosis factor, alpha-induced protein 8-like 1 (TNFAIP8L1), mRNA. |
| CXorf38 | -2.44 | Homo sapiens chromosome X open reading frame 38 (CXorf38), mRNA. |
| NOP56 | -2.45 | Homo sapiens NOP56 ribonucleoprotein homolog (yeast) (NOP56), transcript variant 1, mRNA. |
| CHM | -2.45 | Homo sapiens choroideremia (Rab escort protein 1) (CHM), transcript variant 1, mRNA. |
| BRCC3 | -2.45 | Homo sapiens BRCA1/BRCA2-containing complex, subunit 3 (BRCC3), transcript variant 1, mRNA. |
|  | -2.45 | full-length cDNA clone CS0DA009YB08 of Neuroblastoma of Homo sapiens (human) |
| RP2 | -2.45 | Homo sapiens retinitis pigmentosa 2 (X-linked recessive) (RP2), mRNA. |
| PBRM1 | -2.45 | Homo sapiens polybromo 1 (PBRM1), transcript variant 4, mRNA. |
| ETNK1 | -2.45 | Homo sapiens ethanolamine kinase 1 (ETNK1), transcript variant 1, mRNA. |
| CCDC84 | -2.45 | Homo sapiens coiled-coil domain containing 84 (CCDC84), mRNA. |
| WDR73 | -2.45 | Homo sapiens WD repeat domain 73 (WDR73), mRNA. |
| JMJD1C | -2.46 | Homo sapiens jumonji domain containing 1C (JMJD1C), transcript variant 1, mRNA. |
| NIN | -2.46 | Homo sapiens ninein (GSK3B interacting protein) (NIN), transcript variant 2, mRNA. |
| LRRC17 | -2.46 | Homo sapiens leucine rich repeat containing 17 (LRRC17), transcript variant 2, mRNA. |
| NUP155 | -2.46 | Homo sapiens nucleoporin 155kDa (NUP155), transcript variant 1, mRNA. |
| RBPJ | -2.46 | Homo sapiens recombination signal binding protein for immunoglobulin kappa J region (RBPJ), transcript variant 4, mRNA. |
| GTPBP8 | -2.46 | Homo sapiens GTP-binding protein 8 (putative) (GTPBP8), transcript variant 1, mRNA. |
| C11orf31 | -2.46 | Homo sapiens chromosome 11 open reading frame 31 (C11orf31), mRNA. |
| ALG14 | -2.46 | Homo sapiens asparagine-linked glycosylation 14 homolog (S. cerevisiae) (ALG14), mRNA. |
| MTFR1 | -2.47 | Homo sapiens mitochondrial fission regulator 1 (MTFR1), nuclear gene encoding mitochondrial protein, mRNA. |
| MTUS1 | -2.47 | Homo sapiens mitochondrial tumor suppressor 1 (MTUS1), nuclear gene encoding mitochondrial protein, transcript variant 5, mRNA. |
| OSBPL8 | -2.47 | Homo sapiens oxysterol binding protein-like 8 (OSBPL8), transcript variant 1, mRNA. |
| TSPYL5 | -2.47 | Homo sapiens TSPY-like 5 (TSPYL5), mRNA. |
| STXBP5 | -2.47 | Homo sapiens syntaxin binding protein 5 (tomosyn) (STXBP5), mRNA. |
| LOC648509 | -2.47 | PREDICTED: Homo sapiens similar to plakophilin 4 isoform a (LOC648509), mRNA. |
| PSMD12 | -2.47 | Homo sapiens proteasome (prosome, macropain) 26S subunit, non-ATPase, 12 (PSMD12), transcript variant 1, mRNA. XM_946055 XM_946058 |
| CRLF3 | -2.48 | Homo sapiens cytokine receptor-like factor 3 (CRLF3), mRNA. |
| CTGLF3 | -2.48 | Homo sapiens centaurin, gamma-like family, member 3 (CTGLF3), mRNA. |
| ATL2 | -2.48 | Homo sapiens atlastin GTPase 2 (ATL2), mRNA. |
| DAAM1 | -2.49 | Homo sapiens dishevelled associated activator of morphogenesis 1 (DAAM1), mRNA. |
| SUSD1 | -2.49 | Homo sapiens sushi domain containing 1 (SUSD1), mRNA. |
| ENOX2 | -2.49 | Homo sapiens ecto-NOX disulfide-thiol exchanger 2 (ENOX2), transcript variant 1, mRNA. |
| FLJ38482 | -2.5 | Homo sapiens hypothetical protein FLJ38482 (FLJ38482), mRNA. |
| C14orf102 | -2.5 | Homo sapiens chromosome 14 open reading frame 102 (C14orf102), transcript variant 1, mRNA. |
| PKD1 | -2.5 | Homo sapiens polycystic kidney disease 1 (autosomal dominant) (PKD1), transcript variant 1, mRNA. |
| LRRFIP1 | -2.51 | Homo sapiens leucine rich repeat (in FLII) interacting protein 1 (LRRFIP1), mRNA. |
| USP48 | -2.51 | Homo sapiens ubiquitin specific peptidase 48 (USP48), transcript variant 1, mRNA. |
| LOC440354 | -2.51 | Homo sapiens PI-3-kinase-related kinase SMG-1 pseudogene (LOC440354), non-coding RNA. |
| C19orf4 | -2.52 | Homo sapiens chromosome 19 open reading frame 4 (C19orf4), mRNA. |
| CYP20A1 | -2.52 | Homo sapiens cytochrome P450, family 20, subfamily A, polypeptide 1 (CYP20A1), transcript variant 1, mRNA. |
| ZNF549 | -2.52 | Homo sapiens zinc finger protein 549 (ZNF549), mRNA. |
| C5orf41 | -2.52 | Homo sapiens chromosome 5 open reading frame 41 (C5orf41), mRNA. |
| LOC653080 | -2.54 | PREDICTED: Homo sapiens similar to Beta-glucuronidase precursor, transcript variant 2 (LOC653080), mRNA. |
| SCN2A | -2.54 | Homo sapiens sodium channel, voltage-gated, type II, alpha subunit (SCN2A), transcript variant 2, mRNA. |
| SNORD13 | -2.54 | Homo sapiens small nucleolar RNA, C/D box 13 (SNORD13), small nucleolar RNA. |
| C9orf102 | -2.54 | Homo sapiens chromosome 9 open reading frame 102 (C9orf102), mRNA. |
| TTC31 | -2.56 | Homo sapiens tetratricopeptide repeat domain 31 (TTC31), mRNA. |
| LINGO4 | -2.56 | Homo sapiens leucine rich repeat and Ig domain containing 4 (LINGO4), mRNA. |
| DENND5B | -2.56 | Homo sapiens DENN/MADD domain containing 5B (DENND5B), mRNA. |
| LOC441087 | -2.56 | Homo sapiens hypothetical gene supported by AK125735 (LOC441087), mRNA. |
| GRIPAP1 | -2.56 | Homo sapiens GRIP1 associated protein 1 (GRIPAP1), transcript variant 2, mRNA. |
| ZNF462 | -2.57 | Homo sapiens zinc finger protein 462 (ZNF462), mRNA. |
| IGF2BP3 | -2.57 | Homo sapiens insulin-like growth factor 2 mRNA binding protein 3 (IGF2BP3), mRNA. |
| MCOLN1 | -2.58 | Homo sapiens mucolipin 1 (MCOLN1), mRNA. |
| SLC35E3 | -2.58 | Homo sapiens solute carrier family 35, member E3 (SLC35E3), mRNA. |
| CHEK2 | -2.58 | Homo sapiens CHK2 checkpoint homolog (S. pombe) (CHEK2), transcript variant 1, mRNA. |
| C14orf138 | -2.58 | Homo sapiens chromosome 14 open reading frame 138 (C14orf138), transcript variant 1, mRNA. |
|  | -2.58 | Homo sapiens cDNA FLJ32174 fis, clone PLACE6001064 |
| OAT | -2.58 | Homo sapiens ornithine aminotransferase (gyrate atrophy) (OAT), nuclear gene encoding mitochondrial protein, mRNA. |
| KCTD12 | -2.58 | Homo sapiens potassium channel tetramerisation domain containing 12 (KCTD12), mRNA. |
| PRR11 | -2.58 | Homo sapiens proline rich 11 (PRR11), mRNA. |
| VTA1 | -2.58 | Homo sapiens Vps20-associated 1 homolog (S. cerevisiae) (VTA1), mRNA. |
| PCDH17 | -2.59 | Homo sapiens protocadherin 17 (PCDH17), mRNA. |
| LRDD | -2.59 | Homo sapiens leucine-rich repeats and death domain containing (LRDD), transcript variant 2, mRNA. |
| ODZ3 | -2.6 | Homo sapiens odz, odd Oz/ten-m homolog 3 (Drosophila) (ODZ3), mRNA. |
| LPPR4 | -2.6 | Homo sapiens plasticity related gene 1 (LPPR4), mRNA. |
| SGIP1 | -2.6 | Homo sapiens SH3-domain GRB2-like (endophilin) interacting protein 1 (SGIP1), mRNA. |
| GAS1 | -2.6 | Homo sapiens growth arrest-specific 1 (GAS1), mRNA. |
| 10-Sep | -2.6 | Homo sapiens septin 10 (SEPT10), transcript variant 2, mRNA. |
| KIF11 | -2.61 | Homo sapiens kinesin family member 11 (KIF11), mRNA. |
| MID1 | -2.62 | Homo sapiens midline 1 (Opitz/BBB syndrome) (MID1), transcript variant 3, mRNA. |
| HOOK1 | -2.62 | Homo sapiens hook homolog 1 (Drosophila) (HOOK1), mRNA. |
| ATP1B2 | -2.63 | Homo sapiens ATPase, Na+/K+ transporting, beta 2 polypeptide (ATP1B2), mRNA. |
| COL11A1 | -2.63 | Homo sapiens collagen, type XI, alpha 1 (COL11A1), transcript variant A, mRNA. |
| GPR56 | -2.63 | Homo sapiens G protein-coupled receptor 56 (GPR56), transcript variant 3, mRNA. |
| ABCB1 | -2.64 | Homo sapiens ATP-binding cassette, sub-family B (MDR/TAP), member 1 (ABCB1), mRNA. |
| LOC648852 | -2.64 | PREDICTED: Homo sapiens hypothetical protein LOC648852 (LOC648852), mRNA. |
| FBXO11 | -2.65 | Homo sapiens F-box protein 11 (FBXO11), transcript variant 2, mRNA. |
| C21orf24 | -2.65 | Homo sapiens chromosome 21 open reading frame 24 (C21orf24), mRNA. |
| DDR2 | -2.66 | Homo sapiens discoidin domain receptor tyrosine kinase 2 (DDR2), transcript variant 2, mRNA. |
| CUTL1 | -2.66 | Homo sapiens cut-like 1, CCAAT displacement protein (Drosophila) (CUTL1), transcript variant 1, mRNA. |
| EPC1 | -2.67 | Homo sapiens enhancer of polycomb homolog 1 (Drosophila) (EPC1), mRNA. |
| SPIN1 | -2.67 | Homo sapiens spindlin 1 (SPIN1), mRNA. |
| DSE | -2.67 | Homo sapiens dermatan sulfate epimerase (DSE), transcript variant 1, mRNA. |
| PHACTR2 | -2.69 | Homo sapiens phosphatase and actin regulator 2 (PHACTR2), transcript variant 1, mRNA. |
| USP33 | -2.7 | Homo sapiens ubiquitin specific peptidase 33 (USP33), transcript variant 2, mRNA. |
| DCN | -2.7 | Homo sapiens decorin (DCN), transcript variant C, mRNA. |
| PDPK1 | -2.71 | Homo sapiens 3-phosphoinositide dependent protein kinase-1 (PDPK1), transcript variant 2, mRNA. |
| GNL3L | -2.71 | Homo sapiens guanine nucleotide binding protein-like 3 (nucleolar)-like (GNL3L), mRNA. |
| MBOAT2 | -2.72 | Homo sapiens membrane bound O-acyltransferase domain containing 2 (MBOAT2), mRNA. |
| IGFBP5 | -2.73 | Homo sapiens insulin-like growth factor binding protein 5 (IGFBP5), mRNA. |
| ENTPD4 | -2.73 | Homo sapiens ectonucleoside triphosphate diphosphohydrolase 4 (ENTPD4), mRNA. |
| DCUN1D5 | -2.73 | Homo sapiens DCN1, defective in cullin neddylation 1, domain containing 5 (S. cerevisiae) (DCUN1D5), mRNA. |
| PCDH20 | -2.73 | Homo sapiens protocadherin 20 (PCDH20), mRNA. |
| GPR27 | -2.76 | Homo sapiens G protein-coupled receptor 27 (GPR27), mRNA. |
| SLC35A5 | -2.76 | Homo sapiens solute carrier family 35, member A5 (SLC35A5), mRNA. |
| PDSS2 | -2.79 | Homo sapiens prenyl (decaprenyl) diphosphate synthase, subunit 2 (PDSS2), mRNA. |
| KIF15 | -2.79 | Homo sapiens kinesin family member 15 (KIF15), mRNA. |
| KCNT2 | -2.8 | Homo sapiens potassium channel, subfamily T, member 2 (KCNT2), mRNA. |
| C14orf153 | -2.8 | Homo sapiens chromosome 14 open reading frame 153 (C14orf153), mRNA. |
| GADD45A | -2.81 | Homo sapiens growth arrest and DNA-damage-inducible, alpha (GADD45A), mRNA. |
| NOL8 | -2.81 | Homo sapiens nucleolar protein 8 (NOL8), mRNA. |
| CEP135 | -2.81 | Homo sapiens centrosomal protein 135kDa (CEP135), mRNA. |
| SRFBP1 | -2.81 | Homo sapiens serum response factor binding protein 1 (SRFBP1), mRNA. |
| C12orf26 | -2.82 | Homo sapiens chromosome 12 open reading frame 26 (C12orf26), mRNA. |
| PHF17 | -2.84 | Homo sapiens PHD finger protein 17 (PHF17), transcript variant S, mRNA. |
| RGS2 | -2.84 | Homo sapiens regulator of G-protein signalling 2, 24kDa (RGS2), mRNA. |
| C18orf56 | -2.84 | Homo sapiens chromosome 18 open reading frame 56 (C18orf56), mRNA. |
| NRAS | -2.87 | Homo sapiens neuroblastoma RAS viral (v-ras) oncogene homolog (NRAS), mRNA. |
| LRBA | -2.87 | Homo sapiens LPS-responsive vesicle trafficking, beach and anchor containing (LRBA), mRNA. |
| NFIX | -2.87 | Homo sapiens nuclear factor I/X (CCAAT-binding transcription factor) (NFIX), mRNA. |
| SAP30 | -2.88 | Homo sapiens Sin3A-associated protein, 30kDa (SAP30), mRNA. |
| TSPAN6 | -2.89 | Homo sapiens tetraspanin 6 (TSPAN6), mRNA. |
| GPATCH1 | -2.89 | Homo sapiens G patch domain containing 1 (GPATCH1), mRNA. |
| SLC35B3 | -2.9 | Homo sapiens solute carrier family 35, member B3 (SLC35B3), mRNA. |
| OPA1 | -2.9 | Homo sapiens optic atrophy 1 (autosomal dominant) (OPA1), nuclear gene encoding mitochondrial protein, transcript variant 1, mRNA. |
| SNAI2 | -2.92 | Homo sapiens snail homolog 2 (Drosophila) (SNAI2), mRNA. |
| BIRC3 | -2.95 | Homo sapiens baculoviral IAP repeat-containing 3 (BIRC3), transcript variant 2, mRNA. |
| DDX51 | -2.97 | Homo sapiens DEAD (Asp-Glu-Ala-Asp) box polypeptide 51 (DDX51), mRNA. |
| HDGF2 | -2.99 | Homo sapiens hepatoma-derived growth factor-related protein 2 (HDGF2), transcript variant 1, mRNA. |
| ADAMTS19 | -3 | Homo sapiens ADAM metallopeptidase with thrombospondin type 1 motif, 19 (ADAMTS19), mRNA. |
| AXIN1 | -3.01 | Homo sapiens axin 1 (AXIN1), transcript variant 2, mRNA. |
| UPF3A | -3.01 | Homo sapiens UPF3 regulator of nonsense transcripts homolog A (yeast) (UPF3A), transcript variant 1, mRNA. |
| TMEM18 | -3.01 | Homo sapiens transmembrane protein 18 (TMEM18), mRNA. |
| C1orf173 | -3.03 | Homo sapiens chromosome 1 open reading frame 173 (C1orf173), mRNA. |
| STIL | -3.03 | Homo sapiens SCL/TAL1 interrupting locus (STIL), transcript variant 2, mRNA. |
| PNN | -3.04 | Homo sapiens pinin, desmosome associated protein (PNN), mRNA. |
| SHROOM4 | -3.07 | Homo sapiens shroom family member 4 (SHROOM4), mRNA. |
| NLGN4X | -3.08 | Homo sapiens neuroligin 4, X-linked (NLGN4X), transcript variant 2, mRNA. |
| SLC36A4 | -3.09 | Homo sapiens solute carrier family 36 (proton/amino acid symporter), member 4 (SLC36A4), mRNA. |
| USP14 | -3.1 | Homo sapiens ubiquitin specific peptidase 14 (tRNA-guanine transglycosylase) (USP14), transcript variant 2, mRNA. |
| FCAR | -3.11 | Homo sapiens Fc fragment of IgA, receptor for (FCAR), transcript variant 9, mRNA. |
| PCYOX1 | -3.11 | Homo sapiens prenylcysteine oxidase 1 (PCYOX1), mRNA. |
| MEPCE | -3.12 | Homo sapiens methylphosphate capping enzyme (MEPCE), mRNA. |
| ID2 | -3.12 | Homo sapiens inhibitor of DNA binding 2, dominant negative helix-loop-helix protein (ID2), mRNA. |
| PRKCQ | -3.13 | Homo sapiens protein kinase C, theta (PRKCQ), mRNA. |
| TMEM17 | -3.15 | Homo sapiens transmembrane protein 17 (TMEM17), mRNA. |
| PRDM8 | -3.18 | Homo sapiens PR domain containing 8 (PRDM8), transcript variant 1, mRNA. |
| GOLPH4 | -3.2 | Homo sapiens golgi phosphoprotein 4 (GOLPH4), mRNA. |
| PAX7 | -3.21 | Homo sapiens paired box 7 (PAX7), transcript variant 1, mRNA. |
| RORB | -3.25 | Homo sapiens RAR-related orphan receptor B (RORB), mRNA. |
| TDRD7 | -3.25 | Homo sapiens tudor domain containing 7 (TDRD7), mRNA. |
| UGCGL1 | -3.27 | Homo sapiens UDP-glucose ceramide glucosyltransferase-like 1 (UGCGL1), transcript variant 1, mRNA. |
| C3orf34 | -3.27 | Homo sapiens chromosome 3 open reading frame 34 (C3orf34), mRNA. |
| BEND6 | -3.27 | Homo sapiens BEN domain containing 6 (BEND6), mRNA. |
| ARHGAP11B | -3.27 | Homo sapiens Rho GTPase activating protein 11B (ARHGAP11B), mRNA. XM_931045 XM_931050 |
| RAD23B | -3.28 | Homo sapiens RAD23 homolog B (S. cerevisiae) (RAD23B), mRNA. |
| TM4SF4 | -3.28 | Homo sapiens transmembrane 4 L six family member 4 (TM4SF4), mRNA. |
| TMEM55A | -3.28 | Homo sapiens transmembrane protein 55A (TMEM55A), mRNA. |
| OXR1 | -3.28 | Homo sapiens oxidation resistance 1 (OXR1), mRNA. |
| MYCBP2 | -3.28 | Homo sapiens MYC binding protein 2 (MYCBP2), mRNA. |
| SEMA3E | -3.29 | Homo sapiens sema domain, immunoglobulin domain (Ig), short basic domain, secreted, (semaphorin) 3E (SEMA3E), mRNA. |
| DCAF16 | -3.3 | Homo sapiens DDB1 and CUL4 associated factor 16 (DCAF16), mRNA. |
| SGK | -3.3 | Homo sapiens serum/glucocorticoid regulated kinase (SGK), mRNA. |
| GAS2L3 | -3.39 | Homo sapiens growth arrest-specific 2 like 3 (GAS2L3), mRNA. |
| PROX1 | -3.4 | Homo sapiens prospero homeobox 1 (PROX1), mRNA. |
| KCTD21 | -3.42 | Homo sapiens potassium channel tetramerisation domain containing 21 (KCTD21), mRNA. |
| MBD4 | -3.42 | Homo sapiens methyl-CpG binding domain protein 4 (MBD4), mRNA. |
| TDRD1 | -3.44 | Homo sapiens tudor domain containing 1 (TDRD1), mRNA. |
| ID2 | -3.48 | Homo sapiens inhibitor of DNA binding 2, dominant negative helix-loop-helix protein (ID2), mRNA. |
| CHRNA5 | -3.49 | Homo sapiens cholinergic receptor, nicotinic, alpha 5 (CHRNA5), mRNA. |
| IGFBP5 | -3.59 | Homo sapiens insulin-like growth factor binding protein 5 (IGFBP5), mRNA. |
| SCN3A | -3.63 | Homo sapiens sodium channel, voltage-gated, type III, alpha subunit (SCN3A), transcript variant 1, mRNA. |
| SIRPG | -3.69 | Homo sapiens signal-regulatory protein gamma (SIRPG), transcript variant 3, mRNA. |
| FLJ41423 | -3.7 | Homo sapiens FLJ41423 protein (FLJ41423), mRNA. |
| ZNF618 | -3.8 | Homo sapiens zinc finger protein 618 (ZNF618), mRNA. |
| PTN | -3.94 | Homo sapiens pleiotrophin (PTN), mRNA. |
| PDLIM7 | -3.97 | Homo sapiens PDZ and LIM domain 7 (enigma) (PDLIM7), transcript variant 1, mRNA. |
| PCBP4 | -3.98 | Homo sapiens poly(rC) binding protein 4 (PCBP4), transcript variant 2, mRNA. |
| C3orf17 | -4.01 | Homo sapiens chromosome 3 open reading frame 17 (C3orf17), transcript variant 2, mRNA. |
| C13orf27 | -4.05 | Homo sapiens chromosome 13 open reading frame 27 (C13orf27), mRNA. |
| CALML4 | -4.09 | Homo sapiens calmodulin-like 4 (CALML4), transcript variant 2, mRNA. |
| HIST1H2BJ | -4.11 | Homo sapiens histone cluster 1, H2bj (HIST1H2BJ), mRNA. |
| GSTCD | -4.19 | Homo sapiens glutathione S-transferase, C-terminal domain containing (GSTCD), transcript variant 1, mRNA. |
| MRPL55 | -4.56 | Homo sapiens mitochondrial ribosomal protein L55 (MRPL55), nuclear gene encoding mitochondrial protein, transcript variant 4, mRNA. |
| FOSB | -4.67 | Homo sapiens FBJ murine osteosarcoma viral oncogene homolog B (FOSB), mRNA. |
| RTN2 | -5.08 | Homo sapiens reticulon 2 (RTN2), transcript variant 1, mRNA. |
| PTTG2 | -5.35 | Homo sapiens pituitary tumor-transforming 2 (PTTG2), mRNA. |
| CYR61 | -5.78 | Homo sapiens cysteine-rich, angiogenic inducer, 61 (CYR61), mRNA. |
| EGR1 | -6.81 | Homo sapiens early growth response 1 (EGR1), mRNA. |
| HTR2A | -7.49 | Homo sapiens 5-hydroxytryptamine (serotonin) receptor 2A (HTR2A), mRNA. |
